
JOURNAL INFORMATION
==============================
NLM Title Abbreviation: Hemasphere
Journal ID: Hemasphere
NLM Journal ID: 101740619
Journal ID: HEM3

HemaSphere

EISSN: 2572-9241
Publisher: Wiley

ARTICLE INFORMATION
==============================
PMCID: PMC13254490
DOI: 10.1002/hem3.70420
Article ID: HEM370420
Article version: 1
Subjects: Abstract Book, Abstract Book, Posters

Poster Sessions

Electronic publication date: 2026 Jun 10
Publication date: 2026 Jun
Volume: 10
Issue: Suppl 1
Electronic Location ID: e70420
Copyright: © 2026 The Author(s). HemaSphere published by John Wiley & Sons Ltd. on behalf of European Hematology Association.
License: This is an open access article under the terms of the http://creativecommons.org/licenses/by/4.0/ License, which permits use, distribution and reproduction in any medium, provided the original work is properly cited.
License URL: https://creativecommons.org/licenses/by/4.0/

Figure count: 0
Table count: 0
Page count: 4709

source-schema-version-number: 2.0
cover-date: June 2026
details-of-publishers-convertor: Converter:WILEY_ML3GV2_TO_JATSPMC version:6.8.0 mode:remove_FC converted:11.06.2026

==============================

eha

HemaSphere

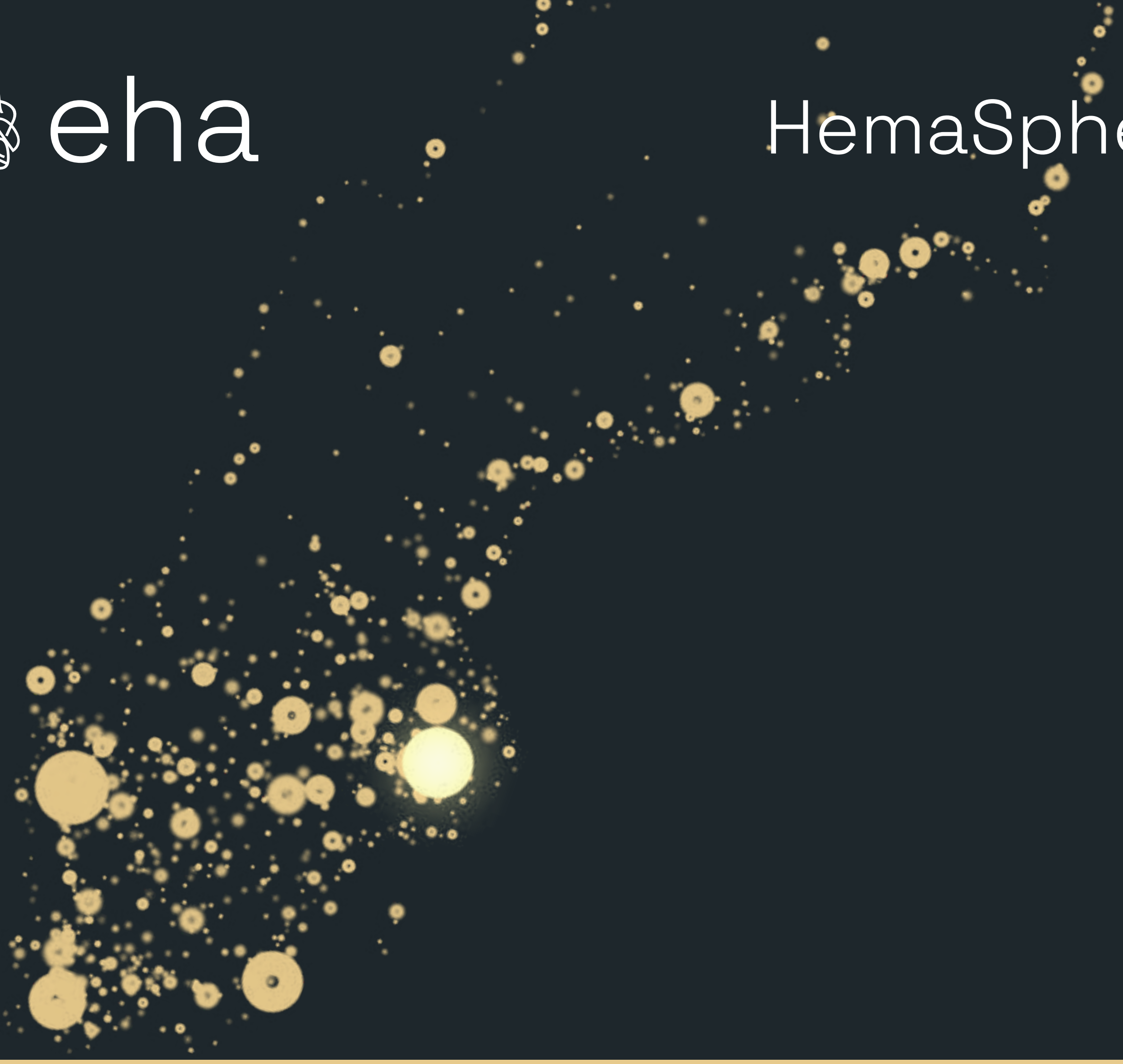

### Abstract Book

EHA2026
Congress

June 11-14
Stockholm, Sweden

Volume 10
Issue S1

www.hemaspherejournal.com

Medical Education

### Attend MM & CLL symposia at EHA2026!

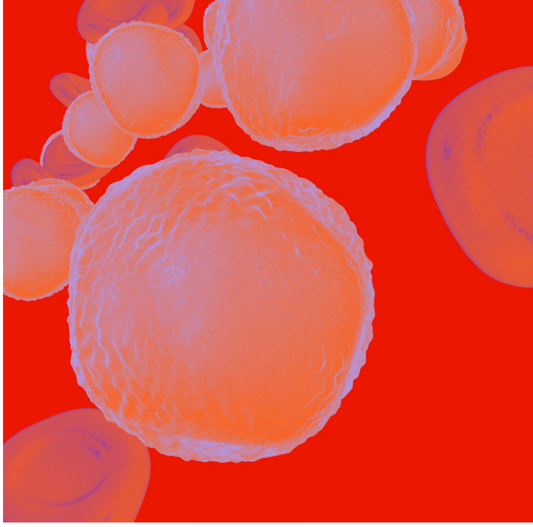

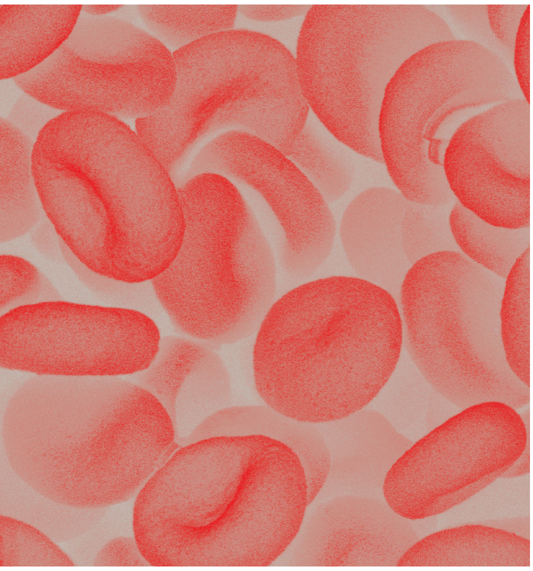

On **Thursday 11$^{th}$, Friday 12$^{th}$ and Saturday 13$^{th}$ June**, follow the red thread with us throughout an exciting programme of satellite symposia supported by Johnson & Johnson and hosted by the esteemed Symposium Committee.

Prepare your burning questions for the expert panel and get ready to participate in stimulating Q&A sessions and interactive polling activities.

Thursday 11$^{th}$, Friday 12$^{th}$ and Saturday 13$^{th}$ June 2026
10:00–11:30 CEST
Nobel Hall, Stockholmsmässan, Stockholm

Thursday 11$^{th}$ June
10:00–11:30 CEST

Weaving the red thread towards cure in newly diagnosed multiple myeloma (NDMM)

Prof. María-Victoria Mateos (Chair & Symposium Committee)
Prof. Philippe Moreau (Symposium Committee)
Prof. Paula Rodriguez-Otero
Dr Charlotte Pawlyn

Friday 12$^{th}$ June
10:00–11:30 CEST

Following the red thread in multiple myeloma (MM): aligning innovation with clinical practice

Prof. María-Victoria Mateos (Chair & Symposium Committee)
Dr Rakesh Popat (Symposium Committee)
Prof. Elena Zamagni
Dr Roberto Mina

Saturday 13$^{th}$ June
10:00-11:30 CEST

Following the red thread of evidence: guiding optimal decision-making in first-line (1L) chronic lymphocytic leukaemia (CLL)

Prof. Martin Trepel (Symposium Committee)
Dr Alessandra Tedeschi (Symposium Committee)
Dr Anna Panovská (Co-chair)
Prof. Paolo Sportoletti (Co-chair)
Dr José-Ángel Hernández-Rivas

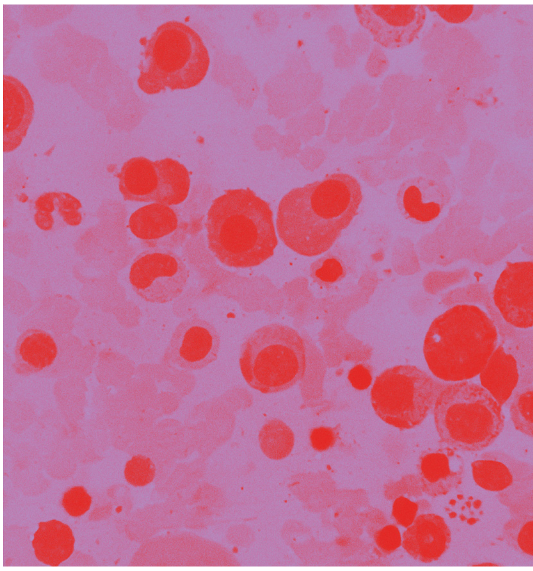

*This symposium is organised and sponsored by Johnson & Johnson and is not part of the official EHA scientific programme.*

Johnson&Johnson

Organised and supported by Janssen Pharmaceutica NV,
a Johnson & Johnson company
Janssen Pharmaceutica NV, Turnhoutseweg 30, 2340 Beerse Belgium
Copyright © 2026 Janssen Pharmaceutica NV
Job code: EM-199756 Date of prep: May 2026

31st Congress of the
European Hematology Association

EHA2026 Annual Congress Edition
June 2026

### ABSTRACT BOOK

### SECTION 02

**Author Information**

***HemaSphere*** is an open access journal powered by the European Hematology Association and dedicated to supporting hematology patient care, research, and education worldwide.

This Abstract Book of the European Hematology Association's 31st Annual Congress - EHA2026 Congress - is published as a supplement of *HemaSphere* and available online at www.hemaspherejournal.com.

Please read our author guidelines for preparing and submitting articles. Manuscripts must be submitted online via the Research Exchange submission portal.

**Article Processing Charges (APC)**

| Article types | EHA members | Non-members |
|---|---|---|
| Tier 1: Original articles, review articles, guidelines | 10%* | €2500 |
| Tier 2: Letters, perspectives | 10%* | €1500 |
| Tier 3: Editorials, comments, HemaTopics, HemaPolicy | Free | Free |
| All invited manuscripts** | Free | Free |

*2026 Promotional Discounts on APCs: 10% discount for EHA members who are lead or co-authors on an accepted submission
**APCs for all contributions on *invitation* by the Editors will be waived

**Copyright Information**
(Online) ISSN: 2572-9241
© 2026 The Author(s). *HemaSphere* published by John Wiley & Sons Ltd on behalf of European Hematology Association. This is an open access Abstract Book distributed under the Attribution-NonCommercial-NoDerivs (CC BY-NC-ND), which allows third parties to download the articles and share them with others as long as they credit the author and the Abstract Book, but they cannot change the content in any way or use them commercially.

**Rights and Permissions**
For reprints and to order copies of the Abstract Book, please email commercialreprints@wiley.com. Questions regarding permissions should be addressed to hemasphere@ehaweb.org.

**Abstract Book Citations:** Authors, Title, *HemaSphere*, 2026;10:(S1):pages.

**DOIs:**
Oral section: 10.1002/hem3.70151
Poster section: 10.1002/hem3.70152
Publication only section: 10.1002/hem3.70153

**Disclaimer**
Articles published in the journal *HemaSphere* exclusively reflect the opinions of the authors. The authors are responsible for all content in their abstracts including accuracy of the facts, statements, citing resources, etc.

HemaSphere

### Editorial Board

### Editor in Chief
**Professor Jan Cools**
*VIB-KU Leuven Center for Cancer Biology, Belgium*

### Deputy Editor in Chief
**Professor Claire Harrison**
*Guy's and St Thomas' NHS Foundation Trust, London, United Kingdom*

### Associate Editors
Simone Ferrero, *University of Torino, Italy*
Adele Fielding, *University of York, Hull York Medical School, York, United Kingdom*
Simon Hallam, *St. Bartholomew's Hospital, Barts Health NHS Trust, London, United Kingdom*
Steffen Koschmieder, *University Medical Center RWTH, Aachen, Germany*
Riitta Lassila, *University of Helsinki, Helsinki, Finland*
Marek Mraz, *CEITEC Masaryk University and University Hospital Brno, Czech Republic*
Paula Rodríguez Otero, *Clínica Universidad de Navarra, Spain*
Jürg Schwaller, *University Hospital Basel, Switzerland*
Francesca Vinchi, *Emory University School of Medicine, Atlanta, Georgia, United States*

### Scientific Editors
Charles de Bock, *Children's Cancer Institute, Sydney, Australia*
Stephen Hibbs, *Queen Mary University of London, United Kingdom*
Mandy Lauw, *Erasmus University MC, Rotterdam, The Netherlands*
Lauren Merz, *University of Michigan, Ann Arbor, Michigan, United States*
Edeghonghon Olayemi, *Vancouver Coastal Health (Richmond Hospital Site), Richmond, British Columbia, Canada*

### Statistics Editor
Robert Hills, *University of Oxford, United Kingdom*

### Editorial Manager, European Hematology Association (EHA)
Jessica Mastrodomenico, *EHA/HemaSphere, The Hague, The Netherlands*

### Editorial Board
Frederic Baron, *University of Liège, GIGA Institute, Liège, Belgium*
Grzegorz Basak, *Medical University of Warsaw, Poland*
Andrea Biondi, *University of Milano-Bicocca, Italy*
Jonathan Bond, *University College Dublin, Ireland*
Dominique Bonnet, *The Francis Crick Institute, London, United Kingdom*
Arndt Borkhardt, *University of Düsseldorf, Germany*
Monika Brüggemann, *University Hospital Schleswig-Holstein, Kiel, Germany*
David Bryder, *Lund University, Sweden*
Ryan Cassaday, *Fred Hutchinson Cancer Center, Seattle, Washington, United States*
Giancarlo Castaman, *Azienda Ospedaliera Universitaria Careggi, Florence, Italy*
Anna Castleton, *The Christie Hospital NHS Foundation Trust, Manchester, United Kingdom*
Nicholas Chiorazzi, *Northwell Health, Manhasset, New York, United States*
Emmanuelle Clappier, *Hôpital Saint-Louis, Paris, France*
Paolo Corradini, *Università degli Studi di Milano, Italy*
Lucia De Franceschi, *University of Verona, Italy*
Kim De Keersmaecker, *University of Leuven, Belgium*

Marianne De Montalembert, *Hôpital Universitaire Necker-Enfants Malades, Paris, France*
Michel Delforge, *Leuven Cancer Institute, Leuven, Belgium*
Catherine Diefenbach, *Perlmutter Cancer Center, New York, United States*
Carlos Fernández De Larrea, *Hospital Clínic Barcelona, Spain*
Francesca Gay, *University of Turin, Italy*
Paolo Ghia, *Università Vita Salute San Raffaele, Milan, Italy*
Domenico Girelli, *Universita degli Studi di Verona, Italy*
Florian Grebien, *University of Veterinary Medicine Vienna, Austria*
Hildegard Greinix, *Medical University of Graz, Austria*
Kirsten Grønbæk, *Rigshospitalet, Copenhagen, Denmark*
Iqbal Hamza, *University of Maryland School of Medicine, Baltimore, Maryland, United States*
Florian Heidel, *University Medical Center Greifswald, Germany*
Olaf Heidenreich, *Princess Máxima Center, Utrecht, The Netherlands*
Brian Huntly, *University of Cambridge, United Kingdom*
Elaine Jaffe, N*ational Cancer Institute, Bethesda, Maryland, United States*
Mats Jerkeman, *Lund University, Sweden*
Efstathios Kastritis, *National and Kapodistrian University of Athens, Greece*
Léon Kautz, *Inserm, Toulouse, France*
David Kent, *University of York, United Kingdom*
Ulf Klein, *University of Leeds, United Kingdom*
Joost Kluiver, *Universitair Medisch Centrum Groningen, The Netherlands*
Daniela Krause, *University of Frankfurt, Germany*
Nicolaus Kröger, *University Hospital Hamburg-Eppendorf, Germany*
Steven Lane, *QIMR Berghofer Medical Research Institute, Australia*
Frank Leebeek, *Erasmus MC Cancer Institute, Rotterdam, The Netherlands*
Florent Malard, *INSERM, Saint-Antoine Hospital, Paris, France*
Maria-Victoria Mateos, *University of Salamanca, Spain*
Francesco Maura, *Memorial Sloan Kettering Cancer Center, New York, United States*
Simon Mendez-Ferrer, *University of Cambridge, United Kingdom*
Thomas Mercher, *Gustave Roussy Institute, Villejuif, France*
Martina Muckenthaler, *Heidelberg University Hospital, Heidelberg, Germany*
Cesar Nombela Arrieta, *University Hospital Zurich, Switzerland*
James O'Donnell, *Royal College of Surgeons in Ireland, Dublin, Ireland*
Kostas Pantopoulos, *McGill University, Montreal, Quebec, Canada*
Jakob Passweg, *University of Basel, Switzerland*
Heike Pfeifer, *University Hospital Frankfurt, Germany*
Marc Raab, *Heidelberg University Hospital, Heidelberg, Germany*
Marc Raaijmakers, *Erasmus Medical Center Cancer Institute, Rotterdam, The Netherlands*
Alejo Rodriguez-Fratelli, *Institute for Research in Biomedicine, Barcelona, Spain*
Richard Rosenquist, *Karolinska Institutet, Stockholm, Sweden*
Deepa Sampath, *MD Anderson Cancer Center, Houston, Texas, United States*
Rebekka Schneider, *Erasmus MC, Rotterdam, The Netherlands*
Marie Scully, *University College London Hospitals, NHS Foundation Trust, London, United Kingdom*
Kostas Stamatopoulos, *CERTH, Centre for Research and Technology Hellas, Thessaloniki, Greece*
Anna Maria Sureda Balari, *Institut Català d'Oncologia, Barcelona, Spain*
Evangelos Terpos, *National and Kapodistrian University of Athens, Greece*
Alexandre Theocharides, *University Hospital Zürich, Switzerland*
Jan Trka, *University of Prague, Czech Republic*
Karen Vanhoorelbeke, *KU Leuven, Laboratory for Thrombosis Research, Kortrijk, Belgium*
Carlo Visco, *University of Verona, Italy*
Clive Zent, *University of Rochester Medical Center, Rochester, New York, United States*

eha

### Borderless Hematology

### Join us, and let’s make a difference together.

Welcome to a passionate, global network that improves patient care, advances scientific discovery, and drives innovation in hematology.

Here, every voice is heard, and every contribution matters. It’s a place where we learn, inspire, and support each other, facing challenges and celebrating victories as one.

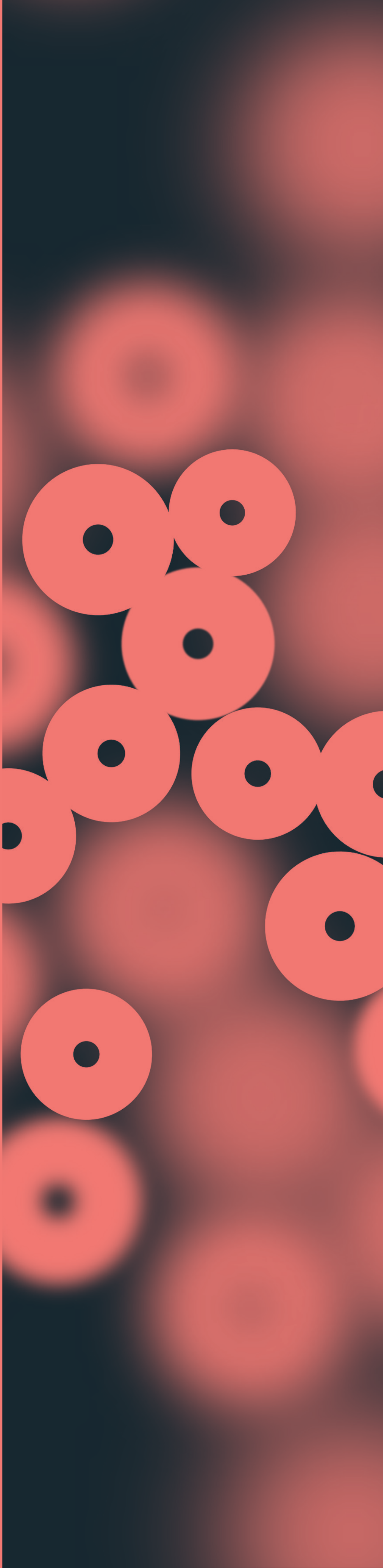

**Learn more about our community**

**EHA Board and Organization EHA2026 Congress**

**Executive Board**
K Döhner, *Germany* (President)
A Almeida, *Portugal* (Past President)
B Huntly, *United Kingdom* (President Elect)
F Leebeek, *The Netherlands* (Treasurer)
K Grønbæk, *Denmark* (Secretary)
M Alberich Jorda, *Czechia* (General member)

**Councillors**
D Bonnet, *United Kingdom*
J Delgado, *Spain*
L De Franceschi, *Italy*
M Guenova, *Bulgaria*
M Hudecek, *Germany*
S Leppä, *Finland*
S Mustjoki, *Finland*
E Terpos, *Greece*
J Trka, *Czechia*

**EHA2026 Congress Scientific Program Committee**
M Alberich Jorda, *Czechia* (Chair)
D Bonnet, *United Kingdom* (Chair Elect)
M Dreyling, *Germany* (Past Chair)
M Carlsten, *Sweden* (Local representative)
V Brousse, *France*
V Buxhofer-Ausch, *Austria*
A Cvejic, *Denmark*
M D'Agostino, *Italy*
K Giannopoulos, *Poland*
M Gomes da Silva, *Portugal*
J Hauer, *Germany*
D Hodson, *United Kingdom*
S Lehmann, *Sweden*
S Leppä, *Finland*
T Masszi, *Hungary*
K Meijer, *The Netherlands*
M Morado, *Spain*
C Papayannidis, *Italy*
D Rea, *France*
S Šaulytė Trakymienė, *Lithuania*
B Skopec, *Slovenia*
E Solomou, *Greece*
C So-Osman, *The Netherlands*
G Vassiliou, *United Kingdom*
MT Voso, *Italy*
G Weiss, *Austria*

**EHA2026 Congress SPC Advisory Board**
L Arranz, *Norway*
Y Aydinok, *Türkiye*
C Bödör, *Hungary*
M Buschbeck, *Spain*
J Caers, *Belgium*
M Della Porta, *Italy*
M Doubek, *Czechia*
F Fioredda, *Italy*
C Fox, *United Kingdom*
S Garciaz, *France*
M Gavriatopoulou, *Greece*
E Giné, *Spain*
A Glenthøj, *Denmark*
C Halsey, *United Kingdom*
V Helgason, *United Kingdom*
C João, *Portugal*
M Martello, *Italy*
J O'Sullivan, *Ireland*
F Pirenne, *France*
C Röllig, *Germany*
S Roulland, *France*
E Sala, *Germany*
M Sobas, *Poland*
A Stathis, *Switzerland*
J Thaler, *Austria*
M Themeli, *The Netherlands*
A Theocharides, *Switzerland*
C Touzeau, *France*
E van Beers, *The Netherlands*
J Werner Hansen, *Denmark*
P Woll, *Sweden*

**EHA2026 Congress Scientific Program Committee - Observers**
N Borges, *United Kingdom (Young EHA Committee)*
S Nier, *Switzerland (Patient Advocacy Committee)*

**EHA Executive Office**
Koninginnegracht 12b
2514 AA The Hague
The Netherlands
Tel: +31 (0)70 345 55 63
Fax: +31 (0)70 392 36 63
E-mail: info@ehaweb.org
Website: www.ehaweb.org

**Abstract Reviewers**
EHA would like to thank the following experts for their time and efforts reviewing abstracts for this Congress

R Advani, United States
A Agathangelidis, Greece
I Age Kos, Germany
J Aguadé-Gorgorió, Spain
S Akyol, Türkiye
A Al-Ani, Iraq
A Alanya, Belgium
J Alberge, France
M Alberich Jorda, Czechia
A Alhajahjeh, Jordan
A Almeida, Portugal
M Almowaled, Saudi Arabia
A Anastasiadi, Greece
A Anastasopoulou, Greece
A Aprile, Italy
S Aras Gürcan, Türkiye
A Armatys, Poland
L Arranz, Norway
F Auer, Germany
I Aurer, Croatia
F Awan, United States
Y Aydinok, Türkiye
E Azzoni, Italy
A Bachev, Bulgaria
E Bachy, France
A Bacquelaine Veloso, Belgium
D Baggio, United Kingdom
A Balduini, Italy
A Barrett, Ireland
A Baruchel, France
K Basso, United States
B Bátai, Hungary
J Batinic, Croatia
A Bazarbachi, Lebanon
S Bea, Spain
R Beekman, Spain
F Beier, Germany
C Bellodi, Denmark
S Bertoli, France
A Bigas, Spain
M Bishton, United Kingdom
N Bizymi, Greece
S Bjelosevic, United States
J Blatny, Czechia
I Bodo, Hungary
C Bödör, Hungary
D Bogdanic, Croatia
C Bonini, Italy
D Bonnet, United Kingdom
F Borzooee, Canada
F Bosch, Spain
C Brierley, United Kingdom
S Bringhen, Italy
J Broseus, France
V Brousse, France
P Bruch, Germany
T Brümmendorf, Germany
V Bücklein, Germany
B Burak, United States
M Buschbeck, Spain
C Buske, Germany
V Buxhofer-Ausch, Austria
J Caers, Belgium
M Callanan, France
V Camus, France
A Candoni, Italy
J Carda, Portugal
B Cardoso, Portugal
M Carlsten, Sweden
M Carpenedo, Italy
M Carter, United Kingdom
F Castagnetti, Italy
H Cavé, France
G Cengiz, Türkiye
C Chabannon, France
T Chatzikonstantinou, Greece
G Cheminet, France
X Chen, China
A Chiappella, Italy
P Christoforou, Greece
G Collins, United Kingdom
A Condoluci, Switzerland
E Crisà, Italy
J Croden, Canada
N Cross, United Kingdom
N Crump, United Kingdom
M Csanadi, Hungary
S Cuartero, Spain
A Cvejic, Denmark
M D'Agostino, Italy
F Damm, Germany
A Davies, United States
S de Botton, France
L de Franceschi, Italy
S de la Iglesia, Spain
C de Larrea, Spain
P de Nully Brown, Denmark
Z DeFilipp, United States
A Degulys, Lithuania
S Delicou, Greece
M Della Porta, Italy
O Demska, Poland
T Devos, Belgium
A Di Rocco, Italy
M Dickinson, Australia
A Dimitrijevic, Denmark
M Dimopoulos, Greece
A Ditadi, Italy
M Długosz-Danecka, Poland
M Doubek, Czechia
M Dreyling, Germany
Ľ Drgoňa, Slovakia
R Duléry, France
A Duminuco, Italy
B Eichhorst, Germany
T Eipe, India

A Eiring, United States
I El Serafi, United Arab Emirates
G Enblad, Sweden
T Eyre, United Kingdom
T Facon, France
B Faganel Kotnik, Slovenia
A Fagnan, France
S Faict, Belgium
L Falchi, United States
M Farina, Italy
B Fattizzo, Italy
G Faulhaber, New Zealand
E Fermo, Italy
H Feys, Belgium
W Fibbe, The Netherlands
F Fioredda, Italy
R Fiskesund, Sweden
S Fleming, Australia
F Forconi, United Kingdom
S Forté, Canada
D Fotiou, Greece
C Fox, United Kingdom
v Frank, The Netherlands
K Freson, Belgium
E Fronkova, Czechia
S Gandhi, United Kingdom
V Garcia, Spain
S Garciaz, France
M Gavriatopoulo, Greece
E Genesca, Spain
H Ghesquières, France
A Ghiaur, Romania
K Giannopoulos, Poland
A Gimbert, Spain
E Giné, Spain
V Gjylameti Cavolli, Kosovo
S Glavey, Ireland
H Gleitz, The Netherlands
A Glenthøj, Denmark
C Glytsou, United States
S Gohil, United Kingdom
A Gołos, Poland
M Gomes da Silva, Portugal
M Gomez Alvarez, Spain
F Gonzales, Switzerland
A Gozzetti, Italy
M Griffin, United Kingdom
E Grignano, Canada
A Gullà, Italy
L Haaksma, The Netherlands
S Haebe, Germany
C Halsey, United Kingdom
E Hatjiharissi, Greece
J Hauer, Germany
O Havranek, Czechia
J Heger, Germany
S Heibl, Austria
A Heine, Austria
J Helby Petersen, Denmark
G Held, Germany
V Helgason, United Kingdom
C Herbaux, France
D Hodson, United Kingdom
I Horvat, Croatia
K Hosokawa, Japan
S Hrabovsky, Czechia
M Hultcrantz, United States
A Hundebøll Torpe, Denmark
M Hutchings, Denmark
G Iacoboni, Spain
V Iacomi, Moldova
G Illerhaus, Germany
J Infante, Portugal
I Ionita, Romania
T Ionova, Russia
M Iqbal, United States
R Irimia, Romania
S Iyengar, United Kingdom
R Jaeger, Austria
F Jardin, France
C João, Portugal
A Jonasova, Czechia
A Jurak Begonja, Croatia
W Jurczak, Poland
B Kalaja, Albania
I Kalashnikov, Finland
T Kalina, Czechia
D Karapınar, Türkiye
A Karow, Austria
I Karsten, Germany
A Kater, The Netherlands
K Keeshan, United Kingdom
W Kern, Germany
C Khadanpour, Germany
A Khan, United Kingdom
A Kittai, United States
R Klaassen, Canada
M Klimiankou, Germany
S Klobuch, The Netherlands
C Koch, Switzerland
S Koschmieder, Germany
F Köseoğlu, Türkiye
A Kramer, The Netherlands
I Krečak, Croatia
V Krishnan, Singapore
M Ku, Australia
M Kulis, Spain
K Kuo, Canada
J Kuruvilla, Canada
H Kuusanmäki, Finland
L Lanikov, Czechia
M Lauw, The Netherlands
S Lehmann, Sweden
D Lekovic, Serbia
J Lengefeld, Sweden
S Leppä, Finland
K Lewandowski, Poland
K Liang, Belgium
H Lindqvist, Sweden
P Ljungman, Sweden
C Lo Celso, United Kingdom
L Lobo de Figueiredo Pontes, Brazil
O Lohi, Finland
C Loke, United Kingdom

C Lopez, United Kingdom
Y López García, Mexico
A Lopez Guillermo, Spain
M Lordkipanidze, Canada
S Loughran, United Kingdom
M Ludvigsen, Denmark
S Luminari, Italy
M Lupo-Stanghellini, Italy
F Lussana, Italy
E Magalhaes Rego, Brazil
M Mahevas, France
A Maiques, Spain
P Malandrakis, Greece
S Manier, France
A Marin Niebla, Spain
O Marques, Portugal
M Martello, Italy
A Martín García-Sancho, Spain
I Martin-Subero, Spain
M Massaia, Italy
T Masszi, Hungary
E McCormack, Norway
H Medyouf, Germany
K Meijer, The Netherlands
M Merz, Germany
M Metzler, Germany
M Miano, Italy
M Minnema, The Netherlands
R Moia, Italy
M Morado, Spain
A Mosquera Orgueira, Spain
D Mott, United Kingdom
I Motta, Italy
C Mueller-Tidow, Germany
A Müller, Austria
A Mussetti, Spain
M Myllymäki, Finland
N Narayan, United Kingdom
R Nelles, Australia
S Nier, Switzerland
S Nier, Switzerland
M Nijland, The Netherlands
O Nnodu, Nigeria
J Nordlund, Sweden
F Onida, Italy
R Origa, Italy
M Ormhoj, Denmark
J O'Sullivan, United Kingdom
C Owen, Canada
I Pagani, Australia
L Pagano, Italy
J Panse, Germany
H Papadaki, Greece
C Papayannidis, Italy
A Pavlovsky, Argentina
E Payne, United Kingdom
Z Peric, Croatia
F Peters, Austria
A Piekarska, Poland
F Piel, United Kingdom
L Pinczes, Hungary
F Pirenne, France
M Pirosa, Switzerland
A Plesa, France
K Plevova, Czechia
I Plo, France
C Pontikoglou, Greece
R Popat, United Kingdom
K Porkka, Finland
L Pour, Czechia
I Preloznik Zupan, Slovenia
A Puissant, France
P Quarello, Italy
A Queirós, Portugal
L Quek, United Kingdom
R Rääty, Finland
M Rab, The Netherlands
J Radocha, Czechia
S Ragaini, Italy
D Rea, France
A Reinisch, Austria
M Reiterová, Czechia
R Renella, Switzerland
S Richardson, United Kingdom
L Rigacci, Italy
I Roberts, United Kingdom
P Rodriguez, Argentina
E Roldan Galvan, United Kingdom
C Röllig, Germany
A Romano, Italy
A Roque, Portugal
S Roulland, France
A Rovó, Switzerland
E Ruggiero, Italy
N Sabljic, Serbia
E Sala, United States
I Salaverria, Spain
E Salido, Spain
J Salmanton-García, Germany
V Sampath, Singapore
M Sanchez, Spain
J Sanz, Spain
A Sarmento, Portugal
B Sarni Sanches, Brazil
J Sarry, France
T Sauer, Germany
S Šaulytė Trakymienė, Lithuania
K Savage, Canada
A Schavgoulidze, France
S Scheding, Sweden
M Schemionek, Germany
C Schinke, United States
L Schmalbrock, United States
C Schneider, United States
P Schneider, United States
A Schurich, United Kingdom
V Šeda, Czechia
L Sehn, Canada
D Serruggia, Austria
C Skedgel, United Kingdom
J Skokowa, Germany
B Skopec, Slovenia
A Slomka, Poland

L Smit, The Netherlands
M Sobas, Poland
E Solomou, Greece
C So-Osman, The Netherlands
T Standal, Norway
A Stathis, Switzerland
U Steidl, United States
F Stella, Italy
M Stelljes, Germany
Y Stepanishyna, Belgium
T Stoklosa, Poland
J Stuchly, Czechia
A Sureda, Spain
M Syed, United Kingdom
M Szopa, Poland
Ł Szukalski, Poland
A Tatic, Romania
E Tausch, Germany
P te Boekhorst, The Netherlands
J Thaler, Austria
M Themeli, The Netherlands
A Theocharides, Switzerland
M Tobiasson, Sweden
D Todorieva, Bulgaria
C Tomuleasa, Romania
T Toubai, Japan
C Touzeau, France
A Trinquand, Ireland
J Trka, Czechia
C Tsioutis, Cyprus
V Turati, Sweden
A Turki, Germany
T Tzenou, Greece
A Ukmeraj, Kosovo
E van Beers, The Netherlands
M Van Duin, The Netherlands
R van Wijk, The Netherlands
G Vassiliou, United Kingdom
L Velten, Spain
P Verhamme, Belgium
K Vettenranta, Finland
A Viardot, Germany
C Visco, Italy
M von Lilienfeld-Toal, Germany
B von Tresckow, Germany
A Voskanyan, Armenia
MT Voso, Italy
E Wagner-Drouet, Germany
Y Wang, Taiwan
U Wartiovaara-Kautto, Finland
V Wauters, Argentina
T Weber, Germany
G Weiss, Austria
J Werner Hansen, Denmark
A Wierzbowska, Poland
M Wilson, United Kingdom
R Winkler, Spain
A Woelfler, Austria
P Woll, Sweden
T Wrobel, Poland
N Yasin, Malaysia
U Yilmaz, Türkiye
S Yilmaz, Türkiye
L Zamora, Spain
S Zanwar, United States
J Zaugg, Switzerland
R Zeiser, Germany
X Zhong, Sweden
J Zubicaray, Spain
A Žučenka, Lithuania
J Zuna, Czechia
S Zver, Slovenia
M Zwaan, The Netherlands eha

### Borderless Careers

### Igniting progress in hematology by enriching your professional journey

Advance your career at every stage with the European Hematology Association. Whether you're a student, an early-career professional, or a seasoned specialist, EHA offers a vibrant community and invaluable resources to support your growth. Join us to connect with peers, access cutting-edge research, and enhance your skills through workshops and events.

Our network is not just a professional association; it is a community that nurtures growth, mentorship, and innovation in a manner that transcends geographical and intellectual borders.

To us, this is Borderless Hematology.

**Find out more about our career path here!**

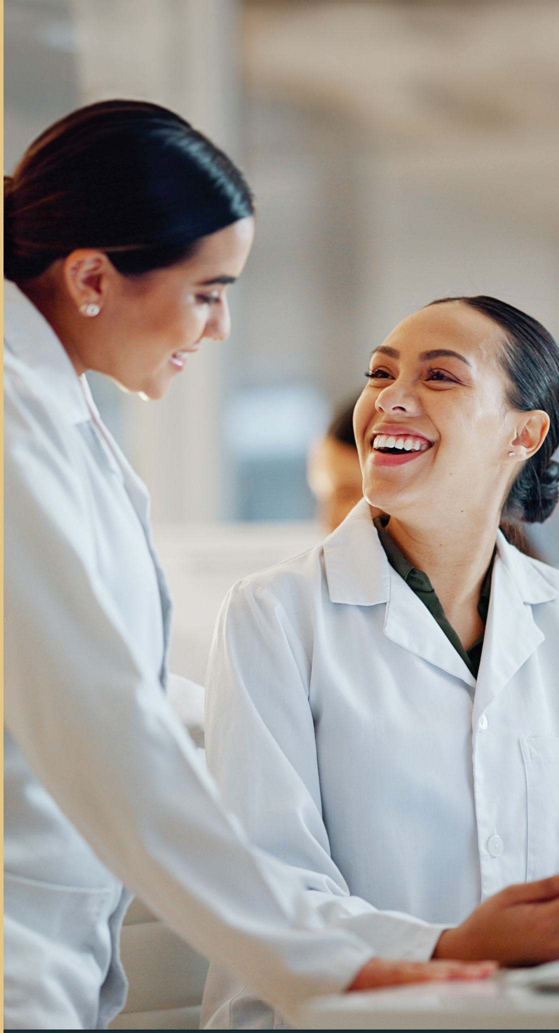

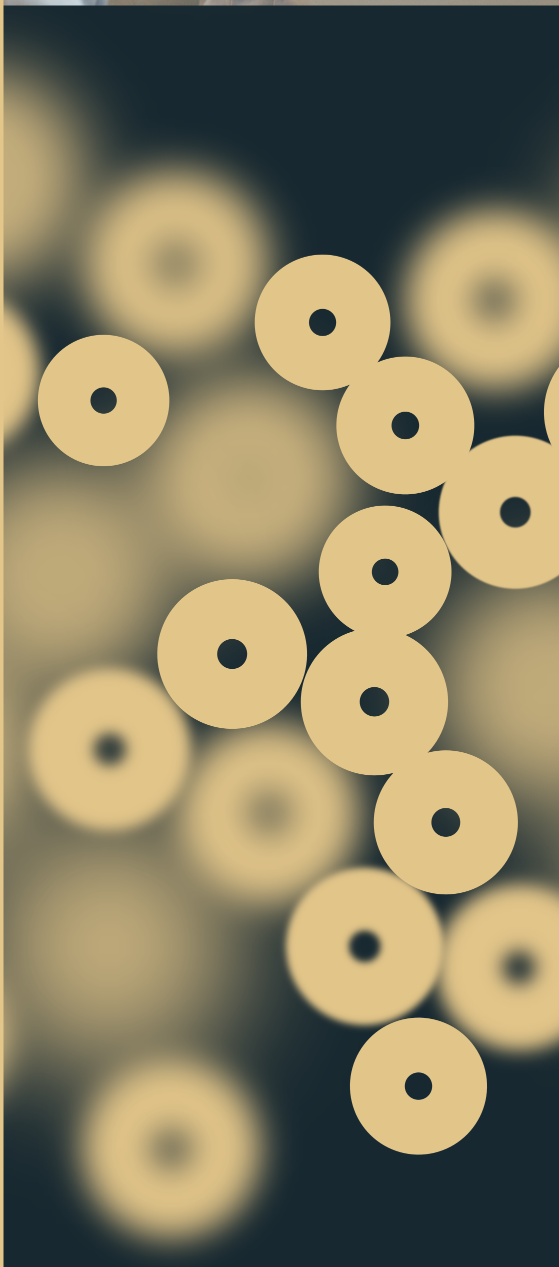

### Words of Welcome

On behalf of the EHA Board and the Scientific Program Committee, we are pleased to present to you this year's Abstract Program for the EHA2026 Congress.

We have received a record number of 4738 abstract submissions from across the globe. After a thorough and careful review process, the Scientific Program Committee has designed a comprehensive program comprising both oral and poster presentations. We are also happy to announce an increased number of Oral Sessions to accommodate the high-quality number of submissions representing all fields of hematology. The highest scoring abstracts will be presented during the Plenary Abstract Session on Saturday, June 13 from 12:00 - 13:30 CEST, one of the highlights of the Congress program.

This year we continue with the new Congress program structure introduced last year with the scientific sessions spread out of 3,5 days (Thursday to Sunday), which also positively impacts the abstract program. This change provides you with ample opportunity to attend many of them and get updated on a broad range of topics in clinical and basic science areas.

We aim to give maximum exposure to scientific work and allow for direct interaction between the authors and the Congress delegates, so do not miss this unique opportunity to discuss and network during the Poster Sessions, which have been increased from one to two! Don't miss the opportunity to attend both on Friday and Saturday. In addition, all accepted posters can be viewed on (e)poster screens in Stockholm and on the Congress platform as of Thursday, June 11 at 09:00 CEST.

Supporting the next generation of hematologists is of the highest priority for EHA and this year we are awarding close to 300 travel grants to junior members who are presenting an abstract. In addition to the grants, EHA is again awarding Young EHA Best Abstract Awards in four categories to trainees with diverse scientific backgrounds and at different stages in their career. The names of all grant winners and awardees can be found on the next pages. EHA warmly congratulates them all.

The Late-Breaking Abstract Program is designed to provide an opportunity for investigators to submit abstracts with novel data that were not yet available at the time of the regular abstract submission deadline. Multiple abstracts of the highest scientific quality have been selected for presentation during the Late-Breaking Oral Session on Sunday, June 14 from 09:15 - 10:45 CEST, so please join us there.

All abstracts are available on the EHA Library and the Congress platform.

On behalf of the EHA Board, Scientific Program Committee & Advisory Board, all reviewers, and others involved in this year's Abstract Program, we trust you will have an interesting and inspiring congress of the European Hematology Association!

Looking forward to a lively conference in Stockholm!

Prof Meritxell Alberich Jorda
Chair Scientific Program Committee of the EHA2026 Congress

### Grant Winners

For this Congress, 300 travel grants have been awarded to junior members of EHA, based on the mean score of their abstracts as well as them being the first, submitting and presenting authors.

EHA congratulates the following grant awardees:

Abarca Rios E, Czechia
Age Kos I, Germany
Aguadé-Gorgorió J, Spain
Akhlaghi T, United States
Almabadi R, United Kingdom
Angotzi F, Italy
Appelman M, The Netherlands
Aquilina C, Italy
Argiro E, The Netherlands
Arnhardt I, Germany
Arrigo G, Italy
Bacarin F, Brazil
Baden D, Germany
Baech J, Denmark
Bähler L, Switzerland
Balantinakis A, Sweden
Barrena N, Spain
Bassani Lins De Miranda L, The Netherlands
Bataller A, United States
Becking AM, The Netherlands
Bertuglia G, Italy
Bhatnagar S, United States
Biosca C, Spain
Bock A, United States
Boldrin E, Sweden
Bonsdorff A, Finland
Booth C, United Kingdom
Boscaro E, Italy
Boudry A, France
Bouhlal J, Finland
Brieva Lopez A, Spain
Bushuieva M, Ukraine
Caduc M, Germany
Campoverde L, United States
Cantera Estefanía R, Spain
Capelletti MM, Italy
Carmody C, Ireland
Caruso M, Belgium
Cavalleri A, Italy
Ceran E, Germany
Chang YS, Taiwan
Chatzikalil E, Greece
Chatzikonstantinou T, Greece
Chen Z, United Kingdom
Cherng HJ, United States
Ciboddo M, Finland
Civettini I, Italy
Colmenero A, Spain
Consoli C, Italy
Corona L, Italy
Corsale AM, Italy
Craig R, United Kingdom
Cristóbal Vargas S, Spain
Croese K, The Netherlands
Croizier C, France
D'Addona M, Italy
Das Gupta D, Germany
de la Mata L, Spain
de Wit F, The Netherlands
Decombis S, Finland
Deyell J, United Kingdom
Doeven T, The Netherlands
Donato E, Germany
Dong M, China
Dreier T, Austria
Dulla D, Czechia
Duminuco A, Italy
Eipe T, India
Ellerington R, United Kingdom
Encinas Mayoral J, United States
Evangelidis P, Greece
Falodia R, India
Faria Zeni P, Czechia
Ferdinandus J, Germany
Fernandez-Luis S, Spain
Flight K, United Kingdom
Foo CY, United States
Foston A, United Kingdom
Galanopoulos A, Germany
Gariazzo C, Italy
Garrote-de-Barros A, Spain
Gil-Alós D, Spain
Giorgi M, United States
Globisch M, Sweden
Godard A, France
Gomez-Echarte N, Spain
Goulart H, United States
Gräßel L, Germany
Grau M, Spain
Grenier J, France
Grosu TI, Ireland
Gupta H, India
Gutierrez-Padilla C, Spain
Hamer L, United Kingdom
Hannuksela N, Finland
Herzberg F, Germany
Hoekx M, United Kingdom
Horváth R, Hungary
Htut TW, United States
Hu S, China
Hundebøll Torpe A, Denmark
Hunzeker Z, United States
Ibraheem A, United Kingdom
Idrizovic A, Spain
Jia Q, United Kingdom
Julia E, France
Kalafati E, Greece
Kamizela A, United Kingdom

Kamperman Z, The Netherlands
Kato J, Japan
Kato S, Japan
Kauer J, Germany
Kaulen LD, Germany
Ketharnathan S, Canada
Khan A, United Kingdom
Khan MA, United States
Kim R, France
Kiracı Şen B, Türkiye
Koca O, Türkiye
Koch C, Switzerland
Kolarik M, Czechia
Kolijn PM, The Netherlands
Kouroutzidou AZ, Greece
Koziel S, Germany
Kreuzbauer T, Australia
Krishnan V, United States
Laaouimir L, Belgium
Lam S, United Kingdom
Larios-Martinez KI, United Kingdom
Leonard E, Sweden
Li E, China
Li Q, China
Li S, United States
Liebaert J, Germany
Liem C, Germany
Lin F, China
Ling V, United Kingdom
Liu X, United Kingdom
Loap A, France
Löffler L, Denmark
Lui D, France
M Steiner T, Australia
MacAldaz M, Canada
MacPhee L, Canada
Maegaki M, Japan
Maeng C, Denmark
Maher N, Italy
Malik A, United Kingdom
MALJI A, India
Mandleywala K, United States
Manfreda L, Italy
Manzano-Muñoz A, Spain
Márquez-López I, Spain
Marugal MA, Spain
Marvin-Peek J, United States
Masnikov D, France
Massaar S, The Netherlands
Mato S, Spain
Mecklenbrauck R, United Kingdom
Medeiros Visentini L, Brazil
Merrion C, United Kingdom
Meskes N, The Netherlands
Mey F, Canada
Michaelou A, Czechia
Miedema SE, The Netherlands
Mikulski D, Poland
Milani M, Italy
Mönnig M, Germany
Montanaro A, Italy
Morcos MNF, Germany
Murali A, Canada
Myint PT, United States
Nasiri A, Saudi Arabia
Natani S, United Kingdom
Nikolaidou K, Greece
Nostroso A, Italy
Nyamondo K, United Kingdom
Ogłoza-Puchowska A, Poland
Ong XJ, Australia
Óskarsson JP, Iceland
Otasevic V, United States
Palmer M, United States
Palo S, Sweden
Palombella L, Switzerland
Pan M, China
Paschoudi K, Greece
Pasqualini G, Italy
Paterson J, United Kingdom
Pearce J, Germany
Pedone GL, Italy
Peek C, United States
Peng L, China
Pereira M, Portugal
Perpinello S, Italy
Perzolli A, The Netherlands
Portaluri M, Italy
Prummel K, Germany
Pula A, United States
Quero-Dotor C, Spain
Ramadani P, Canada
Ramanathan R, United States
Ramirez-Gamero A, United States
Ravattinen L, Finland
Ray G, United States
Renders S, Germany
Reuvekamp T, The Netherlands
Riccardi V, Italy
Richardson A, Iceland
Rigamonti C, Italy
RInkinen O, Finland
Rivero Equiza T, Spain
Rocha AL, Belgium
Rodriguez-Res C, Spain
Roehnert M, Germany
Rosato BE, Italy
Rosun T, United Kingdom
Roussos A, Greece
Ruiz Arenas C, Spain
Russo F, Italy
Salehi M, Switzerland
Sales L, Brazil
Sambruna E, Italy
Sánchez-Castillo A, Belgium
Scaramellini N, Italy
Schaefers C, United States
Schavgoulidze A, France
Schniederjohann C, Germany
Schönung M, Germany
Schovsbo J, Denmark
Schulze N, Germany
Seeling C, Germany
Sevim O, United Kingdom

Shah A, India
Shah P, United States
Shah R, United States
Sharif MI, Canada
Sharma s, India
Shimizu Y, Japan
Shokun N, Ukraine
Shuwaikan R, Spain
Simon F, Germany
Smits F, The Netherlands
Spadea M, Italy
Staehle H, Germany
Štefíková B, Czechia
Steinhäuser S, Germany
Stompór P, Poland
Sun R, Sweden
Swart K, The Netherlands
Szepesi G, Hungary
Tameni A, Italy
Tan M, United Kingdom
Tavakoli Shirazi P, Australia
Tavernari L, Italy
Tharmaseelan H, Germany
Toho M, Japan
Tseng TC, Taiwan
Turkulainen E, Finland
Ullahammer WM, Denmark
Urizar-Compains E, Spain
Vallvé i Oliva M, Spain
Van den Bergh S, Belgium
van der Velde J, The Netherlands
Van Trimpont M, Belgium
Vasileiou E, Germany
Vedenjuoksu J, Finland
Veltmaat N, The Netherlands
Vodicka P, Czechia
Voigt S, Germany
Wan H, Sweden
Wang S, China
Wang W, China
Wannberg F, Sweden
Wei Y, China
Weinhäuser I, The Netherlands
Wencel J, France
Wiechers L, Germany
Wikström F, Sweden
Yamamoto Y, Japan
Yami A, Italy
YANG M, Japan
Yuan Y, Australia
Zaratiegui A, Spain
Zeng A, Canada
Zhang F, China
Zhang X, China
Zhu J, Finland
Zhu J, United Kingdom
Zhu Y, United Kingdom
Žučenka A, Lithuania
Zugasti I, Spain
Zuleeg MR, Germany
Zwolsman M, The Netherlands

### Young EHA Best Abstract Awards

One of the primary missions of the European Hematology Association is to support young hematology clinicians and researchers. For the fourth year after launching in 2017, we are proud to announce the Young EHA Best Abstract Awards for 2024. These are awarded to the highest-ranking abstracts in the following four categories: Clinicians or medical students training for a PhD degree, PhD research students, postdoctoral fellows, and clinical hematology trainees. We are honored that these outstanding Young EHA trainees will be presenting during the EHA Congress - they are the future of hematology!

### CLINICIANS OR MEDICAL STUDENTS TRAINING FOR A PHD DEGREE
W Wang (China)

### POSTDOCTORAL FELLOWS
C Brieghel (Denmark)

### CLINICAL HEMATOLOGY TRAINEES
A Zhang (China)

### PHD RESEARCH STUDENTS
H Bergugnat (France)

abbvie
Join Us
as we share our commitment and passion in treating patients with hematologic malignancies
Visit the AbbVie booth B2.03 in Hall C
Don't miss our AML Product Theater on
Friday, June 12, 09:35 – 09:55
EHA Theater in Hall C
abbvie.com
ALL-ONC-260008 V1.0 Approved 3/2026

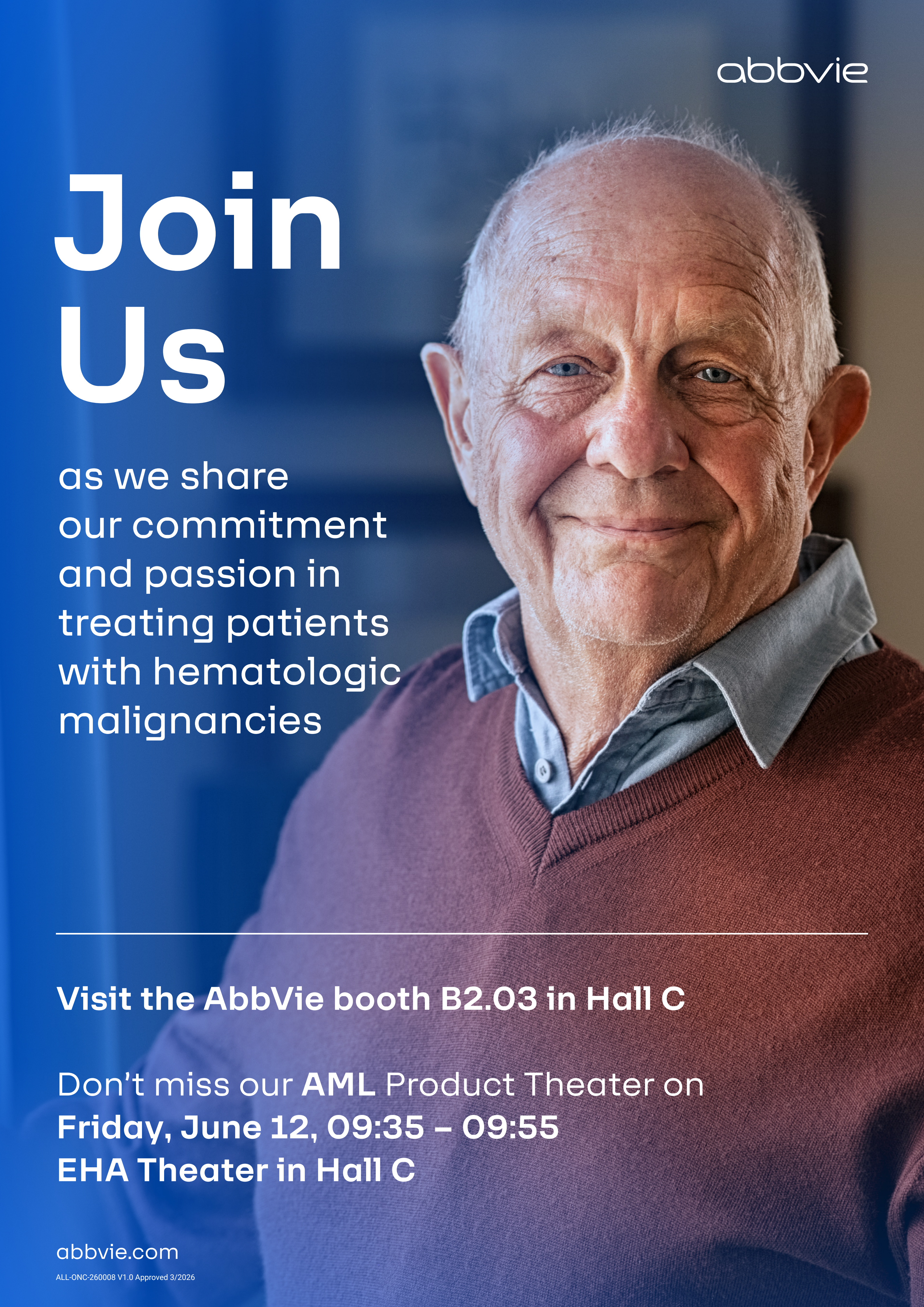

### COME SEE WHAT'S NEW IN MULTIPLE MYELOMA

Stop by the Sanofi booth to learn more sanofi

©2026 Sanofi-aventis Groupe. All rights reserved. Sanofi is a registered trademark of Sanofi or an affiliate.
MAT-GLB-2601772-v2.0-04/2026

### Have questions about multiple myeloma or myelofibrosis treatment options for your patients?

### Visit GSK Booth #E3/F3

Trademarks are owned by or licensed to the GSK group of companies.
©2026 GSK or licensor.
NP-GBL-MML-BNNR-260001 April 2026

GSK

BeOne
It's time to push the limits
of what BCL-2 inhibition can achieve
BeOne is unlocking the true power of BCL-2 inhibition with a more comprehensive development approach.
Speak with a representative at Booth G2.01
Redefine your expectations
Visit BCL2i.com
BCL-2=B-cell lymphoma-2.
BeOne Medicines is a trademark owned by BeOne Medicines I GmbH or its affiliates.
© BeOne Medicines I GmbH, 2026 All Rights Reserved. 0426-NP-PRC-077 6/2026

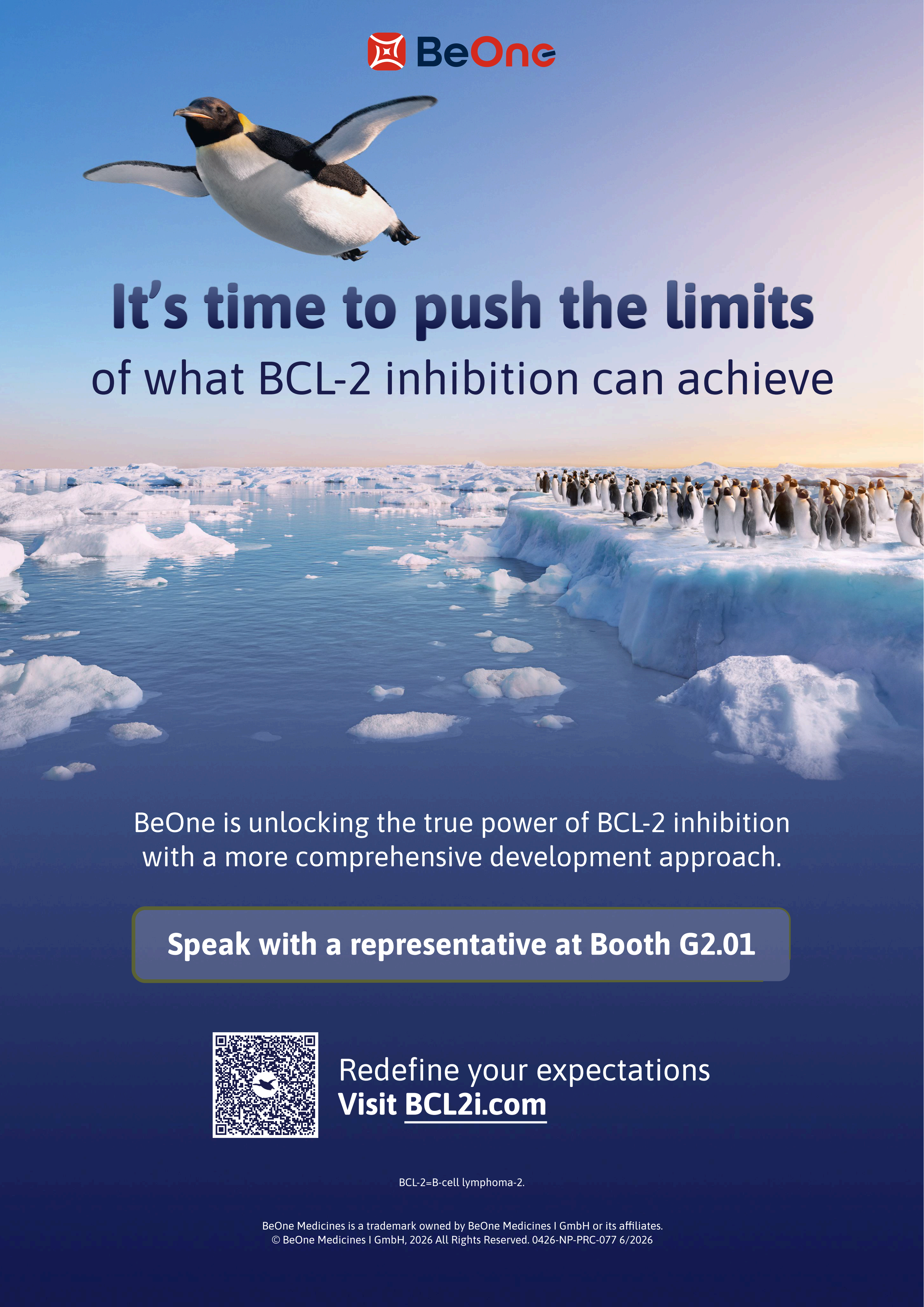

Syndax

### THE LEADER IN
### MENIN CLINICAL RESEARCH

### REVEAL ND NPM1

Revumenib Evaluation in Acute Leukemia (REVEAL-ND NPM1)

A global, actively recruiting phase 3 clinical trial investigating revumenib in combination with intensive chemotherapy for the treatment of patients aged 12 years and older with newly diagnosed AML with an *NPM1* mutation

This study is sponsored by Syndax Pharmaceuticals, Inc.

### Learn more about REVEAL-ND NPM1

**RevealTrialAML.com**

**revealsiteinquiries@syndax.com**
Please do not include any identifiable patient information in your email.
Note: Your message will be directed to a US-based email inbox.

### EVOLVE-2

A global, actively recruiting phase 3 clinical trial of revumenib in combination with azacitidine + venetoclax in adult patients with newly diagnosed *NPM1*-mutated or *KMT2A*-rearranged AML ineligible for intensive chemotherapy

This study is sponsored by HOVON, conducted by HOVON, AMLSG, UKAMLRN, ALFA, FILO, CETLAM, GIMEMA, ALLG, SAKK, Nordic AML Group, and the Beat AML Consortium, in collaboration with and funded by Syndax.

### Learn more about EVOLVE-2

**EUClinicalTrials.EU**
2024-512733-32-00

**HO177@erasmusmc.nl**
Please do not include any identifiable patient information in your email.
Note: Your message will be directed to a non-US-based email inbox.

VISIT US AT **BOOTH #A4.06**

Use of revumenib in this setting is under investigation and has not been approved by any regulatory authority worldwide for the uses under investigation.

This material is for scientific purposes only and is intended solely for healthcare practitioners.
Patient enrollment is dependent on approval by local ethics committees.

AML, acute myeloid leukemia; *KMT2A*, lysine methyltransferase 2A; *NPM1*, nucleophosmin 1.

© 2026 Syndax Pharmaceuticals, Inc. All rights reserved. The Syndax logo is a registered trademark of Syndax. All other referenced trademarks are the property of their respective owners.
MED-REV-260016 5/2026

Learn more at **syndaxmedical.com**

### B.H.D.M

BHDM is a **global initiative confronting inequality in healthcare, research and treatment** access for Black communities affected by underfunded conditions like **Sickle Cell Disease**.
Through research, advocacy and storytelling, **we challenge a system where health innovation follows profit instead of need** — pushing to make equitable healthcare impossible to ignore.

DATA.
DIGNITY.
CHANGE.
NOT OPTIONAL.

**20+ MILLION**
people worldwide live with Sickle Cell Disease.

**<1%**
of NIH funding for SCD compared to diseases with lower mortality.

**MILLIONS**
lack access to adequate care, treatment and safe infrastructure.

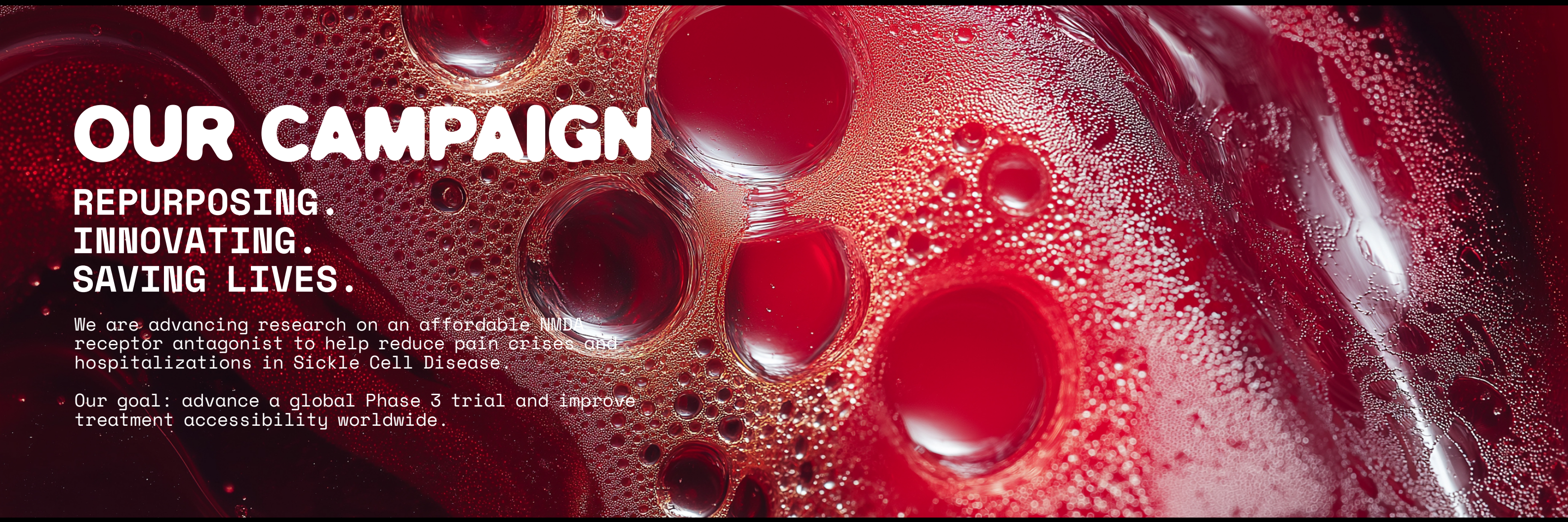

### OUR CAMPAIGN

**REPURPOSING.
INNOVATING.
SAVING LIVES.**

We are advancing research on an affordable NMDA receptor antagonist to help reduce pain crises and hospitalizations in Sickle Cell Disease.

Our goal: advance a global Phase 3 trial and improve treatment accessibility worldwide.

### JOIN the movement

Where research meets action. Join the conversation on advancing toward a Global Phase 3 Trial

### READ the research

Find the evidence recently published in **HemaSphere**, 2026, Jan 11; 10 (1): e70278

### WATCH the documentary

Discover the human stories behind the data. A film about resilience, inequity and hope.

EQUITABLE HEALTHCARE SHOULDN'T BE RADICAL

Click for more!

**SCAN NOW**
to discover what's missing from the conversation

### Table of Contents

### Poster Session 1

| | |
|---|---|
| 01. Acute lymphoblastic leukemia - Biology & translational research | PF336-PF373 |
| 02. Acute lymphoblastic leukemia - Clinical | PF374-PF409 |
| 03. Acute myeloid leukemia - Biology & translational research | PF410-PF487 |
| 04. Acute myeloid leukemia - Clinical | PF488-PF568 |
| 05. Chronic lymphocytic leukemia and related disorders - Biology & translational research | PF569-PF589 |
| 06. Chronic lymphocytic leukemia and related disorders - Clinical | PF590-PF619 |
| 07. Chronic myeloid leukemia - Biology & translational research | PF620-PF624 |
| 08. Chronic myeloid leukemia - Clinical | PF625-PF639 |
| 09. Myelodysplastic syndromes - Biology & translational research | PF640-PF658 |
| 10. Myelodysplastic syndromes - Clinical | PF659-PF678 |
| 11. Bone marrow failure syndromes incl. PNH - Biology & translational research | PF679-PF686 |
| 12. Bone marrow failure syndromes incl. PNH - Clinical | PF687-PF715 |
| 13. Myeloma and other monoclonal gammopathies - Biology & translational research | PF716-PF4568 |
| 14. Myeloma and other monoclonal gammopathies - Clinical | PF762-PF852 |
| 15. Myeloproliferative neoplasms - Biology & translational research | PF853-PF883 |
| 16. Myeloproliferative neoplasms - Clinical | PF884-PF4569 |
| 17. Hodgkin lymphoma - Clinical | PF915-PF930 |
| 18. Indolent and mantle-cell non-Hodgkin lymphoma - Clinical | PF931-PF961 |
| 19. Large B cell lymphomas - Clinical | PF962-PF1028 |
| 20. Other aggressive lymphomas | PF1029-PF1060 |
| 21. Lymphoma biology & translational research | PF1061-PF1107 |
| 22. Stem cell transplantation - Experimental | PF264-PF1114 |
| 23. Stem cell transplantation - Clinical | PF1115-PF1179 |
| 24. Hematopoiesis, stem cells and microenvironment | PF1180-PF1205 |
| 25. Gene therapy, cellular immunotherapy and vaccination - Biology & translational research | PF1206-PF1229 |
| 26. Gene therapy, cellular immunotherapy and vaccination - Clinical | PF1230-PF1257 |
| 27. Sickle cell disease | PF1258-PF1279 |
| 28. Thalassemias | PF1280-PF1289 |
| 29. Enzymopathies, membranopathies and other red blood cell disorders | PF1290-PF1298 |
| 30. Iron metabolism, deficiency and overload | PF1299-PF1307 |
| 31. Infections in hematology (incl. supportive care/therapy) | PF1308-PF1328 |
| 32. Transfusion medicine | PF1329-PF1336 |
| 33. Platelet disorders | PF1337-PF1354 |
| 34. Bleeding disorders (congenital and acquired) | PF1355-PF1366 |
| 35. Thrombosis and vascular biology | PF1367-PF4573 |
| 36. Quality of life, ethics, supportive and palliative care | PF1385-PF1407 |
| 37. Health economics | PF1408-PF1418 |
| 38. Novel technologies, techniques and digital analytical tools in hematology | PF1419-PF1434 |

### Poster Session 2

| | |
|---|---|
| 01. Acute lymphoblastic leukemia - Biology & translational research | PS1435-PS1472 |
| 02. Acute lymphoblastic leukemia - Clinical | PS1473-PS1508 |
| 03. Acute myeloid leukemia - Biology & translational research | PS1509-PS1586 |
| 04. Acute myeloid leukemia - Clinical | PS1587-PS1668 |
| 05. Chronic lymphocytic leukemia and related disorders - Biology & translational research | PS1669-PS1688 |
| 06. Chronic lymphocytic leukemia and related disorders - Clinical | PS1689-PS1718 |
| 07. Chronic myeloid leukemia - Biology & translational research | PS1719-PS1723 |
| 08. Chronic myeloid leukemia - Clinical | PS1724-PS1739 |
| 09. Myelodysplastic syndromes - Biology & translational research | PS1740-PS1757 |
| 10. Myelodysplastic syndromes - Clinical | PS1758-PS1777 |
| 11. Bone marrow failure syndromes incl. PNH - Biology & translational research | PS1778-PS1785 |
| 12. Bone marrow failure syndromes incl. PNH - Clinical | PS1786-PS1815 |
| 13. Myeloma and other monoclonal gammopathies - Biology & translational research | PS1816-PS1861 |
| 14. Myeloma and other monoclonal gammopathies - Clinical | PS1862-PS1952 |
| 15. Myeloproliferative neoplasms - Biology & translational research | PS1953-PS1982 |
| 16. Myeloproliferative neoplasms - Clinical | PS1983-PS2014 |
| 17. Hodgkin lymphoma - Clinical | PS2015-PS2030 |
| 18. Indolent and mantle-cell non-Hodgkin lymphoma - Clinical | PS2031-PS2061 |
| 19. Large B cell lymphomas - Clinical | PS2062-PS2128 |
| 20. Other aggressive lymphomas | PS2129-PS2159 |
| 21. Lymphoma biology & translational research | PS2160-PS2207 |
| 22. Stem cell transplantation - Experimental | PS2208-PS2214 |
| 23. Stem cell transplantation - Clinical | PS2215-PS2279 |
| 24. Hematopoiesis, stem cells and microenvironment | PS2280-PS2305 |
| 25. Gene therapy, cellular immunotherapy and vaccination - Biology & translational research | PS2306-PS2328 |
| 26. Gene therapy, cellular immunotherapy and vaccination - Clinical | PS2329-PS2356 |
| 27. Sickle cell disease | PS2357-PS2378 |
| 28. Thalassemias | PS2379-PS2388 |
| 29. Enzymopathies, membranopathies and other red blood cell disorders | PS2389-PS2398 |
| 30. Iron metabolism, deficiency and overload | PS2399-PS4570 |
| 31. Infections in hematology (incl. supportive care/therapy) | PS2407-PS2428 |
| 32. Transfusion medicine | PS2429-PS2435 |
| 33. Platelet disorders | PS2436-PS2453 |
| 34. Bleeding disorders (congenital and acquired) | PS2454-PS2466 |
| 35. Thrombosis and vascular biology | PS2467-PS2484 |
| 36. Quality of life, ethics, supportive and palliative care | PS2485-PS2507 |
| 37. Health economics | PS2508-PS2518 |
| 38. Novel technologies, techniques and digital analytical tools in hematology | PS2519-PS2534 |

### Poster Session 2

| | |
|---|---|
| 01. Acute lymphoblastic leukemia - Biology & translational research | PS1435-PS1472 |
| 02. Acute lymphoblastic leukemia - Clinical | PS1473-PS1508 |
| 03. Acute myeloid leukemia - Biology & translational research | PS1509-PS1586 |
| 04. Acute myeloid leukemia - Clinical | PS1587-PS1668 |
| 05. Chronic lymphocytic leukemia and related disorders - Biology & translational research | PS1669-PS1688 |
| 06. Chronic lymphocytic leukemia and related disorders - Clinical | PS1689-PS1718 |
| 07. Chronic myeloid leukemia - Biology & translational research | PS1719-PS1723 |
| 08. Chronic myeloid leukemia - Clinical | PS1724-PS1739 |
| 09. Myelodysplastic syndromes - Biology & translational research | PS1740-PS1757 |
| 10. Myelodysplastic syndromes - Clinical | PS1758-PS1777 |
| 11. Bone marrow failure syndromes incl. PNH - Biology & translational research | PS1778-PS1785 |
| 12. Bone marrow failure syndromes incl. PNH - Clinical | PS1786-PS1815 |
| 13. Myeloma and other monoclonal gammopathies - Biology & translational research | PS1816-PS1861 |
| 14. Myeloma and other monoclonal gammopathies - Clinical | PS1862-PS1952 |
| 15. Myeloproliferative neoplasms - Biology & translational research | PS1953-PS1982 |
| 16. Myeloproliferative neoplasms - Clinical | PS1983-PS2014 |
| 17. Hodgkin lymphoma - Clinical | PS2015-PS2030 |
| 18. Indolent and mantle-cell non-Hodgkin lymphoma - Clinical | PS2031-PS2061 |
| 19. Large B cell lymphomas - Clinical | PS2062-PS2128 |
| 20. Other aggressive lymphomas | PS2129-PS2159 |
| 21. Lymphoma biology & translational research | PS2160-PS2207 |
| 22. Stem cell transplantation - Experimental | PS2208-PS2214 |
| 23. Stem cell transplantation - Clinical | PS2215-PS2279 |
| 24. Hematopoiesis, stem cells and microenvironment | PS2280-PS2305 |
| 25. Gene therapy, cellular immunotherapy and vaccination - Biology & translational research | PS2306-PS2328 |
| 26. Gene therapy, cellular immunotherapy and vaccination - Clinical | PS2329-PS2356 |
| 27. Sickle cell disease | PS2357-PS2378 |
| 28. Thalassemias | PS2379-PS2388 |
| 29. Enzymopathies, membranopathies and other red blood cell disorders | PS2389-PS2398 |
| 30. Iron metabolism, deficiency and overload | PS2399-PS4570 |
| 31. Infections in hematology (incl. supportive care/therapy) | PS2407-PS2428 |
| 32. Transfusion medicine | PS2429-PS2435 |
| 33. Platelet disorders | PS2436-PS2453 |
| 34. Bleeding disorders (congenital and acquired) | PS2454-PS2466 |
| 35. Thrombosis and vascular biology | PS2467-PS2484 |
| 36. Quality of life, ethics, supportive and palliative care | PS2485-PS2507 |
| 37. Health economics | PS2508-PS2518 |
| 38. Novel technologies, techniques and digital analytical tools in hematology | PS2519-PS2534 |

### (PF336) CHRONOLOGICAL OUTCOME AND RISK FACTORS FOR CHILDREN AND YOUNG ADULTS DIAGNOSED WITH ACUTE LYMPHOBLASTIC LEUKAEMIA AND HIGH-RISK GENETICS.

**Topic:** 01. Acute lymphoblastic leukemia - Biology & translational research

Anthony Moorman*[1], Jude Gibson[1], Melvin Joy[1], Amy Kirkwood[2], Anna Lawson[3], Pamela Kearns[3], John Moppett[4], Sujith Samarasinghe[5], Ajay Vora[5], Amir Enshaei[1], Christine Harrison[1]

*[1]Newcastle University, Translational and Clinical Research Institute, Newcastle Upon Tyne, United Kingdom; [2]UCL, Cancer Research UK & University College London Cancer Trials Centre, London, United Kingdom; [3]University of Birmingham, Cancer Research UK Clinical Trials Unit (CRCTU), Birmingham, United Kingdom; [4]Bristol Royal Hospital for Sick Children, Haematology, Bristol, United Kingdom; [5]Great Ormond Street Hospital for Children, Haematology, London, United Kingdom;*

### Background

High risk genetic abnormalities (HRG) in children and young adults diagnosed with acute lymphoblastic leukaemia (ALL) are well established and important for treatment stratification. Collectively, they account for 10-15% patients but are individually rare and frequently grouped together for risk stratification and analysis.

### Aims

To determine the outcome of patients with HRG treated on consecutive clinical trials both collectively and individually. To identify additional risk factors associated with specific HRG

### Methods

We analysed individual patient data from 615 patients treated on or according to four consecutive treatment protocols: UKALLXI (n=67), UKALL97 (n=153), UKALL2003 (n=226), UKALL2011 (n=169). The cohort comprised *KMT2A* rearrangements (n=202), iAMP21 (n=164), *BCR::ABL1* (n=75), ABL-class fusions (n=58), low hypodiploidy (LH, n=47), near haploid (NH, n=43),*MYC* rearrangements (n=14) and *TCF3::HLF* (n=12). None of the patients with *BCR::ABL1* or an ABL-fusion in this cohort received a tyrosine kinase inhibitor in frontline therapy. Over the course of these four protocols trial eligibility, risk stratification criteria and routine screening changed. Hence the relative frequency of the abnormalities are not representative. Survival rates are quoted at 5 years with 95% confidence interval (CI).

**Copyright Information:** (Online) ISSN: 2572-9241
©2026 The Author(s). HemaSphere is published by John Wiley & Sons Ltd on behalf of European Hematology Association. This is an open access Abstract Book distributed under the Attribution-NonCommercial-NoDerivs (CC BY-NC-ND), which allows third parties to download the articles and share them with others as long as they credit the author and the Abstract Book, but they cannot change the content in any way or use them commercially.
**Abstract Book Citations:** Authors, Title, HemaSphere, 2026;10:(S1):pages. The abstract book DOIs can be found on the "Author Information" page.
**Disclaimer:** Articles published in the journal HemaSphere exclusively reflect the opinions of the authors. The authors are responsible for all content in their abstracts including accuracy of the facts, statements, citing resources, etc.

**Results**

Between 1990 and 2021 an increasing number of genetic abnormalities were treated as high-risk driving an increase in survival (OS) for patients with HRG from 47% (35-59) to 84% (77-89), p<0.001. Although treatment evolved considerably over this period, we noted a decrease in relapse rate (RR) for patients with HRG treated on ALL97/99, UKALL2003 and UKALL2011 who were allocated to the high-risk arm (regimen C) compared to those who were not treated as high-risk [47% (36-60) to 28% (24-33), p<0.01]. Analysis by individual abnormality showed clear evidence of improved outcome for patients with *KMT2A*-r or iAMP21 but the RR for patients with NH and LH remained high. In the whole cohort and within each protocol there was evidence of outcome heterogeneity by individual genetic abnormality with NH, *MYC*-r and *TCF3::HLF* having higher RR compared with *KMT2A*-r and iAMP21.
Analysis of risk factors per abnormality revealed important observations. Among *KMT2A*-r cases, risk factors for RR included CNS disease (positive 68% v negative 29%, p<0.01) and immunophenotype (B-cell 35% v T-cell 7%, p<0.01) but not age, partner gene or end of induction measurable residual disease (EOI MRD). Similarly, the RR for ABL-class fusion patients varied by immunophenotype [T-cell 10% (1-53) v B-cell 52% (37-68), p=0.03]. We did not observe any risk factors among patients with iAMP21 or NH. Among LH patients, male sex and EOI MRD were associated with inferior outcomes but the number of cases was modest. For both the NH and LH groups, we examined outcome according to the presence and absence of a masked clone. This was not prognostic among NH cases. However, LH patients with both clones visible had an inferior OS compared to LH cases where only one clone was detected (36% (11-63) v 79% (47-93) / 77% (54-90), p=0.01]. This difference could not be explained by changes in cytogenetic analysis over this period.

**Summary/Conclusion**

The outcome of patients with ALL and HRG has improved over the past three decades largely due to treating treatment stratification. Among most patients with HRG, age and EOI MRD were not strong predictors of outcome. The presence of HRG in T-ALL may confer a differential prognostic effect.

**Copyright Information:** (Online) ISSN: 2572-9241
©2026 The Author(s). HemaSphere is published by John Wiley & Sons Ltd on behalf of European Hematology Association. This is an open access Abstract Book distributed under the Attribution-NonCommercial-NoDerivs (CC BY-NC-ND), which allows third parties to download the articles and share them with others as long as they credit the author and the Abstract Book, but they cannot change the content in any way or use them commercially.
**Abstract Book Citations:** Authors, Title, HemaSphere, 2026;10:(S1):pages. The abstract book DOIs can be found on the “Author Information” page.
**Disclaimer:** Articles published in the journal HemaSphere exclusively reflect the opinions of the authors. The authors are responsible for all content in their abstracts including accuracy of the facts, statements, citing resources, etc.

### (PF337) SINGLE-CELL SEQUENCING REVEALS PATHWAYS AND PERTURBATIONS PREDICTIVE OF CENTRAL NERVOUS SYSTEM DISSEMINATION IN B-LINEAGE ACUTE LYMPHOBLASTIC LEUKEMIA

**Topic:** 01. Acute lymphoblastic leukemia - Biology & translational research

Luca Vincenzo Cappelli*[1], Fabio Iannelli[2], Deborah Cardinali[1], Lorenzo Fumagalli[2], Alessandro Natoni[1], Francesca Kaiser[1], Mariangela Di Trani[1], Sabina Chiaretti[1], Roberto Chiarle[2, 3, 4], Stefano Pileri[2], Robin Foà[1]

*[1]Sapienza University of Rome, Department of Translational and Precision Medicine, Rome, Italy;[2]IEO European Institute of Oncology IRCCS, Division of Hematopathology, Milan, Italy; [3]Children's Hospital and Harvard Medical School, Department of Pathology, Boston, United States of America; [4]University of Torino, Department of Molecular Biotechnology and Health Sciences, Turin, Italy;*

### Background

Central nervous system (CNS) dissemination in B-lineage acute lymphoblastic leukemia (B-ALL) is a major unmet need. Molecular predictors of CNS relapse at diagnosis are lacking, and it is unknown whether CNS-tropic leukemic subpopulations are already present in the bone marrow (BM) at disease onset.

### Aims

To define, at single-cell resolution, transcriptional programs and cellular states associated with CNS dissemination (CNS+) in B-ALL at diagnosis and to identify CNS prognostic gene sets to be implemented prospectively.

### Methods

Single-cell RNA sequencing (scRNA-seq) was performed on 15 diagnostic BM samples from B-ALL patients: 4 with isolated CNS relapse, 2 with a BM/CNS relapse, 3 with a BM relapse only, 6 with no relapse. Unsupervised and supervised differential expression analyses were used to derive a “CNS dissemination” gene set. Clustering, pseudotime trajectory inference, and gene set variation analysis (GSVA) were applied to dissect dissemination-specific programs across cellular states.

**Copyright Information:** (Online) ISSN: 2572-9241
©2026 The Author(s). HemaSphere is published by John Wiley & Sons Ltd on behalf of European Hematology Association. This is an open access Abstract Book distributed under the Attribution-NonCommercial-NoDerivs (CC BY-NC-ND), which allows third parties to download the articles and share them with others as long as they credit the author and the Abstract Book, but they cannot change the content in any way or use them commercially.
**Abstract Book Citations:** Authors, Title, HemaSphere, 2026;10:(S1):pages. The abstract book DOIs can be found on the “Author Information” page.
**Disclaimer:** Articles published in the journal HemaSphere exclusively reflect the opinions of the authors. The authors are responsible for all content in their abstracts including accuracy of the facts, statements, citing resources, etc.

**Results**

By contrasting single-cell transcriptomics CNS+ profiles with those from no relapse patients, we identified a 240 gene CNS gene set enriched for pathways and hubs including E2F targets, cell-cycle progression, MYC, MTORC1 and TNFα signaling, together with genes crucial for ALL progression and invasion, including *ZAP70, CTGF, VPREB1, LEF1, ID3, CD99* (Fig. 1A). CNS+ samples displayed an expanded population of immature blasts resembling common lymphoid progenitors (CLP), while no relapse samples were closer to a naïve B-cell state (Fig. 1B-C). Pseudotime analysis confirmed this maturation gradient, with CNS+ samples preferentially represented by early, immature B-cell stages. By GSVA analysis, the CNS gene set was significantly upregulated in blasts within these CLP-like clusters mainly enriched with CNS+ patients' cells. Importantly, this translates into an expanded population of immature blasts characterized by activation, proliferative and migratory programs. When comparing CNS+ and no relapse cells within the same maturation clusters, dissemination-associated transcriptional differences persisted, demonstrating that the CNS gene set reflected leukemic properties rather than differentiation stage alone. Comparative analyses between CNS and BM only relapses further showed that BM+ relapsing cases exhibited intermediate transcriptional profiles between CNS+ and no relapse, whereas CNS+ relapsing patients displayed distinct enrichment of migration-related genes and downregulation of homing molecules such as *CXCR4*, consistent with reduced BM niche dependence and increased dissemination propensity. To derive a clinically tractable model, we generated a 39-genes signature retaining the main features of the CNS gene set. We then validated three candidate genes (*CXCR4, CD99, ZAP70*) by RT–qPCR in an independent cohort of 26 B-ALL patients diagnostic BM samples and built a proof-of-concept model discriminating CNS+ versus no relapse cases (Fig. 1D).

**Summary/Conclusion**

We found that diagnostic BM samples from B-ALL patients who subsequently developed a CNS localization harbor pre-existing immature leukemic subpopulations enriched for proliferative and migratory transcriptional programs, reflecting intrinsic leukemic fitness and tropism for dissemination. These findings refine the biological understanding of CNS+ B-ALL and lay the ground for future prospective risk stratification strategies.

*L.V.C., F.I., D.C. and L.F. contributed equally to this work

**Copyright Information:** (Online) ISSN: 2572-9241
©2026 The Author(s). HemaSphere is published by John Wiley & Sons Ltd on behalf of European Hematology Association. This is an open access Abstract Book distributed under the Attribution-NonCommercial-NoDerivs (CC BY-NC-ND), which allows third parties to download the articles and share them with others as long as they credit the author and the Abstract Book, but they cannot change the content in any way or use them commercially.
**Abstract Book Citations:** Authors, Title, HemaSphere, 2026;10:(S1):pages. The abstract book DOIs can be found on the "Author Information" page.
**Disclaimer:** Articles published in the journal HemaSphere exclusively reflect the opinions of the authors. The authors are responsible for all content in their abstracts including accuracy of the facts, statements, citing resources, etc.

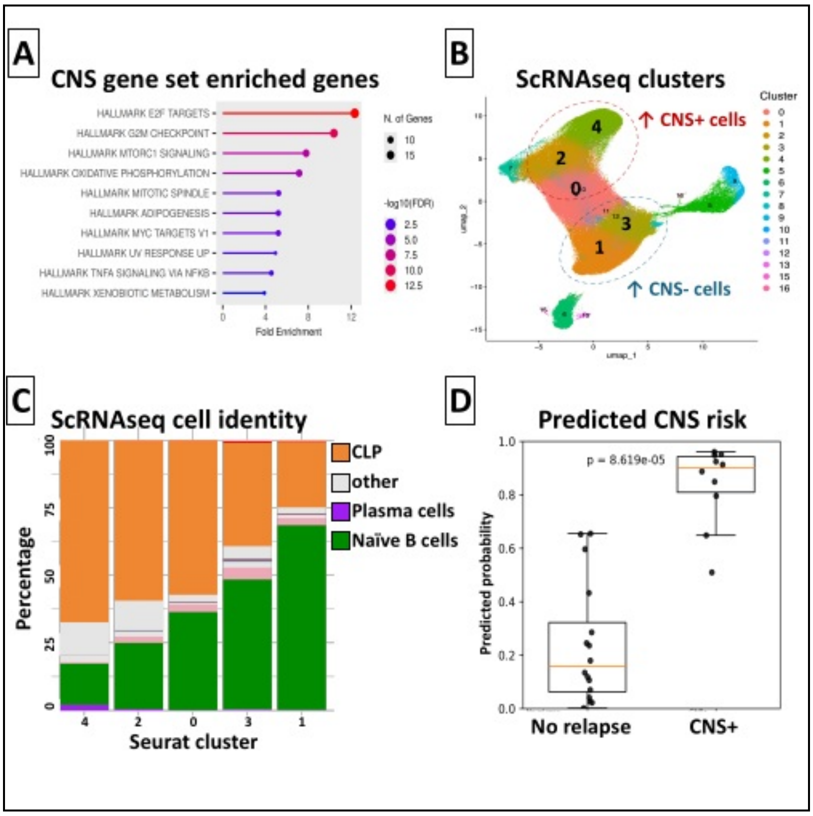

(A) Enrichment scores of genes found to be upregulated in CNS+ ALL samples vs CNS- ones ("CNS gene set"). (B) Uniform Manifold Approximation and Projection (UMAP) reduction of all 15 samples and related clusters, with cells colored by unsupervised clustering (C) Stacked barplot showing the percentage of cells inferred by SingleR from each sample group in each cluster. (D) Box-and-whisker plot showing predicted probability of CNS involvement derived from the three-gene model. CNS+ patients display significantly higher predicted probabilities compared with no relapse cases (Mann–Whitney U test, p<0.0001).

**Copyright Information:** (Online) ISSN: 2572-9241
©2026 The Author(s). HemaSphere is published by John Wiley & Sons Ltd on behalf of European Hematology Association. This is an open access Abstract Book distributed under the Attribution-NonCommercial-NoDerivs (CC BY-NC-ND), which allows third parties to download the articles and share them with others as long as they credit the author and the Abstract Book, but they cannot change the content in any way or use them commercially.
**Abstract Book Citations:** Authors, Title, HemaSphere, 2026;10:(S1):pages. The abstract book DOIs can be found on the "Author Information" page.
**Disclaimer:** Articles published in the journal HemaSphere exclusively reflect the opinions of the authors. The authors are responsible for all content in their abstracts including accuracy of the facts, statements, citing resources, etc.

### (PF338) NATURAL KILLER CELL SURVEILLANCE IS INTACT IN ETV6::RUNX1+ CORD BLOOD AND MAY PROTECT AGAINST PROGRESSION TO OVERT LEUKEMIA

**Topic:** 01. Acute lymphoblastic leukemia - Biology & translational research

Eleni Vasileiou*[1, 2, 3, 4], Nadine Rüchel[1, 2], Tiina Tuononen[5], Rigveda Nitin Bhave[1, 6], Vera Jepsen[1, 2], Julian Schliehe-Diecks[1, 2], Hazel Sahin[2, 3, 4], Sanil Bhatia[1, 2], Katharina Gössling[1], Ulrike Strube[3], Susanne Schmidt[3], Merja Heinäniemi[5], Gesine Kögler[2, 6], Arndt Borkhardt[1, 2], Ute Fischer[1, 2], Aleksandra Pandyra[1, 2, 3, 4]

*[1]Heinrich-Heine-University, Department of Pediatric Oncology, Hematology and Clinical Immunology, Düsseldorf, Germany; [2]Center for Integrated Oncology Aachen Bonn Cologne Düsseldorf (CIO ABCD), Düsseldorf, Germany; [3]University Hospital Bonn, Institute of Clinical Chemistry and Clinical Pharmacology, Bonn, Germany; [4]German Center for Infection Research (DZIF), Partner Site Bonn-Cologne, Bonn, Germany; [5]University of Eastern Finland, Institute of Biomedicine, School of Medicine, Kuopio, Finland; [6]University Hospital Düsseldorf, Institute for Transplantation Diagnostics and Cell Therapeutics, Düsseldorf, Germany;*

**Background**

*ETV6::RUNX1 (E::R)* is the most common, translocation generated fusion gene in children with BCP-ALL and already acquired *in utero*. Since only a fraction of children with this translocation develop leukemia, it has been proposed that dysregulated immune responses lead to acquisition of secondary mutations. Detection of *E::R* in early hematopoietic progenitors in cord blood (CB) allows direct evaluation of the preleukemic immune environment. Previously, we showed that mononuclear cells derived from peripheral blood (PBMCs) from *E::R*+ BCP-ALL children demonstrated cytokine hyperresponsiveness.

**Aims**

We aimed at analyzing the link between immunosurveillance and BCP-ALL development.

**Methods**

We screened a cohort of 1400 CB samples for *E::R* with the DNA-based GIPFEL assay. We immunophenotyped and evaluated the killing potential of *E::R+* and *E::R-* CB by *ex vivo* co-culture with K562 leukemia target cells. We performed scRNA-seq on *E::R+* and *E::R-* CB total nucleated cells (TNCs) (Fig. 1A). We used the NUcleic acid Linked Immuno-Sandwich Assay (NULISA™) platform to assess differences between *E::R*+ CB and serum from children two years after *E::R*+ BCP-ALL treatment. Killing assays, neutralizing antibodies and ELISAs were used for validation (Fig. 1B).

**Results**

Of 1400 unselected CB samples, 102 presented as *E::R*+ (7.28%) and none developed BCP-ALL. Immunophenotyping of *E::R+* and *E::R-* CBs, revealed a pronounced skewing towards NK cells with higher NKG2D expression and increased cytotoxic $CD56^{dim}CD16^{+}$ effector NK cells in the *E::R+* CB (Fig. 1C-D). In *ex vivo* killing assays, NK cells from *E::R+* CB maintained cytotoxicity, exhibiting increased degranulation and IFN⍰ production (Fig. 1E-G). scRNA-seq confirmed higher NK activity and revealed an activation of the TGFβ signaling pathway in *E::R*+ CB NK cells (Fig. 1H).

Proteomic analysis, employing the NULISA platform, revealed differences in immunomodulatory, antiviral, and inflammatory cytokines between *E::R+* CB and serum of children two years after *E::R*+ BCP-ALL treatment (Fig. 1I). Interferons were decreased and CX3CL1 was elevated in *E::R+* BCP-ALL. As the immune modulatory effects of the CX3CL1-CX3CR1 axis can be either pro- or anti-tumorigenic, we tested this in our models using neutralizing antibodies. Short-term inhibition of CX3CL1/CX3CR1 decreased NK cell killing and GZMB release (Fig 1J). However, chronically elevated levels could increase immunosuppressive myeloid cell infiltration and/or NK cell exhaustion leading to immunosurveillance escape.

**Copyright Information:** (Online) ISSN: 2572-9241
©2026 The Author(s). HemaSphere is published by John Wiley & Sons Ltd on behalf of European Hematology Association. This is an open access Abstract Book distributed under the Attribution-NonCommercial-NoDerivs (CC BY-NC-ND), which allows third parties to download the articles and share them with others as long as they credit the author and the Abstract Book, but they cannot change the content in any way or use them commercially.
**Abstract Book Citations:** Authors, Title, HemaSphere, 2026;10:(S1):pages. The abstract book DOIs can be found on the "Author Information" page.
**Disclaimer:** Articles published in the journal HemaSphere exclusively reflect the opinions of the authors. The authors are responsible for all content in their abstracts including accuracy of the facts, statements, citing resources, etc.

**Summary/Conclusion**

Our study demonstrated increased NK cell frequency and robust NK cell cytotoxicity in *E::R+* preleukemic CB. As none of the CB cohort developed BCP-ALL, we hypothesized that intact NK immunosurveillance may help to prevent long-term leukemic transformation. We further suggest, that immunosurveillance may be compromised in children who develop *E::R+* BCP-ALL via pathways that impair NK cell cytotoxicity including interferon signaling and the CX3CL1-CX3CR1 axis.

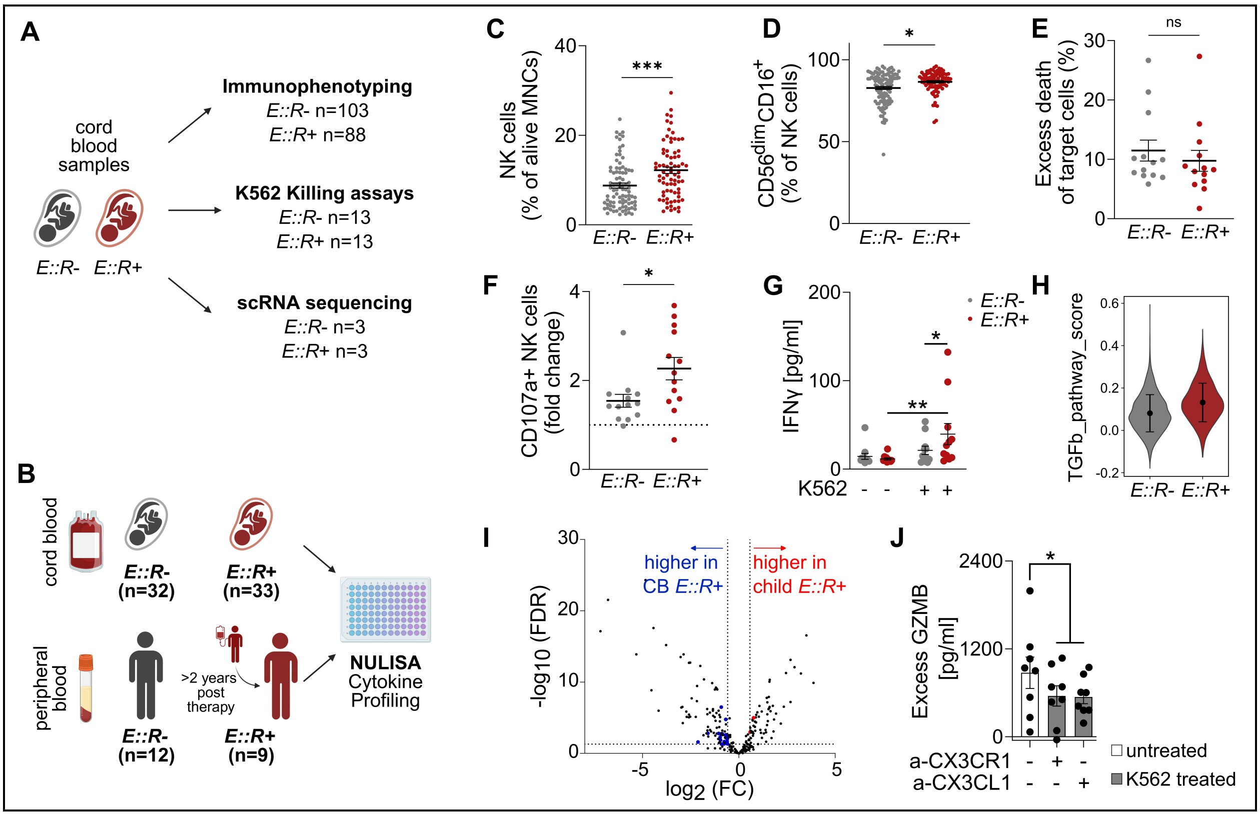

**Copyright Information:** (Online) ISSN: 2572-9241
©2026 The Author(s). HemaSphere is published by John Wiley & Sons Ltd on behalf of European Hematology Association. This is an open access Abstract Book distributed under the Attribution-NonCommercial-NoDerivs (CC BY-NC-ND), which allows third parties to download the articles and share them with others as long as they credit the author and the Abstract Book, but they cannot change the content in any way or use them commercially.
**Abstract Book Citations:** Authors, Title, HemaSphere, 2026;10:(S1):pages. The abstract book DOIs can be found on the "Author Information" page.
**Disclaimer:** Articles published in the journal HemaSphere exclusively reflect the opinions of the authors. The authors are responsible for all content in their abstracts including accuracy of the facts, statements, citing resources, etc.

### (PF339) DECITABINE-INDUCED CD38 UPREGULATION AUGMENTS DARATUMUMAB ACTIVITY IN XENOGRAFT T-ALL

**Topic:** 01. Acute lymphoblastic leukemia - Biology & translational research

Julia Heymann[1], Alexander Wurm[2, 3], Marie-Pauline Thömke[1], Ekaterina Logvinova[2, 3], Denny Takudzwa Mahachi[1], Gina Münch[1], Greta Balow[1], Melissa Cornils[1], Denis Pinkle[1], Patrick Ehm[1], Emily Funke[1], Dorothee Winterberg[4], Carina Gehlert[4], Johanna-Maria Horns[5], Thomas Valerius[5], Martin Schrappe[1], Matthias Peipp[4], Fotini Vogiatzi[1], Gunnar Cario[1], Denis Martin Schewe[2, 3], Lennart Lenk*[1]

*[1]Department of Pediatrics I, ALL-BFM Study Group, Christian-Albrechts University Kiel and University Medical Center Schleswig-Holstein, Kiel, Germany; [2]National Center for Tumor Diseases (NCT), Dresden partner site, Dresden, Germany;[3]Department of Pediatric Hematology and Oncology, University Hospital Dresden, Dresden, Germany; [4]Division of Antibody-Based Immunotherapy, Department of Medicine II, Kiel University and University Medical Center Schleswig-Holstein, Kiel, Germany; [5]Medical Department II, Hematology and Oncology, University Hospital Schleswig-Holstein, Kiel, Germany;*

**Background**

T-cell acute lymphoblastic leukemia (T-ALL) is a biologically heterogeneous hematological malignancy. Prognosis remains poor in patients with relapsed or refractory (r/r) disease and targeted therapeutic options remain limited. The CD38 antibody daratumumab (Dara) has shown antileukemic activity in T-ALL, predominantly through antibody-dependent cellular phagocytosis (ADCP). However, heterogeneous CD38 expression may limit its efficacy and target downregulation is frequently observed under Dara therapy. Thus, approaches aimed at restoring or enhancing CD38 expression may represent a promising strategy to improve treatment responses. Given that T-ALL is frequently characterized by epigenetic dysregulation, hypomethylating agents (HMAs) such as decitabine (DEC) represent attractive candidates to modulate CD38 expression through promoter demethylation.

**Aims**

We aimed to examine the preclinical efficacy of DEC and whether epigenetic priming with DEC enhances CD38 expression and the antileukemic efficacy of Dara in T-ALL models, particularly in r/r disease.

**Methods**

The effects of DEC on CD38 promoter methylation were assessed using EPIC arrays, and CD38 surface expression was analyzed *in vitro* by flow cytometry following treatment with biologically active DEC doses in the T-ALL cell lines MOLT-13, P12 and HSB-2. *In vitro* phagocytosis induced by DEC, Dara, and their combination was assessed in T-ALL cell lines (n=3) and patient-derived xenograft (PDX) cells obtained at initial diagnosis (*de novo*, n=5) or r/r disease (n=5) employing human primary macrophages as effector cells. The *in vivo* efficacy of DEC, Dara and their combination was investigated in a randomized phase II–like PDX trial using *de novo* and r/r PDX samples.

**Copyright Information:** (Online) ISSN: 2572-9241
©2026 The Author(s). HemaSphere is published by John Wiley & Sons Ltd on behalf of European Hematology Association. This is an open access Abstract Book distributed under the Attribution-NonCommercial-NoDerivs (CC BY-NC-ND), which allows third parties to download the articles and share them with others as long as they credit the author and the Abstract Book, but they cannot change the content in any way or use them commercially.
**Abstract Book Citations:** Authors, Title, HemaSphere, 2026;10:(S1):pages. The abstract book DOIs can be found on the "Author Information" page.
**Disclaimer:** Articles published in the journal HemaSphere exclusively reflect the opinions of the authors. The authors are responsible for all content in their abstracts including accuracy of the facts, statements, citing resources, etc.

### Results

DEC induced global DNA demethylation and reduced CD38 promoter methylation in T-ALL cell lines, resulting in a significant upregulation of CD38 surface expression and molecule density in all examined models.

Life-cell imaging revealed markedly enhanced macrophage-mediated phagocytosis in T-ALL cell lines treated with DEC and Dara (1.6-fold; p=0.072 and 1.6-fold; p=0.009, respectively) while the combination of both showed the strongest ADCP induction (3-fold; p<0.0001) compared with controls. Importantly, this effect was also shown for *de novo* and r/r T-ALL PDX samples, where the most significant ADCP induction was shown following DEC/Dara combination treatment (7.9-fold; p=0.0002 and 21.53-fold; p<0.0001, respectively, **Figure 1A**).

In a phase-2-like *in vivo* PDX trial, DEC single treatment for two weeks and Dara treatment both prolonged the median survival of PDX-mice (66d vs 44d; p=0.0452 and 62d vs 44d; p=0.2046). The DEC/Dara combination showed the strongest impact on median survival of PDX-mice (101d vs 44d; p=0.0042 **Figure 1B)**. Notably, two DEC/Dara-treated PDX mice were free of leukemia at the end of the study. Retrospective analysis of CD38 expression showed that CD38-high PDX samples derived the greatest benefit from combination therapy, indicating CD38 expression as a predictive biomarker for DEC/Dara response *in vivo*.

### Summary/Conclusion

Our data show that epigenetic priming with DEC increases CD38 expression and substantially enhances Dara-mediated ADCP in T-ALL models *in vitro* and *in vivo*. These findings provide a strong preclinical rationale for clinical evaluation DEC therapy in patients with r/r T-ALL, particularly in combination with Dara.

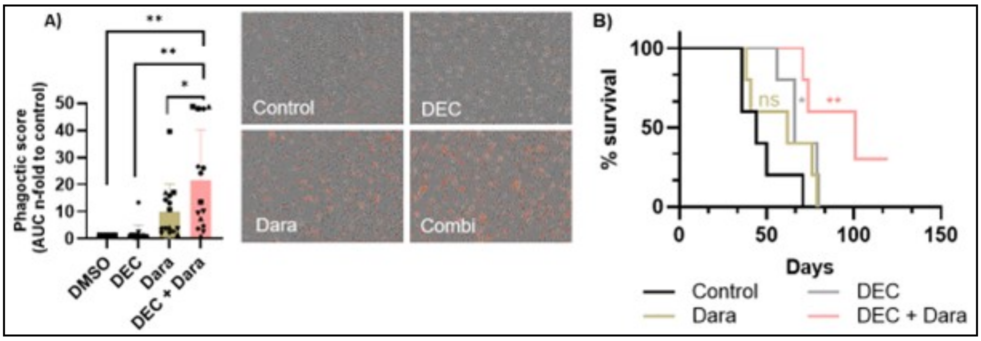

Figure 1: A) T-ALL PDX samples generated from relapsed/refractory (r/r) T-ALL samples were treated with decitabine (DEC), Daratumumab (Dara) or the combination for 48h versus dimethyl sulfoxide (DMSO) control). PDX cells were investigated for phagocytosis/ADCP using primary macrophages from healthy donors as effector cells and IncuCyte live-cell imaging as readout. The number of phagocytosing macrophages is depicted as a normalized phagocytic score. Statistical assessment of n=3 experiments per PDX samples (left) and representative pictures of one experiment (right) are depicted; nested one-way ANOVA. B) Four NSG mice per patient were transplanted with PDX cells amplified from patient samples obtained from initial diagnosis (de novo) or r/r T-ALL. Following engraftment of PDX T-ALL cells, mice were randomized into four treatment groups, receiving either vehicle control (NaCl), Dara alone, DEC alone, or the combination (DEC + Dara). Kaplan-Meier survival analysis of mice transplanted with n=5 CD38-high T-ALL PDX samples.

**Copyright Information:** (Online) ISSN: 2572-9241
©2026 The Author(s). HemaSphere is published by John Wiley & Sons Ltd on behalf of European Hematology Association. This is an open access Abstract Book distributed under the Attribution-NonCommercial-NoDerivs (CC BY-NC-ND), which allows third parties to download the articles and share them with others as long as they credit the author and the Abstract Book, but they cannot change the content in any way or use them commercially.
**Abstract Book Citations:** Authors, Title, HemaSphere, 2026;10:(S1):pages. The abstract book DOIs can be found on the "Author Information" page.
**Disclaimer:** Articles published in the journal HemaSphere exclusively reflect the opinions of the authors. The authors are responsible for all content in their abstracts including accuracy of the facts, statements, citing resources, etc.

### (PF340) COOPERATIVE GENETIC LESIONS INDUCE AEROBIC GLYCOLYSIS IN B-ALL, CREATING A THERAPEUTICALLY EXPLOITABLE METABOLIC VULNERABILITY

**Topic:** 01. Acute lymphoblastic leukemia - Biology & translational research

Dennis Das Gupta*[1], Britta Steer[1], Denis Pinkle[1, 2], Lea Hoeft[1], Lauritz Miarka[3], Nadine Wolgast[1], Katharina Iben[1], Thomas Beder[1], Miriam Denker[1], Theodor Hutter[1], Carla Pieper[1], Lennart Lenk[2], Lars Fransecky[1], Claudia Baldus[1], Lorenz Bastian[1]

*[1]University Medical Center Schleswig-Holstein, Campus Kiel, Internal Medicine II, Hematology and Oncology, Kiel, Germany; [2]University Medical Center Schleswig-Holstein, Campus Kiel, Pediatrics, Kiel, Germany; [3]University Medical Center Freiburg, Institute of Neuropathology, Freiburg, Germany;*

### Background

Despite high response rates in first-line therapy, survival in relapsed B-cell acute lymphoblastic leukemia (B-ALL) remains poor. Recently, increased glycolytic activity at diagnosis has been shown to be a hallmark of relapse-associated leukemia cells (Liu *et al.*, Blood 2026). Whereas metabolic reprogramming towards aerobic glycolysis (i.e. Warburg effect) confers therapy resistance in myeloid leukemia, its implication in B-ALL is insufficiently characterized.

### Aims

To characterize the cooperative genomic-metabolic reprogramming in high-risk B-ALL and evaluate therapeutic targeting of this dependency.

### Methods

We generated a stepwise leukemogenesis model in murine Pre-B cells using retroviral overexpression of constitutively active (ca)*STAT5* or *BCR::ABL1* combined with CRISPR/Cas9 knockout of *Ikzf1* or *Cdkn2a*. SCENITH-assay assessed metabolic profiling. Single-nucleus (sn)RNA-seq of *BCR::ABL1*$^{+}$ *Cdkn2a*-ko transplanted mice characterized gene regulation. An RNA-based Warburg Index was established from 15 diagnostic B-ALL samples versus healthy B-cell progenitor subsets and validated in 515 diagnostic samples (IntegrateALL cohort, spanning 17 ICC/WHO subtypes). Dichloroacetic acid (DCA) (0.1-10 mM) and venetoclax (1 nM-10 µM) effects were evaluated in murine cells and human B-ALL cell lines (SUP-B15, SD-1, 697, NALM-6). BH3 profiling assessed mitochondrial priming.

### Results

Stepwise leukemic transformation induced progressive metabolic reprogramming toward glycolysis. Enrichment of HALLMARK glycolysis signature (NES 1.77, FDR $1.06\times10^{-4}$) was observed in both preleukemia and leukemia stages compared to normal or ca*STAT5*$^{+}$ Pre-B cells. However, near-complete mitochondrial independence (SCENITH) required cooperative interaction of *BCR::ABL1* with *Ikzf1* or *Cdkn2a* deletion, confirming OXPHOS-independent aerobic glycolysis as unique to fully transformed leukemia. snRNA-seq of leukemia-transplanted mice confirmed enhanced glycolysis programs in leukemic blasts versus normal hematopoiesis, with marked upregulation of *Pdk1*, a master regulator of aerobic glycolysis, specifically in leukemia cells. In human B-ALL, our RNA-based "Warburg Index" readily separated 515 diagnostic B-ALL samples from healthy precursors (F1=1.0, AUC=1.0) and robustly correlated with *PDK1* expression ($\rho=0.33$, $p<0.0001$), establishing high Warburg Index as a pan-B-ALL feature. Accordingly, targeting *PDK1* with its clinical-grade inhibitor DCA to force OXPHOS recoupling preferentially induced apoptosis in murine leukemia models versus normal Pre-B cells. Finally, DCA treatment increased cellular dependence on BCL-2 and BCL-XL, resulting in robust synergy with venetoclax across four B-ALL cell lines (mean Bliss scores: 3.26-22.65).

**Copyright Information:** (Online) ISSN: 2572-9241
©2026 The Author(s). HemaSphere is published by John Wiley & Sons Ltd on behalf of European Hematology Association. This is an open access Abstract Book distributed under the Attribution-NonCommercial-NoDerivs (CC BY-NC-ND), which allows third parties to download the articles and share them with others as long as they credit the author and the Abstract Book, but they cannot change the content in any way or use them commercially.
**Abstract Book Citations:** Authors, Title, HemaSphere, 2026;10:(S1):pages. The abstract book DOIs can be found on the "Author Information" page.
**Disclaimer:** Articles published in the journal HemaSphere exclusively reflect the opinions of the authors. The authors are responsible for all content in their abstracts including accuracy of the facts, statements, citing resources, etc.

### Summary/Conclusion

Fusion oncogenes cooperate with secondary alterations to induce aerobic glycolysis during B-ALL leukemogenesis, representing a therapeutically exploitable vulnerability. Targeting the Warburg regulator *PDK1* via DCA induces mitochondrial apoptosis preferentially in fully transformed leukemia cells and robustly potentiates venetoclax across multiple genomic drivers, supporting combined metabolic-BCL2 directed therapy to target relapse-associated glycolytic B-ALL cells.

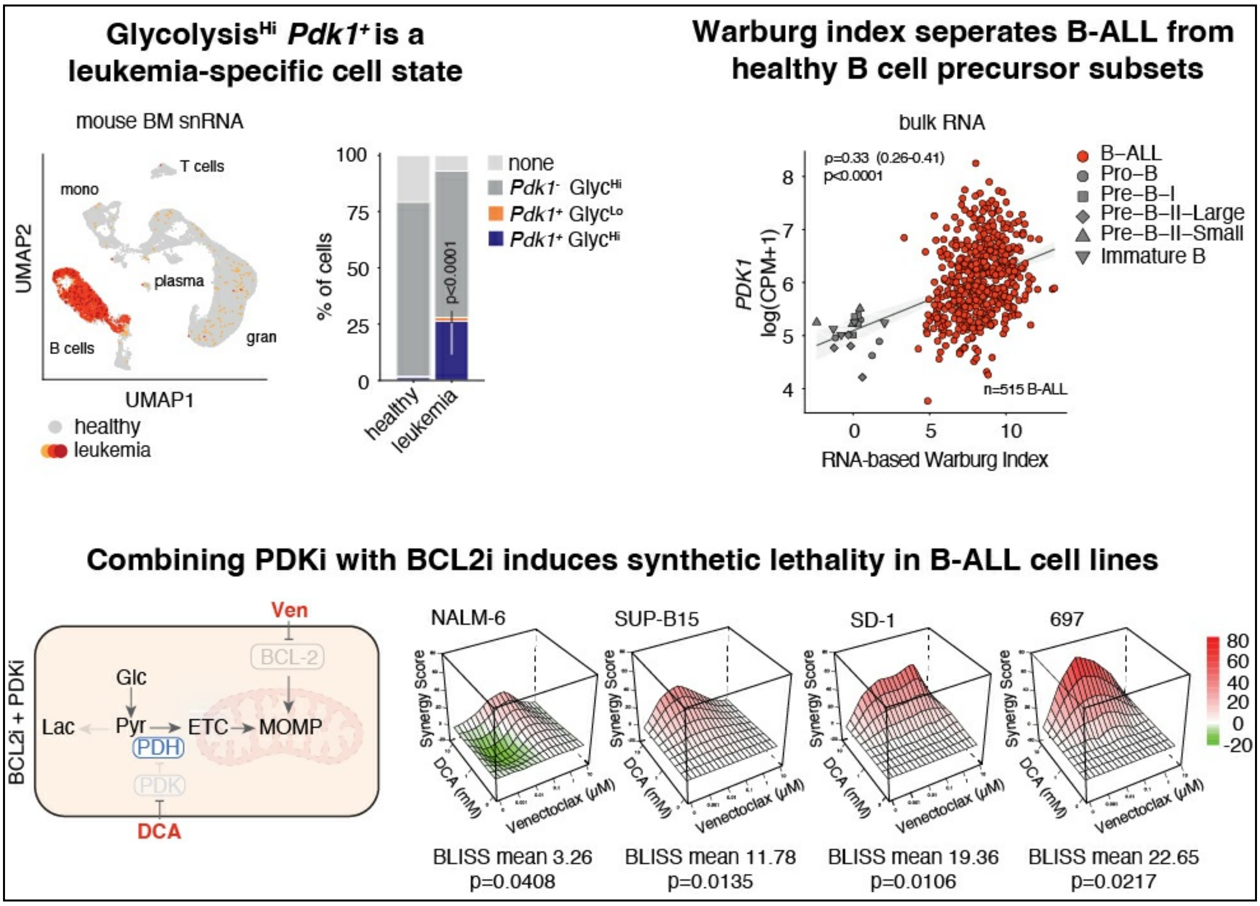

**Copyright Information:** (Online) ISSN: 2572-9241
©2026 The Author(s). HemaSphere is published by John Wiley & Sons Ltd on behalf of European Hematology Association. This is an open access Abstract Book distributed under the Attribution-NonCommercial-NoDerivs (CC BY-NC-ND), which allows third parties to download the articles and share them with others as long as they credit the author and the Abstract Book, but they cannot change the content in any way or use them commercially.
**Abstract Book Citations:** Authors, Title, HemaSphere, 2026;10:(S1):pages. The abstract book DOIs can be found on the "Author Information" page.
**Disclaimer:** Articles published in the journal HemaSphere exclusively reflect the opinions of the authors. The authors are responsible for all content in their abstracts including accuracy of the facts, statements, citing resources, etc.

### (PF341) INTEGRATED MULTI-OMIC PROFILING OF MRD IDENTIFIES PLASTICITY-DRIVEN RESISTANCE PROGRAMS IN B-ALL

**Topic:** 01. Acute lymphoblastic leukemia - Biology & translational research

Riccardo Scribano[1], Adrian Delgado[2], Pablo Domizi[2], Martina Basilico[1], Chiara Buracchi[3], Astraea Jager[2], Abhishek Koladiya[2], Timothy Keyes[2], Joseph Kang[2], Cristina Bugarin[3], Erin Craig[4], Giuseppe Gaipa[3], Kara Davis[2], Jolanda Sarno*[1]

*[1]University of Milano Bicocca, Medicine and Surgery, Monza, Italy; [2]Stanford University, Pediatrics, Stanford, United States of America; [3]Tettamanti Research Center, Fondazione IRCCS San Gerardo Dei Tintori, Monza, Italy; [4]University of Michigan, Department of Biostatistics, Ann Arbor, United States of America;*

### Background

Relapsed B-cell Acute Lymphoblastic Leukemia (B-ALL) remains an unmet clinical need for pediatric patients. Minimal Residual Disease (MRD) detection is a strong prognostic predictor, however the mechanisms underlying treatment resistance in MRD cells are still not well understood. To improve relapsed ALL outcomes, it is critical to identify cellular populations and features causing treatment failure at very early stages of the disease.

### Aims

Our study aims at revealing cellular features predictive of relapse at early MRD timepoints with the final goal to identify a gene/protein signature that can be used to further refine risk stratification and to identify targetable pathways of resistant cells.

### Methods

To do so, we performed single-cell analyses on longitudinal samples of 48 pediatric B-ALL patients enrolled on the AIEOP-BFM ALL 2009 clinical trial (NCT01117441) collected at different timepoints (diagnosis, day 8, day 15 and relapse) for a total of 146 samples analyzed. These samples were profiled using a 42-parameter CyTOF panel and leukemic blasts were aligned to their closest healthy counterpart before being used to train a LASSO logistic regression model for multiple instance learning (MMIL). In addition, we performed CITE-seq on a subset of the longitudinal samples (n=26) collected from 11 patients. Cell types were annotated using the BoneMarrowMap dataset and molecular subtype classification using the ALLCatchR classifier. Finally, we integrated CyTOF and CITEseq data using common markers (n=15) measured across the two technologies.

### Results

To identify populations predictive of the outcome we performed developmental B-cell classification of all the leukemic blasts profiled by CyTOF and we identified that relapsing patients (25% of our cohort) have an increased proportion of Pre-B cells compared to CR patients at day 15. To investigate the contribution of individual cells to patient outcome, we used MMIL, a classification model based on multiple instance learning.
The model identified significantly higher abundance of relapse-associated cells and higher mean resistance probabilities in relapsing patients ($p < 0.01$), independently of their clinical risk status (standard, intermediate or high).
Analysis of the model's coefficients suggest that relapse-predictive cells are phenotypically plastic with a quiescent stem-like phenotype, characterized by higher expression of progenitor markers (CD34 and CD304) and lower expression of signaling molecules (pSYK, pBTK).
In addition, integrated transcriptomic data showed that MRD cells of relapsing patients exhibit a metabolic rewiring from glycolysis towards oxidative phosphorylation and that resistant cells identified by MMIL are associated with an immature phenotype suggesting lineage infidelity as a potential hallmark of treatment resistance.

**Copyright Information:** (Online) ISSN: 2572-9241
©2026 The Author(s). HemaSphere is published by John Wiley & Sons Ltd on behalf of European Hematology Association. This is an open access Abstract Book distributed under the Attribution-NonCommercial-NoDerivs (CC BY-NC-ND), which allows third parties to download the articles and share them with others as long as they credit the author and the Abstract Book, but they cannot change the content in any way or use them commercially.
**Abstract Book Citations:** Authors, Title, HemaSphere, 2026;10:(S1):pages. The abstract book DOIs can be found on the "Author Information" page.
**Disclaimer:** Articles published in the journal HemaSphere exclusively reflect the opinions of the authors. The authors are responsible for all content in their abstracts including accuracy of the facts, statements, citing resources, etc.

**Summary/Conclusion**

In this study we successfully integrated CyTOF and CITE-seq data to uncover the transcriptional profiles of functionally distinct phenotypes linked to early treatment resistance in pediatric B-ALL. MMIL classification identified exceedingly rare cell populations in early MRD timepoints of which the abundance recapitulates patients' outcomes. These findings lay the ground for a more integrative model of early resistance that takes into account the developmental classification, functional state and transcriptional programs of resistant cells. Such approach could be leveraged to inform the development of novel therapeutic strategies aimed at preventing relapse and outcomes.

**Copyright Information:** (Online) ISSN: 2572-9241
©2026 The Author(s). HemaSphere is published by John Wiley & Sons Ltd on behalf of European Hematology Association. This is an open access Abstract Book distributed under the Attribution-NonCommercial-NoDerivs (CC BY-NC-ND), which allows third parties to download the articles and share them with others as long as they credit the author and the Abstract Book, but they cannot change the content in any way or use them commercially.
**Abstract Book Citations:** Authors, Title, HemaSphere, 2026;10:(S1):pages. The abstract book DOIs can be found on the "Author Information" page.
**Disclaimer:** Articles published in the journal HemaSphere exclusively reflect the opinions of the authors. The authors are responsible for all content in their abstracts including accuracy of the facts, statements, citing resources, etc.

### (PF342) EXPLORING THE NON-CANONICAL FUNCTION OF EZH2 IN T-CELL ACUTE LYMPHOBLASTIC LEUKEMIA

**Topic:** 01. Acute lymphoblastic leukemia - Biology & translational research

Stef Van den Bergh*[1, 2], Nicole Mentens[1, 2], Lien Boogaerts[1, 2], Olga Gielen[1, 2], Kris Jacobs[1, 2], Krista Verhoeft[1, 2], Marlies Vanden Bempt[3], Guillaume Andrieu[4, 5], Aurore Touzart[4, 5], Sofie Demeyer[1, 2], Alexandra Veloso[6], Jan Cools[1, 2]

*[1]KU Leuven, Laboratory for the Molecular Biology of Leukemia, Department of Human Genetics, Leuven, Belgium,[2]VIB-KU Leuven, Center for Cancer Biology, Leuven, Belgium; [3]KU Leuven, Laboratory of Experimental Hematology, Department of Oncology, Leuven, Belgium; [4]Institut Necker Enfants-Malades, INSERM U1151 CNRS Université Paris Cité, Paris, France; [5]Laboratoire d'Onco-Hématologie, Hôpital Necker-Enfants Malades (APHP), Paris, France;[6]University of Liège, GIGA Institute, Laboratory of Hematology, Liège, Belgium;*

### Background

T-cell acute lymphoblastic leukemia (T-ALL) is a hematological cancer characterized by the presence of (epi)genomic lesions. A subset of T-ALL cases have mutations in *EZH2*, *SUZ12* or *EED*, which belong to the Polycomb Repressive Complex 2 (PRC2). Strikingly, *SUZ12* and *EED* mutations are typically inactivating mutations (large deletions and indels), while *EZH2* more often has missense mutations. These cases commonly harbor activating mutations in the tyrosine kinase JAK3 and are associated with the immature early T-cell precursor subtype. While EZH2 is well-known as the enzymatic subunit of the PRC2 that catalyzes the gene repressive H3K27me3 marker, recent studies also observed a PRC2-independent function of EZH2 as a transcriptional activator. Moreover, JAK3 is known to phosphorylate EZH2, which promotes its release from the PRC2. We hypothesized that non-canonical EZH2 activity might be a major driver in PRC2 and *JAK3* co-altered T-ALL. However, the underlying mechanisms and therapeutic value of non-canonical EZH2 activity in T-ALL is currently unknown.

### Aims

In this study, we aim to uncover if there is a non-canonical function of EZH2 as a transcriptional activator in T-ALL. We therefore characterized the members and target genes of EZH2 transcriptional activator complexes. Moreover, we studied potential therapeutic targets of the non-canonical function of EZH2.

### Methods

To study EZH2's non-canonical function, mouse and PDX models with *Suz12* inactivation and/or *Jak3* activation were generated and characterized. Using these models, members of non-canonical complexes of EZH2 were identified by immunoprecipitation-mass spectrometry (IP-MS). Target genes of the non-canonical EZH2 were identified by ChIP- and RNA-seq. To study potential therapeutic targets, *ex vivo* treatments were performed followed by Annexin V staining.

**Copyright Information:** (Online) ISSN: 2572-9241
©2026 The Author(s). HemaSphere is published by John Wiley & Sons Ltd on behalf of European Hematology Association. This is an open access Abstract Book distributed under the Attribution-NonCommercial-NoDerivs (CC BY-NC-ND), which allows third parties to download the articles and share them with others as long as they credit the author and the Abstract Book, but they cannot change the content in any way or use them commercially.
**Abstract Book Citations:** Authors, Title, HemaSphere, 2026;10:(S1):pages. The abstract book DOIs can be found on the "Author Information" page.
**Disclaimer:** Articles published in the journal HemaSphere exclusively reflect the opinions of the authors. The authors are responsible for all content in their abstracts including accuracy of the facts, statements, citing resources, etc.

**Results**

Mice with *Suz12* inactivation and *Jak3* activation showed significantly shorter disease latency compared to mice with only one of these mutations. Remarkably, this was not the case for *Ezh2* inactivation, indicating the T-ALL development was not due to simple PRC2 inactivation. Moreover, treatment of *Suz12* inactivated cells with EZH2 degraders strongly induced apoptosis, which was much lower in *Suz12* wild-type cells. Together, this indicates that EZH2 is crucial in T-ALL with *Suz12* inactivation. Using these models, IP-MS revealed binding of EZH2 with a set of transcriptional (co-)activators in *Suz12* inactivated cells, including the embryonic stem cell BAF, TIP60 and SAGA complexes, which are main regulators of stem cell identity. In line, we detected by ChIP-seq that EZH2 binds at the promoter regions of genes that activate stem cell activity. Accordingly, RNA-seq on these cells after *ex vivo* treatment with EZH2 degraders showed downregulation of stem cell-associated transcriptional pathways, such as Hedgehog, MYC and Wnt signaling. Finally, we observed that *Jak3* and *Suz12* co-altered leukemic cells displayed a higher sensitivity to several compounds targeting non-canonical EZH2 activity *ex vivo.*

**Summary/Conclusion**

Our data indicates that EZH2 is a dependency factor in SUZ12 inactivated T-ALL and that EZH2 binds a set of transcriptional (co-)activators, that are known stem cell regulators. As such, non-canonical complexes of EZH2 activate transcriptional programs underlying stem cell identity. Targeting non-canonical EZH2 activity might be a potential strategy to treat this subgroup of T-ALL patients.

**Copyright Information:** (Online) ISSN: 2572-9241
©2026 The Author(s). HemaSphere is published by John Wiley & Sons Ltd on behalf of European Hematology Association. This is an open access Abstract Book distributed under the Attribution-NonCommercial-NoDerivs (CC BY-NC-ND), which allows third parties to download the articles and share them with others as long as they credit the author and the Abstract Book, but they cannot change the content in any way or use them commercially.
**Abstract Book Citations:** Authors, Title, HemaSphere, 2026;10:(S1):pages. The abstract book DOIs can be found on the “Author Information” page.
**Disclaimer:** Articles published in the journal HemaSphere exclusively reflect the opinions of the authors. The authors are responsible for all content in their abstracts including accuracy of the facts, statements, citing resources, etc.

### (PF343) DISTINCT REGULATION AND FUNCTION OF THE EXOSC10 RNA EXOSOME IN B CELL ACUTE LYMPHOBLASTIC LEUKAEMIA SUBTYPES

**Topic:** 01. Acute lymphoblastic leukemia - Biology & translational research

Charlotte Booth*[1, 2, 3], Dimitris Lagos[1, 2, 3], Adele Fielding[1, 2, 3], Jesús Gutiérrez-Abril[4], Elli Papaemmanuil[4]

*[1]Hull York Medical School, York, United Kingdom;[2]University of York, York, United Kingdom;[3]York Centre For Blood Research, York, United Kingdom; [4]Memorial Sloan Kettering Cancer Center, New York, United States of America;*

### Background

Precursor B cell acute lymphoblastic leukaemia (B-ALL) is an aggressive haematological malignancy driven by chromosomal abnormalities. Determining molecular hallmarks of B-ALL subtypes including those driven by the *BCR::ABL1* translocation and *KMT2Ar* rearrangements can lead to novel diagnostic and therapeutic approaches. B-ALL is characterised by low mutational burden, with *KMT2Ar* B-ALL having the lowest. Therefore, we searched for co- and post-transcriptional processes that could distinguish B-ALL subtypes.

### Aims

To uncover subtype-specific RNA metabolic hallmarks in B-ALL.

### Methods

We conducted Nanopore long-read RNA sequencing and functional in *vitro* assays on B-ALL cell lines: SEM (*KMT2A::AFF1*) and SD1 (*BCR::ABL1*). Alongside, we analysed diagnostic bulk RNA sequencing from 408 UKALL14 trial patients, focussing on *KMT2Ar* (N = 52) and *BCR::ABL1* (N = 162) groups.

**Copyright Information:** (Online) ISSN: 2572-9241
©2026 The Author(s). HemaSphere is published by John Wiley & Sons Ltd on behalf of European Hematology Association. This is an open access Abstract Book distributed under the Attribution-NonCommercial-NoDerivs (CC BY-NC-ND), which allows third parties to download the articles and share them with others as long as they credit the author and the Abstract Book, but they cannot change the content in any way or use them commercially.
**Abstract Book Citations:** Authors, Title, HemaSphere, 2026;10:(S1):pages. The abstract book DOIs can be found on the "Author Information" page.
**Disclaimer:** Articles published in the journal HemaSphere exclusively reflect the opinions of the authors. The authors are responsible for all content in their abstracts including accuracy of the facts, statements, citing resources, etc.

**Results**

Nanopore RNA sequencing revealed thousands of gene expression and transcript usage differences between *KMT2A::AFF1* and *BCR::ABL1* B-ALL cells. Notably, we found that *KMT2Ar* B-ALL cells are characterised by up-regulation of the nuclear RNA exosome machinery, which regulates ribosomal RNA (rRNA) biogenesis and degrades aberrant transcripts. 6/9 core components of the RNA exosome complex, the nuclear catalytic component EXOSC10, and 6 nuclear exosome cofactors were up-regulated in *KMT2Ar* B-ALL cells compared to *BCR::ABL1*. Differential transcript usage analysis revealed 9 splice variants of EXOSC8 with significant differential abundance between the cell lines. The upregulation of core exosome components and EXOSC10 in *KMT2Ar* cells were validated at the protein-level. Furthermore, we constructed a core exosome gene signature score and found that this was upregulated in B-ALL diagnostic samples from patients with *KMT2Ar* disease in the UKALL14 study compared to all other B-ALL subtypes, including the *BCR::ABL1* group. Menin inhibition by revumenib in the *KMT2Ar* cell line supressed expression of EXOSC10 and core exosome components by over half, but treatment of the *BCR::ABL1* cell line with ponatinib did not have the same effect, suggesting *KMT2Ar*-specific transcriptional coactivation of exosome expression.
Functionally, knockdown of EXOSC10 in *KMT2Ar* cells reduced expression of rRNAs by over half and inhibited cell growth and viability. rRNAs were also up-regulated in *KMT2Ar* cells compared to *BCR::ABL1* cells and in *KMT2Ar* the patient samples. Menin inhibition led to significant downregulation of rRNA expression. In UKALL14 patient samples, expression of the structurally critical core exosome component EXOSC3 correlated (Spearman $r \geq 0.5$, $p \leq 0.05$) with expression of genes involved in ribosome biogenesis in all subgroups but demonstrated *KMT2Ar* subtype-specific correlations with genes involved in DNA repair and the cell cycle. This was also observed for EXOSC10. Notably, *KMT2Ar* cells exhibited an 8-fold greater resistance to the EXOSC10-targeting drug 5-fluorocuracil compared to *BCR::ABL1* cells. Finally, exosome expression in clinical samples demonstrated an association with differentiation status, with high exosome sample demonstrating early myeloid, early lymphoid, and pre-B cell gene signatures.

**Summary/Conclusion**

We reveal that a fundamental RNA metabolism process distinguishes the *KMT2Ar* B-ALL subtype, promoting cell survival and potentially contributing to cell differentiation status. This could reveal novel therapeutic vulnerabilities for *KMT2Ar* B-ALL.

**Copyright Information:** (Online) ISSN: 2572-9241
©2026 The Author(s). HemaSphere is published by John Wiley & Sons Ltd on behalf of European Hematology Association. This is an open access Abstract Book distributed under the Attribution-NonCommercial-NoDerivs (CC BY-NC-ND), which allows third parties to download the articles and share them with others as long as they credit the author and the Abstract Book, but they cannot change the content in any way or use them commercially.
**Abstract Book Citations:** Authors, Title, HemaSphere, 2026;10:(S1):pages. The abstract book DOIs can be found on the "Author Information" page.
**Disclaimer:** Articles published in the journal HemaSphere exclusively reflect the opinions of the authors. The authors are responsible for all content in their abstracts including accuracy of the facts, statements, citing resources, etc.

### (PF344) SCRNA-SEQ REVEALS AN IMMUNE MICROENVIRONMENT AND JUN-MEDIATED NK CELL EXHAUSTION IN RELAPSED T-ALL

**Topic:** 01. Acute lymphoblastic leukemia - Biology & translational research

Yong Liu*[1], Chun Chen[1]

[1] *The Seventh Affiliated Hospital of Sun Yat-Sen University, Pediatric Hematology Laboratory, Division of Hematology/Oncology, Department of Pediatrics, Shenzhen, China;*

### Background

T cell acute lymphoblastic leukemia (T-ALL) is a heterogeneous hematological malignancy associated with a high rate of relapse. A comprehensive understanding of the bone marrow immune microenvironment, particularly in the context of relapse, is crucial for developing novel therapeutic strategies.

### Aims

This study aimed to characterize the cellular composition and immune landscape of the bone marrow microenvironment in patients with T-ALL using single-cell transcriptome analysis. A key objective was to identify immune cell dysfunction and potential therapeutic targets associated with relapsed disease.

### Methods

We performed single-cell transcriptome analysis on bone marrow samples from patients with T-ALL. The cellular composition was deconvoluted to identify major cell populations and their transcriptional states. Findings related to immune dysfunction, particularly in natural killer (NK) cells, were validated using *in vitro* models, including a relapsed T-ALL cell line, and *in vivo* models, including cell-derived tumor xenograft (CDX), patient-derived tumor xenograft (PDX), and NOTCH1-mutant mouse models.

### Results

Analysis identified 13 major cell clusters within the T-ALL bone marrow microenvironment. Patients exhibited abnormal expansion of hematopoietic stem cells (HSCs) and granulocyte-monocyte progenitors (GMPs), along with immunosuppressive features in CD4+ T, CD8+ T, and NK cells. Subdivision of CD4+ T cells revealed two distinct subsets transitioning between T helper (Th)1/Th2 states: ANXA1−GATA3− and ANXA1+GATA3+ cells. Notably, NK cells in patients with relapsed T-ALL displayed an exhausted phenotype, with the transcription factor JUN identified as a critical regulator. JUN was also found to be highly expressed in T-ALL cells and essential for their proliferation. Pharmacological inhibition of JUN effectively targeted leukemic cells and alleviated NK cell exhaustion in both *in vitro* and *in vivo* models of relapsed disease.

### Summary/Conclusion

Our findings provide a detailed map of the bone marrow immune microenvironment in T-ALL, revealing distinct immune dysregulation associated with relapse, including the exhaustion of NK cells driven by JUN. The dual effect of JUN inhibition—suppressing leukemia proliferation and reversing immune exhaustion—highlights its potential as a promising immunotherapeutic target for patients with relapsed T-ALL.

**Copyright Information:** (Online) ISSN: 2572-9241
©2026 The Author(s). HemaSphere is published by John Wiley & Sons Ltd on behalf of European Hematology Association. This is an open access Abstract Book distributed under the Attribution-NonCommercial-NoDerivs (CC BY-NC-ND), which allows third parties to download the articles and share them with others as long as they credit the author and the Abstract Book, but they cannot change the content in any way or use them commercially.
**Abstract Book Citations:** Authors, Title, HemaSphere, 2026;10:(S1):pages. The abstract book DOIs can be found on the “Author Information” page.
**Disclaimer:** Articles published in the journal HemaSphere exclusively reflect the opinions of the authors. The authors are responsible for all content in their abstracts including accuracy of the facts, statements, citing resources, etc.

### (PF345) METABOLIC STATES DEFINE CHEMOTHERAPY RESPONSE AND RESISTANCE IN PEDIATRIC BCP-ALL PATIENT-DERIVED XENOGRAFTS

**Topic:** 01. Acute lymphoblastic leukemia - Biology & translational research

Julia Starkova*[1, 2, 3], Srdjan Grusanovic[4], Pavel Jakoube[5], Nadia Alaei[1, 2], Anna Sachova[1, 2], Matus Kolarik[1, 2, 6], Bryan Marzullo[7], Daniel Tennant[7], Miroslava Adamcova[4], Jakub Rohlena[5], Jan Trka[1, 2, 3], Meritxell Alberich Jorda[1, 4], Katerina Rohlenova[5]

*[1]Department of Pediatric Hematology and Oncology, Second Faculty of Medicine, Charles University, Prague, Czechia;[2]CLIP - Childhood Leukemia Investigation Prague, Prague, Czechia; [3]University Hospital Motol and Homolka, Prague, Czechia; [4]Laboratory of Hemato-oncology, Institute of Molecular Genetics of the Czech Academy of Sciences, Prague, Czechia; [5]Laboratory of Cellular Metabolism, Institute of Biotechnology of the Czech Academy of Sciences, BIOCEV, Vestec, Prague-west, Czechia; [6]First Faculty of Medicine, Charles University, Prague, Czechia;[7]Institute of Metabolism and Systems Research, College of Medical and Dental Sciences, University of Birmingham, Birmingham, United Kingdom;*

### Background

Despite advances in treating childhood acute lymphoblastic leukemia (ALL), 15-20% of patients still experience relapse, and current risk stratification cannot fully explain why some patients fail therapy. Understanding the mechanisms underlying treatment resistance could enable improved patient stratification and therapeutic strategies.

### Aims

To identify molecular mechanisms of treatment resistance, we established patient-derived xenograft (PDX) models that recapitulate clinical treatment responses and enable molecular characterization of therapy-resistant cells.

### Methods

We established ten PDX models from BCP-ALL patients: five standard-risk (SR, favorable prognosis; ETV6::RUNX1, TCF3::PBX1) and five high-risk (HR, poor response; KMT2A-r, B-other) according to AIEOP-BFM-ALL criteria. Secondary recipients from HR PDX models were treated with VXLD (vincristine, dexamethasone, L-asparaginase, daunorubicin) for four weeks, simulating induction phase of therapy. Post-treatment, we sorted leukemic cells and performed scRNA-seq on untreated controls and VXLD-treated samples, measured extracellular metabolic flux using Seahorse XFp Analyzer and performed stable isotope tracing (SIT) with [U-13C]-glucose.

**Copyright Information:** (Online) ISSN: 2572-9241
©2026 The Author(s). HemaSphere is published by John Wiley & Sons Ltd on behalf of European Hematology Association. This is an open access Abstract Book distributed under the Attribution-NonCommercial-NoDerivs (CC BY-NC-ND), which allows third parties to download the articles and share them with others as long as they credit the author and the Abstract Book, but they cannot change the content in any way or use them commercially.
**Abstract Book Citations:** Authors, Title, HemaSphere, 2026;10:(S1):pages. The abstract book DOIs can be found on the "Author Information" page.
**Disclaimer:** Articles published in the journal HemaSphere exclusively reflect the opinions of the authors. The authors are responsible for all content in their abstracts including accuracy of the facts, statements, citing resources, etc.

**Results**

HR PDX engrafted more rapidly (8.8 weeks) compared to SR PDX (18.4 weeks), reflecting the more aggressive biology of high-risk disease. VXLD treatment mirrored clinical responses in which SR PDX achieved near-complete cell eradication while HR PDX retained significant resistant populations.
Integrated transcriptome analysis across all patients identified metabolic signatures specific for each risk group, and associated with therapy resistance. Untreated cells from HR PDX displayed a hypermetabolic state with elevated expression of genes involved in bioenergetics, pentose phosphate pathway (PPP), antioxidant defence, and fatty acid metabolism compared to cells from SR PDX. scRNA-seq cluster analysis revealed two resistance patterns in HR PDX models. 3/5 showed overlapping transcriptomic profiles between untreated and treated cells, indicating pre-existing resistant clones present already at diagnosis. 2/5 showed dramatic transcriptomic changes, suggesting therapy-induced modifications. Resistant clusters from both patterns showed consistent metabolic adaptations including bioenergetic rewiring toward fatty acid oxidation (FAO), antioxidant defence pathways and enhanced niche interaction signaling.
Importantly, functional studies validated key aspects of this metabolic rewiring. Seahorse analysis confirmed untreated HR cells exhibited higher glycolytic function (ECAR) than SR cells, while VXLD-treated resistant cells showed decreased ECAR. SIT demonstrated glucose redirection: decreased pyrimidine de novo synthesis with increased PPP flux, generating NADPH for glutathione-mediated antioxidant defence and validating the transcriptomic signatures.

**Summary/Conclusion**

Metabolic state determines ALL treatment response, with hypermetabolic activity at diagnosis characterizing HR patients while low metabolic activity characterizes SR patients. Resistant cells employ metabolic rewiring strategies by redirecting bioenergetics and enhance antioxidant defence to survive chemotherapy. Metabolic profiling could identify HR patients for upfront treatment modification, while discovered metabolic dependencies represent therapeutic targets for overcoming resistance.

Supported by Czech Health Research Agency NW24-03-00376, Charles University Research Centre program No. UNCE/24/MED/003,

**Copyright Information:** (Online) ISSN: 2572-9241
©2026 The Author(s). HemaSphere is published by John Wiley & Sons Ltd on behalf of European Hematology Association. This is an open access Abstract Book distributed under the Attribution-NonCommercial-NoDerivs (CC BY-NC-ND), which allows third parties to download the articles and share them with others as long as they credit the author and the Abstract Book, but they cannot change the content in any way or use them commercially.
**Abstract Book Citations:** Authors, Title, HemaSphere, 2026;10:(S1):pages. The abstract book DOIs can be found on the "Author Information" page.
**Disclaimer:** Articles published in the journal HemaSphere exclusively reflect the opinions of the authors. The authors are responsible for all content in their abstracts including accuracy of the facts, statements, citing resources, etc.

### (PF346) EPIGENETIC REWIRING DRIVES CHEMORESISTANCE BY DUAL MECHANISMS IN POLYCOMB REPRESSIVE COMPLEX 2-ALTERED T-ALL

**Topic:** 01. Acute lymphoblastic leukemia - Biology & translational research

Theodora-Ioana Grosu*[1, 2], Cosmin Tudose[1, 2, 3], Thomas Lefeivre[1, 2], Marie-Claire Fitzgerald[1, 2], Fernanda Schäfer Hackenhaar[4], Kieran Wynne[1, 2], Theresa Leon[5], Marc Mansour[5, 6], Sofie Degerman[4, 7], Jonathan Bond[1, 2, 8]

*[1]Systems Biology Ireland, Dublin, Ireland; [2]School of Medicine, University College Dublin, Dublin, Ireland; [3]The SFI Centre for Research Training in Genomics Data Science, Galway, Ireland; [4]Department of Medical Biosciences, Umeå University, Umeå, Sweden; [5]UCL Cancer Institute, University College London, Department of Haematology, London, United Kingdom; [6]Division of Developmental Biology and Cancer, Great Ormond Street Institute of Child Health, London, United Kingdom; [7]Department of Clinical Microbiology, Umeå University, Umeå, Sweden; [8]Children's Health Ireland at Crumlin, Dublin, Ireland;*

### Background

Loss-of-function alterations in Polycomb Repressive Complex 2 (PRC2) core components (EZH2, SUZ12, EED) are found in ~25% of T-Acute Lymphoblastic Leukemia (T-ALL) cases, with enrichment in the phenotypically immature/early thymic precursor (ETP-ALL) subtype. PRC2 alterations and expression of immature transcriptional signatures are each linked to poor T-ALL treatment responses. We have previously shown that PRC2 depletion decreases sensitivity to asparaginase, but the molecular mechanisms underpinning general chemoresistance are unclear.

### Aims

To comprehensively characterise the epigenomic mechanisms that underpin chemoresistance in PRC2-depleted T-ALL.

### Methods

We used an isogenic EZH2-altered T-ALL model to catalogue PRC2-dependent gene expression (RNA-seq), histone modifications (CUT&RUN), chromatin accessibility (ATAC-seq), DNA methylation, and PRC2 complex composition (Immunoprecipitation with Mass Spectrometry, IP-MS). Results were validated using PRC2 chemical degraders in orthogonal T-ALL models and transcriptional analysis of PRC2-altered primary T-ALL cases.

**Copyright Information:** (Online) ISSN: 2572-9241
©2026 The Author(s). HemaSphere is published by John Wiley & Sons Ltd on behalf of European Hematology Association. This is an open access Abstract Book distributed under the Attribution-NonCommercial-NoDerivs (CC BY-NC-ND), which allows third parties to download the articles and share them with others as long as they credit the author and the Abstract Book, but they cannot change the content in any way or use them commercially.
**Abstract Book Citations:** Authors, Title, HemaSphere, 2026;10:(S1):pages. The abstract book DOIs can be found on the "Author Information" page.
**Disclaimer:** Articles published in the journal HemaSphere exclusively reflect the opinions of the authors. The authors are responsible for all content in their abstracts including accuracy of the facts, statements, citing resources, etc.

**Results**

Integrative analysis revealed two predominant mechanisms of epigenetic rewiring in EZH2-depleted leukemia cells. First, we identified a core set of 95 upregulated genes, including the ETP-ALL drivers *MEF2C* and *NCAM1* (CD56), which lost the repressive H3K27me3 mark and gained promoter accessibility and active H3K27ac marks. Moreover, EZH2 knockout also led to upregulation of the BMP-17 geneset described to drive chemoresistance in T-ALL, including within the immature ETP-ALL cohort. These changes, including surface CD56 expression, were reversed by EZH2 complementation *in vitro*, directly linking PRC2 function to ETP-ALL-related transcriptional reprogramming.
Secondly, and counterintuitively, we also observed globally reduced chromatin accessibility, with over 18000 regions becoming more compact in EZH2-deficient cells relative to wildtype. This chromatin compaction was found at 336 downregulated loci enriched for T-cell differentiation genes, including *CD1A*. The *CD1A* gene promoter had concurrent loss of the H3K27ac mark, explaining the negative CD1A status of the EZH2-deficient clones that we detected by flow cytometry.
IP-MS of endogenous PRC2 core components revealed that in the absence of EZH2, its paralog EZH1 is incorporated into the PRC2 complex. This compensatory EZH1:PRC2 assembly was validated in both CRISPR-engineered clones and independent T-ALL lines treated with the EZH2 PROTAC MS1943, alongside CUT&RUN experiments. Since EZH1 exhibits preferential compaction activity relative to EZH2, aberrant EZH1 incorporation into PRC2 provides a potential mechanistic explanation for these accessibility changes.

**Summary/Conclusion**

We report that EZH2 loss triggers marked changes in chemoresistance-linked immature transcriptional programs linked to altered chromatin accessibility and histone modification patterns. We further identify EZH1 compensation as a potential mechanistic explanation for repression of specific differentiation genes through chromatin compaction, thereby locking the cells in a chemoresistant, stem-like state. These data challenge the paradigm that EZH2 loss is solely a “loss-of-repression” event in T-ALL, and rather that epigenetic dysregulation in these cells is complex and multifactorial.

**Copyright Information:** (Online) ISSN: 2572-9241
©2026 The Author(s). HemaSphere is published by John Wiley & Sons Ltd on behalf of European Hematology Association. This is an open access Abstract Book distributed under the Attribution-NonCommercial-NoDerivs (CC BY-NC-ND), which allows third parties to download the articles and share them with others as long as they credit the author and the Abstract Book, but they cannot change the content in any way or use them commercially.
**Abstract Book Citations:** Authors, Title, HemaSphere, 2026;10:(S1):pages. The abstract book DOIs can be found on the “Author Information” page.
**Disclaimer:** Articles published in the journal HemaSphere exclusively reflect the opinions of the authors. The authors are responsible for all content in their abstracts including accuracy of the facts, statements, citing resources, etc.

### (PF347) AUGMENTER OF LIVER REGENERATION (ALR) PROMOTES CHEMORESISTANCE IN T-CELL ACUTE LYMPHOBLASTIC LEUKEMIA BY SUPPRESSING FERROPTOSIS VIA THE AMPK/SREBP1/FADS2 AXIS

**Topic:** 01. Acute lymphoblastic leukemia - Biology & translational research

Shiyi Yuan*[1], Shifeng Lou[1], Yan Shen[1], Ying Zhou[1], Rongsen Xu[1]

[1]*The Second Affiliated Hospital of Chongqing Medical University, Department of Hematology, Chongqing, China;*

### Background

T-cell acute lymphoblastic leukemia (T-ALL) is an aggressive hematologic malignancy with frequent relapse driven by therapy resistance. Augmenter of liver regeneration (ALR) is a mitochondrial protein we previously identified as an anti-apoptotic oncogene in T-ALL; however, its role in metabolic adaptation and alternative cell death pathways remains elusive.

### Aims

This study aims to investigate whether and how ALR regulates ferroptosis, a newly recognized form of regulated cell death, and to elucidate the underlying molecular mechanisms and therapeutic implications in T-ALL.

### Methods

Transcriptomic profiling and network analysis identified FADS2 as the central ferroptosis-related lipid metabolic hub in ALR-manipulated T-ALL cells. Genetic and pharmacological modulation of ALR, AMPK, SREBP1, and FADS2 was performed in cell lines and primary patient samples, assessing ferroptotic markers (lipid ROS, $Fe^{2+}$, lipid peroxidation, AA levels, mitochondrial morphology, GPX4/ACSL4). In vivo, xenograft mice bearing control or ALR-knockdown Jurkat cells received PBS, SC26196, chemotherapy (dexamethasone+methotrexate), or combinations; leukemia burden and survival were evaluated.

### Results

Transcriptomic analysis revealed that ALR loss activates a coordinated transcriptional program of polyunsaturated fatty acid (PUFA) biosynthesis, with fatty acid desaturase 2 (FADS2) identified as the central hub. Mechanistically, ALR maintains AMPK activity, which phosphorylates SREBP1 at Ser372 to restrain its transcriptional activity. ALR depletion inactivates AMPK, releases SREBP1 inhibition, and drives FADS2-dependent PUFA synthesis, leading to lipid peroxide accumulation and ferroptotic cell death. Genetic or pharmacological restoration of the AMPK-SREBP1 axis, or direct FADS2 inhibition, abrogates ALR deficiency-induced ferroptosis. Clinically, this ALR-driven signaling signature defines a T-ALL subset with inherent ferroptosis resistance and poor prognosis. ALR knockdown in patient-derived primary cells recapitulates ferroptosis sensitization, which is targetable by FADS2 inhibitor SC26196. In vivo, ALR depletion suppresses leukemic progression in xenograft models, an effect largely reversed by FADS2 inhibition. Strikingly, ALR knockdown synergizes with conventional chemotherapy, and this synergy is abolished by FADS2 inhibition.

### Summary/Conclusion

Our findings establish the ALR-AMPK-SREBP1-FADS2 axis as a previously unrecognized metabolic checkpoint coupling mitochondrial signaling to ferroptosis defense. This work positions ALR as a dual guardian against apoptosis and ferroptosis, and provides a compelling rationale for targeting FADS2 as a ferroptosis-inducing and chemosensitizing strategy in T-ALL.

**Copyright Information:** (Online) ISSN: 2572-9241
©2026 The Author(s). HemaSphere is published by John Wiley & Sons Ltd on behalf of European Hematology Association. This is an open access Abstract Book distributed under the Attribution-NonCommercial-NoDerivs (CC BY-NC-ND), which allows third parties to download the articles and share them with others as long as they credit the author and the Abstract Book, but they cannot change the content in any way or use them commercially.
**Abstract Book Citations:** Authors, Title, HemaSphere, 2026;10:(S1):pages. The abstract book DOIs can be found on the “Author Information” page.
**Disclaimer:** Articles published in the journal HemaSphere exclusively reflect the opinions of the authors. The authors are responsible for all content in their abstracts including accuracy of the facts, statements, citing resources, etc.

### (PF348) CEBP AND ZEB2 ALTERATIONS DEFINE THREE DISTINCT SUBTYPES OF B-CELL ACUTE LYMPHOBLASTIC LEUKEMIA

**Topic:** 01. Acute lymphoblastic leukemia - Biology & translational research

Rathana Kim*[1, 2], Marie Passet[1, 2], Hugo Bergugnat[1], Loïc Vasseur[1, 2], Thibaut Leguay[3], Francoise Huguet[4], Mathilde Hunault[5], Tom Sexton[6], Melha Benlebna[1], Julie Renard[2], Emmanuel Raffoux[2], Cedric Pastoret[7], Cathie Erb[6], Laureen Chat[1, 2], Eric Delabesse[4], Stephanie Gachet[1], Caroline Bonmati[8], Marie Balsat[9], Thorsten Braun[10], Audrey Bidet[3], Nicolas Duployez[11], Carlos Graux[12], Yves Chalandon[13], Philippe Rousselot[14], Patrice Chevallier[15], Jean Soulier[1, 2], Véronique Lhéritier[16], Hervé Dombret[1, 2], Nicolas Boissel[1, 2], Emmanuelle Clappier[1, 2]

*[1]Université Paris Cité, Inserm UMR 1342, Institut de Recherche Saint-Louis (IRSL), Paris, France;[2]AP-HP, hopital Saint-Louis, Hematology department, Paris, France; [3]CHU de Bordeaux, hôpital du Haut-Levêque, Hematology departement, Bordeaux, France; [4]Institut Universitaire de Cancer Toulouse-Oncopole, Hematology department, Toulouse, France; [5]CHU Angers, Hematology Department, Angers, France; [6]Université de Strasbourg, Institute of Genetics and Molecular and Cellular Biology (IGBMC), Illkirch, France; [7]Centre Hospitalier Universitaire de Rennes, Hematology laboratory, Rennes, France; [8]CHU de Nancy, Hematology department, Nancy, France; [9]Hospices Civils de Lyon, Hôpital Lyon Sud, Hematology department, Pierre Benite, France; [10]AP-HP hopital Avicenne, Hematology department, Bobigny, France; [11]Centre Hospitalier Régional Universitaire de Lille, Hematology laboratory, Lille, France; [12]CHU UCL Namur Godinne, Hematology department, Yvoir, Belgium; [13]Geneva University Hospitals and Faculty of Medicine, Division of Hematology, Department of Oncology, Geneva, Switzerland; [14]Centre Hospitalier de Versailles, Hematology departement, Versailles, France; [15]Hôtel-Dieu - CHU de Nantes, Hematology department, Nantes, France; [16]Group for Research on Adult Acute Lymphoblastic Leukemia GRAALL, Centre Hospitalier Universitaire Lyon Sud, Lyon, France;*

### Background

Comprehensive molecular profiling has revealed marked heterogeneity in B-cell acute lymphoblastic leukemia (B-ALL), leading to the identification of over 20 genetic subtypes with distinct clinical outcomes. However, a subset of cases remains unclassified. Chromosomal translocations involving *CEBP* genes were reported nearly two decades ago as rare recurrent lesions in B-ALL, yet their biological and clinical significance remains unclear. More recently, a subtype was defined by the presence of *IGH::CEBPE* rearrangements or the recurrent *ZEB2* p.H1038R mutation and provisionally recognized in the International Consensus Classification (ICC) as “mutated ZEB2 (p.H1038R)/IGH::CEBPE”. However, the boundaries and genomic basis of this putative entity remain insufficiently resolved.

### Aims

To comprehensively characterize B-ALL with alterations of CEBP family genes and/or ZEB2.

### Methods

The study included all Philadelphia-negative (Ph-) B-ALL from adults enrolled in the GRAALL-2005, GRAALL-2014 and EWALL-INO trials with available material and molecular annotation (n=965). Additional cases with *CEBP* or *ZEB2* alterations (age 16–64 years) were included (n=27). Whole-transcriptome sequencing was performed in 538 cases, including all cases lacking subtype-defining molecular drivers. Unresolved cases underwent optical genome mapping and capture-based sequencing of *CEBP* loci. Whole-genome or whole-exome sequencing data were available for some cases through the French Genomic Medicine Initiative (PFMG2025).

**Copyright Information:** (Online) ISSN: 2572-9241
©2026 The Author(s). HemaSphere is published by John Wiley & Sons Ltd on behalf of European Hematology Association. This is an open access Abstract Book distributed under the Attribution-NonCommercial-NoDerivs (CC BY-NC-ND), which allows third parties to download the articles and share them with others as long as they credit the author and the Abstract Book, but they cannot change the content in any way or use them commercially.
**Abstract Book Citations:** Authors, Title, HemaSphere, 2026;10:(S1):pages. The abstract book DOIs can be found on the “Author Information” page.
**Disclaimer:** Articles published in the journal HemaSphere exclusively reflect the opinions of the authors. The authors are responsible for all content in their abstracts including accuracy of the facts, statements, citing resources, etc.

**Results**

Integrated genomic analyses of 992 adult Ph- B-ALL identified 61 cases with alterations deregulating a *CEBP* family gene. These included *IGH* fusions involving *CEBPA* (n=19), *CEBPB* (n=13), *CEBPD* (n=12), and *CEBPE* (n=5), as well as rare non-*IGH* fusions, ie *LINC00426::CEBPB* (n=3), *ZCCHC7::CEBPB* (n=1) and *IL7R::CEBPA* (n=1). All cases displayed high expression of the corresponding *CEBP* gene. In 7 cases with high *CEBPA* expression but no detectable fusion, targeted sequencing identified insertions or duplications located 2.3 kb downstream of *CEBPA,* suggesting novel non-coding regulatory activity at this locus.
Gene expression profiling across the cohort revealed that different *CEBP* family genes did not define distinct transcriptional signatures, supporting a convergent oncogenic program. Integrative genomic and transcriptomic analyses delineated 3 subgroups with *CEBP* and/or *ZEB2* alterations: (1) *CEBP* alteration only (n=42, *CEBP*alt) frequently associated with *IKZF1* deletions (62%) and *FLT3* mutations (50%); (2) combined *CEBP* alteration and *ZEB2* p.H1038R mutation (n=18, *CEBP/ZEB2*); (3) isolated *ZEB2* p.H1038R mutation with a second alteration targeting *ZEB2* (n=15, ZEB2alt).
Clinical analyses supported the existence of distinct entities. Patients with CEBPalt showed a trend toward higher cumulative incidence of relapse (CIR) compared with other GRAALL B-ALL (SHR 1.55, 95%CI[0.90-2.54], P=0.11). CEBP/ZEB2 cases had intermediate outcomes. In contrast, ZEB2alt patients had dismal outcomes despite young age, with significantly increased CIR (specific hazard ratio SHR 3.37, 95%CI[1.39-8.17], p=0.007) and inferior event-free survival (HR 2.27, 95%CI[1.01-5.09], p=0.046).

**Summary/Conclusion**

We provide a comprehensive characterization of adult B-ALL with *CEBP* and/or *ZEB2* alterations and define three biologically and clinically distinct subtypes, providing a rationale for subtype-specific risk stratification.

**Copyright Information:** (Online) ISSN: 2572-9241
©2026 The Author(s). HemaSphere is published by John Wiley & Sons Ltd on behalf of European Hematology Association. This is an open access Abstract Book distributed under the Attribution-NonCommercial-NoDerivs (CC BY-NC-ND), which allows third parties to download the articles and share them with others as long as they credit the author and the Abstract Book, but they cannot change the content in any way or use them commercially.
**Abstract Book Citations:** Authors, Title, HemaSphere, 2026;10:(S1):pages. The abstract book DOIs can be found on the "Author Information" page.
**Disclaimer:** Articles published in the journal HemaSphere exclusively reflect the opinions of the authors. The authors are responsible for all content in their abstracts including accuracy of the facts, statements, citing resources, etc.

### (PF349) PRECLINICAL IN VIVO AND EX VIVO ACTIVITY OF DASATINIB IN ABL1-REARRANGED T-ACUTE LYMPHOBLASTIC LEUKEMIA PATIENT-DERIVED XENOGRAFT MODELS

**Topic:** 01. Acute lymphoblastic leukemia - Biology & translational research

Diego Barcenas Lopez*[1], Jackson Dardis[1], Satoshi Yoshimura[2], Camilo Arenas Merizalde[1], Andishe Attarbaschi[3], Jun J Yang[2], Sarah Elitzur[2], Sarah Tasian[1, 4]

*[1]Children's Hospital of Philadelphia, Oncology and Center for Childhood Cancer Research, Philadelphia, United States of America; [2]St. Jude Children's Research Hospital, Pharmacy and Pharmaceutical Sciences, Memphis, United States of America; [3]St. Anna Children's Cancer Research Institute, Pediatric Hematology/Oncology and Clinical Trials Unit, Vienna, Austria; [4]University of Pennsylvania, University of Pennsylvania School of Medicine and Abramson Cancer Center, Philadelphia, United States of America;*

### Background

Despite excellent outcomes of pediatric patients with acute lymphoblastic leukemia (ALL) treated with modern multi-agent chemotherapy, prognosis of children with specific genetic subtypes remains suboptimal. Approximately 5-6% of T-ALL cases harbor *ABL1* rearrangements, most commonly *NUP214*::*ABL1* fusions, which may confer similar sensitivity to tyrosine kinase inhibitors (TKIs) to that of *BCR::ABL1* (Ph+) B-ALL. While activity of TKIs (eg, imatinib, dasatinib, nilotinib) has been reported in preclinical models of ABL-class Ph-like B-ALL (I van Outersterp Blood 2024), limited data exist regarding potential efficacy in T-ALL (TH Tran ASH 2025 annual meeting).

### Aims

To elucidate the *in vivo* and *ex vivo* preclinical activity of dasatinib in *ABL1*-rearranged T-ALL.

### Methods

Patient-derived xenograft (PDX) models were generated by intravenous injection of primary T-ALL (n=8) or B-ALL (n=4) samples into NSG mice with serial transplantation for experimental studies. *ABL1* rearrangements and co-occurring variants were confirmed in PDX models via RNA- and DNA-based next generation sequencing (**Table**). Cohorts of PDX mice (n=10/arm) were randomized to treatment with vehicle or dasatinib 20 mg/kg per day via oral gavage for five days per week for 3-6 weeks depending upon rate of ALL progression in control animals. T-ALL cells were enumerated weekly in murine blood and in end-study spleens by quantitative flow cytometry as previously described (SK Tasian Blood 2017a). High-throughput *ex vivo* drug response profiling (DRP) was also performed via co-culture of PDX-origin ALL cells with mesenchymal stem cells and fluorescence imaging-based viability (SHR Lee Nat Med 2023).

**Copyright Information:** (Online) ISSN: 2572-9241
©2026 The Author(s). HemaSphere is published by John Wiley & Sons Ltd on behalf of European Hematology Association. This is an open access Abstract Book distributed under the Attribution-NonCommercial-NoDerivs (CC BY-NC-ND), which allows third parties to download the articles and share them with others as long as they credit the author and the Abstract Book, but they cannot change the content in any way or use them commercially.
**Abstract Book Citations:** Authors, Title, HemaSphere, 2026;10:(S1):pages. The abstract book DOIs can be found on the "Author Information" page.
**Disclaimer:** Articles published in the journal HemaSphere exclusively reflect the opinions of the authors. The authors are responsible for all content in their abstracts including accuracy of the facts, statements, citing resources, etc.

**Results**

Dasatinib monotherapy robustly inhibited *in vivo* leukemia proliferation with near-curative effects seen in all tested *ABL1*-rearranged T-ALL PDX models (representative data in **Figure**). Effects on ABL class B-ALL models were also statistically significant, but more moderate. DRP of harvested viable PDX-origin T-ALL cells showed variable *ex vivo* sensitivity to dasatinib, while Ph+ and ABL class B-ALL were uniformly highly sensitive.

| PDX model | Lineage | Fusion | Other alterations |
|---|---|---|---|
| I_TALL1 | T | *BCR::ABL1* | *LEF1* del |
| I_TALL2 | T | *NUP214::ABL1,STIL::TAL1* | *LEF1* del |
| I_TALL3 | T | *NUP214::ABL1,STIL::TAL1* | *LEF1* del, *ABL1* gain |
| I_TALL5 | T | *NUP214::ABL1* | *NRAS* G13V, *WT1* V376_P377delinsR* |
| A_TALL6 | T | *NUP214::ABL1* | *NOTCH1* V1578del, *WT1* V376Mfs*15 |
| A_TALL7 | T | *NUP214::ABL1* | *NOTCH1* V1726_F1736dup, *NRAS* G12D |
| A_TALL8 | T | *NUP214::ABL1,ETV6::ABL1* | *DNM2* K562del, *PHF6* Y301* |
| A_TALL9 | T | *ZBTB16::ABL1* | *NOTCH1* L1593P |
| I_BALL1 | B | *ETV6::ABL1* | *IKZF1* del, *CDK2NA/B* del |
| I_BALL6 | B | *ETV6::ABL1* | *ABL1* gain |
| PANSFD | B | *ETV6::ABL1* | *PAX5* del |
| PAKVKK | B | *NUP214::ABL1* | *PAX5* G24R |

**Copyright Information:** (Online) ISSN: 2572-9241
©2026 The Author(s). HemaSphere is published by John Wiley & Sons Ltd on behalf of European Hematology Association. This is an open access Abstract Book distributed under the Attribution-NonCommercial-NoDerivs (CC BY-NC-ND), which allows third parties to download the articles and share them with others as long as they credit the author and the Abstract Book, but they cannot change the content in any way or use them commercially.
**Abstract Book Citations:** Authors, Title, HemaSphere, 2026;10:(S1):pages. The abstract book DOIs can be found on the "Author Information" page.
**Disclaimer:** Articles published in the journal HemaSphere exclusively reflect the opinions of the authors. The authors are responsible for all content in their abstracts including accuracy of the facts, statements, citing resources, etc.

**Summary/Conclusion**

*ABL1*-rearranged T-ALL PDX models with various fusion partners demonstrated exquisite *in vivo* sensitivity to dasatinib with remarkable single-agent efficacy, while *ex vivo* DRP activity was more heterogeneous. Conversely, dasatinib induced potent *ex vivo* and more variable *in vivo* effects against Ph+ and ABL class Ph-like B-ALL models. Further studies are needed to ascertain potential etiologies of such differences, including intra-tumoral heterogeneity that may influence therapeutic responses. Nonetheless, dasatinib clearly has strong anti-leukemia activity in *NUP214*::*ABL1* fusion and other *ABL1*-rearranged T-ALL subtypes and may improve clinical outcomes of patients. To this end, the current ALLTogether-1 study (NCT04307576) is adding imatinib to multi-agent chemotherapy for patients with *ABL1*-rearranged T-ALL with results eagerly anticipated.

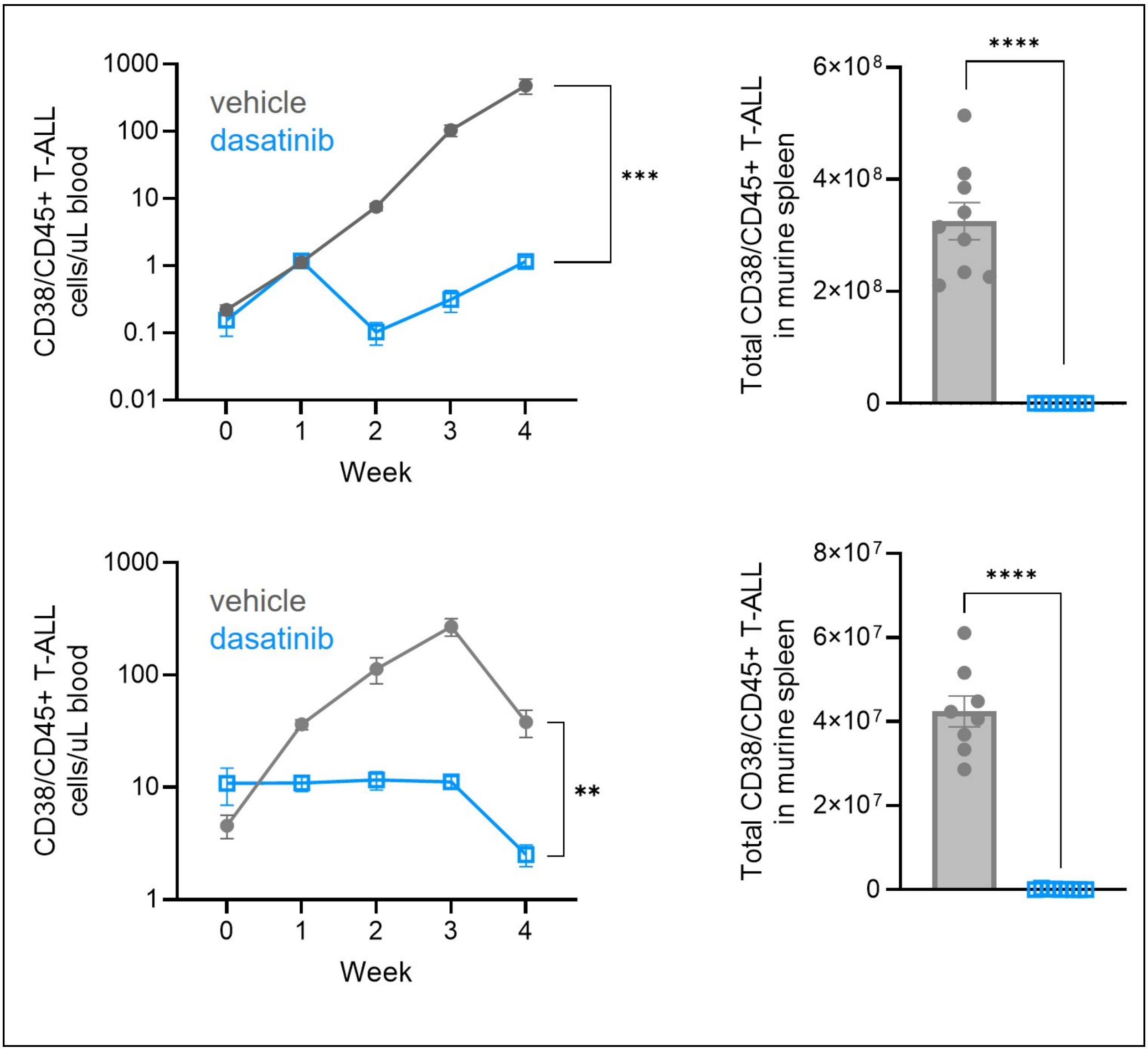

Dasatinib effects in ABL-rearranged T-ALL patient-derived xenograft (PDX) models. Flow cytometry quantification showing leukemia progression in murine blood (left column) and total leukemia in terminal spleens (right column) after dasatinib treatment. Significance values (**p<0.01, ***p<0.001, ****p<0.0001) indicate results from unpaired T-tests with Welch's correction

**Copyright Information:** (Online) ISSN: 2572-9241
©2026 The Author(s). HemaSphere is published by John Wiley & Sons Ltd on behalf of European Hematology Association. This is an open access Abstract Book distributed under the Attribution-NonCommercial-NoDerivs (CC BY-NC-ND), which allows third parties to download the articles and share them with others as long as they credit the author and the Abstract Book, but they cannot change the content in any way or use them commercially.
**Abstract Book Citations:** Authors, Title, HemaSphere, 2026;10:(S1):pages. The abstract book DOIs can be found on the "Author Information" page.
**Disclaimer:** Articles published in the journal HemaSphere exclusively reflect the opinions of the authors. The authors are responsible for all content in their abstracts including accuracy of the facts, statements, citing resources, etc.

### (PF350) THERAPEUTIC EFFECT OF CX-4945 COMBINED WITH XPO1 INHIBITOR BY TARGETING AMINO ACID METABOLISM IN T-CELL ACUTE LYMPHOBLASTIC LEUKEMIA

**Topic:** 01. Acute lymphoblastic leukemia - Biology & translational research

Qi Han[1], Tong Wu[1], Chunhua Song[2], Zheng Ge*[1]

*[1]Zhongda Hospital Southeast University, Institute of Hematology Southeast University, Department of Hematology, Nanjing, China; [2]Division of Hematology, The Ohio State University Wexner Medical Center, The James Cancer Hospital, Columbus, United States of America;*

### Background

T-cell acute lymphoblastic leukemia (T-ALL) is an aggressive hematological malignancy with limited treatment choices and has an extremely poor prognosis. Amino acid metabolism is essential for the development and progression of human cancers. We reported that targeting Casein Kinase II (CK2) by CK2 inhibitor CX-4945 demonstrated therapeutic efficacy in acute leukemia. XPO1 is frequently highly expressed in T-ALL. However, the effect of CX-4945 combined with XPO1 inhibitor KPT-330 in T-ALL has not been explored.

### Aims

To investigate the synergistic effect of the combination of XPO1 inhibitor KPT-330 with CK2 inhibitor CX-4945 and the potential underlying mechanisms of this novel drug combination in T-ALL.

### Methods

CCK-8 assay and apoptosis assay with Annexin V staining were performed to analyze the synergistic effect of KPT-330 combined with CX-4945 in T-ALL cell lines and patient samples. Whole transcriptome with RNA-seq was performed in CEM T-ALL cells upon the treatment of KPT-330, CX-4945, and vehicle control. Patient-derived xenograft (PDX) mouse models were used to explore the in vivo efficacy of the drugs.

### Results

XPO1 inhibitor KPT-330 combined with CK2 inhibitor CX-4945, exhibited a significant effect on cell proliferation arrest in T-ALL patient primary cells compared to the KPT-330 only control ($P<0.001$) (Fig. 1A), and Calcusyn analysis indicated that they had a significant synergistic effect (Fig. 1B). KPT-330 combined with CX-4945 also significantly induced apoptosis in the patient samples compared to either single drug control ($P<0.001$) (Fig. 1C). RNA-seq analysis identified the differentially expressed genes (DEGs) in CEM cells treated with either CX-4945 or KPT-330 (Fig. 1D, E), and KEGG analysis showed that metabolic pathways are the top 1 significantly enriched ones. Heatmap showed the SLC7A5, responsible for Leucine, Isoleucine, and Valine transport, is significantly downregulated upon the treatment of CX-4945 and KPT-330. Combination of KPT-330 with CX-4945 remarkably down-regulates the SLC7A5 in mRNA and protein levels in T-ALL patient samples compared with either single drug controls ($P<0.001$) (Fig. 1F, G). Amino acid metabolomics analysis confirmed that both CX-4945 and KPT-330 significantly decreased the Leucine, Isoleucine, and Valine levels in T-ALL cells (Fig. 1H). The *in vivo* PDX model showed that the combination of CX-4945 and KPT-330 significantly reduced the spleen sizes (Fig. 1I), % of human $CD45^{+}CD7^{+}$ T-ALL cells ($P<0.001$) (Fig. 1J), and prolonged mouse survival ($P<0.001$) (Fig. 1K) compared with either single drug controls. Western blot results revealed that SLC7A5 is remarkably decreased in the combination vs single drug controls (Fig.1L).

**Copyright Information:** (Online) ISSN: 2572-9241
©2026 The Author(s). HemaSphere is published by John Wiley & Sons Ltd on behalf of European Hematology Association. This is an open access Abstract Book distributed under the Attribution-NonCommercial-NoDerivs (CC BY-NC-ND), which allows third parties to download the articles and share them with others as long as they credit the author and the Abstract Book, but they cannot change the content in any way or use them commercially.
**Abstract Book Citations:** Authors, Title, HemaSphere, 2026;10:(S1):pages. The abstract book DOIs can be found on the "Author Information" page.
**Disclaimer:** Articles published in the journal HemaSphere exclusively reflect the opinions of the authors. The authors are responsible for all content in their abstracts including accuracy of the facts, statements, citing resources, etc.

### Summary/Conclusion

Our data showed that XPO1 inhibitor KPT-330, combined with CK2 inhibitor CX-4945 have synergistic efficacy in T-ALL by suppressing SLC7A5 expression and the amino acid metabolism. Targeting amino acid metabolism by KPT-330 and CX-4945 may be a potential therapeutic approach for T-ALL patients.

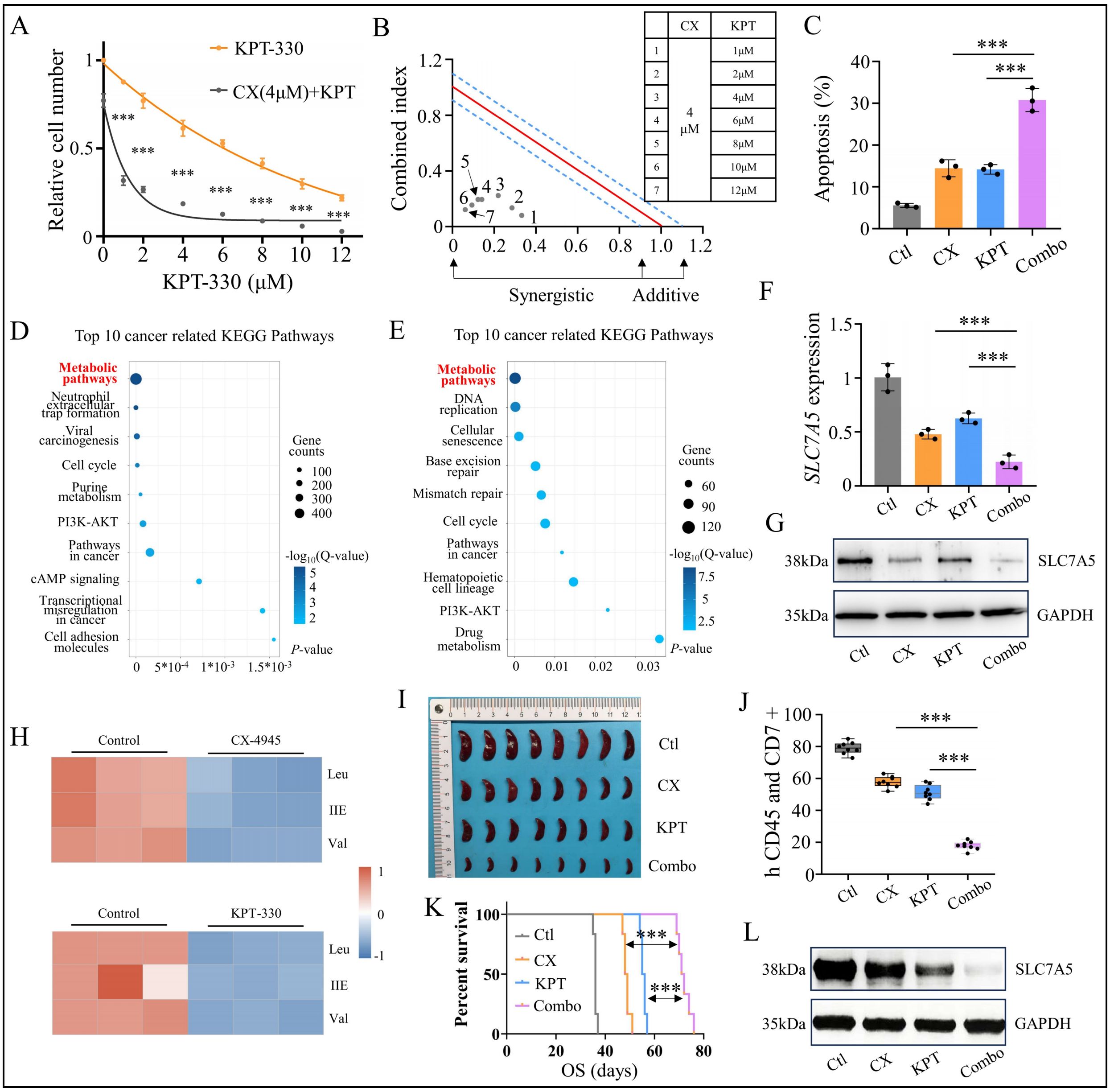

Fig 1. Therapeutic effect on amino acid metabolism with CX-4945 and XPO1 inhibitor in T-ALL. (A-B) Effect (A) and synergistic analysis (B) of various KPT-330 doses combined with CX-4945 on cell proliferation in T-ALL patient samples. (C) Effect of the combination of KPT-330 with CX-4945 on apoptosis compared with single drug controls in the patient samples. (D-E) KEGG analysis of DEGs upon the treatment of CX-4945 (D) and KPT-330 (E). (F-G) Expression of SLC7A5 in mRNA (F) and protein (G) levels upon the indicated treatment. (H) Leucine, Isoleucine, and Valine expression in CEM cells treated with either KPT-330 or CX-4945. (I-K) Spleen image (I), % of human CD45+CD7+ positive T-ALL cells (J), and the survival (K) of the PDX model upon the indicated treatment for 28 days. (L) SLC7A5 expression in the mouse spleen upon the treatment for 28 days. *** P<0.001.

**Copyright Information:** (Online) ISSN: 2572-9241
©2026 The Author(s). HemaSphere is published by John Wiley & Sons Ltd on behalf of European Hematology Association. This is an open access Abstract Book distributed under the Attribution-NonCommercial-NoDerivs (CC BY-NC-ND), which allows third parties to download the articles and share them with others as long as they credit the author and the Abstract Book, but they cannot change the content in any way or use them commercially.
**Abstract Book Citations:** Authors, Title, HemaSphere, 2026;10:(S1):pages. The abstract book DOIs can be found on the "Author Information" page.
**Disclaimer:** Articles published in the journal HemaSphere exclusively reflect the opinions of the authors. The authors are responsible for all content in their abstracts including accuracy of the facts, statements, citing resources, etc.

### (PF351) TRANSCRIPTIONAL HETEROGENEITY IN PAEDIATRIC PHILADELPHIA-POSITIVE ACUTE LYMPHOBLASTIC LEUKAEMIA IDENTIFIES A PRIMITIVE POOR-RISK SUBGROUP

**Topic:** 01. Acute lymphoblastic leukemia - Biology & translational research

Rachel Craig*[1], Amy Muir[1], Jin Tongsom[1], Gillian Horne[1]

[1] *University of Glasgow, Glasgow, United Kingdom;*

### Background

Ph+ALL is characterised by the presence of the Philadelphia chromosome (Ph+), resulting in the BCR::ABL1 oncoprotein with enhanced tyrosine kinase activity. It is found in approximately 5% of childhood and up to 30% of adult acute lymphoblastic leukaemia (ALL). Despite targeted treatment approaches, it is associated with frequent relapses, particularly CNS relapse in the paediatric population. We and others have demonstrated that Ph+ALL can be further sub-divided into a true lymphoid leukaemia with acquisition of *BCR::ABL1* in committed B-cell progenitors and a 'primitive' Ph+ALL with *BCR::ABL1* arising in haematopoietic stem cells (HSC) and multipotent progenitors (MPP). Current methods rely on detecting discordance between *Ig/TCR* and *BCR::ABL1* at the end of induction. Earlier identification of these subgroups at diagnosis may enable improved risk stratification and therapeutic strategies.

### Aims

We aimed to identify transcriptomic differences in paediatric Ph+ ALL at bulk and single-cell resolution and to develop improved diagnostic strategies.

### Methods

Paediatric Ph+ALL bone marrow mononuclear cell (BMNC) samples (n=34) were sorted into stem and progenitor populations by fluorescence-activated cell sorting (FACS). *BCR::ABL1* fusion was identified by fluorescence *in situ* hybridisation (FISH) on FACS-sorted populations. Bulk RNA sequencing (n=13) was performed and analysed using DESeq2 and ClusterProfiler R packages. Single cell RNA sequencing (scRNA-Seq) was performed on viable cells using the 10x Chromium Single-Cell 3' v3 Gel Bead Kit and Library (10x Genomics) (n=5). scRNA-Seq data were processed using the Cell Ranger (v8.0.0; 10x Genomics) pipeline. Quality control, data integration, normalisation, dimension reduction and cell clustering was performed using Seurat and SCTransform.

### Results

Using bulk RNAseq we identified two transcriptionally distinct populations within our paediatric Ph+ALL cohort with evidence of a survival disadvantage, defined by overall survival and MRD persistence through *IgTCR*, within one population. Gene set enrichment analysis (GSEA) identified this same population to be enriched for cell adhesion and HSC proliferation pathways, consistent with a more primitive and migratory phenotype. Further characterisation of these samples using scRNA-Seq identified higher proportions of cells with a primitive or non-B cell phenotype within this poor-risk population. *BCR::ABL1* was detected in HSC/MPP populations of patients in both clusters indicating that *BCR::ABL1* alone does not distinguish poor-risk disease. Integration of bulk and single-cell transcriptomic data enabled the development of a predictive RNA-sequencing gene expression panel, which is currently undergoing validation in an independent cohort.

**Copyright Information:** (Online) ISSN: 2572-9241
©2026 The Author(s). HemaSphere is published by John Wiley & Sons Ltd on behalf of European Hematology Association. This is an open access Abstract Book distributed under the Attribution-NonCommercial-NoDerivs (CC BY-NC-ND), which allows third parties to download the articles and share them with others as long as they credit the author and the Abstract Book, but they cannot change the content in any way or use them commercially.
**Abstract Book Citations:** Authors, Title, HemaSphere, 2026;10:(S1):pages. The abstract book DOIs can be found on the "Author Information" page.
**Disclaimer:** Articles published in the journal HemaSphere exclusively reflect the opinions of the authors. The authors are responsible for all content in their abstracts including accuracy of the facts, statements, citing resources, etc.

**Summary/Conclusion**

These results identify a primitive, poor-risk subgroup of paediatric Ph+ ALL that cannot be distinguished using current markers. A transcriptome-based diagnostic panel offers a strategy for early identification of these patients and may support risk-adapted therapeutic strategies.

**Copyright Information:** (Online) ISSN: 2572-9241
©2026 The Author(s). HemaSphere is published by John Wiley & Sons Ltd on behalf of European Hematology Association. This is an open access Abstract Book distributed under the Attribution-NonCommercial-NoDerivs (CC BY-NC-ND), which allows third parties to download the articles and share them with others as long as they credit the author and the Abstract Book, but they cannot change the content in any way or use them commercially.
**Abstract Book Citations:** Authors, Title, HemaSphere, 2026;10:(S1):pages. The abstract book DOIs can be found on the "Author Information" page.
**Disclaimer:** Articles published in the journal HemaSphere exclusively reflect the opinions of the authors. The authors are responsible for all content in their abstracts including accuracy of the facts, statements, citing resources, etc.

### (PF352) COMBINATION MENIN (ZIFTOMENIB) AND BCL-2 (VENETOCLAX) INHIBITION ENHANCES ANTI-LEUKEMIC ACTIVITY IN PRECLINICAL MODELS OF HUMAN KMT2AR ACUTE LYMPHOBLASTIC LEUKEMIA

**Topic:** 01. Acute lymphoblastic leukemia - Biology & translational research

Marianna Giorgi*[1], Leslie Martinez-Gutierrez[1], Karina Gill[1], Spencer Rosario[2], Eunice Wang[1, 3]

*[1]Roswell Park Comprehensive Cancer Center, Leukemia Service, Buffalo, United States of America;[2]Roswell Park Comprehensive Cancer Center, Medicine, Buffalo, United States of America; [3]University at Buffalo South Campus, Buffalo, United States of America;*

### Background

The presence of KMT2A gene rearrangements (KMT2Ar) in adult and pediatric acute lymphoblastic leukemia (ALL) confer worse outcomes following chemotherapy. Menin inhibitors have demonstrated promising clinical efficacy in heavily pretreated patients with relapsed/refractory KMT2Ar acute leukemias. However, low response rates, short survival, and rapid development of resistance to single agent menin inhibitor therapy have prompted exploration of combinatorial approaches. Venetoclax (Ven) has exhibited clinical activity in patients with relapsed/refractory ALL. We hypothesized that combining the menin inhibitor Ziftomenib (Zifto) and Ven may result in synergistic anti-leukemic effects on human KMT2Ar ALL cells.

### Methods

Human KMT2Ar ALL cells (SEM, RS4;11) were continuously treated *in vitro* with Zifto and Ven alone and in combination (Zifto+Ven) for 4-7 days and assessed for viability, apoptosis, cell cycle, and lymphoid differentiation via flow cytometry. Dose dependent growth to single agent inhibition was observed ($IC_{50}$ values: Zifto=1-24µM, Ven= 21-115nM). Combining Zifto (0.1-1µM) with Ven (10-50nM) further enhanced anti-leukemic activity with a combination index (CI) <1 consistent with synergistic effects in both cell lines. Combination therapy significantly enhanced apoptosis and decreased cell cycling with fewer cells in S-phase (60% to 25%, p=0.0225) as compared with monotherapy. Moreover, combination treated ALL cells exhibited increased senescence and induction of lymphoid differentiation via altered expression of B-cell markers (higher CD20 and lower CD19) versus Zifto or Ven alone. Marrow cells from KMT2Ar ALL patients cultured in Methocult were treated with vehicle, Zifto, Ven, and Zifto+Ven followed by enumeration of colony formation units (CFUs). Significantly reduced CFUs were noted following combination therapy vs monotherapy or vehicle. RNA sequencing and gene set enrichment analysis demonstrated that Zifto+Ven significantly altered genes involved in metabolomic pathways as compared with Zifto or Ven alone in ALL cell lines.
Immunodeficient mice were systematically engrafted with luciferase-labeled SEM cells via tail vein injection and treated with vehicle, Zifto (75mg/kg), Ven (100mg/kg), or Zifto+Ven (same doses) for 3 weeks. Tumor burden, toxicity, and survival were determined by weekly whole animal bioluminescent imaging, animal weights, and time to morbidity, respectively. Mice did not experience significant weight loss. As shown (Figure 1), leukemic burden (via total bioluminescent flux) was significantly reduced in combination treated animals (p<0.01) as compared to monotherapy groups. Mice treated with dual therapy also experienced significantly prolonged overall survival (p<0.05) compared to other treatments.

**Copyright Information:** (Online) ISSN: 2572-9241
©2026 The Author(s). HemaSphere is published by John Wiley & Sons Ltd on behalf of European Hematology Association. This is an open access Abstract Book distributed under the Attribution-NonCommercial-NoDerivs (CC BY-NC-ND), which allows third parties to download the articles and share them with others as long as they credit the author and the Abstract Book, but they cannot change the content in any way or use them commercially.
**Abstract Book Citations:** Authors, Title, HemaSphere, 2026;10:(S1):pages. The abstract book DOIs can be found on the "Author Information" page.
**Disclaimer:** Articles published in the journal HemaSphere exclusively reflect the opinions of the authors. The authors are responsible for all content in their abstracts including accuracy of the facts, statements, citing resources, etc.

### Summary/Conclusion

Addition of Ven to Zifto synergistically improved the anti-leukemic activity of menin inhibition in human B-ALL cells via induction of apoptosis, decreased cell cycle and proliferation, increased differentiation, and senescence. Combination Zifto +Ven significantly reduced colony formation propagation of primary ALL cells. *In vivo* therapy with Zifto+Ven in human ALL xenografted mice markedly reduced systemic leukemic disease burden and prolonged overall survival over single agent or vehicle therapy. These studies support the development of future clinical trials exploring menin inhibition combined with Venetoclax for relapsed/refractory KMT2Ar ALL.

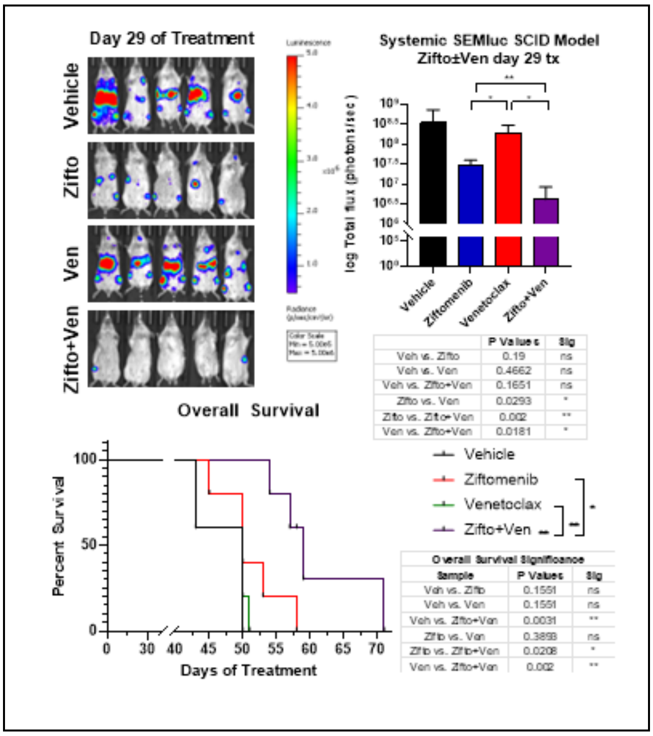

Figure 1 Systemic human ALL mouse model. 1e6 SEMluc cells were inoculated into 20 SCID mice via tail vein injection. After confirmation of engraftment by whole body bioluminescent imaging, treatment of vehicle, Zifto (75mg/kg 5 days/week), Ven (100mg/kg 5 days/week), or the dual therapy began. At day 29 of treatment, combination treated mice exhibited significantly reduced burden when compared to both single agent treatments as well as the vehicle treatment groups. Dual treated mice also exhibited a significantly longer overall survival time when compared to both single agent groups and vehicle treated mice.

**Copyright Information:** (Online) ISSN: 2572-9241
©2026 The Author(s). HemaSphere is published by John Wiley & Sons Ltd on behalf of European Hematology Association. This is an open access Abstract Book distributed under the Attribution-NonCommercial-NoDerivs (CC BY-NC-ND), which allows third parties to download the articles and share them with others as long as they credit the author and the Abstract Book, but they cannot change the content in any way or use them commercially.
**Abstract Book Citations:** Authors, Title, HemaSphere, 2026;10:(S1):pages. The abstract book DOIs can be found on the "Author Information" page.
**Disclaimer:** Articles published in the journal HemaSphere exclusively reflect the opinions of the authors. The authors are responsible for all content in their abstracts including accuracy of the facts, statements, citing resources, etc.

### (PF353) DYNAMIC BH3 PROFILING IMPROVES CLINICAL RESPONSE IN RELAPSED AND REFRACTORY PEDIATRIC ACUTE LYMPHOBLASTIC LEUKEMIA PATIENTS

**Topic:** 01. Acute lymphoblastic leukemia - Biology & translational research

Albert Manzano-Muñoz*[1, 2], Pablo Velasco[3, 4, 5], Clara Alcon[1, 2], Adela Escudero[6], Elisa Izquierdo[6], Nerea Vega-Garcia[7], Mireia Camos Guijosa[7], Vicky Martínez[8], Ajla Idrizovic[4, 5], José Manuel Vagace[9], Berta González Martínez[6], Laura Arqués[7], Rosa Adán[10], Miren Oscoz[11], Marina García Morín[12], Vanesa Pérez Alonso[13], María Tasso Cereceda[14], Blanca Herrero[15], Águeda Molinos-Quintana[16], Josep Samitier[1, 17], Clara Bueno[18], Pablo Menendez[1, 18, 19, 20], José Luis Fuster[8], Joan Montero[1, 2]

*[1]University of Barcelona, Barcelona, Spain;[2]Institut d'Investigacions Biomèdiques August Pi i Sunyer (IDIBAPS), Barcelona, Spain; [3]Hospital Vall d'Hebrón, Barcelona, Spain; [4]Universitat Autonoma de Barcelona, Barcelona, Spain;[5]Vall d'Hebron Research Institute (VHIR), Barcelona, Spain; [6]Hospital Universitario La Paz, Madrid, Spain; [7]Hospital Sant Joan de Déu Barcelona, Barcelona, Spain; [8]Hospital Clínico Universitario Virgen de la Arrixaca, Murcia, Spain;[9]Hospital Materno Infantil Badajoz, Badajoz, Spain; [10]Hospital Universitario de Cruces, Barakaldo, Spain;[11]Complejo Hospitalario de Navarra, Pamplona, Spain; [12]Hospital Universitario Gregorio Marañón, Madrid, Spain; [13]Hospital Universitario 12 de Octubre, Madrid, Spain; [14]Hospital General Universitario Dr Balmis, Alicante, Spain; [15]Hospital Niño Jesús, Barcelona, Spain; [16]Hospital Universitario Virgen del Rocío, Sevilla, Spain; [17]Institut de Bioenginyeria de Catalunya (IBEC), Barcelona, Spain;[18]Josep Carreras Leukemia Research Institute, Barcelona, Spain; [19]Institut de Recerca Hospital Sant Joan de Déu-Pediatric Cancer Center Barcelona, Barcelona, Spain; [20]Institució Catalana de Recerca i Estudis Avançats (ICREA), Barcelona, Spain;*

### Background

Pediatric Relapsed and Refractory Acute Lymphoblastic Leukemia (R/R ALL) remains one of the leading causes of cancer-related death in children. Different therapeutic strategies exist for these types of patients (intensive chemotherapy, targeted drugs and immunotherapy), but effective biomarkers to assign therapy are needed. Functional Precision Medicine (FPM) solves this problem by testing how a patient's own cancer cells respond to anti-cancer drugs, uncovering the treatments most likely to work for each individual. Dynamic BH3 Profiling (DBP) is a rapid functional assay that measures mitochondrial apoptotic priming using pro-apoptotic peptides, enabling early identification of cytotoxic drug responses in primary patient samples within 48 hours — a clinically actionable timeframe.

### Aims

In this study, we used a FPM approach and applied DBP to support therapeutic decision-making in pediatric R/R ALL. The primary objective was to prospectively validate the predictive capacity of DBP for treatment response. A secondary objective was to evaluate whether DBP-guided therapy translated into improved clinical outcomes.

### Methods

First, DBP was performed in pediatric ALL cell lines to validate chemotherapy and targeted drugs efficacy predictions. Then, patient samples were treated ex vivo with drug candidates and analyzed by DBP to predict treatment efficacy, giving treating clinicians a functional report as a new diagnostic layer for these patients. This data was complemented with genetic screening for each patient. Follow-up of the cohort was performed and differences in objective response rate, event-free survival and overall survival between DBP-positive and negative patients were assessed.

**Copyright Information:** (Online) ISSN: 2572-9241
©2026 The Author(s). HemaSphere is published by John Wiley & Sons Ltd on behalf of European Hematology Association. This is an open access Abstract Book distributed under the Attribution-NonCommercial-NoDerivs (CC BY-NC-ND), which allows third parties to download the articles and share them with others as long as they credit the author and the Abstract Book, but they cannot change the content in any way or use them commercially.
**Abstract Book Citations:** Authors, Title, HemaSphere, 2026;10:(S1):pages. The abstract book DOIs can be found on the "Author Information" page.
**Disclaimer:** Articles published in the journal HemaSphere exclusively reflect the opinions of the authors. The authors are responsible for all content in their abstracts including accuracy of the facts, statements, citing resources, etc.

### Results

We first validated DBP as a functional predictor in ALL cell lines and retrospective cryopreserved samples from newly diagnosed patients, correctly stratifying patients' response to induction chemotherapy. Prospectively, DBP was able to accurately predict treatment efficacy in R/R ALL patients and provide actionable personalized therapy recommendations for the treating clinicians, while avoiding ineffective and potentially toxic drugs. DBP's was able to give efficacy predictions in 90% of the samples received, and 39 patients were reviewed in a multidisciplinary tumor board integrating clinical, genomic, and DBP data. After follow-up, patients treated according to DBP predictions achieved a clear improvement in overall response rates (75% in patients treated with predicted effective regimes vs 9.1% in non-guided patients) and prolonged event-free survival (median of 262 days), demonstrating the clinical advantage of using FPM to guide treatment.

### Summary/Conclusion

To our knowledge, this is the first prospective study demonstrating DBP's exceptional worth as a functional biomarker to foster precision medicine and significantly improve outcomes in advanced pediatric leukemia.

**Copyright Information:** (Online) ISSN: 2572-9241
©2026 The Author(s). HemaSphere is published by John Wiley & Sons Ltd on behalf of European Hematology Association. This is an open access Abstract Book distributed under the Attribution-NonCommercial-NoDerivs (CC BY-NC-ND), which allows third parties to download the articles and share them with others as long as they credit the author and the Abstract Book, but they cannot change the content in any way or use them commercially.
**Abstract Book Citations:** Authors, Title, HemaSphere, 2026;10:(S1):pages. The abstract book DOIs can be found on the "Author Information" page.
**Disclaimer:** Articles published in the journal HemaSphere exclusively reflect the opinions of the authors. The authors are responsible for all content in their abstracts including accuracy of the facts, statements, citing resources, etc.

### (PF354) L-ASPARAGINASE REMODELS REDOX HOMEOSTASIS VIA AUTOPHAGY IN ACUTE LYMPHOBLASTIC LEUKEMIA

**Topic:** 01. Acute lymphoblastic leukemia - Biology & translational research

Matus Kolarik*[1, 2], Bryan Marzullo[3], Nadia Alaei[1], Daniel Tennant[3], Katerina Hlozkova[1], Julia Starkova[1, 4]

*[1]Second Faculty of Medicine, Charles University, CLIP, Department of Pediatric Hematology and Oncology, Prague, Czechia; [2]First Faculty of Medicine, Charles University, Prague, Czechia;[3]College of Medical and Dental Sciences, University of Birmingham, Institute of Metabolism and Systems Research, Birmingham, United Kingdom; [4]University Hospital Motol and Homolka, Prague, Czechia;*

### Background

Oxidative stress is a recognized hallmark of various cancers which enhances antioxidant defences leading to chemo-resistance development. In acute lymphoblastic leukemia (ALL), the expression level of the cystine (CC) transporter and ferroptosis regulator SLC7A11, responsible for delivering a key substrate for the main antioxidant glutathione, has been shown to negatively correlate with resistance to cytostatic drug, L-asparaginase (ASNase) (Ferguson et al. 2022).

### Aims

This study investigates how CC availability shapes redox homeostasis and ASNase sensitivity in ALL, and identifies metabolic survival mechanisms that may contribute to treatment resistance

### Methods

ALL cell lines (NALM6, REH, RS4;11, Jurkat and MOLT4) were treated with ASNase, CC depletion and combination treatment. Full RPMI was used as a control. Cell viability was assessed by absolute cell counting with viability staining using flow cytometry. Reactive oxygen species (ROS) and lipid peroxidation (LP) were measured using $H_2$DCF-DA and Bodipy581/591 C11 dyes, respectively. Metabolic fluxes were mapped using LC-MS-based stable isotope tracing (SIT) with $^{13}C_3$-serine and $^{13}C_6$,$^{15}N_2$-cystine in two ALL cell lines and four primary patient ALL blasts.

### Results

CC depletion was lethal in all five tested ALL cell lines, preceded by elevated ROS and LP levels, hallmarks of ferroptosis. Ferroptosis was confirmed by complete rescue with ferroptosis inhibitor Ferrostatin-1 or antioxidant tocopherol. Strikingly, ASNase treatment fully prevented cell death under CC depletion and simultaneously suppressed ROS and LP levels, revealing that cystine-depleted conditions render ALL cells resistant to ASNase-induced cytotoxicity through activation of redox rescue mechanisms.
SIT with $^{13}C_3$-serine revealed that CC depletion caused a marked drop in total and *de novo* synthesized GSH, which was partially restored by ASNase co-treatment, both in the MOLT4 cell line and in primary leukemic cells from 3 of 4 patients at diagnosis. Notably, no labelled cysteine was detected via the reverse transsulfuration pathway in any model, excluding this alternative cysteine synthesis pathway as a compensatory mechanism.
To determine the cysteine source for ASNase-mediated GSH synthesis, we investigated autophagy, a well-established effect of ASNase. Cells were pre-treated with $^{13}C_6$,$^{15}N_2$-cystine to enrich cellular proteins with labelled cysteine; after tracer removal, free labelled cysteine reflecting release from protein degradation was undetectable in untreated controls. However, either ASNase treatment or CC depletion alone induced measurable release of labelled cysteine, and their combination produced a synergistic increase. Furthermore, pharmacological autophagy inhibition abolished the pro-survival effect of ASNase under CC depletion, establishing autophagy as the key mechanism sustaining cysteine supply.

**Copyright Information:** (Online) ISSN: 2572-9241
©2026 The Author(s). HemaSphere is published by John Wiley & Sons Ltd on behalf of European Hematology Association. This is an open access Abstract Book distributed under the Attribution-NonCommercial-NoDerivs (CC BY-NC-ND), which allows third parties to download the articles and share them with others as long as they credit the author and the Abstract Book, but they cannot change the content in any way or use them commercially.
**Abstract Book Citations:** Authors, Title, HemaSphere, 2026;10:(S1):pages. The abstract book DOIs can be found on the "Author Information" page.
**Disclaimer:** Articles published in the journal HemaSphere exclusively reflect the opinions of the authors. The authors are responsible for all content in their abstracts including accuracy of the facts, statements, citing resources, etc.

**Summary/Conclusion**

We demonstrate that ASNase activates a pro-survival axis in ALL cells under cystine-depleted conditions by stimulating autophagy, restoring GSH, and ferroptosis suppression. This study highlights that redox homeostasis and autophagy are tightly connected and may provide a survival benefits under metabolically challenging conditions. Identifying patients with low SLC7A11 expression at diagnosis could enable better treatment stratification and offer new therapeutic strategy based on autophagy inhibition and induction of ferroptosis alongside standard therapy.

Supported by GAUK179624 and Charles University research centre UNCE/24/MED/003

**Copyright Information:** (Online) ISSN: 2572-9241
©2026 The Author(s). HemaSphere is published by John Wiley & Sons Ltd on behalf of European Hematology Association. This is an open access Abstract Book distributed under the Attribution-NonCommercial-NoDerivs (CC BY-NC-ND), which allows third parties to download the articles and share them with others as long as they credit the author and the Abstract Book, but they cannot change the content in any way or use them commercially.
**Abstract Book Citations:** Authors, Title, HemaSphere, 2026;10:(S1):pages. The abstract book DOIs can be found on the “Author Information” page.
**Disclaimer:** Articles published in the journal HemaSphere exclusively reflect the opinions of the authors. The authors are responsible for all content in their abstracts including accuracy of the facts, statements, citing resources, etc.

### (PF356) PTEN–AKT2 SIGNALING-MEDIATED GLYCOLYSIS DRIVES GLUCOCORTICOID RESISTANCE IN T-CELL ACUTE LYMPHOBLASTIC LEUKEMIA

**Topic:** 01. Acute lymphoblastic leukemia - Biology & translational research

Mixue Xie*[1], Sufen Cheng[1], Hongyan Tong[1]

[1]*Department of Hematology\uFFFDThe First Affiliated Hospital, Zhejiang University School of Medicine, Hangzhou, China;*

### Background

Glucocorticoids (GCs) represent a cornerstone of chemotherapeutic regimens for T-cell acute lymphoblastic leukemia (T-ALL). However, up to 25% of T-ALL patients develop resistance to GCs, which is strongly associated with poor clinical outcomes. Despite their clinical significance, the molecular mechanisms underlying GC resistance and leukemia relapse remain poorly understood.

### Aims

Emerging evidence indicates that GC resistance is tightly linked to metabolic reprogramming, especially enhanced glycolysis. Identifying the key metabolic drivers of GC resistance and targeting their downstream mediators may facilitate the development of novel therapeutic strategies.

### Results

To identify genes modulating GC resistance, we performed a genome-wide CRISPR-Cas9 screen in T-ALL (Jurkat) cells under dexamethasone (DEX) treatment (A). Loss of PTEN exerted the stronger protective effect against DEX-induced cytotoxicity, identifying PTEN as a critical regulator of GC resistance (B). We then assessed aerobic glycolysis by measuring the extracellular acidification rate (ECAR) in four T-ALL cell lines (Hut78, CCRF, Jurkat, Molt4) with differential GC sensitivity. GC-resistant cells displayed elevated basal and maximum glycolytic capacity, accompanied by reduced PTEN expression (C-E). Lentivirus-mediated PTEN overexpression in resistant cells restored GC-induced apoptosis and suppressed glycolysis.
Given that PTEN negatively regulates Akt signaling, we investigated the roles of Akt isoforms (Akt1 and Akt2) in GC resistance. Western blot analysis revealed that Akt2, but not Akt1, was markedly upregulated in resistant cells (Jurkat, Molt4) compared with GC-sensitive cells (Hut78). Notably, PTEN overexpression downregulated Akt2 expression.

We performed pathway enrichment analysis of the top 500 genes from CRISPR-Cas9 negative selection using Metascape. Phosphorylation-related signaling pathways were significantly enriched (F), and the PI3K pathway was particularly associated with GC resistance. Further screening based on negative selection scores identified PFKFB2 as the most critical glycolytic kinase downstream of the PI3K/Akt2 signaling axis. Upregulation of PTEN expression or inhibition of Akt2 activity significantly reduced the phosphorylation level of PFKFB2 at Ser483 in T-ALL cells (G). Co-immunoprecipitation assays verified a direct physical interaction between Akt2 and PFKFB2. Mechanistically, PFKFB2 functions as a key regulatory kinase that promotes the synthesis of the allosteric activator F2,6BP and enhances the activity of PFK1, the second rate-limiting enzyme of glycolysis.
To evaluate therapeutic potential, we treated resistant cells with isoform-specific Akt inhibitors (A674563 for Akt1; CCT128930 for Akt2). Strikingly, the Akt2 inhibitor more potently suppressed glycolysis, enhanced DEX-mediated cell death, and reversed GC resistance than the Akt1 inhibitor, both in vitro and in vivo.

**Copyright Information:** (Online) ISSN: 2572-9241
©2026 The Author(s). HemaSphere is published by John Wiley & Sons Ltd on behalf of European Hematology Association. This is an open access Abstract Book distributed under the Attribution-NonCommercial-NoDerivs (CC BY-NC-ND), which allows third parties to download the articles and share them with others as long as they credit the author and the Abstract Book, but they cannot change the content in any way or use them commercially.
**Abstract Book Citations:** Authors, Title, HemaSphere, 2026;10:(S1):pages. The abstract book DOIs can be found on the "Author Information" page.
**Disclaimer:** Articles published in the journal HemaSphere exclusively reflect the opinions of the authors. The authors are responsible for all content in their abstracts including accuracy of the facts, statements, citing resources, etc.

### Summary/Conclusion

PTEN deficiency drives GC resistance in T-ALL by hyperactivating the Akt2/PFKFB2 axis to promote glycolysis. Selective inhibition of Akt2 overcomes GC resistance, representing a promising precision therapeutic strategy for refractory T-ALL.

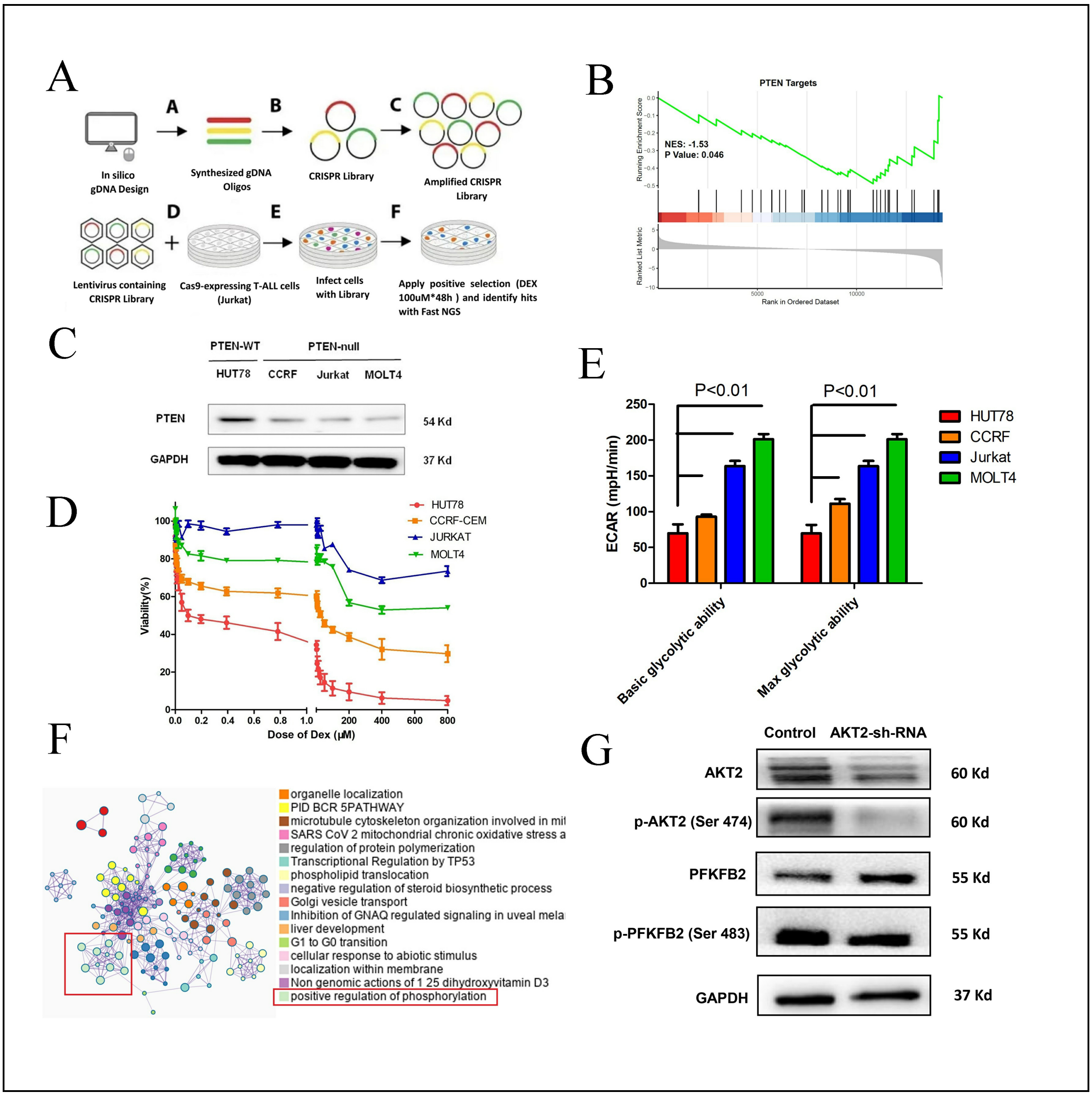

A: Flow chart of genome-wide CRISPR-Cas9 screening in T-ALL cells; B: GSEA enrichment analysis based on CRISPR-Cas9 screening data; C: Western blot analysis of PTEN expression in multiple T-ALL cell lines; D: Glucocorticoid sensitivity of various T-ALL cell lines determined by MTT assay; E: Aerobic glycolysis in multiple T-ALL cell lines measured by Seahorse assay; F: Pathway enrichment analysis of the top 500 negatively selected genes; G: Western blot analysis of Akt2/PFKFB2 phosphorylation pathway in Akt2-knockdown T-ALL cells.

**Copyright Information:** (Online) ISSN: 2572-9241
©2026 The Author(s). HemaSphere is published by John Wiley & Sons Ltd on behalf of European Hematology Association. This is an open access Abstract Book distributed under the Attribution-NonCommercial-NoDerivs (CC BY-NC-ND), which allows third parties to download the articles and share them with others as long as they credit the author and the Abstract Book, but they cannot change the content in any way or use them commercially.
**Abstract Book Citations:** Authors, Title, HemaSphere, 2026;10:(S1):pages. The abstract book DOIs can be found on the "Author Information" page.
**Disclaimer:** Articles published in the journal HemaSphere exclusively reflect the opinions of the authors. The authors are responsible for all content in their abstracts including accuracy of the facts, statements, citing resources, etc.

### (PF357) THE CIRCULATING BLOOD PROTEOME OF CHILDHOOD ACUTE LEUKEMIA

**Topic:** 01. Acute lymphoblastic leukemia - Biology & translational research

Anna Pia Enblad[1, 2, 3], Maria Globisch[1, 2], Dea Gogishvili*[1, 2], Tiina Tuononen[4], Olga Krali[1, 2], Anders Lundmark[1, 2], Laura Oksa[5, 6], Charlotte Hjort[7, 8], Linda Holmfeldt[9], Mikael Åberg[1, 2], Josefine Palle[3], Signe Modvig[7, 8], Olli Lohi[5, 6], Merja Heinäniemi[4], Arja Harila[3], Jessica Nordlund[1, 2]

*[1]Uppsala University, Medical Sciences, Uppsala, Sweden; [2]Uppsala University, SciLifeLab, Uppsala, Sweden; [3]Uppsala University, Department of Women's and Children's Health, Uppsala, Sweden; [4]University of Eastern Finland, Institute of Biomedicine, School of Medicine, Kuopio, Finland; [5]Tampere University, Tampere Center for Child, Adolescent, and Maternal Health Research, Faculty of Medicine and Health Technology, Tampere, Finland; [6]Tampere University Hospital, Tays Cancer Centre, Tampere, Finland; [7]Copenhagen University Hospital Rigshospitalet, Department of Clinical Immunology, Copenhagen, Denmark; [8]Copenhagen University, Department of Clinical Medicine, Faculty of Health and Medical Sciences, Copenhagen, Denmark; [9]Uppsala University, Department of Immunology, Genetics and Pathology, Uppsala, Sweden;*

### Background

Acute leukemia is the most common childhood malignancy worldwide. While genomics-based diagnostics have transformed classification, these approaches provide only a leukemia cell-intrinsic view. Patient outcomes are also shaped by systemic immune responses, which remain poorly characterized. The circulating proteome offers a minimally invasive readout capturing both tumor biology and host immune activity, yet remains underexplored in pediatric leukemia across immunophenotypic and molecular subtypes.

### Aims

We aimed to identify circulating proteomic signatures that distinguish immunophenotypic and molecular subtypes while revealing both tumor-intrinsic biology and systemic host responses.

### Methods

We profiled 3,072 proteins in diagnostic serum from 54 children with ALL (33 BCP-ALL, 21 T-ALL), 21 with AML, and 12 healthy controls using the Olink Proximity Extension Assay. Differential abundance was assessed using limma with FDR-adjusted $p < 0.05$ and $|Log2FC| > 1.5$, adjusting for age and sex. Key findings were validated in an independent cohort and integrated with external RNA-seq and mass spectrometry datasets.

### Results

We observed profound alterations in circulating protein levels in leukemia patients compared to controls and identified immunophenotype-specific protein markers, including SIGLEC15 in BCP-ALL, NOTCH1 in T-ALL, and CEBPA in AML. AML and T-ALL shared the highest number of differentially abundant circulating proteins. Within BCP-ALL, molecular subtypes might influence the circulating proteome; DSC2 and PTPRK were elevated *ETV6*::*RUNX1*-positive cases, while IL-6R and LAT2 were elevated in High Hyperdiploid cases. Examination of protein abundance levels for top discriminating proteins revealed that even in samples with low (<20%) or no peripheral blood blasts, protein levels remained elevated, indicating their potential utility as diagnostic markers. We observed a reduction in angiogenic proteins in circulation across immunophenotypes, suggesting changes in the peripheral vasculature. We also noted elevated circulating EPO across all immunophenotypes, likely reflecting compensatory responses to anemia and bone marrow dysfunction.

**Copyright Information:** (Online) ISSN: 2572-9241
©2026 The Author(s). HemaSphere is published by John Wiley & Sons Ltd on behalf of European Hematology Association. This is an open access Abstract Book distributed under the Attribution-NonCommercial-NoDerivs (CC BY-NC-ND), which allows third parties to download the articles and share them with others as long as they credit the author and the Abstract Book, but they cannot change the content in any way or use them commercially.
**Abstract Book Citations:** Authors, Title, HemaSphere, 2026;10:(S1):pages. The abstract book DOIs can be found on the "Author Information" page.
**Disclaimer:** Articles published in the journal HemaSphere exclusively reflect the opinions of the authors. The authors are responsible for all content in their abstracts including accuracy of the facts, statements, citing resources, etc.

**Summary/Conclusion**

In conclusion, the circulating proteome reflects both immunophenotypic and genetic diversity in pediatric leukemia, encompassing tumor-intrinsic programs as well as systemic physiological responses. This is particularly relevant given the increasing need for scalable, less complex approaches for diagnosis and monitoring. Blood proteomics offers distinct advantages for this purpose: minimally invasive sampling, particularly suitable in a pediatric setting, and feasibility for repeated measurements.

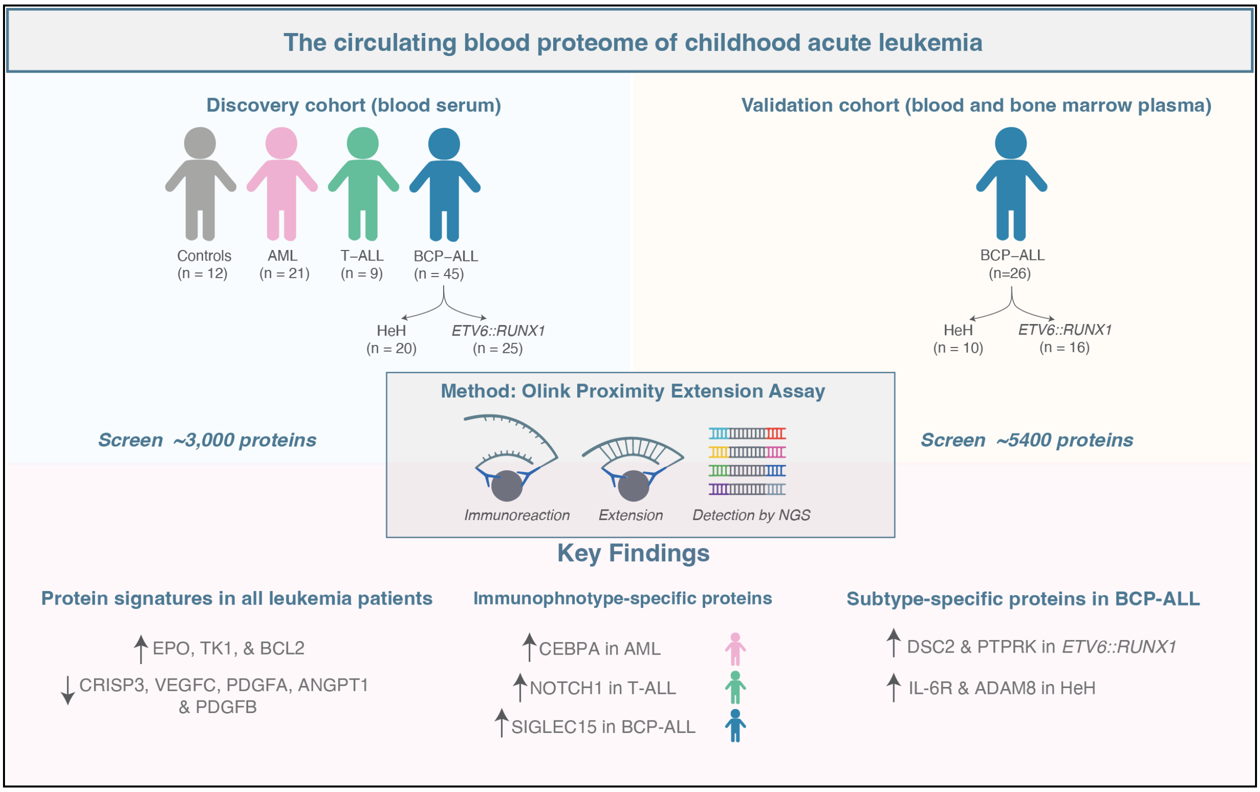

**Copyright Information:** (Online) ISSN: 2572-9241
©2026 The Author(s). HemaSphere is published by John Wiley & Sons Ltd on behalf of European Hematology Association. This is an open access Abstract Book distributed under the Attribution-NonCommercial-NoDerivs (CC BY-NC-ND), which allows third parties to download the articles and share them with others as long as they credit the author and the Abstract Book, but they cannot change the content in any way or use them commercially.
**Abstract Book Citations:** Authors, Title, HemaSphere, 2026;10:(S1):pages. The abstract book DOIs can be found on the "Author Information" page.
**Disclaimer:** Articles published in the journal HemaSphere exclusively reflect the opinions of the authors. The authors are responsible for all content in their abstracts including accuracy of the facts, statements, citing resources, etc.

### (PF358) PROTAC-BASED TARGETING OF ETV5 IN PH+B-ALL

**Topic:** 01. Acute lymphoblastic leukemia - Biology & translational research

Trudy Glaser*[1], Efthymios Gavriil[2], Thomas Jackson[2], Adam Thomas[2], Gege Pei[2], Vanessa Stöcker[2], Daisy Smith[2], Viacheslav Trush[3], Han Leng Ng[1], Edward Tate[2], Niklas Feldhahn[1]

*[1]Imperial College London, Immunology & Inflammation, Centre for Haematology, London, United Kingdom;[2]Imperial College London, Chemistry, London, United Kingdom; [3]Imperial College London, LMS MRC, Drug Discovery Hub, London, United Kingdom;*

### Background

Ph+B-ALL, driven by the oncogenic BCR::ABL1 kinase, is the most common B-ALL subtype in adults and historically has a poor prognosis. Two previous studies using Ph+B-ALL mouse models have implicated transcription factor (TF) ETV5 (E26 transformation specific (ETS) transcription variant 5) as crucial for this leukaemia. ETV5 is part of the ETS related family of TFs and activated through RAS/MEK/ERK signalling originally induced by BCR::ABL1. ETS family TFs are characterised by their highly conserved ETS DNA binding domain that recognises up to nine consecutive DNA bases consisting of a central core sequence GGA(A/T). ETV5, like many TFs, lack an active site, making them difficult to target by traditional inhibitors. We would like to target ETV5 by using PROTAC (PROteolysis TArgeting Chimera) technology. PROTACs are bifunctional modules that bring a target protein in close proximity to an E3 ligase, allowing for the transfer of ubiquitin and proteasomal degradation of the target. We would specifically target the ETS domain of ETV5, which is the only structured domain of the protein.

### Aims

We aim to assess the contribution of ETV5 in driving the oncogenic progression in Ph+B-ALL, as well therapeutically target ETV5 in Ph+B-ALL.

### Methods

To investigate the role ETV5 plays in the transcriptional regulation in Ph+B-ALL, we used RNA interference to knockdown ETV5 and performed RNA-Sequencing on selected Ph+B-ALL leukaemic cell lines. A dTAG/FKBP12-based inducible protein degradation model was used to determine whether ETV5 is susceptible to proteasomal degradation. To identify a binder against the ETS domain, we screened different non-covalent and covalent compound libraries using a variety of methods, including thermal shift assay, fluorescence polarisation (FP), DNA-encoded library (DEL) and intact protein mass spectrometry (MS). Hits were validated using orthogonal methods and short-listed for structure-activity-relationship (SAR) studies.

### Results

We show that ETV5 knockdown results in reduced cell viability in selected Ph+B-ALL cell lines and link this dependency to signalling pathway differences recently suggested for individual Ph+B-ALL subgroups. We hypothesise that ETV5 knockdown has the most effect in cell lines sensitive to RAS/MEK/ERK perturbation and tested this using pathway specific inhibitors. Regarding its potential therapeutic targeting, we provide evidence that using a dTAG/FKBP12-based model, ETV5 can be proteasomally degraded in a Ph+B-ALL cell line, supporting our endeavour to develop an ETV5 targeting PROTAC. We synthesised the most promising covalent hits and formation of covalent adduct was observed by intact MS. SAR studies were performed and binding affinities of analogues were tested. At top concentration, the most potent compounds had over 85% adduct formation. Hits with significant binding were subject to a time- and dose- dependent assay for the calculation of $K_{inact}/K_i$.

**Copyright Information:** (Online) ISSN: 2572-9241
©2026 The Author(s). HemaSphere is published by John Wiley & Sons Ltd on behalf of European Hematology Association. This is an open access Abstract Book distributed under the Attribution-NonCommercial-NoDerivs (CC BY-NC-ND), which allows third parties to download the articles and share them with others as long as they credit the author and the Abstract Book, but they cannot change the content in any way or use them commercially.
**Abstract Book Citations:** Authors, Title, HemaSphere, 2026;10:(S1):pages. The abstract book DOIs can be found on the "Author Information" page.
**Disclaimer:** Articles published in the journal HemaSphere exclusively reflect the opinions of the authors. The authors are responsible for all content in their abstracts including accuracy of the facts, statements, citing resources, etc.

**Summary/Conclusion**

Our results suggest that ETV5 is of particular relevance for a defined subset of Ph+B-ALL patients. Based on the evidence that ETV5 can be proteasomally degraded, we identified covalent compound binders against cysteine 449 in the ETS domain for the development of an ETV5 targeting PROTAC drug. SAR studies were initiated to improve the binding affinity of the most promising hit compounds and analogues were characterised in various biochemical assays.

**Copyright Information:** (Online) ISSN: 2572-9241
©2026 The Author(s). HemaSphere is published by John Wiley & Sons Ltd on behalf of European Hematology Association. This is an open access Abstract Book distributed under the Attribution-NonCommercial-NoDerivs (CC BY-NC-ND), which allows third parties to download the articles and share them with others as long as they credit the author and the Abstract Book, but they cannot change the content in any way or use them commercially.
**Abstract Book Citations:** Authors, Title, HemaSphere, 2026;10:(S1):pages. The abstract book DOIs can be found on the “Author Information” page.
**Disclaimer:** Articles published in the journal HemaSphere exclusively reflect the opinions of the authors. The authors are responsible for all content in their abstracts including accuracy of the facts, statements, citing resources, etc.

### (PF359) PROK1 IS A SUSCEPTILITY LOCUS FOR ANTHRACYCLINE-RELATED CARDIOMYOPATHY IN LONG-TERM CHILDHOOD ACUTE LEUKEMIA SURVIVORS

**Topic:** 01. Acute lymphoblastic leukemia - Biology & translational research

Mathilde Veneziano Broccia[1, 2], Gerard Michel[1], Julie Berbis[3], Jean-François Deleuze[4], Robert Olaso[4], Gaelle Munsch[2], Marine Germain[2], Caroline Meguerditchian[2], Aurélie Phulpin[5], Marilyne Poiree Dupuy[6], Justyna Kanold[7], Corinne Armari Alla[8], Carine Halfon-Domenech[9], Guy Leverger[10], Marie-Dominique Tabone[10], Sandrine Thouvenin-Doulet[11], Andre Baruchel[12], Virginie Gandemer[13], Nicolas Sirvent[14], Jean-Hugues Dalle[15], Stéphane Ducassou[16], Catherine Paillard[17], Isabelle Pellier[18], Laura Olivier[19], Pascal Auquier[3], David-Alexandre Trégouët[2], Paul Saultier*[1]

*[1]APHM, Service d'Immuno-Hémato-Oncologie Pédiatrique, Marseille, France; [2]BPH, Bordeaux Population Health Research Center, UMR 1219, ELEANOR, Bordeaux, France; [3]APHM, Service d'Épidémiologie et d'Économie de la Santé, Unité de Recherche Clinique, Direction de la Recherche en Santé, marseille, France; [4]CEA, Centre National de Recherche en Génomique Humaine, Université Paris-Saclay, Paris, France; [5]CHRU Nancy, Hôpitaux de Brabois, Onco-hématologie pédiatrique, Nancy, France; [6]CHU de Nice, Hôpital de l'Archet, unité Onco hématologie pédiatrique, Nice, France;[7]CHU de Clermont-Ferrand, Service Hématologie et oncologie pédiatrique, Clermont-Ferrand, France; [8]CHU de Grenoble, Service Hémato et Oncologie pédiatrique, Grenoble, France; [9]Hospices Civils de Lyon, Service Hémato et Oncologie pédiatrique, Lyon, France; [10]APHP, Hôpital Armand Trousseau, Service Hémato-immuno-oncologie, Paris, France; [11]CHU Saint-Etienne, Service Hématologie - Oncologie pédiatrique, Saint-Etienne, France; [12]APHP, Hôpital Paris Saint-Louis, Service Immuno, Hémato et Oncologie pédiatrique, Paris, France; [13]CHU Rennes, Service Onco-hématologie pédiatrique, Rennes, France; [14]CHU Montpellier, Service Onco-hématologie pédiatrique, Montpellier, France; [15]APHP, Hôpital Paris Robert Debré, Service Immuno, Hémato et Oncologie pédiatrique, Paris, France; [16]CHU Bordeaux, Service Hématologie et cancérologie pédiatrique, Bordeaux, France; [17]CHU Strasbourg, Service Onco-hématologie pédiatrique, Strasbourg, France; [18]CHU Angers, Service Immunologie Hématologie et Oncologie pédiatriques, Angers, France; [19]CHU Toulouse, Service Hématologie, immunologie et oncologie pédiatrique, Toulouse, France;*

### Background

Survival rates for childhood acute leukemia now reach approximately 70% for acute myeloid leukemia and 90% for acute lymphoblastic leukemia. However, survivors remain at risk of treatment-related late effects. Anthracycline-related cardiomyopathy is a severe complication that impacts quality of life and survival. Despite known risk factors, such as relapse treatment or allogeneic hematopoietic stem cell transplantation (HSCT), substantial inter-individual variability in the development of cardiomyopathy persists, suggesting a critical role for genetic susceptibility. While several studies have investigated genetic factors underlying anthracycline-related cardiomyopathy in childhood cancer survivors, to date no genome-wide association study (GWAS) has focused specifically on homogeneous cohorts of pediatric leukemia survivors.

### Aims

This study aims to identify genetic determinants associated with cardiomyopathy in a homogeneous cohort of pediatric leukemia survivors from the French LEA program.

**Copyright Information:** (Online) ISSN: 2572-9241
©2026 The Author(s). HemaSphere is published by John Wiley & Sons Ltd on behalf of European Hematology Association. This is an open access Abstract Book distributed under the Attribution-NonCommercial-NoDerivs (CC BY-NC-ND), which allows third parties to download the articles and share them with others as long as they credit the author and the Abstract Book, but they cannot change the content in any way or use them commercially.
**Abstract Book Citations:** Authors, Title, HemaSphere, 2026;10:(S1):pages. The abstract book DOIs can be found on the "Author Information" page.
**Disclaimer:** Articles published in the journal HemaSphere exclusively reflect the opinions of the authors. The authors are responsible for all content in their abstracts including accuracy of the facts, statements, citing resources, etc.

**Methods**

LEA is the French national program dedicated to the study of long-term complications following treatment for childhood leukemia. It includes over 8,000 patients treated since 1980 across 19 French pediatric oncology centers. Cardiomyopathy was systematically assessed through clinical evaluation and echocardiography during dedicated long-term follow-up visits occurring every two to four years, and was defined by a left ventricular fractional shortening <28%, a left ventricular ejection fraction <55%, or treatment initiation. LEA biobank was established in 2018 to collect biological samples. For this project, 794 samples with sufficient DNA quantity and quality were typed for genome-wide common single nucleotide polymorphisms (SNPs). Genotyping was performed with Illumina Global Screening Array Multi-disease (GSA-MD V3.0) at the French national center for human genome research (CNRGH). Genotype data were imputed with Minimac4 using the 1000 Genomes phase 3 version 5 reference panel. After standard quality control, we conducted a GWAS on 605 patients who had not undergone HSCT using a Cox proportional hazards model considering delay since leukemia diagnosis as time scale adjusted for sex, age at leukemia diagnosis, leukemia type and the first four principal components derived from genetic data.

**Results**

We analyzed 7015704 SNPs with minor allele frequency (MAF) >5% and imputation quality score (Rsq) >0.3 in 605 leukemia survivors (30 with cardiomyopathy, 575 controls). Variant rs74117657 was significantly associated with cardiomyopathy (Hazard Ratio (HR)=6.48, $p=3.93x10^{-9}$). This variant is located 7 kb downstream PROK1 gene and is not in linkage disequilibrium (r2>0.80) with other nearby SNPs. PROK1 encodes Prokineticin-1, a protein involved in angiogenesis and cardiovascular homeostasis, which has been involved in cardioprotective mechanisms in response to stress (Nebigil et al., 2009), including anthracycline-induced cardiotoxicity (Gasser et al., 2019). Interestingly, risk allele of this variant was significantly associated with lower circulating levels of Prokineticin-1 ($p=9.17x10^{-9}$) in UK Biobank study (Sun et al., 2023). In a preliminary analysis conducted in a subset of 105 patients, we observed a trend for lower plasma PROK1 levels measured by ELISA in carriers of the risk alle compared to non carriers ( (60 vs 147 pg/mL, p = 0.15).

**Summary/Conclusion**

This study suggests PROK1 as a new locus for anthracycline-related cardiomyopathy in long-term childhood acute leukemia survivors.

**Copyright Information:** (Online) ISSN: 2572-9241
©2026 The Author(s). HemaSphere is published by John Wiley & Sons Ltd on behalf of European Hematology Association. This is an open access Abstract Book distributed under the Attribution-NonCommercial-NoDerivs (CC BY-NC-ND), which allows third parties to download the articles and share them with others as long as they credit the author and the Abstract Book, but they cannot change the content in any way or use them commercially.
**Abstract Book Citations:** Authors, Title, HemaSphere, 2026;10:(S1):pages. The abstract book DOIs can be found on the "Author Information" page.
**Disclaimer:** Articles published in the journal HemaSphere exclusively reflect the opinions of the authors. The authors are responsible for all content in their abstracts including accuracy of the facts, statements, citing resources, etc.

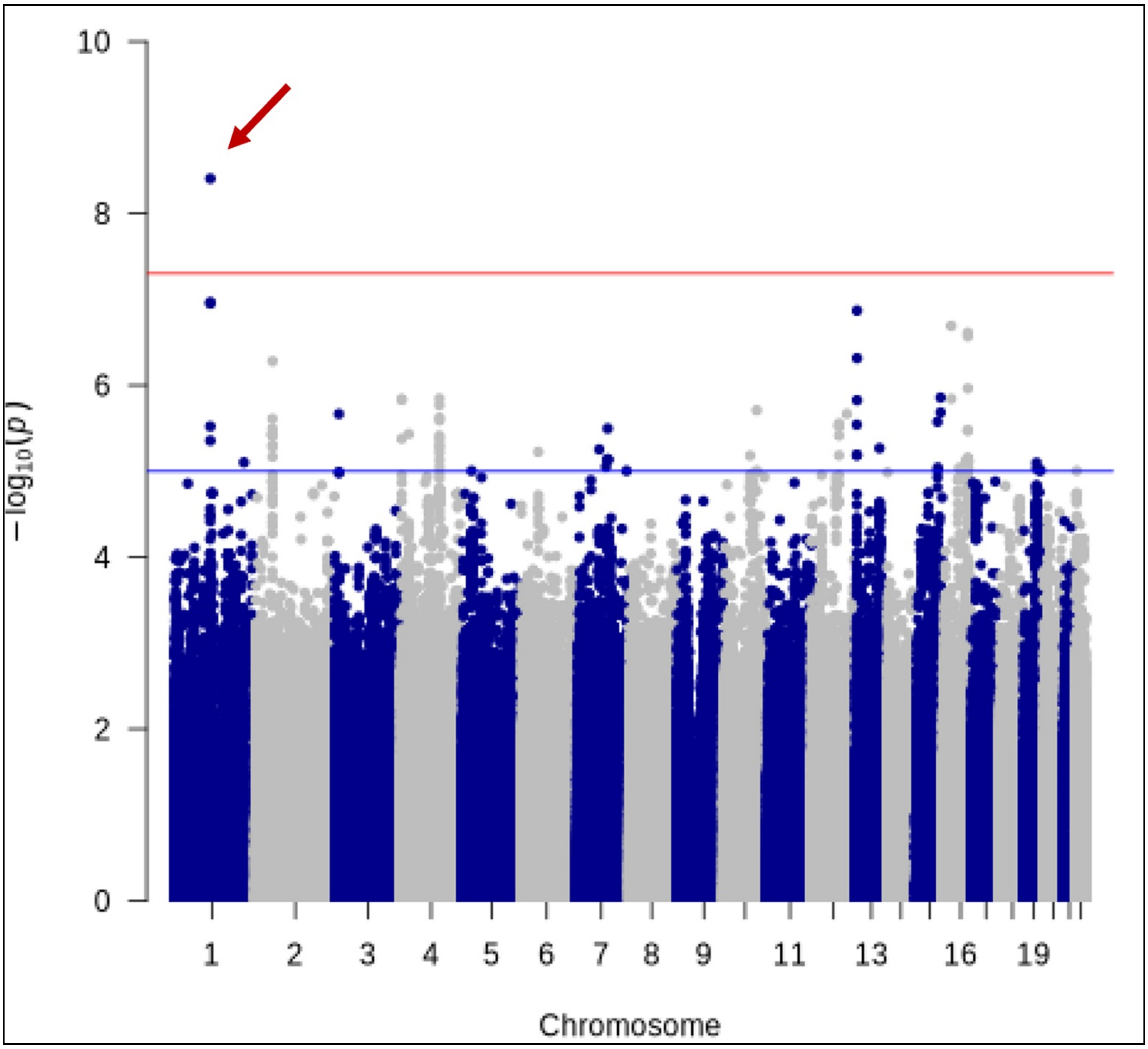

Manhattan Plot showing results of Genome Wide Association Study on Anthracycline-related cardiomyopathy

**Copyright Information:** (Online) ISSN: 2572-9241
©2026 The Author(s). HemaSphere is published by John Wiley & Sons Ltd on behalf of European Hematology Association. This is an open access Abstract Book distributed under the Attribution-NonCommercial-NoDerivs (CC BY-NC-ND), which allows third parties to download the articles and share them with others as long as they credit the author and the Abstract Book, but they cannot change the content in any way or use them commercially.
**Abstract Book Citations:** Authors, Title, HemaSphere, 2026;10:(S1):pages. The abstract book DOIs can be found on the "Author Information" page.
**Disclaimer:** Articles published in the journal HemaSphere exclusively reflect the opinions of the authors. The authors are responsible for all content in their abstracts including accuracy of the facts, statements, citing resources, etc.

### (PF360) RESOLVING CLONAL HETEROGENEITY IN KMT2A-REARRANGED PEDIATRIC MIXED PHENOTYPE ACUTE LEUKEMIA BY SINGLE-CELL SEQUENCING

**Topic:** 01. Acute lymphoblastic leukemia - Biology & translational research

Varsha Singh[1], Mari Lahnalampi*[1], Mattias Pilheden[1], Helena Sturesson[1], Louise Ahlgren[1], Ton Falqués-Costa[1], Carl Sandén[1], Cornelis Jan Pronk[2], Anders Castor[2], Mats Ehinger[3], Veronica Gonzalez-Pena[4], Charles Gawad[4], Anna K. Hagström-Andersson[1, 5]

*[1]Division of Clinical Genetics, Department of Laboratory Medicine, Lund University, Lund, Sweden,[2]Childhood Cancer Center, Skåne University Hospital, Lund, Sweden; [3]Department of Clinical Sciences, Pathology, Skåne University Hospital, Lund University, Lund, Sweden; [4]Division of Pediatric Hematology/Oncology, Stanford University, School of Medicine, Stanford, CA, United States of America; [5]Center for Translational Genomics, Lund University, Lund, Sweden;*

**Background**

Mixed-phenotype acute leukemia (MPAL) with *KMT2A*-rearrangements (*KMT2A*-r), is a rare subtype with a poor prognosis that exhibits lineage plasticity and expression of myeloid and lymphoid markers. The molecular mechanisms driving this lineage plasticity is not known.

**Aims**

To understand the mechanisms underlying lineage plasticity and clonal evolution from initial diagnosis of acute lymphoblastic leukemia (ALL) to early treatment response at day 16 in a child with *KMT2A::AFF1*. At day16, MPAL was identified following the emergence of a myeloid cell population. At this time, 48% of cells were leukemic with 27% being myeloid and 21% lymphoid.

**Methods**

Cells from four diagnostic populations were isolated by flow cytometry including the 1) lymphoid main clone (97%, $CD34^-CD19^+CD33^-CD13^-CD20^-CD58^+$), 2) lymphoid minor population (0.2%, $CD34^+CD19^+CD33^-CD13^+$), 3) lymphoid/myeloid population (0.01%, $CD34^-CD19^+CD33^+CD13^-$), and 4) myeloid blasts (0.1%, $CD34^-CD19^-CD33^+CD13^-CD14^-$), and myeloid and lymphoid cells from d16. DNA was amplified by primary template-directed amplification. 92 mutations were re-sequenced on around 220 diagnostic and 80 d16 cells (300x). The mutations included the *KMT2A*::*AFF1*-fusion, 45 personal mutations from prior WGS, and remaining were known cancer mutations. Around 60 cells were also studied by WES and low pass WGS (33 and 0.76x, respectively). scRNA-sequencing was performed at diagnosis and d16.

**Copyright Information:** (Online) ISSN: 2572-9241
©2026 The Author(s). HemaSphere is published by John Wiley & Sons Ltd on behalf of European Hematology Association. This is an open access Abstract Book distributed under the Attribution-NonCommercial-NoDerivs (CC BY-NC-ND), which allows third parties to download the articles and share them with others as long as they credit the author and the Abstract Book, but they cannot change the content in any way or use them commercially.
**Abstract Book Citations:** Authors, Title, HemaSphere, 2026;10:(S1):pages. The abstract book DOIs can be found on the "Author Information" page.
**Disclaimer:** Articles published in the journal HemaSphere exclusively reflect the opinions of the authors. The authors are responsible for all content in their abstracts including accuracy of the facts, statements, citing resources, etc.

**Results**

Mutations were detected in 36 targets from the personal pool. To delineate the sequence of genetic events that occurred during leukemia formation, we performed clonal analysis of the diagnostic and d16 cells with ≥80% of targets covered at >10x. Three diagnostic subclones were identified, contributing 28%, 34%, and 38% of the total cell population with a stepwise increase in mutational burden (22, 25, and 33 mutations, respectively), indicating branching evolution from a common ancestor. Importantly, evolution was independent of the cellular immunophenotype, highlighting the plasticity of the cells. Mutation counts ranged from 0-33 per cell, with one diagnostic myeloid cell carrying the *KMT2A*-r only, indicating that the *KMT2A*-r target cell was a myeloid progenitor. Analysis of the fraction of each sorted population within each clone showed a relatively balanced composition of the four populations in clone 1, with the lymphoid main clone expanding over time. However, by d16 the clonal dynamics shifted, with clone 1 decreasing in size, clone 2 expanding, and clone 3 remaining stable. Further, while the earliest clones at d16 were dominated by lymphoid cells, 77% of cells in clone 3 were myeloid. This shift in clonal composition from diagnosis to d16 demonstrates the selective therapeutic pressure on clonal evolution. Further, the proportion of cells harboring only the *KMT2A*-r increased from <1% at diagnosis to 10% at d16. These findings demonstrate that treatment-imposed selective pressures differentially shape the evolutionary trajectories of individual clones. Moreover, the scRNA-data revealed a small population of diagnostic cells that shared cellular states with the expanded myeloid and lymphoid cells at d16.

**Summary/Conclusion**

Our findings suggest that the *KMT2A*-rearranged leukemia originated in a myeloid progenitor and subsequently underwent branching evolution. Notably, clonal evolution was independent of cellular immunophenotype, underscoring the intrinsic lineage plasticity of the *KMT2A*-r leukemic cells.

**Copyright Information:** (Online) ISSN: 2572-9241
©2026 The Author(s). HemaSphere is published by John Wiley & Sons Ltd on behalf of European Hematology Association. This is an open access Abstract Book distributed under the Attribution-NonCommercial-NoDerivs (CC BY-NC-ND), which allows third parties to download the articles and share them with others as long as they credit the author and the Abstract Book, but they cannot change the content in any way or use them commercially.
**Abstract Book Citations:** Authors, Title, HemaSphere, 2026;10:(S1):pages. The abstract book DOIs can be found on the "Author Information" page.
**Disclaimer:** Articles published in the journal HemaSphere exclusively reflect the opinions of the authors. The authors are responsible for all content in their abstracts including accuracy of the facts, statements, citing resources, etc.

### (PF361) LONGITUDINAL TRANSCRIPTOMIC PROFILING REVEALS MYELOID REMODELLING OF THE BONE MARROW MICROENVIRONMENT AFTER INDUCTION CHEMOTHERAPY IN ADULT B-ACUTE LYMPHOBLASTIC LEUKAEMIA

**Topic:** 01. Acute lymphoblastic leukemia - Biology & translational research

Rodothea Amerikanou*[1, 2], Emily Cutler[1], Hermione Allen[1], Aditi Dey[2], Jenny Chatzigerou[1], Emma Lloyd[1], Lucas Wing[2], Juma Ward[2], Charlotte Booth[2], Lottie Plunkett-Jones[2], Clare Bennett[1], Adele Fielding[2]

*[1]University College London, Haematology, London, United Kingdom; [2]University of York, York Biomedical Research Institute, London, United Kingdom;*

### Background

The bone marrow (BM) microenvironment is a critical determinant of survival and therapy resistance in B-ALL. We and others have shown that chemotherapy-induced remodelling of stromal cells promotes niche-dependent protection of leukaemic cells. In solid tumours, therapy-driven recruitment and polarisation of myeloid cells contribute to tumour-protective niches and relapse. Unlike stromal cells, myeloid populations are dynamically depleted and reconstituted following chemotherapy. However, whether and how post-induction myeloid reconstitution shapes the persistence or eradication of minimal residual disease in B-ALL remains unknown.

### Aims

To determine the impact of treatment on the BM myeloid compartment, we characterised myelopoiesis in matched samples from diagnosis and after the 1$^{st}$ and 2$^{nd}$ courses of B-ALL chemotherapy using transcriptomic profiling.

### Methods

Bulk and single cell RNA sequencing (bulk-RNAseq and sc-RNAseq) were performed on cryopreserved BM mononuclear cells collected at diagnosis and after the 1$^{st}$ (C1, dexamethasone, daunorubicin, vincristine, and asparaginase) and 2$^{nd}$ (C2, cytarabine, mercaptopurine, and cyclophosphamide) courses of UKALL14 chemotherapy; (bulk-RNAseq n=3 patients, sc-RNAseq n=4 different patients). Data were analysed using voom-limma with gene set enrichment analysis (GSEA). Single cell data were integrated with publicly available normal BM data using SCTransform normalisation and Harmony for batch correction.

**Copyright Information:** (Online) ISSN: 2572-9241
©2026 The Author(s). HemaSphere is published by John Wiley & Sons Ltd on behalf of European Hematology Association. This is an open access Abstract Book distributed under the Attribution-NonCommercial-NoDerivs (CC BY-NC-ND), which allows third parties to download the articles and share them with others as long as they credit the author and the Abstract Book, but they cannot change the content in any way or use them commercially.
**Abstract Book Citations:** Authors, Title, HemaSphere, 2026;10:(S1):pages. The abstract book DOIs can be found on the "Author Information" page.
**Disclaimer:** Articles published in the journal HemaSphere exclusively reflect the opinions of the authors. The authors are responsible for all content in their abstracts including accuracy of the facts, statements, citing resources, etc.

**Results**

Sequencing of single cells from matched samples revealed the dynamics of myeloid cell reconstitution after successive treatments. Pseudo-time analysis supported the initial expansion of haematopoietic stem cells and differentiation of granulocyte-biased progenitors post-C1 with little contribution to more mature myeloid cell populations at this time point. By comparison, after C2 we observed a reduced granulocytic bias and relative monocytic enrichment, with parallel expansion of type 2 dendritic cells.
Examination of differentiated monocytes at a higher resolution from diagnosis to C1 and C2 demonstrated loss of monocytes associated with antigen presentation and effector functions compared to healthy BM, and expansion of a distinct subcluster enriched in heat shock proteins. Concordantly, GSEA suggested enrichment of oxidative phosphorylation and MYC/translation programmes in addition to the reactive oxygen species (ROS) and the p53 checkpoint pathways after C2.
Our findings were validated using bulk-RNAseq from a different patient group. CibersortX deconvolution of bulk-RNA seq data showed a striking increase in monocytes after C2, supporting the monocytosis revealed by single cell sequencing. Principal Component Analysis demonstrated transition of samples from different patients in the same direction from diagnosis to C2, supporting a common treatment-associated transcriptional change in BM cells. Analysis of the pathways that described these changes again showed upregulation of the ROS pathway and p53 pathways indicate persistent oxidative and genotoxic stress within the regenerating marrow following C2 treatment.

**Summary/Conclusion**

In summary, our data demonstrate that the myeloid compartment remains persistently remodelled following C2 treatment, characterised by expansion of metabolically active yet genotoxically stressed monocytes in the context of ROS-inducing chemotherapy. Ongoing studies are aimed at defining how these reprogrammed monocytes influence ALL cell survival and persistence.

**Copyright Information:** (Online) ISSN: 2572-9241
©2026 The Author(s). HemaSphere is published by John Wiley & Sons Ltd on behalf of European Hematology Association. This is an open access Abstract Book distributed under the Attribution-NonCommercial-NoDerivs (CC BY-NC-ND), which allows third parties to download the articles and share them with others as long as they credit the author and the Abstract Book, but they cannot change the content in any way or use them commercially.
**Abstract Book Citations:** Authors, Title, HemaSphere, 2026;10:(S1):pages. The abstract book DOIs can be found on the "Author Information" page.
**Disclaimer:** Articles published in the journal HemaSphere exclusively reflect the opinions of the authors. The authors are responsible for all content in their abstracts including accuracy of the facts, statements, citing resources, etc.

### (PF362) CDK8 AT THE MITOTIC SPINDLE: A NOVEL VULNERABILITY IN LEUKEMIA

**Topic:** 01. Acute lymphoblastic leukemia - Biology & translational research

Elisabeth Gamper*[1], Alessia Schirripa[1], Mark Steinlechner[1], Sebastian Kollmann[1], Jonatan Kendler[2], Thorsten Klampfl[1], Christoph Stifter[1], Jana Trifinopoulos[1], Thomas Rülicke[3], Veronika Sexl[4], Karoline Kollmann[1]

*[1]University of Veterinary Medicine, Pharmacology and Toxicology, Vienna, Austria; [2]Center for Cancer Research, Medical University of Vienna, Vienna, Austria; [3]LBI for Hematology and Oncology, Medical University of Vienna, Vienna, Austria; [4]University of Innsbruck, Vienna, Austria;*

### Background

Cyclin-dependent kinase 8 (CDK8) is being explored as a therapeutic target across multiple cancer types. In B cell acute lymphoblastic leukemia (B-ALL), kinase-independent functions of CDK8 are critical for leukemic maintenance[1]. In contrast, pharmacological inhibition of CDK8 kinase activity shows pronounced anti-proliferative effects in acute myeloid leukemia (AML)[2]. This highlights a possible context-dependent and divergent role of CDK8 in leukemia, underscoring the need for a better understanding of CDK8's functions and novel therapeutic strategies with CDK8 inhibitors.

### Aims

The aim of this study is to dissect kinase-dependent and kinase-independent functions and to identify novel roles of CDK8 in leukemia, by mapping the CDK8 interactome as well as transcriptomic patterns of cells with CDK8 inhibition and deletion. Ultimately, this work aims to provide a rational for mechanism-based combination strategies targeting CDK8 and its functional network in leukemia.

### Methods

The generation of a novel mouse model expressing wild-type CDK8 (wt), kinase-inactive CDK8$^{D173A}$ or lacking CDK8 (ko) allowed us to establish bone-marrow derived stable leukemic cell lines expressing BCR-ABL$^{p185+}$. The impact of CDK8 on cell proliferation and disease latency was analysed by *in vitro* and *in vivo* experiments. The combination of RNA sequencing and CDK8 co-immunoprecipitation (Co-IP) followed by mass spectrometry (MS) and Western Blot was used to unravel CDK8 downstream mechanisms and identified novel CDK8 interactors. Findings were validated in human AML cell lines and combinatorial drug treatments.

### Results

The lack of significant effects of CDK8$^{D173A}$ expression and of CDK8 loss on normal hematopoiesis supports the feasibility of therapeutically targeting CDK8, suggesting selective vulnerability of malignant cells while sparing normal hematopoietic functions. Notably, CDK8$^{D173A}$ and CDK8 ko BCR-ABL$^{p185+}$ cell lines displayed reduced proliferative capacity and resulted in a significantly prolonged disease latency *in vivo*. RNA sequencing of stable BCR-ABL$^{p185+}$ cell lines uncovered both kinase-dependent and -independent functions of CDK8. Integration of CDK8 Co-IP MS and transcriptomic data revealed a novel direct involvement of CDK8 in mitotic spindle organization and centrosome function, validated in human AML cell lines and underlining a general novel CDK8 kinase dependent mechanism. Pharmacologic agents targeting mitotic processes in combination with CDK8 inhibition highlighted anti-proliferative and apoptotic effects in our models.

**Copyright Information:** (Online) ISSN: 2572-9241
©2026 The Author(s). HemaSphere is published by John Wiley & Sons Ltd on behalf of European Hematology Association. This is an open access Abstract Book distributed under the Attribution-NonCommercial-NoDerivs (CC BY-NC-ND), which allows third parties to download the articles and share them with others as long as they credit the author and the Abstract Book, but they cannot change the content in any way or use them commercially.
**Abstract Book Citations:** Authors, Title, HemaSphere, 2026;10:(S1):pages. The abstract book DOIs can be found on the "Author Information" page.
**Disclaimer:** Articles published in the journal HemaSphere exclusively reflect the opinions of the authors. The authors are responsible for all content in their abstracts including accuracy of the facts, statements, citing resources, etc.

**Summary/Conclusion**

This project advances our understanding of CDK8's versatile roles by establishing a mechanistic link between the transcriptional CDK and centrosome regulation, using novel leukemia models. Our findings support combinatorial strategies that pair CDK8 inhibition with agents targeting mitotic processes, with applicability extending beyond BCR-ABL$^{p185+}$ disease to AML subtypes driven by diverse oncogenic programs.

1. Menzl, I. *et al.* A kinase-independent role for CDK8 in BCR-ABL1+ leukemia. *Commun.* **10**, 1–15 (2019).
2. Lee, J. C., Liu, S., Wang, Y., Liang, Y. & Jablons, D. M. MK256 is a novel CDK8 inhibitor with potent antitumor activity in AML through downregulation of the STAT pathway. *Oncotarget* **13**, 1217–1236 (2022).

**Copyright Information:** (Online) ISSN: 2572-9241
©2026 The Author(s). HemaSphere is published by John Wiley & Sons Ltd on behalf of European Hematology Association. This is an open access Abstract Book distributed under the Attribution-NonCommercial-NoDerivs (CC BY-NC-ND), which allows third parties to download the articles and share them with others as long as they credit the author and the Abstract Book, but they cannot change the content in any way or use them commercially.
**Abstract Book Citations:** Authors, Title, HemaSphere, 2026;10:(S1):pages. The abstract book DOIs can be found on the "Author Information" page.
**Disclaimer:** Articles published in the journal HemaSphere exclusively reflect the opinions of the authors. The authors are responsible for all content in their abstracts including accuracy of the facts, statements, citing resources, etc.

### (PF363) INTEGRATIVE SINGLE-CELL AND EX VIVO PROFILING IDENTIFIES REGULATORY DRIVERS OF DRUG RESPONSES IN PEDIATRIC B-CELL ACUTE LYMPHOBLASTIC LEUKEMIA

**Topic:** 01. Acute lymphoblastic leukemia - Biology & translational research

Arghavan Alizadeh Fekejvar*[1], Anna Pia Enblad[2, 3], Henrik Gezelius[2, 3], Sanni Moisio[1], Martin Åberg[2], Claes Andersson[2], Arja Harila[4], Jessica Nordlund[2, 3], Merja Heinäniemi[1]

*[1]University of Eastern Finland, Institute of Biomedicine, Kuopio, Finland;[2]Uppsala University, Department of Medical Sciences, Uppsala, Sweden; [3]Uppsala University, Science for Life Laboratory, Uppsala, Sweden; [4]Uppsala University, Department of Women's and Children's Health, Uppsala, Sweden;*

### Background

B-cell acute lymphoblastic leukemia (B-ALL) is the most common pediatric cancer. The *ETV6::RUNX1* (E::R) fusion is the most frequent translocation in B-ALL and has been associated with relapse and slow treatment response. *Ex vivo* drug screening, particularly when combined with transcriptomic profiling, has emerged as a promising functional precision-medicine strategy for predicting therapy response. In addition, the limited number of relapse-associated genetic mutations in ALL has shifted attention toward non-genetic regulatory mechanisms that enable leukemic cells to persist after therapy. A deeper understanding of these regulatory programs that shape drug sensitivity at diagnosis is critical for informing more effective strategies and guiding treatment intensity.

### Aims

We hypothesized that variability in treatment response is driven by non-genetic regulation of key transcription factors (TFs). To investigate the molecular basis of these differential responses, we integrated clinical data from pediatric B-ALL patients with single-cell RNA sequencing (scRNA-seq) and matched *ex vivo* drug-sensitivity profiles.

### Methods

*Ex vivo* drug responses to induction therapy drugs in E::R B-ALL cases were assessed at the bulk level using the fluorometric microculture cytotoxicity assay (FMCA) in 162 diagnostic samples. Four representative cases were subsequently subjected to scRNA-seq and FMCA across 13 approved and novel therapeutic agents. Differentially expressed genes (DEGs) between response groups for each drug were identified using limma. TF activity was quantified using SCENIC, based on regulon gene sets previously derived from ALL samples versus healthy control comparisons.

**Copyright Information:** (Online) ISSN: 2572-9241
©2026 The Author(s). HemaSphere is published by John Wiley & Sons Ltd on behalf of European Hematology Association. This is an open access Abstract Book distributed under the Attribution-NonCommercial-NoDerivs (CC BY-NC-ND), which allows third parties to download the articles and share them with others as long as they credit the author and the Abstract Book, but they cannot change the content in any way or use them commercially.
**Abstract Book Citations:** Authors, Title, HemaSphere, 2026;10:(S1):pages. The abstract book DOIs can be found on the "Author Information" page.
**Disclaimer:** Articles published in the journal HemaSphere exclusively reflect the opinions of the authors. The authors are responsible for all content in their abstracts including accuracy of the facts, statements, citing resources, etc.

**Results**

Bulk FMCA profiles showed that 45% of the 162 E::R B-ALL cases were uniformly either resistant or sensitive to all induction drugs (dexamethasone, prednisolone and vincristine). Among the remaining 89 cases, 75% displayed opposing response patterns to glucocorticoids (GCs) and vincristine. To further explore this, we compared four of these cases at single-cell resolution and observed a similar *ex vivo* response pattern, with cases tending to segregate into two groups based on GC sensitivity. This binary segregation extended beyond induction drugs, revealing a mutually exclusive drug-response pattern across a broader panel of commonly used anti-leukemic drugs. DEG analysis identified 334 genes distinguishing GC-resistant from -sensitive cases. This was complemented by pathway enrichment analysis linking these transcriptional signatures to downregulation of interferon-response and upregulation of metabolic pathways, such as oxidative phosphorylation, in GC-sensitive donors. We previously identified two regulons (GATA2 and MAX) associated with a higher level of minimal residual disease (MRD) at the end of induction. Both regulons showed higher activity scores (p-value $< 0.05$) in the GC-resistant group. Based on the expression levels of GATA2 regulon targets, GC-resistant cases formed a more transcriptionally homogeneous group than GC-sensitive cases.

**Summary/Conclusion**

We report a subgroup of E::R B-ALL cases exhibiting opposing GC response states with reciprocal multidrug vulnerabilities, a pattern with potential clinical relevance for tailoring therapy to enhance efficacy while minimizing unnecessary toxicity.

**Copyright Information:** (Online) ISSN: 2572-9241
©2026 The Author(s). HemaSphere is published by John Wiley & Sons Ltd on behalf of European Hematology Association. This is an open access Abstract Book distributed under the Attribution-NonCommercial-NoDerivs (CC BY-NC-ND), which allows third parties to download the articles and share them with others as long as they credit the author and the Abstract Book, but they cannot change the content in any way or use them commercially.
**Abstract Book Citations:** Authors, Title, HemaSphere, 2026;10:(S1):pages. The abstract book DOIs can be found on the "Author Information" page.
**Disclaimer:** Articles published in the journal HemaSphere exclusively reflect the opinions of the authors. The authors are responsible for all content in their abstracts including accuracy of the facts, statements, citing resources, etc.

### (PF364) COMBINATION OF OPTICAL GENOME MAPPING (OGM) AND RNA SEQUENCING REFINES CLASSIFICATION OF ADULT T-CELL ACUTE LYMPHOBLASTIC LEUKEMIA (T-ALL)

**Topic:** 01. Acute lymphoblastic leukemia - Biology & translational research

Idoia Vázquez*[1], Celia González Gil[1], Estefania Mancini[1], Thaysa Lopes[1], Mireia Morgades[1], Vaclava Polivkova[2], Jitka Křížková[2], Katerina MacHova Polakova[2], Jorge García[3], Mar Mallo[1], Isabel Granada[4], Jordi Ribera[1], Esperanza Such[5], Jesus Maria Hernandez Rivas[6], Manuel Ramirez Orellana[3], Francesc Sole Ristol[1], Alberto Orfao[7], Cyril Salek[2], Jose Maria Ribera[1], Eulalia Genesca[1]

*[1]Josep Carreras Leukaemia Research Institute, ICO-Germans Trias i Pujol Campus, Autonomous University of Barcelona, Badalona, Spain; [2]Institute of Hematology and Blood Transfusion, Prague, Czechia;[3]Hospital Infantil Universitario Niño Jesús, Department of Paediatric Haematology and Oncology, Madrid, Spain; [4]ICO-Hospital Germans Trias i Pujol, Department of Haematology, Badalona, Spain; [5]Health Research Institute Hospital La Fe, Department of Haematology, Valencia, Spain; [6]Hospital Universitario de Salamanca, Department of Haematology, Salamanca, Spain;[7]Universidad de Salamanca, IBSAL, CIBERONC, Research Centre (IBMCC-CSIC/USAL) and Department of Medicine, Salamanca, Spain;*

### Background

T-ALL molecular classification remains challenging due to its marked genetic heterogeneity. The current ICC classification recognizes several molecular subgroups defined primarily by gene expression profiles in pediatric T-ALL cohorts. These entities, considered provisional in the ICC classification, were not mentioned in the last WHO classification (2022).

The implementation of genomic techniques, such as Optical Genome Mapping (OGM), may facilitate the detection of structural alterations underlying these subgroups and support their integration into routine diagnostic workflows.

### Aims

(1) To validate OGM technique for T-ALL molecular classification according to the ICC criteria. (2) To validate the pediatric-based ICC molecular classification in an adult T-ALL cohort. (3) To determine the added value of RNA-seq for improving classification accuracy.

### Methods

The study cohort included 141 T-ALL cases. Samples were processed using the Saphyr®™ system (Bionano Genomics®) and analyzed with Access software (v1.7.2, v1.8.2). OGM results were compared with karyotyping and SNP-array data (CytoScan® HD, ThermoFisher). Expression data was obtained via RNA-seq (Ilumina, NOVOGENE). Overall survival (OS) was calculated for patients treated under the AR11, LAL19 (PETHEMA) and ALL CELL Junior 2012 protocols using SPSS v.26 (IBM).

**Copyright Information:** (Online) ISSN: 2572-9241
©2026 The Author(s). HemaSphere is published by John Wiley & Sons Ltd on behalf of European Hematology Association. This is an open access Abstract Book distributed under the Attribution-NonCommercial-NoDerivs (CC BY-NC-ND), which allows third parties to download the articles and share them with others as long as they credit the author and the Abstract Book, but they cannot change the content in any way or use them commercially.
**Abstract Book Citations:** Authors, Title, HemaSphere, 2026;10:(S1):pages. The abstract book DOIs can be found on the "Author Information" page.
**Disclaimer:** Articles published in the journal HemaSphere exclusively reflect the opinions of the authors. The authors are responsible for all content in their abstracts including accuracy of the facts, statements, citing resources, etc.

**Results**

In a technical comparison subset (n=50), OGM demonstrated 78% concordance with SNP-array results. Compared with conventional karyotyping, OGM identified 58% additional structural alterations, highlighting its superior resolution, whereas 18% of abnormalities were detected exclusively by karyotyping, mainly in samples with low leukemic blast infiltration (<20%), reflecting limitation of OGM sensitivity.

Among T-ALL cases with both OGM and RNA-seq data available (n=111), OGM alone enabled assignment of 78/111 cases (70.3%) to ICC molecular subgroups. The remaining 29.7% (33/111) were classified only after integration of RNA-seq data, resulting in complete classification of all cases. RNA-seq data confirmed the OGM-based classification in 58/78 cases (74.3%), refined subgroup assignment in 10.3% (8/78), and led to reclassification in 15.4% (12/78), underscoring the complementary value of transcriptomics profiling **(Fig. 1A).**

Using the refined-RNA-seq classification, 14 molecular subgroups were identified and further consolidated into three preliminary risk categories based on one-year OS data: (i) low risk patients with 100% one-year OS rates, including TAL1a/BCL11Ba, NKX2-1a, NKX2-2a, NKX2-5a, TLX1a, and TLX3a/LMO2a cases (n=28); (ii) intermediate risk group with 70% one-year OS (95% CI: 57–82.9%), including TAL1a/LMO2a, TLX3a, LMO2a, LMO1a, and HOXAa patients (n=56); and (iii) high risk group with one-year OS rates of only 18.8% (95% CI: 0–49.8%), including HOXAa/BCL11Ba, NKX2-2a/5a, and TAL1a/LMO1a cases (n=8) **(Fig. 1B)**.

**Summary/Conclusion**

The integrated analysis of OGM and RNA-seq data enables comprehensive classification of T-ALL cases according to the pediatric-based ICC criteria. The overall distribution of molecular subgroups in this adult cohort recapitulated the pediatric-derived ICC classification. However, differences were observed in subgroup frequencies and subgroup combinations. The incorporation of additional genomic information (i.e. point mutations) may further refine RNA-based subgrouping and facilitate routine implementation.

**Acknowledgments**
ISCIII (PI22/01880, PI25/00827); Leukemia Stiftung (DJCLS 08 R/2022).

**Copyright Information:** (Online) ISSN: 2572-9241
©2026 The Author(s). HemaSphere is published by John Wiley & Sons Ltd on behalf of European Hematology Association. This is an open access Abstract Book distributed under the Attribution-NonCommercial-NoDerivs (CC BY-NC-ND), which allows third parties to download the articles and share them with others as long as they credit the author and the Abstract Book, but they cannot change the content in any way or use them commercially.
**Abstract Book Citations:** Authors, Title, HemaSphere, 2026;10:(S1):pages. The abstract book DOIs can be found on the “Author Information” page.
**Disclaimer:** Articles published in the journal HemaSphere exclusively reflect the opinions of the authors. The authors are responsible for all content in their abstracts including accuracy of the facts, statements, citing resources, etc.

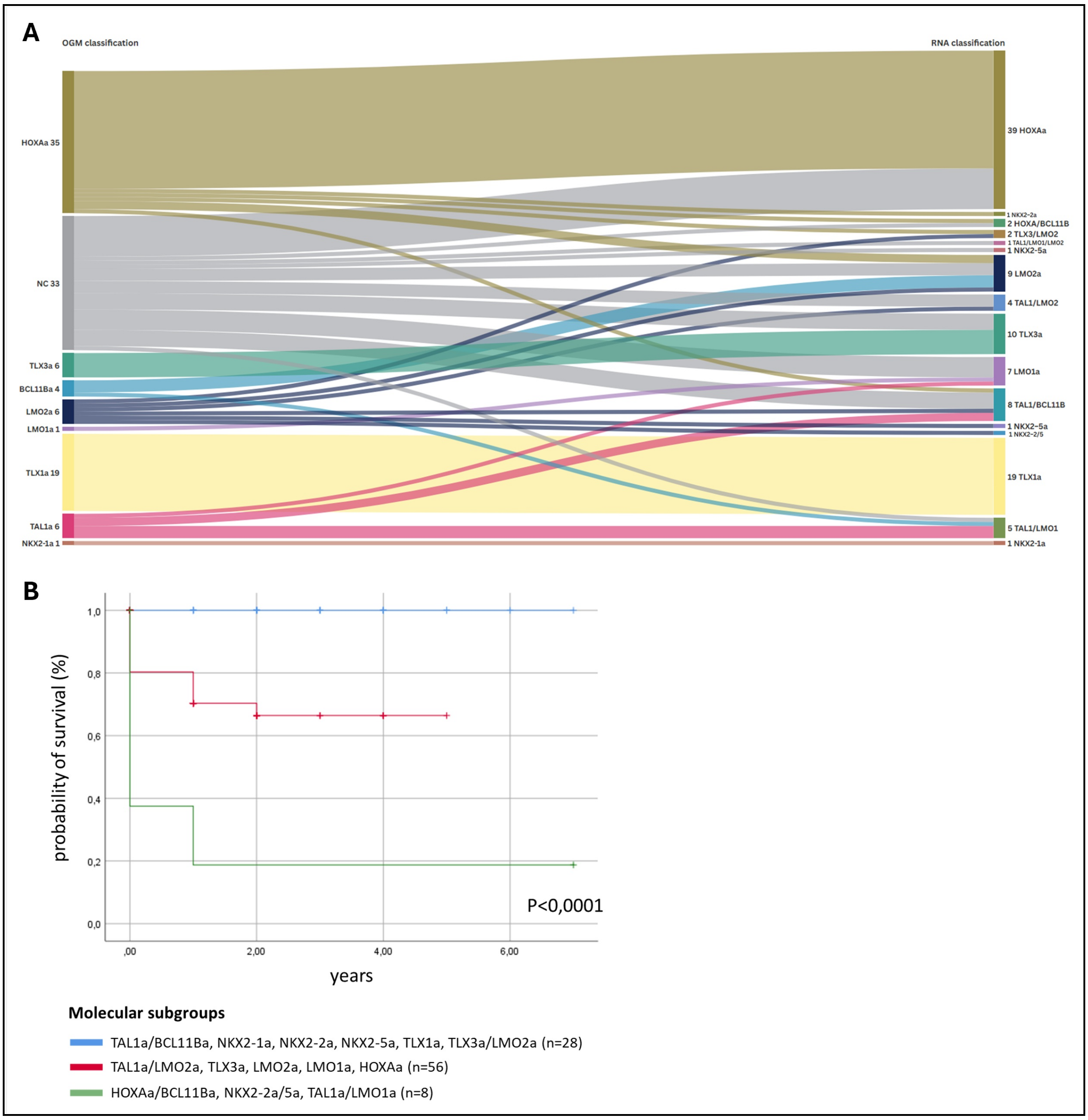

Figure 1. Refined T-ALL classification and overall survival of RNA-seq molecular groups. (A) Alluvial diagram showing the classification of T-ALL cases according to the ICC-defined groups, based on OGM and RNA-seq results (n=111). (B) Overall survival for the different molecular subgroups based on RNA-seq data in 92 adult T-ALL patients.

**Copyright Information:** (Online) ISSN: 2572-9241
©2026 The Author(s). HemaSphere is published by John Wiley & Sons Ltd on behalf of European Hematology Association. This is an open access Abstract Book distributed under the Attribution-NonCommercial-NoDerivs (CC BY-NC-ND), which allows third parties to download the articles and share them with others as long as they credit the author and the Abstract Book, but they cannot change the content in any way or use them commercially.
**Abstract Book Citations:** Authors, Title, HemaSphere, 2026;10:(S1):pages. The abstract book DOIs can be found on the "Author Information" page.
**Disclaimer:** Articles published in the journal HemaSphere exclusively reflect the opinions of the authors. The authors are responsible for all content in their abstracts including accuracy of the facts, statements, citing resources, etc.

### (PF365) CHARACTERIZING THE 3D-SURFACE PROTEOME OF BLASTS EXPOSED TO CHEMOTHERAPY IN PEDIATRIC ACUTE LYMPHOBLASTIC LEUKEMIA

**Topic:** 01. Acute lymphoblastic leukemia - Biology & translational research

Maria Globisch*[1, 2], Mouhamad Al Moussawy[3], Tiina Tuononen[3], Anders Lundmark[1, 2], Martin Åberg[1, 4], Claes Andersson[1, 4], Olli Lohi[5, 6], Merja Heinäniemi[3], Jessica Nordlund[1, 2]

*[1]Department of Medical Sciences, Uppsala University, Uppsala, Sweden;[2]SciLifeLab, Uppsala University, Uppsala, Sweden; [3]The Institute of Biomedicine, University of Eastern Finland, Kuopio, Finland;[4]Department of Clinical Chemistry and Pharmacology, Uppsala University Hospital, Uppsala, Sweden; [5]Tampere Center for Child, Adolescent, and Maternal Health Research, Faculty of Medicine and Health Technology, Tampere, Finland; [6]Tampere University Hospital, Tays Cancer Centre, Tampere, Finland;*

### Background

Intensive multi-agent chemotherapy has increased survival rates for pediatric acute lymphoblastic leukemia (ALL) to >90% (Toft *et al*., 2018 *Leukemia*) yet, it may induce short- and long-term toxicity (Björk-Eriksson *et al*., 2022 *JAMA*) or select for resistant clones. Emerging immunotherapies (e.g. CART-cell) also require preconditioning chemotherapy (e.g. fludarabine) for the most optimal response (Fabrizio *et al*., 2022 *Blood Advances*), therefore, understanding the molecular impact of chemotherapy on blasts and the immune landscape remains critical. Trascriptomic profiles of chemotherapy-exposed blasts have been well characterized, yet functional protein networks and their evolution during chemotherapy remain poorly understood.

### Aims

The overarching aim of this study is to characterize the abundance and dynamic remodeling of surface proteins on leukemic blasts at single-cell resolution during chemotherapy exposure.

### Methods

We applied Molecular Pixelation (MPX, *Karlsson et al. Nat Methods 2024*) to the *ETV6::RUNX1*+ B-ALL cell-line Reh after a 72-hour exposure to cladribine, daunorubicin, melphalan, and vincristine. MPX quantified protein abundance, spatial distribution, and colocalization of 76 proteins which were computationally arranged from DNA-sequencing reads by inferring the relative locations of antibody oligo conjugates generating >1,000 spatially connected 3D-neighborhoods per single cell. We also employed the Proximity Network Assay (PNA, Karlsson et al 2025 *bioRxiv*) to characterize the surface proteome in two pre-B ALL cases (Philadelphia-like (Ph-like) and *KMT2A*-rearranged) at diagnosis (peripheral blood and bone marrow) and 2 days post-induction chemotherapy (peripheral blood). With an estimated spatial resolution of ~50 nm, PNA generated 3D spatial interaction networks for 155 proteins in single cells capturing abundance, clustering, and colocalization of proteins on patient-derived leukemic blasts.

**Copyright Information:** (Online) ISSN: 2572-9241
©2026 The Author(s). HemaSphere is published by John Wiley & Sons Ltd on behalf of European Hematology Association. This is an open access Abstract Book distributed under the Attribution-NonCommercial-NoDerivs (CC BY-NC-ND), which allows third parties to download the articles and share them with others as long as they credit the author and the Abstract Book, but they cannot change the content in any way or use them commercially.
**Abstract Book Citations:** Authors, Title, HemaSphere, 2026;10:(S1):pages. The abstract book DOIs can be found on the "Author Information" page.
**Disclaimer:** Articles published in the journal HemaSphere exclusively reflect the opinions of the authors. The authors are responsible for all content in their abstracts including accuracy of the facts, statements, citing resources, etc.

**Results**

The tetraspainin CD82 increased in abundance and polarity in Reh cells exposed to cladribine, melphalan, and daunorubicin. This aligned with our previous observations where the preconditioning drug fludarabine increased the abundance, spatial distribution, and colocalization of CD82 and CD53; results which were validated using immunocytochemistry and high-resolution microscopy (Globisch *et al.*, 2024 *bioRxiv*). In the Ph-like pre-B ALL case, the blast surface proteome underwent remodeling two days post-induction chemotherapy. The transmembrane proteins CD27 and CD127 increased in abundance, while tetraspanins CD81 and CD37 as well as MHC class II molecules decreased in abundance. Despite this, tetraspanin-enriched microdomains (notably CD81, CD82, CD9, and CD53) increased in polarization and co-localization. Similarly, in the *KMT2A*-rearranged pre-B ALL subtype circulating blasts showed enhanced formation of tetraspanin-rich domains, with increased clustering and colocalization with CD19, which maintained stable expression levels.

**Summary/Conclusion**

Our findings reveal that chemotherapy induces remodeling of the blast surface proteome, particularly in tetraspanin-organized membrane domains. These spatial rearrangements occur independently of, and in some cases despite, stable or reduced protein expression levels, highlighting the importance of protein organization beyond abundance. This dynamic reorganization may play key roles in mediating treatment response and resistance in ALL, offering new insights into leukemia biology and potential therapeutic vulnerabilities.

**Copyright Information:** (Online) ISSN: 2572-9241
©2026 The Author(s). HemaSphere is published by John Wiley & Sons Ltd on behalf of European Hematology Association. This is an open access Abstract Book distributed under the Attribution-NonCommercial-NoDerivs (CC BY-NC-ND), which allows third parties to download the articles and share them with others as long as they credit the author and the Abstract Book, but they cannot change the content in any way or use them commercially.
**Abstract Book Citations:** Authors, Title, HemaSphere, 2026;10:(S1):pages. The abstract book DOIs can be found on the “Author Information” page.
**Disclaimer:** Articles published in the journal HemaSphere exclusively reflect the opinions of the authors. The authors are responsible for all content in their abstracts including accuracy of the facts, statements, citing resources, etc.

### (PF366) CHARACTERISING THE MITOCHONDRIA OF B-ALL CELLS WITH DIFFERENT PRIMARY GENETIC TRANSLOCATIONS.

**Topic:** 01. Acute lymphoblastic leukemia - Biology & translational research

Jenny Chatzigerou*[1, 2], Lucas Wing[1], Emily Cutler[3], Juma Ward[1], Aditi Dey[1], Ian Hitchcock[1], Adele Fielding[1, 2]

*[1]University of York, Centre for Blood Research, York, United Kingdom;[2]Hull York Medical School, York, United Kingdom;*
*[3]University College London, Cancer Institute, London, United Kingdom;*

### Background

B-ALL outcome is strongly linked to the primary genetic lesion. We previously showed that cytarabine (AraC), commonly used in chemotherapy for B-ALL, induces a chemoprotective niche, stimulating mesenchymal stromal cells to become cancer-associated fibroblasts (CAFs) which donate mitochondria (Mt) to leukaemia cells via tunneling nanotubes leading to chemoresistance (Burt et al, 2019). We also observed non-nanotube dependent Mt transfer from the ALL to stromal cells (Burt, Dey at al, 2024). This was 3 times greater from the high reactive oxygen species (ROS), CAF-inducing *BCR::ABL1* cell line, SD1, than from the low ROS, non-CAF-inducing *KMT2A::AFF1* cell line SEM, suggesting an association with either ROS levels or the ALL driver lesion.

### Aims

To establish differences in Mt dynamics and function in ALL cell lines and patient-derived xenografts (PDXs) at baseline and post treatment with AraC, a mitotoxicant.

### Methods

We assessed intracellular ATP levels, Mt superoxide, membrane potential and mass using flow cytometry. Galactose culture was used to increase Mt metabolism. Mt biogenesis was assessed using mtDNA copy number quantification. MtDNA fragmentation (indicator of damage), was evaluated by the short:long fragment ratio of the *MT-CO3* gene. We used three ALL lines with a range of sensitivities to AraC (most to least resistant: SD1 (*BCR::ABL1)*, SEM (*KMT2A::AFF1)*, REH (*ETV6::RUNX1))* and PDXs.

### Results

At baseline, there were no differences in Mt superoxide levels across the cell lines. Galactose culture did not reduce viability or ATP levels. However, SD1 cells had higher baseline ATP than SEM cells and higher Mt mass than SEM and REH cells. AraC treatment significantly increased Mt mass in all 3 cell lines. However, in the chemosensitive cells, REH and SEM, it resulted in a loss of Mt membrane potential and a significant increase in mtDNA fragmentation in REH cells (AraC vs PBS control, 39.6:4.7 short:long fragment). MtDNA copy number was then assessed in these cell lines, as well as in additional *BCR::ABL1* expressing cells (SUPB15 (*BCR::ABL1)*, NALM6 (*ETV6::PDGFRB),* and NALM6p190 (NALM6 overexpressing *BCR::ABL1*). MtDNA copy number was not associated with *BCR::ABL1* expression, suggesting no genotype-dependence. This was confirmed using the tyrosine kinase inhibitor, ponatinib. This was further evaluated in PDXs expressing *BCR::ABL1* (4)*, KMT2A::AFF1* (3) and *ETV6::RUNX1* (5). No significant differences were observed in MtDNA copy number between the genotypes.

**Copyright Information:** (Online) ISSN: 2572-9241
©2026 The Author(s). HemaSphere is published by John Wiley & Sons Ltd on behalf of European Hematology Association. This is an open access Abstract Book distributed under the Attribution-NonCommercial-NoDerivs (CC BY-NC-ND), which allows third parties to download the articles and share them with others as long as they credit the author and the Abstract Book, but they cannot change the content in any way or use them commercially.
**Abstract Book Citations:** Authors, Title, HemaSphere, 2026;10:(S1):pages. The abstract book DOIs can be found on the “Author Information” page.
**Disclaimer:** Articles published in the journal HemaSphere exclusively reflect the opinions of the authors. The authors are responsible for all content in their abstracts including accuracy of the facts, statements, citing resources, etc.

**Summary/Conclusion**

We showed that B-ALL cell lines have functional mitochondria capable of oxidative metabolism following galactose culture. Our data suggests that B-ALL cells retain mitochondrial function and oxidative metabolism, at baseline, suggesting they do not employ aerobic glycolysis (Warburg effect). AraC was shown to clearly act as a mitotoxicant in our *in vitro* model. AraC-resistant cells suffered less mitochondrial damage, as indicated by the membrane potential and the MtDNA fragmentation index, suggesting a potential link between mitochondrial resilience and susceptibility to AraC. This was further suggested by the increase in mitochondrial mass in the residual live cells post-AraC treatment, suggesting this could represent a rescue mechanism. We have not identified a link between genotype and MtDNA copy number. This is of interest given emerging evidence linking mtDNA quantity and chemoresistance.

**Copyright Information:** (Online) ISSN: 2572-9241
©2026 The Author(s). HemaSphere is published by John Wiley & Sons Ltd on behalf of European Hematology Association. This is an open access Abstract Book distributed under the Attribution-NonCommercial-NoDerivs (CC BY-NC-ND), which allows third parties to download the articles and share them with others as long as they credit the author and the Abstract Book, but they cannot change the content in any way or use them commercially.
**Abstract Book Citations:** Authors, Title, HemaSphere, 2026;10:(S1):pages. The abstract book DOIs can be found on the "Author Information" page.
**Disclaimer:** Articles published in the journal HemaSphere exclusively reflect the opinions of the authors. The authors are responsible for all content in their abstracts including accuracy of the facts, statements, citing resources, etc.

### (PF367) CHARACTERIZING AND TARGETING METABOLIC REWIRING IN PEDIATRIC PAX5-REARRANGED B-ALL PDX-DERIVED MODELS

**Topic:** 01. Acute lymphoblastic leukemia - Biology & translational research

Rebecca Rapisarda*[1, 2], Alessia Curto[1, 2], Luigia Valsecchi[2], Manuel Quadri[2], Michela Bardini[2], Jolanda Sarno[1, 2], Giovanni Cazzaniga[1, 2], Grazia Fazio[1, 2]

*[1]School of Medicine and Surgery, University of Milano Bicocca, Milan, Italy;[2]Tettamanti Center, Fondazione IRCCS San Gerardo dei Tintori, Monza, Italy;*

### Background

B-cell Precursor Acute Lymphoblastic Leukemia (BCP-ALL) is the most common pediatric cancer, with cure rates exceeding 85%. However, relapsed patients face worse outcomes, with survival rates ranging from 20% to 45%. Among high-risk subgroups, Philadelphia-like ALL has unfavorable prognosis and around 30% of Ph-like cases harbor alterations in the *PAX5* gene. *PAX5* encodes a transcription factor essential for B-cell development and identity, and it has also been shown to modulate cellular metabolism by enforcing a state of chronic energy deprivation, associated with low glycolytic rate. When *PAX5* is altered as a consequence of chromosomal rearrangements (*PAX5r*), its regulatory function is compromised, leading to enhanced glycolytic flux and a metabolic rewiring supporting cell proliferation. However, most available evidence derives from established cell lines. Whether metabolic rewiring represents a targetable feature in pediatric *PAX5r* B-ALL is poorly explored in patient-derived models.

### Aims

To characterize metabolic alterations in pediatric *PAX5r* BCP-ALL and to assess targetable metabolic vulnerabilities.

### Methods

Metabolic profiling was performed in PAX5r PDX samples, in the NALL-1 cell line (harboring the *PAX5::ETV6* translocation) and in the 697 cell line (negative for *PAX5* alterations) by CyTOF, comparing metabolic enzyme expression with healthy bone marrow B-cells. Viability was assessed with FACS analysis of apoptotic cells using AnnexinV/7-AAD staning after *in-vitro* and *ex-vivo* treatment with 2-deoxy-D-glucose. Functional metabolism was analyzed by Seahorse assays in untreated and 2-DG treated cell lines and PDX-derived cells.

**Copyright Information:** (Online) ISSN: 2572-9241
©2026 The Author(s). HemaSphere is published by John Wiley & Sons Ltd on behalf of European Hematology Association. This is an open access Abstract Book distributed under the Attribution-NonCommercial-NoDerivs (CC BY-NC-ND), which allows third parties to download the articles and share them with others as long as they credit the author and the Abstract Book, but they cannot change the content in any way or use them commercially.
**Abstract Book Citations:** Authors, Title, HemaSphere, 2026;10:(S1):pages. The abstract book DOIs can be found on the "Author Information" page.
**Disclaimer:** Articles published in the journal HemaSphere exclusively reflect the opinions of the authors. The authors are responsible for all content in their abstracts including accuracy of the facts, statements, citing resources, etc.

**Results**

CyTOF analysis revealed upregulated glycolytic activity, characterized by increased expression of GLUT1 and MCT1, and higher levels of glycolytic enzymes, like GAPDH, ENO1, HK2, G6PD, and LDHA, compared with healthy bone marrow. Mitochondrial protein expression was also significantly elevated, with strong upregulation of CytC, ATP5A, BCL2, and OPA1.
2-DG significantly reduced cell viability both *in-vitro* and *ex-vivo*: the NALL-1 cell line showed sensitivity to 2-DG with an IC50 of 0.5 mM. In contrast, the 697 cell line exhibited a higher IC50 (1.11 mM). To confirm whether this effect is specific for the PAX5 genetic alteration, further experiments are ongoing in additional cell lines.
Seahorse glycolytic stress test showed a reduction in glycolytic function upon 2-DG treatment in the NALL-1 cell line, as shown by a 3-fold decrease in glycolytic flux ($P<0.0001$) and a 4-fold decrease in glycolytic capacity ($P<0.0001$). Results were confirmed in PAX5r PDX cells, showing 1.7-fold decrease in glycolytic flux ($P<0.05$) and 2-fold decrease in glycolytic capacity ($P<0.0005$).
Mitochondrial stress test on the NALL-1 cell line revealed a 1.3-fold increase in maximal respiration in 2-DG treated cells ($P=0.0009$), suggesting compensatory upregulation of oxidative phosphorylation in response to glycolysis inhibition. Treated cells showed 2-fold higher spare respiratory capacity compared to untreated cells ($P<0.0001$). Mitochondrial stress test results will however need further confirmation and other replicates are ongoing.

**Summary/Conclusion**

PAX5r BCP-ALL PDX models exhibit metabolic rewiring with enhanced glycolytic and mitochondrial activity compared to healthy controls; glycolytic inhibition using 2-DG significantly impairs leukemic cell survival. Transcriptomic analyses of treated and untreated cells are ongoing.

**Copyright Information:** (Online) ISSN: 2572-9241
©2026 The Author(s). HemaSphere is published by John Wiley & Sons Ltd on behalf of European Hematology Association. This is an open access Abstract Book distributed under the Attribution-NonCommercial-NoDerivs (CC BY-NC-ND), which allows third parties to download the articles and share them with others as long as they credit the author and the Abstract Book, but they cannot change the content in any way or use them commercially.
**Abstract Book Citations:** Authors, Title, HemaSphere, 2026;10:(S1):pages. The abstract book DOIs can be found on the "Author Information" page.
**Disclaimer:** Articles published in the journal HemaSphere exclusively reflect the opinions of the authors. The authors are responsible for all content in their abstracts including accuracy of the facts, statements, citing resources, etc.

### (PF368) FUNCTIONAL STUDY OF CIRCRNAS WITH ECTOPIC EXPRESSION IN T-ALL AND CIRCRNA-DRIVEN T-ALL SUBTYPE CLASSIFICATION

**Topic:** 01. Acute lymphoblastic leukemia - Biology & translational research

Alberto Caregari[1, 2], Saman Habibi Anjedani[1, 2, 3], Enrico Gaffo[1], Alice Cani[3], Giulia Veltri[3], Elisa Rampazzo[4], Antonella Teramo[4], Shiyun Hu[5], Joost Kluiver[5], Anke van den Berg[5], Barbara Buldini[3, 6], Valentina Serafin[3, 7], Silvia Bresolin[3], Stefania Bortoluzzi*[1]

*[1]University of Padova, Department of Surgery, Oncology and Gastroenterology, Computational Medicine Group, Padova, Italy; [2]University of Padova, Department of Biology, Padova, Italy;[3]Pediatric Research Institute, Città della Speranza, Padova, Italy; [4]University of Padova, Department of Medicine, Hematology and Clinical Immunology Branch, Padova, Italy;[5]University of Groningen, Department of Pathology and Medical Biology, Groningen, The Netherlands; [6]University of Padova, Maternal and Child Health Department, Pediatric Hematology, Oncology and Stem Cell Transplant Division, Padova, Italy; [7]University of Modena and Reggio Emilia, Department of Biomedical, Metabolic and Neural Sciences, Modena, Italy;*

### Background

Omics studies in T cell acute lymphoblastic leukemia (T-ALL) are uncovering disease mechanisms and explaining the disease heterogeneity. Circular RNAs (circRNAs) have gained traction in cancer research as regulators of cellular activities, drivers of oncogenic axes and biomarkers.

### Aims

In T-ALL, data on dysregulation and pathogenetic relevance of circRNAs prompted us to study circRNAs' functional roles and improve T-ALL molecular subtype classification by including circRNA information.

### Methods

We considered three cohorts of pediatric patients with T-ALL profiled by RNA-seq, comprising 25 (TALL25), 264 (TALL264) and 1300 (TALL1300) cases at diagnosis and 19 samples of immature thymocytes from healthy donors. We quantified circRNA expression using CirComPara2, followed by data normalization and batch-effect removal. Differentially expressed circRNAs were assessed using DESeq2 and Limma (p-adj <0.01). Unsupervised analysis and UMAP used the Leiden algorithm from Seurat and the uwot package, respectively. For the T-ALL classifier, a tree-based model (XGBoost) was trained by iteratively comparing each class to all remaining cases across 100 bootstrap-generated datasets to mitigate training-set bias. The most discriminant circRNAs were selected according to the Gain score.
Target circRNA expression was quantified in a panel of 10 T-ALL and 11 other acute leukemia cell lines by RT-qPCR using divergent primers designed with CircPrimer2. The back-splicing junctions were confirmed by Sanger sequencing. CircRNA silencing was achieved using lentiviral vectors from VectorBuilder. RNA pull-down experiments were performed in triplicate using ElementZero probes, followed by RNA-seq.

**Copyright Information:** (Online) ISSN: 2572-9241
©2026 The Author(s). HemaSphere is published by John Wiley & Sons Ltd on behalf of European Hematology Association. This is an open access Abstract Book distributed under the Attribution-NonCommercial-NoDerivs (CC BY-NC-ND), which allows third parties to download the articles and share them with others as long as they credit the author and the Abstract Book, but they cannot change the content in any way or use them commercially.
**Abstract Book Citations:** Authors, Title, HemaSphere, 2026;10:(S1):pages. The abstract book DOIs can be found on the "Author Information" page.
**Disclaimer:** Articles published in the journal HemaSphere exclusively reflect the opinions of the authors. The authors are responsible for all content in their abstracts including accuracy of the facts, statements, citing resources, etc.

### Results

Comparison of TALL25 and TALL264 with normal thymocytes identified 1009 and 551 circRNAs with significantly altered expression, respectively, 85% overexpressed in malignant cells. Combining the results, we further identified a robust set of 256 circRNAs concordantly dysregulated in T-ALL. The quantification in the cell line panel of 14 circRNAs among the most upregulated and abundant in T-ALL defined and prioritized candidates for functional study. One circRNA upregulated and highly expressed has been efficiently knocked down, and experimental studies are ongoing. We isolated RNAs interacting with this circRNA using RNA pull-down, achieving high efficiency and reproducibility.
CircRNA expression in TALL1300 has been considered in relation to the 15 main annotated molecular subtypes, characterized by typical lesions and gene expression profiles, and associated with T cell maturation stages. The unsupervised clustering based on circRNA expression mostly matched the known subgroups (Fig. 1A). The classification model achieved an F1 score >70% and an accuracy >90% (Fig. 1B) in all subgroups. The interpretability analysis highlighted the circRNAs most discriminating for each group (Fig. 1C) and defined a minimal set of molecules whose expression can efficiently distinguish T-ALL subtypes.

### Summary/Conclusion

We identified and validated several circRNAs that were markedly upregulated in T-ALL and conducted functional investigations using RNA pull-down experiments and in vitro circRNA silencing. The development of a machine learning model for circRNA-based T-ALL subtype classification is a novelty. It has the potential to identify the most informative dysregulated circRNA for each molecular subgroup, thereby increasing understanding of disease heterogeneity and indicating new disease players in T-ALL.

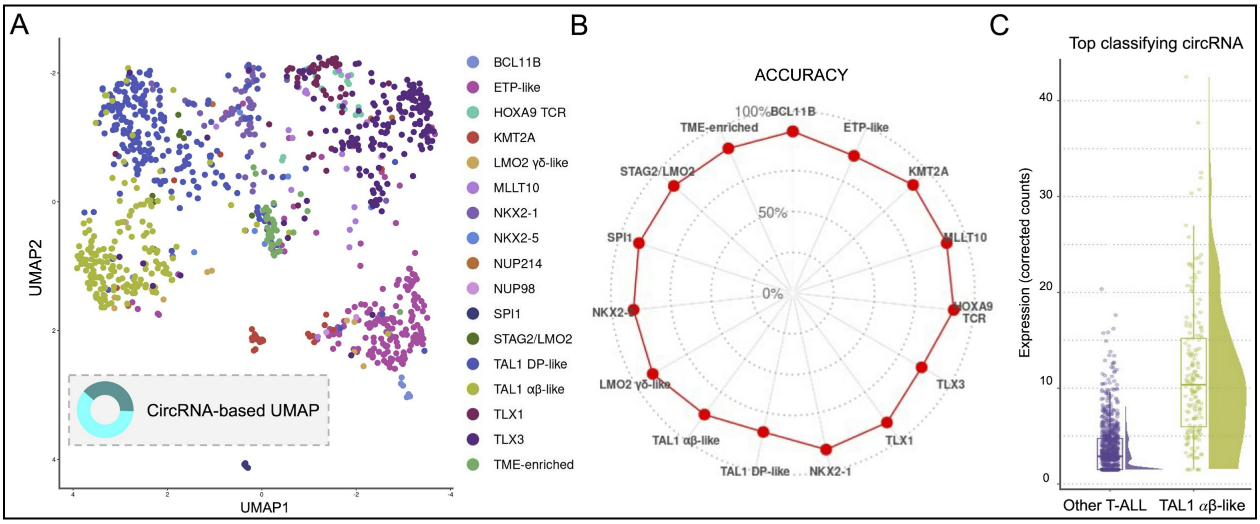

Figure 1. CircRNA-based classification of T-ALL subtypes. A) UMAP of TALL1300 samples based on circRNA expression profiles; B) Accuracy of the developed classifier for each of the 15 evaluated subtypes; C) Expression of one of the most classifying circRNAs in the target group and in other T-ALL cases.

**Copyright Information:** (Online) ISSN: 2572-9241
©2026 The Author(s). HemaSphere is published by John Wiley & Sons Ltd on behalf of European Hematology Association. This is an open access Abstract Book distributed under the Attribution-NonCommercial-NoDerivs (CC BY-NC-ND), which allows third parties to download the articles and share them with others as long as they credit the author and the Abstract Book, but they cannot change the content in any way or use them commercially.
**Abstract Book Citations:** Authors, Title, HemaSphere, 2026;10:(S1):pages. The abstract book DOIs can be found on the “Author Information” page.
**Disclaimer:** Articles published in the journal HemaSphere exclusively reflect the opinions of the authors. The authors are responsible for all content in their abstracts including accuracy of the facts, statements, citing resources, etc.

### (PF369) A NOVEL TRISPECIFIC ANTIBODY KT209 TARGETING CD19, CD20, AND CD3 (CD19XCD20XCD3) DEMONSTRATED POTENT ACTIVITY IN B-CELL DISEASES WITH LOWER CYTOKINE RELEASES

**Topic:** 01. Acute lymphoblastic leukemia - Biology & translational research

John Wang[1], Min Bao[1, 1], Thomas Sidders[1], Jay Zhao[1], Weihao Xu*[1]

*[1]Kali Therapeutics, San Mateo, United States of America;*

**Background**

Bispecific T-cell engagers (BiTEs) targeting CD19 or CD20 have shown potent efficacy in B cell diseases, but treatment failure due to low antigen (CD19 or CD20 -/low or loss) remains a major limitation. Dual-antigen targeting may prevent escape and improve outcomes. KT209, a novel trispecific antibody (tsAb) co-engaging CD19, CD20, and CD3 was developed to redirect endogenous T cells against B cells with reduced cytokine releases.

**Aims**

A Novel Trispecific Antibody Targeting CD19, CD20, and CD3 (CD19xCD20xCD3)

**Methods**

The CD19xCD20xCD3 tsAb was engineered with a 2:1:1 format. In vitro efficacy was assessed using T cells against a panel of B leukemia cell lines with heterogeneous expression. Cytotoxicity, T-cell activation, and cytokine release were measured. Pilot safety study was performed in cynomolgus monkey.

**Results**

The tsAb induced potent, specific cytotoxicity against the leukemia cells, irrespective of single antigen density. Critically, it effectively lysed CD19-low or CD20-low variants more potent than Blinatumomab or Glofitamab. T-cell activation was robust but with a lower cytokine profile compared to Blinatumomab or Glofitamab.  In cynomolgus monkeys, KT209 induced rapid and deep B-cell depletion in a dose responsive manner with minimal cytokine releases observed.

**Summary/Conclusion**

The CD19xCD20xCD3 trispecific antibody demonstrated enhanced preclinical potency and effectively eliminates leukemia cells with heterogeneous antigen expression, mitigating a key resistance mechanism. By simultaneously targeting two B-cell antigens, this approach may improve depth and duration of response. These data support the clinical development of KT209 for patients with broad relapsed/refractory B cell diseases.

**Copyright Information:** (Online) ISSN: 2572-9241
©2026 The Author(s). HemaSphere is published by John Wiley & Sons Ltd on behalf of European Hematology Association. This is an open access Abstract Book distributed under the Attribution-NonCommercial-NoDerivs (CC BY-NC-ND), which allows third parties to download the articles and share them with others as long as they credit the author and the Abstract Book, but they cannot change the content in any way or use them commercially.
**Abstract Book Citations:** Authors, Title, HemaSphere, 2026;10:(S1):pages. The abstract book DOIs can be found on the “Author Information” page.
**Disclaimer:** Articles published in the journal HemaSphere exclusively reflect the opinions of the authors. The authors are responsible for all content in their abstracts including accuracy of the facts, statements, citing resources, etc.

### (PF370) COMPREHENSIVE WHOLE-GENOME SEQUENCING REVEALS UNRECOGNIZED GENOMIC SUBTYPES IN PEDIATRIC B-CELL ACUTE LYMPHOBLASTIC LEUKEMIA

**Topic:** 01. Acute lymphoblastic leukemia - Biology & translational research

Miwako Toyohara*[1], Nao Takasugi[1], Aiko Sato-Otsubo[1], Shota Kato[1], Yuka Iijima-Yamashita[2], Masashi Sanada[2], Yasuhiro Okamoto[3], Toshihiko Imamura[4], Akiko Kada[5], Akiko M Saito[2], Seiya Imoto[6], Kentaro Oki[7], Kentaro Watanabe[1], Kenichi Yoshida[8], Yuichi Shiraishi[9], Katsuyoshi Koh[10], Motohiro Kato[1]

*[1]Department of Pediatrics, Graduate School of Medicine, the University of Tokyo, Tokyo, Japan;[2]Clinical Research Center, NHO Nagoya Medical Center, Aichi, Japan; [3]Department of Pediatrics, Kagoshima University Graduate School of Medical and Dental Sciences, Kagoshima, Japan; [4]Department of Pediatrics, Kyoto Prefectural University of Medicine, Kyoto, Japan;[5]Translational Research Seeds Exploration Center, Fujita Health University, Aichi, Japan; [6]Human Genome Center, The Institute of Medical Science, The University of Tokyo, Tokyo, Japan; [7]Division of Molecular Pathology, Department of Pediatric Hematology and Oncology Research, National Center for Child Health and Development, Tokyo, Japan; [8]Division of Cancer Evolution, National Cancer Center Research Institute, Tokyo, Japan; [9]Division of Genome Analysis Platform Development, National Cancer Center Research Institute, Tokyo, Japan; [10]Department of Hematology/Oncology, Saitama Children's Medical Center, Saitama, Japan;*

### Background

B-cell acute lymphoblastic leukemia (B-ALL) is the most common subtype of childhood acute lymphoblastic leukemia and comprises genetically diverse groups with distinct biological and clinical features. Advances in next-generation sequencing have refined genomic classification and enabled identification of diagnostically and therapeutically relevant subtypes. Risk-adapted treatment strategies based on genomic subtype are increasingly incorporated into clinical trials. However, conventional diagnostic approaches such as chromosomal karyotyping and targeted fusion gene screening do not fully characterize all cases. These methods may miss cryptic rearrangements, low-abundance leukemic clones, or novel genomic lesions, resulting in incomplete subtype assignment. Whole-genome sequencing (WGS) provides comprehensive genomic profiling and may overcome these limitations.

### Aims

To perform short-read WGS in a large pediatric B-ALL cohort and compare WGS-based subtype classification with conventional clinical subtyping, focusing on improving resolution in previously unclassified cases.

### Methods

A total of 246 pediatric B-ALL samples enrolled in the Genome research in CAncers and Rare Diseases (G-CARD) program were analyzed. Short-read WGS detected small variants, structural variations, and copy number variations. Among these, 234 cases were also registered in the JPLSG ALL-B12 clinical trial and had undergone central diagnostic review including chromosomal analysis and fusion gene screening. These 234 cases were directly compared to assess subtype classification between WGS and conventional approaches.

**Copyright Information:** (Online) ISSN: 2572-9241
©2026 The Author(s). HemaSphere is published by John Wiley & Sons Ltd on behalf of European Hematology Association. This is an open access Abstract Book distributed under the Attribution-NonCommercial-NoDerivs (CC BY-NC-ND), which allows third parties to download the articles and share them with others as long as they credit the author and the Abstract Book, but they cannot change the content in any way or use them commercially.
**Abstract Book Citations:** Authors, Title, HemaSphere, 2026;10:(S1):pages. The abstract book DOIs can be found on the "Author Information" page.
**Disclaimer:** Articles published in the journal HemaSphere exclusively reflect the opinions of the authors. The authors are responsible for all content in their abstracts including accuracy of the facts, statements, citing resources, etc.

**Results**

Short-read WGS enabled high-resolution genomic classification and identified the following subtypes: hyperdiploid (n = 82), *ETV6::RUNX1* (n = 44), *TCF3::PBX1* (n = 25), *PAX5*alt (n = 15), *BCR::ABL1* (n = 12), *DUX4* rearrangement (n = 10), *ZNF384* rearrangement (n = 10), Ph-like (n = 9), *MEF2D* rearrangement (n = 7), *KMT2A* rearrangement (n = 3), *NUTM1* rearrangement (n = 2), *PAX5* P80R (n = 2), *HLF* rearrangement (n = 1), iAMP21 (n = 1), near haploid (n = 1),*ZEB2*/*CEBP* altered (n = 1), and B-other (n = 21). Subtypes including *PAX5*alt, *DUX4* rearrangement, *ZNF384* rearrangement, Ph-like, *MEF2D* rearrangement, *NUTM1* rearrangement, *PAX5* P80R, *HLF* rearrangement, iAMP21, near haploid, and *ZEB2*/*CEBP* altered were not detected by central RT-PCR or chromosomal analysis and were newly identified by WGS. Importantly, WGS identified one *TCF3::HLF* case that had remained undiagnosed by conventional methods. Five *ETV6::RUNX1* cases negative on routine fusion screening were also detected. Additional copy number variation analysis enabled diagnosis of 27 further hyperdiploid cases. Within the B-other subgroup, WGS detected additional driver gene alterations in five cases.

**Summary/Conclusion**

In this large pediatric cohort, short-read WGS enabled genomic subtype identification in cases previously classified as unassigned by conventional diagnostic methods, accounting for approximately 38% of the entire cohort. These findings underscore the substantial diagnostic value of WGS and support its integration into clinical workflows. Integration of short-read WGS with established central diagnostic procedures improves genomic classification accuracy and enhances understanding of disease biology and clinical outcomes in pediatric B-ALL. Future application of long-read WGS may further refine risk stratification and facilitate more precise personalized treatment strategies.

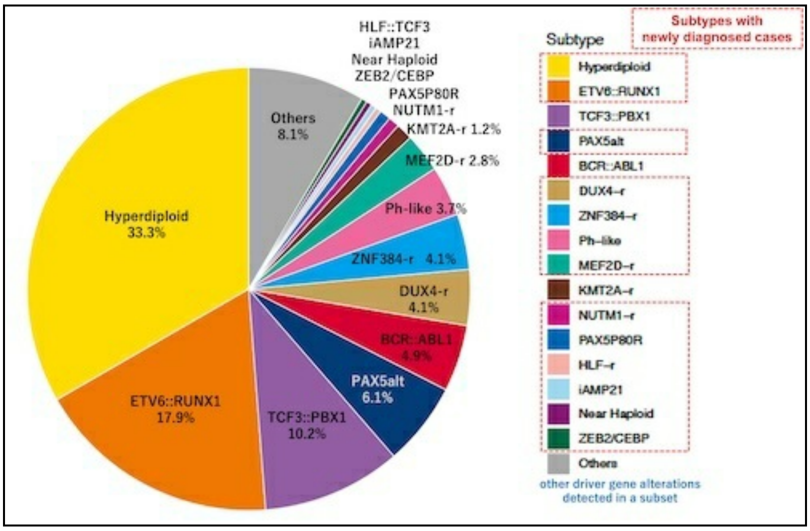

WGS-based subtype classification

**Copyright Information:** (Online) ISSN: 2572-9241
©2026 The Author(s). HemaSphere is published by John Wiley & Sons Ltd on behalf of European Hematology Association. This is an open access Abstract Book distributed under the Attribution-NonCommercial-NoDerivs (CC BY-NC-ND), which allows third parties to download the articles and share them with others as long as they credit the author and the Abstract Book, but they cannot change the content in any way or use them commercially.
**Abstract Book Citations:** Authors, Title, HemaSphere, 2026;10:(S1):pages. The abstract book DOIs can be found on the "Author Information" page.
**Disclaimer:** Articles published in the journal HemaSphere exclusively reflect the opinions of the authors. The authors are responsible for all content in their abstracts including accuracy of the facts, statements, citing resources, etc.

### (PF371) DDX3X DOWNREGULATION BUFFERS JAK-STAT SIGNALING IN CRLF2 REARRANGED ACUTE LYMPHOBLASTIC LEUKEMIA

**Topic:** 01. Acute lymphoblastic leukemia - Biology & translational research

Letizia Marchetti[1], Nefeli Karataraki[1], Stefano Tonin[1], Brynelle Myers[1], Lisa Jones[1], Yvonne Ng[1], Jamie Rutherford[1], Jasper Mogg[1], Kent Fung[1], Helen Blair[1], Rolando Berlinguer-Palmini[1], Olaf Heidenreich[2, 2], Lisa Russell*[1]

*[1]Newcastle University, Newcastle-upon-Tyne, United Kingdom; [2]Princess Maxima Center for Pediatric Oncology, Utrecht, The Netherlands;*

### Background

Dysregulated expression of CRLF2 driven by chromosomal rearrangements is observed in ~50% of patients with*BCR::ABL1*-like/Ph-like acute lymphoblastic leukemia (ALL). Whether driven by the *IGH* locus super-enhancer or *P2RY8* promoter, CRLF2 overexpression leads to the activation of several kinase pathways, including JAK-STAT, PIK3 and mTOR. Whilst the activation of these pathways contributes to transformation, their hyperactivation could be detrimental to cell viability. It may, therefore, be essential that leukemic cells acquire additional abnormalities to lower or buffer the intensity of these pathways to ensure long-term cell survival. Interestingly, we discovered a novel *USP9X::DDX3X* fusion in 19% of BCP-ALL, when characterising the genomic landscape of *CRLF2*-rearranged (*CRLF2*-r) ALL.

### Aims

Having identified this fusion, this study set out to elucidate the role DDX3Xplays in the transformation or maintenance of leukemic blasts that harbour *CRLF2*-r.

### Methods

Western blot was used to assess the presence of the fusion and DDX3X and USP9X levels in*CRLF2*-r patient derived xenograft (PDX) models and primary patient samples. Effects of DDX3X loss were investigated in lentiviral-transduced non-*CRLF2*-r cell line NALM6, and by chemical inhibition of DDX3X in *CRLF2*-r cell line YCUB5 (*JAK2*$^{mut}$) and PDXs using a first-in-class small molecule inhibitor, RK-33. EOAI3402143 was used to inhibit USP9X. RNA Immunoprecipitation (RNA-IP) was employed to confirm DDX3X interaction with specific mRNAs. Immunofluorescence allowed visualisation of DDX3X cellular location under different conditions.

### Results

Whilst we failed to identify the fusion protein in PDXs and primary patient samples, we observed heterogeneous DDX3X and USP9X protein levels, with low or no protein often found in patients with Down syndrome-ALL (3/5) and/or JAK2/JAK1 mutations (3/4). Upon DDX3X knockout in NALM6 (non-*CRLF2*-r), DDX3X and USP9X protein levels were reduced to <20% and <60% at day 4, suggesting USP9X could be a direct target of DDX3X. Similar results were obtained by inhibiting DDX3X with RK-33 in YCUB5 (*CRLF2*-r, *JAK2*$^{mut}$), leading to complete DDX3X loss by 48h. USP9X showed a gradual decline and almost complete loss at 96h. Interestingly, we observed pSTAT5 complete loss by 24h irrespective of total STAT5. Similar observations were seen in *CRLF2*-r PDXs. To investigate if DDX3X was a direct target of USP9X, chemical inhibition of USP9X was achieved with EOAI3402143 but it did not result in altered DDX3X expression. However, pSTAT5 and direct USP9X target MCL1 were both reduced.
RNA-IP confirmed that DDX3X directly binds the mRNA of USP9X, thus regulating its expression, and using immunofluorescence, DDX3X was seen in nuclear and cytoplasmic speckles, which were reduced upon chemical inhibition with RK-33.

**Copyright Information:** (Online) ISSN: 2572-9241
©2026 The Author(s). HemaSphere is published by John Wiley & Sons Ltd on behalf of European Hematology Association. This is an open access Abstract Book distributed under the Attribution-NonCommercial-NoDerivs (CC BY-NC-ND), which allows third parties to download the articles and share them with others as long as they credit the author and the Abstract Book, but they cannot change the content in any way or use them commercially.
**Abstract Book Citations:** Authors, Title, HemaSphere, 2026;10:(S1):pages. The abstract book DOIs can be found on the “Author Information” page.
**Disclaimer:** Articles published in the journal HemaSphere exclusively reflect the opinions of the authors. The authors are responsible for all content in their abstracts including accuracy of the facts, statements, citing resources, etc.

**Summary/Conclusion**

Collectively, this work suggests that DDX3X can regulate the JAK-STAT pathway through its regulation of USP9X, potentially providing the cells with the optimal level of activation to ensure leukemic survival. pSTAT5 loss upon DDX3X inhibition could be explained by previous observations that USP9X binds to JAK2 at a residue near Tyr1007, which phosphorylation is essential for JAK2 activity. Without USP9X, Tyr1007 might not be accessible, leading to reduced pJAK2 and pSTAT5. Altogether, these results support DDX3X as an alternative druggable target for abrogating JAK-STAT signalling.

**Copyright Information:** (Online) ISSN: 2572-9241
©2026 The Author(s). HemaSphere is published by John Wiley & Sons Ltd on behalf of European Hematology Association. This is an open access Abstract Book distributed under the Attribution-NonCommercial-NoDerivs (CC BY-NC-ND), which allows third parties to download the articles and share them with others as long as they credit the author and the Abstract Book, but they cannot change the content in any way or use them commercially.
**Abstract Book Citations:** Authors, Title, HemaSphere, 2026;10:(S1):pages. The abstract book DOIs can be found on the “Author Information” page.
**Disclaimer:** Articles published in the journal HemaSphere exclusively reflect the opinions of the authors. The authors are responsible for all content in their abstracts including accuracy of the facts, statements, citing resources, etc.

### (PF372) GENOMIC LANDSCAPE OF NEWLY DIAGNOSED ADULT ACUTE LYMPHOBLASTIC LEUKEMIA

**Topic:** 01. Acute lymphoblastic leukemia - Biology & translational research

Hidehito Fukushima*[1], Koji Okazaki[2], Kaito Mimura[3], Hiroyuki Takamori[4], Ryunosuke Saiki[2], Yotaro Ochi[2], Kazuaki Yokoyama[1], Kenichi Yoshida[3], Yuichi Shiraishi[5], Miwako Toyohara[6], Shota Kato[6], Kentaro Watanabe[6], Motohiro Kato[6], Akifumi Takaori[7], Nobuhiro Hiramoto[8], Naoki Hosen[9], Nobuhiro Kanemura[10], Fumiya Wada[11], Yasuhiro Murakawa[11, 12], Shoji Watanabe[13], Kazuto Yoshimi[13], Tomoji Mashimo[13], Nobuo Sezaki[14], Kasahara Senji[15], Yasunori Ueda[16], Nobuhiko Uoshima[17], Motoshi Ichikawa[18], Kazunori Imada[19], Kotoe Katayama[20], Seiya Imoto[20, 21], Seishi Ogawa[2, 11, 22], Yasuhito Nannya[1]

*[1]The Institute of Medical Science, The University of Tokyo, Division of Hematopoietic Disease Control, Tokyo, Japan;[2]Graduate School of Medicine, Kyoto University, Department of Pathology and Tumor Biology, Kyoto, Japan; [3]National Cancer Center Research Institute, Division of Cancer Evolution, Tokyo, Japan; [4]Graduate School of Medicine, The University of Osaka, Department of Hematology and Oncology, Osaka, Japan; [5]National Cancer Center Research Institute, Division of Genome Analysis Platform Development, Tokyo, Japan; [6]Graduate School of Medicine, The University of Tokyo, Department of Pediatrics, Tokyo, Japan; [7]Graduate School of Medicine, Kyoto University, Department of Hematology, Kyoto, Japan;[8]Kobe City Medical Center General Hospital, Department of Hematology, Kobe, Japan; [9]Graduate School of Medicine, The University of Osaka, Department of Hematology and Oncology, Suita, Japan; [10]Gifu University Hospital, Department of Hematology and Infectious Diseases, Gifu, Japan; [11]Institute for the Advanced Study of Human Biology (WPI-ASHBi), Kyoto University, Kyoto, Japan; [12]Graduate School of Medicine, Kyoto University, Department of Pathology and Biology of Diseases, Kyoto, Japan;[13]Center for Experimental Medicine and Systems Biology, Institute of Medical Science, The University of Tokyo, Division of Genome Engineering, Tokyo, Japan; [14]Chugoku Central Hospital, Department of Hematology, Fukuyama, Hiroshima, Japan; [15]Gifu Municipal Hospital, Department of Hematology, Gifu, Japan; [16]Kurashiki Central Hospital, Department of Hematology/Oncology, Kurashiki, Japan; [17]Japanese Red Cross Kyoto Daini Hospital, Department of Hematology, Kyoto, Japan;[18]NTT Medical Center Tokyo, Department of Hematology, Tokyo, Japan; [19]Japanese Red Cross Osaka Hospital, Department of Hematology, Osaka, Japan; [20]Human Genome Center, The Institute of Medical Science, Laboratory of Sequence Analysis, Tokyo, Japan;[21]Human Genome Center, The Institute of Medical Science, Division of Health Medical Intelligence, Tokyo, Japan; [22]Faculty of Medicine, Kindai University, Osaka, Japan;*

### Background

The genomic profile of newly diagnosed acute lymphoblastic leukemia (ALL) has not been extensively characterized through whole genome analyses especially for adult cases, which precluded elucidating unbiased landscape of genomic alterations to this group.

### Methods

We analyzed 486 adult ALL cases, including 156 *de novo* cases profiled by short-read whole genome sequencing (WGS) and an additional cohort of 330 cases evaluated by targeted sequencing for validation. The WGS cohort consisted of 126 B-ALL or mixed phenotype acute leukemia (MPAL) and 30 T-ALL cases diagnosed across 18 institutions in Japan (2012–2025) with matched buccal swab germline controls. SNVs, CNVs, and SVs were called using Mutect2, Battenberg, and GRIDSS/GRIPSS, respectively, and significantly mutated genes were inferred with dNdScv. Transcriptomic consequences of SVs were assessed by RNA-seq and long-read PacBio Iso-Seq. Genomic features were compared with those of publicly available pediatric ALL cohort from St. Jude Children's Research Hospital.

**Copyright Information:** (Online) ISSN: 2572-9241
©2026 The Author(s). HemaSphere is published by John Wiley & Sons Ltd on behalf of European Hematology Association. This is an open access Abstract Book distributed under the Attribution-NonCommercial-NoDerivs (CC BY-NC-ND), which allows third parties to download the articles and share them with others as long as they credit the author and the Abstract Book, but they cannot change the content in any way or use them commercially.
**Abstract Book Citations:** Authors, Title, HemaSphere, 2026;10:(S1):pages. The abstract book DOIs can be found on the "Author Information" page.
**Disclaimer:** Articles published in the journal HemaSphere exclusively reflect the opinions of the authors. The authors are responsible for all content in their abstracts including accuracy of the facts, statements, citing resources, etc.

**Results**

WGS identified over 450,000 SNVs across the WGS cohort (n=156), confirming established drivers and revealing novel candidate drivers in adults (including *PRKD2* and *HDAC7*). Subtypes more commonly associated with pediatric ALL (*ETV6::RUNX1*, hyperdiploid, hypodiploid) were rare. Mutational signature analysis revealed a substantial involvement of SBS18 in adult B-ALL, consistent with oxidative stress–associated mutagenesis. SV analysis identified recurrent deletions affecting *ADD3* (18%) and *XBP1* (15%), as well as deletions upstream of *ZNG1B* (28%), in addition to the canonical lesions (*IKZF1*, *CDKN2A*, *PAX5*). These events were also detected in the validation cohort and showed a trend toward inferior event-free survival (EFS), although not statistically significant (p = 0.21, 0.082, and 0.16, respectively). Each of these SV events exhibited a distinct pattern of coexistence with known genetic alterations. *ZNG1B* upstream deletions clustered near *IGKV1OR2-108* and nearby RAG recombination signal sequences, implicating aberrant RAG-mediated recombination. Integrative expression analyses linked *ZNG1B* upstream deletions to increased expression of B-lineage differentiation genes (*PAX5, EBF1*), repression of *HOXA9/10*, and elevated *MDM2* expression, suggesting attenuation of the p53 axis. When compared with pediatric ALL cases, adult cases were featured with a higher number of driver alterations per case, and more significant involvement of the PI3K–AKT–mTOR pathway in "B-other" subgroup. In addition, one-fourth of T-ALL harbored structural alterations involving *ZFP36L2*, frequently co-occurring with *JAK3* and *NOTCH1* mutations.

**Summary/Conclusion**

Adult ALL is characterized by adult-specific mutational processes and recurrent SVs with measurable transcriptional consequences and subtype-associated pathway alterations. Incorporating SV-based markers may refine adult risk stratification and inform precision medicine trial design.

**Copyright Information:** (Online) ISSN: 2572-9241
©2026 The Author(s). HemaSphere is published by John Wiley & Sons Ltd on behalf of European Hematology Association. This is an open access Abstract Book distributed under the Attribution-NonCommercial-NoDerivs (CC BY-NC-ND), which allows third parties to download the articles and share them with others as long as they credit the author and the Abstract Book, but they cannot change the content in any way or use them commercially.
**Abstract Book Citations:** Authors, Title, HemaSphere, 2026;10:(S1):pages. The abstract book DOIs can be found on the "Author Information" page.
**Disclaimer:** Articles published in the journal HemaSphere exclusively reflect the opinions of the authors. The authors are responsible for all content in their abstracts including accuracy of the facts, statements, citing resources, etc.

### (PF373) SPATIAL ANALYSIS OF MICRO-NICHES IN TDT+ ACUTE LEUKEMIA IDENTIFIES FEATURES ASSOCIATED WITH OVERALL SURVIVAL

**Topic:** 01. Acute lymphoblastic leukemia - Biology & translational research

Karin Huber*[1, 2], Julia Richter[3], Karin Schmitz[4], Philipp Altrock[1, 2], Malwine J. Barz[1, 2], Alina Marie Hartmann[1, 2], Thomas Beder[1, 2], Lorenz Bastian[1, 2], Lars Fransecky[1], Ralf Trappe[1], Wolfram Klapper[2, 3], Claudia Baldus[1, 2], Friedrich Stölzel[1, 2]

*[1]University Hospital Schleswig-Holstein, Campus Kiel, Department for Hematology and Oncology, Kiel, Germany;[2]Clinical Research Unit “CATCH ALL” (KFO 5010/1), Kiel, Germany; [3]University Hospital Schleswig-Holstein, Campus Kiel, Department of Pathology, Hematopathology Section and Lymph Node Registry, Kiel, Germany; [4]DIAKO Hospital Bremen, Department for Hematology and Oncology, Bremen, Germany;*

### Background

In acute leukemias, the immune microenvironment is receiving increasing attention. However, a profound understanding of how immune cell composition and interaction within the niche influence disease progression and treatment response is still lacking. This is partially due to peripheral blood and bone marrow aspirates being the most accessible biomaterial sources; these differ in overall cellular composition from intact bone marrow trephines and have lost the original spatial context.

### Aims

We aim to characterize the composition and interaction of immune cells in the preserved leukemic niche and within distinct micro-niches. By correlating spatial metrics with clinical data, we investigate features predictive of disease progression and treatment response.

### Methods

A cohort of n=27 FFPE sections from patients with TdT$^+$ acute leukemia (24 B-cell precursor ALL, two acute leukemia of ambiguous lineage, one T-ALL) at initial diagnosis (n=24) or relapse (n=3) was collected. All patients were treated according to GMALL protocols at the academic and collaborating center. Sections were stained using an oligoplexed immunofluorescence panel marking TdT$^+$ lymphoid blasts, CD4$^+$ T-helper cells, CD8$^+$ cytotoxic T-cells, CD68$^+$ macrophages and CD34$^+$ vasculature. Segmentation and classification steps were performed using QuPath (v0.5.1), and spatial cell and neighborhood metrics were calculated using Scimap (v2.1.1). Correlation with clinical data was conducted using the R packages “survival” and “survminer”.

**Copyright Information:** (Online) ISSN: 2572-9241
©2026 The Author(s). HemaSphere is published by John Wiley & Sons Ltd on behalf of European Hematology Association. This is an open access Abstract Book distributed under the Attribution-NonCommercial-NoDerivs (CC BY-NC-ND), which allows third parties to download the articles and share them with others as long as they credit the author and the Abstract Book, but they cannot change the content in any way or use them commercially.
**Abstract Book Citations:** Authors, Title, HemaSphere, 2026;10:(S1):pages. The abstract book DOIs can be found on the “Author Information” page.
**Disclaimer:** Articles published in the journal HemaSphere exclusively reflect the opinions of the authors. The authors are responsible for all content in their abstracts including accuracy of the facts, statements, citing resources, etc.

**Results**

The median overall frequency of immune cells ($CD4^+$ T-helper cells, $CD8^+$ cytotoxic T-cells and $CD68^+$macrophages) was 1.8% (IQR 1.3% - 3.5%), while $TdT^+$ blasts accounted for 61.9% (IQR 52.7% - 88.7%), and other non-classified cells for 33.9% (IQR 10.6% - 44.3%). Immune cells were heterogeneously distributed across the trephines and formed cellular neighborhoods (CNs). Three distinct CNs were defined by k-means clustering of the neighborhood matrix, characterized by decreasing immune cell infiltration (CN I++, CN I+, CN I-).
For spatial analysis, cell type frequencies and spatial interaction metrics were calculated per section and CN. These were tested for association with overall survival using exploratory univariate Cox regression; features with p<0.05 were selected for heatmap clustering. Based on dendrogram splits, three clusters were identified, primarily driven by interaction metrics between $TdT^+$ blasts and $CD8^+$ cytotoxic T cells, as well as between T-cell subsets in CN I- and CN I+. Survival analysis using these clusters resulted in clear separation of Kaplan-Meier curves (p=0.008).

**Summary/Conclusion**

In $TdT^+$ acute leukemia, immune cells are non-randomly distributed throughout the bone marrow, leading to the formation of micro-niches. Interaction patterns of immune cells within these niches may be indicative of functional states and are potentially associated with overall survival.

**Copyright Information:** (Online) ISSN: 2572-9241
©2026 The Author(s). HemaSphere is published by John Wiley & Sons Ltd on behalf of European Hematology Association. This is an open access Abstract Book distributed under the Attribution-NonCommercial-NoDerivs (CC BY-NC-ND), which allows third parties to download the articles and share them with others as long as they credit the author and the Abstract Book, but they cannot change the content in any way or use them commercially.
**Abstract Book Citations:** Authors, Title, HemaSphere, 2026;10:(S1):pages. The abstract book DOIs can be found on the “Author Information” page.
**Disclaimer:** Articles published in the journal HemaSphere exclusively reflect the opinions of the authors. The authors are responsible for all content in their abstracts including accuracy of the facts, statements, citing resources, etc.

**Figure 1: Correlation of spatial features with clinical data indicates a potential association with clinical course.**

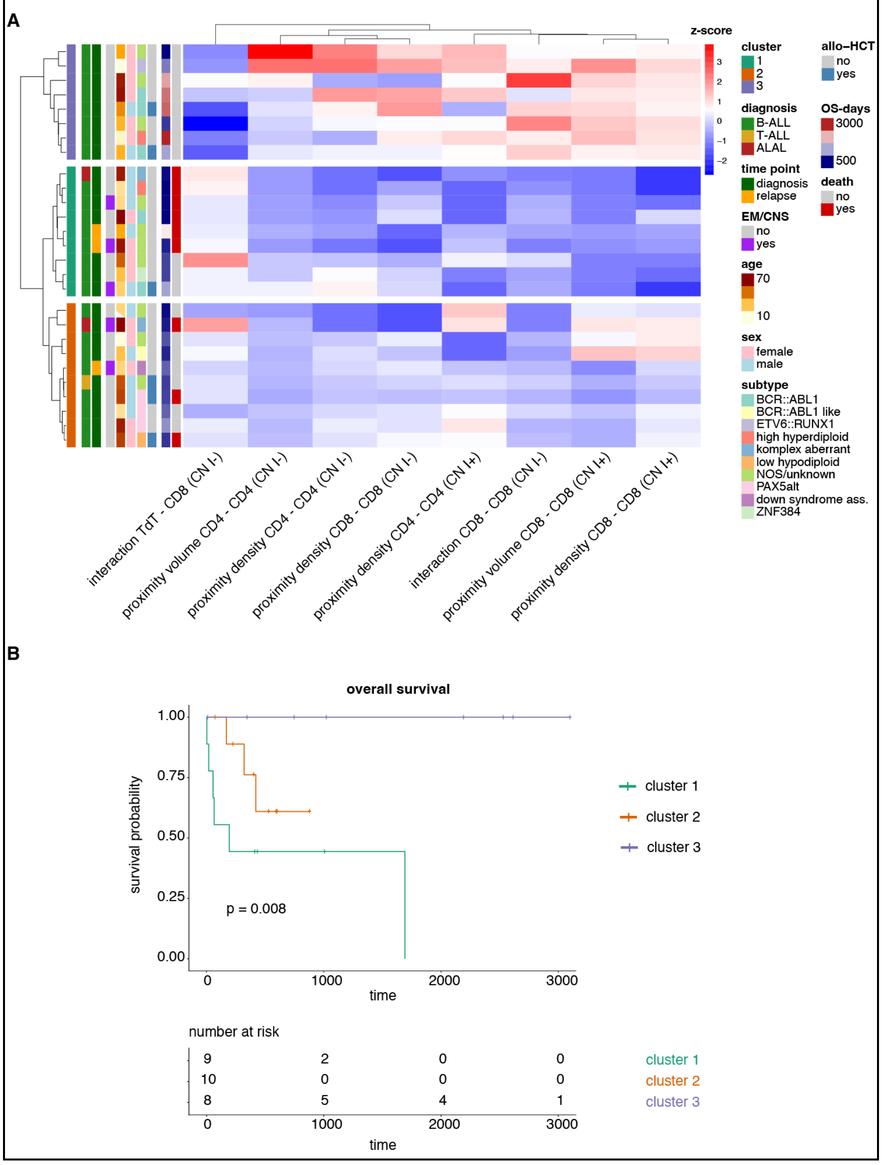

Figure 1: Correlation of spatial features with clinical data indicates a potential association with clinical course. A) Heatmap clustering of spatial features with p<0.05 in exploratory univariate Cox regression with clinical annotations. B) Kaplan–Meier curve showing overall survival stratified by three clusters defined by dendrogram-based splitting.

**Copyright Information:** (Online) ISSN: 2572-9241
©2026 The Author(s). HemaSphere is published by John Wiley & Sons Ltd on behalf of European Hematology Association. This is an open access Abstract Book distributed under the Attribution-NonCommercial-NoDerivs (CC BY-NC-ND), which allows third parties to download the articles and share them with others as long as they credit the author and the Abstract Book, but they cannot change the content in any way or use them commercially.
**Abstract Book Citations:** Authors, Title, HemaSphere, 2026;10:(S1):pages. The abstract book DOIs can be found on the "Author Information" page.
**Disclaimer:** Articles published in the journal HemaSphere exclusively reflect the opinions of the authors. The authors are responsible for all content in their abstracts including accuracy of the facts, statements, citing resources, etc.

### (PF374) LONG-TERM OUTCOMES OF ALLO-HSCT FOR RELAPSED/REFRACTORY B-CELL ACUTE LYMPHOBLASTIC LEUKEMIA WITH NON-REMISSION OR MINIMAL RESIDUAL DISEASE POSITIVITY AFTER CHIMERIC ANTIGEN RECEPTOR T-CELL THERAPY

**Topic:** 02. Acute lymphoblastic leukemia - Clinical

Yanzhi Song*[1], Zhihui Li[1], Yongqiang Zhao[1], Erhui Yuan[1], Zhanxiang Liu[1], Qi Wang[1], Tong Wu[1]

[1]*Beijing Boren Hospital, Bone Marrow Transplantation, Beijing, China;*

### Background

**Background:** Relapsed/refractory (r/r) B-cell acute lymphoblastic leukemia (B-ALL) patients who fail to achieve complete remission (CR) following chimeric antigen receptor T-cell (CAR-T) therapy carry an extremely poor prognosis. Salvage allogeneic hematopoietic stem cell transplantation (allo-HSCT) represents the only potential curative treatment option for this patient population.

### Aims

To evaluate the efficacy and safety of allo-HSCT in r/r B-ALL patients who were unable to achieve CR or minimal residual disease (MRD) negativity after CAR-T cell therapy.

### Methods

Between January 2018 and February 2026, a total of 82 consecutive patients with r/r B-ALL were enrolled. All patients had non-remission (NR, *n*=23) or MRD positivity (*n*=59) following CAR-T therapy; among the MRD-positive patients, 18 were identified by flow cytometry (FCM), and 41 were FCM-negative with MRD detected via fusion gene and gene mutation assays. All patients underwent allo-HSCT at our institution. The median age was 25.5 years (1–61 years), with a female-to-male ratio of 37:45. Prior to allo-HSCT, 44 patients (53.7%) had received at least two lines of CAR-T therapy. Myeloablative conditioning regimens were administered, including total body irradiation (fractionated, total dose: 8–10 Gy) combined with etoposide (200 mg/m² for 3 days) or teniposide (100 mg/m² for 3 days), fludarabine (30 mg/m² for 5 days) or cladribine (5 mg/m² for 5 days), alternatively, cyclophosphamide (1.8 g/m² for 2 days) combined with rabbit anti-T-cell globulin. Graft-versus-host disease (GVHD) prophylaxis comprised cyclosporine, mycophenolate mofetil, and short-course methotrexate. Additionally, some patients received targeted maintenance therapy for up to 2 years post-transplant, tailored to their individual fusion gene and gene mutation profiles.

### Results

All patients achieved durable engraftment. Patients who had achieved CR prior to allo-HSCT had significantly superior 2-year overall survival (OS, 66.1% vs. 36.2%) and leukemia-free survival (LFS, 63.4% vs. 33.2%) compared with those with NR, as shown in Figure 1. The cumulative relapse rates were 56.5% in the NR group and 30.5% in the CR group, respectively. For the 23 NR patients before transplantation, the median follow-up duration was 17.6 months (range, 1.53–81.6 months), with seven patients (7/23, 30.4%) in this group achieving a 2-year LFS. Among CR patients with MRD positivity, the median follow-up duration was 30 months (range, 0.77–90 months); notably, those with FCM negativity exhibited better 2-year OS (71.4% vs. 55.6%) and LFS (58.6% vs. 50.0%) than their FCM-positive counterparts. Nine patients (9/82, 11.0%) died of transplant-related events (TREs), including 2 in NR group and 7 in CR group. Acute aGVHD of grade Ⅲ–Ⅳ developed in 20 patients, and 8 patients presented with extensive chronic GVHD (cGVHD). Cox multivariate regression analysis showed that PH positive (+) B-ALL and the number of CAR-T therapy lines were associated with LFS. The 2-year LFS in PH + patients versus non-PH + patients was 71.9% vs. 59.2%.

**Copyright Information:** (Online) ISSN: 2572-9241
©2026 The Author(s). HemaSphere is published by John Wiley & Sons Ltd on behalf of European Hematology Association. This is an open access Abstract Book distributed under the Attribution-NonCommercial-NoDerivs (CC BY-NC-ND), which allows third parties to download the articles and share them with others as long as they credit the author and the Abstract Book, but they cannot change the content in any way or use them commercially.
**Abstract Book Citations:** Authors, Title, HemaSphere, 2026;10:(S1):pages. The abstract book DOIs can be found on the “Author Information” page.
**Disclaimer:** Articles published in the journal HemaSphere exclusively reflect the opinions of the authors. The authors are responsible for all content in their abstracts including accuracy of the facts, statements, citing resources, etc.

### Summary/Conclusion

Our findings demonstrate that salvage allo-HSCT confers a marked survival benefit in patients with r/r B-ALL who have failed both conventional chemotherapy and CAR-T therapy. Per our institutional treatment protocol, transplant-related mortality was notably low; even in this heavily pretreated cohort of r/r B-ALL, allo-HSCT remains a safe and effective therapeutic option. In particular, among PH + patients, LFS can exceed 70%.

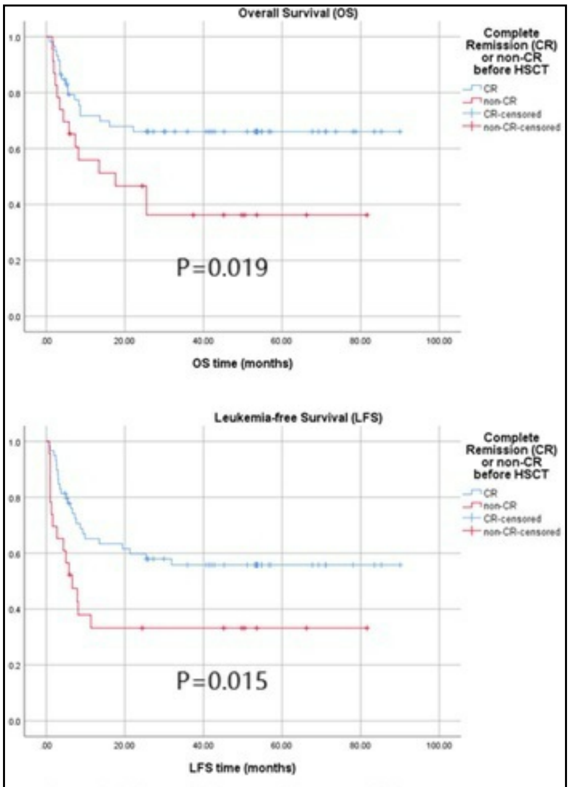

**Figure 1. LFS and OS Curves: CR vs. non-CR Groups**

**Copyright Information:** (Online) ISSN: 2572-9241
©2026 The Author(s). HemaSphere is published by John Wiley & Sons Ltd on behalf of European Hematology Association. This is an open access Abstract Book distributed under the Attribution-NonCommercial-NoDerivs (CC BY-NC-ND), which allows third parties to download the articles and share them with others as long as they credit the author and the Abstract Book, but they cannot change the content in any way or use them commercially.
**Abstract Book Citations:** Authors, Title, HemaSphere, 2026;10:(S1):pages. The abstract book DOIs can be found on the "Author Information" page.
**Disclaimer:** Articles published in the journal HemaSphere exclusively reflect the opinions of the authors. The authors are responsible for all content in their abstracts including accuracy of the facts, statements, citing resources, etc.

### (PF375) FRONTLINE CD19-DIRECTED CAR T-CELL THERAPY AS CONSOLIDATION IN HIGH-RISK B-ALL: A REAL-WORLD SINGLE-CENTER EXPERIENCE

**Topic:** 02. Acute lymphoblastic leukemia - Clinical

Hasmukh Jain[1], Manju Sengar[1], Alok Shetty[1], Lingaraj Nayak[1], Anupa John[1], Yash Shah[1], Atharva Karulkar[2], Thomas Eipe*[1], Neha Sharma[1], Yuktam Yadav[2], Devanshi Kalra[2], Smrithi Ravikumar[2], Bhausaheb Bagal[1], Rahul Purwar[3]

*[1]Tata Memorial Centre, Mumbai, India;[2]Immunoadoptive Cell Therapy Private Limited (ImmunoACT), Navi Mumbai, India;* *[3]Indian Institute of Technology (IIT) Bombay, Mumbai, India;*

### Background

MRD positivity post induction therapy is the most important adverse prognostic factor in B-ALL. Patients (pts) unable to complete standard chemotherapy due to significant treatment-related toxicities similarly represent a population at high risk of treatment failure. In settings without access to blinatumomab and with low transplant rates, CD19-directed CAR T-cell therapy may serve as a potential consolidation option.

### Aims

To evaluate the safety and efficacy of Talicabtagene autoleucel (Tali-cel) as consolidation in high-risk B-ALL.

### Methods

This single-center real-world study included pts ≥15 years with B-ALL who received Tali-cel between January 2024 and November 2025 in the front-line setting. Eligible pts were defined as high-risk, due to persistent MRD (>0.01%) on Day 64 induction (including blasts <10%) or inability to tolerate further intensive chemotherapy. All received Flu-Cy conditioning prior to Tali-cel infusion at ≥5 × $10^6$ CAR-T cells/kg.

**Copyright Information:** (Online) ISSN: 2572-9241
©2026 The Author(s). HemaSphere is published by John Wiley & Sons Ltd on behalf of European Hematology Association. This is an open access Abstract Book distributed under the Attribution-NonCommercial-NoDerivs (CC BY-NC-ND), which allows third parties to download the articles and share them with others as long as they credit the author and the Abstract Book, but they cannot change the content in any way or use them commercially.
**Abstract Book Citations:** Authors, Title, HemaSphere, 2026;10:(S1):pages. The abstract book DOIs can be found on the “Author Information” page.
**Disclaimer:** Articles published in the journal HemaSphere exclusively reflect the opinions of the authors. The authors are responsible for all content in their abstracts including accuracy of the facts, statements, citing resources, etc.

### Results

30 pts met criteria for high-risk disease and were planned for CAR-T cell therapy. 1 had manufacturing failure and 29 received Tali-cel infusion, and were included in this analysis. The median age was 25 years (range 15–54), and 23 (79%) were male. At baseline, median blast was 84% (range 30–95) and CSF was involved by FCM +/- morphology in 7 pts (24%). All pts underwent FISH for cytogenetics and NGS was performed in 20, based on which ALL classification was assigned as B-ALL NOS (n=11); TP53 alterations (near triploid n=1; hypodiploidy n=2); IKZF1 N159 mutation (n=1); TCF3::PBX1 fusion (n=1); ZNF384 rearrangement (n=1); BCR::ABL1 fusion (n=7); and BCR::ABL1-like features (n=5).

All pts received conventional chemotherapy for induction, only 1 received inotuzumab during phase 2 induction. Among the 29 pts, indication for CAR-T cell therapy was day 64 MRD positivity for 24 pts, including 4 with morphological disease (<10% blasts). 5 pts were considered due to inability to tolerate further intensive treatment secondary to MDR sepsis, fungal infections, or poor performance status. Bridging therapy was given at physician discretion; 1 pt received mini-HyperCVD plus Inotuzumab, and others received conventional chemotherapy. Prior to CAR-T, 12 pts were in CR with MRD negativity, 11 were in CR with MRD positivity, and 6 had low disease burden with MRD positivity. All pts were CSF negative at the time of infusion. The median vein-to-vein time was 28 days (range 22–65).

On day 28 response assessment, all pts achieved CR with MRD clearance. CRS occurred in 19 pts (66%), with grade 2–3 events in 17%. Tocilizumab was administered in 17 pts (90%) who developed CRS, with a median dose of 1 (range 1–3); vasopressors were required in 2 pts. IEC-HS was observed in 9 (31%), all of whom received anakinra, and 4 also received ruxolitinib. No ICANS were reported, and none required ICU-level care. IVIg for hypogammaglobulinemia was given in 16 pts (55%). At data cutoff, 5 pts had relapsed with ongoing B-cell aplasia in 19 of 24 pts. No consolidation alloSCT was performed. After a median follow-up of 13 months, the 1-year EFS was 83.5% (95% CI 61.6–93.5) and the 1-year OS was 90.4% (95% CI 66.5–97.5).

### Summary/Conclusion

This real-world analysis demonstrates that Tali-cel is a safe and effective consolidation strategy in high-risk and chemotherapy-ineligible B-ALL. Although longer follow-up is warranted, our findings support the need for prospective evaluation of CAR T-cell therapy as consolidation in this setting.

**Copyright Information:** (Online) ISSN: 2572-9241
©2026 The Author(s). HemaSphere is published by John Wiley & Sons Ltd on behalf of European Hematology Association. This is an open access Abstract Book distributed under the Attribution-NonCommercial-NoDerivs (CC BY-NC-ND), which allows third parties to download the articles and share them with others as long as they credit the author and the Abstract Book, but they cannot change the content in any way or use them commercially.
**Abstract Book Citations:** Authors, Title, HemaSphere, 2026;10:(S1):pages. The abstract book DOIs can be found on the "Author Information" page.
**Disclaimer:** Articles published in the journal HemaSphere exclusively reflect the opinions of the authors. The authors are responsible for all content in their abstracts including accuracy of the facts, statements, citing resources, etc.

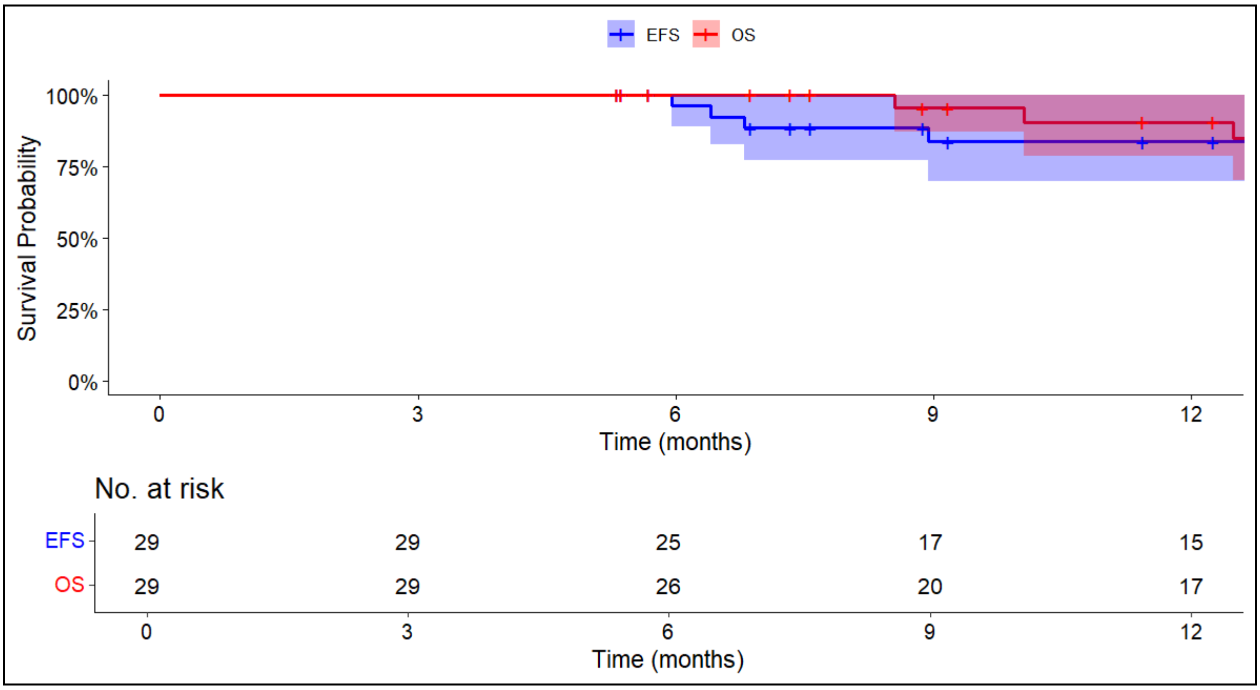

Kaplan-Meier curves showing event-free survival and overall survival

**Copyright Information:** (Online) ISSN: 2572-9241
©2026 The Author(s). HemaSphere is published by John Wiley & Sons Ltd on behalf of European Hematology Association. This is an open access Abstract Book distributed under the Attribution-NonCommercial-NoDerivs (CC BY-NC-ND), which allows third parties to download the articles and share them with others as long as they credit the author and the Abstract Book, but they cannot change the content in any way or use them commercially.
**Abstract Book Citations:** Authors, Title, HemaSphere, 2026;10:(S1):pages. The abstract book DOIs can be found on the "Author Information" page.
**Disclaimer:** Articles published in the journal HemaSphere exclusively reflect the opinions of the authors. The authors are responsible for all content in their abstracts including accuracy of the facts, statements, citing resources, etc.

### (PF376) EARLY USE OF INOTUZUMAB OZOGAMICIN IN FIRST REMISSION ACHIEVES DEEP AND DURABLE MRD ERADICATION IN ADULTS WITH PHILADELPHIA CHROMOSOME-NEGATIVE B-ALL: A PROSPECTIVE PHASE II STUDY

**Topic:** 02. Acute lymphoblastic leukemia - Clinical

Runxia Gu*[1], Yuntao Liu[1], Shuning Wei[1], Ying Wang[1], Xiaoyuan Gong[2], Yan Li[1], Benfa Gong[1], Dong Lin[1], Kaiqi Liu[1], Chunlin Zhou[1], Guangji Zhang[2], Qiuyun Fang[2], Shouyun Li[2], Shaowei Qiu[1], Xibin MA[1], Bingcheng Liu[1], Hui Wei[1], Yingchang Mi[1], Ying Wang[1], Jianxiang Wang[1]

*[1]State Key Laboratory of Experimental Hematology, National Clinical Research Center for Blood Diseases, Haihe Laboratory of Cell Ecosystem, Institute of Hematology and Blood Diseases Hospital, Chinese Academy of Medical Sciences and Peking Union Medical College, Tianjin, China; [2]State Key Laboratory of Experimental Hematology, National Clinical Research Center for Blood Diseases, Haihe Laboratory of Cell Ecosystem, Institute of Hematology and Blood Diseases Hospital, Chinese Academy of Medical Sciences and Peking Union Medical College, The International Medical Center, Tianjin, China;*

### Background

While targeting CD22 with the antibody-drug conjugate inotuzumab ozogamicin (InO) has proven effective in relapsed/refractory B-ALL, evidence guiding its earlier application to eradicate measurable residual disease (MRD) in first complete remission (CR1) remains limited. Specifically, the optimal regimen to maximize MRD clearance while minimizing toxicity in this setting is undefined. Here, we present the results of a phase 2 study evaluating the safety and efficacy of early InO administration for MRD clearance in adults with Philadelphia chromosome-negative B-ALL (Ph− B-ALL) in CR1.

### Methods

This single-arm, open-label, prospective phase II trial enrolled adult patients with Ph− B-ALL in CR1 who failed to achieve MRD negativity after frontline therapy or developed MRD recurrence, defined as ≥0.01% by multiparameter flow cytometry (FCM). InO was administered at a total dose of 1.5 mg/m² per 28-day cycle, given as 0.5 mg/m² on days 1, 8, and 15, for up to two cycles in the absence of disease progression or unacceptable toxicity. The primary endpoint was achievement of MRD negativity (defined as <0.01%) by FCM after the first cycle of InO.

### Results

Between May 2023 and Oct. 2025, 31 patients were enrolled and all included in the efficacy and safety analyses. The median age was 35 years (range 18 to 69). At baseline, 30 patients had persistent MRD following initial therapy, and 1 patient experienced MRD recurrence in CR1. High-risk molecular features included Ph-like disease (n=5), ZNF384 fusion (n=11), IKZF1 alteration (n=9), and TP53 mutations (n=2).

Following one cycle of InO treatment, 87% (27/31) of evaluable patients achieved MRD negativity as assessed by flow cytometry. Three patients remained MRD-positive, two of whom harbored a ZNF384 fusion and one an IKZF1 alteration. One additional patient who withdrew from the study was classified as treatment failure due to nonevaluable efficacy. All three patients with residual MRD received a second cycle of InO and subsequently achieved MRD negativity. Consequently, the overall MRD clearance rate was 97% (30/31).

Regarding safety, the toxicities of greatest concern were thrombocytopenia and liver toxicities, consistent with the known safety profile of InO. Thrombocytopenia occurred in 29% (9/31) of patients, all of which were grade 1–2. Transient liver function abnormalities were observed in 16% (5/31) of patients, also limited to grade 1–2. Importantly, no cases of sinusoidal obstruction syndrome/veno-occlusive disease (SOS/VOD) or hemorrhagic events were reported, including among patients who proceeded to allogeneic stem cell transplantation.

**Copyright Information:** (Online) ISSN: 2572-9241
©2026 The Author(s). HemaSphere is published by John Wiley & Sons Ltd on behalf of European Hematology Association. This is an open access Abstract Book distributed under the Attribution-NonCommercial-NoDerivs (CC BY-NC-ND), which allows third parties to download the articles and share them with others as long as they credit the author and the Abstract Book, but they cannot change the content in any way or use them commercially.
**Abstract Book Citations:** Authors, Title, HemaSphere, 2026;10:(S1):pages. The abstract book DOIs can be found on the "Author Information" page.
**Disclaimer:** Articles published in the journal HemaSphere exclusively reflect the opinions of the authors. The authors are responsible for all content in their abstracts including accuracy of the facts, statements, citing resources, etc.

**Summary/Conclusion**

Early administration of inotuzumab ozogamicin in CR1 results in rapid, deep, and clinically meaningful MRD eradication in adult patients with Ph-negative B-ALL, including those with high-risk molecular features. This approach demonstrates a favorable safety profile and does not appear to increase the risk of post-transplant SOS/VOD. These findings support the incorporation of InO as an effective and safe MRD-directed strategy in earlier lines of therapy for Ph-negative B-ALL.

**Copyright Information:** (Online) ISSN: 2572-9241
©2026 The Author(s). HemaSphere is published by John Wiley & Sons Ltd on behalf of European Hematology Association. This is an open access Abstract Book distributed under the Attribution-NonCommercial-NoDerivs (CC BY-NC-ND), which allows third parties to download the articles and share them with others as long as they credit the author and the Abstract Book, but they cannot change the content in any way or use them commercially.
**Abstract Book Citations:** Authors, Title, HemaSphere, 2026;10:(S1):pages. The abstract book DOIs can be found on the “Author Information” page.
**Disclaimer:** Articles published in the journal HemaSphere exclusively reflect the opinions of the authors. The authors are responsible for all content in their abstracts including accuracy of the facts, statements, citing resources, etc.

### (PF377) A RISK-STRATIFIED TREATMENT STRATEGY COMBINING CHEMOTHERAPY AND CAR-T CELL THERAPY FOR PEDIATRIC RELAPSED/REFRACTORY T-LYMPHOBLASTIC LYMPHOMA

**Topic:** 02. Acute lymphoblastic leukemia - Clinical

Ying Liu*[1], Yang Liu[1], Qing Zhu[1], Pei Zhang[1], Yali Peng[1], Guomin Wang[1], Kai Wang[1], Qinlong Zheng[1], Chunju Zhou[2, 2], Biping Deng[3], Yonghong Zhang[1]

*[1]Beijing Gobroad Boren Hospital, Department of Pediatric Lymphoma, Beijing, China;[2]Beijing Gobroad Boren Hospital, Department of Pathology, Beijing, China; [3]Beijing Gobroad Boren Hospital, Cytology Laboratory, Beijing, China;*

### Background

Risk-stratified chemotherapy followed by hematopoietic stem cell transplantation (HSCT) is the standard treatment for pediatric relapsed or refractory (R/R)  ALL. Protocols such as the UKALLR3 and ALL-REZ BFM 2002 have achieved long-term EFS of 60%–70% in patients with intermediate-risk relapsed ALL, predominantly B-ALL (over 80%). However, the prognosis for high-risk patients, particularly those with R/R T-ALL/LBL, remains dismal. With survival rates of approximately 30-50%, this population presents a substantial therapeutic challenge in the second-line setting.

### Aims

This study aims to improve remission rates and long-term outcomes for pediatric R/R TLBL by implementing a risk-stratified, comprehensive treatment framework that integrates second-line chemotherapy with targeted agents, CAR-T cell therapy, and allogeneic HSCT.

### Methods

A total of 23 patients aged up to 18 years with R/R TLBL were enrolled between January 1, 2022, and December 31, 2024 with a follow-up cutoff date of December 31, 2025. Treatment was guided by a dynamic risk stratification system based on time and site of relapse, high-risk cytogenetics, BM MRD, and tumor response following each induction phase. The backbone therapy comprised the UKALLR3 induction regimen, augmented with bortezomib, venetoclax, and CAR-T cell therapy. Following Induction I, patients were assigned to transplant or non-transplant groups based on risk stratification and response. Specifically, patients in the standard-and intermediate-risk groups who achieved CR were allocated to the non-transplant group, whereas all high-risk patients, along with intermediate-risk patients not attaining CR, were assigned to the transplant group. Treatment allocation after Induction II was determined by BM MRD and tumor response. Patients with BM MRD >1% or tumor reduction <50% were switched from the UKALLR3 regimen to CAR-T cell therapy; all others proceeded to Induction III. Patients in the transplant group who achieved BM MRD <0.01% and radiographic CR after three cycles of induction or CAR-T cell therapy subsequently proceeded to allogeneic HSCT.

**Copyright Information:** (Online) ISSN: 2572-9241
©2026 The Author(s). HemaSphere is published by John Wiley & Sons Ltd on behalf of European Hematology Association. This is an open access Abstract Book distributed under the Attribution-NonCommercial-NoDerivs (CC BY-NC-ND), which allows third parties to download the articles and share them with others as long as they credit the author and the Abstract Book, but they cannot change the content in any way or use them commercially.
**Abstract Book Citations:** Authors, Title, HemaSphere, 2026;10:(S1):pages. The abstract book DOIs can be found on the “Author Information” page.
**Disclaimer:** Articles published in the journal HemaSphere exclusively reflect the opinions of the authors. The authors are responsible for all content in their abstracts including accuracy of the facts, statements, citing resources, etc.

### Results

Among the 23 enrolled patients, 1 was classified as intermediate-risk and 22 as high-risk. This cohort included 7 patients with large mediastinal masses, 10 with BM involvement, 4 with central nervous system involvement, 2 with early T-cell precursor phenotype, 4 with PTEN mutations, and 4 with NOTCH1 fusions. Following UKALLR3 induction chemotherapy, 12 patients achieved CR. Among them, the one intermediate-risk patient remained in continuous CR after completing consolidation and maintenance therapy without transplantation. The remaining 11 patients proceeded to allogeneic HSCT, with 5 maintaining CR post-transplant. Nine patients achieved CR after CAR-T therapy following induction and subsequently underwent allogeneic HSCT, with 8 maintaining CR post-transplant. Two patients who failed to achieve CR after both induction chemotherapy and CAR-T therapy underwent salvage transplantation, but both experienced disease progression. CR2 rate for the entire cohort was 91.3% (21/23), and the estimated EFS and OS at 24 months were 0.56 (95% CI, 0.34–0.74) and 0.66 (95% CI, 0.40 –0.82), respectively.

.

### Summary/Conclusion

PTEN mutations and NOTCH1 fusions are common genetic alterations of poor prognosis in pediatric TLBL. A risk-stratified approach combining induction chemotherapy with CAR-T cell therapy may increase CR rates and offers the potential to improve long-term outcomes in children with R/R TLBL.

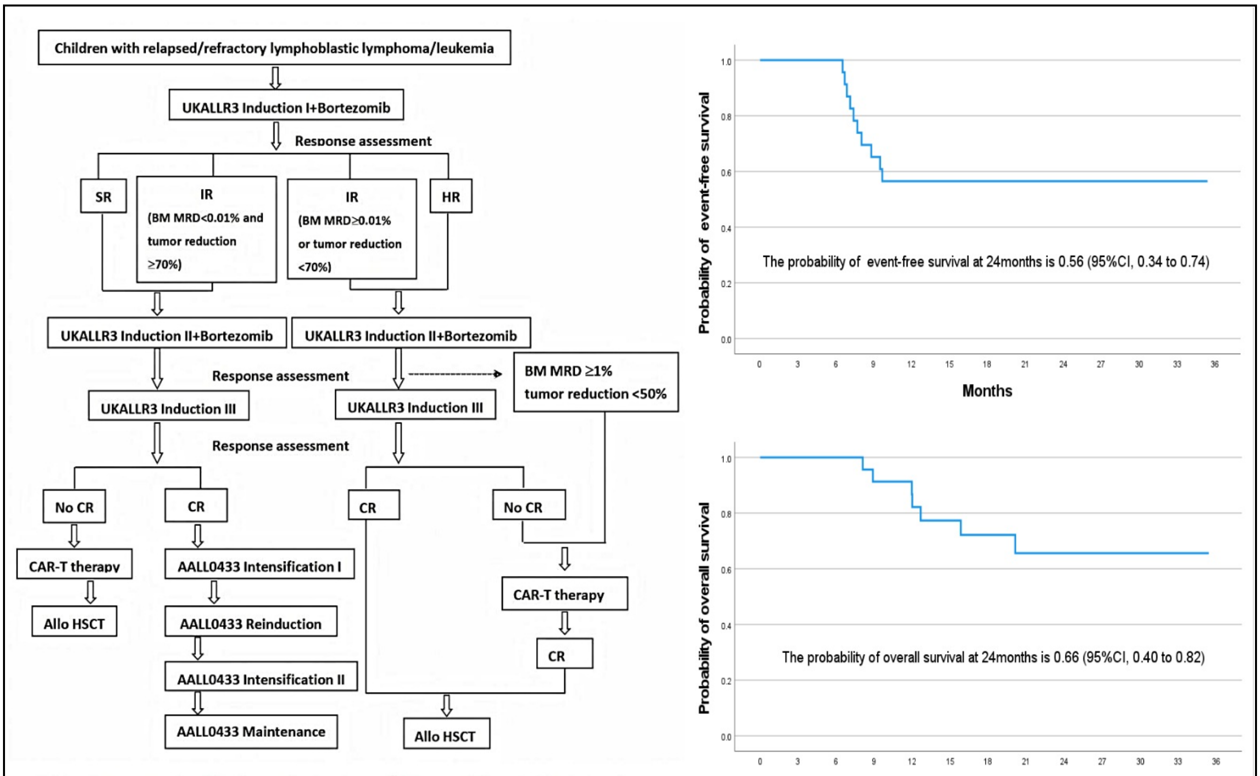

Table : Treatment algorithm for pediatric relapsed/refractory T-lymphoblastic lymphoma

Copyright Information: (Online) ISSN: 2572-9241
©2026 The Author(s). HemaSphere is published by John Wiley & Sons Ltd on behalf of European Hematology Association. This is an open access Abstract Book distributed under the Attribution-NonCommercial-NoDerivs (CC BY-NC-ND), which allows third parties to download the articles and share them with others as long as they credit the author and the Abstract Book, but they cannot change the content in any way or use them commercially.
Abstract Book Citations: Authors, Title, HemaSphere, 2026;10:(S1):pages. The abstract book DOIs can be found on the “Author Information” page.
Disclaimer: Articles published in the journal HemaSphere exclusively reflect the opinions of the authors. The authors are responsible for all content in their abstracts including accuracy of the facts, statements, citing resources, etc.

### (PF378) GENOMIC ARCHITECTURE OVERRIDES SINGLE-LOCUS RISK SIGNALS IN T-ALL: PROGNOSTIC UTILITY OF A MULTI-GENE NGS CLASSIFIER INDEPENDENT OF MEASURABLE RESIDUAL DISEASE

**Topic:** 02. Acute lymphoblastic leukemia - Clinical

Neha Singh*[1, 2], Deepali Saxena[1, 2], Anil Singh[2, 3], Ankita Pal[2, 4], Nilesh Dhole[1, 2], Rohit Kori[1, 2], Snehal Jaiswar[1, 2], Seema Biswas[1, 2], Bal Mishra[2, 3]

*[1]TATA MEMORIAL CENTRE (HBCH & MPMMCC), HEMATOPATHOLOGY, VARANASI, India;[2]HOMI BHABHA NATIONAL INSTITUTE, MUMBAI, Mumbai, India; [3]TATA MEMORIAL CENTRE (HBCH & MPMMCC), ADULT HEMATOLYMPHOID UNIT, VARANASI, India; [4]TATA MEMORIAL CENTRE (HBCH & MPMMCC), BIOSTATISTICS AND EPIDEMIOLOGY, VARANASI, India;*

**Background**

T-cell acute lymphoblastic leukemia (T-ALL) demonstrates significant molecular heterogeneity with variable treatment responses. The advent of next-generation sequencing (NGS) has enabled simultaneous interrogation of multiple oncogenic drivers, offering a platform to construct composite mutation classifiers with superior prognostic resolution compared to single-gene approaches.

**Aims**

This study aimed to evaluate the prognostic performance of an NGS-derived mutation classifier in a cohort of T-ALL patients, specifically assessing its capacity to stratify relapse-free survival (RFS) and overall survival (OS), and to determine its relationship with measurable residual disease (MRD) clearance following induction therapy.

**Methods**

We conducted a retrospective analysis of 100 adult T-ALL patients (median age 24.5 years, 82% male) treated at a single center. All patients underwent comprehensive NGS-based genomic profiling at diagnosis. Based on mutation landscape, patients were allocated to one of four classifier categories: Good risk (NOTCH1/FBXW7/PHF6 mutations; n = 39), Poor risk (IDH1/2, PTEN, NRAS/KRAS, TP53, DNMT3A, or IKZF1 alterations; n = 15), Both (co-occurrence of favorable and unfavorable mutations; n = 25), or None (no classifier mutations detected; n = 21). Survival outcomes were estimated using the Kaplan-Meier method and compared across groups with the log-rank test.

**Results**

ETP-ALL and non-ETP T-ALL subtypes were equally distributed (50% each). Post-induction MRD negativity was achieved in 66% of patients, with 34% remaining MRD-positive. NOTCH1/FBXW7 co-mutations were the most prevalent alterations, followed by PHF6, JAK kinase pathway mutations, NRAS/KRAS, WT1, and IDH1/2. The NGS classifier demonstrated statistically significant prognostic discrimination for both RFS (log-rank $p = 0.005$) and OS (log-rank $p = 0.04$). The Good-risk category achieved superior long-term outcomes, with a mean RFS of 45.1 months and a 1-year RFS rate of 88%. In contrast, the Both group—harboring concurrent favorable and unfavorable mutations—exhibited the most adverse prognosis, with a mean RFS of only 13.3 months and a 1-year RFS rate of 35%, suggesting that co-occurring adverse mutations functionally override the protective effect of NOTCH1/FBXW7 alterations. A binary reclassification into favorable (Good + None) versus unfavorable (Poor + Both) strata yielded even more pronounced separation (RFS $p = 0.002$; OS $p = 0.01$), with mean RFS values of 41.9 versus 19.4 months, respectively. Despite a trend toward higher MRD negativity in Good-category patients (76.9%), the overall correlation between classifier assignment and MRD status was weak and did not reach statistical significance (Cramer's $V = 0.23$; $p = 0.15$), indicating that the classifier's prognostic value likely extends beyond early MRD kinetics.

**Copyright Information:** (Online) ISSN: 2572-9241
©2026 The Author(s). HemaSphere is published by John Wiley & Sons Ltd on behalf of European Hematology Association. This is an open access Abstract Book distributed under the Attribution-NonCommercial-NoDerivs (CC BY-NC-ND), which allows third parties to download the articles and share them with others as long as they credit the author and the Abstract Book, but they cannot change the content in any way or use them commercially.
**Abstract Book Citations:** Authors, Title, HemaSphere, 2026;10:(S1):pages. The abstract book DOIs can be found on the "Author Information" page.
**Disclaimer:** Articles published in the journal HemaSphere exclusively reflect the opinions of the authors. The authors are responsible for all content in their abstracts including accuracy of the facts, statements, citing resources, etc.

### Summary/Conclusion

An NGS-based composite mutation classifier provides robust, independent prognostic stratification in T-ALL. Notably, the co-occurrence of favorable and unfavorable mutations defines a high-risk subgroup whose outcomes rival those of purely adverse profiles, underscoring the importance of multi-gene rather than single-locus assessment. The classifier's prognostic signal persists independently of MRD status, suggesting complementary mechanisms through which genomic architecture shapes long-term disease behavior.

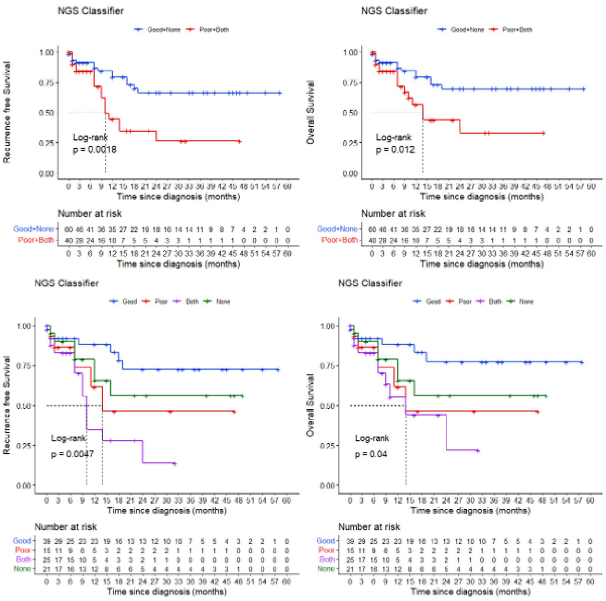

**Figure 1.** Kaplan–Meier curves depicting RFS and OS according to NGS classifier, demonstrating a significant prognostic stratification for both RFS and OS.

**Copyright Information:** (Online) ISSN: 2572-9241
©2026 The Author(s). HemaSphere is published by John Wiley & Sons Ltd on behalf of European Hematology Association. This is an open access Abstract Book distributed under the Attribution-NonCommercial-NoDerivs (CC BY-NC-ND), which allows third parties to download the articles and share them with others as long as they credit the author and the Abstract Book, but they cannot change the content in any way or use them commercially.
**Abstract Book Citations:** Authors, Title, HemaSphere, 2026;10:(S1):pages. The abstract book DOIs can be found on the “Author Information” page.
**Disclaimer:** Articles published in the journal HemaSphere exclusively reflect the opinions of the authors. The authors are responsible for all content in their abstracts including accuracy of the facts, statements, citing resources, etc.

### (PF379) PREDICTING CNS RELAPSE IN CHILDREN AND YOUNG ADULTS DIAGNOSED WITH ACUTE LYMPHOBLASTIC LEUKAEMIA (ALL)

**Topic:** 02. Acute lymphoblastic leukemia - Clinical

Anthony Moorman*[1], Melvin Joy[1], Ilaria Di Fazio[2], Amy Kirkwood[3], Anna Lawson[4], Pamela Kearns[4], John Moppett[5], Sujith Samarasinghe[6], Ajay Vora[6], Christine Harrison[1], Amir Enshaei[1], Chris Halsey[2]

*[1]Newcastle University, Translational and Clinical Research Institute, Newcastle Upon Tyne, United Kingdom; [2]University of Glasgow, School of Cancer Sciences, Glasgow, United Kingdom; [3]UCL, Cancer Research UK & University College London Cancer Trials Centre, London, United Kingdom; [4]University of Birmingham, Cancer Research UK Clinical Trials Unit (CRCTU), Birmingham, United Kingdom; [5]Bristol Royal Hospital for Sick Children, Haematology, Bristol, United Kingdom;[6]Great Ormond Street Hospital for Children, Haematology, London, United Kingdom;*

### Background

Central Nervous System (CNS) relapse occurs in ~5% patients diagnosed with ALL. Blinatumomab significantly reduces the risk of BM relapse but not CNS relapse. This presents challenges when blinatumomab is used to de-escalate systemic agents with known CNS-penetration. Identifying children at high and low risk of CNS relapse is important to prevent both undertreatment and overtreatment of the CNS.

### Aims

To identify demographic, clinical and genomic determinants of CNS relapse. To develop a risk model which can be used in treatment protocols using blinatumomab.

### Methods

Our primary cohort comprised 2,436 patients (1-24 years) diagnosed and treated on UKALL2011. The median follow-up time was 7.3 years (IQR 6.0-8.9) during which 139 relapses involving the CNS were recorded: 84 isolated and 55 combined relapses. We report cumulative incidence of CNS relapse (CIR-CNS), isolated CNS relapse (CIR-ISO_CNS) and combined CNS relapse (CIR-CNS-COMBO) rates at 5 years with 95% confidence intervals. Non-CNS relapses and non-relapse deaths were counted as competing events.

**Copyright Information:** (Online) ISSN: 2572-9241
©2026 The Author(s). HemaSphere is published by John Wiley & Sons Ltd on behalf of European Hematology Association. This is an open access Abstract Book distributed under the Attribution-NonCommercial-NoDerivs (CC BY-NC-ND), which allows third parties to download the articles and share them with others as long as they credit the author and the Abstract Book, but they cannot change the content in any way or use them commercially.
**Abstract Book Citations:** Authors, Title, HemaSphere, 2026;10:(S1):pages. The abstract book DOIs can be found on the “Author Information” page.
**Disclaimer:** Articles published in the journal HemaSphere exclusively reflect the opinions of the authors. The authors are responsible for all content in their abstracts including accuracy of the facts, statements, citing resources, etc.

**Results**

The overall CIR-CNS rate was 5.4% and was higher in T-cell patients (8.4%) than in B-cell patients (4.9%) (p<0.02) (Table). Isolated CNS relapse was more common than combined CNS relapse (Table). Among B-cell patients, CNS2/3, 1-year olds, WBC, iAMP21, *CRLF2*-r, *IKZF1* deletions, IKZF1plus and end of induction (EOI) MRD were associated with an increased CIR-CNS rate. While, *ETV6::RUNX1* and high hyperdiploidy (HeH) were associated with lower CIR-CNS rates.
We modelled two CNS risk stratification scenarios in B-cell ALL: firstly, using baseline risk factors and secondly factoring in EOI MRD. Our baseline model included age, CNS2/3, WBC, *ETV6::RUNX1*, HeH and iAMP21. Whereas our EOI model included age, CNS2/3, *ETV6::RUNX1*, HeH and MRD. The output of each model was a continuous risk score. The top 10% patients (i.e. those with the highest risk scores) had CIR-CNS rates that were 3 times higher than the whole cohort [baseline model 15.3% (10.5-20.9), EOI model 14.6% (9.6-20.5)]. In both models, ~55% patients with CNS2/3 had risk scores in the top 10% and they had more than 3-fold increased CIR-CNS rate compared to CNS2/3 cases with lower risk scores: baseline model 17% (9-27) v 4% (1-12) (p=0.03), EOI model 19% (10-31) v 5% (1-14) (p=0.03). Next, we calculated the proportion of cases with a CICR of <2%. Using the baseline model, the bottom 20% cases had a CIR-CNS rate of 1.8% (0.9-3.7) whereas using the EOI model it was 27% cases [1.9% (0.9-3.5)]. Given the overlap between the models in terms of variables, some patients were captured in the top 10% or low risk groups of both models but there were differences indicating that the addition of MRD to the EOI model was selecting different patients. Although our models were built using any CNS relapse as the event of interest, the results apply equally to isolated and combined CNS relapses (Table). We successfully validated these models using data from patients treated on UKALL2003.

**Summary/Conclusion**

The risk of CNS relapse is not uniform across key clinical and genomic subtypes of B-cell ALL. Our integrated risk models can identify patients with the lowest and highest CIR-CNS rate. Moreover, as they are based on standard-of-care information they can be readily incorporated into stratification algorithms.

**Cumulative incidence of any CNS, isolated CNS and combined CNS relapse in B-cell ALL**

| | CIR-CNS | CIR-ISO_CNS | CIR-COMB_CNS |
|---|---|---|---|
| All cases | 5.4% (4.5 - 6.3) | 3.4% (2.7 - 4.2) | 2% (1.5 - 2.6) |
| T-cell | 8.4% (5.6 - 11.9) | 6.0% (3.7 - 9.1) | 2.4% (1.1 - 4.6) |
| B-cell | 4.9% (4.1-5.9) | 3.0% (2.3 - 3.8) | 1.9% (1.4 - 2.6) |
| Baseline model | | | |
| Top 10% | 15.3% (10.5 - 20.9) | 8.7% (5.2 - 13.3) | 6.6% (3.6 - 10.9) |
| Low Risk Group | 1.8% (0.9 - 3.7) | 1.1% (0.4 - 2.6) | 0.8% (0.2 - 2.2) |
| EOI Model | | | |
| Top 10% | 14.6% (9.6 - 20.5) | 8.2% (4.6 - 13.1) | 6.4% (3.3 - 10.9) |
| Low Risk Group | 1.9% (0.9 - 3.5) | 0.9% (0.3 - 2.2) | 0.9% (0.3 - 2.2) |

**Copyright Information:** (Online) ISSN: 2572-9241
©2026 The Author(s). HemaSphere is published by John Wiley & Sons Ltd on behalf of European Hematology Association. This is an open access Abstract Book distributed under the Attribution-NonCommercial-NoDerivs (CC BY-NC-ND), which allows third parties to download the articles and share them with others as long as they credit the author and the Abstract Book, but they cannot change the content in any way or use them commercially.
**Abstract Book Citations:** Authors, Title, HemaSphere, 2026;10:(S1):pages. The abstract book DOIs can be found on the "Author Information" page.
**Disclaimer:** Articles published in the journal HemaSphere exclusively reflect the opinions of the authors. The authors are responsible for all content in their abstracts including accuracy of the facts, statements, citing resources, etc.

### (PF380) EFFICACY AND GENETIC CORRELATES OF VENETOCLAX-BASED IMMUNOCHEMOTHERAPY IN OLDER/UNFIT B-ALL: A PHASE 2 STUDY IN THE ERA OF NOVEL COMBINATIONS: RESULTS A PHASE 2 MULTI-CENTER CLINICAL TRIAL

**Topic:** 02. Acute lymphoblastic leukemia - Clinical

Runxia Gu*[1], Zhao Xingli[2], Shaowei Qiu[1], Delin Lu[1], Haizhou Xing[3], Zhang Hao[4], Baohong Wang[5], Yihao Wang[6], Yingqi Shao[1], Shuning Wei[1], Jianxiang Wang[1], Ying Wang[1]

*[1]National Clinical Research Center for Blood Diseases, State Key Laboratory of Experimental Hematology, Haihe Laboratory of Cell Ecosystem, Institute of Hematology & Blood Diseases Hospital, Chinese Academy of Medical Sciences &Peking Union Medical College, Tianjin, China; [2]Department of Hematology, Oncology Center, Tianjin Union Medical Center, The First Affiliated Hospital of Nankai University, Tianjin, China; [3]Department of Hematology, The First Affiliated Hospital of Zhengzhou University, Zhengzhou, China; [4]Department of Hematology, Affiliated Hospital of Jining Medical University, Jining Medical University, Jining, China; [5]Department of Hematology, Weifang People's Hospital, Shandong Second Medical University, Weifang, China; [6]Department of Hematology, Tianjin Medical University General Hospital, Tianjin, China;*

### Background

Most existing prognostic markers for B-cell acute lymphoblastic leukemia (B-ALL) were established in the context of conventional, often intensive, chemotherapy. The shift toward lower-intensity chemo-immunotherapy combinations necessitates a re-evaluation of predictive factors for treatment response in this evolving landscape.

### Aims

We conducted a phase 2, multicenter trial to assess an integrated therapeutic strategy combining molecular targeted therapy (venetoclax), immunotherapy (dose-reduced inotuzumab ozogamicin, InO), and reduced-intensity chemotherapy. In addition to evaluating treatment efficacy for older or unfit adults with Philadelphia chromosome-negative (Ph-negative) B-ALL, the study aimed to explore potential correlations between genetic profiles and clinical outcomes.

### Methods

We prospectively enrolled previously untreated adults with Ph-negative B-ALL who were either older than 60 years or deemed intolerant to intensive chemotherapy. The AVCDV induction regimen consisted of vincristine (1.4 mg/m² on days 1 and 8), cyclophosphamide (150 mg/m² every 12 hours on days 1-3), dexamethasone (20 mg on days 1-4 and 11-14), venetoclax (100 mg on day 6, 200 mg on day 7, and 400 mg on days 8-14), and inotuzumab ozogamicin (1 mg on day 4, with a second optional dose on day 15 if bone marrow blasts exceeded 5% on day 14). Treatment response was assessed by bone marrow morphology and measurable residual disease (MRD; 0.01% sensitivity) using multiparameter flow cytometry on day 28 post-induction, with relapse monitored throughout. Baseline genomic profiling by targeted next-generation sequencing and RNA sequencing enabled mutation analysis, fusion detection, and expression-based subclassification for correlation with clinical outcomes.

**Copyright Information:** (Online) ISSN: 2572-9241
©2026 The Author(s). HemaSphere is published by John Wiley & Sons Ltd on behalf of European Hematology Association. This is an open access Abstract Book distributed under the Attribution-NonCommercial-NoDerivs (CC BY-NC-ND), which allows third parties to download the articles and share them with others as long as they credit the author and the Abstract Book, but they cannot change the content in any way or use them commercially.
**Abstract Book Citations:** Authors, Title, HemaSphere, 2026;10:(S1):pages. The abstract book DOIs can be found on the "Author Information" page.
**Disclaimer:** Articles published in the journal HemaSphere exclusively reflect the opinions of the authors. The authors are responsible for all content in their abstracts including accuracy of the facts, statements, citing resources, etc.

**Results**

Between June 2024 and February 2026, 36 patients were enrolled. The median age was 55 years (range, 18-82), and 47.2% (17/36) were male. The median bone marrow blast percentage was 81.5% (range, 6%-96.5%). Among 33 patients with evaluable molecular genetic data, notable high-risk genetic alterations included TP53 mutations (11 patients, 30.6%), KMT2A rearrangements (5 patients, 13.9%), IKZF1 alterations (5 patients, 13.9%), PAX5 alterations (non-P80R; 9 patients, 25.0%), Ph-like genotype (4 patients, 11.1%), and ZNF384 rearrangements (2 patients, 5.6%).

The AVCDV induction regimen achieved a complete remission (CR) rate of 97% (35/36), with no treatment-related deaths. One patient with a Ph-like genotype (JAK2::THRAP rearrangement) did not respond. Among the patients who achieved CR, 82.9% (29/35) were MRD-negative by flow cytometry on day 28. Among the six MRD-positive patients, one harbored concurrent KMT2A rearrangement and TP53 mutation, three carried ZNF-related fusion genes (ZNF362::SMARCA4, CREBBP::ZNF384, and EP300::ZNF384), and two had no identifiable high-risk genetic alterations. The median MRD level was 0.28%. Notably, all patients with ZNF384-related fusions were MRD-positive, whereas all other patients with TP53 mutations (without concurrent KMT2A rearrangement) achieved MRD-negative remission. Additionally, 50% (2/4) of patients with partial CD22 expression were MRD-positive.

**Summary/Conclusion**

This integrated regimen shows promising efficacy as first-line induction therapy for older or chemotherapy-unfit patients with Ph-negative B-ALL. However, the presence of ZNF-related fusions or partial CD22 expression may identify a subgroup with higher risk of residual disease.

**Copyright Information:** (Online) ISSN: 2572-9241
©2026 The Author(s). HemaSphere is published by John Wiley & Sons Ltd on behalf of European Hematology Association. This is an open access Abstract Book distributed under the Attribution-NonCommercial-NoDerivs (CC BY-NC-ND), which allows third parties to download the articles and share them with others as long as they credit the author and the Abstract Book, but they cannot change the content in any way or use them commercially.
**Abstract Book Citations:** Authors, Title, HemaSphere, 2026;10:(S1):pages. The abstract book DOIs can be found on the “Author Information” page.
**Disclaimer:** Articles published in the journal HemaSphere exclusively reflect the opinions of the authors. The authors are responsible for all content in their abstracts including accuracy of the facts, statements, citing resources, etc.

### (PF381) LINEAGE PLASTICITY–MEDIATED RESISTANCE TO CD7-DIRECTED CAR-T THERAPY IN RELAPSED/REFRACTORY T-ALL/LBL

**Topic:** 02. Acute lymphoblastic leukemia - Clinical

Jiahua Niu*[1], Kun Wang[2], Fang Xiang[3], Haopeng Wang[4], Xianmin Song[5]

*[1]Shanghai General hospital, Shanghai, China;[2]School of Life Science and Technology, ShanghaiTech University, Shanghai, China; [3]Hrain Biotechnology Co., Ltd, Shanghai, China;[4]School of Life science and Technology, ShanghaiTech University; Changping Laboratory,Beijing, Shanghai, China; [5]Shanghai General Hospital, Shanghai Jiaotong University School of Medicine, Shanghai, China;*

### Background

CD7-directed chimeric antigen receptor (CAR) T-cell therapy has shown promising efficacy in relapsed or refractory (R/R) T-cell acute lymphoblastic leukemia and lymphoblastic lymphoma (T-ALL/LBL). However, relapse remains a major obstacle limiting long-term benefit.

### Aims

To evaluate its safety and efficacy of anti-CD7 CAR T therapy in patients with R/R T-ALL/LBL (NCT05290155). The primary objective was to assess the safety of CD7-targeted CAR-T cells for R/R T-ALL/LBL and the secondary objective was to assess its efficacy.

### Methods

We generated a recyclable CD7 CAR-T cells (CD7 $CAR^{Recyclable}$) and conducted an open, phase I clinical trial. Traditional 1+3+3+3 dose escalation was performed to determine the optimal single dose of CAR-T cells, which corresponded to four dose levels (DLs) of $0.25 \times 10^6$ cells/kg, $0.5 \times 10^6$ cells/kg, $1 \times 10^6$ cells/kg and $2 \times 10^6$ cells/kg, respectively. CD7-CAR-T cells were manufactured under GMP conditions, utilizing a retroviral vector-based platform. CAR-T cells were engineered from autologous (patient-derived) or allogeneic (donor-derived) peripheral blood mononuclear cells (PBMCs) through genetic modification. Peripheral blood and bone marrow samples were collected at predefined time points before and after CAR-T cell infusion for longitudinal disease monitoring.

### Results

We engineered a recyclable CD7 CAR which demonstrated superior tumor-killing activity. Fourteen patients (median age 25y; 15-52; M:F=12:2) received CAR-T infusion and were evaluable for safety and response. Immunophenotypic and molecular profiling were performed longitudinally to characterize relapse patterns. Robust CAR-T expansion was observed in 13 of 14 patients (92.9%). Peak CAR-T concentration (Cmax) reached 62,389.69 copies/μg DNA (range: 3,175.94-326,482.67) at median 12 days post-infusion (range: 10-21). All patients experienced hematologic toxicities, and cytokine release syndrome occurred in 71.4% of patients, predominantly grade 1–2. Thirteen patients (92.9%) achieved MRD-negative complete remission in bone marrow, while the objective response rate for extramedullary disease was 78.5%. With a median follow-up of 8.5 months, the estimated 6-month overall survival and progression-free survival were 62.3% and 44.6%, respectively. Notably, lineage switch relapse occurred in two patients with ETP-immunophenotype following CAR-T therapy. Paired analyses demonstrated retention of pre–CAR-T molecular features despite a complete shift in immunophenotype toward a myeloid lineage, consistent with clonal lineage reprogramming rather than therapy-related secondary leukemia.

**Copyright Information:** (Online) ISSN: 2572-9241
©2026 The Author(s). HemaSphere is published by John Wiley & Sons Ltd on behalf of European Hematology Association. This is an open access Abstract Book distributed under the Attribution-NonCommercial-NoDerivs (CC BY-NC-ND), which allows third parties to download the articles and share them with others as long as they credit the author and the Abstract Book, but they cannot change the content in any way or use them commercially.
**Abstract Book Citations:** Authors, Title, HemaSphere, 2026;10:(S1):pages. The abstract book DOIs can be found on the "Author Information" page.
**Disclaimer:** Articles published in the journal HemaSphere exclusively reflect the opinions of the authors. The authors are responsible for all content in their abstracts including accuracy of the facts, statements, citing resources, etc.

**Summary/Conclusion**

While CD7 CAR-T is a promising treatment option for patients with r/r T-ALL/LBL, our cases highlight lineage switch as a important resistance mechanism, particularly relevant for ETP-ALL/LBL with inherent lineage plasticity.

**Copyright Information:** (Online) ISSN: 2572-9241
©2026 The Author(s). HemaSphere is published by John Wiley & Sons Ltd on behalf of European Hematology Association. This is an open access Abstract Book distributed under the Attribution-NonCommercial-NoDerivs (CC BY-NC-ND), which allows third parties to download the articles and share them with others as long as they credit the author and the Abstract Book, but they cannot change the content in any way or use them commercially.
**Abstract Book Citations:** Authors, Title, HemaSphere, 2026;10:(S1):pages. The abstract book DOIs can be found on the "Author Information" page.
**Disclaimer:** Articles published in the journal HemaSphere exclusively reflect the opinions of the authors. The authors are responsible for all content in their abstracts including accuracy of the facts, statements, citing resources, etc.

### (PF382) PRACTICAL RESULTS OF MRD DIRECTED BFM 2000 IN TYA IN COMPARISON TO NON MRD BASED MODIFIED BFM AND HCVAD- ACUTE LYMPHOBLASTIC. A COMPARATIVE ANALYSIS OF 279 PATIENTS WITH LEUKEMIA CONTEXT

**Topic:** 02. Acute lymphoblastic leukemia - Clinical

Misbah Maktoob*[1], Ammara Yasmin[1], Zaira Asad[1], Imran Iftikhar[1], Shehar Bano[1], Areeba Fatima Ghani[1], Ehsan Elahi[1], Bushra Ahsan[1], Usman Ahmad[1], Syed Waqas Iman Bokhari[1]

*[1]Shaukat khanum memorial cancer hospital and research centre, Clinical haematology, Lahore, Pakistan;*

### Background

The treatment of Adolescent and Young Adult (TYA) patients with Acute Lymphoblastic Leukemia (ALL) is still difficult, and the results of adult-based and pediatric-inspired chemotherapy regimens frequently differ greatly. Although MRD-directed therapy is the norm in many settings, real-world data comparing these approaches to intense adult regimens like HCVAD and modified non-MRD-based protocols is essential for maximizing care in situations with limited resources.

### Aims

The purpose of this study was to assess and compare the disease-free survival (DFS) and overall survival (OS) of TYA ALL patients treated with three different protocols: HCVAD, non-MRD-based Modified BFM, and MRD-directed BFM 2000.
Secondary objectives included the assessment of molecular risk factors and treatment-related toxicities.

### Methods

A retrospective analysis was conducted on a cohort of 279 TYA patients (median age 22 years; range 16–47). Patients were stratified by induction protocol: Modified BFM (n=156), HCVAD (n=74), and BFM 2000 (n=49). Molecular and cytogenetic risk factors, including Ph+ and MLL rearrangements, were analyzed. Survival was estimated using Kaplan-Meier curves, and independent prognostic factors were identified through an updated Cox Proportional Hazards model adjusting for age, lineage, and MRD status.

### Results

The cohort was predominantly B-cell lineage (96.4%). High-risk molecular features were identified in 20.1% (n=56) of patients with Ph+ ALL and 1.8% (n=5) with MLL rearrangements. At a median follow-up of 41.5 months, the MRD-directed BFM 2000 protocol demonstrated superior outcomes. Median OS was significantly higher for BFM 2000 (60.0 months) compared to Modified BFM (30.0 months) and HCVAD (8.0 months) ($p < 0.001$). The 5-year OS rates were 47.3%, 42.5%, and 28.6%, respectively.
In the multivariate analysis, protocol choice emerged as the strongest independent predictor of mortality. Compared to BFM 2000, the hazard of death was significantly higher for patients treated with HCVAD (HR 2.77; 95% CI 1.85–4.23; $p < 0.001$) and Modified BFM (HR 1.72; 95% CI 1.07–2.75; $p = 0.024$). Sepsis and infection were the leading causes of non-relapse mortality (50.4%). Asparaginase-related toxicities included hepatotoxicity (4.3%) and pancreatitis (1.4%). After receiving allogeneic HSCT, sixteen patients (5.7%) demonstrated exceptional survival (median OS not attained; median DFS 50 months).

### Summary/Conclusion

In the TYA ALL group, pediatric-inspired, MRD-directed treatments such as BFM 2000 offer a notable survival advantage over both non-MRD-based modified BFM and HCVAD regimens. Although high rates of infection-related mortality underscore the continued need for improved supportive care, the incorporation of MRD-directed intensification is crucial for enhancing long-term results.

**Copyright Information:** (Online) ISSN: 2572-9241
©2026 The Author(s). HemaSphere is published by John Wiley & Sons Ltd on behalf of European Hematology Association. This is an open access Abstract Book distributed under the Attribution-NonCommercial-NoDerivs (CC BY-NC-ND), which allows third parties to download the articles and share them with others as long as they credit the author and the Abstract Book, but they cannot change the content in any way or use them commercially.
**Abstract Book Citations:** Authors, Title, HemaSphere, 2026;10:(S1):pages. The abstract book DOIs can be found on the "Author Information" page.
**Disclaimer:** Articles published in the journal HemaSphere exclusively reflect the opinions of the authors. The authors are responsible for all content in their abstracts including accuracy of the facts, statements, citing resources, etc.

### (PF383) IMPACT OF DELAYED FIRST LUMBAR PUNCTURE AND TRIPLE INTRATHECAL THERAPY ON OUTCOMES OF PEDIATRIC ACUTE LYMPHOBLASTIC LEUKEMIA ACCORDING TO CNS STATUS: THE REPORT OF THE TPOG-ALL-2013 STUDY

**Topic:** 02. Acute lymphoblastic leukemia - Clinical

Hsi-Che Liu*[1], Shiann-Tarng Jou[2], Shih-Hsiang Chen[3], Tang-Her Jaing[3], Tingchi Yeh[1], Jiann-Shiuh Chen[4], Kang-Hsi Wu[5], Shih-Chung Wang[6], Chih-Cheng Hsiao[7], Te-Kau Cheng[2], Meng-Yao Lu[8], Hsiu-Ju Yen[9], Fang-Liang Huang[10], Shu-Huey Chen[11], Shang-Hsien Yang[12], Yen-Lin Liu[13], Shyh-Shin Chiou[14], Chao-Ping Yang[3], Lee-Yung Shih[3], Dong-Tsamn Lin[8]

*[1]MacKay Children's Hospital, Taipei, Taiwan; [2]China Medical University Children's Hospital, Taichung, Taiwan; [3]Chang Gung Memorial Hospital, Taoyuan, Taiwan; [4]Cheng Kung University Hospital, Tainan, Taiwan; [5]Chung Shan Medical University Hospital, Taichung, Taiwan; [6]Changhua Christian Hospital, Changhua, Taiwan; [7]Chang Gung Memorial Hospital-Kaohsiung, Kaohsiung, Taiwan; [8]National Taiwan University Hospital, Taipei, Taiwan; [9]Taipei Veterans General Hospital, Taipei, Taiwan; [10]Taichung Veterans General Hospital, Taichung, Taiwan; [11]Taipei Medical University–Shuang Ho Hospital, Taipei, Taiwan; [12]Buddhist Tzu Chi General Hospital, Hualien, Taiwan; [13]Taipei Medical University Hospital, Taipei, Taiwan; [14]Kaohsiung Medical University Hospital, Kaohsiung, Taiwan;*

### Background

The CNS-directed therapy of Taiwan Pediatric Oncology Group (TPOG)-Acute Lymphoblastic Leukemia (ALL)-2013 protocol (TPOG-2013), following our previous report (Yeh et al., Cancer 2018), omitted cranial radiation for all newly diagnosed ALL patients and delayed the diagnostic lumbar puncture (LP) and the first triple intrathecal therapy (TIT) until circulating lymphoblasts had cleared from the peripheral blood.

### Aims

The study aimed to evaluate the impact of delaying the first LP and TIT on the outcomes of pediatric ALL according to CNS status.

### Methods

The delayed LP and first TIT were recommended to be performed within 10 days of initiating induction therapy. The TPOG-2013 protocol was conducted from January 1, 2013 to December 31, 2020. A total of 870 children aged ≧1 year with newly diagnosed ALL were enrolled.

**Copyright Information:** (Online) ISSN: 2572-9241
©2026 The Author(s). HemaSphere is published by John Wiley & Sons Ltd on behalf of European Hematology Association. This is an open access Abstract Book distributed under the Attribution-NonCommercial-NoDerivs (CC BY-NC-ND), which allows third parties to download the articles and share them with others as long as they credit the author and the Abstract Book, but they cannot change the content in any way or use them commercially.
**Abstract Book Citations:** Authors, Title, HemaSphere, 2026;10:(S1):pages. The abstract book DOIs can be found on the "Author Information" page.
**Disclaimer:** Articles published in the journal HemaSphere exclusively reflect the opinions of the authors. The authors are responsible for all content in their abstracts including accuracy of the facts, statements, citing resources, etc.

### Results

As of October 31, 2025, the 5-year event-free survival (EFS) and overall survival (OS) were 87.1% and 97.2%, respectively. And the 5-year cumulative incidences of isolated CNS relapse and any CNS (isolated plus combined) relapse were 1.2% and 2.3%, respectively.

Following the delayed LP, 93.2%, 5.4% and 1.1% of patients were classified as CNS-1, CNS-2 and CNS-3 status, respectively; two additional patients had traumatic LP without detectable blasts. The proportion of CNS-1 appeared higher than that reported in some cooperative group studies. In contrast, the incidence of CNS-2 was substantially lower than that observed in most groups, whereas the incidence of CNS-3 was comparable (Thastrup et al., Leukemia, 2022).

In subsequent analysis, patients with CNS-2 showed a trend toward superior EFS compared with those with CNS-1 status (95.5% vs. 87.2%, P=0.07). No significant differences were observed between the CNS-2 and CNS-1 groups in OS (95.7% vs. 91.3%), isolated CNS relapse (0.9% vs. 0%) or any CNS relapse (1.9% vs. 0%) (Fig. 1).

In the CNS-1 cohort, univariate Cox proportional hazards regression analysis demonstrated that high-risk or very-high-risk group, NCI high-risk classification, age ≧10 years, initial WBC ≧$50\times10^9$/L, T-lineage, absence of hyperdiploidy, absence of *ETV6::RUNX1* fusion; presence of *BCR::ABL1* fusion and positive measurable residual disease (MRD) at the end of induction (EOI) were significantly associated with inferior EFS. In multivariate analysis, only high-risk or very-high-risk group (hazard ratio [HR]: 2.3, P=0.034), age ≧10 years (HR: 2.1, P=0.016), initial WBC ≧$50\times10^9$/L (HR: 2.7, P=0.002), presence of *BCR::ABL1* transcript (HR: 2.3, P=0.01), and positive EOI MRD (HR: 1.9, P=0.005) remained independent predictors of inferior EFS. Accordingly, patients of very-high-risk (56.9%), age ≧10 years (74.0%), WBC ≧$50\times10^9$/L (74.9%), positive *BCR::ABL1* (66.3%), and positive EOI MRD (72.7%) had significantly worst EFS.

### Summary/Conclusion

Delaying the diagnostic LP and first TIT may alter the distribution of CNS status at diagnosis of pediatric ALL but did not compromise survival or CNS control. CNS outcomes were comparable between patients with CNS-1 and CNS-2 status. The results of TPOG-2013 suggest that further improvement in survival among patients with CNS-1 status should be achieved through integration of precision diagnostics, immunotherapy, and targeted therapy in subsequent TPOG-ALL protocols rather than reintroducing first-day TIT or cranial irradiation.

**Copyright Information:** (Online) ISSN: 2572-9241
©2026 The Author(s). HemaSphere is published by John Wiley & Sons Ltd on behalf of European Hematology Association. This is an open access Abstract Book distributed under the Attribution-NonCommercial-NoDerivs (CC BY-NC-ND), which allows third parties to download the articles and share them with others as long as they credit the author and the Abstract Book, but they cannot change the content in any way or use them commercially.
**Abstract Book Citations:** Authors, Title, HemaSphere, 2026;10:(S1):pages. The abstract book DOIs can be found on the "Author Information" page.
**Disclaimer:** Articles published in the journal HemaSphere exclusively reflect the opinions of the authors. The authors are responsible for all content in their abstracts including accuracy of the facts, statements, citing resources, etc.

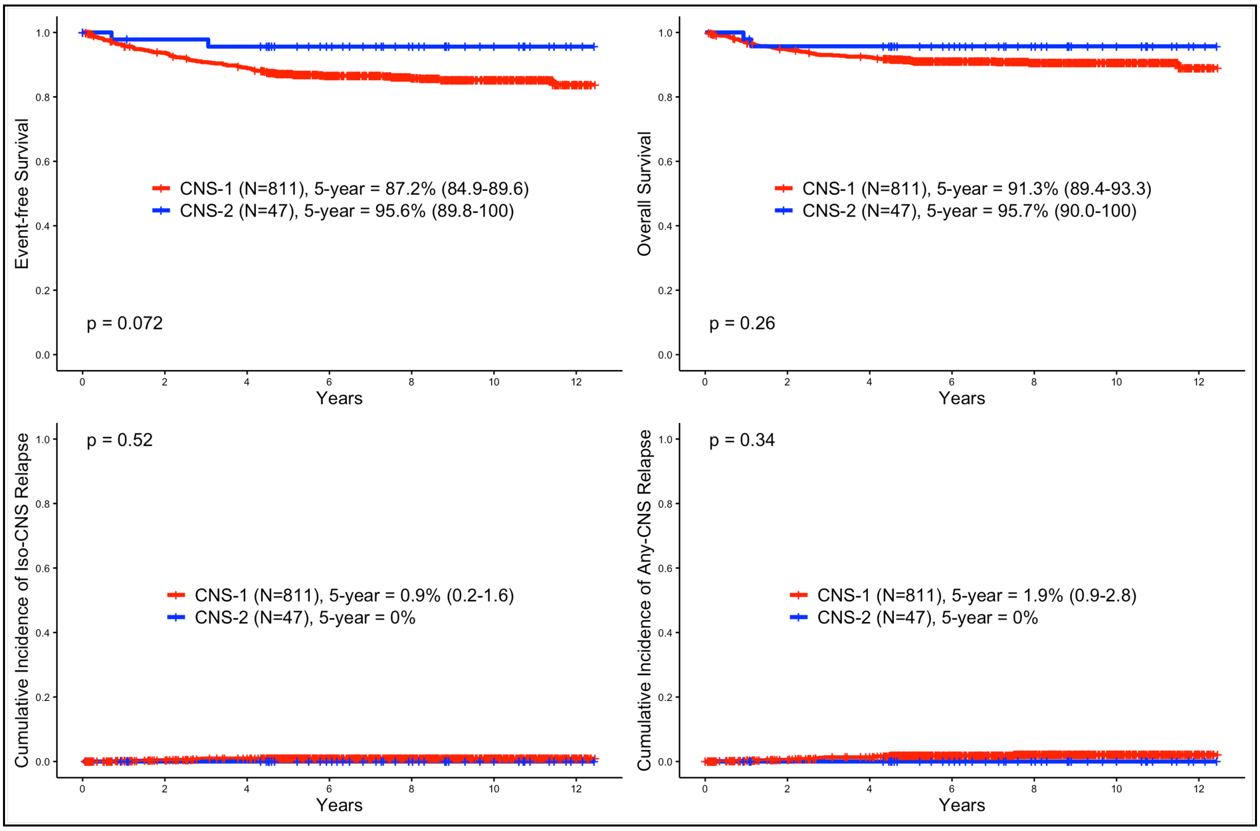

Figure 1. Comparison of Survival Outcomes Between Patients with CNS-1 and CNS-2 Status in the TPOG-ALL-2013 Protocol . Iso, isolated; Any, isolated plus combined.

**Copyright Information:** (Online) ISSN: 2572-9241
©2026 The Author(s). HemaSphere is published by John Wiley & Sons Ltd on behalf of European Hematology Association. This is an open access Abstract Book distributed under the Attribution-NonCommercial-NoDerivs (CC BY-NC-ND), which allows third parties to download the articles and share them with others as long as they credit the author and the Abstract Book, but they cannot change the content in any way or use them commercially.
**Abstract Book Citations:** Authors, Title, HemaSphere, 2026;10:(S1):pages. The abstract book DOIs can be found on the "Author Information" page.
**Disclaimer:** Articles published in the journal HemaSphere exclusively reflect the opinions of the authors. The authors are responsible for all content in their abstracts including accuracy of the facts, statements, citing resources, etc.

### (PF384) ADDITION OF VENETOCLAX ENHANCES MINIMAL RESIDUAL DISEASE RESPONSE IN CRLF2-REARRANGED PHILADELPHIA-LIKE ALL

**Topic:** 02. Acute lymphoblastic leukemia - Clinical

Ibrahim Aldoss*[1], Jianying Zhang[2], Vaibhav Agrawal[1], Marjorie Robbins[3], Ahmed Aribi[1], Hoda Pourhassan[1], Paul Koller[1], Haris Ali[1], Amanda Blackmon[1], Salman Otoukesh[1], Karamjeet S. Sandhu[1], Brian J Ball[1], Andrew Artz[1], Idoroenyi Amanam[1], Sunmin Park[1], Jose Tinajero[4], Monzr Al Malki[1], Amandeep Salhotra[1], Zhaohui Gu[5], Angelo Ong[6], Pamela S. Becker[1], Ryotaro Nakamura[1], Caner Saygin[7], Wendy Stock[7], Stephen Forman[1], Anthony Stein[1], Guido Marcucci[1], Vinod Pullarkat[1]

*[1]City of Hope, Department of Hematology & Hematopoietic Cell Transplantation, Duarte, United States of America;[2]City of Hope, Department of Biostatistics, Duarte, United States of America; [3]City of Hope, Department of Clinical Translational Project Development, Duarte, United States of America; [4]City of Hope, Department of Pharmacy, Duarte, United States of America;[5]City of Hope, Beckman Research Institute, Duarte, United States of America; [6]City of Hope, Clinical Trials Office, Duarte, United States of America; [7]University of Chicago, Department of Medicine-Hematology and Oncology, Chicago, United States of America;*

### Background

Ph-like acute lymphoblastic leukemia (ALL) is a high-risk subtype with substantial unmet therapeutic need, characterized by increased rates of induction failure and persistent measurable residual disease (MRD), with historical MRD- rates post-induction (IND) of 20% and post-consolidation (CON) of 40%. We previously reported the safety and activity of incorporating a 2-week course of venetoclax (VEN) during IND and CON therapy in 24 newly diagnosed adult patients (pts) with Ph-negative B-ALL (Aldoss I., Haematologica, 2025). Notably, this cohort included 12 pts with Ph-like ALL, among whom the MRD- rate after CON was 92%. Building on these promising results, we have expanded the study to enroll an additional 12 pts with CRLF2-rearranged Ph-like ALL.

### Aims

To further evaluate the efficacy and safety of VEN in combination with the C10403 regimen in adults with Ph-like ALL.

### Methods

VEN was administered for the first 2 weeks during IND and CON of the C10403 regimen backbone, with a ramp-up dosing during IND. MRD- was defined as <0.01% by flow and <0.0001% by clonoSEQ.

**Copyright Information:** (Online) ISSN: 2572-9241
©2026 The Author(s). HemaSphere is published by John Wiley & Sons Ltd on behalf of European Hematology Association. This is an open access Abstract Book distributed under the Attribution-NonCommercial-NoDerivs (CC BY-NC-ND), which allows third parties to download the articles and share them with others as long as they credit the author and the Abstract Book, but they cannot change the content in any way or use them commercially.
**Abstract Book Citations:** Authors, Title, HemaSphere, 2026;10:(S1):pages. The abstract book DOIs can be found on the “Author Information” page.
**Disclaimer:** Articles published in the journal HemaSphere exclusively reflect the opinions of the authors. The authors are responsible for all content in their abstracts including accuracy of the facts, statements, citing resources, etc.

### Results

As of data cutoff on Feb 24, 2026, accrual was completed with a total of 36 pts, in which 24 pts had Ph-like B-ALL and the focus of this analysis **(Table 1).** The majority (96%) had CRLF2-rearrangement, with only one pt having a JAK2-fusion. Half of the pts had concurrent JAK2 mutations. The median age was 32 (range: 18-53) yrs, of which 19/24 (79%) were males, 23/24 (96%) were Hispanics, and the median body mass index (BMI) was 30 (range: 22-42).All 24 (100%) Ph-like ALL pts achieved CR/CRi following IND ± extended IND, with only 3/24 (13%) requiring extended IND due to partial response post IND. One pt was taken off the study post IND due to fungemia and transitioned to blinatumomab. The MRD- response rate post IND+ extended IND was 38% by flow and 13% by clonoSEQ. Among 23 pts who started consolidation, MRD- response rate was 91% (21/23) by flow and 55% (11/20) by clonoSEQ. Among Ph-like ALL pts, 21/24 (88%) and 11/24 (46%) received blinatumomab in remission and underwent transplant in CR1, respectively. The median follow is 11 (2-38) months for Ph-like pts and 26 (1-47) months for all pts. Only one Ph-like pt relapsed at 5 months and another patient died of venoocclusive disease posttransplant in CR (EFS 94.4%; see **Figure**). The most common grade ≥3 treatment (VEN or C10403) related adverse events were leukopenia (71%), anemia (67%), neutropenia (63%), thrombocytopenia (54%), lymphopenia (42%), elevated ALT (38%), hyperbilirubinemia (29%), and sepsis (25%).

### Summary/Conclusion

The addition of 14 days of VEN at 400 mg daily dose to a pediatric-inspired regimen (C10403) during IND and CON was highly effective in inducing MRD- remission state post CON in adults with newly diagnosed Ph-like B-ALL.

**Table 1.** Patient Characteristics and Responses

| | **All patients, N (%)** | **Ph-like, N (%)** |
|---|---|---|
| **Number** | 36 | 24 |
| **Median age (range), yrs**<br>Age ≥ 40 yrs | 32 (18-53)<br>12 (33) | 32 (18-53)<br>8 (33) |
| **Sex**<br>Male<br>Female | 28 (78)<br>8 (22) | 19 (79)<br>5 (21) |
| **Ethnicity**<br>Hispanic<br>White<br>Pacific Islander | 34 (94)<br>1 (3)<br>1 (3) | 23 (96)<br>0<br>1 (4) |
| **BMI (range)**<br>BMI ≥ 30 | 30 (19-54)<br>20 (56) | 30 (22-42)<br>13 (54) |
| **WBC at diagnosis (range), K/uL** | 5.3 (0.19-276) | 23 (2.2-276) |
| **CR after IND/extended IND** | 35/35 (100) | 24 (100) |
| **Patients requiring extended IND** | 3 (8) | 3 (12.5) |
| **MRD- by flow (<0.01%) after IND** | 15/35 (43) | 9/24 (38) |
| **MRD- by clonoseq (<0.0001%) after IND** | 9/35 (26) | 3/24 (13) |
| **MRD- by flow (<0.01%) after CON** | 30/33 (91) | 21/23 (91) |
| **MRD- by clonoseq (<0.0001%) after CON** | 19/30 (63) | 11/20 (55) |

**Copyright Information:** (Online) ISSN: 2572-9241
©2026 The Author(s). HemaSphere is published by John Wiley & Sons Ltd on behalf of European Hematology Association. This is an open access Abstract Book distributed under the Attribution-NonCommercial-NoDerivs (CC BY-NC-ND), which allows third parties to download the articles and share them with others as long as they credit the author and the Abstract Book, but they cannot change the content in any way or use them commercially.
**Abstract Book Citations:** Authors, Title, HemaSphere, 2026;10:(S1):pages. The abstract book DOIs can be found on the "Author Information" page.
**Disclaimer:** Articles published in the journal HemaSphere exclusively reflect the opinions of the authors. The authors are responsible for all content in their abstracts including accuracy of the facts, statements, citing resources, etc.

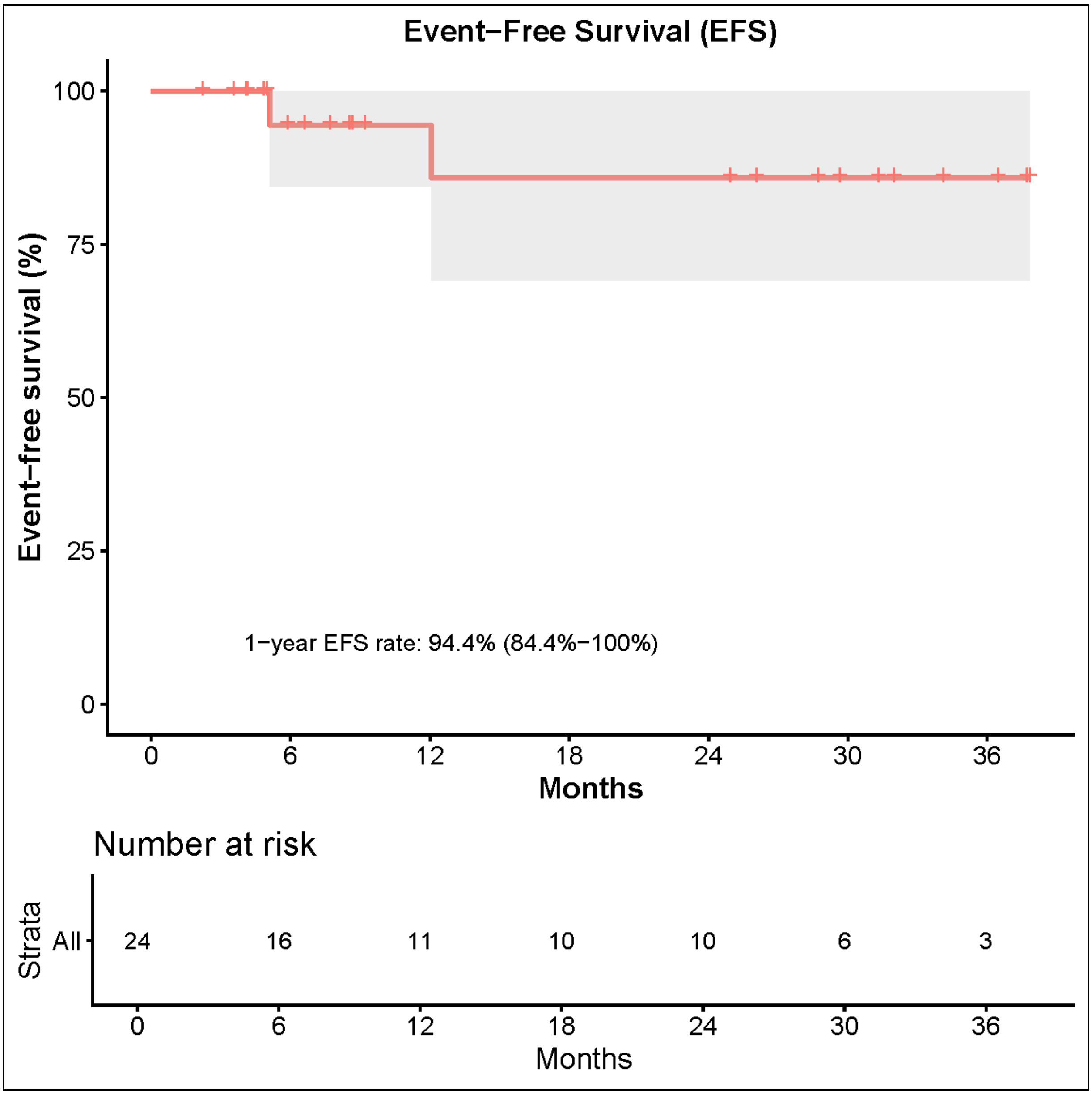

**Copyright Information:** (Online) ISSN: 2572-9241
©2026 The Author(s). HemaSphere is published by John Wiley & Sons Ltd on behalf of European Hematology Association. This is an open access Abstract Book distributed under the Attribution-NonCommercial-NoDerivs (CC BY-NC-ND), which allows third parties to download the articles and share them with others as long as they credit the author and the Abstract Book, but they cannot change the content in any way or use them commercially.
**Abstract Book Citations:** Authors, Title, HemaSphere, 2026;10:(S1):pages. The abstract book DOIs can be found on the "Author Information" page.
**Disclaimer:** Articles published in the journal HemaSphere exclusively reflect the opinions of the authors. The authors are responsible for all content in their abstracts including accuracy of the facts, statements, citing resources, etc.

### (PF385) PREDICTING RELAPSE AFTER FRONTLINE BLINATUMOMAB IN CHILDREN AND YOUNG PEOPLE WITH B-ALL

**Topic:** 02. Acute lymphoblastic leukemia - Clinical

Miguel Vieira Martins*[1, 2], Amir Enshaei[3], David O'connor[1, 4], Rachael Hough[5], John Moppett[6], Ajay Vora[1], Sujith Samarasinghe[1], Anthony Moorman[3]

*[1]Great Ormond Street Hospital for Children NHS Trust, Haematology, London, United Kingdom;[2]Princess Maxima Center for Pediatric Oncology, Research - Pieters Group, Utrecht, The Netherlands; [3]Leukaemia Research Cytogenomics Group, Translational and Clinical Research Institute, Newcastle University, Newcastle, United Kingdom; [4]University College London, Cancer Institute, London, United Kingdom; [5]University College London Hospital NHS Trust, Haematology, London, United Kingdom; [6]Bristol Royal Hospital for Children, Bristol, United Kingdom;*

**Background**

Blinatumomab improves survival when added to chemotherapy for B-precursor acute lymphoblastic leukaemia (B-ALL). However, whether this benefit is uniform across biological subgroups, particularly when used as a chemotherapy-replacement strategy, remains uncertain.

**Aims**

To develop a novel blinatumomab-specific risk model derived from outcomes of chemotherapy-intolerant or resistant patients treated within a standardised chemotherapy replacement pathway.

**Methods**

Children and young people aged 1–24 years, in the United Kingdom (UK) and Ireland diagnosed with B-ALL between 2018 and 2025 who were chemotherapy-intolerant or resistant received blinatumomab in place of selected components of the frontline chemotherapy backbone. Outcomes were analysed according to conventional prognostic variables, genetic abnormalities, and response to blinatumomab to identify determinants of relapse. To benchmark this cohort and contextualise the results, we compared the outcome of patients in this cohort to an equivalent cohort of patients treated on the UKALL2003 trial.

**Copyright Information:** (Online) ISSN: 2572-9241
©2026 The Author(s). HemaSphere is published by John Wiley & Sons Ltd on behalf of European Hematology Association. This is an open access Abstract Book distributed under the Attribution-NonCommercial-NoDerivs (CC BY-NC-ND), which allows third parties to download the articles and share them with others as long as they credit the author and the Abstract Book, but they cannot change the content in any way or use them commercially.
**Abstract Book Citations:** Authors, Title, HemaSphere, 2026;10:(S1):pages. The abstract book DOIs can be found on the “Author Information” page.
**Disclaimer:** Articles published in the journal HemaSphere exclusively reflect the opinions of the authors. The authors are responsible for all content in their abstracts including accuracy of the facts, statements, citing resources, etc.

**Results**

Among 225 patients, 195 received chemotherapy following blinatumomab (Blin-CT) and 30 underwent first-remission HSCT. The survival rates of the cases and controls at 2 years was almost identical: Relapse rate 8% v 8%, event-free survival 89% v 87% and overall survival 94% v 90% respectively

In the Blin-CT cohort, traditional risk factors, including age, white cell count, high-risk genetics, and pre-blinatumomab end-of-induction measurable residual disease (MRD) did not predict relapse. On univariable analysis, relapse risk was increased with detectable MRD after blinatumomab cycle 1 (C1-END; hazard ratio (HR) 6.61 (95% confidence interval (CI) 2.46-17.70), p<0.001), IKZF1plus 3.64 (1.09-12.21), p=0.04), JAK-STAT abnormalities (3.56 (1.01-12.53), p=0.05), and *DUX4* rearrangements (4.24 (1.20-15.05), p=0.03). In multivariable modelling, only C1-END MRD and IKZF1plus remained independently associated with relapse, whereas *DUX4*-rearranged cases were strongly associated with persistent MRD at C1-END. An integrated model – UKBlinPredict incorporating C1-END MRD, IKZF1plus, and *DUX4*-r defined a high-risk subgroup (33% cases) with a 18% 2-year RR versus 0% in remaining patients (log-rank *p*-value < 0.001; yielding a 2-year HR12.82 (95% CI 2.86-57.39; *P* = 0.001) . This risk model also discriminated central nervous system (CNS) relapse, with 2-year CNS relapse rates of 0% in the good-risk group versus 6% in the high-risk group, (log-rank *p*-value <0.02, yielding a 2-year HR of 8.73 (95%CI, 0.97-78.26, *p*=0.05). Patients in the UKBlinPredict low-risk group had a non-significant lower 2-year RR risk compared with their matched UKALL2003 controls [0% v 6% (95% CI 4-9), p=0.16]. In contrast, patients in the UKBlinPredict high-risk group had a higher RR compared with their matched UKALL2003 controls [21% (95% CI 11-38) v 8% (4-14), p=0.004].

**Summary/Conclusion**

In our study, relapse following frontline blinatumomab was not predicted by conventional chemotherapy-derived factors. Conversely, early post-blinatumomab MRD response, particularly at C1-END, combined with selected genetic features such as IKZF1plus and *DUX4*-r defined relapse risk more accurately. These findings support a paradigm shift toward blinatumomab-specific risk stratification, in which early response and genetics can guide the intensity of subsequent therapy.

**Copyright Information:** (Online) ISSN: 2572-9241
©2026 The Author(s). HemaSphere is published by John Wiley & Sons Ltd on behalf of European Hematology Association. This is an open access Abstract Book distributed under the Attribution-NonCommercial-NoDerivs (CC BY-NC-ND), which allows third parties to download the articles and share them with others as long as they credit the author and the Abstract Book, but they cannot change the content in any way or use them commercially.
**Abstract Book Citations:** Authors, Title, HemaSphere, 2026;10:(S1):pages. The abstract book DOIs can be found on the "Author Information" page.
**Disclaimer:** Articles published in the journal HemaSphere exclusively reflect the opinions of the authors. The authors are responsible for all content in their abstracts including accuracy of the facts, statements, citing resources, etc.

### (PF386) VENETOCLAX, CLADRIBINE, AND LOW-DOSE CYTARABINE FOR THE TREATMENT OF PH-RELAPSE/REFRACTORY ACUTE LYMPHOBLASTIC LEUKEMIA: AN ANALYSIS OF A PHASE 2 TRIAL

**Topic:** 02. Acute lymphoblastic leukemia - Clinical

Fang-Tong Liu[1], Si-Man Huang[1], Haixia Zhou[1], Mingzhu Xu[1], Zheng Li[1], Zhen Yao[1], Haiping Dai[1], Shengli Xue*[1]

*[1]The First Affiliated Hospital of Soochow University, Department of Hematology, Suzhou, China;*

### Background

Acute lymphoblastic leukaemia(ALL) is a heterogeneous haematological malignancy. Frontline chemotherapy fails in up to two-thirds of high-risk adult B-ALL patients, and while salvage regimens enable allo-HSCT, many patients remain ineligible with poor prognosis. Purine analogs like clofarabine show activity in relapsed/refractory ALL but achieve only 20-40% complete remission rates with remission lasting approximately six months. The selective BCL-2 inhibitor venetoclax is approved for chronic lymphocytic leukemia and acute myeloid leukemia, with promising activity in lymphoid malignancies. Based on shared mechanisms where lymphoid tumors overexpress BCL-2, venetoclax demonstrates activity in ALL. However, monotherapy resistance necessitates combination strategies, leading to increasing incorporation of venetoclax into ALL induction and consolidation regimens.

### Aims

This study was to evaluate the efficiency and safety of venetoclax in combination with cladribine, and low dose cytarabine(CAV) regimen in the treatment of Ph- R/R B-ALL.

### Methods

Between February 2021 and December 2025, 30 patients with relapsed/refractory Ph- B-cell acute lymphoblastic leukemia (R/R B-ALL) were enrolled from multiple centers. Refractory ALL was defined as failure to achieve CR following induction therapy, while relapsed ALL was defined by the reappearance of blasts in peripheral blood, bone marrow (>5%), or any extramedullary site after achieving CR. The CAV regimen consisted of cladribine 5 mg/m²/day intravenously on d1-d5, cytarabine 20 mg subcutaneously every 12 hours on d1-d10, and venetoclax 100 mg orally d1, 200mg d2, and 400mg d3-21. Response assessment was performed according to the 2023 National Comprehensive Cancer Network (NCCN) guidelines.

### Results

A total of 30 patients with a median age of 39 years (range, 15–67) were enrolled. Following one cycle of CAV therapy, the ORR was 70% (21/30), with 18 patients (60%) achieving CR/CRh/CRi and 3 (10%) achieving MLFS and 8 (26.7%) achieved NR. Additionally, among the 3 patients with extramedullary relapse, 2 achieved CR and 1 achieved PR by PET-CT after one cycle of CAV. Among responders, the MFC MRD negative rate was 28.6% (6/21). Of the 8 non-responders to CAV, 1 subsequently achieved remission with chimeric antigen receptor T-cell (CART) therapy, 2 with salvage allogeneic hematopoietic stem cell transplantation (allo-HSCT), 3 received blinatumomab/inotuzumab ozogamicin and 2 received supportive care. Overall, 13 patients underwent allo-HSCT after CAV; 4 experienced post-transplant relapse, of whom 1 underwent a second allo-HSCT, 1 achieved remission with blinatumomab, and 2 received supportive care. The 1-year OS and EFS rates were 61.9% and 56.0%, respectively. With a median follow-up of 18 months (range, 2–45), median OS was not reached and median EFS was 32months. Grade 3–4 hematologic toxicities included granulocytopenia (76.7%), thrombocytopenia (70.0%), and anemia (53.3%). The main grade 3–4 non-hematologic adverse event was infection (10%); grade 1–2 events were predominantly gastrointestinal symptoms (33.3%). No treatment-related mortality occurred. Following CAV, 18 patients remained in sustained remission. Ten patients died: 1 from cerebral hemorrhage, 1 from septicemia and 8 from disease progression. Two patients were lost to follow-up.

**Copyright Information:** (Online) ISSN: 2572-9241
©2026 The Author(s). HemaSphere is published by John Wiley & Sons Ltd on behalf of European Hematology Association. This is an open access Abstract Book distributed under the Attribution-NonCommercial-NoDerivs (CC BY-NC-ND), which allows third parties to download the articles and share them with others as long as they credit the author and the Abstract Book, but they cannot change the content in any way or use them commercially.
**Abstract Book Citations:** Authors, Title, HemaSphere, 2026;10:(S1):pages. The abstract book DOIs can be found on the “Author Information” page.
**Disclaimer:** Articles published in the journal HemaSphere exclusively reflect the opinions of the authors. The authors are responsible for all content in their abstracts including accuracy of the facts, statements, citing resources, etc.

**Summary/Conclusion**

The CAV regimen appears to be safe and effective in patients with Ph- R/R B-ALL, warranting further clinical validation through larger-scale, randomized controlled trials.

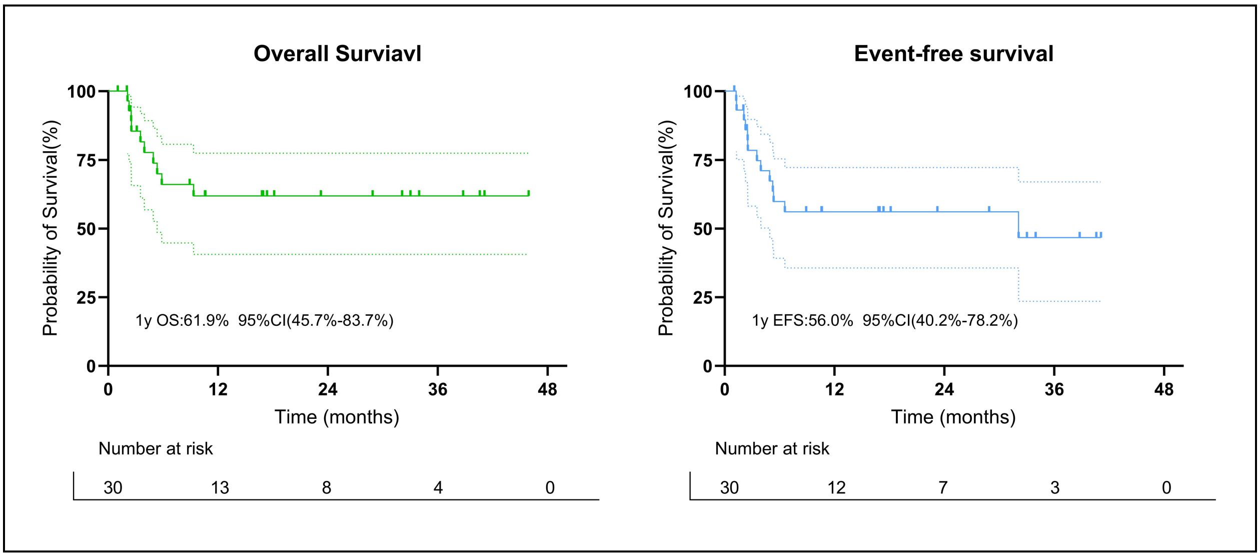

**Copyright Information:** (Online) ISSN: 2572-9241
©2026 The Author(s). HemaSphere is published by John Wiley & Sons Ltd on behalf of European Hematology Association. This is an open access Abstract Book distributed under the Attribution-NonCommercial-NoDerivs (CC BY-NC-ND), which allows third parties to download the articles and share them with others as long as they credit the author and the Abstract Book, but they cannot change the content in any way or use them commercially.
**Abstract Book Citations:** Authors, Title, HemaSphere, 2026;10:(S1):pages. The abstract book DOIs can be found on the "Author Information" page.
**Disclaimer:** Articles published in the journal HemaSphere exclusively reflect the opinions of the authors. The authors are responsible for all content in their abstracts including accuracy of the facts, statements, citing resources, etc.

### (PF387) PREGNANCY-ASSOCIATED ACUTE LYMPHOBLASTIC LEUKEMIA: A FRENCH NATIONWIDE MULTICENTER RETROSPECTIVE COHORT

**Topic:** 02. Acute lymphoblastic leukemia - Clinical

Elise Bandet*[1], Francoise Huguet[2], Marie Balsat[3], Céline Berthon[4], Alice Garnier[5], Jacques Vargaftig[6], Florence Pasquier[7], Carlos Felipe Suarez[8], Pierre Arnautou[9], Laure Farnault/De Lassus[10], Emmanuel Gyan[11], Magalie Joris[12], Celia Salanoubat[13], Marion Simonet Boissard[14], Emmanuelle Tavernier Tardy[15], Madalina Uzunov[16], Anouk Giulianelli[1], Ania Ryngaert[1], Justine De Croocq[1], Véronique Lhéritier[17], Nicolas Boissel[18], Rudy Birsen[1], Florian Chevillon[18]

*[1]Hôpital Cochin APHP, Department of Hematology, Paris, France;[2]Centre Hospitalier Universitaire de Toulouse, Department of Hematology, Toulouse, France; [3]Centre Hospitalier Universitaire Lyon Sud, Department of Hematology, Lyon, France;[4]Centre Hospitalier Universitaire de Lille, Department of Hematology, Lille, France; [5]Centre Hospitalier Universitaire de Nantes, Department of Hematology, Nantes, France; [6]Institut Curie, Department of Hematology, Saint Cloud, France;[7]Gustave Roussy, Department of Hematology, Villejuif, France; [8]Hôpital Necker Enfants Malades, AP-HP, Department of Hematology, Paris, France; [9]Hôpital Percy, Department of Hematology, Clamart, France; [10]Conception University Hospital, Department of Hematology, Marseille, France; [11]Centre Hospitalier Régional Universitaire Bretonneau, Department of Hematology, Tours, France; [12]Centre Hospitalier Universitaire d'Amiens, Department of Hematology, Amiens, France; [13]Centre Hospitalier Sud Francilien, Department of Hematology, Corbeil-Essones, France; [14]Department of Hematology, Centre Hospitalier Universitaire de Besançon, Besançon, France; [15]Department of Hematology, Centre Hospitalier Universitaire de Saint Etienne, Saint Etienne, France;[16]La Pitié Salpetrière, AP-HP, Department of Hematology, Paris, France; [17]Centre Hospitalier Universitaire Lyon Sud, Group for Research on Adult Acute Lymphoblastic Leukemia, Lyon, France; [18]Hôpital Saint Louis, AP-HP, Department of Adolescent and Young Adult Hematology, Paris, France;*

### Background

Pregnancy-associated cancer is defined as malignancy diagnosed during pregnancy or within one year after delivery. Despite major physiologic changes, their impact on cancer biology and outcomes remains unclear. Pregnancy-associated acute lymphoblastic leukemia (ALL) is rare, requires complex multidisciplinary management, and is poorly characterized due to the exclusion of pregnant patients from clinical trials.

### Aims

To describe the clinical characteristics, management, and outcomes of women diagnosed with pregnancy-associated ALL.

### Methods

We conducted a retrospective multicentric cohort study of pregnancy-associated ALL within the GRAALL network. Baseline characteristics, treatment, response, pregnancy course and outcomes were extracted from medical records. Overall survival (OS) and progression-free survival (PFS) were estimated using Kaplan-Meier method.

**Copyright Information:** (Online) ISSN: 2572-9241
©2026 The Author(s). HemaSphere is published by John Wiley & Sons Ltd on behalf of European Hematology Association. This is an open access Abstract Book distributed under the Attribution-NonCommercial-NoDerivs (CC BY-NC-ND), which allows third parties to download the articles and share them with others as long as they credit the author and the Abstract Book, but they cannot change the content in any way or use them commercially.
**Abstract Book Citations:** Authors, Title, HemaSphere, 2026;10:(S1):pages. The abstract book DOIs can be found on the "Author Information" page.
**Disclaimer:** Articles published in the journal HemaSphere exclusively reflect the opinions of the authors. The authors are responsible for all content in their abstracts including accuracy of the facts, statements, citing resources, etc.

**Results**

A total of 34 patients from 14 GRAALL-affiliated centers were identified including 20 diagnosed during pregnancy and 14 within one year postpartum. Diagnosis occurred between 2005 and 2024. The median age at diagnosis was 30 years (range, 18-47). Among patients diagnosed during pregnancy, 2 were diagnosed in the first trimester, 12 in the second trimester, and 8 during the third trimester. Patients diagnosed postpartum were diagnosed at a median of 6.5 months (range, 0.5-12) after delivery.

Patients diagnosed during pregnancy more frequently presented with asymptomatic disease likely reflecting increased routine laboratory monitoring (p=0.011). Twenty-three patients had B-cell ALL. Oncogenetic data were available for 12 patients: 4 had *BCR::ABL1* rearrangement, 2 a *BCR::ABL1*-like profile, 2 *KMT2A* rearrangement, 2 *TCF3::PBX1* rearrangement, 1 hypodiploidy, and 1 hyperdiploidy. Ten patients had T-cell ALL, including 1 early T-cell precursor ALL. According to the four-gene classifier (*NOTCH1, FBXW7, RAS* and *PTEN*), prognosis was favorable in 4 patients, unfavorable in 3, and not available in 3. One patient had mixed-phenotype acute leukemia (T/myeloid) with *KMT2A* rearrangement.

Both patients diagnosed during the first trimester and 4 diagnosed during the second trimester (before 24 weeks of gestation) underwent pregnancy termination prior to chemotherapy initiation. Fourteen patients (8 diagnosed during the second trimester after 24 weeks of gestation and 6 during the third trimester) had live births, including 12 preterm deliveries before 36 weeks, and 4 before 32 weeks. In utero exposure to chemotherapy (daunorubucin and/or vincristin) occurred in 6 cases during the second and third trimester, and 2 fetuses were exposed to tyrosine kinase inhibitors. No congenital malformations were reported. Four women who received L-asparaginase within the first 2 months postpartum developed venous thromboembolism.

Treatment outcomes were consistent with those expected in younger adult ALL populations, with a complete response rate of 94.1% following induction. At 2 years, OS and PFS rates were 79.6% and 70.6% respectively. There was no significant difference in survival between patients diagnosed during pregnancy and those diagnosed postpartum (Figure).

**Summary/Conclusion**

Pregnancy-associated ALL is a rare and challenging condition. In this nationwide cohort, outcomes were comparable to those expected in young adults, with no unexpected fetal safety signals among women diagnosed after 24 weeks of gestation. These findings provide valuable data to inform multidisciplinary care.

**Copyright Information:** (Online) ISSN: 2572-9241
©2026 The Author(s). HemaSphere is published by John Wiley & Sons Ltd on behalf of European Hematology Association. This is an open access Abstract Book distributed under the Attribution-NonCommercial-NoDerivs (CC BY-NC-ND), which allows third parties to download the articles and share them with others as long as they credit the author and the Abstract Book, but they cannot change the content in any way or use them commercially.
**Abstract Book Citations:** Authors, Title, HemaSphere, 2026;10:(S1):pages. The abstract book DOIs can be found on the "Author Information" page.
**Disclaimer:** Articles published in the journal HemaSphere exclusively reflect the opinions of the authors. The authors are responsible for all content in their abstracts including accuracy of the facts, statements, citing resources, etc.

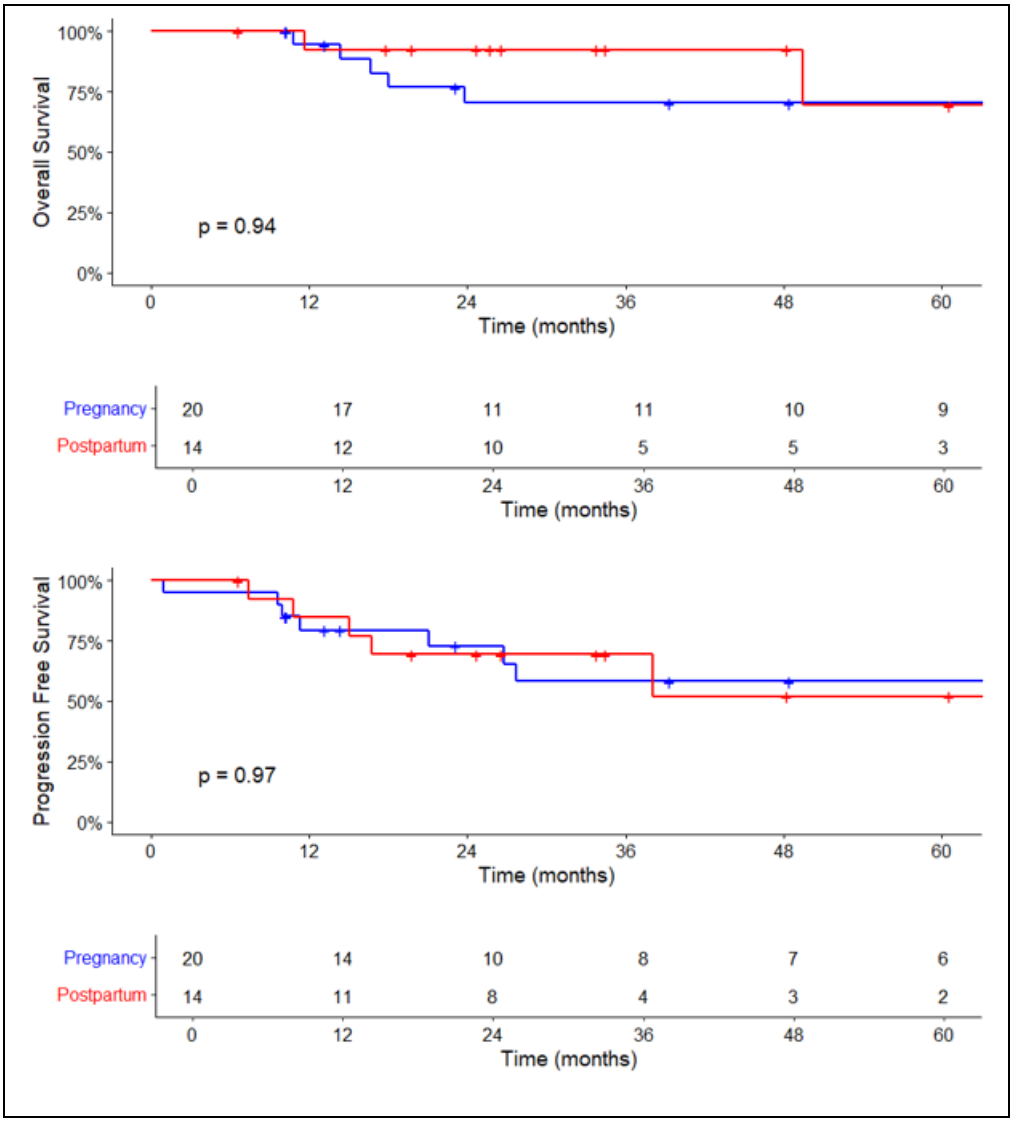

OS and PFS of patients diagnosed during pregnancy and postpartum

**Copyright Information:** (Online) ISSN: 2572-9241
©2026 The Author(s). HemaSphere is published by John Wiley & Sons Ltd on behalf of European Hematology Association. This is an open access Abstract Book distributed under the Attribution-NonCommercial-NoDerivs (CC BY-NC-ND), which allows third parties to download the articles and share them with others as long as they credit the author and the Abstract Book, but they cannot change the content in any way or use them commercially.
**Abstract Book Citations:** Authors, Title, HemaSphere, 2026;10:(S1):pages. The abstract book DOIs can be found on the "Author Information" page.
**Disclaimer:** Articles published in the journal HemaSphere exclusively reflect the opinions of the authors. The authors are responsible for all content in their abstracts including accuracy of the facts, statements, citing resources, etc.

### (PF388) TREATMENT RESPONSE, SURVIVAL AND PROGNOSTIC FACTORS IN ADULT PATIENTS WITH LYMPHOBLASTIC LYMPHOMA TREATED ACCORDING TO PETHEMA ALL PROTOCOLS IN SPAIN

**Topic:** 02. Acute lymphoblastic leukemia - Clinical

Vitor Botafogo*[1], Mireia Morgades[1], Anna Torrent[1], Pau Montesinos[2], Laura Torres Miñana[2], Jose Gonzalez Campos[3], Susana Barrera[4], Mar Tomo[5], Ana Vicent[6], Cristina Gil Cortes[7], Eulalia Genesca[8], Antonio Garcia Guiñon[9], Irene Garcia Cadenas[10], Pere Barba[11], Sergi Camarillas[11], Jordi Ribera[8], Ferran Vall-Llovera Calmet[12], Nuria Martinez-Cibrian[13], Marta Sitges Arriaga[14], Teresa Artola[15], Clara Maluquer[16], Marta Valero[17], Maria Carmen Mateos Rodriguez[18], Jorge Labrador[19], Marta Mateo-Morales[20], Lorenzo Algarra[21], Bernardo Javier González[22], Lucía Gómez-Pérez[23], Maria Angeles Foncillas[24], Ignacio Alberto Gómez Centurión[25], Mónica Cabrero[26], Maria Sole Rodríguez[27], Carlos Grande García[28], Rafael Flores-Cornejo[29], Laura Parrilla[30], Alberto Orfao[4], Jose Maria Ribera[1]

*[1]Institut Català d´Oncologia - Badalona, Hematologia Clínica, Badalona, Spain;[2]Hospital Universitari i Politècnic La Fe, Valencia, Spain; [3]Hospital Universitario Virgen del Rocío, Sevilla, Spain;[4]Servicio De Citometría (NUCLEUS) and Department of Medicine, Centro De Investigación Del Cáncer (IBMCC, University of Salamanca-CSIC) and IBSAL, Salamanca, Spain., Salamanca, Spain; [5]Hospital Clínico Universitario de Valencia, Valencia, Spain; [6]Institut Català d´Oncologia - Tarragona, Tarragona, Spain;[7]Hospital General Universitario Dr. Balmis, Alicante, Spain; [8]Josep Carreras Leukaemia Research Institute (IJC), Universitat Autònoma de Barcelona, Badalona, Spain; [9]Hospital Arnau de Vilanova, Lleida, Spain; [10]Hospital de la Santa Creu i Sant Pau, Barcelona, Spain; [11]Hospital Universitario Vall d´Hebron, Barcelona, Spain; [12]Hospital Universitari Mútua de Terrassa, Terrassa, Spain; [13]Hospital Clínic de Barcelona, Barcelona, Spain;[14]Institut Català d´Oncologia - Girona, Girona, Spain; [15]Hospital Universitario de Donostia, Donostia, Spain; [16]Institut Català d´Oncologia - L´Hospitalet de Llobregat, Barcelona, Spain;[17]Hospital Arnau de Villanova-Llíria, Valencia, Spain; [18]Hospital Universitario de Navarra, Pamplona, Spain; [19]Hospital Universitario de Burgos, Burgos, Spain; [20]Hospital Clínico San Carlos, Madrid, Spain; [21]Hospital General Universitario de Albacete, Albacete, Spain; [22]Hospital Universitario de Canarias, Santa Cruz de Tenerife, Spain;[23]Hospital del Mar, Barcelona, Spain; [24]Hospital Universitario Infanta Leonor, Madrid, Spain; [25]Hospital General Universitario Gregorio Marañón, Madrid, Spain;[26]Hospital Clinico Universitario de Salamanca, Salamanca, Spain; [27]Hospital Universitario Juan Ramón Jiménez, Huelva, Spain;[28]Clinica Universidad de Navarra - Madrid, Madrid, Spain; [29]Hospital Virgen de la Macarena, Sevilla, Spain;[30]Hospital Universitario de Toledo, Toledo, Spain;*

### Background

Lymphoblastic lymphoma (LL) is rare in adults, and most patients are treated according to acute lymphoblastic leukemia (ALL) protocols. Responses are comparable to ALL, but few studies have specifically addressed the outcomes of those patients.

### Aims

To analyze treatment response, survival and prognosis of adult patients with LL treated with three consecutive PETHEMA protocols.

### Methods

We included 105 patients with LL diagnosed between 2003 and 2024 and treated in 40 hospitals in Spain with the national pediatric-inspired PETHEMA ALL HR-03 (n=13), HR-11 (n=37) and ALL-19 (n=55) protocols. Kaplan-Meier, log-rank test and Cox regression (univariate/multivariable) were used for statistical analysis.

**Copyright Information:** (Online) ISSN: 2572-9241
©2026 The Author(s). HemaSphere is published by John Wiley & Sons Ltd on behalf of European Hematology Association. This is an open access Abstract Book distributed under the Attribution-NonCommercial-NoDerivs (CC BY-NC-ND), which allows third parties to download the articles and share them with others as long as they credit the author and the Abstract Book, but they cannot change the content in any way or use them commercially.
**Abstract Book Citations:** Authors, Title, HemaSphere, 2026;10:(S1):pages. The abstract book DOIs can be found on the "Author Information" page.
**Disclaimer:** Articles published in the journal HemaSphere exclusively reflect the opinions of the authors. The authors are responsible for all content in their abstracts including accuracy of the facts, statements, citing resources, etc.

**Results**

Of the 105 patients included, 94 had T-LL (89.5%). Median age (range) at diagnosis was 28 years (20-53) for B-LL and 34 years (15-61) for T-LL. Most patients were male (63.6% in B-LL; 74.5% in T-LL). Median LDH (range in U/L) values for B-LL and T-LL at diagnosis were 234 (140-611) and 344 (120-1853) (*p=0.09*), respectively. T-LL patients showed mediastinal mass in 88.2% of cases vs. 10% in B-LL (*p<0.001*). Bone marrow (BM) infiltration (median %; range) was found in 70% of B-LL (2%; 0-8) and 56.7% of T-LL (1%; 0-19) patients. In T-LL, 6.6% of patients had CNS involvement vs. none in B-LL. Serous involvement was found in 71.4% of T-LL and 12.5% of B-LL patients (*p=0.003*).
After induction-1 (Ind1), B-LL and T-LL patients achieved complete response (CR) by PET scan and/or BM cytology in 72.7% (8/11) and 77.7% (73/94) of cases, respectively. BM MRD by next-generation flow cytometry after Ind-1 was below 0.01% in 90.5% (38/42) of T-LL and 66.7% (4/6) of B-LL patients. PET scan showed metabolic CR for 80% (44/55) of T-LL and 57.1% (4/7) of B-LL after Ind1. Eleven patients with T-LL and 2 with B-LL received a second induction with FLAG-IDA due to positive MRD or failure to Ind1. Overall, CR was achieved in 91.5% of T-LL and 90.9% of B-LL.
The median (range) follow up was 1.4 years (0.2-15.5) with 2-year overall survival (OS) and disease-free survival (DFS) rates of 74% (95%CI: 62-83) and 67% (95%CI: 54 – 77), respectively. According to treatment protocol, 2y OS rates were of 54% (25-76) for HR-03; 64% (41-80) for HR-11 and 92% (79-97) for ALL-19 (*p=0.023*). DFS at 2y was 31% (10-55) for HR-03; 71% (46-86) for HR-11 and 81% (64-91) for ALL-19 (*p=0.002*) (**Figure 1**). There were no survival differences between B-LL and T-LL. In the univariate analysis, only for patients with T-LL, age ≥30y was associated with inferior OS (HR: 3.75; 95%CI: 1.10-12.79) (*p=0.035*) and DFS (HR: 3.25; 1.10-9.61) (*p=0.033*) as well as the use of HR-03 protocol (HR: 5.26; 1.56-17.73, *p=0.007* for OS; and HR: 3.71; 1.43-9.61, *p=0.007* for DFS). Patients without metabolic CR by PET scan after Ind1 had inferior OS (HR: 5.69; 95%CI 1.56-20.69) (*p=0.008*), although the number of patients with available PET data was limited (n=56). Multivariable analysis confirmed the inferior prognostic impact of older age and protocol HR-03 in OS and DFS.

**Summary/Conclusion**

Patients with LL treated with three consecutive PETHEMA ALL protocols showed an improvement in OS and DFS over time, due to lower relapse rate, decreased treatment-related toxicities and protocol changes. For T-LL, young age (<30y) and the use of more recent protocols had a favorable prognostic impact. Metabolic response by PET scan after Ind1 also had prognostic impact despite the low number of cases.

**Copyright Information:** (Online) ISSN: 2572-9241
©2026 The Author(s). HemaSphere is published by John Wiley & Sons Ltd on behalf of European Hematology Association. This is an open access Abstract Book distributed under the Attribution-NonCommercial-NoDerivs (CC BY-NC-ND), which allows third parties to download the articles and share them with others as long as they credit the author and the Abstract Book, but they cannot change the content in any way or use them commercially.
**Abstract Book Citations:** Authors, Title, HemaSphere, 2026;10:(S1):pages. The abstract book DOIs can be found on the “Author Information” page.
**Disclaimer:** Articles published in the journal HemaSphere exclusively reflect the opinions of the authors. The authors are responsible for all content in their abstracts including accuracy of the facts, statements, citing resources, etc.

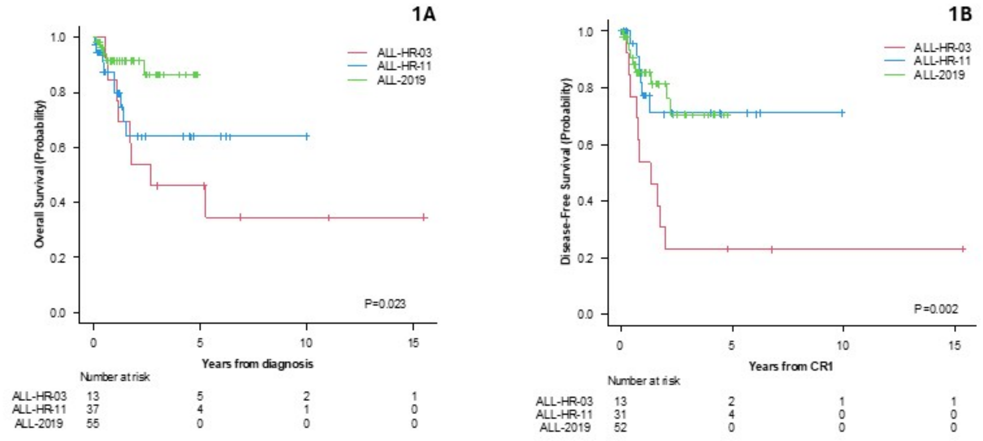

Figure 1: Overall survival (1A) and Disease-Free survival (1B) of LL patients (n=105) according to different PETHEMA ALL treatment protocols.

**Copyright Information:** (Online) ISSN: 2572-9241
©2026 The Author(s). HemaSphere is published by John Wiley & Sons Ltd on behalf of European Hematology Association. This is an open access Abstract Book distributed under the Attribution-NonCommercial-NoDerivs (CC BY-NC-ND), which allows third parties to download the articles and share them with others as long as they credit the author and the Abstract Book, but they cannot change the content in any way or use them commercially.
**Abstract Book Citations:** Authors, Title, HemaSphere, 2026;10:(S1):pages. The abstract book DOIs can be found on the "Author Information" page.
**Disclaimer:** Articles published in the journal HemaSphere exclusively reflect the opinions of the authors. The authors are responsible for all content in their abstracts including accuracy of the facts, statements, citing resources, etc.

### (PF389) EFFICACY AND SAFETY OF OLVEREMBATINIB COMBINED WITH LISAFTOCLAXIN PEDIATRIC PATIENTS WITH RELAPSED/REFRACTORY PHILADELPHIA CHROMOSOME–POSITIVE ACUTE LYMPHOBLASTIC LEUKEMIA: A PHASE 1B STUDY

**Topic:** 02. Acute lymphoblastic leukemia - Clinical

Jingliao Zhang*[1], Huanshan Guo[2], Zi Chen[2], Xiaojuan Chen[1], Yao Zou[1], Wenyu Yang[2], Yifan Zhai[2], Xiaofan Zhu[1]

*[1]State Key Laboratory of Experimental Hematology, Institute of Hematology & Blood Diseases Hospital, Chinese Academy of Medical Sciences & Peking Union Medical College, Tianjin, China; [2]Guangzhou Healthquest Pharma Co., Ltd., Guangzhou, China;*

### Background

Philadelphia chromosome–positive acute lymphoblastic leukemia (Ph+ ALL) in pediatric patients remains a high-risk subtype with limited treatment options following tyrosine kinase inhibitor (TKI) failure. Olverembatinib is a potent third-generation TKI with activity against *BCR::ABL1* genetic variants, including T315I. Lisaftoclax, a selective BCL-2 inhibitor, has shown encouraging activity in hematologic malignancies.

### Aims

This phase 1b study evaluates the safety, tolerability, preliminary efficacy, and pharmacokinetics (PK) of olverembatinib combined with lisaftoclax in pediatric patients with relapsed/refractory (R/R) Ph+ ALL.

### Methods

This open-label, dose-escalation and expansion study (NCT05495035) enrolled patients aged <18 years with R/R Ph+ ALL resistant or intolerant to ≥1 prior TKI using a 3+3 design. Patients received olverembatinib monotherapy (40 mg adult-equivalent dose [AED] every other day, days 1–14), followed by combination therapy with lisaftoclax (200, 400, or 600 mg AED daily, with 3-day dose ramp-up) and dexamethasone (6 mg/m²/day, days 15–42). Primary endpoints included safety, overall response rate (ORR), cytogenetic and molecular responses, cytometric measurable residual disease (MRD) negativity, and PK. Data are reported through February 4, 2025.

**Copyright Information:** (Online) ISSN: 2572-9241
©2026 The Author(s). HemaSphere is published by John Wiley & Sons Ltd on behalf of European Hematology Association. This is an open access Abstract Book distributed under the Attribution-NonCommercial-NoDerivs (CC BY-NC-ND), which allows third parties to download the articles and share them with others as long as they credit the author and the Abstract Book, but they cannot change the content in any way or use them commercially.
**Abstract Book Citations:** Authors, Title, HemaSphere, 2026;10:(S1):pages. The abstract book DOIs can be found on the "Author Information" page.
**Disclaimer:** Articles published in the journal HemaSphere exclusively reflect the opinions of the authors. The authors are responsible for all content in their abstracts including accuracy of the facts, statements, citing resources, etc.

### Results

Seventeen patients received treatment (200 mg, n=4; 400 mg, n=4; 600 mg, n=9). Median age was 13.0 years (range 9–15). Most patients (93.3%) had p190 transcripts. 40% (6/15) harbored *ABL1* kinase domain variants, including T315I (n=4). Sixteen patients received lisaftoclax combination therapy and completed two treatment courses.
Grade ≥3 treatment-related adverse events (TRAEs) occurred in 64.7% (n=11). The most common grade ≥3 TRAEs were neutrophil count decreased (41.2%), anemia (17.6%), and platelet count decreased (17.6%). Hepatic function abnormalities occurred in 41.2% (any grade), with one serious adverse event reported (5.9%). Two patients (11.8%) discontinued due to adverse events (one seizure on C1D1; one hepatic toxicity). No treatment-related deaths occurred.
Among nine patients evaluable for morphologic response, ORR (complete remission [CR] + CR with incomplete count recovery [CRi]) was 55.6% at the end of olverembatinib monotherapy (EOM). By end of combination therapy (C2D27), ORR increased to 88.9% (8/9; all CR). Complete cytogenetic remission (by FISH) increased from 40.0% (4/10) at EOM to 88.9% (8/9) at C2D15. Cytometric MRD negativity increased from 25.0% (3/12) at EOM to 66.7% (8/12) at C2D15 and C2D28. By quantitative PCR, major molecular response or better was achieved in 93.3% (14/15) by C2D28, with complete molecular response in 20.0% (3/15). Responses occurred across dose levels and in patients with baseline *ABL1* variants.
PK data demonstrated comparable exposure to adult profiles for both agents, predictable exposure without significant drug-drug interactions or accumulation. Both agents were detectable in cerebrospinal fluid, indicating CNS penetration.

### Summary/Conclusion

Olverembatinib combined with lisaftoclax is a chemotherapy-free regimen that induces rapid, deep, and high-quality remissions in pediatric R/R Ph+ ALL with manageable toxicity. The profound molecular response rates—including activity against *ABL1* variants and evidence of CNS penetration—position this fully oral dual-targeted regimen as a highly promising strategy for this high-risk population.

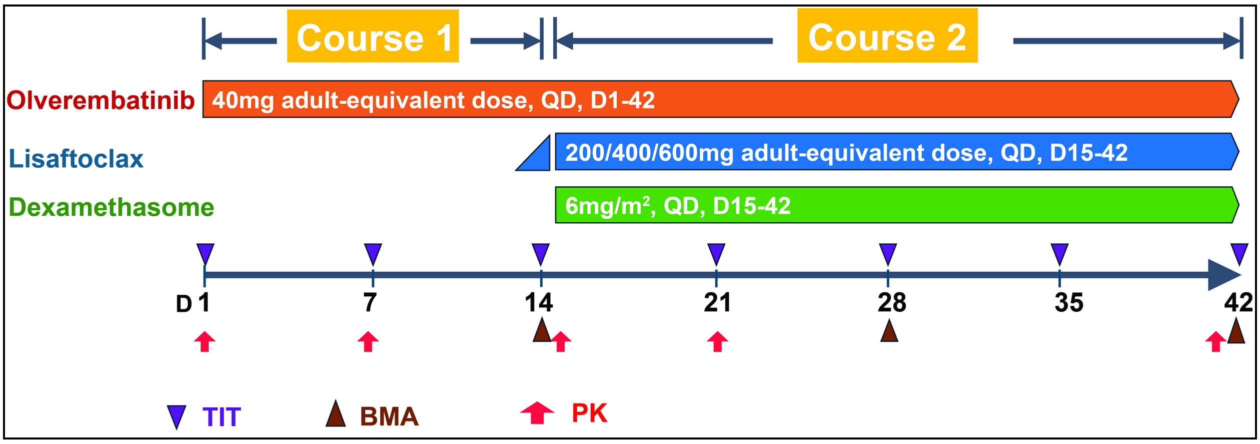

Schematic Protocol of HQP1351ACE01 Trial

Copyright Information: (Online) ISSN: 2572-9241
©2026 The Author(s). HemaSphere is published by John Wiley & Sons Ltd on behalf of European Hematology Association. This is an open access Abstract Book distributed under the Attribution-NonCommercial-NoDerivs (CC BY-NC-ND), which allows third parties to download the articles and share them with others as long as they credit the author and the Abstract Book, but they cannot change the content in any way or use them commercially.
Abstract Book Citations: Authors, Title, HemaSphere, 2026;10:(S1):pages. The abstract book DOIs can be found on the "Author Information" page.
Disclaimer: Articles published in the journal HemaSphere exclusively reflect the opinions of the authors. The authors are responsible for all content in their abstracts including accuracy of the facts, statements, citing resources, etc.

### (PF390) CHEMOTHERAPY-FREE REGIMEN FOLLOWING PHALLCON CYTOREDUCTION IN ADULTS WITH NEWLY DIAGNOSED PHILADELPHIA POSITIVE ACUTE LYMPHOBLASTIC LEUKEMIA

**Topic:** 02. Acute lymphoblastic leukemia - Clinical

Jose Tinajero[1], Paul Koller[1], Dubi Yoo[1], Tamer Othman[1], Vaibhav Agrawal[1], Hoda Pourhassan[1], Dat Ngo[1], Yazeed Samara[1], Amanda Blackmon[1], Pamela S. Becker[1], Lindsey Murphy[1], Andrew Artz[1], Amandeep Salhotra[1], Monzr Al Malki[1], Stephen Forman[1], Anthony Stein[1], Guido Marcucci[1], Vinod Pullarkat[1], Ibrahim Aldoss*[1]

*[1]City of Hope, Duarte, United States of America;*

### Background

Chemotherapy-free regimens have demonstrated encouraging safety and efficacy in newly diagnosed adults with Ph+ ALL. Unlike established approaches that administer blinatumomab (blina) and ponatinib either upfront without sufficient cytoreduction (Kantarjian et al. JCO 2024) or only after three months of ponatinib plus steroids (Chiaretti et al ASH 2025), our strategy introduces blinatumomab following a single cycle of low-intensity induction consistent with PhALLCON induction. This approach aims to reduce toxicity in the setting of active disease while ensuring that effective immunotherapy is initiated early without unnecessary delay.

### Aims

Describe the safety and efficacy of integrating blinatumomab and TKI consolidation following PhALLCON cytoreduction

### Methods

This retrospective analysis included adult patients ≥18 years (yrs) with newly diagnosed Ph+ B-ALL who were treated according to the PhALLCON induction and initiated blina as consolidation therapy in CR1 at the City of Hope between February 2020 until December 2025. The study objectives include the safety of sequential PhALLCON and blina, and to describe early survival outcomes, relapse-rate, MRD and molecular MRD negativity, and allogeneic transplant (HCT) rate in CR1. MRD negativity was defined as achieving <0.01% leukemic cells of total nucleated cells by flow cytometry and/or PCR (molecular CR).

### Results

Forty-four adults were treated at our institution. The median age was 56 (range: 25-81) yrs, including 41% ≥ 60 yrs. 27% (n=12) had IKZF1plus and 8 (18%) had VPREB1 deletion. The majority (86%) of patients received one cycle of PhALLCON induction. Median WBC at presentation was 27.5k and 36% had WBC >50k. The median number of administered IT chemo was 9 (range: 3-16) and 9 (21%) patients had intermediate dose systemic methotrexate. The CR/CRi after PhALLCON induction was 100% with molecular and flow MRD negativity rates at 36% and 57%, respectively. Three (7%) patients changed from ponatinib to another TKI due to treatment toxicities after induction. Molecular and flow MRD negativity rates after the first cycle of blina were 74% and 100% respectively with no grade ≥3 cytokine release syndrome (CRS) or neurotoxicity. All grade CRS and neurotoxicity were 9% and 2% respectively, with no patients permanently discontinued blina. 36% of patients underwent HCT in CR1. With a median follow up of 16.7 months, the estimated median overall survival was 41.3 months (95% CI: 41.3 – NR), with one death occurring post HCT and one relapse (2.3%). The only relapse was in the marrow and manifested with preexisting CRLF2::IGH translocation without BCR::ABL1 after alloHCT.

**Copyright Information:** (Online) ISSN: 2572-9241
©2026 The Author(s). HemaSphere is published by John Wiley & Sons Ltd on behalf of European Hematology Association. This is an open access Abstract Book distributed under the Attribution-NonCommercial-NoDerivs (CC BY-NC-ND), which allows third parties to download the articles and share them with others as long as they credit the author and the Abstract Book, but they cannot change the content in any way or use them commercially.
**Abstract Book Citations:** Authors, Title, HemaSphere, 2026;10:(S1):pages. The abstract book DOIs can be found on the “Author Information” page.
**Disclaimer:** Articles published in the journal HemaSphere exclusively reflect the opinions of the authors. The authors are responsible for all content in their abstracts including accuracy of the facts, statements, citing resources, etc.

**Summary/Conclusion**

Our early experience indicates that incorporating blina shortly after induction using a lower intensity PhALLCON regimen is safe and effective. This approach allows early introducing blina, facilitates rapid MRD eradication in adults with Ph+ ALL and minimizes blina-related toxicity.

**Table 1. Patient, Disease and Treatment Characteristics**

| | (n=44) |
|---|---|
| **Gender, n (%)**<br>Male | <br>20 (45.5) |
| **Median (Range) Age at Baseline (years)**<br>≥60 years old, n (%) | 55.5 (25-81)<br>18 (40.9) |
| **Race/Ethnicity, n (%)**<br>Hispanic<br>Caucasian<br>Asian<br>Other | <br>31 (70.5)<br>11 (25)<br>2 (4.5)<br>1 (2.3) |
| **Median (range) BMI at Baseline**<br>BMI ≥ 30, n (%) | 27.9 (18-45)<br>19 (43.2) |
| **Median (range) White Blood Cell Count at Diagnosis**<br>WBC > 50k/uL | 27.5 (0.61-482)<br>16 (36.4) |
| **AlloHCT in CR1, n (%)** | 16 (36.4) |
| **Relapse Rate, n (%)** | 1 (2.3) |
| **Median Cycles of PhaLLCON induction (range)**<br>PhALLCON for One Cycle | 1 (1-3)<br>38 (86.4) |
| **Molecular (BCR-ABL < 0.01%) Negativity after Induction** | 16 (36.4) |
| **Measurable Residual Disease Negativity after Induction** | 25 (56.8) |
| **Median Cycles of Blinatumomab (range)** | 3 (1-5) |
| **Received Blinatumomab as First Consolidation After Induction** | 40 (90.9) |
| **Molecular (BCR-ABL < 0.01%) Negativity after Blinatumomab** | 17/23 (73.9) |
| **Measurable Residual Disease Negativity after Blinatumomab** | 17/17 (100) |
| **All Grade CRS from Blinatumomab**<br>Grade 3 or higher | 4 (9.1)<br>0 (0) |
| **All Grade Neurotoxicity from Blinatumomab**<br>Grade 3 or higher | 1 (2.3)<br>0 (0) |

**Copyright Information:** (Online) ISSN: 2572-9241
©2026 The Author(s). HemaSphere is published by John Wiley & Sons Ltd on behalf of European Hematology Association. This is an open access Abstract Book distributed under the Attribution-NonCommercial-NoDerivs (CC BY-NC-ND), which allows third parties to download the articles and share them with others as long as they credit the author and the Abstract Book, but they cannot change the content in any way or use them commercially.
**Abstract Book Citations:** Authors, Title, HemaSphere, 2026;10:(S1):pages. The abstract book DOIs can be found on the "Author Information" page.
**Disclaimer:** Articles published in the journal HemaSphere exclusively reflect the opinions of the authors. The authors are responsible for all content in their abstracts including accuracy of the facts, statements, citing resources, etc.

### (PF391) DYNAMIC TRAJECTORIES AND INFLUENCING FACTORS OF CANCER-RELATED FATIGUE IN PATIENTS WITH ACUTE LEUKEMIA DURING INDUCTION CHEMOTHERAPY: A LONGITUDINAL STUDY

**Topic:** 02. Acute lymphoblastic leukemia - Clinical

Lu Tang*[1], Xin Du[1]

[1]*Xinqiao Hospital of Army Medical University, Chong, China;*

### Background

Acute leukemia is a malignant clonal disease originating from hematopoietic stem cells, with an increasing incidence year by year. Induction chemotherapy is a key treatment phase for patients with acute leukemia to achieve complete remission, but intense chemotherapy regimens often lead to a significant symptom burden. Cancer-related fatigue (CRF) is one of the most common symptoms in patients with acute leukemia, with an incidence rate of over 80%, severely affecting their functional status and quality of life. However, current research on the dynamic changes and influencing factors of fatigue in acute leukemia patients during induction chemotherapy is still insufficient, especially lacking systematic exploration based on longitudinal design.

### Aims

To describe the dynamic changes in cancer-related fatigue in patients with acute leukemia during induction chemotherapy, identify different subgroups of fatigue development, and explore the sociodemographic, clinical characteristics, and psychosocial factors affecting the fatigue trajectory.

### Methods

A prospective longitudinal study design was adopted, conveniently selecting acute leukemia patients who first received induction chemotherapy in the hematology departments of two tertiary hospitals from July 2024 to May 2025. Follow-up surveys will be conducted at five time points: before chemotherapy (T0), during the first week of chemotherapy (T1), the second week (T2), the third week (T3), and before discharge (T4). A general information questionnaire was used to collect demographic and clinical information, and the Brief Fatigue Inventory was used to assess cancer-related fatigue, along with evaluations of anxiety, depression, sleep quality, and social support levels. Using latent variable growth mixture modeling to identify heterogeneous trajectories of fatigue, and employing multivariate logistic regression to analyze the predictive factors of different trajectory subgroups.

### Results

A total of 275 patients with acute leukemia were included, with a mean age of 56.02±13.96 years . CRF scores showed an initial increase followed by a decrease during induction chemotherapy, peaking at the second week and then gradually relieving. Latent growth mixture modeling identified three heterogeneous fatigue trajectories: persistent high fatigue group (30.7%), moderate fatigue relief group (38.6%), and low fatigue stable group (30.7%). Multivariate analysis showed that female gender (OR=2.34, 95%CI: 1.28-4.28), hemoglobin level <80g/L at initial treatment (OR=3.12, 95%CI: 1.67-5.83), anxiety symptoms (OR=2.78, 95%CI: 1.52-5.09), and sleep disturbance (OR=2.45, 95%CI: 1.36-4.42) were independent predictors of the persistent high fatigue trajectory (all P<0.05), while high social support level (OR=0.31, 95%CI: 0.15-0.64) was a protective factor for fatigue relief.

**Copyright Information:** (Online) ISSN: 2572-9241
©2026 The Author(s). HemaSphere is published by John Wiley & Sons Ltd on behalf of European Hematology Association. This is an open access Abstract Book distributed under the Attribution-NonCommercial-NoDerivs (CC BY-NC-ND), which allows third parties to download the articles and share them with others as long as they credit the author and the Abstract Book, but they cannot change the content in any way or use them commercially.
**Abstract Book Citations:** Authors, Title, HemaSphere, 2026;10:(S1):pages. The abstract book DOIs can be found on the "Author Information" page.
**Disclaimer:** Articles published in the journal HemaSphere exclusively reflect the opinions of the authors. The authors are responsible for all content in their abstracts including accuracy of the facts, statements, citing resources, etc.

**Summary/Conclusion**

CRF in patients with acute leukemia during induction chemotherapy exhibits heterogeneous developmental trajectories, with approximately one-third of patients experiencing persistently high levels of fatigue. Female gender, anemia, anxiety, and sleep disturbance are risk factors for fatigue, while social support plays a protective role. Clinical nursing should focus on early identification of high-risk populations, strengthen symptom management during the fatigue peak at the second week of chemotherapy, and provide targeted psychological support and sleep interventions to reduce fatigue burden and improve patients' quality of life.

**Copyright Information:** (Online) ISSN: 2572-9241
©2026 The Author(s). HemaSphere is published by John Wiley & Sons Ltd on behalf of European Hematology Association. This is an open access Abstract Book distributed under the Attribution-NonCommercial-NoDerivs (CC BY-NC-ND), which allows third parties to download the articles and share them with others as long as they credit the author and the Abstract Book, but they cannot change the content in any way or use them commercially.
**Abstract Book Citations:** Authors, Title, HemaSphere, 2026;10:(S1):pages. The abstract book DOIs can be found on the "Author Information" page.
**Disclaimer:** Articles published in the journal HemaSphere exclusively reflect the opinions of the authors. The authors are responsible for all content in their abstracts including accuracy of the facts, statements, citing resources, etc.

### (PF392) OUTCOME OF SECOND-LINE THERAPY IN ADULT B-CELL PHILADELPHIA CHROMOSOME NEGATIVE ACUTE LYMPHOBLASTIC LEUKEMIA

**Topic:** 02. Acute lymphoblastic leukemia - Clinical

Wei-Ying Jen*[1], Elias Jabbour[1], Eitan Kugler[1], Wei Qiao[1], Nitin Jain[1], Nicholas Short[1], Rebecca Garris[1], Sherry Pierce[1], Partow Kebriaei[1], Sa Wang[1], Fadi Haddad[1], Farhad Ravandi[1], Xuelin Huang[1], Hagop Kantarjian[1]

[1]*The University of Texas MD Anderson Cancer Center, Houston, United States of America;*

### Background

Historic data show that the outcomes of relapsed B-ALL are associated with complete response (CR) rates of 40% and 3y overall survival (OS) rates of <10%. How novel therapies have influenced these outcomes has not been defined.

### Aims

Define the predictors of prognosis and outcome of Ph-neg B-ALL in first salvage, in the era of novel therapies.

### Methods

This was a retrospective, single center cohort study of pts aged ≥18y treated from 2000-2024. Pts with relapsed/refractory (RR) Ph-neg B-ALL were identified and included if they had one prior line of therapy. Logistic regression was used to determine the odds of CR, while the Cox proportional hazards model was used for the hazard ratio (HR) of death. Variables with a *p* value <0.1 on univariate analysis were included in a multivariate model.

### Results

372 pts were included, median age 40y (range,18-87) with 20% aged ≥60y. Karyotype was 31% diploid, 25% adverse (complex (≥5 abn), low hypodiploidy/near-triploidy or *KMT2A*r), 44% others. 14% were primary refractory and 34% had a CR1 duration of <12mo. Salvage therapy was chemo only in 43%, chemo + inotuzumab (ino) ± blinatumomab (blina, ChemoInoBlina) in 43%, ino ± blina (InoBlina) in 14% and others in 11%.
The overall CR rate was 70%. Type of therapy was significantly associated with improved odds of CR (CR rates: ChemoInoBlina 96%, chemo 66%, InoBlina 55%, others 23%, *p*<0.0001). Univariate analysis identified the following variables to be associated with worse OR for CR: age ≥40, ECOG performance status (PS) 2-4, adverse karyotype, platelets (plts) <$20x10^9$/L, peripheral blood (PB) blasts >20%, LDH >ULN and CR1 duration <12mo. On multivariate analysis, thrombocytopenia and adverse karyotype were independently adverse. Importantly, ChemoInoBlina was the most important independent variable associated with higher odds of CR (OR for ChemoInoBlina 4.6 (95%CI, 2. 5-9.1) vs chemo, *p* <0.0001).
The median OS was 12 months, with a 3-year OS rate of 30%. 3y OS rates were 40% for pts age <40y, 21% for 40-59y, 21% for 60-69y, and 17% for age ≥70y. Overall survival stratified by type of therapy is shown in the **Figure**. Pts treated with ChemoInoBlina had the best 3y OS of 52%, compared with 22% for chemo, 20% for InoBlina and 13% for others, *p*<0.0001. Type of therapy was strongly associated with the time period of therapy and with patient age. On univariate analysis, the same variables which were associated with a higher odds of CR were associated with improved OS. On multivariate analysis, age ≥40y (HR 1.72), CR1 <12mo or refractory disease (HR 2.01) and adverse karyotype (HR 1.83 vs diploid) were independently associated with a higher hazard of death. Treatment with ChemoInoBlina was independently associated with a lower hazard of death (HR 0.35 [95%CI, 0.25-0.49], *p*<0.001 vs chemo). This was strongly correlated with improved OS rates over time, with 3y OS rates of 18% in 2000-2009, 27% in 2010-2016, and 46% in 2017-2024.

**Copyright Information:** (Online) ISSN: 2572-9241
©2026 The Author(s). HemaSphere is published by John Wiley & Sons Ltd on behalf of European Hematology Association. This is an open access Abstract Book distributed under the Attribution-NonCommercial-NoDerivs (CC BY-NC-ND), which allows third parties to download the articles and share them with others as long as they credit the author and the Abstract Book, but they cannot change the content in any way or use them commercially.
**Abstract Book Citations:** Authors, Title, HemaSphere, 2026;10:(S1):pages. The abstract book DOIs can be found on the "Author Information" page.
**Disclaimer:** Articles published in the journal HemaSphere exclusively reflect the opinions of the authors. The authors are responsible for all content in their abstracts including accuracy of the facts, statements, citing resources, etc.

**Summary/Conclusion**

The approval of ino and blina and its incorporation with multiagent chemo has been associated with improved outcomes in RR Ph-neg B-ALL. This is independent of traditional prognostic factors like karyotype and CR1 duration. This study establishes important benchmarks against which future therapies and expectations can be assessed.

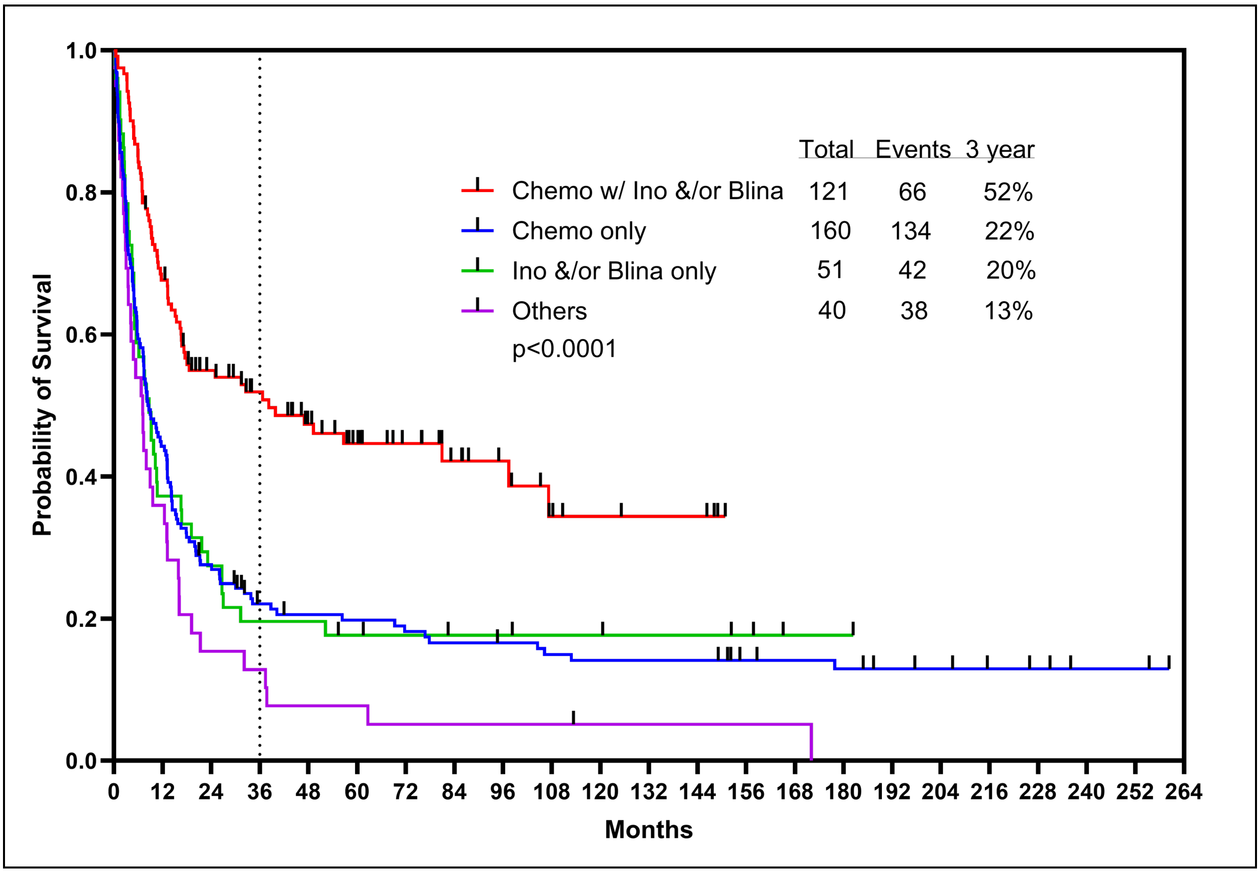

**Copyright Information:** (Online) ISSN: 2572-9241
©2026 The Author(s). HemaSphere is published by John Wiley & Sons Ltd on behalf of European Hematology Association. This is an open access Abstract Book distributed under the Attribution-NonCommercial-NoDerivs (CC BY-NC-ND), which allows third parties to download the articles and share them with others as long as they credit the author and the Abstract Book, but they cannot change the content in any way or use them commercially.
**Abstract Book Citations:** Authors, Title, HemaSphere, 2026;10:(S1):pages. The abstract book DOIs can be found on the "Author Information" page.
**Disclaimer:** Articles published in the journal HemaSphere exclusively reflect the opinions of the authors. The authors are responsible for all content in their abstracts including accuracy of the facts, statements, citing resources, etc.

### (PF393) UPDATED RESULTS FROM THE PHASE II STUDY OF DUAL EPIGENETIC TARGETING WITH CHIDAMIDE AND AZACITIDINE IN PATIENTS WITH HIGH-RISK ACUTE MYELOID LEUKEMIA AFTER ALLO-HSCT

**Topic:** 02. Acute lymphoblastic leukemia - Clinical

Yaxue Wu[1], Depei Wu[1], Shengli Xue[1], Xiao-Hui Hu*[1]

[1] *The First Affiliated Hospital of Soochow University, Soochow, China;*

### Background

Allogeneic hematopoietic stem cell transplantation (allo-HSCT) is potentially the only curative option for high-risk acute myeloid leukemia (AML) patients. However, disease relapse remains the principal cause of treatment failure of these patients, and outcomes of salvage treatments are poor. This research seeks to evaluate the efficacy and safety of a dual epigenetic targeting maintenance therapy with chidamide and azacitidine (AZA) in patients with high-risk AML post-allo-HSCT.

### Aims

To evaluate the efficacy and safety of a dual epigenetic targeting maintenance therapy with chidamide and azacitidine (AZA) in patients with high-risk AML post-allo-HSCT.

### Methods

This multicenter, open-label, phase 2 prospective clinical trial (ChiCTR2300067593) recruited and followed up 52 patients diagnosed with high-risk AML post-allo-HSCT from 3 hospitals in China from November 2021 to October 2024. Chidamide (5 mg) was administered orally once daily for 5 days, combined with AZA (75 mg/m2 ) subcutaneously daily for 5 days, respectively. Treatment started as early as 3 months after transplantation. All patients were in complete remission before each maintenance cycle. A total of 6 cycles was recommended.

### Results

The median age of the patients was 39 years (range, 17-60).  As of the cutoff date for the final survival analysis on January 8, 2026, the median follow-up time was 38.8 (12.6-56.5) months, the 3-year CIR was 7.7 (2.6-9.0) %, the 3-year OS and RFS rates were 91.4 (87.2-95.6)% and 90.4 (86.3-94.5) %, respectively. Four patients relapsed: one declined intensive treatment and died; two relapsed post-second transplantation and died; one remains alive in remission following second transplantation. The most common AE was hematological toxicity, with an incidence of 86.5% (45/52). However, most patients experienced only mild bone marrow suppression, which required either outpatient administration of hematopoietic growth factors or simple observation without medication. Only 11 (21.2%) patients received platelet transfusions, and 1 (1.9%) patient received red blood cell transfusions. Among the 6 infected patients, 4 had pulmonary infections, including 1 patient with EB viremia reactivation. Additionally, 1 patient had an intestinal infection following unclean eating, and 1 had cytomegaloviremia. All AEs were tolerable with active treatment and did not affect subsequent therapy. No grade>3 AEs were noted. Overall, 15 (28.8%) patients developed cGVHD and only 1 of them developed aGVHD. One patient died of lung GVHD, and non-relapse mortality (NRM) was 1.9 (1/52) %.

### Summary/Conclusion

The results of our study suggested that dual epigenetic targeting maintenance treatment with CHI-AZA has an acceptable toxicity profile and might potentially be effective to prevent relapse after allo HSCT.

**Copyright Information:** (Online) ISSN: 2572-9241
©2026 The Author(s). HemaSphere is published by John Wiley & Sons Ltd on behalf of European Hematology Association. This is an open access Abstract Book distributed under the Attribution-NonCommercial-NoDerivs (CC BY-NC-ND), which allows third parties to download the articles and share them with others as long as they credit the author and the Abstract Book, but they cannot change the content in any way or use them commercially.
**Abstract Book Citations:** Authors, Title, HemaSphere, 2026;10:(S1):pages. The abstract book DOIs can be found on the "Author Information" page.
**Disclaimer:** Articles published in the journal HemaSphere exclusively reflect the opinions of the authors. The authors are responsible for all content in their abstracts including accuracy of the facts, statements, citing resources, etc.

### (PF394) OUTCOME OF SECOND-LINE THERAPY IN ADULT B-CELL PHILADELPHIA CHROMOSOME POSITIVE ACUTE LYMPHOBLASTIC LEUKEMIA

**Topic:** 02. Acute lymphoblastic leukemia - Clinical

Wei-Ying Jen*[1], Elias Jabbour[1], Eitan Kugler[1], Rebecca Garris[1], Sherry Pierce[1], Nitin Jain[1], Nicholas Short[1], Issa Khouri[1], Sanam Loghavi[1], Fadi Haddad[1], Farhad Ravandi[1], Hagop Kantarjian[1]

[1] *The University of Texas MD Anderson Cancer Center, Houston, United States of America;*

### Background

The approval of *BCR::ABL* tyrosine kinase inhibitors (TKIs) and blinatumomab (blina) have improved outcomes in Ph-positive B-ALL. However, a proportion of patients still relapse, and optimal salvage therapies have yet to be defined.

### Aims

Define the contemporary predictors of prognosis and outcome of Ph-pos B-ALL in first salvage.

### Methods

This was a retrospective, single center cohort study of pts aged ≥16y treated from 1992-2025. Pts with relapsed/refractory (RR) Ph-pos B-ALL were identified and included if they had one prior line of therapy. Logistic regression was used to determine the odds of CR, while the Cox proportional hazards model was used for the hazard ratio (HR) of death. Variables with a *p* value <0.1 on univariate analysis were included in a multivariate model.

### Results

166 patients were identified and included. The median age was 47y (range, 17-78); 24% were age ≥60y. Baseline white blood cell count (WBC) was ≥50x10$^9$/L in 14%, while 51% had additional cytogenetic abnormalities. The predominant *BCR::ABL* transcript was p190 in 74% and p210 in 26%. 10% of pts were primary refractory, 34% had CR1 duration <12mo, with the remaining 56% having CR1 >12mo or refractory disease. 7% of pts were received salvage containing a first-generation (1G) imatinib, 46% with a second-generation TKI (nilotinib, dasatinib or bosutinib, 2G) and 26% with a third-generation TKI (ponatinib, olverambatinib or asciminib, 3G). the remaining 14% were treated with salvage regimens without TKI.

The overall CR rate was 80%. By univariate analysis bone marrow (BM) blasts ≥50% (OR 0.4 *p*=0.03), platelets (plt) <20x10$^9$/L (OR 0.32 vs plt >100x10$^9$/L, *p*=0.02), CR1 duration <12mo (OR 0.49 vs CR1 >12mo or refractory. *p*=0.03) were associated with a lower odds of CR. The addition of TKI was associated with the strongest odds of attaining CR (OR 66.0 for 1G, 66.0 for 2G and 72.6 for 3G, *p*<0.0001). By multivariate analysis, only the addition of TKI remained independently associated with better odds of CR compared with no TKI (1G OR 65.0, *p*=0.0012; 2G OR 81.2, *p*<0.0001, 3G OR 84.9, *p*<0.0001).

The median overall survival (OS) was 15mo; the 3y OS rate was 31%. 3y OS was 58% with 3G TKI combinations, 38% with 2G TKIs, 8% with 1G TKIs, and 0% without TKIs (**Figure**). Survival was also worse with older age (3y OS 57% for age <40 vs 31% for age 40-59, 25% for age 60-69, 32% age ≥70, *p*=0.008), presenting WBC ≥50x10$^9$/L and CR1 duration <12mo. By multivariate analysis only two variables were independently predictive of worse survival: no TKI therapy and WBC ≥50x10$^9$/L (HR1.86, *p*=0.02). Compared with no TKI therapy, TKIs were associated with reduced hazards of death: HR 0.37 (95%CI, 0.17-0.83, *p*=0.016) for 1G, HR 0.19 (95%CI, 0.11-0.34, *p*<0.001) for 2G, and HR 0.10 (95%CI, 0.05-0.20, *p*<0.001) for 3G. This correlated with improved 3y OS rates over time: 0% before 2000, 11% from 2000-2009, 39% from 2010-2019, and 71% from 2020-2025.

**Copyright Information:** (Online) ISSN: 2572-9241
©2026 The Author(s). HemaSphere is published by John Wiley & Sons Ltd on behalf of European Hematology Association. This is an open access Abstract Book distributed under the Attribution-NonCommercial-NoDerivs (CC BY-NC-ND), which allows third parties to download the articles and share them with others as long as they credit the author and the Abstract Book, but they cannot change the content in any way or use them commercially.
**Abstract Book Citations:** Authors, Title, HemaSphere, 2026;10:(S1):pages. The abstract book DOIs can be found on the "Author Information" page.
**Disclaimer:** Articles published in the journal HemaSphere exclusively reflect the opinions of the authors. The authors are responsible for all content in their abstracts including accuracy of the facts, statements, citing resources, etc.

**Summary/Conclusion**

The approval of *BCR::ABL* TKIs has transformed the landscape of Ph+ ALL. We demonstrate that newer TKIs are associated with improved odds of CR and reduced hazards of death, independently of other traditional risk factors. WBC ≥50x10$^{9}$/L remains an independent adverse prognostic factor, suggesting that such patients may require incorporation of chemoimmunotherapy to improve outcomes.

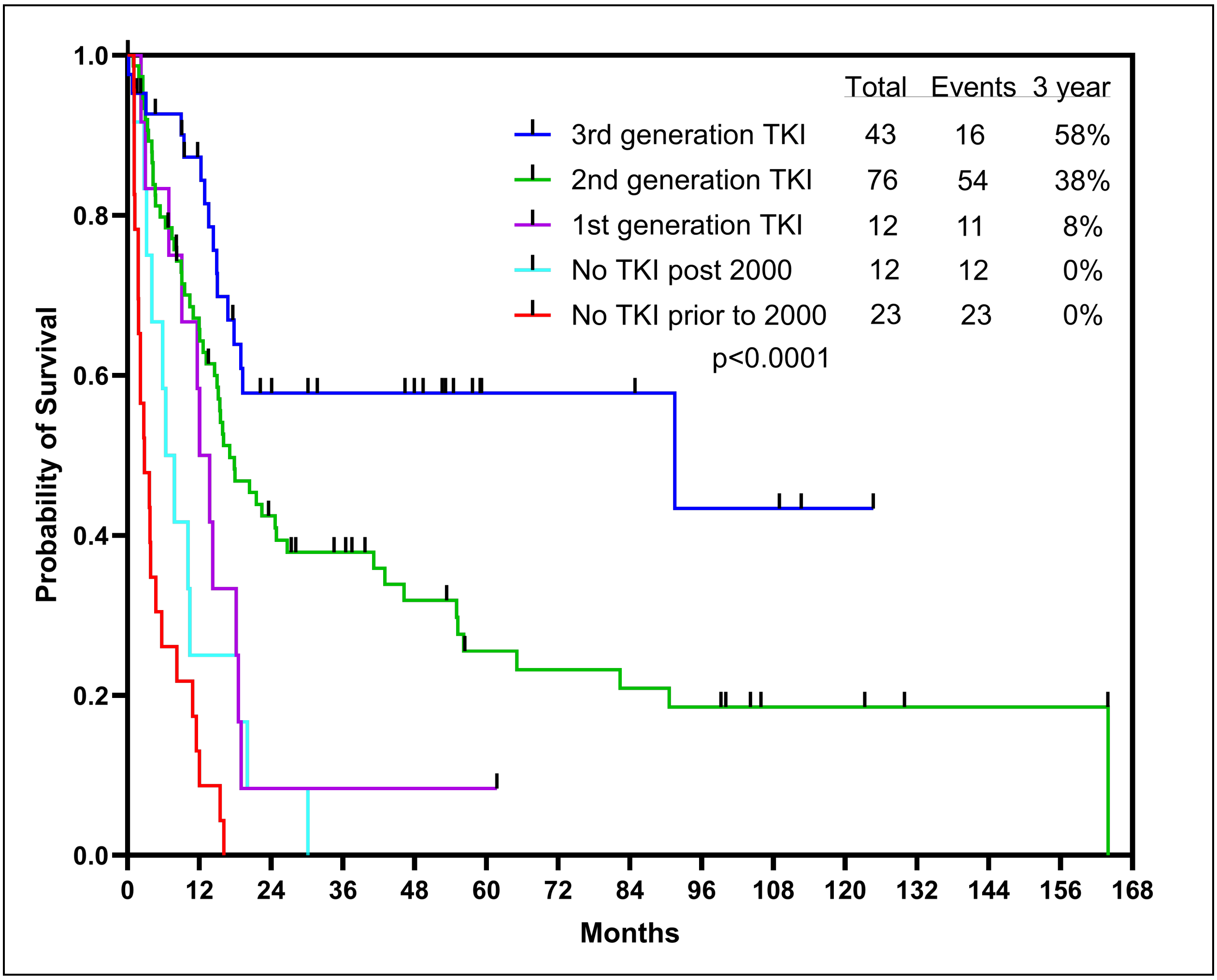

**Copyright Information:** (Online) ISSN: 2572-9241
©2026 The Author(s). HemaSphere is published by John Wiley & Sons Ltd on behalf of European Hematology Association. This is an open access Abstract Book distributed under the Attribution-NonCommercial-NoDerivs (CC BY-NC-ND), which allows third parties to download the articles and share them with others as long as they credit the author and the Abstract Book, but they cannot change the content in any way or use them commercially.
**Abstract Book Citations:** Authors, Title, HemaSphere, 2026;10:(S1):pages. The abstract book DOIs can be found on the "Author Information" page.
**Disclaimer:** Articles published in the journal HemaSphere exclusively reflect the opinions of the authors. The authors are responsible for all content in their abstracts including accuracy of the facts, statements, citing resources, etc.

### (PF395) ADULT T-ALL OUTCOMES AND THE PROGNOSTIC IMPACT OF EARLY T-CELL PRECURSOR TREATED WITH PEDIATRIC-INSPIRED REGIMEN

**Topic:** 02. Acute lymphoblastic leukemia - Clinical

Yael Morgenstern*[1], Mark Turingan[2], Hong Chang[2], Dawn Maze[1], Karen Yee[1], Aniket Bankar[1], Marta Davidson[1], Guillaume Richard-Carpentier[1], Steven M Chan[1], Aaron Schimmer[1], Andre Schuh[1], Mark Minden[1], Vikas Gupta[1], Hassan, Sibai[1], Maria Agustina Perusini[1]

[1]*Princess Margaret Cancer Center, Toronto, Canada, Toronto, Canada;*[2]*Toronto General Hospital, Toronto, Canada;*

### Background

T-cell acute lymphoblastic leukemia (T-ALL) is a biologically heterogeneous malignancy. Early T-cell precursor ALL (ETP-ALL) is a distinct subtype historically associated with inferior outcomes, although this has not been consistently observed in pediatric cohorts treated with intensive regimens. Data on adult patients (pts) treated with pediatric-inspired approaches remain limited.

### Aims

To evaluate overall survival (OS) and leukemia-free survival (LFS) in adultpts with T-ALL treated with a pediatric-inspired regimen, and to determine the prognostic impact of the ETP phenotype inthis setting.

### Methods

We performed a retrospective cohort study of adult pts (≥18 years) diagnosed with T-ALL between 2001 and 2024 and treated with a pediatric-inspired regimen (dose-adjusted for pts >60 years) that included peg-asparaginase at Princess Margaret Hospital. ETP classification was confirmed by expert hematopathologists using multiparameter flow cytometry per WHO criteria.

### Results

The study included 146 pts, of whom 24 (17%) had ETP-ALL and 122 (83%) had non-ETP T-ALL. The median age was 35 years (IQR 25–50), with 15% (n=22) of pts aged >60 years. Extramedullary disease (EMD) was present in 74% (n=107) of pts, and central nervous system (CNS) involvement in 9% (n=12). A total of 86% of pts achieved complete remission (CR). After a median follow-up of 3.9 years (IQR 1.4–8.5), the 3-year overall survival (OS) rate was 73% (95% CI 70.8–75.5), and the 3-year leukemia-free survival (LFS) rate was 77% (95% CI 75.3–79.4). Advanced age was correlated with poorer OS, with 3-year OS rates of 81%, 67%, and 47% for pts aged <40, 40–60, and >60 years, respectively (p=0.005). In patients achieving CR, 3-year cumulative incidence of relapse of 22% (95% CI 14.1–28.9). Among pts with relapse, the prognosis remained poor, with a 3-year OS of 39% (95% CI 23.8–62.5). Given the historically poor prognosis associated with ETP-ALL, outcomes were compared between pts with ETP and non-ETP phenotype. Pts with ETP-ALL were older, with a median age of 40 (IQR 32.5–48.5) versus 32.5 (IQR 24.0–49.8) years; p=0.05, with no other baseline differences observed. No significant difference in OS or LFS between ETP and non-ETP cohorts, with 3-year OS of 74% versus 68% (p=0.71) and 3-year LFS of 76% in both groups (p=0.70). Allogeneic stem cell transplantation (allo-SCT) in first complete remission (CR1) was performed in 1 pt (0.04%) with ETP-ALL and 13 pts (11%) with non-ETP ALL. To account for transplant-related effects, analyses were censored at time of allo-SCT. With censoring, the 3-year OS for the overall cohort was 80% (95% CI 73.2–87.4), with no significant difference between ETP and non-ETP pts (71.7% vs 81.8%; p=0.40), indicating that comparable survival was not driven by transplant rates. In multivariable analysis, age >60 years emerged as the only factor independently associated with inferior OS (HR 2.56, 95% CI 1.23–5.34; p=0.012), while ETP immunophenotype was not associated with worse OS (HR 1.64, 95% CI 0.72–3.72; p=0.238). No association with OS was observed for WBC count, EMD, CNS involvement, or allo-SCT.

**Copyright Information:** (Online) ISSN: 2572-9241
©2026 The Author(s). HemaSphere is published by John Wiley & Sons Ltd on behalf of European Hematology Association. This is an open access Abstract Book distributed under the Attribution-NonCommercial-NoDerivs (CC BY-NC-ND), which allows third parties to download the articles and share them with others as long as they credit the author and the Abstract Book, but they cannot change the content in any way or use them commercially.
**Abstract Book Citations:** Authors, Title, HemaSphere, 2026;10:(S1):pages. The abstract book DOIs can be found on the "Author Information" page.
**Disclaimer:** Articles published in the journal HemaSphere exclusively reflect the opinions of the authors. The authors are responsible for all content in their abstracts including accuracy of the facts, statements, citing resources, etc.

**Summary/Conclusion**

Pediatric-inspired therapy was associated with favorable long-term survival in adults with T-ALL, with no adverse prognostic impact of the ETP immunophenotype. In contrast, post-relapse outcomes remained dismal, emphasizing the critical need for enhanced predictive tools and innovative therapeutic approaches for this high-risk population.

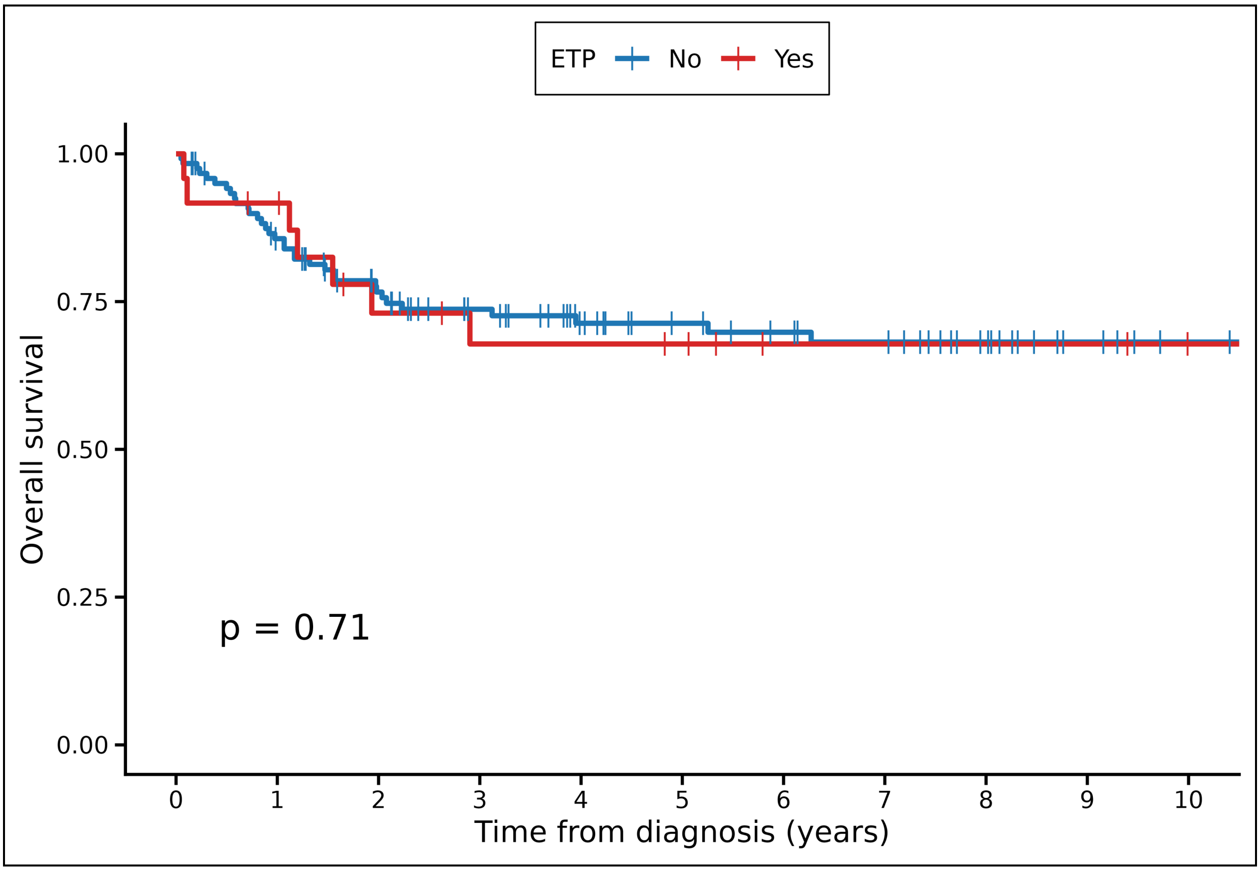

Figure 1 Overall survival of pediatric-inspired-regimen-treated T-ALL patients stratified by subtype classification as ETP versus non-ETP

**Copyright Information:** (Online) ISSN: 2572-9241
©2026 The Author(s). HemaSphere is published by John Wiley & Sons Ltd on behalf of European Hematology Association. This is an open access Abstract Book distributed under the Attribution-NonCommercial-NoDerivs (CC BY-NC-ND), which allows third parties to download the articles and share them with others as long as they credit the author and the Abstract Book, but they cannot change the content in any way or use them commercially.
**Abstract Book Citations:** Authors, Title, HemaSphere, 2026;10:(S1):pages. The abstract book DOIs can be found on the "Author Information" page.
**Disclaimer:** Articles published in the journal HemaSphere exclusively reflect the opinions of the authors. The authors are responsible for all content in their abstracts including accuracy of the facts, statements, citing resources, etc.

### (PF396) INOTUZUMAB OZOGAMICIN AS SALVAGE THERAPY FOR HEAVILY PRETREATED R/R B-ALL: BRIDGING TO TRANSPLANTATION DRIVES SURVIVAL BENEFIT

**Topic:** 02. Acute lymphoblastic leukemia - Clinical

Gailing Zhang*[1], Jing Long[2], Yan-Li Zhao[1], Yue Lu[1], Zhijie Wei[1], Xian Zhang[1]

[1]*Hebei Yanda Lu Daopei Hospital, Langfang, China;*[2]*Beijing Lu Daopei Hospital, Beijing, China;*

### Background

Relapsed or refractory (R/R) B-ALL following allogeneic transplantation or immunotherapies such as CAR-T and BiTEs carries poor prognosis and limited therapeutic options. Inotuzumab ozogamicin (INO), an anti-CD22 antibody–drug conjugate, induces high remission rates and serves as an effective bridge to allo-HSCT. However, real-world evidence regarding its efficacy and safety in these heavily pretreated patients remains limited.

### Aims

To assess INO as salvage therapy in heavily pretreated R/R B-ALL, evaluating response, transplant bridging, VOD, and survival.

### Methods

We retrospectively analyzed 90 patients with R/R B-ALL treated with INO at Hebei Yanda Lu Daopei Hospital during 2022 to 2025, evaluating response, bridging to allo-HSCT, VOD/SOS incidence, and survival outcomes. OS and PFS were estimated using the Kaplan–Meier method and compared using the log-rank test.

**Copyright Information:** (Online) ISSN: 2572-9241
©2026 The Author(s). HemaSphere is published by John Wiley & Sons Ltd on behalf of European Hematology Association. This is an open access Abstract Book distributed under the Attribution-NonCommercial-NoDerivs (CC BY-NC-ND), which allows third parties to download the articles and share them with others as long as they credit the author and the Abstract Book, but they cannot change the content in any way or use them commercially.
**Abstract Book Citations:** Authors, Title, HemaSphere, 2026;10:(S1):pages. The abstract book DOIs can be found on the “Author Information” page.
**Disclaimer:** Articles published in the journal HemaSphere exclusively reflect the opinions of the authors. The authors are responsible for all content in their abstracts including accuracy of the facts, statements, citing resources, etc.

**Results**

A total of 92 patients with R/R B-ALL were enrolled; two were excluded due to infection or prophylactic INO use, leaving 90 for analysis (56 males, 34 females; median age 21 years, range 2–75). Patients were heavily pretreated, including relapse after allo-HSCT (24, 26.7%; mostly after one transplant, 87.5%), CAR-T therapy failure/relapse (13, 14.4%), relapse after BiTE therapy (9, 10.0%), and relapse following conventional chemotherapy (44, 48.9%). Before INO, 43 patients (47.8%) had persistent BM blasts (median 85%, range 5–99.5), whereas 47 (52.2%) had BM blasts <5% but were FCM-MRD positive (median 0.6%, range 0.01–15.7). INO exposure included one vial in 27 patients (30.0%), two vials in 41 (45.6%), and ≥3 vials in 22 (24.4%).

Response was assessed within 7 days after INO completion. Overall, 72 patients (80.0%) achieved CR, including MRD+CR in 11 (12.2%) and MRD−CR in 61 (67.8%), while 18 (20.0%) were non-responders (NR). Among patients with persistent BM blasts (n=43), 25 (58.1%) achieved CR (MRD+CR 6; MRD−CR 19), whereas 18 remained refractory. Among FCM-MRD–positive patients (n=47), 42 (89.4%) achieved MRD negativity and 5 (10.6%) remained MRD positive. VOD occurred in 5/90 patients (5.6%), all successfully managed without VOD-related mortality.

Among 72 CR patients, 43 (59.7%) proceeded to consolidative allo-HSCT at a median of 34 days (range 9–123), while 28 (40.3%) received non-transplant therapy. With a median follow-up of 392 days (range 73–1198), the allo-HSCT cohort showed superior 2-year LFS (58.9% vs. 31.9%, p=0.01) and a trend toward improved 2-year OS (66.6% vs. 44.7%, p=0.06). MRD+CR patients (n=6) had lower 2-year OS and LFS than MRD−CR patients (n=37), without statistical significance (OS 44.4% vs. 70.9%; LFS 44.4% vs. 62.5%; both p>0.05).

Among 18 NR patients, 6 underwent salvage allo-HSCT: three relapsed and died within one year, whereas three (50%) achieved disease-free survival >1.5 years. Outcomes were markedly inferior in NR patients without salvage transplantation (n=12; two lost to follow-up), with 9/10 deaths within 100 days after INO and only one patient remaining disease-free at 690 days; 1-year OS and LFS were significantly improved with salvage transplantation (log-rank p<0.01).

**Summary/Conclusion**

INO achieved high remission rates with manageable toxicity in heavily pretreated R/R B-ALL. Survival benefit was primarily observed in patients proceeding to allo-HSCT, while salvage transplantation enabled durable disease control even in non-responders.

**Copyright Information:** (Online) ISSN: 2572-9241
©2026 The Author(s). HemaSphere is published by John Wiley & Sons Ltd on behalf of European Hematology Association. This is an open access Abstract Book distributed under the Attribution-NonCommercial-NoDerivs (CC BY-NC-ND), which allows third parties to download the articles and share them with others as long as they credit the author and the Abstract Book, but they cannot change the content in any way or use them commercially.
**Abstract Book Citations:** Authors, Title, HemaSphere, 2026;10:(S1):pages. The abstract book DOIs can be found on the “Author Information” page.
**Disclaimer:** Articles published in the journal HemaSphere exclusively reflect the opinions of the authors. The authors are responsible for all content in their abstracts including accuracy of the facts, statements, citing resources, etc.

### (PF397) DYNAMIC MEASURABLE RESIDUAL DISEASE KINETICS AND CONDITIONAL TRANSPLANTATION BENEFIT IN ADULT ACUTE LYMPHOBLASTIC LEUKEMIA: A GRELAL CHILE REAL-WORLD STUDY

**Topic:** 02. Acute lymphoblastic leukemia - Clinical

Marcela Espinoza[1, 2], Miguel López Cáceres[3], Edgar Zapata[4], Joaquin Jerez[4, 5], Ignacio Dominguez[6, 6], Marcelo Abarca[6], Nicolás Rodriguez[7, 8], Dayanara Serrano Lemus[8], Gonzalo Guerrero[9], Fabián Ruiz[10], Nicolás Quinteros[11], Javier Rojas[12], Torres Vivianne[13], Yaima Gutierrez Torrado[14], Yorman Flores*[15], Verónica Contreras[16, 16], Veronica Perez[17], Andrés Rojas Verasay[18], Maria Constanza Flores[18], José Tomás Gazmuri[1], Marcelo Navarrete[19]

*[1]Clínica Dávila, Hematology, Santiago, Chile; [2]Pontificia Universidad Católica, Programa Doctorado Ciencias Médicas, Santiago, Chile; [3]Clínica Alemana de Santiago, Santiago, Chile;[4]Fundación Arturo López Pérez, Hematology, Santiago, Chile; [5]Universidad de Los Andes, Programa de Doctorado en Biomedicina, Santiago, Chile; [6]Complejo Asistencial Dr Sótero del Río, Hematology, Santiago, Chile; [7]Hospital Barros Luco Trudeau, Hematology, Santiago, Chile;[8]Universidad de Chile, Departamento de Medicina Interna Sur, Universidad de Chile., Santiago, Chile; [9]Clínica Red Salud Vitacura, Programa Doctorado Ciencias Médicas, Santiago, Chile; [10]Clínica Red Salud Vitacura, Santiago, Chile; [11]Hospital Clínico San Borja de Arriarán, Hematology, Santiago, Chile; [12]Hospital Regional de Talca, Talca, Chile; [13]Hospital Base Valdivia, Valdivia, Chile; [14]Hospital Hernán Henríquez Aravena, Temuco, Chile; [15]Hospital UC Christus, Santiago, Chile; [16]Hospital Carlos Van Buren, Valparaíso, Chile; [17]Hospital Juan Noe Crevani, Arica, Chile; [18]Hospital San Juan de Dios de La Serena, La Serena, Chile; [19]Universidad de Magallanes, Punta Arenas, Chile;*

### Background

Measurable residual disease (MRD) is the most powerful prognostic biomarker in adult acute lymphoblastic leukemia (ALL). However, whether transplantation benefit is biologically conditioned by MRD kinetics  rather than static MRD measurement remains incompletely defined, particularly in real-world adult populations treated outside prospective trials.

### Aims

To evaluate the prognostic impact of MRD kinetics and the conditional benefit of transplantation in a large, real-world cohort of adults with ALL.

### Methods

We analyzed 147 consecutive adults with newly diagnosed ALL treated across multiple centers, with a median follow-up of 36 months. MRD negativity is defined as bone marrow blasts <0.01%, measured by flow cytometry, It was assessed after induction and pre-transplant. Multivariable Cox modeling, formal interaction testing, landmark analysis, and dynamic trajectory classification were performed to evaluate the relationship between MRD status, allogeneic hematopoietic stem cell transplantation (allo-HSCT) in first complete remission (CR1), and survival outcomes.

**Copyright Information:** (Online) ISSN: 2572-9241
©2026 The Author(s). HemaSphere is published by John Wiley & Sons Ltd on behalf of European Hematology Association. This is an open access Abstract Book distributed under the Attribution-NonCommercial-NoDerivs (CC BY-NC-ND), which allows third parties to download the articles and share them with others as long as they credit the author and the Abstract Book, but they cannot change the content in any way or use them commercially.
**Abstract Book Citations:** Authors, Title, HemaSphere, 2026;10:(S1):pages. The abstract book DOIs can be found on the "Author Information" page.
**Disclaimer:** Articles published in the journal HemaSphere exclusively reflect the opinions of the authors. The authors are responsible for all content in their abstracts including accuracy of the facts, statements, citing resources, etc.

### Results

Patient Characteristics and Treatment: The cohort had a median age at diagnosis of 35.7 years (IQR: 21.9–52.0). B-lineage ALL was the most prevalent subtype (92.3%), with BCR::ABL1 positivity identified in 26.8% of these cases. At presentation, the median white blood cell count was 14,500 cells x mm3, and 62.0% of patients exhibited elevated lactate dehydrogenase levels. Regarding consolidation therapy, 77.7% of the patients underwent allogeneic hematopoietic stem cell transplantation (allo-HSCT) in first complete remission (CR1).

Survival Outcomes: With a total of 48 deaths and 51 progression events recorded, the median progression-free survival (PFS) and overall survival (OS) for the entire cohort were 7.5 years. The OS rates at 1, 3, and 5 years were 77.1%, 61.3%, and 59.2%, respectively; corresponding PFS rates were 73.0%, 60.0%, and 58.3%. In multivariate analysis, allo-HSCT in CR1 was identified as an independent protective factor for both OS (HR: 0.51) and PFS (HR: 0.57).

Prognostic Impact of MRD Dynamics: Post-induction MRD positivity was a robust independent predictor of inferior outcomes, associated with a significantly higher hazard for OS (HR: 2.68; 95% CI: 1.69–4.25) and PFS (HR: 2.46; 95% CI: 1.59–3.83). Dynamic MRD trajectory modeling demonstrated a stepwise divergence in survival: median survival reached 11 years for patients with sustained negativity, 7.4 years for those achieving molecular conversion, and decreased to 1 year for the persistent MRD-positive group. These associations remained consistent across age strata (<40 vs. ≥40 years) and Philadelphia chromosome status, with no significant interaction effects observed. Sensitivity analyses excluding non-intensive treatment strategies confirmed the robustness of these findings, yielding materially unchanged results.

### Summary/Conclusion

This large multicenter analysis demonstrates that MRD kinetics function as a biologically coherent and clinically actionable axis of risk in adult ALL. Persistent molecular disease following induction therapy identifies patients with markedly inferior survival independent of age and transplantation status.
Importantly, transplantation benefit appears aligned with molecular disease state. The protective association observed for allo-HSCT in CR1 is most biologically plausible when interpreted within the context of residual tumor burden and clonal susceptibility to graft-versus-leukemia effects.

**Copyright Information:** (Online) ISSN: 2572-9241
©2026 The Author(s). HemaSphere is published by John Wiley & Sons Ltd on behalf of European Hematology Association. This is an open access Abstract Book distributed under the Attribution-NonCommercial-NoDerivs (CC BY-NC-ND), which allows third parties to download the articles and share them with others as long as they credit the author and the Abstract Book, but they cannot change the content in any way or use them commercially.
**Abstract Book Citations:** Authors, Title, HemaSphere, 2026;10:(S1):pages. The abstract book DOIs can be found on the “Author Information” page.
**Disclaimer:** Articles published in the journal HemaSphere exclusively reflect the opinions of the authors. The authors are responsible for all content in their abstracts including accuracy of the facts, statements, citing resources, etc.

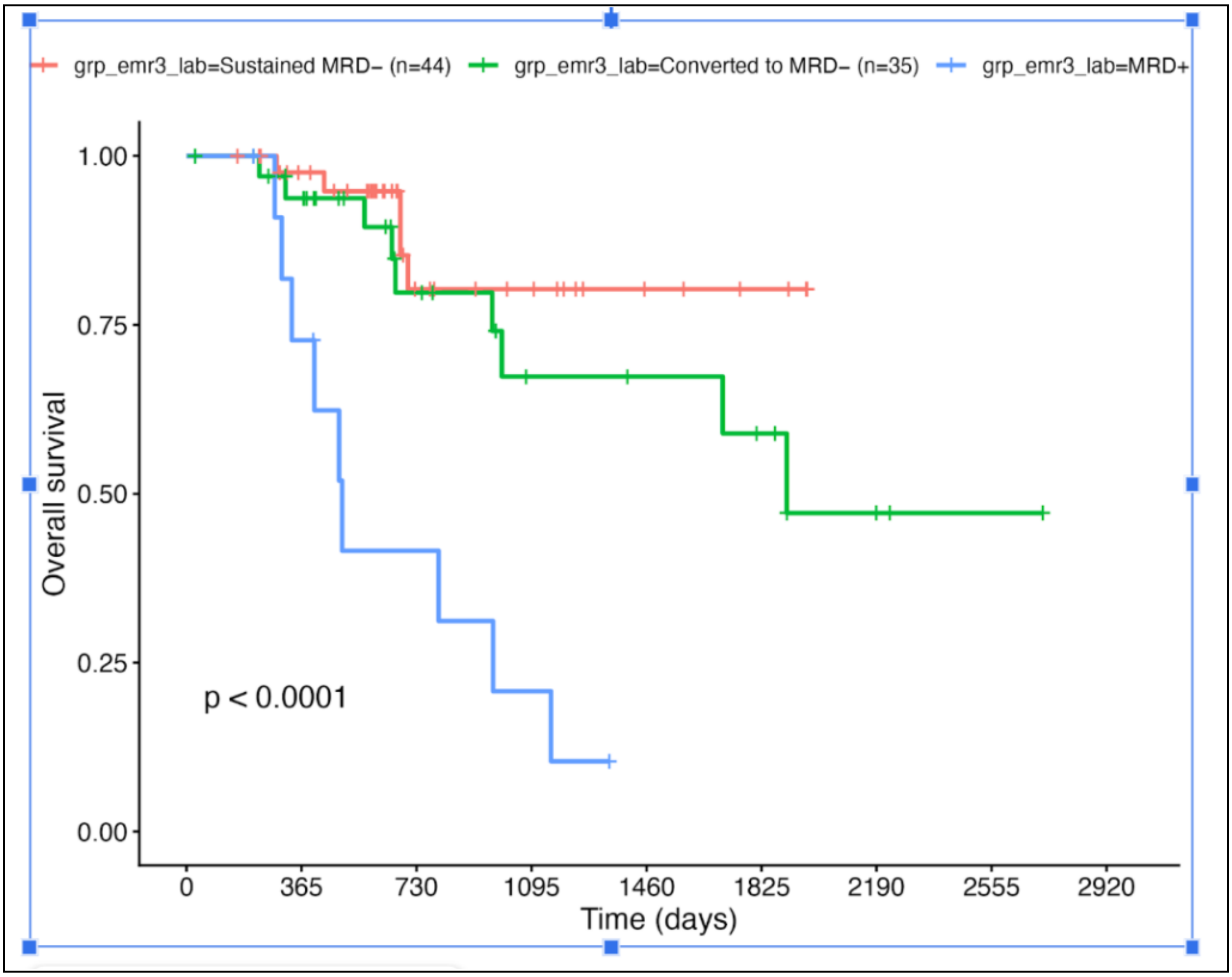

Dynamic classification revealed stepwise survival divergence across sustained negativity, molecular conversion, and persistent positivity groups. Persistent MRD positivity conferred the highest hazard ratios in age-adjusted models, exceeding tenfold relative to sustained negativity.

**Copyright Information:** (Online) ISSN: 2572-9241
©2026 The Author(s). HemaSphere is published by John Wiley & Sons Ltd on behalf of European Hematology Association. This is an open access Abstract Book distributed under the Attribution-NonCommercial-NoDerivs (CC BY-NC-ND), which allows third parties to download the articles and share them with others as long as they credit the author and the Abstract Book, but they cannot change the content in any way or use them commercially.
**Abstract Book Citations:** Authors, Title, HemaSphere, 2026;10:(S1):pages. The abstract book DOIs can be found on the “Author Information” page.
**Disclaimer:** Articles published in the journal HemaSphere exclusively reflect the opinions of the authors. The authors are responsible for all content in their abstracts including accuracy of the facts, statements, citing resources, etc.

### (PF398) IMMUNO-TARGETED THERAPIES VERSUS ALLOGENEIC HEMATOPOIETIC CELL TRANSPLANTATION IN ADULT PATIENTS WITH PHILADELPHIA CHROMOSOME-POSITIVE ACUTE LYMPHOBLASTIC LEUKEMIA: A SINGLE-CENTER EXPERIENCE

**Topic:** 02. Acute lymphoblastic leukemia - Clinical

Chenying Li[1], Hongyan Tong[1], Liping Mao[1], Huafeng Wang[1], Yanling Ren[1], Jiejin Qian[1], Xinping Zhou[1], Gaixiang Xu[1], Liangshun You[1], Jie Jin[1], Yinjun Lou*[1]

[1]*Department of Hematology, Leukemia Center, First Affiliated Hospital of Zhejiang University,, HZNGZHOU, China;*

### Background

Philadelphia chromosome–positive (Ph+) acute lymphoblastic leukemia (ALL) accounts for 20–30% of adult B-cell ALL cases—and up to 50% among patients aged >60 years. Tyrosine kinase inhibitor (TKI)– and corticosteroid-based induction therapy achieves complete remission (CR) in >95% of patients. Historically, allogeneic hematopoietic cell transplantation (allo-HCT) has been recommended as first-line consolidation for fit Ph+ ALL patients with a suitable donor. More recently, third-generation TKIs (e.g., ponatinib, olverembatinib, asciminib) combined with novel immunotherapies—including blinatumomab, inotuzumab ozogamicin, and CD19-directed chimeric antigen receptor T-cell (CAR-T) therapy—have emerged as promising frontline alternatives. However, robust comparative data on TKI plus immuno-targeted therapy versus TKI plus allo-HCT or TKI plus conventional chemotherapy remain scarce.

### Aims

To compare efficacy and long-term outcomes across these three consolidation strategies in adults with newly diagnosed Ph+ ALL.

### Methods

We conducted a retrospective cohort study of 336 consecutive adult patients (≥18 years) with newly diagnosed Ph+ ALL treated at a tertiary academic center between October 2019 and March 2025. Primary endpoints were 3-year overall survival (OS) and event-free survival (EFS).

### Results

Early death rate was 1.2% (4/336). CR was achieved in 332 patients (98.8%). Patients were stratified into: (1) TKI + allo-HCT (n = 160), (2) TKI + immuno-targeted therapy (n = 49), and (3) TKI + chemotherapy alone (n = 123). At 3 years, OS was 84.6%, 80.0%, and 61.7% ($P < 0.001$), respectively; EFS was 72.4%, 75.4%, and 40.7% ($P < 0.001$), respectively. Notably, no statistically significant difference in OS ($P = 0.32$) or EFS ($P = 0.68$) was observed between the TKI + allo-HCT and TKI + immuno-targeted therapy groups.

### Summary/Conclusion

TKI-based immuno-targeted therapy is a highly effective, transplant-sparing strategy for adults with Ph+ ALL—especially for older or transplant-ineligible patients. These findings support prospective validation of immunotherapy-intensified, allo-HCT–deferred regimens.

**Copyright Information:** (Online) ISSN: 2572-9241
©2026 The Author(s). HemaSphere is published by John Wiley & Sons Ltd on behalf of European Hematology Association. This is an open access Abstract Book distributed under the Attribution-NonCommercial-NoDerivs (CC BY-NC-ND), which allows third parties to download the articles and share them with others as long as they credit the author and the Abstract Book, but they cannot change the content in any way or use them commercially.
**Abstract Book Citations:** Authors, Title, HemaSphere, 2026;10:(S1):pages. The abstract book DOIs can be found on the "Author Information" page.
**Disclaimer:** Articles published in the journal HemaSphere exclusively reflect the opinions of the authors. The authors are responsible for all content in their abstracts including accuracy of the facts, statements, citing resources, etc.

### (PF399) INOTUZUMAB OZOGAMICIN AS SALVAGE AND BRIDGING THERAPY AFTER CAR-T FAILURE IN RELAPSED/REFRACTORY B-CELL ACUTE LYMPHOBLASTIC LEUKEMIA

**Topic:** 02. Acute lymphoblastic leukemia - Clinical

Jiujiang He*[1], Gangwei Zhang[1], Yan Wu[1], Yunxiao Yang[1], Min Zhang[1], Qian Yang[2], Junfang Yang[1], Jing Long[1]

*[1]Hebei Yanda Lu Daopei hospital, Langfang, China;[2]Gao Beidian Hospital, Baoding, China;*

### Background

Relapse or resistance after CAR-T therapy represents a major therapeutic challenge in relapsed/refractory B-cell acute lymphoblastic leukemia (R/R B-ALL), particularly in heavily pretreated patients (pts) with prior transplantation, antigen escape, and high-risk genetic features. Outcomes remain poor and effective salvage options are limited.

### Aims

To assess the efficacy and safety of inotuzumab ozogamicin (INO) as a salvage and bridging strategy to transplantation in pts with R/R B-ALL after CAR-T therapy failure.

### Methods

We retrospectively analyzed 14 consecutive pts with R/R B-ALL who relapsed or were refractory after CAR-T therapy and received INO at Hebei Yanda Lu Daopei Hospital between July 2022 and July 2025.Responses, survival outcomes, and adverse events were assessed. Survival was estimated using the Kaplan–Meier method, with follow-up censored on February 10, 2026.

### Results

Among 14 pts (male/female: 8/6), the median age was 13 years (range, 6–53). Relapse occurred after CD19/CD22 dual-target CAR-T therapy in 8 pts (57.1%), including 4 previously treated with CD19 CAR-T therapy. Five pts (35.7%) relapsed after CD22 CAR-T therapy, all with prior CD19 CAR-T exposure. One patient relapsed after sequential CD19-, CD22-, and CD72-targeted CAR-T therapies.
Four pts (28.6%) experienced post-transplant relapse, including two after second allo-HSCT. Complex karyotypes were observed in 4 pts (28.6%). Genetic data were evaluable in 12 pts, of whom 10 (83.3%) harbored molecular abnormalities. The most frequent alterations included ***ETV6::RUNX1*** (25%), and *IKZF1* deletion was detected in 16.7% of patients, while *CREBBP, TP53, and KRAS* mutations were each identified in one case. Before INO treatment, 11 pts had NR disease with a median BM blasts of 84.5% (range, 11–99.5), while 3 pts were in CR with persistent MRD positivity. CD22 expression was preserved in all patients, whereas 3 patients (21.4%) were CD19-negative.
The complete remission (CR) rate was 81.8% (9/11) within one week after completion of INO therapy, including MRD-negative CR in 8 pts and MRD-positive CR in 1 patient; two pts (18.2%) remained NR. Among 3 MRD-positive pts treated with INO, two converted to MRD-negative CR, while one patient with prior dual-target CAR-T failure remained MRD-positive.
Among 12 pts achieving CR, 11 (91.7%) proceeded to consolidative allo-HSCT at a median of 15 days (range, 7–47). With a median follow-up of 375 days (range, 97–1115), the 1- and 2-year overall survival (OS) rates were both 62.3%, and corresponding leukemia-free survival (LFS) rates were 63.6%. Three pts experienced CD22-positive relapse after HSCT and subsequently died. One patient who did not undergo transplantation achieved a durable CR approaching 2 years. Of the two pts refractory to INO, one discontinued treatment and one achieved durable CR lasting 17 months after salvage allo-HSCT. Notably, no cases of hepatic sinusoidal obstruction syndrome (SOS/VOD) were observed.

**Copyright Information:** (Online) ISSN: 2572-9241
©2026 The Author(s). HemaSphere is published by John Wiley & Sons Ltd on behalf of European Hematology Association. This is an open access Abstract Book distributed under the Attribution-NonCommercial-NoDerivs (CC BY-NC-ND), which allows third parties to download the articles and share them with others as long as they credit the author and the Abstract Book, but they cannot change the content in any way or use them commercially.
**Abstract Book Citations:** Authors, Title, HemaSphere, 2026;10:(S1):pages. The abstract book DOIs can be found on the "Author Information" page.
**Disclaimer:** Articles published in the journal HemaSphere exclusively reflect the opinions of the authors. The authors are responsible for all content in their abstracts including accuracy of the facts, statements, citing resources, etc.

**Summary/Conclusion**

In this ultra–high-risk, heavily pretreated population after CAR-T failure, INO induced high remission rates with favorable safety and enabled successful bridging to allo-HSCT, suggesting that INO may represent a feasible salvage strategy in a setting with otherwise limited treatment options.

**Copyright Information:** (Online) ISSN: 2572-9241
©2026 The Author(s). HemaSphere is published by John Wiley & Sons Ltd on behalf of European Hematology Association. This is an open access Abstract Book distributed under the Attribution-NonCommercial-NoDerivs (CC BY-NC-ND), which allows third parties to download the articles and share them with others as long as they credit the author and the Abstract Book, but they cannot change the content in any way or use them commercially.
**Abstract Book Citations:** Authors, Title, HemaSphere, 2026;10:(S1):pages. The abstract book DOIs can be found on the "Author Information" page.
**Disclaimer:** Articles published in the journal HemaSphere exclusively reflect the opinions of the authors. The authors are responsible for all content in their abstracts including accuracy of the facts, statements, citing resources, etc.

### (PF400) CLINICAL PROFILES AND RISK STRATIFICATION IN ELDERLY PATIENTS WITH NEWLY DIAGNOSED BCR::ABL1-POSITIVE ACUTE LYMPHOBLASTIC LEUKEMIA

**Topic:** 02. Acute lymphoblastic leukemia - Clinical

Weiyang Liu*[1, 2], Cheng Wang[1, 2], Li-Jun Peng[1, 2], Jiayi Ren[1, 2], Yongmei Zhu[1, 2], Xiangqin Weng[1, 2], Jie Hao[3], Yuanfang Liu[1, 2], Weiping Zhang[1, 2], Duohui Jing[1, 2], Jian-qing Mi[1, 2], Jin Wang[1, 2]

*[1]Shanghai Institute of Hematology, State Key Laboratory of Medical Genomics, National Research Centre for Translational Medicine at Shanghai, Ruijin Hospital Affiliated to Shanghai Jiao Tong University School of Medicine, Shanghai, China; [2]Shanghai Key Laboratory of Gene Editing and Cell-based Immunotherapy for Hematological Diseases, Ruijin Hospital, Shanghai jiao Tong University School of Medicine, Shanghai, China; [3]Department of Hematology, Shanghai Jing'an District Beizhan Hospital, Shanghai, China;*

### Background

For adult patients (pts) (≤ 65 years) with *BCR::ABL1*-positive acute lymphoblastic leukemia (*BCR::ABL1*+ ALL), we previously established a refined risk stratification integrating the *IKZF1*$^{plus}$ genotype with 3-month MRD assessment. This model enabled classification of pts into three risk groups and facilitated precise therapeutic decisions (*Wang C, et al. Blood Cancer J. 2024;14(1):71*). However, its applicability in elderly pts remains unclear.

### Aims

Herein, we evaluated this prognostic model in newly diagnosed pts aged ≥ 65 years, demonstrating its potential to guide treatment decisions.

### Methods

In this study, newly diagnosed *BCR::ABL1*+ ALL pts aged ≥ 65 years were analyzed. All pts received the 2$^{nd}$ generation TKI, flumatinib (600mg/day), and VP-based (Vincristine/Prednisone) chemotherapy. No pts underwent allo-HSCT. MRD was monitored by MFC at a sensitivity of 0.01% and RT-qPCR at a sensitivity of 0.001%. CMR was defined as the absence of quantifiable *BCR::ABL1* transcripts. *IKZF1*$^{plus}$ genotyping was identified by multiplex ligation-dependent probe amplification (MLPA). AEs were assessed according to CTCAE v5.0.

**Copyright Information:** (Online) ISSN: 2572-9241
©2026 The Author(s). HemaSphere is published by John Wiley & Sons Ltd on behalf of European Hematology Association. This is an open access Abstract Book distributed under the Attribution-NonCommercial-NoDerivs (CC BY-NC-ND), which allows third parties to download the articles and share them with others as long as they credit the author and the Abstract Book, but they cannot change the content in any way or use them commercially.
**Abstract Book Citations:** Authors, Title, HemaSphere, 2026;10:(S1):pages. The abstract book DOIs can be found on the "Author Information" page.
**Disclaimer:** Articles published in the journal HemaSphere exclusively reflect the opinions of the authors. The authors are responsible for all content in their abstracts including accuracy of the facts, statements, citing resources, etc.

**Results**

From January 2021 to January 2026, 38 pts with de novo *BCR::ABL1*+ ALL received flumatinib combined with VP-based chemotherapy. The median age was 68.5 years (range, 65-78), and 52.6% were females. The median WBC count was 49.7×$10^9$/L (IQR, 13.3-97.7). *BCR::ABL1* p190 transcript was detected in 27 pts, while 8 exhibited co-expression of p190 and p210 isoforms. Based on MLPA analysis of 36 evaluable pts, 12 were classified as *IKZF1*$^{plus}$.
The ORR was 100% (CR: 81.6%, 31/38; CRi: 18.4%,7/38) following induction therapy. MRD flow negativity and CMR rates were 60.5% (23/38) and 10.8% (4/37) post-induction, respectively, which improved to 69.7% (23/33) and 29.0% (9/31) at 3 months. With a median follow-up of 28.9 months (range: 0.8-64.4), the 2-year OS and PFS rates were 78.7 and 64.5%, respectively.
Among 32 pts with available data for the refined risk stratification, 12 were classified as high-risk (*IKZF1*$^{plus}$), 14 as intermediate-risk (non-*IKZF1*$^{plus}$/MRD+), and 6 as low-risk (non-*IKZF1*$^{plus}$/MRD-). The low-risk group exhibited superior survival outcomes (2-year OS and PFS rates were both 100%) compared to the intermediate/high-risk groups (2-year OS and PFS rates were 72.9% and 53.9%, respectively). Of the 37 evaluable pts for ABL1 mutations, 18 (48.6%) harbored at least 1 mutation, with *T315I* most prevalent (66.7%). Pts with *T315I* mutation had significantly inferior outcomes compared to non-*T315I* carriers (OS: p=0.001; PFS: p=0.002). Besides, the *T315I* mutation rate was higher in the intermediate/high-risk groups than in the low-risk group (45.8% vs. 16.7%). Four pts received CAR-T therapy: 3 from intermediate/high-risk groups and 2 with *T315I* mutation. During a post-CAR-T follow-up of 9 to 13 months, all remained alive and recurrence-free.
The safety profile was acceptable, with no flumatinib-related deaths. Most non-hematological AEs were grade 1-2. Grade 3 AEs, including paroxysmal atrial fibrillation (n=3) and cerebral infarction (n=1), were all resolved with appropriate management. Additionally, hematologic toxicity was mild, generally grading 1-2.

**Summary/Conclusion**

This study validates our prognostic system in elderly pts with BCR::ABL1+ ALL, revealing a previously unrecognized subgroup (non-*IKZF1*$^{plus}$/MRD-) with a favorable prognosis. Furthermore, for high-risk pts in this poor-outcome population, CAR-T therapy may offer a viable treatment strategy.

**Copyright Information:** (Online) ISSN: 2572-9241
©2026 The Author(s). HemaSphere is published by John Wiley & Sons Ltd on behalf of European Hematology Association. This is an open access Abstract Book distributed under the Attribution-NonCommercial-NoDerivs (CC BY-NC-ND), which allows third parties to download the articles and share them with others as long as they credit the author and the Abstract Book, but they cannot change the content in any way or use them commercially.
**Abstract Book Citations:** Authors, Title, HemaSphere, 2026;10:(S1):pages. The abstract book DOIs can be found on the "Author Information" page.
**Disclaimer:** Articles published in the journal HemaSphere exclusively reflect the opinions of the authors. The authors are responsible for all content in their abstracts including accuracy of the facts, statements, citing resources, etc.

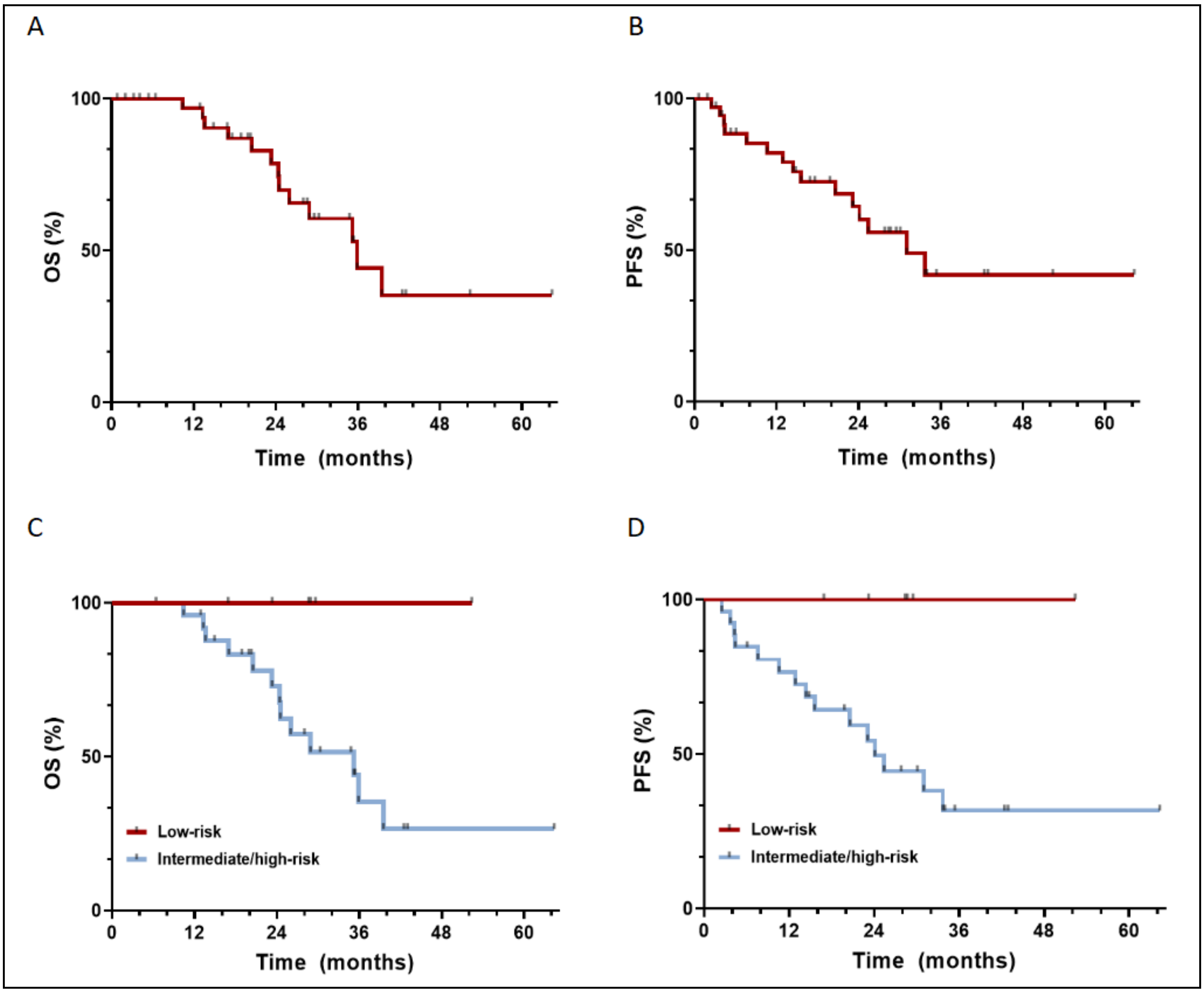

Figure 1. Survival outcomes and subgroup analysis (low-risk vs. Intermediate/high-risk)

**Copyright Information:** (Online) ISSN: 2572-9241
©2026 The Author(s). HemaSphere is published by John Wiley & Sons Ltd on behalf of European Hematology Association. This is an open access Abstract Book distributed under the Attribution-NonCommercial-NoDerivs (CC BY-NC-ND), which allows third parties to download the articles and share them with others as long as they credit the author and the Abstract Book, but they cannot change the content in any way or use them commercially.
**Abstract Book Citations:** Authors, Title, HemaSphere, 2026;10:(S1):pages. The abstract book DOIs can be found on the "Author Information" page.
**Disclaimer:** Articles published in the journal HemaSphere exclusively reflect the opinions of the authors. The authors are responsible for all content in their abstracts including accuracy of the facts, statements, citing resources, etc.

### (PF401) EFFECTS OF IMMUNOGLOBULIN REPLETION IN ADULTS WITH B CELL ACUTE LYMPHOBLASTIC LEUKEMIA RECEIVING BLINATUMOMAB

**Topic:** 02. Acute lymphoblastic leukemia - Clinical

Emma Matzinger*[1], Rosa Ancaya[2], David Swoboda[2], Daniel Kerr[2], Nikesh Shah[2]

*[1]University of South Florida Morsani College of Medicine, Tampa, United States of America;[2]Tampa General Hospital Cancer Institute, Tampa, United States of America;*

### Background

Blinatumomab (blin) has improved survival outcomes for patients with B-cell acute lymphoblastic leukemia (B-ALL); however, infectious complications remain a challenge, with some prospective trials reporting upwards of 30% of patients developing infections. There is data supporting the use of intravenous immunoglobulin (IVIG) repletion for patients with hypogammaglobulinemia after CAR-T therapy and BCMA-directed bispecific antibodies; however, there is minimal evidence on the role of IVIG for blin-associated hypogammaglobulinemia.

### Aims

In this study, we aim to retrospectively review oursingle-center population treated with blin for B-ALL and determine rates of infection and whether any difference exists for those who received IVIG repletion during the duration of treatment

### Methods

This is a retrospective cohort study reviewing patients with B-ALL treated at a tertiary care center in the United States from 2021-2025. Patients completing at least one cycle of blin with adequate toxicity data were included for further analysis.Statistical analysis was completed on SPSSv31. Chi square tests were performed to determine association between IVIG repletion and infection rates, as well as infection-related hospitalization/ER visit rates.

### Results

Among the 55 patients with B-ALL who received blin, 15 (27%) received IVIG. 33 (60%) patients developed infections of any grade. 22 (40%) patients developed grade 3 or higher infections requiring emergency room (ER) visits or hospitalization. Of the 15 patients who received IVIG, 12 (80%) had infections, 9 (60%) with grade 3 or higher. Of the 40 patients who did not receive IVIG, 21 (52.5%) had infections, 12 (30%) with grade 3 or higher. Of the 15 patients who received IVIG, 8 (53%) received as primary prophylaxis and 7 (47%) received as secondary prophylaxis. Of the patients who received IVIG primary prophylaxis, 5 (62.5%) developed infections, out of which 4 (50%) developed infections necessitating an ER visit/hospitalization. There was no significant association between IVIG replacement and total infection rates (p=0.064), or grade 3 or higher infection rates leading to ER visit or hospitalization (p=0.064). Of the total 33 patients who developed infections, 9 (27.3%) developed URI, 6 (18.2%) developed UTI, 6 (18.2%) developed GI infections, 6 (18.2%) developed COVID-19, 6 (18.2%) developed bacteremia, 3 (9%) developed pneumonia, and 10 (30%) developed other infections, including endocarditis, CMV viremia, CNS infection, joint replacement infection, port infection, and oral thrush. 13 (40%) developed multiple infections. Two (3.6%) patients died with active infection with concurrent leukemia progression.

**Copyright Information:** (Online) ISSN: 2572-9241
©2026 The Author(s). HemaSphere is published by John Wiley & Sons Ltd on behalf of European Hematology Association. This is an open access Abstract Book distributed under the Attribution-NonCommercial-NoDerivs (CC BY-NC-ND), which allows third parties to download the articles and share them with others as long as they credit the author and the Abstract Book, but they cannot change the content in any way or use them commercially.
**Abstract Book Citations:** Authors, Title, HemaSphere, 2026;10:(S1):pages. The abstract book DOIs can be found on the "Author Information" page.
**Disclaimer:** Articles published in the journal HemaSphere exclusively reflect the opinions of the authors. The authors are responsible for all content in their abstracts including accuracy of the facts, statements, citing resources, etc.

**Summary/Conclusion**

This is the largest retrospective study to our knowledge assessing the impact of IVIG repletion on infections in adult B-ALL patients receiving blin. There was no difference in rates of any grade infection, or grade 3 or higher infection with IVIG replacement. This is consistent with findings by Wo et al, though both studies are limited by their retrospective nature and small sample size. In contrast, studies have shown benefits of IVIG repletion for hypogammaglobulinemia after other T-cell-engaging therapies in myeloma and lymphoma, and a prospective study in pediatric B-ALL patients showed decreased hospitalization for fever and decreased antibiotic use with IVIG primary prophylaxis. Ultimately, a randomized prospective trial may be warranted to elucidate the role of IVIG in post-blin hypogammaglobulinemia.

**Copyright Information:** (Online) ISSN: 2572-9241
©2026 The Author(s). HemaSphere is published by John Wiley & Sons Ltd on behalf of European Hematology Association. This is an open access Abstract Book distributed under the Attribution-NonCommercial-NoDerivs (CC BY-NC-ND), which allows third parties to download the articles and share them with others as long as they credit the author and the Abstract Book, but they cannot change the content in any way or use them commercially.
**Abstract Book Citations:** Authors, Title, HemaSphere, 2026;10:(S1):pages. The abstract book DOIs can be found on the “Author Information” page.
**Disclaimer:** Articles published in the journal HemaSphere exclusively reflect the opinions of the authors. The authors are responsible for all content in their abstracts including accuracy of the facts, statements, citing resources, etc.

### (PF402) THE FIRST CZECH REAL-WORLD EVIDENCE OF BREXUCABTAGENE AUTOLEUCEL TREATMENT OUTCOMES AND TOXICITY IN ADULT ACUTE LYMPHOBLASTIC LEUKEMIA

**Topic:** 02. Acute lymphoblastic leukemia - Clinical

Stepan Hrabovsky*[1], Frantisek Folber[1], Jan Horacek[2, 3], Jiří Šrámek[4], Barbora Čemusová[5], Barbora Dluhosova[6], Adam Laznicka[5, 7], Ondrej Susol[6], Michal Karas[4], Zdenek Koristek[6], Tomáš Szotkowski[8], Hana Halamova[1], Barbara Dvorackova[1], Zuzana Vrzalova[1, 9], Jana Kotaskova[1], Vaclava Polivkova[5, 7], Katerina MacHova Polakova[5, 7], Pavel Zak[2], Michael Doubek[1, 9], Cyril Salek[5, 7]

*[1]Faculty of Medicine, Masaryk University and University Hospital Brno, Department of Internal Medicine, Hematology and Oncology, Brno, Czechia; [2]Faculty of Medicine in Hradec Kralove, Charles University and University Hospital Hradec Kralove, 4th Department of Internal Medicine – Haematology, Hradec Kralove, Czechia; [3]Military Faculty of Medicine, University of Defence, Department of Military Internal Medicine and Military Hygiene, Hradec Kralove, Czechia; [4]Faculty of Medicine in Plzen, Charles University and University Hospital Plzen, Department of Hemato-oncology, Plzen, Czechia; [5]Institute of Hematology and Blood Transfusion, Prague, Czechia; [6]Faculty of Medicine, University of Ostrava and University Hospital Ostrava, Department of Hematooncology, Ostrava, Czechia; [7]First Faculty of Medicine, Charles University, Prague, Czechia;[8]Faculty of Medicine, Palacky University Olomouc and University Hospital Olomouc, Department of Hemato-oncology, Olomouc, Czechia; [9]Central European Institute of Technology (CEITEC), Brno, Czechia;*

### Aims

Brexucabtagene autoleucel (brexu-cel) has emerged as an important therapeutic option for adult patients with B-cell precursor acute lymphoblastic leukemia (B-ALL), particularly following prior exposure to blinatumomab and inotuzumab ozogamicin. The objective of this study was to evaluate the efficacy and safety of brexu-cel in Czech patients and to compare these findings with outcomes reported from international clinical trials and real-world cohorts.

### Methods

We conducted an analysis of all adult patients with B-ALL who were indicated for brexu-cel therapy in Czechia between December 2022 and September 2025. Collected data included baseline demographic and disease characteristics prior to apheresis, details of bridging therapy, treatment response after infusion, adverse events, and survival parameters. The results were compared with published data from the ZUMA-3 clinical trial and real-world evidence (RWE) registries ROCCA, CIBMTR, and DESCAR-T.

**Copyright Information:** (Online) ISSN: 2572-9241
©2026 The Author(s). HemaSphere is published by John Wiley & Sons Ltd on behalf of European Hematology Association. This is an open access Abstract Book distributed under the Attribution-NonCommercial-NoDerivs (CC BY-NC-ND), which allows third parties to download the articles and share them with others as long as they credit the author and the Abstract Book, but they cannot change the content in any way or use them commercially.
**Abstract Book Citations:** Authors, Title, HemaSphere, 2026;10:(S1):pages. The abstract book DOIs can be found on the "Author Information" page.
**Disclaimer:** Articles published in the journal HemaSphere exclusively reflect the opinions of the authors. The authors are responsible for all content in their abstracts including accuracy of the facts, statements, citing resources, etc.

### Results

Overall, 27 patients were indicated for brexu-cel; 25 proceeded to apheresis and 23 ultimately received the infusion. The median cohort follow-up was 11.2 months. Among the 23 infused patients, 3 (13%) harbored the *BCR::ABL*1 fusion, 2 had a *KMT2A* rearrangement, 1 had the *TCF3::PBX1* fusion, 2 had the *BCR::ABL1-like* profile, and 2 had the *IKZF1*plus profile. Five of these 23 patients (22%) had been previously exposed to blinatumomab, 19/23 (83%) to inotuzumab ozogamicin (mostly as bridging therapy) and 12/23 (52%) had undergone allogeneic hematopoietic stem cell transplantation (alloSCT) before brexu-cel infusion. Following bridging therapy, which incorporated inotuzumab ozogamicin in 14/25 (56%) cases, 20/22 (91%) evaluable patients achieved complete hematologic remission (CR). Complete molecular remission (CMR) was achieved in 17/23 patients (74%) at 3 months after brexu-cel administration, while another 3/23 (13%) patients achieved CR with positive measurable residual disease (MRD). Severe adverse events (CRS or ICANS grade ≥3) were both documented in 2/23 (9%) of patients. Two patients (9%) died within 28 days after the infusion (septic shock, invasive fungal infection). Peak CAR-T cell expansion was variable among the cohort, spanning 0.3–1500 cells/μL of blood and had no statistically significant impact on survival. Loss of detectable CAR-T cells occurred at 1.0–17.0 months (median 3.0), and loss of B-cell aplasia at 1.9–14.5 months (median 3.0) after the infusion. No statistically significant difference in EFS or OS was observed between patients with early (≤3 months) vs. late (>3 months) loss of detectable CAR-T cells or B-cell aplasia. Eight patients (35%) subsequently underwent alloSCT between 3 and 6 months after brexu-cel infusion. The estimated 12-month event-free survival (EFS) and overall survival (OS) were 61% and 74%, respectively, while the corresponding 24-month EFS and OS were 34% and 49%.

### Summary/Conclusion

This is the first Czech real-world analysis of brexu-cel treatment outcomes in B-ALL. In comparison with previously published cohorts, we observed a lower incidence of severe ICANS. The survival outcomes in our cohort were comparable to those reported in ZUMA-3 and RWE datasets. These findings may be attributable to the more frequent use of inotuzumab ozogamicin as bridging therapy—resulting in lower disease burden at the time of brexu-cel infusion—as well as to the systematic use of anakinra in ICANS prophylaxis at our centers.

This work was supported by the Czech Ministry of Health (AZV NU22-03-00210).

**Copyright Information:** (Online) ISSN: 2572-9241
©2026 The Author(s). HemaSphere is published by John Wiley & Sons Ltd on behalf of European Hematology Association. This is an open access Abstract Book distributed under the Attribution-NonCommercial-NoDerivs (CC BY-NC-ND), which allows third parties to download the articles and share them with others as long as they credit the author and the Abstract Book, but they cannot change the content in any way or use them commercially.
**Abstract Book Citations:** Authors, Title, HemaSphere, 2026;10:(S1):pages. The abstract book DOIs can be found on the "Author Information" page.
**Disclaimer:** Articles published in the journal HemaSphere exclusively reflect the opinions of the authors. The authors are responsible for all content in their abstracts including accuracy of the facts, statements, citing resources, etc.

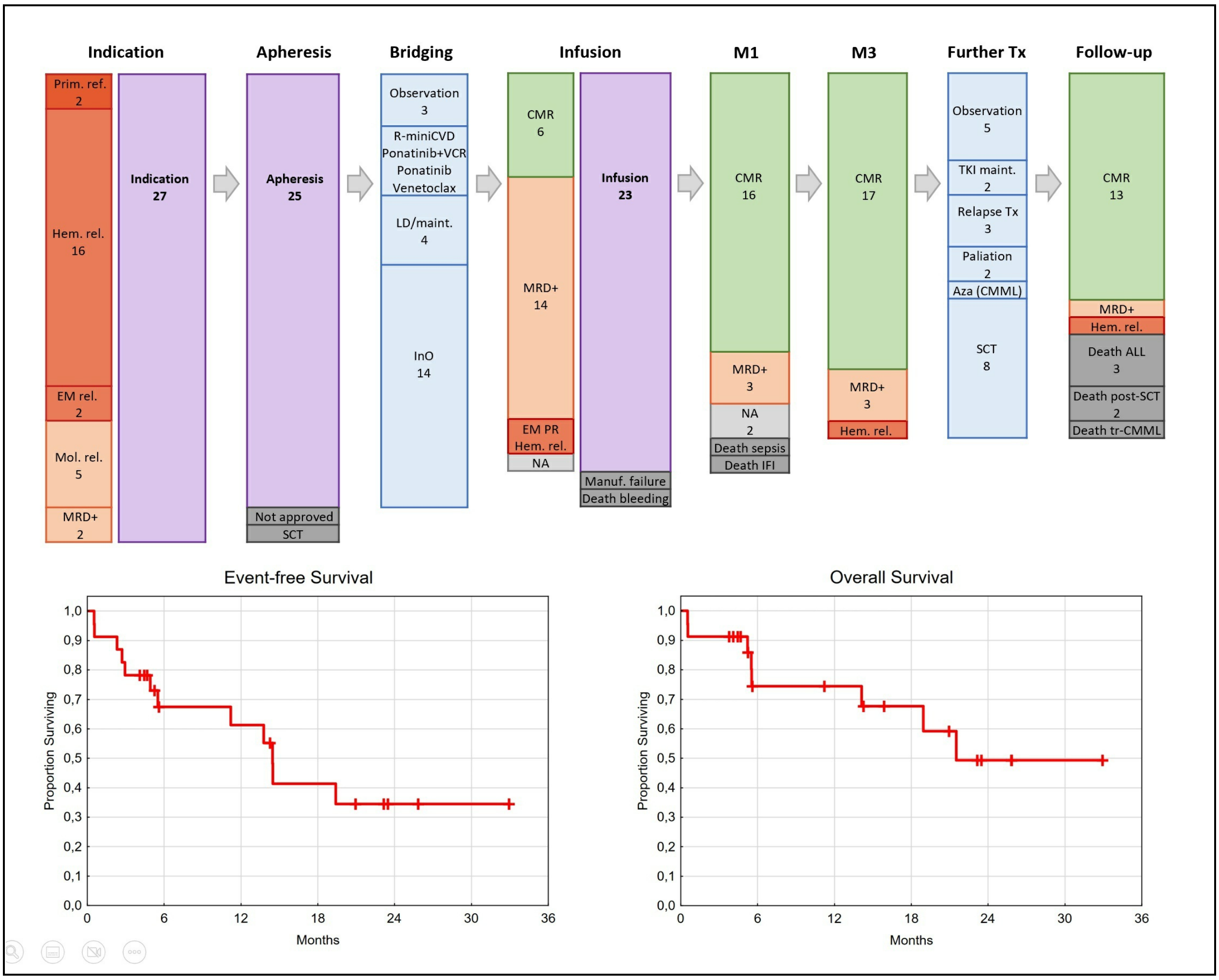

**Copyright Information:** (Online) ISSN: 2572-9241
©2026 The Author(s). HemaSphere is published by John Wiley & Sons Ltd on behalf of European Hematology Association. This is an open access Abstract Book distributed under the Attribution-NonCommercial-NoDerivs (CC BY-NC-ND), which allows third parties to download the articles and share them with others as long as they credit the author and the Abstract Book, but they cannot change the content in any way or use them commercially.
**Abstract Book Citations:** Authors, Title, HemaSphere, 2026;10:(S1):pages. The abstract book DOIs can be found on the "Author Information" page.
**Disclaimer:** Articles published in the journal HemaSphere exclusively reflect the opinions of the authors. The authors are responsible for all content in their abstracts including accuracy of the facts, statements, citing resources, etc.

### (PF403) PEGASPARGASE TOXICITY ACROSS DIFFERENT INDUCTION DOSING SCHEMES IN ADULT ACUTE LYMPHOBLASTIC LEUKEMIA: A COMPREHENSIVE RETROSPECTIVE ANALYSIS

**Topic:** 02. Acute lymphoblastic leukemia - Clinical

Stepan Hrabovsky*[1, 2], Cyril Vojtech Precechtel[3], Lenka Radova[2, 4], Frantisek Folber[1, 2], Hana Halamova[1, 2], Barbara Dvorackova[1, 2], Zuzana Vrzalova[1, 2, 4], Jana Kotaskova[1, 2], Michael Doubek[1, 2, 4]

*[1]University Hospital Brno, Department of Internal Medicine, Hematology and Oncology, Brno, Czechia; [2]Faculty of Medicine, Masaryk University, Brno, Czechia; [3]Faculty of Pharmacy, Masaryk University, Brno, Czechia; [4]Central European Institute of Technology (CEITEC), Brno, Czechia;*

### Background

Pegylated asparaginase (pegaspargase) belongs to the cornerstones of intensive treatment for acute lymphoblastic leukemia/lymphoma (ALL/LBL). Although its toxicity profile is well recognized, adult ALL treatment protocols differ substantially in the number and dosing of pegaspargase administrations during induction and consolidation, and comparative toxicity data across regimens remain limited.

### Aims

To describe and compare pegaspargase-associated toxicity across different dosing schemes in adult ALL/LBL patients treated with intensive and non-intensive protocols.

### Methods

We retrospectively analyzed adult ALL/LBL patients treated consecutively at our center between 2006 and 2025 within 5 protocol cohorts: three intensive regimens with 0, 1, or 2 doses of pegaspargase during two induction cycles (group I0, I1, I2) and another 6 doses during the consolidation cycles; and two non-intensive regimens administering pegaspargase either in consolidation only (N0) or induction only (N1). Predisposing factors (obesity, hepatic steatosis, liver impairment, prior thromboembolism) were recorded. For each cycle, the administered dose and the worst post-dose laboratory values were collected (bilirubin, liver enzymes, amylase, coagulation, D-dimer, albumin). Clinical events included thromboembolism, pancreatitis, and allergic reactions.

### Results

A total of 129 patients were included (106 intensive; 23 non-intensive). Within intensive protocols, patients in the I2 cohort received overall lower pegaspargase doses and exhibited lower ALT, AST, amylase, aPTT, and fewer thromboembolic events after Induction I. When comparing Induction I versus Induction II within the I2 cohort, the second pegaspargase dose was associated with higher ALT, AST, ALP, D-dimer and lower fibrinogen. However, these laboratory changes did not translate into increased rates of thrombosis, pancreatitis, or allergic reactions. Patients with *BCR::ABL1*+ B-ALL demonstrated higher ALT and AST and lower albumin and fibrinogen during induction compared with *BCR::ABL1*– B-ALL and T-ALL/LBL. Across consolidation cycles, laboratory differences among I0, I1, and I2 groups were inconsistent, without a clear dose–toxicity relationship. In pooled comparisons, non-intensive protocols were associated with lower albumin and fibrinogen levels despite lower cumulative doses, but without a higher incidence of thromboembolism, pancreatitis, or allergic reactions. Among predisposing factors, obesity and hepatic steatosis were associated with higher amylase levels, female sex with lower albumin, while prior thromboembolism correlated with higher fibrinogen levels after induction I. Liver impairment and hepatomegaly did not significantly influence post-asparaginase toxicity.

**Copyright Information:** (Online) ISSN: 2572-9241
©2026 The Author(s). HemaSphere is published by John Wiley & Sons Ltd on behalf of European Hematology Association. This is an open access Abstract Book distributed under the Attribution-NonCommercial-NoDerivs (CC BY-NC-ND), which allows third parties to download the articles and share them with others as long as they credit the author and the Abstract Book, but they cannot change the content in any way or use them commercially.
**Abstract Book Citations:** Authors, Title, HemaSphere, 2026;10:(S1):pages. The abstract book DOIs can be found on the “Author Information” page.
**Disclaimer:** Articles published in the journal HemaSphere exclusively reflect the opinions of the authors. The authors are responsible for all content in their abstracts including accuracy of the facts, statements, citing resources, etc.

### Summary/Conclusion

With rigorous patient-specific dose tailoring, administration of a second pegaspargase dose during induction was safe and, despite laboratory abnormalities, did not translate into a higher incidence of thromboembolism, pancreatitis, or allergy compared with a single induction dose. *BCR::ABL1*+ B-ALL patients displayed more pronounced toxicity, likely reflecting concomitant tyrosine kinase inhibitor exposure. Pegaspargase was safe also for fit elderly patients receiving non-intensive regimens without increased clinical toxicity. Interpretation of consolidation-phase toxicity was limited by the retrospective design and variable laboratory monitoring frequency and warrants further research.
This work was supported by the Czech Ministry of Health (AZV NU22-03-00210).

| **Patient characteristics** | **Intensive** N = 106 | **Non-intensive** N = 23 |
|---|---|---|
| **Sex, male** | 70 (66%) | 13 (56%) |
| **Age (median)** | 18–63 (35) | 51–77 (65) |
| **ALL subtype** | | |
| *BCR::ABL1*– B-ALL | 60 (57%) | 9 (39%) |
| *BCR::ABL1*+ B-ALL | 16 (15%) | 8 (35%) |
| T-ALL | 20 (19%) | 5 (22%) |
| T-LBL | 10 (9%) | 1 (4%) |
| **Treatment protocol** | | |
| I0 | 16 (15%) | - |
| I1 | 77 (73%) | - |
| I2 | 13 (12%) | - |
| N0 | - | 8 (35%) |
| N1 | - | 15 (65%) |
| **Predisposing factors** | | |
| Obesity BMI ≥35 | 23 (22%) | 7 (30%) |
| Liver steatosis | 20 (19%) | 6 (26%) |
| Liver enzymes/bili gr. ≥2 | 21 (20%) | 1 (4%) |
| Hepatomegaly | 17 (16%) | 0 |
| Thrombembolism in history | 7 (7%) | 4 (17%) |

**Treatment protocols pegaspargase standard dosing (U/m²)**

| Cohort | Protocol | Induction | Consolidation |
|---|---|---|---|
| I0 | BlinCELL trial (NCT04554485) | 0 | 6x 2000 |
| I1 | ALL CELL 2012 Junior | 2000 | 6x 2000 |
| I2 | ALL CELL 2023 Junior | 2000 + 1000 | 6x 2000 |
| N0 | ALL CELL 2023 Elderly | 0 | 3x 500–1000 |
| N1 | ALL CELL 2012 Elderly | 500–1000 | 0 |

**Induction I (group I1 vs. I2)**

| Parameter | p-value* |
|---|---|
| **IND1_PEGASP dose** | **0.031** |
| IND1_Bilirubin_max | 0.172 |
| **IND1_ALT_max** | **0.002** |
| **IND1_AST_max** | **0.002** |
| IND1_GGT_max | 0.245 |
| IND1_ALP_max | 0.753 |
| IND1_Albumin_min | 0.273 |
| **IND1_AMS_max** | **0.003** |
| IND1_PT_max | 1 |
| IND1_Fibrinogen_min | 0.600 |
| **IND1_aPTT_max** | **0.008** |
| IND1_Antithrombin_min | 0.112 |
| IND1_Ddimer_max | 0.156 |
| **IND1_Thromboembolism** | **0.059** |
| IND1_Pancreatitis | 1 |
| IND1_Allergy | 1 |

**Induction I (intensive vs. non-intensive)**

| Parameter | p-value* |
|---|---|
| **IND1_PEGASP dose** | **<0.001** |
| IND1_Bilirubin_max | 0.681 |
| IND1_ALT_max | 0.853 |
| IND1_AST_max | 0.941 |
| IND1_GGT_max | 0.630 |
| IND1_ALP_max | 0.387 |
| **IND1_Albumin_min** | **<0.001** |
| IND1_AMS_max | 0.093 |
| IND1_PT_max | 0.828 |
| **IND1_Fibrinogen_min** | **0.061** |
| IND1_aPTT_max | 0.315 |
| IND1_Antithrombin_min | 0.392 |
| IND1_Ddimer_max | 0.120 |
| IND1_Thromboembolism | 1 |
| IND1_Pancreatitis | 0.466 |
| IND1_Allergy | 1 |

**Predisposing factors effect on Induction I toxicity***

| Parameter | Obesity | Steatosis | Liver enzymes or bili ≥2 | Hepatomegaly | Thrombembolism in history | Sex | *BCR::ABL1*+ B-ALL vs. others |
|---|---|---|---|---|---|---|---|
| IND1_Bilirubin_max | 0.234 | **0.042** | 0.094 | 0.641 | 0.748 | 0.619 | 0.186 |
| IND1_ALT_max | 0.308 | 0.734 | 0.075 | 0.377 | 0.333 | **0.021** | **0.003** |
| IND1_AST_max | 0.746 | 0.758 | 0.298 | 0.217 | 0.121 | 0.949 | **0.022** |
| IND1_GGT_max | 0.530 | 0.329 | 0.363 | 0.673 | 0.593 | 0.393 | 0.307 |
| IND1_ALP_max | 0.950 | 0.921 | 0.684 | 0.843 | 0.279 | 0.504 | 0.349 |
| IND1_Albumin_min | 0.390 | 0.473 | 0.534 | 0.325 | 0.361 | **0.002** | **0.021** |
| IND1_AMS_max | **0.008** | **0.018** | 0.584 | 0.305 | 0.403 | 0.950 | 0.103 |
| IND1_PT_max | 0.062 | 0.206 | 0.408 | 0.729 | 0.393 | 0.356 | 0.386 |
| IND1_Fibrinogen_min | 0.074 | **0.048** | 0.808 | 0.503 | **0.028** | 0.242 | **0.004** |
| IND1_aPTT_max | 0.195 | **0.048** | 0.111 | 0.420 | 0.172 | 0.271 | 0.125 |
| IND1_Antithrombin_min | 0.487 | 0.940 | 0.353 | 0.098 | 0.895 | 0.893 | 0.645 |
| IND1_Ddimer_max | 0.363 | 0.965 | 0.815 | 0.304 | 0.399 | 0.089 | 0.120 |

*Fisher exact, Mann-Whitney

**Copyright Information:** (Online) ISSN: 2572-9241
©2026 The Author(s). HemaSphere is published by John Wiley & Sons Ltd on behalf of European Hematology Association. This is an open access Abstract Book distributed under the Attribution-NonCommercial-NoDerivs (CC BY-NC-ND), which allows third parties to download the articles and share them with others as long as they credit the author and the Abstract Book, but they cannot change the content in any way or use them commercially.
**Abstract Book Citations:** Authors, Title, HemaSphere, 2026;10:(S1):pages. The abstract book DOIs can be found on the "Author Information" page.
**Disclaimer:** Articles published in the journal HemaSphere exclusively reflect the opinions of the authors. The authors are responsible for all content in their abstracts including accuracy of the facts, statements, citing resources, etc.

### (PF404) ASCIMINIB EFFECTIVENESS IN PATIENTS WITH PHILADELPHIA CHROMOSOME-POSITIVE ACUTE LYMPHOBLASTIC LEUKEMIA (PH+ ALL) IN THE REAL WORLD: THE ASCERTAIN STUDY

**Topic:** 02. Acute lymphoblastic leukemia - Clinical

Marlise Luskin*[1], Gaurav Sutrave[2, 3], Mathilde Chanut[4, 5], Yok Lam Kwong[6], Elad Jacoby[7, 8], Piero Galieni[9], Anita Rijneveld[10], Raheel Iftikhar[11], Łukasz Bołkun[12], Javier Ortiz Martin[13], Deborah Yallop[14], Sibel Gunes[15], Germano Ferreira[15], Biswajit Kumar[16], Himanshu Pokhriyal[17], Philippe Rousselot[4, 5]

*[1]Dana-Farber Cancer Institute, Department of Medical Oncology, Boston, United States of America;[2]The University of Sydney, Sidney, Australia; [3]Westmead Hospital, Department of Haematology, Sidney, Australia;[4]Centre Hospitalier de Versailles Site André Mignot, Hematology, Le Chesnay-Rocquencourt, France; [5]University of Versailles Paris-Saclay, Versailles, France;[6]Queen Mary Hospital, Department of Medicine, Hong Kong, Hong Kong; [7]The Edmond and Lily Safra Children's Hospital, Sheba Medical Center, Division of Pediatric Hematology and Oncology, Tel Hashomer, Israel; [8]Tel Aviv University, Faculty of Medical & Health Sciences, Tel Aviv, Israel; [9]Ospedale C. E G. Mazzoni, UOC Ematologia e Terapia Cellulare, Ascoli Piceno, Italy; [10]Erasmus University Medical Center Cancer Institute, Department of Haematology, Rotterdam, The Netherlands;[11]National University of Medical Sciences, Rawalpindi, Pakistan; [12]Medical University of Bialystok, Department of Hematology, Bialystok, Poland; [13]Hospital Universitario de La Princesa, Servicio de Hematología y Hemoterapia, Madrid, Spain;[14]King's College Hospital NHS Foundation Trust, Department of Haematological Medicine, London, United Kingdom; [15]Novartis Pharma AG, Basel, Switzerland; [16]Novartis Pharmaceuticals Corporation, East Hanover, United States of America; [17]Novartis Healthcare Private Ltd, Hyderabad, India;*

### Background

ATP-competitive tyrosine kinase inhibitors (TKIs) used in combination with chemo- or immunotherapy have improved outcomes in Ph+ ALL; however, relapse still occurs despite appropriate frontline therapy, resulting in limited treatment options. Asciminib is approved for Ph+ chronic myeloid leukemia (CML) in chronic phase. The asciminib managed access program (MAP) allowed adults with Ph+ ALL resistant/refractory or intolerant to available treatment to receive asciminib as monotherapy or in combination upon individual request when clinicians deemed the potential benefit to outweigh the risk.

### Aims

This chart review study collected existing data from the medical charts of patients (pts) enrolled in the MAP to better understand the effectiveness and safety of asciminib in a real-world setting.

### Methods

This was a non-interventional, multi-country, retrospective chart review in eligible pts aged ≥18 years with Ph+ ALL who had relapsed or were refractory/resistant or intolerant to available treatment and who received ≥1 dose of asciminib within the MAP. The date of the first administration of asciminib (index date) occurred between 1 March 2019 and 30 June 2023. Existing data were extracted from index date up to 13 months, or until discontinuation, death or last visit, whichever occurred first. The primary endpoint was complete hematologic remission (CR) and CR with incomplete count recovery (CRi) anytime in the first 3 months of treatment based on peripheral blood assessments (circulating lymphoblasts and blood counts).

### Results

A total of 37 pts received asciminib. At treatment start, 25 pts (67.6%) received asciminib as monotherapy. Median asciminib dose intensity was 400 mg/day (range 71.6–400.0). Pts were heavily pretreated and almost all had previously received ponatinib (**Table**).

**Copyright Information:** (Online) ISSN: 2572-9241
©2026 The Author(s). HemaSphere is published by John Wiley & Sons Ltd on behalf of European Hematology Association. This is an open access Abstract Book distributed under the Attribution-NonCommercial-NoDerivs (CC BY-NC-ND), which allows third parties to download the articles and share them with others as long as they credit the author and the Abstract Book, but they cannot change the content in any way or use them commercially.
**Abstract Book Citations:** Authors, Title, HemaSphere, 2026;10:(S1):pages. The abstract book DOIs can be found on the "Author Information" page.
**Disclaimer:** Articles published in the journal HemaSphere exclusively reflect the opinions of the authors. The authors are responsible for all content in their abstracts including accuracy of the facts, statements, citing resources, etc.

Overall, 23 pts (62.2%) achieved CR/CRi within 3 months (95% CI: 44.8, 77.6). Median duration of response was 7.4 months. Median overall survival among all pts was 7.7 months. Nineteen pts were assessed for minimal residual disease (MRD) at time of CR/CRi and 7 (36.8%) were MRD negative. Of the 13 pts with T315I mutation, 10 achieved CR/CRi at any time during follow-up. Thirty-three pts (89.2%) permanently discontinued asciminib; reasons for permanent discontinuation included disease progression (n=16) and adverse events (AEs, n=5). Any-grade treatment-emergent AEs were reported in 73.0% of pts (Grade ≥3, 56.8%); the most common any-grade AE was neutropenia (21.6%). No new safety signals were observed. Most deaths (22/28) were Ph+ ALL related.

**Table.** Patient baseline characteristics

**Copyright Information:** (Online) ISSN: 2572-9241
©2026 The Author(s). HemaSphere is published by John Wiley & Sons Ltd on behalf of European Hematology Association. This is an open access Abstract Book distributed under the Attribution-NonCommercial-NoDerivs (CC BY-NC-ND), which allows third parties to download the articles and share them with others as long as they credit the author and the Abstract Book, but they cannot change the content in any way or use them commercially.
**Abstract Book Citations:** Authors, Title, HemaSphere, 2026;10:(S1):pages. The abstract book DOIs can be found on the "Author Information" page.
**Disclaimer:** Articles published in the journal HemaSphere exclusively reflect the opinions of the authors. The authors are responsible for all content in their abstracts including accuracy of the facts, statements, citing resources, etc.

| | Patients<br>N=37 |
|---|---|
| Median age at index date (range), years | 53 (19–84) |
| Disease status (bone marrow blasts), n (%)<br>≥5%<br><5%<br>Unknown | <br>25 (67.6)<br>9 (24.3)<br>3 (8.1) |
| Prior treatment lines, n (%)<br>1<br>2<br>3<br>≥4 | <br>1 (2.7)<br>12 (32.4)<br>8 (21.6)<br>16 (43.2) |
| Ponatinib pretreatment, n (%) | 35 (94.6) |
| T315I mutation, n (%) | 13 (35.1) |
| Reason for starting asciminib, n (%)<br>Relapse<br>Specific mutations<br>Resistance<br>Intolerance<br>Other | <br>25 (67.6)<br>10 (27.0)<br>9 (24.3)<br>7 (18.9)<br>5 (13.5) |

**Copyright Information:** (Online) ISSN: 2572-9241
©2026 The Author(s). HemaSphere is published by John Wiley & Sons Ltd on behalf of European Hematology Association. This is an open access Abstract Book distributed under the Attribution-NonCommercial-NoDerivs (CC BY-NC-ND), which allows third parties to download the articles and share them with others as long as they credit the author and the Abstract Book, but they cannot change the content in any way or use them commercially.
**Abstract Book Citations:** Authors, Title, HemaSphere, 2026;10:(S1):pages. The abstract book DOIs can be found on the "Author Information" page.
**Disclaimer:** Articles published in the journal HemaSphere exclusively reflect the opinions of the authors. The authors are responsible for all content in their abstracts including accuracy of the facts, statements, citing resources, etc.

**Summary/Conclusion**

Adults with Ph+ ALL that was relapsed, resistant/refractory or intolerant to previous treatment achieved high rates of CR/CRi with asciminib as monotherapy or in combination with chemotherapy/immunotherapy, despite most pts being heavily pretreated and/or harboring T315I. The safety profile was consistent with that of previous CML studies. Results, while positive, should be cautiously interpreted given the retrospective nature of the study and heterogenous pt population.

© 2026 American Society of Clinical Oncology, Inc. Reused with permission. This abstract was accepted and previously presented at the 2026 ASCO Annual Meeting. All rights reserved.

**Copyright Information:** (Online) ISSN: 2572-9241
©2026 The Author(s). HemaSphere is published by John Wiley & Sons Ltd on behalf of European Hematology Association. This is an open access Abstract Book distributed under the Attribution-NonCommercial-NoDerivs (CC BY-NC-ND), which allows third parties to download the articles and share them with others as long as they credit the author and the Abstract Book, but they cannot change the content in any way or use them commercially.
**Abstract Book Citations:** Authors, Title, HemaSphere, 2026;10:(S1):pages. The abstract book DOIs can be found on the "Author Information" page.
**Disclaimer:** Articles published in the journal HemaSphere exclusively reflect the opinions of the authors. The authors are responsible for all content in their abstracts including accuracy of the facts, statements, citing resources, etc.

### (PF405) REAL-WORLD INCIDENCE AND MANAGEMENT OF CRS AND ICANS IN ADULTS WITH NEWLY DIAGNOSED PHILADELPHIA CHROMOSOME–POSITIVE B-CELL ACUTE LYMPHOBLASTIC LEUKEMIA TREATED WITH FRONTLINE TKI PLUS BLINATUMOMAB

**Topic:** 02. Acute lymphoblastic leukemia - Clinical

Rosa Ancaya*[1], Emma Matzinger[1], David Swoboda[1], Daniel Kerr[1], Nikesh Shah[1]

[1]*Tampa General Hospital Cancer Institute, Hematology Oncology, Tampa, United States of America;*

### Background

Tyrosine kinase inhibitors (TKI) with blinatumomab (blina) are a new standard for Philadelphia chromosome–positive B-cell acute lymphoblastic leukemia (Ph+ B-ALL); however, cytokine release syndrome (CRS) and immune effector cell-associated neurotoxicity syndrome (ICANS) remain concerns. Data on the incidence and management of these toxicities in real-world practice are limited. Characterizing their safety profile is critical to inform routine clinical use.

### Aims

To evaluate the real-world incidence, severity, and management of CRS and ICANS in adults with newly diagnosed (1L) Ph+ B-ALL treated with frontline TKI-based therapy followed by blina. Secondary objectives included treatment interruption or discontinuation due to immune-related toxicities.

### Methods

We performed a retrospective analysis of adult patients with 1L Ph+ B-ALL treated at a single institution from 2021 to 2025. Patients received frontline TKI, with or without one cycle of cytoreductive chemotherapy (chemo) followed by blina. CRS and ICANS were graded according to ASTCT consensus criteria. The primary endpoints were incidence and severity of CRS and ICANS. Secondary endpoints included management strategies, treatment interruption, and permanent discontinuation due to immune-related toxicities.

### Results

In our cohort of 99 patients with 1L B-ALL, 46 (46.5%) had Ph+ disease; 30 (30.3%) patients treated with TKI plus blina were included for analysis, others were excluded for receipt of alternative regimens. Median age was 63 years (30-84). Twenty-nine patients (97%) received ponatinib and one received asciminib due to cardiac comorbidities. Twenty-nine patients (97%) received one cycle of chemo prior to blina (17% hyperCVAD, 33% mini-CVD, 46% vincristine/dexamethasone), and one (3%) received steroids plus TKI alone. Median cycles of blincyto were 4 (1-4). All patients who received chemo with induction started blina at 28mcg/day on D1 with no ramp-up. CRS occurred in 12 patients (40%): grade 1 in 9 (30%) and grade 2 in 3 (10%) and no grade 3 or 4 events. Median time to CRS onset was 2 days (1-16), with 75% occurring by cycle 1, day 3. All events resolved within < 24 hours with supportive care ± corticosteroids; no patients required tocilizumab or permanent treatment discontinuation. ICANS occurred in 1 patient (3%), was grade 1, occurred concurrently with CRS, and resolved with supportive care. Infections were observed in 13 patients (43%), with 9 episodes (30%) requiring emergency department evaluation or hospitalization No treatment-related mortality was observed, and all patients were able to proceed with planned therapy. 2y OS was 100%, compared to 42.9% for 16 patients not receiving a blina-based regimen. CRS and ICANS did not impact OS.

**Copyright Information:** (Online) ISSN: 2572-9241
©2026 The Author(s). HemaSphere is published by John Wiley & Sons Ltd on behalf of European Hematology Association. This is an open access Abstract Book distributed under the Attribution-NonCommercial-NoDerivs (CC BY-NC-ND), which allows third parties to download the articles and share them with others as long as they credit the author and the Abstract Book, but they cannot change the content in any way or use them commercially.
**Abstract Book Citations:** Authors, Title, HemaSphere, 2026;10:(S1):pages. The abstract book DOIs can be found on the "Author Information" page.
**Disclaimer:** Articles published in the journal HemaSphere exclusively reflect the opinions of the authors. The authors are responsible for all content in their abstracts including accuracy of the facts, statements, citing resources, etc.

**Summary/Conclusion**

In this real-world single-institution cohort, frontline TKI-based therapy followed by blina was associated with a low incidence of high-grade immune-related toxicities. Though CRS and infections were more common than in prospective trials, this may reflect an older population with comorbidities excluded in trials.[1,2] CRS was limited to grade 1–2 and brief in duration, while ICANS was rare and mild. All patients were able to continue therapy, and no treatment-related mortality was observed. These findings support the feasibility and tolerability of TKI with blina in adults with 1L Ph+ B-ALL with appropriate monitoring and intervention, and may guide future monitoring strategies.

1. Jabbour, et al. Lancet Haematology, 2023.
2. Foa, et al. NEJM, 2020.

**Copyright Information:** (Online) ISSN: 2572-9241
©2026 The Author(s). HemaSphere is published by John Wiley & Sons Ltd on behalf of European Hematology Association. This is an open access Abstract Book distributed under the Attribution-NonCommercial-NoDerivs (CC BY-NC-ND), which allows third parties to download the articles and share them with others as long as they credit the author and the Abstract Book, but they cannot change the content in any way or use them commercially.
**Abstract Book Citations:** Authors, Title, HemaSphere, 2026;10:(S1):pages. The abstract book DOIs can be found on the "Author Information" page.
**Disclaimer:** Articles published in the journal HemaSphere exclusively reflect the opinions of the authors. The authors are responsible for all content in their abstracts including accuracy of the facts, statements, citing resources, etc.

### (PF406) TKI TREATMENT IN THE ELDERLY PH+ ALL. A CAMPUS ALL RETROSPECTIVE STUDY IN PATIENTS AGED 70 YEARS AND OLDER

**Topic:** 02. Acute lymphoblastic leukemia - Clinical

Francesca Kaiser*[1], Marco Antonacci[1], Loredana Elia[1], Michela Ansuinelli[1], Gianluca Cavallaro[2], Erika Borlenghi[3], Monia Lunghi[4], Fabio Forghieri[5], Ilaria Tanasi[6], Benedetta Cambo'[7], Sabina Chiaretti[1], Robin Foà[1]

*[1]Sapienza Università di Roma, Hematology, Department of Translational and Precision Medicine, Roma, Italy;[2]ASST Ospedale Papa Giovanni XXIII, Hematology and Bone Marrow Transplant Unit, Bergamo, Italy; [3]Azienda Ospedaliera Spedali Civili-Brescia, Hematology, Brescia, Italy; [4]Università del Piemonte Orientale and AOU Maggiore della Carità di Novara, Division of Hematology, Department of Translational Medicine, Novara, Italy; [5]Università degli Studi di Modena e Reggio Emilia, Section of Hematology, Department of Medical and Surgical Sciences, Reggio nell'Emilia, Italy; [6]Azienda Ospedaliera Universitaria Integrata di Verona, Hematology Unit, Department of Medicine, Verona, Italy; [7]University of Parma, Department of Medicine and Surgery, Hematology and BMT Unit, University of Parma, Parma, Italy., Parma, Italy;*

### Background

Philadelphia-positive acute lymphoblastic leukemia (Ph+ ALL), previously considered as the most unfavorable ALL subgroup, has shown improved outcomes since the introduction of tyrosine kinase inhibitors (TKIs) since 2000. The incidence of Ph+ ALL increases with age, being >50% in patients from the 6$^{th}$ decade onward. Indeed, in the elderly, the administration of a targeted therapy has allowed an effective treatment while markedly reducing chemotherapy-related toxicities.

### Aims

Thus, we assessed the real world outcome of elderly pts treated with toxicity-sparing strategies.

### Methods

We retrospectively collected, within the Campus ALL network in Italy, 69 cases of Ph+ ALL diagnosed in patients (pts) aged ≥70 between 2006 and 2025. All pts were treated only with TKIs (and steroids) in induction and consolidation. Pts who received chemotherapy after first relapse were also included.

**Copyright Information:** (Online) ISSN: 2572-9241
©2026 The Author(s). HemaSphere is published by John Wiley & Sons Ltd on behalf of European Hematology Association. This is an open access Abstract Book distributed under the Attribution-NonCommercial-NoDerivs (CC BY-NC-ND), which allows third parties to download the articles and share them with others as long as they credit the author and the Abstract Book, but they cannot change the content in any way or use them commercially.
**Abstract Book Citations:** Authors, Title, HemaSphere, 2026;10:(S1):pages. The abstract book DOIs can be found on the "Author Information" page.
**Disclaimer:** Articles published in the journal HemaSphere exclusively reflect the opinions of the authors. The authors are responsible for all content in their abstracts including accuracy of the facts, statements, citing resources, etc.

**Results**

The median age of the 69 pts was 77.2 years (range 70-90), median ECOG was 1 (0-4) and the median white blood cell count (WBC) at diagnosis was 18.7 x$10^9$/L (1.5-379 x$10^9$/L). The p190 isoform was detected in 43 pts, p210 in 24, and both isoforms were present in 2 cases. All pts were treated frontline with a TKI: 51 with imatinib, 15 with dasatinib and 3 with ponatinib. Complete hematologic remission (CHR) at d+70 was achieved in 57 pts (82.6%), 7 were refractory/early relapsed, 4 died prior to d+28, while in 1 response could not be assessed due to early deterioration. A complete molecular remission (CMR) was recorded in 29% of pts (n=20). Among the 57 who achieved a CHR, 33 (56%) switched to another TKI due to intolerance (n=10) or failure (n=23, 16 hematologic relapses and 7 molecular persistence), after a median time of 8 months. Two refractory pts switched to another TKI before d+70 due to intolerance and molecular persistence respectively, while the remaining 5 changed therapy after the established timepoint. Twenty-nine pts (42%) experienced a hematologic relapse at a median time of 13 months (mo); 2 relapsed after TKI discontinuation due to personal reasons. The median number of lines of treatment was 2 (1-6), with 9 pts being treated in third or further line with immunotherapy (either blinatumomab or inotuzumab). After a median follow-up of 52.9 mo (0.7-192), the median overall survival (mOS) was 26.3 mo (95% CI 19-34.4) and the median disease-free survival (mDFS) 17 mo (95% CI: 12–25). Pts aged 70-80 had a significantly better survival compared to pts aged ≥80: mOS 27.8 vs 13.2 mo, $p<0.05$ (Fig. 1A-). Overall, 48 pts died, 27 (56%) due to disease progression, 6 for senectus, 5 for cardio-vascular events, 4 for infections, 3 for non-hematologic malignancies, 2 for brain bleeding and 1 for unknown reasons. Univariate and multivariate analyses were performed including the following variables: age, gender, WBC, isoform, ECOG, frontline TKI and response at d+70. By univariate analysis, age and ECOG >2 were associated with a worse OS. By multivariate analysis ECOG >2 was confirmed as the main factor, together with WBC at diagnosis (WBC>70 x$10^9$/L was associated with a greater than 2-fold increased risk of death, HR 3.6, p=0.004).

**Summary/Conclusion**

Treatment with a TKI alone (plus steroids) in elderly (³70) Ph+ ALL pts allows to achieve very high rates of remission after induction. An early CMR appears to have a limited impact on survival probability. A personalized approach considering pre-existing comorbidities, TKI dose-adjustments and early toxicity management is crucial in this frail population.

**Copyright Information:** (Online) ISSN: 2572-9241
©2026 The Author(s). HemaSphere is published by John Wiley & Sons Ltd on behalf of European Hematology Association. This is an open access Abstract Book distributed under the Attribution-NonCommercial-NoDerivs (CC BY-NC-ND), which allows third parties to download the articles and share them with others as long as they credit the author and the Abstract Book, but they cannot change the content in any way or use them commercially.
**Abstract Book Citations:** Authors, Title, HemaSphere, 2026;10:(S1):pages. The abstract book DOIs can be found on the "Author Information" page.
**Disclaimer:** Articles published in the journal HemaSphere exclusively reflect the opinions of the authors. The authors are responsible for all content in their abstracts including accuracy of the facts, statements, citing resources, etc.

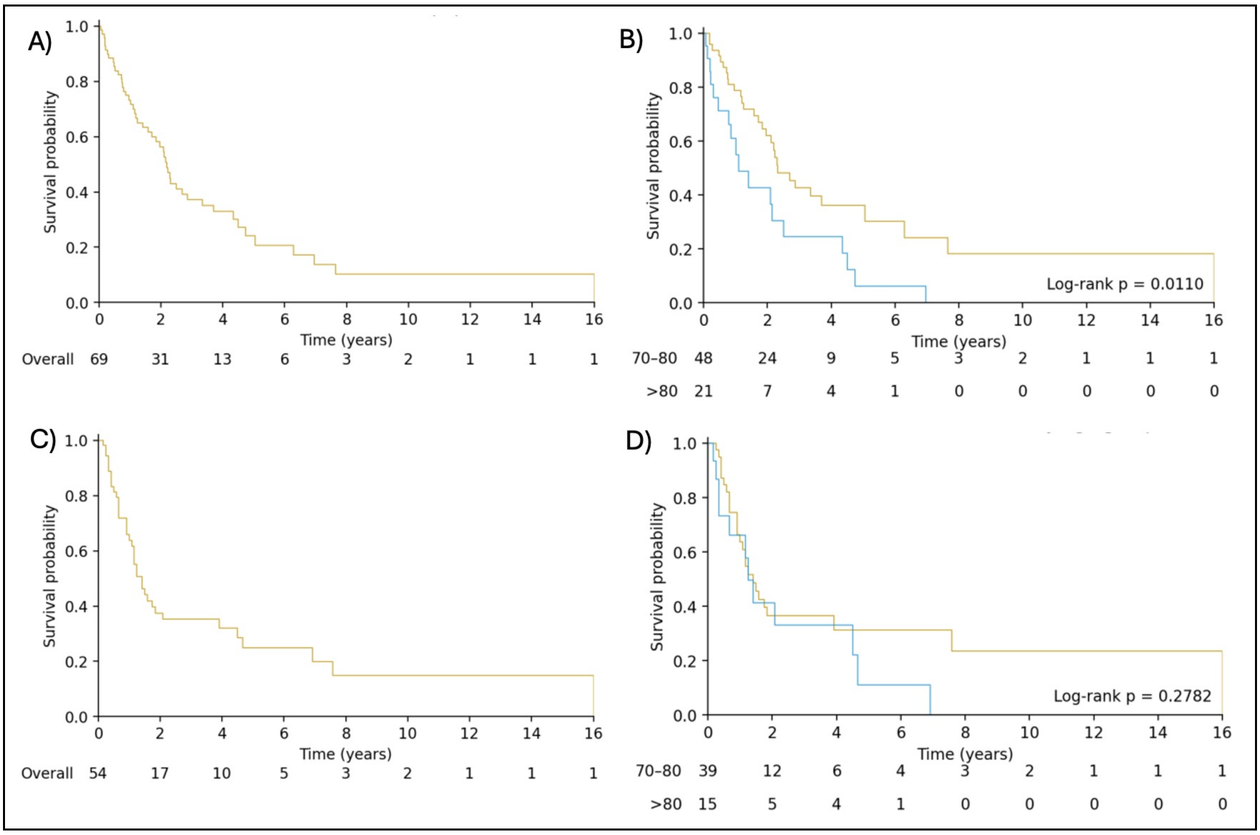

1A) mOS of pts, expressed in years. 1B) mOS of pts stratified by age (70-80 yrs vs >80 yrs). 1C) mDFS of pts, expressed in years. 1D) mDFS of ptS stratified by age (70-80 yrs vs >80 yrs).

**Copyright Information:** (Online) ISSN: 2572-9241
©2026 The Author(s). HemaSphere is published by John Wiley & Sons Ltd on behalf of European Hematology Association. This is an open access Abstract Book distributed under the Attribution-NonCommercial-NoDerivs (CC BY-NC-ND), which allows third parties to download the articles and share them with others as long as they credit the author and the Abstract Book, but they cannot change the content in any way or use them commercially.
**Abstract Book Citations:** Authors, Title, HemaSphere, 2026;10:(S1):pages. The abstract book DOIs can be found on the "Author Information" page.
**Disclaimer:** Articles published in the journal HemaSphere exclusively reflect the opinions of the authors. The authors are responsible for all content in their abstracts including accuracy of the facts, statements, citing resources, etc.

### (PF407) EFFICACY OF THROMBOPROPHYLAXIS IN EARLY-PHASE ACUTE LYMPHOBLASTIC LEUKEMIA THERAPY: AN UPDATED META-ANALYSIS OF 14 CLINICAL STUDIES

**Topic:** 02. Acute lymphoblastic leukemia - Clinical

Ahmed Elaraby*[1], Ali Bakr[1], Youssef Nader[1]

[1]*Al-Azhar University, Faculty of Medicine, Cairo, Egypt;*

### Background

Asparaginase is essential in the initial management of acute lymphoblastic leukemia (ALL) but it leads to venous thromboembolism (VTE). While the use of anticoagulants for primary VTE prevention has been extensively studied, a standard consensus on their routine use and overall efficacy has historically been mixed.

### Aims

To evaluate the overall efficacy of primary pharmacological thromboprophylaxis compared to standard of care in preventing VTE during early-phase ALL therapy.

### Methods

We conducted a systematic literature search across PubMed, Scopus, and Web of Science for studies evaluating primary pharmacological thromboprophylaxis during early-phase asparaginase therapy in patients with ALL. this updated analysis includes a total of 14 studies. A random-effects meta-analysis was performed using the Mantel-Haenszel method to calculate the pooled risk ratio of VTE, comparing these active pharmacological interventions directly against standard of care (no systemic anticoagulation) across all age groups.

### Results

Our updated meta-analysis of 14 studies showed that pharmacological thromboprophylaxis significantly decreases the risk of VTE by 33% compared to standard of care (RR, 0.67; 95% CI, 0.53-0.85; $p = 0.00$). Notably, the updated pooling of these studies resolved previous statistical inconsistencies, resulting in zero heterogeneity across the included trials ($I^2 = 0.00\%$, $p = 0.53$), demonstrating a highly consistent protective effect across different patient populations and pharmacological agents.

### Summary/Conclusion

Primary pharmacological thromboprophylaxis provides a statistically significant and highly consistent reduction in VTE risk for patients undergoing early-phase ALL therapy. These findings strongly support the integration of prophylactic anticoagulation into standard leukemia management protocols to avoid thrombotic complications.

**Copyright Information:** (Online) ISSN: 2572-9241
©2026 The Author(s). HemaSphere is published by John Wiley & Sons Ltd on behalf of European Hematology Association. This is an open access Abstract Book distributed under the Attribution-NonCommercial-NoDerivs (CC BY-NC-ND), which allows third parties to download the articles and share them with others as long as they credit the author and the Abstract Book, but they cannot change the content in any way or use them commercially.
**Abstract Book Citations:** Authors, Title, HemaSphere, 2026;10:(S1):pages. The abstract book DOIs can be found on the "Author Information" page.
**Disclaimer:** Articles published in the journal HemaSphere exclusively reflect the opinions of the authors. The authors are responsible for all content in their abstracts including accuracy of the facts, statements, citing resources, etc.

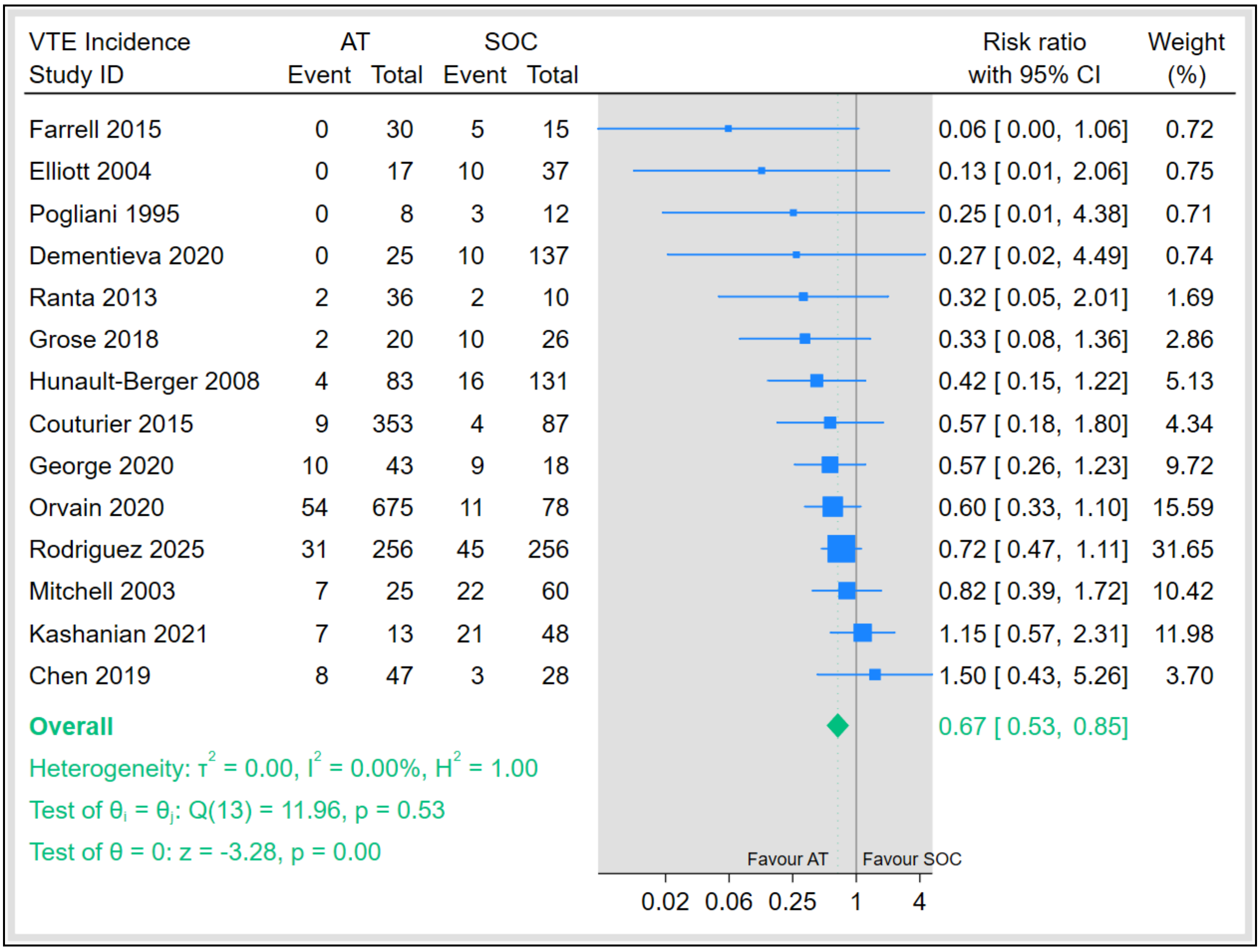

Figure 1. Forest plot comparing the effect of primary pharmacological thromboprophylaxis versus standard of care on the incidence of VTE in patients with acute lymphoblastic leukemia.

**Copyright Information:** (Online) ISSN: 2572-9241
©2026 The Author(s). HemaSphere is published by John Wiley & Sons Ltd on behalf of European Hematology Association. This is an open access Abstract Book distributed under the Attribution-NonCommercial-NoDerivs (CC BY-NC-ND), which allows third parties to download the articles and share them with others as long as they credit the author and the Abstract Book, but they cannot change the content in any way or use them commercially.
**Abstract Book Citations:** Authors, Title, HemaSphere, 2026;10:(S1):pages. The abstract book DOIs can be found on the “Author Information” page.
**Disclaimer:** Articles published in the journal HemaSphere exclusively reflect the opinions of the authors. The authors are responsible for all content in their abstracts including accuracy of the facts, statements, citing resources, etc.

### (PF408) TREATMENT-FREE REMISSION IN NON-TRANSPLANTED ADULT PATIENTS WITH PHILADELPHIA CHROMOSOME-POSITIVE ACUTE LYMPHOBLASTIC LEUKEMIA IN CR1

**Topic:** 02. Acute lymphoblastic leukemia - Clinical

Eitan Kugler*[1], Niranjan Khaire[1], Elias Jabbour[1], Nicholas Short[1], Tapan Kadia[1], Fadi Haddad[1], Koji Sasaki[1], Abhishek Maiti[1], Koichi Takahashi[1], Jan Burger[1], Alessandra Ferrajoli[1], Rashmi Kanagal-Shamanna[1], Rebecca Garris[1], Guillermo Garcia-Manero[1], Farhad Ravandi[1], Hagop Kantarjian[1], Nitin Jain[1]

[1]*MD Anderson Cancer Center, Leukemia, Houston, Tx, United States of America;*

### Background

**BCR::ABL1 tyrosine kinase inhibitors (TKIs) have significantly improved outcomes of patients (pts) with Philadelphia chromosome-positive acute lymphoblastic leukemia (Ph+ ALL). However, the optimal duration of TKI therapy for pts in molecular response who do not undergo allogeneic stem cell transplantation (allo-SCT) remains unclear.**

### Aims

**To report updated outcomes of pts with Ph+ ALL in CR1 who discontinued TKI after remaining in complete molecular response (CMR) for varying durations.**

### Methods

**In this retrospective analysis, we included pts with newly diagnosed Ph+ ALL who received first-line therapy at our institution between 2001 and 2023. We selected pts who did not undergo allo-SCT and discontinued TKI therapy after achieving a CMR (BCR::ABL1 transcripts undetectable by PCR at sensitivity of 1 in 10^-5 in blood or marrow). Pts who discontinued TKI due to noncompliance, pregnancy-related issues, or as a temporary measure to manage side effects were excluded. Molecular relapse was defined as the loss of CMR. Treatment-free remission (TFR) was assessed from the time of TKI discontinuation to molecular/clinical relapse, last follow-up, or death.**

**Copyright Information:** (Online) ISSN: 2572-9241
©2026 The Author(s). HemaSphere is published by John Wiley & Sons Ltd on behalf of European Hematology Association. This is an open access Abstract Book distributed under the Attribution-NonCommercial-NoDerivs (CC BY-NC-ND), which allows third parties to download the articles and share them with others as long as they credit the author and the Abstract Book, but they cannot change the content in any way or use them commercially.
**Abstract Book Citations:** Authors, Title, HemaSphere, 2026;10:(S1):pages. The abstract book DOIs can be found on the “Author Information” page.
**Disclaimer:** Articles published in the journal HemaSphere exclusively reflect the opinions of the authors. The authors are responsible for all content in their abstracts including accuracy of the facts, statements, citing resources, etc.

Results

We identified 17 pts with Ph+ ALL in CR1 who achieved CMR and subsequently discontinued TKI therapy. Ten pts (59%) were male. The median age at diagnosis was 60 years (20–82). Twelve pts (71%) harbored the p190 transcript and 5 (29%) had a p210 transcript. Induction therapy included hyper-CVAD + imatinib (n=2), hyper-CVAD + dasatinib (n=7), hyper-CVAD + ponatinib (n=5), and blinatumomab + ponatinib (n=3). Five pts transitioned to a different TKI for adverse events while still in CR1 prior to therapy discontinuation. Reasons for TKI discontinuation included vascular complications (n=5), pleural effusion (n=4), pulmonary arterial hypertension (n=2), cytopenia (n=1), pancreatitis (n=1), skin rash (n=1), and patient choice (n=1). Overall, the median duration of TKI therapy was 65.2 months (range, 31.3–124.8) with a median continuous CMR duration of 52.2 months (range, 2.7–121.4) prior to TKI discontinuation.
With a median follow-up of 47.9 months after TKI discontinuation, 4/17 pts (24%) (p190, n=3; p210, n=1) experienced molecular relapse (including 1 pt with clinical relapse); 13 pts (76%) remained in TFR. Prior to TKI discontinuation, the pts with molecular relapse were on TKI therapy for 42.4, 43.1, 32.5, and 59.3 months and in continuous CMR for 40.5, 36.8, 31.2, and 2.7 months, respectively. The median time to molecular relapse was 6.8 months (range, 4.4–16.2) from TKI discontinuation. Three pts regained molecular response upon resuming TKI (including 1 pt with clinical relapse who received blinatumomab + TKI), while the fourth pt has not yet restarted therapy.
Notably, none of the 8 pts with a CMR duration of more than 4 years prior to TKI discontinuation experienced molecular/clinical relapse. Additionally, 6 pts were NGS MRD undetectable (10^-6 sensitivity) at the time of discontinuation; 5 were in CMR for >4 years and the sixth for 33.3 months. None of these pts relapsed. One pt had NGS MRD detectable below the assay's lower limit, had CMR duration of 31.2 months before discontinuation, and developed molecular relapse 7.3 months later.

Summary/Conclusion

Our findings suggest that TKI discontinuation may be safe for a highly selected group of pts with Ph+ ALL in CR1 who are in long-term continuous CMR. CMR duration of more than 4 years was associated with durable remissions off therapy.

**Copyright Information:** (Online) ISSN: 2572-9241
©2026 The Author(s). HemaSphere is published by John Wiley & Sons Ltd on behalf of European Hematology Association. This is an open access Abstract Book distributed under the Attribution-NonCommercial-NoDerivs (CC BY-NC-ND), which allows third parties to download the articles and share them with others as long as they credit the author and the Abstract Book, but they cannot change the content in any way or use them commercially.
**Abstract Book Citations:** Authors, Title, HemaSphere, 2026;10:(S1):pages. The abstract book DOIs can be found on the "Author Information" page.
**Disclaimer:** Articles published in the journal HemaSphere exclusively reflect the opinions of the authors. The authors are responsible for all content in their abstracts including accuracy of the facts, statements, citing resources, etc.

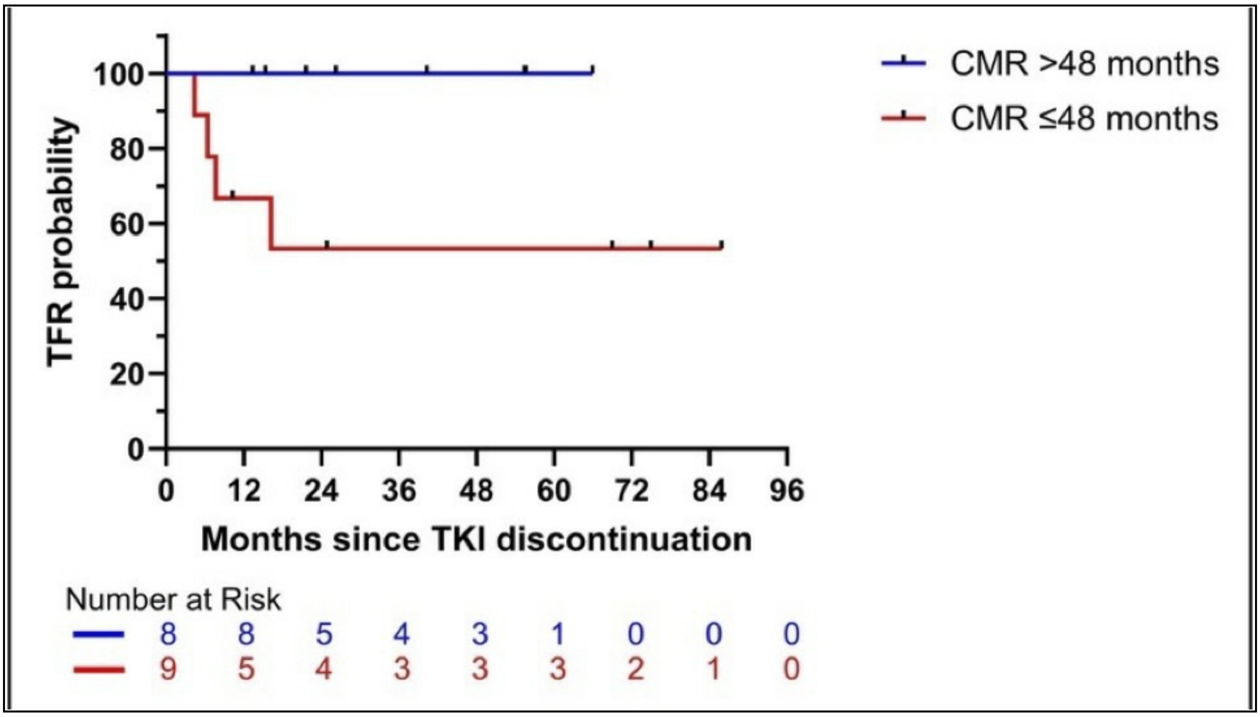

TFR probability following TKI discontinuation stratified by duration of prior CMR. TFR, treatment-free remission; CMR, complete molecular response

**Copyright Information:** (Online) ISSN: 2572-9241
©2026 The Author(s). HemaSphere is published by John Wiley & Sons Ltd on behalf of European Hematology Association. This is an open access Abstract Book distributed under the Attribution-NonCommercial-NoDerivs (CC BY-NC-ND), which allows third parties to download the articles and share them with others as long as they credit the author and the Abstract Book, but they cannot change the content in any way or use them commercially.
**Abstract Book Citations:** Authors, Title, HemaSphere, 2026;10:(S1):pages. The abstract book DOIs can be found on the "Author Information" page.
**Disclaimer:** Articles published in the journal HemaSphere exclusively reflect the opinions of the authors. The authors are responsible for all content in their abstracts including accuracy of the facts, statements, citing resources, etc.

### (PF409) SURVIVAL OUTCOMES AND DISEASE CHARACTERISTICS OF ADULT T-ALL WITH OR WITHOUT ALLOGENEIC TRANSPLANTATION: A SINGLE-CENTER COHORT

**Topic:** 02. Acute lymphoblastic leukemia - Clinical

Xiao Xia Zhang*[1], Reyna Altook[1, 2], Yasser Abou Mourad[1, 2], David Sanford[1, 2], Donna Forrest[1, 2], Hannah Cherniawsky[1, 2], Florian Kuchenbauer[1, 2], Deepesh Lad[1, 2], Stephen Nantel[1, 2], Sujaatha Bhatt[1, 2], Thomas Nevill[1, 2], Judith Rodrigo[1, 2], Claudie Roy[1, 2], Kevin Song[1, 2], Ryan Stubbins[1, 2], Cynthia Toze[1, 2], Jennifer White[1, 2], Eric Zhao[1, 2], Shanee Chung[1, 2]

*[1]University of British Columbia, Medicine, Vancouver, Canada; [2]Leukemia/Bone Marrow Transplant Program of British Columbia, Vancouver, Canada;*

### Background

Adult T-cell acute lymphoblastic leukemia (T-ALL) is an aggressive hematologic malignancy with inferior outcomes compared to B-ALL. The role of allogeneic hematopoietic cell transplantation (allo-HCT) remains to be fully defined.

### Aims

To describe clinical characteristics, treatment outcomes and survival between adult T-ALL patients treated with or without allo-HCT at a single tertiary center.

### Methods

We conducted a retrospective cohort study of 46 adults with T-ALL at a single tertiary center (Vancouver General Hospital) between January 2014 and January 2024 (Non-BMT n=22; BMT n=24). Baseline characteristics were summarized and compared using Mann-Whitney U and Fisher's exact tests. Overall survival (OS) and event-free survival (EFS) were estimated using Kaplan-Meier. To mitigate immortal time bias, a landmark analysis and a time-dependent Cox regression model were performed.

### Results

Baseline characteristics were largely balanced (table 1). Median follow-up was 42.9 months in non-BMT, and 79.0 months in BMT. In the overall cohort, 1- and 3-year OS were 0.67 (0.50–0.79) and 0.42 (0.27–0.56); corresponding EFS were 0.58 (0.42–0.71) and 0.41 (0.26–0.56). In landmark analysis restricted to patients alive and in remission 90 days post-CR (non-BMT n=13; BMT n=23), 1- and 2-year OS were both 0.62 (95% CI 0.32–0.82) in the non-BMT group versus 0.52 (0.31–0.70) and 0.43 (0.23–0.62) in the BMT group. Corresponding 1- and 2-year EFS were 0.68 (0.36–0.87) and 0.60 (0.29–0.81) versus 0.48 (0.27–0.66) and 0.43 (0.23–0.62), respectively. No statistically significant differences were observed. In a time-dependent Cox model, allo-HCT was not significantly associated with EFS (HR 1.50, 95% CI 0.57–3.95, p=0.42).

Table 1:

| Parameters<br>median [IQR] (range) OR n (%) | Non-BMT (n=22) | BMT (n=24) | p-value (Non-BMT vs BMT) |
|---|---|---|---|
| **Demographics** | | | |
| Age at diagnosis (years) | 33 [24-49] (17-71) | 35 [25-52] (17-64) | 0.64 |

**Copyright Information:** (Online) ISSN: 2572-9241
©2026 The Author(s). HemaSphere is published by John Wiley & Sons Ltd on behalf of European Hematology Association. This is an open access Abstract Book distributed under the Attribution-NonCommercial-NoDerivs (CC BY-NC-ND), which allows third parties to download the articles and share them with others as long as they credit the author and the Abstract Book, but they cannot change the content in any way or use them commercially.
**Abstract Book Citations:** Authors, Title, HemaSphere, 2026;10:(S1):pages. The abstract book DOIs can be found on the "Author Information" page.
**Disclaimer:** Articles published in the journal HemaSphere exclusively reflect the opinions of the authors. The authors are responsible for all content in their abstracts including accuracy of the facts, statements, citing resources, etc.

| | | | |
|---|---|---|---|
| **Sex** | | | 0.29 |
| Male | 19 (86) | 17 (71) | |
| Female | 3 (14) | 7 (29) | |
| **Disease characteristics at diagnosis** | | | |
| Hemoglobin | 96 [75-130] (18-150) | 113 [77-128] (68-148) | 0.76 |
| Platelet count | 59 [37-115] (7-335) | 140 [57-203] (24-382) | 0.06 |
| WBC count | 19 [8-55] (2-245) | 9 [5-14] (1-552) | 0.10 |
| Bone marrow involvement % | 90 [80-95] (30-100) | 89 [34-94] (10-100) | 0.25 |
| **Cytogenetics** | | | |
| Normal | 10 (45) | 9 (37) | |
| Abnormal | 10 (45) | 11 (46) | |
| Unknown | 2 (10) | 4 (17) | |
| CNS involvement present | 6 (27) | 3 (13) | 0.28 |
| Mediastinal mass present | 8 (36) | 10 (42) | 0.76 |
| **Induction protocol** | | | |
| ALL 89-01 | 9 (41) | 9 (37) | |
| ALL 13-01 | 7 (32) | 11 (46) | |
| Other | 6 (27) | 4 (17) | |
| **Remission status at end of induction** | | | |
| Primary refractory disease | 7 (32) | 2 (8) | 0.07 |
| CR, MRD negative | 9 (41) | 6 (25) | 0.35 |
| CR, MRD positive | 1 (5) | 7 (29) | 0.04 |
| CR, MRD unknown | 4 (18) | 9 (38) | 0.20 |
| Unknown | 1 (5) | | |
| **BMT treatment characteristics** | | | |
| **Conditioning regime** | | | |
| Cy/TBI +/- ATG | | 9 (38) | |
| Flu/Bu based | | 7 (29) | |
| Bu/Cy +/- ATG | | 3 (13) | |
| non-Cy backbone + TBI | | 3 (13) | |
| Thiotepa-based | | 2 (8) | |
| **Donor type** | | | |
| Matched sibling | | 7 (29) | |
| Matched VUD | | 9 (38) | |
| Mismatched VUD | | 2 (8) | |

**Copyright Information:** (Online) ISSN: 2572-9241
©2026 The Author(s). HemaSphere is published by John Wiley & Sons Ltd on behalf of European Hematology Association. This is an open access Abstract Book distributed under the Attribution-NonCommercial-NoDerivs (CC BY-NC-ND), which allows third parties to download the articles and share them with others as long as they credit the author and the Abstract Book, but they cannot change the content in any way or use them commercially.
**Abstract Book Citations:** Authors, Title, HemaSphere, 2026;10:(S1):pages. The abstract book DOIs can be found on the "Author Information" page.
**Disclaimer:** Articles published in the journal HemaSphere exclusively reflect the opinions of the authors. The authors are responsible for all content in their abstracts including accuracy of the facts, statements, citing resources, etc.

| | | | |
|---|---|---|---|
| Haploidentical | | 5 (21) | |
| Chord blood | | 1 (4) | |

**Summary/Conclusion**

In this single-center cohort, the transplant cohort demonstrated a higher proportion of MRD positivity, consistent with selection of higher-risk disease. After adjustment for immortal time bias, no significant difference in OS or EFS was observed between patients treated with or without allo-HCT. Notably, comparable outcomes despite higher MRD positivity in the transplant group suggest that allo-HCT may mitigate adverse disease biology in select high-risk patients. These findings underscore the need for improved risk stratification and more effective post-remission strategies to enhance outcomes in this population.

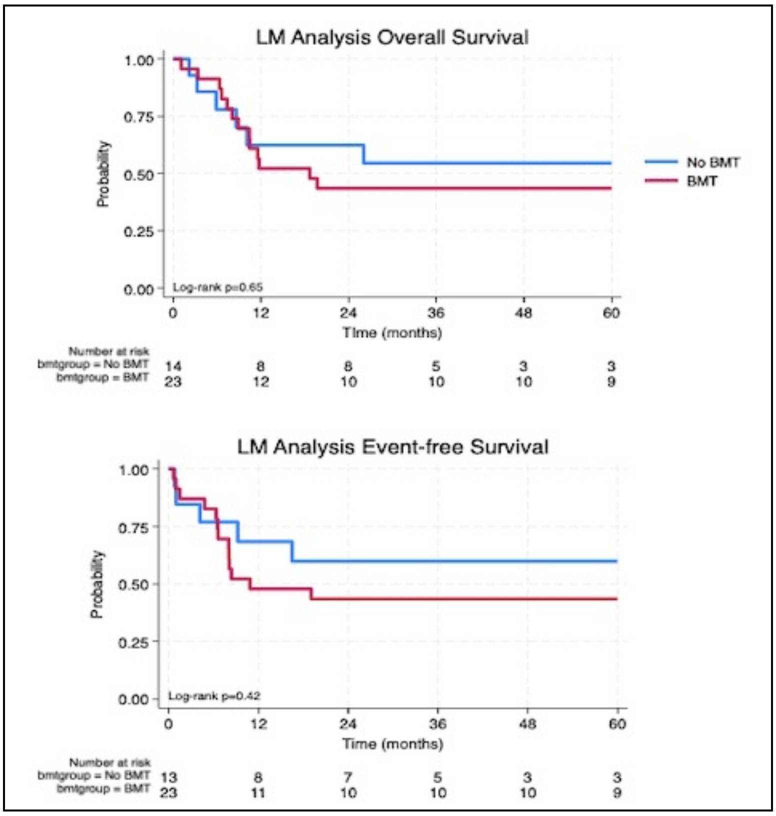

Figure 1. Landmark analysis (day 90 post-CR) of overall survival and event-free survival by BMT status

**Copyright Information:** (Online) ISSN: 2572-9241
©2026 The Author(s). HemaSphere is published by John Wiley & Sons Ltd on behalf of European Hematology Association. This is an open access Abstract Book distributed under the Attribution-NonCommercial-NoDerivs (CC BY-NC-ND), which allows third parties to download the articles and share them with others as long as they credit the author and the Abstract Book, but they cannot change the content in any way or use them commercially.
**Abstract Book Citations:** Authors, Title, HemaSphere, 2026;10:(S1):pages. The abstract book DOIs can be found on the "Author Information" page.
**Disclaimer:** Articles published in the journal HemaSphere exclusively reflect the opinions of the authors. The authors are responsible for all content in their abstracts including accuracy of the facts, statements, citing resources, etc.

### (PF411) RESNET TO THE RESCUE: AN AUTOMATED APPROACH FOR THE DETECTION OF MEASURABLE RESIDUAL DISEASE IN PATIENTS WITH ACUTE MYELOID LEUKEMIA

**Topic:** 03. Acute myeloid leukemia - Biology & translational research

Maximilian Roehnert*[1], Jonas Schadt[1, 2], Lars Thielecke[3], Chiara Löffler[1, 2], Michael Kramer[4], Christoph Röllig[1], Friedemann Uschner[3], Martin Bornhauser[1, 5], Uta Oelschlaegel[1], Malte von Bonin[1], Ingmar Glauche[3]

*[1]Department of Internal Medicine I, Faculty of Medicine and University Hospital Carl Gustav Carus TUD Dresden, University of Technology, Dresden, Germany; [2]Else Kroener Fresenius Center for Digital Health, Technical University Dresden, Dresden, Germany; [3]Institute for Medical Informatics and Biometry (IMB), Faculty of Medicine and University Hospital Carl Gustav Carus TUD Dresden, Dresden, Germany; [4]AvenCell Europe GmbH, Dresden, Germany; [5]National Center for Tumor Disease (NCT), Dresden, Germany;*

### Background

Measurable residual disease (MRD) is one of the strongest prognostic factors in acute myeloid leukemia (AML). Multicolor flow cytometry (MFC) enables MRD assessment in nearly all patients but remains limited by complex workflows, lack of standardization and expert-dependent interpretation. Machine learning (ML) approaches improve reproducibility and scalability, but MRD detection by ML is challenging due to low disease burden, phenotypic heterogeneity and uncertain ground truth.

### Aims

To develop and validate an automated ML-based framework for MRD detection in AML using standardized MFC data and to benchmark its performance against expert-based assessment and clinical outcomes.

### Methods

We analyzed 1,361 raw MFC files from the AML Registry of the Study Alliance Leukemia (SAL), including diagnostic (n=765), post-induction (n=474), pre-alloHCT (n=32) samples, and leukemia-free controls (n=90). Manual MRD assessment followed an established strategy (Röhnert et al., Leukemia 2022), incorporating all ELN-recommended core antigens as well as baseline LAIP tracking and different-from-normal detection. Event-level annotations from 545 expert-gated samples were reconstructed to generate a supervised reference UMAP embedding. Raw MFC data were projected into this fixed 2D space and transformed into standardized density images. A ResNet34 was trained to classify samples as MRD-positive or MRD-negative using repeated stratified 4-fold cross-validation (16 repetitions). Model performance was evaluated by sensitivity, specificity, F1-score and ROC analysis. Prognostic relevance was assessed using overall survival (OS).

**Copyright Information:** (Online) ISSN: 2572-9241
©2026 The Author(s). HemaSphere is published by John Wiley & Sons Ltd on behalf of European Hematology Association. This is an open access Abstract Book distributed under the Attribution-NonCommercial-NoDerivs (CC BY-NC-ND), which allows third parties to download the articles and share them with others as long as they credit the author and the Abstract Book, but they cannot change the content in any way or use them commercially.
**Abstract Book Citations:** Authors, Title, HemaSphere, 2026;10:(S1):pages. The abstract book DOIs can be found on the "Author Information" page.
**Disclaimer:** Articles published in the journal HemaSphere exclusively reflect the opinions of the authors. The authors are responsible for all content in their abstracts including accuracy of the facts, statements, citing resources, etc.

### Results

Using repeated stratified 4-fold cross-validation (16 repetitions) with soft-voting, the optimized ResNet34 achieved a mean sensitivity of 0.912 (95% CI 0.9116–0.9126), specificity of 0.896 (95% CI 0.8945–0.8969), and F1-score of 0.942 (95% CI 0.9416–0.9423). The confusion matrix (Table 1) showed 1006/1103 correctly classified MRD-positive samples (91%) and 232/259 correctly classified MRD-negative samples (90%), corresponding to 97 false negatives (9%) and 27 false positives (10%).
When benchmarked against independent human evaluators (Table 2), performance metrics were comparable: Expert 2 achieved a sensitivity of 0.90 and specificity of 0.96, Expert 3 0.88 and 0.89, respectively, while the ResNet34 achieved 0.91 and 0.90.
ML-based MRD classification stratified overall survival in patients ≤60 years after induction therapy, with survival separation comparable to expert-based MRD assessment (Figure).

| Rater | Sensitivity | Specificity | F1-score |
|---|---|---|---|
| Expert 2 | 0.90 | 0.96 | 0.93 |
| Expert 3 | 0.88 | 0.89 | 0.89 |
| ResNet34 | 0.91 | 0.90 | 0.94 |

**Table 1.** Confusion matrix of the ResNet34 model compared to the reference expert (Expert 1) as ground truth. Percentages are column-based and refer to the proportion within each ground-truth category.

| | $MRD^{pos}$ (ground truth) | $MRD^{neg}$ (ground truth) |
|---|---|---|
| $MRD^{pos}$ (prediction) | 1006 (91%) | 27 (10%) |
| $MRD^{neg}$ (prediction) | 97 (9%) | 232 (90%) |

**Table 2.** Performance metrics of two independent human experts and the trained ResNet34, evaluated against the selected gold standard “Expert 1”.

### Summary/Conclusion

An image-based machine learning approach applied to standardized MFC data enables accurate and reproducible MRD detection in AML with expert-level performance and preserved prognostic relevance. This framework may support standardized MRD assessment and facilitate scalable implementation in routine clinical practice.

**Copyright Information:** (Online) ISSN: 2572-9241
©2026 The Author(s). HemaSphere is published by John Wiley & Sons Ltd on behalf of European Hematology Association. This is an open access Abstract Book distributed under the Attribution-NonCommercial-NoDerivs (CC BY-NC-ND), which allows third parties to download the articles and share them with others as long as they credit the author and the Abstract Book, but they cannot change the content in any way or use them commercially.
**Abstract Book Citations:** Authors, Title, HemaSphere, 2026;10:(S1):pages. The abstract book DOIs can be found on the “Author Information” page.
**Disclaimer:** Articles published in the journal HemaSphere exclusively reflect the opinions of the authors. The authors are responsible for all content in their abstracts including accuracy of the facts, statements, citing resources, etc.

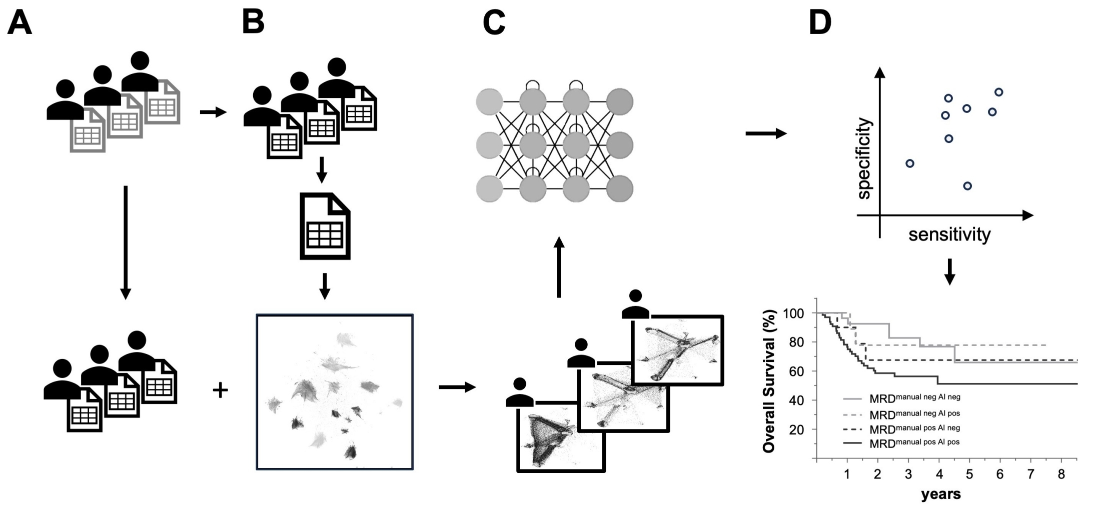

**Figure**: ML pipeline development process, starting with (A) data preprocessing and transformation, (B) generation of the artificial dataset for the *reference embedding,* (C) model training and (D) model selection and validation.

**Copyright Information:** (Online) ISSN: 2572-9241
©2026 The Author(s). HemaSphere is published by John Wiley & Sons Ltd on behalf of European Hematology Association. This is an open access Abstract Book distributed under the Attribution-NonCommercial-NoDerivs (CC BY-NC-ND), which allows third parties to download the articles and share them with others as long as they credit the author and the Abstract Book, but they cannot change the content in any way or use them commercially.
**Abstract Book Citations:** Authors, Title, HemaSphere, 2026;10:(S1):pages. The abstract book DOIs can be found on the “Author Information” page.
**Disclaimer:** Articles published in the journal HemaSphere exclusively reflect the opinions of the authors. The authors are responsible for all content in their abstracts including accuracy of the facts, statements, citing resources, etc.

### (PF412) THE ROLE AND MECHANISM OF HISTONE H4K8 LACTYLATION IN ACUTE MYELOID LEUKEMIA STEM CELLS

**Topic:** 03. Acute myeloid leukemia - Biology & translational research

Deyang Shi*[1], Mengke Li[1], Hongchen Di[1], Shiwei Yang[1], Xiangli Chen[1], Zunmin Zhu[1]

[1]*Henan Provincial People's Hospital, Zhengzhou, China;*

### Background

Leukemia stem cells (LSCs) are the primary drivers of relapse and drug resistance in AML. Unlike HSCs and leukemia cells (Blasts), which predominantly utilize glycolysis for energy production, LSCs rely on oxidative phosphorylation. However, whether the metabolic disparity between LSCs and HSCs/Blasts impacts histone lactylation modification, thereby influencing LSC function, remains unexplored.

### Aims

This study aimed to investigate whether metabolic differences between LSCs and HSCs/blasts influence histone lactylation modifications and to elucidate the role and molecular mechanism of these modifications in regulating LSC functional maintenance.

### Methods

We first characterized glycolysis-related gene expression by integrating public data with samples from AML patients and healthy donors. Using an MLL-AF9 AML mouse model, we delineated the glycolytic metabolic landscape in LSCs and compared histone lactylation between AML LSCs versus blasts. These findings were extended to clinical samples, where Western blot quantified lactylation levels in AML LSCs, AML blasts, normal HSCs, and MNCs. To investigate the functional role of the identified lactylation mark, we performed CUT&Tag sequencing on LSCs, blasts, HSCs, and MNCs, enabling genome-wide identification of downstream targets, including enriched binding regions, associated signaling pathways, and key target genes.

### Results

Public AML transcriptome data revealed LDHA was upregulated in AML LSCs versus blasts. To validate this, we sorted LSCs and blasts from four diagnosed AML patients and assessed glycolysis-related gene expression, with healthy donor HSCs and MNCs as controls. Consistent with the transcriptome data, LDHA, PFKP, PDK1 were significantly elevated in AML LSCs. Detection of histone lactylation in AML patient PBMCs revealed broad upregulation of multiple marks, including H4K5la, H4K8la, H4K12la, H4K16la, H2bK16la, and H3K9la, indicating widespread epigenetic reprogramming in AML. Using an MA9 AML mouse model, we sorted c-Kit$^{+}$ LSPCs and c-Kit$^{-}$ blasts and found that H4K8la was specifically elevated in MA9 LSPCs compared to both wild-type controls and MA9 blasts, while other lactylation marks remained unchanged. This specific enrichment of H4K8la in LSCs was further validated in primary human AML samples. Collectively, these results identify H4K8la as a distinct epigenetic feature of AML LSCs. In one AML patient, CUT&Tag analysis of HSCs, LSCs, blasts, and MNCs revealed increased H4K8la enrichment at promoter regions of VEGF signaling pathway genes in LSCs compared to blasts, suggesting a potential role for H4K8la in regulating LSCs via this pathway. Additionally, we identified genes upregulated in LSCs versus HSCs with differential peaks and ranked them by signal intensity. NRAS emerged as the top hit. Further examination revealed that this patient's bone marrow cells carried NRAS p.G12D and p.G13D mutations. These findings raise the possibility that upon acquisition of a "second hit" in HSC, H4K8la may contribute to sustained aberrant activation of mutant RAS in LSCs. Validation in larger cohorts and functional studies are warranted.

**Copyright Information:** (Online) ISSN: 2572-9241
©2026 The Author(s). HemaSphere is published by John Wiley & Sons Ltd on behalf of European Hematology Association. This is an open access Abstract Book distributed under the Attribution-NonCommercial-NoDerivs (CC BY-NC-ND), which allows third parties to download the articles and share them with others as long as they credit the author and the Abstract Book, but they cannot change the content in any way or use them commercially.
**Abstract Book Citations:** Authors, Title, HemaSphere, 2026;10:(S1):pages. The abstract book DOIs can be found on the "Author Information" page.
**Disclaimer:** Articles published in the journal HemaSphere exclusively reflect the opinions of the authors. The authors are responsible for all content in their abstracts including accuracy of the facts, statements, citing resources, etc.

### Summary/Conclusion

AML patients show broad upregulation of histone lactylation in bone marrow cells, with H4K8la specifically enriched in LSCs. As a distinct epigenetic mark, H4K8la potentially sustains LSCs via VEGF signaling and mutant RAS activation. These findings offer a rationale for targeting histone lactylation in relapsed/refractory AML.

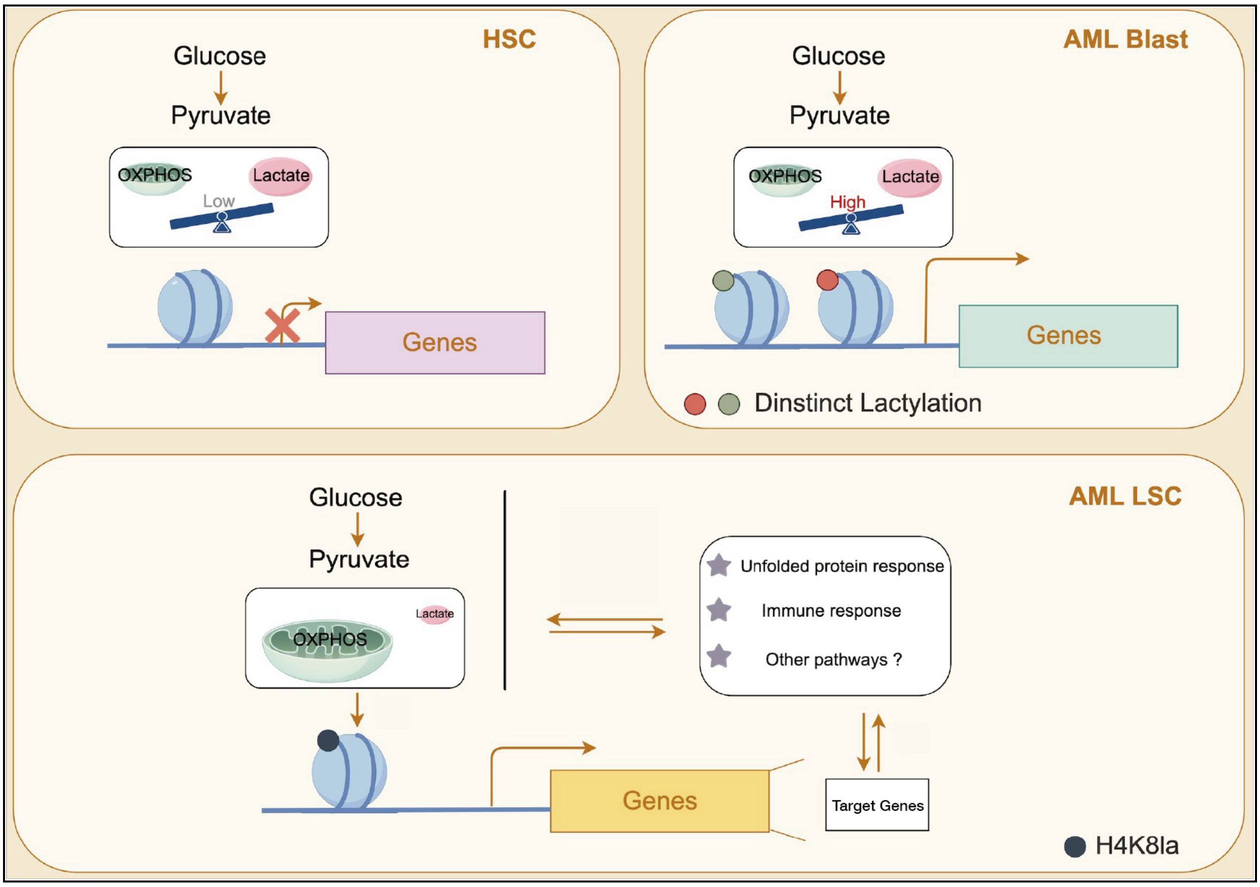

Copyright Information: (Online) ISSN: 2572-9241
©2026 The Author(s). HemaSphere is published by John Wiley & Sons Ltd on behalf of European Hematology Association. This is an open access Abstract Book distributed under the Attribution-NonCommercial-NoDerivs (CC BY-NC-ND), which allows third parties to download the articles and share them with others as long as they credit the author and the Abstract Book, but they cannot change the content in any way or use them commercially.
Abstract Book Citations: Authors, Title, HemaSphere, 2026;10:(S1):pages. The abstract book DOIs can be found on the "Author Information" page.
Disclaimer: Articles published in the journal HemaSphere exclusively reflect the opinions of the authors. The authors are responsible for all content in their abstracts including accuracy of the facts, statements, citing resources, etc.

### (PF413) SP1 REGULATION ACUTE MYELOID LEUKEMIA STEMNESS VIA PHASE SEPARATION-MEDIATED SUPER-ENHANCER ACTIVATION OF HNRNPUL1

**Topic:** 03. Acute myeloid leukemia - Biology & translational research

Yuchen Wen*[1], Zefan Du[1], Chunmou Li[1], Ran Yao[1], Liang Li[1], Yun Chen[1], Cheng Ouyang[1], Chun Chen[1]

[1] *The Seventh Affiliated Hospital of Sun Yat-Sen University, Shen Zhen, China;*

### Background

Acute myeloid leukemia (AML) is a heterogeneous malignancy with high relapse rates, particularly among poor-prognosis subtypes. Transcription factor SP1 has been implicated in AML pathogenesis, but the underlying molecular mechanisms remain elusive. Emerging evidence suggests that liquid-liquid phase separation (LLPS) governs transcriptional regulation, yet whether SP1 leverages this biophysical process to drive AML progression is unknown.

### Aims

To investigate whether SP1 undergoes phase separation and contributes to AML progression, and to explore the therapeutic potential of targeting SP1 condensates.

### Methods

Public datasets were analyzed for SP1 expression and prognostic significance. Phase separation capacity was assessed by immunofluorescence, FRAP assays, and 1,6-hexanediol treatment. Targeted mutagenesis and structure-function rescue experiments were performed to identify the minimal region required for SP1 condensation. Loss- and gain-of-function studies were conducted in vitro and in multiple in vivo models, including cell line-derived xenograft (CDX), patient-derived xenograft (PDX), and MLL-AF9 retroviral-induced AML models. CUT&RUN sequencing with SP1 and H3K27ac antibodies was performed to map genome-wide binding and super-enhancer occupancy. Candidate target genes were validated by qRT-PCR, Western blot, and dual-luciferase reporter assays. A interfering peptide targeting SP1 condensates was designed and evaluated for anti-leukemia efficacy in PDX models.

### Results

SP1 was significantly upregulated in AML and correlated with poor prognosis. SP1 formed nuclear condensates via phase separation dependent on its intrinsically disordered region (aa 674-708). Disruption of SP1 condensation abolished its oncogenic functions, impairing AML cell proliferation, stemness, and disease progression across CDX, PDX, and MLL-AF9 models. Mechanistically, SP1 preferentially occupied super-enhancer regions, and its condensation capacity directly modulated super-enhancer activity. Among SP1-associated super-enhancer targets, HNRNPUL1 emerged as a key oncogenic effector whose expression was tightly coupled to SP1 condensation status. A rationally designed interfering peptide (Pep1) effectively disrupted SP1 condensates, bound directly to SP1, and exhibited potent anti-leukemia efficacy in PDX models.

### Summary/Conclusion

Our findings establish that SP1 leverages phase separation to assemble transcriptional condensates that drive super-enhancer-mediated oncogene expression in AML. Targeting SP1 condensation represents a promising therapeutic strategy for high-risk and relapsed AML.

**Copyright Information:** (Online) ISSN: 2572-9241
©2026 The Author(s). HemaSphere is published by John Wiley & Sons Ltd on behalf of European Hematology Association. This is an open access Abstract Book distributed under the Attribution-NonCommercial-NoDerivs (CC BY-NC-ND), which allows third parties to download the articles and share them with others as long as they credit the author and the Abstract Book, but they cannot change the content in any way or use them commercially.
**Abstract Book Citations:** Authors, Title, HemaSphere, 2026;10:(S1):pages. The abstract book DOIs can be found on the “Author Information” page.
**Disclaimer:** Articles published in the journal HemaSphere exclusively reflect the opinions of the authors. The authors are responsible for all content in their abstracts including accuracy of the facts, statements, citing resources, etc.

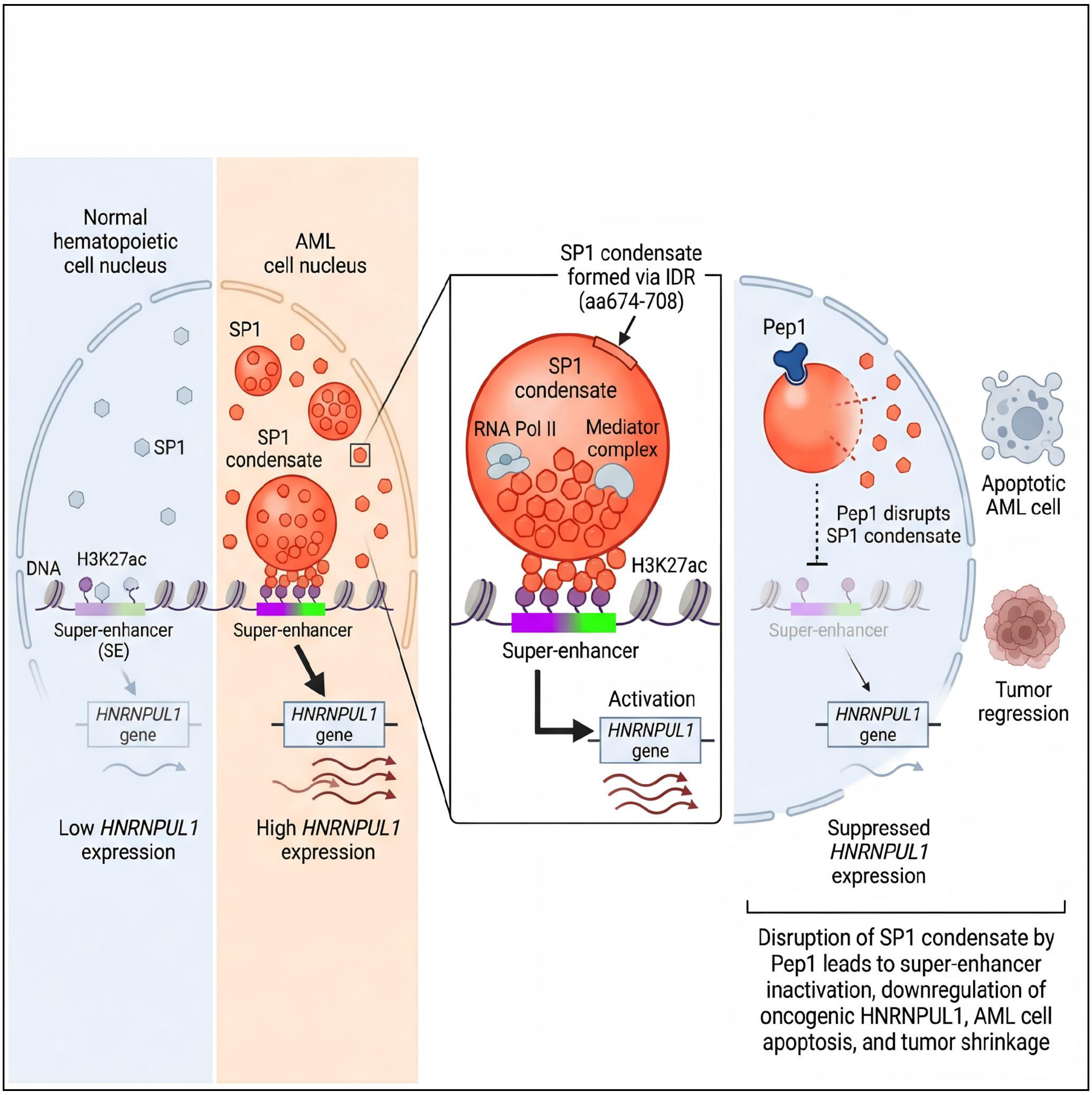

**Copyright Information:** (Online) ISSN: 2572-9241
©2026 The Author(s). HemaSphere is published by John Wiley & Sons Ltd on behalf of European Hematology Association. This is an open access Abstract Book distributed under the Attribution-NonCommercial-NoDerivs (CC BY-NC-ND), which allows third parties to download the articles and share them with others as long as they credit the author and the Abstract Book, but they cannot change the content in any way or use them commercially.
**Abstract Book Citations:** Authors, Title, HemaSphere, 2026;10:(S1):pages. The abstract book DOIs can be found on the "Author Information" page.
**Disclaimer:** Articles published in the journal HemaSphere exclusively reflect the opinions of the authors. The authors are responsible for all content in their abstracts including accuracy of the facts, statements, citing resources, etc.

### (PF414) STING ACTIVATION SYNERGIZES WITH SORAFENIB TO INDUCE PYROPTOSIS IN ACUTE MYELOID LEUKEMIA

**Topic:** 03. Acute myeloid leukemia - Biology & translational research

Danqi Pan[1], Juan Du[1], Xingcheng Liu[1], Quan Wu[2], Yizhen Li[3], Min Dai[4], Hui Zeng*[2]

*[1]The First Affiliated Hospital of Jinan University, Guangzhou, China;[2]Guangdong Provincial People's Hospital (Guangdong Academy of Medical Sciences), Southern Medical University, Guangzhou, China; [3]Guangzhou Hospital of Integrated Traditional and Western Medicin, Guangzhou, China; [4]Nanfang Hospital, Guangzhou, China;*

### Background

Acute myeloid leukemia (AML) is the most common adult leukemia, and approximately 30% of patients harbor FLT3 mutations. Although FLT3 inhibitors have significantly improved outcomes in this subgroup, their efficacy as monotherapy is limited by rapid relapse and resistance. Therefore, novel combination strategies are urgently needed to improve the long-term prognosis of FLT3-mutated AML. The STING (stimulator of interferon genes) pathway is a key cytosolic DNA–sensing mechanism with emerging antitumor potential, yet its role in AML, particularly in FLT3-mutated disease, remains poorly understood.

### Aims

To investigate the biological role of STING in FLT3-mutated AML and to evaluate the therapeutic efficacy and mechanism of combining the STING agonist diABZI with the FLT3 inhibitor Sorafenib.

### Methods

AML cell lines, primary patient samples, and mouse leukemia models were used to evaluate the anti-leukemic effects of diABZI and Sorafenib, alone or in combination. DNA damage repair, mitochondrial DNA (mtDNA) release, and cytosolic dsDNA accumulation were assessed. Molecular mechanisms were investigated using immunoblotting, genetic and pharmacologic inhibition, and functional cell death assays.

### Results

Higher STING expression correlated with superior overall survival in AML patients. STING agonists, particularly diABZI, exerted potent anti-proliferative effects in AML cell lines and primary samples. FLT3 mutations enhanced DNA damage repair, whereas Sorafenib suppressed this process and promoted mtDNA leakage into the cytosol, leading to dsDNA accumulation and further STING activation. Consequently, diABZI and Sorafenib exhibited strong synergistic anti-leukemic activity in vitro and in vivo, significantly inhibiting leukemia progression and prolonging survival in mouse models.
Mechanistically, STING activation selectively induced the inflammasome sensor NLRP1. NLRP1 activated both Caspase-1 and Caspase-8, revealing a previously unrecognized inflammasome–caspase crosstalk. Caspase-1 and Caspase-8 redundantly cleaved GSDME, establishing a dual-caspase–driven, GSDME-dependent pyroptotic program in AML cells.

### Summary/Conclusion

In summary, this study identifies STING activation increase the sensitivity of AML cells to Sorafenib and establishes a novel anti-AML mechanism, in which STING activation triggers NLRP1 induction and subsequent Caspase-1/Caspase-8-mediated GSDME cleavage to execute pyroptosis. These findings provide a mechanistic basis for the synergistic therapeutic effects of diABZI and Sorafenib, providing a new targeted therapeutic strategy for AML and paving the way for the pyroptosis-induction treatment in hematologic malignancies.

**Copyright Information:** (Online) ISSN: 2572-9241
©2026 The Author(s). HemaSphere is published by John Wiley & Sons Ltd on behalf of European Hematology Association. This is an open access Abstract Book distributed under the Attribution-NonCommercial-NoDerivs (CC BY-NC-ND), which allows third parties to download the articles and share them with others as long as they credit the author and the Abstract Book, but they cannot change the content in any way or use them commercially.
**Abstract Book Citations:** Authors, Title, HemaSphere, 2026;10:(S1):pages. The abstract book DOIs can be found on the “Author Information” page.
**Disclaimer:** Articles published in the journal HemaSphere exclusively reflect the opinions of the authors. The authors are responsible for all content in their abstracts including accuracy of the facts, statements, citing resources, etc.

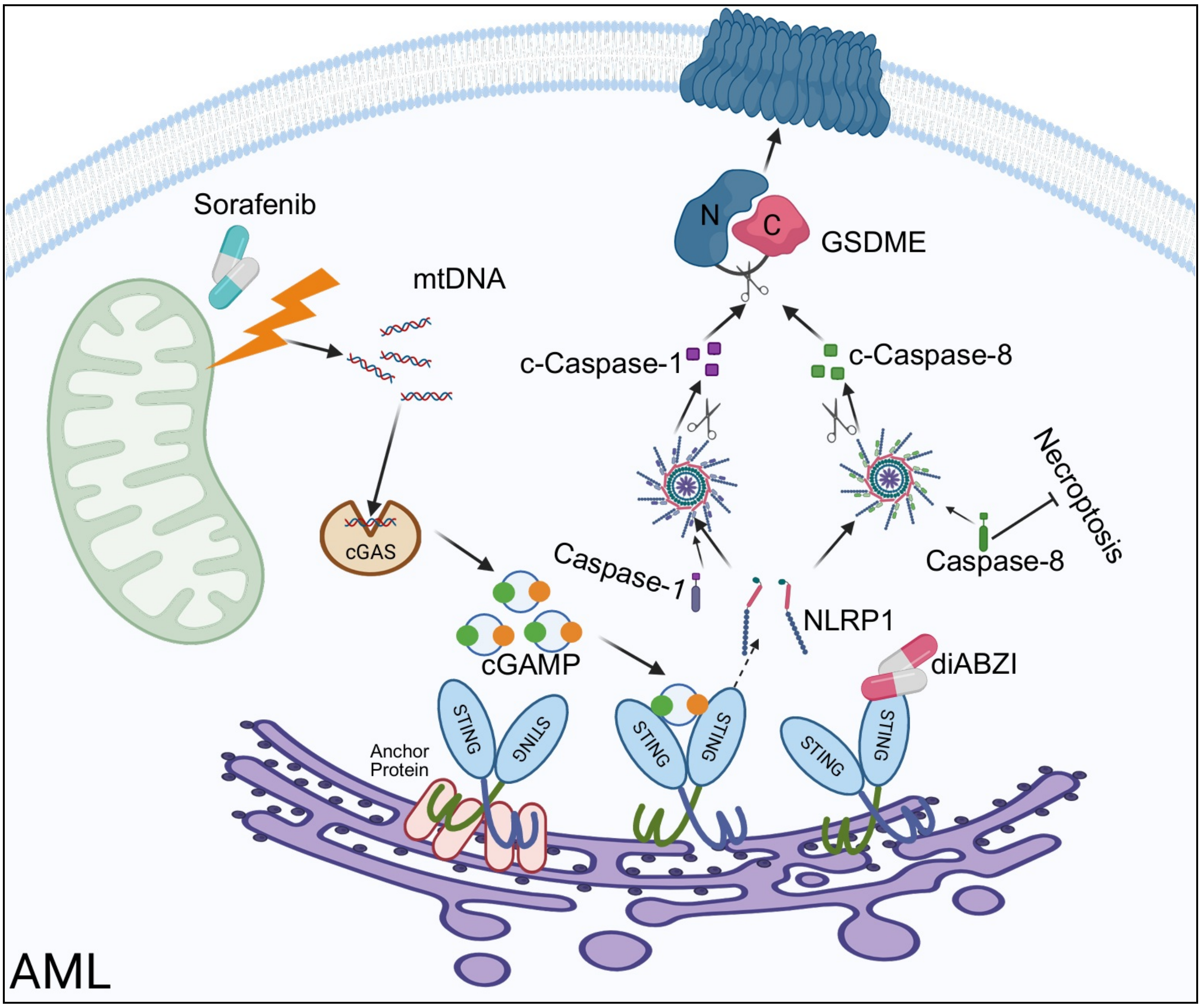

**Copyright Information:** (Online) ISSN: 2572-9241
©2026 The Author(s). HemaSphere is published by John Wiley & Sons Ltd on behalf of European Hematology Association. This is an open access Abstract Book distributed under the Attribution-NonCommercial-NoDerivs (CC BY-NC-ND), which allows third parties to download the articles and share them with others as long as they credit the author and the Abstract Book, but they cannot change the content in any way or use them commercially.
**Abstract Book Citations:** Authors, Title, HemaSphere, 2026;10:(S1):pages. The abstract book DOIs can be found on the "Author Information" page.
**Disclaimer:** Articles published in the journal HemaSphere exclusively reflect the opinions of the authors. The authors are responsible for all content in their abstracts including accuracy of the facts, statements, citing resources, etc.

### (PF415) HDAC8 DEGRADER SYNERGIZES WITH MENIN-KMT2A INHIBITORS ACROSS DIFFERENT GENETIC ACUTE MYELOID LEUKEMIA SUBTYPES.

**Topic:** 03. Acute myeloid leukemia - Biology & translational research

Jesse van der Velde*[1, 2], Diego A Pereira-Martins[1], Luise Albuquerque-Simões[1], Isabel Weinhauser[1], Livia Bassani[1], Chunlong Zhao[2], Alvan Shalas[2], Gerwin Huls[1], Frank J. Dekker[2], Jan Jacob Schuringa[1]

*[1]UMCG groningen, Experimental Hematology, Groningen, The Netherlands;[2]Rijksuniveristeit groningen, Chemical Pharmaceutical Biology, Groningen, The Netherlands;*

**Background**

Differentiation arrest of is a hallmark of acute myeloid leukemia (AML). Histone deacetylase (HDAC) inhibitors have emerged as epigenetic modulators in AML that impact on differentiation. HDAC8 belongs to the class I family of histone deacetylases and besides deacetylating histones, HDAC8 is also able to deacetylate several non-histone proteins, including TP53 and the cohesion component SMC3, although little is known about its targets in AML.

**Aims**

To evaluate efficacy of HDAC8 degradation in combination with Menin/KMT2A inhibitors across different genetic AML subtypes and whether combination treatment could overcome resistance.

**Methods**

We developed a selective proteolysis-targeting chimera (PROTAC), Z16, to degrade HDAC8. Acetylome studies were performed to identify the HDAC8-controlled acetylome. Z16 was tested in combination with Menin inhibitors in several AML models for effects on differentiation, viability, the transcriptome and epigenome.

**Copyright Information:** (Online) ISSN: 2572-9241
©2026 The Author(s). HemaSphere is published by John Wiley & Sons Ltd on behalf of European Hematology Association. This is an open access Abstract Book distributed under the Attribution-NonCommercial-NoDerivs (CC BY-NC-ND), which allows third parties to download the articles and share them with others as long as they credit the author and the Abstract Book, but they cannot change the content in any way or use them commercially.
**Abstract Book Citations:** Authors, Title, HemaSphere, 2026;10:(S1):pages. The abstract book DOIs can be found on the “Author Information” page.
**Disclaimer:** Articles published in the journal HemaSphere exclusively reflect the opinions of the authors. The authors are responsible for all content in their abstracts including accuracy of the facts, statements, citing resources, etc.

### Results

Z16 selectively degraded HDAC8 in AML cells at low nanomolar, non-cytotoxic concentrations. Across a panel of AML cell lines (n=10) and primary blasts (n=18), Z16 robustly induced myeloid differentiation, as evidenced by increased HLA-DR, CD14 and CD11b levels. Z16 responders included *KMT2A*-rearranged (KMT2Ar), *NPM1*cyt and *NUP98*-upregulated subtypes, which have recently been shown to respond to Menin inhibition. Therefore, Z16 efficacy was tested in combination with the Menin inhibitor Revumenib (Rev). The combination synergistically enhanced myeloid differentiation in Menin-responsive, but also in Menin non-responsive models alike, including samples without KMT2Ar or *NPM1*cyt, indicating that HDAC8 degradation can broaden the differentiation response beyond classical Menin-sensitive genotypes. In the Menin non-responsive *MLL-AF9*/*NRAS*$^{G12D}$ THP1 model, RNAseq showed no major transcriptional effects of Rev alone. In contrast, Z16 single treatment, and most notably the combination with Rev, induced profound downregulation of KMT2A targets (*MEF2C*, *PBX3*, *JMJD1C*) and strong upregulation of differentiation-associated genes (*CD14*, *HLA-DR*), while stem/self-renewal programs were downregulated. CRISPR-Cas9-mediated *CIITA* knockout models showed that *CIITA* is required for HLA-DR induction by Z16+Rev. Intriguingly, H3K27ac was not altered at the *CIITA* locus upon combination treatment, but H3K4me3 levels were increased. We therefore hypothesized that H3K27 might not be the direct target of HDAC8, but rather that KMT2A machinery would be affected. We performed pan-acetylated lysine antibody-mediated pullouts followed by LC-MS/MS to uncover the HDAC8-controlled acetylome in AML and identified several non-histone targets. Some of these control KMT2A function such as the PHD finger proteins PHF14/5, potentially relevant for condensate formation via which KMT2A/Menin is considered to drive oncogenic gene transcription, as well as the O-linked N-acetylglucosamine transferase OGT, known to catalyze the O-GlcNAcylation of target proteins, and their role in mediating the synergy between HDAC8 and Menin inhibition will be discussed.

### Summary/Conclusion

Selective HDAC8 degradation induces myeloid differentiation across a broad spectrum of genetically distinct AML subtypes and synergizes with Menin inhibition, potentially not only involving acetylome changes on histones but also on non-histone HDAC8 targets. These findings position HDAC8 degradation as a promising strategy to enhance and extend Menin-based differentiation therapies in AML.

**Copyright Information:** (Online) ISSN: 2572-9241
©2026 The Author(s). HemaSphere is published by John Wiley & Sons Ltd on behalf of European Hematology Association. This is an open access Abstract Book distributed under the Attribution-NonCommercial-NoDerivs (CC BY-NC-ND), which allows third parties to download the articles and share them with others as long as they credit the author and the Abstract Book, but they cannot change the content in any way or use them commercially.
**Abstract Book Citations:** Authors, Title, HemaSphere, 2026;10:(S1):pages. The abstract book DOIs can be found on the "Author Information" page.
**Disclaimer:** Articles published in the journal HemaSphere exclusively reflect the opinions of the authors. The authors are responsible for all content in their abstracts including accuracy of the facts, statements, citing resources, etc.

### (PF416) TNIK EXPANDS LEUKEMIC STEM CELLS BY ACTIVATING NF-ΚB SIGNALING AND REPRESENTS A NOVEL THERAPEUTIC TARGET IN ACUTE MYELOID LEUKEMIA

**Topic:** 03. Acute myeloid leukemia - Biology & translational research

Luca Palombella*[1, 2, 3], Elias Bührer[1, 2], Michael Amrein[1, 2], Adrian Wegmüller[1, 2, 3], Bofei Wang[4], Hussein Abbas[4, 5], Ramin Radpour[1, 2, 3], Carsten Riether[1, 2], Adrian Ochsenbein[1, 2]

*[1]Inselspital, University Hospital of Bern, Department of Medical Oncology, Bern, Switzerland;[2]University of Bern, Department of BioMedical Research, Tumorimmunolgy, Bern, Switzerland; [3]University of Bern, Graduate School for Cellular and Biomedical Sciences, Bern, Switzerland; [4]University of Texas MD Anderson Cancer Center, Department of Leukemia, Houston, United States of America; [5]University of Texas MD Anderson Cancer Center, Department of Genomic Medicine, Houston, United States of America;*

### Background

TRAF2- and NCK-interacting kinase (TNIK) is a serine/threonine kinase best known as a regulator of Wnt/β-catenin signaling and cytoskeletal organization. Beyond Wnt signaling, TNIK integrates MAPK, JNK, NF-κB, and small GTPase pathways, positioning it as a signaling hub controlling stemness, proliferation, differentiation, and survival. Dysregulation of stem cell maintenance programs is a hallmark of leukemogenesis, particularly in acute myeloid leukemia (AML), which is sustained by chemotherapy-resistant leukemic stem cells (LSCs). While TNIK activation has been implicated in solid tumors and selective TNIK inhibitors are emerging as precision oncology agents, its role in normal hematopoiesis and LSC biology is unknown.

### Aims

To define the role of TNIK in hematopoiesis and hematopoietic stem cell (HSC) function and to determine its contribution to LSC maintenance and disease progression in myeloid leukemias.

### Methods

TNIK expression was analyzed in primary human AML samples and correlated with clinical outcome. Functional studies were performed in AML and chronic myeloid leukemia (CML) mouse models driven by MLL-AF9 and BCR-ABL, as well as in AML cell lines and AML patient-derived LSCs. Pharmacologic TNIK inhibition (INS018-055) was used to evaluate the effects and therapeutic potential of TNIK blockade on LSC function and leukemia progression.
A hematopoietic-specific TNIK knockout (KO) mouse model (Vav-iCre) was generated to assess TNIK function in normal hematopoiesis. Flow cytometry was used to analyze HSCs, progenitor populations, and differentiated blood cells under steady-state conditions. HSC function was evaluated by cell cycle analysis, in vitro colony-forming assays, limiting dilution analysis, and competitive bone marrow transplantation.

**Copyright Information:** (Online) ISSN: 2572-9241
©2026 The Author(s). HemaSphere is published by John Wiley & Sons Ltd on behalf of European Hematology Association. This is an open access Abstract Book distributed under the Attribution-NonCommercial-NoDerivs (CC BY-NC-ND), which allows third parties to download the articles and share them with others as long as they credit the author and the Abstract Book, but they cannot change the content in any way or use them commercially.
**Abstract Book Citations:** Authors, Title, HemaSphere, 2026;10:(S1):pages. The abstract book DOIs can be found on the “Author Information” page.
**Disclaimer:** Articles published in the journal HemaSphere exclusively reflect the opinions of the authors. The authors are responsible for all content in their abstracts including accuracy of the facts, statements, citing resources, etc.

**Results**

Elevated TNIK expression was associated with inferior clinical outcome in AML. Genetic TNIK ablation significantly delayed leukemia progression in AML and CML models and reduced LSC frequency both immunophenotypically and functionally by impairing proliferation and clonogenic capacity. Consistently, TNIK knockdown suppressed leukemia cell line growth and reduced colony formation of patient-derived AML LSCs. Mechanistically, TNIK promoted LSC self-renewal and survival through activation of NF-κB signaling. Pharmacologic inhibition with INS018-055 selectively targeted AML LSCs while sparing normal HSCs, resulting in reduced leukemic burden and delayed disease progression in vivo.
Despite detectable TNIK expression in HSCs and progenitors, TNIK deletion did not significantly affect HSC frequency, lineage composition, colony-forming capacity, or competitive repopulation ability.

**Summary/Conclusion**

TNIK is a critical regulator of LSC self-renewal, proliferation, and survival in myeloid leukemias, acting in part through NF-κB activation. Targeting TNIK selectively impairs leukemic stem cells while preserving normal hematopoiesis, identifying TNIK inhibition as a promising therapeutic strategy to improve outcomes in AML.

**Copyright Information:** (Online) ISSN: 2572-9241
©2026 The Author(s). HemaSphere is published by John Wiley & Sons Ltd on behalf of European Hematology Association. This is an open access Abstract Book distributed under the Attribution-NonCommercial-NoDerivs (CC BY-NC-ND), which allows third parties to download the articles and share them with others as long as they credit the author and the Abstract Book, but they cannot change the content in any way or use them commercially.
**Abstract Book Citations:** Authors, Title, HemaSphere, 2026;10:(S1):pages. The abstract book DOIs can be found on the “Author Information” page.
**Disclaimer:** Articles published in the journal HemaSphere exclusively reflect the opinions of the authors. The authors are responsible for all content in their abstracts including accuracy of the facts, statements, citing resources, etc.

### (PF417) INTEGRATED MOLECULAR PROFILING REVEALS CRYPTIC FUSIONS AND BLAST-INDEPENDENT RISK STRATIFICATION IN DDX41-ASSOCIATED AML

**Topic:** 03. Acute myeloid leukemia - Biology & translational research

Ira Kraft*[1], Rebecca Alexander[1], Yi Liu[2], Jeremy Scutenaire[1, 3], Trinh Pham[4], Kathy McGraw[1], Srinivas Tantravahi[5], Caner Saygin[6], Liqiang Xi[4], Mark Raffeld[4], Markus Hafner[3], Sung-Yun Pai[1], Daniel Larson[1]

*[1]National Institutes of Health, National Cancer Institute, Bethesda, United States of America;[2]National Institutes of Health, Genetics Branch, Center for Cancer Research, Bethesda, United States of America; [3]National Institutes of Health, National Institute of Arthritis Musculoskeletal and Skin Diseases, Bethesda, United States of America; [4]National Institutes of Health, Molecular Diagnostics Section, Center for Cancer Research, Bethesda, United States of America; [5]University of Utah, Salt Lake City, United States of America; [6]University of Chicago, Chicago, United States of America;*

### Background

Deleterious germline variants in DEAD-box helicase 41 (*DDX41*) predispose to myelodysplastic syndrome (MDS) and acute myeloid leukemia (AML). The optimal management of carriers and molecular events driving leukemogenesis are not defined.

### Aims

To characterize the progression and molecular features of *DDX41* germline predisposition syndrome (DDX41-GPS) via longitudinal clinical review, next-generation sequencing (DNA/RNA-seq), and quantitative proteomics (TMT-MS).

### Methods

We launched a prospective cohort study (NCT07019155) enrolling participants into four groups: *DDX41* with MDS/AML (1), *DDX41* without MDS/AML (2), other germline variants (3), or without germline variants (4) (Fig. 1a). Participants undergo longitudinal clinical assessment, genetic counseling, integrated DNA/RNA-seq and TMT-MS, and multidisciplinary reviews to inform tailored surveillance and therapy. Enrollment opened 07/24/2025; data are summarized as of 02/27/2026.

**Copyright Information:** (Online) ISSN: 2572-9241
©2026 The Author(s). HemaSphere is published by John Wiley & Sons Ltd on behalf of European Hematology Association. This is an open access Abstract Book distributed under the Attribution-NonCommercial-NoDerivs (CC BY-NC-ND), which allows third parties to download the articles and share them with others as long as they credit the author and the Abstract Book, but they cannot change the content in any way or use them commercially.
**Abstract Book Citations:** Authors, Title, HemaSphere, 2026;10:(S1):pages. The abstract book DOIs can be found on the "Author Information" page.
**Disclaimer:** Articles published in the journal HemaSphere exclusively reflect the opinions of the authors. The authors are responsible for all content in their abstracts including accuracy of the facts, statements, citing resources, etc.

### Results

At data cutoff, 32 of 58 referred individuals were enrolled (1: N=5; 2: N=13; 3: N=3; 4: N=2; pending germline testing: N=9). Median time enrolled on study was 2.7 months (range 0.6-7.3). Study review modified the diagnosis of two Cohort 1 participants leading to changes in therapeutic recommendations. No Cohort 2 participants developed MDS/AML, and clonal hematopoiesis (*DNMT3A*) was detected in one individual under close surveillance. All enrolled participants reported a personal or family history of solid tumors, suggesting broader cancer predisposition within *DDX41* families.
Archival tumor tissue from patients with germline *DDX41*-mutated AML were available for RNA-seq (N=9) and TMT-MS (N=11) and compared to healthy controls (N=8) (Fig. 1b). Somatic *DDX41* mutations occurred in 31% (N=4/13), and 62% (N=8/13) harbored somatic AML-associated mutations. Unsupervised clustering of RNA-seq and TMT-MS proteomics provided complementary stratification and identified molecularly defined AML subtypes discordant with blast percentage (Fig. 1c-d). Partial cross-platform overlap was noted in samples with both RNA-seq and TMT-MS data available (Fig. 1e, $\chi^2$=12.5, p=0.0517, Cramer's V=0.884, N=8). Karyotypically cryptic, in-frame PICALM-MLLT10 and RUNX1-FOXJ2 fusions (N=2) were identified through transcriptomic fusion analysis. These molecular programs defined RNA Cluster 1 (N=5, median blasts 50%, range 7-92%) and showed favorable-risk GSEA signatures: HOXA9-low (NES=-2.32), functional p53 (NES=-1.52), and suppressed proliferation (all FDR<0.05) (Fig. 1f). RNA Cluster 3 with low blast burden (median 2.5%, range 0-5%, n=4) exhibited more aggressive features: KRAS-hyperactive (NES=+1.85), JAK2-active (NES=+1.49), YAP-high (NES=-2.13), and p53-dysfunctional (NES=-1.56, all FDR<0.05) (Fig. 1f). RNA and proteomics enrichment signatures showed pathway concordance for RNA Cluster 1 and Protein Cluster 4 (Spearman ρ = 0.379, p = 1.12e-07, n = 23) but did not demonstrate significant overlap in RNA Cluster 3 and Protein Cluster 3, highlighting post-transcriptional molecular heterogeneity within low-blast *DDX41*-AML.

### Summary/Conclusion

*DDX41*-AML comprises of biologically distinct subtypes that do not reflect blast percentage requiring molecularly informed stratification. Cryptic in-frame fusions were associated with favorable-risk molecular programs independent of blast percentage challenging reliance on morphologic blast burden in DDX41-GPS. Ongoing longitudinal follow-up will define pre-leukemic and AML trajectories refining surveillance and therapeutic strategies.

**Copyright Information:** (Online) ISSN: 2572-9241
©2026 The Author(s). HemaSphere is published by John Wiley & Sons Ltd on behalf of European Hematology Association. This is an open access Abstract Book distributed under the Attribution-NonCommercial-NoDerivs (CC BY-NC-ND), which allows third parties to download the articles and share them with others as long as they credit the author and the Abstract Book, but they cannot change the content in any way or use them commercially.
**Abstract Book Citations:** Authors, Title, HemaSphere, 2026;10:(S1):pages. The abstract book DOIs can be found on the "Author Information" page.
**Disclaimer:** Articles published in the journal HemaSphere exclusively reflect the opinions of the authors. The authors are responsible for all content in their abstracts including accuracy of the facts, statements, citing resources, etc.

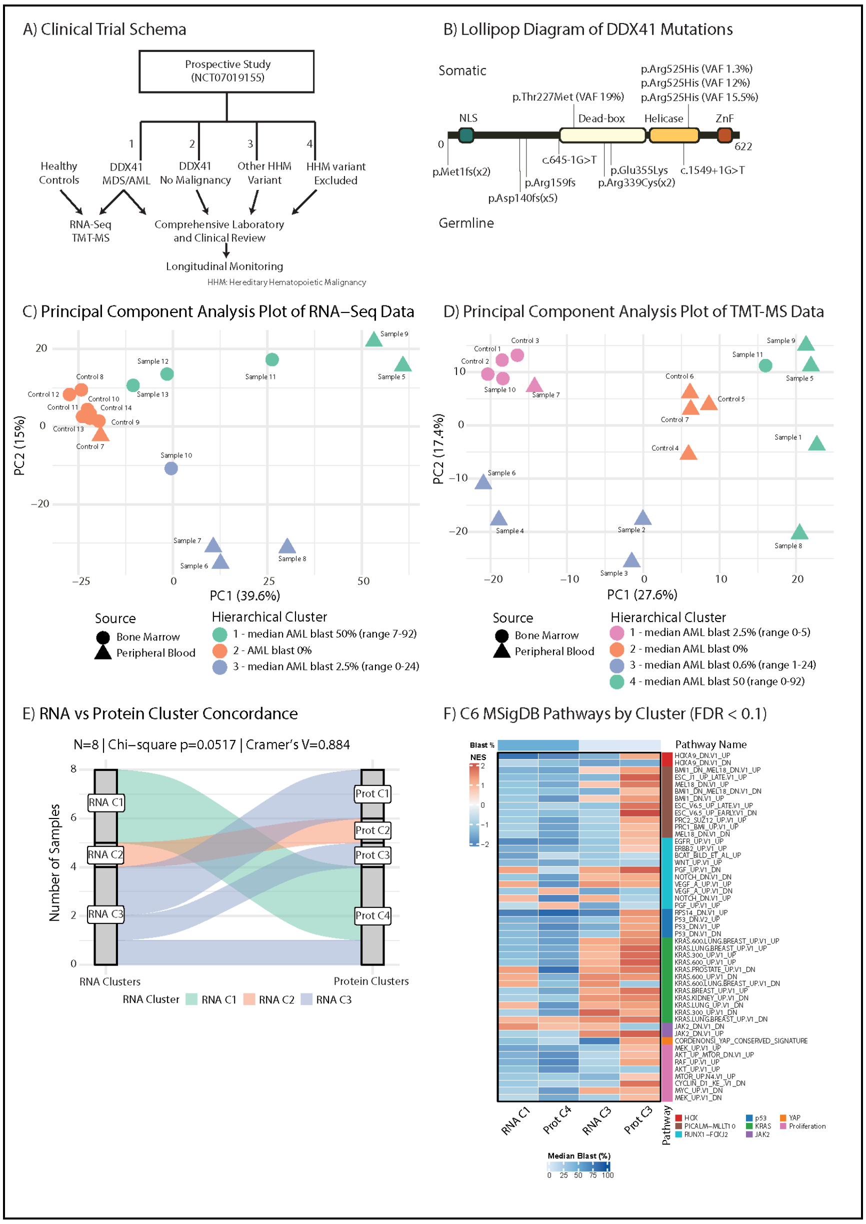

**Copyright Information:** (Online) ISSN: 2572-9241
©2026 The Author(s). HemaSphere is published by John Wiley & Sons Ltd on behalf of European Hematology Association. This is an open access Abstract Book distributed under the Attribution-NonCommercial-NoDerivs (CC BY-NC-ND), which allows third parties to download the articles and share them with others as long as they credit the author and the Abstract Book, but they cannot change the content in any way or use them commercially.
**Abstract Book Citations:** Authors, Title, HemaSphere, 2026;10:(S1):pages. The abstract book DOIs can be found on the "Author Information" page.
**Disclaimer:** Articles published in the journal HemaSphere exclusively reflect the opinions of the authors. The authors are responsible for all content in their abstracts including accuracy of the facts, statements, citing resources, etc.

### (PF418) METABOLIC NICHE REMODELING BY BONE MARROW ADIPOCYTES IN PEDIATRIC ACUTE MYELOID LEUKEMIA

**Topic:** 03. Acute myeloid leukemia - Biology & translational research

Sara Perpinello*[1], Maddalena Benetton[1], Ambra Da Ros[1], Katia Polato[1], Veronica Zampieri[2], Giorgia Longo[1], Claudia Tregnago[1], Martina Pigazzi[1, 3], Franco Locatelli[4, 5]

*[1]University of Padova, Department of Women's and Children's Health, Padova, Italy;[2]Azienda Ospedale-Università Padova, Padova, Italy; [3]Istituto di Ricerca Pediatrica (IRP) - Città della Speranza, Pediatric Hematology, Oncology, Hematopoietic Cell and Gene Therapy Research Area, Padua, Italy; [4]IRCCS Bambino Gesù Children's Hospital, Catholic University of the Sacred Heart, Pediatric Haematology/Oncology and Cell and Gene Therapy, Rome, Italy; [5]Catholic University of the Sacred Heart, Department of Life Sciences, Rome, Italy;*

### Background

Increasing evidence indicates that cell crosstalk within the bone marrow (BM) niche critically influences pediatric Acute Myeloid Leukemia (AML). Bone marrow adipocytes (BMAds), key regulators of lipid metabolism and energy supply, may provide metabolic substrates and functional signals that support leukemic cell survival, yet their role in pediatric AML has not been clearly defined.

### Aims

The project aimed to determine whether aberrant adipogenesis influences BM niche to fuel AML metabolic plasticity and chemotherapy resistance.

### Methods

Primary MSCs derived from the BM of pediatric AML patients (AML-MSCs) or healthy donors (h-MSCs) were differentiated into BMAds and analyzed functionally and transcriptionally in two-dimensional (2D) and biomimetic three-dimensional (3D) BM models. BMAds were cultured with primary AML blasts to assess proliferation, metabolic rewiring, lipid transfer, therapy response, and stemness properties of AML cells.

**Copyright Information:** (Online) ISSN: 2572-9241
©2026 The Author(s). HemaSphere is published by John Wiley & Sons Ltd on behalf of European Hematology Association. This is an open access Abstract Book distributed under the Attribution-NonCommercial-NoDerivs (CC BY-NC-ND), which allows third parties to download the articles and share them with others as long as they credit the author and the Abstract Book, but they cannot change the content in any way or use them commercially.
**Abstract Book Citations:** Authors, Title, HemaSphere, 2026;10:(S1):pages. The abstract book DOIs can be found on the "Author Information" page.
**Disclaimer:** Articles published in the journal HemaSphere exclusively reflect the opinions of the authors. The authors are responsible for all content in their abstracts including accuracy of the facts, statements, citing resources, etc.

### Results

Transcriptomic analysis revealed a distinct expression profile in AML-MSCs (n=21) compared to h-MSCs (n=6), with a significant enrichment of genes related to fat cell differentiation (NES=1.90), white adipocyte differentiation (NES=2.06), and lipid storage (NES=2.00). Functionally, AML-MSCs showed enhanced adipogenic differentiation compared to h-MSCs in 2D (68.6% in AML-MSCs vs 43.2% in h-MSCs; n≥7, *p*=0.006). However, canonical adipogenesis genes (*ADIPOQ*, *FABP4,* and *CEBPA*) were found not to be differentially expressed between AML- and h-MSCs, indicating activation of alternative, leukemia-driven adipogenic pathways. In a 3D niche model, mature BMAds were generated from AML-MSCs (n=4), whereas h-MSCs exhibited minimal or absent adipogenesis, suggesting a potential role of spatial architecture and extracellular matrix in leukemic BM niche reprogramming. In co-cultures, BMAds significantly promoted AML proliferation compared to MSCs in both 2D (n=7, *p*=0.011) and 3D (n=3, *p*=0.048). Moreover, BMAds conferred resistance to treatments (live cells with AraC: 70.4% on MSCs vs 89.1% on BMAds, *p*=0.090; Venetoclax: 43.6% on MSCs vs 72.3% on BMAds, *p*=0.058; n=5). When co-cultured with BMAds, AML cells showed significant uptake of total free fatty acids (FFAs, n=6, *p*=0.007) or palmitate (n=6, *p*=0.012). This lipid transfer induced a robust AML metabolic shift toward fatty acid oxidation (by Scenith, n=5, *p*=0.030) and an increased mitochondrial membrane potential (n=8, *p*=0.024) and ROS production (n=4, *p*=0.032), supporting an active BMAds-derived lipid utilization to sustain AML metabolic demands. Under nutrient-deprived conditions, primary AML (n=3) preferentially metabolized palmitate at 40h (*p*=0.034) and 48h (*p*=0.046). Notably, exposure to palmitate enhanced AML clonogenic capacity (n=2, *p*=0.002) even under cytotoxic and mitochondrial stress, without affecting ATP production, proliferation, and viability, indicating that BMAds-derived lipids specifically sustained therapy tolerance and stem-like features rather than simply driving expansion. These findings were validated *in vivo* by implanting a fully-human 3D AML niche with MSCs+AML and BMAds+AML (n=2). In this model, BMAds provided higher support to AML proliferation compared to MSCs during and after 7 days of chemotherapeutic treatment (AraC 40mg/kg, 21 days).

### Summary/Conclusion

AML actively reprograms MSCs within the BM to generate a lipid-rich environment that fuels leukemic cells and promotes chemotherapy resistance, identifying BMAds-AML crosstalk as an actionable therapeutic vulnerability.

**Copyright Information:** (Online) ISSN: 2572-9241
©2026 The Author(s). HemaSphere is published by John Wiley & Sons Ltd on behalf of European Hematology Association. This is an open access Abstract Book distributed under the Attribution-NonCommercial-NoDerivs (CC BY-NC-ND), which allows third parties to download the articles and share them with others as long as they credit the author and the Abstract Book, but they cannot change the content in any way or use them commercially.
**Abstract Book Citations:** Authors, Title, HemaSphere, 2026;10:(S1):pages. The abstract book DOIs can be found on the "Author Information" page.
**Disclaimer:** Articles published in the journal HemaSphere exclusively reflect the opinions of the authors. The authors are responsible for all content in their abstracts including accuracy of the facts, statements, citing resources, etc.

### (PF419) BONE MARROW STROMAL CELLS IMPAIR NATURAL KILLER (NK) CELL ACTIVATION AND PROMOTE RESISTANCE OF ACUTE MYELOID LEUKEMIA CELLS TO NK CELL CYTOTOXICITY THROUGH CYTOKINE SIGNALLING

**Topic:** 03. Acute myeloid leukemia - Biology & translational research

Salome Decombis*[1, 2, 3], Johannes Smolander[1, 2, 3], Jonas Bouhlal[1, 2, 3], Hanna Duàn[1, 2, 3], Emmi Jokinen[1, 2, 3], Konstantin Matjusinski[1, 2, 3], Jay Klievink[1, 2, 3], Hanna Lähteenmäki[1, 2, 3], Linh Lin[2, 3], Hanna Laitinen[2, 3], Mikko Myllymäki[1, 2, 3], Heidi Haikala[2, 3], Sara Gandolfi[1, 2, 3], Satu Mustjoki[1, 2, 3]

*[1]Hematology Research Unit Helsinki, University of Helsinki and Helsinki University Hospital, HELSINKI, Finland;[2]Translational Immunology Research Program, University of Helsinki, HELSINKI, Finland; [3]iCAN Digital Precision Cancer Medicine Flagship, HELSINKI, Finland;*

### Background

Natural killer (NK) cells are emerging as a promising therapeutic option in hematological malignancies including acute myeloid leukemia (AML). However, the complete eradication of AML cells from the bone marrow (BM) represents a major clinical challenge. The BM stromal microenvironment plays an important role in AML cells' survival and resistance to chemotherapy, whereas the impact of stromal cells on NK cell function remains unclear.

### Aims

To decipher the molecular modulations that occur in the BM stromal niche inducing AML cell resistance to NK cell attack, we performed functional co-cultures of NK, AML and stromal cells.

### Methods

We designed a co-culture system mimicking the BM stromal compartment using HS-5 stromal cells and either AML (MOLM-14, OCI-M1, THP-1) and CML (K562) cell lines or BM samples from AML patients at diagnosis (n=10). After culturing leukemia cells with HS-5 cells for 24h, primary expanded NK cells were added for 24h. To understand NK-AML cells interactions within the stromal microenvironment, we performed flow cytometry, single-cell RNA sequencing (scRNA-seq) and soluble factors analysis. In addition, genome-scale loss-of-function CRISPR screen was performed using THP-1 cells.

**Copyright Information:** (Online) ISSN: 2572-9241
©2026 The Author(s). HemaSphere is published by John Wiley & Sons Ltd on behalf of European Hematology Association. This is an open access Abstract Book distributed under the Attribution-NonCommercial-NoDerivs (CC BY-NC-ND), which allows third parties to download the articles and share them with others as long as they credit the author and the Abstract Book, but they cannot change the content in any way or use them commercially.
**Abstract Book Citations:** Authors, Title, HemaSphere, 2026;10:(S1):pages. The abstract book DOIs can be found on the "Author Information" page.
**Disclaimer:** Articles published in the journal HemaSphere exclusively reflect the opinions of the authors. The authors are responsible for all content in their abstracts including accuracy of the facts, statements, citing resources, etc.

**Results**

We integrated all scRNA-seq co-culture conditions and performed unsupervised clustering to distinguish expanded NK, leukemia, and HS-5 cells. K562 co-culture induced an activated NK cell phenotype (*TNFRSF9/18, HAVCR2, TIGIT*), whereas in the co-culture with MOLM-14 cells, NK cells maintained a resting state. OCI-M1 and THP-1 promoted a type I interferon NK phenotype (*OAS1/2, MX1/2, ISG15*). Co-culture with primary AML samples also showed heterogeneous effects on NK cells: six samples induced either activated or type I interferon NK phenotypes, whereas four maintained NK cells in a resting state. Stromal cell co-culture decreased both activated and type I interferon NK phenotypes and triggered a stromal associated NK cell state characterized by decreased *NKp30* expression and increased *TIM3*, *TIGIT* and *LAG3*.
In K562, THP-1 and MOLM-14 cells interacting with NK cells, we observed an increase of the expression of genes related to MHC class I (*B2M, TAP1, HLA-A/B/C/E*), JAK-STAT signalling (*STAT1, IRF1*) and immunoproteasome (*PSMB8/9, PSME1/2*). Stromal cell addition in the co-culture decreased the expression of the NK-induced genes and downregulated MHC class II in the leukemia cells. Stromal cells induced a distinct leukemia phenotype characterized by the increase of the expression of JAK-STAT/interferon signalling (*CISH, CSF3R, SOCS3, CST7, IFITM2*) and IL-1β signalling (*IL1RAP, LRG1*).
The analysis of soluble dialog during NK-AML cell interactions in the stromal niche revealed an increase of IL-1β, IL-6, IL-10, G-CSF and GM-CSF secretion involved in AML survival and a decrease of CCL3, CCL4, CCL5, and TNFα secretion, involved in NK cytotoxicity. Reduced IFNγ secretion was observed, and concordantly, the genome-scale CRISPR screen identified the loss of *SOCS3* and *SBNO2*, involved in interferon /STAT3 pathway, as sensitizers of THP-1 cells to NK cell mediated-cytotoxicity.

**Summary/Conclusion**

Collectively, at single-cell resolution, our findings reveal the dynamic transcriptomic changes and soluble dialogue of tumor and NK cells interacting within the stromal BM niche. Stromal cells impair NK cell activation and promote AML resistance through cytokine signalling, including reduced IFNγ secretion and SOCS3-dependent suppression of cytotoxicity, supporting cytokine-targeted strategies in AML.

**Copyright Information:** (Online) ISSN: 2572-9241
©2026 The Author(s). HemaSphere is published by John Wiley & Sons Ltd on behalf of European Hematology Association. This is an open access Abstract Book distributed under the Attribution-NonCommercial-NoDerivs (CC BY-NC-ND), which allows third parties to download the articles and share them with others as long as they credit the author and the Abstract Book, but they cannot change the content in any way or use them commercially.
**Abstract Book Citations:** Authors, Title, HemaSphere, 2026;10:(S1):pages. The abstract book DOIs can be found on the "Author Information" page.
**Disclaimer:** Articles published in the journal HemaSphere exclusively reflect the opinions of the authors. The authors are responsible for all content in their abstracts including accuracy of the facts, statements, citing resources, etc.

### (PF420) DYSREGULATED INTRACELLULAR CHOLESTEROL MEDIATES CHEMOTHERAPY RESISTANCE IN PERSISTENT LEUKEMIA STEM CELLS IN VIVO

**Topic:** 03. Acute myeloid leukemia - Biology & translational research

Elisa Donato*[1, 2, 3], Roberto Würth[1, 2], Maria Puschhof[1, 2], Nadia Correia[1, 2], Carolin Andresen[1, 2], Darja Karpova[1, 2, 3], Nesrine Aroua[1, 2], Michelle Neßling[1], Karsten Richter[1], Christopher Manring[4], Deedra Nicolet[4], Halvard Bonig[3], Sebastian Stasik[5], Christoph Röllig[5], Romina Ludwig[6], Irmela Jeremias[6], Anna Dolnik[7], Lars Bullinger[7], Frank Buchholz[5], Christian Thiede[5], Daniel Hübschmann[1, 2], Ann-Kathrin Eisfeld[4], Andreas Trumpp[1, 2]

*[1]German Cancer Research Center (DKFZ), Division of Stem Cells and Cancer, Heidelberg, Germany;[2]Heidelberg Institute for Stem Cell Technology and Experimental Medicine (HI-STEM gGmbH), Heidelberg, Germany; [3]German Red Cross Blood Service Baden-Württemberg-Hessen, Cell Therapy/GMP/ATMP, Frankfurt am Main, Germany; [4]The Ohio State University Comprehensive Cancer Center, Columbus, United States of America; [5]University Hospital Carl Gustav Carus, Technische Universität (TU) Dresden, Dresden, Germany; [6]Helmholtz Center Munich, Munich, Germany; [7]Charité – Universitätsmedizin, Berlin, Germany;*

### Background

Relapse in acute myeloid leukemia (AML) is predominantly driven by a reservoir of chemoresistant leukemia stem cells (LSCs) that survive initial therapy and fuel disease re-emergence. These cells exhibit remarkable phenotypic plasticity, enabling dynamic adaptation to therapeutic pressure. We hypothesized that this cellular flexibility is accompanied by metabolic plasticity, creating context-specific metabolic dependencies that could be therapeutically exploited.

### Aims

In this study, we profiled the transcriptomic and metabolic landscape of *DNMT3A/NPM1*-mutated AML to characterize the molecular programs that enable LSCs to evade chemotherapy and to uncover actionable metabolic vulnerabilities aiming to prevent or target relapse.

### Methods

We performed an integrated transcriptomic and functional analysis of 46 *DNMT3A/NPM1*-mutated AML patient samples, stratified in two groups based on clinical outcome (early relapse vs long term remission). To isolate the drivers of resistance, we utilized patient-derived xenograft (PDX) models subjected to *in vivo* treatment with intensive chemotherapy (AraC+Daunorubicin), the OxPHOS complex I inhibitor IACS-010759 (IACS), or combination of the two. Residual LSCs surviving these therapy pressures were harvested and analyzed using single-cell RNA sequencing (CITE-seq) to map the "persister" cell landscape.

**Copyright Information:** (Online) ISSN: 2572-9241
©2026 The Author(s). HemaSphere is published by John Wiley & Sons Ltd on behalf of European Hematology Association. This is an open access Abstract Book distributed under the Attribution-NonCommercial-NoDerivs (CC BY-NC-ND), which allows third parties to download the articles and share them with others as long as they credit the author and the Abstract Book, but they cannot change the content in any way or use them commercially.
**Abstract Book Citations:** Authors, Title, HemaSphere, 2026;10:(S1):pages. The abstract book DOIs can be found on the "Author Information" page.
**Disclaimer:** Articles published in the journal HemaSphere exclusively reflect the opinions of the authors. The authors are responsible for all content in their abstracts including accuracy of the facts, statements, citing resources, etc.

**Results**

Initial functional analyses revealed a pronounced dependency on oxidative phosphorylation (OxPHOS) as a significant metabolic vulnerability in aggressive LSCs. However, *in vivo* inhibition of the Electron Transport Chain (ETC) with IACS in AML-PDX models led only to a transient reduction of phenotypic and functional LSCs during active treatment. Upon drug withdrawal, rapid leukemia regrowth occurred mirroring clinical relapse suggesting that ETC inhibition suppresses, but does not eradicate, the LSC pool.
To resolve this escape mechanism, we performed CITE-seq on residual cells from mice treated with Chemo, IACS, or combination therapy. This high-resolution mapping identified a distinct, pre-existing "persister" LSC subpopulation that selectively survives across all treatment arms. These cells exhibit a paradoxical molecular profile: they showed a significant downregulation of cholesterol homeostasis-related genes, despite displaying the highest baseline LSC17 stemness scores. Mechanistically, this "low-cholesterol" transcriptional signature coincides with the physical sequestration of intracellular cholesterol within cytoplasmic multivesicular bodies. Pharmacological perturbation using statins confirmed that this dysregulated intracellular cholesterol trafficking serves as a protective metabolic state, allowing the most primitive LSCs to survive therapeutic stress.

**Summary/Conclusion**

Our findings demonstrate that although elevated OxPHOS is closely linked to chemoresistance, it represents only a transient vulnerability that LSCs can ultimately withstand. Instead, our data suggest, that LSC persistence appears to be driven by intracellular dysregulation of cholesterol metabolism. Finally, we will present data on the relapse-free survival of AML patients stratified by statins intake.
Targeting cholesterol sequestration may provide a novel rational dual-targeting strategy to eradicate the persistent LSC reservoir and prevent or overcome LSC-mediated relapse in AML.

**Copyright Information:** (Online) ISSN: 2572-9241
©2026 The Author(s). HemaSphere is published by John Wiley & Sons Ltd on behalf of European Hematology Association. This is an open access Abstract Book distributed under the Attribution-NonCommercial-NoDerivs (CC BY-NC-ND), which allows third parties to download the articles and share them with others as long as they credit the author and the Abstract Book, but they cannot change the content in any way or use them commercially.
**Abstract Book Citations:** Authors, Title, HemaSphere, 2026;10:(S1):pages. The abstract book DOIs can be found on the "Author Information" page.
**Disclaimer:** Articles published in the journal HemaSphere exclusively reflect the opinions of the authors. The authors are responsible for all content in their abstracts including accuracy of the facts, statements, citing resources, etc.

### (PF421) SINGLE-CELL TRANSCRIPTOMIC ANALYSIS UNCOVERS MOLECULAR MECHANISMS AND IDENTIFIES POTENTIAL THERAPEUTIC TARGETS IN REFRACTORY/RELAPSED IN ACUTE MYELOID LEUKEMIA

**Topic:** 03. Acute myeloid leukemia - Biology & translational research

Jianbiao Zhou*[1, 2, 3], Henry Yang[2], Keir Audrey Mendoza Bono[1], Cuithbert Jun Rong Lim[1], Yi Chai[2], Sabrina Hui-Min Toh[1], Melissa G. Ooi[2, 4], Wee Joo Chng[2, 3, 4]

*[1]National University of Singapore, Cancer Science Institute of Singapore, Singapore, Singapore;[2]National University of Singapore, Department of Medicine, Yong Loo Lin School of Medicine, Singapore, Singapore; [3]National University of Singapore, NUS Centre for Cancer Research (N2CR), Singapore, Singapore; [4]The National University Health System (NUHS), Department of Hematology-Oncology, National University Cancer Institute of Singapore (NCIS), Singapore, Singapore;*

### Background

Approximately 20% of patients with acute myeloid leukemia (AML) exhibit primary resistance to induction chemotherapy, while an additional 40–60% develop secondary resistance, ultimately leading to relapse and refractory disease (RR-AML). Although diverse molecular mechanisms underlying drug resistance and the RR phenotype have been proposed, the timing of relapse and the patients most likely to develop resistance remain largely unpredictable. Single-cell (sc) RNA sequencing has transformed our understanding of tumor heterogeneity and clonal evolution in AML.

### Aims

We aimed to comparatively decipher the cellular ecosystem among patients with RR-AML versus AML patients who achieved continuous complete remission (CCR) more than 4 years, as well as heathy controls. Our objectives were to elucidate the molecular mechanisms underlying the pathogenesis of RR-AML and identify potential therapeutic targets for RR-AML.

### Methods

Frozen bone marrow (BM) or peripheral blood (PB) samples at diagnosis (pretreatment) obtained from the National Cancer Institute of Singapore (NCIS) were viably thawed and processed using 10x Genomics Single Cell 3' Library Kit v3. A total of 13 BM or PB samples were processed from six RR-AML patients and seven CCR patients. Four sorted, normal CD34+ BM and two normal whole BM samples were processed in parallel as controls. Various in vitro experiments including cell proliferation, colony formation unit (CFU) assay, and in vivo mouse experiments were performed to validate the scRNA-seq findings.

**Copyright Information:** (Online) ISSN: 2572-9241
©2026 The Author(s). HemaSphere is published by John Wiley & Sons Ltd on behalf of European Hematology Association. This is an open access Abstract Book distributed under the Attribution-NonCommercial-NoDerivs (CC BY-NC-ND), which allows third parties to download the articles and share them with others as long as they credit the author and the Abstract Book, but they cannot change the content in any way or use them commercially.
**Abstract Book Citations:** Authors, Title, HemaSphere, 2026;10:(S1):pages. The abstract book DOIs can be found on the “Author Information” page.
**Disclaimer:** Articles published in the journal HemaSphere exclusively reflect the opinions of the authors. The authors are responsible for all content in their abstracts including accuracy of the facts, statements, citing resources, etc.

**Results**

After preprocessing, alignment, and quality control, including the removal of low-quality reads and cells, we obtained 31,149 cells from RR-AML patients (n = 6), 30,565 cells from CCR-AML patients (n = 7), 37,047 cells from sorted CD34⁺ cells from healthy donors (n = 4), and 23,515 cells from whole bone marrow samples of healthy donors (n = 2). Unsupervised clustering identified 11 major cell populations and 35 subpopulations. We focused on the HSC-like and HSPC-like subpopulations, which are enriched for leukemia stem cells (LSCs). To identify RR-enriched markers, we systematically evaluated all combinations of HSC-, GMP-, and CMP-like clusters, comparing RR-AML with CCR-AML and healthy controls. This analysis identified an optimal cluster combination (clusters 3, 16, and 29). The significantly upregulated genes in these three clusters included *FLT3, CD34, ADGRE5, PROM1, CD9, CLEC9A, CLEC1B, BAALC, CALCRL,* and *HMGA2*, among others. Many of these genes have been previously reported as LSC-associated markers. CD9 has been identified as a cancer stem cell (CSC) marker in both AML and pancreatic cancer. Sorted CD9⁺ AML cells, or ectopic overexpression of CD9, promoted cell migration, enhanced colony-forming capacity, and conferred chemoresistance. Xenograft models further demonstrated that CD9 contributes to drug resistance and extramedullary AML in vivo. However, CD9 is also highly expressed on platelets and certain immune cell subsets. To minimize on-target off-tumor toxicity, we are investigating a second cell surface antigen co-expressed with CD9 in RR-LSCs to rationally develop dual-target immunotherapeutic strategies.

**Summary/Conclusion**

Our integrative scRNA-seq and validation analyses identify a CD9-high LSC subpopulation as a potential driver of RR-AML. Co-targeting CD9 with a co-expressed antigen may therefore provide an effective strategy to eradicate chemoresistant, relapse-driving LSCs.

**Copyright Information:** (Online) ISSN: 2572-9241
©2026 The Author(s). HemaSphere is published by John Wiley & Sons Ltd on behalf of European Hematology Association. This is an open access Abstract Book distributed under the Attribution-NonCommercial-NoDerivs (CC BY-NC-ND), which allows third parties to download the articles and share them with others as long as they credit the author and the Abstract Book, but they cannot change the content in any way or use them commercially.
**Abstract Book Citations:** Authors, Title, HemaSphere, 2026;10:(S1):pages. The abstract book DOIs can be found on the “Author Information” page.
**Disclaimer:** Articles published in the journal HemaSphere exclusively reflect the opinions of the authors. The authors are responsible for all content in their abstracts including accuracy of the facts, statements, citing resources, etc.

### (PF422) ARE SIGNALING GENE MUTATIONS OTHER THAN FLT3-ITD PROGNOSTIC MRD MARKERS IN AML?

**Topic:** 03. Acute myeloid leukemia - Biology & translational research

Isabell Arnhardt*[1], Christian Vonk[2], Cheng-Hong Tsai[3], Felicitas Thol[1], Maximilian Bader[1], Carolin Funke[1], Wen-Chien Chou[3], Hsin-An Hou[3], Razif Gabdoulline[4], Florian Heidel[1], Hwei-Fang Tien[3, 5], Jae-Sook Ahn[6], Dennis Kim[7], Michael Heuser[4], Petrus Valk[2]

*[1]Hannover Medical School, Department of Hematology, Hemostasis, Oncology and Stem Cell Transplantation, Hannover, Germany; [2]Erasmus MC Cancer Institute, University Medical Center Rotterdam, Department of Hematology, Rotterdam, The Netherlands; [3]Department of Internal Medicine, National Taiwan University Hospital, Division of Hematology, Taipei, Taiwan; [4]University Hospital Halle (Saale), Martin-Luther-University Halle-Wittenberg, Department of Internal Medicine IV, Halle, Germany; [5]Far-Eastern Memorial Hospital, Department of Internal Medicine, New Taipei, Taiwan; [6]Chonnam National University Hwasun Hospital, Chonnam National University, Hematology-Oncology, Hwasun, South Korea; [7]Princess Margaret Cancer Centre, University Health Network, Toronto, Canada;*

### Background

Measurable residual disease (MRD) assessment by next-generation sequencing (NGS) is an established prognostic marker in acute myeloid leukemia (AML). While *FLT3* internal tandem duplication (ITD) is currently recommended for NGS-based MRD (NGS-MRD) monitoring, less is known about the prognostic value of MRD defined by mutations in genes related to intracellular signaling pathways.

### Aims

We aimed to evaluate the prognostic impact of NGS-MRD based on mutations in other signaling pathway genes than*FLT3*-ITD in intensively treated AML patients.

### Methods

We pooled patient-level data from four published studies, resulting in an overall cohort of 1,157 patients. 461 AML patients (40%) with signaling pathway gene mutations including *NRAS, FLT3*-TKD*, KIT, PTPN11, KRAS, CBL, JAK2* and *CSF3R* were analyzed. MRD was assessed by NGS in CR/CRi samples after intensive chemotherapy using study-specific cutoffs. Survival was analyzed using univariate and multivariate Cox models, CIR with Fine-Gray regression, and landmark analyses at 4 months with interaction terms for allogeneic hematopoietic cell transplantation (alloHCT). The studies were approved by the local ethics committee; data sharing was performed under a data sharing agreement.

**Copyright Information:** (Online) ISSN: 2572-9241
©2026 The Author(s). HemaSphere is published by John Wiley & Sons Ltd on behalf of European Hematology Association. This is an open access Abstract Book distributed under the Attribution-NonCommercial-NoDerivs (CC BY-NC-ND), which allows third parties to download the articles and share them with others as long as they credit the author and the Abstract Book, but they cannot change the content in any way or use them commercially.
**Abstract Book Citations:** Authors, Title, HemaSphere, 2026;10:(S1):pages. The abstract book DOIs can be found on the "Author Information" page.
**Disclaimer:** Articles published in the journal HemaSphere exclusively reflect the opinions of the authors. The authors are responsible for all content in their abstracts including accuracy of the facts, statements, citing resources, etc.

### Results

The median age of the 461 AML patients was 46 years (18-84), 51% were female. 428 patients (93%) were diagnosed with de novo AML, and 260 (56%) were considered favorable risk according to the ELN 2017 risk classification. Overall, 58% patients received alloHCT in first CR. The average number of mutations per patient at diagnosis was 3 (range: 1-9), and the most frequently mutated genes were *NRAS*, *NPM1* and *DNMT3A*. All patients achieved CR/CRi with intensive induction chemotherapy.

Among the 461 AML patients, 98 (21%) were signaling gene NGS-MRD$^{pos}$ after induction. 303 (66%) were *NPM1* wild type and *FLT3*-ITD negative at diagnosis, highlighting its relevance as an additional MRD marker. Baseline characteristics were comparable. MRD positivity was associated with sAML and a significantly higher fraction of ELN 2017 intermediate and adverse risk disease. AlloHCT was performed more frequently in MRD$^{pos}$ patients.

In univariate analysis, signaling gene MRD$^{pos}$ was significantly associated with increased CIR (3-year CIR: 63% vs 50%, hazard ratio [HR]: , 95% Cl [1.23-2.25]; p<0.001), inferior RFS (3-year RFS: 38% vs 56% HR: 1.77 [1.36-2.29]; p<0.001), and inferior OS compared to MRD$^{neg}$ (3-year OS: 49% vs 70%, HR: 1.93 [1.42-2.60]; p<0.001). In multivariate analysis, mutant signaling gene MRD$^{pos}$ remained a single prognostic factor for worse outcome for CIR, RFS and OS. Gene-specific analyses showed that mutations in *FLT3*-TKD and *NRAS* contributed most to this association. Landmark analysis suggested that alloHCT was associated with reduced CIR in MRD$^{pos}$ patients (2-year CIR in MRD$^{pos}$ patients with alloHCT 33% vs no alloHCT 46%, p=0.061), while RFS and OS were similar with and without alloHCT (2-year RFS 56% vs 46% p=0.42, 2-year OS 63% vs 60%, p=0.38).

### Summary/Conclusion

MRD monitoring defined by residual mutations in signaling pathway genes provides relevant prognostic information in AML. Mutant signaling gene MRD may extend the use of NGS-MRD to additional patients without established molecular MRD markers and may support transplantation decision-making in patients in CR/CRi after intensive chemotherapy.

Shared first authors: Isabell Arnhardt, Christian M Vonk
Shared senior authorship: Michael Heuser, Peter J M Valk

**Copyright Information:** (Online) ISSN: 2572-9241
©2026 The Author(s). HemaSphere is published by John Wiley & Sons Ltd on behalf of European Hematology Association. This is an open access Abstract Book distributed under the Attribution-NonCommercial-NoDerivs (CC BY-NC-ND), which allows third parties to download the articles and share them with others as long as they credit the author and the Abstract Book, but they cannot change the content in any way or use them commercially.
**Abstract Book Citations:** Authors, Title, HemaSphere, 2026;10:(S1):pages. The abstract book DOIs can be found on the "Author Information" page.
**Disclaimer:** Articles published in the journal HemaSphere exclusively reflect the opinions of the authors. The authors are responsible for all content in their abstracts including accuracy of the facts, statements, citing resources, etc.

### (PF424) COMBINED INHIBITION OF HSP90 AND CDK9 OVERCOMES VENETOCLAX-RESISTANCE IN AML

**Topic:** 03. Acute myeloid leukemia - Biology & translational research

Marie Kemkes*[1], Melina Vogt[1], Julian Schliehe-Diecks[1], Jia-Wey Tu[1], Aden Aderajew[1], Sophie Maria Evertz[1], Danielle Brandes[1], Jonas Fullin[2], Aleksandra Pandyra[1, 3, 4], Steffen Boettcher[2], Irmela Jeremias[5, 6], Arndt Borkhardt[1, 7], Sanil Bhatia[1, 7]

*[1]Heinrich Heine University Düsseldorf, Pediatric Oncology, Hematology and Clinical Immunology, Düsseldorf, Germany; [2]University of Zurich and University Hospital Zurich, Department for Medical Oncology and Hematology, Zurich, Switzerland; [3]University Hospital Bonn, Institute of Clinical Chemistry and Clinical Pharmacology, Bonn, Germany;[4]Partner Site Bonn-Cologne, German Center for Infection Research (DZIF), Bonn, Germany; [5]Helmholtz Center Munich, Research Unit Apoptosis in Hematopoietic Stem Cells, Munich, Germany; [6]DKFZ and University Hospital LMU Munich, German Cancer Consortium (DKTK), Munich, Germany; [7]Partner site Essen/Düsseldorf, German Cancer Consortium (DKTK), Düsseldorf, Germany;*

### Background

Venetoclax (VEN), combined with chemotherapy or hypomethylating agents, has shown therapeutic benefit against acute myeloid leukemia (AML), yet resistance frequently emerges from the upregulation of BCL-2 family members (BCL-xL, MCL-1), including in *TP53*-mutant AML. The molecular chaperone HSP90 sustains leukemic cell survival by stabilizing multiple oncogenic client proteins involved in apoptotic regulation, thereby contributing to stress adaptation and therapeutic resistance. CDK9 inhibition suppresses RNAPII-dependent transcription of short-lived pro-survival factors, such as MCL-1, uncovering a rational novel strategy to overcome VEN resistance. We previously demonstrated that RNAPII inhibition potentiates HSP90 inhibitor activity in therapy-resistant leukemia (*Vogt et al., Cell Death Dis., 2023*).

### Aims

Given that *TP53* inactivation increases the dependence of AML cells on HSP90-mediated chaperone activity (*Carter et al., Blood, 2023*) and since HSP90 stabilizes key BCL-2 family proteins, we hypothesize that combined HSP90 and CDK9 targeting would synergistically disrupt AML survival networks and overcome VEN resistance.

### Methods

*Ex vivo* drug screening was performed across a panel of 33 cell lines and 37 PDX samples from patients with initial or relapsed AML/ALL. VEN resistance was modeled in (HL60 and SKNO-1) AML cells via enforced MCL-1 overexpression or chronic VEN exposure. Mechanistic analyses were conducted using integrated transcriptomic (bulk RNA-seq) and (mass spectrometry-based) proteomic profiling. The combination was further tested on isogenic *TP53*-mutant (R248Q, R273H, +/- or -/-) MOLM13 AML models. Therapeutic efficacy was evaluated *in vivo* using preclinical NSG mouse models inoculated with AML-PDX cells.

**Copyright Information:** (Online) ISSN: 2572-9241
©2026 The Author(s). HemaSphere is published by John Wiley & Sons Ltd on behalf of European Hematology Association. This is an open access Abstract Book distributed under the Attribution-NonCommercial-NoDerivs (CC BY-NC-ND), which allows third parties to download the articles and share them with others as long as they credit the author and the Abstract Book, but they cannot change the content in any way or use them commercially.
**Abstract Book Citations:** Authors, Title, HemaSphere, 2026;10:(S1):pages. The abstract book DOIs can be found on the "Author Information" page.
**Disclaimer:** Articles published in the journal HemaSphere exclusively reflect the opinions of the authors. The authors are responsible for all content in their abstracts including accuracy of the facts, statements, citing resources, etc.

### Results

*Ex vivo* drug screening identified a selective (p = 0.009) synergistic interaction between HSP90 and CDK9 inhibition in AML cells compared to ALL, demonstrating a favorable therapeutic window with minimal impact on healthy PBMCs (Fig. 1A). Notably, the combination demonstrated synergistic activity (Fig. 1B) and enhanced apoptosis induction in VEN-resistant isogenic AML models. Integrated multi-omics profiling revealed that dual inhibition profoundly suppressed key pro-survival pathways, including the downregulation of MCL-1. Importantly, *in vivo* administration of the HSP90i (PU-H71): CDK9i (Enitociclib, Lead Discovery Centre GmbH) combination was well tolerated in preclinical NSG mouse models. In *KMT2A-r* and *NPM1*-mutated relapsed/refractory AML-PDX models, the treatment induced a significant ($p < 0.05$) reduction in leukemia burden and prolonged survival (Fig. 1C). Correlative transcriptomic analyses were performed against *ex vivo* drug sensitivity, revealing a significant ($p < 0.05$) inverse association between *TP53* expression and treatment response, with *TP53*-deficient AML cells exhibiting enhanced sensitivity. Isogenic *TP53*-mutant models (MOLM13 R248Q and R273H) and those with mono-allelic (*TP53*$^{+/-}$) and bi-allelic (*TP53*$^{-/-}$) loss exhibited comparable or enhanced synergistic responses compared to respective *TP53*-WT controls. Consistently, *in vivo* evaluation using the *TP53*-null (HL60) AML model demonstrated a significant ($p < 0.05$) reduction in leukemia progression with the combination compared to monotherapies or the vehicle group.

### Summary/Conclusion

Combined HSP90 and CDK9 inhibition synergistically overcomes VEN resistance by disrupting key survival networks, particularly in *TP53*-deficient AML, offering a promising therapeutic strategy for high-risk AML.

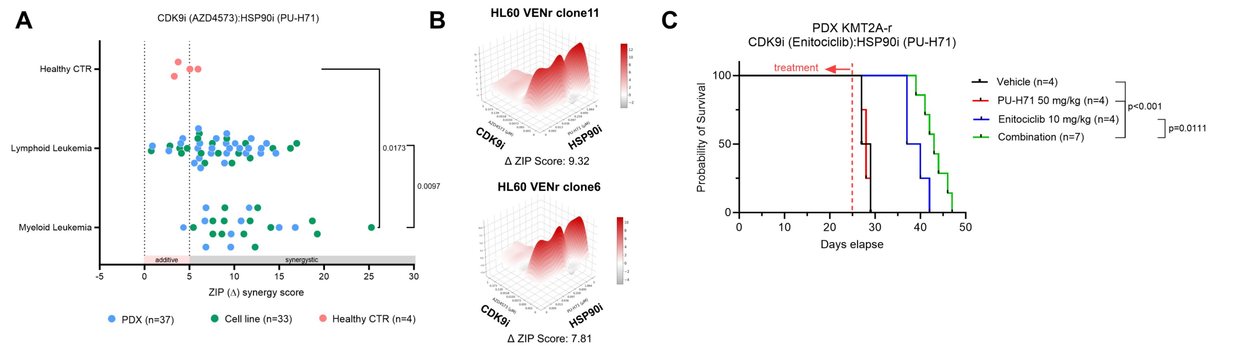

**Figure 1: Dual HSP90 and CDK9 inhibition induced a significant synergistic response in AML cell lines and PDX-AML models as compared to ALLs, as well as in venetoclax resistant cells and prolongs survival in AML mouse model. (A)** High throughput drug screening was performed on 74 samples, including healthy PBMCs, cell lines and PDX samples. The delta ZIP synergy scores were calculated from 8 × 8 dose response matrices with Synergyfinder software and plotted for each sample. Data points represent individual samples. Statistical analysis was performed using a student's t-test (unpaired, two-tailed). **(B)** Three-dimensional synergy landscapes of venetoclax resistant cells confirm strong combinatorial activity. **(C)** Kaplan-Meier survival curves of NSG AML-PDX model treated with CDK9i and HSP90i. Treatment significantly reduces leukemia burden and prolongs survival in NSG AML-PDX model compared to monotherapy or vehicle control. Survival curves were compared using the log-rank test (Mantel-Cox Test).

**Copyright Information:** (Online) ISSN: 2572-9241
©2026 The Author(s). HemaSphere is published by John Wiley & Sons Ltd on behalf of European Hematology Association. This is an open access Abstract Book distributed under the Attribution-NonCommercial-NoDerivs (CC BY-NC-ND), which allows third parties to download the articles and share them with others as long as they credit the author and the Abstract Book, but they cannot change the content in any way or use them commercially.
**Abstract Book Citations:** Authors, Title, HemaSphere, 2026;10:(S1):pages. The abstract book DOIs can be found on the "Author Information" page.
**Disclaimer:** Articles published in the journal HemaSphere exclusively reflect the opinions of the authors. The authors are responsible for all content in their abstracts including accuracy of the facts, statements, citing resources, etc.

### (PF425) CLINICAL AND MOLECULAR LANDSCAPES OF CSF3R-MUTATIONS IN ACUTE MYELOID LEUKEMIA - INSIGHTS FROM A MULTICENTER COHORT INCLUDING THE HARMONY PLATFORM

**Topic:** 03. Acute myeloid leukemia - Biology & translational research

Lara Wiechers*[1], Ángela Villaverde Ramiro[2], Rabea Mecklenbrauck[3], Brian Huntly[4], Ian Thomas[5], Klaus Metzeler[6], Christian Thiede[7], Christoph Röllig[7], Guadalupe Oñate[8], Jorge Sierra Gil[8], Petrus Valk[9], Jurjen Versluis[9], Ken Mills[10], Amin Turki[11], Walter Fiedler[12], Franziska Westendorf[12], Michael Heuser[13], Arnold Ganser[1], Florian Heidel[1], Lars Bullinger[14], Felicitas Thol[1]

*[1]Hannover Medical School, Department of Haematology, Haemostaseology, Oncology and Stem Cell Transplantation, Hannover, Germany; [2]HARMONY Alliance Foundation, Salamanca, Spain; [3]MRC Weatherall Institite Of Molecular Medicine, Oxford, United Kingdom; [4]Wellcome-MRC Cambridge Stem Cell Institute, Department of Hematology, Cambridge, United Kingdom; [5]Cardiff University, Centre for Trials Research, College of Biomedical and Life Sciences, Cardiff, United Kingdom; [6]University Leipzig Medical Center, Department for Hematology, Cell Therapy and Hemostaseology, Leipzig, Germany; [7]University Hospital Carl Gustav Carus Dresden, Department for Hematology, Cell Therapy and Medical Oncology, Dresden, Germany; [8]Hospital de la Santa Creu i Sant Pau, Clinic for Hematology, Barcelona, Spain; [9]Erasmus Medical Centre, Department of Hematology, Rotterdam, The Netherlands; [10]Queens University Belfast, Centre for Cancer Research and Cell Biology, Belfast, United Kingdom; [11]University Hospital Essen, Clinic for Hematology and Stem Cell Transplantation, Essen, Germany; [12]University Medical Center Hamburg-Eppendorf, Department of Oncology, Hematology and Bone Marrow Transplantation with Section Pneumology, Hamburg, Germany; [13]University Medicine Halle, Department of Hematology and Oncology, Halle, Germany; [14]Charité - Universitätsmedizin Berlin, Department of Hematology, Oncology and Cancer Immunology, Berlin, Germany;*

### Background

Mutations in the *colony-stimulating factor-3 receptor* (*CSF3R*) are a hallmark of chronic neutrophilic leukemia (CNL) and are rare in acute myeloid leukemia (AML). Due to their low frequency, *CSF3R*-mutations in AML are poorly characterized and their clinical and prognostic relevance remains unclear.

### Aims

We aimed to systematically investigate the clinical and comutational features of *CSF3R*-mutations in AML cohorts Hannover Medical School and the HARMONY Platform.

### Methods

Adult patients with newly diagnosed, non-APL AML harboring *CSF3R*-mutations were identified in two independent cohorts from Hannover Medical School and the HARMONY Platform. Variants were annotated using variant databases. Only pathogenic or likely pathogenic *CSF3R*-variants were included. Characteristics were compared between cohorts and against a *CSF3R* wild-type (wt) cohort from Hannover Medical School with available clinical and molecular data.

**Copyright Information:** (Online) ISSN: 2572-9241
©2026 The Author(s). HemaSphere is published by John Wiley & Sons Ltd on behalf of European Hematology Association. This is an open access Abstract Book distributed under the Attribution-NonCommercial-NoDerivs (CC BY-NC-ND), which allows third parties to download the articles and share them with others as long as they credit the author and the Abstract Book, but they cannot change the content in any way or use them commercially.
**Abstract Book Citations:** Authors, Title, HemaSphere, 2026;10:(S1):pages. The abstract book DOIs can be found on the "Author Information" page.
**Disclaimer:** Articles published in the journal HemaSphere exclusively reflect the opinions of the authors. The authors are responsible for all content in their abstracts including accuracy of the facts, statements, citing resources, etc.

### Results

Among 7,869 AML cases across cohorts, *CSF3R*-mutations were identified in 1.6% of cases (n=120, Fig. A). *CSF3R*-mutations predominantly involved the membrane-proximal region (55%) and truncating mutations of the cytoplasmic domain (45%), consistent with the canonical mutational spectrum observed in CNL, with T618I constituting the dominant hotspot variant (29%, Fig. B). The cohorts were demographically comparable with a median age of 53 years (range 19 – 80) and gender distribution (57% male) across both cohorts. The cohorts differed significantly in AML subtype distribution with secondary AML (41% vs. 10%, p<0.001) and ELN 2022 adverse risk classification (72% vs. 43%, p<0.001) being significantly more frequent in the Hannover cohort, likely reflecting a trial-based bias of the HARMONY cohort. Cytogenetic analyses revealed no major differences in proportion of complex karyotypes between cohorts. *CSF3R*-mutations constituted the dominant clone in one quarter of patients and were associated with a significantly higher comutational burden compared with *CSF3R*-wt AML (mean 4.2 vs. 3.6, p<0.001). Recurrent comutations involved *RUNX1* in both cohorts (36% and 21% vs. 16%, p=0.006), with additional enrichment of myelodysplasia-related genes such as *ASXL1* (18%), *SRSF2* (15%) and *STAG2* (14%) as well as *TET2*-mutations (29%) in the Hannover cohort (each p<0.001 vs. comparison cohort, Fig. C). In the HARMONY cohort alone, ELN 2022 risk stratification remained the dominant prognostic determinant, and *CSF3R*-mutation status did not significantly affect overall survival. However, upon inclusion of the real-world Hannover cohort, *CSF3R*-mutated cases showed superior overall survival compared to *CSF3R*-wt within the adverse-risk category (p=0.007, Fig. D).

### Summary/Conclusion

We establish the largest *CSF3R*-mutated AML cohort to date. Our findings demonstrate that *CSF3R*-mutations are rare in AML, occurring in 1.6% of patients. They exhibit hotspot clustering similar to CNL and are associated with increased comutational complexity. In one fifth of patients, the *CSF3R*-mutation was the dominant clone. We identified associations between *CSF3R*-mutations and myelodysplasia-related gene mutations as well as *TET2*-mutations. Despite their biological distinctiveness and comutational characteristics, *CSF3R*-mutations do not consistently confer independent prognostic impact beyond ELN 2022 risk stratification in clinical trial-based cohorts. However, in real-world settings enriched for secondary AML, *CSF3R*-mutations may identify patients with superior outcomes within the ELN adverse-risk category.

**Copyright Information:** (Online) ISSN: 2572-9241
©2026 The Author(s). HemaSphere is published by John Wiley & Sons Ltd on behalf of European Hematology Association. This is an open access Abstract Book distributed under the Attribution-NonCommercial-NoDerivs (CC BY-NC-ND), which allows third parties to download the articles and share them with others as long as they credit the author and the Abstract Book, but they cannot change the content in any way or use them commercially.
**Abstract Book Citations:** Authors, Title, HemaSphere, 2026;10:(S1):pages. The abstract book DOIs can be found on the "Author Information" page.
**Disclaimer:** Articles published in the journal HemaSphere exclusively reflect the opinions of the authors. The authors are responsible for all content in their abstracts including accuracy of the facts, statements, citing resources, etc.

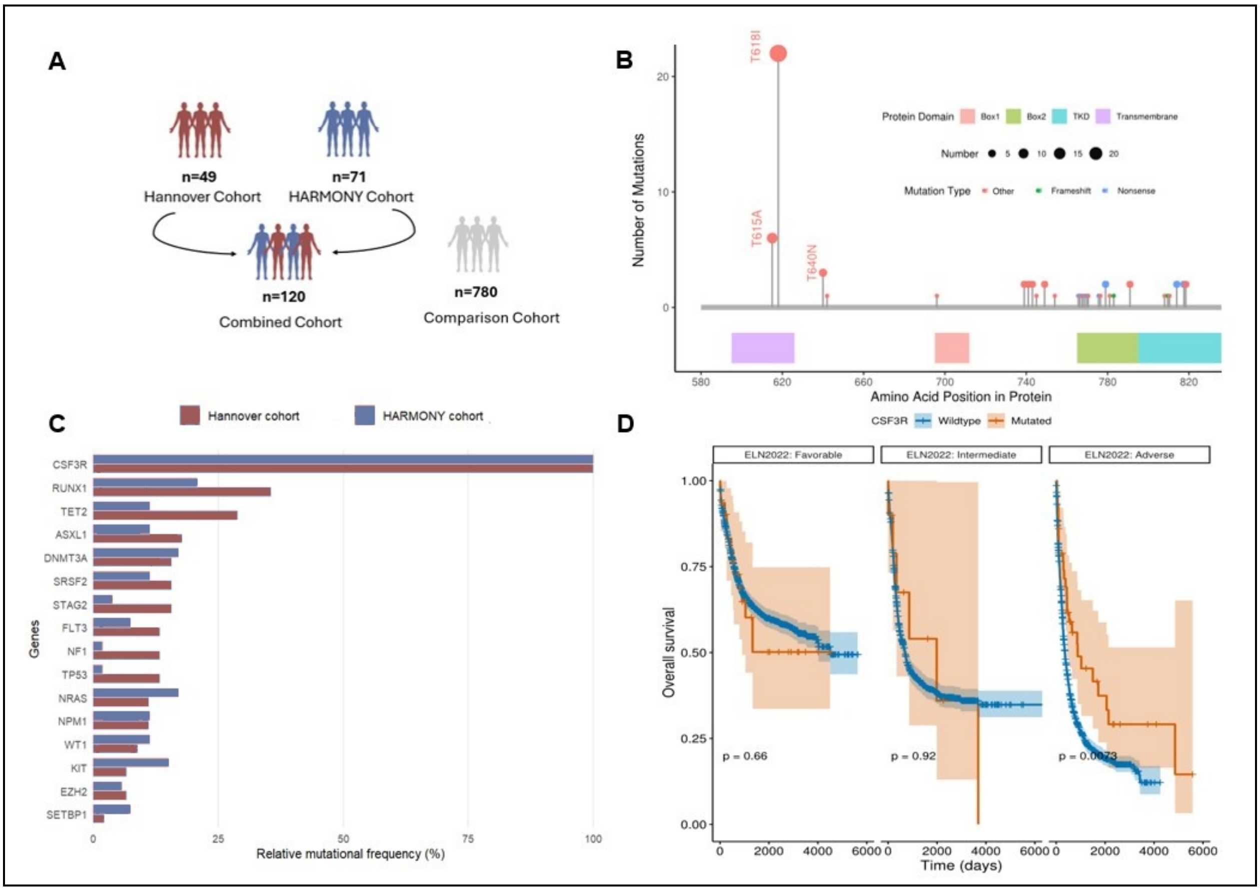

Figure: Cohort composition, CSF3R-mutation distribution, comutational profile, and cohort survival analysis of CSF3R-mutated AML in a multicenter cohort.(A) Overview of the Hannover (n = 49) and HARMONY (n = 71) cohorts combined into a multicenter CSF3R-mutated cohort (n = 120) and compared with a CSF3R wild-type comparison cohort (n = 780). (B) Lollipop plot illustrating the distribution and types of CSF3R-mutations. (C) Bar plot showing the relative mutational frequency of recurrent comutations in the Hannover and HARMONY cohorts. (D) Kaplan-Meier curves for overall survival (OS) stratified by CSF3R-mutation status within ELN2022 risk groups. In the favorable and intermediate ELN2022 groups, OS did not differ between CSF3R-mutated and CSF3R wild-type cases (p = 0.66 and p = 0.92 respectively). Within the adverse ELN2022 risk group, CSF3R mutation status was associated with significantly different OS (p = 0.0073), indicating prognostic separation specifically in this high-risk subgroup.

**Copyright Information:** (Online) ISSN: 2572-9241
©2026 The Author(s). HemaSphere is published by John Wiley & Sons Ltd on behalf of European Hematology Association. This is an open access Abstract Book distributed under the Attribution-NonCommercial-NoDerivs (CC BY-NC-ND), which allows third parties to download the articles and share them with others as long as they credit the author and the Abstract Book, but they cannot change the content in any way or use them commercially.
**Abstract Book Citations:** Authors, Title, HemaSphere, 2026;10:(S1):pages. The abstract book DOIs can be found on the “Author Information” page.
**Disclaimer:** Articles published in the journal HemaSphere exclusively reflect the opinions of the authors. The authors are responsible for all content in their abstracts including accuracy of the facts, statements, citing resources, etc.

### (PF426) MITIGATING MCL-1-DRIVEN CARDIOTOXICITY: PRECLINICAL CHARACTERIZATION OF THE FIRST-IN-CLASS MCL-1 DEGRADER

**Topic:** 03. Acute myeloid leukemia - Biology & translational research

Agata Śnieżewska*[1], Klaudia Bielak[1], Tomasz Tomczyk[1], Jose Arencibia[1], Monika Milewicz[1], Klaudia Poniatowska[1], Jolanta Skalska[1], Andrzej Tracz[1], Diana Trębicka[1], Karolina Wójcik[1], Sylvain Cottens[1], Piotr Kowalczyk[1], Paweł Dobrzański[1], Michał Biśta[1], Katarzyna Brach[1], Toshimitsu Takagi[1], Martyna Pastok[1], Justyna Adamczyk[1], Iwona Mames[1], Michal Walczak[1]

*[1]Captor Therapeutics Inc., Wrocław, Poland;*

### Background

MCL-1 is a key anti-apoptotic regulator, the overexpression of which drives survival and therapeutic resistance across diverse hematological and solid malignancies. Unlike conventional inhibitors that require high concentrations for target saturation, Targeted Protein Degradation (TPD) facilitates protein depletion at sub-stoichiometric dosages. This approach mitigates dose-limiting cardiac toxicities inherent to occupancy-based inhibition, significantly expanding the therapeutic window. Here, we describe the preclinical evaluation of a highly potent, first-in-class MCL-1 degrader engineered for the selective ablation of MCL-1 in hematological malignancies. Our MCL-1 degrader demonstrates rapid and robust apoptosis induction across multiple *in vitro* and *in vivo* models, translating enhanced selectivity and sustained pharmacodynamic responses into a broadened therapeutic window for MCL-1 targeting.

### Aims

To evaluate the pharmacological profile of our MCL-1 degrader and establish its therapeutic potential as a cardiosafe monotherapy or venetoclax-combination treatment for R/R AML.

### Methods

Ternary complex formation between the degrader, CRBN and MCL-1 was validated through biophysical assays, while degradation efficiency and cytotoxicity were assessed via Western blot and CellTiter-Glo assay across a panel of cancer cell lines, including MV-4-11, OPM2, NCI-H838 and DMS114. Comparative cardiotoxicity was evaluated in human iPSC-derived cardiomyocytes by monitoring MCL-1 levels and viability. *In vivo* efficacy was studied in MV-4-11 AML xenograft model, both as monotherapy and in combination with low-dose venetoclax. Finally, the PK/PD profile and systemic safety were characterized in cynomolgus macaques.

### Results

Biophysical assays confirmed degrader-mediated ternary complex formation (CRBN/MCL-1). In MV-4-11, OPM2, and DMS114 cells, our MCL-1 degrader demonstrated potent anti-proliferative activity ($IC_{50}$ = 0.99 ± 0.57 nM for MV-4-11; 10.17 ± 7.1 nM for OPM-2 cells; 31.6 ± 17.3 nM for DMS-114 cells) and robust MCL-1 degradation, with a 1-hour treatment sufficient to trigger irreversible apoptosis. In human iPSC-derived cardiomyocytes, our degrader demonstrated no toxicity, avoiding the persistent MCL-1 accumulation typically observed with small-molecule inhibitors, following washout. In AML xenograft models, our degrader demonstrated potent anti-tumor activity both as a monotherapy and in combination. Notably, the combination with low-dose venetoclax promoted tumor regression in the MV-4-11 model, demonstrating a highly synergistic effect. Studies in non-human primates (NHP) confirmed a favorable PK/PD profile and cardiac safety at exposures significantly exceeding predicted efficacious doses.

**Copyright Information:** (Online) ISSN: 2572-9241
©2026 The Author(s). HemaSphere is published by John Wiley & Sons Ltd on behalf of European Hematology Association. This is an open access Abstract Book distributed under the Attribution-NonCommercial-NoDerivs (CC BY-NC-ND), which allows third parties to download the articles and share them with others as long as they credit the author and the Abstract Book, but they cannot change the content in any way or use them commercially.
**Abstract Book Citations:** Authors, Title, HemaSphere, 2026;10:(S1):pages. The abstract book DOIs can be found on the "Author Information" page.
**Disclaimer:** Articles published in the journal HemaSphere exclusively reflect the opinions of the authors. The authors are responsible for all content in their abstracts including accuracy of the facts, statements, citing resources, etc.

**Summary/Conclusion**

Our first-in-class MCL-1 degrader was designed to overcome the cardiotoxicity typical of traditional inhibitors, which are often constrained by toxic protein accumulation. By achieving irreversible target depletion and demonstrating a superior safety profile in NHP models, our degrader represents a potent therapeutic strategy for relapsed/refractory AML. The compound is currently in IND/CTA-enabling studies, with a First-in-Human Phase 1 trial expected to commence by the end of 2026 to evaluate its efficacy as monotherapy and in combination with venetoclax.

**Copyright Information:** (Online) ISSN: 2572-9241
©2026 The Author(s). HemaSphere is published by John Wiley & Sons Ltd on behalf of European Hematology Association. This is an open access Abstract Book distributed under the Attribution-NonCommercial-NoDerivs (CC BY-NC-ND), which allows third parties to download the articles and share them with others as long as they credit the author and the Abstract Book, but they cannot change the content in any way or use them commercially.
**Abstract Book Citations:** Authors, Title, HemaSphere, 2026;10:(S1):pages. The abstract book DOIs can be found on the “Author Information” page.
**Disclaimer:** Articles published in the journal HemaSphere exclusively reflect the opinions of the authors. The authors are responsible for all content in their abstracts including accuracy of the facts, statements, citing resources, etc.

### (PF427) ACU SMALL MOLECULE STING AGONISTS DEMONSTRATE TP53-INDEPENDENT ANTI-LEUKAEMIC ACTIVITY AND VENETOCLAX SYNERGY IN ACUTE MYELOID LEUKAEMIA

**Topic:** 03. Acute myeloid leukemia - Biology & translational research

Yin Yuan*[1, 2, 3], Fiona Brown[1, 4], Veronique Litalien[1, 4], Rhiannon Morris[1, 4], Geoffrey Matthews[5], Ben Morrow[5], Mark Devlin[5], Andrew Roberts[1, 4, 6], Andrew Wei[1, 4, 6]

*[1]Walter and Eliza Hall Institute of Medical Research, Melbourne, Australia;[2]The Princess Alexandra Hospital, Brisbane, Australia; [3]QIMR Berghofer Medical Research Institute, Brisbane, Australia;[4]University of Melbourne, Melbourne, Australia;[5]Aculeus Therapeutics, Melbourne, Australia;[6]Peter MacCallum Cancer Centre and Royal Melbourne Hospital, Melbourne, Australia;*

### Background

Patients with acute myeloid leukaemia (AML) face a poor prognosis, particularly those with high-risk disease such as *TP53* mutations. Despite recent therapeutic advances, sustaining long-term remission remains a major challenge that must be met. Drug activators of the "stimulator of interferon genes" (STING) pathway have been explored for their potential as an immunotherapy in the solid tumour setting. We previously reported a novel cell-intrinsic mechanism of action of the STING agonist diABZi in AML (Cancer Cell 2024). STING activation induced apoptosis potently in AML cells, with diABZI demonstrating combinatorial activity with the pro-apoptotic BCL-2 inhibitor venetoclax (VEN) in AMLs with and without *TP53* mutation. Here we report the preclinical activity of a new series of systemically deliverable, synthetic non-cyclic dinucleotide small molecule STING agonists in AML.

### Aims

To investigate the preclinical activity of ACU STING agonists (ACU2086 and ACU0943) against AML.

### Methods

ACU STING agonists were provided by Aculeus Therapeutics. *In vitro* assays were performed using ACU2086 and *in vivo* experiments with the clinical grade compound ACU0943. VEN was purchased from Chemgood. Drug sensitivity assays were performed on human AML cell lines and primary patient samples. Cell death was assessed by PI/DAPI staining and flow cytometry. Cell proliferation was assessed by CellTitreGlow (CTG). For *in vivo* testing, MOLM-13 cells were transplanted into NOD-*scid*IL2Rgamma$^{null}$ (NSG) mice. Mice received no treatment (control), VEN (50mg/kg orally 5 days/week), ACU0943 (1.5mg/kg IV biweekly), or combination VEN/ACU0943 for 2 weeks (n=5 per group). Leukaemic burden (%human CD45+ cells) in peripheral blood (PB), bone marrow (BM) and spleen was measured at end of therapy. Groups were compared using Welch's ANOVA with Dunnett's multiple comparison test.

**Copyright Information:** (Online) ISSN: 2572-9241
©2026 The Author(s). HemaSphere is published by John Wiley & Sons Ltd on behalf of European Hematology Association. This is an open access Abstract Book distributed under the Attribution-NonCommercial-NoDerivs (CC BY-NC-ND), which allows third parties to download the articles and share them with others as long as they credit the author and the Abstract Book, but they cannot change the content in any way or use them commercially.
**Abstract Book Citations:** Authors, Title, HemaSphere, 2026;10:(S1):pages. The abstract book DOIs can be found on the "Author Information" page.
**Disclaimer:** Articles published in the journal HemaSphere exclusively reflect the opinions of the authors. The authors are responsible for all content in their abstracts including accuracy of the facts, statements, citing resources, etc.

**Results**

*In vitro*, ACU2086 monotherapy demonstrated activity in 6/7 AML cell lines tested (including 3/4 with *TP53* mutations). Using CRISPR-modified cell lines, cell death induced by ACU2086 was confirmed to be via STING dependent apoptosis, with killing blocked by knockout of either *STING* or *BAX* and *BAK*. In contrast, cell death was independent of TP53, with isogenic *TP53* knockout (KO) cells demonstrating equivalent sensitivity to wildtype (WT) cells. Although *BAX/BAK* KO cells were resistant to STING-induced cell death, CTG assays demonstrated their growth was inhibited by ACU2086. Growth arrest was also seen in *TP53/BAX/BAK* KO cells, suggesting the anti-proliferative effect of ACU2086 is also TP53 independent. Combination ACU2086 and VEN was additive to synergistic in MOLM-13 and OCI-AML3 cells (Bliss Synergy Scores 11.1 and 8.9, respectively). Across 14 primary samples tested, the average $IC_{50}$ for VEN was >10 μM, 3.3μM (95% CI 2.2-5.1μM) for ACU2086 and 0.33μM (95% CI 0.26-0.41μM) for VEN/ACU2086 combination.

*In vivo*, mice treated with ACU0943 had approximately 10-fold lower blast burden in PB, BM and spleen compared with control and VEN treated mice (Figure 1). Combination VEN/ACU0943 was most effective, with further log-fold reduction in blast burden compared with ACU0943 alone, providing clear evidence of combinatorial activity.

**Summary/Conclusion**

ACU STING agonists exert potent cell-intrinsic, *TP53*-independent activity against AML, with efficacy enhanced by combination with VEN. These data highlight the potential of ACU STING agonists as a novel treatment for AML and provide preclinical evidence supporting a planned phase 1 clinical trial.

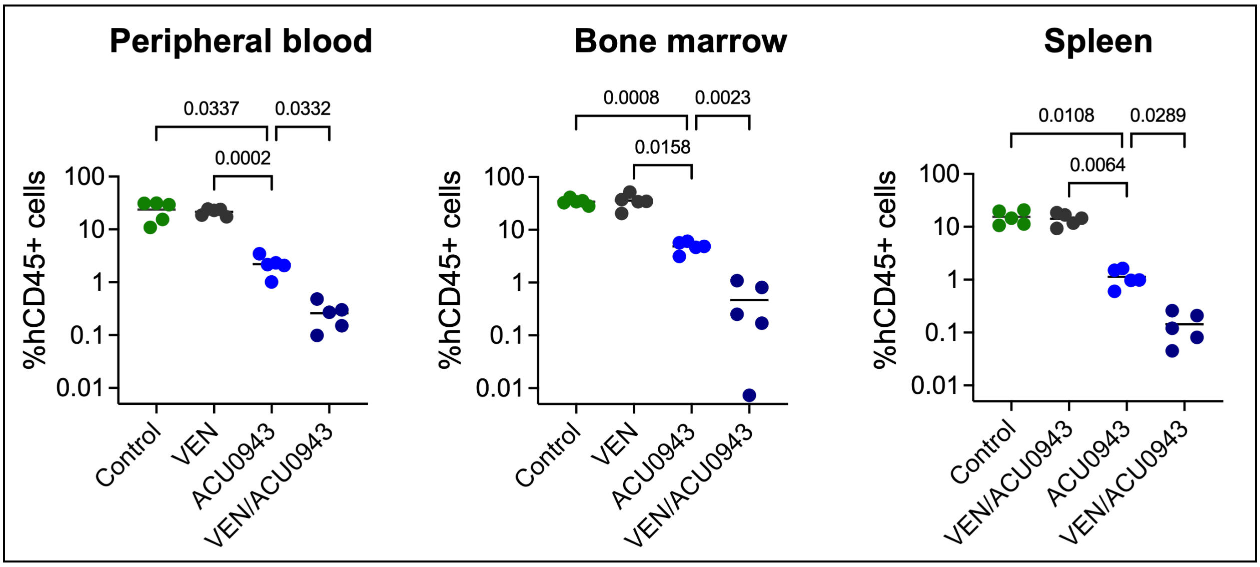

Figure 1

**Copyright Information:** (Online) ISSN: 2572-9241
©2026 The Author(s). HemaSphere is published by John Wiley & Sons Ltd on behalf of European Hematology Association. This is an open access Abstract Book distributed under the Attribution-NonCommercial-NoDerivs (CC BY-NC-ND), which allows third parties to download the articles and share them with others as long as they credit the author and the Abstract Book, but they cannot change the content in any way or use them commercially.
**Abstract Book Citations:** Authors, Title, HemaSphere, 2026;10:(S1):pages. The abstract book DOIs can be found on the "Author Information" page.
**Disclaimer:** Articles published in the journal HemaSphere exclusively reflect the opinions of the authors. The authors are responsible for all content in their abstracts including accuracy of the facts, statements, citing resources, etc.

### (PF428) ZMYND8 READS H3K14AC TO ACTIVATE TFAM TRANSCRIPTION AND PROMOTES ACUTE MYELOID LEUKEMIA PROGRESSION BY SUSTAINING MITOCHONDRIAL OXIDATIVE PHOSPHORYLATION

**Topic:** 03. Acute myeloid leukemia - Biology & translational research

Hui Feng*[1], Runyu Yang[2], Peng-Cheng He[3], Fan Niu[3]

*[1]The First Affiliated Hospital of Xi'an Jiaotong University, Department of Hematology, Xi'an, China;[2]The First Affiliated Hospital of Xi'an Jiaotong University, Department of Hematology, xi"an, China; [3]The First Affiliated Hospital of Xi'an Jiaotong University, xi"an, China;*

### Background

Acute myeloid leukemia (AML) is an aggressive hematologic malignancy with complex pathogenesis, and current therapeutic strategies still face challenges of relapse and drug resistance. ZMYND8, as a multifunctional histone reader, recognizes acetylation modifications and participates in transcriptional regulation. However, its specific role in mitochondrial metabolic remodeling in AML remains unclear.

### Aims

This study aims to investigate whether ZMYND8 regulates the transcription of TFAM, a key factor in mitochondrial function, by recognizing H3K14ac modifications, thereby sustaining oxidative phosphorylation (OXPHOS) activity and promoting AML cell proliferation and disease progression.

### Methods

Public databases were first analyzed to assess ZMYND8 expression levels in AML and their correlation with patient prognosis. AML cell lines with ZMYND8 knockdown were established, and the effects on cell proliferation and *in vivo* tumorigenicity were evaluated using CCK-8 assays and xenograft mouse models. Transcriptome sequencing (RNA-seq) was performed on knockdown and control groups, followed by GSEA and GO analysis to identify enriched pathways. CUT&Tag technology was employed to map the genome-wide binding profile of ZMYND8 and its co-localization with histone modifications H3K14ac and H3K27ac. A dual-luciferase reporter vector containing the TFAM promoter was constructed and co-transfected to validate the regulatory effect of ZMYND8 on TFAM transcriptional activity.

### Results

Bioinformatics analysis revealed that ZMYND8 is highly expressed in AML and significantly associated with poor prognosis. Knockdown of ZMYND8 markedly inhibited AML cell proliferation *in vitro* and tumor growth *in vivo*. RNA-seq analysis showed that downregulated genes upon ZMYND8 knockdown were significantly enriched in pathways related to the respiratory electron transport chain, oxidative phosphorylation, and mitochondrial ATP synthesis, with the top 13 most significantly decreased genes being mitochondrial DNA (mtDNA)-encoded genes. CUT&Tag analysis revealed substantial co-localization of ZMYND8 with H3K14ac and H3K27ac at the TFAM promoter region, suggesting that ZMYND8 may directly regulate TFAM transcription by recognizing H3K14ac. Dual-luciferase reporter assays confirmed that ZMYND8 overexpression significantly enhanced TFAM promoter activity.

**Copyright Information:** (Online) ISSN: 2572-9241
©2026 The Author(s). HemaSphere is published by John Wiley & Sons Ltd on behalf of European Hematology Association. This is an open access Abstract Book distributed under the Attribution-NonCommercial-NoDerivs (CC BY-NC-ND), which allows third parties to download the articles and share them with others as long as they credit the author and the Abstract Book, but they cannot change the content in any way or use them commercially.
**Abstract Book Citations:** Authors, Title, HemaSphere, 2026;10:(S1):pages. The abstract book DOIs can be found on the "Author Information" page.
**Disclaimer:** Articles published in the journal HemaSphere exclusively reflect the opinions of the authors. The authors are responsible for all content in their abstracts including accuracy of the facts, statements, citing resources, etc.

### Summary/Conclusion

This study demonstrates that ZMYND8, functioning as a reader of H3K14ac, sustains mitochondrial oxidative phosphorylation by binding to and activating the TFAM promoter, thereby promoting AML cell proliferation and disease progression. This finding reveals a critical link between epigenetic regulation and mitochondrial metabolic remodeling, providing a theoretical basis for targeting ZMYND8 in AML therapy.

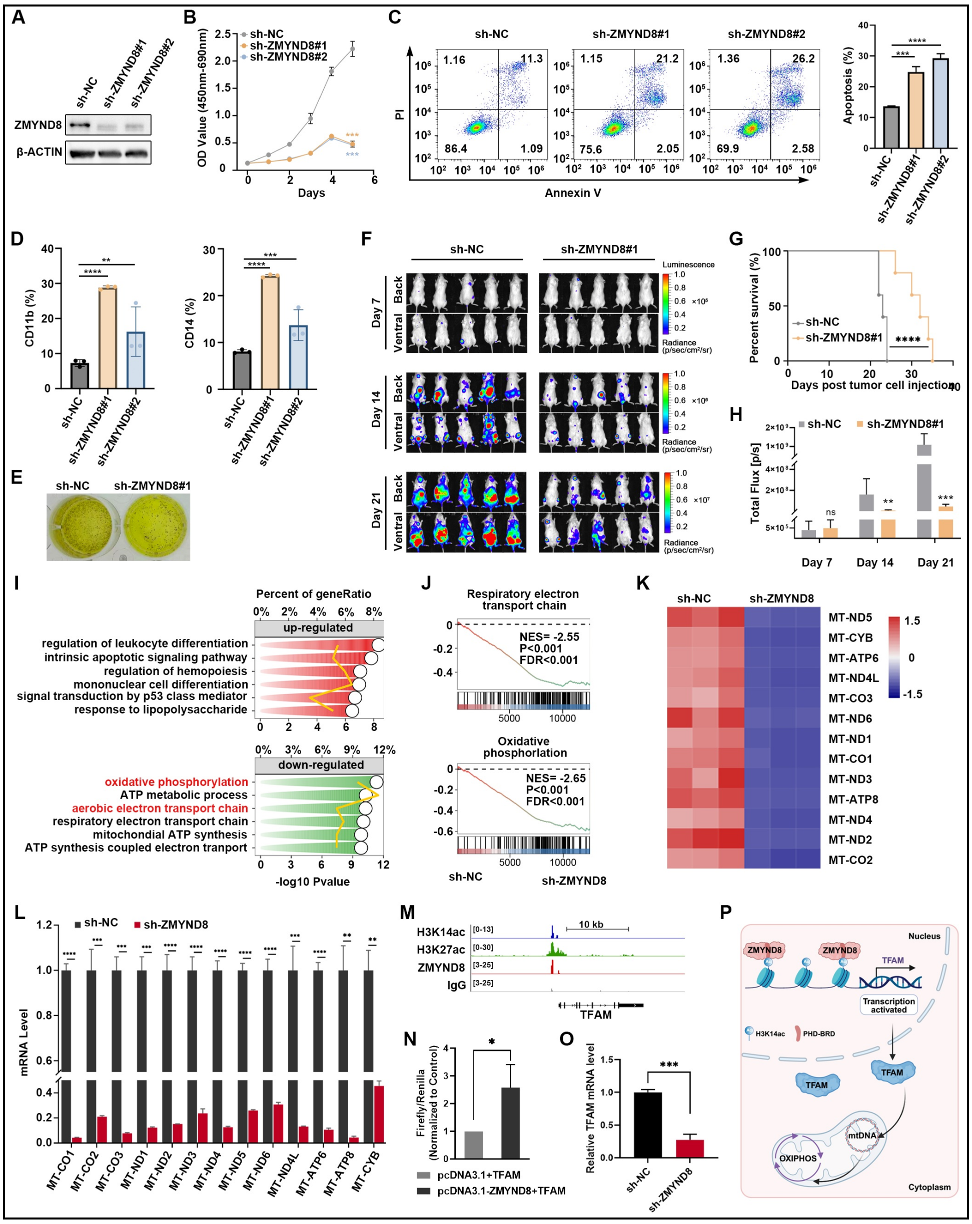

**Copyright Information:** (Online) ISSN: 2572-9241
©2026 The Author(s). HemaSphere is published by John Wiley & Sons Ltd on behalf of European Hematology Association. This is an open access Abstract Book distributed under the Attribution-NonCommercial-NoDerivs (CC BY-NC-ND), which allows third parties to download the articles and share them with others as long as they credit the author and the Abstract Book, but they cannot change the content in any way or use them commercially.
**Abstract Book Citations:** Authors, Title, HemaSphere, 2026;10:(S1):pages. The abstract book DOIs can be found on the "Author Information" page.
**Disclaimer:** Articles published in the journal HemaSphere exclusively reflect the opinions of the authors. The authors are responsible for all content in their abstracts including accuracy of the facts, statements, citing resources, etc.

### (PF429) ACUTE MYELOID LEUKEMIA WITH ABERRANT MONOCYTES: IS IT A DISTINCT CLINICAL ENTITY AND WHAT IS THE RELEVANCE OF MEASURABLE RESIDUAL DISEASE?

**Topic:** 03. Acute myeloid leukemia - Biology & translational research

Tom Reuvekamp*[1, 2, 3], Lukas Haaksma[1, 2], Angèle Kelder[1, 2], Willemijn Scholten[1, 2], Willemijn Hobo[4], Jennichjen Slomp[5], Vincent van der Velden[6], Yvonne Oussoren-Brockhoff[1, 2], Kasper Croese[1, 2], Patrycja Gradowska[7, 8], Bob Löwenberg[7], Gert Ossenkoppele[1, 2], Adriano Venditti[9], Arjan Van de Loosdrecht[1, 2], Costa Bachas[1, 2], Petrus Valk[7], David de Leeuw[1, 2], Francesco Buccisano[9], Jacqueline Cloos[1, 2]

*[1]Amsterdam UMC location Vrije Universiteit Amsterdam, Department of Hematology, Amsterdam, The Netherlands;[2]Cancer Center Amsterdam, Imaging and Biomarkers, Amsterdam, The Netherlands; [3]Amsterdam UMC location Universiteit van Amsterdam, Department of Hematology, Amsterdam, The Netherlands; [4]Radboud University Medical Center, Department of Laboratory Medicine-Laboratory of Hematology, Nijmegen, The Netherlands; [5]Medisch Spectrum Twente/Medlon, Department of Clinical Chemistry, Enschede, The Netherlands; [6]Erasmus MC, University Medical Center Rotterdam, Department of Immunology, Rotterdam, The Netherlands; [7]Erasmus MC Cancer Institute, Department of Hematology, Rotterdam, The Netherlands;[8]HOVON Foundation, Rotterdam, The Netherlands; [9]University of Rome "Tor Vergata", Department of Biomedicine and Prevention, Rome, Italy;*

### Background

Acute myeloid leukemia (AML) is often considered a stem cell disease. In a subset of patients, there is monocytic differentiation. The role of these cells and the clinical behavior, genetic background and relevance for measurable residual disease (MRD) assessment is less clear.

### Aims

To investigate if AML with aberrant monocytes is a distinct clinical entity compared to primitive AML and the clinical relevance of monocytic MRD assessment.

### Methods

We included patients with AML or high-risk myelodysplastic syndrome (MDS) from HOVON-SAKK trials that received intensive treatment (HO42A, HO81, HO92, HO102, HO103, and HO132). Presence of aberrant monocytes at diagnosis was defined using the MRD panel as ≥20% of CD45-expressing cells (WBC) in the monocytic region (CD45+SSCint; in gating helped by the expression of CD14 or CD11b), with ≥10% of these monocytic cells showing a leukemia associated immunophenotype (LAIP, for example bright expression of CD56). A primitive phenotype is defined as at least 10% CD34+, CD117+ or CD133+ LAIP in the CD45dim blast compartment.

**Copyright Information:** (Online) ISSN: 2572-9241
©2026 The Author(s). HemaSphere is published by John Wiley & Sons Ltd on behalf of European Hematology Association. This is an open access Abstract Book distributed under the Attribution-NonCommercial-NoDerivs (CC BY-NC-ND), which allows third parties to download the articles and share them with others as long as they credit the author and the Abstract Book, but they cannot change the content in any way or use them commercially.
**Abstract Book Citations:** Authors, Title, HemaSphere, 2026;10:(S1):pages. The abstract book DOIs can be found on the "Author Information" page.
**Disclaimer:** Articles published in the journal HemaSphere exclusively reflect the opinions of the authors. The authors are responsible for all content in their abstracts including accuracy of the facts, statements, citing resources, etc.

**Results**

At diagnosis, we included a total of 2490 patients of which 2249 (90%) had an AML with an aberrant primitive phenotype, 98 (4%) with only aberrant monocytes, and 143 (6%) with an aberrant primitive phenotype and aberrant monocytes. In patients with AML with presence of aberrant monocytes (termed monocytic AML), *NPM1*, *DNMT3A*, *TET2*, and *NRAS* mutations were more frequently observed, while primitive AML showed more frequent *IDH1*, *IDH2*, *FLT3*-ITD and *TP53* mutations (Figure 1A). To determine the prognostic relevance of mutations within monocytic AML we performed survival analysis and found that, in particular, an *NPM1* mutation was a strong prognostic factor (HR: 3.1 (2.0-5.0) [Mono *NPM1*mut vs Mono *NPM1*wt]; Figure 1B), even more so than in primitive AML (HR: 1.5 (1.3-1.8) [NonMono *NPM1*mut vs NonMono *NPM1*wt]).
We investigated the difference in level of MRD after two cycles of therapy for both primitive and monocytic LAIPs in patients with aberrant monocytes at diagnosis and found that monocytic MRD (monoMRD; based on LAIPs in the monocytic compartment) is commonly detected at higher percentages than immature MRD (imMRD; CD34+, CD117+ or CD133+). Stratifying patients based on MRD using a cut-off of 0.1%, we found that monoMRD did not result in a prognostic difference (HR: 1.96 (0.42-2.2); Figure 1C), whereas imMRD was highly prognostic (HR: 3.1 (1.2-7.3); Figure 1D). Five patients had PCR NPM1 MRD available, all were NPM1-MRDpos. Validation in the GIMEMA AML1310 trial confirmed the lack of prognostic value of monoMRD.
When investigating the CD34+CD38- leukemia stem cell (LSC) population, we found that LSC were present in lower frequencies in monocytic AML compared to primitive AML. However, 24% (25/104) of monocytic AML patients were LSCpos (>0.03% LSC of WBC), with shorter overall survival compared to LSCneg (<0.03% LSC of WBC) and CD34neg (<1% CD34 of WBC and absence of LSC) monocytic AML patients (Figure 1E). Interestingly, we found a predominance of the immature clone in the relapse sample, compared with the dominant monocytic clone at diagnosis.

**Summary/Conclusion**

Monocytic AML has a distinct genetic landscape with NPM1 mutations carrying pronounced prognostic significance, supporting its classification as separate entity. MRD assessment of the monocytic cell population does not have prognostic value, whereas MRD based on the immature fraction is highly prognostic and shows enrichment at relapse.

**Copyright Information:** (Online) ISSN: 2572-9241
©2026 The Author(s). HemaSphere is published by John Wiley & Sons Ltd on behalf of European Hematology Association. This is an open access Abstract Book distributed under the Attribution-NonCommercial-NoDerivs (CC BY-NC-ND), which allows third parties to download the articles and share them with others as long as they credit the author and the Abstract Book, but they cannot change the content in any way or use them commercially.
**Abstract Book Citations:** Authors, Title, HemaSphere, 2026;10:(S1):pages. The abstract book DOIs can be found on the "Author Information" page.
**Disclaimer:** Articles published in the journal HemaSphere exclusively reflect the opinions of the authors. The authors are responsible for all content in their abstracts including accuracy of the facts, statements, citing resources, etc.

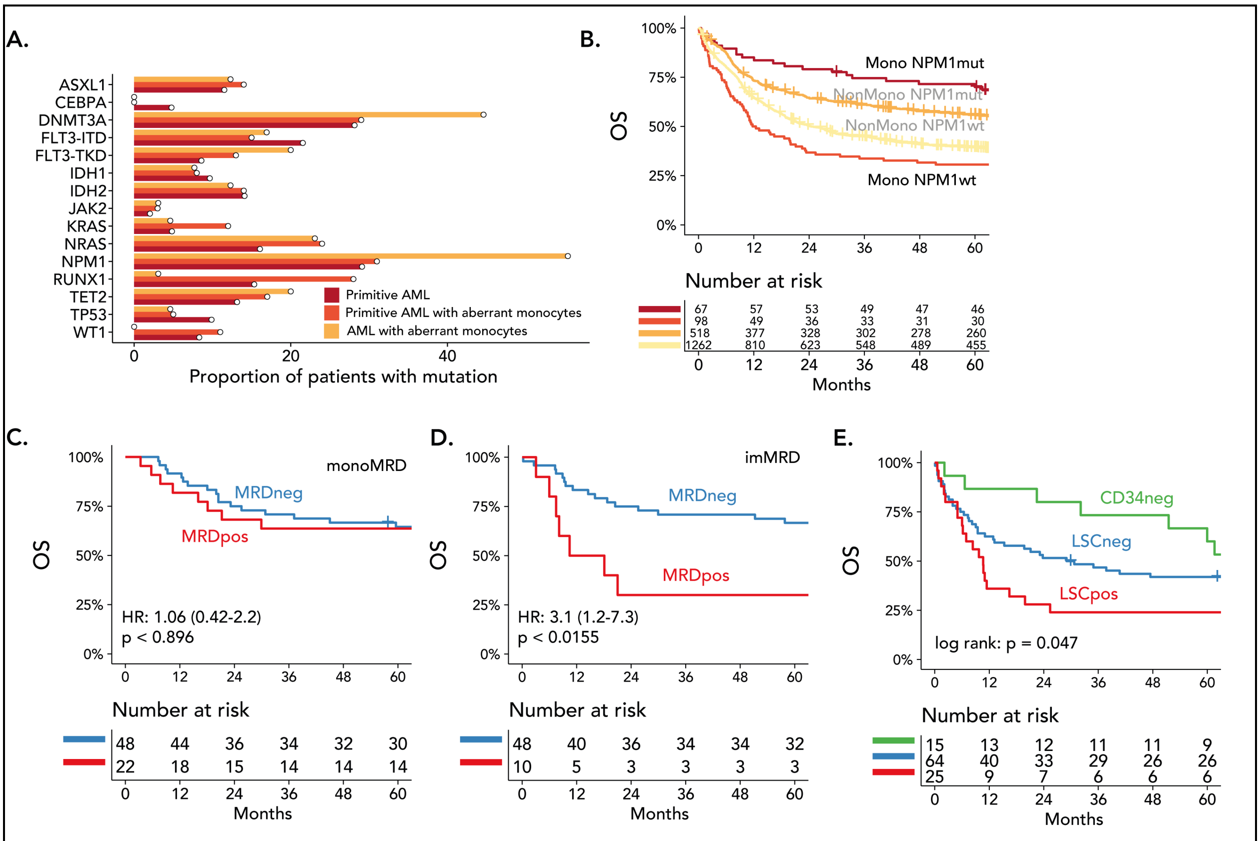

**Figure 1.** Characterization of AML with aberrant monocytes treated with intensive chemotherapy. **A)** genetic landscape of primitive AML compared to AML with aberrant monocytes. **B)** Overall survival stratified for presence of aberrant monocytes and NPM1 mutation (start time analysis: time of diagnosis). **C)** Overall survival stratified by MRD status after two cycles based on the monocytic cell fraction (start time analysis: time of MRD measurement). **D)** Overall survival stratified by MRD status after two cycles based on the immature cell fraction (CD34+, CD117+, or CD133+) in monocytic AML (start time analysis: time of MRD measurement). **E)** Overall survival of leukemia stem cell status at diagnosis in monocytic AML (start time analysis: time of diagnosis).

**Copyright Information:** (Online) ISSN: 2572-9241
©2026 The Author(s). HemaSphere is published by John Wiley & Sons Ltd on behalf of European Hematology Association. This is an open access Abstract Book distributed under the Attribution-NonCommercial-NoDerivs (CC BY-NC-ND), which allows third parties to download the articles and share them with others as long as they credit the author and the Abstract Book, but they cannot change the content in any way or use them commercially.
**Abstract Book Citations:** Authors, Title, HemaSphere, 2026;10:(S1):pages. The abstract book DOIs can be found on the "Author Information" page.
**Disclaimer:** Articles published in the journal HemaSphere exclusively reflect the opinions of the authors. The authors are responsible for all content in their abstracts including accuracy of the facts, statements, citing resources, etc.

### (PF430) DEVELOPMENT OF A NOVEL T-CELL IMMUNOTHERAPY FOR NPM1-MUTATED AML

**Topic:** 03. Acute myeloid leukemia - Biology & translational research

Xiaoxuan Liu*[1], Ommar Omarjee[2], Qunwei Wang[2], Hanif Ali[2], Aneta Skrodzka[2], Hadyn Prosser[2], Laura Bond[1], Aristi Damaskou[1], Hannes Ponstingl[2], Yizhou Yu[1], Jayne Colebrook[2], Wei Wang[2], Allan Bradley[2], George Vassiliou[1]

[1]*Cambridge Stem Cell Institute, Cambridge, United Kingdom;* [2]*T-Therapeutics, Cambridge, United Kingdom;*

### Background

Acute Myeloid Leukaemia (AML) is an aggressive blood cancer characterised by the clonal expansion of immature myeloid blasts that infiltrate the bone marrow and cause haematopoietic failure. Standard treatments such as chemotherapy and allogeneic bone marrow transplantation can cure up to 30-40% of cases, but most patients still succumb to the disease, revealing an urgent need for new treatments. The most common subtype of AML, representing ~30% of cases, is defined by mutations in *NPM1*, the gene encoding the nuclear phosphoprotein Nucleophosmin 1. The mutant NPM1 protein (NPM1c) represents an appealing target for immunotherapy, as it is essential to AML cells and harbours an 11 amino acid C-terminal neoantigen capable of eliciting T-cell immune responses.

### Aims

Using a novel state-of-the-art mouse model expressing the complete human T-cell receptor (hTCR) genome and HLA-A*02, we aim to develop a TCR-based therapy specifically targeting NPM1c, for use in combination with existing anti-AML treatments to improve patient survival and reduce the toxicities of conventional therapies.

### Methods

We immunised hTCR mice with subcutaneously injected NPM1c-derived neopeptide 9mers and isolated peptide-specific TCRs using NPM1c/HLA-A*02 multimers. These TCRs were individually sequenced, cloned, and transgenically expressed in a Jurkat T-cell reporter line to test their ability to recognise NPM1c neopeptides presented on HLA-A*02. The candidate TCRs were then expressed in primary human T cells by lentiviral transduction and functionally assessed *in vitro* against peptide-loaded T2 cells (HLA-A*02 +ve), AML cell lines, and primary AML blasts. Computational modelling was used to predict TCR-pHLA 3D structures and guide affinity optimisation strategies for lead TCRs. *AAD* mice carrying human HLA-A*02 were crossed with *Npm1*$^{flox\text{-}cA/+}$*; Mx1-Cre*$^{+}$ mice to establish a model for evaluating the *in vivo* cytotoxic efficacy of lead TCRs.

### Results

NPM1c neopeptides 9mer-1 (AIQDLCLAV) and 9mer-6 (CLAVEEVSL) induced robust HLA-A*02-restricted T-cell responses in hTCR mice. Hundreds of TCRs were isolated and sequenced; the top 34 were cloned and expressed in Jurkat cells. Two TCRs demonstrated specific reactivity against the NPM1c 9mer-1/HLA-A*02 complex in the Jurkat reporter assay. Both TCRs exhibited cytotoxicity against peptide-loaded T2 and OCI-AML3 cells, with one also showing activity against a primary AML blast. Structural modelling identified two key amino acid residues that will be engineered into the lead TCRs to improve binding affinity.

**Copyright Information:** (Online) ISSN: 2572-9241
©2026 The Author(s). HemaSphere is published by John Wiley & Sons Ltd on behalf of European Hematology Association. This is an open access Abstract Book distributed under the Attribution-NonCommercial-NoDerivs (CC BY-NC-ND), which allows third parties to download the articles and share them with others as long as they credit the author and the Abstract Book, but they cannot change the content in any way or use them commercially.
**Abstract Book Citations:** Authors, Title, HemaSphere, 2026;10:(S1):pages. The abstract book DOIs can be found on the “Author Information” page.
**Disclaimer:** Articles published in the journal HemaSphere exclusively reflect the opinions of the authors. The authors are responsible for all content in their abstracts including accuracy of the facts, statements, citing resources, etc.

### Summary/Conclusion

By immunisation of state-of-the-art hTCR mice with NPM1c neopeptides 9mers, we developed two novel first-in-class TCR therapeutics targeting the NPM1c 9mer-1/HLA-A*02 complex. Our approach bypassed the challenges associated with *ex vivo* TCR isolation from AML patients or healthy donors to generate these novel immunotherapies that have the potential to improve the treatment of patients with *NPM1*-mutated AML.

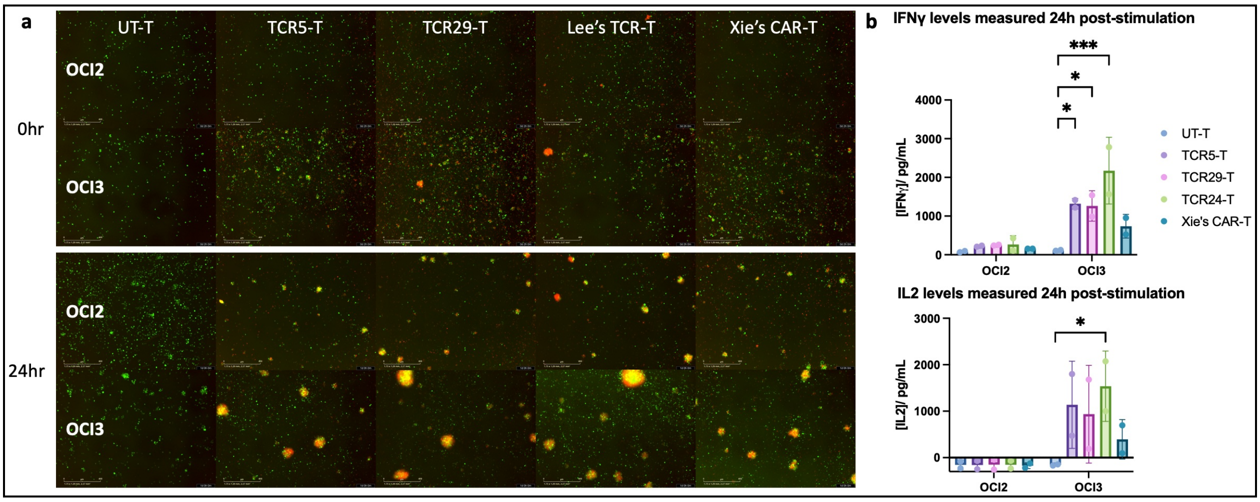

NPM1c 9mer-1-specific TCR5 and TCR29 isolated from immunised hTCR mice demonstrate cytotoxicity against OCI-AML3 cells. a) Representative IncuCyte images of co-cultures of primary human TCR-T cells (mCherry+) and OCI-AML cells (GFP+) (2:1 E:T) at 0 and 24 hours. b) IFNγ and IL2 release measured 24 hours post TCR-T stimulation (n=2 biological replicates). Error bars represent SD. *, p≤0.05; ***, p≤0.001 (two-way ANOVA with Dunnett's multiple comparisons test).

**Copyright Information:** (Online) ISSN: 2572-9241
©2026 The Author(s). HemaSphere is published by John Wiley & Sons Ltd on behalf of European Hematology Association. This is an open access Abstract Book distributed under the Attribution-NonCommercial-NoDerivs (CC BY-NC-ND), which allows third parties to download the articles and share them with others as long as they credit the author and the Abstract Book, but they cannot change the content in any way or use them commercially.
**Abstract Book Citations:** Authors, Title, HemaSphere, 2026;10:(S1):pages. The abstract book DOIs can be found on the "Author Information" page.
**Disclaimer:** Articles published in the journal HemaSphere exclusively reflect the opinions of the authors. The authors are responsible for all content in their abstracts including accuracy of the facts, statements, citing resources, etc.

### (PF431) METABOLIC REWIRING VIA METFORMIN RESTORES VENETOCLAX SENSITIVITY IN AML

**Topic:** 03. Acute myeloid leukemia - Biology & translational research

Mai Fujita*[1], Hiroshi Ureshino[1], Ryo Yanagiya[1], Keisuke Kidoguchi[1], Yuta Yamamoto[1], Moe Kawasaki[1, 2], Shinya Kimura[1]

*[1]Saga University, Saga, Japan; [2]OHARA Pharmaceutical Co., Ltd., Shiga, Japan;*

### Background

Venetoclax (VEN)–based therapy has significantly improved outcomes in acute myeloid leukemia (AML). However, resistance frequently emerges and limits long-term efficacy. Increasing evidence suggests that metabolic and epigenetic reprogramming contributes to drug resistance, yet the mechanistic interplay between these processes in VEN-resistant (VEN-R) AML remains poorly understood.

### Aims

To elucidate the metabolic–epigenetic mechanisms underlying VEN resistance in AML and to identify therapeutic strategies capable of restoring antileukemia efficacy.

### Methods

VEN-R AML cell lines (MOLM13, MOLM14, HL-60, KG-1) were established through continuous VEN exposure. Metabolomic and transcriptomic profiling was performed in parental and VEN-R lines. VEN-R MOLM14 cells were treated with metformin (MET) to assess metabolic restoration. Protein expression was assessed by Western blot and flow cytometry. The DNMT1-targeting hypomethylating agent OR-2100 (decitabine prodrug; jRCT2071220035) was evaluated alone or in combination with MET. Public RNA-sequencing datasets (GSE270621, GSE183329, GSE97346) were analyzed for clinical and transcriptional/epigenetic validations.

### Results

VEN-R cells exhibited high basal MCL1 expression, which decreased under glucose-free conditions, indicating that glucose-dependent metabolic reprogramming supports MCL1 overexpression in VEN-R AML cells. Metabolome showed that VEN-R cells demonstrated coordinated activation of glycolysis, fatty acid oxidation (FAO), and oxidative phosphorylation (OXPHOS), generating a reductive intracellular state conducive to resistance. Consistent with these observations, two independent patient-derived RNA-seq datasets confirmed similar upregulation of these metabolic pathways in clinically VEN-R AML samples. Furthermore, metabolites involved in ROS scavenging, including NADPH, fumarate, and glutathione, were increased in the resistant cells. Consistent with these metabolic alterations, flow cytometric analysis demonstrated a reduction in intracellular ROS levels in the resistant cells. Comparative RNA sequencing analyses between parental and resistant cells in MOLM13, MOLM14, HL60, and KG1a consistently showed lower BAX expression in the resistant cells. These findings suggest that resistant cells evade VEN-induced cell death by suppressing BAX expression, at least in part through attenuation of intracellular ROS levels. Based on RNA sequencing data (GSE97346), MET treatment in MOLM14 and HL60 cells downregulated glycolysis, OXPHOS, and FAO, indicating MET can reprogram aberrant cellular metabolism in VEN-R AML cells. Furthermore, MET induced up-regulation of the AMPK pathway. Western blot showed MET induced a BAX upregulation via pAMPK up-regulation. Combined MET and VEN treatment markedly enhanced apoptosis, indicating that MET overcame VEN resistance. Moreover, triple therapy with the DNA hypomethylating agent OR-2100, MET and VEN substantially promoted cell apoptosis through increased ROS induction.

**Copyright Information:** (Online) ISSN: 2572-9241
©2026 The Author(s). HemaSphere is published by John Wiley & Sons Ltd on behalf of European Hematology Association. This is an open access Abstract Book distributed under the Attribution-NonCommercial-NoDerivs (CC BY-NC-ND), which allows third parties to download the articles and share them with others as long as they credit the author and the Abstract Book, but they cannot change the content in any way or use them commercially.
**Abstract Book Citations:** Authors, Title, HemaSphere, 2026;10:(S1):pages. The abstract book DOIs can be found on the “Author Information” page.
**Disclaimer:** Articles published in the journal HemaSphere exclusively reflect the opinions of the authors. The authors are responsible for all content in their abstracts including accuracy of the facts, statements, citing resources, etc.

### Summary/Conclusion

VEN resistance in AML is primarily driven by upregulation of glycolysis, FAO, and OXPHOS that maintain MCL1 and suppress BAX through ROS attenuation. MET reprograms metabolic rewiring leading to downregulation of MCL1 and upregulation of BAX. The combination treatment of VEN, MET, and OR-2100 may constitute a clinically feasible metabolic-priming approach.

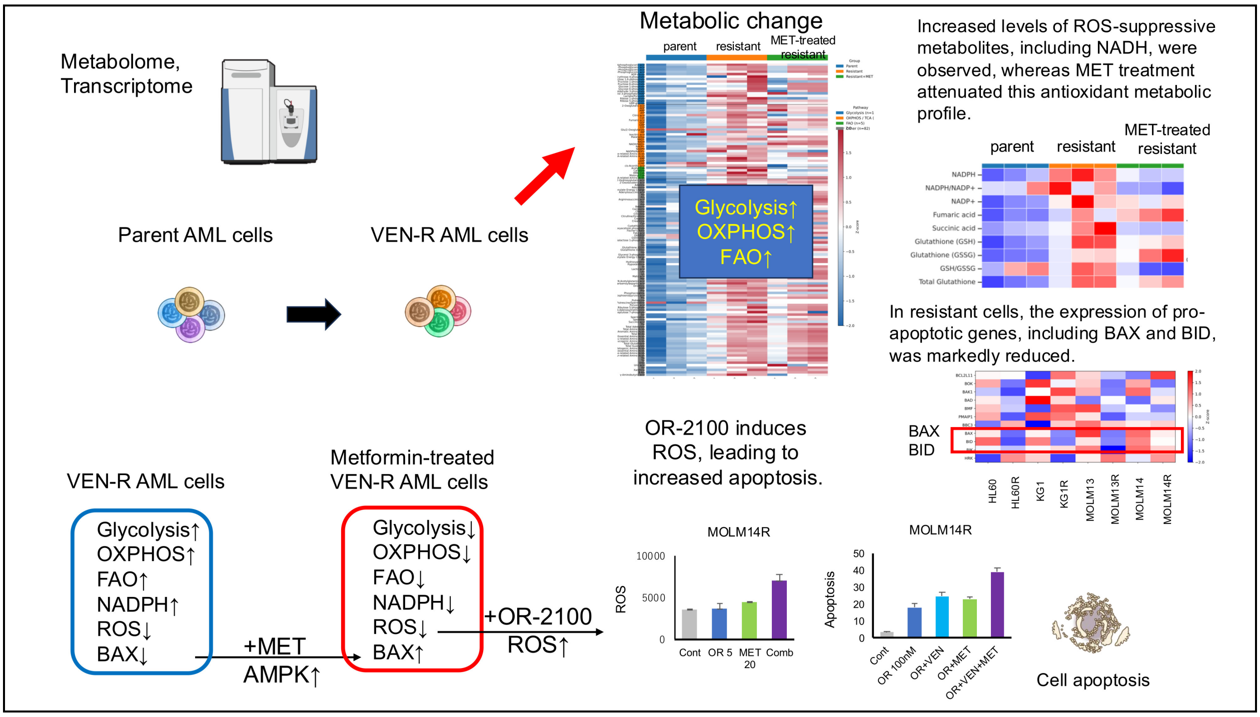

**Copyright Information:** (Online) ISSN: 2572-9241
©2026 The Author(s). HemaSphere is published by John Wiley & Sons Ltd on behalf of European Hematology Association. This is an open access Abstract Book distributed under the Attribution-NonCommercial-NoDerivs (CC BY-NC-ND), which allows third parties to download the articles and share them with others as long as they credit the author and the Abstract Book, but they cannot change the content in any way or use them commercially.
**Abstract Book Citations:** Authors, Title, HemaSphere, 2026;10:(S1):pages. The abstract book DOIs can be found on the "Author Information" page.
**Disclaimer:** Articles published in the journal HemaSphere exclusively reflect the opinions of the authors. The authors are responsible for all content in their abstracts including accuracy of the facts, statements, citing resources, etc.

### (PF432) SINGLE CELL CHARTING OF DNMT3A-MUTANT LEUKEMIC EVOLUTION

**Topic:** 03. Acute myeloid leukemia - Biology & translational research

Sanne Massaar*[1], Dennis Bosch[1], Gregory van Beek[1], Claire van Dijk-Christiaanse[1], Eric Bindels[1], Marisa Westers[2], Angèle Kelder[2], Pedro Moura[3], Petrus Valk[1], Mikko Myllymäki[4], Arjan Van de Loosdrecht[2], Jacqueline Cloos[2], Eva Hellström Lindberg[3], Bas Wouters[1], Jurjen Versluis[1], Marc Raaijmakers[1], Mathijs Sanders[1]

*[1]ErasmusMC Cancer Institute, Department of Hematology, Rotterdam, The Netherlands; [2]AmsterdamUMC, Department of Hematology, Amsterdam, The Netherlands; [3]Karolinska Institutet, Department of Medicine, Stockholm, Sweden; [4]Helsinki University Hospital Comprehensive Cancer Center, Hematology Research Unit Helsinki, Helsinki, Finland;*

### Background

*DNMT3A*, encoding a de novo methyltransferase, is the most frequently mutated gene in myeloid malignancies. *DNMT3A*-mutant hematopoietic stem and progenitor cells (HSPCs) have been associated with inflammation and the remodeling of the microenvironment. Inflammatory remodeling of the bone marrow (BM) niche supports leukemic transformation and growth. Inflammatory remodeling combined with the acquisition of additional driver mutations increases the risk of progression to myelodysplastic syndrome (MDS), including the low-risk (LR) and high-risk (HR) subtypes, and ultimately acute myeloid leukemia (AML). Hence, there is an urgent need to better understand what happens in the BM microenvironment during progression of *DNMT3A*-mutant myeloid malignancies.

### Aims

We aimed to understand how *DNMT3A*-mutant HSPCs and the BM microenvironment co-evolve during progression from healthy BM to LR-MDS to HR-MDS to AML. As *DNMT3A*-mutant HSPCs have previously been associated with inflammation, we hypothesize that increased inflammation contributed to the progression across these disease stages.

### Methods

Single-cell RNA sequencing with genotyping, to discern genotype-phenotype relationships, was performed on 17 primary BM samples harboring *DNMT3A* mutations (6 LR-MDS, 6 HR-MDS and 5 AML). MDS disease state was determined with the IPSS-M risk system. Four in-house normal bone marrow (NBM) samples served as controls. A fluorescence-activated cell sorting strategy was used to enrich for typically underrepresented cell populations and construct a comprehensive cellular taxonomy. In total 4 populations were sorted and pooled for sequencing: 1. non-hematopoietic/niche cells and endothelial cells, 2. hematopoietic stem and progenitor cells, 3. myeloid fraction and 4. non-myeloid fraction. Genotyping was performed by a customized capture on 10x cDNA libraries, followed by full-length Nanopore sequencing. Pseudobulk analyses on *DNMT3A* mutant versus wildtype HSPCs, followed by gene set enrichment analyses (GSEA) were performed to identify gene-expression profiles unique to *DNMT3A*-mutant HPSCs.

**Copyright Information:** (Online) ISSN: 2572-9241
©2026 The Author(s). HemaSphere is published by John Wiley & Sons Ltd on behalf of European Hematology Association. This is an open access Abstract Book distributed under the Attribution-NonCommercial-NoDerivs (CC BY-NC-ND), which allows third parties to download the articles and share them with others as long as they credit the author and the Abstract Book, but they cannot change the content in any way or use them commercially.
**Abstract Book Citations:** Authors, Title, HemaSphere, 2026;10:(S1):pages. The abstract book DOIs can be found on the “Author Information” page.
**Disclaimer:** Articles published in the journal HemaSphere exclusively reflect the opinions of the authors. The authors are responsible for all content in their abstracts including accuracy of the facts, statements, citing resources, etc.

### Results

HR-MDS and AML samples were enriched for a distinct HSPC subset (P = 0.021, Figure 1), characterized by a transcriptional signature associated with BM exfiltration and aggressive disease in solid tumors and multiple myeloma. GSEA indicated that these cells were enriched for gene expression profiles associated with inflammation, hypoxia and migration. Disease progression correlated with increased frequencies of plasmacytoid dendritic cells (pDCs, P = 0.027), a subtype of dendritic cells from the lymphoid lineage, associated with high levels of inflammation and production of type I interferons. This enrichment for pDCs was accompanied by depletion of their progenitors (P = 0.003), naïve CD8 T-cells (P = 0.030) and plasma cells (P = 0.025). *DNMT3A* mutations were predominantly detected in HSPC and myeloid progenitor subsets. Notably, *DNMT3A*-mutant HSPCs displayed distinct gene-expression profiles and altered cell-cell communication compared with their wildtype counterparts. Within the mesenchymal stromal cell (MSC) compartment we observed a shift from normal MSCs towards a more inflammatory subtype during disease progression.

### Summary/Conclusion

We have generated a unique single-cell dataset capturing BM evolution from normal hematopoiesis through successive MDS stages to AML in the context of *DNMT3A* mutations, revealing cellular and molecular features associated with inflammatory remodeling and disease progression.

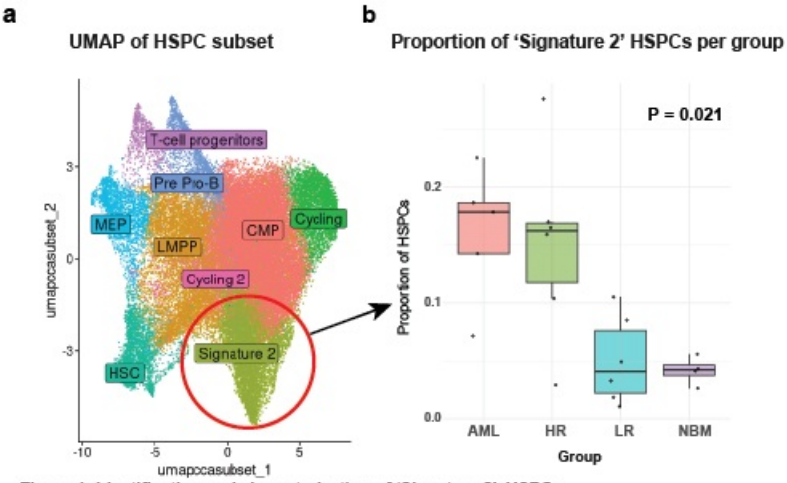

**Figure 1: Identification and characterization of 'Signature 2' HSPCs.**
(a) UMAP visualizing the HSPC subset of the single-cell dataset.
(b) Boxplots showing the proportion of HSPCs belonging to the 'Signature 2' cluster per disease state.

**Copyright Information:** (Online) ISSN: 2572-9241
©2026 The Author(s). HemaSphere is published by John Wiley & Sons Ltd on behalf of European Hematology Association. This is an open access Abstract Book distributed under the Attribution-NonCommercial-NoDerivs (CC BY-NC-ND), which allows third parties to download the articles and share them with others as long as they credit the author and the Abstract Book, but they cannot change the content in any way or use them commercially.
**Abstract Book Citations:** Authors, Title, HemaSphere, 2026;10:(S1):pages. The abstract book DOIs can be found on the "Author Information" page.
**Disclaimer:** Articles published in the journal HemaSphere exclusively reflect the opinions of the authors. The authors are responsible for all content in their abstracts including accuracy of the facts, statements, citing resources, etc.

### (PF433) APOPTOTIC PRIMING DEFINES EFFECTIVE COMBINATION STRATEGIES WITH THIOGUANINE IN AML

**Topic:** 03. Acute myeloid leukemia - Biology & translational research

Lucia Rico Pizarro*[1, 2], Nasim Pouryaghoub[1, 2], Nona Struyf[1, 2], Sean Rudd[1, 2], Martin Jädersten[3, 4], Miriam Selle[5, 6], Bernhard Schmierer[5, 6], Olli Kallioniemi[1, 2, 7], Brinton Seashore-Ludlow[1, 2], Tom Erkers[1, 2]

*[1]Karolinska Institutet, Department of Oncology-Pathology, Stockholm, Sweden;[2]SciLifeLab, Stockholm, Sweden; [3]Karolinska Institutet, Department of Medicine Huddinge, Stockholm, Sweden; [4]Karolinska University Hospital, Stockholm, Sweden; [5]Karolinska Institutet, Department of Medical Biochemistry and Biophysics, Stockholm, Sweden;[6]SciLifeLab, CRISPR Functional Genomics, Stockholm, Sweden; [7]Institute for Molecular Medicine Finland, University of Helsinki, Helsinki, Finland;*

### Background

6-Thioguanine (6-Tg) is a purine analogue approved for the treatment of acute myeloid leukemia (AML) and is occasionally used in relapsed or refractory settings. However, rational combination strategies remain undefined, and deeper mechanistic insight into its cytotoxic activity is lacking. This knowledge gap limits its optimized clinical use with effective therapeutic combinations.

### Aims

We aim to identify rational drug combinations with 6-Tg and to delineate the molecular mechanisms underlying its activity in AML.

### Methods

To unbiasedly identify synergistic partners, we performed high-throughput combinatorial screening of 6-Tg with a library of 526 oncology compounds across multiple AML cell lines. Twenty-eight candidate compounds were selected for validation using 8x8 dose-response matrix synergy testing across AML models, including sequential treatment schedules. Mechanistic dependencies were interrogated using a genome-wide CRISPR-Cas9 knockout screen and mitochondrial priming and apoptotic dependencies after thioguanine treatment was assessed by BH3 profiling.

### Results

Synergistic interactions with 6-Tg were predominantly observed with apoptosis-modulating agents, including BH3 mimetics (e.g., the BCL-2 inhibitor venetoclax and the BCL-XL inhibitor A-1155463), PARP inhibitors, and HDAC inhibitors. In contrast, other cytotoxic and antimetabolite agents, including cytarabine, demonstrated limited or antagonistic interactions.
Synergy validation in 8x8 dose matrices confirmed these findings and revealed that sequential pretreatment with 6-Tg further enhanced the activity of apoptosis-modulating agents, consistent with a 6-Tg induced apoptotic priming effect.
Genome-wide CRISPR screening identified genes involved in thioguanine uptake and activation (SLC43A3, HPRT1), mismatch repair components (MSH2, MSH6), and intrinsic apoptotic mediators (BAX, BIM, APAF1, CYCS, CASP9, CASP3) as essential for 6-Tg cytotoxicity. These findings indicate that 6-Tg-induced lethality depends on mismatch repair-mediated DNA damage signaling converging on mitochondrial apoptosis, aligning with the synergistic drug partners founded. Notably, synergistic partners differed between AML models according to their anti-apoptotic dependency profiles, which was functionally confirmed by BH3 profiling.

**Copyright Information:** (Online) ISSN: 2572-9241
©2026 The Author(s). HemaSphere is published by John Wiley & Sons Ltd on behalf of European Hematology Association. This is an open access Abstract Book distributed under the Attribution-NonCommercial-NoDerivs (CC BY-NC-ND), which allows third parties to download the articles and share them with others as long as they credit the author and the Abstract Book, but they cannot change the content in any way or use them commercially.
**Abstract Book Citations:** Authors, Title, HemaSphere, 2026;10:(S1):pages. The abstract book DOIs can be found on the “Author Information” page.
**Disclaimer:** Articles published in the journal HemaSphere exclusively reflect the opinions of the authors. The authors are responsible for all content in their abstracts including accuracy of the facts, statements, citing resources, etc.

**Summary/Conclusion**

Altogether, our integrated functional profiling identifies apoptosis-directed therapy as the dominant strategy to potentiate 6-Tg in AML. Our data suggest a mechanistic rational linking 6-Tg metabolic activation, DNA incorporation, and mismatch repair-dependent stress to intrinsic mitochondrial apoptosis. This create a therapeutically exploitable primed state that can be leveraged through rational combination strategies.

**Copyright Information:** (Online) ISSN: 2572-9241
©2026 The Author(s). HemaSphere is published by John Wiley & Sons Ltd on behalf of European Hematology Association. This is an open access Abstract Book distributed under the Attribution-NonCommercial-NoDerivs (CC BY-NC-ND), which allows third parties to download the articles and share them with others as long as they credit the author and the Abstract Book, but they cannot change the content in any way or use them commercially.
**Abstract Book Citations:** Authors, Title, HemaSphere, 2026;10:(S1):pages. The abstract book DOIs can be found on the "Author Information" page.
**Disclaimer:** Articles published in the journal HemaSphere exclusively reflect the opinions of the authors. The authors are responsible for all content in their abstracts including accuracy of the facts, statements, citing resources, etc.

### (PF434) TARGETING POST-TRANSCRIPTIONAL ACTIVITY OF BETA-CATENIN IN ACUTE MYELOID LEUKAEMIA

**Topic:** 03. Acute myeloid leukemia - Biology & translational research

Okan Sevim*[1], Megan Wagstaff[1], Rebecca Ling[2], Aaron Goff[1], David Palmer[3, 4], Hyun Park[3], Leandro Castellano[1, 5], Sarah Newbury[3], Ben Towler[1], Anindita Roy[2, 6], Rhys Morgan[1]

*[1]School of Life Sciences, University of Sussex, Brighton, United Kingdom;[2]Department of Paediatrics, University of Oxford, Oxford, United Kingdom; [3]Brighton & Sussex Medical School, University of Sussex, Brighton, United Kingdom;[4]University Hospitals Sussex NHS Foundation NHS Trust, Sussex, United Kingdom; [5]Department of Surgery and Cancer, Division of Cancer, Imperial College London, Imperial Centre for Translational and Experimental Medicine (ICTEM), London, United Kingdom; [6]MRC Molecular Haematology Unit, MRC Weatherall Institute of Molecular Medicine, University of Oxford, Oxford, United Kingdom;*

### Background

Wnt/β-catenin signalling is frequently dysregulated across multiple haematological malignancies, including acute myeloid leukaemia (AML). Efforts to therapeutically target the central mediator β-catenin have been limited by an incomplete understanding of its haematopoietic-specific molecular interactions. Our previous exploration of the β-catenin interactome in leukaemia cells revealed a dense RNA-binding protein (RBP) interaction network, and we further discovered abundant β-catenin:mRNA interactions through β-catenin RNA-immunoprecipitation coupled to RNA-sequencing (RIP-seq). These molecular interactions imply β-catenin has important post-transcriptional roles in leukaemia which remain incompletely characterised.

### Aims

Given β-catenin lacks a canonical RNA-binding motif and is unlikely to bind RNA directly, we explored β-catenin:RBP interactions in this study which could facilitate post-transcriptional activity in this setting with a focus on LIN28B; a fetal haematopoiesis and *let*-7 miRNA regulating RBP. We aimed to characterise the molecular and functional consequences of this interaction and investigate whether the β-catenin:LIN28B axis could represent a novel therapeutic vulnerability for AML cells.

### Methods

Co-immunoprecipitation was used to confirm β-catenin:LIN28B interaction in immortalised cell lines and primary cells. Wnt signalling output was assessed by TCF/LEF reporters in lentivirally transduced myeloid cell lines. Immunoblotting, RT-qPCR and miRNA sequencing were used to evaluate regulatory crosstalk between β-catenin and LIN28B. β-catenin and LIN28B were targeted in AML cells using combined genetic and pharmacological inhibition approaches. The impact of β-catenin on LIN28B's RNA-binding activity was assessed using organic orthogonal phase separation (OOPS) coupled to immunoblotting.

**Copyright Information:** (Online) ISSN: 2572-9241
©2026 The Author(s). HemaSphere is published by John Wiley & Sons Ltd on behalf of European Hematology Association. This is an open access Abstract Book distributed under the Attribution-NonCommercial-NoDerivs (CC BY-NC-ND), which allows third parties to download the articles and share them with others as long as they credit the author and the Abstract Book, but they cannot change the content in any way or use them commercially.
**Abstract Book Citations:** Authors, Title, HemaSphere, 2026;10:(S1):pages. The abstract book DOIs can be found on the "Author Information" page.
**Disclaimer:** Articles published in the journal HemaSphere exclusively reflect the opinions of the authors. The authors are responsible for all content in their abstracts including accuracy of the facts, statements, citing resources, etc.

**Results**

Motif enrichment analysis of β-catenin-associated mRNAs identified the GGAG motif (the canonical LIN28B binding sequence) as one of the most frequently enriched RBP recognition elements. β-Catenin:LIN28B interaction was confirmed in myeloid and lymphoid cell lines, as well as primary human CD34$^{+}$ fetal liver haematopoietic stem cells, where both proteins are abundantly expressed. Modulation of LIN28B positively regulated Wnt signalling output by influencing expression of the Wnt signalling transcription factor LEF-1. Mechanistically, this effect was primarily mediated through LIN28B-dependent regulation of *let*-7 miRNAs, as demonstrated using two LIN28:*let*-7 interaction inhibitors; LIN28-C1632 and ln268. miRNA sequencing of LIN28B-depleted and β-catenin-depleted myeloid cell lines revealed potential cooperative and antagonistic patterns of miRNA regulation, supporting functional interplay between these proteins. OOPS execution in β-catenin-depleted myeloid cells further revealed a decreased LIN28B presence on RNA with no overall change in LIN28B level, suggesting β-catenin can influence the RNA-binding activity of its RBP partners. Finally, dual targeting of LIN28B and β-catenin through either genetic and/or pharmacological inhibition significantly decreased AML cell viability.

**Summary/Conclusion**

This study indicates crosstalk between β-catenin and LIN28B in the post-transcriptional landscape of haematopoietic cells that may represent a novel synthetically lethal relationship in AML. This axis could be therapeutically exploitable in rare AML subsets where LIN28B expression becomes reactivated and β-catenin is expressed.

**Copyright Information:** (Online) ISSN: 2572-9241
©2026 The Author(s). HemaSphere is published by John Wiley & Sons Ltd on behalf of European Hematology Association. This is an open access Abstract Book distributed under the Attribution-NonCommercial-NoDerivs (CC BY-NC-ND), which allows third parties to download the articles and share them with others as long as they credit the author and the Abstract Book, but they cannot change the content in any way or use them commercially.
**Abstract Book Citations:** Authors, Title, HemaSphere, 2026;10:(S1):pages. The abstract book DOIs can be found on the “Author Information” page.
**Disclaimer:** Articles published in the journal HemaSphere exclusively reflect the opinions of the authors. The authors are responsible for all content in their abstracts including accuracy of the facts, statements, citing resources, etc.

### (PF435) GLOBAL ALTERATIONS IN 3D DNA ARCHITECTURE DRIVE UNIQUE LEUKEMOGENIC PROFILES IN IDH1- AND DNMT3A-MUTANT ACUTE MYELOID LEUKEMIA

**Topic:** 03. Acute myeloid leukemia - Biology & translational research

Sophie Steinhäuser*[1, 2], Varun K. A. Sreenivasan[3], David Baden[1, 2], Patricia Silva[4], Thomas Beder[1, 2], Kathrin Richter[1, 2], Miriam Denker[1, 2], Verónica Yumiceba[3], Kristin Schultz[3], Alina Marie Hartmann[1, 2], Nadine Wolgast[1, 2], Simone Lipinski[1, 2], Lorenz Bastian[1, 2], Sonja Bendig[1, 2, 5], Monika Brüggemann[1, 2, 5], Lars Fransecky[1, 2], Martin Neumann[1, 2, 5], Christoph Röllig[6], Malte Spielmann[2, 3, 7, 8, 9], Claudia Baldus[1, 2, 5]

*[1]University Hospital Schleswig-Holstein, Hematology/Oncology, Kiel, Germany; [2]University Cancer Center Schleswig-Holstein (UCCSH), Kiel, Germany; [3]University Hospital Schleswig-Holstein, Institute of Human Genetics, Lübeck, Germany; [4]Charité University Hospital, Hematology/Oncology, Berlin, Germany; [5]Clinical Research Unit CATCH ALL (KFO 5010), Kiel, Germany; [6]University Hospital TU Dresden, Dresden, Germany; [7]German Center for Cardiovascular Research, Lübeck, Germany; [8]Max Planck Institute for Molecular Genetics, Berlin, Germany; [9]Charité University Hospital, Institute for Medical and Human Genetics, Berlin, Germany;*

### Background

Acute myeloid leukemia (AML) is characterized by genomic alterations and mutations in epigenetic regulators such as *IDH1* and *DNMT3A* that are associated with aberrant DNA methylation and transcriptional dysregulation. While recent therapeutic advances have improved outcome, resistance and relapse remain a major challenge, and their driving mechanisms are still not fully understood. We recently showed that *IDH1* mutation associates with changes in DNA looping, leading to oncogene upregulation in AML. However, comprehensive studies addressing the global impact of altered 3D-DNA architecture in AML with epigenetic mutations are lacking.

### Aims

Here, we assessed 3D-DNA alterations in *IDH1*- and *DNMT3A*-mutated (mut) AML as global driver of leukemic signaling on a genome-wide scale to identify novel therapeutic vulnerabilities.

### Methods

Using a CRISPR model of KG1a with either *IDH1* p.R132H or *DNMT3A* p.R882H mutation and primary AML samples with or without *IDH1/DNMT3A* mutation, we analyzed mutation-specific 3D-DNA architecture by chromatin-conformation-capture (HiC) and transcription by RNA-sequencing. Paired-end sequencing data of HiC libraries were analyzed using HiC Pro and DNA loops were called using Peakachu loop caller at 10 kb resolution. Loops were categorized as “lost” or “gained” based on mean Peakachu loop score within a genotype (> 0.97 = loop present, < 0.97 = loop absent). Differential gene expression (DE) of the same samples was analyzed using Limma.

**Copyright Information:** (Online) ISSN: 2572-9241
©2026 The Author(s). HemaSphere is published by John Wiley & Sons Ltd on behalf of European Hematology Association. This is an open access Abstract Book distributed under the Attribution-NonCommercial-NoDerivs (CC BY-NC-ND), which allows third parties to download the articles and share them with others as long as they credit the author and the Abstract Book, but they cannot change the content in any way or use them commercially.
**Abstract Book Citations:** Authors, Title, HemaSphere, 2026;10:(S1):pages. The abstract book DOIs can be found on the “Author Information” page.
**Disclaimer:** Articles published in the journal HemaSphere exclusively reflect the opinions of the authors. The authors are responsible for all content in their abstracts including accuracy of the facts, statements, citing resources, etc.

**Results**

Overall, HiC identified shared and distinct changes in active (A) vs. inactive (B) compartments and underlying loop formation in both *IDH1*- and *DNMT3A*-mut KG1a and patient samples compared to wildtype (wt). Comparative analysis of DE vs. non-DE genes revealed enrichment of upregulated DE- compared to non-DE genes among B-to-A-shifted compartments in both *IDH1*-mut and *DNMT3A*-mut KG1a while downregulated DE genes were enriched in A-to-B-shifted compartments. Despite moderate overlap of shifted compartments between KG1a and patient samples likely reflecting patient heterogeneity, DE gene enrichment patterns exhibited a similar direction, underlining the biological relevance of 3D-DNA changes as driver of transcription changes associated with either mutation. This was supported by DE genes in both *IDH1*-mut and *DNMT3A*-mut cells residing significantly more often at anchors of lost/gained as opposed to non-altered loops, and by loops hosting at least one DE gene showing a higher magnitude of loop score change. Pathway analysis of DE altered-loop genes in *IDH1*-mut vs. -wt cell and patient samples revealed enrichment of immune response genes, DNA binding- and signaling regulators including *EXT1, CHD7* or *IGF1R.* DE altered-loop genes in *DNMT3A*-mut AML were enriched for transcription factors, cell adhesion and immune regulators such as *IKZF3*, *ETS1*, *SEPTIN9* or *IRS2.* While *IGF1R* upregulation corresponded to *in-vitro* sensitivity of *IDH1*-mut KG1a to IGF1R inhibitor BMS-754807 as mono- and combinational therapy with ivosidenib tested by WST1 assay, loop-mediated transcriptional dysregulation of trafficking regulator *TBC1D16* in *DNMT3A*-mut AML was associated with sensitivity to the p38 MAKP inhibitor ralimetinib.

**Summary/Conclusion**

Taken together, our data demonstrate genotype-specific 3D-genome reorganization that alters transcription and signaling in *IDH1*- or *DNMT3A*-mut AML and identified targetable candidates for both entities, highlighting the role of 3D-DNA alteration as global driver of leukemogenesis in AML with epigenetic mutations.

**Copyright Information:** (Online) ISSN: 2572-9241
©2026 The Author(s). HemaSphere is published by John Wiley & Sons Ltd on behalf of European Hematology Association. This is an open access Abstract Book distributed under the Attribution-NonCommercial-NoDerivs (CC BY-NC-ND), which allows third parties to download the articles and share them with others as long as they credit the author and the Abstract Book, but they cannot change the content in any way or use them commercially.
**Abstract Book Citations:** Authors, Title, HemaSphere, 2026;10:(S1):pages. The abstract book DOIs can be found on the "Author Information" page.
**Disclaimer:** Articles published in the journal HemaSphere exclusively reflect the opinions of the authors. The authors are responsible for all content in their abstracts including accuracy of the facts, statements, citing resources, etc.

### (PF436) TRANSCRIBED CIS-REGULATORY ELEMENT PROFILING REVEALS THE MUTATION-DRIVEN MOLECULAR, CELLULAR AND PROGNOSTIC HETEROGENEITY IN CYTOGENETICALLY NORMAL ACUTE MYELOID LEUKEMIA

**Topic:** 03. Acute myeloid leukemia - Biology & translational research

Manli Yang*[1], Chi-Wai Yip[2], Miki Kojima[2], Jonathan Moody[1], Xiangfu Zhong[3], Sofia Bengtzen[3], Cheng-Tsung Pan[3], Anna Bohlin[3], Erik Arner[4], Sören Lehmann[3], Andreas Lennartsson[3], Chung-Chau Hon[1]

*[1]RIKEN Center for Integrative Medical Sciences, Laboratory for Regulatory Genomics,, Yokohama, Kanagawa, Japan;[2]RIKEN Center for Integrative Medical Sciences, Laboratory for Transcriptome Technology, Yokohama, Kanagawa, Japan; [3]Karolinska Institutet,, Department of Medicine, Center for Hematology and Regenerative Medicine, Stockholm, Sweden; [4]RIKEN Center for Integrative Medical Sciences, Yokohama, Japan;*

### Background

Cytogenetically normal acute myeloid leukemia (CN-AML) is a heterogeneous subtype of AML defined by the absence of recurrent chromosomal abnormalities. Instead, the disease is driven by somatic mutations in approximately a dozen recurrently mutated genes that collectively shape the heterogeneous behavior and prognosis.

### Aims

We aim to investigate the effects of these somatic mutations on gene expression and their associations to the molecular, cellular and prognostic heterogeneity in CN-AML.

### Methods

We performed 5'-based RNA-sequencing in both bulk (Cap Analysis of Gene Expression, n=88) and single-cell (n=16) resolutions on bone marrow mononuclear cells from CN-AML patients in a Swedish cohort, with panel sequencing revealing 35 recurrently mutated genes among those profiled.

**Copyright Information:** (Online) ISSN: 2572-9241
©2026 The Author(s). HemaSphere is published by John Wiley & Sons Ltd on behalf of European Hematology Association. This is an open access Abstract Book distributed under the Attribution-NonCommercial-NoDerivs (CC BY-NC-ND), which allows third parties to download the articles and share them with others as long as they credit the author and the Abstract Book, but they cannot change the content in any way or use them commercially.
**Abstract Book Citations:** Authors, Title, HemaSphere, 2026;10:(S1):pages. The abstract book DOIs can be found on the "Author Information" page.
**Disclaimer:** Articles published in the journal HemaSphere exclusively reflect the opinions of the authors. The authors are responsible for all content in their abstracts including accuracy of the facts, statements, citing resources, etc.

**Results**

By capturing transcripts at transcription starting sites, both methods enable the identification of transcribed *cis*-regulatory elements (tCREs), including coding and non-coding genomic regions such as promoters and enhancers. We focused on the tCRE-based rather than gene-based transcriptome to better understand gene expression regulation and identified potential prognostic tCRE biomarkers by profiling tCRE transcriptional activity across patients and cell populations. Based on bulk tCRE profiles, we classified four molecular subtypes characterized by distinct cellular compositions, clinical outcomes, and associations with distinct subsets of somatic mutations. Based on the single-cell data, we investigated cell type–specific effects of somatic mutations by comparing tCRE profiles between cells carrying mutant versus wild-type alleles of the corresponding genes. Using such information from single-cell data to validate a deconvolution approach applied to bulk data, we obtained the differentially active tCREs across various cell types for major 10 somatic mutations. To better understand the regulatory roles of these tCREs, we defined 62 tCRE co-activity modules that underlies distinct biological functions. These include cell-type identity modules, such as those involved in monocyte chemotaxis, as well as activity modules, including those associated with DNA methylation and interferon response. Notably, we found several modules significantly enriched in somatic mutation-associated tCREs and associated with patient survival, highlighting the potential clinical relevance of these modules. After identifying the key transcription factors and super enhancers within each module, we constructed tCRE-based transcriptional regulatory networks and assessed regulatory importance in regulator-centric subnetworks using composite centrality and path strength scores. Applying this approach to more than 200 AML core genes, we elucidated the regulatory mechanisms linking these genes to their associated modules. Finally, we identified drug-targetable genes within shared pathways of coordinated modules across distinct core-gene nexuses, revealing common therapeutic strategies that may be effective despite diverse mutational origins. Together, these findings provide a framework for understanding how somatic mutations shape regulatory programs and drive heterogeneity, uncovering targetable pathways that may inform new therapeutic strategies to overcome the effects of diverse mutations.

**Summary/Conclusion**

**Copyright Information:** (Online) ISSN: 2572-9241
©2026 The Author(s). HemaSphere is published by John Wiley & Sons Ltd on behalf of European Hematology Association. This is an open access Abstract Book distributed under the Attribution-NonCommercial-NoDerivs (CC BY-NC-ND), which allows third parties to download the articles and share them with others as long as they credit the author and the Abstract Book, but they cannot change the content in any way or use them commercially.
**Abstract Book Citations:** Authors, Title, HemaSphere, 2026;10:(S1):pages. The abstract book DOIs can be found on the "Author Information" page.
**Disclaimer:** Articles published in the journal HemaSphere exclusively reflect the opinions of the authors. The authors are responsible for all content in their abstracts including accuracy of the facts, statements, citing resources, etc.

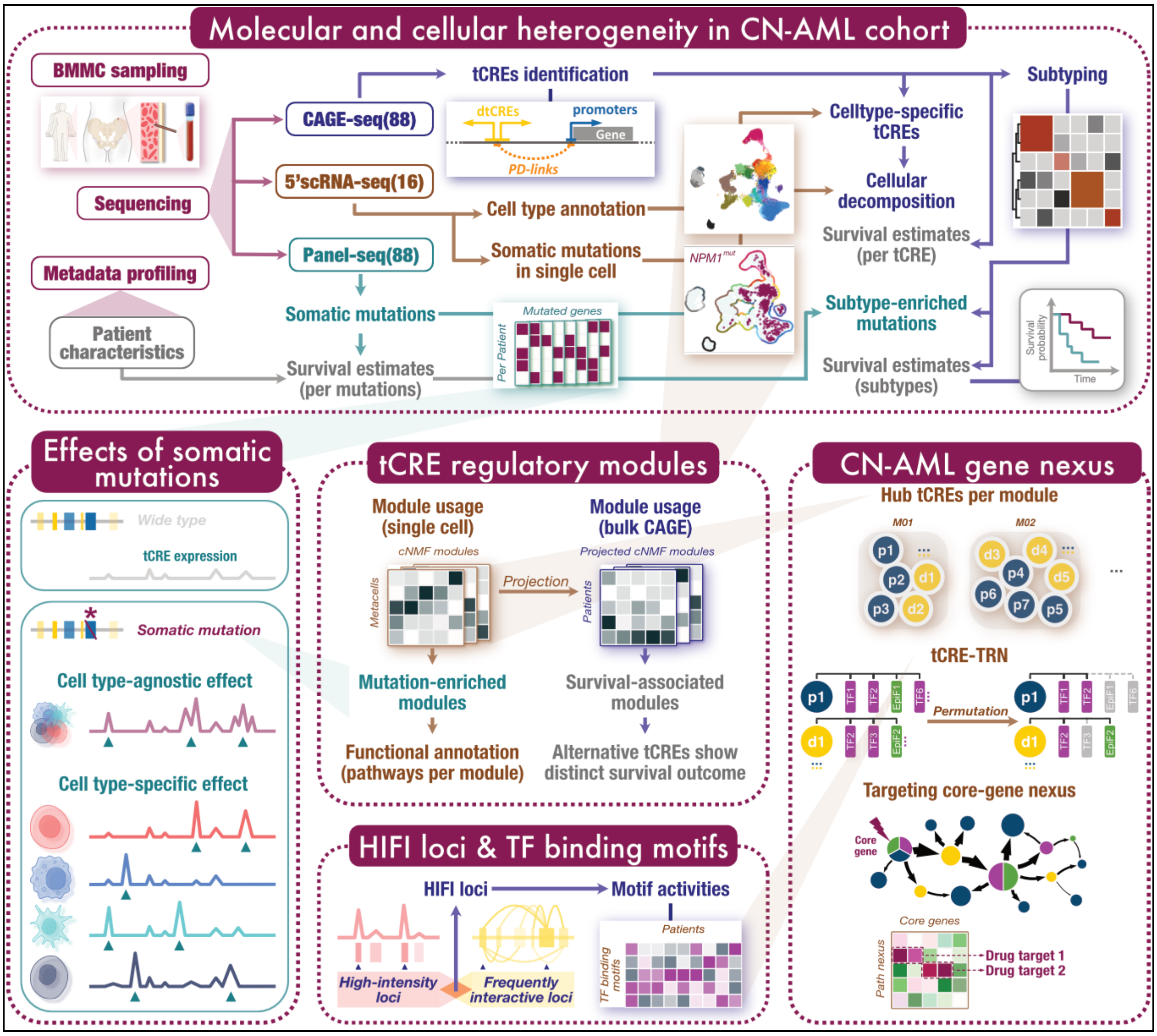

**Copyright Information:** (Online) ISSN: 2572-9241
©2026 The Author(s). HemaSphere is published by John Wiley & Sons Ltd on behalf of European Hematology Association. This is an open access Abstract Book distributed under the Attribution-NonCommercial-NoDerivs (CC BY-NC-ND), which allows third parties to download the articles and share them with others as long as they credit the author and the Abstract Book, but they cannot change the content in any way or use them commercially.
**Abstract Book Citations:** Authors, Title, HemaSphere, 2026;10:(S1):pages. The abstract book DOIs can be found on the "Author Information" page.
**Disclaimer:** Articles published in the journal HemaSphere exclusively reflect the opinions of the authors. The authors are responsible for all content in their abstracts including accuracy of the facts, statements, citing resources, etc.

### (PF437) HUTCL1A MICE DEVELOP MYELOID MALIGNANCY

**Topic:** 03. Acute myeloid leukemia - Biology & translational research

Isak Tengesdal*[1], Thais Aljentera[1], Raghda Fouda[1], Richard Lam[1], Benjamin Rolles[1], Debasmita Paul[1], Ann Mullally[1], Siddhartha Jaiswal[2]

*[1]Stanford University, Pathology, Stanford, United States of America; [2]Stanford University, Institute for Stem Cell Biology & Regenerative Medicine, Stanford, United States of America;*

### Background

All blood cells arise from a common pool of hematopoietic stem cells (HSCs). Some HSCs acquire mutations which afford ability for clonal expansion at greater rates in the stem cell pool over non-mutated HSCs, this phenomena describes a pre-malignant state for blood cancers termed clonal hematopoiesis of indeterminate potential (CHIP). Our group recently uncovered that the clonal expansion associated with CHIP is driven by de-repressed expression of the gene *TCL1A* in HSCs. Although CHIP is a pre-malignant state, it may also evolve into blood cancers. Additionally, many of the driver mutations in CHIP are also common in myeloid malignancies. Whether TCL1A expression in HSCs plays a role in the development of these malignant states is currently unknown.

### Aims

The *TCL1A* promoter is poorly conserved between mice and humans, suggesting existing mouse models of CHIP driver mutations do not fully recapitulate human CHIP. Therefore, we developed mice expressing hu*TCL1A*. By crossing hu*TCL1A* mice with mice expressing Vav1-Cre and Tal1-Cre (tamoxifen inducible) we sought to model the effect *TCL1A* has on HSCs and determine if *TCL1A* expression in HSCs can promote development of blood cancers.

### Results

HSPC populations from the bone marrow (BM) of 3 month old hu*TCL1A Vav1-Cre*, hu*TCL1A Tal1-Cre* (both referred to as hu*TCL1A* mice) and Cre control mice was assessed by flow cytometry and ex-vivo functional assays were performed. hu*TCL1A* mice exhibited 2-fold more LT-HSCs, and progenitor populations in BM compared to Cre controls (A). Functional HSC assays revealed LT-HSCs from hu*TCL1A* expanded at a greater rate ex vivo than LT-HSCs from Cre control mice. Additionally, cKit$^+$ cells from hu*TCL1A* bone marrow exhibit reduced apoptosis ex vivo, as measured by Annexin V/7-AAD flow cytometry, and increased cell cycling in an in vivo BrDU assay versus Cre controls. Shockingly, all hu*TCL1A* mice died within one year, whereas no Cre control mice died in our first survival cohort ($P$<0.0001) (B). In follow up survival cohorts bone marrow, spleen, and liver were analyzed in moribund and littermate Cre control mice by flow cytometry and immunohistochemistry (IHC). Half of moribund hu*TCL1A* mice exhibit splenomegaly and hepatomegaly accompanied by stark infiltration of CD34$^+$/CD11b$^+$/MPO+ cells as determined by flow cytometry, suggesting development of myeloid malignancy which was validated by IHC. The remaining moribund hu*TCL1A* mice develop two distinct phenotypes: 25% develop a chronic lymphocytic leukemia (CLL)-like phenotype characterized by aggressive expansion of CD19+/IgG κ or λ restricted/CD5$^+$ cells in the spleen and liver and the remaining 25% of mice develop a mix phenotype displaying concurrent myeloid and CLL-like characteristics. Single-cell RNA-seq analysis on BM from a moribund hu*TCL1A* with a myeloid malignancy phenotype corroborated the findings above. Lastly, BM from a healthy control or moribund hu*TCL1A* mouse with an expanded CD34$^+$/cKit$^+$/CD11b$^+$ (myeloid malignancy) population was transplanted into lethally irradiated mice. At 6 months, all moribund hu*TCL1A* recipient mice died, whereas no healthy control recipients died.

**Copyright Information:** (Online) ISSN: 2572-9241
©2026 The Author(s). HemaSphere is published by John Wiley & Sons Ltd on behalf of European Hematology Association. This is an open access Abstract Book distributed under the Attribution-NonCommercial-NoDerivs (CC BY-NC-ND), which allows third parties to download the articles and share them with others as long as they credit the author and the Abstract Book, but they cannot change the content in any way or use them commercially.
**Abstract Book Citations:** Authors, Title, HemaSphere, 2026;10:(S1):pages. The abstract book DOIs can be found on the "Author Information" page.
**Disclaimer:** Articles published in the journal HemaSphere exclusively reflect the opinions of the authors. The authors are responsible for all content in their abstracts including accuracy of the facts, statements, citing resources, etc.

**Summary/Conclusion**

In conclusion, the development of myeloid malignancy in our hu*TCL1A* mice suggests that *TCL1A* expression in HSCs acts as an oncogenic switch, positioning aberrant *TCL1A* expression in HSCs as a contributing component of CHIP progression to myeloid malignancy.

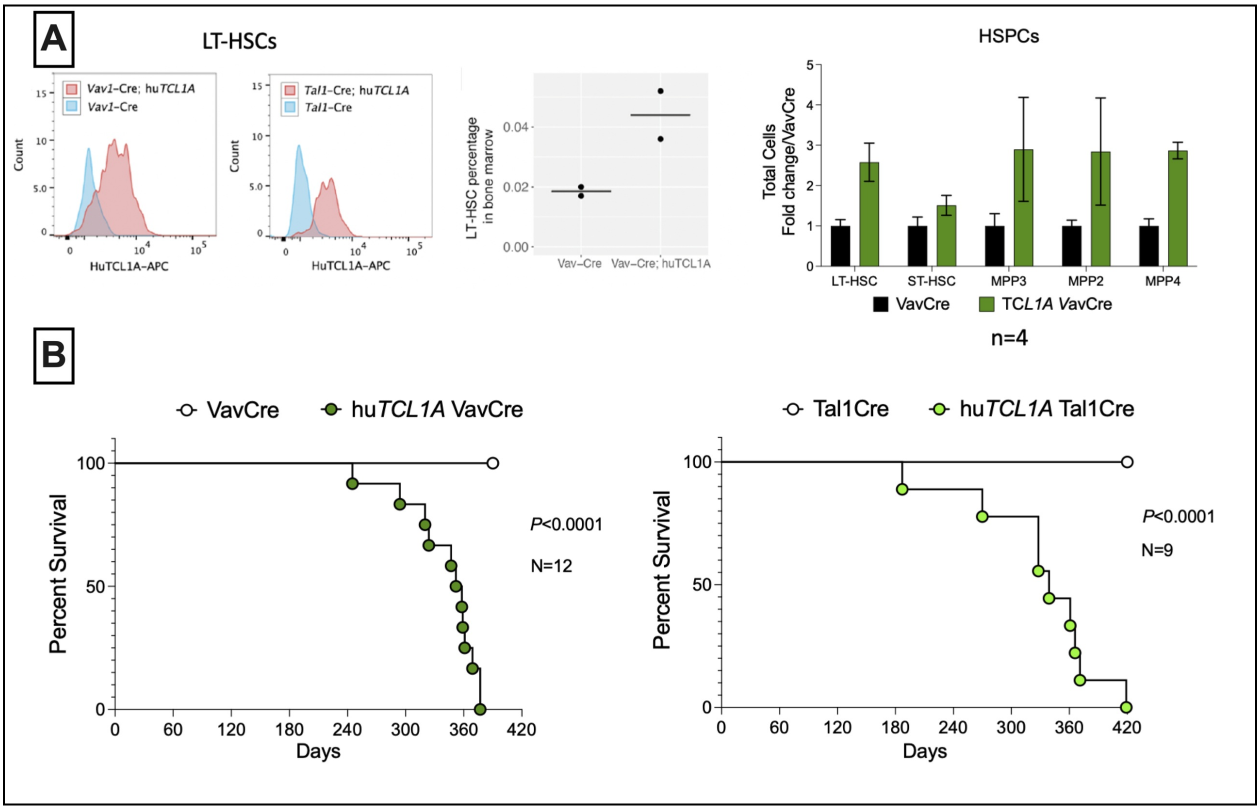

**Copyright Information:** (Online) ISSN: 2572-9241
©2026 The Author(s). HemaSphere is published by John Wiley & Sons Ltd on behalf of European Hematology Association. This is an open access Abstract Book distributed under the Attribution-NonCommercial-NoDerivs (CC BY-NC-ND), which allows third parties to download the articles and share them with others as long as they credit the author and the Abstract Book, but they cannot change the content in any way or use them commercially.
**Abstract Book Citations:** Authors, Title, HemaSphere, 2026;10:(S1):pages. The abstract book DOIs can be found on the "Author Information" page.
**Disclaimer:** Articles published in the journal HemaSphere exclusively reflect the opinions of the authors. The authors are responsible for all content in their abstracts including accuracy of the facts, statements, citing resources, etc.

### (PF438) MORIN OVERCOMES VENETOCLAX RESISTANCE IN AML BY MODULATING THE USP15/HSP70/BCL-2 AXIS

**Topic:** 03. Acute myeloid leukemia - Biology & translational research

Zi-Yuan Nie*[1], Dongfeng Zeng[2], Yan Qin[3]

*[1]Department of Hematology, The Second Hospital of Hebei Medical University, Shijiazhuang, China;[2]Department of Haematology, Daping Hospital, Army Medical University, Chongqing, China; [3]Affiliated Hospital of Hebei University, Baoding, China;*

### Background

Venetoclax (VEN) combined regimens are first-line therapies for acute myeloid leukemia (AML), but acquired resistance limits clinical efficacy. Morin, a natural flavonoid, has various bioactivities. We previously reported that Morin sensitizes drug-resistant CML cells to TKIs. However, its role in VEN-resistant AML remains unclear.

### Aims

To investigate the anti-leukemia effects and sensitizing activity of Morin in VEN-resistant AML cells and elucidate the underlying molecular mechanisms.

### Methods

VEN-resistant AML cell lines (U937R, MV4-11R) were treated with Morin. Cell viability and VEN sensitivity were assessed by CCK-8 assay. Apoptosis was detected by flow cytometry and Wright staining. The in vivo effect was evaluated in a xenograft mouse model. Transcriptomic analysis was performed to identify key pathways. Expression of USP15 and related proteins was detected by qRT-PCR, Western blot, and immunohistochemistry. The interaction between USP15 and HSP70 was validated by Co-IP and immunofluorescence. Rescue experiments were conducted by knocking down or overexpressing USP15/HSP70. The therapeutic efficacy was further validated in an MLL-AF9 murine leukemia model.

### Results

Morin inhibited proliferation of VEN-resistant AML cells in a dose- and time-dependent manner (IC50: 79.3 μM for U937R; 73.04 μM for MV4-11R) and significantly re-sensitized cells to VEN. Morin induced apoptosis in vitro and reduced tumor growth, decreased Ki-67, and increased TUNEL positivity in vivo. Transcriptome analysis revealed that Morin downregulated the deubiquitinating enzyme USP15. USP15 was highly expressed in VEN-resistant patients and cell lines, correlating with poor prognosis. Knockdown of USP15 mimicked the effects of Morin, inhibiting proliferation, inducing apoptosis, downregulating BCL-2/MCL-1, upregulating BAX, and enhancing VEN sensitivity. Mechanistically, USP15 interacted with and deubiquitinated HSP70, thereby stabilizing it. Knockdown of USP15 promoted HSP70 ubiquitination and degradation. Overexpression of HSP70 partially reversed the effects of USP15 knockdown. In line with this, Morin downregulated both USP15 and HSP70, and the anti-leukemia effects of Morin were partially abrogated by USP15 overexpression. In the MLL-AF9 mouse model, Morin treatment prolonged survival, reduced leukemic burden, and downregulated the USP15/HSP70/BCL-2 axis.

**Copyright Information:** (Online) ISSN: 2572-9241
©2026 The Author(s). HemaSphere is published by John Wiley & Sons Ltd on behalf of European Hematology Association. This is an open access Abstract Book distributed under the Attribution-NonCommercial-NoDerivs (CC BY-NC-ND), which allows third parties to download the articles and share them with others as long as they credit the author and the Abstract Book, but they cannot change the content in any way or use them commercially.
**Abstract Book Citations:** Authors, Title, HemaSphere, 2026;10:(S1):pages. The abstract book DOIs can be found on the “Author Information” page.
**Disclaimer:** Articles published in the journal HemaSphere exclusively reflect the opinions of the authors. The authors are responsible for all content in their abstracts including accuracy of the facts, statements, citing resources, etc.

### Summary/Conclusion

Morin overcomes VEN resistance in AML by downregulating USP15, which promotes ubiquitination of its substrate HSP70, leading to modulation of the BCL-2 family, inhibition of proliferation, induction of apoptosis, and restoration of VEN sensitivity. Our findings suggest Morin as a potential therapeutic strategy for VEN-resistant AML by targeting the USP15/HSP70/BCL-2 signaling pathway.

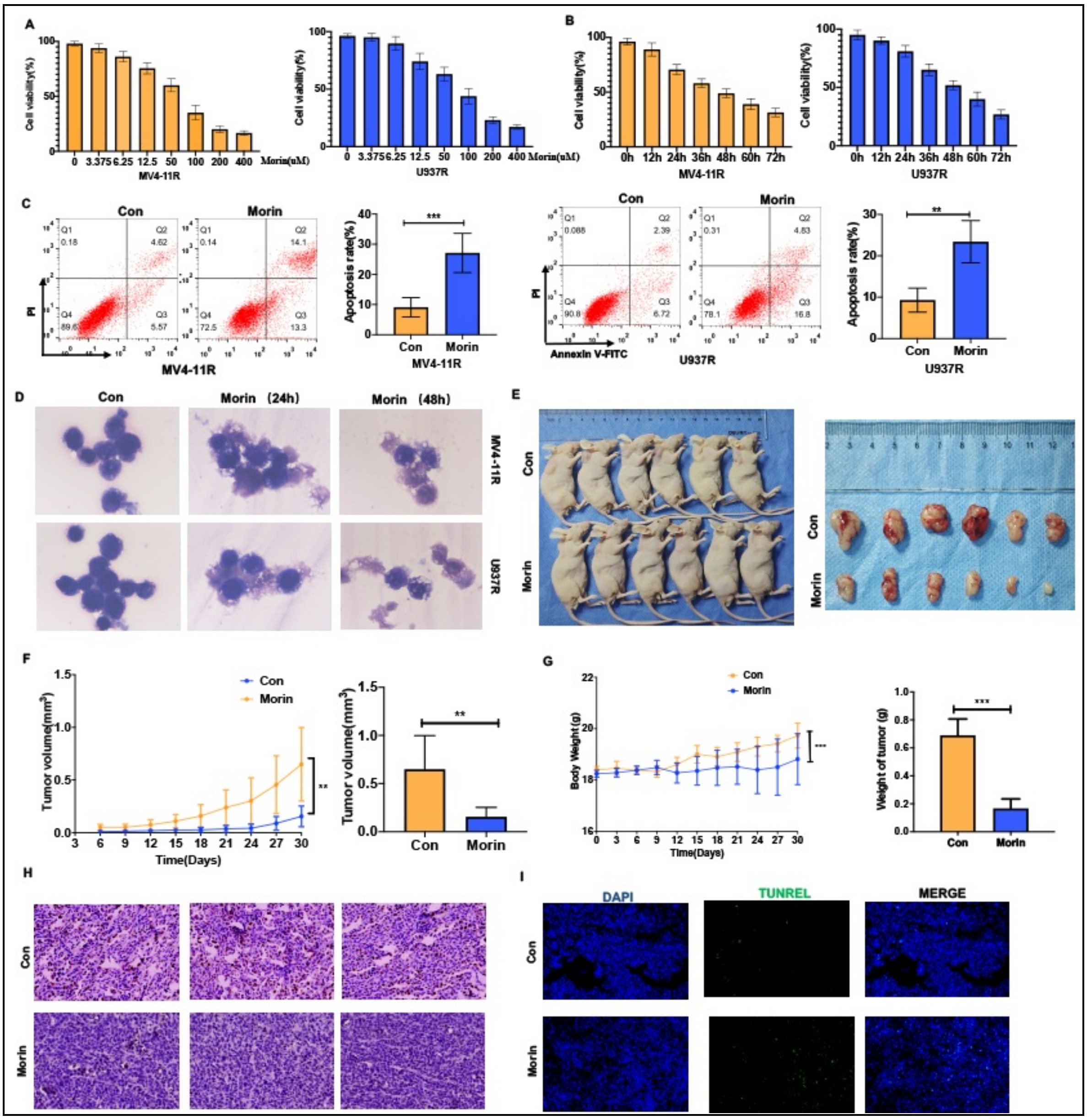

**Copyright Information:** (Online) ISSN: 2572-9241
©2026 The Author(s). HemaSphere is published by John Wiley & Sons Ltd on behalf of European Hematology Association. This is an open access Abstract Book distributed under the Attribution-NonCommercial-NoDerivs (CC BY-NC-ND), which allows third parties to download the articles and share them with others as long as they credit the author and the Abstract Book, but they cannot change the content in any way or use them commercially.
**Abstract Book Citations:** Authors, Title, HemaSphere, 2026;10:(S1):pages. The abstract book DOIs can be found on the "Author Information" page.
**Disclaimer:** Articles published in the journal HemaSphere exclusively reflect the opinions of the authors. The authors are responsible for all content in their abstracts including accuracy of the facts, statements, citing resources, etc.

### (PF439) SINGLE-CELL SURFACE PROTEOMICS AND LONG-READ METHYLOMICS IDENTIFY DETERMINANTS OF HMA RESPONSE AND RESISTANCE IN RELAPSED AML

**Topic:** 03. Acute myeloid leukemia - Biology & translational research

Yi Chen[1], Rong Deng[1], Varun Sudunagunta[1], Edna Stewart[1], Stefan Petkov[2], Sunil Iyer[1], Michele Simonetti[2], Simon Fredriksson[2], Joseph Jurcic[1], Todd Rosenblat[1], Aaron Viny*[1]

*[1]Columbia University, Department of Medicine, Herbert Irving Comprehensive Cancer Center, New York, United States of America; [2]Pixelgen Technologies, Stockholm, Sweden;*

### Background

Resistance to hypomethylating agents (HMAs) remains a major clinical challenge in relapsed/refractory (R/R) myelodysplastic syndrome (MDS) and acute myeloid leukemia (AML), where response rates remain 10–20% and median survival <6 months. Although HMAs are presumed to promote DNA demethylation and differentiation, precise molecular mechanisms underlying response remain poorly defined. In a phase I trial of decitabine plus thioguanine (6TG), we observed a 30% response rate in R/R AML. We applied integrated single-cell proteomics and long-read methylomics to define cellular and epigenetic correlates of response and resistance.

### Aims

To define cellular, immunologic, and epigenetic determinants of therapeutic response to HMA/6TG through integrated single-cell surface proteomics and long-read methylation profiling.

### Methods

Paired pre- and on-therapy bone-marrow samples were collected from patients treated with HMA/6TG and classified as complete responders (CR) or non-responders (NR). Pixelgen Proximity Network Assay quantified per-cell abundance, polarity, clustering, and colocalization across surface antigens (CR n=5; NR n=11). Whole-genome Oxford Nanopore (ONT) sequencing profiled 5mC/5hmC states in paired samples (CR n=3; NR n=3), focusing on differentially methylated and hydroxymethylated regions (DMRs).

### Results

Pre-treatment NR samples demonstrated elevated, polarized CD44 with strong HLA/β2M co-localization, while CR samples showed low CD44, uniform antigen distribution, and expanded effector T-cell pools. Post-treatment, NR samples retained CD44-anchored adhesion signatures, whereas CR samples displayed increased CD36 expression and restoration of normal stem-cell antigen patterns. Pixelgen profiling revealed that dynamic changes in CD34 surface protein topology discriminate hematopoietic recovery from refractory disease. Late-timepoint CD34+ cells in CR patients exhibited healthy HSC-like spatial signatures, including reduced aberrant polarity and normalized proximity to GPI-anchored proteins, while NR patients maintained blast-like CD34 colocalization patterns despite cytopenias.
Differential timepoint comparisons showed that CR marrow transitioned toward increasingly structured and less polarized CD34+ topologies, whereas NR marrow failed to reorganize, preserving a disease-associated proximity signature. A derived CD34 "health score" accurately distinguished regenerating marrow from residual leukemia, even in the context of post-ablative samples.
Long-read ONT methylome profiling revealed that CR patients exhibited significantly more enhancer-linked DMRs and enriched 5hmC activation signatures involving immune, WNT, and autophagy/trafficking pathways, whereas NR samples preserved stable, unremodeled methylation blocks.

**Copyright Information:** (Online) ISSN: 2572-9241
©2026 The Author(s). HemaSphere is published by John Wiley & Sons Ltd on behalf of European Hematology Association. This is an open access Abstract Book distributed under the Attribution-NonCommercial-NoDerivs (CC BY-NC-ND), which allows third parties to download the articles and share them with others as long as they credit the author and the Abstract Book, but they cannot change the content in any way or use them commercially.
**Abstract Book Citations:** Authors, Title, HemaSphere, 2026;10:(S1):pages. The abstract book DOIs can be found on the "Author Information" page.
**Disclaimer:** Articles published in the journal HemaSphere exclusively reflect the opinions of the authors. The authors are responsible for all content in their abstracts including accuracy of the facts, statements, citing resources, etc.

**Summary/Conclusion**

Integrated single-cell spatial proteomics and long-read methyl-omics identify convergent immunologic, adhesive, stem-cell, and epigenetic determinants of HMA response. Beyond CD44-anchored adhesion resistance, CD34 spatial topology emerges as a powerful discriminator between hematopoietic regeneration and refractory disease, with CR patients demonstrating a shift toward healthy HSC-like CD34 architecture and NR patients retaining blast-like CD34 proximity networks. Coupled with responder-specific enhancer methylation remodeling, these multi-omic features offer a biologically grounded framework for response assessment, risk stratification, and mechanistic understanding of HMA-based therapy.

**Copyright Information:** (Online) ISSN: 2572-9241
©2026 The Author(s). HemaSphere is published by John Wiley & Sons Ltd on behalf of European Hematology Association. This is an open access Abstract Book distributed under the Attribution-NonCommercial-NoDerivs (CC BY-NC-ND), which allows third parties to download the articles and share them with others as long as they credit the author and the Abstract Book, but they cannot change the content in any way or use them commercially.
**Abstract Book Citations:** Authors, Title, HemaSphere, 2026;10:(S1):pages. The abstract book DOIs can be found on the “Author Information” page.
**Disclaimer:** Articles published in the journal HemaSphere exclusively reflect the opinions of the authors. The authors are responsible for all content in their abstracts including accuracy of the facts, statements, citing resources, etc.

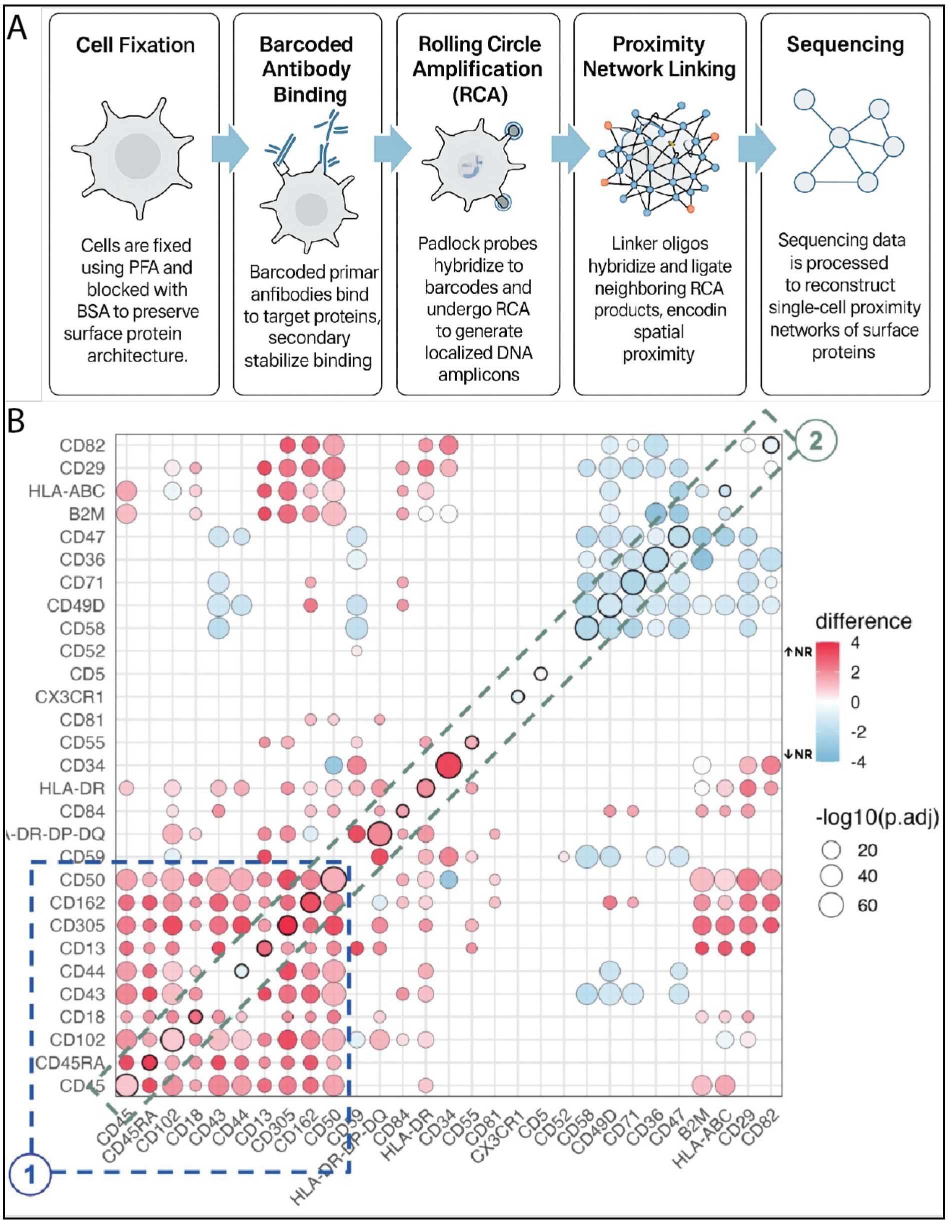

Figure. Single-cell mapping of disease-specific surface proximity networks enables spatially gated immune targeting. (A) PNA workflow: Fixed cells are labeled with DNA-barcoded antibodies; proximity ligation converts nanoscale protein co-localization into sequenced DNA relationships for network reconstruction. (B) Differential CD34+ cell proximity landscape: Bubble plots show rewiring of surface protein neighborhoods between disease (1) and normal (2) states, highlighting network-level changes.

Copyright Information: (Online) ISSN: 2572-9241
©2026 The Author(s). HemaSphere is published by John Wiley & Sons Ltd on behalf of European Hematology Association. This is an open access Abstract Book distributed under the Attribution-NonCommercial-NoDerivs (CC BY-NC-ND), which allows third parties to download the articles and share them with others as long as they credit the author and the Abstract Book, but they cannot change the content in any way or use them commercially.
Abstract Book Citations: Authors, Title, HemaSphere, 2026;10:(S1):pages. The abstract book DOIs can be found on the "Author Information" page.
Disclaimer: Articles published in the journal HemaSphere exclusively reflect the opinions of the authors. The authors are responsible for all content in their abstracts including accuracy of the facts, statements, citing resources, etc.

### (PF440) RUNX1 MUTATIONS ACTIVATING BCAT1 IDENTIFIED BY LENTIVIRAL LIBRARY AS A THERAPEUTIC VULNERABILITY IN AML

**Topic:** 03. Acute myeloid leukemia - Biology & translational research

Siyang Wang*[1]

[1] *The First Affiliated Hospital of Xiamen University, Hematology, Xiamen, China;*

### Background

*RUNX1* mutations define a high-risk subgroup of acute myeloid leukemia (AML) characterized by poor prognosis and a 5-year survival rate below 30%. Despite therapeutic advances, patients frequently experience relapse and resistance. Direct pharmacologic inhibition of RUNX1 remains formidable due to its lack of intrinsic catalytic activity, its essential role in normal hematopoiesis (leading to on-target toxicity), and the profound biological heterogeneity driven by over 1,600 reported variants. Identifying mutation-specific downstream drivers is therefore critical for developing precise therapeutic interventions.

### Aims

Current treatment strategies for RUNX1-mutated AML largely rely on non-specific venetoclax-based regimens and are associated with limited durability. This study aimed to identify mutation-specific downstream vulnerabilities in RUNX1-mutated AML and to explore their potential as adjunctive therapeutic targets to improve treatment efficacy.

### Methods

*We integrated transcriptomic data from TCGA and the BEAT-AML database to identify differentially expressed leukemia driver genes across AML subtypes. Meanwhile, to resolve the functional impact of RUNX1 heterogeneity within the RUNX1-mutant AML cohort, we developed a high-throughput "variant-to-function" screening platform. A lentiviral cDNA library comprising ClinVar-annotated pathogenic RUNX1 variants, each tagged with a unique 20-bp barcode, was introduced into RUNX1-deficient AML cells. Single-cell RNA sequencing (scRNA-seq) was then employed to map specific mutations to their respective transcriptional profiles. Candidate mutations were validated for sensitivity to the BCAT1 inhibitor gabapentin, both as a monotherapy and in combination with venetoclax, using in vitro assays and in vivo xenograft models.*

### Results

*Integrated transcriptomic analysis identified BCAT1 (branched-chain amino acid aminotransferase 1) as significantly upregulated in RUNX1-mutated AML. Our scRNA-seq screen further pinpointed seven specific pathogenic RUNX1 mutations that directly drive BCAT1 overexpression at both mRNA and protein levels. Functional assays revealed that AML cells harboring these "BCAT1-activating" mutations exhibited markedly increased sensitivity to gabapentin compared to wild-type cells or those carrying other RUNX1 variants. Furthermore, gabapentin demonstrated potent synergy with venetoclax, significantly enhancing anti-leukemic activity, reducing tumor burden, and prolonging survival in animal models.*

**Copyright Information:** (Online) ISSN: 2572-9241
©2026 The Author(s). HemaSphere is published by John Wiley & Sons Ltd on behalf of European Hematology Association. This is an open access Abstract Book distributed under the Attribution-NonCommercial-NoDerivs (CC BY-NC-ND), which allows third parties to download the articles and share them with others as long as they credit the author and the Abstract Book, but they cannot change the content in any way or use them commercially.
**Abstract Book Citations:** Authors, Title, HemaSphere, 2026;10:(S1):pages. The abstract book DOIs can be found on the "Author Information" page.
**Disclaimer:** Articles published in the journal HemaSphere exclusively reflect the opinions of the authors. The authors are responsible for all content in their abstracts including accuracy of the facts, statements, citing resources, etc.

### Summary/Conclusion

Our results identify *BCAT1* as a mutation-specific vulnerability in a distinct subset of *RUNX1*-mutated AML. This study provides a rationale for a precision medicine approach, suggesting that targeting the BCAT1 axis—particularly in combination with venetoclax—may improve clinical outcomes for this high-risk patient population.

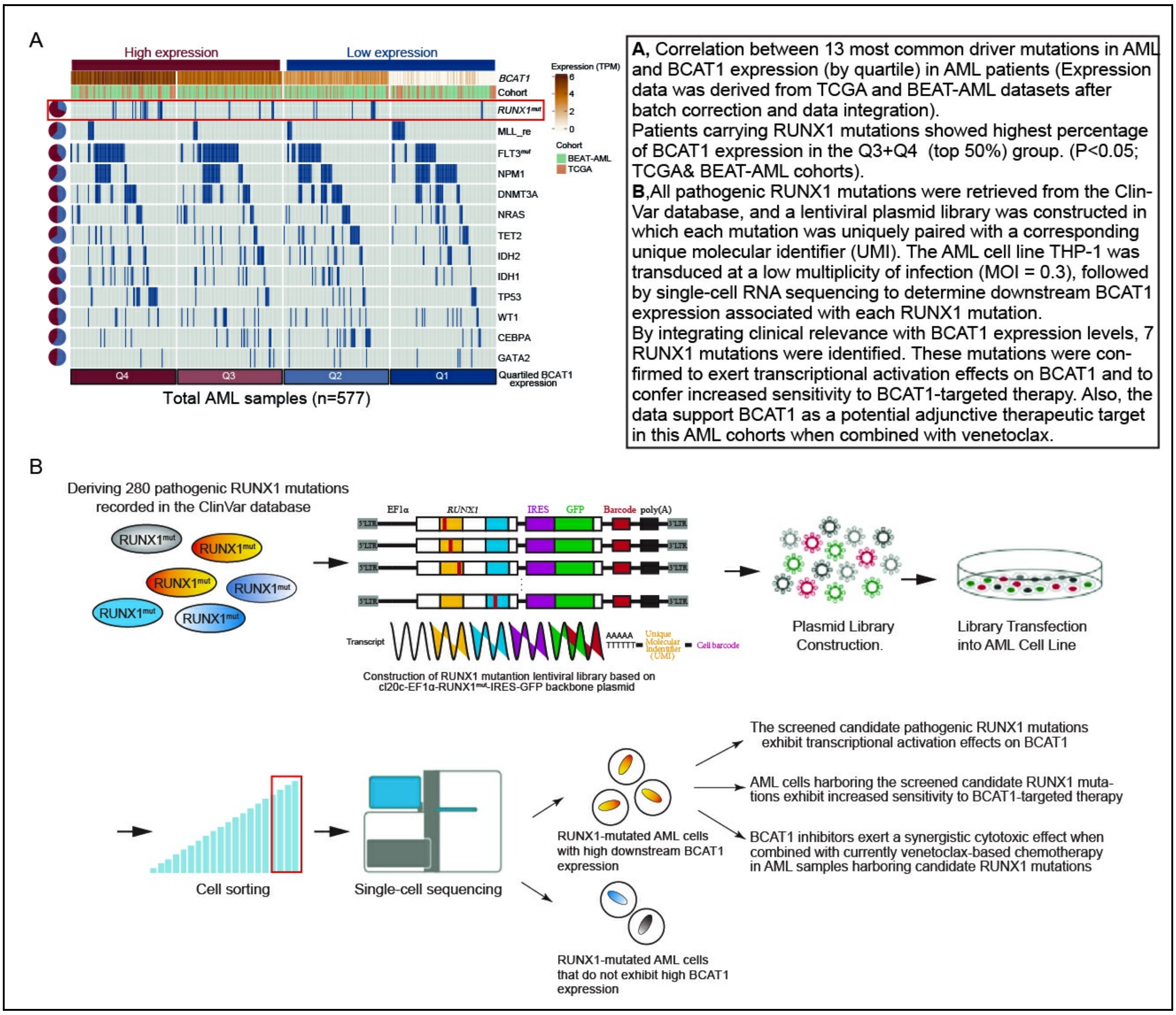

**Copyright Information:** (Online) ISSN: 2572-9241
©2026 The Author(s). HemaSphere is published by John Wiley & Sons Ltd on behalf of European Hematology Association. This is an open access Abstract Book distributed under the Attribution-NonCommercial-NoDerivs (CC BY-NC-ND), which allows third parties to download the articles and share them with others as long as they credit the author and the Abstract Book, but they cannot change the content in any way or use them commercially.
**Abstract Book Citations:** Authors, Title, HemaSphere, 2026;10:(S1):pages. The abstract book DOIs can be found on the “Author Information” page.
**Disclaimer:** Articles published in the journal HemaSphere exclusively reflect the opinions of the authors. The authors are responsible for all content in their abstracts including accuracy of the facts, statements, citing resources, etc.

### (PF441) UNCOVERING HYPOXIA-INDUCED METABOLIC AND REDOX REPROGRAMMING DRIVING THERAPY RESISTANCE AND FERROPTOSIS EVASION IN AML

**Topic:** 03. Acute myeloid leukemia - Biology & translational research

Lorenzo Manfreda*[1], Marta Penco[1], Martina Basilico[1], Riccardo Scribano[1], Martina Maria Capelletti[1], Martina Failla[1], Jolanda Sarno[1, 2], Angela Maria Savino[1]

*[1]University of Milano-Bicocca, 1School of Medicine and Surgery, Monza, Italy;[2]Fondazione IRCCS San Gerardo dei Tintori, Monza, Italy;*

### Background

Treatment of Acute Myeloid Leukaemia (AML) remains a clinical challenge. The combination of azacitidine and venetoclax (aza/ven) may improve treatment outcomes but still fails in a significant fraction of patients, with relapse occurring in the bone marrow (BM), as approximately 30–35% exhibit primary resistance and more than half of responders eventually relapse. The BM microenvironment protects leukemic cells from chemotherapy and has been extensively characterized over the past years, revealing complex crosstalk with niche components such as mesenchymal stromal cells, contributing significantly to chemoresistance. The bone marrow contains hypoxic regions, as extensively demonstrated by the Bonnet and Silberstein groups; however, how hypoxia drives metabolic adaptations that promote resistance to conventional therapies in the context of haematological malignancies remains incompletely understood. Moreover, the reduced apoptotic priming and the shift in apoptotic dependency in response to aza/ven suggest that engaging alternative cell death pathways, such as ferroptosis, may represent a promising strategy to overcome resistance.

### Aims

To elucidate hypoxia-driven therapy adaptations to identify metabolic vulnerabilities that can be therapeutically targeted.

### Methods

To model the hypoxic BM niche, AML cells were cultured under low oxygen conditions (2% $O_2$). Metabolic reprogramming was analysed using a 34-antibody CyTOF panel, complemented by Western blotting, RT-qPCR, and immunofluorescence. Drug sensitivity was assessed through dose–response curves. Hypoxia was assessed using pimonidazole staining.

### Results

By modelling hypoxia in vitro, we found that low oxygen promoted resistance to venetoclax and was also associated with increased resistance to two well-known ferroptosis inducers, RSL-3 and IKE, suggesting an adaptive enhancement of antioxidant defences. To identify potential mediators of this phenotype, we re-analysed two independent datasets from AML cell lines (HL-60 and MV4-11) cultured under hypoxia. Gene Set Enrichment Analysis identified cholesterol homeostasis as the top upregulated Hallmark gene set, alongside hypoxia, epithelial-to-mesenchymal transition, inflammatory response, and other metabolism-related pathways. In parallel, we applied a targeted single-cell CyTOF panel of metabolic markers showing that AML cells rapidly adapt to hypoxia by upregulating glycolytic pathways and redox-associated proteins, including G6PD, whose expression correlates with venetoclax resistance and increased NADPH-dependent redox capacity. Guided by both proteomic and transcriptomic data, we focused on the potential mechanism underlying increased ferroptosis resistance. GPX4 protein levels were reduced under hypoxia, indicating a shift from GPX4-dependent detoxification toward lipidomic reprogramming characterized by decreased ACSL4 and increased SCD1 (PUFA-to-MUFA switch), identifying SCD1 as a metabolic vulnerability. Functionally, treatment of hypoxic cells with the clinical-grade SCD1 inhibitor SSI-4 showed synergistic effects in combination with RSL-3, restoring ferroptosis sensitivity.

**Copyright Information:** (Online) ISSN: 2572-9241
©2026 The Author(s). HemaSphere is published by John Wiley & Sons Ltd on behalf of European Hematology Association. This is an open access Abstract Book distributed under the Attribution-NonCommercial-NoDerivs (CC BY-NC-ND), which allows third parties to download the articles and share them with others as long as they credit the author and the Abstract Book, but they cannot change the content in any way or use them commercially.
**Abstract Book Citations:** Authors, Title, HemaSphere, 2026;10:(S1):pages. The abstract book DOIs can be found on the “Author Information” page.
**Disclaimer:** Articles published in the journal HemaSphere exclusively reflect the opinions of the authors. The authors are responsible for all content in their abstracts including accuracy of the facts, statements, citing resources, etc.

**Summary/Conclusion**

Overall, hypoxia drives coordinated metabolic and redox adaptations that promote drug resistance in AML. These findings suggest that specific hypoxic regions in the BM could represent protective niches for AML blasts against pro-apoptotic and ferroptotic drugs, and that targeting specific pathways (G6PD and SCD1 inhibitors) could represent a strategy to resensitize them to therapy.

**Copyright Information:** (Online) ISSN: 2572-9241
©2026 The Author(s). HemaSphere is published by John Wiley & Sons Ltd on behalf of European Hematology Association. This is an open access Abstract Book distributed under the Attribution-NonCommercial-NoDerivs (CC BY-NC-ND), which allows third parties to download the articles and share them with others as long as they credit the author and the Abstract Book, but they cannot change the content in any way or use them commercially.
**Abstract Book Citations:** Authors, Title, HemaSphere, 2026;10:(S1):pages. The abstract book DOIs can be found on the "Author Information" page.
**Disclaimer:** Articles published in the journal HemaSphere exclusively reflect the opinions of the authors. The authors are responsible for all content in their abstracts including accuracy of the facts, statements, citing resources, etc.

### (PF442) PIEZO1 AS A KEY REGULATOR OF HEMATOPOIETIC STEM CELL QUIESCENCE AND ACUTE MYELOID LEUKEMIA PROGRESSION

**Topic:** 03. Acute myeloid leukemia - Biology & translational research

Lucia de la Mata*[1], Miguel Ángel Navarro Aguadero[1], Miguel Angel Marugal[1], Pablo Blanco-Carlon[2], Alvaro Otero Sobrino[1], Cristina Aragoneses[1], Ricardo Sánchez[1], Miguel Gallardo[1], Joaquín Martínez-Lopez[1], Maria Velasco Estevez[2]

[1]*Instituto de Investigación Sanitaria Hospital 12 de Octubre (Imas12), Madrid, Spain;*[2]*Cnio - Centro Nacional de Investigaciones Oncologicas, Madrid, Spain;*

### Background

Relapse in acute myeloid leukemia (AML) is largely driven by the persistence and expansion of leukemic stem cells (LSCs), a rare subpopulation characterized by self-renewal capacity, quiescence, and resistance to conventional therapies. LSCs share key functional properties with normal hematopoietic stem cells (HSCs), including long-term repopulation ability and stemness programs, which enable them to survive treatment and reinitiate disease. While molecular pathways regulating LSC biology have been extensively studied, the contribution of biophysical signals and mechanosensing mechanisms to LSC maintenance remains poorly understood. PIEZO1 is a mechanosensitive ion channel implicated in HSC regulation and myeloid expansion; however, its role in HSC and LSC biology as well as AML progression has not yet been explored

### Aims

To investigate the impact of Piezo1 overexpression on hematopoietic differentiation and its contribution to stemness and disease progression in acute myeloid leukemia (AML).

### Methods

We used two transgenic murine models with ubiquitous (Piezo1$^{Tg/UBCcreERT2}$) or hematopoietic-specific (Piezo1$^{Tg/VaV1\text{-}iCre}$) overexpression of Piezo1. Hematopoietic populations were analyzed by flow cytometry in the VaV1 model, and the HSCs were also cultured in methylcellulose to evaluate their proliferation and differentiation capacity under pharmacological regulation of Piezo1. *PIEZO1* expression in AML was analyzed using public datasets (BloodSpot, GEPIA2, MILE) and validated by qPCR in CD34$^{+}$ cells from both AML patients and healthy donors. In addition, we assessed the effects of Piezo1 overexpression in HL60 cells through RNAseq and corroborated changes in cell cycle progression and stemness by flow cytometry, qPCR and Western-Blot.

### Results

Piezo1$^{Tg/UBCcreERT2}$ mice showed significant hematological alterations, including expansion of immature hematopoietic compartments and increased myeloid progenitors, with 40% of animals developing leukemia. Conditional overexpression in HSCs resulted in enrichment of LSK, both ST-HSC and LT-HSC populations and myeloid progenitors, indicating impaired differentiation and enhanced stem-like maintenance in the Piezo1$^{Tg/VaV1\text{-}iCre}$ mice. Database analyses revealed increased *PIEZO1* expression across all AML subtypes, which was confirmed in our patient cohort from the Hospital 12 de Octubre (Madrid, Spain). In HL60 cells, Piezo1 overexpression promoted G0 cell cycle arrest and upregulation of stemness markers OCT3/4 and NANOG at both mRNA and protein levels

**Copyright Information:** (Online) ISSN: 2572-9241
©2026 The Author(s). HemaSphere is published by John Wiley & Sons Ltd on behalf of European Hematology Association. This is an open access Abstract Book distributed under the Attribution-NonCommercial-NoDerivs (CC BY-NC-ND), which allows third parties to download the articles and share them with others as long as they credit the author and the Abstract Book, but they cannot change the content in any way or use them commercially.
**Abstract Book Citations:** Authors, Title, HemaSphere, 2026;10:(S1):pages. The abstract book DOIs can be found on the "Author Information" page.
**Disclaimer:** Articles published in the journal HemaSphere exclusively reflect the opinions of the authors. The authors are responsible for all content in their abstracts including accuracy of the facts, statements, citing resources, etc.

### Summary/Conclusion

Piezo1 overexpression drives the accumulation of immature hematopoietic cells and myeloid progenitors *in vivo*, while promoting a stem-like, quiescent phenotype in AML cells. These findings identify Piezo1 as a key regulator of hematopoietic stemness and a potential contributor to leukemic progression

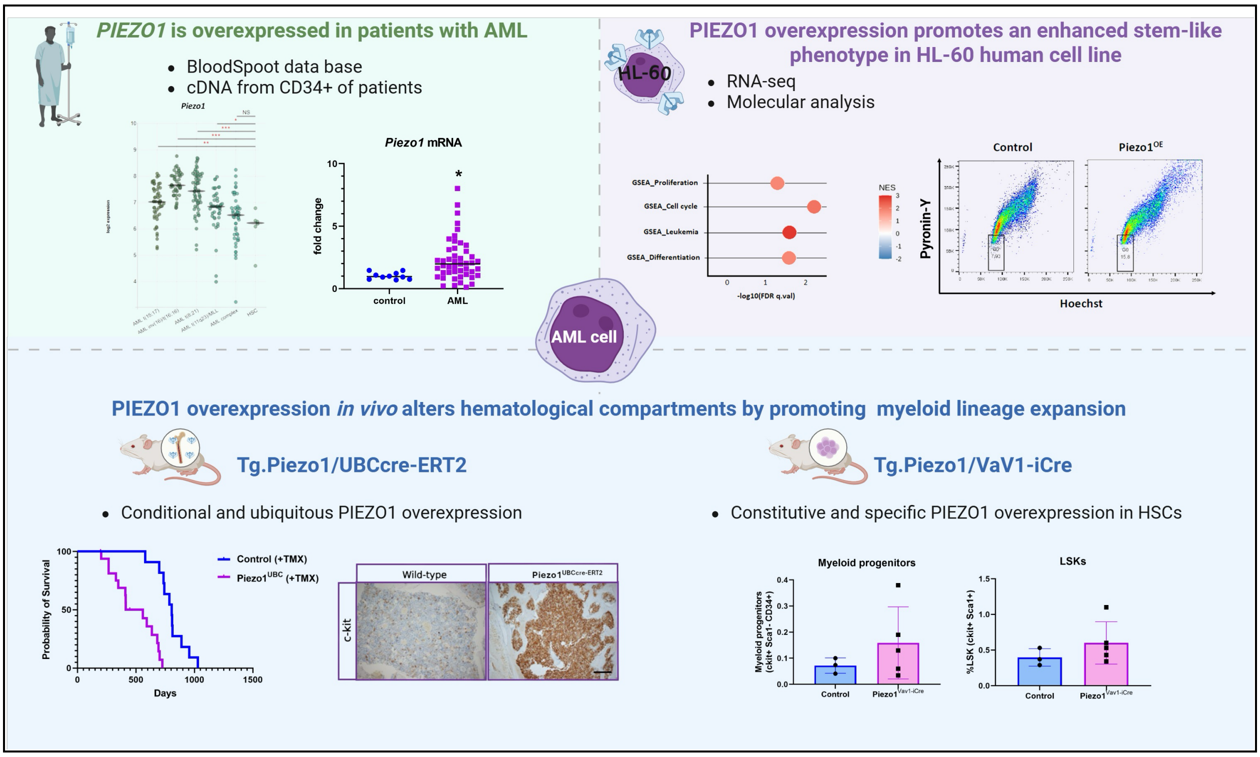

**Copyright Information:** (Online) ISSN: 2572-9241
©2026 The Author(s). HemaSphere is published by John Wiley & Sons Ltd on behalf of European Hematology Association. This is an open access Abstract Book distributed under the Attribution-NonCommercial-NoDerivs (CC BY-NC-ND), which allows third parties to download the articles and share them with others as long as they credit the author and the Abstract Book, but they cannot change the content in any way or use them commercially.
**Abstract Book Citations:** Authors, Title, HemaSphere, 2026;10:(S1):pages. The abstract book DOIs can be found on the "Author Information" page.
**Disclaimer:** Articles published in the journal HemaSphere exclusively reflect the opinions of the authors. The authors are responsible for all content in their abstracts including accuracy of the facts, statements, citing resources, etc.

### (PF443) LOOKING BEYOND VARIANT ALLELE FREQUENCY (VAF) FOR THE MONOALLELIC/BIALLELIC DETERMINATION OF TP53 MUTATIONS (MUTS) IN ACUTE MYELOID LEUKEMIA (AML) AND ASSOCIATIONS WITH OTHER GENOMIC ABERRATIONS

**Topic:** 03. Acute myeloid leukemia - Biology & translational research

Jonathan Gerber*[1], Russell Madison[2], Candice Francheska Tambaoan[2], Caleb Ho[2], Gerald Li[2], Bernard Fendler[2], Dexter Jin[2], Lauren Scarpetti[3, 4], Shyam Patel[1], Noah Littmann[1], Karl Simin[1], Richard Huang[2], Amaya Gasco[2], Lincoln Pasquina[2]

*[1]University of Massachusetts Chan Medical School, Worcester, United States of America;[2]Foundation Medicine, Inc., Boston, United States of America; [3]Perlmutter Cancer Center, NYU Langone Health, New York, United States of America;[4]NYU Grossman School of Medicine, New York, United States of America;*

### Background

*TP53*-mutant AML represents a distinct genomic subtype associated with adverse risk per ELN criteria and diminished therapeutic efficacy. Variant allele frequency (VAF) with zygosity may further risk-stratify patients (pts) with *TP53*-mutant AML.

### Aims

Here, we explore the landscape of *TP53* muts in a large genomic database to elucidate mechanisms of *TP53* inactivation in AML.

### Methods

DNA- and RNA-based next generation sequencing results from bone marrow or blood were analyzed from 2916 unique pts with AML profiled with FoundationOne Heme, a clinical laboratory developed test. Tumor purity, the fraction of DNA from blasts in the sample, was derived from aneuploidy in the copy number (CN) profile and minor allele frequencies of ~10,000 loci. Zygosity was determined using the somatic, germline, zygosity (SGZ) algorithm as previously described (Sun *et al* PMID 29415044) incorporating coverage ratios, CN model parameters, and VAFs. Muts with VAF less than 10% of the specimen purity were considered subclonal. Loss of heterozygosity (LOH) via deviation from median CN was defined as unbalanced copy number loss (uBL). LOH with no deviation from median CN (copy neutral) was defined as balanced loss (BL). Specimens with chromosomal aberrations (CA) had significant CA (SCA) for: 3+ distinct CA or rearrangements (RE); 2+ uBL; or 1 uBL with co-occurring RE.

**Copyright Information:** (Online) ISSN: 2572-9241
©2026 The Author(s). HemaSphere is published by John Wiley & Sons Ltd on behalf of European Hematology Association. This is an open access Abstract Book distributed under the Attribution-NonCommercial-NoDerivs (CC BY-NC-ND), which allows third parties to download the articles and share them with others as long as they credit the author and the Abstract Book, but they cannot change the content in any way or use them commercially.
**Abstract Book Citations:** Authors, Title, HemaSphere, 2026;10:(S1):pages. The abstract book DOIs can be found on the “Author Information” page.
**Disclaimer:** Articles published in the journal HemaSphere exclusively reflect the opinions of the authors. The authors are responsible for all content in their abstracts including accuracy of the facts, statements, citing resources, etc.

### Results

*TP53* alterations (alts) were detected in 465 (16%) specimens, mostly coding muts (98%, 578/588 total variants). See Figure for alt class frequency, homozygous (hmz) vs heterozygous (het) status and clonality. Median VAF for *TP53* hmz muts was 67% (IQR: 46-83%). 31% (72/235) had VAF <50%. Conversely, het muts had a median VAF of 39% (IQR: 26-45%). 97% (144/149) had VAF <50%.

The *TP53* locus was also assessed for CN change. For samples with hmz muts, uBL of *TP53* was observed in 156/221 (71%) specimens and BL occurred in 65 (29%). SCA was most prevalent in cases with hmz *TP53* muts (87%), followed by cases with multiple het muts (83%), then single het mut (50%), and rarely in cases with only subclonal (5%) muts. For cases with hmz muts, SCA was observed at similar rates across all quantiles of max VAF (<25%: 16/18=89%; 25-50%: 36/41=88%; >50%: 141/162=87%). In cases with multiple het muts SCA was more frequently observed in samples with a higher max *TP53* VAF (<25%: 8/14=57%; ≥25: 44/51=86%) and similarly in those with a single het mut (<25%: 3/9=33%; ≥25: 8/13=62%).

We assessed known actionable alts for co-occurrence with *TP53* muts. *NPM1* muts were rarely observed in specimens with either hmz uBL, hmz BL or multiple het muts (1/284) but occurred at similar rates to wild-type specimens (17%), specimens with a single het (18%), or subclonal mut (16%). *FLT3* (33%) and *IDH2* (33%) muts and *KMT2A* RE (11%) often co-occurred with subclonal muts but were seen less frequently vs. wild-type in all other *TP53* subgroups.

### Summary/Conclusion

In AML, *TP53* muts are most frequently hmz, but het and subclonal muts are observed. While higher VAF was associated with hmz muts, 31% of biallelic inactivations had VAF <50%, demonstrating that VAF alone inadequately captures zygosity. Three distinct mechanisms of biallelic inactivation of *TP53* (uBL, BL, multi-hit) were observed, all of which were associated with adverse cytogenetic features and low rates of co-occurring actionable alts. These findings demonstrate that integrated analysis of zygosity, LOH, and CN is essential for accurate *TP53* risk stratification, with direct implications on transplant decions.

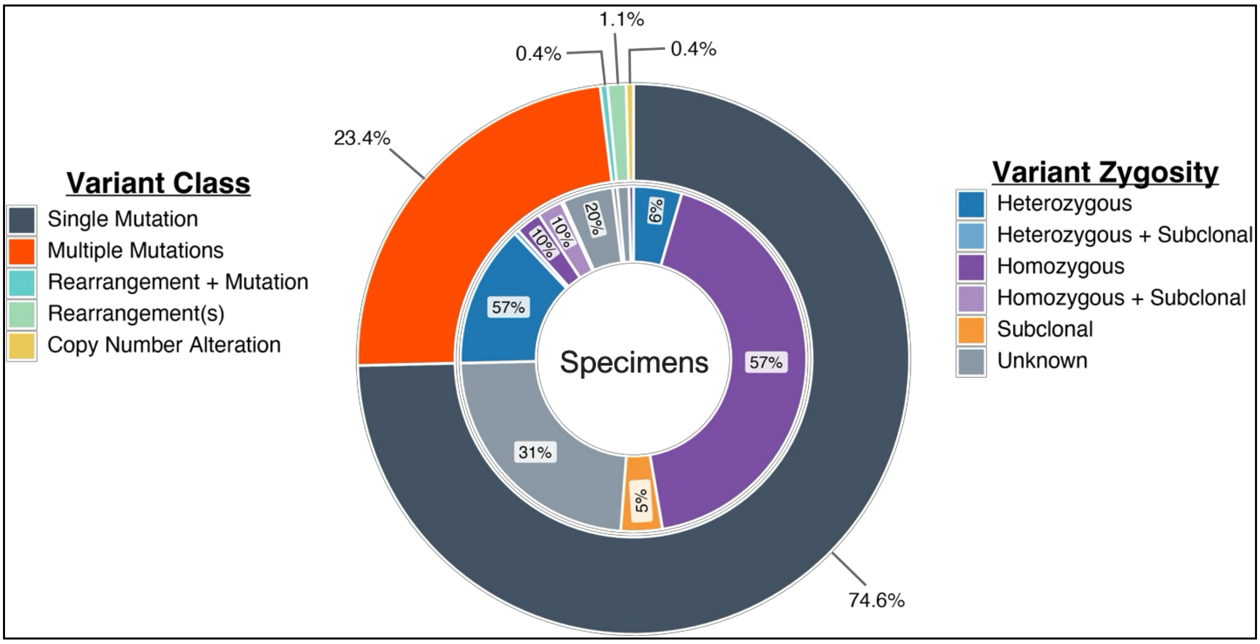

**Copyright Information:** (Online) ISSN: 2572-9241
©2026 The Author(s). HemaSphere is published by John Wiley & Sons Ltd on behalf of European Hematology Association. This is an open access Abstract Book distributed under the Attribution-NonCommercial-NoDerivs (CC BY-NC-ND), which allows third parties to download the articles and share them with others as long as they credit the author and the Abstract Book, but they cannot change the content in any way or use them commercially.
**Abstract Book Citations:** Authors, Title, HemaSphere, 2026;10:(S1):pages. The abstract book DOIs can be found on the "Author Information" page.
**Disclaimer:** Articles published in the journal HemaSphere exclusively reflect the opinions of the authors. The authors are responsible for all content in their abstracts including accuracy of the facts, statements, citing resources, etc.

### (PF444) VISTA SUPPORTS PROLIFERATION AND MAINTENANCE OF ACUTE MYELOID LEUKEMIA STEM CELLS IN A CELL-INTRINSIC MANNER

**Topic:** 03. Acute myeloid leukemia - Biology & translational research

Lukas Bähler*[1, 2, 3], Noah Schnüriger[1, 2], Hugo Legenne[1, 2, 3], Adrian Ochsenbein[1, 2], Carsten Riether[1, 2]

*[1]Inselspital, Department of Medical Oncology, Bern, Switzerland; [2]University of Bern, Department for Biomedical Research, Bern, Switzerland; [3]Graduate School of Cellular and Biomedical Sciences, University of Bern, Bern, Switzerland;*

### Background

Acute myeloid leukemia (AML) is a group of hematological malignancies driven by chemotherapy-resistant leukemia stem cells (LSC) which frequently cause relapse. V-domain Ig suppressor of T cell activation (VISTA) is a negative immune checkpoint predominantly expressed on myeloid cells. Although its immunoregulatory functions have already been characterized in AML, its cell-intrinsic role in the regulation of AML LSCs remains incompletely understood.

### Aims

We aimed to define VISTA expression patterns in AML, assess its association with clinical outcome, and determine its immune-independent functional role in LSCs and blasts.

### Methods

VISTA expression was analyzed by flow cytometry of primary AML samples and by interrogation of publicly available TCGA and single-cell RNA sequencing datasets (GSE116256, GSE239721). Soluble VISTA levels were measured in patient sera by ELISA and correlated with patient survival. VISTA knockdown cell lines were generated using shRNA. Proliferation was assessed in vitro, and in vivo following xenograft transplantation into NSGS mice. Therapeutic targeting was evaluated using an anti-VISTA blocking monoclonal antibody in colony-forming assays with patient-derived xenograft (PDX) cells.

### Results

Bulk RNA sequencing of AML bone marrow revealed VISTA expression, with higher levels predicting inferior survival. To focus our investigation on LSCs, we analyzed single-cell RNA sequencing data of AML bone marrow (n = 20) and found VISTA to be expressed by LSCs, where elevated expression again showed a trend towards worse survival (p = 0.059). Furthermore, high serum levels of soluble VISTA, which is likely a proxy for VISTA signaling activity, were also correlated to adverse outcome. Flow cytometry confirmed VISTA expression in around two-thirds of AML samples, whereas VISTA was not detectable in healthy hematopoietic stem cells (HSCs). Functionally, VISTA knockdown in THP-1 and HEL cells significantly impaired their proliferation in vitro, an effect which was further amplified when injected into NSGS mice, which lack a functional immune system, indicating a cell–intrinsic role of VISTA. Moreover, antibody-mediated VISTA blockade reduced the colony-forming capacity of PDX cells in vitro without affecting HSCs.

**Copyright Information:** (Online) ISSN: 2572-9241
©2026 The Author(s). HemaSphere is published by John Wiley & Sons Ltd on behalf of European Hematology Association. This is an open access Abstract Book distributed under the Attribution-NonCommercial-NoDerivs (CC BY-NC-ND), which allows third parties to download the articles and share them with others as long as they credit the author and the Abstract Book, but they cannot change the content in any way or use them commercially.
**Abstract Book Citations:** Authors, Title, HemaSphere, 2026;10:(S1):pages. The abstract book DOIs can be found on the “Author Information” page.
**Disclaimer:** Articles published in the journal HemaSphere exclusively reflect the opinions of the authors. The authors are responsible for all content in their abstracts including accuracy of the facts, statements, citing resources, etc.

**Summary/Conclusion**

These findings demonstrate that VISTA promotes leukemic cell proliferation and maintenance in a cell-intrinsic manner. VISTA is selectively expressed on LSCs compared to HSCs, and its targeting preferentially disrupts LSCs while sparing healthy hematopoiesis. Furthermore, we plan to investigate the cellular mechanisms of VISTA signaling and to define molecular subtypes of AML that could benefit from VISTA blockade. Together with its established immunomodulatory function, these findings position VISTA as an attractive dual-mechanism therapeutic target in AML.

**Copyright Information:** (Online) ISSN: 2572-9241
©2026 The Author(s). HemaSphere is published by John Wiley & Sons Ltd on behalf of European Hematology Association. This is an open access Abstract Book distributed under the Attribution-NonCommercial-NoDerivs (CC BY-NC-ND), which allows third parties to download the articles and share them with others as long as they credit the author and the Abstract Book, but they cannot change the content in any way or use them commercially.
**Abstract Book Citations:** Authors, Title, HemaSphere, 2026;10:(S1):pages. The abstract book DOIs can be found on the “Author Information” page.
**Disclaimer:** Articles published in the journal HemaSphere exclusively reflect the opinions of the authors. The authors are responsible for all content in their abstracts including accuracy of the facts, statements, citing resources, etc.

### (PF445) TARGETING ACLY AUGMENTS VENETOCLAX SENSITIVITY IN ACUTE MYELOID LEUKEMIA THROUGH FSP1-CASPASE3-GSDME AXIS

**Topic:** 03. Acute myeloid leukemia - Biology & translational research

Yingying Wu[1], Fangshu Liu[2], Xingcheng Liu[1], Yizhen Li[3], Hai Lan[4], Juan Du[1], Hui Zeng*[5]

*[1]The First Affiliated Hospital of Jinan University, Guangzhou, China;[2]Zhuzhou Hospital Affiliated to Xiangya School of Medicine, Zhuzhou, China; [3]Guangzhou Hospital of Integrated Traditional and Western Medicin, Guangzhou, China;[4]Shunde Affiliated Hospital of Guangzhou University of Chinese Medicine, Guangzhou, China; [5]Guangdong Provincial People's Hospital (Guangdong Academy of Medical Sciences), Southern Medical University, Guangzhou, China;*

### Background

Acute myeloid leukemia (AML) is a malignant hematologic neoplasm. Although treatment with the BCL-2 inhibitor venetoclax (VEN) has significantly improved clinical outcomes in patients with AML, relapse and primary resistance remain major bottlenecks limiting its long-term efficacy. Mounting evidence has demonstrated that metabolic reprogramming, particularly enhanced lipid metabolism, plays a critical role in mediating chemoresistance in tumor cells. ATP-citrate lyase (ACLY), a key enzyme linking glucose metabolism and lipid synthesis, has been shown in multiple solid tumors to enhance chemosensitivity upon inhibition. However, whether ACLY can serve as an effective target to augment VEN sensitivity in AML remains unclear.

### Aims

To investigate the effects of ACLY inhibition on the proliferation and survival of AML cells, evaluate the synergistic antileukemic effects of ACLY inhibitors in combination with venetoclax, and further elucidate the underlying molecular mechanisms.

### Methods

We used AML cell lines and primary AML cells derived from clinical patients as research objects and treated them with the ACLY-specific small molecule inhibitor NDI-091143 alone or in combination with VEN. The cytotoxic effects of the combined treatment on leukemic cells were evaluated by CCK-8 assay, Annexin VPI staining and LDH release assay. An xenograft mouse model derived from AML cell lines (CDX) was established to verify the anti-leukemic efficacy and safety of the combined treatment with NDI-091143 and VEN in vivo. The specific molecular mechanisms underlying cell death induced by the combined treatment were investigated by Western blot analysis, detection of reactive oxygen species, lipid peroxidation detection and malondialdehyde (MDA) content measurement. Furthermore, the critical roles of these molecules in this mechanism were confirmed by knockout of GSDME and Caspase-3, as well as overexpression of FSP1.

### Results

In AML patients, high ACLY expression is associated with shorter overall survival. In the BeatAML cohort, higher ACLY expression correlated with lower sensitivity to VEN. Inhibition of ACLY can suppress the proliferation of AML cells. Combination of the ACLY inhibitor NDI-091143 with VEN synergistically inhibits the growth of AML cell lines and patient-derived primary cells. Most importantly, the combined therapy exhibited anti-AML efficacy in vivo. And, this combination induces ferroptosis and pyroptosis in leukemia cells through the FSP1/Caspase-3/GSDME axis.

**Copyright Information:** (Online) ISSN: 2572-9241
©2026 The Author(s). HemaSphere is published by John Wiley & Sons Ltd on behalf of European Hematology Association. This is an open access Abstract Book distributed under the Attribution-NonCommercial-NoDerivs (CC BY-NC-ND), which allows third parties to download the articles and share them with others as long as they credit the author and the Abstract Book, but they cannot change the content in any way or use them commercially.
**Abstract Book Citations:** Authors, Title, HemaSphere, 2026;10:(S1):pages. The abstract book DOIs can be found on the "Author Information" page.
**Disclaimer:** Articles published in the journal HemaSphere exclusively reflect the opinions of the authors. The authors are responsible for all content in their abstracts including accuracy of the facts, statements, citing resources, etc.

### Summary/Conclusion

Our research results confirmed that ACLY is a metabolic vulnerability in AML cells, and this defect can effectively overcome venetoclax resistance, thus proposing a promising combined treatment strategy and expanding the understanding of the interactions between cell death pathways in the context of lipid peroxidation.

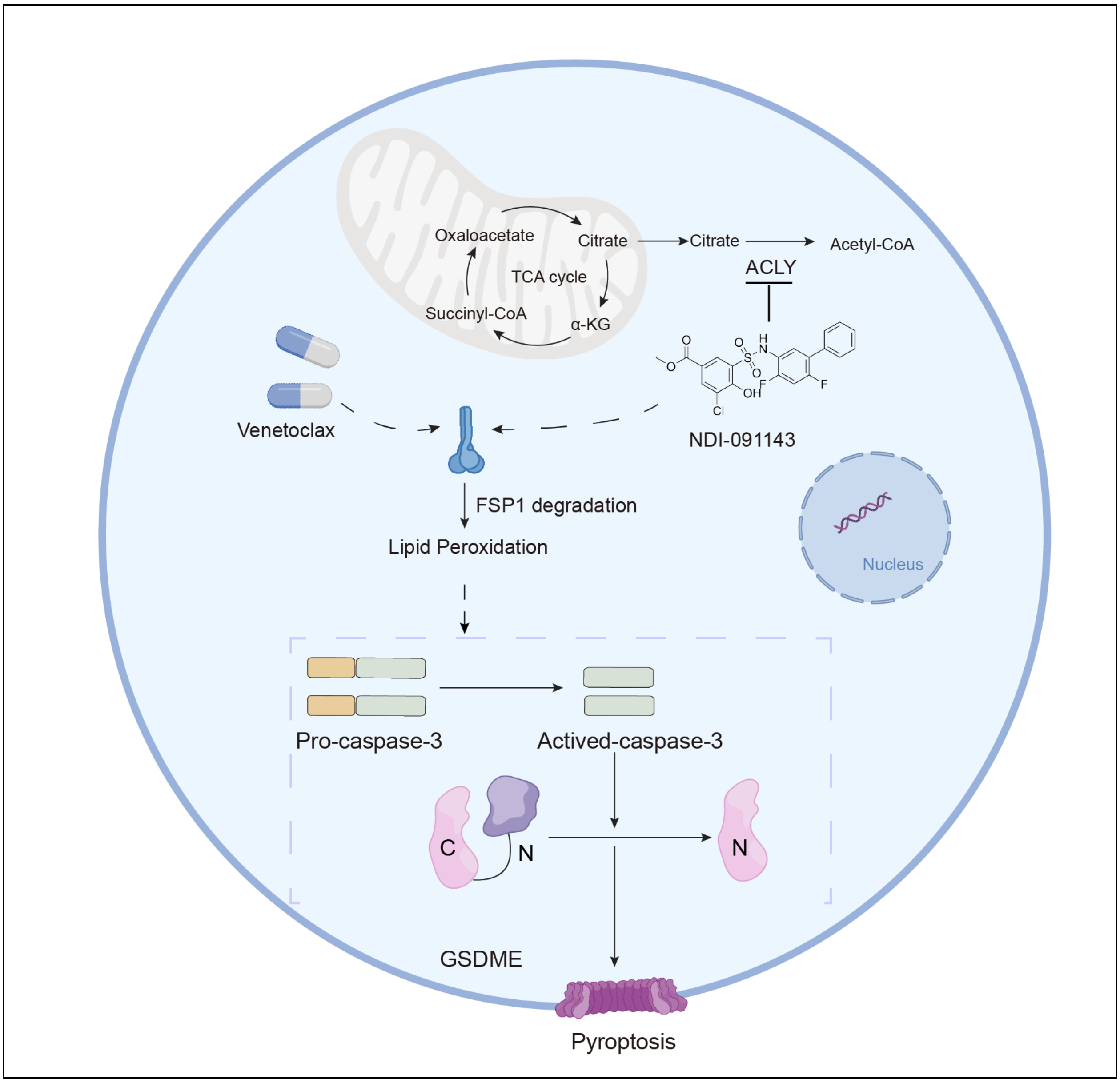

**Copyright Information:** (Online) ISSN: 2572-9241
©2026 The Author(s). HemaSphere is published by John Wiley & Sons Ltd on behalf of European Hematology Association. This is an open access Abstract Book distributed under the Attribution-NonCommercial-NoDerivs (CC BY-NC-ND), which allows third parties to download the articles and share them with others as long as they credit the author and the Abstract Book, but they cannot change the content in any way or use them commercially.
**Abstract Book Citations:** Authors, Title, HemaSphere, 2026;10:(S1):pages. The abstract book DOIs can be found on the "Author Information" page.
**Disclaimer:** Articles published in the journal HemaSphere exclusively reflect the opinions of the authors. The authors are responsible for all content in their abstracts including accuracy of the facts, statements, citing resources, etc.

### (PF447) NON-CANONICAL MITOCHONDRIAL FUNCTIONS OF TP53 R248 DRIVE LINEAGE-SPECIFIC METABOLIC VULNERABILITIES IN AML

**Topic:** 03. Acute myeloid leukemia - Biology & translational research

Livia Bassani Lins De Miranda*[1, 2], Diego A Pereira-Martins[1, 3], Dominique Sternadt[1], Isabel Weinhauser[1, 3], Luise Albuquerque-Simões[1], Rafael Andrade[1], Jesse van der Velde[1], Prodromos Chatzikyriakou[3], Gerwin Huls[1], João Agostinho MacHado Neto[2], Lynn Quek[3], Jan Jacob Schuringa[1]

*[1]University Medical Center Groningen, Department of Hematology, Groningen, The Netherlands;[2]Instituto de Ciências Biomédicas da Universidade de São Paulo, Department of Pharmacology, São Paulo, Brazil; [3]King's College London, Myeloid Leukaemia Genomics and Biology Group, School of Cancer and Pharmaceutical Sciences, London, United Kingdom;*

### Background

Clonal hematopoiesis (CH) and myeloid malignancies with *TP53* mutations (*TP53*mut), particularly at codon R248, are associated with therapy resistance and poor prognosis. We previously showed in CH that *TP53*mut cells, especially in *TP53*$^{R248}$, display higher oxidative stress and distinct metabolic features, suggesting mutation-specific metabolic rewiring may underlie their clonal fitness.

### Aims

To identify how *TP53*$^{R248}$ shapes metabolism across leukemic lineages and whether this creates actionable vulnerabilities.

### Methods

*TP53*$^{R248}$ prevalence was assessed in myeloid malignancies by analysing sequencing data from 17 studies of MDS, MPN and AML (14007 cases). We then investigated *TP53*$^{R248}$-associated metabolic adaptations using two AML models representing distinct hematopoietic compartments: MV4-11 (monocytic, homozygous R248W by clonal selection) and K562 (erythroid, heterozygous R248Q by gene editing) compared to controls retaining ≥1 *TP53*wt allele. To distinguish mutation specific from loss of function effects, we generated *TP53* knockdown MV4-11 cells (shTP53).

**Copyright Information:** (Online) ISSN: 2572-9241
©2026 The Author(s). HemaSphere is published by John Wiley & Sons Ltd on behalf of European Hematology Association. This is an open access Abstract Book distributed under the Attribution-NonCommercial-NoDerivs (CC BY-NC-ND), which allows third parties to download the articles and share them with others as long as they credit the author and the Abstract Book, but they cannot change the content in any way or use them commercially.
**Abstract Book Citations:** Authors, Title, HemaSphere, 2026;10:(S1):pages. The abstract book DOIs can be found on the "Author Information" page.
**Disclaimer:** Articles published in the journal HemaSphere exclusively reflect the opinions of the authors. The authors are responsible for all content in their abstracts including accuracy of the facts, statements, citing resources, etc.

**Results**

Among 14007 myeloid malignancy cases, 1307 harboured *TP53*mut, with 7% affecting R248. In both MV4-11 and K562 *TP53*$^{R248W/Q}$ models, western blot showed that MDM2 inhibition failed to induce canonical p53 targets p21 and PUMA, confirming non-canonical effects. *TP53*$^{R248}$ cells in both lineages exhibited enhanced proliferation and basal mitochondrial respiration relative to controls. However, downstream metabolic adaptation was strongly context dependent. In MV4-11 *TP53*$^{R248}$ cells, oxidative phosphorylation (OXPHOS) activation was accompanied by increased glycolytic reserve but modest reduction in spare respiratory capacity (SRC), indicating limited mitochondrial stress adaptability. K562 *TP53*$^{R248}$ cells, in turn, displayed higher SRC with modest glycolytic increase, suggesting more flexible mitochondrial adaptation. Notably, shTP53 did not phenocopy the R248 pattern. MV4-11 shTP53 cells showed increased basal respiration and SRC together with higher glycolytic reserve, indicating that metabolic rewiring in *TP53*$^{R248}$ mutants is not explained by *TP53* loss of function alone. Cellular fractionation revealed pronounced cytoplasmic accumulation of mutant *TP53*$^{R248}$ protein compared to controls. Co-immunoprecipitation of p53 identified mitochondrial complex V (ATP synthase) as an interaction partner in *TP53*$^{R248}$ mutants, supporting non-transcriptional role for *TP53*mut directly modulating mitochondrial function. To exploit this rewiring therapeutically, we performed metabolic inhibitor screening. In K562, *TP53*$^{R248}$ conferred increased resistance to glycolytic and OXPHOS inhibitors, but higher sensitivity to the GPX4 inhibitor RSL3 and the statin atorvastatin, suggesting that ferroptosis induction as a vulnerability. On the other hand, MV4-11 *TP53*$^{R248}$ cells showed broader sensitivity to mitochondrial inhibitors, atorvastatin and RSL3, which was not observed on shTP53 cells. This suggests lineage and possibly mutational co-occurrence dependent vulnerabilities (MV4-11, KMT2Ar vs K562, BCR-ABL1) despite shared OXPHOS activation.

**Summary/Conclusion**

*TP53*mut drives metabolic rewiring, characterized by increased mitochondrial respiration and non-transcriptional engagement of mitochondrial proteins. However, the balance between metabolic changes and drug responses is lineage-specific. Our data support context specific metabolic targeting in *TP53*mut malignancies and highlight a cytoplasmic, non-canonical role of mutant p53 in metabolic reprogramming.

**Copyright Information:** (Online) ISSN: 2572-9241
©2026 The Author(s). HemaSphere is published by John Wiley & Sons Ltd on behalf of European Hematology Association. This is an open access Abstract Book distributed under the Attribution-NonCommercial-NoDerivs (CC BY-NC-ND), which allows third parties to download the articles and share them with others as long as they credit the author and the Abstract Book, but they cannot change the content in any way or use them commercially.
**Abstract Book Citations:** Authors, Title, HemaSphere, 2026;10:(S1):pages. The abstract book DOIs can be found on the “Author Information” page.
**Disclaimer:** Articles published in the journal HemaSphere exclusively reflect the opinions of the authors. The authors are responsible for all content in their abstracts including accuracy of the facts, statements, citing resources, etc.

### (PF448) HIGH DIMENSIONAL SPECTRAL IMMUNE PROFILING REVEALS PROGNOSTIC AML SUBGROUPS AND A METABOLICALLY ACTIVE CD56+ MONOCYTE POPULATION LINKED TO POOR OUTCOME

**Topic:** 03. Acute myeloid leukemia - Biology & translational research

Nisha van der Meer[1], Shanna Hogeling*[1], Gerwin Huls[1], Diego A Pereira-Martins[1], Jan Jacob Schuringa[1]

[1] *UMCG, Experimental Hematology, Groningen, The Netherlands;*

### Background

Current treatment strategies for acute myeloid leukemia (AML) are often inadequate, in part because they largely ignore the tumor microenvironment. A better understanding of the dynamic immune landscape may enable more precise risk stratification and patient-specific therapies.

### Aims

Using multiparameter spectral flow cytometry–based immunophenotyping on primary AML samples, we aimed to define immunophenotypic clusters with prognostic value beyond classical risk stratification.

### Methods

We performed 40-colour spectral flow cytometry on 91 AML patients, quantifying 27 leukemic and immune populations by high-dimensional spectral gating. To link phenotypes to transcriptional programs, we carried out single-cell RNA-seq combined with surfaceome profiling (166 surface markers) on 15 AML samples (62657 cells).

### Results

Unsupervised clustering of the 27 populations identified five AML immune–leukemic clusters independently of mutational landscape: (1) patients with an M2-rich, monocytic-dominated microenvironment; (2) patients with an M1-rich, monocytic-dominated microenvironment; (3) GMP-like CD34$^+$ blast enriched samples; (4) patients enriched for adaptive immune cells; and (5) patients with increased LSC-like CD34$^+$ blasts. Considering only intensively treated patients, cluster 5 had the worst overall survival (OS), whereas cluster 4 had the best OS. Across all samples, high frequencies of LSC-like blasts and M2-like macrophages were associated with inferior clinical responses, with concomitant T-regulatory and exhausted T cells, indicative of impaired immune surveillance. In contrast, higher infiltration of early NK cells, CD8$^+$ memory T cells and gamma delta T cells correlated with superior clinical responses. In our proteogenomic studies combining the surfaceome with the scRNA transcriptome we identified novel AML-specific monocyte/macrophage subpopulations marked by differential expression of the adhesion protein CD56 (*NCAM1*) and the fatty acid importer CD36. High CD56 expression on monocytic cells was associated with inferior overall survival, independent of conventional M1/M2 immunophenotypic classification, suggesting that CD56 marks a particularly adverse monocyte state that refines risk beyond classical polarization. CD56$^{high}$ monocyte/macrophages were enriched for mitochondrial complex components (*MT-ND4*, *MT-CYTB*), ribosomal genes and E-cadherin-related regulators. This transcriptional profile indicates enhanced translational capacity, increased reliance on oxidative phosphorylation (OXPHOS) and augmented cell–cell interaction potential, features that are consistent with metabolically fitness. Patients with high expression of CD36 in monocyte/macrophages segregated into two distinct subtypes, whereby CD36$^{high}$/CD56$^{high}$ cells were linked to high OXPHOS states, potentially linked to fatty acid driven mitochondrial metabolism, while CD36$^{high}$/CD56$^{low}$ monocyte/macrophages were associated with inflammatory programs, previously associated with an M1 polarized state.

**Copyright Information:** (Online) ISSN: 2572-9241
©2026 The Author(s). HemaSphere is published by John Wiley & Sons Ltd on behalf of European Hematology Association. This is an open access Abstract Book distributed under the Attribution-NonCommercial-NoDerivs (CC BY-NC-ND), which allows third parties to download the articles and share them with others as long as they credit the author and the Abstract Book, but they cannot change the content in any way or use them commercially.
**Abstract Book Citations:** Authors, Title, HemaSphere, 2026;10:(S1):pages. The abstract book DOIs can be found on the "Author Information" page.
**Disclaimer:** Articles published in the journal HemaSphere exclusively reflect the opinions of the authors. The authors are responsible for all content in their abstracts including accuracy of the facts, statements, citing resources, etc.

**Summary/Conclusion**

Comprehensive immunophenotyping stratifies AML into distinct immune subtypes linked to clinical outcomes and metabolic programs, underscoring the value of incorporating the immune landscape into diagnostic workflows and treatment decision-making. These data suggest that targeting strategies not only aimed at leukemic blasts but also at metabolically rewired AML-specific monocytes/macrophage populations might further improve treatment strategies in AML.

**Copyright Information:** (Online) ISSN: 2572-9241
©2026 The Author(s). HemaSphere is published by John Wiley & Sons Ltd on behalf of European Hematology Association. This is an open access Abstract Book distributed under the Attribution-NonCommercial-NoDerivs (CC BY-NC-ND), which allows third parties to download the articles and share them with others as long as they credit the author and the Abstract Book, but they cannot change the content in any way or use them commercially.
**Abstract Book Citations:** Authors, Title, HemaSphere, 2026;10:(S1):pages. The abstract book DOIs can be found on the "Author Information" page.
**Disclaimer:** Articles published in the journal HemaSphere exclusively reflect the opinions of the authors. The authors are responsible for all content in their abstracts including accuracy of the facts, statements, citing resources, etc.

### (PF449) IMPROVED SINGLE-CELL LONG-READ GENOTYPING DEFINES CLONAL ARCHITECTURES, LINEAGE COMMITMENT AND TRANSCRIPTIONAL PROGRAMS IN SRSF2MUT AML

**Topic:** 03. Acute myeloid leukemia - Biology & translational research

Angelos Galanopoulos*[1, 2], Jonas Berger[1, 2], Priyanka Venkatesh[1], Kenneth Livak[3, 4], Catherine Wu[3], Felicitas Thol[5], Florian Heidel[5], Jana Ihlow[6, 7], Jörg Westermann[1], Lars Bullinger[1], Livius Penter[1, 7]

*[1]Charité – Universitätsmedizin Berlin, Department of Hematology, Oncology, and Cancer Immunology, Berlin, Germany;[2]Freie Universität Berlin, Department of Biology, Chemistry and Pharmacy, Berlin, Germany; [3]Dana-Farber Cancer Institute, Department of Medical Oncology, Boston, United States of America; [4]Dana-Farber Cancer Institute, Translational Immunogenomics Laboratory, Boston, United States of America; [5]Hannover Medical School, Department of Hematology, Hemostasis, Oncology and Stem Cell Transplantation, Hannover, Germany; [6]Charité - Universitätsmedizin Berlin, Institute of Pathology, Berlin, Germany; [7]Berlin Institute of Health, Berlin Institute of Health Charité Digital Clinician Scientist Program, Berlin Institute of Health Biomedical Innovation Academy, Berlin, Germany;*

### Background

*SRSF2*$^{mut}$ AML carries poor prognosis and frequent relapse, yet its differentiation states and lineage-specific transcriptional programs remain incompletely defined. Linking genotype to phenotype at single-cell resolution is essential to dissect clonal architecture, malignant transformation, and therapy resistance. However, reliable identification of leukemic cells in scRNA-seq data remains challenging, even with targeted long-read genotyping. We hypothesized that systematic incorporation of patient-specific private mutations as leukemic barcodes would markedly improve malignant cell annotation and clonal resolution.

### Aims

We streamlined and scaled our scRNA-seq genotyping workflow (*nanoranger v2*) to characterize the cellular and transcriptional landscape of *SRSF2*$^{mut}$ AML.

### Methods

Somatic mutations were selected by whole-exome sequencing based on expression at 5′ transcript ends in hematopoietic cells. Target transcripts were amplified in a 2-step PCR followed by Oxford Nanopore sequencing. Genotypes were integrated with scRNA-seq profiles to assign mutations to individual cells and transcriptional states.

**Copyright Information:** (Online) ISSN: 2572-9241
©2026 The Author(s). HemaSphere is published by John Wiley & Sons Ltd on behalf of European Hematology Association. This is an open access Abstract Book distributed under the Attribution-NonCommercial-NoDerivs (CC BY-NC-ND), which allows third parties to download the articles and share them with others as long as they credit the author and the Abstract Book, but they cannot change the content in any way or use them commercially.
**Abstract Book Citations:** Authors, Title, HemaSphere, 2026;10:(S1):pages. The abstract book DOIs can be found on the “Author Information” page.
**Disclaimer:** Articles published in the journal HemaSphere exclusively reflect the opinions of the authors. The authors are responsible for all content in their abstracts including accuracy of the facts, statements, citing resources, etc.

### Results

The *nanoranger v2* workflow replaces RNase H-dependent PCR with TaqMan-based preamplification for simultaneous interrogation of large mutation panels. Genotyping efficiency was on par with the original protocol ( *r*=0.74, p<0.001), while substantially reducing cost and technical complexity.

We profiled 17 *SRSF2*$^{mut}$ AML cases at diagnosis, identifying a median of 9 (range 7-16) genotypable mutations per case and obtaining genotyping information in 71% (range 59-92%) of cells. To define clonal architectures, we analyzed single-cell co-mutational status, which revealed that 94.1% of private mutations were acquired after *SRSF2*$^{mut}$ and could therefore serve as robust leukemic markers. We found pre-leukemic clonal expansion in only 2 AML cases, where *STAG2*$^{S377fs}$ or *CBL*$^{END386del}$ frequently occurred in *SRSF2*$^{wt}$ cells. None of the AML-associated mutations were mutually exclusive, consistent with linear evolution. In sum, *SRSF2*$^{mut}$ is an early driver of secondary AML, which is dominated by highly selected leukemic clones.

We mapped differentiation hierarchies and demonstrated extensive AML involvement of progenitor compartments, with HSC, GMP and MEP populations being 81% (61-95%), 76% (65-96%) and 73% (66-93%) leukemic. In 75% of cases, full differentiation potential along myeloid and erythroid lineages was retained, which were 69% (50-96%) and 74% (6-93%) leukemic. Lymphoid progenitors were also frequently leukemia-derived (76%, 65-93%), yet differentiation into mature lymphocytes was heterogeneous across T (10%, 3-30%), NK (38%, 0-77%), or B cells (22%, 0-92%). Together, residual non-leukemic hematopoiesis is highly lineage-specific, despite near-total leukemic involvement of hematopoietic progenitors.

Finally, we defined a leukemic transcriptional signature based on 24,879 *SRSF2*$^{mut}$ vs. 11,321 *SRSF2*$^{wt}$ cells, revealing enrichment for core spliceosome components, DNA replication, cell cycle regulators, and chromatin-associated genes, consistent with enhanced RNA processing, proliferative capacity, and replicative stress. This signature was consistently higher in *SRSF2*$^{mut}$ cells across patients, indicating a conserved, transcriptional program.

### Summary/Conclusion

We define single-cell differentiation hierarchies and conserved transcriptional programs of *SRSF2*$^{mut}$ AML, and establish a framework for interrogating leukemic evolution, relapse biology, and therapeutic resistance at scale.

**Copyright Information:** (Online) ISSN: 2572-9241
©2026 The Author(s). HemaSphere is published by John Wiley & Sons Ltd on behalf of European Hematology Association. This is an open access Abstract Book distributed under the Attribution-NonCommercial-NoDerivs (CC BY-NC-ND), which allows third parties to download the articles and share them with others as long as they credit the author and the Abstract Book, but they cannot change the content in any way or use them commercially.
**Abstract Book Citations:** Authors, Title, HemaSphere, 2026;10:(S1):pages. The abstract book DOIs can be found on the "Author Information" page.
**Disclaimer:** Articles published in the journal HemaSphere exclusively reflect the opinions of the authors. The authors are responsible for all content in their abstracts including accuracy of the facts, statements, citing resources, etc.

### (PF450) MOLECULAR AND CLINICAL CHARACTERISTICS OF HEMATOLOGIC MALIGNANCIES HARBORING NUP98 REARRANGEMENTS

**Topic:** 03. Acute myeloid leukemia - Biology & translational research

Bingqing Huang*[1], Yujiao Jia[1], Haoxu Wang[1], Yiran Cui[1], Li Qin[1], Hui Wei[1], Xiaofan Zhu[1], Jianxiang Wang[1], Qi Sun[1], Yuxuan Liu[1], Zhijian Xiao[1], Lihong Shi[1]

*[1]State Key Laboratory of Experimental Hematology, National Clinical Research Center for Blood Diseases, Haihe Laboratory of Cell Ecosystem, Institute of Hematology & Blood Diseases Hospital, Chinese Academy of Medical Sciences & Peking Union Medical Colleg, Tianjin, China;*

**Background**

Translocations involving nucleoporin 98 (NUP98) and over 40 fusion partner genes are well-characterized drivers of hematologic malignancies and have long been associated with poor prognosis. Although progress has been made in understanding the molecular mechanisms underlying NUP98 translocations, the literature primarily focuses on pediatric AML, leaving a gap in understanding their clinical significance in adult AML cohorts. Here, we analyzed the molecular and clinical characteristics of 153 NUP98 fusion-positive patients (including 108 adults).

**Aims**

This study aims to elucidate the molecular subtypes of NUP98-rearranged hematologic malignancies. By utilizing whole-transcriptome and DNA-seq data, we sought to identify potential therapeutic targets for each subtype, thereby providing a theoretical foundation for the precision treatment of patients with NUP98-rearranged hematologic malignancies.

**Methods**

All patients underwent routine testing and risk assessment, including analysis of age, WBC count, cellular morphological and pathology, cytogenetic, immunophenotyping, measurable residual disease (MRD), targeted DNA sequencing (364 genes), and whole-transcriptome sequencing (WTS) or targeted-transcriptome sequencing.

**Copyright Information:** (Online) ISSN: 2572-9241
©2026 The Author(s). HemaSphere is published by John Wiley & Sons Ltd on behalf of European Hematology Association. This is an open access Abstract Book distributed under the Attribution-NonCommercial-NoDerivs (CC BY-NC-ND), which allows third parties to download the articles and share them with others as long as they credit the author and the Abstract Book, but they cannot change the content in any way or use them commercially.
**Abstract Book Citations:** Authors, Title, HemaSphere, 2026;10:(S1):pages. The abstract book DOIs can be found on the “Author Information” page.
**Disclaimer:** Articles published in the journal HemaSphere exclusively reflect the opinions of the authors. The authors are responsible for all content in their abstracts including accuracy of the facts, statements, citing resources, etc.

**Results**

RNA-seq analysis identified 153 patients with NUP98 fusions involving 24 partner genes. Among these, 19 were known partners (e.g., NSD1, HOXA9, RAP1GDS1, PRRX2, DDX10), and 5 were novel (BRWD3, HOXA7, MLLT1, WT1, ZNF215). Abnormal karyotypes were present in 50.1% of patients with NUP98 rearrangements, primarily in non-NSD1 cases. Male predominance was observed in NUP98::NSD1-positive patients (62.5% vs. 37.5%; $p<0.05$). Breakpoints in NUP98 were predominantly in intron 12 (113/153), preserving the N-terminal FG-rich domain.
The most common co-mutated genes in NUP98 rearrangement-positive patients were FLT3 (56.0%) and WT1 (41.3%), particularly in the NUP98::NSD1 group (FLT3: 65.8%, $p<0.001$; WT1: 51.9%, $p<0.01$). CEBPA mutations occurred almost exclusively in the NUP98::NSD1 group (19.1% vs. 3%, $p<0.01$). After one cycle of conventional chemotherapy, the complete remission rate was significantly lower in the NUP98::NSD1 group than in the non-NSD1 group (60.3% vs. 73.7%, $p<0.05$). Transcriptomic data revealed that NUP98::NSD1-positive patients specifically exhibited high expression of the HOXB family, whereas NUP98::HOXA9-positive patients specifically exhibited high expression of the HOXA family. Among NUP98::NSD1-positive patients, two distinct groups with high and low expression of FLT3 and WT1 were identified, and the expression levels of FLT3 and WT1 were significantly higher in the group with FLT3 or WT1 gene mutations compared to the non-mutated group. Additionally, all NUP98::NSD1-positive patients showed high expression of PRDM16, but high expression of CCNA1 was observed only in the NUP98::NSD1-positive subgroup with high FLT3 and WT1 expression. NUP98::HOXA9-positive patients specifically exhibited high expression of MECOM.

**Summary/Conclusion**

Our data indicate that NUP98 rearrangements confer a poor prognosis, particularly in NUP98::NSD1-positive patients, attributable to distinct mutational spectra and gene expression profile compared to non-NSD1 group. These specific target genes (CCNA1 and MECOM) represent potential therapeutic targets for each respective patient group.

**Copyright Information:** (Online) ISSN: 2572-9241
©2026 The Author(s). HemaSphere is published by John Wiley & Sons Ltd on behalf of European Hematology Association. This is an open access Abstract Book distributed under the Attribution-NonCommercial-NoDerivs (CC BY-NC-ND), which allows third parties to download the articles and share them with others as long as they credit the author and the Abstract Book, but they cannot change the content in any way or use them commercially.
**Abstract Book Citations:** Authors, Title, HemaSphere, 2026;10:(S1):pages. The abstract book DOIs can be found on the "Author Information" page.
**Disclaimer:** Articles published in the journal HemaSphere exclusively reflect the opinions of the authors. The authors are responsible for all content in their abstracts including accuracy of the facts, statements, citing resources, etc.

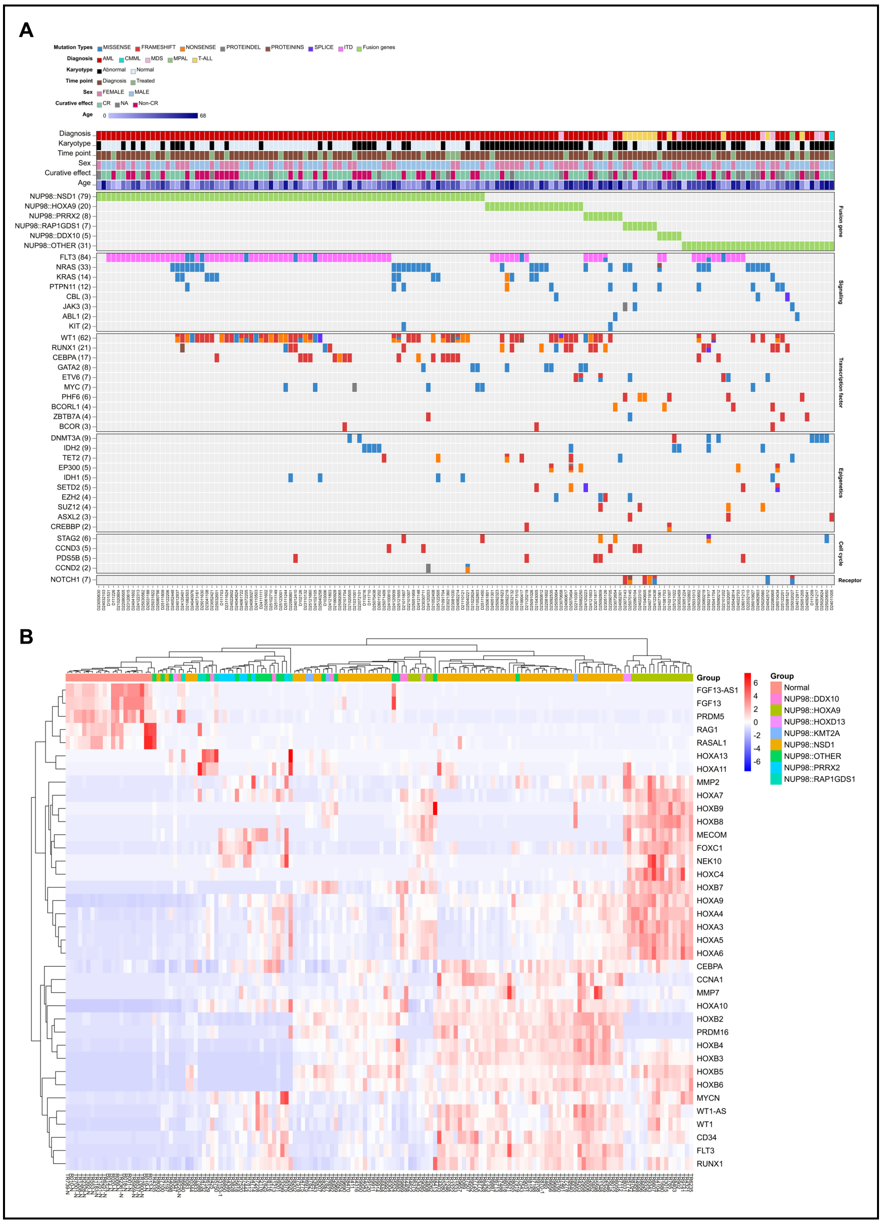

**Copyright Information:** (Online) ISSN: 2572-9241
©2026 The Author(s). HemaSphere is published by John Wiley & Sons Ltd on behalf of European Hematology Association. This is an open access Abstract Book distributed under the Attribution-NonCommercial-NoDerivs (CC BY-NC-ND), which allows third parties to download the articles and share them with others as long as they credit the author and the Abstract Book, but they cannot change the content in any way or use them commercially.
**Abstract Book Citations:** Authors, Title, HemaSphere, 2026;10:(S1):pages. The abstract book DOIs can be found on the "Author Information" page.
**Disclaimer:** Articles published in the journal HemaSphere exclusively reflect the opinions of the authors. The authors are responsible for all content in their abstracts including accuracy of the facts, statements, citing resources, etc.

### (PF451) LIPID METABOLIC REWIRING DRIVES VENETOCLAX RESISTANCE IN ACUTE MYELOID LEUKEMIABEYOND BCL2 DEPENDENCY

**Topic:** 03. Acute myeloid leukemia - Biology & translational research

Federico De Marchi[1], Giulia Ciotti*[1], Michele Gottardi[2]

*[1]Istituto Oncologico veneto (IOV), Oncohematology, Castelfranco Veneto, Italy;[2]Istituto Oncologico veneto (IOV), Castelfranco Veneto, Italy;*

### Background

Venetoclax-based regimens have improved outcomes in acute myeloid leukemia (AML); however, primary resistance remains frequent, particularly in monocytic subtypes. Resistance is classically attributed to altered apoptotic priming, while the contribution of upstream metabolic adaptations remains incompletely understood.

### Aims

To systematically identify lipid metabolic programs associated with venetoclax resistance in AML and define their relationship with monocytic differentiation.

### Methods

RNA-sequencing data from 300 adult AML patients from the BeatAML cohort with matched ex vivo venetoclax sensitivity (area under the curve, AUC) were analyzed. A curated panel of 33 genes regulating lipid uptake, fatty acid activation, mitochondrial import, β-oxidation, lipid biosynthesis, and apoptosis was examined. Associations with drug response were assessed using Pearson correlation and cross-validated Random Forest machine learning. Samples were stratified by venetoclax sensitivity and monocytic status (FAB M4/M5).

### Results

Machine learning identified lipid metabolic genes as key predictors of venetoclax resistance, exceeding canonical apoptotic regulators including BCL2. Low-density lipoprotein receptor–related protein 1 (LRP1) emerged as the strongest single predictor of resistance and showed the most robust correlation with venetoclax AUC ($R = 0.68$, $p < 1 \times 10^{-40}$), exceeding the direct drug target BCL2 ($R = -0.54$, $p < 1 \times 10^{-23}$). Resistant AML samples demonstrated coordinated upregulation of lipid uptake receptors (LRP1; CD36, $R = 0.46$, $p < 1 \times 10^{-16}$) and fatty acid activation enzymes (ACSL1, $R = 0.52$, $p < 1 \times 10^{-22}$; ACSL4, $R = 0.35$, $p < 1 \times 10^{-9}$). In contrast, genes governing de novo lipid synthesis (FASN, $R = -0.35$) and mitochondrial fatty acid oxidation (CPT1A, $R = -0.45$) were significantly downregulated. This configuration defines a distinct lipid "metabolic scavenging" phenotype characterized by accumulation and activation of exogenous lipids without engagement of mitochondrial oxidation. Canonical ferroptosis defense genes (GPX4, SLC7A11) showed no association with venetoclax response. This lipid scavenging signature was significantly enriched in monocytic AML (FAB M4/M5) but was also present in a subset of non-monocytic resistant cases.

**Copyright Information:** (Online) ISSN: 2572-9241
©2026 The Author(s). HemaSphere is published by John Wiley & Sons Ltd on behalf of European Hematology Association. This is an open access Abstract Book distributed under the Attribution-NonCommercial-NoDerivs (CC BY-NC-ND), which allows third parties to download the articles and share them with others as long as they credit the author and the Abstract Book, but they cannot change the content in any way or use them commercially.
**Abstract Book Citations:** Authors, Title, HemaSphere, 2026;10:(S1):pages. The abstract book DOIs can be found on the "Author Information" page.
**Disclaimer:** Articles published in the journal HemaSphere exclusively reflect the opinions of the authors. The authors are responsible for all content in their abstracts including accuracy of the facts, statements, citing resources, etc.

**Summary/Conclusion**

Venetoclax resistance in AML is characterized by an LRP1-dominated lipid scavenging program marked by increased lipid uptake and activation alongside suppressed biosynthesis and mitochondrial oxidation. This metabolic rewiring extends resistance mechanisms beyond apoptotic priming and suggests a state of lipid dependency associated with potential redox vulnerability. Ongoing studies are evaluating therapeutic strategies aimed at disrupting this lipid scavenging dependency in venetoclax-resistant AML.

**Copyright Information:** (Online) ISSN: 2572-9241
©2026 The Author(s). HemaSphere is published by John Wiley & Sons Ltd on behalf of European Hematology Association. This is an open access Abstract Book distributed under the Attribution-NonCommercial-NoDerivs (CC BY-NC-ND), which allows third parties to download the articles and share them with others as long as they credit the author and the Abstract Book, but they cannot change the content in any way or use them commercially.
**Abstract Book Citations:** Authors, Title, HemaSphere, 2026;10:(S1):pages. The abstract book DOIs can be found on the "Author Information" page.
**Disclaimer:** Articles published in the journal HemaSphere exclusively reflect the opinions of the authors. The authors are responsible for all content in their abstracts including accuracy of the facts, statements, citing resources, etc.

### (PF452) DISTINCT CLINICAL AND MOLECULAR PROFILES OF AML DEFINED BY MUTATIONS IN IDH1, IDH2 AND TET2

**Topic:** 03. Acute myeloid leukemia - Biology & translational research

Fanny Asbaghi*[1], Erik Håkansson[2], Vladimir Lazarevic[2], Gunnar Juliusson[2], Ravindra Majeti[3], Niklas Landberg[1]

*[1]Lund University, Department of Laboratory Medicine - Division of Clinical Genetics, Lund, Sweden;[2]Skåne University Hospital, Department of Hematology, Oncology and Radiation Physics, Lund, Sweden; [3]Institute for Stem Cell Biology and Regenerative Medicine, Stanford, United States of America;*

**Background**

Acute myeloid leukaemia (AML) is a heterogenous disease driven by recurrent somatic mutations, often in epigenetic regulators. Mutations in *IDH1, IDH2*, and *TET2* are thought to converge on a shared pathway by disrupting TET-mediated DNA demethylation, either directly or indirectly through (R)-2-hydroxyglutarate (2-HG). Whether this biochemical convergence translates into similar disease biology remains unclear.

**Aims**

To compare clinical and molecular differences between *IDH1, IDH2* and *TET2*-mutated AML patients, by integrating population-based data from the Swedish AML Registry with transcriptomic and 5-hydroxymethylcytosine (5hmC) profiling of primary AML blasts.

**Methods**

Clinical data on AML with genetic information available were extracted from the Swedish AML registry. Survival was compared in multivariate Cox regression analyses. The mRNA and 5hmC-directed pull down sequencing were performed on FACS isolated AML blasts from twelve highly selected patient samples. Bioinformatic analyses were performed using R (v.4.5.1), DEseq2 (v.1.49.4), and Gene Set Enrichment Analysis using MSigDB Hallmark gene sets.

**Copyright Information:** (Online) ISSN: 2572-9241
©2026 The Author(s). HemaSphere is published by John Wiley & Sons Ltd on behalf of European Hematology Association. This is an open access Abstract Book distributed under the Attribution-NonCommercial-NoDerivs (CC BY-NC-ND), which allows third parties to download the articles and share them with others as long as they credit the author and the Abstract Book, but they cannot change the content in any way or use them commercially.
**Abstract Book Citations:** Authors, Title, HemaSphere, 2026;10:(S1):pages. The abstract book DOIs can be found on the "Author Information" page.
**Disclaimer:** Articles published in the journal HemaSphere exclusively reflect the opinions of the authors. The authors are responsible for all content in their abstracts including accuracy of the facts, statements, citing resources, etc.

### Results

Among 2,471 AML patients with genetic data available, 204 had *IDH1* (8%), 392 *IDH2* (16%), and 548 *TET2* (22%) mutations, which were largely mutually exclusive. Samples were compared to AML without these mutations (*Other*, n=1327, 53%). *TET2*-mutated AML patients were significantly older (median 73 compared to 68–71 years) and had a more pronounced thrombocytopenia ($57\times10^9$/L compared to $93\times10^9$/L), while *IDH1/IDH2*-mutated patients showed more pronounced neutropenia (0.3–$0.7\times10^9$/L vs. $2.4\times10^9$/L). Co-mutations differed significantly between groups, *DNMT3A* was more frequent in the *IDH1/IDH2*-group compared to *TET2* and *Other* (40-45% compared to 21-27%). SRSF2 mutations were most frequent in *IDH2*-mutant AML (34%) but prevalent in *TET2*-mutant (27%) and *IDH1*-mutant (19%) AML, compared to 9% in *Other*. *TP53*-mutations were rare in all three groups compared to Other AML (5-8% compared to 22.8%). *IDH1/IDH2*-mutations were associated with significantly better overall survival compared to *TET2*-mutated AML, both in univariate Kaplan-Meier analysis and in multivariate Cox regression adjusted for age, treatment intensity and stem cell transplantation (Figure 1A–B).
Transcriptomic profiling revealed pronounced heterogeneity across samples, but direct comparison between *IDH1/IDH2*- and *TET2*-mutated AML showed transcriptional differences with 434 upregulated and 762 downregulated genes with enrichment of inflammatory and metabolic pathways in *TET2*-mutant cases. 5hmC pull-down sequencing revealed distinct epigenetic profiles between mutational subgroups (Figure 1C), with clear separation of the *IDH1/IDH2* and *TET2*-groups and overall reduced 5hmC levels in all three groups compared to *Other* AML. Pathway-level differences were observed with TNF-α signaling and apoptosis enriched in *IDH1/IDH2*, and oxidative phosphorylation enriched in *TET2*-mutant samples. Integrated analysis of RNA and 5hmC revealed a modest correlation ($\rho = 0.269$, $p < 0.001$), with 150 genes showing concordant regulation in both modalities (Figure 1D).

### Summary/Conclusion

Despite a shared link to disrupted DNA de-methylation, AML with mutations in *IDH1, IDH2,* or *TET2* represents biologically and clinically distinct subgroups, with substantial uncoupling between 5hmC and transcription, underscoring the importance of mutation-based disease stratification in AML.

**Copyright Information:** (Online) ISSN: 2572-9241
©2026 The Author(s). HemaSphere is published by John Wiley & Sons Ltd on behalf of European Hematology Association. This is an open access Abstract Book distributed under the Attribution-NonCommercial-NoDerivs (CC BY-NC-ND), which allows third parties to download the articles and share them with others as long as they credit the author and the Abstract Book, but they cannot change the content in any way or use them commercially.
**Abstract Book Citations:** Authors, Title, HemaSphere, 2026;10:(S1):pages. The abstract book DOIs can be found on the “Author Information” page.
**Disclaimer:** Articles published in the journal HemaSphere exclusively reflect the opinions of the authors. The authors are responsible for all content in their abstracts including accuracy of the facts, statements, citing resources, etc.

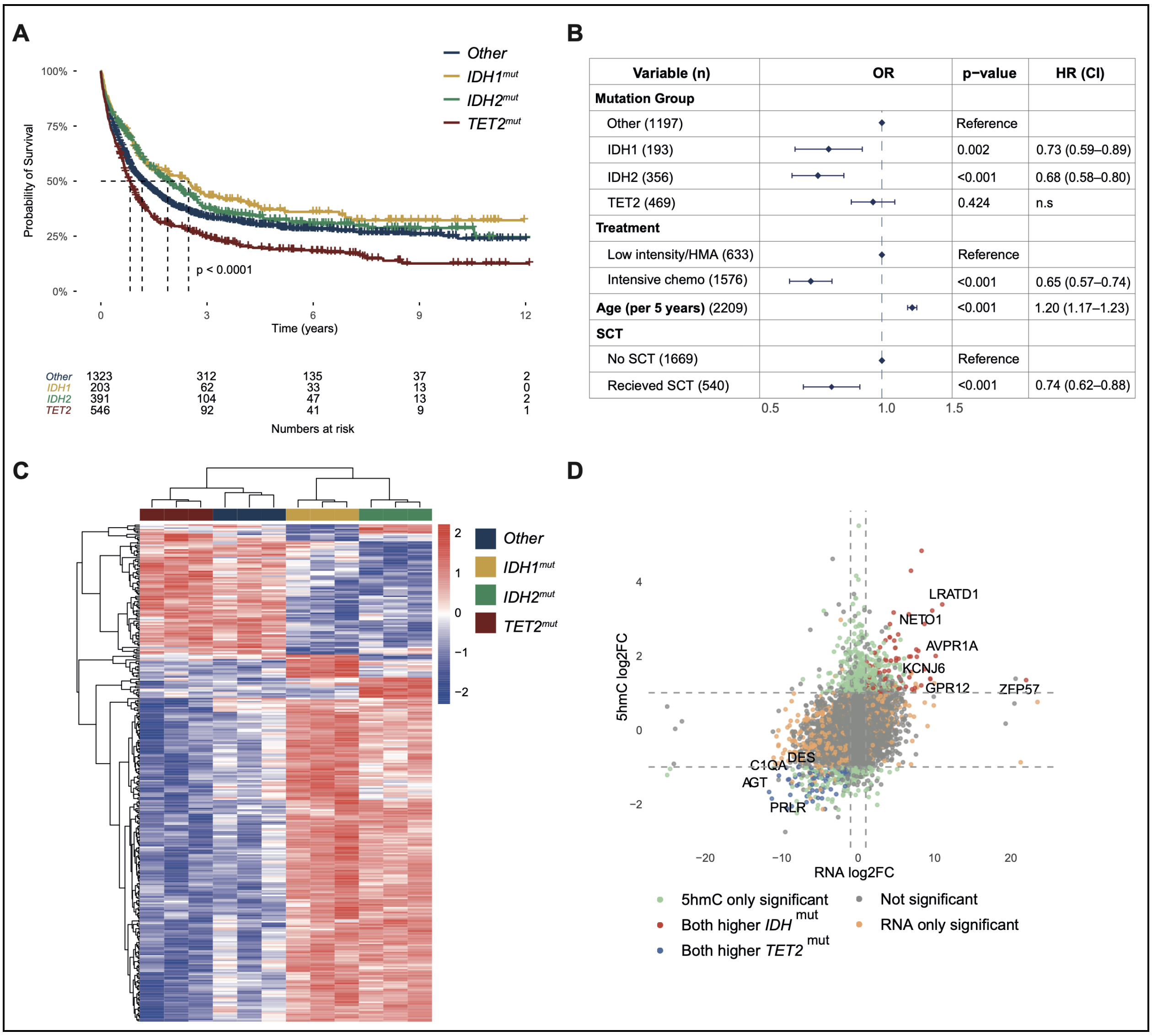

Figure 1. A) Kaplan-Meier survival curves show significant differences in overall survival between mutational groups. B) Multivariate Cox regression for overall survival, adjusting for age (5-year increments), treatment intensity, and stem cell transplantation. C) Heatmap of genes with the most significantly different 5hmC-levels based on multi-group ANOVA (4 groups IDH1, IDH2, TET2, and Other; FDR < 0.10). D) Scatter plot comparing differential gene expression and 5hmC enrichment by mutational groups.

**Copyright Information:** (Online) ISSN: 2572-9241
©2026 The Author(s). HemaSphere is published by John Wiley & Sons Ltd on behalf of European Hematology Association. This is an open access Abstract Book distributed under the Attribution-NonCommercial-NoDerivs (CC BY-NC-ND), which allows third parties to download the articles and share them with others as long as they credit the author and the Abstract Book, but they cannot change the content in any way or use them commercially.
**Abstract Book Citations:** Authors, Title, HemaSphere, 2026;10:(S1):pages. The abstract book DOIs can be found on the “Author Information” page.
**Disclaimer:** Articles published in the journal HemaSphere exclusively reflect the opinions of the authors. The authors are responsible for all content in their abstracts including accuracy of the facts, statements, citing resources, etc.

### (PF453) SINGLE-CELL PROFILING OF CSF1R EXPRESSION AND M-CSF/IL-34 SIGNALING IN ACUTE MYELOID LEUKEMIA (AML)

**Topic:** 03. Acute myeloid leukemia - Biology & translational research

Oriol Castells*[1, 2], Sturla Grøndal[1, 2, 3], Kristine Sletta[2], Tara Helén Dowling[2], Reidun-Kristin Kopperud[2], Monica Hellesøy[3], Jørn Skavland[1], Vibeke Andresen[1, 2], Sonia Gavasso[4], Gerwin Huls[5], Jan Jacob Schuringa[5], Stein-Erik Gullaksen[1, 2, 3], Bjørn T. Gjertsen[1, 2, 3]

*[1]CCBIO, Centre for Cancer Biomarkers, Department of Clinical Science, University of Bergen, Bergen, Norway;[2]K.G. Jebsen Centre for Myeloid Blood Cancer, Department of Clinical Science, University of Bergen, Bergen, Norway; [3]Department of Internal Medicine, Hematology section, Haukeland University Hospital, Bergen, Norway; [4]Neuro-SysMed Centre of Clinical Treatment Research, Department of Clinical Science, University of Bergen, Bergen, Norway; [5]Department of Hematology, Cancer Research Centre Groningen, University Medical Center Groningen, University of Groningen, Groningen, The Netherlands;*

**Background**

The colony-stimulating factor 1 receptor (CSF1R) on tumor-supportive monocyte-derived cells has been suggested as a key mediator of AML protective effects. However, because AML arises from myeloid progenitors that phenotypically overlap with healthy counterparts, CSF1R expression alone is difficult to interpret. We hypothesize that combining M-CSF/IL-34 stimulation with antibody-based detection could provide a functional readout of CSF1R activity across both leukemic and healthy myeloid populations.

**Aims**

To examine single-cell M-CSF/IL-34 signaling profiling together with CSF1R mapping to functionally identify CSF1R-expressing cells.

**Methods**

For the present study, we used patient samples from the HOVON 135 phase II clinical trial (EudraCT: 2015-002855-85), which evaluated the hypomethylating agent decitabine (± ibrutinib) in older patients with AML unfit for standard chemotherapy. A single-cell mass cytometry workflow interrogated CSF1R signaling across cryopreserved peripheral blood (PB) and bone marrow (BM) mononuclear samples from 117 patients enrolled in the HOVON-135 trial, alongside healthy donor controls (6 PB and 7 BM). Cells were stimulated in vitro with 7 stimulants and profiled using a 43-parameter panel that captured surface phenotype and intracellular signaling responses (Figure A). Focusing on the myeloid compartment alone in the pretreatment samples, encompassing 45 million cells, we integrated baseline and stimulated measurements to map CSF1R expression and downstream phosphorylation dynamics across differentiation states and disease contexts (Figure B).

**Copyright Information:** (Online) ISSN: 2572-9241
©2026 The Author(s). HemaSphere is published by John Wiley & Sons Ltd on behalf of European Hematology Association. This is an open access Abstract Book distributed under the Attribution-NonCommercial-NoDerivs (CC BY-NC-ND), which allows third parties to download the articles and share them with others as long as they credit the author and the Abstract Book, but they cannot change the content in any way or use them commercially.
**Abstract Book Citations:** Authors, Title, HemaSphere, 2026;10:(S1):pages. The abstract book DOIs can be found on the “Author Information” page.
**Disclaimer:** Articles published in the journal HemaSphere exclusively reflect the opinions of the authors. The authors are responsible for all content in their abstracts including accuracy of the facts, statements, citing resources, etc.

**Results**

High-resolution immune profiling of pretreatment samples identified 18 myeloid populations (45 million single-cell events), ranging from the most immature (HSPC-like, CD45loCD34+CD38-CD117+) to the most differentiated (classical monocytes, CD45+CD14+CD64+). Our results show heterogeneous CSF1R expression across the myeloid lineage, with clear upregulation in AML compared to healthy individuals in both immature and mature populations. Interestingly, several AML-specific differences were restricted to the bone marrow-derived cells, suggesting microenvironment-dependent upregulation of this receptor. Moreover, M-CSF stimulation linked CSF1R expression to pS6 signaling in HSPC-like cells from the bone marrow (CD45loCD34+CD38-CD117+, Figure C), linking receptor abundance to downstream signaling capacity. Ongoing analyses aim to determine whether functionally active CSF1R associates with genetic subgroups or clinical outcomes.

**Summary/Conclusion**

We generated a large-scale single-cell signaling map of AML that highlights substantial heterogeneity of CSF1R expression across myeloid differentiation states. CSF1R enrichment in specific AML populations, particularly within the bone marrow, suggests context-dependent regulation of this receptor. By integrating receptor expression with ligand-induced signaling responses, our data provide a functional view of CSF1R-associated signaling states in AML and offer a framework to investigate how microenvironmental cues shape leukemic cell behavior. These findings suggest that CSF1R-positive cells may represent a biologically distinct leukemic cellular subset, although further studies are required to clarify their developmental origins and potential relevance to therapeutic response and disease persistence.

**Copyright Information:** (Online) ISSN: 2572-9241
©2026 The Author(s). HemaSphere is published by John Wiley & Sons Ltd on behalf of European Hematology Association. This is an open access Abstract Book distributed under the Attribution-NonCommercial-NoDerivs (CC BY-NC-ND), which allows third parties to download the articles and share them with others as long as they credit the author and the Abstract Book, but they cannot change the content in any way or use them commercially.
**Abstract Book Citations:** Authors, Title, HemaSphere, 2026;10:(S1):pages. The abstract book DOIs can be found on the "Author Information" page.
**Disclaimer:** Articles published in the journal HemaSphere exclusively reflect the opinions of the authors. The authors are responsible for all content in their abstracts including accuracy of the facts, statements, citing resources, etc.

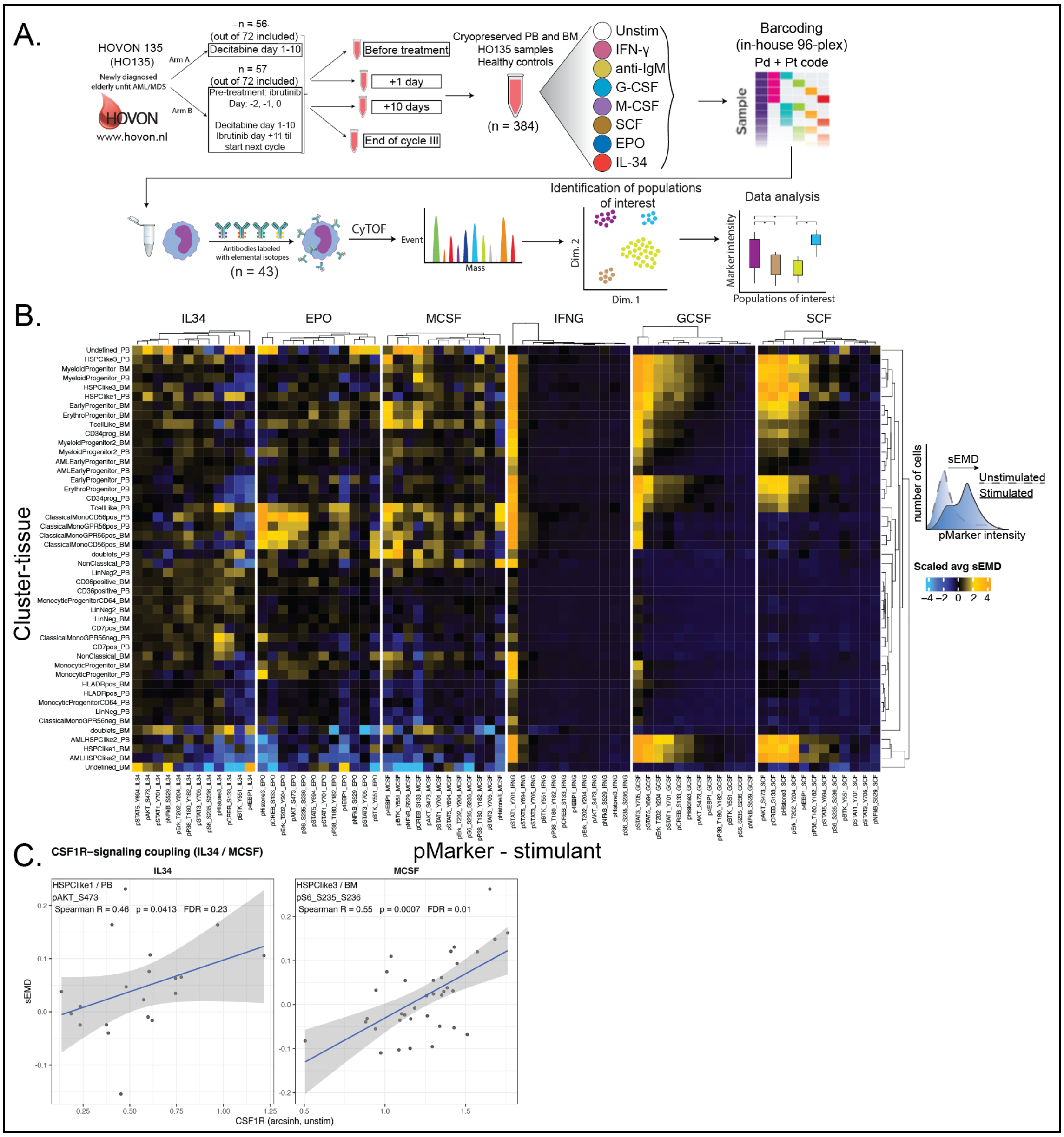

**Copyright Information:** (Online) ISSN: 2572-9241
©2026 The Author(s). HemaSphere is published by John Wiley & Sons Ltd on behalf of European Hematology Association. This is an open access Abstract Book distributed under the Attribution-NonCommercial-NoDerivs (CC BY-NC-ND), which allows third parties to download the articles and share them with others as long as they credit the author and the Abstract Book, but they cannot change the content in any way or use them commercially.
**Abstract Book Citations:** Authors, Title, HemaSphere, 2026;10:(S1):pages. The abstract book DOIs can be found on the "Author Information" page.
**Disclaimer:** Articles published in the journal HemaSphere exclusively reflect the opinions of the authors. The authors are responsible for all content in their abstracts including accuracy of the facts, statements, citing resources, etc.

### (PF454) INTEGRATED SINGLE-CELL PROFILING IDENTIFIES RELAPSE-ASSOCIATED CLONAL DYNAMICS AND REFINES MRD RISK STRATIFICATION IN AML

**Topic:** 03. Acute myeloid leukemia - Biology & translational research

Enise Ceran*[1], Anne Merbach[1], Saurabh Parikh[2], Adam Sciambi[2], Christian Rohde[1], Robert Durruthy-Durruthy[2], Zivjena Vucetic[2], Michelle Lotze[1], Sonia Jaramillo Segura[1], Richard Schlenk[1], Christoph Röllig[3], Lutz Peter Müller[4], Christian Junghanss[5], Claudia Baldus[6], Uwe Platzbecker[7], Hubert Serve[8], Martin Bornhauser[9], Carsten Müller-Tidow[1]

*[1]Heidelberg University Hospital, Department of Internal Medicine V, Heidelberg, Germany; [2]Mission Bio, South San Francisco, United States of America; [3]University Hospital Carl Gustav Carus Dresden, Department of Internal Medicine I, Dresden, Germany; [4]Martin-Luther-Universität Halle-Wittenberg, Department of Internal Medicine IV, Halle (Saale), Germany; [5]University Medicine Rostock, Clinic and Polyclinic for Hematology, Hemostaseology, Oncology, Stem Cell Therapy and Palliative Care, Rostock, Germany; [6]Universitätsklinikum Schleswig-Holstein Campus Kiel, Kiel, Germany; [7]University Leipzig Medical Center, Department of Hematology, Cellular Therapy, Hemostaseology and Infectious Diseases, Leipzig, Germany; [8]Goethe-University Frankfurt, University Hospital, Department of Medicine, Hematology/Oncology, Frankfurt am Main, Germany; [9]National Center for Tumor Diseases (NCT/UCC) Dresden, Dresden, Germany;*

### Background

Although measurable residual disease (MRD) is a major prognostic factor in AML, current MRD strategies do not resolve the clonal architecture or phenotypic state of residual leukemic populations. Single-cell multiomic profiling enables direct genotype-phenotype coupling and may uncover relapse-driving subclones that remain undetected by conventional approaches.

### Aims

The aim of this study was to determine whether integrated single-cell DNA and protein profiling improves MRD-based relapse prediction in AML.

### Methods

We analyzed bone marrow samples from 43 patients after induction chemotherapy enrolled in the BLAST trial (NCT02502968) using targeted single-cell DNA and surface protein profiling (Mission Bio Tapestri). Clones were defined by pathogenic variants. A pathogenicity score was developed integrating the number of pathogenic clones with a high-risk protein signature (CD34+ phenotype, monocytic differentiation, absence from mature T-cell compartments, immune activation markers, and treatment-dependent CXCR4 expression). Relapse-free survival (RFS) was assessed using ROC analysis and Kaplan-Meier estimates. Phylogenetic trees were reconstructed for 10 paired screening-relapse samples.

### Results

The integrated DNA-protein pathogenicity score robustly predicted relapse. In the overall cohort, the score predicted RFS with an area under the curve (AUC) of 0.75; patients above the optimized threshold demonstrated markedly inferior RFS (HR 6.9, $p<0.01$). In the placebo arm (n=21), discrimination further improved (AUC 0.77), with high-pathogenicity patients exhibiting inferior RFS (HR 8.9, $p<0.01$). Phylogenetic analyses revealed branched evolution with selective expansion of high-pathogenicity subclones at relapse, indicating that relapse originates from biologically distinct residual populations identifiable at single-cell resolution.

**Copyright Information:** (Online) ISSN: 2572-9241
©2026 The Author(s). HemaSphere is published by John Wiley & Sons Ltd on behalf of European Hematology Association. This is an open access Abstract Book distributed under the Attribution-NonCommercial-NoDerivs (CC BY-NC-ND), which allows third parties to download the articles and share them with others as long as they credit the author and the Abstract Book, but they cannot change the content in any way or use them commercially.
**Abstract Book Citations:** Authors, Title, HemaSphere, 2026;10:(S1):pages. The abstract book DOIs can be found on the "Author Information" page.
**Disclaimer:** Articles published in the journal HemaSphere exclusively reflect the opinions of the authors. The authors are responsible for all content in their abstracts including accuracy of the facts, statements, citing resources, etc.

**Summary/Conclusion**

In this AML cohort, single-cell integration of clonal architecture and immunophenotypic context refines MRD assessment and enables biologically informed risk stratification for relapse. These findings support further validation of multiomic approaches to improve AML risk assessment.

**Copyright Information:** (Online) ISSN: 2572-9241
©2026 The Author(s). HemaSphere is published by John Wiley & Sons Ltd on behalf of European Hematology Association. This is an open access Abstract Book distributed under the Attribution-NonCommercial-NoDerivs (CC BY-NC-ND), which allows third parties to download the articles and share them with others as long as they credit the author and the Abstract Book, but they cannot change the content in any way or use them commercially.
**Abstract Book Citations:** Authors, Title, HemaSphere, 2026;10:(S1):pages. The abstract book DOIs can be found on the "Author Information" page.
**Disclaimer:** Articles published in the journal HemaSphere exclusively reflect the opinions of the authors. The authors are responsible for all content in their abstracts including accuracy of the facts, statements, citing resources, etc.

### (PF455) CD36 AS A POTENTIAL MARKER AND THERAPEUTIC TARGET OF LEUKEMIC STEM CELLS IN ACUTE MYELOID LEUKEMIA

**Topic:** 03. Acute myeloid leukemia - Biology & translational research

Narges Aghaallaei*[1, 2], Daniel Ivanov[1, 2], Nadine Witzeneder[1, 2], Karin Bauer[1, 2], Katharina Slavnitsch[1, 3], Karoline Gleixner[2], Maik Dahlhoff[3], Peter Bettelheim[4], Wolfgang Sperr[1, 2], Peter Valent[1, 2]

*[1]Ludwig Boltzmann Institute for Hematology and Oncology, Vienna, Austria; [2]Medical University of Vienna, Department of Internal Medicine I, Vienna, Austria; [3]University of Veterinary Medicine, Department of Biological Sciences and Pathobiology, Laboratory Animal Medicine, Vienna, Austria; [4]Ordensklinikum Linz, Elisabethinen, Department of Hematology, Linz, Austria;*

**Background**

A major focus of current AML research is the eradication of leukemic stem cells (LSC), a small population of AML cells with *in vivo* leukemia-initiating and sustained self-renewal capacity. AML LSC typically reside in $CD34^+/CD38^-$ and sometimes also in $CD34^+/CD38^+$ subfractions of the malignant clone. While several surface antigens are expressed on LSC, most of these targets are also present on normal hematopoietic stem cells (HSC). Therefore, the identification of more specific LSC-associated markers and targets is critical for the development of curative AML therapies. We identified CD36 as a surface marker expressed in LSC-containing AML subsets. CD36 is a lipid uptake receptor that has been implicated in AML evolution and drug resistance. These observations suggest that CD36 may represent a promising marker and therapeutic target in AML.

**Aims**

The aims of the present study were to explore expression of CD36 on neoplastic cells in various subsets of AML, to investigate the mechanisms contributing to the expression of CD36 in AML cells, and to examine whether CD36 can be developed as a novel LSC marker and target in AML.

**Methods**

Surface CD36 expression was analyzed in primary AML cells and AML-derived cell lines (U937, MOLM13, MV4-11, THP-1) by monoclonal antibodies and flow cytometry. The target pathways contributing to CD36 expression were examined by specific signal transduction inhibitors and other anti-neoplastic agents. The engraftment capacity of primary $CD36^+$ AML cells and $CD36^+$ U937 cells and the effects of the CD36-related lipid uptake inhibitor sulfosuccinimidyl oleate (SSO) on *in vivo* engraftment of AML cells was evaluated in NSGS mice.

**Copyright Information:** (Online) ISSN: 2572-9241
©2026 The Author(s). HemaSphere is published by John Wiley & Sons Ltd on behalf of European Hematology Association. This is an open access Abstract Book distributed under the Attribution-NonCommercial-NoDerivs (CC BY-NC-ND), which allows third parties to download the articles and share them with others as long as they credit the author and the Abstract Book, but they cannot change the content in any way or use them commercially.
**Abstract Book Citations:** Authors, Title, HemaSphere, 2026;10:(S1):pages. The abstract book DOIs can be found on the “Author Information” page.
**Disclaimer:** Articles published in the journal HemaSphere exclusively reflect the opinions of the authors. The authors are responsible for all content in their abstracts including accuracy of the facts, statements, citing resources, etc.

**Results**

CD36 was found to be expressed on $CD34^+/CD38^-$ and $CD34^+/CD38^+$ AML cells in various forms of AML, whereas normal HSC did not express CD36. Sorted CD36+ bone marrow (BM) mononuclear cells derived from 4 AML patients also engrafted in NSGS mice, confirming that AML LSC express CD36 (14.4±23.7 percent engraftment of human CD45+ cells). Furthermore, the CD36-related fatty acid uptake inhibitor sulfosuccinimidyl oleate (SSO) was found to induce apoptosis in primary $CD34^+/CD38^-$ AML LSC (percent of Annexin V/DAPI-positive $CD34^+/CD38^-$ cells, control [n=8]: 31.6±14%, versus SSO, 100 µM [n=8]: 67.6±21%, $p<0.05$). Correspondingly, purified (sorted) $CD36^+$ U937 cells successfully engrafted NSGS mice, and SSO treatment (1000 µM at 37°C for 1 hour) reduced engraftment compared to untreated controls (controls: 46.2±19.3% [n=13] versus SSO: 29.1±20.5% [n=14]). Moreover, blocking CD36-mediated fatty acid uptake by SSO significantly decreased the growth and survival of U937 cells. Interestingly, vitamin D also suppressed CD36 expression as well as growth in various AML cell lines including U937. We also found that the PI3 kinase blocker BEZ235 and the BRD4-targeting drugs JQ1 and dBET6 counteract CD36 expression in AML cells, indicating that CD36 is regulated by oncogenic signaling pathways.

**Summary/Conclusion**

CD36 is expressed in LSC-containing BM AML fractions ($CD34^+/CD38^-$ and $CD34^+/CD38^+$) but not in normal HSC. Drugs targeting PI3 kinase or BRD4/MYC and vitamin D suppressed the expression of CD36 in AML cells. The CD36-related fatty acid uptake blocker SSO reduced cell growth/survival in U937 cells and primary AML cells including LSC, and suppressed engraftment of U937 cells in NSGS mice. Whether CD36 can be developed far enough to reach clinical application in AML, remains to be determined.

**Copyright Information:** (Online) ISSN: 2572-9241
©2026 The Author(s). HemaSphere is published by John Wiley & Sons Ltd on behalf of European Hematology Association. This is an open access Abstract Book distributed under the Attribution-NonCommercial-NoDerivs (CC BY-NC-ND), which allows third parties to download the articles and share them with others as long as they credit the author and the Abstract Book, but they cannot change the content in any way or use them commercially.
**Abstract Book Citations:** Authors, Title, HemaSphere, 2026;10:(S1):pages. The abstract book DOIs can be found on the “Author Information” page.
**Disclaimer:** Articles published in the journal HemaSphere exclusively reflect the opinions of the authors. The authors are responsible for all content in their abstracts including accuracy of the facts, statements, citing resources, etc.

### (PF456) EZH2 MODULATES HELLS EXPRESSION THROUGH EPIGENETIC ENHANCER ACTIVATION IN ACUTE MYELOID LEUKEMIA

**Topic:** 03. Acute myeloid leukemia - Biology & translational research

Chan Yang[1], Chunhua Song[2], Zheng Ge*[1]

*[1]Department of Hematology, Zhongda Hospital, School of Medicine, Southeast University, Institute of Hematology Southeast University, Nanjing, China; [2]Division of Hematology, The Ohio State University Wexner Medical Center, the James Cancer Hospital, Columbus, OH 43210, United States of America;*

### Background

Dysregulated epigenetic mechanisms play a crucial role in the pathogenesis of acute myeloid leukemia (AML), thereby conferring a therapeutic vulnerability, as these epigenetic abnormalities are frequently reversible by inhibitors of histone-modifying proteins. Helicase Lymphoid Specific (HELLS), a unique member of the SWI/SNF2 family, has been historically associated with DNA methylation and DNA damage repair. However, the role of HELLS in AML remains largely undefined. Enhancer of zeste homolog 2 (EZH2) is the core subunit of Polycomb Repressive Complex 2 (PRC2), which canonically catalyzes H3K27me3 to mediate gene silencing. Emerging evidence has suggested that EZH2 interacts with the SWI /SNF complex in a PRC2-independent manner to activate target genes.

### Aims

This study aims to investigate the role of HELLS and the regulatory effect of EZH2 on HELLS gene expression in AML.

### Methods

A total of 98 AML patients and 70 healthy donors were enrolled at our institution following the approval of the Ethics Committee. Kaplan–Meier analysis was conducted to compared the overall survival between high- and low- HELLS group. The expression of genes were determined by western blot and qPCR. The correlation between EZH2 and HELLS expression was estimated by Pearson correlation coefficient analysis in AML cohorts from the OHSU and GSE13159. RNA-seq was performed in U937 cells treated with 2μM DZNeP or vehicle for 48 h. ChIP-seq data for anti-EZH2, H3K27me3 and H3K27ac in AML were retrieved from the GSE180448, and enrichment of these epigenetic regulators at the HELLS enhancer region was validated by CUT&RUN assay.

**Copyright Information:** (Online) ISSN: 2572-9241
©2026 The Author(s). HemaSphere is published by John Wiley & Sons Ltd on behalf of European Hematology Association. This is an open access Abstract Book distributed under the Attribution-NonCommercial-NoDerivs (CC BY-NC-ND), which allows third parties to download the articles and share them with others as long as they credit the author and the Abstract Book, but they cannot change the content in any way or use them commercially.
**Abstract Book Citations:** Authors, Title, HemaSphere, 2026;10:(S1):pages. The abstract book DOIs can be found on the "Author Information" page.
**Disclaimer:** Articles published in the journal HemaSphere exclusively reflect the opinions of the authors. The authors are responsible for all content in their abstracts including accuracy of the facts, statements, citing resources, etc.

**Results**

We examined the HELLS expression in 98 AML patients versus 70 health donors. Data showed that the mRNA level of HELLS was significantly higher in AML patients than that of healthy controls (Fig A), and elevated HELLS expression was related to shorter overall survival (Fig B). HELLS has been implicated in the DNA damage response (DDR), leading us to hypothesize that HELLS transcriptionally regulates DDR genes. We performed overexpression and knockdown of HELLS in MOLM13 AML cells (Fig C). Notably, the expression of DDR genes (FANCI, FEN1, RRM2, POLE2, POLA1) was significantly upregulated upon HELLS overexpression, whereas these genes were markedly downregulated following HELLS knockdown (Fig D). Bioinformatic analyses identified a significant positive correlation between HELLS and EZH2 expression (Fig E). To explore the relationship of EZH2 and HELLS, RNA-seq analysis revealed that HELLS expression was markedly decreased following treatment with the EZH2 inhibitor DZNeP compared with the vehicle control (Fig F), and significant downregulation of both EZH2 and HELLS was observed upon pharmacological inhibition of EZH2 in AML cells (Fig GH). Besides, EZH2 overexpression upregulated HELLS, while EZH2 knockdown significantly reduced HELLS expression at both the mRNA and protein levels in AML cells (Fig IJ). Reanalysis of ChIP-seq data found prominent binding peaks of EZH2 and H3K27ac at the enhancer region of HELLS, with no detectable H3K27me3 enrichment (Fig K), which was further validated by CUT&RUN experiments in AML cell lines (Fig L).

**Summary/Conclusion**

Our results reveal the oncogenic role of HELLS in AML by governing the downstream genes implicated in DNA damage repair. Furthermore, HELLS is epigenetically regulated by EZH2 via co-occupancy with H3K27ac at its enhancer region independent of H3K27me3, and targeting EZH2 can effectively repress the expression of HELLS.

**Copyright Information:** (Online) ISSN: 2572-9241
©2026 The Author(s). HemaSphere is published by John Wiley & Sons Ltd on behalf of European Hematology Association. This is an open access Abstract Book distributed under the Attribution-NonCommercial-NoDerivs (CC BY-NC-ND), which allows third parties to download the articles and share them with others as long as they credit the author and the Abstract Book, but they cannot change the content in any way or use them commercially.
**Abstract Book Citations:** Authors, Title, HemaSphere, 2026;10:(S1):pages. The abstract book DOIs can be found on the "Author Information" page.
**Disclaimer:** Articles published in the journal HemaSphere exclusively reflect the opinions of the authors. The authors are responsible for all content in their abstracts including accuracy of the facts, statements, citing resources, etc.

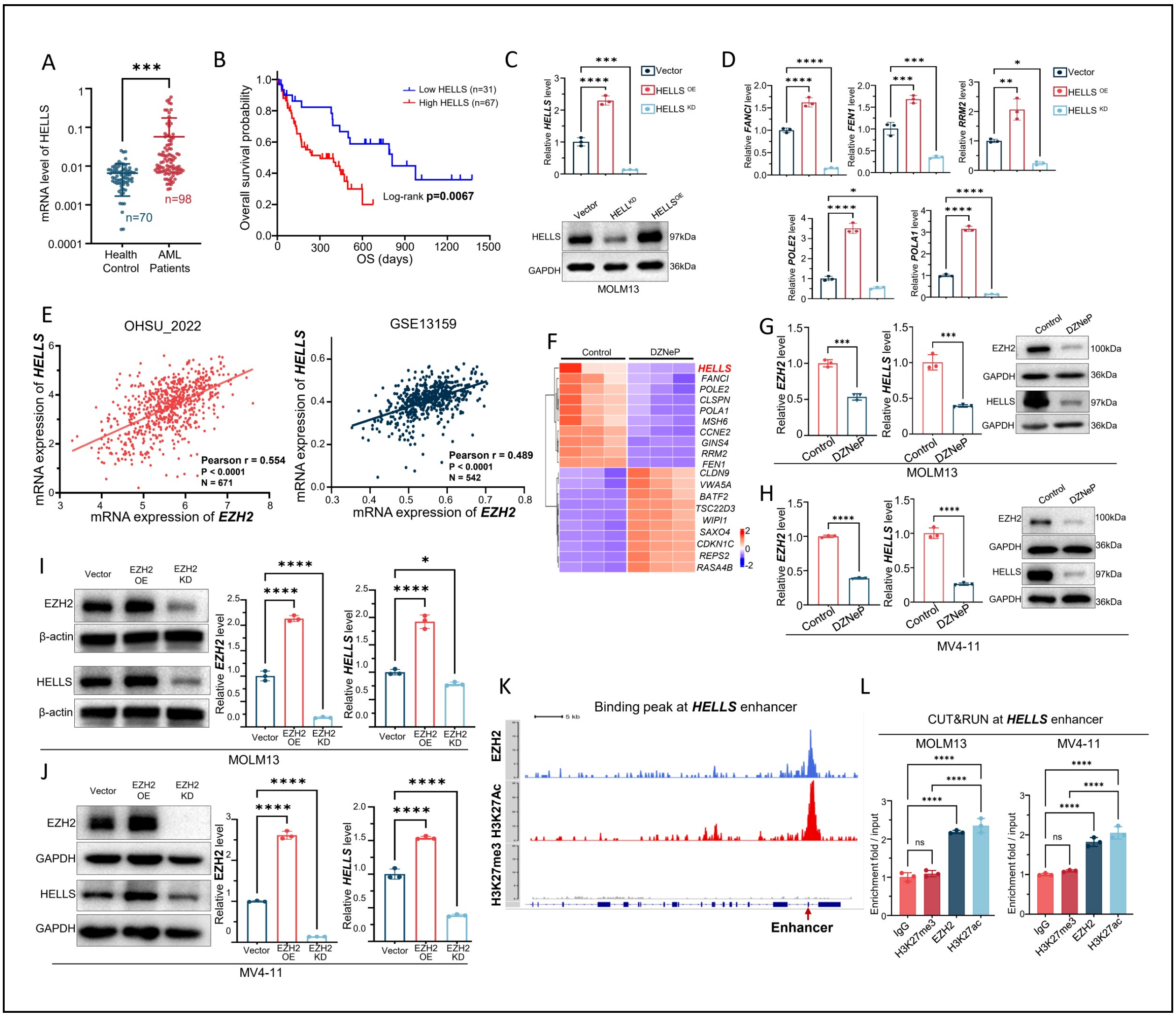

**Copyright Information:** (Online) ISSN: 2572-9241
©2026 The Author(s). HemaSphere is published by John Wiley & Sons Ltd on behalf of European Hematology Association. This is an open access Abstract Book distributed under the Attribution-NonCommercial-NoDerivs (CC BY-NC-ND), which allows third parties to download the articles and share them with others as long as they credit the author and the Abstract Book, but they cannot change the content in any way or use them commercially.
**Abstract Book Citations:** Authors, Title, HemaSphere, 2026;10:(S1):pages. The abstract book DOIs can be found on the "Author Information" page.
**Disclaimer:** Articles published in the journal HemaSphere exclusively reflect the opinions of the authors. The authors are responsible for all content in their abstracts including accuracy of the facts, statements, citing resources, etc.

### (PF457) RIBOSOMAL VULNERABILITIES IN HNRNPK-HAPLOINSUFFICIENT ACUTE MYELOID LEUKEMIA UNCOVER POTENTIAL THERAPEUTIC TARGETS

**Topic:** 03. Acute myeloid leukemia - Biology & translational research

Sean Post*[1], Ashley Rodriguez[1], Marisa Aitken[2], Oscar Benitez[1], Yu-An Kuo[1], Yutong Sun[1], Hussein Abbas[1], Miguel Gallardo[3], Prerna Malaney[4]

*[1]MD Anderson Cancer Center, Houston, United States of America;[2]University of Michigan, Ann Arbor, United States of America; [3]Fundación Investigación Biomedical Hospital 12 De Octubre Centro De Actividades Ambulatorias 6º Planta Bloque D, Madrid, Spain; [4]Dartmouth, Hanover, United States of America;*

### Background

Loss-of-function and missense mutations in *HNRNPK* contribute to AML initiation and progression. Moreover, monoallelic deletion chromosome 9q, particularly del(9q), encompassing the minimally deleted region 9q21.32 that harbors the *HNRNPK* locus, represents a recurrent cytogenetic abnormality in AML. Given that *HNRNPK* genetic ablation is observed in ~5–6% of AML cases, a deeper investigation of this multifunctional RNA-binding protein in leukemogenesis is warranted.

### Aims

*HNRNPK* haploinsufficiency disrupts myeloid lineage-specificity through aberrant C/EBPα and PU.1 expression, impairing differentiation while promoting expansion of myeloid progenitors via dysregulation of the p53/21 axis —recapitulating aspects of del(9q) AML.  Our recent studies further demonstrated that *HNRNPK* deficiency disrupts the mTOR pathway, leading to impaired ribosomal protein S6 signaling, expression changes in translation initiation and elongation factors, and protein translation defects. To elucidate how *HNRNPK* alterations contribute AML, particularly in the context of del(9q), we have taken molecular approaches to define its impact on critical regulators of ribosome biogenesis and translational control.

### Methods

We are currently interrogating the impact of *HNRNPK* haploinsufficiency on rRNA synthesis, pre-ribosomal complex formation, and ribosomal maturation, and ribosomal recycling. Here, we observed that reduced hnRNP K expression impaired rRNA processing, evidenced by diminished 47S precursor levels and defective maturation of rRNA intermediates. These alterations disrupted 40S and 60S subunit assembly, led to accumulation of stalled 80S ribosomes and reduced polysomes formation. Cellular fractionation and density gradient centrifugation revealed hnRNP K-dependent alterations in pre-ribosomal complex formation, while ribosomal profiling (Ribo-Seq) uncovered hnRNP K mediated changes in open reading frame usage, start site initiation defects, translational pausing, and ribosomal recycling. Collectively, these findings suggest that AML cases harboring *HNRNPK* deficiencies (like del9q), represent a previously uncharacterized ribosomopathy driven by impaired ribosome biogenesis and translational control.

### Results

To exploit *HNRNPK* loss in malignancy, we performed genome-wide CRISPR-Cas9 dropout screens in*HNRNPK*-deficient cells to identify synthetic lethal genetic interactions. These screens uncovered targetable vulnerabilities in ribosome biogenesis and translation initiation. Given that *HNRNPK* is an essential for survival, further inhibition of its functions in a haploinsufficient context may selectively eradicate leukemic cells. To examine this, we have identified small molecules that inhibit hnRNP K RNA binding activities and are evaluating their efficacy in primary cells harboring *HNRNPK* deletions and genetically engineered models. By leveraging the essential nature of HNRNPK, this approach holds the potential to eliminate therapy-resistance population, including leukemic stem cells.

**Copyright Information:** (Online) ISSN: 2572-9241
©2026 The Author(s). HemaSphere is published by John Wiley & Sons Ltd on behalf of European Hematology Association. This is an open access Abstract Book distributed under the Attribution-NonCommercial-NoDerivs (CC BY-NC-ND), which allows third parties to download the articles and share them with others as long as they credit the author and the Abstract Book, but they cannot change the content in any way or use them commercially.
**Abstract Book Citations:** Authors, Title, HemaSphere, 2026;10:(S1):pages. The abstract book DOIs can be found on the "Author Information" page.
**Disclaimer:** Articles published in the journal HemaSphere exclusively reflect the opinions of the authors. The authors are responsible for all content in their abstracts including accuracy of the facts, statements, citing resources, etc.

**Summary/Conclusion**

These studies provide evidence that hnRNP K is a key mediator of ribosomal biogenesis and protein translation. Critical to AML, these findings suggest that del(9q) AML is a previously unrecognized ribosomopathy driven by *HNRNPK* haploinsufficiency. Importantly, reduced hnRNP K expression creates an exploitable synthetic vulnerability, offering a potentially therapeutic avenue for patients with HNRNPK-deficient AML.

**Copyright Information:** (Online) ISSN: 2572-9241
©2026 The Author(s). HemaSphere is published by John Wiley & Sons Ltd on behalf of European Hematology Association. This is an open access Abstract Book distributed under the Attribution-NonCommercial-NoDerivs (CC BY-NC-ND), which allows third parties to download the articles and share them with others as long as they credit the author and the Abstract Book, but they cannot change the content in any way or use them commercially.
**Abstract Book Citations:** Authors, Title, HemaSphere, 2026;10:(S1):pages. The abstract book DOIs can be found on the "Author Information" page.
**Disclaimer:** Articles published in the journal HemaSphere exclusively reflect the opinions of the authors. The authors are responsible for all content in their abstracts including accuracy of the facts, statements, citing resources, etc.

### (PF458) 1Q AMPLIFICATION AS A NOVEL MECHANISM OF RESISTANCE TO VENETOCLAX IN AML PATIENTS

**Topic:** 03. Acute myeloid leukemia - Biology & translational research

Laura Beckmann*[1, 2], Daniel Thomalla[1, 2], Leonie Breull-Wierschem[1, 2], Dirk Schmitz[1], Olaf Merkel[1, 2], Prerana Wagle[2], Martin Peifer[2, 3], Karl-Anton Kreuzer[1], Michael Hallek[1, 2, 4], Vinod Pullarkat[5], Christian Schrenk[1], Stuart Blakemore[1, 2], Lukas Frenzel[1, 2, 4]

*[1]Department I of Internal Medicine, Center for Integrated Oncology Aachen, Faculty of Medicine and Cologne University Hospital, University of Cologne, Cologne, Germany; [2]Cologne Cluster of Excellence on Cellular Stress Responses in Aging-Associated Diseases, Cologne, Germany; [3]Center for Molecular Medicine Cologne (CMMC), University of Cologne, Cologne, Germany; [4]Faculty of Medicine and University Hospital of Cologne, Center for Molecular Medicine Cologne, University of Cologne, Cologne, Germany; [5]Department of Hematology and Hematopoietic Cell Transplantation and Gehr Family Center for Leukemia Research, City of Hope National Medical Center, Duarte, United States of America;*

### Background

Venetoclax (VEN) containing therapies have significantly improved outcomes of acute myeloid leukemia (AML) patients unfit for intensive chemotherapy. However, despite high overall response rates, treatment failure due to emerging VEN resistance remains a major clinical challenge. While FISH analysis is routinely performed in newly diagnosed AML, cytogenetic testing at relapse is not yet standard, as cytogenetic aberrations would not impact treatment decisions in elderly or unfit patients.

### Aims

We aimed to identify novel mechanisms of acquired VEN resistance in AML cell lines and patient samples including the role of cytogenetic aberrations and their impact on therapeutic strategies.

### Methods

AML cell lines with acquired resistance to VEN and primary bone marrow samples from AML patients including relapsed/refractory AML (n=40) and newly diagnosed AML (n=40, control cohort) were analyzed through bulk RNA-sequencing (cell lines), karyotyping and fluorescence-in-situ hybridization (FISH) analysis (cell lines and primary samples).

**Copyright Information:** (Online) ISSN: 2572-9241
©2026 The Author(s). HemaSphere is published by John Wiley & Sons Ltd on behalf of European Hematology Association. This is an open access Abstract Book distributed under the Attribution-NonCommercial-NoDerivs (CC BY-NC-ND), which allows third parties to download the articles and share them with others as long as they credit the author and the Abstract Book, but they cannot change the content in any way or use them commercially.
**Abstract Book Citations:** Authors, Title, HemaSphere, 2026;10:(S1):pages. The abstract book DOIs can be found on the "Author Information" page.
**Disclaimer:** Articles published in the journal HemaSphere exclusively reflect the opinions of the authors. The authors are responsible for all content in their abstracts including accuracy of the facts, statements, citing resources, etc.

### Results

Among the top-upregulated genes in VEN-resistant cell lines were *MCL1* (logFC 1.69, p<0.01), *PSMD4* (logFC 1.40, p<0.01), *ENSA* (logFC=1.36, p<0.01) and *VPS72* (logFC=1.28, p<0.01), *ANP32E* (logFC=1.07, p<0.01). Strikingly, these top upregulated genes are located on the long arm of chromosome 1, specifically at 1q23.1 (*VPS72, PSMD4, ENSA*) or 1q21.2 (*MCL1, ANP32E*). 1q21.3 (p<0.01) and 1q22-23.1 (p=0.02) were among the top chromosomal locations for genes enriched in resistant cell lines. FISH analysis with two probes targeting *CKS1B* and flanking regions (1q21-22) and *MCL1* (1q21.3), revealed amplification of the 1q region containing *MCL1* in AML cell lines upon VEN resistance. Testing commercially available inhibitors of 1q-associated genes, we demonstrate that VEN resistance in cell lines can be overcome by inhibition of PSMD4. Furthermore, VEN resistant cell lines were highly MCL1 dependent and significantly more susceptible to MCL1 inhibition than their parental cell line. Given the ongoing clinical efforts to target CDK9, a key regulator of *MCL1*, we show that CDK9 inhibition can overcome VEN resistance but may not be as effective as MCL1 inhibition *in vitro.*
To validate the clinical significance of this finding, we performed FISH analysis on bone marrow samples from AML patients. 1q amplification was detected in six AML patients after VEN exposure. One patient, after receiving VEN treatment for ~40 months, had an aberration of chromosome 1 as the only genetic alteration (46,XY, dup(1)(q21q32)[13]/46,XY,+1,der(1;22)(q10;q10)[1]/46,XY[1]) resulting in *MCL1* gain (**Figure 1**). The patient was treated with a CDK9 inhibitor (NCT05665530) with good short-term clinical response, with normalization of leukocytosis and a ~50% reduction of *MCL1* amplified cells by FISH. *In vitro* treatment of AML blasts from the same patient demonstrated susceptibility to both MCL1 and CDK9 inhibition, but resistance to VEN.

### Summary/Conclusion

In summary, we demonstrated amplification of 1q as a potential novel mechanism of acquired VEN resistance in AML, resulting in MCL1 dependency and providing potential novel therapeutic opportunities. To understand the impact of VEN treatment on chromosomal aberrations, we are currently investigating larger cohorts. Based on our results, we recommend implementation of cytogenetic analyses in routine diagnostics of relapsed/refractory AML patients.

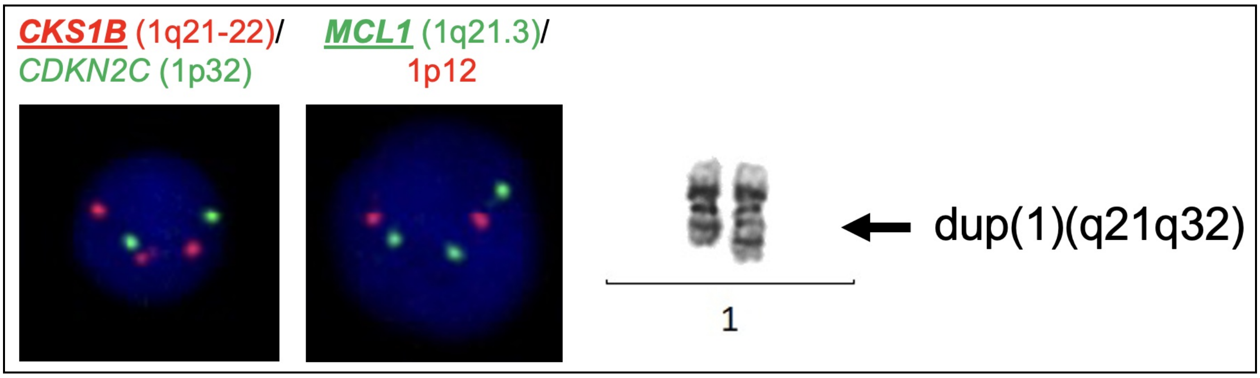

Figure 1. FISH analysis for CKS1B (left FISH image, red) and MCL1 (right FISH image, green) and excerpt from karyogram (right) in an AML patient with acquired venetoclax resistance

**Copyright Information:** (Online) ISSN: 2572-9241
©2026 The Author(s). HemaSphere is published by John Wiley & Sons Ltd on behalf of European Hematology Association. This is an open access Abstract Book distributed under the Attribution-NonCommercial-NoDerivs (CC BY-NC-ND), which allows third parties to download the articles and share them with others as long as they credit the author and the Abstract Book, but they cannot change the content in any way or use them commercially.
**Abstract Book Citations:** Authors, Title, HemaSphere, 2026;10:(S1):pages. The abstract book DOIs can be found on the "Author Information" page.
**Disclaimer:** Articles published in the journal HemaSphere exclusively reflect the opinions of the authors. The authors are responsible for all content in their abstracts including accuracy of the facts, statements, citing resources, etc.

### (PF459) THE PICALM::MLLT10 FUSION COOPERATES WITH MUTATIONS IN CBL AND LEADS TO LEUKEMIA IN A MURINE MODEL

**Topic:** 03. Acute myeloid leukemia - Biology & translational research

Rhea Desai*[1], Alyona Oryshchuk[1], Peter Browett[1], Purvi M Kakadia[1], Stefan Bohlander[1]

[1]*Blood Cancer Research Unit, Department of Molecular Medicine and Pathology, Faculty of Medical and Health Sciences, The University of Auckland,, Auckland, New Zealand;*

### Background

Acute myeloid leukemia (AML) is a genetically complex disease caused by combinations of somatic mutations that act synergistically. The specific co-occurring mutations in a patient most likely impact the phenotype, prognosis, and therapy response. To develop better targeted therapies for AML, it is imperative that we not only understand which mutations co-occur but also their relative contribution to the disease.

The t(10;11)(p13;q14), which gives rise to the *PICALM/MLLT10* or *CALM/AF10* (C/A) fusion gene, is a rare but recurring chromosomal aberration observed in AML, acute lymphoblastic leukemia and malignant lymphoma. We have established knock-in (KI) and murine bone marrow transplantation leukemia models driven by C/A. Whole-exome sequencing of leukemic mice from both models revealed additional mutations in signaling genes, including *Cbl*, *Ptpn11*, *Flt3* and *Kras*. We thus hypothesized that C/A requires the cooperation of mutations in signal transduction genes to lead to leukemia.

### Aims

To investigate if C/A cooperates with mutations in genes involved in activated signaling.

### Methods

In our C/A-driven KI leukemia model (CD45.2$^{+}$, C57BL/6 background), C/A is expressed in the entire hematopoietic compartment and results in leukemia with 100% penetrance and a median latency of 1 year. To investigate whether C/A cooperates with mutations in signaling genes, we isolated bone marrow (BM) cells from 8–12-week-old C/A expressing mice (long before leukemia onset). The isolated BM cells were genetically modified using CRISPR/Cas9-mediated gene editing to introduce mutations in the ring finger domain of *Cbl*.

The edited cells were transplanted into sub-lethally irradiated congenic (CD45.1$^{+}$) recipient mice ($CA^{+}/Cbl^{mut}$, n=3). Two control groups were included: one group received C/A positive BM cells not genetically modified for *Cbl* ($CA^{+}/Cbl^{WT}$; n=3), while the second group received C/A negative cells that were modified for *Cbl* ($CA^{-}/Cbl^{mut}$; n=5). Each recipient mouse received 1x10$^{6}$ BM cells. Peripheral blood (PB) was collected from transplanted mice at 4–8-week intervals and analyzed for engraftment of CD45.2$^{+}$ cells by flow cytometry and for gene editing efficiency by next generation sequencing (NGS). All transplanted mice were monitored for leukemia development.

**Copyright Information:** (Online) ISSN: 2572-9241
©2026 The Author(s). HemaSphere is published by John Wiley & Sons Ltd on behalf of European Hematology Association. This is an open access Abstract Book distributed under the Attribution-NonCommercial-NoDerivs (CC BY-NC-ND), which allows third parties to download the articles and share them with others as long as they credit the author and the Abstract Book, but they cannot change the content in any way or use them commercially.
**Abstract Book Citations:** Authors, Title, HemaSphere, 2026;10:(S1):pages. The abstract book DOIs can be found on the “Author Information” page.
**Disclaimer:** Articles published in the journal HemaSphere exclusively reflect the opinions of the authors. The authors are responsible for all content in their abstracts including accuracy of the facts, statements, citing resources, etc.

**Results**

Of the 11 transplanted mice, 10 engrafted successfully, with mean CD45.2$^{+}$ cell engraftment of 82% at 8 weeks post-transplant. One *CA$^{+}$/Cbl$^{mut}$* mouse did not engraft and was excluded from further analysis. NGS analysis of PB samples from *CA$^{-}$/Cbl$^{mut}$* mice at 16 weeks post-transplant revealed a mean editing efficiency of 62.34% at the *Cbl* target site (n=5).

Strikingly, the two *CA$^{+}$/Cbl$^{mut}$* mice both developed leukemia with latencies of 72 and 86 days, while none of the control mice developed leukemia (**Fig1A**). Interestingly, the two *CA$^{+}$/Cbl$^{mut}$* mice (Leu1 and Leu3) showed different leukemia phenotypes (**Fig1B**). Leu3 predominantly exhibited expansion of CD11b$^{+}$ myeloid cells. In Leu1, in addition to increased expression of myeloid markers CD11b and Gr1, a population of cells co-expressing CD11b and the B-cell marker B220 was observed, suggesting a biphenotypic leukemia.

**Summary/Conclusion**

Our results indicate that the C/A fusion cooperates with mutations in *Cbl*, leading to aggressive leukemia, and highlight the role of mutational interactions in AML pathogenesis. This model provides a clinically relevant platform to study cooperating oncogenic mechanisms and inform the development of more precise therapeutic strategies in AML.

**Copyright Information:** (Online) ISSN: 2572-9241
©2026 The Author(s). HemaSphere is published by John Wiley & Sons Ltd on behalf of European Hematology Association. This is an open access Abstract Book distributed under the Attribution-NonCommercial-NoDerivs (CC BY-NC-ND), which allows third parties to download the articles and share them with others as long as they credit the author and the Abstract Book, but they cannot change the content in any way or use them commercially.
**Abstract Book Citations:** Authors, Title, HemaSphere, 2026;10:(S1):pages. The abstract book DOIs can be found on the "Author Information" page.
**Disclaimer:** Articles published in the journal HemaSphere exclusively reflect the opinions of the authors. The authors are responsible for all content in their abstracts including accuracy of the facts, statements, citing resources, etc.

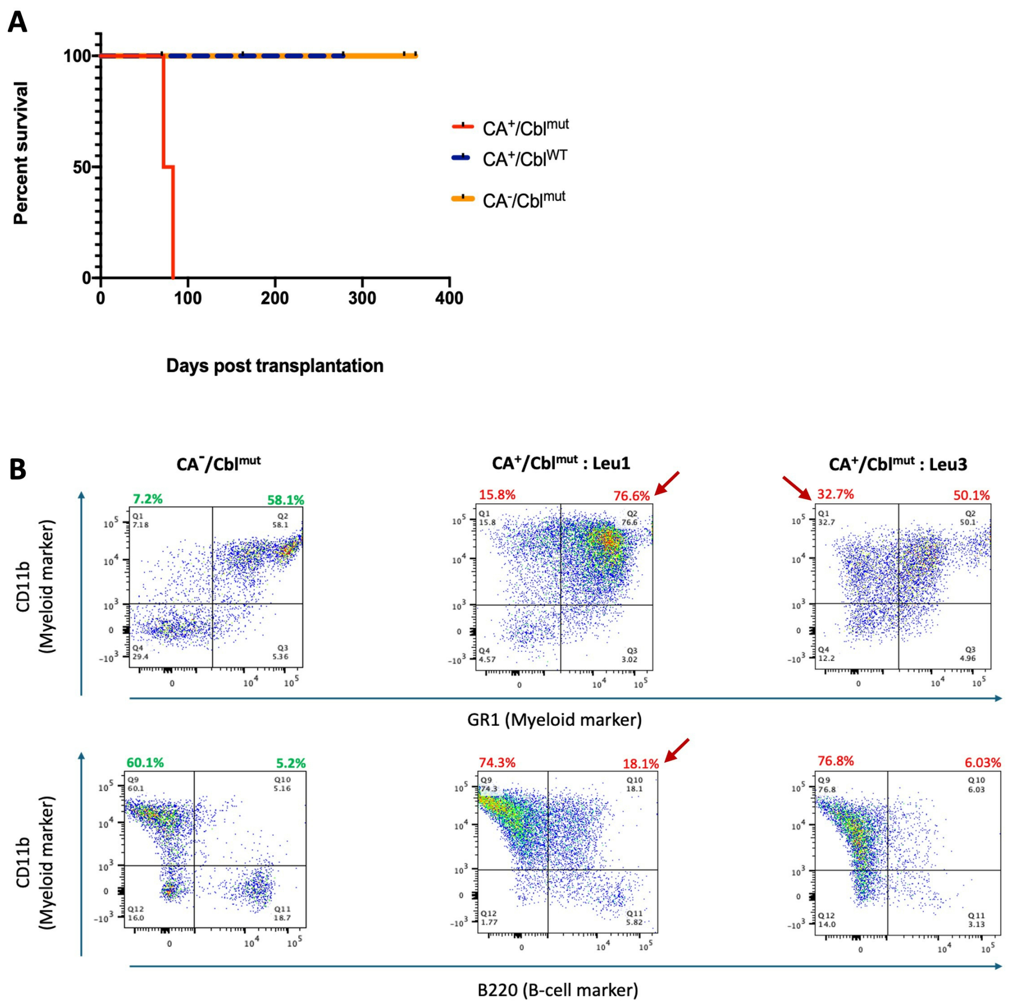

**Figure 1:** (A) Survival of CA+/Cbl$^{mut}$ mice compared to CA+/Cbl$^{WT}$ and CA-/Cbl$^{mut}$ controls
(B) Flow cytometry plots depicting the immunophenotype of a CA-/Cbl$^{mut}$ control mouse and two CA+/Cbl$^{mut}$ leukemic mice

**Copyright Information:** (Online) ISSN: 2572-9241
©2026 The Author(s). HemaSphere is published by John Wiley & Sons Ltd on behalf of European Hematology Association. This is an open access Abstract Book distributed under the Attribution-NonCommercial-NoDerivs (CC BY-NC-ND), which allows third parties to download the articles and share them with others as long as they credit the author and the Abstract Book, but they cannot change the content in any way or use them commercially.
**Abstract Book Citations:** Authors, Title, HemaSphere, 2026;10:(S1):pages. The abstract book DOIs can be found on the "Author Information" page.
**Disclaimer:** Articles published in the journal HemaSphere exclusively reflect the opinions of the authors. The authors are responsible for all content in their abstracts including accuracy of the facts, statements, citing resources, etc.

### (PF460) UNFOLDED PROTEIN RESPONSE AND AUTOPHAGY FACILITATE SURVIVAL OF RESIDUAL SENESCENT-LIKE ACUTE MYELOID LEUKEMIA CELLS ARISING AFTER CHEMOTHERAPY

**Topic:** 03. Acute myeloid leukemia - Biology & translational research

Anna Skwarska*[1], Gowri Poigaialwar[1], Varun Gupta[1], Yen B Huynh[2], Stefano Tiziani[3], Maria Sosa[4], Julio Aguirre-Ghiso[5], Marina Konopleva[6]

*[1]Albert Einstein College of Medicine, Department of Oncology, New York, United States of America;[2]The University of Texas at Austin, Department of Nutritional Sciences, Austin, United States of America; [3]The University of Texas at Austin, Department of Nutritional Sciences, Dell Medical School, Center for Molecular Carcinogenesis and Toxicology, Austin, United States of America; [4]Albert Einstein College of Medicine, Department of Microbiology and Immunology, Department of Oncology, Cancer Dormancy Institute, New York, United States of America; [5]Albert Einstein College of Medicine, Department of Cell Biology, Cancer Dormancy Institute, Gruss-Lipper Biophotonics Center, New York, United States of America; [6]Albert Einstein College of Medicine, Department of Cell Biology, Department of Oncology, New York, United States of America;*

### Background

Outcomes of relapsed/refractory acute myeloid leukemia (AML) remain poor. Durable disease control requires effective eradication of both bulk leukemic blasts and residual cells that survive initial therapy. The unfolded protein response (UPR) and autophagy are critical mechanisms that enable tumor adaptation to proliferative stress, increased protein synthesis and chemotherapy. UPR-mediated pro-survival signaling is linked to hematopoietic stem cells (HSCs) function and self-renewal. Activation of PERK–eIF2α–ATF4 axis supports survival of both HSCs and leukemia stem cells under amino acid deprivation (*van Galen et al., Cell Rep 2018*). AML cells also exhibit enhanced autophagy, a catabolic process that degrades damaged cellular components and promotes chemoresistance under conditions mimicking hypoxic bone marrow (*Wang et al. bioRxiv 2024*). Cytarabine (AraC) induces senescence-like resilient cells capable of initiating AML recurrence (*Duy et al., Cancer Discovery, 2021*). We recently showed that these residual senescent-like cells upregulate canonical PERK signaling (via eIF2α/ATF4) and non-canonical PERK–NRF2 pathways, and that PERK inhibition reduces their survival (*Skwarska et al. ASH 2024*).

### Aims

To determine whether post-AraC senescent-like AML cells invoke both UPR and autophagy for survival and to delineate the mechanistic interplay between these adaptive pathways.

### Methods

We generated doxycycline-inducible H2B-GFP MOLM14 AML cells to track non-proliferating cells. After doxycycline withdrawal, non-dividing cells retain H2B histone-associated GFP fluorescence, while proliferating cells dilute GFP signal with division. After 3 days of doxycycline induction, cells were treated with 0.1 µM AraC for 10 days. GFP-positive cells were FACS-sorted and analyzed for senescence, UPR and autophagy markers by RNAseq, RT-PCR and western blotting.

**Copyright Information:** (Online) ISSN: 2572-9241
©2026 The Author(s). HemaSphere is published by John Wiley & Sons Ltd on behalf of European Hematology Association. This is an open access Abstract Book distributed under the Attribution-NonCommercial-NoDerivs (CC BY-NC-ND), which allows third parties to download the articles and share them with others as long as they credit the author and the Abstract Book, but they cannot change the content in any way or use them commercially.
**Abstract Book Citations:** Authors, Title, HemaSphere, 2026;10:(S1):pages. The abstract book DOIs can be found on the "Author Information" page.
**Disclaimer:** Articles published in the journal HemaSphere exclusively reflect the opinions of the authors. The authors are responsible for all content in their abstracts including accuracy of the facts, statements, citing resources, etc.

**Results**

We previously showed that MOLM14 cells treated with Ara-C for 3 days exhibited a senescence-like phenotype, characterized by high senescence-associated β-galactosidase (SA-β-Gal) activity, upregulation of p21 and BCL-xL and induction of senescence-associated secretory phenotype (SASP) factors (IL-8, IL-1β, CCL5), accompanied by elevated PERK expression and phosphorylation and upregulation of ATF4 and NRF2 effectors. Notably, following prolonged treatment (10 days), a small population (~7%) of viable, non-proliferating cells persisted, retaining senescence features (high SA-β-Gal, loss of Ki67 and nuclear lamin B1, sustained SASP). RNA-seq confirmed enrichment of senescence-associated gene signatures and depletion of cell cycle/mitosis programs. Unexpectedly, expression of UPR-related genes (PERK, ATF4, CHOP, eIF2S1, NRF2) was partially reduced. In contrast, gene set enrichment analysis revealed upregulation of lysosomal and autophagy pathways. Consistently, western blotting revealed increased LC3-I to LC3-II conversion, supporting increased autophagosome formation and activation of autophagy.

**Summary/Conclusion**

AraC induces a senescence-like program accompanied by early activation of the UPR as part of the acute stress response. However, UPR signaling is not sustained in persistent senescent-like AML cells. Instead, prolonged survival of these cells is associated with enhanced autophagy and lysosomal activity, suggesting a shift from UPR-driven stress adaptation to autophagy-dependent survival mechanisms. This adaptive transition may represent a new therapeutic vulnerability in residual AML arising after chemotherapy.

**Copyright Information:** (Online) ISSN: 2572-9241
©2026 The Author(s). HemaSphere is published by John Wiley & Sons Ltd on behalf of European Hematology Association. This is an open access Abstract Book distributed under the Attribution-NonCommercial-NoDerivs (CC BY-NC-ND), which allows third parties to download the articles and share them with others as long as they credit the author and the Abstract Book, but they cannot change the content in any way or use them commercially.
**Abstract Book Citations:** Authors, Title, HemaSphere, 2026;10:(S1):pages. The abstract book DOIs can be found on the “Author Information” page.
**Disclaimer:** Articles published in the journal HemaSphere exclusively reflect the opinions of the authors. The authors are responsible for all content in their abstracts including accuracy of the facts, statements, citing resources, etc.

### (PF461) THE ROLE OF THE HUMAN BONE MARROW NICHE IN DENDRITIC CELL REGULATION AND ITS IMPLICATIONS FOR ACUTE MYELOID LEUKEMIA IMMUNOTHERAPY

**Topic:** 03. Acute myeloid leukemia - Biology & translational research

Laura Rabanal Cajal*[1, 2, 3], Aurélie Baudet[1, 2, 3], Ervin Ascic[2, 3, 4], Malavika Nair[2, 3, 4], Sara González Antón[1, 2, 3], Camille Sauter[1, 2, 3], Léa Chicoisne[2, 3, 5], Dimitra Zacharaki[1, 2, 3], Camila Consiglio[2, 3, 5], Filipe Pereira[2, 3, 4], Paul Bourgine[1, 2, 3]

*[1]Cell, Tissue & Organ engineering laboratory, Biomedical Centre (BMC), Department of Clinical Sciences Lund, Lund University, Lund, Sweden; [2]Wallenberg Centre for Molecular Medicine, Lund University, Lund, Sweden; [3]Lund Stem Cell Centre, Lund University, Lund, Sweden; [4]Cellular Reprogramming and Immunity laboratory, Biomedical Centre (BMC), Department of Molecular Medicine and Gene Therapy, Lund, Sweden; [5]Systems Immunology Lab, Biomedical Centre (BMC), Division of Molecular Hematology, Department of Laboratory Medicine, Lund University, Lund, Sweden;*

### Background

The bone marrow (BM) hosts mesenchymal stem/stromal cells (MSCs) and their progeny, comprising osteogenic, adipogenic, stromal and perivascular populations. Altogether, they compose the BM mesenchymal niche (BM niche), which holds a pivotal role in supporting hematopoiesis. However, its capacity to regulate immune functions remains cryptic.
Among immune cells, conventional type 1 DCs (cDC1s) are essential orchestrators of antitumoral immunity. While the BM niche is known to restrict DC differentiation and dampen their immunostimulatory capacity, how mesenchymal populations regulate DC in health and disease contexts remains unclear. This is particularly relevant in acute myeloid leukemia (AML), where cellular immunotherapies have shown limited success. This may be partly explained by the rewiring of the BM niche by inflammatory cues that enhance its immune suppression and favor tumor immune evasion.
We hypothesize that the BM niche plays a central role in regulating DC function, impacting the efficacy of DC-based immunotherapies against AML.

### Aims

- Evidence the regulatory role of the human BM niche in DC differentiation at homeostasis
- Determine how distinct mesenchymal populations modulate DC reprogramming immunotherapy in AML
- Assess the impact of the whole human BM niche on DC reprogramming of AML in vivo

**Copyright Information:** (Online) ISSN: 2572-9241
©2026 The Author(s). HemaSphere is published by John Wiley & Sons Ltd on behalf of European Hematology Association. This is an open access Abstract Book distributed under the Attribution-NonCommercial-NoDerivs (CC BY-NC-ND), which allows third parties to download the articles and share them with others as long as they credit the author and the Abstract Book, but they cannot change the content in any way or use them commercially.
**Abstract Book Citations:** Authors, Title, HemaSphere, 2026;10:(S1):pages. The abstract book DOIs can be found on the "Author Information" page.
**Disclaimer:** Articles published in the journal HemaSphere exclusively reflect the opinions of the authors. The authors are responsible for all content in their abstracts including accuracy of the facts, statements, citing resources, etc.

**Methods**

We used humanized ossicles (hOss), consisting of human bone and BM organs grown ectopically in immunodeficient mice. These hOss fully recapitulate the human BM niche and support robust human hematopoietic and AML engraftment[1,2].
To study DC regulation at homeostasis, hOss-bearing mice were humanized by intravenous transplantation of human cord blood-derived CD34+ cells and reconstituted for 17 weeks. Murine bones and hOss were immunophenotyped by flow cytometry.
To evaluate the impact of the human BM niche in DC-based immunotherapies against AML, we focused on DC reprogramming, which directly converts tumor cells into cDC1-like cells to induce antitumoral immunity[3,4]. We established 2D and 3D cocultures of AML cells undergoing DC reprogramming with stromal, osteogenic, and adipogenic MSCs, and assessed cDC1 markers (HLA-DR, Clec9A, CD141, CD40) by flow cytometry. Finally, AML was established in hOss-bearing mice in vivo, DC reprogramming therapy was induced, and cDC1 markers were assessed by flow cytometry.

[1]Pigeot et al, Adv Mat, 2021

[2]Grigoryan et al, STM, 2022

[3]Zimmermannova et al, Sci Immunol, 2023

[4]Ascic et al, Science, 2024

**Results**

At homeostasis, hOss selectively impaired cDC1 differentiation, while remaining permissive to cDC2s and plasmacytoid DCs.

In AML setting, stromal MSCs reduced reprogramming efficiency (% of HLA-DR$^+$ Clec9A$^+$ cells) and decreased HLA-DR median fluorescence intensity (MFI) in this population, suggesting impaired function. Osteogenic and adipogenic MSCs permitted higher reprogramming efficiency but showed an even higher reduction in HLA-DR MFI than stromal MSCs cocultures. In vivo, the human BM niche increased reprogramming efficiency but was associated with ~50% reduction in HLA-DR MFI and fewer CD40$^+$ cells compared to murine bone, consistent with in vitro findings.

**Summary/Conclusion**

These findings position the human BM niche as a critical regulator of cDC1 development at homeostasis and of DC reprogramming efficacy in AML. By modulating reprogramming efficiency and impairing HLA-DR upregulation in reprogrammed DCs, the human BM niche may limit their therapeutic potential, thus becoming a key target for improving DC-immunotherapy efficacy in AML.

**Copyright Information:** (Online) ISSN: 2572-9241
©2026 The Author(s). HemaSphere is published by John Wiley & Sons Ltd on behalf of European Hematology Association. This is an open access Abstract Book distributed under the Attribution-NonCommercial-NoDerivs (CC BY-NC-ND), which allows third parties to download the articles and share them with others as long as they credit the author and the Abstract Book, but they cannot change the content in any way or use them commercially.
**Abstract Book Citations:** Authors, Title, HemaSphere, 2026;10:(S1):pages. The abstract book DOIs can be found on the "Author Information" page.
**Disclaimer:** Articles published in the journal HemaSphere exclusively reflect the opinions of the authors. The authors are responsible for all content in their abstracts including accuracy of the facts, statements, citing resources, etc.

### (PF462) PASSENGER VARIANTS AS PAN-CLONAL MARKERS OF PEDIATRIC AML: IMPLICATIONS FOR MOLECULAR MRD

**Topic:** 03. Acute myeloid leukemia - Biology & translational research

Ruth Shiloh*[1], Dana Dayan[2], Ifat Geron[1], Maor Cohen[3], Tamar Feuerstein[1], Jorge Cano-Nistal[2], Nurit Gal-Mark[1], Shlomit Barzilai[1], Dafna Gaash[1], Michal Hameiri-Grossman[1], Jamal Mahajna[3], Nino Oniashvili[1], Hava Rosenfeld[1], Dan Scharf[2], Keren Shichrur[1], Elena Shinderman-Maman[1], Sarah Elitzur[1], Yehudit Birger[1], Shai Izraeli[1], Yosef Maruvka[2]

*[1]Schneider Children's Medical Center, The Rina Zaizov Division of Pediatric Hematology-Oncology, Petach Tikva, Israel; [2]Technion, Haifa, Israel; [3]Tel Hai Academic College, Qiryat Shemona, Israel;*

### Background

Measurable residual disease (MRD) detection is the most powerful prognostic indicator for relapse and overall survival in pediatric acute lymphoid and myeloid leukemias (ALL & AML). However, the biological and genetic heterogeneity of AML presents challenges for MRD assessment. Unlike specific V(D)J rearrangements in ALL, there are no universal pan-clonal molecular markers for MRD detection in AML, and MRD assessment therefore relies largely on flow cytometry (FACS). Notably, most relapses occur in cases classified as FACS-MRD negative. Molecular detection of specific driver aberrations is applicable to only a subset of patients. Passenger mutations acquired during cell division typically have no biological effect but can serve as markers for a specific cell and its progeny. Since leukemia arises from a single transformed cell, passenger variants present at transformation are inherited by all leukemic daughter cells and may form stable molecular signatures shared across the malignant population. We therefore hypothesized that non-coding passenger variants could serve as universal MRD markers.

### Aims

To develop a sensitive molecular MRD detection strategy that is applicable to all patients, by shifting focus from pathogenic driver mutations to pan-clonal non-coding passenger variants.

### Methods

Whole-genome sequencing (WGS) of diagnostic bone marrow DNA was used to identify a set of patient-specific, somatic pan-clonal non-coding variants, which we term 'Leukemia-Specific Passenger Variants' (LSPVs). Single-cell DNA proteogenomics was performed to assess the specificity and distribution of LSPVs within leukemic cell populations. Longitudinal samples were analyzed by ddPCR to evaluate LSPV stability over the disease course. A dedicated computational pipeline (DAISY-MRD) was developed to enable MRD quantification by WGS (100x), by aggregating all LSPV reads and determining a collective LSPV variant allelic frequency (VAF).

### Results

LSPVs were identified in all patients, with a median of ~300 patient-specific variants identified per patient. Single-cell analyses demonstrated that LSPVs serve as pan-clonal markers of leukemic cells, as they are shared across the entire leukemic population at both diagnosis and relapse and absent from normal cell populations. Highly sensitive ddPCR analysis of longitudinal samples showed that LSPVs accurately reflected disease burden from diagnosis to relapse. LSPV VAF at early MRD time points was prognostic and discriminated between relapsed and non-relapsed patients. ROC analysis of relapse prediction using DAISY-MRD scores yielded an AUC of 0.90, indicating excellent discriminatory performance.

**Copyright Information:** (Online) ISSN: 2572-9241
©2026 The Author(s). HemaSphere is published by John Wiley & Sons Ltd on behalf of European Hematology Association. This is an open access Abstract Book distributed under the Attribution-NonCommercial-NoDerivs (CC BY-NC-ND), which allows third parties to download the articles and share them with others as long as they credit the author and the Abstract Book, but they cannot change the content in any way or use them commercially.
**Abstract Book Citations:** Authors, Title, HemaSphere, 2026;10:(S1):pages. The abstract book DOIs can be found on the "Author Information" page.
**Disclaimer:** Articles published in the journal HemaSphere exclusively reflect the opinions of the authors. The authors are responsible for all content in their abstracts including accuracy of the facts, statements, citing resources, etc.

**Summary/Conclusion**

LSPVs are accurate and stable markers of disease burden, which can be identified in all patients. These properties make LSPVs highly suitable for MRD monitoring, overcoming limitations of current commonly used methods. Leveraging LSPVs, we present DAISY-MRD - a streamlined method for MRD assessment using WGS. This approach does not rely on the presence of specific trackable genetic aberrations and does not require tailored assay design, making it simple, efficient and feasible in clinical settings. While this study is focused on pediatric AML, this approach could be potentially applicable to other hematological malignancies.

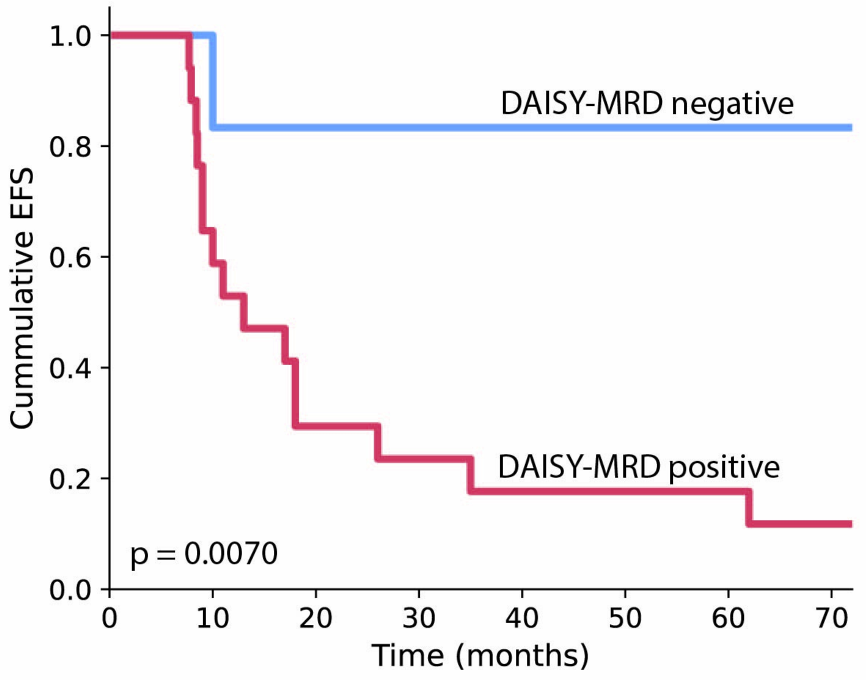

**DAISY-MRD shows potential prognostic value for relapse prediction.**
Cumulative event-free survival of patients stratified by DAISY-MRD level of 5x10-4.
Statistical significance was calculated using log-rank test.

**Copyright Information:** (Online) ISSN: 2572-9241
©2026 The Author(s). HemaSphere is published by John Wiley & Sons Ltd on behalf of European Hematology Association. This is an open access Abstract Book distributed under the Attribution-NonCommercial-NoDerivs (CC BY-NC-ND), which allows third parties to download the articles and share them with others as long as they credit the author and the Abstract Book, but they cannot change the content in any way or use them commercially.
**Abstract Book Citations:** Authors, Title, HemaSphere, 2026;10:(S1):pages. The abstract book DOIs can be found on the "Author Information" page.
**Disclaimer:** Articles published in the journal HemaSphere exclusively reflect the opinions of the authors. The authors are responsible for all content in their abstracts including accuracy of the facts, statements, citing resources, etc.

### (PF463) CD71-TARGETED AND ROS-RESPONSIVE MICELLES FOR HOMOHARRINGTONINE DELIVERY TO ENHANCE THERAPEUTIC EFFICIENCY AGAINST FLT3-ITD ACUTE MYELOID LEUKEMIA

**Topic:** 03. Acute myeloid leukemia - Biology & translational research

Yuqian Tang*[1], Yuping Gong[1]

[1]*West China Hospital, Sichuan University, Department of Hematology, Chengdu, China;*

### Background

The FLT3-ITD (FMS-like tyrosine kinase 3- internal tandem duplication) subtype of acute myeloid leukemia (AML) is associated with poor clinical outcomes. Homoharringtonine (HHT), a natural protein inhibitor, has shown strong activity against FLT3-ITD AML. However, its clinical use is limited by rapid clearance and systemic toxicity.

### Aims

To address these limitations, a CD71-targeted, ROS-responsive micelle (TDTP/HHT) was developed for precise and efficient HHT delivery.

### Methods

CD71 expression was assessed in AML patient samples and cell lines. TDTP/HHT was prepared by self-assembly using a D-peptide ligand ($^{D}$T7) for CD71 targeting and a ROS-cleavable linker for stimuli-responsive drug release. Cellular uptake, cytotoxicity and signaling pathway inhibition were evaluated in vitro across AML cell lines with distinct FLT3 statuses. The therapeutic efficacy of TDTP/HHT was further validated in a disseminated AML xenograft mouse model.

### Results

Cytotoxicity analysis revealed that FLT3-ITD AML is intrinsically sensitive to HHT. Analysis of patient samples and cell lines revealed high CD71 expression and elevated ROS levels in AML, most predominantly in the FLT3-ITD subtype. The designed TDTP/HHT showed prolonged circulation time, ROS-responsive drug release, and stable targeting capacity. It did not compete with endogenous transferrin for binding sites but instead demonstrated transferrin-promoted cellular uptake. Both *in vitro* and *in vivo* studies confirmed that TDTP/HHT significantly inhibited the growth of FLT3-ITD AML cells. Mechanistically, TDTP/HHT suppressed multiple key downstream signaling proteins of FLT3-ITD.

### Summary/Conclusion

This study presents a novel targeted nanodrug that exploits the intrinsic HHT sensitivity of FLT3-ITD AML cells and their characteristic high CD71 expression and elevated ROS levels. By enabling specific intracellular accumulation of HHT and multitarget inhibition of FLT3-ITD signaling pathways, this system achieves superior anti-AML efficacy both *in vitro* and *in vivo*, offering strong potential for future clinical translation.

**Copyright Information:** (Online) ISSN: 2572-9241
©2026 The Author(s). HemaSphere is published by John Wiley & Sons Ltd on behalf of European Hematology Association. This is an open access Abstract Book distributed under the Attribution-NonCommercial-NoDerivs (CC BY-NC-ND), which allows third parties to download the articles and share them with others as long as they credit the author and the Abstract Book, but they cannot change the content in any way or use them commercially.
**Abstract Book Citations:** Authors, Title, HemaSphere, 2026;10:(S1):pages. The abstract book DOIs can be found on the "Author Information" page.
**Disclaimer:** Articles published in the journal HemaSphere exclusively reflect the opinions of the authors. The authors are responsible for all content in their abstracts including accuracy of the facts, statements, citing resources, etc.

### (PF464) THERAPEUTIC ROLE OF GLV8 BY TARGETING GPR84 IN VENETOCLAX-RESISTANT ACUTE MYELOID LEUKEMIA

**Topic:** 03. Acute myeloid leukemia - Biology & translational research

Yangyang Ding[1], Shan Gao[2], Zheng Ge*[1]

*[1]Zhongda Hospital Southeast University, Institute of Hematology Southeast University, Department of Hematology, Nanjing, China; [2]Zhongda Hospital, School of Life Sciences and Technology, Advanced Institute for Life and Health, Southeast University, Nanjing, China;*

### Background

Venetoclax–resistant acute myeloid leukemia (AML) has a poor prognosis, necessitating the development of novel therapeutic strategies and molecular targets. The antitumor effects and mechanisms of GLV8, a novel synthetic flavonoid, in venetoclax-resistant AML remain unclear; furthermore, the clinical value of its potential downstream target, G protein-coupled receptor 84 (GPR84), a pro-inflammatory receptor that responds to medium-chain fatty acids, in AML also needs to be elucidated.

### Aims

This study aimed to investigate the therapeutic potential of GLV8 and to elucidate the clinical significance of its downstream target GPR84 in AML.

### Methods

Venetoclax–resistant cell lines were established, and the functional effects of GLV8 were evaluated using cell viability, colony formation, and apoptosis assays. Venetoclax–resistant Molm-13 cells were subjected to GLV8 intervention and divided into four groups: venetoclax-sensitive, venetoclax-resistant, venetoclax-sensitive + GLV8 treatment, and venetoclax-resistant + GLV8 treatment. RNA sequencing was conducted to identify differentially expressed genes, followed by analysis and visualization using the R2 Genomics Analysis and Visualization Platform. GPR84 mRNA levels were measured by quantitative PCR (qPCR). Kaplan–Meier survival analysis was performed using an interactive gene expression profiling platform.

### Results

Compared with parental venetoclax–sensitive cells, venetoclax–resistant AML cell lines exhibited a markedly increased $IC_{50}$ (>80-fold) and elevated MCL1 expression (Fig A, B), indicating successful establishment of resistance. GLV8 significantly inhibited cell proliferation in both venetoclax–sensitive and resistant AML cells in a dose-dependent manner and induced cell apoptosis (Fig C-E). It also markedly suppressed venetoclax–resistant AML cell colony formation (Fig F), accompanied by dose-dependent downregulation of MCL1 and BCL2 (Fig G), suggesting a potential therapeutic effect of GLV8 in AML. RNA sequencing was performed on venetoclax–sensitive and venetoclax–resistant cells with or without GLV8 treatment (Fig H). Compared with sensitive cells, resistant cells exhibited 2,909 upregulated and 3,108 downregulated genes (fold change >1.5, FDR <0.05). After GLV8 treatment, 241 genes were downregulated and 352 upregulated in resistant cells, and 1,272 genes were downregulated and 1,790 upregulated in sensitive cells (Fig I). Integrative analysis identified 56 genes consistently regulated by GLV8, among which GPR84 showed the most significant change (Fig J). qPCR confirmed that GPR84 was highly expressed in resistant cells and significantly decreased after GLV8 treatment (Fig K). Analysis of public datasets further demonstrated that GPR84 is overexpressed in AML and is associated with poor prognosis (Fig L). Collectively, these findings suggest that GLV8 and its downstream target GPR84 may be potential drug candidates and therapeutic targets for the treatment of venetoclax-resistant AML.

**Copyright Information:** (Online) ISSN: 2572-9241
©2026 The Author(s). HemaSphere is published by John Wiley & Sons Ltd on behalf of European Hematology Association. This is an open access Abstract Book distributed under the Attribution-NonCommercial-NoDerivs (CC BY-NC-ND), which allows third parties to download the articles and share them with others as long as they credit the author and the Abstract Book, but they cannot change the content in any way or use them commercially.
**Abstract Book Citations:** Authors, Title, HemaSphere, 2026;10:(S1):pages. The abstract book DOIs can be found on the “Author Information” page.
**Disclaimer:** Articles published in the journal HemaSphere exclusively reflect the opinions of the authors. The authors are responsible for all content in their abstracts including accuracy of the facts, statements, citing resources, etc.

### Summary/Conclusion

GLV8 markedly inhibited cell proliferation, colony formation, and promoted apoptosis in venetoclax-resistant AML cells by down-regulation of GPR84. Our data also indicates that GPR84 may be a new oncogenic gene in AML. Our results reveal that GLV8, by suppressing GPR84 expression, may have therapeutic potential in venetoclax-resistant AML.

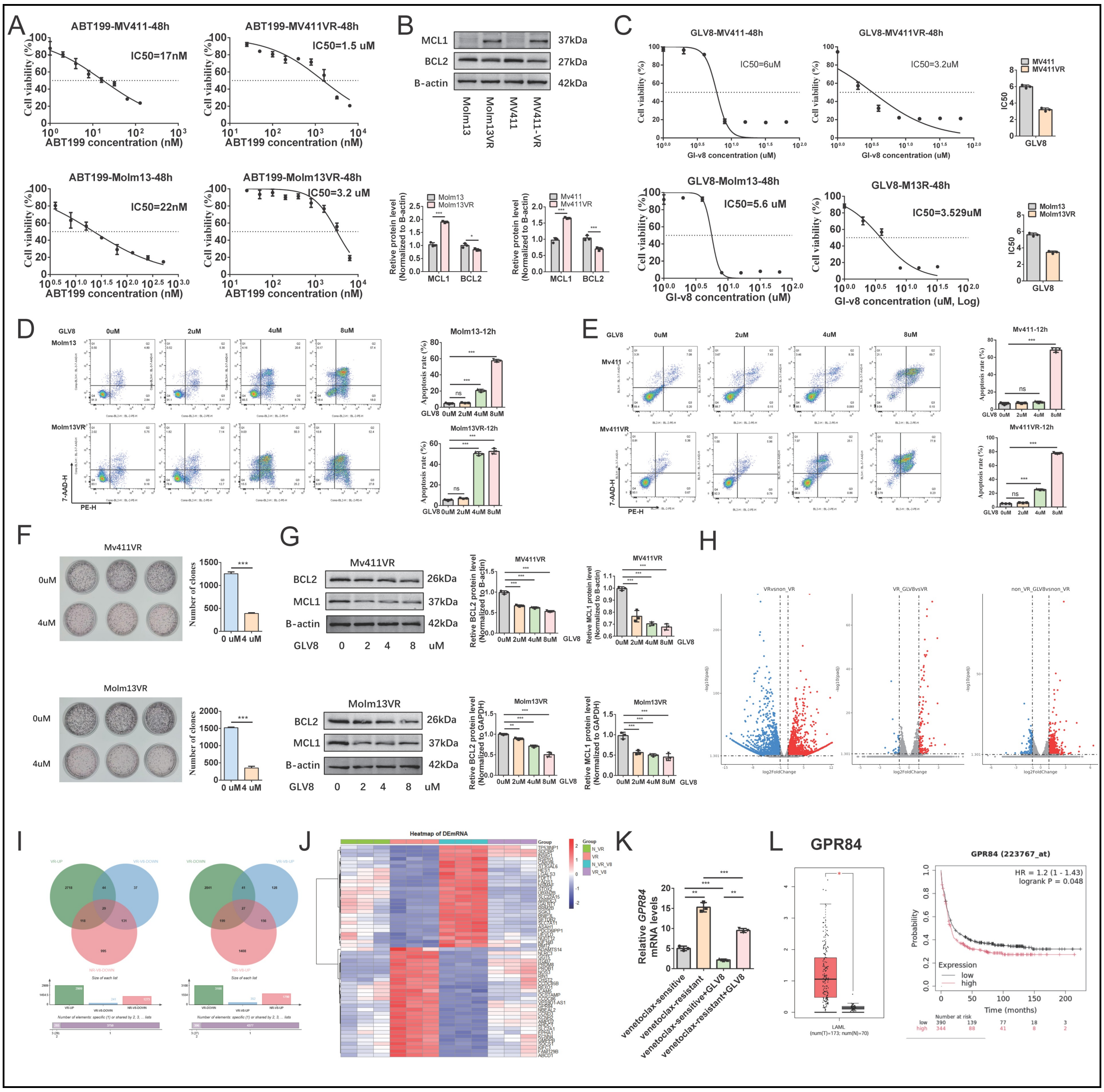

**Copyright Information:** (Online) ISSN: 2572-9241
©2026 The Author(s). HemaSphere is published by John Wiley & Sons Ltd on behalf of European Hematology Association. This is an open access Abstract Book distributed under the Attribution-NonCommercial-NoDerivs (CC BY-NC-ND), which allows third parties to download the articles and share them with others as long as they credit the author and the Abstract Book, but they cannot change the content in any way or use them commercially.
**Abstract Book Citations:** Authors, Title, HemaSphere, 2026;10:(S1):pages. The abstract book DOIs can be found on the "Author Information" page.
**Disclaimer:** Articles published in the journal HemaSphere exclusively reflect the opinions of the authors. The authors are responsible for all content in their abstracts including accuracy of the facts, statements, citing resources, etc.

### (PF465) GM-CSF NEUTRALISATION EFFECTIVE IN VENETOCLAX RESISTANT AML WITH RAS MUTATIONS

**Topic:** 03. Acute myeloid leukemia - Biology & translational research

Kelly Lim*[1, 2], Monika Kutyna[2, 3], Akash Kalro[3], Devendra Hiwase[1, 2, 3], Daniel Thomas[1, 2, 3]

[1]*Adelaide University, School of Medicine, College of Health, Adelaide, Australia;*[2]*South Australian Health and Medical Research Institute, Blood Cancer Group, Precision Cancer Medicine Theme, Adelaide, Australia;* [3]*Central Adelaide Local Health Network, Department of Haematology, Adelaide, Australia;*

### Background

Venetoclax (VEN) plus azacitidine (AZA) is the current standard-of-care for acute myeloid leukaemia (AML); however, outcomes remain poor in AML arising from antecedent myelodysplastic syndromes (MDS), particularly chronic myelomonocytic leukaemia (CMML).  Data from published reports and our South Australian MDS/AML Registry revealed inferior median overall survival (OS) in CMML-transformed AML (3.5-3.7 months) and lower CR/CRi rates (0-33%) compared with newly-diagnosed, previously untreated AML receiving VEN/AZA (median OS 14.7 months; CR/CRi 66.4%). Granulocyte-macrophage colony stimulating factor (GM-CSF) hypersensitivity is a hallmark of CMML, and augmented spontaneous myeloid colony formation in semisolid medium has been proposed as a surrogate parameter for RAS pathway hyperactivation in CMML. We recently observed durable responses to the humanised anti-GM-CSF antibody, Lenzilumab (LENZ) in high blast, RAS pathway mutant CMML at increased risk of AML transformation in the PREcision Approach to CHronic Myelomonocytic Leukaemia (PREACH-M; ACTRN 1262100022383) clinical trial.

### Aims

To investigate the cytokine dependency of RAS-mutant CMML-transformed AML and evaluate sensitivity to antibody-directed GM-CSF neutralisation, alone and in combination with VEN/AZA.

### Methods

Mononuclear cells from RAS-mutant CMML-transformed AML were assayed for viability, colony formation and drug sensitivity in the presence of receptor-blocking or ligand-neutralising antibodies and VEN/AZA.

### Results

In the absence of exogenous cytokines, CMML-transformed AML (CD45+ myeloid cells) demonstrated robust spontaneous growth in vitro. Receptor blockade and cytokine neutralisation established that this spontaneous growth was uniquely dependent on GM-CSF. In colony-forming assays, spontaneous colonies were inhibited by 20 µg/mL LENZ (mean 67.1 ± 7.8% reduction *vs.* IgG control, $P$=0.003), indicative of an autocrine GM-CSF loop amenable to antibody blockade.
CMML-transformed AML exhibited marked resistance to VEN ($IC_{50}$ >500 nM), consistent with clinical experience. Notably, VEN-resistant colonies were significantly suppressed by LENZ, alone and in combination with VEN/AZA. Preliminary mechanistic data suggest that GM-CSF neutralisation downregulates MCL-1 protein expression but not Bcl-2.
Clinically, a patient with CMML-transformed AML refractory to 8 cycles of VEN/AZA was treated with VEN/AZA/LENZ triple therapy (100 mg PO VEN D1-D28, AZA 75 mg/$m^2$ D1-D7, LENZ 552 mg D1 and D15; 35-day cycle). The patient achieved a durable response following 15 cycles of triple therapy, with bone marrow blasts <20%, improved haemoglobin and platelet counts. Triple therapy was well-tolerated and the patient remained transfusion-independent with no acute admissions and reported meaningful improvements in weight and quality of life.

**Copyright Information:** (Online) ISSN: 2572-9241
©2026 The Author(s). HemaSphere is published by John Wiley & Sons Ltd on behalf of European Hematology Association. This is an open access Abstract Book distributed under the Attribution-NonCommercial-NoDerivs (CC BY-NC-ND), which allows third parties to download the articles and share them with others as long as they credit the author and the Abstract Book, but they cannot change the content in any way or use them commercially.
**Abstract Book Citations:** Authors, Title, HemaSphere, 2026;10:(S1):pages. The abstract book DOIs can be found on the "Author Information" page.
**Disclaimer:** Articles published in the journal HemaSphere exclusively reflect the opinions of the authors. The authors are responsible for all content in their abstracts including accuracy of the facts, statements, citing resources, etc.

**Summary/Conclusion**

Spontaneous growth in VEN-resistant RAS mutant, CMML-transformed AML may be driven by autocrine production of the pro-inflammatory cytokine, GM-CSF, revealing a therapeutically targetable vulnerability. GM-CSF neutralisation with LENZ limited MCL-1 expression and suppressed VEN-resistant colonies. LENZ/VEN/AZA triple therapy in a VEN-refractory AML patient produced a well-tolerated and durable response, supporting further clinical investigation.

**Copyright Information:** (Online) ISSN: 2572-9241
©2026 The Author(s). HemaSphere is published by John Wiley & Sons Ltd on behalf of European Hematology Association. This is an open access Abstract Book distributed under the Attribution-NonCommercial-NoDerivs (CC BY-NC-ND), which allows third parties to download the articles and share them with others as long as they credit the author and the Abstract Book, but they cannot change the content in any way or use them commercially.
**Abstract Book Citations:** Authors, Title, HemaSphere, 2026;10:(S1):pages. The abstract book DOIs can be found on the "Author Information" page.
**Disclaimer:** Articles published in the journal HemaSphere exclusively reflect the opinions of the authors. The authors are responsible for all content in their abstracts including accuracy of the facts, statements, citing resources, etc.

### (PF466) UNRAVELING HOW TREATMENT SHAPES CLONAL EVOLUTION AND RESISTANCE IN MURINE KMT2A-REARRANGED LEUKEMIA WITH SUBCLONAL KRASG12D

**Topic:** 03. Acute myeloid leukemia - Biology & translational research

Ton Falqués-Costa*[1], Mattias Pilheden[1], Larissa Moura Castro[1], Qirui Zhang[1], Louise Ahlgren[1], Helena Sturesson[1], Kajsa Paulsson[1], Axel Hyrenius-Wittsten[1], Anna K. Hagström-Andersson[1]

[1]*Department Of Laboratory Medicine, Clinical Genetics, Lund, Sweden;*

### Background

Our understanding of how individual mutations, whether present in all or just a fraction of leukemia cells, affect cellular responses to therapy is limited. Leukemia mouse models provide unique possibilities to explore how therapy affects the evolution of genetically distinct clones and to identify resistance mechanisms with translational relevance.

### Aims

To investigate how different therapies influence survival, clonal evolution, and resistance patterns in murine*KMT2A::MLLT3* leukemia harboring subclonal *Kras*$^{G12D}$.

### Methods

Bone marrow (BM) from a leukemia expressing *KMT2A::MLLT3-mCherry* in all cells and a de *novo Kras*$^{G12D}$ in 38% of cells was re-transplanted into sublethally irradiated recipients. Upon engraftment, treatment was initiated with either chemotherapy mimicking AML induction (cytarabine for 5 days + doxorubicin for 3 days, n=4), the MEK inhibitor Trametinib for 28 days (n=7), or chemotherapy followed by Trametinib (n=6); controls received vehicle. Targeted sequencing was used to track *Kras*$^{G12D}$ clonal dynamics. RNA sequencing (n=19) was performed to assess transcriptional changes associated with treatment and resistance.

### Results

All treatments significantly prolonged survival compared to controls (median latency: 25 days chemotherapy, 27 days Trametinib, 33.5 days combination vs 20 days control). At relapse, most BM cells expressed KMT2A::MLLT3-mCherry and mice developed splenomegaly and leukocytosis.

In control mice, *Kras*$^{G12D}$-containing cells expanded to clonal dominance. Chemotherapy maintained*Kras*$^{G12D}$ frequency at ~40%, indicating suppression without eradication. However, after tertiary transplantation and repeated chemotherapy, *Kras*$^{G12D}$ clones expanded to dominance. Across other treatment groups, most mice relapsed with *Kras*$^{G12D}$-dominant disease, except in two mice receiving combination therapy where Kras-mutant frequency was reduced or maintained.

In 37% of mice, *Kras*$^{G12D}$ VAF exceeded 0.5, consistent with allelic imbalance at the*Kras* locus, particularly in mice receiving targeted therapy, suggesting selective pressure for increased Ras dosage during MEK inhibition. Previous data have shown selective gain of chromosome 6 or Robertsonian translocations involving chromosome 6 under similar pressure. Ongoing analyses will determine whether comparable mechanisms operate in this cohort.

RNA sequencing identified three major clusters corresponding to untreated, Trametinib-treated (± chemotherapy), and chemotherapy-treated mice. Chemotherapy resistance was associated with upregulation of fatty acid metabolism and ROS signaling, whereas Trametinib resistance involved activation of JAK/STAT, TP53, and TNFα pathways, indicating treatment-specific resistance programs.

**Copyright Information:** (Online) ISSN: 2572-9241
©2026 The Author(s). HemaSphere is published by John Wiley & Sons Ltd on behalf of European Hematology Association. This is an open access Abstract Book distributed under the Attribution-NonCommercial-NoDerivs (CC BY-NC-ND), which allows third parties to download the articles and share them with others as long as they credit the author and the Abstract Book, but they cannot change the content in any way or use them commercially.
**Abstract Book Citations:** Authors, Title, HemaSphere, 2026;10:(S1):pages. The abstract book DOIs can be found on the "Author Information" page.
**Disclaimer:** Articles published in the journal HemaSphere exclusively reflect the opinions of the authors. The authors are responsible for all content in their abstracts including accuracy of the facts, statements, citing resources, etc.

### Summary/Conclusion

Distinct therapeutic strategies differentially shape clonal evolution and *Kras*$^{G12D}$ expansion in *KMT2A::MLLT3* leukemia. Targeted sequencing revealed allelic imbalance at the Kras locus under MEK inhibition, suggesting dosage-driven resistance. Transcriptomic profiling identified treatment-specific resistance pathways, highlighting potential strategies to target residual disease.

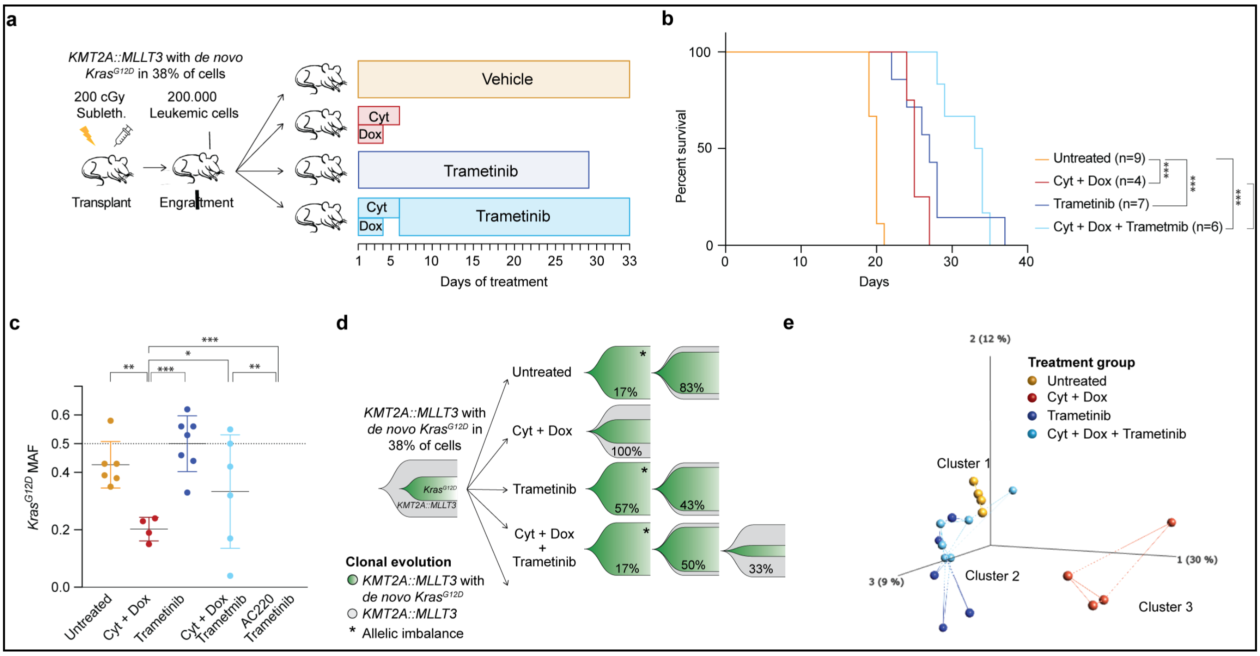

(a) Experimental design and treatment schedule in a murine KMT2A::MLLT3 leukemia model harboring subclonal KrasG12D. (b) Kaplan–Meier survival curves demonstrating prolonged latency upon treatment. (c) KrasG12D variant allele frequency (VAF) at relapse. (d) Clonal evolution patterns, including allelic imbalance at the Kras locus. (e) RNA-seq analysis showing treatment-associated clustering and distinct transcriptional programs at resistance.

**Copyright Information:** (Online) ISSN: 2572-9241
©2026 The Author(s). HemaSphere is published by John Wiley & Sons Ltd on behalf of European Hematology Association. This is an open access Abstract Book distributed under the Attribution-NonCommercial-NoDerivs (CC BY-NC-ND), which allows third parties to download the articles and share them with others as long as they credit the author and the Abstract Book, but they cannot change the content in any way or use them commercially.
**Abstract Book Citations:** Authors, Title, HemaSphere, 2026;10:(S1):pages. The abstract book DOIs can be found on the "Author Information" page.
**Disclaimer:** Articles published in the journal HemaSphere exclusively reflect the opinions of the authors. The authors are responsible for all content in their abstracts including accuracy of the facts, statements, citing resources, etc.

### (PF467) MUTATION-SPECIFIC REMODELING OF THE BONE MARROW MICROENVIRONMENT IN HIGH-RISK AML

**Topic:** 03. Acute myeloid leukemia - Biology & translational research

Manja Wobus*[1], Anna-Lena Baumann[1], Kristin Möbus[1], Heidi Altmann[1], Matt Steel[2], Lynn Quek[2], Martin Bornhäuser[1]

*[1]University Hospital Carl Gustav Carus, Technische Universität, Dresden, Department of Medicine 1, Dresden, Germany;[2]King's College London, Department of Haematology, Cancer and Pharmaceutical Sciences, London, United Kingdom;*

### Background

High-risk AML subtypes, including *TP53*-mutated and *inv(3)(q21q26)/MECOM*-rearranged cases, are associated with chemoresistance, aggressive disease, and poor survival. While leukemic cell-intrinsic factors drive malignancy, the bone marrow microenvironment actively supports leukemic persistence and therapy resistance. Mesenchymal stromal cells (MSCs) provide structural support, metabolic resources, and immune regulatory signals, yet how high-risk genetic lesions reprogram MSC function and niche interactions remains poorly defined. Understanding mutation-specific niche alterations may uncover novel targets to disrupt leukemia–stroma crosstalk.

### Aims

We aimed to characterize mutation-dependent alterations in MSCs from high-risk AML (*TP53*, *inv(3)*) compared with MSCs from favorable-prognosis *NPM1*-mutated AML, focusing on immune, metabolic, and functional regulators within the stromal compartment.

### Methods

Bone marrow mononuclear cells were obtained from AML patients with defined mutations. MSCs were expanded *in vitro* and analyzed by bulk RNA sequencing to assess transcriptional and pathway-level differences. Functional characterization included adipogenic differentiation assays, measurement of CD36 and CD146 expression, quantification of reactive oxygen species (ROS), and assessment of mitochondrial oxygen consumption rates (OCR) using Seahorse technology.

### Results

Transcriptomic profiling revealed pronounced mutation-specific MSC signatures. Compared with *NPM1*, *inv(3)* MSCs displayed 23 up- and 104 down-regulated genes, whereas *TP53* MSCs exhibited 54 up- and 214 down-regulated genes. Both high-risk subtypes showed down-regulation of innate immune system pathways, which may contribute to create an immunosuppressive niche that facilitates immune evasion and blast survival. *Inv(3)* MSCs showed strong upregulation of GABA receptor signaling, consistent with an inhibitory role in the microenvironment, while *TP53* MSCs were enriched for lysine catabolism, a metabolic pathway that feeds into epigenetic regulation and energy production in leukemic cells. Further MSC characterization showed enhanced adipogenic differentiation, demonstrated by about 180-fold expression of PPARg, in *TP53* MSCs associated with significantly elevated CD36 expression (600-fold). ROS levels and OCR were increased in both *inv(3)* and *TP53* MSCs relative to *NPM1*, reflecting elevated oxidative and metabolic activity. Reduced CD146 expression in *TP53* MSCs indicated structural remodeling of the stromal niche.

**Copyright Information:** (Online) ISSN: 2572-9241
©2026 The Author(s). HemaSphere is published by John Wiley & Sons Ltd on behalf of European Hematology Association. This is an open access Abstract Book distributed under the Attribution-NonCommercial-NoDerivs (CC BY-NC-ND), which allows third parties to download the articles and share them with others as long as they credit the author and the Abstract Book, but they cannot change the content in any way or use them commercially.
**Abstract Book Citations:** Authors, Title, HemaSphere, 2026;10:(S1):pages. The abstract book DOIs can be found on the “Author Information” page.
**Disclaimer:** Articles published in the journal HemaSphere exclusively reflect the opinions of the authors. The authors are responsible for all content in their abstracts including accuracy of the facts, statements, citing resources, etc.

**Summary/Conclusion**

High-risk AML subtypes drive mutation-specific remodeling of the surrounding bone marrow microenvironment, affecting immune regulation, metabolism, oxidative stress, and MSC differentiation. Our findings revealed stromal mechanisms that may sustain leukemic persistence and highlight niche-directed pathways as potential therapeutic targets to complement leukemia-focused therapies.

**Copyright Information:** (Online) ISSN: 2572-9241
©2026 The Author(s). HemaSphere is published by John Wiley & Sons Ltd on behalf of European Hematology Association. This is an open access Abstract Book distributed under the Attribution-NonCommercial-NoDerivs (CC BY-NC-ND), which allows third parties to download the articles and share them with others as long as they credit the author and the Abstract Book, but they cannot change the content in any way or use them commercially.
**Abstract Book Citations:** Authors, Title, HemaSphere, 2026;10:(S1):pages. The abstract book DOIs can be found on the "Author Information" page.
**Disclaimer:** Articles published in the journal HemaSphere exclusively reflect the opinions of the authors. The authors are responsible for all content in their abstracts including accuracy of the facts, statements, citing resources, etc.

### (PF468) TARGETING MITOCHONDRIAL TRANSFER WITH LIMK INHIBITION AND METFORMIN OVERCOMES MACROPHAGE-DRIVEN THERAPY RESISTANCE IN ACUTE MYELOID LEUKAEMIA

**Topic:** 03. Acute myeloid leukemia - Biology & translational research

Mark Williams*[1], Ebubechukwu Nwarunma[1], Aikaterini Miari[2], Athanasia Papadopoulou[3], Stefan Corrandini[1], Glen Watt[1], Samantha Hurwitz[4], Tatiana Fourfouris[4], Ki Jun Lee[4], Xenia Bubnova[2], Rebecca Briggs[5], Carl Goodyear[6], Theodoros Simakou[6], Marcus Doohan[6], Lucy MacDonald[6], Mariola Kurowska-Stolarska[6], Timothy Humpton[1], Victoria Campbell[7], Lesley Forrester[8], Ken Mills[5], Katrina Lappin[5], Valerie Ferro[9], Yong-Mi Kim[4], Helen Wheadon[2]

*[1]Glasgow Caledonian University, Biological and Biomedical Sciences, Glasgow, United Kingdom; [2]University of Glasgow, School of Cancer Sciences, Glasgow, United Kingdom; [3]The Francis Crick Institute, London, United Kingdom; [4]Children's Hospital Los Angeles, Los Angeles, United States of America; [5]Queen's University Belfast, Belfast, United Kingdom; [6]University of Glasgow, Glasgow, United Kingdom; [7]Western General Hospital, Edinburgh, United Kingdom; [8]The University of Edinburgh, Edinburgh, United Kingdom; [9]University of Stathclyde, Glasgow, United Kingdom;*

### Background

Interactions between acute myeloid leukaemia (AML) cells and the bone marrow microenvironment (BME) contribute to treatment failure and disease persistence. Macrophages (Mφs) represent a key cellular component of the BME in AML patients and have been implicated in supporting leukaemic cell survival. In addition to previously described Mφ driven contact-dependent protective effects (Miari et al., HemaSphere 2023), we have also shown that Mφs transfer mitochondria to AML cells via horizontal mitochondrial transfer (HMT) (Nwarunma et al., Blood 2024). However, the functional consequences, regulatory signalling pathways, and therapeutic potential of targeting HMT remain unclear.

### Aims

Our aim was to investigate the signalling pathways governing Mφ-to-AML HMT and determine whether targeting these pathways can disrupt Mφ-driven therapy resistance.

### Methods

Primary human CD14⁺ monocyte-derived M2-like Mφs were co-cultured with AML cell lines for 24 h. Metabolic activity was assessed using the Seahorse platform. Tandem Mass Tag (TMT)-based mass spectrometry was performed to identify proteins and pathways associated with mitochondrial transfer. Activation of the LIMK1/2-cofilin pathway following daunorubicin (DNR) exposure was assessed by phospho-cofilin immunoblotting in the presence or absence of the LIMK1/2 inhibitor TH-257 (10 µM). Mitochondrial transfer was quantified using MitoTracker-based flow cytometry. Apoptosis following DNR treatment with TH-257 (10 µM) or Metformin (5 mM) was assessed by Annexin V/FVD staining.

**Copyright Information:** (Online) ISSN: 2572-9241
©2026 The Author(s). HemaSphere is published by John Wiley & Sons Ltd on behalf of European Hematology Association. This is an open access Abstract Book distributed under the Attribution-NonCommercial-NoDerivs (CC BY-NC-ND), which allows third parties to download the articles and share them with others as long as they credit the author and the Abstract Book, but they cannot change the content in any way or use them commercially.
**Abstract Book Citations:** Authors, Title, HemaSphere, 2026;10:(S1):pages. The abstract book DOIs can be found on the "Author Information" page.
**Disclaimer:** Articles published in the journal HemaSphere exclusively reflect the opinions of the authors. The authors are responsible for all content in their abstracts including accuracy of the facts, statements, citing resources, etc.

**Results**

Co-culture with M2-like Mφs enhanced AML mitochondrial function, with increased basal and maximal respiration, spare respiratory capacity, and ATP production compared to monoculture. Proteomic analysis identified upregulation of RhoC, which was associated with inferior overall survival in AML patient datasets (TCGA). Given the role of the RhoC-LIMK-cofilin pathway in actin remodelling and tunnelling nanotube formation, we investigated its involvement in HMT. DNR induced peak cofilin phosphorylation at 12 h indicating potential activation of LIMK-dependent signalling. Pharmacological inhibition of LIMK1/2 with TH-257 reduced both basal and DNR-induced phospho-cofilin levels and significantly impaired HMT. This was accompanied by loss of Mφ-mediated chemoprotection and increased AML sensitivity to DNR, without affecting Mφ viability. Metformin similarly reduced mitochondrial transfer and restored chemosensitivity under co-culture conditions, with no impact on Mφ survival.

**Summary/Conclusion**

These findings identify a RhoC-LIMK-cofilin-driven axis as a key regulator of Mφ-to-AML mitochondrial transfer and therapy resistance. Targeting this pathway, either directly via LIMK inhibition or indirectly with Metformin, disrupts HMT and restores chemosensitivity. Mφ-driven HMT therefore represents a promising and therapeutically actionable vulnerability in AML.

**Copyright Information:** (Online) ISSN: 2572-9241
©2026 The Author(s). HemaSphere is published by John Wiley & Sons Ltd on behalf of European Hematology Association. This is an open access Abstract Book distributed under the Attribution-NonCommercial-NoDerivs (CC BY-NC-ND), which allows third parties to download the articles and share them with others as long as they credit the author and the Abstract Book, but they cannot change the content in any way or use them commercially.
**Abstract Book Citations:** Authors, Title, HemaSphere, 2026;10:(S1):pages. The abstract book DOIs can be found on the “Author Information” page.
**Disclaimer:** Articles published in the journal HemaSphere exclusively reflect the opinions of the authors. The authors are responsible for all content in their abstracts including accuracy of the facts, statements, citing resources, etc.

### (PF469) UNMASKING CRYPTIC GENOMIC ALTERATIONS IN THERAPY-RELATED MYELOID NEOPLASMS (T-MN) USING OPTICAL GENOME MAPPING (OGM)

**Topic:** 03. Acute myeloid leukemia - Biology & translational research

Sara García Ávila*[1, 2, 3], Lucía Gómez-Pérez[1, 2], Anna Puiggros[4, 5], Blanca Espinet Sola[4, 5], María del Mar Rodríguez Rivera[4, 5], Patricia Vélez[1, 2], Maria Alicia Senin Magan[1, 2], Ramón Diez-Feijóo[1, 2, 3], Clara Nadal Pellise[1], Thalia Guiulfo Knell[1], Lierni Fernández Ibarrondo[2], Concepción Fernández-Rodriguez[2, 4], Barbara Ymaine[2, 4], Silvia Saumell[4, 5], Xavier Calvo[4, 5], Leonor Arenillas[6], Christelle Ferrà[1, 2], Beatriz Bellosillo[3, 4, 5], Marta Salido[4, 5]

*[1]Hospital del Mar, Hematology, Barcelona, Spain;[2]Hospital Del Mar Research Institute, Group of Applied Clinical Research in Hematology, Barcelona, Spain; [3]Pompeu Fabra University, Barcelona, Spain;[4]Hospital del Mar, Pathology, Barcelona, Spain; [5]Hospital Del Mar Research Institute, Translational research group on hematological neoplasms, Barcelona, Spain;[6]Hospital Clinic, Pathology, Barcelona, Spain;*

### Background

Therapy-related myeloid neoplasms (t-MN) represent 10–20% of all myeloid neoplasms and are a severe late complication of cytotoxic chemotherapy and/or radiotherapy. Compared with de novo disease, t-MN are characterized by a higher burden of adverse cytogenetic abnormalities, including deletions or monosomies of chromosomes 5 and 7, and a markedly increased frequency of complex karyotypes (CK). Moreover, t-MN frequently harbor *TP53* alterations, which are closely associated with chromosomal instability. Complex or cryptic structural variants that may be inadequately resolved by chromosome banding analysis (CBA) remaining relevant in t-MN. Optical genome mapping (OGM) enables genome-wide, high-resolution detection of balanced and unbalanced structural variants and complex rearrangements.

### Aims

To improve the characterization of cytogenetic alterations in t-MN using OGM and to assess its added value over standard cytogenetic methods for risk stratification.

### Methods

Patients consecutively diagnosed with t-MN since 2023 to date in our institution were included. CBA, OGM and NGS were performed on diagnostic bone marrow samples. CBA was performed following routinely operating procedures. Ultra–high molecular weight DNA from bone marrow was analyzed by OGM using the Bionano Saphyr® system (Bionano Genomics). Genome-wide structural variants (≥5 kb) and copy number alterations were identified using Rare Variant Analysis pipeline and prioritized based on relevance to myeloid neoplasms using specific BED-files. NGS was performed on DNA extracted from diagnostic samples using QIAseq targeted DNA custom panel (QIAGEN) or the Oncomine Myeloid Assay GX v2 (Thermo Fisher Scientific).

**Copyright Information:** (Online) ISSN: 2572-9241
©2026 The Author(s). HemaSphere is published by John Wiley & Sons Ltd on behalf of European Hematology Association. This is an open access Abstract Book distributed under the Attribution-NonCommercial-NoDerivs (CC BY-NC-ND), which allows third parties to download the articles and share them with others as long as they credit the author and the Abstract Book, but they cannot change the content in any way or use them commercially.
**Abstract Book Citations:** Authors, Title, HemaSphere, 2026;10:(S1):pages. The abstract book DOIs can be found on the "Author Information" page.
**Disclaimer:** Articles published in the journal HemaSphere exclusively reflect the opinions of the authors. The authors are responsible for all content in their abstracts including accuracy of the facts, statements, citing resources, etc.

### Results

A total of 15 patients were identified including 7 Acute myeloid leukemia (AML), 5 myelodisplastic syndrome (MDS) and 3 chronic myelomonocytic leukemia (CMML). CBA revealed cytogenetic alterations in 66.7% of patients, most commonly involving CK (n = 5, 33.3%). OGM detected additional cryptic alterations not resolved by CBA, refining genomic complexity in 60% of patients. Moreover, OGM provided a change in CBA risk stratification by identifying clinically relevant abnormalities in 3 patients [del(7q) involving *CUX* locus, t(4;7)(p15.1;q31.31) and monosomy 7]. )]. Other new alterations identified by OGM included cryptic balanced rearrangements involving genes fusions: *NACA::RUNX1*, *RUNX1::PRDM16, NUP153::AP1M2*, and *EPS15::KMT2A,* and a cryptic cytogenetic unbalanced rearrangement involving chromosomes 7 and 13, resulting in loss of 7q and gain of 13q. Notably, OGM enabled detailed characterization of highly complex rearrangements, including chromoanagenesis events in 2 patients. *TP53*-mutated cases showed the highest degree of structural complexity by CBA and OGM, supporting the association between *TP53* alterations and genomic instability in t-MN neoplasms (Figure).

### Summary/Conclusion

OGM is a highly concordant and informative complement to CBA in t-MN, particularly in cases withapparently normal karyotypes. By identifying cryptic balanced rearrangements, novel fusion genes, and complex structural variants not resolved by conventional cytogenetics, OGM refines genomic architecture and enhances detection of clinically relevant abnormalities. In *TP53*-mutated cases, it further delineates extensive structural complexity, including chromoanagenesis events, supporting its added value for a more comprehensive biological and cytogenomic characterization.

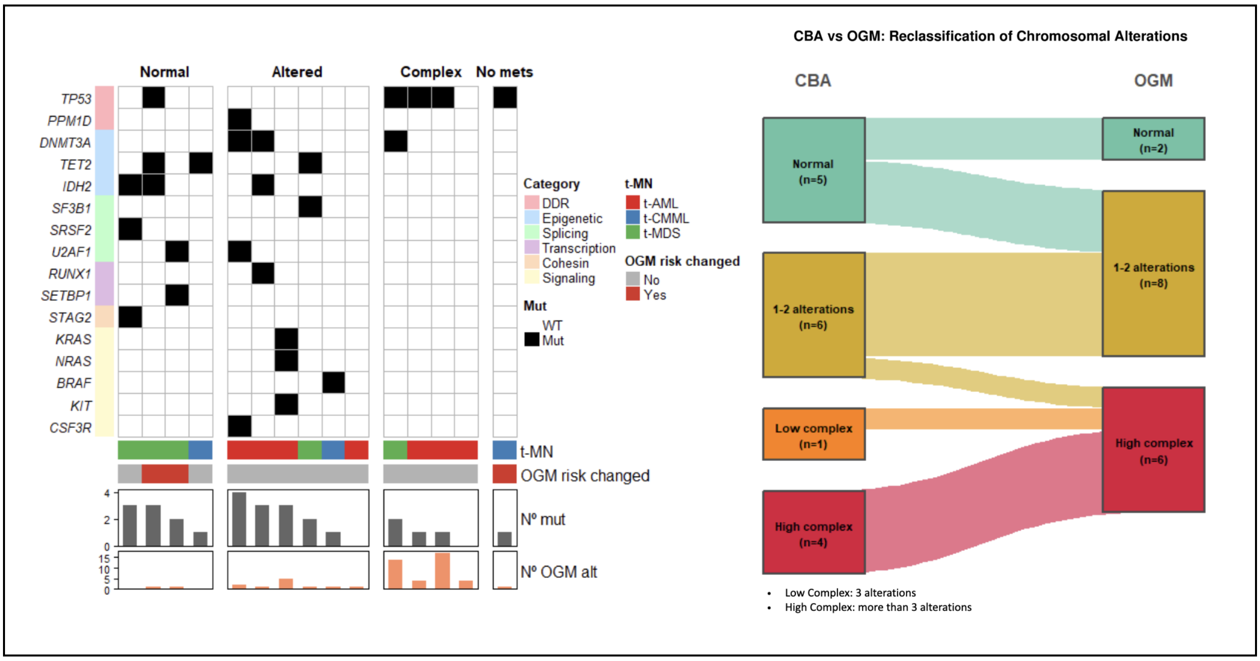

Integrated genomic profiling of therapy-related myeloid neoplasms (t-MN) by CBA, OGM and NGS. Left panel: Distribution of recurrent gene mutations across cytogenetic categories. Right panel: Sankey diagram illustrating reclassification of chromosomal alterations from CBA to OGM.

**Copyright Information:** (Online) ISSN: 2572-9241
©2026 The Author(s). HemaSphere is published by John Wiley & Sons Ltd on behalf of European Hematology Association. This is an open access Abstract Book distributed under the Attribution-NonCommercial-NoDerivs (CC BY-NC-ND), which allows third parties to download the articles and share them with others as long as they credit the author and the Abstract Book, but they cannot change the content in any way or use them commercially.
**Abstract Book Citations:** Authors, Title, HemaSphere, 2026;10:(S1):pages. The abstract book DOIs can be found on the "Author Information" page.
**Disclaimer:** Articles published in the journal HemaSphere exclusively reflect the opinions of the authors. The authors are responsible for all content in their abstracts including accuracy of the facts, statements, citing resources, etc.

### (PF470) HUMAN RETROVIRUS K ENVELOPE PROTEIN ACTIVATION REGULATES THE BIOLOGICAL BEHAVIOR OF AML CELLS AND THE UNDERLYING MECHANISM

**Topic:** 03. Acute myeloid leukemia - Biology & translational research

Ying Xu*[1], Hongyan Tong[1]

[1] *The First Affiliated Hospital, Zhejiang University School of Medicine, Hangzhou, China;*

### Background

Emerging evidence implicates viruses in the pathogenesis of human hematologic malignancies. Human endogenous retroviruses (HERVs), which constitute approximately 8% of the human genome, include the HERV-K family—the most transcriptionally active and the only family containing human-specific members. The HERV-K envelope protein (K-Env), a conserved membrane protein, is highly expressed in various human cancers. Notably, K-Env is markedly upregulated in most hematologic malignancy cell lines and patient samples but minimally expressed in healthy individuals. Its expression significantly decreases in leukemia patients following chemotherapy-induced remission, suggesting a potential role in acute leukemia pathogenesis. Nevertheless, the precise function of K-Env in leukemogenesis remains to be fully elucidated.

### Aims

To elucidate the function of K-Env activation in AML cells and the associated molecular mechanism.

### Methods

Clinical Samples and Gene Expression Analysis： RNA-seq data from bone marrow cells (BMCs) of patients with myeloid hematological malignancies and healthy donors were analyzed to assess *K-Env* expression levels. CD34+ and CD34- cell populations were isolated using anti-CD34 magnetic beads, and *K-Env* expression was quantified by real-time PCR.

In Vitro Functional Assays： The effects of K-Env on AML cell proliferation, cell cycle, apoptosis, and stem cell self-renewal were evaluated using CCK-8 assays, colony formation assays, flow cytometry, and Ki-67 staining.

In Vivo AML Mouse Models： Luciferase-expressing AML cell lines with K-Env overexpression were injected into NSG mice via the tail vein to establish AML models. General condition and overall survival were monitored, and tumor burden was assessed using an *in vivo* imaging system (IVIS). Tumor infiltration in the spleen and bone marrow was evaluated by flow cytometry (hCD45+ cells), H&E staining, and immunohistochemistry.

Mechanistic Studies：RNA-seq data from AML patients and MOLM-13 cells were analyzed for c-Myc pathway enrichment and MYC Targets Hallmark signatures. c-Myc mRNA and protein levels in K-Env-overexpressing AML cells were detected by RT-qPCR and Western blot. mRNA stability was assessed using actinomycin D chase assays, and the effect of K-Env on c-Myc promoter activity was examined by dual-luciferase reporter assays. Key signaling pathways, including PI3K/AKT, MAPK/ERK, Wnt/β-catenin, JAK/STAT, and TGF-β/SMAD, were evaluated by Western blot and immunofluorescence.

**Copyright Information:** (Online) ISSN: 2572-9241
©2026 The Author(s). HemaSphere is published by John Wiley & Sons Ltd on behalf of European Hematology Association. This is an open access Abstract Book distributed under the Attribution-NonCommercial-NoDerivs (CC BY-NC-ND), which allows third parties to download the articles and share them with others as long as they credit the author and the Abstract Book, but they cannot change the content in any way or use them commercially.
**Abstract Book Citations:** Authors, Title, HemaSphere, 2026;10:(S1):pages. The abstract book DOIs can be found on the "Author Information" page.
**Disclaimer:** Articles published in the journal HemaSphere exclusively reflect the opinions of the authors. The authors are responsible for all content in their abstracts including accuracy of the facts, statements, citing resources, etc.

**Results**

The expression level of the *k-env* gene in bone marrow cells (BMCs) from AML patients was 1.98-fold higher than that in healthy donors. Notably, *k-env* expression was significantly elevated in CD34+ cells compared to CD34- cells, suggesting a potential role for K-Env in maintaining the biological functions of stem cells. Consistently, knockdown of K-Env resulted in a 60.7% reduction in colony formation by MOLM-13 cells relative to controls. Furthermore, high K-Env expression promoted cell proliferation by increasing the proportion of cells in the S phase of the cell cycle. *In vivo*, K-Env enhanced tumor growth and shortened survival in two independent AML mouse models. Mechanistically, K-Env activated the PI3K/AKT signaling pathway and enhanced the transcriptional activity of the c-Myc promoter, leading to upregulated c-Myc expression.

**Summary/Conclusion**

This study reveals that K-Env drives AML pathogenesis by activating the PI3K/AKT/c-Myc axis, establishing it as a potential therapeutic target.

**Copyright Information:** (Online) ISSN: 2572-9241
©2026 The Author(s). HemaSphere is published by John Wiley & Sons Ltd on behalf of European Hematology Association. This is an open access Abstract Book distributed under the Attribution-NonCommercial-NoDerivs (CC BY-NC-ND), which allows third parties to download the articles and share them with others as long as they credit the author and the Abstract Book, but they cannot change the content in any way or use them commercially.
**Abstract Book Citations:** Authors, Title, HemaSphere, 2026;10:(S1):pages. The abstract book DOIs can be found on the "Author Information" page.
**Disclaimer:** Articles published in the journal HemaSphere exclusively reflect the opinions of the authors. The authors are responsible for all content in their abstracts including accuracy of the facts, statements, citing resources, etc.

### (PF471) MAGNETIC-INDUCED CELL-PATIENT-DERIVED XENOGRAFT (MAGIC-PDX) MODEL AS PARALLEL STUDIES FOR PERSONALIZED TREATMENT IN HEMATOLOGICAL MALIGNANCIES

**Topic:** 03. Acute myeloid leukemia - Biology & translational research

Taosong Liu[1], Zizhan Wang[2], Yufang Lv[1], Nan Ding[3], Rakesh Kumar Raut[3], Shiying Lu[1], Hongfei Zhu[1], Luxia Tang[1], Jingyi Guo[1], Sihan Wei[1], Lihan Dai[1], Qinglan Lin[1], Jiaxing Zhang[2], Baowen Liang[2], Yiting Li[2], Qianli Jiang*[3, 4]

*[1]Southern Medical University, Guangzhou, China;[2]Guangzhou Maishi Biotechnology Co., Ltd., Guangzhou, China;[3]Nanfang Hospital, Southern Medical University, Department of Hematology, Guangzhou, China; [4]Nanfang Hospital, Southern Medical University, Clinical Trial Center, Guangzhou, China;*

### Background

Establishing PDX models of hematological malignancies continues to be a major challenge, which has hindered its clinical application for decades. The main bottlenecks include low success rates (10%-30%), long modeling times (3-6 months), high costs, and difficulties in standardization. Our team utilizes MagIC (Magnetically Induced Cell) technology to magnetize tumor cells and apply a precise magnetic field locally, guiding the tumor cells to into the bone marrow, and accelerating its colonization, thereby allowing the establishment of various models of primary bone tumors or metastatic bone cancer.

### Aims

To test the effectiveness of MagIC-PDX modeling using fresh clinical samples and cell lines from hematological malignancies.

### Methods

(1) Comparison of Modeling Methods: Thirty-six NSG mice were randomized into four groups (n=9) tail vein (IV) group, intra-bone marrow injection (IF) group, and femur MagIC (MagIC) group, and PBS control. Each mice received 1×10^6 of RPMI8226-GL myeloma cells labeled with GFP and luciferase. After stable bioluminescence (BL) detection, drug testing was performed. (2) Personalized PDX: Bone marrow mononuclear cells from 11 patients with hematological malignancies (AML, MM and ALL) were injected using MagIC method into the right femur of 11 NSG mice, 1×10^7 cells per mouse. Mice's body weight and blood routine tests were monitored. Bone marrow smears, flow cytometry, FISH, RT-qPCR, and pathology were performed at different time points. This study was approved by the institutional ethics committees.

**Copyright Information:** (Online) ISSN: 2572-9241
©2026 The Author(s). HemaSphere is published by John Wiley & Sons Ltd on behalf of European Hematology Association. This is an open access Abstract Book distributed under the Attribution-NonCommercial-NoDerivs (CC BY-NC-ND), which allows third parties to download the articles and share them with others as long as they credit the author and the Abstract Book, but they cannot change the content in any way or use them commercially.
**Abstract Book Citations:** Authors, Title, HemaSphere, 2026;10:(S1):pages. The abstract book DOIs can be found on the “Author Information” page.
**Disclaimer:** Articles published in the journal HemaSphere exclusively reflect the opinions of the authors. The authors are responsible for all content in their abstracts including accuracy of the facts, statements, citing resources, etc.

### Results

(1) On +35d, based on the BL signals, the success rates of Group IV, Group IF, and Group MagIC were 0, 33.33%, and 77.78%, respectively (p = 0.0046). In +3d, the BL signal in MagIC was stable, strong and consistent, enabling drug testing. From +5d, the blasts were visible in the bone marrow smears of MagIC mouse, whereas none were observed in Group IV on +28d. MRI and micro-CT revealed osteolytic bone destruction of the right femur in Group MagIC on +28d.
(2): Table 1 displays the information of 11 patients and the success rate based on the blasts (>5%) in bone marrow smears or the blasts (>1%) by flow cytometry. The overall success rate is 72.7%. The tumor fusion genes RT-qPCR and FISH results of PDX were consistent with clinical findings. Figure 1 shows the blood routine test.
Tab.1 Basic information of 11 patients and their PDX models

| Patient | Sex | Age | Types of diseases | Bone marrow smear | FISH | Modeling successful | Tumorigenesis days | Survival days |
|---|---|---|---|---|---|---|---|---|
| 1 | F | 26 | AML-M3 | +/- | PML/RARα⊠+⊠ | + | 30d | 30d |
| 2 | F | 58 | AML-NR | +/+ | - | + | 40d | 40d |
| 3 | M | 63 | MM | - | IGH::CCND1⊠+⊠ | - | - | 27d |
| 4 | M | 45 | AML-M5 | +/+ | - | + | 33d | 37d |
| 5 | F | 43 | AML-NR | + | - | + | 24d | 25d |
| 6 | M | 24 | AML-M3 | - | PML/RARα⊠+⊠ | - | - | 96d |
| 7 | M | 72 | MM | + | IGH::MAF⊠+⊠ | + | 59d | 82d |
| 8 | M | 45 | AML-M5 | + | - | + | 20d | 20d |
| 9 | M | 38 | ALL | - | CDKN2A⊠-⊠ | - | - | 62d |
| 10 | M | 72 | AML-M2 | +/- | - | + | 23d | 36d |
| 11 | M | 72 | AML-M2 | +/+ | - | + | 38d | 38d |

### Summary/Conclusion

The MagIC method for modeling is significantly superior than the classical tail vein injection method. The success rate of 72.7% in 11 batches of MagIC-PDX is much higher than the literature. The model can successfully reproduce clinical conditions and meet the needs of personalized medicine. We will conduct parallel cohort studies between PDX and clinical patients. In the era of AI, such model will accelerate the development of new drugs.

**Copyright Information:** (Online) ISSN: 2572-9241
©2026 The Author(s). HemaSphere is published by John Wiley & Sons Ltd on behalf of European Hematology Association. This is an open access Abstract Book distributed under the Attribution-NonCommercial-NoDerivs (CC BY-NC-ND), which allows third parties to download the articles and share them with others as long as they credit the author and the Abstract Book, but they cannot change the content in any way or use them commercially.
**Abstract Book Citations:** Authors, Title, HemaSphere, 2026;10:(S1):pages. The abstract book DOIs can be found on the "Author Information" page.
**Disclaimer:** Articles published in the journal HemaSphere exclusively reflect the opinions of the authors. The authors are responsible for all content in their abstracts including accuracy of the facts, statements, citing resources, etc.

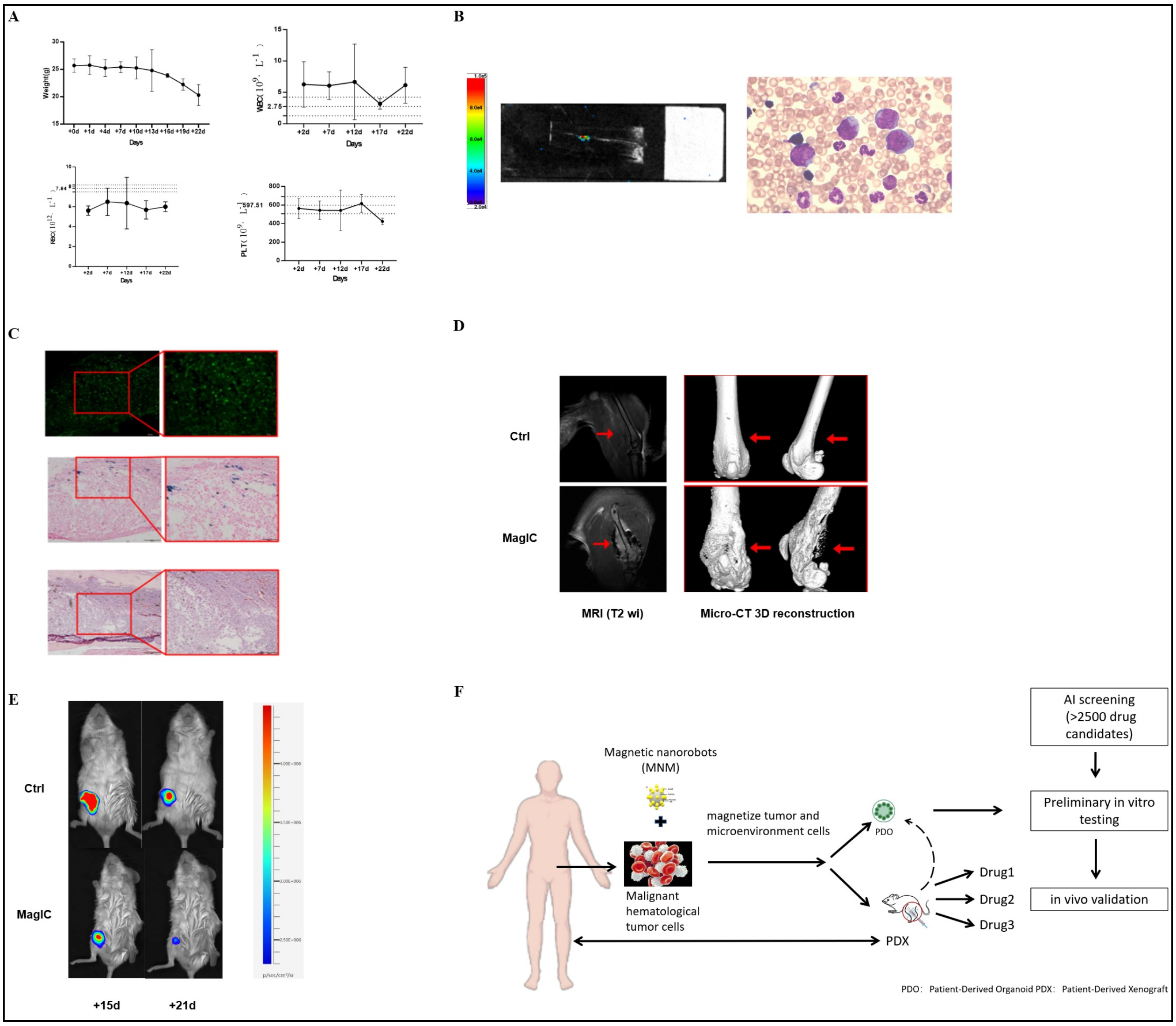

A: Changes in body weight and blood routine examination of PDX mice. (n=6) B: Bioluminescence images of bone marrow smears from mice in the MagIC group (+5d) and microscopic bone marrow images after Wright-Giemsa staining. C: GFP fluorescence images, Prussian blue iron staining images, and HE staining images of tumor tissue from the right femur of mice in the MagIC group. D: MRI and Micro-CT images of the right femur of mice in the Ctrl and MagIC groups. E: Bioluminescence images of mice in the MagIC group before and after combined treatment with thalidomide and dexamethasone (THA/DEX) and CD4/TGF-β bispecific antibody. F: Technical roadmap of the MagIC-PDX platform.

**Copyright Information:** (Online) ISSN: 2572-9241
©2026 The Author(s). HemaSphere is published by John Wiley & Sons Ltd on behalf of European Hematology Association. This is an open access Abstract Book distributed under the Attribution-NonCommercial-NoDerivs (CC BY-NC-ND), which allows third parties to download the articles and share them with others as long as they credit the author and the Abstract Book, but they cannot change the content in any way or use them commercially.
**Abstract Book Citations:** Authors, Title, HemaSphere, 2026;10:(S1):pages. The abstract book DOIs can be found on the “Author Information” page.
**Disclaimer:** Articles published in the journal HemaSphere exclusively reflect the opinions of the authors. The authors are responsible for all content in their abstracts including accuracy of the facts, statements, citing resources, etc.

### (PF472) RAPID PLASMA PROTEOMIC STRATIFICATION OF TP53 AND FLT3 POSITIVE ACUTE MYELOID LEUKEMIA

**Topic:** 03. Acute myeloid leukemia - Biology & translational research

Albin Osterroos*[1], Klara Hernandez Svedevall[1], Gunnar Larfors[1], Gunilla Enblad[2], Anna Robelius[1]

[1]*Uppsala University, Department of Medical Sciences, Uppsala, Sweden;*[2]*Uppsala University, Department of Immunology, Genetics & Pathology, Uppsala, Sweden;*

### Background

Risk stratification of acute myeloid leukemia (AML) increasingly relies on the rapid identification of molecular aberrations, most notably in *TP53* and *FLT3*. The latency of next-generation sequencing forces clinicians to initiate induction therapy without critical genomic insights. Plasma-based proteomic signatures offer an accelerated readout, potentially circumventing these diagnostic delays.

### Aims

To characterize the mutation-specific proteomic landscapes of *TP53+* and *FLT3+* AML and develop high-certainty protein signatures as rapid diagnostic surrogates.

### Methods

Diagnostic pre-treatment plasma samples from 99 adult AML patients and 60 healthy controls (HCs) were analyzed using multiplex proximity extension assay for 183 proteins (Olink panels Oncology II and CVD III). Differential expression was assessed via an age- and sex-adjusted linear modeling framework (limma). Functional landscapes were mapped through Hallmark pathway enrichment and transcription factor (TF) activity inference. Diagnostic signatures were distilled using bootstrapped elastic net regression, with weights calibrated via unpenalized logistic regression and optimal cutoffs determined by Youden's J Index to obtain area under the curve (AUC) estimates.

### Results

Our cohort included 15 (15%) TP53+ and 29 (29%) FLT3+ AML patients with median age 65 years (range 18-87). We observed substantial protein upregulations in *TP53*+ and *FLT3*+ samples compared to HCs with 95 and 92 significantly upregulated proteins, respectively. Functional enrichment of these upregulated proteins highlighted Hallmark pathways including inflammation, IL2-STAT5 signaling and apoptosis for both *TP53+* and *FLT3+*. Next, when comparing *TP53+* and *FLT3+* cases to their wild-type counterparts, we observed 6 and 22 upregulated proteins, respectively, with no overlap. Of note, top hits included described targetable proteins such as delta-like non-canonical notch ligand 1 (DLK1) in *TP53+* and E-selectin (SELE) in *FLT3+*. TF activity inference resulted in two distinct patterns where *TP53*+ AML is characterized by a loss of myeloid differentiation (inactivation of PU.1/SPI1) and a compensatory survival drive via TFAP2A, whereas *FLT3+* AML is driven by a stress-response program centered on JUN.

We applied a bootstrapped pipeline to distill these proteome differences into two high-performance signatures based on weighted diagnostic likelihood scores for the five most stable proteins. The likelihood scores for *TP53+* included MCP-1, CPA1, DLK-1, CD207 and TGF-alpha and for *FLT3+* the proteins SELE, VEGFR-3, IL-1RT1, hK8 and LDL-R. Individual patient risk scores, visualized through waterfall plots (Fig. 1), demonstrated diagnostic separation with an obtained AUC of 0.84 (95% confidence interval (CI) 0.72-0.95) and 0.88 (95% CI 0.80-0.96) using a threshold of 1.29 and 1.96 for *TP53*+ and *FLT3*+, respectively. Performance metric assessment resulted in high negative predictive values of 0.94 and 0.91 for *TP53*+ and *FLT3*+, respectively, suggesting these models as potent clinical rule-out tools.

**Copyright Information:** (Online) ISSN: 2572-9241
©2026 The Author(s). HemaSphere is published by John Wiley & Sons Ltd on behalf of European Hematology Association. This is an open access Abstract Book distributed under the Attribution-NonCommercial-NoDerivs (CC BY-NC-ND), which allows third parties to download the articles and share them with others as long as they credit the author and the Abstract Book, but they cannot change the content in any way or use them commercially.
**Abstract Book Citations:** Authors, Title, HemaSphere, 2026;10:(S1):pages. The abstract book DOIs can be found on the "Author Information" page.
**Disclaimer:** Articles published in the journal HemaSphere exclusively reflect the opinions of the authors. The authors are responsible for all content in their abstracts including accuracy of the facts, statements, citing resources, etc.

### Summary/Conclusion

These findings highlight *TP53+* and *FLT3+* proteomic signatures in AML that can be detected in plasma providing clinicians with immediate genomic insights. In addition, TF inference identified TFAP2A as a therapeutic vulnerability in *TP53+* AML. External validation in an independent cohort is currently underway.

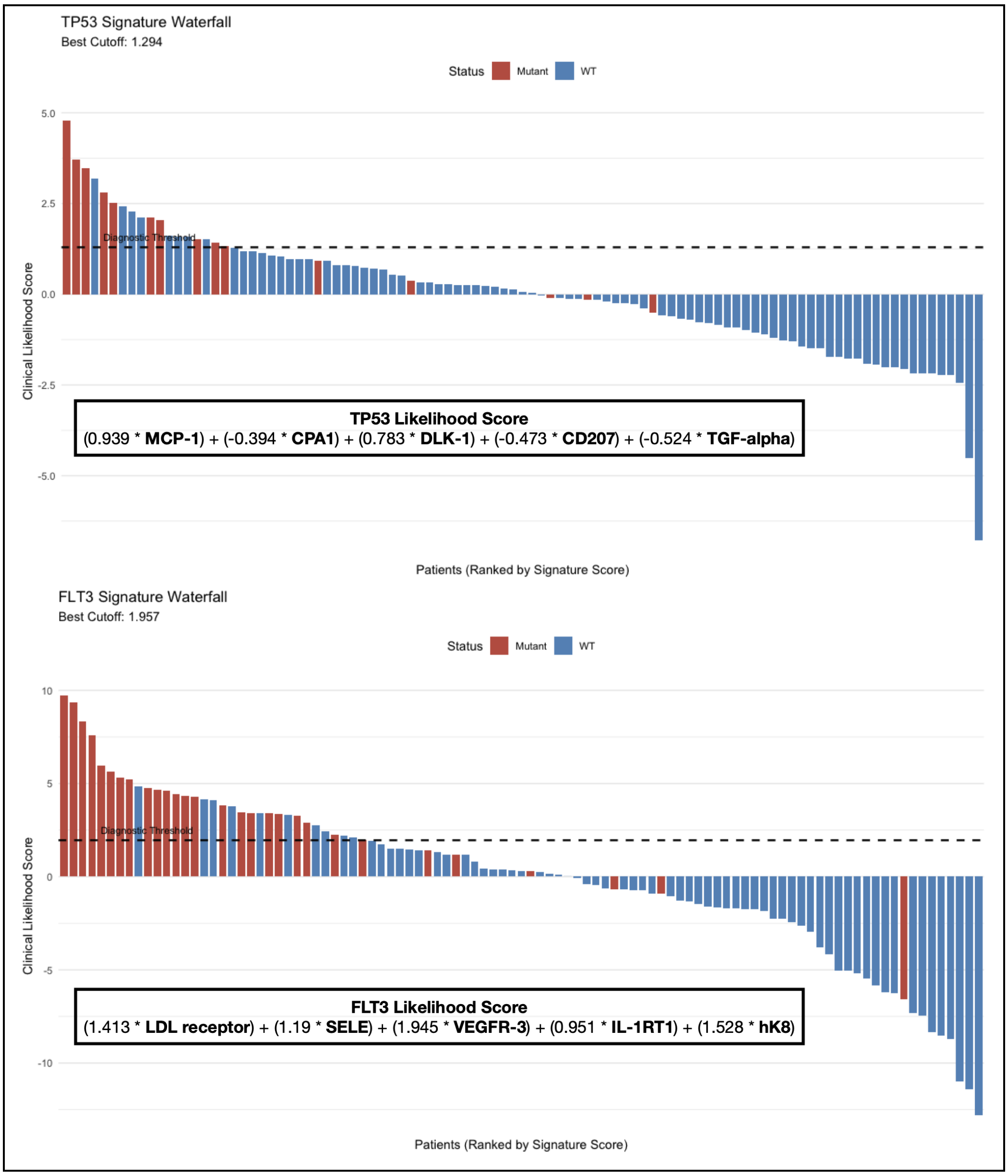

**Copyright Information:** (Online) ISSN: 2572-9241
©2026 The Author(s). HemaSphere is published by John Wiley & Sons Ltd on behalf of European Hematology Association. This is an open access Abstract Book distributed under the Attribution-NonCommercial-NoDerivs (CC BY-NC-ND), which allows third parties to download the articles and share them with others as long as they credit the author and the Abstract Book, but they cannot change the content in any way or use them commercially.
**Abstract Book Citations:** Authors, Title, HemaSphere, 2026;10:(S1):pages. The abstract book DOIs can be found on the "Author Information" page.
**Disclaimer:** Articles published in the journal HemaSphere exclusively reflect the opinions of the authors. The authors are responsible for all content in their abstracts including accuracy of the facts, statements, citing resources, etc.

### (PF473) RAD51 INHIBITION SYNERGIZES WITH MLN4924 BY DISABLING HOMOLOGOUS RECOMBINATION AND ACTIVATING CGAS–STING–MEDIATED INNATE IMMUNITY IN ACUTE MYELOID LEUKEMIA

**Topic:** 03. Acute myeloid leukemia - Biology & translational research

Yu Zhu*[1, 2], Xiao Tang[1], Guozhi Wu[1], Yin Wang[1], Yuancheng Guo[3], Yali Zhang[1], Haiping Liang[1], Bei Liu[2, 3]

*[1]The First Clinical Medical College of Lanzhou University, Lanzhou, China;[2]Gansu Provincial Clinical Medical Research Center for Molecular Diagnosis and Treatment of Hematological Diseases, Lanzhou, China; [3]The First Hospital of Lanzhou University, Department of Hematology, Lanzhou, China;*

### Background

The NEDD8-activating enzyme inhibitor MLN4924 (pevonedistat) has shown robust preclinical activity in acute myeloid leukemia (AML) but did not translate into improved outcomes in the PANTHER phase III trial, suggesting adaptive resistance mechanisms. Identifying compensatory pathways may provide a rationale for combination strategies.

### Aims

To investigate the therapeutic potential of combining MLN4924 with the RAD51 inhibitor RI-1 in AML.

### Methods

Transcriptomic and proteomic profiling were performed with validation by qPCR and western blotting. Cell viability, apoptosis, and cell-cycle distribution were assessed by CCK-8 and flow cytometry. DNA damage, cGAS–STING activation, type I interferon (IFN-I) transcripts, and PD-L1 cell surface expression were evaluated by western blotting, qPCR, and flow cytometry. STING-knockout MOLM13 cells were generated by CRISPR/Cas9. Antitumor efficacy, tolerability, and immune phenotyping were examined in AML cell line–derived xenografts and PBMC–reconstituted xenograft models.

### Results

Multi-omics analyses and validation experiments revealed that MLN4924 induced RAD51 upregulation and homologous recombination repair (HRR) activation in AML cells. Pharmacologic RAD51 inhibition (RI-1) synergistically enhanced MLN4924-induced cytotoxicity, significantly reducing viability, increasing apoptosis, and inducing cell-cycle arrest with marked accumulation of DNA double-strand breaks. The combination activated cGAS–STING signaling, induced a type I interferon (IFN-I) transcriptional program, and increased PD-L1 surface expression. In vivo, MLN4924/RI-1 suppressed AML xenograft growth more effectively than either monotherapy without overt toxicity and increased T-cell infiltration and cytotoxic phenotypes in PBMC-reconstituted xenografts; these effects were attenuated in STING-deficient settings.

### Summary/Conclusion

RAD51 inhibition potentiates MLN4924 by concurrently disabling HRR-mediated DNA repair and activating cGAS–STING–driven innate immune signaling, providing a mechanistic and translational rationale for this combination strategy in AML.

**Copyright Information:** (Online) ISSN: 2572-9241
©2026 The Author(s). HemaSphere is published by John Wiley & Sons Ltd on behalf of European Hematology Association. This is an open access Abstract Book distributed under the Attribution-NonCommercial-NoDerivs (CC BY-NC-ND), which allows third parties to download the articles and share them with others as long as they credit the author and the Abstract Book, but they cannot change the content in any way or use them commercially.
**Abstract Book Citations:** Authors, Title, HemaSphere, 2026;10:(S1):pages. The abstract book DOIs can be found on the "Author Information" page.
**Disclaimer:** Articles published in the journal HemaSphere exclusively reflect the opinions of the authors. The authors are responsible for all content in their abstracts including accuracy of the facts, statements, citing resources, etc.

### (PF474) SELINEXOR-INDUCED GLYCOLYTIC SUPPRESSION REVEALS A METABOLIC ACHILLES' HEEL IN ACUTE MYELOID LEUKEMIA

**Topic:** 03. Acute myeloid leukemia - Biology & translational research

Xuntao Lai[1], Donghua Zhang[2], Anqiao Li[1], Xinliang Mao[3, 4], Jia Wei[2], Longlong Liu[1], Zhenqian Huang*[1]

*[1]The First Affiliated Hospital of Guangzhou Medical University, Hematology, Guangzhou, China;[2]Tongji Hospital, Tongji Medical College, Huazhong University of Science and Technology, Hematology, Wuhan, China; [3]The First Affiliated Hospital of Guangzhou Medical University, Key Laboratory of Advanced Interdisciplinary Studies, Guangzhou, China; [4]School of Basic Medical Sciences, Guangzhou Medical University, Guangdong Provincial Key Laboratory of Protein Modification and Diseases, Guangzhou, China;*

### Background

Metabolic heterogeneity in acute myeloid leukemia (AML) shapes drug sensitivity and underlies resistance. Selinexor, an XPO1 inhibitor that restores nuclear tumor suppressors, has shown clinical activity but variable responses. Emerging evidence links nuclear-export blockade to metabolic reprogramming; whether selinexor shifts carbon-metabolism dependencies and creates exploitable vulnerabilities is unknown.

### Aims

To explore whether selinexor shifts carbon-metabolism dependencies and creates exploitable vulnerabilities

### Methods

We interrogated BEAT-AML2 and FPMTB transcriptomes to compare metabolic pathway enrichment between selinexor-sensitive and -resistant AML samples. Unbiased quantitative proteomics on multiple AML cell lines profiled protein-level pathway changes after selinexor exposure. Functional phenotyping used ECAR (glycolytic flux) and OCR (mitochondrial respiration) assays. Antiproliferative effects of selinexor alone and combined with the glycolysis inhibitor 2-deoxy-D-glucose (2-DG) were quantified; drug interactions were assessed by synergy metrics.

### Results

Cohort analyses consistently showed enrichment of glycolysis-related gene sets in selinexor-resistant specimens, defining a transcriptional "glycolysis-high" resistance state. Proteomics revealed coordinated downregulation of core glycolytic enzymes following selinexor treatment, indicating suppression of glycolytic machinery. Functionally, most AML lines displayed a marked, convergent reduction in ECAR after selinexor exposure, while OCR responses were heterogeneous—some lines increased OXPHOS, others decreased or remained unchanged. Crucially, selinexor plus 2-DG produced reproducible, strong synergy across diverse AML cell lines, transforming selinexor's mainly cytostatic effect into potent antiproliferative and cytotoxic responses.

### Summary/Conclusion

Integrated cohort, proteomic and functional data define a novel metabolic paradigm: selinexor resistance is associated with a glycolysis-high phenotype, whereas selinexor treatment suppresses glycolysis and unmasks sensitivity to glycolytic inhibition. Heterogeneous OXPHOS adaptation likely modulates combination efficacy, but the consistent glycolytic suppression provides a rationale for combining XPO1 and glycolysis inhibitors in AML. These results support preclinical optimization and biomarker-guided clinical evaluation of this combination strategy.

**Copyright Information:** (Online) ISSN: 2572-9241
©2026 The Author(s). HemaSphere is published by John Wiley & Sons Ltd on behalf of European Hematology Association. This is an open access Abstract Book distributed under the Attribution-NonCommercial-NoDerivs (CC BY-NC-ND), which allows third parties to download the articles and share them with others as long as they credit the author and the Abstract Book, but they cannot change the content in any way or use them commercially.
**Abstract Book Citations:** Authors, Title, HemaSphere, 2026;10:(S1):pages. The abstract book DOIs can be found on the "Author Information" page.
**Disclaimer:** Articles published in the journal HemaSphere exclusively reflect the opinions of the authors. The authors are responsible for all content in their abstracts including accuracy of the facts, statements, citing resources, etc.

### (PF475) NANOBODY-BASED CHIMERIC ANTIGEN RECEPTOR T CELLS TARGETING TYROBP IN PEDIATRIC ACUTE MYELOID LEUKEMIA

**Topic:** 03. Acute myeloid leukemia - Biology & translational research

Laurens Van Camp*[1, 2, 3], Jolien Vanhooren[1, 2, 3], Stijn De Munter[3, 4], Larissa Deneweth[2, 3], Mattias Hofmans[3, 4, 5], Christophe Chantrain[6], Laurence Dedeken[7], An Van Damme[8], Anne Uyttebroeck[9], Bart Vandekerckhove[3, 4], Jan Gettemans[3, 10], Tim Lammens[1, 2, 3], Barbara De Moerloose[1, 2, 3]

*[1]Ghent University, Department of Internal Medicine and Pediatrics, Ghent, Belgium;[2]Ghent University Hospital, Department of Pediatric Hemato-Oncology and Stem Cell Transplantation, Ghent, Belgium; [3]Cancer Research Institute Ghent (CRIG), Ghent, Belgium; [4]Ghent University, Department of Diagnostic Sciences, Ghent, Belgium;[5]Ghent University Hospital, Laboratory Medicine, Ghent, Belgium; [6]Centre Hospitalier Chrétien (CHC), Liège, Belgium; [7]Hôpital Universitaire des Enfants Reine Fabiola, Brussels, Belgium; [8]Cliniques universitaires Saint-Luc, Université catholique de Louvain, Brussels, Belgium;[9]University Hospital Gasthuisberg, Leuven, Belgium; [10]Ghent University, Department of Biomolecular Medicine, Ghent, Belgium;*

### Background

Overall survival in primary pediatric acute myeloid leukemia (pedAML) has plateaued at ~75%, with 30–40% of patients experiencing relapse, largely attributed to therapy-resistant leukemic stem cells (LSCs). Efforts to improve outcomes through LSC-targeted therapies, including chimeric antigen receptor T (CAR-T) cells, are hindered by the lack of leukemia-specific antigens that spare healthy hematopoietic stem cells (HSCs) and avoid off-tumor toxicity. Targeting molecules shared by LSCs and leukemic blasts, but absent on HSCs, may offer a novel therapeutic strategy. Tyrosine kinase binding protein (TYROBP), an immunoreceptor tyrosine-based activation motif (ITAM)- containing adaptor involved in immune signaling and inflammation, has been implicated in the function of tumor-associated macrophages and disease progression in other contexts. Interestingly, we have recently shown that *TYROBP* is overexpressed in LSCs and leukemic blasts in both pedAML and adult AML patients, while being absent on healthy HSCs.

### Aims

In this study, we explored the role of TYROBP in AML and developed and evaluated nanobody-based anti-TYROBP CAR-T cells (nanoCARs).

### Methods

Associations between *TYROBP* expression and clinical and molecular features, as well as survival, were analyzed using the TARGET database, including a multivariate Cox regression analysis. TYROBP expression was assessed by RT-qPCR and flow cytometry in hematological cell lines, pedAML samples, healthy control samples, and cord blood samples, including stimulated T cells. Knockout and overexpression AML cell line models were generated using CRISPR/Cas9 ribonucleoprotein electroporation. Impact on proliferation, cell cycle, and drug sensitivity was assessed. RNA sequencing was performed to identify differentially expressed genes upon knock-out or overexpression and to explore the involved pathways. TYROBP-specific nanobodies were developed by injecting a llama with TYROBP protein and used to generate nanoCARs. Cytotoxic lysing capacity of the different anti-TYROBP nanoCARs was evaluated in a co-culture assay with luciferase-expressing hematological cell lines. Cytokine release was measured by ELISA.

**Copyright Information:** (Online) ISSN: 2572-9241
©2026 The Author(s). HemaSphere is published by John Wiley & Sons Ltd on behalf of European Hematology Association. This is an open access Abstract Book distributed under the Attribution-NonCommercial-NoDerivs (CC BY-NC-ND), which allows third parties to download the articles and share them with others as long as they credit the author and the Abstract Book, but they cannot change the content in any way or use them commercially.
**Abstract Book Citations:** Authors, Title, HemaSphere, 2026;10:(S1):pages. The abstract book DOIs can be found on the "Author Information" page.
**Disclaimer:** Articles published in the journal HemaSphere exclusively reflect the opinions of the authors. The authors are responsible for all content in their abstracts including accuracy of the facts, statements, citing resources, etc.

### Results

High *TYROBP* expression was significantly associated with the presence of *KMT2A*-rearrangement and inv(16) ($p<0.001$) and independently predicted inferior event-free and overall survival ($p=0.003$ and $p=0.017$) by a multivariate Cox regression analysis. Cumulative incidence of relapse was also higher in patients with a higher *TYROBP* expression (hazard ratio 1.29, 95% confidence interval 1.09-1.53, $p = 0.0027$). *TYROBP* was highly expressed in AML cell lines, while low or absent in other hematological cell lines. Flow cytometry showed TYROBP expression in 7/9 pedAML samples, while absent on B cells and T cells, including stimulated T cells. *TYROBP* knockout reduced proliferation and increased sensitivity to chemotherapy, while overexpression had the opposite effect. TYROBP-specific nanoCARs (Nb1, Nb2, Nb9, and Nb13) selectively lysed antigen-positive THP-1 cells, while sparing antigen-low (HL-60) and -negative cell lines (SUP-B15) (Figure 1). Similarly, IFN-γ secretion *in vitro* was selectively observed for THP-1 cells treated with anti-TYROBP nanoCARs.

### Summary/Conclusion

TYROBP is a novel prognostic biomarker and oncogene in pedAML. Our *in vitro* results show that TYROBP is a promising immunotherapeutic target for nanobody-based CAR-T cell strategies with confirmed cytotoxicity against AML cell lines. *In vivo* experiments are currently ongoing.

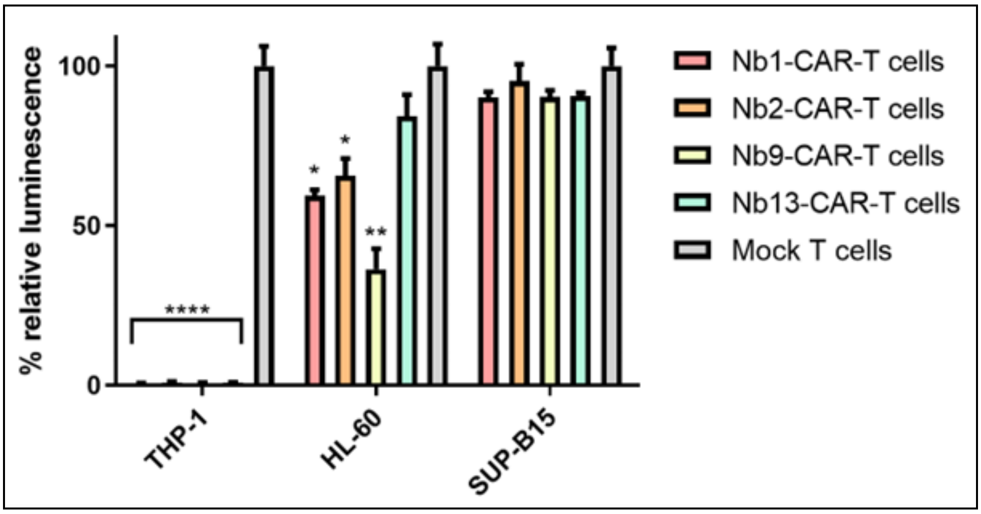

Figure 1. Antigen-specific cytotoxicity of anti-TYROBP nanoCAR-T cells, using luciferase positive antigen-high (THP-1), antigen-low (HL-60), and antigen-negative (SUP-B15) cells in a co-culture for 48h with an E:T ratio of 1:1.

**Copyright Information:** (Online) ISSN: 2572-9241
©2026 The Author(s). HemaSphere is published by John Wiley & Sons Ltd on behalf of European Hematology Association. This is an open access Abstract Book distributed under the Attribution-NonCommercial-NoDerivs (CC BY-NC-ND), which allows third parties to download the articles and share them with others as long as they credit the author and the Abstract Book, but they cannot change the content in any way or use them commercially.
**Abstract Book Citations:** Authors, Title, HemaSphere, 2026;10:(S1):pages. The abstract book DOIs can be found on the "Author Information" page.
**Disclaimer:** Articles published in the journal HemaSphere exclusively reflect the opinions of the authors. The authors are responsible for all content in their abstracts including accuracy of the facts, statements, citing resources, etc.

### (PF476) DIAGNOSTIC IMPACT OF OPTICAL GENOME MAPPING IN ACUTE MYELOID LEUKEMIA

**Topic:** 03. Acute myeloid leukemia - Biology & translational research

Anna Bekő*[1, 2], Balázs Kardos[1, 2], Borbála Péterffy[1, 2], Alex Hughes[1, 2], Botond Timár[1, 2], Alex Jenei[1, 2], Gergő Papp[1, 2], Lajos Hegyi[1, 2], Donát Alpár[1, 2], Csaba Bödör[1, 2]

*[1]Semmelweis University, MTA-SE Lendület Molecular Oncohematology Research Group, Department of Pathology and Experimental Cancer Research, Budapest, Hungary; [2]Semmelweis University, HCEMM-SE Molecular Oncohematology Research Group, Department of Pathology and Experimental Cancer Research, Budapest, Hungary;*

### Background

Comprehensive and reliable detection of clinically relevant structural variants (SVs), copy number alterations (CNAs), and gene mutations is essential for accurate classification, risk assessment and patient stratification in adult acute myeloid leukemia (AML). Conventional cytogenetics and targeted molecular assays identify recurrent abnormalities but may fail to resolve cryptic or complex genomic alterations. Optical genome mapping (OGM) enables genome-wide detection of SVs and CNAs larger than 500 basepairs in a single assay, providing high-resolution structural profiling.

### Aims

To assess the feasibility and added value of OGM in the diagnostics of adult AML, and to identify clinically relevant patterns of co-occurrence and mutual exclusivity among genomic alterations.

### Methods

Diagnostic bone marrow samples from 100 adults with AML (median age: 67 years, range: 19-89 years) were analyzed using karyotyping and FISH. Molecular testing included standard screening for *NPM1*, *FLT3*-ITD, *FLT3*-TKD, *CEBPA* and *IDH1* mutations, with targeted DNA-seq (DNA-based, panel) performed in a subset of patients, covering 68 genes. OGM was conducted for all 100 cases, using the Saphyr system (Bionano Genomics, San Diego, USA). Results were integrated across platforms. Statistical analyses, including co-occurrence and mutual exclusivity, were performed in GraphPad Prism v.8.0.2 and Python v.3.12.5 ($p<0.05$).

### Results

OGM was successful in all 100 cases and generated a digital karyotype, also including 25 patients with failed G-banding. Integration of OGM with targeted NGS refined the WHO classification in 13 patients, identifying 3 cases with *MECOM* rearrangements, 9 AML with myelodysplasia-related changes, and one case with *NUP98* rearrangement. According to the ELN 2022 criteria (excluding *PML*::*RARA* cases), risk assessment based stratification changed in 15 patients (15%), predominantly shifting from intermediate to adverse risk due to the identification of myelodysplasia-related changes. *KMT2A* partial tandem duplications (*KMT2A*-PTD) were detected in eight cases and significantly co-occurred with AML defined by differentiation ($p=0.004$). *KMT2A*-PTD was also observed in three AML with myelodysplasia-related cases. In AML with *RUNX1*::*RUNX1T1*, *KRAS* mutations were significantly enriched ($p=0.017$), and were also associated with focal deletions of 9q22 ($p=0.017$). Additionally, a novel t(1;3)(p36.32;q21.3) translocation, resulting in conferring *PRDM16*::*GATA2* fusion, was identified in a patient presenting with marked thrombocytosis, dysplastic megakaryopoiesis, marrow fibrosis, and aggressive clinical course, sharing phenotypic features with *MECOM*-rearranged AML.

**Copyright Information:** (Online) ISSN: 2572-9241
©2026 The Author(s). HemaSphere is published by John Wiley & Sons Ltd on behalf of European Hematology Association. This is an open access Abstract Book distributed under the Attribution-NonCommercial-NoDerivs (CC BY-NC-ND), which allows third parties to download the articles and share them with others as long as they credit the author and the Abstract Book, but they cannot change the content in any way or use them commercially.
**Abstract Book Citations:** Authors, Title, HemaSphere, 2026;10:(S1):pages. The abstract book DOIs can be found on the "Author Information" page.
**Disclaimer:** Articles published in the journal HemaSphere exclusively reflect the opinions of the authors. The authors are responsible for all content in their abstracts including accuracy of the facts, statements, citing resources, etc.

**Summary/Conclusion**

OGM enables comprehensive detection of cryptic and complex structural alterations that may be missed by conventional methods. Integration of OGM with targeted NGS refined WHO classification in more than 10% of cases and altered the ELN 2022 risk stratification in 15% of patients. Beyond diagnostic refinement, OGM identified *KMT2A*-PTD in 8% of the cases, and demonstrated its ability to detect novel gene fusions with potential clinical significance.

**Copyright Information:** (Online) ISSN: 2572-9241
©2026 The Author(s). HemaSphere is published by John Wiley & Sons Ltd on behalf of European Hematology Association. This is an open access Abstract Book distributed under the Attribution-NonCommercial-NoDerivs (CC BY-NC-ND), which allows third parties to download the articles and share them with others as long as they credit the author and the Abstract Book, but they cannot change the content in any way or use them commercially.
**Abstract Book Citations:** Authors, Title, HemaSphere, 2026;10:(S1):pages. The abstract book DOIs can be found on the "Author Information" page.
**Disclaimer:** Articles published in the journal HemaSphere exclusively reflect the opinions of the authors. The authors are responsible for all content in their abstracts including accuracy of the facts, statements, citing resources, etc.

### (PF477) BEYOND THE BONE MARROW: ARE WE MISSING GENE MUTATIONS IN EXTRAMEDULLARY ACUTE MYELOID LEUKEMIA?

**Topic:** 03. Acute myeloid leukemia - Biology & translational research

Fien Compernolle*[1], Alexander Schauwvlieghe[1], Helena Devos[1], Barbara Cauwelier[1]

[1]*AZ Sint-Jan, Brugge, Belgium;*

### Background

Extramedullary acute myeloid leukemia (eAML) refers to leukemic infiltration of tissues. The disease is classified as synchronous when accompanied by bone marrow involvement, otherwise termed isolated eAML. The genomic landscape of eAML remains poorly defined in current literature. Consequently, the added value of performing next-generation sequencing (NGS) on extramedullary (EM) tissue in addition to bone marrow samples warrants further evaluation. As reimbursement for NGS analysis on both sample types is currently not provided in Belgium, such evaluation has important clinical and policy implications.

### Aims

The aim of this study is to assess the benefit of performing NGS on EM samples in addition to bone marrow samples in eAML.

### Methods

A single-center retrospective study at AZ Sint-Jan Brugge included 13 patients with eAML and 1 with chronic myelomonocytic leukemia (CMML) with EM involvement, diagnosed between 2022 and 2025. Two patients presented with isolated eAML. NGS was performed on EM tissue and bone marrow using a 44-gene targeted myeloid NGS panel. Only (probable) pathogenic variants were included.

**Copyright Information:** (Online) ISSN: 2572-9241
©2026 The Author(s). HemaSphere is published by John Wiley & Sons Ltd on behalf of European Hematology Association. This is an open access Abstract Book distributed under the Attribution-NonCommercial-NoDerivs (CC BY-NC-ND), which allows third parties to download the articles and share them with others as long as they credit the author and the Abstract Book, but they cannot change the content in any way or use them commercially.
**Abstract Book Citations:** Authors, Title, HemaSphere, 2026;10:(S1):pages. The abstract book DOIs can be found on the “Author Information” page.
**Disclaimer:** Articles published in the journal HemaSphere exclusively reflect the opinions of the authors. The authors are responsible for all content in their abstracts including accuracy of the facts, statements, citing resources, etc.

### Results

The most common EM tissue localization was the skin (50,0%), followed by the gastrointestinal tract (21,4%) - including spleen, liver and small intestine. Less frequent localizations were pleural fluid (14,3%), lymph node and testis (each 7,1%). Isolated eAML cases presented on gastro-intestinal biopsy and pleural fluid samples.

A total of 14 patients were analyzed: 12 with synchronous eAML and 2 with isolated eAML. Among the patients with synchronous eAML, 7 showed no additional EM-specific mutations, whereas 5 harbored additional mutations (see patients 1-5 Table 1). The EM-specific mutations included ASXL1, BCOR, NRAS and ZRSR2. NGS results of 2 patients with isolated eAML are shown as patients 6 and 7 in Table 1.

The identification of additional EM mutations may enable the possibility of targeted therapies, including IDH1/2 and FLT3 inhibitors. However, no such targetable mutations could be identified in this cohort.

In one patient with a DNMT3A and NPM1 mutation in the bone marrow, detection of an additional EM-specific ASXL1 mutation resulted in a change in ELN risk classification from intermediate to adverse-risk AML, thereby supporting the indication for allogeneic hematopoietic stem cell transplantation (aHSCT). However, the patient failed to reach transplantation due to death following consolidation therapy. Patients harboring additional BCOR, NRAS and ZRSR2 mutations remained within the adverse risk category.

| | Non-EM site NGS | EM site NGS |
|---|---|---|
| Patient 1 | ASXL1, RUNX1, TET2, U2AF1 | ASXL1, RUNX1, TET2, U2AF1, **NRAS** |
| Patient 2 | ASXL1, NPM1, JAK2 | ASXL1, NPM1, **ZRSR2** |
| Patient 3 | U2AF1, RUNX1, FLT3-ITD | U2AF1, RUNX1, **BCOR**, **NRAS** |
| Patient 4 | IDH1, TP53* | IDH1, TP53, **PTPN11** |
| Patient 5 | DNMT3A, NPM1 | DNMT3A, NPM1, **ASXL1** |
| Patient 6 | Not applicable | **PTPN11**, **TP53** |
| Patient 7 | Not applicable | **CBL**, **TP53**, **U2AF1** |

*The original NGS panel performed on bone marrow did not include PTPN11.

### Summary/Conclusion

Site-specific mutational heterogeneity was observed between bone marrow and EM samples. The detection of additional EM-specific mutations may have direct implications for risk stratification and therapeutic decision-making, including eligibility for aHSCT or targeted therapies. These findings highlight the clinical relevance of performing NGS analysis on both bone marrow and EM sites and provide a rationale for reimbursement. Study limitations include the small cohort size, highlighting the need for larger studies.

**Copyright Information:** (Online) ISSN: 2572-9241
©2026 The Author(s). HemaSphere is published by John Wiley & Sons Ltd on behalf of European Hematology Association. This is an open access Abstract Book distributed under the Attribution-NonCommercial-NoDerivs (CC BY-NC-ND), which allows third parties to download the articles and share them with others as long as they credit the author and the Abstract Book, but they cannot change the content in any way or use them commercially.
**Abstract Book Citations:** Authors, Title, HemaSphere, 2026;10:(S1):pages. The abstract book DOIs can be found on the "Author Information" page.
**Disclaimer:** Articles published in the journal HemaSphere exclusively reflect the opinions of the authors. The authors are responsible for all content in their abstracts including accuracy of the facts, statements, citing resources, etc.

### (PF478) GLYCOLYTIC ENZYMES ARE PART OF AN ONCOGENIC NETWORK IN AML

**Topic:** 03. Acute myeloid leukemia - Biology & translational research

Stefan Nagel*[1], Corinna Meyer[1], Meina Neumann-Schaal[1, 2], Claudia Pommerenke[1, 3]

*[1]Leibniz-Institute DSMZ, Human and Animal Cell Lines, Braunschweig, Germany;[2]Leibniz-Institute DSMZ, Metabolomics, Braunschweig, Germany; [3]Leibniz-Institute DSMZ, Bioinformatics, Braunschweig, Germany;*

### Background

Deregulated differentiation is a general feature of cancer and a major factor in leukemogenesis. Normal hematopoiesis is mainly controlled at the transcriptional level. Therefore, aberrant activity of developmental transcription factors frequently underlies differentiation defects in leukemia. Glucose and glycolytic enzymes are central players in the cellular metabolism, generating energy and biosynthetic building blocks. This highly orchestrated metabolic system is frequently disturbed in cancer including leukemia. Erythroid acute myeloid leukemia (AML) cell line OCI-M2 expresses a particular oncogenic network: IRF6 in concert with ETV2 and HEY1 aberrantly activates NKL homeobox gene NKX2-4, which in turn represses megakaryocytic lineage factor FLI1, deregulating differentiation. Interestingly, in keratinocytes IRF6 is able to bind glucose which promotes IRF6-dimerization and thus alters its binding site selection.

### Aims

Here, we used OCI-M2 as a model to investigate the role of glucose level and IRF6 in leukemogenesis.

### Results

Treatment of OCI-M2 with high glucose or its stable derivate 2-deoxy-glucose resulted in downregulation of IRF6 and NKX2-4, and upregulation of FLI1, indicating that glucose-mediated dimerization of IRF6 altered its reported autoactivation. Screening of this cell line via RNA-seq for genes encoding glycolytic enzymes identified aberrant overexpression of glucose-6-phosphate isomerase (GPI) and phosphofructokinase L (PFKL), which was confirmed by RQ-PCR and Western blot analysis. Both genes were targeted by genomic amplification and chromothripsis, respectively, as analyzed by genomic profiling, underlying their enhanced activity. Furthermore, knockdown studies showed that GPI was activated by NKX2-4 and ETV2, and PFKL by ETV2. SiRNA-mediated downregulation of PFKL resulted in elevated intra-cellular glucose levels, as quantified by gas-chromatography mass-spectrometric analysis, and suppressed expression of IRF6 and NKX2-4, and activated FLI1. Finally, TCGA-data analysis of AML patients showed that elevated PFKL expression is associated with reduced overall survival, supporting its clinical significance in this malignancy.

### Summary/Conclusion

We have connected an aberrant gene regulatory network with deregulated glycolytic enzymes and glucose metabolism, thereby establishing a new in vitro model to develop novel therapeutic avenues in AML subsets.

**Copyright Information:** (Online) ISSN: 2572-9241
©2026 The Author(s). HemaSphere is published by John Wiley & Sons Ltd on behalf of European Hematology Association. This is an open access Abstract Book distributed under the Attribution-NonCommercial-NoDerivs (CC BY-NC-ND), which allows third parties to download the articles and share them with others as long as they credit the author and the Abstract Book, but they cannot change the content in any way or use them commercially.
**Abstract Book Citations:** Authors, Title, HemaSphere, 2026;10:(S1):pages. The abstract book DOIs can be found on the “Author Information” page.
**Disclaimer:** Articles published in the journal HemaSphere exclusively reflect the opinions of the authors. The authors are responsible for all content in their abstracts including accuracy of the facts, statements, citing resources, etc.

### (PF479) HIGH PTP4A3 EXPRESSION IS ASSOCIATED WITH ADVERSE PROGNOSIS AND PRO-LEUKEMOGENIC SIGNALING PATHWAYS IN ACUTE MYELOID LEUKEMIA

**Topic:** 03. Acute myeloid leukemia - Biology & translational research

Natália Parducci[1], Maria Fernanda Carvalho[1], Isabelle Santos[1], Letícia Bertoline[1], Givanna Castro[2], Juan Luiz Coelho-Silva*[3], Tiago Almeida[2], Peter Wipf[4], João Agostinho MacHado Neto[1]

*[1]University of São Paulo - USP, Pharmacology and Physiology, São Paulo, Brazil; [2]Federal University of São Carlos (UFSCar), Department of Computer Science, Sorocaba, Brazil; [3]University of Pernambuco, Pernambuco, Brazil; [4]University of Pittsburgh, Department of Chemistry, Pittsburgh, United States of America;*

### Background

*PTP4A3* expression is strongly associated with poor prognosis and therapy failure in acute myeloid leukemia (AML), as demonstrated by machine learning analyses of publicly available datasets. Despite its established role in regulating apoptosis, angiogenesis, and metastatic progression in multiple malignancies, and its overexpression in nearly 50% of AML cases, its functional contribution to the disease pathobiology remains poorly defined. Given the genetic heterogeneity and aggressive clinical course of AML, elucidating the role of *PTP4A3* is critical to determining its viability as a novel therapeutic target.

### Aims

To define the functional relevance of *PTP4A3* in the maintenance of AML using *in vitro* models, and to characterize the molecular networks associated with its expression and pharmacological inhibition.

### Methods

RNA sequencing data, clinical characteristics, and outcomes were retrieved from AML cohorts available in cBioPortal. Samples were stratified according to high or low *PTP4A3* expression, and differential expression analyses were performed using pre-ranked RNA-seq transcripts. Survival curves were assessed using Cox proportional hazards regression models, complemented by correlation, association, and group comparison analyses. The relative proportions of immune cell subsets were estimated using CIBERSORT from bulk RNA-seq data. Pre-ranked gene set enrichment analysis and gene ontology enrichment analyses were conducted to identify signaling pathways associated with *PTP4A3. In vitro*, the effects of *PTP4A3* inhibition were evaluated in 14 AML cell lines using MTT assays to determine cell viability and calculate dependence scores. *PTP4A3* expression was confirmed by PCR, and responses to the treatments with JMS-053 and EJR-925 (PRL inhibitors) were analyzed by apoptosis, cell cycle distribution, Western blotting, and clonogenic assays.

**Copyright Information:** (Online) ISSN: 2572-9241
©2026 The Author(s). HemaSphere is published by John Wiley & Sons Ltd on behalf of European Hematology Association. This is an open access Abstract Book distributed under the Attribution-NonCommercial-NoDerivs (CC BY-NC-ND), which allows third parties to download the articles and share them with others as long as they credit the author and the Abstract Book, but they cannot change the content in any way or use them commercially.
**Abstract Book Citations:** Authors, Title, HemaSphere, 2026;10:(S1):pages. The abstract book DOIs can be found on the "Author Information" page.
**Disclaimer:** Articles published in the journal HemaSphere exclusively reflect the opinions of the authors. The authors are responsible for all content in their abstracts including accuracy of the facts, statements, citing resources, etc.

**Results**

High *PTP4A3* expression was significantly associated with inferior overall and disease-free survival, along with adverse molecular risk. Elevated *PTP4A3* levels were observed in cases harboring trisomy 8, complex karyotype, and *FLT3* mutations, suggesting an association with cytogenetic instability. *PTP4A3* expression was enriched in quiescent and primed leukemic stem and progenitor cells (LSPCs), indicating a link with immature leukemic compartments. Pathway enrichment analyses and gene ontology revealed activation of proliferative, inflammatory, and oncogenic signaling programs in the *PTP4A3*-high group. *In vitro*, JMS-053 and EJR-925 exhibited $IC_{50}$ values ranging from 0.93 to 12.73 µM and 1.20 to 39.61 µM, respectively. Sensitivity to JMS-053 correlated with *PTP4A3* dependency scores across AML cells. Transcript profiling identified NB4, U-937, and HL-60 as the highest *PTP4A3*-expressing models. Treatment of THP-1, U-937, and HL-60 cells with PRL inhibitors significantly increased apoptosis and Sub-$G_1$ fractions, accompanied by reduced clonogenicity. Both inhibitors reduced total and cleaved ERK1/2 protein levels. JMS-053 decreased phosphorylated STMN1 and increased phosphorylated ERM, γ-H2AX, and cleaved PARP1, indicating interference in MAPK/ERK signaling, cytoskeletal remodeling, DNA damage, and apoptosis.

**Summary/Conclusion**

High *PTP4A3* expression is linked to poor prognosis, adverse molecular features, and immature leukemic compartments in AML. Its pharmacological inhibition reduces viability and clonogenicity, suppresses MAPK/ERK signaling, and induces DNA damage and apoptosis, supporting *PTP4A3* as a potential therapeutic target.

**Copyright Information:** (Online) ISSN: 2572-9241
©2026 The Author(s). HemaSphere is published by John Wiley & Sons Ltd on behalf of European Hematology Association. This is an open access Abstract Book distributed under the Attribution-NonCommercial-NoDerivs (CC BY-NC-ND), which allows third parties to download the articles and share them with others as long as they credit the author and the Abstract Book, but they cannot change the content in any way or use them commercially.
**Abstract Book Citations:** Authors, Title, HemaSphere, 2026;10:(S1):pages. The abstract book DOIs can be found on the "Author Information" page.
**Disclaimer:** Articles published in the journal HemaSphere exclusively reflect the opinions of the authors. The authors are responsible for all content in their abstracts including accuracy of the facts, statements, citing resources, etc.

### (PF480) ZBTB16::RARA DISRUPTS ANTIOXIDANT HOMEOSTASIS AND CREATES AN EXPLOITABLE REDOX VULNERABILITY IN ACUTE PROMYELOCYTIC LEUKEMIA

**Topic:** 03. Acute myeloid leukemia - Biology & translational research

Alessandra Zaza*[1], Elisabetta De Marinis[2], Martina Gentile[2], Gloria Pasqualini[1], Eliezer Espina Barroso[1], Emiliano Fabiani[3], Daniela F. Angelini[4], Salvatore Perrone[5], Adriano Venditti[3], Maria Teresa Voso[3], Gianfranco Catalano[3], Clara Nervi[6], Nelida I. Noguera[3]

*[1]Fondazione Santa Lucia IRCCS, Neuro-oncology laboratory, Rome, Italy; [2]Sapienza University of Rome, Department of Medical-Surgical Sciences and Biotechnologies, Latina, Italy; [3]University of Rome Tor Vergata, Rome, Italy; [4]Fondazione Santa Lucia IRCCS, Rome, Italy; [5]Santa Maria Goretti Hospital, Latina, Italy; [6]Sapienza University of Rome, Rome, Italy;*

### Background

The ZBTB16::RARa fusion defines a rare and therapy-resistant subtype of acute promyelocytic leukemia (APL), yet its impact on cellular redox homeostasis remains poorly characterized. Antioxidant activity is essential in normal hematopoiesisan is regulated by key factors: NRF2 (erythroid 2–related factor 2), a master regulator of cellular stress response; G6PDH (glucose-6-phosphate dehydrogenase), the rate-limiting enzyme in the pentosephosphate pathway and major NADPH source; and FOXO3a, a transcription factor involved in stress resistance .

### Aims

To elucidate the impact of ZBTB16::RARa on the antioxidant system in order to individuate new therapeutic targets

### Methods

Primary blasts from ZBTB16::RARa APL patients and a $ZnSO_4$-inducible ZBTB16::RARa [U937-B412/control U937-MT] cell lines were studied. ROS were measured by MitoSOX; gene and protein expression by qRT-PCR and Western blot; Protein interactions by proximity ligation assay (PLA); $NADP^+$/NADPH by NADP/NADPH-Glo™. Apoptosis by mitochondrial membrane potential (ΔΨm) and Caspase-3/PARP cleavage

**Copyright Information:** (Online) ISSN: 2572-9241
©2026 The Author(s). HemaSphere is published by John Wiley & Sons Ltd on behalf of European Hematology Association. This is an open access Abstract Book distributed under the Attribution-NonCommercial-NoDerivs (CC BY-NC-ND), which allows third parties to download the articles and share them with others as long as they credit the author and the Abstract Book, but they cannot change the content in any way or use them commercially.
**Abstract Book Citations:** Authors, Title, HemaSphere, 2026;10:(S1):pages. The abstract book DOIs can be found on the "Author Information" page.
**Disclaimer:** Articles published in the journal HemaSphere exclusively reflect the opinions of the authors. The authors are responsible for all content in their abstracts including accuracy of the facts, statements, citing resources, etc.

**Results**

ZBTB16::RARa expression impaired antioxidant balance and increased oxidative susceptibility. After 3mM Ascorbate (ASC, a ROS inductor) ROS were higher in B412 vs MT (8058±568 vs 5037±575). Rotenone-Antimycin further amplified ROS (38148±1756 vs 19589±2761) (Fig.1A) indicating defective buffering capacity. NRF2 protein was markedly reduced after induction (MT 1.2±0.3 vs B412 24h: 0.3±0.1; p=0.008) (Fig.1B), whereas mRNA was unchanged, suggesting post-translational destabilization. PLA showed nuclear colocalization between ZBTB16::RARa and NRF2, supporting functional inhibiton, further supported by reduced HO-1 protein in B412 cells. In primary blasts NRF2 was lower in ZBTB16::RARa-APL (n=5: 0.5±0.5) vs PML::RARa APLs (n=15: 4.8±2.2; p =0.004), AML (n=14: 8.9±19; p=0.02), and NBM (n=5:1±0.4). G6PDH mRNA and protein were reduced in B412 cells ( 1.8±0.5 vs 3.1±0.6; p=0.04) (Fig.1C). NADPH was higher in MT, whereas NADP+ accumulated in B412(Fig.1D) indicating impaired pentose phosphate pathway activity. Primary ZBTB16::RARa blasts also show lower G6PDH (n=5 0.7±0.5) vs NBM (n=5: 1.6±1.2). FOXO3a protein decreased after induction( MT 1.9±0.4 vs B412 0.4±0.2; p=0.01) despite stable mRNA. Downstream antioxidant targets of FOXO3a were compromised: SOD1 protein was reduced (MT: 0.86 ± 0.28 vs. B412: 0.49 ± 0.08) (p=0.05) and catalase activity declined by ~57%. These data demonstrate coordinated repression of key antioxidant regulators. . High-dose ascorbate (1–3 mM) induced dose-dependent apoptosis in B412 cells and primary blasts, further enhanced by azacitidine (AZA), as shown by mitochondrial depolarization and caspase-3/PARP cleavage (Fig.1E). Building on this, the BCL2 inhibitor venetoclax (VEN) combined with AZA markedly induced mitochondrial depolarization and apoptosis in B412 cells (AZA: 24%; VEN: 8%; AZA+VEN: 31%) with minimal effects in MT controls, confirmed by caspase-3/PARP cleavage and synergistic inhibition of proliferation (ZIP synergy 11.0)(Fig.1F).

**Summary/Conclusion**

ZBTB16::RARA ZBTB16::RARα represses NRF2, G6PDH, and FOXO3a, disrupting redox homeostasis and reducing NADPH-dependent defense. This creates intrinsic oxidative vulnerability that can be therapeutically exploited. ASC-based strategies and VEN–AZA exploits mitochondrial and redox offering a promising strategy to overcome resistance in this rare AML subtype.

**Copyright Information:** (Online) ISSN: 2572-9241
©2026 The Author(s). HemaSphere is published by John Wiley & Sons Ltd on behalf of European Hematology Association. This is an open access Abstract Book distributed under the Attribution-NonCommercial-NoDerivs (CC BY-NC-ND), which allows third parties to download the articles and share them with others as long as they credit the author and the Abstract Book, but they cannot change the content in any way or use them commercially.
**Abstract Book Citations:** Authors, Title, HemaSphere, 2026;10:(S1):pages. The abstract book DOIs can be found on the “Author Information” page.
**Disclaimer:** Articles published in the journal HemaSphere exclusively reflect the opinions of the authors. The authors are responsible for all content in their abstracts including accuracy of the facts, statements, citing resources, etc.

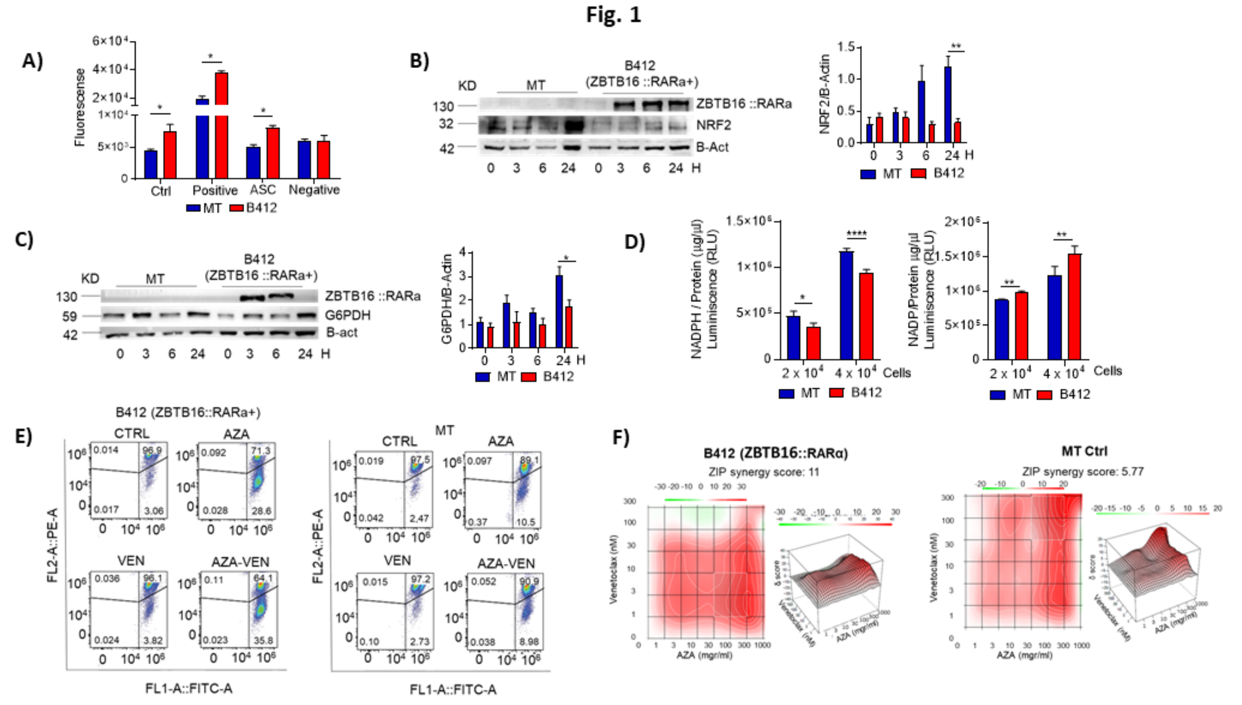

**Fig. 1. A)** ROS levels were measured in B412 expressing ZBTB16::RARa and control cells, using Midium with SO4Zn(Ctrl), Rotenone/Antimicyn (positive control) and high dose ascorbate (ASC, ROS inducer). In all conditions, ZBTB16::RARa expressing cells exhibited significantly higher ROS levels compared with MT cells; **B)** NRF2 protein level increased and persisted higher in the 24 h after SO4Zn treatment in MT cells, but not in B412 cells expressing ZBTB16::RARa; **C)** G6PDH protein level decreases in B412 cells due to the presence of ZBTB16::RARa; **D)** NADPH /NADP ratio was measured in MT and B412 cells over a 6 h time course after $SO4Zn_4$ induction. **E)** Cytofluorometer analysis of the mitochondrial membrane potential of B412 and MT cells after 48 h of exposure to 100 AZA ng/ml and 10nM VEN; **F)** Reduced growth of B412 cells measured by MTT assay.

**Copyright Information:** (Online) ISSN: 2572-9241
©2026 The Author(s). HemaSphere is published by John Wiley & Sons Ltd on behalf of European Hematology Association. This is an open access Abstract Book distributed under the Attribution-NonCommercial-NoDerivs (CC BY-NC-ND), which allows third parties to download the articles and share them with others as long as they credit the author and the Abstract Book, but they cannot change the content in any way or use them commercially.
**Abstract Book Citations:** Authors, Title, HemaSphere, 2026;10:(S1):pages. The abstract book DOIs can be found on the "Author Information" page.
**Disclaimer:** Articles published in the journal HemaSphere exclusively reflect the opinions of the authors. The authors are responsible for all content in their abstracts including accuracy of the facts, statements, citing resources, etc.

### (PF481) DJ-1 REGULATES NECROPTOSIS THROUGH THE TNFAIP3–RIPK1 AXIS IN FLT3-ITD ACUTE MYELOID LEUKEMIA

**Topic:** 03. Acute myeloid leukemia - Biology & translational research

Xi Quan*[1], Zhiming Luo[1], Nan Zhang[1], Jian-Chuan Deng[1], Shifeng Lou[1]

[1] *The Second Affiliated Hospital of Chongqing Medical University, Department of hematology, Chongqing, China;*

### Background

Resistance to cell death remains a major obstacle in FLT3-ITD acute myeloid leukemia (AML). Although apoptosis has been extensively investigated in AML, alternative programmed cell death pathways may represent underexplored therapeutic vulnerabilities. A systematic evaluation of distinct cell death programs in FLT3-ITD AML has not yet been performed.

### Aims

To define dominant programmed cell death programs in FLT3-ITD AML and identify targetable regulators of necroptosis.

### Methods

Large-scale RNA-sequencing datasets from FLT3-ITD AML cohorts were analyzed to model the activity of 15 programmed cell death pathways. Candidate regulators were identified through integrative transcriptomic analyses. Functional validation was performed in FLT3-ITD AML cell lines using genetic silencing of DJ-1 and pathway-specific pharmacologic inhibitors. Necroptosis was evaluated by cell viability assays, LDH release, and immunoblotting of phosphorylated RIPK1, RIPK3, and MLKL. RNA sequencing following DJ-1 knockdown was conducted to identify downstream mediators. Co-immunoprecipitation assays were performed to assess protein interactions, and ubiquitination assays were used to evaluate RIPK1 ubiquitin modification. In vivo efficacy of the DJ-1 inhibitor DJ-1-IN-1 was tested in a murine MLL-AF9 AML model.

### Results

Comparative pathway modeling revealed necroptosis as the most prominently dysregulated programmed cell death program in FLT3-ITD AML. Transcriptomic integration identified DJ-1 (PARK7) as a candidate survival regulator associated with suppression of necroptotic signaling. Genetic silencing or pharmacologic inhibition of DJ-1 selectively enhanced necroptotic cell death in FLT3-ITD AML cells, as evidenced by increased phosphorylation of RIPK1, RIPK3, and MLKL, together with enhanced membrane damage, whereas inhibition of apoptosis or ferroptosis did not reproduce this phenotype. RNA sequencing of DJ-1–depleted cells demonstrated significant downregulation of TNFAIP3, encoding the ubiquitin-editing enzyme A20. Reduced A20 expression following DJ-1 loss was accompanied by augmented RIPK1 activation. Co-immunoprecipitation assays demonstrated an interaction between DJ-1 and A20, and ubiquitination assays revealed that DJ-1 depletion altered RIPK1 ubiquitin modification, consistent with enhanced RIPK1 activation. Collectively, these data establish a DJ-1–TNFAIP3–RIPK1 signaling axis that constrains necroptotic execution in FLT3-ITD AML. Importantly, pharmacologic inhibition of DJ-1 using DJ-1-IN-1 significantly delayed leukemia progression in vivo in the MLL-AF9 AML model.

### Summary/Conclusion

DJ-1 maintains necroptotic resistance in FLT3-ITD AML by sustaining TNFAIP3 expression and regulating RIPK1 ubiquitin signaling. Targeting DJ-1 unleashes RIPK1-dependent necroptosis and exposes a novel therapeutic vulnerability in high-risk AML.

**Copyright Information:** (Online) ISSN: 2572-9241
©2026 The Author(s). HemaSphere is published by John Wiley & Sons Ltd on behalf of European Hematology Association. This is an open access Abstract Book distributed under the Attribution-NonCommercial-NoDerivs (CC BY-NC-ND), which allows third parties to download the articles and share them with others as long as they credit the author and the Abstract Book, but they cannot change the content in any way or use them commercially.
**Abstract Book Citations:** Authors, Title, HemaSphere, 2026;10:(S1):pages. The abstract book DOIs can be found on the "Author Information" page.
**Disclaimer:** Articles published in the journal HemaSphere exclusively reflect the opinions of the authors. The authors are responsible for all content in their abstracts including accuracy of the facts, statements, citing resources, etc.

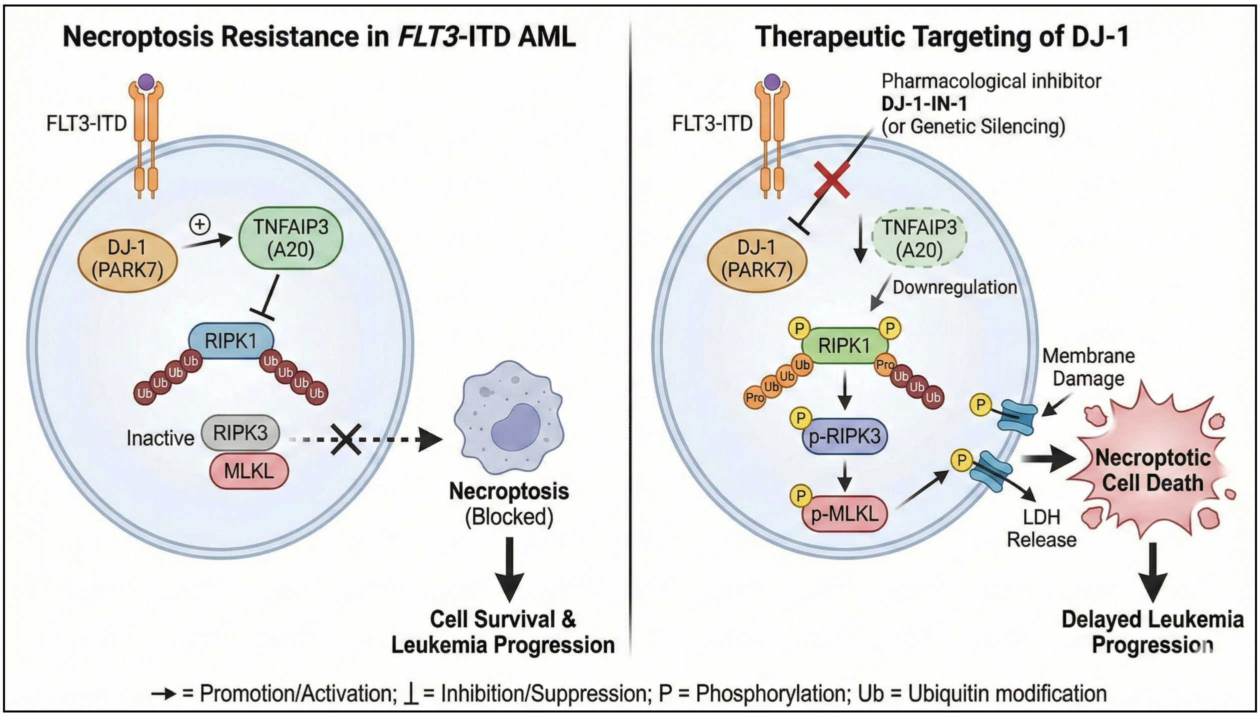

DJ-1 sustains TNFAIP3 (A20) expression, maintaining RIPK1 ubiquitin homeostasis and restraining RIPK1-dependent necroptotic signaling, thereby promoting leukemia cell survival in FLT3-ITD AML. Pharmacologic inhibition or genetic silencing of DJ-1 reduces TNFAIP3 expression, enhances RIPK1 kinase activation and downstream RIPK3/MLKL phosphorylation, leading to membrane disruption, LDH release, and necroptotic cell death. Targeting DJ-1 delays leukemia progression in vivo. Abbreviations: DJ-1 (PARK7); TNFAIP3 (A20); RIPK1, receptor-interacting serine/threonine-protein kinase 1; RIPK3; MLKL; Ub, ubiquitin; P, phosphorylation; LDH, lactate dehydrogenase.

**Copyright Information:** (Online) ISSN: 2572-9241
©2026 The Author(s). HemaSphere is published by John Wiley & Sons Ltd on behalf of European Hematology Association. This is an open access Abstract Book distributed under the Attribution-NonCommercial-NoDerivs (CC BY-NC-ND), which allows third parties to download the articles and share them with others as long as they credit the author and the Abstract Book, but they cannot change the content in any way or use them commercially.
**Abstract Book Citations:** Authors, Title, HemaSphere, 2026;10:(S1):pages. The abstract book DOIs can be found on the "Author Information" page.
**Disclaimer:** Articles published in the journal HemaSphere exclusively reflect the opinions of the authors. The authors are responsible for all content in their abstracts including accuracy of the facts, statements, citing resources, etc.

### (PF482) LACTATE REGULATORY GENE LSP1 LEADING TO BCL-2 INHIBITOR RESISTANCE THROUGH DUAL EFFECTS BY PROMOTING AML CELL INVASION AND IMMUNE ESCAPE

**Topic:** 03. Acute myeloid leukemia - Biology & translational research

Zhibo Guo*[1], Shengjin Fan[1], Xin Hai[1]

[1]*First Affiliated Hospital of Harbin Medical University, Harbin, China;*

### Background

The overall survival rate of acute myeloid leukemia (AML) remains below 30%. Despite continuous advancements in BCL-2-based targeted combination chemotherapy, drug resistance and relapse remain the primary causes of death in AML. Metabolic reprogramming of leukemia cells, such as the Warburg effect, enables them to adapt to the microenvironment and thereby develop. Normal leukemic cells rely on oxidative phosphorylation for energy supply, while some cells convert to glycolysis upon exposure to BCL-2 inhibitors to sustain survival, producing large amounts of lactic acid, which promotes leukemic cell persistence and immunosuppression.

### Aims

Elucidating the landscape of lactate regulated gene in AML helps clarify the mechanism of BCL-2 inhibitor resistance from the perspective of metabolic reprogramming and identify possibilities for optimizing current treatment modalities.

### Methods

Seahorse cell energy metabolism analysis was applied to compare metabolic signatures between primary cells from patients with de nove and relapsed AML. CCK8 and CTG were used to evaluate the effects of lactate and LDH inhibitors, either alone or in combination with BCL-2 inhibitors, on the viability of AML cell lines. Western blot was employed to detect the level of pan-lactation, while flow cytometry was utilized to assess cell cycle and apoptosis. Mechanistically, multiple bioinformatics approaches were employed to explore downstream of lactate regulation. The Seurat and limma package algorithms, along with Weighted Gene Coexpression Network Analysis, were utilized to identify candidate lactylation-related genes (LRGs) based on GEO and TCGA data. Univariate Cox regression and machine learning algorithms (LASSO-logistic, SVM-RFE and Boruta narrowed the range of LRGs. We evaluated the associations between LRGs and AML using a two-sample Mendelian randomization analysis. ChIP-PCR confirmed the targeted relationship between H3K18la modification and LSP1. Short-hairpin RNA vectors were used to construct overexpression and knockdown of LSP1 cell models. THP-1 cells were induced to dendritic cells (DCs) and co-cultured with primitive T cells to investigate the induction of Treg cell differentiation.

### Results

Primary AML cells primarily derive energy through oxidative phosphorylation, whereas relapsed/resistant AML cells switch to glycolysis. Lactate promotes AML cells proliferation, inhibits apoptosis, and advances the cell cycle into the S phase, and LDH inhibitors exhibit the opposite effects. LSP1 were identified as key LRG in AML. THP-1 cells exhibit low LSP1 expression, while OCI-AML3 cells show high LSP1 expression, thus we established LSP1 overexpression and knockdown models. LSP1 overexpression promotes AML cell proliferation, inhibits apoptosis, enhances colony formation, and promotes BCL-2 resistance, and LSP1 knockdown suppresses these processes. Single-cell level analysis revealed that LSP1 overexpression is associated with AML cells and DCs. THP-1 cells overexpressing LSP1 were further induced to differentiate into LSP1+DCs and demonstrated that LSP1+DCs promote Treg differentiation and mediate immune evasion.

**Copyright Information:** (Online) ISSN: 2572-9241
©2026 The Author(s). HemaSphere is published by John Wiley & Sons Ltd on behalf of European Hematology Association. This is an open access Abstract Book distributed under the Attribution-NonCommercial-NoDerivs (CC BY-NC-ND), which allows third parties to download the articles and share them with others as long as they credit the author and the Abstract Book, but they cannot change the content in any way or use them commercially.
**Abstract Book Citations:** Authors, Title, HemaSphere, 2026;10:(S1):pages. The abstract book DOIs can be found on the "Author Information" page.
**Disclaimer:** Articles published in the journal HemaSphere exclusively reflect the opinions of the authors. The authors are responsible for all content in their abstracts including accuracy of the facts, statements, citing resources, etc.

**Summary/Conclusion**

We established lactate as a critical regulator of AML, and LSP1 was identified as lactylation-related clinical modeling indicators. LSP1 leads to BCL-2 inhibitor resistance through dual effects by promoting AML cell invasion and immune escape. Targeting lactate metabolism or inhibiting LSP1 could be potential therapeutic targets for BCL-2 inhibitor resistance in AML.

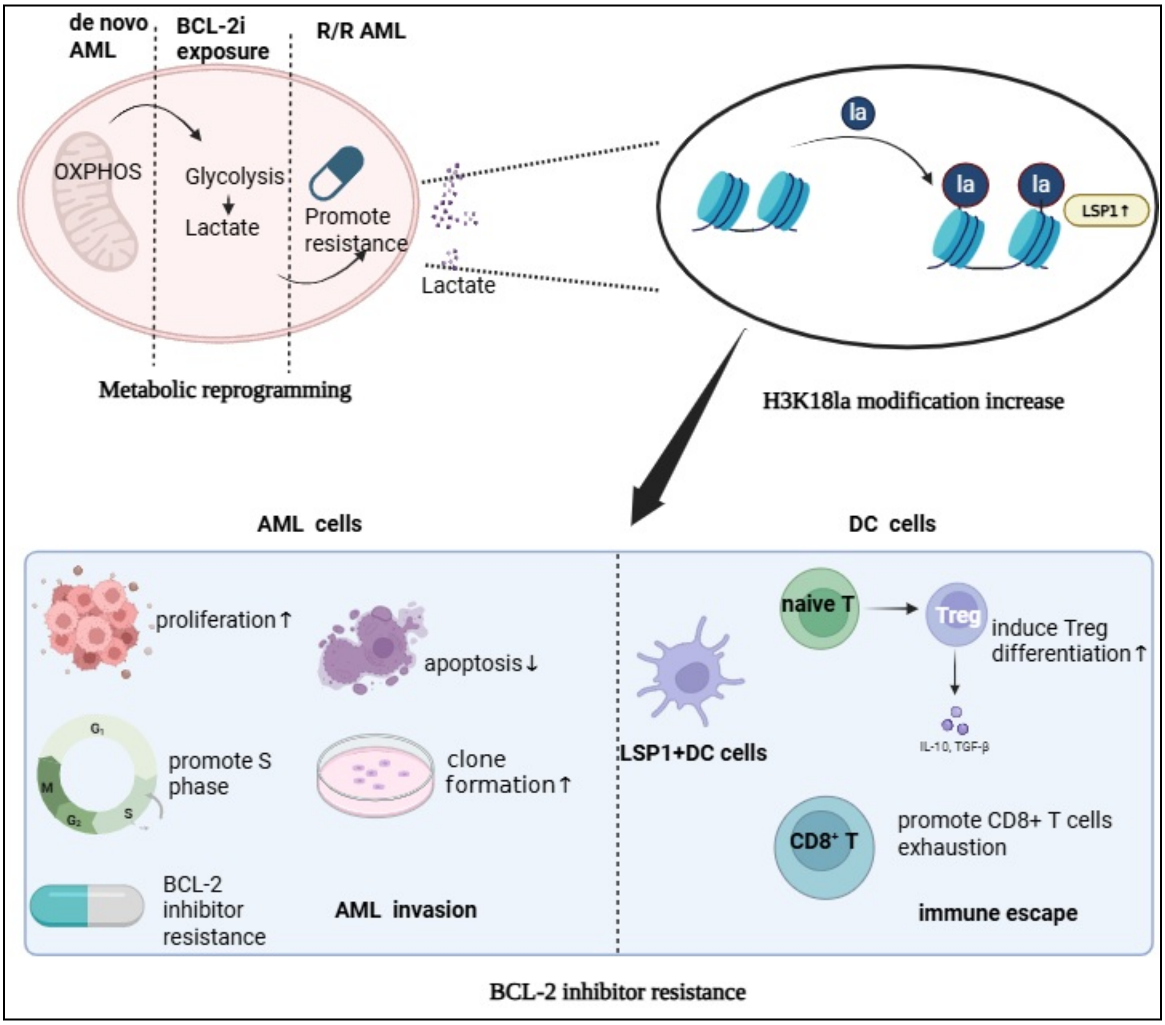

**Copyright Information:** (Online) ISSN: 2572-9241
©2026 The Author(s). HemaSphere is published by John Wiley & Sons Ltd on behalf of European Hematology Association. This is an open access Abstract Book distributed under the Attribution-NonCommercial-NoDerivs (CC BY-NC-ND), which allows third parties to download the articles and share them with others as long as they credit the author and the Abstract Book, but they cannot change the content in any way or use them commercially.
**Abstract Book Citations:** Authors, Title, HemaSphere, 2026;10:(S1):pages. The abstract book DOIs can be found on the "Author Information" page.
**Disclaimer:** Articles published in the journal HemaSphere exclusively reflect the opinions of the authors. The authors are responsible for all content in their abstracts including accuracy of the facts, statements, citing resources, etc.

### (PF483) LDLR AND KLF2 ARE CRITICAL MEDIATORS IN STATIN-INDUCED CELL DEATH IN AML

**Topic:** 03. Acute myeloid leukemia - Biology & translational research

Yuta Fukui*[1], Yohei Yasuda[1], Ken Morita[1], Muzhapaer Mubalizi[1], Michiaki Sato[1], Hiroki Hayashida[1], Yosuke Masamoto[2], Mineo Kurokawa[1]

*[1]The University of Tokyo Hospital, Department of Hematology and Oncology, Tokyo, Japan;[2]The University of Tokyo Hospital, Department of Cell Therapy and Transplantation Medicine, Tokyo, Japan;*

### Background

Statins are widely used HMG-CoA reductase inhibitors for cardiovascular disease prevention. Increasing evidence indicates that statins exert anti-tumor effects in several malignancies, including acute myeloid leukemia (AML). However, the molecular mechanisms determining statin sensitivity in AML remain poorly understood.

### Aims

To clarify the molecular mechanisms underlying the anti-leukemic effects of statins and to identify determinants of statin sensitivity in AML.

### Methods

A genome-wide CRISPR-Cas9 knockout screen was performed in the AML cell line THP-1 under statin treatment to identify genes regulating statin sensitivity. Functional validation was conducted using gene knockout and doxycycline-inducible overexpression systems. RNA sequencing was performed to evaluate transcriptional responses to statin treatment. The involvement of cholesterol availability and the mevalonate pathway was assessed by extracellular cholesterol depletion or supplementation and by GGPP rescue experiments.

### Results

The CRISPR screen identified the low-density lipoprotein receptor (LDLR) as a critical determinant of statin sensitivity. LDLR knockout markedly reduced statin-induced cell death and conferred resistance to statin treatment.
RNA sequencing revealed that statin treatment significantly induced the expression of the transcription factor Krüppel-like factor 2 (KLF2) in LDLR wild-type cells, whereas this induction was strongly attenuated in LDLR knockout cells. Doxycycline-inducible overexpression of KLF2 suppressed cell proliferation, induced apoptosis, and caused G0/G1 cell cycle arrest, closely phenocopying statin treatment, indicating that KLF2 functions as a key downstream effector of statin-induced AML cell death.
Gene set enrichment analysis showed that genes downregulated in LDLR wild-type cells compared with LDLR knockout cells under statin treatment were significantly enriched for SREBP target pathways. It is known that statin-induced intracellular cholesterol decline activates SREBP2, thereby promoting production of geranylgeranyl pyrophosphate (GGPP), a growth-promoting intermediate of the mevalonate pathway that counteracts statin cytotoxicity. In line with this, depletion of extracellular cholesterol enhanced statin resistance and suppressed KLF2 induction, whereas supplementation with exogenous cholesterol restored statin sensitivity and KLF2 expression. Furthermore, GGPP supplementation abrogated statin-induced KLF2 expression and cell death.

**Copyright Information:** (Online) ISSN: 2572-9241
©2026 The Author(s). HemaSphere is published by John Wiley & Sons Ltd on behalf of European Hematology Association. This is an open access Abstract Book distributed under the Attribution-NonCommercial-NoDerivs (CC BY-NC-ND), which allows third parties to download the articles and share them with others as long as they credit the author and the Abstract Book, but they cannot change the content in any way or use them commercially.
**Abstract Book Citations:** Authors, Title, HemaSphere, 2026;10:(S1):pages. The abstract book DOIs can be found on the "Author Information" page.
**Disclaimer:** Articles published in the journal HemaSphere exclusively reflect the opinions of the authors. The authors are responsible for all content in their abstracts including accuracy of the facts, statements, citing resources, etc.

**Summary/Conclusion**

These findings demonstrate that LDLR-mediated cholesterol uptake promotes statin-induced AML cell death through KLF2 activation by restraining SREBP2 activation and subsequent GGPP production. Our findings reveal a previously unrecognized LDLR–KLF2 axis underlying the anti-leukemic effects of statins and highlight cholesterol metabolism–dependent vulnerabilities as a potential therapeutic target in AML.

**Copyright Information:** (Online) ISSN: 2572-9241
©2026 The Author(s). HemaSphere is published by John Wiley & Sons Ltd on behalf of European Hematology Association. This is an open access Abstract Book distributed under the Attribution-NonCommercial-NoDerivs (CC BY-NC-ND), which allows third parties to download the articles and share them with others as long as they credit the author and the Abstract Book, but they cannot change the content in any way or use them commercially.
**Abstract Book Citations:** Authors, Title, HemaSphere, 2026;10:(S1):pages. The abstract book DOIs can be found on the "Author Information" page.
**Disclaimer:** Articles published in the journal HemaSphere exclusively reflect the opinions of the authors. The authors are responsible for all content in their abstracts including accuracy of the facts, statements, citing resources, etc.

### (PF484) COMPUTATIONAL ASSESSMENT OF CD34+CD38- ACUTE MYELOID LEUKEMIA STEM CELL FREQUENCY AT DIAGNOSIS

**Topic:** 03. Acute myeloid leukemia - Biology & translational research

Felix Zwolle*[1, 2], Tom Reuvekamp[1, 2, 3], Tim Mocking[1, 2, 4], Sven de Regt[1, 2], Daphne den Hartog[1, 2], Angèle Kelder[1, 2], Dimitri Breems[5], Thomas Fischer[6], Bjørn T. Gjertsen[7], Laimonas Griškevičius[8], Gunnar Juliusson[9], Johan Maertens[10], Markus G. Manz[11, 12], Thomas Pabst[12, 13], Jakob Passweg[12, 14], Kimmo Porkka[15], Patrycja Gradowska[16, 17], Bob Löwenberg[16], Gert Ossenkoppele[1, 2], Arjan Van de Loosdrecht[1, 2], David de Leeuw[1, 2], Jacqueline Cloos[1, 2], Costa Bachas[1, 2]

*[1]Amsterdam UMC, Department of Hematology, Amsterdam, The Netherlands;[2]Cancer Center Amsterdam, Amsterdam, The Netherlands; [3]Amsterdam UMC location Universiteit van Amsterdam, Department of Hematology, Amsterdam, The Netherlands; [4]Center for Translational Immunology (CTI), Utrecht, The Netherlands;[5]Ziekenhuis aan de Stroom, Department of Hematology, Antwerpen, Belgium; [6]Otto von Guericke University Hospital Magdeburg, Magdeburg, Germany;[7]Haukeland University Hospital, Bergen, Norway; [8]Vilnius University Hospital Santaros Klinikos and Vilnius University, Vilnius, Lithuania;[9]Skanes University Hospital, Lund, Sweden; [10]University Hospital Gasthuisberg, Leuven, Belgium; [11]University Hospital, Zurich, Switzerland; [12]Swiss Group for Clinical Cancer Research, Bern, Switzerland; [13]University Hospital Inselspital, Bern, Switzerland; [14]University Hospital, Basel, Switzerland; [15]Helsinki University Hospital Cancer Center, Helsinki, Finland; [16]Erasmus Medical Center Cancer Institute, Rotterdam, The Netherlands; [17]HOVON Foundation, Rotterdam, The Netherlands;*

### Background

Acute myeloid leukemia (AML) relapse is thought to be driven by leukemia stem cells (LSC). Immunophenotypically, these cells are defined as CD34+CD38− with the addition of an aberrant marker (e.g., CD45RA, CD123). Both at diagnosis and during treatment, a higher LSC burden confers adverse prognosis. Quantification of LSC using multiparameter flow cytometry (MFC) is challenging due to their low frequency and phenotypic overlap with hematopoietic stem cells (HSC). Gating is labor-intensive and prone to inter-operator variability. We adapted our previously developed Gaussian mixture model (GMM)-based approach (Mocking et al., Nature Communications, 2024) to automate LSC detection and improve reproducibility.

### Aims

To develop a computational pipeline to detect LSC and compute computational stratified LSC proportions (csLSC) from MFC data.

### Methods

Samples with varying degrees of LSC burden were obtained from AML patients at diagnosis and relapse enrolled in the HOVON-SAKK 132 trial and measured using the one-tube LSC assay (Zeijlemaker et al., Leukemia 2016). CD34+CD38− cells were labeled as LSC or non-LSC by manual gating. We trained a 3-component GMM using 5-fold cross-validation with patient-level grouping, classifying individual cells for LSC versus non-LSC. A linear regression model was trained to predict total white blood cell (WBC) counts from the GMM-derived non-LSC components, allowing estimation of LSC burden relative to WBC. WBC model performance was evaluated using leave-one-out cross-validation and assessed with Spearman correlation. To assess prognostic relevance, the models were applied to independent samples at diagnosis from HOVON-SAKK 132 patients. Survival analyses included Kaplan Meier and Cox regression for overall survival (OS), and Fine–Gray regression for cumulative incidence of relapse (CIR), with death as a competing event.

**Copyright Information:** (Online) ISSN: 2572-9241
©2026 The Author(s). HemaSphere is published by John Wiley & Sons Ltd on behalf of European Hematology Association. This is an open access Abstract Book distributed under the Attribution-NonCommercial-NoDerivs (CC BY-NC-ND), which allows third parties to download the articles and share them with others as long as they credit the author and the Abstract Book, but they cannot change the content in any way or use them commercially.
**Abstract Book Citations:** Authors, Title, HemaSphere, 2026;10:(S1):pages. The abstract book DOIs can be found on the "Author Information" page.
**Disclaimer:** Articles published in the journal HemaSphere exclusively reflect the opinions of the authors. The authors are responsible for all content in their abstracts including accuracy of the facts, statements, citing resources, etc.

**Results**

The model was trained on 93 samples (n=47 at diagnosis; n=46 at relapse) from 47 AML patients, displaying a wide range of LSC frequencies. Figure 1A shows an example patient of predicted LSC events. Predicted WBC counts strongly correlated with manually gated counts (ρ=0.98). Predicted LSC counts showed high agreement with ground truth (ρ=0.87). In samples with low LSC frequencies, the model tended to overestimate LSC burden compared to manual gating. In 527 HOVON-SAKK 132 patients (excluding the 47 patients on which the model was trained), predicted csLSC percentages strongly correlated with manually gated LSC percentages (mgLSC) (ρ=0.82; Figure 1B). Classifier performance was strongest in samples with higher LSC frequencies, whereas performance decreased in LSC-negative and CD34-negative samples, where csLSC counts were higher than in manual gated analysis. For survival analysis, a csLSC cutoff at diagnosis of 0.5% was selected to have one third csLSC-positive patients. Using this cutoff, csLSC-positive patients had significantly shorter OS and higher CIR compared to csLSC-negative patients (Figure 1C&D).

**Summary/Conclusion**

Our computational pipeline accurately estimates LSC burden and csLSC percentages, with strong concordance to manual gating in both training and unseen validation cohorts. Higher csLSC percentages were associated with adverse outcome. Future work will further evaluate prognostic performance and refine modeling strategies to reduce misclassification and false-positive rates, particularly in LSC-negative cases.

**Copyright Information:** (Online) ISSN: 2572-9241
©2026 The Author(s). HemaSphere is published by John Wiley & Sons Ltd on behalf of European Hematology Association. This is an open access Abstract Book distributed under the Attribution-NonCommercial-NoDerivs (CC BY-NC-ND), which allows third parties to download the articles and share them with others as long as they credit the author and the Abstract Book, but they cannot change the content in any way or use them commercially.
**Abstract Book Citations:** Authors, Title, HemaSphere, 2026;10:(S1):pages. The abstract book DOIs can be found on the “Author Information” page.
**Disclaimer:** Articles published in the journal HemaSphere exclusively reflect the opinions of the authors. The authors are responsible for all content in their abstracts including accuracy of the facts, statements, citing resources, etc.

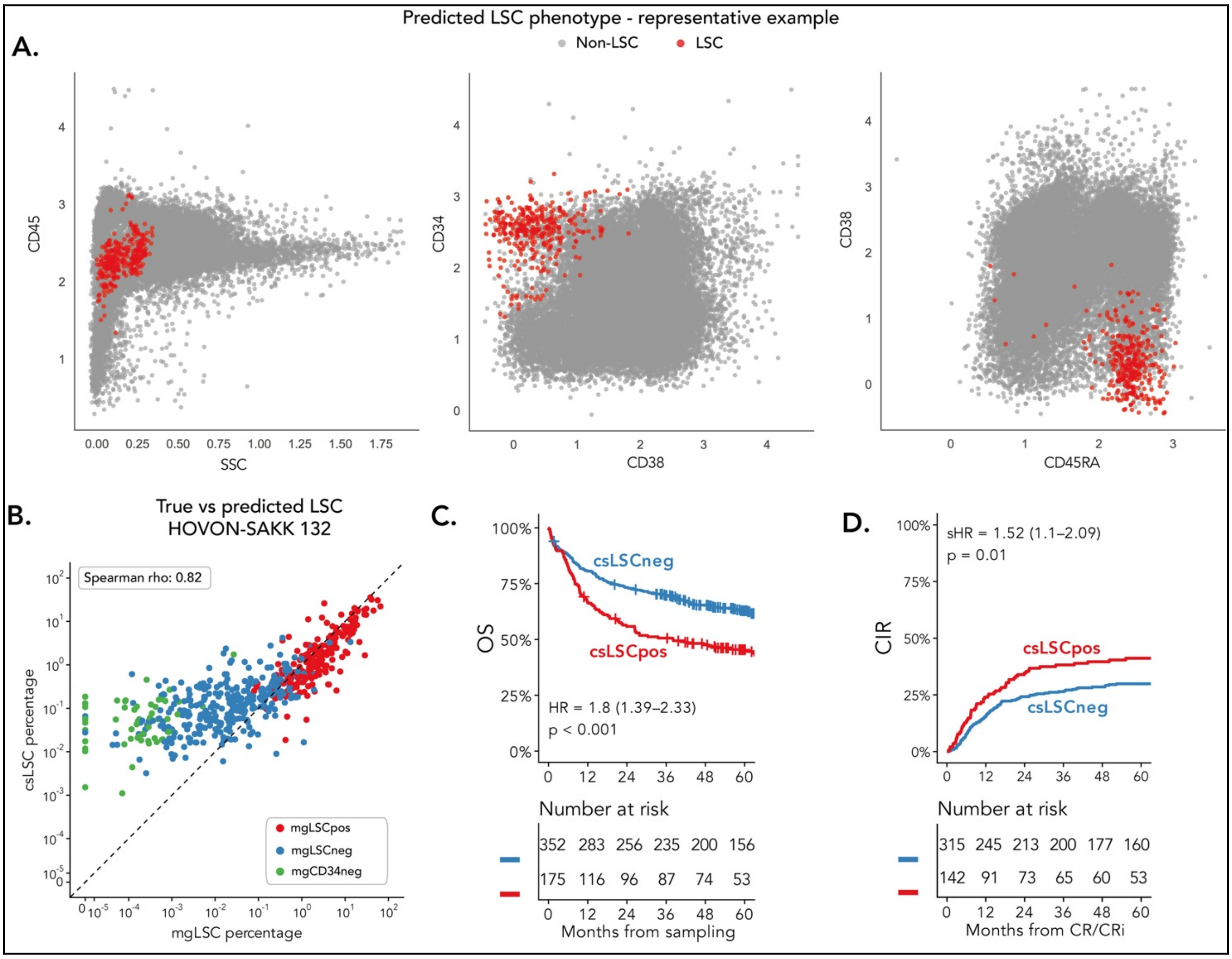

Figure 1. Computational assessment and clinical validation of CD34+CD38- leukemia stem cells at diagnosis. A) Representative example of the LSC model, showing predicted LSC in red. B) computer stratified LSC (csLSC) percentage vs the manual gated LSC percentage (mgLSC) in the HOVON-SAKK 132 clinical trial with a spearman rho of 0.82. C) Overall survival of computer csLSC at diagnosis with cutoff of 0.5% of predicted white blood cells (WBC) from date of sampling at diagnosis. D) Cumulative incidence of relapse based on csLSC at diagnosis with a cutoff of 0.5% of WBC in patients with complete remission (CR) or CR with incomplete blood counts (CRi).

Copyright Information: (Online) ISSN: 2572-9241
©2026 The Author(s). HemaSphere is published by John Wiley & Sons Ltd on behalf of European Hematology Association. This is an open access Abstract Book distributed under the Attribution-NonCommercial-NoDerivs (CC BY-NC-ND), which allows third parties to download the articles and share them with others as long as they credit the author and the Abstract Book, but they cannot change the content in any way or use them commercially.
Abstract Book Citations: Authors, Title, HemaSphere, 2026;10:(S1):pages. The abstract book DOIs can be found on the “Author Information” page.
Disclaimer: Articles published in the journal HemaSphere exclusively reflect the opinions of the authors. The authors are responsible for all content in their abstracts including accuracy of the facts, statements, citing resources, etc.

### (PF485) DISCOVERY AND VALIDATION OF A ROBUST 11-GENE PROGNOSTIC SIGNATURE VIA INTEGRATIVE MULTI-OMICS PROFILING IN VD-CAG-TREATED NON-M3 AML

**Topic:** 03. Acute myeloid leukemia - Biology & translational research

Jirui Tang*[1], Yuping Gong[1]

[1]*West China Hospital, Sichuan University, Department of Hematology, Chengdu, China;*

### Background

Acute myeloid leukemia (AML), particularly non-M3 subtypes, presents significant therapeutic challenges characterized by heterogeneous prognoses and high relapse rates despite intensive chemotherapy. While the venetoclax-based VD-CAG regimen (venetoclax, decitabine, cytarabine, aclarubicin, granulocyte colony-stimulating factor) demonstrates promising efficacy, the underlying molecular response mechanisms and prognostic biomarkers remain poorly understood.

### Aims

This study aimed to elucidate the molecular alterations associated with the VD-CAG response and to identify and validate robust prognostic biomarkers for non-M3 AML using an integrative multi-omics approach.

### Methods

Integrative multi-omics profiling, comprising RNA-sequencing (RNA-seq) and data-independent acquisition (DIA) proteomics, was conducted on bone marrow aspirate samples from 15 non-M3 AML patients obtained pre- and post-VD-CAG induction. Weighted gene co-expression network analysis (WGCNA) was utilized to identify gene modules correlated with composite complete remission (CRc). Prognostic signatures were derived via Least Absolute Shrinkage and Selection Operator (LASSO) regression using the TCGA-LAML cohort for discovery and the Beat AML cohort for validation.

### Results

RNA-seq analysis identified differentially expressed genes enriched in MAPK signaling, cytokine interactions, and cell adhesion pathways. WGCNA confirmed five modules correlated with CRc. From the Chocolate4 module, a robust 11-gene signature (*TLN1, ARL15, PDZD2, ACER2, IGF2BP3, TMEM200A, DOCK1, SYTL4, CPNE8, FNDC3B*, and *MANBA*) was derived that effectively stratified overall survival (OS). The signature demonstrated strong predictive value in the TCGA cohort (HR=3.60, *p*=7.6e-10; 3-year AUC=0.79) and the Beat AML validation cohort (HR=2.07, *p*=3.5e-4; 3-year AUC=0.83). Cox regression confirmed the signature's independent prognostic value. DIA proteomics quantified 6830 proteins, identifying 447 differentially expressed proteins enriched in metabolic and signaling pathways. Notably, FNDC3B, a key protein within the prognostic signature, showed significant downregulation post-VD-CAG treatment.

### Summary/Conclusion

This study unveils specific molecular alterations related to the VD-CAG response and offers a validated 11-gene prognostic signature for non-M3 AML. These findings enhance the understanding of molecular response mechanisms and provide a potential tool for improved prognostic stratification.

**Copyright Information:** (Online) ISSN: 2572-9241
©2026 The Author(s). HemaSphere is published by John Wiley & Sons Ltd on behalf of European Hematology Association. This is an open access Abstract Book distributed under the Attribution-NonCommercial-NoDerivs (CC BY-NC-ND), which allows third parties to download the articles and share them with others as long as they credit the author and the Abstract Book, but they cannot change the content in any way or use them commercially.
**Abstract Book Citations:** Authors, Title, HemaSphere, 2026;10:(S1):pages. The abstract book DOIs can be found on the "Author Information" page.
**Disclaimer:** Articles published in the journal HemaSphere exclusively reflect the opinions of the authors. The authors are responsible for all content in their abstracts including accuracy of the facts, statements, citing resources, etc.

### (PF486) INDOLE-3-ACETIC ACID REVERSES VENETOCLAX RESISTANCE IN ACUTE MYELOID LEUKEMIA VIA THE JAK2/STAT3 SIGNALING PATHWAY

**Topic:** 03. Acute myeloid leukemia - Biology & translational research

Haiping Liang*[1], Bei Liu[1, 2]

*[1]The First Clinical Medical College of Lanzhou University, Lanzhou, China;[2]The First Hospital of Lanzhou University, Department of Hematology, Lanzhou, China;*

### Background

Venetoclax-based combination regimens have emerged as a first-line treatment option for AML patients unfit for intensive chemotherapy, owing to their high response rates and favorable tolerability. However, with the widespread application of these regimens, the issue of venetoclax resistance has become increasingly prominent, representing a key challenge in current clinical practice. Therefore, elucidating the mechanisms underlying venetoclax resistance has become a pressing clinical need.

### Aims

This study aimed to investigate the effect of the tryptophan metabolite indole-3-acetic acid (3-IAA) on reversing venetoclax resistance in acute myeloid leukemia (AML) cells and to untangle its underlying molecular mechanisms.

### Methods

To elucidate the molecular mechanisms underlying venetoclax resistance in AML, this study integrated clinical sample analysis with cellular model validation. Targeted metabolomics and transcriptomics were employed to delineate the metabolic profile of venetoclax-resistant AML and to identify significantly altered metabolic pathways and metabolites. Concurrently, venetoclax-resistant cell lines were established via gradient induction, and the half-maximal inhibitory concentration (IC50) of resistant cells was assessed using the CCK-8 proliferation assay. In functional experiments, the effects of venetoclax and 3-IAA, used alone or in combination, on the proliferation of resistant cells were evaluated using the CCK-8 assay, EdU staining, and colony formation assays. The level of apoptosis was assessed via Annexin V/PI double staining. Furthermore, Western blot analysis was utilized to evaluate the regulatory effects of venetoclax monotherapy or its combination with 3-IAA on the expression of JAK2-STAT3 pathway proteins and the signaling activity in resistant cells.

### Results

This study found that targeted metabolomics analysis of peripheral blood mononuclear cells from venetoclax-sensitive and -resistant AML patients revealed abnormalities in the tryptophan metabolic pathway in resistant AML, particularly a reduction in 3-IAA levels in resistant patients. Venetoclax-resistant cell lines were established through gradient induction. Treatment with 3-IAA alone or in combination with venetoclax inhibited the proliferation, DNA replication, and colony formation of the resistant cells, while promoting apoptosis. Furthermore, Western blot analysis confirmed that 3-IAA inhibited the activation of the JAK2-STAT3 pathway. The combination treatment suppressed STAT3 transcriptional activity and downregulated MCL1 expression. Notably, activation of the JAK2/STAT3 signaling pathway could partially reverse these effects and restore drug sensitivity in the resistant cells. These results indicate that 3-IAA may be involved in the development of venetoclax resistance in AML by modulating the JAK2/STAT3/MCL-1 signaling axis.

**Copyright Information:** (Online) ISSN: 2572-9241
©2026 The Author(s). HemaSphere is published by John Wiley & Sons Ltd on behalf of European Hematology Association. This is an open access Abstract Book distributed under the Attribution-NonCommercial-NoDerivs (CC BY-NC-ND), which allows third parties to download the articles and share them with others as long as they credit the author and the Abstract Book, but they cannot change the content in any way or use them commercially.
**Abstract Book Citations:** Authors, Title, HemaSphere, 2026;10:(S1):pages. The abstract book DOIs can be found on the "Author Information" page.
**Disclaimer:** Articles published in the journal HemaSphere exclusively reflect the opinions of the authors. The authors are responsible for all content in their abstracts including accuracy of the facts, statements, citing resources, etc.

**Summary/Conclusion**

We identified a reduction in indoleacetic acid in venetoclax-resistant AML. The combination of indoleacetic acid and venetoclax effectively improved venetoclax resistance. Supplementation with indoleacetic acid may represent a potential strategy to overcome venetoclax resistance.

**Copyright Information:** (Online) ISSN: 2572-9241
©2026 The Author(s). HemaSphere is published by John Wiley & Sons Ltd on behalf of European Hematology Association. This is an open access Abstract Book distributed under the Attribution-NonCommercial-NoDerivs (CC BY-NC-ND), which allows third parties to download the articles and share them with others as long as they credit the author and the Abstract Book, but they cannot change the content in any way or use them commercially.
**Abstract Book Citations:** Authors, Title, HemaSphere, 2026;10:(S1):pages. The abstract book DOIs can be found on the "Author Information" page.
**Disclaimer:** Articles published in the journal HemaSphere exclusively reflect the opinions of the authors. The authors are responsible for all content in their abstracts including accuracy of the facts, statements, citing resources, etc.

### (PF487) ESTABLISHMENT OF TRANSLATIONAL 3D AML BONE MARROW-ON-CHIP MODELS TO STUDY BONE MARROW NICHE INTERACTION AND DRUG SENSITIVITY

**Topic:** 03. Acute myeloid leukemia - Biology & translational research

Nurbanu Erölmez*[1, 2], Amr Othman[1], Linda Gijzen[1], Henriëtte Lanz[1], Karla Queiroz[1]

[1]*Mimetas - Organ-On-A-Chip, Oegstgeest, The Netherlands;* [2]*Amsterdam UMC location Vrije Universiteit Amsterdam, Department of Hematology, Amsterdam, The Netherlands;*

### Background

Acute myeloid leukemia (AML) is an aggressive cancer of the myeloid cells, characterized by rapid and uncontrolled clonal expansion of myeloblasts. Despite high initial remission rates, AML is still associated with a high relapse rate and poor prognosis. Relapse in AML is driven by residual therapy-resistant leukemic cells that survive frontline treatment and minimal residual disease (MRD) is a major predictor of relapse and reflects persistence of leukemic cells below morphologic detection. The bone marrow microenvironment (BMME) plays a crucial role in the onset, progression and relapse of the disease as cell-cell and cell-ECM interactions provide pro-survival cues to AML cells. Current 2D cultures and animal models cannot replicate the complexity of the human BMME, poorly predicting the biological performance of drug candidates during human clinical trials. Therefore, there is a need for 3D *in vitro* models that recapitulate the AML-BMME interactions to investigate AML pathologies and drug responses and to study how therapy-resistant AML cells survive, adapt, and respond to therapy.

### Aims

Our aim is to develop a human 3D AML-bone marrow niche model that (1) recreates key niche interactions under controlled perfusion, (2) enables quantitative assessment of leukemic-stromal-endothelial interactions, and (3) supports evaluation of niche-driven therapy response and resistance in AML.

### Methods

A membrane-free 3-lane microfluidic culture plate (OrganoPlate, 40 independent cultures per plate) was used; each chip comprises three channels for ECM loading and medium perfusion. This design enables barrier-free co-culture, controlled perfusion, and high-resolution imaging, allowing leukemic cells to interact with the microenvironment in a physiologically relevant manner. To mimic the BMME, primary human mesenchymal stem cells (MSCs) were cultured in a collagen I ECM gel, optionally enriched with laminin, fibronectin, and hyaluronic acid to increase the relevance of the model. AML cell lines (HL-60, THP-1, OCI-AML3) were introduced either together with MSCs (to model direct niche contact) or in an adjacent channel (to study chemotaxis/mobilization under compartmentalized conditions). Endothelial cells (HMEC-1, HUVEC) were added to the bottom perfusion channel, where they proliferate and form a perfusable tubule.

### Results

We established a reproducible 3D microfluidic tri-culture on-a-chip model in OrganoPlate combining AML cells embedded in ECM (collagen I) with perfused stromal and endothelial compartments. Using a sequential seeding workflow, microvascular endothelial cells formed a continuous $CD31^+$ tubule in the bottom perfusion channel, while MSCs populated in the top perfusion channel, generating a multicellular niche adjacent to the AML-containing ECM channel. Confocal imaging shows spatial compartmentalization and visualization of leukemic cell localization relative to stromal and endothelial structures.

**Copyright Information:** (Online) ISSN: 2572-9241
©2026 The Author(s). HemaSphere is published by John Wiley & Sons Ltd on behalf of European Hematology Association. This is an open access Abstract Book distributed under the Attribution-NonCommercial-NoDerivs (CC BY-NC-ND), which allows third parties to download the articles and share them with others as long as they credit the author and the Abstract Book, but they cannot change the content in any way or use them commercially.
**Abstract Book Citations:** Authors, Title, HemaSphere, 2026;10:(S1):pages. The abstract book DOIs can be found on the "Author Information" page.
**Disclaimer:** Articles published in the journal HemaSphere exclusively reflect the opinions of the authors. The authors are responsible for all content in their abstracts including accuracy of the facts, statements, citing resources, etc.

**Summary/Conclusion**

Altogether, we established a perfusable, compartmentalized 3D microfluidic AML tri-culture niche model and we will further expand it to additional AML genotypes and patient-derived AML cells; incorporating other relevant cellular BM niche components and applying it to drug-response benchmarking and MRD studies. Thereby, this model represents a significant step towards high-throughput drug screening and personalized medicine for AML and other hematological disorders.

**Copyright Information:** (Online) ISSN: 2572-9241
©2026 The Author(s). HemaSphere is published by John Wiley & Sons Ltd on behalf of European Hematology Association. This is an open access Abstract Book distributed under the Attribution-NonCommercial-NoDerivs (CC BY-NC-ND), which allows third parties to download the articles and share them with others as long as they credit the author and the Abstract Book, but they cannot change the content in any way or use them commercially.
**Abstract Book Citations:** Authors, Title, HemaSphere, 2026;10:(S1):pages. The abstract book DOIs can be found on the "Author Information" page.
**Disclaimer:** Articles published in the journal HemaSphere exclusively reflect the opinions of the authors. The authors are responsible for all content in their abstracts including accuracy of the facts, statements, citing resources, etc.

### (PF488) CLINICAL FEATURES, TREATMENT PATTERNS AND OUTCOMES OF 4,351 ADULT PATIENTS WITH SECONDARY ACUTE LEUKEMIA: A PETHEMA STUDY

**Topic:** 04. Acute myeloid leukemia - Clinical

Pilar Lloret-Madrid[1], Rosa María Ayala Díaz[2], Josefina Serrano Lopez[3], Eduardo Rodríguez-Arbolí[4], Marisa Calabuig[5], Mar Tomo[5], Isabel Cano Ferri[1], Cristina Gil Cortes[6], Juan Miguel Bergua Burgues[7], Fernanda Trigo[8], Olga Salamero[9], José Luis López-Lorenzo[10], Jorge Labrador[11], Carlos Rodriguez Medina[12], Teresa Bernal[13], Susana Vives Polo[14], Armando Mena Durán[15], Lorenzo Algarra[16], Maria de Las Mercedes Colorado[17], Maria Luz Amigo[18], Pilar Herrera[19], Emilia Nobre Barata Roxo Cortesao[20], Raimundo Garcia Boyero[21], Aitor Abuín Blanco[22], Olga Arce[23], Marta Valero[24], Bernardo Javier González González[25], Luis Felipe Casado Montero[26], Ana Oliva Hernández[27], Maria Jose Sayas Lloris[28], Mayte Olave[29], Agata Almela Gallego[30], Celina Benavente[31], Victor Noriega Concepción[32], Beatriz De Rueda Ciller[33], Gabriela Rodríguez MacÍas[34], Irene Sánchez Vadillo[35], Ana Alfonso Pierola[36], Maria-Angeles Foncillas[37], Maria Dolores Madrigal Toscano[38], Maria Garcia Fortes[39], Marta Callejas[40], María Solé[41], Claudia Sossa-Melo[42], Monica Alejandra Romero Riquelme[43], Nidia Paulina Zapata Canto[44], Pau Montesinos*[1]

*[1]Hospital Universitari I Politècnic La Fe, València, Spain; [2]Hospital Universitario 12 de Octubre/IMAS12, Madrid, Spain; [3]Hospital Universitario Reina Sofía/IMIBIC/UCO, Hematology, Córdoba, Spain; [4]Hospital Universitario Virgen del Rocío/IBIS/CSIC, Sevilla, Spain; [5]Hospital Clínico Universitario de Valencia / INCLIVA, València, Spain;[6]Hospital General Universitario de Alicante, Alicante, Spain; [7]Hospital San Pedro de Alcantara, Cáceres, Spain;[8]Centro Hospitalar Universitário de São João, Porto, Portugal; [9]Vall d'Hebron University Hospital / VHIO, Barcelona, Spain;[10]Hospital Universitario Fundación Jiménez Díaz, Madrid, Spain; [11]Hospital Universitario de Burgos / IBioBURGOS, Burgos, Spain;[12]Hospital Universitario de Gran Canaria Doctor Negrin, Las Palmas de Gran Canaria, Spain; [13]Hospital Universitario Central de Asturias / ISPA / IUOPA, Oviedo, Spain;[14]ICO Badalona – Hospital Germans Trias i Pujol, Badalona, Spain; [15]Hospital General Universitario de Valencia, Valencia, Spain; [16]Hospital Universitario General de Albacete, Albacete, Spain; [17]Hospital Universitario Marqués de Valdecilla, Santander, Spain;[18]Hospital Morales Meseguer, Murcia, Spain; [19]Hospital Universitario Ramón y Cajal, Madrid, Spain;[20]Centro Hospitalar e Universitário de Coimbra, Coimbra, Portugal; [21]Hospital General Universitario de Castellón, Castellón, Spain;[22]Hospital Universitario Lucus Augusti, Lugo, Spain; [23]Hospital Universitario Basurto, Bilbao, Spain;[24]Hospital Arnau de Vilanova, Valencia, Spain;[25]Hospital Universitario de Canarias, Santa Cruz de Tenerife, Spain; [26]Hospital HM Sanchinarro CIOCC, Madrid, Spain;[27]Hospital Universitario Nuestra Señora de Candelaria, Tenerife, Spain; [28]Hospital Universitario Doctor Peset, Valencia, Spain;[29]Hospital Clínico Universitario Lozano Blesa, Zaragoza, Spain;[30]Hospital Universitario de León, León, Spain;[31]Hospital Clínico San Carlos, Madrid, Spain; [32]Hospital Universitario de A Coruña, A Coruña, Spain;[33]Hospital Universitario Miguel Servet, Zaragoza, Spain; [34]Hospital General Universitario Gregorio Marañón, Madrid, Spain;[35]Hospital Universitario La Paz, Madrid, Spain; [36]Clínica Universidad de Navarra (CUN), Pamplona, Spain;[37]Hospital Universitario Infanta Leonor, Madrid, Spain;[38]Hospital Universitario Virgen de la Macarena, Sevilla, Spain; [39]Hospital Universitario Virgen de la Victoria, Málaga, Spain;[40]Hospital Universitario Príncipe de Asturias, Alcalá de Henares, Spain; [41]Hospital Universitario Juan Ramón Jiménez, Huelva, Spain; [42]Clínica FOSCAL, Bucaramanga, Colombia;[43]Hospital Guillermo Grant Benavente, Concepción, Chile;[44]Instituto Nacional de Cancerología, Ciudad de México, Mexico;*

### Background

Secondary acute myeloid leukemia (sAML) encompasses AML arising after prior cytotoxic therapy (t-AML) and after antecedent hematologic disease (AHD-AML) such as myeloproliferative neoplasms (MPN) or myelodysplastic syndromes (MDS), and is associated with poor prognosis.

### Aims

To analyze the epidemiology, characteristics, and outcomes of patients with sAML.

**Copyright Information:** (Online) ISSN: 2572-9241
©2026 The Author(s). HemaSphere is published by John Wiley & Sons Ltd on behalf of European Hematology Association. This is an open access Abstract Book distributed under the Attribution-NonCommercial-NoDerivs (CC BY-NC-ND), which allows third parties to download the articles and share them with others as long as they credit the author and the Abstract Book, but they cannot change the content in any way or use them commercially.
**Abstract Book Citations:** Authors, Title, HemaSphere, 2026;10:(S1):pages. The abstract book DOIs can be found on the "Author Information" page.
**Disclaimer:** Articles published in the journal HemaSphere exclusively reflect the opinions of the authors. The authors are responsible for all content in their abstracts including accuracy of the facts, statements, citing resources, etc.

### Methods

Patients with sAML reported between 1980 and 2025 in the multinational PETHEMA registry.

### Results

Among 15,872 AML pts with available information on AML type, 11,295 (71.2%) had de novo AML and 4,577 (28.8%) sAML. Among pts with data to further characterize sAML (n=4,351), 2,610 (60%) were AHD-AML and 1,741 (40%) t-AML. The incidence of sAML increased over time, 21.1% before 2000, 21.9% 2000-2009, 29.9% 2010-2020, and 34.3% 2021-2025. Compared with de novo AML, sAML pts were older (median 70 vs 64 years), had more adverse cytogenetics (38.8% vs 24.2%), less NPM1 (13.6% vs 33%) and less FLT3-ITD mutations (10.1% vs 21.5%) (all p<0.001). Among sAML, AHD-AML pts were older (71 vs 69 years), had higher WBC counts (6.8 vs 4.9 $x10^9$/L) and extramedullary disease (21.8% vs 14.1%), while adverse cytogenetic risk was more frequent in t-AML (41.2% vs 37.1%) (all p<0.001). NPM1 and TP53 mutations were more frequent in t-AML vs AHD-AML (18.2% vs 9.2%, and 28.7% vs 22.3%, respectively) (p<0.001 and p=0.002, respectively). Among AHD-AML, 62.6% were MDS-AML, 20.4% MPN-AML, and 17% MDS/MPN-AML. Hyperleukocytosis (≥20 $x10^9$/L) was present in 55.1% vs 35.9% vs 21.1% for MDS/MPN-AML, MPN-AML, and MDS-AML, respectively. MPN-AML showed more extramedullary disease compared with MDS/MPN-AML and MDS-AML (36.8% vs 26.3% vs 15.2%, respectively), more adverse cytogenetic risk (56.7% vs 21.5% vs 34.8%, respectively), and TP53 mutations (34.2% vs 7.3% vs 23.5%, respectively). In the whole cohort, mOS was worse in sAML than in de novo AML (median 5.7 vs 13.2 months; p<0.001), and AHD-AML had inferior OS compared with t-AML (Figure 1A). Sustained mOS increase occurred per decade (3.2 months 80´s, 7.7 in 90´s, 9.1 in 00´s, 10.7 in 10´s, and 14.3 in 20´s; p<0.001). mOS was 11.4 months with intensive vs 6.3 months with non-intensive regimens (p<0.001). Among 4,104 pts with frontline treatment data, 20% received supportive care, 35.6% low-intensity, and 44.4% intensive regimens, most commonly 3+7 (58.3%), followed by FLAG-based (15.4%), CPX-351 (10.9%), and other intensive schedules (15.4%). CR/CRi rates were 57.9% after 3+7; 64.6% after 3+7+etoposide; CPX-351 53.2%; FLAG-based 54.4%; other intensive 39.2%. Of patients in first CR/CRi (n=887), 41.5% underwent allo-HSCT, 7.1% auto-HSCT, and 51.4% were not transplanted. Allo-HSCT showed a significant survival benefit (HR 0.52; p<0.001), while auto-HSCT showed a non-significant trend (HR 0.75; p=0.09) (Figure 1B). For non-intensive therapy, HMA predominated (51.4%), followed by low-intensity FLAG (19.8%), HMA-VEN (16.1%), other low (10.7%) and LDAC (2%). CR/CRi rates were 20.4% after FLAG-low; HMA 16.6%; HMA-VEN 44.4%; LDAC 6.9%, and other low 29.2%. mOS was 7.5 months with HMA-VEN, 6.8 months with HMA, 4.6 months with FLAG-low, 3.1 months with LDAC, and 7.7 months with other low (p<0.001).

### Summary/Conclusion

Despite sustained improvements, sAML remains a heterogeneous and aggressive disease. Clinical and prognostic features differ between AHD-AML and t-AML and should be considered distinct entities. There is an urgent medical need for this increasing AML population.

**Copyright Information:** (Online) ISSN: 2572-9241
©2026 The Author(s). HemaSphere is published by John Wiley & Sons Ltd on behalf of European Hematology Association. This is an open access Abstract Book distributed under the Attribution-NonCommercial-NoDerivs (CC BY-NC-ND), which allows third parties to download the articles and share them with others as long as they credit the author and the Abstract Book, but they cannot change the content in any way or use them commercially.
**Abstract Book Citations:** Authors, Title, HemaSphere, 2026;10:(S1):pages. The abstract book DOIs can be found on the “Author Information” page.
**Disclaimer:** Articles published in the journal HemaSphere exclusively reflect the opinions of the authors. The authors are responsible for all content in their abstracts including accuracy of the facts, statements, citing resources, etc.

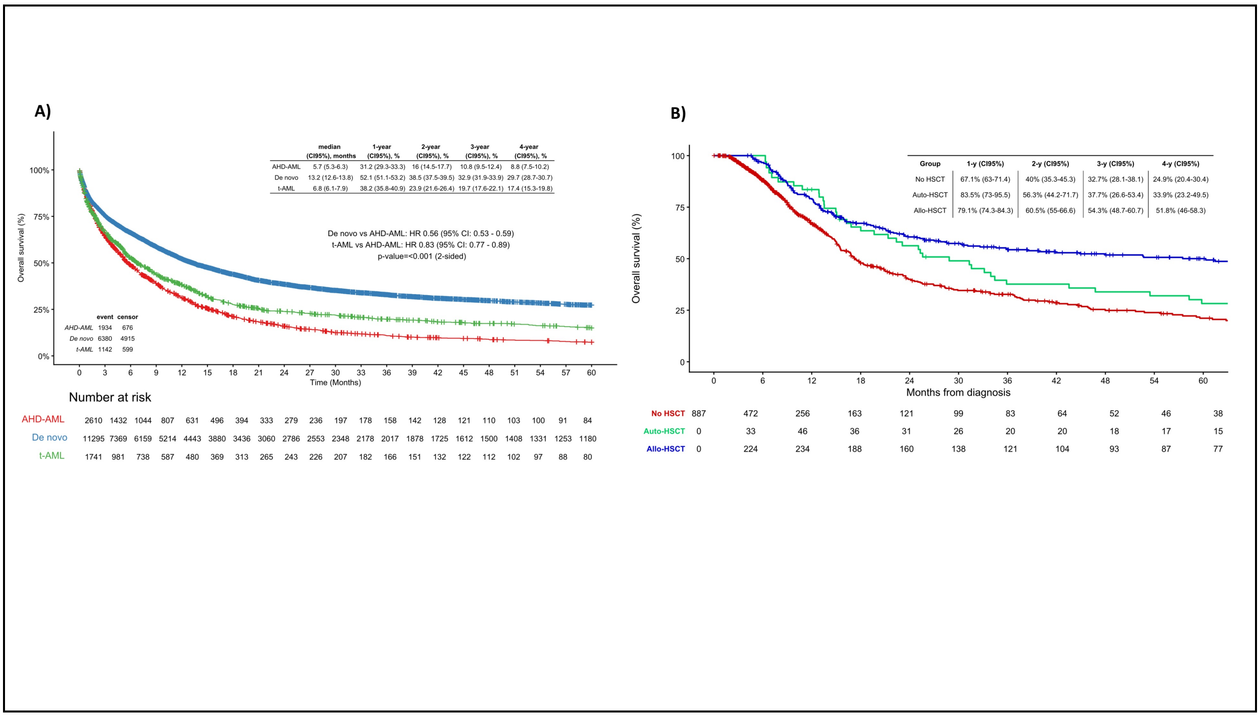

Figure 1. (A) Overall survival in de novo AML versus sAML subtypes (AHD-AML and t-AML). (B) Simon-Makuch plot of overall survival in fit sAML patients according to HSCT status in first CR/CRi.

Copyright Information: (Online) ISSN: 2572-9241
©2026 The Author(s). HemaSphere is published by John Wiley & Sons Ltd on behalf of European Hematology Association. This is an open access Abstract Book distributed under the Attribution-NonCommercial-NoDerivs (CC BY-NC-ND), which allows third parties to download the articles and share them with others as long as they credit the author and the Abstract Book, but they cannot change the content in any way or use them commercially.
Abstract Book Citations: Authors, Title, HemaSphere, 2026;10:(S1):pages. The abstract book DOIs can be found on the "Author Information" page.
Disclaimer: Articles published in the journal HemaSphere exclusively reflect the opinions of the authors. The authors are responsible for all content in their abstracts including accuracy of the facts, statements, citing resources, etc.

### (PF489) REVUMENIB + INTENSIVE CHEMOTHERAPY FOR NEWLY DIAGNOSED ACUTE MYELOID LEUKEMIA HARBORING GENETIC ALTERATIONS IN KMT2A, NPM1, OR NUP98: UPDATED PHASE 1 RESULTS FROM SNDX-5613-0708

**Topic:** 04. Acute myeloid leukemia - Clinical

Ibrahim Aldoss*[1], Carl T. Shultz[2], Bradley D. Hunter[3], David Swoboda[4], Pau Montesinos[5], Sarit Assouline[6], Shaun Fleming[7], Carolyn Grove[8], Andre Schuh[9], David Taussig[10], Karla Malloy[11], Enoch Cobbina[11], Jingshan Zhang[11], Jessica Clement[11], Eytan Stein[12], John Dipersio[13]

*[1]City of Hope National Medical Center, Duarte, CA, United States of America;[2]Transplant and Cellular Therapy/Hematologic Malignancies, Department of Medical Oncology, West Virginia University, Morgantown, WV, United States of America; [3]Intermountain Health, Salt Lake City, UT, United States of America;[4]Tampa General Hospital Cancer Institute, Tampa, FL, United States of America; [5]Hospital Universitari i Politècnic La Fe, Valencia, Spain; [6]Division of Hematology, Department of Oncology, McGill University, Montreal, QC, Canada; [7]The Alfred Hospital, Melbourne, VIC, Australia;[8]Sir Charles Gairdner Hospital, PathWest, and The University of Western Australia, Nedlands, WA, Australia; [9]Princess Margaret Cancer Centre and University of Toronto, Toronto, ON, Canada; [10]The Royal Marsden NHS Foundation Trust, The Institute of Cancer Research, London, United Kingdom; [11]Syndax Pharmaceuticals, Inc., New York, NY, United States of America; [12]Memorial Sloan Kettering Cancer Center, New York, NY, United States of America; [13]Washington University School of Medicine, St Louis, MO, United States of America;*

### Background

Acute leukemias (AL) harboring *KMT2A* or *NUP98* rearrangements (*KMT2A*r or *NUP98*r) are associated with poor clinical outcomes, and adults with *NPM1*-mutated (*NPM1*m) acute myeloid leukemia (AML) lacking a concurrent *FLT3* mutation relapse in ~50% of cases. These genomic alterations promote leukemogenesis by sustaining the menin-KMT2A interaction, which blocks hematopoietic differentiation. Revumenib, a first-in-class, oral, potent, and selective menin-KMT2A inhibitor, is used for the treatment (tx) of relapsed/refractory (R/R) AL harboring a *KMT2A* translocation or R/R AML with *NPM1* mutation in adult and pediatric patients (pts) 1 year and older.

### Aims

To report findings from the dose-escalation phase 1 study of revumenib + intensive chemotherapy (IC) in newly diagnosed (ND) AML with a *KMT2A*r, *NPM1*m, or *NUP98*r.

### Methods

SNDX-5613-0708 (NCT06226571) is a multicenter, open-label, dose-escalation/expansion study enrolling pts 18–75 years with ND AML harboring *KMT2A*r, *NPM1*m, or *NUP98*r who are eligible for IC. Two dose levels (DL) of revumenib were evaluated (DL1: 110 mg/220 mg ± strong CYP3A4 inhibitor [CYP3A4i]; DL2: 160 mg/270 mg ± strong CYP3A4i) in combination with cytarabine plus daunorubicin or idarubicin. Pts achieving complete remission (CR) or CR with incomplete hematologic recovery received consolidation therapy consisting of revumenib + high-dose cytarabine, followed by optional hematopoietic stem cell transplant (HSCT) and revumenib maintenance. Primary endpoints are dose-limiting toxicities (DLTs) and safety. Additional endpoints are overall response rate (ORR), CR rate, composite CR (CRc) rate, and measurable residual disease–negative (MRD–) CR rate (assessed locally).

**Copyright Information:** (Online) ISSN: 2572-9241
©2026 The Author(s). HemaSphere is published by John Wiley & Sons Ltd on behalf of European Hematology Association. This is an open access Abstract Book distributed under the Attribution-NonCommercial-NoDerivs (CC BY-NC-ND), which allows third parties to download the articles and share them with others as long as they credit the author and the Abstract Book, but they cannot change the content in any way or use them commercially.
**Abstract Book Citations:** Authors, Title, HemaSphere, 2026;10:(S1):pages. The abstract book DOIs can be found on the “Author Information” page.
**Disclaimer:** Articles published in the journal HemaSphere exclusively reflect the opinions of the authors. The authors are responsible for all content in their abstracts including accuracy of the facts, statements, citing resources, etc.

### Results

As of 02Oct2025, 13 pts were treated at DL1 and 18 at DL2; 12 and 13, respectively, were evaluable for DLTs per protocol criteria (received ≥75% planned dosing or experienced a DLT during the evaluation period). Median (range) age was 43.0 y (20.0–71.0) for DL1 and 57.5 y (19.0–68.0) for DL2. *KMT2A*r was detected in 92% (12/13) of pts in DL1 and 39% (7/18) in DL2;*NPM1*m was detected in 8% (1/13) of pts in DL1 and 56% (10/18) in DL2. Median (range) tx duration was 73.0 days (1.0–170.0) for DL1 and 34.0 days (1.0–156.0) for DL2. One DLT occurred (grade 3 QTcF prolongation) in DL1. Tx-emergent adverse events are summarized in the **Table**. No pt developed differentiation syndrome. Two pts in DL1 (15% of DL1 pts, 6% of all pts) experienced any-grade QTcF prolongation. Among response-evaluable pts, ORR was 100% (12/12) in DL1 and 92.9% (13/14) in DL2; CRc rate was 100% (12/12) and 85.7% (12/14); and CR rate was 91.7% (11/12) and 78.6% (11/14), respectively. MRD– CR was achieved in 100% (8/8) of tested pts in DL1 and 70.0% (7/10) in DL2. Seven pts proceeded to HSCT (all in DL1, which had longer follow-up than DL2), and 5 resumed revumenib post HSCT. From induction Cycle 1, median (range) time to neutrophil recovery (≥1000 cells/μL) was 29.0 days (19.0–42.0, DL1) and 29.0 days (22.0–46.0, DL2); platelet recovery (≥100,000 cells/μL) was 28.0 days (22.0–31.0, DL1) and 29.0 days (25.0–47.0, DL2).

### Summary/Conclusion

Revumenib + IC demonstrated a manageable safety profile across both DLs, comparable to IC alone, with no new safety signals. Preliminary efficacy data suggest deep responses with high MRD– CR rates. These findings support continued evaluation of revumenib + IC in the phase 3 REVEAL-ND NPM1 trial in fit pts with *NPM1*m AML (NCT07211958). Updated results will be presented.

**Table. Summary of TEAEs**

| Parameter, n (%) | DL1 (n=13) | DL2 (n=18) | Total (N=31) |
|---|---|---|---|
| **Any TEAE** | **13 (100)** | **18 (100)** | **31 (100)** |
| Revumenib-related | 9 (69) | 10 (56) | 19 (61) |
| **Grade ≥3 TEAEs[a]** | **11 (85)** | **16 (89)** | **27 (87)** |
| Febrile neutropenia | 6 (46) | 10 (56) | 16 (52) |
| Platelet count decreased | 5 (38) | 4 (22) | 9 (29) |
| Anemia | 3 (23) | 6 (33) | 9 (29) |
| White blood cell count decreased | 4 (31) | 4 (22) | 8 (26) |
| Neutrophil count decreased | 4 (31) | 3 (17) | 7 (23) |
| Thrombocytopenia | 1 (8) | 5 (28) | 6 (19) |
| Neutropenia | 1 (8) | 4 (22) | 5 (16) |
| **Grade ≥3 revumenib-related TEAEs[b]** | **5 (38)** | **8 (44)** | **13 (42)** |
| Anemia | 3 (23) | 3 (17) | 6 (19) |
| Neutrophil count decreased | 3 (23) | 1 (6) | 4 (13) |
| **Serious TEAEs** | **6 (46)** | **8 (44)** | **14 (45)** |
| **TEAEs leading to dose modifications** | | | |
| Reduction | 2 (15) | 0 | 2 (6) |
| Discontinuation | 3 (23) | 1 (6) | 4 (13) |
| **TEAEs leading to death** | **0** | **1 (6)** | **1 (3)** |

[a]Events that occurred in ≥15% of total population. [b]Events that occurred in ≥10% of total population. DL1, dose level 1; DL2, dose level 2; TEAE, treatment-emergent adverse event.

Copyright Information: (Online) ISSN: 2572-9241
©2026 The Author(s). HemaSphere is published by John Wiley & Sons Ltd on behalf of European Hematology Association. This is an open access Abstract Book distributed under the Attribution-NonCommercial-NoDerivs (CC BY-NC-ND), which allows third parties to download the articles and share them with others as long as they credit the author and the Abstract Book, but they cannot change the content in any way or use them commercially.
Abstract Book Citations: Authors, Title, HemaSphere, 2026;10:(S1):pages. The abstract book DOIs can be found on the “Author Information” page.
Disclaimer: Articles published in the journal HemaSphere exclusively reflect the opinions of the authors. The authors are responsible for all content in their abstracts including accuracy of the facts, statements, citing resources, etc.

### (PF490) CHIDAMIDE PLUS VENETOCLAX AND AZACITIDINE (VAC) VERSUS VA REGIMEN IN NEWLY DIAGNOSED IMMUNOPHENOTYPICALLY MATURE MONOCYTIC AML: AN UPDATED ANALYSIS FROM A MULTICENTER RANDOMIZED STUDY

**Topic:** 04. Acute myeloid leukemia - Clinical

Kaiwen Tan*[1], Shanshan Pei[2], Miao Wang[1], Lan Dai[1], Hao Zhang[3], Chunyan Chen[4], Zhenqi Huang[5], Depei Wu[1], Zheng Li[1], Haiping Dai[1], Shengli Xue[1]

*[1]Department of Hematology, The First Affiliated Hospital of Soochow University, Jiangsu Institute of Hematology, National Clinical Research Center for Hematologic Diseases., Suzhou, China; [2]Bone Marrow Transplantation Center of The First Affiliated Hospital & Liangzhu Laboratory, Zhejiang University School of Medicine, Hangzhou, China; [3]Department of Hematology, Affiliated Hospital of Jining Medical University, Jining, China; [4]Department of Hematology, Qilu Hospital of Shandong University, Jinan, China; [5]Department of Hematology, The First Affiliated Hospital of Anhui Medical University, Hefei, China;*

### Background

While the combination of Venetoclax and Azacitidine (VA) has become the first-line induction regimen for newly diagnosed AML patients who are ineligible or decline intensive chemotherapy, resistance remains a critical challenge. Mature monocytic differentiation has been identified as a key driver of resistance to BCL-2 inhibition (Pei S et al. *Cancer Discov*, 2020), suggesting the need to target the specific subgroup the mature monocytic AML subgroup.  Since monocytic lineage leukemia cells show susceptibility to histone deacetylase inhibitors (HDACi), we hypothesized that incorporating Chidamide, a selective HDACi, into the VA regimen (VAC regimen) could overcome resistance.

### Aims

To evaluate the updated efficacy and safety of VAC versus VA regimens in a larger cohort of unfit patients with immunophenotypic monocytic AML.

### Methods

In this multicenter study (NCT05566054), we screened and enrolled patients with newly diagnosed Im-Mono AML, defined by strict flow cytometry criteria that promonocytes positive for more than 2 monocytic markers (CD11b, CD14, CD36 and CD64) and negative for CD34 or CD117. Patients were treated with either the VA regimen (Azacitidine 75mg/m$^2$ on d1-d7 and Venetoclax at an escalated dose of 100mg, 200mg and 400mg by day 28) or the VAC regimen (VA + Chidamide 10mg/d for d1-d7). This analysis compares the updated clinical outcomes between the two arms. The primary endpoint was the composite complete remission rate (CRc). Secondary endpoints included MRD negativity (defined as $<1\times10^{-3}$ by flow cytometry), overall survival (OS), and adverse events (AE).

**Copyright Information:** (Online) ISSN: 2572-9241
©2026 The Author(s). HemaSphere is published by John Wiley & Sons Ltd on behalf of European Hematology Association. This is an open access Abstract Book distributed under the Attribution-NonCommercial-NoDerivs (CC BY-NC-ND), which allows third parties to download the articles and share them with others as long as they credit the author and the Abstract Book, but they cannot change the content in any way or use them commercially.
**Abstract Book Citations:** Authors, Title, HemaSphere, 2026;10:(S1):pages. The abstract book DOIs can be found on the "Author Information" page.
**Disclaimer:** Articles published in the journal HemaSphere exclusively reflect the opinions of the authors. The authors are responsible for all content in their abstracts including accuracy of the facts, statements, citing resources, etc.

### Results

Clinical data cutoff was September 2025, a total of 80 patients with ND Im-Mono AML disease by flow criteria were enrolled. 40 patients received the VAC regimen and 40 patients received the VA regimen. There were no significant difference in baseline characteristics between the two groups of patients (P>0.1). At the end of 1 cycle of treatments, the CRc in the VAC group was 70.0%, which was significantly higher than the 47.5% in the VA group (P=0.041).

The MRD-negative CR rate in the VAC group was 82.1% and the VA group was 73.7% (P=0.740). At a median follow-up of 12.6 months (range, 0.8-32.1), median OS was not reached in either group. The 2-year OS rates were 63.9% for VAC versus 56.4% for VA. Importantly, the addition of Chidamide did not increase toxicity, with comparable incidences of grade ≥3 neutropenia, thrombocytopenia, and transfusion dependence between arms (all P>0.05). *In vitro*, dual BCL-2/HDAC inhibition synergistically targeted the distinct metabolic dependencies of monocytic blasts.

### Summary/Conclusion

This updated analysis substantiates the clinical benefit of the VAC regimen over standard VA therapy for Im-Mono AML. By specifically targeting the monocytic differentiation resistance mechanism, the triple combination significantly enhances remission rates and response depth without compromising safety. These findings advocate for differentiating AML subtypes by immunophenotype to guide precision combination therapies.

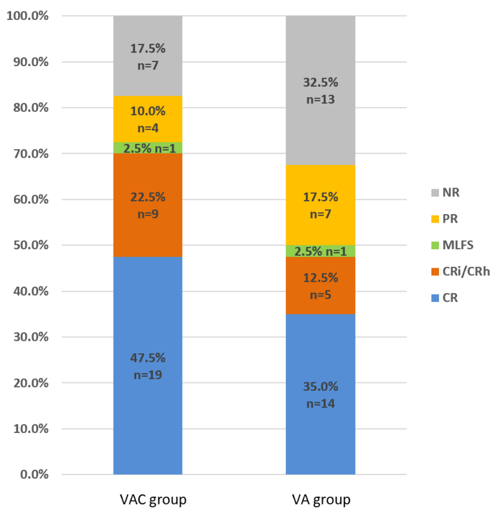

**Figure 1. Efficacy of VAC versus VA regimen.**

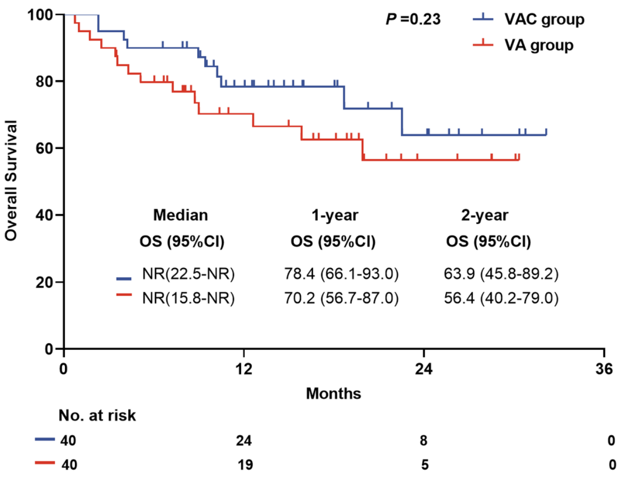

**Figure 2. Overall survival of of patients treated with VAC regimen versus VA regimen.**

Copyright Information: (Online) ISSN: 2572-9241
©2026 The Author(s). HemaSphere is published by John Wiley & Sons Ltd on behalf of European Hematology Association. This is an open access Abstract Book distributed under the Attribution-NonCommercial-NoDerivs (CC BY-NC-ND), which allows third parties to download the articles and share them with others as long as they credit the author and the Abstract Book, but they cannot change the content in any way or use them commercially.
Abstract Book Citations: Authors, Title, HemaSphere, 2026;10:(S1):pages. The abstract book DOIs can be found on the "Author Information" page.
Disclaimer: Articles published in the journal HemaSphere exclusively reflect the opinions of the authors. The authors are responsible for all content in their abstracts including accuracy of the facts, statements, citing resources, etc.

### (PF491) NOVEL RANDOM SURVIVAL FOREST MODEL FOR PRECISION RISK STRATIFICATION IN NPM1-MUTATED ACUTE MYELOID LEUKEMIA

**Topic:** 04. Acute myeloid leukemia - Clinical

Yiyi Yao*[1, 2], Lijie Wang[3], Yu Zhu[4], Zhen Yao[5], Xucan Lou[1], Ruixin Li[6], Yile Zhou[1], Mingyuan Zhang[1], Shasha Zhang[3], Wanzhuo Xie[1], Haitao Meng[1], Yinjun Lou[1], Liping Mao[1], Hongyan Tong[1], Jiejing Qian[1], Min Yang[1], Wenjuan Yu[1], De Zhou[1], Jie Jin[1], Shengli Xue[5], Erlie Jiang[6], Zhaoxiang Wang[3], Huafeng Wang[1]

*[1]The First Affiliated Hospital, Zhejiang University School of Medicine, Hangzhou, China;[2]Sir Run Run Shaw Hospital, Zhejiang University School of Medicine, Hangzhou, China; [3]Zhejiang lab, Hangzhou, China; [4]The First Affiliated Hospital of Nanjing Medical University, Jiangsu Province Hospital, Nanjing, China; [5]The First Affiliated Hospital of Soochow University, Suzhou, China; [6]Institute of Hematology & Blood Diseases Hospital, Chinese Academy of Medical Sciences & Peking Union Medical College, Tianjin, China;*

### Background

*NPM1*-mutated acute myeloid leukemia (AML) is the most common molecular subtype in adult AML. While the European LeukemiaNet (ELN) 2022 primarily assigned “favorable” risk to *NPM1*-mutated AML lacking adverse cytogenetics and *FLT3-ITD* mutation, our previous research indicated that additional high frequency co-mutations—particularly *DNMT3A* or *TET2* mutations—abolished this survival advantage, whereas *IDH1/2* or *PTPN11* mutations suggested relatively better outcome. Thus, stratification based primarily on *FLT3-ITD* status is inadequate to capture the complex interactions of co-mutations. Moreover, AML’s molecular heterogeneity demands development of subtype-specific tools. Machine learning algorithms like random survival forest (RSF) excel in handling nonlinear correlations and complex feature interactions, providing more precise risk stratification. No studies have yet developed RSF models for *NPM1*-mutated AML.

### Aims

We integrated co-mutation profiles in adult *NPM1*-mutated AML to develop a novel RSF model, aiming to overcome limitations of traditional risk stratification and provide accurate risk assessment.

### Methods

Among 3654 consecutive newly diagnosed AML patients screened from January 2019 to December 2024, 349 eligible with *NPM1* mutation were enrolled in the training cohort, with an external validation cohort of 172 multicenter patients. Targeted next-generation sequencing covering 88 myeloid malignancy-associated genes was performed. After comparing RSF with Cox regression and DeepSurv through 20 repeated internal random split validations, we constructed the NMG-17 model using RSF with core prognostic features identified by permutation importance and informational gradient decay analysis. Model performance was evaluated by C-index, time-dependent AUC, and calibration curves. SHapley Additive exPlanations (SHAP) analysis and RNA-seq were performed for model interpretation and biological exploration.

**Copyright Information:** (Online) ISSN: 2572-9241
©2026 The Author(s). HemaSphere is published by John Wiley & Sons Ltd on behalf of European Hematology Association. This is an open access Abstract Book distributed under the Attribution-NonCommercial-NoDerivs (CC BY-NC-ND), which allows third parties to download the articles and share them with others as long as they credit the author and the Abstract Book, but they cannot change the content in any way or use them commercially.
**Abstract Book Citations:** Authors, Title, HemaSphere, 2026;10:(S1):pages. The abstract book DOIs can be found on the “Author Information” page.
**Disclaimer:** Articles published in the journal HemaSphere exclusively reflect the opinions of the authors. The authors are responsible for all content in their abstracts including accuracy of the facts, statements, citing resources, etc.

**Results**

Among 349 eligible patients (median age 60 years; IQR 50-69), median follow-up was 27.8 months (95% CI 24.2-31.3), with median overall survival (OS) not reached. The RSF model selected 17 features including: mutations in *FLT3-ITD*, *DNMT3A*, *TET2*, *IDH2*, *FLT3-TKD*, *PTPN11*, *ASXL1*, *NRAS*, *IDH1*, *SRSF2*, *KMT2D*, *WT1*, *RAD21*; *NPM1* subtype (A/non-ABD); and cytogenetic features (adverse abnormalities, no metaphases). RSF demonstrated superior performance (mean C-index 0.696) compared to Cox (0.656, P < 0.001) and DeepSurv (0.608, P < 0.001). NMG-17 stratified patients into three distinct risk groups with significantly different OS (favorable: 89.5%, intermediate: 47.4%, adverse: 14.5%; all pairwise P < 0.001). Two novel high-risk co-mutation patterns were identified: *FLT3-ITD*/*DNMT3A*/*TET2* triple mutation and *TET2*/*ASXL1* double mutation. Venetoclax-based therapy failed to overcome NMG-17-defined high risk, and allogeneic hematopoietic stem cell transplantation showed no survival benefit for intermediate-risk patients. External validation confirmed model robustness (C-index 0.699; 3-year AUC 0.780). Transcriptomic analysis revealed downregulation of cell adhesion pathways and upregulation of immune-inflammatory pathways in the adverse-risk group. A web-based tool was developed for clinical application.

**Summary/Conclusion**

NMG-17 is the first RSF-based precision prognostic model for *NPM1*-mutated AML. By integrating multi-gene co-mutation interactions, it refines ELN 2022 risk stratification and provides crucial insights for clinical decision-making and development of novel therapeutic strategies.

**Copyright Information:** (Online) ISSN: 2572-9241
©2026 The Author(s). HemaSphere is published by John Wiley & Sons Ltd on behalf of European Hematology Association. This is an open access Abstract Book distributed under the Attribution-NonCommercial-NoDerivs (CC BY-NC-ND), which allows third parties to download the articles and share them with others as long as they credit the author and the Abstract Book, but they cannot change the content in any way or use them commercially.
**Abstract Book Citations:** Authors, Title, HemaSphere, 2026;10:(S1):pages. The abstract book DOIs can be found on the "Author Information" page.
**Disclaimer:** Articles published in the journal HemaSphere exclusively reflect the opinions of the authors. The authors are responsible for all content in their abstracts including accuracy of the facts, statements, citing resources, etc.

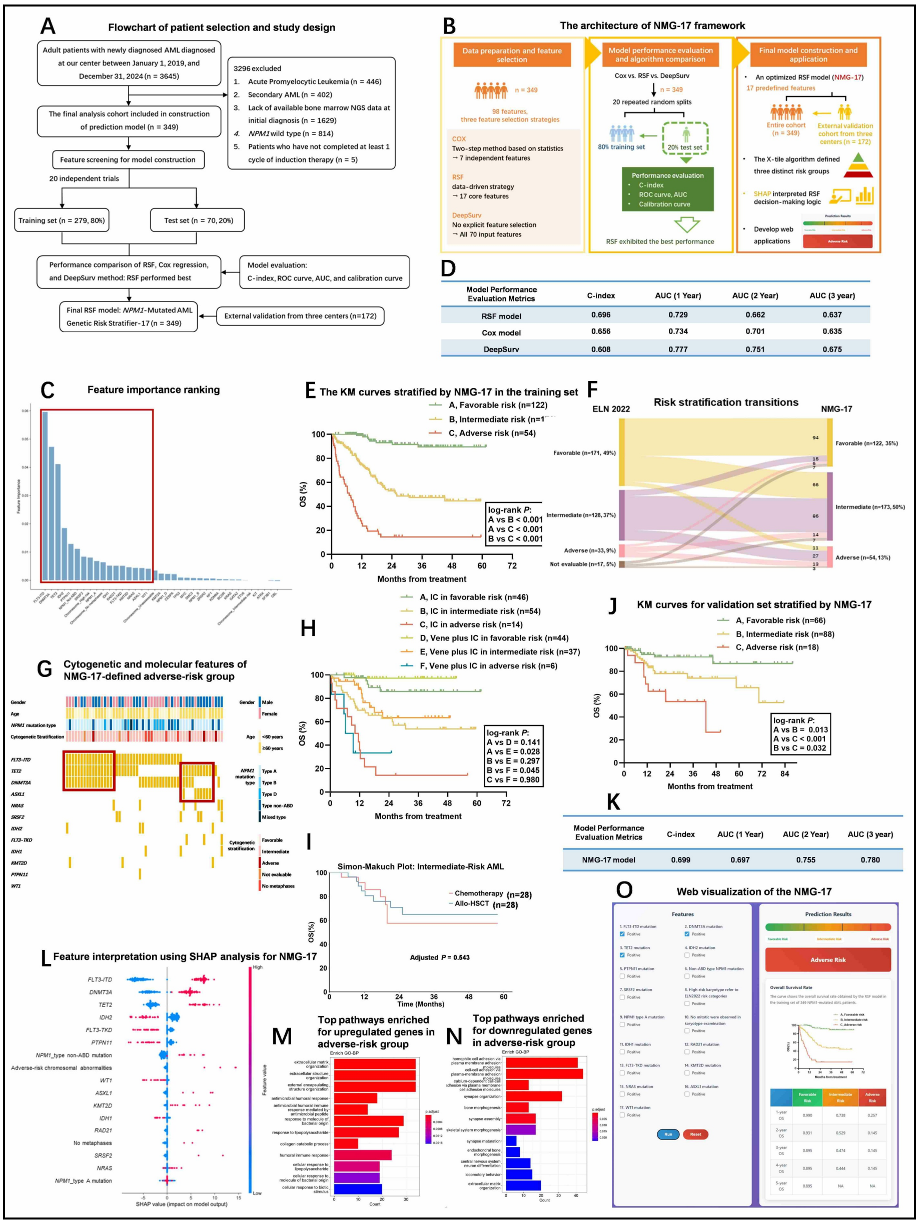

**Copyright Information:** (Online) ISSN: 2572-9241
©2026 The Author(s). HemaSphere is published by John Wiley & Sons Ltd on behalf of European Hematology Association. This is an open access Abstract Book distributed under the Attribution-NonCommercial-NoDerivs (CC BY-NC-ND), which allows third parties to download the articles and share them with others as long as they credit the author and the Abstract Book, but they cannot change the content in any way or use them commercially.
**Abstract Book Citations:** Authors, Title, HemaSphere, 2026;10:(S1):pages. The abstract book DOIs can be found on the "Author Information" page.
**Disclaimer:** Articles published in the journal HemaSphere exclusively reflect the opinions of the authors. The authors are responsible for all content in their abstracts including accuracy of the facts, statements, citing resources, etc.

### (PF492) HEAD-TO-HEAD COMPARISON OF SIX CONTEMPORARY PROGNOSTIC MODELS IN NON-INTENSIVE FRONTLINE ACUTE MYELOID LEUKEMIA TREATED IN THE VENETOCLAX ERA: A MULTICENTER CETLAM COOPERATIVE GROUP STUDY

**Topic:** 04. Acute myeloid leukemia - Clinical

Tomas Rivero Equiza*[1, 2], Mireia Morgades[1, 2], Pablo Castillo Jimenez[1, 2], Daniel Esteban Corredera[1, 2], Carlos Jimenez Vicente[3], Ana Garrido Diaz[4], Marta Gomez Nuñez[5], Ferran Vall-Llovera Calmet[6], Montserrat Arnan Sangerman[7], Mar Tomo[8], Sara Garcia Ávila[9], Joan Bargay[10, 11], Albert Altes Hernandez[12], Marta Cervera Calvo[7], Marina Diaz Beya[3], Lurdes Zamora Plana[1, 2], Guadalupe Oñate[4], Laura Segura De La Torre[5], Maria Pilar Pilamunga Toala[6], Helena Pomares[7], Marta Pratcorona[13], Marta Gomez Hernando[1, 2], Monica Lopez-Guerra[3], Neus Ruiz-Xivillé[1, 2], Dolors Costa[3], Jordi Esteve Reyner[3], Susana Vives Polo[1, 2]

*[1]Institut Català d'Oncologia Badalona- Hospital Germans Trias i Pujol, Badalona, Spain;[2]Josep Carreras Research Institute., Badalona, Spain; [3]Hospital Clínic, Barcelona, Spain; [4]Hospital Santa Creu i Sant Pau, Barcelona, Spain;[5]Hospital Universitario Parc Taulí de Sabadell, Sabadell, Spain; [6]Hospital Universitari Mútua de Terrassa, Terrassa, Spain;[7]Institut Català d'Oncologia Hospitalet, Barcelona, Spain; [8]Hospital Clínico Universitario de Valencia, INCLIVA, Valencia, Spain; [9]Hospital del Mar, Barcelona, Spain; [10]Hospital Universitario Son Llatzer, Palma de Mallorca, Spain; [11]Institut d' investigacio Illes Balears (IdISBa), Palma de Mallorca, Spain; [12]Hospital Sant Joan de Déu, Althaia Xarxa Assistencial Universitària de Manresa, Manresa, Spain; [13]Hospital de Sant Pau, Barcelona, Spain;*

### Background

Several prognostic models have been developed for acute myeloid leukemia (AML) patients (pts) treated with non-intensive therapies (non-IT) in the venetoclax era. However, no study has compared these models head-to-head within the same cohort.

### Aims

To compare six contemporary prognostic models for overall survival (OS) in AML pts treated with frontline non-IT within the CETLAM multicenter registry.

### Methods

Adult AML patients treated with first-line non-IT (2019-2025) and available NGS data were analyzed. Six models were evaluated: 4-gene model (H Döhner, Blood 2024), Beat-AML (FH Hoff, Blood Neoplasia 2025), Mayo model (M Gangat, Am J Hematol 2025), refined ELN 2024 (CA Lachowiez, Blood 2025), molecular ontogeny (S Shimony, Leukemia 2024) and PRISM (CA Lachowiez,Blood 2025). The discriminative ability of prognostic scores for OS was assessed using Cox proportional hazards models and Harrell's C-index.

**Copyright Information:** (Online) ISSN: 2572-9241
©2026 The Author(s). HemaSphere is published by John Wiley & Sons Ltd on behalf of European Hematology Association. This is an open access Abstract Book distributed under the Attribution-NonCommercial-NoDerivs (CC BY-NC-ND), which allows third parties to download the articles and share them with others as long as they credit the author and the Abstract Book, but they cannot change the content in any way or use them commercially.
**Abstract Book Citations:** Authors, Title, HemaSphere, 2026;10:(S1):pages. The abstract book DOIs can be found on the "Author Information" page.
**Disclaimer:** Articles published in the journal HemaSphere exclusively reflect the opinions of the authors. The authors are responsible for all content in their abstracts including accuracy of the facts, statements, citing resources, etc.

### Results

A total of 206 patients from 11 centers were included (mean age 75 years; 56% male), 103/199 (51.8%) were diagnosed with *de novo* AML and 71/197 (36%) had adverse risk karyotype (ELN2022). The majority of patients (190/206, 92%) received venetoclax-azacitidine. CR/CRi rate with venetoclax-azacitidine was 61.6%.
With a median follow-up of 11 months, median OS was 1.06 years (95% CI 0.89-1.42). All six models significantly stratified OS (Figure 1). Discriminatory performance was modest and largely overlapping, with the highest C-index observed for the 4-gene model (0.630), followed by PRISM (0.623), Beat-AML and refined ELN 2024 (0.612), molecular ontogeny (0.603) and Mayo (0.597) (Table 1).

| Score | N | OS, HR (95% CI) | p | C-index (95% CI) |
|---|---|---|---|---|
| **4 gene model** | | | | |
| Favorable | 120 | Reference | | |
| Intermediate | 45 | 1.611 (1.026 – 2.530) | 0.038 | **0.630** (0.575 – 0.685) |
| Adverse | 41 | 2.765 (1.794 – 4.262) | <0.001 | |
| **Beat AML** | | | | |
| Favorable | 69 | Reference | | |
| Intermediate | 85 | 1.620 (1.013 – 2.593) | 0.044 | 0.612 (0.555 – 0.669) |
| Adverse | 52 | 2.846 (1.730 – 4.681) | <0.001 | |
| **Mayo model** | | | | |
| Favorable | 27 | Reference | | |
| Intermediate | 95 | 1.094 (0.580 – 2.064) | 0.782 | 0.597 (0.544 – 0.650) |
| Adverse | 82 | 1.959 (1.046 – 3.668) | 0.036 | |
| **Refined ELN 2024** | | | | |
| Favorable | 61 | Reference | | |
| Intermediate | 79 | 1.309 (0.812 – 2.110) | 0.270 | 0.612 (0.555 – 0.669) |
| Adverse | 66 | 2.253 (1.408 – 3.607) | 0.001 | |
| **Genetic ontogeny** | | | | |
| De novo | 53 | Reference | | |
| Secondary | 115 | 1.893 (1.147 – 3.123) | 0.013 | 0.603 (0.546 – 0.660) |
| TP53 | 38 | 3.248 (1.829 – 5.770) | <0.001 | |
| **PRISM** | | | | |
| Low | 68 | Reference | | |
| Moderate | 60 | 1.658 (1.004 – 2.737) | 0.048 | **0.623** (0.572 – 0.674) |
| High | 76 | 2.766 (1.746 – 4.382) | <0.001 | |

### Summary/Conclusion

In this multicenter real-world, venetoclax-dominant cohort, contemporary prognostic models significantly stratify survival in non-IT AML. Nevertheless, discrimination between risk groups remains modest, and no model clearly outperforms the others. These findings underscore the need for refined, therapy-adapted prognostic strategies in venetoclax-based frontline AML.

**Copyright Information:** (Online) ISSN: 2572-9241
©2026 The Author(s). HemaSphere is published by John Wiley & Sons Ltd on behalf of European Hematology Association. This is an open access Abstract Book distributed under the Attribution-NonCommercial-NoDerivs (CC BY-NC-ND), which allows third parties to download the articles and share them with others as long as they credit the author and the Abstract Book, but they cannot change the content in any way or use them commercially.
**Abstract Book Citations:** Authors, Title, HemaSphere, 2026;10:(S1):pages. The abstract book DOIs can be found on the “Author Information” page.
**Disclaimer:** Articles published in the journal HemaSphere exclusively reflect the opinions of the authors. The authors are responsible for all content in their abstracts including accuracy of the facts, statements, citing resources, etc.

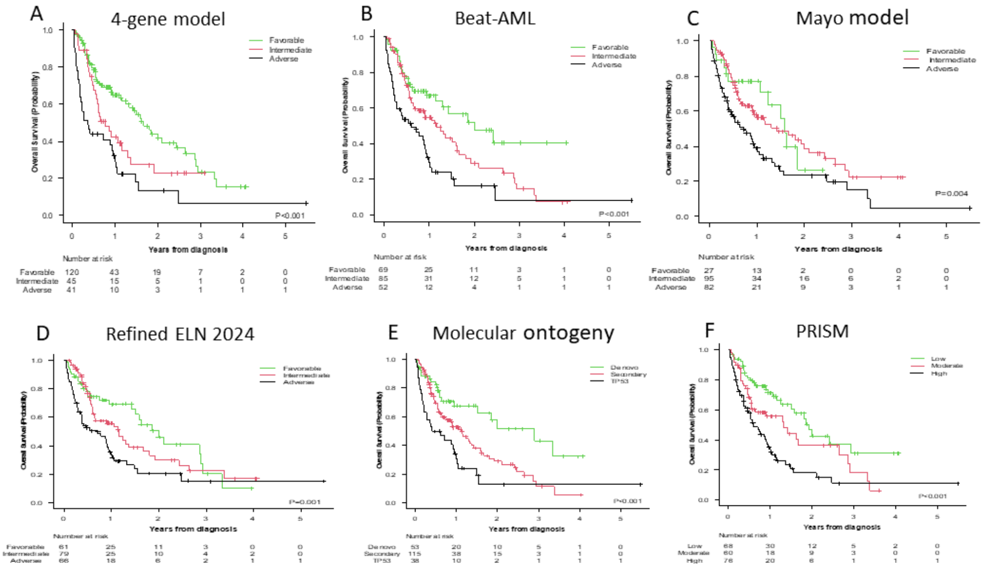

**Copyright Information:** (Online) ISSN: 2572-9241
©2026 The Author(s). HemaSphere is published by John Wiley & Sons Ltd on behalf of European Hematology Association. This is an open access Abstract Book distributed under the Attribution-NonCommercial-NoDerivs (CC BY-NC-ND), which allows third parties to download the articles and share them with others as long as they credit the author and the Abstract Book, but they cannot change the content in any way or use them commercially.
**Abstract Book Citations:** Authors, Title, HemaSphere, 2026;10:(S1):pages. The abstract book DOIs can be found on the "Author Information" page.
**Disclaimer:** Articles published in the journal HemaSphere exclusively reflect the opinions of the authors. The authors are responsible for all content in their abstracts including accuracy of the facts, statements, citing resources, etc.

### (PF493) CD7 CAR T-CELL THERAPY IN RELAPSED OR REFRACTORY ACUTE MYELOID LEUKEMIA

**Topic:** 04. Acute myeloid leukemia - Clinical

Yuehui Lin[1], Yurong Yan[2], Biyi Li[1], Shuang You Liu[1], Biping Deng[1], Chunrong Tong[1], Tong Wu[1], Fengyan Jin[2], Zhao Defeng*[1]

*[1]Department of Hematology, Beijing Boren Hospital, Beijing, China;[2]Department of Hematology, the First Hospital of Jilin University, Hematology, Changchun, China;*

### Background

Relapsed or refractory acute myeloid leukemia (R/R AML) is associated with dismal clinical outcomes, and no standard salvage therapy has been established. CD7-directed chimeric antigen receptor (CAR) T-cell therapy has shown promising activity in CD7-positive T-cell hematologic malignancies; however, clinical evidence in R/R AML remains limited.

### Aims

To evaluate the efficacy, safety, and cellular kinetics of anti-CD7 CAR T-cell therapy in patients with R/R AML.

### Methods

This study included 11 R/R AML patients treated at Beijing Boren Hospital between August 2023 and August 2025. The median age was 35 years (range, 10–65), and 7 patients (63.6%) were male. Six patients (54.5%) had extramedullary disease. Patients had received a median of three prior lines of therapy (range, 1–8), and 6 (54.5%) had previously undergone allogeneic hematopoietic stem cell transplantation (HSCT), including one patient with two prior transplants.
This single-center observational study included 11 patients with CD7-positive (≥70% by flow cytometry) R/R AML treated with anti-CD7 CAR T cells. Patients received lymphodepleting chemotherapy with fludarabine and cyclophosphamide prior to CAR T-cell infusion. Clinical responses, minimal residual disease (MRD), adverse events, and CAR T-cell expansion were assessed according to standard criteria.

### Results

All 11 patients with R/R AML received CD7 CAR T-cell therapy.At day 14 post-infusion, 6 of 11 patients (54.6%) achieved confirmed remission, including 5 patients with MRD-negative complete remission with incomplete hematologic recovery (CRi) and 1 patient with MRD-negative complete remission (CR). Bone marrow MRD negativity was observed in an additional patient with prior double HSCT and extramedullary disease; however, extramedullary lesions could not be evaluated due to early death from grade 4 immune effector cell–associated neurotoxicity syndrome (ICANS), and this patient was not considered fully evaluable for overall response. Four patients showed no response, including one patient without detectable CAR T-cell expansion and three patients who exhibited loss of CD7 expression on leukemic blasts, suggesting antigen escape as a mechanism of resistance.With a median follow-up of 129days (95% CI, 103–155), five responding patients proceeded to consolidation allogeneic HSCT and all maintained MRD-negative remission at last follow-up. One patient who achieved MRD-negative CR but did not undergo HSCT remained in continuous remission with maintenance therapy. Among non-responders, all underwent salvage HSCT; two achieved MRD-negative CR, while two died from post-transplant infectious complications.Cytokine release syndrome (CRS) occurred in 10 patients, including grade 3 CRS in two patients and grade 4 CRS in one patient. One patient developed fatal grade 4 ICANS. The most common grade ≥3 adverse events were hematologic toxicities. CAR T-cell expansion peaked at a median of 14.5 days post-infusion, and inflammatory biomarker dynamics differed between patients with and without CAR T-cell expansion.

**Copyright Information:** (Online) ISSN: 2572-9241
©2026 The Author(s). HemaSphere is published by John Wiley & Sons Ltd on behalf of European Hematology Association. This is an open access Abstract Book distributed under the Attribution-NonCommercial-NoDerivs (CC BY-NC-ND), which allows third parties to download the articles and share them with others as long as they credit the author and the Abstract Book, but they cannot change the content in any way or use them commercially.
**Abstract Book Citations:** Authors, Title, HemaSphere, 2026;10:(S1):pages. The abstract book DOIs can be found on the "Author Information" page.
**Disclaimer:** Articles published in the journal HemaSphere exclusively reflect the opinions of the authors. The authors are responsible for all content in their abstracts including accuracy of the facts, statements, citing resources, etc.

**Summary/Conclusion**

1、Anti-CD7 CAR T-cell therapy demonstrates meaningful antileukemic activity in R/R AML, inducing rapid and deep MRD-negative remissions in a subset of patients.
2、Consolidation with allogeneic HSCT appears critical for durable disease control.
3、Antigen escape and severe neurotoxicity highlight the narrow therapeutic window of this approach in heavily pretreated patients and underscore the need for optimized patient selection and treatment strategies

**Copyright Information:** (Online) ISSN: 2572-9241
©2026 The Author(s). HemaSphere is published by John Wiley & Sons Ltd on behalf of European Hematology Association. This is an open access Abstract Book distributed under the Attribution-NonCommercial-NoDerivs (CC BY-NC-ND), which allows third parties to download the articles and share them with others as long as they credit the author and the Abstract Book, but they cannot change the content in any way or use them commercially.
**Abstract Book Citations:** Authors, Title, HemaSphere, 2026;10:(S1):pages. The abstract book DOIs can be found on the “Author Information” page.
**Disclaimer:** Articles published in the journal HemaSphere exclusively reflect the opinions of the authors. The authors are responsible for all content in their abstracts including accuracy of the facts, statements, citing resources, etc.

### (PF494) A FIRST-IN-HUMAN STUDY OF THE ORAL CLK INHIBITOR BH-30236 IN ADULTS WITH RELAPSED/REFRACTORY ACUTE MYELOID LEUKEMIA OR HIGHER-RISK MYELODYSPLASTIC SYNDROME: MONOTHERAPY AND VENETOCLAX COMBINATION

**Topic:** 04. Acute myeloid leukemia - Clinical

Eytan Stein*[1], Gabriel Mannis[2], Justin Watts[3], Alice Mims[4], Farhad Ravandi[5], Zhuoer Xie[6], Jessica K. Altman[7], Gary Schiller[8], Brian J Ball[9], Hongtao Liu[10], Eunice Wang[11], Jacob Appelbaum[12], Jean Cui[13], Jihao Zhou[13], Jessi Vlcek[13], Diane Wang[13], Gregory Sims[13], Aditya Kulkarni[13], Zachary Zimmerman[13], Stephen Strickland[14]

*[1]Memorial Sloan Kettering Cancer Center, New York, NY, United States of America;[2]Stanford Cancer Institute, Stanford, CA, United States of America; [3]Sylvester Comprehensive Cancer Center, Miami, FL, United States of America; [4]The Ohio State University, Columbus, OH, United States of America; [5]The University Of Texas MD Anderson Cancer Center, Houston, TX, United States of America; [6]H Lee Moffitt Cancer Center, Tampa, FL, United States of America; [7]Northwestern University, Chicago, IL, United States of America; [8]University of California Los Angeles, Los Angeles, CA, United States of America;[9]City of Hope National Medical Center, Duarte, CA, United States of America; [10]University of Wisconsin at Madison, Madison, WI, United States of America; [11]Roswell Park Comprehensive Cancer Center, Buffalo, NY, United States of America; [12]University of Washington/Fred Hutchinson Cancer Center, Seattle, WA, United States of America; [13]BlossomHill Therapeutics, San Diego, CA, United States of America; [14]Sarah Cannon Research Institute, Nashville, TN, United States of America;*

### Background

Relapsed/refractory (R/R) acute myeloid leukemia (AML) and higher-risk myelodysplastic syndromes (HR-MDS) have limited options after intensive chemotherapy or hypomethylating agent/venetoclax-based (HMA/VEN) standard of care (SOC). Aberrant alternative splicing (AS) promotes leukemic survival and therapy resistance. CDC-like kinases (CLKs) regulate AS by modulating serine/arginine rich splicing factor phosphorylation leading to DNA repair and apoptosis pathway modulation. BH-30236 is an orally bioavailable, macrocyclic CLK inhibitor which shows activity in preclinical models including robust synergy with VEN. This is the initial clinical report of the first-in-human dose escalation study of BH-30236 in adults with R/R AML and HR-MDS as monotherapy or in combination with VEN.

### Aims

(1) Evaluate safety/tolerability and identify recommended dose(s) for expansion of BH-30236; (2) Characterize single agent and combination pharmacokinetics (PK); (3) Assess preliminary anti-leukemic activity.

### Methods

BH-30236-01 (NCT06501196) is an open-label phase 1/1b BOIN dose escalation/expansion study in adults with R/R AML or HR-MDS as monotherapy or in combination with VEN (≤ 5 prior lines; prior VEN allowed). BH-30236 is dosed orally once daily (QD) in continuous 28-day cycles. Combination cohorts add oral VEN (target dose 400mg QD, 15-28 days/cycle). Responses are assessed per 2022 ELN (AML) and 2023 IWG (MDS) criteria.

**Copyright Information:** (Online) ISSN: 2572-9241
©2026 The Author(s). HemaSphere is published by John Wiley & Sons Ltd on behalf of European Hematology Association. This is an open access Abstract Book distributed under the Attribution-NonCommercial-NoDerivs (CC BY-NC-ND), which allows third parties to download the articles and share them with others as long as they credit the author and the Abstract Book, but they cannot change the content in any way or use them commercially.
**Abstract Book Citations:** Authors, Title, HemaSphere, 2026;10:(S1):pages. The abstract book DOIs can be found on the "Author Information" page.
**Disclaimer:** Articles published in the journal HemaSphere exclusively reflect the opinions of the authors. The authors are responsible for all content in their abstracts including accuracy of the facts, statements, citing resources, etc.

### Results

As of 23 Jan 2026, 28 patients (23 AML; 5 HR-MDS) received BH-30236 at 5–120mg as monotherapy. Median age was 71 years (23-85); median 2 prior lines of therapy; 86% (24/28) had prior VEN; median duration of treatment 1.4 months (0.2-7.6). PK exposure increased with dose with no accumulation observed. BH-30236 was generally well tolerated up to 90mg as a continuous daily dose. One DLT of grade 3 diarrhea occurred at 120mg. Treatment-related AEs (TRAEs) occurred in 15/28 (53.6%) patients; mostly ≤ Grade 2, most commonly (≥10%) diarrhea, nausea and vomiting. Grade ≥3 TRAEs occurring in ≥10% included diarrhea. Two cases of possible differentiation syndrome were observed, both managed with corticosteroids and no dose interruption. Of 17 monotherapy patients with post-baseline marrow assessments, 5 (29%) had ≥50% blast count reduction during treatment, including one HR-MDS patient treated at 60mg with sustained blast count reduction with duration of treatment 7.6 months. 11 patients (9 AML; 2 HR-MDS) received BH-30236 at 20-60mg in combination with VEN. Median age was 61 years (range 27-80); median 3 prior lines of therapy; 91% (10/11) had prior VEN; median duration of treatment 1.9 months (0.4-2.9). No apparent drug interaction was observed between BH-30236 and VEN. TRAEs were consistent with a VEN-based regimen, occurring in 6/11 (54.5%) patients, most commonly (≥10%) decreased appetite, fatigue, neutrophil count decreased, platelet count decreased and white blood cell count decreased. Among 9 evaluable patients at 20-30mg, 5 (55%) had ≥50% blast count reduction during treatment including one AML patient refractory to all prior therapy including VEN achieving MRD-negative complete response (CR) and coming off study to undergo transplant.

### Summary/Conclusion

BH-30236 is the first CLK inhibitor to demonstrate tolerability with once daily dosing and in combination with VEN. Preliminary anti-leukemic activity includes a CR observed early in VEN combination dose escalation, which is ongoing in the US and aiming to further validate the preclinical synergy seen with VEN.

**Copyright Information:** (Online) ISSN: 2572-9241
©2026 The Author(s). HemaSphere is published by John Wiley & Sons Ltd on behalf of European Hematology Association. This is an open access Abstract Book distributed under the Attribution-NonCommercial-NoDerivs (CC BY-NC-ND), which allows third parties to download the articles and share them with others as long as they credit the author and the Abstract Book, but they cannot change the content in any way or use them commercially.
**Abstract Book Citations:** Authors, Title, HemaSphere, 2026;10:(S1):pages. The abstract book DOIs can be found on the "Author Information" page.
**Disclaimer:** Articles published in the journal HemaSphere exclusively reflect the opinions of the authors. The authors are responsible for all content in their abstracts including accuracy of the facts, statements, citing resources, etc.

### (PF495) PHASE 1/2 STUDY OF THE ALL-ORAL COMBINATION OF REVUMENIB (SNDX-5613) WITH DECITABINE/CEDAZURIDINE (ASTX727) AND VENETOCLAX (SAVE) IN RELAPSED/REFRACTORY AML

**Topic:** 04. Acute myeloid leukemia - Clinical

Branko Cuglievan*[1], Wei-Ying Jen[1], Nicholas Short[1], Alex Bataller[1], Courtney Dinardo[1], Sanam Loghavi[1], Naval Daver[1], Georgina El Hajjar[1], Aziz Farhat[1], David McCall[1], Amber Gibson[1], Miriam Garcia[1], Cesar Nunez[1], Michael Roth[1], Tapan Kadia[1], Alexandre Bazinet[1], Elias Jabbour[1], Guillermo Montalban-Bravo[1], Farhad Ravandi[1], Guillermo Garcia-Manero[1], Michael Andreeff[1], Hagop Kantarjian[1], Ghayas Issa[1]

*[1]The University of Texas MD Anderson Cancer Center, Leukemia, Houston, United States of America;*

### Background

Revumenib is an oral, selective inhibitor of the menin–KMT2A interaction, a critical dependency in *KMT2Ar*, *NUP98t*, and *NPM1mt* acute leukemias. Dual inhibition of menin and BCL2 shows synergistic activity in preclinical models.

### Aims

To describe the safety and efficacy of the all-oral combination of revumenib, ASTX727, and venetoclax in children and adults with relapsed/refractory (R/R) AML (NCT05360160).

### Methods

Patients (pts) aged ≥12 years with R/R AML or myeloid MPAL were enrolled. Phase 1 followed a 3+3 design. ASTX727 (35/100 mg) was given PO on days 1–5, venetoclax 400 mg PO on days 1–14, and revumenib 113 mg (DL0) or 163 mg (DL1) PO Q12h on days 1–28 with posaconazole or voriconazole prophylaxis. Revumenib monotherapy was resumed post-HSCT for 1-year of maintenance. A protocol amendment allowed holding revumenib after day 21 if day 14 bone marrow blasts were <5%. Measurable residual disease (MRD) analysis was done by multicolor flow cytometry (MFC, sen $10^{-4}$). Mutational analysis to assess resistance, and molecular MRD at $10^{-5}$ were performed.

**Copyright Information:** (Online) ISSN: 2572-9241
©2026 The Author(s). HemaSphere is published by John Wiley & Sons Ltd on behalf of European Hematology Association. This is an open access Abstract Book distributed under the Attribution-NonCommercial-NoDerivs (CC BY-NC-ND), which allows third parties to download the articles and share them with others as long as they credit the author and the Abstract Book, but they cannot change the content in any way or use them commercially.
**Abstract Book Citations:** Authors, Title, HemaSphere, 2026;10:(S1):pages. The abstract book DOIs can be found on the "Author Information" page.
**Disclaimer:** Articles published in the journal HemaSphere exclusively reflect the opinions of the authors. The authors are responsible for all content in their abstracts including accuracy of the facts, statements, citing resources, etc.

**Results**

As of the data cutoff (Feb 2026), 42 pts with R/R AML were enrolled. The median age was 40 years (range, 12–82), including 5 adolescents (12%). Seventeen pts (40%) had *KMT2Ar*, 16 (38%) *NPM1mt*, and 9 (21%) *NUP98r*. Pts were heavily pretreated (median 2 prior lines, range 1–5); 60% had prior venetoclax, 50% prior hypomethylating agents, 32% prior HSCT, and 2 pts (5%) had prior menin inhibitor. The recommended phase 2 dose of revumenib was 163 mg PO Q12h with strong CYP3A4 inhibitors. The most common all-grade adverse events were nausea (60%), vomiting (52%), QTc prolongation (50%), and hypokalemia (45%), mostly grade 1–2. Grade ≥3 events were most frequently febrile neutropenia (35%), thrombocytopenia (22%), lung infection (20%), and respiratory failure (15%). QTc prolongation grade ≥3 occurred in 2 pts (5%). Differentiation syndrome occurred in 4 pts (10%) [grade ≥3, in 2 pts (5%)], all resolved with steroids. There were no grade 5 treatment-related toxicities or early (60-day) mortality.

The overall response rate (CR+CRh+CRi+PR+MLFS) was 88% (37/42 pts), including 100% (17/17) in *KMT2Ar*, 75% (12/16) in *NPM1mt, and* 89% (8/9) in *NUP98r*. The CR/CRh rate was 60% (25 pts), with CR in 43% (18 pts), CRh in 17% (7 pts), CRi in 10% (4 pts), CRp in 2% (1 pt), MLFS in 14% (6 pts), and PR in 2% (1 pt). Median time to best response was 1 cycle (range, 1–3), and MRD negativity by MFC in responders was 64% (24/37), and 80% (20/25) in those with CR/CRh. Twenty pts (48%) proceeded to HSCT, with (71%, 12/17), in *KMT2Ar,* 38% (6/16) in *NPM1mt* and 22% (2/9) in *NUP98r*.

With a median follow-up of 14 months, 18-mo overall survival (OS) was 43% (95% CI, 29–63) for all, with an 18-months OS of 47% (95% CI, 28–78) for *KMT2Ar*, 44% (95% CI, 17–100) for *NUP98r*, and 36% (95% CI, 18–76) for *NPM1mt*. Among pts achieving CR/CRh, 18-months OS was 61% (95% CI, 43–87), and 50% (95% CI, 32–77) remained in remission at 18 months. Median duration of response in pts with CR/CRh was not reached for *KMT2Ar*, 10 months (95% CI, 7.5–NR) for *NPM1mt*, and 5.9 months (95% CI, 4.1–NR) for *NUP98r* (**Figure**). Mutational analysis to assess resistance and molecular MRD assessments are ongoing, and results will be presented.

**Summary/Conclusion**

The all-oral combination of revumenib, venetoclax, and decitabine yields high remission rates in R/R AML with *KMT2Ar*, *NPM1mt*, or *NUP98r*, with durable responses in patients achieving deep remission.

**Copyright Information:** (Online) ISSN: 2572-9241
©2026 The Author(s). HemaSphere is published by John Wiley & Sons Ltd on behalf of European Hematology Association. This is an open access Abstract Book distributed under the Attribution-NonCommercial-NoDerivs (CC BY-NC-ND), which allows third parties to download the articles and share them with others as long as they credit the author and the Abstract Book, but they cannot change the content in any way or use them commercially.
**Abstract Book Citations:** Authors, Title, HemaSphere, 2026;10:(S1):pages. The abstract book DOIs can be found on the "Author Information" page.
**Disclaimer:** Articles published in the journal HemaSphere exclusively reflect the opinions of the authors. The authors are responsible for all content in their abstracts including accuracy of the facts, statements, citing resources, etc.

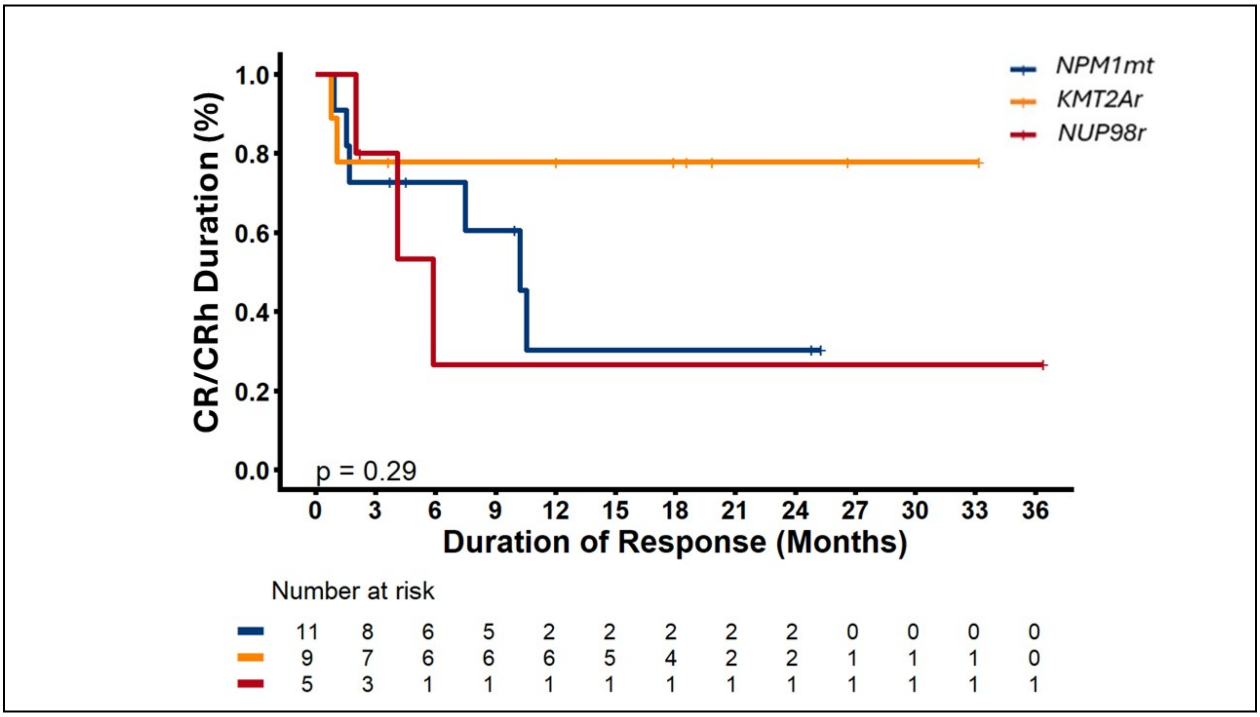

Fig.1: Duration of response by genotype

**Copyright Information:** (Online) ISSN: 2572-9241
©2026 The Author(s). HemaSphere is published by John Wiley & Sons Ltd on behalf of European Hematology Association. This is an open access Abstract Book distributed under the Attribution-NonCommercial-NoDerivs (CC BY-NC-ND), which allows third parties to download the articles and share them with others as long as they credit the author and the Abstract Book, but they cannot change the content in any way or use them commercially.
**Abstract Book Citations:** Authors, Title, HemaSphere, 2026;10:(S1):pages. The abstract book DOIs can be found on the "Author Information" page.
**Disclaimer:** Articles published in the journal HemaSphere exclusively reflect the opinions of the authors. The authors are responsible for all content in their abstracts including accuracy of the facts, statements, citing resources, etc.

### (PF496) VENETOCLAX PLUS ‘2 + 5’ MODIFIED INTENSIVE CHEMOTHERAPY WITH DAUNORUBICIN AND CYTARABINE IN FIT ELDERLY PATIENTS WITH UNTREATED DE NOVO ACUTE MYELOID LEUKEMIA: A MULTICENTER PROSPECTIVE ANALYSIS

**Topic:** 04. Acute myeloid leukemia - Clinical

Jiajia Pan*[1], Shengli Xue[2], Liping Mao[1], Min Yang[1], Dengju Li[3], Wanzhuo Xie[1], Chunmei Yang[1], De Zhou[1], Huaiyu Wang[4], Yinjun Lou[1], Juying Wei[1], Liya MA[1], Hongyan Tong[1], Jie Jin[1], Huafeng Wang[1]

*[1]the First Affiliated Hospital, Zhejiang University School of Medicine, Department of Hematology, Hangzhou, China;[2]the First Affiliated Hospital of Soochow University, Suzhou, China; [3]Department of Hematology, Tongji Hospital, Tongji Medical College, Huazhong University of Science and Technology, wuhan, China; [4]The First Affiliated Hospital of Xi'an Jiaotong University, Xi'an, China;*

### Background

The optimal therapeutic strategy for fit AML patients aged ≥60 years remains undefined. Our previous results showed promising clinical outcomes in 25 untreated, fit elderly AML patients who received modified DAV induction regimen (venetoclax plus ‘2 + 5’ daunorubicin-cytarabine), with an overall complete remission (CR) rate of 92.0% and a minimal residual disease (MRD) negativity rate of 95.7% among responders. We therefore conducted a prospective, multicenter clinical study to evaluate the efficacy and safety of the modified DAV regimen in the treatment of elderly, untreated AML patients. This trail was registered as ChiCTR2300073897.

### Aims

To assess the efficacy and safety of modified DAV regimen in fit elderly patients with de novo AML.

### Methods

Patients with AML aged between 60-75 years old who were eligible for intensive chemotherapy were included and received modified DAV regimen induction chemotherapy from August 2023 to August 2025. The primary endpoint was the composite complete remission (CRc) rate after one cycle of the modified DAV regimen, the secondary endpoints included CRc rate after two cycles of modified DAV regimen, adverse events (AEs), MRD negativity rate, overall survival (OS), event free survival (EFS) and relapse-free survival (RFS).

**Copyright Information:** (Online) ISSN: 2572-9241
©2026 The Author(s). HemaSphere is published by John Wiley & Sons Ltd on behalf of European Hematology Association. This is an open access Abstract Book distributed under the Attribution-NonCommercial-NoDerivs (CC BY-NC-ND), which allows third parties to download the articles and share them with others as long as they credit the author and the Abstract Book, but they cannot change the content in any way or use them commercially.
**Abstract Book Citations:** Authors, Title, HemaSphere, 2026;10:(S1):pages. The abstract book DOIs can be found on the “Author Information” page.
**Disclaimer:** Articles published in the journal HemaSphere exclusively reflect the opinions of the authors. The authors are responsible for all content in their abstracts including accuracy of the facts, statements, citing resources, etc.

### Results

A total of 33 patients were enrolled from August 2023 to August 2025 from 4 centers, with a median age of 63.5 years (range, 60–71 years); 19 were male (57.6%) and 14 were female (42.4%). According to the European Leukemia Net risk stratification criteria, patients were stratified into the favorable-risk (n=10, 30.3%), intermediate-risk (n=12, 36.4%), and adverse-risk groups (n=10, 33.3%). Following one cycle of induction therapy, 24 patients (72.7%) achieved CR and 3 patients (9.1%) attained partial remission (PR); 2 PR patients subsequently achieved CR after a second cycle of the same induction regimen. 5 patients (15.2%) failed to achieve remission after induction therapy, and 1 patient died during induction therapy. Overall, the CR rate reached 81.8% after two cycles of induction therapy. With a median follow-up of 17.5 months, the OS, EFS, and RFS had not yet been reached. The estimated 1-year OS, EFS, and RFS rates for the entire cohort were 72.6% (95% confidence interval [CI], 52.3%–85.3%), 59.2% (95% CI, 40.2%–74.0%), and 87.6% (95% CI, 66.3%–95.8%), respectively. The estimated 2-year OS, EFS, and RFS rates for the entire cohort were 67.4% (95% CI, 46.5%-82.0%), 59.2% (95% CI, 40.2%–74.0%), and 74.0% (95% CI, 46.6%–88.8%), respectively.
During induction therapy, the most frequent grade ≥3 hematological AEs were neutropenia (100%), anemia (96.9%), thrombocytopenia (100%). For patients who responded to the first induction cycle, the median time (interquartile range) to hematological recovery (absolute neutrophil count $\geq 1\times10^9$/L and platelet count $\geq 50\times10^9$/L) was 20.0 (16.0–22.0) days. The most frequent non-hematological grade ≥3 AEs pneumonia (48.5%), and febrile neutropenia (39.4%).

### Summary/Conclusion

Our study showed promising efficacy and safety of the modified DAV regimen in fit elderly patients with de novo AML.Future prospective multi-center randomized controlled trials are warranted to further elucidate and optimize the therapeutic strategies for this population.

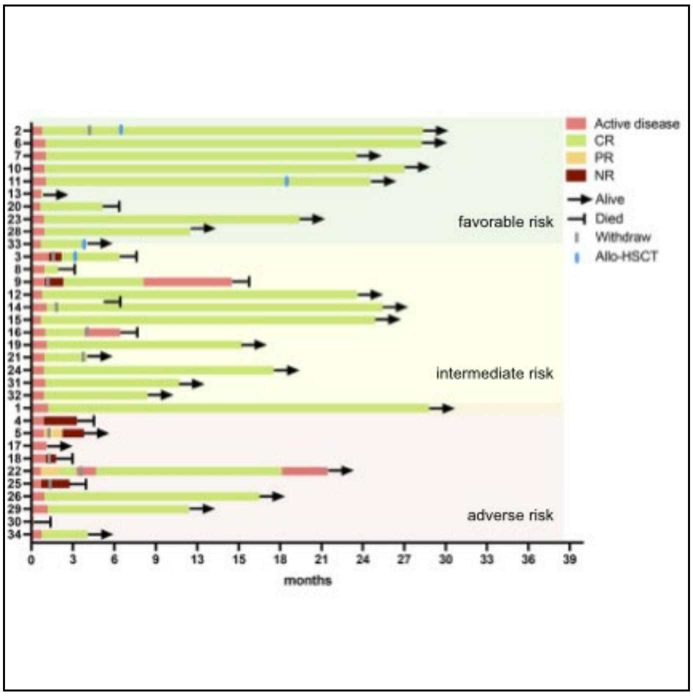

Copyright Information: (Online) ISSN: 2572-9241
©2026 The Author(s). HemaSphere is published by John Wiley & Sons Ltd on behalf of European Hematology Association. This is an open access Abstract Book distributed under the Attribution-NonCommercial-NoDerivs (CC BY-NC-ND), which allows third parties to download the articles and share them with others as long as they credit the author and the Abstract Book, but they cannot change the content in any way or use them commercially.
Abstract Book Citations: Authors, Title, HemaSphere, 2026;10:(S1):pages. The abstract book DOIs can be found on the "Author Information" page.
Disclaimer: Articles published in the journal HemaSphere exclusively reflect the opinions of the authors. The authors are responsible for all content in their abstracts including accuracy of the facts, statements, citing resources, etc.

### (PF497) GENOMIC SIGNATURE AND CLINICAL FEATURES OF DHX15 MUTATED RUNX1::RUNX1T1 AML.

**Topic:** 04. Acute myeloid leukemia - Clinical

Eleonora Boscaro*[1], Eric Sträng[2], Ann-Kathrin Wagner[3], Nuria Tubio Santamaria[2], Monika Szelest[4], Bernhard Gillissen[2], Krzysztof Giannopoulos[4], Frederik Damm[2], Ekaterina Jahn[5], Nikolaus Jahn[6], Jeffery Klco[7], Konstanze Döhner[5], Lars Bullinger[2], Marco Cerrano[1], Anna Dolnik[2]

*[1]AOU Città della Salute e della Scienza, Torino, Italy;[2]Charité - Universitätsmedizin Berlin, Berlin, Germany;[3]Max Delbrück Center for Molecular Medicine, Berlin, Germany; [4]Medical University of Lublin, Lublin, Poland;[5]Universitätsklinikum Ulm, Ulm, Germany; [6]Stanford Medicine, Stanford, United States of America; [7]St. Jude Children's Research Hospital, Memphis, United States of America;*

### Background

Core binding factor AML with *RUNX1::RUNX1T1* rearrangement is considered a favorable-risk entity, yet relapse remains a major cause of treatment failure. Recent studies have identified recurrent mutations in the RNA helicase *DHX15*, mainly hotspot R222G, that are associated with *RUNX1::RUNX1T1* AML, though their biological and clinical impact remains unclear.

### Aims

To determine the clinical trajectories and genomic signature of *DHX15*-mutated (-mut) leukemia in *RUNX1::RUNX1T1* AML.

### Methods

We performed an integrated genomic, transcriptomic, and clinical analysis across a large cohort of publicly available *RUNX1::RUNX1T1* AML (n = 754), including 41 cases harboring *DHX15* mutations, collected across clinical trials and registry data.
An intersected panel of 41 genes (n = 510, of which 41*DHX15*-mut) was used to estimate co-occurrence of gene classes mutations using one-tailed Fisher exact test. Bradley Terry model was applied to estimate clonal hierarchy in leukemogenesis. RNAseq data were collected from the TARGET-AML cohort and analyzed with Deseq2 and GSEA, and compared to a *DHX15* knocked-out (*DHX15* KO) HeLa cell line model.
For a subset of cases, clinical data were available through collaborative studies from AMLSG, HOVON, MRC/NCI and German register (n = 550, of which 33 *DHX15*-mut); Kaplan Meyer analysis and cumulative incidence of relapse (CIR) function were applied for definition of overall survival (OS) and CIR respectively; log-rank and Gray's test were used for comparison and Cox and Fine-Gray were applied for regression models and competitive risk analysis.

**Copyright Information:** (Online) ISSN: 2572-9241
©2026 The Author(s). HemaSphere is published by John Wiley & Sons Ltd on behalf of European Hematology Association. This is an open access Abstract Book distributed under the Attribution-NonCommercial-NoDerivs (CC BY-NC-ND), which allows third parties to download the articles and share them with others as long as they credit the author and the Abstract Book, but they cannot change the content in any way or use them commercially.
**Abstract Book Citations:** Authors, Title, HemaSphere, 2026;10:(S1):pages. The abstract book DOIs can be found on the "Author Information" page.
**Disclaimer:** Articles published in the journal HemaSphere exclusively reflect the opinions of the authors. The authors are responsible for all content in their abstracts including accuracy of the facts, statements, citing resources, etc.

### Results

*DHX15* mutations represent early clonal events in leukemogenesis, and are mutually exclusive with mutations in epigenetic regulators (P = .005) suggesting alternative leukemogenic trajectories compared to *DHX15*-wild type (WT) *RUNX1::RUNX1T1* AML. Transcriptome profiling of *DHX15*-mut cases showed enrichment of pathways related to MYC activation and ribosome biogenesis, partially recapitulated in KO model.

*DHX15*-mut patients presented with higher white blood cell counts (P = .03), while other clinical variables, rate of recurring mutations and cytogenetic abnormalities were similarly distributed between groups. While all pts considered in the clinical analysis underwent high dose anthracycline-based chemotherapy, median OS was 75 months in *DHX15*-mut patients and not reached in WT cases, showing no statistical impact (P = .31) with a median follow-up of 78 months. Older age, *JAK2*-mut, *FLT3*-ITD and +8 were independently associated with inferior OS (P < .05). CIR was significantly lower in *DHX15*-mut pts at 3 years (9.5% vs 31.5%, P=.01), with a persisting difference at 6 years (9.7% vs 31.5%, P = .06) attenuated by increasing censoring (see figure); also loss of sex chromosome was confirmed protective for relapse (P = .004). Inferior relapse rate may be related to an increased chemosensitivity of *DHX15*-mut leukemia due to its proliferative nature.

### Summary/Conclusion

*DHX15* mutations are associated with unique co-mutational patterns and increased proliferation, possibly resulting in increased chemosensitivity and lower relapse risk. Our data suggest a silencing effect of the mutations on DHX15 activity and a compensatory upregulation in ribosome metabolism and cell growth, likely prompting disease aggressiveness. While these findings raise the possibility that *DHX15* status may define a distinct subset of *RUNX1::RUNX1T1* AML, future studies are warranted to confirm the impact of *DHX15* mutations on clinical outcome.

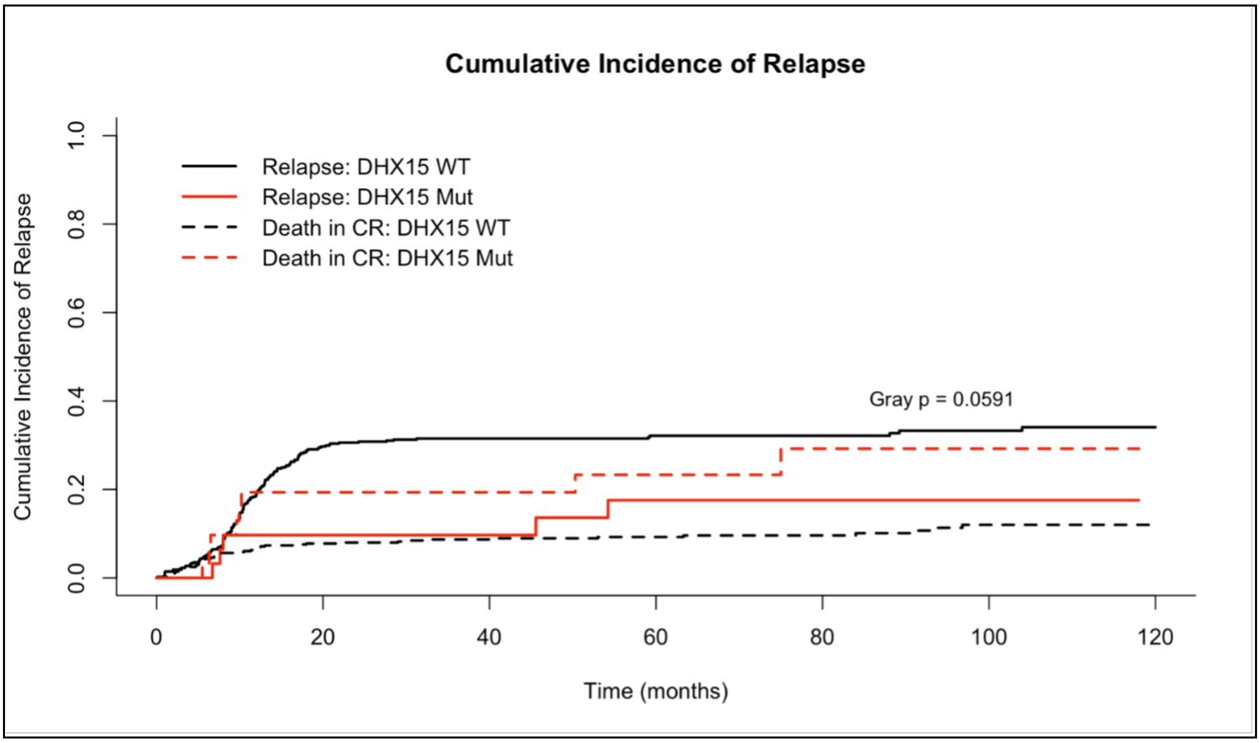

Figure. Six years cumulative incidence of relapse. WT, wild type; Mut, mutated; CR, complete response.

**Copyright Information:** (Online) ISSN: 2572-9241
©2026 The Author(s). HemaSphere is published by John Wiley & Sons Ltd on behalf of European Hematology Association. This is an open access Abstract Book distributed under the Attribution-NonCommercial-NoDerivs (CC BY-NC-ND), which allows third parties to download the articles and share them with others as long as they credit the author and the Abstract Book, but they cannot change the content in any way or use them commercially.
**Abstract Book Citations:** Authors, Title, HemaSphere, 2026;10:(S1):pages. The abstract book DOIs can be found on the "Author Information" page.
**Disclaimer:** Articles published in the journal HemaSphere exclusively reflect the opinions of the authors. The authors are responsible for all content in their abstracts including accuracy of the facts, statements, citing resources, etc.

### (PF498) BIOLOGICAL FEATURES AND CLINICAL CHARACTERISTICS OF TP53-MUTATED AML: INSIGHTS FROM THE HARMONY PLATFORM

**Topic:** 04. Acute myeloid leukemia - Clinical

Rabea Mecklenbrauck*[1, 2], Luca Guarnera[3], Angela Villaverde Rimero[4, 5], Magdalena Karasek[6], Eric Sträng[7], Klaus Metzeler[8], Amin Turki[9], Jesse Tettero[10], Alberto Hernandez-Sanchez[11], Ian Thomas[12], Peter Valk[13], Brian Huntly[14], Christian Thiede[15], Ken Mills[16], Guadalupe Oñate[17], Florian Heidel[2], Torsten Haferlach[18], Sören Lehmann[19], Hartmut Döhner[20], Konstanze Döhner[20], Maria Teresa Voso[3], Michael Heuser[21], Jesus Maria Hernandez Rivas[11], Lars Bullinger[7], Marta Sobas[22]

*[1]MRC Weatherall Institute of Molecular Medicine, Oxford, United Kingdom;[2]Hannover Medical School, Department of Hematology, Hemostasis, Oncology and Stem Cell Transplantation, Hannover, Germany; [3]University of Tor Vergata, Rome, Italy; [4]Institute of Biomedical Research of Salamanca (IBSAL), Salamanca, Spain;[5]Fundación HARMONY Alliance Foundation (HAF), Salamanca, Spain; [6]Wrocław Medical University, Wrocław, Poland; [7]Charité Universitätsmedizin, Berlin, Germany; [8]University of Leipzig, Department of Hematology, Cell Therapy, Hemostaseology and Infectious Diseases, Leipzig, Germany; [9]Ruhr-University Bochum, Department of Hematology and Oncology, Marienhospital University Hospital, Bochum, Germany; [10]Amsterdam University Medical Centers, Amsterdam, The Netherlands; [11]University of Salamanca, Department of Medicine, Salamanca, Spain; [12]Cardiff University, Centre for Trials Research, Cardiff, United Kingdom; [13]Erasmus University Medical Center Cancer Institute, Rotterdam, The Netherlands; [14]Wellcome-MRC Cambridge Stem Cell Institute, Cambridge, United Kingdom; [15]Gustav-Carus University, Department of Hematology, Dresden, Germany; [16]Queens University Belfast, Belfast, United Kingdom; [17]Hospital Santa Creu i Sant Pau, Barcelina, Spain;[18]MLL Munich Leukemia Laboratory, Munich, Germany; [19]Karolinska Institutet, Stockholm, Sweden; [20]University Hospital Ulm, Department of Internal Medicine III, Ulm, Germany; [21]University Hospital Halle (Saale), Department of Internal Medicine IV, Halle (Saale), Germany; [22]Nicolaus Copernicus University, Torun, Poland;*

### Background

*TP53* mutations occur in approximately 10% of patients diagnosed with acute myeloid leukemia (AML) and are enriched in older individuals and those with therapy-related disease. They frequently co-occur with complex or monosomal karyotypes and define a biologically distinct subgroup with a highly adverse prognosis. Patients with *TP53*-mutated (TP53-mut) AML exhibit low complete remission (CR) rates and markedly inferior relapse-free survival (RFS) and overall survival (OS). Despite recent therapeutic advances, outcomes in this subgroup remain poor, highlighting the urgent need to better define the disease biology and to optimize post-induction strategies.

### Aims

To identify baseline clinical, cytogenetic, and molecular characteristics associated with outcome in intensively-treated*TP53*-mut AML and to evaluate the impact of post-induction treatment strategies, with particular focus on the prognostic relevance of *TP53* allelic status and the role of allogeneic hematopoietic cell transplantation (alloHCT).

**Copyright Information:** (Online) ISSN: 2572-9241
©2026 The Author(s). HemaSphere is published by John Wiley & Sons Ltd on behalf of European Hematology Association. This is an open access Abstract Book distributed under the Attribution-NonCommercial-NoDerivs (CC BY-NC-ND), which allows third parties to download the articles and share them with others as long as they credit the author and the Abstract Book, but they cannot change the content in any way or use them commercially.
**Abstract Book Citations:** Authors, Title, HemaSphere, 2026;10:(S1):pages. The abstract book DOIs can be found on the "Author Information" page.
**Disclaimer:** Articles published in the journal HemaSphere exclusively reflect the opinions of the authors. The authors are responsible for all content in their abstracts including accuracy of the facts, statements, citing resources, etc.

### Methods

We retrospectively analysed 706 intensively treated *TP53*-mut AML patients from the HARMONY Alliance database. RFS was calculated from CR to relapse, death, or last follow-up, and OS from diagnosis to death or last follow-up. Survival was analysed using the Kaplan–Meier method and Cox proportional hazards models with multivariable analyses based on multiple imputation. AlloHCT was modelled as a time-dependent covariate using a Simon–Makuch approach. Multihit *TP53* was defined according to allelic state (≥2 mutations with variant allele frequency (VAF) >10%, one *TP53* mutation with VAF >50%, indicating copy-neutral loss of heterozygosity, or with concurrent del(17p)).

### Results

Among 8,096 intensively treated AML patients, 706 (9%) harboured *TP53* mutations. *TP53*-mut patients were older (median 62 vs 55 y, p < 0.001) and had a substantially lower likelihood of achieving CR (45% vs 70%, p < 0.001) compared to *TP53* wildtype patients. Both RFS and OS were significantly inferior (RFS: HR 2.98, 95% CI 2.64–3.36; OS: HR 3.55, 95% CI 3.26–3.86; both p < 0.001). In multivariable analysis, OS in *TP53*-mut AML was independently associated with age, white blood cell count, and the presence of additional adverse cytogenetic abnormalities, while alloHCT was independently associated with improved OS (HR 0.66, 95% 0.61 – 0.71, p < 0.001).
A subset of 312 *TP53*-mut patients (44%) with complete cytogenetic and molecular data was evaluable for *TP53* allelic status. 219 (70%) were multihit and 93 (30%) single hit *TP53*. Multihit *TP53* was strongly associated with complex (92% vs 54%, p < 0.001) and monosomal karyotypes (85% vs 54%, p < 0.001). Although RFS did not differ significantly between single hit and multihit *TP53* AML, multihit status predicted inferior OS (HR 1.52, 95% CI 1.17–1.97; p=0.002) and retained its prognostic impact in a multivariable model including age, white blood cell count, and *NPM1* mutation status. Eighty-five patients underwent alloHCT (46 in CR1). Modelled as a time-dependent variable, alloHCT remained a protective factor (HR 0.49, 95%CI 0.42 – 0.58, p < 0.001), whereas multihit *TP53*, high white blood cell count, and older age remained adverse.

### Summary/Conclusion

In this large multicenter dataset, multihit *TP53* identifies a subgroup with particularly poor survival. Time-dependent modelling demonstrates that alloHCT confers a significant survival benefit and remains a key therapeutic strategy for all patients with *TP53*-mut AML including – multihit *TP53* mutant patients.

**Copyright Information:** (Online) ISSN: 2572-9241
©2026 The Author(s). HemaSphere is published by John Wiley & Sons Ltd on behalf of European Hematology Association. This is an open access Abstract Book distributed under the Attribution-NonCommercial-NoDerivs (CC BY-NC-ND), which allows third parties to download the articles and share them with others as long as they credit the author and the Abstract Book, but they cannot change the content in any way or use them commercially.
**Abstract Book Citations:** Authors, Title, HemaSphere, 2026;10:(S1):pages. The abstract book DOIs can be found on the "Author Information" page.
**Disclaimer:** Articles published in the journal HemaSphere exclusively reflect the opinions of the authors. The authors are responsible for all content in their abstracts including accuracy of the facts, statements, citing resources, etc.

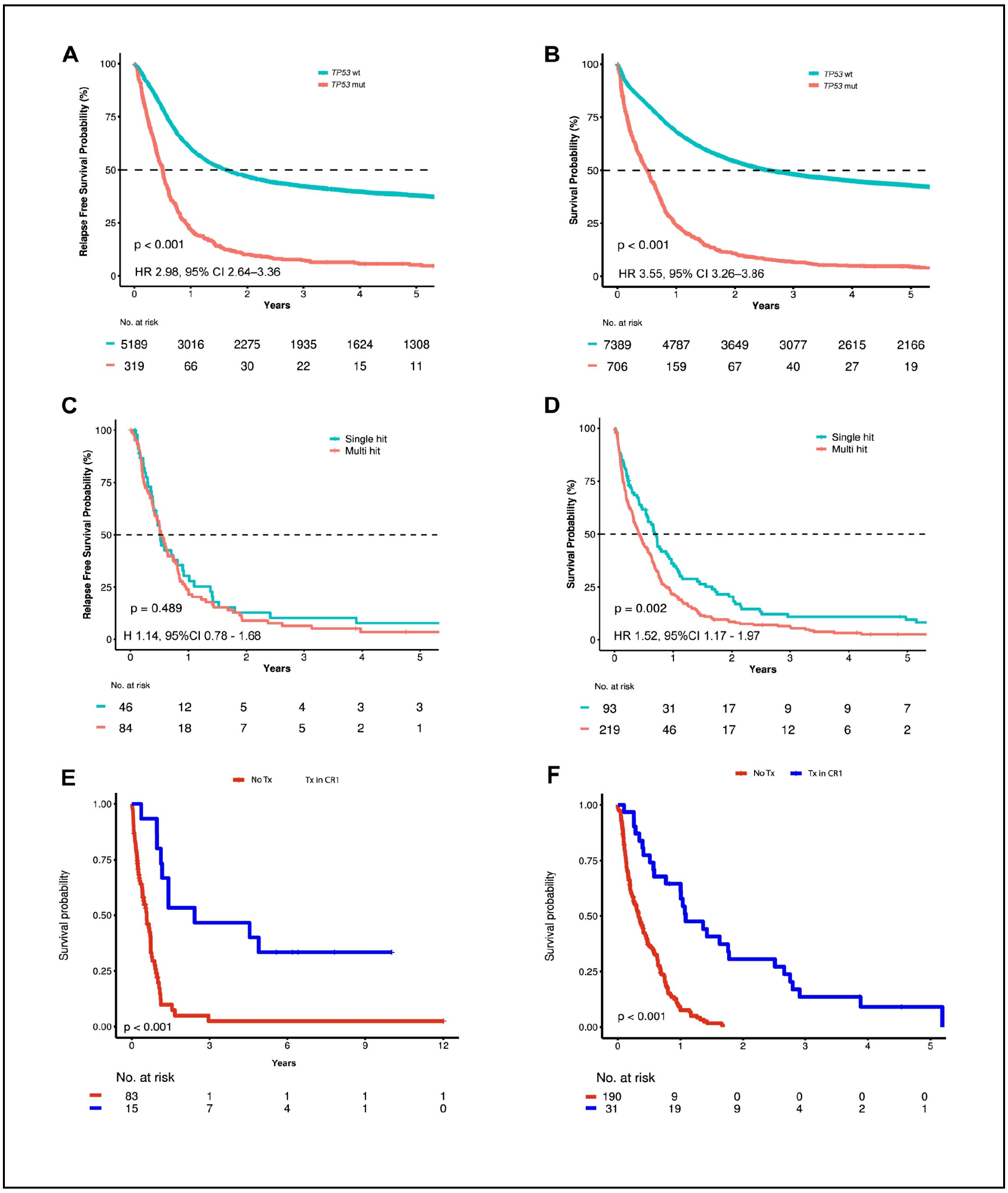

Figure: RFS (A) and OS (B) of TP53-mut patients compared with TP53 wt patients. RFS (C) and OS (D) of single hit and multihit TP53-mut AML. Simon Makuch Model showing benefit of undergoing alloHCT in CR1 for both single (E) and multihit (F) TP53-mut patients.

**Copyright Information:** (Online) ISSN: 2572-9241
©2026 The Author(s). HemaSphere is published by John Wiley & Sons Ltd on behalf of European Hematology Association. This is an open access Abstract Book distributed under the Attribution-NonCommercial-NoDerivs (CC BY-NC-ND), which allows third parties to download the articles and share them with others as long as they credit the author and the Abstract Book, but they cannot change the content in any way or use them commercially.
**Abstract Book Citations:** Authors, Title, HemaSphere, 2026;10:(S1):pages. The abstract book DOIs can be found on the “Author Information” page.
**Disclaimer:** Articles published in the journal HemaSphere exclusively reflect the opinions of the authors. The authors are responsible for all content in their abstracts including accuracy of the facts, statements, citing resources, etc.

### (PF499) POOR RESPONSE OF VENETOCLAX AND AZACITIDINE IN MONOCYTIC ACUTE MYELOID LEUKEMIA IS ASSOCIATED WITH GENETIC PATTERNS AND IS IMPROVED BY ADDITION OF HOMOHARRINGTONINE, A MULTI-CENTER COHORT STUDY

**Topic:** 04. Acute myeloid leukemia - Clinical

Guopan Yu*[1, 1], Rong Hu[1], Yishan Chen[1], Zhao Yin[1], Dandan Chen[1], Dan Xu[1], Qifa Liu[1], Hua Jin[1], Jiacheng Xu[1], Zhao Weihua[2], Nie Danian[3], Guangyang Weng[4], Ziwen Guo[5], Dongjun Lin[6], Li Xuan[1]

*[1]Nanfang Hospital, Southern Medical University, Guangzhou, China;[2]First Affiliated Hospital of Guangxi Medical University, Nanning, China; [3]Sun Yat-Sen Memorial Hospital, Sun Yat-Sen University, Guangzhou, China; [4]Shenzhen Second People's Hospital, Shenzhen, China; [5]Zhongshan City People's Hospital, Zhongshan, China; [6]The Seventh Affiliated Hospital of Sun Yat-Sen University, Shenzhen, China;*

### Background

Monocytic acute myeloid leukemia (AML) responds poorly to venetoclax and azacitidine (VA) treatment, yet the mechanism remains not totally clear. Our previous studies revealed homoharringtonine (HHT) enhances the anti-leukemia effect of VA. Whether HHT could increase the response of VA in monocytic AML is unknown.

### Aims

In this study we explored the factors impacting on the response of VA in monocytic AML and whether addition of HHT could enhance the response.

### Methods

321 patients with relapsed/refractory (RR) AML from our previous study (NCT05456048), being treated with VA or VA combined with HHT (VAH), were analyzed. Patients with AML-M4/M5 were classified into monocytic group, while those with non-M4/M5 were defined as nonmonocytic group.

### Results

There were 139 patients with monocytic AML and 182 in non-monocytic group, of whom 149 were treated with VA and 172 with VAH. In the cohort with VA treatment, the patients with monocytic AML had a significantly lower response than those with nonmonocytic AML, presenting as overall response rate (ORR) of 27/57(47.4%) vs. 60/92(65.2%) (P=0.032). Meanwhile, in the cohort with VAH treatment, monocytic AML patients had comparable response to those with nonmonocytic AML. In addition, in the patients with monocytic AML, VAH significantly improved the response compared to VA (ORR, 27/57(47.4%), P=0.001; CRc rate, 21/57(36.8%), P=0.002). Also, the patients with monocytic AML had better survival with VAH than those with VA treatment (median overall survival (mOS), no reach vs. 12.4 months, P=0.087; median event-free survival (mEFS), 14.0 vs. 2.0 months, P<0.001). The poor response of VA in monocytic AML was associated with the disparate impact of mutant FLT3, DNMT3A and MLL rearrangement in the monocytic versus nonmonocytic AML, which might be partly due to the different co-mutations. The poor influence of FLT3, RAS, DNMT3A and NPM1 mutations in monocytic AML was significantly overcome by VAH.

### Summary/Conclusion

Poor response of VA in monocytic AML is associated with genotype-phenotype. Addition of HHT to VA significantly improves the response and mitigates the negative impact of the adverse genotype.

**Copyright Information:** (Online) ISSN: 2572-9241
©2026 The Author(s). HemaSphere is published by John Wiley & Sons Ltd on behalf of European Hematology Association. This is an open access Abstract Book distributed under the Attribution-NonCommercial-NoDerivs (CC BY-NC-ND), which allows third parties to download the articles and share them with others as long as they credit the author and the Abstract Book, but they cannot change the content in any way or use them commercially.
**Abstract Book Citations:** Authors, Title, HemaSphere, 2026;10:(S1):pages. The abstract book DOIs can be found on the "Author Information" page.
**Disclaimer:** Articles published in the journal HemaSphere exclusively reflect the opinions of the authors. The authors are responsible for all content in their abstracts including accuracy of the facts, statements, citing resources, etc.

### (PF500) THE EFFICACY OF PIVEKIMAB SUNIRINE (PVEK) IN PATIENTS (PTS) WITH BLASTIC PLASMACYTOID DENDRITIC CELL NEOPLASM (BPDCN) WITH BASELINE SKIN INVOLVEMENT IN THE CADENZA STUDY

**Topic:** 04. Acute myeloid leukemia - Clinical

Naveen Pemmaraju*[1], Giovanni Marconi[2], Pau Montesinos[3], Andrew Lane[4], Luca Mazzarella[5], David Sallman[6], Matthew Ulrickson[7], Gary Schiller[8], Harry Erba[9], Eunice Wang[10], Roland Walter[11], Eric Deconinck[12], Ahmed Aribi[13], Ollivier Legrand[14], Delphine Lebon[15], Giovanni Martinelli[2], Daniel J. Deangelo[4], Yining Du[16], Jalaja Potluri[16], Naval Daver[1]

*[1]MD Anderson Cancer Center, Houston, United States of America;[2]IRCCS Istituto Romagnolo per lo Studio dei Tumori "Dino Amadori" IRST S.r.l., Meldola, Emilia-Romagna, Italy; [3]Hospital Universitari i Politècnic La Fe, Valencia, Spain; [4]Dana-Farber Cancer Institute, Boston, MA, United States of America; [5]European Institute of Oncology IRCCS, Milano, Italy;[6]Moffitt Cancer Center and Research Institute, Tampa, United States of America; [7]Banner MD Ander Cancer Center, Gilbert, United States of America; [8]University of California, Los Angeles, Los Angeles, United States of America;[9]Duke University, Durham, United States of America; [10]Roswell Park Comprehensive Cancer Center, Buffalo, United States of America; [11]Fred Hutchinson Cancer Center, Seattle, United States of America; [12]CHU Besançon-Hématologie, Besançon, France; [13]City of Hope, Duarte, United States of America; [14]Hospital St. Antoine, Paris, France; [15]CHU Picardie, Amiens, France; [16]AbbVie, Inc, North Chicago, United States of America;*

### Background

BPDCN, a rare and aggressive hematologic malignancy characterized by CD123 (IL-3Rα) overexpression, primarily involves skin, bone marrow (BM), and lymph nodes. PVEK is a first-in-class antibody-drug conjugate comprised of a high-affinity anti-CD123 antibody, a cleavable linker, and an indolinobenzodiazepine (IGN) pseudodimer payload that alkylates DNA and causes single-strand breaks without crosslinking, demonstrating less toxicity to normal marrow progenitors compared with other DNA-targeting payloads. In the primary analysis of CADENZA (NCT03386513), pts with first-line (1L) BPDCN treated with PVEK achieved high (70%) and durable composite complete response (CR) (CR + clinical CR [CR with minimal skin abnormality or CRc]), with a manageable safety profile.

### Aims

We report a post-hoc analysis of PVEK efficacy in pts with 1L BPDCN stratified by baseline skin involvement from the CADENZA study.

### Methods

In the open-label, international, multicenter, Phase 1/2 CADENZA study, adults with BPDCN received PVEK 0.045 mg/kg intravenously (<30-min infusion) on Day 1 of a 21-day cycle. Baseline skin involvement was assessed by photographs and a Modified Severity-Weighted Assessment Tool (mSWAT) ≤28 days prior to first dose of PVEK. Pts were stratified based on a median mSWAT score of 9.25 (low mSWAT <9.25 and high mSWAT ≥9.25 subgroups, with higher scores associated with increased skin involvement). Outcomes included CR+CRc rate, duration of CR+CRc, overall response rate (ORR), overall survival (OS), and percentage of pts bridged to posttreatment stem cell transplant (SCT). Informed consent was obtained.

**Copyright Information:** (Online) ISSN: 2572-9241
©2026 The Author(s). HemaSphere is published by John Wiley & Sons Ltd on behalf of European Hematology Association. This is an open access Abstract Book distributed under the Attribution-NonCommercial-NoDerivs (CC BY-NC-ND), which allows third parties to download the articles and share them with others as long as they credit the author and the Abstract Book, but they cannot change the content in any way or use them commercially.
**Abstract Book Citations:** Authors, Title, HemaSphere, 2026;10:(S1):pages. The abstract book DOIs can be found on the "Author Information" page.
**Disclaimer:** Articles published in the journal HemaSphere exclusively reflect the opinions of the authors. The authors are responsible for all content in their abstracts including accuracy of the facts, statements, citing resources, etc.

**Results**

Of 33 1L pts, 30 (91%) had skin involvement and an mSWAT score at baseline, with a median (range) mSWAT score of 9.25 (0.5–116.0). Median (range) age was similar between pts with low (n=15) vs high (n=15) mSWAT scores (73 [63–84] vs 74 [59–84] years). Of 30 pts with skin involvement at baseline, 14 (46%) pts had both skin and BM involvement at baseline; in the total 1L population, 16 (48%) had BM involvement at baseline. In the low vs high mSWAT groups, 5 (33%) vs 9 (60%) pts had BM involvement at baseline. For pts with low vs high mSWAT, 11 (73%) vs 9 (60%) achieved CR+CRc; of responders, 9/11 vs 6 /9 pts achieved CR (100% skin clearance); ORR was 80% vs 87%. For low vs high mSWAT groups, median (95% CI) duration of CR+CRc was not reached (NR [4.4–NR]) vs 8.7 (1.6–12.8) months; 8/15 pts (53%) vs 4/15 (27%) were bridged to SCT; and median (95% CI) OS was 24.3(5.8–NR) vs 13.7 (5.1–NR) months. Safety was assessed among all 30 pts and no capillary leak syndrome events were reported.

**Summary/Conclusion**

Despite high burden of skin involvement among 1L pts with BPDCN, PVEK demonstrated high and durable response rates across pts with both high and low mSWAT, enabling eligible pts to proceed to transplant. These results demonstrate PVEK as a potential new therapeutic option for this pt subgroup.

**Copyright Information:** (Online) ISSN: 2572-9241
©2026 The Author(s). HemaSphere is published by John Wiley & Sons Ltd on behalf of European Hematology Association. This is an open access Abstract Book distributed under the Attribution-NonCommercial-NoDerivs (CC BY-NC-ND), which allows third parties to download the articles and share them with others as long as they credit the author and the Abstract Book, but they cannot change the content in any way or use them commercially.
**Abstract Book Citations:** Authors, Title, HemaSphere, 2026;10:(S1):pages. The abstract book DOIs can be found on the "Author Information" page.
**Disclaimer:** Articles published in the journal HemaSphere exclusively reflect the opinions of the authors. The authors are responsible for all content in their abstracts including accuracy of the facts, statements, citing resources, etc.

### (PF501) SAFETY AND EFFICACY OF IADADEMSTAT PLUS GILTERITINIB IN THE FRIDA EXPANSION COHORT OF FLT3-MUTATED RELAPSED/REFRACTORY ACUTE MYELOID LEUKEMIA

**Topic:** 04. Acute myeloid leukemia - Clinical

Sangeetha Venugopal[1], Guru Subramanian Guru Murthy[2], Neil Palmisiano[3], Stephen Strickland[4], Douglas Tremblay[5], Sharad Khurana[6], Ambuga Badari[7], Theodore Braun[8], Harry Erba[9], Beatriz Bruno[10], Sonia Gutierrez[10], Douglas Faller[11], Ana Limon[11, 11], Amir Fathi*[12]

*[1]Sylvester Comprehensive Cancer Center, Miami, United States of America;[2]Medical College of Wisconsin, Milwaukee, United States of America; [3]Rutgers Cancer Institute of New Jersey, New Brunswick, United States of America;[4]Sara Cannon Research Institute, Nashville, United States of America; [5]5Icahn School of Medicine at Mount Sinai, New Yok, United States of America; [6]The University of Arizona Cancer Center, Tucson, United States of America;[7]Banner M.D. Anderson Cancer Center, Gilbert, United States of America; [8]Oregon Health and Science University, Portland, United States of America;[9]Duke Cancer Center, Durham, United States of America; [10]Oryzon Genomics, Hospitalet de Llobregat, Spain;[11]Oryzon Genomics, Boston, United States of America; [12]Massachusetts General Hospital, Boston, United States of America;*

### Background

Fms-related tyrosine kinase 3 mutations (FLT3mut) occur in ~30–40% of patients (pts) with acute myeloid leukemia (AML), where FLT3 inhibitors (FLT3is) provide clinical benefit. Gilteritinib is approved for R/R FLT3mut AML and showed a CR+CRh of 34%, 2.8 months (mo) EFS and 9.3 mo of OS (Admiral trial). However, in contemporary real-world cohorts enriched for heavily pre-treated patients (including HSCT, venetoclax, FLT3i), responses to gilteritinib are substantially lower (CR+CRi 28%; mOS 7.1 mo), highlighting the urgent need for novel agents in this setting. Up to 70% of recurrent AML mutations affect epigenetic regulators of gene expression, resulting in impaired myeloid differentiation and leukemic stem cell proliferation. Iadademstat (iada) is a specific, potent, oral covalent inhibitor of the epigenetic enzyme lysine-specific demethylase 1 (LSD1/KDM1A). Preclinical studies show that iada is synergistic with gilteritinib in FLT3mut AML cells, including models resistant to venetoclax, azacitidine, or other FLT3is. In the ALICE study, iada+azacitidine safely produced rapid, deep, and durable responses in ND-AML pts, including those with FLT3mut.

### Aims

The FRIDA Phase 1b study (NCT05546580) was designed to evaluate the safety and tolerability of iada+gilteritinib and to establish the reccomended Phase 2 dose (RP2D) of the regimen in pts with FLT3mut R/R AML. Here, we report safety and preliminary efficacy results from 23 patients treated in the expansion phase.

### Methods

Adult pts with ≤ 2 prior lines of therapy (including quizartinib or gilteritinib if not refractory) received iada PO at doses of 50 to 150 µg, on a 5 days ON-2 days OFF (5+2) schedule for 3 or 4 wks in 28-day cycles with gilteritinib 120 mg/day PO in the escalation phase. In the expansion phase, up to 20 evaluable pts at the selected safe and pharmacologically active iada dose were enrolled. Primary endpoints are treatment-emergent adverse events (TEAEs) and RP2D determination. Secondary endpoints include EFS, OS, responses, and transfusion rate. Exploratory endpoints include measurable residual disease and gene mutational analysis.

**Copyright Information:** (Online) ISSN: 2572-9241
©2026 The Author(s). HemaSphere is published by John Wiley & Sons Ltd on behalf of European Hematology Association. This is an open access Abstract Book distributed under the Attribution-NonCommercial-NoDerivs (CC BY-NC-ND), which allows third parties to download the articles and share them with others as long as they credit the author and the Abstract Book, but they cannot change the content in any way or use them commercially.
**Abstract Book Citations:** Authors, Title, HemaSphere, 2026;10:(S1):pages. The abstract book DOIs can be found on the “Author Information” page.
**Disclaimer:** Articles published in the journal HemaSphere exclusively reflect the opinions of the authors. The authors are responsible for all content in their abstracts including accuracy of the facts, statements, citing resources, etc.

**Results**

Iada at 75 µg PO on a (5+2) schedule for 4 wks, plus gilteritinib SoC, was selected for expansion and 23 pts signed ICF and were dosed (7 females and 16 males) with median age 59.5 years (range 20-82). 52% had adverse risk (ELN 2022); 52% had AML with myelodysplastic-related genes; at least 26% had received prior transplant, 43% prior venetoclax, 43% FLT3i therapy and 57% were refractory to prior therapy. Most common ≥G3 TEAEs considered related to iada were platelet count decrease and neutrophil count decrease (both 28%), white blood cell decrease (10%) and febrile neutropenia and anemia (both 7%). 5 serious AEs were considered related to iada (febrile neutropenia (2), pneumonia (1), dizziness (1) and myocarditis (1)). 3 pts died on study (pneumonia, respiratory failure and disease progression), none related to treatment. Among 20 response-evaluable pts, 18 harbored canonical FLT3mut (14 ITD, 3 TKD-D835, 1 both); of those 12 (67%) achieved composite complete remission (CCR: 3CR, 4CRh and 5CRi), all by the end of Cycle 2, and 6 did not respond. 2 pts with the non-canonical N676Kmut did not respond. Additional data will be presented at the conference.

**Summary/Conclusion**

In a heavily pre-treated and refractory FLT3mut AML population, iadademstat+gilteritinib demonstrated a favorable safety profile and high CCR (67%), supporting further clinical development of this combination.

**Copyright Information:** (Online) ISSN: 2572-9241
©2026 The Author(s). HemaSphere is published by John Wiley & Sons Ltd on behalf of European Hematology Association. This is an open access Abstract Book distributed under the Attribution-NonCommercial-NoDerivs (CC BY-NC-ND), which allows third parties to download the articles and share them with others as long as they credit the author and the Abstract Book, but they cannot change the content in any way or use them commercially.
**Abstract Book Citations:** Authors, Title, HemaSphere, 2026;10:(S1):pages. The abstract book DOIs can be found on the "Author Information" page.
**Disclaimer:** Articles published in the journal HemaSphere exclusively reflect the opinions of the authors. The authors are responsible for all content in their abstracts including accuracy of the facts, statements, citing resources, etc.

### (PF502) PROSPECTIVE VALIDATION OF MAC-SCORING IN AML ALLOWS SAME DAY PREDICTION OF HMA/VENETOCLAX RESPONSE

**Topic:** 04. Acute myeloid leukemia - Clinical

Simon Renders*[1], Alexander Waclawiczek[2], Ricarda Schwab[3], Aino-Maija Leppä[2], Julia Unglaub[3], Stefan A. Klein[4], Tim Sauer[3], Carsten Müller-Tidow[3], Michael Hundemer[3], Andreas Trumpp[2]

*[1]DKFZ/ Hi-STEM gGMBH / University hospital Heidelberg, Heidelberg, Germany;[2]DKFZ / HI-STEM gGMBH, Heidelberg, Germany; [3]University hospital Heidelberg, Heidelberg, Germany; [4]University hospital Mannheim, Mannheim, Germany;*

### Background

The addition of the BCL-2 inhibitor Venetoclax (VEN) to hypomethylating agents (HMA) has become standard for unfit acute myeloid leukemia (AML) patients with remission rates of 66%. This combination challenges induction chemotherapy in first-line, in younger, fit patients with similar response rates but a fewer side effects (Lu/ Fathi, 2025). Established genomic risk scores perform poorly for HMA/VEN therapy. To overcome this, the 4-gene score was developed for HMA/VEN risk stratificaty (Doehner, 2024), but NGS-results have turnaround time of >10 days. We recently introduced the "*Mediators of Apoptosis combinatorial*" ("MAC")-Score, a flowcytometry-based assay quantifying the BCL-2 family protein ratio (BCL-2 / [BCL-xL + MCL-1]) in GPR56+ leukemic stem cells (LSCs). MAC-S. predicts response and event-free survival (EFS) with HMA/VEN therapy in retrospective samples (Waclawiczek, 2023).

### Aims

We aimed to implement and prospectively validate MAC-Scoring in our diagnostic laboratory to predict AML outcome with HMA/VEN.

### Methods

From 2023 to 2026, 130 later confirmed newly diagnosed AML cases were MAC-Scored in parallel to the standard ALOT-tube. MAC-S. was compared with response to chemotherapy or HMA/VEN, genetics and risk scores. For selected cases, MAC-S. was repeated at relapse.

**Copyright Information:** (Online) ISSN: 2572-9241
©2026 The Author(s). HemaSphere is published by John Wiley & Sons Ltd on behalf of European Hematology Association. This is an open access Abstract Book distributed under the Attribution-NonCommercial-NoDerivs (CC BY-NC-ND), which allows third parties to download the articles and share them with others as long as they credit the author and the Abstract Book, but they cannot change the content in any way or use them commercially.
**Abstract Book Citations:** Authors, Title, HemaSphere, 2026;10:(S1):pages. The abstract book DOIs can be found on the "Author Information" page.
**Disclaimer:** Articles published in the journal HemaSphere exclusively reflect the opinions of the authors. The authors are responsible for all content in their abstracts including accuracy of the facts, statements, citing resources, etc.

**Results**

The MAC-S. of the 130 prospectively assessed fresh samples was close to 0,4, similar to our retrospective cohort. Technically, MAC-Scores were reproducible, stable for up to 48 hours and comparable between bone marrow or peripheral blood blasts.
Of 130 patients, 50 received HMA/VEN-based first-line treatment. Only one patient of the upper MAC-S. half but 8 in the lower were refractory to HMA/VEN. Within the high MAC-S. group, this remains the only event before day 100. Median EFS was 89 days (low) and 346 (high) with a median follow-up of 145 days, with a Hazard Ratio (HR) of 3.41.
Similar results were obtained after including 14 patients receiving HMA/VEN as a salvage regimen after induction failure (8: MAC-S. low; 6: high). While MAC-S. did not predict response to AraC-based induction, every R/R patient with a high MAC-S. at diagnosis went into remission with salvage HMA/VEN.
Low MAC-S. correlated with poor risk features like complex karyotyp or *TP53*, and high MAC-S. with *IDH1/2* or *NPM1* mutations. However, MAC-S. identified individual patients defying genetic predictors: Among the 18 *NPM1-mut.* HMA/VEN patients, those with low MAC-S. had a median EFS of only 106 days compared to not reached for MAC-S. high patients (HR 10.8). Similarly, in 14 *RUNX1*-mut. patients EFS was 61 days in low MAC-S. vs. not reached (HR 4.7). MAC-Scoring also identified high and low-risk patients within the different 4-gene score subgroups.
At relapse, MAC-Scores were generally lower. For some longitudinal cases, small BCL-2 low subpopulations present at diagnosis grew out, while in most BCL-xL increased.

**Summary/Conclusion**

We prospectively validate MAC-S. as an high-turnover, easily implementable and cost-effective tool for risk stratification of AML in a real-world clinical setting. High MAC-S. predicted response rates >95% and a >3-fold increase in EFS for HMA/VEN. MAC-S. identified high/low-risk patients within mutational subgroups, providing a truly individualized biomarker. All patients refractory to induction chemotherapy with a high MAC-S. went into remission with HMA/VEN treatment. Therefor, MAC-S. stratification may allow safe avoidance of induction chemotherapy side-effects in fit patients.

**Copyright Information:** (Online) ISSN: 2572-9241
©2026 The Author(s). HemaSphere is published by John Wiley & Sons Ltd on behalf of European Hematology Association. This is an open access Abstract Book distributed under the Attribution-NonCommercial-NoDerivs (CC BY-NC-ND), which allows third parties to download the articles and share them with others as long as they credit the author and the Abstract Book, but they cannot change the content in any way or use them commercially.
**Abstract Book Citations:** Authors, Title, HemaSphere, 2026;10:(S1):pages. The abstract book DOIs can be found on the "Author Information" page.
**Disclaimer:** Articles published in the journal HemaSphere exclusively reflect the opinions of the authors. The authors are responsible for all content in their abstracts including accuracy of the facts, statements, citing resources, etc.

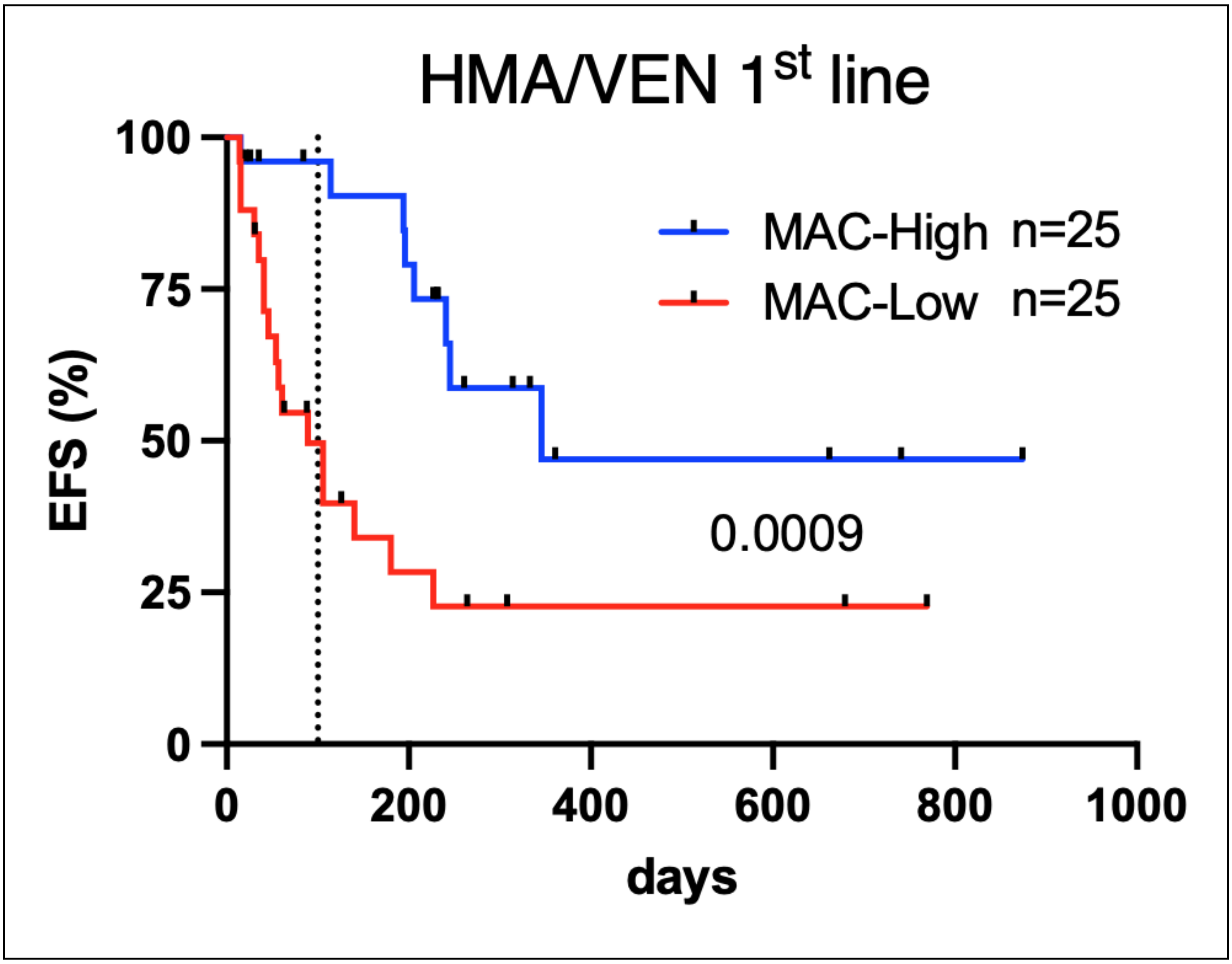

Legend: EFS of MAC-Score high (n=25) vs. low (n=25) patients, after HMA/VEN-based induction treatment.

**Copyright Information:** (Online) ISSN: 2572-9241
©2026 The Author(s). HemaSphere is published by John Wiley & Sons Ltd on behalf of European Hematology Association. This is an open access Abstract Book distributed under the Attribution-NonCommercial-NoDerivs (CC BY-NC-ND), which allows third parties to download the articles and share them with others as long as they credit the author and the Abstract Book, but they cannot change the content in any way or use them commercially.
**Abstract Book Citations:** Authors, Title, HemaSphere, 2026;10:(S1):pages. The abstract book DOIs can be found on the “Author Information” page.
**Disclaimer:** Articles published in the journal HemaSphere exclusively reflect the opinions of the authors. The authors are responsible for all content in their abstracts including accuracy of the facts, statements, citing resources, etc.

### (PF503) LONG-TERM OUTCOMES WITH VENETOCLAX PLUS "7+3" (DAV) INDUCTION IN NEWLY DIAGNOSED ACUTE MYELOID LEUKEMIA

**Topic:** 04. Acute myeloid leukemia - Clinical

Huafeng Wang*[1], Xiaona Zheng[1], Hongyan Tong[1], Liping Mao[1], Min Yang[1], Wanzhuo Xie[1], Ying Lu[2], De Zhou[1], Haitao Meng[1], Jiejing Qian[1], Liya MA[1], Juying Wei[1], Jian Huang[1, 3], Yinjun Lou[1], Wenyuan Mai[1], Gaixiang Xu[1], Honghu Zhu[1], Jie Jin[1]

*[1]The First Affiliated Hospital, Zhejiang University School of Medicine, Hangzhou, China;[2]The Affiliated People's Hospital of Ningbo University, Ningbo, China; [3]The Fourth Affiliated Hospital, Zhejiang University School of Medicine, Hangzhou, China;*

### Background

The "7+3" regimen remains the standard induction therapy for newly diagnosed acute myeloid leukemia (AML), yet outcomes are often suboptimal, with 5-year overall survival hovering around 40%. While Venetoclax (VEN) has revolutionized treatment for unfit patients, its integration into intensive chemotherapy for fit populations is still being explored. Despite high response rates, VEN-plus-intensive regimens (e.g., FLAG-IDA, CLIA) are often limited by severe myelosuppression and infectious complications. Building on our pilot DAV regimen (hypomethylating agent, cytarabine, and VEN), which yielded a promising 91% CR rate and manageable safety, we conducted this expanded study with longer follow-up to evaluate whether combining VEN with the classic "7+3" backbone could further enhance long-term efficacy while maintaining a tolerable toxicity profile.

### Aims

This study aimed to evaluate the long-term efficacy and safety of Venetoclax combined with the classic "7+3" regimen in an expanded cohort of 64 patients with newly diagnosed AML.

### Methods

This study was a prospective, multicenter, single-arm, phase 2 trial. The primary endpoint was the composite complete remission (CRc) rate after one cycle of induction. Secondary endpoints included overall response rate (ORR), measurable residual disease (MRD) in bone marrow assessed by flow cytometry, disease-free survival (DFS), event-free survival (EFS), overall survival (OS), and adverse events. The trial was registered with the Chinese Clinical Trial Registry (ChiCTR2000041509).

### Results

Between Dec 25, 2020 and Sept 20, 2024, 64 patients were enrolled. With one discontinuation due to infection, 63 patients were assessable for the primary endpoint, and all 64 were included in the secondary endpoint analyses. Among 63 response-evaluable patients, the ORR was 90.5% (95%CI 80.7-95.6) and the CRc rate was 88.9% (95%CI 78.8-94.5), with all responses being CR. Of these patients who achieved CR, 93.7% attained flow cytometry-negative MRD status. With a median follow-up of 46.3 (IQR 26.8-52.9) months for the entire cohort (N = 64), the estimated 4-year OS and EFS rates were 70.0% (95%CI 56.0-80.3) and 67.5% (95%CI 54.2-77.7), respectively. The estimated 4-year DFS in CR patients was 69.0% (95%CI 55.0-79.5). The most common grade 3 or higher adverse events were neutropenia in 64 (100%) of 64 patients, thrombocytopenia in 64 (100%), anaemia in 64 (100%), febrile neutropenia in 42 (65.6%), lung infection in 17 (26.6%), and sepsis in 8 (12.5%). Both the 30-day and 60-day mortality rates were 1.6% (n = 1), with the single death attributed to infection.

**Copyright Information:** (Online) ISSN: 2572-9241
©2026 The Author(s). HemaSphere is published by John Wiley & Sons Ltd on behalf of European Hematology Association. This is an open access Abstract Book distributed under the Attribution-NonCommercial-NoDerivs (CC BY-NC-ND), which allows third parties to download the articles and share them with others as long as they credit the author and the Abstract Book, but they cannot change the content in any way or use them commercially.
**Abstract Book Citations:** Authors, Title, HemaSphere, 2026;10:(S1):pages. The abstract book DOIs can be found on the "Author Information" page.
**Disclaimer:** Articles published in the journal HemaSphere exclusively reflect the opinions of the authors. The authors are responsible for all content in their abstracts including accuracy of the facts, statements, citing resources, etc.

### Summary/Conclusion

This expanded cohort confirms that the DAV regimen is a highly effective and safe induction therapy for newly diagnosed AML. These findings validate its role as a practical and readily implementable strategy, successfully integrating venetoclax into the internationally standard "7+3" backbone.

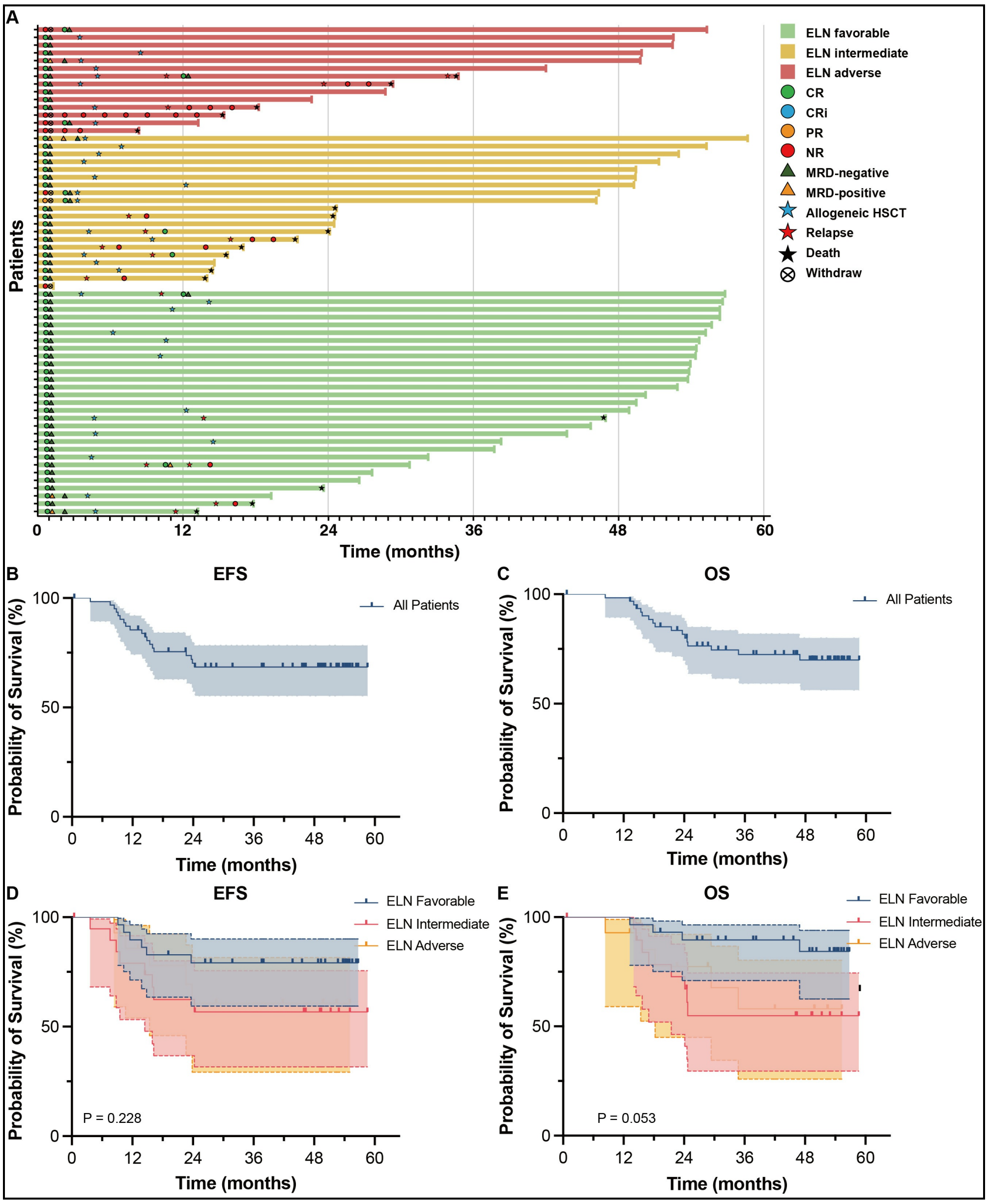

Figure 1. Clinical outcomes and survival analysis of VEN plus "7+3" regimen. (A) Swimmer plot depicting the clinical course and key events for 63 response-evaluable patients. Each bar represents an individual patient, illustrating milestones including response achievement, MRD status, timing of allo-HSCT, relapse, and survival status. (B–C) Kaplan-Meier estimates of event-free survival (EFS) and overall survival (OS) for the entire cohort (N = 64). (D–E) EFS and OS stratified by ELN 2022 genetic risk categories.

**Copyright Information:** (Online) ISSN: 2572-9241
©2026 The Author(s). HemaSphere is published by John Wiley & Sons Ltd on behalf of European Hematology Association. This is an open access Abstract Book distributed under the Attribution-NonCommercial-NoDerivs (CC BY-NC-ND), which allows third parties to download the articles and share them with others as long as they credit the author and the Abstract Book, but they cannot change the content in any way or use them commercially.
**Abstract Book Citations:** Authors, Title, HemaSphere, 2026;10:(S1):pages. The abstract book DOIs can be found on the "Author Information" page.
**Disclaimer:** Articles published in the journal HemaSphere exclusively reflect the opinions of the authors. The authors are responsible for all content in their abstracts including accuracy of the facts, statements, citing resources, etc.

### (PF504) MITOXANTRONE LIPOSOME, SUBCUTANEOUS CYTARABINE AND G-CSF COMBINED WITH VENETOCLAX AS FIRST-LINE TREATMENT IN SECONDARY AML PATIENTS: A MULTICENTER, PROSPECTIVE CLINICAL TRIAL WITH CONCURRENT CONTROL

**Topic:** 04. Acute myeloid leukemia - Clinical

Xiaoqian Xu[1], Ying Lu[2], Weiying Bao[3], Xiuhua Han[4], Zhiyin Liu[1], Haimin Sun[1], Ran An[1], Jie Tian[1], Xing-Ying Li[1], Ying Wang[1], Li Chen[1], Sujiang Zhang*[1]

*[1]Shanghai Jiao Tong University School of Medicine Ruijin Hospital, Shanghai, China;[2]People's Hospital Affiliated to Ningbo University, Ningbo, China; [3]Songjiang Hospital Affiliated to Shanghai Jiao Tong University School of Medicine, Shanghai, China; [4]Jiading District Central Hospital, Shanghai, China;*

### Background

The prognosis of secondary acute myeloid leukemia (sAML) including AML-myelodysplastic related (AML-MR), myeloproliferative neoplasm in blast phase (MPN-BP)and AML post-cytotoxic therapy (AML-pCT) is still very poor, overlapping with elderly AML, although CPX-351 showing OS advantage in these patients' group. Considering the unavailable of CPX-351 in China, and the toxicities of standard azacytidine combined with venetoclax (AZA+VEN) regimen, the optimal treatment in sAML is still being investigated.

### Aims

The aim of this study was to investigate the activity and safety of mitoxantrone liposome, subcutaneous injection cytarabine and G-CSF combined with Venetoclax(CMG+VEN)in patients with sAML.

### Methods

The CMG+VEN regimen was mitoxantrone liposome 15mg/m2 on day 1, cytarabine 10mg/m2, subcutaneous injection, q12h, on days 1-7, G-CSF 5ug/kg from d0 until the WBC count of patients was above 20×109/L. Venetoclax was also added on day2 100 mg, 200mg day3, 400mg from day4 to 10. The primary endpoint was composite complete remission. The secondary endpoint was overall response, measurable residual disease (MRD) by flow cytometry, event-free survival, overall survival, and safety. This trial was registered with ClinicalTrials.gov, NCT06329999.

### Results

A total of 37 patients were enrolled, including 18 males and 19 females, with a median age of 62 (25-75) years old. Among them, there were 26 AML-MR patients, 5 MPN-BP and 6 AML-pCT respectively. According to the European Leukemia Net (ELN) 2022 risk stratification, the favorable prognosis group, the intermediate prognosis group and the adverse prognosis group were 5.4% (2/37), 13.5% (5/37) and 81.1% (30/37), respectively. The overall response rate (ORR) after induction therapy was 63.9% in all patients group. The composite complete remission rate (CRc) and MRD negative rate were 68.2% and 61.1%, respectively. Grade 3 or 4 adverse events were mainly hematological toxicities. Comparing with the matched 37 patients including age and ELN risk stratification during the same period who using standard AZA+VEN regimen as induction therapy, no statistically significant difference in CRc and ORR between two groups was found. However, MRD negative rate in CMG+VEN group was significantly higher than AZA+VEN group (*P*=0.047), and hospitalization duration in CMG+VEN group was significantly shorter than AZA+VEN group (*P*=0.015). With a median follow-up of 13 months (IQR 11–14.5), the estimated 1-year overall survival was 65.9% and 1-year event-free survival (EFS) and relapse-free survival was 36.1% and 43.8% respectively.

**Copyright Information:** (Online) ISSN: 2572-9241
©2026 The Author(s). HemaSphere is published by John Wiley & Sons Ltd on behalf of European Hematology Association. This is an open access Abstract Book distributed under the Attribution-NonCommercial-NoDerivs (CC BY-NC-ND), which allows third parties to download the articles and share them with others as long as they credit the author and the Abstract Book, but they cannot change the content in any way or use them commercially.
**Abstract Book Citations:** Authors, Title, HemaSphere, 2026;10:(S1):pages. The abstract book DOIs can be found on the "Author Information" page.
**Disclaimer:** Articles published in the journal HemaSphere exclusively reflect the opinions of the authors. The authors are responsible for all content in their abstracts including accuracy of the facts, statements, citing resources, etc.

**Summary/Conclusion**

CMG+VEN represents a safer alternative regimen in sAML patients, showing higher MRD negative rate and deserving further investigation.

**Copyright Information:** (Online) ISSN: 2572-9241
©2026 The Author(s). HemaSphere is published by John Wiley & Sons Ltd on behalf of European Hematology Association. This is an open access Abstract Book distributed under the Attribution-NonCommercial-NoDerivs (CC BY-NC-ND), which allows third parties to download the articles and share them with others as long as they credit the author and the Abstract Book, but they cannot change the content in any way or use them commercially.
**Abstract Book Citations:** Authors, Title, HemaSphere, 2026;10:(S1):pages. The abstract book DOIs can be found on the "Author Information" page.
**Disclaimer:** Articles published in the journal HemaSphere exclusively reflect the opinions of the authors. The authors are responsible for all content in their abstracts including accuracy of the facts, statements, citing resources, etc.

### (PF505) VENETOCLAX PLUS CLADRIBINE AND 5-AZACITIDINE (VCAZA) AS INDUCTION THERAPY IN NEWLY DIAGNOSED OLDER, UNFIT OR YOUNG HIGH-RISK ACUTE MYELOID LEUKEMIA: A PROSPECTIVE, SINGLE-ARM, PHASE II STUDY

**Topic:** 04. Acute myeloid leukemia - Clinical

Cong Wang*[1], Qiuju Liu[1], Yehui Tan[1], Jingnan Sun[1], Sujun Gao[1]

[1] *The first hospital of Jilin University, Changchun, China;*

### Background

Approximately 45% of patients with newly diagnosed acute myeloid leukemia (AML) are ineligible for standard "7+3" induction chemotherapy due to advanced age or poor performance status. Furthermore, high-risk patients derive limited benefit from conventional chemotherapy, with complete remission rates of only 30-40%. The combination of venetoclax and azacitidine is safe and effective in elderly or unfit patients, yielding a composite complete remission (CRc) rate of nearly 70% after two cycles. However, the median response duration is 1.3 months, with a CRc rate of only 43.4% and an measurable residual disease (MRD) negativity rate of just 23.4% after the first cycle.

### Aims

To improve the initial remission rate and shorten the time to response in this population, we evaluated the efficacy and safety of a novel triplet regimen comprising venetoclax, cladribine and azacitidin (VCAza) as induction therapy.

### Methods

This prospective, single-arm, phase II trial was conducted at The First Hospital of Jilin University in China. Newly diagnosed AML patients were eligible if they met one of the following criteria (1) elderly (≥60 years); (2) considered unfit for intensive chemotherapy by the treating physician; or (3) young (<60 years) with adverse-risk disease. Patients received 1-2 cycles of VCAza induction: venetoclax (100mg D1, 200mg D2, 400mg D3-14,orally), cladribine (5mg/m² D1-5,IV), and azacitidine (100mg/m² D1-7,subcutaneously). Responders could receive subsequent consolidation based on risk stratification. The primary endpoint was the CRc rate, MRD negativity (assessed by flow cytometry, 0.1%) rate and time to response after induction therapy. Secondary endpoints included overall survival (OS), event-free survival (EFS), time to hematologic recovery, and safety. The trial is registered at the Chinese Clinical Trial Registry (ChiCTR2300074386).

### Results

From Sep 18, 2023, to Nov 25, 2025, 38 patients were enrolled. The median age was 64 years (range, 34-82), and 42% (16/38) were male. With a median follow-up of 12.3 months (IQR, 5.3-24.6), the CRc rate after one cycle of induction was 94.7% (36/38). Among the 36 patients achieving CRc, the MRD negativity rate was 83.3% (30/36). The median time to initial response was 23 days (range, 9-57). Adverse-risk patients comprised 31.6% (12/38) of the cohort; in this subgroup, the CRc rate was 83.3% (10/12), and the MRD negativity rate among responders was 100% (10/10). The 1-year OS and EFS rates were 89% and 76%, respectively. No treatment-related deaths occurred. Grade ≥3 adverse events included neutropenia (100%, 38/38), thrombocytopenia (89.5%, 34/38), anemia (92.1%, 35/38), and pneumonia (39.5%, 15/38). The median time to platelet recovery >50×10⁹/L was 16 days, and to neutrophil recovery >0.5×10⁹/L was 25 days. The most common genetic alterations were *FLT3* (31.6%, 12/38), *NPM1* (28.9%, 11/38), *DNMT3A* (23.7%, 9/38), and *NRAS* (23.7%, 9/38) mutations. Notably, both patients who were refractory to induction harbored *NRAS* mutations.

**Copyright Information:** (Online) ISSN: 2572-9241
©2026 The Author(s). HemaSphere is published by John Wiley & Sons Ltd on behalf of European Hematology Association. This is an open access Abstract Book distributed under the Attribution-NonCommercial-NoDerivs (CC BY-NC-ND), which allows third parties to download the articles and share them with others as long as they credit the author and the Abstract Book, but they cannot change the content in any way or use them commercially.
**Abstract Book Citations:** Authors, Title, HemaSphere, 2026;10:(S1):pages. The abstract book DOIs can be found on the "Author Information" page.
**Disclaimer:** Articles published in the journal HemaSphere exclusively reflect the opinions of the authors. The authors are responsible for all content in their abstracts including accuracy of the facts, statements, citing resources, etc.

**Summary/Conclusion**

The triplet combination of venetoclax, azacitidine, and cladribine (VCAza) is a safe and highly effective first-line induction regimen for newly diagnosed elderly, unfit, or young high-risk AML. It achieves high rates of composite remission and MRD negativity, with rapid time to response and promising early survival outcomes. The association between *NRAS* mutations and primary resistance to this regimen warrants further investigation as a potential biomarker.

**Copyright Information:** (Online) ISSN: 2572-9241
©2026 The Author(s). HemaSphere is published by John Wiley & Sons Ltd on behalf of European Hematology Association. This is an open access Abstract Book distributed under the Attribution-NonCommercial-NoDerivs (CC BY-NC-ND), which allows third parties to download the articles and share them with others as long as they credit the author and the Abstract Book, but they cannot change the content in any way or use them commercially.
**Abstract Book Citations:** Authors, Title, HemaSphere, 2026;10:(S1):pages. The abstract book DOIs can be found on the "Author Information" page.
**Disclaimer:** Articles published in the journal HemaSphere exclusively reflect the opinions of the authors. The authors are responsible for all content in their abstracts including accuracy of the facts, statements, citing resources, etc.

### (PF506) QUIZARTINIB WITH REINDUCTION CHEMOTHERAPY AND AS SINGLE-AGENT MAINTENANCE IN CHILDREN AND ADOLESCENTS WITH RELAPSED/REFRACTORY FLT3-ITD+ AML: A PHASE 1/2 MULTICENTER STUDY

**Topic:** 04. Acute myeloid leukemia - Clinical

C. Michel Zwaan*[1, 2], Carmelo Rizzari[3], Alba Rubio-San-Simon[4], Jonas Abrahamsson[5], Nicoletta Bertorello[6], Carine Domenech[7], Barbara De Moerloose[8], Aerang Kim[9], Franco Locatelli[10], Karsten Nysom[11], Antonio Pérez-Martínez[12], Arnaud Petit[13], Yvonne Duong[14], Jenna Yejin Park[14], Yuan Xu[14], James Jin[14], Kristy Burns[14], Akash Nahar[14], Noha Biserna[14], Alok Kothari[15]

*[1]Prinses Máxima Centrum voor Kinderoncologie, Utrecht, The Netherlands;[2]Erasmus MC Sophia Children's Hospital, Rotterdam, Rotterdam, The Netherlands; [3]Pediatric Hematology-Oncology Unit, IRCCS San Gerardo dei Tintori, Monza, Italy; [4]Hospital Infantil Universitario Niño Jesus, Madrid, Spain; [5]Sahlgrenska Universitetssjukhuset - Drottning Silvias Barn- och Ungdomssjukhus, Gothenburg, Sweden; [6]Ospedale Infantile Regina Margherita, Turin, Italy; [7]IHOPe, Hospices Civils de Lyon, UCBL1, Lyon, France; [8]Universitair Ziekenhuis Gent, Gent, Belgium; [9]Children's National Hospital, Washington, DC, United States of America; [10]IRCCS Bambino Gesù Children's Hospital, Catholic University of the Sacred Heart, Rome, Italy; [11]Rigshospitalet Copenhagen University Hospital, Copenhagen, Denmark; [12]Hospital Universitario La Paz, Madrid, Spain; [13]Hôpital Armand-Trousseau, APHP, Sorbonne Université, Paris, France; [14]Daiichi Sankyo, Inc., Basking Ridge, NJ, United States of America; [15]Phoenix Children's Hospital, Phoenix, AZ, United States of America;*

### Background

*FLT3*-ITD mutations occur in approximately 10–15% of pediatric AML cases and are often associated with a poor prognosis and unfavorable genotypes. Quizartinib (quiz) is an FLT3 inhibitor with single-agent activity in relapsed/refractory (R/R) AML, and is approved in combination with chemotherapy, followed by maintenance monotherapy, for adult patients (pts) with newly diagnosed *FLT3*-ITD$^+$ AML. Quiz demonstrated tolerability and activity in pediatric pts with R/R AML in a COG-sponsored phase (Ph) 1 trial (NCT01411267), warranting further investigation.

### Aims

To report results from a Ph 1/2 study of quiz in combination with reinduction chemotherapy and as single-agent maintenance in children and adolescents with R/R *FLT3*-ITD$^+$ AML.

### Methods

This is a global, multicenter, Ph 1/2 dose-escalation and expansion trial (NCT03793478). Pts received 1–2 reinduction cycles of fludarabine 30 mg/m$^2$/day (d) + cytarabine 2000 mg/m$^2$/d + quiz (starting at 40 mg/m$^2$/d or 60 mg/d for pts with body surface area [BSA] <1.5m$^2$ or ≥1.5 m$^2$, respectively, in Ph 1, or at the recommended Ph 2 dose [RP2D] in Ph 2). Following reinduction, pts could receive optional allo-HCT if eligible (or consolidation therapy if allo-HCT was not immediately available), and 1 year of single-agent quiz maintenance at reinduction dose (Ph 1) or RP2D (Ph 2). Primary endpoints were composite complete remission (CRc) rate, safety and tolerability, PK of quiz and AC886 (primary metabolite), and determination of quiz RP2D (Ph 1 only). Dose-limiting toxicities (DLTs) were evaluated in reinduction Cycle 1 (C1) during Ph 1 for the determination of quiz RP2D. Safety was assessed in pts who received ≥1 dose of quiz. Efficacy assessment included pts with centrally confirmed *FLT3*-ITD$^+$ who received ≥75% of prescribed quiz doses during reinduction C1 and had ≥1 follow-up bone marrow evaluation.

**Copyright Information:** (Online) ISSN: 2572-9241
©2026 The Author(s). HemaSphere is published by John Wiley & Sons Ltd on behalf of European Hematology Association. This is an open access Abstract Book distributed under the Attribution-NonCommercial-NoDerivs (CC BY-NC-ND), which allows third parties to download the articles and share them with others as long as they credit the author and the Abstract Book, but they cannot change the content in any way or use them commercially.
**Abstract Book Citations:** Authors, Title, HemaSphere, 2026;10:(S1):pages. The abstract book DOIs can be found on the "Author Information" page.
**Disclaimer:** Articles published in the journal HemaSphere exclusively reflect the opinions of the authors. The authors are responsible for all content in their abstracts including accuracy of the facts, statements, citing resources, etc.

### Results

Overall, 16 pts were enrolled (dose escalation n=9, dose expansion n=7;**Figure**). All pts entered C1, 6 (38%) received C2 reinduction, 1 (6%) received consolidation chemotherapy, and 9 (56%) received allo-HCT and quiz maintenance treatment (Tx). Quiz RP2Ds were 40 mg/m$^2$/d (BSA <1.5m$^2$) and 60 mg/d (BSA ≥1.5 m$^2$); 1 pt experienced G3 thrombocytopenia that initially qualified as a DLT but was found not to be drug related. Of 13 pts eligible for efficacy evaluation (*FLT3*-ITD$^+$ centrally confirmed), 3 (23%) achieved CRc, 8 (62%) achieved a morphologic leukemia-free state (MLFS), and 2 (13%) did not respond. Durations of CRc were 32.4 mo (death after CRi), 22.4 mo (relapse after CR), and 13.4 mo (relapse after CRi). Flow response data were not available.
All pts had ≥1 Tx-emergent adverse event (TEAE), most commonly anemia (n=9 [56%]) and vomiting (n=8 [50%]). Furthermore, 14 pts (88%) experienced one or more Grade ≥3 TEAE, with cytopenias being the most common (thrombocytopenia, n=7 [44%]; anemia, n=6 [38%]; neutropenia, n=6 [38%]). Ten pts (63%) had serious TEAEs, most commonly febrile neutropenia (n=4 [25%]). Two TEAEs led to Tx discontinuation: QTc prolongation (n=1) and thrombocytopenia (n=1). Noncompartmental analysis showed that pediatric PK for quiz and AC886 was comparable to adult PK, with $C_{max}$ values 1.03- and 1.09-fold, and AUC values 0.79- and 0.88-fold of adult values, respectively.

### Summary/Conclusion

Quiz demonstrated a manageable safety profile and clinical activity in combination with reinduction chemotherapy and as single-agent maintenance in pediatric pts with R/R *FLT3*-ITD$^+$ AML.

**Figure.** Baseline demographics and disease characteristics.

| | Safety analyses set (N=16) |
|---|---|
| Age, years, median (range) | 13.5 (3–17) |
| Weight, kg, median (range) | 51.1 (17–84) |
| BSA, m$^2$, median (range) | 1.54 (0.7–2) |
| Relapsed AML, n (%) | 5 (31) |
| Refractory AML, n (%) | 11 (69) |
| Time from AML diagnosis to R/R, weeks, median (range) | 7.0 (3–75) |
| Risk status with specific cytogenetic patterns, n (%) | |
| Intermediate | 1 (6) |
| Unfavorable | 13 (81) |
| Unknown | 2 (13) |
| AML subtype, n (%) | |
| *AML with recurrent genetic abnormalities* | |
| AML with mutated *RUNX1* | 1 (6) |
| Unknown | 1 (6) |
| *AML not otherwise categorized* | |
| AML with minimal differentiation | 2 (13) |
| AML without maturation | 2 (13) |
| AML with maturation | 5 (31) |
| Acute monoblastic/monocytic leukemia | 3 (19) |
| Unknown | 2 (13) |
| Prior allogeneic transplant, n (%) | 3 (19) |

AML, acute myeloid leukemia; BSA, body surface area; R/R, relapsed/refractory.

Copyright Information: (Online) ISSN: 2572-9241
©2026 The Author(s). HemaSphere is published by John Wiley & Sons Ltd on behalf of European Hematology Association. This is an open access Abstract Book distributed under the Attribution-NonCommercial-NoDerivs (CC BY-NC-ND), which allows third parties to download the articles and share them with others as long as they credit the author and the Abstract Book, but they cannot change the content in any way or use them commercially.
Abstract Book Citations: Authors, Title, HemaSphere, 2026;10:(S1):pages. The abstract book DOIs can be found on the “Author Information” page.
Disclaimer: Articles published in the journal HemaSphere exclusively reflect the opinions of the authors. The authors are responsible for all content in their abstracts including accuracy of the facts, statements, citing resources, etc.

### (PF507) RESULTS FROM THE PHASE 1 MONOTHERAPY DOSE ESCALATION OF APL-4098, AN ORAL GCN2 INHIBITOR, IN RELAPSED/REFRACTORY ACUTE MYELOID LEUKEMIA (R/R AML) AND MYELODYSPLASTIC SYNDROMES (MDS)/AML PATIENTS

**Topic:** 04. Acute myeloid leukemia - Clinical

Shaun Fleming[1, 2], Shuh Ying Tan[3], Mridula Mokoonlall[1, 2], Andrew McQuillan[4], Paul Yeh[5, 6], Hun Chuah[7], Rueben Amofa[8], David Taussig[9], Laurie Green[10], Misba Beerahee[11], Brian Hamilton[12], Nadine Clemo*[13], Sanjay Aggarwal[10], Richard Dillon[14, 15]

*[1]Alfred Hospital, Department of Clinical Haematology, Melbourne, Australia;[2]Monash University, Australian Centre for Blood Diseases, Melbourne, Australia; [3]St. Vincent's Hospital, Melbourne, Australia; [4]Hollywood Private Hospital , University of Notre Dame, Perth, Australia; [5]Monash University, Department of Medicine, School of Clinical Sciences at Monash Health, Clayton, Australia; [6]Monash Health, Monash Haematology, Clayton, Australia;[7]Royal Perth Hospital, Haematology Service, Perth, Australia; [8]Sarah Cannon Research Institute UK, London, United Kingdom;[9]Royal Marsden, London, United Kingdom; [10]Apollo Therapeutics Ltd, Boston, United States of America; [11]MBPharmaco Consulting Ltd, London, United Kingdom; [12]BioPharm Solutions, Inc, Newton, MA, United States of America; [13]Apollo Therapeutics Ltd, Cambridge, United Kingdom; [14]Guy's Hospital, London, United Kingdom; [15]Kings College London, London, United Kingdom;*

### Background

There remains an unmet medical need for new effective therapies to manage R/R AML and MDS/AML. General control nondepressible 2 (GCN2) is an evolutionarily conserved kinase and a pivotal regulator of the integrated stress response (ISR). Activation of GCN2 in response to amino acid deprivation results in the attenuation of global protein synthesis and an orchestrated transcriptional rescue response. GCN2 activation is a mechanism by which tumor cells, including leukemic cells, can survive under nutrient stress. GCN2 inhibitors may thus represent a novel class of therapeutic agents with anti-leukemic activity. APL-4098 is a potent small molecule inhibitor of GCN2. APL-4098 inhibits AML leukemic cell viability *ex vivo* and reduces AML tumor burden in non-clinical *in vivo* models where APL-4098 depletes AML patient-derived (PDX) leukemic stem cells (LSCs) and acts synergistically with the BCL-2 inhibitor venetoclax.

### Aims

The purpose of this Phase 1 study (NCT06372717) was to establish the Recommended Phase 2 Dose (RP2D) for APL-4098. Additional objectives included assessment of preliminary efficacy and pharmacodynamics (PD) of APL-4098.

### Methods

The study included APL-4098 dose escalation as monotherapy. Dose escalation was performed initially using an Accelerated Titration Design (ATD) and then the Bayesian Optimal Interval (BOIN) design. The study enrolled patients with R/R AML and MDS/AML with no available therapies known to provide clinical benefit. APL-4098 was administered orally once daily in 28-day cycles until disease progression, unacceptable toxicity, or patient withdrawal from the study.

**Copyright Information:** (Online) ISSN: 2572-9241
©2026 The Author(s). HemaSphere is published by John Wiley & Sons Ltd on behalf of European Hematology Association. This is an open access Abstract Book distributed under the Attribution-NonCommercial-NoDerivs (CC BY-NC-ND), which allows third parties to download the articles and share them with others as long as they credit the author and the Abstract Book, but they cannot change the content in any way or use them commercially.
**Abstract Book Citations:** Authors, Title, HemaSphere, 2026;10:(S1):pages. The abstract book DOIs can be found on the "Author Information" page.
**Disclaimer:** Articles published in the journal HemaSphere exclusively reflect the opinions of the authors. The authors are responsible for all content in their abstracts including accuracy of the facts, statements, citing resources, etc.

**Results**

28 patients have been treated with APL-4098 monotherapy across 5 dose levels ranging from 20 to 150 mg. The median age was 73 years (range: 37-84). 26 (93%) patients had R/R AML of which 21 (81%) had adverse genetics; 2 patients had MDS/AML. The median prior lines of therapy was 2 (range: 1-6). The median duration of treatment was 41 days (range: 9-165). APL-4098 was well tolerated up to 100 mg. Dose limiting toxicities (DLTs) of diarrhoea, nausea and a reversible increase in alanine aminotransferase levels (not associated with bilirubin increase) were observed in the 150 mg dose level in 2 patients. Treatment-emergent adverse events (TEAEs) have been predominantly grades 1 and 2. Most frequently observed TEAEs suspected to be related to APL-4098 include nausea (57% any grade; 21% grade 3) and diarrhoea (46% any grade; 14% grade 3). A reduction in AML blasts was observed in 8 of 19 evaluable patients. 7 of these had high risk cytogenetics including 2 with *TP53* mutations. 3 patients (including 2 with adverse cytogenetics) achieved >50% reduction in bone marrow blast count. The PK profile of APL-4098 showed linear and dose-proportional characteristics. An exposure-dependent induction in mRNA expression of CHAC-1, a pro-apoptotic stress response gene, was observed indicative of APL-4098 therapeutic activity.

**Summary/Conclusion**

APL-4098 is a potent, selective GCN2 inhibitor that has demonstrated excellent non-clinical potency and efficacy properties. Dose escalation of APL-4098 monotherapy in R/R AML and MDS/AML patients is now complete, confirming a favourable tolerability and safety profile. APL-4098 has shown predictable, dose-proportionate PK supporting once daily oral dosing and has demonstrated exposure-driven PD activity. APL-4098 is currently being evaluated in combination studies with azacitidine and venetoclax / azacitidine in R/R AML and newly diagnosed AML patients.

**Copyright Information:** (Online) ISSN: 2572-9241
©2026 The Author(s). HemaSphere is published by John Wiley & Sons Ltd on behalf of European Hematology Association. This is an open access Abstract Book distributed under the Attribution-NonCommercial-NoDerivs (CC BY-NC-ND), which allows third parties to download the articles and share them with others as long as they credit the author and the Abstract Book, but they cannot change the content in any way or use them commercially.
**Abstract Book Citations:** Authors, Title, HemaSphere, 2026;10:(S1):pages. The abstract book DOIs can be found on the “Author Information” page.
**Disclaimer:** Articles published in the journal HemaSphere exclusively reflect the opinions of the authors. The authors are responsible for all content in their abstracts including accuracy of the facts, statements, citing resources, etc.

### (PF508) LONG-TERM FOLLOW-UP OF PEDIATRIC/YOUNG ADULT PATIENTS WITH RELAPSED/REFRACTORY KMT2AR ACUTE LEUKEMIA TREATED WITH REVUMENIB IN AUGMENT-101

**Topic:** 04. Acute myeloid leukemia - Clinical

Branko Cuglievan*[1], Neerav Shukla[2], Nicole Karras[3], Bill Chang[4], Jeffrey Magee[5], David M. Loeb[6], Adam S. Duvall[7], James A. Whitlock[8], Lincy Thomas[9], Li Yu[9], C. Michel Zwaan[10]

*[1]The University of Texas MD Anderson Cancer Center, Houston, TX, United States of America;[2]Memorial Sloan Kettering Cancer Center, New York, NY, United States of America; [3]City of Hope National Medical Center, Duarte, CA, United States of America; [4]Knight Cancer Institute, Oregon Health & Science University, Portland, OR, United States of America;[5]Washington University School of Medicine, St Louis, MO, United States of America; [6]The Children's Hospital at Montefiore, Bronx, NY, United States of America; [7]Department of Medicine, University of Chicago, Chicago, IL, United States of America;[8]University of Toronto, The Hospital for Sick Children, Toronto, Canada; [9]Syndax Pharmaceuticals, Inc., New York, NY, United States of America; [10]Princess Máxima Center, Utrecht, and Erasmus MC Sophia Children's Hospital, Rotterdam, The Netherlands;*

### Background

*KMT2A* rearrangements (*KMT2A*r) are frequently present in patients (pts) with acute leukemia (AL) and are associated with aggressive disease and high relapse rates. In pediatric pts, 5-y event-free survival (EFS) ranges from 29%–61%, and 44%–65% for overall survival (OS). Revumenib, a first-in-class, oral, potent, and selective inhibitor of the menin-KMT2A interaction, is used for the treatment (tx) of relapsed/refractory (R/R) AL harboring a *KMT2A* translocation or R/R acute myeloid leukemia (AML) with *NPM1* mutation in adult and pediatric pts 1 year and older.

### Aims

To report long-term follow-up data for pediatric/young adult pts with R/R *KMT2A*r AL treated with revumenib monotherapy in AUGMENT-101 (NCT04065399).

### Methods

AUGMENT-101 is an ongoing phase 1/2, open-label, dose-escalation/-expansion study of revumenib in pts with R/R *KMT2A*r and *NPM1*-mutated AL. Pts received revumenib 160 mg q12h orally (95 mg/m$^2$ if <40 kg) with a strong CYP3A4 inhibitor, or 270 mg (160 mg/m$^2$ if <40 kg) without, in 28-day cycles. Primary endpoints were rate of complete remission (CR)+CR with partial hematologic recovery (CRh) and safety/tolerability. Secondary endpoints were overall response rate (ORR), duration of response, time to response, EFS, and OS. This pooled phase 1/2 data analysis evaluated long-term efficacy and safety of revumenib in pts 30 days to <21 y old. Efficacy outcomes are shown by AL subtype in the efficacy population (pts with *KMT2A*r [local testing], treated at recommended phase 2 dose, with ≥5% baseline morphological blasts).

**Copyright Information:** (Online) ISSN: 2572-9241
©2026 The Author(s). HemaSphere is published by John Wiley & Sons Ltd on behalf of European Hematology Association. This is an open access Abstract Book distributed under the Attribution-NonCommercial-NoDerivs (CC BY-NC-ND), which allows third parties to download the articles and share them with others as long as they credit the author and the Abstract Book, but they cannot change the content in any way or use them commercially.
**Abstract Book Citations:** Authors, Title, HemaSphere, 2026;10:(S1):pages. The abstract book DOIs can be found on the "Author Information" page.
**Disclaimer:** Articles published in the journal HemaSphere exclusively reflect the opinions of the authors. The authors are responsible for all content in their abstracts including accuracy of the facts, statements, citing resources, etc.

### Results

As of 26Feb2025, 38 *KMT2A*r pts were in the safety population (27 AML, 8 acute lymphoblastic leukemia [ALL], 3 mixed-phenotype acute leukemia [MPAL]); median (range) age was 6.0 y (1.3–20.0), 2.5 y (0.6–20.0), and 1.5 y (1.0–2.0), respectively. In the efficacy population (N=36), CR+CRh rate was 30.8% (8/26) in AML, 14.3% (1/7) in ALL, and 33.3% (1/3) in MPAL pts; measurable residual disease negativity for CR+CRh was achieved by 6/7 pts with AML and 1/1 with MPAL. All subgroups demonstrated responses in ~1 mo (range, 0.9–1.8) that were durable (**Table**). Median (95% CI) EFS was 4.5 mo (2.3–11.7) for AML, 1.9 mo (0.3–not reached [NR]) for ALL, and 5.0 mo (0.9–NR) for MPAL; median (95% CI) OS was 6.9 mo (2.9–NR), 3.9 mo (0.3–NR), and 5.7 mo (0.9–NR), respectively. Among pts included in ORR, 11 pts (10 AML, 1 MPAL) proceeded to hematopoietic stem cell transplant (HSCT) while in remission; 3 AML pts resumed revumenib post HSCT.

Grade ≥3 tx-emergent adverse events (TEAEs) occurred in 92% of pts (tx-related AE [TRAE] in 37% of pts). Three (8%) pts with AML had a TEAE that led to tx discontinuation (febrile neutropenia, sepsis, and septic shock, n=1 each). No deaths were tx related. Differentiation syndrome (DS) occurred in 13 (34%) pts (4 grade ≥3; prophylaxis was not protocol mandated); 11 (85%) pts recovered/resolved with steroid or hydroxyurea tx; none discontinued tx due to DS. QTcF prolongation occurred in 8 (21%) pts (2 grade 3; no grade ≥4); all pts recovered/resolved; none discontinued tx due to QTcF prolongation (**Table**).

### Summary/Conclusion

Revumenib demonstrated clinically meaningful responses in pediatric/young adult pts with R/R*KMT2A*r AML, ALL, and MPAL. The safety profile of revumenib was consistent with previously reported data and remained generally manageable, with no new safety signals identified. These findings support revumenib as an effective tx option for pediatric pts with R/R *KMT2A*r AL across multiple leukemia types.

**Table. Efficacy and safety of revumenib in pediatric/young adult patients with relapsed/refractory *KMT2A*r acute leukemia**

| | Efficacy population (N=36) | | |
|---|---|---|---|
| | AML (n=26) | ALL (n=7) | MPAL (n=3) |
| **ORR,[a] n (%)** | 15 (57.7) | 3 (42.9) | 2 (66.7) |
| 95% CI | 36.9–76.6 | 9.9–81.6 | 9.4–99.2 |
| **Duration of ORR, mo (95% CI)** | 4.8 (2.0–NR) | 1.4 (0.9–NR) | NR (3.6–NR) |
| **Time to ORR, mo, median (range)** | 0.99 (0.9–1.8) | 0.99 (0.9–1.8) | 1.23 (1.1–1.4) |
| **CR+CRh, n (%)** | 8 (30.8) | 1 (14.3) | 1 (33.3) |
| 95% CI | 14.3–51.8 | 0.4–57.9 | 0.8–90.6 |
| **MRD-negative status,[b,c] n/N (%)** | | | |
| CR+CRh | 6/7 (85.7) | 0 | 1/1 (100) |
| **Response in pts proceeded to HSCT, n/N (%)** | | | |
| CR+CRh | 6/10 (60.0) | 0 | 0/1 (0) |
| | Safety population (N=38) | | |
| | AML (n=27) | ALL (n=8) | MPAL (n=3) |
| **TEAEs leading to dose modification, n (%)** | 7 (26) | 3 (38) | 2 (67) |
| Treatment-related | 2 (7) | 1 (13) | 1 (33) |
| **Differentiation syndrome, n (%)** | 11 (41) | 1 (13) | 1 (33) |
| Time to onset, days, median (range) | 7.0 (3.0–41.0) | 9.0 (9.0–9.0) | 3.0 (3.0–3.0) |
| Duration, days, median (range) | 12.5 (4.0–30.0) | 25.0 (25.0–25.0) | 19.0 (19.0–19.0) |
| **QTcF prolongation, n (%)** | 7 (26) | 1 (13) | 0 |
| Time to onset, days, median (range) | 4.0 (1.0–56.0) | 4.0 (4.0–4.0) | NA |
| Duration, days, median (range) | 1.0 (1.0–8.0) | 18.0 (18.0–18.0) | NA |

[a]ORR=CR+CRh+CRi+CRp+MLFS+PR divided by number of evaluable patients. [b]Among those with MRD status available. [c]Determined by flow cytometry or PCR, assessed locally. ALL, acute lymphoblastic leukemia; AML, acute myeloid leukemia; CI, confidence interval; CR, complete remission; CRh, CR with partial hematologic recovery; CRi, CR with incomplete hematologic recovery; CRp, CR with incomplete platelet recovery; MLFS, morphologic leukemia-free state; mo, months; MPAL, mixed-phenotype acute leukemia; MRD, measurable residual disease; NA; not applicable; NR, not reached; ORR, overall response rate; PR, partial response.

**Copyright Information:** (Online) ISSN: 2572-9241
©2026 The Author(s). HemaSphere is published by John Wiley & Sons Ltd on behalf of European Hematology Association. This is an open access Abstract Book distributed under the Attribution-NonCommercial-NoDerivs (CC BY-NC-ND), which allows third parties to download the articles and share them with others as long as they credit the author and the Abstract Book, but they cannot change the content in any way or use them commercially.
**Abstract Book Citations:** Authors, Title, HemaSphere, 2026;10:(S1):pages. The abstract book DOIs can be found on the "Author Information" page.
**Disclaimer:** Articles published in the journal HemaSphere exclusively reflect the opinions of the authors. The authors are responsible for all content in their abstracts including accuracy of the facts, statements, citing resources, etc.

### (PF509) TRIPLET THERAPY WITH QUIZARTINIB, DECITABINE, AND VENETOCLAX IN NEWLY DIAGNOSED AND RELAPSED/REFRACTORY FLT3-ITD ACUTE MYELOID LEUKEMIA

**Topic:** 04. Acute myeloid leukemia - Clinical

Musa Yilmaz*[1], Muharrem Muftuoglu[1], Courtney Dinardo[1], Tapan Kadia[1], Marina Konopleva[1], Onyee Chan[2], Gautam Borthakur[1], Naveen Pemmaraju[1], Nicholas Short[1], Yesid Alvarado[1], Abhishek Maiti[1], Lucia Masarova[1], Guillermo Montalban-Bravo[1], Sanam Loghavi[1], David Sallman[2], Elias Jabbour[1], Guillermo Garcia-Manero[1], Farhad Ravandi[1], Michael Andreeff[1], Naval Daver[1]

*[1]MD Anderson Cancer Center, Houston, United States of America;[2]Moffitt Cancer Center, Tampa, United States of America;*

### Background

Venetoclax (VEN) combined with hypomethylating agents (HMAs; azacitidine or decitabine) is standard therapy for newly diagnosed AML patients ineligible for intensive chemotherapy (IC). Outcomes remain suboptimal in FLT3-ITD–mutated disease, which is associated with resistance and inferior survival. Quizartinib (QUIZ), a selective FLT3 inhibitor, may enhance the efficacy of HMA+VEN in this molecular subset.

### Aims

The primary objectives were to determine the recommended phase 2 dose (RP2D) and optimal schedule of DAC + VEN + Quiz in R/R or newly diagnosed FLT3m AML.

### Methods

The study included newly diagnosed pts ineligible for IC and R/R pts who had received ≤ 6 prior treatments for AML. On cycle 1 Day 14, all pts underwent bone marrow (BM) evaluation, and VEN (400 mg/day or equivalent with azole) was discontinued on cycle 1 Day 14 for pts with BM blasts ≤ 5% (or marrow aplasia/hypocellularity). Pts with cycle 1 Day 14 BM blast >5% continued VEN for 21 days. Pts received 10 days of DAC (20 mg/m2) for induction, followed by 5-day DAC in subsequent cycles. Quiz (26.5 or 35.4 mg/day) was continuously administered daily.

**Copyright Information:** (Online) ISSN: 2572-9241
©2026 The Author(s). HemaSphere is published by John Wiley & Sons Ltd on behalf of European Hematology Association. This is an open access Abstract Book distributed under the Attribution-NonCommercial-NoDerivs (CC BY-NC-ND), which allows third parties to download the articles and share them with others as long as they credit the author and the Abstract Book, but they cannot change the content in any way or use them commercially.
**Abstract Book Citations:** Authors, Title, HemaSphere, 2026;10:(S1):pages. The abstract book DOIs can be found on the “Author Information” page.
**Disclaimer:** Articles published in the journal HemaSphere exclusively reflect the opinions of the authors. The authors are responsible for all content in their abstracts including accuracy of the facts, statements, citing resources, etc.

**Results**

A total of 88 patients were treated (frontline, n=42; R/R, n=46). QUIZ 26.5 mg daily was established as the RP2D.
Frontline cohort:
Median age was 70 years (range, 62–85), with 36% ≥75 years. AML was de novo in 60%, secondary in 28%, and therapy-related in 12%. Frequent co-mutations included DNMT3A (43%), NPM1 (33%), and RUNX1 (31%). CR, CRi, and MLFS rates were 72% (n=29), 17% (n=7), and 2% (n=1), respectively. At end of cycle 1 (EOC1), MRD negativity by multicolor flow cytometry (MFC; sensitivity 0.01%) was 58% (19/32), and FLT3-ITD clearance by NGS ($5\times10^{-5}$ sensitivity) was 27% (5/18). Best MRD negativity rates were 68% (23/34) by MFC and 83% (15/18) by FLT3-NGS.
Median time to ANC >500, ANC >1000, and platelets >50K was 40 (19–72), 41 (14–72), and 35 (16–71) days. After a median follow-up of 14.5 months, median relapse-free survival (RFS) was 24.7 months and median overall survival (OS) was 36.5 months. Fifteen patients (36%) underwent allogeneic stem cell transplantation (ASCT) in CR1. OS was not reached in transplanted patients versus 36.6 months in non-transplanted patients (p=0.65; landmark analysis). Patients received a median of 3 cycles (range, 1–39); 11 remain on study. Treatment discontinuation (n=31) occurred due to ASCT (15), relapse (9), physician/patient decision (3), induction death (1), death in CR (1), death with active disease (1), and hospice (1).
Grade ≥3 non-hematologic adverse events occurring in >5% included febrile neutropenia (37%), pneumonia (33%), infections (17%), pain (10%), ALT elevation (10%), fracture (10%), sepsis (9%), hypertension (7%), hypotension (7%), hyponatremia (7%), gait disturbance (7%), oral mucositis (7%), and pleural effusion (7%).
Relapsed/Refractory cohort:
CR/CRi was achieved in 28% (n=13), with MLFS in 33% (n=15). Seventeen patients (37%) proceeded to ASCT. Median OS was 6.3 months.

**Summary/Conclusion**

The triplet of QUIZ, decitabine, and VEN demonstrated substantial CR/CRi rates, deep MRD clearance, and encouraging survival in newly diagnosed FLT3-ITD AML unfit for intensive therapy.

© 2026 American Society of Clinical Oncology, Inc. Reused with permission. This abstract was accepted and previously presented at the 2026 ASCO Annual Meeting. All rights reserved.

**Copyright Information:** (Online) ISSN: 2572-9241
©2026 The Author(s). HemaSphere is published by John Wiley & Sons Ltd on behalf of European Hematology Association. This is an open access Abstract Book distributed under the Attribution-NonCommercial-NoDerivs (CC BY-NC-ND), which allows third parties to download the articles and share them with others as long as they credit the author and the Abstract Book, but they cannot change the content in any way or use them commercially.
**Abstract Book Citations:** Authors, Title, HemaSphere, 2026;10:(S1):pages. The abstract book DOIs can be found on the “Author Information” page.
**Disclaimer:** Articles published in the journal HemaSphere exclusively reflect the opinions of the authors. The authors are responsible for all content in their abstracts including accuracy of the facts, statements, citing resources, etc.

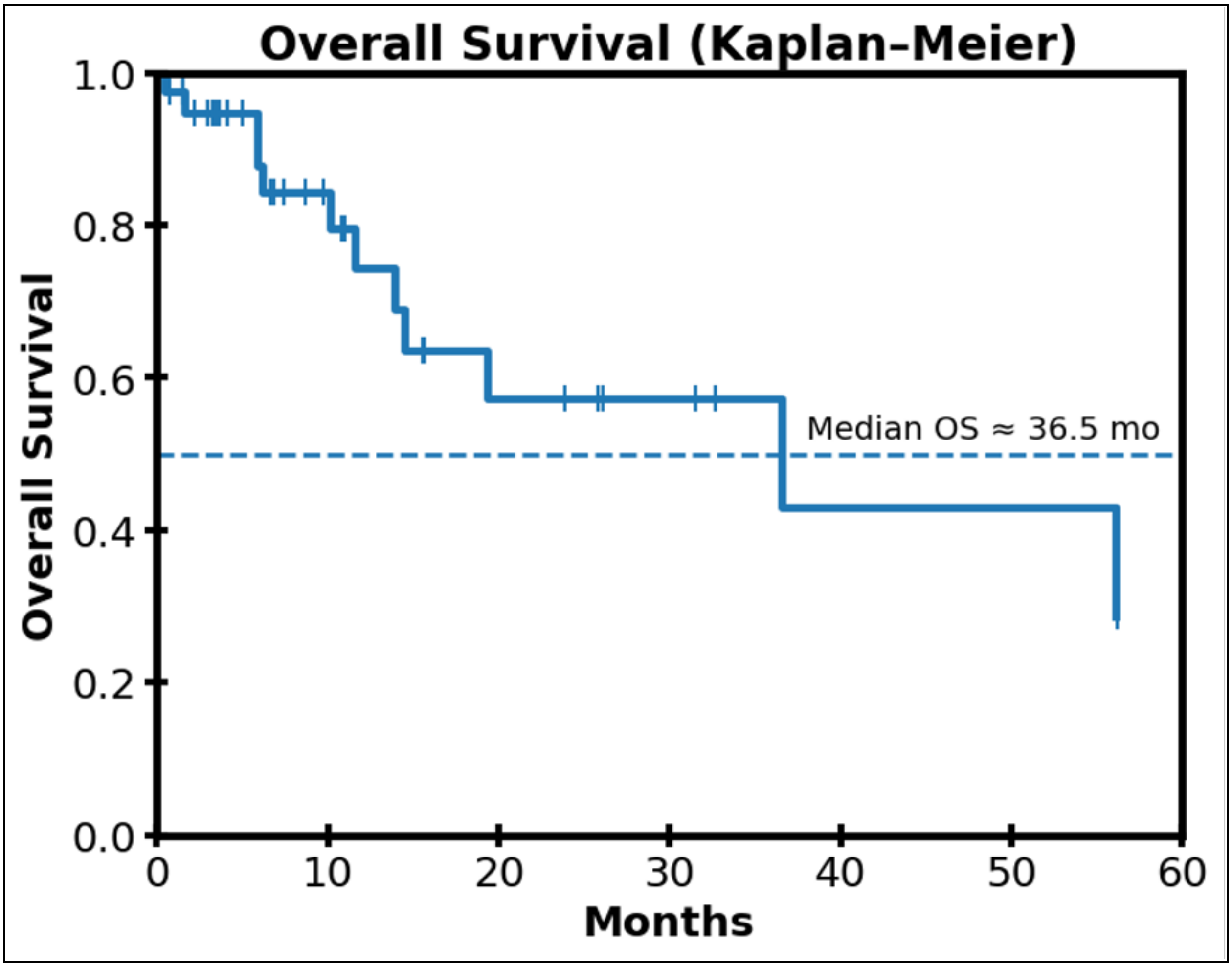

Figure. Overall Survival of Patients with Newly Diagnosed FLT3-ITD Mutated AML

**Copyright Information:** (Online) ISSN: 2572-9241
©2026 The Author(s). HemaSphere is published by John Wiley & Sons Ltd on behalf of European Hematology Association. This is an open access Abstract Book distributed under the Attribution-NonCommercial-NoDerivs (CC BY-NC-ND), which allows third parties to download the articles and share them with others as long as they credit the author and the Abstract Book, but they cannot change the content in any way or use them commercially.
**Abstract Book Citations:** Authors, Title, HemaSphere, 2026;10:(S1):pages. The abstract book DOIs can be found on the "Author Information" page.
**Disclaimer:** Articles published in the journal HemaSphere exclusively reflect the opinions of the authors. The authors are responsible for all content in their abstracts including accuracy of the facts, statements, citing resources, etc.

### (PF510) HYPOMETHYLATING AGENTS PLUS VENETOCLAX IN YOUNGER AML: META-ANALYSIS OF A SHIFTING TREATMENT PARADIGM

**Topic:** 04. Acute myeloid leukemia - Clinical

Matteo Molica*[1], Laura De Fazio[1], Sebastian Monachetti[2], Salvatore Perrone[2]

*[1]AOU RENATO DULBECCO, CATANZARO, Italy; [2]Polo Universitario Pontino, LATINA, Italy;*

### Background

Intensive chemotherapy (IC) followed by consolidation and/or allogeneic hematopoietic stem cell transplantation (HSCT) remains the standard of care for younger, fit patients with acute myeloid leukemia (AML). However, IC is associated with significant toxicity and prolonged hospitalization, and outcomes remain heterogeneous across molecular subgroups. The combination of hypomethylating agents (HMA) and venetoclax has transformed the management of older or unfit AML patients, yielding high complete remission (CR) rates and measurable residual disease (MRD) negativity. Its role in younger, potentially transplant-eligible patients is not yet clearly defined.

### Aims

To systematically evaluate efficacy outcomes of HMA plus venetoclax in AML patients with median age <70 years, focusing on response rates, MRD negativity, survival, and feasibility of bridging to HSCT.

### Methods

This systematic review and meta-analysis was conducted according to PRISMA guidelines and registered in PROSPERO. MEDLINE and the Cochrane Library were searched through February 2026, including major hematology conference proceedings. Eligible studies enrolled AML patients treated with HMA plus venetoclax and reported a median age <70 years. Primary endpoints were CR/CRi rate, MRD negativity, 1-year overall survival (OS), 1-year event-free survival (EFS), and HSCT rate. Pooled estimates were calculated using random-effects models. Heterogeneity was assessed using Cochran's Q and $I^2$ statistics. Meta-regression explored the impact of age and HMA backbone (azacitidine vs decitabine).

### Results

Eight studies (2 randomized trials, 2 phase II trials, 4 real-world studies) including 673 patients were analyzed. Mean age was 54 years (range of study medians: 39–68). Three studies used decitabine, three azacitidine, and two mixed regimens.

The pooled CR/CRi rate (6 studies, n=609) was 66% (95% CI, 48–85%), with substantial heterogeneity ($I^2$=93%). Among evaluable studies, the pooled MRD-negative rate was 69% (95% CI, 49–90%; $I^2$=83%), indicating frequent deep remissions.

The pooled 1-year EFS (5 studies, n=530) was 59% (95% CI, 53–65%) with minimal heterogeneity ($I^2$=3%), representing the most consistent endpoint across datasets. The pooled 1-year OS (5 studies, n=537) was 75% (95% CI, 63–86%; $I^2$=74%), comparing favorably with historical population-based cohorts reporting approximately 60–65% 1-year OS in similarly aged patients treated with conventional approaches.

Across all studies, 66% of patients (95% CI, 48–84%) proceeded to allogeneic HSCT, supporting the feasibility of HMA/venetoclax as an effective bridge-to-transplant strategy. Meta-regression suggested a trend toward improved EFS and OS in studies employing decitabine rather than azacitidine, and superior EFS in cohorts with median age ≤45 years, generating hypotheses for future prospective evaluation.

**Copyright Information:** (Online) ISSN: 2572-9241
©2026 The Author(s). HemaSphere is published by John Wiley & Sons Ltd on behalf of European Hematology Association. This is an open access Abstract Book distributed under the Attribution-NonCommercial-NoDerivs (CC BY-NC-ND), which allows third parties to download the articles and share them with others as long as they credit the author and the Abstract Book, but they cannot change the content in any way or use them commercially.
**Abstract Book Citations:** Authors, Title, HemaSphere, 2026;10:(S1):pages. The abstract book DOIs can be found on the "Author Information" page.
**Disclaimer:** Articles published in the journal HemaSphere exclusively reflect the opinions of the authors. The authors are responsible for all content in their abstracts including accuracy of the facts, statements, citing resources, etc.

**Summary/Conclusion**

In younger AML patients, HMA plus venetoclax is associated with high remission rates, frequent MRD negativity, encouraging 1-year survival, and substantial transition to HSCT. Notably, EFS outcomes were consistent despite heterogeneous study designs, reinforcing the robustness of disease control with this approach. While IC remains the current standard, these data provide a strong biological and clinical rationale for prospective randomized trials directly comparing HMA/venetoclax with IC-based regimens within risk-adapted, molecularly informed treatment algorithms.

**Copyright Information:** (Online) ISSN: 2572-9241
©2026 The Author(s). HemaSphere is published by John Wiley & Sons Ltd on behalf of European Hematology Association. This is an open access Abstract Book distributed under the Attribution-NonCommercial-NoDerivs (CC BY-NC-ND), which allows third parties to download the articles and share them with others as long as they credit the author and the Abstract Book, but they cannot change the content in any way or use them commercially.
**Abstract Book Citations:** Authors, Title, HemaSphere, 2026;10:(S1):pages. The abstract book DOIs can be found on the "Author Information" page.
**Disclaimer:** Articles published in the journal HemaSphere exclusively reflect the opinions of the authors. The authors are responsible for all content in their abstracts including accuracy of the facts, statements, citing resources, etc.

### (PF511) OUTCOMES AND RISK STRATIFICATION WITH HIGH-DOSE CYTARABINE, MITOXANTRONE, AND VENETOCLAX IN RELAPSED OR REFRACTORY ACUTE MYELOID LEUKEMIA

**Topic:** 04. Acute myeloid leukemia - Clinical

Andrew Berdel*[1], Christoph Röllig[2], Clara Burkhard-Meier[3], Marco Tembrink[4], Julia Unglaub[5], Tim Sauer[5], Martina Crysandt[6], Edgar Jost[6], Paul Jaeger[7], Christoph Kiefel[8], Lars Fransecky[8], Krischan Braitsch[9], Katharina Götze[9], Kai Wille[10], Kerstin Schäfer-Eckart[11], Klaus Metzeler[12], Sara Hilber[13], Julian Ronnacker[1], Marcel Kemper[1], Nardos Hölscher[14], Klaus Wethmar[1], Thomas Schröder[4], Sylvia Herold[15], David Poitz[16], Björn Steffen[17], Andreas Rank[18], Mathias Hänel[19], Jan Moritz Middeke[2], Martin Kaufmann[20], Andreas Burchert[21], Johannes Schetelig[2], Uwe Platzbecker[2], Christian Thiede[2], Matthias Stelljes[1], Georg Lenz[1], Hubert Serve[17], Claudia Baldus[8], Martin Bornhäuser[2], Carsten Müller-Tidow[5], Jan-Henrik Mikesch[1], Lukas Frenzel[3, 22, 23], Maher Hanoun[4], Leo Ruhnke[2], Christoph Schliemann[1]

*[1]University Hospital Münster, Department of Medicine A, Münster, Germany;[2]University Hospital TU Dresden, Department of Internal Medicine I, Dresden, Germany; [3]University of Cologne, Faculty of Medicine and Cologne University Hospital, Department I of Internal Medicine and Center for Integrated Oncology (CIO) Aachen, Bonn, Cologne, Düsseldorf (ABCD), Cologne, Germany; [4]University of Duisburg-Essen, University Hospital Essen, Department of Hematology, Essen, Germany;[5]Medical Faculty, University of Heidelberg and Department of Medicine, Hematology, Oncology and Rheumatology, University Hospital Heidelberg, Heidelberg, Germany; [6]Clinic for Hematology, Oncology, Hemostaseology and Stem Cell Transplantation University Hospital Aachen & Center for Integrated Oncology CIO ABCD, Aachen, Germany; [7]University of Düsseldorf, Medical Faculty, Department of Hematology, Oncology and Clinical Immunology, Düsseldorf, Germany; [8]University Hospital Schleswig-Holstein, Department of Hematology and Oncology, Campus Kiel, Kiel, Germany; [9]TUM University Hospital Munich, Department of Medicine III, Munich, Germany; [10]University Clinic for Hematology, Oncology, Hemostaseology and Palliative Care, Johannes Wesling Medical Center Minden, University Hospital of the University of Bochum, Minden, Germany; [11]Paracelsus Medical University, Nuremberg Hospital North, Department of Internal Medicine V, Nuremberg, Germany; [12]University of Leipzig Medical Center, Department for Hematology, Cell Therapy and Hemostaseology, Leipzig, Germany; [13]Department of Internal Medicine III, Heinrich-Braun-Klinikum gGmbH, Zwickau, Germany; [14]University Hospital of Münster, Hospital Pharmacy, Münster, Germany; [15]Institute of Pathology, Molecular Pathology, University Hospital Dresden, TUD Dresden University of Technology, Dresden, Germany; [16]Institute of Clinical Chemistry and Laboratory Medicine, University Hospital Dresden, TUD Dresden University of Technology, Dresden, Germany; [17]University Hospital Frankfurt, Department of Internal Medicine II, Frankfurt, Germany; [18]Department of Internal Medicine II, Hematology and Oncology, University Hospital Augsburg, Augsburg, Germany; [19]Department of Internal Medicine III, Klinikum Chemnitz, Chemnitz, Germany;[20]Department of Hematology, Oncology and Palliative Care, Robert-Bosch-Krankenhaus, Stuttgart, Germany; [21]Department of Internal Medicine, Hematology, Oncology and Immunology, University Hospital Marburg, Marburg, Germany; [22]CECAD Cologne Cluster of Excellence on Cellular Stress Responses in Aging-Associated Diseases, Cologne, Germany; [23]Faculty of Medicine and University Hospital of Cologne, Center for Molecular Medicine Cologne (CMMC), Cologne, Germany;*

### Background

Relapsed or refractory acute myeloid leukemia (R/R AML) remains a therapeutic challenge, with high-dose cytarabine-based salvage regimens achieving CR rates of 40-55%. In the phase I/II RELAX trial of the Study Alliance Leukemia (SAL), adding venetoclax (VEN) to high-dose cytarabine and mitoxantrone (HAM) was safe and resulted in CR/CRi in 75% of patients (pts).

### Aims

To evaluate response and survival outcomes in adult pts with R/R AML treated with HAM plus VEN in a real-world setting and to develop a pragmatic prognostic classifier.

**Copyright Information:** (Online) ISSN: 2572-9241
©2026 The Author(s). HemaSphere is published by John Wiley & Sons Ltd on behalf of European Hematology Association. This is an open access Abstract Book distributed under the Attribution-NonCommercial-NoDerivs (CC BY-NC-ND), which allows third parties to download the articles and share them with others as long as they credit the author and the Abstract Book, but they cannot change the content in any way or use them commercially.
**Abstract Book Citations:** Authors, Title, HemaSphere, 2026;10:(S1):pages. The abstract book DOIs can be found on the "Author Information" page.
**Disclaimer:** Articles published in the journal HemaSphere exclusively reflect the opinions of the authors. The authors are responsible for all content in their abstracts including accuracy of the facts, statements, citing resources, etc.

**Methods**

Clinicopathological data of 128 adult pts treated with HAM (cytarabine 1000 mg/m² IV BID days 3-5; mitoxantrone 10 mg/m² IV days 5-7) plus VEN (400 mg days 1-14) between March 2023 and June 2025 were collected under SAL registry or local ethics approval. Real-world data were merged with the RELAX cohort to develop a prognostic model (n = 183). Variable selection used random survival forests (Boruta, randomForestSRC in R), followed by multivariable Cox regression (backward selection, $P < 0.20$) to define the final model. Weighted scores were assigned to generate risk groups stratified by OS.

**Results**

Median age was 60 years (range, 20-74). Thirty-eight percent of pts were primary refractory, 14% had refractory relapsed disease, and 48% had untreated relapse. Median prior therapy lines were 1 (range, 1-5); 38% had prior allogeneic HCT and 25% had prior VEN exposure. By ELN 2022 criteria, genetic risk was favorable, intermediate, and adverse in 27%, 26%, and 47%. Overall CR/CRi rate was 69% (78% in pts with <2 prior lines; 76% in VEN-naïve pts). Sixty-eight percent proceeded to allogeneic HCT. At a median follow-up of 11.5 months (0.3-28), median RFS and OS were not reached; 1-year RFS and OS were 59% and 58%. In transplanted pts, median OS was not reached, with a 1-year OS of 67%. In the combined real-world and RELAX dataset, CR/CRi was 71%. Univariable analyses identified *TP53*, *SF3B1*, or *ASXL1* mutations, complex karyotype (cKT), AML post cytotoxic therapy, antecedent MDS or MDS/MPN, and refractory disease as associated with a lower likelihood of achieving CR/CRi, whereas younger age, one prior line, and no prior VEN exposure predicted higher remission rates. With a median follow-up of 13.7 months (range, 0.3-52.3), median OS was 25 months, with a 1-year OS rate of 60%. A prognostic model identified *TP53* mutation, cKT, ≥2 prior lines, and ECOG PS ≥2 as predictors of inferior outcomes. Pts with none of the variables were classified as favorable risk (score 0, 52% of pts). Pts with any single or combination of the predictors cKT, ≥2 prior lines of therapy, and/or Eastern Cooperative Oncology Group performance status (ECOG PS) ≥2 were classified as intermediate risk (score 1-5, 40%; there were no pts with *TP53* mutation only and no additional risk factor). Pts with *TP53*-mutated AML in combination with any other predictor were classified as poor risk (score ≥6, 8%). One-year OS was 73%, 56%, and 0% for favorable-, intermediate-, and poor-risk pts. Corresponding CR/CRi rates were 85%, 58%, and 36%, and 30-day mortality rates 3%, 8%, and 15%.

**Summary/Conclusion**

HAM plus VEN represents an effective salvage regimen for pts with R/R AML, with robust real-world efficacy and high rates of transition to allogeneic HCT. Survival outcomes varied according to clinical fitness, prior treatment exposure, and disease biology, indicating that careful patient selection may optimize benefit while limiting early toxicity.

**Copyright Information:** (Online) ISSN: 2572-9241
©2026 The Author(s). HemaSphere is published by John Wiley & Sons Ltd on behalf of European Hematology Association. This is an open access Abstract Book distributed under the Attribution-NonCommercial-NoDerivs (CC BY-NC-ND), which allows third parties to download the articles and share them with others as long as they credit the author and the Abstract Book, but they cannot change the content in any way or use them commercially.
**Abstract Book Citations:** Authors, Title, HemaSphere, 2026;10:(S1):pages. The abstract book DOIs can be found on the “Author Information” page.
**Disclaimer:** Articles published in the journal HemaSphere exclusively reflect the opinions of the authors. The authors are responsible for all content in their abstracts including accuracy of the facts, statements, citing resources, etc.

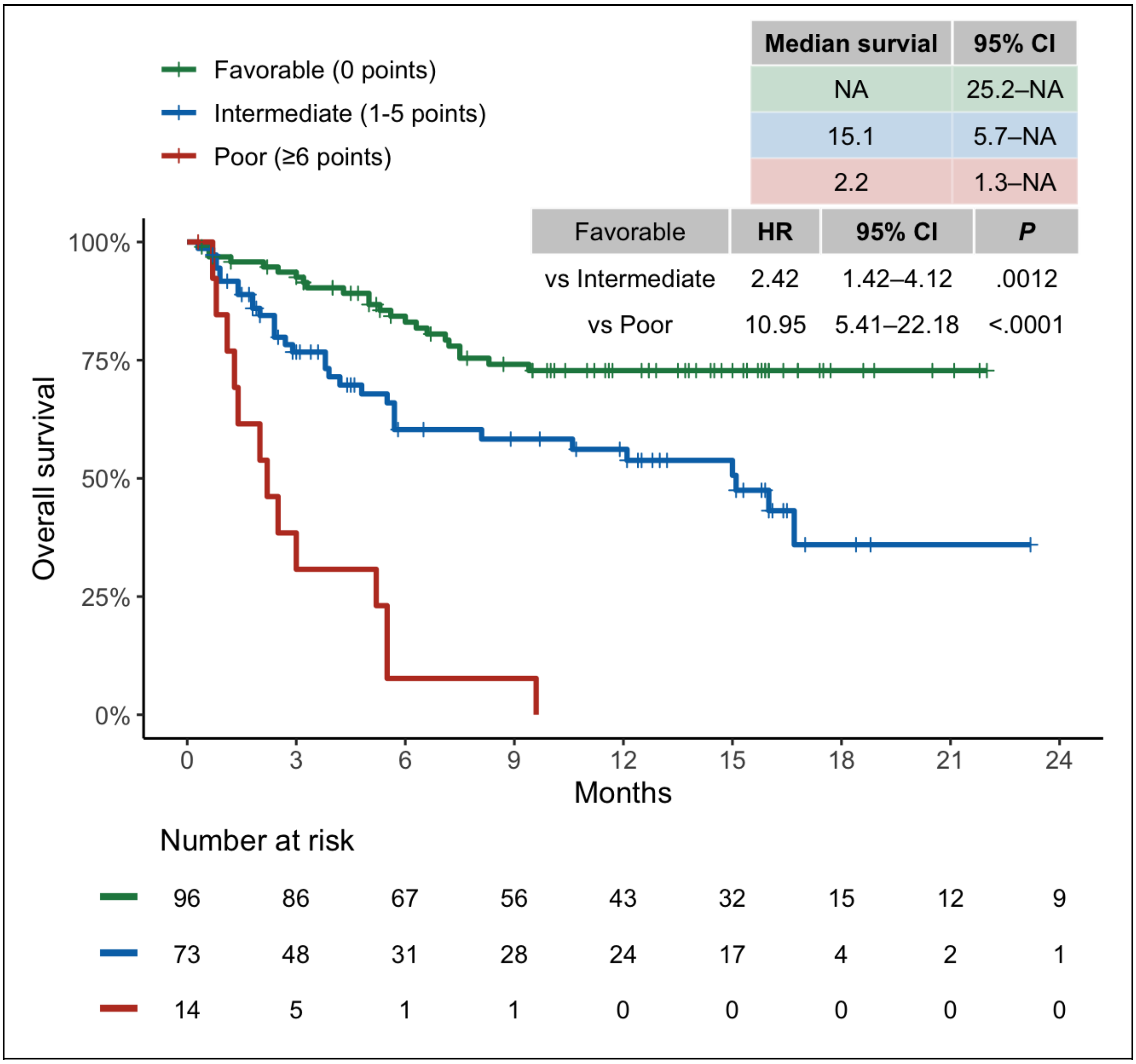

**Copyright Information:** (Online) ISSN: 2572-9241
©2026 The Author(s). HemaSphere is published by John Wiley & Sons Ltd on behalf of European Hematology Association. This is an open access Abstract Book distributed under the Attribution-NonCommercial-NoDerivs (CC BY-NC-ND), which allows third parties to download the articles and share them with others as long as they credit the author and the Abstract Book, but they cannot change the content in any way or use them commercially.
**Abstract Book Citations:** Authors, Title, HemaSphere, 2026;10:(S1):pages. The abstract book DOIs can be found on the "Author Information" page.
**Disclaimer:** Articles published in the journal HemaSphere exclusively reflect the opinions of the authors. The authors are responsible for all content in their abstracts including accuracy of the facts, statements, citing resources, etc.

### (PF512) EFFICACY OF THE TRIPLET THERAPY WITH VENETOCLAX, AZACITIDINE AND GILTERITINIB FOR THE TREATMENT OF FLT3-ITD MUTATED R/R AML PATIENTS BRIDGING TO ALLOGENEIC STEM CELL TRANSPLANTATION

**Topic:** 04. Acute myeloid leukemia - Clinical

Chen Liang*[1], Donglin Yang[1], Yi He[1], Aiming Pang[1], Rongli Zhang[1], Sizhou Feng[1], Erlie Jiang[1]

[1] *Institute of Hematology & Blood Diseases Hospital, Chinese Academy of Medical Sciences & Peking Union Medical College, Tianjin, China;*

### Background

Patients with relapsed or refractory (R/R) acute myeloid leukemia (AML) with FLT3-Internal Tandem Duplication (ITD) mutation have a poor prognosis and high risk of relapse.Triplet therapy with venetoclax[VEN], azacitidine[AZA] and gilteritinib is effective and significantly improves response rates and survival outcomes in this high-risk patient population.

### Aims

We aimed to evaluate the efficacy and safety of the triplet regimen including VEN, AZA and gilteritinib in FLT3-ITD mutated AML patients with R/R disease or persistent minimal residual disease (MRD), as a bridge to allogeneic hematopoietic stem cell transplantation (allo-HSCT).

### Methods

We analyzed 48 FLT3-ITD mutated AML patients in a retrospective cohort study. Patients with R/R disease or persistent MRD received triplet therapy (VEN, AZA and gilteritinib) and bridged to allo-HSCT between August 2021 and June 2024. Patients received myeloablative conditioning regimens followed by post-transplant gilteritinib maintenance therapy. The therapeutic process and clinical outcomes were retrospectively analyzed.

**Copyright Information:** (Online) ISSN: 2572-9241
©2026 The Author(s). HemaSphere is published by John Wiley & Sons Ltd on behalf of European Hematology Association. This is an open access Abstract Book distributed under the Attribution-NonCommercial-NoDerivs (CC BY-NC-ND), which allows third parties to download the articles and share them with others as long as they credit the author and the Abstract Book, but they cannot change the content in any way or use them commercially.
**Abstract Book Citations:** Authors, Title, HemaSphere, 2026;10:(S1):pages. The abstract book DOIs can be found on the “Author Information” page.
**Disclaimer:** Articles published in the journal HemaSphere exclusively reflect the opinions of the authors. The authors are responsible for all content in their abstracts including accuracy of the facts, statements, citing resources, etc.

**Results**

48 adult patients were enrolled in this study, 27 (56.2%) were female and 21 (43.8%) were male. The median age was 41 (range 15-61). Based on prognostic stratification defined by the 2022 European LeukemiaNet (ELN) guidelines, 38 (79.2%) patients were categorized in the Intermediate-risk group and 10 (20.8%) were categorized in the adverse-risk group at diagnosis. At the time of enrollment, 38 (79.2%) patients had R/R disease, of whom 36 cases failed to achieve remission with prior chemotherapy and two cases attained complete remission with MRD positivity. The remaining 10 patients (20.8%) were in CR1 but were detected with persistent positive MRD during chemotherapy.
After one cycle of triplet therapy (VEN, AZA and gilteritinib), 39 (81.3%) patients reached CR/CRi (16 were MRD negative and 23 were MRD positive). The remaining 9 (18.7%) patients showed no treatment response and proceeded to transplantation with active disease. No treatment-related mortality (TRM) was observed. Patients received myeloablative conditioning with a modified Bu/Cy/Cla/Ara-c (busulfan/cyclophosphamide/cladribine/cytarabine) or MCBC (melphalan/cladribine/busulfan/cyclophosphamide) preparative regimen. All patients engrafted successfully, with hematopoietic recovery within 60 days of transplantation. Gilteritinib maintenance therapy started between day +30 to latest day +90 after allo-HSCT.
At the time of this analysis, 40 (83.3%) patients remained alive, while disease relapse had occurred in 9 patients (18.8%). The median follow-up of alive patients was 16.6 months (11-43.2). The 1-year overall survival (OS) and relapse-free survival (RFS) were 83.3% (95% CI 72.7-93.9%) and 75.0% (95% CI 62.7-87.3%), respectively. The leading causes of death were leukemia relapse (n=4), followed by grade IV aGVHD (n=3) and severe infection (n=1). The 1-year incidences of relapse and non-relapse mortality (NRM) were 14.6% (95% CI 6.3-26.1%) and 10.4% (95% CI 3.8-21.0%) , respectively. 19 (39.6%) patients experienced aGVHD and 15 (31.3%) experienced cGVHD.

**Summary/Conclusion**

The triplet therapy of VEN, AZA and gilteritinib resulted in high rates of CR/CRi and enhanced the feasibility of HSCT in FLT3-ITD mutated AML patients with R/R disease or persistent MRD. Sequential allo-HSCT followed by gilteritinib maintenance therapy may significantly improve the long-term prognosis in this high-risk group of patients.

**Copyright Information:** (Online) ISSN: 2572-9241
©2026 The Author(s). HemaSphere is published by John Wiley & Sons Ltd on behalf of European Hematology Association. This is an open access Abstract Book distributed under the Attribution-NonCommercial-NoDerivs (CC BY-NC-ND), which allows third parties to download the articles and share them with others as long as they credit the author and the Abstract Book, but they cannot change the content in any way or use them commercially.
**Abstract Book Citations:** Authors, Title, HemaSphere, 2026;10:(S1):pages. The abstract book DOIs can be found on the "Author Information" page.
**Disclaimer:** Articles published in the journal HemaSphere exclusively reflect the opinions of the authors. The authors are responsible for all content in their abstracts including accuracy of the facts, statements, citing resources, etc.

### (PF513) INDIRECT COMPARISON OF IVOSIDENIB + VENETOCLAX + AZACITIDINE VERSUS DOUBLET REGIMENS IN INTENSIVE CHEMOTHERAPY-INELIGIBLE IDH1-MUTATED ACUTE MYELOID LEUKEMIA

**Topic:** 04. Acute myeloid leukemia - Clinical

Jennifer Marvin-Peek*[1], Weilong Li[2], Tarun Bhagnani[2], Assaf Vestin[2], Anne Angiolillo[2], Emma K. MacKay[3], Yuting Kuang[4], Guillermo Garcia-Manero[1], Courtney Dinardo[1]

*[1]MD Anderson Cancer Center, Houston, United States of America;[2]Servier Pharmaceuticals, Boston, United States of America;*
*[3]IQVIA, Mississauga, Canada;[4]IQVIA, San Mateo, United States of America;*

### Background

For patients with newly diagnosed *IDH1*-mutated (m*IDH1*) AML who are intensive chemotherapy-ineligible (ICIE), azacitidine plus ivosidenib (IVO+AZA) and azacitidine plus venetoclax (VEN+AZA) are established frontline regimens. A prior indirect treatment comparison (ITC) showed favorable overall survival (OS) with IVO+AZA relative to other therapies, consistent with evidence supporting the clinical relevance of *IDH1*-targeted therapy. Emerging data suggest the triplet regimen of ivosidenib, venetoclax, and azacitidine (IVO+VEN+AZA) may further improve outcomes. As no randomized head-to-head trials have yet reported on this regimen compared to either doublet, an ITC helps contextualize comparative efficacy.

### Aims

To assess OS and response outcomes associated with the triplet vs IVO+AZA and VEN+AZA.

### Methods

An unanchored ITC was conducted using individual patient data from the triplet (NCT03471260; n=27) and IVO+AZA (AGILE; n=73), and published aggregate *IDH1*-subgroup data from VEN+AZA (VIALE-A and a prior phase Ib study; n=33). OS was defined as time from treatment initiation to death or last follow-up. Response outcomes included composite complete remission (CRc: CR, CRi, CRh) and CR.
Unadjusted comparisons were performed for the triplet vs IVO+AZA and VEN+AZA. The comparison vs IVO+AZA was additionally evaluated using inverse probability of treatment weighting (IPTW), adjusting for age and ECOG performance status. Cox proportional hazards models and logistic regression were used to estimate hazard ratios (HRs) and odds ratios.
Due to limited baseline covariate reporting for the *IDH1*-mutated subgroup, analyses vs VEN+AZA relied on unadjusted comparisons, supported by matching-adjusted indirect comparison (MAIC) sensitivity analyses using aggregate baseline characteristics from the combined *IDH1/2*-mutated population.

**Copyright Information:** (Online) ISSN: 2572-9241
©2026 The Author(s). HemaSphere is published by John Wiley & Sons Ltd on behalf of European Hematology Association. This is an open access Abstract Book distributed under the Attribution-NonCommercial-NoDerivs (CC BY-NC-ND), which allows third parties to download the articles and share them with others as long as they credit the author and the Abstract Book, but they cannot change the content in any way or use them commercially.
**Abstract Book Citations:** Authors, Title, HemaSphere, 2026;10:(S1):pages. The abstract book DOIs can be found on the “Author Information” page.
**Disclaimer:** Articles published in the journal HemaSphere exclusively reflect the opinions of the authors. The authors are responsible for all content in their abstracts including accuracy of the facts, statements, citing resources, etc.

### Results

In unadjusted analyses, the triplet was associated with significantly longer OS vs IVO+AZA (HR = 0.244; 95% CI: 0.087–0.685) and VEN+AZA (HR = 0.149; 95% CI: 0.048–0.455). The triplet demonstrated higher CRc rates vs IVO+AZA (92.6% vs 54.2%, *P*=0.002) and VEN+AZA (92.6% vs 66.7%, *P*=0.026). CR rates numerically favored the triplet vs IVO+AZA (66.7% vs 47.2%) and were significantly higher vs VEN+AZA (66.7% vs 27.3%, *P*=0.003).
For the VEN+AZA comparison, MAIC sensitivity analyses yielded consistent OS estimates (HR range: 0.066–0.120). Results remained robust when censoring triplet patients at the time of stem-cell transplant (HR = 0.150; 95% CI: 0.043–0.526).
In the IPTW-adjusted comparison vs IVO+AZA, the triplet remained associated with longer OS (HR = 0.257; 95% CI: 0.090–0.735) and higher CRc and CR rates (92.6% vs 48.8%, *P*=0.002; 66.7% vs 43.5%). Findings were consistent in sensitivity analyses excluding favorable-risk patients (HR = 0.321; 95% CI: 0.113–0.908) and censoring at transplant (HR = 0.276; 95% CI: 0.084–0.907).

### Summary/Conclusion

Triplet regimen was associated with improved OS and higher CRc and CR rates compared to doublet regimens in ICIE patients with m*IDH1* AML. Findings demonstrate the benefit of a triplet-based approach and, together with prior evidence on improved survival with IVO-based therapy vs other therapies, underscore the effectiveness of IVO across treatment regimens. Collectively, the data support consideration of IVO as a backbone for a broad range of patients with m*IDH1* AML. Limitations inherent to an ITC with small sample sizes should be considered when interpreting results.

Figure. Overall Survival Across Indirect Comparisons

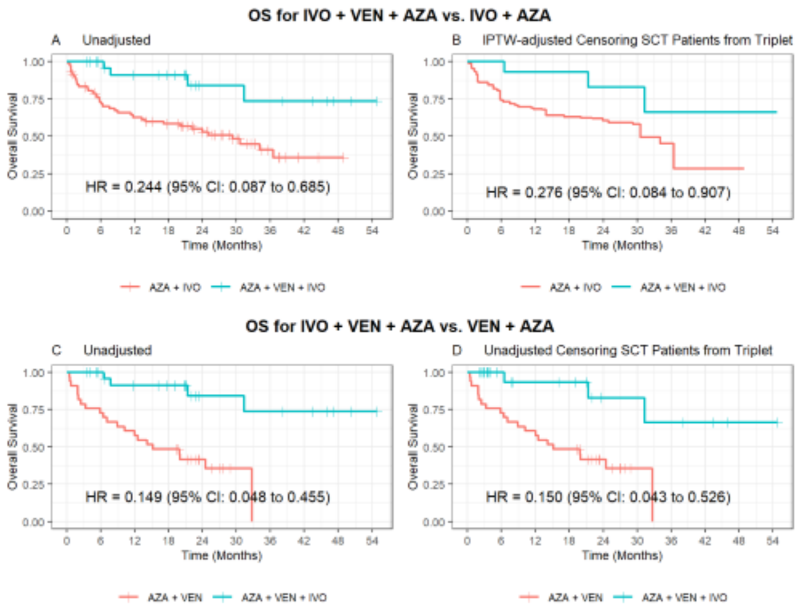

AML, Acute myeloid leukemia; AZA, Azacitidine; CI, Confidence interval; HR, Hazard ratio; IVO, Ivosidenib; IPTW, Inverse probability of treatment weighting; OS, Overall survival; SCT, Stem-cell transplant; VEN, Venetoclax.

**Copyright Information:** (Online) ISSN: 2572-9241
©2026 The Author(s). HemaSphere is published by John Wiley & Sons Ltd on behalf of European Hematology Association. This is an open access Abstract Book distributed under the Attribution-NonCommercial-NoDerivs (CC BY-NC-ND), which allows third parties to download the articles and share them with others as long as they credit the author and the Abstract Book, but they cannot change the content in any way or use them commercially.
**Abstract Book Citations:** Authors, Title, HemaSphere, 2026;10:(S1):pages. The abstract book DOIs can be found on the "Author Information" page.
**Disclaimer:** Articles published in the journal HemaSphere exclusively reflect the opinions of the authors. The authors are responsible for all content in their abstracts including accuracy of the facts, statements, citing resources, etc.

### (PF514) EFFICACY OF REVUMENIB IN ACUTE MYELOID LEUKEMIA HARBORING NPM1-MUTATED CO-MUTATIONS: POST HOC ANALYSIS OF AUGMENT-101

**Topic:** 04. Acute myeloid leukemia - Clinical

James S. Blachly*[1], Eytan Stein[2], Ghayas Issa[3], Lincy Thomas[4], Li Yu[4], Anthony Zembillas[4], Jessica Piel[4], Michael Thirman[5], John Dipersio[6], Gabriel Mannis[7], Alexander Perl[8], Mael Heiblig[9], Stéphane De Botton[10], Andre Schuh[11], Carolyn Grove[12], Andrius Žučenka[13], Martha L Arellano[14]

*[1]The Ohio State University, Columbus, OH, United States of America; [2]Memorial Sloan Kettering Cancer Center, New York, NY, United States of America; [3]The University of Texas MD Anderson Cancer Center, Houston, TX, United States of America; [4]Syndax Pharmaceuticals, Inc., New York, NY, United States of America; [5]The University of Chicago Medicine, Chicago, IL, United States of America; [6]Washington University School of Medicine, St. Louis, MO, United States of America; [7]Stanford University School of Medicine, and Stanford Cancer Institute, Stanford, CA, United States of America; [8]University of Pennsylvania, Philadelphia, PA, United States of America; [9]Centre Hospitalier Lyon Sud, Lyon, France; [10]Institut Gustave Roussy, Villejuif, France; [11]Princess Margaret Cancer Centre, and University of Toronto, Toronto, ON, Canada; [12]Sir Charles Gairdner Hospital, PathWest and the University of Western Australia, Nedlands, WA, Australia; [13]Vilnius University, Faculty of Medicine and Vilnius University Hospital Santaros Klinikos, National Cancer Center, Hematology, Oncology, and Transfusion Medicine Center, Vilnius, Lithuania; [14]Winship Cancer Institute of Emory University School of Medicine, Atlanta, GA, United States of America;*

### Background

*NPM1*-mutated (*NPM1*m) acute myeloid leukemia (AML) is molecularly complex; newly diagnosed patients (pts) have a median of 4 co-mutations (range, 0–11). Co-mutations in *DNMT3A*, *FLT3*, *WT1*, and *IDH1* are associated with poor prognosis, while the impact of *IDH2* is variable. Revumenib, a first-in-class, oral, potent, and selective inhibitor of the menin-KMT2A interaction, is used for the treatment of relapsed/refractory (R/R) acute leukemias harboring a *KMT2A* translocation or R/R AML with *NPM1* mutation in adult and pediatric pts 1 year and older. In an updated efficacy analysis from AUGMENT-101 (NCT04065399) evaluating revumenib monotherapy in pts with R/R *NPM1*m AML, the efficacy-evaluable population (N=77) demonstrated an overall response rate (ORR) of 48.1%, complete remission (CR)+CR with partial hematologic recovery (CRh) rate of 26.0%, composite CR (CRc) rate of 32.5%, and measurable residual disease negativity (MRD−) rate of 63.2% among CR+CRh responders with MRD status available.

### Aims

To report the efficacy of revumenib monotherapy in pts with R/R AML harboring *NPM1*m co-mutations that are potentially prognostic of adverse outcomes.

### Methods

AUGMENT-101 is an ongoing phase 1/2, open-label, dose-escalation/-expansion study of revumenib in pts with R/R *KMT2A*-rearranged and *NPM1*m AL. Pts received revumenib 160 mg q12h orally with a strong CYP3A4 inhibitor (270 mg without) in 28-day cycles. Primary endpoints were CR+CRh rate and safety/tolerability. Key secondary endpoints were CRc rate and ORR. Pts with centrally confirmed *NPM1*m and next-generation sequencing results were included in this post hoc analysis.

**Copyright Information:** (Online) ISSN: 2572-9241
©2026 The Author(s). HemaSphere is published by John Wiley & Sons Ltd on behalf of European Hematology Association. This is an open access Abstract Book distributed under the Attribution-NonCommercial-NoDerivs (CC BY-NC-ND), which allows third parties to download the articles and share them with others as long as they credit the author and the Abstract Book, but they cannot change the content in any way or use them commercially.
**Abstract Book Citations:** Authors, Title, HemaSphere, 2026;10:(S1):pages. The abstract book DOIs can be found on the "Author Information" page.
**Disclaimer:** Articles published in the journal HemaSphere exclusively reflect the opinions of the authors. The authors are responsible for all content in their abstracts including accuracy of the facts, statements, citing resources, etc.

### Results

This analysis included 54 adults with *NPM1*m AML with confirmed co-mutation results. Median (range) age was 65.0 y (19.0–84.0); 59% were white; 57% were female; median (range) prior lines of tx was 2 (1–7); 74% had prior venetoclax; 26% had prior hematopoietic stem cell transplant. Median (range) co-mutations was 3 (1–8). Co-mutation subgroup results are shown in the **Figure**. As of 26Feb2025, CR+CRh, CRc, and MRD– rates (from responders with MRD status available) were as follows:
*DNMT3A+FLT3* (n=15): CR+CRh rate was 26.7% (95% CI 7.8–55.1) and 4/4 pts achieved MRD–. CRc rate was 40.0% (95% CI 16.3–67.7) and 5/5 pts achieved MRD–.
*FLT3* (n=23): CR+CRh rate was 21.7% (95% CI 7.5–43.7) and 5/5 pts achieved MRD–. CRc rate was 30.4% (95% CI 13.2–52.9) and 6/6 pts achieved MRD–.
*DNMT3A+IDH1/2* (n=9): CR+CRh and CRc rates were both 55.6% (95% CI 21.2–86.3) and 5/5 pts achieved MRD–.
*IDH1/2* (n=14): CR+CRh and CRc rates were both 64.3% (95% CI 35.1–87.2) and 8/9 pts achieved MRD–.
*FLT3+IDH1/2* (n=8): CR+CRh and CRc rates were both 62.5% (95% CI 24.5–91.5) and 5/5 pts achieved MRD–.
Other AML co-mutations (n=19): CR+CRh rate was 26.3% (95% CI 9.1–51.2) and 3/5 pts achieved MRD–. CRc rate was 36.8% (95% CI 16.3–61.6) and 4/6 pts achieved MRD–.
Spliceosome co-mutations (n=8): CR+CRh rate was 50.0% (95% CI 15.7–84.3) and 3/4 pts achieved MRD–. CRc rate was 62.5% (95% CI 24.5–91.5) and 4/5 pts achieved MRD–.
*TET2* (n=17): CR+CRh rate was 23.5% (95% CI 6.8–49.9) and 3/4 pts achieved MRD–. CRc rate was 29.4% (95% CI 10.3–56.0) and 3/4 pts achieved MRD–.
*TP53* (n=4): CR+CRh and CRc rates were both 25.0% (95% CI 0.6–80.6) and 0/1 pt achieved MRD–.

### Summary/Conclusion

Revumenib demonstrated deep, clinically meaningful responses in pts with high-risk co-mutation profiles across the broad and heterogeneous *NPM1*m AML population, including those associated with adverse outcomes.

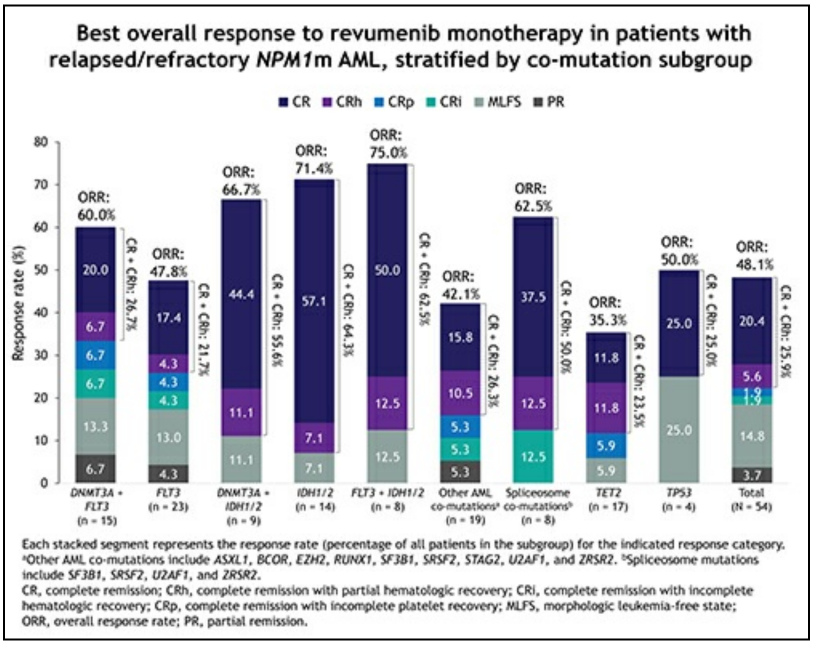

**Copyright Information:** (Online) ISSN: 2572-9241
©2026 The Author(s). HemaSphere is published by John Wiley & Sons Ltd on behalf of European Hematology Association. This is an open access Abstract Book distributed under the Attribution-NonCommercial-NoDerivs (CC BY-NC-ND), which allows third parties to download the articles and share them with others as long as they credit the author and the Abstract Book, but they cannot change the content in any way or use them commercially.
**Abstract Book Citations:** Authors, Title, HemaSphere, 2026;10:(S1):pages. The abstract book DOIs can be found on the "Author Information" page.
**Disclaimer:** Articles published in the journal HemaSphere exclusively reflect the opinions of the authors. The authors are responsible for all content in their abstracts including accuracy of the facts, statements, citing resources, etc.

### (PF515) OPTIMIZED CD33(FL33) CAR-T CELLS IN RELAPSED/REFRACTORY ACUTE MYELOID LEUKEMIA: A PHASE 1/2CLINICAL TRIAL

**Topic:** 04. Acute myeloid leukemia - Clinical

Jing Pan*[1, 2], Bing Liu[3], Ang Zhang[4], Chen Liu[5], Yandong Gong[3], Wenjing Zhang[2], Shengnan Zhou[2]

*[1]State Key Laboratory of Experimental Hematology, National Clinical Research Center for Blood Diseases, Haihe Laboratory of Cell Ecosystem, Institute of Hematology & Blood Diseases Hospital, Chinese Academy of Medical College, Sciences & Peking Union Medical College, Beijing, China; [2]Beijing Gobroad Hospital, Beijing, China; [3]State Key Laboratory of Experimental Hematology, Haihe Laboratory of Cell Ecosystem, Senior Department of Hematology, Fifth Medical Center, Medical Innovation Research Department, Chinese PLA General Hospital, Beijing, China; [4]Department of Comprehensive Basic Experiment, The Ninth Medical Center of Chinese PLA General Hospital, Beijing, China; [5]State Key Laboratory of Experimental Hematology, National Clinical Research Center for Blood Diseases, Haihe Laboratory of Cell Ecosystem, Institute of Hematology & Blood Diseases Hospital, Chinese Academy of Medical Sciences & Peking Union Medical College, Tianjin, China;*

### Background

Despite our previous optimized CD33 CAR-T therapy(FL33-01, J Pan, et al.Nature Communications, 2024) showed improved anti-leukemia efficacy in relapsed/refractory acute myeloid leukemia (r/r AML), grade 4 cytokine release syndrome (CRS) were identified, associated with elevated serum IL-6, IFN-γ, and TNF-α unresponsive to tocilizumab or corticosteroids. To address these safety issues, we upgraded our CD33 CAR structure (FL33-02 and FL33-03), adjusted lymphodepletion protocols,and defined bridging hematopoietic stem cell transplantation (HSCT)timing in a new trial.

### Aims

Explore the safety and efficacy of optimized FL33 CAR-T therapy for r/r AML.

### Methods

A multi-center, single-arm, open-label phase 1/2 trial (NCT06326021) of optimized CD33(FL-33) CAR-T cells (FL33-02 and FL33-03) was conducted in post-transplant and transplant-naïve r/r AML patients.Patients were assigned to three cohorts by CAR-T source: autologous (A),pre-transplant donor-derived(B), or new donor-derived(C).Using the BOIN12 design,phase I determined the optimal biological dose (OBD) via escalation,followed by phase II expansion. two dose levels ($5.0\times10^5$ and $1.0\times10^6$cells/kg) were explored. Cohorts A/B received standard lymphodepletion (Bu 0.8-1.2mg/kg q6h×1d, VP-16 100mg/m²×1d, Fludarabine 30mg/m²×3d, Cyclophosphamide 250mg/m²×3d); Cohort C received enhanced lymphodepletion (Cyclophosphamide 30mg/kg×3d instead of 250mg/m² 3d). HSCT was guided by treatment response, remission, and recurrence. Primary endpoint: safety; secondary: efficacy.

**Copyright Information:** (Online) ISSN: 2572-9241
©2026 The Author(s). HemaSphere is published by John Wiley & Sons Ltd on behalf of European Hematology Association. This is an open access Abstract Book distributed under the Attribution-NonCommercial-NoDerivs (CC BY-NC-ND), which allows third parties to download the articles and share them with others as long as they credit the author and the Abstract Book, but they cannot change the content in any way or use them commercially.
**Abstract Book Citations:** Authors, Title, HemaSphere, 2026;10:(S1):pages. The abstract book DOIs can be found on the "Author Information" page.
**Disclaimer:** Articles published in the journal HemaSphere exclusively reflect the opinions of the authors. The authors are responsible for all content in their abstracts including accuracy of the facts, statements, citing resources, etc.

### Results

Seven patients were enrolled (5 males, 2 females, median age 13 years, range 7-46), with 3-7prior therapies and median baseline bone marrow tumor burden 20.44% (3.69%-86%). 1, 2, 4 patients were enrolled in cohort A, B, C, respectively, all received $0.5x10^6$(+20%) kg CAR-T cells. First 6 consecutive patients received FL33-02 with accepted safety profiles: 100% grade 1-2 CRS (median onset: day 6 post-infusion, range 1-8; median duration: 6.5days, range 3-12). 100% grade 3-4 neutropenia, onocytopenia and thrombocytopenia and obvious serum TNF-a increased (median peak: 62.89pg/ml, range 16.21-271.82), potentially due to CD33-expressing monocyte-macrophage activation, with median recovery 8.5 days after etanercept. CAR-T expansion was robust (median peak count: 2323 cells/ul,range0.8-313.6; median peak transgene: 3690.5 copies/ug, range 817-171145). Patient 7 received FL33-03. On the 7th day after infusion, patient 7 developed grade 2 CRS, which lasted for 5 days. The serum level of TNF-a slightly increased to 5.49 pg/ml, and the recovery time was 7 days. The expansion of CAR- T cells was even more significant (peak cell count: 103.79 cells per microliter, peak median transgenecopy number: 215,281 copies per microgram) All7 (100%) achieved CRi, 5/7 (71.4%) achieved MRD-Six responders underwent bridging HSCT (median 17.5 days post-infusion, range 12-35), one refused HSCT and died of Trichosporon asahii infection on day30. One patient died during pre-transplant conditioning. As of February 28, 2026, DFS rate was 71.4% (median follow-up 10.9 months, range0.6-15).

### Summary/Conclusion

Optimized CD33(FL33-02 and FL33-03) CAR-T cells showed robust proliferation and potent anti-tumor activity in r/ AML FL33-03 structure have potent anti-tumor effects while also having the function of down-regulating TNFa and improving safety. However, infection remains a prominent safety concern, early bridging HSCT may reduce infection-related risks.

**Copyright Information:** (Online) ISSN: 2572-9241
©2026 The Author(s). HemaSphere is published by John Wiley & Sons Ltd on behalf of European Hematology Association. This is an open access Abstract Book distributed under the Attribution-NonCommercial-NoDerivs (CC BY-NC-ND), which allows third parties to download the articles and share them with others as long as they credit the author and the Abstract Book, but they cannot change the content in any way or use them commercially.
**Abstract Book Citations:** Authors, Title, HemaSphere, 2026;10:(S1):pages. The abstract book DOIs can be found on the "Author Information" page.
**Disclaimer:** Articles published in the journal HemaSphere exclusively reflect the opinions of the authors. The authors are responsible for all content in their abstracts including accuracy of the facts, statements, citing resources, etc.

### (PF516) MEASURABLE RESIDUAL DISEASE MONITORING IN PATIENTS WITH ACUTE MYELOID LEUKEMIA PRE- AND POST-ALLOGENEIC HEMATOPOIETIC CELL TRANSPLANTATION TO ASSESS RISK OF RELAPSE USING 16 KEY AML-ASSOCIATED GENES

**Topic:** 04. Acute myeloid leukemia - Clinical

Sean Glenn*[1], Blake Burgher[1], Jesse Luce[1], Vincent Giamo[1], Kiersten Marie Miles[1], Carl Morrison[1], Phillip Galbo[1]

[1]*Roswell Park Comprehensive Cancer Center, Department of Pathology, Buffalo, United States of America;*

### Background

Measurable Residual Disease (MRD) has become an essential prognostic marker for assessing how patients with Acute Myeloid Leukemia (AML) are responding to therapy. Currently, limitations in MRD testing include the paucity of key mutations for monitoring, as well as a lack of highly sensitive comprehensive assays that can efficiently test many patients in a single workflow. There is an imminent need to increase the number of targets that can be identified and utilized for monitoring progression of disease pre- and post- allogenic hematopoietic cell transplantation (alloHCT) in AML, as many patients cannot be monitored due to lack of mutations currently tracked by MRD.  To this end, we have utilized an NGS-based MRD assay to evaluate a retrospective cohort of serially collected bone marrow specimens (post-induction, pre- and post- alloHCT) from AML patients to assess the ability of this assay to better identify risk of relapse.

### Aims

Utilizing a sensitive and comprehensive NGS-based assay the two key aims of this study are, to first predict risk of recurrence when monitoring pre-alloHCT bone marrow specimens and second to monitor serially collected post-alloHCT bone marrow specimens for identifying recurrence earlier than current methodologies. Both aims focus on the identification of MRD with the future goal of using this assay to modify patient treatment and outcomes.

### Methods

Two distinct AML cohorts, one with known relapse post-alloHCT (n=26) and one non-relapse cohort (n=23), were identified and serial bone marrow (BM) specimens (pre and post alloHCT) were sequenced (n=166). NGS-based MRD assessment was carried out using the SureSeq™ Myeloid MRD panel (OGT, United Kingdom) which consists of 53 exon hotspot regions covering 16 genes frequently mutated in AML. To evaluate relapse prediction post-induction, pre-alloHCT BM samples were analyzed to establish variant allele frequency (VAF) thresholds associated with relapse risk.  Post-alloHCT serial samples were analyzed to assess whether tumor-informed mutation VAF monitoring correlated with relapse. NGS-based MRD monitoring was compared to standard in the field multiparameter flow cytometry (MFC) to assess whether NGS is more sensitive leading to sooner detection of relapse in this patient cohort.

### Results

By expanding the gene target list utilized for MRD testing 75% of our diagnostic patient cohort contained a mutation within the 16-gene panel. Using these tumor-informed variants the pre-alloHCT samples were highly informative for risk of relapse even when monitoring VAFs as low as 0.1%. Serial monitoring of post-alloHCT samples demonstrated a strong correlation between increased VAF and recurrence leading to identification of relapse significantly earlier than MCF (86-day vs. 176-day lead time, $p = 1.2 \times 10^{-2}$). Monitoring pre- and post-alloHCT bone marrows with defined VAF thresholds identified relapse with high sensitivity and specificity when compared to clinical outcomes (relapse vs. no relapse cohorts).

**Copyright Information:** (Online) ISSN: 2572-9241
©2026 The Author(s). HemaSphere is published by John Wiley & Sons Ltd on behalf of European Hematology Association. This is an open access Abstract Book distributed under the Attribution-NonCommercial-NoDerivs (CC BY-NC-ND), which allows third parties to download the articles and share them with others as long as they credit the author and the Abstract Book, but they cannot change the content in any way or use them commercially.
**Abstract Book Citations:** Authors, Title, HemaSphere, 2026;10:(S1):pages. The abstract book DOIs can be found on the “Author Information” page.
**Disclaimer:** Articles published in the journal HemaSphere exclusively reflect the opinions of the authors. The authors are responsible for all content in their abstracts including accuracy of the facts, statements, citing resources, etc.

**Summary/Conclusion**

Utilizing a multigene NGS-based MRD assay allows for a greater number of AML patients to be monitored with high sensitivity during their course of treatment. Testing the post-induction, pre-alloHCT specimen for VAFs >0.1% has defined a highly informed risk of relapse in this cohort. Furthermore, post-alloHCT monitoring using this NGS platform has identified relapse significantly sooner than standard MFC.

**Copyright Information:** (Online) ISSN: 2572-9241
©2026 The Author(s). HemaSphere is published by John Wiley & Sons Ltd on behalf of European Hematology Association. This is an open access Abstract Book distributed under the Attribution-NonCommercial-NoDerivs (CC BY-NC-ND), which allows third parties to download the articles and share them with others as long as they credit the author and the Abstract Book, but they cannot change the content in any way or use them commercially.
**Abstract Book Citations:** Authors, Title, HemaSphere, 2026;10:(S1):pages. The abstract book DOIs can be found on the "Author Information" page.
**Disclaimer:** Articles published in the journal HemaSphere exclusively reflect the opinions of the authors. The authors are responsible for all content in their abstracts including accuracy of the facts, statements, citing resources, etc.

### (PF517) ISOCITRATE DEHYDROGENASE-1 TESTING AND FIRST-LINE TREATMENT IN PATIENTS WITH EUROPEAN LEUKAEMIA NETWORK-DEFINED ACUTE MYELOID LEUKAEMIA INELIGIBLE FOR INTENSIVE THERAPY: A MULTI-COUNTRY SURVEY

**Topic:** 04. Acute myeloid leukemia - Clinical

Alessandro Isidori*[1], Ana Alfonso Pierola[2], Emma Searle[3], Maria Teresa Voso[4], Adolfo De La Fuente[5], Mael Heiblig[6], Felicitas Thol[7], Carolina Ricarte Iniesta[8], Stéphane De Botton[9]

*[1]Hematology and Stem Cell Transplant Center, san Salvatore Hospital, AST PU, Pesaro, Italy;[2]Clínica Universidad de Navarra, Pamplona, Spain; [3]The Christie NHS Foundation Trust and University of Manchester, Manchester, United Kingdom;[4]Università degli Studi di Roma "Tor Vergata", Rome, Italy; [5]MD Anderson Cancer Center Madrid, Madrid, Spain; [6]Service d'Hématologie Clinique, Hôpital Lyon Sud, Hospices Civils de Lyon, Oullins-Pierre-Bénite, France; [7]Department of Hematology, Hemostaseology, Oncology and Stem Cell Transplantation Hannover Medical School, Hannover, Germany; [8]IQVIA, Courbevoie, France; [9]Service d'Hématologie Clinique, Institut Gustave Roussy, Paris, France;*

### Background

Many patients with acute myeloid leukaemia (AML) are ineligible for intensive chemotherapy due to age, Eastern Cooperative Oncology Group performance status (ECOG PS), or existing comorbidities. Isocitrate dehydrogenase-1 (IDH1) testing has become increasingly relevant to guide treatment choices for these patients; however, real-world data on testing practices are limited.

### Aims

The primary objective was to describe real-world IDH1 testing practices in patients with AML ineligible for intensive therapy and receiving first-line (1L) treatment in France, Germany, Italy, Spain, and the UK. Secondary objectives included testing technologies, 1L regimens, and physician satisfaction.

### Methods

This retrospective analysis used data from the IQVIA Oncology Dynamics multi-country survey, which captures physician-reported information. The study analysed data recorded between July 2024 and June 2025. Eligible patients were those with AML receiving 1L therapy and classified as ineligible for intensive chemotherapy according to European Leukaemia Network (ELN)-based criteria incorporating age, ECOG PS, and comorbidity burden. Outcomes assessed were IDH1 testing rates, clinical setting of testing (academic vs non-academic centres), use of next-generation sequencing (NGS), IDH1 positivity, and treatment regimens. Physicians rated treatment satisfaction from less (1–3) to more (5–7) satisfied, with 4 being neutral. To avoid biasing responses, no guidance was given for the ratings. To reflect real-world clinical practice, patients who received intensive chemotherapy treatment despite being classified as ineligible per ELN criteria were not excluded. A sensitivity analysis was conducted excluding these patients.

**Copyright Information:** (Online) ISSN: 2572-9241
©2026 The Author(s). HemaSphere is published by John Wiley & Sons Ltd on behalf of European Hematology Association. This is an open access Abstract Book distributed under the Attribution-NonCommercial-NoDerivs (CC BY-NC-ND), which allows third parties to download the articles and share them with others as long as they credit the author and the Abstract Book, but they cannot change the content in any way or use them commercially.
**Abstract Book Citations:** Authors, Title, HemaSphere, 2026;10:(S1):pages. The abstract book DOIs can be found on the “Author Information” page.
**Disclaimer:** Articles published in the journal HemaSphere exclusively reflect the opinions of the authors. The authors are responsible for all content in their abstracts including accuracy of the facts, statements, citing resources, etc.

**Results**

A total of 1065 patients were included (Italy, 333; Spain, 260; France, 182; Germany, 159; UK, 131). IDH1 mutation was tested (**Figure**) in 851 (79.9%) patients (France, 93.4%; UK, 86.3%; Germany, 84.9%; Spain, 83.8%; Italy, 64.6%). The main setting for testing varied by country, with patients predominantly tested at academic centres in Spain (90.4%), the UK (69.9%), and Germany (63.0%), and at non-academic centres in France (55.9%) and Italy (54.9%). NGS was used in 532 (62.5%) patients tested for IDH1 mutation (UK, 86.7%; Spain, 78.9%; France, 65.9%; Germany, 43.7%; Italy, 42.3%). Of the 782 (73.4%) patients with available IDH1 test results, 119 (15.2%) tested positive for IDH1 mutation (UK, 24.0%; Spain, 17.6%; Italy, 16.9%; Germany, 11.4%; France, 8.0%). The most common treatments (>10%) among 119 patients with positive IDH1 status receiving 1L, were venetoclax plus azacitidine (n=56, 47.1%), ivosidenib plus azacitidine (n=19, 16.0%), and azacitidine alone (n=15, 12.6%). Physicians reported they were most satisfied with treatment for 30 (53.6%) patients on venetoclax plus azacitidine and 18 patients on ivosidenib plus azacitidine (94.7%). Intensive chemotherapy treatment was received by 160 (15%) patients who met non-intensive criteria, reflecting physicians' discretion in real-world clinical practice. The results of analyses excluding these patients were consistent with those of the overall population.

**Summary/Conclusion**

This large real-world survey highlights substantial variability in IDH1 testing and treatment patterns among patients with ELN-defined AML ineligible for intensive therapy. The findings underscore opportunities to optimise molecular testing strategies and treatment decision-making across routine clinical practice.

**Figure:** IDH1 testing, overall and by country

**Copyright Information:** (Online) ISSN: 2572-9241
©2026 The Author(s). HemaSphere is published by John Wiley & Sons Ltd on behalf of European Hematology Association. This is an open access Abstract Book distributed under the Attribution-NonCommercial-NoDerivs (CC BY-NC-ND), which allows third parties to download the articles and share them with others as long as they credit the author and the Abstract Book, but they cannot change the content in any way or use them commercially.
**Abstract Book Citations:** Authors, Title, HemaSphere, 2026;10:(S1):pages. The abstract book DOIs can be found on the "Author Information" page.
**Disclaimer:** Articles published in the journal HemaSphere exclusively reflect the opinions of the authors. The authors are responsible for all content in their abstracts including accuracy of the facts, statements, citing resources, etc.

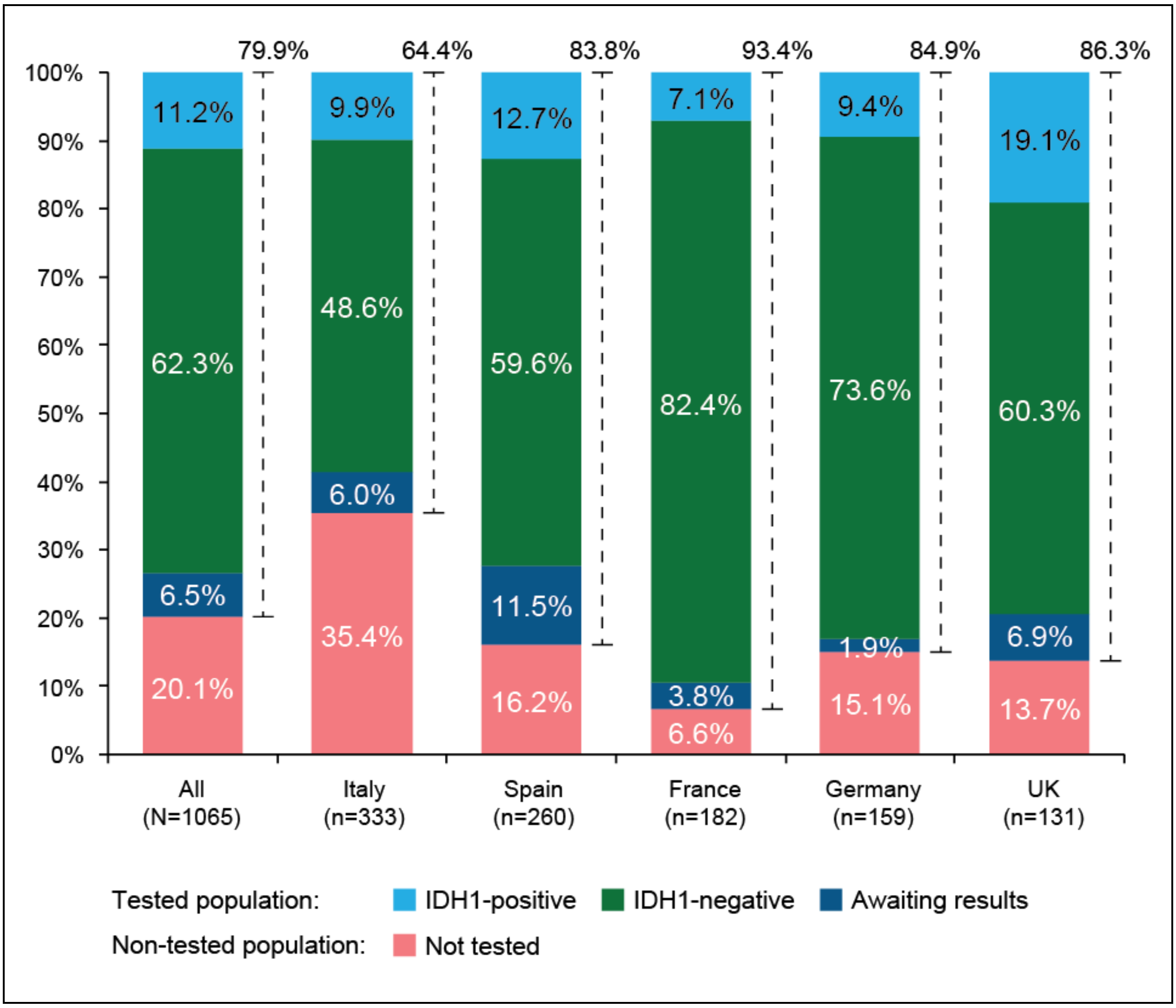

**Copyright Information:** (Online) ISSN: 2572-9241
©2026 The Author(s). HemaSphere is published by John Wiley & Sons Ltd on behalf of European Hematology Association. This is an open access Abstract Book distributed under the Attribution-NonCommercial-NoDerivs (CC BY-NC-ND), which allows third parties to download the articles and share them with others as long as they credit the author and the Abstract Book, but they cannot change the content in any way or use them commercially.
**Abstract Book Citations:** Authors, Title, HemaSphere, 2026;10:(S1):pages. The abstract book DOIs can be found on the "Author Information" page.
**Disclaimer:** Articles published in the journal HemaSphere exclusively reflect the opinions of the authors. The authors are responsible for all content in their abstracts including accuracy of the facts, statements, citing resources, etc.

### (PF518) COMPREHENSIVE LANDSCAPE OF CODING AND NON-CODING DRIVER ALTERATIONS REVEALED BY DEEP WHOLE-GENOME SEQUENCING OF MYELOID NEOPLASMS

**Topic:** 04. Acute myeloid leukemia - Clinical

Koji Okazaki*[1, 2], Ryunosuke Saiki[1], Yotaro Ochi[1, 3], Kenichi Yoshida[4], Kotoe Katayama[5], Hidehito Fukushima[6], Kaito Mimura[4], Masahiro Nakagawa[1], Tetsuichi Yoshizato[1], Masanori Motomura[1], Akinori Yoda[1, 3], Rurika Okuda[1, 3], Zhao Lanying[1, 3], Motohiro Kato[7], Tatsuki Ogasawara[8], Shigeo Fuji[9], Lee-Yung Shih[10], Uoshima Nobuhiko[11], Mitsumasa Watanabe[12], Yasushi Miyazaki[13], Motoshi Ichikawa[14], Takeshi Maeda[15], Toshiyuki Kitano[16], Satoshi Yoshihara[17], Kazunori Imada[18], Kinuko Mitani[19], Joop Jansen[20], Mamiko Sakata-Yanagimoto[21], Luca Malcovati[22, 23], Akihiko Gotoh[24], Hitoshi Kiyoi[25], Nobuhiro Kanemura[26], Nobuo Sezaki[27], Nobuhiro Hiramoto[28], Senji Kasahara[29, 30], Seiya Imoto[5, 31], Akifumi Takaori[2], Yasuhito Nannya[6], Seishi Ogawa[1, 32]

*[1]Kyoto University, Department of Pathology and Tumor Biology, Graduate School of Medicine, Kyoto, Japan;[2]Kyoto University, Department of Hematology, Graduate School of Medicine, Kyoto, Japan; [3]Kyoto University, Institute for the Advanced Study of Human Biology, Kyoto, Japan; [4]National Cancer Center Research Institute, Division of Cancer Evolution, Tokyo, Japan;[5]The University of Tokyo, Laboratory of Sequence Analysis, Institute of Medical Science, Tokyo, Japan; [6]The University of Tokyo, Division of Hematopoietic Disease Control, Institute of Medical Science, Tokyo, Japan; [7]The University of Tokyo, Department of Pediatrics, Tokyo, Japan; [8]Kyushu University, Department of Medicine and Bioregulatory Science, Fukuoka, Japan;[9]Osaka International Cancer Institute, Department of Hematology, Osaka, Japan; [10]Chang Gung University, Division of Hematology-Oncology, Chang Gung Memorial Hospital-Linkou, School of Medicine, Taoyuan, Taiwan; [11]Japanese Red Cross Kyoto Daini Hospital, Department of Hematology, Kyoto, Japan; [12]Hyogo Prefectural Amagasaki General Medical Center, Department of Hematology, Hyogo, Japan; [13]Nagasaki University, Department of Hematology, Unit for Radiation Effects on Humans, Atomic Bomb Disease Institute, Nagasaki, Japan; [14]NTT Medical Center Tokyo, Department of Hematology, Tokyo, Japan;[15]Kurashiki Central Hospital, Department of Hematology/Oncology, Kurashiki, Japan; [16]Medical Research Institute Kitano Hospital, Hematology, Osaka, Japan; [17]Hyogo Medical University, Department of Respiratory Medicine and Hematology, Nishinomiya, Japan; [18]Japanese Red Cross Osaka Hospital, Department of Hematology, Osaka, Japan;[19]Dokkyo Medical University, Department of Medicine, Tochigi, Japan; [20]Radboud University Medical Center, Laboratory of Hematology, Department of Laboratory Medicine, Nijmegen, The Netherlands; [21]University of Tsukuba, Department of Hematology, Institute of Medicine, Ibaraki, Japan; [22]Istituto di Ricovero e Cura a Carattere Scientifico (IRCCS) Fondazione Policlinico San Matteo, Department of Hematology Oncology, Pavia, Italy; [23]University of Pavia, Department of Molecular Medicine, Pavia, Italy; [24]Tokyo Medical University, Department of Hematology, Tokyo, Japan; [25]Nagoya University, Department of Hematology and Oncology, Graduate School of Medicine, Nagoya, Japan; [26]Gifu University Hospital, Department of Hematology and Infectious Disease, Gifu, Japan; [27]Chugoku Central Hospital,, Department of Hematology, Sezaki, Japan;[28]Department of Hematology, Department of Hematology, Hyogo, Japan; [29]Gifu Pharmaceutical University, Laboratory of Pharmaceutical Health Care and Promotion, Gifu, Japan; [30]Gifu Municipal Hospital, Department of Hematology, Gifu, Japan;[31]The University of Tokyo, Division of Health Medical Intelligence, Institute of Medical Science, Tokyo, Japan; [32]Kindai University, Department of Innovative Medicine, Faculty of Medicine, Osaka, Japan;*

### Background

During the past two decades, our knowledge about the molecular pathogenesis of myeloid neoplasms (MNs), including acute myeloid leukemia (AML) and myelodysplastic syndromes (MDS), has dramatically improved through the identification of major driver alterations using next-generation sequencing. However, due to the lack of large-scale whole-genome sequencing (WGS) analyses with sufficient sequencing depths, the contribution of non-coding alterations, structural variants (SVs), and somatic copy-number alterations (CNA) remains incompletely characterized. The etiology of mutagenic processes also remains poorly understood.

**Copyright Information:** (Online) ISSN: 2572-9241
©2026 The Author(s). HemaSphere is published by John Wiley & Sons Ltd on behalf of European Hematology Association. This is an open access Abstract Book distributed under the Attribution-NonCommercial-NoDerivs (CC BY-NC-ND), which allows third parties to download the articles and share them with others as long as they credit the author and the Abstract Book, but they cannot change the content in any way or use them commercially.
**Abstract Book Citations:** Authors, Title, HemaSphere, 2026;10:(S1):pages. The abstract book DOIs can be found on the "Author Information" page.
**Disclaimer:** Articles published in the journal HemaSphere exclusively reflect the opinions of the authors. The authors are responsible for all content in their abstracts including accuracy of the facts, statements, citing resources, etc.

**Aims**

In the current study, we therefore conducted large-scale deep WGS to address these issues.

**Methods**

We performed paired tumor–normal WGS in 1,123 MN cases (564 AML, 559 MDS). Mean sequencing depth was 105.9× for tumors and 30.1× for matched normals. Targeted deep sequencing of 446 known/putative driver genes and RNA-seq were performed for validation and functional interpretation.

**Results**

We identified 1,545,936 single-nucleotide variants (SNVs) and 97,955 short insertions/deletions (sInDels). Median mutation burdens were 0.46 SNVs/Mb and 0.030 sInDels/Mb in AML, and 0.39 SNVs/Mb and 0.022 sInDels/Mb in MDS. Targeted validation demonstrated a 95% positive predictive value and 78.1% sensitivity for known driver mutations.
Among these mutations, clock-like signatures, SBS1 and SBS5, predominated. Therapy-related signatures (SBS25, SBS31, SBS99) were enriched in therapy-related MNs. SBS18, associated with oxidative stress, was significantly enriched in AML cases carrying t(8;21) and inv(16), but rarely observed in MDS, highlighting the role of oxidative stress in core-binding-factor leukemia. We also detected rare yet novel signatures (SBS24, SBS39, SBS40b) in approximately 1% of cases.
Driver analysis in coding regions identified 94 significantly mutated genes, including 21 novel coding candidates such as *SMARCB1* and *PTEN*. Intronic mutations predicted to alter splicing in known drivers (e.g., *TET2*, *DNMT3A*) were detected in 3.9% of cases. Mutations in non-coding RNAs, including *miR-142*, were detected in 3.3%.
We detected 11,050 SV events. SVs disrupted tumor suppressor genes in 12.9% of cases, including *RUNX1*, *ETV6*, *CBL*, and *TP53*. Chromothripsis occurred in 5.3% of cases, strongly associated with *TP53* alterations, affecting a median of 4 chromosomes in a single event, frequently involving regions relevant to MNs (e.g., *EPOR*, *ERG*, *ETV6*). A total of 340 gene fusions were identified (22.6% of cases), including 105 recurrent events. In *TP53*–wild-type cases, 5.4% harbored rare in-frame fusions, including *KAT6A::NUTM1*. SV-mediated enhancer hijackings were also observed.
High-resolution CNA analysis revealed recurrent focal events, including known targets of gain (e.g., *MYC*, *ETS1*, *EPOR*) and deletion (e.g., *FOXP1*, *DLEU1/2*). Notably, we identified recurrent focal gains of *CCND2*, frequently arising through complex SVs and strongly enriched in *TP53*-mutated cases.

**Summary/Conclusion**

Through deep WGS of a large cohort of MN cases, we have delineated a comprehensive landscape of driver mutations and detected new mutational signatures and candidates for driver mutations, including those affecting non-coding regions. Our analysis underscored the importance of WGS for better understanding of MN pathogenesis.

**Copyright Information:** (Online) ISSN: 2572-9241
©2026 The Author(s). HemaSphere is published by John Wiley & Sons Ltd on behalf of European Hematology Association. This is an open access Abstract Book distributed under the Attribution-NonCommercial-NoDerivs (CC BY-NC-ND), which allows third parties to download the articles and share them with others as long as they credit the author and the Abstract Book, but they cannot change the content in any way or use them commercially.
**Abstract Book Citations:** Authors, Title, HemaSphere, 2026;10:(S1):pages. The abstract book DOIs can be found on the “Author Information” page.
**Disclaimer:** Articles published in the journal HemaSphere exclusively reflect the opinions of the authors. The authors are responsible for all content in their abstracts including accuracy of the facts, statements, citing resources, etc.

### (PF519) EXTENSIVE OVERLAP BETWEEN POST-CYTOTOXIC AND DE NOVO CBFB-MYH11-POSITIVE ACUTE MYELOID LEUKEMIA: A MULTICENTER STUDY IN CHINA

**Topic:** 04. Acute myeloid leukemia - Clinical

Linya Wang*[1], Feifei Tang[1]

[1]*Peking University People's Hospital, Peking University Institute of Hematology, Beijing, China;*

### Background

Acute myeloid leukemia post-cytotoxic therapy (AML-pCT) generally confers poorer outcomes than de novo AML. Although the CBFB-MYH11 fusion is classified as favorable-risk per the 2022 European LeukemiaNet guidelines, the clinical characteristics and prognosis of CBFB-MYH11+ AML-pCT remain heterogeneous.

### Aims

In this study, we aimed to characterize the clinicopathologic, cytogenetic, molecular, and prognostic features of CBFB-MYH11+ AML-pCT, and compare them with those of CBFB-MYH11+ de novo AML and CBFB-MYH11– AML-pCT.

### Methods

This multicenter retrospective study analyzed 580 adult AML patients diagnosed from 2015 to 2024 across 14 Chinese centers. We compared the genetics, treatment responses, and survival outcomes of CBFB-MYH11+ de novo AML (n = 303), CBFB-MYH11+ AML-pCT (n = 21), and CBFB-MYH11– AML-pCT (n = 256). Cytogenetic/molecular analyses used standard methods and targeted next-generation sequencing (NGS).

### Results

The median age of CBFB-MYH11+ AML-pCT (49 years) was higher than CBFB-MYH11+ de novo AML (38 years; P = 0.012), but comparable to CBFB-MYH11– AML-pCT (53 years; P = 0.479). Mutational profiling revealed that CBFB-MYH11+ AML-pCT and de novo AML shared overlapping genetic features. KIT mutations were highly prevalent in CBFB-MYH11+ AML-pCT (41.2%) and de novo AML (49.0%), but rare in CBFB-MYH11– AML-pCT (8.2%; P = 0.028 and P = 0.043, respectively). Conversely, epigenetic mutations (DNMT3A, IDH1/2, TET2) were significantly less frequent in CBFB-MYH11+ AML-pCT (11.8%) and de novo AML (19.1%) compared to CBFB-MYH11– AML-pCT (37.4%; P = 0.039 and P = 0.048, respectively). The composite complete remission (CRc) rate for CBFB-MYH11+ AML-pCT was intermediate between CBFB-MYH11+ de novo AML and CBFB-MYH11– AML-pCT (61.9% vs. 83.2% , P = 0.013; 61.9% vs. 45.3%, P = 0.043; respectively). However, the CBFB-MYH11 MRD-negativity rate following two cycles of consolidation in CBFB-MYH11+ AML-pCT was comparable to that in CBFB-MYH11+ de novo AML (69.2% vs. 75.4%; P = 0.742). Survival outcomes were remarkably similar between CBFB-MYH11+ AML-pCT and de novo AML. The 3-year overall survival (OS) and disease-free survival (DFS) rates for both CBFB-MYH11+ AML-pCT and CBFB-MYH11+ de novo AML were significantly superior to those of CBFB-MYH11– AML-pCT (3-year OS: 85.4% and 92.0% vs. 60.7%, P < 0.001; 3-year DFS: 95.0% and 73.8% vs. 63.9%, P < 0.001). The survival advantage of CBFB-MYH11+ AML-pCT and de novo AML over CBFB-MYH11– AML-pCT was evident regardless of the presence of KIT mutations (3-year OS: 71.4% and 74.0% vs. 53.3%, P = 0.011) and signaling pathway gene (FLT3, RAS, KIT, PTPN11, NF1) mutations (75.0% and 70.0% vs. 48.6%, P < 0.0001).

**Copyright Information:** (Online) ISSN: 2572-9241
©2026 The Author(s). HemaSphere is published by John Wiley & Sons Ltd on behalf of European Hematology Association. This is an open access Abstract Book distributed under the Attribution-NonCommercial-NoDerivs (CC BY-NC-ND), which allows third parties to download the articles and share them with others as long as they credit the author and the Abstract Book, but they cannot change the content in any way or use them commercially.
**Abstract Book Citations:** Authors, Title, HemaSphere, 2026;10:(S1):pages. The abstract book DOIs can be found on the “Author Information” page.
**Disclaimer:** Articles published in the journal HemaSphere exclusively reflect the opinions of the authors. The authors are responsible for all content in their abstracts including accuracy of the facts, statements, citing resources, etc.

### Summary/Conclusion

The clinical and biological features of CBFB-MYH11+ AML-pCT and de novo AML significantly overlap. Given the highly similar mutational landscape, robust MRD responses, and excellent long-term survival, CBFB-MYH11+ AML-pCT should be managed as a single favorable-risk entity.

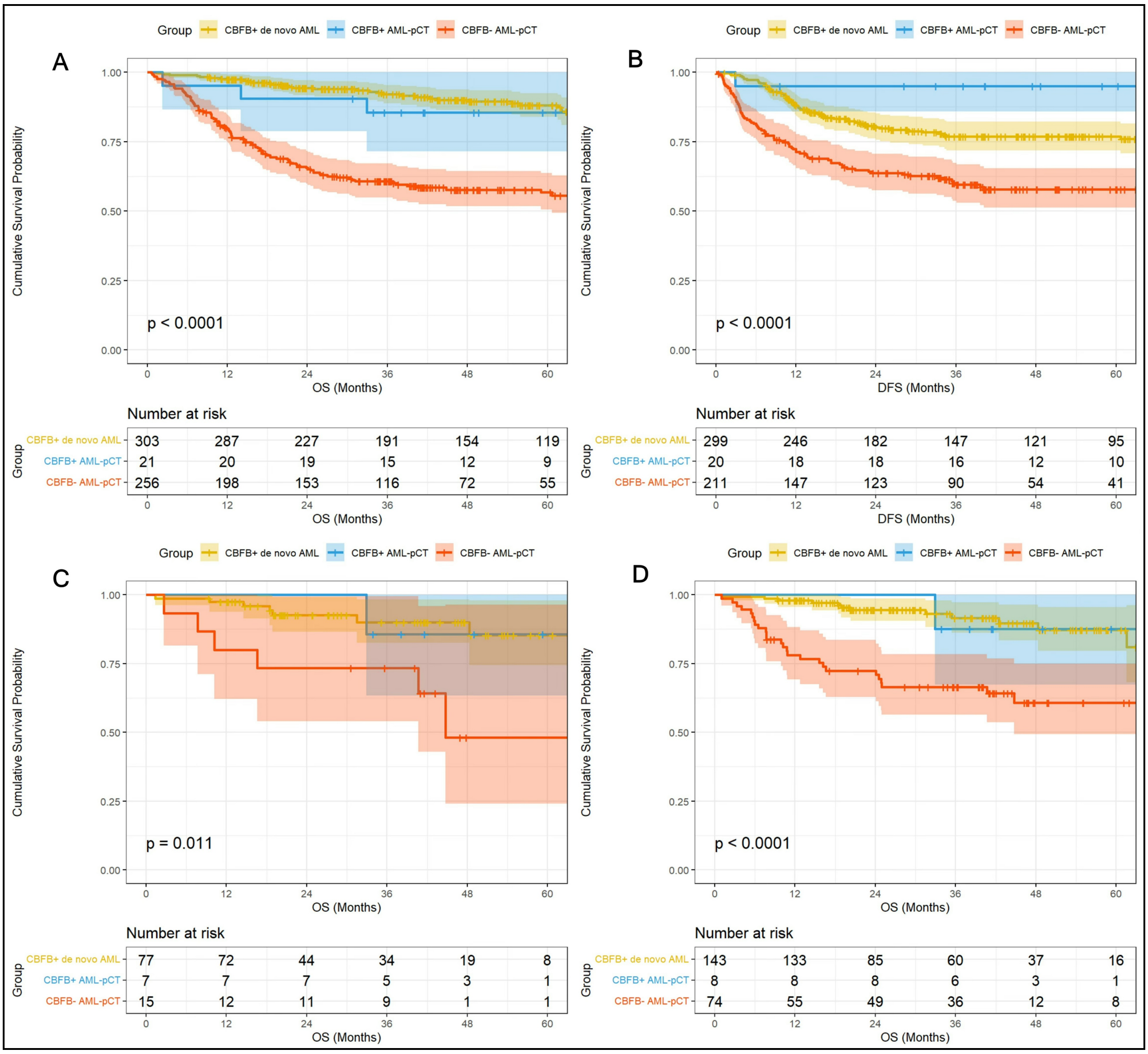

Kaplan-Meier estimates of overall survival (OS) and disease-free survival (DFS) in CBFB-MYH11+ AML-pCT, CBFB-MYH11+ de novo AML and CBFB-MYH11– AML-pCT. (A) OS and (B) DFS in the overall cohort. (C) OS in the patient subgroup harboring KIT mutations. (D) OS in the patient subgroup harboring signaling pathway gene (FLT3, RAS, KIT, PTPN11, NF1) mutations.

**Copyright Information:** (Online) ISSN: 2572-9241
©2026 The Author(s). HemaSphere is published by John Wiley & Sons Ltd on behalf of European Hematology Association. This is an open access Abstract Book distributed under the Attribution-NonCommercial-NoDerivs (CC BY-NC-ND), which allows third parties to download the articles and share them with others as long as they credit the author and the Abstract Book, but they cannot change the content in any way or use them commercially.
**Abstract Book Citations:** Authors, Title, HemaSphere, 2026;10:(S1):pages. The abstract book DOIs can be found on the "Author Information" page.
**Disclaimer:** Articles published in the journal HemaSphere exclusively reflect the opinions of the authors. The authors are responsible for all content in their abstracts including accuracy of the facts, statements, citing resources, etc.

### (PF520) A PHASE II TRIAL OF TAGRAXOFUSP, HYPER-CVAD, AND VENETOCLAX FOR PATIENTS WITH NEWLY DIAGNOSED OR RELAPSED/REFRACTORY BPDCN

**Topic:** 04. Acute myeloid leukemia - Clinical

Hannah Goulart*[1], Marina Konopleva[2], Nitin Jain[1], Naval Daver[1], Courtney Dinardo[1], Tapan Kadia[1], Farhad Ravandi[1], Yesid Alvarado[1], Fadi Haddad[1], Koichi Takahashi[1], Gautam Borthakur[1], Sherry Pierce[1], Susan Wahl[1], Allison Pike[1], Elias Jabbour[1], Sanam Loghavi[1], Muzaffar H. Qazilbash[1], Guillermo Garcia-Manero[1], Naveen Pemmaraju[1]

*[1]The University of Texas MD Anderson Cancer Center, Houston, United States of America;[2]Montefiore Einstein, Leukemia, New York, United States of America;*

### Background

Blastic plasmacytoid dendritic cell neoplasm (BPDCN) is a rare, clinically aggressive hematologic malignancy with historically poor outcomes. Tagraxofusp (TAG), a CD123-directed cytotoxin, led to a median overall survival (OS) of 15.8 months in newly diagnosed (ND) patients (pts) (Pemmaraju et al *JCO* 2022) thereby establishing itself as the first approved therapy and the backbone for future combination regimens in BPDCN. Venetoclax (VEN) has demonstrated activity in BPDCN (Montero et al *Cancer Disc* 2017), and intensified chemotherapy may further improve outcomes. However, the safety and efficacy of TAG in combination with intensive chemotherapy has not yet been elucidated in BPDCN.

### Aims

In order to further improve upon the outcomes in BPDCN, we conducted an investigator-initiated phase II trial (**NCT04216524**) of TAG, Hyper-CVAD, and VEN in newly ND or relapsed/refractory (R/R) BPDCN.

### Methods

Adults (≥18 years [yrs]) w/ ND or R/R BPDCN received: Cycle [C] 1: TAG days [d] 1-5; C2, 4, 6, 8: TAG d1 – 5 w/ VEN d2 – 14 (C2) and d1 – 7 (C4, 6, 8); C3, 7: VEN d1 – 7 w/ Hyper-CVAD or mini-hyper-CVD; C5: VEN d1 – 7 w/ high-dose methotrexate/cytarabine or mini-methotrexate/cytarabine. Cycles were 28 days for up to 8 cycles, followed by POMP, VEN, and TAG maintenance. Pts received ≥8 intrathecals.

**Copyright Information:** (Online) ISSN: 2572-9241
©2026 The Author(s). HemaSphere is published by John Wiley & Sons Ltd on behalf of European Hematology Association. This is an open access Abstract Book distributed under the Attribution-NonCommercial-NoDerivs (CC BY-NC-ND), which allows third parties to download the articles and share them with others as long as they credit the author and the Abstract Book, but they cannot change the content in any way or use them commercially.
**Abstract Book Citations:** Authors, Title, HemaSphere, 2026;10:(S1):pages. The abstract book DOIs can be found on the "Author Information" page.
**Disclaimer:** Articles published in the journal HemaSphere exclusively reflect the opinions of the authors. The authors are responsible for all content in their abstracts including accuracy of the facts, statements, citing resources, etc.

**Results**

Nineteen pts were enrolled (14 ND; 5 R/R). The median age was 61 yrs (range, 20 – 79) with 79% male. Disease sites included 84% skin, 63% bone marrow, 16% CNS, 16% extramedullary. Co-occurring mutations were *TET2* (60%), *ASXL1* (33%), *DNMT3A* (20%), *NRAS* (13%), *KRAS* (13%). Two pts had prior/concomitant hematologic malignancies (n= 2 with myelodysplastic syndrome).

For ND, the overall response rate (ORR; complete remission [CR] plus CR w/ incomplete count recovery [CRi] plus partial response [PR]) was 93% (13/14, all CR/CRi). Pts received a median of 2 (1 – 6) cycles w/ 2 (1 – 3) cycles to best response. **The 30- and 60-day mortality was 0%.** After a 55-month median follow-up, (19.5 – not reached), median OS was 15 months with 24-month OS of 42%. Median event-free survival (EFS) was 14.6 months with 24-month EFS of 42%. After a median of 3 (2 – 5) cycles, 9 pts (65%) went to stem cell transplant (SCT) (of these, 2 died in CR, 6 remain alive in CR of which, 3 remain alive in CR almost 5 yrs; with only 1 post-SCT relapse). In the 4 responding pts who did not go to SCT, 3 relapsed, and 1 came off therapy in CR after 2 cycles to prepare for SCT.

For R/R, ORR was 100%; CR/CRi of 50%. Pts received a median of 2 (2 – 4) prior lines; 3 (60%) had prior SCT. Pts received a median of 3 (1 – 7) cycles w/ 3 (1 – 3) to best response. Two pts went to SCT (1 auto, 1 allo); 1 pt is alive on active treatment. The 30- and 60-day mortality was 0% and 20%. With f/u of 1.4 – 21.8 months, median OS and EFS is 8.9 months; 12-month OS and EFS were both 30%. **Figure 1** depicts OS in ND (A) and R/R (B) pts.

In all responding pts, causes of death were: progression n = 4; died in CR n = 5 (3 ND, 2 R/R; 1 post SCT, 1 unknown, 3 sepsis). The most common adverse events (AE) of any grade were: edema (n = 13) and fatigue (n = 11). There were 2 grade 5 AE (progression, respiratory failure). Any grade capillary leak syndrome (CLS) was seen in 2 pts (10%); both grade 3.

**Summary/Conclusion**

As frontline therapy, TAG with Hyper-CVAD, and VEN shows promising safety with no early mortality or high-grade CLS, with high response rates enabling frequent SCT consolidation and no CNS relapse.

© 2026 American Society of Clinical Oncology, Inc. Reused with permission. This abstract was accepted and previously presented at the 2026 ASCO Annual Meeting. All rights reserved.

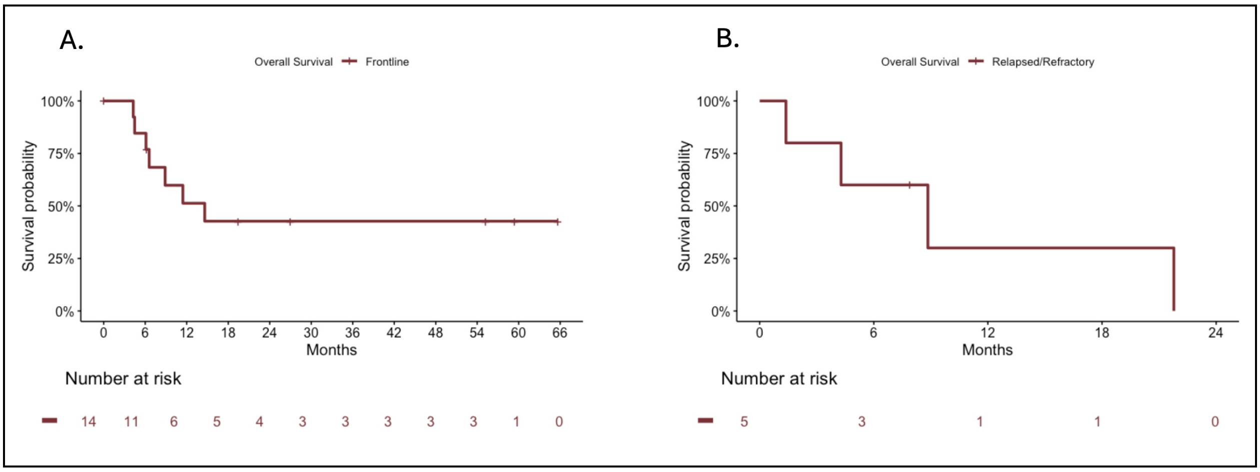

Overall Survival in Frontline (A) and Relapsed/Refractory (B)

**Copyright Information:** (Online) ISSN: 2572-9241
©2026 The Author(s). HemaSphere is published by John Wiley & Sons Ltd on behalf of European Hematology Association. This is an open access Abstract Book distributed under the Attribution-NonCommercial-NoDerivs (CC BY-NC-ND), which allows third parties to download the articles and share them with others as long as they credit the author and the Abstract Book, but they cannot change the content in any way or use them commercially.
**Abstract Book Citations:** Authors, Title, HemaSphere, 2026;10:(S1):pages. The abstract book DOIs can be found on the "Author Information" page.
**Disclaimer:** Articles published in the journal HemaSphere exclusively reflect the opinions of the authors. The authors are responsible for all content in their abstracts including accuracy of the facts, statements, citing resources, etc.

### (PF521) IMPACT OF PRIOR INDUCTION CYCLE NUMBER ON OUTCOMES WITH GILTERITINIB IN RELAPSED/REFRACTORY FLT3-MUTATED AML: A POOLED POST-HOC ANALYSIS OF ADMIRAL AND COMMODORE STUDIES

**Topic:** 04. Acute myeloid leukemia - Clinical

Jianxiang Wang[1], Alexander Perl[2], Hee-Je Kim[3], Je-Hwan Lee[4], Vladimir Ivanov[5], Hisayuki Yokoyama[6], Naoko Hosono[7], Ulas Durgut[8], Jamie An[9], Dina Elsouda[10], Mark Levis*[11]

*[1]State Key Laboratory of Experimental Hematology, National Clinical Research Center for Blood Diseases, Institute of Hematology and Blood Diseases Hospital, Chinese Academy of Medical Sciences and Peking Union Medical College, Tianjin, China; [2]Division of Hematology-Oncology, Department of Medicine, University of Pennsylvania, Philadelphia, PA, United States of America; [3]Catholic Hematology Hospital, Seoul St. Mary's Hospital, College of Medicine, The Catholic University of Korea, Seoul, South Korea; [4]Department of Hematology, Asan Medical Center, University of Ulsan College of Medicine, Seoul, South Korea; [5]Personalized Medicine Center, Almazov National Medical Research Center, St. Petersburg, Russia;[6]Division of Hematology and Cell Therapy, Department of Internal Medicine III, Yamagata University Graduate School of Medicine, Yamagata University, Yamagata, Japan; [7]Department of Hematology and Oncology, Faculty of Medical Sciences, University of Fukui, Fukui, Japan; [8]Astellas Pharma ilaç Ticaret ve Sanayi A.Ş., Istanbul, Türkiye;[9]Astellas Pharma, Inc., Singapore, Singapore; [10]Astellas Pharma Inc., Northbrook, IL, United States of America; [11]Division of Hematologic Malignancies, Johns Hopkins University, Baltimore, MD, United States of America;*

### Background

In frontline therapy of acute myeloid leukemia (AML), achieving remission after 1 vs multiple anthracycline-based induction therapy cycles has been associated with differences in relapse risk and survival. However, it is unknown if the number of induction cycles required before relapsed or refractory (R/R) disease influences outcomes with gilteritinib, the established standard of care for R/R *FMS*-like tyrosine kinase 3-mutated (*FLT3*$^{\text{mut+}}$) AML. Understanding this relationship may inform expectations regarding prognosis at R/R *FLT3*$^{\text{mut+}}$ AML diagnosis or gilteritinib response.

### Aims

To describe the association between response outcome and number of cycles of anthracycline-containing regimens received as front-line induction therapy in gilteritinib-treated patients (pts) with R/R *FLT3*$^{\text{mut+}}$ AML.

### Methods

This post hoc analysis used pooled data from the gilteritinib arms of the ADMIRAL and COMMODORE trials. Pts were grouped by prior induction exposure (1 or ≥2 prior induction cycles). Baseline and clinical characteristics, composite complete remission (CRc; defined as the sum of complete remission [CR], CR with incomplete hematologic recovery, or CR with incomplete platelet recovery), overall survival (OS), changes in transfusion independence, and safety were evaluated by exposure groups.

**Copyright Information:** (Online) ISSN: 2572-9241
©2026 The Author(s). HemaSphere is published by John Wiley & Sons Ltd on behalf of European Hematology Association. This is an open access Abstract Book distributed under the Attribution-NonCommercial-NoDerivs (CC BY-NC-ND), which allows third parties to download the articles and share them with others as long as they credit the author and the Abstract Book, but they cannot change the content in any way or use them commercially.
**Abstract Book Citations:** Authors, Title, HemaSphere, 2026;10:(S1):pages. The abstract book DOIs can be found on the “Author Information” page.
**Disclaimer:** Articles published in the journal HemaSphere exclusively reflect the opinions of the authors. The authors are responsible for all content in their abstracts including accuracy of the facts, statements, citing resources, etc.

### Results

Of 338 gilteritinib-treated pts, 257 (76.0%) received 1 prior induction cycle and 81 (24.0%) received ≥2 prior induction cycles. At baseline, in 1 vs ≥2 cycle groups, Eastern Cooperative Oncology Group performance status score 0–1 (83.7% vs 81.5%, *P*=0.999), Asian Race (52.9% vs 50.6%, Chi-square test [CST] *P*=0.631), prior FLT3 inhibitor use (14.0% vs 11.1%, CST *P*=0.999), and prior consolidation treatment rate (16.7% vs 12.3%, CST *P*=0.998) were comparable. There were numerical differences in mean (SD) age (53.7 [15.4] vs 48.8 [15.6] years, CST *P*=0.013) and intermediate cytogenic risk status (76.7% vs 63.0%, CST *P*=0.072). Median gilteritinib treatment duration (95% confidence interval [CI]) was 3.6 (3.2–4.0) and 3.8 (3.2–4.6) months in 1 and ≥2 cycle groups, respectively. Median OS and median follow-up times were comparable between groups (**Figure**). In 1 and ≥2 cycle groups, respectively, the CRc rate was 54.5% and 58.0% (*P*=0.999), and CR rate was 23.3% and 25.9% (*P*=0.999). Among pts with primary refractory AML without hematopoietic stem cell transplantation (HSCT) in the 1 (n=113) and ≥2 (n=36) cycle groups, respectively, median (95% CI) OS was 10.4 (8.8–12.7) and 11.1 (8.0–13.0) months (hazard ratio 0.87 [95% CI: 0.52–1.45]; *P*=0.591), and the CRc rates were 55.8% and 58.3%. HSCT rates in frontline AML (14.8% vs 16.0%) and on-study (23.0% vs 24.7%) were comparable between groups. Transfusion independence (63.8% vs 60.0%) and conversion rates (37.7% vs 43.3%) were comparable between groups (CST *P*=0.527). The grade ≥3 treatment-emergent adverse events (TEAE) of special interest rate was 44.4% in the 1 cycle group and 32.1% in the ≥2 cycle group. The treatment discontinuation rate due to TEAEs was 21.0% in the 1 cycle group and 17.3% in the ≥2 cycle group.

### Summary/Conclusion

There was no evidence that prior anthracycline-based induction history (1 vs ≥2 cycles) was associated with differences in outcomes in gilteritinib-treated pts with R/R *FLT3*$^{mut+}$ AML. These findings support gilteritinib use irrespective of prior induction burden and suggest induction history alone should not guide treatment selection. Limitations include the retrospective nature of this analysis and potential baseline imbalances.

**Figure. Kaplan–Meier plot of OS**

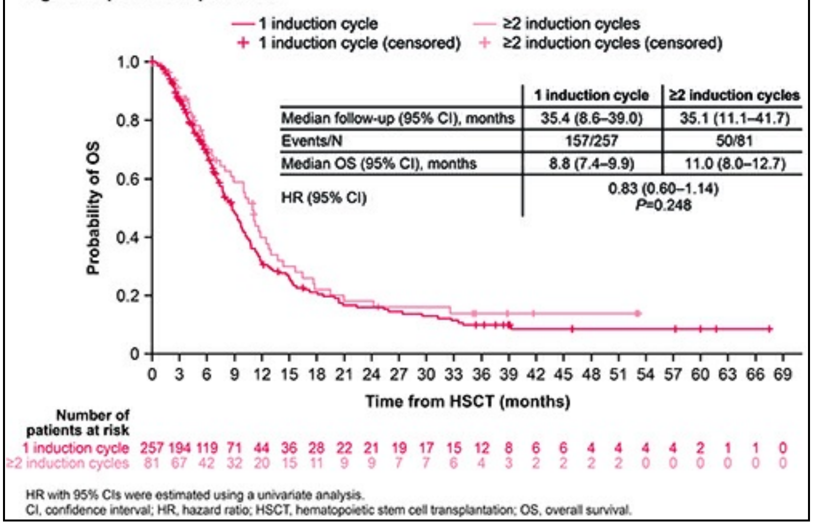

**Copyright Information:** (Online) ISSN: 2572-9241
©2026 The Author(s). HemaSphere is published by John Wiley & Sons Ltd on behalf of European Hematology Association. This is an open access Abstract Book distributed under the Attribution-NonCommercial-NoDerivs (CC BY-NC-ND), which allows third parties to download the articles and share them with others as long as they credit the author and the Abstract Book, but they cannot change the content in any way or use them commercially.
**Abstract Book Citations:** Authors, Title, HemaSphere, 2026;10:(S1):pages. The abstract book DOIs can be found on the "Author Information" page.
**Disclaimer:** Articles published in the journal HemaSphere exclusively reflect the opinions of the authors. The authors are responsible for all content in their abstracts including accuracy of the facts, statements, citing resources, etc.

### (PF522) GEMTUZUMAB OZOGAMICIN (GO) IN COMBINATION WITH CYTARABINE (ARA-C) IN RELAPSED ACUTE MYELOID LEUKEMIA (R-AML)

**Topic:** 04. Acute myeloid leukemia - Clinical

Giulia Arrigo*[1], Marie Fourmont[2], Lionel Ades[3], Florence Rabian[4], Emmanuelle Clappier[5], Raphael Itzykson[6], Régis Peffault de Latour[7], Nathalie Dhedin[4], Etienne Lengline[6], Nicolas Boissel[8], Marco Cerrano[9], Sofiane Fodil[6], Emmanuel Raffoux[6]

*[1]U.O.C Oncoematologia, AO Ospedali Riuniti Villa-Sofia Cervello, Palermo, Italy; [2]Hôtel Dieu CHU Nantes, Hématologie adulte, Nantes, France; [3]Hôpital Saint Louis, Service Hématologie Seniors, Paris, France; [4]Hôpital Saint Louis, Hématologie Adolescents Jeunes Adultes, Paris, France; [5]Hôpital Saint Louis, Laboratoire d'Hématologie, Paris, France; [6]Hôpital Saint Louis, Hématologie adulte, Paris, France; [7]Hôpital Saint Louis, Service d'hématologie greffe, Paris, France; [8]Hôpital Saint Louis, Département d'hématologie, Paris, France; [9]Azienda Ospedaliera Città della Salute e della Scienza, Torino, Italy;*

### Background

Although the prognosis of patients with AML has improved in recent years, relapse occurs in up two-thirds of patients. Patients with R-AML have poor outcomes and the 5-year survival rate is about 10%. Treatment is challenging and generally aims to reach a second complete remission (CR2) and to bridge eligible patients to allogenic hematopoietic stem cells transplantation (HSCT). Currently, there is no standard salvage chemotherapy regimen, and treatment choices are based on multiple factors, including center experience. GO is an anti-CD33 monoclonal antibody linked to calicheamicin, approved as first-line AML treatment in combination with daunorubicin and Ara-C. While several studies have shown the GO activity also in R-AML, the combination GO + Ara-C need further investigations.

### Aims

To evaluate the efficacy and safety of GO + Ara-C to treat AML patients in first relapse.

### Methods

We conducted a retrospective observational study in Saint-Louis Hospital (Paris, France) between January 2008 and June 2024. All consecutive patients with AML in first relapse treated with GO + Ara-C (high/intermediate doses, in bolus or in continuous infusion) were included. Statistical analyses were performed using NCSS 2024.

**Copyright Information:** (Online) ISSN: 2572-9241
©2026 The Author(s). HemaSphere is published by John Wiley & Sons Ltd on behalf of European Hematology Association. This is an open access Abstract Book distributed under the Attribution-NonCommercial-NoDerivs (CC BY-NC-ND), which allows third parties to download the articles and share them with others as long as they credit the author and the Abstract Book, but they cannot change the content in any way or use them commercially.
**Abstract Book Citations:** Authors, Title, HemaSphere, 2026;10:(S1):pages. The abstract book DOIs can be found on the "Author Information" page.
**Disclaimer:** Articles published in the journal HemaSphere exclusively reflect the opinions of the authors. The authors are responsible for all content in their abstracts including accuracy of the facts, statements, citing resources, etc.

**Results**

94 patients with a median age at relapse of 47 years (range,17-77) were included. Median time to relapse (TTR) was 11 months (range, 3-140). 5 patients (5%) had been transplanted in CR1. According to ELN-2010 classification, 46 patients (49%) were favorable risk, 33 (35%) intermediate and 15 (16%) adverse. Median percentage of CD33 expression on blast cells was 93% (range, 22-100). Median follow-up was 7.8 years (range, 0-16.7). Overall response rate (ORR) was 84% (N=79) with 46 patients (49%) achieving CR, 29 (31%) CR with incomplete count recovery (CRi), 2 (2%) morphologic leukemia free state (MLFS) and 2 (2%) partial response (PR). Factors significantly associated with increased ORR were TTR >12 months (p=0.0074) and CD33 expression >70% (p=0.037). Among the responders, 38 patients (48%) received a consolidation containing GO ± Ara-C, 22 patients (28%) with Ara-C alone. Median time between re-induction and first consolidation was 48 days (range, 28-105). 41 patients (44%) were bridged to allo-HSCT. 9 patients (10%) experienced a veno-occlusive disease (VOD), with 2/9 after HSCT. 1-year non-relapse mortality was 12% (95%, CI 2-33) with 4 infections, 3 severe bleedings, 2 VOD and 2 unknown causes. The 3-years cumulative incidence of relapse (CIR) was 57% (95% CI, 47-68). In univariate and multivariate analysis factors influencing CIR were TTR <12 months (HR=5.079, 95% CI, [2.05-12.57], p= 0.0004), minimal residual disease (MRD) positivity (HR=4.591, 95% CI, [1.83-11.49], p=0.001) and allo-HSCT in CR2 (HR=0.265, 95% CI [0.10-0.64] p=0.003). The 3-year overall survival (OS) was 38% (95% CI, 28-48). In univariate and multivariate analysis, factors influencing OS were TTR <12 months (HR=4.502, 95% CI [1.80-11.23], p= 0.0013), MRD+ (HR= 4.216, 95% CI [1.57-11.28] p=0.004) and allo-HSCT (HR=0.417, 95% CI [0.17-1], p=0.05). In patients bridged to allo-HSCT, 3-years OS was 60% (95% CI, 43-73).

**Summary/Conclusion**

GO+Ara-C is a safe and efficient regimen as a bridge to allo-HSCT in R-AML, especially in patients with a TTR >12 months and MRD negativity.

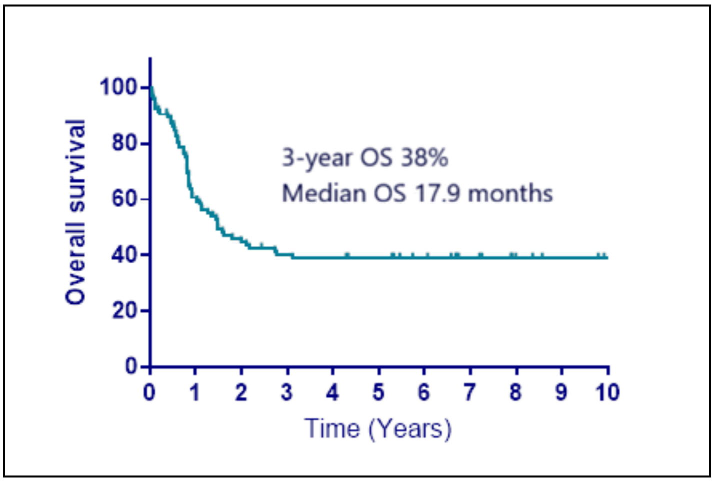

**Copyright Information:** (Online) ISSN: 2572-9241
©2026 The Author(s). HemaSphere is published by John Wiley & Sons Ltd on behalf of European Hematology Association. This is an open access Abstract Book distributed under the Attribution-NonCommercial-NoDerivs (CC BY-NC-ND), which allows third parties to download the articles and share them with others as long as they credit the author and the Abstract Book, but they cannot change the content in any way or use them commercially.
**Abstract Book Citations:** Authors, Title, HemaSphere, 2026;10:(S1):pages. The abstract book DOIs can be found on the "Author Information" page.
**Disclaimer:** Articles published in the journal HemaSphere exclusively reflect the opinions of the authors. The authors are responsible for all content in their abstracts including accuracy of the facts, statements, citing resources, etc.

### (PF523) OUTCOMES AFTER ALLOGENEIC STEM CELL TRANSPLANTATION IN ACUTE MYELOID LEUKEMIA TREATED WITH INTENSIVE CHEMOTHERAPY WITH VENETOCLAX

**Topic:** 04. Acute myeloid leukemia - Clinical

Leticia Campoverde*[1], Wei-Ying Jen[1], Alexandre Bazinet[1], Uday R. Popat[1], Ian Bouligny[1], Naval Daver[1], Guillermo Garcia-Manero[1], Nicholas Short[1], Ghayas Issa[1], Abhishek Maiti[1], Jayastu Senapati[1], Musa Yilmaz[1], Betul Oran[1], Amin Majid Alousi[1], Rohtesh Mehta[1], Samer A. Srour[1], Jin Im[1], Elizabeth J. Shpall[1], Farhad Ravandi[1], Hagop Kantarjian[1], Courtney Dinardo[1], Tapan Kadia[1]

[1]*MD Anderson Cancer Center, Houston, United States of America;*

**Background**

Allogeneic stem cell transplantation (SCT) remains the most effective consolidative strategy for pts with newly diagnosed (ND) acute myeloid leukemia (AML). Effective disease control and adequate fitness prior to SCT are key determinants of post-SCT outcomes and cure rate. Induction therapy with intensive chemotherapy (IC) plus venetoclax (VEN) has been shown to produce high rates of complete remission (CR), undetectable measurable residual disease (MRDu), and short time to best response.

**Aims**

Describe the characteristics and outcomes of pts with ND AML treated with IC+VEN who underwent SCT in first remission (CR1).

**Methods**

We included pts with ND AML treated on clinical trials of CLIA+VEN (NCT02115295) or FLAG-IDA+VEN (NCT03214562) from 2019 to 2025 who underwent CR1 SCT. Pts were risk-stratified according to ELN 2022 criteria. We analyzed pre-treatment and pre-SCT patient characteristics, response dynamics, as well as features of SCT and post-SCT course and long-term outcomes. Time-to-event outcomes were calculated from the date of SCT. Relapse and transplant-related, non-relapse mortality (NRM) were analyzed as competing risks and compared using Gray's test.

**Copyright Information:** (Online) ISSN: 2572-9241
©2026 The Author(s). HemaSphere is published by John Wiley & Sons Ltd on behalf of European Hematology Association. This is an open access Abstract Book distributed under the Attribution-NonCommercial-NoDerivs (CC BY-NC-ND), which allows third parties to download the articles and share them with others as long as they credit the author and the Abstract Book, but they cannot change the content in any way or use them commercially.
**Abstract Book Citations:** Authors, Title, HemaSphere, 2026;10:(S1):pages. The abstract book DOIs can be found on the "Author Information" page.
**Disclaimer:** Articles published in the journal HemaSphere exclusively reflect the opinions of the authors. The authors are responsible for all content in their abstracts including accuracy of the facts, statements, citing resources, etc.

### Results

Among 203 pts treated with IC+VEN, 140 (69%) underwent SCT in CR1. Of these pts, the median age was 47 years (range, 19–67). 117 (84%), 22 (16%), and 10 (7%) were classified as *de novo*, secondary, and therapy-related AML, respectively. 30 (21%) were favorable (FAV, primarily *NPM1*$^{mut}$ and *CEBPA*$^{bZIP}$), 48 (34%) intermediate (INT), and 62 (44%) adverse (ADV) risk. 15 (11%) pts had *FLT3*-ITD, all with VAF <0.1. Pts received a median of 2 cycles (range, 1–5) prior to SCT; best response was CR in 86%, CRh in 1%, CRi in 9%, PR in 1%, and MLFS in 3% prior to SCT. Before SCT, 126 (90%) pts were MRDu by multiparameter flow cytometry.
Data on transplant characteristics were available for 129/140 (92%) pts. Of these, 55 pts (43%) underwent matched unrelated donor, 8 (6%) mismatched unrelated donor, 47 (36%) matched related donor, and 19 (15%) haploidentical SCT. Conditioning was myeloablative (MAC) in 101 pts (78%) and reduced-intensity (RIC) in 28 (22%). 117 pts (91%) received post-SCT cyclophosphamide, and GVHD prophylaxis included tacrolimus (97%), mycophenolate mofetil (70%), and methotrexate (1%). NRM within the first 100 days was 5% and 7% among MAC and RIC, respectively.
Median follow-up post-SCT was 36 months. The median OS and RFS were not reached for any risk group. 3y OS was 81 % (95%CI, 75-89); 3-yr OS was 92% (95%CI, 83-100) in FAV, 86% (95%CI, 76-97) in INT, and 72% (95%CI, 60-86) in ADV risk. 3y RFS overall was 82% (95% CI, 76-90): 93% (95% CI, 84-100), 86% (95% CI, 76 -97), and 74% (95% CI, 63-87) among FAV, INT, and ADV, respectively.
The cumulative incidence of relapse (CIR) was 9.5%, while the cumulative incidence of NRM was 8% at 3 years. These differed by risk groups: FAV (3.6% vs. 3.3%); INT (2.4% vs. 11%); and ADV (14% vs. 11%), respectively. The difference was statistically significant for CIR ($p$=0.0162), but not NRM ($p$=0.49).

### Summary/Conclusion

Pts undergoing SCT in CR1 after IC + VEN had excellent outcomes, with 3-yr OS of 81% and RFS of 82%.

**Copyright Information:** (Online) ISSN: 2572-9241
©2026 The Author(s). HemaSphere is published by John Wiley & Sons Ltd on behalf of European Hematology Association. This is an open access Abstract Book distributed under the Attribution-NonCommercial-NoDerivs (CC BY-NC-ND), which allows third parties to download the articles and share them with others as long as they credit the author and the Abstract Book, but they cannot change the content in any way or use them commercially.
**Abstract Book Citations:** Authors, Title, HemaSphere, 2026;10:(S1):pages. The abstract book DOIs can be found on the “Author Information” page.
**Disclaimer:** Articles published in the journal HemaSphere exclusively reflect the opinions of the authors. The authors are responsible for all content in their abstracts including accuracy of the facts, statements, citing resources, etc.

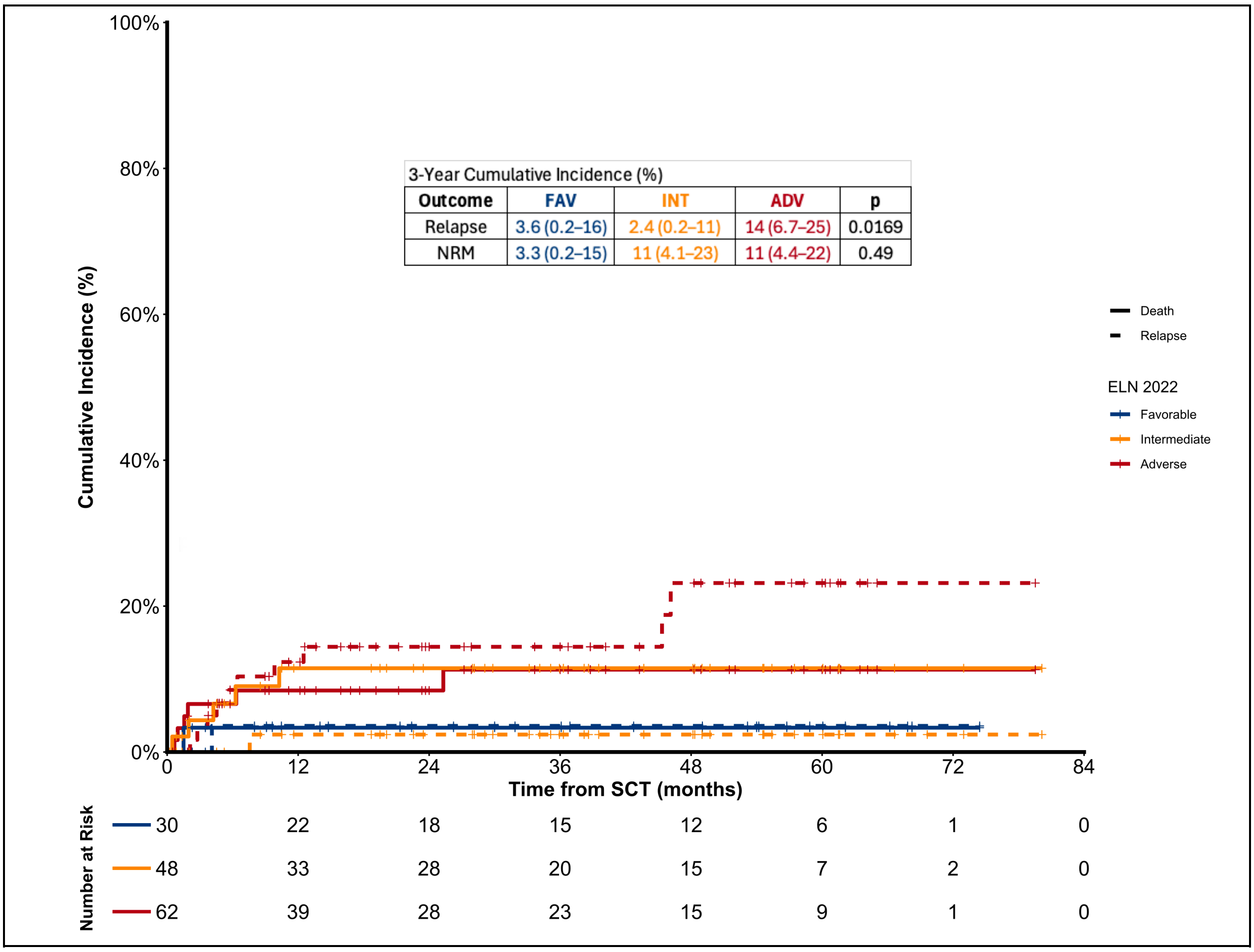

Cumulative incidence of relapse and NRM from time of SCT in transplanted patients, stratified by ELN 2022 risk.

Copyright Information: (Online) ISSN: 2572-9241
©2026 The Author(s). HemaSphere is published by John Wiley & Sons Ltd on behalf of European Hematology Association. This is an open access Abstract Book distributed under the Attribution-NonCommercial-NoDerivs (CC BY-NC-ND), which allows third parties to download the articles and share them with others as long as they credit the author and the Abstract Book, but they cannot change the content in any way or use them commercially.
Abstract Book Citations: Authors, Title, HemaSphere, 2026;10:(S1):pages. The abstract book DOIs can be found on the "Author Information" page.
Disclaimer: Articles published in the journal HemaSphere exclusively reflect the opinions of the authors. The authors are responsible for all content in their abstracts including accuracy of the facts, statements, citing resources, etc.

### (PF524) COMBINED FADS1 AND FADS2 EXPRESSION IS ASSOCIATED WITH PROGNOSIS AND RISK CLASSIFICATION IN PEDIATRIC ACUTE MYELOID LEUKEMIA

**Topic:** 04. Acute myeloid leukemia - Clinical

Yijun Wu*[1], Li Gao[1], Shaoyan Hu[1]

[1]*Children's Hospital of Soochow University, Department of Hematology and Oncology, Suzhou, China;*

### Background

Fatty acid desaturase 1 (FADS1) and fatty acid desaturase 2 (FADS2) are key enzymes in polyunsaturated fatty acid (PUFA) biosynthesis. Dysregulated PUFA metabolism has been linked to tumor progression and treatment resistance. While high FADS1 expression predicts poor outcomes in adult acute myeloid leukemia (AML), its prognostic value in pediatric AML remains unclear.

### Aims

This retrospective study aimed to investigate the association between FADS1 and FADS2 expression levels and clinical outcomes of pediatric AML. We further explored their potential biological relevance and clinical value in risk stratification.

### Methods

We retrospectively analyzed clinical data from 148 pediatric AML patients in our center to evaluate the association between FADS1 and FADS2 expression levels and AML prognosis. External validation was performed using the TARGET AML cohort. We further assessed their relevance in refining risk stratification for pediatric AML.

### Results

FADS1 and FADS2 expression levels were significantly positively correlated ($p < 0.001$). Concurrent high expression of FADS1 and FADS2 was significantly associated with AML-M7, CEBPA bZIP mutations, and FLT3-ITD positivity, whereas RUNX1::RUNX1T1 fusion was more frequent in the low-expression group ($P < 0.05$). Patients with high FADS1 expression had significantly inferior 5-year overall survival (OS) (55.6 ± 8.7% vs. 93.3 ± 2.9%, $p < 0.001$) and event-free survival (EFS) (53.6 ± 6.2% vs. 71.2 ± 5.9%, $p = 0.018$) compared with the low-expression group. Stratification based on combined FADS1 and FADS2 expression showed that patients with concurrent high expression (H–H) had significantly poorer outcomes than those with concurrent low expression (L–L). The 5-year OS rates were 52.8 ± 10.0% vs. 91.7 ± 3.6% ($p < 0.001$), and the 5-year EFS rates were 51.9 ± 6.9% vs. 73.8 ± 5.9% ($p = 0.022$), respectively. These findings were validated in the TARGET cohort, where the H–H group demonstrated significantly lower 5-year OS (54.6 ± 2.2% vs. 65.9 ± 2.1%, $p < 0.001$) and EFS (35.1 ± 2.0% vs. 38.9 ± 2.0%, $p = 0.027$). Higher FADS1 and FADS2 expression was also associated with poor response to induction therapy, defined by bone marrow blasts >5% and measurable residual disease (MRD) >0.01% after the first and second induction cycles. Incorporation of combined FADS1 and FADS2 expression into the existing risk classification system improved prognostic discrimination. Following reclassification, patients with concurrent high expression were assigned to the high-risk group and those with concurrent low expression to the intermediate/low-risk group. The 5-year OS rates for the high-risk versus intermediate/low-risk groups were 60.9 ± 9.5% vs. 83.1 ± 4.8% ($p = 0.0293$) under the original classification and 56.8 ± 8.7% vs. 91.1 ± 3.2% ($p = 0.0004$) under the revised classification incorporating dual-gene expression. Similarly, the 5-year EFS) rates were 49.5 ± 7.5% vs. 69.8 ± 5.3% ($p = 0.0125$) before reclassification and 48.9 ± 7.0% vs. 73.9 ± 5.2% ($p = 0.0061$) after reclassification. Overall, risk classification based on combined gene expression achieved better separation of survival curves and improved prognostic accuracy.

**Copyright Information:** (Online) ISSN: 2572-9241
©2026 The Author(s). HemaSphere is published by John Wiley & Sons Ltd on behalf of European Hematology Association. This is an open access Abstract Book distributed under the Attribution-NonCommercial-NoDerivs (CC BY-NC-ND), which allows third parties to download the articles and share them with others as long as they credit the author and the Abstract Book, but they cannot change the content in any way or use them commercially.
**Abstract Book Citations:** Authors, Title, HemaSphere, 2026;10:(S1):pages. The abstract book DOIs can be found on the “Author Information” page.
**Disclaimer:** Articles published in the journal HemaSphere exclusively reflect the opinions of the authors. The authors are responsible for all content in their abstracts including accuracy of the facts, statements, citing resources, etc.

**Summary/Conclusion**

Combined FADS1 and FADS2 expression levels are strongly associated with prognosis in pediatric AML and may provide additional value for risk stratification and therapeutic decision-making.

**Copyright Information:** (Online) ISSN: 2572-9241
©2026 The Author(s). HemaSphere is published by John Wiley & Sons Ltd on behalf of European Hematology Association. This is an open access Abstract Book distributed under the Attribution-NonCommercial-NoDerivs (CC BY-NC-ND), which allows third parties to download the articles and share them with others as long as they credit the author and the Abstract Book, but they cannot change the content in any way or use them commercially.
**Abstract Book Citations:** Authors, Title, HemaSphere, 2026;10:(S1):pages. The abstract book DOIs can be found on the "Author Information" page.
**Disclaimer:** Articles published in the journal HemaSphere exclusively reflect the opinions of the authors. The authors are responsible for all content in their abstracts including accuracy of the facts, statements, citing resources, etc.

### (PF525) POPULATION-BASED STUDY OF ACUTE PROMYELOCYTIC LEUKEMIA IN GERMANY: INCIDENCE AND SURVIVAL OUTCOMES

**Topic:** 04. Acute myeloid leukemia - Clinical

David Baden*[1], Nadine Wolgast[1], Leo Ruhnke[2], Klaus Metzeler[3], Thomas Beder[1], Alexander Pohlmann[4], Sophie Steinhäuser[1], Jacqueline Müller-Nordhorn[5], Alexander Katalinic[1], Maria Teresa Voso[6], Claudia Baldus[1], Lars Fransecky[1]

*[1]University Hospital Schleswig-Holstein, Campus Kiel, Kiel, Germany; [2]University Clinic Carl Gustav Carus, Department of Hematology, Dresden, Germany; [3]University of Leipzig, Department of Hematology, Cell Therapy, Hemostaseology and Infectious Diseases, Comprehensive Cancer Center Central Germany (CCCG), Leipzig, Germany; [4]University Hospital Münster, Department of Medicine A, Münster, Germany; [5]Bavarian Cancer Registry, Nuremberg, Germany; [6]Tor Vergata University, Department of Biomedicine and Prevention, Rome, Italy;*

### Background

Acute promyelocytic leukemia (APL) is a rare subtype of acute myeloid leukemia characterized by the t(15;17) translocation and the PML::RARA fusion protein, which impairs differentiation and apoptosis. Rapid diagnosis is critical because APL is often associated with severe bleeding or thrombosis, leading to fatal complications. The current standard of care combining all-trans retinoic acid (ATRA) and arsenic trioxide (ATO) has transformed APL into a highly curable disease in clinical trials. However, it remains unclear how advances from clinical trials translate to population-level outcomes as much population-based survival data predates ATO/ATRA and therefore reflects current results only insufficiently.

### Aims

In this study, we performed a population-based analysis to investigate the epidemiological characteristics and survival patterns of patients diagnosed with APL in Germany, aiming to provide a comprehensive overview of incidence and outcomes in a real-world national setting.

### Methods

Nationwide data on APL diagnosed between 2016 and 2021 were obtained from all German cancer registries. Cases were identified by ICD-10 code C92.4 and ICD-O morphology 9866/3.

### Results

Out of 26,190 AML cases, 649 (2.5%) were APL, representing a mean age-standardized incidence rate of 1.27 per 1,000,000 inhabitants and roughly 108 cases per year. The median age at diagnosis was 55.6 years (range 9–87 years), and incidence increased markedly with advancing age. Additional survival data were available for 450 patients with a median follow-up of 35.5 months. Three- and five-year OS were 76.7% and 75.2%, respectively, without sex differences. Survival declined markedly with age, with 5-year OS of 87.9% in patients 0–59 years, 70.8% in those 60–74 years, and 25.7% in those over 75 years. ATO/ATRA was the most common treatment and was associated with the best outcomes (3-year OS 87.3%). Outcomes were inferior when patients underwent intensified therapy with additional chemotherapy alongside ATO and/or ATRA (3-y OS 77.4%) and particularly poor with chemotherapy alone (3-y OS 49.2%). In multivariate analysis, age and treatment type independently predicted survival, whereas sex did not. Early mortality within 60 days occurred in 14.4% overall and increased steeply with age, reaching 37.3% in patients older than 75 years. Early death was lowest with ATO/ATRA and highest with intensive chemotherapy.

**Copyright Information:** (Online) ISSN: 2572-9241
©2026 The Author(s). HemaSphere is published by John Wiley & Sons Ltd on behalf of European Hematology Association. This is an open access Abstract Book distributed under the Attribution-NonCommercial-NoDerivs (CC BY-NC-ND), which allows third parties to download the articles and share them with others as long as they credit the author and the Abstract Book, but they cannot change the content in any way or use them commercially.
**Abstract Book Citations:** Authors, Title, HemaSphere, 2026;10:(S1):pages. The abstract book DOIs can be found on the "Author Information" page.
**Disclaimer:** Articles published in the journal HemaSphere exclusively reflect the opinions of the authors. The authors are responsible for all content in their abstracts including accuracy of the facts, statements, citing resources, etc.

### Summary/Conclusion

In summary, APL incidence in Germany is stable and comparable to other Western countries. Modern ATO/ATRA therapy translates into favorable real-world survival, but outcomes remain worse than in trials, largely due to early deaths and poor results in the elderly. Strengthening rapid diagnostic pathways, early supportive care, and collaboration between specialized and community centers may further reduce early mortality and improve outcomes in this curable leukemia.

**Figure 1: Overall survival of patients with acute promyelocytic leukemia stratified by age groups**

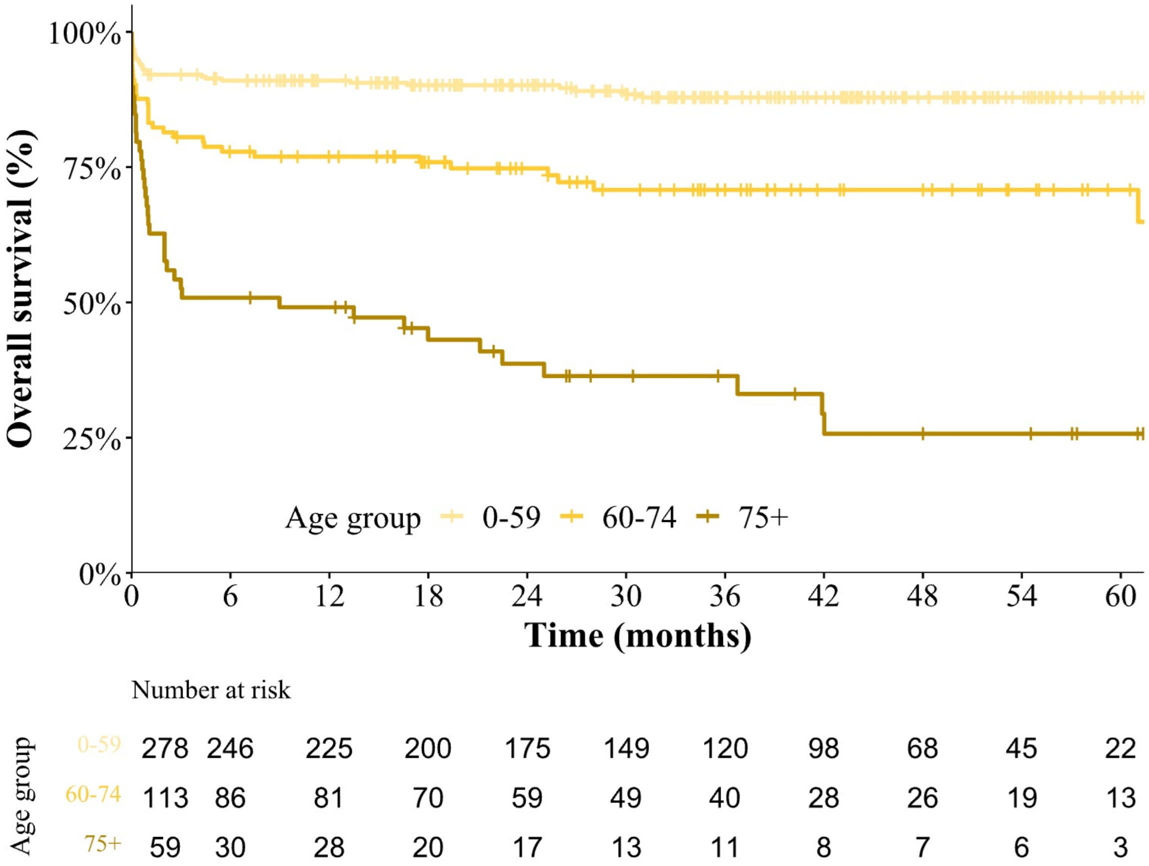

Figure 1: Overall survival in acute promyelocytic leukemia according to age group. Five-year overall survival was 87.9% for patients ≤59 years, 70.8% for patients 60–74 years, and 25.7% for patients ≥75 years.

**Copyright Information:** (Online) ISSN: 2572-9241
©2026 The Author(s). HemaSphere is published by John Wiley & Sons Ltd on behalf of European Hematology Association. This is an open access Abstract Book distributed under the Attribution-NonCommercial-NoDerivs (CC BY-NC-ND), which allows third parties to download the articles and share them with others as long as they credit the author and the Abstract Book, but they cannot change the content in any way or use them commercially.
**Abstract Book Citations:** Authors, Title, HemaSphere, 2026;10:(S1):pages. The abstract book DOIs can be found on the “Author Information” page.
**Disclaimer:** Articles published in the journal HemaSphere exclusively reflect the opinions of the authors. The authors are responsible for all content in their abstracts including accuracy of the facts, statements, citing resources, etc.

### (PF526) ZEFAMENIB COMBINED WITH CHEMOTHERAPY OR TARGETED AGENTS IN PATIENTS WITH ACUTE MYELOID LEUKEMIA: PRELIMINARY RESULT OF AN OPEN-LABEL, PHASE I/II STUDY

**Topic:** 04. Acute myeloid leukemia - Clinical

Haiping Dai*[1], Depei Wu[1], Suning Chen[1]

[1]*Jiangsu Institute of Hematology, Suzhou, China;*

### Background

Zefamenib is a potent small molecule inhibitor targeting menin, which has demonstrated anti-leukemia efficacy and safety in relapsed/refractory (R/R) acute leukemia patients in a phase 1 study. However, the safety and efficacy of zefamenib combined with intensive chemotherapy or venetoclax and azacitidine in AML remains unknown.

### Aims

To evaluate the safety and efficacy of zefamenib combined with intensive chemotherapy or venetoclax and azacitidine (VA regimen) in AML.

### Methods

This is an open-label, multicenter, phase I/II study (NCT06746519). Eligible patients should be AML, aged 18 years or older, harboring *KMT2A/NUP98* rearrangement or *NPM1* mutation with intermediate or adverse risk. This study comprised the following 3 groups. Group A enrolled untreated AML patients who were fit for intensive chemotherapy. Group B enrolled untreated AML patients unfit for intensive chemotherapy. Group C enrolled R/R AML patients who showed resistance to zefamenib monotherapy. Patients in group A received induction with zefamenib and 7+3, followed by high-dose cytarabine consolidation for a maximum of 4 cycles. Patients in group B and C received induction and cosolidation therapy with zefamenib, venetoclax and azacitidine. Allo-HSCT can be implemented after CR. In all 3 groups, zefamenib was planned to be given at a 3+3 dose escalation of 200mg; 400mg or 600mg BID since day 4 of cycle 1 and day 1 of the following cycles. Primary endpoints were safety and recommended phase 2 dose. Secondary endpoints included composite CR rate, CR rate, duration of remission and overall survival.

### Results

Since Nov, 2024, a total of 27 patients were screened. Twenty-six patients were enrolled and one screened failure. The median age of the patients were 40.5 (range: 30-58) years old and 12 patients (46.2%) were male. Of whom, 24 (92.3%) were primary AML and 2 (7.7%) were secondary AML. Twenty patients (76.9%) were newly diagnosed AML, 2 (7.7%) were relapsed AML and 4 (15.4%) were refractory AML.  Four (15.4%) had previously menin inhibitor exposure history. Patient baseline characteristics was shown in Table 1.Twenty-five (96.2%) patients were evaluable for treatment response. Of whom, 21 patients (80.8%) achieved CRc, all were CR. Four patients (15.4%) should no response. The CRc rates in untreated AML and R/R AML patients were 90% (18/20) and 50% (3/6), respectively, and all were CR. The CRc and CR rate in patients received zeftamenib plus chemotherapy and zeftamenib plus venetoclax and azacitidine (VA) were both 87.5% (17/18) and 77.8% (14/18), respectively (Table 2). 12/25 patients (46.2%, 95% CI: 26.59-66.63) achieved measurable residual disease (MRD) negative remission. Of the 21 patients with *KMT2A* rearrangement, 16 (76.2%) achieved CR, and MRD negative remission was observed in 9 patients (42.9%, 95% CI: 21.82-65.99) (Table 3). The 9-months rate of duration of remission was 72% (95% CI: 23.79-92.76). At the date of data cutoff (Dec 20, 2025), only one patient (3.8%) was dead. The 12-months overall survival rate was 96% (95% CI: 74.84-99.43) (Figure 1, 2). Grade 3 or higher infections were observed in 7 patients (26.9 %), including 37.5% and 22.2% of patients treated with zeftamenib plus chemotherapy and VA, respectively. Sepsis were observed in one patient (12.5%) treated with zeftamenib and chemotherapy.

**Copyright Information:** (Online) ISSN: 2572-9241
©2026 The Author(s). HemaSphere is published by John Wiley & Sons Ltd on behalf of European Hematology Association. This is an open access Abstract Book distributed under the Attribution-NonCommercial-NoDerivs (CC BY-NC-ND), which allows third parties to download the articles and share them with others as long as they credit the author and the Abstract Book, but they cannot change the content in any way or use them commercially.
**Abstract Book Citations:** Authors, Title, HemaSphere, 2026;10:(S1):pages. The abstract book DOIs can be found on the "Author Information" page.
**Disclaimer:** Articles published in the journal HemaSphere exclusively reflect the opinions of the authors. The authors are responsible for all content in their abstracts including accuracy of the facts, statements, citing resources, etc.

### Summary/Conclusion

Zefamenib-based combination therapies demonstrated a high response rate in treatment-naïve AML patients, and also exhibited notable efficacy in patients with zefamenib resistance.

Table 1: Baseline characteristics of patients

| | Group A-200mg (N=6) n(%) | Group A-400mg (N=2) n(%) | Combined with Chemotherapy (N=8) n(%) | Group B-200mg (N=8) n(%) | Group B-400mg (N=4) n(%) | Group C-200mg (N=5) n(%) | Group C-400mg (N=1) n(%) | Combined with VA (N=18) n(%) | Total (N=26) n(%) |
|---|---|---|---|---|---|---|---|---|---|
| Disease Subtype | | | | | | | | | |
| Primary AML | 6 (100.0) | 2 (100.0) | 8 (100.0) | 8 (100.0) | 4 (100.0) | 4 ( 80.0) | 0 ( 0.0) | 16 ( 88.9) | 24 ( 92.3) |
| Secondary AML | 0 ( 0.0) | 0 ( 0.0) | 0 ( 0.0) | 0 ( 0.0) | 0 ( 0.0) | 1 ( 20.0) | 1 (100.0) | 2 ( 11.1) | 2 ( 7.7) |
| Unknown | 0 ( 0.0) | 0 ( 0.0) | 0 ( 0.0) | 0 ( 0.0) | 0 ( 0.0) | 0 ( 0.0) | 0 ( 0.0) | 0 ( 0.0) | 0 ( 0.0) |
| FAB Classification | | | | | | | | | |
| AML-M0 | 0 ( 0.0) | 0 ( 0.0) | 0 ( 0.0) | 0 ( 0.0) | 0 ( 0.0) | 0 ( 0.0) | 0 ( 0.0) | 0 ( 0.0) | 0 ( 0.0) |
| AML-M1 | 0 ( 0.0) | 0 ( 0.0) | 0 ( 0.0) | 0 ( 0.0) | 0 ( 0.0) | 0 ( 0.0) | 0 ( 0.0) | 0 ( 0.0) | 0 ( 0.0) |
| AML-M2 | 2 ( 33.3) | 0 ( 0.0) | 2 ( 25.0) | 1 ( 12.5) | 0 ( 0.0) | 0 ( 0.0) | 0 ( 0.0) | 1 ( 5.6) | 3 ( 11.5) |
| AML-M3 | 0 ( 0.0) | 0 ( 0.0) | 0 ( 0.0) | 0 ( 0.0) | 0 ( 0.0) | 0 ( 0.0) | 0 ( 0.0) | 0 ( 0.0) | 0 ( 0.0) |
| AML-M4 | 0 ( 0.0) | 1 ( 50.0) | 1 ( 12.5) | 1 ( 12.5) | 1 ( 25.0) | 1 ( 20.0) | 1 (100.0) | 4 ( 22.2) | 5 ( 19.2) |
| AML-M5 | 3 ( 50.0) | 0 ( 0.0) | 3 ( 37.5) | 5 ( 62.5) | 2 ( 50.0) | 2 ( 40.0) | 0 ( 0.0) | 9 ( 50.0) | 12 ( 46.2) |
| AML-M6 | 0 ( 0.0) | 0 ( 0.0) | 0 ( 0.0) | 0 ( 0.0) | 0 ( 0.0) | 0 ( 0.0) | 0 ( 0.0) | 0 ( 0.0) | 0 ( 0.0) |
| AML-M7 | 0 ( 0.0) | 0 ( 0.0) | 0 ( 0.0) | 0 ( 0.0) | 0 ( 0.0) | 0 ( 0.0) | 0 ( 0.0) | 0 ( 0.0) | 0 ( 0.0) |
| Unknown | 1 ( 16.7) | 1 ( 50.0) | 2 ( 25.0) | 1 ( 12.5) | 1 ( 25.0) | 2 ( 40.0) | 0 ( 0.0) | 4 ( 22.2) | 6 ( 23.1) |
| Disease Status | | | | | | | | | |
| Newly Diagnosed Disease | 6 (100.0) | 2 (100.0) | 8 (100.0) | 8 (100.0) | 4 (100.0) | 0 ( 0.0) | 0 ( 0.0) | 12 ( 66.7) | 20 ( 76.9) |
| Relapsed Disease | 0 ( 0.0) | 0 ( 0.0) | 0 ( 0.0) | 0 ( 0.0) | 0 ( 0.0) | 1 ( 20.0) | 1 (100.0) | 2 ( 11.1) | 2 ( 7.7) |
| Refractory Disease | 0 ( 0.0) | 0 ( 0.0) | 0 ( 0.0) | 0 ( 0.0) | 0 ( 0.0) | 4 ( 80.0) | 0 ( 0.0) | 4 ( 22.2) | 4 ( 15.4) |
| Has previously Menin inhibitor therapy been received | | | | | | | | | |
| Yes | 0 ( 0.0) | 0 ( 0.0) | 0 ( 0.0) | 0 ( 0.0) | 0 ( 0.0) | 3 ( 60.0) | 1 (100.0) | 4 ( 22.2) | 4 ( 15.4) |
| No | 0 ( 0.0) | 0 ( 0.0) | 0 ( 0.0) | 0 ( 0.0) | 0 ( 0.0) | 2 ( 40.0) | 0 ( 0.0) | 2 ( 11.1) | 2 ( 7.7) |
| Unknown | 0 ( 0.0) | 0 ( 0.0) | 0 ( 0.0) | 0 ( 0.0) | 0 ( 0.0) | 0 ( 0.0) | 0 ( 0.0) | 0 ( 0.0) | 0 ( 0.0) |

Note: Percentages are calculated with the number of patients in each group of the mITT population as the denominator.
FAB: The French-American-British. ELN: European LeukemiaNet.

Table 2: Results of overall efficacy evaluation

| Efficacy Indicators | Group A-200mg (N=6) n(%) | Group A-400mg (N=2) n(%) | Combined with Chemotherapy (N=8) n(%) | Group B-200mg (N=8) n(%) | Group B-400mg (N=4) n(%) | Group C-200mg (N=5) n(%) | Group C-400mg (N=1) n(%) | Combined with VA (N=18) n(%) | Total (N=26) n(%) |
|---|---|---|---|---|---|---|---|---|---|
| Best Overall Response | | | | | | | | | |
| CR | 6 (100.0) | 1 ( 50.0) | 7 ( 87.5) | 8 (100.0) | 3 ( 75.0) | 3 ( 60.0) | 0 ( 0.0) | 14 ( 77.8) | 21 ( 80.8) |
| CRh | 0 ( 0.0) | 0 ( 0.0) | 0 ( 0.0) | 0 ( 0.0) | 0 ( 0.0) | 0 ( 0.0) | 0 ( 0.0) | 0 ( 0.0) | 0 ( 0.0) |
| CRi | 0 ( 0.0) | 0 ( 0.0) | 0 ( 0.0) | 0 ( 0.0) | 0 ( 0.0) | 0 ( 0.0) | 0 ( 0.0) | 0 ( 0.0) | 0 ( 0.0) |
| MLFS | 0 ( 0.0) | 0 ( 0.0) | 0 ( 0.0) | 0 ( 0.0) | 0 ( 0.0) | 0 ( 0.0) | 0 ( 0.0) | 0 ( 0.0) | 0 ( 0.0) |
| PR | 0 ( 0.0) | 0 ( 0.0) | 0 ( 0.0) | 0 ( 0.0) | 0 ( 0.0) | 0 ( 0.0) | 0 ( 0.0) | 0 ( 0.0) | 0 ( 0.0) |
| No Response | 0 ( 0.0) | 1 ( 50.0) | 1 ( 12.5) | 0 ( 0.0) | 1 ( 25.0) | 1 ( 20.0) | 1 (100.0) | 3 ( 16.7) | 4 ( 15.4) |
| Haematologic Relapse | 0 ( 0.0) | 0 ( 0.0) | 0 ( 0.0) | 0 ( 0.0) | 0 ( 0.0) | 0 ( 0.0) | 0 ( 0.0) | 0 ( 0.0) | 0 ( 0.0) |
| Not Evaluable | 0 ( 0.0) | 0 ( 0.0) | 0 ( 0.0) | 0 ( 0.0) | 0 ( 0.0) | 1 ( 20.0) | 0 ( 0.0) | 1 ( 5.6) | 1 ( 3.8) |
| ORR | | | | | | | | | |
| n (%) | 6 (100.0) | 1 ( 50.0) | 7 ( 87.5) | 8 (100.0) | 3 ( 75.0) | 3 ( 60.0) | 0 ( 0.0) | 14 ( 77.8) | 21 ( 80.8) |
| 95% CI | 54.07, 100.00 | 1.26, 98.74 | 47.35, 99.68 | 63.06, 100.00 | 19.41, 99.37 | 14.66, 94.73 | 0.00, 97.50 | 52.36, 93.59 | 60.65, 93.45 |
| CR and CR with Partial Hematologic Recovery | | | | | | | | | |
| n (%) | 6 (100.0) | 1 ( 50.0) | 7 ( 87.5) | 8 (100.0) | 3 ( 75.0) | 3 ( 60.0) | 0 ( 0.0) | 14 ( 77.8) | 21 ( 80.8) |
| 95% CI | 54.07, 100.00 | 1.26, 98.74 | 47.35, 99.68 | 63.06, 100.00 | 19.41, 99.37 | 14.66, 94.73 | 0.00, 97.50 | 52.36, 93.59 | 60.65, 93.45 |
| Composite Complete Remission (CRc) | | | | | | | | | |
| n (%) | 6 (100.0) | 1 ( 50.0) | 7 ( 87.5) | 8 (100.0) | 3 ( 75.0) | 3 ( 60.0) | 0 ( 0.0) | 14 ( 77.8) | 21 ( 80.8) |
| 95% CI | 54.07, 100.00 | 1.26, 98.74 | 47.35, 99.68 | 63.06, 100.00 | 19.41, 99.37 | 14.66, 94.73 | 0.00, 97.50 | 52.36, 93.59 | 60.65, 93.45 |

Note: Objective Response Rate (ORR), Defined as investigator-assessed complete remission (CR), complete remission with partial hematologic recovery (CRh), complete remission with incomplete hematologic recovery (CRi), morphologic leukemia-free state (MLFS), or partial remission (PR). The formula is as follows: ORR=n1/N, n1=CR+PR+CRh+CRi+MLFS.

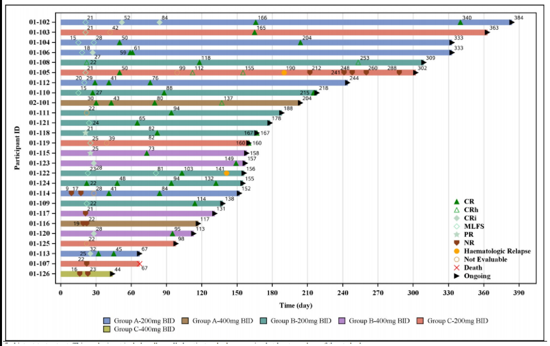

Figure 1: Swimming plot of the patients

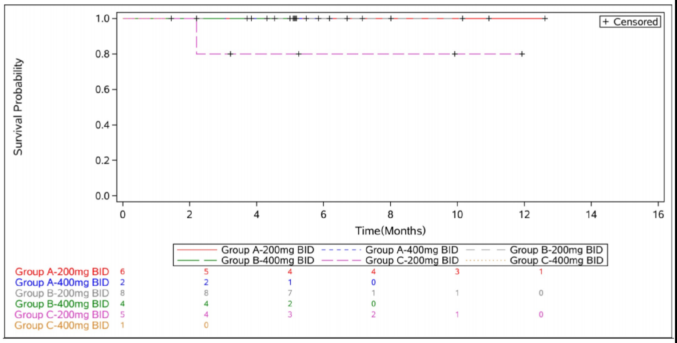

Figure 2: Overall survival curve

Copyright Information: (Online) ISSN: 2572-9241
©2026 The Author(s). HemaSphere is published by John Wiley & Sons Ltd on behalf of European Hematology Association. This is an open access Abstract Book distributed under the Attribution-NonCommercial-NoDerivs (CC BY-NC-ND), which allows third parties to download the articles and share them with others as long as they credit the author and the Abstract Book, but they cannot change the content in any way or use them commercially.
Abstract Book Citations: Authors, Title, HemaSphere, 2026;10:(S1):pages. The abstract book DOIs can be found on the "Author Information" page.
Disclaimer: Articles published in the journal HemaSphere exclusively reflect the opinions of the authors. The authors are responsible for all content in their abstracts including accuracy of the facts, statements, citing resources, etc.

### (PF527) FLAG-MITOXANTRONE-VENETOCLAX (7 DAYS) IS AN EFFICIENT SALVAGE THERAPY IN RELAPSED/REFRACTORY AML, WITH AN IMPACT OF NGS ON RESPONSE, AND AN EXCELLENT BRIDGE TO ALLOGENEIC STEM CELL TRANSPLANTATION

**Topic:** 04. Acute myeloid leukemia - Clinical

Ahmad Ibrahim*[1], Kamal Zahran[2], Ahmad Khalil[3], Jad Ibrahim[4], Eliana Kachaamy[3], Ali Youssef[5], Issam Chehadeh[6], Muhammad Abbas[6], Rima Moghnieh[7], Charbel Khalil[3]

*[1]University of Balamand: Middle East Institute of Health, Lebanese and Beirut Arab Universities: Makassed Hospital, Beirut, Lebanon, Beirut, Lebanon; [2]University of Balamand: Middle East Institute of Health, Beirut, Lebanon; Lebanese University, Beirut, Lebanon; [3]University of Balamand: Middle East Institute of Health, Beirut, Lebanon, Beirut, Lebanon;[4]American University of Beirut, Beirut, Lebanon, Beirut, Lebanon; [5]Beirut Arab University: Makassed Hospital, Beirut, Lebanon; Lebanese University, Beirut, Lebanon; [6]Beirut Arab University: Makassed Hospital, Beirut, Lebanon, Beirut, Lebanon;[7]Lebanese American University, Beirut, Lebanon, Beirut, Lebanon;*

### Background

Allogeneic hematopoietic stem cell transplantation (AHSCT) is a curative option for relapsed/refractory (R/R) acute myeloblastic leukemia (AML). FLAG Mitox (G-CSF, Fludarabine, Cytarabine, Mitoxantrone) is an effective salvage regimen for these patients (pts) (Hatsum N. 2019). FLAG-Idarubicin (Ida) combined to Venetoclax (Ven) induced deeper remissions and high rate of transition to successful AHSCT in R/R AML. Shortening the duration of Ven administration (14d) in FLAG- Ida-Ven led to lower toxicities (Di Nardo. 2025 – Shahswar 2024).

### Aims

This is a pilot study involving pts with R/R AML fit for intensive chemotherapy treated with a salvage therapy associating FLAG Mitox and Ven given within a short period of time (7 days) and followed by AHSCT when feasible. The objectives included rate of composite CR (CRc) and toxicity due to salvage therapy, in addition to OS and RFS after AHSCT.

### Methods

AML pts (≥ 18yo) in first relapse or refractory to induction therapy were included. FLAG-Mitox-Ven combined G-CSF (5µg/kg/d) on d1-7, Fludarabine (30mg/m2/d IV) and Cytarabine (1.5g/m2/d IV) on d2-5, Mitox (12mg/m2/d IV) d2,4 and Ven (100mg PO daily) given with voriconazole d2-8. Responding pts planned for AHSCT received 1 to 2 cycles of consolidation therapy, while other responding pts received 3 cycles. In consolidation therapy, G-CSF was given on d1-5, while Fludarabine and Cytarabine were given on d2-4. Anti FLT3 therapy was given to patients with FLT3 mutations. Measurable residual disease (MRD) was assessed by flow cytometry.

**Copyright Information:** (Online) ISSN: 2572-9241
©2026 The Author(s). HemaSphere is published by John Wiley & Sons Ltd on behalf of European Hematology Association. This is an open access Abstract Book distributed under the Attribution-NonCommercial-NoDerivs (CC BY-NC-ND), which allows third parties to download the articles and share them with others as long as they credit the author and the Abstract Book, but they cannot change the content in any way or use them commercially.
**Abstract Book Citations:** Authors, Title, HemaSphere, 2026;10:(S1):pages. The abstract book DOIs can be found on the “Author Information” page.
**Disclaimer:** Articles published in the journal HemaSphere exclusively reflect the opinions of the authors. The authors are responsible for all content in their abstracts including accuracy of the facts, statements, citing resources, etc.

### Results

Between June 2020 and December 2025, 59 pts (33 females) were included. Median age was 43y (25-67). First line therapy consisted of 7+3 regimen for 41 pts, FLAG-Ida for 15 pts, and 5-azacytidine+Ven for 3pts. AHSCT was performed for 13 pts. Anti FLT3 therapy was included in first line therapy for 14 pts. Median interval between diagnosis and salvage therapy was 28m (1-75). Anti FTL3 was included in the salvage therapy of 14 pts. After salvage therapy, all pts experienced CTCAE grade3 cytopenia. All the pts included developed febrile neutropenia. Pneumonia occurred in 10%. The 30-day mortality was 1.66% (1 pt). Of the evaluable 59 pts for response, 48 (81%) obtained CRc. MRD negativity rate was 76%. 38 pts (63%) underwent AHSCT. However, 22 did not undergo AHSCT because of early toxic death (1), refractory disease (11), multiple comorbidities (4), and economic reasons (6). AHSCT was performed from an HLA identical sibling (conditioning associating Fludarabine (Fluda), Busilvex (Bu) and Thymoglobulins) in 20 pts, and from a familial haploidentical donor (conditioning associating Thiotepa, Bu and Fluda with PTCy) in 18 pts. The 3y-OS and RFS of the transplanted pts were 66% and 62% respectively, significantly higher than the 3y-OS and RFS of the non-transplanted pts which were 22% (p=0.025) and 18% (p=0.002) respectively.
At relapse, we performed next generation sequencing in all the pts. Among the 48-responding pts,DNMT3A (12), TP53 (4), KIT (7), RUNX1 (7), IDH2 (6), and KRAS (3) were the most common identified mutations. Non-responding patients to salvage therapy (11) were significantly more likely to harbor TP53 (7) mutations.

### Summary/Conclusion

FLAG-Mitox-Ven (7d) followed by AHSCT was highly effective for R/R AML, leading to at least similar CR rate with lower toxicity compared to recent reported salvage regimens including Ven for at least 14d (Di Nardo C. 2022 – Rahme R. 2024). This regimen constituted a strong bridge to successful AHSCT.

**Copyright Information:** (Online) ISSN: 2572-9241
©2026 The Author(s). HemaSphere is published by John Wiley & Sons Ltd on behalf of European Hematology Association. This is an open access Abstract Book distributed under the Attribution-NonCommercial-NoDerivs (CC BY-NC-ND), which allows third parties to download the articles and share them with others as long as they credit the author and the Abstract Book, but they cannot change the content in any way or use them commercially.
**Abstract Book Citations:** Authors, Title, HemaSphere, 2026;10:(S1):pages. The abstract book DOIs can be found on the “Author Information” page.
**Disclaimer:** Articles published in the journal HemaSphere exclusively reflect the opinions of the authors. The authors are responsible for all content in their abstracts including accuracy of the facts, statements, citing resources, etc.

### (PF528) DNMT3A MUTANTS COMBINED WITH POST-CONSOLIDATION MRD STATUS IMPROVES RISK STRATIFICATION IN ACUTE MYELOID LEUKEMIA WITHOUT RECEIVING ALLOGENEIC HEMATOPOIETIC STEM CELL TRANSPLANTATION

**Topic:** 04. Acute myeloid leukemia - Clinical

Jiayan Gu*[1], Chenhui Bao[1], Huafeng Wang[1], Jie Jin[1], Hongyan Tong[1]

*[1]the First Affiliated Hospital, Zhejiang University School of Medicine, Hangzhou, China, Department of Hematology, Hanghzou, China;*

### Background

DNMT3A mutations are frequent in acute myeloid leukemia but their prognostic impact remains controversial, particularly in the era of widely used allogeneic hematopoietic stem cell transplantation (allo-HSCT). The interplay between DNMT3A Mutation status, sequential MRD, and allo-HSCT in risk stratification has not been systematically evaluated.

### Aims

To evaluate the prognostic significance of DNMT3A mutations and sequential flow cytometry-based MRD (FCM-MRD) at two timepoints (post-induction [MRD1] and post-consolidation [MRD2]), and to assess their integrated utility for risk stratification in AML patients, particularly focusing on the patients without receiving allo-HSCT.

### Methods

This retrospective study included 835 de novo AML patients (≥14 years) diagnosed between 2015–2023. DNMT3A mutation status was determined by sequencing. FCM-MRD was assessed at post-induction MRD status (MRD1) and MRD status after consolidation therapy (MRD2), with positivity defined as ≥0.1%. Survival analyses (Kaplan-Meier, Cox models) evaluated relapse-free survival (RFS) and overall survival (OS), stratified by DNMT3A mutation, MRD status and allo-HSCT.

### Results

DNMT3A mutations (RFS: 16 vs. 57 months, P<0.001; OS: 34 months vs. not reached, P<0.001), MRD2P (RFS: 15 vs. 37 months, P<0.001; OS: 24 months vs. 107 months, P<0.001) and without allo-HSCT (OS: 36 vs median not reached, P<0.001) were associated with inferior outcomes in the entire cohort. The adverse effect of DNMT3A mutations was exclusively observed in non-received allo-HSCT patients (RFS: 13 vs. 29 months; OS: 19 vs. 82 months; both P<0.001) and abrogated in those receiving allo-HSCT. Multivariable analysis confirmed DNMT3A mutation (RFS:HR=1.55,P<0.001;OS:HR=1.50,P<0.001) and MRD2 positivity (RFS:HR=1.69,P=0.002;OS:HR=1.87,P<0.001) as independent adverse predictors in non-transplanted patients. Within DNMT3A-mutated non-transplanted patients, only MRD2—not MRD1—retained independent prognostic significance (RFS:HR=1.77,P=0.023;OS:HR=1.78,P=0.021) .

**Copyright Information:** (Online) ISSN: 2572-9241
©2026 The Author(s). HemaSphere is published by John Wiley & Sons Ltd on behalf of European Hematology Association. This is an open access Abstract Book distributed under the Attribution-NonCommercial-NoDerivs (CC BY-NC-ND), which allows third parties to download the articles and share them with others as long as they credit the author and the Abstract Book, but they cannot change the content in any way or use them commercially.
**Abstract Book Citations:** Authors, Title, HemaSphere, 2026;10:(S1):pages. The abstract book DOIs can be found on the "Author Information" page.
**Disclaimer:** Articles published in the journal HemaSphere exclusively reflect the opinions of the authors. The authors are responsible for all content in their abstracts including accuracy of the facts, statements, citing resources, etc.

### Summary/Conclusion

DNMT3A mutations (RFS: 16 vs. 57 months, P<0.001; OS: 34 months vs. not reached, P<0.001), MRD2P (RFS: 15 vs. 37 months, P<0.001; OS: 24 months vs. 107 months, P<0.001) and without allo-HSCT (OS: 36 vs median not reached, P<0.001) were associated with inferior outcomes in the entire cohort. The adverse effect of DNMT3A mutations was exclusively observed in non-received allo-HSCT patients (RFS: 13 vs. 29 months; OS: 19 vs. 82 months; both P<0.001) and abrogated in those receiving allo-HSCT. Multivariable analysis confirmed DNMT3A mutation (RFS:HR=1.55,P<0.001;OS:HR=1.50,P<0.001) and MRD2 positivity (RFS:HR=1.69,P=0.002;OS:HR=1.87,P<0.001) as independent adverse predictors in non-transplanted patients. Within DNMT3A-mutated non-transplanted patients, only MRD2—not MRD1—retained independent prognostic significance (RFS:HR=1.77,P=0.023;OS:HR=1.78,P=0.021)

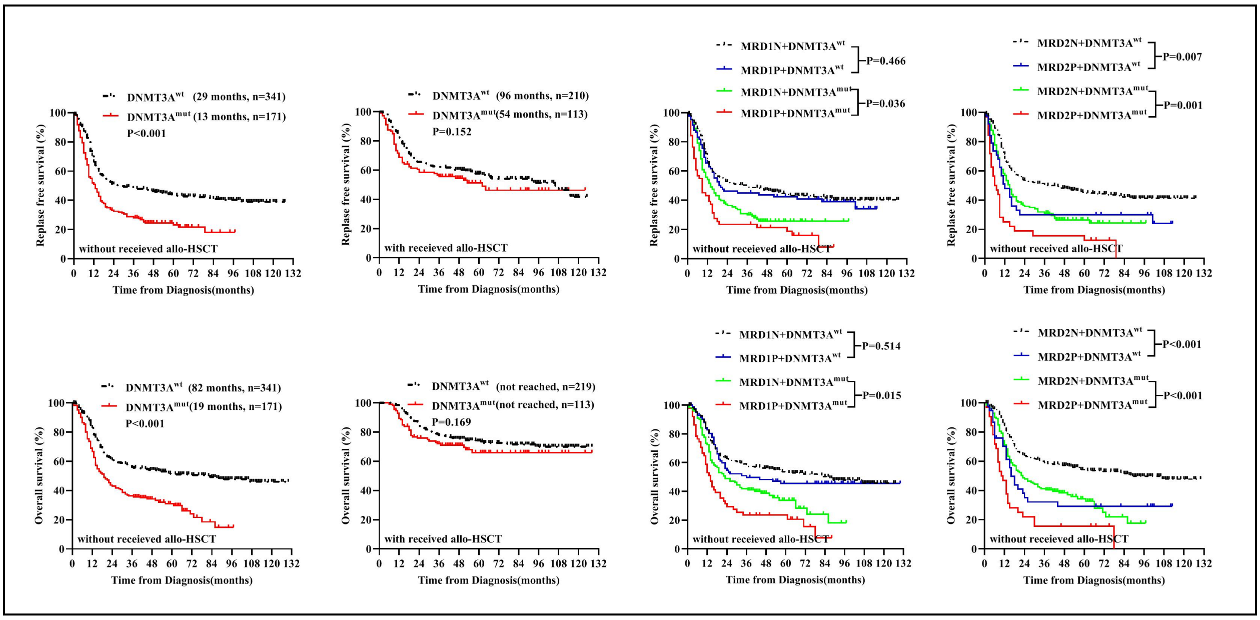

Figure . Prognostic analysis stratified by DNMT3A mutations, MRD status and allo-HSCT.

**Copyright Information:** (Online) ISSN: 2572-9241
©2026 The Author(s). HemaSphere is published by John Wiley & Sons Ltd on behalf of European Hematology Association. This is an open access Abstract Book distributed under the Attribution-NonCommercial-NoDerivs (CC BY-NC-ND), which allows third parties to download the articles and share them with others as long as they credit the author and the Abstract Book, but they cannot change the content in any way or use them commercially.
**Abstract Book Citations:** Authors, Title, HemaSphere, 2026;10:(S1):pages. The abstract book DOIs can be found on the "Author Information" page.
**Disclaimer:** Articles published in the journal HemaSphere exclusively reflect the opinions of the authors. The authors are responsible for all content in their abstracts including accuracy of the facts, statements, citing resources, etc.

### (PF529) UPDATED FINDINGS FROM THE PHASE 2 RIVER-81 STUDY OF ROMACICLIB (RVU120) COMBINED WITH VENETOCLAX IN ACUTE MYELOID LEUKEMIA AFTER FIRST-LINE VENETOCLAX AND HYPOMETHYLATING AGENT FAILURE

**Topic:** 04. Acute myeloid leukemia - Clinical

Pau Montesinos*[1], Maria Benedetta Giannini[2], Thomas Cluzeau[3], Krzysztof Madry[4], Piotr Centkowski[5], Witold Prejzner[6], Cristina Papayannidis[7], Oleksandr Moybenko[8], Milena Mazan[8], Urszula Pakulska[8], Urszula Glowniak[8], Jan Kulis[8], Diego Coelho[8], Marta Obacz[8], Nemanja Nikic[8], Malgorzata Garscia[8], Kinga Michalik[8], Agnieszka Sroka-Porada[8], Gary Wilkinson[8], Renata Windak[8], Renata Dudziak[8], Chadi Saba[8], Hendrik Nogai[8], Juan Miguel Bergua Burgues[9], Sylvain Garciaz[10], Giovanni Marconi[11], Jarosław Dybko[12], Aleksandra Butrym[13], Sara Garcia Ávila[14], Ernesta Audisio[15], Adolfo De La Fuente[16]

*[1]Hospital La Fe Valencia, University of Valencia, Valencia, Spain; [2]IRCCS Istituto Romagnolo per lo Studio dei Tumori Dino Amadori, Meldola, Italy; [3]Centre Hospitalier Universitaire (CHU) de Nice - Hopital L'Archet, Nice, France;[4]MTZ Clinical Research powered by Pratia, Warsaw, Poland; [5]Wojewódzki Szpital Specjalistyczny w Białej Podlaskiej, Biała Podlaska, Poland; [6]Uniwersyteckie Centrum Kliniczne, Klinika Hematologii i Transplantologii, Gdańsk, Poland; [7]University of Bologna Policlinico Sant'Orsola, Bologna, Italy; [8]Ryvu Therapeutics, Kraków, Poland; [9]Hospital General San Pedro De Alcántara, Caceres, Spain; [10]Institut Paoli-Calmettes, Marseille, France; [11]University of Bologna, Ospedale Santa Maria Delle Croci, Ravenna, Italy; [12]Dolnośląskie Centrum Onkologii, Wrocław, Poland; [13]Department of Hematology and Oncology, Wroclaw Medical University, Branch in Walbrzych, Wałbrzych, Poland; [14]Hospital del Mar, Barcelona, Spain; [15]AOU Città della Salute e della Scienza di Torino, Turin, Italy; [16]MD Anderson Cancer Center Madrid, Madrid, Spain;*

### Background

Venetoclax (VEN) plus hypomethylating agents (HMA) is the established first-line standard for older AML patients ineligible for intensive chemotherapy; however, ~70% develop relapsed/refractory (R/R) disease with median OS <3 months. Romaciclib (RVU120), a selective CDK8/CDK19 inhibitor, has demonstrated single-agent activity in R/R AML (NCT04021368) and preclinical synergy with VEN in resistant AML models. Mechanistically, romaciclib enhances apoptotic signaling and suppresses transcriptional programs implicated in therapeutic resistance (IL6/JAK/STAT3, PI3K/AKT/mTOR). The Phase 2 RIVER-81 study (NCT06191263) evaluates romaciclib plus VEN in AML progressing after frontline VEN+HMA.

### Aims

The primary objective of Part 1 is safety and dose determination; the primary endpoint of Part 2 is composite complete remission rate (CRc: CR, CRh, CRi ± MRD). Secondary endpoints include duration of response (DoR), OS, transfusion independence, HSCT bridging, PK, and pharmacodynamic assessment of STAT5 phosphorylation.

### Methods

RIVER-81 is a multicenter study employing a 3+3 dose-escalation design. Initial cohorts evaluated romaciclib 125–250 mg QOD (Days 1–13) with VEN 100–400 mg QD (14 days). Based on safety and PK findings, QD dosing (150, 200, 250 mg) with VEN 400 mg was explored.

**Copyright Information:** (Online) ISSN: 2572-9241
©2026 The Author(s). HemaSphere is published by John Wiley & Sons Ltd on behalf of European Hematology Association. This is an open access Abstract Book distributed under the Attribution-NonCommercial-NoDerivs (CC BY-NC-ND), which allows third parties to download the articles and share them with others as long as they credit the author and the Abstract Book, but they cannot change the content in any way or use them commercially.
**Abstract Book Citations:** Authors, Title, HemaSphere, 2026;10:(S1):pages. The abstract book DOIs can be found on the "Author Information" page.
**Disclaimer:** Articles published in the journal HemaSphere exclusively reflect the opinions of the authors. The authors are responsible for all content in their abstracts including accuracy of the facts, statements, citing resources, etc.

**Results**

As of February 9, 2026, 58 patients were treated (median age 76; 32 with high-risk mutations). No DLTs occurred at lower dose levels; one Grade 3 nausea DLT was observed at 250 mg QD. PK demonstrated dose-proportional exposure, and pSTAT5 inhibition confirmed robust target engagement. The most compelling activity was observed at 150 mg QD + VEN 400 mg, achieving CRc 43% (3/7), including 2 CRs and 1 CRi, with durable responses lasting 169, 95, and 106 days, respectively. Across other dose levels (n=51), 6 CRh/CRi responses were reported. The most common AEs were nausea (61%), vomiting (44%), constipation (20%), asthenia (19%), thrombocytopenia (18%), and anemia (18%).

**Summary/Conclusion**

Romaciclib plus VEN demonstrates clinically meaningful and durable activity in R/R AML following VEN+HMA failure. The 150 mg QD regimen achieved the most consistent responses and was selected for expansion based on integrated safety, PK, and efficacy findings. These data support continued clinical development of this combination in a population with limited therapeutic options.

**Copyright Information:** (Online) ISSN: 2572-9241
©2026 The Author(s). HemaSphere is published by John Wiley & Sons Ltd on behalf of European Hematology Association. This is an open access Abstract Book distributed under the Attribution-NonCommercial-NoDerivs (CC BY-NC-ND), which allows third parties to download the articles and share them with others as long as they credit the author and the Abstract Book, but they cannot change the content in any way or use them commercially.
**Abstract Book Citations:** Authors, Title, HemaSphere, 2026;10:(S1):pages. The abstract book DOIs can be found on the "Author Information" page.
**Disclaimer:** Articles published in the journal HemaSphere exclusively reflect the opinions of the authors. The authors are responsible for all content in their abstracts including accuracy of the facts, statements, citing resources, etc.

### (PF530) UPDATED RESULT OF SELINEXOR-BASED NON-INTENSIVE CHEMOTHERAPY REGIMENS ADJUSTED ACCORDING TO EARLY RESPONSES ACHIEVE HIGH EARLY REMISSION IN NEWLY DIAGNOSED AML OR MDS

**Topic:** 04. Acute myeloid leukemia - Clinical

Shiyuan Zhang[1], Yi Zhu[1], Bin Xu[1], Yuling Wan[1], Shuai Su[1], Xiwen Tong[1, 2], Andie Fu[1], Zhuoxin Wen[1], Longlong Liu[3], Min Xiao[1], Zhiqiong Wang[1], Zhenqian Huang[3], Jia Wei[1], Donghua Zhang*[1]

*[1]Tongji Hospital, Tongji Medical College, Huazhong University of Science and Technology, Hematology, Wuhan, China;[2]Sir Run Run Shaw Hospital, Zhejiang University School of Medicine, Hematology and Cell Therapy, Hangzhou, China; [3]The First Affiliated Hospital of Guangzhou Medical University, Hematology, Guangzhou, China;*

### Background

Some newly diagnosed (ND) acute myeloid leukemia (AML) or higher-risk myelodysplastic syndrome (MDS) patients (pts) are ineligible for or unwilling to receive intensive chemotherapy. These pts usually receive low-intensity regimens. We explored selinexor-based chemo-free or low-dose chemotherapy regimens and presented the preliminary findings at 2025 EHA (PB2563). Here we present updated results.

### Aims

To investigate the efficacy and safety of selinexor based non-intensive chemotherapy for the ND AML or MDS, and evaluate the timing for early adjustment of the regimens.

### Methods

Pts who refused or were ineligible for intensive chemotherapy were enrolled and received the following XAB regimens: selinexor was initially dosed at 35 mg/m$^2$ twice a week or three times a week (60mg BIW or TIW). Azacitidine 75 mg/m$^2$/d for 5 to 7 days, venetoclax was administered at 100 mg, 200 mg, and 400 mg on d1-3, respectively, followed by 400 mg daily days 3 to 14.
Bone marrow puncture was performed every week during the treatment. According to whether CR/CRi was achieved in the first and second weeks, subsequent treatments were adjusted. Pts who achieved CR/CRi subsequently received allo-HSCT if they were fit for or willing to undergo it. Other pts were treated with low-dose cytarabine 50 mg/d on the basis of XAB regimen for 5 days, followed consolidation and maintenance therapy.

**Copyright Information:** (Online) ISSN: 2572-9241
©2026 The Author(s). HemaSphere is published by John Wiley & Sons Ltd on behalf of European Hematology Association. This is an open access Abstract Book distributed under the Attribution-NonCommercial-NoDerivs (CC BY-NC-ND), which allows third parties to download the articles and share them with others as long as they credit the author and the Abstract Book, but they cannot change the content in any way or use them commercially.
**Abstract Book Citations:** Authors, Title, HemaSphere, 2026;10:(S1):pages. The abstract book DOIs can be found on the "Author Information" page.
**Disclaimer:** Articles published in the journal HemaSphere exclusively reflect the opinions of the authors. The authors are responsible for all content in their abstracts including accuracy of the facts, statements, citing resources, etc.

**Results**

As of January 2026, 43 pts were enrolled, median age of 58, comprising 29 males. 27 pts with AML, 16 pts with MDS. Risk stratification was as follows: AML: 12 adverse, 9 intermediate, 5 favor, 1 unknown (owing to the absence of available mutation or chromosomal information); MDS: 5 very high, 7 high, 4 intermediate.
After one week of XAB treatment, 25 pts (58.1%) achieved CR/CRi, 9 (20.9%) PR, and 9 (20.9%) NR. After the second week of treatment, 32 pts (74.4%) CR, 7 (16.3%) PR, 4 (9.3%) NR (5 of NR and 2 of PR acquired CR/CRi). 12 pts subsequently received allo-HSCT. The median follow-up time was 108 days, and 35 pts (81.4%) were survived.
Following the observed acceleration in blast cell decline with the increased selinexor dosing frequency from BIW to TIW, all subsequently enrolled patients (22 pts) were transitioned to the TIW regimen. The proportion of pts with bone marrow blast cells ≤ 5% in TIW group was 50% in the first week and 78.6% in the second week, while in the BIW were 46.2% and 61.5% respectively. The minimal residual disease (MRD)-negative rate was much higher in the TIW group (13.6% of pts in the first week and 40.9% in the second week), compared with the BIW (4.8% and 9.5% respectively).
After 2 weeks of treatment, among pts evaluable for safety, the incidence of grade 4 neutropenia was 71.4% (15/21) in BIW group, with a median recovery time of 20 (range, 5 to 35) days. In TIW group, the incidence was 90.9% (20/22), and the median recovery time was 15 (range, 10 to 31) days. The incidence of grade 4 thrombocytopenia was 33.3% (7/21) in BIW group, with a median recovery time of 27 (range, 16 to 60) days. In TIW group, the incidence was 50% (11/22), and the median recovery time was 27 (range, 17 to 52) days.

**Summary/Conclusion**

A high rate of early remission could be achieved in ND AML/MDS pts treated with non-intensive regimen containing Selinexor, and administration of selinexor TIW was associated with higher rate of early remission and MRD-negative compared with BIW schedule. Selinexor-containing regimens provide a new treatment option for these pts.

**Copyright Information:** (Online) ISSN: 2572-9241
©2026 The Author(s). HemaSphere is published by John Wiley & Sons Ltd on behalf of European Hematology Association. This is an open access Abstract Book distributed under the Attribution-NonCommercial-NoDerivs (CC BY-NC-ND), which allows third parties to download the articles and share them with others as long as they credit the author and the Abstract Book, but they cannot change the content in any way or use them commercially.
**Abstract Book Citations:** Authors, Title, HemaSphere, 2026;10:(S1):pages. The abstract book DOIs can be found on the "Author Information" page.
**Disclaimer:** Articles published in the journal HemaSphere exclusively reflect the opinions of the authors. The authors are responsible for all content in their abstracts including accuracy of the facts, statements, citing resources, etc.

### (PF531) EFFICACY AND SAFETY OF VENETOCLAX COMBINED WITH CAG REGIMEN AS INDUCTION THERAPY IN ELDERLY PATIENTS WITH NEWLY DIAGNOSED ACUTE MYELOID LEUKEMIA: A MULTICENTER PHASE II STUDY

**Topic:** 04. Acute myeloid leukemia - Clinical

Mei Sun*[1], Bin He[1], Xiaoyan Xie[1], Fangfang Wang[1], Lingling Qian[1], Miao Zhu[1], Xiao Wang[1], Honghu Zhu[2]

*[1]Northern People's Hospital affiliated to Yangzhou University, Yangzhou, China;[2]Beijing Chao-Yang Hospital, Department of Hematology, Beijing, China;*

### Background

Elderly patients with newly diagnosed acute myeloid leukemia (AML) often have poor tolerance to intensive chemotherapy and unfavorable clinical outcomes. Venetoclax-based low-intensity regimens have demonstrated promising efficacy in this population. This phase II, single-arm study aimed to evaluate the efficacy and safety of venetoclax combined with the CAG regimen (cytarabine, aclarubicin, and G-CSF) as induction therapy in elderly AML patients.

### Aims

The aim of this phase II, single-arm study was to evaluate both the efficacy and safety of venetoclax combined with the CAG regimen (cytarabine, aclarubicin, and G-CSF) as induction therapy in elderly patients with AML.

### Methods

Patients aged ≥60 years with newly diagnosed, previously untreated AML were enrolled. Venetoclax was administered orally at 400 mg daily on days 1–7 (dose-adjusted to 100 mg when combined with azole antifungals), together with aclarubicin (20 mg, days 1–4), cytarabine (20 mg/m$^2$, subcutaneously every 12 h, days 1–7), and G-CSF (5 µg/kg/day, days 0–7). Consolidation therapy consisted of up to six cycles of the same regimen. The primary endpoint was complete remission (CR) rate after 1–2 cycles. Secondary endpoints included safety, composite CR (CRc), minimal residual disease (MRD), event-free survival (EFS), overall survival (OS), and cumulative incidence of relapse (CIR).

### Results

A total of 11 patients were enrolled, with a median age of 66 years (range, 56–77). According to cytogenetic and molecular risk stratification, 2 patients were classified as favorable risk, 3 as intermediate risk, and 6 as adverse risk. After induction therapy, 10 patients achieved CR or CRi, resulting in a CRc rate of 90.9%. One patient with adverse-risk disease achieved CR after two cycles, while one patient discontinued treatment after a single cycle due to lack of response. Treatment-emergent adverse events were mainly hematologic and infectious. Grade 3–4 pneumonia occurred in 73% of patients, and one patient (9%) developed sepsis. Febrile events were common but manageable. No treatment-related mortality was observed during induction.

### Summary/Conclusion

Venetoclax combined with the CAG regimen demonstrated encouraging efficacy with a high remission rate in elderly patients with newly diagnosed AML, including those with adverse-risk features. Although infectious complications were frequent, the overall safety profile was acceptable and manageable. This regimen may represent an effective induction option for elderly AML patients, warranting further investigation in larger prospective studies.

**Copyright Information:** (Online) ISSN: 2572-9241
©2026 The Author(s). HemaSphere is published by John Wiley & Sons Ltd on behalf of European Hematology Association. This is an open access Abstract Book distributed under the Attribution-NonCommercial-NoDerivs (CC BY-NC-ND), which allows third parties to download the articles and share them with others as long as they credit the author and the Abstract Book, but they cannot change the content in any way or use them commercially.
**Abstract Book Citations:** Authors, Title, HemaSphere, 2026;10:(S1):pages. The abstract book DOIs can be found on the “Author Information” page.
**Disclaimer:** Articles published in the journal HemaSphere exclusively reflect the opinions of the authors. The authors are responsible for all content in their abstracts including accuracy of the facts, statements, citing resources, etc.

### (PF532) DECITABINE/CEDAZURIDINE WITH OR WITHOUT A SECOND TARGETED AGENT (VENETOCLAX, GILTERITINIB, ENASIDENIB, OR IVOSIDENIB) AS FULLY ORAL PERSONALIZED MAINTENANCE THERAPY IN ACUTE MYELOID LEUKEMIA

**Topic:** 04. Acute myeloid leukemia - Clinical

Alexandre Bazinet*[1], Guillermo Garcia-Manero[1], Naval Daver[1], Farhad Ravandi[1], Maro Ohanian[1], Nicholas Short[1], Koji Sasaki[1], Musa Yilmaz[1], Kelly Chien[1], Danielle Hammond[1], Elias Jabbour[1], Abhishek Maiti[1], Courtney Dinardo[1], Fadi Haddad[1], Jo Ishizawa[1], Nitin Jain[1], Eitan Kugler[1], Lucia Masarova[1], William Wierda[1], Gautam Borthakur[1], Hagop Kantarjian[1], Tapan Kadia[1]

[1]*MD Anderson Cancer Center, Houston, United States of America;*

### Background

Relapse is the major cause of morbidity/mortality in acute myeloid leukemia (AML). Maintenance therapy consists of prolonged administration of lower intensity therapy with the goal of delaying or preventing relapse.

### Aims

Evaluate the safety and preliminary efficacy of oral targeted maintenance regimens in AML.

### Methods

This was a phase 1b study of oral decitabine/cedazuridine (ASTX727) +/- physician's choice of targeted agent based on molecular profile. We included patients (pts) ≥ 18 years with AML in first complete remission (CR) or CR with incomplete blood count recovery (CRi) unable to complete standard therapy and not immediate candidates for stem cell transplantation (SCT). At least 2 courses of intensive chemotherapy (standard/high-dose cytarabine) or 3 courses of low-intensity therapy (HMA or low-dose cytarabine-based) were required prior to enrollment. Therapy consisted of ASTX727 35/100 mg PO on D1-3 alone (arm A) or in combination with venetoclax 400 mg PO on D1-5 (arm B), gilteritinib 120mg PO on D1-28 (arm C), enasidenib 100 mg PO on D1-28 (arm D), or ivosidenib 500 mg PO on D1-28 (arm E), for up to 24 cycles. A 3+3 dose de-escalation design was used for the safety lead-in cohort in each arm. The primary objective was safety/tolerability. Secondary objectives included relapse-free survival (RFS) and overall survival (OS) from time of enrollment. Pts were censored at the time of SCT. ClinicalTrials.gov NCT05010772.

**Copyright Information:** (Online) ISSN: 2572-9241
©2026 The Author(s). HemaSphere is published by John Wiley & Sons Ltd on behalf of European Hematology Association. This is an open access Abstract Book distributed under the Attribution-NonCommercial-NoDerivs (CC BY-NC-ND), which allows third parties to download the articles and share them with others as long as they credit the author and the Abstract Book, but they cannot change the content in any way or use them commercially.
**Abstract Book Citations:** Authors, Title, HemaSphere, 2026;10:(S1):pages. The abstract book DOIs can be found on the "Author Information" page.
**Disclaimer:** Articles published in the journal HemaSphere exclusively reflect the opinions of the authors. The authors are responsible for all content in their abstracts including accuracy of the facts, statements, citing resources, etc.

**Results**

Between 11/2021 and 11/2025, 43 pts were treated (9 on arm A, 25 on arm B, 3 on arm C, 4 on arm D, and 2 on arm E). Induction therapy was intensive in 19 (44%) pts and low-intensity in 24 (56%) pts. The median age was 68 years (26-85) and 23/43 (53%) were male. By ELN 2022, 15 (35%) were favorable, 3 (7%) were intermediate, and 25 (58%) were adverse. Two (5%) pts were *TP53*-mutated (1 in arm A, 1 in arm B) and 5 (12%) had complex cytogenetics. At enrollment, 28/41 (68%) were MRD-negative and 13/41 (32%) were MRD-positive.
No dose-limiting toxicities occurred in cycle 1. The most common grade 3/4 adverse events were leukopenia (84%), neutropenia (81%), thrombocytopenia (60%), and anemia (42%). Neutropenic fever occurred in 14% of pts. Thirteen grade 3/4 infections (8 pneumonia, 2 Covid-19, 2 colitis, and 1 sepsis) occurred in 11 (26%) unique pts. Only 1 death occurred while on maintenance therapy (from sepsis). All other deaths occurred after relapse or following transition to SCT.
The median follow-up time was 25.9 months (m). The median RFS was 24 m for the full cohort. By arm, RFS was 22.4 m in arm A, not reached (NR) in arm B, 10.5 m in arm C, NR in arm D, and NR in arm E (Fig A). Pts who received intensive induction had a median RFS of NR compared to 22.4 m in those who received low-intensity induction (p=0.16). By ELN 2022 risk, the median RFS was NR in favorable risk, 24 m in intermediate, and 11.7 m in adverse (p=0.13). MRD-positive pts had significantly shorter RFS compared to pts who were MRD-negative (6.6 m vs NR; p=0.003). Only 2/13 (15%) MRD-positive pts cleared their MRD on maintenance. Six pts went off protocol to receive SCT.
The median OS was 30.8 m for the full cohort, NR in the pts who received intensive induction, and 22.4 m in those who received low-intensity induction. By arm, the median OS was 40.2 m in arm A, 33.7 m in arm B, 30.7 m in arm C, NR in arm D, and NR in arm E.

**Summary/Conclusion**

Personalized oral maintenance therapy in AML was feasible/safe and demonstrated encouraging RFS.

**Copyright Information:** (Online) ISSN: 2572-9241
©2026 The Author(s). HemaSphere is published by John Wiley & Sons Ltd on behalf of European Hematology Association. This is an open access Abstract Book distributed under the Attribution-NonCommercial-NoDerivs (CC BY-NC-ND), which allows third parties to download the articles and share them with others as long as they credit the author and the Abstract Book, but they cannot change the content in any way or use them commercially.
**Abstract Book Citations:** Authors, Title, HemaSphere, 2026;10:(S1):pages. The abstract book DOIs can be found on the "Author Information" page.
**Disclaimer:** Articles published in the journal HemaSphere exclusively reflect the opinions of the authors. The authors are responsible for all content in their abstracts including accuracy of the facts, statements, citing resources, etc.

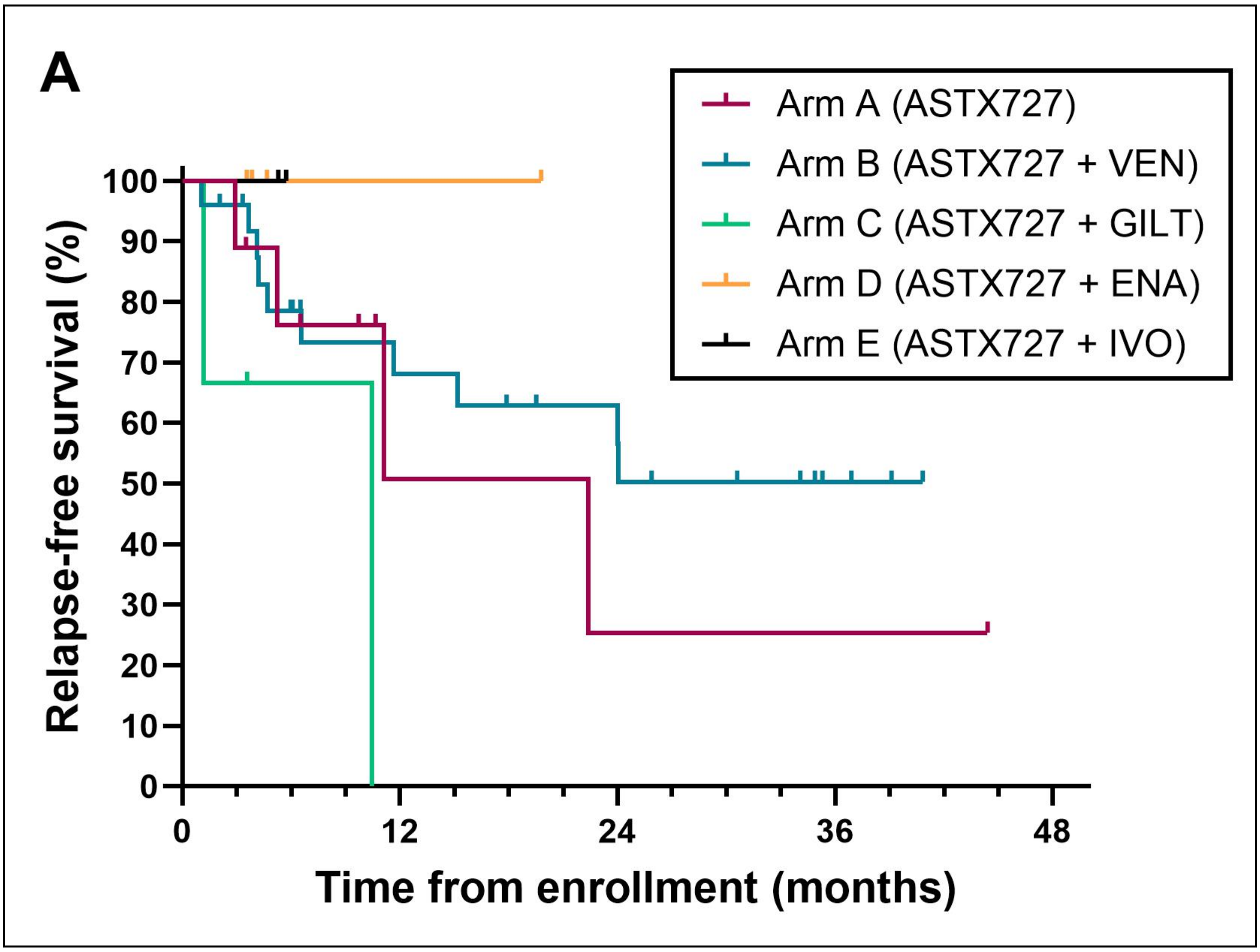

**Copyright Information:** (Online) ISSN: 2572-9241
©2026 The Author(s). HemaSphere is published by John Wiley & Sons Ltd on behalf of European Hematology Association. This is an open access Abstract Book distributed under the Attribution-NonCommercial-NoDerivs (CC BY-NC-ND), which allows third parties to download the articles and share them with others as long as they credit the author and the Abstract Book, but they cannot change the content in any way or use them commercially.
**Abstract Book Citations:** Authors, Title, HemaSphere, 2026;10:(S1):pages. The abstract book DOIs can be found on the "Author Information" page.
**Disclaimer:** Articles published in the journal HemaSphere exclusively reflect the opinions of the authors. The authors are responsible for all content in their abstracts including accuracy of the facts, statements, citing resources, etc.

### (PF533) AUTOMATIC AND EXPLAINABLE ANALYSIS OF BONE MARROW SMEARS USING MACHINE LEARNING METHODS : AN INNOVATIVE MODEL FOR THE DIAGNOSIS OF ACUTE MYELOID LEUKEMIA

**Topic:** 04. Acute myeloid leukemia - Clinical

Jules Morata*[1, 2, 3], Valentin Andral[1, 4, 5], Philippe Leleux[1, 2, 3], Pierre Brousset[1, 5, 6], Christian Recher[1, 5, 6], Alban Canali[6], Jean-Baptiste Rieu[6], Sandrine Mouysset[1, 4, 5], Sarah Bertoli[1, 5, 6]

*[1]Centre de Recherche en Cancerologie de Toulouse, Toulouse, France;[2]Laboratoire d'Analyse et d'Architecture des Systèmes - CNRS, Toulouse, France; [3]Institut National des Sciences Appliquées, Toulouse, France; [4]Institut de Recherches en Informatique de Toulouse, Toulouse, France; [5]Université de Toulouse, Toulouse, France; [6]Centre Hospitalier Universitaire de Toulouse, Toulouse, France;*

### Background

Acute Myeloid Leukemia (AML) diagnosis relies on cytological analysis of bone marrow smears and the evaluation of the percentage of blasts. At the moment, the vast majority of diagnosis methods using machine learning are based on approches often referred to as « black-box » (the model's diagnosis can't be justified). We have developed an automatic pipeline which classifies bone marrow cells and identifies blasts to assist in AML diagnosis.

### Methods

We have digitized 84 bone marrow smears, including 69 AML and 15 from patients without AML. These smears allowed us to validate our pipeline while 12,650 cells annotated by expert cytologists have been used to train our models.
Using several machine learning methods, we have developed an original tool for digitized bone marrow smears analysis, which is able to segment and classify up to 5,000 cells in a smear. This system relies on three successive steps. The first one consists in selecting on each smear the optimal zones of interest for morphological analysis of the cells. It relies on an algorithmic clustering method to regroup images in three classes, based on the color of their pixels, allowing the identification of regions which are neither too dense nor too sparse in terms of cell density, thus selecting the optimal zones.
The second step, called segmentation, aims to detect nucleated elements using a Deep Learning model, Mask R-CNN, initially designed for automatic object detection and classification in images. Here it is used to automatically isolate and cut out each nucleated element.
The final step allows cut out cells classification into two classes : blasts or differentiated hematopoietic cells. This classification is made using a model that uses decision trees, using visual features extracted from each cell (size, shape, texture of nucleus and cytoplasm).
In order to compare the performances of our pipeline to state of the art models, we have also trained and optimized two deep learning models called DINOv3 and DinoBloom.

**Copyright Information:** (Online) ISSN: 2572-9241
©2026 The Author(s). HemaSphere is published by John Wiley & Sons Ltd on behalf of European Hematology Association. This is an open access Abstract Book distributed under the Attribution-NonCommercial-NoDerivs (CC BY-NC-ND), which allows third parties to download the articles and share them with others as long as they credit the author and the Abstract Book, but they cannot change the content in any way or use them commercially.
**Abstract Book Citations:** Authors, Title, HemaSphere, 2026;10:(S1):pages. The abstract book DOIs can be found on the "Author Information" page.
**Disclaimer:** Articles published in the journal HemaSphere exclusively reflect the opinions of the authors. The authors are responsible for all content in their abstracts including accuracy of the facts, statements, citing resources, etc.

### Results

Our model presents better diagnostic performances (AML vs non-AML) than DINOv3 and DinoBloom (respectively 92.4%, 86.9% and 83.7%). Moreover, it correctly identifies 94% of AML patients, against 83% for DINOv3 and 78% for DinoBloom. The average difference between the blast percentage predicted by our model and the one from the microscopic exam of an expert cytologist is 16.52 (standard deviation : 18.90) compared to 30.50 (std : 21.45) for DINOv3 and 27.88 (std : 17.28) for DinoBloom, also indicating a more reliable estimation of the blast percentages.
By allowing the analysis of the most influential features on the prediction, our model provides some explainability of the results. For example, it highlights the importance of a high nucleus/cytoplasm ratio for a cell to be a blast, which is coherent with the cytological criteria used in practice.

### Summary/Conclusion

With its strong performance and the explainability of its results, our model is a relevant approach for bone marrow smear evaluation and offers promising perspectives for the diagnosis and monitoring of patients suffering from AML.

**Copyright Information:** (Online) ISSN: 2572-9241
©2026 The Author(s). HemaSphere is published by John Wiley & Sons Ltd on behalf of European Hematology Association. This is an open access Abstract Book distributed under the Attribution-NonCommercial-NoDerivs (CC BY-NC-ND), which allows third parties to download the articles and share them with others as long as they credit the author and the Abstract Book, but they cannot change the content in any way or use them commercially.
**Abstract Book Citations:** Authors, Title, HemaSphere, 2026;10:(S1):pages. The abstract book DOIs can be found on the "Author Information" page.
**Disclaimer:** Articles published in the journal HemaSphere exclusively reflect the opinions of the authors. The authors are responsible for all content in their abstracts including accuracy of the facts, statements, citing resources, etc.

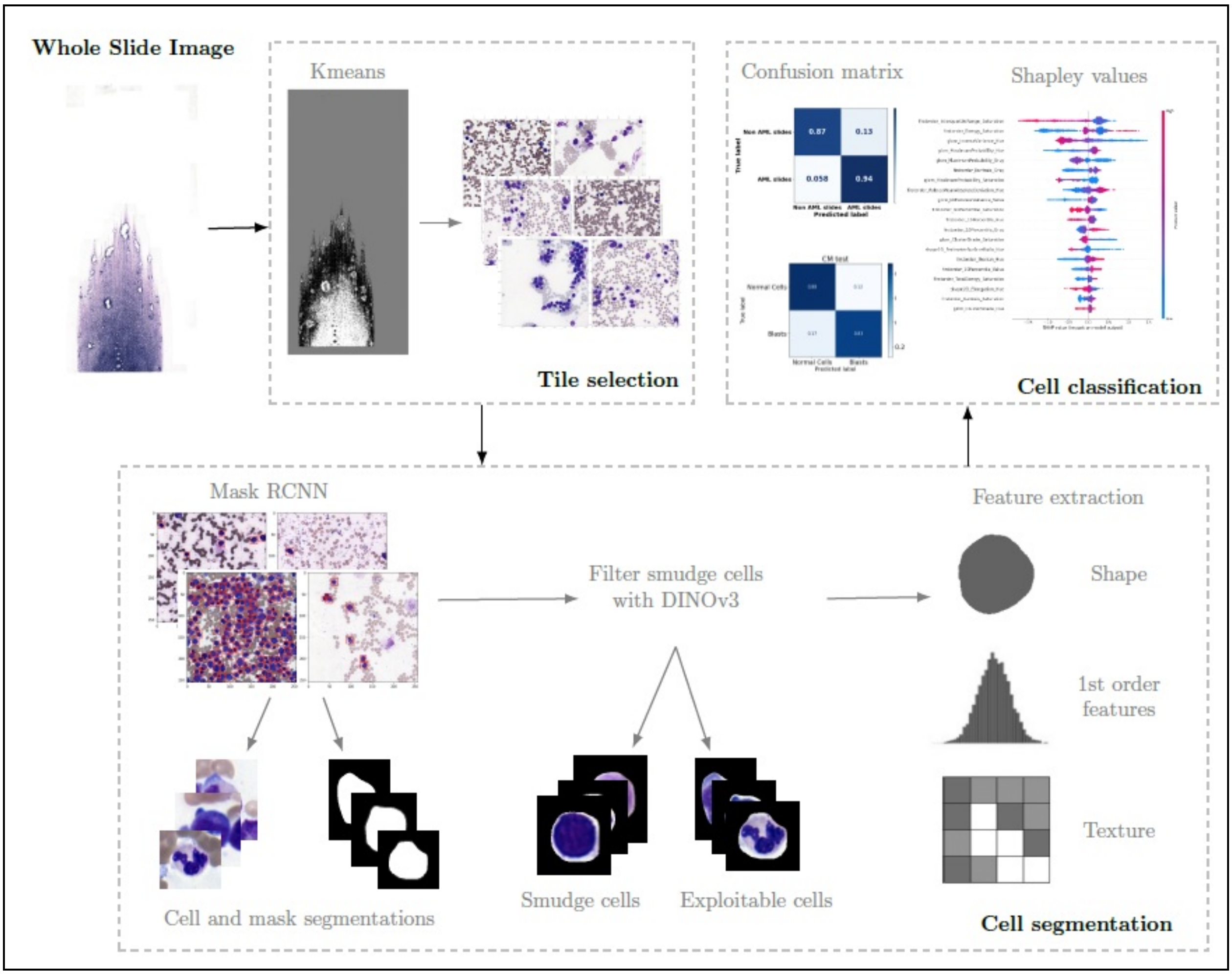

Schematic representation of the pipeline

**Copyright Information:** (Online) ISSN: 2572-9241
©2026 The Author(s). HemaSphere is published by John Wiley & Sons Ltd on behalf of European Hematology Association. This is an open access Abstract Book distributed under the Attribution-NonCommercial-NoDerivs (CC BY-NC-ND), which allows third parties to download the articles and share them with others as long as they credit the author and the Abstract Book, but they cannot change the content in any way or use them commercially.
**Abstract Book Citations:** Authors, Title, HemaSphere, 2026;10:(S1):pages. The abstract book DOIs can be found on the "Author Information" page.
**Disclaimer:** Articles published in the journal HemaSphere exclusively reflect the opinions of the authors. The authors are responsible for all content in their abstracts including accuracy of the facts, statements, citing resources, etc.

### (PF534) FEASIBILITY AND CLINICAL IMPACT OF CYTARABINE CONSOLIDATION FOR AML IN A FULL OUTPATIENT SETTING

**Topic:** 04. Acute myeloid leukemia - Clinical

Marina Vallvé I Oliva*[1], Ana Garrido Diaz[1], Irene Garcia Cadenas[1], Rodrigo Martino[1], Albert Esquirol Sanfeliu[1], Sara Redondo[1], Annalisa Paviglianti[1], Jordi Lopez Pardo[1], Ángela Sánchez[1], Silvana Daniela Saavedra Gerosa[1], Miguel Argüello de Tomás[1], Ana Muntañola Prat[1], Anna Bosch[1], Ana Carolina Caballero Gonzalez[1], Javier Briones Meijide[1], Guadalupe Oñate[1]

[1]*Hospital de Sant Pau, Hematology, Barcelona, Spain;*

### Background

Acute myeloid leukemia (AML) in fit patients is treated with intensive induction chemotherapy and 1-3 consolidation (Cons) cycles with high-dose cytarabine (HiDAC). Classical inpatient (IP) treatment administration, to ensure infection monitoring and supportive treatment, is being replaced by hybrid programs where patients are discharged after HiDAC. The SARS-CoV-2 pandemic forced hematologists to adapt protocols to optimize resources and decrease infection risks. Here, we report the results of a full outpatient (OP) protocol for AML-Cons cycles.

### Aims

To compare the outcomes of IP and OP administration of HiDAC consolidations to determine the feasibility and safety of the full OP management.

### Methods

All consecutive fit adult AML patients diagnosed at our institution from January 2018 to December 2024 were retrospectively analyzed. From 2018-2020, patients <60 years received HiDAC 3g/m2/12h days 1, 3, and 5 (CETLAM-12). Since 2020, they received 1.5g/m2/12h days 1-3 (CETLAM-22). In both protocols, patients >60 years received a reduced dose of 1g/m2/12h. Protocol included pegfilgrastim on day 4 and triple anti-infectious prophylaxis.

Since the SARS-CoV-2 pandemic, all patients were considered for the OP program unless selected cases with >1h distance from the hospital, caregiver unavailability, limiting comorbidities, platelet refractoriness and previous severe complications. Patients receiving 3g/m2/12g were also IP due to higher toxicity.

OP cycles were administered at our day-care unit,starting at 7am, with q12 dose starting at 5pm, and patients were followed in the outpatient clinic 1-3 times a week. IP patients received HiDAC treatment hospitalized, were di scharged after finishing teratment on day 5 and were followed similarly in the outpatient clinic. Fluoroquinolones were used as antibacterial prophylaxis.

**Copyright Information:** (Online) ISSN: 2572-9241
©2026 The Author(s). HemaSphere is published by John Wiley & Sons Ltd on behalf of European Hematology Association. This is an open access Abstract Book distributed under the Attribution-NonCommercial-NoDerivs (CC BY-NC-ND), which allows third parties to download the articles and share them with others as long as they credit the author and the Abstract Book, but they cannot change the content in any way or use them commercially.
**Abstract Book Citations:** Authors, Title, HemaSphere, 2026;10:(S1):pages. The abstract book DOIs can be found on the "Author Information" page.
**Disclaimer:** Articles published in the journal HemaSphere exclusively reflect the opinions of the authors. The authors are responsible for all content in their abstracts including accuracy of the facts, statements, citing resources, etc.

**Results**

A total of 91 patients were included in the study, 46 IP and 45 OP. Baseline characteristics were similar in both groups: median age (55 vs 56y, p=0,69), female gender (51.1% vs 48.9%, p=0.754), and ELN-22 risk category (favorable 52.2% vs 60%, intermediate 23.9% vs 22.2%, and adverse 23.9% vs 17.8%, p=0.7).

Regarding HiDAC dosing, although we found an increased RFS with 1.5g/m2 compared to 3g/m2 (2yr RFS 45% vs 64 p=0.02, respectively) we found no differences in OS between doses (2yr OS 70% and 68% respectively, p=0.5).

Re-admission rates were higher in the IP group in all cycles: 71.8% vs. 28.2% in Cons-1 (p=0.03), 84% vs. 16% in Cons-2(p=0.001) and 63.6% vs. 36.4% in Cons-3 (p=0.24). The main cause of admission was febrile neutropenia, with a higher incidence of resistant bacterial infections in the IP group (Cons-1 10.7% vs. 0% p=0.28) (Cons-2 14.3% vs. 0% p=0.42). ICU rate was for each Cons 1 and 2: 17.9% and 14.3% in IP vs. 10% and 25% in OP (p=0.55; p= 0.59) with similar median hospitalization days (10.8, 7-14). OP presented a HR for OS of 0.52 (0.25-1.08 p=0.08) with a 2y OS 80,9% vs. 64% for OP and IP, respectively (p=0.07).

**Summary/Conclusion**

In this study, full OP administration of HiDAC in AML was feasible and safe in selected cases, with fewer re-admissions and a trend in resistant bacterial infections. These findings support the OP approach as a viable alternative, potentially reducing resource needs and nosocomial complications.

**Copyright Information:** (Online) ISSN: 2572-9241
©2026 The Author(s). HemaSphere is published by John Wiley & Sons Ltd on behalf of European Hematology Association. This is an open access Abstract Book distributed under the Attribution-NonCommercial-NoDerivs (CC BY-NC-ND), which allows third parties to download the articles and share them with others as long as they credit the author and the Abstract Book, but they cannot change the content in any way or use them commercially.
**Abstract Book Citations:** Authors, Title, HemaSphere, 2026;10:(S1):pages. The abstract book DOIs can be found on the "Author Information" page.
**Disclaimer:** Articles published in the journal HemaSphere exclusively reflect the opinions of the authors. The authors are responsible for all content in their abstracts including accuracy of the facts, statements, citing resources, etc.

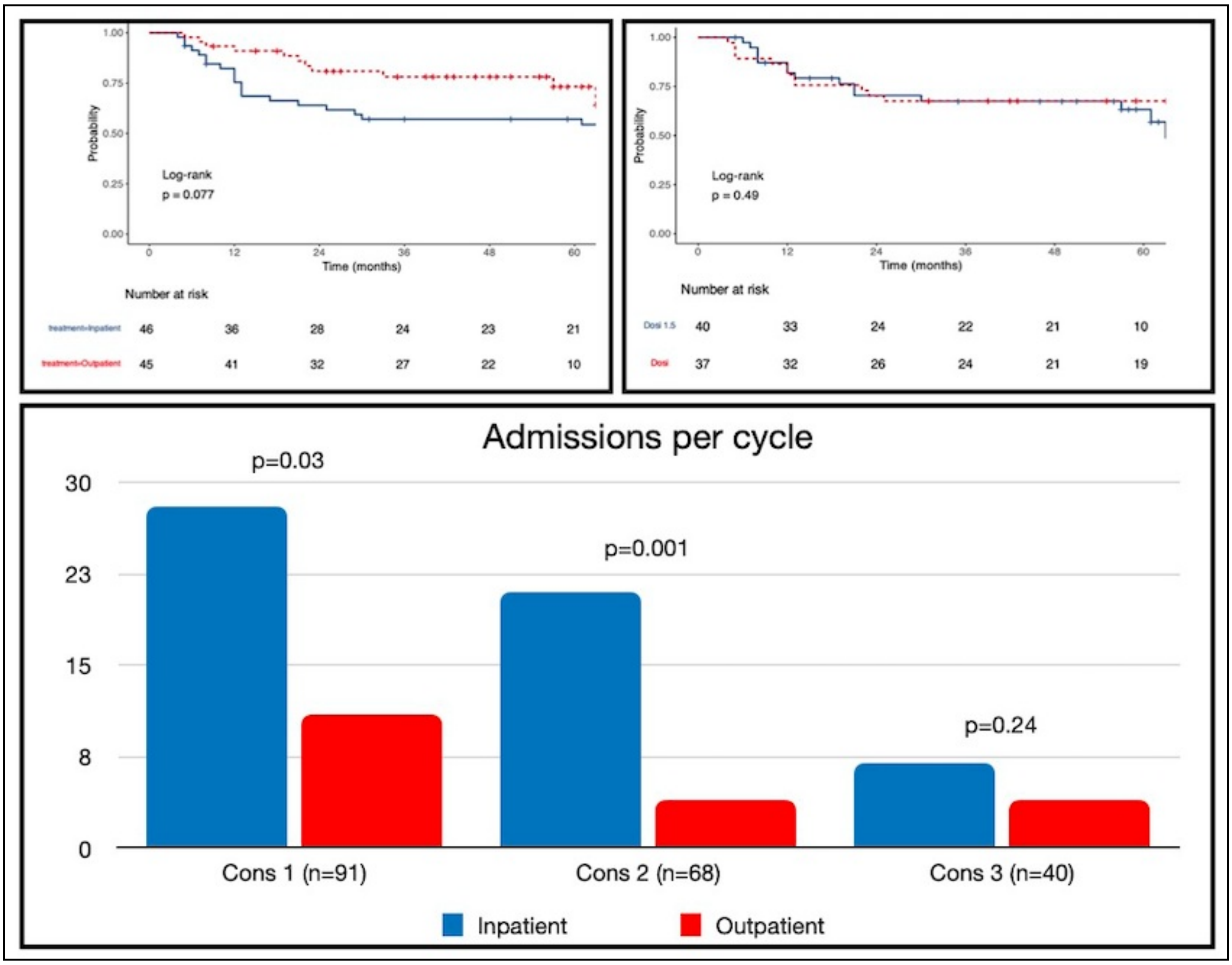

OS between IP and OP, HiDAC doses and hospital admissions

**Copyright Information:** (Online) ISSN: 2572-9241
©2026 The Author(s). HemaSphere is published by John Wiley & Sons Ltd on behalf of European Hematology Association. This is an open access Abstract Book distributed under the Attribution-NonCommercial-NoDerivs (CC BY-NC-ND), which allows third parties to download the articles and share them with others as long as they credit the author and the Abstract Book, but they cannot change the content in any way or use them commercially.
**Abstract Book Citations:** Authors, Title, HemaSphere, 2026;10:(S1):pages. The abstract book DOIs can be found on the "Author Information" page.
**Disclaimer:** Articles published in the journal HemaSphere exclusively reflect the opinions of the authors. The authors are responsible for all content in their abstracts including accuracy of the facts, statements, citing resources, etc.

### (PF535) DNMT3A R882 HOTSPOT MUTATION DEFINES A HIGH-RISK SUBGROUP IN TREATMENT-NAIVE AML IRRESPECTIVE OF CO-MUTATION STATUS

**Topic:** 04. Acute myeloid leukemia - Clinical

Mingyu Zhao[1], Xin Yan[1], Zheng Ge*[1]

[1]*Zhongda Hospital Southeast University, Institute of Hematology Southeast University, Department of Hematology, Nanjing, China;*

### Background

DNMT3A R882 hotspot mutation accounts for half of DNMT3A mutations, which occur in 20–30% of acute myeloid leukemia (AML) patients. While R882 is linked to poor prognosis, the clinical relevance of non-R882 mutations and co-mutations remains unclear. Their impact on hypomethylating agent (HMA) efficacy is also poorly defined.

### Aims

To evaluate the prognostic value of DNMT3A R882 hotspot vs. non-R882 and co-mutations, and their association with response to HMA-based therapy in newly diagnosed AML patients.

### Methods

We retrospectively analyzed 138 treatment-naïve AML patients who received standard chemotherapy or HMA regimens. DNMT3A mutations were detected by next-generation sequencing (NGS). Patients were stratified by mutation status: wild-type (WT), R882 hotspot, and non-R882 mutations. Co-mutations was defined as DNMT3A mutations with additional gene mutations including TP53, NPM1, FLT3 and IDH1/2. Baseline characteristics, complete remission/ complete remission with incomplete count recovery (CR/CRi), and overall survival (OS) were compared. Univariate Cox regression identified prognostic factors. Median age was 67 years (IQR 58-74). Data processing and analysis were performed using R 4.3.3, R studio, along with Zstats 1.0.

### Results

DNMT3A mutation was found in 84 patients (60.9%), including 27 R882 (19.6%) and 57 non-R882 (41.3%). Co-mutations occurred in 62 patients (51.7%). Baseline characteristics (age, sex, WBC, Hb, PLT, bone marrow blasts, HMA exposure) were similar between WT and mutated groups (P>0.05), except for karyotype distribution (P=0.001).
DNMT3A R882 hotspot mutation was associated with significantly inferior OS (P=0.036) and lower CR/CRi rates (48% vs. 70% in WT, P=0.03). DNMT3A mutations (including DNMT3A R882 hotspot and non-R882 mutations) showed CR/CRi comparable to WT (P=0.11), but it strongly predicts inferior OS(P=0.0053). Although the co-mutation showed marginal statistical significance for CR/CRi, we find that co-mutation status did not impact OS (HR=0.81, P=0.53). In this real-world study, no statistically significant difference for OS or CR/CRi was observed in whether patients were exposed to HMA (P>0.05),. Karyotype and bone marrow blast percentage were not significant in univariate analysis.

### Summary/Conclusion

DNMT3A R882 hotspot mutation is a powerful adverse prognostic biomarker in treatment-naïve AML, independent of co-mutation patterns and associated with poor response to standard therapy. Non-R882 mutations do not alter clinical outcomes. These findings support routine subtyping of DNMT3A mutations for risk stratification, suggest that DNMT3A R882-mutated AML may require alternative or intensified therapeutic approaches beyond conventional HMA and further observation is going on.

**Copyright Information:** (Online) ISSN: 2572-9241
©2026 The Author(s). HemaSphere is published by John Wiley & Sons Ltd on behalf of European Hematology Association. This is an open access Abstract Book distributed under the Attribution-NonCommercial-NoDerivs (CC BY-NC-ND), which allows third parties to download the articles and share them with others as long as they credit the author and the Abstract Book, but they cannot change the content in any way or use them commercially.
**Abstract Book Citations:** Authors, Title, HemaSphere, 2026;10:(S1):pages. The abstract book DOIs can be found on the "Author Information" page.
**Disclaimer:** Articles published in the journal HemaSphere exclusively reflect the opinions of the authors. The authors are responsible for all content in their abstracts including accuracy of the facts, statements, citing resources, etc.

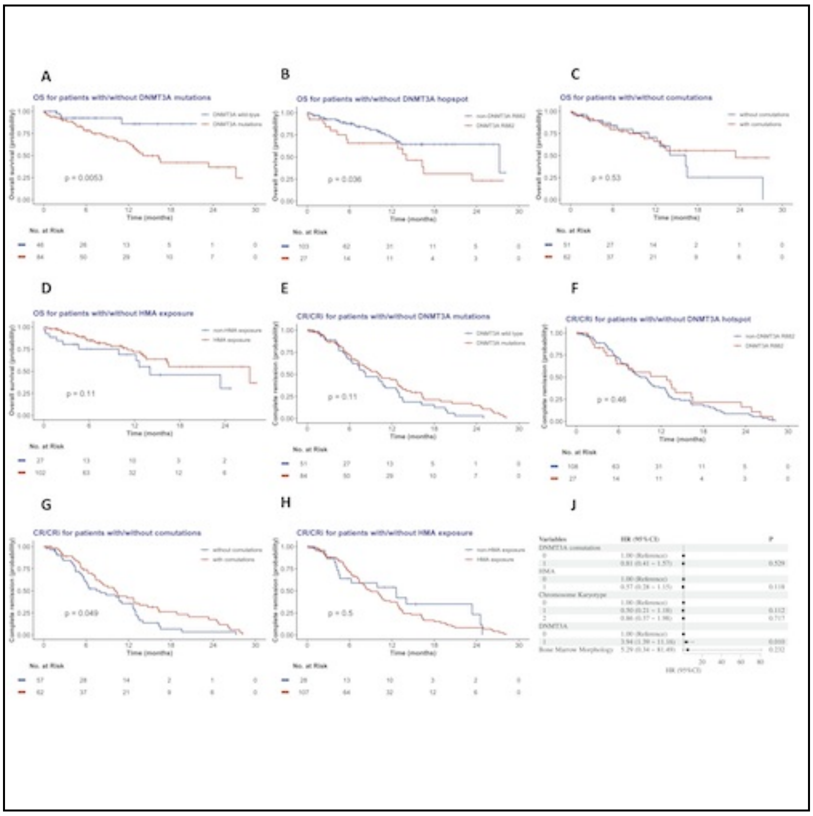

**Copyright Information:** (Online) ISSN: 2572-9241
©2026 The Author(s). HemaSphere is published by John Wiley & Sons Ltd on behalf of European Hematology Association. This is an open access Abstract Book distributed under the Attribution-NonCommercial-NoDerivs (CC BY-NC-ND), which allows third parties to download the articles and share them with others as long as they credit the author and the Abstract Book, but they cannot change the content in any way or use them commercially.
**Abstract Book Citations:** Authors, Title, HemaSphere, 2026;10:(S1):pages. The abstract book DOIs can be found on the "Author Information" page.
**Disclaimer:** Articles published in the journal HemaSphere exclusively reflect the opinions of the authors. The authors are responsible for all content in their abstracts including accuracy of the facts, statements, citing resources, etc.

### (PF536) REAL-WORLD MANAGEMENT PRACTICES WITH VENETOCLAX-BASED THERAPY FOR AML - RESULTS FROM THE PROSPECTIVE REVIVE STUDY

**Topic:** 04. Acute myeloid leukemia - Clinical

Yakir Moshe*[1], Ofir Wolach[2, 3], Itai Levi[4], Boaz Nachmias[5, 6], Sigal Tavor[7], Irina Amitai[8], Yishai Ofran[9, 10], Chezi Ganzel[9, 10], Tsila Zuckerman[11, 12], Doaa Okasha[13], Ilana Nili Hellmann[14], Tamar Tadmor[15], Najib Dally[16, 17], Moran Barak[18], Pazit Lindenberg[18], Jenia Berelovich[18], Alexandra Bleterman[18], Brit Vidmayer[18], Galia Stemer[16, 19]

*[1]Tel Aviv Sourasky Medical Center, Department of Hematology, Tel Aviv, Israel;[2]Rabin Medical Center, Beilinson Hospital., institute of Hematology, Davidoff Cancer Center, Petach Tikva, Israel; [3]Tel Aviv University, Gray Faculty of Medical and Health Sciences, Tel Aviv, Israel; [4]Soroka Medical Center, Hematology Institute, Beer-sheba, Israel; [5]Hadassah Medical Center, Leukemia Service, Department of Hematology, Jerusalem, Israel; [6]Hebrew University of Jerusalem, Faculty of Medicine, Jerusalem, Israel; [7]Assuta Medical Center, Department of Hematology, Tel Aviv, Israel;[8]Chaim Sheba Medical Center, Hematology Division, Ramat Gan, Israel; [9]Shaare Zedek Medical Center, The Hematology and Bone Marrow Transplantation Department, Jerusalem, Israel; [10]The Hebrew University of Jerusalem, The Eisenberg Research and Development Authority, Jerusalem, Israel; [11]Rambam Health Care Campus, Department of Hematology and Bone Marrow Transplantation, Haifa, Israel; [12]Technion, Israel Institute of Technology, Bruce Rappaport Faculty of Medicine, Haifa, Israel;[13]Ha'Emek Medical Center, Department of Hematology, Afula, Israel; [14]Meir Medical Center, Department of Hematology, Kfar Saba, Israel;[15]Bnai Zion Medical Center, Hematology Unit, Haifa, Israel; [16]Bar-Ilan University, Azrieli Faculty of Medicine, Safed, Israel;[17]Ziv Medical Center, Hematology Unit, Safed, Israel; [18]AbbVie Inc., Hod-Hasharon, Israel; [19]Galilee Medical Center, Hematology Unit, Nahariya, Israel;*

### Background

Venetoclax (Ven) + hypomethylating agents (HMA) is the standard of care for newly diagnosed (ND) acute myeloid leukemia (AML) patients (pts) unfit for intensive chemotherapy. Real-world treatment management in elderly, unfit patients involves considerations of infection prevention, post remission cytopenia management and treatment setting.

### Aims

To examine the possible effect of supportive care decisions on the safety and effectiveness of Ven-HMA in real-world ND AML patients.

### Methods

The REVIVE study enrolled 207 ND AML patients treated with Ven-HMA at 12 Israeli centers (Aug 2019–Dec 2022); data cutoff for this analysis was Feb 2026. Treatment management followed local label and physician discretion, resulting in varying practices across institutions. We examined the effect of antimicrobial prophylaxis (AMP), post-remission administration of G-CSF and treatment initiation setting on safety and effectiveness outcomes, using Wilcoxon rank-sum, chi-square and Fisher's exact tests.

**Copyright Information:** (Online) ISSN: 2572-9241
©2026 The Author(s). HemaSphere is published by John Wiley & Sons Ltd on behalf of European Hematology Association. This is an open access Abstract Book distributed under the Attribution-NonCommercial-NoDerivs (CC BY-NC-ND), which allows third parties to download the articles and share them with others as long as they credit the author and the Abstract Book, but they cannot change the content in any way or use them commercially.
**Abstract Book Citations:** Authors, Title, HemaSphere, 2026;10:(S1):pages. The abstract book DOIs can be found on the "Author Information" page.
**Disclaimer:** Articles published in the journal HemaSphere exclusively reflect the opinions of the authors. The authors are responsible for all content in their abstracts including accuracy of the facts, statements, citing resources, etc.

**Results**

AMP- AMP was given to 76/207 pts (37%), pts receiving AMP were treated more often as inpatients than outpatients (48% vs 18%, p<0.001) had a lower incidence of a history of MPN (11% vs 31%) and a higher incidence of stem cell transplantation (SCT) (28% vs 9.3%, p<0.001). AMP was not associated with higher complete response (CR/CRi) rates, reduced early mortality or infection rates. Median overall survival (OS) was 18.3 months for AMP recipients and 11.2 months for non-recipients (p=0.11). In a multivariable analysis (MVA) in overall population including AMP status, history of MPN and SCT, both SCT (HR 0.43, p=0.023) and MPN (HR 1.88, p=0.015), but not AMP status, were significantly associated with survival.
When tested separately, neither antifungal nor antibacterial prophylaxis were associated with CR, early mortality, infection or survival outcomes.
G-CSF- G-CSF was given post-remission to 53/132 pts (40%) in CR/CRi; recipients had lower baseline WBC and more comorbidities but were otherwise similar to non-recipients. In patients with CR/CRi mOS was 22.7 months overall, 30.2 months with G-CSF use and 20.7 months without (p=0.16). A MVA in patients achieving CR (including G-CSF use, ELN risk category, AML ontogeny, SCT, and ECOG) found only SCT to be significantly associated with OS (HR=0.47, p=0.038).
Treatment setting- 71 pts (34%) initiated treatment as outpatients. Compared to inpatients, these patients had lower WBC, lower ECOG scores, lower baseline transfusion dependence, and were more likely to have secondary AML, especially previously treated myelodysplastic syndromes (64%). Rates of CR/CRi, tumor lysis syndrome, and neutropenic fever were similar between initiation settings. 30-day mortality was numerically higher in inpatients (8.8% vs 1.4%, p=0.074), but median OS was similar (11.5 vs 12.5 months, p=0.27), and confirmed in a MVA including treatment setting, AML ontogeny, ECOG, and transfusion dependence.

**Summary/Conclusion**

In a real-world prospective study of ND AML patients receiving Ven-HMA, antimicrobial prophylaxis had no notable effect on outcomes. Survival in patients receiving G-CSF in CR/CRi responders was numerically higher but not statistically significant, possibly due to a low sample size.
Outpatient treatment initiation in selected patients was safe and yielded outcomes comparable to inpatient care. These results support individualized, risk-adapted management strategies for Ven-HMA therapy in clinical practice.

**Copyright Information:** (Online) ISSN: 2572-9241
©2026 The Author(s). HemaSphere is published by John Wiley & Sons Ltd on behalf of European Hematology Association. This is an open access Abstract Book distributed under the Attribution-NonCommercial-NoDerivs (CC BY-NC-ND), which allows third parties to download the articles and share them with others as long as they credit the author and the Abstract Book, but they cannot change the content in any way or use them commercially.
**Abstract Book Citations:** Authors, Title, HemaSphere, 2026;10:(S1):pages. The abstract book DOIs can be found on the "Author Information" page.
**Disclaimer:** Articles published in the journal HemaSphere exclusively reflect the opinions of the authors. The authors are responsible for all content in their abstracts including accuracy of the facts, statements, citing resources, etc.

**REVIVE study baseline characteristics by antimicrobial prophylaxis**

| Median (IQR); n (%); n/N Non-missing (%) | **Overall** N = 205 | **Prophylaxis** N = 76 | **No prophylaxis** N = 129 | **p-value**[1] |
|---|---|---|---|---|
| **Age (years)** | 75 (71, 81) | 74 (71, 81) | 76 (71, 81) | 0.5 |
| **Male sex** | 113 (55%) | 45 (59%) | 68 (53%) | 0.4 |
| **BMI (Kg/m$^2$)** | 26.6 (24.1, 29.5) | 26.0 (24.0, 29.4) | 26.9 (24.3, 29.7) | 0.4 |
| **ECOG** | | | | 0.5 |
| 0-1 | 135/201 (67%) | 49/76 (64%) | 86/125 (69%) | |
| 2-4 | 66/201 (33%) | 27/76 (36%) | 39/125 (31%) | |
| **ELN risk category** | | | | 0.10 |
| Favorable | 19/170 (11%) | 12/71 (17%) | 7/99 (7.1%) | |
| Intermediate | 78/170 (46%) | 28/71 (39%) | 50/99 (51%) | |
| Poor | 73/170 (43%) | 31/71 (44%) | 42/99 (42%) | |
| **Secondary AML** | | | | **0.006** |
| History of MDS | 51/113 (45%) | 15/38 (39%) | 36/75 (48%) | |
| History of MPN | 27/113 (24%) | 4/38 (11%) | 23/75 (31%) | |
| Therapy-related AML | 32/113 (28%) | 17/38 (45%) | 15/75 (20%) | |
| Unknown | 3/113 (2.7%) | 2/38 (5.3%) | 1/75 (1.3%) | |
| **Modified Charlson Comorbidity Index** | 4 (3, 4) | 4 (3, 4) | 4 (3, 4) | 0.7 |
| **Stem cell transplantation** | 33 (16%) | 21 (28%) | 12 (9.3%) | **<0.001** |
| **Care setting** | | | | **<0.001** |
| Inpatient | 134 (65%) | 63 (83%) | 71 (55%) | |
| Outpatient | 71 (35%) | 13 (17%) | 58 (45%) | |

[1]Wilcoxon rank sum test; Pearson's Chi-squared test; Fisher's exact test. Prophylaxis administration: antibacterial (18%), antifungal (11%), both (9%), none (62%). Antibacterial treatments: Ciprofloxacin (93%), Trimehoprim-sulphamethoxazole (7%). Antifungal treatments: Fluconazole (85%), Voriconazole (10%), both (5%). AML- acute myeloid leukemia, BMI- body mass index, ECOG- performance status scale, ELN- European leukemia net.

**Copyright Information:** (Online) ISSN: 2572-9241
©2026 The Author(s). HemaSphere is published by John Wiley & Sons Ltd on behalf of European Hematology Association. This is an open access Abstract Book distributed under the Attribution-NonCommercial-NoDerivs (CC BY-NC-ND), which allows third parties to download the articles and share them with others as long as they credit the author and the Abstract Book, but they cannot change the content in any way or use them commercially.
**Abstract Book Citations:** Authors, Title, HemaSphere, 2026;10:(S1):pages. The abstract book DOIs can be found on the “Author Information” page.
**Disclaimer:** Articles published in the journal HemaSphere exclusively reflect the opinions of the authors. The authors are responsible for all content in their abstracts including accuracy of the facts, statements, citing resources, etc.

### (PF537) EXPOSURE-RESPONSE ANALYSIS OF ZIFTOMENIB COMBINED WITH VENETOCLAX/AZACITIDINE OR CYTARABINE/DAUNORUBICIN IN NEWLY DIAGNOSED AND RELAPSED/REFRACTORY NPM1-M OR KMT2A-R ACUTE MYELOID LEUKEMIA

**Topic:** 04. Acute myeloid leukemia - Clinical

Amitava Mitra*[1], Xiaoyan Yang[1], Martine Allard[1], Roberto Heredia Ortiz[2], Claudia Jomphe[2], Mollie Leoni[1], Nathalie Gosselin[2]

*[1]Kura Oncology, Inc., San Diego, United States of America;[2]Certara Drug Development Solutions, Montreal, Canada;*

### Background

Ziftomenib is a potent, selective, oral menin inhibitor that is U.S. FDA approved as monotherapy for adults with relapsed/refractory *NPM1*-m AML at 600 mg QD. It has also shown clinical activity and tolerability in combination for adults with newly diagnosed (ND) and relapsed/refractory (R/R) *NPM1*-m or *KMT2A*-r AML. KOMET-007 (NCT05735184) is an ongoing, multi-cohort, open-label, Ph1a/b (dose-escalation and expansion) study of ziftomenib combined with standard chemotherapies, including venetoclax/azacitidine (Ven/Aza) or cytarabine/daunorubicin (7+3), in adults with *NPM1*-m or *KMT2A*-r AML.

### Aims

Here we developed population pharmacokinetic (popPK) and exposure-response (ER) models to describe ziftomenib PK and assess ER relationships between PK and efficacy/safety endpoints for ND and R/R *NPM1*-m or *KMT2A*-r patients treated with ziftomenib in combination with Ven/Aza or 7+3.

### Methods

A ziftomenib monotherapy popPK model was updated based on PK data as of 16Jan2026 from ND and R/R patients treated with ziftomenib/Ven/Aza or ND patients treated with ziftomenib/7+3 in KOMET-007. A thorough covariate analysis was conducted to identify potential sources of variability. Finally, ER analyses were conducted using logistic regression models for 6 efficacy endpoints and 16 safety endpoints based on data as of 16Jan2026.

### Results

Ziftomenib PK was best described using a 2-compartment model with first-order elimination, and with first-order absorption with lag time. In the final popPK model, the significant covariates on absorption parameters were food and proton pump inhibitors, and on clearance were strong CYP3A inhibitors and health status. These analyses demonstrated that co-administration of Ven/Aza or 7+3 and patient mutational status (ie, *NPM1*-m or *KMT2A*-r) had no impact on ziftomenib PK. ER analyses were performed on efficacy and safety data collected from a total of 260 patients. There was no relationship between steady-state area under the curve (AUC) and probability of overall response rate (MLFS, CRp, CRi, CRh, CR, or $CR_{MRD-}$) or Gr≥4 treatment emergent adverse event (TEAE), when ziftomenib was dosed at 200 mg, 400 mg, and 600 mg once daily in combination with Ven/Aza in ND and R/R *NPM1*-m or *KMT2A*-r patients (**Figure**). In addition, no correlation was observed for maximum and trough concentrations of ziftomenib. ER analysis for ORR could not be performed for ziftomenib/7+3 in *NPM1*-m patients because ORR was >90% across the evaluated exposure range, resulting in an efficacy ceiling effect that precluded reliable estimation of an ER relationship in this subgroup. However, no ER relationship was observed between ziftomenib AUC and ORR in *KMT2A*-r patients. Similarly, there was no ER relationship observed for any Gr≥4 TEAE for ziftomenib/7+3 in *NPM1*-m or *KMT2A*-r patients.

**Copyright Information:** (Online) ISSN: 2572-9241
©2026 The Author(s). HemaSphere is published by John Wiley & Sons Ltd on behalf of European Hematology Association. This is an open access Abstract Book distributed under the Attribution-NonCommercial-NoDerivs (CC BY-NC-ND), which allows third parties to download the articles and share them with others as long as they credit the author and the Abstract Book, but they cannot change the content in any way or use them commercially.
**Abstract Book Citations:** Authors, Title, HemaSphere, 2026;10:(S1):pages. The abstract book DOIs can be found on the "Author Information" page.
**Disclaimer:** Articles published in the journal HemaSphere exclusively reflect the opinions of the authors. The authors are responsible for all content in their abstracts including accuracy of the facts, statements, citing resources, etc.

### Summary/Conclusion

There was no statistically significant relationship between ziftomenib exposure and efficacy/safety responses for doses ranging from 200–600 mg QD in combination with Ven/Aza or 7+3 in *NPM1*-m or *KMT2A*-r patients, demonstrating wide therapeutic margin for ziftomenib. The lack of drug-drug interaction supported coadministration of all agents, without any required dose adjustments. These results along with the totality of clinical safety/efficacy, PK, and pharmacodynamic data support ziftomenib 600 mg QD as the optimal dose in combination with standard-of-care doses of Ven/Aza or 7+3, providing the best benefit/risk outcomes in *NPM1*-m or *KMT2A*-r patients.

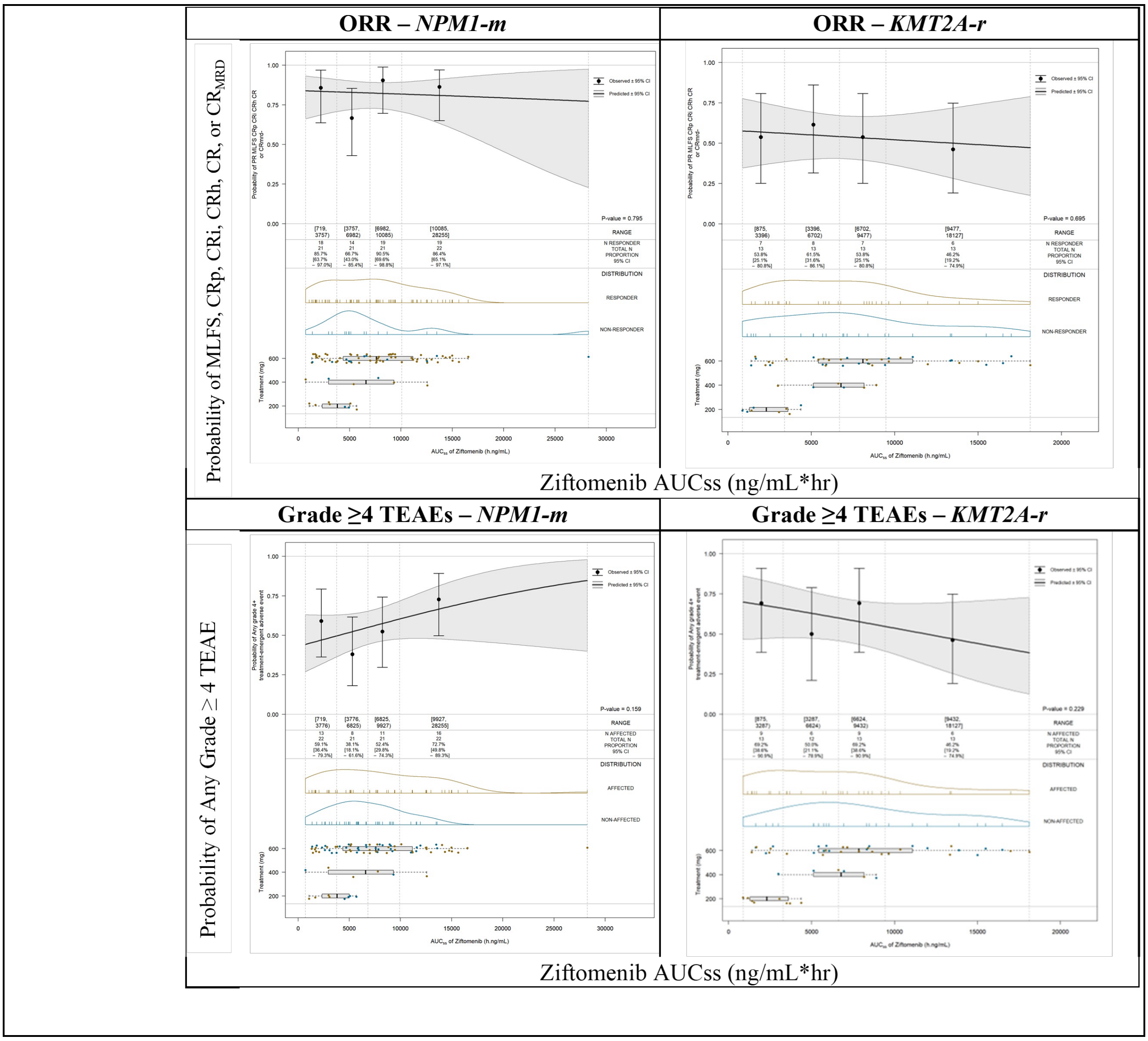

Figure. ER profiles for ziftomenib and venetoclax/azacitidine combination in ND and R/R pa-tients

**Copyright Information:** (Online) ISSN: 2572-9241
©2026 The Author(s). HemaSphere is published by John Wiley & Sons Ltd on behalf of European Hematology Association. This is an open access Abstract Book distributed under the Attribution-NonCommercial-NoDerivs (CC BY-NC-ND), which allows third parties to download the articles and share them with others as long as they credit the author and the Abstract Book, but they cannot change the content in any way or use them commercially.
**Abstract Book Citations:** Authors, Title, HemaSphere, 2026;10:(S1):pages. The abstract book DOIs can be found on the "Author Information" page.
**Disclaimer:** Articles published in the journal HemaSphere exclusively reflect the opinions of the authors. The authors are responsible for all content in their abstracts including accuracy of the facts, statements, citing resources, etc.

### (PF538) MACROPHAGE ACTIVATION-LIKE SYNDROME IN ACUTE MYELOID LEUKEMIA

**Topic:** 04. Acute myeloid leukemia - Clinical

Veronica Zimmer*[1, 2], Martin Henkes[1], Sabine Hensler[1], Angela Sermersheim[1], Susanne Mertins[1], Gunnar Lachmann[3], Robert Zeiser[4], Paul La Rosée[1, 5]

*[1]Cancer Center Schwarzwald-Baar-Heuberg, Klinik für Innere Medizin II, Villingen-Schwenningen, Germany;[2]Albert-Ludwigs-Universität Freiburg, Faculty of Medicine, Freiburg im Breisgau, Germany; [3]Berlin Institute of Health at Charité - Universitätsmedizin Berlin, Department of Anesthesiology and Intensive Care Medicine, Berlin, Germany; [4]University Medical Center, Department of Hematology, Oncology, and Stem Cell Transplantation, Freiburg im Breisgau, Germany; [5]Universitätsklinikum Jena, Faculty of Medicine, Jena, Germany;*

### Background

Macrophage activation-like syndrome (MALS) is a hyperinflammatory condition within the spectrum of hyperferritinaemic hyperinflammatory disorders, with hemophagocytic lymphohistiocytosis (HLH) being the most severe manifestation. It is characterized by uncontrolled activation of macrophages and cytotoxic T-cells, leading to cytokine storm with potentially lethal multiorgan failure. In acute myeloid leukemia (AML), MALS remains underdiagnosed due to its features overlapping with those of sepsis and chemotherapy toxicity; validated MALS-specific diagnostic criteria remain to be established.

### Aims

The aim of this study is to analyze the incidence, clinical characteristics and outcomes of MALS in adult AML patients, and to identify predictive patterns for the development of MALS.

### Methods

This retrospective single-center study (2016–2025) included 222 adult AML patients (pats). MALS was diagnosed using two parallel approaches: (1) the optimized HLH-index (OHI), a scoring system optimized for adult pats with modified thresholds for ferritin (>1,000 µg/L) and sIL-2R (>3,900 U/mL), and (2) modified HLH-2024 criteria (≥4/7 criteria, applying a ferritin threshold of ≥4,420 µg/L as defined by Kyriazopoulou et al.). Pats were stratified by treatment intensity: high-intensity (7+3) versus low-intensity (hypomethylating agents +/- venetoclax). Ferritin kinetics and sIL-2R levels were compared between MALS and non-MALS pats. Kaplan-Meier analysis with log-rank testing compared overall survival (OS). Multivariable Cox regression (n=183 with complete ferritin data) assessed age, sex, ferritin peak, and log-ferritin slope as predictors of OS.

### Results

Of 222 AML pats (59% male; 19% aged 18–59, 42% aged 60–74, 39% aged ≥75), 30 (13.5%) developed MALS (67% male). Fifteen fulfilled OHI criteria, 24 fulfilled modified HLH-2024 criteria, and nine met both, indicating limited diagnostic overlap. Median peak ferritin was significantly higher in MALS versus non-MALS pats (9,873 vs. 2,320 µg/L; p<0.001), with accelerated kinetics: doubling time 3.1 vs. 11.1 days (p<0.001) and peak daily rise 464 vs. 0 µg/L/day (p<0.001). Median sIL-2R was 4,987 vs. 1,063 U/mL for MALS versus non-MALS pats (p<0.001; n=70). Of the MALS cohort, 11 pats received high-intensity 7+3 therapy and 19 low-intensity regimens; MALS-directed immunomodulatory treatment was administered in 17 pats (57%). Median OS was 4.3 months (range 0.2–52.0) vs. 6.4 months (range 0.0–116.4) for MALS versus non-MALS pats (six-month OS 37.9% vs. 53.2%; log-rank p=0.24). Thirty-day mortality after MALS diagnosis was 43% (13/30). On multivariable Cox regression, all four predictors were independently associated with inferior OS: age (HR 1.08 per year, p<0.005), male sex (HR 1.51, p=0.03), ferritin peak (HR 1.02 per 1,000 µg/L, p<0.005), and log-ferritin slope (HR 1.06 per 0.01/day, p<0.005); concordance index 0.74.

**Copyright Information:** (Online) ISSN: 2572-9241
©2026 The Author(s). HemaSphere is published by John Wiley & Sons Ltd on behalf of European Hematology Association. This is an open access Abstract Book distributed under the Attribution-NonCommercial-NoDerivs (CC BY-NC-ND), which allows third parties to download the articles and share them with others as long as they credit the author and the Abstract Book, but they cannot change the content in any way or use them commercially.
**Abstract Book Citations:** Authors, Title, HemaSphere, 2026;10:(S1):pages. The abstract book DOIs can be found on the "Author Information" page.
**Disclaimer:** Articles published in the journal HemaSphere exclusively reflect the opinions of the authors. The authors are responsible for all content in their abstracts including accuracy of the facts, statements, citing resources, etc.

**Summary/Conclusion**

MALS occurred in 13.5% of AML pats, with a 30-day mortality of over 40%. Peak ferritin and log-ferritin slope were independent predictors of OS, suggesting ferritin kinetics may serve as prognostic markers beyond their diagnostic utility. The limited overlap between OHI and modified HLH-2024 criteria highlights the necessity of combined or AML-specific diagnostic frameworks. Systematic monitoring of ferritin kinetics in AML pats with presumed sepsis should be evaluated prospectively to detect MALS, stratify risk, and guide life-saving immunosuppression in those pats.

**Copyright Information:** (Online) ISSN: 2572-9241
©2026 The Author(s). HemaSphere is published by John Wiley & Sons Ltd on behalf of European Hematology Association. This is an open access Abstract Book distributed under the Attribution-NonCommercial-NoDerivs (CC BY-NC-ND), which allows third parties to download the articles and share them with others as long as they credit the author and the Abstract Book, but they cannot change the content in any way or use them commercially.
**Abstract Book Citations:** Authors, Title, HemaSphere, 2026;10:(S1):pages. The abstract book DOIs can be found on the "Author Information" page.
**Disclaimer:** Articles published in the journal HemaSphere exclusively reflect the opinions of the authors. The authors are responsible for all content in their abstracts including accuracy of the facts, statements, citing resources, etc.

### (PF539) DETERMINANTS OF SURVIVAL IN 356 PATIENTS FAILING FRONTLINE VENETOCLAX–HMA FOR NEWLY DIAGNOSED ACUTE MYELOID LEUKEMIA

**Topic:** 04. Acute myeloid leukemia - Clinical

Mahnoor Fatima[1], Sudhesh Kumar[1], Momna Warraich[1], Kristen McCullough[1], Aref Al-Kali[1], Hassan Alkhateeb[1], Kebede Begna[1], Abhishek Mangaonkar[1], Antoine Saliba[1], Mark Litzow[1], Animesh Pardanani[1], Mithun Shah[1], William Hogan[1], Mrinal Patnaik[1], Jeanne Palmer[2], Talha Badar[3], James M Foran[3], Cecilia Arana Yi[2], Ayalew Tefferi[1], Naseema Gangat*[1]

*[1]Mayo Clinic, Division of Hematology, Rochester,MN, United States of America; [2]Mayo Clinic, Division of Hematology, Scottsdale,AZ, United States of America; [3]Mayo Clinic, Division of Hematology, Jacksonville,FL, United States of America;*

### Background

Venetoclax (Ven) in combination with hypomethylating agents (HMA) is FDA-approved frontline therapy for elderly or unfit newly-diagnosed acute myeloid leukemia (ND-AML) patients; however, many relapse or are refractory (*DiNardo NEJM 2020*). In the current study, we examined outcomes after frontline Ven-HMA failure to define salvage therapy efficacy and identify clinical and molecular survival predictors.

### Methods

ND-AML patients receiving frontline Ven+HMA with treatment failure defined as failure to achieve CR/CRi (refractory) or loss of CR/CRi (relapse), excluding post-transplant relapse were retrospectively studied. Response evaluated per European Leukemia Net 2022 criteria (*Dohner Blood 2022*).

**Copyright Information:** (Online) ISSN: 2572-9241
©2026 The Author(s). HemaSphere is published by John Wiley & Sons Ltd on behalf of European Hematology Association. This is an open access Abstract Book distributed under the Attribution-NonCommercial-NoDerivs (CC BY-NC-ND), which allows third parties to download the articles and share them with others as long as they credit the author and the Abstract Book, but they cannot change the content in any way or use them commercially.
**Abstract Book Citations:** Authors, Title, HemaSphere, 2026;10:(S1):pages. The abstract book DOIs can be found on the "Author Information" page.
**Disclaimer:** Articles published in the journal HemaSphere exclusively reflect the opinions of the authors. The authors are responsible for all content in their abstracts including accuracy of the facts, statements, citing resources, etc.

### Results

Among 356 ND-AML patients (median age 75y; 65% male; 55% secondary/therapy-related) failing Ven+HMA (median 3 cycles, 1-26), 219 (62%) were refractory and 137 (38%) relapsed. Complex karyotype was present in 142/347 (41%). Mutations: *TP53* 29%, *ASXL1* 19%, *RUNX1* 17%, *TET2* 17%, *SRSF2* 15%, *K/NRAS* 13%, *DNMT3A* 9%, *IDH2* 7%, *FLT3-ITD* 7%, *NPM1* 6%, *IDH1* 5%. Refractory disease was enriched for *TP53* mutations (34% vs 22%, p=0.02), while relapsed disease showed higher frequencies of *TET2, ASXL1, DNMT3A, SRSF2,* and *STAG2* mutations (all p<0.05).
At treatment failure, complex karyotype was present in 72/180 (40%), including 55 from baseline. In 137 patients with paired NGS, mutations persisted in *TP53* (88%), *IDH1* (86%), *IDH2* (78%), *NPM1* (75%), *K/NRAS* (73%), *RUNX1* (70%), *FLT3-ITD* (65%) mutations. Mutations were acquired in a minority [*IDH1/IDH2* (2% each) *TP53* (3%), *NPM1* (4%), *FLT3-ITD* (5%) *K/NRAS* (6%)]. Clearance of mutations were infrequent for *TP53* (12%) and *IDH1*(14%).
At median follow-up 4 months (0-80) from relapse/refractory disease, 320 (90%) patients died; median OS was 4 months (1-/2-/3-yr survival 19%/8%/5%). Multivariate analysis of diagnostic variables identified peripheral blasts >20% (HR 1.6), complex karyotype (HR 1.8), and wild-type *IDH1* (HR 2.3) as independent predictors of inferior survival; ASCT improved survival (HR 0.27). A 3-point model (peripheral blasts ≥20%, complex karyotype, wild-type *IDH1*) stratified patients into low-, intermediate-, and high-risk groups with median survival of 7, 3, and 2 months, respectively (p<0.01) (Fig.1).
ASCT was performed in 13 patients (median survival 22.5 mo; 3-yr survival 33%) in low-(n=10) or intermediate-risk(n=3) groups. The prediction model held true when applied to non-transplanted patients. In separate analysis of genetic variables at relapse/refractory disease, complex karyotype (HR 2.0) and wild-type *IDH2* (HR 2.9) emerged as independent predictors.
Salvage therapy (n=170, 48%) yielded CR/CRi of 31% for Ven+HMA rechallenge (n=36), 54% intensive chemotherapy (n=26), 50% FLT3 inhibitors (i) (n=22), 37% IDH1/2i (n=19), 25% other regimens (n=69). Median survival was similar: Ven+HMA 8 mo, intensive chemotherapy 10 mo, FLT3i 6.5 mo, IDH1/2i 14 mo, other regimens 6 mo, but inferior with supportive care (2 mo; p<0.01).

### Summary/Conclusion

The current study identifies peripheral blasts ≥20%, complex karyotype, and wild-type *IDH1* as predictors of inferior survival in ND-AML patients relapsed or refractory to front-line Ven+HMA, proposes a three-tiered survival model, and shows that while no specific salvage therapy conferred superior outcomes, ASCT was indispensable for long-term survival.

**Copyright Information:** (Online) ISSN: 2572-9241
©2026 The Author(s). HemaSphere is published by John Wiley & Sons Ltd on behalf of European Hematology Association. This is an open access Abstract Book distributed under the Attribution-NonCommercial-NoDerivs (CC BY-NC-ND), which allows third parties to download the articles and share them with others as long as they credit the author and the Abstract Book, but they cannot change the content in any way or use them commercially.
**Abstract Book Citations:** Authors, Title, HemaSphere, 2026;10:(S1):pages. The abstract book DOIs can be found on the "Author Information" page.
**Disclaimer:** Articles published in the journal HemaSphere exclusively reflect the opinions of the authors. The authors are responsible for all content in their abstracts including accuracy of the facts, statements, citing resources, etc.

**Figure 1.** **Survival outcome after frontline Venetoclax-HMA failure among 356 patients with newly diagnosed AML**
*Salvage therapy (48%) (N=170), allogeneic stem cell transplant (ASCT) 4% (N=13)*
*Predictors of survival: peripheral blasts ≥20%; complex karyotype; wild-type IDH1 mutation, ASCT*

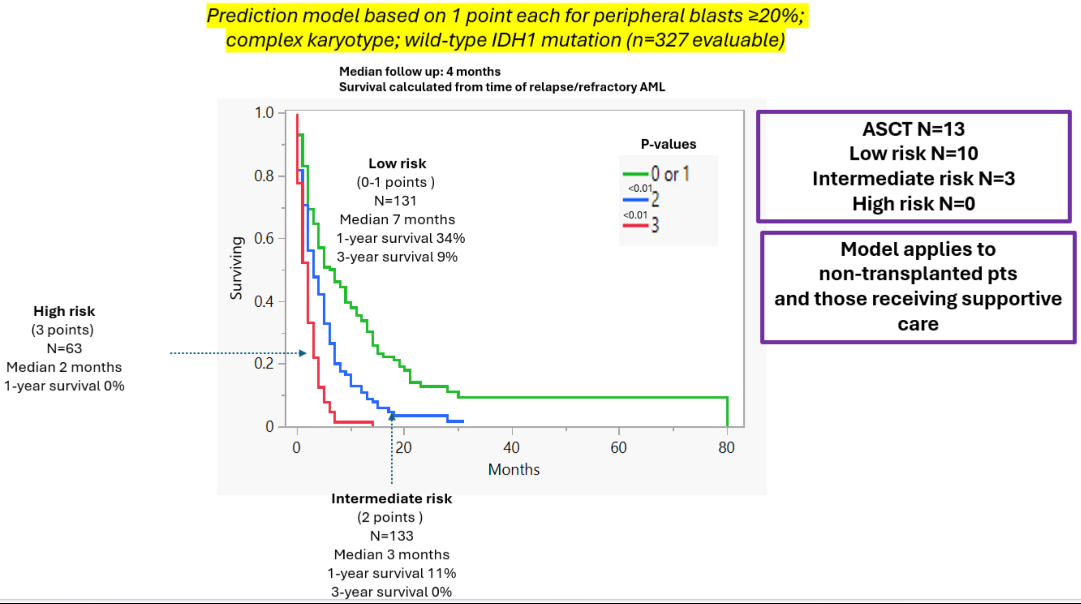

**Copyright Information:** (Online) ISSN: 2572-9241
©2026 The Author(s). HemaSphere is published by John Wiley & Sons Ltd on behalf of European Hematology Association. This is an open access Abstract Book distributed under the Attribution-NonCommercial-NoDerivs (CC BY-NC-ND), which allows third parties to download the articles and share them with others as long as they credit the author and the Abstract Book, but they cannot change the content in any way or use them commercially.
**Abstract Book Citations:** Authors, Title, HemaSphere, 2026;10:(S1):pages. The abstract book DOIs can be found on the “Author Information” page.
**Disclaimer:** Articles published in the journal HemaSphere exclusively reflect the opinions of the authors. The authors are responsible for all content in their abstracts including accuracy of the facts, statements, citing resources, etc.

### (PF540) REAL-WORLD INSIGHTS INTO SALVAGE CHEMOTHERAPY FOR FIT FAVORABLE-RISK AML PATIENTS AT FIRST RELAPSE

**Topic:** 04. Acute myeloid leukemia - Clinical

Elisa Seubert*[1], Felix Klingler[1], Francis Ayuk[2], Carsten Bokemeyer[1], Walter Fiedler[1], Franziska Westendorf[1]

*[1]University Medical Center Hamburg-Eppendorf, Department of Oncology, Hematology and Bone Marrow Transplantation with Section Pneumology, Hamburg, Germany; [2]University Medical Center Hamburg-Eppendorf, Department of Stem Cell Transplantation, Hamburg, Germany;*

### Background

Recent data from the ASAP trial has prompted a re-evaluation of salvage therapy in fit patients (pts) with relapsed acute myeloid leukemia (AML). The benefit of intensive salvage regimens prior to allogeneic hematopoietic stem cell transplantation (HSCT) remains to be defined.

### Aims

This study aims to characterize pts with relapsed favorable-risk AML, to identify those who might benefit from intensive salvage chemotherapy, and to assess the potential harm associated with these therapies.

### Methods

Eligible pts were those treated between January 2012 and November 2025 at the University Medical Center Hamburg-Eppendorf with favorable-risk AML according to the 2022 European LeukemiaNet (ELN) classification with *NPM1* mutation, or core-binding factor rearrangements *t(8;21)* and *inv(16)*. They were required to have a relapse after completing intensive first-line therapy, and to have received salvage chemotherapy with intent to proceed to HSCT.

**Copyright Information:** (Online) ISSN: 2572-9241
©2026 The Author(s). HemaSphere is published by John Wiley & Sons Ltd on behalf of European Hematology Association. This is an open access Abstract Book distributed under the Attribution-NonCommercial-NoDerivs (CC BY-NC-ND), which allows third parties to download the articles and share them with others as long as they credit the author and the Abstract Book, but they cannot change the content in any way or use them commercially.
**Abstract Book Citations:** Authors, Title, HemaSphere, 2026;10:(S1):pages. The abstract book DOIs can be found on the "Author Information" page.
**Disclaimer:** Articles published in the journal HemaSphere exclusively reflect the opinions of the authors. The authors are responsible for all content in their abstracts including accuracy of the facts, statements, citing resources, etc.

**Results**

Among 152 fit AML pts harboring *NPM1* mutation, *t(8;21)*, or *inv(16)* at initial diagnosis, 40 experienced relapse and received intensive salvage chemotherapy. Of these, 21 (52.5%) were male, with a median age at initial diagnosis of 52 years (IQR 41-63). Among relapsed patients, 28 (70%) had *NPM1* mutation, 3 (7.5%) had *t(8;21)*, and 9 (22.5%) had *inv(16)*. The median time from initial diagnosis to relapse was 1.27 years (IQR 0.85-2.01). Of the 40 pts, 13 (32.5%) experienced a molecular relapse (molR), whereas 27 (67.5%) presented with a hematological relapse (hemR). Salvage therapies included MEC (15), FLAG-Ida +/- Venetoclax (off-label) (23), cytarabine in combination with liposomal daunorubicin (1), and SAIL study protocol (1). Overall, salvage therapy showed high efficacy. Only 2 pts (5%) did not respond, and no deaths were observed during the first salvage cycle. 28 pts (70%) achieved composite complete remission, 7 pts (17.5%) morphologic leukemia-free state, and 3 pts (7.5%) were not evaluable. Regarding measurable residual disease (MRD) assessment, a substantial reduction in MRD burden was observed from the time of relapse to post-salvage chemotherapy. The median MRD log-level reduction after salvage was 3.03 (Figure 1). Despite the relapse setting, overall survival (OS) remained favorable, with a 10-year survival probability of 69.5% from the time of initial diagnosis. In subgroup analyses by mutational status, pts with *inv(16)* showed the highest survival, followed by those with *NPM1* mutation and *t(8;21)*, without any statistically significant difference between groups. During salvage therapy, pts with molR exhibited shorter median durations of both thrombocytopenia and leukocytopenia compared to those with hemR. Specifically, thrombocytopenia lasted 13 vs. 17 days (IQR 11-18 vs. 11-21, p=0.4), and leukocytopenia lasted 16 vs. 20 days (IQR 13-20 vs. 16-25, p=0.069). Notably, when these cytopenias were prolonged (>19 days for thrombocytopenia or >25 days for leukocytopenia), they coincided with inferior OS, likely indicating reduced hematopoietic regeneration potential. Despite this, early mortality during salvage therapy remained unaffected.

**Summary/Conclusion**

Overall, the study demonstrates that in this cohort of fit, favorable-risk AML pts experiencing relapse, an encouraging long-term OS, a low treatment-related mortality, and substantial MRD-log reduction can be achieved during salvage chemotherapy. However, patient numbers remain small, warranting larger multicenter studies.

**Copyright Information:** (Online) ISSN: 2572-9241
©2026 The Author(s). HemaSphere is published by John Wiley & Sons Ltd on behalf of European Hematology Association. This is an open access Abstract Book distributed under the Attribution-NonCommercial-NoDerivs (CC BY-NC-ND), which allows third parties to download the articles and share them with others as long as they credit the author and the Abstract Book, but they cannot change the content in any way or use them commercially.
**Abstract Book Citations:** Authors, Title, HemaSphere, 2026;10:(S1):pages. The abstract book DOIs can be found on the "Author Information" page.
**Disclaimer:** Articles published in the journal HemaSphere exclusively reflect the opinions of the authors. The authors are responsible for all content in their abstracts including accuracy of the facts, statements, citing resources, etc.

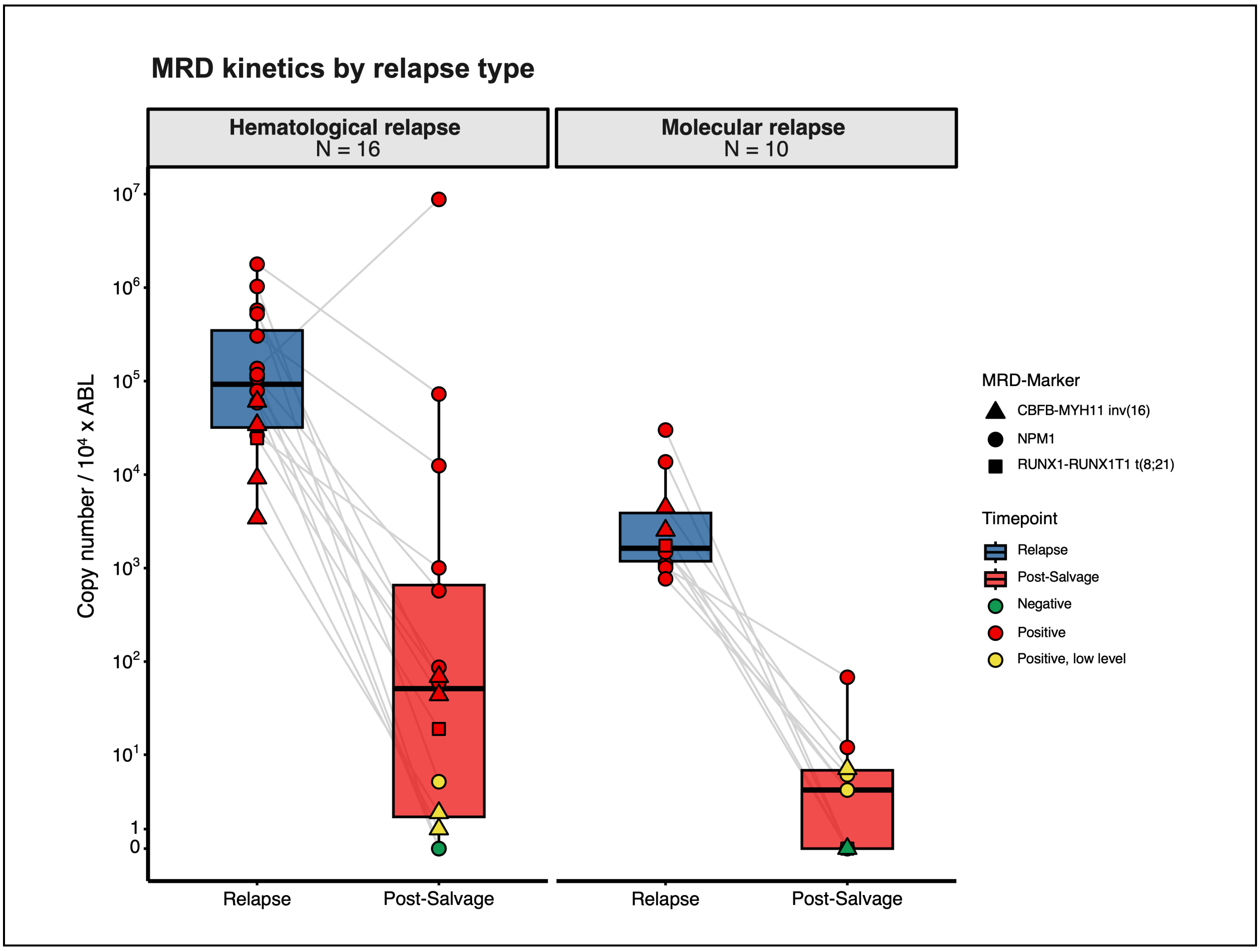

MRD kinetics by relapse type. All patients with available paired pre- and post-salvage MRD samples (N = 26) are shown in the plot. If the report stated "0 copies / 1E4 x ABL", copy number was assumed to be the limit of detection (1E-5 for all tests). Y-Scale is linear at the bottom and logarithmic at the top.

**Copyright Information:** (Online) ISSN: 2572-9241
©2026 The Author(s). HemaSphere is published by John Wiley & Sons Ltd on behalf of European Hematology Association. This is an open access Abstract Book distributed under the Attribution-NonCommercial-NoDerivs (CC BY-NC-ND), which allows third parties to download the articles and share them with others as long as they credit the author and the Abstract Book, but they cannot change the content in any way or use them commercially.
**Abstract Book Citations:** Authors, Title, HemaSphere, 2026;10:(S1):pages. The abstract book DOIs can be found on the "Author Information" page.
**Disclaimer:** Articles published in the journal HemaSphere exclusively reflect the opinions of the authors. The authors are responsible for all content in their abstracts including accuracy of the facts, statements, citing resources, etc.

### (PF541) LOW-DOSE INDUCTION CHEMOTHERAPY IS ASSOCIATED WITH VERY LOW EARLY MORTALITY IN PEDIATRIC ACUTE MEGAKARYOBLASTIC LEUKEMIA: A PROSPECTIVE MULTICENTER STUDY

**Topic:** 04. Acute myeloid leukemia - Clinical

Shengqin Cheng*[1], Yixin Hu[2], Xiuli Ju[3], Ninning Wang[4], Hua Jiang[5], Xiaowen Zhai[6], Yunyan He[7], Minghua Yang[8], Liangchun Yang[9], Qi An[10], Shaoyan Hu[11], Raul Ribeiro[12]

*[1]Children's Hospital of Soochow University, Department of Hematology and Oncology, Suzhou, China;[2]Children's Hospital of Soochow University, Suzhou, China; [3]Qilu Hospital of Shandong University, Qilu, China;[4]The Second Affiliated Hospital of Anhui Medical University, Hefei, China; [5]Guangzhou Women and Children's Medical Center, Guangzhou, China;[6]Children's Hospital of Fudan University, Shanghai, China; [7]The First Affiliated Hospital of Guangxi Medical University, Guangxi, China;[8]The Third Xiangya Hospital of Central South University, Hunan, China; [9]Xiangya Hospital, Hunan, China; [10]Xuzhou Children's Hospital, Xuzhou, China; [11]Children's Hospital of Soochow University, Soochow, China; [12]St.Jude Children's Research Hospital, Memphis, United States of America;*

### Background

Pediatric non–Down syndrome acute megakaryoblastic leukemia (non-DS AMKL) is a rare, highly aggressive subtype of acute myeloid leukemia (AML) with poor outcomes despite intensive therapy. Early death and treatment-related mortality (TRM) are major barriers to improving survival, particularly in high-risk genetic cases, underscoring the need for improved induction regimens.

### Aims

This study aimed to evaluate the safety and efficacy of low-dose HAG (homoharringtonine, low-dose cytarabine, and G-CSF priming) as an induction therapy for children with newly diagnosed non-DS AMKL. The primary goal was to assess the complete remission (CR) rate after two induction courses. Secondary objectives included overall survival (OS), event-free survival (EFS), cumulative incidence of relapse (CIR), and the role of measurable residual disease (MRD) in predicting outcomes. Additionally, the study evaluated the safety of low-dose HAG and its potential to reduce early mortality and TRM.

### Methods

This was a prospective, phase II, multicenter clinical trial (CALD-AMKL-18) conducted across 9 pediatric centers in China. Between June 2018 and December 2022, 51 children aged <18 years with newly diagnosed non-DS AMKL were enrolled. Patients received a low-dose chemotherapy regimen (HAG: homoharringtonine, low-dose cytarabine, and G-CSF priming) as induction therapy, followed by risk-adapted consolidation and post-remission therapy. The primary endpoint was complete remission (CR) after two induction courses, and secondary endpoints included OS, EFS, CIR, MRD status, and treatment-related toxicities.

**Copyright Information:** (Online) ISSN: 2572-9241
©2026 The Author(s). HemaSphere is published by John Wiley & Sons Ltd on behalf of European Hematology Association. This is an open access Abstract Book distributed under the Attribution-NonCommercial-NoDerivs (CC BY-NC-ND), which allows third parties to download the articles and share them with others as long as they credit the author and the Abstract Book, but they cannot change the content in any way or use them commercially.
**Abstract Book Citations:** Authors, Title, HemaSphere, 2026;10:(S1):pages. The abstract book DOIs can be found on the "Author Information" page.
**Disclaimer:** Articles published in the journal HemaSphere exclusively reflect the opinions of the authors. The authors are responsible for all content in their abstracts including accuracy of the facts, statements, citing resources, etc.

**Results**

The median age at diagnosis was 40 months (range 2–114), with 26 (51%) male patients. Twenty-seven patients underwent allogeneic hematopoietic stem cell transplantation (allo-HSCT). No early deaths occurred during induction, and only one patient (2%) died due to severe infection during consolidation. After the second induction, the CR rate was 76.5% (26/34). The two-year overall survival (OS) was 48.7%, and event-free survival (EFS) was 49.0%, with a 2-year cumulative incidence of relapse (CIR) of 31.3%. Fusion-negative and *KMT2A*-rearranged patients demonstrated relatively favorable survival, while *CBFA2T3::GLIS2* or *NUP98::KDM5A* fusions were associated with poor long-term outcomes. MRD ≥0.1% after Induction II identified a high-risk subgroup with significantly worse OS (12.5% vs 64.5%, P < 0.01) and EFS (12.5% vs 63.0%, P < 0.01). MRD ≥0.1% after Induction II, but not after Induction I, was strongly associated with inferior outcomes. Pre-transplant MRD negativity predicted better post-HSCT outcomes.

**Summary/Conclusion**

Low-dose HAG induction reduces early mortality and significantly lowers TRM, providing a well-tolerated backbone for risk-adapted strategies. This approach supports the incorporation of targeted therapies guided by fusion profiles and MRD status, offering a promising treatment for pediatric non-DS AMKL. Further studies are needed to validate these findings and explore the long-term benefits of combining low-dose chemotherapy with targeted therapies in high-risk populations.

**Copyright Information:** (Online) ISSN: 2572-9241
©2026 The Author(s). HemaSphere is published by John Wiley & Sons Ltd on behalf of European Hematology Association. This is an open access Abstract Book distributed under the Attribution-NonCommercial-NoDerivs (CC BY-NC-ND), which allows third parties to download the articles and share them with others as long as they credit the author and the Abstract Book, but they cannot change the content in any way or use them commercially.
**Abstract Book Citations:** Authors, Title, HemaSphere, 2026;10:(S1):pages. The abstract book DOIs can be found on the “Author Information” page.
**Disclaimer:** Articles published in the journal HemaSphere exclusively reflect the opinions of the authors. The authors are responsible for all content in their abstracts including accuracy of the facts, statements, citing resources, etc.

### (PF542) REVUMENIB IN THE REAL WORLD: INTERIM FINDINGS FROM THE ROAR STUDY IN RELAPSED/REFRACTORY ACUTE LEUKEMIA

**Topic:** 04. Acute myeloid leukemia - Clinical

Shai Shimony*[1], Joseph Fleming[1], Kuo-Kai Chin[2], Jose Tinajero[3], Matthew Rees[4], Sneha S. Kelkar[5], Lorena Lopez-Gonzalez[6], Shweta Kamat[5], Craig W. Freyer[6], Kyle Roney[5], Huan Huang[6], Marlise Luskin[1], Ibrahim Aldoss[3], Aaron Goldberg[2]

*[1]Dana-Farber Cancer Institute, Boston, MA, United States of America; [2]Memorial Sloan Kettering Cancer Center, New York, NY, United States of America; [3]City of Hope National Medical Center, Duarte, CA, United States of America;[4]St. Jude Children's Research Hospital, Memphis, TN, United States of America; [5]OPEN Health, New York, NY, United States of America;[6]Syndax Pharmaceuticals, Inc., New York, NY, United States of America;*

### Background

Revumenib, a first-in-class, oral, selective menin inhibitor, is used for the treatment of relapsed/refractory (R/R) acute leukemia harboring a *KMT2A* translocation or R/R acute myeloid leukemia (AML) with *NPM1* mutation (*NPM1*m) in adult and pediatric patients 1 year and older based on the AUGMENT-101 trial (NCT04065399). Real-world evidence offers critical insights into drug safety, effectiveness, and treatment decisions across broader patient populations beyond controlled trials.

### Aims

To describe treatment patterns, effectiveness, and tolerability among patients with R/R acute leukemia treated with revumenib in the real-world setting.

### Methods

ROAR (Real-world Outcomes for Acute leukemia treated with Revumenib) is an ongoing 3-year multicenter, longitudinal real-world study in the US. Consecutive patients with R/R acute leukemia who received ≥1 dose of revumenib in the real-world setting are included. Patients are followed from date of revumenib initiation until data cutoff date or death. The overall response rate (ORR) includes complete remission (CR), CR with partial hematologic recovery (CRh), CR with incomplete hematologic recovery (CRi), morphologic leukemia-free state (MLFS), and partial response. Composite CR (CRc) includes CR, CRh, and CRi. This abstract presents the first interim results with a data cutoff of 23Jan2026.

**Copyright Information:** (Online) ISSN: 2572-9241
©2026 The Author(s). HemaSphere is published by John Wiley & Sons Ltd on behalf of European Hematology Association. This is an open access Abstract Book distributed under the Attribution-NonCommercial-NoDerivs (CC BY-NC-ND), which allows third parties to download the articles and share them with others as long as they credit the author and the Abstract Book, but they cannot change the content in any way or use them commercially.
**Abstract Book Citations:** Authors, Title, HemaSphere, 2026;10:(S1):pages. The abstract book DOIs can be found on the "Author Information" page.
**Disclaimer:** Articles published in the journal HemaSphere exclusively reflect the opinions of the authors. The authors are responsible for all content in their abstracts including accuracy of the facts, statements, citing resources, etc.

### Results

As of 23Jan2026, 13 patients (12 with AML; 1 with acute lymphoblastic leukemia) were identified from 1 center with a median follow-up of 6 months (mo) (range, 2–11) from revumenib initiation. Eleven patients had a *KMT2A* translocation while 2 had an *NPM1*m. Median age was 41 yrs (range, 28–80); 76.9% were White; 69.2% were female. Prior to revumenib treatment, patients received a median of 2 prior lines of therapy (range, 1–5); 69.2% had prior venetoclax and 46.2% had a prior hematopoietic stem cell transplant (HSCT). Median duration of revumenib treatment was 3.2 mo (range, 0.4–9.7) with 8 patients treated with revumenib monotherapy and 5 patients treated with revumenib combination therapy.

Of the 11 patients evaluable for efficacy (monotherapy [n=7]/combination therapy [n=4]), the ORR was 81.8% (9/11) and CRc rate was 54.5% (6/11). Best overall response was CR in 36.4% (4/11), CRh in 18.2% (2/11), and MLFS in 27.3% (3/11). Median time to first and best response was 1.1 mo (range, 0.7–3.9) and 2.0 mo (range, 0.8–4.2), respectively. Median overall survival was 9.3 mo (95% CI, 9.3–NE) (**Figure**). Among the 9 responding patients, median relapse-free survival was 8.2 mo (95% CI, 4.9–NE). Three patients (33.3%) proceeded to HSCT (one as second HSCT); 2/3 patients resumed revumenib as post-HSCT maintenance.

In safety evaluable patients (N=13), 1 patient experienced grade 3 QTcF prolongation which resolved after discontinuation of concomitant QTc-prolonging medication without any dose reduction, interruption, or discontinuation of revumenib. Two patients experienced grade 2 differentiation syndrome (DS); no patient experienced grade 1 or grade ≥3 DS.

### Summary/Conclusion

In this first ROAR interim analysis, revumenib, as monotherapy and in combination, demonstrated real-world effectiveness and was generally well tolerated in patients with R/R acute leukemia harboring a *KMT2A* translocation or *NPM1*m. Safety findings were consistent with the known profile. High response rates, rapid time to response, and the ability to bridge patients to HSCT highlight the clinical utility of revumenib in a heavily pretreated population.

**Copyright Information:** (Online) ISSN: 2572-9241
©2026 The Author(s). HemaSphere is published by John Wiley & Sons Ltd on behalf of European Hematology Association. This is an open access Abstract Book distributed under the Attribution-NonCommercial-NoDerivs (CC BY-NC-ND), which allows third parties to download the articles and share them with others as long as they credit the author and the Abstract Book, but they cannot change the content in any way or use them commercially.
**Abstract Book Citations:** Authors, Title, HemaSphere, 2026;10:(S1):pages. The abstract book DOIs can be found on the “Author Information” page.
**Disclaimer:** Articles published in the journal HemaSphere exclusively reflect the opinions of the authors. The authors are responsible for all content in their abstracts including accuracy of the facts, statements, citing resources, etc.

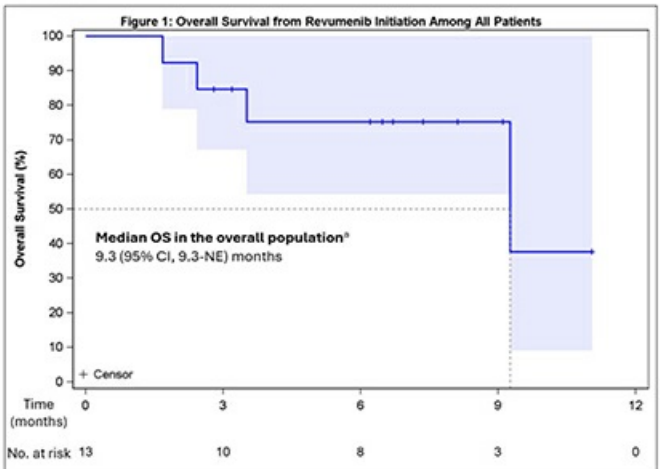

| Key Outcomes Among Those Evaluable for Response | All (N = 11) | Monotherapy (N = 7) | Combination (N = 4) |
|---|---|---|---|
| ORR, N (%) | 9 (81.8%) | 6 (85.7%) | 3 (75.0%) |
| CRc, N (%) | 6 (54.5%) | 4 (57.1%) | 2 (50.0%) |
| CR, N (%) | 4 (36.4%) | 2 (28.6%) | 2 (50.0%) |
| CRh, N (%) | 2 (18.2%) | 2 (28.6%) | 0 (0.0%) |
| MLFS, N (%) | 3 (27.3%) | 2 (28.6%) | 1 (25.0%) |
| No Response, N (%) | 2 (18.2%) | 1 (14.3%) | 1 (25.0%) |
| Relapse, N (%) | 3 (27.3%) | 2 (28.6%) | 1 (25.0%) |
| HSCT after Revumenib Initiation | 3 (27.3%) | 1 (14.3%) | 2 (50.0%) |
| Time to first response from revumenib initiation (months), median (Range) | 1.1 (0.7-3.9) | 1.9 (1.0-3.9) | 0.8 (0.7-1.1) |
| Time to best response from revumenib initiation (months), median (Range) | 2.0 (0.8-4.2) | 2.0 (1.0-3.9) | 1.0 (0.8-4.2) |

**Copyright Information:** (Online) ISSN: 2572-9241
©2026 The Author(s). HemaSphere is published by John Wiley & Sons Ltd on behalf of European Hematology Association. This is an open access Abstract Book distributed under the Attribution-NonCommercial-NoDerivs (CC BY-NC-ND), which allows third parties to download the articles and share them with others as long as they credit the author and the Abstract Book, but they cannot change the content in any way or use them commercially.
**Abstract Book Citations:** Authors, Title, HemaSphere, 2026;10:(S1):pages. The abstract book DOIs can be found on the "Author Information" page.
**Disclaimer:** Articles published in the journal HemaSphere exclusively reflect the opinions of the authors. The authors are responsible for all content in their abstracts including accuracy of the facts, statements, citing resources, etc.

### (PF544) A LARGE MULTICENTER REAL-WORLD STUDY DEMONSTRATES SUPERIOR OUTCOMES WITH VENETOCLAX-BASED REGIMENS IN ACUTE MYELOID LEUKEMIA HARBORING EZH2 MUTATIONS

**Topic:** 04. Acute myeloid leukemia - Clinical

Yaru Wang[1], Xiangli Chen*[2], Yijie Wang[1], Tiantian Dai[3], Xiaoting Xu[1], Wenyi Lu[2], Pingfan Lu[2], Fei Ge[2], Xiaona Niu[2]

*[1]Henan Provincial People's Clinical Medical School of Zhengzhou University, Department of Hematology, Zhengzhou City, China; [2]Henan Provincial People's Hospital, Department of Hematology, Zhengzhou City, China; [3]People's Hospital of Henan University, Department of Hematology, Zhengzhou City, China;*

### Background

EZH2 mutations confer an independent poor prognosis in acute myeloid leukemia (AML). However, the optimal therapeutic approach for this subset remains undefined. While venetoclax-based regimens are standard for older/unfit AML, robust real-world evidence regarding their efficacy in EZH2-mutant AML is scarce.

### Aims

This large-scale, multicenter real-world study aimed to characterize EZH2-mutant AML and, crucially, to compare the efficacy and safety of venetoclax-based low-intensity therapy (VEN) versus intensive chemotherapy (IC) as first-line induction in this population.

### Methods

We analyzed 1227 newly diagnosed non-APL AML patients from four tertiary centers (Jan 2018-Dec 2023), constituting one of the largest real-world cohorts on this topic. Based on next-generation sequencing, 53 (4.3%) patients with EZH2 mutations were identified and stratified by first-line therapy: VEN-group (n=18, venetoclax + HMA/LDAC) and IC-group (n=35, “7+3” etc.). The primary endpoint was composite complete remission (CRc) rate after first induction. Key secondary endpoints included overall response rate (ORR), overall survival (OS), relapse-free survival (RFS), and safety.

### Results

EZH2-mutant patients exhibited aggressive features: higher WBC, lower platelets, higher co-mutation burden (all P<0.01), and 94.3% were classified as ELN 2022 adverse risk. Critically, VEN-group demonstrated markedly superior efficacy over IC-group: CRc (83.3% vs. 42.9%, P=0.008) and ORR (100% vs. 57.1%, P<0.001). The survival advantage was profound: median OS was 28.7 months for VEN-group versus 7.3 months for IC-group (Hazard Ratio [HR]=0.35, 95% CI 0.16-0.76; P=0.006). Median RFS was also significantly longer (25.7 vs. 11.7 months, P=0.044). The incidence of grade III-IV toxicities was comparable between groups.

### Summary/Conclusion

This real-world study confirms that EZH2-mutant AML responds poorly to IC with dismal outcomes. Venetoclax-based therapy fundamentally alters the prognosis, delivering deep responses and a significant survival benefit with a manageable safety profile. These findings establish a new and effective first-line standard of care for this high-risk AML subset, with immediate clinical implications.

**Copyright Information:** (Online) ISSN: 2572-9241
©2026 The Author(s). HemaSphere is published by John Wiley & Sons Ltd on behalf of European Hematology Association. This is an open access Abstract Book distributed under the Attribution-NonCommercial-NoDerivs (CC BY-NC-ND), which allows third parties to download the articles and share them with others as long as they credit the author and the Abstract Book, but they cannot change the content in any way or use them commercially.
**Abstract Book Citations:** Authors, Title, HemaSphere, 2026;10:(S1):pages. The abstract book DOIs can be found on the “Author Information” page.
**Disclaimer:** Articles published in the journal HemaSphere exclusively reflect the opinions of the authors. The authors are responsible for all content in their abstracts including accuracy of the facts, statements, citing resources, etc.

### (PF545) PROOF-OF-CONCEPT: DEEP LEARNING-BASED PREDICTION OF MEASURABLE RESIDUAL DISEASE IN ACUTE MYELOID LEUKEMIA WITHOUT REFERENCE VALUES

**Topic:** 04. Acute myeloid leukemia - Clinical

Maximilian Roehnert*[1], Chiara Löffler[1, 2, 3], Nepomuk Linsenbarth[2], Christoph Röllig[1], Martin Bornhäuser[1, 3], Uta Oelschlaegel[1], Malte von Bonin[1], Jakob Kather[1, 2, 4], Jonas Schadt[1, 2]

*[1]Department of Internal Medicine I, Faculty of Medicine and University Hospital Carl Gustav Carus TUD Dresden, University of Technology, Dresden, Germany; [2]Else Kroener Fresenius Center for Digital Health, Technical University Dresden, Dresden, Germany; [3]National Center for Tumor Disease (NCT), Dresden, Germany;[4]Medical Oncology, National Center for Tumor Diseases (NCT), University Hospital Heidelberg, Heidelberg, Germany;*

### Background

Measurable residual disease (MRD) assessment by multiparametric flow cytometry (MFC) is a key prognostic factor in acute myeloid leukemia (AML), yet its clinical implementation is limited by institution-specific protocols, reliance on cut-offs derived from leukemia-free controls (reference measurements), and expert-dependent manual interpretation causing interobserver variability. This dependency hampers reproducibility and cross-center standardization. Whether MRD can be inferred from patient-derived flow cytometry data without reference measurements using deep learning remains unclear.

### Aims

To establish a proof-of-concept deep learning framework for reference value-free AML-MRD prediction by MFC using Random Forest or Tabular Prior-Data Fitted Networks (TabPFN), and to evaluate its prognostic relevance.

### Methods

We manually analyzed 329 MFC samples after completion of induction therapy (post-IT, n = 296) and before allogeneic hematopoietic cell transplantation (pre-alloHCT, n = 33) cohorts. The manual analysis served as ground truth and used an established protocol with fixed gates to reduce interobserver variability (Röhnert et al., Leukemia 2022). Frequencies of 32 immunophenotypically aberrant populations, as determined by manual analysis, were extracted into a tabular feature matrix for model input. We compared two supervised learning approaches analyzing these tables: Random Forest as a classical machine learning benchmark and TabPFN, a pretrained transformer architecture. Models were trained on the post-induction cohort and externally validated on the independent pre-alloHCT cohort with no overlapping patients or repeated measurements between training and validation datasets.

### Results

TabPFN achieved high discriminatory performance regarding MRD status in the external pre-alloHCT cohort (AUROC 0.98), outperforming Random Forest (AUROC 0.94). Importantly, MRD prediction was accomplished without the need for reference values or predefined cut-offs. TabPFN-based MRD classification also stratified patients by overall survival, yielding effect sizes comparable to expert-based MRD assessment (HR 5.6, 95% CI 0.7-43.2, p=0.10 vs. 6.2, 95% CI 0.8-48.1, p=0.08). Model performance remained stable across distinct clinical settings, supporting generalizability.

**Copyright Information:** (Online) ISSN: 2572-9241
©2026 The Author(s). HemaSphere is published by John Wiley & Sons Ltd on behalf of European Hematology Association. This is an open access Abstract Book distributed under the Attribution-NonCommercial-NoDerivs (CC BY-NC-ND), which allows third parties to download the articles and share them with others as long as they credit the author and the Abstract Book, but they cannot change the content in any way or use them commercially.
**Abstract Book Citations:** Authors, Title, HemaSphere, 2026;10:(S1):pages. The abstract book DOIs can be found on the "Author Information" page.
**Disclaimer:** Articles published in the journal HemaSphere exclusively reflect the opinions of the authors. The authors are responsible for all content in their abstracts including accuracy of the facts, statements, citing resources, etc.

### Summary/Conclusion

This study provides proof-of-concept that deep learning can predict clinically meaningful MRD status directly from tabular feature matrices derived from manual flow cytometry analysis, without reference measurements. By decoupling MRD prediction from laboratory-specific controls, this approach opens the possibility to train standardized, outcome-driven MRD models across centers and potentially extend this paradigm to other MRD methodologies, provided a robust ground truth is available.

| | Predicted MRD– (TabPFN) | Predicted MRD+ (TabPFN) |
|---|---|---|
| Manual MRD– (Ground Truth) | 7 | 1 |
| Manual MRD+ (Ground Truth) | 2 | 23 |

**Table:** Confusion matrix of TabPFN-based MRD prediction (TabPFN) versus ground truth by manual MRD assessment (GT).

**Figure:** Deep learning-based MRD prediction pipeline from flow cytometry data in AML.

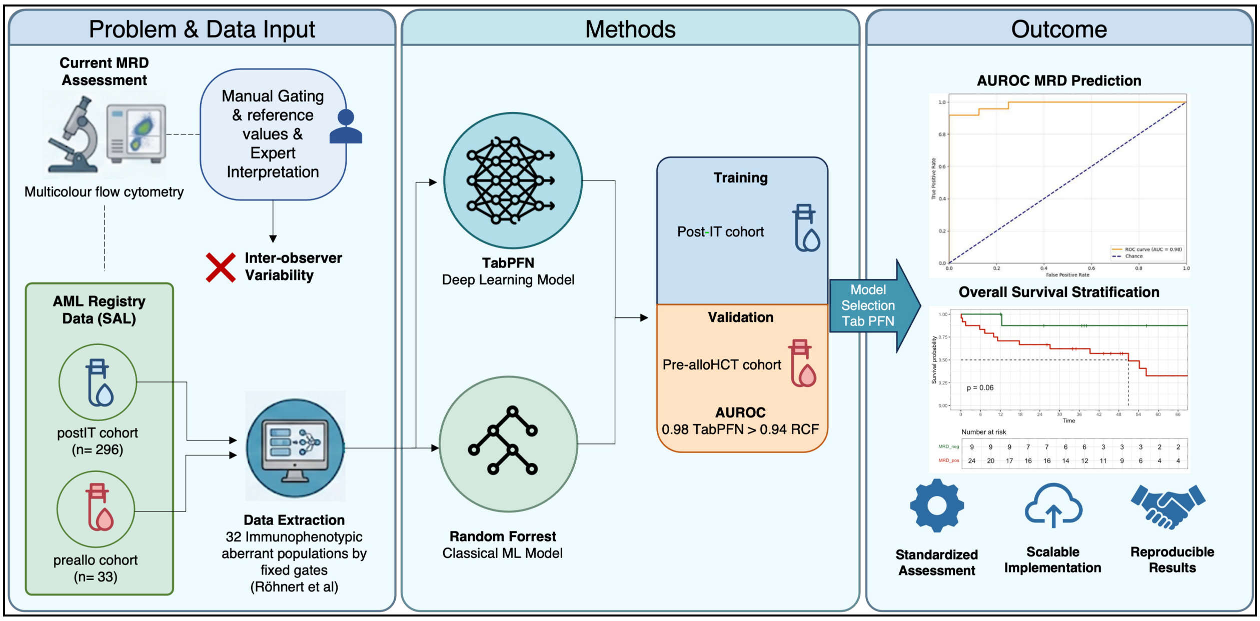

**Copyright Information:** (Online) ISSN: 2572-9241
©2026 The Author(s). HemaSphere is published by John Wiley & Sons Ltd on behalf of European Hematology Association. This is an open access Abstract Book distributed under the Attribution-NonCommercial-NoDerivs (CC BY-NC-ND), which allows third parties to download the articles and share them with others as long as they credit the author and the Abstract Book, but they cannot change the content in any way or use them commercially.
**Abstract Book Citations:** Authors, Title, HemaSphere, 2026;10:(S1):pages. The abstract book DOIs can be found on the "Author Information" page.
**Disclaimer:** Articles published in the journal HemaSphere exclusively reflect the opinions of the authors. The authors are responsible for all content in their abstracts including accuracy of the facts, statements, citing resources, etc.

### (PF546) FLAG-MITOXANTRONE WITH 7 DAYS VENETOCLAX IS A HIGHLY EFFICIENT REGIMEN IN NEWLY DIAGNOSED ACUTE MYELOBLASTIC LEUKEMIA AND AN EFFECTIVE BRIDGE TO ALLOGENEIC HEMATOPOIETIC STEM CELL TRANSPLANTATION

**Topic:** 04. Acute myeloid leukemia - Clinical

Ahmad Ibrahim*[1], Ahmad Khalil[2], Kamal Zahran[2], Jad Ibrahim[3], Eliana Kachaamy[2], Issam Chehadeh[4], Ali Youssef[4], Muhammad Abbas[4], Rima Moghnieh[5], Charbel Khalil[6], Tamima Jisr[7]

*[1]University of Balamand: Middle East Institute of Health, Lebanese and Beirut Arab Universities: Makassed Hospital, Beirut, Lebanon, Beirut, Lebanon; [2]University of Balamand: Middle East Institute of Health, Beirut, Lebanon; Lebanese University, Beirut, Lebanon; [3]American University of Beirut, Beirut, Lebanon, Beirut, Lebanon;[4]Beirut Arab University: Makassed Hospital, Beirut, Lebanon; Lebanese University, Beirut, Lebanon; [5]Lebanese American University, Beirut, Lebanon, Beirut, Lebanon;[6]University of Balamand: Middle East Institute of Health, Beirut, Lebanon, Beirut, Lebanon; [7]Beirut Arab University: Makassed Hospital, Beirut, Lebanon, Beirut, Lebanon;*

### Background

FLAG (G-CSF, Fludarabine, Cytarabine) combined with either Idarubicin (Ida) or Mitoxantrone (Mitox) are effective and well tolerated in newly diagnosed (ND) Acute Myeloblastic Leukemia (AML) (Burnett et al. 2018 – Muhlneck et al. 2022). FLAG-Ida with Venetoclax (Ven) induced deep remissions and high rates of transition to allogeneic hematopoietic stem cell transplantation (AHSCT) in patients (pts) with ND AML (DiNardo 2022). Lowering the Cytarabine dose (to 1.5mg/m2/d for 5d) and shortening the duration of Ven administration (14d) in FLAG-Ida+Ven led to lower hematological and infectious toxicities (DiNardo 2025 – Shahswar 2024).

### Aims

This is a pilot study involving pts with ND AML fit for intensive chemotherapy treated with FLAG-Mitox and Ven given within a short period of time (7d) and followed by AHSCT when indicated. The objectives included rate and composite CR (CRc) and toxicity, in addition to overall survival and EFS.

### Methods

Our study included AML pts aged 18 to 60 y.o fit for intensive chemotherapy. FLAG-Mitox +Ven combined G-CSF (5µg/kg/d) on d1-7, Fludarabine (30mg/m2/d IV) and Cytarabine (1.5g/m2/d IV) on d2-5, Mitox (10mg/m2/d IV) d2,4 and Ven (100mg PO daily) given with voriconazole d2-8. Responding pts planned for AHSCT received 1 to 2 cycles of consolidation, while responding pts non-planned for AHSCT received 3 cycles of consolidation. In consolidation, Fludarabine and Cytarabine were given on d2-4 and G-CSF on d1-5, while Mitox and Ven were given at the same schedule. Anti-FLT3 was given to pts with FLT3 mutations. Measurable residual disease (MRD) was assessed by flow cytometry. Endpoints included rate of composite CR (CRc), OS, EFS and toxicity due to our induction therapy regimen.

### Results

Between 6/2020 and 12/2025, 60 pts (32 females and 28 males) were included. Median age was 43yo (18–60). According to the ELN 2022 risk stratification, 12 (20%), 19 (32%), and 29 (48%) pts were favorable, intermediate, and adverse, respectively. Sixty percent underwent AHSCT. The CRc rate after the first induction therapy was 91%, with a flow MRD-negativity of 86%. The 3-y OS and 3-y EFS rates were 69% and 64% respectively. Outcomes were not significantly different across ELN 2022 risk groups. The most frequent adverse events (grade ≥3) were hematologic, infectious (pneumonia), and gastrointestinal. The mortality rates at 30 and 60 days of FLAG-Mitox+Ven were 0% and 3.3% respectively.

**Copyright Information:** (Online) ISSN: 2572-9241
©2026 The Author(s). HemaSphere is published by John Wiley & Sons Ltd on behalf of European Hematology Association. This is an open access Abstract Book distributed under the Attribution-NonCommercial-NoDerivs (CC BY-NC-ND), which allows third parties to download the articles and share them with others as long as they credit the author and the Abstract Book, but they cannot change the content in any way or use them commercially.
**Abstract Book Citations:** Authors, Title, HemaSphere, 2026;10:(S1):pages. The abstract book DOIs can be found on the "Author Information" page.
**Disclaimer:** Articles published in the journal HemaSphere exclusively reflect the opinions of the authors. The authors are responsible for all content in their abstracts including accuracy of the facts, statements, citing resources, etc.

**Summary/Conclusion**

FLAG–Mitox+Ven (7d) is a highly effective and well tolerated regimen for remission induction and MRD negativity in ND AML pts. High survival outcomes were demonstrated across ELN 2022 risk groups. This regimen is also an effective bridge to AHSCT.

**Copyright Information:** (Online) ISSN: 2572-9241
©2026 The Author(s). HemaSphere is published by John Wiley & Sons Ltd on behalf of European Hematology Association. This is an open access Abstract Book distributed under the Attribution-NonCommercial-NoDerivs (CC BY-NC-ND), which allows third parties to download the articles and share them with others as long as they credit the author and the Abstract Book, but they cannot change the content in any way or use them commercially.
**Abstract Book Citations:** Authors, Title, HemaSphere, 2026;10:(S1):pages. The abstract book DOIs can be found on the "Author Information" page.
**Disclaimer:** Articles published in the journal HemaSphere exclusively reflect the opinions of the authors. The authors are responsible for all content in their abstracts including accuracy of the facts, statements, citing resources, etc.

### (PF547) VENETOCLAX PLUS AZACITIDINE FOR NEWLY DIAGNOSED LOW-RISK NPM1-MUTATED ACUTE MYELOID LEUKEMIA: A PROSPECTIVE STUDY

**Topic:** 04. Acute myeloid leukemia - Clinical

Xueqing Dou*[1], Suning Chen[1]

[1] *The First Affiliated Hospital of Soochow University, Jiangsu Institute of Hematology, National Clinical Research Center for Hematologic Diseases, SUZHOU, China;*

### Background

The NPM1 mutation, a common genetic alteration in acute myeloid leukemia (AML), is associated with favorable prognosis and promising sensitivity to venetoclax. This has raised the question of whether intensive chemotherapy is necessary for these patients. However, no prospective trial has evaluated a purely non-intensive, venetoclax-based regimen in this specific population.

### Aims

This study aims to evaluate the efficacy of venetoclax plus azacitidine (VA) as an alternative to intensive chemotherapy in patients with newly diagnosed, favorable-risk NPM1-mutated AML.

### Methods

We conducted a multicenter, single-arm, phase II study of venetoclax plus azacitidine (VA) in patients with newly diagnosed, favorable-risk NPM1-mutated AML. Patients received one cycle of induction and ten cycles of consolidation with VA. After completing 11 cycles, patients entered the follow-up phase.

### Results

From January 2024 to December 2025, a total of 38 patients (17 males, 21 females) with newly diagnosed AML were enrolled. The median age was 55 years (range, 15–80); 28 patients were considered fit, and 10 were unfit for intensive chemotherapy. Regarding NPM1 mutation subtypes, 28 patients (74%) harbored type A transcripts, 4 (11%) type B, and 6 (16%) rare subtypes. Most patients (81%) had normal karyotype; none had high-risk cytogenetics. The most frequently co-occurring mutations were DNMT3A (45%) and NRAS (21%). Additionally, 9 patients (24%) had concurrent myelodysplasia-related gene mutations.

Of the 38 enrolled patients, 34 were evaluable for response to induction therapy as of the data cutoff; 3 patients were still undergoing induction treatment, and 1 died of sepsis before response assessment. All 34 (100%) achieved composite complete remission (CRc) after one course of VA. The median duration of neutropenia during induction was 23 days (range, 0–38 days). The median number of red blood cell units and platelet therapeutic doses transfused were 8.0 (range, 0–13.5) and 3.0 (range, 0–9.5), respectively. The most common adverse event during induction was pneumonia (29%), followed by upper respiratory tract infection (13%) and febrile neutropenia (8%). Other infectious events included sepsis (2.6%), appendicitis (2.6%), and perianal abscess (2.6%).

As of the data cutoff on December 10, 2025, the median follow-up was 8.9 months (range, 0.6–22.3 months), with a median of 4 treatment cycles completed (range, 0–11). Nineteen patients achieved NPM1 negativity (assessed by qPCR), requiring a median of 2 treatment cycles (range, 1–8). All patients achieved NPM1 negativity within 8 treatment cycles, with more than half (63%) achieving negativity within the first 3 cycles.

Among the 35 evaluable patients, 4 (11%) experienced disease relapse, including 2 molecular relapses (defined as a ≥1-log increase in NPM1 transcript levels) during VA treatment and 2 hematologic relapses after entering the follow-up phase. Of the 19 patients who had achieved NPM1 negativity, 3 (16%) subsequently relapsed (2 hematologic, 1 molecular). No FLT3-ITD mutations were detected in any patient, including those who relapsed.

**Copyright Information:** (Online) ISSN: 2572-9241
©2026 The Author(s). HemaSphere is published by John Wiley & Sons Ltd on behalf of European Hematology Association. This is an open access Abstract Book distributed under the Attribution-NonCommercial-NoDerivs (CC BY-NC-ND), which allows third parties to download the articles and share them with others as long as they credit the author and the Abstract Book, but they cannot change the content in any way or use them commercially.
**Abstract Book Citations:** Authors, Title, HemaSphere, 2026;10:(S1):pages. The abstract book DOIs can be found on the "Author Information" page.
**Disclaimer:** Articles published in the journal HemaSphere exclusively reflect the opinions of the authors. The authors are responsible for all content in their abstracts including accuracy of the facts, statements, citing resources, etc.

**Summary/Conclusion**

The VA regimen induced rapid, deep molecular responses in favorable-risk *NPM1*-mutated AML with manageable toxicity, challenging the need for intensive chemotherapy in this population. These findings support VA as a potential frontline non-intensive standard; longer follow-up is needed to confirm survival benefits.

**Copyright Information:** (Online) ISSN: 2572-9241
©2026 The Author(s). HemaSphere is published by John Wiley & Sons Ltd on behalf of European Hematology Association. This is an open access Abstract Book distributed under the Attribution-NonCommercial-NoDerivs (CC BY-NC-ND), which allows third parties to download the articles and share them with others as long as they credit the author and the Abstract Book, but they cannot change the content in any way or use them commercially.
**Abstract Book Citations:** Authors, Title, HemaSphere, 2026;10:(S1):pages. The abstract book DOIs can be found on the "Author Information" page.
**Disclaimer:** Articles published in the journal HemaSphere exclusively reflect the opinions of the authors. The authors are responsible for all content in their abstracts including accuracy of the facts, statements, citing resources, etc.

### (PF548) REAL-WORLD TREATMENT PATTERNS AND OUTCOMES OF PATIENTS WITH RELAPSED/REFRACTORY IDH1-MUTATED ACUTE MYELOID LEUKEMIA TREATED WITH IVOSIDENIB

**Topic:** 04. Acute myeloid leukemia - Clinical

Vanessa Kennedy*[1], Pierantonio Russo[2], Tarun Bhagnani[3], Weilong Li[3], Assaf Vestin[3], Anne Angiolillo[3], Ramaa Nathan[2], Amer M. Zeidan[4]

*[1]Stanford University, Palo Alto, United States of America; [2]Eversana, Overland Park, United States of America; [3]Servier Pharmaceuticals, Boston, United States of America; [4]Yale University, New Haven, United States of America;*

### Background

Ivosidenib (IVO) is an oral targeted inhibitor of isocitrate dehydrogenase 1 mutation (mIDH1), approved for the treatment of relapsed/refractory (R/R) mIDH1 acute myeloid leukemia (AML). In the pivotal AG120-C-001 trial (NCT02074839) of patients with R/R mIDH1 AML, patients receiving IVO demonstrated a complete remission (CR+CRh) rate of 32.8%, and a 12-month overall survival (OS) rate of 35%. However, real-world data describing treatment patterns and outcomes remain limited.

### Aims

To characterize real-world clinical outcomes and treatment patterns among patients with R/R mIDH1 AML treated with IVO.

### Methods

In this retrospective cohort study, adult patients (≥18 years) with mIDH1 AML with an R/R event between May 2018 and April 2025 were identified from Loopback Analytics, an electronic medical records database that aggregates data from >30 health systems, covering 220+ hospitals and >50 million patients across the US. Eligible patients received ≥14 days of IVO as single agent or in combination any time after an R/R event. Patients were excluded if they received IVO as a maintenance treatment, had participated in a clinical trial, or had received another IDH1-targeted therapy. Outcomes included OS, composite complete remission (CR+CRi), and frequency of stem cell transplant (SCT). Subgroup analyses were conducted in patients who received IVO as monotherapy (IVO MONO). Descriptive statistics were used to summarize baseline characteristics and outcomes. OS was assessed using Kaplan–Meier methods.

### Results

Of 282 eligible patients with R/R mIDH1 AML, 48 received an IVO-based regimen. After applying other inclusion/exclusion criteria, 24 patients were included in the main final cohort, among whom 20 received IVO MONO and 4 received IVO in combination with a hypomethylating agent (IVO+HMA). Median follow-up time was 306 days. In the main final cohort, the median age was 68.5 years; 70.9% had ECOG score 0–1; 62.5% were female, 75% were White; 62.5% had primary AML, and 87.5% had adverse risk per 2022 European LeukemiaNet (ELN) or cytogenetic risk classification. The cohort was heavily pretreated, with 50% receiving ≥3 prior lines of treatment (LOT) and 25% receiving IVO after second relapse. CR+CRi was achieved in 7 (29.2%) patients; 12-month OS was 45.8% (Figure), and 20.8% of patients received SCT post IVO treatment. Among those receiving IVO MONO (N=20), CR+CRi was achieved in 5 (25%) patients, and 12-month OS was 45% (Figure). 25% of patients received SCT after IVO MONO treatment (Table).

**Copyright Information:** (Online) ISSN: 2572-9241
©2026 The Author(s). HemaSphere is published by John Wiley & Sons Ltd on behalf of European Hematology Association. This is an open access Abstract Book distributed under the Attribution-NonCommercial-NoDerivs (CC BY-NC-ND), which allows third parties to download the articles and share them with others as long as they credit the author and the Abstract Book, but they cannot change the content in any way or use them commercially.
**Abstract Book Citations:** Authors, Title, HemaSphere, 2026;10:(S1):pages. The abstract book DOIs can be found on the "Author Information" page.
**Disclaimer:** Articles published in the journal HemaSphere exclusively reflect the opinions of the authors. The authors are responsible for all content in their abstracts including accuracy of the facts, statements, citing resources, etc.

**Summary/Conclusion**

Compared with the AG120-C-001 trial population, IVO treated patients in this real-world cohort were heavily pre-treated (median prior LOT: 3 vs. 2 in the trial), had higher-risk disease (87.5% adverse risk vs. 27% in the trial) and initiated IVO after their second or later relapse (25% vs. 15% in the trial). Despite this high-risk clinical profile, observed outcomes were consistent with results of the registrational trial, supporting the effectiveness of IVO for R/R mIDH1 AML in real-world practice.

**Table**. Characteristics and outcomes of patients with R/R mIDH1 AML treated with IVO

| **Characteristic/Outcome** | **Overall (N=24)** | **IVO MONO (N=20)** |
|---|---|---|
| Median age, yrs | 68.5 | 68.5 |
| Female, % | 62.5 | 55 |
| Primary AML, % | 62.5 | 55 |
| ECOG 0–1, % | 70.9 | 75 |
| Adverse ELN risk, % | 87.5 | 85 |
| ≥3 prior lines of treatment, % | 50 | 45 |
| ≥2 R/R events prior to IVO, % | 25 | 25 |
| CR+CRi, % | 29.2 | 25 |
| 12-month OS, % | 45.8 | 45 |
| SCT after IVO treatment, % | 20.8 | 25 |

**Copyright Information:** (Online) ISSN: 2572-9241
©2026 The Author(s). HemaSphere is published by John Wiley & Sons Ltd on behalf of European Hematology Association. This is an open access Abstract Book distributed under the Attribution-NonCommercial-NoDerivs (CC BY-NC-ND), which allows third parties to download the articles and share them with others as long as they credit the author and the Abstract Book, but they cannot change the content in any way or use them commercially.
**Abstract Book Citations:** Authors, Title, HemaSphere, 2026;10:(S1):pages. The abstract book DOIs can be found on the "Author Information" page.
**Disclaimer:** Articles published in the journal HemaSphere exclusively reflect the opinions of the authors. The authors are responsible for all content in their abstracts including accuracy of the facts, statements, citing resources, etc.

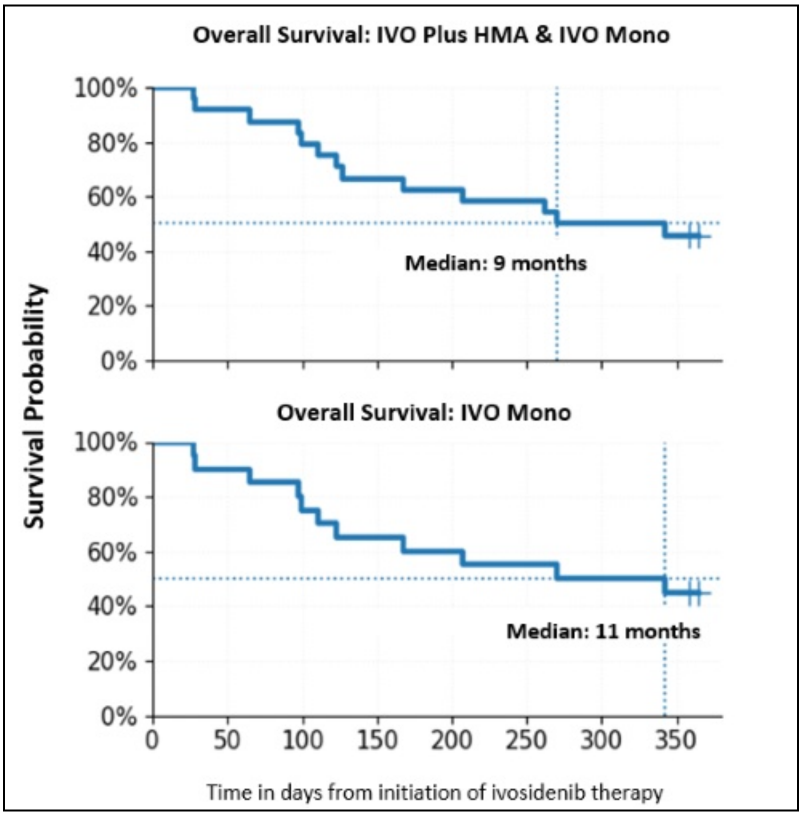

**Copyright Information:** (Online) ISSN: 2572-9241
©2026 The Author(s). HemaSphere is published by John Wiley & Sons Ltd on behalf of European Hematology Association. This is an open access Abstract Book distributed under the Attribution-NonCommercial-NoDerivs (CC BY-NC-ND), which allows third parties to download the articles and share them with others as long as they credit the author and the Abstract Book, but they cannot change the content in any way or use them commercially.
**Abstract Book Citations:** Authors, Title, HemaSphere, 2026;10:(S1):pages. The abstract book DOIs can be found on the “Author Information” page.
**Disclaimer:** Articles published in the journal HemaSphere exclusively reflect the opinions of the authors. The authors are responsible for all content in their abstracts including accuracy of the facts, statements, citing resources, etc.

### (PF549) CLADRIBINE COMBINED WITH LOW-DOSE CYTARABINE AND G-CSF IN UNFIT REFRACTORY/RELAPSED ACUTE MYELOID LEUKEMIA

**Topic:** 04. Acute myeloid leukemia - Clinical

Qiaoyi Zhou*[1], Hong Liu[1], Lihua Wu[1], Chunhua Liu[1], Xiyan Wang[1], Zhenzhen Wang[1], Runxia Gu[1], Shuning Wei[1], Ying Wang[1]

[1]*Institute of Hematology and Blood Diseases Hospital, Chinese Academy of Medical Sciences, Tianjin, China;*

### Background

CLAG, consisting of cladribine, cytarabine and granulocyte colony-stimulating factor(G-CSF), is recommended by National Comprehensive Cancer Network (NCCN) in 2025 as one of the first-line salvage treatments for R/R AML patients who are eligible for intensive chemotherapy.The common protocol under this circumstances is stipulated as infusion of 5mg/m$^2$ of cladribine and 2g/m$^2$ of cytarabine for 5 days with G-CSF 300ug/m$^2$ subcutaneously given. However, the toxicity limits its usage in older and unfit patients.Cladribine with decreasing-dose cytarabine(200-400mg/m$^2$/day) for 5 days infusion executed as salvage therapy in R/R AML children showed the rate of CR/CR with incomplete hematopoietic recovery (CRi) was up to 63.64% and the overall CR/CRi rate in older patients with new diagnosis AML under the regimen of cladribine +low-dose cytarabine (LDAC) was 62% with 1% 4-week mortality. The latter administrated cladribine 5mg/m$^2$ for 5 days and cytarabine 20mg twice daily for 10 days as induction treatment.

### Aims

Inspired by these, we tried to explore a suitable regimen with insurable efficiency and safety for unfit adults R/R AML by using LDAC+cladribine+G-CSF(miniCLAG) with longer administration duration.

### Methods

We retrospectively analyzed 16 unfit R/R AML patients treated with the miniCLAG regimen.Informed consent was obtained from all patients. All of them received at least one course of miniCLAG. The regimen was comprised by administrating cladribine 5mg/m$^2$ from day 1 to day 5 intravenously, cytarabine 20mg q12h from day1 to day10 subcutaneously and G-CSF 300μg/d from day0 to day 10, omitting when white cell count >20×10$^9$/L.

### Results

The median age of our cohort was 61 years old with 50% aged 60 or older. The CR/CRi rate was 37.5%, without treatment-related death. The median overall survival wasn't reached and the estimated 1-year overall survival was 85.94% (95%CI: 69.49%-100%). The median relapse-free survival was 293 days (95%CI:246 days-NA). Patients without CBF fusion protein gained more survival benefit from the miniCLAG with EFS prolonged. (P=0.041).

### Summary/Conclusion

The miniCLAG regimen may supply considerable efficiency and toxicity to the refractory/relapsed unfit AML patients, especially for those without core-binding factor fusion protein.

**Copyright Information:** (Online) ISSN: 2572-9241
©2026 The Author(s). HemaSphere is published by John Wiley & Sons Ltd on behalf of European Hematology Association. This is an open access Abstract Book distributed under the Attribution-NonCommercial-NoDerivs (CC BY-NC-ND), which allows third parties to download the articles and share them with others as long as they credit the author and the Abstract Book, but they cannot change the content in any way or use them commercially.
**Abstract Book Citations:** Authors, Title, HemaSphere, 2026;10:(S1):pages. The abstract book DOIs can be found on the "Author Information" page.
**Disclaimer:** Articles published in the journal HemaSphere exclusively reflect the opinions of the authors. The authors are responsible for all content in their abstracts including accuracy of the facts, statements, citing resources, etc.

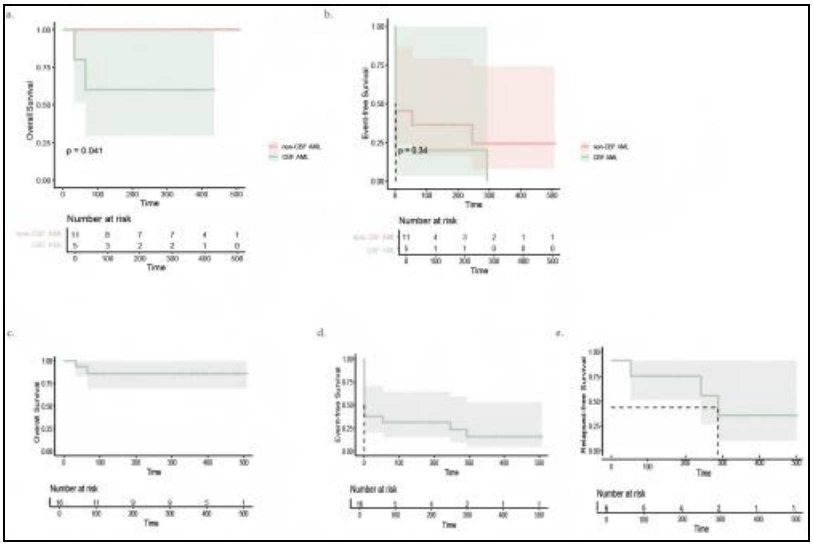

a: The OS between the CBF AML group and the non-CBF AML group. b: The EFS between the CBF AML group and the non-CBF AML group. c: The OS for all the patients. d: The EFS for all the patients. e: The RFS for the patients reached CR/CRi.

**Copyright Information:** (Online) ISSN: 2572-9241
©2026 The Author(s). HemaSphere is published by John Wiley & Sons Ltd on behalf of European Hematology Association. This is an open access Abstract Book distributed under the Attribution-NonCommercial-NoDerivs (CC BY-NC-ND), which allows third parties to download the articles and share them with others as long as they credit the author and the Abstract Book, but they cannot change the content in any way or use them commercially.
**Abstract Book Citations:** Authors, Title, HemaSphere, 2026;10:(S1):pages. The abstract book DOIs can be found on the "Author Information" page.
**Disclaimer:** Articles published in the journal HemaSphere exclusively reflect the opinions of the authors. The authors are responsible for all content in their abstracts including accuracy of the facts, statements, citing resources, etc.

### (PF550) A PHASE I/II STUDY OF GILTERITINIB AND MOMELOTINIB IN ADULTS WITH RELAPSED OR REFRACTORY FLT3-MUTATED ACUTE MYELOID LEUKEMIA

**Topic:** 04. Acute myeloid leukemia - Clinical

Leticia Campoverde[1], Maria Hachem[1], Farhad Ravandi[1], Guillermo Garcia-Manero[1], Gautam Borthakur[1], Naval Daver[1], Guillermo Montalban-Bravo[1], Ian Bouligny[1], Musa Yilmaz[1], Danielle Hammond[1], Steven Kornblau[1], Mahesh Swaminathan[1], Courtney Dinardo[1], Wei Qiao[1], Regina Abramova[1], Brianna Kammerer[1], Hussein Abbas[1], Nicholas Short*[1]

[1]*University of Texas MD Anderson Cancer Center, Leukemia, Houston, United States of America;*

### Background

Although gilteritinib improves outcomes in relapsed/refractory (R/R) *FLT3*-mutated acute myeloid leukemia (AML), responses are often short-lived. Preclinical data suggest that cytokine-mediated activation of the JAK/STAT pathway contributes to resistance to FLT3 inhibitors. Emerging evidence also supports a role for ACVR1/SMAD signaling in adaptive resistance in *FLT3*-mutated AML. We therefore hypothesized that momelotinib, an oral inhibitor of JAK1/2 and ACVR1, may suppress compensatory survival signaling and enhance the efficacy of FLT3 targeted therapy. Momelotinib has also demonstrated potential FLT3 inhibitory activity in preclinical models, providing a complementary mechanistic rationale for its use in *FLT3*-mutated AML.

### Aims

This is a phase I/II study evaluating the safety and efficacy of the combination of momelotinib + gilteritinib in adults ≥18 years of age with R/R *FLT3*-mutated AML

### Methods

Adult patients (pts) with R/R *FLT3*-mutated AML, including ITD or TKD (D835/836) mutations, were eligible, without restriction on prior therapies. In the phase I portion, pts receive momelotinib at a dose of 200mg, 400mg, or 600mg orally daily combined with gilteritinib 120mg orally daily. In cycle 1, pts receive a 7-day momelotinib monotherapy lead-in, followed by continuous combination therapy (given in 28-day cycles). The primary endpoint of the phase I study is the minimum safe and biologically effective dose of momelotinib in combination with gilteritinib.

**Copyright Information:** (Online) ISSN: 2572-9241
©2026 The Author(s). HemaSphere is published by John Wiley & Sons Ltd on behalf of European Hematology Association. This is an open access Abstract Book distributed under the Attribution-NonCommercial-NoDerivs (CC BY-NC-ND), which allows third parties to download the articles and share them with others as long as they credit the author and the Abstract Book, but they cannot change the content in any way or use them commercially.
**Abstract Book Citations:** Authors, Title, HemaSphere, 2026;10:(S1):pages. The abstract book DOIs can be found on the "Author Information" page.
**Disclaimer:** Articles published in the journal HemaSphere exclusively reflect the opinions of the authors. The authors are responsible for all content in their abstracts including accuracy of the facts, statements, citing resources, etc.

### Results

As of February 2026, 13 pts have been treated. Median age was 67 years (range, 31–84). Eleven pts (85%) had a *FLT3*-ITD mutation. The median *FLT3* VAF was 9% (range, 3-59%). Pts were heavily pretreated, with a median of 3 prior therapies (range, 1–6) and a median of 2 prior FLT3 inhibitors (range, 0-3), including 8 (62%) with prior quizartinib exposure, and 12 (92%) with prior gilteritinib exposure.

Three pts received momelotinib 200mg, 3 received 400mg, and 7 received 600mg. No dose-limiting toxicities have been observed at any dose level. The most common grade ≥3 non-hematologic adverse events were infection in 7 pts (54%), sepsis in 4 pts (31%), and acute kidney injury in 3 pts (23%). One patient experienced transient grade 2 orthostatic hypotension within 24h of starting momelotinib (600mg), a phenomenon previously described with higher doses of momelotinib.
The median number of cycles was 2 (range, 1-5). As of the data cutoff, 12 pts were evaluable for response. Three pts (25%) achieved CRc (CRi in 2 pts and MLFS in 1 pt). All 3 responding pts had received prior gilteritinib and quizartinib. Responses were observed at the 400mg (n=1) and 600mg (n=2) dose levels and only in the *FLT3*-ITD pts. Two additional pts, both with prior gilteritinib exposure, achieved ≥50% reduction in bone marrow blasts but did not meet formal response criteria. 5/9 pts (56%) with baseline peripheral blood (PB) blasts had PB blast reduction ≥50% during the momelotinib lead-in phase, suggesting possible monotherapy activity of momelotinib; 2 of these pts achieved formal response (CRi and MLFS).

### Summary/Conclusion

The combination of gilteritinib and momelotinib (up to 600mg daily) was safe and demonstrates encouraging preliminary activity in heavily pretreated R/R *FLT3*-mutated AML, including in pts with prior gilteritinib and quizartinib exposure. The study continues to accrue pts, and correlative studies are ongoing to better understand the mechanism of response and resistance to this novel regimen.

**Copyright Information:** (Online) ISSN: 2572-9241
©2026 The Author(s). HemaSphere is published by John Wiley & Sons Ltd on behalf of European Hematology Association. This is an open access Abstract Book distributed under the Attribution-NonCommercial-NoDerivs (CC BY-NC-ND), which allows third parties to download the articles and share them with others as long as they credit the author and the Abstract Book, but they cannot change the content in any way or use them commercially.
**Abstract Book Citations:** Authors, Title, HemaSphere, 2026;10:(S1):pages. The abstract book DOIs can be found on the "Author Information" page.
**Disclaimer:** Articles published in the journal HemaSphere exclusively reflect the opinions of the authors. The authors are responsible for all content in their abstracts including accuracy of the facts, statements, citing resources, etc.

### (PF551) IMPACT OF TIME TO TREATMENT ON OUTCOMES IN HYPERPROLIFERATIVE ACUTE MYELOID LEUKEMIA

**Topic:** 04. Acute myeloid leukemia - Clinical

Benjamin McCormick*[1], Kendall Diebold[1], Supriya Maheshwari[1], William Mudd[1], Aditi Sharma[1], Pankit Vachhani[1], Sravanti Rangaraju[1], Kimo Bachishvili[1], Manuel Espinoza Gutarra[1], Razan Mohty[1], Jorge Cortes[1], Ravi Bhatia[1], Omer Jamy[1]

[1]*University of Alabama at Birmingham, Division of Hematology/Oncology, Birmingham, United States of America;*

### Background

The optimal timing of treatment initiation in newly diagnosed acute myeloid leukemia (AML) remains debated. In unselected AML populations, modest delays have not consistently been associated with early mortality or overall survival (OS), and short delays for molecular or cytogenetic evaluation are often considered acceptable. However, patients with hyperproliferative AML, characterized by marked leukocytosis and early complications, are underrepresented in prior studies, and whether similar findings apply remains unclear.

### Aims

To evaluate the association between time from diagnosis to treatment (TDT) and early clinical outcomes in newly diagnosed hyperproliferative AML.

### Methods

We conducted a retrospective cohort study of adults from 2018-2025 with newly diagnosed hyperproliferative AML, defined as white blood cell count (WBC) ≥25×10⁹/L prior to cytoreduction/induction. TDT was defined as days from definitive AML diagnosis (peripheral blood or marrow) to initiation of AML-directed therapy (cytoreduction excluded). Patients (pts) were categorized by TDT ≤48 vs >48 hours (hrs). The primary endpoint was early adverse outcomes – composite of 30-day mortality or failure to initiate induction despite treatment intent (to mitigate immortal time/survival bias). Induction response and OS were analyzed.

### Results

Among 285 pts, median age was 64 years (range, 19-94); 56% were male. AML was de novo in 78%, secondary/treatment-related in 23%. FLT3 mutations were present in 37.5%. ELN 2022 risk was favorable in 25%, intermediate in 26%, and adverse in 45%. Induction was intended in 256 patients (90%), of whom 226 initiated therapy and 30 never received treatment; pts who never initiated induction experienced high 30-day mortality (96.7%). These pts were critically ill at admission (97% requiring ICU care). Among treated pts, 58% received intensive induction. Among treated pts with TDT >48 hrs, median TDT was 5 days (IQR 4–6; range 3–34).

Among pts for whom induction was intended, those with TDT >48 hrs experienced higher rates of early adverse outcomes compared to TDT ≤ 48 hrs (32.7% vs 8.7%; OR 5.07, 95% CI 2.36–10.87). This association persisted after adjusting for age, peak WBC, and ICU admission prior to induction (adjusted OR 3.80, 95% CI 1.59–9.08) (Figure 1), and in sensitivity analyses excluding pts admitted to the ICU. Sensitivity analyses at higher WBC thresholds (≥50k–≥100k) demonstrated a consistent and progressively stronger association with delayed treatment (WBC ≥100k, OR 18.02, 95% CI 2.68–121.22).

TDT >48 hrs was associated with lower remission rates (50.4% vs 66%, p=0.026) and shorter median OS (8.2 vs 17 months; p=0.023). Stratification by ELN 2022 risk demonstrated the association between treatment delay and inferior remission rates persisted across groups and was most pronounced in intermediate/adverse-risk. After adjusting for ELN risk and age, induction intensity was not associated with 30-day mortality. Findings remained significant after excluding pts receiving HMA/Ven with planned cytoreduction.

**Copyright Information:** (Online) ISSN: 2572-9241
©2026 The Author(s). HemaSphere is published by John Wiley & Sons Ltd on behalf of European Hematology Association. This is an open access Abstract Book distributed under the Attribution-NonCommercial-NoDerivs (CC BY-NC-ND), which allows third parties to download the articles and share them with others as long as they credit the author and the Abstract Book, but they cannot change the content in any way or use them commercially.
**Abstract Book Citations:** Authors, Title, HemaSphere, 2026;10:(S1):pages. The abstract book DOIs can be found on the "Author Information" page.
**Disclaimer:** Articles published in the journal HemaSphere exclusively reflect the opinions of the authors. The authors are responsible for all content in their abstracts including accuracy of the facts, statements, citing resources, etc.

### Summary/Conclusion

In newly diagnosed hyperproliferative AML, TDT >48 hrs was associated with substantially worse early outcomes, including higher early mortality and lower remission rates. The adverse impact of this delay persists across ELN strata, suggesting deferring induction for cytogenetic/molecular risk stratification may be harmful. These findings support prompt induction initiation in hyperproliferative AML, incorporating targeted agents as results become available.

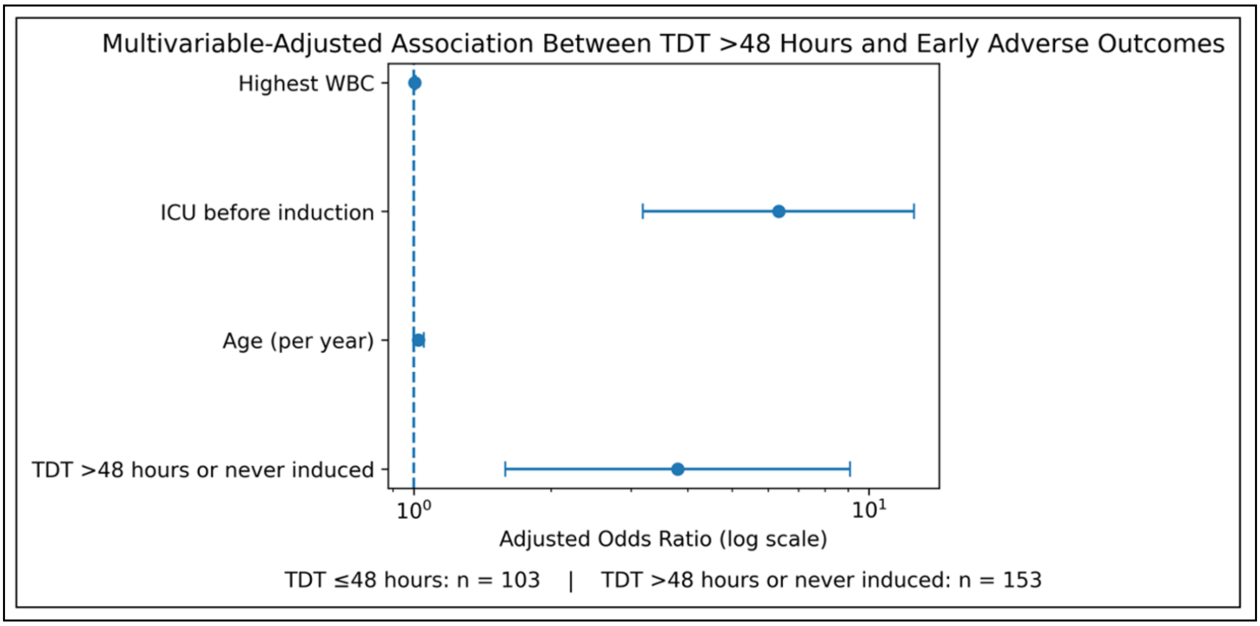

Figure 1. Multivariable-adjusted association between time from diagnosis to treatment (TDT) and early adverse outcomes (30-day mortality or failure to initiate induction) in newly diagnosed hyperproliferative acute myeloid leukemia.

**Copyright Information:** (Online) ISSN: 2572-9241
©2026 The Author(s). HemaSphere is published by John Wiley & Sons Ltd on behalf of European Hematology Association. This is an open access Abstract Book distributed under the Attribution-NonCommercial-NoDerivs (CC BY-NC-ND), which allows third parties to download the articles and share them with others as long as they credit the author and the Abstract Book, but they cannot change the content in any way or use them commercially.
**Abstract Book Citations:** Authors, Title, HemaSphere, 2026;10:(S1):pages. The abstract book DOIs can be found on the "Author Information" page.
**Disclaimer:** Articles published in the journal HemaSphere exclusively reflect the opinions of the authors. The authors are responsible for all content in their abstracts including accuracy of the facts, statements, citing resources, etc.

### (PF552) HEALTHY HUMAN STUDY OF A NOVEL ORAL BCL-2 INHIBITOR LONITOCLAX (ZE50-0134) AND EARLY RESULTS OF A PHASE 1/B STUDY OF LONITOCLAX TOGETHER WITH AZACITIDINE IN RELAPSED AND REFRACTORY AML

**Topic:** 04. Acute myeloid leukemia - Clinical

John Byrd*[1, 2, 3], Vadim Doronin[4], Sergey Voloshin[5], Brian Ledwith[1], Fraser Pickersgill[1], Kate Dokukina[1], Ruben Karapetian[1], Chia Sharpe[2], Erin Hertlein[2], Megan Johnstone[2], Alexei Pushechnikov[6], Christopher Argent[7], Nikolay Savchuk[1], Iain Dukes[1], Amy Burd[1]

*[1]Lomond Therapeutics, Dover, United States of America; [2]University of Cincinnati, Internal Medicine, Cincinnati, United States of America; [3]UPMC Hillman Cancer Center and University of Pittsburgh, Pittsburgh, United States of America;[4]S.P. Botkin Moscow Multidisciplinary Scientific and Clinical Center, Moscow, Russia; [5]Leningrad Regional Clinical Hospital, Saint Petersburg, Russia; [6]ChemDiv Inc, San Diego, United States of America; [7]Scientia Clinical Research, Sydney, Australia;*

### Background

B-cell lymphoma 2 (Bcl-2) is a member of the anti-apoptotic Bcl-2 family, Sensitivity to pharmaceutical disruption of Bcl-2 BH3 domain interactions with pro-apoptotic proteins characterizes diseases overtly responsive to this treatment. Combination treatments of venetoclax (V) together with azacitidine (A) have greatly impacted the outcome of unfit acute myeloid leukemia (AML). However, hematologic toxicity with the approved continuous dosing of V in the VA regimen is often difficult to manage and efforts to add additional treatments to VA have similarly been limited by prolonged hematologic toxicity. To mitigate the hematologic effects, immune suppression and drug-drug interactions (CYP3A4) observed with V, we developed lonitoclax (L), a structurally unique chemotype with a shorter half-life and reduced CYP3A4 inhibition. Pre-clinical studies in a syngeneic model demonstrated L and azacitidine (LA) had minimal immunosuppressive activity on B cells, CD8 T cells, and NK cells and was more effective than VA.[1] Herein we report both completed results from our phase 1 healthy volunteer study (HVS) of L and combination study of LA in relapsed AML.

### Methods

A single-center, prospective, randomized, double-blind placebo-controlled study of L in HVS (NCT06787131) was performed. The primary objective was pharmacokinetic (PK) evaluation of L; secondary objective was safety, and exploratory objective was pharmacodynamics (immune cell changes and plasma apoptosis assay [PAA]). The ongoing RR AML study (NCT07303660) LA is given BID for 28 days.

**Copyright Information:** (Online) ISSN: 2572-9241
©2026 The Author(s). HemaSphere is published by John Wiley & Sons Ltd on behalf of European Hematology Association. This is an open access Abstract Book distributed under the Attribution-NonCommercial-NoDerivs (CC BY-NC-ND), which allows third parties to download the articles and share them with others as long as they credit the author and the Abstract Book, but they cannot change the content in any way or use them commercially.
**Abstract Book Citations:** Authors, Title, HemaSphere, 2026;10:(S1):pages. The abstract book DOIs can be found on the “Author Information” page.
**Disclaimer:** Articles published in the journal HemaSphere exclusively reflect the opinions of the authors. The authors are responsible for all content in their abstracts including accuracy of the facts, statements, citing resources, etc.

**Results**

In the HVS, a total of 48 healthy subjects have been treated with L in 8 SAD cohorts (100, 200, 400 mg, 800 mg, 400 mg BID, 400 mg with food, 200 mg with CYP3A4 inhibition, and 400 mg with a proton pump inhibitor). L was well tolerated with no significant treatment-related safety signals reported in any of the cohorts. The PK of L was linear with mean plasma exposure ($AUC_{0\text{-}inf}$) values that increased proportionally with dose escalation. There was a rapid absorption with a $T_{max}$ at approximately 2 hours and a half-life of approximately 12 hours. In the PAA, apoptosis was confirmed in a dose-dependent and plasma concentration-dependent manner. There was no change in B-cells, CD8 T-cells, or NK cells with L treatment. PK $AUC_{0\text{-}inf}$ was increased (3.5-fold) with food but minimally influenced by itraconazole (+3%) or PPI administration (-27%). In the heavily pretreated RR AML study of LA, enrollment of two cohorts (100 mg and 200 mg BID L, n=6 pts) has been completed. The initial results have demonstrated LA to be well tolerated with no DLTs and a decrease in blood and bone marrow blasts in both cohorts, with 1 patient in Cohort 2 having an MLFS after cycle 1. PK of L in the RR AML study was similar to that observed in the HVS. Enrollment to this AML trial continues and clinical, PK, and pharmacodynamic results will be updated at the meeting.

**Summary/Conclusion**

Lonitoclax has favorable PK and safety profiles when administered orally daily at doses of up to 800 mg in HV, with dose-proportional increases in systemic exposure, positive food effect, and no DDI or CYP3A4 effect, with target engagement demonstrated using PAA, and no significant treatment-related adverse events. The Phase 1B study in R/R AML patients was initiated with early evidence of efficacy with no DLTs and will be updated.

**Copyright Information:** (Online) ISSN: 2572-9241
©2026 The Author(s). HemaSphere is published by John Wiley & Sons Ltd on behalf of European Hematology Association. This is an open access Abstract Book distributed under the Attribution-NonCommercial-NoDerivs (CC BY-NC-ND), which allows third parties to download the articles and share them with others as long as they credit the author and the Abstract Book, but they cannot change the content in any way or use them commercially.
**Abstract Book Citations:** Authors, Title, HemaSphere, 2026;10:(S1):pages. The abstract book DOIs can be found on the "Author Information" page.
**Disclaimer:** Articles published in the journal HemaSphere exclusively reflect the opinions of the authors. The authors are responsible for all content in their abstracts including accuracy of the facts, statements, citing resources, etc.

### (PF553) EARLY RESULTS OF THE ONGOING PHASE II MAVEN STUDY: VENETOCLAX-AZACITIDINE-LIPOSOMAL MITOXANTRONE (VAM) IN MYELOID SARCOMA AND SECONDARY ACUTE MYELOID LEUKEMIA

**Topic:** 04. Acute myeloid leukemia - Clinical

Xinjie Chen*[1], Yubing Zhao[1], Hongming Zhu[1], Wen Wu[1], Yang Shen[1]

*[1]Shanghai Institute of Hematology, State Key Laboratory of Medical Genomics, National Research Center for Translational Medicine at Shanghai, Ruijin Hospital[illegible]Shanghai Jiao Tong University School of Medicine, Shanghai, China;*

### Background

Patients with myeloid sarcoma have unmet therapeutic needs, as mainstream induction regimens (systemic chemotherapy, targeted therapy) inadequately address the unique biological characteristics of extramedullary lesions. Additionally, the superior efficacy of CPX351 over conventional regimens in secondary acute myeloid leukemia (sAML) has demonstrated the advantages of liposomal formulations in this AML subtype. Thus, we designed a prospective, phase II study (NCT07028086, shorten for MAVEN) to explore the efficacy and safety of VAM regimen (venetoclax + azacitidine + liposomal mitoxantrone) in two cohorts: myeloid sarcoma (cohort 1) and sAML (cohort 2).

### Methods

As of manuscript submission, 24 patients had been enrolled. The regimen was administered as follows: liposomal mitoxantrone 20 mg/m$^2$ d1, AZA 75 mg/m$^2$ d1-7, VEN 100 mg d1, 200 mg d2, 400 mg d3-14. The primary objective of the MAVEN study was to assess the composite complete remission (CRc: CR+CRi) rate after two cycles of VAM treatment. The secondary objective included the minimal residual disease (MRD)-negative remission rate, treatment-related adverse events, and overall safety profile. The duration of CR, overall survival and event-free survival rate will be further assessed as follow-up matures.

### Results

The median age of currently enrolled patients was 43 (31-68) years old. Detailed baseline characteristics were summarized in Table 1. CRc was achieved in 79.2% (19/24) of intention-to-treat (ITT) population after two cycles of VAM regimen, with 85.7% (12/14) in cohort 1 and 70% (7/10) in cohort 2, respectively (Figure A). In cohort 1, 13/14 participants bridged to allogeneic hematopoietic stem cell transplantation (allo-HSCT), only one patient shift to other regimen because of unavailable donors. Although two patients got partial remission (PR) after two cycles of VAM regimen, they received CAG regimen for the third cycle and achieved MRD-negative remission before allo-HSCT. In cohort 2, one patient got PR, with 5.8% blasts remained; two patients withdrew during the first cycle of treatment: one for poor therapeutic effect at day 14 assessment of cycle 1, the other withdrew informed consent due to personal reasons. The most frequent grade 3/4 non-hematologic adverse events (according to CTCAE version 5.0) were febrile neutropenia (grade 3, n = 8; grade 4, n = 2), pneumonia (grade 3, n = 3) and sepsis (grade 3, n = 2; grade 4, n = 1) (Table 2). Only one patient in cohort 1 got tumor lysis syndrome in grade 4. However, after emergency dialysis and supportive care, the patient completed the trial as protocoled, successfully proceeded to allo-HSCT and was discharged post-engraftment. Long-term follow-up is ongoing.

### Summary/Conclusion

The VAM regimen shows promising efficacy in achieving high composite complete remission rates and successful bridging to allo-HSCT in patients with myeloid sarcoma and secondary AML. The safety profile is manageable, supporting further investigation of this regimen in larger prospective studies with prolonged follow-up.

**Copyright Information:** (Online) ISSN: 2572-9241
©2026 The Author(s). HemaSphere is published by John Wiley & Sons Ltd on behalf of European Hematology Association. This is an open access Abstract Book distributed under the Attribution-NonCommercial-NoDerivs (CC BY-NC-ND), which allows third parties to download the articles and share them with others as long as they credit the author and the Abstract Book, but they cannot change the content in any way or use them commercially.
**Abstract Book Citations:** Authors, Title, HemaSphere, 2026;10:(S1):pages. The abstract book DOIs can be found on the "Author Information" page.
**Disclaimer:** Articles published in the journal HemaSphere exclusively reflect the opinions of the authors. The authors are responsible for all content in their abstracts including accuracy of the facts, statements, citing resources, etc.

**Table 1. Baseline characteristics**

| | |
|---|---|
| **Age in years, median (range)** | 43 (31 - 68) |
| **Gender(n,%)** | |
| Male | 9 (37.5%) |
| Female | 15 (62.5%) |
| **WBC (*10^9/L), median (range)** | 3.56 (0.89 - 98.8) |
| **Hb (g/L), median (range)** | 92 (60 - 148) |
| **PLT (*10^9/L), median (range)** | 81 (4 - 358) |
| **BM blasts (%), median (range)** | 28.75 (0 - 85) |
| < 30 | 12 (50.0%) |
| 30-50 | 5 (20.8%) |
| > 50 | 7 (29.1%) |
| **Disease subtype** | |
| Myeloid sarcoma (cohort 1) | 14 |
| secondary AML (cohort 2) | 10 |
| **ELN, karyotype (n,%)** | |
| Favorable | 9 (37.5%) |
| Intermediate | 5 (20.8%) |
| Adverse | 7 (29.2%) |

**Table 2. Response assessment**

| | One-cycle (N = 24) | Two-cycle (N = 22) |
|---|---|---|
| **Overall response** | 22 (91.7%) | 22 (100.0%) |
| Composite complete response | 19 (86.3%) | 19 (86.3%) |
| Partial response | 3 (13.6%) | 3 (13.6%) |
| **No response** | 2 (8.3%) | / |

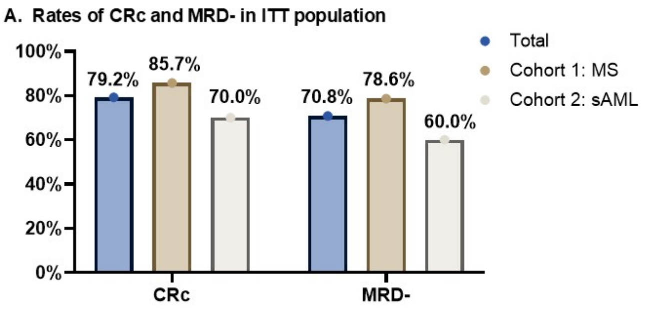

**Copyright Information:** (Online) ISSN: 2572-9241
©2026 The Author(s). HemaSphere is published by John Wiley & Sons Ltd on behalf of European Hematology Association. This is an open access Abstract Book distributed under the Attribution-NonCommercial-NoDerivs (CC BY-NC-ND), which allows third parties to download the articles and share them with others as long as they credit the author and the Abstract Book, but they cannot change the content in any way or use them commercially.
**Abstract Book Citations:** Authors, Title, HemaSphere, 2026;10:(S1):pages. The abstract book DOIs can be found on the "Author Information" page.
**Disclaimer:** Articles published in the journal HemaSphere exclusively reflect the opinions of the authors. The authors are responsible for all content in their abstracts including accuracy of the facts, statements, citing resources, etc.

### (PF554) ABSOLUTE LYMPHOCYTE COUNT AFTER INDUCTION CHEMOTHERAPY IS A PREDICTIVE BIOMARKER IN ACUTE MYELOID LEUKEMIA PATIENTS AND RELATES WITH POST-THERAPY CHANGES IN IMMUNE TRANSCRIPTOMIC BONE MARROW PROFILE.

**Topic:** 04. Acute myeloid leukemia - Clinical

Gianluca Cristiano[1], Federico Zingarelli*[2, 3], Jayakumar Vadakekolathu[3], Jacopo Nanni[2], Chiara Sartor[4], Martina Barone[4], Agnese Patuelli[2], Lorenza Bandini[2], Carmen Baldazzi[4], Emanuela Ottaviani[4], Lucia Catani[2, 2], Stefania Paolini[4], Cristina Papayannidis[4], Sergio Rutella[5, 6], Antonio Curti[4]

*[1]Fondazione ANT ETS, Bologna, Italy; [2]Dipartimento di Scienze Mediche e Chirurgiche, Università di Bologna, Bologna, Italy; [3]John van Geest Cancer Research Centre, Nottingham Trent University, Nottingham, United Kingdom;[4]IRCCS Azienda Ospedaliero-Universitaria di Bologna, Istituto di Ematologia "Seràgnoli", Bologna, Italy; [5]Insigneo Institute, University of Sheffield, Sheffield, United Kingdom; [6]Division of Clinical Medicine, School of Medicine and Population Health, University of Sheffield, Sheffield, United Kingdom;*

### Background

In recent years, interest in the bone marrow (BM) tumor microenvironment (TME) of acute myeloid leukemia (AML) has been rapidly growing. Absolute lymphocyte count (ALC) is known to be an independent prognostic factor in many neoplasms; in AML, enhanced ALC recovery after intensive chemotherapy (IC) has been associated with superior overall survival (OS). However,data correlating ALC recovery and immune-related prognostic factors in the era of novel therapies are still lacking.

### Aims

To evaluate ALC recovery as an outcome predictor in AML patients achieving complete response (CR) to IC and to explore the role of TME in mediating ALC.

### Methods

This is a monocenter retrospective study evaluating 188 AML patients who achieved CR after IC. IC was divided in "3+7" cohort and fludarabine(fluda)-based cohort. From IC start, 4 ALC time-points (TPs) were established and ALC recovery was defined as >500/mm$^3$.ALC kinetics was analysed at single TPs and dynamically by stratification in 2 subgroups: group A ALC≥500/mm$^3$ at all 4 TPs and group B ALC<500/mm$^3$ in at least one TP .
PanCancer Immune Profiling (PCI) panel and the nCounter platform (NanoString) were used to explore the immune transcriptomic variation before and after IC, stratifying patients according to ALC kinetics. Overall, 48 BM samples collected from 35 AML patients, including 13 after therapy were analyzed.

**Copyright Information:** (Online) ISSN: 2572-9241
©2026 The Author(s). HemaSphere is published by John Wiley & Sons Ltd on behalf of European Hematology Association. This is an open access Abstract Book distributed under the Attribution-NonCommercial-NoDerivs (CC BY-NC-ND), which allows third parties to download the articles and share them with others as long as they credit the author and the Abstract Book, but they cannot change the content in any way or use them commercially.
**Abstract Book Citations:** Authors, Title, HemaSphere, 2026;10:(S1):pages. The abstract book DOIs can be found on the "Author Information" page.
**Disclaimer:** Articles published in the journal HemaSphere exclusively reflect the opinions of the authors. The authors are responsible for all content in their abstracts including accuracy of the facts, statements, citing resources, etc.

### Results

Overall, data from 188 AML patients were collected; 97 (51.6%) received "3+7" and 91 (48.4%) fluda-based IC. Median patient age was 55 years. No significant differences were observed among patients' groups regarding biologic features at diagnosis. Median follow-up was 51.4 months.

In patients receiving "3+7" regimen, a statistically significant correlation with OS in patients in group A (25,5% of all patients) compared to group B (26%) was observed (p=0.02). This finding was similar on day 15 ALC evaluation (ALC-15), comparing patients with ≥500/mm$^3$ ALC (31,9%) vs those under the cut-off (19,6%). In contrast, in the fluda-based cohort, ALC kinetics did not correlate with OS. We, then, stratified whole population into those who received allogeneic transplantation (HSCT; 66%) and those who did not (34%). OS was not affected by ALC recovery in HSCT cohort, whereas, in non-HSCT patients, an inferior OS group was identified in group B, whereas group A performed as transplanted patients. Notably, within "3+7" patients who achieved measurable residual disease (MRD) negativity (neg), group A experienced superior OS. Furthermore, overall MRDneg before HSCT was superior in group A vs group B (62.2 vs 47.2%, p=0.05).

NanoString analysis was performed in 35 patients, 13 (37%) and 22 (63%) belonged to group A and group B, respectively, with no significant differences at baseline between the two groups. Notably, after IC, the BM of patients in group A was enriched in immune genes, includingmediators of T-cell activation, immune checkpoints, and Tregs genes (Figure 1a). Conversely, Group B samples exhibited gene expression patterns consistent with the activation of inflammatory and dysfunctional myeloid cells, without evidence of an immune regulatory TME (Figure 1b).

### Summary/Conclusion

Our study demonstrates that ALC kinetics during IC is a promising biomarker to refine prognostic stratification of AML patients. The immune transcriptomic profile of BM samplesvaries according to ALC kinetics and supports a biological significance of immune microenvironment in guiding ALC recovery.

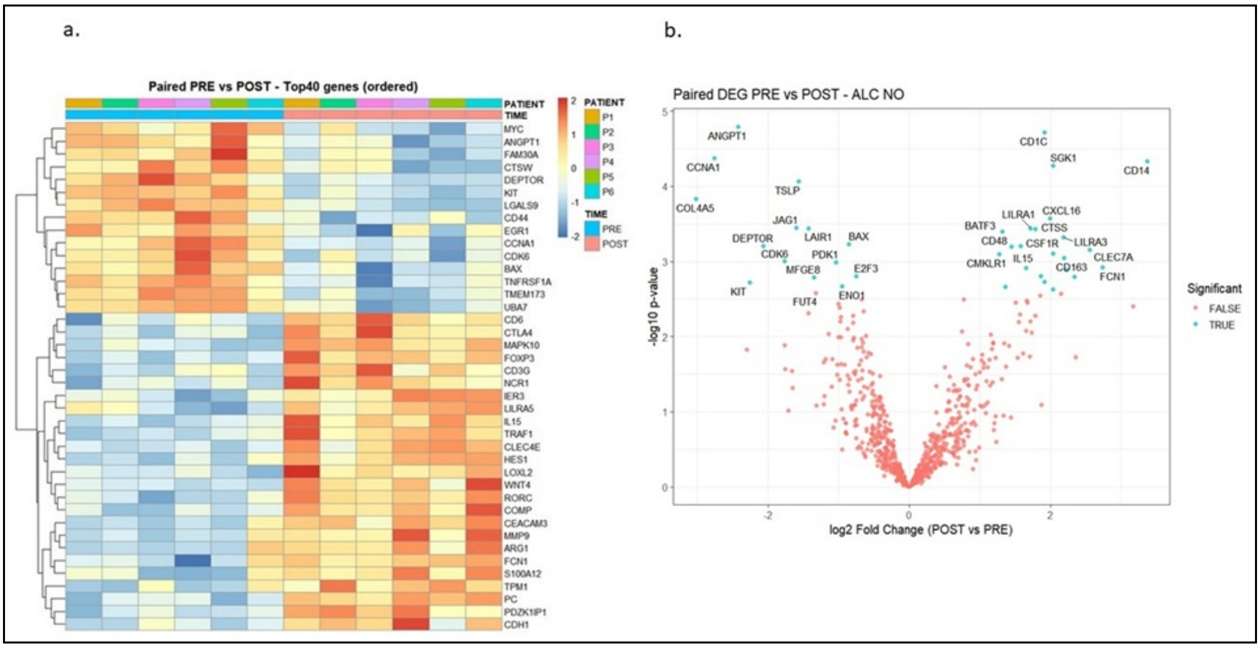

Figure 1 | Differential gene expression according to ALCkinetics a, Heatmap of the top 40 differentially expressed genes (DEGs) identified by NanoString analysis in paired bone marrow samples collected pre- and post-therapy from AML patients who achieved absolute lymphocyte count (ALC) recovery. b, Volcano plot of differential gene expression (post- vs pre-therapy) in AML patients who did not achieve ALC recovery.

**Copyright Information:** (Online) ISSN: 2572-9241
©2026 The Author(s). HemaSphere is published by John Wiley & Sons Ltd on behalf of European Hematology Association. This is an open access Abstract Book distributed under the Attribution-NonCommercial-NoDerivs (CC BY-NC-ND), which allows third parties to download the articles and share them with others as long as they credit the author and the Abstract Book, but they cannot change the content in any way or use them commercially.
**Abstract Book Citations:** Authors, Title, HemaSphere, 2026;10:(S1):pages. The abstract book DOIs can be found on the "Author Information" page.
**Disclaimer:** Articles published in the journal HemaSphere exclusively reflect the opinions of the authors. The authors are responsible for all content in their abstracts including accuracy of the facts, statements, citing resources, etc.

### (PF555) NO EVIDENCE OF QTC INTERVAL PROLONGATION WITH THE MENIN INHIBITOR BLEXIMENIB WHEN GIVEN AS MONOTHERAPY OR IN COMBINATION WITH AML-DIRECTED THERAPIES FOR KMT2A OR NPM1 ALTERED AML

**Topic:** 04. Acute myeloid leukemia - Clinical

Maher Abdul-Hay[1], Sameem Abedin[2], Ibrahim Aldoss[3], Ana Alfonso Pierola[4], Juan Manuel Alonso Dominguez[5], Nikki Daskalakis[6], Hartmut Döhner[7], Amber D'souza[6], Neil Dunavin[3], Jordi Esteve Reyner[8], Matthew Exum[6], Amir Fathi[9], Pasquale Fedele[10], Lucille Ferrante[6], Sylvain Garciaz[11], Ana Garrido Diaz[12], Emmanuel Gyan[13], Nahor Haddish-Berhane[6], Elias Jabbour[14], Madlen Jentzsch[15], Marina Konopleva[16], Jan Krönke[17], Anita Kumar[6], Dandan Luo[6], Christina Loefgren[6], Oliver Lomas[18], Valentina Mancini[19], Ioannis Mantzaris[16], Anna Mitselos[20], Daniel Morillo[5], Teng Fong Ng[21], Marianna Norata[22], Jenny O'nions[23], Kathryn Packman[24], Cristina Papayannidis[25], Ulrike Philippar[20], Giorgos Psarros[20], Christian Recher[26], Christoph Röllig[27], Naa Sakey[6], Olga Salamero[28], Tim Sauer[29], Emma Searle[30], Prathap Nagarajahastri[31], Richard Stone[32], Peter Tan[33], Tao Sun[34], Trevor Tucker[6], Lachlin Vaughan[35], Gala Vega[5], Paresh Vyas[36], Andrew H. Wei[37], Marie Luise Hütter-Krönke*[17]

*[1]Laura and Isaac Perlmutter Cancer Center at New York University Langone Health, New York, United States of America; [2]Medical College Of Wisconsin, Milwaukee, United States of America;[3]City of Hope National Medical Center, Duarte, United States of America; [4]Clínica Universidad de Navarra, Navarra, Spain;[5]START Madrid-FJD Early Phase Unit, University Hospital Fundación Jiménez Díaz, IIS-FJD, Madrid, Spain; [6]Johnson & Johnson Innovative Medicine, Spring House, United States of America; [7]Universitätsklinikum Ulm, Ulm, Germany; [8]Hospital Clínic de Barcelona, Barcelona, Spain;[9]Massachusetts General Hospital, Harvard Medical School, Boston, United States of America; [10]Monash Health and School of Clinical Sciences at Monash Health, Monash University, Clayton, Australia; [11]Aix-Marseille Université, Inserm, CNRS, Institut Paoli-Calmettes, Centre de Recherche en Cancérologie de Marseille, Marseille, France; [12]Hospital de la Santa Creu i Sant Pau, Barcelona, Spain;[13]Centre Hospitalier Universitaire de Tours, CIC INSERM U1415, Tours, France; [14]MD Anderson Cancer Center, University of Texas, Texas, United States of America; [15]Universitätsklinikum Leipzig, Leipzig, Germany; [16]Montefiore Einstein Comprehensive Cancer Center, Bronx, United States of America; [17]Charité – Universitätsmedizin Berlin, Berlin, Germany; [18]Janssen Research & Development, LLC, High Wycombe, United Kingdom; [19]ASST Grande Ospedale Metropolitano Niguarda, Milan, Italy; [20]Janssen Research & Development, LLC, Beerse, Belgium; [21]Griffith University and Gold Coast University Hospital, Southport, Australia; [22]IRCCS Istituto Romagnolo per lo Studio de Tuori (IRST) "Dino Amadori", Meldola, Italy;[23]University College London Hospitals NHS Foundation Trust and UCL/NIHR Clinical Research Facility, London, United Kingdom; [24]Johnson & Johnson Innovative Medicine, Cambridge, United States of America; [25]Sant'Orsola-Malpighi Polyclinic, Bologna, Italy;[26]University Cancer Institute Toulouse Oncopole, Toulouse, France; [27]Universitätsklinikum TU Dresden, Dresden, Germany; [28]University Hospital Vall d'Hebron, and Experimental Hematology, Vall d'Hebron Institute of Oncology, Barcelona, Spain; [29]Department of Internal Medicine V, Heidelberg University Hospital, Heidelberg, Germany; [30]The Christie NHS Foundation Trust and University of Manchester, Manchester, United Kingdom; [31]Johnson & Johnson Innovative Medicine, Spring House, Pennsylvania, United States of America; [32]Dana-Farber Cancer Institute, Boston, United States of America;[33]Royal Perth Hospital, Perth, Australia; [34]Johnson & Johnson Innovative Medicine, Raritan, United States of America; [35]Westmead Hospital and University of Sydney, Sydney, Australia; [36]Weatherall Institute of Molecular Medicine, Radcliffe Department of Medicine, Oxford University and Department of Haematology, Oxford University Hospitals NHS Trust, Oxford, United Kingdom; [37]Peter MacCallum Cancer Centre, Royal Melbourne Hospital, Walter and Eliza Hall Institute of Medical Research and University of Melbourne, Melbourne, Australia;*

**Copyright Information:** (Online) ISSN: 2572-9241
©2026 The Author(s). HemaSphere is published by John Wiley & Sons Ltd on behalf of European Hematology Association. This is an open access Abstract Book distributed under the Attribution-NonCommercial-NoDerivs (CC BY-NC-ND), which allows third parties to download the articles and share them with others as long as they credit the author and the Abstract Book, but they cannot change the content in any way or use them commercially.
**Abstract Book Citations:** Authors, Title, HemaSphere, 2026;10:(S1):pages. The abstract book DOIs can be found on the "Author Information" page.
**Disclaimer:** Articles published in the journal HemaSphere exclusively reflect the opinions of the authors. The authors are responsible for all content in their abstracts including accuracy of the facts, statements, citing resources, etc.

### Background

Corrected QT interval (QTc) prolongation is a known risk for fatal cardiac arrythmias and emerged as a safety signal with currently approved menin inhibitors (all-grade: 12%–36%; high-grade: 8–17%). QTc prolongation may limit dose intensity of available menin inhibitor therapy and complicate management of acute myeloid leukemia (AML) by preventing combination with anti-leukemic therapies and use of critical supportive care medicines. Bleximenib is being evaluated in late-stage clinical studies in relapsed/refractory or newly diagnosed *KMT2A*-rearranged or *NPM1*-mutated AML. We report cardiac safety analyses from two Phase 1 trials evaluating bleximenib as monotherapy (ALE1001 [NCT04811560]) or in combination with AML-directed therapies (ALE1002 [NCT05453903]).

### Methods

As of Oct 2025, the safety population included 141 participants (pts) in ALE1001 and 193 pts in ALE1002. Pts were excluded from enrolment based on QTc to Fridericia's formula (QTcF) ≥450ms for males or ≥470ms for females, or with family history of long QT syndrome. Pts received oral bleximenib continuously on 28-day cycles at dose levels from 50–100mg daily (QD), or 15–150mg twice daily (BID), with various step-up doses, as a monotherapy, or from 15–150mg BID in combination with venetoclax (VEN)/azacitidine (AZA), and 30–100 mg BID in combination with intensive chemotherapy ('7+3'). Cardiac safety monitoring included collection of 12-lead triplicate ECGs at baseline and during treatment on Day 1 of each cycle (or as clinically indicated) with central cardiology review. Adverse events (AEs) were evaluated according to CTCAE v5.0. Coupled with intensive pharmacokinetic sampling and mean ΔQTcP, an exposure-response analysis using ΔQTcF as the dependent variable, and time-matched concentration of bleximenib as the explanatory variable, was performed in 139 pts enrolled in ALE1001 with central ECG data available.

### Results

Of 334 pts receiving bleximenib across both studies, only one (0.3%) high grade (Grade [Gr] 3) event of QTc prolongation was observed in a pt receiving bleximenib 100mg QD as monotherapy with baseline comorbidities of ongoing cardiomyopathy and hypokalemia. At the recommended Phase 2 dose (RP2D) in ALE1001, a single (1.6%) Gr 1 QTcF prolongation treatment emergent AE (TEAE) occurred that resolved without dose reduction or interruption. In ALE1002, minimal QTcF prolongation TEAEs were reported in pts receiving bleximenib 15–150mg BID in combination with VEN, VEN+AZA, or '7+3'; 8 (4.1%) experienced a QTcF prolongation event. All were low-grade (7 Gr1, 1 Gr2), non-serious, and did not result in bleximenib dose modifications. No dose-dependent trend in QTcF interval increases were apparent across analyses in either study.
In ALE1001 central ECG data (n=139), time course of mean change in heart rate (HR) was comparable across different doses. No increase of >10 bpm was observed, with no QTc prolongation seen up to the highest dose explored (2-fold higher exposure vs RP2D). Observations from ALE1002 were similar, with no dose-dependent effects on QTc prolongation. ALE1001 exposure-response analysis based on time-matched central ECG data indicated no clinically relevant effect of bleximenib on cardiac repolarization following bleximenib monotherapy at all doses explored. Bleximenib had no clinically significant impact on HR.

### Summary/Conclusion

Administration of bleximenib as monotherapy or in combination with AML-directed therapies did not prolong QTc interval and no cardiac safety signal was identified.

**Copyright Information:** (Online) ISSN: 2572-9241
©2026 The Author(s). HemaSphere is published by John Wiley & Sons Ltd on behalf of European Hematology Association. This is an open access Abstract Book distributed under the Attribution-NonCommercial-NoDerivs (CC BY-NC-ND), which allows third parties to download the articles and share them with others as long as they credit the author and the Abstract Book, but they cannot change the content in any way or use them commercially.
**Abstract Book Citations:** Authors, Title, HemaSphere, 2026;10:(S1):pages. The abstract book DOIs can be found on the "Author Information" page.
**Disclaimer:** Articles published in the journal HemaSphere exclusively reflect the opinions of the authors. The authors are responsible for all content in their abstracts including accuracy of the facts, statements, citing resources, etc.

### (PF556) VENETOCLAX-AZACITIDINE-CHIDAMIDE PLUS CAG AS INDUCTION CHEMOTHERAPY FOR MYELODYSPLASIA-RELATED ACUTE MYELOID LEUKEMIA

**Topic:** 04. Acute myeloid leukemia - Clinical

Lei Lv[1], Jingjing Yang[1], Qingyang Liu*[1], Sai Huang[1], Liping Dou[1]

[1] *The Fifth Medical Center of Chinese PLA General Hospital, Beijing, China;*

### Background

Myelodysplasia-related acute myeloid leukemia (AML-MR) exhibits poor outcomes with intensive chemotherapy. This retrospective study was conducted to evaluate the clinical efficacy and safety of the venetoclax-azacitidine-chidamide plus CAG (CACAG-VEN) regimen in AML-MR patients.

### Aims

This retrospective study was conducted to evaluate the clinical efficacy and safety of the venetoclax-azacitidine-chidamide plus CAG (CACAG-VEN) regimen in AML-MR patients.

### Methods

We included 14–75-year-old AML-MR patients (n = 29) treated with CACAG-VEN (aclarubicin, azacitidine, cytarabine, chidamide, and venetoclax plus G-CSF) between December 1, 2022 and October 31, 2024 and compared this cohort with patients (n = 39) who received the standard “3+7” regimen (daunorubicin plus cytarabine) from January 1, 2011 to January 31, 2020. The primary endpoint was the overall response rate (ORR) after one cycle of induction.

### Results

In this retrospective analysis of 29 patients treated with CACAG-VEN, the median age was 60 (range, 27–73) years. The CACAG-VEN achieved a high ORR (96.6%, 95% CI, 82.8%–99.8%) and composite complete response (CRc, 93.1% [95% CI, 78.0%–98.8%]). The 1-year overall survival and relapse incidences were 84.0% (95% CI: 62.6–93.7) and 25.6% (95% CI: 9.8–44.9), respectively. No significant differences in OS (1-year: 74.7% [non-HSCT] vs. 92.3% [HSCT]) or CIR (1-year: 44.6% [non-HSCT] vs. 8.3% [HSCT]) were observed, regardless of whether patients underwent HSCT. The most frequent grade 1–4 adverse events were febrile neutropenia (72.4%), sepsis (24.1%), and pneumonia (48.3%). The median time to recovery was 15 days for platelet counts ≥20,000/μL and 19 days for absolute neutrophil counts ≥1,000 cells/μL after induction therapy. The outcomes in the “3+7” group were inferior to those of patients who received the CACAG-VEN regimen (ORR: 71.8%, $p = 0.009$; CRc: 51.9%, $p < 0.001$; 1-year OS: 70.3%, $p = 0.049$).

### Summary/Conclusion

The CACAG-VEN regimen is a potential frontline therapy for AML-MR, yielding superior response rates and longer survival durations than the standard “3+7” chemotherapy

**Copyright Information:** (Online) ISSN: 2572-9241
©2026 The Author(s). HemaSphere is published by John Wiley & Sons Ltd on behalf of European Hematology Association. This is an open access Abstract Book distributed under the Attribution-NonCommercial-NoDerivs (CC BY-NC-ND), which allows third parties to download the articles and share them with others as long as they credit the author and the Abstract Book, but they cannot change the content in any way or use them commercially.
**Abstract Book Citations:** Authors, Title, HemaSphere, 2026;10:(S1):pages. The abstract book DOIs can be found on the “Author Information” page.
**Disclaimer:** Articles published in the journal HemaSphere exclusively reflect the opinions of the authors. The authors are responsible for all content in their abstracts including accuracy of the facts, statements, citing resources, etc.

### (PF557) SAFETY RUN-IN RESULTS OF THE CHIP-AML22 PHASE II TRIAL EVALUATING QUIZARTINIB COMBINED WITH CHEMOTHERAPY IN PEDIATRIC PATIENTS WITH NEWLY DIAGNOSED FLT3-ITD–POSITIVE, NPM1–WILD-TYPE AML

**Topic:** 04. Acute myeloid leukemia - Clinical

Daria Valerio[1], Sae Ishimaru[1], Jonas Abrahamsson[2], Nira Arad-Cohen[3], Renske Benedictus[1], Noha Biserna[4], Vitor Costa[5], Jose Fernandez Navarro[6], Kristian Juul-Dam[7], Maarja Karu[8], Sunitha Kodidela[4], Zanna Kovalova[9], Maaike Luesink[1], Barbara De Moerloose[10], Monica Munthe-Kaas[11], Stefan Nierkens[1], Ellen van Opstal-Wijbrans[1], Sauli Palmu[12], Cornelis Jan Pronk[13], Nastassja Scheidegger[14], Indre Tamuliene[15], Harm van Tinteren[1], Dominik Turkiewicz[13], Gauti Vilbergsson[16], C. Michel Zwaan[1], Gertjan Kaspers*[1]

*[1]Princess Máxima Center for Pediatric Oncology, Utrecht, The Netherlands;[2]Children's Cancer Center, Sahlgrenska University Hospital, Goteborg, Sweden; [3]Rambam Health Care Campus, Department of Pediatric Hemato-Oncology, Haifa, Israel;[4]Daiichi Sankyo, Inc., Basking Ridge, NJ, United States of America; [5]Instituto Português de Oncologia, Porto, Portugal;[6]Hospital Universitario y Politécnico La Fe, Department of Pediatric Hemato-Oncology, Valencia, Spain; [7]Aarhus University Hospital, Department of Pediatrics and Adolescent Medicine, Aarhus, Denmark; [8]New Children's Hospital, Pediatric Research Center, University of Helsinki and Helsinki University Hospital, Helsinki, Finland; [9]Children's Clinical University Hospital, Riga, Latvia; [10]Ghent University Hospital, Department of Pediatric Hematology-Oncology, Gent, Belgium; [11]Oslo University Hospital, Department of Pediatric Oncology and Hematology, Oslo, Norway; [12]Tampere University Hospital, Tampere, Finland; [13]Skåne University Hospital, Department of Pediatric Oncology and Hematology, Lund, Sweden; [14]University Children's Hospital Zurich, Department of Oncology, Zurich, Switzerland; [15]Center for Pediatric Oncology and Hematology, Vilnius University Hospital Santaros Klinikos, Vilnius, Lithuania; [16]Children's Hospital, Landspitali University Hospital, Reykjavik, Iceland;*

### Background

FLT3-ITD–positive, NPM1–wild-type acute myeloid leukemia (AML) represents a high-risk pediatric AML subgroup with historical event-free survival of 30-50%. FLT3 inhibitors showed efficacy in adults with FLT3-ITD–positive AML and the FLT3 inhibitor quizartinib is approved with standard chemotherapy and as maintenance monotherapy in adults, including in the USA and EU. However, no FLT3 inhibitors are approved for pediatric AML.

### Aims

This multinational, open-label, single-arm phase II study aims to assess safety, efficacy, and pharmacokinetics of quizartinib in combination with standard-of-care chemotherapy in newly diagnosed pediatric patients with FLT3-ITD–positive, NPM1–wild-type AML. It includes a safety run-in and expansion phase. This study is sponsored by the Princess Máxima Center and is conducted within a Pediatric Investigational Plan.

### Methods

The CHIP-AML22/Quizartinib trial is a sub-trial of the CHIP-AML22 protocol (EU CT: 023-504999-25-00), a frontline treatment protocol for patients aged ≥1 month to ≤18 years with newly diagnosed AML within the NOPHO-DB-SHIP consortium. The safety run-in aimed to confirm the recommended phase II dose (RP2D), based on safety and tolerability during 2 induction courses in the first 6-12 dose-limiting toxicity (DLT)-evaluable patients, using a Rolling-6 design. Patients were considered DLT-evaluable if they received ≥75% of the prescribed quizartinib dose or experienced a DLT after ≥1 dose. Both courses included cytarabine, etoposide and intrathecal methotrexate, plus mitoxantrone (induction 1) or daunorubicin (induction 2). The quizartinib starting dose level (DL1) was bodyweight (BW)-adjusted,  to achieve exposure comparable to the adult RP2D established in the phase III QuANTUM-First trial. De-escalation to DL-1 was planned if ≥2 DLTs occurred. At DL1, patients with BW ≥40 kg received quizartinib 40 mg once daily (while those <40kg received 0.7mg/kg to 30mg based on BW) for 14 days following each chemotherapy course.

Copyright Information: (Online) ISSN: 2572-9241
©2026 The Author(s). HemaSphere is published by John Wiley & Sons Ltd on behalf of European Hematology Association. This is an open access Abstract Book distributed under the Attribution-NonCommercial-NoDerivs (CC BY-NC-ND), which allows third parties to download the articles and share them with others as long as they credit the author and the Abstract Book, but they cannot change the content in any way or use them commercially.
Abstract Book Citations: Authors, Title, HemaSphere, 2026;10:(S1):pages. The abstract book DOIs can be found on the "Author Information" page.
Disclaimer: Articles published in the journal HemaSphere exclusively reflect the opinions of the authors. The authors are responsible for all content in their abstracts including accuracy of the facts, statements, citing resources, etc.

**Results**

As of August 2025, 6 patients were enrolled and had completed 2 induction courses; 3 had also completed consolidation followed by allogeneic hematopoietic stem cell transplant. One DLT occurred (delayed platelet regeneration, possibly related to quizartinib) at DL1, confirming DL1 as RP2D. Fourteen serious adverse events (SAEs) occurred in 5 patients during induction; 6 events in 3 patients (febrile neutropenia [2], lung infection [2], sepsis [1], nausea [1]) were considered at least possibly related to protocol treatment (chemotherapy and quizartinib). All SAEs resolved. Quizartinib was not interrupted or dose-reduced due to toxicity. No adverse events of special interest (grade $\geq$2 QTcF prolongation or Hy's law) were observed and no deaths occurred. Four patients (67%) achieved early Minimal Residual Disease negativity (<0.1% by flow cytometry before start of induction 2), whereas 2 (33%) were refractory and discontinued treatment.

**Summary/Conclusion**

In this safety run-in, quizartinib combined with intensive chemotherapy showed a manageable safety profile in pediatric patients with newly diagnosed FLT3-ITD–positive, NPM1–wild-type AML. DL1 was confirmed as RP2D for the expansion phase. Study enrollment is ongoing. EU CT: 2023-505000-27-01.

**Copyright Information:** (Online) ISSN: 2572-9241
©2026 The Author(s). HemaSphere is published by John Wiley & Sons Ltd on behalf of European Hematology Association. This is an open access Abstract Book distributed under the Attribution-NonCommercial-NoDerivs (CC BY-NC-ND), which allows third parties to download the articles and share them with others as long as they credit the author and the Abstract Book, but they cannot change the content in any way or use them commercially.
**Abstract Book Citations:** Authors, Title, HemaSphere, 2026;10:(S1):pages. The abstract book DOIs can be found on the “Author Information” page.
**Disclaimer:** Articles published in the journal HemaSphere exclusively reflect the opinions of the authors. The authors are responsible for all content in their abstracts including accuracy of the facts, statements, citing resources, etc.

### (PF559) SHORTER TREATMENT, FASTER DISCHARGE. THE IMPACT OF DAY 14 ASSESSMENT IN AML PATIENTS ON AZA-VEN: A SINGLE CENTER EXPERIENCE.

**Topic:** 04. Acute myeloid leukemia - Clinical

Felix Klingler*[1], Elisa Seubert[1], Clara Philine Wienecke[1], Saskia Pfaff[1], Annika Holzapfel[1], Florian Kruse[1], Carsten Bokemeyer[1], Thomas Stübig[1], Walter Fiedler[1], Franziska Westendorf[1]

*[1]University Medical Center Hamburg-Eppendorf, Department of Medicine II: Hematology, Oncology, Bone Marrow Transplantation with Section Pneumology, Hamburg, Germany;*

### Background

The standard of care for older patients with newly diagnosed AML (Acute myeloid leukemia) lacking targeted therapy options is Azacitidine (AZA) 75 mg/m² for 7 days combined with Venetoclax (VEN) 400 mg for 28 days. However, as this regimen frequently leads to sometimes severe complications, the feasibility of shortening AZA-VEN therapy requires investigation.

### Aims

Investigation and characterization of patients achieving rapid blast clearance during the first cycle of AZA-VEN.

### Methods

All newly diagnosed, unfit AML patients (pts) treated with first-cycle AZA-VEN at the University Medical Center Hamburg-Eppendorf since 2022, who underwent bone marrow evaluation between days 12 and 17, were included. Data for this retrospective analysis was extracted from electronic medical records.

### Results

The analysis included 36 patients, 28 of whom were male. Median age at diagnosis was 82 years, and 70% of patients had a favorable risk profile per ELN 2024 criteria. At the first response assessment (R1), performed at a median of day 13, 20 patients (56%) achieved blast clearance (BC), and four patients (11%) achieved a composite complete remission (CRc). Patients with BC received venetoclax for a median of 17 days, compared to a median of 27 days for those without blast clearance (nBC). Additionally, patients with a short Venetoclax duration (defined as ≤ 17 days) had significantly shorter hospital stays (26 vs. 35 days, p=0.015; see Figure 1). The rate of bloodstream infections was lower in the BC group than in the nBC group (5% vs. 25%), with no difference in the duration of severe leukopenia or thrombocytopenia, or the incidence of fever.
Median overall survival (OS) for the entire cohort was 422 days, consistent with previously reported data. When analyzing factors influencing overall survival, age at initial diagnosis emerged as the strongest parameter. Patients > 82 years had a median overall survival of 168 days, compared to 523 days in the younger cohort (≤ 82 years; p=0.03). Conversely, stratifying by Venetoclax duration revealed no significant difference (median 331 days for short vs. 422 days for long duration; p=0.88).
Among patients achieving early blast clearance, there was a higher frequency of prior MDS/MPN or therapy-related AML, whereas patients with myelodysplasia-related karyotypic abnormalities more frequently lacked early BC. IDH2 or NPM1 mutations were more common in BC patients (25% (n=5) vs. 6.2% for IDH2; 20% (n=4) vs. 12.5% for NPM1), while DNMT3A, DDX41, or SF3B1 mutations occurred more frequently in nBC patients (25% (n=4) vs. 15% for DNMT3A; 12.5% (n=2) vs. 0% for DDX41 and SF3B1).

**Copyright Information:** (Online) ISSN: 2572-9241
©2026 The Author(s). HemaSphere is published by John Wiley & Sons Ltd on behalf of European Hematology Association. This is an open access Abstract Book distributed under the Attribution-NonCommercial-NoDerivs (CC BY-NC-ND), which allows third parties to download the articles and share them with others as long as they credit the author and the Abstract Book, but they cannot change the content in any way or use them commercially.
**Abstract Book Citations:** Authors, Title, HemaSphere, 2026;10:(S1):pages. The abstract book DOIs can be found on the “Author Information” page.
**Disclaimer:** Articles published in the journal HemaSphere exclusively reflect the opinions of the authors. The authors are responsible for all content in their abstracts including accuracy of the facts, statements, citing resources, etc.

### Summary/Conclusion

The primary goal of AZA-VEN therapy is to prolong survival while maintaining the highest possible quality of life. The option to discontinue Venetoclax early is beneficial for patient comfort, especially when patients can be discharged from hospital faster. This initial insight into our data demonstrates that many patients achieve blast clearance after approximately two weeks of therapy, experience fewer bloodstream infections, and that shortening VEN has no impact on survival. Further data collection is required to better identify which patients are suitable candidates for shortened VEN therapy.

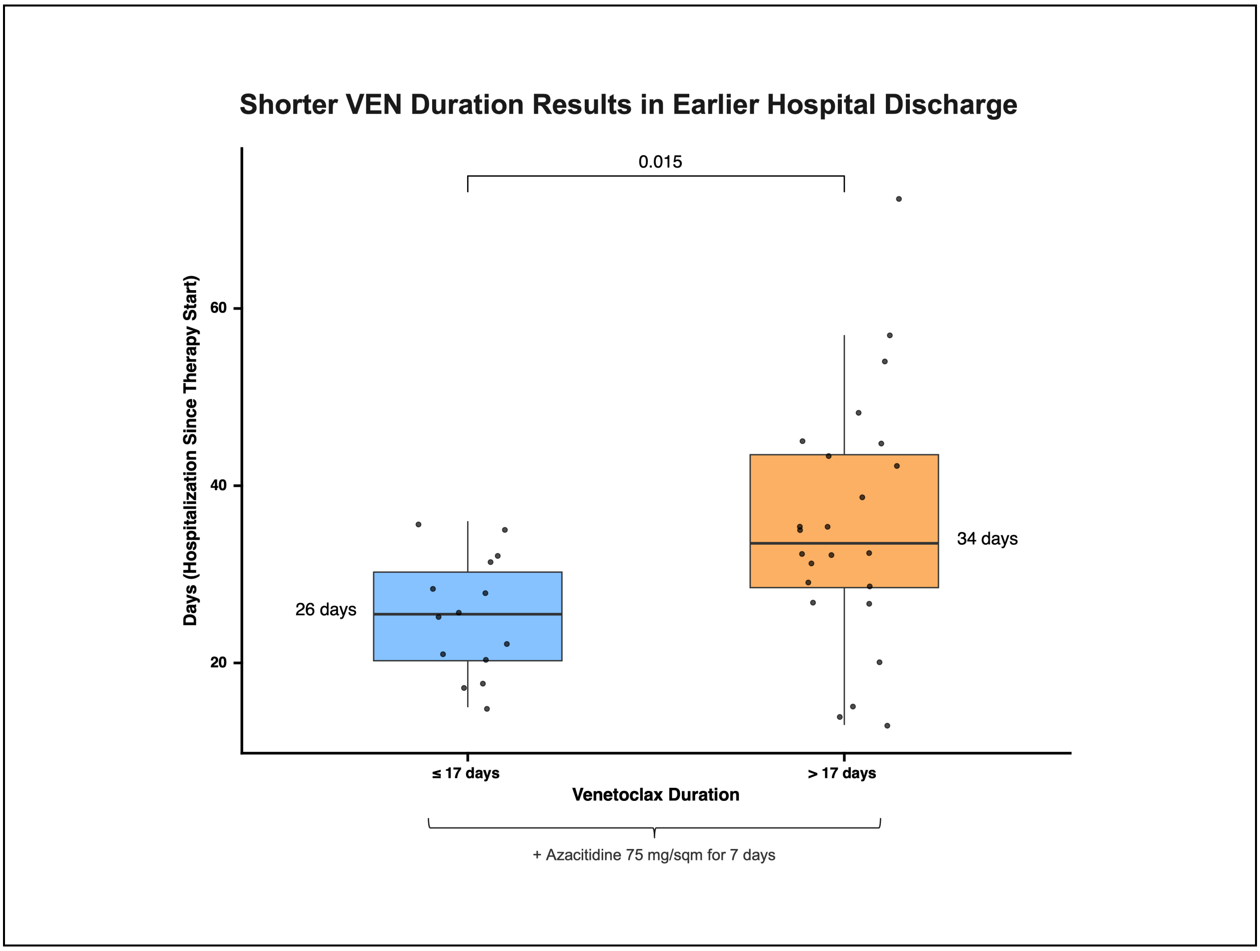

Correlation between duration of hospitalization and duration of Venetoclax intake. Patients with a longer Venetoclax intake were admitted to the hospital for 8 days more.

**Copyright Information:** (Online) ISSN: 2572-9241
©2026 The Author(s). HemaSphere is published by John Wiley & Sons Ltd on behalf of European Hematology Association. This is an open access Abstract Book distributed under the Attribution-NonCommercial-NoDerivs (CC BY-NC-ND), which allows third parties to download the articles and share them with others as long as they credit the author and the Abstract Book, but they cannot change the content in any way or use them commercially.
**Abstract Book Citations:** Authors, Title, HemaSphere, 2026;10:(S1):pages. The abstract book DOIs can be found on the "Author Information" page.
**Disclaimer:** Articles published in the journal HemaSphere exclusively reflect the opinions of the authors. The authors are responsible for all content in their abstracts including accuracy of the facts, statements, citing resources, etc.

### (PF560) NEWER AML RISK SCORES IMPROVE RISK STRATIFICATION IN CPX-351 TREATED S-AML PATIENTS COMPARED TO ELN2022, BUT A TAILORED PROGNOSTIC MODEL IS STILL NEEDED

**Topic:** 04. Acute myeloid leukemia - Clinical

Carola Riva*[1], Alessandro Cacioppo[2], Stefano Nicoletta[2], Paola Minetto[1], Sofia Schiavetti[2], Martina Occelli[2], Enrico Carminati[1], Clara Nurra[2], Michele Cea[1, 2], Roberto M. Lemoli[1, 2], Fabio Guolo[1, 2]

*[1]IRCCS Azienda Ospedaliera Metropolitana, Genoa, Italy; [2]University of Genoa, Genoa, Italy;*

### Background

The increasing use of new drugs highlights the need for treatment-specific prognostic models in acute myeloid leukemia (AML). For unfit patients receiving hypomethylating agents plus venetoclax (HMA-VEN) that are not adequately stratified by traditional risk classifications developed for 7+3 chemotherapy, new scores have recently been proposed.
CPX-351 has replaced standard 7+3 chemotherapy as the preferred regimen for fit patients with secondary AML (sAML), a high-risk entity characterized by high-risk mutations. In this context, the prognostic discrimination provided by ELN 2022 classification is limited, as the majority of sAML patients are assigned to the adverse-risk group, and no dedicated score exists for CPX-351–treated patients.

### Aims

To evaluate if the newer HMA-VEN risk scores improve prognostic stratification compared to ELN 2022 in sAML patients treated with CPX-351.

### Methods

96 s-AML patients (median age 69, range 38-77) received CPX-351.
NGS was performed on bone marrow with the Myeloid Solution panel by SOPHiA Genetics, targeting 34 critical genes, using Illumina MiSeq platform and SOPHiA DDM® Software.
Patients were stratified using the ELN 2022 and 2024 classification, the Mayo Clinic genetic risk model (Gangat N. Am J Hematol 2025), and the Beat-AML 2024 prognostic model (Hoff FW. Blood Adv 2024).

**Copyright Information:** (Online) ISSN: 2572-9241
©2026 The Author(s). HemaSphere is published by John Wiley & Sons Ltd on behalf of European Hematology Association. This is an open access Abstract Book distributed under the Attribution-NonCommercial-NoDerivs (CC BY-NC-ND), which allows third parties to download the articles and share them with others as long as they credit the author and the Abstract Book, but they cannot change the content in any way or use them commercially.
**Abstract Book Citations:** Authors, Title, HemaSphere, 2026;10:(S1):pages. The abstract book DOIs can be found on the "Author Information" page.
**Disclaimer:** Articles published in the journal HemaSphere exclusively reflect the opinions of the authors. The authors are responsible for all content in their abstracts including accuracy of the facts, statements, citing resources, etc.

**Results**

As expected, with ELN 2022 the majority of patients fell in the adverse risk group (79/96, 82.3%), whereas 10 (10.4%) and 7 (7.3%) were assigned to the favorable and intermediate risk group, respectively.
With ELN 2024 criteria, 54 (56.3%), 17 (17.7%) and 25 (26%) were considered as low, intermediate and high risk, respectively.
Based on the Mayo Clinic model, 9 (9.4%), 31 (32.3%) and 56 (58.3%) patients were classified as low, intermediate and high risk, respectively.
According to the Beat-AML classification, 17 (17.7%), 40 (41.7%) and 39 (40.6%) were stratified as low, intermediate and high risk, respectively.
After cycle 1, 77/96 patients (80.2%) achieved CR.
ELN 2022, Mayo, and Beat-AML did not correlate with CR, whereas favorable ELN 2024 score predicted higher CR probability (91.8% vs 62.5% and 75.0%; p=0.019).
Median follow-up was 43 months and the median overall survival (OS) was 14 months.
ELN 2022 and Mayo Clinic models did not stratify OS, whereas ELN 2024 and Beat-AML significantly discriminated outcomes (median OS of 20 months vs 10 and 8 months with ELN 2024 and 43 months vs 12 and 10 months with Beat-AML model, for low, intermediate and high risk group, respectively, p<0.05, Fig 1).
Allogeneic hematopoietic stem cell transplantation (HSCT) was performed in 29/77 CR patients (37.7%) and was the strongest predictor of OS in a landmark model (p<0.05). Among HSCT patients, no model stratified OS, whereas among non-HSCT patients, both ELN2024 and Beat-AML retained prognostic value (median OS of 28 months versus 13 and 8 months in the ELN 2024 and 38 months versus 17 and 9 months in the Beat AML model, for low, intermediate and high risk group, respectively, p<0.05, Fig. 1).

**Summary/Conclusion**

ELN 2022 poorly stratifies sAML patients treated with CPX-351, assigning most to adverse risk. ELN 2024 and Beat-AML identify a favorable-risk subset and effectively stratify non-transplanted patients, but perform poorly for intermediate/high-risk and transplanted patients.
While these newer models outperform ELN 2022 in this challenging population, the findings underscore the need for a prognostic score specifically tailored for sAML patients treated with CPX-351.

**Copyright Information:** (Online) ISSN: 2572-9241
©2026 The Author(s). HemaSphere is published by John Wiley & Sons Ltd on behalf of European Hematology Association. This is an open access Abstract Book distributed under the Attribution-NonCommercial-NoDerivs (CC BY-NC-ND), which allows third parties to download the articles and share them with others as long as they credit the author and the Abstract Book, but they cannot change the content in any way or use them commercially.
**Abstract Book Citations:** Authors, Title, HemaSphere, 2026;10:(S1):pages. The abstract book DOIs can be found on the “Author Information” page.
**Disclaimer:** Articles published in the journal HemaSphere exclusively reflect the opinions of the authors. The authors are responsible for all content in their abstracts including accuracy of the facts, statements, citing resources, etc.

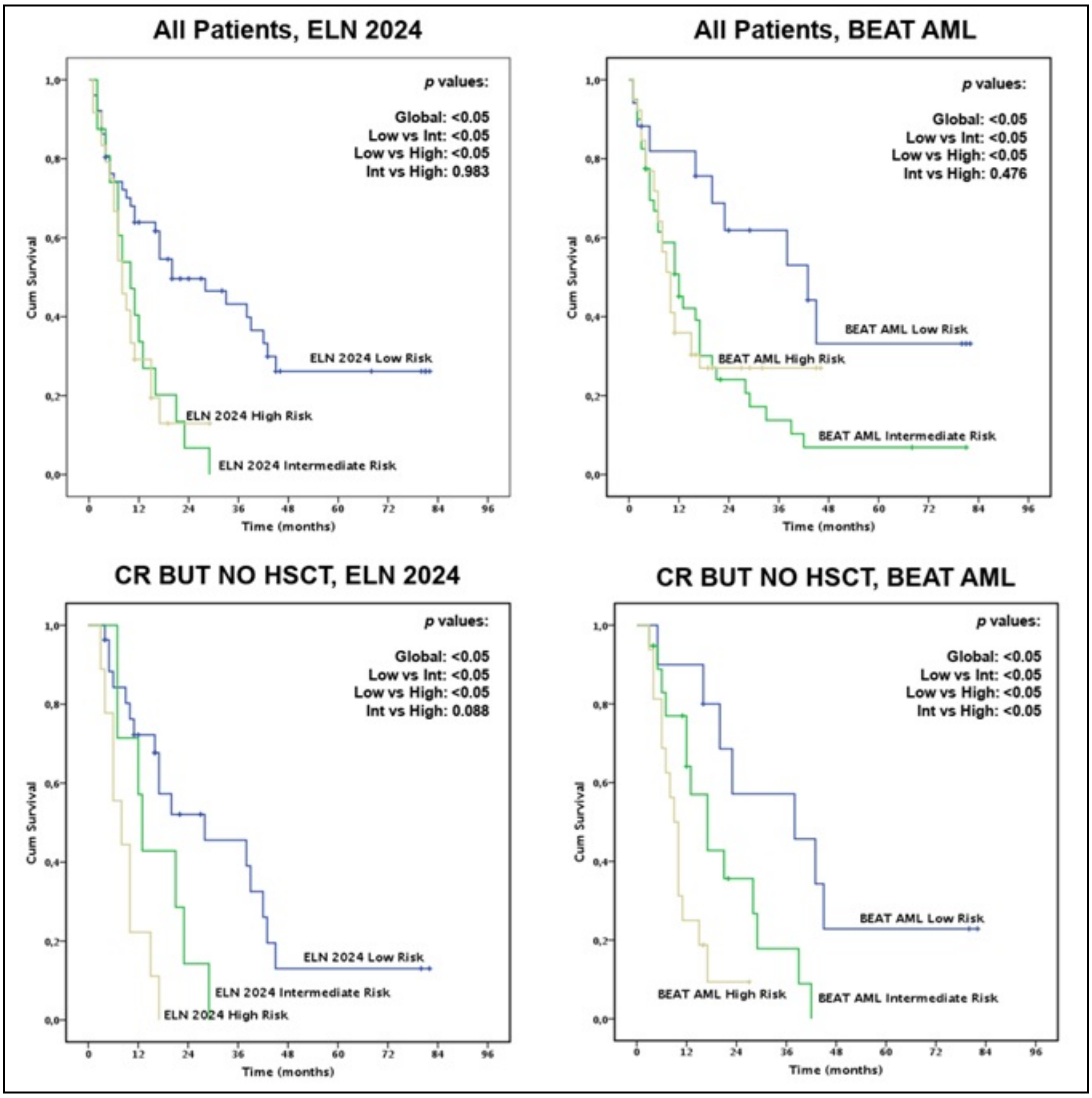

Figure 1: Overall Survival According to ELN 2024 and BEAT AML risk score in all patients (panel A and B) or in CR patients not receiving HSCT (panel C and D)

**Copyright Information:** (Online) ISSN: 2572-9241
©2026 The Author(s). HemaSphere is published by John Wiley & Sons Ltd on behalf of European Hematology Association. This is an open access Abstract Book distributed under the Attribution-NonCommercial-NoDerivs (CC BY-NC-ND), which allows third parties to download the articles and share them with others as long as they credit the author and the Abstract Book, but they cannot change the content in any way or use them commercially.
**Abstract Book Citations:** Authors, Title, HemaSphere, 2026;10:(S1):pages. The abstract book DOIs can be found on the "Author Information" page.
**Disclaimer:** Articles published in the journal HemaSphere exclusively reflect the opinions of the authors. The authors are responsible for all content in their abstracts including accuracy of the facts, statements, citing resources, etc.

### (PF561) EFFICACY OF VENETOCLAX-BASED REGIMENS AS SALVAGE THERAPY, FOLLOWED BY ALLOGENEIC TRANSPLANTATION, IN RELAPSED/REFRACTORY AML PATIENTS.

**Topic:** 04. Acute myeloid leukemia - Clinical

Matteo Fanin*[1], Davide Lazzarotto[1], Carla Fili[1], Erica Simeone[1], Maria Elena Zannier[1], Giulia Battaglia[1], Gian Luca Morelli[1], Luca Tosoni[1], Chiara Callegari[1], Melissa Bergnach[1], Francesca Patriarca[1], Daniela Damiani[1], Renato Fanin[1], Mario Tiribelli[1]

*[1]Division of Hematology and BMT, Azienda Sanitaria Universitaria Friuli Centrale and Department of Medicine, University of Udine, Udine, Italy;*

### Background

Despite recent advances, the only curative approach for most acute myeloid leukemia (AML) patients remains allogeneic hematopoietic cell transplant (HCT). Results of HCT are superior when performed in complete remission (CR), and prognosis of patients with refractory or relapsed (R/R) AML remains dismal. In recent years venetoclax (VEN)-based combinations emerged as a standard of care in older/unfit patients, but VEN favorable efficacy and safety profile prompted its use also in R/R patients and the inclusion of VEN in induction chemotherapy for younger patients deemed candidate to transplant. However, to date little is known on the role of VEN-based therapy as a salvage therapy, followed by allogeneic HCT, in the real-life setting.

### Aims

The aim of this study is to retrospectively analyze the efficacy of VEN-based therapy, in association with HMAs, as a bridge to transplant when used as a salvage regimen for R/R AML and it's role in reducing the disease burden and obtaining cytological and molecular CRs in patients candidate to recieve allogeneic HCT.

### Methods

Data was gathered from 25 AML patients treated at our center, from 2020 to 2026, who received VEN in association with hypomethylating agents Azacitidine (AZA) or Decitabine (DAC), as a salvage therapy, after failure of intensive chemotherapy regimens, as a bridge to allogeneic hematopoietic cell transplant with the aim of obtaining a first or second remission and lowering the disease burden. Among patients with R/R AML, 20 (80%) received VEN as 2nd line therapy while 5 (20%) as 3rd or subsequent lines. Median age at AML diagnosis was 58 years (range: 25-73), ELN 2022 risk, where determinable, was favorable in 6 patients (24%), intermediate in 6 (24%) and advanced in 10 (40%). VEN was administered for a median of 2 cycles (range: 1-6) with AZA or DAC in 18 (72%) and 7 (28%) patients, respectively. Prior to VEN, patients received multiple intensive regimens including "3+7" alone or with midostaurin/GO (44%), CPX-351 (36%), FLAI (32%), MEC (8%) or other (4%). At the VEN-HMA therapy timepoint, 18/25 patients (72%) had active AML, 3 (12%) were in partial response (PR) and 4 (16%) in molecular relapse. Regarding allogeneic HCT, among the 25 patients, median HCT-CI score was 2 (range: 0-4), and donors were HLA-identical siblings in 3 cases (12%), matched unrelated in 11 (44%), mismatched unrelated in 1 (4%) and haploidentical in 10 (40%).

### Results

After VEN, 6 patients (24%) attained molecular CR, 12 (48%) cytological CR, 4 (16%) PR while 3 patients (12%) still had active disease. After HCT 23 patients (92%) are alive, with 16 in molecular/cytological CR and 4 with relapsed AML while 4 (16%) died for TRM (2 while in CR). According to Kaplan-Meier estimates, overall survival (OS) at 12 months is 79.2%, lowering to 72% at the 36-month timepoint while progression free survival (PFS) at 12 months was 74.3%, decreasing to 49% at the 36-month timepoint.

**Copyright Information:** (Online) ISSN: 2572-9241
©2026 The Author(s). HemaSphere is published by John Wiley & Sons Ltd on behalf of European Hematology Association. This is an open access Abstract Book distributed under the Attribution-NonCommercial-NoDerivs (CC BY-NC-ND), which allows third parties to download the articles and share them with others as long as they credit the author and the Abstract Book, but they cannot change the content in any way or use them commercially.
**Abstract Book Citations:** Authors, Title, HemaSphere, 2026;10:(S1):pages. The abstract book DOIs can be found on the "Author Information" page.
**Disclaimer:** Articles published in the journal HemaSphere exclusively reflect the opinions of the authors. The authors are responsible for all content in their abstracts including accuracy of the facts, statements, citing resources, etc.

**Summary/Conclusion**

With the limit of a small sample size, our data suggests that VEN-based regimens are an effective strategy to induce response before transplant. VEN bridging therapy in R/R AML, even in heavily pre-treated patients, grants high rates of CR, also molecular, allowing to perform allogeneic HCT with a low leukemia burden. Cytological and molecular CRs thus obtained tend to be maintained and improved after transplant.

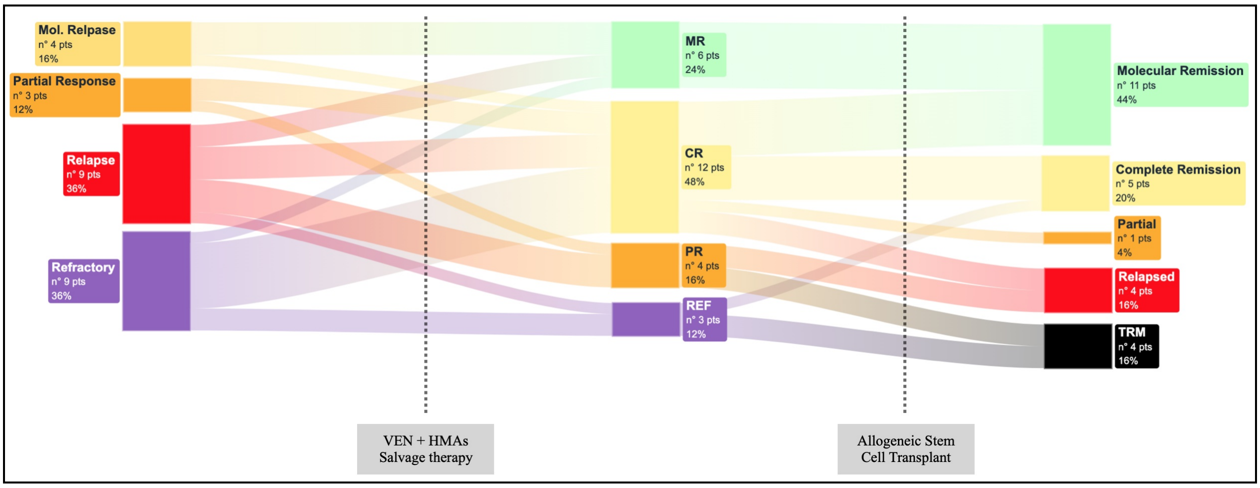

Figure 1. Response obtained with salvage bridge therapy and HSCT respectively. MR Molecular response, CR Complete response, PR Partial response, REF Refractory.

**Copyright Information:** (Online) ISSN: 2572-9241
©2026 The Author(s). HemaSphere is published by John Wiley & Sons Ltd on behalf of European Hematology Association. This is an open access Abstract Book distributed under the Attribution-NonCommercial-NoDerivs (CC BY-NC-ND), which allows third parties to download the articles and share them with others as long as they credit the author and the Abstract Book, but they cannot change the content in any way or use them commercially.
**Abstract Book Citations:** Authors, Title, HemaSphere, 2026;10:(S1):pages. The abstract book DOIs can be found on the “Author Information” page.
**Disclaimer:** Articles published in the journal HemaSphere exclusively reflect the opinions of the authors. The authors are responsible for all content in their abstracts including accuracy of the facts, statements, citing resources, etc.

### (PF562) REAL-WORLD EFFICACY AND SAFETY OF LISAFTOCLAX IN MYELOID NEOPLASMS: A MULTICENTER STUDY

**Topic:** 04. Acute myeloid leukemia - Clinical

Chen Cao*[1], Ruijuan Wang[2], Zhaoxia Liu[3], Changrong Wei[4], Qing Meng[1], Xinwen Jiang[5], Qingli Ji[1], Dongmei Hu[1], Minran Zhou[1], Ran Wang[1], Sai MA[1], Chunyan Chen[1]

*[1]Qilu Hospital of Shandong University, Jinan, China;[2]Nanyang Central Hospital, Nanyang, China;[3]Affiliated Hospital of Shandong University of Traditional Chinese Medicine, Jinan, China; [4]Linyi People's Hospital, Linyi, China;[5]Qilu Hospital, Shandong University, Jinan, China;*

### Background

Lisaftoclax, a novel BCL-2 inhibitor, has shown promising anti-tumor activity in preclinical studies and early-phase clinical trials for hematologic malignancies. However, real-world data regarding its efficacy and safety in patients with acute myeloid leukemia (AML), myelodysplastic syndromes (MDS), and chronic myelomonocytic leukemia (CMML) remain limited.

### Aims

Therefore, this multicenter retrospective study aimed to evaluate the real-world efficacy and safety of lisaftoclax in patients with these myeloid neoplasms.

### Methods

This retrospective study enrolled AML, MDS, or CMML patients receiving lisaftoclax across four Chinese centers. All met 2022 WHO criteria. Data collected included baseline characteristics, mutations, responses, and adverse events. Efficacy endpoints: CR, CRi, MRD negativity. AML risk stratified by ELN 2024. Adverse events graded per CTCAE v5.0. Descriptive statistics used.

### Results

4.1 Baseline Characteristics
Thirty patients enrolled: median age 63 years (26–77); 60% male. AML: 25 (83%), MDS: 3 (10%), CMML: 2 (7%). AML risk: low 15, intermediate 9, high 1. Firstline: 12 (48%); laterline: 13 (52%). Common mutations: DNMT3A (33%), RUNX1 (30%), ASXL1 (27%), NPM1 (20%), FLT3 (20%). HMR mutations: 43%.
4.2 AML Efficacy
CR/CRi rate was 72% (18/25): 12 CR, 6 CRi, 6 NR. Lowrisk: 87% (13/15), intermediaterisk: 67% (6/9), highrisk: no response. MRD negativity: 61% (11/18). Firstline: 75% (9/12), laterline: 69% (9/13). Among 5 lowrisk patients with pretreatment CRi or MRD positivity, 2 (40%) achieved deeper responses (CRi to CR, MRD positivity to MRD negativity).
4.3 MDS/CMML Efficacy
MDS: 2/3 achieved CRi (67%). CMML: 1 stable disease, 1 progression.
4.4 Mutation Analysis
NPM1mutated (n=6): 100% CR/CRi. IDH2mutated (n=6): 83%. DNMT3Amutated (n=10): 70%. FLT3mutated (n=6): 67%. RUNX1mutated (n=9): 56%. ASXL1mutated (n=8): 50%. NRASmutated (n=5): 60%.
4.5 Safety
Anygrade TEAEs: 87% (26/30). Hematologic: thrombocytopenia (57%), anemia (53%), neutropenia (47%). Grade≥3: 47% (14/30): thrombocytopenia (27%), anemia (23%), neutropenia (20%). Nonhematologic: febrile pneumonia (27%), liver dysfunction (23%), renal impairment (17%). Two AML patients transplanted; one death.

**Copyright Information:** (Online) ISSN: 2572-9241
©2026 The Author(s). HemaSphere is published by John Wiley & Sons Ltd on behalf of European Hematology Association. This is an open access Abstract Book distributed under the Attribution-NonCommercial-NoDerivs (CC BY-NC-ND), which allows third parties to download the articles and share them with others as long as they credit the author and the Abstract Book, but they cannot change the content in any way or use them commercially.
**Abstract Book Citations:** Authors, Title, HemaSphere, 2026;10:(S1):pages. The abstract book DOIs can be found on the “Author Information” page.
**Disclaimer:** Articles published in the journal HemaSphere exclusively reflect the opinions of the authors. The authors are responsible for all content in their abstracts including accuracy of the facts, statements, citing resources, etc.

**Summary/Conclusion**

Lisaftoclax showed 72% CR/CRi in AML, with lowrisk patients benefiting most (87%). MRD negativity (61%) correlated with response depth; 40% of pretreatment CRi or MRD positivity patients achieved deeper responses. MDS showed 67% CRi rate. NPM1 and IDH2 mutations predicted high response; RUNX1 and ASXL1 mutations had lower responses. Safety was manageable, with expected hematologic toxicities. Limitations: small sample, short followup, retrospective design.Lisaftoclax demonstrates promising efficacy (72% CR/CRi in AML) and manageable safety. ELN lowrisk and NPM1mutated patients derived greatest benefit. The swimmer plot supports its clinical utility.

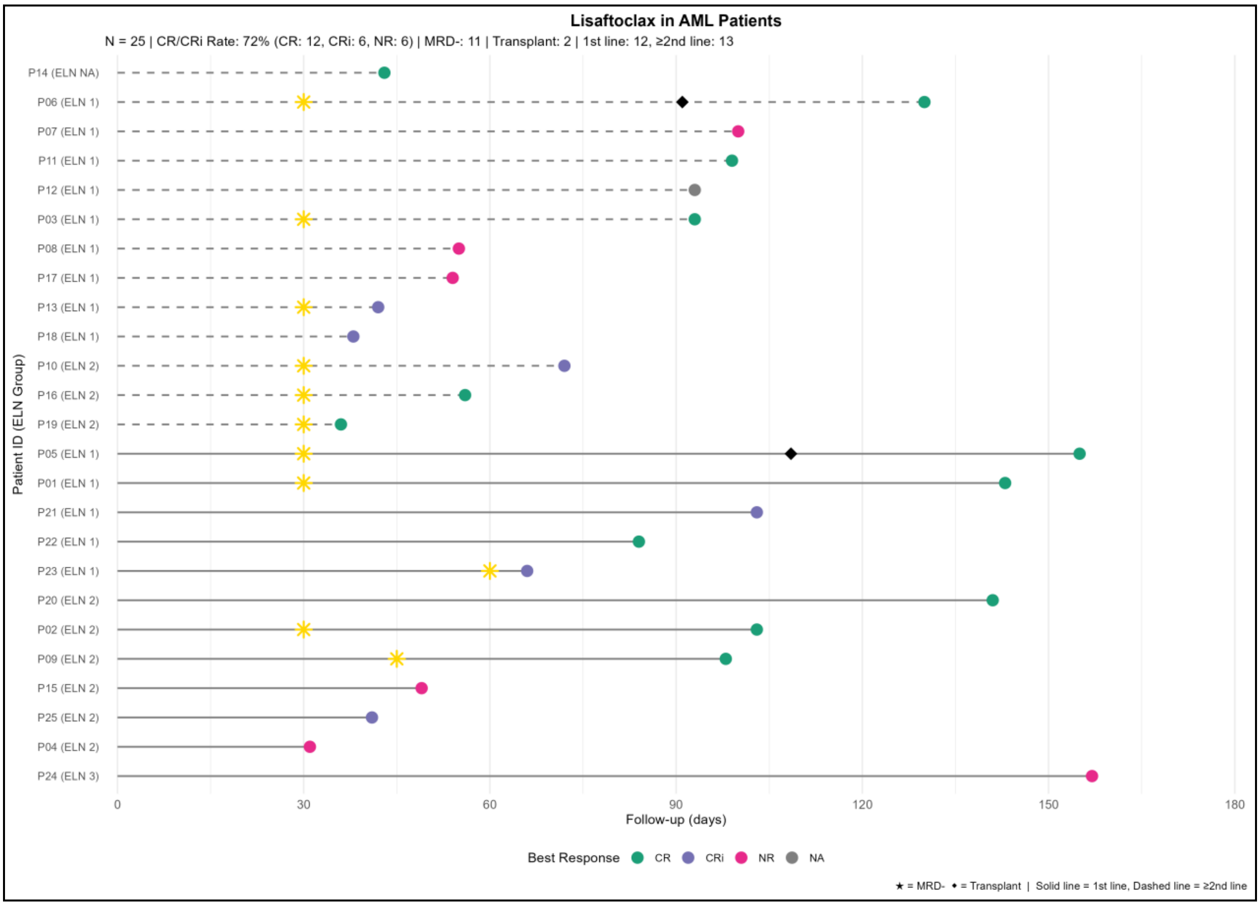

**Copyright Information:** (Online) ISSN: 2572-9241
©2026 The Author(s). HemaSphere is published by John Wiley & Sons Ltd on behalf of European Hematology Association. This is an open access Abstract Book distributed under the Attribution-NonCommercial-NoDerivs (CC BY-NC-ND), which allows third parties to download the articles and share them with others as long as they credit the author and the Abstract Book, but they cannot change the content in any way or use them commercially.
**Abstract Book Citations:** Authors, Title, HemaSphere, 2026;10:(S1):pages. The abstract book DOIs can be found on the "Author Information" page.
**Disclaimer:** Articles published in the journal HemaSphere exclusively reflect the opinions of the authors. The authors are responsible for all content in their abstracts including accuracy of the facts, statements, citing resources, etc.

### (PF563) CLINICAL OUTCOMES AFTER FAILURE OF AZACITIDINE PLUS VENETOCLAX FOR FRONTLINE TREATMENT OF ADULT PATIENTS WITH ACUTE MYELOID LEUKEMIA

**Topic:** 04. Acute myeloid leukemia - Clinical

Zahra Alhaj Issa*[1], Aarya Murali[1], Cristiano MacHado De Freitas[1], Aniket Bankar[1], Hassan, Sibai[1], Maria Agustina Perusini[1], Steven M Chan[1], Aaron Schimmer[1], Karen Yee[1], Andre Schuh[1], Vikas Gupta[1], Dawn Maze[1], Marta Davidson[1], Guillaume Richard-Carpentier[1]

[1]*Princess Margaret Cancer Center, Toronto, Canada;*

### Background

Azacitidine plus venetoclax (Aza-Ven) has transformed frontline therapy for older or unfit patients (pts) with newly diagnosed acute myeloid leukemia (AML), improving remission rates and overall survival (OS) versus Aza alone. However, relapse and primary resistance remain common, and most pts ultimately die of progressive disease. As Aza-Ven becomes standard frontline therapy even beyond unfit pts with AML, defining real-world post-failure survival and predictors of outcome is increasingly important.

### Aims

To assess survival after Aza-Ven failure in AML and identify predictors of post-failure OS.

### Methods

We retrospectively analyzed pts with newly diagnosed AML treated with frontline Aza-Ven at Princess Margaret Cancer Centre (2017–2024). Responses were defined per ELN 2022 criteria (CR, CRi, CRh, MLFS). Treatment failure included primary refractory disease or relapse after prior morphologic remission. Post-failure OS (OS-PF) was measured from confirmation of refractory disease or relapse. Baseline variables included age, secondary AML, ELN 2024 risk, cytogenetics, and gene mutations. OS was estimated using the Kaplan–Meier method and Cox regression was used to identify factors associated with OS-PF.

**Copyright Information:** (Online) ISSN: 2572-9241
©2026 The Author(s). HemaSphere is published by John Wiley & Sons Ltd on behalf of European Hematology Association. This is an open access Abstract Book distributed under the Attribution-NonCommercial-NoDerivs (CC BY-NC-ND), which allows third parties to download the articles and share them with others as long as they credit the author and the Abstract Book, but they cannot change the content in any way or use them commercially.
**Abstract Book Citations:** Authors, Title, HemaSphere, 2026;10:(S1):pages. The abstract book DOIs can be found on the "Author Information" page.
**Disclaimer:** Articles published in the journal HemaSphere exclusively reflect the opinions of the authors. The authors are responsible for all content in their abstracts including accuracy of the facts, statements, citing resources, etc.

**Results**

Among 175 pts treated with frontline Aza-Ven, 89 (51%) had treatment failure: 35 (39%) refractory disease and 54 (61%) relapse. Five (6%) pts relapsed after allogeneic stem cell transplant (HSCT) in first complete remission (CR1) at a median 5.3 months post-transplant. High-risk features were common, including secondary AML (81%), complex karyotype (29%), and *TP53* mutation (25%).
Following treatment failure, 42 (47%) pts received second-line therapy: intensive chemotherapy (IC) in 13 (7+3, n=3; FLAG-based, n=10); low-intensity regimen (LIT) in 15 (Aza-Ven–based, n=6; LDAC-Ven, n=4; HMA n=5); and targeted therapy in 10 (gilteritinib n=9; enasidenib n=1). Four pts participated in clinical-trials and the remainder 47(53%) pts received best supportive care (BSC). Among treated pts (n=42), 8 (19%) achieved second CR (CR2): 5/13 (39%) with IC, 2/15 (13%) with LIT, and 1/9 (11%) with gilteritinib. Among pts who relapsed post-HSCT, 1 pt (20%) achieved CR2 with Aza and proceeded to a second HSCT.
Among all pts, the median OS-PF was 4.1 months and estimated 6-, 12-, and 24-month OS-PF rates were 37%, 16%, and 7%, respectively. OS-PF was longer in refractory versus relapsed pts (4.9 vs 3.1 months; HR 1.92, p=0.01). Receipt of second-line therapy improved OS-PF (5.7 vs 3.0 months; HR 0.46, p<0.01) and achieving remission was associated with superior OS-PF (23.5 vs 5.1 months; HR 0.30, p=0.03). HSCT after post-failure remission (n=3) showed a trend toward improved OS-PF (HR 0.28, p=0.09). *TP53* mutation was significantly associated with inferior OS-PF (3.6 vs 5.3 months; HR 2.05, p=0.01). Increasing age was also associated with inferior OS-PF (HR 1.03 per year, p=0.04). In multivariable analysis, *TP53* mutation (HR 2.42, p<0.01) and relapse (HR 2.30, p<0.01) independently predicted inferior OS-PF, whereas receipt of second-line therapy was independently associated with improved OS-PF (HR 0.55, p=0.03).

**Summary/Conclusion**

Outcomes after frontline Aza-Ven failure were poor, particularly among patients unable to receive salvage therapy. *TP53* mutation and relapse predicted inferior survival, whereas second-line therapy and remission were associated with improved outcomes, underscoring the need for more effective post–Aza-Ven failure treatment strategies.

**Copyright Information:** (Online) ISSN: 2572-9241
©2026 The Author(s). HemaSphere is published by John Wiley & Sons Ltd on behalf of European Hematology Association. This is an open access Abstract Book distributed under the Attribution-NonCommercial-NoDerivs (CC BY-NC-ND), which allows third parties to download the articles and share them with others as long as they credit the author and the Abstract Book, but they cannot change the content in any way or use them commercially.
**Abstract Book Citations:** Authors, Title, HemaSphere, 2026;10:(S1):pages. The abstract book DOIs can be found on the "Author Information" page.
**Disclaimer:** Articles published in the journal HemaSphere exclusively reflect the opinions of the authors. The authors are responsible for all content in their abstracts including accuracy of the facts, statements, citing resources, etc.

### (PF564) PHARMACOKINETIC ASSESSMENT OF REVUMENIB IN PATIENTS WITH RELAPSED/REFRACTORY ACUTE LEUKEMIAS HARBORING A KMT2A REARRANGEMENT OR NPM1 MUTATION: IMPACT OF FOOD AND CONCOMITANT MEDICATIONS

**Topic:** 04. Acute myeloid leukemia - Clinical

Enoch Cobbina[1], Joshua Stewart[1], Yuwei Lin[2], Angela Smith[1], Ophelia Yin[1], Ghayas Issa*[3]

*[1]Syndax Pharmaceuticals, Inc., New York, NY, United States of America;[2]Certara Drug Development Solutions, Princeton, NJ, United States of America; [3]The University of Texas MD Anderson Cancer Center, Houston, TX, United States of America;*

### Background

Revumenib is a first-in-class, oral, potent, and selective inhibitor of the menin-KMT2A interaction, which disrupts multiple key drivers of leukemogenesis (*KMT2A* rearrangement [*KMT2A*r], *NPM1* mutation [*NPM1*m], and *NUP98* rearrangement). Revumenib is used for the treatment of relapsed/refractory (R/R) acute leukemias harboring a *KMT2A* translocation or R/R acute myeloid leukemia with *NPM1* mutation in adult and pediatric patients 1 year and older. Given the severity of disease, frequent polypharmacy, and variable clinical status in this population, dosing flexibility is essential to support consistent treatment delivery and optimize outcomes.

### Aims

To characterize the pharmacokinetics (PK) of revumenib and its major metabolite (M1), with emphasis on factors that may influence dosing flexibility, including food and concomitant medications such as CYP3A4 inhibitors (CYP3A4i) and gastric acid–reducing agents such as antacids, H2 blockers (H2Bs), and proton pump inhibitors (PPIs).

### Methods

PK data were obtained from 286 adult (≥18 years) and 49 pediatric (<18 years) patients with *KMT2A*r or *NPM1*m acute leukemia enrolled in the phase 1/2 AUGMENT-101 trial (NCT04065399) following single and multiple doses of revumenib. Non-compartmental analyses were performed in selected subgroups, while population PK modeling was conducted using data from all patients to systematically quantify PK variability and evaluate covariate effects.

### Results

As compared to fasted state, a low-fat meal (400–500 calories with <25% fat content) reduced revumenib $C_{max}$ and AUC by 27% and 12%, respectively, and M1 $C_{max}$ and AUC by 14% and 18%, respectively, although exposure ranges overlapped under fasted and fed (low-fat meal) conditions. Median $T_{max}$ was unchanged for revumenib and delayed by 1.8 hours for M1.
Co-administration with strong CYP3A4i (eg, voriconazole, posaconazole, itraconazole) resulted in ~2-fold increase in revumenib $C_{max}$ and AUC, whereas moderate CYP3A4i (eg, fluconazole, isavuconazole) had no meaningful effect. M1 exposure was unaffected by strong or moderate CYP3A4i. Approximately 52% of patients received antacids, H2Bs, or PPIs; revumenib and M1 $C_{max}$ and AUC were not altered, consistent with the high solubility of revumenib across physiological pH range (1.2–6.8).

Copyright Information: (Online) ISSN: 2572-9241
©2026 The Author(s). HemaSphere is published by John Wiley & Sons Ltd on behalf of European Hematology Association. This is an open access Abstract Book distributed under the Attribution-NonCommercial-NoDerivs (CC BY-NC-ND), which allows third parties to download the articles and share them with others as long as they credit the author and the Abstract Book, but they cannot change the content in any way or use them commercially.
Abstract Book Citations: Authors, Title, HemaSphere, 2026;10:(S1):pages. The abstract book DOIs can be found on the "Author Information" page.
Disclaimer: Articles published in the journal HemaSphere exclusively reflect the opinions of the authors. The authors are responsible for all content in their abstracts including accuracy of the facts, statements, citing resources, etc.

**Summary/Conclusion**

Revumenib exposure increases ~2-fold with strong CYP3A4i, supporting dose reduction in this setting, while M1 exposure remains unaffected. Low-fat meals do not cause clinically meaningful changes in revumenib or M1 PK. Similarly, antacids, H2Bs, and PPIs, which are frequently used in this patient population, had no impact on revumenib or M1 PK. These findings reinforce the flexible administration of revumenib that may support treatment adherence and patient convenience.

© 2026 American Society of Clinical Oncology, Inc. Reused with permission. This abstract was accepted and previously presented at the 2026 ASCO Annual Meeting. All rights reserved.

**Copyright Information:** (Online) ISSN: 2572-9241
©2026 The Author(s). HemaSphere is published by John Wiley & Sons Ltd on behalf of European Hematology Association. This is an open access Abstract Book distributed under the Attribution-NonCommercial-NoDerivs (CC BY-NC-ND), which allows third parties to download the articles and share them with others as long as they credit the author and the Abstract Book, but they cannot change the content in any way or use them commercially.
**Abstract Book Citations:** Authors, Title, HemaSphere, 2026;10:(S1):pages. The abstract book DOIs can be found on the “Author Information” page.
**Disclaimer:** Articles published in the journal HemaSphere exclusively reflect the opinions of the authors. The authors are responsible for all content in their abstracts including accuracy of the facts, statements, citing resources, etc.

### (PF565) GILTERITINIB OR IVOSIDENIB IN COMBINATION WITH VENETOCLAX-BASED THERAPIES FOR FLT3- OR IDH1-MUTATED ACUTE MYELOID LEUKEMIA

**Topic:** 04. Acute myeloid leukemia - Clinical

Andrius Žučenka*[1, 2], Tautvilė Smalinskaitė[1, 2], Milda Mikalonytė[1, 2], Guoda Daukėlaitė[1, 2], Milvydė Tamutytė-Jankauskė[1, 2], Regina Pileckyte[1, 2], Linas Davainis[1], Lukas Kevlicius[1], Tomas Daukšas[1], Andrius Degulys[1, 2], Sima Pakstyte Daneliene[1], Agnė Šiaudinienė[1, 2], Roberta Petrauskaite[1], Lina Kryžauskaitė[1, 2], Vilmante Vaitekenaite[1], Rūta Semaškevičienė[1], Adomas Bukauskas[1], Skirmante Cernauskiene[1, 2], Birute Davainiene[1], Indrė Klimienė[1], Austeja Dapkeviciute Purliene[1], Karolis Šablauskas[1], Igoris Trociukas[1], Renata Jucaitienė[1], Artūras Slobinas[1], Inga Šlepikienė[1], Justė Strumskytė[1], Gabrielė Buikauskienė[1], Vaida Jaraminė[1], Rita Cekauskiene[1, 1], Antanas Griškevičius[1], Artūras Jakubauskas[1], Kazimieras Maneikis[1], Mingaudas Busevičius[1], Valdas Pečeliūnas[1, 2], Laimonas Griškevičius[1, 2]

*[1]Vilnius University Hospital Santaros Klinikos, National Cancer Center, Hematology, Oncology, and Transfusion Medicine Center, Vilnius, Lithuania; [2]Vilnius University, Faculty of Medicine, Hematology and Oncology Department, Vilnius, Lithuania;*

### Background

Early-phase clinical trials demonstrate promising antileukemic efficacy of triplet regimens in *FLT3*- or *IDH*-mutated acute myeloid leukemia (*FLT3*m or *IDH*m AML). Herein, we demonstrate the real-world outcomes of Gilteritinib or Ivosidenib added to Venetoclax-based regimens in patients with *FLT3*m or *IDH1*m AML.

### Aims

The aim of the study was to evaluate the outcomes of *FLT3*m *or IDH1*m AML patients who received Gilteritinib / Ivosidenib in combination with Venetoclax-based backbone therapies.

### Methods

This was a retrospective, single-center study. The patients were ≥ 18 years old, had *FLT3*m or *IDH1*m AML, and received gilteritinib or ivosidenib in combination with venetoclax-based regimens. Treatment and data collection were approved by the Vilnius University Hospital Santaros Klinikos Hematology Board and the Vilnius Regional Biomedical Research Ethics Committee.
Patients with newly diagnosed (ND) AML received VenHMA+Gilt (*FLT3*m) or VenHMA+Ivo (*IDH1*m) regimens. VenHMA consisted of azacitidine 75 mg/m$^2$ (days 1-7) or decitabine 20 mg/m$^2$ (days 1-5) + venetoclax 400 mg (days 1-14). Gilteritinib 80 mg was given on days 1-14, whereas ivosidenib 500mg was given on days 1-28.
Treatment regimens for relapsed/refractory (R/R) AML were ACTIVE+Gilt (*FLT3*m) or ACTIVE+Ivo (*IDH1*m). ACTIVE consisted of actinomycin D 12.5 mcg/kg (days 1-3 or 1-2), low-dose cytarabine 20mg/m$^2$ (days 1-10), and venetoclax 600 mg (days 1-14, or continued up to day 21/28, if MLFS was not confirmed on day 14). Gilteritinib 80 mg or 120 mg was given on days 1-14 (or continued up to day 28, if MLFS was not confirmed on day 14), whereas ivosidenib 500 mg was given on days 1-14 or 1-28.
Responders proceeded to alloSCT, whereas transplant-ineligible patients went on maintenance with individually dosed triplets every 6-8 weeks.
Data collection included patient and AML-related characteristics, treatment schedules, CR, CRp, MLFS, and MRD negativity rates, early mortality, OS, and DoR.

**Copyright Information:** (Online) ISSN: 2572-9241
©2026 The Author(s). HemaSphere is published by John Wiley & Sons Ltd on behalf of European Hematology Association. This is an open access Abstract Book distributed under the Attribution-NonCommercial-NoDerivs (CC BY-NC-ND), which allows third parties to download the articles and share them with others as long as they credit the author and the Abstract Book, but they cannot change the content in any way or use them commercially.
**Abstract Book Citations:** Authors, Title, HemaSphere, 2026;10:(S1):pages. The abstract book DOIs can be found on the "Author Information" page.
**Disclaimer:** Articles published in the journal HemaSphere exclusively reflect the opinions of the authors. The authors are responsible for all content in their abstracts including accuracy of the facts, statements, citing resources, etc.

**Results**

53 patients (58% female) were enrolled. Their baseline characteristics, treatment details, response, and early mortality data are summarized in Table 1.

The CR+CRp rate in the *FLT3*m AML subgroup was 73% (77% in ND and 72% in R/R patients) and 100% in the *IDH1*m subgroup.

Overall survival data are summarized in Figure 1. The median duration of response in the *FLT3*m AML subgroup was 13 months (not reached in ND and 8.3 months in R/R cases). No relapses were confirmed in the *IDH1*m AML subgroup.

| **Characteristics** | **ND FLT3m AML (13)** | **R/R FLT3m AML (32)** | **ND IDH1m AML (5)** | **R/R IDH1m AML (3)** |
|---|---|---|---|---|
| Age, median (range) | 79 (65-88) | 64 (34-87) | 65 (48-82) | 67 (58-75) |
| ECOG, median (range) | 2 (0-3) | 1 (0-3) | 1 (0-2) | 1 (0-1) |
| Adverse risk group (ELN 2022) | 54% | 34% | 100% | 67% |
| FLT3-ITD | 69% | 66% | 0 | 0 |
| FLT3-TKD | 23% | 22% | 0 | 0 |
| FLT3-ITD + FLT3-TKD | 8% | 12% | 0 | 0 |
| **Response evaluation** | | | | |
| CR | 62% | 47% | 60% | 100% |
| CRp | 15% | 25% | 40% | 0 |
| MLFS | 0 | 22% | 0 | 0 |
| MRD negativity in CR/CRp patients (MFC) | 70% | 57% | 80% | 100% |
| FLT3 or IDH1 molecular clearance in CR/CRp patients | 50% | 52% | 40% | 67% |
| Consolidation with alloSCT | 8% | 47% | 40% | 33% |
| Consolidation with alloSCT in eligible patients at baseline | 50% | 79% | 100% | 100% |
| **Treatment details** | | | | |
| Number of treatment cycles, median (range) | 2 (1-22) | 2 (1-12) | 4 (1-6) | 4 (1-11) |
| Treatment stopped | 54% (1 alloSCT, 4 RD/relapse, 2 died in CR). | 97% (15 alloSCT, 12 RD/relapse, 4 died in MLFS/CR) | 40% (2 alloSCTs) | 33% (1 alloSCT) |
| **Early all-cause mortality** | | | | |
| Day 30 mortality | 0 | 6% | 0 | 0 |
| Day 60 mortality | 8% | 16% | 0 | 0 |

**Copyright Information:** (Online) ISSN: 2572-9241
©2026 The Author(s). HemaSphere is published by John Wiley & Sons Ltd on behalf of European Hematology Association. This is an open access Abstract Book distributed under the Attribution-NonCommercial-NoDerivs (CC BY-NC-ND), which allows third parties to download the articles and share them with others as long as they credit the author and the Abstract Book, but they cannot change the content in any way or use them commercially.
**Abstract Book Citations:** Authors, Title, HemaSphere, 2026;10:(S1):pages. The abstract book DOIs can be found on the "Author Information" page.
**Disclaimer:** Articles published in the journal HemaSphere exclusively reflect the opinions of the authors. The authors are responsible for all content in their abstracts including accuracy of the facts, statements, citing resources, etc.

### Summary/Conclusion

Our real-world study demonstrates high antileukemic efficacy of venetoclax + gilteritinib/ivosidenib combination regimens for *FLT3*m or *IDH1*m AML, both in ND and R/R settings.

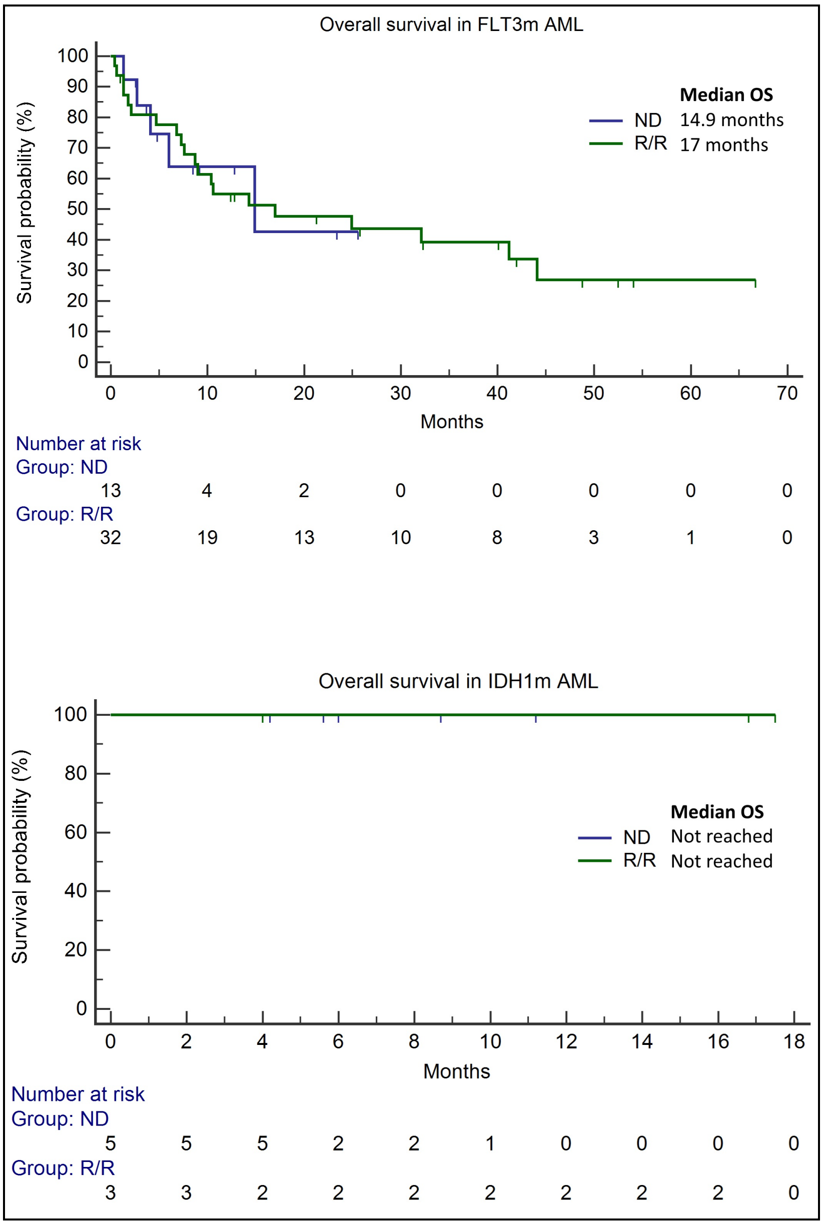

Figure 1. Overall survival

**Copyright Information:** (Online) ISSN: 2572-9241
©2026 The Author(s). HemaSphere is published by John Wiley & Sons Ltd on behalf of European Hematology Association. This is an open access Abstract Book distributed under the Attribution-NonCommercial-NoDerivs (CC BY-NC-ND), which allows third parties to download the articles and share them with others as long as they credit the author and the Abstract Book, but they cannot change the content in any way or use them commercially.
**Abstract Book Citations:** Authors, Title, HemaSphere, 2026;10:(S1):pages. The abstract book DOIs can be found on the "Author Information" page.
**Disclaimer:** Articles published in the journal HemaSphere exclusively reflect the opinions of the authors. The authors are responsible for all content in their abstracts including accuracy of the facts, statements, citing resources, etc.

### (PF567) ORAL AZACITIDINE MAINTENANCE IN FAVORABLE-RISK AML: ON BEHALF OF THE ISRAEL ACUTE LEUKEMIA GROUP (IALG)

**Topic:** 04. Acute myeloid leukemia - Clinical

Noa Hurvitz*[1], Irina Amitai[2], Ilana Levy Yurkovski[3], Ariella Tvito[4], Yishai Ofran[4], Tzvika Porges[5], Ofir Wolach[6], Adi Sherban[6], Noa Gross Even Zohar[1], Vladimir Vainstein[1], Arnon Haran[1], Shlomzion Aumann[1], Robert Michashvili[1], Avraham Frisch[7], Boaz Nachmias[1]

*[1]Hadassah Medical Center and Faculty of Medicine, Hebrew University of Jerusalem, Department of Hematology, jerusalem, Israel; [2]Chaim Sheba Medical Center, Faculty of Medicine, Tel Aviv University, Department of Hematology, Ramat Gan, Israel; [3]Bnai Zion medical canter, Department of Hematology, Haifa, Israel;[4]Shaare Zedek Medical Center, Hematology and Stem Cell Transplantation Department and the Eisenberg RD Authority, jerusalem, Israel; [5]Soroka university Medical Center, Department of Hematology, jerusalem, Israel; [6]Rabin medical center, Department of Hematology, Petah Tikva, Israel;[7]Rambam Health Care Campus, Department of Hematology and Bone Marrow Transplantation, Haifa, Israel;*

### Background

Oral azacitidine improves survival in patients with AML in first complete remission who are not candidates for allogeneic transplantation. Most favorable-risk AML patients harbor molecular markers enabling sensitive measurable residual disease (MRD) monitoring. While MRD is a well-established prognostic factor in AML, the clinical significance of MRD dynamics during maintenance therapy remains incompletely defined.

### Aims

To evaluate real-world outcomes of oral azacitidine maintenance in patients with favorable- and intermediate-risk AML, with a particular focus on the prognostic impact of measurable residual disease (MRD) status and MRD dynamics during therapy

### Methods

We conducted a multicenter, real-world retrospective study of 30 patients with AML, predominantly ELN favorable-risk, treated with oral azacitidine maintenance following intensive induction-consolidation. MRD was assessed longitudinally using standardized molecular and flow-based assays. Outcomes were analyzed according to MRD status at maintenance initiation and subsequent MRD conversion.

### Results

At initiation of oral azacitidine, 47% of patients were MRD-positive. MRD conversion to negativity occurred in 64% of these patients during maintenance. Relapse-free survival (RFS) was comparable between patients who were MRD-negative at baseline and those who converted to MRD negativity (24-months restricted median RFS 577 vs. 638 days), whereas patients with persistent MRD positivity experienced early relapse (24-months restricted median RFS 63 days). Despite inferior RFS, overall survival did not significantly differ between MRD-defined groups. Patients relapsing after oral azacitidine retained sensitivity to subsequent therapies, including venetoclax–azacitidine–based regimens, with high rates of molecular response.

**Copyright Information:** (Online) ISSN: 2572-9241
©2026 The Author(s). HemaSphere is published by John Wiley & Sons Ltd on behalf of European Hematology Association. This is an open access Abstract Book distributed under the Attribution-NonCommercial-NoDerivs (CC BY-NC-ND), which allows third parties to download the articles and share them with others as long as they credit the author and the Abstract Book, but they cannot change the content in any way or use them commercially.
**Abstract Book Citations:** Authors, Title, HemaSphere, 2026;10:(S1):pages. The abstract book DOIs can be found on the "Author Information" page.
**Disclaimer:** Articles published in the journal HemaSphere exclusively reflect the opinions of the authors. The authors are responsible for all content in their abstracts including accuracy of the facts, statements, citing resources, etc.

**Summary/Conclusion**

Oral azacitidine was associated with high rates of MRD conversion, conferring RFS comparable to patients who were MRD-negative at treatment initiation. Persistent MRD identified patients at high-risk for early relapse, while effective salvage therapies mitigated overall survival differences. These findings support prospective evaluation of MRD-guided maintenance strategies in favorable-risk AML.

**Copyright Information:** (Online) ISSN: 2572-9241
©2026 The Author(s). HemaSphere is published by John Wiley & Sons Ltd on behalf of European Hematology Association. This is an open access Abstract Book distributed under the Attribution-NonCommercial-NoDerivs (CC BY-NC-ND), which allows third parties to download the articles and share them with others as long as they credit the author and the Abstract Book, but they cannot change the content in any way or use them commercially.
**Abstract Book Citations:** Authors, Title, HemaSphere, 2026;10:(S1):pages. The abstract book DOIs can be found on the "Author Information" page.
**Disclaimer:** Articles published in the journal HemaSphere exclusively reflect the opinions of the authors. The authors are responsible for all content in their abstracts including accuracy of the facts, statements, citing resources, etc.

### (PF568) PTPN11 MUTATED ACUTE MYELOID LEUKEMIA: EVALUATION OF DOMAIN-SPECIFIC MUTATIONS AND THEIR POTENTIAL IMPACT ON CO-MUTATIONAL PROFILES AND CLINICAL OUTCOMES

**Topic:** 04. Acute myeloid leukemia - Clinical

Francesca Crupi*[1, 2, 3], Francesco Grimaldi[4], Barbara Izzo[5, 6], Gaia Ciolli[1, 2], Matteo Piccini[2], Barbara Scappini[2], Mara Memoli[4], Roberta Visconti[6], Mario Annunziata[7], Jessica Caroprese[1, 2, 3], Andrea Pasquini[1, 2], Niccolò Bartalucci[1, 3], Alessio Enderti[3], Chiara MacCari[3], Giada Rotunno[2, 3], Fabiana Pancani[3], Leonardo Signori[1, 3], Paola Guglielmelli[1, 2, 3], Alessandro Maria Vannucchi[1, 2, 3], Francesco Mannelli[1, 2, 3]

*[1]Università di Firenze, Medicina Sperimentale e Clinica, Florence, Italy;[2]AOU Careggi, Hematology, Florence, Italy;[3]AOU Careggi, Centro di Ricerca e Innovazione Malattie Mieloproliferative (CRIMM), Florence, Italy; [4]University of Naples, "Federico II", Hematology Division, Department of Clinical Medicine and Surgery, Naples, Italy; [5]University of Naples, "Federico II", Department of Molecular Medicine and Medical Biotechnology, Naples, Italy; [6]CEINGE, Biotecnologie Avanzate Franco Salvatore,, Naples, Italy; [7]AORN Cardarelli, Division of Hematology, Naples, Italy;*

### Background

*PTPN11* mutations can be found in 1.5%–12% of acute myeloid leukemia (AML) cases. They have been reported to cluster mainly in the N-terminal Src homology region 2 (N-SH2) and phosphatase (PTP) domains. The protein's catalytic activity is normally auto-regulated through intramolecular binding of the N-terminal SH2 domain to the phosphatase domain, which blocks the active site. Mutations in either domain disrupt this autoinhibition, resulting in a gain-of-function state that activates the proto-oncogene and triggers downstream pathways, including RAS/MAPK. The prognostic role of *PTPN11* is still controversial: although several studies showed an association with poor prognosis in intensively treated patients (pts), *PTPN11* mutations were not included in the ELN 2022 risk stratification. Conversely, the recently refined ELN 2024 guidelines have classified *PTPN11*-mutated (mut) pts as having a poor benefit from treatment with hypomethylating agents (HMA) plus venetoclax (ven).

### Aims

To investigate interactions between mutations N-SH2 and PTP domains of *PTPN11* gene with baseline characteristics and outcomes.

### Methods

Retrospective analysis of *PTPN11*$^{mut}$ AML pts from the Florence cohort and the CEINGE Campania registry

**Copyright Information:** (Online) ISSN: 2572-9241
©2026 The Author(s). HemaSphere is published by John Wiley & Sons Ltd on behalf of European Hematology Association. This is an open access Abstract Book distributed under the Attribution-NonCommercial-NoDerivs (CC BY-NC-ND), which allows third parties to download the articles and share them with others as long as they credit the author and the Abstract Book, but they cannot change the content in any way or use them commercially.
**Abstract Book Citations:** Authors, Title, HemaSphere, 2026;10:(S1):pages. The abstract book DOIs can be found on the "Author Information" page.
**Disclaimer:** Articles published in the journal HemaSphere exclusively reflect the opinions of the authors. The authors are responsible for all content in their abstracts including accuracy of the facts, statements, citing resources, etc.

**Results**

From 2011 to 2026, 78 *PTPN11*$^{mut}$ AML pts were observed at study sites of whom 57 (73.1%) with *PTPN11*-N-SH2 domain, 19 (24.4%) with PTP domain mutations, and 2 (2.5%) with mutations in both domains (*PTPN11*$^{dm}$). Characteristics of patients are detailed in Table 1 (Fig. 1A; for primary comparative analysis, PTPN11$^{dm}$ were excluded). No significant differences were observed according to mutations in different domains in baseline characteristics (age, WBC, platelets, LDH, ELN 2022 risk stratification). Regarding co-occurring mutations, *PTPN11*-PTP$^{mut}$ pts showed a trend toward a higher incidence of *NPM1*$^{mut}$ (58% vs 37%, p=0.118) and a significantly higher incidence of *DNMT3A*$^{mut}$ (53% vs 26%, p=0.049). Conversely, *K/NRAS*$^{mut}$ were significantly more frequent in *PTPN11*-N-SH2$^{mut}$ pts (44% vs 16%, p=0.031). We also studied whether mutations in different domains of the *PTPN11* gene affected outcomes. For this purpose, we separated pts according to treatment approaches. In patients receiving intensive treatment (n=31 N-SH2$^{mut}$ and n=10 PTP$^{mut}$), initial responses were not significantly different, although a trend for *PTPN11*-N-SH2$^{mut}$ pts having lower CR rate (55% vs 80%, p=0.263) and MRD negativity rate (50% vs 87.5%, p=0.099) were observed. EFS (12.36 vs 67.34 months, p=0.193) and OS (18.9 vs NR, p=0.073, Fig. 1B) were also worse in this subgroup, not reaching statistical significance likely due to sample size. In less-intensively treated patients (n=20 N-SH2$^{mut}$ and n=8 PTP$^{mut}$) response rates were low in both groups (29.4% N-SH2$^{mut}$ and 37.5% PTP$^{mut}$ pts, p=1.0) and survival estimates were superimposable (OS 12 vs 10 months respectively in N-SH2$^{mut}$ and PTP$^{mut}$, p=0.682).

**Summary/Conclusion**

Although limited by the number of cases, our findings suggest the involved *PTPN11* domain to be associated with distinct leukemogenesis, as suggested by the different co-mutational patterns. In the setting of intensive treatment, pts with *PTPN11*-N-SH2 domain showed a trend toward worse survival compared with those harbouring PTP-domain mutations. Studies on larger cohorts are warranted to elucidate a potential distinct effect on response and outcome that could support an adapted indication to treatment modalities and HSCT in these patients.

**Copyright Information:** (Online) ISSN: 2572-9241
©2026 The Author(s). HemaSphere is published by John Wiley & Sons Ltd on behalf of European Hematology Association. This is an open access Abstract Book distributed under the Attribution-NonCommercial-NoDerivs (CC BY-NC-ND), which allows third parties to download the articles and share them with others as long as they credit the author and the Abstract Book, but they cannot change the content in any way or use them commercially.
**Abstract Book Citations:** Authors, Title, HemaSphere, 2026;10:(S1):pages. The abstract book DOIs can be found on the "Author Information" page.
**Disclaimer:** Articles published in the journal HemaSphere exclusively reflect the opinions of the authors. The authors are responsible for all content in their abstracts including accuracy of the facts, statements, citing resources, etc.

**Figure 1 A: Baseline characteristics of $PTPN11^{mut}$ patients**

| | Overall | N-SH2 domain | PTP domain | P value |
|---|---|---|---|---|
| | **78** | **57 (75%)** | **19 (25%)** | |
| **Age yrs (median, IQR)** | 63.50 (53 – 73) | 64 (46 – 73) | 58 (54 -70) | 0.791 |
| **WBC $10^9$/L (median, IQR)** | 26.50 (5.3 – 70) | 22 (5.7 – 60.3) | 37 (2.7 -108) | 0.784 |
| **Platelets $10^9$/L (median, IQR)** | 55 (33 – 121) | 57 (38 – 136) | 55 (29 – 100) | 0.459 |
| **LDH U/L (median, IQR)** | 470 (269 – 763) | 431 (245 – 741) | 528 (277 – 898) | 0.369 |
| **ELN 2022** | | | | |
| Favorable | 19 (25%) | 14 (25%) | 5 (26%) | 0.838 |
| Intermediate | 21 (28%) | 15 (26%) | 6 (32%) | |
| High | 36 (47%) | 28 (49%) | 8 (42%) | |
| **Karyotype** | | | | |
| Poor | 16 (21%) | 11 (19.3%) | 5 (26%) | 0.809 |
| Complex | 5 (6.6%) | 4 (7%) | 1 (5%) | |
| **Co-mutations:** | | | | |
| N° of mut | 3 (2-4) | 3 (2-4) | 3 (2-3) | 0.455 |
| *NPM1* | 32 (42%) | 21 (37%) | 11 (58%) | 0.118 |
| *FLT3*-ITD | 15 (19.7%) | 11 (19%) | 4 (21%) | 1 |
| *DNMT3A* | 25 (33%) | 15 (26%) | 10 (53%) | **0.049** |
| *IDH1-2* | 12 (15.8) | 9 (15.8) | 3 (15.8) | 1 |
| *K/NRAS* | 28 (36.8%) | 25 (44%) | 3 (16%) | **0.031** |
| *ASXL1* | 14 (18.4%) | 12 (21%) | 2 (10.5%) | 0.496 |
| *RUNX1* | 8 (10.5%) | 8 (14%) | 0 | 0.189 |
| *SRSRF2* | 8 (10.5%) | 7 (12.3%) | 1 (5.3%) | 0.671 |
| *TET2* | 7 (9.2%) | 7 (12.3%) | 0 | 0.182 |
| *TP53* | 6 (8%) | 4 (7%) | 2 (10.5%) | 0.636 |
| **VAF%** | 10.8 (6 – 27) | 12 (6 -31) | 9 (5 -24) | 0.495 |
| **Induction:** | | | | |
| Chemotherapy | 43 (57.3%) | 32 (57%) | 11 (58%) | 0.677 |
| Less intensive tp | 28 (37.3%) | 20 (35.7%) | 8 (42.1%) | |
| **HSCT** | 30 (50.7%) | 28 (49%) | 10 (55.6%) | 0.788 |

**Figure 1 B: Overall Survival in intensively treated patients**

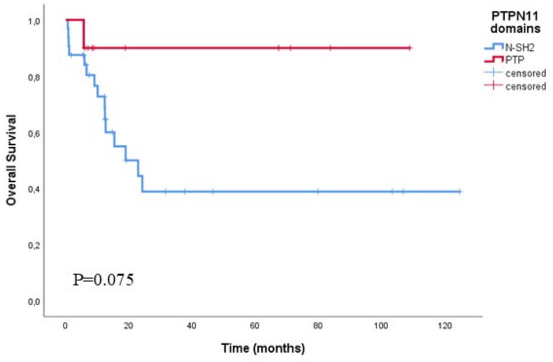

| PTPN11 Type | N° of cases | N° of events | Censored N | Censored Percentage |
|---|---|---|---|---|
| N-SH2 | 32 | 15 | 17 | 53,1% |
| PTP | 10 | 1 | 9 | 90,0% |
| Overall | 42 | 16 | 26 | 61,9% |

| PTPN11 Type | Median Estimate | Median 95%IC Lower limit | Median 95%IC Upper limit |
|---|---|---|---|
| NSH-2 | 18,918 | 4,531 | 33,305 |
| PTP | - | - | - |
| Overall | 24,197 | - | - |

Figure 1 A Baseline characteristics of PTPN11 mutated patients; Figure 1 B Kaplan-Meier curves of overall survival in intensively treated patients

**Copyright Information:** (Online) ISSN: 2572-9241
©2026 The Author(s). HemaSphere is published by John Wiley & Sons Ltd on behalf of European Hematology Association. This is an open access Abstract Book distributed under the Attribution-NonCommercial-NoDerivs (CC BY-NC-ND), which allows third parties to download the articles and share them with others as long as they credit the author and the Abstract Book, but they cannot change the content in any way or use them commercially.
**Abstract Book Citations:** Authors, Title, HemaSphere, 2026;10:(S1):pages. The abstract book DOIs can be found on the "Author Information" page.
**Disclaimer:** Articles published in the journal HemaSphere exclusively reflect the opinions of the authors. The authors are responsible for all content in their abstracts including accuracy of the facts, statements, citing resources, etc.

### (PF569) STAT1 SIGNALING DRIVES MICROENVIRONMENT-MEDIATED VENETOCLAX RESISTANCE AND PROLIFERATION IN CLL

**Topic:** 05. Chronic lymphocytic leukemia and related disorders - Biology & translational research

Miroslav Boudny*[1], Erika Bajusova[1], Pedro Faria Zeni[1], Daniel Filip[1], Laura Ondrisova[1], Kvetoslava Matulova[2], Josef Veceřa[1], Krystof Hlavac[1], Vaclav Seda[1], Sarka Pospisilova[2], Anantharamanan Rajamani[3], Othman Al-Sawaf[3], Kirsten Fischer[3], Leos Kren[2], Anna Panovská[2], Michael Doubek[2], Marek Mraz[1]

*[1]University Hospital Brno and CEITEC Masaryk University, Brno, Czechia; [2]University Hospital Brno, Brno, Czechia; [3]University Hospital of Cologne, Cologne, Germany;*

### Background

Venetoclax-based therapy has substantially improved CLL outcomes; however, resistance occurs and is mainly driven by the tumor microenvironment (TME) in lymph nodes (LNs) (Thijssen et al. 2015, 2022). The key signaling pathways sustaining CLL proliferation and venetoclax resistance remain incompletely defined.

### Aims

To identify TME-derived signaling pathways driving venetoclax resistance and CLL proliferation, and to evaluate JAK-STAT inhibition as a strategy to restore venetoclax sensitivity.

### Methods

Primary CLL cells (n=32) were stimulated with TME factors or stromal co-culture (model by Hoferkova et al. 2024), and in presence of venetoclax ± JAK inhibitors. CRISPR was performed in primary CLL cells using Cas9-gRNA transfection.

**Copyright Information:** (Online) ISSN: 2572-9241
©2026 The Author(s). HemaSphere is published by John Wiley & Sons Ltd on behalf of European Hematology Association. This is an open access Abstract Book distributed under the Attribution-NonCommercial-NoDerivs (CC BY-NC-ND), which allows third parties to download the articles and share them with others as long as they credit the author and the Abstract Book, but they cannot change the content in any way or use them commercially.
**Abstract Book Citations:** Authors, Title, HemaSphere, 2026;10:(S1):pages. The abstract book DOIs can be found on the “Author Information” page.
**Disclaimer:** Articles published in the journal HemaSphere exclusively reflect the opinions of the authors. The authors are responsible for all content in their abstracts including accuracy of the facts, statements, citing resources, etc.

**Results**

Re-analysis of transcriptomic data from CLL samples obtained at relapse post venetoclax (n=7 pairs, Ghia et al. 2022; n=29 pairs, Al-Sawaf et al. 2023) revealed strong enrichment of JAK-STAT and IFNγ signaling (IPA, GSEA) with STAT1 among the top predicted transcriptional regulators in 2 independent datasets (**Fig.A,B**). To determine whether this pathway also contributes to LN-driven proliferation/survival, we performed transcriptomic analysis of CXCR4/CD5 intraclonal CLL subpopulations (n=10 pairs). JAK-STAT signaling was markedly activated in proliferative CXCR4$^{dim}$CD5$^{brigh}$ cells, with STAT1 and its target IRF1 showing the highest regulatory activity (**Fig.C**). Increased STAT1 activity was independently confirmed in LN-resident CLL cells (n=29 pairs, Sun et al. 2023). Clinically, higher *STAT1* expression was associated with shorter time-to-first-treatment and OS across 2 independent CLL cohorts (n=191, n=106, **Fig.D**). Functional screening of 16 TME stimuli identified IFNγ and IL21 as the main inducers of STAT1 phosphorylation in primary CLL cells (**Fig.E**). CLL-derived T cells (CD3/28-activated) produced both IFNγ (312 ng/ml) and IL21 (1 ng/ml), induced STAT1 activation, and promoted CLL cell proliferation. Despite the immunologically "cold" nature of CLL LNs, immunohistochemistry confirmed IFNγ cytokine expression in CLL LNs (n=8; **Fig.F**). IFNγ stimulation significantly enhanced CLL cell viability (**Fig.G**) and cooperated with other TME stimuli to boost proliferation (**Fig.H**), while IL21 strongly promoted CLL proliferation in the co-culture model (10% vs. 49%, n=16, P<0.05). Notably, JAK inhibition (ruxolitinib, AZD1480; **Fig.I**) or *STAT1* knockout in primary CLL cells (CRISPR; **Fig.J**) impaired TME-driven proliferation. ChIP-seq for STAT1 revealed regulation of multiple pro-survival and pro-proliferative genes in CLL cells following IFNγ or IL21 treatment or co-culture. IFNγ stimulation effectively rescued CLL cells from venetoclax-induced apoptosis (**Fig.K**), whereas IL21 had no significant effect. This was accompanied by upregulation of the anti-apoptotic MCL1 and BCLxL by IFNγ. Importantly, JAK inhibition (ruxolitinib or AZD1480) restored venetoclax sensitivity across multiple *in vitro* models, including IFNγ stimulation, T-cell-conditioned media, and stromal co-culture (**Fig.K,L**).

**Summary/Conclusion**

We identify STAT1 signaling as a key TME–driven pathway promoting CLL cell proliferation, survival, and TME-mediated resistance to venetoclax. JAK inhibition consistently restored venetoclax sensitivity across TME models, providing a strong rationale for combining JAK inhibitors with venetoclax.

Supported by the Ministry of Health of the Czech Republic (NU22-03-00117, NW24-03-00369), Czech Science Foundation (25-15368X), MH CZ-DRO (FNBr,65269705), MUNI/A/1733/2025.

**Copyright Information:** (Online) ISSN: 2572-9241
©2026 The Author(s). HemaSphere is published by John Wiley & Sons Ltd on behalf of European Hematology Association. This is an open access Abstract Book distributed under the Attribution-NonCommercial-NoDerivs (CC BY-NC-ND), which allows third parties to download the articles and share them with others as long as they credit the author and the Abstract Book, but they cannot change the content in any way or use them commercially.
**Abstract Book Citations:** Authors, Title, HemaSphere, 2026;10:(S1):pages. The abstract book DOIs can be found on the "Author Information" page.
**Disclaimer:** Articles published in the journal HemaSphere exclusively reflect the opinions of the authors. The authors are responsible for all content in their abstracts including accuracy of the facts, statements, citing resources, etc.

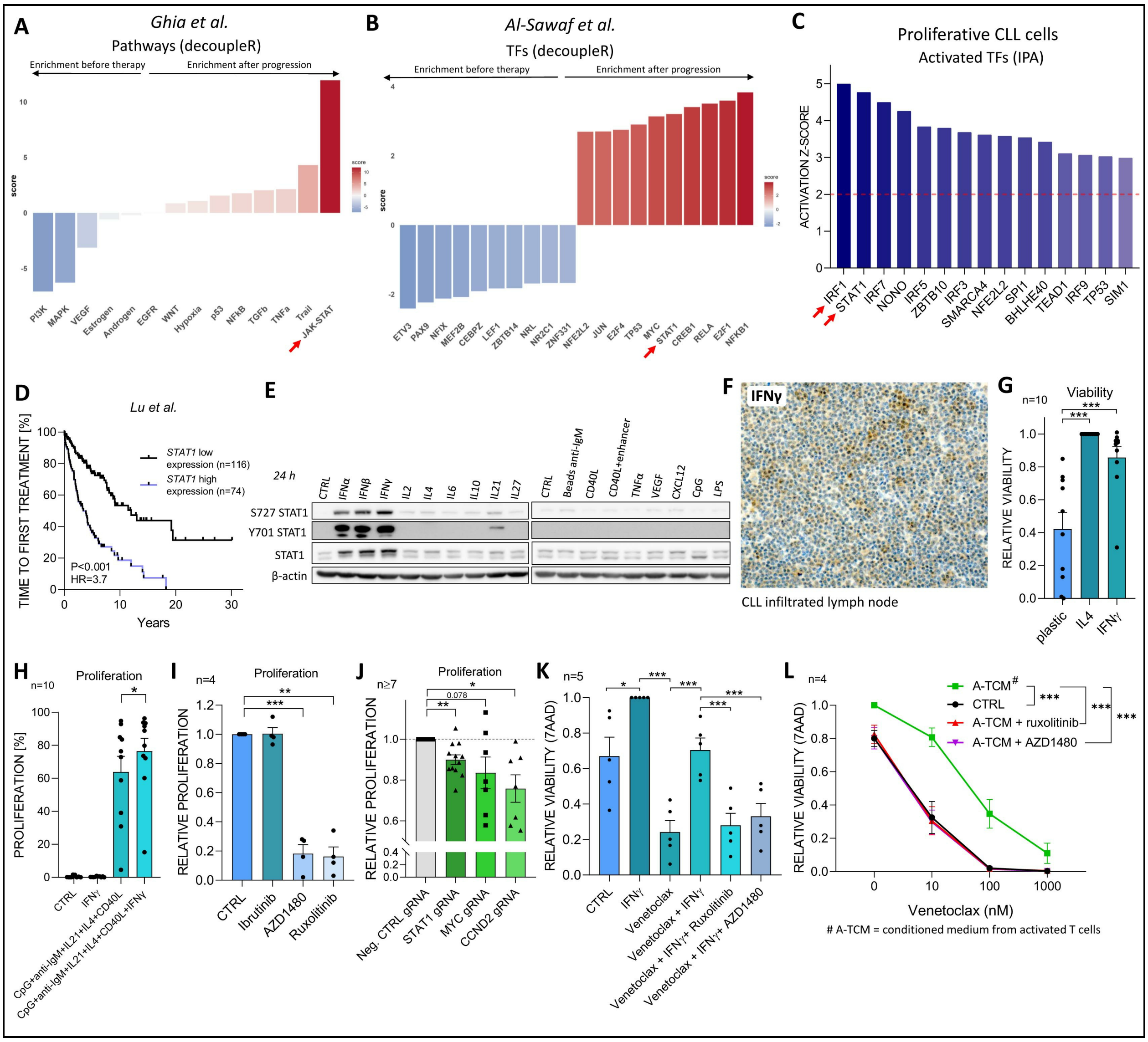

**Copyright Information:** (Online) ISSN: 2572-9241
©2026 The Author(s). HemaSphere is published by John Wiley & Sons Ltd on behalf of European Hematology Association. This is an open access Abstract Book distributed under the Attribution-NonCommercial-NoDerivs (CC BY-NC-ND), which allows third parties to download the articles and share them with others as long as they credit the author and the Abstract Book, but they cannot change the content in any way or use them commercially.
**Abstract Book Citations:** Authors, Title, HemaSphere, 2026;10:(S1):pages. The abstract book DOIs can be found on the "Author Information" page.
**Disclaimer:** Articles published in the journal HemaSphere exclusively reflect the opinions of the authors. The authors are responsible for all content in their abstracts including accuracy of the facts, statements, citing resources, etc.

### (PF570) CEBPB COUPLES B-CELL RECEPTOR SIGNALING TO CLL CELL PROLIFERATION AND SURVIVAL

**Topic:** 05. Chronic lymphocytic leukemia and related disorders - Biology & translational research

Josef Vecera*[1, 2], Lucie Rolkova[2, 3], Miroslav Boudny[1, 2], Daniel Filip[1, 2], Krystof Hlavac[1, 2, 3], Michaela Medkova[1, 2, 3], Sarka Pospisilova[1], Kvetoslava Matulova[4], Leos Kren[4], Anna Panovská[1], Michael Doubek[1], Marek Mraz[1, 2]

*[1]University Hospital Brno, Dept of Internal Medicine, Hematology and Oncology, Brno, Czechia;[2]Masaryk University, Central European Institute of Technology, Brno, Czechia; [3]Masaryk University, Faculty of Science, Brno, Czechia;[4]University Hospital Brno, Dept of Pathology, Brno, Czechia;*

### Background

BCR signaling in CLL microenvironment is one of the key drivers of proliferation and survival. However, it is not fully known how BCR activation triggers massive gene expression changes resulting in CLL cells' proliferation and survival.

### Aims

To identify a key transcription factor (TF) involved in signaling pathways essential for the survival and/or proliferation of CLL cells in the microenvironment and test potential of its therapeutic inhibition.

### Methods

ChIP-seq and RNA-seq analysis from BCR-stimulated primary CLL cells (bead-bound anti-IgM, 250 ng/10e6 cells). Gene editing and pharmacological inhibition of CEBPB performed in proliferating CLL cells using Cas9-gRNA ribonucleoprotein complex and antagonistic peptide lucicebtide, respectively.

### Results

Re-analysis of transcriptomic data from CLL samples isolated from peripheral blood (PB) and lymph nodes (LNs) (n=29 pairs, Sun et al., 2023) revealed CEBPB as the most significant transcriptional regulator of genes activated in the LN microenvironment (IPA, **Fig. A**). Notably, the increased expression of the mRNAs regulated by CEBPB associated with worse prognosis in CLL (**Fig. B**). The analysis of CXCR/CD5 intraclonal CLL subpopulations (n=10 pairs) showed enrichment of transcriptionally active p-CEBPB (Thr235) in proliferative CXCR4$^{dim}$CD5$^{bright}$ cells (n=8; **Fig. C**), and p-CEBPB protein levels were clearly detected in CLL pseudofollicles in LNs (IHC, **Fig. D**). We tested a panel of 7 microenvironmental factors (anti-IgM, CD40L, IL4, IL6, IFNa/g, CpG-ODN), revealing that BCR activation (bead-bound anti-IgM) was the strongest inducer of CEBPB phosphorylation and expression (**Fig. E)**. CEBPB overexpression in primary CLL cells (via transfection of plasmid) induced expression of genes activated in LN-resident CLL cells and involved in CLL proliferation and survival (MYC, NFKB-P50, BCL2 family; at both mRNA and protein levels; **Fig. F top, G**). ChIPseq for CEBPB revealed its direct binding to the regulatory regions of such genes, especially after BCR activation (6 hrs, **Fig. F bottom**). Notably, *CEBPB* silencing (siRNA in primary CLL, n=5) demonstrated that BCR-induced C-Myc expression is dependent on CEBPB (**Fig. H**), suggesting that CEBPB directly regulates this key cell proliferation driver. Furthermore, we observed that *CEBPB* knockdown (transfection of Cas9-gRNA ribonucleoprotein complex) in proliferating primary CLL cells (after pro-proliferative stimulation with anti-IgM, CD40L, IL4, IL21, CpG) leads to a decrease in C-Myc (n=6), impaired proliferation (n=10), and reduced viability (n=11; **Fig. I, J**). Finally, we evaluated blocking CEBPB function with the antagonist peptide lucicebtide, which is clinically tested in glioblastoma (trial NCT04478279). Lucicebtide strongly reduced CLL cell proliferation (n=10) and viability in dose dependent manner (n=11; **Fig. K**) and decreased C-Myc, NFKB-p50, and BCL2 family proteins (n=3 for each;**Fig. L**).

**Copyright Information:** (Online) ISSN: 2572-9241
©2026 The Author(s). HemaSphere is published by John Wiley & Sons Ltd on behalf of European Hematology Association. This is an open access Abstract Book distributed under the Attribution-NonCommercial-NoDerivs (CC BY-NC-ND), which allows third parties to download the articles and share them with others as long as they credit the author and the Abstract Book, but they cannot change the content in any way or use them commercially.
**Abstract Book Citations:** Authors, Title, HemaSphere, 2026;10:(S1):pages. The abstract book DOIs can be found on the “Author Information” page.
**Disclaimer:** Articles published in the journal HemaSphere exclusively reflect the opinions of the authors. The authors are responsible for all content in their abstracts including accuracy of the facts, statements, citing resources, etc.

### Summary/Conclusion

We identify CEBPB as a transcriptional factor activated by BCR signaling in CLL cells. CEBPB is a key driver of gene expression differences in lymph node-resident CLL cells and is involved in their proliferation and survival by inducing C-Myc, BCL2 family, and NFkB activity. CEBPB can be inhibited by antagonist peptide lucicebtide in therapeutically-relevant concentrations.

Supported by the Ministry of Health of the Czech Republic (NU22-03-00117, NW24-03-00369), Czech Science Foundation (25-15368X), MH CZ-DRO (FNBr,65269705), MUNI/A/1733/2025.

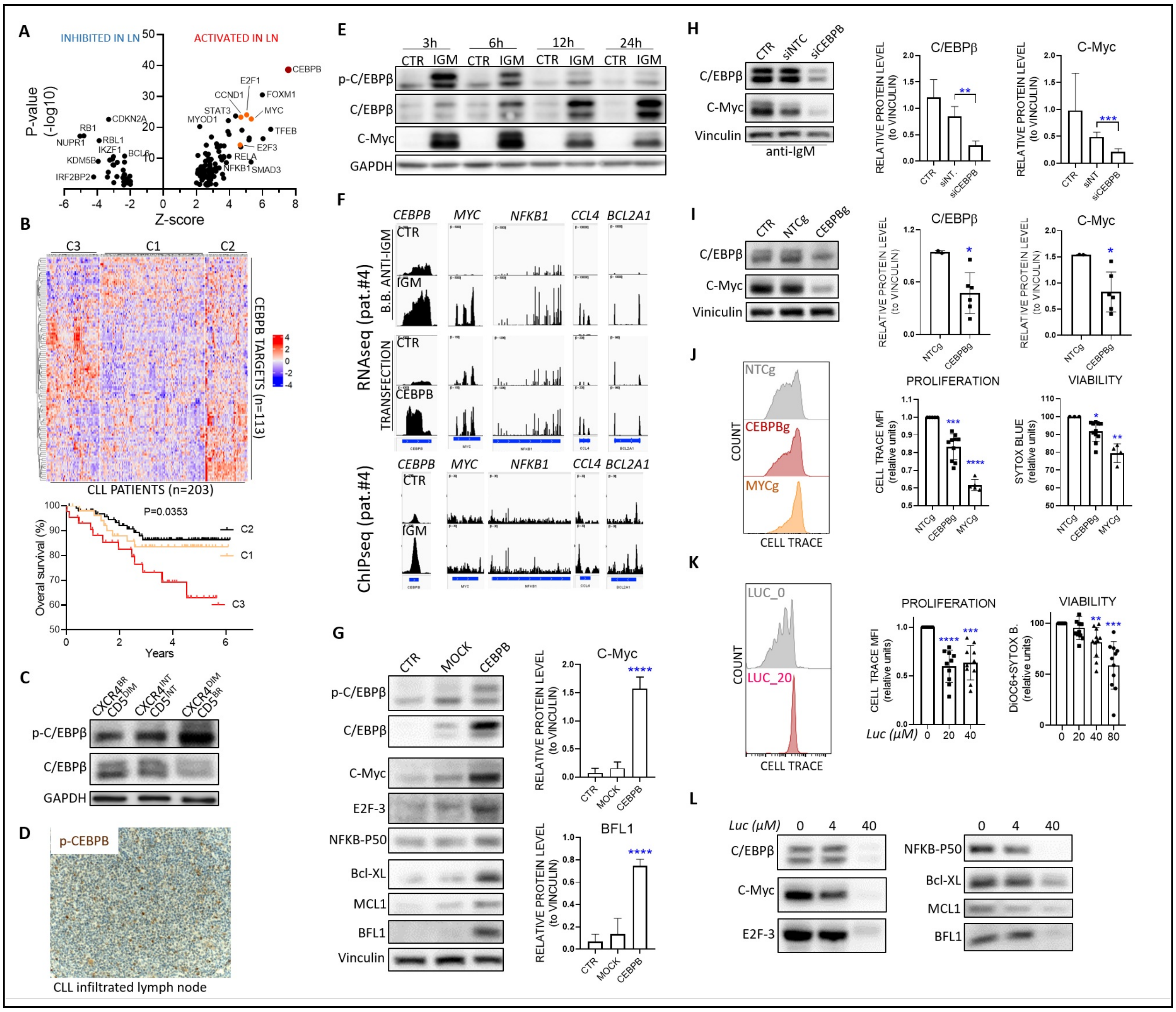

**Copyright Information:** (Online) ISSN: 2572-9241
©2026 The Author(s). HemaSphere is published by John Wiley & Sons Ltd on behalf of European Hematology Association. This is an open access Abstract Book distributed under the Attribution-NonCommercial-NoDerivs (CC BY-NC-ND), which allows third parties to download the articles and share them with others as long as they credit the author and the Abstract Book, but they cannot change the content in any way or use them commercially.
**Abstract Book Citations:** Authors, Title, HemaSphere, 2026;10:(S1):pages. The abstract book DOIs can be found on the "Author Information" page.
**Disclaimer:** Articles published in the journal HemaSphere exclusively reflect the opinions of the authors. The authors are responsible for all content in their abstracts including accuracy of the facts, statements, citing resources, etc.

### (PF571) MYC OVEREXPRESSION DRIVES POOR PROGNOSIS AND DIFFERENTIAL SENSITIVITY TO TREATMENTS ACCORDING TO TP53 STATUS IN CHRONIC LYMPHOCYTIC LEUKEMIA

**Topic:** 05. Chronic lymphocytic leukemia and related disorders - Biology & translational research

Alba Garrote-de-Barros*[1, 2], Juan Pérez-Fernández[2], Andrés Arroyo[1], Irene Bragado-García[2], Roberto Garcia-Vicente[1], Raquel Ancos[1, 2], Maria Velasco Estevez[3], María Linares[1, 2, 4], Joaquín Martinez-Lopez[1, 3, 4, 5], María Hernández-Sánchez[1, 2, 4]

*[1]Instituto de Investigación Hospital 12 de Octubre, Hematological Malignancies Clinical Research Unit H12O-CNIO, Madrid, Spain; [2]Universidad Complutense de Madrid, Department of Biochemistry and Molecular Biology, Pharmacy School, Madrid, Spain; [3]Cnio - Centro Nacional de Investigaciones Oncologicas, Hematological Malignancies Clinical Research Unit H12O-CNIO, Madrid, Spain; [4]Co-Senior authors, Madrid, Spain; [5]Universidad Complutense de Madrid, Department of Medicine, Medicine School, Madrid, Spain;*

### Background

*MYC* and *TP53* are well-established individual drivers in chronic lymphocytic leukemia (CLL), linked to poor prognosis and a higher risk of Richter Transformation. However, the effects of the concurrence of *MYC* overexpression and *TP53* dysfunction in CLL and their impact on drug response remain poorly understood.

### Aims

To evaluate the clinical and biological impact of *MYC* activation according to *TP53* status in CLL patients as well as to analyze the sensitivity to drugs using cellular CLL models harboring these alterations.

### Methods

We analyzed 566 CLL patients (CLLmap database) with clinical and transcriptomic and genomic data. The patients were stratified by *MYC* expression (MaxStat). Regression models were performed to analyze the correlation of MYC activation and prognostic variables. Overall survival (OS) and time to first treatment (TFT) were evaluated in the whole cohort as well as for those with *TP53* alteration (mutation and/or deletion) and *TP53* wild-type (WT).

*MYC* overexpression ($MYC^{OE}$) was induced using CRISPR/SAM in CLL cell lines: MEC-1 ($TP53^{del/mut}$) and PGA-1 ($TP53^{WT}$). RNA-seq was performed to assess *MYC*-driven transcriptomic changes in both contexts. Alternatively, both alterations ($MYC^{cOE}$ by cDNA and $TP53^{KD}$ by shRNA) were introduced in PGA-1 to validate findings. Mechanistic validation was performed by qPCR, western blot (WB) and proliferation assays. Drug sensitivity to the current therapies (BTK or BCL2 inhibitors) as well as a library of 203 compounds were also performed in these cellular models.

**Copyright Information:** (Online) ISSN: 2572-9241
©2026 The Author(s). HemaSphere is published by John Wiley & Sons Ltd on behalf of European Hematology Association. This is an open access Abstract Book distributed under the Attribution-NonCommercial-NoDerivs (CC BY-NC-ND), which allows third parties to download the articles and share them with others as long as they credit the author and the Abstract Book, but they cannot change the content in any way or use them commercially.
**Abstract Book Citations:** Authors, Title, HemaSphere, 2026;10:(S1):pages. The abstract book DOIs can be found on the “Author Information” page.
**Disclaimer:** Articles published in the journal HemaSphere exclusively reflect the opinions of the authors. The authors are responsible for all content in their abstracts including accuracy of the facts, statements, citing resources, etc.

**Results**

High levels of *MYC* expression (*MYC*$^{high}$) were associated with poor prognosis in terms of TFT and OS (p=0.003 and p=0.0027, respectively), and was significantly correlated with *TP53* alterations (mutation and/or deletion) (p<0.001). Interestingly, within the *TP53*$^{WT}$ group, *MYC*$^{high}$ was associated with shorter OS and TFT (p=0.006 and p=0.018, respectively). Of note, multivariate analysis revealed *MYC*$^{high}$ was independently associated with increased risk of OS and TFT in *TP53*$^{WT}$ CLLs (p=0.006 and p=0.056, respectively). In contrast, *MYC*$^{high}$ was not associated with inferior OS or TFT in *TP53*$^{altered}$ patients.
Clinical differences could be related to increased proliferation rate when *MYC* is overexpressed in *TP53*$^{WT}$ CRISPR/SAM and cDNA/shRNA models (p=0.047 and p=0.0001). These results were in line with an overexpression of *CCNE1* (p=0.04) and downregulation of *CDKN1C* (p<0.001) as well as activation of ERK and AKT/mTOR pathways. Interestingly, we also observed an overexpression of *MECOM*, which has been associated with an activation of these biological processes, in *MYC*$^{high}$/*TP53*$^{WT}$ patients and *MYC*$^{OE}$/*TP53*$^{WT}$ *in vitro* models (p=0.016).
PGA-1-*MYC*$^{OE}$/*TP53*$^{KD}$ showed resistance to ibrutinib and venetoclax. These results were validated in MEC1-*MYC*$^{OE}$. The high-throughput screening of drugs revealed that *MYC*$^{OE}$/*TP53*$^{KD}$ models were sensitive to glycolysis inhibitors, that can be related with a significant enrichment of glycolysis pathway in MEC1-*MYC*$^{OE}$.

**Summary/Conclusion**

*MYC* overexpression is linked to poor prognosis in CLL and serves an independent prognostic factor in *TP53*$^{WT}$ cases. These features may be driven by an activation of AKT/mTOR and *MECOM* overexpression, leading to increased proliferation. Notably, resistance to treatments such as ibrutinib or venetoclax observed in *MYC*$^{OE}$/*TP53*$^{altered}$ models could be overcome by metabolism modulation.

Funding: 2024/27 (CRIS Cancer), PID2023-149241OA-I00 (AEI); GdB, A (PIPF-2023/SAL-GL-31039)

**Copyright Information:** (Online) ISSN: 2572-9241
©2026 The Author(s). HemaSphere is published by John Wiley & Sons Ltd on behalf of European Hematology Association. This is an open access Abstract Book distributed under the Attribution-NonCommercial-NoDerivs (CC BY-NC-ND), which allows third parties to download the articles and share them with others as long as they credit the author and the Abstract Book, but they cannot change the content in any way or use them commercially.
**Abstract Book Citations:** Authors, Title, HemaSphere, 2026;10:(S1):pages. The abstract book DOIs can be found on the "Author Information" page.
**Disclaimer:** Articles published in the journal HemaSphere exclusively reflect the opinions of the authors. The authors are responsible for all content in their abstracts including accuracy of the facts, statements, citing resources, etc.

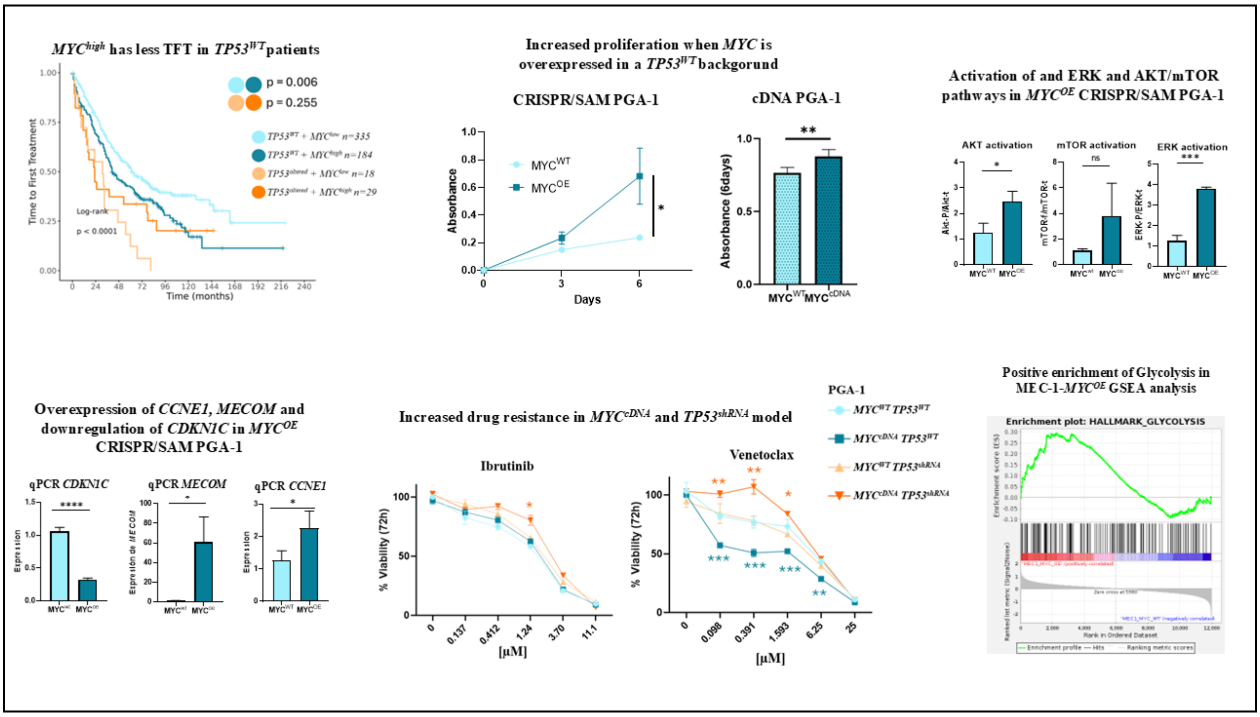

**Copyright Information:** (Online) ISSN: 2572-9241
©2026 The Author(s). HemaSphere is published by John Wiley & Sons Ltd on behalf of European Hematology Association. This is an open access Abstract Book distributed under the Attribution-NonCommercial-NoDerivs (CC BY-NC-ND), which allows third parties to download the articles and share them with others as long as they credit the author and the Abstract Book, but they cannot change the content in any way or use them commercially.
**Abstract Book Citations:** Authors, Title, HemaSphere, 2026;10:(S1):pages. The abstract book DOIs can be found on the “Author Information” page.
**Disclaimer:** Articles published in the journal HemaSphere exclusively reflect the opinions of the authors. The authors are responsible for all content in their abstracts including accuracy of the facts, statements, citing resources, etc.

### (PF572) BIOLOGICAL DETERMINANTS OF CLL MRD RESPONSE KINETICS WITH IBRUTINIB-VENETOCLAX IN THE FLAIR TRIAL

**Topic:** 05. Chronic lymphocytic leukemia and related disorders - Biology & translational research

Andy Rawstron*[1], Surita Dalal[1, 2], Sean Girvan[3], David Cairns[3], Nichola Webster[1, 2], Jane Shingles[1], Ruth Detute[1], Charlotte Evans[1, 2], Darren Newton[2], Sue Bell[3], Natasha Greatorex[3], Sharon Jackson[3], David Stones[3], David Allsup[4], Adrian Bloor[5, 6], Anita Sarma[1], Abraham Varghese[7], Piers Patten[8, 9], Peter Hillmen[7], Talha Munir[7]

*[1]Haematological Malignancy Diagnostic Service, Leeds Cancer Centre, Leeds Teaching Hospitals NHS Trust, Leeds, United Kingdom; [2]Leeds Institute of Medical Research, University of Leeds, Leeds, United Kingdom;[3]Leeds Cancer Research UK Clinical Trials Unit, University of Leeds, Leeds, United Kingdom; [4]Hull University Teaching Hospitals NHS Trust, Hull, United Kingdom; [5]The Christie Hospital NHS Foundation Trust, Manchester, United Kingdom;[6]University of Manchester, Manchester, United Kingdom; [7]Leeds Cancer Centre, Leeds Teaching Hospitals NHS Trust, Leeds, United Kingdom;[8]Comprehensive Cancer Centre, King's College London, London, United Kingdom; [9]King's College Hospital NHS Foundation Trust, London, United Kingdom;*

### Background

The FLAIR (ISRCTN01844152) phase III trial showed that MRD-guided ibrutinib-venetoclax (I+V) significantly improved response, progression-free survival, and overall survival compared with ibrutinib or FCR. Treatment duration was individualised, with end-of-treatment (EoT) defined as twice the time from randomisation to reaching uMRD4(<0.0.1%) in peripheral blood (PB) with confirmatory bone-marrow. Outcomes and response kinetics varied according to by immunoglobulin heavy chain variable region (IGHV) gene mutation status.

### Aims

To assess biological characteristics including IGHV mutation status, targeted gene mutations, and CLL immunophenotype to identify patients who respond rapidly versus those benefitting from extended treatment.

### Methods

260 participants received MRD-guided I+V. Baseline immunophenotyping (8-colour flow, 32 markers) in this analysis defines "atypical" as one or more ERIC-confirmed MRD markers overlapping with normal B-cell expression level.

Targeted sequencing of 33 recurrently mutated genes was performed using Illumina MiSeq with in-house analysis pipeline. IGHV status and subset assignment used IGHV leader PCR and bidirectional Sanger sequencing with IMGT/V-Quest and ARResT/AssignSubsets. MRD was assessed every 6 months by ERIC-compliant flow cytometry (up to 001%/uMRD5 sensitivity).

**Copyright Information:** (Online) ISSN: 2572-9241
©2026 The Author(s). HemaSphere is published by John Wiley & Sons Ltd on behalf of European Hematology Association. This is an open access Abstract Book distributed under the Attribution-NonCommercial-NoDerivs (CC BY-NC-ND), which allows third parties to download the articles and share them with others as long as they credit the author and the Abstract Book, but they cannot change the content in any way or use them commercially.
**Abstract Book Citations:** Authors, Title, HemaSphere, 2026;10:(S1):pages. The abstract book DOIs can be found on the "Author Information" page.
**Disclaimer:** Articles published in the journal HemaSphere exclusively reflect the opinions of the authors. The authors are responsible for all content in their abstracts including accuracy of the facts, statements, citing resources, etc.

**Results**

Among 260 I+V participants, 39.2% achieved PB uMRD4 by 9 months, 66.4% by 12–18 months, and 83.1% by 36 months. Response kinetics correlated with IGHV-mutation status: rapid (<9 months from baseline) PB uMRD4 occurred in 52% with 100% identity to IGHV germline (IGHV-unmutated), 42.9% with 98.0–99.99% (IGHV-unmutated), 42.1% with 97.0–97.99% (borderline-mutated), while only 27.5% with <97% IGHV-germline identity (mutated) achieved early uMRD4. Delayed (12-18 month) uMRD4 responses occurred in ~30-40% across all IGHV strata. Sub-optimal (>36 month) MRD responses were uncommon in IGHV-unmutated CLL (9.6%) and highest in IGHV-mutated disease (32.5%). Stereotyped subset #2 (n=16) mirrored IGHV-unmutated kinetics. CLL immunophenotype (typical vs atypical) and TP53/ATM/SF3B1/NOTCH1 abnormalities were not associated with differences in MRD response kinetics. MYD88-mutated cases (6/8) showed sub-optimal clearance, but most were also IGHV-mutated. BIRC3- and BRAF-mutated disease demonstrated rapid responses (>50% within 9 months), with BRAF linked to unmutated IGHV, while responses in BIRC3-mutated disease appeared independent of IGHV status.

**Summary/Conclusion**

Time to uMRD response with MRD-guided I+V is strongly influenced by IGHV mutation status. There is no association with TP53/ATM mutation status or immunophenotype. Unmutated IGHV, subset #2, borderline-mutated IGHV, and BIRC3-mutated disease appear best suited to MRD-guided strategies. These data support biologically informed personalisation of treatment duration in future CLL trials.

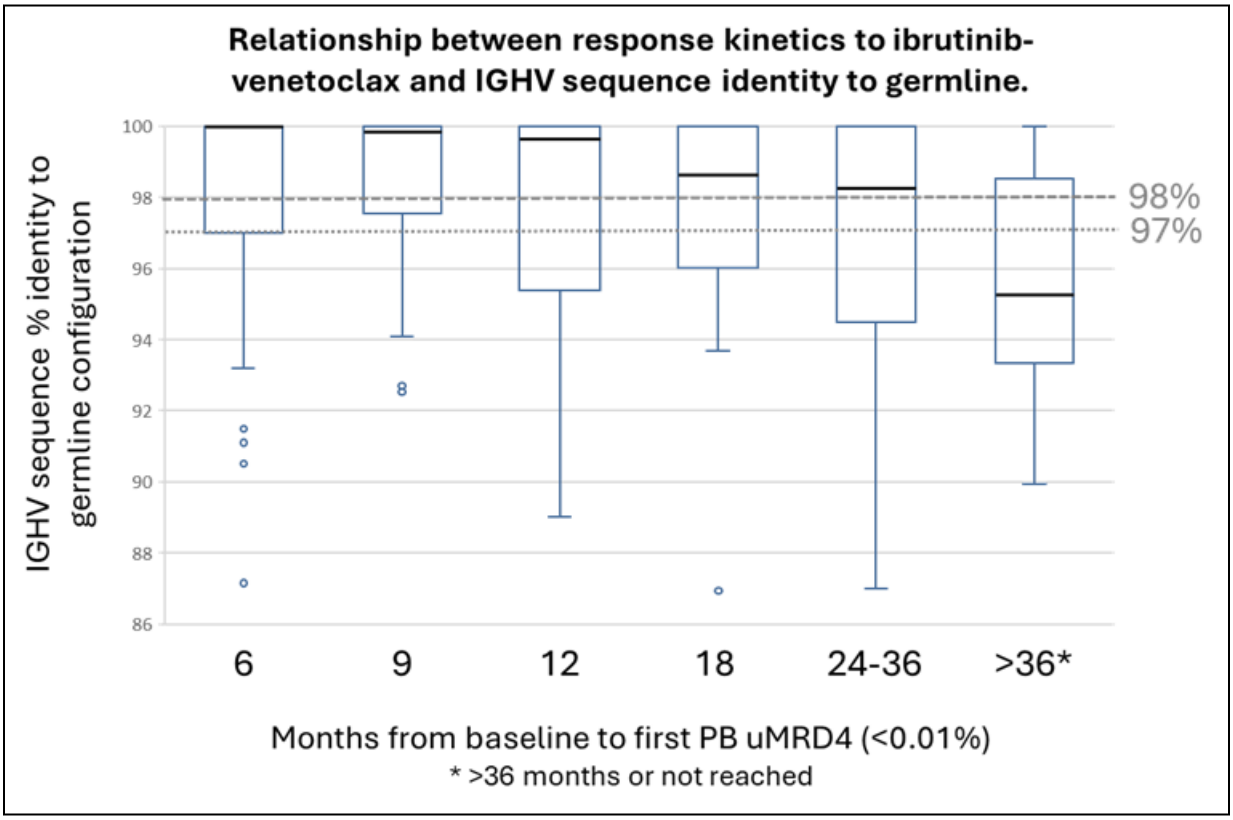

**Copyright Information:** (Online) ISSN: 2572-9241
©2026 The Author(s). HemaSphere is published by John Wiley & Sons Ltd on behalf of European Hematology Association. This is an open access Abstract Book distributed under the Attribution-NonCommercial-NoDerivs (CC BY-NC-ND), which allows third parties to download the articles and share them with others as long as they credit the author and the Abstract Book, but they cannot change the content in any way or use them commercially.
**Abstract Book Citations:** Authors, Title, HemaSphere, 2026;10:(S1):pages. The abstract book DOIs can be found on the "Author Information" page.
**Disclaimer:** Articles published in the journal HemaSphere exclusively reflect the opinions of the authors. The authors are responsible for all content in their abstracts including accuracy of the facts, statements, citing resources, etc.

### (PF573) UNCOVERING THE CHARACTERISTICS OF ACCELERATED CLL THROUGH CLINICAL AND SINGLE-CELL RNA-SEQ ANALYSIS

**Topic:** 05. Chronic lymphocytic leukemia and related disorders - Biology & translational research

Enrico Gaffo[1], Francesco Angotzi*[2], Alessandra Carpanese[1], Chiara Adele Cavarretta[2], Federico Scarmozzino[3], Alessandro Cellini[2], Andrea Serafin[2], Arianna Bevilacqua[2], Nicolò Danesin[2], Giovanni Leone[2], Federica Frezzato[2], Monica Facco[2], Elisa Pagnin[2], Annalisa Martines[4], Laura Bonaldi[4], Marco Pizzi[3], Francesco Piazza[2], Livio Trentin[2], Stefania Bortoluzzi[1], Andrea Visentin[2]

*[1]University of Padova, Computational Genomics Group, Department of Surgery, Oncology and Gastroenterology, Padova, Italy; [2]Hematology Unit, University of Padova, Department of Medicine (DIMED), Padova, Italy; [3]Pathology Unit, University of Padova, Department of Medicine (DIMED), Padova, Italy; [4]Veneto Institute of Oncology IOV-IRCCS, Immunology and Molecular Oncology Unit, Padova, Italy;*

### Background

Accelerated chronic lymphocytic leukaemia (aCLL) is a clinically and biologically aggressive form of CLL, positioned between classic CLL (cCLL) and Richter transformation (RT).

### Aims

We aimed to characterise the clinical and molecular features of aCLL relative to those of CLL and RT.

### Methods

We enrolled 203 patients: 32 (16%) with aCLL, 16 (8%) with RT, and 155 (76%) with cCLL, having clinical and histological data retrieved from medical records. All aCLL and RT cases were confirmed by histologic review. We performed single-cell RNA-seq (10x Genomics) of lymph nodes from 10 aCLL patients, 3 cCLL patients, and 3 RT patients. The Seurat R package was used for data analysis.

**Copyright Information:** (Online) ISSN: 2572-9241
©2026 The Author(s). HemaSphere is published by John Wiley & Sons Ltd on behalf of European Hematology Association. This is an open access Abstract Book distributed under the Attribution-NonCommercial-NoDerivs (CC BY-NC-ND), which allows third parties to download the articles and share them with others as long as they credit the author and the Abstract Book, but they cannot change the content in any way or use them commercially.
**Abstract Book Citations:** Authors, Title, HemaSphere, 2026;10:(S1):pages. The abstract book DOIs can be found on the “Author Information” page.
**Disclaimer:** Articles published in the journal HemaSphere exclusively reflect the opinions of the authors. The authors are responsible for all content in their abstracts including accuracy of the facts, statements, citing resources, etc.

**Results**

Compared with cCLL, aCLL was enriched for both aggressive clinical features (lymphadenopathies >5cm, constitutional symptoms, extranodal disease) and adverse prognostic molecular markers, such as U-IGHV status and TP53 disruption. Accordingly, aCLL also showed a shorter OS than cCLL (median 63 vs 177 months; p=0.002), but still longer than RT (median 7.5 months; p <0.001; Fig. A). Among patients treated with BTKi, aCLL displayed shorter survival than cCLL (median 7.98 months vs NR; HR: 3.43; 95% CI: 1.2-9.8; p=0.01).
Regarding gene expression, aCLL lymph node samples showed a distinct expression profile, intermediate between that of cCLL and RT. After unsupervised clustering of single-cell data, we identified 26 distinct cell subpopulations, of which 12 were significantly enriched in a single condition (L2FC >2; p <0.001; Fig. B). RT was characterized by three germinal center B-cell clusters accounting for 20%, 17%, and 6% of total RT cells, which showed high expression of BCL2-family genes. aCLL was enriched in three memory-B-cell clusters, one naïve-B-cell cluster, and a distinct immature-B-cell cluster marked by high BTK expression, accounting for 27% of the overall aCLL cells. cCLL was mainly enriched in a cluster of naive B cells (26% cCLL cells) and one of memory B cells (0.8%). RT B-cell clusters showed the highest fraction of proliferating cells (~75% in G2M/S phase), compared with those of aCLL (~40%) and cCLL (<25%).
Notable differences between the three conditions were also observed in the microenvironmental composition, with a progressive increase in aCLL and RT compared to cCLL of T-CD4+ central memory cells (3%, 6%, 8%), T-γδ (0.3%, 1%, 2%), and of T-CD8+ (0.1%, 0.2%, 4%). Monocytes were instead significantly enriched in aCLL (0.7%).
Disease-specific cell cluster hallmark gene set enrichment further supported a trajectory across the tree groups. cCLL showed significant enrichment limited to the unfolded protein response and TNFα–NF-κB signalling, consistent with low-grade proteostatic stress without broad proliferative engagement. aCLL exhibited instead dominant interferon, inflammatory, and cytokine-driven programs, indicative of heightened microenvironmental stimulation and immune activation. RT shifted toward MYC/E2F-driven proliferation, oxidative metabolism, and stress-adaptation circuits, reflecting the behaviour of high-grade lymphoma.

**Summary/Conclusion**

Our integrated clinical, pathological, and molecular analyses highlight aCLL as a distinct entity along the spectrum from cCLL to RT. Its unique expression profile and evolving microenvironment suggest a role in disease transformation, providing a basis for improved therapies and deeper insight into pathways driving aCLL progression.

**Copyright Information:** (Online) ISSN: 2572-9241
©2026 The Author(s). HemaSphere is published by John Wiley & Sons Ltd on behalf of European Hematology Association. This is an open access Abstract Book distributed under the Attribution-NonCommercial-NoDerivs (CC BY-NC-ND), which allows third parties to download the articles and share them with others as long as they credit the author and the Abstract Book, but they cannot change the content in any way or use them commercially.
**Abstract Book Citations:** Authors, Title, HemaSphere, 2026;10:(S1):pages. The abstract book DOIs can be found on the "Author Information" page.
**Disclaimer:** Articles published in the journal HemaSphere exclusively reflect the opinions of the authors. The authors are responsible for all content in their abstracts including accuracy of the facts, statements, citing resources, etc.

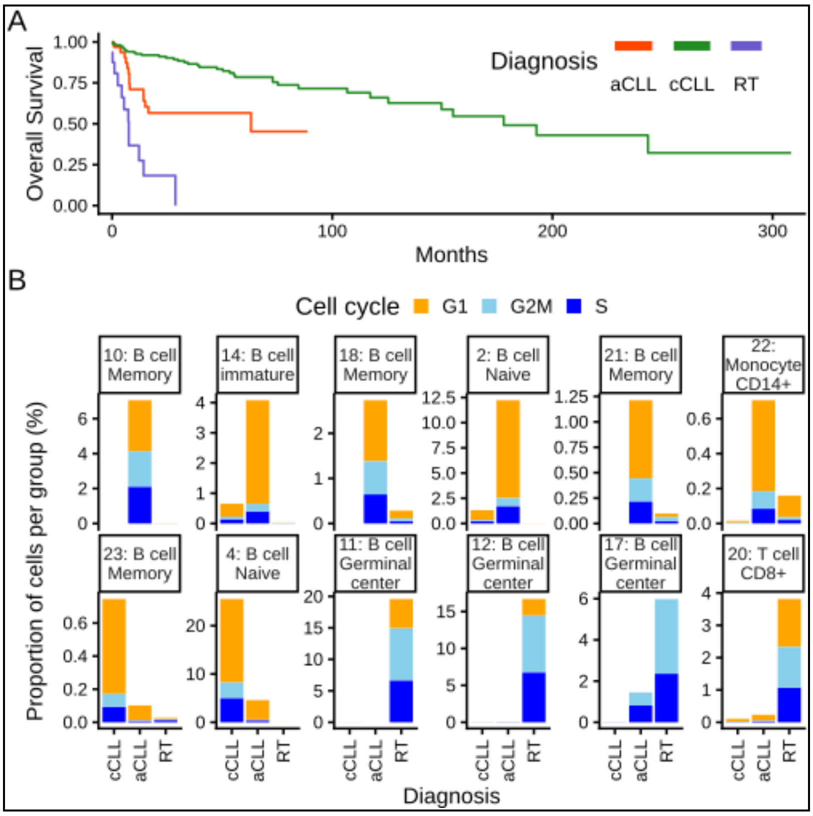

Figure 1: A) Kaplan-Meier curves for OS for the three conditions; B) Cell-cycle inference for each cell cluster unique of every condition, depicting the proportion of cells in various stages of the cell cycle

**Copyright Information:** (Online) ISSN: 2572-9241
©2026 The Author(s). HemaSphere is published by John Wiley & Sons Ltd on behalf of European Hematology Association. This is an open access Abstract Book distributed under the Attribution-NonCommercial-NoDerivs (CC BY-NC-ND), which allows third parties to download the articles and share them with others as long as they credit the author and the Abstract Book, but they cannot change the content in any way or use them commercially.
**Abstract Book Citations:** Authors, Title, HemaSphere, 2026;10:(S1):pages. The abstract book DOIs can be found on the "Author Information" page.
**Disclaimer:** Articles published in the journal HemaSphere exclusively reflect the opinions of the authors. The authors are responsible for all content in their abstracts including accuracy of the facts, statements, citing resources, etc.

### (PF575) 3D CLL SPHEROID MODEL RECAPITULATES LYMPH NODE PHENOTYPES AND IN VIVO RESPONSES TO IBRUTINIB; A SINGLE-CELL CHARACTERIZATION

**Topic:** 05. Chronic lymphocytic leukemia and related disorders - Biology & translational research

Marco Haselager[1], Danial Lashgari[1], Aldo Jongejan[2], Jamie Lee Roos[1], Julie Dubois[1], Fleur Peters[1], Arnon Philip Kater[1], Perry Moerland[1], Eric Eldering*[1]

*[1]Amsterdam UMC, Experimental Immunology & Hematology, Amsterdam, The Netherlands;[2]Amsterdam UMC, Epidemiology and Data Science, Amsterdam, The Netherlands;*

### Background

We recently developed the first in vitro 3D chronic lymphocytic leukemia (CLL) culture model that also incorporates T cells and myeloid cells in a spatially organized, lymph node (LN)-mimicking context (Haselager et al., In Vitro 3D Spheroid Culture System Displays Sustained T Cell-dependent CLL Proliferation and Survival, 2023). This model uniquely allows parallel investigation of CLL proliferation and drug response/resistance. Ibrutinib is clinically successful, however patients frequently experience relapse, suggesting that residual tumor cells may be protected at LN sites and subsequently drive disease progression. An unanswered question is what happens in the LN-residing CLL cells upon ibrutinib treatment. Another drawback is that many signals that drive CLL activation, proliferation and drug resistance are not recapitulated in standard in vitro models that are based on peripheral blood (PB) sampling.

### Aims

In the current study, we aimed to bridge the gap between in vitro and in vivo studies by investigating to what extent the 3D model mimics the in vivo LN and response to ibrutinib.

### Methods

We performed single-cell RNA sequencing (scRNA-seq) on 8 patient-derived CLL spheroids and paired PB and LN samples. First, we directly compared the 3D CLL model and corresponding ibrutinib response to matched patient samples and their in vivo response to ibrutinib. Next, we utilize the 3D model to investigate the previously established 'activated' and proliferating CLL fractions and their response to ibrutinib. Third, we investigate the divergent ibrutinib responses observed in the 3D model between PB and LN counterparts and characterize T cells and their functional implications.

### Results

Comparison of the in vitro ibrutinib response in spheroids to the ibrutinib response in patients demonstrated that key molecular features were are also reflected in vitro. CLL spheroids amplified the proportion of the clinically relevant LN niches – resting, activated, proliferating – making these more amenable to molecular characterization. Furthermore, these three known CLL fractions showed distinct ibrutinib responses. Ibrutinib treatment shifted resting and proliferating cells towards a more resting phenotype, yet activated CLL cells showed upregulation of TLR9-MALT1-MYC signaling, suggesting emergent resistance mechanisms. Lastly, 11 T cell subsets were identified, collectively demonstrating a T cell-dependent role in promoting CLL proliferation, even in the presence of ibrutinib.

**Copyright Information:** (Online) ISSN: 2572-9241
©2026 The Author(s). HemaSphere is published by John Wiley & Sons Ltd on behalf of European Hematology Association. This is an open access Abstract Book distributed under the Attribution-NonCommercial-NoDerivs (CC BY-NC-ND), which allows third parties to download the articles and share them with others as long as they credit the author and the Abstract Book, but they cannot change the content in any way or use them commercially.
**Abstract Book Citations:** Authors, Title, HemaSphere, 2026;10:(S1):pages. The abstract book DOIs can be found on the "Author Information" page.
**Disclaimer:** Articles published in the journal HemaSphere exclusively reflect the opinions of the authors. The authors are responsible for all content in their abstracts including accuracy of the facts, statements, citing resources, etc.

**Summary/Conclusion**

The current 3D spheroid system represents key features of the CLL LN setting, using PB cells as source. The comprehensive single-cell dataset reveals dynamic transitions between resting, activated, and proliferating CLL phenotypes and identifies the activated CLL fraction as inherently ibrutinib-insensitive niche. Its unique transcriptional adaptations may be leveraged to guide future therapeutic interventions overcoming microenvironment-mediated resistance. A promising new option is to apply AI-driven morphological analyses of spheroids under distinct drug regimens.

**Copyright Information:** (Online) ISSN: 2572-9241
©2026 The Author(s). HemaSphere is published by John Wiley & Sons Ltd on behalf of European Hematology Association. This is an open access Abstract Book distributed under the Attribution-NonCommercial-NoDerivs (CC BY-NC-ND), which allows third parties to download the articles and share them with others as long as they credit the author and the Abstract Book, but they cannot change the content in any way or use them commercially.
**Abstract Book Citations:** Authors, Title, HemaSphere, 2026;10:(S1):pages. The abstract book DOIs can be found on the “Author Information” page.
**Disclaimer:** Articles published in the journal HemaSphere exclusively reflect the opinions of the authors. The authors are responsible for all content in their abstracts including accuracy of the facts, statements, citing resources, etc.

### (PF576) REVISITING COMPLEX KARYOTYPE IN CHRONIC LYMPHOCYTIC LEUKEMIA PATIENTS TREATED WITH CONTINUOUS BTK INHIBITION: A MULTICENTER ERIC STUDY

**Topic:** 05. Chronic lymphocytic leukemia and related disorders - Biology & translational research

Anna Puiggros*[1], Thomas Chatzikonstantinou[2], Persefoni Talimtzi[2], Ahmad Alhuraiji[3], Aseel Alsouqi[4], Maria Angelopoulou[5], Darko Antic[6], Anastasia Athanasiadou[7], Amneet Bajwa[4], Sandra Bašić Kinda[8], Leonidas Benetatos[9], Jennifer R. Brown[10], Elise Chapiro[11], Anastasia Chatzidimitriou[2], Sofia Chatzileontiadou[12], Rosa Collado Nieto[13], Dolors Costa[14], Marta Crespo Maull[15], Antonio Cuneo[16], Ana Carla De Oliveira[17], Maria Dimou[18], Michael Doubek[19], José Joaquin Duarte[20], Maria Efstathopoulou[21], Lucia Farina[22], Anna Maria Frustaci[23], José Antonio García Vela[24], Rocío García-Serra[25], Gloria Gaudio[26], Eva Gimeno[27], Isabel Teresa González Gascón Y Marín[28], Isabel Granada[29], Evdoxia Chatzicharisi[30], Eleftheria Hatzimichael[9], José-Angel Hernández-Rivas[28], Marta Julia Arenas[31], Joanna Kamaso[1], Elżbieta Kalicińska[32], Maria Kislova[33], Dorota Koczkodaj[34], Ioannis Kotsianidis[35], Andreas-Epameinondas Koumpis[9], Anjana Kumar Seri[20], Ioannis Kyriakou[7], Alexandros MacHairas[5], Francesca Martini[36], Kiyomi Mashima[10], Bargoin Maxence[37], Emine Merve Savas[38], Cecelia Miller[39], Eva Minga[2], Marta Morawska[40], Pablo Javier Mozas Fernandez[41], Florence Nguyen-Khac[42], Alessandro Noto[23], Eva Ondrouskova[43], Margarita Ortega[15], MacArena Ortiz Pareja[20], Gerassimos Pangalis[44], Sarka Pavlova[45], Ioannis Papadopoulos[7], Juan A. Piñeyroa[41], Helena Podgornik[46], Sarka Pospisilova[45], Steven Richebourg[47], Gian Matteo Rigolin[48], Ilaria Romano[49], Davide Rossi[49], Fazla Saheed[50], Marta Salido[1], Alessandro Sanna[51], Lydia Scarfò[52], Angel Serna Pareja[15], Irma Slavutsky[53], Basile Stamatopoulos[54], Niki Stavrogianni[7], Isolde Summerer[55], Tamar Tadmor[56], Samantha Teierle[39], Kristina Tomic[57], Fernanda Tosin[58], Livio Trentin[59], Theodoros Vassilakopoulos[5], Lauren Veronese[60], Andrea Visentin[59], Georgios Vrachiolias[35], Ewa Wasik-Szczepanek[61], Jennifer Woyach[4], Seyma Yildiz[38], Paolo Ghia[52], Kostas Stamatopoulos[2], Blanca Espinet Sola[1]

*[1]Molecular Cytogenetics Laboratory, Pathology Department, Hospital del Mar and Translational Research on Hematological Neoplasms Group, Hospital del Mar Research Institute (HMRIB), Barcelona, Spain; [2]Institute of Applied Biosciences, Centre for Research and Technology Hellas, Thessaloniki, Greece; [3]Department of Translational research, Dasman Diabetes Institute, Kuwait, Kuwait, Kuwait; [4]Division of Hematology, The Ohio State University, Ohio, United States of America;[5]Department of Haematology, University of Athens, Laikon General Hospital, Athens, Greece; [6]Clinic for Hematology, University Clinical Center of Serbia, Faculty of Medicine University of Belgrade, Belgrade, Serbia; [7]Hematology Department and HCT Unit, G. Papanicolaou Hospital, Thessaloniki, Greece; [8]Divison of Hematology, Department of Internal Medicine, University Hospital Centre Zagreb, School of Medicine, University of Zagreb, Zagreb, Croatia; [9]Department of Haematology, Faculty of Medicine, University of Ioannina, Ioannina, Greece; [10]Department of Medicine, Dana-Farber Cancer Institute, Boston, United States of America; [11]Pitié-Salpêtrière University Hospital (AP-HP), Paris, France; [12]Hematology Unit, 1st Dept of Internal Medicine, AUTH, AHEPA Hospital, Thessaloniki, Greece, Thessaloniki, Greece; [13]Servicio de Hematología, Consorcio Hospital General Universitario de Valencia. Fundación de Investigación Hospital General Universitario de Valencia, Valencia, Spain; [14]Hematopatology section, Department of Pathology, Hospital Clínic Barcelona, Barcelona, Spain; [15]Department of Hematology, Vall d'Hebron University Hospital, Experimental Hematology, Vall d'Hebron Institute of Oncology (VHIO), Vall d'Hebron Barcelona Hospital Campus, and Department of Medicine, Universitat Autònoma de Barcelona, Barcelona, Spain; [16]Hematology-Department of Medical Sciences, University of Ferrara, Ferrara, Italy, Ferrara, Italy; [17]Department of Clinical Hematology, Lymphoma Unit, ICO-Hospital Duran i Reynals, IDIBELL, Barcelona, Spain; [18]Department of Haematology, University of Athens, Laikon General Hospital, Athens, Greece, Athens, Greece; [19]Department of Internal Medicine, Hematology and Oncology, University Hospital Brno, and Centre for Molecular Medicine, Central European Institute of Technology, Masaryk University, Brno, Czechia; [20]Department of Hematology and Hemotherapy, Regional University Hospital of Málaga, Malaga, Spain; [21]Department of Haematology, Athens Medical Center, Athens, Greece, Athens, Greece; [22]Fondazione IRCCS Istituto nazionale dei tumori, Milan, Italy; [23]ASST Grande Ospedale Metropolitano Niguarda - Niguarda Cancer Center, Milan, Italy; [24]Hospital Universitario Puerta de Hierro, Madrid, Spain; [25]Servicio de Hematología, Consorcio Hospital General Universitario de Valencia, Fundación de Investigación Hospital General Universitario de Valencia, Valencia, Spain; [26]Medical Genetics Department, EOLAB, Ente Ospedaliero Cantonale (EOC), Bellinzona, Switzerland; [27]Hematology Department, Hospital del Mar, Barcelona, Spain; [28]Department of Hematology, Hospital*

**Copyright Information:** (Online) ISSN: 2572-9241
©2026 The Author(s). HemaSphere is published by John Wiley & Sons Ltd on behalf of European Hematology Association. This is an open access Abstract Book distributed under the Attribution-NonCommercial-NoDerivs (CC BY-NC-ND), which allows third parties to download the articles and share them with others as long as they credit the author and the Abstract Book, but they cannot change the content in any way or use them commercially.
**Abstract Book Citations:** Authors, Title, HemaSphere, 2026;10:(S1):pages. The abstract book DOIs can be found on the "Author Information" page.
**Disclaimer:** Articles published in the journal HemaSphere exclusively reflect the opinions of the authors. The authors are responsible for all content in their abstracts including accuracy of the facts, statements, citing resources, etc.

Universitario Infanta Leonor, Universidad Complutense, Madrid, Madrid, Spain; [29]Department of hematology, Institut Català d'Oncologia (ICO)-Hospital Germans Trias i Pujol, and Institut de Recerca Contra la Leucèmia Josep Carreras (IJC), ICO-Hospital Germans Trias i Pujol, Universitat Autònoma de Barcelona, Badalona, Spain; [30]Hematology Unit, 1st Dept of Internal Medicine, AUTH, AHEPA Hospital, Thessaloniki, Greece; [31]Department of hematology,Institut Català d'Oncologia (ICO)-Hospital Germans Trias i Pujol, Badalona, Spain; [32]Department of Hematology, Cell Therapies, and Internal Diseases, Wroclaw Medical University, Wroclaw, Poland; [33]Botkin hospital, Moscow, Russia; [34]Department of Cancer Genetics with Cytogenetics Laboratory, Medical University of Lublin, Lublin, Poland; [35]Department of Hematology, University Hospital of Alexandroupolis, Democritus University of Thrace, Alexandroupolis, Greece; [36]Strategic Research Program on CLL, Comprehensive Cancer Center, IRCCS Ospedale San Raffaele, Milano, Italy., Milan, Italy; [37]Clermont-Ferrand University Hospital (CHU), Clermont-Ferrand, France; [38]Gazi Universtiy, Gazi, Türkiye; [39]Department of Pathology , The Ohio State University, Ohio, United States of America; [40]Experimental Hematooncology Department, Medical University of Lublin, Lublin, Poland;[41]Department of Hematology, Hospital Clínic Barcelona, Barcelona, Spain; [42]Sorbonne Université, Service d'Hématologie Clinique, Hôpital Pitié-Salpêtrière, APHP, Paris, France; [43]Department of Internal Medicine, Hematology and Oncology, University Hospital Brno and Medical Faculty, Masaryk University, Brno, Czechia; [44]Department of Haematology, Athens Medical Center, Psychikon Branch, Athens, Greece, Athens, Greece; [45]Department of Internal Medicine, Hematology and Oncology, University Hospital Brno and Medical Faculty, Masaryk University, and Institute of Medical Genetics and Genomics, University Hospital Brno and Medical Faculty, Masaryk University, and Central European Institute of Technology (CEITEC), Masaryk University, Brno, Czechia; [46]Department of Haematology, University Medical Centre Ljubljana, Ljubljana, Slovenia; [47]Brest University Hospital, Brest, France; [48]Department of Hematology, University of Ferrara, Ferrara, Italy; [49]Oncology Institute of Southern Switzerland, Bellinzona, Switzerland; [50]Kuwait Cancer Control Center (KCCC), Kuwait, Kuwait;[51]Azienda Ospedaliero-Universitaria Careggi (AOU Careggi), Firenze, Italy; [52]Strategic Research Program on CLL, Comprehensive Cancer Center, IRCCS Ospedale San Raffaele, and School of Medicine, Università Vita Salute San Raffaele, Milan, Italy; [53]Instituto de Medicina Experimental (IMEX), CONICET-Academia Nacional de Medicina (ANM), Buenos Aires, Argentina; [54]Translational Hematology and Oncology Research laboratory, Université Libre de Bruxelles (ULB), Jules Bordet Institute, Brussels, Belgium; [55]MLL Munich Leukemia Laboratory, Munich, Germany; [56]Hematology, Bnai Zion Medical Center and the Ruth and Bruce Rappaport Faculty of Medicine, Haifa, Israel, Haifa, Israel; [57]Clinic for Hematology, University Clinical Center of Serbia, Belgrade, Serbia;[58]Servicio de Hematologia, Hospital El Cruce, Buenos Aires, Argentina; [59]Hematology unit, Department of Medicine, University of Padova, Padova, Italy;[60]Department of Cytogenetics, Clermont-Ferrand University Hospital (CHU) and EA7453 CHELTER, Clermont Auvergne University, Clermont-Ferrand, France; [61]Department of Hematooncology and Bone Marrow Transplantation, Medical University in Lublin, Lublin, Poland;

### Background

Complex karyotype (CK; ≥3 abnormalities, abn) is a recognised adverse prognostic factor in patients with chronic lymphocytic leukemia (CLL) treated with chemoimmunotherapy (CIT). High-CK (≥5 abn) identifies patients with particularly poor outcomes, while those with 3-4 abn are clinically heterogeneous. The prognostic weight of CK in the context of targeted agents remains uncertain, and most available studies have not specifically addressed the biological and clinical heterogeneity within the CK. Therefore, the clinical relevance of CK in patients treated with BTK inhibitors (BTKi) warrants further clarification.

### Aims

1. To assess the impact of CK in patients with CLL treated with BTKi in front line (1L); 2. To evaluate whether the number (3-4 vs ≥5) and the type of cytogenetic abnormalities influence clinical outcomes.

Copyright Information: (Online) ISSN: 2572-9241
©2026 The Author(s). HemaSphere is published by John Wiley & Sons Ltd on behalf of European Hematology Association. This is an open access Abstract Book distributed under the Attribution-NonCommercial-NoDerivs (CC BY-NC-ND), which allows third parties to download the articles and share them with others as long as they credit the author and the Abstract Book, but they cannot change the content in any way or use them commercially.
Abstract Book Citations: Authors, Title, HemaSphere, 2026;10:(S1):pages. The abstract book DOIs can be found on the “Author Information” page.
Disclaimer: Articles published in the journal HemaSphere exclusively reflect the opinions of the authors. The authors are responsible for all content in their abstracts including accuracy of the facts, statements, citing resources, etc.

**Methods**

This is a retrospective international multicentre study conducted by ERIC, the European Research Initiative on CLL. Patients treated with BTKi in 1L and available pre-treatment karyotype were included. CK was defined as ≥3 chromosomal abn and further stratified into Low/int-CK (3-4) and High-CK (≥5). The impact of CK and the presence of unbalanced abnormalities (U-abn) [structural unbalanced alterations excluding isolated deletions (Rigolin et al, 2018)] on time to next treatment or death (TTNT) and overall survival (OS) was assessed.

**Results**

Overall, 1051 patients were included [median age 68y; male (659, 62.7%); IGHV-unmutated (IGHV-UM, 631/900, 70.1%), del(13q) (444/963, 46.1%), trisomy 12 (226/963, 23.5%), del(11q) (225/1005, 22.4%), del(17p) (215/1035, 20.8%) and *TP53* mutation (163/757, 21.5%)]. BTKis mainly concerned ibrutinib (729, 69.4%) and acalabrutinib (270, 25.7%), while only 52 patients (4.9%) received zanubrutinib or pirtobrutinib. Median follow-up from BTKi initiation was 50 months (IQR: 28-74), 177 patients (16.8%) required 2L treatment and 187 (17.8%) died. Regarding cytogenetic results, 247 cases (23.5%) had CK, including 133 (12.7%) Low/int-CK and 114 (10.5%) High-CK. Both CK groups showed significantly higher IGHV-UM rates compared to non-CK group (78% and 83%, respectively, vs 68%; P<0.01). Additionally, *TP53* aberrations (*TP53*$^{aber}$, deletions and/or mutations) were detected in 20% of non-CK patients and increased progressively with genomic complexity, being 37% in Low/int-CK and 75% in High-CK (P<0.01). Low/int-CK and High-CK were significantly associated with inferior outcomes compared with non-CK, with High-CK exhibiting the poorest TTNT (median 50 m) and OS (median 8 m). Similarly, the presence of U-abn, detected in 319 patients (30.4%) who also wew enriched in *TP53*$^{aber}$ (63%), was significantly associated with shorter TTNT and OS compared with patients lacking such alterations (**Figure**). In multivariable analysis for OS including age, sex, IGHV gene status, del(11q), High-CK, U-abn, and *TP53*$^{aber}$, the number of cytogenetic abnormalities lost statistical significance, whereas independent prognostic impact was retained for U-abn (HR 1.99, 95% CI 1.35–2.92; P<0.01) and *TP53*$^{aber}$ (HR 1.59, 95% CI 1.12–2.25; P=0.01) (Figure).

**Summary/Conclusion**

CK remains a significant prognostic factor in patients with CLL treated with continuous BTKi. However, beyond the number of abnormalities, the specific nature of cytogenetic alterations, particularly unbalanced abnormalities, may carry greater clinical weight, supporting the need for a more refined cytogenetic risk stratification in the targeted therapy era.

**Copyright Information:** (Online) ISSN: 2572-9241
©2026 The Author(s). HemaSphere is published by John Wiley & Sons Ltd on behalf of European Hematology Association. This is an open access Abstract Book distributed under the Attribution-NonCommercial-NoDerivs (CC BY-NC-ND), which allows third parties to download the articles and share them with others as long as they credit the author and the Abstract Book, but they cannot change the content in any way or use them commercially.
**Abstract Book Citations:** Authors, Title, HemaSphere, 2026;10:(S1):pages. The abstract book DOIs can be found on the “Author Information” page.
**Disclaimer:** Articles published in the journal HemaSphere exclusively reflect the opinions of the authors. The authors are responsible for all content in their abstracts including accuracy of the facts, statements, citing resources, etc.

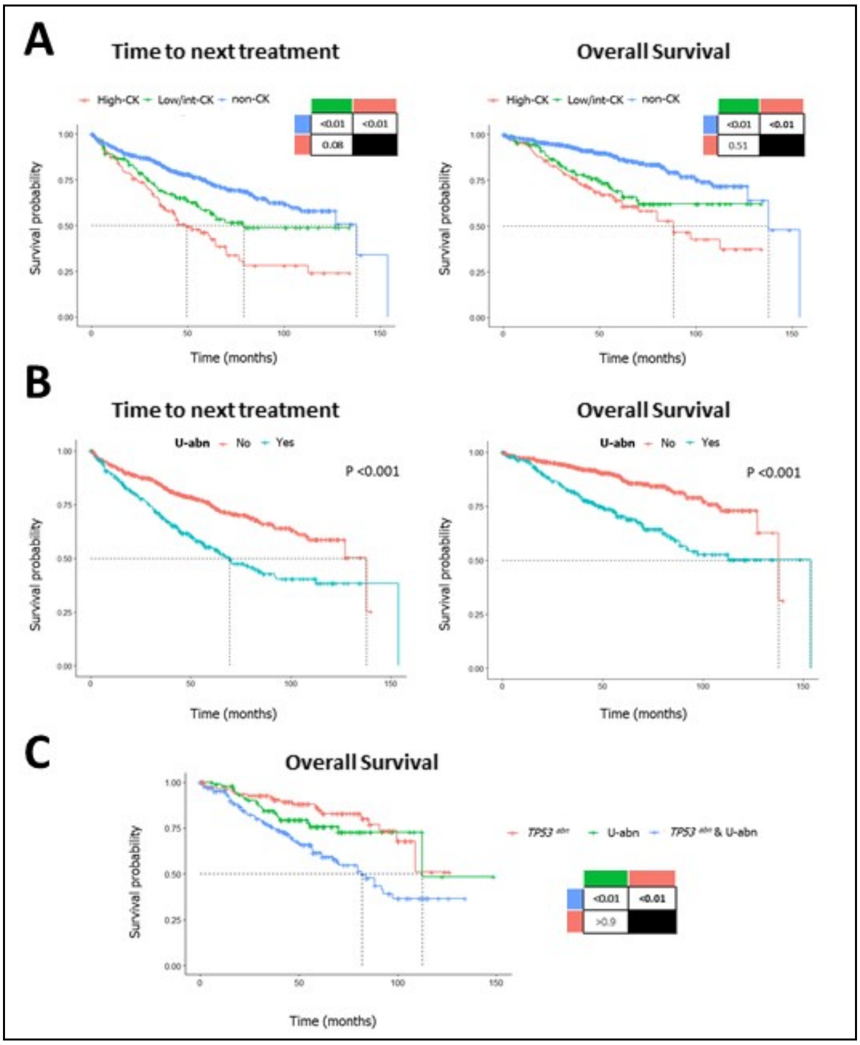

Figure. Kaplan-Meier curves for Time to next treatment and Overall survival. Patients were classified according the detection of (A) complex karyotype or (B) unbalanced abnormalities in the karyotype. Patients showing multiple trisomies (+12,+19,+other, n=7) were excluded from survival analyses based on complexity.

**Copyright Information:** (Online) ISSN: 2572-9241
©2026 The Author(s). HemaSphere is published by John Wiley & Sons Ltd on behalf of European Hematology Association. This is an open access Abstract Book distributed under the Attribution-NonCommercial-NoDerivs (CC BY-NC-ND), which allows third parties to download the articles and share them with others as long as they credit the author and the Abstract Book, but they cannot change the content in any way or use them commercially.
**Abstract Book Citations:** Authors, Title, HemaSphere, 2026;10:(S1):pages. The abstract book DOIs can be found on the "Author Information" page.
**Disclaimer:** Articles published in the journal HemaSphere exclusively reflect the opinions of the authors. The authors are responsible for all content in their abstracts including accuracy of the facts, statements, citing resources, etc.

### (PF577) MCL1 COPY NUMBER GAIN IS A RECURRENT MARK OF VENETOCLAX RESISTANCE IN PATIENTS WITH CHRONIC LYMPHOCYTIC LEUKEMIA

**Topic:** 05. Chronic lymphocytic leukemia and related disorders - Biology & translational research

Carolyne Croizier*[1, 2], Lauren Veronese[2, 3], Stéphanie Poulain[4], Loic Ysebaert[5], Sylvain Carras[6], Damien Roos-Weil[7], Anne Quinquenel[8], Anne-Sophie Michallet[9], Emmanuelle Tchernonog[10], Pierre Morel[11], Adrien Chauchet[12], Pierre Feugier[13], Natalia Dmytruk[14], Morgane Nudel Ortmans[15], Elise Chapiro[16], Grégory Lazarian[17], Olivier Tournilhac[1, 3], Marc Berger[2, 18], Audrey Bidet[19], Bruno Pereira[20], Ludivine Paris[1], Inès Ramos[20], Nathan Papon[21], Andreï Tchirkov[2, 3], Manon Garcia[1, 3], Romain Guieze[1, 3]

*[1]CHU Estaing, thérapie cellulaire et hématologie clinique adulte, Clermont-Ferrand, France;[2]UCA, EA 7453 CHELTER, Clermont-Ferrand, France; [3]CHU Estaing, cytogénétique médicale, Clermont-Ferrand, France; [4]CHU Lille, Laboratoire hématologie, Lille, France; [5]Oncopole, Hématologie clinique, Toulouse, France; [6]CHU Grenoble, hématologie clinique, Grenoble, France; [7]APHP La Pitié Salpétrière, Hématologie clinique, Paris, France; [8]CHU Reims, Hématologie clinique, Reims, France; [9]Centre Leon Berard, Hématologie clinique, Lyon, France; [10]CHU Montpellier, Hématologie clinique, Montpellier, France; [11]CHU Amiens, Hématologie clinique, Amiens, France; [12]CHU Besançon, Hématologie clinique, Besançon, France; [13]CHU Nancy, Hématologie clinique, Nancy, France; [14]CHU Limoges, Hématologie clinique, Limoges, France; [15]CHU Lille, Hématologie clinique, Lille, France; [16]APHP La Pitié Salpétrière, Laboratoire Cytogénétique, Paris, France; [17]APHP Avicenne Bobigny, Laboratoire hématologie, Paris, France; [18]CHU Estaing, Laboratoire hématologie, Clermont-Ferrand, France; [19]CHU Bordeaux, Laboratoire hématologie, Bordeaux, France; [20]CHU Gabriel Montpied, Département médico-économique et bio-statistique, DRCI, Clermont-Ferrand, France; [21]CHU Gabriel Montpied, Plateforme de biologie moléculaire, Clermont-Ferrand, France;*

### Background

The BCL2 inhibitor venetoclax (VEN) is now widely used for patients (pts) with chronic lymphocytic leukemia (CLL), although resistance to this agent develops in a subset of pts. VEN resistance may arise from mutations (mut) affecting its binding site on the BCL2 protein, which are usually screened in post-VEN clinical trials and in routine practice when VEN retreatment is considered. Furthermore, the overexpression of the anti-apoptotic protein MCL1 may also confer VEN resistance with a very limited series of 4 cases harboring *MCL1* copy number gain (+1q21), a finding that remained to be confirmed in larger cohorts.

### Aims

In this study, we report on the incidence of +1q21 in a large cohort of CLL pts relapsing after VEN (RAVEN study, NCT05246345).

### Methods

We assembled a cohort of CLL relapsing on or after VEN therapy, either as CLL or Richter transformation (RT). Tumor samples had to be available at time to relapse, and pre-VEN samples were also collected when available. *BCL2* mut were investigated using a targeted NGS panel and ddPCR, and +1q21 by FISH. Optical genome mapping (OGM) assays was conducted in selected cases.

**Copyright Information:** (Online) ISSN: 2572-9241
©2026 The Author(s). HemaSphere is published by John Wiley & Sons Ltd on behalf of European Hematology Association. This is an open access Abstract Book distributed under the Attribution-NonCommercial-NoDerivs (CC BY-NC-ND), which allows third parties to download the articles and share them with others as long as they credit the author and the Abstract Book, but they cannot change the content in any way or use them commercially.
**Abstract Book Citations:** Authors, Title, HemaSphere, 2026;10:(S1):pages. The abstract book DOIs can be found on the “Author Information” page.
**Disclaimer:** Articles published in the journal HemaSphere exclusively reflect the opinions of the authors. The authors are responsible for all content in their abstracts including accuracy of the facts, statements, citing resources, etc.

### Results

Between March 2022 and August 2025, 76 pts were included across 12 centers (median age 74 years). IGHV status was unmutated in 94% of pts, and *TP53* aberration (prior to VEN) was seen in 56% of pts. The median number of prior lines was 3 [1–6] with 4 pts received VEN as frontline. Forty-eight pts (61%) had been exposed to BTK inhibitors (BTKi), and 60 (79%) to chemo-immunotherapy. VEN was used as monotherapy in 28 pts (37%) and in combination with anti-CD20 antibody in 45 pts (59%). The median duration of VEN was 23 months [1–67]. Relapse was as CLL in 62 pts (82%) and as RT in 14 pts (18%). Relapses occurred on VEN for 33 pts (43%), and after the end of VEN for the remaining 43 pts (57%). A total of 134 tumor samples were analyzed, including 90 at VEN relapse and 44 pre-VEN. At relapse, 58 *BCL2* mut were detected in 23/72 pts (31.9%), with a median VAF of 2.2% [0.02%–30%]. *MCL1* gain was identified in 26/71 pts (36.6%), with a median proportion of affected interphase nuclei of 54% [0.5–100%]. None had +1q21 before VEN. Only 5 pts harbored concomitant *BCL2* mut and +1q21. Relapses associated with *+1q21* occurred significantly earlier than those with *BCL2* mut (median time from VEN initiation: 34.5 months [95% CI, 27–41] vs 42 months [95% CI, 30–52]; *P* = 0.0258). At relapse, 42/64 pts (65.6%) harbored *TP53* aberration, and 29/50 pts (58%) had a highly complex karyotype (≥5 abnormalities). Among the 4 pts relapsing after VEN as frontline therapy, 3 had +1q21 and none had *BCL2* mut. None of the pts relapsing as RT had *BCL2* mut, while 3 exhibited *+1q21* in the RT compartment. Among the 44 BTKi pre-exposed pts, 15 (34%) had *BTK* mut, and 4 (9%) *PLCG2* mut. OGM (performed in 16 pts) revealed translocations of interest involving immunoglobulin gene loci with *MCL1* locus in 3 pts, and with *BCL2L1* locus in 2 pts. These resulted in functional transcriptional upregulation of *MCL1* and *BCL2L1* genes. Median OS from VEN relapse was 31 months (95% CI, 17.5–NA), with no differential impact of *BCL2* mut or +1q21.

### Summary/Conclusion

This RAVEN study identifies +1q21 as a recurrent mark of resistance to VEN in CLL and occurred mostly in patients without*BCL2* mut. Detection of +1q21 should be integrated into the characterization of patients in clinical trials and its role in routine clinical practice should be investigated.

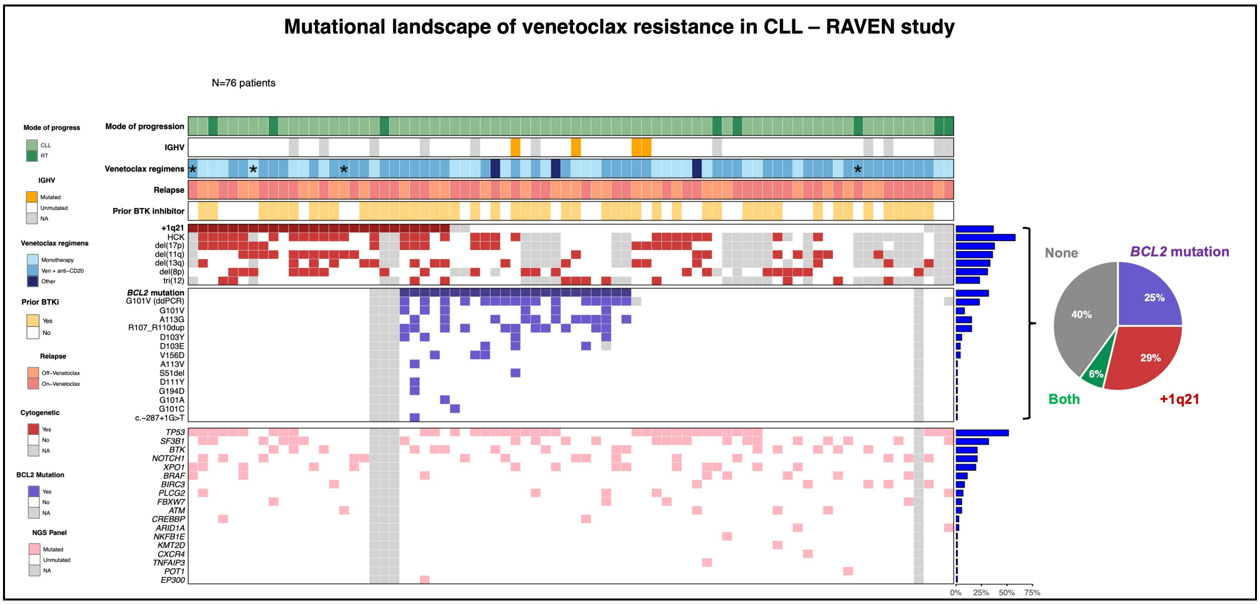

**Copyright Information:** (Online) ISSN: 2572-9241
©2026 The Author(s). HemaSphere is published by John Wiley & Sons Ltd on behalf of European Hematology Association. This is an open access Abstract Book distributed under the Attribution-NonCommercial-NoDerivs (CC BY-NC-ND), which allows third parties to download the articles and share them with others as long as they credit the author and the Abstract Book, but they cannot change the content in any way or use them commercially.
**Abstract Book Citations:** Authors, Title, HemaSphere, 2026;10:(S1):pages. The abstract book DOIs can be found on the "Author Information" page.
**Disclaimer:** Articles published in the journal HemaSphere exclusively reflect the opinions of the authors. The authors are responsible for all content in their abstracts including accuracy of the facts, statements, citing resources, etc.

### (PF578) FERROPTOSIS AS A THERAPEUTIC TARGET IN CHRONIC LYMPHOCYTIC LEUKEMIA

**Topic:** 05. Chronic lymphocytic leukemia and related disorders - Biology & translational research

Martin Boettcher*[1, 2], Lea Reemts[1], Paul Hengeveld[3], Romy Boettcher-Loschinski[1], Vikas Bhuria[1, 2], Junyan Lu[4, 5], Silvia Materna-Reichelt[6], Durdam Das[6], Natasa Stojanovic Guzvic[6], Heiko Bruns[7], Wolfgang Huber[4], Thorsten Zenz[8], Denny Schanze[9], Martin Zenker[9], Sascha Dietrich[10], Anton Langerak[3], Dimitrios Mougiakakos[1, 2]

*[1]University Hospital Magdeburg, Dpt. of Hematology, Oncology and Cell Therapy, Magdeburg, Germany;[2]Otto-von-Guericke University, Healthcampus of Immunology, Inflammation and Infectiology, Magdeburg, Germany; [3]Erasmus MC, University Medical Centre Rotterdam, Dpt. of Immunology, Rotterdam, The Netherlands; [4]European Molecular Biology Laboratory, Genome Biology Unit, Heidelberg, Germany; [5]Heidelberg University, Medical Faculty Heidelberg, Heidelberg, Germany; [6]Fraunhofer Institute for Toxicology and Experimental Medicine ITEM-R, Regensburg, Germany; [7]Friedrich-Alexander-Universität Erlangen-Nürnberg and University Hospital Erlangen, Department of Medicine 5, Hematology and Oncology, Erlangen, Germany; [8]University Hospital Zurich, Department of Medical Oncology and Hematology, Zurich, Switzerland; [9]University Hospital Magdeburg, Institute of Human Genetics, Magdeburg, Germany; [10]Medical Faculty and University Hospital Düsseldorf, Heinrich-Heine-University Düssel-dorf, Department of Hematology, Oncology and Clinical Immunology, Düsseldorf, Germany;*

### Background

Chronic lymphocytic leukemia (CLL) is the most common adult leukemia in the Western world. The disease is characterized by the accumulation of clonal B cells, mainly due to impaired cell death rather than increased proliferation. Although targeted therapies such as Ibrutinib (Bruton's tyrosine kinase, BTK inhibitor) and Venetoclax (BCL-2 inhibitor) have greatly advanced treatment outcomes, resistance mechanisms persist, especially in patients with high-risk genetic profiles such as *TP53* mutations and unmutated immunoglobulin heavy-chain variable region (IGHV) genes (U-CLL). Ferroptosis, a regulated, iron-dependent form of cell death, may represent an exploitable vulnerability in CLL. However, its mechanisms and clinical role remain poorly understood.

### Aims

Therefore, this study aimed to elucidate the clinical relevance and therapeutic potential of ferroptosis in CLL with a particular focus on high-risk patients and incorporating microenvironmental features.

### Methods

The ferroptotic vulnerability/potential of CLL cells was characterized in human CLL cell lines, patient-derived PBMCs, as well as murine samples from the Eμ-TCL1 transgenic model compared to healthy or non-transgenic controls. Additionally, the role of the bone marrow (BM) niche was evaluated using an *in* vitro niche model with a human BM stroma cell line (HS-5). Furthermore, identified targetable nodes were validated *in vivo* using the Eμ-TCL1 adoptive transfer model.

**Copyright Information:** (Online) ISSN: 2572-9241
©2026 The Author(s). HemaSphere is published by John Wiley & Sons Ltd on behalf of European Hematology Association. This is an open access Abstract Book distributed under the Attribution-NonCommercial-NoDerivs (CC BY-NC-ND), which allows third parties to download the articles and share them with others as long as they credit the author and the Abstract Book, but they cannot change the content in any way or use them commercially.
**Abstract Book Citations:** Authors, Title, HemaSphere, 2026;10:(S1):pages. The abstract book DOIs can be found on the "Author Information" page.
**Disclaimer:** Articles published in the journal HemaSphere exclusively reflect the opinions of the authors. The authors are responsible for all content in their abstracts including accuracy of the facts, statements, citing resources, etc.

**Results**

We demonstrate that CLL cells, which exhibit elevated baseline levels of labile iron and peroxidized lipids, are more susceptible to ferroptosis compared to healthy B cells. However, stromal interaction reduces ferroptosis sensitivity through enhanced cystine import and glutathione synthesis. BTK inhibition sensitizes CLL cells to ferroptosis by upregulating the transferrin receptor (TFRC, CD71) and thereby increasing the intracellular $Fe^{2+}$ pool. The therapeutic and prognostic relevance is supported by the association of higher TFRC expression with improved patient survival in two independent CLL cohorts. The combination of Ibrutinib with the GPX4 inhibitor RSL3, a ferroptosis inducer, further enhanced its therapeutic efficacy *in vivo*. U-CLL cells exhibit lower TFRC/CD71 expression and, accordingly, reduced $Fe^{2+}$ levels which renders them more resistant to ferroptosis than IGHV-mutated CLL (M-CLL). This resistance can be overcome by Ibrutinib-mediated TFRC induction or via metabolic targeting of fatty acid metabolism. Notably, ACSL1 is selectively upregulated in U-CLL cells and represents a targetable metabolic enhancer of ferroptosis sensitivity, as shown *in vivo*.

**Summary/Conclusion**

Our findings establish ferroptosis as a novel therapeutic vulnerability in CLL. Although TFRC and ACSL1 are functionally distinct, they represent targetable nodes governing ferroptosis sensitivity in CLL patients and may guide novel therapeutic strategies to overcome therapy resistance and improve outcomes particularly for high-risk patients.

**Copyright Information:** (Online) ISSN: 2572-9241
©2026 The Author(s). HemaSphere is published by John Wiley & Sons Ltd on behalf of European Hematology Association. This is an open access Abstract Book distributed under the Attribution-NonCommercial-NoDerivs (CC BY-NC-ND), which allows third parties to download the articles and share them with others as long as they credit the author and the Abstract Book, but they cannot change the content in any way or use them commercially.
**Abstract Book Citations:** Authors, Title, HemaSphere, 2026;10:(S1):pages. The abstract book DOIs can be found on the “Author Information” page.
**Disclaimer:** Articles published in the journal HemaSphere exclusively reflect the opinions of the authors. The authors are responsible for all content in their abstracts including accuracy of the facts, statements, citing resources, etc.

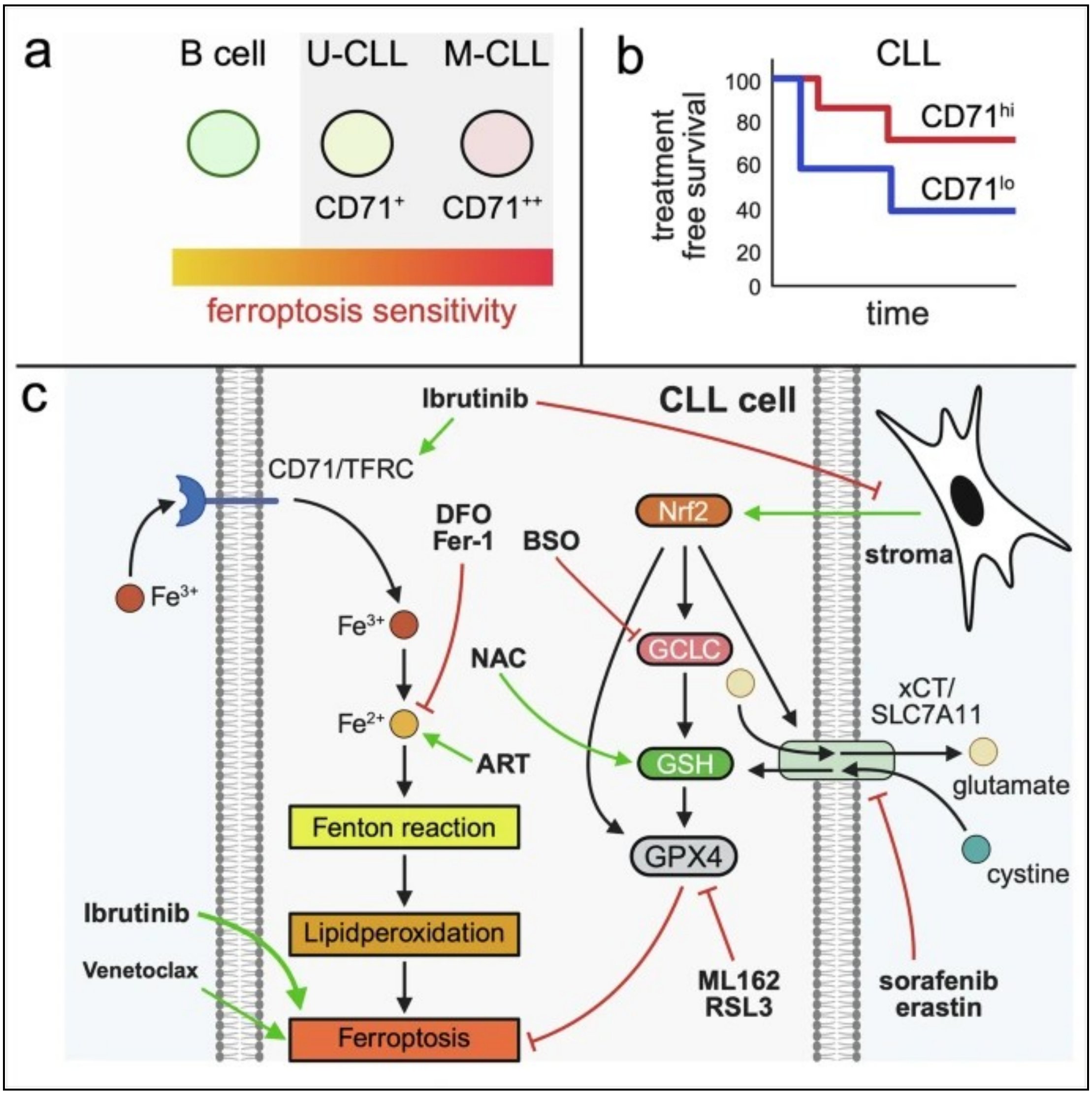

**Copyright Information:** (Online) ISSN: 2572-9241
©2026 The Author(s). HemaSphere is published by John Wiley & Sons Ltd on behalf of European Hematology Association. This is an open access Abstract Book distributed under the Attribution-NonCommercial-NoDerivs (CC BY-NC-ND), which allows third parties to download the articles and share them with others as long as they credit the author and the Abstract Book, but they cannot change the content in any way or use them commercially.
**Abstract Book Citations:** Authors, Title, HemaSphere, 2026;10:(S1):pages. The abstract book DOIs can be found on the "Author Information" page.
**Disclaimer:** Articles published in the journal HemaSphere exclusively reflect the opinions of the authors. The authors are responsible for all content in their abstracts including accuracy of the facts, statements, citing resources, etc.

### (PF579) DISTINCT STEREOTYPED SUBSETS OF CHRONIC LYMPHOCYTIC LEUKEMIA REMODEL METABOLISM THROUGH A WARBURG-LIKE EFFECT

**Topic:** 05. Chronic lymphocytic leukemia and related disorders - Biology & translational research

John Mollstedt*[1], Blaž Oder[1], Anastasia Iatrou[2], Leily Rabbani[1], Larry Mansouri[1], Lesley-Ann Sutton[1], Diego Cortese[1], Andreas Agathangelidis[2, 3], Karla Plevova[4, 5, 6], Lydia Scarfò[7, 8], Marine Armand[9], Frederic Davi[9], Paolo Ghia[7, 8], Davide Rossi[10], Neil Kay[11, 12], Tait Shanafelt[13], Sarka Pospisilova[4, 5, 6], Anton Langerak[14], Anastasia Chatzidimitriou[1, 2], Cecilia Österholm[1], Kostas Stamatopoulos[1, 2], Daniel Hägerstrand[1], Richard Rosenquist[1, 15]

*[1]Karolinska Institutet, Department of Molecular Medicine and Surgery, Stockholm, Sweden; [2]Institute of Applied Biosciences, Centre for Research and Technology Hellas, Thessaloniki, Greece; [3]National and Kapodistrian University of Athens, Department of Biology, School of Science, Athens, Greece; [4]Masaryk University and University Hospital Brno, Department of Internal Medicine, Hematology and Oncology, Medical Faculty, Brno, Czechia; [5]Masaryk University and University Hospital Brno, Institute of Medical Genetics and Genomics, Medical Faculty, Brno, Czechia; [6]Masaryk University, Central European Institute of Technology, Brno, Czechia; [7]Università Vita-Salute San Raffaele, Milan, Italy; [8]San Raffaele Scientific Institute, Comprehensive Cancer Center, IRCCS, Milan, Italy; [9]Hospital Pitie-Salpetriere, Sorbonne University, Department of Hematology, Paris, France; [10]Oncology Institute of Southern Switzerland and Institute of Oncology Research, Department of Hematology, Bellinzona, Switzerland; [11]Mayo Clinic, Division of Hematology, Department of Internal Medicine, Rochester, MN, United States of America; [12]Mayo Clinic, Department of Immunology, Rochester, MN, United States of America; [13]Stanford University Medical Center, Division of Hematology, Department of Medicine, Stanford, CA, United States of America; [14]University Medical Center Rotterdam, Department of Immunology, Erasmus MC, Rotterdam, The Netherlands; [15]Karolinska University Hospital, Clinical Genetics and Genomics, Karolinska University Laboratory, Stockholm, Sweden;*

### Background

More than 40% of patients with chronic lymphocytic leukemia (CLL) express near identical, or stereotyped, B-cell receptor (BcR) immunoglobulins that define biologically and clinically distinct subsets. Among major stereotyped subsets, subset #1 (IGHV1/5/7/IGKV1-39) and subset #2 (IGHV3-21/IGLV3-21) exhibit inferior clinical outcomes, whereas subset #4 (IGHV4-34/IGKV2-30) demonstrates favorable prognosis and rarely require treatment. Despite their well-established clinical heterogeneity, the underlying molecular mechanisms remain incompletely understood.

### Aims

This study aimed to examine differences in gene expression and metabolic programs across major CLL stereotyped subsets #1, #2 and #4 and to identify distinct subset-specific molecular signatures that may underlie differences in clinical behaviour.

### Methods

Total RNA-sequencing was performed in 74 patients with CLL belonging to subsets #1 (n=25), #2 (n=35) and #4 (n=14). Validation with in-depth proteomic analysis was conducted in two independent cohorts (n=13/n=30). Critical findings were further validated in primary CLL samples (subsets #1, n=8; #2, n=6; #4, n=6) through targeted protein and metabolite profiling.

**Copyright Information:** (Online) ISSN: 2572-9241
©2026 The Author(s). HemaSphere is published by John Wiley & Sons Ltd on behalf of European Hematology Association. This is an open access Abstract Book distributed under the Attribution-NonCommercial-NoDerivs (CC BY-NC-ND), which allows third parties to download the articles and share them with others as long as they credit the author and the Abstract Book, but they cannot change the content in any way or use them commercially.
**Abstract Book Citations:** Authors, Title, HemaSphere, 2026;10:(S1):pages. The abstract book DOIs can be found on the "Author Information" page.
**Disclaimer:** Articles published in the journal HemaSphere exclusively reflect the opinions of the authors. The authors are responsible for all content in their abstracts including accuracy of the facts, statements, citing resources, etc.

**Results**

Unsupervised gene expression analysis demonstrated that each subset represents a distinct transcriptional entity; however, clinically aggressive subsets #1 and #2 showed convergent metabolic programs. Pathway analysis revealed enrichment of MYC target genes (including MYC), mTORC1 signaling, and oxidative phosphorylation (OXPHOS) in subsets #1 and #2, whereas subset #4 was enriched for WNT and β-catenin signaling pathways. Specifically, genes involved in oxygen sensing and α-ketoglutarate (α-KG)–dependent metabolism were upregulated in subsets #1 and #2, including the prolyl hydroxylase EGLN3, which regulates HIF-1α stability, and the metabolic regulators BCAT1 and SLC7A5, which control glutamine flux and intracellular α-KG availability. Alternative splicing analysis further supported enrichment of MYC- and mTORC1-associated genes in subsets #1 and #2. Additionally, MYC target genes and OXPHOS enrichment was validated by proteomics. Network analysis revealed functional interactions converging on pyruvate and lactate metabolism as well as hypoxia-related pathways. Functional validation confirmed increased mTORC1 activity and elevated protein expression of SLC7A5 (#1 vs #4, p=0.007; #2 vs #4, p=0.005) and BCAT1 (#1 vs #4, p=0.04; #2 vs #4, ns) in subsets #1 and #2, accompanied by altered metabolite levels, including elevated glutamate (#1 vs #4, p=0.02; #2 vs #4, p=0.02), reduced branched-chain amino acids (#1 vs #4, p=0.04; #2 vs #4, ns) and glutamine (#1 vs #4, p=0.02; #2 vs #4, p=0.02). These metabolic changes correlated with altered EGLN3 activity and increased HIF-1α levels in subset #1 ($p<0.05$ for all comparisons), consistent with pseudohypoxia, while subset #2 displayed an intermediate phenotype.

**Summary/Conclusion**

We uncovered glutamine-dependent metabolic reprogramming in clinically aggressive CLL subsets #1 and #2 relative to subset #4, specifically involving α-KG-dependent EGLN3 activity promoting pseudohypoxia and a Warburg-like metabolic state that supports adaptive survival programs. Furthermore, we identified potential metabolic biomarkers in CLL subsets associated with poor prognosis, with implications for precision medicine strategies.

**Copyright Information:** (Online) ISSN: 2572-9241
©2026 The Author(s). HemaSphere is published by John Wiley & Sons Ltd on behalf of European Hematology Association. This is an open access Abstract Book distributed under the Attribution-NonCommercial-NoDerivs (CC BY-NC-ND), which allows third parties to download the articles and share them with others as long as they credit the author and the Abstract Book, but they cannot change the content in any way or use them commercially.
**Abstract Book Citations:** Authors, Title, HemaSphere, 2026;10:(S1):pages. The abstract book DOIs can be found on the "Author Information" page.
**Disclaimer:** Articles published in the journal HemaSphere exclusively reflect the opinions of the authors. The authors are responsible for all content in their abstracts including accuracy of the facts, statements, citing resources, etc.

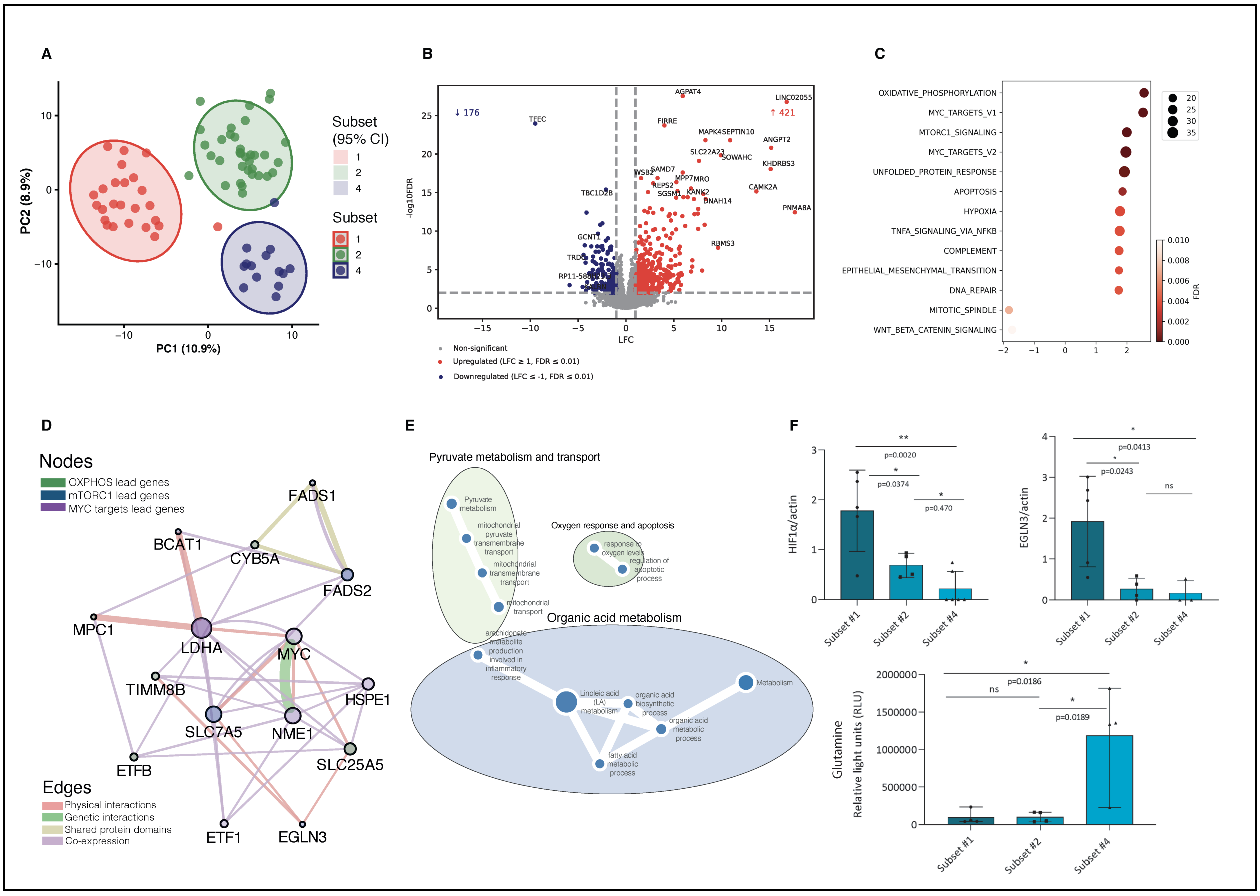

A–F: Transcriptomic analysis and functional validation of subsets #1, #2 and #4. (A) PCA of RNA-seq profiles. (B) Differential gene expression for subset #1 vs subset #4 (volcano plot). (C) GSEA of subset #1 vs subset #4 (dot plot). (D) Functional interaction network of top 5 lead genes driving the enriched main pathways in subset #1 vs subset #4. (E) Enrichment map of (D) with affinity-propagation clustering. (F) Functional validation by immunoblotting of selected proteins and luminescence-based metabolite assays.

**Copyright Information:** (Online) ISSN: 2572-9241
©2026 The Author(s). HemaSphere is published by John Wiley & Sons Ltd on behalf of European Hematology Association. This is an open access Abstract Book distributed under the Attribution-NonCommercial-NoDerivs (CC BY-NC-ND), which allows third parties to download the articles and share them with others as long as they credit the author and the Abstract Book, but they cannot change the content in any way or use them commercially.
**Abstract Book Citations:** Authors, Title, HemaSphere, 2026;10:(S1):pages. The abstract book DOIs can be found on the “Author Information” page.
**Disclaimer:** Articles published in the journal HemaSphere exclusively reflect the opinions of the authors. The authors are responsible for all content in their abstracts including accuracy of the facts, statements, citing resources, etc.

### (PF580) ERIC TP53 VARIANT DATABASE IN CHRONIC LYMPHOCYTIC LEUKEMIA (CLL): A COMMUNITY-DRIVEN RESOURCE FOR FUNCTIONAL AND CLINICAL INTERPRETATION

**Topic:** 05. Chronic lymphocytic leukemia and related disorders - Biology & translational research

Sarka Pavlova[1, 2, 3], Thomas Chatzikonstantinou[4], Barbara Dvorackova[1], Frank Dicker[5], Lea Rodriguez[6], Silvia Petrezselyova[7], Libor MacUrek[7], Jana Kopecna[1], Mark Catherwood[8], Fanny Baran Marszak[9, 10], Grégory Lazarian[9, 10], Jack Cowland[11], Mette Andersen[11], Maria Karypidou[4], Eva Zapletalova[1], Audrey Bidet[12], Bella Biderman[13], Andrey Sudarikov[13], Riccardo Moia[14], Gianluca Gaidano[14], Helena Podgornik[15, 16], Sandra Sucurovic[15], Iwona Solarska[17], Malwina Hut-Bogołębska[17], Bartosz Puła[18, 19], María J. Larráyoz[20], María José Calasanz Abinzano[20], Margarida Coucelo[21], Rita Costa e Sousa[21], Arnon Philip Kater[22], Clemens Mellink[23], Anne-Marie van der Kevie-Kersemaekers[24], Jonathan Strefford[25], Helen Parker[25], Zadie Davis[26], Amy Christian[26], Monica Lopez-Guerra[27, 28, 29], Juan A. Piñeyroa[30, 31, 32], Martin Simkovic[33], Paula Gameiro[34], Maria Ramos Lopes Gomes Da Silva[34], Andrea Visentin[35], Andrea Serafin[35], Livio Trentin[35], Rossana Maffei[36], Roberto Marasca[37], Blanca Ferrer Lores[38], Alicia Serrano Alcala[38], Eugen Tausch[39], Stephan Stilgenbauer[39], Miguel Alcoceba Sánchez[40], Blanca Espinet[41], Anna Puiggros[41], Ana Balanzategui[40], Concepción Fernández-Rodriguez[41], Krzysztof Giannopoulos[42], Agnieszka Karczmarczyk[42], Marta Morawska[42], Rosa Collado Nieto[43], Tomasz Wrobel[44], Elżbieta Kalicińska[45], Bozena Jazwiec[46], Irma Slavutsky[47], Carmen Stanganelli[48], Ezzat El Hassadi[49], Lisa Bonello[50], Ivana Jeziskova[1], Martina Sopkovicova[51], Silvia Zibellini[52], Marzia Varettoni[52], Mirjana Suver Stević[53], Anna Panovská[1], Michael Doubek[1, 2, 3], Sarka Pospisilova[1, 2, 3], Paolo Ghia[54, 55], Kostas Stamatopoulos[4], Thierry Soussi[56, 57], Jitka Malcikova*[1, 2, 3]

*[1]University Hospital Brno and Medical Faculty, Masaryk University, Department of Internal Medicine, Hematology and Oncology, Brno, Czechia; [2]Central European Institute of Technology, Masaryk University, Brno, Czechia;[3]University Hospital Brno and Medical Faculty, Masaryk University, Institute of Medical Genetics and Genomics, Brno, Czechia; [4]Institute of Applied Biosciences, Centre for Research and Technology Hellas, Thessaloniki, Greece; [5]MLL Munich Leukemia Laboratory, Munich, Germany; [6]Sorbonne Université, INSERM, Centre de Recherche Saint-Antoine, CRSA, UMRS_938 Hematopoietic and Leukemic Development, AP-HP, SIRIC CURAMUS, Paris, France; [7]Cancer Cell Biology, Institute of Molecular Genetics of the Czech Academy of Sciences, Prague, Czechia; [8]Belfast City Hospital, Haematology Department, Belfast, United Kingdom;[9]HUPSSD Hôpital Avicenne AP-HP, Bobigny, France; [10]INSERM U1345 Université Sorbonne Paris Nord, Bobigny, France; [11]Copenhagen University Hospital, Department of Clinical Genetics, Copenhagen, Denmark; [12]Laboratoire d'Hématologie Biologique, CHU Bordeaux, Bordeaux, France; [13]National Medical Research Center for Hematology, Novy Zykovski Lane, 4a, 125167, Moscow, Russia; [14]Division of Hematology, Department of Translational Medicine University of Eastern Piedmont, Novara, Italy; [15]University medical center, Department of Haematology, Ljubljana, Slovenia; [16]Faculty of Pharmacy, University of Ljubljana, Ljubljana, Slovenia; [17]Center for Oncology and Traumatology M. Kopernika w Łodzi, Department of Laboratory Diagnostics, Lódź, Poland; [18]Medical University of Lódź, 90-364 Lódź, Department of Haematology, Lódź, Poland;[19]Copernicus Memorial, 93-513 Lódź, Department of General Haematology and Internal Medicine, Lódź, Poland; [20]CIMA LAB Diagnostics-Clínica Universidad de Navarra, Pamplona, Navarra Institute for Health Research (IdiSNA), Pamplona, Spain; [21]Clinical Hematology Department, Unidade Local de Saúde de Coimbra, Coimbra, Portugal; [22]Department of Hematology, Cancer Center Amsterdam, Amsterdam University Medical Centers, University of Amsterdam, also on behalf of HOVON, Amsterdam, The Netherlands; [23]Genome Diagnostics Laboratory, Amsterdam University Medical Centers, University of Amsterdam, Amsterdam, The Netherlands; [24]A.M.F. van der Kevie-Kersemaekers, Department of Genetics, Genome diagnostics, University Medical Center Utrecht, Utrecht, The Netherlands; [25]Cancer Genomics, Cancer Sciences, Faculy of Medicine, University of Southampton, Southampton, United Kingdom; [26]University Hospitals Dorset, Molecular Pathology Department, Bournemouth, United Kingdom; [27]Hematopathology Section, Pathology Department, Hospital Clinic, Barcelona, Spain;[28]Institut d'Investigacions Biomèdiques August Pi I Sunyer (IDIBAPS), Barcelona, Spain; [29]Centro de Investigación Biomédica en Red de Cáncer (CIBERONC), Madrid, Spain; [30]Facultat de Medicina i ciències de la salut, Universitat de Barcelona, Barcelona, Spain;[31]Servei d'Hematologia, Hospital Clínic de Barcelona, Barcelona, Spain; [32]Terapias experimentales en neoplasias linfoides, Institut d'Investigacions Biomèdiques August Pi i Sunyer (IDIBAPS), Barcelona, Spain; [33]Faculty of Medicine in Hradec Králové, University Hospital and*

**Copyright Information:** (Online) ISSN: 2572-9241
©2026 The Author(s). HemaSphere is published by John Wiley & Sons Ltd on behalf of European Hematology Association. This is an open access Abstract Book distributed under the Attribution-NonCommercial-NoDerivs (CC BY-NC-ND), which allows third parties to download the articles and share them with others as long as they credit the author and the Abstract Book, but they cannot change the content in any way or use them commercially.
**Abstract Book Citations:** Authors, Title, HemaSphere, 2026;10:(S1):pages. The abstract book DOIs can be found on the "Author Information" page.
**Disclaimer:** Articles published in the journal HemaSphere exclusively reflect the opinions of the authors. The authors are responsible for all content in their abstracts including accuracy of the facts, statements, citing resources, etc.

Charles University, Hradec Králové, 4th Department of Internal Medicine-Hematology, Hradec Králové, Czechia; [34]Haematology Unit, Instituto Português de Oncologia de Lisboa Francisco Gentil, Lisbon, Portugal; [35]Hematology Unit, Department of Medicine, University of Padova, Padova, Italy; [36]Policlinico di Modena, Modena, Italy; [37]University of Modena and RE, Dept of Medical Sciences, Section of Hematology, Modena, Italy; [38]Hospital Clínico Universitario-INCLIVA Valencia, Hematology Department, Valencia, Spain; [39]Division of CLL, Department of Internal Medicine III, Ulm University, Ulm, Germany; [40]University Hospital of Salamanca (HUS/IBSAL), CIBERONC and Cancer Research Institute of Salamanca-IBMCC (USAL-CSIC), Salamanca, Spain; [41]Pathology Department, Hospital del Mar, Barcelona, Spain; Hospital del Mar Research Institute, Barcelona, Spain; [42]Experimental Hematooncology Department, Medical University of Lublin, Lublin, Poland; [43]Consorcio Hospital General Universitario de Valencia, Department of Hematology, Valencia, Spain; [44]Clinical Department of Hematology, Cell Therapies and Internal Diseases, Faculty of Medicine, Wroclaw Medical University, Wroclaw, Poland; [45]Department of Hematology, Cell Therapies and Internal Diseases, Wrocław Medical University, Wrocław, Poland; [46]Department of Hematology, Blood Neoplasms and Bone Marrow Transplantation, Medical University of Wrocław, Wrocław, Poland; [47]Laboratorio Genética de Neoplasias Linfoides, Instituto de Medicina Experimental, CONICET-Academia Nacional de Medicina, Buenos Aires, Argentina; [48]División Patología Molecular, Instituto de Investigaciones Hematológicas, Academia Nacional de Medicina, Buenos Aires, Argentina; [49]Department of Hematology, University Hospital Waterford, Waterford, Ireland; [50]San Giovanni Battista University Hospital, Molecular Pathology Unit, 10126, Torino, Italy; [51]Department of Medical Genetics, National Cancer Institute, Slovakia, Bratislava, Slovakia; [52]Department of Hematology Oncology,IRCCS S. Matteo Hospital Foundation, Pavia, Italy; [53]Clinical Institute for Transfusion Medicine, University Hospital Center Osijek, HR-31000, Osijek, Croatia; [54]Medical School, Università Vita-Salute San Raffaele and IRCCS Ospedale San Raffaele, Milan, Italy; [55]Comprehenzive Cancer Center, IRCCS Ospedale San Raffaele, Milan, Italy; [56]Centre de Recherche Saint-Antoine UMRS_938, INSERM, Sorbonne Université, Paris, France; [57]Department of Immunology, Genetics and Pathology, Science for Life Laboratory, Uppsala University, Clinical Genetics, Uppsala University Hospital, Uppsala, Sweden;

**Background**

The clinical relevance of *TP53* mutations has driven their integration into current treatment algorithms. The *TP53* Network of the European Research Initiative on CLL (ERIC) aims to maintain high standards of *TP53* diagnostics in CLL by addressing challenges encountered by diagnostic laboratories. The increasing number and heterogeneity of *TP53* variants detected in routine practice pose challenges for accurate interpretation, particularly in cases of rare variants. A particularly challenging class of variants comprises in-frame deletions and insertions, for which interpretative guidance and functional data remain limited.

**Aims**

The aim is to leverage routine *TP53* testing in CLL to establish a comprehensive, curated database of *TP53* variants detected in diagnostics, thereby supporting harmonized interpretation of molecular results. A specific objective is to improve the interpretation of in-frame *TP53* variants.

**Methods**

*TP53* variants are continuously collected from collaborating ERIC-certified laboratories. All entries have been curated using Variant Validator to ensure HGVS-compliant descriptions. Data from The *TP53* Database, the UMD (Universal Mutation Database), and ClinGen *TP53* VCEP classifications have been extracted. Ambiguous variants have been selected for functional analysis and tested using a yeast assay supplemented by functional testing in human cells.

**Copyright Information:** (Online) ISSN: 2572-9241
©2026 The Author(s). HemaSphere is published by John Wiley & Sons Ltd on behalf of European Hematology Association. This is an open access Abstract Book distributed under the Attribution-NonCommercial-NoDerivs (CC BY-NC-ND), which allows third parties to download the articles and share them with others as long as they credit the author and the Abstract Book, but they cannot change the content in any way or use them commercially.
**Abstract Book Citations:** Authors, Title, HemaSphere, 2026;10:(S1):pages. The abstract book DOIs can be found on the "Author Information" page.
**Disclaimer:** Articles published in the journal HemaSphere exclusively reflect the opinions of the authors. The authors are responsible for all content in their abstracts including accuracy of the facts, statements, citing resources, etc.

**Results**

To date, we have gathered 5,895 *TP53* variants detected in 4,016 CLL patients from 28 centers across 16 countries. The majority of the variants were classified as pathogenic/likely pathogenic (94%), but we also identified difficult-to-interpret variants, including hypomorphic variants (2%). Benign/likely benign variants were rare (2%) and were mostly detected at VAFs around 50%, suggesting a germline origin. The variant spectrum largely aligned with that observed across all tumor entities, with a few exceptions: notably, the CLL-specific variant p.Arg209LysfsTer6 constituted 2.5% of all variants, and was significantly more frequent than in general cancer datasets (0.2% in the UMD and 0.05% in The TP53 Database; p<0.0001). Other CLL-enriched variants involved: p.Tyr234Cys, p.His214Arg, p.Val157Phe, p.Ile195Thr, and c.673–2A>C. The functional analysis of selected variants is ongoing, and all the existing data, including their occurrence in CLL, will be integrated into the UMD TP53 Database.
As part of the project, we analyzed 60 samples with 54 distinct in-frame *TP53* variants. Initial classification attempts using existing guidelines and databases were inconclusive. We performed transactivation analysis using a yeast-based assay and, for seven variants, additional testing in RPE1 *TP53*-knockout cells. Forty-nine variants within the core DNA-binding domain (DBD) exhibited complete loss of function, indicating pathogenicity. Five variants located downstream of codon 287 displayed variable activity depending on their localization and the assay system: the two variants at the DBD boundary retained 70% and 63% of wild-type p53 activity, respectively, whereas three variants in the oligomerization domain were nonfunctional.

**Summary/Conclusion**

The ERIC *TP53* database is a growing, community-driven effort that collects diagnostic data on *TP53* variants in CLL. The dedicated analysis of in-frame *TP53* variants indicated that those in the core DNA-binding domain are likely pathogenic, whereas those outside this region require a multifactorial approach to interpretation.
Supported by FNBr, 65269705, NW24-03-00114, MUNI/A/1733/2025

**Copyright Information:** (Online) ISSN: 2572-9241
©2026 The Author(s). HemaSphere is published by John Wiley & Sons Ltd on behalf of European Hematology Association. This is an open access Abstract Book distributed under the Attribution-NonCommercial-NoDerivs (CC BY-NC-ND), which allows third parties to download the articles and share them with others as long as they credit the author and the Abstract Book, but they cannot change the content in any way or use them commercially.
**Abstract Book Citations:** Authors, Title, HemaSphere, 2026;10:(S1):pages. The abstract book DOIs can be found on the "Author Information" page.
**Disclaimer:** Articles published in the journal HemaSphere exclusively reflect the opinions of the authors. The authors are responsible for all content in their abstracts including accuracy of the facts, statements, citing resources, etc.

### (PF581) INTRACLONAL DIVERSIFICATION IN IMMUNOGLOBULIN LIGHT CHAIN GENES REFINES PROGNOSTICATION IN EARLY-STAGE CHRONIC LYMPHOCYTIC LEUKEMIA

**Topic:** 05. Chronic lymphocytic leukemia and related disorders - Biology & translational research

Jana Nabki*[1], Filippo Vit[2], Bashar Al Deeban[1], Samir Mouhssine[1], Mohammad Almasri[1], Nawar Maher[1], Silvia Rasi[1], Fatemeh Bokaei[1], Chiara Cosentino[1], Luca Cividini[1], Matteo Bellia[1], Riccardo Dondolin[1], Joseph Ghanej[1], Lorenzo Maraschi[1], Dana Ghalayiny[1], Francesca Maiellaro[1], Eleonora Secomandi[1], Sreekar Kogila[1], Mohammad Reshad Nawabi[1], Antonella Zucchetto[2], Tamara Bittolo[2], Riccardo Bomben[2], Gianluca Gaidano[1], Valter Gattei[2], Riccardo Moia[1]

*[1]University of Eastern Piedmont, Department of translational medicine, Novara, Italy;[2]Centro di Riferimento Oncologico di Aviano (CRO), Clinical and Experimental Onco-Hematology Unit, Aviano, Italy;*

### Background

Intraclonal diversification (ID) is defined as the process of ongoing somatic hypermutation (SHM) that generates subclonal heterogeneity within a single B-cell chronic lymphocytic leukemia (CLL) clone. When studied within immunoglobulin heavy chain variable genes (IGHV), ID identified a subgroup of M-CLL patients with high-ID [inverse Simpson Index (iSI)>1.2] with more favorable clinical outcomes (Vit et al, *Leukemia*, 2025). The introduction of a 99% identity cut-off for light chain (LC) genes further refined the prognostic value of IGHV mutational status (Nabki, Al Deeban et al, *Leukemia*, 2025), although LC-ID has been scarcely examined and its impact on a large consecutive CLL cohort remains unexplored.

### Aims

To investigate ID levels in LC genes in CLL and assess the possible synergistic role of IGHV and LC ID on the prognosis of CLL patients.

### Methods

An amplicon-based NGS approach was implemented to analyze ID in both IGHV and LC genes. An original stringent bioinformatic pipeline (Patent #102022000027138) was applied to identify subclones that were considered different from the dominant clonotypic rearrangement by at least one SHM event across the variable domains. iSI was implemented to quantify ID with time-to-first treatment (TTFT) as clinical endpoint.

**Copyright Information:** (Online) ISSN: 2572-9241
©2026 The Author(s). HemaSphere is published by John Wiley & Sons Ltd on behalf of European Hematology Association. This is an open access Abstract Book distributed under the Attribution-NonCommercial-NoDerivs (CC BY-NC-ND), which allows third parties to download the articles and share them with others as long as they credit the author and the Abstract Book, but they cannot change the content in any way or use them commercially.
**Abstract Book Citations:** Authors, Title, HemaSphere, 2026;10:(S1):pages. The abstract book DOIs can be found on the "Author Information" page.
**Disclaimer:** Articles published in the journal HemaSphere exclusively reflect the opinions of the authors. The authors are responsible for all content in their abstracts including accuracy of the facts, statements, citing resources, etc.

### Results

A consecutive real-life cohort of 636 CLL patients referring at a single institution was sequenced for IGHV and LC genes. After bioinformatic filtering, recursive partitioning (Maxstat) in Binet A CLL identified 1.288 and 1.007 for IGHV (N=428) and LC (N=415), respectively, as optimal iSI cut-offs values predicting TTFT. Using these thresholds, High-ID patients (IGHV, N=259; LC, N=240) had significantly prolonged TTFT compared with Low-ID patients (IGHV, N=169; LC, N=175; both p<0.001).
The incorporation of the mutational status with ID levels provided further prognostic value. For IGHV, High-ID patients had significantly longer TTFT than Low-ID patients within the M-IGHV (p=0.003) and U-IGHV (p=0.003) subgroups (Fig.1A). For LC, High-ID refined risk stratification primarily within the unmutated subgroup, where U-light High-ID patients showed significantly longer TTFT compared with U-light Low-ID cases (p=0.003), although a trend toward longer TTFT was observed also for High-ID patients within the M-light subgroup (p=0.07; Fig.1B). In multivariable Cox analysis, High-ID in either chain independently associated with longer TTFT after adjustment for the International Prognostic Score for Early stage CLL (IPS-E) variables with the addition of the light chain mutational status (Fig.1C; 1D).
Parallel assessment of IGHV and LC genes revealed that, overall, LC genes displayed lower ID documented by lower iSI levels than their IGHV counterparts in Binet A (N=376; p<0.001; Fig.1E). Considering the stratification of ID, concordant High-ID status in both chains (N=136) associated with significantly longer TTFT compared to concordant Low-ID status (N=79; p<0.001) and discordant groups [IGHV High-ID + Light Low-ID (N=87; p=0.003) or IGHV Low-ID + Light High-ID (N=74; p<0.001)] in Binet A CLL (Fig.1F; 1G). Concordant High-ID patients also retained independent prognostic value in multivariate analysis after adjustment for IPS-E variables and light chain mutational status (Fig.1H).

### Summary/Conclusion

These findings highlight the first evidence that ID in IG LC genes may provide additional prognostic value for early-stage CLL patients.

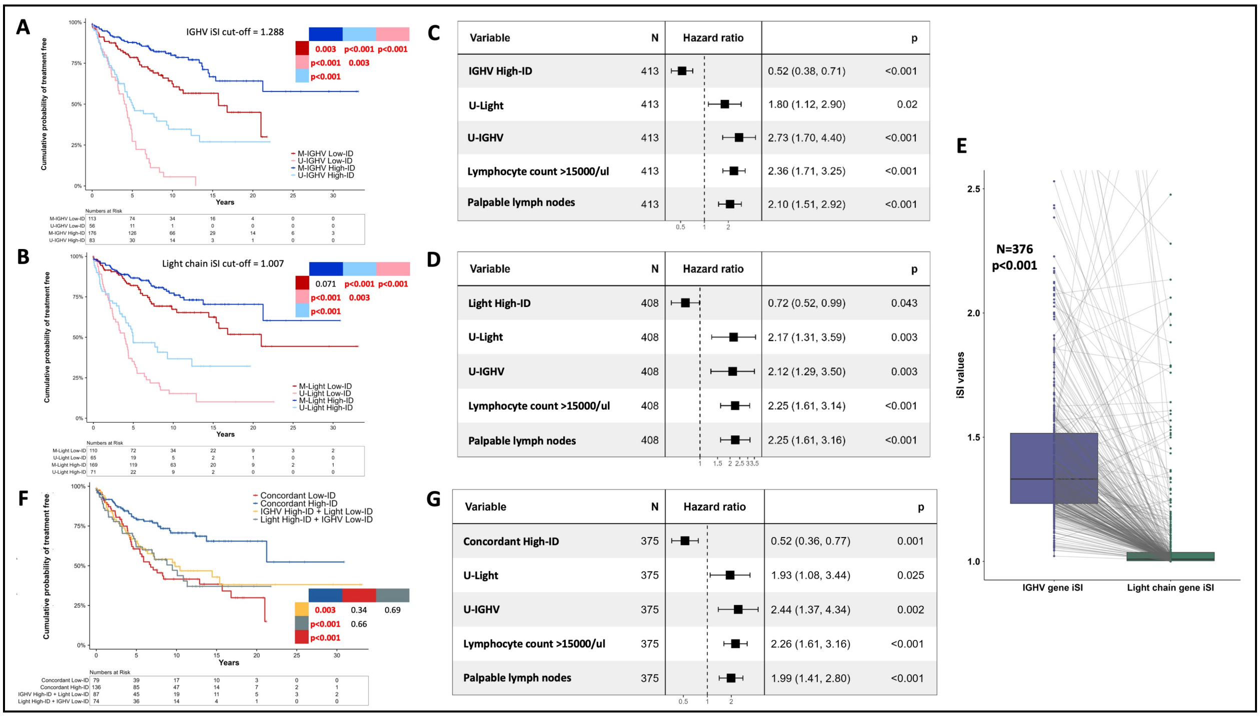

**Copyright Information:** (Online) ISSN: 2572-9241
©2026 The Author(s). HemaSphere is published by John Wiley & Sons Ltd on behalf of European Hematology Association. This is an open access Abstract Book distributed under the Attribution-NonCommercial-NoDerivs (CC BY-NC-ND), which allows third parties to download the articles and share them with others as long as they credit the author and the Abstract Book, but they cannot change the content in any way or use them commercially.
**Abstract Book Citations:** Authors, Title, HemaSphere, 2026;10:(S1):pages. The abstract book DOIs can be found on the "Author Information" page.
**Disclaimer:** Articles published in the journal HemaSphere exclusively reflect the opinions of the authors. The authors are responsible for all content in their abstracts including accuracy of the facts, statements, citing resources, etc.

### (PF582) SINGLE-CELL RNA AND BCR SEQUENCING REVEALS CLONAL EVOLUTION AND DISTINCT MICROENVIRONMENTAL HETEROGENEITY IN RICHTER TRANSFORMATION

**Topic:** 05. Chronic lymphocytic leukemia and related disorders - Biology & translational research

Xiaozhong Shen[1], Jiaojiao Zhang*[1, 2], Jin Wang[1, 2], Lu Jiang[1], Jian-qing Mi[1, 2]

*[1]Shanghai Institute of Hematology, State Key Laboratory of Medical Genomics, National Research Center for Translational Medicine at Shanghai, Ruijin Hospital, Shanghai Jiao Tong University School of Medicine, Shanghai, China; [2]Shanghai Key Laboratory of Gene Editing and Cell-based Immunotherapy for Hematological Diseases, Ruijin Hospital, Shanghai jiao Tong University School of Medicine, Shanghai, China;*

### Background

Richter transformation (RT), the clonal evolution of chronic lymphocytic leukemia (CLL) into diffuse large B-cell lymphoma (DLBCL), is associated with an extremely poor prognosis. The mechanisms driving clonal evolution and the differential microenvironmental influences across anatomical compartments remain poorly characterized at single-cell resolution.

### Aims

To elucidate tumor clonal evolution and microenvironmental heterogeneity in RT through integrated single-cell RNA sequencing (scRNA-seq) and single cell B cell receptor sequencing (scBCR-seq).

### Methods scRNA-seq and BCR sequencing were performed on paired lymph node (LN) and peripheral blood (PB) samples from a high-risk RT patient. Bioinformatics analyses included Harmony for multi-sample integration, scRepertoire/IgBlast for BCR clonotype and IGHV alignment, and Dynamo for RNA velocity-based trajectory inference. Additionally, sciCSR, InferCNV, SCENIC, and CellChat were employed to profile class-switch recombination, copy number alterations (CNAs), transcription factor activity, and cell-cell communication, respectively.

### Results

Integrated analysis identified 14 transcriptionally distinct clusters, including 7 B-cell populations and 7 microenvironmental cell types. Single-cell BCR profiling revealed a dominant monoclonal B-cell clone with concordant clonotypes across LN and PB, confirming clonal continuity between CLL and DLBCL. IGHV alignment identified an unmutated status (U-CLL) and a non-GCB DLBCL phenotype. Differential expression of *NME1* and *STMN1* demarcated CLL from DLBCL; the latter was further resolved into DLBCL-G2M and DLBCL-S subpopulations. Dynamo trajectory inference identified "CLL-Precursor" as the cell of origin. "CLL-3", uniquely expressing the RT-associated gene *PLEKHG1*, represented the most terminally differentiated CLL state, characterized by progressive S-phase accumulation. InferCNV revealed a significantly higher CNA burden in DLBCL compared to CLL.Anatomically, the LN was enriched for advanced CLL-3 and DLBCL, while the PB predominantly harbored early-stage CLL. The LN tumor microenvironment (TME) uniquely contained cancer-associated fibroblasts (CAFs), endothelial cells (ECs), macrophages, and elevated T/DNT cell proportions. CellChat analysis identified DLBCL as the primary signal emitter. Specifically, CXCL signaling from ECs and CD8+ T cells targeted CLL subsets but not DLBCL. CAF-derived IGF ligands targeted CLL-Precursors, potentially licensing differentiation, while coordinated MK signaling from ECs and CAFs promoted global tumor proliferation. Furthermore, EC-derived IGFBP signaling appeared to reinforce DLBCL growth.

**Copyright Information:** (Online) ISSN: 2572-9241
©2026 The Author(s). HemaSphere is published by John Wiley & Sons Ltd on behalf of European Hematology Association. This is an open access Abstract Book distributed under the Attribution-NonCommercial-NoDerivs (CC BY-NC-ND), which allows third parties to download the articles and share them with others as long as they credit the author and the Abstract Book, but they cannot change the content in any way or use them commercially.
**Abstract Book Citations:** Authors, Title, HemaSphere, 2026;10:(S1):pages. The abstract book DOIs can be found on the "Author Information" page.
**Disclaimer:** Articles published in the journal HemaSphere exclusively reflect the opinions of the authors. The authors are responsible for all content in their abstracts including accuracy of the facts, statements, citing resources, etc.

**Summary/Conclusion**

Our findings demonstrate clonal continuity and a hierarchical evolutionary trajectory from CLL-Precursor to DLBCL in RT. We also highlight markedly distinct TME landscapes between LN and PB compartments. The identification of niche-specific signaling networks (CXCL, IGF, MK, and IGFBP) suggests that the LN microenvironment is a critical driver of the aggressive biology and clonal progression in RT.

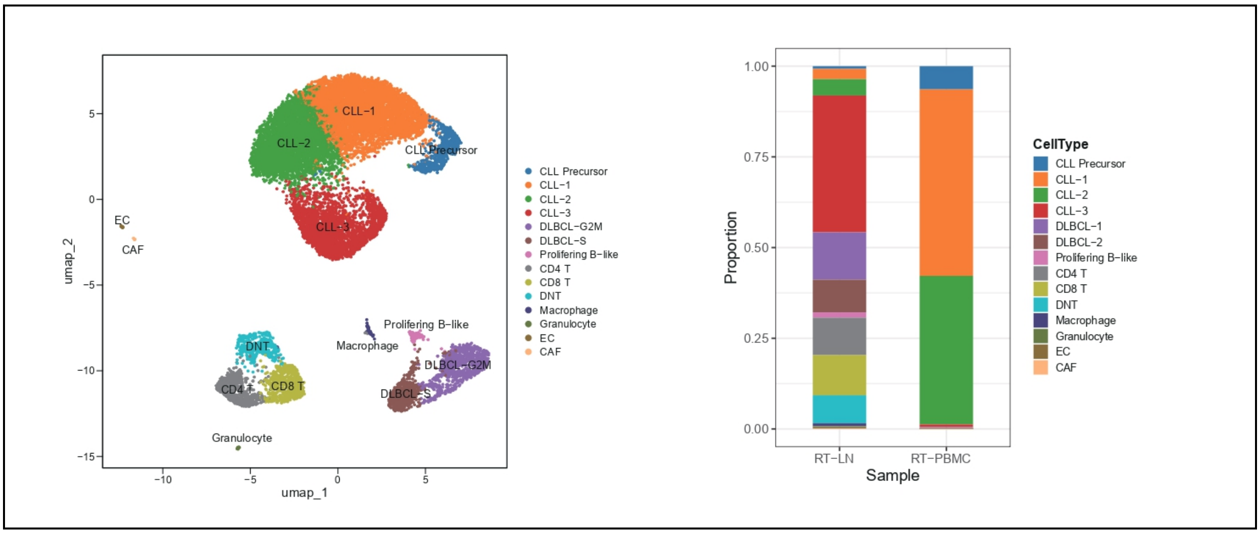

(Left) UMAP visualization of single cell RNAseq dataset, colored and labeled by cell types.(Right) Stacked bar chart showing the relative proportions of each identified cell type across the RT-LN and RT-PBMC samples.

**Copyright Information:** (Online) ISSN: 2572-9241
©2026 The Author(s). HemaSphere is published by John Wiley & Sons Ltd on behalf of European Hematology Association. This is an open access Abstract Book distributed under the Attribution-NonCommercial-NoDerivs (CC BY-NC-ND), which allows third parties to download the articles and share them with others as long as they credit the author and the Abstract Book, but they cannot change the content in any way or use them commercially.
**Abstract Book Citations:** Authors, Title, HemaSphere, 2026;10:(S1):pages. The abstract book DOIs can be found on the "Author Information" page.
**Disclaimer:** Articles published in the journal HemaSphere exclusively reflect the opinions of the authors. The authors are responsible for all content in their abstracts including accuracy of the facts, statements, citing resources, etc.

### (PF583) DISSECTION OF BIALLELIC NOTCH1 DISRUPTION IN CHRONIC LYMPHOCYTIC LEUKEMIA REVEALS CHROMOSOME 9 COPY NEUTRAL-LOSS OF HETEROZYGOSITY

**Topic:** 05. Chronic lymphocytic leukemia and related disorders - Biology & translational research

Giulia Ianna*[1], Francesca Maria Rossi[1], Filippo Vit[1], Tamara Bittolo[1], Robel Papotti[1], Mario Ballerini[2], Francesco Zaja[2], Luca Laurenti[3], Jacopo Olivieri[4], Annalisa Chiarenza[5], Fabiana Esposito[6], Massimiliano Postorino[6], Roberta Laureana[7], Maria Ilaria DEL Principe[7], Antonella Zucchetto[1], Riccardo Bomben[1], Valter Gattei[1], Federico Pozzo[1]

*[1]Centro di Riferimento Oncologico di Aviano (CRO), IRCCS, Aviano, Italy;[2]UCO Ematologia, Azienda Sanitaria Universitaria Giuliano-Isontina, Trieste, Italy; [3]Institute of Hematology, Fondazione Policlinico Universitario Agostino Gemelli IRCCS, Roma, Italy; [4]Azienda Sanitaria Universitaria Friuli Centrale (ASU FC), Udine, Italy; [5]Division of Haematology, Ospedale Ferrarotto, Catania, Italy; [6]Department of Biomedicine and Prevention, University of Rome tor Vergata, Roma, Italy;[7]Ospedale Sant'Eugenio, University of Tor Vergata, Roma, Italy;*

### Background

*NOTCH1* is among the most frequently mutated genes in CLL, with an incidence of 12–25%, and shorter TTFT and OS compared to wild-type cases, with subclonal mutations also exerting a residual prognostic impact. However, the prognostic significance of NOTCH1 disruption with biallelic involvement (VAF > 50%) has not yet been specifically evaluated.

### Aims

To investigate the molecular features of High-VAF *NOTCH1* mutation.

### Methods

A total of 4,603 peripheral blood samples from 3,564 CLL patients were screened for *NOTCH1* mutations by NGS (exon 34 and part of 3′UTR). Copy number analysis of *NOTCH1* was performed using TaqMan probes for exon 34 (Hs00124234_cn) by ddPCR, or FISH on fixed nuclei. Whole-exome sequencing (WES) was conducted on sorted cell fractions from frozen samples. The CNVkit package was used for copy number estimation.

**Copyright Information:** (Online) ISSN: 2572-9241
©2026 The Author(s). HemaSphere is published by John Wiley & Sons Ltd on behalf of European Hematology Association. This is an open access Abstract Book distributed under the Attribution-NonCommercial-NoDerivs (CC BY-NC-ND), which allows third parties to download the articles and share them with others as long as they credit the author and the Abstract Book, but they cannot change the content in any way or use them commercially.
**Abstract Book Citations:** Authors, Title, HemaSphere, 2026;10:(S1):pages. The abstract book DOIs can be found on the "Author Information" page.
**Disclaimer:** Articles published in the journal HemaSphere exclusively reflect the opinions of the authors. The authors are responsible for all content in their abstracts including accuracy of the facts, statements, citing resources, etc.

**Results**

*NOTCH1* mutations were present in 1,056 samples (23%); of these, 116 (11%) had a high-VAF mutation ≥ 50%. We performed copy number analysis to verify the biallelic involvement of *NOTCH1* mutation and to exclude chr9 deletions. Unexpectedly, ddPCR revealed a 2n diploid status in virtually all high-burden samples, with only 3 cases showing chr9 monosomy. Independent FISH analysis confirmed these findings. However, NGS of *NOTCH1* exon 34 in most high-VAF samples revealed a peculiar polymorphic unbalancing of the rs2229974 D2185D SNP in heterozygous samples, with allelic frequencies between 0.1-0.4 or 0.6-0.9.
We investigated the hypothesis of tumor-restricted allelic imbalance using FACS-sorted CD19+/CD5+ (tumor) and CD3+ (germline) fractions from 19 high-VAF cases. NGS showed that both the *NOTCH1* mutation and D2185D unbalancing were confined to CLL cells. Complete allelic loss occurred in 12 samples and partial loss in 7. Matched copy number analysis confirmed a diploid (2n) status in both fractions.
WES was performed to assess the chr9 status on FACS-sorted CD19+/5+ and CD3+ fractions of 27 CLL cases, plus 30 unsorted cases (with CD19+/5+ purity >80%), with varying *NOTCH1* mutation burdens. WES revealed strong allelic imbalance on 9q in high-VAF cases (panel A), suggesting chromosomal deletions, yet CNVkit predicted diploid status in all cases (except one deleted control; panel B). Independent ddPCR and FISH on 9q22 confirmed the diploid status. WES also detected alterations on other chromosomes (chr8q gain/loss, del11q, del13q, del22), all confirmed by FISH, suggesting a mechanism of 9q deletion and subsequent allelic duplication resulting in copy-neutral loss of heterozygosity (cn-LOH). Functionally, patients with high-VAF mutation present higher levels of stabilized NOTCH1 protein and, likely, increased Notch signalling and possibly increased survival. Of note, 6 cases with serial samples available showed over-time increase of 9q cn-LOH, hinting to clonal evolution dynamics. Overall NGS data suggest presence of a D2185D imbalance and 9q alterations in 102 (4.5%) out of 2,234 D2185D heterozygous samples, independently of *NOTCH1* mutations; accounting for the ~45% expected heterozygosity of rs2229974, the extrapolated overall incidence of 9q cn-LOH in CLL may reach 10%

**Summary/Conclusion**

This study describes, for the first time in CLL, a non-rare cn-LOH mechanism on chromosome 9, where high-burden*NOTCH1* disruption coexists with a normal (2n) copy number, and are likely caused by an allelic duplication event on 9q. Ongoing analyses aim to clarify the clinical implications of cn-LOH in both *NOTCH1*-mutated and wild-type CLL cases.

**Copyright Information:** (Online) ISSN: 2572-9241
©2026 The Author(s). HemaSphere is published by John Wiley & Sons Ltd on behalf of European Hematology Association. This is an open access Abstract Book distributed under the Attribution-NonCommercial-NoDerivs (CC BY-NC-ND), which allows third parties to download the articles and share them with others as long as they credit the author and the Abstract Book, but they cannot change the content in any way or use them commercially.
**Abstract Book Citations:** Authors, Title, HemaSphere, 2026;10:(S1):pages. The abstract book DOIs can be found on the “Author Information” page.
**Disclaimer:** Articles published in the journal HemaSphere exclusively reflect the opinions of the authors. The authors are responsible for all content in their abstracts including accuracy of the facts, statements, citing resources, etc.

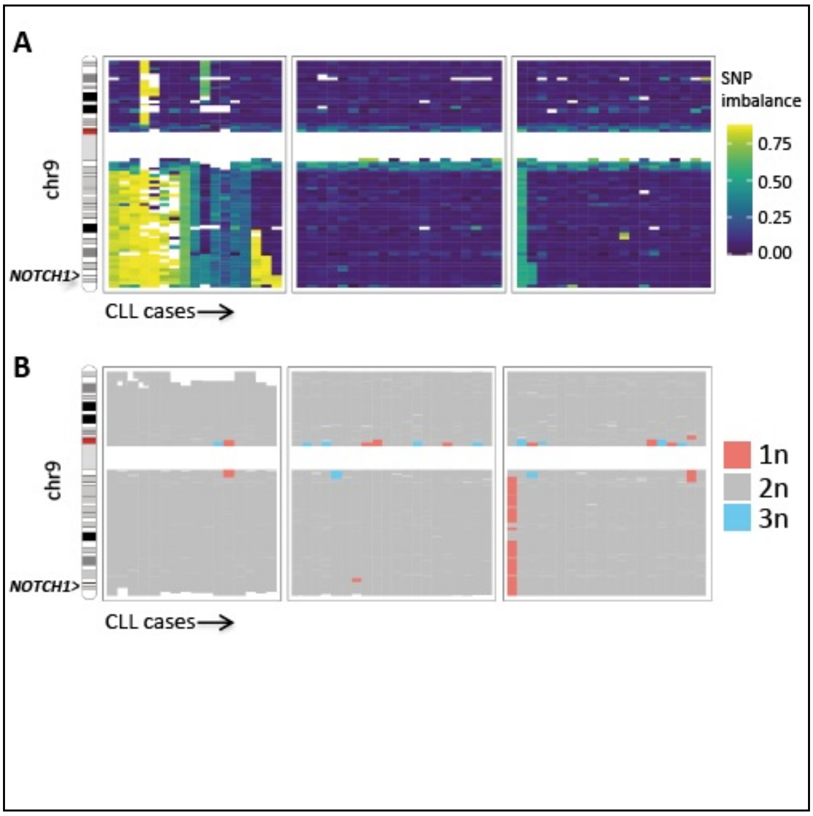

Copyright Information: (Online) ISSN: 2572-9241
©2026 The Author(s). HemaSphere is published by John Wiley & Sons Ltd on behalf of European Hematology Association. This is an open access Abstract Book distributed under the Attribution-NonCommercial-NoDerivs (CC BY-NC-ND), which allows third parties to download the articles and share them with others as long as they credit the author and the Abstract Book, but they cannot change the content in any way or use them commercially.
Abstract Book Citations: Authors, Title, HemaSphere, 2026;10:(S1):pages. The abstract book DOIs can be found on the "Author Information" page.
Disclaimer: Articles published in the journal HemaSphere exclusively reflect the opinions of the authors. The authors are responsible for all content in their abstracts including accuracy of the facts, statements, citing resources, etc.

### (PF584) PTPN2 INHIBITION RESTORES T-CELL FUNCTION AND POTENTIATES T-CELL ENGAGER ACTIVITY IN CHRONIC LYMPHOCYTIC LEUKEMIA

**Topic:** 05. Chronic lymphocytic leukemia and related disorders - Biology & translational research

Liron Yitzhak[1], Yamit Shorer Arbel[1], Ben-Zion Katz[1, 2], Yair Herishanu*[1, 2]

*[1]Tel Aviv Sourasky University Medical Center, Hematology, Tel Aviv, Israel;[2]Gray Faculty of Medicine and Health Sciences, Tel Aviv University, Tel Aviv, Israel;*

### Background

Chronic lymphocytic leukemia (CLL) is characterized by profound T-cell dysfunction that limits the efficacy of emerging immunotherapies, including CD20×CD3 bispecific antibodies. Protein tyrosine phosphatase non-receptor type 2 (PTPN2) is a central intracellular negative regulator of T-cell activation, restraining both proximal TCR signaling and cytokine-driven JAK/STAT pathways. Preclinical studies show that genetic deletion or pharmacologic inhibition of PTPN2 enhances T-cell activation, proliferation, cytokine secretion, effector differentiation, and antitumor activity.

### Aims

This study aimed to investigate the potential of PTPN2 inhibition in combination with the bispecific antibody mosunetuzumab as a therapeutic approach for CLL. We hypothesized that inhibition of PTPN2 in CLL-derived T-cells will restore functional competence and synergize with mosunetuzumab to produce potent cytotoxicity against malignant B-cells.

### Methods

To evaluate this hypothesis, we conducted several assays such as flow cytometry, confocal microscopy, ELISA and Incucyte Live-cell Analysis system. CLL patients-derived PBMCs were used in our experiments (n=18).

### Results

Our results show that pretreatment with the PTPN2 inhibitor AC-484 enhances T-cell activation following stimulation with CD3/CD28 beads. T-cell activation in PBMCs was assessed by flow-cytometric analysis of surface CD69 and CD25 expression. AC-484 pretreatment led to increased CD69 expression in both $CD4^+$ and $CD8^+$ T-cell subsets and significantly upregulated CD25 expression in $CD8^+$ T cells. Using confocal microscopy, we show morphological changes reflected in an increased CD8+ T-cell size, induction and release of granzyme B, formation of large CLL-CD8+ T cells clusters and strong CD8+ T-cell: CLL immune synapses in samples treated with mosunetuzumab +/- PTPN2 inhibitor. In parallel, cytotoxicity and T-cell proliferation assays were conducted in co-cultures of purified T cells and B-CLL cells using Incucyte™ time-lapse imaging over 7 days. Our findings indicate a modest increase in T-cell mediated cytotoxicity (day 3) and enhanced proliferative responses following PTPN2 inhibition in combination with mosunetuzumab. Similar to the findings observed with the confocal microscopy, the treatment also induced CLL-T-cell clustering. Using ELISA-based assay platform, we show increased levels of granzyme B in sampled treated with AC-484 and mosunetuzumab. In comparisons between samples of immunoglobulin heavy chain gene (IGHV)-mutated CLL (M-CLL) and IGHV-unmutated CLL (U-CLL), the effects are more pronounced in the M-CLL group.

**Copyright Information:** (Online) ISSN: 2572-9241
©2026 The Author(s). HemaSphere is published by John Wiley & Sons Ltd on behalf of European Hematology Association. This is an open access Abstract Book distributed under the Attribution-NonCommercial-NoDerivs (CC BY-NC-ND), which allows third parties to download the articles and share them with others as long as they credit the author and the Abstract Book, but they cannot change the content in any way or use them commercially.
**Abstract Book Citations:** Authors, Title, HemaSphere, 2026;10:(S1):pages. The abstract book DOIs can be found on the "Author Information" page.
**Disclaimer:** Articles published in the journal HemaSphere exclusively reflect the opinions of the authors. The authors are responsible for all content in their abstracts including accuracy of the facts, statements, citing resources, etc.

**Summary/Conclusion**

In conclusion, in this study we demonstrate the potential of combining PTPN2 inhibitor with the bispecific antibody mosunetuzumab to enhance antitumor T-cell activity in patient-derived CLL samples. Using this combination, we showed increased expression levels of surface activation markers,T cells proliferation, morphological changes that were compatible with T-cell activation, increased granzyme B secretion and improved anti-CLL T- cell cytotoxicity. The findings of our research may contribute to the development of novel approaches that use PTPN2 inhibition to enhance cellular therapies in CLL, potentially leading to better clinical outcomes.

**Copyright Information:** (Online) ISSN: 2572-9241
©2026 The Author(s). HemaSphere is published by John Wiley & Sons Ltd on behalf of European Hematology Association. This is an open access Abstract Book distributed under the Attribution-NonCommercial-NoDerivs (CC BY-NC-ND), which allows third parties to download the articles and share them with others as long as they credit the author and the Abstract Book, but they cannot change the content in any way or use them commercially.
**Abstract Book Citations:** Authors, Title, HemaSphere, 2026;10:(S1):pages. The abstract book DOIs can be found on the "Author Information" page.
**Disclaimer:** Articles published in the journal HemaSphere exclusively reflect the opinions of the authors. The authors are responsible for all content in their abstracts including accuracy of the facts, statements, citing resources, etc.

### (PF585) MULTI-OMIC INTEGRATION OF WGS, WTS, AND MIRNA PROFILES ELUCIDATES THE MOLECULAR BASIS OF CLL HETEROGENEITY

**Topic:** 05. Chronic lymphocytic leukemia and related disorders - Biology & translational research

Wencke Walter*[1], Veronika Ecker[1], Torsten Haferlach[1], Anna Stengel[1]

[1]*MLL Munich Leukemia Laboratory, Munich, Germany;*

### Background miRNAs, small non-coding RNAs regulating gene expression, are key players in cancer biology, including leukemia where they influence hematopoietic cell gene expression, pathogenesis, progression, and therapy resistance. Specific miRNA profiles offer precise diagnostic and prognostic biomarkers for leukemia subtype classification and survival prediction, underscoring their importance for precision medicine advancement.

### Aims

To elucidate the role of miRNAs in CLL pathogenesis across various cytogenetic subgroups.

### Methods

We performed an integrated analysis of whole genome sequencing (WGS), whole transcriptome sequencing (WTS), and miRNA profiles from 72 patients with typical CLL immunophenotypes across six cytogenetic subgroups (19 del(13q), 10 del(11q), 9 del(6q), 10 trisomy 12, 14 normal karyotype, and 10 rare t(14;18)), using peripheral blood or bone marrow samples. WGS copy number variations (CNVs) were identified using GATK (v4.0.8.1), WTS gene expression profiles were generated by Kallisto, and miRNA sequencing data were processed with Nextflow's smrnaseq pipeline.

### Results

876 miRNAs were detected across all samples, of which 89 miRNAs had sufficiently large counts to be retained for statistical analyses. miR-29a expression (exp), identified as a CLL marker, correlated strongly with the size of the mature B-cell population (correlation coefficient (cc) 0.59, p-value < 0.0001). High miR-29a exp was associated with significantly worse overall survival compared to low exp (10.1 vs. 17.5 years, p-value = 0.005), regardless of cytogenetic subgroup, *TP53* mutation status, IGHV mutation status, as well as *ZAP70* and *CD38* gene exp levels. Integration of WGS CNV results with miRNA exp profiles showed a strong correlation between the copy number state of region chr13:50622001-50926000 and the exp of miR-15a and miR-16-1 (cc 0.83 and cc 0.91, both p-value < 0.0001), confirming the loss of this miRNA cluster in cases with del(13q). For CLL with del(11q), we found a significant aberrant exp of miR-148a (up-regulation) and miR-150 (down-regulation). miR-150 acts as a potent tumor suppressor in leukemia and upregulated miR-148a exp enhances cell survival by inhibiting pro-apoptotic factors. Hence, both miRNAs might contribute to the more aggressive clinical behaviour of CLL cases with del(11q). Interestingly, miR-150 was up-regulated in CLL cases with t(14;18). We identified significant differences in the expression levels of miR-26a, let-7i and miR-331 between CLL cases with trisomy 12 and other cytogenetic subgroups. miR-331 is predicted to target the 3'UTR of *TMEM64*, a key regulator of the Wnt/B-catenin signalling pathway. *TMEM64* was down-regulated in CLLs with trisomy 12 compared to the other groups (lfc = -0.74, p-value < 0.0001). CLL patients with trisomy 12 or del(6q) showed lower levels of miR-146a, which is known to regulate the IL-23 receptor complex and associated with an adverse prognosis in CLL. Patients with del(6q) also showed reduced miR-29c expression, which similar to higher miR-29a exp, has been associated with worse outcome.

**Copyright Information:** (Online) ISSN: 2572-9241
©2026 The Author(s). HemaSphere is published by John Wiley & Sons Ltd on behalf of European Hematology Association. This is an open access Abstract Book distributed under the Attribution-NonCommercial-NoDerivs (CC BY-NC-ND), which allows third parties to download the articles and share them with others as long as they credit the author and the Abstract Book, but they cannot change the content in any way or use them commercially.
**Abstract Book Citations:** Authors, Title, HemaSphere, 2026;10:(S1):pages. The abstract book DOIs can be found on the "Author Information" page.
**Disclaimer:** Articles published in the journal HemaSphere exclusively reflect the opinions of the authors. The authors are responsible for all content in their abstracts including accuracy of the facts, statements, citing resources, etc.

**Summary/Conclusion**

Our study underscores the importance of integrating genomic, transcriptomic, and miRNA data to unravel CLL pathogenesis. It highlights the potential of miRNA biomarkers in enhancing disease diagnosis and informing precision medicine strategies, demonstrating the critical need for a comprehensive 'omic' perspective in understanding cancer biology and improving patient care.

**Copyright Information:** (Online) ISSN: 2572-9241
©2026 The Author(s). HemaSphere is published by John Wiley & Sons Ltd on behalf of European Hematology Association. This is an open access Abstract Book distributed under the Attribution-NonCommercial-NoDerivs (CC BY-NC-ND), which allows third parties to download the articles and share them with others as long as they credit the author and the Abstract Book, but they cannot change the content in any way or use them commercially.
**Abstract Book Citations:** Authors, Title, HemaSphere, 2026;10:(S1):pages. The abstract book DOIs can be found on the "Author Information" page.
**Disclaimer:** Articles published in the journal HemaSphere exclusively reflect the opinions of the authors. The authors are responsible for all content in their abstracts including accuracy of the facts, statements, citing resources, etc.

### (PF587) MULTIOMIC ANALYSIS OF NORMAL B CELLS IN CLL AND IBRUTINIB-TREATED PATIENTS DEMONSTRATES OLIGOCLONALITY, REDUCED POTENTIAL FOR T CELL INTERACTION AND GC FORMATION, AND BTK BYPASS USING PI3K AND NF-KB

**Topic:** 05. Chronic lymphocytic leukemia and related disorders - Biology & translational research

Amelia Fisher*[1, 2], Matthew Care[3], Peter Hillmen[1], Darren Newton[1]

*[1]University of Leeds, Leeds, United Kingdom;[2]Leeds Teaching Hospitals NHS Trust, Leeds, United Kingdom;[3]University of York, York, United Kingdom;*

### Background

Chronic lymphocytic leukaemia (CLL) has a footprint of immunosuppression, associated with hypogammaglobulinaemia and resulting in problematic infections and poor vaccine responses for many patients. Normal B cell counts in this context are low, and treatment using BTK inhibitors (BTKi), for example ibrutinib as monotherapy or in combination, can suppress counts further. Despite the central role of normal B cells in humoral immune responses, they have not previously undergone focused multiomic study in CLL disease and treatment due to low numbers.

### Aims

To assess how normal B cell subtypes and transcriptomes are impacted by CLL and BTKi treatment, and evaluate whether this provides opportunities to improve immune function.

### Methods

CLL patients (n=30) and age-matched control (n=10) samples were obtained with informed consent. Peripheral blood mononuclear cells (PBMCs) were enriched for normal B cells using magnetic and FACS cell sorting. Single cell libraries were created to include proteomic antibody oligonucleotides (IgD/IgM/CD27), B cell receptor (BCR) and targeted mRNA sequencing.
Targeted mRNA panel was designed using parsimonious gene correlation network analysis and literature informed additions.
Data was analysed using Scirpy, Scanpy and Cellismo, with novel pipeline development to enable removal of residual CLL cells from the final analysis.

**Copyright Information:** (Online) ISSN: 2572-9241
©2026 The Author(s). HemaSphere is published by John Wiley & Sons Ltd on behalf of European Hematology Association. This is an open access Abstract Book distributed under the Attribution-NonCommercial-NoDerivs (CC BY-NC-ND), which allows third parties to download the articles and share them with others as long as they credit the author and the Abstract Book, but they cannot change the content in any way or use them commercially.
**Abstract Book Citations:** Authors, Title, HemaSphere, 2026;10:(S1):pages. The abstract book DOIs can be found on the “Author Information” page.
**Disclaimer:** Articles published in the journal HemaSphere exclusively reflect the opinions of the authors. The authors are responsible for all content in their abstracts including accuracy of the facts, statements, citing resources, etc.

**Results**

Residual CLL cells were removed from the analysis bioinformatically, 50,573 normal B cells were included in the final analysis.
B cell subtypes (naïve, unswitched memory, class-switched memory) defined by antibody-oligonucleotide analysis demonstrated a significantly higher proportion of class-switched normal B cells in CLL pre-treatment (CLLPreRx) cases (p<0.0001), and fewer naïve cells (p<0.0001) compared to controls. There was a shift towards control proportions during BTKi-based treatment, but differences remained significant for both naïve (p<0.01) and class-switched (p<0.01) subtypes. Double negative B cells (senescence-associated) were increased compared to controls in CLLPreRx (p<0.001) cases.
BCR analysis demonstrated increased oligoclonality (adjusted for the number of B cells) in CLLPreRx cases compared to controls (p=0.01).
Single cell cluster analysis further identified a 'naïve activated' subtype. Differential expression analysis revealed the following consistent features across B cell subtypes: in CLLPreRx compared to controls there is a) lower potential for T cell interaction and germinal center (GC) formation; b) reduction in non-canonical NF-kB signalling; c) increased potential for auto-antibody production. In ibrutinib-treated patients compared to controls there is: a) improved potential for T cell interaction; b) bypass of BTKi reduction of BCR signalling initiated by PI3k and maintained through upregulation of non-canonical NF-kB pathways; c) fewer 'CLL-like' features suggestive of microenvironmental changes associated with reduced CLL disease burden. There were not any significant changes in genes associated with cellular senescence.

**Summary/Conclusion**

As the first multiomic study of isolated normal B cells from CLLPreRx and BTKi treated patients, this study provides novel insight into this vital cell population. Results reveal potential for BTKi bypass mechanisms in the normal B cells and also provide a focus for target pathways that could enhance immune responsiveness, such as the non-canonical NF-kB pathway and optimization of cell trafficking to GCs.

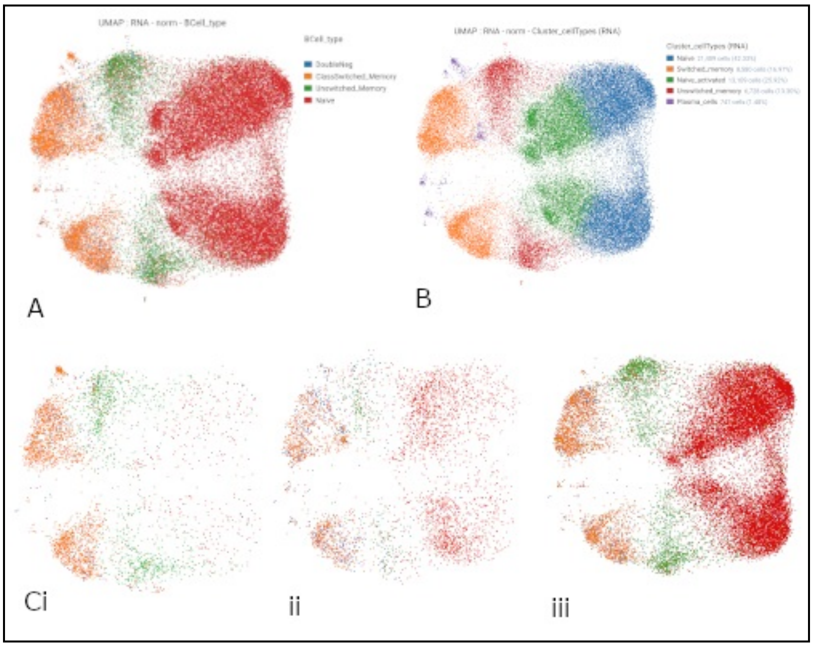

Figure 1. A. B cell subtypes defined by antibody oligonucleotides overlayed on UMAP. B. UMAP cluster analysis identified an additional cluster of activated B cells. C. UMAP distribution of cell types i. CLL pre-treatment, ii. on ibrutinib treatment, iii. controls.

**Copyright Information:** (Online) ISSN: 2572-9241
©2026 The Author(s). HemaSphere is published by John Wiley & Sons Ltd on behalf of European Hematology Association. This is an open access Abstract Book distributed under the Attribution-NonCommercial-NoDerivs (CC BY-NC-ND), which allows third parties to download the articles and share them with others as long as they credit the author and the Abstract Book, but they cannot change the content in any way or use them commercially.
**Abstract Book Citations:** Authors, Title, HemaSphere, 2026;10:(S1):pages. The abstract book DOIs can be found on the "Author Information" page.
**Disclaimer:** Articles published in the journal HemaSphere exclusively reflect the opinions of the authors. The authors are responsible for all content in their abstracts including accuracy of the facts, statements, citing resources, etc.

### (PF588) THE IMPACT OF FIRST-LINE THERAPY ON T CELL FITNESS IN CHRONIC LYMPHATIC LEUKEMIA

**Topic:** 05. Chronic lymphocytic leukemia and related disorders - Biology & translational research

Giulia Benintende*[1], Idanna Innocenti[2], Alessia Crocione[1], Florenzia Vuono[2], Clara Di Mario[3], Franziska Gsottberger[1], Richard Pelzl[1], Lisa Mellenthin[1], Barbara Tolusso[4], Elisa Gremese[4], Julia Katharina Scholz[1], Simon Völkl[1], Andreas MacKensen[1], Krystelle Nganou-Makamdop[5], Luca Laurenti[6], Fabian Müller[1]

*[1]Universitätsklinikum Erlangen, Department of Internal Medicine 5, Hematology and Oncology, Erlangen, Germany;[2]Fondazione Policlinico Universitario A. Gemelli IRCCS, Department of Laboratory and Haematological Sciences, Haematology Area, Rome, Italy; [3]Fondazione Policlinico Universitario A. Gemelli IRCCS, Immunology Research Core Facility, Gemelli Science and Technology Park, Rome, Italy; [4]IRCCS Humanitas Research Hospital, Milan, Italy; [5]Universitätsklinikum Erlangen, Department of Internal Medicine 3, Rheumatology and Immunology, Erlangen, Germany; [6]Fondazione Policlinico Universitario A. Gemelli, IRCCS - Università Cattolica del Sacro Cuore, Dipartimento di Scienze di Laboratorio ed Ematologiche, Area Ematologia, Rome, Italy;*

### Background

Chronic lymphocytic leukemia (CLL) cells activate, exhaust, and immune suppress T cell subsets (Pelzl, 2025; Jitschin 2014). Today, the previous standard of immuno-chemotherapy (Chemo-R) has been mostly replaced by Bruton Tyrosine kinase (BTK) or B cell leukemia 2 (Bcl2) inhibitors which shows superior efficacy. While BTKi has been shown to partly restore T cell fitness, very little data exist for Bcl2i or R-Chemo

### Aims

To characterize the impact of distinct first-line therapies on T cell fitness in CLL.

### Methods

Patients from the two university hospitals in Rome and Erlangen were retrospectively compared. Patient groups encompassed CLL in watch and wait (w&w, n= 32 patients), patients in complete remission (CR) by iwCLL criteria after first-line therapy with continuous BTKi (n=25), Bcl2i and anti-CD20 antibody (n=16) or Chemo-R (n=15). T cell subsets (CD4, CD8, naïve, CM, EM, TEMRA) including their activation (HLA-DR) and exhaustion (PD1) were measured by flow cytometry. Gene expression and clonality of CD4/CD8 subsets were analyzed by bulk RNA and TCR sequencing. Cytokine profiling was conducted using the Olink® Target 96 Immuno-Oncology Panel. Single- and repeated stimulation was tested in vitro through SEB or a bispecific antibody (BsAb) assay against the mantle cell lymphoma cell line Jeko-1.

**Copyright Information:** (Online) ISSN: 2572-9241
©2026 The Author(s). HemaSphere is published by John Wiley & Sons Ltd on behalf of European Hematology Association. This is an open access Abstract Book distributed under the Attribution-NonCommercial-NoDerivs (CC BY-NC-ND), which allows third parties to download the articles and share them with others as long as they credit the author and the Abstract Book, but they cannot change the content in any way or use them commercially.
**Abstract Book Citations:** Authors, Title, HemaSphere, 2026;10:(S1):pages. The abstract book DOIs can be found on the "Author Information" page.
**Disclaimer:** Articles published in the journal HemaSphere exclusively reflect the opinions of the authors. The authors are responsible for all content in their abstracts including accuracy of the facts, statements, citing resources, etc.

**Results**

The median time from treatment start to sample collection was 51 months for the BTKi (range 12-90), 19 mo for the Bcl2i (8-54), and 42 mo for the Chemo-R group (17-73). The absolute number of total and naïve CD4 T cells were lower than normal in w&w, were similar in BTKi-treated patients, non-significantly reduced by Bcl2i and significantly reduced following Chemo-R. Moreover, CD8 TEMRA expansion in w&w was normalized in CR similarly by all three therapies. In line, CD4 activation and CD8 exhaustion was higher than normal in w&w and normalized by all three therapies. Gene set enrichment analysis of sequenced CD4 and CD8 cells confirmed reduced lymphocyte activation combined with increased antiviral response. Reduced inflammation was further supported by serum inflammasome which showed marked down-regulation of T cell inflammatory cytokines including TNF, CD27, CD70, CD40L. In the TCR sequencing of CD4 and CD8 cells we found an increase of clonality in w&w, correlating with a relative expansion of the top 10 clonotypes in active CLL. Interestingly, the Chemo-R group showed the lowest clonality, possibly due to lymphodepletion-derived immune reset of the T cell compartment. Functionally, T cell stimulation assays showed higher activation potential in BTKi and Bcl2i treated patients compared with Chemo-R and w&w. In line with higher T cell fitness in short-term activation, BTKi and Bcl2i samples showed more T cell expansion and significantly superior long-term tumor control in repeated exposure assays.

**Summary/Conclusion**

In summary, our findings suggest that the absence of CLL achieved by any of the three therapies normalizes hyperactivation of T cells and proinflammation. Nevertheless, BTKi and Bcl2i significantly improve T cell function, possibly offering an advantage for cellular therapies or BsAb, while Chemo-R induces profound depletion and dysfunction of T cells. In line with clinical practice, these immunological data support BTKi or Bcl2i as first-line treatment and provide additional arguments against conventional chemotherapy in CLL.

**Copyright Information:** (Online) ISSN: 2572-9241
©2026 The Author(s). HemaSphere is published by John Wiley & Sons Ltd on behalf of European Hematology Association. This is an open access Abstract Book distributed under the Attribution-NonCommercial-NoDerivs (CC BY-NC-ND), which allows third parties to download the articles and share them with others as long as they credit the author and the Abstract Book, but they cannot change the content in any way or use them commercially.
**Abstract Book Citations:** Authors, Title, HemaSphere, 2026;10:(S1):pages. The abstract book DOIs can be found on the “Author Information” page.
**Disclaimer:** Articles published in the journal HemaSphere exclusively reflect the opinions of the authors. The authors are responsible for all content in their abstracts including accuracy of the facts, statements, citing resources, etc.

### (PF589) SESHAT II AND THE NEW UMD TP53 ERA: TURNING NOISY TP53 SEQUENCING INTO CLEAN, CLINICALLY ACTIONABLE REPORTS.

**Topic:** 05. Chronic lymphocytic leukemia and related disorders - Biology & translational research

Lea Rodriguez[1], Bernard Leroy[1], Peter Freeman[2], Sarka Pavlova[3], Jitka Malcikova[3], Grégory Lazarian[4], Fanny Baran Marszak[4], Panagiotis Baliakas[5], François Delhommeau[1], Thierry Soussi*[5]

*[1]Sorbonne Université, Centre de Recherche Saint-Antoine, Paris, France;[2]University of Manchester, Division of Informatics, Imaging & Data Science,, Manchester, United Kingdom; [3]University Hospital Brno, Department of Internal Medicine, Brno, Czechia; [4]Hôpital Avicenne HUPSSD APHP Bobigny, Bobigny, France;[5]Uppsala University, Department of Immunology, Genetics and Pathology,, Uppsala, Sweden;*

### Background

Accurate interpretation of TP53 variants is critical for prognostication and treatment selection, particularly in hematologic malignancies such as CLL and AML. Interpretation remains challenging because of extreme allelic heterogeneity, transcript complexity, technical artifacts, and the coexistence of highly pathogenic mutations with rare benign or low-penetrance germline variants.

### Aims

The new UMD_TP53 database and Seshat II were developed to provide a unified, TP53-specific framework that (i) delivers robust pathogenicity/oncogenicity classification of somatic and germline variants, (ii) resolves splice and isoform annotation and nomenclature issues in strict accordance with HGVS recommendations, and (iii) translates this information into clinically actionable reports that can be directly used in routine diagnostics and clinical decision-making.

### Methods

The updated UMD TP53 resource aggregates ~270,000 TP53-mutated cancer cases, including ~25,000 hematologic malignancies, and integrates large-scale multiplex functional assays (>9,000 variants), refined splice predictions (SpliceAI plus tumor RNA stability data), population databases, and curated clinical–pathologic correlations, followed by re-classification using TP53-tailored ACMG/ClinGen and contemporary somatic-variant criteria. Seshat II builds on this backbone to automatically process single variants or NGS files, standardize HGVS-compliant genomic, coding, and protein descriptions on a stable TP53 reference, and return structured variant-level reports including function, splice impact, frequencies, and a final pathogenicity label aligned with international TP53/ERIC recommendations.

**Copyright Information:** (Online) ISSN: 2572-9241
©2026 The Author(s). HemaSphere is published by John Wiley & Sons Ltd on behalf of European Hematology Association. This is an open access Abstract Book distributed under the Attribution-NonCommercial-NoDerivs (CC BY-NC-ND), which allows third parties to download the articles and share them with others as long as they credit the author and the Abstract Book, but they cannot change the content in any way or use them commercially.
**Abstract Book Citations:** Authors, Title, HemaSphere, 2026;10:(S1):pages. The abstract book DOIs can be found on the “Author Information” page.
**Disclaimer:** Articles published in the journal HemaSphere exclusively reflect the opinions of the authors. The authors are responsible for all content in their abstracts including accuracy of the facts, statements, citing resources, etc.

**Results**

The combined system substantially refines the boundary between pathogenic and benign TP53 variation by reclassifying numerous recurrent missense SNPs as benign or likely benign, including several ethnic-specific polymorphisms, and by improving prediction of splice-altering variants through concordant in silico and RNA-based evidence supported by quantitative functional readouts. A key advance is that Seshat II actively identifies and corrects technical and annotation problems that commonly affect TP53 testing: it enforces HGVS rules (including “most 3′” representation and consistent transcript/reference use), resolves discordant c./p. notations across isoforms, flags internal inconsistencies within input files, and highlights likely sequencing artefacts or low-quality calls that are incompatible with curated UMD TP53 experience. By mapping each submitted variant onto a unique, HGVS-standardized TP53 event and cross-checking against a very large, functionally annotated catalogue, Seshat II reduces false-positive somatic calls driven by artefacts or rare germline SNPs, facilitates recognition of multi-hit versus mono-allelic disease and therapy-associated patterns, and underpins ERIC-style recommendations that treat clearly function-impairing TP53 variants as highly actionable lesions irrespective of VAF.

**Summary/Conclusion**

The updated UMD TP53 database, coupled with Seshat II as an automated, HGVS-compliant expert annotator, transforms TP53 testing from heterogeneous, gene-agnostic pipelines into a standardized, error-controlled interpretation process. It enables laboratories to report all TP53 variants above their validated detection limit with consistent nomenclature and TP53-specific classification, and provides clinicians with concise, reliable information directly translatable into treatment decisions and risk stratification.

**Copyright Information:** (Online) ISSN: 2572-9241
©2026 The Author(s). HemaSphere is published by John Wiley & Sons Ltd on behalf of European Hematology Association. This is an open access Abstract Book distributed under the Attribution-NonCommercial-NoDerivs (CC BY-NC-ND), which allows third parties to download the articles and share them with others as long as they credit the author and the Abstract Book, but they cannot change the content in any way or use them commercially.
**Abstract Book Citations:** Authors, Title, HemaSphere, 2026;10:(S1):pages. The abstract book DOIs can be found on the “Author Information” page.
**Disclaimer:** Articles published in the journal HemaSphere exclusively reflect the opinions of the authors. The authors are responsible for all content in their abstracts including accuracy of the facts, statements, citing resources, etc.

### (PF590) OUTCOMES AFTER 1L TREATMENT OF PATIENTS WITH CHRONIC LYMPHOCYTIC LEUKEMIA BELONGING TO STEREOTYPED SUBSET #2, AN INTERNATIONAL MULTICENTER STUDY CONDUCTED BY ERIC.

**Topic:** 06. Chronic lymphocytic leukemia and related disorders - Clinical

Thomas Chatzikonstantinou*[1], Andreas Agathangelidis[2], Persefoni Talimtzi[1], Eva Minga[1], Alyssa Adlaon[3], Panagiotis Baliakas[4], Fanny Baran Marszak[5, 6], Beatriz Bellosillo[7], Bella Biderman[8], Riccardo Bomben[9], András Bors[10], Christian Brieghel[11], Aleksandra Butrym[12], Martin Čermák[13], Li Yuan Chan[14], Annalisa Chiarenza[15], Amy Christian[16], Rosa Collado Nieto[17], Marta Crespo Maull[18], Maria Ilaria Del Principe[19], Maria Dimou[20], Erica Dorillo[21], Elżbieta Drab-Urbanek[22], Jarosław Dybko[23], Fabiana Esposito[24], Alberto Fresa[25], Paula Gameiro[26], Rocío García-Serra[27], José Antonio García Vela[28], Ritu Gupta[29], Eleftheria Hatzimichael[30], Thure Haunstrup[31], Yair Herishanu[32], Thor Høyer[33], Francesco Iadevaia[34], Vanessa Innao[35], Idanna Innocenti[36], Paula Jabłonowska-Babij[37], Emilia Jaskula[38, 39], Elżbieta Kalicińska[40], Teodora Karan-Đurašević[41], Lukas Kazianka[42], Sabina Kersting[43], Maria Kislova[44], Andreas-Epameinondas Koumpis[30], Ioannis Kyriakou[45], Nicole Larmonie[46], Tamás László[47], Luca Laurenti[48], Mark-David Levin[49], Vincent Levy[50], Irene López-Oreja[51, 52], Bogusław MacHaliński[53], Francesca Martini[54], Kiyomi Mashima[55], Zoltan Istvan Matrai[56], Joanne Milligan[16], Vanyo Mitev[57, 58], Riccardo Moia[59], Samir Mouhssine[60], Elisabetta Novella[61], Ana Catarina Oliveira[62], Jacopo Olivieri[63], Irina Panovska-Stavridis[64], Miguel Arturo Pavlovsky[65], Juan A. Piñeyroa[66, 67, 68], Karla Plevova[69, 70], Helena Podgornik[71, 72], Anna Puiggros[73, 74], Bartosz Puła[75, 76], Gian Matteo Rigolin[77], Sotirios Sachanas[78], Benjamin Sale[79], Yorick Sandberg[80], Uday Aditya Sarkar[55], Marta Sebastião[26], Angel Serna Pareja[81], Martin Simkovic[82], Svetlana Smirnova[83], Lukáš Smolej[84], Paolo Sportoletti[85], Basile Stamatopoulos[86], Andrey B. Sudarikov[87], Wojciech Szlasa[23], Tamar Tadmor[88, 89], Albena Todorova[58], Matthias Vanderkerken[90], Katrina Vanura[42], Andrea Visentin[91], Niki de Winter[92], Marzena Wojtaszewska[93], Joy Yan[94], Angelina Yosifova[95, 96], Debora Zdrojewska[53], Antonella Zucchetto[9], Inhye E. Ahn[55], Csaba Bödör[47], Francisco Bosch Albareda[81], Jennifer R. Brown[55], Mark Catherwood[97], Nicholas Chiorazzi[98], Zadie Davis[16], Michael Doubek[70, 99], Blanca Espinet[100], Francesco Forconi[3, 101], Gianluca Gaidano[59], Valter Gattei[9], Sunil Iyengar[14], Arnon Philip Kater[102], Anton Langerak[92], Carsten Niemann[11], Eugene Nikitin[44], Gerassimos Pangalis[78], Sarka Pavlova[103], Davide Rossi[104, 105, 106], Niki Stavrogianni[45], Livio Trentin[107], Tomasz Wróbel[108], Loic Ysebaert[109], Lydia Scarfò[110, 111], Anastasia Chatzidimitriou[1], Paolo Ghia[110, 111], Kostas Stamatopoulos[1]

*[1]Institute of Applied Biosciences, Centre for Research and Technology Hellas, Thessaloniki, Greece;[2]Department of Biology, School of Science, National and Kapodistrian University of Athens, Athens, Greece; [3]Cancer Sciences, Faculty of Medicine, University of Southampton, Southampton, United Kingdom; [4]Department of Immunology, Genetics and Pathology, Uppsala University, Uppsala, Sweden; [5]GHUPSSD Hôpital Avicenne AP-HP, Bobigny, France; [6]INSERM U1349 Université Sorbonne Paris Nord, Bobigny, France; [7]Pathology Service, Hospital del Mar, Barcelona, Spain; [8]National Medical Research Center for Hematology, Novy Zykovski Lane, 4a, 125167, Moscow, Russia; [9]Clinical and Experimental Onco-Hematology Unit, Centro di Riferimento Oncologico di Aviano (CRO) IRCCS, Aviano, Italy; [10]Laboratory of Molecular Genetics, Central Hospital of Southern Pest, Budapest, Hungary; [11]Department of Hematology, Rigshospitalet, Copenhagen University Hospital, Copenhagen, Denmark; [12]Wroclaw Medical University, Wroclaw, Poland; [13]Department of Genetics, The National Institute of Oncology, Bratislava, Slovakia; [14]Department of Haematology, The Royal Marsden Hospital, London, United Kingdom;[15]Division of Haematology, Ferrarotto Hospital, Catania, Italy; [16]Molecular Pathology, UHD Royal Bournemouth Hospital, Bournemouth, United Kingdom; [17]Servicio de Hematología, Consorcio Hospital General Universitario de Valencia, Fundación de Investigación Hospital Genera, Valencia, Spain; [18]Experimental Hematology, Vall d'Hebron Institute of Oncology, Barcelona, Spain;[19]Hematology, Department of Biomedicine and Prevention, University of Rome Tor Vergata, Rome, Italy; [20]Department of Hematology and Bone Marrow Transplantation Unit, National and Kapodistrian University of Athens, Laikon General, Athens, Greece; [21]Institute of Hematology-Centro di Ricerche Emato-Oncologiche (CREO), University of Perugia, Perugia, Italy; [22]Department of Haematooncology and Bone Marrow Transplantation, Medical University of Lublin, Lublin, Poland; [23]Hematology and Transplantology Centre of Lower Silesia Centre for Oncology, Pulmonology and Hematology in Wrocław, Wrocław, Poland; [24]Department of Biomedicine and Prevention, University of Rome tor Vergata, Rome, Italy;[25]Hematology-Oncology and Stem*

**Copyright Information:** (Online) ISSN: 2572-9241
©2026 The Author(s). HemaSphere is published by John Wiley & Sons Ltd on behalf of European Hematology Association. This is an open access Abstract Book distributed under the Attribution-NonCommercial-NoDerivs (CC BY-NC-ND), which allows third parties to download the articles and share them with others as long as they credit the author and the Abstract Book, but they cannot change the content in any way or use them commercially.
**Abstract Book Citations:** Authors, Title, HemaSphere, 2026;10:(S1):pages. The abstract book DOIs can be found on the "Author Information" page.
**Disclaimer:** Articles published in the journal HemaSphere exclusively reflect the opinions of the authors. The authors are responsible for all content in their abstracts including accuracy of the facts, statements, citing resources, etc.

Cell Transplantation Unit, National Cancer Institute - IRCCS - Fondazione "G. Pascale", Naples, Italy; [26]Haematology Unit, Instituto Português de Oncologia de Lisboa Francisco Gentil, Lisbon, Portugal; [27]Servicio de Hematología, Consorcio Hospital General Universitario de Valencia, Fundación de Investigación Hospital General, Valencia, Spain; [28]Servicio de Hematologia, Laboratorio de Citometria de Flujo Hospital Universitario Puerta de Hierro, Majadahonda, Madrid, Spain; [29]Laboratory Oncology Unit, Dr. B.R.A. IRCH, All India Institute of Medical Sciences (AIIMS), New Delhi, India; [30]Faculty of Medicine, Department of Haematology, School of Health Sciences, University of Ioannina, Stavros Niarchos Avenue, Ioannina, Greece; [31]Department of Hematology, Aalborg University Hospital, Aalborg, Denmark; [32]Department of Henatology, Tel Aviv Sourasky Medical Center and Gray Faculty of Medical & Health Sciences, Tel Aviv, Israel; [33]Department of Hematology, Aalborg University Hospital, Aalborg, Denmark; [34]Sezione di Ematologia, Dipartimento di Scienze Radiologiche ed Ematologiche, Università Cattolica del Sacro Cuore, Rome, Italy; [35]UOC Ematologia, ARNAS Garibaldi, Catania, Italy; [36]Department of Laboratory and Hematological Sciences, Hematology Area. Fondazione Policlinico Universitario A. Gemelli, IRCCS, Rome, Italy; [37]Wroclaw Medical University, Faculty of Nursing and Midwifery, Department of Nursing, Wroclaw, Poland; [38]L. Hirszfeld Institute of Immunology and Experimental Therapy, Polish Academy of Sciences, Rudolf Weigl 12,53-114,, Wroclaw, Poland; [39]Lower Silesian Oncology Hematology and Pulmonology Center, Ludwik Hirszfeld square 12, 53-413, Wroclaw, Poland; [40]Department and Clinic of Hematology Blood Neoplasms and Bone Marrow Transplantation Wroclaw Medical University, Wroclaw, Poland; [41]Laboratory for Molecular Biomedicine, Institute of Molecular Genetics and Genetic Engineering, University of Belgrade, 11042, Belgrade, Serbia; [42]Department of Medicine I, Division of Hematology and Hemostaseology, Medical University of Vienna, Waehringer Guertel 18-20, 1090 Vienna, Austria, Vienna, Austria; [43]Dept. Hematology, Haga Hospital, The Hague, The Netherlands; [44]Botkin Hospital, RMACPE, Moscow, Russia; [45]Hematology Department and HCT Unit, G. Papanicolaou Hospital, Thessaloniki, Greece; [46]Dept. Hematology, Erasmus MC, University Medical Center, Rotterdam, The Netherlands; [47]MTA-SE Lendulet Molecular Oncohematology Research Group, Department of Pathology and Experimental Cancer Research, Semmelweis University, Budapest, Hungary; [48]Department of Laboratory and Hematological Sciences, Hematology Area. Fondazione Policlinico Universitario A. Gemelli, IRCCS, Rome, Italy - Università Cattolica del Sacro Cuore, Rome, Italy; [49]Dept. Internal Medicine, Albert Schweitzer Hospital, Dordrecht, The Netherlands; [50]ECSTRRA, 1, avenue Claude Vellefaux, Paris 75010, France; Ďepartement de Recherche Clinique, Hôpital Avicenne, Bobigny, France; [51]Department of Hematopathology, Hospital Clínic, Barcelona, Spain;[52]Institut d'Investigacions Biomèdiques August Pi i Sunyer (IDIBAPS), Barcelona, Spain; [53]Department of General Pathology, Pomeranian Medical University, Szczecin, Poland; [54]Strategic Research Program on CLL, Comprehensive Cancer Center, IRCCS Ospedale San Raffaele, Milan, Italy; [55]Department of Medical Oncology, Dana-Farber Cancer Institute, Boston, United States of America; [56]Central Hospital of Southern Pest, National Institute for Haematology and Infectology, Budapest, Hungary;[57]Research Institute of Innovative Medical Science, Sofia, Bulgaria; [58]Medical Chemistry and Biochemistry, Medical University Sofia, Sofia, Bulgaria; [59]Division of Hematology, Department of Translational Medicine, University of Eastern Piedmont, Novara, Italy;[60]Division of Hematology, Department of Translational Medicine, Università del Piemonte Orientale and Azienda Ospedaliero-Universitaria Maggiore della Carità, Novara, Italy; [61]Departmental Unit for Genetic and Genomic Diagnostics, San Bortolo Hospital (Aulss 8 Veneto), Vicenza, Italy; [62]Department of Clinical Hematology, ICO, Hospital Duran i Reynals, IDIBELL, Barcelona, Spain; [63]Hematology Clinic, ASUFC, Udine, Italy; [64]Medical Faculty, University Clinic of Hematology, University Ss. Cyril and Methodius, Skopje, Macedonia; [65]Fundaleu, Buenos Aires, Argentina; [66]Facultat de Medicina i ciències de la salut, Universitat de Barcelona, Barcelona, Spain; [67]Servei d'Hematologia, Hospital Clínic de Barcelona, Barcelona, Spain;[68]Terapias experimentales en neoplasias linfoides, Institut d'Investigacions Biomèdiques August Pi i Sunyer (IDIBAPS), Barcelona, Spain; [69]Center of Molecular Medicine, Central European Institute of Technology, Masaryk University, Brno, Czechia; [70]Department of Internal Medicine - Hematology and Oncology, University Hospital Brno and Faculty of Medicine, Masaryk University, Brno, Czechia; [71]University medical center, Ljubljana, Ljubljana, Slovenia; [72]Faculty of Pharmacy, University of Ljubljana, Ljubljana, Slovenia;[73]Molecular Cytogenetics Laboratory, Pathology Department, Hospital del Mar, Barcelona, Spain; [74]Translational Research on Hematological Neoplasms Group, Hospital del Mar Research Institute (IMIM), Barcelona, Spain; [75]Department of Hematology, Medical University of Lodz, Lodz, Poland; [76]Department of General Hematology and Internal Medicine, Copernicus Memorial Hospital, Lodz, Poland; [77]St. Anna University Hospital, Ferrara, Italy;[78]Department of Haematology, Athens Medical Center-Psychikon Branch, Athens, Greece; [79]School of Cancer Sciences, University of Southampton, Southampton, United Kingdom;[80]Dep. Internal Medicine, Maasstad Hospital, Rotterdam, The Netherlands; [81]Department of Haematology, University Hospital Vall d'Hebron, Autonomous University, Barcelona, Spain; [82]4th Department of Internal Medicine-Hematology, Faculty of Medicine in

**Copyright Information:** (Online) ISSN: 2572-9241
©2026 The Author(s). HemaSphere is published by John Wiley & Sons Ltd on behalf of European Hematology Association. This is an open access Abstract Book distributed under the Attribution-NonCommercial-NoDerivs (CC BY-NC-ND), which allows third parties to download the articles and share them with others as long as they credit the author and the Abstract Book, but they cannot change the content in any way or use them commercially.
**Abstract Book Citations:** Authors, Title, HemaSphere, 2026;10:(S1):pages. The abstract book DOIs can be found on the "Author Information" page.
**Disclaimer:** Articles published in the journal HemaSphere exclusively reflect the opinions of the authors. The authors are responsible for all content in their abstracts including accuracy of the facts, statements, citing resources, etc.

*Hradec Králové, University Hospital and Charles University, Hradec Králové, Czechia; [83]Consultative Hematology Department with a Day Hospital for Intensive High-Dose Chemotherapy, National Medical Research Center for Hematology, Moscow, Russia; [84]4th Department of Internal Medicine-Haematology, University Hospital and Faculty of Medicine, Hradec Králové, Czechia; [85]Institute of Hematology and Center for Hemato-Oncology Research, University of Perugia and Santa Maria della Misericordia Hospital, Perugia, Italy; [86]Translational Hematology and Oncology Research laboratory, Université Libre de Bruxelles (ULB), Jules Bordet Institute, Brussels, Belgium; [87]National Medical Research Center for Hematology, Moscow, Russia;[88]Hematology, Bnai-Zion Medical Center, Haifa, Israel; [89]Ruth and Bruce Rappaport Faculty of Medicine, Technion, Haifa, Israel; [90]Antwerp University Hospital, Antwerp, Belgium; [91]Hematology Unit, Department of Medicine, University of Padova, Padova, Italy; [92]Laboratory Medical Immunology, Dept. Immunology, Erasmus MC, University Medical Center, Rotterdam, The Netherlands; [93]Department of Hematology, Frederic Chopin Provincial Specialist Hospital, Rzeszów, Poland;[94]Chemical Biology and Molecular Medicine Program, H. Lee Moffitt Cancer Center and Research Institute, Tampa, FL, United States of America; [95]Independent Medico-Diagnostic Laboratory "Genome Center Bulgaria", Sofia, Bulgaria; [96]Department of Medical Chemistry and Biochemistry, Medical University Sofia, Sofia, Bulgaria; [97]Clinical Haematology, Belfast City Hospital, Belfast, United Kingdom; [98]Feinstein Institute for Medical Research, Manhasset, NY, United States of America;[99]Central European Institute of Technology (CEITEC), Masaryk University, Brno, Czechia; [100]Molecular Cytogenetics Laboratory, Pathology Department, Hospital del Mar and Translational Research on Hematological, Barcelona, Spain; [101]Haematology Department, Cancer Care Directorate, University Hospital Southampton NHS Foundation Trust, Southampton, United Kingdom; [102]Dept of Hematology, Cancer Center Amsterdam, Amsterdam University Medical Centers, University of Amsterdam, Amsterdam, The Netherlands; [103]Department of Internal Medicine, Hematology and Oncology, University Hospital Brno and Medical Faculty, Masaryk University, Brno, Czechia; [104]Oncology Institute of Southern Switzerland (IOSI), Ente Ospedaliero Cantonale (EOC), Bellinzona, Switzerland; [105]Institute of Oncology Research (IOR), Bellinzona, Switzerland; [106]Faculty of Biomedical Sciences, Università della Svizzera Italiana (USI), Lugano, Switzerland; [107]Hematology and Clinical Immunology Unit, Department of Medicine, University of Padova, Padova, Italy; [108]Clinical Department of Hematology, Cell Therapies and Internal Diseases, Faculty of Medicine, Wroclaw Medical University, Wroclaw, Poland; [109]Department of Hematology, Institut Universitaire du Cancer Toulouse-Oncopole, Toulouse, France; [110]Medical School, Università Vita-Salute San Raffaele and IRCCS Ospedale San Raffaele, Milan, Italy; [111]Comprehenzive Cancer Center, IRCCS Ospedale San Raffaele, Milan, Italy;*

**Background**

Patients (pts) with chronic lymphocytic leukemia (CLL) belonging to stereotyped subset #2 (defined by the IGHV3-21/IGLV3-21 combination with a short VH CDR3 of 9 amino acids) experience suboptimal outcomes when treated with 1$^{st}$ line (1L) chemoimmunotherapy (CIT) despite carrying mutated immunoglobulin heavy variable (IGHV) genes in most cases. Limited data are available on the outcomes of these pts when treated with targeted drugs.

**Aims**

We aimed to describe the clinicobiological characteristics and outcomes of pts with CLL belonging to subset #2 and treated with different treatment paradigms [CIT, chemotherapy (CTx), continuous BTK inhibitors (BTKi) and fixed-duration (FD) targeted therapies].

**Methods**

This is a retrospective international multicenter study conducted by the European Research Initiative on CLL. Pts treated for CLL belonging to subset #2 who have received at least 1 line of CLL-directed treatment were included in the study. The IGHV gene sequences of all cases were centrally reviewed to confirm subset #2 assignment.

**Copyright Information:** (Online) ISSN: 2572-9241
©2026 The Author(s). HemaSphere is published by John Wiley & Sons Ltd on behalf of European Hematology Association. This is an open access Abstract Book distributed under the Attribution-NonCommercial-NoDerivs (CC BY-NC-ND), which allows third parties to download the articles and share them with others as long as they credit the author and the Abstract Book, but they cannot change the content in any way or use them commercially.
**Abstract Book Citations:** Authors, Title, HemaSphere, 2026;10:(S1):pages. The abstract book DOIs can be found on the "Author Information" page.
**Disclaimer:** Articles published in the journal HemaSphere exclusively reflect the opinions of the authors. The authors are responsible for all content in their abstracts including accuracy of the facts, statements, citing resources, etc.

### Results

Data from 445 pts at 66 sites in 25 countries were analyzed. Most pts were males (256, 57.5%), and the majority had CLL (420, 94.4%), while 25 (5.6%) were diagnosed with SLL. The median age at 1L treatment was 69 years [interquartile range (IQR) 62-75]. The median follow-up from 1L treatment was 52.9 (IQR 24-89.7) months.
The majority of pts had mutated IGHV genes (268, 60.2%). Similar to previous studies, only a minority of pts had *TP53* aberrations (34/394, 8.6%) and complex karyotype with ≥5 abnormalities without *TP53* aberrations (3/72, 4.1%). del(13q), trisomy 12 and del(11q) were present in 239/351, (68.1%), 6/344 (1.7%) and 87/368 (23.6%), respectively.
In total, 187 pts received CIT (65 BR, 63 FCR, 35 chlorambucil plus anti-CD20 and 24 other regimens), 109 CTx (66 chlorambucil monotherapy) and 138 targeted agents-based treatments. Of the latter group, 84 cases were treated with a continuous BTKi (43 ibrutinib, 35 acalabrutinib, 4 zanubrutinib and 2 pirtobrutinib), 51 with FD regimens (29 venetoclax(ven)-obinutuzumab, 15 ven-ibrutinib, 6 ven-rituximab and 1 ven-acalabrutinib-obinutuzumab) and 3 with ven monotherapy.
The median age of pts receiving BTKis, FD regimens, CIT and CTx was 72 (IQR:66-77), 68 (IQR:56-74), 68 (IQR:62-74) and 70 (IQR:62-76) years, respectively. *TP53* aberrations were present in 10/80 (12.5%), 2/33 (6%), and 6/165 (3.6%) of pts treated with BTKis, FD regimens, and CIT, respectively.
The 3-year time to next therapy or death (TTNTD) was significantly improved for pts treated with BTKis and FD regimens compared with CIT (HR: 0.34, 95% CI:0.17-0.69) and CTx (HR: 0.30, 95% CI:0.09, 0.95, respectively). Pts treated with CTx had worse 3-year TTNTD than those treated with CIT (HR: 1.72, 95% CI: 1.2-2.46). The median TTNTD was reached only for pts treated with CIT (50 months, 95% CI=41.5-59) and CTx (33.7 months, 95% CI=27-43). The 3-year-OS for BTKis, FD regimens, CIT and CTx was 89.1% (95% CI:81.6%-97.3%), 91.2% (95% CI:81.8%-100%), 87.3% (95% CI:82.5%-92.4%) and 75.3% (95% CI:67.5%-84%), respectively.

### Summary/Conclusion

In the largest cohort of pts with CLL belonging to subset #2, TTNTD after 1L was significantly improved with the use of targeted agents compared to CIT. In this analysis, the 3-year-TTNTD was comparable between continuous BTKis and FD regimens.

**Copyright Information:** (Online) ISSN: 2572-9241
©2026 The Author(s). HemaSphere is published by John Wiley & Sons Ltd on behalf of European Hematology Association. This is an open access Abstract Book distributed under the Attribution-NonCommercial-NoDerivs (CC BY-NC-ND), which allows third parties to download the articles and share them with others as long as they credit the author and the Abstract Book, but they cannot change the content in any way or use them commercially.
**Abstract Book Citations:** Authors, Title, HemaSphere, 2026;10:(S1):pages. The abstract book DOIs can be found on the “Author Information” page.
**Disclaimer:** Articles published in the journal HemaSphere exclusively reflect the opinions of the authors. The authors are responsible for all content in their abstracts including accuracy of the facts, statements, citing resources, etc.

### (PF591) SECOND PRIMARY MALIGNANCIES IN PATIENTS WITH CHRONIC LYMPHOCYTIC LEUKEMIA TREATED WITH TARGETED THERAPIES IN THREE PHASE-III TRIALS OF THE GERMAN CLL STUDY GROUP

**Topic:** 06. Chronic lymphocytic leukemia and related disorders - Clinical

Ellinor Goergen*[1], Rudy Ligtvoet[1], Sandra Robrecht[1], Othman Al-Sawaf[1], Moritz Fürstenau[1], Nadine Kutsch[1], Mark-David Levin[2], Caspar Da Cunha-Bang[3], Carsten Niemann[3], Arnon Philip Kater[4], Paula Cramer[1], Christof Schneider[5], Eugen Tausch[5], Kirsten Fischer[1], Stephan Stilgenbauer[5], Michael Hallek[1], Barbara Eichhorst[1], Petra Langerbeins[1]

*[1]Faculty of Medicine and University Hospital Cologne, Department I of Internal Medicine, Center for Integrated Oncology Aachen Bonn Cologne Duesseldorf; German CLL Study Group, University of Cologne, Cologne, Germany; [2]Department of Internal Medicine, Albert Schweitzer Hospital, Dordrecht, The Netherlands; [3]Department of Hematology, Rigshospitalet, Copenhagen University Hospital, Copenhagen, Denmark; [4]Department of Hematology, Cancer Center Amsterdam, Amsterdam University Medical Centers, University of Amsterdam, Amsterdam, The Netherlands; [5]Department III of Internal Medicine, University Hospital Ulm, Ulm, Germany;*

### Background

Despite advancements in targeted therapies, second primary malignancies (SPM) remain a major challenge for patients (pts) with chronic lymphocytic leukemia (CLL) contributing to morbidity.

### Aims

The aim of this pooled analysis is to identify risk factors for the development of SPM in pts with treatment-naïve CLL treated in phase-III trials of the German CLL Study Group.

### Methods

Data from phase-III trials (CLL12, CLL13, CLL14) were pooled in 5 cohorts: venetoclax-based (ven) (n=677), Bruton tyrosine kinase inhibitor (BTKi) (n=170), BTKi-ven-based (n=231), chemoimmunotherapy (CIT) (n=430) or untreated (n=320). SPM (including benign neoplasias) were reported until end of study. Follow-up adjusted incidence rates (FUAIR) of SPM were calculated based on time from study entry to first SPM or last observation while alive and reported per 1,000 patient-months (pt-mo). Multivariable logistic regression was used to identify prognostic factors for SPM development. Overall survival was assessed using Cox regression with SPM as a time-dependent covariate with onset at the time of first SPM from study entry.

**Copyright Information:** (Online) ISSN: 2572-9241
©2026 The Author(s). HemaSphere is published by John Wiley & Sons Ltd on behalf of European Hematology Association. This is an open access Abstract Book distributed under the Attribution-NonCommercial-NoDerivs (CC BY-NC-ND), which allows third parties to download the articles and share them with others as long as they credit the author and the Abstract Book, but they cannot change the content in any way or use them commercially.
**Abstract Book Citations:** Authors, Title, HemaSphere, 2026;10:(S1):pages. The abstract book DOIs can be found on the “Author Information” page.
**Disclaimer:** Articles published in the journal HemaSphere exclusively reflect the opinions of the authors. The authors are responsible for all content in their abstracts including accuracy of the facts, statements, citing resources, etc.

### Results

With a median observation time of 68.4 months (mo) (IQR 58.6 – 78.5 mo) a total of 453 SPMs were reported in 349 of 1,828 pts (19.1%). 260 (57.4%) SPMs were CTC grade ≥3, including 41 (9.1%) fatal SPM (26 solid, 9 RTs, 3 hematological; hem, and 3 metastasized non-melanoma skin cancers; NMSC). Of 453 SPM, 172 were NMSC (38%), 150 solid (33%), 67 benign (15%), 39 RT (9%) and 21 hem (5%).
Age >65 years (OR 2.21, 95% CI 1.74 – 2.82, p<0.001), male gender (OR 1.82, 95% CI 1.38 – 2.40, p<0.001), and *TP53* aberration (OR 1.68, 95% CI 1.01 – 2.79, p=0.045) were risk factors for SPM development. For RT (n=39) development, risks included male gender (OR 3.62, 95% CI 1.28 – 10.3, p=0.016), unmutated IGHV (OR 2.60, 95% CI 1.20 – 5.62, p=0.015), and β2-microglobulin ≥ 3.5 mg/L (OR 2.18, 95% CI 1.05 – 4.49, p=0.036). Risk factors for each cohort are depicted in **Fig. 1**.
When evaluating different treatment modalities, median time to first SPM was 19.7 in the ven-cohort, 30.6 mo in the BTKi-cohort, 19.2 mo in the BTKi-ven-cohort, 20.7 mo in the CIT cohort and 25.7 mo in the untreated cohort. Details on FUAIR and number of SPM for each cohort are listed in **Table 1.**

**Table 1:** Details on FUAIR and SPM frequency per treatment cohort

| Cohort | Total SPM | Pts with SPM (%) | Overall FUAIR (per 1,000 pt-mo) | Solid | NMSC | Benign | RT | Hem |
|---|---|---|---|---|---|---|---|---|
| **ven** | 163 | 133 (19.6%) | 3.5 | 1.3 | 1.2 | 0.6 | 0.4 | 0.1 |
| **BTKi** | 35 | 30 (17.6%) | 3.1 | 1.3 | 0.5 | 1.1 | 0.1 | 0.0 |
| **BTKi-ven** | 50 | 40 (17.3%) | 3.1 | 1.3 | 0.6 | 0.7 | 0.2 | 0.6 |
| **CIT** | 133 | 89 (20.7%) | 3.9 | 1.4 | 1.7 | 0.4 | 0.5 | 0.3 |
| **untreated** | 72 | 57 (17.8%) | 3.0 | 1.3 | 1.0 | 0.4 | 0.3 | 0.1 |

Overall survival was significantly shorter for pts with vs pts without SPM in all cohorts (ven HR 4.37, 95% CI 2.72 – 7.04, p<0.001), BTKi (HR 5.33, 95% CI 1.53 – 18.61, p=0.009), BTKi-ven (HR 9.20, 95% CI 2.91 – 29.07, p<0.001), the CIT (HR 2.67, 95% CI 1.70 – 4.21, p<0.001), untreated (HR 11.74, 95% CI 4.65 – 29.63, p<0.001).

### Summary/Conclusion

In this pooled analysis of 1,828 CLL pts treated within 3 phase-III trials, SPM occurred in 19.1% of pts with FUAIR ranging from 3.1 to 3.9 per 1,000 pt-mo across various treatment cohorts with the highest incidence in the CIT cohort, and 3.0 in the untreated cohort, suggesting a comparable SPM rate in the untreated and treated cohorts. Significant risk factors for SPM included higher age, *TP53* aberration, and male gender. With a mortality rate of 9.1%, SPMs remain a challenge in the era of targeted therapies and a continuous surveillance for SPM is recommended.

**Copyright Information:** (Online) ISSN: 2572-9241
©2026 The Author(s). HemaSphere is published by John Wiley & Sons Ltd on behalf of European Hematology Association. This is an open access Abstract Book distributed under the Attribution-NonCommercial-NoDerivs (CC BY-NC-ND), which allows third parties to download the articles and share them with others as long as they credit the author and the Abstract Book, but they cannot change the content in any way or use them commercially.
**Abstract Book Citations:** Authors, Title, HemaSphere, 2026;10:(S1):pages. The abstract book DOIs can be found on the “Author Information” page.
**Disclaimer:** Articles published in the journal HemaSphere exclusively reflect the opinions of the authors. The authors are responsible for all content in their abstracts including accuracy of the facts, statements, citing resources, etc.

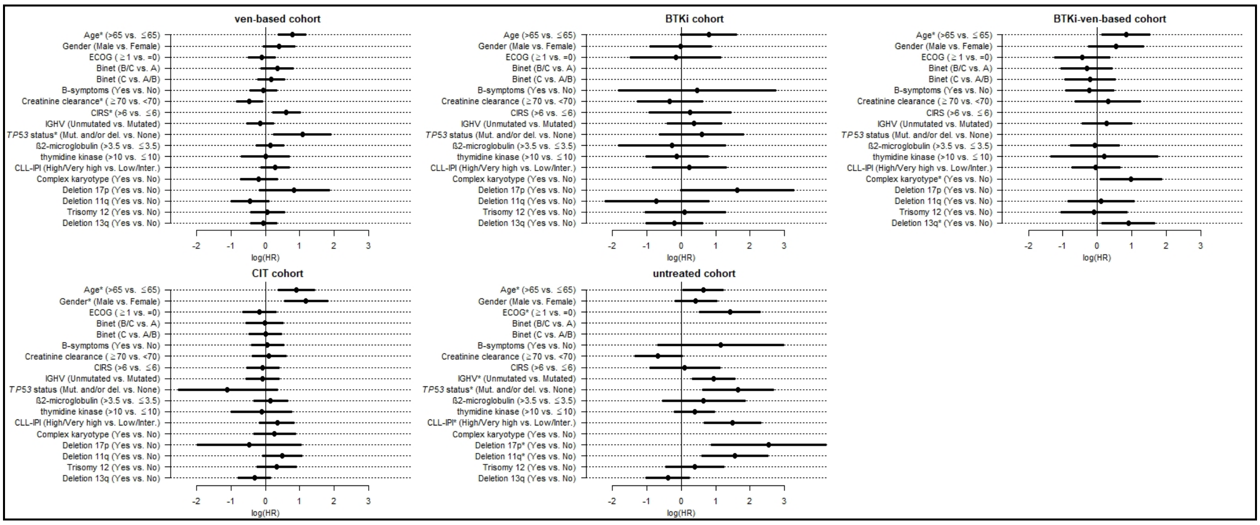

Figure 1: Forest plots of risk factors for each treatment cohort verified analyzed by univariable analysis. ECOG: Eastern Cooperative Oncology Group; CLL-IPI: international prognostic index for patients with chronic lymphocytic leukemia; *: significant predictors in multivariable analysis.

**Copyright Information:** (Online) ISSN: 2572-9241
©2026 The Author(s). HemaSphere is published by John Wiley & Sons Ltd on behalf of European Hematology Association. This is an open access Abstract Book distributed under the Attribution-NonCommercial-NoDerivs (CC BY-NC-ND), which allows third parties to download the articles and share them with others as long as they credit the author and the Abstract Book, but they cannot change the content in any way or use them commercially.
**Abstract Book Citations:** Authors, Title, HemaSphere, 2026;10:(S1):pages. The abstract book DOIs can be found on the "Author Information" page.
**Disclaimer:** Articles published in the journal HemaSphere exclusively reflect the opinions of the authors. The authors are responsible for all content in their abstracts including accuracy of the facts, statements, citing resources, etc.

### (PF592) FIGHT TRIAL: FIXED-DURATION IBRUTINIB PLUS OBINUTUZUMAB AS FIRST-LINE THERAPY FOR TP53-INTACT CHRONIC LYMPHOCYTIC LEUKEMIA (CLL)

**Topic:** 06. Chronic lymphocytic leukemia and related disorders - Clinical

Emanuela Sant'antonio*[1], Silvia Heltai[2], Lydia Scarfò[1, 2], Daniele Sorcini[3], Paolo Sportoletti[3], Gaetano Vaudo[4], Andrea Visentin[5, 6], Marika Porrazzo[7], Marina Motta[8], Giulia Quaresmini[9], Alessandro Gozzetti[10], Eloise Scarano[1], Teresa Musco[2], Catalina Combi[1], Maria Colia[1], Elisa Albi[1], Francesca Martini[1], Ilaria Del Giudice[11], Ludovica Calvo[11], Antonio Cuneo[12], Gian Matteo Rigolin[12], Maura Nicolosi[13], Paolo Ghia[1, 2]

*[1]IRCCS Ospedale San Raffaele, Strategic Research Program on CLL, Milano, Italy; [2]Università Vita-Salute San Raffaele, Milano, Italy; [3]University of Perugia, Department of Medicine and Surgery, Institute of Hematology Centro di Ricerca Emato-Oncologica (CREO), Perugia, Italy; [4]Terni University Hospital, Unit of Internal Medicine, Terni, Italy; [5]Azienda Ospedale Università, Hematology Unit, Padova, Italy; [6]University of Padova, Department of Medicine, Padova, Italy; [7]Azienda Ospedaliera Villa Sofia Cervello, Department of Oncohematology, Palermo, Italy; [8]ASST Spedali Civili, S.C. Ematologia, Brescia, Italy; [9]Hematology Unit, ASST Papa Giovanni XXIII, Department of Oncology and Hematology, Bergamo, Italy; [10]University of Siena, Department of Medical Science, Surgery and Neuroscience, Hematology, Siena, Italy; [11]Sapienza University, Department of Translational and Precision Medicine, Hematology, Rome, Italy; [12]St. Anna University Hospital, Hematology Section, Ferrara, Italy; [13]A.O.U. Città della Salute e della Scienza di Torino, Hematology Division, Torino, Italy;*

### Background

Frontline CLL therapy has progressively shifted from continuous covalent BTK inhibitor (cBTKi) monotherapy toward fixed-duration (FD) combinations, mainly venetoclax with cBTKi or anti-CD20 antibodies. These combinations can lead to clinically meaningful treatment-free (TF) intervals, especially in *TP53*-intact patients (pts), while limiting cumulative toxicity, drug exposure and costs. Though single agent cBTKi rarely induce deep responses, in the iLLUMINATE trial, ibrutinib (IBR) plus obinutuzumab (OBI) induced undetectable measurable residual disease <$10^{-4}$ (uMRD4) in up to 38% of treatment-naïve (TN) high-risk pts. Moreover, the IcICLLe study suggested that delayed OBI after ≥1y of IBR may further deepen responses (50% bone marrow (BM), 60% peripheral blood (PB) uMRD4).

### Aims

To evaluate the efficacy of a 2y FD regimen of IBR + delayed OBI in TN *TP53*-intact CLL. Primary endpoint is BM uMRD4 rate at 30 days post-treatment completion (FU30D).

### Methods

Phase 2, national, multicenter trial. IBR was administered for 24 cycles (C); OBI was added in C13–18, infused as per standard schedule. IBR could be resumed upon progressive disease (PD).

**Copyright Information:** (Online) ISSN: 2572-9241
©2026 The Author(s). HemaSphere is published by John Wiley & Sons Ltd on behalf of European Hematology Association. This is an open access Abstract Book distributed under the Attribution-NonCommercial-NoDerivs (CC BY-NC-ND), which allows third parties to download the articles and share them with others as long as they credit the author and the Abstract Book, but they cannot change the content in any way or use them commercially.
**Abstract Book Citations:** Authors, Title, HemaSphere, 2026;10:(S1):pages. The abstract book DOIs can be found on the "Author Information" page.
**Disclaimer:** Articles published in the journal HemaSphere exclusively reflect the opinions of the authors. The authors are responsible for all content in their abstracts including accuracy of the facts, statements, citing resources, etc.

**Results**

54 pts were enrolled from Dec21 to Jul25 (table 1). Treatment indication (iwCLL 2018) was mainly cytopenia due to BM infiltration (41.7%) and bulky adenopathy (29.2%). At data cut-off (18Feb26), 10 pts are still on treatment.
After 1y of IBR, before starting OBI, 27/28 evaluable pts had a partial response (PR, 96.4%) and 1 stable disease (SD, 3.6%). 26 pts were not evaluable: 5 had not reached this timepoint, 2 discontinued early and 19 due to missing data.
After completing the 2y therapy, at FU30D, 7/25 evaluable pts were in complete response (28%), 14 in PR (56%), yielding an ORR of 84%, while 4 (16%) had PD. 29 were not evaluable: 10 had not yet reached the endpoint, 5 for early discontinuation (1 drop out, 1 PD, 2 for AEs, 1 for clinical decision) and 14 for missing data.
21/34 evaluable pts (61.8%) achieved BM uMRD4, 11 of them were uMRD5 (32.4%); 26/37 (70.3%) achieved PB uMRD4, 12 pts (32.4%) were uMRD5 (fig 1).
No new safety signals emerged; there was a single grade 2 infusion-related reaction to OBI, confirming improved tolerability of the delayed schedule. 18 SAEs were reported, including 2 secondary malignancies (liposarcoma, pancreatic adenocarcinoma), 2 atrial fibrillations, 1 atrial flutter, 1 NSTEMI, 1 acute heart failure. 5 SAEs were deemed possibly related to study drugs. One pt discontinued due to treatment-emergent sensorineural hearing loss (IBR-related), not meeting criteria for SAE.
5 pts required dose reduction: 1 for drug-drug interaction, 4 for AEs, including two grade 3 hypertension.
After a median follow up of 32 months (range 2-48), 7 pts (12.9%) had PD: 1 occurred during C17 and received Venetoclax+Rituximab, 2 PD at FU30D and IBR was resumed. The remaining 4 pts had asymptomatic PD not requiring therapy. PFS and TTNT at 30 months were 75.1% and 87.8%, respectively.

| **Pt characteristics** | **Result** |
|---|---|
| Sex (n=51) | 22 F, 29 M |
| Age, median | 65.4y (39.7-86.1) |
| Rai stage (n=50) | I: 12, 24%<br>II: 13, 26%<br>III: 17, 34%<br>IV: 8, 16% |
| Binet stage (n=50) | A: 4, 8%<br>B: 26, 52%<br>C: 20, 40% |
| IGHV (n=47) | mutated 15, 32%<br>unmutated 32, 68% |
| FISH (n=43) | normal: 6, 14%<br>del13q: 13, 30%<br>trisomy 12: 14, 33%<br>del11q: 10, 23% |
| CIRS (n=49) | mean 3, median 2 (0-10) |

**Copyright Information:** (Online) ISSN: 2572-9241
©2026 The Author(s). HemaSphere is published by John Wiley & Sons Ltd on behalf of European Hematology Association. This is an open access Abstract Book distributed under the Attribution-NonCommercial-NoDerivs (CC BY-NC-ND), which allows third parties to download the articles and share them with others as long as they credit the author and the Abstract Book, but they cannot change the content in any way or use them commercially.
**Abstract Book Citations:** Authors, Title, HemaSphere, 2026;10:(S1):pages. The abstract book DOIs can be found on the "Author Information" page.
**Disclaimer:** Articles published in the journal HemaSphere exclusively reflect the opinions of the authors. The authors are responsible for all content in their abstracts including accuracy of the facts, statements, citing resources, etc.

**Summary/Conclusion**

2-year FD IBR+OBI appears to achieve deep responses and allow safe treatment discontinuation in a significant proportion of TN CLL pts.

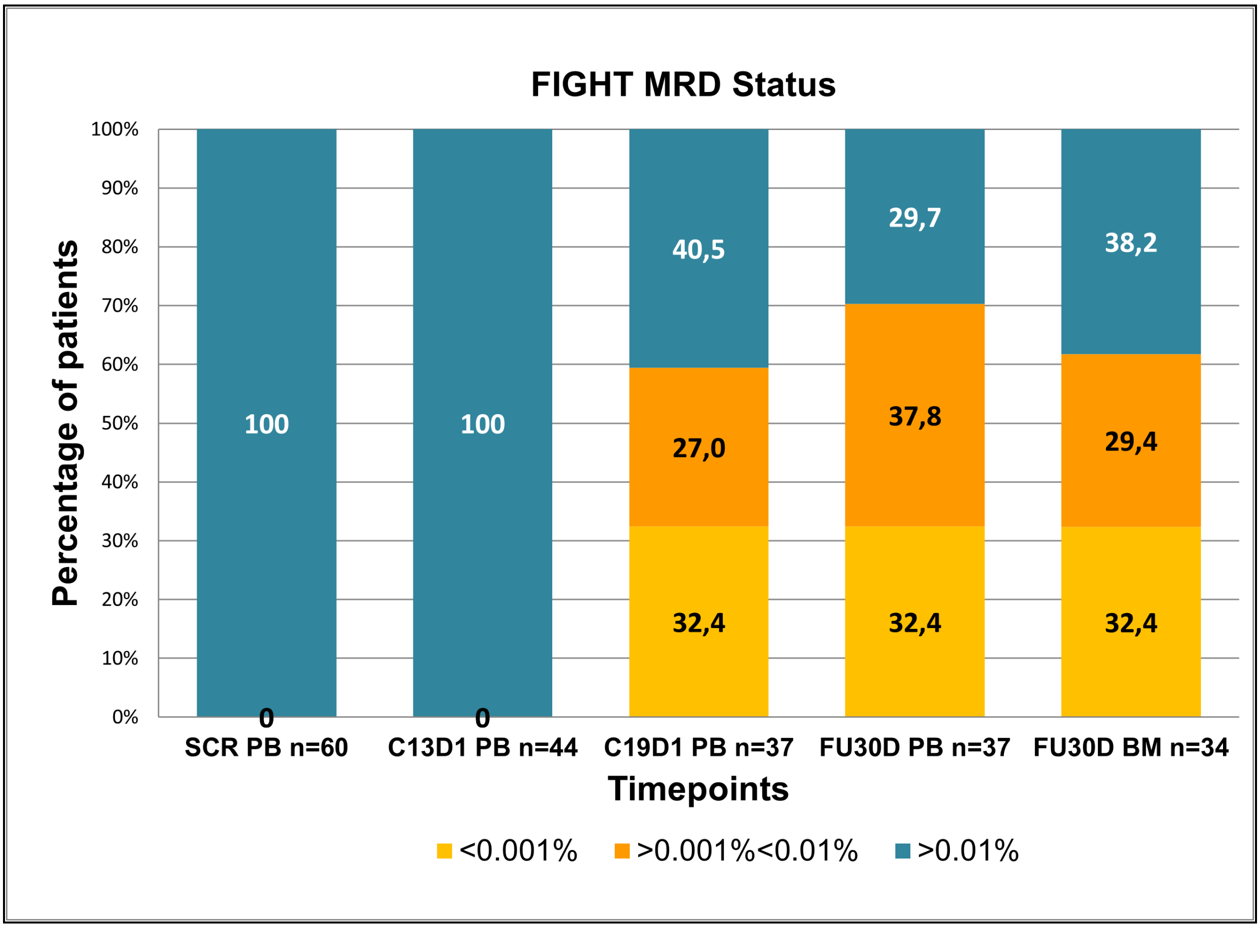

**Copyright Information:** (Online) ISSN: 2572-9241
©2026 The Author(s). HemaSphere is published by John Wiley & Sons Ltd on behalf of European Hematology Association. This is an open access Abstract Book distributed under the Attribution-NonCommercial-NoDerivs (CC BY-NC-ND), which allows third parties to download the articles and share them with others as long as they credit the author and the Abstract Book, but they cannot change the content in any way or use them commercially.
**Abstract Book Citations:** Authors, Title, HemaSphere, 2026;10:(S1):pages. The abstract book DOIs can be found on the "Author Information" page.
**Disclaimer:** Articles published in the journal HemaSphere exclusively reflect the opinions of the authors. The authors are responsible for all content in their abstracts including accuracy of the facts, statements, citing resources, etc.

### (PF594) VENETOCLAX AND DELAYED RITUXIMAB WITH IBRUTINIB CONSOLIDATION AIMING AT UNDETECTABLE MINIMAL RESIDUAL DISEASE IN TREATMENT-NAÏVE (TN) PATIENTS WITH CHRONIC LYMPHOCYTIC LEUKEMIA (CLL): VALUABLE TRIAL

**Topic:** 06. Chronic lymphocytic leukemia and related disorders - Clinical

Francesca Martini*[1], Silvia Heltai[2], Riccardo Moia[3], Andrea Visentin[4], Giulia Quaresmini[5], Marzia Varettoni[6], Gioacchino Catania[7], Andrea Ferrario[8], Lydia Scarfò[1, 2], Elisa Albi[1], Emanuela Sant'antonio[1], Eloise Scarano[1], Paolo Sportoletti[9], Lucia Farina[10], Marina Motta[11], Luca Laurenti[12, 13], Francesca Maria Quaglia[14], Anna Maria Frustaci[15], Ilaria Del Giudice[16], Ludovica Calvo[16], Antonio Cuneo[17], Gian Matteo Rigolin[17], Teresa Musco[2], Catalina Combi[1], Maria Colia[1], Paolo Ghia[1, 2]

*[1]IRCCS Ospedale San Raffaele, Strategic Research Program on CLL, Comprehensive Cancer Center, Milan, Italy;[2]Università Vita e Salute San Raffaele, Milan, Italy; [3]Università del Piemonte Orientale and AOU Maggiore della Carità di Novara, Division of Hematology, Department of Translational Medicine, Novara, Italy; [4]Hematology unit, Azienda Ospedale - Università di Padova, Department of Medicine, Padova, Italy; [5]Azienda Socio Sanitaria Territoriale Papa Giovanni XXIII Bergamo, USC Ematologia, Bergamo, Italy; [6]Fondazione IRCCS Policlinico San Matteo, SC Ematologia 1, Pavia, Italy;[7]AOU SS Antonio e Biagio e C. Arrigo, Alessandria, Italy; [8]Azienda Socio Sanitaria Territoriale dei Sette Laghi, Ospedale di Circolo di Varese, Varese, Italy;[9]University of Perugia, Santa Maria della Misericordia Hospital, Department of Medicine and Surgery, Institute of Hematology and Center for Hemato-Oncology Research (CREO), Perugia, Italy; [10]Fondazione IRCCS Istituto Nazionale dei Tumori, Hematology Dep, Milan, Italy; [11]S.C. Ematologia, ASST Spedali Civili, Brescia, Italy; [12]Fondazione Policlinico Universitario A Gemelli, IRCCS, Dipartimento di Scienze di Laboratorio ed Ematologiche, Area Ematologia, Rome, Italy; [13]Università Cattolica del Sacro Cuore, Rome, Italy; [14]Azienda Ospedaliera Universitaria Integrata Verona, U.O.C. di Ematologia, Verona, Italy; [15]ASST Grande Ospedale Metropolitano Niguarda, Milan, Italy; [16]Sapienza University, Hematology, Department of Translational and Precision Medicine, Rome, Italy; [17]Università degli Studi di Ferrara, Ferrara, Italy;*

### Background

In CLL, achieving undetectable minimal residual disease (uMRD4, i.e. <$10^{-4}$) with venetoclax (ven)-based therapy (tx) correlates with longer progression free survival (PFS) and overall survival (OS) and MRD has been explored to guide tx duration. In randomized trials, ven plus obinutuzumab (obi) led to higher uMRD rates and longer PFS compared to ven plus rituximab (R), but with an increased risk of infection including fatal cases. Sequential tx approach with ven, delayed R and sequential ibrutinib (ibr) may be helpful to maximize efficacy and reduce toxicity.

### Aims

To show an increase in uMRD4 rate by delaying the use of R in combination with ven for 12 months (mo) and by adding sequential ibr in patients (pts) who did not achieve uMRD4 at the end of 12 mo of Ven+delayed R treatment. Since the expected uMRD4 rate for treatment naive (TN) CLL pts treated with ven + obi is approximately 56% in the CLL-14 trial, an increase in uMRD4 rate from 56 to 75% is considered relevant.

### Methods

In this phase 2 multicentre study TN pts ≤ 65 y were treated with ven mono at standard ramp-up schedule up to 400 mg QD for 6 cycles, adding delayed R at standard CLL dose for 6 cycles (C7-C12). Pts with uMRD4 by the ERIC 6-color flow cytometric analysis in peripheral blood (PB) in 3 consecutive tests and also in bone marrow (BM) at C13D1 discontinued tx, otherwise continued ven up to 24 cycles adding Ibr 420 mg QD for up to 12 cycles, until confirmed uMRD4, progressive disease (PD) or unacceptable toxicity. Pts with unconfirmed uMRD4 at the end of C12, continued ibr mono as per standard. Target sample size is 55 pts.

**Copyright Information:** (Online) ISSN: 2572-9241
©2026 The Author(s). HemaSphere is published by John Wiley & Sons Ltd on behalf of European Hematology Association. This is an open access Abstract Book distributed under the Attribution-NonCommercial-NoDerivs (CC BY-NC-ND), which allows third parties to download the articles and share them with others as long as they credit the author and the Abstract Book, but they cannot change the content in any way or use them commercially.
**Abstract Book Citations:** Authors, Title, HemaSphere, 2026;10:(S1):pages. The abstract book DOIs can be found on the "Author Information" page.
**Disclaimer:** Articles published in the journal HemaSphere exclusively reflect the opinions of the authors. The authors are responsible for all content in their abstracts including accuracy of the facts, statements, citing resources, etc.

### Results

At data cut off 36 pts were enrolled (Table 1). Median follow-up was 24 mo (range, 0-42). Median time to first PB uMRD4 was 8 mo (range, 3-24). At C13D1, 20/28 evaluable pts (71.4%) discontinued tx due to confirmed uMRD4, 8 pts added Ibr. 3/8 subsequently met uMRD4 criteria and discontinued Ibr+ven after a median time of 11 mo (range 6-12), 4 pts did not achieve confirmed uMRD4 after 12 cycles of Ibr-ven and 1 pt was still on Ibr-ven. Overall, 23/28 pts (82.12%) achieved a confirmed BM uMRD4 at C13D1 or afterwards (Fig 1), meeting the study primary endpoint. Overall response rate (ORR) at C13D1 was 100%: 58.4% complete (CR), 41.6% partial remissions (PR). The most common grade ≥ 3 (G3+) adverse event (AE) was neutropenia (33.3%). Other G3+ AEs included: transaminitis, diarrhea, hypertension, thrombocytopenia, anemia with 2 cases of warm haemolytic autoimmune anemia (wAIHA) on ven mono, 1 laboratory tumor lysis syndrome (TLS), but no clinical TLS. 4 serious AE occurred (11.1%): wAIHA, ankle fracture, lung cancer G5, lower limb paresthesia. No tx-related deaths were reported.

| **Pts characteristics** | **Results** |
|---|---|
| Median age (n=35) | 59 y (48-65) |
| Sex (n=35) | F 10 (28.6 %), M 25 (71.4%) |
| Del 17p (n=29) | NO 24, 82%, YES 5,18% |
| *TP53* mutation (n=28) | NO 24, 85.7%, YES 4. 14.3% |
| IGHV (n=30) | Unm 26 (86.6%), Mut 4 (13.3%) |
| RAI stage (n=36) | 0, 1 (2.7 %), I, 3 (8.3 %). II, 8 (22.2 %) III, 19 (52.7%) IV, 5 (13.8 %) |
| TLS baseline risk (N=28) | Low/medium, 15 (53.5%), High 13 (46 %) |

### Summary/Conclusion

This first prospective trial showed that delayed R administration following ven lead-in yields higher BM uMRD rates exceeding historical ven–R and, more importantly, ven-obi data, which are further improved with MRD guided sequential ibr intensification with no reported G3+ infections, confirming the higher tolerability of R vs obi combination. Updated data will be presented at the conference.

**Copyright Information:** (Online) ISSN: 2572-9241
©2026 The Author(s). HemaSphere is published by John Wiley & Sons Ltd on behalf of European Hematology Association. This is an open access Abstract Book distributed under the Attribution-NonCommercial-NoDerivs (CC BY-NC-ND), which allows third parties to download the articles and share them with others as long as they credit the author and the Abstract Book, but they cannot change the content in any way or use them commercially.
**Abstract Book Citations:** Authors, Title, HemaSphere, 2026;10:(S1):pages. The abstract book DOIs can be found on the "Author Information" page.
**Disclaimer:** Articles published in the journal HemaSphere exclusively reflect the opinions of the authors. The authors are responsible for all content in their abstracts including accuracy of the facts, statements, citing resources, etc.

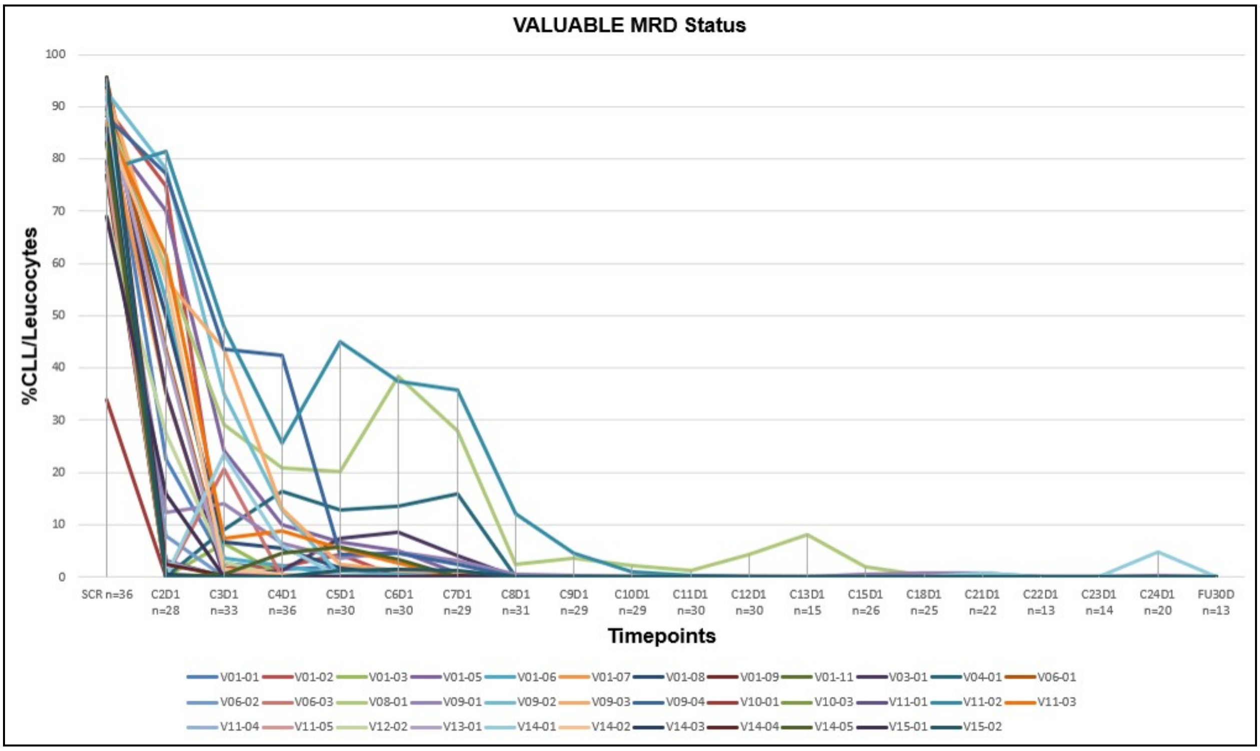

**Copyright Information:** (Online) ISSN: 2572-9241
©2026 The Author(s). HemaSphere is published by John Wiley & Sons Ltd on behalf of European Hematology Association. This is an open access Abstract Book distributed under the Attribution-NonCommercial-NoDerivs (CC BY-NC-ND), which allows third parties to download the articles and share them with others as long as they credit the author and the Abstract Book, but they cannot change the content in any way or use them commercially.
**Abstract Book Citations:** Authors, Title, HemaSphere, 2026;10:(S1):pages. The abstract book DOIs can be found on the "Author Information" page.
**Disclaimer:** Articles published in the journal HemaSphere exclusively reflect the opinions of the authors. The authors are responsible for all content in their abstracts including accuracy of the facts, statements, citing resources, etc.

### (PF595) THE CLINICAL AND BIOLOGICAL CHARACTERISTICS AND OUTCOMES OF ACCELERATED CHRONIC LYMPHOCYTIC LEUKEMIA AND RICHTER TRANSFORMATION

**Topic:** 06. Chronic lymphocytic leukemia and related disorders - Clinical

Ziyuan Zhou[1], Luomengjia Dai[1], Dan Yang[1], Hanyi Ouyang[1], Wen Chen[2], Chongyang Ding[3], Jianyong Li[1], Huayuan Zhu*[1]

*[1]The First Affiliated Hospital with Nanjing Medical University, Department of Hematology, Nanjing, China;[2]The First Affiliated Hospital with Nanjing Medical University, Department of Pathology, Nanjing, China; [3]The First Affiliated Hospital with Nanjing Medical University, Department of Nuclear Medicine, Nanjing, China;*

### Background

Accelerated chronic lymphocytic leukemia (aCLL) is a rare subtype of chronic lymphocytic leukemia (CLL) characterized by expanded and highly active proliferation centers. Richter transformation (RT) is defined as the histologic progression of CLL into an aggressive subtype, associated with extremely poor prognosis. However, the differences of clinical and biological characteristics between aCLL and RT remain incompletely defined.

### Aims

To characterize the clinical and biological features and outcomes of aCLL and RT.

### Methods

42 aCLL and 68 RT patients with histologically proven between 2014 and 2025 were included. The diagnosis of aCLL is based on the criteria published by Giné et al. The clinical and biological data were analyzed. Plasma circulating tumor DNA (ctDNA) analysis using a 571-gene panel were performed in 27 aCLL and 23 RT, with additionally 23 CLL as controls. Progression-free survival (PFS) is defined as the time from diagnosis to first disease progression or death, or last follow-up. Overall survival (OS) is defined as the time from diagnosis to death or last follow-up.

### Results

The median age of aCLL and RT were 57 and 60 years old. 26 aCLL and 59 RT were previously diagnosed with CLL. Among 23 aCLL and 51 RT who had previously received treatment for CLL, the median time from initiation of last-line treatment to disease progression was 28.6 months and 9.8 months ($p$<0.001). Patients with aCLL were less frequently presented with B symptoms, anemia, thrombocytopenia, bulky disease (≥5 cm), extranodal disease, and with lower serum LDH levels, SUVmax (median 7.1 vs. 12.6, $p$<0.001) and TMTV (median 61.4 vs. 571.3, $p$<0.001). Interestingly, the absolute lymphocyte counts did not show a significant increase both in aCLL and RT patients. Ki-67 expression in aCLL was predominantly confined to expanded/confluent proliferation centers, with a lower positive index compared to RT (median 50% vs. 70%, $p$<0.001). Moreover, RT exhibited more frequently loss of typical CLL markers, such as CD5 (63.8% vs. 88.1% positive, $p$=0.012), CD23 (67.9% vs. 87.8% positive, $p$=0.044), and LEF-1 (71% vs. 97.3% positive, $p$=0.005). 55.9% and 34.6% patients were with complex karyotype in aCLL and RT. Both aCLL and RT were enriched in IGHV unmutated status, and the most frequently utilization of IGHV segments were 3-48 and 4-39 in aCLL and RT, respectively. Plasma ctDNA analysis revealed that the most frequently gene mutations in aCLL were NOTCH1 (33.3%), SF3B1 (29.6%), TP53 (25.9%), ATM (22.2%) and BRAF (18.5%), whereas in RT were TP53 (60.9%), NOTCH1 (29.1%), PLCG2 (30.4%), BTG1 (30.4%) and PCLO (26.1%). Moreover, ctDNA burden increased in parallel with histologically aggressiveness. With a median follow-up of 10.6 months, the median OS and PFS of aCLL patients were 55.7 and 43.9 months, both significantly superior to RT (the median OS and PFS were 14.7 and 6.6 months). Plasma ctDNA burden (log10 hGE/mL) was an important biomarker related to tumor burden, and was a potential prognostic index in both aCLL and RT.

**Copyright Information:** (Online) ISSN: 2572-9241
©2026 The Author(s). HemaSphere is published by John Wiley & Sons Ltd on behalf of European Hematology Association. This is an open access Abstract Book distributed under the Attribution-NonCommercial-NoDerivs (CC BY-NC-ND), which allows third parties to download the articles and share them with others as long as they credit the author and the Abstract Book, but they cannot change the content in any way or use them commercially.
**Abstract Book Citations:** Authors, Title, HemaSphere, 2026;10:(S1):pages. The abstract book DOIs can be found on the “Author Information” page.
**Disclaimer:** Articles published in the journal HemaSphere exclusively reflect the opinions of the authors. The authors are responsible for all content in their abstracts including accuracy of the facts, statements, citing resources, etc.

### Summary/Conclusion aCLL represents an aggressive entity intermediate between indolent CLL and RT, characterized by a distinct histological feature and mutational landscape. Although its prognosis is superior to RT, disease-specific therapeutic strategies still need to be explored. Plasma ctDNA may serve as a valuable biomarker for reflecting tumor burden and predicting clinical outcomes in both aCLL and RT.

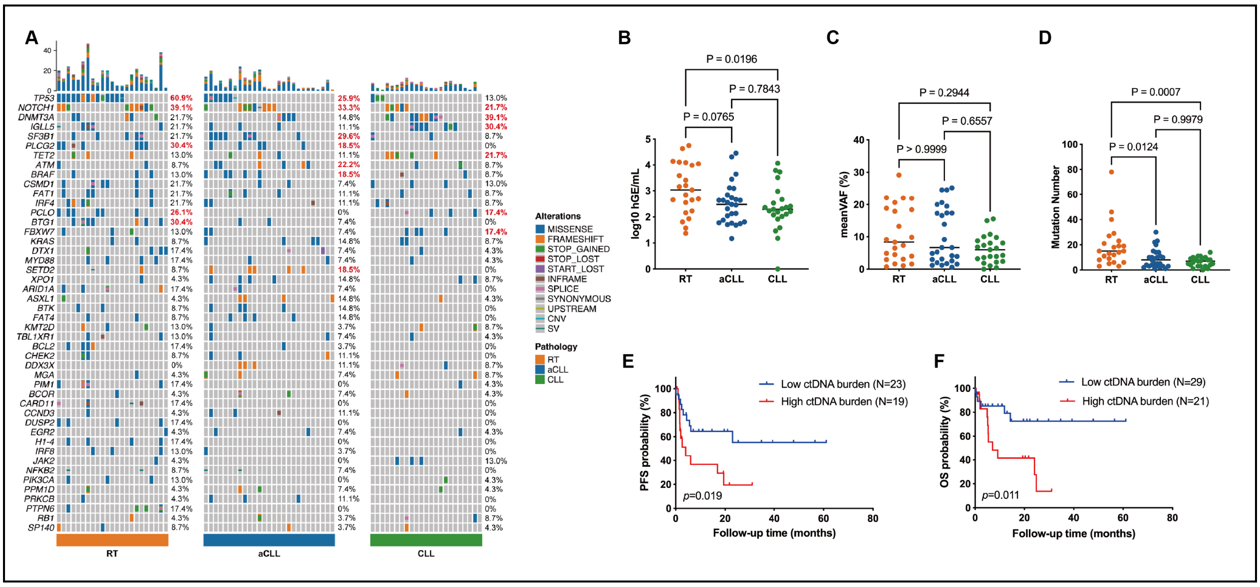

**Copyright Information:** (Online) ISSN: 2572-9241
©2026 The Author(s). HemaSphere is published by John Wiley & Sons Ltd on behalf of European Hematology Association. This is an open access Abstract Book distributed under the Attribution-NonCommercial-NoDerivs (CC BY-NC-ND), which allows third parties to download the articles and share them with others as long as they credit the author and the Abstract Book, but they cannot change the content in any way or use them commercially.
**Abstract Book Citations:** Authors, Title, HemaSphere, 2026;10:(S1):pages. The abstract book DOIs can be found on the "Author Information" page.
**Disclaimer:** Articles published in the journal HemaSphere exclusively reflect the opinions of the authors. The authors are responsible for all content in their abstracts including accuracy of the facts, statements, citing resources, etc.

### (PF596) IMPACT OF SEX ON EFFICACY, TOLERABILITY AND PATIENT-REPORTED OUTCOMES IN FIRST-LINE THERAPY FOR CHRONIC LYMPHOCYTIC LEUKEMIA: RESULTS FROM THE CLL13/GAIA STUDY

**Topic:** 06. Chronic lymphocytic leukemia and related disorders - Clinical

Julia von Tresckow*[1, 2], Moritz Fürstenau[2], Sandra Robrecht[2], Adam Giza[2], Can Zhang[2], Michael Gregor[3], Patrick Thornton[4], Philipp Staber[5, 6], Tamar Tadmor[7, 8], Vesa Lindström[9], Gunnar Juliusson[10], Ann Janssens[11], Caspar Da Cunha-Bang[12], Mark-David Levin[13], Christof Schneider[14], Florian Simon[2], Anna-Maria Fink[2], Kirsten Fischer[2], Emily Holmes[2], Karl-Anton Kreuzer[2], Matthias Ritgen[15], Eugen Tausch[14], Stephan Stilgenbauer[14], Michael Hallek[2], Arnon Kater[16], Carsten Niemann[12], Barbara Eichhorst[2]

*[1]Department for hematology and oncology with palliative care, St. Josef Hospital Bochum, Ruhr-University Bochum, Bochum, Germany; [2]Department I of Internal Medicine and Center of Integrated Oncology Cologne-Bonn, German CLL Study Group, University Hospital Cologne, Cologne, Germany; [3]Department of Hematology, Luzerner Kantonsspital, Luzern, Switzerland; [4]Cancer Clinical Trials Unit, Beaumont Hospital, Dublin, Ireland; [5]Department of Medicine I, Division of Hematology and Hemostaseology, Medical University, Vienna, Austria; [6]Saarland University Medical School, Saarbrücken, Germany; [7]Hematology Unit, Bnai Zion Medical Center, Haifa, Israel; [8]The Ruth and Bruce Rappaport Faculty of Medicine, Technion, Haifa, Israel; [9]Comprehensive Cancer Center, Department of Hematology, Helsinki University Hospital and University of Helsinki, Helsinki, Finland; [10]Department of Hematology, Skåne University Hospital, Lund, Sweden; [11]Universitaire Ziekenhuizen Leuven, Leuven, Belgium; [12]Department of Hematology, Rigshospitalet, Copenhagen University Hospital, Copenhagen, Denmark; [13]Department of Internal Medicine, Albert Schweitzer Hospital, Dordrecht, The Netherlands; [14]Department III of Internal Medicine, University Hospital Ulm, Ulm, Germany; [15]Department of Hematology and Oncology, University Hospital Schleswig Holstein, Kiel, Germany; [16]Department of Hematology, Cancer Center Amsterdam, University Medical Center, Amsterdam, The Netherlands;*

### Background

Venetoclax (V) and obinutuzumab (GA101, G), with or without ibrutinib (I), has demonstrated superiority over chemoimmunotherapy (C) with fludarabine, cyclophosphamide (FC) and rituximab (R) or R-Bendamustine as first-line treatment in fit patients (pts) with chronic lymphocytic leukemia (CLL). Furthermore, biological sex influences time to relapse after R-, but not G-based C (Al Sawaf, Leukemia 2017).

### Aims

To investigate the impact of sex on clinical and pts-reported outcomes (PRO) in fit CLL pts without *TP53* aberrations receiving C or V-based first-line therapy.

### Methods

CLL13/GAIA is a randomized, open-label phase III trial enrolling fit CLL pts without *TP53* aberrations. Pts received six cycles of C or 12 cycles of RV, GV or GIV, with I discontinued according to undetectable minimal residual disease (uMRD, < $10^{-4}$) status. Efficacy and PRO were post-hoc analyzed in all randomized pts and safety in all pts with ≥1 dose of study treatment, grouped by sex.

**Copyright Information:** (Online) ISSN: 2572-9241
©2026 The Author(s). HemaSphere is published by John Wiley & Sons Ltd on behalf of European Hematology Association. This is an open access Abstract Book distributed under the Attribution-NonCommercial-NoDerivs (CC BY-NC-ND), which allows third parties to download the articles and share them with others as long as they credit the author and the Abstract Book, but they cannot change the content in any way or use them commercially.
**Abstract Book Citations:** Authors, Title, HemaSphere, 2026;10:(S1):pages. The abstract book DOIs can be found on the "Author Information" page.
**Disclaimer:** Articles published in the journal HemaSphere exclusively reflect the opinions of the authors. The authors are responsible for all content in their abstracts including accuracy of the facts, statements, citing resources, etc.

**Results**

A total of 926 pts were randomized: 229 to C (28.8% F), 237 to RV (26.2% F), 229 to GV (25.3 F) and 231 to GIV (31.6% F). Overall, 912 pts (98.5%) received ≥ 1 dose of study treatment. Median age was 62 years in F and 61 years in M. Binet stage A (34.7 vs 23.3%), a longer median time between first diagnosis and randomization (32.2 vs 27.5 months), a body mass index < 25 kg/$m^2$ (58.3 vs 44.4%) and a lower frequency of high CLL-IPI (45.5 vs 53.2%) were more common in F.
At month 15, overall response rates were comparable between F and M (C 84.8 vs 79.1%, RV 96.8 vs 92.0%, GV 96.6 vs 95.9%, GIV 97.3 vs 93.0%) whereas significantly higher complete response rates (CRR) were observed in F in the V-containing arms (RV 64.5 vs 42.3%, p=0.003; GV 67.2 vs 52.0%, p=0.044; GIV 75.3 vs 51.9%, p<0.001).
uMRD rates were comparable in F and M (C 57.6 vs 49.7%, RV 59.7 vs 56.0%, GIV 94.5 vs 91.1%), except for the GV arm, where uMRD rates were significantly higher in F (94.8 vs 83.6%, p=0.031).
Progression free and overall survival did not differ significantly between F and M (Figure 1).
Early treatment discontinuations were comparable in F and M across treatment arms (C 21.0 vs 17.5%, RV 8.1 vs 7.4%, GV 5.2 vs 6.5%, GIV 11.0 vs 15.2%).
While the proportion of pts with ≥1 adverse event (AE) was similar by sex (C 100 vs 98.7%, RV 100 vs 97.1%, GV 100 vs 98.2%, GIV 100 vs 99.4%), F had a significantly higher median number of AE in the RV (10.5 vs 8.5, p=0.049) and GIV arms (16 vs 12, p<0.001).
F had a lower follow-up adjusted incidence of secondary primary malignancies (SPM) in the C arm (1.4 vs 5.0 cases per 1000 pts-months, p=0.003), with no additional sex-specific differences in SPM or Richter transformation across treatment arms.
Sex-related differences in PRO were treatment-specific, with F reporting lower physical functioning in the RV and GIV arms and higher symptom burden in the RV arm during early treatment phases, while no consistent differences were observed in C and GV arms. Global health status was unaffected by sex.

**Summary/Conclusion**

In the CLL13/GAIA trial, F experienced a higher number of toxicities and reported worse PRO during early treatment phases in selected scales in the RV and GIV arms. In addition, F achieved higher CRR in V-containing arms and a higher uMRD rate in the GV arm, respectively. These findings suggest that sex may influence treatment tolerability, depth of remission and pts experience in CLL, warranting further investigation regarding treatment optimization according to sex.

**Copyright Information:** (Online) ISSN: 2572-9241
©2026 The Author(s). HemaSphere is published by John Wiley & Sons Ltd on behalf of European Hematology Association. This is an open access Abstract Book distributed under the Attribution-NonCommercial-NoDerivs (CC BY-NC-ND), which allows third parties to download the articles and share them with others as long as they credit the author and the Abstract Book, but they cannot change the content in any way or use them commercially.
**Abstract Book Citations:** Authors, Title, HemaSphere, 2026;10:(S1):pages. The abstract book DOIs can be found on the “Author Information” page.
**Disclaimer:** Articles published in the journal HemaSphere exclusively reflect the opinions of the authors. The authors are responsible for all content in their abstracts including accuracy of the facts, statements, citing resources, etc.

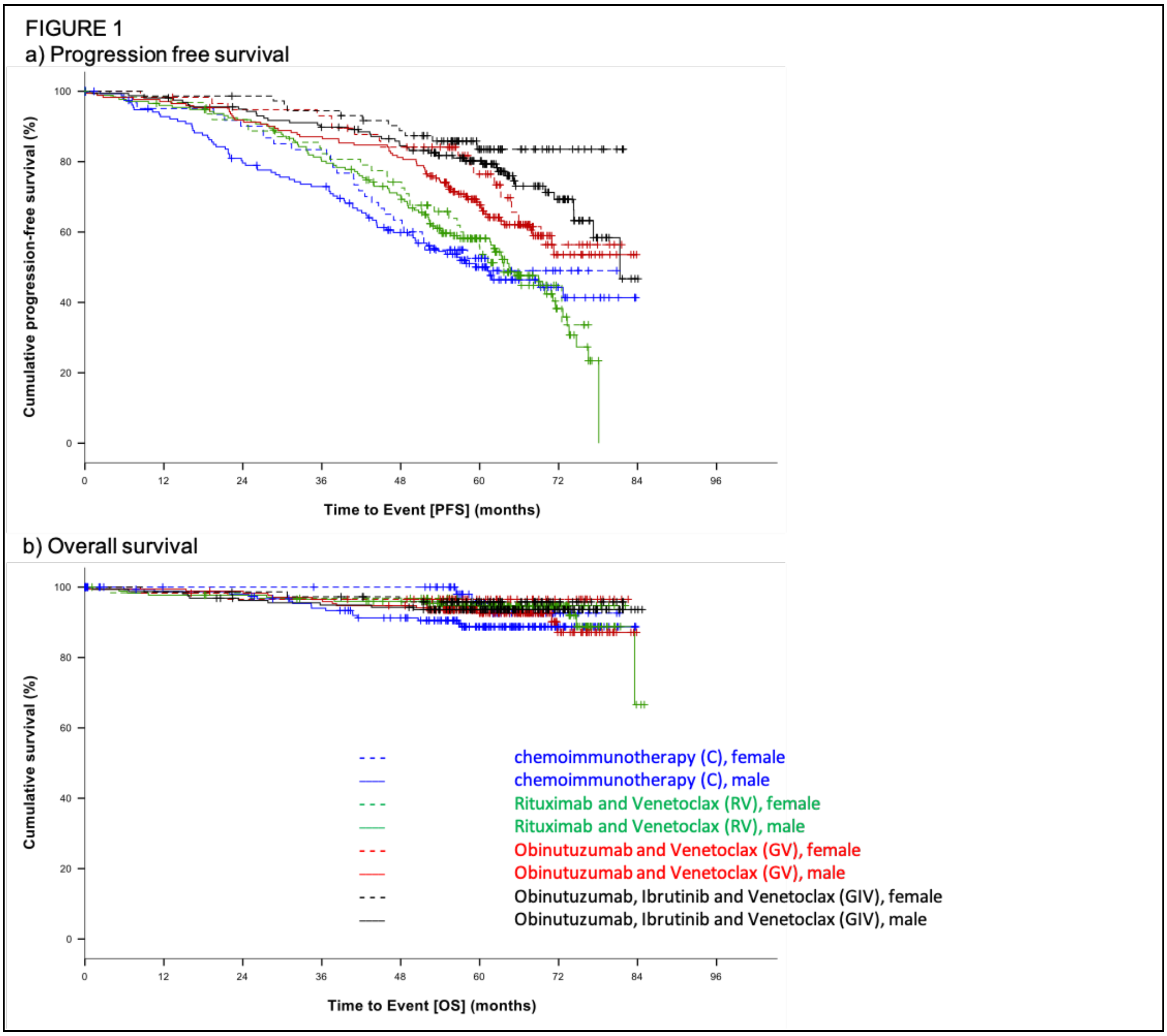

Figure 1 shows a) progression free survival and b) overall survival according to sex in patients treated with chemoimmunotherapy, rituximab and venetoclax, obinutuzumab and venetoclax as well as obinutzumab, venetoclax and ibrutinib as first-line treatment in chronic lymphocytic leukemia.

**Copyright Information:** (Online) ISSN: 2572-9241
©2026 The Author(s). HemaSphere is published by John Wiley & Sons Ltd on behalf of European Hematology Association. This is an open access Abstract Book distributed under the Attribution-NonCommercial-NoDerivs (CC BY-NC-ND), which allows third parties to download the articles and share them with others as long as they credit the author and the Abstract Book, but they cannot change the content in any way or use them commercially.
**Abstract Book Citations:** Authors, Title, HemaSphere, 2026;10:(S1):pages. The abstract book DOIs can be found on the "Author Information" page.
**Disclaimer:** Articles published in the journal HemaSphere exclusively reflect the opinions of the authors. The authors are responsible for all content in their abstracts including accuracy of the facts, statements, citing resources, etc.

### (PF597) DUAL MAPK PATHWAY INHIBITION, ALONE OR COMPLEMENTED BY ANTI-CD20 IMMUNOTHERAPY, IN PREVIOUSLY TREATED HAIRY CELL LEUKEMIA/HCL: A HIGHLY ACTIVE TARGETED STRATEGY OF FIXED AND SHORT DURATION

**Topic:** 06. Chronic lymphocytic leukemia and related disorders - Clinical

Luca Decarolis[1], Monia Capponi[1], Alessandro Mancini*[1], Flavio Falcinelli[1], Caterina Stelitano[2], Alessandro Pulsoni[3], Edoardo Simonetti[1], Alessandra Romano[4], Gianna Maria D'elia[5], Jacopo Olivieri[6], Maria Paola Martelli[1], Pier Luigi Zinzani[7], Marzia Varettoni[8], Simone Ferrero[9], Massimo Pini[10], Robin Foà[5], Elisa Lucchini[11], Maurizio Frezzato[12], Brunangelo Falini[1], Enrico Tiacci[1]

*[1]Institute of Hematology, University and Hospital of Perugia, Perugia, Italy, Perugia, Italy;[2]Hematology, Hospital of Reggio Calabria, Reggio Calabria, Italy, Reggio Calabria, Italy; [3]Hematology, Sapienza University of Rome and Hospital of Latina, Latina, Italy, Latina, Italy; [4]Hematology, University and Hospital of Catania, Catania, Italy, Catania, Italy; [5]Hematology, Sapienza University and Hospital Umberto I of Rome, Rome, Italy, Rome, Italy; [6]Hematology, University and Hospital of Udine, Udine, Italy, Udine, Italy; [7]Hematology, University of Bologna and Sant'Orsola Hospital, Bologna, Italy, Bologna, Italy;[8]Hematology, IRCCS S. Matteo, Pavia, Italy, Pavia, Italy; [9]Hematology, University of Turin and Hospital "Città della Salute e della Scienza" of Turin, Turin, Italy, Turin, Italy; [10]Hematology, S. Giovanni Bosco Hospital, Turin, Italy, Turin, Italy;[11]Hematology, Hospital of Trieste, Trieste, Italy, Trieste, Italy; [12]Hematology, Hospital of Vicenza, Vicenza, Italy, Vicenza, Italy;*

### Background

HCL, a rare chronic B-cell neoplasm caused by the BRAF-V600E kinase-activating mutation (Tiacci et al. NEJM 2011), is very sensitive to chemotherapy, but ~50% of patients (pts) relapse. A short course of the oral BRAF inhibitor vemurafenib (V) for a few months led to frequent responses in relapsed/refractory (R/R) pts, but complete remissions (CR) were a minority (35%) and minimal residual disease (MRD) was always present, resulting in a progression-free survival (PFS) of ~1 year considering all pts; persistent ERK phosphorylation (pERK+) was frequently observed in residual bone marrow (BM) HCL cells despite ongoing BRAF inhibition by V, suggesting bypass MEK-ERK reactivation downstream of BRAF as a resistance mechanism (Tiacci, Park et al. NEJM 2015). Dual MAPK pathway inhibition of BRAF and MEK with dabrafenib+trametinib for an indefinite duration (median ~3 years) yielded a CR rate of 66% in R/R HCL, but MRD clearance was rare (16% of pts); such continuous therapy sustained a high PFS at 2 years (94%) but led to frequent drug-related serious adverse events (SAE; 35% of pts) (Kreitman et al. Blood 2023). Adding rituximab (R; 8 doses) to a short V course (8 weeks) produced in R/R HCL 87% CR, frequent MRD clearance (60%), 78% PFS at ~3 years, and few clinically relevant toxicities (3% of pts - Tiacci et al. NEJM 2021).

### Aims

To evaluate in R/R HCL the efficacy and safety of a brief course of oral dual MAPK pathway inhibition with V plus the MEK inhibitor cobimetinib (C), first alone and then combined with obinutuzumab (O; possibly more active than R).

### Methods

We enrolled 19 R/R HCL pts (median age: 58 years; median prior treatments: 3) in a phase-2 investigator-initiated multicenter trial of 2 to 3 cycles (1 cycle=28 days) of V (960 mg twice daily; days 1-28 of each cycle) and C (60 mg daily; days 1-21 of each cycle). If cytopenia(s) persisted or relapsed, pts were to receive 2 to 3 additional cycles of V+C combined with O (1000 mg i.v.; 8 doses over 6 cycles, starting with V+C).

Copyright Information: (Online) ISSN: 2572-9241
©2026 The Author(s). HemaSphere is published by John Wiley & Sons Ltd on behalf of European Hematology Association. This is an open access Abstract Book distributed under the Attribution-NonCommercial-NoDerivs (CC BY-NC-ND), which allows third parties to download the articles and share them with others as long as they credit the author and the Abstract Book, but they cannot change the content in any way or use them commercially.
Abstract Book Citations: Authors, Title, HemaSphere, 2026;10:(S1):pages. The abstract book DOIs can be found on the “Author Information” page.
Disclaimer: Articles published in the journal HemaSphere exclusively reflect the opinions of the authors. The authors are responsible for all content in their abstracts including accuracy of the facts, statements, citing resources, etc.

**Results**

After V+C, 1/19 pts resolved cytopenias, but had a non-evaluable BM biopsy. 13/18 pts (72%) achieved CR (all but one MRD+). Persistent pERK+ BM HCL cells were detected in only 1/9 pts evaluated in this regard (11%), suggesting that adding C to V can counteract resistance to sole BRAF inhibition. Indeed, at a median follow-up of 36 months, median PFS was 38.4 months (indirectly longer than 12.5 months with V alone; Fig. 1A), and a sizable pts subset (4/19, 21%) remained progression-free at >5 years. Toxicity was as expected and manageable, mostly of low grade and always reversible, allowing high drug dose intensities (median: 91% for V, 92% for C). 3/19 pts (16%) had drug-related SAE.
Of 12 pts with cytopenia persistence (n=2) or relapse (n=10), 3 did not receive further therapy for various reasons. The other 9 pts were treated with V+C+O after a median of 41 months following V+C, and all (100%) achieved MRD- CR, with no unexpected toxicities; 1/9 pts (11%) had a drug-related SAE. PFS was 100% at a median follow-up of 33 months, comparing well with PFS recorded in the same 9 pts after their prior V+C course (median reached at 35.9 months; Fig. 1B), and also with the PFS of V+R observed in our previous trial (Fig. 1C).

**Summary/Conclusion**

In R/R HCL, a short course of dual MAPK pathway blockade with V+C, particularly when combined with anti-CD20 immunotherapy with O, is safe and highly effective, comparing favorably with other approaches in this setting.
Cofirst authors LDC, MC, AM
Colast authors BF, ET

**Copyright Information:** (Online) ISSN: 2572-9241
©2026 The Author(s). HemaSphere is published by John Wiley & Sons Ltd on behalf of European Hematology Association. This is an open access Abstract Book distributed under the Attribution-NonCommercial-NoDerivs (CC BY-NC-ND), which allows third parties to download the articles and share them with others as long as they credit the author and the Abstract Book, but they cannot change the content in any way or use them commercially.
**Abstract Book Citations:** Authors, Title, HemaSphere, 2026;10:(S1):pages. The abstract book DOIs can be found on the "Author Information" page.
**Disclaimer:** Articles published in the journal HemaSphere exclusively reflect the opinions of the authors. The authors are responsible for all content in their abstracts including accuracy of the facts, statements, citing resources, etc.

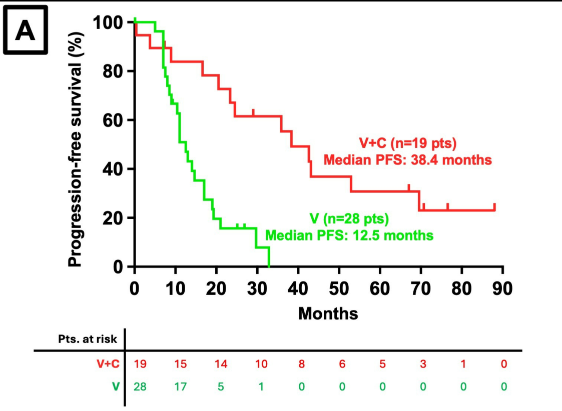

***Panel A:** Indirect cross-trial comparison of PFS curves among pts treated with V alone (green curve) or with V+C (red curve).*

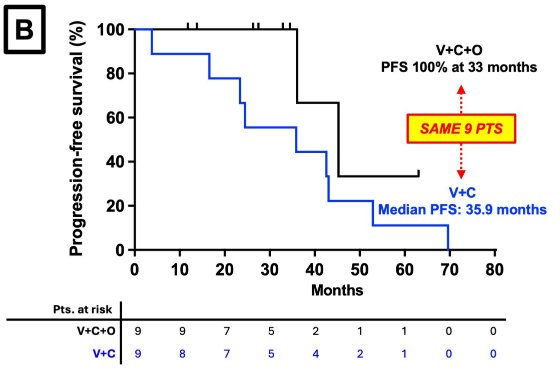

***Panel B:** PFS curves of the same 9 pts after initial treatment with V+C (blue curve), and after subsequent treatment with V+C+O (black curve) for cytopenia persistence or relapse after V+C.*

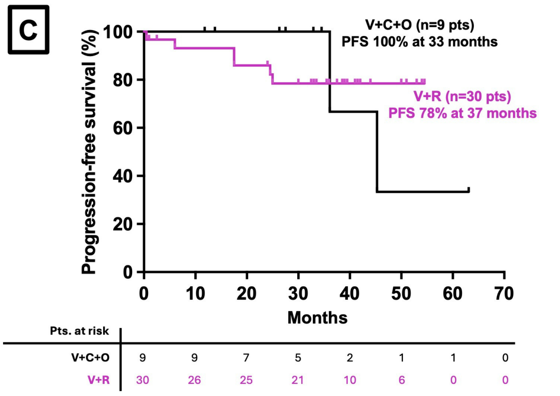

***Panel C:** Indirect cross-trial comparison of PFS curves among pts treated with V+C+O (black curve) or V+R (purple curve).*

**Copyright Information:** (Online) ISSN: 2572-9241
©2026 The Author(s). HemaSphere is published by John Wiley & Sons Ltd on behalf of European Hematology Association. This is an open access Abstract Book distributed under the Attribution-NonCommercial-NoDerivs (CC BY-NC-ND), which allows third parties to download the articles and share them with others as long as they credit the author and the Abstract Book, but they cannot change the content in any way or use them commercially.
**Abstract Book Citations:** Authors, Title, HemaSphere, 2026;10:(S1):pages. The abstract book DOIs can be found on the “Author Information” page.
**Disclaimer:** Articles published in the journal HemaSphere exclusively reflect the opinions of the authors. The authors are responsible for all content in their abstracts including accuracy of the facts, statements, citing resources, etc.

### (PF598) FINAL RESULTS FROM ASSURE: A PHASE 3B STUDY OF THE SAFETY AND EFFICACY OF ACALABRUTINIB IN PATIENTS WITH CHRONIC LYMPHOCYTIC LEUKEMIA

**Topic:** 06. Chronic lymphocytic leukemia and related disorders - Clinical

Stephen Opat*[1], Laura Fogliatto[2], Olga Samoilova[3], Eugene Nikitin[4], Bryone Kuss[5], Milton Aranha[6], Srinivas Jujjavarapu[7], Javier De La Serna[8], Jason Hart[9], Versha Banerji[10], Hoa T. T. Tran[11], Maria Novikova[12], Caroline Dartigeas[13], Ki-Seong Eom[14], Michele Merli[15], Eve Deacon[16], Jiefen Munley[17], Susana Wargo[18], Richard Hermann[18], Farrukh Awan[19], Carsten Niemann[20]

*[1]Monash University and Monash Health, Melbourne, Australia;[2]Hospital de Clinicas de Porto Alegre, Porto Alegre, Brazil; [3]Regional Clinical Hospital, Nizhny Novgorod, Russia; [4]Botkin Hospital and RMACPE, Moscow, Russia; [5]Flinders University and Medical Centre, SA Pathology Laboratories, Bedford Park, Australia; [6]Instituto de Ensino e Pesquisa São Lucas, São Paulo, Brazil; [7]Illinois CancerCare, Peoria, United States of America; [8]Hospital Universitario 12 de Octubre, Madrid, Spain;[9]BC Cancer-Victoria, Victoria, Canada; [10]University of Manitoba and Paul Albrechtsen Research Institute, CancerCare Manitoba, Manitoba, Canada; [11]Akershus University Hospital, Lørenskog, Norway; [12]Uromed Ltd, Smolensk, Russia; [13]CHRU Tours, Tours, France; [14]Catholic Hematology Hospital, Seoul St. Mary's Hospital, College of Medicine, the Catholic University of Korea, Seoul, South Korea; [15]Fondazione IRCCS CA Granda Ospedale Maggiore Policlinico, Milan, Italy; [16]AstraZeneca, Macclesfield, United Kingdom; [17]AstraZeneca, Wilmington, United States of America; [18]AstraZeneca, Gaithersburg, United States of America; [19]University of Texas Southwestern Medical Center, Dallas, United States of America;[20]Rigshospitalet Copenhagen University Hospital, University of Copenhagen, and Danish Cancer Society, Copenhagen, Denmark;*

### Background

Acalabrutinib (acala), a second-generation Bruton tyrosine kinase inhibitor, is approved for the treatment (tx) of tx-naive (TN) and relapsed/refractory (R/R) chronic lymphocytic leukemia (CLL). Interim results from ASSURE (NCT04008706), a phase 3b study evaluating acala monotherapy in patients (pts) with CLL, demonstrated that the safety profile of acala in a real-world clinical practice setting was consistent with prior clinical trials (Opat S, et al. EHA 2024. Abstract P684).

### Aims

To report final safety and efficacy results from ASSURE.

### Methods

This global, open-label, single-arm study enrolled pts aged ≥18 y with CLL requiring tx per iwCLL criteria and ECOG performance status ≤2 into 1 of 3 cohorts (TN, R/R, or ibrutinib intolerant [IbrI; ibrutinib discontinued for any reason except disease progression]). Acala 100 mg was given twice daily for 48 cycles (28 d each; pts could continue beyond 48 cycles if still benefitting) or until disease progression or unacceptable toxicity. The primary endpoint was to evaluate all adverse events (AEs). Secondary endpoints were investigator-assessed overall response rate (ORR) after 1 y of tx, duration of response (DOR; time from first response to disease progression or death), and progression-free survival (PFS).

**Copyright Information:** (Online) ISSN: 2572-9241
©2026 The Author(s). HemaSphere is published by John Wiley & Sons Ltd on behalf of European Hematology Association. This is an open access Abstract Book distributed under the Attribution-NonCommercial-NoDerivs (CC BY-NC-ND), which allows third parties to download the articles and share them with others as long as they credit the author and the Abstract Book, but they cannot change the content in any way or use them commercially.
**Abstract Book Citations:** Authors, Title, HemaSphere, 2026;10:(S1):pages. The abstract book DOIs can be found on the "Author Information" page.
**Disclaimer:** Articles published in the journal HemaSphere exclusively reflect the opinions of the authors. The authors are responsible for all content in their abstracts including accuracy of the facts, statements, citing resources, etc.

### Results

Overall, 552 pts received acala (TN, n=310; R/R, n=202; IbrI, n=40); median age was 69.5 y, 17% of pts had del(17p) and/or mutated *TP53* (*TP53*m), and 63% had unmutated IGHV (uIGHV). The median number of prior lines of therapy was 1 in the R/R cohort and 2 in the IbrI cohort. As of 4 November 2025, median time on study was 51.5 mo (TN, 54.3; R/R, 45.8; IbrI, 31.6). Overall, 42% of pts discontinued tx (TN, 36%; R/R, 48%; IbrI, 63%); the most common reason for discontinuation was AEs (TN, 18%; R/R, 29%; IbrI, 23%) followed by disease progression (TN, 5%; R/R, 13%; IbrI, 23%). Overall, the most common tx-emergent AEs (TEAEs; any grade, ≥20% or grade ≥3, ≥5%) were COVID-19 (any grade: 46%, grade ≥3: 14%), headache (43%, 1%), diarrhea (33%, 1%), contusion (26%, 0%), arthralgia (22%, 1%), anemia (15%, 5%), pneumonia (15%, 9%), COVID-19 pneumonia (7%, 5%), and neutropenia (7%, 5%). Serious TEAEs occurred in 51% of pts overall (TN, 49%; R/R, 58%; IbrI, 30%), most commonly (≥5% overall) COVID-19 (13%), pneumonia (8%), and COVID-19 pneumonia (5%). TEAEs of special interest occurred in 1% of pts overall (TN, 1%; R/R, 0%; IbrI, 3%; all ventricular extrasystoles). Among TEAEs of clinical interest, grade ≥3 infections occurred in 35% of pts overall.
The ORR was 78% overall, 82% for TN, 76% for R/R, and 55% for IbrI. Complete response (CR) rates (including CR with incomplete marrow recovery) and partial response rates, respectively, were 3% and 75% overall, 4% and 79% for TN, 1% and 74% for R/R, and 0% and 55% for IbrI. Median DOR was 53.5 mo overall, not reached for TN, 47.2 mo for R/R, and 33.3 mo for IbrI. An estimated 77% of pts remained progression free at 42 mo (TN, 86%; R/R, 66%; IbrI, 61%; **Figure**). Among pts with del(17p) and/or *TP53*m and pts with uIGHV, 42-mo PFS estimates were 73% and 72% overall, 80% and 87% for TN, 73% and 57% for R/R, and 43% and 53% for IbrI, respectively.

### Summary/Conclusion

Final results of ASSURE demonstrated that the safety profile of acala was maintained with >4 y of follow-up in pts with CLL treated in a real-world clinical setting. PFS overall and in pts with high-risk disease characteristics reported in this real-world trial was similar to PFS reported in clinical trials.

**Figure. Progression-free survival for patients treated with acalabrutinib for chronic lymphocytic leukemia.**
CI, confidence interval; IbrI, ibrutinib intolerant; mo, months; PFS, progression-free survival; R/R, relapsed/refractory; TN, treatment naive.

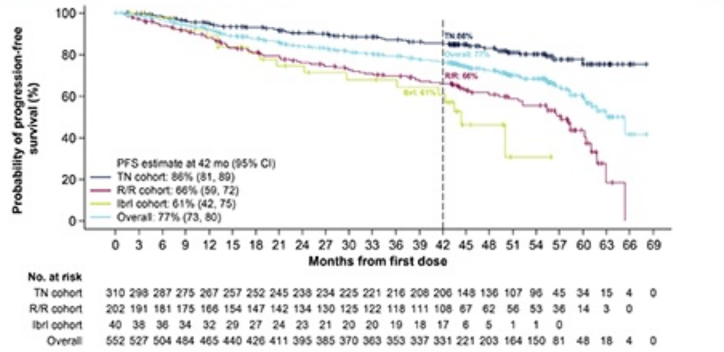

**Copyright Information:** (Online) ISSN: 2572-9241
©2026 The Author(s). HemaSphere is published by John Wiley & Sons Ltd on behalf of European Hematology Association. This is an open access Abstract Book distributed under the Attribution-NonCommercial-NoDerivs (CC BY-NC-ND), which allows third parties to download the articles and share them with others as long as they credit the author and the Abstract Book, but they cannot change the content in any way or use them commercially.
**Abstract Book Citations:** Authors, Title, HemaSphere, 2026;10:(S1):pages. The abstract book DOIs can be found on the "Author Information" page.
**Disclaimer:** Articles published in the journal HemaSphere exclusively reflect the opinions of the authors. The authors are responsible for all content in their abstracts including accuracy of the facts, statements, citing resources, etc.

### (PF599) PRIMARY ENDPOINT ANALYSIS OF A PHASE II STUDY USING MINIMAL RESIDUAL DISEASE STATUS TO GUIDE VENETOCLAX DURATION IN PATIENTS WITH CHRONIC LYMPHOCYTIC LEUKEMIA

**Topic:** 06. Chronic lymphocytic leukemia and related disorders - Clinical

Meghan C. Thompson*[1], Anthony R. Mato[2, 3], Chaitra Ujjani[4], Nicole Lamanna[5], Andriy Derkach[1], David Nemirovsky[1], Philip Caron[1], Michael Silvestrone[1], Monica Shah[1], Leora Boussi[1], Mark Blaine Geyer[1], Andrew Zelenetz[1], Prioty Islam[1], Meira Yisraeli Salman[1], Clare Sun[1], Pallawi Torka[1], Andrew Lipsky[5], Eytan Stein[1], Brian Chernak[1], Miranda Harris[1], Dhara Soni[1], Megha Karnam[1], Amanda Morales[1], Lauren Nogan[1], Megan Morrison[5], Jamila Brutus[1], Colleen Dorsey[1], Anna Lincke[1], Dianna Tyznar[1], Jennifer Howgate-Klingaman[1], Mikelle Lasorsa[1], Ajay Gopal[4], Mazyar Shadman[4], Joshua Dizon[4], Ryan Lynch[4], Stephen Smith[4], Lindsey Roeker*[6]

*[1]Memorial Sloan Kettering Cancer Center, New York, United States of America;[2]Cayuga Medical Center, Ithaca, United States of America; [3]AstraZeneca, New York, United States of America; [4]Fred Hutchinson Cancer Center, University of Washington, Seattle, United States of America; [5]Columbia University Irving Medical Center, New York, United States of America;[6]Mayo Clinic, Rochester, United States of America;*

### Background

Minimal residual disease (MRD) at completion of venetoclax (ven)-based treatment (tx) predicts progression-free survival (PFS) in CLL. Although MRD-guided discontinuation (dc) has been explored with Bruton tyrosine kinase inhibitor (BTKi) and ven combinations, limited data exist to support MRD-adapted cessation of ven ± anti-CD20 monoclonal antibodies (CD20 mAb).

### Aims

We sought to evaluate the durability of tx-free remission after time-limited ven ± CD20 mAb therapy guided by peripheral blood MRD status. Specifically, we aimed to determine the proportion of patients (pts) remaining off CLL-directed tx at 12 cycles (C, 1C = 28 days) after dc ven, irrespective of prior tx duration.

### Methods

This ongoing, multicenter, phase II investigator-initiated clinical trial (NCT04419519) enrolled pts with CLL/SLL treated with ≥ 6 months of ven ± CD20 mAb in the frontline (1L) or relapsed/refractory (R/R) setting. Pts underwent pre-screening with peripheral blood (PB) MRD testing (Clonoseq, $10^{-5}$ sensitivity). Pts with undetectable MRD (uMRD5) on 2 samples ≥28 days apart and iwCLL partial or complete response were eligible to dc ven and enter tx free observation (TFO). The primary endpoint is the proportion of patients remaining tx-free 12C after ven dc. Secondary endpoints include durability of uMRD response and PFS. Pts are followed in TFO through C36 including MRD testing every 3C.

**Copyright Information:** (Online) ISSN: 2572-9241
©2026 The Author(s). HemaSphere is published by John Wiley & Sons Ltd on behalf of European Hematology Association. This is an open access Abstract Book distributed under the Attribution-NonCommercial-NoDerivs (CC BY-NC-ND), which allows third parties to download the articles and share them with others as long as they credit the author and the Abstract Book, but they cannot change the content in any way or use them commercially.
**Abstract Book Citations:** Authors, Title, HemaSphere, 2026;10:(S1):pages. The abstract book DOIs can be found on the "Author Information" page.
**Disclaimer:** Articles published in the journal HemaSphere exclusively reflect the opinions of the authors. The authors are responsible for all content in their abstracts including accuracy of the facts, statements, citing resources, etc.

**Results**

80 pts dc ven and entered TFO. Baseline characteristics at TFO start included: median age 67 years (range 37, 85), 80% male, 89% White, 7.5% Other/Unknown race, 3.8% Black. Ven-based regimens included: ven + obinutuzumab (84%), ven + rituximab (11%), ven monotherapy (5%). Ven regimen preceding enrollment was initial tx for 55 pts (68.8%) and in the R/R setting for 25 pts (31.2%, median of 1 prior tx). Prior tx included: CIT (n=14), BTKi (n=9), ven (n=3). Median duration of ven prior to dc was 10.1 months (range 6.2-25.8). For pts receiving ven as initial tx, median ven duration was 9.4 months (range 6.2-21.4) vs 12 months (range 7.8-25.8) for R/R pts.
At a median follow-up of 30 months of TFO (95% CI: 27, 32), PFS estimates from C1D1 of TFO were: 12-month PFS: 97% (95% CI: 94%, 100%), 24-month PFS: 87% (95% CI: 78%, 96%, Figure 1a). 73 pts reached C12 and were evaluable for the primary endpoint. 97.3% of pts (71/73 pts; 95% CI: 90.5 -99.7) had not required CLL tx by C12.
The median time to detectable MRD (dMRD5) during TFO was 21 months (95% CI: 18, 31, Figure 1b). Estimated 12- and 24-month dMRD5-free-survival were 68% (95% CI: 58, 79) and 48% (95% CI: 37%, 61%).

**Summary/Conclusion**

This study met its primary endpoint with 97.3% of patients not requiring CLL tx at C12 following MRD-guided ven dc. Estimated 24-month PFS from start of TFO was 87%. Personalized ven tx duration based on uMRD5 status leads to durable tx free remissions while minimizing ven exposure.

**Copyright Information:** (Online) ISSN: 2572-9241
©2026 The Author(s). HemaSphere is published by John Wiley & Sons Ltd on behalf of European Hematology Association. This is an open access Abstract Book distributed under the Attribution-NonCommercial-NoDerivs (CC BY-NC-ND), which allows third parties to download the articles and share them with others as long as they credit the author and the Abstract Book, but they cannot change the content in any way or use them commercially.
**Abstract Book Citations:** Authors, Title, HemaSphere, 2026;10:(S1):pages. The abstract book DOIs can be found on the “Author Information” page.
**Disclaimer:** Articles published in the journal HemaSphere exclusively reflect the opinions of the authors. The authors are responsible for all content in their abstracts including accuracy of the facts, statements, citing resources, etc.

### (PF600) PERSISTENT UNDETECTABLE MINIMAL RESIDUAL DISEASE AND LONG-TERM REMISSIONS IN PATIENTS WITH CLL AFTER TREATMENT WITH OBINUTUZUMAB-VENETOCLAX

**Topic:** 06. Chronic lymphocytic leukemia and related disorders - Clinical

Karen Swart*[1, 2], Kazem Nasserinejad[2], Joyce Schilperoord-Vermeulen[2], Paul Hengeveld[1, 2], Julie Dubois[3], Arnon Philip Kater[3], Anton Langerak[2], Mark-David Levin[1]

*[1]Albert Schweitzer Ziekenhuis, Dordrecht, The Netherlands; [2]ErasmusMC, Rotterdam, The Netherlands; [3]Amsterdam UMC, Amsterdam, The Netherlands;*

### Background

The fixed-duration regimen combining the BCL2 inhibitor venetoclax (Ven) with the anti-CD20 antibody obinutuzumab (Ven-O) is a standard first-line treatment for chronic lymphocytic leukemia (CLL). In the FLAIR study (doi: 10.1056/NEJMoa2504341), MRD-guided combination treatment cessation of Ven with ibrutinib (Ven-I) showed favorable outcomes, including an OS benefit. Whether this holds true for sequential first-line Ven-O treatment followed by MRD-guided or fixed-duration Ven consolidation, remains unknown. The randomized phase 2 HOVON-139/GiVe trial enrolled previously untreated CLL patients considered unfit for FCR and evaluated the efficacy of Ven-O with MRD-guided Ven consolidation and longitudinal MRD monitoring.

### Aims

This long follow-up analysis of the HOVON-139 trial aims to report up to 7 years of post-registration of the deep molecular MRD and clinical outcomes. The kinetics of MRD with a lower limit of detection (LoD) of $10^{-5}$ will be associated with survival endpoints and patient baseline characteristics.

### Methods

Eligible patients (N=67) deemed unfit for FCR with untreated CLL received Ven-O (2 cycles O, 6 cycles Ven-O, 6 cycles Ven) (doi: 10.1016/s2352-3026(22)00034-5). After this initial treatment, open-label randomization (R) was initiated for 62 patients, of whom 32 received 12 cycles of Ven (arm A), and 30 MRD-guided Ven (arm B), using $10^{-4}$ as the cut-off MRD value.
MRD-NGS was measured with the IGHV leader-based assay. The early-stage serial MRD analysis (doi: 10.1038/s41408-023-00870-2), was extended with the post-treatment MRD approximately 5, 6 and 7 years after registration (medians: 61.8, 73.8, and 85.2 months; IQRs: 60.9-63.8, 72.9-75.1, and 83.9-86.5, respectively).

**Copyright Information:** (Online) ISSN: 2572-9241
©2026 The Author(s). HemaSphere is published by John Wiley & Sons Ltd on behalf of European Hematology Association. This is an open access Abstract Book distributed under the Attribution-NonCommercial-NoDerivs (CC BY-NC-ND), which allows third parties to download the articles and share them with others as long as they credit the author and the Abstract Book, but they cannot change the content in any way or use them commercially.
**Abstract Book Citations:** Authors, Title, HemaSphere, 2026;10:(S1):pages. The abstract book DOIs can be found on the “Author Information” page.
**Disclaimer:** Articles published in the journal HemaSphere exclusively reflect the opinions of the authors. The authors are responsible for all content in their abstracts including accuracy of the facts, statements, citing resources, etc.

**Results**

5/67 patients dropped out of the trial before randomization. Among the randomized patients, the 7-year PFS rates (95% CI) were 53% (35-68%) vs. 53% (34-69%) and the 7-year OS rates (95% CI) were 81% (63-91%) vs. 90% (72-97%) for arm A (all patients received 12 cycles of Ven maintenance) and arm B (1 patient received 3 cycles of Ven and 1 patient 9 cycles of Ven, the others none), respectively.
In 46/67 (68.7%) patients, ≥1 MRD value was measured post-treatment up to 7-years follow-up. At approximately 3, 4, and 5 years after end of treatment (EoT) (See Figure), the numbers of patients with progressive disease (PD) or who had detectable MRD above $10^{-5}$, were 41/67 (61.2%), 42/67 (62.7%), and 44/67 (65.7%), respectively.
In spite of missing MRD data, we could establish that in 13/67 (19.4%) of patients (7 for all 3 timepoints, 3 for 2 timepoints and 3 for 1 timepoint) uMRD5 persisted during the follow-up phase. Additionally, 14 patients (8 from arm A and 6 from arm B) who were uMRD5 at 1≥ follow-up timepoint, remained progression-free at a median follow-up of 86 months (IQR 84.8-89.9).

**Summary/Conclusion**

Overall, MRD-guided Ven-O leads to long-term disease control in the HOVON-139 study, irrespective of the Ven maintenance arm. Late-stage clinical follow-up and PB MRD analysis shows increasing re-emergence of MRD. However, a minority of patients show persistent uMRD5 without disease progression up to 7 years of follow-up.

**Copyright Information:** (Online) ISSN: 2572-9241
©2026 The Author(s). HemaSphere is published by John Wiley & Sons Ltd on behalf of European Hematology Association. This is an open access Abstract Book distributed under the Attribution-NonCommercial-NoDerivs (CC BY-NC-ND), which allows third parties to download the articles and share them with others as long as they credit the author and the Abstract Book, but they cannot change the content in any way or use them commercially.
**Abstract Book Citations:** Authors, Title, HemaSphere, 2026;10:(S1):pages. The abstract book DOIs can be found on the “Author Information” page.
**Disclaimer:** Articles published in the journal HemaSphere exclusively reflect the opinions of the authors. The authors are responsible for all content in their abstracts including accuracy of the facts, statements, citing resources, etc.

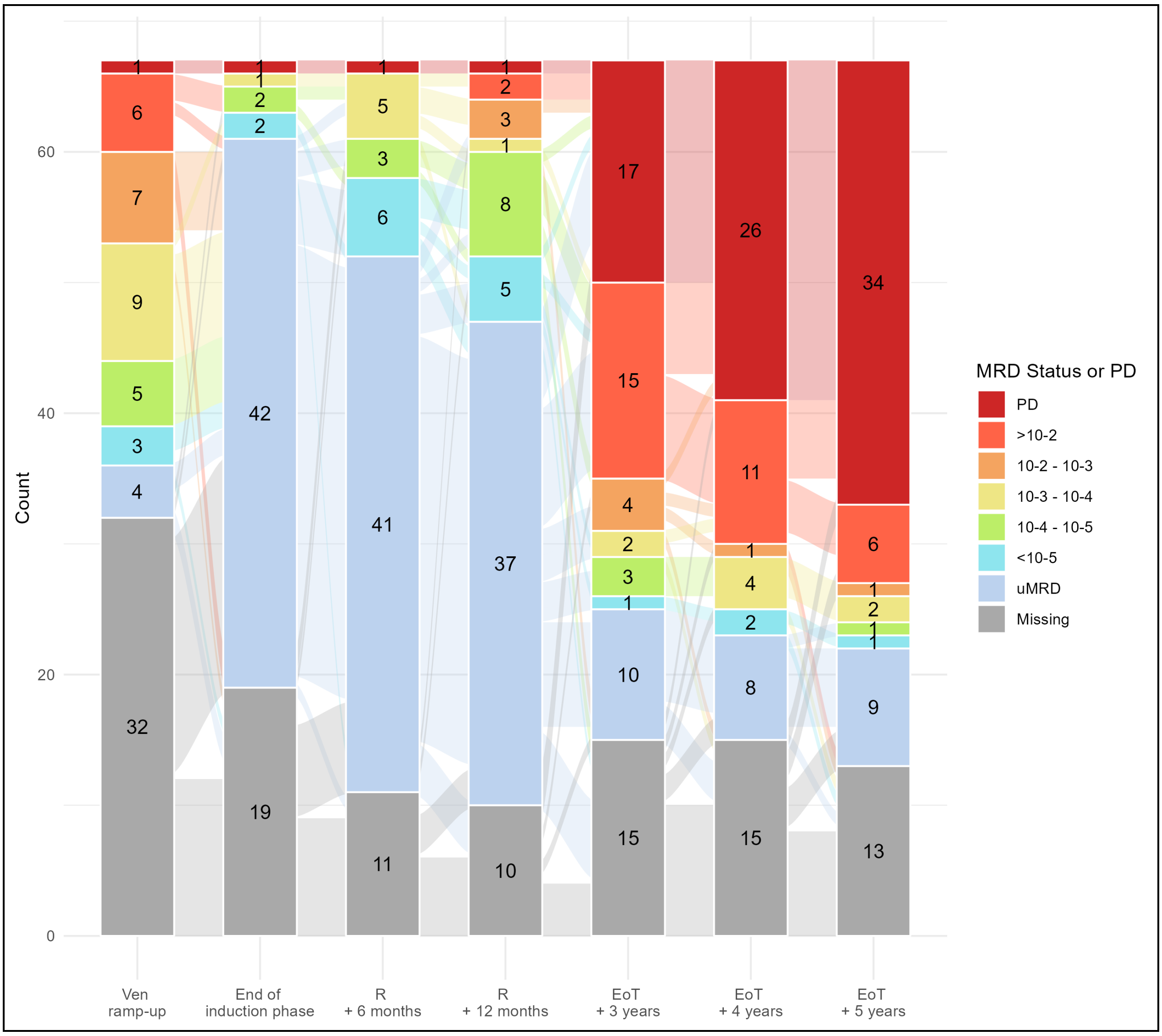

Figure: Longitudinal MRD depth and PFS events (progressive disease/death) over time for the whole cohort. Randomization (R) was initiated at the end of the induction phase. End of treatment (EoT) is defined as 12 months post-R.

**Copyright Information:** (Online) ISSN: 2572-9241
©2026 The Author(s). HemaSphere is published by John Wiley & Sons Ltd on behalf of European Hematology Association. This is an open access Abstract Book distributed under the Attribution-NonCommercial-NoDerivs (CC BY-NC-ND), which allows third parties to download the articles and share them with others as long as they credit the author and the Abstract Book, but they cannot change the content in any way or use them commercially.
**Abstract Book Citations:** Authors, Title, HemaSphere, 2026;10:(S1):pages. The abstract book DOIs can be found on the "Author Information" page.
**Disclaimer:** Articles published in the journal HemaSphere exclusively reflect the opinions of the authors. The authors are responsible for all content in their abstracts including accuracy of the facts, statements, citing resources, etc.

### (PF601) SUBSEQUENT THERAPIES AND TIME TO SECOND PROGRESSION-FREE SURVIVAL EVENTS IN CHRONIC LYMPHOCYTIC LEUKEMIA/SMALL LYMPHOCYTIC LYMPHOMA PREVIOUSLY TREATED WITH ZANUBRUTINIB OR BENDAMUSTINE-RITUXIMAB

**Topic:** 06. Chronic lymphocytic leukemia and related disorders - Clinical

Constantine S. Tam*[1], Brad Kahl[2], Talha Munir[3], Tadeusz Robak[4], Jennifer R. Brown[5], Paolo Ghia[6, 7], Stephen Opat[8], Patricia Walker[9], Masa Lasica[10], Ian W. Flinn[11], Wojciech Jurczak[12], Jiayi Shen[13], Marcus Lefebure[14], Jamie Hirata[13], Mazyar Shadman[15, 16]

*[1]Alfred Hospital and Monash University, Melbourne, VIC, Australia;[2]Siteman Cancer Center, Washington University School of Medicine, St. Louis, MO, United States of America; [3]Leeds Teaching Hospitals NHS Trust, Leeds, United Kingdom;[4]Copernicus Memorial Hospital, Medical University of Lodz, Lodz, Poland; [5]Dana-Farber Cancer Institute, Boston, MA, United States of America; [6]Università Vita-Salute San Raffaele, Milano, Italy; [7]Comprehensive Cancer Center, IRCCS Ospedale San Raffaele, Milano, Italy; [8]Lymphoma Research Group, School of Clinical Sciences at Monash Health, Monash University, Clayton, VIC, Australia; [9]Peninsula Health and Peninsula Private Hospital, Melbourne, VIC, Australia; [10]St Vincent's Hospital Melbourne, Melbourne, VIC, Australia; [11]Tennessee Oncology/OneOncology, Nashville, TN, United States of America; [12]Maria Sklodowska-Curie National Research Institute of Oncology, Kraków, Poland; [13]BeOne Medicines, Ltd, San Carlos, CA, United States of America; [14]BeOne Medicines, Ltd, London, United Kingdom; [15]Fred Hutchinson Cancer Center, Seattle, WA, United States of America; [16]University of Washington, Seattle, WA, United States of America;*

### Background

Cohort 1 of the phase 3 SEQUOIA trial assessed zanubrutinib, a next-generation Bruton tyrosine kinase inhibitor (BTKi), compared with bendamustine-rituximab (BR) in treatment-naive chronic lymphocytic leukemia/small lymphocytic lymphoma (TN CLL/SLL) without del(17p). Zanubrutinib previously demonstrated sustained superiority in progression-free survival (PFS) and time to next treatment (TTNT) vs BR. However, evidence regarding outcomes on subsequent therapies after progressive disease (PD) on zanubrutinib remains limited. Here, we present results on follow-up therapies after zanubrutinib, including second progression-free survival events (PFS2).

### Methods

Patients without del(17p) were randomized to receive continuous zanubrutinib or six cycles of BR. Patients who received BR could crossover to receive zanubrutinib after PD. All patients were followed post-PD for details on any subsequent anti-CLL treatments. This analysis evaluated PFS, TTNT, TTNT or death (TTNT-D) and PFS2. *P*-values are descriptive.

**Copyright Information:** (Online) ISSN: 2572-9241
©2026 The Author(s). HemaSphere is published by John Wiley & Sons Ltd on behalf of European Hematology Association. This is an open access Abstract Book distributed under the Attribution-NonCommercial-NoDerivs (CC BY-NC-ND), which allows third parties to download the articles and share them with others as long as they credit the author and the Abstract Book, but they cannot change the content in any way or use them commercially.
**Abstract Book Citations:** Authors, Title, HemaSphere, 2026;10:(S1):pages. The abstract book DOIs can be found on the "Author Information" page.
**Disclaimer:** Articles published in the journal HemaSphere exclusively reflect the opinions of the authors. The authors are responsible for all content in their abstracts including accuracy of the facts, statements, citing resources, etc.

### Results

In total 479 patients were randomized; 241 to zanubrutinib and 238 to BR. Baseline demographics and disease characteristics were well balanced between the arms. As of 31 October 2025, median follow-up was 78.7 months (range, 0.0-96.0). Sustained PFS superiority with zanubrutinib vs BR was observed (HR, 0.28; 95% CI, 0.21-0.38; *P*<.0001) with 78-month PFS estimates of 70.9% (95% CI, 64.2-76.6) for zanubrutinib and 28.6% (21.7-35.8) for BR. After adjustment for COVID, PFS estimates were 73.7% (67.0, 79.3) for zanubrutinib and 29.0% (22.1, 36.3) for BR. Overall, 211/241 (87.6%) of patients treated with zanubrutinib had not received subsequent therapy and 34 patients had died without subsequent therapy. TTNT and TTNT-D favored zanubrutinib over BR (HR, 0.23 [95% CI, 0.15- 0.35; *P*<.0001] and 0.37 [0.27-0.50; *P*<.0001], respectively). Overall, 24/241 (10.0%) zanubrutinib patients and 82/238 (34.0%) BR patients had PD after study treatment and received subsequent therapy. Of the 24 zanubrutinib patients receiving subsequent therapy after PD, 13 (54.2%) received B-cell lymphoma 2 inhibitor (BCL2i)-based regimens, 8 (33.3%) received chemoimmunotherapy (CIT) (including anti-CD20 antibody monotherapy), and 3 (12.5%) received BTKi. Of the 82 BR patients receiving subsequent therapy after PD, 77 (93.9%) received BTKi (including 71 crossover patients), 3 (3.7%) received BCL2i-based regimens and 2 (2.4%) received CIT. Median time from PD following first therapy to initiation of next therapy was 2.1 months for zanubrutinib (range 0-25.7) and 7.4 months (0.3-48.0) for BR. Zanubrutinib patients who had PD and received subsequent BCL2i-based therapy had a median follow-up of 33.5 months from initiation of next-line therapy. Among these patients, 10 remain alive and without PD, two had died, and one had PD. Overall, PFS2 significantly favored zanubrutinib over BR (HR, 0.66; 95% CI, 0.45-0.98; *P*<.05) with 78-month PFS2 estimates of 81.3% (95% CI, 75.6-85.8) vs 73.8% (67.2-79.3), respectively (**Figure**).

### Summary/Conclusion

PFS2 remained superior with zanubrutinib despite broad use of novel subsequent therapy (including crossover) in BR patients. Importantly, BCL2i-based salvage after initial zanubrutinib achieved high PFS rates at almost 3 years of follow-up. Together, these findings support that initial zanubrutinib provides durable benefit while preserving sensitivity to effective subsequent therapy.

**Figure.** Time to Second PFS Event

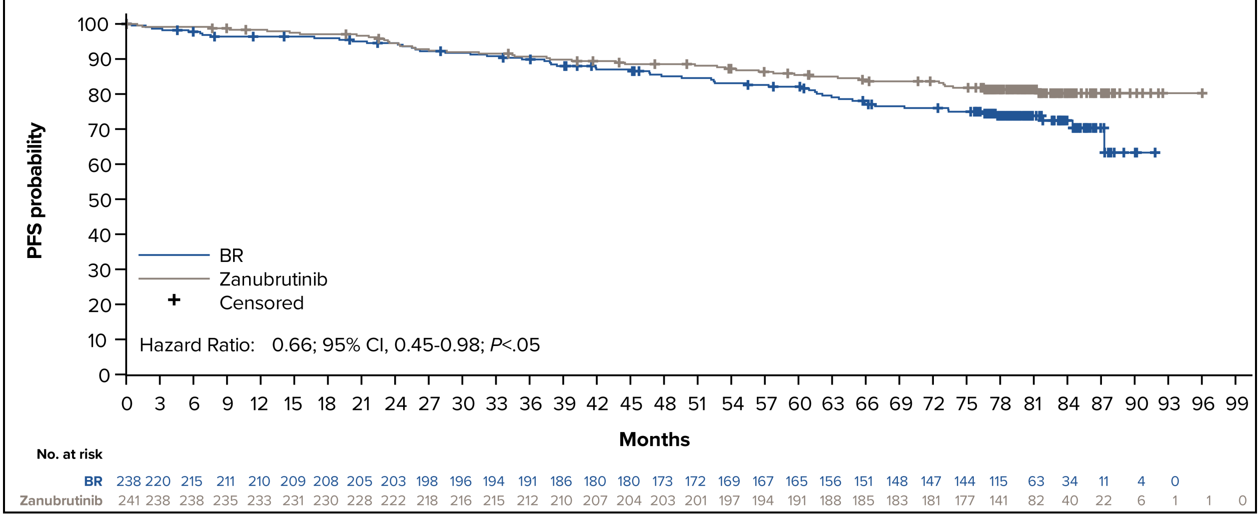

**Copyright Information:** (Online) ISSN: 2572-9241
©2026 The Author(s). HemaSphere is published by John Wiley & Sons Ltd on behalf of European Hematology Association. This is an open access Abstract Book distributed under the Attribution-NonCommercial-NoDerivs (CC BY-NC-ND), which allows third parties to download the articles and share them with others as long as they credit the author and the Abstract Book, but they cannot change the content in any way or use them commercially.
**Abstract Book Citations:** Authors, Title, HemaSphere, 2026;10:(S1):pages. The abstract book DOIs can be found on the "Author Information" page.
**Disclaimer:** Articles published in the journal HemaSphere exclusively reflect the opinions of the authors. The authors are responsible for all content in their abstracts including accuracy of the facts, statements, citing resources, etc.

### (PF602) A LONGITUDINAL COMPREHENSIVE GERIATRIC ASSESSMENT TO DEFINE FITNESS IN FIRST-LINE CHRONIC LYMPHOCYTIC LEUKEMIA PATIENTS AGED ≥65 YEARS: A PROSPECTIVE MULTICENTER ITALIAN STUDY (CLYF TRIAL)

**Topic:** 06. Chronic lymphocytic leukemia and related disorders - Clinical

Anna Maria Frustaci*[1], Arianna Zappaterra[1], Andrea Galitzia[2], Ester Lovato[3], Candida Vitale[4], Maura Nicolosi[5], Chiara Borella[6], Vittorio Bolis[7], Giulia Quaresmini[8], Massimo Gentile[9], Sara Marinoni[10], Lorena Appio[11], Annamaria Giordano[12], Andrea Visentin[13], Giuliana Farina[14], Valentina Rossi[15], Filippo Bagnoli[16], Elia Boccellato[17], Enrico Lista[18], Alfredo Molteni[19], Gloria Turri[20], Vittorio Del Fabro[21], Marina Deodato[1], Francesca Perutelli[22], Alessandro Noto[1], Marina Motta[3], Roberta Murru[2], Andrea Ferrario[23], Alessandra Tedeschi[1]

*[1]ASST Grande Ospedale Metropolitano Niguarda, Niguarda Cancer Center, Hematology, Milan, Italy; [2]Hematology and Stem Cell Transplantation Unit Ospedale Oncologico A. Businco ARNAS “G. Brotzu”, Cagliari, Italy; [3]Hematology ASST Spedali Civili, Brescia, Italy; [4]Hematology Section, Department of Molecular Biotechnology and Health Sciences, University of Torino; Division of Hematology, A.O.U. Città della Salute e della Scienza di Torino, Torino, Italy; [5]Hematology Division A.O.U. Città della Salute e della Scienza di Torino, Torino, Italy; [6]Hematology Division, IRCCS San Gerardo dei Tintori Hospital, Monza, Italy; [7]Hematology, Fondazione IRCCS Istituto Nazionale dei Tumori; School Of Medicine, University of Milan, Milano, Italy; [8]SC Ematologia ASST Papa Giovanni XXIII, Bergamo, Italy; [9]Hematology Unit AO of Cosenza, Cosenza; Department of Pharmacy, Health and Nutritional Sciences, University of Calabria, Rende, Cosenza, Italy; [10]UOC Ematologia, ASST Ovest milanese, Ospedale di Legnano, Legnano, Italy; [11]ASST Valle Olona, S.C. Ematologia, Ospedale di Busto Arsizio, Busto Arsizio, Italy;[12]Department of precision and regenerative medicine and Ionian area, Aldo Moro University, School of Medicine, Bari; Unit of Hematology and Stem Cell Transplantation, AOUC Policlinico Bari, Bari, Italy; [13]Hematology unit Department of Medicine University of Padova, Padova, Italy; [14]Hematology, Hospital; Sant Anna e San Sebastiano, Caserta, Italy; [15]SC Ematologia, ASST Fatebenefratelli Sacco, Polo Universitario Luigi Sacco, Milano, Italy; [16]Department of Oncology and Hematology, IRCCS Humanitas Research Hospital, Rozzano, Italy; [17]Division of Hematology, Santa Croce e Carle Hospital, Cuneo, Italy;[18]UO Ematologia, Ospedale Santa Chiara, APSS, Trento, Italy; [19]Hematology, ASST Cremona, Cremona, Italy; [20]Hematology and Bone Marrow Transplant Center, Central Hospital of Bolzano, Bolzano, Italy; [21]Faculty of Medicine and Surgery, "Kore" University of Enna, Enna, Italy; [22]Hematology Section, Department of Molecular Biotechnology and Health Sciences, University of Torino, Torino, Italy; Division of Hematology, A.O.U. Città della Salute e della Scienza di Torino, Torino, Italy; [23]Hematology, ASST dei Sette Laghi-Ospedale di Circolo, Varese, Italy;*

### Background

Frailty affects treatment choice and outcomes in chronic lymphocytic leukemia (CLL), however it remains undefined and still based on chemotherapy-derived parameters such as Creatinine Clearance (CrCl) <70 and/or CIRS>6 (traditional fitness).

### Aims

In the era of targeted agents, we aim to evaluate whether an integrated geriatric and quality of life (QoL) approach could better define fitness and guide treatment.

**Copyright Information:** (Online) ISSN: 2572-9241
©2026 The Author(s). HemaSphere is published by John Wiley & Sons Ltd on behalf of European Hematology Association. This is an open access Abstract Book distributed under the Attribution-NonCommercial-NoDerivs (CC BY-NC-ND), which allows third parties to download the articles and share them with others as long as they credit the author and the Abstract Book, but they cannot change the content in any way or use them commercially.
**Abstract Book Citations:** Authors, Title, HemaSphere, 2026;10:(S1):pages. The abstract book DOIs can be found on the “Author Information” page.
**Disclaimer:** Articles published in the journal HemaSphere exclusively reflect the opinions of the authors. The authors are responsible for all content in their abstracts including accuracy of the facts, statements, citing resources, etc.

### Methods

In this ongoing prospective, multicenter, observational cohort study, consecutive pts ≥65 years receiving first line CLL treatment completed the ASCO-validated Practical Geriatric Assessment (PGA, *Dale et al., JCO, 2023*) and EORTC QLQ-C30 (*Aaronson et al., J Natl Cancer Inst, 1993*) questionnaires before treatment start (baseline) and at 6, 12 and 18 months. PGA domains included: physical, functional, psychological and cognitive function, nutritional status, social support and self-perceived comorbidities. Primary objective: defining frailty in CLL by PGA metrics. Secondary: feasibility; impact of clinical/disease characteristics on PGA domains; distribution of PGA/EORTC QLQ-C30 global health status (GHS) among treatment choices and longitudinal changes. Here we report preliminary data at baseline and +6 months.

### Results

From March 2025 to January 2026, 167 pts were included: 58% on BTKi, 33.5% on ibrutinib-venetoclax, 8.4% on venetoclax-obinutuzumab. Compared with fixed-duration regimens, BTKi pts were older (*p<.001*), unfit by traditional fitness (*p<.001*), had higher ECOG-PS (*p.036*), more concomitant medications (*p<.001*) and more frequently carried *TP53*-aberrations (*p.005*). Overall, 98% had ≥1 altered PGA domain (median 3). BTKi pts showed higher PGA burden (*p.003*), driven by impaired physical function (*p.001*). Median GHS for the whole cohort was 67 (range 0-100) with no differences among treatment groups. When comparing pts stratification by traditional fitness with respect to PGA burden and treatment choice, traditional fitness and PGA were discordant only in the fit group, with higher PGA burden in BTKi-treated pts. Moreover, of 115 pts (68%) with no anamnestic severe comorbidities recorded, 84.3% reported self-perception of relevant coexisting conditions. Age was the only baseline factor independently predicting higher PGA burden (*p.002*). Figure 1 shows multivariate analysis on relation between baseline characteristics and single PGA domains. Low platelets (*p=.006*) and musculoskeletal CIRS (*p=.010*) worsened GHS. Declined psychological (*p<.001*), cognitive (*p.025*) and functional status (*p.013*) were also detrimental to GHS.
At 6 months 90 pts were re-evaluated, showing a statistically significant improvement of PGA burden (median 5 versus 4,*p.001*) with a benefit in nutritional status (*p.003*). Treatment choice did not impact on 6-months PGA burden. No differences were recorded in GHS at 6 months, with no treatment effect. Median PGA+QLQ-C30 completion time was 20 minutes for pts and 5 for physicians.

### Summary/Conclusion

Here we present the interim analysis on the first prospective cohort of CLL pts assessed employing the PGA, a tool already widely applied in oncology. In our cohort, PGA proved feasible and was able to capture changes occurring during targeted therapies. Notably, even among pts classified as fit by traditional fitness criteria, PGA identified frailer individuals for whom fixed-duration regimens were not considered appropriate by physicians. These preliminary findings support the integration of dynamic PGA and QoL assessment into routine CLL management.

**Copyright Information:** (Online) ISSN: 2572-9241
©2026 The Author(s). HemaSphere is published by John Wiley & Sons Ltd on behalf of European Hematology Association. This is an open access Abstract Book distributed under the Attribution-NonCommercial-NoDerivs (CC BY-NC-ND), which allows third parties to download the articles and share them with others as long as they credit the author and the Abstract Book, but they cannot change the content in any way or use them commercially.
**Abstract Book Citations:** Authors, Title, HemaSphere, 2026;10:(S1):pages. The abstract book DOIs can be found on the "Author Information" page.
**Disclaimer:** Articles published in the journal HemaSphere exclusively reflect the opinions of the authors. The authors are responsible for all content in their abstracts including accuracy of the facts, statements, citing resources, etc.

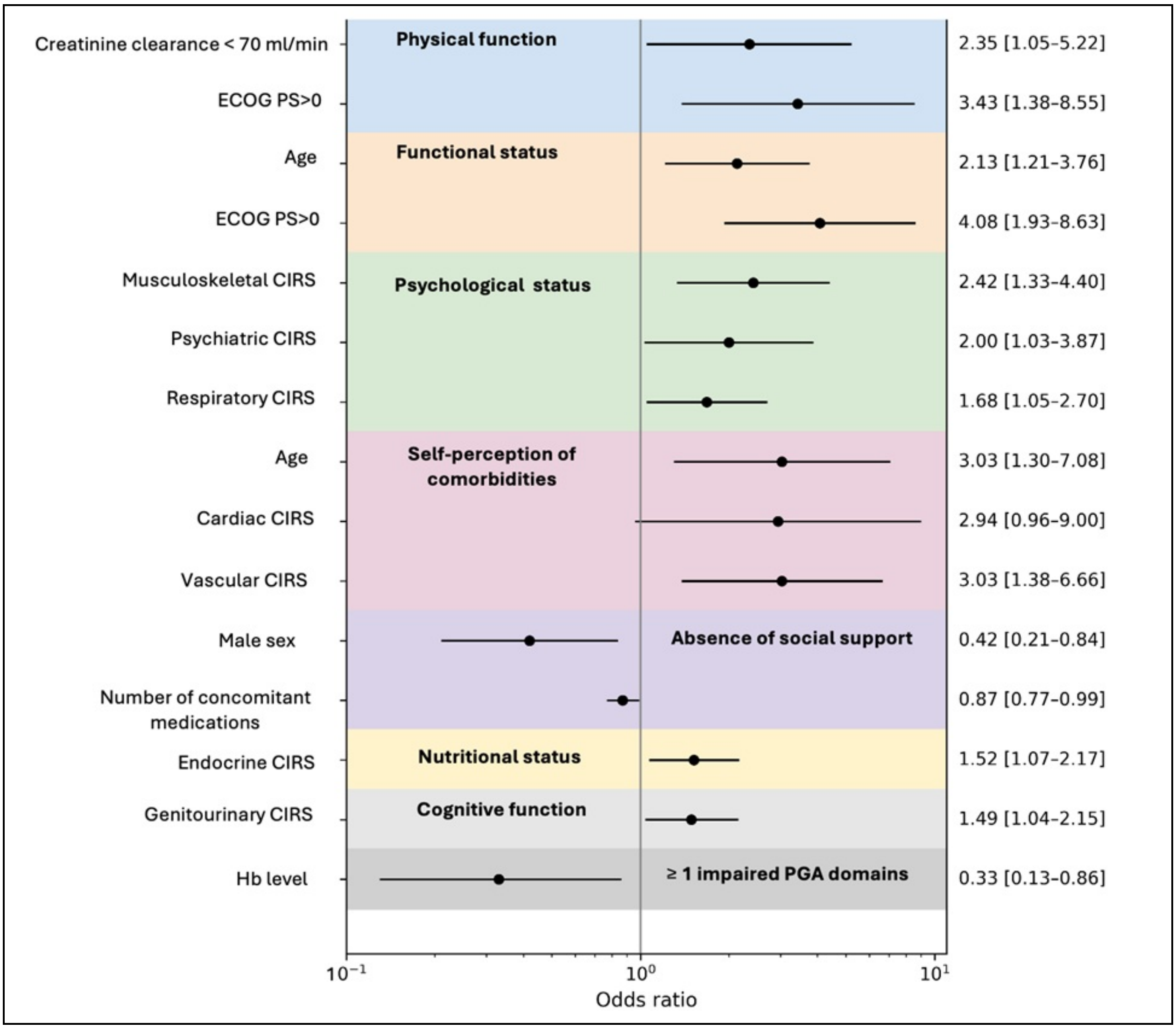

Figure 1. Multivariable model on factors associated with impairments in PGA domains

Copyright Information: (Online) ISSN: 2572-9241
©2026 The Author(s). HemaSphere is published by John Wiley & Sons Ltd on behalf of European Hematology Association. This is an open access Abstract Book distributed under the Attribution-NonCommercial-NoDerivs (CC BY-NC-ND), which allows third parties to download the articles and share them with others as long as they credit the author and the Abstract Book, but they cannot change the content in any way or use them commercially.
Abstract Book Citations: Authors, Title, HemaSphere, 2026;10:(S1):pages. The abstract book DOIs can be found on the “Author Information” page.
Disclaimer: Articles published in the journal HemaSphere exclusively reflect the opinions of the authors. The authors are responsible for all content in their abstracts including accuracy of the facts, statements, citing resources, etc.

### (PF603) A PRE-TREATMENT PROGNOSTIC SCORE FOR HAIRY CELL LEUKEMIA TREATED WITH 2CDA: DEVELOPMENT AND INTERNAL VALIDATION IN A MULTICENTRIC COHORT OF 513 PATIENTS

**Topic:** 06. Chronic lymphocytic leukemia and related disorders - Clinical

Francesco Angotzi*[1], Marianna Criscuolo[2], Maria Tosti[3], Alessandro Broccoli[4, 5], Marzia Varettoni[6], Alessio Maria Edoardo Maraglino[7], Antonella Anastasia[8], Maria Cantonetti[9], Luca Guarnera[9], Massimiliano Postorino[9], Alessandro Sanna[10], Sofia Kovalchuk[10], Maura Nicolosi[11], Marina Deodato[12], Angelica Spolzino[13, 14], Raffaella Pasquale[15], Ombretta Annibali[16], Cristiana Gasbarrino[17], Sergio Storti[18], Caterina Stelitano[19], Francesco Marchesi[20], Sonia Morè[21], Laura Maracci[21], Alessandro Cellini[1], Nicolò Danesin[1], Brunangelo Falini[22], Alessandro Pulsoni[23], Ilaria Del Giudice[23], Enrico Tiacci[22], Pier Luigi Zinzani[4, 5], Livio Trentin[1], Livio Pagano[2, 24]

*[1]Hematology Unit, University of Padova, Department of Medicine (DIMED), Padova, Italy; [2]Fondazione Policlinico Universitario A. Gemelli IRCCS, Dipartimento di Diagnostica per Immagini, Radioterapia Oncologica ed Ematologia, Roma, Italy; [3]Istituto Superiore di Sanità, Roma, Italy; [4]IRCCS Azienda Ospedaliero-Universitaria di Bologna, Istituto di Ematologia "Seragnoli", Bologna, Italy; [5]Università di Bologna, Dipartimento di Medicina Specialistica, Diagnostica e Sperimentale, Bologna, Italy; [6]Fondazione IRCCS Policlinico San Matteo, Ematologia 1, Pavia, Italy; [7]IEO European Institute of Oncology IRCCS, Oncohematology Division, Milan, Italy; [8]ASST Spedali Civili, Department of Hematology, Brescia, Italy; [9]Università Tor Vergata, Dipartimento di Oncoematologia Policlinico Tor Vergata, Roma, Italy; [10]AOU Careggi, SOD C Ematologia, Firenze, Italy; [11]AOU Città della Salute e della Scienza di Torino, S.C. Ematologia, Turin, Italy; [12]ASST Grande Ospedale Metropolitano Niguarda, Divisione di Ematologia, Milano, Italy; [13]Università degli Studi di Parma, Dipartimento di Medicina e Chirurgia, Parma, Italy; [14]Istituto Oncologico Veneto IOV-IRCCS, Oncoematologia, Castelfranco Veneto, Italy; [15]Azienda Sanitaria Universitaria Friuli Centrale, Clinica Ematologica, Udine, Italy; [16]Campus Bio-Medico University, Hematology and Stem Cell Transplantation Unit, Rome, Italy; [17]Fondazione di Ricerca e Cura Giovanni Paolo II-Università Cattolica del Sacro Cuore, UOC Oncoematologia, Campobasso, Italy; [18]Fondazione di Ricerca e Cura Giovanni Paolo II, Campobasso—Università Cattolica del Sacro Cuore, UOC Oncoematologia, Campobasso, Italy; [19]Azienda Ospedaliera "Bianchi Melacrino Morelli", Divisione di Ematologia, Reggio Calabria, Italy; [20]IRCCS Regina Elena National Cancer Institute, Haematology and Stem Cell Transplantation Unit, Rome, Italy; [21]AOU delle Marche, Clinica di Ematologia, Ancona, Italy;[22]Institute of Hematology and Center for Hemato-Oncology Research, University of Perugia, Santa Maria della Misericordia Hospital, Department of Medicine and Surgery, Perugia, Italy; [23]Sapienza University of Rome, Divisione di Ematologia, Department of Translational and Precision Medicine, Rome, Italy; [24]Università Cattolica del Sacro Cuore, Sezione di Ematologia, Dipartimento di Scienze Radiologiche ed Ematologiche, Rome, Italy;*

### Background

Patients with Hairy Cell Leukemia (HCL) typically achieve durable remissions after first-line cladribine (2CDA) often exceeding 10 years. However, a fraction of patients experiences early relapses and ultimately require second-line treatments with progressive shorter remissions. While identification of these patients is paramount, there are no available prognostic scoring systems for HCL.

### Aims

To develop a pre-treatment prognostic scoring system to stratify patients with HCL treated with 2CDA according to progression-free survival (PFS).

**Copyright Information:** (Online) ISSN: 2572-9241
©2026 The Author(s). HemaSphere is published by John Wiley & Sons Ltd on behalf of European Hematology Association. This is an open access Abstract Book distributed under the Attribution-NonCommercial-NoDerivs (CC BY-NC-ND), which allows third parties to download the articles and share them with others as long as they credit the author and the Abstract Book, but they cannot change the content in any way or use them commercially.
**Abstract Book Citations:** Authors, Title, HemaSphere, 2026;10:(S1):pages. The abstract book DOIs can be found on the "Author Information" page.
**Disclaimer:** Articles published in the journal HemaSphere exclusively reflect the opinions of the authors. The authors are responsible for all content in their abstracts including accuracy of the facts, statements, citing resources, etc.

### Methods

We leveraged a multicentric cohort of patients with HCL (n=513) who underwent first line treatment with 2CDA. Baseline clinical characteristics were collected before treatment and included: demographics, ECOG PS, complete blood counts, comorbidities, presence of active infection, hepatomegaly, splenomegaly and/or lymphadenopathies. Missing values were imputed by the missForest imputation algorithm. Variable selection was performed using random survival forests selecting variables with variable importance values (VIMP) ≥0.002, followed by multivariate Cox regression. Significant variables ($p < 0.05$) were used to generate a point-based score from beta-coefficients. Continuous variables were dichotomized with maximally selected rank statistics for score simplicity. The primary outcome was PFS, defined as the time from the start of therapy until relapse, death or last follow-up whichever came first. Internal validation was performed using bootstrap resampling with 500 iterations to estimate optimism-corrected discrimination by Harrel's C-index.

### Results

Median age was 54 years; 94 patients were females (18.3%). The median follow-up in the whole cohort was 6.8 years (interquartile range: 3.4 – 12.5). Five variables were selected for multivariate analysis: white blood cell count (WBC; VIMP = 0.21), age (VIMP = 0.02), platelet count (PLTs; VIMP = 0.007), hemoglobin (Hb; VIMP = 0.006), and hepatomegaly (VIMP = 0.002). Comorbidities and conventional scores (ECOG PS, CIRS) were instead not associated with PFS. In the cox model, 4 variables remained independently associated with PFS and were used to derive the final score: age >71 years (HR: 2.04; $p<0.001$; 1.5 points), WBC ≥6000/uL (HR: 2.11; $p<0.001$; 1.5 points), PLTs <67000/uL (HR: 1.5; $p=0.01$; 1 point) and Hb <130 (HR: 1.55; $p=0.01$; 1 point). Patients were categorized as high-risk (≥2.5 points), intermediate (1.5 points) and low-risk (0-1 points). A total of 93 (18%), 129 (25%), and 291 patients (57.6%) were classified as high, intermediate and low-risk respectively. During follow-up, 174 PFS events were recorded. Median PFS were 5.5 years, 8.3, and 19.5 years for high, intermediate and low-risk patients categories ($p < 0.001$) (Figure). The original C-index was 0.624 and optimism-corrected C-index after internal validation 0.623, indicating modest but stable discriminative performance.

### Summary/Conclusion

Our prognostic model was able to stratify patients with HCL into three distinct risk categories according to PFS prior to treatment with 2CDA. Although discrimination was modest, performance remained stable after internal validation. High-risk patients, unlikely to derive durable benefit from 2CDA may thus be considered for alternative first-line treatments or inclusion in clinical trials. Future work will focus on increasing the model's discriminative capacity and on validation in external cohorts.

**Copyright Information:** (Online) ISSN: 2572-9241
©2026 The Author(s). HemaSphere is published by John Wiley & Sons Ltd on behalf of European Hematology Association. This is an open access Abstract Book distributed under the Attribution-NonCommercial-NoDerivs (CC BY-NC-ND), which allows third parties to download the articles and share them with others as long as they credit the author and the Abstract Book, but they cannot change the content in any way or use them commercially.
**Abstract Book Citations:** Authors, Title, HemaSphere, 2026;10:(S1):pages. The abstract book DOIs can be found on the "Author Information" page.
**Disclaimer:** Articles published in the journal HemaSphere exclusively reflect the opinions of the authors. The authors are responsible for all content in their abstracts including accuracy of the facts, statements, citing resources, etc.

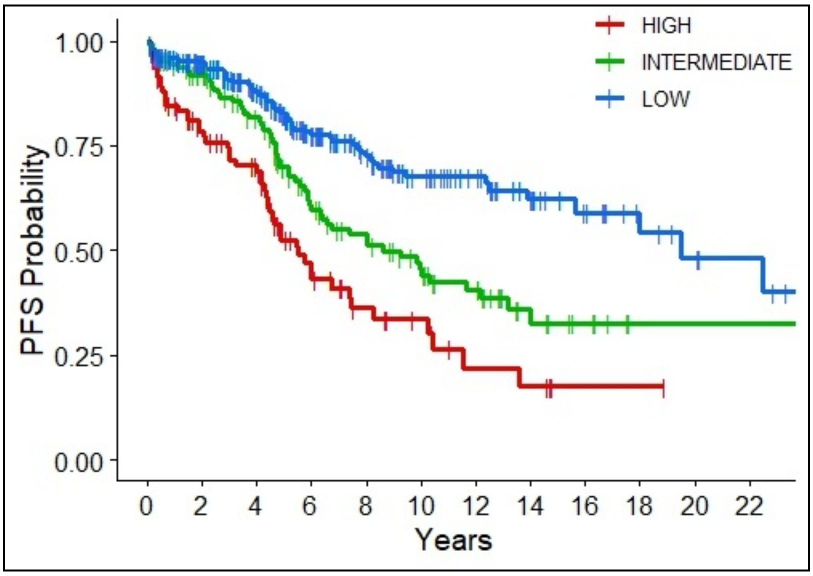

Figure: Kaplan-Meier curves for PFS in patients with HCL stratified by our scoring system

**Copyright Information:** (Online) ISSN: 2572-9241
©2026 The Author(s). HemaSphere is published by John Wiley & Sons Ltd on behalf of European Hematology Association. This is an open access Abstract Book distributed under the Attribution-NonCommercial-NoDerivs (CC BY-NC-ND), which allows third parties to download the articles and share them with others as long as they credit the author and the Abstract Book, but they cannot change the content in any way or use them commercially.
**Abstract Book Citations:** Authors, Title, HemaSphere, 2026;10:(S1):pages. The abstract book DOIs can be found on the "Author Information" page.
**Disclaimer:** Articles published in the journal HemaSphere exclusively reflect the opinions of the authors. The authors are responsible for all content in their abstracts including accuracy of the facts, statements, citing resources, etc.

### (PF604) LONG-TERM FOLLOW-UP RESULTS OF A PHASE 2 STUDY OF VENETOCLAX ADDED TO IBRUTINIB TO ELIMINATE RESISTANCE MUTATIONS IN CHRONIC LYMPHOCYTIC LEUKEMIA (CLL)

**Topic:** 06. Chronic lymphocytic leukemia and related disorders - Clinical

Kerry Rogers*[1], Eric McLaughlin[2], Lai Wei[2], Aseel Alsouqi[1], Amneet Bajwa[1], Seema Ali Bhat[1], Savannah Butt[3], Cara Grantier[3], Michael Grever[1], Corinne Hoffman[3], Daniel Jones[4], Adam Kittai[5], Gerard Lozanski[4], Jordan Lundberg[6], Cecelia Miller[4], John Byrd[7], Jennifer Woyach[1]

*[1]The Ohio State University, Division of Hematology, Columbus, OH, United States of America;[2]The Ohio State University, Center for Biostatistics, Columbus, OH, United States of America; [3]The Ohio State University, Comprehensive Cancer Center, Columbus, OH, United States of America; [4]The Ohio State University, Department of Pathology, Columbus, OH, United States of America; [5]Icahn School of Medicine at Mount Sinai, New York, NY, United States of America;[6]The Ohio State University, Department of Pharmacy, Columbus, OH, United States of America; [7]University of Pittsburgh, UPMC Hillman Cance Cancer and Department of Medicine, Pittsburgh, PA, United States of America;*

**Background**

Resistance to BTK inhibitors (BTKi) is an important mechanism of disease progression in CLL. Mutations associated with BTKi-resistance develop in BTK and PLCG2 and can be detected prior to progressive disease (PD). As a novel strategy of overcoming resistance to the first-in-class BTKi, ibrutinib (Ibr), we conducted a phase 2 study adding venetoclax (Ven) to Ibr. We have previously shown this strategy improves progression-free survival (PFS) relative to treatment with Ven monotherapy and can allow discontinuation. Here we report long-term results of this clinical trial (NCT03513562).

**Aims**

To describe outcomes after Ven was added to Ibr in patients with acquired mutations associated with Ibr resistance, including PFS, overall survival (OS), time to next treatment (TTNT), and change in CLL proportion with mutation (VAF).

**Methods**

CLL patients on Ibr who developed a detectable mutation associated with Ibr resistance with or without PD were eligible. Patients continued Ibr and added Ven. Ibr/Ven combination was given for up to 24 cycles (C) and stopped if a complete remission with undetectable measurable residual disease (MRD) was achieved after C12 or C24. If this was not achieved after C24, Ven alone was continued. MRD was measured to a level of $<1x10^{-4}$ by flow cytometry. BTK C481X mutations were detected down to ≥0.5% VAF by NGS or ≥0.1% VAF by PCR on sorted CD19+ B-cells. Kaplan Meier methods were used to assess PFS and OS. All statistical analyses were performed using SAS version 9.4 (SAS Institute, Inc., Cary, NC). Trial data are as of 2/9/2026.

**Copyright Information:** (Online) ISSN: 2572-9241
©2026 The Author(s). HemaSphere is published by John Wiley & Sons Ltd on behalf of European Hematology Association. This is an open access Abstract Book distributed under the Attribution-NonCommercial-NoDerivs (CC BY-NC-ND), which allows third parties to download the articles and share them with others as long as they credit the author and the Abstract Book, but they cannot change the content in any way or use them commercially.
**Abstract Book Citations:** Authors, Title, HemaSphere, 2026;10:(S1):pages. The abstract book DOIs can be found on the "Author Information" page.
**Disclaimer:** Articles published in the journal HemaSphere exclusively reflect the opinions of the authors. The authors are responsible for all content in their abstracts including accuracy of the facts, statements, citing resources, etc.

**Results**

28 patients were enrolled between 3/2019 and 2/2022. Median age was 62 (range 41-82) years, 29% (8/28) were women, and 96% (27/28) were white. Median treatments prior to Ibr was 2 (range 0-13). The CLL was IGHV unmutated in 75% (21/28) and 43% (12/28) had del(17p). At a median follow-up of 45.9 (range 2.8-79.1) months 1 patient remains on Ven, 4 remain in follow-up, 17 are off study, and 6 died. The best overall response rate was 89.3%. Treatment was discontinued for observation in 50% (14/28) of patients (C12=5, C19=1, C25=6, post-C25=2) and in 1 due to AE (neutropenia and diarrhea). The median PFS from time Ven was added was 40.7 (95% CI: 33.4-51.8) months and the 1- and 2- year cumulative incidence of PD were 3.6% (95% CI: 0.2-15.7%) and 14.3% (95% CI: 4.4-29.8%), respectively. The median OS was 79.2 (95% CI: 53.9-not reached) months (Figure). The median PFS from addition of Ven was 51.8 (95% CI 35.9-not reached) and 31.7 (95% CI: 22.1-47.1) months for those who discontinued in remission compared to those who did not, respectively. For the 14 patients who discontinued treatment in remission, 7 started a new treatment at a median of 22.0 (range 17.1-46.6) months after discontinuation. 16 total patients started a new treatment, 12 for CLL (BTKi=7, Ven=2, CAR-T=2, and intrathecal methotrexate=1) and 4 for Richter's. A BTK C481X mutation was present and tracked serially in 96.4% (27/28) of patients. This became undetectable in 74.1% (20/27) of patients at a median of 7.1 (range 2.0-25.3) months. Median time to return of detectable mutation was 27.3 (range 5.3-56.2) months. Adverse events were similar to our initial report.

**Summary/Conclusion**

Adding Ven to Ibr after development of BTKi-resistance is an effective strategy resulting in mutation regression, prolonging PFS, and enabling treatment discontinuation. This approach should be explored using drugs that better target mutant BTK.

**Copyright Information:** (Online) ISSN: 2572-9241
©2026 The Author(s). HemaSphere is published by John Wiley & Sons Ltd on behalf of European Hematology Association. This is an open access Abstract Book distributed under the Attribution-NonCommercial-NoDerivs (CC BY-NC-ND), which allows third parties to download the articles and share them with others as long as they credit the author and the Abstract Book, but they cannot change the content in any way or use them commercially.
**Abstract Book Citations:** Authors, Title, HemaSphere, 2026;10:(S1):pages. The abstract book DOIs can be found on the "Author Information" page.
**Disclaimer:** Articles published in the journal HemaSphere exclusively reflect the opinions of the authors. The authors are responsible for all content in their abstracts including accuracy of the facts, statements, citing resources, etc.

Figure. PFS and OS After Adding Venetoclax to Ibrutinib

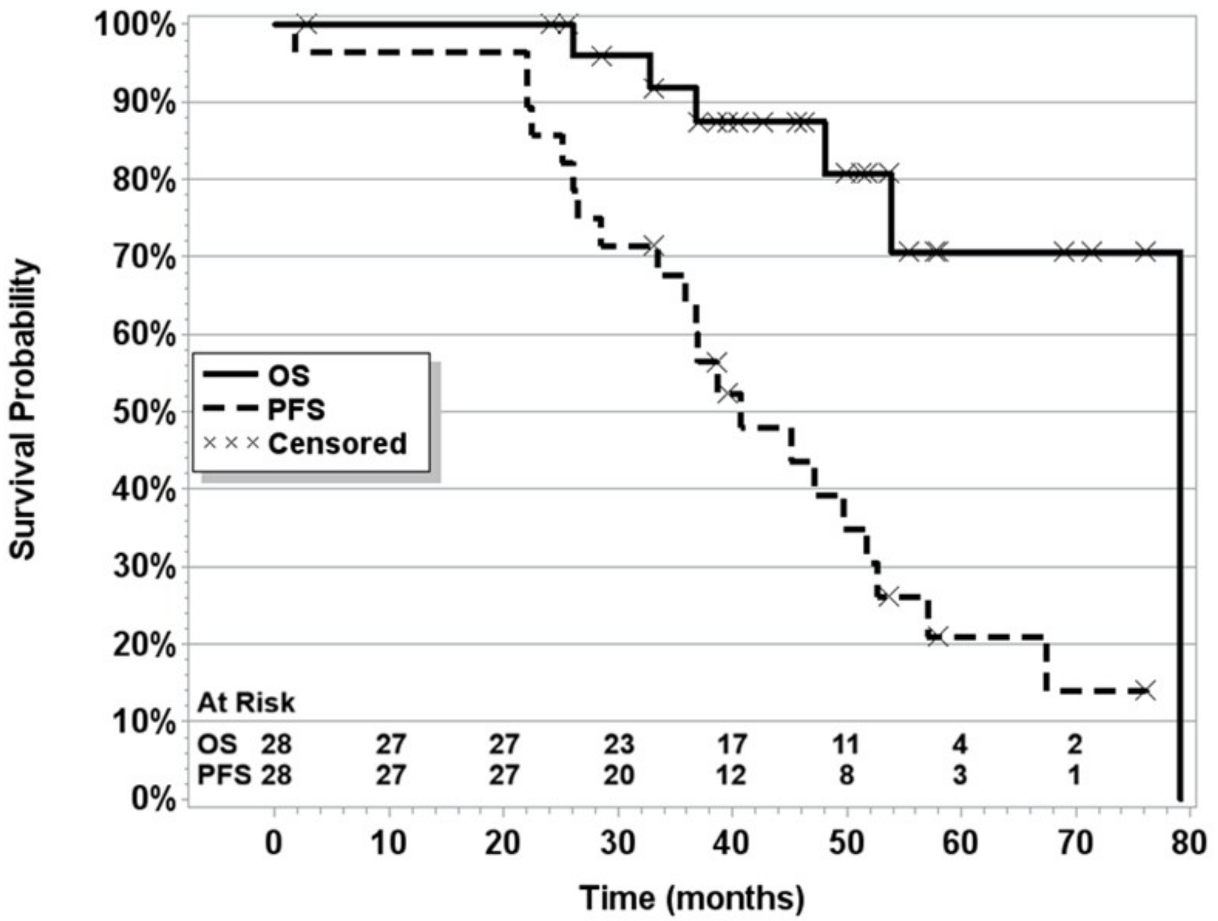

Copyright Information: (Online) ISSN: 2572-9241
©2026 The Author(s). HemaSphere is published by John Wiley & Sons Ltd on behalf of European Hematology Association. This is an open access Abstract Book distributed under the Attribution-NonCommercial-NoDerivs (CC BY-NC-ND), which allows third parties to download the articles and share them with others as long as they credit the author and the Abstract Book, but they cannot change the content in any way or use them commercially.
Abstract Book Citations: Authors, Title, HemaSphere, 2026;10:(S1):pages. The abstract book DOIs can be found on the "Author Information" page.
Disclaimer: Articles published in the journal HemaSphere exclusively reflect the opinions of the authors. The authors are responsible for all content in their abstracts including accuracy of the facts, statements, citing resources, etc.

### (PF605) TREATMENT BURDEN AMONG PATIENTS (PTS) AGED 75 OR OLDER WITH CHRONIC LYMPHOCYTIC LEUKEMIA AND SMALL LYMPHOCYTIC LYMPHOMA (CLL/SLL)

**Topic:** 06. Chronic lymphocytic leukemia and related disorders - Clinical

Daniel Ermann*[1], Aseel Alsouqi[2], Xiaoliang Wang[3], Dong Yuan[3], G. Rhys Williams[3], Erlene Seymour[3], Derrick Van Beuge[3], Joanna Rhodes[4]

*[1]Huntsman Cancer Institute, University of Utah School of Medicine, Salt Lake City, UT, United States of America;[2]The Ohio State University Comprehensive Cancer Center, Columbus, OH, United States of America; [3]BeOne Medicines, Ltd, San Carlos, CA, United States of America; [4]Rutgers Cancer Institute, New Brunswick, NJ, United States of America;*

### Background

CLL is the most common leukemia among adults in the US, with median age at diagnosis of 70 y and median age at death of 82 y. Novel therapies have improved survival outcomes among pts with CLL; however, evidence in older pts is limited due to low clinical trial enrollment.

### Aims

This study describes treatment burden, defined as likelihood of receiving next line of therapy, among pts aged ≥75 y overall and by subgroups.

### Methods

This retrospective, observational cohort study utilized the US nationwide Flatiron Health electronic health record–derived, deidentified database. Pts were included if they were aged ≥75 y at diagnosis and had received first-line (1L) therapy for CLL between Jan 2011-Oct 2025. Primary outcome was the cumulative probability of starting next treatment, including death as a competing risk. Pts were followed from 1L until the earliest of next treatment/second-line (2L) start, end of follow-up, death, or study end. Cumulative incidence function was used to summarize overall pts, age groups (75-79, ≥80 y), del(17p)/*TP53* status (positive, negative, not tested) and immunoglobulin heavy chain variable region (IGHV) status (unmutated, mutated, not tested). Secondary outcomes included a descriptive summary of the most common 1L treatment by time period and age group, and frequencies of Richter transformation during follow-up.

**Copyright Information:** (Online) ISSN: 2572-9241
©2026 The Author(s). HemaSphere is published by John Wiley & Sons Ltd on behalf of European Hematology Association. This is an open access Abstract Book distributed under the Attribution-NonCommercial-NoDerivs (CC BY-NC-ND), which allows third parties to download the articles and share them with others as long as they credit the author and the Abstract Book, but they cannot change the content in any way or use them commercially.
**Abstract Book Citations:** Authors, Title, HemaSphere, 2026;10:(S1):pages. The abstract book DOIs can be found on the "Author Information" page.
**Disclaimer:** Articles published in the journal HemaSphere exclusively reflect the opinions of the authors. The authors are responsible for all content in their abstracts including accuracy of the facts, statements, citing resources, etc.

**Results**

In total, 3234 pts were included, with a median age of 79 y at diagnosis; 60% were male, and 73% were non-Hispanic White. Overall, 27% and 33% had baseline Eastern Cooperative Oncology Group performance status (ECOG PS) of 0 and 1, respectively; 13% had del(17p)/*TP53* mutation, and 14% had unmutated IGHV. Median time from diagnosis to 1L treatment was 10.4 mo (IQR, 1.7-29.5). Among all pts, 1901 (59%) were aged 75-79 y at diagnosis, 1215 (38%) were 80-84 y, and 118 (4%) were ≥85 y. Overall median follow-up was 24.7 mo (IQR, 8.9-48.1) with decreasing follow-up observed across increasing age strata (median 35.8 mo in 75-79 y, 16.2 mo in 80-84 y, and 1.7 mo in ≥85 y). Using competing-risk analysis, the probability of receiving 2L increased through the follow-up period: 12.4% (95% CI, 11.3-13.6) at 6 mo, 28.5% (26.8-30.1) at 24 mo, 37.9% (36.1-39.8) at 48 mo, and 46.5% (44.2-48.6) at 96 mo, and did not plateau. The likelihood of receiving 2L was higher in pts aged 75-79 y with 53.3% (95% CI, 50.3-56.1) of pts receiving 2L by the end of follow-up compared with 34.4% (95% CI, 31.4-37.4) in pts aged ≥80 y (Figure). The likelihood of receiving 2L was also consistently higher among those not tested for del(17p)/*TP53* during follow-up (positive subgroup, 42.0%; negative subgroup, 46.8%; not tested at 96 mo, 48.4%). The results were similar by IGHV mutation status until 60 months or later. For secondary outcomes, Bruton tyrosine kinase inhibitor monotherapy was the most common 1L treatment since 2014 overall and across age groups. In addition, 55 pts (1.7%) had Richter transformation and 52.3% of patients died during follow-up (median age at death, 85 y).

**Summary/Conclusion**

The treatment burden for pts aged ≥75 y at CLL diagnosis continues to increase throughout a pt's journey, with more than half receiving next treatment among pts aged 75-79 (majority of pts in this elderly cohort) and over one-third among pts aged ≥80 y. The data also suggest it would continue to increase with longer follow-up. These results emphasize the need to consider longitudinal treatment sequencing when choosing front-line treatment for most older pts.

**Copyright Information:** (Online) ISSN: 2572-9241
©2026 The Author(s). HemaSphere is published by John Wiley & Sons Ltd on behalf of European Hematology Association. This is an open access Abstract Book distributed under the Attribution-NonCommercial-NoDerivs (CC BY-NC-ND), which allows third parties to download the articles and share them with others as long as they credit the author and the Abstract Book, but they cannot change the content in any way or use them commercially.
**Abstract Book Citations:** Authors, Title, HemaSphere, 2026;10:(S1):pages. The abstract book DOIs can be found on the "Author Information" page.
**Disclaimer:** Articles published in the journal HemaSphere exclusively reflect the opinions of the authors. The authors are responsible for all content in their abstracts including accuracy of the facts, statements, citing resources, etc.

**Figure. Cumulative Incidence of Next Treatment by Age Groups**

| Incidence (95% CI), % | Follow-up time, months: 12 | 24 | 36 | 48 | 60 | 72 | 84 | 96 |
|---|---|---|---|---|---|---|---|---|
| 75-79 y at diagnosis | 19.8 (18.0-21.7) | 29.9 (27.8-32.1) | 36.3 (34.0-38.6) | 41.1 (38.7-43.5) | 45.0 (42.5-47.5) | 49.1 (46.4-51.7) | 52.0 (49.1-54.7) | 53.3 (50.3-56.1) |
| ≥80 y at diagnosis | 17.2 (15.2-19.4) | 26.4 (23.8-28.9) | 30.7 (28.0-33.5) | 33.0 (30.2-35.9) | 34.1 (31.1-37.1) | 34.4 (31.4-37.4) | 34.4 (31.4-37.4) | 34.4 (31.4-37.4) |
| Overall | 18.8 (17.4-20.2) | 28.5 (26.8-30.1) | 34.0 (32.3-35.8) | 37.9 (36.1-39.8) | 40.8 (38.9-42.8) | 43.7 (41.6-45.7) | 45.6 (43.4-47.7) | 46.5 (44.2-48.6) |

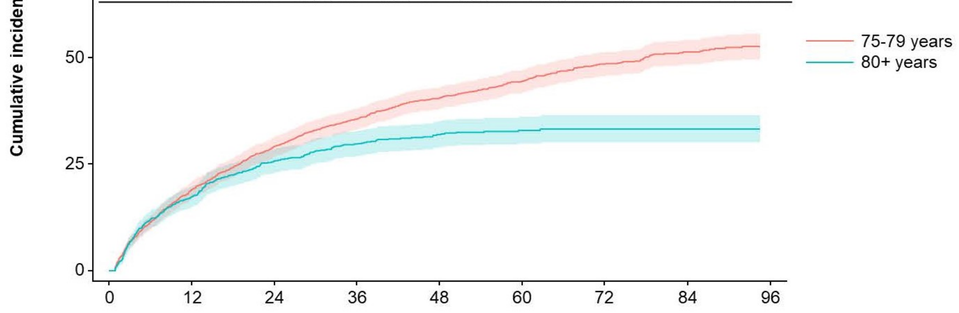

| At risk | 0 | 12 | 24 | 36 | 48 | 60 | 72 | 84 | 96 |
|---|---|---|---|---|---|---|---|---|---|
| 75-79 years | 1899 | 1239 | 891 | 633 | 453 | 291 | 171 | 91 | 40 |
| 80+ years | 1331 | 678 | 365 | 180 | 85 | 35 | 7 | 3 | 3 |

**Copyright Information:** (Online) ISSN: 2572-9241
©2026 The Author(s). HemaSphere is published by John Wiley & Sons Ltd on behalf of European Hematology Association. This is an open access Abstract Book distributed under the Attribution-NonCommercial-NoDerivs (CC BY-NC-ND), which allows third parties to download the articles and share them with others as long as they credit the author and the Abstract Book, but they cannot change the content in any way or use them commercially.
**Abstract Book Citations:** Authors, Title, HemaSphere, 2026;10:(S1):pages. The abstract book DOIs can be found on the "Author Information" page.
**Disclaimer:** Articles published in the journal HemaSphere exclusively reflect the opinions of the authors. The authors are responsible for all content in their abstracts including accuracy of the facts, statements, citing resources, etc.

### (PF606) SAFETY AND EFFICACY OF DOSE-MODIFIED APPROACHES OF FIXED-DURATION (FD) IBRUTINIB+VENETOCLAX (I+V) IN PATIENTS WITH PREVIOUSLY UNTREATED CLL: PRIMARY ANALYSIS OF THE PROSPECTIVE PHASE 2 TAILOR STUDY

**Topic:** 06. Chronic lymphocytic leukemia and related disorders - Clinical

Paolo Ghia*[1, 2], Jan Novák[3], Jana Mihályová[4], Michael Doubek[5], Mitul Gandhi[6], Agnieszka Giza[7], Zsolt Lazar[8], Laszlo Rejto[9], Luca Laurenti[10], Alessandro Sanna[11], Celeste Bremer[12], Paolo Sportoletti[13], Mary Salib[14], Jacqueline Barrientos[15], Katherine Sue Robinson[16], Francisco Bosch Albareda[17], Lucrecia Yáñez[18], Jan Burger[19], Mark Hoffman[20], Brian Koffman[21], Gabriel Krigsfeld[22], Christopher Abbazio[22, 23], Erin Franceschini[22], Magdalena Uhart[22], Brooke Patterson[24], Irina Usankova[25], John Loffredo[26], Zacharias Anastasiou[27], Huiling Pei[28], Christoph Tapprich[29], Qing Li[30], Barbara Pinto[31], Sowmya Srikanthan[32], Wasiulla Khan[33], Boo Messahel[30], Claire Kavanagh[34], Lori Parisi[30], Mark Wildgust[30], Jeff P. Sharman[35]

*[1]Università Vita-Salute San Raffaele, Milan, Italy; [2]Comprehensive Cancer Center, IRCCS Ospedale San Raffaele, Milan, Italy; [3]University Hospital Kralovske Vinohrady and Third Faculty of Medicine, Prague, Czechia;[4]University Hospital Ostrava, Department of Hematooncology, Ostrava, Czechia; [5]University Hospital Brno, Brno, Czechia; [6]Virginia Cancer Specialists, Fairfax, United States of America; [7]Jagiellonian University Medical College, Kraków, Poland; [8]Petz Aladár County Teaching Hospital, Győr, Hungary; [9]Szabolcs-Szatmár-Bereg County Teaching Hospital, Nyíregyháza, Hungary; [10]Fondazione Policlinico Universitario A. Gemelli IRCCS, Rome, Italy; [11]Azienda Ospedaliero Universitaria Careggi, Florence, Italy; [12]Virginia Oncology Associates, Virginia Beach, United States of America; [13]Institute of Hematology and Center for Hemato-Oncology Research (CREO), University of Perugia, Department of Medicine and Surgery, Perugia, Italy; [14]Walker Family Cancer Centre, St. Catharines, Canada; [15]Mount Sinai Comprehensive Cancer Center, Miami Beach, United States of America; [16]The Queen Elizabeth II Health Sciences Centre, Halifax, Canada; [17]Vall D'Hebron University Hospital, Department of Hematology and Hemotherapy, Barcelona, Spain; [18]University Hospital Marqués de Valdecilla-IDIVAL, University of Cantabria, Santander, Spain; [19]University of Texas MD Anderson Cancer Center, Houston, United States of America;[20]University of California, San Diego, United States of America; [21]CLL Society Inc., Nashville, United States of America;[22]AbbVie, North Chicago, United States of America; [23]Pharmacyclics LLC, an AbbVie Company, North Chicago, United States of America; [24]Johnson & Johnson, Iowa City, United States of America; [25]Johnson & Johnson, Allschwil, Switzerland; [26]Johnson & Johnson, Spring House, United States of America; [27]Johnson & Johnson, Athens, Greece; [28]Johnson & Johnson, Titusville, United States of America; [29]Johnson & Johnson, Neuss, Germany; [30]Johnson & Johnson, Raritan, United States of America; [31]Johnson & Johnson, Porto Salvo, Portugal; [32]Johnson & Johnson, Somerset, United States of America; [33]Johnson & Johnson, Horsham, United States of America; [34]Johnson & Johnson, Dublin, Ireland; [35]SCRI, Willamette Valley Cancer Institute, Eugene, United States of America;*

### Background

FD I+V has transformed first-line (1L) treatment (tx) of patients (pts) with chronic lymphocytic leukemia (CLL), with benefit demonstrated in >1200 pts in phase 2/3 studies with up to 5.7y median follow-up (FU) and in >500 pts in RW studies. Furthermore, in a recent analysis, physicians indicated all-oral administration, quality of life advantages, and patient preference as key factors for FD I+V initiation (Giza 2025). Updated ibrutinib (Ibr) prescribing information (PI) from May 2024 recommended dose adjustments (DA) for adverse events (AEs) to improve tolerability. TAILOR is the first study to prospectively evaluate outcomes using 2 clinical strategies to improve tolerability: reactive DA per updated PI and proactive DA.

### Aims

We present results from the FD I+V cohorts of TAILOR, assessing efficacy and safety of reactive DA per PI and proactive DA approaches in 1L CLL.

**Copyright Information:** (Online) ISSN: 2572-9241
©2026 The Author(s). HemaSphere is published by John Wiley & Sons Ltd on behalf of European Hematology Association. This is an open access Abstract Book distributed under the Attribution-NonCommercial-NoDerivs (CC BY-NC-ND), which allows third parties to download the articles and share them with others as long as they credit the author and the Abstract Book, but they cannot change the content in any way or use them commercially.
**Abstract Book Citations:** Authors, Title, HemaSphere, 2026;10:(S1):pages. The abstract book DOIs can be found on the "Author Information" page.
**Disclaimer:** Articles published in the journal HemaSphere exclusively reflect the opinions of the authors. The authors are responsible for all content in their abstracts including accuracy of the facts, statements, citing resources, etc.

### Methods

Pts were randomized 1:1 into reactive (1A) and proactive (1B) DA cohorts and dosed per approved label for 12 cycles (C) of FD I+V following a 3C lead-in (LI) with Ibr 420mg QD. Venetoclax step-up and dosing was per label. Pts in 1B had Ibr dose proactively reduced to 280mg QD after Ibr LI. The primary endpoint was best overall response rate (ORR) tested individually for each cohort vs a benchmark of >75% representing a clinically meaningful difference from 90% based on GLOW and CAPTIVATE studies. Secondary endpoints included progression-free survival (PFS), overall survival (OS), and safety. TAILOR was not powered for comparisons between cohorts. Safety results are summarized using descriptive measures.

### Results

171 pts were randomized to 1A (n=86) and 1B (n=85). Median age was 63y. In 1A/B, 36.0%/41.2% had unmutated IGHV status (3.5% unknown in both cohorts), 16.3%/9.4% had *TP53*/del(17p) aberration, 4.7%/8.2% had CIRS >6, 43.0%/51.8% were high risk, respectively. Median FU was 14.6mo.
1A: ORR was statistically significant at 94.2% (95% CI, 87-98%). 12-mo PFS/OS rates were both 98.8%; 1 pt had progressive disease (PD). Treatment emergent AEs (TEAE) (any grade [Gr] [after C3]/Gr ≥3 [after C3]) were 95.3% (91.8%)/48.2% (41.2%). Any Gr/Gr ≥3 atrial fibrillation (AF) was 4.7%/2.4%, hypertension 10.6%/2.4%, arthralgia 21.2%/2.4%, infections 51.8%/2.4%, neutropenia 38.8%/30.6%. AEs led to Ibr discontinuation (DC) in 2 (2.4%) pts during LI and 2 (2.4%) pts after C3, and Ibr DA in 4 (4.7%) pts during LI and 13 (15.3%) after C3. 1 death due to intracranial hemorrhage.
1B: ORR was statistically significant at 89.4% (95% CI, 81-95%). 12-mo PFS/OS rates were 97.6%/98.8%; 1 pt had PD. TEAE (any Gr [after C3]/Gr ≥3 [after C3]) were 94.1% (83.5%)/50.6% (42.4%). Any Gr/Gr ≥3 AF was 8.2%/7.1%, hypertension 16.5%/4.7%, arthralgia 17.6%/1.2%, infections 31.8%/8.2%, neutropenia 23.5%/15.3%. AEs led to Ibr DC in 5 (5.9%) pts during LI and 5 (5.9%) after C3, and Ibr DA in 4 (4.7%) pts during LI and 6 (7.1%) after C3. 1 death due to pneumonia.
There were no cardiac deaths. MRD data will be presented.

### Summary/Conclusion

In this prospective study, reactive and proactive DA I+V cohorts met the primary endpoint with a statistically significant improvement in ORR vs historical controls. When treated per DA guidance in the updated PI, pts had low rates of AF and low-Gr AEs. Pts with proactive DA had a similar safety profile and showed a trend toward lower rates of arthralgia, infections, and neutropenia. Results from TAILOR further strengthen the clinical profile and dosing flexibility of I+V in 1L CLL.

**Copyright Information:** (Online) ISSN: 2572-9241
©2026 The Author(s). HemaSphere is published by John Wiley & Sons Ltd on behalf of European Hematology Association. This is an open access Abstract Book distributed under the Attribution-NonCommercial-NoDerivs (CC BY-NC-ND), which allows third parties to download the articles and share them with others as long as they credit the author and the Abstract Book, but they cannot change the content in any way or use them commercially.
**Abstract Book Citations:** Authors, Title, HemaSphere, 2026;10:(S1):pages. The abstract book DOIs can be found on the "Author Information" page.
**Disclaimer:** Articles published in the journal HemaSphere exclusively reflect the opinions of the authors. The authors are responsible for all content in their abstracts including accuracy of the facts, statements, citing resources, etc.

### (PF607) FEASIBILITY AND SAFETY OF VENETOCLAX–OBINUTUZUMAB IN FIT AND UNFIT CHRONIC LYMPHOCYTIC LEUKEMIA PATIENTS: COMPARISON OF CLL13 AND CLL14 POOLED ANALYSIS WITH AN ITALIAN CLINICAL PRACTICE COHORT

**Topic:** 06. Chronic lymphocytic leukemia and related disorders - Clinical

Arianna Zappaterra*[1], Sandra Robrecht[2], Adam Giza[2], Andrea Galitzia[3], Othman Al-Sawaf[2], Marina Deodato[1], Alessandro Noto[1], Lorena Appio[4], Luca Laurenti[5], Mariano Lucignano[6], Jacopo Olivieri[7], Caterina Patti[8], Alessandro Sanna[9], Luana Schiattone[10], Eugen Tausch[11], Mark-David Levin[12], Caspar Da Cunha-Bang[13], Stephan Stilgenbauer[11], Arnon Philip Kater[14], Carsten Niemann[13], Michael Hallek[2], Alessandra Tedeschi[1], Kirsten Fischer[2], Barbara Eichhorst[2], Anna Maria Frustaci[1], Moritz Fürstenau[2]

*[1]ASST Grande Ospedale Metropolitano Niguarda, Hematology Department, Niguarda Cancer Center, Milano, Italy; [2]University of Cologne, Department I of Internal Medicine, Faculty of Medicine and University Hospital of Cologne, Center for Integrated Oncology Aachen Bonn Cologne Duesseldorf, German Chronic Lymphocytic Leukemia Study Group, Cologne, Germany; [3]Ospedale Oncologico A. Businco, ARNAS G. Brotzu, Hematology and Stem Cell Transplantation Unit, Cagliari, Italy; [4]ASST Valle Olona, Ospedale Busto Arsizio, S.C. Ematologia, Busto Arsizio, Italy; [5]Fondazione Policlinico Universitario A Gemelli UCSC, Rome, Italy; [6]Andrea Tortora Hospital ASL Salerno, Pagani, Italy; [7]Azienda Sanitaria Universitaria Integrata di Udine, Clinica Ematologica, Centro Trapianti e Terapie Cellulari "Carlo Melzi", Udine, Italy; [8]Azienda Ospedali Riunita Villa Sofia-Cervello, Division of Hematology, Pelrmo, Italy; [9]AOU Careggi, Hematology Unit, Department of Oncolgy, Florence, Italy; [10]Ospedale Civile S. Spirito Pescara, Ematologia Clinica, Pescara, Italy; [11]University Hospital Ulm, Division of CLL, Department III of Internal Medicine, Ulm, Germany; [12]Albert Schweitzer Ziekenhuis, Dordrecht, The Netherlands; [13]Rigshospitalet, Copenhagen University Hospital, Copenhagen, Denmark; [14]University of Amsterdam, Department of Hematology, Cancer Center Amsterdam, Hemato-Oncology Foundation for Adults Netherlands (HOVON), Amsterdam University Medical Centers, Amsterdam, The Netherlands;*

### Background

Based on the CLL13 and CLL14 clinical trials (ClTr), fixed-duration venetoclax–obinutuzumab (VO) became a standard first-line treatment for chronic lymphocytic leukemia (CLL) patients (pts), both fit and with comorbidities. However, longitudinal data in clinical practice (CP) on its feasibility and outcomes remains largely unexplored.

### Aims

To compare the feasibility and safety of fixed-duration VO in CP versus ClTr.

### Methods

We performed descriptive analyses separately for the pooled CLL13/CLL14 ClTr pts and a multicenter, retrospective Italian CP cohort (55 university/community hospitals, from May 2022 to June 2023; *Frustaci et al, Haematologica, 2025).* Pts were grouped by fitness status (unfit:CIRS>6 and/or CrCL<70 ml/min; fit:CIRS≤6 and CrCl≥70 ml/min). Toxicity and schedule modifications were analyzed across early combination (EaC;cycles, C1–2), combination (COMB;C3–6) and maintenance phases (MAINT;C7 to end of treatment +28 days). Adverse events (AE) were prospectively graded per Common Terminology Criteria for AE in ClTr and retrospectively collected in CP cohort. Pts visits in CP followed site-specific standards of care.

**Copyright Information:** (Online) ISSN: 2572-9241
©2026 The Author(s). HemaSphere is published by John Wiley & Sons Ltd on behalf of European Hematology Association. This is an open access Abstract Book distributed under the Attribution-NonCommercial-NoDerivs (CC BY-NC-ND), which allows third parties to download the articles and share them with others as long as they credit the author and the Abstract Book, but they cannot change the content in any way or use them commercially.
**Abstract Book Citations:** Authors, Title, HemaSphere, 2026;10:(S1):pages. The abstract book DOIs can be found on the "Author Information" page.
**Disclaimer:** Articles published in the journal HemaSphere exclusively reflect the opinions of the authors. The authors are responsible for all content in their abstracts including accuracy of the facts, statements, citing resources, etc.

### Results

In total, 711 pts received ≥1 treatment dose and were therefore included in the analysis: 440 in ClTr (257 unfit, 182 fit, 1 with missing fitness status) and 271 in CP (133 unfit, 138 fit). Median observation was 72.3 months (interquartile range [IQR] 60.9–78.7) for ClTr and 29.3 months (IQR 28.3–30.3) for CP pts. Median age was 67 years in ClTr (unfit 71, range 43-89; fit 58, range 31-79) and 65.8 in CP (unfit 72.7, range 41.6-89.3; fit 59.9, range 34.1-80.4). More ClTr pts had ECOG≥1 (42.5% in ClTr vs 29.5% in CP) and CIRS>6 (41.6% vs 20.7%). Unmutated IGHV status was more common in ClTr pts (59% vs 51.2%).
Overall, 86.6% of ClTr pts completed treatment (81.7% unfit, 93.4% fit) and 89.3% in CP (88% unfit, 90.6% fit). Toxicity-drive discontinuations occurred in 8% of ClTr pts (10.5% unfit, 4.4% fit) and 10% of CP pts (11.3% unfit, 8.7% fit). V dose intensity<70% was observed in ClTr cohort in 22.7%, 18.9% and 22.9% during EaC, COMB and MAINT, compared with 7%, 10.4% and 11.9% in CP pts, respectively. O was not administered at full dose in 11.8% and 9.5% of ClTr pts during EaC and COMB phases, and in 13.5% and 8.4% of CP pts.
Grade (G)≥3 AE were recorded in 59.3%, 40.9% and 37.3% during EaC, COMB and MAINT in ClTr, compared to 38.4%, 21.2% and 14.7% in CP pts, respectively. G≥3 infections occurred in 4.5%, 3.8% and 7% during EaC, COMB and MAINT in ClTr, and in 6.3%, 4.6% and 5.2% in CP, respectively. Figure1 reports rates stratified by fitness status. Tumor lysis syndrome was reported in 6.6% ClTr and 5.9% CP pts, the majority O-related, while infusion related reactions occurred in 48.6% and 36.9% pts, respectively. Table shows time-to-event exploratory endpoints at 24 months.

| | ClTr unfit% | ClTr fit% | CP unfit% | CP fit% |
|---|---|---|---|---|
| **Progression-free survival** | 88.7 | 92.8 | 87.7 | 96.3 |
| **Overall survival** | 92.5 | 99.4 | 89.2 | 98.5 |
| **Time to next treatment** | 95.9 | 97.8 | 97.7 | 97.8 |
| **Treatment-free survival** | 89.7 | 97.2 | 87.7 | 96.3 |

### Summary/Conclusion

In this large comparative analysis, VO appeared consistent with trials and feasible in clinical practice. Although G≥3 AE and V dose intensity reductions were numerically higher in ClTr cohort, rates of severe infections and discontinuations due to toxicity were consistent between cohorts. However, in spite of the more systematic reporting in clinical trials and the short FU time of the CP data, the analysis supports VO use as frontline treatment in routine clinical practice.

**Copyright Information:** (Online) ISSN: 2572-9241
©2026 The Author(s). HemaSphere is published by John Wiley & Sons Ltd on behalf of European Hematology Association. This is an open access Abstract Book distributed under the Attribution-NonCommercial-NoDerivs (CC BY-NC-ND), which allows third parties to download the articles and share them with others as long as they credit the author and the Abstract Book, but they cannot change the content in any way or use them commercially.
**Abstract Book Citations:** Authors, Title, HemaSphere, 2026;10:(S1):pages. The abstract book DOIs can be found on the "Author Information" page.
**Disclaimer:** Articles published in the journal HemaSphere exclusively reflect the opinions of the authors. The authors are responsible for all content in their abstracts including accuracy of the facts, statements, citing resources, etc.

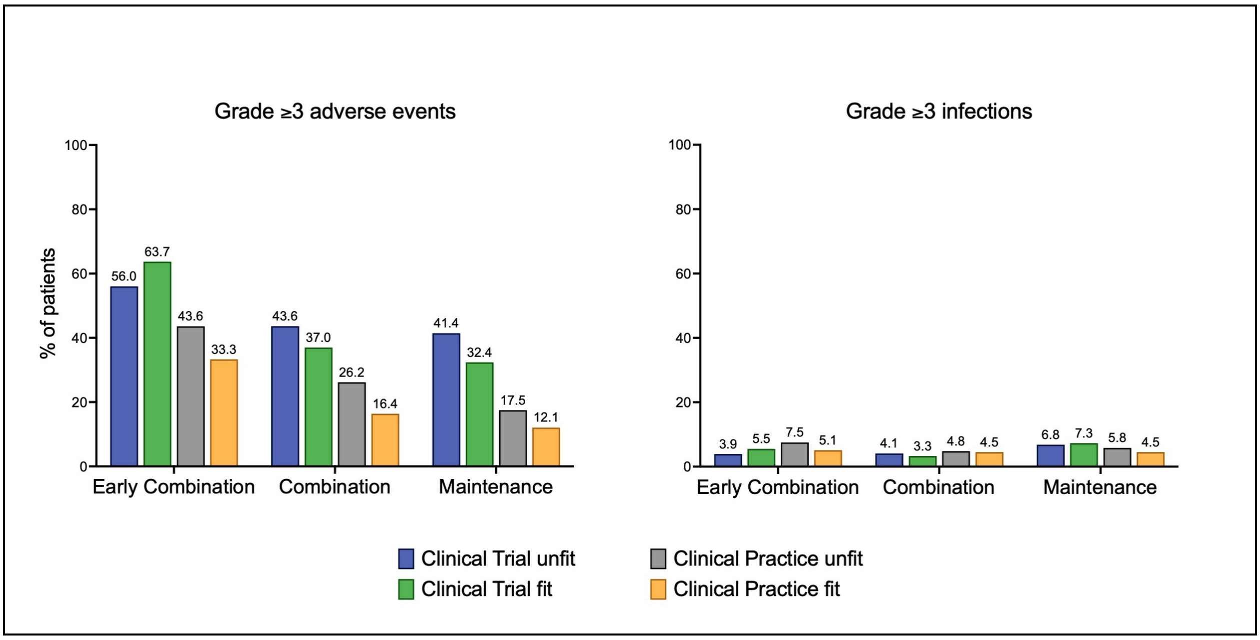

Figure 1 Grade ≥3 adverse events and infections on the whole population according to cohorts and fitness category

**Copyright Information:** (Online) ISSN: 2572-9241
©2026 The Author(s). HemaSphere is published by John Wiley & Sons Ltd on behalf of European Hematology Association. This is an open access Abstract Book distributed under the Attribution-NonCommercial-NoDerivs (CC BY-NC-ND), which allows third parties to download the articles and share them with others as long as they credit the author and the Abstract Book, but they cannot change the content in any way or use them commercially.
**Abstract Book Citations:** Authors, Title, HemaSphere, 2026;10:(S1):pages. The abstract book DOIs can be found on the "Author Information" page.
**Disclaimer:** Articles published in the journal HemaSphere exclusively reflect the opinions of the authors. The authors are responsible for all content in their abstracts including accuracy of the facts, statements, citing resources, etc.

### (PF608) REAL-WORLD TREATMENT AND SURVIVAL OUTCOMES FOR ZANUBRUTINIB AND ACALABRUTINIB MONOTHERAPY AMONG TREATMENT-NAÏVE PATIENTS WITH CHRONIC LYMPHOCYTIC LEUKEMIA IN THE UNITED STATES

**Topic:** 06. Chronic lymphocytic leukemia and related disorders - Clinical

Ryan Jacobs*[1], Manasi Suryavanshi[2], Gregory Maglinte[2], Divya Nagpal[3], Nishit Jain[3], Varun Gupta[3], Catherine Fu[2], G. Rhys Williams[2], Daniel Ermann[4]

*[1]Atrium Health Levine Cancer Institute, Wake Forest University School of Medicine, Charlotte, NC, United States of America; [2]BeOne Medicines Ltd, San Carlos, CA, United States of America; [3]ZS Associates, Gurugram, Haryana, India; [4]Huntsman Cancer Institute, University of Utah School of Medicine, Salt Lake City, UT, United States of America;*

### Background

Bruton tyrosine kinase (BTK) inhibitors are central to the management of chronic lymphocytic leukemia/small lymphocytic lymphoma (CLL/SLL). Acalabrutinib (acala) was approved for CLL in the United States (US) in November 2019, followed by zanubrutinib (zanu) in January 2023. Despite their widespread use, real-world evidence comparing therapy effectiveness is limited.

### Aims

This US-based study compared the real-world effectiveness of zanu and acala monotherapy as first-line treatment for patients with CLL based on overall survival (OS) and time to next treatment (TTNT).

### Methods

This retrospective analysis utilized the Komodo claims database between January 2015 and August 2025. Eligible patients were adults (aged ≥18 years) in the US with ≥2 diagnoses of CLL or SLL, who were treatment naïve for CLL and had an index claim for monotherapy with zanu (January 2023 to August 2025) or acala (November 2019 to August 2025). The index date was defined as the date of the first observed claim for the respective therapy. Patients were required to have continuous enrollment or activities within 1 year prior to and 3 months after the index date. Patients with evidence of clinical trial participation, prior CLL/SLL treatment, or a diagnosis of mantle cell lymphoma between January 2015 and the index date were excluded. Outcomes included OS (measured from index claim to all-cause mortality) and TTNT (measured from index claim to initiation of subsequent therapy or death). If death did not occur, patients were censored at the date of last activity or the enrollment end date. For OS analysis, Komodo data captured the death date, but not the cause of mortality. Survival analyses were conducted using Kaplan-Meier estimates and Cox proportional hazards models. Inverse probability of treatment weighting (IPTW) was adjusted for age, sex, US region, treatment initiation year, and Charlson Comorbidity Index (CCI).

### Results

Among 16,788 patients (zanu, n=5819; acala, n=10,969), median age was higher in zanu (73.3 years) than acala (71.8 years) cohorts. Most patients were male (acala, 62%; zanu, 59%), non-Hispanic white (acala, 73%; zanu, 72%), and the median CCI was 2 in both cohorts. Median follow-up was 12.8 and 16.4 months in zanu and acala cohorts, respectively. Median TTNT and OS were not reached for either cohort. With the unadjusted model, zanu had a longer TTNT (unadjusted hazard ratio [HR]=0.88; 95% CI, 0.79-0.97; $P$=.009) and OS (HR=0.72; 95% CI, 0.62-0.82; $P$<.001; **Table**). After IPTW adjustment, zanu was associated with a significantly improved OS (IPTW-adjusted HR=0.75; 95% CI, 0.65-0.86; $P$<.001) and TTNT (IPTW-adjusted HR=0.89; 95% CI, 0.80-0.98; $P$=.02) compared with acala.

**Copyright Information:** (Online) ISSN: 2572-9241
©2026 The Author(s). HemaSphere is published by John Wiley & Sons Ltd on behalf of European Hematology Association. This is an open access Abstract Book distributed under the Attribution-NonCommercial-NoDerivs (CC BY-NC-ND), which allows third parties to download the articles and share them with others as long as they credit the author and the Abstract Book, but they cannot change the content in any way or use them commercially.
**Abstract Book Citations:** Authors, Title, HemaSphere, 2026;10:(S1):pages. The abstract book DOIs can be found on the "Author Information" page.
**Disclaimer:** Articles published in the journal HemaSphere exclusively reflect the opinions of the authors. The authors are responsible for all content in their abstracts including accuracy of the facts, statements, citing resources, etc.

### Summary/Conclusion

In this real-world analysis, zanu monotherapy demonstrated significantly longer TTNT and significantly improved OS compared with acala monotherapy in patients with CLL/SLL. These findings suggest that zanu may offer superior outcomes in the real-world setting.

**Table. TTNT and OS Landmark Rates and HRs**

| | | Zanu | Acala (ref) |
|---|---|---|---|
| **TTNT** | 12 m, % (95% CI) | 91.3 (90.4-92.0) | 90.1 (89.4-90.7) |
| | 18 m, % (95% CI) | 88.3 (87.2-89.3) | 86.8 (86.0-87.6) |
| | 24 m, % (95% CI) | 85.3 (83.8-86.6) | 83.8 (82.9-84.6) |
| | Unadjusted HR (95% CI) | 0.88 (0.79-0.97) | |
| | Adjusted HR (95% CI) | 0.89 (0.80-0.98) | |
| **OS** | 12 m, % (95% CI) | 96.0 (95.4-96.5) | 94.5 (94.0-95.0) |
| | 18 m, % (95% CI) | 94.2 (93.4-94.9) | 92.0 (91.3-92.6) |
| | 24 m, % (95% CI) | 92.5 (91.3-93.5) | 89.4 (88.7-90.1) |
| | Unadjusted HR (95% CI) | 0.72 (0.62-0.82) | |
| | Adjusted HR (95% CI) | 0.75 (0.65-0.86) | |

**Copyright Information:** (Online) ISSN: 2572-9241
©2026 The Author(s). HemaSphere is published by John Wiley & Sons Ltd on behalf of European Hematology Association. This is an open access Abstract Book distributed under the Attribution-NonCommercial-NoDerivs (CC BY-NC-ND), which allows third parties to download the articles and share them with others as long as they credit the author and the Abstract Book, but they cannot change the content in any way or use them commercially.
**Abstract Book Citations:** Authors, Title, HemaSphere, 2026;10:(S1):pages. The abstract book DOIs can be found on the "Author Information" page.
**Disclaimer:** Articles published in the journal HemaSphere exclusively reflect the opinions of the authors. The authors are responsible for all content in their abstracts including accuracy of the facts, statements, citing resources, etc.

### (PF609) REAL-LIFE DATA ON FIRST LINE AND RELAPSED/REFRACTORY CLL ACALABRUTINIB TREATMENT: UPDATED 3Y RESULTS OF THE LARGE NAOS OBSERVATIONAL STUDY IN FRANCE, INCLUDING HIGH-RISK AND IBRUTINIB-EXPOSED PATIENTS

**Topic:** 06. Chronic lymphocytic leukemia and related disorders - Clinical

Anne Quinquenel*[1], Stephane Lepretre[2], Axelle Dautresire[3], Amir Lahouegue[3], Loic Ysebaert[4], Vincent Levy[5]

*[1]Hôpital Robert Debré, REIMS, France; [2]CLCC Henri Becquerel, ROUEN, France; [3]AstraZeneca, Courbevoie, France; [4]IUCT Oncopole, Toulouse, France; [5]Avicenne Hospital, Bobigny, France;*

### Background

Acalabrutinib, a second generation Bruton tyrosine kinase (BTK) inhibitor, is approved for chronic lymphocytic leukaemia (CLL) as continuous treatment in first (L1) and ≥second (≥L2) lines and as fixed duration combination with venetoclax +/- obinutuzumab in L1, based on its respective demonstrated efficacy and safety in clinical trials (1-4). NAOS, a large real-world (rw) evidence study conducted in France, complements existing continuous regimens data.

### Aims

To assess rw effectiveness and safety of acalabrutinib in CLL patients treated in L1 and ≥L2, overall and in predefined subgroups.

### Methods

NAOS is a retrospective non-interventional, 3-year longitudinal study in 59 sites in France, enrolling adults who initiated acalabrutinib for CLL in 2021 or 2022 in routine practice. Data were collected from treatment start to end of 2024, (median follow-up of 34.2 months). Rw effectiveness was evaluated using Kaplan-Meier estimates to assess time to discontinuation (TTD), progression-free survival (PFS) and overall survival (OS). Log-rank tests were used for comparisons. Outcomes were assessed by treatment line (L1/≥L2), cardiovascular (CV) history (Yes/No), cytogenetics (With/Without del(17p) and/or TP53 mutation [del17p/TP53m]), and prior ibrutinib exposure.

**Copyright Information:** (Online) ISSN: 2572-9241
©2026 The Author(s). HemaSphere is published by John Wiley & Sons Ltd on behalf of European Hematology Association. This is an open access Abstract Book distributed under the Attribution-NonCommercial-NoDerivs (CC BY-NC-ND), which allows third parties to download the articles and share them with others as long as they credit the author and the Abstract Book, but they cannot change the content in any way or use them commercially.
**Abstract Book Citations:** Authors, Title, HemaSphere, 2026;10:(S1):pages. The abstract book DOIs can be found on the “Author Information” page.
**Disclaimer:** Articles published in the journal HemaSphere exclusively reflect the opinions of the authors. The authors are responsible for all content in their abstracts including accuracy of the facts, statements, citing resources, etc.

### Results

Among 485 patients, 285 received acalabrutinib in L1 and 200 in ≥L2; median age was 75y (75.7%≥70y), 60% had CV history. High-risk features included del(17p)/TP53m in 25.1%, complex karyotype ≥3 in 33.8%, and unmutated IGHV in 67.4%. At M0, 74 patients had prior ibrutinib exposure (L1:3; ≥L2:71) of whom 57 (77%) discontinued due to intolerance including 6 for cardiac adverse event (AE) (4 atrial fibrillation).
In L1, discontinuation (DC), rwPFS and rwOS rates remained stable between M24 and M36, with rates at 33.5% (95% CI, 28.1-39.1) and 39% (95% CI, 32.6-45.3) for DC, at 85.1% (80.3-88.8) and 80.1% (74-85) for rwPFS and 89.1% (84.8-92.3) and 86.5% (81.4-90.3) for OS, respectively (Fig1A). L1 rwPFS did not differ by del17p/TP53m status (p=0.4), with M30 rwPFS rates of 84.4% (69.4–92.4) and 84.1% (78.2–88.5), respectively (Fig1B-C). RwPFS was also similar in L1 patients with vs without CV history at M30 (82.0% vs 86.5%, p=0.3), although DC rate was higher in those with CV history (41.5% vs 27.9%, p=0.05). In ≥L2, DC, rwPFS and rwOS rates at M36 were 63.2% (54-71.1), 58.8% (50.0-66.6) and 67.3% (59.2-74.2) respectively. At M36, rwPFS was 51.4% (37.7–63.5) in the 74 patients with prior ibrutinib and 57.1% (41.3–70.2) in those intolerant to ibrutinib (n=57) (Fig1D).
Dose reduction occurred in 10.5% (L1) and 11.5% (≥L2) of patients, AEs leading to treatment changes in 24.2% (L1) and 32.0% (≥L2) including 9.9% grade 3/4 AEs (L1: 10.2% ; L2: ≥9.5%) and 1.4% grade 3/4 cardiac AE. Median time to first AE was 3.5 (IQR 0.8–10.5) and 5.6 months (IQR 0.9–13.3) in L1 and ≥L2. Among the 74 patients with prior ibrutinib, 37.8% had AEs and 24.3% AE leading to acalabrutinib DC (Fig1E); these were mainly gastrointestinal (n=6) and general disorders (n=6), with no reported cardiac AEs.

### Summary/Conclusion

NAOS provides results on acalabrutinib use in a diverse CLL population. It highlights on the sustained outcomes (rwPFS and rwOS) in clinical practice even in high-risk subgroups and in patients with CV comorbidities and the low need for dose changes to manage AEs. Acalabrutinib appears suitable in patients previously intolerant to ibrutinib, with no cardiac AEs observed on acalabrutinib after switching due to ibrutinib-related cardiac AEs.

**Copyright Information:** (Online) ISSN: 2572-9241
©2026 The Author(s). HemaSphere is published by John Wiley & Sons Ltd on behalf of European Hematology Association. This is an open access Abstract Book distributed under the Attribution-NonCommercial-NoDerivs (CC BY-NC-ND), which allows third parties to download the articles and share them with others as long as they credit the author and the Abstract Book, but they cannot change the content in any way or use them commercially.
**Abstract Book Citations:** Authors, Title, HemaSphere, 2026;10:(S1):pages. The abstract book DOIs can be found on the "Author Information" page.
**Disclaimer:** Articles published in the journal HemaSphere exclusively reflect the opinions of the authors. The authors are responsible for all content in their abstracts including accuracy of the facts, statements, citing resources, etc.

A/ B/ C/

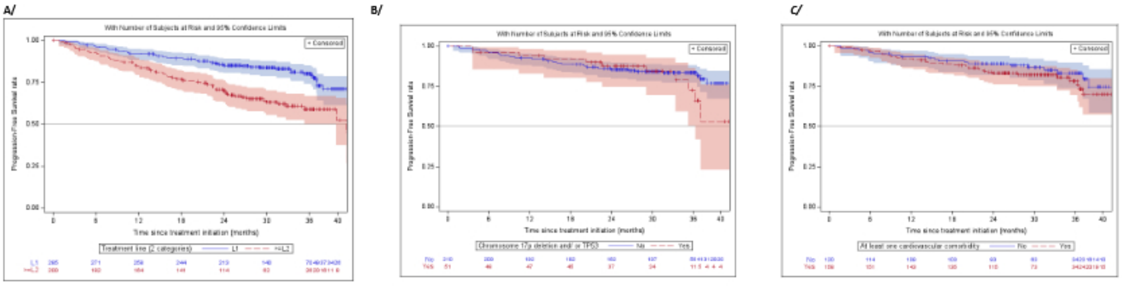

D/

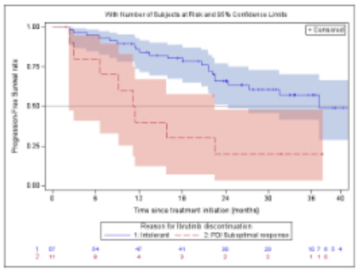

E/

| In patients priorly exposed to ibrutinib (n=74) | | Patients with AE | | | |
|---|---|---|---|---|---|
| (MedDRA v28.0) | | All | | leading to discontinuation | |
| SOC | PT | n | % | n | % |
| Total Patients | | 28 | 37.8 | 18 | 24.3 |
| Gastrointestinal | Diarrhoea | 6 | 8.1 | 4 | 5.4 |
| | Dyspepsia | 1 | 1.4 | 1 | 1.4 |
| General disorders and administration site conditions | Asthenia | 2 | 2.7 | 2 | 2.7 |
| | General physical health deterioration | 2 | 2.7 | 1 | 1.4 |
| | Disease progression | 1 | 1.4 | 1 | 1.4 |
| | Fatigue | 1 | 1.4 | . | . |
| Blood and lymphatic system | Neutropenia | 2 | 2.7 | . | . |
| | Anaemia | 1 | 1.4 | 1 | 1.4 |
| | Haematotoxicity | 1 | 1.4 | . | . |
| Infections and infestations | Corona virus infection | 2 | 2.7 | . | . |
| | Actinomycosis | 1 | 1.4 | 1 | 1.4 |
| | Pneumonia | 1 | 1.4 | 1 | 1.4 |
| Respiratory, thoracic and mediastinal | Bronchitis | 1 | 1.4 | 1 | 1.4 |
| | Cough | 1 | 1.4 | 1 | 1.4 |
| | Respiratory failure | 1 | 1.4 | 1 | 1.4 |
| Nervous system | Headache | 2 | 2.7 | 2 | 2.7 |
| Vascular | Haematoma | 1 | 1.4 | 1 | 1.4 |
| | Haemorrhage | 1 | 1.4 | . | . |
| Injury, poisoning and procedural complications | Fall | 1 | 1.4 | 1 | 1.4 |
| Musculoskeletal and connective tissue | Back pain | 1 | 1.4 | 1 | 1.4 |
| Skin and subcutaneous tissue disorders | Eczema | 1 | 1.4 | . | . |

***References:*** *1/ Ghia P et al. ASCEND: Phase III, Randomized Trial of Acalabrutinib Versus Idelalisib Plus Rituximab or Bendamustine Plus Rituximab in Relapsed or Refractory Chronic Lymphocytic Leukemia. J Clin Oncol. 2020;38(25):2849-61. 2/ Sharman et al. Acalabrutinib with or without obinutuzumab versus chlorambucil and obinutuzumab for treatment-naive chronic lymphocytic leukaemia (ELEVATE-TN): a randomised, controlled, phase 3 trial. Lancet Lond Engl. 1 2020;395(10232):1278-91. 3/ Byrd JC, Hillmen P, Ghia P, et al. Acalabrutinib versus ibrutinib in previously treated chronic lymphocytic leukemia: results of the first randomized phase 3 trial. J Clin Oncol. 2021;39(31):3441–3452 4/ Brown JR et al... Fixed-Duration Acalabrutinib Combinations in Untreated Chronic Lymphocytic Leukemia. N Engl J Med. 2025 Feb 20;392(8):748-762.*

**Figure 1 : A/ rwPFS in L1 and ≥L2 patients initiating acalabrutinib (Kaplan Meier); B/ rwPFS in L1 patients with and without del17p/TP53m (Kaplan Meier); C/ rwPFS in L1 patients with and without cardiovascular history (Kaplan Meier); D/ rwPFS in priorly-ibrutinib-exposed patients with ibrutinib discontinuation for AE or for progression or suboptimal response ; E/ Incidence of AEs leading to treatment changes by SOC and PT classes in patients priorly exposed to ibrutinib (n=74)**

**Copyright Information:** (Online) ISSN: 2572-9241
©2026 The Author(s). HemaSphere is published by John Wiley & Sons Ltd on behalf of European Hematology Association. This is an open access Abstract Book distributed under the Attribution-NonCommercial-NoDerivs (CC BY-NC-ND), which allows third parties to download the articles and share them with others as long as they credit the author and the Abstract Book, but they cannot change the content in any way or use them commercially.
**Abstract Book Citations:** Authors, Title, HemaSphere, 2026;10:(S1):pages. The abstract book DOIs can be found on the "Author Information" page.
**Disclaimer:** Articles published in the journal HemaSphere exclusively reflect the opinions of the authors. The authors are responsible for all content in their abstracts including accuracy of the facts, statements, citing resources, etc.

### (PF610) ORELABRUTINIB FOR THE TREATMENT OF CHRONIC LYMPHOCYTIC LEUKEMIA/SMALL LYMPHOCYTIC LYMPHOMA: SAFETY AND EFFICACY RESULTS FROM A GLOBAL PHASE 1/2 STUDY

**Topic:** 06. Chronic lymphocytic leukemia and related disorders - Clinical

Yucai Wang*[1], James D'olimpio[2], Bertrand Anz[3], Ian W. Flinn[4], David Gallinson[5], Bipinkumar Amin[6], Arvind Chaudhry[7], Nashat Gabrail[8], Steven Dunder[9], Faithlore Gardner[10], Jing-Zhou Hou[11], David Morrison[12], Guofeng Liu[13], Jie Ding[13], Renbin Zhao[13], Michael L. Wang[14]

*[1]Mayo Clinic, Rochester, MN, United States of America;[2]Clinical Research Alliance, Westbury, NY, United States of America; [3]Chattanooga Oncology and Hematology Associates, Tennessee Oncology, Chattanooga, TN, United States of America; [4]Tennessee Oncology/OneOncology, Nashville, TN, United States of America;[5]Summit Medical Group, Florham Park Campus, Florham Park, NJ, United States of America; [6]Prairie Lakes Healthcare System, Watertown, SD, United States of America; [7]Medical Oncology Associates, P.S., Spokane Valley, WA, United States of America;[8]Gabrail Cancer Research Center, Canton, OH, United States of America; [9]Cancer Partners of Nebraska, Lincoln, NE, United States of America;[10]Florida Cancer Specialists and Research Institute, Fort Myers, FL, United States of America; [11]Hillman Cancer Center, University of Pittsburgh, Pittsburgh, Fort Myers, FL, United States of America; [12]Tulane University School of Medicine, New Orleans, LA, United States of America; [13]Beijing InnoCare Pharma Tech Co., Ltd., Beijing, China; [14]MD Anderson Cancer Center, Houston, TX, United States of America;*

### Background

Orelabrutinib is a highly selective, covalent Bruton's tyrosine kinase (BTK) inhibitor. A study in 80 Chinese patients with relapsed/refractory (R/R) chronic lymphocytic leukemia/small lymphocytic lymphoma (CLL/SLL) showed promising efficacy with an overall response rate (ORR) of 92.5%, a complete response (CR) rate of 21.3%, and 30-month progression-free survival (PFS) and overall survival (OS) rates of 70.9% and 81.3%, respectively.

### Aims

To evaluate the safety and efficacy of orelabrutinib in patients with CLL/SLL from the United States and Europe.

### Methods

This global study (NCT04014205) comprised phase 1 (dose escalation) and phase 2 (dose expansion). Patients received oral orelabrutinib (100 mg or 150 mg once daily in phase 1 and 150 mg once daily in phase 2) until disease progression or unacceptable toxicity. The primary endpoint was incidence of does limiting toxicities (DLTs) in phase 1 and ORR in phase 2.

**Copyright Information:** (Online) ISSN: 2572-9241
©2026 The Author(s). HemaSphere is published by John Wiley & Sons Ltd on behalf of European Hematology Association. This is an open access Abstract Book distributed under the Attribution-NonCommercial-NoDerivs (CC BY-NC-ND), which allows third parties to download the articles and share them with others as long as they credit the author and the Abstract Book, but they cannot change the content in any way or use them commercially.
**Abstract Book Citations:** Authors, Title, HemaSphere, 2026;10:(S1):pages. The abstract book DOIs can be found on the "Author Information" page.
**Disclaimer:** Articles published in the journal HemaSphere exclusively reflect the opinions of the authors. The authors are responsible for all content in their abstracts including accuracy of the facts, statements, citing resources, etc.

**Results**

Thirty-nine patients (median age 67 years) were enrolled and received at least one dose of orelabrutinib. No DLT was observed in phase 1 and 150 mg once daily was the recommended phase 2 dose. Treatment-emergent adverse events (TEAEs) occurred in 97.4% (38/39) of patients, mostly Grade 1-2. Common (≥20%) TEAEs included cough, COVID-19, diarrhoea, arthralgia, contusion, haematuria, and headache. Grade ≥3 TEAEs included neutropenia (10.3%), COVID-19 (7.7%), and anaemia, hypertension, weight increased, and diarrhoea (5.1% each). Serious AEs occurred in 10 (25.6%) patients, with 4 assessed as treatment related. No treatment-related bleeding or atrial fibrillation was observed.

In 19 efficacy-evaluable treatment-naïve (TN) CLL/SLL patients (median follow-up 38.1 months), ORR and CR rate was 100% and 15.8%, respectively. Median duration of response (DOR) and PFS were not reached, with 24-month rates at 94.4% for both; 24-month OS rate was 100%. In 15 efficacy-evaluable R/R CLL/SLL patients (median follow-up 36.8 months), ORR was 86.7% and CR rate was 13.3%. Median DOR and PFS were not reached, with 24-month rates of 83.9% and 85.7%, respectively; 24-month OS rate was 100%.

**Summary/Conclusion**

Orelabrutinib demonstrated a favorable safety profile and excellent efficacy in this study, with durable responses in both TN and R/R patients. These results are consistent with the prior report in Chinese patients, confirming the efficacy and safety of orelabrutinib for CLL/SLL in a global population.

**Copyright Information:** (Online) ISSN: 2572-9241
©2026 The Author(s). HemaSphere is published by John Wiley & Sons Ltd on behalf of European Hematology Association. This is an open access Abstract Book distributed under the Attribution-NonCommercial-NoDerivs (CC BY-NC-ND), which allows third parties to download the articles and share them with others as long as they credit the author and the Abstract Book, but they cannot change the content in any way or use them commercially.
**Abstract Book Citations:** Authors, Title, HemaSphere, 2026;10:(S1):pages. The abstract book DOIs can be found on the "Author Information" page.
**Disclaimer:** Articles published in the journal HemaSphere exclusively reflect the opinions of the authors. The authors are responsible for all content in their abstracts including accuracy of the facts, statements, citing resources, etc.

### (PF611) REAL-WORLD OUTCOMES IN PATIENTS WITH CLL TREATED WITH FIRST-LINE IBRUTINIB OR ACALABRUTINIB AND UNDERGOING DOSE REDUCTIONS: A CHART REVIEW STUDY

**Topic:** 06. Chronic lymphocytic leukemia and related disorders - Clinical

Mazyar Shadman*[1], Lindsey Roeker[2], Javier Pinilla[3], Bhavini Srivastava[4], Kavita Sail[4], Christopher Abbazio[4], Erin Franceschini[4], Chadi Saifan[4], Siddharth Karanth[5], Ravi K Goyal[5], Julien Rombi[5], Deborah Stephens[6]

*[1]Fred Hutchinson Cancer Center, Seattle, WA, United States of America;[2]Memorial Sloan Kettering Cancer Center, New York, NY, United States of America; [3]Moffitt Cancer Center, Tampa, FL, United States of America;[4]AbbVie, North Chicago, IL, United States of America; [5]RTI Health Solutions, Durham, NC, United States of America;[6]University of North Carolina, Chapel Hill, NC, United States of America;*

### Background

Adverse events (AEs) occurring during first-line (1L) treatment (tx) with the Bruton tyrosine kinase inhibitors ibrutinib (ibr) and acalabrutinib (acala) in chronic lymphocytic leukemia (CLL) often lead to tx discontinuation. Real-world data suggest that ibr dose reductions (DRs) can mitigate AEs without compromising tx efficacy, potentially reducing discontinuation rates.

### Aims

To evaluate real-world progression-free survival (PFS) and duration of treatment (DOT) in patients (pts) with CLL undergoing DR with ibr or acala.

### Methods

In a retrospective US chart review, data for adult pts (aged ≥18 years) with CLL who initiated 1L ibr (420 mg/day) or acala (100 mg twice daily) between October 1, 2017, and October 31, 2023, and experienced DRs were abstracted by participating hematologist-oncologists. DOT was defined as the time from tx initiation to discontinuation or death among pts with DR due to AE (DR-AE). PFS was measured as time from tx start to the documented disease progression or death in all pts with DR and DR-AE. The Kaplan-Meier (KM) method was used; pts without events were censored at last follow-up. Multivariable Cox regressions adjusting for baseline characteristics were used to assess time-to-event outcomes; adjusted hazard ratio (HR) and 95% confidence intervals (CIs) were estimated. Restricted mean survival time (RMST) was estimated to address potential violation of the proportional hazard assumption.

**Copyright Information:** (Online) ISSN: 2572-9241
©2026 The Author(s). HemaSphere is published by John Wiley & Sons Ltd on behalf of European Hematology Association. This is an open access Abstract Book distributed under the Attribution-NonCommercial-NoDerivs (CC BY-NC-ND), which allows third parties to download the articles and share them with others as long as they credit the author and the Abstract Book, but they cannot change the content in any way or use them commercially.
**Abstract Book Citations:** Authors, Title, HemaSphere, 2026;10:(S1):pages. The abstract book DOIs can be found on the "Author Information" page.
**Disclaimer:** Articles published in the journal HemaSphere exclusively reflect the opinions of the authors. The authors are responsible for all content in their abstracts including accuracy of the facts, statements, citing resources, etc.

**Results**

The analysis included 231 pts treated with ibr (median age, 65 years; male, 58.4%; White, 69.3%; del(17p), 16.0%; stage I-II, 70.1%) and 169 pts treated with acala (median age, 64 years; male, 60.4%; White, 58.6%; del(17p), 26.6%; stage I-II, 34.9%). DR-AEs occurred in 158 and 149 pts treated with ibr and acala, respectively. The median Charlson Comorbidity Index score was 1 (range, 0-5) in both cohorts. Median follow-up duration from tx initiation was 30.5 months (mo) for ibr (DR-AE, 28.5 mo) and 23.5 mo for acala (DR-AE, 22.6 mo). The median time to first DR was 6.8 mo (DR-AE, 5.1 mo) for ibr and 5.1 mo (DR-AE, 4.7 mo) for acala cohorts. In pts with DR-AE, the estimated median DOT from the KM analysis was 38.4 mo (95% CI, 29.3-44.3) for ibr, and not estimable (NE) for acala; adjusted HR for time to discontinuation or death (ibr vs acala) was 0.78 (95% CI, 0.50-1.22; *P*=0.272). In the RMST analysis, mean DOT among pts with DR-AE was 28.4 mo for ibr and 28.5 mo for acala (*P*=0.945). Among all pts with DR, median PFS in the KM analysis was NE with both ibr and acala; 36-mo PFS was 92.2% for ibr and 73.5% for acala; adjusted HR for time to PFS event (ibr vs acala) was 0.23 (95% CI, 0.10-0.52; *P*≤0.001). Among pts with DR-AE, PFS was NE for both ibr and acala, with 36-mo PFS at 90.7% and 78.7%, respectively; adjusted HR for time to PFS event (ibr vs acala) was 0.36 (95% CI, 0.14-0.95; *P*=0.038). In the RMST analysis, mean PFS among all pts with DR was 36.0 mo for ibr versus 33.9 mo for acala (*P*=0.006); likewise, mean PFS among pts with DR-AE was 34.6 mo for ibr versus 33.4 mo for acala (*P*=0.073).

**Summary/Conclusion**

This real-world study demonstrates that pts with CLL treated with 1L ibr and undergoing DR had similar DOT and a longer time to progression compared with pts treated with 1L acala with DR. These results indicate that DRs during ibr tx potentially lead to favorable PFS outcomes compared with DRs during acala tx, thus offering a valuable strategy for managing AEs while maintaining tx efficacy.

**Copyright Information:** (Online) ISSN: 2572-9241
©2026 The Author(s). HemaSphere is published by John Wiley & Sons Ltd on behalf of European Hematology Association. This is an open access Abstract Book distributed under the Attribution-NonCommercial-NoDerivs (CC BY-NC-ND), which allows third parties to download the articles and share them with others as long as they credit the author and the Abstract Book, but they cannot change the content in any way or use them commercially.
**Abstract Book Citations:** Authors, Title, HemaSphere, 2026;10:(S1):pages. The abstract book DOIs can be found on the "Author Information" page.
**Disclaimer:** Articles published in the journal HemaSphere exclusively reflect the opinions of the authors. The authors are responsible for all content in their abstracts including accuracy of the facts, statements, citing resources, etc.

### (PF612) DURABLE RESPONSES AFTER TIME-LIMITED THERAPY WITH ZANUBRUTINIB PLUS RITUXIMAB IN PATIENTS WITH TREATMENT-NAÏVE CHRONIC LYMPHOCYTIC LEUKEMIA

**Topic:** 06. Chronic lymphocytic leukemia and related disorders - Clinical

Jan Burger*[1], Ekaterina Kim[1], Gitanjali Jayachandran[1], Thomas Mathew[1], Yesid Alvarado[1], Danielle Hammond[1], Fadi Haddad[1], Nitin Jain[1], William Wierda[1], Alessandra Ferrajoli[1]

[1] *The University of Texas MD Anderson Cancer Center, Department of Leukemia, Houston, United States of America;*

### Background

Long-term Bruton's tyrosine kinase inhibitor (BTKi) monotherapy induces durable remissions in patients with chronic lymphocytic leukemia (CLL). The necessity for long-term BTKi monotherapy has been challenged, because patients who discontinue frontline BTKi therapy can have surprisingly durable remissions (24 or 25 months in the Alliance A041202 or ECOG-E1912 studies). Here, we report early results from a phase II study of time-limited therapy with zanubrutinib in combination with anti-CD20 antibody rituximab in 24 treatment-naïve patients with CLL (NCT04458610).

### Aims

The primary objective is to determine the proportion of patients who have treatment-free remission (TFR) 6 months after zanubrutinib discontinuation. Secondary objectives are length of TFR, and identify factors associated with durable remissions.

### Methods

Patients with treatment-naïve CLL requiring therapy per iwCLL criteria received zanubrutinib 160 mg orally twice daily and rituximab (375 mg/m$^2$ intravenously) on days 1, 8, 15, 22 of cycle 1, and then on day 1 of cycles 2 to 6 (each cycle is 28 days). We used a response-guided treatment design in which response was assessed after 6 and 12 cycles of therapy. Patients who achieve a complete remission (CR) after 6 or 12 cycles received another 6 cycles of zanubrutinib monotherapy, before discontinuing treatment after 12 or 18 cycles, respectively. Patients who achieved a partial remission (PR) or stable disease (SD) after 6 cycles of therapy received another 6 cycles of combination therapy, and then 6 cycles of zanubrutinib as monotherapy if they achieved a CR, or 12 more cycles of Zanubrutinib as monotherapy if they achieved a PR/SD.

### Results

The median age of patients was 68 years (range, 41 – 82 years), 37% had 13q deletion, 21% trisomy 12, 21% 11q deletion, 42% had unmutated IGHV. The baseline absolute lymphocyte count was 50.9 x 10$^9$/L (range, 1.54 – 231 x 10$^9$/L). Twenty-one patients completed treatment and transitioned to observation, 1 patient after 12 cycles, 1 after 18 cycles, and 19 patients after 24 cycles. With a median follow-up of 13 months (range: 0 – 40 months) after treatment discontinuation, 15 patients remain in remission, 6 experienced disease progression, but only 3 patients required re-treatment (one patient with unmutated IGHV and +12, two patients mutated IGHV with del11q). The 6- and 12-month TFR is 100%, with time-to-next treatment being 13, 13, and 34 months in patients that required to resumed therapy. At this time, the shortest time to progression after treatment discontinuation was 11 months and the longest progression-free remission after treatment discontinuation is 40 months. Among the 6 patients with the longest TFR five were IGHV mutated, two had del13q, three with normal FISH, and one had del11q. After a median follow-up of 34 months, all patients are alive, and the estimated two-year progression-free (PFS) and overall survival (OS) are 100% and the estimated median PFS is 38 months. Treatment was well tolerated, with arterial hypertension (n=4) and neutropenia (n=3) as the only adverse events Grade 3 or higher occurring in more than one patient.

**Copyright Information:** (Online) ISSN: 2572-9241
©2026 The Author(s). HemaSphere is published by John Wiley & Sons Ltd on behalf of European Hematology Association. This is an open access Abstract Book distributed under the Attribution-NonCommercial-NoDerivs (CC BY-NC-ND), which allows third parties to download the articles and share them with others as long as they credit the author and the Abstract Book, but they cannot change the content in any way or use them commercially.
**Abstract Book Citations:** Authors, Title, HemaSphere, 2026;10:(S1):pages. The abstract book DOIs can be found on the "Author Information" page.
**Disclaimer:** Articles published in the journal HemaSphere exclusively reflect the opinions of the authors. The authors are responsible for all content in their abstracts including accuracy of the facts, statements, citing resources, etc.

### Summary/Conclusion

Time-limited therapy with zanubrutinib and rituximab can induce durable remissions. The duration of treatment-free remissions ranges from 11 to 40 months at the time of data cutoff. After a median follow-up of 13 months after therapy discontinuation, 15/21 (71%) of patients remain off therapy and in remission.

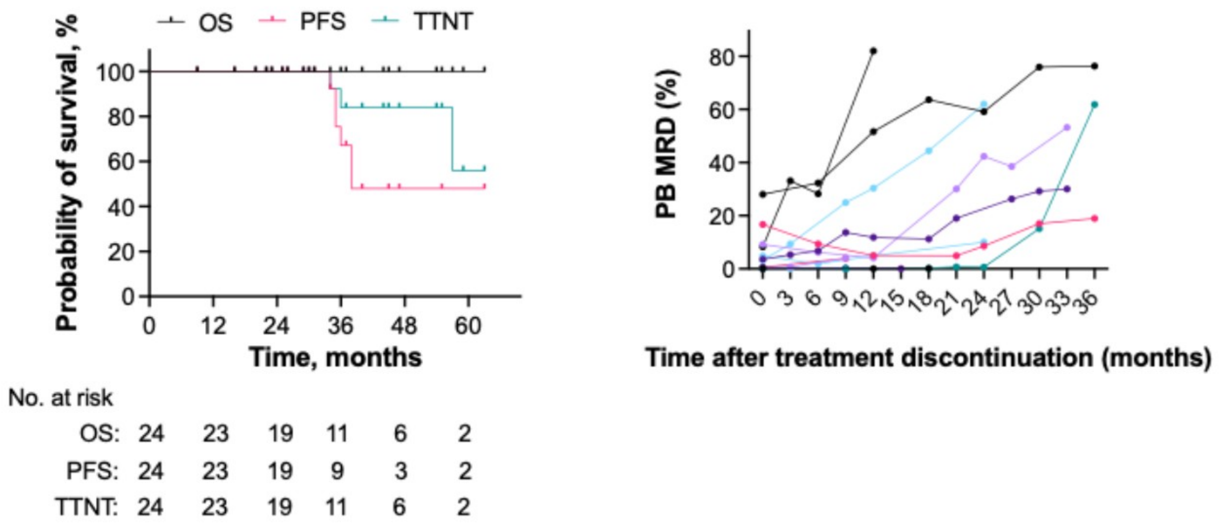

Kaplan-Meier estimates of progression-free (PFS), overall survival (OS), and time-to-next treatment (TTNT) (left panel). Peripheral blood MRD levels by flow cytometry after therapy discontinuation (right panel).

**Copyright Information:** (Online) ISSN: 2572-9241
©2026 The Author(s). HemaSphere is published by John Wiley & Sons Ltd on behalf of European Hematology Association. This is an open access Abstract Book distributed under the Attribution-NonCommercial-NoDerivs (CC BY-NC-ND), which allows third parties to download the articles and share them with others as long as they credit the author and the Abstract Book, but they cannot change the content in any way or use them commercially.
**Abstract Book Citations:** Authors, Title, HemaSphere, 2026;10:(S1):pages. The abstract book DOIs can be found on the "Author Information" page.
**Disclaimer:** Articles published in the journal HemaSphere exclusively reflect the opinions of the authors. The authors are responsible for all content in their abstracts including accuracy of the facts, statements, citing resources, etc.

### (PF613) REAL-WORLD FIRST-LINE FIXED-DURATION (FD) IBRUTINIB+VENETOCLAX (IBR+VEN) TREATMENT (TX) IN PATIENTS WITH CHRONIC LYMPHOCYTIC LEUKEMIA (CLL): ANALYSIS FROM THE PROSPECTIVE REALITY-WW STUDY

**Topic:** 06. Chronic lymphocytic leukemia and related disorders - Clinical

Catherine Thieblemont*[1], Adrien Caillet[2], Vanessa Innao[3], Alessandro Sanna[4], David Allsup[5], Talha Munir[6], Oliver Miles[7], Danielle Leão[8], Adriana Alves De Souza Scheliga[9], Ahmed Absi[10], Galia Stemer[11], Tamar Tadmor[12], Mark Hoffman[13], Brian Koffman[14], Boo Messahel[15], Sowmya Srikanthan[16], Anat Raz[17], Christopher Abbazio[17], Zacharias Anastasiou[18], Huiling Pei[19], Qing Li[15], Jose Manuel De Jesus Antunes[20], Barbara Pinto[20], Claire Kavanagh[21], Christoph Tapprich[22], Mohamed Morsy[23], Lori Parisi[15], Mark Wildgust[15], Paolo Ghia[24, 25]

*[1]Assistance Publique & Hôpitaux de Paris (APHP), Hôpital Saint-Louis, Hémato-oncologie, Université de Paris, Paris, France; [2]CHU Besançon, Hematology Department, Besançon, France; [3]ARNAS Garibaldi, Catania, Italy; [4]AOU Careggi, Hematology Unit, Firenze, Italy; [5]Hull York Medical School, Centre for Biomedicine, Hull, United Kingdom;[6]St James's University Hospital, Leeds, United Kingdom; [7]Musgrove Park Hospital, Taunton, United Kingdom; [8]A Beneficência Portuguesa de São Paulo, São Paulo, Brazil; [9]Instituto Oncoclinicas, Rio de Janeiro, Brazil; [10]National Guard Health Affairs: King Abdulaziz Medical City, Riyadh, Saudi Arabia; [11]Hematology Institute, Galilee Medical Center, Naharyia, Israel; [12]Bnai Zion Medical Center, and the Ruth and Bruce Rappaport Faculty of Medicine, Hematology Unit, Haifa, Israel; [13]University of California, San Diego, United States of America; [14]CLL Society, Inc., Nashville, United States of America;[15]Johnson & Johnson, Raritan, United States of America; [16]Johnson & Johnson, Somerset, United States of America; [17]Pharmacyclics LLC, an AbbVie Company, North Chicago, United States of America; [18]Johnson & Johnson, Athens, Greece; [19]Johnson & Johnson, Titusville, United States of America; [20]Johnson & Johnson, Porto Salvo, Portugal; [21]Johnson & Johnson, Dublin, Ireland; [22]Johnson & Johnson, Neuss, Germany; [23]Johnson & Johnson Middle East FZ-LLC, Dubai, United Arab Emirates; [24]Università Vita-Salute San Raffaele, Milan, Italy; [25]Comprehensive Cancer Center, IRCCS Ospedale San Raffaele, Milan, Italy;*

### Background

FD Ibr+Ven has demonstrated noninferiority to continuous Ibr with similar 3-year progression-free survival and overall survival (OS) rates in patients (pts) with previously untreated (CLL)/small lymphocytic lymphoma (SLL) and a generally favorable safety profile. A real-world (RW) survival advantage for Ibr vs other BTKis has also been reported. FD Ibr+Ven also achieves OS rates comparable to an age-matched general European population. Limited RW data have been reported on first-line FD Ibr+Ven use and outcomes in the 78 countries in which it is approved. Previously, pooled analysis from the prospective REALITY-WW and REALITY-2 studies indicated FD Ibr+Ven has a manageable safety profile and is effective in clinical practice.

### Aims

Here we present the interim analysis of REALITY-WW with all pts fully enrolled.

### Methods

Pts aged ≥18 years with CLL/SLL per iwCLL 2018 guidelines were enrolled over 63 sites in Europe, the Middle East, and Latin America. Pts received a 3-cycle lead-in of Ibr (420mg once daily), followed by 12 cycles of FD Ibr+Ven (with Ven dose ramp-up from 20 to 400mg over 5 weeks). Data were collected every ~12 weeks during routine clinic follow-up visits until subsequent Tx or study completion. The primary endpoint was best overall response rate (ORR) by the end of 15 Tx cycles; ORR included complete response (CR), CR with incomplete hematological recovery (CRi), partial response (PR), and PR with lymphocytosis (PR-L). Secondary endpoints included duration of response, safety, and patient-reported outcomes.

**Copyright Information:** (Online) ISSN: 2572-9241
©2026 The Author(s). HemaSphere is published by John Wiley & Sons Ltd on behalf of European Hematology Association. This is an open access Abstract Book distributed under the Attribution-NonCommercial-NoDerivs (CC BY-NC-ND), which allows third parties to download the articles and share them with others as long as they credit the author and the Abstract Book, but they cannot change the content in any way or use them commercially.
**Abstract Book Citations:** Authors, Title, HemaSphere, 2026;10:(S1):pages. The abstract book DOIs can be found on the "Author Information" page.
**Disclaimer:** Articles published in the journal HemaSphere exclusively reflect the opinions of the authors. The authors are responsible for all content in their abstracts including accuracy of the facts, statements, citing resources, etc.

### Results

A total of 200 pts received FD Ibr+Ven (as of Dec 2025 data cutoff) with a median time on study of 7.1mo. Median age was 65.5 (range, 39-87) years, 53.5%/16.0% of pts were aged ≥65/≥75 years, 65.5% were male, and 11.3% had a CIRS score >6. Median duration of exposure was 6.8mo, with 185 pts still on Tx. Unmutated/mutated IGHV (42.7%/30.3%; 24.9% not available), unmutated/mutated *TP53* (84.8%/3.8%; 11.4% not available), and present/absent del17p (13.0%/65.9%; 21.1% not available) were reported. Among the 135 treated pts who had ≥1 post-baseline disease assessments, ORR by the end of 15 Tx cycles was 91.1% (95% CI, 86.3-95.9). ORR by the end of 3 Tx cycles (Ibr lead-in) was 80.0%, increasing to 90.4% after 6 cycles, and stabilizing at 91.1% from 9 cycles onward. ORR by the end of 15 Tx cycles included a CR in 34.8% of pts, CRi in 1.5%, PR in 43%, PR-L in 11.9%. FD Ibr+Ven was well tolerated with 92.5% of pts still on therapy. 12 pts discontinued Tx, 5 due to adverse events (AEs). Tx-emergent AEs (TEAEs) and grade 3/4 TEAEs were reported in 60.0% and 11.5% of pts, respectively. The most common TEAEs were diarrhea (16.0%), neutropenia (11.0%), and muscle spasms (9.5%); the most common grade 3/4 TEAE was neutropenia (4.5%). Any-grade cardiac disorders were reported in 10.0% of pts, including atrial fibrillation (AF) (3.5%) and palpitations (1.5%); hypertension was documented in 4.0%. At baseline, pts with low, intermediate, and high tumor lysis syndrome (TLS) risk were 14.0%, 32.5%, and 25.5%, shifting to 18.0%, 20.5%, and 13.5% after 3 Tx cycles (Ibr lead-in). No cases of clinical/laboratory TLS were reported; no pts had an increase in TLS risk between baseline and Cycle 4.

### Summary/Conclusion

Results from the prospective REALITY-WW study demonstrate that FD Ibr+Ven is effective and well tolerated with low rates of AF and no TLS, including in elderly pts and those with high risks features, in an RW setting. Longer-term follow-up data will be presented.

**Copyright Information:** (Online) ISSN: 2572-9241
©2026 The Author(s). HemaSphere is published by John Wiley & Sons Ltd on behalf of European Hematology Association. This is an open access Abstract Book distributed under the Attribution-NonCommercial-NoDerivs (CC BY-NC-ND), which allows third parties to download the articles and share them with others as long as they credit the author and the Abstract Book, but they cannot change the content in any way or use them commercially.
**Abstract Book Citations:** Authors, Title, HemaSphere, 2026;10:(S1):pages. The abstract book DOIs can be found on the “Author Information” page.
**Disclaimer:** Articles published in the journal HemaSphere exclusively reflect the opinions of the authors. The authors are responsible for all content in their abstracts including accuracy of the facts, statements, citing resources, etc.

### (PF614) SAFETY OF FIXED-DURATION (FD) ACALABRUTINIB-VENETOCLAX COMBINATIONS IN PATIENTS (PTS) WITH CHRONIC LYMPHOCYTIC LEUKEMIA (CLL) STRATIFIED BY AGE: POST HOC ANALYSIS OF THE PHASE 3 AMPLIFY TRIAL

**Topic:** 06. Chronic lymphocytic leukemia and related disorders - Clinical

John F. Seymour*[1], Andrew Aw[2], Vincent Ribrag[3], Guillaume Cartron[4], Arnon Philip Kater[5], Ki-Seong Eom[6], Alan Skarbnik[7], Alessandra Tedeschi[8], Malgorzata Wach[9], Jing-Zhou Hou[10], Arpad Illes[11], Batul Suterwala[12], Jiefen Munley[13], Ellie John[14], Toshifumi Fujimori[14], Jennifer R. Brown[15]

*[1]Peter MacCallum Cancer Centre, Royal Melbourne Hospital, The University of Melbourne, Melbourne, Australia;[2]University of Ottawa, Ottawa, Canada; [3]Institut Gustave Roussy, Villejuif, France; [4]Centre Hospitalier Universitaire de Montpellier UMR5535, Montpellier, France; [5]Department of Hematology, Cancer Center Amsterdam, Amsterdam University Medical Centers, University of Amsterdam, Amsterdam, The Netherlands; [6]Catholic Hematology Hospital, Seoul St. Mary's Hospital, College of Medicine, the Catholic University of Korea, Seoul, South Korea; [7]Novant Health Cancer Institute, Charlotte, United States of America;[8]ASST Grande Ospedale Metropolitano Niguarda, Niguarda Cancer Center, Milan, Italy; [9]Medical University of Lublin, Lublin, Poland; [10]University of Pittsburgh Medical Center Hillman Cancer Center, Pittsburgh, United States of America;[11]University of Debrecen, Debrecen, Hungary; [12]AstraZeneca, South San Francisco, United States of America; [13]AstraZeneca, Wilmington, United States of America; [14]AstraZeneca, Cambridge, United Kingdom; [15]Dana-Farber Cancer Institute, Boston, United States of America;*

### Background

Novel FD combinations are emerging as standard-of-care frontline CLL treatment (tx). GLOW demonstrated efficacy with FD ibrutinib + venetoclax in older/comorbid pts, but cardiac adverse events (AEs) are a consideration. AMPLIFY evaluated FD acalabrutinib + venetoclax (AV) or AV + obinutuzumab (AVO) vs chemoimmunotherapy (CIT) in fit pts with tx-naive (TN) CLL. AV/AVO prolonged progression-free survival (PFS) vs CIT with manageable safety (Brown et al. *NEJM* 2025).

### Aims

To evaluate FD AV and AVO safety stratified by age.

### Methods

AMPLIFY (NCT03836261) is a phase 3 trial in adults with TN CLL, ECOG PS ≤2, and no del(17p) or *TP53* mutation. Pts were randomized 1:1:1 to AV (A, cycles [C] 1–14; V, C3–14), AVO (AV as above; O, C2–7), or CIT (fludarabine-cyclophosphamide-rituximab or bendamustine-rituximab; C1–6). Descriptive statistics were used to evaluate AEs and discontinuations in pts <65 or ≥65 y who received AV or AVO.

### Results

Pts who received ≥1 dose of AV or AVO were included. At baseline, older pts (≥65 y) had reduced renal function and higher mean comorbidity scores (**Table**). Incidence of overall AEs, as well as infections and hemorrhage (events of clinical interest [ECIs]), were generally similar across age and tx groups. Incidence of grade ≥3 AEs and serious AEs (SAEs) were numerically increased in the AVO arm and among older pts. SAEs with fatal outcome were numerically increased in pts ≥65 y, largely COVID-19–related (mostly in unvaccinated pts [Brown et al. EHA 2025]), and more frequent in the AVO arm. Tx discontinuations due to AEs were higher in older pts; COVID-19–related AEs were the most common reason. PFS improvement with AV/AVO vs CIT was observed in subgroups of pts <65 y (AV vs CIT: HR 0.84 [95% CI 0.58–1.22]; AVO vs CIT: 0.42 [0.27–0.64]) and ≥65 y (AV vs CIT: 0.47 [0.29–0.76]; AVO vs CIT: 0.47 [0.28–0.79]), consistent with the overall population (Brown et al. *NEJM* 2025).

**Copyright Information:** (Online) ISSN: 2572-9241
©2026 The Author(s). HemaSphere is published by John Wiley & Sons Ltd on behalf of European Hematology Association. This is an open access Abstract Book distributed under the Attribution-NonCommercial-NoDerivs (CC BY-NC-ND), which allows third parties to download the articles and share them with others as long as they credit the author and the Abstract Book, but they cannot change the content in any way or use them commercially.
**Abstract Book Citations:** Authors, Title, HemaSphere, 2026;10:(S1):pages. The abstract book DOIs can be found on the "Author Information" page.
**Disclaimer:** Articles published in the journal HemaSphere exclusively reflect the opinions of the authors. The authors are responsible for all content in their abstracts including accuracy of the facts, statements, citing resources, etc.

### Summary/Conclusion

Safety was comparable across age groups, with modest increases, particularly in AE-related discontinuations, in pts aged ≥65 y, with COVID-19 as the leading cause. No new safety signals were identified.

**Table. Characteristics, AEs, and discontinuations**

| | AV (N=291) | | AVO (N=284) | |
|---|---|---|---|---|
| | <65 y (n=194) | ≥65 y (n=97) | <65 y (n=198) | ≥65 y (n=86) |
| Follow-up, mo, median (min, max) | 40.5 (0.0, 58.5) | 42.3 (0.4, 53.8) | 41.1 (1.9, 57.7) | 42.2 (0.5, 57.1) |
| **Baseline characteristics** | | | | |
| Age, y, median (min, max) | 56.0 (31, 64) | 69.0 (65, 84) | 57.0 (29, 64) | 70.0 (65, 81) |
| ECOG PS ≤1, % | 91.8 | 86.6 | 94.9 | 95.3 |
| CrCl <60 mL/min,[a] % | 4.6 | 29.9 | 5.6 | 32.6 |
| CIRS-G score,[b] mean (SD) | 2.2 (1.9) | 3.7 (1.9) | 2.4 (1.9) | 3.5 (2.3) |
| **AEs, %** | | | | |
| Any grade | 93.8 | 90.7 | 95.5 | 93.0 |
| Grade ≥3 | 51.0 | 58.8 | 67.2 | 74.4 |
| SAE[c] | 23.7 | 26.8 | 36.4 | 43.0 |
| COVID-19 pneumonia | 5.7 | 6.2 | 12.6 | 8.1 |
| COVID-19 | 2.6 | 4.1 | 5.6 | 7.0 |
| SAE with fatal outcome[d] | 2.6 | 5.2 | 3.5 | 11.6 |

**Copyright Information:** (Online) ISSN: 2572-9241
©2026 The Author(s). HemaSphere is published by John Wiley & Sons Ltd on behalf of European Hematology Association. This is an open access Abstract Book distributed under the Attribution-NonCommercial-NoDerivs (CC BY-NC-ND), which allows third parties to download the articles and share them with others as long as they credit the author and the Abstract Book, but they cannot change the content in any way or use them commercially.
**Abstract Book Citations:** Authors, Title, HemaSphere, 2026;10:(S1):pages. The abstract book DOIs can be found on the “Author Information” page.
**Disclaimer:** Articles published in the journal HemaSphere exclusively reflect the opinions of the authors. The authors are responsible for all content in their abstracts including accuracy of the facts, statements, citing resources, etc.

| | | | | |
|---|---|---|---|---|
| COVID-19 pneumonia | 1.0 | 4.1 | 2.5 | 5.8 |
| COVID-19 | 1.0 | 0 | 1.0 | 3.5 |
| ECI[e] | | | | |
| Cardiac events | 8.8 | 10.3 | 9.6 | 17.4 |
| Atrial fibrillation | 0 | 2.1 | 1.5 | 3.5 |
| Ventricular arrhythmias (tachycardia and extrasystoles) | 1.0 | 0 | 1.0 | 1.2 |
| Hypertension | 5.2 | 2.1 | 5.1 | 1.2 |
| Infections | 53.6 | 45.4 | 56.6 | 47.7 |
| Hemorrhage | 29.9 | 37.1 | 30.3 | 30.2 |
| Major hemorrhage | 0.5 | 2.1 | 1.5 | 5.8 |
| **Discontinuations, %** | | | | |
| Due to AE[c] | 5.7 | 12.4 | 14.6 | 32.6 |
| COVID-19 pneumonia | 1.0 | 4.1 | 3.5 | 3.5 |
| COVID-19 | 1.0 | 0 | 5.1 | 5.8 |

CIRS-G, Cumulative Illness Rating Scale-Geriatric; CrCl, creatinine clearance.

[a]Pts with estimated CrCl <50 mL/min were excluded.

[b]Pts aged >65 y with CIRS-G score >6 were excluded.

[c]Event/reason reported in >5% of pts in any subgroup.

[d]Event reported in >3% of pts in any subgroup.

[e]Select ECIs; hypertension and hemorrhage were evaluated using Standardized MedDRA Queries.

**Copyright Information:** (Online) ISSN: 2572-9241
©2026 The Author(s). HemaSphere is published by John Wiley & Sons Ltd on behalf of European Hematology Association. This is an open access Abstract Book distributed under the Attribution-NonCommercial-NoDerivs (CC BY-NC-ND), which allows third parties to download the articles and share them with others as long as they credit the author and the Abstract Book, but they cannot change the content in any way or use them commercially.
**Abstract Book Citations:** Authors, Title, HemaSphere, 2026;10:(S1):pages. The abstract book DOIs can be found on the "Author Information" page.
**Disclaimer:** Articles published in the journal HemaSphere exclusively reflect the opinions of the authors. The authors are responsible for all content in their abstracts including accuracy of the facts, statements, citing resources, etc.

### (PF615) EFFECT OF A STRUCTURED, SEMI-SUPERVISED EXERCISE INTERVENTION IN CHRONIC LYMPHOCYTIC LEUKEMIA (CLL): FITNESS, CLINICAL OUTCOMES, AND CIRCULATING IMMUNE REPERTOIRE

**Topic:** 06. Chronic lymphocytic leukemia and related disorders - Clinical

McKayla Deisz**[1], Lindsey Roeker*[2], Sameer Parikh[2], Elijah Blonigen[2], Kari Rabe[3], Jessica Johnston[4], Briana Ziegler[4], Rebekah McCoy[5], Dongwook Yeo[4], Emmanuel Contreras Guzman[2], Weiguo Han[2], Jacob Ray[2], Paul Hampel[2], Saad Kenderian[2], Yucai Wang[2], Amy Behnken[2], Amber Koehler[2], Catherine Wagner[2], Susan Slager[6], Bruce Johnson[4], Michael Gustafson[7], Neil Kay[8], Courtney Wheatley-Guy[5]

*[1]Mayo Clinic, Department of Medicine, Rochester, United States of America;[2]Mayo Clinic, Department of Medicine, Division of Hematology, Rochester, United States of America; [3]Mayo Clinic, Department of Quantitative Health Sciences, Division of Clinical Trials and Biostatistics, Rochester, United States of America; [4]Mayo Clinic, Department of Cardiovascular Diseases, Rochester, United States of America; [5]Mayo Clinic, Department of Cardiovascular Diseases, Scottsdale, United States of America; [6]Mayo Clinic, Department of Medicine, Division of Hematology and Department of Quantitative Health Sciences, Division of Computational Biology, Rochester, United States of America; [7]Mayo Clinic, Department of Laboratory Medicine and Pathology, Scottsdale, United States of America; [8]Mayo Clinic, Department of Medicine, Division of Hematology and Department of Immunology, Rochester, United States of America;*

### Background

Patients (pts) with CLL experience significant morbidity, including increased risks of infection and second malignancies. CLL cells engage the tumor microenvironment to promote immune dysfunction affecting innate and adaptive immunity. Exercise is increasingly recognized as a biologically active intervention with potential to improve survival, reduce immunosenescence, enhance immune function, and support anti-tumor activity.

### Aims

To evaluate the effects of a 12-week semi-supervised exercise program (EX12) on aerobic fitness and clinical outcomes. To examine exercise-associated changes in peripheral immune repertoire and *ex vivo* drug sensitivity in pts with CLL.

### Methods

Pts with CLL/SLL were eligible for EX12: a personalized, progressive, vigorous interval-based aerobic program via mobile application, consisting of three 30-40 min sessions/week for 12 weeks including 8 remotely supervised sessions. Aerobic fitness was assessed by peak oxygen consumption ($VO_{2PEAK}$) during maximal cardiopulmonary exercise testing (CPET) at enrollment (E0) and after completion of EX12. Clinical outcomes, including infections and progression requiring therapy (tx), were obtained from medical record review. A subset of untreated pts < 5 years from diagnosis (dx) provided peripheral blood samples before and immediately after CPET at E0 and completion of EX12. All pts provided consent.

**Copyright Information:** (Online) ISSN: 2572-9241
©2026 The Author(s). HemaSphere is published by John Wiley & Sons Ltd on behalf of European Hematology Association. This is an open access Abstract Book distributed under the Attribution-NonCommercial-NoDerivs (CC BY-NC-ND), which allows third parties to download the articles and share them with others as long as they credit the author and the Abstract Book, but they cannot change the content in any way or use them commercially.
**Abstract Book Citations:** Authors, Title, HemaSphere, 2026;10:(S1):pages. The abstract book DOIs can be found on the "Author Information" page.
**Disclaimer:** Articles published in the journal HemaSphere exclusively reflect the opinions of the authors. The authors are responsible for all content in their abstracts including accuracy of the facts, statements, citing resources, etc.

**Results**

Twenty-seven pts (n=25 tx naïve, n=2 previously treated) enrolled between 6/2023 and 9/2025. Median age at CLL dx was 64 years (range 44-73), 11 (41%) were men, and median time from dx to E0 was 22 months (range 2-76). Disease characteristics: 9/26 (35%) *IGHV* unmutated, 1/21 *TP53* mutated, FISH testing found 14 sole del13q, 1 tri12, 4 del11q, 1 del17p.
85% completed EX12; 4 pts did not complete EX12 (1 due to infection, 3 withdrew consent). The cohort demonstrated average aerobic fitness based on age- and sex-matched normative values at E0 (mean $VO_{2PEAK}$: 23.8 ± 5.5 mL/min/kg, 106% ± 23% predicted). EX12 improved $VO_{2PEAK}$ ($\Delta VO_{2PEAK}$: 2.0 mL/min/kg, 9% predicted) and improved exercise tolerance with later anaerobic threshold onset.
With a median follow-up of 9 months (range 0-31; n=26), 13 pts had an infection (1 required hospitalization, 12 with minor infection, outpatient oral antibiotics). Of 25 tx naïve pts, 6 initiated CLL-directed tx after E0; 12-month treatment-free survival rate was 75%.
In a subset of tx naïve pts < 5 years of CLL dx at E0 (n=18), circulating NK (CD16+/CD56+) and T cells (CD3+) consistently increased after CPET relative to pre-exercise levels at E0 and EX12. CPET increased the circulating CLL B cell count in 10/13 evaluable pts at E0 and 17/18 pts after EX12. Drug sensitivity testing was performed at E0 and after EX12 (n=2). IC50 levels were significantly lower after EX12 as compared to E0 for acalabrutinib (mean IC50: 62.2 uM vs. 363.7 uM, $p$=0.0047) and venetoclax (mean IC50: 0.63 nM vs 1.30 nM, $p$=0.012). Further nonlinear regression and extra sum-of-squares F test analysis showed significant differences in killing curves between E0 and after EX12 (acalabrutinib $p$=0.0021, venetoclax $p$=0.039, ibrutinib $p$=0.025, rocbrutinib $p$=0.038) indicating enhanced drug sensitivity after EX12.

**Summary/Conclusion**

EX12 is feasible and improves $VO_{2PEAK}$ in CLL pts. Acute exercise mobilizes key circulating immune subsets including leukemic cells. Serious infections were uncommon during follow-up. Clinical implications of exercise-induced immune mobilization in CLL warrant further investigation.

*contributed equally

**Copyright Information:** (Online) ISSN: 2572-9241
©2026 The Author(s). HemaSphere is published by John Wiley & Sons Ltd on behalf of European Hematology Association. This is an open access Abstract Book distributed under the Attribution-NonCommercial-NoDerivs (CC BY-NC-ND), which allows third parties to download the articles and share them with others as long as they credit the author and the Abstract Book, but they cannot change the content in any way or use them commercially.
**Abstract Book Citations:** Authors, Title, HemaSphere, 2026;10:(S1):pages. The abstract book DOIs can be found on the "Author Information" page.
**Disclaimer:** Articles published in the journal HemaSphere exclusively reflect the opinions of the authors. The authors are responsible for all content in their abstracts including accuracy of the facts, statements, citing resources, etc.

### (PF616) IMPACT OF IGHV MUTATIONAL STATUS ON OUTCOME IN CHRONIC LYMPHOCYTIC LEUKEMIA

**Topic:** 06. Chronic lymphocytic leukemia and related disorders - Clinical

Christof Schneider*[1], Eugen Tausch[1], Sandra Robrecht[2], Adam Giza[2], Kim Fritsch[1], Theresa Laile[1], Billy Chelliah Jebaraj[1], Moritz Fürstenau[2], Carsten Niemann[3], Arnon Philip Kater[4], Valentin Goede[5], Paula Cramer[2], Othman Al-Sawaf[2], Kirsten Fischer[2], Hartmut Döhner[1], Michael Hallek[3], Barbara Eichhorst[2], Stephan Stilgenbauer[1]

*[1]University hospital Ulm, Department of Internal Medicine III, Division of CLL, Ulm, Germany;[2]University Hospital Cologne, Department I of Internal Medicine and Center of Integrated Oncology Aachen Bonn Cologne Duesseldorf, Cologne, Germany; [3]Copenhagen University Hospital, Department of Hematology, Rigshospitalet, Copenhagen, Denmark;[4]Cancer Center Amsterdam, Amsterdam University Medical Centers, University of Amsterdam, Department of Hematology, Amsterdam, The Netherlands; [5]Cellitinnen-Hospital St. Marien, Department of Oncogeriatrics, Cologne, Germany;*

### Background

The somatic hypermutation status of the rearranged immunoglobulin heavy variable (IGHV) genes is crucial for stratifying chronic lymphocytic leukemia (CLL) patients into risk groups. Specific immunogenetic "stereotyped" subsets further refine prognosis beyond the IGHV mutated/unmutated distinction.

### Aims

We investigated the prognostic impact of the IGHV mutational status and stereotyped subsets across clinical trials with distinct treatment types.

### Methods

From 2000 to 2025, we analyzed more than 10,000 CLL samples from 40 clinical trials. IGHV mutation status was determined according to ERIC recommendations. Outcome correlations were performed on a cohort of 3,834 previously untreated patients from the prospective multicenter German CLL Study Group trials CLL4, 5, 8, 10, 11, 13, 14, GIVE and the BXX series receiving either chemo(immuno)therapy (CIT; n=2,794) or targeted therapy (TT; n=1,040). The optimal cut-off for determination of IGHV mutational status was suggested by minimization of the Akaike information criterion for Cox proportional hazard regression modelling of PFS.

**Copyright Information:** (Online) ISSN: 2572-9241
©2026 The Author(s). HemaSphere is published by John Wiley & Sons Ltd on behalf of European Hematology Association. This is an open access Abstract Book distributed under the Attribution-NonCommercial-NoDerivs (CC BY-NC-ND), which allows third parties to download the articles and share them with others as long as they credit the author and the Abstract Book, but they cannot change the content in any way or use them commercially.
**Abstract Book Citations:** Authors, Title, HemaSphere, 2026;10:(S1):pages. The abstract book DOIs can be found on the "Author Information" page.
**Disclaimer:** Articles published in the journal HemaSphere exclusively reflect the opinions of the authors. The authors are responsible for all content in their abstracts including accuracy of the facts, statements, citing resources, etc.

### Results

We analyzed IGHV mutational status in 12,012 patients and found 12,324 productive rearrangements. 59% showed an unmutated IGHV (uIGHV) with germline identity greater than 98%, while 41% exhibited a mutated IGHV (mIGHV). Stereotyped B-cell receptors sequences were found in 14% of cases when using the ARResT algorithm.
In the frontline cohort (n=3,834), after a median observation time of 71.3 months across all treatment groups, patients with uIGHV demonstrated shorter PFS (hazard ratio [HR] 2.09 [95%-confidence interval 1.91-2.28], fig. 1A), TTNT (HR 2.78 [2.47-3.13]) and OS (HR 2.11 [1.84-2.42]). This negative prognostic effect persisted when patients treated with CIT (PFS: HR 2.04 [1.85-2.25]; TTNT: HR 2.77 [2.44-3.14]; OS: HR 2.13 [1.84-2.46]) or TT (PFS: HR 2.57 [2.03-3.25]; TTNT: HR 3.43 [2.39-4.92]; OS: HR 1.74 [1.12-2.73]) were analyzed separately. A slightly lower threshold (≥97.57%) was suggested as the optimal cut-off for defining uIGHV (PFS: HR 2.19 [1.96-2.45]; TTNT: HR 2.98 [2.56-3.47]; OS: HR 2.21 [1.85-2.65]).
Further refinement of the mIGHV group, based on germline identity thresholds, showed that patients with homology <97% experienced better outcomes than patients with 97-97.99% homology (PFS: HR 0.70 [0.57-0.85], fig. 1B; TTNT: HR 0.56 [0.44-0.72]; OS: HR 0.62 [0.46-0.83]). However, in subgroup analyses, this effect was limited to the CIT cohort (PFS: HR 0.68 [0.56-0.84]; TTNT: HR 0.54 [0.41-0.70]; OS: HR 0.59 [0.44-0.81]) and not observed in the TT cohort (PFS: HR 0.70 [0.42-1.16]; TTNT: HR 0.62 [0.28-1.36]; OS: HR 0.81 [0.31-2.13]).
Subset analyses of the four major stereotyped groups #1 (n=96), #2 (n=160), #4 (n=26), and #8 (n=31) showed heterogeneous prognoses, with subset #8 patients showing the poorest as compared to subset #4 showing the most favorable PFS (HR 5.48 [2.47-12.13], fig. 1C). Consistent PFS patterns were observed in both treatment cohorts (CIT: HR 4.03 [1.62-10.05]; TT: HR 14.94 [2.93-76.25]).

### Summary/Conclusion

In this large series derived from frontline, prospective clinical trials, IGHV mutational status retained prognostic significance across treatment eras. Unmutated IGHV was consistently associated with shorter PFS, TTNT, and OS. These data underscore IGHV mutational status as a robust, prognostic marker and support its continued use for risk stratification in CLL clinical practice and trial design.

**Copyright Information:** (Online) ISSN: 2572-9241
©2026 The Author(s). HemaSphere is published by John Wiley & Sons Ltd on behalf of European Hematology Association. This is an open access Abstract Book distributed under the Attribution-NonCommercial-NoDerivs (CC BY-NC-ND), which allows third parties to download the articles and share them with others as long as they credit the author and the Abstract Book, but they cannot change the content in any way or use them commercially.
**Abstract Book Citations:** Authors, Title, HemaSphere, 2026;10:(S1):pages. The abstract book DOIs can be found on the "Author Information" page.
**Disclaimer:** Articles published in the journal HemaSphere exclusively reflect the opinions of the authors. The authors are responsible for all content in their abstracts including accuracy of the facts, statements, citing resources, etc.

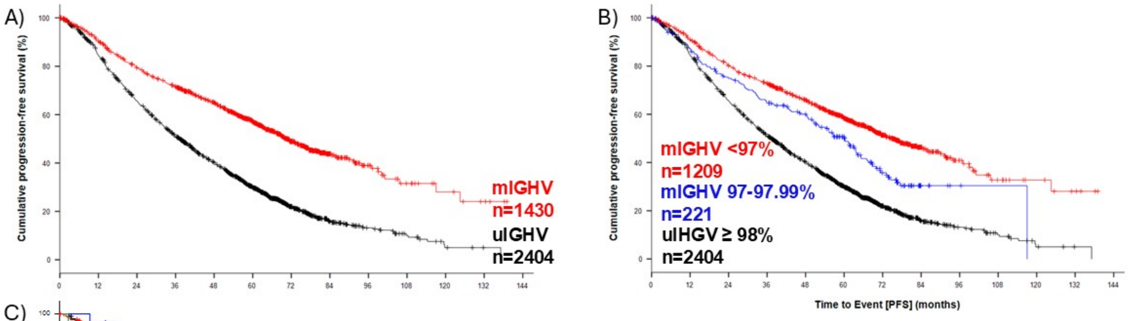

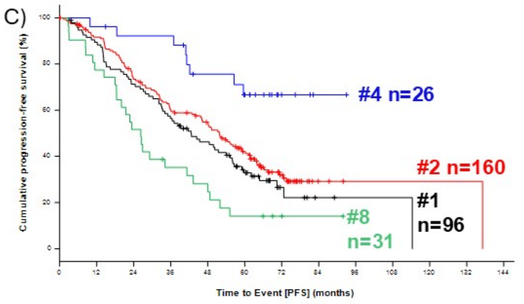

Figure 1: Progression free survival frontline clinical trial cohort (n=3834)

a) according to mutated- or unmutated-IGHV status
b) mutated IGHV separated in two groups <97% homology and 97-97.99% homology
c) according to stereotyped subsets

**Copyright Information:** (Online) ISSN: 2572-9241
©2026 The Author(s). HemaSphere is published by John Wiley & Sons Ltd on behalf of European Hematology Association. This is an open access Abstract Book distributed under the Attribution-NonCommercial-NoDerivs (CC BY-NC-ND), which allows third parties to download the articles and share them with others as long as they credit the author and the Abstract Book, but they cannot change the content in any way or use them commercially.
**Abstract Book Citations:** Authors, Title, HemaSphere, 2026;10:(S1):pages. The abstract book DOIs can be found on the “Author Information” page.
**Disclaimer:** Articles published in the journal HemaSphere exclusively reflect the opinions of the authors. The authors are responsible for all content in their abstracts including accuracy of the facts, statements, citing resources, etc.

### (PF617) FIXED-DURATION IBRUTINIB PLUS VENETOCLAX IN FRONTLINE CLL/SLL: UPDATED REAL-WORLD OUTCOMES FROM THE SPANISH LI+VE STUDY

**Topic:** 06. Chronic lymphocytic leukemia and related disorders - Clinical

José-Angel Hernández-Rivas*[1], Ana Muntañola Prat[2], Esther Sancho Ponce[3], Ángeles Medina[4], Rafael Lluch García[5], Ana Carla De Oliveira[6], Alicia Rodriguez Fernandez[7], Joan Alfons Soler Campos[8], Maria Jose Terol Castera[9], Patricia MacHado-MacHado[10], Manuel Mateo Perez Encinas[11], Rafael Romero Carmona[12], Lucrecia Yáñez[13], Paloma García Martín[14], Clara Mompo[15], Celia Bolumburu Camara[15], Elena Zubillaga Marbán[15]

*[1]Hospital Universitario Infanta Leonor, Madrid, Spain; [2]Hospital De La Santa Creu I Sant Pau, Barcelona, Spain; [3]Hospital General de Granollers, Barcelona, Spain; [4]Hospital Universitario Costa del Sol, Málaga, Spain; [5]Hospital Universitario de la Ribera, Valencia, Spain; [6]Instituto Catalán de Oncología (ICO) Hospitalet, Barcelona, Spain; [7]Hospital Universitario Virgen Macarena, Sevilla, Spain; [8]Hospital Universitario Parc Taulí, Barcelona, Spain; [9]Hospital Clínico Universitario de Valencia, Va, Spain; [10]Hospital Universitario de Canarias, Santa Cruz de Tenerife, Spain; [11]Hospital Clínico Universitario de Santiago, Santiago de Compostela, Spain; [12]Hospital Universitario Montecelo, Pontevedra, Spain; [13]Hospital Universitario Marqués de Valdecilla, Cantabria, Spain; [14]Hospital Universitario Clínico San Cecilio, Granada, Spain; [15]Janssen-Cilag, S.A., Madrid, Spain;*

### Background

Ibrutinib plus venetoclax (I+V) is the first fixed-duration (FDT), once-daily, oral, chemotherapy-free regimen approved by EMA for first-line (1L) treatment of chronic lymphocytic leukemia/small lymphocytic lymphoma (CLL/SLL). Real-world evidence (RWE) is emerging, but longer follow-up is needed.

### Aims

LI+VE study provided the first global RWE on I+V FDT, representing the dataset with the longest follow-up to date.

### Methods

LI+VE is a multicenter, ambispective, observational study in Spain evaluating I+V with retrospective baseline data and 2-year follow-up. This 1-year interim analysis summarizes baseline characteristics, management, effectiveness, and tolerability. Data shown as n/N (n=pts with available data; N=total pts).

**Copyright Information:** (Online) ISSN: 2572-9241
©2026 The Author(s). HemaSphere is published by John Wiley & Sons Ltd on behalf of European Hematology Association. This is an open access Abstract Book distributed under the Attribution-NonCommercial-NoDerivs (CC BY-NC-ND), which allows third parties to download the articles and share them with others as long as they credit the author and the Abstract Book, but they cannot change the content in any way or use them commercially.
**Abstract Book Citations:** Authors, Title, HemaSphere, 2026;10:(S1):pages. The abstract book DOIs can be found on the "Author Information" page.
**Disclaimer:** Articles published in the journal HemaSphere exclusively reflect the opinions of the authors. The authors are responsible for all content in their abstracts including accuracy of the facts, statements, citing resources, etc.

**Results**

At cut-off date (22/10/2025), 93 pts were included (CLL: 88.0%, SLL: 12.0%). Median age was 63 years (range: 41-84) (29.0% ≥70 years) 67.7% were male, 80.5% had Rai stage 0–II, and 97.0% had an ECOG 0–1. Cardiovascular (CV) risk factors were present in 70.3%, mainly hypertension (60.9%). 58.2% were at medium/high CV risk. Comorbidities were reported in 65.2% of which 51.7% had ≥2. IGHV status was unmutated in 59.8% of pts and del(17p)/*TP53* mutations were reported in 5.5%.
At the time of the analysis 65/93 (69.9%) pts had completed visit 3 and 62/93 (66.7%) had completed I+V FDT with a median time on treatment of 14.4 months. Of these, 3 have started a 2L (2 for Richter's transformation-RT). V ramp-up was completed in 83/87 (95.4%) (only 10 pts required hospitalization, for tumor lysis syndrome (TLS) prophylaxis/premedication). No TLS cases were reported.
After a median follow-up of 21.2 months, among 79 pts with data, 56 (70.9%) showed complete response, 21 (26.6%) partial response, 1 (1.3%) stable disease, and 1 pt progressed. The 30-months OS and PFS rates were 97.4% and 94.5%, respectively.
The most common all-grade (G) adverse events (AEs) were diarrhea (34.4%), neutropenia (30.1%), and upper respiratory tract infection (19.4%). AEs were G1-2 in most pts (86.0%) with neutropenia being the most common G≥3 AE (16.1%). G-CSF was used in 28.9% of pts. Cardiac events were infrequent (**Table 1**). Two deaths occurred (intestinal sepsis; unknown cause), both unrelated to treatment.
Dose reductions occurred in 3/93 pts (3.2%) for I - due to thrombocytopenia, neutropenia and atrial fibrillation and in 13 pts (14.0%) for V, mainly for diarrhea (6). AEs leading to dose modification resolved or improved in all cases for I and in 85.7% for V. Treatment was temporarily interrupted in 39/93 (41.9%) (I in 27, V in 14 and both in 14 cases), mainly due to neutropenia (23.1%) and infections (12.8%). All AEs leading to treatment interruptions were resolved (59.0%) or partially resolved (15.4%); 46.2% were not toxicity related. Treatment was permanently discontinued in 7/93 pts [(7,5%; RT, glioblastoma, intestinal perforation, pyoderma gangrenosum, hypertransaminasemia, death, and 1 sequential I→V stop (lymphocytosis and palpitations G1)], none due to CV toxicity.

**Summary/Conclusion**

LI+VE observational study reinforces that FD I+V is effective and well-tolerated in clinical practice, including elderly and comorbid patients. With THRIVE and REALITY studies, the cumulative real-world experience adds clinically relevant information to guide management and may help inform decision-making in clinical practice. I+V is the only all-oral FDT with clinical-trial and real-world evidence across all patient profiles.

**Copyright Information:** (Online) ISSN: 2572-9241
©2026 The Author(s). HemaSphere is published by John Wiley & Sons Ltd on behalf of European Hematology Association. This is an open access Abstract Book distributed under the Attribution-NonCommercial-NoDerivs (CC BY-NC-ND), which allows third parties to download the articles and share them with others as long as they credit the author and the Abstract Book, but they cannot change the content in any way or use them commercially.
**Abstract Book Citations:** Authors, Title, HemaSphere, 2026;10:(S1):pages. The abstract book DOIs can be found on the "Author Information" page.
**Disclaimer:** Articles published in the journal HemaSphere exclusively reflect the opinions of the authors. The authors are responsible for all content in their abstracts including accuracy of the facts, statements, citing resources, etc.

**Table 1. Adverse events in patients treated with ibrutinib + venetoclax (N=93)**

| AEs | Any grade | Grade =3 |
|---|---|---|
| **Patients with any AE**, n (%) | 85 (91.4) | 35 (37.6) |
| **Most common AEs (=5%)**, n (%) | | |
| Diarrhea | 32 (34.4) | 3 (3.2) |
| Neutropenia | 28 (30.1) | 15 (16.1) |
| Upper respiratory tract infection | 18 (19.4) | 2 (2.2) |
| Arthralgia | 13 (14.0) | 0 (0.0) |
| Bleeding (including contusion) | 12 (12.9) | 1 (1.1) |
| Thrombocytopenia | 12 (12.9) | 2 (2.2) |
| Nausea | 9 (9.7) | 0 (0.0) |
| Asthenia | 7 (7.5) | 0 (0.0) |
| Fatigue | 7 (7.5) | 1 (1.1) |
| Headache | 6 (6.5) | 0 (0.0) |
| Hypertension | 6 (6.5) | 1 (1.1) |
| Lymphocytosis | 5 (5.4) | 2 (2.2) |
| Rash | 5 (5.4) | 1 (1.1) |
| Aphthous ulcer | 5 (5.4) | 0 (0.0) |
| Constipation | 5 (5.4) | 0 (0.0) |
| Urinary tract infection | 6 (6.5) | 1 (1.1) |
| Skin infection | 5 (5.4) | 0 (0.0) |
| **Other AEs of clinical interest**, n (%) | | |
| Atrial fibrillation | 3 (3.2) | 0 (0.0) |
| Arrhythmia | 1 (1.1) | 1 (1.1) |
| Palpitations | 1 (1.1) | 0 (0.0) |
| TLS | 0 (0.0) | 0 (0.0) |
| Second primary malignancy | 6 (6.5) | 2 (2.2) |
| **SAEs**, n (%) | 23 (24.7) | 19 (20.4) |

Data expressed as n (%) AE, adverse event; TLS, tumor lysis syndrome; SAE; severe adverse event

**Copyright Information:** (Online) ISSN: 2572-9241
©2026 The Author(s). HemaSphere is published by John Wiley & Sons Ltd on behalf of European Hematology Association. This is an open access Abstract Book distributed under the Attribution-NonCommercial-NoDerivs (CC BY-NC-ND), which allows third parties to download the articles and share them with others as long as they credit the author and the Abstract Book, but they cannot change the content in any way or use them commercially.
**Abstract Book Citations:** Authors, Title, HemaSphere, 2026;10:(S1):pages. The abstract book DOIs can be found on the "Author Information" page.
**Disclaimer:** Articles published in the journal HemaSphere exclusively reflect the opinions of the authors. The authors are responsible for all content in their abstracts including accuracy of the facts, statements, citing resources, etc.

### (PF618) REAL-WORLD ZANUBRUTINIB TREATMENT PATTERNS IN CLL/SLL AMONG US COMMUNITY ONCOLOGY PATIENTS WITH PRIOR ACALABRUTINIB THERAPY

**Topic:** 06. Chronic lymphocytic leukemia and related disorders - Clinical

Jing-Zhou Hou[1], Rushir Choksi*[1], Gregory Maglinte[2], Xiaoliang Wang[2], Anna Rui[3], Manasi Suryavanshi[2], Beverly Chigarira[3], Lindsay Aton[3], Melissa Hagan[2], Lisa Morere[3], G. Rhys Williams[2], Daniel Ermann[4]

*[1]University of Pittsburgh Medical Center, Pittsburgh, PA, United States of America;[2]BeOne Medicines Ltd, San Carlos, CA, United States of America; [3]IntegraConnect PrecisionQ, West Palm Beach, FL, United States of America;[4]University of Utah Huntsman Cancer Institute, Salt Lake City, UT, United States of America;*

### Background

Bruton tyrosine kinase inhibitors (BTKis) have demonstrated clinical efficacy in treating chronic lymphocytic leukemia/small lymphocytic lymphoma (CLL/SLL). Zanubrutinib (zanu) is a highly specific and potent next-generation BTKi designed to maximize BTK occupancy and limit off-target kinase binding. Recent clinical data suggest that patients with CLL who were intolerant of ibrutinib (ibru) or acalabrutinib (acala) and switched to zanu experienced continued or improved clinical benefit (Shadman *Blood Adv.* 2025). Additionally, most of the ibru and acala intolerance adverse events did not recur during zanu treatment. There is limited real-world evidence describing the patient characteristics and treatment journey for patients with CLL/SLL who initiate acala and subsequently receive zanu (Krackeler *Cancer Medicine* 2025; Warner *Blood* [supplement] 2025).

### Aims

This study describes the characteristics, treatment patterns, and reasons for discontinuation among patients who had previously discontinued acala and changed to zanu in a real-world setting.

### Methods

The IntegraConnect PrecisionQ database, which contains electronic health records from >3 million de-identified cancer patients across >500 care sites, was used to create a retrospective cohort of CLL/SLL patients who initiated and discontinued acala and then initiated zanu between 11/21/2019 and 11/30/2024. Patients were followed through 2/28/2025. A medical record review was conducted to identify dates of treatment initiation and discontinuation and reasons for acala and zanu discontinuation.

### Results

A total of 806 patients initiated acala and subsequently received another therapy; of these, 121 patients subsequently received zanu; 102 (84.3%) changed directly from acala to zanu, and 19 (15.7%) changed from acala to another therapy before initiating zanu. The majority of patients received prior acala monotherapy (n=111, 91.7%). Of the 102 patients who changed directly from acala to zanu, 74 (72.6%) received acala in the first line of therapy after the start date and 28 (27.5%) received acala in the second line or later. The median duration of acala therapy prior to zanu treatment was 5.6 months (interquartile range [IQR]: 2.1, 16.5). The median duration of zanu therapy after having received acala was 10.7 months (IQR: 4.1, 18.8), and 59 (57.8%) patients remained on zanu at the end of the follow-up period. Overall, 53 (52.0%) patients discontinued acala within 6 months and 68 (66.7%) discontinued within 1 year. The reasons for acala discontinuation were toxicity (68.6%), other (11.8%), or disease progression (6.9%). A total of 43 (42.2%) patients who changed from acala to zanu discontinued zanu at the time of data cutoff. The reasons for zanu discontinuation were toxicity (58.1%), other (7.0%), or disease progression (2.3%).

**Copyright Information:** (Online) ISSN: 2572-9241
©2026 The Author(s). HemaSphere is published by John Wiley & Sons Ltd on behalf of European Hematology Association. This is an open access Abstract Book distributed under the Attribution-NonCommercial-NoDerivs (CC BY-NC-ND), which allows third parties to download the articles and share them with others as long as they credit the author and the Abstract Book, but they cannot change the content in any way or use them commercially.
**Abstract Book Citations:** Authors, Title, HemaSphere, 2026;10:(S1):pages. The abstract book DOIs can be found on the "Author Information" page.
**Disclaimer:** Articles published in the journal HemaSphere exclusively reflect the opinions of the authors. The authors are responsible for all content in their abstracts including accuracy of the facts, statements, citing resources, etc.

**Summary/Conclusion**

In this real-world US community oncology setting, most zanu-treated patients with prior acala therapy had discontinued acala within 1 year. The primary reason for acala discontinuation was toxicity. Consistent with previous research, real-world data from across the US have demonstrated that zanu was well tolerated and maintained effectiveness in patients with CLL who had received a prior BTKi.

**Copyright Information:** (Online) ISSN: 2572-9241
©2026 The Author(s). HemaSphere is published by John Wiley & Sons Ltd on behalf of European Hematology Association. This is an open access Abstract Book distributed under the Attribution-NonCommercial-NoDerivs (CC BY-NC-ND), which allows third parties to download the articles and share them with others as long as they credit the author and the Abstract Book, but they cannot change the content in any way or use them commercially.
**Abstract Book Citations:** Authors, Title, HemaSphere, 2026;10:(S1):pages. The abstract book DOIs can be found on the "Author Information" page.
**Disclaimer:** Articles published in the journal HemaSphere exclusively reflect the opinions of the authors. The authors are responsible for all content in their abstracts including accuracy of the facts, statements, citing resources, etc.

### (PF619) AUTOIMMUNE COMPLICATIONS IN CHRONIC LYMPHOCYTIC LEUKAEMIA – A SINGLE INSTITUTION STUDY OVER 30 YEARS

**Topic:** 06. Chronic lymphocytic leukemia and related disorders - Clinical

Jasmine Ong[1], Yandong Shen[2], Ian Kerridge[3], Luke Coyle[3], Naomi MacKinley[3], Christopher Ward[3], Keith Fay[3], Matthew Greenwood[3], Grace Gifford[3], William Stevenson[3], David Kliman[3], Stephen Mulligan*[3]

*[1]University of Sydney, Camperdown, Australia;[2]Royal North Shore Hospital, Sydney, Australia;[3]Royal North Shore Hospital, Haematology, Sydney, Australia;*

### Background

The incidence of autoimmune complications (AICs) in chronic lymphocytic leukaemia (CLL), especially haematological autoimmune cytopenias (Haem-AICs) such as autoimmune haemolytic anaemia (AIHA), immune thrombocytopenia (ITP), autoimmune neutropenia (AIN) and pure red cell aplasia (PRCA) vary widely, with reported incidences of 5-25%. Many series are reported from clinical trials and collaborative groups with ascertainment bias for those requiring therapy, leading to a paucity of data for those whose CLL never requires treatment.

### Aims

Ascertaining an accurate, population-based incidence of Haem-AICs in CLL, and characterising those who developed Haem-AICs.

### Methods

We performed a retrospective single-institution cohort study with a geographically localised referral base.

**Copyright Information:** (Online) ISSN: 2572-9241
©2026 The Author(s). HemaSphere is published by John Wiley & Sons Ltd on behalf of European Hematology Association. This is an open access Abstract Book distributed under the Attribution-NonCommercial-NoDerivs (CC BY-NC-ND), which allows third parties to download the articles and share them with others as long as they credit the author and the Abstract Book, but they cannot change the content in any way or use them commercially.
**Abstract Book Citations:** Authors, Title, HemaSphere, 2026;10:(S1):pages. The abstract book DOIs can be found on the "Author Information" page.
**Disclaimer:** Articles published in the journal HemaSphere exclusively reflect the opinions of the authors. The authors are responsible for all content in their abstracts including accuracy of the facts, statements, citing resources, etc.

### Results

A total of 487 CLL patients with a minimum 3 years sequential annual follow-up were individually analysed from 1995-2025, of whom 78 (16.0%) developed a Haem-AIC. These were 51.3% AIHA (47.4% warm antibody {Ab}; 3.8% cold Ab); 51.3% ITP (42.3% severe {<50}; 9.0% mild {50-100}); 11.5% AIN, and 6.4% PRCA (some patients had more than 1 AIC). An additional 80 patients (17.9%) had ≥1 positive DAT with no AIHA. The average time from diagnosis of CLL to AIC was for AIHA 7.3 years (yrs) (range 0-28), severe ITP 6.5 yrs (0-27), AIN 4.9 yrs (0-19), and PRCA 9.5 yrs (1-17).
Overall, 47.0% of all CLL patients were hypogammaglobulinemic (HYPOG) (IgG<6 g/L) at some point in their disease course, compared to 44.3% of patients who never developed Haem-AICs, and 61.5% of patients who did develop Haem-AICs with 72.5% in AIHA; 52.5% ITP; 66.7% AIN, and 80.0% PRCA. The average IgM and IgG levels at CLL diagnosis were 0.69 and 8.84 g/L respectively in those without Haem-AICs, compared to 0.46 and 8.19 g/L with Haem-AICs. IgG nadirs averaged 6.41 and 5.30 g/L without and with Haem-AICs respectively.
Overall, 82.0% of the patients who developed Haem-AICs required treatment for their AIC. Steroids ± intravenous immunoglobulin (IVIg) alone provided clinical control for 18 Haem-AIC patients (23.1%), with 10 AIHA (55.6%), 10 severe ITP (55.6%), 1 AIN (5.6%), and 2 PRCA (11.1%).
Within the overall cohort, 47.4% of patients never required CLL-directed treatment, compared to only 28.2% of Haem-AICs. By AIC type, no CLL-directed therapy was required for 20.0% of AIHA; 40.0% of ITP; 22.2% of AIN, and 20.0% of PRCA. Of the Haem-AICs who did receive CLL-directed therapy, development of the Haem-AIC was mostly unrelated to therapy (occurring prior to, or >6 months after cessation of therapy). Overall, 37.3% of Haem-AICs developed the AIC prior to CLL-directed treatment, and 25.4% >6 months after (Figure 1). There was no correlation between CLL-directed alkylating agent, fludarabine-based immunochemotherapy, or targeted therapies and Haem-AIC development.

### Summary/Conclusion

A significant proportion (28.2%) with indolent CLL who never receive CLL-directed therapy develop a Haem-AIC. Although most patients with Haem-AICs have progressive CLL, AIC events occurred mostly prior to, or >6 months after CLL-directed therapy. AIHA and ITP had the same incidence, representing a higher incidence of ITP than typically reported. Patients who developed Haem-AICs had lower levels of both IgM and IgG at CLL diagnosis, with lower IgG nadirs than those without, consistent with Haem-AICs as a manifestation of their CLL-related immunological dysfunction.

**Copyright Information:** (Online) ISSN: 2572-9241
©2026 The Author(s). HemaSphere is published by John Wiley & Sons Ltd on behalf of European Hematology Association. This is an open access Abstract Book distributed under the Attribution-NonCommercial-NoDerivs (CC BY-NC-ND), which allows third parties to download the articles and share them with others as long as they credit the author and the Abstract Book, but they cannot change the content in any way or use them commercially.
**Abstract Book Citations:** Authors, Title, HemaSphere, 2026;10:(S1):pages. The abstract book DOIs can be found on the "Author Information" page.
**Disclaimer:** Articles published in the journal HemaSphere exclusively reflect the opinions of the authors. The authors are responsible for all content in their abstracts including accuracy of the facts, statements, citing resources, etc.

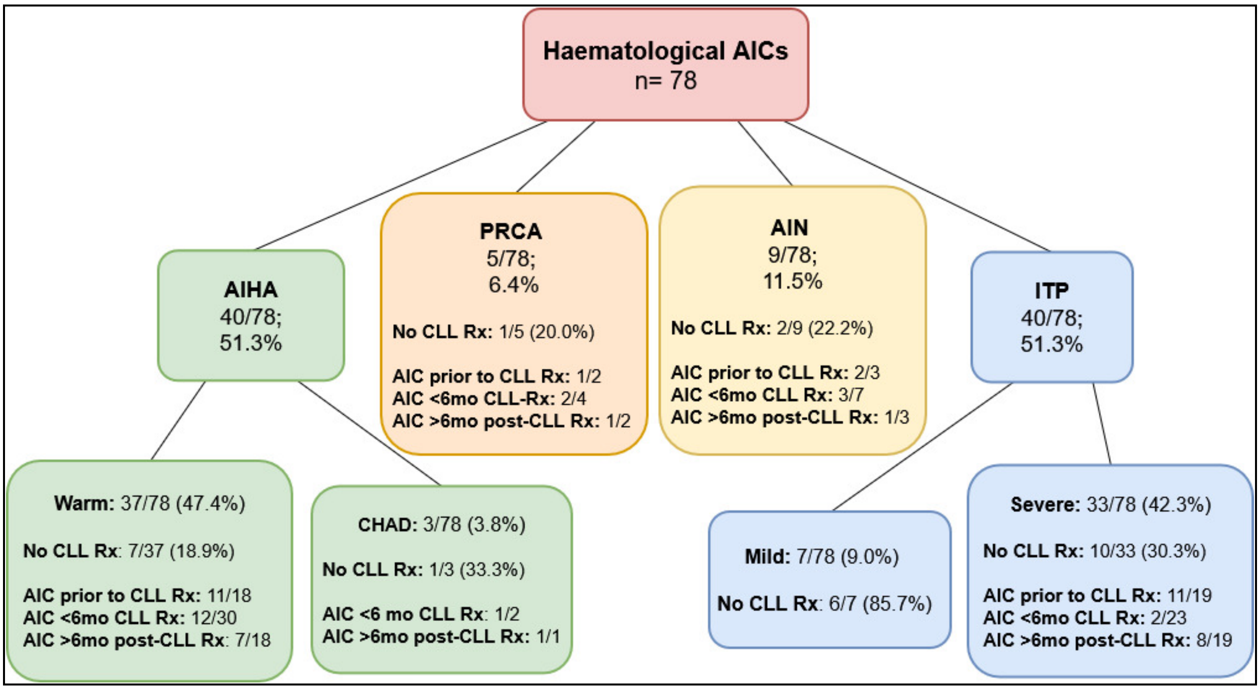

CLL Haematological Autoimmune Complications incidence & characteristics

**Copyright Information:** (Online) ISSN: 2572-9241
©2026 The Author(s). HemaSphere is published by John Wiley & Sons Ltd on behalf of European Hematology Association. This is an open access Abstract Book distributed under the Attribution-NonCommercial-NoDerivs (CC BY-NC-ND), which allows third parties to download the articles and share them with others as long as they credit the author and the Abstract Book, but they cannot change the content in any way or use them commercially.
**Abstract Book Citations:** Authors, Title, HemaSphere, 2026;10:(S1):pages. The abstract book DOIs can be found on the "Author Information" page.
**Disclaimer:** Articles published in the journal HemaSphere exclusively reflect the opinions of the authors. The authors are responsible for all content in their abstracts including accuracy of the facts, statements, citing resources, etc.

### (PF620) EARLY PREDICTION OF NILOTINIB RESPONSE IN CHRONIC MYELOID LEUKEMIA BY DRUG PLASMA CONCENTRATION AND SINGLE-CELL SIGNALING PROFILING

**Topic:** 07. Chronic myeloid leukemia - Biology & translational research

Stein-Erik Gullaksen*[1, 2, 3], Christoph Seger[4, 5], Jørn Skavland[3], Sieghart Sopper[6], Mathias Brevik[2, 3, 7], Stine Haugstvedt[1, 2, 3], Andrea Griesmacher[8], Leif Stenke[9], Jesper Stentoft[10], Satu Mustjoki[11, 12], Henrik Hjorth-Hansen[13], Andreas Hochhaus[14], Dominik Wolf[6], Bjørn T. Gjertsen[1, 2, 3]

*[1]Haukeland University Hospital, Department of medicine, Bergen, Norway;[2]University of Bergen, K.G. Jebsen Centre for Myeloid Blood Cancer CMYC, Department of Clinical Science, Bergen, Norway; [3]University of Bergen, Centre of Cancer Biomarkers CCBIO, Department of Clinical Science, Bergen, Norway; [4]University of Innsbruck, Institute of Pharmacy/Pharmacognosy, CCB-Centrum of Chemistry and Biomedicine, Innsbruck, Austria; [5]Labordiagnostic St. Gallen West AG, St. Gallen, Switzerland; [6]Innsbruck Medical University, Internal Medicine V, Hematology and Oncology, Innsbruck, Austria; [7]University of Bergen, Department of Chemistry, Bergen, Norway; [8]Innsbruck University Hospital, Central Institute for Medical and Chemical Laboratory Diagnosis, Innsbruck, Austria; [9]Karolinska Institutet and Karolinska University Hospital, Stockholm, Sweden; [10]Aarhus University Hospital, Department of Hematology, Aarhus, Denmark; [11]University of Helsinki, Translational Immunology Research Program and Department of Clinical Chemistry and Hematology, Helsinki, Finland; [12]University of Helsinki and Helsinki University Hospital Comprehensive Cancer Center, Hematology Research Unit Helsinki, Helsinki, Finland; [13]Norwegian University of Science and Technology, University IKOM, Trondheim, Norway; [14]Jena University Hospital, Jena, Germany;*

### Background

Approximately 40% of patients with chronic myeloid leukemia (CML) are required to switch TKI due to insufficient efficacy or intolerance. Evaluation of response is performed months after starting treatment, leaving non-responders vulnerable to ineffective treatments with harmful side effects. Early identification of suboptimal responders could enable timely dose optimization or TKI switching, improving outcomes and reducing toxicity.

### Aims

To investigate whether nilotinib pharmacokinetics and pharmacodynamics, assessed by drug plasma concentration and single-cell signaling profiling of peripheral blood leukocytes, can predict long-term molecular response within days of treatment initiation.

### Methods

We analyzed nilotinib plasma concentration by LC-MS in samples collected from 54 chronic phase CML patients in the ENEST1st clinical trial (NCT01061177) treated with frontline nilotinib (300 mg BID) as shown in **Fig-1a**. BCR::ABL1-related signaling was assessed in blood leukocytes by Flow cytometry (18 phosphoproteins and 5 cell surface markers) and mass cytometry (15 intracellular targets and 29 cell surface markers) at baseline, 3 hours, and days 7 and 28. We focused on modulation of BCR::ABL1-related signaling from before to after 7 days of nilotinib.

**Copyright Information:** (Online) ISSN: 2572-9241
©2026 The Author(s). HemaSphere is published by John Wiley & Sons Ltd on behalf of European Hematology Association. This is an open access Abstract Book distributed under the Attribution-NonCommercial-NoDerivs (CC BY-NC-ND), which allows third parties to download the articles and share them with others as long as they credit the author and the Abstract Book, but they cannot change the content in any way or use them commercially.
**Abstract Book Citations:** Authors, Title, HemaSphere, 2026;10:(S1):pages. The abstract book DOIs can be found on the "Author Information" page.
**Disclaimer:** Articles published in the journal HemaSphere exclusively reflect the opinions of the authors. The authors are responsible for all content in their abstracts including accuracy of the facts, statements, citing resources, etc.

### Results

Nilotinib plasma concentration reached a median peak of 405.7 ng/ml at 3 hours, reaching a plateau after 6 to 12 months of treatment at ~1000 ng/ml, but with substational variation between paients. We found that the nilotinib trough concentration after 3 months is negatively correlated with BCR::ABL$^{IS}$ levels the first 18-21 months of treatment (**Fig-1b**). The concentration of nilotinib was significantly higher in patients achieving MR4 (wilcoxon, p=0.00689). Compared to monocytes and lymphocytes, the population of predominantly mature and immature neutrophils (CD163$^{neg}$CD5$^{neg}$CD45$^{dim}$SSC$^{high}$) showed the most profound nilotinib-induced signaling inhibition, with significant downregulation of pAbl1 Y245, pGab2 Y452, pS6 S240/244, and pSTAT5 Y694 after 7 days. In this population, changes in pSTAT3 Y705, pSTAT3 S727, and pS6 S235/236 after 7 days of nilotinib were negatively correlated with long-term BCR::ABL1$^{IS}$ (**Fig-1c**). Furthermore, longitudinal analysis showed distinct signaling trajectories between responder groups, with a delayed increase in the patients without MR4. We collected mass cytometry data from the same set of PB samples. After single cell gating we identified all immune cell populations present in PB, including various maturation states of neutrophils, basophils and CD34$^{+}$ stem and progenitor-like cells. Integrative analysis linking these high-dimensional single-cell profiles to molecular responses is ongoing.

### Summary/Conclusion

Combined PK/PD analysis enables early prediction of nilotinib response in CML. Three-month nilotinib plasma concentration stratifies patients by molecular outcome, while single-cell signaling changes at day 7, particularly pSTAT3 and pS6, predict deep molecular response. Mass cytometry profiling of the same samples has resolved the full peripheral blood immune landscape at high resolution, and integrative analysis is ongoing. These findings support the feasibility of early pharmacological assessment to guide personalized TKI dosing and therapy optimization.

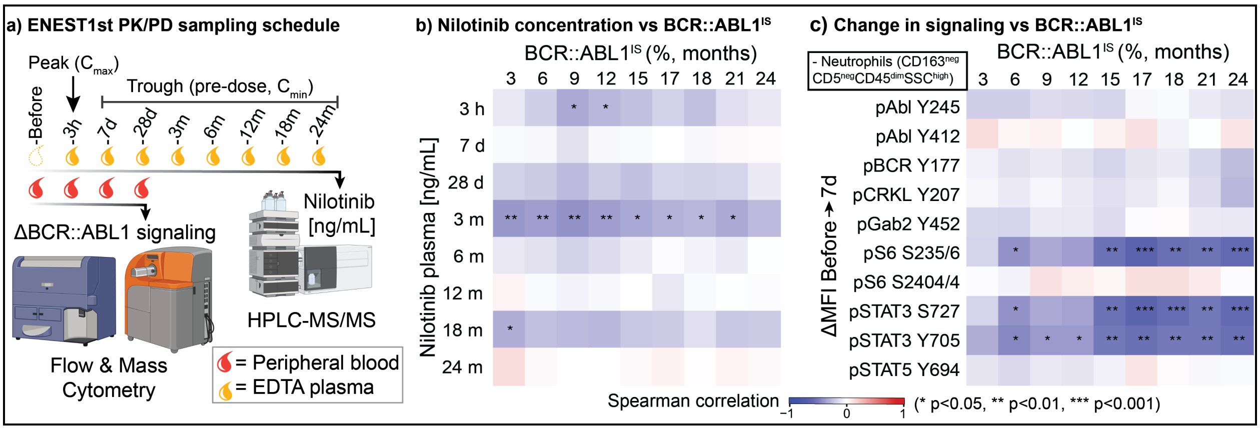

Figure 1- Pharmacokinetics and dynamics correlate with BCR::ABLIS. a) Sampling schedule for patients in the PD/PK sub-study of the ENEST1st clinical trial. b) Spearman correlation between nilotinib plasma concentration and BCR::ABL1IS across all measured time points. c) Spearman correlation of change in the MFI of BCR::ABL1-related signaling after 7 days of nilotinib treatment in neutrophils (CD163negCD5negCD45dimSSChigh) and BCR::ABL1IS. h = hours; d = days; m = months; IS = International Scale.

**Copyright Information:** (Online) ISSN: 2572-9241
©2026 The Author(s). HemaSphere is published by John Wiley & Sons Ltd on behalf of European Hematology Association. This is an open access Abstract Book distributed under the Attribution-NonCommercial-NoDerivs (CC BY-NC-ND), which allows third parties to download the articles and share them with others as long as they credit the author and the Abstract Book, but they cannot change the content in any way or use them commercially.
**Abstract Book Citations:** Authors, Title, HemaSphere, 2026;10:(S1):pages. The abstract book DOIs can be found on the "Author Information" page.
**Disclaimer:** Articles published in the journal HemaSphere exclusively reflect the opinions of the authors. The authors are responsible for all content in their abstracts including accuracy of the facts, statements, citing resources, etc.

### (PF621) EMERGENCE OF HIGHLY ASCIMINIB-RESISTANT CML VIA MINOR BCR::ABL1 SWITCH AND MUTATIONS

**Topic:** 07. Chronic myeloid leukemia - Biology & translational research

Barbora Štefíková*[1, 2], Nikola Čuřík[1, 3], Jitka Křížková[1], Vaclava Polivkova[1], Adela Benesova[1], Pavla Suchankova[1], Katerina Rajnisova[1], Adam Laznicka[1, 3, 4], Sarka Ransdorfova[1], Daniela Kužílková[4, 5], Adela Vavrova[4, 5], Tomas Kalina[4, 5], Katerina MacHova Polakova[1, 3]

*[1]Institute of Hematology and Blood Transfusion, Department of Molecular Genetics, Prague, Czechia;[2]First Faculty of Medicine, Charles University, Prague, Czechia; [3]Institute of Pathological Physiology, First Faculty of Medicine, Charles University, Prague, Czechia; [4]Second Faculty of Medicine, Charles University, Prague, Czechia;[5]CLIP (Childhood Leukaemia Investigation Prague), Second Faculty of Medicine, Charles University, Department of Pediatric Haematology and Oncology, Prague, Czechia;*

### Background

Asciminib is a next-generation, highly selective inhibitor of BCR::ABL1 and has emerged as a promising therapy for chronic myeloid leukemia (CML). However, data on resistance development following prior TKI failure are still limited, and strategies to prevent disease progression under asciminib treatment remain to be defined.

### Aims

This work aimed to assess the efficacy of clinically available drugs following resistance development during sequential TKI treatment with first-line imatinib followed by asciminib.

### Methods

Cross-resistant imatinib/asciminib (IM/A-R) clones were generated from the CML myeloid blast cell line KCL-22. Two imatinib-resistant clones harbouring BCR::ABL1 mutations T315I and Y253H were single-cell sorted and exposed to escalating asciminib concentrations up to 1000 nM. Three T315I-derived and four Y253H-derived clones proliferated at the highest concentration. Mutational status was analyzed using next-generation sequencing (NGS) and whole-exome sequencing (WES). Protein levels were evaluated by mass cytometry (CyTOF). Drug sensitivity and $IC_{50}$ values were determined by flow cytometry and WST assays.

### Results

All IM/A-R clones remained sensitive to ponatinib (median $IC_{50}$ 2.59 nM, range 1.78–5.49 nM), whereas most clones were resistant to venetoclax. The highest venetoclax resistance ($IC_{50}$ 9.7 µM) was observed in an Y253H-derived clone, in which the Y253H mutation progressively declined during asciminib exposure and became undetectable by major BCR::ABL1 NGS analysis at 1000 nM. WES identified newly acquired mutations in SETD2 (p.D1169N), NOTCH1 (p.R2327Q, p.Q1722X), and CREBBP (p.Q2342H, p.G1028R). WES also detected ABL1 mutations Y253H and P465Q, discordant with the negative NGS BCR::ABL1 analysis. Cytogenetic analysis revealed the presence of three BCR::ABL1 fusions in this clone, one of them with the minor rearrangement. Mutational analysis was subsequently performed on the minor (e1a2) transcript. This revealed a striking transcriptional switch, with the minor BCR::ABL1 transcript increasing from 0.05% to 100%, accompanied by the emergence of Y253H and P465Q mutations reaching 100% variant allele frequency at the 1000 nM asciminib. Expression of the major BCR::ABL1 transcript remained stable. CyTOF demonstrated reduced STAT5 signalling and markedly decreased BCL2 and BIM expression. Despite venetoclax resistance, combined treatment with ponatinib (0.1 nM) and venetoclax (1 µM) showed strong *in vitro* synergy, suggesting that inhibition of mutated p190 BCR::ABL1 and additional ponatinib targets (SRC-family kinases Lyn, Hck, and FLT3) restored sensitivity to BCL2 inhibition.

**Copyright Information:** (Online) ISSN: 2572-9241
©2026 The Author(s). HemaSphere is published by John Wiley & Sons Ltd on behalf of European Hematology Association. This is an open access Abstract Book distributed under the Attribution-NonCommercial-NoDerivs (CC BY-NC-ND), which allows third parties to download the articles and share them with others as long as they credit the author and the Abstract Book, but they cannot change the content in any way or use them commercially.
**Abstract Book Citations:** Authors, Title, HemaSphere, 2026;10:(S1):pages. The abstract book DOIs can be found on the “Author Information” page.
**Disclaimer:** Articles published in the journal HemaSphere exclusively reflect the opinions of the authors. The authors are responsible for all content in their abstracts including accuracy of the facts, statements, citing resources, etc.

**Summary/Conclusion**

We demonstrate *de novo* emergence and clonal selection of a highly asciminib-resistant CML blast clone driven by a transcriptional switch toward e1a2 BCR::ABL1 carrying at protein level Y253H and P465Q mutations. While Y253H in p210 confers asciminib resistance only as compound mutation, substantially higher resistance has been reported for solo Y253H-mutated p190, consistent with our findings (Lucini et al., Blood 2024). These results highlight the need for careful use of asciminib in patients with p190 BCR::ABL1 after previous failure on other TKI. Ponatinib retained potent activity, and its combination with venetoclax showed marked synergy, suggesting a potential therapeutic strategy for therapy-refractory CML blast phase with p190 BCR::ABL1.
**Support:** NW24-03-00056, EUTOS 2024, MH CZ – DRO (IHBT, 00023736)

**Copyright Information:** (Online) ISSN: 2572-9241
©2026 The Author(s). HemaSphere is published by John Wiley & Sons Ltd on behalf of European Hematology Association. This is an open access Abstract Book distributed under the Attribution-NonCommercial-NoDerivs (CC BY-NC-ND), which allows third parties to download the articles and share them with others as long as they credit the author and the Abstract Book, but they cannot change the content in any way or use them commercially.
**Abstract Book Citations:** Authors, Title, HemaSphere, 2026;10:(S1):pages. The abstract book DOIs can be found on the “Author Information” page.
**Disclaimer:** Articles published in the journal HemaSphere exclusively reflect the opinions of the authors. The authors are responsible for all content in their abstracts including accuracy of the facts, statements, citing resources, etc.

### (PF622) FUNCTIONAL DETECTION OF TKI RESISTANCE IN LIVING CML CELLS USING THE FRET BIOSENSOR PICKLES

**Topic:** 07. Chronic myeloid leukemia - Biology & translational research

Tatsuaki Mizutani*[1, 2], Aya Satoh[1], Mari Fujioka[2], Shizuka Sakurazawa[2], Takeshi Kondo[3], Maho Amano[2], Yusuke Ohba[1, 2]

[1]*Hokkaido University, Cell physiology, Sapporo, Japan;* [2]*Horizon Illuination Lab Optics, Co. Ltd., Sapporo, Japan;* [3]*Aiiku Hospital, Sapporo, Japan;*

### Background

Recent advances in molecularly targeted therapies, particularly BCR::ABL1 tyrosine kinase inhibitors (TKIs), have markedly improved clinical outcomes in patients with chronic phase chronic myeloid leukemia (CML-CP). However, there are currently no established criteria for selecting the most appropriate TKI for individual patients. Moreover, real-world studies in Japan show that approximately half of patients discontinue first-line TKIs due to resistance and/or intolerance. Therefore, the development of a rapid and reliable laboratory test for optimal drug selection, as well as the identification of novel therapeutic strategies for TKI-resistant CML, remains an urgent clinical need.

### Aims

We have developed an opto-diagnostic biosensor, Pickles, based on the principle of Förster resonance energy transfer (FRET). Pickles enables real-time monitoring of BCR::ABL1 kinase activity in living CML cells and may allow prediction of future TKI responses in individual patients.

### Methods

K562 CML cell lines harboring clinically relevant point mutations in the BCR::ABL1 gene (Y253F, Y253H, V299L, T315I, P465S, and Y253H/T315I) were generated using CRISPR/Cas9 genome editing. Pickles expression plasmids were introduced into each mutant cell line, and their sensitivities to six different TKIs were evaluated by opto-diagnostic analysis. In addition, mixed populations of TKI-sensitive and TKI-resistant cells at defined proportions were prepared to assess the sensitivity of detecting resistant cells. Furthermore, we applied this biosensor to primary CML-CP samples obtained from patients and evaluated its ability to predict therapeutic outcomes, including BCR::ABL1 mRNA halving time and deep molecular response (DMR).

### Results

The TKI sensitivity profiles determined by the Pickles-based opto-diagnostic assay were highly consistent with those obtained using conventional cell proliferation assays. Importantly, two distinct patterns of Y253 mutations in BCR::ABL1 (Y253F and Y253H) were clearly discriminated based on their differential sensitivity to nilotinib. In addition, treatment with high-dose ponatinib effectively reduced the FRET/CFP ratio in T315I-mutant K562 cells, indicating robust inhibition of kinase activity even in this clinically challenging mutation. Notably, Pickles enabled the detection of drug-resistant subclones at frequencies below 1% within heterogeneous cell populations, demonstrating its high sensitivity in identifying minor resistant subpopulations. Clinical evaluation using primary CML-CP samples is ongoing, and preliminary findings support the feasibility and clinical relevance of Pickles-based functional prediction.

**Copyright Information:** (Online) ISSN: 2572-9241
©2026 The Author(s). HemaSphere is published by John Wiley & Sons Ltd on behalf of European Hematology Association. This is an open access Abstract Book distributed under the Attribution-NonCommercial-NoDerivs (CC BY-NC-ND), which allows third parties to download the articles and share them with others as long as they credit the author and the Abstract Book, but they cannot change the content in any way or use them commercially.
**Abstract Book Citations:** Authors, Title, HemaSphere, 2026;10:(S1):pages. The abstract book DOIs can be found on the “Author Information” page.
**Disclaimer:** Articles published in the journal HemaSphere exclusively reflect the opinions of the authors. The authors are responsible for all content in their abstracts including accuracy of the facts, statements, citing resources, etc.

**Summary/Conclusion**

Pickles enables accurate functional assessment of TKI sensitivity in CML cells harboring various BCR::ABL1 mutations and allows highly sensitive detection of drug-resistant subclones. Importantly, this system can identify resistant cells regardless of known mutation status, highlighting its potential to uncover novel resistance mechanisms. By integrating Pickles-based functional diagnostics with live single-cell isolation followed by transcriptomic profiling, this platform may accelerate the discovery of new drug response/resistance mechanisms in CML. Furthermore, given its high predictive performance in real-world patient samples, Pickles represents a promising clinical tool for guiding both first-line and subsequent TKI selection, ultimately contributing to personalized treatment strategies in CML.

**Copyright Information:** (Online) ISSN: 2572-9241
©2026 The Author(s). HemaSphere is published by John Wiley & Sons Ltd on behalf of European Hematology Association. This is an open access Abstract Book distributed under the Attribution-NonCommercial-NoDerivs (CC BY-NC-ND), which allows third parties to download the articles and share them with others as long as they credit the author and the Abstract Book, but they cannot change the content in any way or use them commercially.
**Abstract Book Citations:** Authors, Title, HemaSphere, 2026;10:(S1):pages. The abstract book DOIs can be found on the "Author Information" page.
**Disclaimer:** Articles published in the journal HemaSphere exclusively reflect the opinions of the authors. The authors are responsible for all content in their abstracts including accuracy of the facts, statements, citing resources, etc.

### (PF623) TARGETING EPIGENETIC–METABOLIC CROSSTALK IN CML VIA BUTYRATE-INDUCED HDAC INHIBITION

**Topic:** 07. Chronic myeloid leukemia - Biology & translational research

Rita Vacca*[1], Alessandro Ferrando[1], Frolli Antonio[1], Selene Grano[1], Simone Rocco[1], Alice Danzero[1], Afshin Bagherzadeh[1], Valentina Schiavo[1], Cristina Panuzzo[1], Daniela Cilloni[1], Barbara Pergolizzi[1], Carmen Fava[1]

[1]*Università degli studi di Torino, Department of Clinical and Biological Sciences, Torino, Italy;*

### Background

Tyrosine kinase inhibitors (TKIs) have greatly improved outcomes in Chronic Myeloid Leukemia (CML), yet some patients develop resistance, often linked to metabolic and epigenetic adaptations. In this context, *ASXL1* mutations are the most frequent additional alteration, present in 9–20% of chronic-phase CML at diagnosis. These mutations identify patients with poorer prognosis, more aggressive disease, and lower chances of achieving major molecular response (MMR). ASXL1 acts as a transcriptional and epigenetic regulator, and C-terminally truncated mutants. As previously described, retain BAP1 binding but sequester it from functional chromatin complexes, impairing BAP1 activity and deregulating tumor suppressors controlling glycolysis and hypoxia response. This promotes leukemic survival and growth partially independent of BCR::ABL1, contributing to TKI resistance. Butyrate, a short-chain fatty acid from gut microbiota as an HDAC inhibitor, induces cell death, promotes differentiation, reduces inflammation, and suppresses cancer stem cell proliferation.

### Aims

This study aimed to investigate the effects of butyrate on proliferation, differentiation, epigenetic regulation, metabolism, and TKI sensitivity in ASXL1-mutated versus wild-type CML cells.

### Methods

K562 (heterozygous ASXL1 Y591Y/X) and LAMA-84 (ASXL1-WT) cells were treated with butyrate (0.048–100 mM) and imatinib (0.0048–20 µM). For mutation-focused experiments, cells were pre-treated with 1 mM butyrate for 24 h before exposure to imatinib (0.0048–20 µM). Viability was measured by resazurin assay, erythroid differentiation by FACS (CD235a), transcripts of BCR::ABL1, HDAC1, HIF1α, and GLUT1 by qRT-PCR, and protein levels by Western blot. Mitochondria and HIF-1α were analyzed by immunofluorescence and quantified using Fiji (ImageJ).

### Results

Butyrate slowed proliferation in both K562 and LAMA-84 cells, but responses differed. In K562, it induced erythroid differentiation, reduced HDAC1 transcripts and protein, increased histone H4 acetylation, suppressed HIF-1α and GLUT1, decreased glycolytic intermediates, and shifted metabolism toward OXPHOS; immunofluorescence confirmed near-complete loss of nuclear HIF-1α. LAMA-84 showed minimal changes in HDAC1, HIF-1α, GLUT1, glycolysis, or OXPHOS. Despite similar BCR::ABL1 reduction, butyrate sensitized K562 to imatinib (IC50 1.90 → 0.36 µM) but rendered LAMA-84 more resistant (IC50 1.90 → 7.55 µM). These results indicate selective epigenetic and metabolic remodeling in ASXL1-mutant K562 enhances TKI sensitivity, while wild-type LAMA-84 remain largely unaffected.

**Copyright Information:** (Online) ISSN: 2572-9241
©2026 The Author(s). HemaSphere is published by John Wiley & Sons Ltd on behalf of European Hematology Association. This is an open access Abstract Book distributed under the Attribution-NonCommercial-NoDerivs (CC BY-NC-ND), which allows third parties to download the articles and share them with others as long as they credit the author and the Abstract Book, but they cannot change the content in any way or use them commercially.
**Abstract Book Citations:** Authors, Title, HemaSphere, 2026;10:(S1):pages. The abstract book DOIs can be found on the "Author Information" page.
**Disclaimer:** Articles published in the journal HemaSphere exclusively reflect the opinions of the authors. The authors are responsible for all content in their abstracts including accuracy of the facts, statements, citing resources, etc.

**Summary/Conclusion**

In conclusion, our findings highlight butyrate as a versatile anti-leukemic agent in CML, capable of modulating proliferation, differentiation, epigenetic regulation, and metabolism. Notably, ASXL1-mutated cells exhibit pronounced sensitivity to TKI following butyrate pre-treatment, associated with epigenetic remodeling and enhanced oxidative phosphorylation, whereas wild-type cells show minimal response. These results underscore the potential of butyrate as a complementary therapeutic strategy, particularly for patients harboring ASXL1 mutations or in situations where standard TKI therapy is limited, offering a safe and mechanistically multifaceted approach to improve treatment outcomes.

**Copyright Information:** (Online) ISSN: 2572-9241
©2026 The Author(s). HemaSphere is published by John Wiley & Sons Ltd on behalf of European Hematology Association. This is an open access Abstract Book distributed under the Attribution-NonCommercial-NoDerivs (CC BY-NC-ND), which allows third parties to download the articles and share them with others as long as they credit the author and the Abstract Book, but they cannot change the content in any way or use them commercially.
**Abstract Book Citations:** Authors, Title, HemaSphere, 2026;10:(S1):pages. The abstract book DOIs can be found on the "Author Information" page.
**Disclaimer:** Articles published in the journal HemaSphere exclusively reflect the opinions of the authors. The authors are responsible for all content in their abstracts including accuracy of the facts, statements, citing resources, etc.

### (PF624) PERIPHERAL BLOOD NEUTROPHIL QUANTITY AND MATURATION PREDICT TREATMENT-FREE REMISSION IN CHRONIC MYELOID LEUKEMIA

**Topic:** 07. Chronic myeloid leukemia - Biology & translational research

Tatsuya Mihashi*[1], Hiroshi Ureshino[1], Shinya Kimura[1]

[1]*Saga University, Saga, Japan;*

### Background

Treatment-free remission (TFR) has become a key therapeutic goal in chronic myeloid leukemia (CML). However, only 40–60% of patients who achieve deep molecular response (DMR) can maintain remission after tyrosine kinase inhibitor (TKI) discontinuation. Although treatment duration and depth of molecular response are established predictors of TFR, clinically applicable biological markers that can be readily assessed in routine practice remain insufficient. Emerging evidence suggests that neutrophils play an important role in antitumor immunity in CML, highlighting their potential relevance to sustained remission after TKI cessation.

### Aims

To evaluate whether peripheral blood neutrophil quantity and maturation status are associated with successful TFR in patients with CML.

### Methods

We conducted a retrospective single-center analysis of patients with chronic-phase CML treated and followed at our institution. Patients who attempted TKI discontinuation after achieving sustained DMR were included. Peripheral blood neutrophil counts at the time of TKI discontinuation were evaluated in the entire cohort, irrespective of the type of TKI administered. In addition, routine peripheral blood differential counts obtained at diagnosis were analyzed to assess neutrophil maturation status, defined by the proportion of band and segmented neutrophils, as well as the percentage of circulating blasts. TFR was analyzed using Kaplan–Meier methods, and prognostic factors were evaluated using Cox proportional hazards models.

### Results

In our single-center cohort, 53 patients were evaluated for the association between peripheral blood neutrophil counts at the time of TKI discontinuation and TFR. A higher neutrophil count at discontinuation was significantly associated with successful TFR across the entire patient population, with patients having neutrophil counts ≥ 2439/μL showing a markedly lower risk of molecular relapse (hazard ratio 0.325, 95% confidence interval 0.137–0.772, p = 0.011). Among these patients, 45 treated at our institution had peripheral blood differential counts available at diagnosis and were therefore eligible for further analysis of neutrophil maturation based on manual microscopic examination. Analysis of diagnostic peripheral blood smears demonstrated that patients with impaired neutrophil maturation—defined as mature neutrophils <64%—in combination with circulating blasts ≥0.5% had significantly inferior TFR compared with other patients (hazard ratio 2.815, 95% confidence interval 1.040–7.617, p = 0.0416). These findings suggest that both neutrophil quantity at TKI discontinuation and qualitative maturation status at diagnosis reflect the biological capacity to maintain remission after TKI cessation.

**Copyright Information:** (Online) ISSN: 2572-9241
©2026 The Author(s). HemaSphere is published by John Wiley & Sons Ltd on behalf of European Hematology Association. This is an open access Abstract Book distributed under the Attribution-NonCommercial-NoDerivs (CC BY-NC-ND), which allows third parties to download the articles and share them with others as long as they credit the author and the Abstract Book, but they cannot change the content in any way or use them commercially.
**Abstract Book Citations:** Authors, Title, HemaSphere, 2026;10:(S1):pages. The abstract book DOIs can be found on the "Author Information" page.
**Disclaimer:** Articles published in the journal HemaSphere exclusively reflect the opinions of the authors. The authors are responsible for all content in their abstracts including accuracy of the facts, statements, citing resources, etc.

**Summary/Conclusion**

Peripheral blood neutrophil parameters—higher neutrophil counts at the time of TKI discontinuation and preserved neutrophil maturation with minimal circulating blasts—are associated with successful TFR in CML. These readily available and cost-effective hematological markers, assessed using routine clinical data at a single institution, may complement molecular criteria and aid clinical decision-making for TKI discontinuation.

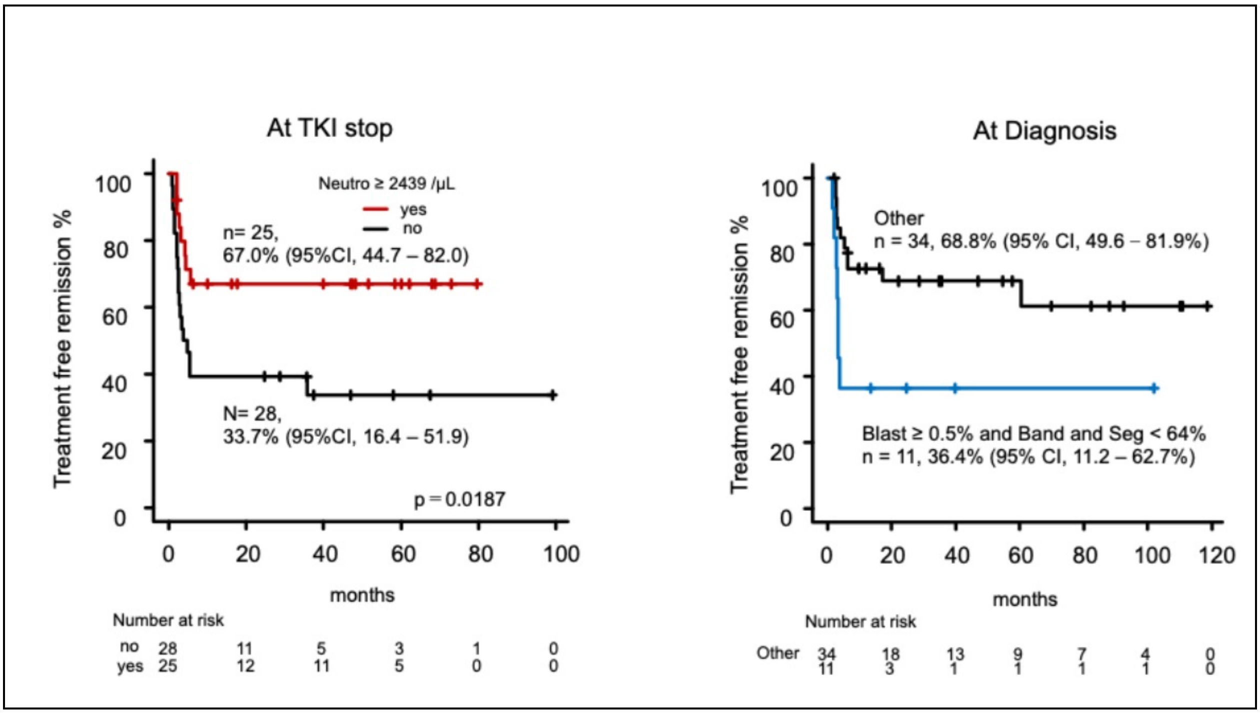

**Copyright Information:** (Online) ISSN: 2572-9241
©2026 The Author(s). HemaSphere is published by John Wiley & Sons Ltd on behalf of European Hematology Association. This is an open access Abstract Book distributed under the Attribution-NonCommercial-NoDerivs (CC BY-NC-ND), which allows third parties to download the articles and share them with others as long as they credit the author and the Abstract Book, but they cannot change the content in any way or use them commercially.
**Abstract Book Citations:** Authors, Title, HemaSphere, 2026;10:(S1):pages. The abstract book DOIs can be found on the "Author Information" page.
**Disclaimer:** Articles published in the journal HemaSphere exclusively reflect the opinions of the authors. The authors are responsible for all content in their abstracts including accuracy of the facts, statements, citing resources, etc.

### (PF625) FRONTLINE ASCIMINIB FOR CHRONIC PHASE CML: LONG TERM SAFETY AND EFFICACY IN THE ALLG CM13 ASCEND TRIAL

**Topic:** 08. Chronic myeloid leukemia - Clinical

David Yeung*[1, 2, 3], Naranie Shanmuganathan[1, 2, 3], Jake Shortt[4, 5], Agnes Yong[6, 7], Lynette Chee[8, 9], John Reynolds[5, 10], Nicholas Viiala[11, 12], Ilona Cunningham[13, 14], David Ross[3, 15], Alwyn D'souza[16], Matthew Wright[7, 17], Rosemary Harrup[18, 19], Cecily Forsyth[20], Robin Filshie[9, 21], Steven Lane[22, 23], Peter Browett[24, 25], Carolyn Grove[7, 26], Sher Gul Gazdar[27], Mannu Walia[27], Susan Branford[3, 28], Andrew Grigg[9, 29], Timothy Hughes[1, 2, 3]

*[1]Royal Adelaide Hospital, Adelaide, Australia;[2]SAHMRI, Adelaide, Australia; [3]Adelaide University, Adelaide, Australia; [4]Monash Medical Centre, Melbourne, Australia; [5]Monash University, Melbourne, Australia; [6]Royal Perth Hospital (RPH), Perth CBD, Australia; [7]University of Western Australia, Nedlands, Australia;[8]The Royal Melbourne Hospital, Parkville, Australia;[9]University of Melbourne, Parkville, Australia; [10]Australian Centre for Blood Diseases, The Alfred Hospital, Melbourne, Australia; [11]Liverpool Hospital, Liverpool, Australia; [12]University of New South Wales, Kensington, Australia; [13]Concord Repatriation General Hospital, Concord West, Australia; [14]University of Sydney, Camperdown, Australia; [15]Flinders Medical Centre, Bedford Park, Australia; [16]Wellington Blood & Cancer Centre, Wellington, New Zealand; [17]Fiona Stanley Hospital, Murdoch, Australia; [18]Royal Hobart Hospital, Hobart CBD, Australia; [19]University of Tasmania-Sandy Bay Campus, Sandy Bay, Australia;[20]Gosford Hospital, Gosford, Australia; [21]St Vincents Hospital, Collingwood, Australia;[22]Queensland Institute of Medical Research, Herston, Australia; [23]Royal Brisbane & Womens Hospital, Herston, Australia;[24]University of Auckland Faculty of Medical & Health Sciences, Grafton, New Zealand; [25]Auckland City Hospital, Grafton, New Zealand;[26]Sir Charles Gairdner Hospital, Nedlands, Australia; [27]Australasian Leukaemia & Lymphoma Group, Richmond, Australia;[28]SA Pathology, Adelaide, Australia;[29]Austin Hospital, Heidelberg, Australia;*

### Background

ASCEND is a phase II study of Asciminib (ASC), a myristoyl pocket BCR::ABL1 inhibitor in newly diagnosed CML-CP patients. We report 3 year outcomes.

### Methods

All patients (pts) started ASC 40mg twice a day at diagnosis and switched to 80 mg daily after 12 months (mos). Pts with treatment failure (*BCR::ABL1* >10% at 3 or 6 mos; *BCR::ABL1* >1% at 12 or 18 mos) continued ASC, and either imatinib, dasatinib or nilotinib were added, according to physician preference.

**Copyright Information:** (Online) ISSN: 2572-9241
©2026 The Author(s). HemaSphere is published by John Wiley & Sons Ltd on behalf of European Hematology Association. This is an open access Abstract Book distributed under the Attribution-NonCommercial-NoDerivs (CC BY-NC-ND), which allows third parties to download the articles and share them with others as long as they credit the author and the Abstract Book, but they cannot change the content in any way or use them commercially.
**Abstract Book Citations:** Authors, Title, HemaSphere, 2026;10:(S1):pages. The abstract book DOIs can be found on the "Author Information" page.
**Disclaimer:** Articles published in the journal HemaSphere exclusively reflect the opinions of the authors. The authors are responsible for all content in their abstracts including accuracy of the facts, statements, citing resources, etc.

### Results

The median follow up was 48 (mos) (36-61) for 100 patients in 15 Australasian centres. Median age at diagnosis was 55 years (19-88). The co-primary endpoints of EMR and MMR by 12 mos, were achieved in 93% and 79% respectively. The cumulative incidence of MR4 and MR4.5 was 73% and 65% respectively, & 42% of patients have achieved and maintained MR4.5 for >24 mos by 48 mos. No deaths were reported. Two pts progressed to blastic phase – 1 on study and 1 at 33 mos after last ASC dose.
The most common adverse events (AEs) were infections, skin rashes, myalgia/arthralgia and fatigue (28-44%, 1-2% grade (gr) 3/4). Increased lipase occurred in 25%, 10% grade 3/4). Thrombocytopenia, neutropenia and anaemia were reported at 15%, 8% and 9% respectively, (gr 3/4 at 5%, 2% & 7%). One 1 haematological toxicity - gr 4 neutropenia - was reported after 24 mos. Five arterial occlusive events (AOEs) occurred (Table), with a rate of 1.4 events per 100 patient years of ASC treatment. Twenty-four pts have discontinued ASC: 6 for intolerance (2 cytopenia, 3 lipase elevation, 1 pancreatitis); 8 for other reasons (3 lost to follow up, 5 withdrawn consent); 10 for resistance (2 EMR failure without mutation, 1 progression to lymphoid blast crisis and 7 loss of response – all had *BCR::ABL1* mutations.
Lipid profiles and blood glucose were reported at study entry (M0), & at months (M) 6, 12, 24 and 36. The median total cholesterol rose from 4.7mM at M0, to 4.9mM at M6 (p=0.03), then remained between 4.5 – 4.9mM afterwards. The median LDL cholesterol was 2.6mM at M0, and between 2.7 – 2.9mM thereafter. Triglyceride levels fell within the first 6 mos of treatment (median 1.9mM at M0, vs 1.2-1.4mM between M6 and M36; p<0.005 when comparing baseline with any later timepoint). The median fasting glucose was unchanged after treatment (5.2-5.3mM between M0 and M36).

| | | | | |
|---|---|---|---|---|
| CML13 Loss of response events | | | | |
| Month | Event | BCR::ABL1 Mutation | Most recent response and treatment | |
| 6 | LBC | A337T/V, P465S | MR4 after transplant | |
| 10 | Lost MMR | A337T | MR4.5, DAS | |
| 10 | Lost MMR | M244V, A337T, L387V | MR4.5, NIL | |
| 12 | Lost MR2 | V506L | MR4, DAS | |
| 13 | Lost MR4.5 | T315I / M244V | MR4.5, OLV | |
| 18 | Lost MR4 | A337T | MR4.5, DAS | |
| 24 | Lost MR4.5 | M244V | MMR, DAS | |
| 41 | Lost MR4.5 | M244V | MMR, DAS | |
| CML13 Arterial Occlusive Events | | | | |
| Month | Age at Dx | Past History | Month | Event |
| 27 | 73 | PAOD/CVA/IHD/HTN | 27 | Ongoing claudication |
| 16-28 | 72 | HTN/dyslipidemia/DM | 16-28 | 4 x CVA/TIA, no sequelae |
| 28 | 62 | None | 28 | IHD |
| 46 | 64 | Nil significant | 46 | IHD / CABG |
| 36 | 88 | HTN/Dyslipidemia/AS | 36 | IHD |
| DAS - Dasatinib, NIL - Nilotinib, OLV – Olverembatinib<br>HTN – hypertension; DM – Diabetes Mellitus, CVA – Stroke, IHD – Ischaemic heart disease, CABG – Coronary artery bypass graft, AS – aortic stenosis, PAOD – peripheral arterial occlusive disease | | | | |

### Summary/Conclusion

ASC in newly diagnosed CP-CML leads to high rates of stable MR4.5 – a platform for treatment free remission. Loss of response was mostly driven by BCR::ABL1 mutations and TKI salvage were effective. AOE risk factors and events occurred at the expected rate for patients with this demographic. Ongoing follow is required to ascertain long term tolerability and TFR rate.

**Copyright Information:** (Online) ISSN: 2572-9241
©2026 The Author(s). HemaSphere is published by John Wiley & Sons Ltd on behalf of European Hematology Association. This is an open access Abstract Book distributed under the Attribution-NonCommercial-NoDerivs (CC BY-NC-ND), which allows third parties to download the articles and share them with others as long as they credit the author and the Abstract Book, but they cannot change the content in any way or use them commercially.
**Abstract Book Citations:** Authors, Title, HemaSphere, 2026;10:(S1):pages. The abstract book DOIs can be found on the "Author Information" page.
**Disclaimer:** Articles published in the journal HemaSphere exclusively reflect the opinions of the authors. The authors are responsible for all content in their abstracts including accuracy of the facts, statements, citing resources, etc.

### (PF626) GENOMIC AND TRANSCRIPTOMIC FEATURES OF TRANSFORMED CHRONIC MYELOID LEUKEMIA IN BLAST PHASE

**Topic:** 08. Chronic myeloid leukemia - Clinical

Mei Bao*[1], Xiaoshuai Zhang[1], Zongru Li[1], Lu Yu[1], Shasha Zhao[2], Qian Jiang[1, 2]

*[1]Peking University People's Hospital, Beijing, China; [2]Peking University People's Hospital,, Qingdao, China;*

**Background**

Chronic myeloid leukemia in blast phase (CML-BP) has dismal outcomes and a poorly understood biological landscape. Comprehensive characterization of genomic and transcriptomic features is not uncovered yet.

**Aims**

To explore the genomic and transcriptomic features of transformed CML-BP and their impact on clinical outcomes.

**Methods**

Data of 124 patients with CML-BP defined by 2022 WHO criteria receiving TKI plus chemotherapy or followed by transplant who had genomic and transcriptomic information were retrospectively analyzed. Event-free survival (EFS) was the interval from TKI therapy start to lack of hematological response by 4-week, loss of response or death. Cox regression model was used to identify covariates associated with outcomes.

**Copyright Information:** (Online) ISSN: 2572-9241
©2026 The Author(s). HemaSphere is published by John Wiley & Sons Ltd on behalf of European Hematology Association. This is an open access Abstract Book distributed under the Attribution-NonCommercial-NoDerivs (CC BY-NC-ND), which allows third parties to download the articles and share them with others as long as they credit the author and the Abstract Book, but they cannot change the content in any way or use them commercially.
**Abstract Book Citations:** Authors, Title, HemaSphere, 2026;10:(S1):pages. The abstract book DOIs can be found on the "Author Information" page.
**Disclaimer:** Articles published in the journal HemaSphere exclusively reflect the opinions of the authors. The authors are responsible for all content in their abstracts including accuracy of the facts, statements, citing resources, etc.

**Results**

124 patients with myeloid BP (MBP, n = 83, 67%) or lymphoid BP (LBP, n = 41, 33%) receiving TKI ± chemotherapy were included. Median age was 46 years (IQR, 34-58). 78 (63%) patients including 47 (57%) MBP and 31 (76%) LBP achieved major hematologic response by 4-week ($p$ = 0.040). 37 (30%) patients underwent transplant. 36 (46%) experienced relapse, 71 died of relapse (n = 62), treatment-related complications (n = 6) or transplant-related mortality (n = 3). With a median follow-up of 19 months (IQR, 7-45), 3-year probabilities of EFS and OS were 20% (95%CI, 12, 28%) and 33% (23, 42%). Compared to patients with LBP, those with MBP had lower 3-year probabilities of EFS (15% *vs.* 31%, $p$ = 0.033) and OS (27% *vs.* 46%, $p$ = 0.031).

*ABL1* (n = 56, 45%) and/or *non-ABL1* (n = 108, 87%) mutations were detected in 114 (92%) patients. The most frequently *ABL1* mutation was *T315I* (n = 36, 29%), followed by *E255K* (n = 35, 28%), *Y253H* and *F359I* (each n = 5, 4%). *Y253H* (10% *vs.* 1%, $p$ = 0.013) mutation was more common in patients with LBP. The most frequently *non-ABL1* mutation was *ASXL1* (n = 36, 29%), followed by *RUNX1* (n = 35, 28%), *GATA2* (n = 14, 11%) and *WT1* (n = 12, 10%). *ASXL1* (36% *vs.* 15%, $p$ = 0.013), *GATA2* (17% *vs.* 0%, $p$ = 0.005) and *KMT2C* (11% *vs.* 0%, $p$ = 0.029) mutations were more common in those with MBP. In multivariate analyses, *KARS* mutation was significantly-associated with worse EFS (HR = 2.6 [1.1, 6.2], $p$ = 0.033) and OS (HR = 7.6 [2.9, 20.0], $p < 0.001$); *ASXL1* (HR = 1.7 [1.0, 3.0], $p$ = 0.061) and *TP53* (HR = 2.5 [1.0, 6.5], $p$ = 0.059) mutations, worse OS. In addition, a longer interval from diagnosis to CML-BP, prior TKI-therapy line ≥ 3, poor response to prior TKIs, and/or non-transplant were significantly-associated with worse EFS and/or OS. However, BP type (MBP or LBP), TKI used, or chemotherapy type had no impact on outcomes.

In the RNA-seq data from 86 patients, MBP exhibited activation of cancer- (MYC, E2F, MTORC1, G2M checkpoint), metabolic- (heme, adipogenesis, fatty acid), structural (EMT, apical junction) and oxidative phosphorylation pathways by GSEA analyses and enriched with Th2, mast and CD56bright NK cells by immune profiling analyses. However, LBP was enriched with T/B and neutrophil cells by immune profiling analyses.

**Summary/Conclusion**

The genomic and transcriptomic features differ significantly between transformed MBP and LBP. *KRAS*, *ASXL1* and *TP53* mutations were identified as predictors of poor outcomes beyond certain clinical characteristics.

**Copyright Information:** (Online) ISSN: 2572-9241
©2026 The Author(s). HemaSphere is published by John Wiley & Sons Ltd on behalf of European Hematology Association. This is an open access Abstract Book distributed under the Attribution-NonCommercial-NoDerivs (CC BY-NC-ND), which allows third parties to download the articles and share them with others as long as they credit the author and the Abstract Book, but they cannot change the content in any way or use them commercially.
**Abstract Book Citations:** Authors, Title, HemaSphere, 2026;10:(S1):pages. The abstract book DOIs can be found on the "Author Information" page.
**Disclaimer:** Articles published in the journal HemaSphere exclusively reflect the opinions of the authors. The authors are responsible for all content in their abstracts including accuracy of the facts, statements, citing resources, etc.

### (PF627) PROGNOSTIC IMPACT OF ADDITIONAL SOMATIC MUTATIONS ON TKD DEVELOPMENT, MOLECULAR RESPONSE AND SURVIVAL IN CHRONIC MYELOID LEUKEMIA PATIENTS TREATED WITH TYROSINE KINASE INHIBITORS

**Topic:** 08. Chronic myeloid leukemia - Clinical

Ruth Stuckey*[1], Adrián Segura-Díaz[1], Cristina Bilbao Sieyro[1], Yanira Florido[1], Guillermo Santana[1], Jose David Gonzalez San Miguel[2], Santiago Sánchez-Sosa[1], Carol Herrera Hernández[1], Juan Francisco López Rodríguez[1], Maria Teresa Gomez Casares[1]

*[1]Hospital Universitario de Gran Canaria Dr. Negrín/Instituto de Investigación Sanitaria de Canarias (IISC), Las Palmas de Gran Canaria, Spain; [2]Complejo Hospitalario Materno-Insular de Gran Canaria, Hematology and Hemotherapy, Las Palmas de Gran Canaria, Spain;*

### Background

Outcomes of chronic myeloid leukemia (CML) have markedly improved with tyrosine kinase inhibitors (TKIs), yet a subset of patients show suboptimal response or develop resistance. While *ABL1* kinase domain (TKD) mutations are a recognized mechanism of resistance, molecular studies have revealed that additional somatic mutations at diagnosis can also influence treatment response and progression.

### Aims

Evaluate the association between somatic mutations and additional chromosomal abnormalities (ACA) at diagnosis with subsequent development of TKD mutations, molecular response and overall survival (OS).

### Methods

Genomic DNA from consecutively diagnosed CML patients was retrospectively analyzed by targeted next-generation sequencing (NGS) using myeloid gene panels. Pathogenic/probably pathogenic variants with VAF≥2% and MAF<1% were included. ACA were assessed by bone marrow karyotype at diagnosis. TKD mutations ( *ABL1* Gly227– Gly514) were analyzed by NGS (NexteraXT) in patients with BCR::ABL1 ≥0.1%. Associations with early molecular response (EMR, BCR::ABL1 <10% at 3 months), major molecular response (MMR) at 12 months, number of TKI lines and OS were evaluated.

**Copyright Information:** (Online) ISSN: 2572-9241
©2026 The Author(s). HemaSphere is published by John Wiley & Sons Ltd on behalf of European Hematology Association. This is an open access Abstract Book distributed under the Attribution-NonCommercial-NoDerivs (CC BY-NC-ND), which allows third parties to download the articles and share them with others as long as they credit the author and the Abstract Book, but they cannot change the content in any way or use them commercially.
**Abstract Book Citations:** Authors, Title, HemaSphere, 2026;10:(S1):pages. The abstract book DOIs can be found on the “Author Information” page.
**Disclaimer:** Articles published in the journal HemaSphere exclusively reflect the opinions of the authors. The authors are responsible for all content in their abstracts including accuracy of the facts, statements, citing resources, etc.

### Results

The cohort comprised 161 patients (158 chronic, 7 advanced/blast phase) with a median age at diagnosis of 52 years [5.6–98] and median follow-up of 126 months (Table 1).
NGS was performed for 118 patients at diagnosis. 28% of patients harbored ≥1 pathogenicsomatic mutation; 12.7% had ≥2. The most frequently mutated genes were *ASXL1* (14.8%), *TET2* (8.9%), *JAK2* and *RUNX1* (6.7% each). ACA were detected in 9.4% of evaluable patients.
TKD mutations developed in 35.6% of patients with suboptimal TKI response (73.3% received imatinib, 20% dasatinib and 6.7% nilotinib). No association was found between development of TKD and first-line TKI (individual agents or first- vs. second-generation).
Patients with ACA at diagnosis did not show increased additional mutations, TKD acquisition or inferior OS, although mutations were more frequent in ACA cases (28.6% vs. 12.7%, not significant).
The presence of any mutation at diagnosis was associated with higher mortality (p=0.020), advanced phase at diagnosis (mutations detected in 85.7% accelerated/blast vs. 35.1% chronic phase, p=0.013) and subsequent TKD development (p=0.038), but not with older age. A trend toward lower EMR rates was observed in mutated patients (46.2% vs. 66.7%, p=0.086). Patients with ≥2 mutations were more likely to require ≥3 TKI lines (p=0.042) and were less likely to achieve MMR at 12 months (33.3% vs. 68.6%, p=0.034). In contrast, *ASXL1* mutations alone were not significantly associated with EMR, MMR, TKD development or OS. Importantly, failure to achieve EMR or MMR by 12 months were strongly associated with need for ≥3 TKI lines (p=0.001 and p<0.001, respectively) and death (p=0.022; Figure 1).

### Summary/Conclusion

Over a quarter of CML patients harbored somatic mutations at diagnosis. Their presence was associated with inferior molecular response and reduced OS. The cumulative mutational burden, rather than isolated *ASXL1* mutations or ACA, appears clinically relevant. NGS at diagnosis identifies patients less likely to achieve EMR and MMR, and these early responses significantly impact survival. Early molecular characterization may refine risk stratification and supports prospective studies to determine whether patients with somatic mutations benefit from more potent first-line TKIs.

**Copyright Information:** (Online) ISSN: 2572-9241
©2026 The Author(s). HemaSphere is published by John Wiley & Sons Ltd on behalf of European Hematology Association. This is an open access Abstract Book distributed under the Attribution-NonCommercial-NoDerivs (CC BY-NC-ND), which allows third parties to download the articles and share them with others as long as they credit the author and the Abstract Book, but they cannot change the content in any way or use them commercially.
**Abstract Book Citations:** Authors, Title, HemaSphere, 2026;10:(S1):pages. The abstract book DOIs can be found on the "Author Information" page.
**Disclaimer:** Articles published in the journal HemaSphere exclusively reflect the opinions of the authors. The authors are responsible for all content in their abstracts including accuracy of the facts, statements, citing resources, etc.

Table 1

| Patient characteristics | | N (%) |
|---|---|---|
| Phase at diagnosis | | |
| | Chronic phase | 154 (95.7) |
| | Accelerated phase | 5 (3.1) |
| | Blast crisis | 2 (1.2) |
| Male | | 52.8% |
| Transcript | e13a2 | 52 (32.3) |
| | e14a2 | 70 (43.5) |
| | (p210) | 36 (22.4) |
| | e1a2 | 2 (1.2) |
| | e19a2 | 1 (0.6) |
| First-line TKI | Imatinib | 100 (62.1) |
| | Nilotinib | 33 (20.5) |
| | Dasatinib | 23 (14.3) |
| | Bosutinib | 1 (0.6) |

Figure 1

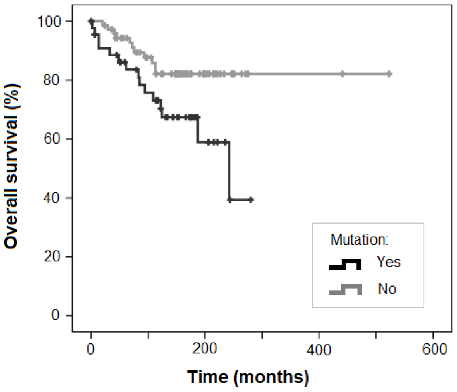

Table 1. Patient characteristics. Figure 1. Kaplan-Meier overall survival curve stratified according to presence of any somatic myeloid mutation (p=0.022, Log-rank test)..

Copyright Information: (Online) ISSN: 2572-9241
©2026 The Author(s). HemaSphere is published by John Wiley & Sons Ltd on behalf of European Hematology Association. This is an open access Abstract Book distributed under the Attribution-NonCommercial-NoDerivs (CC BY-NC-ND), which allows third parties to download the articles and share them with others as long as they credit the author and the Abstract Book, but they cannot change the content in any way or use them commercially.
Abstract Book Citations: Authors, Title, HemaSphere, 2026;10:(S1):pages. The abstract book DOIs can be found on the "Author Information" page.
Disclaimer: Articles published in the journal HemaSphere exclusively reflect the opinions of the authors. The authors are responsible for all content in their abstracts including accuracy of the facts, statements, citing resources, etc.

### (PF628) EFFICACY AND SAFETY OF PONATINIB IN PEDIATRIC PATIENTS WITH BCR::ABL–POSITIVE CHRONIC PHASE CHRONIC MYELOID LEUKEMIA (CP-CML): PRELIMINARY RESULTS FROM THE INCB84344-102 STUDY

**Topic:** 08. Chronic myeloid leukemia - Clinical

Franco Locatelli*[1], Bram De Wilde[2], Franca Fagioli[3], Alba Rubio-San-Simón[4], Marion Strullu[5], Natasha van Eijkelenburg[6], Robert Stratford[7], Nan Nan[7], Russell Schilder[7], Helena Adamis[7], C. Michel Zwaan[8, 9]

*[1]IRCCS Ospedale Pediatrico Bambino Gesù, Catholic University of the Sacred Heart, Department of Pediatric Hematology and Oncology, Rome, Italy; [2]Ghent University Hospital, Department of Pediatric Hematology-Oncology and Stem Cell Transplantation, Ghent, Belgium; [3]Regina Margherita Children's Hospital, City of Health and Science of Turin, Pediatric Onco-Hematology Division, Turin, Italy; [4]Hospital Infantil Universitario Niño Jesús, Department of Pediatric Hematology-Oncology, Madrid, Spain; [5]Hôpital Robert Debré, Department of Pediatric Hemato-Immunology, Paris, France;[6]Princess Máxima Center for Pediatric Oncology, Heidelberglaan, Utrecht, The Netherlands; [7]Incyte Corporation, Wilmington, DE, United States of America;[8]Princess Máxima Center for Pediatric Oncology, Department of Pediatric Hematology-Oncology, Utrecht, The Netherlands; [9]Erasmus MC Sophia Children's Hospital, Rotterdam, The Netherlands;*

### Background

CML is relatively rare in children (2% of leukemias in children aged <15 years; 9% in children aged 15–19 years in the US). CML is typically caused by a mutant BCR::ABL fusion protein, which is the target of first- and second-generation TKIs. In children, imatinib, dasatinib, nilotinib, and bosutinib are approved. However, *T315I* mutations, present in up to 20% of patients (pts) resistant to imatinib, can make these TKIs ineffective. Ponatinib, a TKI targeting BCR::ABL that is efficacious against the *T315I* mutant, is approved in adults with CML resistant or intolerant to prior TKI treatment (tx) or with a *T315I* mutation.

### Aims

To evaluate the efficacy and safety of ponatinib in pediatric pts with CP-CML in the phase 1/2 INCB84344-102 study (NCT03934372).

### Methods

INCB84344-102 enrolled pediatric pts with advanced leukemia, lymphoma, or solid tumors in phase 1 (dose escalation) and pts with CP-CML resistant or intolerant to prior BCR::ABL–targeted tx or with a *T315I* mutation (phase 2 dose expansion). Phase 2 included pts with additional tumor types (data not presented). Pts were stratified based on age (1–5 yr, 6–11 yr, 12–17 yr). Dose escalation initiated with a weight-adjusted ponatinib dose of 20 mg (≥45 kg) or 15 mg (30–44 kg) daily. Phase 1 determined the RP2D based on DLTs. The primary endpoint of phase 2 CP-CML dose expansion was major cytogenetic response (MCyR), defined as complete (CCyR) or partial CyR by 12 months. Secondary endpoints included complete hematologic response (CHR) at 6 months, major molecular response (MMR) at 12 months, safety, and PK. In phase 2, dose reduction upon achievement of MCyR was permitted for pts with CP-CML per investigator's judgment.

**Copyright Information:** (Online) ISSN: 2572-9241
©2026 The Author(s). HemaSphere is published by John Wiley & Sons Ltd on behalf of European Hematology Association. This is an open access Abstract Book distributed under the Attribution-NonCommercial-NoDerivs (CC BY-NC-ND), which allows third parties to download the articles and share them with others as long as they credit the author and the Abstract Book, but they cannot change the content in any way or use them commercially.
**Abstract Book Citations:** Authors, Title, HemaSphere, 2026;10:(S1):pages. The abstract book DOIs can be found on the "Author Information" page.
**Disclaimer:** Articles published in the journal HemaSphere exclusively reflect the opinions of the authors. The authors are responsible for all content in their abstracts including accuracy of the facts, statements, citing resources, etc.

**Results**

As of January 25, 2025, 61 pts received ponatinib, including 32 pts in phase 1 (CP-CML, n=1), and 29 patients in phase 2 expansion (10 with CP-CML [6–11 years, n=2; 12–17 years, n=8]; 19 with other tumor types). All pts with CP-CML had received prior BCR::ABL–targeted TKIs (1 line, n=6; 2 lines, n=5). No DLTs were observed in phase 1, leading to a weight-adjusted daily RP2D of 45 mg (≥45 kg), 30 mg (30–44 kg), 15 mg (15–29 kg), or 5 mg (5–14 kg), based on an adult-equivalent 45 mg dose. CP-CML pts in phase 2 had a median ponatinib exposure of 559 days (range 72–1100). In total, 90.0% achieved MCyR by 12 months, all with a best CyR of CCyR (**Table**). The CHR rate at 6 months was 90.0% and the MCyR rate at 12 months was 50.0%. Cytogenetic responses were observed at 3 months (60.0%, n=6), 6 months (80.0%, n=8), and 9 months (90.0%, n=9). The MMR rate at 12 months was 60.0%. One pt had MCyR and had their dose reduced (45 mg to 15 mg) and remained in remission at data cutoff. Across all pts with CP-CML, 10 (90.9%) had TEAEs, 4 (36.4%) had grade ≥3 TEAEs, and 3 (27.3%) had serious TEAEs. There were no fatal TEAEs. The most common TEAEs were pyrexia (n=5) and headache (n=3). There were no arterial occlusive or vascular thrombotic events reported. Accounting for lower body weight of pts, PK parameters were similar to those found in adults and were used to optimize weight-based doses that achieved overlapping exposures with adults.

**Summary/Conclusion**

No DLTs were observed in pediatric pts treated with ponatinib in dose escalation. Dose expansion showed preliminary efficacy of ponatinib in children with CP-CML. No additional safety signals were observed compared with the safety profile of ponatinib in adult pts, which is well established. INCB84344-102 is ongoing and recruiting pts to the CP-CML expansion phase.

**Copyright Information:** (Online) ISSN: 2572-9241
©2026 The Author(s). HemaSphere is published by John Wiley & Sons Ltd on behalf of European Hematology Association. This is an open access Abstract Book distributed under the Attribution-NonCommercial-NoDerivs (CC BY-NC-ND), which allows third parties to download the articles and share them with others as long as they credit the author and the Abstract Book, but they cannot change the content in any way or use them commercially.
**Abstract Book Citations:** Authors, Title, HemaSphere, 2026;10:(S1):pages. The abstract book DOIs can be found on the “Author Information” page.
**Disclaimer:** Articles published in the journal HemaSphere exclusively reflect the opinions of the authors. The authors are responsible for all content in their abstracts including accuracy of the facts, statements, citing resources, etc.

**Table. Efficacy Results in Phase 2 CP-CML Dose Expansion Overall and by Age**

| Endpoint | Phase 2 CP-CML Expansion 6–11 yr (n=2) | Phase 2 CP-CML Expansion 12–17 yr (n=8) | Total (n=10) |
|---|---|---|---|
| MCyR rate by 12 mo, n (%) | 2 (100) | 7 (87.5) | 9 (90.0) |
| 95% CI | 15.8, 100 | 47.4, 99.7 | 55.5, 99.8 |
| Best CyR by 12 mo, n (%) | | | |
| Complete (CCyR) | 2 (100) | 7 (87.5) | 9 (90.0) |
| Minimal | 0 (0) | 1 (12.5) | 1 (10.0) |
| HR at 6 mo, n (%) | | | |
| CHR | 2 (100) | 7 (87.5) | 9 (90.0) |
| Not assessed* | 0 (0) | 1 (12.5) | 1 (10.0) |
| CyR at 12 mo, n (%) | | | |
| Complete (CCyR) | 1 (50.0) | 4 (50.0) | 5 (50.0) |
| Not assessed* | 1 (50.0) | 4 (50.0) | 5 (50.0) |
| MR at 12 mo, n (%) | | | |
| MR4-5† | 0 (0) | 2 (25.0) | 2 (20.0) |
| MR3‡ | 1 (50.0) | 3 (37.5) | 4 (40.0) |
| Not assessed* | 1 (50.0) | 3 (37.5) | 4 (40.0) |

*No post baseline response at the relevant time point was available. †MR4-5: ≤0.0032% cells with mutant BCR-ABL. ‡MR3: ≤0.1% cells with mutant BCR-ABL (or MMR). CCyR, complete cytogenetic response; CI, confidence interval; CHR, complete hematologic response; CP-CML, chronic phase chronic myeloid leukemia; CyR, cytogenetic response; HR, hematologic response; MCyR, major cytogenetic response; MMR, major molecular response; MR, molecular response; mo, months; yr, years

**Copyright Information:** (Online) ISSN: 2572-9241
©2026 The Author(s). HemaSphere is published by John Wiley & Sons Ltd on behalf of European Hematology Association. This is an open access Abstract Book distributed under the Attribution-NonCommercial-NoDerivs (CC BY-NC-ND), which allows third parties to download the articles and share them with others as long as they credit the author and the Abstract Book, but they cannot change the content in any way or use them commercially.
**Abstract Book Citations:** Authors, Title, HemaSphere, 2026;10:(S1):pages. The abstract book DOIs can be found on the "Author Information" page.
**Disclaimer:** Articles published in the journal HemaSphere exclusively reflect the opinions of the authors. The authors are responsible for all content in their abstracts including accuracy of the facts, statements, citing resources, etc.

### (PF629) LONG-TERM OUTCOMES OF VERY LOW-DOSE DASATINIB IN OLDER PATIENTS WITH NEWLY DIAGNOSED CHRONIC-PHASE CHRONIC MYELOID LEUKEMIA: EXTENDED FOLLOW-UP OF THE DAVLEC PHASE 2 TRIAL

**Topic:** 08. Chronic myeloid leukemia - Clinical

Kazunori Murai*[1], Hiroshi Ureshino[2], Takashi Kumagai[3], Tatsuji Mino[4], Kaichi Nishiwaki[5], Satoshi Wakita[6], Tomoharu Takeoka[7], Masayuki Mita[8], Chikashi Yoshida[9], Uoshima Nobuhiko[10], Toshihiro Fukushima[11], Junya Kuroda[12], Takeshi Kondo[13], Masahiro Chiba[14], Takuya Miyazaki[15], Katsuhiro Io[16], Tomoaki Ueda[17], Takaaki Ono[18], Kensuke Usuki[19], Takahiro Yamauchi[20], Katsumichi Fujimaki[21], Takayuki Ikezoe[22], Atsushi Kawaguchi[23], Shinya Kimura[2]

*[1]Iwate Prefectural Central Hospital, Department of Hematology, Morioka, Japan;[2]Saga University, Division of Hematology, Respiratory Medicine and Oncology, Department of Internal Medicine, Faculty of Medicine, Saga, Japan; [3]Ome Municipal General Hospital, Ome, Japan; [4]Hiroshima City Asa Hospital,, Hiroshima, Japan; [5]The Jikei University Kashiwa Hospital, Kashiwa, Japan; [6]Nippon Medical School, Tokyo, Japan; [7]Otsu Red Cross Hospital, Otsu, Japan; [8]Shirakawa Kosei General Hospital, Shirakawa, Japan; [9]National Hospital Organization Mito Medical Center, Mito, Japan; [10]Japanese Red Cross Kyoto Daini Hospital, Kyoto, Japan; [11]Kanazawa Medical University, Kanazawa, Japan; [12]Kyoto Prefectural University of Medicine, Kyoto, Japan; [13]Aiiku Hospital, Sapporo, Japan; [14]Asahikawa City Hospital, Asahikawa, Japan; [15]Yokohama City University Medical Center, Yokohama, Japan; [16]Kansai Electric Power Hospital, Osaka, Japan; [17]The University of Osaka Graduate School of Medicine, Suita, Japan; [18]Hamamatsu University Hospital, Hamamatsu, Japan; [19]NTT Medical Center Tokyo, Tokyo, Japan; [20]University of Fukui, Fukui, Japan; [21]Fujisawa City Hospital, Fujisawa, Japan;[22]Fukushima Medical University, Fukushima, Japan; [23]Saga University, Education and Research Center for Community Medicine, Faculty of Medicine, Saga, Japan;*

### Background

Second-generation BCR::ABL1 tyrosine kinase inhibitors (TKIs) achieve rapid and deep molecular responses in chronic-phase chronic myeloid leukemia (CML-CP). However, in older patients, standard-dose TKI therapy is frequently complicated by comorbidities, polypharmacy, and treatment-related adverse events, leading to treatment discontinuation. The DAVLEC trial previously demonstrated that very low-dose dasatinib (20 mg/day) is feasible and effective as front-line therapy in patients aged ≥ 70 years. Long-term efficacy, safety, and the feasibility of treatment-free remission (TFR) with this dose-optimized strategy remain unclear.

### Aims

To evaluate the long-term molecular responses, survival outcomes, safety, and feasibility of TFR in older patients with newly diagnosed CML-CP treated with frontline very low-dose dasatinib.

### Methods

DAVLEC was a multicentre, single-arm, phase 2 trial enrolling patients aged ≥ 70 years with newly diagnosed CML-CP. Patients received dasatinib at an initial dose of 20 mg/day. If the treatment was assessed as optimal response at 3 months, 6 months, and 9 months and adverse events were grade 2 or better (according to the NCI Common Toxicity Criteria v 4.0), the same dose was continued. If response was suboptimal and adverse events were grade 2 or better, the dose was increased by 20 mg/day. This analysis represents an extended long-term follow-up after completion of the original 12-month protocol. Cumulative molecular responses (MMR, MR4.0, MR4.5), overall survival (OS), adverse events (AEs), treatment discontinuation, and TFR outcomes were evaluated. Associations between clinical parameters, early molecular response, immune cell profiles, and treatment efficacy were explored.

**Copyright Information:** (Online) ISSN: 2572-9241
©2026 The Author(s). HemaSphere is published by John Wiley & Sons Ltd on behalf of European Hematology Association. This is an open access Abstract Book distributed under the Attribution-NonCommercial-NoDerivs (CC BY-NC-ND), which allows third parties to download the articles and share them with others as long as they credit the author and the Abstract Book, but they cannot change the content in any way or use them commercially.
**Abstract Book Citations:** Authors, Title, HemaSphere, 2026;10:(S1):pages. The abstract book DOIs can be found on the "Author Information" page.
**Disclaimer:** Articles published in the journal HemaSphere exclusively reflect the opinions of the authors. The authors are responsible for all content in their abstracts including accuracy of the facts, statements, citing resources, etc.

**Results**

Between October 1, 2024, and December 31, 2025, 48 patients with chronic-phase CML (CML-CP) who had previously participated in the phase 2 DAVLEC trial (conducted between November 1, 2016, and October 30, 2019) were enrolled from 22 centers in Japan. The 5-year cumulative incidences of MMR, MR4.0, and MR4.5 were 89.3%, 69.6%, and 56.7%, respectively. The median follow-up was 68.1 months. The median dasatinib doses at the time of achieving MMR, MR4.0, and MR4.5 are 20mg for each point. OS at 5 years was 85.5%, with only one CML-related death. In multivariate analyses, high ELTS score, ECOG performance status of 1–2 at diagnosis, and failure to achieve MMR at 12 months were independently associated with overall survival (HR 34.14, $p = 0.041$; HR 1.43, $p = 0.014$; and HR 73.26, $p = 0.008$, respectively). Achievement of MMR at 12 months was a strong predictor of subsequent deep molecular responses (MR4.0 and MR4.5). Higher baseline natural killer (NK) cell proportions were associated with favorable molecular outcomes. Grade ≥ 3 AEs occurred in 25.0% of patients, and treatment discontinuation was mainly driven by tolerability rather than disease progression. Six patients attempted TFR after dasatinib discontinuation at a median age of 78.5 years, and all maintained TFR without loss of MMR during follow-up.

**Summary/Conclusion**

Very low-dose dasatinib (20 mg/day) provides durable molecular efficacy with acceptable long-term safety in older patients with CML-CP. Deep molecular responses and successful TFR can be achieved even in elderly patients when appropriate response milestones are met. These findings support dose-optimized second-generation TKI strategies as a rational long-term treatment approach for older patients with CML.

**Copyright Information:** (Online) ISSN: 2572-9241
©2026 The Author(s). HemaSphere is published by John Wiley & Sons Ltd on behalf of European Hematology Association. This is an open access Abstract Book distributed under the Attribution-NonCommercial-NoDerivs (CC BY-NC-ND), which allows third parties to download the articles and share them with others as long as they credit the author and the Abstract Book, but they cannot change the content in any way or use them commercially.
**Abstract Book Citations:** Authors, Title, HemaSphere, 2026;10:(S1):pages. The abstract book DOIs can be found on the "Author Information" page.
**Disclaimer:** Articles published in the journal HemaSphere exclusively reflect the opinions of the authors. The authors are responsible for all content in their abstracts including accuracy of the facts, statements, citing resources, etc.

### (PF630) THE SAFETY, TOLERABILITY, AND EFFICACY OF HS-10382 COMBINED WITH FLUMATINIB IN PATIENTS WITH NEWLY DIAGNOSED PH+ CML: A PHASE 1B STUDY

**Topic:** 08. Chronic myeloid leukemia - Clinical

Weiming Li[1], Xiao-Dong Wang[2], Yanli Zhang[3], Sujun Gao[4], Zhenfang Liu[5], Zunmin Zhu[6], Haiping Yang[7], Wei Yang[8], Yang Yu[9], Linlin Sun[9], Rili Jiang[9], Xiaoqing Zhang[9], Yu Hu*[1]

*[1]Union Hospital Tongji Medical college Huazhong University of Science and Technology, Wuhan, China;[2]Sichuan Provincial People's Hospital, Chengdu, China; [3]Henan Cancer Hospital, Zhengzhou, China;[4]The First Hospital of Jilin University, Changchun, China; [5]The First Affiliated Hospital of Guangxi Medical University, Nanning, China;[6]The First Affiliated Hospital of Henan University of Science and Technology, Zhengzhou, China; [7]The First Affiliated Hospital, and College of Clinical Medicine of Henan University of Science and Technology, Luoyang, China; [8]Shengjing Hospital of China Medical University, Shenyang, China; [9]Shanghai Hansoh BioMedical Co., Ltd, Shanghai, China;*

### Background

Adenosine triphosphate (ATP)-competitive tyrosine kinase inhibitors targeting the *BCR::ABL1* ATP-binding site have extended the life expectancy of patients with chronic phase chronic myeloid leukemia (CML-CP), but sustained deep molecular response is still limited. HS-10382 is a novel *BCR::ABL1* allosteric inhibitor that specifically targets the ABL myristoyl pocket, with encouraging preclinical data showing synergy with ATP-competitive tyrosine kinase inhibitors. Here, we report results from patients treated with HS-10382 in combination with flumatinib in a phase 1b study (CTR20242299).

### Aims

To assess the safety, tolerability, and preliminary efficacy of HS-10382 in combination with flumatinib as first-line therapy in patients with newly diagnosed Philadelphia chromosome-positive (Ph+) CML-CP.

### Methods

In this single-arm, open-label, multicenter study in China, adults with newly diagnosed *BCR::ABL1*-positive CML-CP (confirmed within 6 months) received oral HS-10382 120 mg twice daily and flumatinib 400 mg once daily in 28-day cycles. The primary objective was to evaluate safety and tolerability.

**Copyright Information:** (Online) ISSN: 2572-9241
©2026 The Author(s). HemaSphere is published by John Wiley & Sons Ltd on behalf of European Hematology Association. This is an open access Abstract Book distributed under the Attribution-NonCommercial-NoDerivs (CC BY-NC-ND), which allows third parties to download the articles and share them with others as long as they credit the author and the Abstract Book, but they cannot change the content in any way or use them commercially.
**Abstract Book Citations:** Authors, Title, HemaSphere, 2026;10:(S1):pages. The abstract book DOIs can be found on the "Author Information" page.
**Disclaimer:** Articles published in the journal HemaSphere exclusively reflect the opinions of the authors. The authors are responsible for all content in their abstracts including accuracy of the facts, statements, citing resources, etc.

### Results

**Patient Demographics and Disposition:** From December 2024 to January 2025, 11 patients with newly diagnosed CML-CP were enrolled in the study. The median age was 54.0 years (range 22-69), 54.5% (6/11) were male, and all patients had ECOG performance status 0-1. According to ELTS risk evaluation at baseline, 72.7% (8/11) of patients were classified as low risk, 27.3% (3/11) as intermediate, and none as high. As of the data cutoff (January 12, 2026), 10 patients had completed the 48-week follow-up period, while 1 patient withdrew after 1 month. The median follow-up time was 11.1 months (range 1.0-12.2). **Safety:** In the safety-evaluable population (n=11), the majority of treatment-emergent adverse events (TEAEs) were Grade 1-2. Grade ≥3 TEAEs were observed in 54.5% of patients (6/11). The most common TEAEs (incidence ≥ 10%) included alanine aminotransferase elevation (18.2%, 2/11) and lipase elevation (18.2%, 2/11). TEAEs leading to treatment discontinuation were reported in 18.2% (2/11). **Efficacy:** In the efficacy-evaluable population (n=10), 8 patients achieved complete cytogenetic response (80.0%, 95% CI: 44.4%-97.5%) at Week 48. Major molecular response (MMR, *BCR::ABL1* ≤ 0.1% on the International scale, IS) was achieved by 7 patients (70.0%; 95% CI: 34.75%-93.33%) at Week 24 and by 8 patients (80.0%; 95% CI: 44.4%-97.5%) at Week 48. Additionally, 3 patients (30.0%, 95% CI: 6.7%-65.3%) achieved MR4.5 (*BCR::ABL1* ≤ 0.0032%, IS) at Week 48. Cumulative best molecular response rates in the evaluable patients were 100% for MMR and 30% for MR4.5.

### Summary/Conclusion

HS-10382 in combination with flumatinib demonstrated a manageable safety profile and promising preliminary efficacy in patients with newly diagnosed CML-CP, characterized by rapid achievement and deepening of molecular responses. These findings support continued evaluation in an expanded cohort with longer follow-up to confirm the durability of deep molecular responses and long-term safety.

**Copyright Information:** (Online) ISSN: 2572-9241
©2026 The Author(s). HemaSphere is published by John Wiley & Sons Ltd on behalf of European Hematology Association. This is an open access Abstract Book distributed under the Attribution-NonCommercial-NoDerivs (CC BY-NC-ND), which allows third parties to download the articles and share them with others as long as they credit the author and the Abstract Book, but they cannot change the content in any way or use them commercially.
**Abstract Book Citations:** Authors, Title, HemaSphere, 2026;10:(S1):pages. The abstract book DOIs can be found on the “Author Information” page.
**Disclaimer:** Articles published in the journal HemaSphere exclusively reflect the opinions of the authors. The authors are responsible for all content in their abstracts including accuracy of the facts, statements, citing resources, etc.

### (PF631) PHASE II TRIAL OF DASATINIB IN COMBINATION WITH ASTX727 IN PATIENTS WITH NEWLY DIAGNOSED CHRONIC MYELOID LEUKEMIA

**Topic:** 08. Chronic myeloid leukemia - Clinical

Elias Jabbour*[1], Fadi Haddad[1], Yesid Alvarado[1], Musa Yilmaz[1], Nicholas Short[1], Hussein Abbas[1], Guillermo Montalban-Bravo[1], Sara Dellasala[1], Ghayas Issa[1], Hagop Kantarjian[1]

*[1]MD Anderson Cancer Center, Leukemia, Houston, United States of America;*

### Background

Decitabine was found to be effective in patients (pts) with resistant chronic myeloid leukemia (CML). Combining decitabine with dasatinib was found to be safe and synergistic. We hypothesized that the combination of ASTX727 (oral decitabine/cedazuridine) with dasatinib could induce a higher rate of sustained deep molecular response (DMR) that can translate into improvement of treatment-free remission (TFR) rates.

### Aims

To evaluate the DMR rate with the combination of dasatinib and ASTX727.

### Methods

In this phase II trial, pts with newly diagnosed CML-CP received dasatinib 50 mg/day for 3 cycles with the addition of ASTX727 as of Cycle 4 given at a fixed combination dose of 100 mg cedazuridine/35mg decitabine daily on Days 1-3 of each treatment cycle for a 3-year period. After 22 pts were treated, 2 (10%) had developed Grade(G) 4 neutropenia and thrombocytopenia requiring treatment interruption. To avoid treatment interruption, the protocol was amended to reduce ASTX727 to Days 1-2 once a DMR was obtained. The primary endpoint was the rate of MR4 after 6 months of combination.

**Copyright Information:** (Online) ISSN: 2572-9241
©2026 The Author(s). HemaSphere is published by John Wiley & Sons Ltd on behalf of European Hematology Association. This is an open access Abstract Book distributed under the Attribution-NonCommercial-NoDerivs (CC BY-NC-ND), which allows third parties to download the articles and share them with others as long as they credit the author and the Abstract Book, but they cannot change the content in any way or use them commercially.
**Abstract Book Citations:** Authors, Title, HemaSphere, 2026;10:(S1):pages. The abstract book DOIs can be found on the "Author Information" page.
**Disclaimer:** Articles published in the journal HemaSphere exclusively reflect the opinions of the authors. The authors are responsible for all content in their abstracts including accuracy of the facts, statements, citing resources, etc.

**Results**

38 pts were treated. The median age was 45 years (range, 21-68); 28 (74%) were male. Sokal score was low-intermediate in 37 pts (97%). With a median follow-up of 35 months, the cumulative best overall response was CCyR in 37 pts (97%), MMR in 30 (79%), MR4 in 22 (58%), and MR4.5 in 18 (47%). The MR4 rate by 6 months of combination or 9 months from the start of therapy (primary endpoint) was 32%.
By 3 months of combination or 6 months of therapy, 32 pts (84%) achieved CCyR, 17 (45%) MMR, 7 (18%) MR4, and 2 (5%) MR4.5. By 9 months of combination or 12 months of therapy, among 36 evaluable pts, the CCyR rate was 92%, MMR rate 61%, MR4 rate 39%, and MR4.5 rate 25%. The MR4 rate by 24 months was 55%. Thirteen pts (34%) achieved and maintained a sustained MR4 for a median duration of 25.6 months (range, 13.8-45.4).
The most frequent adverse events possibly related to the study drugs included neutropenia (G2, n=2; G3-4, n=16), thrombocytopenia (G2, n=1; G3-4, n=4), pleural effusion (G1-2, n=2), fatigue (G1-2, n=3), arthralgia G2 in 5 pts, and ALT elevation G2, anemia G2, heart failure G3, skin rash G1, in 1 pt each.
9 of 38 pts (24%) discontinued therapy, including 4 due to adverse events (recurrent pleural effusions in 1 pt; neutropenia and thrombocytopenia in 1 pt; fatigue and arthralgia in 1 pt; heart failure in 1 pt). One pt achieved CCyR as best response but lost MCyR after 15.3 months of therapy possibly due to drug-drug interaction with semaglutide and had rising *BCR::ABL1* transcripts despite increasing dasatinib to 100 mg/day, and another pt achieved MMR as best response but lost MCyR after 26.7 months of therapy while remaining on dasatinib 50 mg/day. No *ABL1* kinase domain mutations were detected. Treatment was discontinued due to progression to blast phase, pregnancy, and financial constraints, in 1 pt each. The 3-year rates of FFS, EFS, TFS, and OS were 76%, 90%, 97%, 97%, respectively.
With the caveat of across trial comparison, the molecular response rates with this combination were not statistically different from those achieved with dasatinib alone or with dasatinib + venetoclax.

**Summary/Conclusion**

The combination of dasatinib and ASTX727 was safe and effective. Further follow-up is critical to assess the rates of sustained DMR that can translate into higher TFR rates.

**Copyright Information:** (Online) ISSN: 2572-9241
©2026 The Author(s). HemaSphere is published by John Wiley & Sons Ltd on behalf of European Hematology Association. This is an open access Abstract Book distributed under the Attribution-NonCommercial-NoDerivs (CC BY-NC-ND), which allows third parties to download the articles and share them with others as long as they credit the author and the Abstract Book, but they cannot change the content in any way or use them commercially.
**Abstract Book Citations:** Authors, Title, HemaSphere, 2026;10:(S1):pages. The abstract book DOIs can be found on the "Author Information" page.
**Disclaimer:** Articles published in the journal HemaSphere exclusively reflect the opinions of the authors. The authors are responsible for all content in their abstracts including accuracy of the facts, statements, citing resources, etc.

### (PF632) ASCIMINIB VERSUS SECOND-GENERATION TKIS IN CHRONIC-PHASE CML AFTER ≥2 PRIOR TKIS: A PROPENSITY SCORE-MATCHED ANALYSIS

**Topic:** 08. Chronic myeloid leukemia - Clinical

Noora Obaidallah*[1, 2], Sihath Singhabahu[2], Ana Flavia Patino[2], Yael Morgenstern[2], Maria Agustina Perusini[2], Dennis Kim[2]

[1]*Sheikh Shakhbout Medical City, Abu Dhabi, United Arab Emirates;*[2]*Princess Margaret Cancer Centre, Toronto, Canada;*

### Background

Asciminib (ASC) is approved for chronic-phase chronic myeloid leukemia (CML-CP) after failure of ≥2 tyrosine kinase inhibitors (TKIs), demonstrating superior outcomes versus bosutinib in ASCEMBL (Rea et al., *Blood* 2021). However, its efficacy compared with second-generation TKIs (2G-TKIs), including nilotinib and dasatinib, remains unclear due to lack of direct comparisons.

### Aims

This retrospective study compared outcomes between ASC and 2G-TKIs using propensity score matching (PSM) to adjust for baseline differences.

### Methods

We retrospectively analyzed CML-CP patients who received third-line or later TKI therapy at Princess Margaret Cancer Centre between September 2010 and October 2024. Patients receiving ASC were compared with those treated with 2G-TKIs (dasatinib, nilotinib, or bosutinib). A total of 106 patients were identified (53 per group). Primary endpoints were 2-year failure-free survival (FFS) and event-free survival (EFS). FFS was calculated from treatment initiation to the earliest occurrence of treatment failure, defined according to ELN 2020 criteria as confirmed loss of MMR in two consecutive assessments, progression to accelerated phase (AP) or blast crisis (BC), or death from any cause. Patients without an event were censored at the date of last assessment. EFS was defined similarly to FFS but additionally included treatment discontinuation due to adverse events (AEs). Secondary endpoints included 1-year molecular responses (MR2 <1% IS; MMR <0.1% IS; MR4 <0.01% IS) and 2-year overall survival (OS). PSM was performed using logistic regression based on age (>60 vs. ≤60), BCR::ABL1 (>10% vs. ≤10%), line of therapy (3rd vs. ≥4th), and prior TKI failure reason (resistance vs. intolerance). After matching, 37 patients per group remained.

### Results

Before PSM, ASC patients were older (median 64 vs. 53 years, p<0.001), more often ≥60 years (57% vs. 32%, p=0.011), and had more prior TKIs (≥4: 34% vs. 15%, p=0.024). Higher baseline BCR::ABL1 (>10%) was more frequent (38% vs. 21%, p=0.055). Resistance was more common with ASC (51% vs. 34%), while intolerance was more frequent with 2G-TKIs (66% vs. 49%, p=0.070). Median follow-up was shorter with ASC (704 vs. 1720 days, p<0.01).
At 2 years, FFS was 82% (95% CI, 66.8–90.8) vs. 74% (95% CI, 60.2–84.4) (HR 0.63, 95% CI 0.27–1.49; p=0.29) and EFS was 84% (95% CI, 69.7–92.4) vs. 74% (95% CI, 60.2–84.4) (HR 0.54, 95% CI 0.22–1.33; p=0.18) for ASC vs. 2G-TKIs. AE-related discontinuation was lower with ASC (5.7% vs. 22.6%, p=0.012). One-year responses were MR2 78% vs. 93% (p=0.19), MMR 63% vs. 71% (p=0.42), and MR4 42% vs. 45% (p=0.66). OS was 92% vs. 98% (p=0.58).
After PSM, baseline characteristics were balanced. At 2 years, FFS was 83% (95% CI, 65.9–91.9) vs. 72% (95% CI, 53.9–83.8) (HR 0.53, 95% CI 0.20–1.41; p=0.20) and EFS was 86% (95% CI, 69.3–93.9) vs. 72% (95% CI, 53.9–83.8) (HR 0.43, 95% CI 0.15–1.23; p=0.12). AE-related discontinuation remained lower with ASC (5.4% vs. 24.3%, p=0.022). Molecular responses and OS were comparable (OS 94% vs. 97%; HR 1.19, 95% CI 0.20–7.10; p=0.85).

**Copyright Information:** (Online) ISSN: 2572-9241
©2026 The Author(s). HemaSphere is published by John Wiley & Sons Ltd on behalf of European Hematology Association. This is an open access Abstract Book distributed under the Attribution-NonCommercial-NoDerivs (CC BY-NC-ND), which allows third parties to download the articles and share them with others as long as they credit the author and the Abstract Book, but they cannot change the content in any way or use them commercially.
**Abstract Book Citations:** Authors, Title, HemaSphere, 2026;10:(S1):pages. The abstract book DOIs can be found on the "Author Information" page.
**Disclaimer:** Articles published in the journal HemaSphere exclusively reflect the opinions of the authors. The authors are responsible for all content in their abstracts including accuracy of the facts, statements, citing resources, etc.

### Summary/Conclusion

In this real-world cohort, ASC showed numerically improved FFS and EFS over 2G-TKI, though not statistically significant, while molecular response rates and OS were comparable. ASC was consistently associated with significantly fewer AE-related discontinuations both before and after PSM, supporting a favorable tolerability profile. These findings suggest that ASC is an effective and well-tolerated later-line option in CML-CP.

**Copyright Information:** (Online) ISSN: 2572-9241
©2026 The Author(s). HemaSphere is published by John Wiley & Sons Ltd on behalf of European Hematology Association. This is an open access Abstract Book distributed under the Attribution-NonCommercial-NoDerivs (CC BY-NC-ND), which allows third parties to download the articles and share them with others as long as they credit the author and the Abstract Book, but they cannot change the content in any way or use them commercially.
**Abstract Book Citations:** Authors, Title, HemaSphere, 2026;10:(S1):pages. The abstract book DOIs can be found on the "Author Information" page.
**Disclaimer:** Articles published in the journal HemaSphere exclusively reflect the opinions of the authors. The authors are responsible for all content in their abstracts including accuracy of the facts, statements, citing resources, etc.

### (PF633) TWO-YEAR OUTCOMES OF LOW-DOSE VERSUS STANDARD-DOSE DASATINIB IN NEWLY DIAGNOSED CHRONIC MYELOID LEUKAEMIA: A RETROSPECTIVE ANALYSIS

**Topic:** 08. Chronic myeloid leukemia - Clinical

Shailendra Verma*[1], Swasti Sinha[1], Aritra Saha[1], Akshay Middinti[1]

[1]*King George's Medical University, Clinical Hematology, Lucknow, India;*

### Background

Chronic myeloid leukaemia (CML) is a myeloid neoplasm conferring near-normal life expectancy with tyrosine kinase inhibitor (TKI) therapy in the majority of patients. Whereas survival prolongation was formerly paramount, frontline second-generation TKIs now prioritise deeper molecular responses and treatment-free remission. Dasatinib 100 mg once daily remains standard, yet recent data confirm comparable efficacy at 50 mg daily—offering reduced financial burden in resource-constrained settings like India. This study was undertaken to assess the efficacy and safety profile of Dasatinib at a lower dose.

### Aims

Our primary objective was to assess and compare the efficacy and safety of standard-dose Dasatinib versus a lower dose as frontline therapy in patients with newly diagnosed chronic myeloid leukaemia in chronic phase (CML-CP). Our secondary objectives were to assess the adverse effect profile and survival.

### Methods

In this hospital-based observational study, after obtaining institutional ethical clearance, treatment outcomes were retrospectively compared between Dasatinib 100 mg once daily (Dasa100) and Dasatinib 50 mg once daily (Dasa50) in newly diagnosed CML-CP patients aged >13 years. The primary endpoint was optimum response at 3, 6, 12 and 24 months (BCR-ABL IS <10%, <1%, <0.1% and <0.1% respectively); the secondary endpoints were assessing the adverse event profile and survival. Failure-free survival (FFS) was defined as the time from therapy initiation to discontinuation of treatment for any reason other than achievement of treatment-free remission (TFR). Event-free survival (EFS) was measured from the start of treatment until loss of major molecular response (MMR). Progression Free Survival (PFS) was measured from the start of treatment until progression to accelerated or blast phase. Overall survival (OS) was determined from the initiation of TKI therapy to the date of death from any cause or last follow-up

### Results

Median age was 35.5 years (IQR 29–55); Male: Female ratio was 2.3:1.  Of 103 patients, 71 received Dasatinib 100 mg/day and 32 received Dasatinib 50 mg/day. Most belonged to intermediate (36%) and high (34%) ELTS risk groups. Major molecular response (MMR) rates were comparable at 12 months (Dasa100 87.3%; Dasa50 71.9%; p=0.103) and 24 months (Dasa100 88.7%; Dasa50 78.1%; p=0.267). Higher doses showed a non-significant trend towards increased toxicity. Haematological adverse events predominated in both arms; diarrhoea was the leading non-haematological toxicity, differing from Western studies. Over 2 years, the FFS (Dasa100 94.4%; Dasa50 87.5%; p=0.673), PFS (Dasa100: 99%, Dasa50: 97%, p= 0.567) and EFS (Dasa100: 93%; Dasa50: 91%; p=0.074) rates were comparable. All the patients were alive at the end of 2 years.

**Copyright Information:** (Online) ISSN: 2572-9241
©2026 The Author(s). HemaSphere is published by John Wiley & Sons Ltd on behalf of European Hematology Association. This is an open access Abstract Book distributed under the Attribution-NonCommercial-NoDerivs (CC BY-NC-ND), which allows third parties to download the articles and share them with others as long as they credit the author and the Abstract Book, but they cannot change the content in any way or use them commercially.
**Abstract Book Citations:** Authors, Title, HemaSphere, 2026;10:(S1):pages. The abstract book DOIs can be found on the "Author Information" page.
**Disclaimer:** Articles published in the journal HemaSphere exclusively reflect the opinions of the authors. The authors are responsible for all content in their abstracts including accuracy of the facts, statements, citing resources, etc.

### Summary/Conclusion

Low-dose Dasatinib (50 mg daily) demonstrates equivalent efficacy with a favourable safety trend versus standard dosing, supporting its adoption as frontline therapy in resource-limited settings such as India, where treatment costs remain prohibitive.

.

*Figure 1: Kaplan–Meier Curve: Failure-free Survival by Dasatinib Dose*

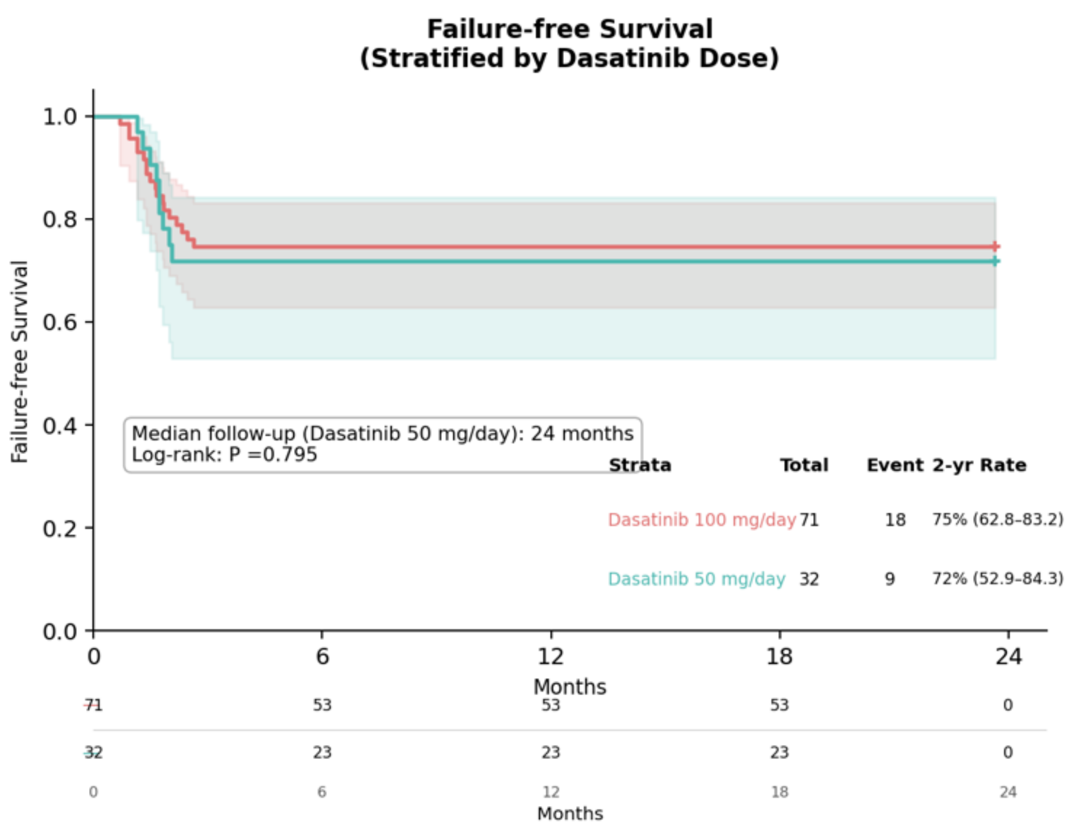

Kaplan–Meier survival analysis was performed for three endpoints: Failure-free Survival (FFS, in days), Progression-free Survival (PFS, in months), and Event-free Survival (EFS, in months). Groups were compared using the log-rank test.

**Copyright Information:** (Online) ISSN: 2572-9241
©2026 The Author(s). HemaSphere is published by John Wiley & Sons Ltd on behalf of European Hematology Association. This is an open access Abstract Book distributed under the Attribution-NonCommercial-NoDerivs (CC BY-NC-ND), which allows third parties to download the articles and share them with others as long as they credit the author and the Abstract Book, but they cannot change the content in any way or use them commercially.
**Abstract Book Citations:** Authors, Title, HemaSphere, 2026;10:(S1):pages. The abstract book DOIs can be found on the "Author Information" page.
**Disclaimer:** Articles published in the journal HemaSphere exclusively reflect the opinions of the authors. The authors are responsible for all content in their abstracts including accuracy of the facts, statements, citing resources, etc.

### (PF634) HIGHER FIRST LINE TKI RESISTANCE IN ADOLESCENTS AND YOUNG ADULTS (AYA) WITH CHRONIC MYELOID LEUKEMIA. EVIDENCE FROM A POPULATION-BASED REGISTRY.

**Topic:** 08. Chronic myeloid leukemia - Clinical

Maria Jose Herranz Marti*[1], Maria Alicia Senin Magan[2], Jasson A Villarreal Hernández[3], Silvana Daniela Saavedra Gerosa[4], Marta Santaliestra Tomas[5], Sara Garrido Paniagua[6], Ana Angona Figueras[7], Montserrat Cortés[8], Karla Vallejo Luna[9], Marta García Pintos[10], Elena Ramila Herrero[11], Blanca Xicoy Cirici[12], Rosario Lopez Sanchez[13], Olga Ramon Planell[14], Xavier Ortín Font[15], Guillermo Orti Pascual[6], Dolors Vela Payan[9], Patricia Velez Tenza[2], Ana Triguero[16], Lurdes Zamora Plana[12], Irene Medina Rangel[6], Rolando Omar Vallansot[17]

*[1]Insititu Català d'Oncologia. Hospital Sant Pau i Santa Tecla., Tarragona, Spain;[2]Parc Salut Mar, Barcelona, Spain;[3]Institut Català d'Oncologia. Hospital Duran i Reynals, Hospitalet, Spain; [4]Hospital de l Santa Creu i Sant Pau, Hematology, Barcelona, Spain; [5]Hospital Universitari Mutua de Terrassa, Hematology, Terrasa, Spain;[6]Hospital Vall d'Hebron, Barcelona, Spain;[7]IDIBGI. Institut Josep Carreras, Girona, Spain; [8]Fundació Althaia, Hematology, Manresa, Spain;[9]Hospital General de Granollers, Granollers, Spain; [10]Hospital de Terrasa, Haematology, Terrassa, Spain; [11]Hospital Parc Taulí, Sabadell, Spain; [12]Institut Català d'oncologia. Hospital Germans Trias i Pujol. Josep Carreras Leukemia Research Insitute., Badalona, Spain; [13]Hospital Universitari Sagrat Cor, Haematology, Barcelona, Spain; [14]Hospital Universitari d'Igualada, Hematology, Igualada, Spain; [15]Institut Català d'Oncologia. Hospital Verge de la Cinta, Hematology, Tortosa, Spain;[16]Hospital Clínic, Barcelona, Spain; [17]Institut Català d'Oncologia. Hospital Joan XXIII. On behalf of Grup Català de Leucèmia Mieloide Crònica, Tarragona, Spain;*

### Background

Adolescent and young adult (AYA) patients with chronic myeloid leukemia (CML) treated in the tyrosine kinase inhibitor (TKI) era have been reported to experience inferior outcomes compared with older patients in some studies. Whether this reflects biological differences or treatment-related factors remains unclear.

### Aims

To characterize the reasons for TKI switching in AYA, and to compare first-line switching patterns with those observed in patients aged ≥40 years.

### Methods

Patients with Philadelphia chromosome-positive (Ph+) CML included in the *Catalan epidemiological registry of CML patients treated with TKI therapy* (initiated in 2020; retrospective and prospective recruitment) were analyzed. Data were extracted in October 2025. Switching was defined as a change from one TKI to another. Reasons for switching were categorized as resistance (primary or secondary according to ELN criteria) or intolerance. Categorical variables were compared using Fisher's exact test.

**Copyright Information:** (Online) ISSN: 2572-9241
©2026 The Author(s). HemaSphere is published by John Wiley & Sons Ltd on behalf of European Hematology Association. This is an open access Abstract Book distributed under the Attribution-NonCommercial-NoDerivs (CC BY-NC-ND), which allows third parties to download the articles and share them with others as long as they credit the author and the Abstract Book, but they cannot change the content in any way or use them commercially.
**Abstract Book Citations:** Authors, Title, HemaSphere, 2026;10:(S1):pages. The abstract book DOIs can be found on the "Author Information" page.
**Disclaimer:** Articles published in the journal HemaSphere exclusively reflect the opinions of the authors. The authors are responsible for all content in their abstracts including accuracy of the facts, statements, citing resources, etc.

### Results

A total of 318 patients were included: 111 AYA and 207 aged ≥40 years. The median follow-up was 118 months in the AYA cohort and 60.5 months in the older patients.
Among AYA patients, 61% had low Sokal risk; 78% received imatinib and 22% second-generation TKIs (2G-TKIs) as 1st line therapy. In older patients, 47% had low Sokal risk; 82% received imatinib and 18% 2G-TKIs.
First-line switching due to resistance or intolerance occurred in 42% of AYA patients and 46% of older patients (p=NS). Resistance accounted for 32% of 1st line switches in AYA patients versus 19% in older patients ($p<0.05$). The median time to switch due to resistance was 17 months for AYA and 14.5 for older patients. Intolerance-driven switching was less frequent in AYA (9.9% vs 24%, $p<0.05$).
In AYA patients, switching remained common in later lines (2nd: 48%; 3rd: 28.5%; 4th: 75%). While resistance predominated in 1st-line switches (intolerance/resistance ratio 0.3), intolerance became the leading cause beyond 2nd line (ratio >2). Upfront use of 2G-TKIs did not appear to reduce resistance-driven 1st line switching (ratio intolerance/resistance 0.8), although subgroup numbers were limited.

### Summary/Conclusion

AYA patients with CML show a significantly higher rate of first-line TKI failure due to resistance compared to older patients, despite similar overall switching rates. Upfront second-generation TKI use does not appear to mitigate this risk. These findings suggest that AYA CML may represent a clinically distinct subgroup warranting prospective evaluation of early optimization strategies and alternative treatment approaches.

**Copyright Information:** (Online) ISSN: 2572-9241
©2026 The Author(s). HemaSphere is published by John Wiley & Sons Ltd on behalf of European Hematology Association. This is an open access Abstract Book distributed under the Attribution-NonCommercial-NoDerivs (CC BY-NC-ND), which allows third parties to download the articles and share them with others as long as they credit the author and the Abstract Book, but they cannot change the content in any way or use them commercially.
**Abstract Book Citations:** Authors, Title, HemaSphere, 2026;10:(S1):pages. The abstract book DOIs can be found on the “Author Information” page.
**Disclaimer:** Articles published in the journal HemaSphere exclusively reflect the opinions of the authors. The authors are responsible for all content in their abstracts including accuracy of the facts, statements, citing resources, etc.

### (PF635) ASCIMINIB FOR NEWLY DIAGNOSED CHRONIC MYELOID LEUKEMIA: INTERIM RESULTS FROM A PHASE II TRIAL

**Topic:** 08. Chronic myeloid leukemia - Clinical

Elias Jabbour*[1], Fadi Haddad[1], Ghayas Issa[1], Lewis Nasr[1], Jayastu Senapati[1], Hannah Goulart[1], Alex Bataller[1], Tapan Kadia[1], Kara Stevens[1], Joseph Castellano[1], Guillermo Montalban-Bravo[1], Naveen Pemmaraju[1], Sara Dellasala[1], Hagop Kantarjian[1]

[1]*MD Anderson Cancer Center, Leukemia, Houston, United States of America;*

### Background

Asciminib is safe and effective across all lines of therapy of chronic myeloid leukemia (CML). Here, we report interim results from an investigator-initiated phase II trial of asciminib in patients (pts) with newly diagnosed CML.

### Aims

To evaluate the efficacy of asciminib in newly diagnosed CML.

### Methods

Asciminib was given at 80 mg/day in pts with newly diagnosed CML in chronic phase or accelerated phase (clonal evolution only). The primary endpoint was the rate of major molecular response (MMR) by 12 months of therapy. AEs were recorded according to the CTCAE version 5.0 criteria. The data cutoff was January 20, 2026.

### Results

A total of 59 pts were treated with a median age at diagnosis of 49 years (range, 21-79); 33 (56%) were male. Sokal score was low in 29 pts (49%), intermediate in 21 (36%), and high in 9 (15%). With a median follow-up of 12 months, the cumulative best overall response was MCyR in 57 pts (97%), CCyR in 54 (92%), MMR in 40 (68%), MR4 in 21 (36%), and MR4.5 in 17 (29%). Among pts who completed at least 1 year of asciminib therapy, the MMR rate by 12 months was 71%.

By 3 months, among 58 evaluable pts, 51 (88%) achieved MCyR or transcript levels below 10% on the international scale (IS), 42 (72%) CCyR or transcript levels below 1% IS, 16 (28%) MMR, 5 (9%) MR4, and 2 (3%) MR4.5. Seven pts (12%) had transcript levels above 10%. By 6 months, among 53 evaluable pts, the rate of CCyR was 85% (45/53), MMR 64% (34/53), MR4 34% (18/53), and MR4.5 26% (14/53). By 12 months, CCyR was achieved in 27 pts (87%), MMR in 22 (71%), MR4 in 13 (42%), and MR4.5 in 10 (32%).

*ASXL1* mutation was detected in 3 of 41 pts (7%) evaluated for cancer gene variants. Of them, two achieved a complete molecular response and one achieved CCyR as best response but later lost it with the acquisition of *A337T* and *F497L*mutations.

The most frequent adverse events related or possibly related to asciminib included elevated lipase (Grade[G]2, n=9; G3, n=3; G4, n=1) and fatigue (G1, n=7; G2, n=2); 3 pts developed pancreatitis (G2, n=1; G3, n=2). A 71-year-old pt with a previous medical history of hypertension developed G5 acute coronary syndrome after 2.5 months of asciminib therapy.

Overall, 8 of 59 pts (14%) discontinued asciminib due to: 1) adverse events in 4 (including 1 fatal acute coronary syndrome); 2) loss of CCyR in 2 (one with the emergence of *A337T* and *F497L* mutations and another with *P465A* and *D381E* mutations); 3) failure to achieve CCyR in 1 patient in the setting of cytopenias and asciminib dose reduction to 40 mg/day; and 4) withdrawal of consent by 1 patient due to inability to return to clinic for treatment due to social constraints and was in MR4 at the time of asciminib discontinuation.

The 12-month rates of failure-free survival, event-free survival, transformation-free survival, and overall survival were 92%, 96%, and 98%, respectively.

**Copyright Information:** (Online) ISSN: 2572-9241
©2026 The Author(s). HemaSphere is published by John Wiley & Sons Ltd on behalf of European Hematology Association. This is an open access Abstract Book distributed under the Attribution-NonCommercial-NoDerivs (CC BY-NC-ND), which allows third parties to download the articles and share them with others as long as they credit the author and the Abstract Book, but they cannot change the content in any way or use them commercially.
**Abstract Book Citations:** Authors, Title, HemaSphere, 2026;10:(S1):pages. The abstract book DOIs can be found on the "Author Information" page.
**Disclaimer:** Articles published in the journal HemaSphere exclusively reflect the opinions of the authors. The authors are responsible for all content in their abstracts including accuracy of the facts, statements, citing resources, etc.

**Summary/Conclusion**

Treatment with asciminib in newly diagnosed CML showed a MMR tare of 71% by 12 months of therapy with no new safety signals. Disease resistance was associated with the emergence of myristoyl pocket mutations.

**Copyright Information:** (Online) ISSN: 2572-9241
©2026 The Author(s). HemaSphere is published by John Wiley & Sons Ltd on behalf of European Hematology Association. This is an open access Abstract Book distributed under the Attribution-NonCommercial-NoDerivs (CC BY-NC-ND), which allows third parties to download the articles and share them with others as long as they credit the author and the Abstract Book, but they cannot change the content in any way or use them commercially.
**Abstract Book Citations:** Authors, Title, HemaSphere, 2026;10:(S1):pages. The abstract book DOIs can be found on the "Author Information" page.
**Disclaimer:** Articles published in the journal HemaSphere exclusively reflect the opinions of the authors. The authors are responsible for all content in their abstracts including accuracy of the facts, statements, citing resources, etc.

### (PF636) TYROSINE KINASE INHIBITOR USE AND THE RISK OF NEURODEGENERATIVE DISEASE: A SWEDISH POPULATION-BASED COHORT STUDY

**Topic:** 08. Chronic myeloid leukemia - Clinical

Felix Wittström*[1], Enoch Chen[1], Iris Blotenberg[2], Torsten Dahlen[1]

*[1]Karolinska Institutet, Stockholm, Sweden; [2]Deutsches Zentrum für Neurodegenerative Erkrankungen, Greifswald, Germany;*

### Background

Effective disease-modifying therapies remain unavailable for Alzheimer's and Parkinson's diseases, the two most prevalent neurodegenerative disorders. Tyrosine kinase inhibitors (TKIs) targeting BCR::ABL1, primarily indicated for chronic myeloid leukemia (CML), have been hypothesized to modulate the pathological mechanisms underlying neurodegenerative diseases. Notably, drugs within this class differ in their kinase target selectivity and capacity to penetrate the blood-brain barrier.

### Aims

To investigate the association between the use of different TKIs and the risk of developing neurodegenerative diseases.

### Methods

CML patients who initiated TKI therapy between 2005 and 2024 were identified from Swedish population-based registers, alongside five age- and sex-matched comparators per patient. Both patients and comparators were followed from the initiation date of the respective TKI using an 'as-treated' follow-up design, stratified into six distinct cohorts. Hazard ratios (HRs) for neurodegenerative disease diagnoses were estimated using Cox proportional hazards models, with adjustments for sex, calendar year at follow-up initiation, age at follow-up initiation, and relevant comorbidities. Sensitivity analyses included an 'as-started' follow-up approach, a lag time after TKI initiation, two alternative outcome definitions, and decubitus ulcer as a negative control outcome.

### Results

A total of 1,681 imatinib, 800 dasatinib, 569 nilotinib, 213 bosutinib, 114 ponatinib, and 41 asciminib users were identified. Adjusted hazard ratios (HRs) comparing each TKI group to their matched comparator populations were 1.00 (95% CI: 0.72–1.37) for imatinib users, 1.18 (95% CI: 0.69–2.04) for dasatinib users, and 0.36 (95% CI: 0.15–0.89) for nilotinib users. The number of events among bosutinib, ponatinib, and asciminib users was insufficient to permit HR estimation. Results remained robust across four sensitivity analyses addressing differential treatment switching, reverse causality, outcome misclassification, and confounding by frailty.

### Summary/Conclusion

These findings indicate a significant reduction in the risk of Alzheimer's and Parkinson's diseases among nilotinib users, in contrast to users of other TKIs. Given that several clinical trials of TKIs have not demonstrated significant effects on neurodegenerative disease symptom progression, despite promising preclinical data, this observational study provides important evidence to support further clinical trials of nilotinib in early-stage neurodegenerative disease, particularly at doses optimized to halt disease progression.

**Copyright Information:** (Online) ISSN: 2572-9241
©2026 The Author(s). HemaSphere is published by John Wiley & Sons Ltd on behalf of European Hematology Association. This is an open access Abstract Book distributed under the Attribution-NonCommercial-NoDerivs (CC BY-NC-ND), which allows third parties to download the articles and share them with others as long as they credit the author and the Abstract Book, but they cannot change the content in any way or use them commercially.
**Abstract Book Citations:** Authors, Title, HemaSphere, 2026;10:(S1):pages. The abstract book DOIs can be found on the "Author Information" page.
**Disclaimer:** Articles published in the journal HemaSphere exclusively reflect the opinions of the authors. The authors are responsible for all content in their abstracts including accuracy of the facts, statements, citing resources, etc.

### (PF637) IMPACT OF ALLOGENEIC HEMATOPOIETIC CELL TRANSPLANTATION ON CLINICAL OUTCOMES IN CHRONIC MYELOID LEUKEMIA IN BLAST PHASE

**Topic:** 08. Chronic myeloid leukemia - Clinical

Rikki Williams*[1], Benjamin McCormick[1], Kendall Diebold[1], Aditi Sharma[1], Razan Mohty[1], Manuel Espinoza Gutarra[1], Sravanti Rangaraju[1], Kimo Bachishvili[1], Pankit Vachhani[1], Antonio Di Stasi[1], Donna Salzman[1], Zaid Al Kadhimi[1], Jorge Cortes[1], Ravi Bhatia[1], Omer Jamy[1]

*[1]University of Alabama at Birmingham, Division of Hematology/Oncology, Birmingham, United States of America;*

### Background

Chronic myeloid leukemia in blast phase (CML-BP) remains associated with poor outcomes despite tyrosine kinase inhibitors (TKIs). Allogeneic hematopoietic cell transplantation (alloHCT) is potentially curative, but comparative outcomes vs non-transplant approaches in the modern TKI era are not well defined.

### Aims

To compare overall survival (OS), leukemia-free survival (LFS), and relapse incidence in patients (pts) with CML-BP treated with alloHCT vs non-transplant strategies in the TKI era.

### Methods

We conducted a single-center retrospective study of 48 treated CML-BP pts (2008–2024); 20 (42%) underwent alloHCT. CML-BP was defined by 2022 WHO criteria (≥20% blasts in blood or marrow or extramedullary blast proliferation). Common reasons for non-transplant included age/comorbidities (n=4), refractory disease (n=9), and socioeconomic factors (n=9). To reduce selection bias, primary analyses were restricted to transplant-eligible pts (n=35; 20 alloHCT, 15 non-alloHCT), retaining those not transplanted due to socioeconomic/donor availability issues or personal choice. OS/LFS were analyzed using time-dependent Cox regression with alloHCT modeled as a time-varying covariate to address immortal time bias. Competing risks methods (Fine–Gray regression) evaluated relapse and non-relapse mortality (NRM).

**Copyright Information:** (Online) ISSN: 2572-9241
©2026 The Author(s). HemaSphere is published by John Wiley & Sons Ltd on behalf of European Hematology Association. This is an open access Abstract Book distributed under the Attribution-NonCommercial-NoDerivs (CC BY-NC-ND), which allows third parties to download the articles and share them with others as long as they credit the author and the Abstract Book, but they cannot change the content in any way or use them commercially.
**Abstract Book Citations:** Authors, Title, HemaSphere, 2026;10:(S1):pages. The abstract book DOIs can be found on the "Author Information" page.
**Disclaimer:** Articles published in the journal HemaSphere exclusively reflect the opinions of the authors. The authors are responsible for all content in their abstracts including accuracy of the facts, statements, citing resources, etc.

### Results

Median follow-up from BP diagnosis was 84 months. *De novo* CML-BP was present in 18 pts. Among transplant-eligible pts, demographics were balanced (median age 45 vs 43 years; p=0.61). Most pts received 2nd- (n=32) or 3rd-generation (n=13) TKIs for CML-BP. Induction consisted of intensive chemotherapy plus TKI in 65%, less-intensive chemotherapy plus TKI in 10%, and TKI alone in 25%. Among alloHCT pts, 74% achieved at least CCyR prior to transplant (37% MMR or deeper); 19/20 were transplanted in CR1 (median 4.6 months from CML-BP to alloHCT).

In the full cohort, mortality was 35% among alloHCT vs 79% in non-alloHCT pts. Among transplant-eligible pts, induction intensity and TKI generation were not independently associated with OS or relapse risk. Although intensive induction was associated with deeper molecular response after adjusting for age and complex cytogenetics, it did not translate into reduced relapse risk once accounting for alloHCT. Myeloid (n=27) and lymphoid (n=21) blast phenotypes were not significantly associated with OS or relapse in the full or alloHCT cohorts.
AlloHCT was associated with a numerically lower hazard of death (HR 0.59, 95% CI 0.19–1.82) and relapse/death (LFS HR 0.43, 95% CI 0.18–1.04). Achieving CCyR or better in CML-BP was independently associated with reduced relapse risk (HR 0.13, p=0.024). Competing risks analysis showed a significantly lower incidence of relapse with alloHCT (subdistribution HR 0.20, 95% CI 0.08–0.54; p=0.001) with 5-year relapse rates of 27% vs 73% without alloHCT. NRM was higher after alloHCT (HR 3.56, 95% CI 0.46–27.6), though not significant. In multivariable Fine–Gray models adjusting for response depth, age, and cytogenetics, alloHCT remained independently associated with reduced relapse (adjusted HR 0.20, p=0.001) (Figure 1).

### Summary/Conclusion

Among transplant-eligible CML-BP pts, alloHCT was associated with a marked reduction in relapse risk. Although OS benefit was attenuated by NRM, alloHCT provided substantial disease control in this high-risk population. These findings support timely transplant evaluation in eligible pts, while highlighting the importance of achieving deep remission prior to alloHCT when feasible.

**Copyright Information:** (Online) ISSN: 2572-9241
©2026 The Author(s). HemaSphere is published by John Wiley & Sons Ltd on behalf of European Hematology Association. This is an open access Abstract Book distributed under the Attribution-NonCommercial-NoDerivs (CC BY-NC-ND), which allows third parties to download the articles and share them with others as long as they credit the author and the Abstract Book, but they cannot change the content in any way or use them commercially.
**Abstract Book Citations:** Authors, Title, HemaSphere, 2026;10:(S1):pages. The abstract book DOIs can be found on the "Author Information" page.
**Disclaimer:** Articles published in the journal HemaSphere exclusively reflect the opinions of the authors. The authors are responsible for all content in their abstracts including accuracy of the facts, statements, citing resources, etc.

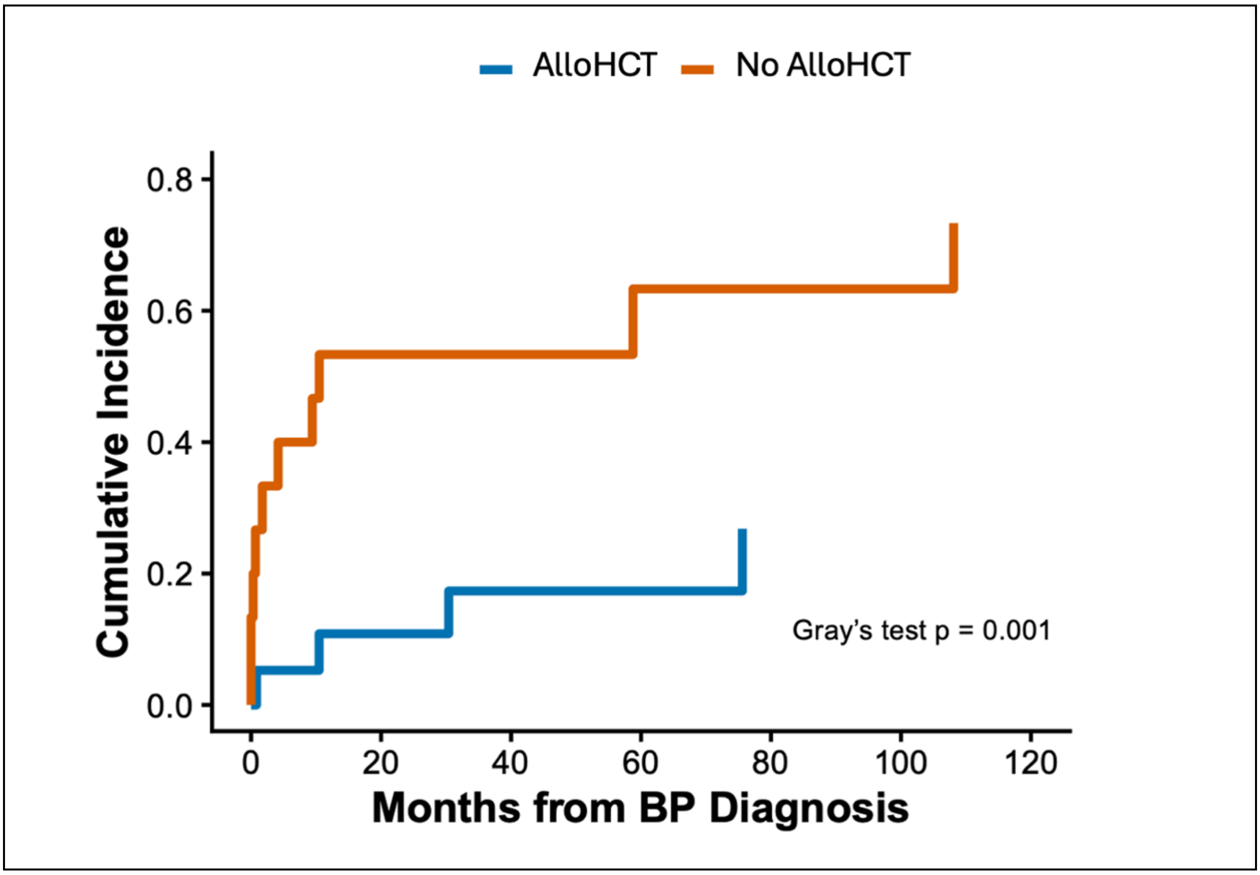

Figure 1. Cumulative incidence of relapse in transplant-eligible CML-BP patients. AlloHCT was associated with a significantly lower relapse risk (subdistribution HR 0.20, 95% CI 0.08–0.54; Gray's test p=0.001) using competing risks analysis.

**Copyright Information:** (Online) ISSN: 2572-9241
©2026 The Author(s). HemaSphere is published by John Wiley & Sons Ltd on behalf of European Hematology Association. This is an open access Abstract Book distributed under the Attribution-NonCommercial-NoDerivs (CC BY-NC-ND), which allows third parties to download the articles and share them with others as long as they credit the author and the Abstract Book, but they cannot change the content in any way or use them commercially.
**Abstract Book Citations:** Authors, Title, HemaSphere, 2026;10:(S1):pages. The abstract book DOIs can be found on the "Author Information" page.
**Disclaimer:** Articles published in the journal HemaSphere exclusively reflect the opinions of the authors. The authors are responsible for all content in their abstracts including accuracy of the facts, statements, citing resources, etc.

### (PF638) A SINGLE-CENTER, CLINICAL STUDY EVALUATING THE EFFICACY AND SAFETY OF HS-10382 IN PATIENTS WITH NEWLY DIAGNOSED PHILADELPHIA-POSITIVE (PH+) CHRONIC MYELOID LEUKEMIA IN THE CHRONIC PHASE (CML-CP)

**Topic:** 08. Chronic myeloid leukemia - Clinical

Li Zhou*[1], Ying Qian[1], Yingli Wu[1], Zhen Jin[1], Zhixiang Shen[1], Jian-qing Mi[1]

*[1]Shanghai Institute of Hematology, State Key Laboratory of Medical Genomics, National Research Center for Translational Medicine at Shanghai, Ruijin Hospital, Shanghai Jiao Tong University School of Medicine, Shanghai, China;*

### Background

Adenosine triphosphate (ATP)-competitive tyrosine kinase inhibitors (TKIs) are main treatment options in newly diagnosed Ph+ chronic myeloid leukemia in chronic phase (CML-CP); however, switching since intolerance is frequent. HS-10382, an investigational, novel, potent and highly selective *BCR::ABL1* TKI, is designed to bind the allosteric myristoyl site on the kinase, circumventing resistance to active site mutations, with potential for synergistic combination with active site TKIs. In preclinical studies, HS-10382 demonstrated greater potency than the currently approved allosteric inhibitor, asciminib, against several *BCR::ABL1* resistance mutations.

### Aims

Assess the efficacy and safety profiles of HS-10382 in patients (pts) with newly diagnosed Ph+ CML-CP.

### Methods

In this single-center, open-label study evaluating the efficacy and safety of HS-10382 in pts with newly diagnosed CML-CP, HS-10382 was administered orally BID at the dose of 160 mg in repeated 28-day cycles.

### Results

As of October 20, 2025, 21 Chinese pts were enrolled and received at least one dose of HS-10382; all were newly diagnosed with CML-CP; the median (range) age was 45.0 (28-69) years; and 57.1% (12/21) were male. All pts were categorized as low or intermediate risk based on the Sokal and ELTS scoring systems. The median (range) follow-up duration was 9.7 (5.6-11.9) months; all pts received ≥3 cycles of treatment, 95.2% (20/21), ≥6 cycles. Two (9.5%) pts discontinued treatment: 1 because of adverse events (AEs, recurrent grade 3 elevation of liver enzymes), and 1, disease progression. HS-10382 showed encouraging antileukemic activity. At Week 12 after treatment, all pts achieved complete hematologic response (CHR), 95.2% (20/21) achieved complete cytogenetic response (CCyR), all achieved early molecular response (EMR), and 38.1% (8/21) achieved major molecular response (MMR). At Week 24 after treatment, 52.4% (11/21) pts achieved MMR, 19.0% (4/21) achieved MR4.0, and 9.5% (2/21) achieved MR4.5. Overall, the cumulative MMR and CCyR rate were 66.7% (14/21) and 100% (21/21) respectively. Twenty (95.2%) pts experienced treatment-emergent AEs (TEAEs). Most TEAEs were grade 1/2 and clinically manageable. Two (9.5%) pts experienced grade ≥3 TEAEs. Four (19.0%) pts experienced TEAEs leading to treatment interruption. No serious AEs were reported. The most common (≥20% incidence) TEAEs were lipase increased (9/21, 42.9%), neutrophil count decreased (7/21, 33.3%), hyperglycaemia (7/21, 33.3%), upper respiratory tract infection (7/21, 33.3%), platelet count decreased (6/21, 28.6%) and hypercholesterolemia (5/21, 23.8%). Grade ≥3 TEAEs were platelet count decreased, alanine aminotransferase increased, aspartate aminotransferase increased and dermatitis psoriasiform (1/21, 4.8% for each).

**Copyright Information:** (Online) ISSN: 2572-9241
©2026 The Author(s). HemaSphere is published by John Wiley & Sons Ltd on behalf of European Hematology Association. This is an open access Abstract Book distributed under the Attribution-NonCommercial-NoDerivs (CC BY-NC-ND), which allows third parties to download the articles and share them with others as long as they credit the author and the Abstract Book, but they cannot change the content in any way or use them commercially.
**Abstract Book Citations:** Authors, Title, HemaSphere, 2026;10:(S1):pages. The abstract book DOIs can be found on the "Author Information" page.
**Disclaimer:** Articles published in the journal HemaSphere exclusively reflect the opinions of the authors. The authors are responsible for all content in their abstracts including accuracy of the facts, statements, citing resources, etc.

**Summary/Conclusion**

In this single-center study, HS-10382 demonstrated promising preliminary efficacy and favorable tolerability and safety at the dose of 160 mg BID in patients with newly diagnosed Ph+ CML-CP. These data support the further development of HS-10382 for the first-line treatment of CML-CP.

**Copyright Information:** (Online) ISSN: 2572-9241
©2026 The Author(s). HemaSphere is published by John Wiley & Sons Ltd on behalf of European Hematology Association. This is an open access Abstract Book distributed under the Attribution-NonCommercial-NoDerivs (CC BY-NC-ND), which allows third parties to download the articles and share them with others as long as they credit the author and the Abstract Book, but they cannot change the content in any way or use them commercially.
**Abstract Book Citations:** Authors, Title, HemaSphere, 2026;10:(S1):pages. The abstract book DOIs can be found on the "Author Information" page.
**Disclaimer:** Articles published in the journal HemaSphere exclusively reflect the opinions of the authors. The authors are responsible for all content in their abstracts including accuracy of the facts, statements, citing resources, etc.

### (PF639) EFFICACY AND SAFETY OF REDUCED-DOSE DASATINIB (50 MG/DAY) AS FIRST-LINE THERAPY IN CHRONIC MYELOID LEUKEMIA: RESULTS FROM A MULTICENTER REAL-WORLD STUDY BY THE SPANISH CML GROUP

**Topic:** 08. Chronic myeloid leukemia - Clinical

Gonzalo Maortua Langdon[1], Maria Raquel De Paz Arias*[2], Ruth Stuckey[3], Guiomar Bautista[4], Adrian Segura Diaz[5], Gonzalo Carreño Gómez-Tarragona[6], Ana Cuervo-Fonseca[6], Pablo Chamorro[7], Ylenia Morales Ruiz[8], Jose David Gonzalez San Miguel[8], Maria Gómez–garcia de Soria[9], Raul Perez Lopez[10], Jasson Andres Villarreal Hernandez[11], Alicia Roldan Perez[12], Marta Santaliestra Tomas[13], Sara Garrido Paniagua[14], Guillermo Ortí[14], María Isabel Mata Vázquez[15], Valentin Garcia Gutierrez[16]

*[1]Hospital Universitario La Paz, Madrid, Spain; [2]Hospital Universitario La Paz, Hematology and Hemotherapy, Madrid, Spain; [3]Hospital Universitario de Gran Canaria Doctor Negrín/Instituto de Investigación Sanitaria de Canarias (IISC),, Las Palmas de Gran Canaria, Spain; [4]Hospital Universitario Puerta de Hierro Majadahonda, Hematology and Hemotherapy, Madrid, Spain; [5]Hospital Universitario de Gran Canaria Doctor Negrín/Instituto de Investigación Sanitaria de Canarias (IISC), Hematology and Hemotherapy, Las Palmas de Gran Canaria, Spain; [6]Hospital Universitario 12 de Octubre, Hematology and Hemotherapy, Madrid, Spain; [7]Hospital Universitario de Cabueñes, Gijón, Spain;[8]Complejo Hospitalario Universitario Insular Materno-Infantil de Gran Canaria, Hematology and Hemotherapy, Las Palmas de Gran Canaria, Spain; [9]Hospital Universitario de la Princesa, Hematology and Hemotherapy, Madrid, Spain; [10]Hospital Clínico Universitario Virgen de la Arrixaca/Instituto Murciano de Investigación Biosanitaria, Hematology and Hemotherapy, Murcia, Spain; [11]Institut Català d'Oncologia Hospitalet, Hematology and Hemotherapy, Barcelona, Spain; [12]Hospital Universitario Infanta Sofía, Hematology and Hemotherapy, Madrid, Spain; [13]Hospital Universitario MútuaTerrasa, Hematology and Hemotherapy, Barcelona, Spain; [14]Hospital Universitario Vall d'Hebron, Hematology and Hemotherapy, Barcelona, Spain; [15]Hospital Universitario Costa del Sol, Hematology and Hemotherapy, Marbella, Spain; [16]Hospital Universitario Ramón y Cajal, Hematology and Hemotherapy, Madrid, Spain;*

### Background

Dasatinib, a second-generation BCR::ABL1 tyrosine kinase inhibitor (TKI), is approved at 100 mg/day as frontline therapy for chronic myeloid leukemia (CML). However, its use may be limited by adverse events (AEs)—most notably pleural effusion—which can compromise treatment adherence. Reduced-dose dasatinib 50 mg/day has emerged to mitigate toxicity while preserving efficacy; yet real-world evidence remains scarce.

### Aims

To assess the efficacy and safety of first-line dasatinib at 50 mg/day in a real-world multicenter cohort of CML patients.

### Methods

We conducted a multicenter retrospective observational study across 13 Spanish hospitals. Fifty-eight newly diagnosed CML patients received dasatinib 50 mg/day as first-line therapy between 2020 and 2025. Dose was based on the treating physician's clinical judgment. Molecular response (MR) was assessed by standardized BCR::ABL1 quantitative PCR at 3, 6, 9, 12, and 48 months.

**Copyright Information:** (Online) ISSN: 2572-9241
©2026 The Author(s). HemaSphere is published by John Wiley & Sons Ltd on behalf of European Hematology Association. This is an open access Abstract Book distributed under the Attribution-NonCommercial-NoDerivs (CC BY-NC-ND), which allows third parties to download the articles and share them with others as long as they credit the author and the Abstract Book, but they cannot change the content in any way or use them commercially.
**Abstract Book Citations:** Authors, Title, HemaSphere, 2026;10:(S1):pages. The abstract book DOIs can be found on the "Author Information" page.
**Disclaimer:** Articles published in the journal HemaSphere exclusively reflect the opinions of the authors. The authors are responsible for all content in their abstracts including accuracy of the facts, statements, citing resources, etc.

### Results

The cohort comprised 58 patients, 30 (51.7%) of whom were male, with a median age of 58.5 years (range 22–88). At diagnosis, 56 patients (96.6%) were in chronic phase and 2 (3.4%) in accelerated phase/blast crisis. Additional cytogenetic abnormalities were detected in 2 patients (3.4%), both with a complex karyotype. All patients harbored p210 transcripts. The SOKAL risk stratification index at diagnosis was available for most patients and was low, intermediate, and high in 28, 23, and 6 patients, respectively.

With a median follow-up (FU) of 17.1 months (range 0.1–48.0), 41 (70.7%) achieved MMR, with a median time to MMR of 6 months (range 3–48). At 3 months, 57 patients were evaluable: 11 (19.3%) achieved at least MMR (MMR: 10; MR4: 1), while 46 (80.7%) had not yet attained MMR. At 6 months (n=46 evaluable), 24 patients (52.2%) reached MMR or deeper (MMR: 13; MR4: 6; MR4.5: 4; MR5: 1). At 12 months (n=33 evaluable), 29 patients (87.9%) had achieved at least MMR (MMR: 9; MR4: 7; MR4.5: 6; MR5: 7). Notably, 3 patients experienced a transient loss of MMR at 9 months. Only 15 patients reached 48 months FU; of these, 14 (93.3%) maintained deep MR (MMR: 3; MR4: 3; MR4.5: 4; MR5: 4).

Treatment was discontinued in 14 patients (24.1%), with a median time to discontinuation of 7.6 months (range, 0.1–25.5): 8 (57.1%) due to intolerance, 1 (7.1%) to resistance, 4 (28.6%) to both, and 1 (7.1%) for other reasons. One patient died during dasatinib treatment from causes not related to CML. Of the 14 patients who discontinued, 13 switched to a second-line TKI (imatinib: 5; nilotinib: 4; asciminib: 2; bosutinib: 1), and 1 underwent dose reduction to dasatinib 20 mg/day, achieving toxicity resolution while maintaining MMR.

AEs were recorded in 25 patients (43.1%): 11 (44%) grade 1–2 and 14 (56%) grade 3–4. Pleural effusion occurred in 7 patients (12.1%), predominantly grade 3 (n = 6; one grade 2), 5 (71.4%) of whom were older than 70 years. Pulmonary arterial hypertension was documented in 1 patient, leading to discontinuation. Additional AEs included musculoskeletal toxicity, hematologic toxicity, heart failure, hearing loss, gynecomastia, and allergic rash.

### Summary/Conclusion

First-line dasatinib at 50 mg/day showed high rates of early and deep molecular responses with a manageable safety profile. Pleural effusion remained frequent in older patients, highlighting the need for careful monitoring in this subgroup. Overall, these findings support reduced-dose dasatinib as an effective frontline dose-optimization strategy in CML.

**Copyright Information:** (Online) ISSN: 2572-9241
©2026 The Author(s). HemaSphere is published by John Wiley & Sons Ltd on behalf of European Hematology Association. This is an open access Abstract Book distributed under the Attribution-NonCommercial-NoDerivs (CC BY-NC-ND), which allows third parties to download the articles and share them with others as long as they credit the author and the Abstract Book, but they cannot change the content in any way or use them commercially.
**Abstract Book Citations:** Authors, Title, HemaSphere, 2026;10:(S1):pages. The abstract book DOIs can be found on the “Author Information” page.
**Disclaimer:** Articles published in the journal HemaSphere exclusively reflect the opinions of the authors. The authors are responsible for all content in their abstracts including accuracy of the facts, statements, citing resources, etc.

### (PF640) LOSS OF HNRNPK DRIVES CMML THROUGH ABERRANT NF1 SPLICING AND RAS ACTIVATION

**Topic:** 09. Myelodysplastic syndromes - Biology & translational research

Miguel Angel Marugal*[1], Miguel Ángel Navarro Aguadero[1], Laura Serrano Ron[2], Sagrario Ortega[2], Cristina Aragoneses[1], Alicia Giménez Sánchez[1], Alvaro Otero Sobrino[1], Pablo Blanco-Carlon[2], Lucia de la Mata[1], Maria Velasco Estevez[2], Joaquín Martínez-Lopez[1, 2], Miguel Gallardo[1]

*[1]Research Institute Hospital 12 de Octubre (imas12), Translational Haematology, Madrid, Spain;[2]Spanish National Cancer Research Center (CNIO), Madrid, Spain;*

### Background

Chronic myelomonocytic leukaemia (CMML) is a clonal myeloid malignancy characterized by monocytosis and limited therapeutic options. Mutations in epigenetic and RNA splicing regulators are highly prevalent, highlighting aberrant RNA processing as a central pathogenic mechanism. Activating mutations in the RAS pathway are also frequently observed, supporting a key role for this signalling axis in CMML biology. HNRNPK is a multifunctional RNA-binding protein involved in alternative splicing regulation and hematopoietic homeostasis, and functions as a tumour suppressor in acute myeloid leukaemia. However, the impact of HNRNPK loss on RNA splicing programs and CMML development remain unknown.

### Aims

To define how loss of the splicing regulator HNRNPK affects hematopoietic stem cell (HSC) homeostasis, leukemic transformation and therapeutic vulnerability.

### Methods

An inducible *Hnrnpk* knockout mouse model (*Hnrnpk*$^{\text{lox-lox/UBC-CreERT2}}$) was generated and disease progression assessed by survival analysis, clinical parameters, peripheral blood counts and immunophenotypic analyses of bone marrow and spleen. Foetal-liver derived HSCs were used for *in vitro* studies. Transcriptomic and splicing alterations were analysed by total RNA sequencing. Gene and protein expression were assessed by RT-qPCR and Western blotting. Drug sensitivity was evaluated using WST-1 viability assays.

### Results

HNRNPK knockout mice showed reduced survival and developed a CMML-like phenotype, with an expansion of myeloid cells ($CD11b^{+}$) and granulocyte-monocyte progenitors (GMP; $cKIT^{+}$ $CD16/32^{+}$). *In vitro, Hnrnpk* loss increased HSC viability, clonogenicity and GMP-like differentiation. RNA sequencing identified aberrant NF1 pseudoexon inclusion upstream exon 32, a known splicing regulatory hotspot, resulting in reduced NF1 protein expression and activation of RAS signalling, with increased ERK and AKT phosphorylation and induction of downstream metabolic, antioxidant and anti-apoptotic programs. In consequence, *Hnrnpk*-deficient HSCs displayed an approximately 100-fold increased sensitivity to the MEK inhibitor trametinib compared to controls.

**Copyright Information:** (Online) ISSN: 2572-9241
©2026 The Author(s). HemaSphere is published by John Wiley & Sons Ltd on behalf of European Hematology Association. This is an open access Abstract Book distributed under the Attribution-NonCommercial-NoDerivs (CC BY-NC-ND), which allows third parties to download the articles and share them with others as long as they credit the author and the Abstract Book, but they cannot change the content in any way or use them commercially.
**Abstract Book Citations:** Authors, Title, HemaSphere, 2026;10:(S1):pages. The abstract book DOIs can be found on the “Author Information” page.
**Disclaimer:** Articles published in the journal HemaSphere exclusively reflect the opinions of the authors. The authors are responsible for all content in their abstracts including accuracy of the facts, statements, citing resources, etc.

### Summary/Conclusion

These findings establish HNRNPK as a critical regulator of haematopoiesis through alternative splicing control. Its loss induces CMML-like disease via aberrant NF1 splicing and consequent RAS activation, uncovering a mechanistic link between spliceosome dysfunction and oncogenic RAS signalling. Our findings identify MEK inhibition as a potential therapeutic strategy in spliceosome-mutant myeloid malignancies. Ongoing validation in our CMML cohort samples aims to confirm this NF1 splicing event, supporting trametinib use in RAS-wild-type patients with splicing factor mutant variants.

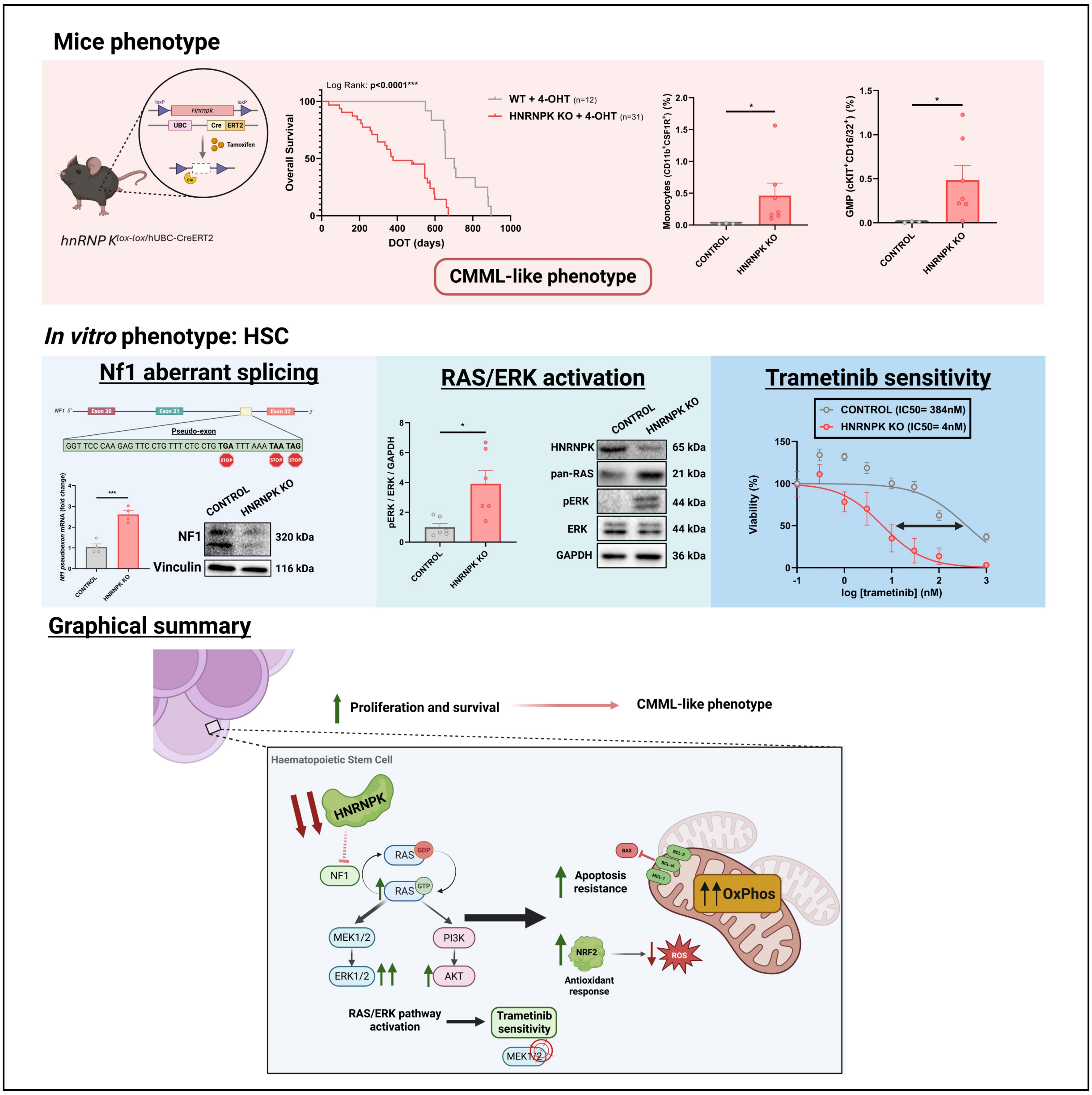

**Copyright Information:** (Online) ISSN: 2572-9241
©2026 The Author(s). HemaSphere is published by John Wiley & Sons Ltd on behalf of European Hematology Association. This is an open access Abstract Book distributed under the Attribution-NonCommercial-NoDerivs (CC BY-NC-ND), which allows third parties to download the articles and share them with others as long as they credit the author and the Abstract Book, but they cannot change the content in any way or use them commercially.
**Abstract Book Citations:** Authors, Title, HemaSphere, 2026;10:(S1):pages. The abstract book DOIs can be found on the "Author Information" page.
**Disclaimer:** Articles published in the journal HemaSphere exclusively reflect the opinions of the authors. The authors are responsible for all content in their abstracts including accuracy of the facts, statements, citing resources, etc.

### (PF641) RNA-GUIDED SELECTIVE DNMT1 INHIBITION RESTORES MYELOID DIFFERENTIATION AND OUTPERFORMS AZACITIDINE IN TET2-MUTANT MYELODYSPLASTIC SYNDROMES

**Topic:** 09. Myelodysplastic syndromes - Biology & translational research

Annalisa Di Ruscio*[1], Lucrezia Rinaldi[2], Davide D'onghia[2], Marcin Kortylewski[3], John Clohessy[1], Vittorio de Franciscis[4], Giovanni Amabile[5], Robert Welner[6]

*[1]Beth Israel Deaconess Medical Center-Harvard Medical School, Medicine, Boston, United States of America;[2]Beth Israel Deaconess Medical Center, Medicine, Bostob, United States of America; [3]Beckman Research Institute, City of Hope, Los Angeles, United States of America; [4]National Research Council (CNR), Institute of Genetic and Biomedical Research (IRGB),, Milan, Italy; [5]Aptadir Therapeutics, Milan, United States of America; [6]O'Neal Comprehensive Cancer Center at The University of Alabama at Birmingham, Birmingham, United States of America;*

### Background

Loss-of-function mutations in *TET2* are among the most frequent genetic alterations in myelodysplastic syndromes (MDS) and drive aberrant DNA methylation and impaired myeloid differentiation. Patients with TET2-mutant MDS commonly receive hypomethylating agents (HMAs), such as azacitidine and decitabine, which broadly inhibit DNMT activity. Although TET2-mutant patients may initially respond, most eventually relapse due to resistance, and treatment is limited by toxicity. This therapeutic gap underscores the need for selective and durable strategies to reverse TET2-driven epigenetic dysregulation. We developed aptaDiR_Ce-49_sh_v1, a DNMT1-selective RNA inhibitor designed to counterbalance TET2 mutation-driven epigenetic rewiring.

### Aims

To determine whether selective DNMT1 inhibition restores myeloid differentiation in TET2-deficient MDS in vivo and and provide advantages over conventional HMAs, specifically azacitidine

### Methods

A syngeneic Tet2/Asxl1 double-knockout hematopoietic transplantation model of myelodysplasia was generated by transplanting Vav-Cre$^+$ Tet2$^{flox/flox}$ Asxl1$^{flox/flox}$ CD45.2$^+$ bone marrow cells with wild-type CD45.1$^+$ support cells into lethally irradiated congenic recipients. Following engraftment, mice developed an MDS-like phenotype characterized by myeloid skewing and HSPC expansion. Three treatment arms (n=3/group) were evaluated: vehicle, azacitidine (1 mg/kg), aptaDiR_Ce-49_sh_v1 (1 mg/kg). RNA molecules were encapsulated in myeloid-targeted lipid nanoparticles and administered intravenously three times weekly for four weeks. Engraftment and differentiation were assessed by flow cytometry, bone marrow cytomorphology, and peripheral white blood cell counts.

### Results

Selective DNMT1 inhibition by aptaDiR_Ce-49_sh_v1 induced robust granulocytic maturation and restored myeloid differentiation in TET2-deficient hematopoiesis, exceeding the effects observed with azacitidine (**Fig.1**). Treatment activated myeloid lineage and innate immune transcriptional programs consistent with differentiation rescue. In contrast to azacitidine, aptaDiR_Ce-49_sh_v1 did not induce cytopenias or reduce total white blood cell counts, indicating superior hematologic tolerability.

**Copyright Information:** (Online) ISSN: 2572-9241
©2026 The Author(s). HemaSphere is published by John Wiley & Sons Ltd on behalf of European Hematology Association. This is an open access Abstract Book distributed under the Attribution-NonCommercial-NoDerivs (CC BY-NC-ND), which allows third parties to download the articles and share them with others as long as they credit the author and the Abstract Book, but they cannot change the content in any way or use them commercially.
**Abstract Book Citations:** Authors, Title, HemaSphere, 2026;10:(S1):pages. The abstract book DOIs can be found on the "Author Information" page.
**Disclaimer:** Articles published in the journal HemaSphere exclusively reflect the opinions of the authors. The authors are responsible for all content in their abstracts including accuracy of the facts, statements, citing resources, etc.

**Summary/Conclusion**

RNA-guided selective DNMT1 inhibition restores myeloid differentiation in vivo in a TET2-mutant MDS model, outperforming conventional HMAs while preserving hematologic integrity. These findings identify selective DNMT1 targeting as a precision therapeutic strategy for TET2-mutant MDS.

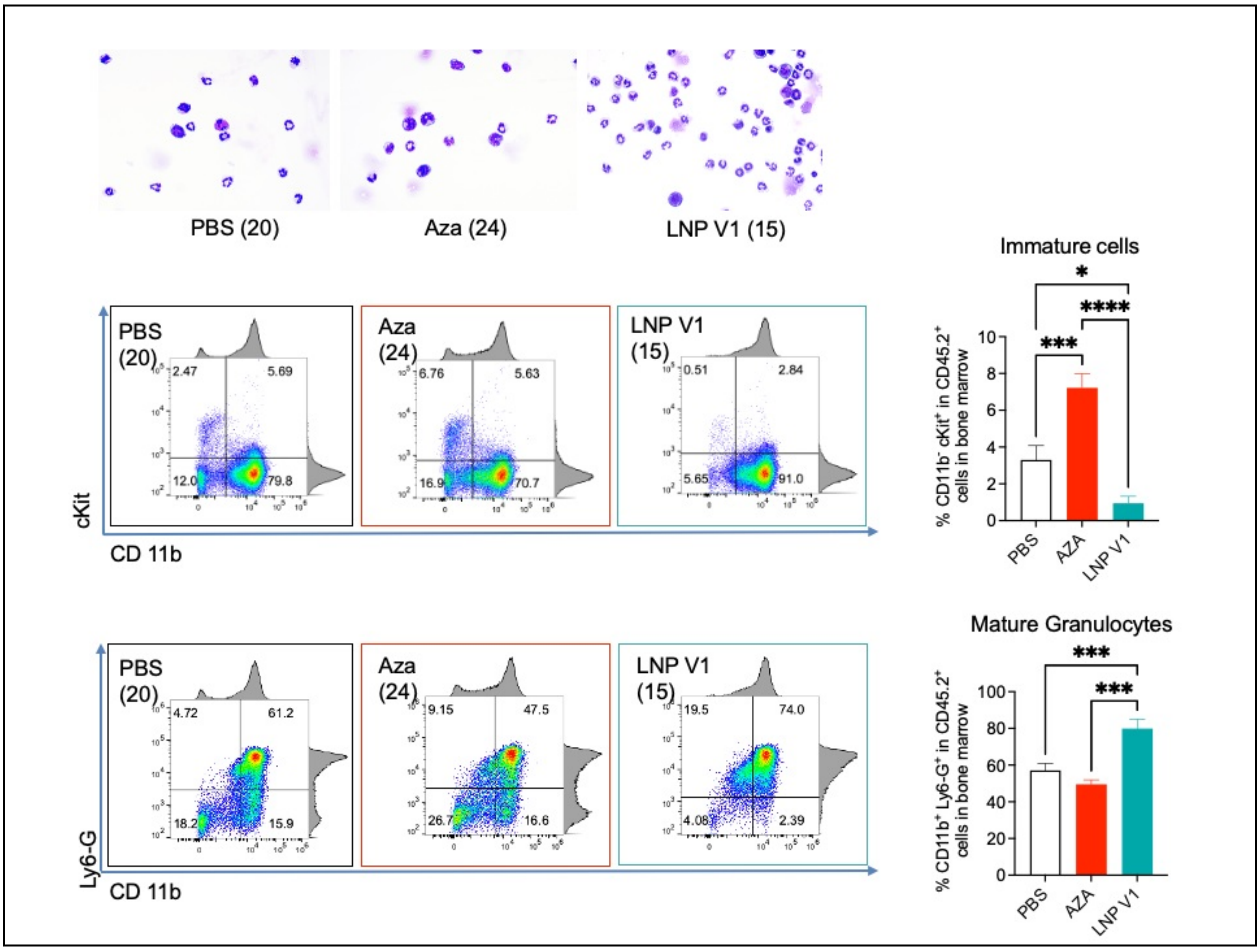

Figure 1. aptaDiR_Ce-49 sh_v1 induces myeloid differentiation. Upper panels: May–Grünwald–Giemsa staining of bone marrow cytospins after 4 weeks of treatment in Vehicle (PBS), azacitidine (1 mg/kg), and aptaDiR_Ce-49_sh_v1 (LNP V1; 1 mg/kg) groups (n=3/group), demonstrating remarkable myeloid maturation with granulocytic differentiation in LNP V1–treated mice. Middle panels: Representative flow cytometry plots showing increased mature myeloid cells ($CD11b^+/cKit^-$) and reduced immature progenitors ($CD11b^-/cKit^+$) following LNP V1 treatment. Quantification is shown on the right. Lower panels: Representative flow cytometry plots demonstrating increased granulocytic differentiation ($CD11b^+/Ly6G^+$) in LNP V1–treated mice, with corresponding quantification. Data are presented as mean ± SD (n=3). Statistical analysis was performed using one-way ANOVA (**p<0.01, ***p<0.001, ****p<0.0001).

Copyright Information: (Online) ISSN: 2572-9241
©2026 The Author(s). HemaSphere is published by John Wiley & Sons Ltd on behalf of European Hematology Association. This is an open access Abstract Book distributed under the Attribution-NonCommercial-NoDerivs (CC BY-NC-ND), which allows third parties to download the articles and share them with others as long as they credit the author and the Abstract Book, but they cannot change the content in any way or use them commercially.
Abstract Book Citations: Authors, Title, HemaSphere, 2026;10:(S1):pages. The abstract book DOIs can be found on the "Author Information" page.
Disclaimer: Articles published in the journal HemaSphere exclusively reflect the opinions of the authors. The authors are responsible for all content in their abstracts including accuracy of the facts, statements, citing resources, etc.

### (PF642) THERAPEUTIC SILENCING OF TMPRSS6 ATTENUATES IRON-DRIVEN INFLAMMASOME ACTIVATION AND DELAYS DISEASE PROGRESSION IN MDS MICE

**Topic:** 09. Myelodysplastic syndromes - Biology & translational research

Jim Vadolas*[1], Shahla Vilcassim[2], Nuttanan Pholngam[3], Rattanawan Thubthed[4], Tiwaporn Nualkaew[5], Saovaros Svasti[6], Pornthip Chaichompoo[7], Kai Kysenius[8], Peter Crouch[8], Alberto Martinez[9], Ute Schaeper[10], George Grigoriadis[1]

*[1]Hudson Institute of Medical Research, Centre of Cancer Research, Melbourne, Australia;[2]Monash University, School of Clinical Sciences at Monash Health,, Melbourne, Australia; [3]Institute of Molecular Biosciences, Mahidol University., Bangkok, Thailand; [4]Mahidol University, Department of Pathobiology, Faculty of Science, Bangkok,, Thailand;[5]Walailak University, Department of Medical Technology, Nakhon Si Thammarat, Thailand; [6]Mahidol University, Institute of Molecular Biosciences, Nakhon Pathom, Thailand; [7]Mahidol University, Department of Pathobiology, Faculty of Science, Bangkok, Thailand;[8]The University of Melbourne, Department of Anatomy & Physiology and Centre for Muscle Research, Melbourne, Australia; [9]Silence Therapeutics PLC, London, United Kingdom; [10]Silence Therapeutics GmbH, Berlin, Germany;*

### Background

Myelodysplastic syndromes (MDS) are clonal hematopoietic malignancies characterized by ineffective hematopoiesis, cytopenias, and risk of transformation to acute myeloid leukemia. Iron overload is increasingly recognized as a pathogenic amplifier of oxidative stress and innate immune activation within the bone marrow (BM) niche, yet strategies to therapeutically modulate iron-driven inflammation remain limited. Hepcidin, encoded by *Hamp*, is a liver-derived peptide hormone that regulates systemic iron homeostasis by binding and degrading the iron exporter ferroportin, thereby limiting dietary absorption and iron release from macrophages and hepatocytes. Hepcidin expression is tightly regulated by the bone morphogenetic protein (BMP)/SMAD signaling whereas the transmembrane serine protease matriptase-2 (*Tmprss6*) acts as a negative regulator by cleaving hemojuvelin, a BMP co-receptor. Targeting the TMPRSS6-hepcidin axis therefore provides a rational strategy to mitigate iron-mediated inflammation and disrupt the pathogenic cycle of MDS.

### Aims

To determine whether hepatocyte-directed Tmprss6 silencing using a GalNAc-conjugated siRNA (SLN124) suppresses iron-driven inflammasome activation and alters disease trajectory in MDS.

### Methods

We utilised a hepatocyte-directed GalNAc-conjugated siRNA (SLN124) to silence *Tmprss6* in vivo. The NUP98–HOXD13 (NHD13) murine model of MDS and wild-type controls received monthly subcutaneous SLN124 (3 mg/kg) or oral deferiprone (1.25 mg/mL). Hepatic *Tmprss6* and *Hamp* transcripts were quantified by RT-qPCR. Spatial distribution of tissue iron was assessed by laser ablation inductively coupled plasma mass spectrometry (LA-ICP-MS), with total iron content determined by bulk ICP-MS. Inflammasome activation was evaluated by immunofluorescent quantification of ASC speck formation in $CD45^+$ BM and splenic cells. Hematological indices and overall survival were monitored longitudinally.

**Copyright Information:** (Online) ISSN: 2572-9241
©2026 The Author(s). HemaSphere is published by John Wiley & Sons Ltd on behalf of European Hematology Association. This is an open access Abstract Book distributed under the Attribution-NonCommercial-NoDerivs (CC BY-NC-ND), which allows third parties to download the articles and share them with others as long as they credit the author and the Abstract Book, but they cannot change the content in any way or use them commercially.
**Abstract Book Citations:** Authors, Title, HemaSphere, 2026;10:(S1):pages. The abstract book DOIs can be found on the "Author Information" page.
**Disclaimer:** Articles published in the journal HemaSphere exclusively reflect the opinions of the authors. The authors are responsible for all content in their abstracts including accuracy of the facts, statements, citing resources, etc.

### Results

Iron accumulation within the hematopoietic niche was most prominent during early disease and coincided with increased ASC speck formation in CD45⁺ cells, consistent with enhanced inflammasome assembly. Quantitative analyses demonstrated a significant positive correlation between tissue iron burden and the frequency of ASC⁺ CD45⁺ hematopoietic cells in both BM and spleen, supporting a mechanistic link between excess iron and innate immune activation. Both SLN124 and deferiprone reduced tissue iron deposition and ASC speck abundance, however, SLN124 achieved a more durable and marked suppression of inflammasome activity. Prolonged SLN124 administration delayed the progression of anemia and significantly extended survival, with 30% of treated mice remaining alive beyond 450 days, whereas complete mortality occurred by day 420 in control and deferiprone-treated cohorts.

### Summary/Conclusion

Hepatocyte-targeted Tmprss6 silencing via SLN124 reduced inflammatory activity in the BM and spleen, and delayed the onset and progression of hematological decline in MDS mice. These findings provide mechanistic insight linking dysregulated iron homeostasis to sustained innate immune activation in MDS and establish proof-of-concept for TMPRSS6 inhibition as a potential disease-modifying therapeutic strategy.

**Copyright Information:** (Online) ISSN: 2572-9241
©2026 The Author(s). HemaSphere is published by John Wiley & Sons Ltd on behalf of European Hematology Association. This is an open access Abstract Book distributed under the Attribution-NonCommercial-NoDerivs (CC BY-NC-ND), which allows third parties to download the articles and share them with others as long as they credit the author and the Abstract Book, but they cannot change the content in any way or use them commercially.
**Abstract Book Citations:** Authors, Title, HemaSphere, 2026;10:(S1):pages. The abstract book DOIs can be found on the “Author Information” page.
**Disclaimer:** Articles published in the journal HemaSphere exclusively reflect the opinions of the authors. The authors are responsible for all content in their abstracts including accuracy of the facts, statements, citing resources, etc.

### (PF643) REFINING MDS CLASSIFICATION: CHARACTERIZATION OF NOVEL STAG2, EZH2, -7, AND TET2 BI-ALLELIC ENTITIES AND THEIR IMPACT ON RISK STRATIFICATION

**Topic:** 09. Myelodysplastic syndromes - Biology & translational research

Carlos Ruiz Arenas*[1], Roberto Sánchez Navarro[1], Marina Diaz Beya[2], Teresa Bernal[3], Montserrat Arnan Sangerman[4], Joaquín Sanchez-García[5], Raúl Azibeiro-Melchor[6], María Luz Amigo[7], Blanca Xicoy Cirici[8], Fernando Ramos[9], Mar Tomo[10, 11], Juan José Bargay Lleonart[12], M Teresa Cedena[13], Leonor Arenillas[14], Jose Falantes[15], Ariane Unamunzaga[16], Lourdes Hermosín Ramos[17], David Valcárcel[18], Maria Diez Campelo[6], Felipe Prosper[19, 20], Irene Gañán Gómez[1, 19, 20], Ana Alfonso Pierola[19, 20], Mikel Hernaez[1, 20, 21]

*[1]CIMA Universidad de Navarra, Instituto de Investigaciones Sanitarias de Navarra (IdiSNA), Cancer Center Clinica Universidad de Navarra (CCUN), Pamplona, Spain; [2]Hospital Clínic de Barcelona, Barcelona, Spain;[3]Hospital Universitario Central de Asturias (HUCA), Oviedo, Spain; [4]Institut Català d'Oncologia, Institut d'Investigació Biomèdica de Bellvitge (IDIBELL),, L'Hospitalet de Llobregat, Spain; [5]Hospital Universitario Reina Sofía, Córdoba, Spain;[6]Hospital Universitario de Salamanca, Salamanca, Spain; [7]Hospital General Universitario Morales Meseguer, Murcia, Spain;[8]Institut Català d'Oncologia (ICO) – Hospital Germans Trias i Pujol,, Badalona, Spain; [9]Hospital de León, León, Spain;[10]Hospital Clínico Universitario de Valencia, Valencia, Spain; [11]Instituto de investigación biomédica INCLIVA, Valencia, Spain; [12]Hospital Universitario Son Llàtzer, Palma de Mallorca, Spain; [13]H. Universitario 12 de Octubre, Madrid, Spain; [14]Hospital del Mar, Barcelona, Spain; [15]Hospital Universitario Virgen del Rocío, Sevilla, Spain; [16]Hospital Universitario de Álava – Txagorritxu, Vitoria-Gasteiz, Spain; [17]Hospital de Jerez de la Frontera, Jerez de la Frontera, Spain; [18]Hospital Universitari Vall D'Hebron, Barcelona, Spain; [19]Clinica Universidad de Navarra, Pamplona, Spain; [20]Centro de Investigacion Biomedica en Red de Cancer (CIBERONC), Madrid, Spain;[21]Center for Data Science and Artificial Intelligence (DATAI), University of Navarra, Pamplona, Spain;*

### Background

Classification of myelodysplastic syndrome (MDS) patients is shifting from morphology to molecular biology. Although the last ICC (International Consensus Classification) defined **3 biological entities**, these only **comprised 30% of the patients,** with the majority still defined only by their blast percentage.

### Aims

To define additional MDS sub-groups based on molecular features, using a data-driven approach.

### Methods

We analyzed 2030 MDS patients (MDS-LB, MDS-IB1, MDS-IB2) from the International Working Group (IWG) (n=1634) and the Spanish Group of MDS (GESMD) (n=396) cohorts. We clustered patients with similar clinical, cytogenetic and mutation features via multiple factor analysis. We identified 10 clusters in the IWG and 11 in GESMD cohorts and mapped them to the most discriminative mutation or karyotype event.

**Copyright Information:** (Online) ISSN: 2572-9241
©2026 The Author(s). HemaSphere is published by John Wiley & Sons Ltd on behalf of European Hematology Association. This is an open access Abstract Book distributed under the Attribution-NonCommercial-NoDerivs (CC BY-NC-ND), which allows third parties to download the articles and share them with others as long as they credit the author and the Abstract Book, but they cannot change the content in any way or use them commercially.
**Abstract Book Citations:** Authors, Title, HemaSphere, 2026;10:(S1):pages. The abstract book DOIs can be found on the "Author Information" page.
**Disclaimer:** Articles published in the journal HemaSphere exclusively reflect the opinions of the authors. The authors are responsible for all content in their abstracts including accuracy of the facts, statements, citing resources, etc.

### Results

Seven mutations or karyotype events identified clusters in both datasets. Three mapped to equivalent ICC sub-groups (del5q-IB, SF3B1-IB, complex karyotype), corresponding to 10% of the patients; four were novel (defined by **EZH2, TET2 bi-allelic, STAG2 mutations, and monosomy 7, or** -7) and **represented 30% of patients**. The remaining 60% of patients were classified based on blast proportions (Fig 1A).
**Our novel sub-groups presented differentiated cytopenic profiles** (Fig 1B). When compared to the largest group (MDS-LB), -7 patients were more likely to present with pancytopenia, STAG2 patients showed higher rates of leukopenia and anemia, while EZH2 patients presented only with anemia. In contrast, TET2 bi-allelic patients had the profile most similar to MDS-LB. Our novel sub-groups also presented with **distinctive mutational profiles**, with EZH2 and STAG2 frequently co-mutated with genes such as *RUNX1* or *ASXL1*, while TET2 bi-allelic mutations were mutually exclusive with mutations in a high number of genes, such as *IDH2* or *U2AF1* (Fig 1C). ICC-equivalent sub-groups also presented distinctive and different cytopenic and mutational profiles.
Our novel sub-groups had consistent risk score predictions by IPSSM (Fig 1D). EZH2, -7 and STAG2 sub-groups predominantly included high-risk patients, while TET2 bi-allelic patients had lower risk. Despite that, we observed key **discrepancies between IPSSM score and prognosis**. For instance, high-risk STAG2 patients (IPSSM from Moderate-High to Very-High) presented a similar overall survival (Fig 1E). These discrepancies can be due to genetic factors having a different effect on prognosis in our sub-groups. Thus, +8 had no impact on overall survival in STAG2 patients (Fig 1F). Remarkably, IPSSM score also underpredicted the risk of acute myeloid leukemia transformation (AMLt) in IPSSM Moderate-Low TET2 bi-allelic patients, who carry a risk comparable to that of high-risk patients (Fig 1G).

### Summary/Conclusion

**Our data-driven approach has identified novel MDS sub-groups with distinct clinical and molecular profiles**, which respond differently to genetic risk factors modelled in the IPSSM score. Current ICC biological entities already fulfill all these characteristics. **Our sub-groups likely represent different biological entities** that may have distinctive responses to treatment, which should be considered in translational studies for drug development. Furthermore, this classification is already relevant for clinical practice in the case of TET2 bi-allelic patients, who have increased risk of AMLt. All in all, our new sub-types set the foundation for the development of improved, patient-tailored strategies for the management of MDS.

**Copyright Information:** (Online) ISSN: 2572-9241
©2026 The Author(s). HemaSphere is published by John Wiley & Sons Ltd on behalf of European Hematology Association. This is an open access Abstract Book distributed under the Attribution-NonCommercial-NoDerivs (CC BY-NC-ND), which allows third parties to download the articles and share them with others as long as they credit the author and the Abstract Book, but they cannot change the content in any way or use them commercially.
**Abstract Book Citations:** Authors, Title, HemaSphere, 2026;10:(S1):pages. The abstract book DOIs can be found on the “Author Information” page.
**Disclaimer:** Articles published in the journal HemaSphere exclusively reflect the opinions of the authors. The authors are responsible for all content in their abstracts including accuracy of the facts, statements, citing resources, etc.

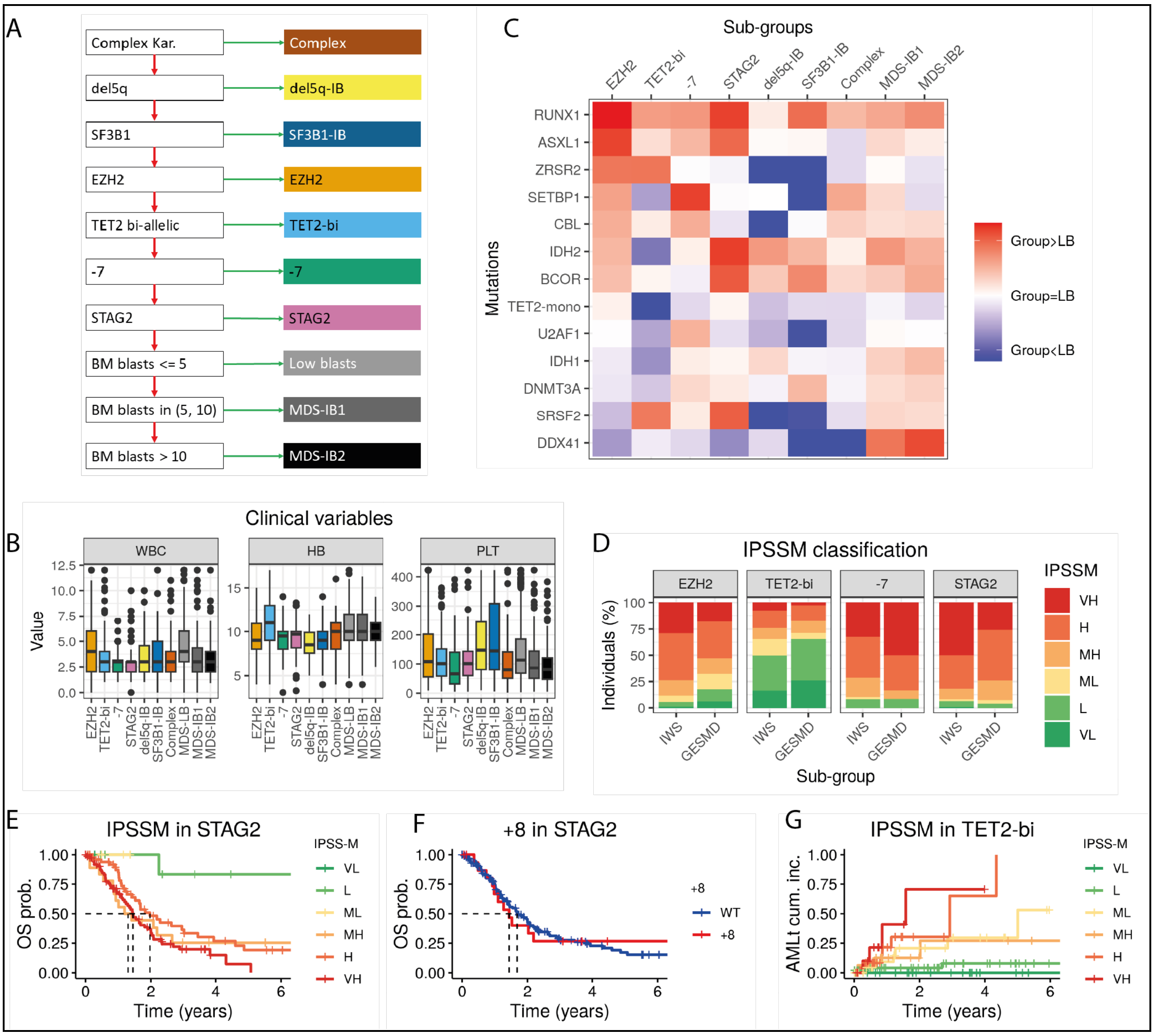

Figure 1. Characterization and Prognostic Impact of Molecular MDS Subgroups. A: Flowchart of MDS subgroup definitions. B: Distribution of clinical variables of MDS subgroups. WBC: White blood cell count; HB: Hemoglobin (g/dL); PLT: Platelet count (G/L). C: Heatmaps representing mutational profiles of MDS subgroups. Mutation frequencies for each subgroup are compared against MDS-LB patients. Red indicates higher values/frequencies and blue indicates lower values/frequencies relative to the MDS-LB cohort. D: Distribution of patients across IPSSM risk categories: VL, Very-Low; L, Low; ML, Moderate-Low; MH, Moderate-High; H, High; VH, Very-High. E: Overall survival of STAG2 patients stratified by IPSSM category. Median OS (years): MH (1.3), H (1.97), VH (1.45). F: Overall survival of STAG2 patients stratified by trisomy 8 (+8) status. G: Cumulative incidence of AML transformation (AMLt) for TET2 bi-allelic patients, stratified by IPSSM risk category.

**Copyright Information:** (Online) ISSN: 2572-9241
©2026 The Author(s). HemaSphere is published by John Wiley & Sons Ltd on behalf of European Hematology Association. This is an open access Abstract Book distributed under the Attribution-NonCommercial-NoDerivs (CC BY-NC-ND), which allows third parties to download the articles and share them with others as long as they credit the author and the Abstract Book, but they cannot change the content in any way or use them commercially.
**Abstract Book Citations:** Authors, Title, HemaSphere, 2026;10:(S1):pages. The abstract book DOIs can be found on the "Author Information" page.
**Disclaimer:** Articles published in the journal HemaSphere exclusively reflect the opinions of the authors. The authors are responsible for all content in their abstracts including accuracy of the facts, statements, citing resources, etc.

### (PF644) RETROTRANSPOSABLE ELEMENT DEREPRESSION DISTINGUISHES DNMT3A- FROM TET2-MUTANT CLONAL HEMATOPOIESIS

**Topic:** 09. Myelodysplastic syndromes - Biology & translational research

Maroof Hasan[1], Richard Thompson[2], Hanae Roussotte[2], Yi-Ting Tsai[2], Nogayhan Seymen[2], Sila Gerlevik[2], Nicholas Bianchini[2], Khadijeh Alishah[2], Merilyn Albuquerque[2], Jen Lewis[2], Laarni Bogaanay[3], Niels Asger Jakobson[4], Bernd Zeisig[2], Sheeba Irshad[2], Paresh Vyas[4], Chi Wai Eric So[2], Alfredo Iacoangeli[5], Xuesen Zheng[2], Sarah MacKie[2], Kai Yi Mok[2], Viktoria Uksaite[2], Louis-Francois Handfield[2], Giorgio Napolitani[2], James Carmichael[1], Ghulam Mufti[2], Anita K. Gandhi[1], Rajasekhar N.V.S. Suragani[1], Lynn Quek[2], Mohammad M Karimi*[2]

*[1]Bristol Myers Squibb, Hematology Translational Medicine, New Jersey, United States of America;[2]King's College London, Comprehensive Cancer Centre, London, United Kingdom; [3]King's College Hospital NHS Foundation Trust, Haematology Department, London, United Kingdom; [4]University of Oxford, MRC Molecular Haematology Unit, Oxford, United Kingdom;[5]King's College London, Department of Basic and Clinical Neuroscience, London, United Kingdom;*

### Background

Clonal hematopoiesis (CH) arises from age-associated somatic mutations in hematopoietic stem cells, most commonly affecting the epigenetic regulators DNA methyltransferase 3α (DNMT3A) and ten-eleven translocation methylcytosine dioxygenase 2 (TET2). These mutations increase the risk of hematologic malignancies and cardiovascular diseases through altered immune and inflammatory signalling. Although both DNMT3A and TET2 mutations are associated with inflammation, the cell-intrinsic mechanisms driving mutation-specific immune phenotypes and clinical heterogeneity in CH remain unclear. Derepression of retrotransposable elements (RTE), a feature of aging and epigenetic instability, represents a potential mechanistic link between most common CH mutations, inflammation, and cellular senescence.

### Aims

To define how DNMT3A and TET2 mutations differentially regulate retrotransposable element expression and to determine how RTE activity relates to clonal burden and downstream inflammatory, senescence, and metabolic signalling in human clonal hematopoiesis.

### Methods

We analysed genomic data from a healthy ageing cohort of 738 individuals (aged >50 years) without hematological malignancy. DNMT3A- and TET2-mutantions and clonal burden were identified by genomic sequencing and variant allele frequency (VAF). Peripheral blood mononuclear cells (PBMCs) RNA-sequencing from 68 individuals (mutation carriers and non-CH controls) was used to quantify RTE expression across classes and families. Associations with mutation site, clonal burden, and inflammatory, interferon, senescence, and senescence-associated secretory phenotype (SASP) pathways were assessed by regression and enrichment analyses and validated in an independent cohort.

**Copyright Information:** (Online) ISSN: 2572-9241
©2026 The Author(s). HemaSphere is published by John Wiley & Sons Ltd on behalf of European Hematology Association. This is an open access Abstract Book distributed under the Attribution-NonCommercial-NoDerivs (CC BY-NC-ND), which allows third parties to download the articles and share them with others as long as they credit the author and the Abstract Book, but they cannot change the content in any way or use them commercially.
**Abstract Book Citations:** Authors, Title, HemaSphere, 2026;10:(S1):pages. The abstract book DOIs can be found on the "Author Information" page.
**Disclaimer:** Articles published in the journal HemaSphere exclusively reflect the opinions of the authors. The authors are responsible for all content in their abstracts including accuracy of the facts, statements, citing resources, etc.

### Results

RTE derepression was strongly associated with DNMT3A mutation status and high clonal burden, whereas TET2-mutant clones showed overall reduced RTE expression. Notably, DNMT3A-mutant clones with high variant allele frequency (VAF >10%) and elevated RTE expression exhibited pronounced TNF-α/NF-κB–driven inflammatory and senescence signatures, whereas low-VAF clones (<10%) with high RTE expression were primarily associated with senescence pathways. In contrast, TET2-mutant clones lacked these RTE-driven signatures and instead showed enrichment of oxidative phosphorylation, reactive oxygen species (ROS), and mechanistic target of rapamycin complex 1 (mTORC1) signalling relative to DNMT3A low-VAF clones. Compared with DNMT3A high-VAF clones, TET2 mutants showed increased ROS signalling and interferon-α responses, consistent with a metabolically driven and redox-associated inflammatory phenotype independent of RTE activation.

### Summary/Conclusion

These findings identified mutation- and clonal burden-specific mechanisms of inflammation in clonal hematopoiesis, with retrotransposon reactivation acting as a cell-intrinsic inflammatory driver in DNMT3A-mutant clones, whereas metabolic and redox dysregulation underlies inflammatory signalling in TET2-mutant clones, supporting the view that epigenetic dysfunction can drive chronic inflammation and clonal expansion through distinct routes. This mechanistic divergence provides a framework for understanding clinical heterogeneity in CH, underscoring mutation-specific therapeutic opportunities and the potential repurposing of antiviral agents.

**Copyright Information:** (Online) ISSN: 2572-9241
©2026 The Author(s). HemaSphere is published by John Wiley & Sons Ltd on behalf of European Hematology Association. This is an open access Abstract Book distributed under the Attribution-NonCommercial-NoDerivs (CC BY-NC-ND), which allows third parties to download the articles and share them with others as long as they credit the author and the Abstract Book, but they cannot change the content in any way or use them commercially.
**Abstract Book Citations:** Authors, Title, HemaSphere, 2026;10:(S1):pages. The abstract book DOIs can be found on the “Author Information” page.
**Disclaimer:** Articles published in the journal HemaSphere exclusively reflect the opinions of the authors. The authors are responsible for all content in their abstracts including accuracy of the facts, statements, citing resources, etc.

### (PF645) TET2 MUTATION IS ASSOCIATED WITH FAVORABLE PROGNOSIS AND SPECIFIC EPIGENETIC CHROMATIN STATE SHIFTS IN CMML

**Topic:** 09. Myelodysplastic syndromes - Biology & translational research

Matilda Kjellander Kynning*[1], Yasuhito Nannya[2], Xiangfu Zhong[1, 3], Yotaro Ochi[2], Tetsuichi Yoshizato[1, 2], Gabriele Todisco[1], Bianca Tesi[1], Andreas Lennartsson[1], Teresa Mortera Blanco[1], Oscar Brück[4], Kazuma Ohyashiki[5], Takayuki Ishikawa[6], Satu Mustjoki[7], Torsten Haferlach[8], Eva Hellström Lindberg[1], Seishi Ogawa[2], Johanna Ungerstedt[3, 9, 10]

*[1]Karolinska Institutet, Center for Hematology and Regenerative Medicine HERM, Department of Medicine, Stockholm, Sweden; [2]Department of Pathology and Tumor biology, Kyoto University, Kyoto, Japan;[3]Department of Biomedical and Clinical Sciences, Linköping University, Linköping, Sweden; [4]Comprehensive Cancer Center, Helsinki University Hospital and Department of Oncology, University of Helsinki, Helsinki, Finland; [5]Department of Hematology, Tokyo Medical University, Tokyo, Japan; [6]Department of Hematology, Kobe City Medical Center General Hospital, Hyogo, Japan;[7]Department of Clinical Chemistry and Haematology, University of Helsinki, Helsinki, Finland; [8]MLL Munich Leukemia Laboratory, Munich, Germany; [9]Hematology Clinic, Linköping University Hospital, Linköping, Sweden; [10]Science for Life Laboratory, Linköping University, Linköping, Sweden;*

### Background

Chronic myelomonocytic leukemia (CMML) is a stem cell disease with adverse prognosis. The pattern of somatic driver mutations has been extensively studied and *TET2* mutation (*TET2*$^{MT}$), the most frequent mutation, is associated with a longer overall survival, especially multihit *TET2*$^{MT}$.

### Aims

Here we use integrative multi-omics of exome, epigenome and expression to improve the understanding of CMML pathobiology.

### Methods

In an international cohort of 153 CMML patients, diagnostic bone marrow samples were collected along with clinical data. Exome sequencing was performed in paired tumour and germline samples, DNA methylation array (EPIC, Illumina) of bone marrow mononuclear cells (BMMNC) and RNA sequencing of CD34+ cells. In a subset of five *TET2*$^{WT}$ and five *TET2*$^{MT}$cases, we also performed ChIP sequencing, ATAC sequencing and RNA sequencing of BMMNC.

**Copyright Information:** (Online) ISSN: 2572-9241
©2026 The Author(s). HemaSphere is published by John Wiley & Sons Ltd on behalf of European Hematology Association. This is an open access Abstract Book distributed under the Attribution-NonCommercial-NoDerivs (CC BY-NC-ND), which allows third parties to download the articles and share them with others as long as they credit the author and the Abstract Book, but they cannot change the content in any way or use them commercially.
**Abstract Book Citations:** Authors, Title, HemaSphere, 2026;10:(S1):pages. The abstract book DOIs can be found on the "Author Information" page.
**Disclaimer:** Articles published in the journal HemaSphere exclusively reflect the opinions of the authors. The authors are responsible for all content in their abstracts including accuracy of the facts, statements, citing resources, etc.

### Results

Unsupervised clustering of exome sequencing data revealed two mutation clusters largely driven by *TET2*$^{MT}$. No novel driver mutations were found. Unsupervised clustering of DNA methylation β-values rendered two clear clusters with profound hypermethylation in one cluster, strongly overlapping with mutation clusters thus the pattern of hypermethylation was largely driven by *TET2*$^{MT}$ (Fig 1a). The *TET2*$^{MT}$ and the hypermethylated cluster was also associated with prolonged survival, higher hemoglobin and lower LDH.

Within hypermethylated DNA regions in the *TET2*$^{MT}$ cluster, the Hallmark Inflammatory Response gene set was significantly enriched.

There was also a significant hypermethylation in enhancer regions in the *TET2*$^{MT}$ cluster, as has been shown before for *TET2*$^{MT}$ in clonal hematopoiesis subjects. Using RNA sequencing data from the same sample, we could show that genes regulated by the hypermethylated enhancers belong to inflammatory gene sets.
Using ATACseq and ChIPseq data and the ChromHMM model we could define chromatin states across the genome, specific for our *TET2*$^{WT}$ and *TET2*$^{MT}$ CMML cases respectively, and clearly demonstrate that the Hallmark Inflammatory Response gene set was significantly overrepresented (Fig 1c) among genomic regions that shift from an active chromatin state in *TET2*$^{WT}$ to a repressed state in *TET2*$^{MT}$ (Fig 1b).

### Summary/Conclusion

In conclusion, genes coding for inflammatory proteins and enhancers regulating genes coding for inflammatory proteins, are hypermethylated in *TET2*$^{MT}$ CMML. In addition, there is a shift in chromatin state for where DNA coding for inflammatory proteins is shifted towards a repressed chromatin state in *TET2*$^{MT}$ CMML.

This implies that *TET2*$^{MT}$ infers a specific epigenetic pattern in CMML by repressing inflammatory response and thus making the *TET2*$^{MT}$ bone marrow more resilient to chronic inflammation.

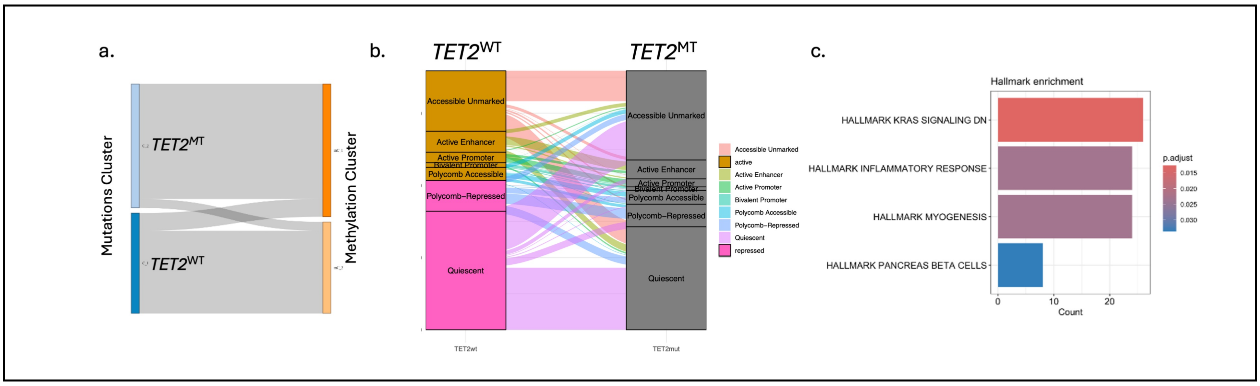

Figure 1. Showing a) strong overlap between clusters based on mutations and clusters based on methylation data, b) the chromatin state of genes in TET2wt and TET2mt and c) enrichment analysis of genes in b) shifting from an active to a repressed chromatin state, where the Hallmark Inflammatory Response gene set is significantly enriched among genes that have shifted to a repressed chromatin state in TET2mt CMML.

**Copyright Information:** (Online) ISSN: 2572-9241
©2026 The Author(s). HemaSphere is published by John Wiley & Sons Ltd on behalf of European Hematology Association. This is an open access Abstract Book distributed under the Attribution-NonCommercial-NoDerivs (CC BY-NC-ND), which allows third parties to download the articles and share them with others as long as they credit the author and the Abstract Book, but they cannot change the content in any way or use them commercially.
**Abstract Book Citations:** Authors, Title, HemaSphere, 2026;10:(S1):pages. The abstract book DOIs can be found on the "Author Information" page.
**Disclaimer:** Articles published in the journal HemaSphere exclusively reflect the opinions of the authors. The authors are responsible for all content in their abstracts including accuracy of the facts, statements, citing resources, etc.

### (PF646) MUTANT SHP2 CONDENSATES IMPAIR MICROTUBULE FUNCTION VIA PAK1 RECRUITMENT TO FOSTER GENOMIC INSTABILITY IN JUVENILE MYELOMONOCYTIC LEUKEMIA

**Topic:** 09. Myelodysplastic syndromes - Biology & translational research

Chunmou Li*[1], Yao Guo[1], Yuming Zhao[1], Lingling MA[1], Dengyang Zhang[1], Liuting Yu[1], Chun Chen[1], Zhizhuang Zhao[2], Yun Chen[1]

*[1]The Seventh Affiliated Hospital of Sun Yat-sen University, Shenzhen, China;[2]University of Oklahoma Health Sciences Center, Oklahoma, United States of America;*

### Background

Juvenile myelomonocytic leukemia (JMML) is a rare but aggressive pediatric myeloproliferative neoplasm driven by hyperactivation of the Ras signaling pathway. SHP2, encoded by *PTPN11*, functions as a critical positive regulator of Ras signaling, and pathogenic gain-of-function mutations in *PTPN11* are the leading drivers in approximately 35% of JMML patients. In our preliminary study, *PTPN11* copy number gain (CNG) occurred in 68.7% of mutant JMML colonies, with significantly higher levels (6.4±3.2) compared to wild-type controls (2.0±0.2). The presence of *PTPN11* CNG within the genome reflects a significant enhancement of genomic instability in JMML cells. While various SHP2 mutant proteins have been found to undergo liquid-liquid phase separation (LLPS), the potential link among this phenomenon, genomic instability and *PTPN11* CNG remains to be elucidated.

### Aims

To investigate the interplay among mutant SHP2 LLPS, genomic instability and *PTPN11* CNG, and to elucidate the impact of such genomic amplification on the pathogenesis and progression of JMML.

### Methods

Phase-separated droplet formation was evaluated using confocal imaging in stable HCD57 cell lines with specific SHP2 mutations (D61Y, E76K, A72V, and G503A). Mutant SHP2-interacting proteins were identified through IP-MS and prioritized by bioinformatic analysis for further mechanistic validation. Then, to experimentally evaluate the pathogenic impact of mutant *PTPN11* gene dosage *in vivo*, we established transgenic mice expressing human SHP2-E76K mutant protein under the hematopoietic-specific *Vav* promoter, with defined transgene copy numbers.

**Copyright Information:** (Online) ISSN: 2572-9241
©2026 The Author(s). HemaSphere is published by John Wiley & Sons Ltd on behalf of European Hematology Association. This is an open access Abstract Book distributed under the Attribution-NonCommercial-NoDerivs (CC BY-NC-ND), which allows third parties to download the articles and share them with others as long as they credit the author and the Abstract Book, but they cannot change the content in any way or use them commercially.
**Abstract Book Citations:** Authors, Title, HemaSphere, 2026;10:(S1):pages. The abstract book DOIs can be found on the "Author Information" page.
**Disclaimer:** Articles published in the journal HemaSphere exclusively reflect the opinions of the authors. The authors are responsible for all content in their abstracts including accuracy of the facts, statements, citing resources, etc.

**Results**

All four stable cell lines exhibited mutant SHP2 LLPS, with SHP2-A72V showing the strongest droplet formation. KEGG enrichment analysis of the differentially expressed proteins (DEPs) identified by IP-MS revealed significant enrichment in microtubule remodeling pathways. Notably, the PAK1-binding protein PAK1ip1 was markedly increased, while the PAK1-interacting protein TFCB, a critical component for maintaining microtubule stability, was significantly decreased. These findings identify PAK1 as a pivotal interactor within mutant SHP2 condensates. Confocal microscopy demonstrated clear spatial co-localization between PAK1 and mutant SHP2 condensates. WB analysis further showed a significant down-regulation of PAK1 in SHP2-mutant HCD57 cells, accompanied by the up-regulation of genomic instability markers γH2AX and p-Chk1/Chk1 ratio. Treatment with the allosteric PAK1 inhibitor NVS-PAK1-1 led to a dose- and time-dependent increase in these genomic instability markers. Conversely, the levels of these proteins gradually declined following inhibitor withdrawal or transient PAK1 overexpression.
Three independent lines were generated, carrying approximately 1, 2, or 6 copies of the SHP2-E76K transgene haploid genome. With the increase in *PTPN11* copy number, the mice exhibited an earlier disease onset and exacerbated symptoms, which more closely recapitulated the clinical features of pediatric JMML patients. Mice from the 6-copy line developed significant thrombocytopenia and anemia as early as 3 weeks of age, consistent with early-onset disease of JMML, and these abnormalities persisted into adulthood.

**Summary/Conclusion**

In summary, mutant SHP2 phase separation sequesters and depletes PAK1, triggering genomic instability that facilitates *PTPN11* CNG and drives JMML progression.

**Copyright Information:** (Online) ISSN: 2572-9241
©2026 The Author(s). HemaSphere is published by John Wiley & Sons Ltd on behalf of European Hematology Association. This is an open access Abstract Book distributed under the Attribution-NonCommercial-NoDerivs (CC BY-NC-ND), which allows third parties to download the articles and share them with others as long as they credit the author and the Abstract Book, but they cannot change the content in any way or use them commercially.
**Abstract Book Citations:** Authors, Title, HemaSphere, 2026;10:(S1):pages. The abstract book DOIs can be found on the “Author Information” page.
**Disclaimer:** Articles published in the journal HemaSphere exclusively reflect the opinions of the authors. The authors are responsible for all content in their abstracts including accuracy of the facts, statements, citing resources, etc.

### (PF647) YOUNG AGE AT DIAGNOSIS OF A MYELOID NEOPLASIA AS A STANDALONE CRITERION FOR GERMLINE INVESTIGATION: RESULTS FROM A PROSPECTIVE COHORT STUDY

**Topic:** 09. Myelodysplastic syndromes - Biology & translational research

Lucy Mathot[1, 2], Berivan Baskin[1, 3], Ahmed Al-Djaber[1, 3], Anna Tranberg[4, 5], Anna Robelius[6], Vladimir Lazarevic[7], Stefan Deneberg[4, 5], Martin Höglund[6], Linda Fogelstrand[8, 9], Johanna Ungerstedt[4, 10], Tatjana Pandzic[1, 3], Magnus Tobiasson[4, 5], Hege Gravdahl Garelius[11, 12], Lena von Bahr[11, 12], Ekaterina Kuchinskaya[13, 14], Fredrik Persson[15], Helena Ågerstam[16, 17], Thoas Fioretos[16, 17], Ann-Charlotte Turesson[1, 3], Sören Lehmann[4, 5], Claes Ladenvall[1, 3], Gisela Barbany[13, 18], Lovisa Vennström[11, 12], Elisabeth Ejerblad[6], Lucia Cavelier[13, 18], Jörg Cammenga[7, 19], Martin Jädersten[4, 5], Eva Hellström Lindberg[4, 5], Bianca Tesi[4, 13], Panagiotis Baliakas*[1, 3]

*[1]Uppsala University, Department of Immunology, Genetics and Pathology, Uppsala, Sweden;[2]Örebro University, Department of Clinical Genetics, Faculty of Medicine and Health, Örebro, Sweden; [3]Uppsala University Hospital, Department of Clinical Genetics, Uppsala, Sweden; [4]Karolinska Institutet, Department of Medicine Huddinge, Center for Hematology and Regenerative Medicine, Stockholm, Sweden; [5]Karolinska University Hospital, Department of Hematology, Stockholm, Sweden;[6]Uppsala University, Department of Medical Sciences, Uppsala, Sweden; [7]Skåne University Hospital, Department of Hematology, Oncology and Radiation Physics, Lund, Sweden; [8]Sahlgrenska University Hospital, Department of Clinical Chemistry,, Gothenburg, Sweden; [9]University of Gothenburg, Department of Laboratory Medicine,, Gothenburg, Sweden;[10]Linköping University Hospital, Department of Hematology, Linköping, Sweden; [11]Sahlgrenska University Hospital, Department of Specialist Medicine, Section of Hematology and Coagulation, Gothenburg, Sweden; [12]Sahlgrenska Academy at Gothenburg University, Institute of Medicine, Gothenburg, Sweden; [13]Karolinska University Hospital, Department of Clinical Genetics and Genomics, Stockholm, Sweden; [14]Linköping University, Department of Clinical Genetics, Linköping, Sweden;[15]Sahlgrenska University Hospital, Department of Clinical Genetics & Genomics, Gothenburg, Sweden; [16]Lund University, Division of Clinical Genetics, Department of Laboratory Medicine, Lund, Sweden; [17]Office for Medical Services, Region Skåne, Department of Clinical Genetics, Pathology and Molecular Diagnostics, Lund, Sweden; [18]Karolinska Institutet, Department of Molecular Medicine and Surgery, Stockholm, Sweden; [19]Lund University, Division of Molecular Medicine and Gene Therapy, Lund, Sweden;*

### Background

Whether young age at the diagnosis of a myeloid neoplasia (MN) in the absence of family/personal history suggestive of a germline background (F/P-H$^{pos}$) should be a standalone criterion for germline investigation, is still under debate. Moreover, consensus regarding the potential age limit has not yet been reached.

### Aims

To prospectively investigate the clinical validity of young age at MN diagnosis as a standalone criterion regarding germline investigation independently of F/P-H$^{pos}$ and investigate the optimal age cut-off.

**Copyright Information:** (Online) ISSN: 2572-9241
©2026 The Author(s). HemaSphere is published by John Wiley & Sons Ltd on behalf of European Hematology Association. This is an open access Abstract Book distributed under the Attribution-NonCommercial-NoDerivs (CC BY-NC-ND), which allows third parties to download the articles and share them with others as long as they credit the author and the Abstract Book, but they cannot change the content in any way or use them commercially.
**Abstract Book Citations:** Authors, Title, HemaSphere, 2026;10:(S1):pages. The abstract book DOIs can be found on the “Author Information” page.
**Disclaimer:** Articles published in the journal HemaSphere exclusively reflect the opinions of the authors. The authors are responsible for all content in their abstracts including accuracy of the facts, statements, citing resources, etc.

**Methods**

In this prospective cohort study, we investigated 88 sequential patients below the age of 50 years, with confirmed or suspected MN [acute myeloid leukemia (AML): n=41, myelodysplastic syndrome (MDS): n=32, thrombocytopenia: n=12, other: n=3] from 13 Swedish institutions, for germline predisposition. Among them, 41 had F/P-H$^{pos}$ (Group A), while 47 did not (F/P-H$^{neg}$, Group B). The F/P-H status was evaluated by trained clinical geneticists with expertise in hereditary cancer. All patients were initially analyzed with a somatic panel (Twist myeloid-panel) including 191 genes recurrently mutated in MNs. Germline investigation [whole exome sequencing including copy number variant analysis and analysis of non-coding regions in genes related to germline predisposition] was performed on genomic DNA from cultured fibroblasts (n=73), T-lymphocytes (n=3), or peripheral blood in remission (n=12).

**Results**

A pathogenic or likely pathogenic (P/LP) germline variant was identified in 17/88 (19%) patients. The involved genes were: *ACTN1* (n=2), *ANKRD26* (n=2), *DDX41* (n=2), *DNAJC21* (n=1), *ERCC6L2* (n=1), *ETV6* (n=1), *FANCA* (n=1), *GATA2* (n=1), *PARN* (n=1), *RPL17* (n=1), *RUNX1* (n=2), *SBDS* (n=1) and *TERT* (n=1). The detection rate of P/LP variants did not differ significantly between the two groups (group A: 11/41, 27% vs group B: 6/47, 13%. P-value=0.82). Germline P/LP variants were more common among patients with MDS (9/32, 28%) compared to those with AML (3/41, 7%) (p=0.03). Five patients (Group A: n=3 and Group B: n=2) carried a P/LP variant in genes mainly associated with predisposition for solid tumors (*BRCA1:* n=2, *CHEK2:* n=1, *PALB2:* n=1 *and PMS2:* n=1). Variants of uncertain significance (VUS) were detected in 8 patients [*TERT* (n=2), *ETV6* (n=1), *SAMD9* (n=1), *RUNX1* (n=1), *PARN* (n=1), *GATA2* (n=1) and *RETL1* (n=1)]. In total 4/88 patients (5% of the cohort and 24% of those with confirmed germline background) were positive for recessive disorders.  Lowering the age limit from 50 to 40 (in line with previous studies) did not alter the outcome of the investigation with no significant difference being observed (Group A: 7/23, 30% vs Group B: 4/26, 15%, p-value=0.83).

**Summary/Conclusion**

This is, to our knowledge, the first prospective study investigating the validity of young age at MN diagnosis as a standalone criterion in relation to germline predisposition. Our data provides an incentive for germline investigation in any patient diagnosed with MN under the age of 50, independent of family history. Moreover, in these patients, clinicians should be alerted to recessive disorders and therefore adjust the diagnostic pipeline accordingly.

**Copyright Information:** (Online) ISSN: 2572-9241
©2026 The Author(s). HemaSphere is published by John Wiley & Sons Ltd on behalf of European Hematology Association. This is an open access Abstract Book distributed under the Attribution-NonCommercial-NoDerivs (CC BY-NC-ND), which allows third parties to download the articles and share them with others as long as they credit the author and the Abstract Book, but they cannot change the content in any way or use them commercially.
**Abstract Book Citations:** Authors, Title, HemaSphere, 2026;10:(S1):pages. The abstract book DOIs can be found on the “Author Information” page.
**Disclaimer:** Articles published in the journal HemaSphere exclusively reflect the opinions of the authors. The authors are responsible for all content in their abstracts including accuracy of the facts, statements, citing resources, etc.

### (PF648) MAPPING AZACITIDINE RESPONSE IN VEXAS WITH PAIRED SINGLE-CELL TRANSCRIPTOMICS AND PLASMA PROTEOMICS

**Topic:** 09. Myelodysplastic syndromes - Biology & translational research

Anish Sharda[1], Mohammed Ali[1], Jan Bewersdorf[1], Luis E. Aguirre[1], Alain Mina[1], Maximilian Stahl[1], Amer M. Zeidan[1], Nikolai Podoltsev[1], Stephanie Halene[1], Alfred Lee[1], Lourdes Mendez*[1]

[1] *Yale University School of Medicine, New Haven, United States of America;*

### Background

VEXAS syndrome is a severe hematoinflammatory disease driven by acquired pathogenic variants in *UBA1*. Patients develop multiorgan inflammation with cytopenias and frequent concomitant MDS. *UBA1*-mutant HSPCs and myeloid cells show heightened innate immune signaling and inflammatory mediators. The hypomethylating agent, azacitadine (Aza), results in hematologic, inflammatory and molecular responses in VEXAS but the cellular programs underlying Aza response remain unclear.

### Aims

To define the molecular pathways and cell populations that mediate response to Aza in VEXAS.

### Methods

Peripheral blood single cell RNA sequencing (scRNAseq; 10X genomics) was performed before (VEXAS-U; n=5) and after Aza (atleast 1 cycle) (VEXAS-A; n=3; paired), and healthy controls (n=3). Cells were annotated into major lineages and subclusters. Within each lineage, paired pseudobulk differential expression (VEXAS-U vs -A) and disease-state contrasts were modeled with multiple-testing correction. Pathway activity was assessed by ranked enrichment and cross-lineage meta-analysis to define core treatment-response gene set. Ligand–receptor inference compared interaction number/strength across conditions. SomaScan plasma proteomics was performed (~4,000 proteins) (5 VEXAS-U; 3 VEXAS-A; 5 matched controls) and integrated with scRNAseq to assign cellular sources and proteo-transcriptomic concordance.

### Results

We resolved 30 clusters spanning 11 immune populations. Compared with controls, VEXAS-U showed broad activation dominated by innate immunity: neutrophils (788 DEGs) and monocytes (300 DEGs) were enriched for TNFα signaling, complement, and inflammatory response programs; NK cells (479 DEGs) demonstrated TNFα, IFN-α and -γ, IL-2/STAT5 signaling; CD8 T cells (470 DEGs) showed Myc Targets V1, IL2-STAT5, and allograft rejection signatures. Aza was associated with state shifts across lineages: neutrophil subclusters moved from TNFα-activated toward interferon-responsive signaling; NK and CD8 subclusters shifted away from G2-M checkpoint/PI3K-AKT-mTOR/protein-secretion programs toward oxidative phosphorylation and Myc/translation signatures. Meta-analysis identified a core Aza treatment signature shared across cell types, implicating cytoplasmic translation (multiple RPL/RPS genes), stress/p53 signaling (*CCND3, ZBTB16, TXNIP*), innate/complement activation (*ITGAM, S100A12/S100A9, CTSS*), IFN-γ response (*M2TA, SP110*), and IL-6/JAK/STAT3-linked signaling (*CSF3R, IL1R2, PIK3R5*). Ligand-receptor analysis suggested treatment-associated remodeling toward communication akin to controls, including increased *BTLA, SELPLG, TGFβ* and *THBS* axes and reduced *ICAM/IL16/APP*-related signaling. Proteomic integration mapped most differential proteins to source cell-type—DEFA1-4/MPO/PRTN3/ELANE to neutrophils; LYZ/FTL/TNFRSF1B to monocytes; ADH4, CCL14, SERPINA11 to NK cells—and treatment-shift proteins highlighted candidate pathways spanning PI3-AKT-mTOR signaling (RET, BAFF, AMBRA1), metabolic reprogramming (GPD1, ACAA1, CPOX), cytokine signaling (LEPROT) and transcription regulation (KAT2A, GCN5).

**Copyright Information:** (Online) ISSN: 2572-9241
©2026 The Author(s). HemaSphere is published by John Wiley & Sons Ltd on behalf of European Hematology Association. This is an open access Abstract Book distributed under the Attribution-NonCommercial-NoDerivs (CC BY-NC-ND), which allows third parties to download the articles and share them with others as long as they credit the author and the Abstract Book, but they cannot change the content in any way or use them commercially.
**Abstract Book Citations:** Authors, Title, HemaSphere, 2026;10:(S1):pages. The abstract book DOIs can be found on the “Author Information” page.
**Disclaimer:** Articles published in the journal HemaSphere exclusively reflect the opinions of the authors. The authors are responsible for all content in their abstracts including accuracy of the facts, statements, citing resources, etc.

### Summary/Conclusion

A shared core Aza treatment signature and ligand-receptor remodeling support the hypothesis that Aza modulates inflammatory/stress states in VEXAS across innate and adaptive immune compartments potentially through metabolic/translational reprogramming. These observations warrant further validation and correlation with clinical responses.

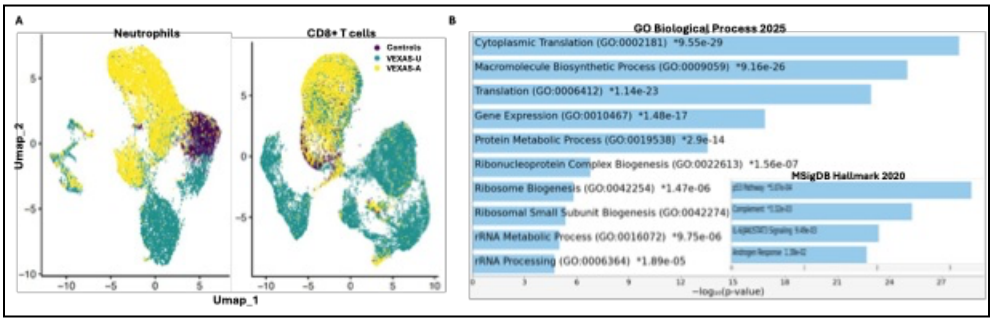

Post-azacitidine neutrophil and CD8+ T cell subclusters and shared core transcriptional programs in VEXAS. (A) UMAPs of neutrophil subclusters (left) and CD8+ T-cell subclusters (right) from peripheral blood leukocyte scRNAseq, colored by subcluster identity to highlight the predominant states enriched in VEXAS-U versus VEXAS-A (paired) and healthy controls. (B) Pathway enrichment of the core azacitidine treatment–associated gene set (meta-analyzed across cell types from paired VEXAS-A vs VEXAS-U), showing the top significantly altered pathways, including ribosomal biogenesis/cytoplasmic translation (GO Biological Processes) and p53 signaling and complement pathways (Hallmark).

Copyright Information: (Online) ISSN: 2572-9241
©2026 The Author(s). HemaSphere is published by John Wiley & Sons Ltd on behalf of European Hematology Association. This is an open access Abstract Book distributed under the Attribution-NonCommercial-NoDerivs (CC BY-NC-ND), which allows third parties to download the articles and share them with others as long as they credit the author and the Abstract Book, but they cannot change the content in any way or use them commercially.
Abstract Book Citations: Authors, Title, HemaSphere, 2026;10:(S1):pages. The abstract book DOIs can be found on the "Author Information" page.
Disclaimer: Articles published in the journal HemaSphere exclusively reflect the opinions of the authors. The authors are responsible for all content in their abstracts including accuracy of the facts, statements, citing resources, etc.

### (PF649) DIVERGENT ROLES OF STAT3 AND STAT5 IN LEUKEMIC TRANSFORMATION

**Topic:** 09. Myelodysplastic syndromes - Biology & translational research

Eirini Sofia Fasouli[1], Leonid Bystrykh[2], Anthi Bouchla[3], Giannis Vatsellas[4], Charikleia Makri[1], Vasiliki Pappa[3], Eleni Katsantoni*[1]

*[1]Biomedical Research Foundation, Academy of Athens, Basic Research Center, Athens, Greece;[2]European Research Institute for the Biology of Ageing (ERIBA), Groningen, The Netherlands; [3]National and Kapodistrian University of Athens, Medical School, University General Hospital "Attikon", Second Department of Internal Medicine and Research Institute, Hematology Unit, Athens, Greece; [4]Biomedical Research Foundation, Academy of Athens, Greek Genome Center, Athens, Greece;*

**Background**

Myelodysplastic Syndrome (MDS) refers to a heterogeneous group of clonal hematologic disorders characterized by hematopoietic dysfunctions and impaired differentiation in the hematopoietic stem and progenitor cell compartment. MDS is commonly referred to as a pre-leukemic state due to the increased risk of progression to Acute Myeloid Leukemia (AML), which is an aggressive hematologic malignancy characterized by the accumulation of immature myeloblasts in the bone marrow and peripheral blood. Signal transducers and activators of transcription 3 and 5 (STAT3 and STAT5) are important regulators of several cellular processes and have been linked with both normal and leukemic hematopoiesis. Abnormal STAT3 and STAT5 signaling has been implicated in various hematologic malignancies, including MDS and AML, rendering them appealing as biomarkers or targets for the development of novel therapeutic strategies.

**Aims**

To delineate the role of STAT3, STAT5A and STAT5B factors and their target gene transcriptional networks in MDS to AML transformation, with the long-term goal of identifying novel biomarkers and therapeutic targets to improve patient stratification and treatment strategies.

**Methods**

STAT3, STAT5A or STAT5B knock-downs (KDs) and no-target controls have been generated in*ex vivo* cultured CD34+ cells isolated from bone marrow biopsies of 14 high-risk MDS and 14 secondary (MDS-related) AML unpaired patients, using shRNAs through lenti-viral delivery. The levels of KDs have been confirmed by reverse transcriptase real-time PCR. mRNA-sequencing libraries have been constructed by using the mRNA Magnetic Isolation Module and the NEBNext Ultra II Directional RNA Library Prep Kit with Beads (NEB) in a total of 112 samples. Sequencing has been performed on a NovaSeq 6000 platform (Illumina). The sequenced reads have been checked for quality with FastQC and trimmed using Cutadapt v5.0. GRCh38 primary assembly version 47 has been used for alignment with the STAR aligner version 2.7.9a. RNA abundance was estimated with MGCount. Further analysis included principal component analysis, distance-based hierarchical clustering, and differential expression analysis. For differential expression analysis, different protocols have been compared, and the most optimal for the data (glmQLFit/Test edgeR protocol) has been applied.

**Copyright Information:** (Online) ISSN: 2572-9241
©2026 The Author(s). HemaSphere is published by John Wiley & Sons Ltd on behalf of European Hematology Association. This is an open access Abstract Book distributed under the Attribution-NonCommercial-NoDerivs (CC BY-NC-ND), which allows third parties to download the articles and share them with others as long as they credit the author and the Abstract Book, but they cannot change the content in any way or use them commercially.
**Abstract Book Citations:** Authors, Title, HemaSphere, 2026;10:(S1):pages. The abstract book DOIs can be found on the “Author Information” page.
**Disclaimer:** Articles published in the journal HemaSphere exclusively reflect the opinions of the authors. The authors are responsible for all content in their abstracts including accuracy of the facts, statements, citing resources, etc.

**Results**

To elucidate the role of STAT3 and STAT5 in MDS to AML transformation, changes in STAT3 and STAT5 target gene networks have been identified. mRNA sequencing data from STAT3, STAT5A or STAT5B KDs in MDS and AML *ex vivo* cultured CD34+ cells revealed distinct groups of differentially expressed genes upon KD of each of the three factors in each state (MDS and AML). The differential gene expression profiles in MDS and AML reciprocally suggested distinct functions for each factor. Potential leukemia-promoting gene networks and the involvement of STAT3 and STAT5 pathways in the leukemic transformation have been revealed. The results are currently integrated using machine learning methods with data generated in our laboratory from MDS and AML cell lines at the transcriptomic, chromatin accessibility and transcription factor binding level.

**Summary/Conclusion**

Our results show a distinct role of the two STAT5 factors (STAT5A and STAT5B) and STAT3 in MDS and AML conditions and define the STAT3, STAT5A and STAT5B target gene networks in leukemic transformation, thus providing novel biomarkers for patient stratification and targets for therapeutic management of MDS and AML.

**Copyright Information:** (Online) ISSN: 2572-9241
©2026 The Author(s). HemaSphere is published by John Wiley & Sons Ltd on behalf of European Hematology Association. This is an open access Abstract Book distributed under the Attribution-NonCommercial-NoDerivs (CC BY-NC-ND), which allows third parties to download the articles and share them with others as long as they credit the author and the Abstract Book, but they cannot change the content in any way or use them commercially.
**Abstract Book Citations:** Authors, Title, HemaSphere, 2026;10:(S1):pages. The abstract book DOIs can be found on the "Author Information" page.
**Disclaimer:** Articles published in the journal HemaSphere exclusively reflect the opinions of the authors. The authors are responsible for all content in their abstracts including accuracy of the facts, statements, citing resources, etc.

### (PF650) LANDSCAPE OF METABOLIC DYSREGULATION WITHIN THE MYELODYSPLASTIC BONE MARROW MICROENVIRONMENT.

**Topic:** 09. Myelodysplastic syndromes - Biology & translational research

Aikaterini Poulaki*[1], Theodora Katsila[2], Maria Garofalaki[3], Stamatios Hatzis[1], Emilia S Hatziyannis[4], Michael Voulgarelis[5], Stavroula Giannouli[1]

*[1]National and Kapodistrian University of Athens, 2nd Dept of Internal Medicine, Athens, Greece;[2]National Hellenic Research Foundation, Biomarker Discovery & Translational of the Research Institute of Chemical Biology, Athens, Greece; [3]Evaggelismos Hospital, Molecular Hematology Laboratory, Department of Hematology-Lymphomas and BMT Unit, Athens, Greece; [4]National and Kapodistrian University of Athens, Second Department of Internal Medicine and Research Laboratory, School of Medicine, Athens, Greece; [5]National and Kapodistrian University of Athens, Department of Pathophysiology, School of Medicine, Athens, Greece;*

### Background

Emerging evidence suggests that metabolic reprogramming plays a central role in the pathogenesis of myelodysplastic syndrome (MDS). Alterations in key bioenergetic pathways, including amino acid and lipid metabolism, may contribute to dysregulated hematopoiesis, oxidative stress, the remodeling of the bone marrow microenvironment and the establishment of a proinflammatory niche. Such perturbations not only reflect the cellular adaptations of malignant progenitors but may also create a permissive background that sustains disease progression and impacts therapeutic response.

### Aims

- To identify key metabolites that differentiate MDS bone marrow microenvironment  from healthy controls.
- To investigate dysregulated pathways: Focus on lipid and amino acid metabolism.
- To identify potential biomarkers and therapeutic targets.

### Methods

Bone marrow (BM) plasma from 19 previously untreated MDS patients (WHO 2022, Low (<5%) intra bone marrow blasts or LB#9, IB1#8, IB2#2 and 10 age-sex matched healthy controls (CTRLs) were subjected to untargeted LC-MS metabolomics. Data analysis took place using Proteowizard and MetaboAnalyst 5.0 coupled to our biostatistics pipeline (R language) and the Mummichog computational algorithm (gamma adjusted p-value <0,001). The mutational burden of the cohort was assessed using a 51-gene expanded NGS panel as per routine risk stratification. 1 out of 2 CTRLs bore at least one mutation consistent with CHIP.  No samples with *idh or sf3b1* mutations were included.

**Copyright Information:** (Online) ISSN: 2572-9241
©2026 The Author(s). HemaSphere is published by John Wiley & Sons Ltd on behalf of European Hematology Association. This is an open access Abstract Book distributed under the Attribution-NonCommercial-NoDerivs (CC BY-NC-ND), which allows third parties to download the articles and share them with others as long as they credit the author and the Abstract Book, but they cannot change the content in any way or use them commercially.
**Abstract Book Citations:** Authors, Title, HemaSphere, 2026;10:(S1):pages. The abstract book DOIs can be found on the "Author Information" page.
**Disclaimer:** Articles published in the journal HemaSphere exclusively reflect the opinions of the authors. The authors are responsible for all content in their abstracts including accuracy of the facts, statements, citing resources, etc.

**Results**

Heat map analyses incorporating the top 25 identified metabolites enabled accurate classification of samples as either CTRL or MDS cases. This discriminatory capacity was observed both in the comprehensive analysis including all CTRL and MDS samples and in subgroup comparisons. Most significant features corresponded to lipid species, primarily long-chain fatty acids, that were differentially regulated. The effect was more pronounced in the IB2 subgroup and less evident in the low-blast vs CTRL comparison. Glutamine and glutamate emerged as the most significantly altered metabolites in our analysis, with the IB vs CTRL comparison demonstrating a 5x increase in glutamate levels accompanied by a corresponding elevation in the glutamate/glutamine ratio. Functional metabolomic analysis revealed a distinct metabolic evolution in MDS by means of BM plasma pathway enrichment analysis. In low-blast states, enrichment of xenobiotic, Vitamin D3, and tryptophan pathways identified a "stress-signature" driven by P450-mediated regulatory disruption. Conversely, the transition to increased blasts was marked by a highly "wired" network of central carbon and nitrogen transfer, where glycolysis, BCAA degradation, and urea cycle activity were found to be prioritized to meet the energetic and pyrimidine demands of clonal expansion. Also see Figure-bubble diameter is indicative of significant compound hits.

**Summary/Conclusion**

Our findings suggest a profound microenvironmental bioenergetic disruption in MDS that largely reflects the major disease phenotypes. MDS progression is defined by a shift from early P450-mediated niche stress to a terminal, lipid-driven mitochondrial wiring, punctuated by a five-fold glutamate surge that fuels the pyrimidine and energetic demands of clonal expansion. This dramatic metabolic metamorphosis—from a stressed regulatory hub to a high-octane glutamate-fueled factory—suggests mitochondrial reprogramming as the critical checkpoint and a potent diagnostic biomarker for leukemic transformation.

**Copyright Information:** (Online) ISSN: 2572-9241
©2026 The Author(s). HemaSphere is published by John Wiley & Sons Ltd on behalf of European Hematology Association. This is an open access Abstract Book distributed under the Attribution-NonCommercial-NoDerivs (CC BY-NC-ND), which allows third parties to download the articles and share them with others as long as they credit the author and the Abstract Book, but they cannot change the content in any way or use them commercially.
**Abstract Book Citations:** Authors, Title, HemaSphere, 2026;10:(S1):pages. The abstract book DOIs can be found on the "Author Information" page.
**Disclaimer:** Articles published in the journal HemaSphere exclusively reflect the opinions of the authors. The authors are responsible for all content in their abstracts including accuracy of the facts, statements, citing resources, etc.

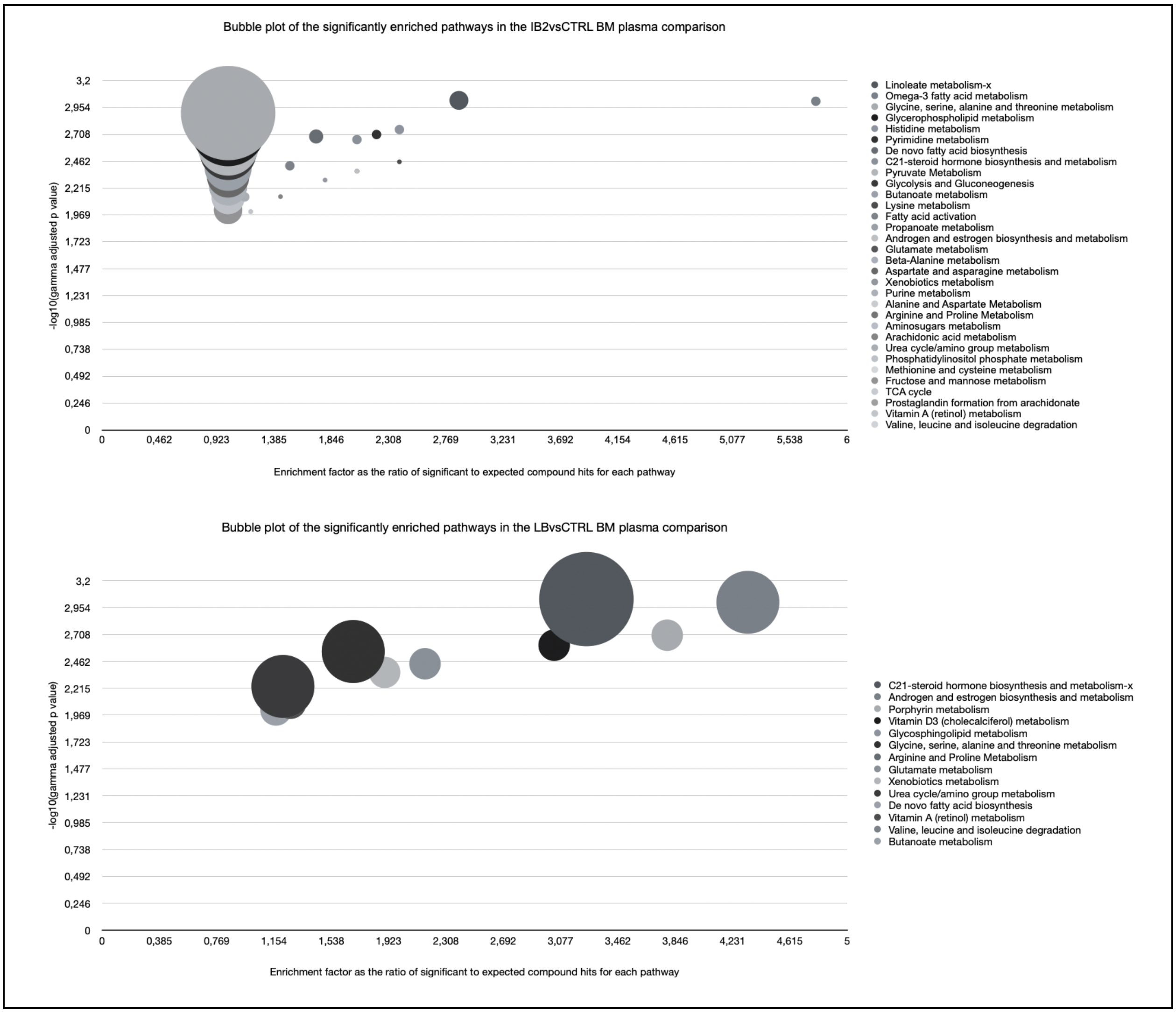

**Copyright Information:** (Online) ISSN: 2572-9241
©2026 The Author(s). HemaSphere is published by John Wiley & Sons Ltd on behalf of European Hematology Association. This is an open access Abstract Book distributed under the Attribution-NonCommercial-NoDerivs (CC BY-NC-ND), which allows third parties to download the articles and share them with others as long as they credit the author and the Abstract Book, but they cannot change the content in any way or use them commercially.
**Abstract Book Citations:** Authors, Title, HemaSphere, 2026;10:(S1):pages. The abstract book DOIs can be found on the "Author Information" page.
**Disclaimer:** Articles published in the journal HemaSphere exclusively reflect the opinions of the authors. The authors are responsible for all content in their abstracts including accuracy of the facts, statements, citing resources, etc.

### (PF651) CLK INHIBITOR CTX-712 POTENTIATES THE ACTIVITY OF ABT-199 AND AZACYTIDINE IN MYELOID LEUKEMIA

**Topic:** 09. Myelodysplastic syndromes - Biology & translational research

Hui Yang[1], Zhihong Fang[1], Yu-An Kuo[1], Elisabeth Rebboah[1], Bofei Wang[1], Hussein Abbas[1], Xiaoping Su[1], Yue Wei[1], Marcos Roberto Estecio[1], Guillermo Montalban-Bravo[1], Kelly Chien[1], Guillermo Garcia-Manero*[1]

[1] *The University of Texas Md Anderson Cancer Center, Houston, United States of America;*

### Background

Alternative splicing (AS) is an essential mechanism in post-transcriptional mRNA processing and produces various mature mRNAs with different structures and functions. Dysregulation of AS plays a critical role in myeloid malignancy progression through abnormal expression or mutation of splicing factors. CDC2-like kinases (CLKs) are key regulators of AS via phosphorylation of serine/arginine (SR) domain rich spicing factors, including SRSF2, U2AF1 and ZRSR2, which are frequently mutated in myelodysplastic syndromes (MDS) and a subset of acute myeloid leukemia (AML). Therefore, we hypothesis that CLK might be a potential target for the treatment of MDS and AML.

### Aims

To study the effect of CLK inhibitor CTX-712 alone and in combination with ABT-199 and/or azacytidine on MDS and AML.

### Methods

Human bone marrow samples were obtained from MD Anderson Cancer Center tissue bank following institutional guidelines. CD34+ cells were selected using CD34 MicroBead kit. StemSpan SFEM II was used for CD34+ cell in vitro culture. RNA sequencing was used for RNA expression and alternative splicing analysis. Flow cytometry was used for apoptosis analysis. Western blot was used for protein analysis.

**Copyright Information:** (Online) ISSN: 2572-9241
©2026 The Author(s). HemaSphere is published by John Wiley & Sons Ltd on behalf of European Hematology Association. This is an open access Abstract Book distributed under the Attribution-NonCommercial-NoDerivs (CC BY-NC-ND), which allows third parties to download the articles and share them with others as long as they credit the author and the Abstract Book, but they cannot change the content in any way or use them commercially.
**Abstract Book Citations:** Authors, Title, HemaSphere, 2026;10:(S1):pages. The abstract book DOIs can be found on the "Author Information" page.
**Disclaimer:** Articles published in the journal HemaSphere exclusively reflect the opinions of the authors. The authors are responsible for all content in their abstracts including accuracy of the facts, statements, citing resources, etc.

### Results

We observed significantly increased CLK2 (p<0.05) and CLK4 (p<0.001) mRNA expression in CD34+ cells from 55 prior untreated MDS patients compared with 20 healthy donors. CTX-712 is a potent pan-CLK inhibitor. To investigate the therapeutic potential of CTX-712, primary CD34+ cells from 3 MDS patients were expand in vitro and then treated with CTX-712 for 72 hours. The IC-50s for the 3 patients were: pt1 (CREBBP, SF3B1, SMC3) 26.8nM; pt2 (TERT, IKZF1) 119.9nM; pt3 (ASXL1, KRAS, SRSF2, TET2, STAG2) 45nM. Induced apoptosis was observed in a dose dependent manner.
AML cell lines MOLM3 and MOLM13-AZA (azacytidine resistant derivatives) were then treated with CTX-712 for 72 hours. The IC-50s were 35.5nM and 34.3nM respectively with no difference between the two cell lines. By western blot analysis, decreased phosphorylation of SRSF4 and SRSF6 were observed in a dose dependent manner in MOLM13 cells at 6 hours of CTX-712 treatment.
MOLM13 cells were then treated with CTX-712 combined with BCL-2 inhibitor ABT-199 and /or azacytidine for 72 hours. Synergistic apoptotic induction effect was observed: 40% of apoptosis was induced with 60nM of CTX-712 treatment, 41% and 56% with 3nM and 10nM of ABT-199 treatment alone; 86% and 94% were observed for the combination treatment. Synergistic apoptotic induction effect was also observed using CTX-712 combined azacytidine with or without ABT-199: 48% of apoptosis was induced with 60nM CTX-712 treatment, 27% with 300nM of azacitidine, 31% with 3nM of ABT-199 treatment. 80% of apoptosis was observed for the combination of CTX-712 and azacitidine. 96% for the combination of all the three drugs. To understand the mechanisms of the synergistic effect of these combinations, we performed RNA-seq analysis on MOLM13 cells. We observed CTX-712 treatment decreased the BCL2 and BCL2A1 expression, increased the BAX and BAK1 expression. Splicing analysis showed that CTX-712 alone or in combination induced increased splicing events, and skipped exon was the major event induced by CTX-712. ABT-199 and azacytidine treatment alone had no significant effect on RNA splicing.

### Summary/Conclusion

Taken together, our results suggested that the CLK inhibitor alone or in combination with ABT-199 and/or azacytidine could be a novel therapeutic approach for the treatment of MDS and AML.

**Copyright Information:** (Online) ISSN: 2572-9241
©2026 The Author(s). HemaSphere is published by John Wiley & Sons Ltd on behalf of European Hematology Association. This is an open access Abstract Book distributed under the Attribution-NonCommercial-NoDerivs (CC BY-NC-ND), which allows third parties to download the articles and share them with others as long as they credit the author and the Abstract Book, but they cannot change the content in any way or use them commercially.
**Abstract Book Citations:** Authors, Title, HemaSphere, 2026;10:(S1):pages. The abstract book DOIs can be found on the “Author Information” page.
**Disclaimer:** Articles published in the journal HemaSphere exclusively reflect the opinions of the authors. The authors are responsible for all content in their abstracts including accuracy of the facts, statements, citing resources, etc.

### (PF653) DEFINING THE ERYTHROBLASTIC ISLAND MACROPHAGE AND ITS INVOLVEMENT IN THE PATHOGENESIS AND ANEMIC STATE OF LOW-RISK MYELODYSPLASTIC SYNDROMES

**Topic:** 09. Myelodysplastic syndromes - Biology & translational research

Sadaf Fazeli*[1], Indira Barbosa[1], Ann-Charlotte Björklund[1], Mikaela Hillberg Widfeldt[1], Anna Tranberg[1, 2], Kovi Shrung[3], Julien Papoin[4], Marion Serra[4], Rui Branca[3], Marc Raaijmakers[5], Janne Lehtiö[3], Lionel Blanc[4], Teresa Mortera Blanco[1], Eva Hellström Lindberg[1, 2], Pedro Moura[1]

*[1]Karolinska Institutet, Center for Hematology and Regenerative Medicine, Department of Medicine Huddinge, Stockholm, Sweden; [2]Karolinska University Hospital, Department of Hematology, Huddinge, Stockholm, Sweden; [3]Karolinska Institutet, Department of Oncology-Pathology, Karolinska Institute and Science for Life Laboratory, Stockholm, Sweden; [4]The Feinstein Institutes for Medical Research, Laboratory of Developmental Erythropoiesis, Les Nelkin Memorial Laboratory of Pediatric Oncology, Institute of Molecular Medicine, The Feinstein Institutes for Medical Research, Manhasset, NY, USA, United States of America; [5]Erasmus MC Cancer Institute, Department of Hematology, Rotterdam 3015 CN, The Netherlands;*

### Background

Macrophages display a wide array of tissue-specific functions. Within the bone marrow (BM), macrophages can localize at the center of multierythroblastic niches called erythroblastic islands (EBI), where they support erythroblast hemoglobinization, proliferation and enucleation. EBIs have been well-described in murine erythropoiesis and in *ex vivo* human cell culture; however, the extent to which EBIs contribute to *in vivo* human erythropoiesis remains poorly defined, and it is unclear whether EBI dysfunction drives pathogenesis in anemia. Myelodysplastic syndromes (MDS), where BM failure is driven by mutational takeover of myeloid cell lineages, provide a relevant clinical context to address these questions.

### Aims

In this study, we investigated the phenotype and functional capacity of human EBI macrophages (EBI-M) under physiological conditions and in MDS driven by somatic *SF3B1* mutations (MDS-SF3B1), a setting where refractory anemia is associated with significant clonal BM takeover.

### Methods

Primary monocytes and EBIs were enriched from fresh BM aspirates through density sedimentation with Lymphoprep and bovine serum albumin density gradients, respectively. EBIs were dissociated by mild enzymatic digestion to obtain single-cell suspensions. Cell subsets were purified by fluorescence-assisted cell sorting with canonical monocyte/macrophage markers CD14, CD68, CD163 and CD169. In parallel, BM monocytes were cultured *in vitro* toward unstimulated, pro-inflammatory and EBI-like monocyte-derived macrophages (MDMs). Cells were genotyped for *SF3B1* mutational burden with digital droplet PCR and analyzed through low-cell proteomics (pauciproteomics, $n = 130$ samples).

**Copyright Information:** (Online) ISSN: 2572-9241
©2026 The Author(s). HemaSphere is published by John Wiley & Sons Ltd on behalf of European Hematology Association. This is an open access Abstract Book distributed under the Attribution-NonCommercial-NoDerivs (CC BY-NC-ND), which allows third parties to download the articles and share them with others as long as they credit the author and the Abstract Book, but they cannot change the content in any way or use them commercially.
**Abstract Book Citations:** Authors, Title, HemaSphere, 2026;10:(S1):pages. The abstract book DOIs can be found on the "Author Information" page.
**Disclaimer:** Articles published in the journal HemaSphere exclusively reflect the opinions of the authors. The authors are responsible for all content in their abstracts including accuracy of the facts, statements, citing resources, etc.

### Results

In contrast to all monocyte populations assessed, EBI-M were highly positive for the surface markers MRC2 and VCAM1 (indicating a *bona fide* macrophage) and displayed a protective anti-ferroptosis program. Comparing EBI-M to EBI-like MDMs, traditionally used as a human EBI phenotype model, revealed a distinct cell compartment with maintained immunoregulatory ability (e.g. lacking key MDM molecules AKR1C3/NDRG1, and highly expressing MHC Class II/CD74, among others).
EBI-M were notably morphologically altered in MDS-SF3B1, with a marked iron deposit accumulation which was absent in both healthy EBI-M and EBI-like MDM. This aberrant state was accompanied by marked upregulation of iron metabolism pathways in EBI-M as well as in $CD14^{+}CD163^{+}CD169^{+}(CD68^{+/-})$ monocytes, highlighting a flux of active differentiation from monocyte precursors to EBI-M during anemia.
All MDS cell subsets mirrored the BM *SF3B1* mutation burden, including MDM, supporting *in vitro* MDM culture as a model for the functional investigation of mutational impact to macrophage biology. The MDS monocyte and macrophage proteome was globally impaired, including deficiency of the iron transporter ABCB7 and of MAP2K6, upstream kinase to the key immune regulator MAPK p38α. Potentially associated with this fact, pro-inflammatory MDMs in MDS uniquely cross-polarized to anti-inflammatory phenotypes despite high IFN-γ titers, which did not occur in healthy MDMs.

### Summary/Conclusion

We here define the elusive human EBI-M as a distinct cell type with clear functional roles in normal and anemic conditions. Through *ex vivo* and *in vitro* analysis, we obtain mechanistic insights into macrophage and EBI-M dysfunction in MDS-SF3B1 and highlight a potential role for clonal takeover of the EBI-M compartment in promoting an already severe anemic state.

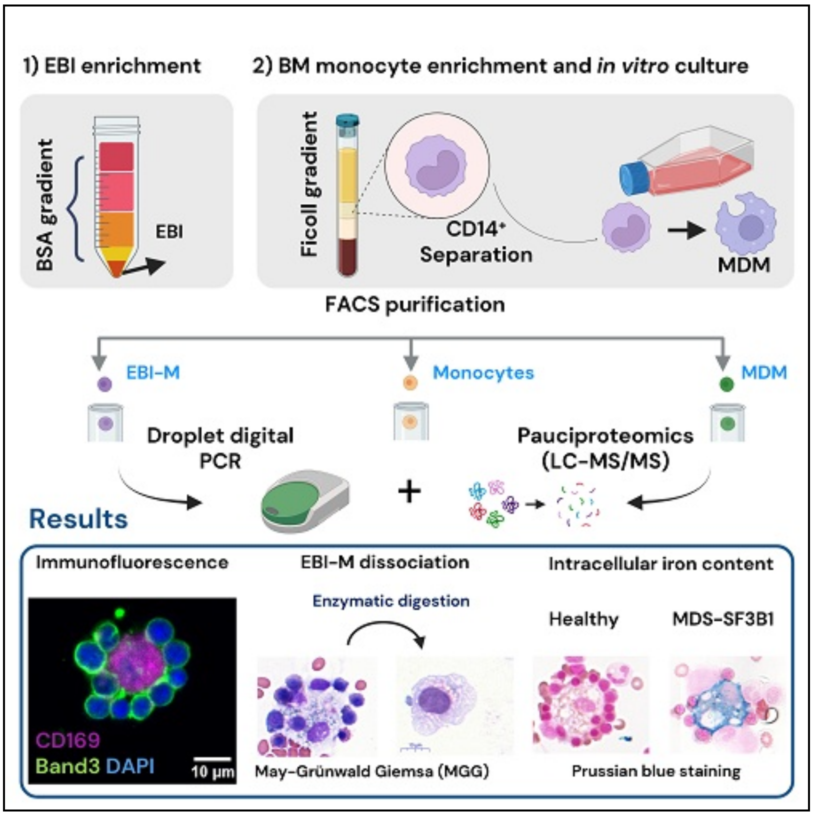

Copyright Information: (Online) ISSN: 2572-9241
©2026 The Author(s). HemaSphere is published by John Wiley & Sons Ltd on behalf of European Hematology Association. This is an open access Abstract Book distributed under the Attribution-NonCommercial-NoDerivs (CC BY-NC-ND), which allows third parties to download the articles and share them with others as long as they credit the author and the Abstract Book, but they cannot change the content in any way or use them commercially.
Abstract Book Citations: Authors, Title, HemaSphere, 2026;10:(S1):pages. The abstract book DOIs can be found on the “Author Information” page.
Disclaimer: Articles published in the journal HemaSphere exclusively reflect the opinions of the authors. The authors are responsible for all content in their abstracts including accuracy of the facts, statements, citing resources, etc.

### (PF654) INCREASED MITOCHONDRIAL ACTIVITY IN HSPCS AND MONOCYTES/MACROPHAGES COMPARTMENT REVEALS METABOLIC VULNERABILITIES IN MYELODYSPLASTIC SYNDROMES

**Topic:** 09. Myelodysplastic syndromes - Biology & translational research

Manuela Albuquerque de Melo*[1], Diego Antonio Pereira-Martins[2], Artur Bernardo Ribeiro Bellomi[1], João Henrique Nogueira[1], Anemari Ramos Dinarte Baccarin[1], Priscila Santos Scheucher[1], Isabel Weinhauser[2], Marjan Geugien[2], Shanna Hogeling[2], Emanuele Ammatuna[2], Gerwin Huls[2], Jan Jacob Schuringa[2], Fabiola Traina[1]

*[1]Department of Medical Imaging, Hematology, and Oncology, University of São Paulo at Ribeirão Preto Medical School, Ribeirão Preto, São Paulo, Brazil; [2]Department of Hematology, University of Groningen, University Medical Center Groningen, Groningen, The Netherlands;*

### Background

Myelodysplastic syndromes (MDS) are heterogeneous myeloid neoplasms characterized by peripheral blood cytopenias, ineffective hematopoiesis and risk of progression to acute myeloid leukemia (AML). Metabolic modulators such as metformin and tamoxifen show anti-tumor activity in hematologic models, but functional studies in MDS are limited by lack of robust primary culture systems and patient-derived models.

### Aims

To establish a soluplus-based culture platform and integrate *ex vivo* and *in vivo* models to functionally interrogate metabolic vulnerabilities in primary MDS.

### Methods

Thirteen primary MDS bone marrow (BM) samples were cultured at 2–5×10⁵ cells/mL for ≥20 days in soluplus-based conditions, with preserved proliferative capacity. In parallel, patient-derived xenografts (PDX) were generated in MISTRG mice to assess engraftment and lineage output. Mitochondrial membrane potential (MMP) was measured in HSPCs (HSC, $CD34^+CD38^-CD45RA^-$; LMPP, $CD34^+CD38^-CD45RA^+$; CMP, $CD34^+CD38^+CD45RA^-CD123^+$; GMP, $CD34^+CD38^+CD45RA^+CD123^+$; MEP, $CD34^+CD38^+CD34RA^-CD123^-$) and immune subsets (monocytes/macrophages; T, B, NK cells) in BM mononuclear cells from healthy donors (n=4), MDS (n=27) and AML (n=6). *Ex vivo* drug screening was performed in MDS (n=5) and healthy BM (n=5) using vehicle, metformin (5 mM) or tamoxifen (5 µM). Clonogenic capacity was assessed after 14 days, and cell frequencies, viability and MMP were analyzed after 24 hours.

**Copyright Information:** (Online) ISSN: 2572-9241
©2026 The Author(s). HemaSphere is published by John Wiley & Sons Ltd on behalf of European Hematology Association. This is an open access Abstract Book distributed under the Attribution-NonCommercial-NoDerivs (CC BY-NC-ND), which allows third parties to download the articles and share them with others as long as they credit the author and the Abstract Book, but they cannot change the content in any way or use them commercially.
**Abstract Book Citations:** Authors, Title, HemaSphere, 2026;10:(S1):pages. The abstract book DOIs can be found on the “Author Information” page.
**Disclaimer:** Articles published in the journal HemaSphere exclusively reflect the opinions of the authors. The authors are responsible for all content in their abstracts including accuracy of the facts, statements, citing resources, etc.

**Results**

In MISTRG mice (4 independent MDS samples), human engraftment was detectable in BM (mean 0.7%, range 0.1-1.5%), liver (17.4%, 2.2-49.2%) and spleen (9.2%, 0.7-55.2%), with higher levels in extramedullary sites, a clear myeloid bias and aberrant expansion of $CD34^{+}CD38^{-}$ cells in all tissues, supporting stem cell–driven disease propagation. Compared with healthy BM, both MDS and AML showed increased MMP in HSPCs (mean MFI: 1,830 healthy vs 4,254 MDS vs 5,745 AML) and in classical monocytes and M2-like macrophages (mean MFI: 4,656 and 3,589 healthy vs 7,489 and 8,272 MDS vs 15,711 and 12,244 AML), suggesting elevated oxidative phosphorylation activity. Moreover, the classical monocytes and M2-like macrophages show expanded frequencies in MDS (4.3% and 3%) relative to healthy donors (2.4% and 1.2%) and to AML (1.3% and 0.2%), highlighting a metabolically active and myeloid-skewed inflammatory niche. In *ex vivo* drug screening, metformin and tamoxifen reduced clonogenic capacity in MDS but not in healthy samples, with a pronounced reduction in monocytic colonies by metformin. After 24 hours, tamoxifen selectively decreased viability in a subset of MDS samples, associated with HSPC depletion. Metformin robustly reduced MMP in MDS HSPCs and across all monocyte subsets, including M2-like macrophages, in both MDS and healthy BM. These data indicate that metformin preferentially targets high-MMP populations and suggest that its anti-tumoral and preventive effects may relate to depletion or functional modulation of the monocytic compartment and attenuation of the associated hyperinflammatory state.

**Summary/Conclusion**

MDS is characterized by increased mitochondrial activity in HSPCs and in the monocytic/macrophage compartment, supporting mitochondrial metabolism as a key therapeutic vulnerability. A soluplus-based culture and a PDX platform enabling functional and metabolic interrogation of primary MDS cells was established, overcoming major technical barriers and providing a clinically relevant system to evaluate metabolic modulators in future translational studies.

**Copyright Information:** (Online) ISSN: 2572-9241
©2026 The Author(s). HemaSphere is published by John Wiley & Sons Ltd on behalf of European Hematology Association. This is an open access Abstract Book distributed under the Attribution-NonCommercial-NoDerivs (CC BY-NC-ND), which allows third parties to download the articles and share them with others as long as they credit the author and the Abstract Book, but they cannot change the content in any way or use them commercially.
**Abstract Book Citations:** Authors, Title, HemaSphere, 2026;10:(S1):pages. The abstract book DOIs can be found on the "Author Information" page.
**Disclaimer:** Articles published in the journal HemaSphere exclusively reflect the opinions of the authors. The authors are responsible for all content in their abstracts including accuracy of the facts, statements, citing resources, etc.

### (PF655) USP15 ALTERNATIVE SPLICING MAY GUIDE TGF-Β PATHWAY HYPERACTIVATION IN SF3B1-K700E LOWER RISK MYELODYSPLASTIC SYNDROMES IMPAIRING ERYTHROPOIESIS.

**Topic:** 09. Myelodysplastic syndromes - Biology & translational research

Emiliano Fabiani*[1], Luca Guarnera[1], Martina Senatore[1], Claudia Fiducioso[2], Hajro Hajrullaj[1], Carmen Iannini[2], Martina Rasicci[1], Giorgia Silvestrini[1], Nicole Lelli[1], Angelo Onorato[1], Antonio Cristiano[1], Marco Pieraccioli[3], Flavia Mallegni[1], Federico Moretti[1], Eleonora Lumia[1], Giulia Falconi[1], Stephan Hutter[4], Niroshan Nadarajah[4], Roberto La Tagliata[5], Torsten Haferlach[4], Claudio Sette[3], Luca Malcovati[2], Martina Sarchi[2], Pamela Bielli[1], Maria Teresa Voso[1]

*[1]University of Tor Vergata, Biomedicine and Prevention, Rome, Italy; [2]Department of Molecular Medicine, University of Pavia, Pavia, Italy; [3]Department of Neuroscience, Section of Human Anatomy, Catholic University of the Sacred Heart, 00168 Rome, Italy; Fondazione Policlinico A. Gemelli IRCCS, Rome, Italy; [4]MLL Munich Leukemia Laboratory, Munich, Germany; [5]UOC Haematology, ASL Viterbo-Santa Rosa Hospital, Vitervo, Italy;*

### Background

Despite the thorough characterization of the mutational landscape of lower risk MDS (LR-MDS), its contribution in the MDS pathogenesis often remain unknown. Mutations in the splicing machinery (SF3B1, SRSF2, U2AF1 and ZRSR2), are among the most frequent drivers in MDS (~50%). SF3B1 mutations are prognostically favorable and associated with long survival. Hyperactivation of TGF-β signaling has been shown to induce apoptosis and cell-cycle arrest in erythroblasts, leading to impairment of erythroid differentiation. USP15 deubiquitinase through interaction with SMAD7 and SMURF2, stabilizes TGF-βRI and enhances TGF-β signaling, contributing to tumor progression. The relevance of TGF-β pathway in the pathogenesis of anemia in LR-MDS is confirmed by the clinical activity of luspatercept.

### Aims

We aimed at characterizing the TGF-β pathway to refine its role as predictive biomarker of response to luspatercept, and as predictor of disease evolution in SF3B1mut LR-MDS.

### Methods

From a retrospective cohort of 300 MDS consecutively enrolled in the “Gruppo Romano Laziale Mielodisplasie (GROM-L)”, we selected 50 pts with paired DNA and RNA samples collected at the time of diagnosis. These samples were used for genome sequencing (WGS and t-NGS), RNA-seq, Q-rt-PCR and alternative splicing (AS) analysis. Cloning assays and in vitro models in K562 SF3B1-K700E and HEK293T USP15 transfected cells were used to confirm the findings.

**Copyright Information:** (Online) ISSN: 2572-9241
©2026 The Author(s). HemaSphere is published by John Wiley & Sons Ltd on behalf of European Hematology Association. This is an open access Abstract Book distributed under the Attribution-NonCommercial-NoDerivs (CC BY-NC-ND), which allows third parties to download the articles and share them with others as long as they credit the author and the Abstract Book, but they cannot change the content in any way or use them commercially.
**Abstract Book Citations:** Authors, Title, HemaSphere, 2026;10:(S1):pages. The abstract book DOIs can be found on the “Author Information” page.
**Disclaimer:** Articles published in the journal HemaSphere exclusively reflect the opinions of the authors. The authors are responsible for all content in their abstracts including accuracy of the facts, statements, citing resources, etc.

### Results

RNA-Seq on 50 LR-MDS vs 64 non-haematologic pts revealed 4221 differentially expressed genes (DEGs), 3921 down- and 300 up-regulated. According to specific SF mutations, SF3B1-mut MDS (n=15) showed the highest number of DEGs (622: 10 down- and 612 up-regulated) when compared to SF-wt pts (n=7).
Gene Ontology analysis identified 37 deregulated pathways in SF3B1-mut vs SF-wt pts (Fig1A). We found 6 DEGs in TGF-β pathway (CHRD, DCN, SMAD9, FST, ID4, PITX2) and 10 in TGF-β modulators (BDNF, BMPER, COL1A1, COL1A2, FN1, GLI2, IGF1, MMP2, NFIB and PLAU) (Fig1B). DEGs were confirmed using Q-rt-PCR assays: DCN (p=0,004), FST (p=0,017), SMAD9 (p=0,030), FN1 (p=0,004), IGF1 (p=0,030) and MMP2 (p= 0,004).
We then investigated the presence of alternative splicing events (ASE) within the TGF-β pathway, identifying an intron 15 retention on the USP15 gene, a ubiquitin specific protease (Fig1C). This yields a truncated protein isoform (USP15-TR), which was preferentially expressed in SF3B1-K700E vs wt MDS, without changes in total mRNA.
USP15-TR was overexpressed in SF3B1-K700E mutated K562 cells ($p<0.05$) as compared to USP15 full length (USP15-FL), without changes in total mRNA (Fig1D). The stability of USP15-TR was confirmed by non-sense mediated decay assays. Western blot (WB) confirmed a reduction of USP15-FL protein (p=0.02), with a relative increase of USP15-TR (Fig1E).
Bioinformatic analysis in MDS showed reduction of SMAD3 and SMAD7 mRNA, confirmed by WB in the K562 SF3B1-K700E cell model ($p<0.01$ and $p<0.05$). SMAD2 protein was reduced, while its phosphorylation was markedly increased, highlighting hyperactivation of the TGF-β pathway in SF3B1-mut cells. This was also confirmed by the ectopic expression of either USP15 isoforms in HEK293T cells, showing an increased SMAD2 phosphorylation in USP15-TR vs -FL engineered cells.

### Summary/Conclusion

Our analysis shows a deregulation of TGF-β pathway at mRNA, ASE and protein levels in SF3B1-mut LR-MDS and engineered cells, suggesting the potential role of USP15 as biomarker and/or actional target in clinical practice.

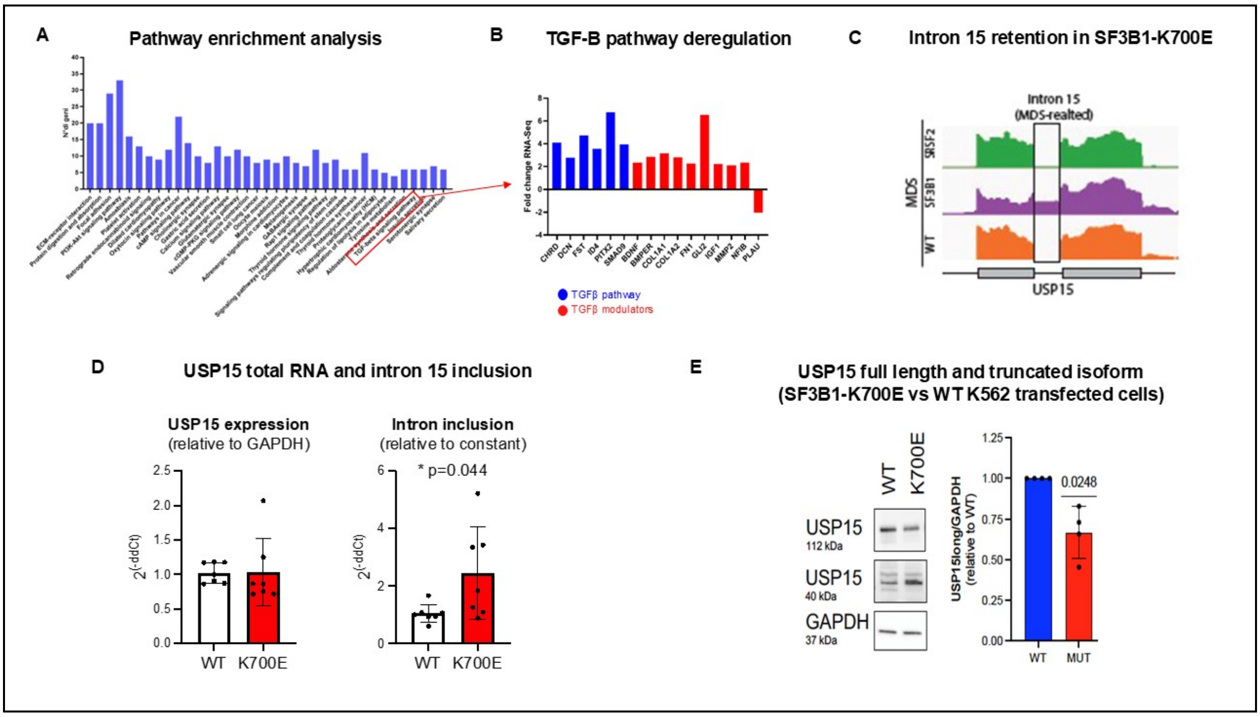

**Copyright Information:** (Online) ISSN: 2572-9241
©2026 The Author(s). HemaSphere is published by John Wiley & Sons Ltd on behalf of European Hematology Association. This is an open access Abstract Book distributed under the Attribution-NonCommercial-NoDerivs (CC BY-NC-ND), which allows third parties to download the articles and share them with others as long as they credit the author and the Abstract Book, but they cannot change the content in any way or use them commercially.
**Abstract Book Citations:** Authors, Title, HemaSphere, 2026;10:(S1):pages. The abstract book DOIs can be found on the "Author Information" page.
**Disclaimer:** Articles published in the journal HemaSphere exclusively reflect the opinions of the authors. The authors are responsible for all content in their abstracts including accuracy of the facts, statements, citing resources, etc.

### (PF656) WHOLE GENOME SEQUENCING REVEALS NOVEL, CLINICALLY RELEVANT BIOMARKER MISSED BY STANDARD OF CARE FOR PATIENTS WITHIN MYELODYSPLASTIC SYNDROME

**Topic:** 09. Myelodysplastic syndromes - Biology & translational research

Alex Bataller*[1], Robert Huether[2], Sanam Loghavi[1], Derick Hoskinson[2], Karl Beutner[2], Adam Hockenberry[2], Charles Koyias[2], Cecile Rose Vibat[2], Victoria Chiou[2], Michael Thompson[2], Kate Sasser[2], Halla Nimeiri[2], Guillermo Garcia-Manero[1]

*[1]The University of Texas MD Anderson Cancer Center, Houston, United States of America;[2]Tempus AI, Inc., Chicago, United States of America;*

### Background

Genomic analysis is critical for accurate diagnosis and risk stratification in myeloid malignancies. Current standard-of-care (SOC) diagnostic tools utilize a fragmented workflow including NGS panels, cytogenetics, and optical genome mapping (OGM), amongst others. This approach may lead to delays or incomplete clinical stratification by missing actionable alterations that fall outside of or below the limit-of-detection for individual assays, potentially compromising patient management. Whole genome sequencing (WGS) provides a consolidated platform that captures the full breadth of genomic alterations in a single assay.

### Aims

We retrospectively evaluated whether Tempus xH, a WGS based assay, could detect clinically impactful genomic alterations that were not detected by SOC from a major academic institution (MDACC).

### Methods

Fifty patient samples were sequenced using the Tempus xH WGS assay. SOC data consisting of karyotyping (+/- FISH), an 81-gene NGS panel, and/or OGM (n=10) served as the source of truth. Sensitivity measurements were evaluated using clinically relevant genomic alterations between the SOC and xH reportable range workflows. Specifically, we excluded mutations with <= 10% VAF and copy number alterations (CNAs) < 5Mb, and combined OGM and cytogenetics (10>= metaphases). Structural variants (SVs) were included if they overlapped with orthogonal truth or were recurrent myeloid fusions.

### Results

WGS identified an average of five clinically-relevant genomic alterations per sample, demonstrating an overall sensitivity of 98% relative to SOC. Sensitivity was 100% for single nucleotide variants (n=133), 95.6% for CNAs (65/68) and 90.9% for SVs (10/11). Notably, the WGS assay identified 34 novel, high confidence variants (26 CNAs, 2 SNVs, and 6 SVs) across 20 specimens (40%) that were not detected by SOC.

Retrospective clinical review determined that 14% (7/50) of patients had novel findings that were clinically significant and may have impacted clinical care. Among these were three *MECOM* rearrangements, a *del(17p)* involving the *TP53* locus, and a *KMT2A-PTD*, potentially missed due to the limited size resolution and sensitivity of SOC techniques. WGS also identified a high VAF (48%) *ASXL1* mutation and a large *RUNX1* deletion in exon 8 (smaller than resolution of observing it in cytogenetics) that codes for part of the transactivation domain—both alterations are associated with poor prognosis in MDS. In 3 out of 7 patients, the additional alterations identified by the Tempus xH WGS assay would have changed their IPSS-M risk: from High to Very High risk (*ASXL1* mutation), from moderate-high to high (*KMT2A*-PTD), and from low to moderate-low (*MECOM* rearrangement). The additional WGS alterations provide molecular insights that refine prognostic accuracy and directly expand the therapeutic and clinical care considerations beyond current SOC.

**Copyright Information:** (Online) ISSN: 2572-9241
©2026 The Author(s). HemaSphere is published by John Wiley & Sons Ltd on behalf of European Hematology Association. This is an open access Abstract Book distributed under the Attribution-NonCommercial-NoDerivs (CC BY-NC-ND), which allows third parties to download the articles and share them with others as long as they credit the author and the Abstract Book, but they cannot change the content in any way or use them commercially.
**Abstract Book Citations:** Authors, Title, HemaSphere, 2026;10:(S1):pages. The abstract book DOIs can be found on the “Author Information” page.
**Disclaimer:** Articles published in the journal HemaSphere exclusively reflect the opinions of the authors. The authors are responsible for all content in their abstracts including accuracy of the facts, statements, citing resources, etc.

**Summary/Conclusion**

WGS demonstrated high concordance (98% sensitivity) with SOC workflows, and detected cryptic clinically relevant alterations in 14% of patients, providing a more comprehensive genomic profile. These data suggest that WGS can aid clinical decision making by consolidating diagnostic workflows and identifying critical prognostic and therapeutic biomarkers that escape traditional testing limits. Further, the breadth of variants detected by WGS assays may facilitate the discovery of novel associations between variants and risk stratification and/or drug targetability in the future.

**Copyright Information:** (Online) ISSN: 2572-9241
©2026 The Author(s). HemaSphere is published by John Wiley & Sons Ltd on behalf of European Hematology Association. This is an open access Abstract Book distributed under the Attribution-NonCommercial-NoDerivs (CC BY-NC-ND), which allows third parties to download the articles and share them with others as long as they credit the author and the Abstract Book, but they cannot change the content in any way or use them commercially.
**Abstract Book Citations:** Authors, Title, HemaSphere, 2026;10:(S1):pages. The abstract book DOIs can be found on the "Author Information" page.
**Disclaimer:** Articles published in the journal HemaSphere exclusively reflect the opinions of the authors. The authors are responsible for all content in their abstracts including accuracy of the facts, statements, citing resources, etc.

### (PF657) LARGE-SCALE MULTI-PHENOTYPE WHOLE SLIDE IMAGING ANALYSIS UNCOVERS SPATIAL NICHE ARCHETYPES ALONG THE MDS/AML CONTINUUM

**Topic:** 09. Myelodysplastic syndromes - Biology & translational research

Mischa Selig*[1], Drazen Papic[2], Stefan Florian[2], Ines Koch[2], Jörg Westermann[1, 3], David Horst[2, 4], Lars Bullinger[1, 2, 3, 4], Jana Ihlow[2, 5], Livius Penter[1, 4, 6]

*[1]Charité - Universitätsmedizin Berlin, Department of Hematology, Oncology and Cancer Immunology, Berlin, Germany;[2]Charité - Universitätsmedizin Berlin, Institute of Pathology, Berlin, Germany; [3]Labor Berlin Charité Vivantes GmbH, Berlin, Germany; [4]German Cancer Consortium (DKTK) and German Cancer Research Center (DKFZ), Berlin, Germany;[5]Berlin Institute of Health at Charité, BIH Biomedical Innovation Academy, BIH Clinician Scientist Program, Berlin, Germany; [6]Berlin Institute of Health at Charité, BIH Biomedical Innovation Academy - Digital Clinician Scientist Program, Berlin, Germany;*

### Background

Myelodysplastic neoplasms (MDS) with low (LR-MDS; <5%) or increased blasts (MDS-IB; >5%) and AML are distinct states along a continuum of abnormal hematopoiesis. While LR-MDS can be driven by immune hyperactivation, MDS-IB/AML associate with immune escape. Yet, as immunotherapies have limited activity, there is a need to better understand the spatial organization of bone marrow (BM) immune cells. Automated imaging analysis of archival BM immunohistochemistry (IHC) data can define the evolving immune microenvironment cost-efficiently and at scale.

### Aims

We leveraged large-scale whole-slide imaging (WSI) analysis on multi-marker IHC data to define progenitor and immune niche archetypes in AML/MDS.

### Methods

We registered serial WSIs (*QuPath*, *VALIS*) into a shared coordinate space, followed by cell segmentation, phenotyping, and density estimation. We quantified niche density, size (effective radius), composition and interaction features, and derived within-niche spatial roles from niche-restricted graphs.

**Copyright Information:** (Online) ISSN: 2572-9241
©2026 The Author(s). HemaSphere is published by John Wiley & Sons Ltd on behalf of European Hematology Association. This is an open access Abstract Book distributed under the Attribution-NonCommercial-NoDerivs (CC BY-NC-ND), which allows third parties to download the articles and share them with others as long as they credit the author and the Abstract Book, but they cannot change the content in any way or use them commercially.
**Abstract Book Citations:** Authors, Title, HemaSphere, 2026;10:(S1):pages. The abstract book DOIs can be found on the "Author Information" page.
**Disclaimer:** Articles published in the journal HemaSphere exclusively reflect the opinions of the authors. The authors are responsible for all content in their abstracts including accuracy of the facts, statements, citing resources, etc.

**Results**

We integrated up to 10 stains (H&E, progenitor: CD34/CD117; myeloid: MPO/CD68/CD163/CD61; immune: CD3/CD20/CD138) from 5 normal controls (NC), 13 LR-MDS, 16 MDS-IB, and 13 AML cases across 443 BM slides, yielding a mean 147,482 single cells per slide (range 13,234–391,850). Macrophage, B and T cell density remained similar across LR-MDS, MDS-IB, and AML. However, their number relative to progenitor cells decreased from 4.39 (T cell), 1.04 (B cell) and 5.6 (macrophage) in LR-MDS to 0.58, 0.18 and 0.95 in AML ($p < 0.01$), consistent with progressive displacement of immune niches by expanding progenitor compartments.

To track organizational BM remodeling, we defined spatial niches as groups of ≥100 cells. Within niches, we classified cells as members of a core (≥10 connected cells), or adjacent, proximal and distant to it. Niche density increased from NC (mean 0.65 niches/mm²) to LR-MDS (1.13), MDS-IB (1.46) and AML (1.07). Based on the ratio of $CD3^+$ and $CD20^+$vs. $CD34^+$ and $CD117^+$ cells (cut-off 0.6), we defined immune and progenitor spatial niches. Immune niche fractions decreased from 80.7% and 78.6% in NC and LR-MDS to 46.2% and 20.9% in MDS-IB and AML ($p < 0.01$). While immune niche size remained largely stable across AML/MDS, progenitor niches showed expansion of mean core areas from NC to AML (0.350 vs. 6.42 mm²; p<0.01) and increased heterogeneity (mean standard deviation 0.153 vs. 7.61 mm²; p<0.01).

Finally, we asked how niche architectures evolved. Pseudotime analysis showed a continuous niche-remodeling across MDS/AML, and composition analysis revealed the most homogeneous immune cell infiltrate in LR-MDS. In immune niches, cell density/mm $^2$ increased for T cells from 188 in NC to 339 in LR-MDS (p = 0.008), 324 in MDS-IB (p = 0.013), and 310 in AML, while it decreased for B cells from 203 in NC to 65 in LR-MDS, 87 in MDS-IB (p = 0.003), and 29 in AML. Macrophage density in progenitor-niche cores decreased from 374 cells/mm² in NC to 204 in AML. By integrating niche architectures, we could classify MDS/AML cases into three niche archetypes: immune-dominated, progenitor-dominated and mixed. These categories associated with blast counts in progenitor- vs. immune-dominated MDS/AML (median 40% vs. 8%, p = 0.043).

**Summary/Conclusion**

Using automated image analysis, we reveal progressive niche remodeling across MDS/AML. This defines spatially interpretable disease archetypes that may inform risk biology and motivate survival outcome and spatial transcriptomic analyses.

**Copyright Information:** (Online) ISSN: 2572-9241
©2026 The Author(s). HemaSphere is published by John Wiley & Sons Ltd on behalf of European Hematology Association. This is an open access Abstract Book distributed under the Attribution-NonCommercial-NoDerivs (CC BY-NC-ND), which allows third parties to download the articles and share them with others as long as they credit the author and the Abstract Book, but they cannot change the content in any way or use them commercially.
**Abstract Book Citations:** Authors, Title, HemaSphere, 2026;10:(S1):pages. The abstract book DOIs can be found on the "Author Information" page.
**Disclaimer:** Articles published in the journal HemaSphere exclusively reflect the opinions of the authors. The authors are responsible for all content in their abstracts including accuracy of the facts, statements, citing resources, etc.

### (PF658) IMMUNE AND NICHE-ASSOCIATED TRANSCRIPTIONAL REMODELING ACROSS MDS AND AML AND ITS RELATIONSHIP TO RECURRENT GENETIC ALTERATIONS

**Topic:** 09. Myelodysplastic syndromes - Biology & translational research

Seon Young Kim*[1], Sooyong Park[2], Yoon Hwan Chang[1], Hyun Kyung Kim[1]

*[1]Seoul National University Hospital, Department of Laboratory Medicine, Seoul, South Korea;[2]Chungnam National University College of Medicine, Department of Laboratory Medicine, Daejeon, South Korea;*

### Background

Myeloid malignancies, including acute myeloid leukemia (AML) and myelodysplastic neoplasms (MDS), are characterized by substantial genetic heterogeneity. Although recurrent mutations have been extensively described, how these genetic alterations relate to immune activation and bone marrow niche–associated transcriptional programs across the disease spectrum from MDS to AML is not fully understood.

### Aims

This study aimed to investigate the relationship between recurrent genetic alterations and immune- and niche-associated transcriptional programs in MDS and AML, and to explore their clinical relevance.

### Methods

A total of 193 bone marrow (BM) samples from 178 patients with myeloid malignancies were analyzed, including 85 AML, 70 MDS, 16 MDS/MPN, and 7 idiopathic cytopenia of uncertain significance (ICUS) cases. Whole-transcriptome sequencing was performed on diagnostic or relapse specimens. Single-sample gene set enrichment analysis (ssGSEA) was applied to curated gene sets representing antigen presentation (APC), interferon (IFN) signaling, TNF/NFκB activation, inflammasome pathways, macrophage polarization (M1-like, M2-like, SPP1-related), cytotoxic T/NK programs, proliferation, oxidative stress (NRF2), senescence-associated secretory phenotype (SASP), and CXCL12-associated niche signatures. Scores were Z-standardized across all samples. Comparisons were performed using non-parametric testing, and survival analyses were conducted using Cox regression models.

### Results

Whole-transcriptome profiling revealed significant associations between recurrent genetic alterations and immune- and niche-related transcriptional programs. In MDS, *SF3B1*-mutated cases (n = 12) were characterized by significantly reduced proliferation signatures ($p = 4.9\times10^{-5}$), lower SASP scores ($p = 0.04$), and decreased myeloid-related programs ($p = 0.018$), whereas *U2AF1*-mutated MDS (n = 8) showed increased proliferation ($p = 0.0057$), elevated SASP signatures ($p = 0.027$), and reduced SPP1_TAM programs ($p = 0.041$). *SRSF2*-mutated MDS (n = 11) demonstrated modest proliferation differences ($p = 0.03$) without broader immune remodeling. Trisomy 8–positive MDS (n = 12) exhibited decreased SPP1-related macrophage programs, while *DDX41*-mutated MDS (n = 6) showed significantly higher CXCL12-associated niche signatures ($p = 0.0014$) with limited changes in inflammatory pathways. In AML, trisomy 8–positive cases (n = 6) displayed increased APC signatures ($p = 0.0041$), whereas CH-like AML (n = 23) was associated with reduced APC and type II interferon signaling. Importantly, within MDS, higher M1-like macrophage and NRF2-associated oxidative stress programs were significantly associated with inferior overall survival and increased risk of leukemic progression, indicating that specific immune and niche-related transcriptional states carry prognostic relevance beyond genetic classification alone.

**Copyright Information:** (Online) ISSN: 2572-9241
©2026 The Author(s). HemaSphere is published by John Wiley & Sons Ltd on behalf of European Hematology Association. This is an open access Abstract Book distributed under the Attribution-NonCommercial-NoDerivs (CC BY-NC-ND), which allows third parties to download the articles and share them with others as long as they credit the author and the Abstract Book, but they cannot change the content in any way or use them commercially.
**Abstract Book Citations:** Authors, Title, HemaSphere, 2026;10:(S1):pages. The abstract book DOIs can be found on the "Author Information" page.
**Disclaimer:** Articles published in the journal HemaSphere exclusively reflect the opinions of the authors. The authors are responsible for all content in their abstracts including accuracy of the facts, statements, citing resources, etc.

**Summary/Conclusion**

Distinct genetic subgroups of AML and MDS exhibited differential immune and niche-associated transcriptional patterns. Rather than uniform inflammatory activation, transcriptional programs varied according to specific cytogenetic and mutational contexts. These findings highlight the heterogeneity of microenvironment-related signaling across myeloid malignancies and underscore the importance of considering genetic background when interpreting immune and niche-associated transcriptomic profiles.

**Copyright Information:** (Online) ISSN: 2572-9241
©2026 The Author(s). HemaSphere is published by John Wiley & Sons Ltd on behalf of European Hematology Association. This is an open access Abstract Book distributed under the Attribution-NonCommercial-NoDerivs (CC BY-NC-ND), which allows third parties to download the articles and share them with others as long as they credit the author and the Abstract Book, but they cannot change the content in any way or use them commercially.
**Abstract Book Citations:** Authors, Title, HemaSphere, 2026;10:(S1):pages. The abstract book DOIs can be found on the "Author Information" page.
**Disclaimer:** Articles published in the journal HemaSphere exclusively reflect the opinions of the authors. The authors are responsible for all content in their abstracts including accuracy of the facts, statements, citing resources, etc.

### (PF659) UPDATED OVERALL SURVIVAL AND LONG-TERM TRANSFUSION INDEPENDENCE IN ESA-NAIVE PATIENTS WITH LOWER-RISK MDS: RESULTS FROM THE PHASE 3 COMMANDS TRIAL

**Topic:** 10. Myelodysplastic syndromes - Clinical

Valeria Santini*[1], Matteo Giovanni Della Porta[2], Amer M. Zeidan[3], Rami S. Komrokji[4], Veronika Pozharskaya[5], Yinzhi Lai[5], Daniel Lopes de Menezes[5], Manuel Ugidos Guerrero[5], Thalia Farazi[5], Dimana Mitev[6], Barkha Aggarwal[5], Sameer Kalsekar[7], David Valcárcel[8], Pierre Fenaux[9], Jake Shortt[10], Uwe Platzbecker[11], Guillermo Garcia-Manero[12]

*[1]MDS Unit, Hematology, University of Florence, Florence, Italy;[2]Cancer Center IRCCS Humanitas Research Hospital, Milan, Italy; Humanitas University, Milan, Italy; [3]Yale University School of Medicine, New Haven, CT, United States of America;[4]Moffitt Cancer Center, Tampa, FL, United States of America; [5]Bristol Myers Squibb, Princeton, NJ, United States of America;[6]Bristol Myers Squibb, Boudry, Switzerland; [7]Bristol Myers Squibb, Hyderabad, Telangana, India; [8]Hospital Universitari Vall d'Hebron, Barcelona, Spain; [9]Hôpital Saint-Louis, Université Paris 7, Paris, France;[10]Monash University and Monash Health, Melbourne, VIC, Australia; [11]University Hospital Leipzig, Leipzig, Germany; [12]University of Texas MD Anderson Cancer Center, Houston, TX, United States of America;*

### Background

In the phase 3 COMMANDS trial (NCT03682536), luspatercept (LUSPA) significantly improved red blood cell-transfusion independence (RBC-TI) ≥12 weeks with concurrent mean hemoglobin (Hb) increase ≥1.5 g/dL during Weeks 1–24 versus epoetin alfa (EA) in erythropoiesis-stimulating agent (ESA)-naive, transfusion-dependent (TD) patients (pts) with lower-risk myelodysplastic syndromes (LR-MDS). Longer follow-up (>2.5 years) showed a favorable overall survival (OS) trend and durable transfusion independence (TI) responses with LUSPA versus EA.

### Aims

To report updated OS, long-term RBC-TI and Hb responses, baseline (BL) biomarkers associated with OS, and safety after an additional 6 months of follow-up (total follow-up >3 years)

### Methods

Eligible pts (≥18 years; ESA-naive; TD; LR-MDS) were stratified by BL transfusion burden (TB), ring sideroblasts (RS), and serum erythropoietin (sEPO), and randomized 1:1 to LUSPA (1.0–1.75 mg/kg; subcutaneous every 3 weeks) or EA (450–1050 IU/kg; subcutaneous weekly) for ≥24 weeks. OS (from randomization), RBC-TI responses and Hb improvement (Weeks 1 to end of treatment), predictive BL biomarkers of OS, and safety were evaluated.

**Copyright Information:** (Online) ISSN: 2572-9241
©2026 The Author(s). HemaSphere is published by John Wiley & Sons Ltd on behalf of European Hematology Association. This is an open access Abstract Book distributed under the Attribution-NonCommercial-NoDerivs (CC BY-NC-ND), which allows third parties to download the articles and share them with others as long as they credit the author and the Abstract Book, but they cannot change the content in any way or use them commercially.
**Abstract Book Citations:** Authors, Title, HemaSphere, 2026;10:(S1):pages. The abstract book DOIs can be found on the "Author Information" page.
**Disclaimer:** Articles published in the journal HemaSphere exclusively reflect the opinions of the authors. The authors are responsible for all content in their abstracts including accuracy of the facts, statements, citing resources, etc.

**Results**

At data cutoff (October 6, 2025), median (min–max) follow-up was 35.9 (1–73) months for LUSPA and 30.8 (0–77) months for EA. Median OS was not reached (NR) with LUSPA and was 46.0 months with EA (hazard ratio [HR] 0.78; 95% CI 0.56–1.09). Similar trends were seen across subgroups (**Table**). RBC-TI ≥12 weeks occurred in 76.4% versus 55.8% of pts (LUSPA vs EA), with 60.4% versus 39.2% achieving concurrent mean Hb ≥10 g/dL. Median cumulative duration (95% CI) of RBC-TI ≥12 weeks was 184.4 (118.4–NE) weeks with LUSPA and 95.1 (74.9–180.1) weeks with EA (HR 0.54; 95% CI 0.36–0.80). RBC-TI ≥2.5 years was achieved by 25.3% versus 10.5% of pts. Mean Hb increase ≥1.5 g/dL occurred in 80.2% versus 58.6% of pts; median duration (95% CI) of Hb increase ≥1.5 g/dL was 72.9 (53.9–89.9) versus 47.9 (35.9–60.0) weeks (HR 0.61; 95% CI 0.44–0.85). Improved OS was associated with BL biomarkers of favorable immune function (LUSPA) and cardiovascular function (both arms), less aggressive disease (EA), and preserved megakaryopoiesis (EA). At cutoff, 17.6% (LUSPA) and 7.8% (EA) of pts remained on treatment; 84.6% and 83.2% had ≥1 dose escalation. No new safety concerns were identified. Deaths (any cause) occurred in 36.3% (LUSPA) and 43.0% (EA); fewer LUSPA pts progressed to higher-risk MDS (3.3% vs 7.3%), and progression to acute myeloid leukemia was comparable (4.9% vs 5.5%).

**Summary/Conclusion**

With long-term follow-up, LUSPA showed sustained responses and improved OS trends versus EA, overall and in subgroups, with no new safety concerns. Taken together, this long-term data reinforces the superior clinical benefit of luspatercept as a first-line treatment in LR-MDS.

© 2026 American Society of Clinical Oncology, Inc. Reused with permission. This abstract was accepted and previously presented at the 2026 ASCO Annual Meeting. All rights reserved.

| Median OS, mos | LUSPA | EA | HR (95% CI) |
|---|---|---|---|
| Overall | (n=182) NR | (n=181) 46.0 | 0.78 (0.56-1.09) |
| Stratification subgroup | | | |
| TB <4 RBC U/8 wks | (n=118) 57.5 | (n=111) 47.2 | 0.87 (0.57-1.33) |
| TB ≥4 RBC U/8 wks | (n=64) NR | (n=70) 39.9 | 0.62 (0.37-1.05) |
| RS+ | (n=133) NR | (n=130) 47.2 | 0.70 (0.47-1.04) |
| RS− | (n=49) 54.8 | (n=50) 46.2 | 0.96 (0.53-1.72) |
| sEPO ≤200 U/L | (n=145) NR | (n=144) 51.4 | 0.81 (0.55-1.20) |
| sEPO >200 U/L | (n=37) 43.0 | (n=37) 35.4 | 0.67 (0.35-1.28) |

**Copyright Information:** (Online) ISSN: 2572-9241
©2026 The Author(s). HemaSphere is published by John Wiley & Sons Ltd on behalf of European Hematology Association. This is an open access Abstract Book distributed under the Attribution-NonCommercial-NoDerivs (CC BY-NC-ND), which allows third parties to download the articles and share them with others as long as they credit the author and the Abstract Book, but they cannot change the content in any way or use them commercially.
**Abstract Book Citations:** Authors, Title, HemaSphere, 2026;10:(S1):pages. The abstract book DOIs can be found on the “Author Information” page.
**Disclaimer:** Articles published in the journal HemaSphere exclusively reflect the opinions of the authors. The authors are responsible for all content in their abstracts including accuracy of the facts, statements, citing resources, etc.

### (PF660) MOLECULAR PREDICTORS OF CLINICAL EFFICACY WITH VENETOCLAX AND HYPOMETHYLATING AGENT IN THE FRONTLINE MANAGEMENT OF HIGHER RISK MYELODYSPLASTIC SYNDROME

**Topic:** 10. Myelodysplastic syndromes - Clinical

Guru Subramanian Guru Murthy*[1], Yasmin Abaza[2], Somedeb Ball[3], Ramzi Abboud[4], Michael Haddadin[5], Jonathan Feld[6], Suresh Balasubramanian[7], Ashwin Kishtagari[8], Adam Cohen Nowak[2], Veronica Guerra[9], Hayley Hawkins[10], Asmita Shukla[11], Virginia Tan[12], Dominika Dukal[13], Reema Tawfiq[14], Emily Dworkin[15], Julian Weiss[16], Amber Feng[16], Jesus D. Gonzalez-Lugo[17], Prajwal Dhakal[18], Jessica Leonard[19], Charles Foucar[20], Talha Badar[14], Rory M. Shallis[21], Annie Im[22], Shyam Patel[23], Chenyu Lin[16], Anand Patel[24], Michael Savona[3], Eric Winer[25], Ehab Atallah[1], Rami S. Komrokji[26]

*[1]Medical College of Wisconsin, Milwaukee, United States of America;[2]Northwestern University, Chicago, United States of America; [3]Vanderbilt University, Nashville, United States of America;[4]Washington University St Louis, St Louis, United States of America; [5]University of Nebraska, Omaha, United States of America;[6]Moffitt Malignant Hematology & Cellular Therapy at Memorial Healthcare System, Tampa, United States of America; [7]Karmanos Cancer Institute, Detroit, United States of America; [8]Vanderbilt Ingram Cancer Center, Nashville, United States of America;[9]Washington University in St Louis, St Louis, United States of America; [10]Vanderbilt-Ingram Cancer Center, Vanderbilt University Medical Center, Nashville, United States of America; [11]Karmanos Cancer Istitute, Detroit, United States of America;[12]Oregon Health and Science University, Portland, United States of America; [13]University of Pittsburgh, Pittsburgh, United States of America;[14]Mayo Clinic, Jacksonville, United States of America; [15]University of Chicago, chicago, United States of America; [16]Duke University, Durham, United States of America; [17]University of Kansas Cancer Center, Kansas, United States of America; [18]University of Iowa, Iowa City, United States of America; [19]OHSU, Oregon, United States of America; [20]University of New Mexico, Albuquerque, United States of America;[21]Yale University, New Haven, United States of America; [22]UPMC, Pittsburgh, United States of America;[23]University of Massachusetts, Worcester, United States of America; [24]University of Chicago, Chicago, United States of America;[25]Dana Farber Cancer Institute, Boston, United States of America; [26]Moffitt Cancer Center, Tampa, United States of America;*

### Background

The Bcl-2 inhibitor venetoclax (VEN) in combination with hypomethylating agent (HMA) has shown encouraging efficacy in early phase clinical trials for higher risk MDS (HR-MDS) (Garcia JS et al. Blood 2025). Despite the lack of survival benefit in the VERONA study (NCT04401748), potential subgroups of patients with HR-MDS patients could derive clinical benefit with Bcl-2 inhibition in the frontline setting. To better understand the impact of baseline mutations on clinical outcomes, we analyzed the genomic data from a large cohort of real-world patients with HR-MDS treated with frontline HMA-VEN or HMA.

### Aims

To determine the association between baseline mutations and outcomes in patients with HR-MDS treated with frontline HMA-VEN therapy.

### Methods

This is a multicenter retrospective analysis of adults (age≥18 years) with treatment naïve HR-MDS (intermediate, high or very high risk by IPSS-R) who received HMA-VEN or HMA monotherapy in United States. Data on clinical characteristics, mutations and outcomes were collected through chart review. Comparison of clinical outcomes based on mutations were performed within HMA-VEN cohort and against HMA monotherapy. Descriptive statistics were used for baseline characteristics. Overall survival (OS) and event free survival (EFS: progression, leukemic transformation, death) analysis was conducted using Kaplan-Meier method. Determinants of outcomes were analyzed using multivariable regression models. All analyses were conducted at a significance level of $p<0.05$.

Copyright Information: (Online) ISSN: 2572-9241
©2026 The Author(s). HemaSphere is published by John Wiley & Sons Ltd on behalf of European Hematology Association. This is an open access Abstract Book distributed under the Attribution-NonCommercial-NoDerivs (CC BY-NC-ND), which allows third parties to download the articles and share them with others as long as they credit the author and the Abstract Book, but they cannot change the content in any way or use them commercially.
Abstract Book Citations: Authors, Title, HemaSphere, 2026;10:(S1):pages. The abstract book DOIs can be found on the "Author Information" page.
Disclaimer: Articles published in the journal HemaSphere exclusively reflect the opinions of the authors. The authors are responsible for all content in their abstracts including accuracy of the facts, statements, citing resources, etc.

**Results**

In a cohort of 1198 patients, 313 received upfront HMA-VEN and 885 received HMA monotherapy. Median age was 70 (63-76) years and the most common mutations observed in at least 10% of patients included *TP53* (34%), *ASXL1* (22%), *DNMT3A* (16%), *RUNX1* (16%), *TET2* (14%), *SRSF2* (13%), and *U2AF1* (11%). Among patients treated with HMA-VEN, similar responses by IWG 2006 and IWG 2023 criteria were seen regardless of the mutation status (Table 1). However, EFS and OS were significantly inferior in patients with *TP53* mutation treated with HMA-VEN (unmutated vs monoallelic *TP53* vs biallelic *TP53*- median EFS (mon)= 21 vs 12 vs 10, p<0.01, median OS (mon)= 21 vs 13 vs 14, p<0.01). When compared to HMA monotherapy, treatment with HMA-VEN was associated with significantly improved EFS and OS across several molecular subgroups except in those with *TP53* mutation (HMA-VEN vs. HMA- monoallelic TP53- median EFS- 7.4 vs 4.1 mon, p=0.11, biallelic TP53-median EFS- 7.5 vs 5.5 mon,p=0.20; median OS- monoallelic TP53- 13 vs 11 mon, p=0.20, biallelic TP53- 12 vs 13 mon, p=0.20). In adjusted multivariable analysis, *TP53* mutation was associated with inferior EFS (monoallelic *TP53* -HR 1.38, 95%CI 1.14-1.66, p<0.01; biallelic *TP53*- HR 1.47, 95%CI 1.25-1.74, p<0.01) and inferior OS (monoallelic *TP53*- HR 1.90, 95%CI 1.54-2.34, p<0.01; biallelic *TP53*- HR 1.89, 95%CI 1.50-2.37, p<0.01).

**Summary/Conclusion**

Somatic mutations are associated with clinical efficacy of Bcl-2 inhibition in patients with HR-MDS treated with HMA-VEN. While the benefit of HMA-VEN treatment was seen across several mutational subgroups, *TP53* mutation (monoallelic and biallelic) was not associated with significant improvement in survival despite similar response rates.

**Copyright Information:** (Online) ISSN: 2572-9241
©2026 The Author(s). HemaSphere is published by John Wiley & Sons Ltd on behalf of European Hematology Association. This is an open access Abstract Book distributed under the Attribution-NonCommercial-NoDerivs (CC BY-NC-ND), which allows third parties to download the articles and share them with others as long as they credit the author and the Abstract Book, but they cannot change the content in any way or use them commercially.
**Abstract Book Citations:** Authors, Title, HemaSphere, 2026;10:(S1):pages. The abstract book DOIs can be found on the “Author Information” page.
**Disclaimer:** Articles published in the journal HemaSphere exclusively reflect the opinions of the authors. The authors are responsible for all content in their abstracts including accuracy of the facts, statements, citing resources, etc.

**Table 1: Response to HMA-VEN therapy in most common mutation subgroups**

| Mutation | IWG 2006 | | | | IWG 2023 | | | | |
|---|---|---|---|---|---|---|---|---|---|
| | CR | Marrow CR | PR | p-value | CR | $CR_h$ | $CR_L$ | PR | p-value |
| *TP53* | | | | 0.23 | | | | | 0.33 |
| None | 19.6% | 55.4% | 8.2% | | 19.6% | 9.1% | 20.5% | 8.2% | |
| Monoallelic | 14.7% | 55.9% | 1.9% | | 16.4% | 11.9% | 10.4% | 1.9% | |
| Biallelic | 8.9% | 57.1% | 10.7% | | 8.9% | 9.6% | 23.1% | 10.7% | |
| *ASXL1* | | | | 0.27 | | | | | 0.72 |
| Mutated | 23.3% | 53.3% | 8.3% | | 23.3% | 7.1% | 21.4% | 8.3% | |
| Unmutated | 15% | 56.3% | 6.9% | | 15.5% | 10.1% | 18.1% | 6.9% | |
| *DNMT3A* | | | | 0.05 | | | | | 0.41 |
| Mutated | 14% | 56% | 4% | | 14.2% | 10.2% | 12.2% | 4% | |
| Unmutated | 17% | 55.6% | 7.8% | | 17.6% | 9.4% | 20% | 7.8% | |
| *RUNX1* | | | | 0.85 | | | | | 0.65 |
| Mutated | 19% | 54% | 9.5% | | 19% | 3.3% | 21.3% | 9.5% | |
| Unmutated | 16% | 56.1% | 6.6% | | 16.3% | 11.2% | 18% | 6.6% | |
| *TET2* | | | | 0.97 | | | | | 0.35 |
| Mutated | 18.2% | 54.5% | 9.1% | | 18.2% | 14.3% | 28.6% | 9.1% | |
| Unmutated | 16.3% | 55.9% | 6.8% | | 16.7% | 8.7% | 17.1% | 6.8% | |
| *SRSF2* | | | | 0.58 | | | | | 0.82 |
| Mutated | 12.2% | 65.9% | 9.8% | | 12.2% | 5.3% | 23.7% | 9.8% | |
| Unmutated | 17.4% | 54% | 6.8% | | 17.4% | 10.2% | 18% | 6.8% | |
| *U2AF1* | | | | 0.79 | | | | | 0.27 |
| Mutated | 17.9% | 46.4% | 10.7% | | 25.9% | 11.1% | 3.7% | 10.7% | |
| Unmutated | 16.5% | 56.5% | 6.8% | | 16.5% | 9.4% | 20.3% | 6.8% | |

Table 1: Response to HMA-VEN therapy in most common mutation subgroups

Copyright Information: (Online) ISSN: 2572-9241
©2026 The Author(s). HemaSphere is published by John Wiley & Sons Ltd on behalf of European Hematology Association. This is an open access Abstract Book distributed under the Attribution-NonCommercial-NoDerivs (CC BY-NC-ND), which allows third parties to download the articles and share them with others as long as they credit the author and the Abstract Book, but they cannot change the content in any way or use them commercially.
Abstract Book Citations: Authors, Title, HemaSphere, 2026;10:(S1):pages. The abstract book DOIs can be found on the "Author Information" page.
Disclaimer: Articles published in the journal HemaSphere exclusively reflect the opinions of the authors. The authors are responsible for all content in their abstracts including accuracy of the facts, statements, citing resources, etc.

### (PF661) WHICH PATIENTS WITH MDS-H AND MDS-LB BENEFIT FROM ALLOGENEIC HEMATOPOIETIC STEM CELL TRANSPLANTATION? EVIDENCE FROM A LARGE SINGLE-CENTER RETROSPECTIVE COHORT OF 636 PATIENTS

**Topic:** 10. Myelodysplastic syndromes - Clinical

Wenjing Guo*[1], Linlin Liu[1], Jia Li[1], Zhijian Xiao[1], Sizhou Feng[1]

*[1]State Key Laboratory of Experimental Hematology, National Clinical Research Center for Blood Diseases, Haihe Laboratory of Cell Ecosystem, Institute of Hematology & Blood Diseases Hospital, Chinese Academy of Medical Sciences & Peking Union Medical College, Tianjin, China;*

### Background

Hypoplastic myelodysplastic neoplasms (MDS-h) and MDS with low blasts (MDS-LB) are characterized by low blasts and absence of defining cytogenetic abnormalities, conferring relatively favorable outcomes. However, which patients may derive the greatest benefit from allogeneic hematopoietic stem cell transplantation (allo-HSCT) in these subtypes remains unclear.

### Methods

We retrospectively analyzed 636 consecutive patients diagnosed with MDS-h (n=146) or MDS-LB (n=490) at our hospital between June 2016 and June 2024. Overall survival (OS) and progression-free survival (PFS, defined as progression to MDS-IB/AML or death from any cause) were estimated using the Kaplan-Meier method. The cumulative incidence of transfusion dependence (TD) was evaluated using competing-risk analysis. Receipt of allo-HSCT was treated as a time-dependent covariate and its survival impact was assessed using Simon-Makuch plots (Mantel-Byar test) and landmark analyses.

### Results

The median age was 52 years and 34.43% were female. At diagnosis,42.41% were TD and 52.08% were classified as Molecular International Prognostic Scoring System (IPSS-M) higher-risk. During follow-up, 32 MDS-h and 80 MDS-LB patients underwent allo-HSCT, with a median interval of 204.5 (range, 19-1838) days from diagnosis. The estimated 5-year PFS and OS for the entire cohort were 62.9% (95% CI: 58.0-68.2) and 60.5% (95% CI: 55.8-65.7), respectively.
TD was associated with inferior survival irrespective of IPSS-M risk category. The 5-year OS was 39.1% and 49.4% in TD patients with higher- and lower-risk disease, respectively, compared with 65.3% and 81.2% in transfusion-independent (TI) patients ($P$<0.001) **(Figure 1A)**. In TD patients, allo-HSCT, modeled as a time-dependent covariate, was independently associated with improved OS (HR=0.513, 95% CI: 0.297-0.886, $P$=0.017) **(Figure 1B)**. The benefit was confined to TD patients with IPSS-M higher-risk disease, in whom 5-year OS was 86.1% after transplantation versus 47.1% without transplantation in a 12-month landmark analysis ($P$=0.004) **(Figure 1C)**. No survival advantage was observed in the lower-risk group ($P$=0.759) **(Figure 1D)**.
Among initially TI patients (n=364), the cumulative incidence of TD during follow-up was 45.6%, independently predicted by IPSS-M higher-risk status (HR=2.413, 95% CI: 1.486-3.917, $P$<0.001) and MDS-LB subtype (HR=2.202, 95% CI: 1.155-4.201, $P$=0.017). In this subgroup, transplantation showed a trend toward benefit in higher-risk patients, whereas excess early mortality was observed in lower-risk patients undergoing allo-HSCT **(Figure 1E-F)**.

**Copyright Information:** (Online) ISSN: 2572-9241
©2026 The Author(s). HemaSphere is published by John Wiley & Sons Ltd on behalf of European Hematology Association. This is an open access Abstract Book distributed under the Attribution-NonCommercial-NoDerivs (CC BY-NC-ND), which allows third parties to download the articles and share them with others as long as they credit the author and the Abstract Book, but they cannot change the content in any way or use them commercially.
**Abstract Book Citations:** Authors, Title, HemaSphere, 2026;10:(S1):pages. The abstract book DOIs can be found on the “Author Information” page.
**Disclaimer:** Articles published in the journal HemaSphere exclusively reflect the opinions of the authors. The authors are responsible for all content in their abstracts including accuracy of the facts, statements, citing resources, etc.

### Summary/Conclusion

In this large cohort, TD refines risk stratification in MDS-h and MDS-LB patients beyond IPSS-M categories. Allo-HSCT confers a clear survival advantage in TD patients with IPSS-M higher-risk disease but not in those with lower-risk disease. Among TI patients, transplantation appears beneficial in the higher-risk subgroup but not the lower-risk subgroup. These findings support a risk-adapted transplantation strategy integrating both molecular risk and transfusion status.

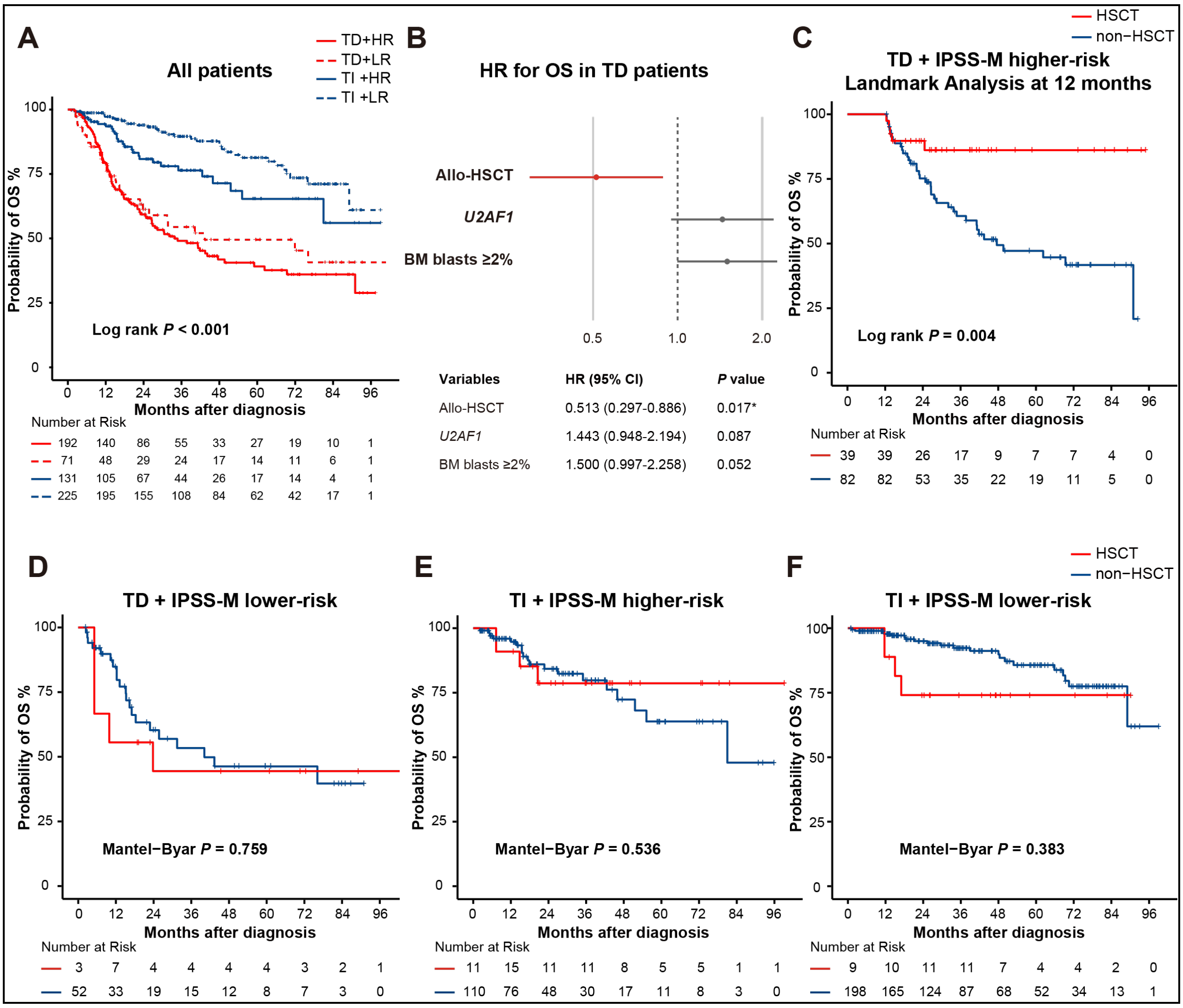

**Copyright Information:** (Online) ISSN: 2572-9241
©2026 The Author(s). HemaSphere is published by John Wiley & Sons Ltd on behalf of European Hematology Association. This is an open access Abstract Book distributed under the Attribution-NonCommercial-NoDerivs (CC BY-NC-ND), which allows third parties to download the articles and share them with others as long as they credit the author and the Abstract Book, but they cannot change the content in any way or use them commercially.
**Abstract Book Citations:** Authors, Title, HemaSphere, 2026;10:(S1):pages. The abstract book DOIs can be found on the "Author Information" page.
**Disclaimer:** Articles published in the journal HemaSphere exclusively reflect the opinions of the authors. The authors are responsible for all content in their abstracts including accuracy of the facts, statements, citing resources, etc.

### (PF662) CYTO-CMML: A MUTATION-INDEPENDENT CYTOGENETIC RISK CLASSIFIER FOR CHRONIC MYELOMONOCYTIC LEUKEMIA

**Topic:** 10. Myelodysplastic syndromes - Clinical

Ayalew Tefferi[1], Matteo Giovanni Della Porta*[2], Muhammad Yousuf[1], Blanca Xicoy Cirici[3], Shraddha Patel[1], Torsten Haferlach[4], Saubia Fathima[1], Laura Palomo[3], Ali Alsugair[1], Giulia Maggioni[2], Animesh Pardanani[1], Ulrich Germing[5], Kaaren Reichard[1], Alessia Campagna[2], Abhishek Mangaonkar[1], Gabriele Todisco[2], Rong He[1], Anthony M. Hunter[6], Naseema Gangat[1], Eric Solary[7], Cinthya Zepeda Mendoza[1], Eric Padron[8], Mrinal Patnaik[1], Luca Lanino[9]

*[1]Mayo Clinic, Rochester, United States of America; [2]Cancer Center IRCCS Humanitas Research Hospital, IRCCS Humanitas University, Milan, Italy; [3]Institut Català d'Oncologia, Hospital Germans Trias i Pujol, Badalona, Josep Carreras Leukaemia Research Institute,, Barelona, Spain; [4]MLL Munich Leukemia Laboratory, Munich, Germany; [5]Department of Hematology, Oncology, and Clinical Immunology, Heinrich-Heine-University,, Düsseldorf, Germany; [6]Emory University, Atlanta, United States of America; [7]INSERM U1287, Gustave Roussy Cancer Center, Villejuif, France; [8]Department of Malignant Hematology, H. Lee Moffitt Cancer Center and Research Institute, Tampa, United States of America; [9]Department of Internal Medicine, Yale School of Medicine, New Haven, United States of America;*

### Background

Allogeneic stem cell transplantation (ASCT) is currently the only treatment in chronic myelomonocytic leukemia (CMML) with the potential to prolong survival or cure the disease. Optimal timing of ASCT requires careful benefit-risk assessment that is best accomplished by applying molecular risk models.

### Aims

The objective of the current study was to refine the prognostic contribution of karyotype to genetic risk prediction in CMML.

### Methods

A retrospective collaborative study was conducted using a Mayo Clinic discovery cohort and multicenter validation cohort. Diagnosis of CMML and its morphologic subcategorization were performed according to the International Consensus Classification (*Blood 2022;140:1200*). Cytogenetic results were reported according to the International System for Human Cytogenetic Nomenclature. Overall survival (OS) and cumulative incidence of blast transformation (BT) calculations were adjusted for ASCT and competing risk, respectively.

**Copyright Information:** (Online) ISSN: 2572-9241
©2026 The Author(s). HemaSphere is published by John Wiley & Sons Ltd on behalf of European Hematology Association. This is an open access Abstract Book distributed under the Attribution-NonCommercial-NoDerivs (CC BY-NC-ND), which allows third parties to download the articles and share them with others as long as they credit the author and the Abstract Book, but they cannot change the content in any way or use them commercially.
**Abstract Book Citations:** Authors, Title, HemaSphere, 2026;10:(S1):pages. The abstract book DOIs can be found on the "Author Information" page.
**Disclaimer:** Articles published in the journal HemaSphere exclusively reflect the opinions of the authors. The authors are responsible for all content in their abstracts including accuracy of the facts, statements, citing resources, etc.

### Results

The discovery cohort included 971 patients (median age 72 years; males 70%) and the validation cohort 1,175 (median age 73 years; males 67%). At median follow-up of 52 and 53 months, respectively, the rates of deaths were 68% vs. 55% and BT 19% vs. 21%. In both study cohorts, multivariable analyses (MVA) revealed OS associated with sole abnormalities of -Y, 5q-, and 7q- was similar to that observed with normal karyotype (*p values 0.3-0.5*). By contrast, OS in patients with complex karyotype aligned with those associated with non-complex abnormalities of -7, +21, i(17)(q10), inv(3)(q21q26.2), and t(1;3)(p36;q21) [*p values 0.3-0.6*]. Accordingly, the former were assigned to "low risk," the latter "high risk," and all other abnormalities "intermediate" risk categories, with respective median OS of 38, 10 (*HR 3.8*), and 22 (*HR 1.5*) months (*p <0.01*). Significant differences in OS between the three cytogenetic risk groups were demonstrated in both study cohorts (*p<0.01 and 0.02*) while only high risk category displayed significantly higher risk of BT with a 1-year risk of 33% vs. 8-9% for the other risk groups (*HR 5.4; p<0.01*).
The independent prognostic contribution of high risk karyotype, for both OS and BT, in the context of other molecular and clinical risk factors was confirmed in both the discovery and validation cohorts while intermediate risk category did not maintain its significance for BT. Similarly, high but not intermediate risk karyotype remained significant in the context of contemporary risk models, including BLAST (*Blood 2025;146:874*), BLAST-mol (*Blood 2025;146:874*), and CPSS-mol (*Blood 2016;128:1408*) [*p<0.01 vs.>0.05, in all instances*]. Accordingly, a two-tiered version, operationally referred to as "Cyto-CMML," combines low and intermediate risk into one "standard" risk category and was more efficient in predicting both OS and risk of BT, in the context of other molecular risk factors (Figure). Cyto-CMML-independent risk factors validated in both cohorts included, for OS, anemia, leukocyte count ≥13 x $10^9$/L, circulating blasts ≥2%, *NPM1*$^{MUT}$, *BCOR*$^{MUT}$, *U2AF1*$^{MUT}$, *ASXL1*$^{MUT}$, and *TET2*$^{MUT}$ and, for BT, CMML-2, *ASXL1*$^{MUT}$, *NPM1*$^{MUT}$, leukocyte count ≥13 x $10^9$/L, and circulating blasts ≥2%.

### Summary/Conclusion

Cyto-CMML provides a high-performing and mutation-independent cytogenetic risk classifier with the prospect of recalibrating the predictive performance of current risk models.

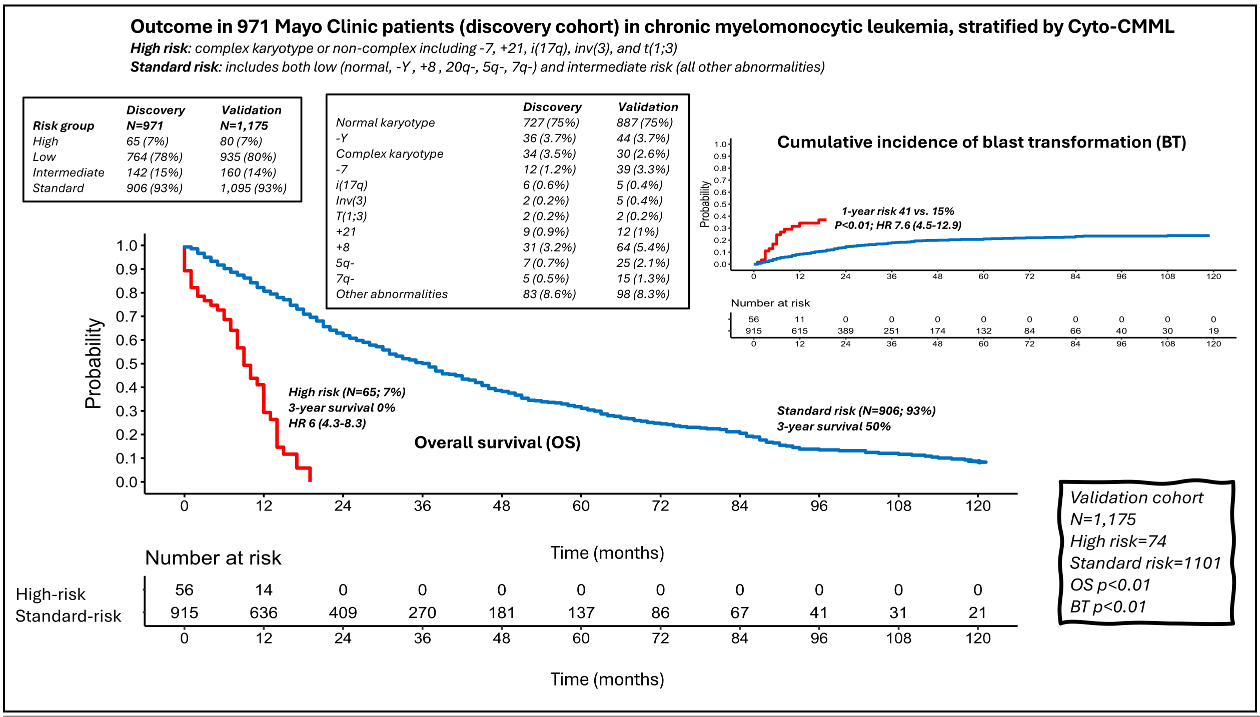

**Copyright Information:** (Online) ISSN: 2572-9241
©2026 The Author(s). HemaSphere is published by John Wiley & Sons Ltd on behalf of European Hematology Association. This is an open access Abstract Book distributed under the Attribution-NonCommercial-NoDerivs (CC BY-NC-ND), which allows third parties to download the articles and share them with others as long as they credit the author and the Abstract Book, but they cannot change the content in any way or use them commercially.
**Abstract Book Citations:** Authors, Title, HemaSphere, 2026;10:(S1):pages. The abstract book DOIs can be found on the "Author Information" page.
**Disclaimer:** Articles published in the journal HemaSphere exclusively reflect the opinions of the authors. The authors are responsible for all content in their abstracts including accuracy of the facts, statements, citing resources, etc.

### (PF663) TRANSLATIONAL INSIGHTS AND CLINICAL EFFICACY WITH BEXMARILIMAB AND AZACITIDINE IN HR-MDS IN THE BEXMAB PHASE 1/2 STUDY

**Topic:** 10. Myelodysplastic syndromes - Clinical

Mika Kontro*[1, 2], Amer M. Zeidan[3], Anthony Stein[4], Jesper Mickos[5], Johanna Rimpiläinen[6], Marja Pyörälä[7], Timo Siitonen Siitonen[8], Mikko Myllymäki[1, 2], Joshua Zeidner[9], Emma Searle[10, 11], Ruth Witherall[12], Petri Bono[13], Aneel Nimba[13], Joab Williamson[13], Sofia Aakko[13], Brian J Ball[4], Naval Daver[14], Maija Hollmén[5, 13]

*[1]Helsinki University Hospital, Comprehensive Cancer Center, Helsinki, Finland;[2]University of Helsinki, Helsinki, Finland;[3]Yale University and Yale Comprehensive Cancer Center, Department of Internal Medicine, New Haven, CT, United States of America;[4]City of Hope Cancer Center, Duarte, United States of America;[5]University of Turku, MediCity Research Laboratory and InFLAMES Flagship, Turku, Finland;[6]Tampere University Hospital, Department of Internal Medicine, Tampere, Finland;[7]Kuopio University Hospital, Kuopio, Finland;[8]Oulu University Hospital, Cancer Center, Oulu, Finland;[9]University of North Carolina, Lineberger Comprehensive Cancer Center, Chapel Hill, United States of America;[10]The Christie NHS Foundation Trust, Manchester, United Kingdom;[11]The University of Manchester, Manchester, United Kingdom;[12]Royal Cornwall Hospitals NHS Trust, Truro, United Kingdom;[13]Faron Pharmaceuticals, Turku, Finland;[14]The University of Texas MD Anderson Cancer Center, Department of Leukemia, Houston, United States of America;*

### Background

Bexmarilimab (BEX) blocks Common lymphatic and vascular endothelial receptor-1 (Clever-1) to enhance macrophage antigen presentation and T cell activation. In the bone marrow (BM), also myeloid blasts express Clever-1. Thus, BEX may change the BM immune environment to anti-leukemic and make the blasts susceptible to other agents, such as hypomethylating agents (HMAs). HMAs are the standard frontline treatment for higher-risk myelodysplastic syndrome (HR-MDS). New treatment options are needed particularly for patients who have especially dismal outcomes with HMAs due to e.g. TP53 mutation (mTP53), (Daver et al., 2022) and after HMA-failure. BEX has a unique mode of action that is potentially synergistic with HMA. The combination of BEX and HMA azacitidine (AZA), has shown promising clinical efficacy in HR-MDS in BEXMAB study (NCT05428969), both in frontline and HMA-failed population. Here, we explore the translational data from the BEX+AZA treated HR-MDS patients.

### Aims

The Phase 1/2 (Ph1/2) BEXMAB clinical study investigates safety, tolerability and preliminary efficacy of BEX in combination with standard-of-care, AZA, in HR-MDS. Immunological and cellular pharmacodynamic parameters related to BEX mechanism of action and clinical activity are studied as an exploratory endpoint.

### Methods

As of 03Nov2025, 21 treatment-naïve and 34 relapsed/refractory (r/r; no response after 4 cycles or disease progression with HMA/HMA containing treatment) HR-MDS patients (IPSS-R>3) were recruited to the BEXMAB study. BEX was administrated weekly in 28-day cycles, in combination with a standard regimen of AZA (75 mg/m$^2$ D1-7 each cycle). BM samples for translational analyses were collected upon response assessments at the end of cycles 1, 3 and 6 and analysed centrally and/or in a translational laboratory with single cell RNA sequencing (scRNAseq, n=5 patients) and multiparameter flow cytometry (all patients).

**Copyright Information:** (Online) ISSN: 2572-9241
©2026 The Author(s). HemaSphere is published by John Wiley & Sons Ltd on behalf of European Hematology Association. This is an open access Abstract Book distributed under the Attribution-NonCommercial-NoDerivs (CC BY-NC-ND), which allows third parties to download the articles and share them with others as long as they credit the author and the Abstract Book, but they cannot change the content in any way or use them commercially.
**Abstract Book Citations:** Authors, Title, HemaSphere, 2026;10:(S1):pages. The abstract book DOIs can be found on the "Author Information" page.
**Disclaimer:** Articles published in the journal HemaSphere exclusively reflect the opinions of the authors. The authors are responsible for all content in their abstracts including accuracy of the facts, statements, citing resources, etc.

**Results**

Preliminary translational data aligns with the hypothesized immune activating mechanism of action of BEX+AZA in MDS, with scRNAseq showing reduction of immunosuppressive monocytes and flow cytometry confirming up to 10-fold increased antigen presenting molecule (HLA-DR) expression on BM monocytes in 2/3 of the MDS patients treated with BEX+AZA. A similar proportion of the MDS patients show activation of adaptive immunity, with increased number of activated cytotoxic BM NK and CD8 T cells with BEX+AZA treatment. In addition, earlier scRNAseq results suggested preferential targeting of blasts that are dependent on oxidative phosphorylation (Mickos et al, 2025). Full results from ongoing confirmatory flow cytometry analyses characterizing the BM immune, blast and progenitor cell changes induced by BEX+AZA will be reported. Relevance of the BEX+AZA molecular mechanism of action is supported by the observed clinical efficacy, highlighted by a 45% CR rate in treatment-naïve HR MDS and a 14.5mo median overall survival in r/r MDS, both surpassing historical comparators with AZA monotherapy.

**Summary/Conclusion**

Translational results show both immune activation and targeting of malignant blasts in the BM upon BEX+AZA treatment in MDS patients. Addition of BEX to AZA results in encouraging clinical efficacy in both treatment-naïve HR-MDS as well as r/r MDS. Translational results for the full study population, including both frontline and r/r MDS, will be reported for the first time.

**Copyright Information:** (Online) ISSN: 2572-9241
©2026 The Author(s). HemaSphere is published by John Wiley & Sons Ltd on behalf of European Hematology Association. This is an open access Abstract Book distributed under the Attribution-NonCommercial-NoDerivs (CC BY-NC-ND), which allows third parties to download the articles and share them with others as long as they credit the author and the Abstract Book, but they cannot change the content in any way or use them commercially.
**Abstract Book Citations:** Authors, Title, HemaSphere, 2026;10:(S1):pages. The abstract book DOIs can be found on the "Author Information" page.
**Disclaimer:** Articles published in the journal HemaSphere exclusively reflect the opinions of the authors. The authors are responsible for all content in their abstracts including accuracy of the facts, statements, citing resources, etc.

### (PF664) CARDIOVASCULAR OUTCOMES IN PATIENTS WITH MYELODYSPLASTIC SYNDROMES AND CHRONIC MYELOMONOCYTIC LEUKEMIA: A NATIONWIDE POPULATION-BASED COHORT STUDY IN TAIWAN

**Topic:** 10. Myelodysplastic syndromes - Clinical

Sin-Syue Li*[1], Pei-An Fu[1], Chen-Yi Yang[2, 3], Tsai-Yun Chen[1], Huang Tz Ou[2, 3]

*[1]Division of Hematology, Department of Internal Medicine, National Cheng Kung University Hospital, College of Medicine, National Cheng Kung University, Tainan City, Taiwan; [2]Institute of Clinical Pharmacy and Pharmaceutical Sciences, College of Medicine, National Cheng Kung University, Tainan City, Taiwan; [3]Department of Pharmacy, College of Medicine, National Cheng Kung University, Tainan City, Taiwan;*

### Background

Patients with myelodysplastic syndromes (MDS) and chronic myelomonocytic leukemia (CMML) are typically elderly and carry a substantial burden of cardiovascular (CV) comorbidities. While chronic inflammation and clonal hematopoiesis are implicated in accelerated atherosclerosis, evidence from nationwide cohorts remains sparse. Whether this risk independently confers excess CV mortality beyond that of other age-matched cancer populations, or varies by subtype and sex, remains incompletely defined. Furthermore, there are currently no specialized guidelines for the primary prevention of CV complications in the MDS/CMML population, representing a critical gap in cardio-oncological care.

### Aims

This study aimed to evaluate the independent risk of major adverse cardiovascular events (MACE) and CV death in patients with MDS/CMML compared to sex-matched solid tumor controls (breast or prostate cancer), while exploring risk variations across disease subtypes, sex-specific patterns, and the influence of baseline antiplatelet therapy.

### Methods

Using the Taiwan Cancer Registry linked to the National Health Insurance Research Database (2013–2022), we identified patients diagnosed with MDS (ICD-O-3: 9980, 9982, 9983, 9985, 9986, 9989, 9991, 9992) or CMML (ICD-O-3: 9945). Female MDS/CMML patients were matched up to 1:5 with breast cancer controls, and male patients with prostate cancer controls based on age and baseline CV risk factors (diabetes, hypertension, dyslipidemia, and atrial fibrillation). The co-primary endpoints were CV death and MACE. Secondary outcomes included individual CV events and the need for invasive procedures. Multivariable Cox proportional hazards models were adjusted for baseline antiplatelet and anticoagulant use, and interaction analyses were performed.

### Results

The cohort included 1,417 females and 2,353 males with MDS/CMML. Compared to controls, MDS/CMML was associated with significantly increased risks of CV death (Females: aHR 3.74; Males: aHR 4.22) and MACE (Females: aHR 2.33; Males: aHR 2.44), all $P < 0.001$. Risks of individual events, including pulmonary embolism, venous thrombosis, stroke, myocardial infarction, and heart failure were all significantly elevated. A distinct risk gradient across subtypes (CMML ≈ High-risk MDS > Low-risk MDS >> Control) was observed exclusively in males, while risks were more uniformly elevated across female subtypes. Despite higher baseline antiplatelet use in MDS/CMML, a significant interaction between disease status and antiplatelet therapy for MACE risk was found only in females ($P_{interaction} < 0.05$).

**Copyright Information:** (Online) ISSN: 2572-9241
©2026 The Author(s). HemaSphere is published by John Wiley & Sons Ltd on behalf of European Hematology Association. This is an open access Abstract Book distributed under the Attribution-NonCommercial-NoDerivs (CC BY-NC-ND), which allows third parties to download the articles and share them with others as long as they credit the author and the Abstract Book, but they cannot change the content in any way or use them commercially.
**Abstract Book Citations:** Authors, Title, HemaSphere, 2026;10:(S1):pages. The abstract book DOIs can be found on the "Author Information" page.
**Disclaimer:** Articles published in the journal HemaSphere exclusively reflect the opinions of the authors. The authors are responsible for all content in their abstracts including accuracy of the facts, statements, citing resources, etc.

### Summary/Conclusion

MDS/CMML is independently associated with a 2- to 4-fold increased risk of MACE and CV death. The sex-specific risk gradients and the differential impact of antiplatelet therapy in women suggest that myeloid-driven CV vulnerability is modulated by complex biological and clinical factors. These findings highlight the urgent need for integrated cardio-oncological monitoring and personalized CV risk management strategies for this high-risk hematologic population.

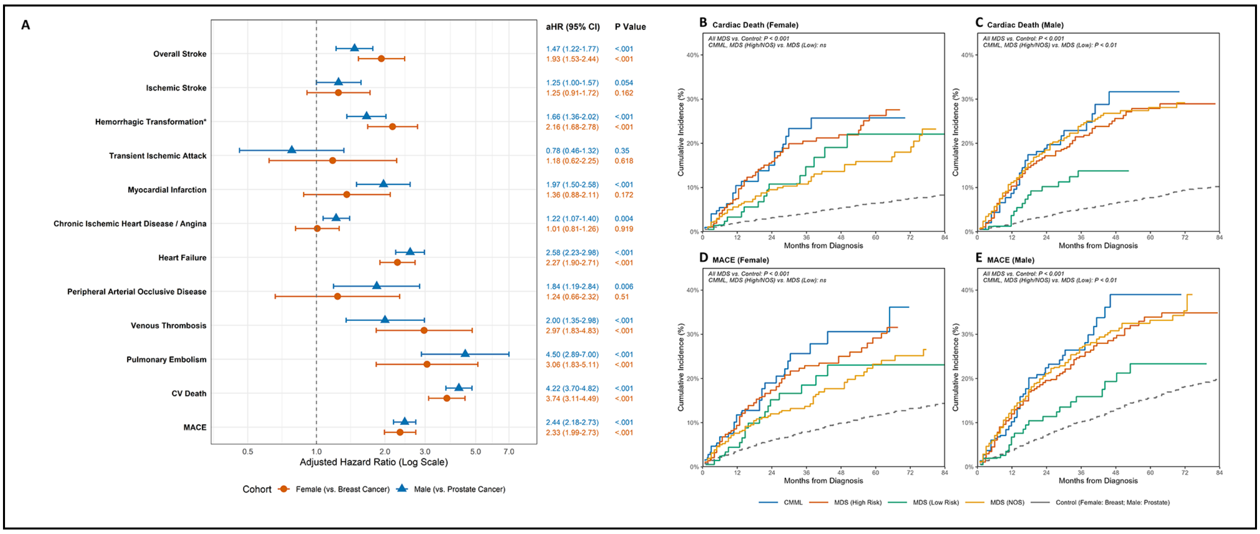

(A) Forest Plot: Adjusted hazard ratios (aHR) for CV outcomes compared to matched cancer controls (Female: Breast; Male: Prostate). aHRs were adjusted for age, comorbidities, and baseline antiplatelet/anticoagulant use. (B–E) Cumulative Incidence Curves: Cardiac Death (B, C) and MACE (D, E) stratified by sex and histological subtypes. Abbreviations: aHR, adjusted hazard ratio; CI, confidence interval; CMML, chronic myelomonocytic leukemia; MACE, major adverse cardiovascular events; MDS, myelodysplastic syndromes; ns, non-significant.

**Copyright Information:** (Online) ISSN: 2572-9241
©2026 The Author(s). HemaSphere is published by John Wiley & Sons Ltd on behalf of European Hematology Association. This is an open access Abstract Book distributed under the Attribution-NonCommercial-NoDerivs (CC BY-NC-ND), which allows third parties to download the articles and share them with others as long as they credit the author and the Abstract Book, but they cannot change the content in any way or use them commercially.
**Abstract Book Citations:** Authors, Title, HemaSphere, 2026;10:(S1):pages. The abstract book DOIs can be found on the "Author Information" page.
**Disclaimer:** Articles published in the journal HemaSphere exclusively reflect the opinions of the authors. The authors are responsible for all content in their abstracts including accuracy of the facts, statements, citing resources, etc.

### (PF665) VALIDATION OF MOLECULAR PROGNOSTIC MODELS FOR CHRONIC MYELOMONOCYTIC LEUKEMIA IN A LARGE MULTICENTER COHORT

**Topic:** 10. Myelodysplastic syndromes - Clinical

Lara Gonzalez Palenzuela*[1], Felicitas Schulz[2], Corinna Strupp[2], Sascha Dietrich[2], Kathrin Nachtkamp[2], Guido Kobbe[2], Mitja Nabergoj[3], Sabine Blum[3], Barbara Hildebrandt[4], Beate Betz[4], Detlef Haase[5], Alexander Streuer[6], Katharina Götze[7], Katja Sockel[8], Ulrich Germing[2]

*[1]Heinrich Heine University Düsseldorf, Haematology, Oncology and Clinical Immunology, Duesseldorf, Germany; [2]University Clinic Duesseldorf, Haematology, Oncology and Clinical Immunology, Duesseldorf, Germany; [3]Centre hospitalier universitaire vaudois, Lausanne, Switzerland; [4]University Clinic Duesseldorf, Genetics, Duesseldorf, Germany; [5]University Clinic Goettingen, Goettingen, Germany; [6]University Clinic Heidelberg, Heidelberg, Germany; [7]TU München, Haematology, Oncology, Munich, Germany; [8]University Clinic Dresden, Haematology, Oncology, Dresden, Germany;*

**Background**

Chronic myelomonocytic leukemia (CMML) is a biologically heterogeneous myeloid neoplasm with challenging risk stratification. Several prognostic scoring systems have been developed, with more recent models incorporating molecular alterations(ASXL1, RUNX1, NRAS, TP53), such as GFM, MMM, CPSSmol, and BLASTmol. However, validation in large real-world cohorts remains limited.

**Aims**

To validate molecular CMML prognostic models for overall survival (OS) and risk of AML transformation in a large multicenter cohort.

**Methods**

A total of 977 CMML patients from the Düsseldorf MDS Registry and collaborating centers of the German–Austrian–Swiss research group (DACH) were analyzed. Clinical parameters, WHO 2022 subtypes, cytogenetics, and molecular features were evaluated. OS and AML transformation were assessed using Kaplan–Meier analyses and multivariable Cox regression. Prognostic systems included GFM, MMM, CPSSmol, and BLASTmol.

**Copyright Information:** (Online) ISSN: 2572-9241
©2026 The Author(s). HemaSphere is published by John Wiley & Sons Ltd on behalf of European Hematology Association. This is an open access Abstract Book distributed under the Attribution-NonCommercial-NoDerivs (CC BY-NC-ND), which allows third parties to download the articles and share them with others as long as they credit the author and the Abstract Book, but they cannot change the content in any way or use them commercially.
**Abstract Book Citations:** Authors, Title, HemaSphere, 2026;10:(S1):pages. The abstract book DOIs can be found on the "Author Information" page.
**Disclaimer:** Articles published in the journal HemaSphere exclusively reflect the opinions of the authors. The authors are responsible for all content in their abstracts including accuracy of the facts, statements, citing resources, etc.

**Results**

Median age was 71 years; median OS was 29 months. AML progression occurred in 21.1% after a median of 25 months. Dysplastic CMML predominated, while proliferative CMML showed markedly inferior OS. Among 911 patients with available treatment data, supportive care was most frequent (72%), while allogeneic transplantation achieved the longest OS (52 months). Interpretation of the induction cohort was limited due to prior AML transformation in most cases.
Clinical factors associated with inferior OS included leukocytes >13×10⁹/L, monocytes >1×10⁹/L, bone marrow blasts >5%, and circulating immature myeloid cells ($p = 0.017$; $p < 0.001$; $p = 0.040$; $p = 0.033$). High-risk cytogenetics were linked to shorter OS (15 vs. 39 months; $p = 0.013$). ASXL1, RUNX1, and TP53 mutations were adverse ($p = 0.007$; $p = 0.019$; $p = 0.035$).
Most molecular models significantly separated risk groups.
MMM demonstrated the strongest and most continuous stratification (106 vs. 64 vs. 35 vs. 20 months; $p < 0.001$).
GFM differentiated low vs. high risk with partial overlap in intermediate groups (57 vs. 85 vs. 35 months; $p < 0.001$).
BLASTmol highlighted pronounced OS decline in high-risk patients (86 vs. 42 vs. 25 months; $p < 0.001$).
CPSSmol showed inferior performance without reaching statistical significance (164 vs. 42 vs. 53 vs. 26 months, $p = 0.31$).
In this cohort, 5-year OS decreased consistently with rising risk across all CMML-specific models, ranging from 36.5% (BLASTmol) to 65.6% (MMM) in low-risk groups and from 16.2% (MMM) to 29.8% (GFM) in high-risk groups.
AML transformation paralleled OS findings.
MMM showed the steepest gradient (3-year AML progression: 12.5% low vs. 8.2% intermediate-1 vs. 18.0% intermediate-2 vs. 40.9% high risk; $p < 0.001$).
BLASTmol significantly differentiated high-risk transformation (16.2% vs. 14.0% vs. 32.9%; $p = 0.05$).
No statistically significant differences were observed for GFM (29.5% vs. 11.9% vs. 24.0%, $p = 0.109$) or for CPSSmol (0.0% vs. 11.0% vs. 0.0% vs. 34.2%; $p = 0.08$).
Model performance was strongest in dysplastic CMML, while proliferative CMML showed overlapping risk categories. In multivariable analyses, RUNX1 mutations and leukocytes >13×10⁹/L independently predicted shorter OS; monocytes >1×10⁹/L, high cytogenetic risk, and RUNX1 mutations independently predicted AML transformation.

**Summary/Conclusion**

Molecular CMML-specific models outperform clinical scoring systems, with MMM showing the most robust discrimination for OS and AML progression, supporting integration of molecular risk profiling into clinical decision-making.

**Copyright Information:** (Online) ISSN: 2572-9241
©2026 The Author(s). HemaSphere is published by John Wiley & Sons Ltd on behalf of European Hematology Association. This is an open access Abstract Book distributed under the Attribution-NonCommercial-NoDerivs (CC BY-NC-ND), which allows third parties to download the articles and share them with others as long as they credit the author and the Abstract Book, but they cannot change the content in any way or use them commercially.
**Abstract Book Citations:** Authors, Title, HemaSphere, 2026;10:(S1):pages. The abstract book DOIs can be found on the "Author Information" page.
**Disclaimer:** Articles published in the journal HemaSphere exclusively reflect the opinions of the authors. The authors are responsible for all content in their abstracts including accuracy of the facts, statements, citing resources, etc.

### (PF666) IMPACT OF U2AF1 PATHOGENIC VARIANTS ON PROGNOSIS OF MYELODYSPLASTIC NEOPLASMS WITH RUNX1 MUTATION

**Topic:** 10. Myelodysplastic syndromes - Clinical

Linlin Liu*[1], Bing Li[1], Tiejun Qin[1], Zefeng Xu[1], Shiqiang Qu[1], Lijuan Pan[1], Qingyan Gao[1], Meng Jiao[1], Zefei Bao[1], Ningning Liu[1], Yujiao Jia[1], Chengwen Li[1], Zhijian Xiao[1]

[1]*State Key Laboratory of Experimental Hematology, National Clinical Research Center for Blood Diseases, Haihe Laboratory of Cell Ecosystem, Institute of Hematology & Blood Diseases Hospital, Chinese Academy of Medical Sciences & Peking Union Medical College, Tianjin, China;*

### Background

*RUNX1* is one of the most commonly mutated genes in myelodysplastic neoplasms (MDS) and essential for haematopoiesis. However, clinical phenotype features of *RUNX1* mutated patients and the interaction between RUNX1 and other genes mutations remains incompletely explored.

### Aims

In this study, we undertook a large single-institution retrospective study to investigate the laboratory, genomic characteristics, and survival of *RUNX1*-mutated patients with primary MDS.

### Methods

We enrolled 1473 consecutive adult primary MDS patients to investigate the laboratory, genomic characteristics and survival of *RUNX1*-mutated MDS subjects.

### Results

*RUNX1* mutations were associated with older age (median, 59 years vs. 56 years, $p$ = 0.007), lower platelet counts (median, 50×10$^9$/L vs. 66×10$^9$/L; p < 0.001), higher bone marrow blasts (median, 6.0% vs. 2.5%, $p$ < 0.001) and a higher proportion of micro-megakaryocytes (median, 26.42% vs. 18.49%, $p$ <0.001). Additionally, the variant allele frequency (VAF) of *RUNX1* mutations was correlated with both platelet counts ($r$ = -0.14, $p$ = 0.069) and the proportion of micro-megakaryocytes ($r$ = 0.189, $p$ = 0.021), suggesting *RUNX1* mutations burden was associated with dysmegakaryopoiesis. *ASXL1*, *U2AF1*, *SRSF2*, *TET2*, and *EZH2* mutations were significantly more frequent in *RUNX1*-mutated patients compared with those without *RUNX1* mutations ($p$ < 0.05). *RUNX1*-mutated patients exhibited poorer overall survival (median OS 18 months vs. 51months, $p$ < 0.001), while *U2AF1* co-mutations were associated with a relatively better prognosis (median OS 34 months vs. 17 months, $p$ = 0.003; Figure A). In subgroups of higher-risk of IPSS-R (score > 3.5, $n$ = 106) and IPSS-M (score > 0, $n$ = 137) in RUNX1-mutated patients, *U2AF1* mutated patients still had a better prognosis (IPSS-R higher: median OS not reached vs. 15 months, $p$ = 0.002; IPSS-M higher: median OS 34 months vs. 17 months, $p$ = 0.004; Figure B-C). Among 156 *RUNX1*-mutated subjects, 45 (28.8%) of them had *U2AF1* mutation, in which *U2AF1* p.S34 ($n$ = 38, 84.4%) was the most common, including 24 (53.3%) subjects of p.S34F and 14 (31.1%) subjects of p.S34Y.

### Summary/Conclusion

*RUNX1* mutations are associated with distinct clinical, genomic features and adverse prognosis in MDS patients. *U2AF1* co-mutations have a potential modifying effect on the outcome of *RUNX1*-mutated patients.

**Copyright Information:** (Online) ISSN: 2572-9241
©2026 The Author(s). HemaSphere is published by John Wiley & Sons Ltd on behalf of European Hematology Association. This is an open access Abstract Book distributed under the Attribution-NonCommercial-NoDerivs (CC BY-NC-ND), which allows third parties to download the articles and share them with others as long as they credit the author and the Abstract Book, but they cannot change the content in any way or use them commercially.
**Abstract Book Citations:** Authors, Title, HemaSphere, 2026;10:(S1):pages. The abstract book DOIs can be found on the “Author Information” page.
**Disclaimer:** Articles published in the journal HemaSphere exclusively reflect the opinions of the authors. The authors are responsible for all content in their abstracts including accuracy of the facts, statements, citing resources, etc.

### (PF668) ROXADUSTAT IMPROVES TRANSFUSION INDEPENDENCE IN LR-MDS PATIENTS WITH ANEMIA AND HIGH TRANSFUSION BURDEN AND IN RING SIDEROBLAST POSITIVE AND NEGATIVE DISEASE: POST-HOC ANALYSIS OF MATTERHORN STUDY

**Topic:** 10. Myelodysplastic syndromes - Clinical

Amer M. Zeidan*[1], Cynthia Wetmore[2], Jianjun Liu[3], Andrew Grigston[3], Carrie Brownstein[2], Moshe Mittelman[4]

*[1]Yale University, New Haven, United States of America;[2]SSI Strategy, Morristown, United States of America;[3]Kyntra Bio, San Diego, United States of America; [4]Tel Aviv University, Tel Aviv, Israel;*

### Background

Lower risk myelodysplastic syndromes (LR-MDS) are associated with anemia, chronic transfusion dependence, iron overload and significant impairment in quality of life. Existing treatment options require parenteral administration and have limited response rates and duration. Roxadustat is an oral inhibitor of hypoxia inducible factor prolyl hydroxylase and is approved for anemia of chronic kidney disease in multiple global markets. MATTERHORN was a placebo-controlled Phase 3 trial that evaluated roxadustat in LR-MDS with anemia (Mittleman et al. 2024). While the study demonstrated a higher proportion of participants achieving 8-week red blood cell transfusion independence (RBC-TI) than observed with placebo, the difference was not statistically significant. This post hoc analysis focuses on participants (pts) with a high transfusion burden (HTB) and those pts with ring sideroblast (RS) data available.

### Methods

The study was conducted in accordance with GCP and the Declaration of Helsinki, approved by IRB/ECs and all pts provided written informed consent. Pts eligible for the Matterhorn Phase 3 study had very low, low or intermediate risk MDS with or without prior ESA exposure and transfusion burden of 1-4 pRBC units per 8 weeks and randomized 3:2 roxadustat vs. placebo (Mittleman et al. 2024). For this post-hoc analysis, the HTB population was defined per IWG 2018 (Platzbecker 2019) as those with baseline transfusion burden of ≥4 units of packed red blood cell units (pRBC) over an 8-week period. Efficacy outcomes were reported in full analysis set (FAS) who received ≥1 dose of study drug and had ≥1 corresponding on-treatment hemoglobin assessment. Determination of ring sideroblast histology was made at local institution and not centrally confirmed.

### Results

In the post hoc analysis, of the 140 pts enrolled, 37 HTB FAS pts were identified (n=22 roxadustat, n=15 placebo). The primary endpoint of at least 8-wk TI in the first 28 weeks was achieved in 8 patients (36.4%) treated with roxadustat vs. 1 (6.7%) with placebo (p=0.041); and 10 (45.5%) vs. 2 (13.3%) over 52 weeks (p=0.043). Similar findings were obtained for longer duration TI endpoints (Table). In the full analysis set for those with RS data available, TI and improvement in hemoglobin were observed in pts with and without RS histology. TEAEs were generally lower grade and managed medically with no new safety signals.

### Summary/Conclusion

Roxadustat induced a durable and clinically meaningful improvement in TI interval and a reduction in transfusion burden with an acceptable safety profile in a cohort of LR-MDS patients with HTB. Improvements were observed in participants with and without ring sideroblasts. Roxadustat addresses the unmet need for additional treatments and an oral treatment option for LR-MDS patients ineligible/refractory to ESA. Based upon these results, a new registrational-intent, global, randomized Phase 3 trial evaluating roxadustat is planned to initiate in the second half of 2026.

**Copyright Information:** (Online) ISSN: 2572-9241
©2026 The Author(s). HemaSphere is published by John Wiley & Sons Ltd on behalf of European Hematology Association. This is an open access Abstract Book distributed under the Attribution-NonCommercial-NoDerivs (CC BY-NC-ND), which allows third parties to download the articles and share them with others as long as they credit the author and the Abstract Book, but they cannot change the content in any way or use them commercially.
**Abstract Book Citations:** Authors, Title, HemaSphere, 2026;10:(S1):pages. The abstract book DOIs can be found on the "Author Information" page.
**Disclaimer:** Articles published in the journal HemaSphere exclusively reflect the opinions of the authors. The authors are responsible for all content in their abstracts including accuracy of the facts, statements, citing resources, etc.

| | FAS Population | | FAS RS positive | | FAS RS negative | | HTB FAS Population | |
|---|---|---|---|---|---|---|---|---|
| | Roxa | Placebo | Roxa | Placebo | Roxa | Placebo | Roxa | Placebo |
| | N=80 | N=57 | N=28 | N=25 | N=52 | N=32 | N=22 | N=15 |
| | n (%) | n (%) | n (%) | n (%) | n (%) | n (%) | n (%) | n (%) |
| **TI over 28 weeks** | | | | | | | | |
| **≥8 week TI** | 38 (48) | 19 (33) | 13 (46) | 10 (40) | 25 (48) | 9 (28) | 8 (36) | 1 (7) |
| | p=0.217 | | p=0.641 | | P=0.072 | | p=0.041 | |
| **TI over 52 weeks** | | | | | | | | |
| **≥8-week TI** | 44 (55) | 23 (40) | 14 (50) | 10 (40) | 30 (58) | 13 (41) | 10 (45) | 2 (13) |
| **≥12-week TI** | 34 (43) | 20 (35) | | | | | 8 (36) | 1 (7) |
| **≥16-week TI** | 22 (28) | 13 (23) | | | | | 4 (18) | 0 |
| **≥20-week TI** | 21 (26) | 13 (23) | | | | | 4 (18) | 0 |

Copyright Information: (Online) ISSN: 2572-9241
©2026 The Author(s). HemaSphere is published by John Wiley & Sons Ltd on behalf of European Hematology Association. This is an open access Abstract Book distributed under the Attribution-NonCommercial-NoDerivs (CC BY-NC-ND), which allows third parties to download the articles and share them with others as long as they credit the author and the Abstract Book, but they cannot change the content in any way or use them commercially.
Abstract Book Citations: Authors, Title, HemaSphere, 2026;10:(S1):pages. The abstract book DOIs can be found on the "Author Information" page.
Disclaimer: Articles published in the journal HemaSphere exclusively reflect the opinions of the authors. The authors are responsible for all content in their abstracts including accuracy of the facts, statements, citing resources, etc.

### (PF669) OUTCOMES AND CHARACTERISTICS OF LOWER-RISK MDS PATIENTS WITH ELEVATED SERUM ERYTHROPOIETIN LEVELS (≥500 IU/L): A MULTICENTER STUDY BY THE POLISH ADULT LEUKEMIA GROUP (PALG)

**Topic:** 10. Myelodysplastic syndromes - Clinical

Piotr Stompór*[1], Michał Dąbrowski[2], Anna Jachalska[3], Paweł Szwedyk[4], Dorota Krochmalczyk[5], Aleksandra Gołos[6], Edyta Subocz[7], Rafal MacHowicz[8], Karol Lis[8], Jagoda Rytel[8], Joanna Drozd-Sokołowska[8], Grzegorz Basak[8], Krysztof Madry[8]

*[1]Medical University of Warsaw, Warsaw, Poland;[2]Computational Biology Group, Institute of Computer Science of the Polish Academy of Sciences 01-248, Warsaw, Poland; [3]Department of Hematology, Biziel Hospital, Bydgoszcz, Poland;[4]Department of Hematology, Rydygier Specialistic Hospital, Kraków, Poland; [5]Department of Hematology, Collegium Medicum, Jagiellonian University, Kraków, Poland; [6]Department of Hematology, Medical University of Lodz, Lodz, Poland;[7]Clinical Hospital of the Ministry of Internal Affairs and Administration with the Warmia-Mazury Oncology Centre in Olsztyn., Department of Hematology, Internal Medicine and BMT, Olsztyn, Poland; [8]Department of Hematology, Transplantation and Internal Medicine, Medical University of Warsaw, Warsaw, Poland;*

### Background

Serum erythropoietin (sEPO) levels are elevated in myelodysplastic syndromes (MDS). While sEPO ≥ 500 IU/L is a well-established predictor of poor response to erythropoiesis-stimulating agents (ESAs), the clinical phenotype and survival outcomes of this specific lower-risk (LR) MDS subpopulation remain inadequately characterized.

### Aims

To evaluate the clinical, laboratory, and prognostic profile of LR-MDS patients with endogenous sEPO ≥ 500 IU/L compared to a control group (sEPO < 500 IU/L).

### Methods

This analysis utilized data collected between 2010 and 2025 within the PALG MDS Registry, representing 50 hematology centers. Out of 435 identified LR-MDS patients with available sEPO data, 56 (12.9%) presented with sEPO ≥500 IU/L. Statistical significance was assessed using the Wilcoxon and Fisher's exact tests. Survival was analized using the Kaplan-Meier method and Cox proportional hazards model was used.

### Results

Patients with sEPO ≥ 500 IU/L were younger (median 69.5 vs. 72 years; p = 0.045) and presented with more severe anemia (median Hb 7.65 vs. 9.1 g/dL; p < 0.001) and higher ferritin concentration (median 601 vs. 457 ng/mL; p = 0.031).
The sEPO >= 500 IU/L group showed a markedly higher prevalence of RBC transfusion dependency (76.8% vs. 43.5%; p < 0.001) and advanced bone marrow fibrosis (13.3% vs. 6.6%; p = 0.02). Additionally, a significantly higher rate of current tobacco exposure (58.9% vs. 44.8%; p = 0.014) was noted in the elevated sEPO group.
The primary endpoint, overall survival (OS), was significantly shorter in the sEPO >= 500 IU/L cohort. The median OS was 33.0 months (95% CI: 27.8–51.3) compared to 48.1 months (95% CI: 38.5–65.3) in the control group (p = 0.02).

**Copyright Information:** (Online) ISSN: 2572-9241
©2026 The Author(s). HemaSphere is published by John Wiley & Sons Ltd on behalf of European Hematology Association. This is an open access Abstract Book distributed under the Attribution-NonCommercial-NoDerivs (CC BY-NC-ND), which allows third parties to download the articles and share them with others as long as they credit the author and the Abstract Book, but they cannot change the content in any way or use them commercially.
**Abstract Book Citations:** Authors, Title, HemaSphere, 2026;10:(S1):pages. The abstract book DOIs can be found on the "Author Information" page.
**Disclaimer:** Articles published in the journal HemaSphere exclusively reflect the opinions of the authors. The authors are responsible for all content in their abstracts including accuracy of the facts, statements, citing resources, etc.

### Summary/Conclusion

Endogenous sEPO ≥ 500 IU/L identifies a distinct high-risk subpopulation within LR-MDS characterized by younger age at diagnosis, severe erythroid failure, higher transfusion burden, and increased bone marrow fibrosis. Marked sEPO elevation is a surrogate for more aggressive disease biology and worse clinical outcome, emphasizing the need for personalized management in this group.

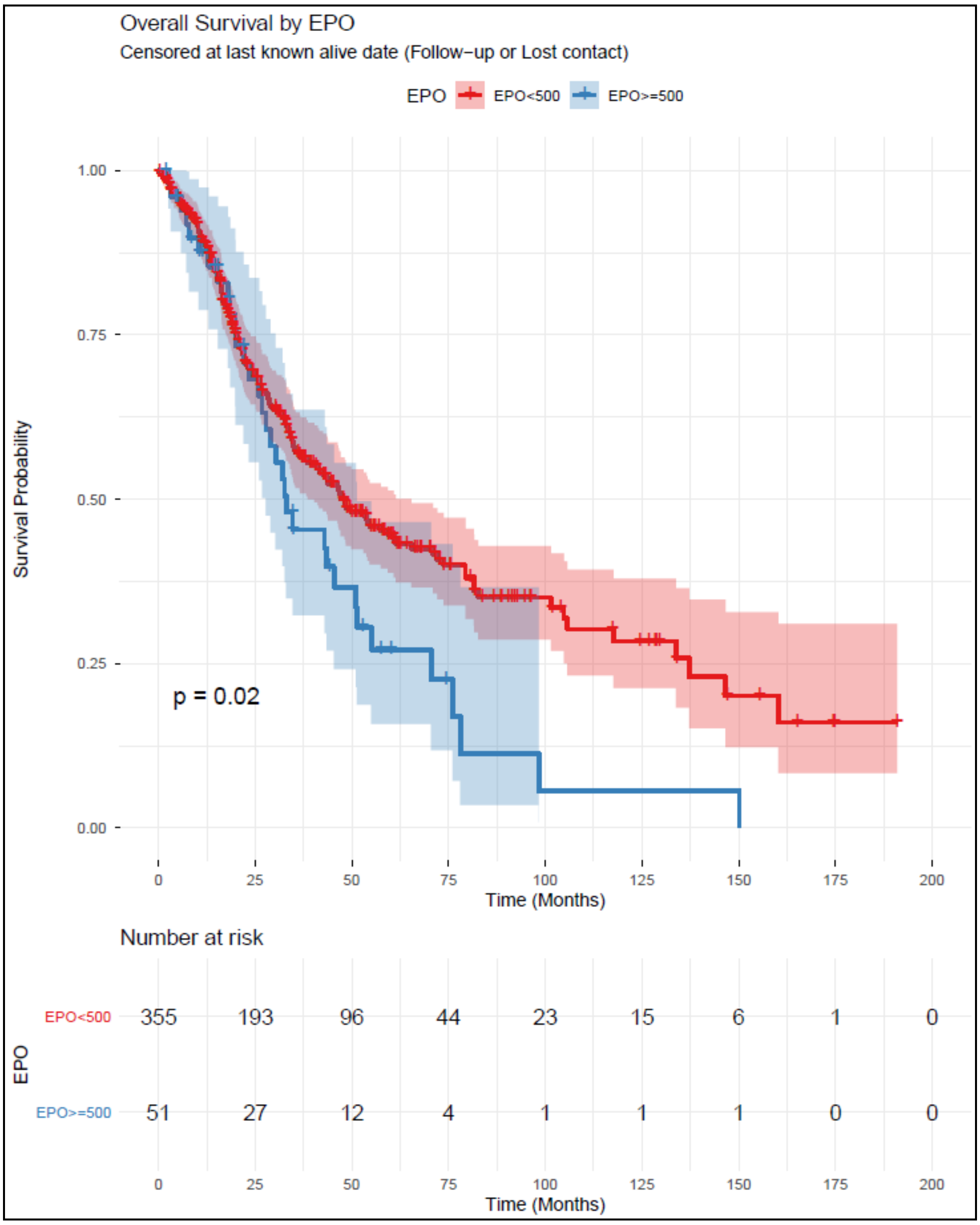

**Copyright Information:** (Online) ISSN: 2572-9241
©2026 The Author(s). HemaSphere is published by John Wiley & Sons Ltd on behalf of European Hematology Association. This is an open access Abstract Book distributed under the Attribution-NonCommercial-NoDerivs (CC BY-NC-ND), which allows third parties to download the articles and share them with others as long as they credit the author and the Abstract Book, but they cannot change the content in any way or use them commercially.
**Abstract Book Citations:** Authors, Title, HemaSphere, 2026;10:(S1):pages. The abstract book DOIs can be found on the "Author Information" page.
**Disclaimer:** Articles published in the journal HemaSphere exclusively reflect the opinions of the authors. The authors are responsible for all content in their abstracts including accuracy of the facts, statements, citing resources, etc.

### (PF670) REAL-WORLD OUTCOMES OF IMETELSTAT: INTERROGATING SAFETY, EFFICACY AND PREDICTORS OF RESPONSE IN HEAVILY PRETREATED LOWER-RISK MDS PATIENTS

**Topic:** 10. Myelodysplastic syndromes - Clinical

Rami S. Komrokji*[1], Najla Al Ali[1], Cyril Patra[1], Julia Fadul[1], Zhuoer Xie[1], Rory M. Shallis[1], Andrew Kuykendall[1], Sam Reynolds[1], Allison Walker[1], Kendra Sweet[1], Brian Sweet[2], Jeffrey Lancet[1], Eric Padron[1], David Sallman[1]

*[1]Moffitt Cancer Center, Tampa, United States of America;[2]Geron, Foster City, United States of America;*

### Background

Imetelstat is a first-in-class, direct and competitive inhibitor of telomerase enzymatic activity approved for lower-risk myelodysplastic syndromes (LR-MDS) based on ~40% ≥8-week RBC transfusion independence (TI) rate with a median duration of 51.6 weeks in patients with ESA failure LR-MDS on the P3 IMerge Trial.

### Aims

We report the first real-world data on efficacy and safety of imetelstat.

### Methods

We analyzed all patients (pts) who received imetelstat at Moffitt Cancer Center since FDA approval. We examined baseline characteristics, details of treatment, response, adverse events, and outcomes.

**Copyright Information:** (Online) ISSN: 2572-9241
©2026 The Author(s). HemaSphere is published by John Wiley & Sons Ltd on behalf of European Hematology Association. This is an open access Abstract Book distributed under the Attribution-NonCommercial-NoDerivs (CC BY-NC-ND), which allows third parties to download the articles and share them with others as long as they credit the author and the Abstract Book, but they cannot change the content in any way or use them commercially.
**Abstract Book Citations:** Authors, Title, HemaSphere, 2026;10:(S1):pages. The abstract book DOIs can be found on the “Author Information” page.
**Disclaimer:** Articles published in the journal HemaSphere exclusively reflect the opinions of the authors. The authors are responsible for all content in their abstracts including accuracy of the facts, statements, citing resources, etc.

### Results

Between July 2024 to October 2025, 40 LR-MDS pts were treated with imetelstat (36 TD at baseline). The median duration of follow up from start of treatment was 14.2 months (95% CI 12.6-15.8). The median age at diagnosis was 69.5 years, majority were Caucasian (38/40) and 58% (23/40) were male. By WHO 2022, 28 patients (70%) were MDS or MDS/MPN *SF3B1* and mean blast was 2%. The mean number of somatic mutations was 2.3 and most common variants were *SF3B1* 80%, *TET-2* 30%, and *ASXL-1* 27.5%. The mean Hb before treatment was 7.4 g/dl (5.5-9.8 g/dl). By IPSS-R, 92.5% were very low and by IPSS-M, 75% were low/moderate low. The mean number of prior RBC transfusion units/16 weeks and 8 weeks was 8.5 (0-21) and 4.7 (0-10), respectively. The median time from diagnosis to start imetelstat treatment was 42.7 months (0.9-173.57). Imetelstat was used as later line of therapy (median as 3$^{rd}$ line; r 1-8), 35 pts (87.5%) had prior luspatercept, 28 pts (70%) had prior ESA, 15 pts (37.5%) had prior hypomethylating agent (HMA), 17 patients (42.5%) had prior lenalidomide (Len) therapy.
The best response in imetelstat-treated patients (RBC-TI > 8 weeks) was 37.5% (15/40) with 7/15 responses ongoing. The median duration of response was 185 days (67-492). The median # of cycles received was significantly increased in responding pts (10 vs 5; p<0.0001). The median maximum hemoglobin in responding patients was 9.6 (r 8.7-14.8). Baseline EPO level, transfusion burden or *SF3B1* mutation were not predictive of response. Among 9 pts with *ASXL-1* mutation, 66.7% (6 pts) responded compared to 29% (9/31) WT, p= 0.04. Baseline platelet count of ≥ 192 was best univariable predictor of response, OR 7.0, AUC 0.68. There was trend for inferior response post HMA and Len use (26.7% vs 44%, p=0.27; 23% vs 28%, p=0.11). There was a trend for better response if imetelstat was used within first 3 lines of therapy (44% vs 26.7% p=0.27). In exploratory predictive model for response, platelet count at baseline, *ASXL-1* mutation, and IPSS-R at diagnosis were the most important predictors of response. The median OS from start of imetelstat was 17.3 months (95% CI 15.7-18.7).
Grade 3/4 neutropenia was observed in 14 pts (35%) after C1 and 9 pts (22.5%) after C2. Grade 3/4 thrombocytopenia was observed in 11 pts (27.5%) after C1 and 8 pts (20%) after C2. No febrile neutropenia episodes occurred. The median time between cycles was 31 days. One and two dose level reductions occurred in 37.5% (n=15) and 2.5% (n=1) pts, respectively. Plt transfusion was required in 5 pts (12.5%) and GCSF in 4 pts (10%).

### Summary/Conclusion

Real world experience identifies safety and clinical efficacy of imetelstat in advanced, heavily transfusion dependent patients with LR-MDS including patients with extensive prior therapies and after luspatercept failure. We report novel predictors of response including baseline platelet count and *ASXL-1* mutation status.

**Copyright Information:** (Online) ISSN: 2572-9241
©2026 The Author(s). HemaSphere is published by John Wiley & Sons Ltd on behalf of European Hematology Association. This is an open access Abstract Book distributed under the Attribution-NonCommercial-NoDerivs (CC BY-NC-ND), which allows third parties to download the articles and share them with others as long as they credit the author and the Abstract Book, but they cannot change the content in any way or use them commercially.
**Abstract Book Citations:** Authors, Title, HemaSphere, 2026;10:(S1):pages. The abstract book DOIs can be found on the “Author Information” page.
**Disclaimer:** Articles published in the journal HemaSphere exclusively reflect the opinions of the authors. The authors are responsible for all content in their abstracts including accuracy of the facts, statements, citing resources, etc.

### (PF671) POOLED PHASE1/2 DATA IN PATIENTS WITH MYELODYSPLASTIC SYNDROMES (MDS) AND MDS/MYELOPROLIFERATIVE NEOPLASMS SUPPORTS A BODY SURFACE AREA (BSA)-BASED DOSING STRATEGY FOR ORAL AZACITIDINE-CEDAZURIDINE

**Topic:** 10. Myelodysplastic syndromes - Clinical

Guillermo Garcia-Manero*[1], Michael Savona[2], Elizabeth A. Griffiths[3], Bart Scott[4], Bonnie Kiner-Strachan[5], Uma Borate[6], Andrew Brunner[7], Yazan F. Madanat[8], Aram Oganesian[9], Yuri Sano[9], Lixia Zhu[9], Gareth Tomlinson[9], Danna Chan[9], Harold Keer[9], James McCloskey[10]

*[1]The University of Texas MD Anderson Cancer Center, Houston, United States of America;[2]Vanderbilt-Ingram Cancer Center, Vanderbilt University School of Medicine, Nashville, United States of America; [3]Roswell Park Comprehensive Cancer Center, Buffalo, United States of America; [4]Fred Hutchinson Cancer Center, Seattle, United States of America; [5]New York University Langone Hospital Long Island, Mineola, United States of America; [6]Ohio State University Comprehensive Cancer Center, Columbus, United States of America; [7]Mass General Brigham Cancer Institute, Boston, United States of America; [8]University of Texas Southwestern Medical Center, Dallas, United States of America; [9]Taiho Oncology, Inc., Pleasanton, United States of America; [10]John Theurer Cancer Center, Hackensack Medical Center, Hackensack, United States of America;*

### Background

The parenteral hypomethylating agent azacitidine (AZA) is a standard-of-care treatment for MDS and MDS/MPN overlap syndromes, including myelodysplastic type chronic myelomonocytic leukemia (CMML-MD). ASTX030 is an oral fixed-dose combination of AZA/cedazuridine (CED) developed to achieve systemic AZA exposure comparable to subcutaneous (SC) AZA. Phase (Ph) 2 pharmacokinetic (PK) results suggested the selection of a body surface area (BSA)–based dosing strategy for Ph 3 development.

### Aims

To report pooled safety, efficacy, and PK results from Ph1 and Ph2 of the ASTX030-01 study and further evaluate BSA-based dosing using a larger dataset.

### Methods

ASTX030-01 included an open-label Ph1 dose-finding study and a randomized, multicenter Ph2 crossover study in adult pts with MDS or MDS/MPN overlap syndromes eligible for AZA therapy. Ph1 evaluated multiple immediate- and delayed-release AZA/CED formulations to determine the recommended Ph2 dose (RP2D), with secondary objectives including safety, tolerability, PK/Pharmacodynamics, and efficacy. In Ph2, pts were randomized 1:1 to Sequence A or B. Sequence A received ASTX030 (140/20 mg) in C1, SC AZA (75 mg/m²) in C2, then ASTX030 from C3 onward; Sequence B received SC AZA in C1 and ASTX030 from C2 onward. The primary objective compared AZA AUC exposure between ASTX030 and SC AZA; secondary objectives included safety, PK, PD, and clinical responses.
The pooled analysis includes pts treated at approximate RP2D levels (136/20 and 144/20 mg) and Ph2 pts treated at RP2D (140/20 mg). Exploratory exposure–response analyses were planned to further characterize the relationship between AZA exposures and clinical outcomes.

**Copyright Information:** (Online) ISSN: 2572-9241
©2026 The Author(s). HemaSphere is published by John Wiley & Sons Ltd on behalf of European Hematology Association. This is an open access Abstract Book distributed under the Attribution-NonCommercial-NoDerivs (CC BY-NC-ND), which allows third parties to download the articles and share them with others as long as they credit the author and the Abstract Book, but they cannot change the content in any way or use them commercially.
**Abstract Book Citations:** Authors, Title, HemaSphere, 2026;10:(S1):pages. The abstract book DOIs can be found on the “Author Information” page.
**Disclaimer:** Articles published in the journal HemaSphere exclusively reflect the opinions of the authors. The authors are responsible for all content in their abstracts including accuracy of the facts, statements, citing resources, etc.

**Results**

As of January 2026, 57 pts (29 in Ph1; 28 in Ph2) including 45 with MDS, 8 with CMML, and 4 with other MDS/MPN. Median age was 72y and median treatment duration was 5.8 months. BSA distribution included 49% (n=28) intermediate (>1.55 and <2.15 $m^2$), 44% (n=25) high (≥2.15 $m^2$), and 7% (n=4) low (≤1.55 $m^2$).
In Ph 2, ASTX030 oral AZA PK was comparable to SC AZA in the intermediate-BSA group with 140/20mg [Geometric mean ratio (GMR) of 0.98; 90% CI 0.85-1.13] but showed lower and higher exposures in high- and low- BSA groups, respectively. Pooled Ph1/2 PK equivalence analysis (n=56) showed GMR of 0.95 (90% CI 0.83-1.08).In pts with MDS, overall response rate (ORR) was 44.4% (20/45) per IWG 2006 criteria, with complete remission (CR) achieved in 20.0%. In pts with CMML or MDS/MPN, ORR was 33.3% (4/12) per IWG 2015, noting the limited size of this subgroup. With a median follow-up of 23.7 months, median progression-free survival was 23.1 months (95% CI, 12.9-NE), and median overall survival was not reached. Among red blood cell (RBC) transfusion–dependent pts at baseline, 31.8% achieved ≥8-week RBC transfusion independence.
Safety findings were consistent with the known AZA profile. Grade ≥3 adverse events (AEs) occurred in 89.5% of pts, most commonly neutropenia (33.3%), thrombocytopenia (31.6%), and anemia (29.8%). Common non-heme AEs were nausea (71.9%), constipation (68.4%), and fatigue (56.1%), mostly Grade 1 or 2.

**Summary/Conclusion**

Pooled Ph1/2 data demonstrate that oral ASTX030 provides equivalent PK AUC exposures to SC AZA and a safety and efficacy profile consistent with that of SC AZA across a broad BSA range. Based on Ph2 PK data, a BSA-based dosing strategy was implemented for the ongoing global Ph3 AZTOUND study.
**Clinical trial registration:** NCT04256317

**Copyright Information:** (Online) ISSN: 2572-9241
©2026 The Author(s). HemaSphere is published by John Wiley & Sons Ltd on behalf of European Hematology Association. This is an open access Abstract Book distributed under the Attribution-NonCommercial-NoDerivs (CC BY-NC-ND), which allows third parties to download the articles and share them with others as long as they credit the author and the Abstract Book, but they cannot change the content in any way or use them commercially.
**Abstract Book Citations:** Authors, Title, HemaSphere, 2026;10:(S1):pages. The abstract book DOIs can be found on the "Author Information" page.
**Disclaimer:** Articles published in the journal HemaSphere exclusively reflect the opinions of the authors. The authors are responsible for all content in their abstracts including accuracy of the facts, statements, citing resources, etc.

### (PF672) PHASE I STUDY OF OP-2100, AN ORALLY BIOAVAILABLE PRODRUG OF DECITABINE, FOR PATIENTS WITH RELAPSED OR REFRACTORY HIGH-RISK MYELODYSPLASTIC SYNDROME AND CHRONIC MYELOMONOCYTIC LEUKEMIA

**Topic:** 10. Myelodysplastic syndromes - Clinical

Senji Kasahara*[1], Sho Okamoto[2], Junya Kuroda[3], Kensuke Usuki[4], Motoshi Ichikawa[5], Tatsuo Ichinohe[6], Takayuki Ikezoe[7], Akihisa Imanishi[8], Kazunari Miyairi[8], Yuka Kagawa[8], Takahiro Yamauchi[9], Shinya Kimura[2]

*[1]Gifu Municipal Hospital, Gifu, Japan; [2]Saga University, Saga, Japan; [3]Kyoto Prefectural University of Medicine, Kyoto, Japan; [4]Mizonokuchi Hospital, Teikyo University School of Medicine, Kanagawa, Japan; [5]NTT Medical Center Tokyo, Tokyo, Japan; [6]Hiroshima University, Hiroshima, Japan; [7]Fukushima Medical University, Fukushima, Japan; [8]Ohara Pharmaceutical Co., Ltd., Shiga, Japan; [9]University of Fukui, Fukui, Japan;*

### Background

Hypomethylating agents (HMAs) are standard therapies for high-risk myelodysplastic syndromes (MDS) and chronic myelomonocytic leukemia (CMML). Decitabine (DEC), an HMA, is incorporated into DNA during S phase; thus, sustained plasma concentrations may enhance its efficacy. OP-2100 is a novel, orally available prodrug of DEC designed to provide a sustained-release pharmacokinetic (PK) profile (Watanabe et al. Blood. 2020; Ureshino et al. Mol Cancer Ther. 2021). Here, we report the safety, PK, and preliminary efficacy from a Phase I study of OP-2100 in patients with relapsed or refractory (R/R) high-risk MDS and CMML.

### Methods

This single-arm, multicenter Phase I study enrolled Japanese patients with IPSS-R intermediate-, high-, or very high-risk MDS or CMML who were relapsed, refractory, or intolerant to standard therapies. The primary objective was to determine the maximum tolerated dose (MTD) using a 3+3 dose-escalation design. OP-2100 was administered orally once daily on days 1–5 of each 28-day cycle. Dose levels were 30, 50, 80, and 120 mg/day, starting at 50 mg/day (jRCT2071220035).

### Results

Eleven patients with MDS were enrolled (50 mg cohort, n=6; 80 mg cohort, n=5). The median age was 71 years (range, 47–79). By IPSS-R, 3 patients (27.3%) were intermediate, 3 (27.3%) high, and 5 (45.5%) very high risk; 10 patients (90.9%) had received prior azacitidine. The median duration of OP-2100 treatment was 89 days (range, 5–490).
Among 9 patients evaluable for dose-limiting toxicities (DLTs), the DLT incidence was 33.3% at 50 mg (2/6 patients: prolonged Grade (Gr) 4 neutropenia and thrombocytopenia [n=1], and prolonged Gr 4 neutropenia [n=1]) and 33.3% at 80 mg (1/3 patients: prolonged Gr 4 neutropenia).
Adverse events (AEs) occurred in all patients. The most frequent Gr ≥3 AEs were related to myelosuppression, including neutropenia (100%), thrombocytopenia (81.8%), and leukopenia (72.7%). Gastrointestinal AEs included constipation (36.4%), nausea (27.3%), diarrhea (18.2%), and gastroesophageal reflux disease (18.2%), were all Gr 1-2.
At 50 mg, OP-2100 exhibited sustained-release characteristics; the area under the curve (AUC) was comparable to the approved intravenous DEC dose (20 mg/m²), while the $C_{max}$ was approximately one-fifth. Based on DLTs, safety, and PK data, 50 mg was determined as the MTD.
Preliminary efficacy included one marrow complete remission (IWG2018). LINE-1 methylation patterns were consistent with conventional HMAs.

**Copyright Information:** (Online) ISSN: 2572-9241
©2026 The Author(s). HemaSphere is published by John Wiley & Sons Ltd on behalf of European Hematology Association. This is an open access Abstract Book distributed under the Attribution-NonCommercial-NoDerivs (CC BY-NC-ND), which allows third parties to download the articles and share them with others as long as they credit the author and the Abstract Book, but they cannot change the content in any way or use them commercially.
**Abstract Book Citations:** Authors, Title, HemaSphere, 2026;10:(S1):pages. The abstract book DOIs can be found on the “Author Information” page.
**Disclaimer:** Articles published in the journal HemaSphere exclusively reflect the opinions of the authors. The authors are responsible for all content in their abstracts including accuracy of the facts, statements, citing resources, etc.

**Summary/Conclusion**

OP-2100 was well tolerated with a safety profile consistent with intravenous DEC, notably without the anticipated severe gastrointestinal toxicity associated with oral HMA administration. At 50 mg, OP-2100 achieved a sustained-release PK profile and an AUC equivalent intravenous DEC. These results support further investigation on the 50 mg dose in Phase II studies.

**Copyright Information:** (Online) ISSN: 2572-9241
©2026 The Author(s). HemaSphere is published by John Wiley & Sons Ltd on behalf of European Hematology Association. This is an open access Abstract Book distributed under the Attribution-NonCommercial-NoDerivs (CC BY-NC-ND), which allows third parties to download the articles and share them with others as long as they credit the author and the Abstract Book, but they cannot change the content in any way or use them commercially.
**Abstract Book Citations:** Authors, Title, HemaSphere, 2026;10:(S1):pages. The abstract book DOIs can be found on the "Author Information" page.
**Disclaimer:** Articles published in the journal HemaSphere exclusively reflect the opinions of the authors. The authors are responsible for all content in their abstracts including accuracy of the facts, statements, citing resources, etc.

### (PF673) A NEW ORAL DOSAGE FORM OF DECITABINE/CEDAZURIDINE DEMONSTRATED CONSISTENT EFFICACY AND FAVORABLE SAFETY PROFILE IN MYELODYSPLASTIC SYNDROMES PATIENTS IN CHINA: FIRST RESULTS FROM A PIVOTAL TRIAL

**Topic:** 10. Myelodysplastic syndromes - Clinical

Xinping Zhou*[1], Wei Yang[2], Hu Zhou[3], Bing Li[4], Sujun Gao[5], Fei Li[6], Miao Miao[7], Songfu Jiang[8], Wei Wang[9], Yu Cao[10], Zhichao Chen[11], Xiaoyu Zhu[12], Li Wang[13], Xin Du[14], Yu Wu[15], Rong Fu[16], Qingchi Liu[17], Ling Jiang[18], Aram Oganesian[19], Harold Keer[19], Yoshitaka Kotobuki[20], Masatoshi Ito[20], Meiyu Wang[21], Jie Jin[1], Hongyan Tong[1]

*[1]The First Affiliated Hospital, Zhejiang University School of Medicine, Department of Hematology, Hangzhou, China;[2]Shengjing Hospital of China Medical University, Department of Hematology, Shenyang, China; [3]Henan Cancer Hospital, Department of Hematology, Zhengzhou, China; [4]Institute of Hematology and Blood Diseases Hospital, Chinese Academy of Medical Sciences & Peking Union Medical College, MDS and MPN Centre, Tianjin, China; [5]The First Hospital of Jilin University, Department of Hematology, Changchun, China; [6]The First Affiliated Hospital of Nanchang University, Department of Hematology, Nanchang, China; [7]The First Affiliated Hospital of Soochow University, Department of Hematology, Suzhou, China;[8]The First Affiliated Hospital of Wenzhou Medical University, Department of Hematology, Wenzhou, China; [9]The Affiliated Hospital of Qingdao University, Department of Hematology, Qingdao, China; [10]The Affiliated Hospital of Qingdao University, Clinical Trial Center, Qingdao, China; [11]Union Hospital, Tongji Medical College, Huazhong University of Science and Technology, Department of Hematology, Wuhan, China; [12]The First Affiliated Hospital of USTC, Department of Hematology, Hefei, China;[13]The First Affiliated Hospital of Chongqing Medical University, Hematology, Chongqing, China; [14]Guangdong Provincial People's Hospital, Department of Hematopathology, Guangzhou, China; [15]West China Hospital, Sichuan University, Department of Hematology, Chengdu, China; [16]Tianjin Medical University General Hospital, Department of Hematology, Tianjin, China;[17]The First Hospital of Hebei Medical University, Department of Hematology, Shijiazhuang, China; [18]Nanfang Hospital, Southern Medical University, Department of Hematology, Guangzhou, China; [19]Taiho Oncology, Inc., Pleasanton, CA, United States of America;[20]Otsuka Pharmaceutical Co., Ltd., Tokyo, Japan; [21]Otsuka Beijing Research Institute, Beijing, China;*

### Background

Hypomethylating agents (HMA) remain the standard of care for higher risk myelodysplastic syndromes (MDS). ASTX727, an oral fixed-dose combination of 35 mg decitabine (DEC) and 100 mg cedazuridine (C), has been approved by FDA and is recommended by NCCN as an oral HMA option alongside parenteral formulation. Cedazuridine inhibits the first-pass elimination of decitabine by the enzyme cytidine deaminase, enabling oral administration of 35 mg to achieve exposure equivalent to that of intravenous (IV) infusion of 20 mg/m$^2$. To extend this indication in Chinese patients, a new dosage form of 30/100 mg (DEC-C) was developed accordingly.

### Aims

To evaluate the efficacy and safety of a new oral dosage form of DEC-C in MDS patients in China.

**Copyright Information:** (Online) ISSN: 2572-9241
©2026 The Author(s). HemaSphere is published by John Wiley & Sons Ltd on behalf of European Hematology Association. This is an open access Abstract Book distributed under the Attribution-NonCommercial-NoDerivs (CC BY-NC-ND), which allows third parties to download the articles and share them with others as long as they credit the author and the Abstract Book, but they cannot change the content in any way or use them commercially.
**Abstract Book Citations:** Authors, Title, HemaSphere, 2026;10:(S1):pages. The abstract book DOIs can be found on the "Author Information" page.
**Disclaimer:** Articles published in the journal HemaSphere exclusively reflect the opinions of the authors. The authors are responsible for all content in their abstracts including accuracy of the facts, statements, citing resources, etc.

### Methods

Eligibility criteria included MDS or chronic myelomonocytic leukemia (CMML) patients at higher risk (IPSS intermediate-1-risk and above). The trial was divided into two parts. Patients enrolled in part 1 were randomized 1:1 to two cohorts to receive DEC-C (one tablet/day on Day 1~5) and IV DEC (20 mg/$m^2$/day on Day 1~5) in a crossover way during the first two cycles for exposure assessment. All patients enrolled in part 1 and part 2 were included in the response assessment in line with the intention-to-treat principle. Starting from cycle 3 in part 1 and all cycles in part 2, patients received DEC-C on Day 1~5 in 28-day cycles, until disease progression or unacceptable toxicity. The trial is registered on ClinicalTrials.gov (NCT06091267) and Chinadrugtrials.org.cn (CTR20233084).

### Results

Between October 2023 and June 2025, 73 patients (part 1 and part 2; median age: 65 yrs [range: 33~82]; 43 [58.9%] male) were enrolled in 15 centers. 70 (95.9%) patients were diagnosed with MDS and 3 (4.1%) were CMML. The baseline characteristics of the patients were shown in the figure 1 below.

The geometric least squares mean ratio of AUC between DEC-C and IV DEC in paired primary population from part 1 fell within the prespecified equivalence range.

By November 2025, 25 patients were still receiving treatment in the trial. After a median of 5 (range: 1~23) cycles of treatment, per IWG 2006, the overall response rate (ORR) was 54.8% (40/73), with complete response (CR) rate of 21.9% (16/73, 95%CI: 13.08%~33.14%) which was consistent with the historical data of ASTX727, mCR (marrow complete response) with HI (hematological improvement) rate of 8.2%, mCR without HI rate of 16.4%, and HI only rate of 8.2%. Additionally, per IWG 2023, the ORR rate was 56.2%, with CR (or equivalent) rate of 20.5%, $CR_{bi}$ rate of 12.3%, $CR_{uni}$ rate of 5.5%, $CR_h$ rate of 1.4%, and HI rate of 16.4%. After a median follow-up of 9 months, the median overall survival (OS) was 19.1 (95%CI: 16.1~not reached [NR]) months, with a 12-month OS rate of 76.3%; the median leukemia free survival (LFS) was 19.1 (95%CI: 15.7~NR) months, with a 12-month LFS rate of 71.0%.

No statistical significance in safety was observed between DEC-C and IV DEC in the first cycle in part 1 (each n=13). Overall, the proportion of treatment emergent adverse events leading to death was 6.8% (5/73), of which 4.1% (3/73) were considered as treatment related (1.4% each for pneumonia, sepsis, and septic shock).

### Summary/Conclusion

We verified consistent efficacy and favorable safety profile of the new oral dosage form of decitabine/cedazuridine, supporting its use as the first oral HMA option alongside parenteral formulations for patients with MDS in China.

**Copyright Information:** (Online) ISSN: 2572-9241
©2026 The Author(s). HemaSphere is published by John Wiley & Sons Ltd on behalf of European Hematology Association. This is an open access Abstract Book distributed under the Attribution-NonCommercial-NoDerivs (CC BY-NC-ND), which allows third parties to download the articles and share them with others as long as they credit the author and the Abstract Book, but they cannot change the content in any way or use them commercially.
**Abstract Book Citations:** Authors, Title, HemaSphere, 2026;10:(S1):pages. The abstract book DOIs can be found on the "Author Information" page.
**Disclaimer:** Articles published in the journal HemaSphere exclusively reflect the opinions of the authors. The authors are responsible for all content in their abstracts including accuracy of the facts, statements, citing resources, etc.

| Characteristic | N=73 |
|---|---|
| Age, median (range), years | 65 (33, 82) |
| Male, n (%) | 43 (58.9) |
| ECOG, n (%) | |
| 0 | 32 (43.8) |
| 1 | 41 (56.2) |
| Karyotype | |
| Normal | 37 (50.7) |
| Abnormal | 34 (46.6) |
| Non-complex | 23 (31.5) |
| Complex | 11 (15.1) |
| Not evaluable | 2 (2.7) |
| FAB classification, n (%) | |
| RA | 9 (12.3) |
| RARS | 1 (1.4) |
| RAEB | 60 (82.2) |
| CMML | 3 (4.1) |
| WHO 2016 classification, n (%) | |
| SLD | 2 (2.9) |
| MLD | 5 (7.1) |
| RS-SLD | 1 (1.4) |
| EB-1 | 39 (55.7) |
| EB-2 | 21 (30.0) |
| U | 2 (2.9) |
| WHO 2022 classification, n (%)† | |
| LB | 6 (10.7) |
| SF3B1 | 1 (1.8) |
| IB1 | 25 (44.6) |
| IB2 | 16 (28.6) |
| biTP53 | 8 (14.3) |

| Characteristic | N=73 |
|---|---|
| IPSS, n (%) | |
| Low | 1 (1.4) |
| Int-1 | 39 (53.4) |
| Int-2 | 23 (31.5) |
| High | 8 (11.0) |
| Unknown* | 2 (2.7) |
| IPSS-R, n (%) | |
| Low | 3 (4.3) |
| Int | 24 (34.3) |
| High | 24 (34.3) |
| Very high | 17 (24.3) |
| Unknown* | 2 (2.9) |
| IPSS-M, n (%)† | |
| Low | 4 (7.1) |
| Moderate low | 3 (5.4) |
| Moderate high | 11 (19.6) |
| High | 12 (21.4) |
| Very high | 25 (44.6) |
| Unknown* | 1 (1.8) |
| Common mutations, n (%)† | |
| ASXL1 | 14 (25.0) |
| U2AF1 | 10 (17.9) |
| TP53 | 9 (16.1) |
| SF3B1 | 6 (10.7) |
| TP53 mutation status‡, n (%)† | |
| WT | 47 (83.9) |
| Single hit | 1 (1.8) |
| Multi hit | 8 (14.3) |

*Unknown for karyotype was unevaluable.
† N=56, NGS samples were available from 56 MDS patients and were tested using corresponding institutional panels.
‡ TP53 mutation status was classified using WHO 2022 definition.

Copyright Information: (Online) ISSN: 2572-9241
©2026 The Author(s). HemaSphere is published by John Wiley & Sons Ltd on behalf of European Hematology Association. This is an open access Abstract Book distributed under the Attribution-NonCommercial-NoDerivs (CC BY-NC-ND), which allows third parties to download the articles and share them with others as long as they credit the author and the Abstract Book, but they cannot change the content in any way or use them commercially.
Abstract Book Citations: Authors, Title, HemaSphere, 2026;10:(S1):pages. The abstract book DOIs can be found on the "Author Information" page.
Disclaimer: Articles published in the journal HemaSphere exclusively reflect the opinions of the authors. The authors are responsible for all content in their abstracts including accuracy of the facts, statements, citing resources, etc.

### (PF674) BEYOND STATIC BASELINES: PREDICTIVE VALUE OF DYNAMIC KARYOTYPE EVOLUTION FOR DISEASE PROGRESSION AND LEUKEMIC TRANSFORMATION IN MYELODYSPLASTIC NEOPLASMS

**Topic:** 10. Myelodysplastic syndromes - Clinical

Ying Wang[1], Xin Yan[1], Zheng Ge*[1]

[1]*Zhongda Hospital Southeast University, Institute of Hematology Southeast University, Department of Hematology, Nanjing, China;*

### Background

Myelodysplastic neoplasms (MDS) are clonal hematopoietic stem cell malignancies characterized by cytopenia, ineffective hematopoiesis, and a high risk of progression to acute myeloid leukemia (AML). Although static cytogenetic risk stratification at diagnosis (e.g., IPSS-R) is widely used, it often fails to accurately predict the risk of progressive disease (PD) or leukemic transformation (LT) triggered by karyotype evolution during longitudinal follow-up.

### Aims

To analyze the patterns of dynamic karyotype evolution and evaluate their impact on disease progression, leukemic transformation, and overall survival (OS) in patients with MDS.

### Methods

We retrospectively reviewed 129 MDS patients diagnosed at Zhongda Hospital affiliated to Southeast University between January 2013 and December 2025, who had at least two documented cytogenetic evaluations. Based on the modified criteria by Julie et al., dynamic karyotypic evolution during follow-up was innovatively stratified into four prognostic classes: Class I (Improvement): ≥20% reduction in abnormal clone size from baseline, fewer abnormalities, or transformation to a normal karyotype. Class II (Stable non-complex): Persistent normal karyotype, or ≤2 abnormalities with no new aberrations and <20% fluctuation in the original clone size. Class III (Stable complex): Persistent ≥3 abnormalities with no new aberrations and <20% fluctuation in the original clone size. Class IV (Progression): Emergence of novel aberrations, or significant clonal expansion (≥20% increase in the original abnormal clone size). Data analysis was performed using SPSS 27.0, and P < 0.05 was considered statistically significant.

### Results

1. Among 129 patients (median age 66 years), 38 experienced PD and 33 experienced LT. The PD/LT group had a significantly higher baseline bone marrow blast percentage (3.6% vs. 0.4%, $P = 0.004$).
2. Baseline cytogenetics showed normal karyotypes in 51.2% ($n = 66$) and complex karyotypes (CK) in 14.7% ($n = 19$). The PD/LT group exhibited higher baseline rates of CK (21.1% vs. 6.9%, $P = 0.026$) and *TP53* mutations (19.7% vs. 3.4%, $P = 0.006$).
3. PD/LT rates differed significantly across the four classes ($P = 0.019$; Fig. A), with Class III (85.7%) and Class IV (70.27%) showing the highest risks.
4. In the non-high-risk IPSS-R subgroup ($n = 107$), PD/LT incidence was significantly higher in Class IV (66.7%) compared to Class II (46.5%) and Class I (22.2%) ($P = 0.0469$; Fig. B). Notably, gain(1q) accounted for 25.9% of Class IV evolutions.
5. Survival analysis showed ClassIII had the worst prognosis (median OS 8.0m), followed by Class IV (32.8m) and Class II (62.6m), while Class I had the best survival (median OS not reached, $P < 0.01$; Fig. C).

**Copyright Information:** (Online) ISSN: 2572-9241
©2026 The Author(s). HemaSphere is published by John Wiley & Sons Ltd on behalf of European Hematology Association. This is an open access Abstract Book distributed under the Attribution-NonCommercial-NoDerivs (CC BY-NC-ND), which allows third parties to download the articles and share them with others as long as they credit the author and the Abstract Book, but they cannot change the content in any way or use them commercially.
**Abstract Book Citations:** Authors, Title, HemaSphere, 2026;10:(S1):pages. The abstract book DOIs can be found on the "Author Information" page.
**Disclaimer:** Articles published in the journal HemaSphere exclusively reflect the opinions of the authors. The authors are responsible for all content in their abstracts including accuracy of the facts, statements, citing resources, etc.

### Summary/Conclusion

Dynamic evolution of chromosomal karyotype during the disease course is an important risk factor for predicting disease outcome and survival prognosis in patients with MDS. Evolution of ClassⅣ and Class Ⅲ acts as a strong warning signal for PD/LT. Clonal evolution such as gain or duplication of 1q may still increase the risk of PD/LT, even among patients with favorable initial karyotype prognosis.

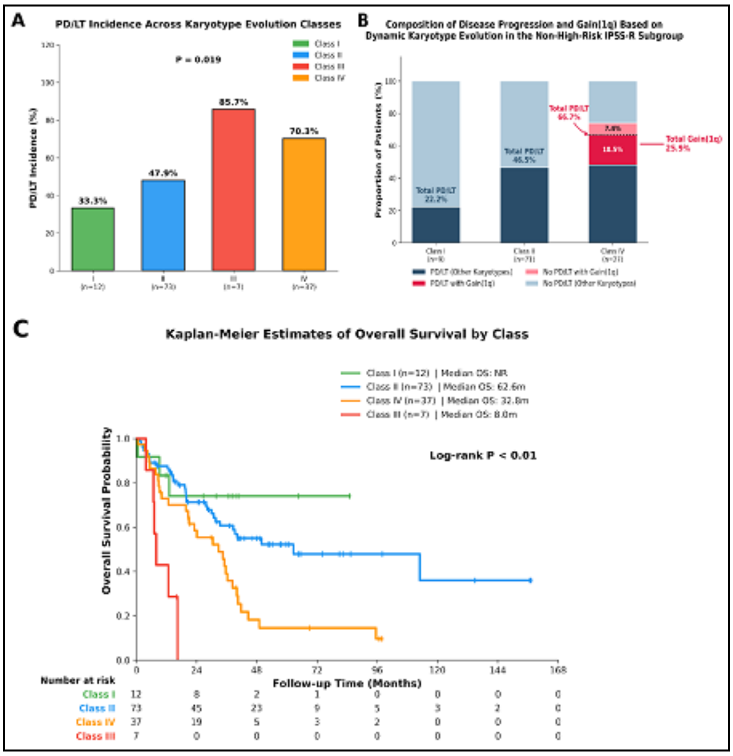

**Copyright Information:** (Online) ISSN: 2572-9241
©2026 The Author(s). HemaSphere is published by John Wiley & Sons Ltd on behalf of European Hematology Association. This is an open access Abstract Book distributed under the Attribution-NonCommercial-NoDerivs (CC BY-NC-ND), which allows third parties to download the articles and share them with others as long as they credit the author and the Abstract Book, but they cannot change the content in any way or use them commercially.
**Abstract Book Citations:** Authors, Title, HemaSphere, 2026;10:(S1):pages. The abstract book DOIs can be found on the "Author Information" page.
**Disclaimer:** Articles published in the journal HemaSphere exclusively reflect the opinions of the authors. The authors are responsible for all content in their abstracts including accuracy of the facts, statements, citing resources, etc.

### (PF675) HEAVY METAL ABNORMALITIES IN UNEXPLAINED CYTOPENIAS AND LOW-BLAST MYELODYSPLASTIC NEOPLASMS: A PROSPECTIVE INTERVENTIONAL COHORT STUDY FROM NORTH INDIA

**Topic:** 10. Myelodysplastic syndromes - Clinical

Kartikey Saini*[1], Mukul Aggarwal[1], Jasmita Dass[1], Rishi Dhawan[1], Ganesh Kumar Viswanathan[1], Richa Chauhan[1], Tulika Seth[1], Manoranjan Mahapatra[1]

*[1]ALL INDIA INSTITUTE OF MEDICAL SCIENCES NEW DELHI, HEMATOLOGY, NEW DELHI, India;*

### Background

Heavy metal deficiency/toxicity is recognized as a etiology of unexplained cytopenias and low-blast myelodysplastic neoplasms (MDS) but is routinely neglected with lack of studies exploring the prevalence and responses in this subset of patients.

### Aims

To determine the prevalence and spectrum of heavy metal abnormalities in patients with unexplained cytopenias and low-blast MDS, and to assess hematologic responses to targeted correction

### Methods

In this single-centre prospective study (February 2024–November 2025), 120 patients with unexplained cytopenias (Hb <10 g/dL, platelets <100×10⁹/L, ANC <1.8×10⁹/L) or WHO 2022 low-blast MDS were included after exclusion of patients with vitamin B12/folate deficiency, active infection, autoimmune disease, blasts ≥5%, or disease-defining cytogenetic/molecular abnormalities. Serum/blood levels of copper, zinc, lead, cadmium, arsenic, mercury, selenium and cobalt were quantified by ICP–MS. Patients with clinically significant abnormalities were offered protocol-directed copper supplementation and/or oral DMSA-based chelation. Hematologic response at 3 months was defined as complete response (CR: Hb ≥10 g/dL, ANC ≥1.0×10⁹/L, platelets ≥100×10⁹/L), partial response (PR: ≥50% reduction in transfusion requirement and/or meaningful count improvement), or no response (NR)

### Results

Median age was 46 years; 50.8% were male and 54.2% had low-blast MDS. Heavy metal abnormalities were identified in 66.7% (80/120) of patients, with 32.4% harbouring ≥2 concurrent abnormalities. Copper deficiency was most frequent (≈65%), while excess zinc, lead and arsenic were the predominant toxic exposures. Ring sideroblasts were observed in 10.6% of marrows and correlated with elevated mercury and zinc. 50 patients had evaluable follow-up. Overall, 70% achieved hematologic response (CR/PR), while 30% were NR. Responders showed a mean haemoglobin increase of ~3 g/dL, with concordant improvements in neutrophils and platelets; among responders, 61.9% achieved transfusion independence and a further 23.8% had ≥50% reduction in transfusion needs. Shorter time from detection of abnormality to treatment initiation (approximately 12 vs 29 months) was significantly associated with response.

### Summary/Conclusion

Heavy metal abnormalities, particularly copper deficiency with coexisting toxic metal excess, are highly prevalent in our cohort of patients with unexplained cytopenias and low-blast MDS and frequently mimic low-risk MDS clinically and morphologically. Early, targeted correction yields substantial hematologic benefit and transfusion reduction in significant number of patients. Systematic heavy metal screening should be integrated into the diagnostic work-up of unexplained cytopenias and low-risk MDS, especially in high-exposure settings

**Copyright Information:** (Online) ISSN: 2572-9241
©2026 The Author(s). HemaSphere is published by John Wiley & Sons Ltd on behalf of European Hematology Association. This is an open access Abstract Book distributed under the Attribution-NonCommercial-NoDerivs (CC BY-NC-ND), which allows third parties to download the articles and share them with others as long as they credit the author and the Abstract Book, but they cannot change the content in any way or use them commercially.
**Abstract Book Citations:** Authors, Title, HemaSphere, 2026;10:(S1):pages. The abstract book DOIs can be found on the "Author Information" page.
**Disclaimer:** Articles published in the journal HemaSphere exclusively reflect the opinions of the authors. The authors are responsible for all content in their abstracts including accuracy of the facts, statements, citing resources, etc.

### (PF676) REAL-WORLD TREATMENT PATTERNS AND OUTCOMES OF LUSPATERCEPT IN PATIENTS WITH LOWER-RISK MYELODYSPLASTIC SYNDROMES (LR-MDS) IN CHINA ('REACH' STUDY)

**Topic:** 10. Myelodysplastic syndromes - Clinical

Zhijian Xiao*[1], Jinsong Jia[2], Zefeng Xu[1], Ling Nie[3], Yajing Xu[3], Jinhai Ren[4], Yuqing Chen[5], Mei Hong[6], Zhiqiang Sun[7], Yu Wu[8], Rong Fu[9], Wenyi Shen[10], Dimana Mitev[11], Barkha Aggarwal[12], Ahmed Hnoosh[13], Jihui Cui[14], Junjie Tang[14], Mengge Zhao[14]

*[1]Institute of Hematology & Blood Diseases Hospital, Chinese Academy of Medical Sciences & Peking Union Medical College, Tianjin, China; [2]Peking University People's Hospital, Beijing, China; [3]Xiangya Hospital, Central South University, Changsha, China; [4]The Second Hospital of Hebei Medical University, Shijiazhuang, China; [5]Henan Provincial People's Hospital, Zhengzhou, China; [6]Union Hospital, Tongji Medical College, Huazhong University of Science and Technology, Wuhan, China; [7]Shenzhen Hospital, Southern Medical University, Shenzhen, China; [8]West China Hospital, Sichuan University, Chengdu, China; [9]General Hospital, Tianjin Medical University, Tianjin, China; [10]Jiangsu Provincial People's Hospital, Nanjing, China; [11]Bristol Myers Squibb, Boudry, Switzerland; [12]Bristol Myers Squibb, Princeton, United States of America; [13]Bristol Myers Squibb, Uxbridge, United Kingdom; [14]Bristol Myers Squibb, Shanghai, China;*

### Background

Lower-risk myelodysplastic syndromes (LR-MDS) patients with anemia mainly rely on RBC transfusions and conventional agents, such as erythropoiesis-stimulating agents (ESAs). In China, RBC transfusions are restricted due to blood supply shortage and are recommended only when Hb < 6 g/dL. The efficacy of ESAs is suboptimal with various limitations.
The efficacy and safety of luspatercept in LR-MDS were demonstrated in the COMMANDS study; the absence of Chinese patients, whose baseline characteristics differ from those in other countries, warrants further evaluation.

### Aims

To describe baseline characteristics, treatment patterns, and clinical outcomes in Chinese ESA-naïve LR-MDS patients treated with luspatercept (NCT07073690).

### Methods

REACH is a real-world, retrospective, multicenter, single-arm study conducted in China. Eligible patients were aged ≥ 18 years with documented LR-MDS with anemia, without del(5q) and ESA-naïve prior to luspatercept treatment, transfusion-dependent (TD, any RBC transfusion within 8 weeks before luspatercept initiation) or non-TD (NTD, no RBC transfusion within 8 weeks before luspatercept initiation), and received luspatercept monotherapy for ≥ 2 cycles. Patients with a history of acute myeloid leukemia or stem cell transplantation were excluded. Electronic medical record data from 10 hospitals were collected for patients initiating luspatercept on or after Jun 15, 2022. The index date was defined as the first luspatercept administration, with a 2-month pre-index period to assess baseline characteristics. Data abstraction was from Apr 15, 2022 to Apr 30, 2025.
The primary endpoints were the rate of RBC-transfusion independence for ≥ 8 weeks in TD patients and a mean Hb increase of ≥ 1.5 g/dL for ≥ 8 weeks in NTD patients during weeks 1–12. Secondary endpoints included hematologic improvement-erythroid (HI-E) per IWG 2006 criteria, mean Hb changes, treatment patterns and safety, etc.

**Copyright Information:** (Online) ISSN: 2572-9241
©2026 The Author(s). HemaSphere is published by John Wiley & Sons Ltd on behalf of European Hematology Association. This is an open access Abstract Book distributed under the Attribution-NonCommercial-NoDerivs (CC BY-NC-ND), which allows third parties to download the articles and share them with others as long as they credit the author and the Abstract Book, but they cannot change the content in any way or use them commercially.
**Abstract Book Citations:** Authors, Title, HemaSphere, 2026;10:(S1):pages. The abstract book DOIs can be found on the "Author Information" page.
**Disclaimer:** Articles published in the journal HemaSphere exclusively reflect the opinions of the authors. The authors are responsible for all content in their abstracts including accuracy of the facts, statements, citing resources, etc.

### Results

A total of 88 patients were included. 52.3% (46/88) were TD patients and 47.7% (42/88) were NTD patients. The median time from initial diagnosis to index date was 1.2 months (IQR 0.3–9.0), median baseline Hb was 6.4 g/dL (IQR 5.1–7.7), in TD patients 5.5 g/dL (IQR 4.6–6.2) and in NTD patients 7.5 g/dL (IQR 6.9–8.5), respectively. 34.4% (22/64) with RS-, and 53.6% (15/28) had baseline erythropoietin (EPO) > 500 U/L. The initial dose of luspatercept was as follows: 36.1% (22/61) at ≥ 0.6–< 1.0 mg/kg, 24.6% (15/61) at 1.0 mg/kg, and 39.3% (24/61) at > 1.0–≤ 1.5 mg/kg. The median duration of treatment was 125 days (95% CI 88–237), with a median of 4 cycles (IQR 2–6).
Primary endpoint was assessed for 76 patients with available data, 41 patients (53.9%) achieved the response (Figure). Among patients with available data, 54.4% (37/68) achieved HI-E during weeks 1–12; 50.0% (38/76) achieved a mean Hb increase of ≥ 1.5 g/dL for ≥ 8 weeks during weeks 1–12, 40.9% (18/44) in TD and 62.5% (20/32) in NTD patients, respectively. The mean increase of Hb from week 1 to end of treatment was 1.83 g/dL (median; IQR 0.55–2.56).
Safety profiles are consistent with COMMANDS study, no new safety signal was observed.

### Summary/Conclusion

Baseline characteristics indicate a severe disease burden in Chinese LR-MDS patients. Nevertheless, these findings demonstrate that luspatercept is an effective therapeutic option for Chinse ESA-naïve LR-MDS patients in a real-world setting, regardless of EPO level or RS status, with greater benefit observed when treatment is initiated earlier.

**Figure. Proportion of patients achieving primary endpoint[a,b]**

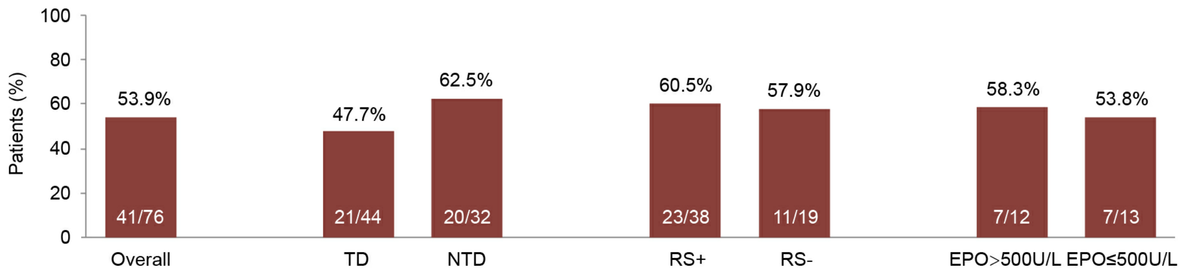

[a] Primary endpoint was defined as the rate of RBC-TI for ≥ 8 weeks in TD patients and a mean Hb increase of ≥ 1.5 g/dL for ≥ 8 weeks in NTD patients (weeks 1–12)

[b] Subgroup analysis of primary endpoint were calculated for pts who had available data

EPO, erythropoietin; NTD, non-transfusion-dependent; RBC-TI, red blood cell-transfusion independence; RS, ring sideroblast; TD, transfusion-dependent

Copyright Information: (Online) ISSN: 2572-9241
©2026 The Author(s). HemaSphere is published by John Wiley & Sons Ltd on behalf of European Hematology Association. This is an open access Abstract Book distributed under the Attribution-NonCommercial-NoDerivs (CC BY-NC-ND), which allows third parties to download the articles and share them with others as long as they credit the author and the Abstract Book, but they cannot change the content in any way or use them commercially.
Abstract Book Citations: Authors, Title, HemaSphere, 2026;10:(S1):pages. The abstract book DOIs can be found on the “Author Information” page.
Disclaimer: Articles published in the journal HemaSphere exclusively reflect the opinions of the authors. The authors are responsible for all content in their abstracts including accuracy of the facts, statements, citing resources, etc.

### (PF677) BLAST DEMONSTRATES HIGHER PROGNOSTIC ACCURACY FOR SURVIVAL THAN CPSS IN CHINESE PATIENTS WITH CHRONIC MYELOMONOCYTIC LEUKEMIA

**Topic:** 10. Myelodysplastic syndromes - Clinical

Zheng Ge*[1], Xin Yan[2], Gege Feng[3], Zhimei Cai[4], Jiale MA[5], Shuqian Xu[6]

*[1]Zhongda Hospital, School of Medicine, Southeast University, Institute of Hematology, Southeast University, Department of Hematology, Nanjing, China; [2]Department of Hematology , Zhongda Hospital, School of Medicine, Southeast University, Institute of Hematology Southeast University, Department of Hematology, Nanjing, China; [3]Department of Hematology, Qilu Hospital of Shandong University, Department of Hematology, Jinan, China; [4]The First People's Hospital of Lianyungang, Department of Hematology, Lianyungang, China; [5]Xuzhou Central Hospital, Southeast University, Department of Hematology, Xuzhou, China; [6]Qilu Hospital of Shandong University, Jinan, China;*

### Background

Recently, the Blasts, Leukocytes, and Anemia-based Survival Tool (BLAST) and BLAST with Molecular Integration (BLAST-mol) have been developed as new prognostic models for CMML. However, these systems have been validated only in Western cohorts, and their performance in Chinese patients remains unclear.

### Aims

We  aims to compare the prognostic predictive performance of the BLAST, BLAST-mol, CPSS, and CPSS-mol scoring systems in this population.

### Methods

A total of 125  with CMML who were diagnosed or reclassified according to the 2022 World Health Organization (WHO) classification between July 20, 2016, and December 1, 2025, across four hospitals were enrolled.

**Copyright Information:** (Online) ISSN: 2572-9241
©2026 The Author(s). HemaSphere is published by John Wiley & Sons Ltd on behalf of European Hematology Association. This is an open access Abstract Book distributed under the Attribution-NonCommercial-NoDerivs (CC BY-NC-ND), which allows third parties to download the articles and share them with others as long as they credit the author and the Abstract Book, but they cannot change the content in any way or use them commercially.
**Abstract Book Citations:** Authors, Title, HemaSphere, 2026;10:(S1):pages. The abstract book DOIs can be found on the "Author Information" page.
**Disclaimer:** Articles published in the journal HemaSphere exclusively reflect the opinions of the authors. The authors are responsible for all content in their abstracts including accuracy of the facts, statements, citing resources, etc.

### Results n multivariable analysis, severe anemia (HR 2.39, 95%CI 1.12–5.11, *P*=0.025) and *TP53* mutation (HR 2.54, 95%CI 1.23–5.22, *P*=0.012) were independent adverse prognostic factors. According to CPSS, 27 cases (21.6%) were assigned to the low-risk group, 40 cases (32.0%) to the intermediate-1 (Int-1) group, 50 cases (40.0%) to the intermediate-2 (Int-2) group, and 8 cases (6.4%) to the high-risk group. The median OS (mOS) was not reached (NR) in the low-risk group, 17 months (95%CI 9.78–24.22) in the Int-1 group, 16 months (95%CI 11.50–20.50) in the Int-2 group, and 8 months (95%CI 0.98–15.02) in the high-risk group. Difference in mOS across all CPSS risk subgroups was statistically significant (*P*=0.0049; **Figure 1A**);Using BLAST, 8.8%, 19.2%, and 72.0% of patients were classified as low-, intermediate-, and high-risk, with median overall survival (mOS) of not reached (NR), 30 months (95% CI 4.25–55.75), and 14 months (95% CI 10.59–17.41), respectively (*P*=0.0005,**Figure 1B**).Using CPSS-mol,10.4%,14.4%,44.0% ,31.2% to the low,int-1,int-2 and high-risk group. The mOS for each group was NR in the low-risk group, 22 months (95% CI 13.60–31.43) in the Int-1 group, 16 months (95% CI 13.01–18.99) in the Int-2 group, and 12 months (95% CI 2.66–21.34) in the high-risk group, respectively. Although overall differences across CPSS-mol categories were significant (*P*=0.0049; **Figure 3C**) Using BLAST-mol, 9.6%, 37.6%, and 52.8% were classified as low-, intermediate-, and high-risk, with mOS of NR, 24 months (95% CI 16.53–31.45), and 12 months (95% CI 9.23–14.77), respectively (*P* < 0.0001). Overall, 76.0% of patients were reclassified from CPSS to BLAST and 45.6% from CPSS-mol to BLAST-mol, with most being upstaged; upgraded patients had significantly shorter OS in both comparisons. The areas under the ROC curve (AUC) for CPSS, CPSS-mol, BLAST, and BLAST-mol at 2 years were 0.578, 0.587, 0.637, 0.664,respectively. At 3 years, the corresponding AUCs were 0.567, 0.596, 0.637, and 0.658, respectively.

### Summary/Conclusion

These findings indicate that BLAST and BLAST-mol provide superior prognostic discrimination compared with CPSS and CPSS-mol in Chinese CMML patients.

**Copyright Information:** (Online) ISSN: 2572-9241
©2026 The Author(s). HemaSphere is published by John Wiley & Sons Ltd on behalf of European Hematology Association. This is an open access Abstract Book distributed under the Attribution-NonCommercial-NoDerivs (CC BY-NC-ND), which allows third parties to download the articles and share them with others as long as they credit the author and the Abstract Book, but they cannot change the content in any way or use them commercially.
**Abstract Book Citations:** Authors, Title, HemaSphere, 2026;10:(S1):pages. The abstract book DOIs can be found on the “Author Information” page.
**Disclaimer:** Articles published in the journal HemaSphere exclusively reflect the opinions of the authors. The authors are responsible for all content in their abstracts including accuracy of the facts, statements, citing resources, etc.

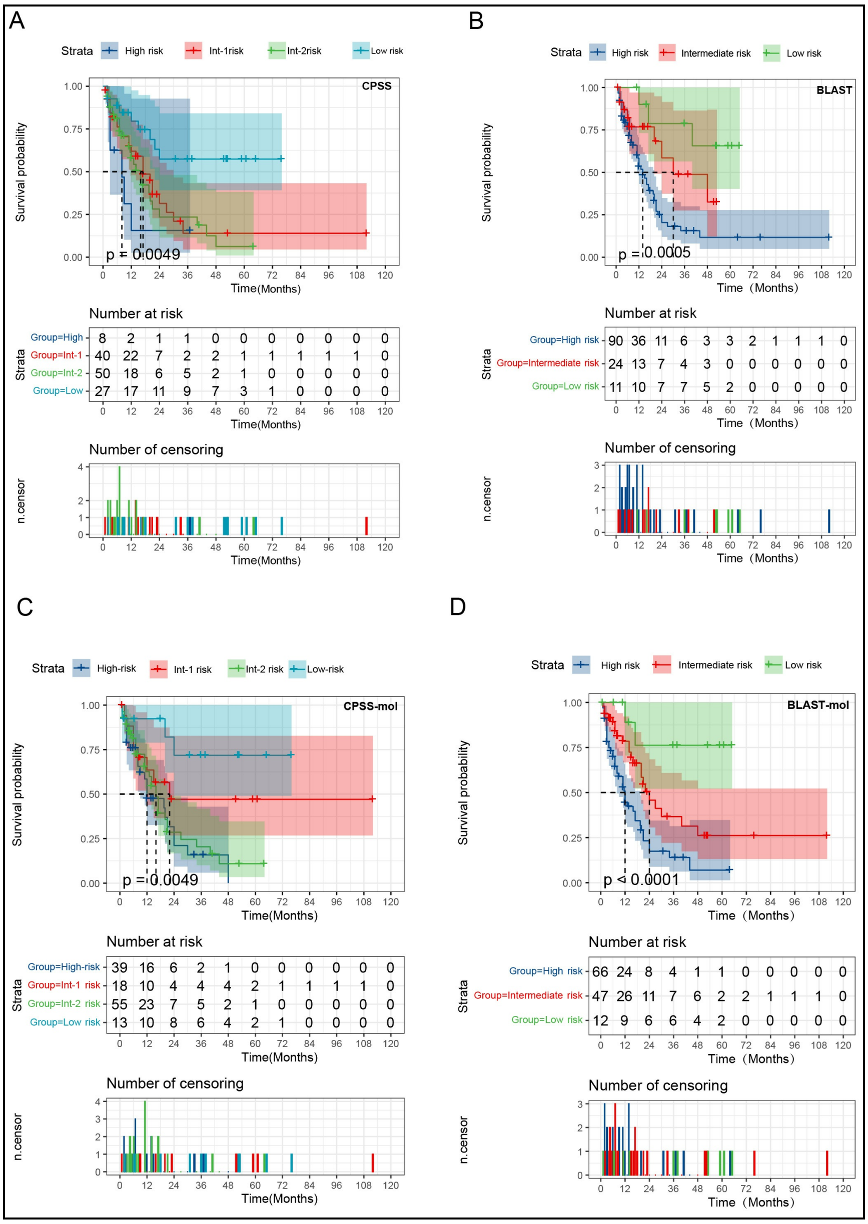

**Copyright Information:** (Online) ISSN: 2572-9241
©2026 The Author(s). HemaSphere is published by John Wiley & Sons Ltd on behalf of European Hematology Association. This is an open access Abstract Book distributed under the Attribution-NonCommercial-NoDerivs (CC BY-NC-ND), which allows third parties to download the articles and share them with others as long as they credit the author and the Abstract Book, but they cannot change the content in any way or use them commercially.
**Abstract Book Citations:** Authors, Title, HemaSphere, 2026;10:(S1):pages. The abstract book DOIs can be found on the "Author Information" page.
**Disclaimer:** Articles published in the journal HemaSphere exclusively reflect the opinions of the authors. The authors are responsible for all content in their abstracts including accuracy of the facts, statements, citing resources, etc.

### (PF678) INTEGRIN Α9 (ITGA9): CLINICAL CORRELATION AND MOLECULAR MECHANISM IN MYELODYSPLASTIC SYNDROMES

**Topic:** 10. Myelodysplastic syndromes - Clinical

Xuanlu Wu*[1], Xiaoxue Wang[1]

[1] *The First Hospital Affiliated to China Medical University, Department of Hematology, Shenyang, China;*

### Background

Myelodysplastic neoplasms (MDS) are clonal hematopoietic malignancies characterized by ineffective hematopoiesis and a high risk of leukemic transformation. Disease pathogenesis is driven by a combination of somatic genetic/epigenetic alterations and critical dysregulation of the bone marrow microenvironment.

### Aims

This study aims to investigate the expression features, clinical prognostic value, and potential molecular mechanism of Integrin α9 (*ITGA9*) in MDS, and to provide novel evidence for risk stratification and targeted therapy of MDS.

### Methods

We mined the GEO database to analyze expression differences of integrin family members in CD34$^{+}$ hematopoietic stem cells between MDS patients and healthy controls, and evaluated their prognostic significance. Subsequently, we determined the optimal *ITGA9* cut-off value via survival analysis to stratify patients into high- and low-expression groups, filtered differentially expressed genes, and detected key pathogenic gene expression in IPSS-M. Then we constructed ITGA9's protein-protein interaction (PPI) network and performed KEGG pathway enrichment analysis using STRING 12.0. In parallel, we measured *ITGA9* expression in bone marrow mononuclear cells from 19 MDS patients and 4 healthy controls, and correlated it with clinical parameters. Finally, we knocked down/overexpressed *ITGA9* in *SKM-1* cells, and assessed proliferation, cycle, and apoptosis. We also verified mRNA levels of key PPI network genes following modulation.

**Copyright Information:** (Online) ISSN: 2572-9241
©2026 The Author(s). HemaSphere is published by John Wiley & Sons Ltd on behalf of European Hematology Association. This is an open access Abstract Book distributed under the Attribution-NonCommercial-NoDerivs (CC BY-NC-ND), which allows third parties to download the articles and share them with others as long as they credit the author and the Abstract Book, but they cannot change the content in any way or use them commercially.
**Abstract Book Citations:** Authors, Title, HemaSphere, 2026;10:(S1):pages. The abstract book DOIs can be found on the "Author Information" page.
**Disclaimer:** Articles published in the journal HemaSphere exclusively reflect the opinions of the authors. The authors are responsible for all content in their abstracts including accuracy of the facts, statements, citing resources, etc.

**Results**

**Integrin family expression and prognostic significance in MDS:** GEO database (GSE4619, GSE19429, GSE58831) showed upregulation of *ITGA9* and *ITGB2-AS* in MDS (high expression related to poor prognosis). *ITGB7* was upregulated (favorable prognosis), whereas *ITGA10/ITGB3* were downregulated (linked to poorer outcomes). Compared with *ITGA9*-low MDS patients, 216 genes (including key pathogenic genes *RUNX1*/*SETBP1*) were upregulated and 305 genes downregulated in *ITGA9*-high patients.

**ITGA9 PPI and pathway enrichment:** STRING identified the ITGA9 PPI network and its functional partners (ITGB1/3/7, TNC, FN1, TNXB, TNR, SPP1, VCAM-1), which were enriched in extracellular matrix (ECM)-receptor interaction, focal adhesion, and PI3K-Akt signaling pathways.

***ITGA9* expression and clinical parameter correlations in the clinical cohort**In our clinical cohort, *ITGA9* was upregulated in 19 MDS patients vs. 4 healthy controls, showed a significant negative correlation with WBC count, and was higher in IPSS-M non-low-risk (score > -0.5) than in low-risk (score ≤ -0.5) groups.

**Effects of *ITGA9* modulation on SKM-1 cell function and downstream gene expression:** *ITGA9* overexpression enhanced proliferation and reduced the proportion of S-phase cells, while *ITGA9* knockdown altered cell cycle (decreased G1, increased S phase) and promoted early apoptosis. Following *ITGA9* overexpression, mRNA levels of *ITGB1* and *TNR* were significantly decreased. Conversely, *ITGA9* knockdown increased *ITGB7/SPP1* mRNA and decreased *TNXB* expression.

**Summary/Conclusion**

*ITGA9* represents a potential prognostic biomarker in MDS, with higher expression detected in IPSS-M non-low-risk patients. It may contribute to MDS initiation and progression by regulating key effector molecules and mediating ECM-associated signaling pathways. Upregulation of *ITGA9* is associated with hematopoietic dysfunction and impaired cell migration and release. It also sustains the survival of malignant clones by enhancing proliferation and coordinately regulating the cell cycle and apoptosis.

**Copyright Information:** (Online) ISSN: 2572-9241
©2026 The Author(s). HemaSphere is published by John Wiley & Sons Ltd on behalf of European Hematology Association. This is an open access Abstract Book distributed under the Attribution-NonCommercial-NoDerivs (CC BY-NC-ND), which allows third parties to download the articles and share them with others as long as they credit the author and the Abstract Book, but they cannot change the content in any way or use them commercially.
**Abstract Book Citations:** Authors, Title, HemaSphere, 2026;10:(S1):pages. The abstract book DOIs can be found on the "Author Information" page.
**Disclaimer:** Articles published in the journal HemaSphere exclusively reflect the opinions of the authors. The authors are responsible for all content in their abstracts including accuracy of the facts, statements, citing resources, etc.

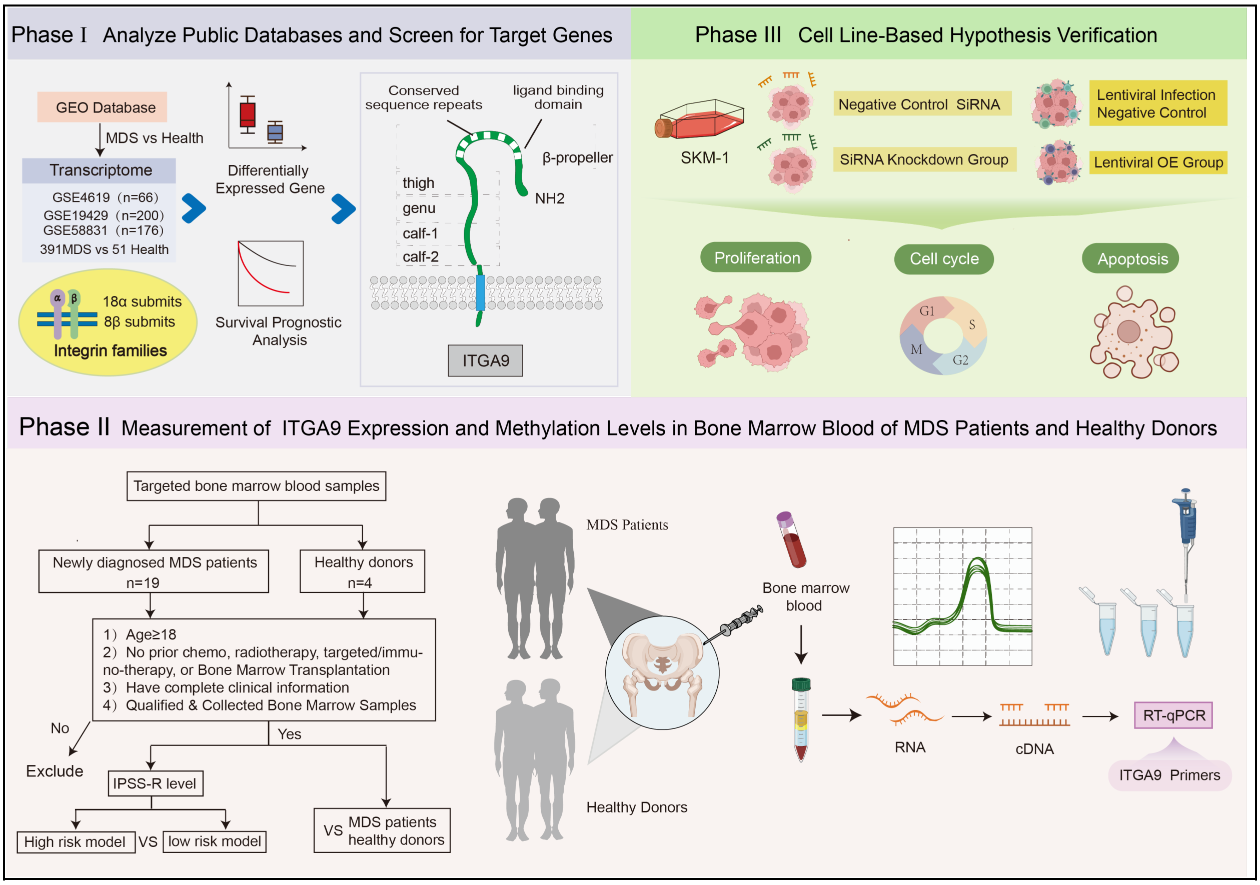

Figure 1. Flowchart of the study design and experimental workflow.

**Copyright Information:** (Online) ISSN: 2572-9241
©2026 The Author(s). HemaSphere is published by John Wiley & Sons Ltd on behalf of European Hematology Association. This is an open access Abstract Book distributed under the Attribution-NonCommercial-NoDerivs (CC BY-NC-ND), which allows third parties to download the articles and share them with others as long as they credit the author and the Abstract Book, but they cannot change the content in any way or use them commercially.
**Abstract Book Citations:** Authors, Title, HemaSphere, 2026;10:(S1):pages. The abstract book DOIs can be found on the "Author Information" page.
**Disclaimer:** Articles published in the journal HemaSphere exclusively reflect the opinions of the authors. The authors are responsible for all content in their abstracts including accuracy of the facts, statements, citing resources, etc.

### (PF679) TARGETING ASH1L-STING AXIS RESTORES NK CELL FUNCTION AND AMELIORATES IMMUNE-MEDIATED BONE MARROW FAILURE DISEASES

**Topic:** 11. Bone marrow failure syndromes incl. PNH - Biology & translational research

Nan Song*[1], Yi Cui[1], Xinrui Zhang[1], Rong Fu[1]

[1] *Tianjin Medical University General Hospital, Tianjin, China;*

**Background**

Aplastic anemia (AA) is an immune-mediated bone marrow failure syndrome involving dysregulated innate and adaptive immunity. Natural killer (NK) cell numerical and functional defects have been implicated in AA pathogenesis, yet the underlying epigenetic mechanisms remain poorly understood.

**Aims**

This study aimed to investigate the role of the histone methyltransferase Ash1L in NK cell dysfunction in AA, elucidate its mechanistic impact on DNA damage repair (DDR) and inflammatory signaling, and evaluate the therapeutic potential of andrographolide (Andro) in restoring NK cell homeostasis and ameliorating immune-mediated bone marrow failure (BMF).

**Methods**

Ash1L expression was quantified in purified bone marrow NK cells from 57 AA patients (20 untreated, 17 complete remission, 20 partial remission) and 30 healthy controls using qRT-PCR. Ash1L was knocked down in NK-92 and YT cells to assess effects on viability, apoptosis, cell cycle, cytokine production (IFN-γ, IL-6, TNF-α), histone modifications (H3K4me3, H3K36me2), DDR efficiency (γH2AX, comet assay, HR/NHEJ reporters, laser microirradiation), and cGAS-STING pathway activation. Andrographolide treatment effects were evaluated in vitro and in an immune-mediated BMF mouse model (4.5 Gy irradiation + allogeneic lymphocyte infusion). Outcomes included peripheral blood counts, bone marrow histology, immune cell subsets, cytokine levels, Ash1L expression, γH2AX, and STING signaling.

**Results**

Ash1L mRNA was significantly downregulated in NK cells from untreated and partially remitted AA patients compared to controls and complete remission cases, with levels negatively correlated to disease severity and IL-6. Ash1L knockdown impaired NK cell viability, induced G1 arrest and apoptosis, increased pro-inflammatory cytokine secretion, reduced H3K4me3/H3K36me2, attenuated ATM-CHK2-p53 activation, caused persistent γH2AX accumulation, compromised HR and NHEJ repair, and triggered aberrant cGAS-STING pathway activation. Andrographolide treatment suppressed STING-TBK1-IRF3 phosphorylation, reduced type I IFN and chemokine production, enhanced DDR efficiency, partially restored Ash1L expression, and attenuated NK cell overactivation in vitro. In the BMF mouse model, Andro (alone or with cyclosporine) significantly improved peripheral blood counts, restored CD4+/CD8+ ratio and Treg proportion, reduced CD8+ T and NK cell cytotoxic markers (IFN-γ, perforin, granzyme B, NKG2D), upregulated NK cell Ash1L, inhibited STING signaling and γH2AX, and markedly improved bone marrow cellularity and hematopoiesis.

**Copyright Information:** (Online) ISSN: 2572-9241
©2026 The Author(s). HemaSphere is published by John Wiley & Sons Ltd on behalf of European Hematology Association. This is an open access Abstract Book distributed under the Attribution-NonCommercial-NoDerivs (CC BY-NC-ND), which allows third parties to download the articles and share them with others as long as they credit the author and the Abstract Book, but they cannot change the content in any way or use them commercially.
**Abstract Book Citations:** Authors, Title, HemaSphere, 2026;10:(S1):pages. The abstract book DOIs can be found on the "Author Information" page.
**Disclaimer:** Articles published in the journal HemaSphere exclusively reflect the opinions of the authors. The authors are responsible for all content in their abstracts including accuracy of the facts, statements, citing resources, etc.

**Summary/Conclusion**

Ash1L acts as a critical epigenetic regulator of genomic stability and functional homeostasis in NK cells. Its downregulation in AA drives DDR defects, persistent DNA damage, and STING-mediated inflammation, exacerbating immune dysregulation and BMF. Andrographolide restores Ash1L expression, enhances DDR, suppresses STING signaling, corrects immune imbalance, and ameliorates BMF, highlighting its potential as a novel therapeutic strategy for AA and related immune-mediated bone marrow failure disorders.

**Copyright Information:** (Online) ISSN: 2572-9241
©2026 The Author(s). HemaSphere is published by John Wiley & Sons Ltd on behalf of European Hematology Association. This is an open access Abstract Book distributed under the Attribution-NonCommercial-NoDerivs (CC BY-NC-ND), which allows third parties to download the articles and share them with others as long as they credit the author and the Abstract Book, but they cannot change the content in any way or use them commercially.
**Abstract Book Citations:** Authors, Title, HemaSphere, 2026;10:(S1):pages. The abstract book DOIs can be found on the "Author Information" page.
**Disclaimer:** Articles published in the journal HemaSphere exclusively reflect the opinions of the authors. The authors are responsible for all content in their abstracts including accuracy of the facts, statements, citing resources, etc.

### (PF680) MODELING PNH EVOLUTION: GENETIC DRIFT AND CLINICAL DETERMINANTS

**Topic:** 11. Bone marrow failure syndromes incl. PNH - Biology & translational research

Ramkarthic Ramanathan*[1], Hussein Awada[2], Valeria Visconte[2], Serhan Unlu[2], Zachary Brady[2], Aashray Mandala[2], Karthik Lakhotiya[2], Jaroslaw P Maciejewski[2], Carmelo Gurnari[2, 3], Arda Durmaz[4]

*[1]Case Western Reserve University, Cleveland, United States of America; [2]Cleveland Clinic Institute, Department of Translational Hematology and Oncology Research, Taussig Cancer Institute, Cleveland, United States of America; [3]Tor Vergata University of Rome, Department of Biomedicine and Prevention, Roma, Italy; [4]Cleveland Clinic Research, Department of Genomic Sciences and Systems Biology, Cleveland, United States of America;*

### Background

Paroxysmal Nocturnal Hemoglobinuria (PNH) is a rare clonal hematopoietic disorder caused by somatic mutations in the PIGA gene, leading to loss of GPI-anchored surface proteins and increased susceptibility to complement-mediated hemolysis. Secondary PNH arises in bone marrow failure syndromes like aplastic anemia (AA), but disease progression varies widely. While *PIGA*-mutant clones are hypothesized to have fitness advantage through immune evasion mechanisms, previous modeling work has demonstrated that neutral evolution can account for PNH clone dynamics without requiring selective advantage, yet clinical observations show substantial variability in progression rates. The mechanisms linking initial clone size, disease severity, and treatment response to clinical outcomes remain incompletely understood.

### Aims

We aimed to investigate the mechanisms driving PNH clonal evolution by developing complementary computational and statistical models. Specifically, we sought to (1) determine whether neutral stochastic drift alone can account for PNH clone dynamics using a cellular automata model of hematopoietic stem cell behavior, and (2) identify clinical determinants of progression to clinically significant PNH (≥20% clone size) in a real-world cohort of aplastic anemia patients using a multivariate Cox proportional hazards model.

### Methods

We developed two complementary models to explore PNH evolution. The first model is represented by a cellular automata (CA) model simulating hematopoietic stem cell dynamics on a 2D lattice under varying bone marrow conditions. The model tracked clone size, diversity, and time to clinically significant progression (defined as ≥20%) based on AA severity-dependent fitness parameters. We compared simulated survival curves by initial clone size (0–5%, 5–10%, >10%) to observed data. Second, we applied a multivariate Cox proportional hazards model to a clinical cohort (n=207), incorporating initial clone size, severity of AA, and treatment response as covariates.

**Copyright Information:** (Online) ISSN: 2572-9241
©2026 The Author(s). HemaSphere is published by John Wiley & Sons Ltd on behalf of European Hematology Association. This is an open access Abstract Book distributed under the Attribution-NonCommercial-NoDerivs (CC BY-NC-ND), which allows third parties to download the articles and share them with others as long as they credit the author and the Abstract Book, but they cannot change the content in any way or use them commercially.
**Abstract Book Citations:** Authors, Title, HemaSphere, 2026;10:(S1):pages. The abstract book DOIs can be found on the “Author Information” page.
**Disclaimer:** Articles published in the journal HemaSphere exclusively reflect the opinions of the authors. The authors are responsible for all content in their abstracts including accuracy of the facts, statements, citing resources, etc.

### Results

The CA model demonstrated that neutral stochastic drift explains most of the early and moderate PNH clone evolution without requiring an inherent fitness advantage. Simulated survival curves for the 10% and 15% initial clone size groups overlapped with the 95% CI of KM estimates (**Fig.1**). However, the 5% group displayed notable divergence, suggesting that additional biological or clinical factors influence early clone behavior in smaller populations. The Cox proportional hazards model achieved strong predictive performance (concordance index = 0.852). As expected, clone size emerged as the strongest independent predictor: clones of 5–10% had a hazard ratio of 4.91 ($p < 0.005$), while clones >10% had a hazard ratio of 7.12 ($p < 0.005$), relative to the 0–5% reference group. Interestingly, AA severity (HR = 0.82, p = 0.36) and treatment response status did not show significant association with progression.

### Summary/Conclusion

Our findings indicate that PNH evolution results from both stochastic cellular dynamics and deterministic clinical factors, challenging the necessity of a fitness advantage to explain clonal expansion and highlighting the role of neutral drift. Integrating these frameworks by extracting CA-derived metrics (growth rates, trajectory patterns) as covariates can provide insights in PNH clone tracking and associated clinical predictors.

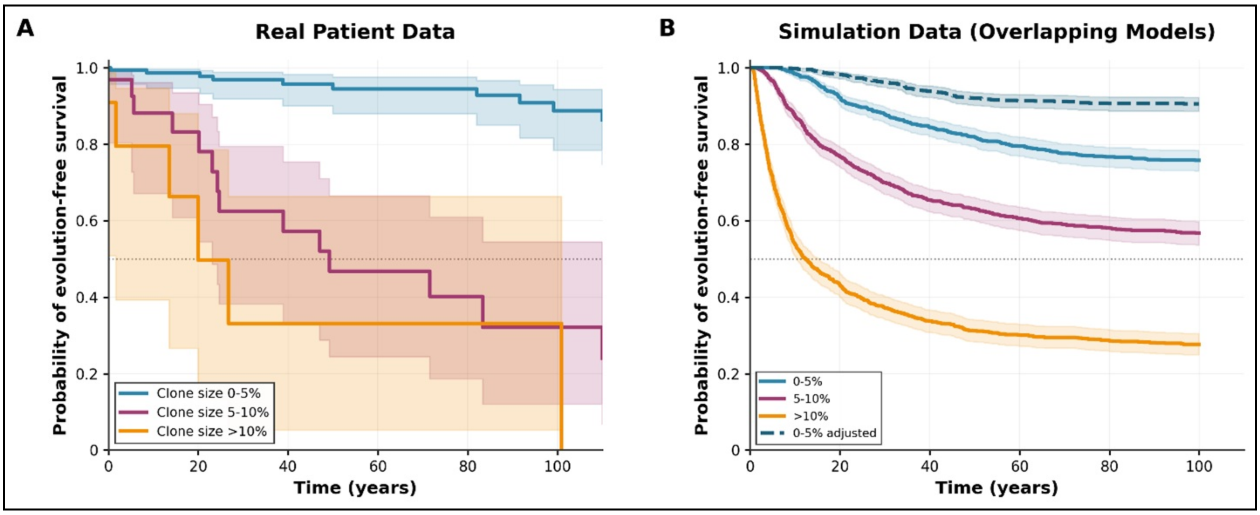

Figure 1: (A) Kaplan-Meier curves showing probability of PNH evolution-free survival stratified by clone size category in patients (n=207). (B) Simulated PNH cellular automata model output showing evolution-free survival trajectories across clone size categories. Shaded regions and error bands represent 95% confidence intervals.

**Copyright Information:** (Online) ISSN: 2572-9241
©2026 The Author(s). HemaSphere is published by John Wiley & Sons Ltd on behalf of European Hematology Association. This is an open access Abstract Book distributed under the Attribution-NonCommercial-NoDerivs (CC BY-NC-ND), which allows third parties to download the articles and share them with others as long as they credit the author and the Abstract Book, but they cannot change the content in any way or use them commercially.
**Abstract Book Citations:** Authors, Title, HemaSphere, 2026;10:(S1):pages. The abstract book DOIs can be found on the "Author Information" page.
**Disclaimer:** Articles published in the journal HemaSphere exclusively reflect the opinions of the authors. The authors are responsible for all content in their abstracts including accuracy of the facts, statements, citing resources, etc.

### (PF681) JDP2-AS1 DRIVES STAT5B-DEPENDENT HDAC11 TRANSCRIPTION TO PROMOTE CD8+ T-CELL HYPERACTIVATION IN APLASTIC ANEMIA

**Topic:** 11. Bone marrow failure syndromes incl. PNH - Biology & translational research

Ling Deng*[1], Xiaorui Meng[1], Rong Fu[1]

[1] *Tianjin Medical University General Hospital, Tianjin, China;*

### Background

Acquired aplastic anemia (AA) is an immune-mediated bone marrow failure syndrome characterized by pathogenic CD8+ T-cell activation. While noncoding regulatory networks are increasingly recognized in autoimmunity, the mechanistic role of long noncoding RNAs (lncRNAs) in AA remains largely undefined.

### Aims

To define the functional and mechanistic role of histone acetylation–associated lncRNA, JDP2-AS1, in CD8+ T-cell–mediated immunopathology in AA.

### Methods

Transcriptomic profiling identified histone acetylation–associated lncRNAs in CD8+ T cells. JDP2-AS1 expression was validated by qRT-PCR. Functional consequences were assessed using gain- and loss-of-function approaches with flow cytometric and cytokine analyses. RNA pull-down and RIP assays identified interacting proteins. RNA-seq, Cut&Tag, ChIP, and luciferase reporter assays delineated downstream regulatory mechanisms. Direct STAT5B binding to the HDAC11 promoter was confirmed by ChIP. Rescue experiments validated pathway dependency. An immune-mediated AA mouse model was used for in vivo therapeutic evaluation.

### Results

JDP2-AS1 was markedly upregulated in AA CD8+ T cells and correlated with disease severity. Proinflammatory cytokines IFN-γ and TNF-α further induced JDP2-AS1 expression, establishing a feed-forward inflammatory circuit. Functionally, JDP2-AS1 enhanced CD8+ T-cell proliferation and effector cytokine production. Mechanistically, JDP2-AS1 potentiated STAT5B transcriptional activity, leading to direct activation of HDAC11. ChIP confirmed STAT5B occupancy at the HDAC11 promoter, and rescue experiments demonstrated that HDAC11 is required for JDP2-AS1–driven T-cell hyperactivation. Pharmacologic inhibition of HDAC11 significantly restored bone marrow cellularity and corrected T-cell subset imbalance in AA mice.

### Summary/Conclusion

We identify JDP2-AS1 as a previously unrecognized epigenetic driver of pathogenic CD8+ T-cell activation in AA through a STAT5B/HDAC11 axis. Targeting HDAC11 represents a rational and therapeutically actionable strategy for immune-mediated bone marrow failure.

**Copyright Information:** (Online) ISSN: 2572-9241
©2026 The Author(s). HemaSphere is published by John Wiley & Sons Ltd on behalf of European Hematology Association. This is an open access Abstract Book distributed under the Attribution-NonCommercial-NoDerivs (CC BY-NC-ND), which allows third parties to download the articles and share them with others as long as they credit the author and the Abstract Book, but they cannot change the content in any way or use them commercially.
**Abstract Book Citations:** Authors, Title, HemaSphere, 2026;10:(S1):pages. The abstract book DOIs can be found on the “Author Information” page.
**Disclaimer:** Articles published in the journal HemaSphere exclusively reflect the opinions of the authors. The authors are responsible for all content in their abstracts including accuracy of the facts, statements, citing resources, etc.

### (PF682) EARLY INFLAMMATORY REPROGRAMMING OF PRIMITIVE HEMATOPOIETIC STEM CELLS DRIVES BONE MARROW FAILURE IN ADENOSINE DEAMINASE 2 DEFICIENCY

**Topic:** 11. Bone marrow failure syndromes incl. PNH - Biology & translational research

Chiara Rigamonti*[1, 2], Guido Pacini[1], Dimitri Bulté[1], Federica Barzaghi[1, 3], Luca Basso-Ricci[1], Emanuela Pettinato[1], Camilla Visconti[3], Pamela Quaranta[1], Michela Lupia[4], Maurizio Miano[4], Silvia Federici[5], Antonella Insalaco[5], Fabrizio de Benedetti[5], Francesca Conti[6], Roberta Caorsi[7], Marco Gattorno[7], Carlo Dufour[4], Serena Scala[1], Alessandro Aiuti[1, 2, 3], Alessandra Mortellaro[1]

*[1]San Raffaele Telethon Institute for Gene Therapy (SR-Tiget), IRCCS San Raffaele Scientific Institute, Milan, Italy;[2]Vita-Salute San Raffaele University, Milan, Italy; [3]Pediatric Immunohematology and Bone Marrow Transplantation Unit, IRCCS San Raffaele Scientific Institute, Milan, Italy; [4]Hematology Unit, IRCCS Istituto Giannina Gaslini, Genoa, Italy; [5]Division of Rheumatology, Bambino Gesù Children's Hospital, Rome, Italy; [6]Pediatric Unit, IRCCS Azienda Ospedaliero-Universitaria di Bologna, Bologna, Italy; [7]UOC Rheumatology and Autoinflammatory Diseases, IRCCS Istituto Giannina Gaslini, Genoa, Italy;*

### Background

Deficiency of adenosine deaminase 2 (DADA2) is a severe autoinflammatory disorder caused by loss-of-function mutations in ADA2. Beyond systemic vasculopathy and immunodeficiency, bone marrow (BM) failure represents a major cause of morbidity and mortality. Although DADA2 is marked by systemic inflammation, it remains unclear whether HSPC dysfunction is a consequence of inflammatory stress or originates from an early, cell-intrinsic defect at the level of primitive stem cells.

### Aims

To define the cellular and molecular basis of BM failure in DADA2, with particular emphasis on the primitive HSPC compartment and inflammation-driven stem cell reprograming.

### Methods

BM samples from pediatric and adult DADA2 patients were analyzed by multidimensional flow cytometry and functional clonogenic assays. Single-cell RNA sequencing (scRNA-seq) was conducted on $CD34^+$ HSPCs to reconstruct transcriptional alterations across the hematopoietic hierarchy and identify pathway-level perturbations.

### Results

We identified a marked quantitative and functional impairment of primitive HSCs and multipotent progenitors, including reduced clonogenic output and defective multilineage differentiation. Strikingly, these abnormalities were detectable even in clinically stable or asymptomatic individuals, indicating subclinical stem cell compromise. ScRNA-seq profiling revealed pervasive inflammatory reprogramming across all HSPC subsets, with enrichment of type I and type II IFN-response pathways. Primitive HSC-enriched clusters exhibited signatures consistent with loss of quiescence and skewed differentiation trajectories, suggesting that chronic inflammatory sensing reshapes stem cell fate decisions at the apex of hematopoiesis. Together, these data indicate that ADA2 deficiency drives early, stem cell–intrinsic dysfunction rather than solely secondary damage from peripheral inflammation.

**Copyright Information:** (Online) ISSN: 2572-9241
©2026 The Author(s). HemaSphere is published by John Wiley & Sons Ltd on behalf of European Hematology Association. This is an open access Abstract Book distributed under the Attribution-NonCommercial-NoDerivs (CC BY-NC-ND), which allows third parties to download the articles and share them with others as long as they credit the author and the Abstract Book, but they cannot change the content in any way or use them commercially.
**Abstract Book Citations:** Authors, Title, HemaSphere, 2026;10:(S1):pages. The abstract book DOIs can be found on the “Author Information” page.
**Disclaimer:** Articles published in the journal HemaSphere exclusively reflect the opinions of the authors. The authors are responsible for all content in their abstracts including accuracy of the facts, statements, citing resources, etc.

**Summary/Conclusion**

Our study provides the first single-cell resolution map of hematopoietic disruption in DADA2 and demonstrates that BM failure originates from early inflammatory reprogramming of primitive HSPCs. These findings position ADA2 as a key regulator of inflammatory–metabolic balance in human stem cells and identify DADA2 as a paradigm of inflammation-driven stem cell failure.

**Copyright Information:** (Online) ISSN: 2572-9241
©2026 The Author(s). HemaSphere is published by John Wiley & Sons Ltd on behalf of European Hematology Association. This is an open access Abstract Book distributed under the Attribution-NonCommercial-NoDerivs (CC BY-NC-ND), which allows third parties to download the articles and share them with others as long as they credit the author and the Abstract Book, but they cannot change the content in any way or use them commercially.
**Abstract Book Citations:** Authors, Title, HemaSphere, 2026;10:(S1):pages. The abstract book DOIs can be found on the "Author Information" page.
**Disclaimer:** Articles published in the journal HemaSphere exclusively reflect the opinions of the authors. The authors are responsible for all content in their abstracts including accuracy of the facts, statements, citing resources, etc.

### (PF683) UMBILICAL CORD MESENCHYMAL STEM CELLS IN COMBINATION WITH FIRST-LINE IMMUNOSUPPRESSIVE THERAPY FOR SEVERE APLASTIC ANEMIA: IMPACT ON EARLY INFECTIOUS COMPLICATIONS AND HEMATOLOGIC RESPONSE DEPTH

**Topic:** 11. Bone marrow failure syndromes incl. PNH - Biology & translational research

Jingnan Sun*[1], Jiaqi Wang[1], Guangsheng He[2], Chuan He[3], Dexiang Ji[4], Yehui Tan[1], Sujun Gao[1]

*[1]Hematology Department，The First Hospital of Jilin University, Changchun, China;[2]The First Affiliated Hospital of Nanjing Medical University,Jiangsu Province Hospital, Nanjing, China; [3]west china hospital, chengdu, China; [4]Hematology Center of the First Affiliated Hospital of Nanchang University, Nanchang, China;*

### Background

Severe aplastic anemia (SAA) is characterized by bone marrow hematopoietic failure and abnormal immune-mediated destruction of hematopoietic cells. Anti-thymocyte globulin (ATG) combined with cyclosporine A (CsA) represents the standard first-line immunosuppressive therapy (IST) for SAA; however, this regimen is still limited by high early infection risk, insufficient complete remission rate (CRR) , and suboptimal efficacy in some patients. Umbilical cord mesenchymal stem cells (UC-MSC) possess multiple biological functions including immunomodulation, anti-inflammation, and bone marrow microenvironment repair, which theoretically provide synergistic effects with IST. This study aimed to investigate the efficacy and safety of UCMSC combined with ATG plus CsA (with hetrombopag or eltrombopag) as first-line therapy in patients with SAA, and to provide evidence for optimizing therapeutic strategies for SAA.

### Methods

This was a prospective multicenter registry study (ChiCTR2100045895). Between January 1, 2022 and December 31, 2024, 15 newly diagnosed adult SAA patients from Chinese Eastern Collaboration Group of Anemia (CECGA) were enrolled in the experimental group, receiving ATG and CsA combined with hetrombopag or eltrombopag, plus additional UCMSC infusion (initiated within 72 hours after ATG treatment, dose: $1\times10^6$/kg, once weekly for a total of 3~4 doses). The control group consisted of 15 contemporaneously matched patients from  CECGA database, receiving the same standard regimen of ATG plus CsA combined with hetrombopag or eltrombopag without UC-MSC. Hetrombopag 15 mg/d or eltrobopag 75mg mg/d was administered  for at least 6 months. ATG was administered intravenously for 5 consecutive days, and CsA 3~5mg/kg/d was given for at least 12 months according to plasma concentration.

### Results

Baseline characteristics were comparable between two groups (Figure 1). At 1 month post-ATG, the infection rate in the experimental group was numerically lower than that in the control group (6.7% vs. 20%, P = 0.605). At 3 months, overall response rate (ORR) was similar between groups (46.7% vs. 40.0%, P = 1.000). At 6 months, ORR remained comparable (73.3% vs. 66.7%, P = 1.000), while CRR in the experimental group was numerically higher (33.3% vs. 26.7%, P = 1.000), without statistical significance. The experimental group had a lower early infection rate (1/15 vs. 3/15) with favorable tolerability, and no severe UC-MSC related adverse events were observed. With a median follow-up of 23.1 months, overall survival and progression-free survival were comparable between groups. Mechanistic analysis at 3 months demonstrated that the proportion and absolute count of peripheral Treg cells were increased and IFN-γ level was decreased in the experimental group, suggesting that UC-MSC could ameliorate immune imbalance and attenuate inflammatory response.

**Copyright Information:** (Online) ISSN: 2572-9241
©2026 The Author(s). HemaSphere is published by John Wiley & Sons Ltd on behalf of European Hematology Association. This is an open access Abstract Book distributed under the Attribution-NonCommercial-NoDerivs (CC BY-NC-ND), which allows third parties to download the articles and share them with others as long as they credit the author and the Abstract Book, but they cannot change the content in any way or use them commercially.
**Abstract Book Citations:** Authors, Title, HemaSphere, 2026;10:(S1):pages. The abstract book DOIs can be found on the "Author Information" page.
**Disclaimer:** Articles published in the journal HemaSphere exclusively reflect the opinions of the authors. The authors are responsible for all content in their abstracts including accuracy of the facts, statements, citing resources, etc.

### Summary/Conclusion

UC-MSC combined with standard ATG plus CsA (with hetrombopag or eltrombopag) as first-line treatment for SAA is safe and feasible. This combined strategy can significantly reduce early infection risk and shows a trend toward improved long-term complete remission rate. The mechanism may be related to the up-regulation of Treg cells, inhibition of IFN-γ-mediated abnormal inflammatory immune response, and remodeling of the bone marrow immune microenvironment. UC-MSC is expected to become a valuable combined modality with standard immunosuppressive therapy for SAA, and larger sample-sized studies are warranted to confirm these findings.

| | ATG+CsA+TPO-RA （n=15) | UC-MSC +ATG+CsA+TPO-RA (n=15) | P |
|---|---|---|---|
| Age,years,range | 45(21,69) | 47（26,71) | 0.458 |
| Severity of aplasia anemia,% | | | |
| SAA | 9(60.0) | 10(67.7) | 0.615 |
| vSAA | 5(40.0) | 5(32.3) | |
| PNH clone,% | 5(33.3) | 5(33.3) | 1.000 |
| CD3,%,IQR | 72.42(68.09,85.50) | 74.15(67.34,82.58) | 0.452 |
| CD4,%,IQR | 46.05(41.20,58.70) | 43.00(34.85,53.80) | 0.592 |
| CD8,%,IQR | 26.35(21.20,33.50) | 28.50(18.55,35.20) | 0.695 |
| Treg,%,IQR | 6.60(5.87,8.95) | 8.05(4.28,9.20) | 0.368 |
| ANC,×109/L,IQR | 0.32（0.14,0.44) | 0.36(0.10,0.62) | 0.455 |
| ALC,×109/L,IQR | 1.30（0.59,1.65) | 1.17(0.66,1.49) | 0.933 |
| HB ,g/L,IQR | 63.00(53.50,69.80) | 61.30(54.60,71.20) | 0.789 |
| RDW-CV,IQR | 15.60(13.70,18.95) | 14.20(11.60,17.65) | 0.765 |
| PLT ,×109/L,IQR | 8.00(5.00,17.50) | 9.00(2.00,11.50) | 0.331 |
| Ret ,×109/L,IQR | 52.00(12.56,128.5) | 36.52(42.6,101.6) | 0.621 |
| Fer ,ng/ml,IQR | 780.50(424.2,1390.8) | 638.50(353.2,1505.12) | 0.235 |
| Infection before IST,%, | 5(33.3) | 6(40.0) | 0.619 |

Figure1. Baseline characteristics of patients between two groups

Copyright Information: (Online) ISSN: 2572-9241
©2026 The Author(s). HemaSphere is published by John Wiley & Sons Ltd on behalf of European Hematology Association. This is an open access Abstract Book distributed under the Attribution-NonCommercial-NoDerivs (CC BY-NC-ND), which allows third parties to download the articles and share them with others as long as they credit the author and the Abstract Book, but they cannot change the content in any way or use them commercially.
Abstract Book Citations: Authors, Title, HemaSphere, 2026;10:(S1):pages. The abstract book DOIs can be found on the “Author Information” page.
Disclaimer: Articles published in the journal HemaSphere exclusively reflect the opinions of the authors. The authors are responsible for all content in their abstracts including accuracy of the facts, statements, citing resources, etc.

### (PF684) PREVALENCE AND CLINICAL IMPACT OF CLONAL HEMATOPOIESIS IN PAROXYSMAL NOCTURNAL HEMOGLOBINURIA: A MULTICENTER STUDY.

**Topic:** 11. Bone marrow failure syndromes incl. PNH - Biology & translational research

Nicole Galli*[1], Marta Lionetti[2, 3], Elisabetta Metafuni[4], Francesco Versino[3], Giacinto Pedone[3, 5], Anna Paola Iori[6], Eloise Beggiato[7], Simona Sica[4], Mara Memoli[8], Loredana Pettine[3], Shreyans Gandhi[9], Akihiro Maeda[3], Marta Bortolotti[3], Paola Bianchi[3], Elisa Fermo[3], Alessio Marella[2], Patrizia Chiusolo[4], Roochi Trikha[9], Joanna Large[9], Deborah Kasmi[6], Ilaria Bersano[7], Niccolò Bolli[2, 3], Wilma Barcellini[3], Francesco Passamonti[2, 3], Austin G. Kulasekararaj[9], Bruno Fattizzo[2, 3]

*[1]U.O.C. di Ematologia e Trapianto di Cellule Staminali Emopoietiche, Ospedale di Legnano - ASST Ovest Milanese, Legnano, Italy; [2]University of Milan, Department of Oncology and Hematology-Oncology, Milan, Italy; [3]Hematology Unit, Foundation IRCCS Ca' Granda Ospedale Maggiore Policlinico, Milan, Italy; [4]Fondazione Policlinico Universitario Agostino Gemelli IRCCS, Rome, Italy; [5]University of Milan, Department of Pathophysiology and Transplantation, Milan, Italy; [6]AOU Policlinico Umberto I, Università La Sapienza, Department of Hematology, Rome, Italy; [7]A.O.U. Città della Salute e della Scienza di Torino, Department of Oncology, Division of Hematology, Torino, Italy; [8]Ematologia dell'Azienda Ospedaliera Universitaria Federico II di Napoli, Napoli, Italy; [9]King's College Hospital, National Institute for Health Research and Wellcome King's Clinical Research Facility and King's College London, Department of Haematological Medicine, London, United Kingdom;*

### Background

Paroxysmal nocturnal hemoglobinuria (PNH) is an acquired clonal hematopoietic stem cell disorder caused by somatic *PIGA* mutations, leading to complement-mediated hemolysis. Although immune selection plays a central role in PNH pathogenesis—particularly in patients with associated aplastic anemia (AA)—the mechanisms underlying clonal expansion and interpatient heterogeneity remain incompletely understood. Recent advances in next-generation sequencing (NGS) have identified clonal hematopoiesis (CH) as a potential modifier of disease evolution in immune-mediated bone marrow failure syndromes.

### Aims

We aimed to assess the prevalence and clinical relevance of CH in PNH, together with a detailed analysis of PIGA mutational profiles and clinical correlates.

### Methods

In this retrospective multicenter study, 108 patients with confirmed PNH underwent myeloid NGS on peripheral blood granulocytes using a targeted panel of genes associated with CH and myeloid neoplasms. CH was defined as somatic mutations with variant allele frequency (VAF) ≥2%, high-risk CH was classified according to previous published studies (Stonestrom et al.; and Weeks et al.). Targeted deep sequencing of *PIGA* was performed in a subgroup (n=41) to characterize mutational burden and clonal complexity. Laboratory and clinical data included flow cytometry clone size, hemolytic markers, thrombosis, treatment lines and responses, and breakthrough hemolysis. Response to complement inhibitor was assessed using European Bone Marrow Transplant Group criteria.

**Copyright Information:** (Online) ISSN: 2572-9241
©2026 The Author(s). HemaSphere is published by John Wiley & Sons Ltd on behalf of European Hematology Association. This is an open access Abstract Book distributed under the Attribution-NonCommercial-NoDerivs (CC BY-NC-ND), which allows third parties to download the articles and share them with others as long as they credit the author and the Abstract Book, but they cannot change the content in any way or use them commercially.
**Abstract Book Citations:** Authors, Title, HemaSphere, 2026;10:(S1):pages. The abstract book DOIs can be found on the “Author Information” page.
**Disclaimer:** Articles published in the journal HemaSphere exclusively reflect the opinions of the authors. The authors are responsible for all content in their abstracts including accuracy of the facts, statements, citing resources, etc.

**Results**

CH was identified in 28/108 patients (26%) and was significantly associated with older age (median 54 vs 35 years; p=0.008). The most frequently mutated genes were *ASXL1* (n=15), *TET2* (n=6), U2AF1 (n=3) and *DNMT3A* (n=3), as shown in Figure 1. CH was more common in classical hemolytic PNH and was associated with a significantly larger granulocyte (median 77% vs 68.5%, p=0.15) and monocyte PNH clone sizes (74.5% vs 67%, p=0.08). In contrast CH was less frequent in AA-associated PNH (15% vs 29% in classical PNH). Thrombosis occurred in 26% of patients and was not associated with NGS positivity, age, or sex. CH did not significantly affect response to C5 inhibitors, however, complete response to pegcetacoplan was lower in NGS-positive vs NGS-negative patients (1/4, 25% vs 6/7, 86%; p=0.03). BTH occurred in 37/80 (46%) and was unrelated to CH (27% vs 30%; p=0.81). Deep PIGA sequencing identified 70 variants in 34/41 tested patients (median VAF 9.6%, range 2–70%); multiple PIGA mutations were common and tended to associate with lower hemoglobin and higher LDH, and truncating variants were enriched among patients requiring two or more therapy lines (9/15, 60% vs 8/19, 42%).

**Summary/Conclusion**

CH is present in approximately one quarter of PNH patients and, more frequently in classical hemolytic PNH, being associated with larger clone size. NGS positivity was associated with lower rate of complete response to proximal complement inhibition in a small pegcetacoplan-treated subset, but not with impaired response to anti-C5. These findings support CH as a potential contributor to clonal dominance in PNH, particularly in classical hemolytic disease. Integration of flow cytometry, myeloid NGS, and deep *PIGA* profiling may improve biological stratification, therapeutic selection, and longitudinal monitoring.

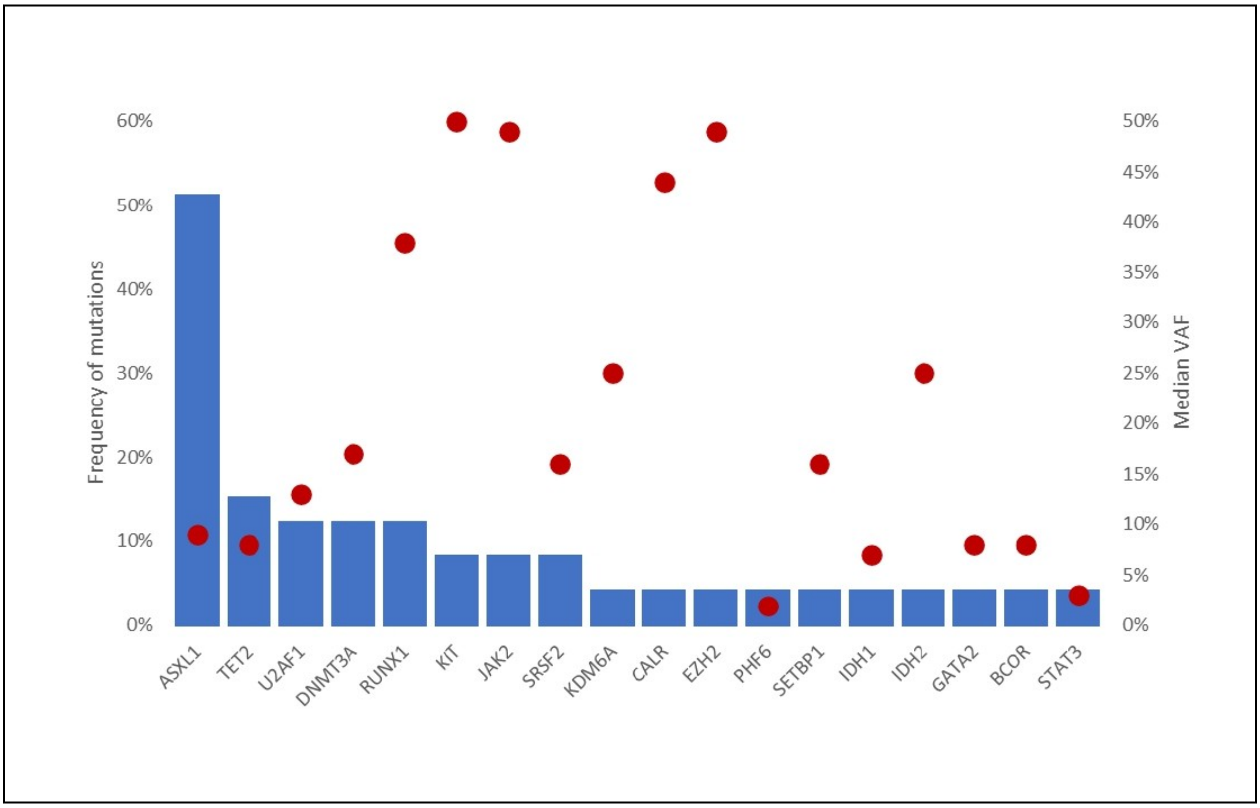

Figure 1. Mutational landscape

**Copyright Information:** (Online) ISSN: 2572-9241
©2026 The Author(s). HemaSphere is published by John Wiley & Sons Ltd on behalf of European Hematology Association. This is an open access Abstract Book distributed under the Attribution-NonCommercial-NoDerivs (CC BY-NC-ND), which allows third parties to download the articles and share them with others as long as they credit the author and the Abstract Book, but they cannot change the content in any way or use them commercially.
**Abstract Book Citations:** Authors, Title, HemaSphere, 2026;10:(S1):pages. The abstract book DOIs can be found on the "Author Information" page.
**Disclaimer:** Articles published in the journal HemaSphere exclusively reflect the opinions of the authors. The authors are responsible for all content in their abstracts including accuracy of the facts, statements, citing resources, etc.

### (PF685) RESTORATION OF RPS19 EXPRESSION IN HUMAN CELLULAR MODELS OF DIAMOND-BLACKFAN ANAEMIA SYNDROME

**Topic:** 11. Bone marrow failure syndromes incl. PNH - Biology & translational research

Joan Case*[1], Pierangela Sabbattini[1], Chrysi Christoduoliduo[1], Josu de la Fuente[1, 2], Irene Roberts[3], Anastasios Karadimitris[1, 4], Deena Iskander[1, 2]

*[1]Imperial College London, Immunology & Inflammation, Centre for Haematology, London, United Kingdom;[2]Imperial College Healthcare Trust, Department of Paediatric Haematology, St Mary's Hospital, London, United Kingdom; [3]University of Oxford, Department of Paediatrics, Oxford, United Kingdom; [4]Imperial College Healthcare Trust, Department of Haematology, Hammersmith Hospital, London, United Kingdom;*

### Background

Diamond Blackfan anaemia syndrome (DBAS) is a rare inherited bone marrow failure syndrome characterised by a severe macrocytic anaemia in childhood, variable congenital abnormalities, and a 5-10-fold elevated risk of malignancies. DBAS is caused by *de novo* or inherited mono-allelic variants in the genes encoding the large and small ribosomal subunits, alongside a select number of ribosomal protein (RP) chaperones. Pathogenic variants are overwhelmingly gene-inactivating, leading to haploinsufficiency and a 50% reduction in functional mRNA. In human and animal models of DBAS, RP haploinsufficiency leads to the loss of 80S ribosomes, aberrant ribosomal RNA (rRNA) processing, and p53 activation due to perturbed free RP stoichiometry. Therapeutic options for DBAS are limited to lifelong blood transfusions, glucocorticoids, or allogeneic stem cell transplantation. Lentiviral gene therapy efforts seek to restore *RSP19* expression by gene transfer or to enhance erythroid commitment by overexpression of the transcription factor GATA-1 in a patient's own haematopoietic stem and progenitor cells (HSPCs). However, more precise manipulation of gene expression may be required to maintain stoichiometry of free RPs, and there is a need to develop alternative gene editing approaches with the potential to correct haploinsufficiency in all tissues.

### Aims

To restore *RPS19* gene expression in haploinsufficient cells by enhancing the mRNA output of the unmutated allele.

### Methods

We generated and characterised monoallelic *RPS19*$^{+/-}$ clones of the erythroid cell line K562 by CRISPR/Cas9 gene editing. To provide proof-of-principle of activating CRISPR (CRISPRa) gene therapy technology, we used lentiviral delivery of the dCas9-VPR/sgRNA construct to enhance transcription of *RPS19*.

**Copyright Information:** (Online) ISSN: 2572-9241
©2026 The Author(s). HemaSphere is published by John Wiley & Sons Ltd on behalf of European Hematology Association. This is an open access Abstract Book distributed under the Attribution-NonCommercial-NoDerivs (CC BY-NC-ND), which allows third parties to download the articles and share them with others as long as they credit the author and the Abstract Book, but they cannot change the content in any way or use them commercially.
**Abstract Book Citations:** Authors, Title, HemaSphere, 2026;10:(S1):pages. The abstract book DOIs can be found on the "Author Information" page.
**Disclaimer:** Articles published in the journal HemaSphere exclusively reflect the opinions of the authors. The authors are responsible for all content in their abstracts including accuracy of the facts, statements, citing resources, etc.

**Results**

We confirmed that *RPS19* expression was reduced to ~50% in *RPS19*$^{+/-}$ clones relative to their unedited counterparts, thus recapitulating haploinsufficiency in DBAS. In RNA-sequencing analysis of *RPS19*$^{+/-}$ knock-out (KO) versus unedited cells (n=2), we observed a significant upregulation of pro-inflammatory and proliferative gene pathways, consistent with findings in DBAS patient bone marrow. Counterintuitively, in puromycin translation assays, *RPS19*$^{+/-}$ KOs maintained translation levels under serum starvation, which was reduced in unedited cells. *RPS19*$^{+/-}$ KOs did not demonstrate any proliferative abnormalities. Furthermore, liquid chromatography mass spectrometry analysis revealed an increase in several tRNA synthetase enzymes in *RPS19*$^{+/-}$ KOs versus unedited controls (n=2). These data suggest a compensatory mechanism of enhanced translation in response to RP haploinsufficiency in a human cellular model of DBAS. Screening of the core *RPS19* promoter with single-guide RNAs (sgRNA) and a dCas9-VPR construct revealed a short ~25 base pair genomic region that, on activation, induced a significant ~1.7-fold increase in *RPS19* expression compared to non-transduced controls (Fig. 1)(n=2).

**Summary/Conclusion**

We provide proof-of-principle that interference at the *RPS19* promoter using CRISPRa technology can enhance transcriptional output and is a potential therapeutic approach in the treatment of haploinsufficient diseases such as DBAS. Ongoing work will test *RPS19* activation in primary DBAS HSPCs and its rescue of the DBAS phenotype.

**Copyright Information:** (Online) ISSN: 2572-9241
©2026 The Author(s). HemaSphere is published by John Wiley & Sons Ltd on behalf of European Hematology Association. This is an open access Abstract Book distributed under the Attribution-NonCommercial-NoDerivs (CC BY-NC-ND), which allows third parties to download the articles and share them with others as long as they credit the author and the Abstract Book, but they cannot change the content in any way or use them commercially.
**Abstract Book Citations:** Authors, Title, HemaSphere, 2026;10:(S1):pages. The abstract book DOIs can be found on the "Author Information" page.
**Disclaimer:** Articles published in the journal HemaSphere exclusively reflect the opinions of the authors. The authors are responsible for all content in their abstracts including accuracy of the facts, statements, citing resources, etc.

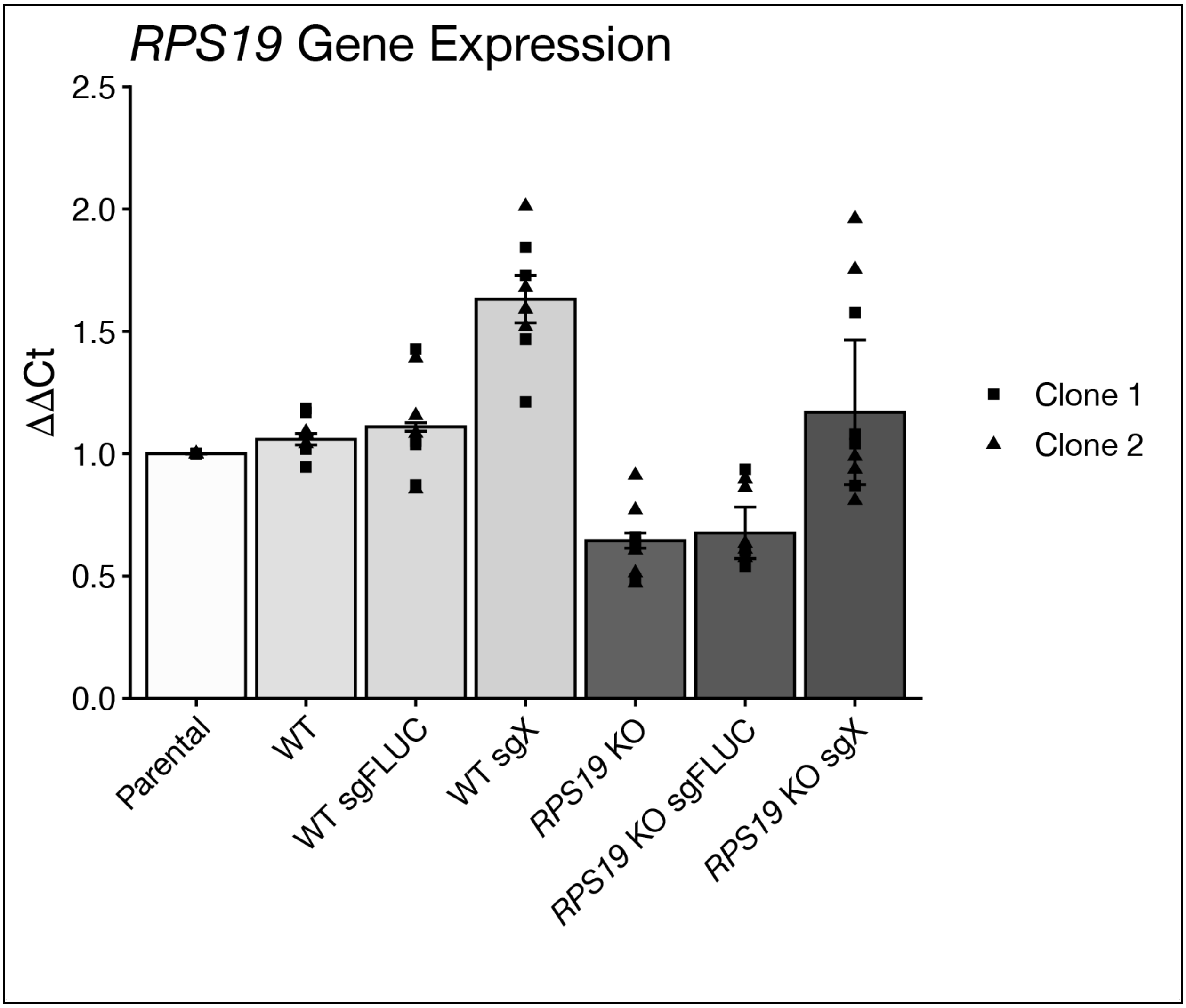

Figure 1: RPS19 expression in CRISPRa activated WT and RPS19+/- KO K562 cells: Average expression of RPS19 mRNA measured by RT-qPCR of parental, unedited (WT), and RPS19+/- KO K562 cell lines (mean ±SD, n=2) transduced with lentiviral dCas9-VPR and sgRNA vector for RPS19 core promoter (sgX) genomic targets or firefly luciferase (FLUC) negative control.

**Copyright Information:** (Online) ISSN: 2572-9241
©2026 The Author(s). HemaSphere is published by John Wiley & Sons Ltd on behalf of European Hematology Association. This is an open access Abstract Book distributed under the Attribution-NonCommercial-NoDerivs (CC BY-NC-ND), which allows third parties to download the articles and share them with others as long as they credit the author and the Abstract Book, but they cannot change the content in any way or use them commercially.
**Abstract Book Citations:** Authors, Title, HemaSphere, 2026;10:(S1):pages. The abstract book DOIs can be found on the "Author Information" page.
**Disclaimer:** Articles published in the journal HemaSphere exclusively reflect the opinions of the authors. The authors are responsible for all content in their abstracts including accuracy of the facts, statements, citing resources, etc.

### (PF686) THE CROSSROADS BETWEEN BONE MARROW STEM CELL DEFECTS AND IMMUNE DYSREGULATION IN ADENOSINE DEAMINASE 2 DEFICIENCY: IMPLICATIONS FOR MONITORING AND HEMATOPOIETIC STEM CELL TRANSPLANTATION

**Topic:** 11. Bone marrow failure syndromes incl. PNH - Biology & translational research

Federica Barzaghi*[1, 2], Camilla Visconti[2, 3], Dimitri Bulté[1], Chiara Rigamonti[1], Valeria Calbi[2], Maurilio Ponzoni[3, 4], Lucia Bongiovanni[3, 4], Raffaella Milani[5], Emanuela Pettinato[1], Francesca Conti[6, 7], Giulia Di Colo[3, 8], Federica Pulvirenti[9], Marco Gattorno[10], Fabio Ciceri[11], Alessandro Aiuti[1, 2, 3], Alessandra Mortellaro[1]

*[1]IRCCS San Raffaele Scientific Institute, San Raffaele Telethon Institute for Gene Therapy (SR-Tiget), Milan, Italy;[2]IRCCS San Raffaele Scientific Institute, Pediatric Immunohematology and Bone Marrow Transplantation Unit, Milan, Italy; [3]Università Vita-Salute San Raffaele, Milan, Italy; [4]IRCCS San Raffaele Scientific Institute, Pathology Unit, Milan, Italy; [5]IRCCS San Raffaele Scientific Institute, Immunohematology and Transfusion Medicine Unit, Milan, Italy; [6]IRCCS Azienda Ospedaliero-Universitaria di Bologna, Pediatric Unit, Bologna, Italy; [7]University of Bologna, Department of Medical and Surgical Sciences (DIMEC), Bologna, Italy; [8]IRCCS San Raffaele Scientific Hospital, Immunology, Rheumatology, Allergy and Rare Disease Unit, Milan, Italy; [9]Sapienza University Hospital Policlinico Umberto I, Reference Centre for Primary Immune Deficiencies, Rome, Italy; [10]IRCCS Istituto Giannina Gaslini, UOC Rheumatology and Autoinflammatory diseases, Genoa, Italy; [11]IRCCS San Raffaele Scientific Institute, Hematology and Bone Marrow Transplantation Unit, Milan, Italy;*

### Background

Deficiency of adenosine deaminase 2 (DADA2) is a monogenic inborn error of immunity caused by loss-of-function mutations in the *ADA2* gene. DADA2 shows a wide clinical spectrum, including systemic vasculitis, immunodeficiency, and hematologic abnormalities: from mild cytopenia to acute severe autoimmune phenomena and bone marrow (BM) failure.

### Aims

This study aims to define the hematologic and BM phenotype of DADA2 through the analysis of a well-characterized clinical cohort, with the goal of informing follow-up strategies and therapeutic decisions.

### Methods

We report a detailed characterization of 11 patients from a single-center cohort who underwent comprehensive peripheral blood (PB) and BM evaluation. Clinical, laboratory, and immunological data were integrated with BM analyses to examine the links between BM alterations, cytopenias, immune dysregulation, and outcomes, using serial samples when available to track disease progression.

**Copyright Information:** (Online) ISSN: 2572-9241
©2026 The Author(s). HemaSphere is published by John Wiley & Sons Ltd on behalf of European Hematology Association. This is an open access Abstract Book distributed under the Attribution-NonCommercial-NoDerivs (CC BY-NC-ND), which allows third parties to download the articles and share them with others as long as they credit the author and the Abstract Book, but they cannot change the content in any way or use them commercially.
**Abstract Book Citations:** Authors, Title, HemaSphere, 2026;10:(S1):pages. The abstract book DOIs can be found on the "Author Information" page.
**Disclaimer:** Articles published in the journal HemaSphere exclusively reflect the opinions of the authors. The authors are responsible for all content in their abstracts including accuracy of the facts, statements, citing resources, etc.

**Results**

DADA2 patients exhibited a significant decrease in BM hematopoietic stem and progenitor cells (HSPCs) numbers, and the remaining HSPCs were functionally compromised compared to age-matched healthy controls. These BM abnormalities were identified in all 11 patients, including children and patients lacking evident hematological manifestations.
Histology was performed on BM biopsies from 9 subjects: 3 patients with non-severe hematological phenotypes, mainly experiencing auto-inflammatory manifestations, 1 patient with severe acute haemolytic anemia and 5 patients with severe cytopenias, candidates for HSCT. Regardless of the hematological severity, all BM biopsies showed evidence of dyshematopoiesis. Patients with non-severe hematological phenotype exhibited dyshematopoiesis with BM hypoplasia and prevalence of mature precursors. The 5 patients with a severe neutropenia presented hypercellular BM, primarily due to a marked increase in T lymphocytes consistent with T-cell large granular lymphocytes. Notably, they displayed a progressive expansion of CD8+ memory T cells, predominantly effector memory T cells re-expressing CD45RA+ (TEMRA), in both PB and BM over the course of the disease, suggesting a potential role for these cells in the advanced stages of BM damage. Two patients with severe acute hemolytic anemia, despite responding to immunosuppressive therapy, showed expansion of T effector memory (TEM) cells on BM flow cytometry, potentially contributing to intramedullary hemolysis. Concomitantly, they also exhibited a reduction or exhaustion of HSPCs, including the most primitive HSCs, likely reflecting sustained erythroid skewing and progressive depletion of an already limited stem cell reservoir.

**Summary/Conclusion**

DADA2 patients reveal significant BM defects, even in clinically silent cases, underscoring the need for close surveillance. PB abnormalities may be late and acute, thus do not identify patients at risk for severe hematological complications: early diagnosis and hematological evaluation can guide follow-up and treatment. Moreover, patients with BM failure syndromes should be tested for DADA2, as appropriate immunosuppression before hematopoietic stem cell transplantation (HSCT) may reduce complications and improve engraftment. Finally, DADA2 patients with acute autoimmune cytopenias deserve close follow-up, since HSCT can have a role not only in severe end-stage BM failure but also in the setting of acute autoimmune phenomena not-compensated by appropriate renewal of HSPCs, with high risk of life-threatening relapses.

**Copyright Information:** (Online) ISSN: 2572-9241
©2026 The Author(s). HemaSphere is published by John Wiley & Sons Ltd on behalf of European Hematology Association. This is an open access Abstract Book distributed under the Attribution-NonCommercial-NoDerivs (CC BY-NC-ND), which allows third parties to download the articles and share them with others as long as they credit the author and the Abstract Book, but they cannot change the content in any way or use them commercially.
**Abstract Book Citations:** Authors, Title, HemaSphere, 2026;10:(S1):pages. The abstract book DOIs can be found on the "Author Information" page.
**Disclaimer:** Articles published in the journal HemaSphere exclusively reflect the opinions of the authors. The authors are responsible for all content in their abstracts including accuracy of the facts, statements, citing resources, etc.

### (PF687) EFFICACY AND SAFETY OF HRS-5965 VERSUS ECULIZUMAB IN PATIENTS WITH PAROXYSMAL NOCTURNAL HEMOGLOBINURIA: A MULTICENTER, RANDOMIZED, OPEN-LABEL, ACTIVE-CONTROLLED PHASE 3 TRIAL

**Topic:** 12. Bone marrow failure syndromes incl. PNH - Clinical

Bing Han*[1], Li Zhang[2], Shunqing Wang[3], Hu Zhou[4], Yingmei Li[5], Rong Fu[6], Chuan He[7], Linghui Xia[8], Shengjin Fan[9], Sujun Gao[10], Yajing Xu[11], Bin Hu[12], Rong Huang[13], Fengkui Zhang[2]

*[1]Peking Union Medical Colleague Hospital, Chinese Academe of Medical Science, Beijing, China;[2]Institute of Hematology & Blood Diseases Hospital, Chinese Academy of Medical Sciences, Tianjin, China; [3]Guangzhou First People's Hospital, Guangzhou, China; [4]Henan Cancer Hospital, Zhengzhou, China; [5]The First Affiliated Hospital of Zhengzhou University, Zhengzhou, China; [6]Tianjin Medical University General Hospital, Tianjin, China;[7]West China Hospital, Chengdu, China; [8]Union Hospital, Tongji Medical College, Huazhong University of Science and Technology, Wuhan, China; [9]The First Affiliated Hospital of Harbin Medical University, Harbin, China; [10]The First Hospital of Jilin University, Changchun, China;[11]Xiangya Hospital of Central South University, Changsha, China; [12]Jiangsu Hengrui Pharmaceuticals Co., Ltd., Beijing, China; [13]Jiangsu Hengrui Pharmaceuticals Co., Ltd., Shanghai, China;*

### Background

Paroxysmal nocturnal hemoglobinuria (PNH) is a rare complement-mediated hemolytic anemia. While the C5 inhibitor eculizumab controls intravascular hemolysis, many patients (pts) remain anemic due to persistent extravascular hemolysis. HRS-5965 is a novel, oral factor B inhibitor of the alternative complement pathway designed to control both hemolytic pathways and improve anemia.

### Aims

The objective was to evaluate the efficacy and safety of HRS-5965 compared with eculizumab in pts with PNH.

### Methods

In this multicenter, randomized, open-label, active-controlled phase 3 trial, complement-inhibitor–naïve PNH pts were randomized (1:1) to oral HRS-5965 (50 mg twice daily) or intravenous eculizumab for 24 weeks. The primary endpoint was the proportion of pts achieving Hb ≥12 g/dL on at least 3 of 4 measurements between Weeks 18 and 24 without red blood cell (RBC) transfusions (after Week 2). The key secondary endpoint was the proportion achieving a ≥2 g/dL Hb increase from baseline under the same conditions.

### Results

Of the 78 randomized pts, 76 received treatment (HRS-5965, n=40; eculizumab, n=36) and were included in efficacy and safety analysis (Table 1). The proportion of pts meeting the primary endpoint was significantly higher with HRS-5965 than with eculizumab (70.0% [28/40] vs. 11.1% [4/36]; rate difference 57.4%, 95% CI: 39.6-75.2; P<0.0001). The key secondary endpoint was also significantly superior for HRS-5965 (95.0% [38/40] vs. 55.6% [20/36]; rate difference 39.2%, 95% CI: 20.9-57.4; P<0.0001). A significantly higher proportion of pts in the HRS-5965 group were transfusion-free after Week 2 (100% vs. 86.1%, P=0.0149). The mean Hb increase from baseline (Weeks 18-24) was greater with HRS-5965 (LS mean difference: 2.61 g/dL, 95% CI: 1.72-3.49; P<0.0001), and FACIT-Fatigue score improvement was superior (LS mean difference: 4.19, P=0.0001). Reticulocyte counts decreased significantly with HRS-5965 versus an increase with eculizumab (LS mean difference: -161.37 x $10^9$/L, P<0.0001). No major adverse vascular events (MAVEs) occurred. Treatment-related adverse events (TRAEs) occurred in 72.5% (HRS-5965) and 55.6% (eculizumab) of pts. Treatment-related serious AEs occurred in 2 patients (5.0%) receiving HRS-5965 (one case each of upper respiratory infection and infectious pneumonia, both moderate in severity).

**Copyright Information:** (Online) ISSN: 2572-9241
©2026 The Author(s). HemaSphere is published by John Wiley & Sons Ltd on behalf of European Hematology Association. This is an open access Abstract Book distributed under the Attribution-NonCommercial-NoDerivs (CC BY-NC-ND), which allows third parties to download the articles and share them with others as long as they credit the author and the Abstract Book, but they cannot change the content in any way or use them commercially.
**Abstract Book Citations:** Authors, Title, HemaSphere, 2026;10:(S1):pages. The abstract book DOIs can be found on the “Author Information” page.
**Disclaimer:** Articles published in the journal HemaSphere exclusively reflect the opinions of the authors. The authors are responsible for all content in their abstracts including accuracy of the facts, statements, citing resources, etc.

### Summary/Conclusion

In pts with PNH, oral HRS-5965 was superior to eculizumab in improving hemoglobin levels, reducing transfusion dependence, and enhancing quality of life, with a manageable safety profile, offering a promising new oral treatment for PNH.

**Table 1. Efficacy and Safety Outcomes**

| | **HRS-5965 (N=40)** | **Eculizumab (N=36)** | **Difference (95% CI)** | **P value** |
|---|---|---|---|---|
| **Primary endpoints** | | | | |
| Hb ≥12 g/dL#, n (%) | 28 (70.0%) | 4 (11.1%) | 57.4% (39.6-75.2) | <0.0001 |
| **Secondary endpoints** | | | | |
| Hb increase ≥2 g/dL#, *, n (%) | 38 (95.0%) | 20 (55.6%) | 39.2% (20.9-57.4) | <0.0001 |
| Transfusion-free (after Wk 2), n (%) | 40 (100%) | 31 (86.1%) | 13.7% (2.4-24.9) | 0.0149 |
| Mean Hb change from baseline#, g/dL, LS Mean (SE) | 5.47 (0.31) | 2.86 (0.32) | 2.61 (1.72-3.49) | <0.0001 |
| FACIT-Fatigue score improvement#, LS Mean (SE) | 8.62 (0.70) | 4.43 (0.74) | 4.19 (2.15-6.23) | 0.0001 |
| Reticulocyte count change from baseline#, x10$^9$/L, LS Mean (SE) | -118.97 (9.41) | 42.40 (9.93) | -161.37 (-188.62, -134.13) | <0.0001 |
| **Safety** | | | | |
| MAVEs, n (%) | 0 | 0 | — | — |
| TRAEs, n (%) | 29 (72.5%) | 20 (55.6%) | — | — |
| Treatment-related serious AEs, n (%) | 2 (5.0%) | 0 | — | — |

#Assessed over Weeks 18-24 in the absence of RBC transfusions after Week 2.
*Hb increase ≥2 g/dL was the key secondary endpoint.

**Copyright Information:** (Online) ISSN: 2572-9241
©2026 The Author(s). HemaSphere is published by John Wiley & Sons Ltd on behalf of European Hematology Association. This is an open access Abstract Book distributed under the Attribution-NonCommercial-NoDerivs (CC BY-NC-ND), which allows third parties to download the articles and share them with others as long as they credit the author and the Abstract Book, but they cannot change the content in any way or use them commercially.
**Abstract Book Citations:** Authors, Title, HemaSphere, 2026;10:(S1):pages. The abstract book DOIs can be found on the “Author Information” page.
**Disclaimer:** Articles published in the journal HemaSphere exclusively reflect the opinions of the authors. The authors are responsible for all content in their abstracts including accuracy of the facts, statements, citing resources, etc.

### (PF688) EFFICACY AND SAFETY OF HRS-5965 IN PAROXYSMAL NOCTURNAL HEMOGLOBINURIA PATIENTS WITH PERSISTENT ANEMIA DESPITE STABLE C5 INHIBITOR THERAPY: A MULTICENTER, SINGLE-ARM, OPEN-LABEL PHASE 3 STUDY

**Topic:** 12. Bone marrow failure syndromes incl. PNH - Clinical

Meili Ge*[1], Hui Liu[2], Shunqing Wang[3], Mei Hong[4], Zenghua Lin[5], Shengjin Fan[6], Yajing Xu[7], Chuan He[8], Rongrong Liu[9], Hu Zhou[10], Xiaoqin Wang[11], Bin Hu[12], Rong Huang[13], Li Zhang[1], Jun Shi[1], Rong Fu[2]

*[1]Institue of Hematology & Blood Disease Hospital, Chinese Academy of Medical Sciences, Tianjin, China;[2]Tianjin Medical University General Hospital, Tianjin, China; [3]Guangzhou First People's Hospital, Guangzhou, China; [4]Union Hospital, Tongji Medical College, Huazhong University of Science and Technology, Wuhan, China; [5]Affiliated Hospital of Nantong University, Nantong, China; [6]The First Affiliated Hospital of Harbin Medical University, Harbin, China;[7]Xiangya Hospital of Central South University, Changsha, China; [8]West China Hospital, Chengdu, China; [9]The First Affiliated Hospital of Guangxi Medical University, Nanning, China; [10]Henan Cancer Hospital, Zhengzhou, China; [11]Huashan Hospital Affiliated to Fudan University, Shanghai, China; [12]Jiangsu Hengrui Pharmaceuticals Co., Ltd., Beijing, China; [13]Jiangsu Hengrui Pharmaceuticals Co., Ltd., Shanghai, China;*

### Background

Paroxysmal Nocturnal Hemoglobinuria (PNH) is a rare, complement-mediated hemolytic disorder. While C5 inhibitors reduce intravascular hemolysis, a significant proportion of patients (pts) remain anemic due to persistent extravascular hemolysis. HRS-5965 is a novel, oral, potent factor B inhibitor targeting the alternative complement pathway, designed to control both hemolytic pathways and improve anemia.

### Aims

This Phase 3 study aimed to evaluate the efficacy and safety of HRS-5965 in PNH pts with persistent anemia despite stable C5 inhibitor therapy.

### Methods

In this multicenter, single-arm, open-label study, eligible pts had confirmed PNH and were anemic (Hemoglobin [Hb] <10 g/dL) despite stable C5 inhibitor (ecucizumab/ kovarimab) therapy for the 6 months prior to enrollment. Pts received HRS-5965 50 mg twice daily for 24 weeks. The primary endpoint was the proportion of pts achieving Hb ≥12 g/dL on at least 3 of 4 measurements between Weeks 18 and 24 without red blood cell (RBC) transfusions (after Week 2). The key secondary endpoint was the proportion achieving a ≥2 g/dL Hb increase from baseline under the same conditions. Efficacy was established if the lower limit of the 95% CI for the response rate exceeded the pre-defined 10% target based on historical controls.

### Results

All 39 enrolled pts completed treatment and were included in efficacy and safety analysis (Table 1). The primary endpoint was achieved by 27/39 pts (69.2%; 95% CI: 52.4-83.0%), and the key secondary endpoint by 36/39 (92.3%; 95% CI: 79.1-98.4%). All pts (100.0%; 95% CI: 91.0-100.0%) were transfusion avoidance after Week 2. Mean Hb increased by 4.95 g/dL, reticulocyte count decreased by a mean of 170.18 x $10^9$/L, and FACIT-Fatigue score improved by a mean of 8.75 points at Weeks 18–24. No major adverse vascular events (MAVEs) were reported. Treatment-related adverse events (TRAEs) occurred in 48.7% of pts, predominantly mild (33.3%) or moderate (12.8%). Treatment-related serious AEs occurred in 2 pts (5.1%), including one case each of febrile infection and thrombocytopenia.

**Copyright Information:** (Online) ISSN: 2572-9241
©2026 The Author(s). HemaSphere is published by John Wiley & Sons Ltd on behalf of European Hematology Association. This is an open access Abstract Book distributed under the Attribution-NonCommercial-NoDerivs (CC BY-NC-ND), which allows third parties to download the articles and share them with others as long as they credit the author and the Abstract Book, but they cannot change the content in any way or use them commercially.
**Abstract Book Citations:** Authors, Title, HemaSphere, 2026;10:(S1):pages. The abstract book DOIs can be found on the "Author Information" page.
**Disclaimer:** Articles published in the journal HemaSphere exclusively reflect the opinions of the authors. The authors are responsible for all content in their abstracts including accuracy of the facts, statements, citing resources, etc.

### Summary/Conclusion

HRS-5965 demonstrated significant efficacy in improving Hb levels, achieving transfusion independence, and enhancing quality of life in PNH pts with persistent anemia despite prior C5 inhibitor therapy, with a favorable safety profile. HRS-5965 represents a promising novel oral treatment for PNH.

**Table 1. Efficacy and Safety Outcomes**

| | **HRS-5965 50mg bid (N = 39)** |
|---|---|
| **Primary endpoints** | |
| Hb ≥12 g/dL#, n (%) (95% CI) | 27 (69.2; 52.4, 83.0) |
| **Secondary endpoints** | |
| Hb increase ≥2 g/dL#, *, n (%) (95% CI) | 36 (92.3; 79.1, 98.4) |
| Transfusion avoidance (after Wk 2), n (%) (95% CI) | 39 (100.0; 91.0-100.0) |
| Mean Hb change from baseline (g/dL)#, LS Mean (SE) (95% CI) | 4.95 (0.14) (4.66, 5.24) |
| FACIT-Fatigue score improvement#, LS Mean (SE) (95% CI) | 8.75 (0.26) (8.23, 9.26) |
| Reticulocyte count change#, x$10^9$/L, LS Mean (SE) (95% CI) | -170.18 (3.52) (-177.13, -163.23) |
| **Safety** | |
| MAVEs, n (%) | 0 |
| Treatment-related AEs, n (%) | 19 (48.7) |
| Treatment-related serious AEs, n (%) | 2 (5.1) |

#Assessed over Weeks 18-24 in the absence of RBC transfusions after Week 2.
*Hb increase ≥2 g/dL was the key secondary endpoint.

**Copyright Information:** (Online) ISSN: 2572-9241
©2026 The Author(s). HemaSphere is published by John Wiley & Sons Ltd on behalf of European Hematology Association. This is an open access Abstract Book distributed under the Attribution-NonCommercial-NoDerivs (CC BY-NC-ND), which allows third parties to download the articles and share them with others as long as they credit the author and the Abstract Book, but they cannot change the content in any way or use them commercially.
**Abstract Book Citations:** Authors, Title, HemaSphere, 2026;10:(S1):pages. The abstract book DOIs can be found on the "Author Information" page.
**Disclaimer:** Articles published in the journal HemaSphere exclusively reflect the opinions of the authors. The authors are responsible for all content in their abstracts including accuracy of the facts, statements, citing resources, etc.

### (PF689) IMPACT OF VARIANTS OF UNCERTAIN SIGNIFICANCE ON TELOMERE BIOLOGY DISORDERS: A SINGLE CENTER EXPERIENCE

**Topic:** 12. Bone marrow failure syndromes incl. PNH - Clinical

Maria Binelli[1], Luca Arcuri*[1], Erika Massaccesi[1], Lucia Vankann[2], Michela Lupia[1], Marina Lanciotti[1], Daniela Guardo[1], Gianluca Dell'orso[1], Maria Carla Giarratana[1], Eugenia Mariani[1], Elena Palmisani[1], Filomena Pierri[1], Michaela Calvillo[1], Maurizio Miano[1], Fabian Beier[2], Tim Brümmendorf[2], Carlo Dufour[1], Francesca Fioredda[1]

*[1]IRCCS- Istituto Giannina Gaslini, Unit of Hematology, Genova, Italy; [2]RWTH Aachen University, Department of Hematology, Oncology, Hemostaseology and Stem Cell Transplantation, Medical Faculty,, Aachen, Germany;*

### Background

Telomere biology disorders (TBDs) are rare multisystem genetic conditions caused by germline pathogenic variants in genes involved in telomere maintenance. The increasing use of multigene panels has led to a higher detection of variants of uncertain significance (VUS), whose clinical interpretation remains a challenge.

### Aims

To assess the impact of VUS in TBD-related genes according to gene category/ function on the biological and clinical aspects of the disorder.

### Methods

Patients carrying i) pathogenic/ likely pathogenic (P/LP) variants in TBD-related genes ii) VUS in TBD-related genes, iii) lymphocyte telomere length (TL) < $10^{th}$ centile without detectable TBD-related variants, followed at the Hematology Unit of IRCCS Istituto Giannina Gaslini, were included. The TBD phenotype was categorized as: A) fully expressed; B) probable TBD (highly suggestive but incomplete phenotype); C) bone marrow failure; D) mono or multilineage cytopenia; E) immunohematological pattern; F family history of TBD. Correlations between genetic groups/TL (very low [VL, <$1^{st}$ centile] and low [L $1^{st}$-$10^{th}$ centile]) and clinical phenotypes were analyzed.

**Copyright Information:** (Online) ISSN: 2572-9241
©2026 The Author(s). HemaSphere is published by John Wiley & Sons Ltd on behalf of European Hematology Association. This is an open access Abstract Book distributed under the Attribution-NonCommercial-NoDerivs (CC BY-NC-ND), which allows third parties to download the articles and share them with others as long as they credit the author and the Abstract Book, but they cannot change the content in any way or use them commercially.
**Abstract Book Citations:** Authors, Title, HemaSphere, 2026;10:(S1):pages. The abstract book DOIs can be found on the "Author Information" page.
**Disclaimer:** Articles published in the journal HemaSphere exclusively reflect the opinions of the authors. The authors are responsible for all content in their abstracts including accuracy of the facts, statements, citing resources, etc.

**Results**

Ninety-eight patients (58 males, 59%; median age at last follow-up 17 years, IQR 10.1–28.4) with confirmed or suspected TBDs were included. The group was stratified into carriers of P/LP variants (23, 24%), VUS (39, 40%), and unknown variants (36, 37%). Genetic variants were isolated or associated (Figure 1).
In the P/LP group TL was VL in 13/20 (65%), L in 5/20 (25%) and normal in 2/20 (10%). In the VUS group, TL was available in 37/39 patients and was VL in 10/37 (27%), L in 6/37 (16.2%) patients and normal in 21/37 (56.8%). In the unknown gene group, 11 /36 (31%) had VL TL and 25/36 (69%) had L TL (*p = 0,001*).
P/LP carriers showed a type A phenotype in 61% of cases, vs 8% in VUS (p<0,001). Among VUS, phenotypes B, C, D, E, and F were observed in 18%, 8%, 28%, 18%, and 20% of cases, respectively. In the Unknown gene group C and D phenotypes were the two most represented (42% and 31%). The hematological phenotype was not significantly different in the three genetic groups while skin, liver and CNS involvement were more frequent in P/LP compared with the other groups (p<0.05). Among the VUS group, 11 variants were longitudinally reclassified as benign. Compared with the 28 patients with confirmed VUS, reclassified cases showed a lower prevalence of short TL (9% vs 53%, p = 0.05) and no extra-hematological organ involvement (0% vs 50%, p = 0.003). No significant differences were observed in hematological manifestations. However, short TL with cytopenia was more frequent in confirmed VUS patients (55% vs 12%, *p*= 0.08).

**Summary/Conclusion**

The present study underlines that TBDs severity is influenced by the presence of P/LP variants on TBD genes as expected; nevertheless, the VUS category doesn't seem neutral. Indeed, some VUS carriers show a fully expressed or likely TBD phenotype and in a considerable portion (50%) are associated with telomere shortening. Whether short TL contributes to cytopenia or represents a consequence of bone marrow dysfunction remains unclear. In conclusion, this study highlights the need to set up dedicated programs of follow-up in VUS carriers with the periodic re-evaluation of their significance too. Moreover, integrated and collaborative approaches are suggested with the aim to enlarge the cohorts and to understand any possible enhancing genetic and epigenetic factors which may have a potentiating role in TBD phenotype.

**Copyright Information:** (Online) ISSN: 2572-9241
©2026 The Author(s). HemaSphere is published by John Wiley & Sons Ltd on behalf of European Hematology Association. This is an open access Abstract Book distributed under the Attribution-NonCommercial-NoDerivs (CC BY-NC-ND), which allows third parties to download the articles and share them with others as long as they credit the author and the Abstract Book, but they cannot change the content in any way or use them commercially.
**Abstract Book Citations:** Authors, Title, HemaSphere, 2026;10:(S1):pages. The abstract book DOIs can be found on the "Author Information" page.
**Disclaimer:** Articles published in the journal HemaSphere exclusively reflect the opinions of the authors. The authors are responsible for all content in their abstracts including accuracy of the facts, statements, citing resources, etc.

| UPN | Gene | Variant classification | Allelic status | Inheritance |
|---|---|---|---|---|
| P01 | *DKC1* | P | H | XLR |
| P02 | *RTEL1/RTEL1/RTEL1* | P | CH | AD/AR |
| P03 | *DKC1* | P | H | XLR |
| P04 | *WRAP53/WRAP53* | P | CH | AR |
| P05 | *TERC* | P | H | AD |
| P06 | *RTEL1* | P | H | AD/AR |
| P07 | *TERT + TERT* | P + VUS | CH | AD/AR |
| P08 | *TERT* | P | H | AD/AR |
| P09 | *TERT + TERT* | P + VUS | H | AD/AR |
| P10 | *TERT* | P | H | AD/AR |
| P11 | *TINF2* | P | H | AD/AR |
| P12 | *TERT/TERT* | P | H | AD/AR |
| P13 | *TINF2* | P | H | AD |
| P14 | *TINF2* | P | H | AD |
| P15 | *PARN* | P | H | AD/AR |
| P16 | *PARN* | P | H | AD/AR |
| P17 | *RTEL1 + RTEL1* | P | H | AD/AR |
| P18 | *RTEL1 + RTEL1* | P | H | AD/AR |
| P19 | *TINF2* | P | H | AD |
| P20 | *DKC1* | P | H | XLR |
| P21 | *TERC* | P | H | AD |
| P22 | *PARN* | P | H | AD/AR |
| P23 | *PARN* | P | H | AD/AR |
| V01 | *TERC* | VUS | H | AD |
| V02 | *TINF2* | VUS | H | AD |
| V03 | *TINF2* | VUS | H | AD |
| V04 | *TERT* | VUS | H | AD/AR |
| V05 | *TERT* | VUS | H | AD/AR |
| V06 | *TINF2* | VUS | H | AD |
| V07 | *RTEL1* | VUS | H | AD/AR |
| V08 | *RTEL1* | VUS | H | AD/AR |
| V09 | *TERT* | VUS | H | AD/AR |
| V10 | *PARN* | VUS | H | AD/AR |
| V11 | *CTC1 + WRAP53* | VUS + VUS | H | AR + AR |
| V12 | *TERT* | VUS | H | AD/AR |
| V13 | *PARN* | VUS | H | AD/AR |
| V14 | *TINF2* | VUS | H | AD |
| V15 | *TINF2* | VUS | H | AD |
| V16 | *NAF1* | VUS | H | AD |
| V17 | *TERT* | VUS | H | AD/AR |
| V18 | *TINF2* | VUS | H | AD |
| V19 | *TERT* | VUS | H | AD/AR |
| V20 | *TERT* | VUS | H | AD/AR |
| V21 | *TERT* | VUS | H | AD/AR |
| V22 | *PARN + WRAP53* | VUS + VUS | H | AD/AR + AR |
| V23 | *RTEL1* | VUS | H | AD/AR |
| V24 | *RTEL1* | VUS | H | AD/AR |
| V25 | *RTEL1* | VUS | H | AD/AR |
| V26 | *ACD* | VUS | H | AD/AR |
| V27 | *TINF2* | VUS | H | AD |
| V28 | *TINF2* | VUS | H | AD |
| V29 | *TINF2* | VUS | H | AD |
| V30 | *PARN + PARN + RTEL1* | VUS + VUS | H | AD/AR |
| V31 | *ACD* | VUS | H | AD/AR |
| V32 | *ACD* | VUS | H | AD/AR |
| V33 | *RTEL1* | VUS | H | AD |
| V34 | *RTEL1* | VUS | H | AD/AR |
| V35 | *RTEL1* | VUS | H | AD/AR |
| V36 | *RTEL1* | VUS | H | AD/AR |
| V37 | *POT1* | VUS | H | AD |
| V38 | *RTEL1* | VUS | H | AD/AR |
| V39 | *ACD* | VUS | H | AD |

Figure 1. Variant associations. Abbreviations: P, pathogenic; VUS, variant of uncertain significance; H, heterozygous; CH, compound heterozygous; XLR, X-linked related; AD, autosomal dominant; AR, autosomal recessive.

**Copyright Information:** (Online) ISSN: 2572-9241
©2026 The Author(s). HemaSphere is published by John Wiley & Sons Ltd on behalf of European Hematology Association. This is an open access Abstract Book distributed under the Attribution-NonCommercial-NoDerivs (CC BY-NC-ND), which allows third parties to download the articles and share them with others as long as they credit the author and the Abstract Book, but they cannot change the content in any way or use them commercially.
**Abstract Book Citations:** Authors, Title, HemaSphere, 2026;10:(S1):pages. The abstract book DOIs can be found on the "Author Information" page.
**Disclaimer:** Articles published in the journal HemaSphere exclusively reflect the opinions of the authors. The authors are responsible for all content in their abstracts including accuracy of the facts, statements, citing resources, etc.

### (PF690) SOVLEPLENIB AMELIORATES APLASTIC ANEMIA THROUGH THE MODULATION OF CGAS-STING-NF-ΚB PATHWAY

**Topic:** 12. Bone marrow failure syndromes incl. PNH - Clinical

Han Wang[1], Tianzi Sheng[2], Hong Liu*[3]

*[1]Affiliated Hospital of Nantong University, China, Department of General Surgery, Nantong, China;[2]Affiliated Hospital of Nantong University, China, Department of Hematology, Nantong, China; [3]Affiliated Hospital of Nantong University, Department of Hematology, Nantong, China;*

### Background

Aplastic anemia (AA) is a bone marrow failure syndrome characterized by pancytopenia.Aberrantly activated T lymphocytes and their excessive secretion of cytokines, particularly interferon-gamma (IFN-γ) and tumor necrosis factor-alpha (TNF-α), are key drivers of this excessive hematopoietic cell apoptosis.Studies have confirmed that combining the thrombopoietin receptor agonists (TPO-RAs) eltrombopag with standard immunosuppressive therapy significantly enhances efficacy, accompanied by a significant recovery in bone marrow CD34+ cell counts and the frequency of early hematopoietic progenitor cells.However,some patients show no or insufficient response or relapse TPO-RAs may also be associated with a risk of clonal evolution, particularly the emergence of high-risk cytogenetic abnormalities.Spleen tyrosine kinase (SYK), a key mediator of immune receptor signal transduction, has garnered considerable attention due to its role in autoimmune diseases. SYK inhibitors restore immune homeostasis by blocking the B-cell receptor (BCR) and Fc receptor signaling pathways, thereby suppressing overactivated T cells, B cells and macrophages. Sovleplenib, a novel and highly selective oral SYK inhibitor, has been approved in China for the treatment of primary immune thrombocytopenia (ITP); however, its efficacy and mechanism of action in AA remain unclear.

The innate immune signaling pathway cGAS-STING plays a central role in sensing cellular damage and pathogen invasion. In AA, apoptotic debris in the bone marrow leads to abnormal accumulation of DNA. In turn, cGAS recognizes cytoplasmic DNA and catalyzes the synthesis of the second messenger cGAMP, which activates the STING protein. Persistent activation of the cGAS-STING pathway has been shown to mediate tissue inflammation and drive hematopoietic stem cell exhaustion.This mechanism provides a molecular explanation for the susceptibility of certain patients with DNA repair disorders to bone marrow failure.Furthermore, it offers a novel theoretical perspective on the immunopathological mechanisms of AA.

### Aims

To investigate the therapeutic potential of sovleplenib, a selective SYK inhibitor, in aplastic anemia (AA), focusing on its effects on immune modulation and the cGAS-STING-NF-κB inflammatory axis.

### Methods

An immune-mediated AA mouse model was established and treated with sovleplenib. Hematopoietic function was assessed via peripheral blood counts and bone marrow mononuclear cell (BMNC) counts. Immune cell profiles were analyzed by flow cytometry. Cytokine levels were measured by ELISA. Underlying mechanisms were explored through proteomics and Western blotting.

### Results

Sovleplenib treatment significantly improved peripheral blood counts (HGB, WBC, PLT) and BMNCs, and reduced bone marrow fat vacuolation. It corrected immune imbalance by increasing the $CD4^+/CD8^+$ T-cell ratio and decreasing the M1/M2 macrophage ratio, promoting an M2 phenotype. Concurrently, it elevated Arg-1 level while suppressing ROS, TNF-α, and IFN-β. Mechanistically, sovleplenib inhibited the activation of key pathway components (cGAS, STING, p-TBK1, p-p65).

**Copyright Information:** (Online) ISSN: 2572-9241
©2026 The Author(s). HemaSphere is published by John Wiley & Sons Ltd on behalf of European Hematology Association. This is an open access Abstract Book distributed under the Attribution-NonCommercial-NoDerivs (CC BY-NC-ND), which allows third parties to download the articles and share them with others as long as they credit the author and the Abstract Book, but they cannot change the content in any way or use them commercially.
**Abstract Book Citations:** Authors, Title, HemaSphere, 2026;10:(S1):pages. The abstract book DOIs can be found on the "Author Information" page.
**Disclaimer:** Articles published in the journal HemaSphere exclusively reflect the opinions of the authors. The authors are responsible for all content in their abstracts including accuracy of the facts, statements, citing resources, etc.

**Summary/Conclusion**

The results demonstrate sovleplenib's dual role in promoting hematopoiesis and immunomodulation. Its efficacy is mechanistically linked to the suppression of the overactivated cGAS-STING-NF-κB pathway, a key driver of inflammation in AA

**Copyright Information:** (Online) ISSN: 2572-9241
©2026 The Author(s). HemaSphere is published by John Wiley & Sons Ltd on behalf of European Hematology Association. This is an open access Abstract Book distributed under the Attribution-NonCommercial-NoDerivs (CC BY-NC-ND), which allows third parties to download the articles and share them with others as long as they credit the author and the Abstract Book, but they cannot change the content in any way or use them commercially.
**Abstract Book Citations:** Authors, Title, HemaSphere, 2026;10:(S1):pages. The abstract book DOIs can be found on the "Author Information" page.
**Disclaimer:** Articles published in the journal HemaSphere exclusively reflect the opinions of the authors. The authors are responsible for all content in their abstracts including accuracy of the facts, statements, citing resources, etc.

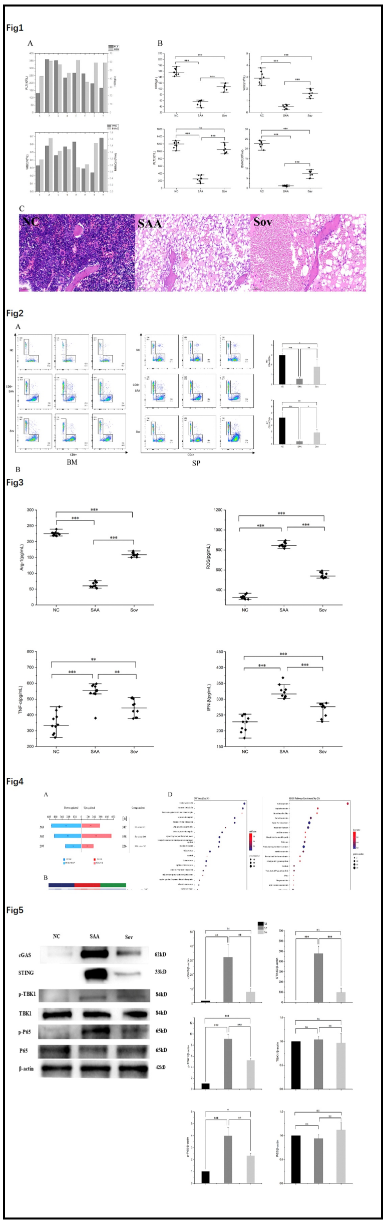

Figure Legends Fig. 1 Sovleplenib treatment significantly improves peripheral blood parameters and bone marrow hematopoietic function in AA mice. (A) Counts of HGB, WBC, PLT, and BMNC in the NC, AA, and Sov groups(n=8) (*P < 0.05, **P < 0.01, ***P < 0.001). (B) Representative sternal bone marrow histology in the NC, AA, and Sov groups (20×, H&E staining). Fig. 2 Sovleplenib promotes bone marrow hematopoietic stem/progenitor cell recovery and modulates immune cell imbalance. (A) Flow cytometric analysis of the CD4⁺/CD8⁺ T cell ratio in bone marrow and spleen of NC, AA, and Sov groups. (B) Flow cytometric analysis of the M1/M2 macrophage ratio in bone marrow and spleen of NC, AA, and Sov groups. (*P < 0.05, **P < 0.01, ***P < 0.001) Fig. 3 Plasma levels of Arg-1, ROS, TNF-α, and IFN-β in mice from each group measured by ELISA. (n=8) (*P < 0.05, **P < 0.01, ***P < 0.001) Fig. 4 Proteomic analysis of NC, AA, and Sov groups. (A) Bar chart showing quantitative differences in protein expression among NC, AA, and Sov groups. (B) Heatmap of differentially expressed proteins across NC, AA, and Sov groups. (C) Volcano plot of differentially expressed proteins between the AA and Sov groups. (D) GO enrichment (Biological Process) and KEGG pathway enrichment bubble plots for all differentially expressed proteins in the Sov versus AA groups. Fig. 5 Western blot analysis of cGAS, STING, p-TBK1, TBK1, p-NF-κB p65, and NF-κB p65 protein expression in femoral bone marrow of mice from each group. (*P < 0.05, **P < 0.01, ***P < 0.001)

**Copyright Information:** (Online) ISSN: 2572-9241
©2026 The Author(s). HemaSphere is published by John Wiley & Sons Ltd on behalf of European Hematology Association. This is an open access Abstract Book distributed under the Attribution-NonCommercial-NoDerivs (CC BY-NC-ND), which allows third parties to download the articles and share them with others as long as they credit the author and the Abstract Book, but they cannot change the content in any way or use them commercially.
**Abstract Book Citations:** Authors, Title, HemaSphere, 2026;10:(S1):pages. The abstract book DOIs can be found on the “Author Information” page.
**Disclaimer:** Articles published in the journal HemaSphere exclusively reflect the opinions of the authors. The authors are responsible for all content in their abstracts including accuracy of the facts, statements, citing resources, etc.

### (PF691) LONG-TERM MUTATIONAL ANALYSIS, CLONAL METRICS AND DYNAMICS IN PATIENTS WITH CHRONIC IDIOPATHIC NEUTROPENIA HIGHLIGHT THE IMPORTANCE OF MOLECULAR MONITORING

**Topic:** 12. Bone marrow failure syndromes incl. PNH - Clinical

Grigorios Tsaknakis[1, 2], Irene Mavroudi[1, 2], Anna Maria Perantonaki[1, 2], Erasmia Boutakoglou[1, 2], Stavros Papadakis[1, 2], Charalampos Pontikoglou[1, 2], Anna Galli[3], Luca Malcovati[3, 4], Helen Papadaki*[1, 2]

*[1]Haemopoiesis Research Laboratory, University of Crete School of Medicine, Heraklion, Greece;[2]Department of Haematology, University Hospital of Heraklion, Heraklion, Greece; [3]Unit of Molecular Hematology and Precision Medicine, IRCCS Fondazione Policlinico San Matteo, Pavia, Italy; [4]Department of Molecular Medicine, University of Pavia, Pavia, Italy;*

### Background

CIN is a rare neutrophil disorder characterized by the prolonged, unexplained reduction in the number of absolute neutrophil counts (ANCs). We have previously shown that CIN adult patients display clonal hematopoiesis (CH) at a percentage of 11.35% and that CIN patients with CH display a 31-fold increased risk for transformation to myelodysplastic neoplasms/acute myeloid leukemia (MDS/AML) compared to non-clonal CIN patients.

### Aims

The aim of the study is to investigate prospectively and longitudinally the occurrence of CH and track the clonal metrics (i.e. mutation patterns and clone size) and dynamics in a large group of well-defined CIN patients, using serial NGS analysis, in association with peripheral blood (PB) counts and disease outcome.

### Methods

256 CIN patients (ANC 1390±410/μL; median 1600, range 100-1700/μL) and 104 patients with follow-up PB samples (median time 32 months, range 12-222 months) were studied, according to previously published criteria. Genomic DNA was extracted from bone marrow or PB samples at baseline and follow-up timepoints, sequencing libraries were prepared and subjected to targeted NGS on an NextSeq2000 Sequencer (Illumina) using a panel of 44 myeloid genes.

### Results

We identified 12.5% CH patients in the CIN patients' group (32/256). The most frequently mutated genes were *DNMT3A* and *TET2* genes followed by *SRSF2*, *IDH1/2*, *ASXL1* and *ZRSR2*, with *SRSF2* and *IDH1/2* emerging as highly mutated genes in our cohort. Most clonal patients carried only one mutation (25/32 patients) and the overall frequency of malignant transformation was 2.34% (5/256). Patients over 60 years and those with VAF over 10% displayed a more diverse mutational profile. Longitudinal tracking of CH incidence in CIN patients by serial NGS analysis showed it was comparable between the initial and follow-up analysis. Only 2 CIN patients acquired CH at follow-up and one patient lost CH. CIN patients with stable disease retained a relatively stable mutational spectrum and clonal size during the time-course. All CH patients who progressed to a hematologic malignancy (n=5), demonstrated acquisition of additional mutations and significant increase of the VAF size of the mutant clone(s) at follow-up compared to baseline. The detection of driver mutations in *IDH1/2* and *SRSF2* at baseline or their acquisition during the course of the disease were associated with the highest risk for malignant transformation (RR of 10.50 and 6.5 respectively), while also displaying the highest clonal expansion. The presence of clonal disease and more than one mutation conferred a substantial RR of 35 and 5.357 for malignant transformation, respectively, compared to non-clonal disease. Moreover, an annual VAF increase (ΔVAF/year)≥7.5% was identified as optimally predicting evolution to malignancy. Notably, PB hematologic parameters were assessed at baseline and longitudinally and found not to display clinically significant changes.

**Copyright Information:** (Online) ISSN: 2572-9241
©2026 The Author(s). HemaSphere is published by John Wiley & Sons Ltd on behalf of European Hematology Association. This is an open access Abstract Book distributed under the Attribution-NonCommercial-NoDerivs (CC BY-NC-ND), which allows third parties to download the articles and share them with others as long as they credit the author and the Abstract Book, but they cannot change the content in any way or use them commercially.
**Abstract Book Citations:** Authors, Title, HemaSphere, 2026;10:(S1):pages. The abstract book DOIs can be found on the "Author Information" page.
**Disclaimer:** Articles published in the journal HemaSphere exclusively reflect the opinions of the authors. The authors are responsible for all content in their abstracts including accuracy of the facts, statements, citing resources, etc.

**Summary/Conclusion**

Our longitudinal data demonstrate that while CH in CIN has a relatively low overall incidence, specific clonal features identify a high-risk subset for malignant transformation. Longitudinal assessment of PB parameters revealed no clinically significant changes, thus failing to provide clinical warning signs of the underlying transformation process. Collectively, these findings underscore the clinical necessity of serial NGS monitoring, as clonal metrics and dynamics provide the only reliable indicator of imminent progression in CIN patients.

**Copyright Information:** (Online) ISSN: 2572-9241
©2026 The Author(s). HemaSphere is published by John Wiley & Sons Ltd on behalf of European Hematology Association. This is an open access Abstract Book distributed under the Attribution-NonCommercial-NoDerivs (CC BY-NC-ND), which allows third parties to download the articles and share them with others as long as they credit the author and the Abstract Book, but they cannot change the content in any way or use them commercially.
**Abstract Book Citations:** Authors, Title, HemaSphere, 2026;10:(S1):pages. The abstract book DOIs can be found on the "Author Information" page.
**Disclaimer:** Articles published in the journal HemaSphere exclusively reflect the opinions of the authors. The authors are responsible for all content in their abstracts including accuracy of the facts, statements, citing resources, etc.

### (PF692) SAFETY OF RAVULIZUMAB USE IN PREGNANCY: INSIGHTS FROM A GLOBAL PHARMACOVIGILANCE ANALYSIS IN PATIENTS WITH PAROXYSMAL NOCTURNAL HEMOGLOBINURIA

**Topic:** 12. Bone marrow failure syndromes incl. PNH - Clinical

Cynthia Carillo Infante[1], Arshad Mujeebuddin[1], Asa Lommele[2], Cory Williams*[3]

*[1]Alexion, AstraZeneca Rare Disease, Boston, MA, United States of America;[2]Alexion, AstraZeneca Rare Disease, Baar, Switzerland; [3]Alexion, AstraZeneca Rare Disease, New Haven, CT, United States of America;*

### Background

Ravulizumab, a second-generation C5 complement inhibitor and an analog of eculizumab, is approved for the treatment of four complement-mediated disorders including paroxysmal nocturnal hemoglobinuria (PNH). Pregnancy is a complement-activating condition which may increase the risk of developing PNH complications (e.g. hemolysis and thrombosis). PNH is associated with high maternal and fetal morbidity in pregnancy when untreated. There are multiple publications on eculizumab use during pregnancy in patients with PNH reporting favorable outcomes and where no new safety signals have been observed. However, data on similar use with ravulizumab are limited. Results from a recent study on ravulizumab use in pregnancy (i.e., 16 PNH patients with 19 pregnancies) suggested that ravulizumab is a safe and effective option for managing PNH during pregnancy, with favorable maternal and neonatal outcomes. All ravulizumab-treated pregnancies resulted in live births with no reported abnormalities.[1]

### Aims

This analysis aims to provide insights into pregnancy-related safety following ravulizumab exposure using data from global post-marketing surveillance in patients with PNH.

### Methods

Here, we analyze all pregnancy cases with PNH reported globally in the post-marketing setting of ravulizumab, with ≥1 documented dose of exposure. Data from both spontaneous and solicited cases recorded in the Alexion pharmacovigilance (PV) database from the initial market approval up to June 26, 2025 were included in the analysis. Descriptive statistics were used to summarize patient demographics, treatment patterns, and pregnancy outcomes.

### Results

Among the 256 identified pregnancy cases with ≥1 dose of ravulizumab exposure in the PV database, PNH (n=117) represented the largest number of cases among approved indications. Detailed information, including ravulizumab exposure and pregnancy outcomes, was available for a subset of 21 PNH cases. For this PNH subset, reported maternal co-morbidities included aplastic anemia (n=3) and Budd-Chiari syndrome (n=1); complications during pregnancy included pre-eclampsia (n=3), HELLP syndrome (n=1), acute renal failure on dialysis (n=2), and anemia requiring blood transfusions (n=2). Pregnancy outcomes for these 21 PNH cases, as reported by exposure category, included: a) 6 cases with full-pregnancy ravulizumab exposure resulted in 6 live births; b) 5 cases with <37 weeks of ravulizumab exposure with a switch to eculizumab resulted in 3 live births and 2 spontaneous abortions; and, c) 10 cases with ≤12 weeks of ravulizumab exposure with a switch to eculizumab resulted in 7 live births and 3 spontaneous abortions (**Table 1**). No maternal deaths or events suggestive of inadequate disease control with ravulizumab during pregnancy (e.g., major thrombotic events) were reported.

**Copyright Information:** (Online) ISSN: 2572-9241
©2026 The Author(s). HemaSphere is published by John Wiley & Sons Ltd on behalf of European Hematology Association. This is an open access Abstract Book distributed under the Attribution-NonCommercial-NoDerivs (CC BY-NC-ND), which allows third parties to download the articles and share them with others as long as they credit the author and the Abstract Book, but they cannot change the content in any way or use them commercially.
**Abstract Book Citations:** Authors, Title, HemaSphere, 2026;10:(S1):pages. The abstract book DOIs can be found on the “Author Information” page.
**Disclaimer:** Articles published in the journal HemaSphere exclusively reflect the opinions of the authors. The authors are responsible for all content in their abstracts including accuracy of the facts, statements, citing resources, etc.

### Summary/Conclusion

This analysis provides real-world insights on the use of ravulizumab in pregnancy for patients with PNH and suggests no unexpected safety signals. These data are consistent with the case series reported in Höchsmann *et al.* as well as with existing evidence on the use of eculizumab in pregnant patients and may support clinical decision-making for ravulizumab use during pregnancy. A global observational study (NCT06312644) evaluating the safety of ravulizumab during pregnancy is currently ongoing to further strengthen the evidence base.

**Table 1**. Ravulizumab exposure and clinical outcomes in a subset of PNH cases with detailed exposure information

| Primary disease | Maternal medical history[a] | Ravulizumab exposure during pregnancy | Pregnancy outcomes |
|---|---|---|---|
| PNH (n=21) | Aplastic anemia, Budd–Chiari, thrombocytopenia, splenomegaly, renal failure, pre-eclampsia | Up to delivery (n=6) | 6 live births |
| | | <37 weeks on ravulizumab when switched to eculizumab (n=5) | 3 live births<br>2 spontaneous abortions |
| | | ≤12 weeks on ravulizumab when switched to eculizumab (n=10) | 7 live births<br>3 spontaneous abortions |

[a]Disease-related complications and comorbidities reported; not all conditions were present in every patient.
PNH, paroxysmal nocturnal hemoglobinuria

**Copyright Information:** (Online) ISSN: 2572-9241
©2026 The Author(s). HemaSphere is published by John Wiley & Sons Ltd on behalf of European Hematology Association. This is an open access Abstract Book distributed under the Attribution-NonCommercial-NoDerivs (CC BY-NC-ND), which allows third parties to download the articles and share them with others as long as they credit the author and the Abstract Book, but they cannot change the content in any way or use them commercially.
**Abstract Book Citations:** Authors, Title, HemaSphere, 2026;10:(S1):pages. The abstract book DOIs can be found on the "Author Information" page.
**Disclaimer:** Articles published in the journal HemaSphere exclusively reflect the opinions of the authors. The authors are responsible for all content in their abstracts including accuracy of the facts, statements, citing resources, etc.

### (PF693) LONG-TERM SAFETY WITH IPTACOPAN TREATMENT IN PATIENTS WITH PAROXYSMAL NOCTURNAL HEMOGLOBINURIA (PNH): POOLED ANALYSIS OF DATA FROM PHASE 2 AND 3 STUDIES AND THE ROLL-OVER EXTENSION PROGRAM

**Topic:** 12. Bone marrow failure syndromes incl. PNH - Clinical

Alexander Röth*[1], Austin G. Kulasekararaj[2, 3, 4, 5], Camilla Frieri[6], Flore Sicre de Fontbrune[2, 7], Phillip Scheinberg[8], Bor-Sheng Ko[9], Anna Gaya[10], Yasutaka Ueda[11], Lily Wong Lee Lee[12], Ilene Weitz[13], Philippe Ferber[14], Silvia Sanz[15], Luca Monaco[14], Richard J. Kelly[16]

*[1]Department of Hematology and Stem Cell Transplantation, West German Cancer Center, University Hospital Essen, University Duisburg-Essen, Essen, Germany; [2]The Severe Aplastic Anemia Working Party of the European Society for Blood and Marrow Transplantation, Barcelona, Spain; [3]King's College Hospital NHS, London, United Kingdom; [4]National Institute for Health and Care Research and Wellcome King's Research Facility, London, United Kingdom; [5]King's College London, London, United Kingdom; [6]U.O.C Ematologia e Terapie cellulari avanzate, AORN San Giuseppe Moscati, Avellino, Italy; [7]French Reference Center for Aplastic Anemia and Paroxysmal Nocturnal Hemoglobinuria, Saint-Louis Hospital, Paris, France; [8]Hospital A Beneficência Portuguesa, São Paolo, Brazil; [9]National Taiwan University Cancer Center, Taipei, Taiwan; [10]Hospital Clinic of Barcelona, Barcelona, Spain; [11]The University of Osaka Graduate School of Medicine, Suita, Japan;[12]Queen Elizabeth Hospital, Kota Kinabalu, Malaysia; [13]USC Norris Cancer Center, Keck-USC School of Medicine, Los Angeles, CA, United States of America; [14]Novartis Pharma AG, Basel, Switzerland; [15]Novartis Farmacéutica, S.A., Barcelona, Spain; [16]St James's University Hospital, Leeds, United Kingdom;*

### Background

Iptacopan demonstrated sustained hematologic control and a favorable safety profile in patients with paroxysmal nocturnal hemoglobinuria (PNH) in phase 2 and 3 studies and the roll-over extension program (PNH-REP); long-term safety data are limited.

### Aims

Assess long-term safety of iptacopan in patients with PNH using pooled data from phase 2 and 3 studies and the PNH-REP.

### Methods

Safety data were pooled from 3 phase 3 studies (APPOINT-PNH [NCT04820530]; APPLY-PNH [NCT04558918] and APPULSE-PNH [NCT05630001]), 2 phase 2 studies (X2201 [NCT03439839] and X2204 [NCT03896152]) and the PNH-REP (NCT04747613). Baseline was defined as day 1 of receiving iptacopan 200 mg twice daily in the parent study. Safety assessments included incidence and exposure-adjusted occurrence rates (OccR, expressed as number of events/100 patient-years [py]) of breakthrough hemolysis (BTH), major adverse vascular events (MAVEs), treatment-emergent adverse events (TEAEs) and serious TEAEs. Total cholesterol was calculated in the whole population; high- (HDL) and low-density lipoprotein (LDL) cholesterol endpoints were calculated in the pooled phase 3 populations only.

**Copyright Information:** (Online) ISSN: 2572-9241
©2026 The Author(s). HemaSphere is published by John Wiley & Sons Ltd on behalf of European Hematology Association. This is an open access Abstract Book distributed under the Attribution-NonCommercial-NoDerivs (CC BY-NC-ND), which allows third parties to download the articles and share them with others as long as they credit the author and the Abstract Book, but they cannot change the content in any way or use them commercially.
**Abstract Book Citations:** Authors, Title, HemaSphere, 2026;10:(S1):pages. The abstract book DOIs can be found on the "Author Information" page.
**Disclaimer:** Articles published in the journal HemaSphere exclusively reflect the opinions of the authors. The authors are responsible for all content in their abstracts including accuracy of the facts, statements, citing resources, etc.

**Results**

The analysis population comprised 223 patients. Total iptacopan exposure was 676.1 py; 126 (56.5%) patients were exposed to iptacopan for ≥3 years and 34 (15.2%) for ≥4 years. At data cut-off, 95 (42.6%) patients had completed study, 106 (47.5%) were on study and 22 (9.9%) had discontinued treatment, including 3 (1.3%) due to adverse events (AEs), 2 (0.9%) due to pregnancy and 8 (3.6%) due to death. BTH occurred in 29 (13.0%) patients (OccR 6.7 events/100 py) and serious BTH events in 6 (2.7%; OccR 0.9 events/100 py). Eight MAVEs were reported in 7 (3.1%) patients (OccR 1.2 events/100 py). These included transient ischemic attack (3 patients); lacunar infarction, unstable angina, thrombophlebitis/deep vein thrombosis (1 patient each); intestinal infarction and portal vein thrombosis (in the same patient). Overall, 210 (94.2%) patients experienced ≥1 TEAE (OccR 329.4 events/100 py). Most frequently reported TEAEs were COVID-19 in 79 (35.4%) patients (OccR 13.8 events/100 py), nasopharyngitis in 50 (22.4%; OccR 11.4 events/100 py) and headache in 47 (21.1%; OccR 11.5 events/100 py). Sixty-seven (30.0%) patients experienced ≥1 serious TEAE (OccR 19.4 events/100 py), of which infections were the most frequent, in 29 (13.0%) patients (OccR 7.0 events/100 py). To evaluate potential lipid alterations, cholesterol levels were assessed. Mean (SD) total cholesterol was 4.9 (1.2) mmol/L at year 3 (n=129) and 5.0 (1.0) mmol/L at year 4 (n=32), with respective mean (SD) elevations of 0.9 (0.9) and 1.1 (0.7) mmol/L from baseline. HDL cholesterol remained stable with mean (SD) change from baseline of 0.0 (0.3) and 0.1 (0.3) mmol/L at years 3 and 4, respectively; LDL cholesterol showed a mean (SD) increase from baseline of 0.8 (0.8) mmol/L at both timepoints. Of 9 reported pregnancies, 4 resulted in elective abortions, 2 in spontaneous abortions, 1 in ectopic pregnancy and 2 in healthy live births.

**Summary/Conclusion**

The safety profile of iptacopan in this pooled analysis of long-term safety data is consistent with that previously reported, with no new safety findings. MAVEs and serious BTH events were infrequent; there were minimal changes in LDL cholesterol levels. Rates of treatment discontinuation for AEs were low. These findings demonstrate that iptacopan is well tolerated in the long-term treatment of PNH.

**Copyright Information:** (Online) ISSN: 2572-9241
©2026 The Author(s). HemaSphere is published by John Wiley & Sons Ltd on behalf of European Hematology Association. This is an open access Abstract Book distributed under the Attribution-NonCommercial-NoDerivs (CC BY-NC-ND), which allows third parties to download the articles and share them with others as long as they credit the author and the Abstract Book, but they cannot change the content in any way or use them commercially.
**Abstract Book Citations:** Authors, Title, HemaSphere, 2026;10:(S1):pages. The abstract book DOIs can be found on the "Author Information" page.
**Disclaimer:** Articles published in the journal HemaSphere exclusively reflect the opinions of the authors. The authors are responsible for all content in their abstracts including accuracy of the facts, statements, citing resources, etc.

### (PF694) PLATELET PNH CLONE ASSESSMENT BY FLOW CYTOMETRY ISFEASIBLE AND PROVIDES A RELIABLE ESTIMATE OF CLONE SIZE AND PLATELET ACTIVATION IN PATIENTS WITH PAROXYSMAL NOCTURNAL HEMOGLOBINURIA

**Topic:** 12. Bone marrow failure syndromes incl. PNH - Clinical

Lucas Sales*[1], Victor Rolim[1], Caio Justino[1], Leticia Paz[1], Ana Rita Fonseca[2], Fabio Pinotti[1], Miriam Rodrigues Barbosa[1], Celso Arrais-Rodrigues[1], Alex Sandes[1]

*[1]Paulista School of Medicine, Federal University of São Paulo (EPM–UNIFESP), São Paulo-SP, Brazil;[2]Hospital Sírio Libanês, São Paulo, Brazil;*

### Background

Paroxysmal nocturnal hemoglobinuria (PNH) results from clonal expansion of hematopoietic stem cells with PIGA mutations, causing deficiency of GPI-anchored proteins. Granulocytes and monocytes are commonly used to assess clone size, while erythrocytes may underestimate disease burden due to hemolysis. Platelets also express CD55 and CD59 and may contribute to thrombosis, but platelet clone assessment remains technically challenging.

### Aims

To develop a flow cytometry method to quantify the PNH platelet clone, assess its correlation with other hematopoietic lineages, and evaluate platelet activation in PNH patients.

### Methods

This cross-sectional observational study was conducted at the PNH outpatient clinic of Hospital São Paulo and included 18 adult patients with prior PNH diagnosis. The cohort comprised 61.1% classic PNH and 38.9% PNH with bone marrow failure; 77.8% were receiving complement inhibitors (eculizumab or crovalimab). An immunophenotypic panel using CD55, CD59, and CD61 was developed for platelet analysis, with FLAER and CD58 also evaluated. Platelet activation was assessed by baseline CD62P expression. Clone sizes across lineages were compared using Pearson correlation and Bland–Altman analysis.

### Results

CD58 and FLAER were not expressed on normal platelets. In contrast, CD55 and CD59 showed low-to-moderate expression on normal platelets, enabling discrimination between type I and type III PNH platelets (type I: CD55 MFI 96.3 ± 47.1, CD59 MFI 294.5 ± 90; type III: CD55 MFI 10 ± 4, CD59 MFI 18 ± 20). Importantly, a single tube containing CD55 and CD59 allowed simultaneous evaluation of platelets and erythrocytes. Median PNH clone sizes were: monocytes 85% (9.7–99.5%), platelets 83.7% (8–99.9%), granulocytes 68.3% (1.14–99.9%), and red blood cells 31.4% (0.17–93.4%). Platelet clone size showed strong correlation with monocytes ($r = 0.931$, $p < 0.001$) and granulocytes ($r = 0.824$, $p < 0.001$), with the monocyte-platelet pair demonstrating the lowest bias (–0.6 ± 11.79) on Bland-Altman analysis. Red blood cell clone was consistently lower across all comparisons, attributable to ongoing hemolysis even under treatment. Discrepant granulocyte clone sizes were significantly associated with neutropenia (mean neutrophil count 1.18 ± 1, vs. $2.5 \pm 0.9 \times 10^9$/L in concordant cases; $p = 0.012$), suggesting that in this context platelet and monocyte clone assessment may better reflect true clone burden. Baseline platelet activation (CD62P expression) was significantly higher in PNH patients on anti-C5 therapy compared to aplastic anemia controls (24.4% vs. 6.15%; $p = 0.045$), indicating residual platelet activation despite complement blockade.

**Copyright Information:** (Online) ISSN: 2572-9241
©2026 The Author(s). HemaSphere is published by John Wiley & Sons Ltd on behalf of European Hematology Association. This is an open access Abstract Book distributed under the Attribution-NonCommercial-NoDerivs (CC BY-NC-ND), which allows third parties to download the articles and share them with others as long as they credit the author and the Abstract Book, but they cannot change the content in any way or use them commercially.
**Abstract Book Citations:** Authors, Title, HemaSphere, 2026;10:(S1):pages. The abstract book DOIs can be found on the "Author Information" page.
**Disclaimer:** Articles published in the journal HemaSphere exclusively reflect the opinions of the authors. The authors are responsible for all content in their abstracts including accuracy of the facts, statements, citing resources, etc.

**Summary/Conclusion**

Platelet clone size closely correlates with granulocyte and monocyte clone burden and may represent a useful complementary or alternative tool, particularly in neutropenic patients. CD55/CD59-based analysis allows simultaneous erythrocyte and platelet evaluation in a single tube, optimizing workflow and potentially reducing costs while preserving diagnostic accuracy. Elevated baseline CD62P expression in treated patients suggests persistent platelet activation despite anti-C5 therapy, supporting further investigation of platelet dysfunction and residual thrombotic risk. Future studies should expand platelet markers, activation analysis, and include treatment-naïve patients.

**Copyright Information:** (Online) ISSN: 2572-9241
©2026 The Author(s). HemaSphere is published by John Wiley & Sons Ltd on behalf of European Hematology Association. This is an open access Abstract Book distributed under the Attribution-NonCommercial-NoDerivs (CC BY-NC-ND), which allows third parties to download the articles and share them with others as long as they credit the author and the Abstract Book, but they cannot change the content in any way or use them commercially.
**Abstract Book Citations:** Authors, Title, HemaSphere, 2026;10:(S1):pages. The abstract book DOIs can be found on the "Author Information" page.
**Disclaimer:** Articles published in the journal HemaSphere exclusively reflect the opinions of the authors. The authors are responsible for all content in their abstracts including accuracy of the facts, statements, citing resources, etc.

### (PF695) 3-YEAR EFFICACY AND SAFETY OF CROVALIMAB IN PATIENTS WITH PAROXYSMAL NOCTURNAL HEMOGLOBINURIA (PNH) WHO SWITCHED FROM ECULIZUMAB: LONG-TERM DATA FROM THE PHASE III COMMODORE 1 TRIAL

**Topic:** 12. Bone marrow failure syndromes incl. PNH - Clinical

Fahri Sahin*[1], Koji Ando[2], Wilma Barcellini[3], Ana Rita Fonseca[4, 5], Gabriela Roncone Gastal[6], Deniz Yilmaz Karapinar[7], Yoojin Lee[8], Vahap Okan[9], Tahiane Soares[10], Sammy Chebon[11], Siobhan Connor-Ahmad[12], Atsue Sawai[13], Sasha Sreckovic[13], Diego Cle[14]

*[1]Ege University Faculty of Medicine, Department of Hematology, Izmir, Türkiye;[2]Atomic Bomb Disease Institute, Nagasaki University, Department of Hematology, Atomic Bomb Disease and Hibakusha Medicine Unit, Nagasaki, Japan; [3]Yonsei University College of Medicine, Severance Hospital, Seoul, South Korea; [4]Universidad Federal de São Paulo, Department of Clinical and Experimental Hematology, São Paulo, Brazil; [5]Hospital Sírio Libanês, São Paulo, Brazil; [6]University of Joinville Region (UNIVILLE), Department of Hematology, Joinville, Brazil; [7]Ege University, Faculty of Medicine, Division of Pediatric Hematology, Department of Pediatrics, İzmir, Türkiye; [8]Ulsan University Hospital, University of Ulsan College of Medicine, Department of Hematology and Oncology, Ulsan, South Korea; [9]Gaziantep University Medical Faculty, Department of Internal Diseases, Division of Hematology, Gaziantep, Türkiye; [10]Hospital de Clínicas de Porto Alegre, Serviço de Hematologia, Porto Alegre, Brazil; [11]F. Hoffman-La Roche Ltd, Basel, Switzerland; [12]Roche Products Ltd., Welwyn Garden City, United Kingdom; [13]Genentech, Inc., South San Francisco, United States of America; [14]Ribeirão Preto School of Medicine, University of São Paulo, Ribeirão Preto, Brazil;*

### Background

Crovalimab (crova; PiaSky®), a novel C5 inhibitor (C5i), enables maintenance subcutaneous injections every 4 weeks (wks). At the time of primary analysis of the global, randomized, Phase III COMMODORE 1 trial (NCT04432584), crova was well tolerated and showed similar efficacy in patients (pts) with PNH who switched from eculizumab (ecu) to crova vs pts who continued ecu (Scheinberg 2024). These results were further supported by data in C5i-naive pts from the COMMODORE 2 trial (NCT04434092; Röth 2024).

### Aims

To report 3-year follow-up data from COMMODORE 1.

### Methods

Adult pts with PNH who received ecu for ≥24 wks with lactate dehydrogenase (LDH) ≤1.5×upper limit of normal (ULN) were randomized 1:1 to switch to crova (Arm A; weight-based tiered dosing) or continue ecu (Arm B) for a 24-wk primary treatment period. Pts who completed the primary treatment period continued crova in Arm A and had the option to switch from ecu to crova in Arm B (Arm B switch) during the extension period. Long-term safety data were pooled across Arms A and B switch and were assessed from the first crova dose up to the clinical cutoff date. Efficacy was assessed from W25–193 in the extension period by evaluating the following endpoints: mean and proportion of pts with hemolysis control (LDH≤1.5×ULN; by visit); proportions of pts with transfusion avoidance (TA), breakthrough hemolysis (BTH), and hemoglobin (Hb) stabilization (all across consecutive 24-wk intervals). Fatigue was assessed by visit (W25–193) using the Functional Assessment of Chronic Illness Therapy (FACIT)-Fatigue score.

**Copyright Information:** (Online) ISSN: 2572-9241
©2026 The Author(s). HemaSphere is published by John Wiley & Sons Ltd on behalf of European Hematology Association. This is an open access Abstract Book distributed under the Attribution-NonCommercial-NoDerivs (CC BY-NC-ND), which allows third parties to download the articles and share them with others as long as they credit the author and the Abstract Book, but they cannot change the content in any way or use them commercially.
**Abstract Book Citations:** Authors, Title, HemaSphere, 2026;10:(S1):pages. The abstract book DOIs can be found on the “Author Information” page.
**Disclaimer:** Articles published in the journal HemaSphere exclusively reflect the opinions of the authors. The authors are responsible for all content in their abstracts including accuracy of the facts, statements, citing resources, etc.

**Results**

Of the 86 pts who received treatment (Arm A: 44; Arm B: 42), 44 pts in Arm A continued crova and 40 pts in Arm B switched to crova after W25 (median treatment duration calculated across both arms: 3.1 years; range, 0.1–4.7). Long-term safety data were pooled across both arms (84 pts; 260.8 pt-years [PY]). The incidence per 100 PY (95% CI) of serious adverse events (AEs) was 29.5 (23.3, 36.9), most commonly BTH, 2.7 (1.1, 5.5); COVID-19, 2.3 (0.8, 5.0); and pneumonia, 1.9 (0.6, 4.5). Fatal AE rate was 1.9 (0.6, 4.5) per 100 PY; all 5 deaths were unrelated to treatment. Transient immune complex reactions (TICRs), one-time events that may occur in pts switching between crova and another C5i, occurred in 18% of pts (15/84). TICR signs or symptoms were mainly mild or moderate skin and joint abnormalities (rash and arthralgia), with no renal manifestations, or life-threatening or fatal events, and resolved without dose modification or interruption of crova treatment. No meningococcal infections occurred.
Mean normalized LDH remained ≤1.5×ULN from W25–193 in both arms; hemolysis control was achieved in 76–93% of pts in Arm A and 86–100% of pts in Arm B switch by visit (W25–193). TA, Hb stabilization, and BTH rates were consistent with 2-year follow-up data (**Table**). Mean FACIT-Fatigue scores remained stable by visit (W25–193), ranging from 40.1 to 46.1 in pts from Arm A and 32.3 to 40.8 in pts from Arm B switch.

**Summary/Conclusion**

Crova was well tolerated during long-term treatment in COMMODORE 1, showing a durable efficacy profile by sustaining disease control over a >3-year median treatment duration in pts who switched from ecu. Crova's safety profile was consistent with that of other C5is, except for the identified risk of typically mild or moderate and manageable TICRs during the switch. These results confirm the favorable long-term benefit–risk profile of crova.

**Table. Range of proportions of patients who achieved endpoints over consecutive 24-wk intervals from W25–193.**

| Endpoint | Arm A (continued crova) | Arm B switch (switched from ecu to crova) |
|---|---|---|
| **TA** | 76%–87% | 77%–86% |
| **Hb stabilization** | 67%–74% | 62%–86% |
| **BTH** | 4%–14% | 0%–12% |

BTH, breakthrough hemolysis; crova, crovalimab; ecu, eculizumab; Hb, hemoglobin; TA, transfusion avoidance.

**Copyright Information:** (Online) ISSN: 2572-9241
©2026 The Author(s). HemaSphere is published by John Wiley & Sons Ltd on behalf of European Hematology Association. This is an open access Abstract Book distributed under the Attribution-NonCommercial-NoDerivs (CC BY-NC-ND), which allows third parties to download the articles and share them with others as long as they credit the author and the Abstract Book, but they cannot change the content in any way or use them commercially.
**Abstract Book Citations:** Authors, Title, HemaSphere, 2026;10:(S1):pages. The abstract book DOIs can be found on the "Author Information" page.
**Disclaimer:** Articles published in the journal HemaSphere exclusively reflect the opinions of the authors. The authors are responsible for all content in their abstracts including accuracy of the facts, statements, citing resources, etc.

### (PF696) COMPARISONS OF TERMINAL COMPLEMENT INHIBITION WITH POZELIMAB + CEMDISIRAN VERSUS RAVULIZUMAB IN PARTICIPANTS WITH PAROXYSMAL NOCTURNAL HEMOGLOBINURIA: PHARMACODYNAMIC RESULTS FROM COHORT A

**Topic:** 12. Bone marrow failure syndromes incl. PNH - Clinical

Morag Griffin*[1], Jun Ho Jang[2], Dipti Pawaskar[3], Kuan-Ju Lin[3], Karoline Meagher[3], Elham Shirvani Dastgerdi[3], Mu Chen[3], Deepak Taneja[3], Eric Bachman[3], Lorah Perlee[3], Umesh Chaudhari[3], Christopher Patriquin[4]

*[1]St James's University Hospital, Leeds, United Kingdom; [2]Samsung Medical Center, Seoul, South Korea; [3]Regeneron Pharmaceuticals, Inc., Tarrytown, United States of America; [4]University Health Network, Toronto General Hospital, Toronto, Canada;*

### Background

Pozelimab is a human monoclonal IgG4P antibody directed against the terminal complement protein C5; cemdisiran is a synthetic siRNA targeting C5 mRNA that inhibits production of C5 protein in the liver. The combination of pozelimab and cemdisiran (combination therapy) is being investigated for the treatment of paroxysmal nocturnal hemoglobinuria (PNH), an ultra-rare disorder caused by dysregulation of the complement system and characterized by hemolysis and/or thrombosis. We report pharmacodynamic (PD) data from Cohort A of a randomized, open-label study of combination therapy compared to ravulizumab.

### Aims

To compare the effect of combination therapy versus ravulizumab on PD complement markers in participants with PNH.

### Methods

In this 26-week, open-label, ravulizumab-controlled study, participants with PNH were randomized (1:1) to receive either combination therapy (single IV loading dose of pozelimab 30 mg/kg plus subcutaneous [SC] pozelimab 400 mg plus cemdisiran 200 mg [Day 1], followed by SC pozelimab 400 mg plus cemdisiran 200 mg every 4 weeks) or ravulizumab (single weight-based IV loading dose [Day 1] followed by weight-based maintenance dose [Day 15] and every 8 weeks thereafter). Key secondary endpoints, assessed using validated assays, included concentration of total C5, and change and percentage change in total complement hemolytic activity assay (CH50) and alternative pathway hemolytic activity (AH50) from baseline to Week 26. Exploratory endpoints included the measurement of complement activation marker (sC5b-9) from baseline to Week 26.

**Copyright Information:** (Online) ISSN: 2572-9241
©2026 The Author(s). HemaSphere is published by John Wiley & Sons Ltd on behalf of European Hematology Association. This is an open access Abstract Book distributed under the Attribution-NonCommercial-NoDerivs (CC BY-NC-ND), which allows third parties to download the articles and share them with others as long as they credit the author and the Abstract Book, but they cannot change the content in any way or use them commercially.
**Abstract Book Citations:** Authors, Title, HemaSphere, 2026;10:(S1):pages. The abstract book DOIs can be found on the “Author Information” page.
**Disclaimer:** Articles published in the journal HemaSphere exclusively reflect the opinions of the authors. The authors are responsible for all content in their abstracts including accuracy of the facts, statements, citing resources, etc.

**Results**

A total of 48 participants were enrolled (n=25 combination therapy; n=23 ravulizumab). Total C5 concentration was reduced rapidly with combination therapy to below the limit of detection and was sustained through Week 26 due to cemdisiran reducing C5 hepatocyte production. By contrast, ravulizumab led to a >2-fold rise in mean total C5 protein concentration by Day 15, reaching steady-state by Week 6 (due to C5 binding to ravulizumab, increasing the half-life; differential pharmacology will be discussed).
CH50 complement activity was completely inhibited by Day 15, with ~100% suppression maintained through Week 26 following combination therapy. With ravulizumab, CH50 was reduced by approximately 90% at Day 15, and sustained suppression of 89.3–97.4% through Week 26, but did not achieve complete inhibition.
Mean AH50 activity reduced by approximately 80% at Week 4 and maintained robust suppression (77.3–83.7%) through Week 26 following combination therapy. Ravulizumab led to a more modest decrease (approximately 45%) at Week 4 and was maintained at 38.8–46.5% through Week 26.
Terminal complement complex (sC5b-9) levels decreased by 76.0% at Day 15 and maintained reductions of 72.9–80.8% through Week 26 following combination therapy. In contrast, sC5b-9 increased by 22.9% at Day 15 and levels were maintained at 0–25% from Week 4 through Week 26 with ravulizumab. The consistent reductions observed with combination therapy indicate less terminal complement activation compared to ravulizumab.

**Summary/Conclusion**

Combination therapy produced more rapid and durable effects across CH50 and AH50 versus ravulizumab in participants with PNH. These data indicate earlier onset and superior complement pathway control with the combination therapy compared to ravulizumab, supporting its potential to provide uninterrupted terminal complement activity suppression over 26 weeks.

**Copyright Information:** (Online) ISSN: 2572-9241
©2026 The Author(s). HemaSphere is published by John Wiley & Sons Ltd on behalf of European Hematology Association. This is an open access Abstract Book distributed under the Attribution-NonCommercial-NoDerivs (CC BY-NC-ND), which allows third parties to download the articles and share them with others as long as they credit the author and the Abstract Book, but they cannot change the content in any way or use them commercially.
**Abstract Book Citations:** Authors, Title, HemaSphere, 2026;10:(S1):pages. The abstract book DOIs can be found on the "Author Information" page.
**Disclaimer:** Articles published in the journal HemaSphere exclusively reflect the opinions of the authors. The authors are responsible for all content in their abstracts including accuracy of the facts, statements, citing resources, etc.

### (PF697) 3-YEAR EFFICACY AND SAFETY OF CROVALIMAB IN PATIENTS WITH PAROXYSMAL NOCTURNAL HEMOGLOBINURIA (PNH) NAIVE TO COMPLEMENT INHIBITORS: LONG-TERM DATA FROM THE PHASE III COMMODORE 2 TRIAL

**Topic:** 12. Bone marrow failure syndromes incl. PNH - Clinical

Naoshi Obara*[1], Narcisa Sonia Comia[2], Ai Sim Goh[3], Chattree Hantaweepant[4], Jun Ho Jang[5], Sharifah Shahnaz Syed Kadir[6], Ponlapat Rojnuckarin[7], Fernando Silva[8], Asad Charania[9], Sammy Chebon[9], Siobhan Connor-Ahmad[10], Sasha Sreckovic[11], Bing Han[12]

*[1]University of Tsukuba Hospital, Department of Hematology, Tsukuba, Japan;[2]Mary Mediatrix Medical Center, Lipa, Philippines; [3]Hospital Pulau Pinang, Georgetown, Malaysia; [4]Mahidol University, Bangkok, Thailand; [5]Samsung Medical Center, Sungkyunkwan University School of Medicine, Seoul, South Korea; [6]Ampang Hospital, Ampang Jaya, Malaysia; [7]Chulalongkorn University and King Chulalongkorn Memorial Hospital, Center of Excellence in Translational Hematology, Bangkok, Thailand; [8]Centro Hospitalar do Baixo Vouga, Medical Science Department of Aveiro University, Aveiro, Portugal;[9]F. Hoffmann-La Roche Ltd, Basel, Switzerland; [10]Roche Product Ltd, Welwyn Garden City, United Kingdom; [11]Genentech, Inc., South San Francisco, United States of America; [12]Peking Union Medical College Hospital, Peking Union Medical College, Chinese Academy of Medical Sciences, Peking, China;*

### Background

Crovalimab (crova; PiaSky®), a novel C5 inhibitor (C5i), enables maintenance subcutaneous (SC) dosing every 4 weeks (wks). The global, randomized, Phase III COMMODORE 2 (NCT04434092) trial demonstrated crova's non-inferior efficacy and comparable safety vs eculizumab (ecu) in patients (pts) with PNH who were C5i-naive (Röth 2024). These findings were supported by data in C5i-experienced COMMODORE 1 pts (NCT04432584; Scheinberg 2024).

### Aims

Report 3-year follow-up data from COMMODORE 2.

### Methods

C5i-naive pts with PNH were randomized 2:1 to crova (Arm A) or ecu (Arm B) for a 24-wk primary treatment period. Pts who continued in the extension period continued crova (Arm A) or switched from ecu to crova (Arm B switch).

Long-term data were evaluated for Arm A and Arm B switch pts who received crova. Efficacy, assessed up to W193, was reported as means and proportions of pts with hemolysis control (LDH ≤1.5×upper limit of normal [ULN]) at all visits, and transfusion avoidance (TA), breakthrough hemolysis (BTH), and hemoglobin (Hb) stabilization over consecutive 24-wk intervals. Fatigue was assessed by visit, using the Functional Assessment of Chronic Illness Therapy (FACIT)-Fatigue scale. Long-term safety, pooled across both arms, was assessed from the first crova dose up to the clinical cutoff date.

**Copyright Information:** (Online) ISSN: 2572-9241
©2026 The Author(s). HemaSphere is published by John Wiley & Sons Ltd on behalf of European Hematology Association. This is an open access Abstract Book distributed under the Attribution-NonCommercial-NoDerivs (CC BY-NC-ND), which allows third parties to download the articles and share them with others as long as they credit the author and the Abstract Book, but they cannot change the content in any way or use them commercially.
**Abstract Book Citations:** Authors, Title, HemaSphere, 2026;10:(S1):pages. The abstract book DOIs can be found on the "Author Information" page.
**Disclaimer:** Articles published in the journal HemaSphere exclusively reflect the opinions of the authors. The authors are responsible for all content in their abstracts including accuracy of the facts, statements, citing resources, etc.

**Results**

Of 204 pts enrolled (Arm A: 135; Arm B: 69), 129 Arm A pts continued crova, and 68 Arm B pts switched from ecu to crova in the extension period after W25. Median treatment duration calculated across both arms was 3.4 yrs (range: <0.1–4.5).

Mean normalized LDH remained ≤1.5×ULN in both arms from W25–193, with 78–94% of Arm A and 74–93% of Arm B switch pts achieving hemolysis control. TA, Hb stabilization, and BTH rates were generally consistent (**Table**). Mean FACIT-Fatigue scores remained stable, ranging from 41.9 to 43.7 in Arm A and 40.1 to 42.7 in Arm B switch, from W25–193.

Long-term safety data during crova treatment were pooled across Arm A and Arm B switch (203 pts; 672.5 pt-y [PY]). The incidence per 100 PY (95% CI) of serious AEs was 25.7 (22.0–29.9), most commonly COVID-19, 1.9 (1.0, 3.3); pneumonia, 1.8 (0.9, 3.1); and pyrexia, 1.0 (0.4, 2.1); for serious AEs of infection the incidence was 9.7 (7.5, 12.3.) per 100 PY. The fatal AE rate was 1.3 (0.6–2.5) per 100 PY; all 9 fatal AEs were unrelated to treatment.

Transient immune complex reactions (TICRs; one-time events which can only occur upon switching between C5is) occurred in 18% of Arm B switch pts (12/68) during the extension period. The median time to first TICR was 1.4 wks (range: 0.3–4.4). Signs or symptoms reported in ≥2 pts were arthralgia, rash and headache; none had renal involvement. Most TICRs were mild or moderate with no life-threatening or fatal events reported. No meningococcal infections were reported.

**Summary/Conclusion**

In COMMODORE 2, crova demonstrated sustained disease control and long-term tolerability over a >3-y median duration. The crova safety profile was consistent with other C5is, excluding the identified and manageable risk of TICRs, usually mild or moderate, when switching between C5is. These findings further confirm the long-term favorable benefit–risk profile of crova in pts with PNH.

**Table. Range of proportions of patients who achieved endpoints over consecutive 24-wk intervals from W25 to W193.**

| Endpoint | Arm A (continued crova) | Arm B switch (switched from ecu to crova) |
|---|---|---|
| TA | 73%–82% | 70%–81% |
| Hb stabilization | 70%–80% | 64%–81% |
| BTH | 2%–7% | 3%–12% |

BTH, breakthrough hemolysis; crova, crovalimab; ecu, eculizumab; Hb, hemoglobin; TA, transfusion avoidance.

Copyright Information: (Online) ISSN: 2572-9241
©2026 The Author(s). HemaSphere is published by John Wiley & Sons Ltd on behalf of European Hematology Association. This is an open access Abstract Book distributed under the Attribution-NonCommercial-NoDerivs (CC BY-NC-ND), which allows third parties to download the articles and share them with others as long as they credit the author and the Abstract Book, but they cannot change the content in any way or use them commercially.
Abstract Book Citations: Authors, Title, HemaSphere, 2026;10:(S1):pages. The abstract book DOIs can be found on the "Author Information" page.
Disclaimer: Articles published in the journal HemaSphere exclusively reflect the opinions of the authors. The authors are responsible for all content in their abstracts including accuracy of the facts, statements, citing resources, etc.

### (PF698) CARDIOVASCULAR COMORBIDITY BURDEN IN VEXAS SYNDROME: GENOTYPE-STRATIFIED, IMAGING AND LONGITUDINAL FINDINGS FROM THE ITALIAN COHORT

**Topic:** 12. Bone marrow failure syndromes incl. PNH - Clinical

Elisa Meddi*[1], Paola Triggianese[1], Giorgia Ranucci[1], Elisa Diral[2], Corrado Campochiaro[3], Marianna Velocci[1], Francesca Romano[1], Vittorio Forte[4], Alessandro Tomelleri[3], Gregorio Maria Bergonzi[2], Agnese MacCioni[1], Jacqueline Ferrari[5], Mariachiara Cuccaro[6], Sara Bindoli[7], Micol Frassi[8], Erika Morsia[9], Cristina Papayannidis[10], Mara Memoli[11], Pellegrino Musto[12], Elena Crisà[13], Arianna Savi[14], Laura Cicconi[15], Gabriele Facchin[16], Giulia Rivoli[17], Antonio Pierini[18], Massimo Breccia[19], Isabella Capodanno[20], Davide Firinu[21], Maria Di Matteo[22], Gabriele Todisco[22], Bruno Fattizzo[23], Antonio Vitale[24], Mario Laudazi[25], Riccardo Morgagni[26], Francesco Garaci[25], Adriano Venditti[1], Nicola Toschi[1], Maria Teresa Voso[1], Carmelo Gurnari[1]

*[1]Policlinico Tor Vergata, Department of Biomedicine and Prevention, University of Rome Tor Vergata, Rome, Italy, Roma, Italy; [2]IRCCS San Raffaele Hospital, Unit of Hematology and Bone Marrow Transplantation, Milan, Italy; [3]IRCCS Ospedale San Raffaele & Vita-Salute San Raffaele University, Unit of Immunology, Rheumatology, Allergy and Rare Diseases (UnIRAR),, Milan, Italy; [4]University of Rome Tor Vergata, Clinical Biochemistry Department of Laboratory Medicine, Rome, Italy; [5]Fondazione IRCCS Policlinico San Matteo, Unit of Molecular Hematology & Precision Medicine, Pavia, Italy; [6]Azienda Ospedaliero Universitaria Senese, University of Siena, Hematology Unit, Siena, Italy; [7]University of Padova, Rheumatology Unit, Department of Medicine, Padua, Italy; [8]Civil Hospital Brescia, Rheumatology and Clinical Immunology Unit, Brescia, Italy; [9]Azienda Ospedaliera Universitaria Ospedali Riuniti di Ancona, Hematology Department, Ancona, Italy; [10]IRCCS Azienda Ospedaliero-Universitaria di Bologna, Istituto di Ematologia "Seràgnoli", Bologna, Italy; [11]University of Naples "Federico II", Hematology, Department of Clinical Medicine and Surgery, Naples, Italy; [12]"Aldo Moro" University, Department of Precision and Regenerative Medicine and Ionian Area, Bari, Italy; [13]Università del Piemonte Orientale and Azienda Ospedaliero-Universitaria Maggiore della Carità, Division of Hematology, Department of Translation Medicine, Novara, Italy; [14]University of Turin, Department of Clinical and Biological Sciences, Turin, Italy; [15]S.M. Goretti Hospital, Department of Hematology, Polo Universitario Pontino,, Latina, Italy; [16]Azienda Sanitaria Universitaria Friuli Centrale, Division of Hematology, S. Maria della Misericordia Hospital, Udine, Italy; [17]IRCCS Ospedale Policlinico San Martino, UO Ematologia e Terapie Cellulari, Genoa, Italy; [18]University of Perugia, Section of Hematology, Department of Medicine and Surgery, Center for Hemato-Oncological Research (CREO), Perugia, Italy; [19]Sapienza University of Rome, Department of Translational and Precision Medicine, Rome, Italy; [20]SOC Ematologia Azienda USL-IRCSS di Reggio Emilia, Reggio Emilia, Italy; [21]University of Cagliari, Department of Medical Sciences and Public Health, Cagliari, Italy; [22]Humanitas University, Department of Biomedical Sciences, Milan, Italy; [23]University of Milan, Department of Oncology and Hemato-Oncology,, Milan, Italy; [24]University of Siena and Azienda Ospedaliero-Universitaria Senese, Rheumatology Unit, Department of Medical Sciences, Siena, Italy; [25]University of Rome "Tor Vergata", Department of Biomedicine and Prevention, Division of Diagnostic Imaging, Rome, Italy; [26]University Tor Vergata, Department of Cardiology and Interventional Cardiology, Rome, Italy;*

### Background

VEXAS syndrome is an adult-onset autoinflammatory syndrome genetically defined by somatic *UBA1*mutations. Cardiovascular (CV) comorbidities are sparsely described in VEXAS, with their overall burden, longitudinal evolution, and potential heterogeneity across *UBA1* M41 hotspot mutations remaining ill-defined.

### Aims

Here, we characterized CV burden, genotype associations, imaging and longitudinal outcomes in a national Italian VEXAS study.

**Copyright Information:** (Online) ISSN: 2572-9241
©2026 The Author(s). HemaSphere is published by John Wiley & Sons Ltd on behalf of European Hematology Association. This is an open access Abstract Book distributed under the Attribution-NonCommercial-NoDerivs (CC BY-NC-ND), which allows third parties to download the articles and share them with others as long as they credit the author and the Abstract Book, but they cannot change the content in any way or use them commercially.
**Abstract Book Citations:** Authors, Title, HemaSphere, 2026;10:(S1):pages. The abstract book DOIs can be found on the "Author Information" page.
**Disclaimer:** Articles published in the journal HemaSphere exclusively reflect the opinions of the authors. The authors are responsible for all content in their abstracts including accuracy of the facts, statements, citing resources, etc.

### Methods

Cardiac involvement was defined as > 1 among heart failure (HF), advanced diastolic dysfunction (DD), valvular heart disease (VHD), pericardial signs, atrial fibrillation (AF), reduced (> -18%, less negative) Global Longitudinal Strain (GLS) on echocardiogram. Genotype-stratified analyses focused on *UBA1* M41 variants. The number of rheumatologic treatment lines served as a marker of cumulative treatment exposure and disease course.

### Results

After merging clinical and cardiac datasets, 93/109 total patients had integrable data, including 79 M41 patients. Traditional CV risk factors were common and not differed meaningfully across M41 variants: hypertension in 40/79 (51%), diabetes in 18/79 (23%), dyslipidemia in 26/79 (33%), smoking history in 31/71 (44%).
At baseline, any CV involvement was present in 42/73 evaluable M41 patients (58%) and did not differ across the 3 canonical M41L, M41T, M41V genotypes (63%, 53%, and 60%, respectively). At last follow-up, any CV involvement was present in 31/59 (53%) evaluated M41 patients, with a borderline overall difference across the three M41 groups (p=0.06). Notably, follow-up involvement was higher in M41T (68% vs 39% in M41L/V, p=0.03; OR 3.34, 95% CI 1.14–9.77). Conversely, M41V was enriched for tricuspid VHD (7/10, 70% vs 0/11, 0% in M41L/T; p<0.01).
Paired baseline–follow-up analyses did not show a significant overall increase in HF, DD, VHD, pericardial signs, AF, or total cardiac features over time (Wilcoxon p≈0.66) at a median follow-up duration of 0.93 years (IQR 0.31–2.04). Baseline cardiac involvement was the strongest predictor of follow-up involvement (OR 4.95, 95% CI 1.44–17.00; p=0.01), with M41T retaining significance after model adjustment (OR 4.35; p=0.03). Among patients without baseline CV disease, a new onset of CV involvement occurred in 27% and was associated with a higher number of treatment lines (median 4 vs 2; p=0.05). Furthermore, each additional treatment line was associated with higher odds of follow-up cardiac involvement (OR 1.51; p=0.03).
Finally, GLS and cardiac MRI at baseline and during disease flares in a subset of cases (n=6) identified subclinical ventricular dysfunction even with preserved ejection fraction (EF). Impaired longitudinal strain, despite normal EF, reflected early systolic dysfunction compensated by circumferential strain. Patients with reduced GLS showed clinical worsening, a finding confirmed by MRI (**Fig.1**).

### Summary/Conclusion

In our Italian VEXAS cohort, CV risk factors and cardiac involvement (clinical and subclinical) were common. M41V was strongly associated with a distinct valvular pattern, whereas follow-up analyses suggested higher cardiac involvement in M41T. While cardiac burden did not clearly increase over time, a greater treatment burden associated with incident and/or follow-up cardiac involvement, likely reflecting cumulative disease severity and/or increased cardiac work-up. Implementation of longitudinal, standardized cardiac assessment integrated with GLS/MRI is ongoing to better define cardiologic trajectories in VEXAS.

**Copyright Information:** (Online) ISSN: 2572-9241
©2026 The Author(s). HemaSphere is published by John Wiley & Sons Ltd on behalf of European Hematology Association. This is an open access Abstract Book distributed under the Attribution-NonCommercial-NoDerivs (CC BY-NC-ND), which allows third parties to download the articles and share them with others as long as they credit the author and the Abstract Book, but they cannot change the content in any way or use them commercially.
**Abstract Book Citations:** Authors, Title, HemaSphere, 2026;10:(S1):pages. The abstract book DOIs can be found on the "Author Information" page.
**Disclaimer:** Articles published in the journal HemaSphere exclusively reflect the opinions of the authors. The authors are responsible for all content in their abstracts including accuracy of the facts, statements, citing resources, etc.

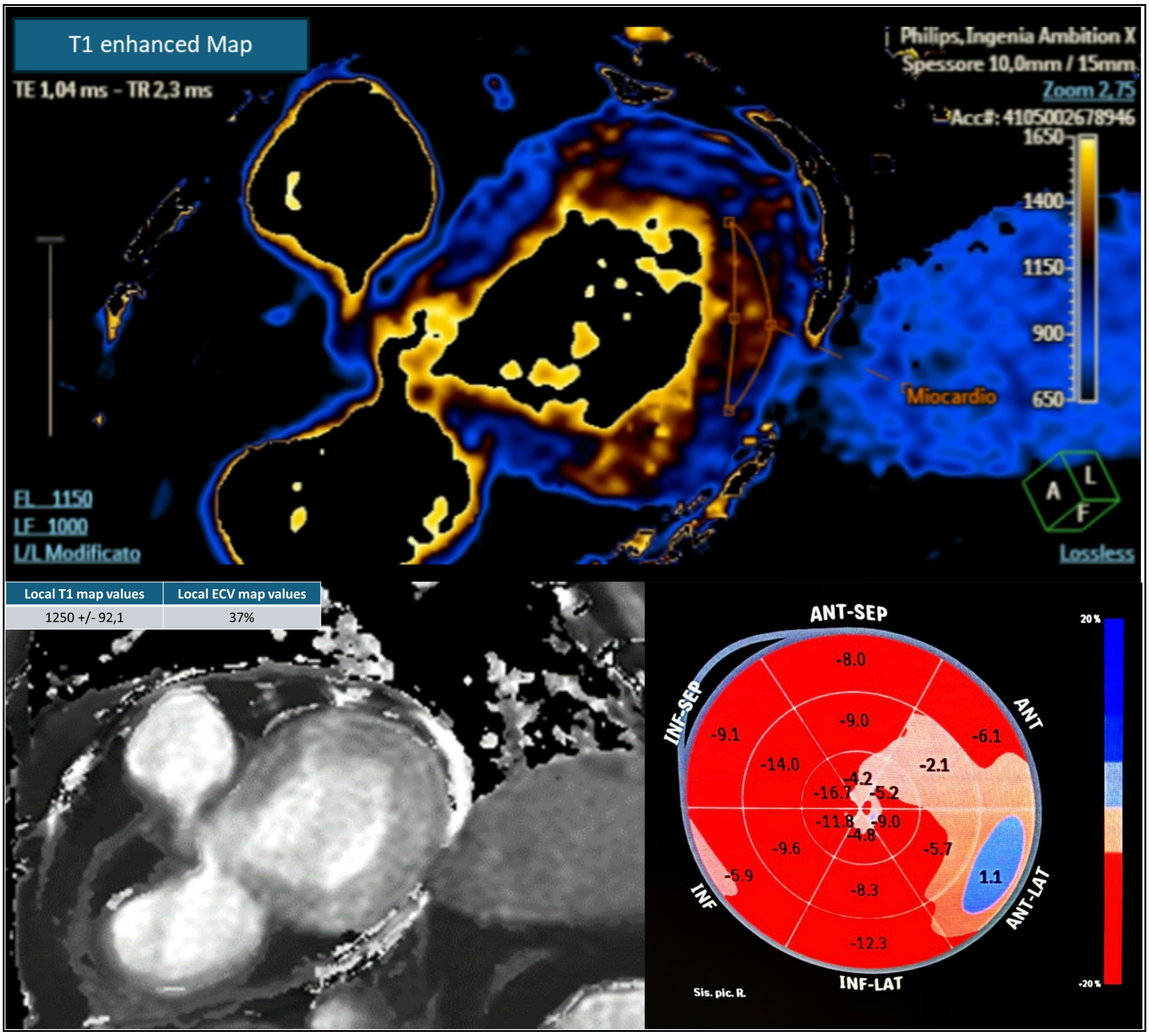

Copyright Information: (Online) ISSN: 2572-9241
©2026 The Author(s). HemaSphere is published by John Wiley & Sons Ltd on behalf of European Hematology Association. This is an open access Abstract Book distributed under the Attribution-NonCommercial-NoDerivs (CC BY-NC-ND), which allows third parties to download the articles and share them with others as long as they credit the author and the Abstract Book, but they cannot change the content in any way or use them commercially.
Abstract Book Citations: Authors, Title, HemaSphere, 2026;10:(S1):pages. The abstract book DOIs can be found on the "Author Information" page.
Disclaimer: Articles published in the journal HemaSphere exclusively reflect the opinions of the authors. The authors are responsible for all content in their abstracts including accuracy of the facts, statements, citing resources, etc.

### (PF699) CONSISTENT AND SUSTAINED EFFICACY AND SAFETY OF IPTACOPAN IN PAROXYSMAL NOCTURNAL HEMOGLOBINURIA REGARDLESS OF PRIOR HISTORY OF MAJOR ADVERSE VASCULAR EVENTS IN APPLY, APPOINT AND EXTENSION PROGRAM

**Topic:** 12. Bone marrow failure syndromes incl. PNH - Clinical

Bing Han*[1], Phillip Scheinberg[2], Yasutaka Ueda[3], Alexander Röth[4], Eros Di Bona[5], Rong Fu[6], Morag Griffin[7], Richard J. Kelly[7], Jens Panse[8, 9], Hubert Schrezenmeier[10, 11], Flore Sicre de Fontbrune[12, 13], Camilla Frieri[14], Philippe Ferber[15], Silvia Sanz[16], Luca Monaco[15], Erik Houtsma[17], Darlington Mapiye[18], Austin G. Kulasekararaj[12, 19, 20, 21]

*[1]Peking Union Medical College Hospital, Chinese Academy of Medical Science and Peking Union Medical College, Beijing, China; [2]Hospital A Beneficência Portuguesa, São Paolo, Brazil; [3]The University of Osaka Graduate School of Medicine, Suita, Japan; [4]Department of Hematology and Stem Cell Transplantation, West German Cancer Center, University Hospital Essen, University Duisburg-Essen, Essen, Germany; [5]UOC Oncoematologia, AULSS7 Pedemontana, Bassano del Grappa, Vicenza, Italy; [6]Tianjin Medical University General Hospital, Tianjin, China; [7]St James's University Hospital, Leeds, United Kingdom; [8]University Hospital RWTH Aachen, Aachen, Germany; [9]Center for Integrated Oncology Aachen Bonn Cologne Düsseldorf, Aachen, Germany; [10]University of Ulm, Institute of Transfusion Medicine and Immunogenetics, Ulm, Germany; [11]German Red Cross Blood Transfusion Service Baden-Württemberg-Hessen and University Hospital Ulm, Ulm, Germany; [12]The Severe Aplastic Anemia Working Party of the European Society for Blood and Marrow Transplantation, Barcelona, Spain; [13]French Reference Center for Aplastic Anemia and Paroxysmal Nocturnal Hemoglobinuria, Saint-Louis Hospital, Paris, France; [14]U.O.C Ematologia e Terapie cellulari avanzate, AORN San Giuseppe Moscati, Avellino, Italy; [15]Novartis Pharma AG, Basel, Switzerland; [16]Novartis Farmacéutica, S.A., Barcelona, Spain; [17]Novartis Ireland Ltd., Dublin, Ireland; [18]IQVIA, Reading, United Kingdom; [19]King's College Hospital NHS, London, United Kingdom; [20]National Institute for Health and Care Research and Wellcome King's Research Facility, London, United Kingdom; [21]King's College London, London, United Kingdom;*

### Background

In the phase 3 APPLY-PNH (NCT04558918) and APPOINT-PNH (NCT04820530) studies and the roll-over extension program (PNH-REP; NCT04747613) iptacopan showed sustained hematologic control and a favorable safety profile in patients with paroxysmal nocturnal hemoglobinuria (PNH). Patients with a prior history of major adverse vascular events (MAVEs) are a clinically important high-risk subgroup.

### Aims

To report the safety and efficacy of iptacopan in patients from APPLY-PNH, APPOINT-PNH and the PNH-REP, according to prior history of MAVEs.

### Methods

All patients received iptacopan monotherapy 200 mg twice daily during the parent studies and the PNH-REP. The first dose was considered baseline. Efficacy assessments included levels of hemoglobin and lactate dehydrogenase (LDH), absolute reticulocyte count, transfusion avoidance, FACIT-fatigue score, breakthrough hemolysis (BTH) rates and prevention of MAVEs. Safety assessments included incidence of treatment-emergent adverse events (TEAEs) and serious adverse events (SAEs).

**Copyright Information:** (Online) ISSN: 2572-9241
©2026 The Author(s). HemaSphere is published by John Wiley & Sons Ltd on behalf of European Hematology Association. This is an open access Abstract Book distributed under the Attribution-NonCommercial-NoDerivs (CC BY-NC-ND), which allows third parties to download the articles and share them with others as long as they credit the author and the Abstract Book, but they cannot change the content in any way or use them commercially.
**Abstract Book Citations:** Authors, Title, HemaSphere, 2026;10:(S1):pages. The abstract book DOIs can be found on the "Author Information" page.
**Disclaimer:** Articles published in the journal HemaSphere exclusively reflect the opinions of the authors. The authors are responsible for all content in their abstracts including accuracy of the facts, statements, citing resources, etc.

### Results

The analysis population comprised 136 patients; 27 (19.9%) had a history of MAVE (HoM) and 109 (80.1%) had no history of MAVE (NHoM). Baseline demographics and clinical characteristics were similar between the subgroups. At data cut-off, study participation was completed or ongoing in 23/27 (85.1%) of HoM patients and 99/109 (90.8%) of NHoM patients. Hemolytic control was consistently sustained with up to 4 years' follow up, regardless of whether patients had a prior history of MAVE (Figure). BTH occurred in 2/27 HoM patients (2.3 events/100 patient-years [py]) and 21/109 NHoM patients (9.7 events/100 py). A total of 6 MAVEs occurred in 5 patients, originating from the APPLY-PNH study (1.3 events/100 py). MAVEs occurred in 2/27 (7.4%) HoM patients. Of these, 1 patient with a history of portal thrombosis had another portal thrombosis (this patient had chosen to discontinue anticoagulation 2 weeks prior to the event) and an intestinal infarction (resulted in death); the other, with history of thrombophlebitis/deep vein thrombosis, had another event of thrombophlebitis/deep vein thrombosis, while not receiving anticoagulation. MAVEs occurred in 3/109 (2.8%) NHoM patients; all had ≥1 cardiovascular or cardiometabolic risk factor. Each had an event of transient ischemic attack. No patient in whom MAVEs occurred had BTH at any time. Overall, the most frequent TEAEs were COVID-19, nasopharyngitis and headache, which occurred in 44.4% and 50.5%, 18.5% and 26.6%, and 3.7% and 28.4% of patients with and without HoM, respectively. One patient in each subgroup had adverse events leading to treatment discontinuation. SAEs occurred in a higher proportion of HoM patients (14 [51.9%]; 33.8 events/100 py) than NHoM patients (36 [33.0%]; 20.3 events/100 py). SAEs related to infections occurred in 6 (22.2%; 13.5 events/100 py) and 18 (16.5%; 7.5 events/100 py) of HoM and NHoM patients, respectively.

### Summary/Conclusion

Hematologic control was consistent and sustained with iptacopan treatment in patients with PNH with or without prior history of MAVE. On-study MAVEs were rare and occurred in patients with pre-study MAVE history or cardiovascular or cardiometabolic risk factors. Iptacopan was well tolerated in both subgroups. These findings suggest durable hematologic control with iptacopan in patients with PNH, accompanied by low incidence of MAVEs, including in patients with prior events.

**Copyright Information:** (Online) ISSN: 2572-9241
©2026 The Author(s). HemaSphere is published by John Wiley & Sons Ltd on behalf of European Hematology Association. This is an open access Abstract Book distributed under the Attribution-NonCommercial-NoDerivs (CC BY-NC-ND), which allows third parties to download the articles and share them with others as long as they credit the author and the Abstract Book, but they cannot change the content in any way or use them commercially.
**Abstract Book Citations:** Authors, Title, HemaSphere, 2026;10:(S1):pages. The abstract book DOIs can be found on the "Author Information" page.
**Disclaimer:** Articles published in the journal HemaSphere exclusively reflect the opinions of the authors. The authors are responsible for all content in their abstracts including accuracy of the facts, statements, citing resources, etc.

**Figure.** Sustained Hematologic and Biomarker Responses From 6 Months to 4 Years in Patients with PNH From APPLY-PNH, APPOINT-PNH and the PNH-REP

| Time point | 6 Months | | 2 Years | | 3 Years | | 4 Years | |
|---|---|---|---|---|---|---|---|---|
| **Prior history of MAVE** | **Yes** | **No** | **Yes** | **No** | **Yes** | **No** | **Yes** | **No** |
| Mean (SD) Hb level, g/dL | 12.48 (1.56) n=26 | 12.53 (1.43) n=101 | 12.58 (2.02) n=25 | 12.71 (2.03) n=99 | 12.92 (1.92) n=20 | 12.83 (1.73) n=84 | 12.18 (1.25) n=5 | 12.33 (1.61) n=15 |
| Hb ≥12.0 g/dL,* n/N (%) | 18/26 (69.2) | 69/101 (68.3) | 19/25 (76.0) | 70/99 (70.7) | 14/20 (70.0) | 62/84 (73.8) | 3/5 (60.0) | 11/15 (73.3) |
| Mean (SD) LDH level, U/L | 260.80 (100.94) n=25 | 279.81 (171.00) n=104 | 280.44 (150.77) n=25 | 305.4 (206.88) n=99 | 279.05 (86.29) n=20 | 271.72 (88.60) n=85 | 268.40 (27.54) n=5 | 273.43 (116.61) n=14 |
| LDH <1.5 ULN, n/N (%) | 24/25 (96.0) | 100/104 (96.2) | 23/25 (92.0) | 87/99 (87.9) | 18/20 (90.0) | 77/85 (90.6) | 5/5 (100) | 13/14 (92.9) |
| Mean (SD) absolute reticulocyte count, $10^9$/L | 71.93 (26.62) n=26 | 72.82 (37.74) n=100 | 83.04 (32.38) n=25 | 77.72 (49.61) n=98 | 70.95 (31.75) n=20 | 75.54 (41.08) n=84 | 47.26 (16.24) n=5 | 61.58 (39.42) n=14 |
| Patients with no RBC transfusions, n/N (%) | 25/27 (92.6)† | 101/109 (92.7)† | 25/27 (92.6) | 98/109 (89.9) | 24/27 (88.9) | 96/109 (88.1) | 23/27 (85.2)‡ | 95/109 (87.2)‡ |
| Mean (SD) FACIT-fatigue score | 40.04 (9.21) n=27 | 41.75 (9.21) n=105 | 42.82 (9.38) n=22 | 42.49 (9.68) n=89 | 42.87 (10.19) n=23 | 42.99 (8.72) n=76 | 45.17 (4.92) n=6 | 41.45 (8.20) n=20 |

*Irrespective of RBC transfusion

†Proportion of patients with no RBC transfusions during Year 1

‡During the entire study

FACIT-fatigue, Functional Assessment of Chronic Illness Therapy - Fatigue Scale; Hb, hemoglobin; LDH, lactate dehydrogenase; MAVE, major adverse vascular event; RBC, red blood cell; SD, standard deviation.

**Copyright Information:** (Online) ISSN: 2572-9241
©2026 The Author(s). HemaSphere is published by John Wiley & Sons Ltd on behalf of European Hematology Association. This is an open access Abstract Book distributed under the Attribution-NonCommercial-NoDerivs (CC BY-NC-ND), which allows third parties to download the articles and share them with others as long as they credit the author and the Abstract Book, but they cannot change the content in any way or use them commercially.
**Abstract Book Citations:** Authors, Title, HemaSphere, 2026;10:(S1):pages. The abstract book DOIs can be found on the "Author Information" page.
**Disclaimer:** Articles published in the journal HemaSphere exclusively reflect the opinions of the authors. The authors are responsible for all content in their abstracts including accuracy of the facts, statements, citing resources, etc.

### (PF700) GOLIDOCITINIB MONOTHERAPY IN HEMOPHAGOCYTIC LYMPHOHISTIOCYTOSIS: A PROSPECTIVE, SINGLE-ARM CLINICAL TRIAL TO EVALUATE EFFICACY AND SAFETY (JACKPOT11)

**Topic:** 12. Bone marrow failure syndromes incl. PNH - Clinical

Jingshi Wang*[1], Leilei Chen[1], Boyu Pi[1], Gaochao Zhang[1], Zhao Wang[1]

[1]*Beijing Friendship Hospital, Capital Medical University, Beijing, China;*

### Background

Hemophagocytic lymphohistiocytosis (HLH) is a hyperinflammatory syndrome resulting from genetic or acquired immune dysregulation. Many key cytokines involved in HLH, including IFN-γ, IL-2, and IL-6, signal through the JAK/STAT pathway. Golidocitinib is a novel, selective JAK1 tyrosine-kinase inhibitor that blocks the phosphorylation and activation of STAT proteins, thereby inhibiting the transcription and release of downstream inflammatory factors. Therefore, we initiated a prospective, single-arm clinical trial to evaluate the efficacy and safety of Golidocitinib monotherapy in patients with HLH.

### Aims

To report preliminary efficacy and safety results of Golidocitinib monotherapy in patients with hemophagocytic lymphohistiocytosis (HLH).

### Methods

This is an ongoing, prospective, single-arm clinical trial (NCT07339345), The trial plans to enroll a total of 28 patients with newly diagnosed or refractory/relapsed HLH.  After enrollment, patients received oral Golidocitinib 150 mg once daily. The dose could be reduced to 150 mg every other day by the investigator based on adverse event severity. Treatment efficacy was assessed at weeks 2, 4, 6, and 8 after initiation. Efficacy endpoints included clinical response rates at weeks 2, 4, 6, and 8 according to international guidelines, as well as survival outcomes (6-month, 1-year, and 2-year survival rates; median survival time). Safety was evaluated per WHO criteria, through monitoring adverse events (e.g., myelosuppression, infection, bleeding, acute pancreatitis, cardiac dysfunction) and tracking hematologic indices, organ function, inflammatory markers (ferritin, sCD25, cytokines), and bone marrow morphology.

### Results

As of the data cutoff date on January 20, 2026, nine patients were enrolled. Diagnoses included lymphoma-associated HLH in 1 patient (11.1%), EBV infection-associated HLH in 2 patients (22.2%), rheumatic autoimmune disease-associated HLH in 5 patients (55.6%), and HLH of unknown origin in 1 patient (11.1%). The median age was 51 years (range 28-68), and 4 patients (44.4%) were male. With golidocitinib treatment, inflammatory markers were significantly reduced after treatment compared to baseline, including neutrophil-to-lymphocyte ratio (NLR), interleukin-6 (IL-6), and tumor necrosis factor-α (TNF-α). The HLH remission rates at weeks 2 and 4 were 44.4% and 66.7%, respectively. Among the 4 patients with baseline Epstein-Barr virus DNA (EBVDNA) positivity, 2 patients with rheumatologic disease-associated HLH achieved negative results after treatment. Treatment-related adverse events (TRAEs) were manageable. The most common TRAE was thrombocytopenia (22.2%). No events of cytomegalovirus reactivation or serious adverse events were observed. Two patients withdrew due to progression of their primary disease.

### Summary/Conclusion

This interim analysis suggests that Golidocitinib monotherapy demonstrates promising early efficacy and favorable safety profile in patients with HLH, supporting further investigation of this targeted approach.

**Copyright Information:** (Online) ISSN: 2572-9241
©2026 The Author(s). HemaSphere is published by John Wiley & Sons Ltd on behalf of European Hematology Association. This is an open access Abstract Book distributed under the Attribution-NonCommercial-NoDerivs (CC BY-NC-ND), which allows third parties to download the articles and share them with others as long as they credit the author and the Abstract Book, but they cannot change the content in any way or use them commercially.
**Abstract Book Citations:** Authors, Title, HemaSphere, 2026;10:(S1):pages. The abstract book DOIs can be found on the “Author Information” page.
**Disclaimer:** Articles published in the journal HemaSphere exclusively reflect the opinions of the authors. The authors are responsible for all content in their abstracts including accuracy of the facts, statements, citing resources, etc.

### (PF701) HLA-IDENTICAL HEMATOPOIETIC STEM CELL TRANSPLANTATION USING A FLUDARABINE-, CYCLOPHOSPHAMIDE-, AND ANTI-LYMPHOCYTE GLOBULIN–BASED CONDITIONING REGIMEN IN FANCONI ANEMIA

**Topic:** 12. Bone marrow failure syndromes incl. PNH - Clinical

Hanane Bouarab[1], Farih Mehdid[1], Baazizi Mounira[1], Nadia Rahmoune[1], Ait Ouali Dina[1], Fouzia Louar[1], Sarah Zerkout[1], Sabrina Akhrouf[1], Hanane Moussaoui[1], Farida Harieche[1], Rose Marie Hamladji[1], Redhouane Ahmed Nacer[1], Malek Benakli*[1]

[1]*Hematology and Bone Marrow Transplantation department, Pierre and Marie Curie Center, Algiers, Algeria;*

### Background

Allogeneic hematopoietic stem cell transplantation (HSCT) from an HLA-identical sibling donor currently represents the only curative treatment for bone marrow failure and prevents progression to myelodysplastic syndrome (MDS) or acute myeloid leukemia (AML) in patients with Fanconi anemia (FA). The introduction of anti-thymoocyte globulin (ATG) into conditioning regimens, in combination with Fludarabine and Cyclophosphamide, has significantly reduced the incidence and severity of graft-versus-host disease (GVHD), improved quality of life, and decreased the risk of secondary malignancies.

### Methods

Our prospective study, conducted from March 2020 to December 2024, included 26 patients aged 5 to 15 years with FA who underwent allogeneic HSCT following conditioning with Fludarabine (120 mg/m²), Cyclophosphamide (40 mg/kg), and reduced-dose ATG (5 mg/kg). At the time of evaluation (December 1, 2025), follow-up ranged from 12 to 69 months. All donors were HLA-identical siblings. Peripheral blood stem cells (PBSC) were used in 21 patients (80.7%), with a mean CD34+ cell dose of 8.84 × $10^6$/kg (range 5.18–16.62). Bone marrow grafts were used in 4 patients, with a mean nucleated cell dose of 2.88 × $10^8$/kg (range 2.2–3.32).

### Results

Engraftment was achieved in 25 patients; one patient experienced primary graft failure and died from sepsis. No cases of acute GVHD were observed. The cumulative incidence of chronic GVHD was 12%, all classified as mild according to NIH criteria. CMV reactivation occurred in 4 patients (16%), and post-engraftment infections were documented in 3 patients (12%). Overall mortality was 3.8%. At 69 months, overall survival (OS) was 96.2%, and graft-versus-host disease–free, relapse-free survival (GRFS) was 88.5%. We compared these outcomes with a historical cohort of 22 FA patients previously transplanted in our center using conditioning with Fludarabine (120 mg/m²) and Cyclophosphamide (40 mg/kg) without ATG. A statistically significant difference in favor of ATG addition was observed for the incidence of acute GVHD (0% vs 18.1%; $p = 0.041$). A reduction in chronic GVHD incidence was also noted (12% vs 28.6%; $p = 0.26$), although not statistically significant. Similarly, improved OS and GRFS at 69 months were observed (96.2% vs 90.2%; $p = 0.86$ and 88.5% vs 63.6%; $p = 0.095$, respectively).

### Summary/Conclusion

Comparison with published literature using ATG doses ranging from 7.5 to 12.5 mg/kg suggests that reduced-dose ATG (5 mg/kg) represents an effective and well-tolerated minimal dose. This strategy reduces both acute and chronic GVHD without increasing infectious risk, thereby contributing to improved overall quality of life.

**Copyright Information:** (Online) ISSN: 2572-9241
©2026 The Author(s). HemaSphere is published by John Wiley & Sons Ltd on behalf of European Hematology Association. This is an open access Abstract Book distributed under the Attribution-NonCommercial-NoDerivs (CC BY-NC-ND), which allows third parties to download the articles and share them with others as long as they credit the author and the Abstract Book, but they cannot change the content in any way or use them commercially.
**Abstract Book Citations:** Authors, Title, HemaSphere, 2026;10:(S1):pages. The abstract book DOIs can be found on the "Author Information" page.
**Disclaimer:** Articles published in the journal HemaSphere exclusively reflect the opinions of the authors. The authors are responsible for all content in their abstracts including accuracy of the facts, statements, citing resources, etc.

### (PF702) A PROGNOSTIC NORMOGRAM TO ESTIMATE THE SEVERE PHENOTYPE IN PATIENTS WITH FANCONI ANEMIA: A SINGLE-CENTER COHORT STUDY

**Topic:** 12. Bone marrow failure syndromes incl. PNH - Clinical

Beril Kiracı Şen*[1], Şule Ünal Cangül[1]

[1]*Hacettepe University Ihsan Dogramaci Children's Hospital, Pediatric Hematology Department, Ankara, Türkiye;*

### Background

**Background:**
Fanconi anemia (FA) is a rare inherited bone marrow failure syndrome characterized by congenital anomalies, progressive cytopenia, and a markedly increased risk of malignancy. Due to its heterogeneous clinical course and multisystem involvement, identifying prognostic factors is essential for individualized risk stratification and optimizing clinical management.

### Aims

**Objective:**
To evaluate demographic, clinical, laboratory, genetic and long-term outcome data in patients with FA and to identify independent predictors of severe phenotype (pancytopenia, leukemia/MDS/solid tumour development, mortality). Additionally, we aimed to develop a prognostic normogram to estimate individualized risk.

### Methods

**Methods:**
This retrospective cohort study included 267 patients diagnosed with FA and followed for at least six months at Hacettepe University Pediatric Hematology Department between January 1975 and August 2025. Demographic, clinical, hematologic, cytogenetic, genetic, and outcome data were analyzed. Genetic testing was available for 95 patients. Overall survival was estimated using the Kaplan–Meier method. Independent predictors of severe phenotype were identified using multivariate logistic regression. A prognostic nomogram was constructed based on significant variables.

**Copyright Information:** (Online) ISSN: 2572-9241
©2026 The Author(s). HemaSphere is published by John Wiley & Sons Ltd on behalf of European Hematology Association. This is an open access Abstract Book distributed under the Attribution-NonCommercial-NoDerivs (CC BY-NC-ND), which allows third parties to download the articles and share them with others as long as they credit the author and the Abstract Book, but they cannot change the content in any way or use them commercially.
**Abstract Book Citations:** Authors, Title, HemaSphere, 2026;10:(S1):pages. The abstract book DOIs can be found on the "Author Information" page.
**Disclaimer:** Articles published in the journal HemaSphere exclusively reflect the opinions of the authors. The authors are responsible for all content in their abstracts including accuracy of the facts, statements, citing resources, etc.

### Results

**Results:**
Among 267 patients (46.8% female), parental consanguinity was present in 76%, including first-cousin marriage in the majority. The most frequent congenital anomalies were dermatologic (95.5%) and skeletal (58.4%), followed by growth retardation and genitourinary abnormalities (35.4%). DEB testing was positive in 89.7% of patients. Molecular genetic analysis identified *FANCA* as the predominant subtype (58.9%), followed by *FANCC* and *FANCG.*
Solid tumors developed in 10.7% of patients, most commonly head and neck squamous cell carcinoma. MDS/AML developed in 30 (11.2%) of the patients. The mean age at hematologic malignancy diagnosis was 14.4 years.
Severe cytopenia developed in 67.9% of patients at a mean age of 107 months. Patients without severe cytopenia development had significantly longer survival compared to those with severe cytopenia (32.4 vs 17 years, $p < 0.05$). Poor prognosis was associated with younger age at diagnosis, significantly worse baseline hematologic parameters, elevated HbF levels, higher chromosomal breakage rates, infection-related hospitalizations ($p < 0.05$).
In multivariate analysis, independent predictors of severe cytopenia included: MCV ≥100 fL; OR ≈ 6.3, p = 0.033) and a history of infection-related hospitalization (OR ≈ 5.5, p = 0.016), while short stature (<–2 SD) remained significantly associated. A prognostic nomogram incorporating these variables generated individualized risk scores corresponding to progressively increasing probabilities of severe course, exceeding 95% at higher scores.

### Summary/Conclusion

**Conclusion:**
This large single center cohort providwes comprehensive long-term outcome data in patients with FA. The proposed prognostic normogram offers a clinically applicable tool for individualized risk prediction and may facilitate optimized surveillance, and improved timing of HSCT.

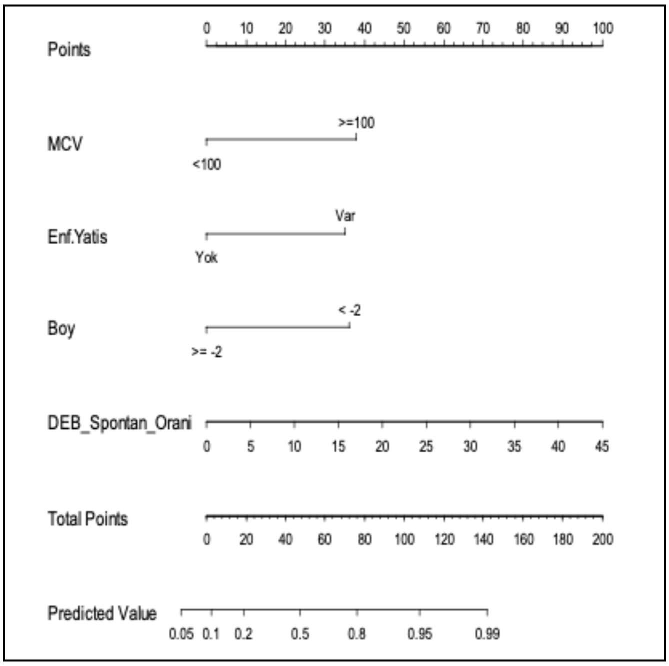

**Copyright Information:** (Online) ISSN: 2572-9241
©2026 The Author(s). HemaSphere is published by John Wiley & Sons Ltd on behalf of European Hematology Association. This is an open access Abstract Book distributed under the Attribution-NonCommercial-NoDerivs (CC BY-NC-ND), which allows third parties to download the articles and share them with others as long as they credit the author and the Abstract Book, but they cannot change the content in any way or use them commercially.
**Abstract Book Citations:** Authors, Title, HemaSphere, 2026;10:(S1):pages. The abstract book DOIs can be found on the “Author Information” page.
**Disclaimer:** Articles published in the journal HemaSphere exclusively reflect the opinions of the authors. The authors are responsible for all content in their abstracts including accuracy of the facts, statements, citing resources, etc.

### (PF703) REAL-WORLD EFFECTIVENESS OF PEGCETACOPLAN IN PATIENTS WITH PAROXYSMAL NOCTURNAL HEMOGLOBINURIA (PNH) AFTER SWITCH FROM C5 INHIBITORS AMONG CEE COUNTRIES

**Topic:** 12. Bone marrow failure syndromes incl. PNH - Clinical

Łukasz Szukalski*[1], Jaroslav Cermak[2], Lenka Mastíková[2], Ana Boban[3, 4], Sławomir Jurek[5], Patrycja Mensah-Glanowska[6], Anna Szmigielska-Kapłon[7], Justyna Kozińska[8], Karla Rener[9], Paweł Bernatowicz[10], Elżbieta Kalicińska[11], Delia Soare[12, 13], Agnieszka Piekarska[14], Anna Kopinska[15], Juraj Sokol[16], Juliana Holasova[17], Miroslav Šimek[18], Marek Hus[8], Jerzy Windyga[5, 19], Marta Sobas[1]

*[1]Department of Hematology, Collegium Medicum in Bydgoszcz, Nicolaus Copernicus University, Torun, Poland;[2]Institute of Hematology and Blood Transfusion, Prague, Czechia; [3]Division of Haematology, Department of Internal Medicine, University Hospital Zagreb, Zagreb, Croatia; [4]School of Medicine, University of Zagreb, Zagreb, Croatia;[5]Department of Hemostasis Disorders and Internal Medicine, Institute of Hematology and Transfusion Medicine, Warsaw, Poland; [6]Department of Hematology, Jagiellonian University, Collegium Medicum in Kraków, Krakow, Poland; [7]Department of Hematology, Medical University of Lodz, Lodz, Poland; [8]Department of Hematooncology and Bone Marrow Transplantation, Medical University of Lublin, Lublin, Poland; [9]Department of Hematology, University Medical Center Ljubljana, Ljubljana, Slovenia; [10]Department of Haematology, Medical University of Bialystok, Bialystok, Poland; [11]Clinical Department of Hematology, Cell Therapies and Internal Diseases, Wroclaw Medical University, Wroclaw, Poland; [12]Bone Marrow Transplantation Unit, University Emergency Hospital Bucharest, Bucharest, Romania; [13]University of Medicine and Pharmacy "Carol Davila", Bucharest, Romania; [14]Department of Hematology, Transplantology and Cellular Therapies, Medical University of Gdansk, Gdansk, Poland; [15]Department of Hematology and Bone Marrow Transplantation, Medical University of Silesia, Katowice, Poland;[16]Department of Hematology and Transfusiology, Jessenius Faculty of Medicine in Martin, Comenius University in Bratislava, University Hospital in Martin, Martin, Slovakia; [17]Hematology Department, University Hospital F.D.Roosevelt, Banska Bystrica, Slovakia; [18]Hematology Outpatient Clinic, Faculty Hospital Nitra, Nitra, Slovakia; [19]Laboratory of Hemostasis and Metabolic Diseases, Institute of Hematology and Transfusion Medicine, Warsaw, Poland;*

### Background

Pegcetacoplan (PEG), a C3/C3b inhibitor, was registered by the FDA (2021) and the EMA (2022) for PNH patients who have suboptimal responses to C5 inhibitors (C5i) due to C3-mediated extravascular hemolysis (EVH). Several RWE analyses of PEG effectiveness in PNH have been published so far, but most are limited by small sample sizes or short follow-up periods.

### Aims

To evaluate the long-term outcomes in a group of adult PNH patients switched to PEG following the C5i therapy.

### Methods

We conducted a retrospective analysis of medical records from haematology departments in 16 hospitals across CEE countries (Poland, Czechia, Slovakia, Slovenia, Croatia, and Romania). The study included adult patients diagnosed with PNH who were routinely treated with PEG as a second-line setting. The collected data included haemoglobin (HGB) levels, absolute reticulocyte count (ARC), lactate dehydrogenase (LDH), indirect bilirubin (BIL), the number of packed red blood cell (pRBC) transfusions, thrombotic or major adverse vascular events (MAVE), changes in the PNH clone, breakthrough hemolysis (BTH) events, and infection occurrences.

**Copyright Information:** (Online) ISSN: 2572-9241
©2026 The Author(s). HemaSphere is published by John Wiley & Sons Ltd on behalf of European Hematology Association. This is an open access Abstract Book distributed under the Attribution-NonCommercial-NoDerivs (CC BY-NC-ND), which allows third parties to download the articles and share them with others as long as they credit the author and the Abstract Book, but they cannot change the content in any way or use them commercially.
**Abstract Book Citations:** Authors, Title, HemaSphere, 2026;10:(S1):pages. The abstract book DOIs can be found on the "Author Information" page.
**Disclaimer:** Articles published in the journal HemaSphere exclusively reflect the opinions of the authors. The authors are responsible for all content in their abstracts including accuracy of the facts, statements, citing resources, etc.

**Results**

38 patients (mean age: 47.7 years; 58% female) received PEG outside clinical trials for a median duration of 19.2 months (range: 2-35.3 months) between 2023 and 2026. Two patients were treated with a combination of a C5i and PEG. The indication for switching from C5i to PEG was EVH leading to anaemia in all cases (eculizumab n=27, ravulizumab n=9, crovalimab n=2). Before the commencement of PEG, the mean HGB concentration was 8.72 g/dL, and the mean ARC was 295 × $10^{9}$/L, 27/38 (71%) of patients required pRBC transfusions in the previous 12 months. After 24, 48, and 80 weeks of PEG therapy, the mean HGB level was 11.91 g/dL (n=33), 11.8 g/dL (n=26), and 11.6 g/dL (n=21) respectively (a mean increase from baseline to 80 weeks of 2.88 g/dL, ***Fig. 1A***). The mean ARC decreased after 24, 48, and 80 weeks to 124 × $10^{9}$/L, 138 × $10^{9}$/L, and 132 × $10^{9}$/L, respectively (mean decrease of 163 × $10^{9}$/L after 80 weeks, ***Fig. 1B***). The median LDH level remained below 1.5x ULN following the commencement of PEG and throughout the follow up, ***Fig. 1C***. 13 out of 38 patients (34.2%) needed at least one pRBC transfusion over a median treatment duration of 19.2 months. All but three patients continued PEG after the 80th week of therapy (one died, one was switched to proximal inhibition, and one underwent alloHSCT). 17/38 patients experienced a total of 27 BTH events with a mean maximum drop in HGB of 4.1g/dL and median maximum rise in LDH of 1692 U/L, with the majority caused by complement-amplifying conditions (CACs). All BTH events were resolved in a mean time of 6.6 days (range 1-28 days). During the PEG treatment, no patients experienced thrombosis or MAVE events. Additionally, none of the patients had infections with *N. meningitidis.*

**Summary/Conclusion**

Patients treated with PEG achieved significant improvement in HGB levels and transfusion avoidance after 80 weeks of therapy, with a decrease in ARC, indicating the resolution of EVH. During almost 20 months of treatment, the median LDH remained below 1.5x ULN, indicating good control of intravascular hemolysis. BTH events on PEG can be severe, but timely interventions in accordance with the guidelines resolve them effectively.

**Copyright Information:** (Online) ISSN: 2572-9241
©2026 The Author(s). HemaSphere is published by John Wiley & Sons Ltd on behalf of European Hematology Association. This is an open access Abstract Book distributed under the Attribution-NonCommercial-NoDerivs (CC BY-NC-ND), which allows third parties to download the articles and share them with others as long as they credit the author and the Abstract Book, but they cannot change the content in any way or use them commercially.
**Abstract Book Citations:** Authors, Title, HemaSphere, 2026;10:(S1):pages. The abstract book DOIs can be found on the "Author Information" page.
**Disclaimer:** Articles published in the journal HemaSphere exclusively reflect the opinions of the authors. The authors are responsible for all content in their abstracts including accuracy of the facts, statements, citing resources, etc.

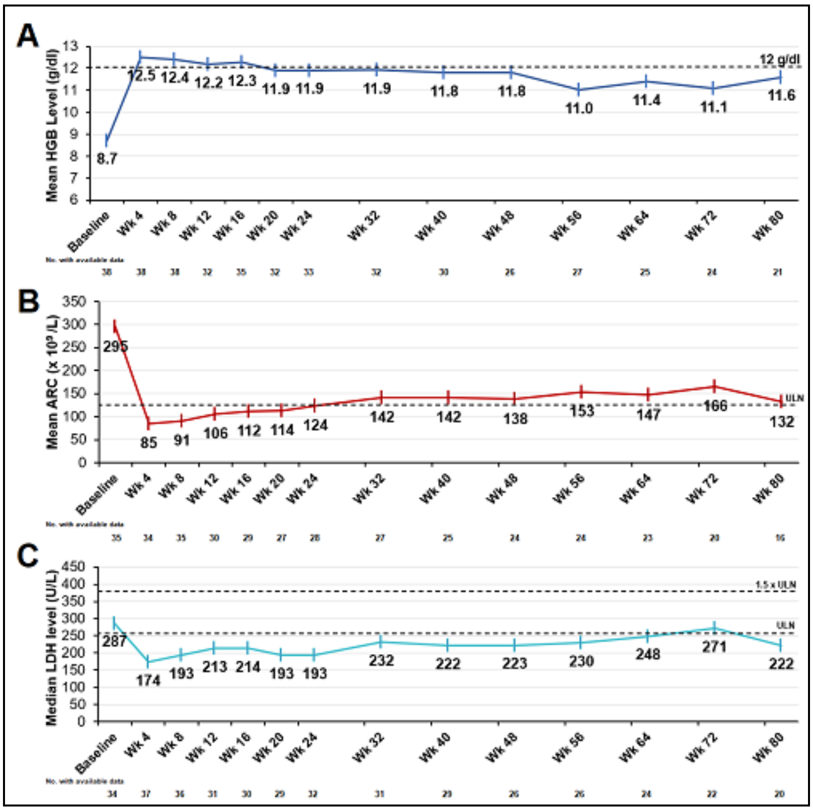

**Copyright Information:** (Online) ISSN: 2572-9241
©2026 The Author(s). HemaSphere is published by John Wiley & Sons Ltd on behalf of European Hematology Association. This is an open access Abstract Book distributed under the Attribution-NonCommercial-NoDerivs (CC BY-NC-ND), which allows third parties to download the articles and share them with others as long as they credit the author and the Abstract Book, but they cannot change the content in any way or use them commercially.
**Abstract Book Citations:** Authors, Title, HemaSphere, 2026;10:(S1):pages. The abstract book DOIs can be found on the "Author Information" page.
**Disclaimer:** Articles published in the journal HemaSphere exclusively reflect the opinions of the authors. The authors are responsible for all content in their abstracts including accuracy of the facts, statements, citing resources, etc.

### (PF704) REAL-WORLD PATIENT-REPORTED TREATMENT BURDEN FROM 106 PATIENTS WITH PAROXYSMAL NOCTURNAL HEMOGLOBINURIA: RESULTS FROM THE VOICE CROSS-SECTIONAL QUANTITATIVE SURVEY

**Topic:** 12. Bone marrow failure syndromes incl. PNH - Clinical

Naoshi Obara[1], Tadakazu Kondo[2], Shikiko Ueno*[3], Takayuki Ikezoe[4], Shin Fujisawa[5], Masatoshi Sakurai[6], Kohei Hosokawa[7], Haruko Tashiro[8], Kaichi Nishiwaki[9], Toshio Kitawaki[10], Tetsuya Nishida[11], Yoshiaki Ogawa[12], Takahiro Suzuki[13], Aiko Kobayashi[14], Masami Yamashita[14], Yuta Fukuoka[15], Tatsunori Murata[15], Kensuke Usuki[16], Tatsuya Kawaguchi[3, 17], Naoki Hosen[18], Yuzuru Kanakura[19, 20], Akihiko Gotoh[21, 22], Jun-Ichi Nishimura[18], Yasutaka Ueda[18]

*[1]University of Tsukuba Hospital, Tsukuba, Japan; [2]Kobe City Medical Center General Hospital, Kobe, Japan; [3]Kumamoto University Hospital, Kumamoto, Japan; [4]Fukushima Medical University, Fukushima, Japan; [5]Yokohama City University Medical Center, Yokohama, Japan; [6]Keio University School of Medicine, Tokyo, Japan; [7]Kanazawa University, Kanazawa, Japan; [8]Teikyo University School of Medicine, Tokyo, Japan; [9]The Jikei University Kashiwa Hospital, Kashiwa, Japan; [10]Kyoto University Hospital, Kyoto, Japan; [11]Japanese Red Cross Aichi Medical Center Nagoya Daiichi Hospital, Nagoya, Japan; [12]Tokai University School of Medicine, Isehara, Japan; [13]Kitasato University School of Medicine, Kanagawa, Japan; [14]Chugai Pharmaceutical Co., Ltd., Tokyo, Japan; [15]CRECON Medical Assessment, Inc., Tokyo, Japan; [16]Mizonokuchi Hospital, Teikyo University School of Medicine, Kawasaki, Japan; [17]Kumamoto Health Science University, Kumamoto, Japan; [18]The University of Osaka Graduate School of Medicine, Osaka, Japan; [19]Japan PNH Study Group, Tokyo, Japan; [20]Sumitomo Hospital, Osaka, Japan; [21]Nerima Hikarigaoka Hospital, Tokyo, Japan; [22]Tokyo Medical University, Tokyo, Japan;*

### Background

Complement inhibitors have markedly improved outcomes for patients (pts) with paroxysmal nocturnal hemoglobinuria (PNH). As treatment options expand, understanding the real-world burden of lifelong therapy is increasingly important for clinical and health policy decisions; however, quantitative pt-reported data in the modern treatment era remain limited.

### Aims

To quantitatively assess PNH treatment burden and to explore pt characteristics associated with selected burdens.

### Methods

VOICE is a non-interventional stepwise study. This quantitative survey of 106 pts with active or previous PNH treatment (from 15 medical centers) was conducted Jan–Nov 2025 following a qualitative survey conducted in 2023 (Ueda et al, *Ann Hematol* 2025). Pts provided demographic information and selected the 3 most burdensome PNH treatment-related factors from a predefined list. Clinical data were collected from their treating physicians. Data were summarized using descriptive statistics; exploratory analyses of selected burdens by pt characteristics included 95% CIs for differences in proportions.

**Copyright Information:** (Online) ISSN: 2572-9241
©2026 The Author(s). HemaSphere is published by John Wiley & Sons Ltd on behalf of European Hematology Association. This is an open access Abstract Book distributed under the Attribution-NonCommercial-NoDerivs (CC BY-NC-ND), which allows third parties to download the articles and share them with others as long as they credit the author and the Abstract Book, but they cannot change the content in any way or use them commercially.
**Abstract Book Citations:** Authors, Title, HemaSphere, 2026;10:(S1):pages. The abstract book DOIs can be found on the “Author Information” page.
**Disclaimer:** Articles published in the journal HemaSphere exclusively reflect the opinions of the authors. The authors are responsible for all content in their abstracts including accuracy of the facts, statements, citing resources, etc.

**Results**

A total of 106 pts participated (median age, 56 years; range, 19–90); 59% were female, 56% were employed, 2% were in school, and 40% were not working or in school. Disease severity at diagnosis was moderate-to-severe in 76% and mild in 22%. Current treatment for most pts was complement inhibitor therapy (86%), most commonly ravulizumab (59%), crovalimab (11%), or iptacopan (10%); 12 pts received danicopan in combination with ravulizumab (n=11) or crovalimab (n=1). Five pts did not receive a complement inhibitor (blood transfusions, n=1; not applicable, n=4); 10 were not currently receiving treatment.
Most pts (86%) reported regular treatment-related hospital visits at least every 8 weeks (45% every 8 weeks, 26% every 4 weeks, 15% every 2 weeks); 1% were weekly and 13% less than every 8 weeks. Mean one-way travel time to the hospital was 1.2 hours (SD, 1.4) and mean total visit duration was 3.8 hours (SD, 1.3); 46% of pts took time off work or school for treatment-related visits.
The 3 most commonly reported PNH treatment burdens reflected attributes often associated with complement inhibitors; others were related to general treatment preferences (**Figure**). Some burden selections differed by pt characteristics. Frequent business travel (≥4 trips/year) was more common among those selecting “lifelong treatment” (74.2% vs 38.6% in others [95% CI: 16.5, 51.4]) and “risk of infection” (77.1% vs 50.7% [95% CI: 6.7, 42.1]). More pts in work or school selected “regular hospital visits” (81.5% vs 49.4% [95% CI: 11.0, 47.0]). Travel time ≥1 hour to the hospital was more common among those selecting “distance to the hospital” (95.5% vs 65.5% [95% CI: 10.4, 41.3]).

**Summary/Conclusion**

Pts reported substantial PNH treatment burden driven by the long-term logistical (frequency/duration of travel for hospital visits) and financial demands of lifelong therapy, with variations based on pt characteristics. This was a notably large study cohort for this rare disease, and responses are applicable to the modern PNH treatment landscape. Though conducted in Japan, the treatment options and management challenges identified in this study are relevant to PNH care in other settings. These findings highlight the importance of incorporating pt-reported burden into treatment selection and policy decisions as the PNH therapeutic landscape evolves.

**Copyright Information:** (Online) ISSN: 2572-9241
©2026 The Author(s). HemaSphere is published by John Wiley & Sons Ltd on behalf of European Hematology Association. This is an open access Abstract Book distributed under the Attribution-NonCommercial-NoDerivs (CC BY-NC-ND), which allows third parties to download the articles and share them with others as long as they credit the author and the Abstract Book, but they cannot change the content in any way or use them commercially.
**Abstract Book Citations:** Authors, Title, HemaSphere, 2026;10:(S1):pages. The abstract book DOIs can be found on the “Author Information” page.
**Disclaimer:** Articles published in the journal HemaSphere exclusively reflect the opinions of the authors. The authors are responsible for all content in their abstracts including accuracy of the facts, statements, citing resources, etc.

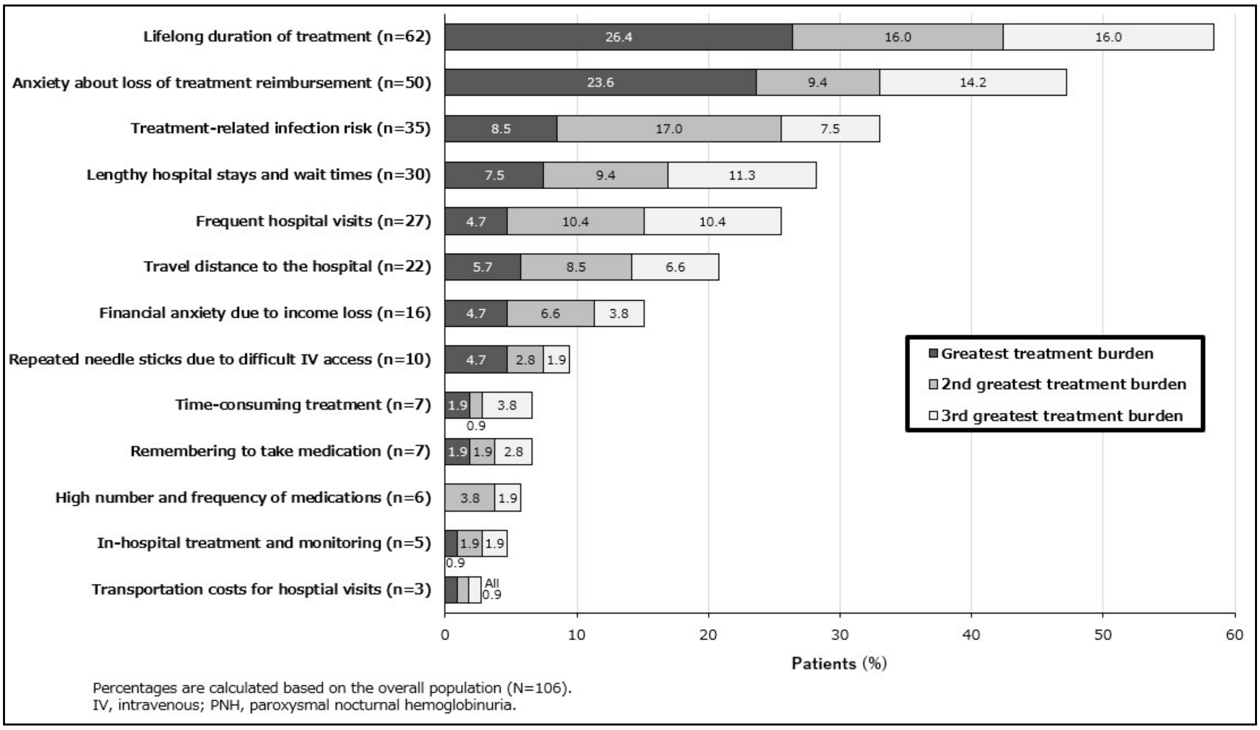

Figure. PNH treatment-related burden (N=106)

**Copyright Information:** (Online) ISSN: 2572-9241
©2026 The Author(s). HemaSphere is published by John Wiley & Sons Ltd on behalf of European Hematology Association. This is an open access Abstract Book distributed under the Attribution-NonCommercial-NoDerivs (CC BY-NC-ND), which allows third parties to download the articles and share them with others as long as they credit the author and the Abstract Book, but they cannot change the content in any way or use them commercially.
**Abstract Book Citations:** Authors, Title, HemaSphere, 2026;10:(S1):pages. The abstract book DOIs can be found on the "Author Information" page.
**Disclaimer:** Articles published in the journal HemaSphere exclusively reflect the opinions of the authors. The authors are responsible for all content in their abstracts including accuracy of the facts, statements, citing resources, etc.

### (PF705) RAVULIZUMAB MAINTAINS OR IMPROVES RENAL FUNCTION IN REAL-WORLD PATIENTS WITH PAROXYSMAL NOCTURNAL HEMOGLOBINURIA

**Topic:** 12. Bone marrow failure syndromes incl. PNH - Clinical

Richard J. Kelly*[1], Hubert Schrezenmeier[2, 3], Ami Patel[4], Benedetta Campolo[5], Jesse Metzger[6], Philippe Gustovic[7], Yasutaka Ueda[8], Jun Ho Jang[9]

*[1]St James's University Hospital, The Leeds Teaching Hospitals NHS Trust, Leeds, United Kingdom;[2]Institute of Transfusion Medicine, University of Ulm, Ulm, Germany; [3]Institute for Clinical Transfusion Medicine and Immunogenetics, German Red Cross Blood Transfusion Service Baden-Württemberg-Hessen and University Hospital Ulm, Ulm, Germany; [4]Alexion, AstraZeneca Rare Disease, Boston, United States of America; [5]Alexion, AstraZeneca Rare Disease, Barcelona, Spain;[6]Parexel, Newton, United States of America; [7]Alexion, AstraZeneca Rare Disease, Baar, Switzerland; [8]Division of Hematology and Oncology, The University of Osaka, Suita, Japan; [9]Department of Hematology-Oncology, Samsung Medical Center, Sungkyunkwan University School of Medicine, Seoul, South Korea;*

### Background

Paroxysmal nocturnal hemoglobinuria (PNH) is a rare hematological disorder driven by uncontrolled terminal complement activity, leading to intravascular hemolysis, thrombosis, organ damage and premature mortality. Patients with PNH have a six-fold greater risk of developing chronic kidney disease (CKD) compared with the general population due to terminal complement activity. Ravulizumab, a second-generation C5 inhibitor (C5i) derived from eculizumab, provides immediate, complete and sustained inhibition of terminal complement activity and is the standard-of-care treatment for patients with PNH, where available. In clinical trials, ravulizumab treatment preserved estimated glomerular filtration rate (eGFR) for up to 3 years ineculizumab-experienced patients with PNH; similar real-world evidence is limited. The International PNH Registry (NCT01374360), the largest global registry of patients with PNH, offers a robust platform to address this evidence gap.

### Aims

To evaluate real-world renal outcomes with ravulizumab treatment in patients enrolled in the International PNH Registry.

### Methods

This retrospective analysis included adults (≥18 years old) with PNH enrolled in the registry as of May 22, 2025, who received ravulizumab for ≥6 months. Patients were stratified as eculizumab-experienced or C5i-naive and by CKD stage (Kidney Disease: Improving Global Outcomes criteria) at baseline (defined as the date of ravulizumab initiation). Eculizumab-experienced patients were those who had received eculizumab and discontinued <6 months before baseline. C5i-naive patients had no prior eculizumab treatment or discontinued eculizumab >6 months before baseline. Outcomes included changes in CKD stage and eGFR (using mixed-effect repeated measures, adjusted for sex and age at baseline), assessed up to 24 months.

**Copyright Information:** (Online) ISSN: 2572-9241
©2026 The Author(s). HemaSphere is published by John Wiley & Sons Ltd on behalf of European Hematology Association. This is an open access Abstract Book distributed under the Attribution-NonCommercial-NoDerivs (CC BY-NC-ND), which allows third parties to download the articles and share them with others as long as they credit the author and the Abstract Book, but they cannot change the content in any way or use them commercially.
**Abstract Book Citations:** Authors, Title, HemaSphere, 2026;10:(S1):pages. The abstract book DOIs can be found on the "Author Information" page.
**Disclaimer:** Articles published in the journal HemaSphere exclusively reflect the opinions of the authors. The authors are responsible for all content in their abstracts including accuracy of the facts, statements, citing resources, etc.

**Results**

Data were available for 205 eculizumab-experienced (53.2% male) and 48 C5i-naive (52.1% male) patients. The distribution of CKD stages at baseline was similar between groups; most patients had CKD-1/-2, and no patients had CKD-5 (eculizumab-experienced vs C5i-naive: CKD-1, 58.5% vs 52.1%; CKD-2, 29.3% vs 27.1%; CKD-3a, 9.3% vs 16.7%; CKD-3b, 1.5% vs 4.2%; CKD-4, 1.5% vs 0.0%). After 24 months of ravulizumab treatment, 89% of eculizumab-experienced patients and 87% of C5i-naive patients maintained or improved CKD stage from baseline. eGFR also remained stable within both groups over 24 months (**Figure**); the least-squares mean (standard deviation) change from baseline eGFR at 24 months did not differ significantly between eculizumab-experienced and C5i-naive patients (–1.2 [1.1] vs –2.6 [2.8] mL/min/1.73 m$^2$; $p$=0.65).

**Summary/Conclusion**

Ravulizumab maintained or improved CKD stage in most patients regardless of baseline renal function or prior eculizumab exposure. eGFR also remained stable in both eculizumab-experienced and C5i-naive patients. These findings reinforce ravulizumab as a first-line treatment option for patients with PNH across varying stages of CKD.

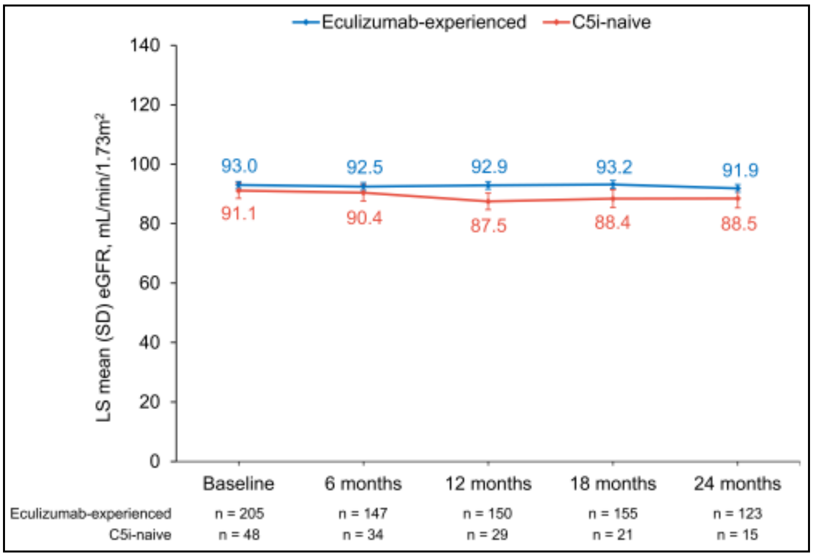

Figure: LS mean eGFR (SD) in eculizumab-experienced and C5i-naive patients during ravulizumab treatment, adjusted for sex and age at baseline. Abbreviations: C5i, complement C5 inhibitor; eGFR, estimated glomerular filtration rate; LS, least-squares; SD, standard deviation.

**Copyright Information:** (Online) ISSN: 2572-9241
©2026 The Author(s). HemaSphere is published by John Wiley & Sons Ltd on behalf of European Hematology Association. This is an open access Abstract Book distributed under the Attribution-NonCommercial-NoDerivs (CC BY-NC-ND), which allows third parties to download the articles and share them with others as long as they credit the author and the Abstract Book, but they cannot change the content in any way or use them commercially.
**Abstract Book Citations:** Authors, Title, HemaSphere, 2026;10:(S1):pages. The abstract book DOIs can be found on the “Author Information” page.
**Disclaimer:** Articles published in the journal HemaSphere exclusively reflect the opinions of the authors. The authors are responsible for all content in their abstracts including accuracy of the facts, statements, citing resources, etc.

### (PF706) LONGITUDINAL EVALUATION OF CLONAL HEMATOPOIESIS IN BONE MARROW FAILURE AND PRIMARY IMMUNE DYSREGULATION DISORDERS: SINGLE CENTER DATA

**Topic:** 12. Bone marrow failure syndromes incl. PNH - Clinical

Luca Arcuri*[1], Anna Galli[2], Maria Carla Giarratana[1], Maurizio Miano[1], Elena Palmisani[1], Eugenia Mariani[1], Daniela Guardo[1], Michaela Calvillo[1], Gianluca Dell'orso[1], Erika Massaccesi[1], Roberta La Starza[3], Caterina Matteucci[3], Luca Malcovati[2], Carlo Dufour[1], Francesca Fioredda[1]

*[1]IRCCS- Istituto Giannina Gaslini, Unit of Hematology, Genova, Italy; [2]University of Pavia, Department of Molecula Medicine, Pavia, Italy; [3]Ospedale Santa Maria della Misericordia, Laboratory of Molecular Medicine and Cytogenetics, Perugia, Italy;*

**Background**

Clonal hematopoiesis (CH) is a well-recognized phenomenon in the general population, with a higher incidence in inherited Bone Marrow Failure (iBMF) due to disruptions in the equilibrium of hematopoietic precursors. Somatic mutations have been observed in patients with chronic cytopenias; however, their role in Primary Immune Dysregulation Disorders (PIRD) remains poorly understood.

**Aims**

To enhance knowledge of somatic “clonal evolution” in a cohort of patients with iBMF and PIRD.

**Methods**

Patients affected by iBMF and chronic cytopenia in the setting of PIRD who underwent regular bone marrow (BM)/peripheral blood (PB) monitoring with a 45 NGS myeloid genes panel were considered evaluable for the present study.

**Results**

From September 2024 to December 2025, 117 samples (93 BM and 24 PB) from 84 patients (42 females, median age 19.9 years [1.5-52.8] were analyzed, 14/84 underwent serial testing. Twenty-seven patients were classified as PIRD due to chronic refractory cytopenia with signs of immune-dysregulation, 43 were diagnosed with iBMF (13 Severe Congenital Neutropenia [SCN]; 11 with Fanconi Anemia [FA]; 9 Telomere Biology Disorders [TBD]; 4 Shwachman-Diamond Syndrome [SDS]; 4 Diamond-Blackfan Anemia [DBA]; and 2 Congenital Thrombocytopenia), and 14 had Acquired Aplastic Anemia, AAA. As shown in Table and Figure 1, 9/84 patients (11%) carried isolated or associated pathogenic (P) or likely pathogenic (LP). CH was more frequent in iBMF (6/43, 14%) than in AAA (1/14, 7%) and PIRD (2/27, 7%), without significant differences among groups (p=0.67). *TP53* variants occurred in 2 SDS patients and in 1 TBD patient without dysplastic features. The latter underwent successful BM transplantation. A SCN patient (UPN5) developed multiple clones and myelodysplasia with monosomy 7. A FA patient with a SF3B1 variant underwent “preemptive” BM transplantation before developing marrow dysplastic changes with favourable outcome. Variants of KRAS were found in a DBA (later transplanted for the severity of cytopenia) and 2 PIRD patients who are currently under surveillance. Among AAA patients only one showed CH with a mutation of KRAS.

**Table 1** Characteristics of the 9 patients carrying P/LP clones

**Copyright Information:** (Online) ISSN: 2572-9241
©2026 The Author(s). HemaSphere is published by John Wiley & Sons Ltd on behalf of European Hematology Association. This is an open access Abstract Book distributed under the Attribution-NonCommercial-NoDerivs (CC BY-NC-ND), which allows third parties to download the articles and share them with others as long as they credit the author and the Abstract Book, but they cannot change the content in any way or use them commercially.
**Abstract Book Citations:** Authors, Title, HemaSphere, 2026;10:(S1):pages. The abstract book DOIs can be found on the “Author Information” page.
**Disclaimer:** Articles published in the journal HemaSphere exclusively reflect the opinions of the authors. The authors are responsible for all content in their abstracts including accuracy of the facts, statements, citing resources, etc.

| UPN | Diagnosis | Germline variants | Somatic variant | BM VAF% PB VAF % | Age of clone appearance (years) | VAF trajectory in bold if on BM (age) |
|---|---|---|---|---|---|---|
| UPN41 | DBA | None | KRAS p.Gly13Asp | 9.7 | 2.9 | **8.2**, 1.6 (3.0) |
| UPN5 | SCN | ELANE (c.583_584insCG) | SF3B1 p.Lys700Glu. | 16.2 (13.8) | 52.9 | **1.5, 0.95** (53.5) |
| | | | ASXL1 p.Arg693* | 29.7 (44.9) | 53.5 | |
| | | | CSFR3 p.Gln741* | 33.6 | 53.5 | |
| | | | SETBP1 p.Ser869Asn | 33.1 (43.1) | 53.5 | |
| UPN66 | AAA | AP3D1 (c.2489-2490del) | GNAS p.Arg201His | 3.2 | 9.4 | |
| UPN68 | SDS | SBDS (c.258+2T>C, c.183_184delinsTA>CT) | TP53 p.Asn239Asp | 6.5 | 1.5 | **7.5**, 6.4 (1.9); **5.2**, 6.2 (2.2) |
| UPN75 | SDS | SBDS (c.258+2T>C, c.128+6T>C) | TP53 p.Gly187Ser | 20.9 | 7.6 | **26.3**, 6.0 (8.2); **31.6**, 12.2 (8.4); **27.3**, 8.9 (8.7); **26.3**, 4.5 (8.9) |
| UPN78 | PIRD | None | KRAS p.Gly12Asp | 44.4 | 26.3 | |
| UPN83 | PIRD | None | KRAS p.Ala146Val | 29 (29.8) | 17.6 | |
| UPN86 | FA | FANCA (c.3971C>T, c.3638_3639delCT) | SF3B1 p.Arg625Cys | (9.3) | 29.0 | |
| UPN87 | TBD | RTEL1 (c.2956C>T, c.3370C>T, c.334G>A) | U2AF1 p.Ser34Phe | 1.2 | 27.3 | **3.4** (28.2) |
| | | | TP53 p.Arg248Trp | 1.7 | 28.2 | |

### Summary/Conclusion

These findings suggest that CH may be more frequent in iBMF compared with AAA and PIRD, and is more common in older individuals with the exception of TP53 in SDS patients. Detection of somatic clones is important to intercept variants that may guide either conservative or interventional treatment strategies. Larger cohort studies of iBMF and PIRD could further refine the clinical interpretation of molecular findings.

**Copyright Information:** (Online) ISSN: 2572-9241
©2026 The Author(s). HemaSphere is published by John Wiley & Sons Ltd on behalf of European Hematology Association. This is an open access Abstract Book distributed under the Attribution-NonCommercial-NoDerivs (CC BY-NC-ND), which allows third parties to download the articles and share them with others as long as they credit the author and the Abstract Book, but they cannot change the content in any way or use them commercially.
**Abstract Book Citations:** Authors, Title, HemaSphere, 2026;10:(S1):pages. The abstract book DOIs can be found on the "Author Information" page.
**Disclaimer:** Articles published in the journal HemaSphere exclusively reflect the opinions of the authors. The authors are responsible for all content in their abstracts including accuracy of the facts, statements, citing resources, etc.

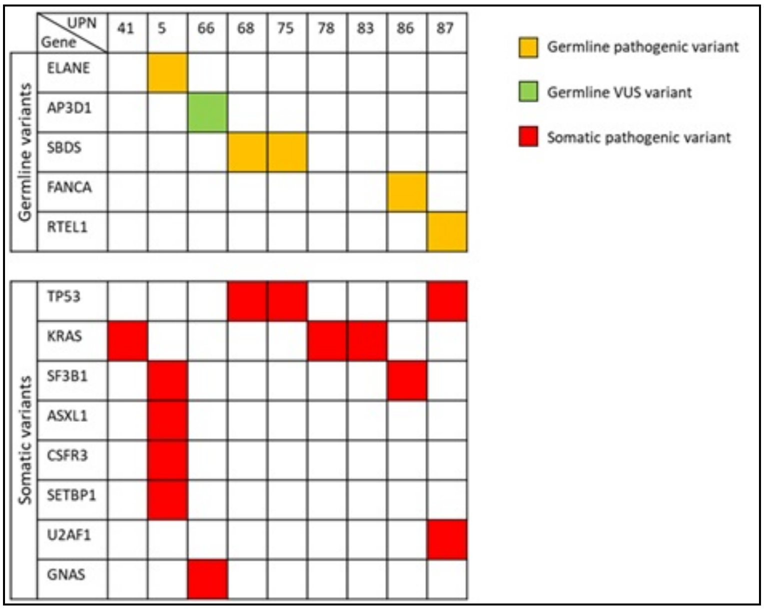

Figure 1 Graphic distribution of the 9 patients carrying P/LP clones

**Copyright Information:** (Online) ISSN: 2572-9241
©2026 The Author(s). HemaSphere is published by John Wiley & Sons Ltd on behalf of European Hematology Association. This is an open access Abstract Book distributed under the Attribution-NonCommercial-NoDerivs (CC BY-NC-ND), which allows third parties to download the articles and share them with others as long as they credit the author and the Abstract Book, but they cannot change the content in any way or use them commercially.
**Abstract Book Citations:** Authors, Title, HemaSphere, 2026;10:(S1):pages. The abstract book DOIs can be found on the "Author Information" page.
**Disclaimer:** Articles published in the journal HemaSphere exclusively reflect the opinions of the authors. The authors are responsible for all content in their abstracts including accuracy of the facts, statements, citing resources, etc.

### (PF707) DANICOPAN ADD-ON THERAPY IN PAROXYSMAL NOCTURNAL HEMOGLOBINURIA: UK EXPERIENCE

**Topic:** 12. Bone marrow failure syndromes incl. PNH - Clinical

Matthew Holt[1, 2], Francesco Versino[3], Pooja Mahapatra[2], Richard J. Kelly[1, 2], Roochi Trikha[3], Petra Muus[2], Shreyans Gandhi[3], Lindsay Mitchell[4], Louise Arnold[2], Sateesh Nagumantry[2, 5], Alexandra Pike[1, 2], Talha Munir[2], Joanna Large[3], Austin G. Kulasekararaj[3], Morag Griffin*[2]

*[1]University of Leeds, Leeds, United Kingdom; [2]St James' University Hospital, Leeds, United Kingdom; [3]King's College Hospital, London, United Kingdom; [4]University Hospital Monklands, Airdrie, United Kingdom; [5]Peterborough City Hospital, Peterborough, United Kingdom;*

### Background

Paroxysmal nocturnal hemoglobinuria (PNH) is a rare disorder with chronic complement-mediated hemolysis. C5 inhibitors (C5i) control intravascular hemolysis and reduce thrombotic risk, but a proportion of patients remain anemic due to extravascular hemolysis (EVH).
Danicopan is an oral Factor D inhibitor which blocks the proximal complement cascade and reduces the risk of EVH. It is approved for use in the UK as add-on therapy with either C5i ravulizumab or eculizumab, in patients who have clinically significant EVH.

### Aims

We present data from all patients with PNH treated with danicopan in the UK.

### Methods

A retrospective study was performed in all patients with PNH in the UK who received danicopan, either through clinical trial or as approved therapy.
Patient demographics, danicopan start date and concurrent C5i were collected. Hemoglobin (Hb), reticulocyte count (ARC) and lactate dehydrogenase (LDH) levels prior to danicopan (baseline) and at 3, 12 and 24 months (m) were collected where available. Transfusion requirements for 6m pre- danicopan and the most recent 6m on treatment were gathered. Outcomes included adverse effects, breakthrough hemolysis (BTH), infections requiring antibiotics, thromboses, treatment discontinuation and death.

**Copyright Information:** (Online) ISSN: 2572-9241
©2026 The Author(s). HemaSphere is published by John Wiley & Sons Ltd on behalf of European Hematology Association. This is an open access Abstract Book distributed under the Attribution-NonCommercial-NoDerivs (CC BY-NC-ND), which allows third parties to download the articles and share them with others as long as they credit the author and the Abstract Book, but they cannot change the content in any way or use them commercially.
**Abstract Book Citations:** Authors, Title, HemaSphere, 2026;10:(S1):pages. The abstract book DOIs can be found on the "Author Information" page.
**Disclaimer:** Articles published in the journal HemaSphere exclusively reflect the opinions of the authors. The authors are responsible for all content in their abstracts including accuracy of the facts, statements, citing resources, etc.

**Results**

34 patients (17 [51.5%] female; median age 55) started danicopan between 18/06/2018 and 04/02/2026. At data collection cut-off, median treatment duration was 254 days (IQR 111-356), with 4 patients on danicopan beyond 24m. Baseline mean PNH clones were 88.8% in granulocytes and 64.1% in erythrocytes. Baseline mean Hb was 98.3g/L, mean ARC 196.9 x$10^9$/L and mean LDH ratio 1.3x upper limit of normal (ULN).
27 patients received at least 3m danicopan at the time of cut-off, with blood results available for 22 patients. Mean Hb at 3m was 113.7g/L, with a mean rise of 21.1g/L. Mean ARC fell to 142.6 x$10^9$/L and mean LDH ratio 1.1x ULN. 7 patients received at least 12m danicopan, with results available for 6 patients. At 12m, mean Hb was 113.3g/L, mean ARC 162.2 x$10^9$/L and mean LDH ratio 1.0x ULN.
15 (45.5%) patients required a blood transfusion in the 6m prior to starting danicopan, falling to 5 patients (15.2%) in the most recent 6m of danicopan therapy; 1 of these patients received transfusion for major hemorrhage unrelated to PNH.
4 of 25 patients (16%) reported adverse reactions; nausea (n=2), headache (n=1) and a transient rise in liver enzymes (n=1). Infections requiring antibiotics occurred in 11 patients (33.3%), 8 requiring hospital admission. Infection sources were chest (n=4), urinary tract (n=2), skin (n=2), biliary (n=1) and unclear (n=2). There were no meningococcal infections.
6 BTH events occurred in 6 patients (18.2%); 5 with infection (3 viral, 2 bacterial) and 1 with no obvious trigger. Mean Hb fall was 25.7g/L with a mean LDH ratio of 2.8x ULN. No blood transfusions were required. 1 patient required an additional dose of C5i.
No thromboses were seen, no patients required danicopan cessation and there were no deaths.

**Summary/Conclusion**

This data shows that danicopan add-on therapy with C5i leads to timely improvements in Hb with a reduction in transfusion requirements. Despite dual complement inhibition, BTH rates are higher than those reported in danicopan clinical trial, and infection remains an important consideration as with all complement inhibitors. Danicopan is well tolerated and remains a good treatment option for patients with PNH and clinically significant EVH on C5i.

**Copyright Information:** (Online) ISSN: 2572-9241
©2026 The Author(s). HemaSphere is published by John Wiley & Sons Ltd on behalf of European Hematology Association. This is an open access Abstract Book distributed under the Attribution-NonCommercial-NoDerivs (CC BY-NC-ND), which allows third parties to download the articles and share them with others as long as they credit the author and the Abstract Book, but they cannot change the content in any way or use them commercially.
**Abstract Book Citations:** Authors, Title, HemaSphere, 2026;10:(S1):pages. The abstract book DOIs can be found on the "Author Information" page.
**Disclaimer:** Articles published in the journal HemaSphere exclusively reflect the opinions of the authors. The authors are responsible for all content in their abstracts including accuracy of the facts, statements, citing resources, etc.

### (PF708) FIRST-LINE HETROMBOPAG COMBINED WITH IMMUNOSUPPRESSIVE THERAPY FOR PEDIATRIC SEVERE APLASTIC ANEMIA: A PROSPECTIVE RANDOMIZED CONTROLLED TRIAL

**Topic:** 12. Bone marrow failure syndromes incl. PNH - Clinical

Lingling Fu*[1], Ruixin Wang[1], Hongmin Li[1], Bixi Yang[1], Jie MA[1], Hui Chen[1]

*[1]Beijing Children's Hospital, Capital Medical University, National Center for Children's Health, Hematology Center, Beijing, China;*

### Background

Hetrombopag (HPAG), an oral thrombopoietin receptor agonist (TPO-RA), has been shown to improve hematologic response without increasing toxicity when combined with standard immunosuppressive therapy (IST) as first- or second-line treatment in adults with severe aplastic anemia (SAA). However, clinical evidence regarding the efficacy of HPAG in pediatric patients with acquired aplastic anemia remains limited and controversial.

### Methods

This single-center, prospective study enrolled 54 children aged ≤18 years with newly diagnosed acquired SAA. Participants were randomized 1:1 to receive either standard IST or HPAG + IST. The primary endpoint was the complete response (CR) rate at 6 months after treatment initiation. Key secondary endpoints included the overall response (OR) rate at 6 months; the OR rate at 12 months, defined as the proportion of patients achieving CR or partial response (PR), and the cumulative incidence of CR; time to response; overall survival (OS); and event-free survival (EFS).

### Results

Among the 54 children with AA, the median age was 6.7 years (IQR 4.3-9.9), with 27 males and 27 females. Twenty-five had SAA and 29 had very severe aplastic anemia (VSAA). The median follow-up was 18.8 months (IQR: 13.3 to 25.1) in the IST+HPAG group and 20.9 months (IQR: 15.6 to 28.7) in the IST group. Baseline characteristics were well-balanced between the two groups. Efficacy: at 3 months, both groups showed identical CR rates (22.2%) and OR rates (85.2%). At 6 months, the HPAG + IST group demonstrated comparable CR rates (59.3% vs 62.9%, P=0.948) and identical OR rates (85.2%) versus the IST group. By 12 months, both groups maintained identical OR rates (87.5%), with CR rates of 74.1% vs 62.9% (P=0.875) in the HPAG + IST and IST groups, respectively. No statistically significant differences were observed in median time to PR (57 vs 50 days, P=0.583) or median time to CR (172 vs 160 days, P=0.421). Age subgroup analysis showed that among children older than 9 years, the HPAG + IST group had a significantly higher 3-month CR rate compared to the IST group (44.4% vs. 27.3%, P = 0.049). The HPAG + IST group reported 8 cases of grade ≥3 adverse events, primarily elevated liver enzymes (5 cases) and hypertriglyceridemia (3 cases), all managed successfully without treatment discontinuation. Clonal evolution occurred in 4/21 evaluable patients (19.0%) in the HPAG + IST group and 5/19 (26.3%) in the IST group, with no significant difference in mutational patterns between groups.

### Summary/Conclusion

The addition of Hetrombopag to standard IST did not significantly improve the 6-month CR rate or OR rate in children with newly diagnosed SAA. Routine upfront combination with Hetrombopag is not necessary for most pediatric SAA patients.

**Copyright Information:** (Online) ISSN: 2572-9241
©2026 The Author(s). HemaSphere is published by John Wiley & Sons Ltd on behalf of European Hematology Association. This is an open access Abstract Book distributed under the Attribution-NonCommercial-NoDerivs (CC BY-NC-ND), which allows third parties to download the articles and share them with others as long as they credit the author and the Abstract Book, but they cannot change the content in any way or use them commercially.
**Abstract Book Citations:** Authors, Title, HemaSphere, 2026;10:(S1):pages. The abstract book DOIs can be found on the "Author Information" page.
**Disclaimer:** Articles published in the journal HemaSphere exclusively reflect the opinions of the authors. The authors are responsible for all content in their abstracts including accuracy of the facts, statements, citing resources, etc.

### (PF709) A MULTICENTER REAL-WORLD STUDY EVALUATING THE EFFICACY AND SAFETY OF IPTACOPAN IN PATIENTS WITH AA-PNH SYNDROME

**Topic:** 12. Bone marrow failure syndromes incl. PNH - Clinical

Zhaoxia Liu*[1], Jinmeng Liu[2], Guanchen Bai[3], Aixia Dou[4], Zhilong Xu[5], Yingying Wang[1], Changyong Yuan[6], Yan Guo[7], Jie Yu[8], Shumin Ding[1], Xiaoqian Liu[2], Linying Li[2]

*[1]Affiliated Hospital of Shandong University of Traditional Chinese Medicine, Shandong, China;[2]School of Clinical Medicine, Shandong University of Traditional Chinese Medicine, Shandong, China; [3]The Affiliated Tai'an City Central Hospital of Qingdao University, Shandong, China; [4]The Second Qilu Hospital of Shandong University Shandong University, Shandong, China; [5]People's Hospital of Lanling County, Shandong, China;[6]Dezhou Hospital Qilu Hospital Shandong University, Shandong, China; [7]The First Affiliated Hospital of Shandong First Medical University & Shandong Provincial Qianfoshan Hospital, Shandong, China; [8]Weihai Municipal Hospital, Cheeloo College of Medicine, Shandong University, Shandong, China;*

### Background

Paroxysmal nocturnal hemoglobinuria (PNH) is a rare hematologic disorder caused by a PIGA gene mutation, leading to GPI-anchored protein loss and increased susceptibility to complement-mediated hemolysis. PNH often coexists with aplastic anemia (AA), forming AA-PNH syndrome, characterized by hemolysis, thrombosis risk, chronic anemia, and bone marrow failure. Iptacopan, an oral complement factor B inhibitor, has shown in phase III studies to significantly improve hemoglobin, reduce LDH, and lower transfusion needs. However, efficacy and safety data in AA-PNH patients, particularly those with concurrent immunosuppressive therapy or transfusion dependence, are still limited.

### Aims

This study aims to evaluate, in a real-world cohort of patients with AA-PNH syndrome, the impact of Iptacopan on hemolysis control, anemia, and transfusion requirements, as well as to assess its safety profile in the setting of concomitant immunosuppressive therapy.

### Methods

This multicenter retrospective observational study included AA-PNH patients receiving iptacopan monotherapy (200 mg, bid) and analyzed hematologic parameters at 3, 6, 9, and 12 months. Concomitant oral immunosuppressive therapy (IST) and transfusion data were recorded as potential confounders for anemia endpoints. The primary endpoints were hemolysis control (LDH ≤1.5 × ULN, ULN=245 U/L) and anemia improvement (Hb ≥12 g/dL, without considering RBC transfusion). Secondary endpoints included transfusion outcomes, platelet response (Plt ≥100 × $10^9$/L), and adverse events (serious infections and thrombotic events). All patients provided informed consent.

**Copyright Information:** (Online) ISSN: 2572-9241
©2026 The Author(s). HemaSphere is published by John Wiley & Sons Ltd on behalf of European Hematology Association. This is an open access Abstract Book distributed under the Attribution-NonCommercial-NoDerivs (CC BY-NC-ND), which allows third parties to download the articles and share them with others as long as they credit the author and the Abstract Book, but they cannot change the content in any way or use them commercially.
**Abstract Book Citations:** Authors, Title, HemaSphere, 2026;10:(S1):pages. The abstract book DOIs can be found on the "Author Information" page.
**Disclaimer:** Articles published in the journal HemaSphere exclusively reflect the opinions of the authors. The authors are responsible for all content in their abstracts including accuracy of the facts, statements, citing resources, etc.

### Results

A total of 21 AA-PNH patients were enrolled, with 20 evaluable (50% male, mean age 45.5 ± 16.8 years). Some patients received concomitant oral immunosuppressive therapy (e.g., cyclosporine/tacrolimus), and some adjusted their IST regimen during treatment. Baseline mean hemoglobin (Hb) was 6.66 ± 2.45 g/dL, and baseline lactate dehydrogenase (LDH) was 6.23 × ULN, with 80% exceeding 1.5 × ULN. The proportion of patients with baseline platelet count ≥100 × $10^9$/L was 25%.
At M3, M6, M9, and M12, mean LDH levels were 0.97 × ULN, 0.96 × ULN, 0.97 × ULN, and 1.17 × ULN, respectively, with no patients exceeding 1.5 × ULN. Hb response (≥12 g/dL) rates were 22.22% (4/18) at M3, 20.00% (2/10) at M6, 55.56% (5/9) at M9, and 66.67% (4/6) at M12. Hb increase of ≥2 g/dL from baseline (without considering RBC transfusion) was seen in 47.06% (8/17) at M3, 88.89% (8/9) at M6, 100% (8/8) at M9, and 100% (6/6) at M12. Transfusion independence rates were 47.06% (8/17) at M3, 88.89% (8/9) at M6, and 100% at M9.
Platelet response (≥100 × $10^9$/L) was 20.00% (3/15) at M3, 10.00% (1/10) at M6, 44.44% (4/9) at M9, and 33.33% (2/6) at M12. Follow-up records indicated a reduction in transfusion requirements or longer intervals between transfusions. One patient developed pneumonia, urinary tract infection, and syncope, and one patient had a rash. No thrombotic events occurred.

### Summary/Conclusion

In a real-world cohort of AA-PNH patients, treatment with Iptacopan resulted in rapid and sustained control of LDH to ≤1.5×ULN, with the proportion of patients achieving hemoglobin levels ≥12 g/dL steadily increasing over time. In the context of concomitant oral immunosuppressive therapy (IST) and transfusion support, Iptacopan effectively controlled hemolysis and improved anemia. However, larger sample sizes and more comprehensive trilineage data are needed to further validate these findings.

**Copyright Information:** (Online) ISSN: 2572-9241
©2026 The Author(s). HemaSphere is published by John Wiley & Sons Ltd on behalf of European Hematology Association. This is an open access Abstract Book distributed under the Attribution-NonCommercial-NoDerivs (CC BY-NC-ND), which allows third parties to download the articles and share them with others as long as they credit the author and the Abstract Book, but they cannot change the content in any way or use them commercially.
**Abstract Book Citations:** Authors, Title, HemaSphere, 2026;10:(S1):pages. The abstract book DOIs can be found on the “Author Information” page.
**Disclaimer:** Articles published in the journal HemaSphere exclusively reflect the opinions of the authors. The authors are responsible for all content in their abstracts including accuracy of the facts, statements, citing resources, etc.

### (PF710) EFFECTIVENESS AND SAFETY OF IPTACOPAN IN CHINESE PATIENTS WITH PAROXYSMAL NOCTURNAL HEMOGLOBINURIA: FIRST REPORT FROM A MULTICENTER AMBISPECTIVE REAL-WORLD COHORT STUDY (IMPACT-PNH CHINA)

**Topic:** 12. Bone marrow failure syndromes incl. PNH - Clinical

Hui Liu*[1], Fankai Meng[2], Mei Hong[3], Ye Baodong[4], Zunmin Zhu[5], Yueheng Qiu[6], Rong Fu[1]

*[1]Tianjin Medical University General Hospital, Tianjin, China;[2]Tongji Hospital, Tongji Medical College, Huazhong University of Science and Technology, Wuhan, China; [3]Union Hospital, Tongji Medical College, Huazhong University of Science and Technology, Wuhan, China; [4]The First Affiliated Hospital of Zhejiang Chinese Medical University, Hangzhou, China;[5]Henan Provincial People's Hospital, Zhengzhou, China; [6]Novartis China, Shanghai, China;*

### Background

Paroxysmal nocturnal hemoglobinuria (PNH) is a rare, life-threatening acquired hematopoietic stem cell disease, affecting about 9.1 per 1,000,000 people in China. Complement inhibitors are the standard of care. Iptacopan, an oral factor B inhibitor, was approved in China in April 2024. Although phase III trials (APPLY and APPOINT) showed favorable efficacy and safety, real-world evidence in Chinese patients (pts) remains limited.

### Aims

To evaluate the real-world effectiveness, safety, adherence, and impact on disease management of iptacopan in adult PNH pts in China.

### Methods

This multicenter, ambispective, non-interventional cohort study (NCT06931691) evaluates disease burden, quality of life, healthcare resource utilization, and treatment regimens over 12 months. 80 adult pts are planned for enrollment: 60 in Cohort 1 (C5i-naïve) and 20 in Cohort 2 (C5i-experienced). This interim analysis included all pts enrolled between April 21, 2025, and January 24, 2026, and focused on the 3-month effectiveness and safety of iptacopan in C5i-naïve PNH pts, as well as baseline characteristics of all pts. Endpoints included baseline characteristics, hematologic response, symptom improvement, and adverse events. Data were summarized using descriptive statistics; where applicable, ANCOVA was used to estimate least squares mean (LSM) changes from baseline for each hematologic indicator (covariates: baseline value, sex, transfusion dependence, and age group [<45 vs. ≥45]).

**Copyright Information:** (Online) ISSN: 2572-9241
©2026 The Author(s). HemaSphere is published by John Wiley & Sons Ltd on behalf of European Hematology Association. This is an open access Abstract Book distributed under the Attribution-NonCommercial-NoDerivs (CC BY-NC-ND), which allows third parties to download the articles and share them with others as long as they credit the author and the Abstract Book, but they cannot change the content in any way or use them commercially.
**Abstract Book Citations:** Authors, Title, HemaSphere, 2026;10:(S1):pages. The abstract book DOIs can be found on the "Author Information" page.
**Disclaimer:** Articles published in the journal HemaSphere exclusively reflect the opinions of the authors. The authors are responsible for all content in their abstracts including accuracy of the facts, statements, citing resources, etc.

### Results

Among 44 pts enrolled, mean (SD) age was 43.8 (15.47) years. Overall, 65.9% were male, and 77.3% were C5i-naïve (N=34). At baseline, 29.5% were transfusion-dependent; mean (SD) hemoglobin (Hb) was 8.0 (2.16) g/dL (Cohort 1: 7.3 (1.79); Cohort 2: 10.3 (1.69)); and 29.5% had a history of aplastic anemia. Prior thrombotic, renal, and cardiac events occurred in 9.1%, 6.8%, and 4.5%, respectively (Table 1). Mean (SD) iptacopan exposure was 84.2 (64.26) days.

Among 22 evaluable C5i-naïve pts at 3 months, mean (SD) Hb increased by 4.3 (2.91) g/dL (LSM [95% CI]: 3.7 [2.50–4.80]) (Figure). Mean (SD) lactate dehydrogenase (LDH) decreased by 1261.2 (970.62) U/L (LSM [95% CI]: -1278.6 [-1332.25, -1224.99]) (Figure). Of these pts, 50.0% achieved hemoglobin normalization (Hb ≥ 12 g/dL), and 90.9% achieved LDH < 1.5 × upper limit of normal (ULN). All pts were transfusion-independent during the 3-month follow-up period. Other biochemical and quality-of-life parameters also improved (Table 2). Trends through 6 months are shown in Figure (follow-up is ongoing).

For safety, all 34 C5i-naïve pts were included. Treatment-emergent adverse events occurred in 23.5%, predominantly mild, and treatment-related adverse events (TRAEs) occurred in 14.7%, with serious TRAEs limited to infections (5.9%). There were no major adverse vascular events; 1 patient experienced breakthrough hemolysis in the context of missed doses (2.9%).

### Summary/Conclusion

This first prospective real-world study in China suggests that iptacopan was associated with rapid control of hemolysis, clinically meaningful Hb improvement, transfusion independence, and fatigue improvement. Iptacopan was well tolerated, with a safety profile consistent with previous reports. Continued follow-up will further define long-term effectiveness, safety, and quality of life outcomes.

**Copyright Information:** (Online) ISSN: 2572-9241
©2026 The Author(s). HemaSphere is published by John Wiley & Sons Ltd on behalf of European Hematology Association. This is an open access Abstract Book distributed under the Attribution-NonCommercial-NoDerivs (CC BY-NC-ND), which allows third parties to download the articles and share them with others as long as they credit the author and the Abstract Book, but they cannot change the content in any way or use them commercially.
**Abstract Book Citations:** Authors, Title, HemaSphere, 2026;10:(S1):pages. The abstract book DOIs can be found on the "Author Information" page.
**Disclaimer:** Articles published in the journal HemaSphere exclusively reflect the opinions of the authors. The authors are responsible for all content in their abstracts including accuracy of the facts, statements, citing resources, etc.

**Table 1: Baseline characteristics**

| | Overall Patients N=44 | Cohort 1 N= 34 | Cohort 2 N=10 |
|---|---|---|---|
| **Age(years)** | | | |
| Mean±SD | 43.8±15.47 | 44.2±16.93 | 42.5±9.51 |
| Median (IQR) | 41.5(23.0) | 42.0 (30.0) | 40.0(16.0) |
| **Sex, n (%)** | | | |
| Male | 29 (65.9) | 24 (70.6) | 5 (50.0) |
| **Blood transfusion dependence (over the last 12 months), n (%)** | | | |
| Yes | 13 (29.5) | 11 (32.4) | 2 (20.0) |
| **Hb level at baseline (g/dL)** | | | |
| Mean±SD | 8.0±2.16 | 7.3±1.79 | 10.3±1.69 |
| Median (IQR) | 7.7 (3.50) | 7.4 (3.10) | 10.4 (1.00) |
| ⩾10, n (%) | 9 (20.5) | 2 (5.9) | 7 (70.0) |
| <10, n (%) | 35 (79.5) | 32 (94.1) | 3 (30.0) |
| **Comorbidities at baseline, n (%)** | | | |
| Thrombosis | 4 (9.1) | 3 (8.8) | 1 (10.0) |
| Kidney disease | 3 (6.8) | 3 (8.8) | 0 (0.0) |
| Heart disease | 2 (4.5) | 2 (5.9) | 0 (0.0) |
| **Aplastic anemia history, n (%)** | 13 (29.5) | 11 (32.4) | 2 (20.0) |

**Table 2: Effectiveness assessment within 3-month follow-up**

| **At 3-month follow-up** | **Cohort 1 N=22** |
|---|---|
| **Hb change from baseline, LSM (95%CI)** | 3.7 (2.50, 4.80) |
| **Hb, LSM (95%CI)** | 10.8 (9.67, 11.97) |
| **Transfusions independence&, n (%)** | 22 (100) |
| **Patients achieving Hb ⩾12 g/dL, n(%)** | 11 (50) |
| **Patients achieving Hb increase ⩾2 g/dL, n(%)** | 16 (72.7) |
| **LDH (U/L) change from baseline, LSM (95%CI)** | -1278.6 (-1332.25, -1224.99) |
| **Patients achieving LDH < 1.5×ULN, n (%)** | 20 (90.9) |
| **Absolute reticulocyte count (10^9/L) change from baseline, LSM (95%CI)** | -54.4 (-83.99, -24.74) |
| **Bilirubin change(μmol/L) from baseline, LSM (95%CI)** | |
| Total bilirubin | -12.8 (-15.96, -9.71) |
| Unconjugated bilirubin | -12.8 (-15.94, -9.58) |
| **FACIT*-Fatigue score change from baseline, LSM (95%CI)** | |
| FACIT-F trial outcome index | 11.0 (2.40, 19.60) |
| FACIT-G total score | 4.8 (-3.40, 12.90) |
| FACIT-F total score | 10.7 (-1.40, 22.80) |

***FACIT-Fatigue score:** The functional assessment of chronic illness therapy – Fatigue score
&**Transfusion independence:** No all-cause transfusions occurred during the follow-up period after the patient's first use of iptacopan.

**Figure. Hb and LDH LSM (95%CI) change from baseline in cohort 1**

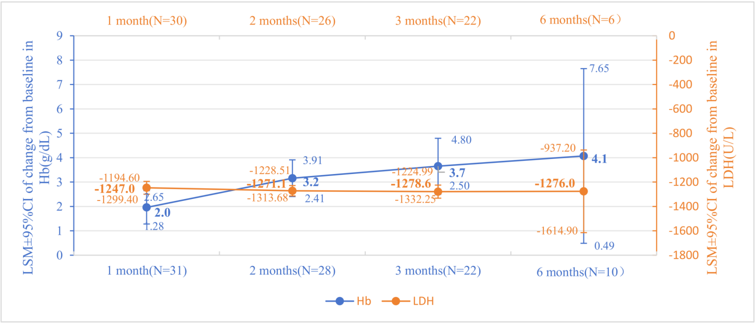

**Copyright Information:** (Online) ISSN: 2572-9241
©2026 The Author(s). HemaSphere is published by John Wiley & Sons Ltd on behalf of European Hematology Association. This is an open access Abstract Book distributed under the Attribution-NonCommercial-NoDerivs (CC BY-NC-ND), which allows third parties to download the articles and share them with others as long as they credit the author and the Abstract Book, but they cannot change the content in any way or use them commercially.
**Abstract Book Citations:** Authors, Title, HemaSphere, 2026;10:(S1):pages. The abstract book DOIs can be found on the "Author Information" page.
**Disclaimer:** Articles published in the journal HemaSphere exclusively reflect the opinions of the authors. The authors are responsible for all content in their abstracts including accuracy of the facts, statements, citing resources, etc.

### (PF711) CLINICAL SPECTRUM AND MUTATIONAL BURDEN OF PIGA VARIANTS DETECTED BY BONE MARROW NGS: REAL-WORLD DIAGNOSTIC IMPLICATIONS AND CORRELATION WITH PNH CLONE SIZE

**Topic:** 12. Bone marrow failure syndromes incl. PNH - Clinical

Min Wu*[1], Han Yan[1], Yongmei Zhu[1], Gang Cai[2], Jianqing Mi[1], Huijin Zhao[1]

*[1]Shanghai Institute of Hematology, State Key Laboratory of Medical Genomics, National Research Center for Translational Medicine at Shanghai, Ruijin Hospital Affiliated to Shanghai Jiao Tong University School of Medicine, Shanghai, China;*
*[2]Department of Laboratory Medicine, Ruijin Hospital, Shanghai JiaoTong University School of Medicine, Shanghai, China;*

### Background

Bone marrow next-generation sequencing (NGS) increasingly identifies incidental PIGA mutations in patients evaluated for cytopenias or suspected hematologic malignancies. The clinical implications of such findings and their relationship to peripheral PNH clone burden remain insufficiently defined, often leading to overlooked diagnoses in routine practice.

### Aims

To characterize the clinicopathologic spectrum of PIGA-mutated cases detected by bone marrow NGS, to assess the association between PIGA mutational burden and granulocyte PNH clone size, and to evaluate current real-world diagnostic workflows.

### Methods

We retrospectively analyzed 4,700 consecutive bone marrow NGS tests performed between October 2020 and August 2025. Cases harboring PIGA variants were identified and clinically reviewed. PNH was confirmed by flow cytometry. Variant types were classified, and mutational burden was defined as maximum variant allele frequency (VAF) and cumulative VAF per patient. Associations with granulocyte clone size were evaluated using Spearman correlation. The utilization rate of diagnostic flow cytometry was also assessed.

### Results

PIGA variants were detected in 25 cases (0.53%). Fifteen cases (60%) were confirmed PNH, including classical PNH (n=10), marrow failure–associated PNH (n=3), and subclinical PNH (n=2). The remaining 10 cases comprised acute myeloid leukemia, myelodysplastic syndrome, myeloproliferative neoplasms, lymphoid malignancies, and unspecified cytopenias. Among these non-PNH cases, diagnostic flow cytometry was utilized in only 3 patients (all negative). Among confirmed PNH patients, median age was 52 years. Median hemoglobin was 80.5 g/L (IQR 62.0–91.25), and median LDH was 1028 U/L (IQR 482–1516.5). Median granulocyte clone size was 51%.
The number of PIGA variants per case ranged from 1 to 5. Variants were predominantly truncating or splice-site alterations, consistent with established loss-of-function patterns in PNH.
Notably, 40% (6/15) of confirmed PNH patients harbored multiple PIGA variants (≥2), and these patients exhibited larger granulocyte clone sizes compared to those with a single variant. In contrast, 100% of the non-PNH cases exclusively harbored a single PIGA variant. The cumulative PIGA VAF demonstrated a significant positive correlation with granulocyte clone size (Spearman $\rho$=0.61, p=0.02), whereas maximum VAF showed only borderline association.
Co-mutations were detected in 9 of 15 confirmed PNH cases, most commonly involving DNMT3A, ASXL1, and TET2. Bone marrow smears frequently showed hypercellularity and erythroid predominance.
Five confirmed PNH patients received complement inhibition (iptacopan n=4; eculizumab n=1). Among iptacopan-treated patients with follow-up (n=3), hemoglobin increased from 86 to 139 g/L and LDH decreased from 2213 to 226 U/L.

**Copyright Information:** (Online) ISSN: 2572-9241
©2026 The Author(s). HemaSphere is published by John Wiley & Sons Ltd on behalf of European Hematology Association. This is an open access Abstract Book distributed under the Attribution-NonCommercial-NoDerivs (CC BY-NC-ND), which allows third parties to download the articles and share them with others as long as they credit the author and the Abstract Book, but they cannot change the content in any way or use them commercially.
**Abstract Book Citations:** Authors, Title, HemaSphere, 2026;10:(S1):pages. The abstract book DOIs can be found on the "Author Information" page.
**Disclaimer:** Articles published in the journal HemaSphere exclusively reflect the opinions of the authors. The authors are responsible for all content in their abstracts including accuracy of the facts, statements, citing resources, etc.

**Summary/Conclusion**

In a real-world bone marrow NGS workflow, most PIGA-mutated cases corresponded to confirmed PNH. Aggregate PIGA mutational burden correlated with peripheral granulocyte clone size, suggesting that cumulative VAF may reflect clonal expansion. The presence of co-mutations and marrow morphological heterogeneity underscores biological complexity in a subset of cases. Integration of NGS findings with flow cytometry may support appropriate diagnostic evaluation. Larger prospective multicenter studies are warranted to further validate these observations.

**Copyright Information:** (Online) ISSN: 2572-9241
©2026 The Author(s). HemaSphere is published by John Wiley & Sons Ltd on behalf of European Hematology Association. This is an open access Abstract Book distributed under the Attribution-NonCommercial-NoDerivs (CC BY-NC-ND), which allows third parties to download the articles and share them with others as long as they credit the author and the Abstract Book, but they cannot change the content in any way or use them commercially.
**Abstract Book Citations:** Authors, Title, HemaSphere, 2026;10:(S1):pages. The abstract book DOIs can be found on the "Author Information" page.
**Disclaimer:** Articles published in the journal HemaSphere exclusively reflect the opinions of the authors. The authors are responsible for all content in their abstracts including accuracy of the facts, statements, citing resources, etc.

### (PF712) LP-005, A BIFUNCTIONAL ANTIBODY, DEMONSTRATES SUSTAINED EFFICACY AND A FAVORABLE SAFETY PROFILE IN PATIENTS WITH PAROXYSMAL NOCTURNAL HEMOGLOBINURIA (PNH): UPDATED RESULTS FROM A PHASE Ⅱ STUDY

**Topic:** 12. Bone marrow failure syndromes incl. PNH - Clinical

Guangsheng He*[1], Yuemin Gong[1], Lei Fan[1], Wenwei Zhu[2], Jingnan Sun[3], Chuan He[4], Cuicui Han[5], Jie Yang[5], Heng Liu[5]

*[1]The First Affiliated Hospital of Nanjing Medical University, Jiangsu Province Hospital, Key Laboratory of Hematology of Nanjing Medical University, Collaborative Innovation Center for Cancer Personalized Medicine, Nanjing, China; [2]Yueyang Hospital of Integrated Traditional Chinese and Western Medical, Shanghai University of Traditional Chinese Medicine, Shanghai, China; [3]The First Hospital of Jilin University, Changchun, China; [4]Guang'an People's Hospital, Guangan, China; [5]LongBio Pharma (Suzhou) Co., Ltd., Shanghai, China;*

### Background

Paroxysmal nocturnal hemoglobinuria (PNH) is a rare, life-threatening hematologic disorder characterized by complement-mediated intravascular and extravascular hemolysis (IVH and EVH). Current complement-targeted therapies have limitations in addressing both pathways of hemolysis, leaving unmet clinical needs for more effective and durable treatment options. LP-005 is a novel bifunctional anti-C5 antibody fusion protein that inhibit both terminal pathway and alternative pathway activation. The molecule was engineered to inhibit pathological complement activation, with initial positive interim efficacy and safety results presented at the 2025 ASH Annual Meeting. Herein, we report updated data from this ongoing study.

### Aims

To present updated efficacy and safety data of LP-005 in PNH patients, focusing on sustained control of hemolysis, improvements in hemoglobin levels, transfusion independence, and tolerability.

### Methods

This was an open-label, single-arm Phase Ⅱ study (NCT07212426) evaluating the efficacy, safety, pharmacokinetics, and pharmacodynamics of LP-005 injection in adult patients with PNH. Patients were randomized to receive intravenous infusions of LP-005 Injection at a dose of 900 mg or 1200 mg once every 4 weeks, with 10 patients per group. Following completion of the 12-week fixed-dose period, doses could be optimized based on efficacy and safety assessments. The primary objective was to assess the effect of LP-005 on LDH and Hb levels.

**Copyright Information:** (Online) ISSN: 2572-9241
©2026 The Author(s). HemaSphere is published by John Wiley & Sons Ltd on behalf of European Hematology Association. This is an open access Abstract Book distributed under the Attribution-NonCommercial-NoDerivs (CC BY-NC-ND), which allows third parties to download the articles and share them with others as long as they credit the author and the Abstract Book, but they cannot change the content in any way or use them commercially.
**Abstract Book Citations:** Authors, Title, HemaSphere, 2026;10:(S1):pages. The abstract book DOIs can be found on the "Author Information" page.
**Disclaimer:** Articles published in the journal HemaSphere exclusively reflect the opinions of the authors. The authors are responsible for all content in their abstracts including accuracy of the facts, statements, citing resources, etc.

### Results

This updated analysis included 20 patients who completed 24 weeks of the ongoing 48-week treatment period. As of the data cutoff date, all the patients in 900 mg group had undergone dose escalation to 1200 mg. Results presented in this abstract are based on intention-to-treat (ITT) analysis per the original randomized treatment assignment. The median (range) age was 38 (20-60) years, and 10 patients (50%) were female. The baseline mean (SD) LDH was 2013.3 (1265.73) U/L in the 900 mg group and 1694.6 (724.34) U/L in the 1200 mg group, with baseline mean (SD) Hb levels of 65.0 (11.84) g/L and 63.5 (10.30) g/L, respectively. By Week 24, substantial clinical improvements were observed in all the patients. Mean (SD) LDH decreased to 959.5 (1930.95) U/L (-68.07%) and 247.6 (109.10) U/L (-82.58%) in the 900 mg and 1200 mg groups, respectively. In the 900 mg group, 5/10 patients (50%) achieved LDH levels below 1.5×ULN, with 3/10 (30%) reaching normal LDH levels. In the 1200 mg group, the corresponding proportions were 9/10 (90%) and 6/10 (60%), respectively. Mean (SD) Hb levels were 118.4 (28.91) g/L and 109.1 (20.60) g/L in the 900 mg and 1200 mg groups, respectively. Hb increases ≥2 g/dL from baseline were observed in 90% of patients in both groups. Moreover, no patients required blood transfusions during the treatment. LP-005 continued to be safe and well tolerated. Treatment-emergent adverse events (TEAEs) occurred in 75% of patients, all of which were mild or moderate. No AEs led to treatment discontinuation, and no treatment-related serious adverse events (SAEs) were reported.

### Summary/Conclusion

Updated data from this Phase Ⅱ study confirm that LP-005 achieves sustained control of complement-mediated hemolysis in patients with PNH, with durable hemoglobin improvements, maintained transfusion independence, and a favorable safety profile. These findings support LP-005 as a promising novel monotherapy for PNH.

**Copyright Information:** (Online) ISSN: 2572-9241
©2026 The Author(s). HemaSphere is published by John Wiley & Sons Ltd on behalf of European Hematology Association. This is an open access Abstract Book distributed under the Attribution-NonCommercial-NoDerivs (CC BY-NC-ND), which allows third parties to download the articles and share them with others as long as they credit the author and the Abstract Book, but they cannot change the content in any way or use them commercially.
**Abstract Book Citations:** Authors, Title, HemaSphere, 2026;10:(S1):pages. The abstract book DOIs can be found on the “Author Information” page.
**Disclaimer:** Articles published in the journal HemaSphere exclusively reflect the opinions of the authors. The authors are responsible for all content in their abstracts including accuracy of the facts, statements, citing resources, etc.

### (PF713) IMPACT OF IPTACOPAN, A COMPLEMENT INHIBITOR, ON CHINESE PATIENTS WITH PAROXYSMAL NOCTURNAL HEMOGLOBINURIA: A MULTICENTER RETROSPECTIVE COHORT STUDY

**Topic:** 12. Bone marrow failure syndromes incl. PNH - Clinical

Shuqian Xu*[1], Guangsheng He[2], Chuan He[3], Jingnan Sun[4], Guanchen Bai[5], Jie Yu[6], Yujie Jiang[7], Li Zhang[8], Zhilong Xu[9]

*[1]Qilu Hospital of Shandong University, Shandong, China; [2]The First Affiliated Hospital of Nanjing Medical University, Nanjing, China; [3]West China Hospital of Sichuan University, Sichuan, China; [4]The First Hospital of Jilin University, Jilin, China; [5]Tai'an Central Hospital, Tai'an, China; [6]Weihai Municipal Hospital, Cheeloo College of Medicine, Shandong University, Shandong, China; [7]Shandong Provincial Hospital Affiliated to Shandong First Medical University, Jinan, China; [8]Liaocheng People's Hospital, Liaocheng, China; [9]Lanling County People's Hospital, Lanling, China;*

### Background

Paroxysmal Nocturnal Hemoglobinuria (PNH) is a rare acquired hematopoietic stem cell disorder characterized by intravascular hemolysis, thrombosis, and hematopoietic failure. It results from PIG-A mutations causing deficiency of complement-regulating proteins CD55 and CD59, leading to chronic hemolysis and complications including anemia and thrombosis (Bravo-Perez, 2023). Complement inhibitors including eculizumab and iptacopan are standard treatments. While eculizumab inhibits C5 activation, it does not fully control intravascular hemolysis (Wu, 2023). Iptacopan, an oral factor B inhibitor, has demonstrated superior efficacy in Phase III trials, achieving greater hemoglobin increases and reduced transfusion needs.

### Aims

This multicenter, retrospective cohort study aimed to assess the effectiveness and safety of complement inhibitors in real-world settings.

### Methods

From March 2025 to February 2026, we included 22 treatment-naïve PNH patients who were treated with Iptacopan at Qilu Hospital of Shandong University, 9 patients treated with Iptacopan at Jilin Hospital, 13 patients treated with Iptacopan and 12 patients treated with Eculizumab at West China Hospital, and 14 patients treated with Iptacopan and 54 patients treated with Eculizumab at Jiangsu Provincial People's Hospital, all located in China. A stable treatment regimen was maintained for at least one month. The study had two primary endpoints: (1) the mean change in hemoglobin levels, and (2) the mean change in lactate dehydrogenase (LDH) levels. The secondary endpoint focused on the proportion of patients with normalized LDH levels and the proportion of patients with LDH levels ≤ 1.5×ULN.

**Copyright Information:** (Online) ISSN: 2572-9241
©2026 The Author(s). HemaSphere is published by John Wiley & Sons Ltd on behalf of European Hematology Association. This is an open access Abstract Book distributed under the Attribution-NonCommercial-NoDerivs (CC BY-NC-ND), which allows third parties to download the articles and share them with others as long as they credit the author and the Abstract Book, but they cannot change the content in any way or use them commercially.
**Abstract Book Citations:** Authors, Title, HemaSphere, 2026;10:(S1):pages. The abstract book DOIs can be found on the "Author Information" page.
**Disclaimer:** Articles published in the journal HemaSphere exclusively reflect the opinions of the authors. The authors are responsible for all content in their abstracts including accuracy of the facts, statements, citing resources, etc.

### Results

A total of 58 iptacopan-treated and 66 eculizumab-treated patients were analyzed. The iptacopan group showed Hb increases of +12.25 g/L (95% CI: 8.42-16.08) at Week 1 and +47.67 g/L (95% CI: 41.20-54.14) at Month 6. The eculizumab group exhibited an initial Hb decline of -5.40 g/L (95% CI: -9.82 to -0.98) at Week 1, followed by gradual recovery to +35.40 g/L (95% CI: 28.93-41.87) at Month 12. For LDH reduction, the iptacopan group achieved 46.45% (95% CI: -52.84 to -40.06) at Week 1 and 90.74% (95% CI: -97.13 to -84.35) at Month 6. The eculizumab group achieved 19.63% (95% CI: -25.38 to -13.88) at Week 1 and 83.73% (95% CI: -90.12 to -77.34) at Month 12. LDH normalization (≤1×ULN) peaked at 79% (95% CI: 0.68-0.87) at Month 2 in the iptacopan group versus 44% (95% CI: 0.32-0.56) at Month 3 in the eculizumab group. For LDH ≤1.5×ULN, the iptacopan group reached 100% (95% CI: 0.94-1.00) by Week 3; the eculizumab group peaked at 89% (95% CI: 0.79-0.95) at Month 3.

| Hb | W1 | W2 | W3 | W4 | M2 | M3 | M6 |
|---|---|---|---|---|---|---|---|
| Iptacopan | 12.25 | 26.71 | 30.20 | 27.07 | 32.44 | 37.92 | 47.67 |

| Hb | W1 | M1 | M3 | M6 | M9 | M12 |
|---|---|---|---|---|---|---|
| Eculizumab | -5.40 | 12.83 | 21.94 | 24.24 | 30.53 | 35.40 |

| LDH | W1 | W2 | W3 | W4 | M2 | M3 | M6 |
|---|---|---|---|---|---|---|---|
| Iptacopan | -46.45 | -70.49 | -71.69 | -63.30 | -66.31 | -77.12 | -90.74 |

| LDH | W1 | M1 | M3 | M6 | M9 | M12 |
|---|---|---|---|---|---|---|
| Eculizumab | -19.63 | -60.55 | -82.32 | -71.12 | -77.91 | -83.73 |

| LDH normalization | W1 | W2 | W3 | W4 | M2 | M3 | M6 |
|---|---|---|---|---|---|---|---|
| Iptacopan | 0.17 | 0.58 | 0.75 | 0.75 | 0.79 | 0.77 | 0.77 |

| LDH normalization | W1 | M1 | M3 | M6 | M9 |
|---|---|---|---|---|---|
| Eculizumab | 0 | 0.37 | 0.44 | 0.44 | 0.38 |

| LDH ≤1.5×ULN | W1 | W2 | W3 | W4 | M2 | M3 |
|---|---|---|---|---|---|---|
| Iptacopan | 0.42 | 0.83 | 1 | 0.89 | 0.96 | 1 |

| LDH ≤1.5×ULN | W1 | M1 | M3 | M6 | M9 |
|---|---|---|---|---|---|
| Eculizumab | 0 | 0.68 | 0.89 | 0.88 | 0.85 |

### Summary/Conclusion

Both iptacopan and eculizumab demonstrated effective disease control in Chinese PNH patients. The iptacopan group showed faster hemoglobin improvement and greater LDH reduction. These findings suggest iptacopan may provide more rapid hemolysis control, though both therapies offered clinically meaningful benefits. Longer follow-up studies are warranted.

**Copyright Information:** (Online) ISSN: 2572-9241
©2026 The Author(s). HemaSphere is published by John Wiley & Sons Ltd on behalf of European Hematology Association. This is an open access Abstract Book distributed under the Attribution-NonCommercial-NoDerivs (CC BY-NC-ND), which allows third parties to download the articles and share them with others as long as they credit the author and the Abstract Book, but they cannot change the content in any way or use them commercially.
**Abstract Book Citations:** Authors, Title, HemaSphere, 2026;10:(S1):pages. The abstract book DOIs can be found on the “Author Information” page.
**Disclaimer:** Articles published in the journal HemaSphere exclusively reflect the opinions of the authors. The authors are responsible for all content in their abstracts including accuracy of the facts, statements, citing resources, etc.

### (PF714) RETHINKING THROMBOTIC RISK IN MINOR PNH CLONES: INSIGHTS BEYOND HEMOLYSIS AND CONVENTIONAL RISK FACTORS

**Topic:** 12. Bone marrow failure syndromes incl. PNH - Clinical

Elena Pirvu*[1], Manuel Marcu[1], Maria Bacanu[1, 2], Diana Grasu[1], Alexandra-Diana Malenda[1, 2], Viola Maria Popov[1], Mihaela Andreescu[1, 2]

*[1]Colentina Clinical Hospital, Hematology, Bucharest, Romania;[2]"Titu Maiorescu" University, Faculty of General Medicine, General Medicine, Bucharest, Romania;*

### Background

Thrombosis represents the leading cause of morbidity and mortality in paroxysmal nocturnal hemoglobinuria (PNH). While thrombotic risk is well established in classical hemolytic disease, the clinical significance of minor PNH clones remains incompletely defined, particularly in the absence of significant biochemical hemolysis and among patients evaluated for cytopenias or thrombotic events.

### Aims

To evaluate thrombotic occurrence in patients with minor PNH clones and explore associations with hemolysis markers, conventional thrombotic risk factors, bone marrow disease overlap, and clone distribution.

### Methods

We conducted a single-center observational study including 35 patients evaluated between 2020 and 2026 for PNH by high-sensitivity flow cytometry using FLAER combined with CD55/CD59 assessment. Patients were stratified into those with minor PNH clones (<10%) and clone-negative controls. The cohort comprised 26 patients with minor clones and 9 controls, with a median age of 55 years, including 19 males (54%) and 16 females (46%).
Clinical data included thrombotic history (deep vein thrombosis, pulmonary embolism, and atypical-site thrombosis), Caprini score, thrombophilia status, hemolysis markers, and bone marrow findings including aplastic anemia and myelodysplastic syndrome. Testing indications included thrombosis, cytopenias, and suspected hemolysis.Clone sizes were assessed across granulocytic, erythroid, and monocytic lineages.
Group comparisons used non-parametric and contingency analyses. Given the limited number of thrombotic events, logistic regression was restricted to clone presence as the primary predictor, with age included in a sensitivity analysis. All statistical analyses were performed using Jamovi (version 2.7.18).

**Copyright Information:** (Online) ISSN: 2572-9241
©2026 The Author(s). HemaSphere is published by John Wiley & Sons Ltd on behalf of European Hematology Association. This is an open access Abstract Book distributed under the Attribution-NonCommercial-NoDerivs (CC BY-NC-ND), which allows third parties to download the articles and share them with others as long as they credit the author and the Abstract Book, but they cannot change the content in any way or use them commercially.
**Abstract Book Citations:** Authors, Title, HemaSphere, 2026;10:(S1):pages. The abstract book DOIs can be found on the “Author Information” page.
**Disclaimer:** Articles published in the journal HemaSphere exclusively reflect the opinions of the authors. The authors are responsible for all content in their abstracts including accuracy of the facts, statements, citing resources, etc.

**Results**

Thrombosis occurred in 50% of clone-positive patients versus 11.1% of controls (OR 8.0, Fisher p=0.056). Atypical thrombotic events (splanchnic, iliac and cerebral thrombosis) were observed exclusively in the clone-positive group (7 patients) and were absent in controls. Hemolysis markers (hemoglobin, reticulocytes, indirect bilirubin, LDH) did not differ between patients with and without thrombosis, and the Caprini score was not predictive (p=0.073), possibly due to limited statistical power and requiring validation in larger cohorts. Clone burden remained low across cellular lineages, with median values of 0.01% in granulocytes, 0.03% in erythrocytes, and 0.00% in monocytes.
In logistic regression analysis, overall PNH clone presence, rather than lineage-specific clone size, was associated with thrombotic risk, while individual clone lineages did not demonstrate independent predictive value.
Age-adjusted logistic regression demonstrated a borderline association between clone presence and thrombosis (OR 7.93, 95% CI 0.85–73.0, p=0.067). Bone marrow disorders were not associated with thrombotic events.

**Summary/Conclusion**

Minor PNH clones were associated with increased thrombotic frequency in the absence of significant biochemical hemolysis and without clear association with conventional risk factors. These observations are consistent with the hypothesis that thrombotic risk in PNH extends beyond classical hemolytic disease and may be present in subclinical clonal states. The findings support clinical surveillance in patients with minor clones and highlight the need for larger prospective cohorts to refine risk stratification and management.

**Copyright Information:** (Online) ISSN: 2572-9241
©2026 The Author(s). HemaSphere is published by John Wiley & Sons Ltd on behalf of European Hematology Association. This is an open access Abstract Book distributed under the Attribution-NonCommercial-NoDerivs (CC BY-NC-ND), which allows third parties to download the articles and share them with others as long as they credit the author and the Abstract Book, but they cannot change the content in any way or use them commercially.
**Abstract Book Citations:** Authors, Title, HemaSphere, 2026;10:(S1):pages. The abstract book DOIs can be found on the “Author Information” page.
**Disclaimer:** Articles published in the journal HemaSphere exclusively reflect the opinions of the authors. The authors are responsible for all content in their abstracts including accuracy of the facts, statements, citing resources, etc.

### (PF715) EFFICACY AND SAFETY OF ROMIPLOSTIM IN NEWLY DIAGNOSED APLASTIC ANEMIA: REAL-WORLD SINGLE CENTRE EXPERIENCE FROM INDIA

**Topic:** 12. Bone marrow failure syndromes incl. PNH - Clinical

Chaitanya H Balakrishnan*[1], Sanjukta S Rao[1], Muhammad Mohsin[2], Kaushik Nagendra[1], Ruthvik Tv[1], Ganapathiearaman Ramakrishnan[1], Cecil Ross[1], Seetharam Anandram[1]

*[1]St. John's Medical College, Clinical Hematology, Bangalore, India;[2]Barnsley Hospital NHS Foundation Trust, Clinical Hematology, Barnsley, United Kingdom;*

### Background

Immunosuppressive therapy (IST) remains the mainstay for aplastic anemia (AA) patients ineligible for transplantation. Thrombopoietin receptor agonists have demonstrated synergistic activity when combined with IST. We evaluated outcomes of a romiplostim-based frontline strategy across AA severity categories.

### Methods

Forty consecutive AA patients (median age 52 years; range 22–78) treated between January 2022 and December 2023 were analyzed. Disease severity included non-severe (NSAA, n=10), severe (SAA, n=19), and very severe (VSAA, n=11). The patients received Romiplostim, Cyclosporine and Danazol but only a subset received ATG (5/40, 2 with SAA and 3 with VSAA) due to financial constraints. Primary endpoint was overall response rate( ORR) at 3, 6, and 12 months which included complete response( Hb>10g/dl, ANC>1000/ul, platelet >1lakh/cu.mm) and partial response(Hb>8g/dl, ANC>500/ul, platelet >20,000/cu.mm). Secondary endpoints included transfusion independence, relapse, clonal evolution, mortality, and hospitalization burden.

### Results

The ORR was 37.5%(15/40) at 3 months, 60%(24/40) at 6 months, and 72.5%(29/40) at 12 months (intention-to-treat). CR rate increased to 47.5%(19/40) in 12 months.
Our outcomes demonstrated that amongst the NSAA cohort(10/40), 100% had ORR by 6 months with 100% CR by 12 months. The SAA cohort(19/40) had an ORR of 36.8%(7/19) at 6 months and 73.7%(14/19) at 12 months. Amongst the VSAA, the ORR was 36.4% (4/11) at 6 months and 45.5%(5/11) by 12 months. Four deaths occurred (10%, 4/40), all in VSAA (three sepsis, one bleeding) while 3 patients relapsed( 7.5%, 3/40) after achieving PR/CR.
RBC and platelet transfusion independence was achieved in 85%(28/33) and 93%(31/33) at 12 months respectively. Among ATG-treated patients (5/40), 80% achieved CR by 6 months.
Clonal evolution occurred in two VSAA patients (5%, 2/40). Twenty-one hospital admissions due to febrile neutropenia or bleeding manifestations occurred in the first six months, >70% among VSAA patients.

### Summary/Conclusion

Romiplostim-based IST achieved high 12-month response rates across severity categories, with particularly favorable outcomes in non-severe and severe AA. Even in patients with VSAA unwilling for ATG, durable responses were still observed in close to half of the patients. These data support romiplostim as a biologically active frontline backbone capable of inducing sustained hematologic recovery even in very severe transplant-ineligible aplastic anemia.

**Copyright Information:** (Online) ISSN: 2572-9241
©2026 The Author(s). HemaSphere is published by John Wiley & Sons Ltd on behalf of European Hematology Association. This is an open access Abstract Book distributed under the Attribution-NonCommercial-NoDerivs (CC BY-NC-ND), which allows third parties to download the articles and share them with others as long as they credit the author and the Abstract Book, but they cannot change the content in any way or use them commercially.
**Abstract Book Citations:** Authors, Title, HemaSphere, 2026;10:(S1):pages. The abstract book DOIs can be found on the “Author Information” page.
**Disclaimer:** Articles published in the journal HemaSphere exclusively reflect the opinions of the authors. The authors are responsible for all content in their abstracts including accuracy of the facts, statements, citing resources, etc.

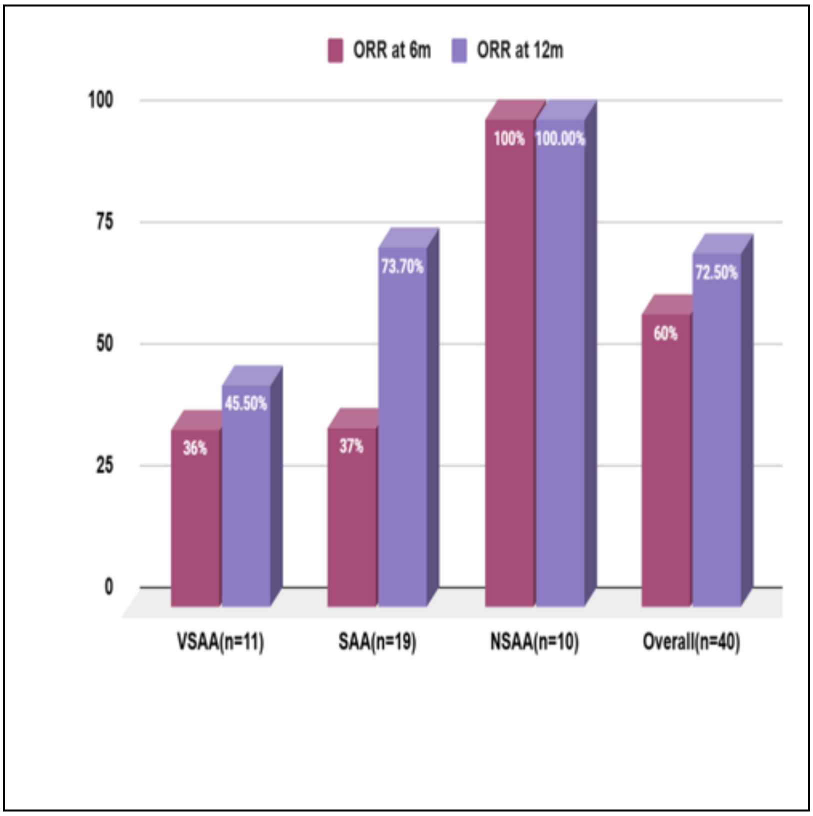

Copyright Information: (Online) ISSN: 2572-9241
©2026 The Author(s). HemaSphere is published by John Wiley & Sons Ltd on behalf of European Hematology Association. This is an open access Abstract Book distributed under the Attribution-NonCommercial-NoDerivs (CC BY-NC-ND), which allows third parties to download the articles and share them with others as long as they credit the author and the Abstract Book, but they cannot change the content in any way or use them commercially.
Abstract Book Citations: Authors, Title, HemaSphere, 2026;10:(S1):pages. The abstract book DOIs can be found on the "Author Information" page.
Disclaimer: Articles published in the journal HemaSphere exclusively reflect the opinions of the authors. The authors are responsible for all content in their abstracts including accuracy of the facts, statements, citing resources, etc.

### (PF716) REPROGRAMMING BCMA-TARGETED CAR-T CELLS THROUGH GAMMA-SECRETASE MODULATION BLOCKS ANTIGEN SHEDDING AND EXTENDS CAR-T LONGEVITY

**Topic:** 13. Myeloma and other monoclonal gammopathies - Biology & translational research

Sheetal Sharma*[1], Varnit Chauhan[2], Amit Verma[1], Tanveer Ahmad[3, 4]

*[1]Jamia Millia Islamia, Department of Biosciences, New Delhi, India;[2]Jamia Millia Islamia, Department of Biotechnology, New Delhi, India; [3]Jamia Millia Islamia, Multidisciplinary centre for advance research and studies, New Delhi, India;[4]Micro Crispr Pvt. Ltd., R&D Divison , New Delhi, New Delhi, India;*

### Background

BCMA is a highly selective therapeutic target in multiple myeloma (MM), as it is largely restricted to malignant plasma cells and supports tumor growth through NF-κB and PI3K–AKT signaling. Although BCMA-directed CAR-T therapies achieve high response rates, relapse remains frequent. Clinical trials of BCMA CAR-T products, idecabtagene vicleucel and ciltacabtagene autoleucel, report overall response rates above 80%, yet median progression-free survival rarely exceeds 12 months, reflecting limited CAR-T persistence. Treatment failure is driven by BCMA shedding, antigen escape, and T-cell senescence. γ-secretase–mediated BCMA cleavage releases soluble BCMA (sBCMA), which impairs CAR engagement, while chronic antigen exposure promotes CAR-T metabolic exhaustion. Thus, strategies that stabilize BCMA while preserving CAR-T cell fitness are urgently needed.

### Aims

To develop a next-generation SHEDLOCK CAR-T cell platform (Shedding Deterrent Locked-in CAR-T cells) capable of overcoming BCMA antigen loss and CAR-T cell functional decline in MM.

### Methods

AI-driven generative modeling and structural MD simulations were used to design peptide inhibitors targeting the γ-secretase cleavage interface of BCMA. Peptide efficacy was validated by ELISA and flow cytometry to assess soluble and membrane BCMA in cell lines, patient-derived myeloma samples, and xenograft models. Humanized anti-BCMA CAR constructs were generated using an AI-guided scFv humanization pipeline. CAR-T cells were engineered to secrete either a synthetic BCMA-targeting peptide (P5; SHEDLOCK-1) or a naturally derived γ-secretase–modulatory peptide (nxP; SHEDLOCK-2). CAR-T function, persistence, exhaustion, metabolism, telomere integrity, and Notch signaling were evaluated using cytotoxicity assays, tumor rechallenge models, metabolic profiling, senescence analyses, and reporter assays. In vivo efficacy, durability, and safety were assessed in NSG xenograft, serial transfer, and patient-derived MM models.

**Copyright Information:** (Online) ISSN: 2572-9241
©2026 The Author(s). HemaSphere is published by John Wiley & Sons Ltd on behalf of European Hematology Association. This is an open access Abstract Book distributed under the Attribution-NonCommercial-NoDerivs (CC BY-NC-ND), which allows third parties to download the articles and share them with others as long as they credit the author and the Abstract Book, but they cannot change the content in any way or use them commercially.
**Abstract Book Citations:** Authors, Title, HemaSphere, 2026;10:(S1):pages. The abstract book DOIs can be found on the “Author Information” page.
**Disclaimer:** Articles published in the journal HemaSphere exclusively reflect the opinions of the authors. The authors are responsible for all content in their abstracts including accuracy of the facts, statements, citing resources, etc.

### Results

Peptide inhibitors effectively blocked γ-secretase–mediated BCMA cleavage, with the lead peptide P5 inducing dose-dependent reduction of sBCMA and sustained mBCMA retention without affecting viability. sBCMA-rich tumor supernatants impaired CAR-T cytotoxicity and cytokine secretion, which was fully rescued by P5. SHEDLOCK-1 CAR-T cells secreting P5 demonstrated enhanced tumor killing, cytokine production, persistence, and improved in vivo tumor control, although prolonged antigen exposure resulted in partial exhaustion. To address CAR-T cell aging, SHEDLOCK-2 CAR-T cells were engineered to secrete nxP, which suppressed BCMA shedding. SHEDLOCK-2 CAR-T cells preserved telomere length and telomerase activity, reduced p21 expression, improved mitochondrial fitness, and shifted toward oxidative phosphorylation, resulting in enhanced central memory differentiation and reduced exhaustion. In xenograft, serial transfer, and patient-derived MM models, SHEDLOCK-2 CAR-T cells achieved durable tumor eradication, long-term persistence, maintained efficacy at reduced doses, and demonstrated a favorable safety profile without systemic γ-secretase–related toxicity.

### Summary/Conclusion

SHEDLOCK-2 CAR-T cells integrate antigen stabilization with metabolic and longevity programming to achieve durable anti-myeloma activity. Localized γ-secretase modulation preserves BCMA surface expression while enhancing CAR-T metabolic fitness, memory phenotype, and persistence, enabling sustained tumor control after repeated antigen exposure.

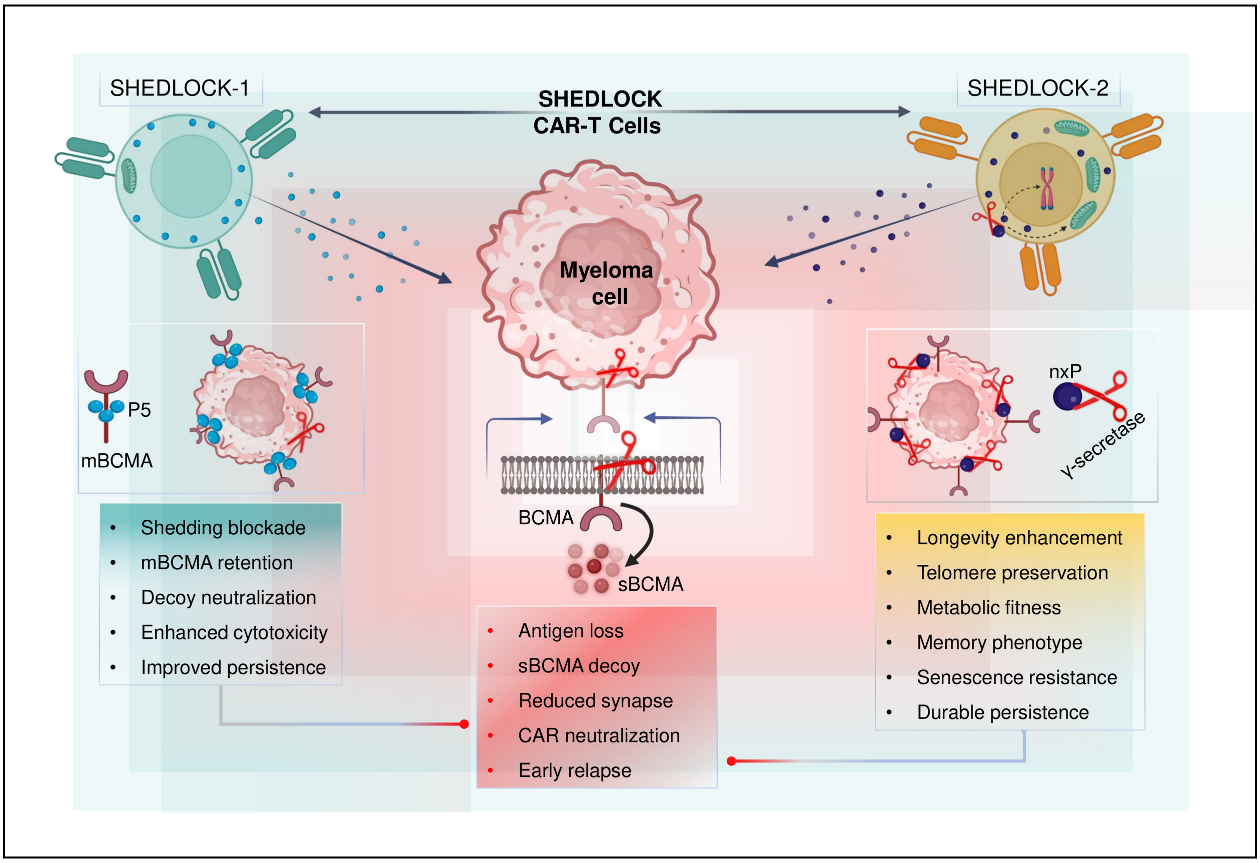

**Copyright Information:** (Online) ISSN: 2572-9241
©2026 The Author(s). HemaSphere is published by John Wiley & Sons Ltd on behalf of European Hematology Association. This is an open access Abstract Book distributed under the Attribution-NonCommercial-NoDerivs (CC BY-NC-ND), which allows third parties to download the articles and share them with others as long as they credit the author and the Abstract Book, but they cannot change the content in any way or use them commercially.
**Abstract Book Citations:** Authors, Title, HemaSphere, 2026;10:(S1):pages. The abstract book DOIs can be found on the "Author Information" page.
**Disclaimer:** Articles published in the journal HemaSphere exclusively reflect the opinions of the authors. The authors are responsible for all content in their abstracts including accuracy of the facts, statements, citing resources, etc.

### (PF717) GENETIC SUSCEPTIBILITY TO ARTERIAL THROMBOEMBOLISM IN NEWLY DIAGNOSED MULTIPLE MYELOMA IN THE UK NCRI MYELOMA XI TRIAL.

**Topic:** 13. Myeloma and other monoclonal gammopathies - Biology & translational research

Sina Beer*[1, 2], David Cairns[3], Molly Went[1], Charlotte Pawlyn[2, 4], Amy Holroyd[1], Elsa Ferris[1], Gordon Cook[3], Mark Drayson[5], Kevin Boyd[1, 2], Faith E Davies[6], Matthew Jenner[7], Gareth Morgan[6], Graham Jackson[8], Richard Houlston[1], Martin Kaiser[1, 2]

*[1]Institute of Cancer Research, Division of Genetics and Epidemiology, London, United Kingdom;[2]The Royal Marsden Hospital, Department of Haematology, London, United Kingdom; [3]Leeds Institute of Clinical Trials Research, Leeds Cancer Research UK Clinical Trials Unit, Leeds, United Kingdom; [4]The Institute of Cancer Research, Division of Cancer Therapeutics, London, United Kingdom; [5]University of Birmingham, Institute of Immunology and Immunotherapy, Birmingham, United Kingdom;[6]NYU Langone Health, Perlmutter Cancer Centre, New York, United States of America; [7]University Hospital Southampton NHS Foundation Trust, Department of Haematology, Southampton, United Kingdom; [8]University of Newcastle, Department of Haematology, Newcastle Upon Tyne, United Kingdom;*

### Background

Immunomodulatory drugs (IMiDs) like lenalidomide (Lena) are key components of multiple myeloma (MM) therapy. However, IMiDs increase the risk of arterial thromboembolism (ATE), such as myocardial infarction and ischemic stroke. As long-term survival in MM improves, understanding treatment-related toxicities and individual susceptibility has become increasingly important for patient quality of life and survivorship.

### Aims

Using a large well-annotated clinical trial dataset, we aimed to interrogate inherited genetic factors not previously investigated for ATE risk in MM.

### Methods

We retrospectively analysed 4,287 newly diagnosed MM patients enrolled in UK NCRI Myeloma-XI. ATE incidence and risk factors were assessed during IMiD-based induction therapy (21,478 cycles; 12 months after diagnosis) and during Lena maintenance in 1,412 patients (9,439 cycles). Risk factors were analysed by time-to-event Cox regression with censoring at death or end of follow-up, incorporating covariates selected from univariable analyses ($P<0.1$). Genome-wide association studies (GWAS) were conducted in 1,836 genotyped patients of European ancestry. Results were restricted to variants with MAF >2%, and genome-wide significance was defined as $P<5\times10^{-8}$.

**Copyright Information:** (Online) ISSN: 2572-9241
©2026 The Author(s). HemaSphere is published by John Wiley & Sons Ltd on behalf of European Hematology Association. This is an open access Abstract Book distributed under the Attribution-NonCommercial-NoDerivs (CC BY-NC-ND), which allows third parties to download the articles and share them with others as long as they credit the author and the Abstract Book, but they cannot change the content in any way or use them commercially.
**Abstract Book Citations:** Authors, Title, HemaSphere, 2026;10:(S1):pages. The abstract book DOIs can be found on the "Author Information" page.
**Disclaimer:** Articles published in the journal HemaSphere exclusively reflect the opinions of the authors. The authors are responsible for all content in their abstracts including accuracy of the facts, statements, citing resources, etc.

**Results**

Among all thromboembolic events (venous and arterial), 8.8% were arterial during induction, whereas 27% were arterial during maintenance. Overall, 85 of 4,287 patients experienced 88 ATE, of which 57 events were induction- and 31 maintenance-related (incidence 1.3 and 2.2%, respectively). 97% of maintenance related ATE were ischemic strokes. The median time to first ATE was 2.3 months (range 0.1-8.3) during induction, compared to 25 months (range 1.1-98.4) after maintenance initiation. Older age independently increased ATE risk during maintenance (HR 1.07 per year, 95% CI 1.0-1.2; *P*=0.04), likely reflecting both longer exposure and age-related increase in baseline ATE risk, although age was not significantly associated with ATE risk during induction (*P*=0.09). ATE risk did not differ by sex, ECOG performance status, paraprotein level or induction regimen.
The GWAS (N=1,836; 36 events) identified a genome-wide significant locus on chromosome 6 with markedly increased ATE risk (OR 4.8, 95% CI 2.9-8.2; $P$=4×10$^{-8}$). The nearest gene to the lead variant rs62417785 (chr6:91034724, G/A) is *MAP3K7*, a regulator of NFκB and MAPK signalling pathways involved in endothelial stress responses. Functional annotation of the risk locus showed overlap with a predicted transcription factor binding site for IKZF1/Ikaros, in a distal active enhancer region. Public ChIPseq support regulatory activity of the locus in aortic endothelial cells. The risk allele was enriched in cases (MAF 27.7%) compared to controls (7.3%). Consistently, in a multivariable Cox model adjusted for age and transplant-eligibility, *MAP3K7* AB/BB was independently associated with increased ATE risk (HR 5.9, 95% CI 3.1-11.5; $P$=9×10$^{-8}$).

**Summary/Conclusion**

For the first time, our analysis identified a genome-wide significant locus associated with increased ATE risk in IMiD-treated MM patients and a potential link to a putative binding site for IKZF1, a key degradation target of IMiDs, which warrants further validation. Our results support a role for common germline variation in ATE susceptibility and may open a perspective on using germline genetics for individualising side effect risk profiles for patients on long-term anti-MM therapy.

**Copyright Information:** (Online) ISSN: 2572-9241
©2026 The Author(s). HemaSphere is published by John Wiley & Sons Ltd on behalf of European Hematology Association. This is an open access Abstract Book distributed under the Attribution-NonCommercial-NoDerivs (CC BY-NC-ND), which allows third parties to download the articles and share them with others as long as they credit the author and the Abstract Book, but they cannot change the content in any way or use them commercially.
**Abstract Book Citations:** Authors, Title, HemaSphere, 2026;10:(S1):pages. The abstract book DOIs can be found on the "Author Information" page.
**Disclaimer:** Articles published in the journal HemaSphere exclusively reflect the opinions of the authors. The authors are responsible for all content in their abstracts including accuracy of the facts, statements, citing resources, etc.

### (PF718) POLYGENIC SCORE PREDICTS MULTIPLE MYELOMA INCIDENCE AND PROGRESSION FROM MGUS ACROSS DIVERSE ANCESTRIES

**Topic:** 13. Myeloma and other monoclonal gammopathies - Biology & translational research

Carl Jannes Neuse*[1, 2], Julian Wanner[1], Henrike Heyne[1, 2], Bernhard Yves Renard[1, 2]

[1]*Hasso Plattner Institute, Data Analytics and Computational Statistics, Potsdam, Germany;* [2]*Icahn School of Medicine at Mount Sinai, Windreich Department of Artificial Intelligence and Human Health at Mount Sinai, New York City, United States of America;*

### Background

Multiple Myeloma (MM) is the second most common hematologic malignancy in the Europe. It arises from the precursor state monoclonal gammopathy of undetermined significance (MGUS). While MM is currently incurable, an early treatment of the disease can slow its progression and improve survival. Identifying individuals at high risk of progression from MGUS to MM, therefore, has high clinical relevance. Current risk models mostly rely on clinical or biochemical parameters. While these models effectively stratify risk for MM in many MGUS patients, they at times fail to identify high-risk patients. Genetic risk for MM is largely driven by common genetic factors, with an estimated heritability of 15-17%. However, this is currently not included in clinical risk prediction models.

### Aims

To address the need to further improve progression risk stratification in MGUS patients, we explored the potential to integrate common genetic factors in the form of polygenic scores into current MM risk prediction models. We derived a MM-specific polygenic score (MM-PGS) and tested its influence on MM incidence and on progression from MGUS to MM.

### Methods

Here, we investigate the effect of a novel MM-PGS (method: PRS-CS), based on the largest available genome-wide association study (GWAS) of MM. We evaluate the association between our MM-PGS and incident MM, as well as progression from MGUS to MM, across 4 cohorts totaling >1.5M individuals in 4 different ancestries. We fit Cox proportional hazard models adjusting for age, sex, batch, and the first 10 principal components of genetic ancestry. In addition, we perform a GWAS to identify genetic factors specifically influencing progression of MGUS to MM.

### Results

Across all cohorts, a higher polygenic burden for MM is associated with a higher lifetime risk of MM (meta-analyzed hazard ratio (mHR) per standard deviation (SD) 1.36, 95% confidence interval (CI) [1.23, 1.49], P=1.39x10-10).
In individuals with a previous MGUS diagnosis, we find that polygenic burden for MM is associated with increased risk of progression to MM (mHR per SD 1.25, 95% CI [1.12, 1.41], P=8.34x10-5). We find that the effect of MM-PGS remains stable across different clinical risk groups defined by the Mayo Clinic MGUS risk model. Further, we identify progression-specific, genome-wide significant loci in a progression GWAS of MGUS to MM. Here, we identify the genomewide significant locus at the *BCAS1* gene (beta = -0.60, P = $9.3 \times 10^{-9}$), a known cancer gene.

**Copyright Information:** (Online) ISSN: 2572-9241
©2026 The Author(s). HemaSphere is published by John Wiley & Sons Ltd on behalf of European Hematology Association. This is an open access Abstract Book distributed under the Attribution-NonCommercial-NoDerivs (CC BY-NC-ND), which allows third parties to download the articles and share them with others as long as they credit the author and the Abstract Book, but they cannot change the content in any way or use them commercially.
**Abstract Book Citations:** Authors, Title, HemaSphere, 2026;10:(S1):pages. The abstract book DOIs can be found on the "Author Information" page.
**Disclaimer:** Articles published in the journal HemaSphere exclusively reflect the opinions of the authors. The authors are responsible for all content in their abstracts including accuracy of the facts, statements, citing resources, etc.

### Summary/Conclusion

Consistent with cancer-specific PGSs in other malignancies, the MM-PGS predicts lifetime risk of MM and, in addition, progression after an MGUS diagnosis. Our results suggest that integrating the influences of common genetic variation into existing clinical models could improve risk stratification and, thus, enable a more personalized risk prediction and follow-up in MM. In addition, we identify a novel locus, within a cancer-associated gene, specifically associated with MGUS-to-MM progression.

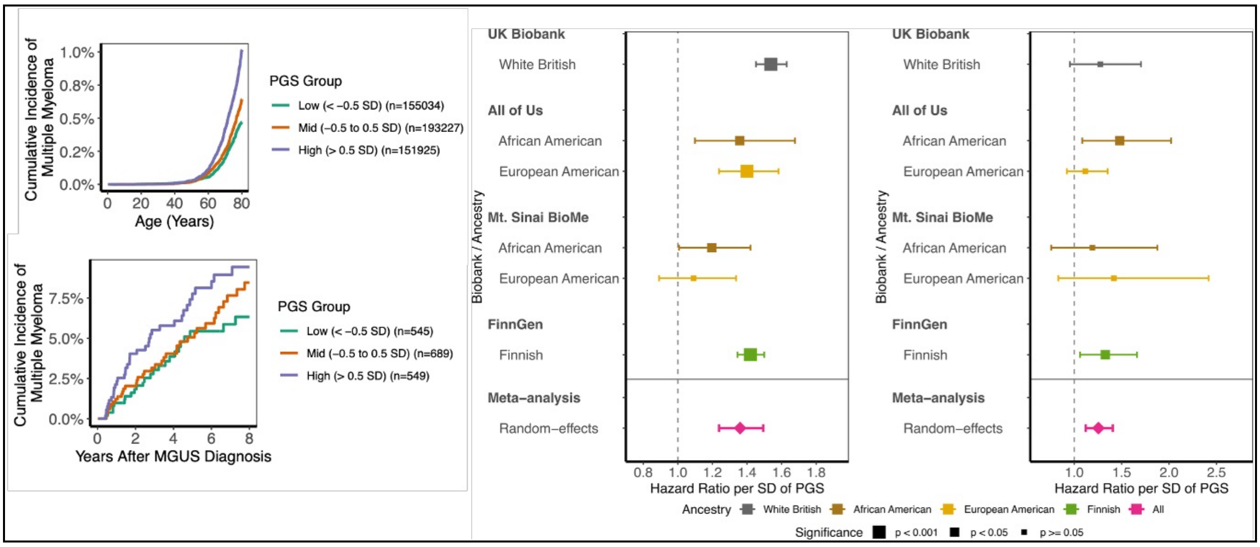

On the left we show two survival plots with the cumulative incidence of Multiple Myeloma across lifetime (top) and after MGUS diagnosis (bottom) in our meta analysis by PGS Group. The plots indicate risk stratification by PGS groups. On the right we show PGS performance (Hazard Ratio per SD of PGS) across the four biobanks and in the Meta-analysis as well as stratification by ancestry. The left plot shows the performance of the PGS across life time, while on the right it shows the performance of the PGS after MGUS diagnosis. We observe that the PGS performs better across lifetime than after MGUS. After MGUS it performs best in cohorts with higher sample size.

**Copyright Information:** (Online) ISSN: 2572-9241
©2026 The Author(s). HemaSphere is published by John Wiley & Sons Ltd on behalf of European Hematology Association. This is an open access Abstract Book distributed under the Attribution-NonCommercial-NoDerivs (CC BY-NC-ND), which allows third parties to download the articles and share them with others as long as they credit the author and the Abstract Book, but they cannot change the content in any way or use them commercially.
**Abstract Book Citations:** Authors, Title, HemaSphere, 2026;10:(S1):pages. The abstract book DOIs can be found on the “Author Information” page.
**Disclaimer:** Articles published in the journal HemaSphere exclusively reflect the opinions of the authors. The authors are responsible for all content in their abstracts including accuracy of the facts, statements, citing resources, etc.

### (PF719) T(8;14) IMPROVES THE IMS/IMWG CONSENSUS GENOMIC STAGING FOR DEFINING HIGH-RISK MULTIPLE MYELOMA PATIENTS

**Topic:** 13. Myeloma and other monoclonal gammopathies - Biology & translational research

Anaïs Schavgoulidze*[1], Lucas Antony[2], Aurore Perrot[1], Titouan Cazaubiel[3], Luka Pavageau[1], Laure Derrier[1], Jean Noël Bastie[4], Pierre Feugier[5], Sabine Brechignac[6], Frederique Orsini Piocelle[7], Jean Marc Schiano De Colella[8], Jean Fontan[9], Didier Bouscary[10], Mamoun Dib[11], Laurence Sanhes[12], Didier Adiko[13], Laurent Pascal[14], Valentine Richez[15], Phillipe Rey[16], Jana Bakala[17], Carine Luttiau-Motard[18], Celine Kennel[19], François Lifermann[20], Karim Belhadj Merzoug[21], Emilie Chalayer[22], Salomon Manier[23], Murielle Roussel[24], Margaret MacRo[25], Mohamad Mohty[26], Xavier Leleu[27], Hervé Avet-Loiseau[1], Jill Corre[1]

*[1]IUCT-Oncopole, Toulouse, France; [2]Université de Toulouse, Toulouse, France; [3]CHU Bordeaux, Bordeaux, France; [4]CHU Dijon, Dijon, France; [5]CHRU Nancy, Nancy, France; [6]Bobigny Hospital, APHP, Paris, France; [7]GH Annecy, Annecy, France; [8]Institut J Paoli I Calmettes, Marseille, France; [9]CHU Besançon, Besançon, France; [10]Hôpital Cochin, APHP, Paris, France; [11]CHU Angers, Angers, France; [12]CH Perpignan, Perpignan, France; [13]CH Libourne, Libourne, France; [14]Groupement des Hôpitaux de l'Institut Catholique de Lille - Hôpital Saint Vincent de Paul, Lille, France; [15]CHU Nice, Nice, France; [16]Centre Léon Bérard, Lyon, France; [17]CH Lens, Lens, France; [18]CH Niort, Niort, France; [19]Centre Hospitalier William Morey, Chalon, France; [20]CH Dax, Dax, France; [21]GH CHENEVIER MONDOR EMILE ROUX, Paris, France; [22]CHU Saint-Etienne, Saint-Etienne, France; [23]CHU Lille, Lille, France; [24]CHU Limoges, Limoges, France; [25]CHU Caen, Caen, France; [26]Sorbonne University, Hôpital Saint-Antoine, and INSERM, Paris, France; [27]Centre Hospitalier Universitaire, Université de Poitiers, Poitiers, France;*

### Background

Multiple myeloma (MM) is a complex disease with very heterogenous outcomes. Recently, the International Myeloma Society / International Myeloma Working Group (IMS/IMWG) released the Consensus Genomic Staging (CGS) for the definition of genomic high risk (HR) MM. The CGS relies mainly on genomic criteria (del(17p) and/or *TP53* mutation, biallelic del(1p32), the combination of intermediate factors among 1q gain, monoallelic del(1p32), or translocations (t(4;14), t(14;16) or t(14;20)). One biochemical criterion was added to the CGS: elevated beta2-microglobulin (≥ 5,5 mg/L) in the setting of normal renal function (creatinine < 1.2 mg/dL).
Translocation t(8;14) with rearrangement of MYC oncogene affects less than 5% of NDMM patients at diagnosis. It has been previously shown to be associated with a poor prognosis but has never been considered as a HR feature that should be systematically assessed at diagnosis.

### Aims

Here we aimed to confirm the adverse impact of t(8;14) and its role for improving the CGS.

### Methods

Clinical data were obtained from 2900 NDMM patients, followed up for ≥ 18 months or having died or progressed within 18 months post treatment. Informed consent was obtained for all included patients. 1350 patients were treated by an intensive therapy. Cytogenetic abnormalities were assessed using our NGS-targeted panel. Follow-up duration was estimated using reverse Kaplan-Meier method. Overall survival (OS) and progression free survival (PFS) curves were estimated using the Kaplan-Meier method and were compared using the log-rank test. Tests were two-sided, and P < .05 was considered significant. All analyses were performed using R version 4.1.1.

**Copyright Information:** (Online) ISSN: 2572-9241
©2026 The Author(s). HemaSphere is published by John Wiley & Sons Ltd on behalf of European Hematology Association. This is an open access Abstract Book distributed under the Attribution-NonCommercial-NoDerivs (CC BY-NC-ND), which allows third parties to download the articles and share them with others as long as they credit the author and the Abstract Book, but they cannot change the content in any way or use them commercially.
**Abstract Book Citations:** Authors, Title, HemaSphere, 2026;10:(S1):pages. The abstract book DOIs can be found on the "Author Information" page.
**Disclaimer:** Articles published in the journal HemaSphere exclusively reflect the opinions of the authors. The authors are responsible for all content in their abstracts including accuracy of the facts, statements, citing resources, etc.

### Results

In our cohort, 136 (4.7%) NDMM patients displayed a t(8;14). Median follow-up was 28 months. The median PFS of patients harboring t(8;14) was significantly shorter than the PFS of patients without t(8;14) (30 vs 49 months, P < 0.0001).
t(8;14) is described as a secondary event. Indeed, most of the t(8;14) were associated with either hyperdiploidy (85/136) or another translocation involving *IGH* (23/136). However, we also found « isolated » t(8;14) (21/136 patients in our cohort, 15%). We wondered if these 3 groups could lead to a different prognosis. Patients harboring t(8;14) with either hyperdiploidy or another translocation had a poorer PFS compared to patients without t(8;14) (respectively 38, 23 and 49 months). Interestingly, the worst survival was observed in patients harboring an « isolated » t(8;14), with a PFS of 14 months. Of note, 5% of the t(8;14) were associated with both hyperdiploidy and another translocation but the effective was too low (7 patients) to draw any conclusion in terms of survival.
We then assessed the PFS for patients with available CGS classification (n = 1779) (Figure 1). Standard risk (SR) patients according to the CGS with a t(8;14) had a lower PFS compared to SR patients without t(8;14) (36 vs 54 months, P < 0.0001). More importantly, PFS of SR patients with t(8;14) was not statistically different from HR patients according to the CGS (36 and 35 months respectively, P = 0.81).

### Summary/Conclusion

Here we have confirmed the pejorative impact of t(8;14) in MM especially when found isolated or associated with other translocations. Moreover, t(8;14) enabled to capture HR patients that were missed by the 2025 Consensus Genomic Staging.

**Copyright Information:** (Online) ISSN: 2572-9241
©2026 The Author(s). HemaSphere is published by John Wiley & Sons Ltd on behalf of European Hematology Association. This is an open access Abstract Book distributed under the Attribution-NonCommercial-NoDerivs (CC BY-NC-ND), which allows third parties to download the articles and share them with others as long as they credit the author and the Abstract Book, but they cannot change the content in any way or use them commercially.
**Abstract Book Citations:** Authors, Title, HemaSphere, 2026;10:(S1):pages. The abstract book DOIs can be found on the "Author Information" page.
**Disclaimer:** Articles published in the journal HemaSphere exclusively reflect the opinions of the authors. The authors are responsible for all content in their abstracts including accuracy of the facts, statements, citing resources, etc.

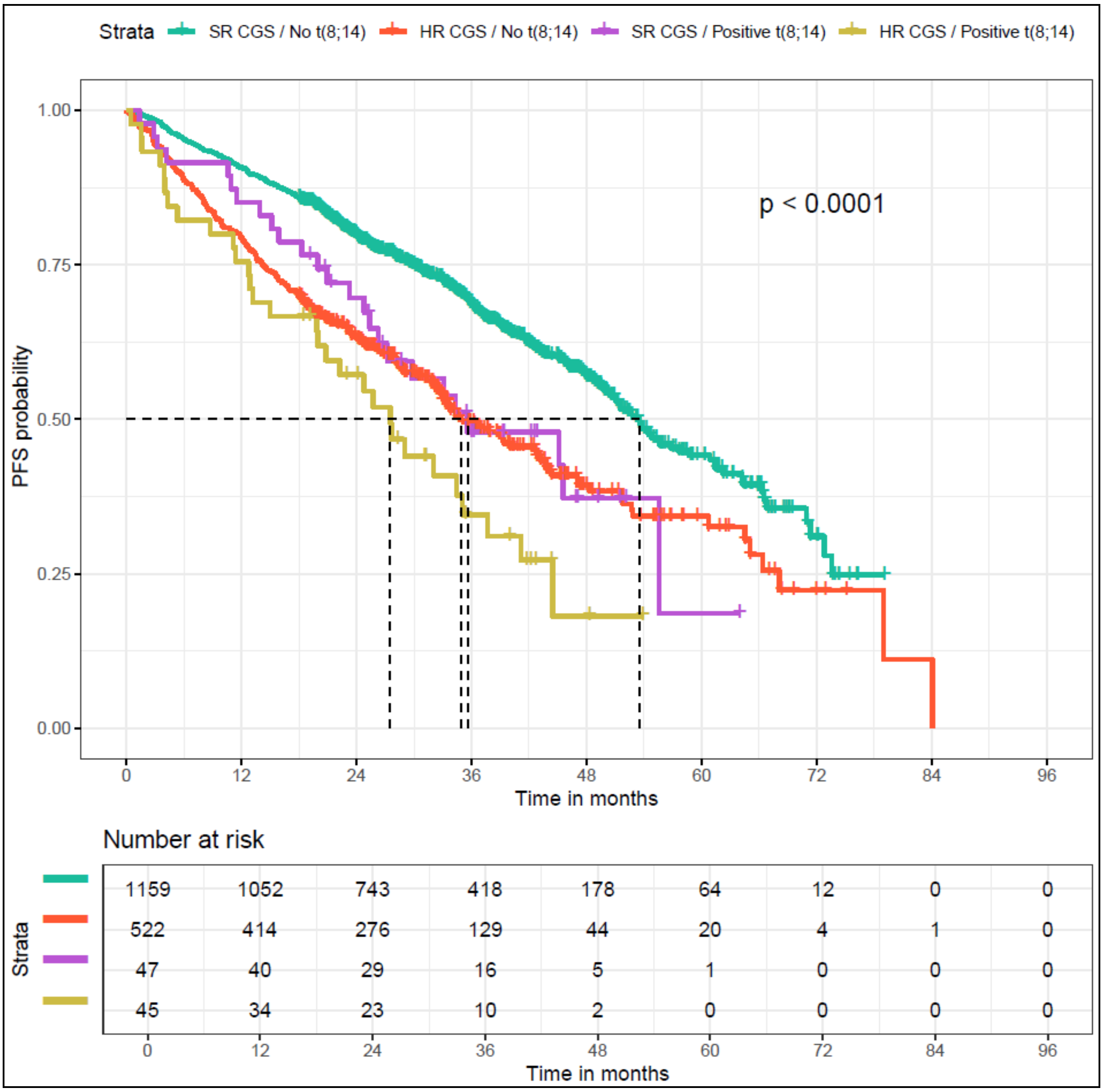

Figure 1. Kaplan–Meier progression-free survival of patients with NDMM according to their risk status defined by the CGS and the presence or absence of t(8;14). The green curve corresponds to standard-risk patients according to the CGS without t(8;14). The purple curve corresponds to standard-risk patients according to the CGS with t(8;14). The red curve corresponds to high-risk patients according to the CGS without t(8;14). The yellow curve corresponds to high-risk patients according to the CGS with t(8;14). P-value is determined by the log-rank test comparison.

**Copyright Information:** (Online) ISSN: 2572-9241
©2026 The Author(s). HemaSphere is published by John Wiley & Sons Ltd on behalf of European Hematology Association. This is an open access Abstract Book distributed under the Attribution-NonCommercial-NoDerivs (CC BY-NC-ND), which allows third parties to download the articles and share them with others as long as they credit the author and the Abstract Book, but they cannot change the content in any way or use them commercially.
**Abstract Book Citations:** Authors, Title, HemaSphere, 2026;10:(S1):pages. The abstract book DOIs can be found on the "Author Information" page.
**Disclaimer:** Articles published in the journal HemaSphere exclusively reflect the opinions of the authors. The authors are responsible for all content in their abstracts including accuracy of the facts, statements, citing resources, etc.

### (PF720) TARGETING GLUTAMINE METABOLISM AS A NEW STRATEGY TO IMPROVE ANTI-BCMA T CELL ENGAGER ACTIVITY IN MYELOMA CELLS.

**Topic:** 13. Myeloma and other monoclonal gammopathies - Biology & translational research

Vincenzo Raimondi[1], Denise Toscani[1, 2], Rosanna Vescovini[1], Oxana Lungu[1], Camilla Sitzia[1], Mattia Dessena[1], Nicolas Thomas Iannozzi[1], Paola Storti[1], Matteo Scita[2], Anna Benedetta Dalla Palma[2], Nicola Giuliani*[1, 2]

*[1]UNIVERSITY OF PARMA, MEDICINE AND SURGERY, PARMA, Italy; [2]Azienda Ospedaliero-Universitaria di Parma, Hemato-Oncology, Parma, Italy;*

### Background

Multiple myeloma (MM) is an hematological malignancy characterized by the high tendency to relapse and to develop drug resistance. The introduction of novel immunotherapeutic treatments, such as CAR-T cells and T cell engagers (TCE) improves the response rate of relapsed MM patients, but the presence of T cell exhaustion and the loose of surface antigens in MM cells may lead to the lack of response to immunotherapy. Growing evidence indicate that MM cells are highly glutamine (Gln)-dependent depleting this nutrient within the bone marrow (BM), creating a metabolic competition that drives immune cells toward alternative substrate. Interestingly it has been recently hypothesized that modulation of Gln metabolism may improve CAR-T cell activity in MM.

### Aims

Based on these data, in this study we aim to explore how altered Gln metabolism impacts immunotherapy response to Elranatamab an anti-BCMA/CD3 TCE. We hypothesized that targeting Gln metabolism may increase MM susceptibility to T cell-mediated cytotoxicity while maintaining T cell effector function.

### Methods

We investigated the effects of Gln restriction on pharmacological modulation of elranatamab activity. Firstly, BCMA expression was assessed in human myeloma cell lines (HMCLs) and $CD138^+$ PCs from 29 MM patients, by flow cytometry analysis. Cytotoxicity and T cell activation (CD69, CD25) were assessed both in co-cultures of $BCMA^+$ HMCLs with healthy donor T cells and in BM mononuclear cells (MNCs) from MM patients. Gln restriction was achieved by culturing HMCLs and BM MNCs in media containing 0.5 mM and 0.2 mM Gln, respectively, for 48 and 72 hours. GLS inhibition was tested using the glutaminase inhibitor CB-839. Single-cell RNA-seq analysis on MM patients bone biopsies and western blotting of purified T cells were also performed.

### Results

We found that elranatamab induced strong anti-HMCLs cytotoxicity and robust T-cell activation, which were preserved under Gln restriction; moreover, combined treatment with elranatamab and CB-839 further enhanced anti-MM cytotoxicity without affecting T-cell activation. Single-cell RNA-seq analysis confirmed that primary MM exhibit high baseline expression of GLS and SLC1A5 along with ASNS and GFPT1, consistent with a strong Gln dependence. Both $CD4^+$ and $CD8^+$ T cells expressed GLS but displayed low expression of ASNS and GFPT1.
In BM MNCs from MM patients, Gln restriction lowered the median $EC_{50}$ of elranatamab and maintained T-cell activation, suggesting MM selective sensitization, while co-treatment with CB-839 synergistically increased cytotoxicity without impairing T-cell activation profile. Lastly, T cells exposed to the combination displayed increased expression of enzymes involved in alternative Gln-utilization pathways (ASNS and O-GlcNAc).

**Copyright Information:** (Online) ISSN: 2572-9241
©2026 The Author(s). HemaSphere is published by John Wiley & Sons Ltd on behalf of European Hematology Association. This is an open access Abstract Book distributed under the Attribution-NonCommercial-NoDerivs (CC BY-NC-ND), which allows third parties to download the articles and share them with others as long as they credit the author and the Abstract Book, but they cannot change the content in any way or use them commercially.
**Abstract Book Citations:** Authors, Title, HemaSphere, 2026;10:(S1):pages. The abstract book DOIs can be found on the "Author Information" page.
**Disclaimer:** Articles published in the journal HemaSphere exclusively reflect the opinions of the authors. The authors are responsible for all content in their abstracts including accuracy of the facts, statements, citing resources, etc.

**Summary/Conclusion**

Overall, these data suggest a rationale to integrate targeting Gln metabolisms with the use of  BCMA/CD3 TCE as a novel strategy to overcome metabolic immunosuppression and enhance immunotherapeutic responses in MM patients.

**Copyright Information:** (Online) ISSN: 2572-9241
©2026 The Author(s). HemaSphere is published by John Wiley & Sons Ltd on behalf of European Hematology Association. This is an open access Abstract Book distributed under the Attribution-NonCommercial-NoDerivs (CC BY-NC-ND), which allows third parties to download the articles and share them with others as long as they credit the author and the Abstract Book, but they cannot change the content in any way or use them commercially.
**Abstract Book Citations:** Authors, Title, HemaSphere, 2026;10:(S1):pages. The abstract book DOIs can be found on the "Author Information" page.
**Disclaimer:** Articles published in the journal HemaSphere exclusively reflect the opinions of the authors. The authors are responsible for all content in their abstracts including accuracy of the facts, statements, citing resources, etc.

### (PF721) SPATIAL IMAGING UNLOCKS THE POTENTIAL OF CHARTING MULTIPLE MYELOMA AND EXTRAMEDULLARY DISEASE

**Topic:** 13. Myeloma and other monoclonal gammopathies - Biology & translational research

Vanessa Desantis[1], Alessandro Andriano[2], Tim Düking[3], Olga Hartwig[3], Giuseppe Ingravallo[4], Marta Biondo[5], Cirino Botta[6], Roberto Ria[2], Angelo Vacca[2], Antonio Giovanni Solimando*[2]

*[1]Department of Precision and Regenerative Medicine and Ionian Area (DiMePRe-J), Section of Pharmacology,University of Bari Aldo Moro Medical School, Department of Precision and Regenerative Medicine and Ionian Area (DiMePRe-J), Section of Pharmacology,, Bari, Italy; [2]Internal Medicine Guido Baccelli, University of Bari Aldo Moro Medical School, Department of Precision and Regenerative Medicine and Ionian Area (DiMePRe-J), Bari, Italy; [3]Miltenyi Biotec B.V. & Co. KG, Bergisch Gladbach, Germany; [4]Pathological Anatomy Unit, Department of Emergency and Organ Transplantation, University of Bari Aldo Moro, Department of Precision and Regenerative Medicine and Ionian Area (DiMePRe-J), Bari, Italy; [5]Department of General Surgery and Medical-Surgical Specialties, University of Catania, Catania, Italy; [6]Department of Health Promotion, Mother and Child Care, Internal Medicine and Medical Specialties, University of Palermo, Palermo, Italy;*

### Background

Multiple myeloma (MM) is a plasma cell (PCs) cancer marked by strong genetic, phenotypic, and microenvironmental heterogeneity. The emergence of extramedullary disease (EMD) signals an aggressive phase linked to treatment resistance and poor outcomes. The mechanisms driving plasma cells to escape the bone marrow and infiltrate extramedullary sites remain unclear.

### Aims

This study uses MACSima™ imaging cyclic staining (MICS), a high-dimensional spatial imaging technology, to map protein expression patterns and cellular interactions, revealing structural and network differences between bone marrow and EMD lesions.

### Methods

Using MICS, we were able to stain 56 biomarkers across carefully prepared BM and EMD tissue samples from 5 newly diagnosed MM patients. Data from 231,186 cells were analysed, with 105,047 annotated as PCs. Computational tools processed and statistically validated the imaging data to generate spatial maps and cellular profiles.

### Results

Distinct clustering patterns of PCs and immune infiltration profiles were identified via spatial distance analyses. This approach allowed for comprehensive 3D mapping of malignant PCs and key tumor microenvironment components, providing an integrated spatial framework for investigating cellular organization and intercellular interactions. MICS technology enabled the identification of 14 distinct cell types in BM and 9 in EMD, showcasing the method's capacity to discern cellular diversity at a single-cell level. EMD lesions displayed disrupted architecture, characterized by a loss of stromal network integrity and diminished cross-talk with regulatory immune cells. Malignant PCs in EMD sites were organized into dense clusters, lacking canonical interactions with $CD8^+$ T cells and dendritic cells, indicative of an immunologically permissive microenvironment (Figure1). PCs at EMD sites exhibited higher expression of drug resistance markers like BCL-2 and EZH2, and reduced CD38 expression, confirmed by immunohistochemistry. The spatial arrangement of immune cells such as T-cells and macrophages, was markedly different in EMD compared to BM. PCs were overall far distant from T-cells in EMD.

**Copyright Information:** (Online) ISSN: 2572-9241
©2026 The Author(s). HemaSphere is published by John Wiley & Sons Ltd on behalf of European Hematology Association. This is an open access Abstract Book distributed under the Attribution-NonCommercial-NoDerivs (CC BY-NC-ND), which allows third parties to download the articles and share them with others as long as they credit the author and the Abstract Book, but they cannot change the content in any way or use them commercially.
**Abstract Book Citations:** Authors, Title, HemaSphere, 2026;10:(S1):pages. The abstract book DOIs can be found on the "Author Information" page.
**Disclaimer:** Articles published in the journal HemaSphere exclusively reflect the opinions of the authors. The authors are responsible for all content in their abstracts including accuracy of the facts, statements, citing resources, etc.

### Summary/Conclusion

This study provides the first evidence that spatial imaging is essential for understanding the structural complexity of MM and its progression to EMD. Three-dimensional reconstruction of cellular networks reveals the loss of immunostromal interactions and the formation of protective niches that sustain highly aggressive plasma cell subclones. This may explain the reduced effectiveness of CD38-targeted and immune-mediated therapies (such as CAR-T and bispecific antibodies) in EMD. Although larger studies are needed to confirm these findings, they offer valuable insights for precision medicine and microenvironment-focused strategies that could transform the treatment of MM with an extramedullary phenotype.

This work was supported by the “Fondo per il Programma Nazionale di Ricerca e Progetti di Rilevante Interesse Nazionale—PRIN” (project n.2022ZKKWLW) to A.G.S.; by the Italian network of excellence for advanced diagnosis (INNOVA), Ministero della Salute (code PNC-E3-2022-23683266 PNC-HLS-DA) to V.D. and A.G.S., and by a grant from “Società Italiana di Medicina Interna-SIMI” 2023 Research Award (CAMEL) to A.G.S. This work was also supported by the Italian Association for Cancer Research (AIRC) within the My First AIRC Grant 2020 (n. 24534, 2021/2025) to C.B.

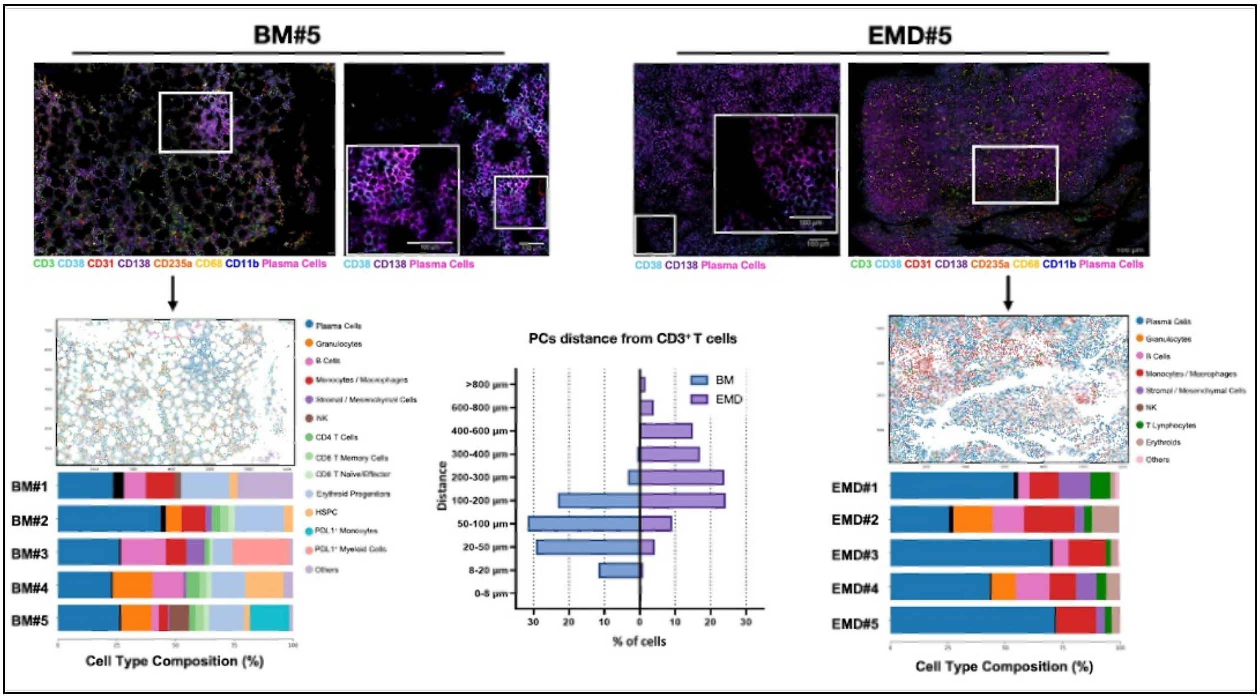

Figure 1: Spatial profiling of the bone marrow (BM) niche and extramedullary disease (EMD) in multiple myeloma (MM) including butterfly plot of distance analysis between plasma cells (PCs) and CD3+ T cells in a representative patient MM#5

**Copyright Information:** (Online) ISSN: 2572-9241
©2026 The Author(s). HemaSphere is published by John Wiley & Sons Ltd on behalf of European Hematology Association. This is an open access Abstract Book distributed under the Attribution-NonCommercial-NoDerivs (CC BY-NC-ND), which allows third parties to download the articles and share them with others as long as they credit the author and the Abstract Book, but they cannot change the content in any way or use them commercially.
**Abstract Book Citations:** Authors, Title, HemaSphere, 2026;10:(S1):pages. The abstract book DOIs can be found on the “Author Information” page.
**Disclaimer:** Articles published in the journal HemaSphere exclusively reflect the opinions of the authors. The authors are responsible for all content in their abstracts including accuracy of the facts, statements, citing resources, etc.

### (PF722) LONGITUDINAL IMMUNE PROFILING REVEALS BASELINE AND ACQUIRED EXTRINSIC DETERMINANTS OF RESISTANCE TO BISPECIFIC ANTIBODIES IN RELAPSED/REFRACTORY MULTIPLE MYELOMA

**Topic:** 13. Myeloma and other monoclonal gammopathies - Biology & translational research

Ondrej Venglar[1, 2], Lucie Broskevičová[1, 2], Eva Radova[1, 2], Marketa Kudelkova[1, 2], Karolina Janochova[3, 4], Zhongfei Tao[1, 2], ,David Žihala[1, 2], Daniel Bilek[1, 2, 5], Tereza Ševčíková[1, 2, 5], Veronika Kapustova[1, 2], Jan Vrana[1], Ludmila Muronova[1, 2], Tereza Popková[1, 2], Sandra Charvátová[1, 2], Hana Plonková[1, 2], Zdenek Svagera[3, 4], Martin Stork[6], Jiri Minarik[7], Jakub Radocha[8], Tomas Jelínek[1, 2], Jana Mihályová*[1, 2], Roman Hájek[1, 2]

*[1]University Hospital Ostrava, Department of Hematooncology, Ostrava, Czechia; [2]University of Ostrava, Department of Hematooncology, Faculty of Medicine, Ostrava, Czechia; [3]University Hospital Ostrava, Department of Clinical biochemistry, Institute of Laboratory Medicine, Ostrava, Czechia; [4]University of Ostrava, Institute of Laboratory Medicine, Faculty of Medicine, Ostrava, Czechia; [5]Unviersity of Ostrava, Department of Biology and Ecology, Faculty of Science, Ostrava, Czechia;[6]University Hospital Brno, Department of Internal Medicine, Hematology and Oncology, Brno, Czechia; [7]Palacky University Olomouc and University Hospital Olomouc, Department of Hematooncology, Faculty of Medicine and Dentistry, Olomouc, Czechia; [8]Charles University and University Hospital in Hradec Kralove, 4th Department of Internal Medicine – Hematology, Faculty of Medicine, Hradec Kralove, Czechia;*

### Background

Bispecific antibodies (bsAbs) are an effective therapeutic option for relapsed/refractory multiple myeloma (RRMM). Tumor-intrinsic and extrinsic mechanisms, including target antigen alterations and T-cell dysfunction, contribute to resistance.

### Aims

To improve understanding of mechanisms of resistance to bsAbs by longitudinal immunoprofiling.

### Methods

Data of 47 RRMM patients treated with anti-GPRC5D Talquetamab (N=9, 19%) and anti-BCMA Teclistamab (N=24, 51%), Elranatamab (N=9, 19%) Etentamig (N=5, 11%) bsabs were analyzed. Peripheral blood samples (N=269) were assessed at baseline (BL) and longitudinally at day 7 (D7), D14, D30, D60, D90, and at progression (PD) by spectral cytometry lymphocyte profiling using OMIP-117 panel and employing conventional gating. 46 serum cytokines were evaluated in 41/47 patients using Bio-Plex MAGPIX multiplex assay (sample N=216).

**Copyright Information:** (Online) ISSN: 2572-9241
©2026 The Author(s). HemaSphere is published by John Wiley & Sons Ltd on behalf of European Hematology Association. This is an open access Abstract Book distributed under the Attribution-NonCommercial-NoDerivs (CC BY-NC-ND), which allows third parties to download the articles and share them with others as long as they credit the author and the Abstract Book, but they cannot change the content in any way or use them commercially.
**Abstract Book Citations:** Authors, Title, HemaSphere, 2026;10:(S1):pages. The abstract book DOIs can be found on the “Author Information” page.
**Disclaimer:** Articles published in the journal HemaSphere exclusively reflect the opinions of the authors. The authors are responsible for all content in their abstracts including accuracy of the facts, statements, citing resources, etc.

### Results

The median age of the cohort was 69 years, and 45% were male. ISS III was present in 30% patients and high-risk cytogenetics in 22%. 68% patients received ≥3 prior lines of therapy. CRS occurred in 31% patients with predominant grade 1. 36% achieved <VGPR (PR/SD/PD) and PD occurred in 39% patients during the follow-up period (10 months).
First, we sought to define the BL immune landscape associated with PD and worse response. Proportion of lymphocytes in leukocytes was reduced in PD group compared to no-PD (median 9 vs 17%, p=0.004). This was driven by T cell loss (p=0.008), without any CD4+/CD8+ shift. CD8+ compartment had reduced terminal effector memory cells (TEM; CD27-CD28-CD45RA-CD197-) in PD patients (6 vs 10%, p=0.047). Reduced proportions of CD39+ CD4+ T cells were detected in <VGPR (p=0.008). Patients with <VGPR showed elevated levels of IL-9, IL-10, IL-15, IFN-α2, and MIP-1α (all p<0.037). PD was associated with increased IL-10 and IL-15 levels (both p<0.047).
Longitudinally, T-cell depletion in PD sustained, reaching significance at D14/D30 (p<0.024) with the same trend across all timepoints. PD-1 expression increased over time in the whole cohort within both CD4+ (D0 vs D90: 44% vs 60%, p=0.004) and CD8+ T cells (38% vs 58%, p<0.001). Baseline reduction of CD8+ TEM cells persisted significant through D14/60/90 (p<0.029), and was accompanied by reduced early-like effector memory cells (CD27-CD28+CD45RA-CD197-) within the CD8+ pool at D30/60/90 (p<0.029). From D14 onward, CD8+ T cell pool showed an increased inhibitory phenotype in PD, including higher frequencies of KIR2DL1+ cells at D14/30/60/90 (p=0.006–0.057) and NKG2A+ cells at D14/30/60 (p<0.044). CXCL9 levels at D30 (p=0.049), CXCL10 at D7/14/30 (p<0.007), and IFN-γ at D30/60/90 (p=0.021-0.057) were reduced in PD. CXCL10 was also reduced at D14/30 in <VGPR (p<0.005). In contrast, MIP-1α was increased at D7/30 in <VGPR (p<0.048), while IL-15 (p=0.052) and IFN-α2 (p=0.007) were elevated at D7.

### Summary/Conclusion

This study suggests that resistance to bsAbs in RRMM accompanied by extrinsic mechanisms might result from both baseline and therapy-acquired factors. Early lymphopenia, depletion of CD8+ TEM T cells, and elevated IL-9, IL-10, and IL-15 indicate impaired T-cell maintenance and predisposition toward T cell dysfunction. Furthermore, progressive enrichment of inhibitory phenotypes in CD8+ T cells, along with reduced levels of key cytokines critical for lymphocyte recruitment and function, indicate evolving immune suppression.

**Copyright Information:** (Online) ISSN: 2572-9241
©2026 The Author(s). HemaSphere is published by John Wiley & Sons Ltd on behalf of European Hematology Association. This is an open access Abstract Book distributed under the Attribution-NonCommercial-NoDerivs (CC BY-NC-ND), which allows third parties to download the articles and share them with others as long as they credit the author and the Abstract Book, but they cannot change the content in any way or use them commercially.
**Abstract Book Citations:** Authors, Title, HemaSphere, 2026;10:(S1):pages. The abstract book DOIs can be found on the "Author Information" page.
**Disclaimer:** Articles published in the journal HemaSphere exclusively reflect the opinions of the authors. The authors are responsible for all content in their abstracts including accuracy of the facts, statements, citing resources, etc.

### (PF723) RAS–MAPK MUTATIONAL PROFILE IS ASSOCIATED WITH MULTI-PATHWAY GENOMIC ALTERATIONSAND A DISTINCT TERMINALLY DIFFERENTIATED/EXHAUSTED T-CELL SIGNATURE IN NEWLY DIAGNOSEDMULTIPLE MYELOMA

**Topic:** 13. Myeloma and other monoclonal gammopathies - Biology & translational research

Paula Piñero*[1, 2], Marina González Martínez[3], Elena Marin Sanchez[3], Vanesa Diaz[3], Javier López[3], Pablo Manresa[3], Lucia Ramon[3], Carmen García[3], Fabian Tarin[2, 3]

*[1]Instituto de Investigación Sanitaria y Biomédica de Alicante (ISABIAL), ALICANTE, Spain;[2]Universidad de Alicante, Departamento de Biotecnología, Alicante, Spain; [3]Hospital Universitario Dr. Balmis, Hematology, Alicante, Spain;*

### Background

Multiple myeloma (MM) is a genetically heterogeneous malignancy characterized by recurrent oncogenic pathway alterations, particularly in the RAS–MAPK axis. While genomic complexity drives clonal evolution, its impact on the immune microenvironment remains poorly understood.

### Aims

To characterize the genomic landscape of MM, focusing the interaction between RAS–MAPK pathway alterations and additional oncogenic events.To evaluate the association between genomic profiles and T-cell immunophenotypic remodeling and whether increasing MAPK genetic complexity correlates with progressive T-cell dysfunction.

### Methods

- Bone marrow (BM) samples of 188 newly diagnosed MM patients
- CD138 magnetic bead enrichment (MACSprep™ CD138 MicroBeads, Miltenyi).
- Flow cytometry: DURAClone IM T-cell Subsets tube (Beckman Coulter). Terminally differentiated/exhausted phenotype was characterized by PD-1 (CD279) and CD57.
- FISH: IGH translocations, del(17p), 1p/1q and ploidy abnormalities
- Capture NGS panel (Sophia CMM)
- Statistics: Mann–Whitney U test (group comparisons). PERMANOVA (differences in overall immune composition). Spearman's rank correlation.

**Copyright Information:** (Online) ISSN: 2572-9241
©2026 The Author(s). HemaSphere is published by John Wiley & Sons Ltd on behalf of European Hematology Association. This is an open access Abstract Book distributed under the Attribution-NonCommercial-NoDerivs (CC BY-NC-ND), which allows third parties to download the articles and share them with others as long as they credit the author and the Abstract Book, but they cannot change the content in any way or use them commercially.
**Abstract Book Citations:** Authors, Title, HemaSphere, 2026;10:(S1):pages. The abstract book DOIs can be found on the "Author Information" page.
**Disclaimer:** Articles published in the journal HemaSphere exclusively reflect the opinions of the authors. The authors are responsible for all content in their abstracts including accuracy of the facts, statements, citing resources, etc.

### Results

1. MAPK is a dominant oncogenic pathway (44% of patients). Mutations were observed in hotspot residues of KRAS and NRAS (G12/G13/Q61), as well as in BRAF and FGFR3. These alterations showed high allele frequencies, consistent with clonal dominance. Mutations in KRAS, NRAS, and BRAF were mutually exclusive VAF (>10%), supporting functional redundancy and biological convergence at the ERK signaling level
2. MAPK alterations are detected across all cytogenetic/molecular groups. MAPK pathway alterations show the highest degree of cross-pathway connectivity, supporting their central position within the multi-pathway genomic architecture (figure 1A).
3. MAPK pathway activation is associated with immune remodeling in the BM microenvironment. Patients harboring MAPK alterations exhibited a distinct T- cell profile characterized by a reduced CD4/CD8 ratio (0.66 vs 1.03; p = 0.006), a lower percentage of $CD4^+$ T cells (37.15% vs 45.59%; p = 0.008), increased of $CD8^+$ T cells (51.80% vs 45.94%; p = 0.015), and expansion of the $CD8^+$ effector memory compartment (27.30% vs 20.38%; p = 0.027). Multivariate analysis confirmed a global difference in immune composition (PERMANOVA p = 0.026), supporting the presence of a distinct MAPK-associated immune signature.
4. Additional mutations are associated with T-cell dysfunction in MAPK-altered cases. Patients harboring MAPK and additional mutated pathways exhibited an increase in CD8 terminally differentiated/exhausted T-cells (p=0.020). Furthermore, this subset increased progressively across ordered subclonal burden groups (0, 1 and >1 mutations with VAF <10%). (ρ = 0.55, p = 0.028) (Figure 1-B).

### Summary/Conclusion

- The MAPK pathway emerges as a central axis in MM, influencing not only tumor proliferation but also immune remodeling in the BM microenvironment.
- The association between genomic complexity and T-cell dysfunction supports a model in which oncogenic architecture contributes to adaptive immune impairment.
- Therapeutic strategies combining MAPK-targeted agents with immunomodulatory approaches may represent a rational avenue for treatment optimization.

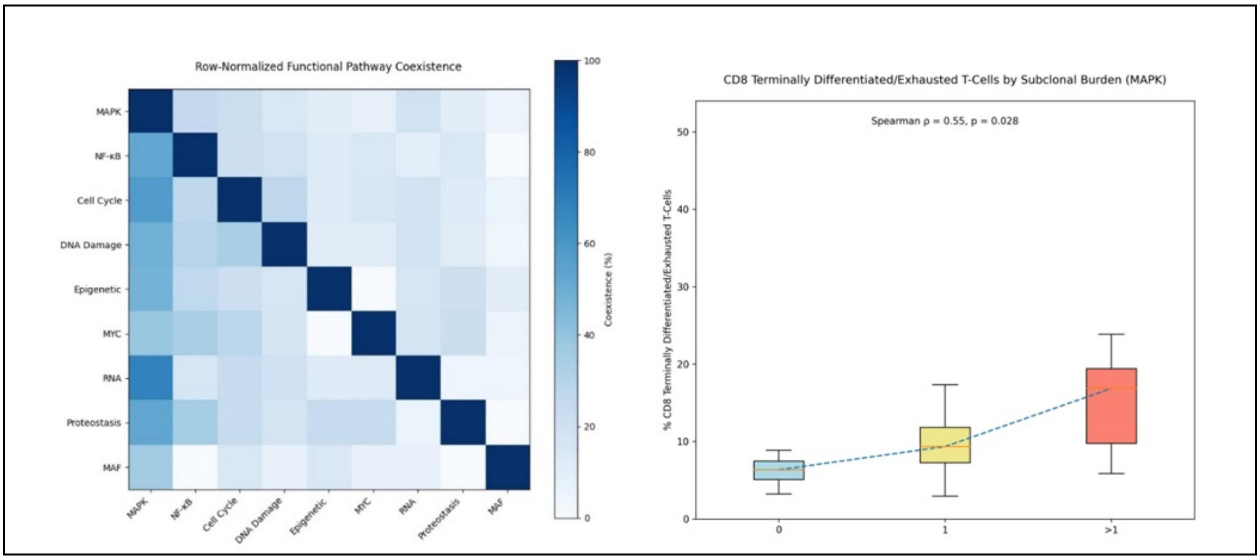

Figure 1. Genomic Architecture and Progressive T-Cell Exhaustion. A) Row-normalized functional pathway coexistence matrix in newly diagnosed MM. B) CD8 Terminally Differentiated/Exhausted T-Cells according to MAPK genetic complexity.

**Copyright Information:** (Online) ISSN: 2572-9241
©2026 The Author(s). HemaSphere is published by John Wiley & Sons Ltd on behalf of European Hematology Association. This is an open access Abstract Book distributed under the Attribution-NonCommercial-NoDerivs (CC BY-NC-ND), which allows third parties to download the articles and share them with others as long as they credit the author and the Abstract Book, but they cannot change the content in any way or use them commercially.
**Abstract Book Citations:** Authors, Title, HemaSphere, 2026;10:(S1):pages. The abstract book DOIs can be found on the "Author Information" page.
**Disclaimer:** Articles published in the journal HemaSphere exclusively reflect the opinions of the authors. The authors are responsible for all content in their abstracts including accuracy of the facts, statements, citing resources, etc.

### (PF725) ABS CATA-001: A NOVEL CD38/AL BISPECIFIC FOR TREATING MULTIPLE MYELOMA

**Topic:** 13. Myeloma and other monoclonal gammopathies - Biology & translational research

Chengcheng Fu*[1], Yue Liu[2], Yun Xu[3], Saroja Putrevu[2], Allison Titong[2], Nicholas Spadoni[2], Bo Wang[2], Matt Hoag[2], Nathan Fay[2], Jianbo Dong[2], Jiazi Zhou[1]

*[1]First Affiliated Hospital of Soochow University, Hematology, Suzhou, China;[2]Ab Studio Inc., Hayward, United States of America;*
*[3]The First Affiliated Hospital of Soochow University, Suzhou, China;*

### Background

In multiple myeloma cases, approximately 15-20% suffer from light chain multiple myeloma (LCMM). Beyond that, clonal plasma cells abnormally secrete free light chain which can evolve to AL amyloidosis leading to amyloid deposition in organs. The treatment goal for LCMM is to control **plasma cell proliferation** and to rapidly reduce **free light chains (FLC)** to prevent or reverse **renal damage** . Current first-line therapy involves a proteasome Inhibitor -based triplet or quadruplet treatment, consisting of a PI +IMiD+ anti-CD38 mAb.

### Aims

LCMM is frequently associated with **higher tumor burden**, **faster renal decline**, and **poorer prognosis** making disease control challenging. These clinical features expose the unmet needs for novel therapeutic strategies that specifically are designed to address both the malignant cancer cells and the pathogenic free light chains driving renal injury. ABS CATA-001 is a bispecific antibody targeting CD38 and free light chain (CD38/AL BsAb), representing a first-in-class approach with dual functionality of MM cell depletion with the unique catalytic capacity to bind and clear circulating free light chain aggregates. This dual mechanism positions ABS CATA-001 as a promising next-gen therapeutic candidate for effective treatment of LCMM.

### Methods

ABS CATA-001was designed with a structurally symmetric bispecific antibody. The CD38 binding arm is a humanized VHH antibody discovered from panning from a computer designed pre-humanized llama VHH library. The anti-AL arm was selected from a preexisting mAb targeting aggregated light chain (2A4). The bispecific antibody was further developed using computer-aided design rationale: detuned CD38 binding to enable better safety; effective CD38 binding to lead to MM cell depletion; built-in catalytic function (computationally optimized) to enable clearance of AL aggregates by both ADPC and catalytic function MOAs. Manufacturability also was optimized by computer-aided design to increase clinical potential backed by on bench validation and clinical POCs.

**Copyright Information:** (Online) ISSN: 2572-9241
©2026 The Author(s). HemaSphere is published by John Wiley & Sons Ltd on behalf of European Hematology Association. This is an open access Abstract Book distributed under the Attribution-NonCommercial-NoDerivs (CC BY-NC-ND), which allows third parties to download the articles and share them with others as long as they credit the author and the Abstract Book, but they cannot change the content in any way or use them commercially.
**Abstract Book Citations:** Authors, Title, HemaSphere, 2026;10:(S1):pages. The abstract book DOIs can be found on the "Author Information" page.
**Disclaimer:** Articles published in the journal HemaSphere exclusively reflect the opinions of the authors. The authors are responsible for all content in their abstracts including accuracy of the facts, statements, citing resources, etc.

**Results**

We selected a CD38 binding arm with a unique epitope that does not compete with Daratumumab. In order to balance safety and efficacy of CD38 affinity, we detuned the CD38 binding arm to retain MM cell depletion function, without affecting NK cells and activated T cells. Functionally, *in vitro* ADPC studies show that ABS CATA-001 can deplete H929 cancer cells in the presence of human PBMCs of a healthy donor. Furthermore, *in vivo* studies showed that ABS CATA-001 can effectively and safely deplete and inhibit MM cells without affecting body weight in a human PBMC reconstituted, and H929 engrafted NDG mouse model. Additionally, the computer designed AL binding arm showed significantly improved catalytic function, implicating therapeutic efficacy against protein aggregates. The *in vitro* assessments studies show that ABS CATA-001 significantly reduces aggregated human serum amyloid A1 (hSAA1). LCMM patient serum treated with ABS CATA-001 showed better reduction of serum light chain aggregates in all five LCMM patient donors when compared to Birtamimab. Computer-aided manufacturability optimization of ABS CATA-001 validated on bench, demonstrated high purity based on SEC-HPLC data and low aggregation potential based on DLS data.

**Summary/Conclusion**

These findings demonstrate that ABS CATA-001 may serve as a next generation therapy for patients with LCMM

**Copyright Information:** (Online) ISSN: 2572-9241
©2026 The Author(s). HemaSphere is published by John Wiley & Sons Ltd on behalf of European Hematology Association. This is an open access Abstract Book distributed under the Attribution-NonCommercial-NoDerivs (CC BY-NC-ND), which allows third parties to download the articles and share them with others as long as they credit the author and the Abstract Book, but they cannot change the content in any way or use them commercially.
**Abstract Book Citations:** Authors, Title, HemaSphere, 2026;10:(S1):pages. The abstract book DOIs can be found on the “Author Information” page.
**Disclaimer:** Articles published in the journal HemaSphere exclusively reflect the opinions of the authors. The authors are responsible for all content in their abstracts including accuracy of the facts, statements, citing resources, etc.

### (PF726) FUNCTIONAL CONSEQUENCES OF TP53 LOSS ACROSS MAJOR IGH TRANSLOCATION SUBTYPES IN MULTIPLE MYELOMA

**Topic:** 13. Myeloma and other monoclonal gammopathies - Biology & translational research

Sara Cristóbal Vargas*[1, 2], Elizabeta A. Rojas[1, 2, 3], David Becerro[1, 3], María García-Álvarez[1, 3], Alejandra K Hachi[1, 2], Ana Balanzategui Echevarría[1], Alejandro Medina Herrera[1, 3], Isabel Isidro[1, 2], Cristina De Ramón Sánchez[1, 2, 3], María-Victoria Mateos[1, 2, 3], Luis A Corchete[1, 2, 3], Myriam Cuadrado[1, 2, 3], Norma Gutierrez Gutierrez[1, 2, 3]

*[1]Department of Hematology, Salamanca University Hospital, IBSAL, Salamanca, Spain;[2]Cancer Research Center- IBMCC (USAL-CSIC), Salamanca, Spain; [3]Biomedical Research Network Center for Cancer (CIBERONC, CB16/12/00233), Salamanca, Spain;*

### Background

Therapeutic advances have improved outcomes in multiple myeloma (MM), enabling long-term disease control in patients with favourable genetic profiles. However, this progress has not extended to MM with high-risk abnormalities, particularly *TP53* loss due to del(17p), which remains strongly associated with poor prognosis and disease progression. Although uncommon at diagnosis, del(17p) emerges in up to 50% of heavily pretreated patients, yet its biological impact remains incompletely understood. We hypothesized that the functional consequences of *TP53* loss might depend on the underlying genetic context defined by the major immunoglobulin heavy chain (*IGH*) translocations.

### Aims

1. To characterize the functional consequences of biallelic *TP53* loss in isogenic MM cell line harboring distinct *IGH*
2. To compare transcriptional programs associated with *TP53* deletion in MM patients according to *IGH* translocation subtype.
3. To identify *IGH* translocation-specific therapeutic vulnerabilities linked to *TP53*

### Methods

Biallelic *TP53* deletion was generated using CRISPR/Cas9 in three *TP53* wild-type MM cell lines: MOLP-8 [t(11;14)], H929 [t(4;14)] and MM1S [t(14;16)]. Transcriptomic profiling was performed by RNA sequencing in isogenic models, and in samples from patients with MM and an *IGH* translocation, with or without *TP53* deletion. Differential expression and pathway enrichment (DESeq2, Biomart and ClusterProfiler; FDR < 0.1) were integrated with functional assays (proliferation, migration, metabolism) and drug sensitivity screening (RealTime-Glo™).

**Copyright Information:** (Online) ISSN: 2572-9241
©2026 The Author(s). HemaSphere is published by John Wiley & Sons Ltd on behalf of European Hematology Association. This is an open access Abstract Book distributed under the Attribution-NonCommercial-NoDerivs (CC BY-NC-ND), which allows third parties to download the articles and share them with others as long as they credit the author and the Abstract Book, but they cannot change the content in any way or use them commercially.
**Abstract Book Citations:** Authors, Title, HemaSphere, 2026;10:(S1):pages. The abstract book DOIs can be found on the "Author Information" page.
**Disclaimer:** Articles published in the journal HemaSphere exclusively reflect the opinions of the authors. The authors are responsible for all content in their abstracts including accuracy of the facts, statements, citing resources, etc.

### Results

*TP53* loss induced distinct, translocation-specific transcriptional and functional alterations. In **t(4;14)**, *TP53* deletion triggered significant metabolic reprogramming in both patients and cell models, with enrichment in energy production and nucleotide metabolism. *TP53*-deficient cells showed increased glucose uptake and ATP production alongside reduced mitochondrial activity, indicating a glycolysis-dependent shift.

Samples from MM patients with **t(11;14)** and *TP53* loss showed enrichment in immune and migration pathways, while isogenic lines exhibited deregulated DNA replication and ribosome biogenesis. Despite these differences, both models converged on cell cycle dysregulation, with accumulation of cells in S phase *in vitro*, consistent with cyclin D1 overexpression inherent to t(11;14) and loss of the p53-dependent G1/S checkpoint.

In **t(14;16)**, *TP53* loss predominantly affected signaling, cytoskeletal organization and migration, confirmed by increased migratory capacity in transwell assays.

Drug sensitivity was equally context dependent. *TP53*-deficient **t(4;14)** cells showed bortezomib resistance, but increased sensitivity to lenalidomide/dexamethasone. In **t(11;14)**, *TP53* loss conferred resistance to bortezomib and selinexor, but marked sensitivity to the PLK1 inhibitor volasertib, indicating cell cycle-related vulnerabilities. Conversely, *TP53* loss in **t(14;16)** was linked to melphalan resistance, but enhanced response to bortezomib and lenalidomide/dexamethasone.

### Summary/Conclusion

Loss of *TP53* in MM does not result in a uniform biological or therapeutic phenotype but instead drives distinct molecular, functional and drug-response programs determined by *IGH* translocation subtype. These findings provide mechanistic insight into the heterogeneity of *TP53*-altered MM and support the development of genetically informed, translocation-adapted therapeutic strategies for high-risk disease.

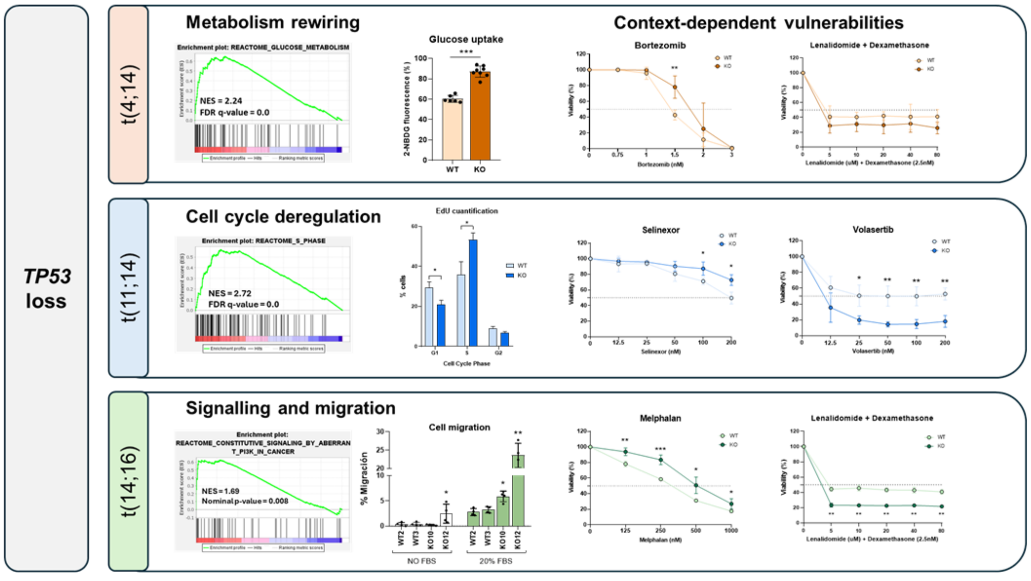

**Impact of *TP53* loss across different cytogenetic subgroups of multiple myeloma.** In **t(4;14)** cells, *TP53* loss triggers metabolic rewiring characterized by increased glucose uptake and confers resistance to bortezomib. In the **t(11;14)** context, loss of *TP53* leads to cell cycle deregulation, specifically an enrichment of S-phase genes and increased sensitivity to volasertib. Finally, in **t(14;16)** models, *TP53* deficiency enhances PI3K signaling and cell migration, while modulating response to melphalan and lenalidomide/dexamethasone. NES: Normalized Enrichment Score; WT: Wild-type; KO: Knock-out.

Copyright Information: (Online) ISSN: 2572-9241
©2026 The Author(s). HemaSphere is published by John Wiley & Sons Ltd on behalf of European Hematology Association. This is an open access Abstract Book distributed under the Attribution-NonCommercial-NoDerivs (CC BY-NC-ND), which allows third parties to download the articles and share them with others as long as they credit the author and the Abstract Book, but they cannot change the content in any way or use them commercially.
Abstract Book Citations: Authors, Title, HemaSphere, 2026;10:(S1):pages. The abstract book DOIs can be found on the "Author Information" page.
Disclaimer: Articles published in the journal HemaSphere exclusively reflect the opinions of the authors. The authors are responsible for all content in their abstracts including accuracy of the facts, statements, citing resources, etc.

### (PF727) A NOVEL HIGH-THROUGHPUT SINGLE-CELL LIPIDOMIC REVEALS POTENTIAL CORRELATION BETWEEN METABOLIC HETEROGENEITY AND VULNERABILITIES WITH CYTOGENETIC ABNORMALITIES IN MULTIPLE MYELOMA

**Topic:** 13. Myeloma and other monoclonal gammopathies - Biology & translational research

Fujing Zhang*[1], Chenxi Cao[2], Peng Liu[3], Xiaoxiao MA[2], Junling Zhuang[1]

*[1]Peking Union Medical College Hospital, Chinese Academy of Medical Sciences, Department of Hematology, Beijing, China; [2]Tsinghua University, State Key Laboratory of Precision Measurement Technology and Instruments, Department of Precision Instrument, Beijing, China; [3]Peking Union Medical College Hospital, Chinese Academy of Medical Sciences, Center for Rare Diseases, State Key Laboratory of Complex, Severe, and Rare Diseases, Beijing, China;*

### Background

Multiple myeloma progresses through a clinical continuum from MGUS and SMM to active MM and PCL. While the genomic landscape is well-mapped, single-cell metabolic heterogeneity and stage-specific lipid remodeling remain poorly characterized. Current lipidomics often fails to resolve lipid isomers in individual cells, leaving these metabolic drivers largely undefined.

### Aims

We utilized a novel high-throughput single-cell lipidomics platform to systematically map the structural lipidomic landscape across the MM disease spectrum. Our objective was to investigate correlations between metabolic heterogeneity and high-risk cytogenetics, specifically uncovering isomer-level vulnerabilities to provide novel insights for precision intervention in MM.

### Methods

Bone marrow CD38+CD138+ plasma cells were purified via fluorescence-activated cell sorting. Our innovation lies in the MMSD-integrated platform, overcoming previous throughput barriers to enable precise lipid identification and isomer-level resolution in single cells. UMAP visualization and Leiden clustering of over 12000 single cells yielded a high-dimensional structural lipidomic atlas, revealing metabolic subclones and stage-specific signatures.

**Copyright Information:** (Online) ISSN: 2572-9241
©2026 The Author(s). HemaSphere is published by John Wiley & Sons Ltd on behalf of European Hematology Association. This is an open access Abstract Book distributed under the Attribution-NonCommercial-NoDerivs (CC BY-NC-ND), which allows third parties to download the articles and share them with others as long as they credit the author and the Abstract Book, but they cannot change the content in any way or use them commercially.
**Abstract Book Citations:** Authors, Title, HemaSphere, 2026;10:(S1):pages. The abstract book DOIs can be found on the "Author Information" page.
**Disclaimer:** Articles published in the journal HemaSphere exclusively reflect the opinions of the authors. The authors are responsible for all content in their abstracts including accuracy of the facts, statements, citing resources, etc.

### Results

17 bone marrow samples from 16 patients, spanning 1 MGUS, 2 SMM, 12 MM, and 2 PCL, were analyzed (Fig.1a,b). UMAP revealed that single cells displayed a highly characteristic spatial distribution pattern. Myeloma cells from the same patient formed distinct, patient-specific clusters, while populations from different individuals remained significantly separated, underscoring profound inter-patient heterogeneity within the lipidomic dimension.

When focusing on MM and PCL cases, lipid phenotypic clustering patterns were intrinsically linked to cytogenetic backgrounds. Samples harboring similar high-risk cytogenetic abnormalities, including 1q21+ and t(4;14), occupied closer topological manifold spaces (Fig 1b), suggesting specific genetic drivers dictate the metabolic architecture of plasma cells.

Beyond species-level analysis, deep isomer-level profiling identified metabolic vulnerabilities in high-risk subtypes (Fig 1c). In a triple-hit patient harboring concurrent 17p- t(4;14) and 1q21+, we observed a marked global upregulation of arachidonic acid (C20:4)-containing phospholipid isomers relative to their C18:2 counterparts. Concurrently, the PE O-16:1/22:5 to PE O-18:2/20:4 ratio was significantly downregulated. Given that C20:4-containing phospholipids are primary executioners of ferroptosis, this isomer profile indicates that MM cells with 17p- and t(4;14) exist in a metabolic state highly primed for ferroptosis sensitivity.

We identified a correlation between metabolic chain-length remodeling and high-risk cytogenetics by comparing isolated 1q21+ vs double-hit t(4;14) and 1q21+. Plasma cells with t(4;14) showed a shift where C20:4 was downregulated and C22:4 was significantly upregulated (Fig 1d). Overall ferroptosis substrate abundance increased,, consistent with ACSL4 and ELOVL5 upregulation in public datasets, constituting a unique metabolic hallmark of this subtype

### Summary/Conclusion

This novel high-throughput single-cell lipidomic study reveals a profound correlation between metabolic heterogeneity and vulnerabilities with cytogenetic abnormalities in MM. By identifying isomer-level metabolic vulnerabilities, particularly ferroptosis priming in high-risk subtypes, this research provides new potential therapeutic targets for MM.

**Copyright Information:** (Online) ISSN: 2572-9241
©2026 The Author(s). HemaSphere is published by John Wiley & Sons Ltd on behalf of European Hematology Association. This is an open access Abstract Book distributed under the Attribution-NonCommercial-NoDerivs (CC BY-NC-ND), which allows third parties to download the articles and share them with others as long as they credit the author and the Abstract Book, but they cannot change the content in any way or use them commercially.
**Abstract Book Citations:** Authors, Title, HemaSphere, 2026;10:(S1):pages. The abstract book DOIs can be found on the "Author Information" page.
**Disclaimer:** Articles published in the journal HemaSphere exclusively reflect the opinions of the authors. The authors are responsible for all content in their abstracts including accuracy of the facts, statements, citing resources, etc.

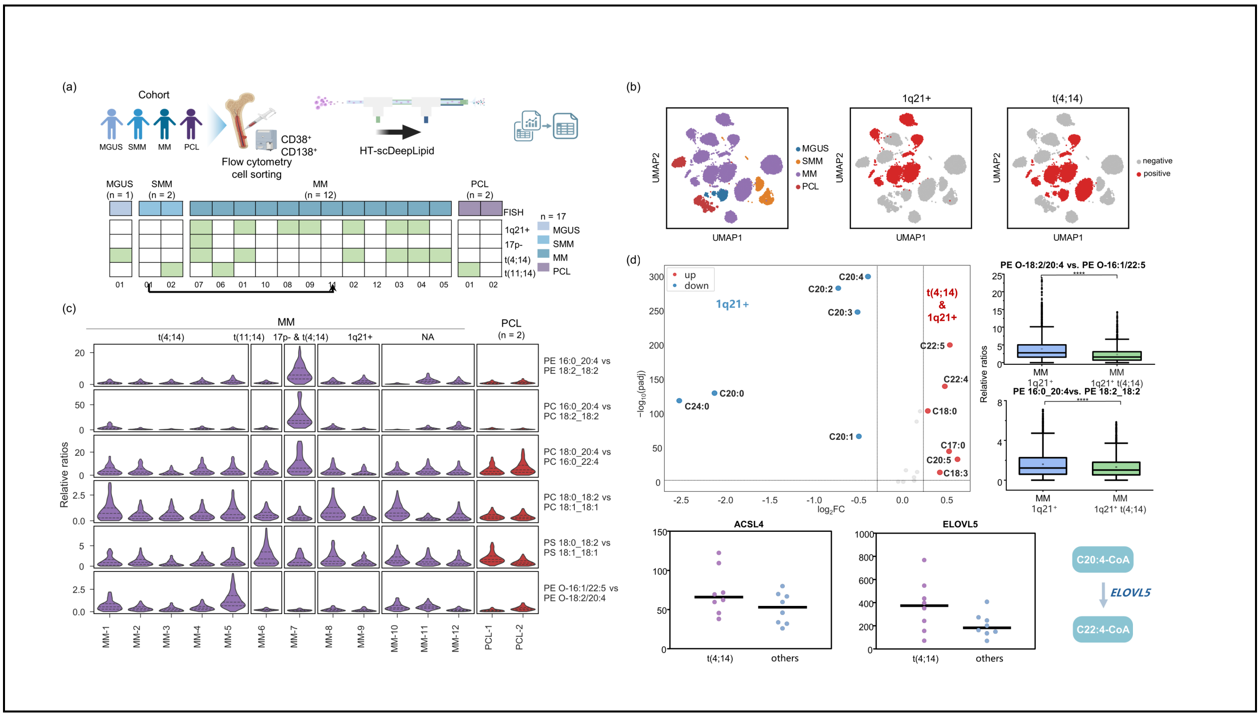

Figure 1. Single-cell structural lipidomic landscape of Multiple Myeloma. (a) Workflow and Cohort: Integration of clinical stages (MGUS, SMM, MM, PCL) with the high-throughput MMSD single-cell lipidomics platform. (b) Heterogeneity and Cytogenetics: UMAP visualization reveals patient-specific metabolic clusters and topological correlation between high-risk cytogenetics (e.g., 1q21+, t(4;14)) and lipid phenotypes. (c) Metabolic Vulnerabilities: Isomer-level profiling identifies a C20:4-rich signature in 17p- and t(4;14) subtypes, indicating increased ferroptosis susceptibility. (d) Subtype Hallmarks: Comparison of t(4;14) cases highlights a specific shift toward C22:4 isomers, consistent with ACSL4 and ELOVL5 expression patterns in public datasets.

**Copyright Information:** (Online) ISSN: 2572-9241
©2026 The Author(s). HemaSphere is published by John Wiley & Sons Ltd on behalf of European Hematology Association. This is an open access Abstract Book distributed under the Attribution-NonCommercial-NoDerivs (CC BY-NC-ND), which allows third parties to download the articles and share them with others as long as they credit the author and the Abstract Book, but they cannot change the content in any way or use them commercially.
**Abstract Book Citations:** Authors, Title, HemaSphere, 2026;10:(S1):pages. The abstract book DOIs can be found on the “Author Information” page.
**Disclaimer:** Articles published in the journal HemaSphere exclusively reflect the opinions of the authors. The authors are responsible for all content in their abstracts including accuracy of the facts, statements, citing resources, etc.

### (PF728) CAR-T CELL THERAPY IN ADVANCED MYELOMA WITH EXTRAMEDULLARY DISEASE – AN IN VIVO IMAGING AND MOLECULAR MONITORING STUDY (CARAMEL): CD8+ T CELL INFILTRATION VISUALISED AT PEAK CILTA-CEL EXPANSION

**Topic:** 13. Myeloma and other monoclonal gammopathies - Biology & translational research

Mark Dowling*[1, 2, 3], Faiza Zuairia Tabassum[2], Jeremy Er[4], Sergio Farina[1], Theresa Connor[2], Christian Wichmann[5], Zhipeng Cao[5], Andrew Scott[5, 6], Michael MacManus[3, 7], Timothy Akhurst[8], Jane Oliaro[2, 3], Simon Harrison[1, 2, 3]

*[1]Peter MacCallum Cancer Centre and Royal Melbourne Hospital, Clinical Haematology, Melbourne, Australia;[2]Peter MacCallum Cancer Centre, Centre of Excellence in Cellular Immunotherapy, Melbourne, Australia; [3]University of Melbourne, Sir Peter MacCallum Department of Oncology, Melbourne, Australia; [4]Walter and Eliza Hall Institute, Inflammation Division, Melbourne, Australia; [5]Olivia Newton John Cancer Research Institute, Melbourne, Australia;[6]Austin Health, Department Of Molecular Imaging And Therapy, Austin Health, Melbourne, Australia; [7]Peter MacCallum Cancer Centre, Radiation Oncology, Melbourne, Australia; [8]Peter MacCallum Cancer Centre, Nuclear Medicine, Australia;*

### Background

Ciltacabtagene autoleucel (cilta-cel), a B-cell maturation antigen (BCMA)–directed CAR T-cell therapy has demonstrated high response rates and durability in heavily pre-treated (³3) myeloma patients (CARTITUDE-1), and superiority compared to standard-of-care in lenalidomide-refractory myeloma patients after 1-3 prior lines of therapy (CARTITUDE-4). Patients with extramedullary myeloma (EMM) appear to have a reduced progression-free survival in subgroup analyses. We have previously reported results of early biodistribution of cilta-cel early post infusion in 4 patients using Copper-64 ($^{64}$Cu) super paramagnetic iron oxide nanoparticles (SPION) and dual PET-CT & PET-MRI, showing tracking to bone-based but not soft-tissue plasmacytomas.

### Aims

We aimed to study the trafficking of CD8+ T cells to sites of medullary and extramedullary myeloma at the time of peak cilta-cel expansion in the blood and during contraction using a novel CD8 PET tracer.

### Methods

$^{89}$Zr-Df-Crefmirlimab Berdoxam ($^{89}$Zr-Crefmirlimab) is a minibody PET tracer that binds to CD8 with high affinity and has no detectable biological effects. Zr-89-Cr can be used to image CD8+ T cell responses in the context of immunotherapy with a half-life of 3.3 days (CD8+ ImmunoPET). Here, we report the results of CD8+ ImmunoPET imaging in the second cohort of 4 patients in the CARAMEL study (CAR-T cell therapy in Advanced Myeloma with Extramedullary disease – an *in vivo* imaging and molecular monitoring study, NCT05666700). Myeloma patients with measurable extramedullary disease and ³2 prior lines of therapy (including a proteasome inhibitor and an immunomodulatory imide drug (IMiD) are eligible. Patients underwent CD8+ ImmunoPET at baseline, pre-lymphodepletion (pre-LD, after bridging at investigator discretion), and then post infusion at peak cilta-cel expansion ($^{89}$Zr-Crefmirlimab D+10, imaging D+11 and D+13) and at contraction ($^{89}$Zr-Crefmirlimab D+32, imaging D+33 and D+35). FDG-PET-CT was performed a baseline, pre-LD and D+28 to assess response of EMM sites.

**Copyright Information:** (Online) ISSN: 2572-9241
©2026 The Author(s). HemaSphere is published by John Wiley & Sons Ltd on behalf of European Hematology Association. This is an open access Abstract Book distributed under the Attribution-NonCommercial-NoDerivs (CC BY-NC-ND), which allows third parties to download the articles and share them with others as long as they credit the author and the Abstract Book, but they cannot change the content in any way or use them commercially.
**Abstract Book Citations:** Authors, Title, HemaSphere, 2026;10:(S1):pages. The abstract book DOIs can be found on the "Author Information" page.
**Disclaimer:** Articles published in the journal HemaSphere exclusively reflect the opinions of the authors. The authors are responsible for all content in their abstracts including accuracy of the facts, statements, citing resources, etc.

**Results**

At baseline, no EMM sites showed CD8 uptake above background. Three out of four patients responded to cilta-cel and a reduction in the size of EMM lesions together with an increase in activity on CD8+ ImmunoPET was seen at the time of peak cilta-cel expansion in all cases. During contraction, the three patients had all achieved a complete response, and therefore residual CD8 uptake was not able to be assessed. In one patient with a soft-tissue plasmacytoma in the mediastinum, there was concurrent increase in uptake in a mediastinal lymph node that was not clearly involved by myeloma in the baseline FDG-PET, Figure 1. In the one patient that did not respond, there was no increase in uptake on CD8+ ImmunoPET above background at peak or contraction. One patient experienced late atypical neurotoxicity, and on review of the peak and contraction CD8+ ImmunoPETs there was no increase in CD8+ uptake in the central nervous system preceding the event. Correlative immunological studies are ongoing to explore the immunological and tumour microenvironment factors associated with CAR-T infiltration and resistance in extramedullary and medullary myeloma.

**Summary/Conclusion**

CD8+ T-cell infiltration into soft-tissue plasmacytomas was able to be visualised post cilta-cel infusion and correlates with early response in this cohort of four patients. Local immune activation may play a role in CAR-T response.

**Copyright Information:** (Online) ISSN: 2572-9241
©2026 The Author(s). HemaSphere is published by John Wiley & Sons Ltd on behalf of European Hematology Association. This is an open access Abstract Book distributed under the Attribution-NonCommercial-NoDerivs (CC BY-NC-ND), which allows third parties to download the articles and share them with others as long as they credit the author and the Abstract Book, but they cannot change the content in any way or use them commercially.
**Abstract Book Citations:** Authors, Title, HemaSphere, 2026;10:(S1):pages. The abstract book DOIs can be found on the "Author Information" page.
**Disclaimer:** Articles published in the journal HemaSphere exclusively reflect the opinions of the authors. The authors are responsible for all content in their abstracts including accuracy of the facts, statements, citing resources, etc.

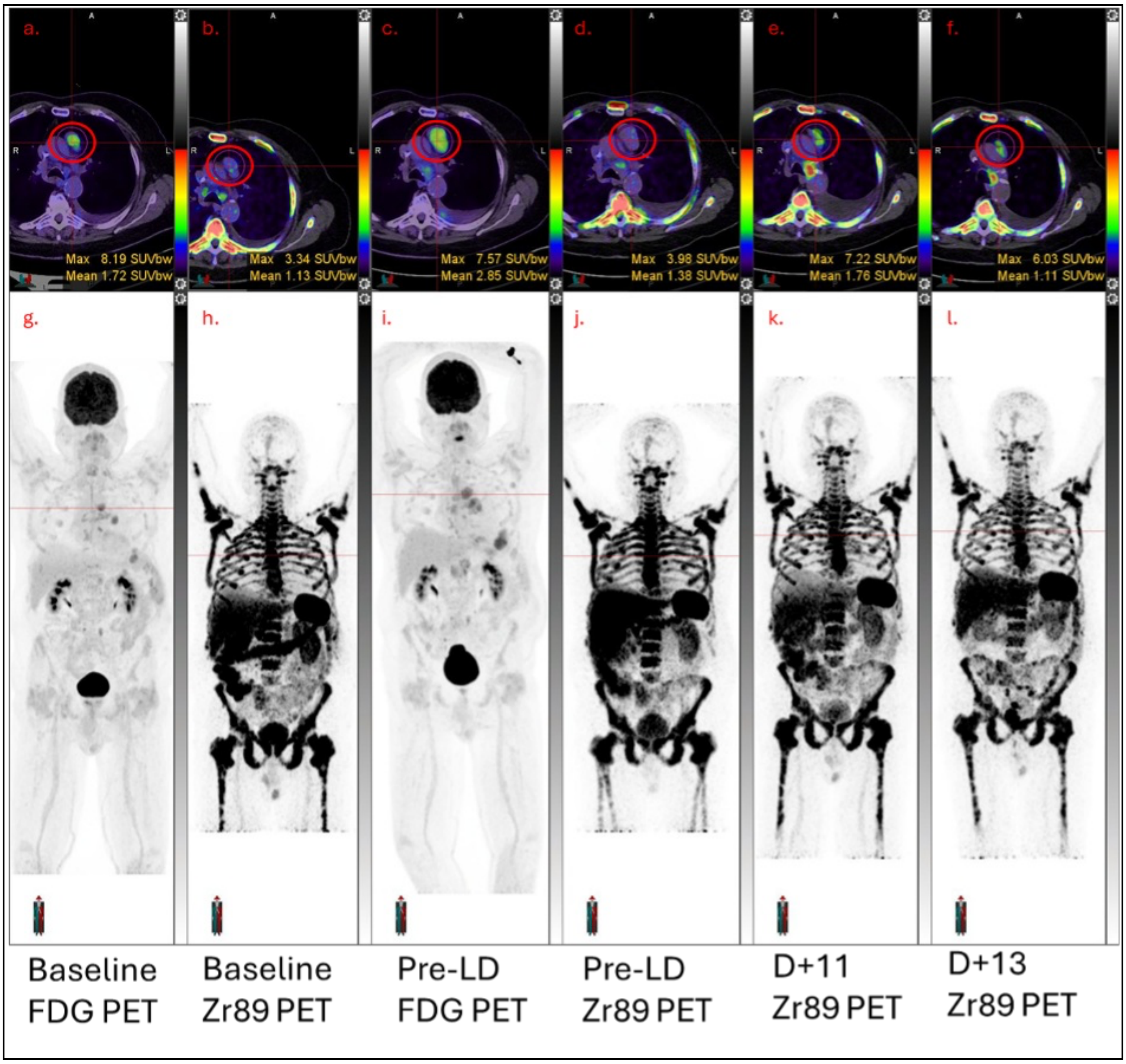

Figure 1: Serial FDG-PET and CD8+ ImmunoPET (Zr89 PET) in one patient from the second cohort of CARAMEL. The mediastinal FDG-avid soft-tissue plasmacytoma (a. and g.) was not active on CD8+ ImmunoPET at baseline (b. and h.), or pre-LD (c., d., i. and j.) but had reduced in size with concurrent increase in CD8 uptake in both the lesion and draining mediastinal lymph node at the time of peak cilta-cel expansion (D+11, e. and k., and D+13, F. and l.).

**Copyright Information:** (Online) ISSN: 2572-9241
©2026 The Author(s). HemaSphere is published by John Wiley & Sons Ltd on behalf of European Hematology Association. This is an open access Abstract Book distributed under the Attribution-NonCommercial-NoDerivs (CC BY-NC-ND), which allows third parties to download the articles and share them with others as long as they credit the author and the Abstract Book, but they cannot change the content in any way or use them commercially.
**Abstract Book Citations:** Authors, Title, HemaSphere, 2026;10:(S1):pages. The abstract book DOIs can be found on the "Author Information" page.
**Disclaimer:** Articles published in the journal HemaSphere exclusively reflect the opinions of the authors. The authors are responsible for all content in their abstracts including accuracy of the facts, statements, citing resources, etc.

### (PF729) COEVOLUTION OF NK AND TUMOR CELL STATES ALONG MULTIPLE MYELOMA PROGRESSION FROM PRECURSOR CONDITIONS

**Topic:** 13. Myeloma and other monoclonal gammopathies - Biology & translational research

Cristina Aquilina*[1], Andrea Romano[2], Anna Maria Corsale[1], Marta Biondo[3], Maria Speciale[2], Elena Tofacchi[1], Marta Di Simone[1], Emilia Gigliotta[1], Costanza Dieli[2], Claudia Avellone[1], Angelo Toscano[4], Lawrence Camarda[5], Alessandra Romano[3], Cambria Daniela[3], Gianluca Giavaresi[4], Lavinia Raimondi[4], Antonino Neri[6], Stefania Campana[7], Nadia Caccamo[8], Francesco Dieli[8], Sergio Siragusa[1], Serena Meraviglia[8], Cirino Botta[1]

*[1]University of Palermo, Health Promotion, Mother and Child Care, Internal Medicine and Medical Specialties, Palermo, Italy; [2]University of Palermo, Department of Surgical, Oncological and Stomatological Disciplines (DICHIRONS), Palermo, Italy; [3]University of Catania, Catania, Italy; [4]IRCCS Rizzoli Orthopedic Institute, Bologna, Italy; [5]University of Palermo, Palermo, Italy; [6]Scientific Directorate, Azienda USL-IRCCS di Reggio Emilia, Reggio Emilia, Italy;[7]University of Messina, Messina, Italy; [8]University of Palermo, Department of Biomedicine, Neuroscience and Advanced Diagnosis (Bi.N.D.), Palermo, Italy;*

### Background

Multiple myeloma (MM) evolves from monoclonal gammopathy of undetermined significance (MGUS) and smoldering MM (SMM) through progressive remodeling of the bone marrow (BM) microenvironment. However, it remains unclear how NK cell functional states change in concert with tumor transcriptional programs across disease stages and which microenvironmental mechanisms sustain immune escape.

### Aims

To define stage-dependent NK cell remodeling during MM evolution and identify coordinated immune–tumor programs, with particular focus on the inhibitory NKG2A–HLA-E checkpoint axis.

### Methods

We integrated nine publicly available BM scRNA-seq datasets (178 subjects: 54 healthy donors [HD], 25 MGUS, 14 SMM, 85 newly diagnosed MM) using standardized quality control and reference-based annotation. Tumor-intrinsic NK sensitivity/resistance gene sets were derived from genome-wide loss-/gain-of-function CRISPR screens in NK–myeloma co-cultures and scored in single-cell plasma cells, while curated NK functional programs were scored in NK cells. Patient-level integration of NK and plasma cell scores enabled unsupervised clustering to capture coordinated immune–tumor variation across conditions. NK cells were subsequently extracted and re-integrated to resolve subset heterogeneity, quantify compositional changes, and perform pseudobulk differential expression and hallmark enrichment (restricted to datasets containing all four conditions). Key findings were validated in an independent BM cohort (6 HD, 12 MGUS, 14 SMM, 20 MM) by flow cytometry.

**Copyright Information:** (Online) ISSN: 2572-9241
©2026 The Author(s). HemaSphere is published by John Wiley & Sons Ltd on behalf of European Hematology Association. This is an open access Abstract Book distributed under the Attribution-NonCommercial-NoDerivs (CC BY-NC-ND), which allows third parties to download the articles and share them with others as long as they credit the author and the Abstract Book, but they cannot change the content in any way or use them commercially.
**Abstract Book Citations:** Authors, Title, HemaSphere, 2026;10:(S1):pages. The abstract book DOIs can be found on the "Author Information" page.
**Disclaimer:** Articles published in the journal HemaSphere exclusively reflect the opinions of the authors. The authors are responsible for all content in their abstracts including accuracy of the facts, statements, citing resources, etc.

### Results

Integrated immune–tumor profiling identified two patient clusters significantly associated with clinical stage ($p=8.789\times10^{-5}$), indicating coordinated shifts in NK functional programs and plasma cell susceptibility/resistance signatures. BM NK cell abundance increased from HD (10.5%) to MGUS (16.6%) and SMM (19.9%; HD vs SMM adj p=0.0035), followed by a non-significant decrease in MM (16.8%). Pseudobulk analyses revealed upregulation of interferon-stimulated genes in MM NK cells, with enrichment of interferon-α/γ response hallmarks. NK re-clustering identified six subsets, including cytotoxic/resting CD56$^{dim}$ and regulatory CD56$^{bright}$ populations. The CD56$^{bright}$ compartment displayed a non-linear compositional trajectory (decrease in MGUS/SMM with partial recovery in MM). Within this subset, stage-dependent modulation of the inhibitory receptor NKG2A (encoded by *KLRC1*) was confirmed by flow cytometry. Its ligand, HLA-E, was most abundantly expressed by CD16$^{+}$ monocytes and followed a distinct non-linear pattern across disease stages ($p<2.2\times10^{-16}$). Sample-level analysis revealed a significant positive correlation between *HLA-E* expression in CD16$^{+}$ monocytes and *KLRC1* expression in CD56$^{bright}$ NK cells ($R=0.51$, $p=4.5\times10^{-8}$), supporting coordinated microenvironmental modulation of this inhibitory axis.

### Summary/Conclusion

MM progression is associated with non-linear changes in BM NK cell abundance and subset composition, alongside coordinated immune–tumor transcriptional remodeling. We identify a microenvironment-associated inhibitory circuit in which HLA-E$^{+}$ CD16$^{+}$ monocytes co-vary with modulation of the corresponding inhibitory receptor in CD56$^{bright}$ NK cells, consistent with progressive restraint of innate anti-tumor surveillance. Clinically, this checkpoint pathway may provide a biomarker framework for disease evolution and supports therapeutic targeting of the NKG2A–HLA-E axis to enhance NK activity in precursor conditions and early MM.

**Copyright Information:** (Online) ISSN: 2572-9241
©2026 The Author(s). HemaSphere is published by John Wiley & Sons Ltd on behalf of European Hematology Association. This is an open access Abstract Book distributed under the Attribution-NonCommercial-NoDerivs (CC BY-NC-ND), which allows third parties to download the articles and share them with others as long as they credit the author and the Abstract Book, but they cannot change the content in any way or use them commercially.
**Abstract Book Citations:** Authors, Title, HemaSphere, 2026;10:(S1):pages. The abstract book DOIs can be found on the "Author Information" page.
**Disclaimer:** Articles published in the journal HemaSphere exclusively reflect the opinions of the authors. The authors are responsible for all content in their abstracts including accuracy of the facts, statements, citing resources, etc.

### (PF730) A MYELOID-DORMANT METABOLIC TRANSCRIPTOME IDENTIFIES A POOR-PROGNOSIS SUBGROUP IN HYPERDIPLOID MULTIPLE MYELOMA WITH TARGETABLE VULNERABILITIES

**Topic:** 13. Myeloma and other monoclonal gammopathies - Biology & translational research

Naroa Barrena*[1], Edurne San Jose-Eneriz[2, 3, 4], Luis V. Valcárcel[1, 2], Danel Olaverri-Mendizabal[1], Leire Garate[3, 4, 5], Estibaliz Miranda[2, 3, 4], Paula Rodriguez-Marquez[2, 4], Romika Kumari[6], Patxi San Martín[2, 3], Juan Roberto Rodriguez-Madoz[2, 4], Leonor Puchades Carrasco[7], Caroline Heckman[6], Paula Rodríguez-Otero[3, 4, 5], Francisco Planes[1], Xabier Agirre[2, 3, 4], Felipe Prosper[2, 3, 4, 5]

*[1]Tecnun, Universidad de Navarra, San Sebastián, Spain;[2]Hemato-Oncology Program, Center for Applied Medical Research (CIMA), Universidad de Navarra, IDISNA, Pamplona, Spain; [3]Cancer Centre Clínica Universidad de Navarra (CCUN), Pamplona, Spain; [4]Centro de Investigación Biomédica en Red Cáncer (CIBERONC), Madrid, Spain;[5]Departamento de Hematología y Terapia Celular, Clínica Universidad de Navarra, Universidad de Navarra, IdISNA, Pamplona, Spain; [6]Institute for Molecular Medicine Finland - FIMM, HiLIFE - Helsinki Institute of Life Science, iCAN Digital Cancer Medicine Flagship, University of Helsinki, Helsinki, Finland; [7]Drug Discovery Unit, Instituto de Investigación Sanitaria La Fe, Valencia, Spain;*

### Background

Aberrant metabolism is a hallmark of cancer and reveals vulnerabilities across human-tumors. The recently available large-scale transcriptomic data sets in multiple myeloma (MM) allow a different approach to study metabolism-related genes by analyzing their expression.

### Aims

The aim of this work is to analyze the transcriptomic alterations of metabolism-related genes (metabolic-transcriptome) in MM so as to identify new metabolic vulnerabilities and biomarkers.

### Methods

We analyzed the transcriptome of 619 MM patients (MMRF-CoMMpass cohort) and focus on the expression data of 2753 metabolic genes (acquired from Possemato et al., 2011). Non-negative matrix factorization was performed to define metabolic profiles.

**Copyright Information:** (Online) ISSN: 2572-9241
©2026 The Author(s). HemaSphere is published by John Wiley & Sons Ltd on behalf of European Hematology Association. This is an open access Abstract Book distributed under the Attribution-NonCommercial-NoDerivs (CC BY-NC-ND), which allows third parties to download the articles and share them with others as long as they credit the author and the Abstract Book, but they cannot change the content in any way or use them commercially.
**Abstract Book Citations:** Authors, Title, HemaSphere, 2026;10:(S1):pages. The abstract book DOIs can be found on the "Author Information" page.
**Disclaimer:** Articles published in the journal HemaSphere exclusively reflect the opinions of the authors. The authors are responsible for all content in their abstracts including accuracy of the facts, statements, citing resources, etc.

**Results**

Principal component analysis revealed that the metabolic transcriptome of plasma cells from MM clustered separate from normal B cell subpopulations (Naive, centroblasts, centrocytes, memory, tonsillar plasma cells and bone marrow plasma cells), also indicating a great inter-patient metabolic transcriptome heterogeneity. The transcriptomic alterations of metabolism-related genes in MM, classifies patients into six metabolic-transcriptional groups with specific metabolic pathways and cytogenetic alterations. MM1 was associated with MM patients harboring t(11;14), t(12;14) or t(6;14), involving genes from the cyclin D family (*CCND1*, *CCND2*, *CCND3*); MM2 group correlated with MM patients with t(4;14); MM3 group was linked to t(14;16), t(14;20) or t(8;14), which encompass genes of the family of transcription factors *MAF* (*MAF*, *MAFA*, *MAFB*); MM4 was associated with hyperdiploidy, amp(1q) and t(8;14), involving the *MYC* gene; and finally, the MM5 and MM6 groups primarily included MM patients with the most relevant characteristic being uniquely the hyperdiploidy. Furthermore, the six MM transcriptional metabolic groups were validated in two independent MM patient cohorts. Interestingly, hyperdiploid patients clustered into 2 groups (MM5 and MM6) with significantly distinct metabolic-transcriptome and MM5 group showing worse Progression Free and Overall Survival. MM5 group, exhibit a significant enrichment in myeloid and dormant cell gene signatures, which might explain the adverse prognosis of these patients. Regulon analysis identified myeloid TFs as regulators of metabolic, myeloid and dormant genes overexpressed in MM5. We demonstrated that metabolic gene *ACSL1* plays a crucial role in MM5 group. *ACSL1* inhibition, decreased cell proliferation by affecting fatty acyl-CoA biosynthesis, cell cycle-related pathways and mitochondrial respiration, sensitizing MM to BCL-2 inhibitor Venetoclax and MCL1 inhibitor S36845, offering a promising therapeutic strategy for targeting high-risk MM5 patients. Finally, we developed a linear model using a binomial family for classification in which the expression of S100A8 gene allows the identification of MM5 patients among MM hyperdiploid patients.

**Summary/Conclusion**

Our study provides a comprehensive metabolic classification of MM and identifies a clinically relevant MM5 subgroup characterized by myeloid and dormant features, metabolic dependency on *ACSL1*, and poor prognosis. These findings offer novel biomarkers and therapeutic opportunities and highlight the importance of integrating metabolic profiling into the clinical framework of MM to enable precision medicine approaches.

**Copyright Information:** (Online) ISSN: 2572-9241
©2026 The Author(s). HemaSphere is published by John Wiley & Sons Ltd on behalf of European Hematology Association. This is an open access Abstract Book distributed under the Attribution-NonCommercial-NoDerivs (CC BY-NC-ND), which allows third parties to download the articles and share them with others as long as they credit the author and the Abstract Book, but they cannot change the content in any way or use them commercially.
**Abstract Book Citations:** Authors, Title, HemaSphere, 2026;10:(S1):pages. The abstract book DOIs can be found on the “Author Information” page.
**Disclaimer:** Articles published in the journal HemaSphere exclusively reflect the opinions of the authors. The authors are responsible for all content in their abstracts including accuracy of the facts, statements, citing resources, etc.

### (PF731) CONCURRENT RAS/RAF MUTATIONS DEFINE AN ULTRA-HIGH-RISK SUBGROUP IN MULTIPLE MYELOMA WITH GAIN OF CHROMOSOME 1Q

**Topic:** 13. Myeloma and other monoclonal gammopathies - Biology & translational research

Xueshi Ye*[1], Jin Zhang[1], Huaping DU[1], Ye Zhang[1], Fengping Zhou[1], Feiteng Huang[1], Shenhe Jin[1], Linlin Shi[1], Xiaochang Chen[1], Cheng Zhang[1]

[1]*Sir Run Run Shaw Hospital School of Medicine, Zhejiang University, Department of Hematology, Hangzhou, China;*

### Background

Gain of chromosome 1q (+1q) is a frequent cytogenetic abnormality in newly diagnosed multiple myeloma (NDMM), occurring in 35–53% of patients. Although associated with adverse outcomes, the prognostic impact of +1q remains inconsistent due to substantial clinical and molecular heterogeneity. Critical molecular determinants stratifying risk within this population remain poorly defined.

### Aims

To identify genetic determinants of prognosis within the +1q subgroup through comprehensive genomic profiling, specifically to determine whether RAS/RAF pathway mutations contribute to heterogeneous outcomes, and to explore potential therapeutic strategies for this high-risk subset.

### Methods

We enrolled 72 consecutive NDMM patients between Nov 2022 and Jun 2025. CD138-enriched plasma cells were analyzed by targeted NGS (254-gene panel, depth >1000×), and cytogenetics were evaluated by FISH (+1q defined as ≥3 copies at 1q21). Progression-free survival (PFS) was estimated by the Kaplan-Meier method, with risk factors assessed using Cox proportional hazards models.

### Results

Among 72 patients (median age 70.8 years), 42 (58.3%) harbored +1q. Targeted NGS identified mutations in 196 genes, with RAS/RAF pathway mutations (NRAS/KRAS/BRAF) being the most prevalent (47.2%). Survival analysis included 67 treated patients; of the 40 with +1q, 15 (37.5%) carried concurrent RAS/RAF mutations.
Isolated +1q status did not significantly impact PFS compared to normal 1q (median: not reached vs. 12.34 months; HR=0.93, 95% CI: 0.41–2.11, P=0.87; Figure 1A). In contrast, RAS/RAF mutations were significantly associated with shorter PFS in the entire cohort (median: 8.0 months vs. not reached; HR=2.25, 95% CI: 1.00–5.06, P=0.045; Figure 1B).
Critically, among +1q patients, those with concurrent RAS/RAF mutations (n=15, 37.5%) constituted a distinct ultra-high-risk subset. These patients exhibited markedly inferior PFS compared to +1q patients with wild-type RAS/RAF (median: 6.57 months vs. not reached; HR=3.78, 95% CI: 1.28–11.14, P=0.01; Figure 1C), representing nearly a fourfold increase in progression risk.
In an exploratory analysis of 28 RAS/RAF-mutated patients, those treated with CD38 antibody-based induction (n=9) showed a favorable trend toward prolonged PFS compared to standard regimens (n=19) (median: not reached vs. 12.4 months; HR=0.57, 95% CI: 0.20–1.68, P=0.31; Figure 1D). Although not statistically significant, the separation of survival curves suggests potential clinical benefit.
Additionally, NUP214 mutations were significantly enriched in the +1q cohort (14.3% vs. 0%, P=0.037) and strongly associated with extramedullary disease (66.7% vs. 18.2%, P=0.02), providing a potential link to the invasive phenotype.

**Copyright Information:** (Online) ISSN: 2572-9241
©2026 The Author(s). HemaSphere is published by John Wiley & Sons Ltd on behalf of European Hematology Association. This is an open access Abstract Book distributed under the Attribution-NonCommercial-NoDerivs (CC BY-NC-ND), which allows third parties to download the articles and share them with others as long as they credit the author and the Abstract Book, but they cannot change the content in any way or use them commercially.
**Abstract Book Citations:** Authors, Title, HemaSphere, 2026;10:(S1):pages. The abstract book DOIs can be found on the “Author Information” page.
**Disclaimer:** Articles published in the journal HemaSphere exclusively reflect the opinions of the authors. The authors are responsible for all content in their abstracts including accuracy of the facts, statements, citing resources, etc.

### Summary/Conclusion

RAS/RAF pathway mutations define a molecularly distinct, ultra-high-risk subgroup within +1q MM, with significantly inferior PFS. Integration of cytogenetic profiling with NGS-based mutational analysis enables more precise risk stratification in +1q patients. Importantly, our data suggest that CD38 antibody-based therapies may partially mitigate the adverse impact of RAS/RAF mutations, providing a rationale for prioritizing these regimens or exploring MEK inhibitors for this specific high-risk population in future clinical trials.

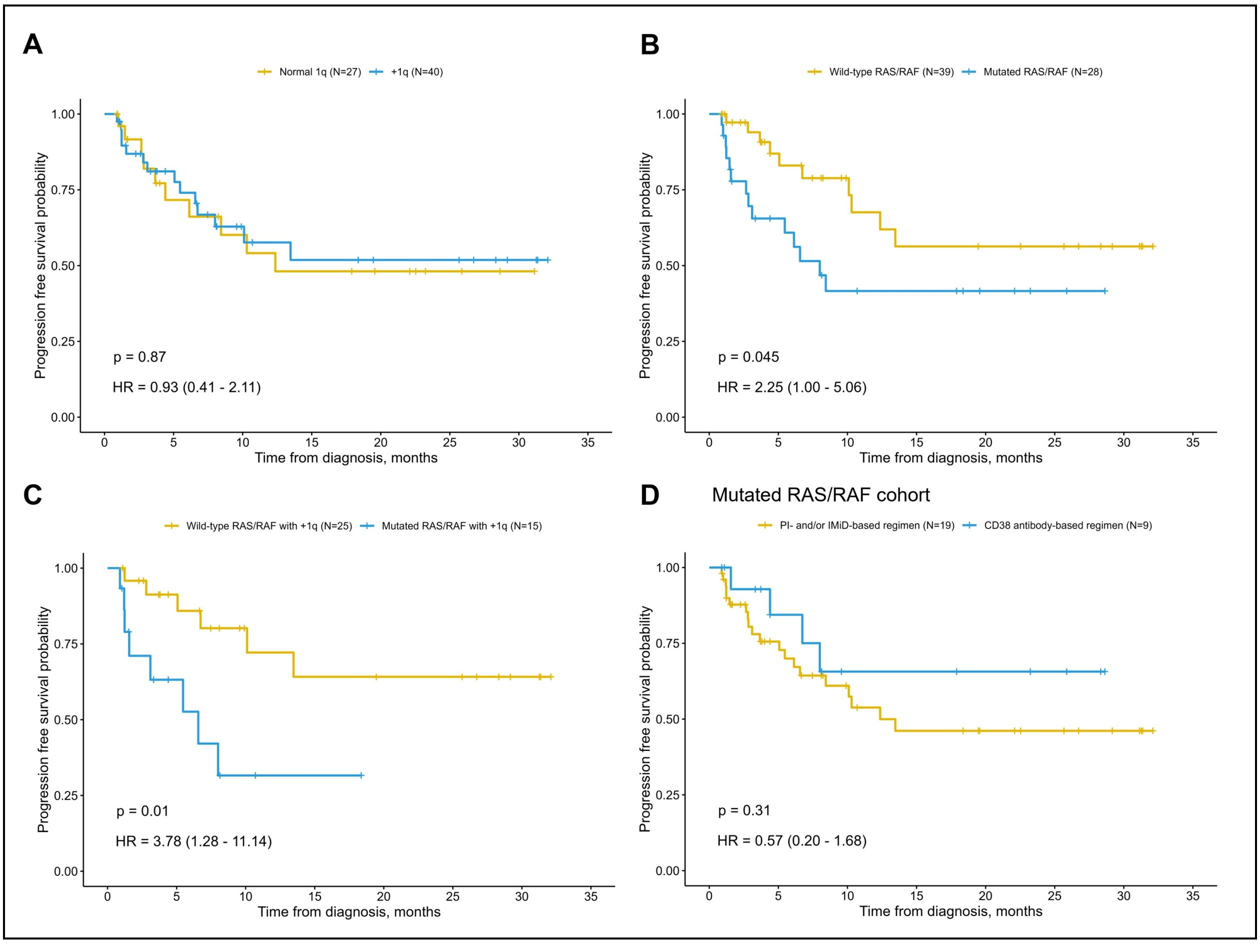

**Copyright Information:** (Online) ISSN: 2572-9241
©2026 The Author(s). HemaSphere is published by John Wiley & Sons Ltd on behalf of European Hematology Association. This is an open access Abstract Book distributed under the Attribution-NonCommercial-NoDerivs (CC BY-NC-ND), which allows third parties to download the articles and share them with others as long as they credit the author and the Abstract Book, but they cannot change the content in any way or use them commercially.
**Abstract Book Citations:** Authors, Title, HemaSphere, 2026;10:(S1):pages. The abstract book DOIs can be found on the "Author Information" page.
**Disclaimer:** Articles published in the journal HemaSphere exclusively reflect the opinions of the authors. The authors are responsible for all content in their abstracts including accuracy of the facts, statements, citing resources, etc.

### (PF732) ONCOGENIC SPLICING OF THE P53-RELATED TUMORSUPPRESSOR ASPP2 PROMOTES DRUG RESISTANCE AND TUMOR PROGRESSION IN MULTIPLE MYELOMA

**Topic:** 13. Myeloma and other monoclonal gammopathies - Biology & translational research

Kasra Jahankhani[1], Marcus Schittenhelm[1], Reka Haraszti[2], Christoph Driessen[3], Kerstin Kampa-Schittenhelm*[1, 2]

*[1]HOCH Health Ostschweiz, St. Gallen, Switzerland;[2]University hospital Tübingen, Tübingen, Germany;[3]Zurich University, Faculty of Medicine, Zuerich, Switzerland;*

### Background

Treatment options for multiple myeloma (MM) have improved significantly in recent years. Nevertheless, therapy resistance occurs in a proportion of patients, presenting a key mortality factor in advanced tumors.
Alterations in the p53 pathway are a hallmark of therapy-refractory MM but the underlying mechanisms that lead to inhibition of *TP53* tumorsuppressor function are only partly understood. We here show that deregulation of members of the ASPP protein family (Apoptosis Stimulating Proteins of TP53) are involved in this process. ASPP2 mediates p53-dependent induction of apoptosis - but also directly interacts with NFkB and BCL-2, putting ASPP2 into a central position to orchestrate major pathways regulating cellular fate.
Importantly, we have identified a novel, oncogenic splicing isoform of *ASPP2*, named *ASPP2κ*, which is characterized by loss of the important C-terminal binding domain for p53. As a result, ASPP2k interferes with central tumor-regulating signaling pathways and promotes all classical hallmarks of cancer.

### Aims

To study the role of oncogenic ASPP2κ in drug resistance and tumor progression in MM.

### Methods standard PCR, transduction, proliferation and apoptosis assays.
Generation of a NSG xenotransplant MM model.

**Copyright Information:** (Online) ISSN: 2572-9241
©2026 The Author(s). HemaSphere is published by John Wiley & Sons Ltd on behalf of European Hematology Association. This is an open access Abstract Book distributed under the Attribution-NonCommercial-NoDerivs (CC BY-NC-ND), which allows third parties to download the articles and share them with others as long as they credit the author and the Abstract Book, but they cannot change the content in any way or use them commercially.
**Abstract Book Citations:** Authors, Title, HemaSphere, 2026;10:(S1):pages. The abstract book DOIs can be found on the "Author Information" page.
**Disclaimer:** Articles published in the journal HemaSphere exclusively reflect the opinions of the authors. The authors are responsible for all content in their abstracts including accuracy of the facts, statements, citing resources, etc.

### Results

A random cohort of newly diagnosed MM patients (n=35) with follow-up data and a healthy bone marrow donor cohort (n=32) were studied to correlate ASSP2κ isoform expression levels with patient outcome data. Notably, the ASPP2 WT isoform is frequently suppressed in CD38+ MM cells, while oncogenic ASPP2κ was found to be overly expressed and functionally active: Isogenic MM isoform-specific shRNA-based cell models (JJN-3, AMO-1 or RPMI-8226 cell lines), stably suppressing ASPP2κ (KD) were established demonstrating significant attenuation of proliferation rates (doubling times decreased by 30% to 40% in all tested models, measured by IncuCyte live cell imager) when compared to EV cell strains. In line, ASPP2κ KD sensitized induction of apoptosis upon treatment with chemotherapy (melphalan +30% at IC50) in all models. Interestingly, treatment with bortezomib led to upregulation of ASPP2κ supporting the notion that ASPP2κ is a target for proteasomal degradation – and inhibition may fuel a resistance mechanism.
Accordingly, ASPP2κ_KD cell strains were more responsive to proteasome inhibition (bortezomib or carfilzomib, average +25% at IC50). Vice versa, overexpression of ASPP2κ rendered cells even more resistant to (chemo)therapy and significantly increased proliferation rates in all models.
An ASPP2κ_KD MM Nod/Scid xenotransplant model confirmes attenuated tumor engraftment and progression as well as longer survival of mice bearing ASPP2κ-KD cell strains compared to respective EV controls.
Consequently, *ASPP2κ*-interference may be an attractive novel therapeutic approach: We established medicinal siRNAs modified for in vivo use – and identified 2 lead siRNAs that significantly inhibit expression of ASPP2κ (30-80%) along with proliferation rates (> 50%) and sensitize MM cells towards (chemo)therapy (+ av. 25%).

### Summary/Conclusion

In summary, we demonstrate that ASPP2κ is overexpressed and functionally active in MM. Expression of oncogenic ASPP2κ thereby directly contributes to tumor biology and associates with a more aggressive phenotype as well as drug resistance. Importantly, ASPP2κ-interference sensitizes MM cells for cytotoxic treatment and proteasome inhibition - thus proofs to be a potential novel therapeutic target, which warrants further exploration.

**Copyright Information:** (Online) ISSN: 2572-9241
©2026 The Author(s). HemaSphere is published by John Wiley & Sons Ltd on behalf of European Hematology Association. This is an open access Abstract Book distributed under the Attribution-NonCommercial-NoDerivs (CC BY-NC-ND), which allows third parties to download the articles and share them with others as long as they credit the author and the Abstract Book, but they cannot change the content in any way or use them commercially.
**Abstract Book Citations:** Authors, Title, HemaSphere, 2026;10:(S1):pages. The abstract book DOIs can be found on the “Author Information” page.
**Disclaimer:** Articles published in the journal HemaSphere exclusively reflect the opinions of the authors. The authors are responsible for all content in their abstracts including accuracy of the facts, statements, citing resources, etc.

### (PF733) A NOVEL CDK9 INHIBITOR, GT-02897, SELECTIVELY INDUCES CYTOTOXIC EFFECTS AND EXHIBITS PRECLINICAL SYNERGISTIC ACTIVITY IN MYELOMA

**Topic:** 13. Myeloma and other monoclonal gammopathies - Biology & translational research

Osman Aksoy*[1]

[1]*Karl Landsteiner University, Department of Molecular Oncology and Hematology,, Krems an der donau, Austria;*

### Background

Despite transformative therapeutic advances with immunomodulatory agents, proteasome inhibitors, monoclonal antibodies, CAR-T, and bispecific antibodies, multiple myeloma (MM) remains incurable, underscoring the need for novel targeted approaches. Dysregulated transcriptional control is a hallmark of MM biology. Cyclin-dependent kinase 9 (CDK9), a key component of the P-TEFb complex, drives RNA polymerase II–mediated transcriptional elongation of short-lived oncogenic survival genes, including *MYC*, *MCL-1*, and *MDM2*, and therefore represents a compelling therapeutic target.

### Aims

To evaluate the therapeutic potential of GT-02897, a novel selective CDK9 degrader, and to define biologically rational combination strategies targeting transcriptional addiction in MM.

### Methods

GT-02897 was systematically evaluated in MM cell lines, patient-derived $CD138^+$ primary cells, bone marrow stromal co-cultures, a dynamic ex vivo 3D system, and a murine in vivo model. Anti-tumor activity was quantified by viability and apoptosis assays. Mechanistic profiling included immunoblot analyses and unbiased quantitative mass spectrometry–based proteomics. In addition, a high-throughput drug screen was performed to identify and validate synergistic combinations with approved and investigational anti-MM agents.

### Results

CDK9 overexpression correlated with inferior clinical outcome, underscoring its pathogenic relevance. GT-02897 demonstrated potent anti-MM activity with $IC_{50}$ values as low as 20–100 nM across MM cell lines and primary samples, with preserved activity under microenvironment-mediated protective conditions. In vivo CDK9 degradation resulted in significant tumor growth suppression. Mechanistically, GT-02897 triggered rapid and sustained collapse of oncogenic transcriptional programs, marked by depletion of MCL-1, MYC, MDM2, and additional survival regulators identified by proteomic profiling. Of note, rational combination strategies demonstrated strong synergy with BCL-2 inhibitors (venetoclax, sonrotoclax), dexamethasone and the MEK inhibitor trametinib.

### Summary/Conclusion

Selective CDK9 degradation dismantles core transcriptional dependencies in MM and represents a mechanistically driven therapeutic strategy. GT-02897 exhibits robust single-agent and combinatorial efficacy, providing compelling evidence for the clinical evaluation of this first-in-class CDK9 degrader in relapsed and high-risk MM.

**Copyright Information:** (Online) ISSN: 2572-9241
©2026 The Author(s). HemaSphere is published by John Wiley & Sons Ltd on behalf of European Hematology Association. This is an open access Abstract Book distributed under the Attribution-NonCommercial-NoDerivs (CC BY-NC-ND), which allows third parties to download the articles and share them with others as long as they credit the author and the Abstract Book, but they cannot change the content in any way or use them commercially.
**Abstract Book Citations:** Authors, Title, HemaSphere, 2026;10:(S1):pages. The abstract book DOIs can be found on the "Author Information" page.
**Disclaimer:** Articles published in the journal HemaSphere exclusively reflect the opinions of the authors. The authors are responsible for all content in their abstracts including accuracy of the facts, statements, citing resources, etc.

### (PF734) SINGLE CELL RNA SEQUENCING REVEALS IMMUNE MICROENVIRONMENT REMODELING IN PRIMARY PLASMA CELL LEUKEMIA: REAL-WORLD EVIDENCE FROM THE KMMWP-2204 STUDY

**Topic:** 13. Myeloma and other monoclonal gammopathies - Biology & translational research

Ho Cheol Jang[1], Mihee Kim[1], Hee Jeong Cho[2], Dae Sik Kim[3], Jongheon Jung[4], Ji Hyun Lee[5], Kihyun Kim[6], Ja Min Byun[7], Dok Hyun Yoon[8], Yoon Seok Choi[9], Jae-Cheol Jo[10], Ho-Young Yhim[11], Myung-Won Lee[12], Sungnam Lim[13], Jae Hoon Lee[14], Sung-Soo Park[15], Sung-Hoon Jung*[1], Chang-Ki Min[16], Je-Jung Lee[1]

*[1]Chonnam National University Hwasun Hospital, Hwasun, South Korea;[2]Kyungpook National University Hospital, Daegu, South Korea; [3]Korea University Guro Hospital, Seoul, South Korea;[4]National Cancer Center, Goyang, South Korea;[5]Dong-A University College of Medicine, Busan, South Korea; [6]Samsung Medical Center, Seoul, South Korea;[7]Seoul National University Hospital, Seoul, South Korea; [8]Asan Medical Center, University of Ulsan College of Medicine, Seoul, South Korea;[9]Korea University College Of Medicine, Seoul, South Korea; [10]Ulsan University Hospital, University of Ulsan College of Medicine, Ulsan, South Korea; [11]Jeonbuk National University Hospital, Jeon ju, South Korea;[12]Chungnam National University Hospital, Daejeon, South Korea; [13]Haeundae Paik Hospital, Busan, South Korea; [14]Gachon University Gil Medical Center, Incheon, South Korea;[15]Catholic Hematology Hospital, Seoul St. Mary's Hospital, Catholic University, Seoul, South Korea; [16]Seoul St. Mary's Hospital, Seoul, South Korea;*

### Background

Multiple myeloma (MM) is a plasma cell disorder that remains medically incurable. Primary plasma cell leukemia (pPCL), defined by ≥5% circulating plasma cells (CPCs), has a markedly worse prognosis, yet the biology and bone marrow (BM) immune microenvironment remain incompletely understood.

### Aims

We conducted integrated clinical and translational analyses using the KMMWP-2204 real-world multicenter study. We aimed to characterize clinical features, outcomes, and prognostic factors in primary plasma cell leukemia, and to interpret these findings through single cell RNA sequencing of the bone marrow immune microenvironment in primary plasma cell leukemia and multiple myeloma.

### Methods

We retrospectively analyzed 127 newly diagnosed pPCL patients from 20 Korean centers (2005–2022) for overall survival (OS), progression-free survival (PFS), and 6-month early mortality using multivariable models, including time-dependent Cox analysis for autologous stem cell transplantation (ASCT). scRNA-seq was performed on BM mononuclear cells from pPCL (n=4), MM (n=2), and healthy donors (n=3), comprising 109,107 cells. Immune subsets were annotated by canonical markers, functional programs were quantified by module scoring and gene set enrichment, receptor linked communication was inferred using CellChat, and activated T cell trajectories were assessed by Monocle.

**Copyright Information:** (Online) ISSN: 2572-9241
©2026 The Author(s). HemaSphere is published by John Wiley & Sons Ltd on behalf of European Hematology Association. This is an open access Abstract Book distributed under the Attribution-NonCommercial-NoDerivs (CC BY-NC-ND), which allows third parties to download the articles and share them with others as long as they credit the author and the Abstract Book, but they cannot change the content in any way or use them commercially.
**Abstract Book Citations:** Authors, Title, HemaSphere, 2026;10:(S1):pages. The abstract book DOIs can be found on the "Author Information" page.
**Disclaimer:** Articles published in the journal HemaSphere exclusively reflect the opinions of the authors. The authors are responsible for all content in their abstracts including accuracy of the facts, statements, citing resources, etc.

**Results**

Patients with 5–19% and ≥20% CPCs showed comparable OS and PFS, supporting the IMWG 2021 ≥5% threshold. Six-month early mortality was 19.7% and was independently associated with elevated lactate dehydrogenase (OR 3.68, P=0.032) and del(17p) (OR 3.49, P=0.050). Median OS and PFS were 27.0 and 13.6 months, respectively. Achieving complete remission after induction was the strongest determinant of OS (HR 0.25, P=0.011), while ASCT appeared consolidative in time-dependent models. scRNA-seq resolved major BM immune compartments. Neutrophils were less frequent in MM and pPCL than in controls, and inflammatory myeloid programs were enriched with greater interferon and myeloid activation in pPCL. M1 macrophages were more frequent in MM and pPCL than in controls. In T cells, $CCR7^{+}$ naive populations contracted in disease and naive maintenance programs, including TCF7 and SELL, were reduced across $CD4^{+}$ and $CD8^{+}$ lineages. Cytotoxicity scores were lower in MM and pPCL, including within activated $CD4^{+}$ and activated $CD8^{+}$ subsets. CellChat analysis demonstrated remodeling of receptor linked signaling between myeloid and lymphoid compartments. Higher myeloid activity correlated with lower cytotoxicity in activated T cells. Monocle pseudotime analysis of activated T cells aligned to controls showed a shared trajectory ordered from controls to MM to pPCL, with GZMA and NKG7 peaking at intermediate positions and declining, while TOX increased toward later positions.

**Summary/Conclusion**

In a large Korean pPCL cohort, early mortality remains substantial and is driven by LDH and del(17p), while deep response is the key survival determinant. Single cell profiling indicates inflammatory myeloid activation accompanied by progressive T cell dysfunction and exhaustion, providing a biological framework for risk stratification and prospective validation of immune-based biomarkers in PCL.

**Copyright Information:** (Online) ISSN: 2572-9241
©2026 The Author(s). HemaSphere is published by John Wiley & Sons Ltd on behalf of European Hematology Association. This is an open access Abstract Book distributed under the Attribution-NonCommercial-NoDerivs (CC BY-NC-ND), which allows third parties to download the articles and share them with others as long as they credit the author and the Abstract Book, but they cannot change the content in any way or use them commercially.
**Abstract Book Citations:** Authors, Title, HemaSphere, 2026;10:(S1):pages. The abstract book DOIs can be found on the “Author Information” page.
**Disclaimer:** Articles published in the journal HemaSphere exclusively reflect the opinions of the authors. The authors are responsible for all content in their abstracts including accuracy of the facts, statements, citing resources, etc.

### (PF735) NUCLEAR DONUT-SHAPED DEFORMATION MARKS MULTIPLE MYELOMA CELL DORMANCY

**Topic:** 13. Myeloma and other monoclonal gammopathies - Biology & translational research

Mengge Pan*[1], Peipei Xu[1]

[1]*Department of Hematology, Nanjing Drum Tower Hospital, Affiliated Hospital of Medical School, Nanjing University, Nanjing, China;*

### Background

Multiple myeloma (MM) remains incurable because most patients eventually relapse. The awakening of dormant myeloma cells is the key to MM relapse, but the regulation mechanisms still remains unclear. We previously found that the knockout of proteolipid protein 2 (PLP2) in myeloma cells induced donut-shaped nuclei and upregulated the dormancy marker AXL (AXL receptor tyrosine kinase). The donut-shaped nuclei are rarely observed in the bone marrow of MM patients. Moreover, PLP2 extracellular vesicles (EVs), abundant in MM bone marrow microenvironment of MM patients, were found to reverse donut-shaped nuclei to normal morphology and downregulated the AXL expression in vitro. Although myeloma cells display abnormal nuclear morphology, whether nuclear deformation directly affects myeloma cell dormancy remains unknown.

### Aims

This study aims to investigate the regulatory mechanisms underlying the dormancy of myeloma cells by examining the deformation of donut-shaped nuclei, thereby developing new strategies to prevent and treat MM relapse.

### Methods

Donut-shaped nuclei deformations were first identified in clinical samples from MM patients. Using optical diffraction tomography (ODT) live cell imaging, we characterized the formation dynamics of donut-shaped nuclei during mitosis following PLP2 knockout. Transcriptome analysis by RNA-seq identified key molecules associated with this phenotype. The functional role of PLP2 in cellular dormancy was further assessed using 3D culture, CAM assays, and an in vivo xenograft mouse MM model. Finally, analysis of a clinical cohort correlated the presence of donut-shaped nuclei with MM progression and prognosis.

### Results

MM cells lacking PLP2 exhibit stable nuclear donut-shaped deformation, which can also be specifically detected in bone marrow biopsies of MM patients. Mechanistically, PLP2 maintains membrane homeostasis and supports normal nuclear envelope remodeling during mitosis, thereby preventing the nuclear donut shaping in MM cells. MM cells with donut-shaped nuclei are susceptible to entering dormancy due to cell-cell competition for membranes. Intrinsic PLP2 prevents and breaks MM cell dormancy. Notably, the nuclear donut-shaped deformation is associated with a reduced risk of MM relapse in patients, establishing it as a morphological marker for dormant myeloma cells.

### Summary/Conclusion

MM cells with donut-shaped nuclei exhibited a dormancy gene signature and positively correlated with the progression-free survival of MM patients, offering a novel perspective for myeloma treatment. This study provides novel insights into the role of nuclear architecture in MM cell function, particularly in relation to dormancy and relapse. By integrating cellular and microenvironmental factors, we propose a morphological framework that informs the development of improved biomarkers and therapeutic strategies for MM.

**Copyright Information:** (Online) ISSN: 2572-9241
©2026 The Author(s). HemaSphere is published by John Wiley & Sons Ltd on behalf of European Hematology Association. This is an open access Abstract Book distributed under the Attribution-NonCommercial-NoDerivs (CC BY-NC-ND), which allows third parties to download the articles and share them with others as long as they credit the author and the Abstract Book, but they cannot change the content in any way or use them commercially.
**Abstract Book Citations:** Authors, Title, HemaSphere, 2026;10:(S1):pages. The abstract book DOIs can be found on the "Author Information" page.
**Disclaimer:** Articles published in the journal HemaSphere exclusively reflect the opinions of the authors. The authors are responsible for all content in their abstracts including accuracy of the facts, statements, citing resources, etc.

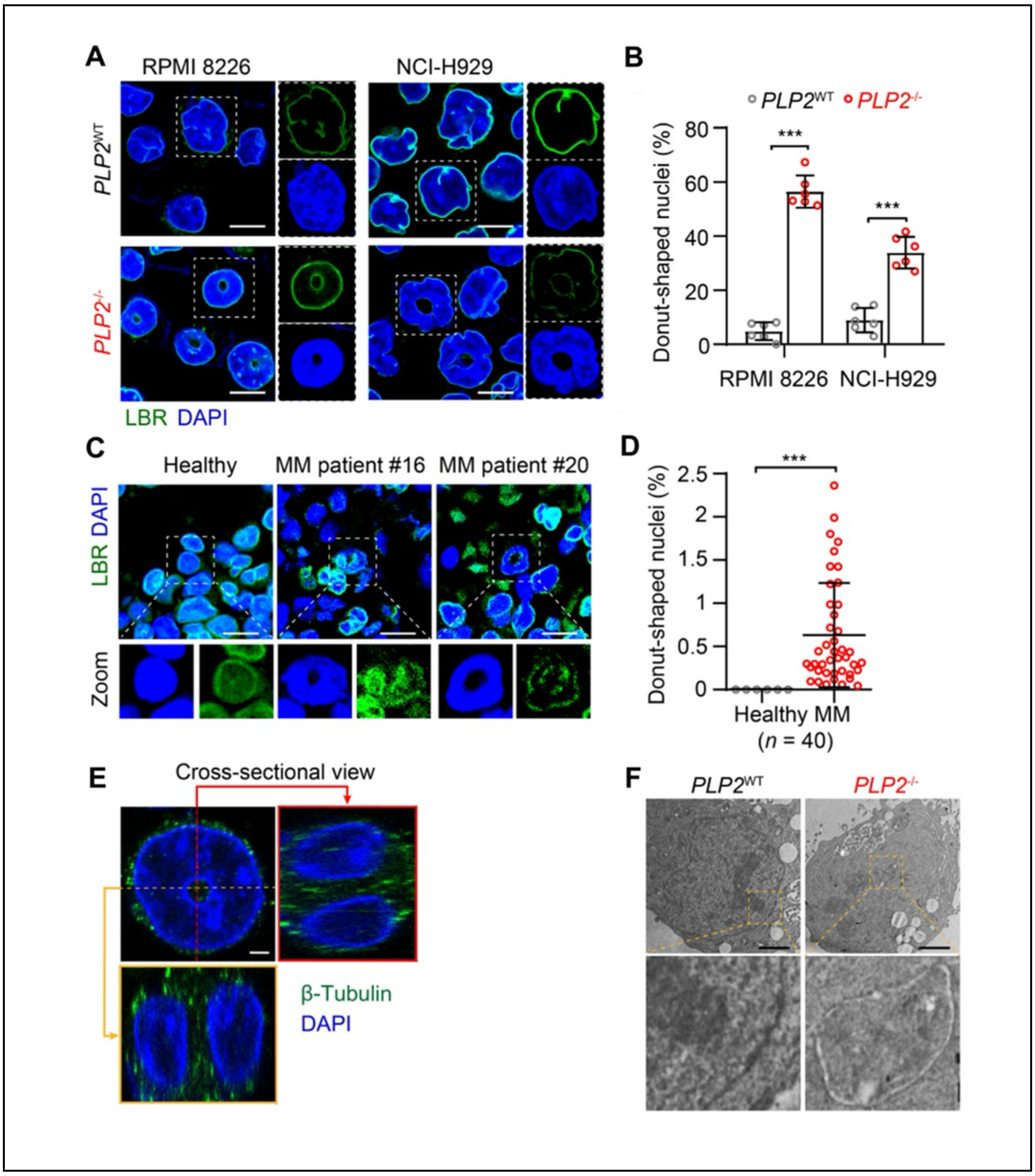

Figure1. Loss of PLP2 induces nuclear donut-shaped deformation in MM cells. (A-B) Immunofluorescence images of PLP2WT or PLP2-/- RPMI 8226 and NCI-H929 cells stained with anti-LBR (green) and DAPI (blue) and the percentage of donut-shaped nuclei. Scale bars, 10 µm. ***P< 0.001. (C-D) Immunofluorescence images of bone marrow sections from MM patients and healthy donors stained with anti-LBR (green) and DAPI (blue) and the percentage of donut-shaped nuclei. Scale bars, 10 µm. ***P< 0.001. (E) 3D imaging of donut-shaped nuclei of RPMI 8226 cells stained with anti-β-Tubulin (green) and DAPI (blue). Scale bar, 2.5 µm. (F) Transmission electron microscope images (scale bars, 2 µm) of PLP2WT and PLP2-/- RPMI 8226 cells (Top). Zoom in nuclear envelope of spherical nucleus and inner hole of donut-shaped nucleus nuclear envelope (Bottom).

**Copyright Information:** (Online) ISSN: 2572-9241
©2026 The Author(s). HemaSphere is published by John Wiley & Sons Ltd on behalf of European Hematology Association. This is an open access Abstract Book distributed under the Attribution-NonCommercial-NoDerivs (CC BY-NC-ND), which allows third parties to download the articles and share them with others as long as they credit the author and the Abstract Book, but they cannot change the content in any way or use them commercially.
**Abstract Book Citations:** Authors, Title, HemaSphere, 2026;10:(S1):pages. The abstract book DOIs can be found on the "Author Information" page.
**Disclaimer:** Articles published in the journal HemaSphere exclusively reflect the opinions of the authors. The authors are responsible for all content in their abstracts including accuracy of the facts, statements, citing resources, etc.

### (PF736) SKP2 INHIBITION INDUCES CELL DEATH IN MULTIPLE MYELOMA IRRESPECTIVE OF P53 STATUS AND SYNERGIZES WITH BORTEZOMIB

**Topic:** 13. Myeloma and other monoclonal gammopathies - Biology & translational research

Hua Bai*[1], Peipei Xu[1], Bing Chen[1]

[1]*Nanjing Drum Tower Hospital, Affiliated Hospital of Medical School, Nanjing University, Department of Hematology, Nanjing, China;*

### Background

Multiple myeloma (MM) remains incurable due to drug resistance and relapse. The oncoprotein SKP2, overexpressed in MM, promotes degradation of cell cycle inhibitors p21/p27 and is associated with poor prognosis. SKP2 also negatively regulates p53, making it an attractive therapeutic target, particularly in p53-deficient MM.

### Aims

To evaluate the anti-myeloma efficacy of quinacrine (QC), identify it as a novel SKP2 inhibitor, and assess its synergistic potential with bortezomib (BTZ) in both p53 wild-type and p53-deficient settings.

### Methods

SKP2 expression and prognosis were analyzed in GEO datasets (GSE5900, GSE2658, GSE31161). Molecular docking and Drug Affinity Responsive Target Stability (DARTS) assays assessed QC-SKP2 interaction. Anti-proliferative effects were evaluated in MM cell lines (H929 p53-wt; ARP-1 p53-deficient) and primary patient samples (NDMM, RRMM) using CCK-8, flow cytometry (apoptosis/cell cycle), and Western blot. Synergy with BTZ was quantified by combination index (CI). In vivo efficacy was tested in H929 xenograft mice.

### Results

SKP2 expression increased progressively from normal plasma cells to active MM ($p<0.001$) and predicted poorer overall survival ($p<0.05$). siRNA-mediated SKP2 knockdown significantly reduced MM cell viability. Molecular docking identified QC as the top SKP2 binder among 13 antiparasitic drugs (-6.54 kcal/mol), with key interactions at Phe163 and Pro149. DARTS confirmed direct QC-SKP2 binding. QC suppressed viability in both H929 and ARP-1 cells ($IC_{50}$ ~6 μM), inducing apoptosis (increased Annexin $V^+$, cleaved PARP, Bax/Bcl-2 ratio) and sub-$G_0/G_1$ arrest regardless of p53 status. QC reduced primary $CD138^+$ MM cell viability in both NDMM and BTZ-resistant RRMM samples, including p53-deficient cases. Mechanistically, QC downregulated SKP2, upregulated p21/p27, and modulated FoxO/cell cycle pathways. QC synergized with BTZ ($CI<1$) across concentrations, with enhanced SKP2 suppression. In RRMM samples, BTZ alone paradoxically increased viable $CD138^+$ cells, while QC overcame this resistance. In vivo, QC (8 mg/kg) significantly reduced tumor volume and weight in H929 xenografts ($p<0.05$), with corresponding SKP2 downregulation by IHC.

### Summary/Conclusion

QC is a novel SKP2 inhibitor with potent p53-independent anti-MM activity, inducing apoptosis via SKP2-p21/p27 axis stabilization. QC synergizes with BTZ and overcomes BTZ resistance in RRMM, including p53-deficient cases. These findings support clinical evaluation of QC alone or in combination with BTZ for MM treatment, particularly in high-risk p53-deficient or relapsed/refractory settings.

**Copyright Information:** (Online) ISSN: 2572-9241
©2026 The Author(s). HemaSphere is published by John Wiley & Sons Ltd on behalf of European Hematology Association. This is an open access Abstract Book distributed under the Attribution-NonCommercial-NoDerivs (CC BY-NC-ND), which allows third parties to download the articles and share them with others as long as they credit the author and the Abstract Book, but they cannot change the content in any way or use them commercially.
**Abstract Book Citations:** Authors, Title, HemaSphere, 2026;10:(S1):pages. The abstract book DOIs can be found on the “Author Information” page.
**Disclaimer:** Articles published in the journal HemaSphere exclusively reflect the opinions of the authors. The authors are responsible for all content in their abstracts including accuracy of the facts, statements, citing resources, etc.

### (PF737) CRBN-INDEPENDENT IKZF1 DEGRADATION VIA TRIM21 BY DNA DEMETHYLATING AGENTS AS A NOVEL THERAPEUTIC MECHANISM FOR MULTIPLE MYELOMA

**Topic:** 13. Myeloma and other monoclonal gammopathies - Biology & translational research

Yuta Yamamoto*[1, 2], Hiroshi Ureshino[1, 2], Moe Kawasaki[2, 3], Keisuke Kidoguchi[1, 2], Mai Fujita[1, 2], Ryo Yanagiya[1, 2], Shinya Kimura[1, 2]

*[1]Saga University, Division of Hematology, saga, Japan;[2]Saga University, Department of Drug Discovery and Biomedical Sciences, saga, Japan; [3]OHARA Pharmaceutical, shiga, Japan;*

### Background

Multiple myeloma (MM) is characterized by uncontrolled proliferation of malignant plasma cells in the bone marrow. Although the prognosis of MM patients has improved markedly with the advent of various therapies, almost all patients eventually relapse and become refractory to treatment.  Hence, MM is still considered an incurable disease, and there is a need to establish more effective therapies.
DNA methylation is an important epigenetic modification that regulates gene expression. Aberrant DNA methylation in promoter regions leads to the silencing of gene expression, a phenomenon observed in many cancers. Localized aberrant DNA methylation has been reported to be related for disease progression and drug resistance in MM. Hence, targeting aberrant DNA methylation presents a promising approach for the treatment of MM.

### Aims

This study aimed to examine the efficacy of a new DNA demethylating agent, OR2100 which we developed as an orally bioavailable decitabine prodrug, on a multiple myeloma.

### Methods

The MM cell lines used were IM9, KMS12BM, KMS11, and RPMI8226. Cell proliferation inhibition was measured using Cell Counting Kit-8. Gene expression was evaluated by qPCR and RNA sequencing. DNA methylation status was determined by bisulfite pyrosequencing.

**Copyright Information:** (Online) ISSN: 2572-9241
©2026 The Author(s). HemaSphere is published by John Wiley & Sons Ltd on behalf of European Hematology Association. This is an open access Abstract Book distributed under the Attribution-NonCommercial-NoDerivs (CC BY-NC-ND), which allows third parties to download the articles and share them with others as long as they credit the author and the Abstract Book, but they cannot change the content in any way or use them commercially.
**Abstract Book Citations:** Authors, Title, HemaSphere, 2026;10:(S1):pages. The abstract book DOIs can be found on the “Author Information” page.
**Disclaimer:** Articles published in the journal HemaSphere exclusively reflect the opinions of the authors. The authors are responsible for all content in their abstracts including accuracy of the facts, statements, citing resources, etc.

### Results

Physiological concentrations of OR2100 suppressed the growth and induced apoptosis of the MM cell lines dose dependent manner *in vitro*. OR2100 reduced DNA methylation levels of long interspersed nuclear element-1 (*LINE-1*), a surrogate marker for global DNA methylation status. OR2100 reduced ikaros family zinc finger 1（IKZF1） protein levels, whereas the mRNA levels of both *IKZF1* and cereblon (*CRBN*) remained unchanged. We focused on *TRIM21*, an E3 ubiquitin ligase whose expression was found to be upregulated by RNA-sequencing–based gene expression profiling. Using a lentiviral vector, we overexpressed TRIM21 and confirmed a consequent decrease in IKZF1 protein levels, accompanied by reduced cell proliferation. Furthermore, TRIM21 knockdown or proteasome inhibition (bortezomib) attenuated the OR2100-induced decrease in IKZF1, with TRIM21 knockout reducing IKZF1 ubiquitination, supporting TRIM21-mediated, ubiquitin–proteasome–dependent degradation. Moreover, knockdown of CRBN did not affect the OR2100-induced decrease in IKZF1 or the growth-inhibitory effects, indicating that this mechanism operates independently of CRBN. Finally, the anti-tumor effect of OR2100 was evaluated using a xenograft MM mouse model for in vivo experiments. In the intravenous inoculation model, the vehicle group had a median survival of 75 days, whereas the OR2100 (3 mg/kg) group had a 1.5-fold survival benefit of 113 days and the OR2100 (4.5 mg/kg) group had a nearly 2-fold survival benefit of 145 days. Hematologic toxicity of OR2100 was tolerable over the experimental period, suggesting OR2100 may be a promising therapeutic strategy for MM.

### Summary/Conclusion

We demonstrated that DNA hypomethylation induced by OR2100 upregulates TRIM21 and reduces IKZF1, an essential survival factor for myeloma cells, thereby exerting antitumor effects in a CRBN-independent manner. We are currently conducting a Phase I study of OR2100 for myelodysplastic syndrome (MDS), with the first step being to obtain approval for the treatment of MDS in Japan (jRCT2071220035), and we hope that OR2100 will be approved for the treatment of MM.

**Copyright Information:** (Online) ISSN: 2572-9241
©2026 The Author(s). HemaSphere is published by John Wiley & Sons Ltd on behalf of European Hematology Association. This is an open access Abstract Book distributed under the Attribution-NonCommercial-NoDerivs (CC BY-NC-ND), which allows third parties to download the articles and share them with others as long as they credit the author and the Abstract Book, but they cannot change the content in any way or use them commercially.
**Abstract Book Citations:** Authors, Title, HemaSphere, 2026;10:(S1):pages. The abstract book DOIs can be found on the "Author Information" page.
**Disclaimer:** Articles published in the journal HemaSphere exclusively reflect the opinions of the authors. The authors are responsible for all content in their abstracts including accuracy of the facts, statements, citing resources, etc.

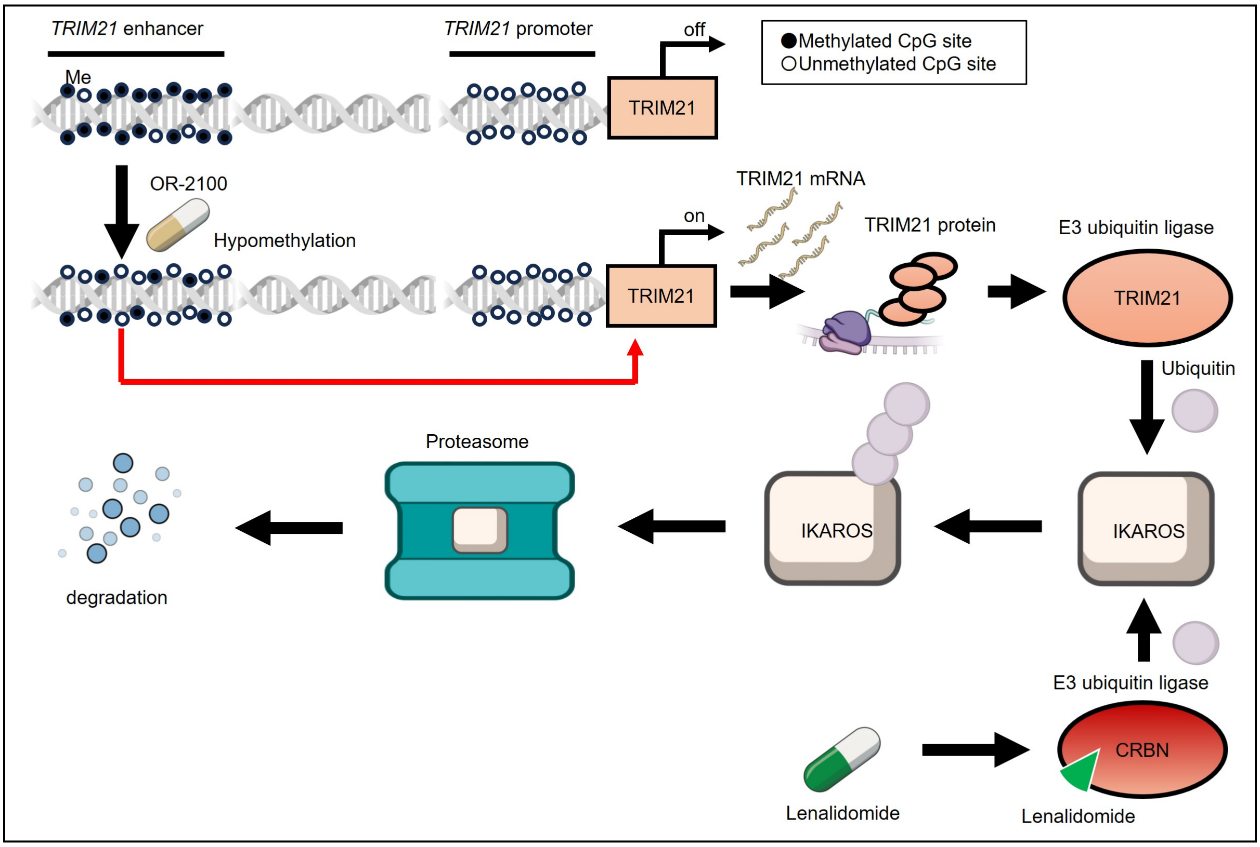

**Copyright Information:** (Online) ISSN: 2572-9241
©2026 The Author(s). HemaSphere is published by John Wiley & Sons Ltd on behalf of European Hematology Association. This is an open access Abstract Book distributed under the Attribution-NonCommercial-NoDerivs (CC BY-NC-ND), which allows third parties to download the articles and share them with others as long as they credit the author and the Abstract Book, but they cannot change the content in any way or use them commercially.
**Abstract Book Citations:** Authors, Title, HemaSphere, 2026;10:(S1):pages. The abstract book DOIs can be found on the "Author Information" page.
**Disclaimer:** Articles published in the journal HemaSphere exclusively reflect the opinions of the authors. The authors are responsible for all content in their abstracts including accuracy of the facts, statements, citing resources, etc.

### (PF738) T CELL MORPHOLOGY PREDICTS OUTCOME IN MULTIPLE MYELOMA USING HIGH-CONTENT IMAGING

**Topic:** 13. Myeloma and other monoclonal gammopathies - Biology & translational research

Frederik Herzberg*[1], Nina Heisterkamp[1], Carin Didoss[1], Alexandra Polici[1], Maximilian Sprechert[1], Jan Krönke[2], Ulrich Keller[1, 3, 4], Stephan Bohl[1]

*[1]Charité - Universitätsmedizin Berlin, Department of Hematology, Oncology and Cancer immunology, Berlin, Germany; [2]University Hospital Greifswald, Clinic for Hematology and Oncology, Greifswald, Germany; [3]German Cancer Consortium (DKTK) Partner Site Berlin, Germany and German Cancer Research Center (DKFZ), Partner Site Berlin, Heidelberg, Germany; [4]Max Delbrück Center for Molecular Medicine, Berlin, Germany;*

### Background

Multiple myeloma (MM) remains an incurable plasma cell malignancy despite major advances in immunotherapy. Growing evidence underscores the role of the immune microenvironment in disease progression and treatment response. While transcriptomic approaches provide important molecular insights, they lack the spatial and functional resolution needed to capture dynamic immune cell behavior. High-content imaging enables scalable, single-cell profiling of immune cell morphology and interactions and may reveal novel functional biomarkers.

### Aims

We investigated whether quantitative single-cell imaging of T cell morphology in primary MM bone marrow mononuclear cells (BMMCs) is associated with clinical characteristics and patient outcome.

### Methods

BMMCs from 34 MM patients were analyzed at treatment initiation using high-content immunofluorescence imaging. Automated single-cell image analysis quantified $CD138^+$ myeloma cells, $CD3^+$ T cells, and T cell morphology. Imaging-derived parameters were correlated with clinical characteristics and progression-free survival (PFS) using Cox regression and non-parametric statistical tests.

### Results

We analyzed BMMCs from 34 patients with MM. 30% of samples were collected at initial diagnosis, while 70% were obtained from patients with relapsed/refractory (r/r) disease. Within the r/r cohort, 80% of patients were triple-class refractory and 50% were penta-class refractory. Using single-cell morphologic profiling of bone marrow–derived T cells, we identified four reproducible T cell morphotypes (TCMs) (Figure 1). Based on characteristic morphologic features, these were annotated as resting, early activated, polarized, and effector. Across the entire cohort, resting (34.6%), early activated (34.1%), and polarized (28.5%) T cells were consistently observed in all samples, whereas effector T cells were infrequent (2.8%). Notably, polarized T cells were significantly enriched in r/r MM compared with newly diagnosed MM ($p = 0.008$). When comparing refractory subgroups, we did not observe significant differences in overall TCM distributions between triple-class and penta-class r/r MM. However, within the triple-class refractory subgroup, a higher combined fraction of polarized and effector T cells was associated with inferior outcomes (HR 2.05, $p = 0.04$). In particular, increased effector T cell fractions were strongly linked to shorter progression-free survival (PFS) (HR 2.77, $p = 0.006$), whereas higher proportions of resting and early activated T cells were associated with improved PFS (HR 0.488, $p = 0.04$). These associations were maintained irrespective of the subsequent treatment regimen. Consistent with this, among patients treated with bispecific T cell engagers (including talquetamab, teclistamab, and elranatamab), enrichment of effector T cells was similarly associated with worse PFS (HR: 2.73, $p = 0.038$), supporting the hypothesis that this TCM may capture an exhausted T cell state and could serve as a clinically relevant biomarker.

**Copyright Information:** (Online) ISSN: 2572-9241
©2026 The Author(s). HemaSphere is published by John Wiley & Sons Ltd on behalf of European Hematology Association. This is an open access Abstract Book distributed under the Attribution-NonCommercial-NoDerivs (CC BY-NC-ND), which allows third parties to download the articles and share them with others as long as they credit the author and the Abstract Book, but they cannot change the content in any way or use them commercially.
**Abstract Book Citations:** Authors, Title, HemaSphere, 2026;10:(S1):pages. The abstract book DOIs can be found on the "Author Information" page.
**Disclaimer:** Articles published in the journal HemaSphere exclusively reflect the opinions of the authors. The authors are responsible for all content in their abstracts including accuracy of the facts, statements, citing resources, etc.

### Summary/Conclusion

High-content imaging identifies four reproducible TCM in primary MM BMMCs. Increased fractions of polarized and effector T cells were associated with adverse clinical outcomes, whereas resting and early activated T cells correlated with improved PFS. These findings establish T cell morphology as a novel functional biomarker of the MM immune microenvironment and suggest its potential value for risk stratification and treatment selection.

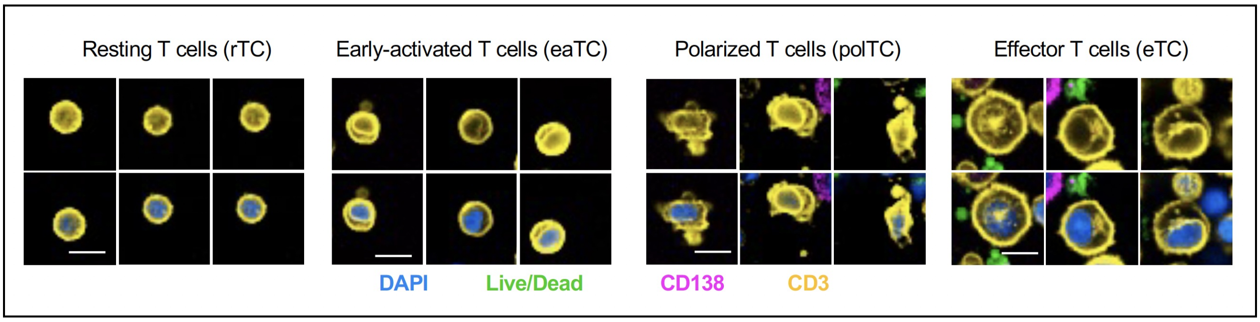

Representative images of the four TCM: rTC, eaTC, polTC, and eTC. CD3⁺ T cells are shown in yellow. Linear contrast settings were applied identically across images. Scale bar, 10 µm.

**Copyright Information:** (Online) ISSN: 2572-9241
©2026 The Author(s). HemaSphere is published by John Wiley & Sons Ltd on behalf of European Hematology Association. This is an open access Abstract Book distributed under the Attribution-NonCommercial-NoDerivs (CC BY-NC-ND), which allows third parties to download the articles and share them with others as long as they credit the author and the Abstract Book, but they cannot change the content in any way or use them commercially.
**Abstract Book Citations:** Authors, Title, HemaSphere, 2026;10:(S1):pages. The abstract book DOIs can be found on the "Author Information" page.
**Disclaimer:** Articles published in the journal HemaSphere exclusively reflect the opinions of the authors. The authors are responsible for all content in their abstracts including accuracy of the facts, statements, citing resources, etc.

### (PF739) GENOMIC LANDSCAPE AND CHARACTERIZATION OF END STAGE MULTIPLE MYELOMA

**Topic:** 13. Myeloma and other monoclonal gammopathies - Biology & translational research

Julia Wiedmeier-Nutor*[1], Karl Franke[2], Surendra Dasari[2], Daniel Bihnam[1], Juana Figueroa Aguirre[1], Megan Weivoda[3], Rafael Fonseca[1], Shaji Kumar[3], Esteban Braggio[4]

*[1]Mayo Clinic Cancer Center, Division Hematology/Oncology, Phoenix, United States of America;[2]Mayo Clinic Cancer Center, Division of Computational Biology, Department of Quantitative Health Sciences, Rochester, United States of America; [3]Mayo Clinic Cancer Center, Department of Internal Medicine, Rochester, United States of America; [4]Mayo Clinic Cancer Center, Department of Cancer Biology, Phoenix, United States of America;*

### Background

To date, genomic and transcriptomic molecular analyses of patients with newly diagnosed multiple myeloma (NDMM) have been well described. An integrative analysis of patients with MM when the disease is refractory, resistant, and/or minimally responsive to myeloma directed therapy, is still fragmented.

### Aims

Here we sought to identify novel molecular drivers in patients with end-stage (ES) MM.

### Methods

ES disease was defined as sample collection within 6 months of death (n=65). Our referent group included untreated newly diagnosed (ND) patient samples defined as collection within 6 months of diagnosis date (n=98). We performed whole exome sequencing (WES), RNAseq, and DNA methylation using the Epic Array on retrospective CD138+ plasma cell samples collected from bone marrow. For each methylation group comparison, all CpGs were filtered with a differential q-value of <=0.05 and an absolute delta methylation difference (b/w the groups) of >=10%.

### Results

There were no significant differences in age, gender, or race between ES and ND. The distribution of cytogenic risk categories (e.g. single-hit, double-hit, triple-hit) differed between the two groups (p=0.03) with a higher prevalence of double-hit and triple-hit in ES. Interestingly, the prevalence of t(11;14) was higher in ES (p=0.01). *TP53* variants and/or del(17p) were highly prevalent in ES (52.3%, n=34), significantly higher than ND (p<0.01). Fourteen ES samples had both del(17p) and *TP53* variants. There was no difference in the prevalence of gain 1q abnormalities between the two groups (p=0.5). GSEA (HALLMARK) of ES compared to ND showed significant enrichment for E2F targets, G2M checkpoint, mitotic spindle, MYC targets, DNA damage repair (DDR), and MTORC pathways. Overwhelmingly, significantly overexpressed genes in ES compared to ND were cancer testes antigens (CTAGs), which have been described in MM and are associated with poor outcomes (Figure 1). Given the striking overexpression of CTAGs in ES and the relationship between CTAG expression and hypomethylation, we evaluated DNA methylation in ES compared to ND. Indeed, we found that CTAG CpG islands were hypomethylated, but we also found significant global DNA hypomethylation in ES compared to ND.  Relevant overexpressed genes in ES compared to NS with transcriptional start site hypomethylation included *CHEK1* and *BIRC5*, both of which are important for MM cell proliferation and/or survival. Next, we used quasi-binomial regression to identify CpG sites associated with shorter time from MM diagnosis to sample collection (within 6 months of death) in ES, identifying 89 CpG sites linked to more rapid disease progression. We also found that ES demonstrated significantly higher epigenetic age scores as measured by the Horvath clock compared to ND samples.

**Copyright Information:** (Online) ISSN: 2572-9241
©2026 The Author(s). HemaSphere is published by John Wiley & Sons Ltd on behalf of European Hematology Association. This is an open access Abstract Book distributed under the Attribution-NonCommercial-NoDerivs (CC BY-NC-ND), which allows third parties to download the articles and share them with others as long as they credit the author and the Abstract Book, but they cannot change the content in any way or use them commercially.
**Abstract Book Citations:** Authors, Title, HemaSphere, 2026;10:(S1):pages. The abstract book DOIs can be found on the "Author Information" page.
**Disclaimer:** Articles published in the journal HemaSphere exclusively reflect the opinions of the authors. The authors are responsible for all content in their abstracts including accuracy of the facts, statements, citing resources, etc.

**Summary/Conclusion**

Our integrated molecular analysis identified key features of ES MM, including *TP53* aberrations in over half of cases. ES samples were enriched for proliferative and DDR pathways and demonstrated high CTAG expression in the setting of global DNA hypomethylation. Horvath methylation clock analysis showed significantly increased epigenetic age in ES compared to ND, suggesting accelerated biological aging in advanced MM. Finally, we identified 89 CpG sites whose methylation status was significantly associated with shorter time to progression, identifying specific epigenetic alterations may serve as biomarkers of aggressive disease.

**Figure 1: Cancer Testes Antigen Gene expression in End Stage versus Newly Diagnosed Multiple Myeloma**

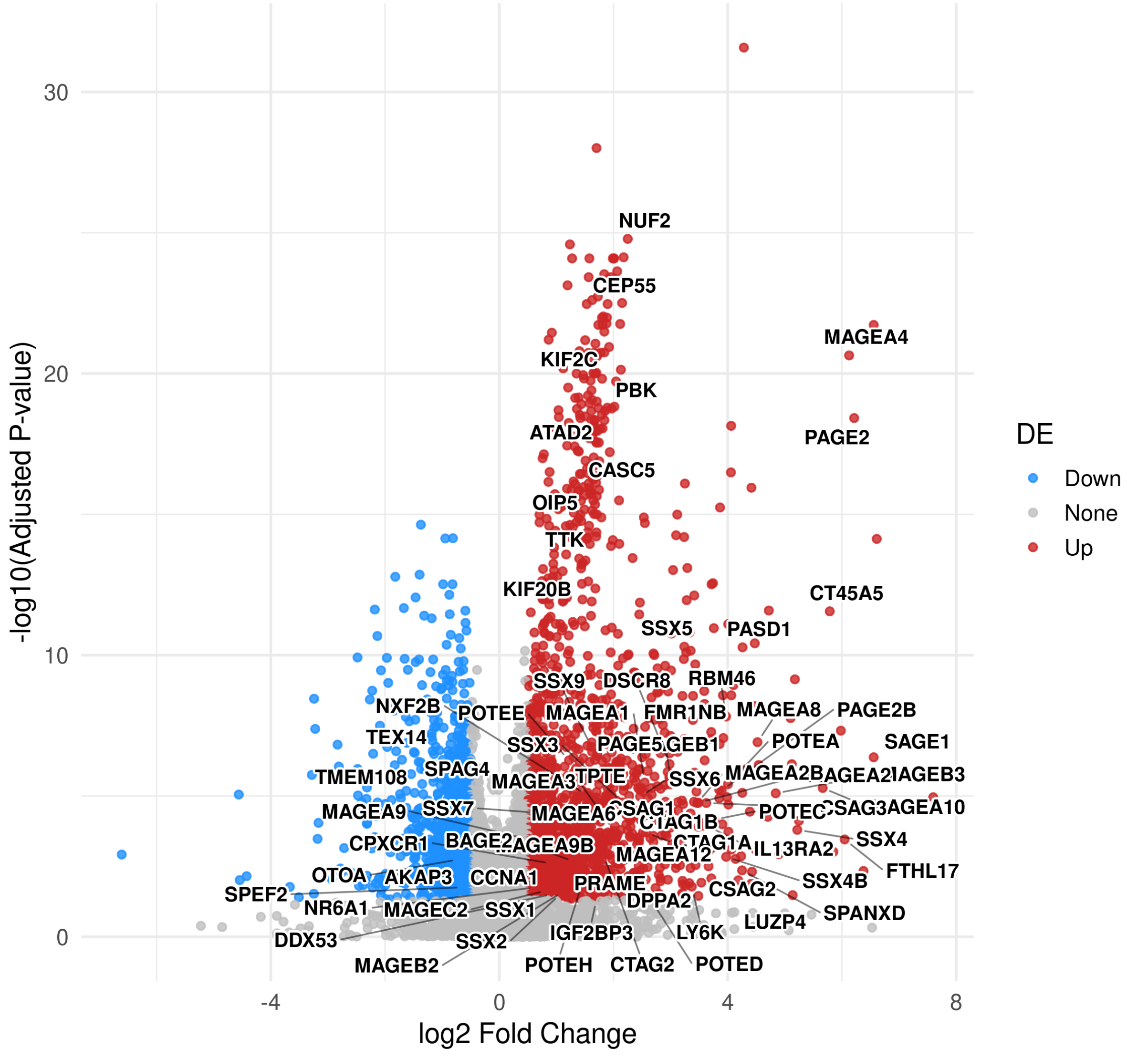

**Copyright Information:** (Online) ISSN: 2572-9241
©2026 The Author(s). HemaSphere is published by John Wiley & Sons Ltd on behalf of European Hematology Association. This is an open access Abstract Book distributed under the Attribution-NonCommercial-NoDerivs (CC BY-NC-ND), which allows third parties to download the articles and share them with others as long as they credit the author and the Abstract Book, but they cannot change the content in any way or use them commercially.
**Abstract Book Citations:** Authors, Title, HemaSphere, 2026;10:(S1):pages. The abstract book DOIs can be found on the "Author Information" page.
**Disclaimer:** Articles published in the journal HemaSphere exclusively reflect the opinions of the authors. The authors are responsible for all content in their abstracts including accuracy of the facts, statements, citing resources, etc.

### (PF740) ANSELAMIMAB TARGETS BOTH CYTOTOXIC SOLUBLE PREFIBRILLAR SPECIES AND INSOLUBLE FIBRILS OF LIGHT CHAIN AMYLOIDOSIS

**Topic:** 13. Myeloma and other monoclonal gammopathies - Biology & translational research

Matt Costello[1], Mike Feulner[1], Brian Meltzer[2], Meera Ramanujam[1], Sahar Usmani-Brown*[1]

*[1]Alexion, AstraZeneca Rare Disease, Translational and Biomarker Research, New Haven, United States of America;[2]Alexion, AstraZeneca Rare Disease, Clinical Development, New Haven, United States of America;*

### Background

Systemic light-chain (AL) amyloidosis arises from a monoclonal plasma cell dyscrasia that drives excess production of free immunoglobulin light chains. These light chains can misfold and aggregate into amyloid fibrils, depositing in tissues and organs and causing substantial morbidity and mortality. Beyond fibrils, soluble pre-fibrillar species are increasingly recognized as pathogenic.

### Aims

In this study, we evaluate the antibody anselamimab (Ch11-1F4, CAEL-101) for its binding to soluble prefibrillar species and insoluble fibrils. We developed a novel whole-blood phagocytosis assay leveraging recombinantly generated fibrils to assess antibody-mediated clearance. Finally, we demonstrate that soluble prefibrillar species exert cytotoxic effects on cardiomyocytes, underscoring the relevance of targeting both fibrillar and prefibrillar forms in AL amyloidosis to have greater impact on disease.

### Methods

SMA is a human kappa IV immunoglobulin light-chain protein originally isolated and sequenced from lymph node-derived amyloid fibrils from an AL amyloidosis patient, and its published variable light chain (VL) sequence was used to generate the recombinant form for further study. Recombinantly generated SMA VL was processed under destabilizing conditions (acidic buffer pH 2.0, with/without incubation) to generate soluble pre-fibrillar species and under native conditions (neutral buffer pH 7.4 with/without incubation) as a control. The recombinant protein was also used to generate insoluble amyloid fibrils. Octet Bio-Layer Interferometry (BLI) was used to assess the binding of anselamimab to soluble SMA VL light chain species and to insoluble SMA fibrils. This was followed by determining the cytotoxic potential of soluble light chain species on iPSC derived cardiomyocytes using a Caspase-Glo 3/7 assay. In addition, a whole blood phagocytosis assay was conducted by incubating healthy donor blood with pHrodo Green-labeled SMA fibrils and anselamimab or isotype antibody, followed by multicolor spectral flow cytometry to quantify fibril uptake by various leukocyte subsets.

### Results

Anselamimab exhibited concentration-dependent binding to soluble SMA VL light chain protein processed under destabilizing conditions. The dissociation constant (KD) was 2.001E-10 M for protein diluted in acidic buffer with brief incubation and 9.724E-10 M for protein diluted in acidic buffer alone and 1.454E-11 M for insoluble fibrils. Anselamimab did not bind to the native form. Isotype control did not bind to any form.
Cytotoxic potential as measured by caspase 3/7 activation was significantly increased in iPSC-derived cardiomyocytes exposed to soluble SMA light chain protein diluted in acidic buffer compared to cells treated with native SMA light chain or vehicle controls ($p<0.05$).
In addition, consistent with previous findings with anselamimab, phagocytosis of amyloid fibrils was demonstrated using a novel whole blood spectral flow cytometric system, demonstrating the role of monocytes, neutrophils and dendritic cells in the clearance of amyloid fibrils.

**Copyright Information:** (Online) ISSN: 2572-9241
©2026 The Author(s). HemaSphere is published by John Wiley & Sons Ltd on behalf of European Hematology Association. This is an open access Abstract Book distributed under the Attribution-NonCommercial-NoDerivs (CC BY-NC-ND), which allows third parties to download the articles and share them with others as long as they credit the author and the Abstract Book, but they cannot change the content in any way or use them commercially.
**Abstract Book Citations:** Authors, Title, HemaSphere, 2026;10:(S1):pages. The abstract book DOIs can be found on the "Author Information" page.
**Disclaimer:** Articles published in the journal HemaSphere exclusively reflect the opinions of the authors. The authors are responsible for all content in their abstracts including accuracy of the facts, statements, citing resources, etc.

**Summary/Conclusion**

Anselamimab demonstrates specific binding to SMA soluble cytotoxic pre-fibrillar species and fibrils. We further characterized its MoA, demonstrating the engagement of myeloid cells in removing insoluble fibrils in a whole blood system. These findings suggest that anselamimab targets misfolded amyloid protein at multiple steps of fibril formation, potentially impacting both early and late stages of AL amyloidosis pathogenesis.

**Copyright Information:** (Online) ISSN: 2572-9241
©2026 The Author(s). HemaSphere is published by John Wiley & Sons Ltd on behalf of European Hematology Association. This is an open access Abstract Book distributed under the Attribution-NonCommercial-NoDerivs (CC BY-NC-ND), which allows third parties to download the articles and share them with others as long as they credit the author and the Abstract Book, but they cannot change the content in any way or use them commercially.
**Abstract Book Citations:** Authors, Title, HemaSphere, 2026;10:(S1):pages. The abstract book DOIs can be found on the "Author Information" page.
**Disclaimer:** Articles published in the journal HemaSphere exclusively reflect the opinions of the authors. The authors are responsible for all content in their abstracts including accuracy of the facts, statements, citing resources, etc.

### (PF741) TRAF2 AND NCK INTERACTING KINASE SIGNALING DRIVES MULTIPLE MYELOMA DEVELOPMENT AND PROGRESSION

**Topic:** 13. Myeloma and other monoclonal gammopathies - Biology & translational research

Adrian Wegmüller*[1, 2, 3], Maxime Boy[1, 2], Luca Palombella[1, 2, 3], Carmen Kalbermatter[3, 4], Branislav Zagrapan[4], Ramin Radpour[1, 2], Carsten Riether[1, 2], Adrian Ochsenbein[1, 2]

*[1]Tumor Immunology, Department for BioMedical Research (DBMR), University of Bern, Bern, Switzerland;[2]Department of Medical Oncology, Inselspital, Bern University Hospital, University of Bern, Bern, Switzerland; [3]Graduate School for Cellular and Biomedical Sciences, University of Bern, Bern, Switzerland; [4]Institute of Tissue Medicine and Pathology (IGMP), University of Bern, Bern, Switzerland;*

### Background

Multiple myeloma (MM) is a genetically and clinically heterogeneous plasma cell malignancy driven by clonal expansion in the bone marrow, causing osteolytic bone disease, anemia, hypercalcemia, and renal impairment. TRAF2- and NCK-interacting kinase (TNIK) is a serine/threonine kinase originally identified as a regulator of Wnt/β-catenin signaling and cytoskeletal dynamics. Beyond its canonical role in Wnt transcriptional control, TNIK integrates MAPK, JNK, NF-κB, and small GTPase signaling pathways, positioning it as a signaling hub that governs stemness, proliferation, differentiation, and survival. Through these functions, TNIK has emerged as a central regulator of tumor initiation and progression, particularly in Wnt-dependent malignancies such as colorectal cancer and chronic myeloid leukemia. However, its role in plasma cell neoplasia remains poorly defined. Therefore, we investigated the functional role and therapeutic relevance of TNIK signaling in the development and maintenance of MM.

### Aims

This project aims to define the functional role of TNIK in the pathogenesis of MM and to evaluate its potential as a therapeutic target.

### Methods

TNIK expression and its clinical relevance were evaluated using RNA-sequencing data from the CoMMpass cohort, comprising purified $CD38^+CD138^+$ bone marrow plasma cells from 766 newly diagnosed MM patients. In addition, TNIK protein expression was quantified by immunohistochemistry in a bone marrow tissue microarray including 342 MM biopsies. TNIK function was investigated through genetic depletion approaches (shRNA knockdown and CRISPR/Cas9 knockout) in MM cell lines and primary patient-derived myeloma cells, as well as by pharmacologic inhibition using small-molecule TNIK inhibitors. Effects on signaling and tumor cell fitness were assessed by transcriptomic profiling, phospho-kinase analyses, and Image Stream based single-cell signaling assays, together with measurements of proliferation, apoptosis, and growth kinetics. In vivo relevance was evaluated in xenograft models using MM cell lines and primary patient samples.

**Copyright Information:** (Online) ISSN: 2572-9241
©2026 The Author(s). HemaSphere is published by John Wiley & Sons Ltd on behalf of European Hematology Association. This is an open access Abstract Book distributed under the Attribution-NonCommercial-NoDerivs (CC BY-NC-ND), which allows third parties to download the articles and share them with others as long as they credit the author and the Abstract Book, but they cannot change the content in any way or use them commercially.
**Abstract Book Citations:** Authors, Title, HemaSphere, 2026;10:(S1):pages. The abstract book DOIs can be found on the “Author Information” page.
**Disclaimer:** Articles published in the journal HemaSphere exclusively reflect the opinions of the authors. The authors are responsible for all content in their abstracts including accuracy of the facts, statements, citing resources, etc.

**Results**

Analysis of the CoMMpass dataset revealed that elevated TNIK expression was associated with inferior overall survival and adverse ISS parameters, implicating TNIK in aggressive disease biology. Bulk RNA sequencing comparing TNIK–wild-type and TNIK-depleted myeloma cells demonstrated enrichment of Wnt/β-catenin and MAPK signaling programs in TNIK-proficient cells. These findings were corroborated by phospho-kinase profiling, which showed increased phosphorylation of multiple MAPK and Wnt pathway components, and by Image Stream single-cell analyses demonstrating enhanced nuclear pERK and active (non-phosphorylated) β-catenin. Pharmacologic TNIK inhibition using INS018-055 reduced proliferation and induced apoptosis in TNIK-proficient MM cells. Consistently, genetic TNIK depletion impaired myeloma cell growth and increased apoptotic activity in vitro, and significantly suppressed tumor growth in xenograft models in vivo, accompanied by enhanced apoptosis.

**Summary/Conclusion**

TNIK acts as a central regulator of multiple myeloma cell growth and survival by promoting MAPK/ERK and Wnt/β-catenin signaling. Genetic depletion or pharmacologic inhibition of TNIK suppresses myeloma cell proliferation and tumor growth in vitro and in vivo, highlighting TNIK as a promising therapeutic target in multiple myeloma.

**Copyright Information:** (Online) ISSN: 2572-9241
©2026 The Author(s). HemaSphere is published by John Wiley & Sons Ltd on behalf of European Hematology Association. This is an open access Abstract Book distributed under the Attribution-NonCommercial-NoDerivs (CC BY-NC-ND), which allows third parties to download the articles and share them with others as long as they credit the author and the Abstract Book, but they cannot change the content in any way or use them commercially.
**Abstract Book Citations:** Authors, Title, HemaSphere, 2026;10:(S1):pages. The abstract book DOIs can be found on the "Author Information" page.
**Disclaimer:** Articles published in the journal HemaSphere exclusively reflect the opinions of the authors. The authors are responsible for all content in their abstracts including accuracy of the facts, statements, citing resources, etc.

### (PF742) MAGNETICALLY INDUCED CELL-PATIENT OR CELL LINE DERIVED XENOGRAFT (MAGIC-PDX/CDX) MODELS FOR THE RAPID ESTABLISHMENT OF MULTIPLE MYELOMA AND DRUG SCREENING

**Topic:** 13. Myeloma and other monoclonal gammopathies - Biology & translational research

Rakesh Kumar Raut[1], Jiaxing Zhang[2], Weishen Zhang[2], Shiying Lu[3], Yiting Li[2], Nan Ding[1], Hongfei Zhu[1], Taosong Liu[1], Zizhan Wang[2], Luxia Tang[1], Yufang Lv[4], Baowen Liang[2], Jingyi Guo[1], Sihan Wei[1], Lihan Dai[1], Qinglan Lin[1], Qianli Jiang*[1, 5, 6]

*[1]Southern Medical University, Guangzhou, China;[2]Guangzhou Maishi Biotechnology Co., Ltd., Guangzhou, China;[3]School of International Education, Southern Medical University, Guangzhou, China; [4]Department of Physiology, Southern Medical University, Guangzhou, China; [5]Department of Hematology, Nanfang Hospital, Guangzhou, China;[6]Drug Clinical Trial center, Nanfang Hospital of Southern Medical University, Guangzhou, China;*

### Background

Personalized therapy for Multiple Myeloma (MM) remains an unmet clinical need. Conventional Patient-Derived or Cell line-Derived Xenograft (PDX or CDX) models are valuable tools but limited by low engraftment rates, slow establishment, and inconsistent clinical replication fidelity. Our team utilizes the Magnetically Induced Cell (MagIC) technology to establish MagIC-PDX/CDX platform, by magnetizing tumor cells and applying localized magnetic field enhancing bone marrow homing, retention, and facilitating rapid and reproducible establishment of various models of bone tumors.
Drugs screened using this platform include the combination therapy thalidomide/dexamethasone (THA/DEX); a first-in-class novel drug MR001, which is a CD4/TGFβ bispecific antibody that reprograms the tumor microenvironment by activating $CD4^+$ T cells and blocking TGFβ signaling (Shenzhen Majory BioTech Co. Ltd.); and IBI3003, a tri-specific antibody targeting GPRC5D and BCMA on MM cells and CD3 on T cells to engage T-cell cytotoxicity (Innovent Biologics (Suzhou) Co. Ltd.).

### Aims

To validate the MagIC-PDX/CDX platform as a rapid and reliable model for multiple myeloma and assess its suitability for accelerating the screening of novel therapeutic agents.

### Methods

The human MM cell line RPMI-8226 was genetically modified to express luciferase (Luc) and Green Fluorescent Protein (GFP) with lentiviral vectors and magnetized using magnetic nanomotors. On day 0, 16 Humanized NSG mice were injected with magnetized RPMI-8226-Luc-GFP cells ($1\times10^6$ in 30 μL PBS) into the marrow cavity of the femur. A precise magnetic field was applied locally overnight to guide and retain cells within the bone marrow niche. Mice were randomly divided into 4 groups (Control, THA/DEX, MR001, and IBI3003). Treatments continued for 28 days. Disease progression was assessed by bioluminescence (BL) imaging on days 3, 7, 15, 21, and 28. General health, body weight, and signs of morbidity were monitored, followed by qPCR, FACS, and pathological examination on day 28.

### Results

By day 3, localized BL signals were present at the injection site, indicating a rapid and high engraftment rate (93.75%). The control group developed progressively MM features with expansion of BL signals (Fig 1) and exhibited the highest burden of MM on morphology and FACS, with strong molecular genetic signals. The THA/DEX combination group displayed rapid tumor growth and lung metastasis, demonstrating resistance to this regimen ($P = 0.785$). In contrast, the MR001 treatment group significantly slowed myeloma progression ($P < 0.001$). The IBI3003 showed the most potent antitumor effect by markedly delaying tumor signal development ($P < 0.001$).

**Copyright Information:** (Online) ISSN: 2572-9241
©2026 The Author(s). HemaSphere is published by John Wiley & Sons Ltd on behalf of European Hematology Association. This is an open access Abstract Book distributed under the Attribution-NonCommercial-NoDerivs (CC BY-NC-ND), which allows third parties to download the articles and share them with others as long as they credit the author and the Abstract Book, but they cannot change the content in any way or use them commercially.
**Abstract Book Citations:** Authors, Title, HemaSphere, 2026;10:(S1):pages. The abstract book DOIs can be found on the "Author Information" page.
**Disclaimer:** Articles published in the journal HemaSphere exclusively reflect the opinions of the authors. The authors are responsible for all content in their abstracts including accuracy of the facts, statements, citing resources, etc.

### Summary/Conclusion

The MagIC-CDX platform enables the rapid establishment of MM models, providing a pivotal preclinical tool for validating novel therapeutic agents and guiding personalized treatment approaches. A parallel study involving MagIC-PDX models and MM patients is ongoing.

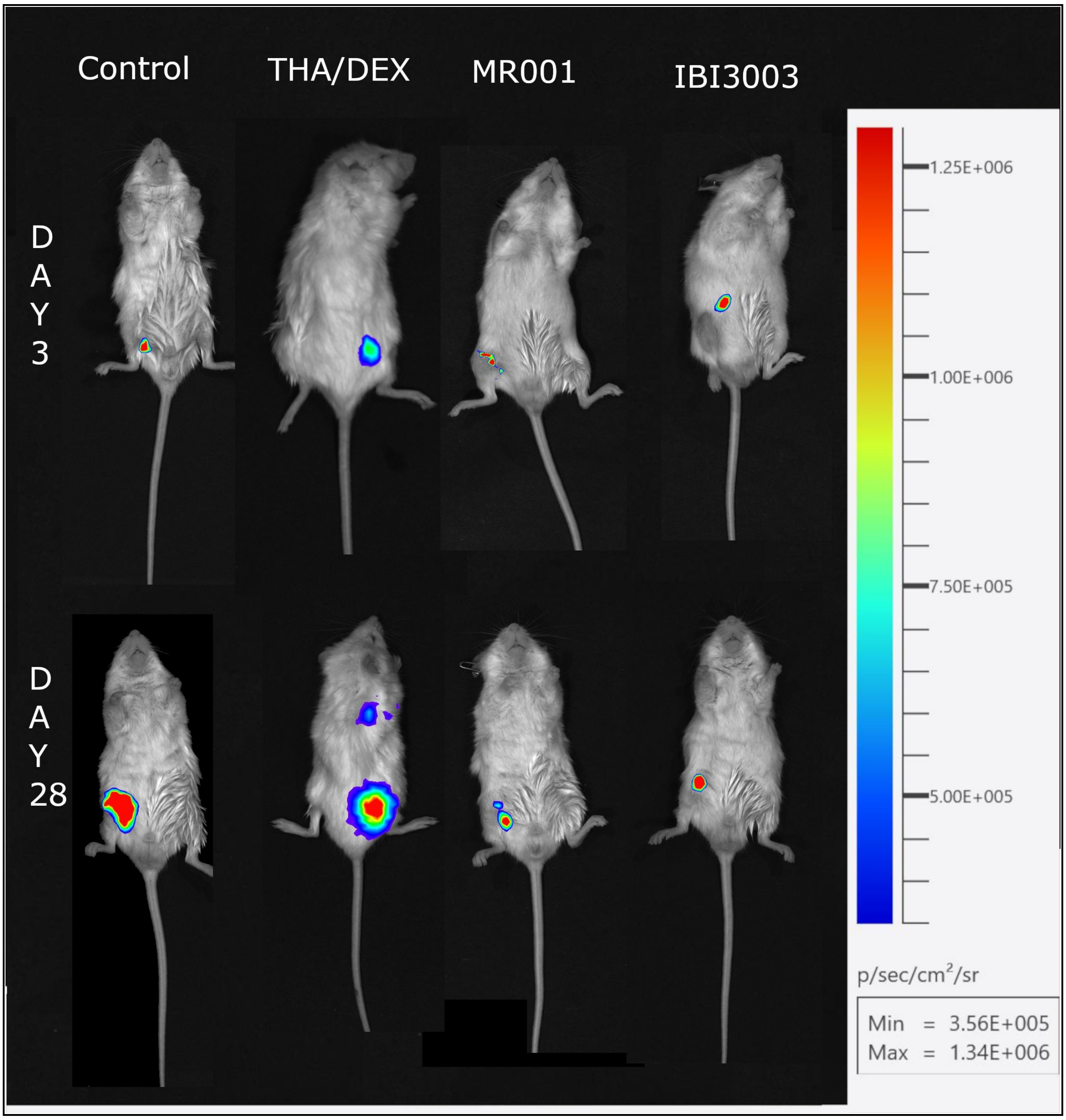

Fig 1: Bioluminescent Signal Comparison Between Day 3 and Day 28 Across Treatment Groups.

**Copyright Information:** (Online) ISSN: 2572-9241
©2026 The Author(s). HemaSphere is published by John Wiley & Sons Ltd on behalf of European Hematology Association. This is an open access Abstract Book distributed under the Attribution-NonCommercial-NoDerivs (CC BY-NC-ND), which allows third parties to download the articles and share them with others as long as they credit the author and the Abstract Book, but they cannot change the content in any way or use them commercially.
**Abstract Book Citations:** Authors, Title, HemaSphere, 2026;10:(S1):pages. The abstract book DOIs can be found on the "Author Information" page.
**Disclaimer:** Articles published in the journal HemaSphere exclusively reflect the opinions of the authors. The authors are responsible for all content in their abstracts including accuracy of the facts, statements, citing resources, etc.

### (PF743) PHOSPHORYLATION REGULATES BCMA ABUNDANCE IN MULTIPLE MYELOMA

**Topic:** 13. Myeloma and other monoclonal gammopathies - Biology & translational research

Sonja Stotz*[1, 2], Kilian Irlinger[1, 2], Nicolas Barbian[1, 2], Melanie Faber[1, 2], Abirami Augsburger[1, 2], Franziska Füchsl[1, 2], Piero Giansanti[2, 3], Bernhard Kuster[3, 4, 5], Leonie Rieger[1, 2], Florian Bassermann[1, 2, 5, 6]

*[1]TUM University Hospital rechts der Isar, Internal Medicine 3, Munich, Germany; [2]TranslaTUM, Center of Translational Cancer Research, Munich, Germany; [3]Bavarian Center for Biomolecular Mass Spectrometry, Munich, Germany; [4]Chair of Proteomics and Bioanalytics, Freising, Germany; [5]Deutsches Konsortium für Translationale Krebsforschung, Heidelberg, Germany; [6]Bavarian Cancer Research Center, Munich, Germany;*

### Background

Immunotherapies targeting B-cell maturation antigen (BCMA), including chimeric antigen receptor (CAR) T cells, T-cell engagers (TCE), and antibody–drug conjugates (ADC), have significantly improved outcomes in multiple myeloma (MM). However, relapse frequently occurs due to reduced or lost BCMA surface expression. While our recent studies demonstrated that the ubiquitin–proteasome system regulates BCMA membrane abundance, additional post-translational mechanisms controlling BCMA expression remain poorly defined.

### Aims

To investigate whether phosphorylation regulates BCMA surface expression in MM and to identify kinases involved in this process, with potential implications for BCMA-directed immunotherapies.

### Methods

BCMA phosphorylation was analyzed in MM cell lines using mass spectrometry. Identified phosphorylation sites were mutated (serine-to-alanine substitutions) and overexpressed in MM cell lines to assess effects on protein turnover and surface expression. Proximity-based interactome screening was performed to identify BCMA-associated kinases and phosphatases. Candidate interactions were validated by co-immunoprecipitation and immunofluorescence microscopy. BCMA surface abundance was quantified following kinase inhibition.

### Results

Mass spectrometry confirmed BCMA phosphorylation in MM cell lines and identified two phosphorylation motifs. Mutation of serine 94 to alanine significantly reduced BCMA turnover, indicating functional relevance of this site. The impact of this mutation on ligand-induced regulation by APRIL is currently under investigation. Using proximity-based interactome screening for BCMA to systematically identify kinases, MARK2 was identified as a potential kinase interacting with BCMA. Co-immunoprecipitation confirmed the MARK2–BCMA interaction, and immunofluorescence analyses indicated co-localization at the plasma membrane. Moreover, suppression of MARK2 increased BCMA surface abundance in MM cell lines. Ongoing studies are evaluating the impact of MARK2 suppression on the efficacy of BCMA-directed CAR T-cell therapy.

**Copyright Information:** (Online) ISSN: 2572-9241
©2026 The Author(s). HemaSphere is published by John Wiley & Sons Ltd on behalf of European Hematology Association. This is an open access Abstract Book distributed under the Attribution-NonCommercial-NoDerivs (CC BY-NC-ND), which allows third parties to download the articles and share them with others as long as they credit the author and the Abstract Book, but they cannot change the content in any way or use them commercially.
**Abstract Book Citations:** Authors, Title, HemaSphere, 2026;10:(S1):pages. The abstract book DOIs can be found on the "Author Information" page.
**Disclaimer:** Articles published in the journal HemaSphere exclusively reflect the opinions of the authors. The authors are responsible for all content in their abstracts including accuracy of the facts, statements, citing resources, etc.

**Summary/Conclusion**

These findings propose MARK2 as a novel regulator of BCMA surface expression in MM. Phosphorylation-driven regulation of BCMA stability influences the availability of the target antigen. Targeting MARK2 may enhance BCMA surface retention and improve responses to BCMA-directed immunotherapies. This approach offers a promising strategy to overcome resistance mechanisms associated with antigen loss in MM.

**Copyright Information:** (Online) ISSN: 2572-9241
©2026 The Author(s). HemaSphere is published by John Wiley & Sons Ltd on behalf of European Hematology Association. This is an open access Abstract Book distributed under the Attribution-NonCommercial-NoDerivs (CC BY-NC-ND), which allows third parties to download the articles and share them with others as long as they credit the author and the Abstract Book, but they cannot change the content in any way or use them commercially.
**Abstract Book Citations:** Authors, Title, HemaSphere, 2026;10:(S1):pages. The abstract book DOIs can be found on the "Author Information" page.
**Disclaimer:** Articles published in the journal HemaSphere exclusively reflect the opinions of the authors. The authors are responsible for all content in their abstracts including accuracy of the facts, statements, citing resources, etc.

### (PF744) ROLE AND MECHANISM OF PIM-2 KINASE INHIBITOR-INDUCED IMMUNOGENIC CELL DEATH IN MULTIPLE MYELOMA

**Topic:** 13. Myeloma and other monoclonal gammopathies - Biology & translational research

Hongli Shen[1], Zhaoyun Liu[1], Rong Fu*[1]

[1] *Tianjin Medical University General Hospital, Tianjin, China;*

### Background

Immune dysfunction is a major component in the pathogenesis of multiple myeloma (MM). Specific antitumor T cell activation provides a new direction for the treatment of multiple myeloma.

### Aims

To investigate the effect of Pim-2 kinase inhibitor on inducing immunogenic cell death (ICD) in multiple myeloma (MM) cells and to investigate the effect and mechanism of SMI-16a treated MM cells-mediated activation of dendritic cells (DCs) and T lymphocytes in MM patients, which enhance anti-MM effect.

### Methods

Perform single-cell sequencing on MM patients(MGUS=4, MM_standard_risk=4, MM_High_risk=4). Commpass analysis of the relationship between Pim-2 gene expression and the prognosis of multiple myeloma(MMRF-COMMPASS, N=787). The expression of DAMPs and ROS level in MM cells were detected by flow cytometry(FCM). MM cells(Pim-2 kinase inhibitor treated), DCs, and pan-T cells were co-cultured in vitro and the expression of activated molecules on DCs and T cells were examined by FCM. The IRE1-XBP1 signaling pathway protein were detected by western blot. The MM mouse model was developed to evaluate the activation of immune cells and its antitumor effect.

### Results

In our study, pim-2 gene is upregulated in multiple myeloma patients, especially high-risk patients, and is associated with poor prognosis by analysis of the single-cell sequencing and MMRF-COMMPASS dataset(Fig.A-C). In the vitro experiment, Pim-2 kinase inhibitor(SMI-16a) promoted the efflux of CALR and secretion of HMGB1 and HSP70 by causing an increase in ROS levels in MM cells(Fig.D-F). In the co-culture system, Pim-2 kinase inhibitor can upregulate the expression of activated molecules of DCs, promote CD3+CD8+ T cells differentiation from Naïve T cells to effector memory T cells, and reduce the expression of CD57 and PD-1 of CD3+CD8+ T cells(Fig.G-H). In vivo, SMI-16a can inhibit tumor growth, and the combination of SMI-16a and CD38 antibody (daratumumab) significantly enhance its antitumor effect and led to further DC and T lymphocyte activation(Fig.I-L). Mechanistically, Pim-2 kinase inhibitor upregulated the expression of the IRE1-XBP1s signaling pathway, manifested as high expression of GRP78, p-IRE1, XBP1, and CHOP(Fig.M).

### Summary/Conclusion

Pim-2 kinase inhibitor induced immunogenic cell death by promoting ROS production to lead endoplasmic reticulum (ER) stress which ultimately mediate specific antitumor T cell activation in MM.

**Copyright Information:** (Online) ISSN: 2572-9241
©2026 The Author(s). HemaSphere is published by John Wiley & Sons Ltd on behalf of European Hematology Association. This is an open access Abstract Book distributed under the Attribution-NonCommercial-NoDerivs (CC BY-NC-ND), which allows third parties to download the articles and share them with others as long as they credit the author and the Abstract Book, but they cannot change the content in any way or use them commercially.
**Abstract Book Citations:** Authors, Title, HemaSphere, 2026;10:(S1):pages. The abstract book DOIs can be found on the "Author Information" page.
**Disclaimer:** Articles published in the journal HemaSphere exclusively reflect the opinions of the authors. The authors are responsible for all content in their abstracts including accuracy of the facts, statements, citing resources, etc.

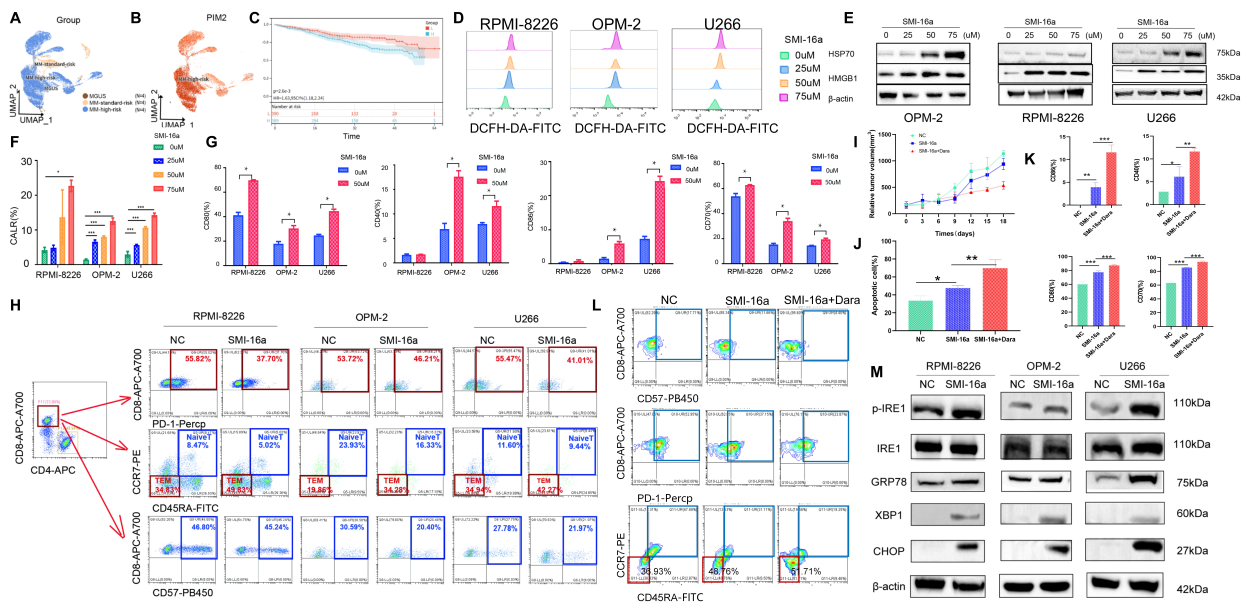

*Figure(A)Cluster analysis of CD138+cells identified according to the disease status of the sample(MGUS=4, MM_standard_risk=4, MM_High_risk=4). (B)Unified manifold approximation and projection visualization of Pim-2 gene expression. (C) Commpass analysis of the relationship between Pim-2 gene expression and the prognosis of multiple myeloma(N=787, PIM-2 gene high expression=394, PIM-2 gene low expression=393, * Compared with normal control, $p < 0.05$).(D) The intracellular ROS levels in MM are determined using flow cytometry (FCM).(E). Pim-2 kinase inhibitors induce increased DAMP secretion in MM cells.(F) CALR expression on the cell surface of MM cells.(G) SMI-16a–treated MM cells induce the expression of DC surface-activating molecules in patients with MM.(H) CD8+ T lymphocyte differentiation and surface PD-1/CD57 expression of CD8+ T lymphocytes in the coculture system.(I)Tumor growth curves in different groups.(J) The apoptotic levels of RPMI-8226 cells in mouse tumor tissues following different treatments. (K)The surface expression of functional molecules on mice peripheral blood DCs measured using FCM. (L)Distribution of different CD8+ T cells in the mice peripheral blood in these groups. (M) ER stress-related protein expression in MM cells following SMI-16a treatment.*

**Copyright Information:** (Online) ISSN: 2572-9241
©2026 The Author(s). HemaSphere is published by John Wiley & Sons Ltd on behalf of European Hematology Association. This is an open access Abstract Book distributed under the Attribution-NonCommercial-NoDerivs (CC BY-NC-ND), which allows third parties to download the articles and share them with others as long as they credit the author and the Abstract Book, but they cannot change the content in any way or use them commercially.
**Abstract Book Citations:** Authors, Title, HemaSphere, 2026;10:(S1):pages. The abstract book DOIs can be found on the "Author Information" page.
**Disclaimer:** Articles published in the journal HemaSphere exclusively reflect the opinions of the authors. The authors are responsible for all content in their abstracts including accuracy of the facts, statements, citing resources, etc.

### (PF745) PHF19 DRIVES LENALIDOMIDE RESISTANCE BY EPIGENETICALLY REPROGRAMMING THE IRF4/MYC AXIS IN MULTIPLE MYELOMA

**Topic:** 13. Myeloma and other monoclonal gammopathies - Biology & translational research

Xiyue Sun*[1], Tengteng Yu[1], Runlin An[1], Runchun Huang[1], Hao Sun[1], Teng Fang[1], Xiaoyu Zhang[1], Zhen Yu[1], Lanting Liu[1], Gang An[1], Lugui Qiu[1], Mu Hao[1]

*[1]State Key Laboratory of Experimental Hematology, National Clinical Research Center for Blood Diseases, Haihe Laboratory of Cell Ecosystem, Institute of Hematology & Blood Diseases Hospital, Chinese Academy of Medical Sciences & Peking Union Medical College, Tianjin, China;*

### Background

Immunomodulatory drugs (IMiDs) constitute the therapeutic backbone of multiple myeloma (MM), yet resistance inevitably limits durable remission. Although epigenetic dysregulation is a defining feature of MM progression, whether specific chromatin regulators actively reprogram oncogenic dependency to enforce IMiD resistance remains unclear.

### Aims

To delineate the role of PHF19 in IMiD resistance and elucidate its molecular mechanisms.

### Methods

We performed integrative transcriptomic profiling of primary CD138$^+$ plasma cells from MM patients with divergent responses to IMiD-based quadruplet induction, followed by functional and epigenomic interrogation using shRNA-mediated PHF19 knockdown in human MM cell lines, including an acquired lenalidomide-resistant model. Chromatin architecture was characterized by ChIP-seq (EZH2, H3K27me3) and ATAC-seq, and protein interactions were assessed by co-immunoprecipitation and chromatin fractionation. Survival analyses were conducted in independent clinical cohorts.

**Copyright Information:** (Online) ISSN: 2572-9241
©2026 The Author(s). HemaSphere is published by John Wiley & Sons Ltd on behalf of European Hematology Association. This is an open access Abstract Book distributed under the Attribution-NonCommercial-NoDerivs (CC BY-NC-ND), which allows third parties to download the articles and share them with others as long as they credit the author and the Abstract Book, but they cannot change the content in any way or use them commercially.
**Abstract Book Citations:** Authors, Title, HemaSphere, 2026;10:(S1):pages. The abstract book DOIs can be found on the "Author Information" page.
**Disclaimer:** Articles published in the journal HemaSphere exclusively reflect the opinions of the authors. The authors are responsible for all content in their abstracts including accuracy of the facts, statements, citing resources, etc.

**Results**

RNA-seq analysis of primary myeloma cells from IMiD non-responders revealed significant activation of MYC, E2F, and G2M checkpoint programs. Cross-cohort integration with published datasets of D-KRd non-responders identified PHF19 as a top-ranked epigenetic regulator associated with treatment failure. Elevated PHF19 expression strongly correlated with IRF4/MYC transcriptional dependency and independently predicted inferior progression-free survival (PFS) and overall survival (OS). Importantly, high PHF19 levels were significantly associated with poor response to IMiD-based therapy, supporting its value as a predictive biomarker of treatment failure.

Functionally, PHF19 depletion induced cell cycle arrest and apoptosis while markedly suppressing myeloma proliferation. Transcriptomic profiling of PHF19-deficient cells demonstrated coordinated collapse of MYC targets, E2F signaling, G2M checkpoint pathways, and IRF4-dependent transcription, effectively reversing the resistant gene signature. Consistently, IRF4 and MYC were reduced at both mRNA and protein levels upon PHF19 loss.

Mechanistically, integrative epigenomic mapping revealed that PHF19 deficiency reprogrammed chromatin architecture at the MYC and IRF4 loci, enforcing a closed chromatin state characterized by diminished accessibility and enhanced EZH2-mediated H3K27me3 deposition. Notably, PHF19 depletion did not destabilize the core PRC2 complex (EZH2/SUZ12/EED), but instead altered locus-specific recruitment and catalytic precision, indicating that PHF19 functions as a chromatin-guiding determinant that shields the IRF4/MYC circuitry from PRC2-mediated silencing. Importantly, PHF19 targeting sensitized intrinsically resistant cells and re-sensitized acquired lenalidomide-resistant MM.1S cells by dismantling compensatory IRF4/MYC stabilization during IMiD exposure.

**Summary/Conclusion**

These findings establish PHF19 as a central epigenetic driver that enforces lenalidomide resistance through reprogramming of the IRF4/MYC axis. Beyond its mechanistic role, PHF19 expression predicts clinical outcome and IMiD response, positioning the PHF19–IRF4/MYC axis as both a therapeutic vulnerability and a biomarker-guided strategy to overcome drug resistance in refractory multiple myeloma.

**Copyright Information:** (Online) ISSN: 2572-9241
©2026 The Author(s). HemaSphere is published by John Wiley & Sons Ltd on behalf of European Hematology Association. This is an open access Abstract Book distributed under the Attribution-NonCommercial-NoDerivs (CC BY-NC-ND), which allows third parties to download the articles and share them with others as long as they credit the author and the Abstract Book, but they cannot change the content in any way or use them commercially.
**Abstract Book Citations:** Authors, Title, HemaSphere, 2026;10:(S1):pages. The abstract book DOIs can be found on the "Author Information" page.
**Disclaimer:** Articles published in the journal HemaSphere exclusively reflect the opinions of the authors. The authors are responsible for all content in their abstracts including accuracy of the facts, statements, citing resources, etc.

### (PF746) A NOVEL CIRCULAR RNA PRODUCED BY THE "NUCLEAR FACTOR OF ACTIVATED T-CELLS, CYTOPLASMIC 3" GENE (CIRCNFATC3), DISCOVERED BY NANOPORE SEQUENCING, HOLDS SIGNIFICANT PROGNOSTIC VALUE IN MULTIPLE MYELOMA

**Topic:** 13. Myeloma and other monoclonal gammopathies - Biology & translational research

Maria Papatsirou[1], Christos Kontos[1], Ioannis Ntanasis-Stathopoulos[2], Christine Liacos[2], Panagiotis Tsiakanikas[1], Maria Gavriatopoulou[2], Efstathios Kastritis[2], Meletios A. Dimopoulos[2], Jérôme Moreaux[3, 4, 5], Andreas Scorilas[1], Evangelos Terpos*[2]

*[1]National and Kapodistrian University of Athens, Faculty of Biology, Department of Biochemistry and Molecular Biology, Athens, Greece; [2]National and Kapodistrian University of Athens, School of Medicine, Department of Clinical Therapeutics, Athens, Greece; [3]UMR CNRS-UM, Institute of Human Genetics, Montpellier, France;[4]CHU Montpellier, Department of Biological Hematology, Montpellier, France; [5]University of Montpellier, UFR Medicine, Montpellier, France;*

### Background

Circular RNAs (circRNAs) belong to a distinct class of non-coding RNAs with multifaceted roles and an emerging potential as non-invasive molecular biomarkers in cancer, including multiple myeloma (MM). To date, the complex landscape of circRNAs in MM remains largely unknown.

### Aims

We sought to unravel the identity and expression patterns of circRNAs in MM and to assess the prognostic potential of selected circRNAs, using the cutting-edge technology of nanopore sequencing.

### Methods

Ten (10) human MM cell lines were propagated. Following total RNA isolation, ribosomal RNA (rRNA) depletion, and incubation with RNase R, cDNA was synthesized via rolling-circle amplification. Next, nanopore sequencing libraries were prepared and sequenced, with high accuracy basecalling. All sequencing reads were aligned against human genome GRCh38, and data analysis was performed using the CIRI-long 1.1.0 pipeline along with *in-house–built* algorithms. Additionally, plasma cells were enriched via CD138+ magnetic selection from bone marrow aspirates (BMA) obtained from 175 adult patients diagnosed with plasma cell dyscrasias. After total RNA extraction and first-strand cDNA synthesis, quantitative expression analysis of 4 circRNAs that were highly abundant in the 10 MM cell lines studied was performed in the 175 samples, by highly sensitive and specific qPCR assays, using divergent primers.

**Copyright Information:** (Online) ISSN: 2572-9241
©2026 The Author(s). HemaSphere is published by John Wiley & Sons Ltd on behalf of European Hematology Association. This is an open access Abstract Book distributed under the Attribution-NonCommercial-NoDerivs (CC BY-NC-ND), which allows third parties to download the articles and share them with others as long as they credit the author and the Abstract Book, but they cannot change the content in any way or use them commercially.
**Abstract Book Citations:** Authors, Title, HemaSphere, 2026;10:(S1):pages. The abstract book DOIs can be found on the "Author Information" page.
**Disclaimer:** Articles published in the journal HemaSphere exclusively reflect the opinions of the authors. The authors are responsible for all content in their abstracts including accuracy of the facts, statements, citing resources, etc.

**Results**

In the 10 MM cell lines, 94,048 distinct full-length circRNA sequences were identified. Remarkably, these circRNAs originated from 14,313 different genes, highlight the extensive alternative splicing and back-splicing in MM, with features such as novel exon inclusion, intron retention, and usage of 5′ and 3′ cryptic splice sites of already annotated exons. Moreover, 4 circRNAs, namely circNFATC3, circMTO1, circATAD2, and circSEC24B, were constantly expressed at high levels in all cell lines. To the best of our knowledge, these alternative circNFATC3 and circMTO1 variants are both novel. Thus, new qPCR protocols were established to measure the expression levels of the aforementioned circRNAs in patients' BMA CD138+ plasma cells. The cohorts of patients included 110 newly diagnosed symptomatic MM cases, 35 smoldering MM (SMM) cases, and 30 cases with monoclonal gammopathy of undetermined significance (MGUS). After circRNA quantification, only circNFATC3 (among those 4 circRNAs) emerged as a promising biomarker, expressed in all MM patients' samples; the other 3 samples were sporadically expressed in small numbers of samples. circNFATC3 expression in CD138+ plasma cells differed among the 3 cohorts, showing a gradual increase from MGUS to SMM and to MM ($P$=0.014). Kaplan-Meier survival analysis uncovered the favorable prognostic value of circNFATC3 expression in MM patients, with regard to overall survival ($P$=0.034).

**Summary/Conclusion**

A great diversity of circRNAs in MM was uncovered in 10 MM cell lines. Besides identifying already annotated human circRNAs, we discovered thousands of circular transcripts, produced either by known genes heavily involved in MM or by genomic regions that were previously considered as transcriptionally silenced. Expression analysis of particular circRNAs in cohorts of patients with MGUS, SMM or MM highlighted a novel circRNA produced from the *NFATC3* (*nuclear factor of activated T-cells, cytoplasmic 3*) gene, namely circNFATC3, as a molecular biomarker with promising potential regarding the prediction of MM patients' overall survival.

**Copyright Information:** (Online) ISSN: 2572-9241
©2026 The Author(s). HemaSphere is published by John Wiley & Sons Ltd on behalf of European Hematology Association. This is an open access Abstract Book distributed under the Attribution-NonCommercial-NoDerivs (CC BY-NC-ND), which allows third parties to download the articles and share them with others as long as they credit the author and the Abstract Book, but they cannot change the content in any way or use them commercially.
**Abstract Book Citations:** Authors, Title, HemaSphere, 2026;10:(S1):pages. The abstract book DOIs can be found on the "Author Information" page.
**Disclaimer:** Articles published in the journal HemaSphere exclusively reflect the opinions of the authors. The authors are responsible for all content in their abstracts including accuracy of the facts, statements, citing resources, etc.

### (PF747) A SYSTEMIC LIPID SIGNATURE DISTINGUISHES AL AMYLOIDOSIS AND SUGGESTS METABOLIC CROSSTALK ACROSS BONE MARROW, CIRCULATION, AND PERIPHERAL TISSUES

**Topic:** 13. Myeloma and other monoclonal gammopathies - Biology & translational research

Tal Zvida-Bloch[1, 2], Maya Aslan[3], Ifat Abramovich[4], Eli Muchtar[5, 6], Bella Agranovich[4], Mali Salmon-Divon1[1, 7], Benjamin Bender[8], Adrian Duek[9, 10], Merav Leiba[9, 10], Moshe Gatt[11], Wilson Gonsalves[5], May Basood[12], Angela Dispenzieri[5], Ofer Shpilberg[2, 7, 12], Inbal Mor[1], Moran Yadid[3], Oshrat Hershkovitz-Rokah*[1, 2]

*[1]Ariel University, Department of Molecular Biology, Faculty of Natural Sciences, Ariel, Israel;[2]Assuta Medical Center, Translational Research Lab, Tel-Aviv, Israel; [3]Bar Ilan University, The Azrieli Faculty of Medicine, Safed, Israel;[4]Technion-Israel Institute of Technology, Laura and Isaac Perlmutter Metabolomics Center, B. Rappaport Faculty of Medicine, Haifa, Israel; [5]Mayo Clinic, Division of Hematology, Rochester, MN, United States of America; [6]Chaim Sheba Medical Center, Tel Hashomer, affiliated with the Faculty of Medicine, Tel Aviv University,, Institute of Hematology, Tel Aviv, Israel, Israel; [7]Ariel University, Adelson School of Medicine, Ariel, Israel; [8]Assuta Medical Center, Orthopedic Department, Tel-Aviv, Israel;[9]Assuta Medical Center, Institute of Hematology, Ashdod, Israel; [10]Ben-Gurion University of the Negev, Faculty of Health Science, Beer Sheva, Israel; [11]Hadassah Hebrew University Medical Center, Department of Hematology, Jerusalem, Israel; [12]Assuta Medical Center, Institute of Hematology, Tel-Aviv, Israel;*

### Background

Systemic light chain (AL) amyloidosis originates from a clonal plasma cell disorder in the bone marrow (BM), yet organ dysfunction reflects systemic consequences extending beyond the BM niche. While light chain deposition is central to pathogenesis, we have previously shown the involvement of the MAPK signaling pathway, mediated by microRNA dysregulation, in AL amyloidosis. However, the contribution of lipid-derived inflammatory mediators across biological compartments remains incompletely understood.

### Aims

To characterize systemic fatty acid imbalance and associated inflammatory–lipid mediator signatures in AL amyloidosis across BM, peripheral blood, and cardiac tissue compartments.

### Methods

Fatty acids profiling from plasma samples was performed using LC-MS in AL patients (n=30), multiple myeloma (MM; n=30), and healthy controls (HC; n=15). Cytokine profiling was quantified in serum and BM CD138+ cells (n=10/group). COX2 (PTGS2) mRNA expression was assessed by qPCR in CD138⁺ BM plasma cells (n=30/group). To explore functional relevance to cardiac involvement, human iPSC-derived cardiomyocytes were exposed to AL and MM patient’s serum, followed by untargeted metabolomics analysis. Finally, based on our previous findings, ALMC-1 cells were treated with a MEK inhibitor and fatty acids profiles and cytokine secretion were analyzed.

**Copyright Information:** (Online) ISSN: 2572-9241
©2026 The Author(s). HemaSphere is published by John Wiley & Sons Ltd on behalf of European Hematology Association. This is an open access Abstract Book distributed under the Attribution-NonCommercial-NoDerivs (CC BY-NC-ND), which allows third parties to download the articles and share them with others as long as they credit the author and the Abstract Book, but they cannot change the content in any way or use them commercially.
**Abstract Book Citations:** Authors, Title, HemaSphere, 2026;10:(S1):pages. The abstract book DOIs can be found on the “Author Information” page.
**Disclaimer:** Articles published in the journal HemaSphere exclusively reflect the opinions of the authors. The authors are responsible for all content in their abstracts including accuracy of the facts, statements, citing resources, etc.

**Results**

AL plasma demonstrated depletion of polyunsaturated fatty acid (PUFAs), including omega-3 and omega-6 (e.g., DHA/EPA/DPA, linoleic acid and AA) species consistent with disruption of fatty acid homeostasis and altered omega-3/omega-6 balance. In parallel, AL samples showed a pro-inflammatory cytokine profile in both BM and peripheral blood, supporting immune activation across compartments. Consistent with a lipid mediator-linked inflammatory program, COX2 mRNA expression was significantly increased in $CD138^+$ BM plasma cells from AL patients, suggesting enhanced prostaglandin biosynthetic potential at the level of the malignant clone. Pathway enrichment analysis of metabolites in cardiomyocyte-conditioned media following exposure to serum from AL patients demonstrated significant enrichment of linoleate metabolism, omega-3 fatty acid metabolism, and prostaglandin biosynthesis, indicating activation of prostaglandin-related metabolic pathways in peripheral tissues. This effect was not observed when the cardiomyocytes were incubated with serum derived from MM patients. In ALMC-1 cells, pharmacologic MEK inhibition restored PUFA levels and decreased cytokines expression, linking MAPK pathway activity to lipid remodeling. Collectively, these findings support the presence of a multicompartment lipid-inflammatory program linking BM plasma cells, circulating lipid imbalance, and peripheral tissue metabolic remodeling in AL amyloidosis.

**Summary/Conclusion**

AL amyloidosis is characterized by systemic omega-3/omega-6 disequilibrium accompanied by coordinated pro-inflammatory cytokine production and increased COX2 expression in BM plasma cells. Enrichment of prostaglandin-related metabolic pathways in peripheral tissue suggests that lipid mediator signaling may contribute to disease pathogenesis beyond the marrow niche. These findings support a marrow-to-heart metabolic-inflammatory relay and upstream MAPK regulation as therapeutic target in AL amyloidosis.

**Copyright Information:** (Online) ISSN: 2572-9241
©2026 The Author(s). HemaSphere is published by John Wiley & Sons Ltd on behalf of European Hematology Association. This is an open access Abstract Book distributed under the Attribution-NonCommercial-NoDerivs (CC BY-NC-ND), which allows third parties to download the articles and share them with others as long as they credit the author and the Abstract Book, but they cannot change the content in any way or use them commercially.
**Abstract Book Citations:** Authors, Title, HemaSphere, 2026;10:(S1):pages. The abstract book DOIs can be found on the “Author Information” page.
**Disclaimer:** Articles published in the journal HemaSphere exclusively reflect the opinions of the authors. The authors are responsible for all content in their abstracts including accuracy of the facts, statements, citing resources, etc.

### (PF748) M6A METHYLATION MEDIATES GUICAOBAIDU DECOCTION-INDUCED MITOPHAGY TO ATTENUATE CARFILZOMIB-INDUCED CARDIOTOXICITY

**Topic:** 13. Myeloma and other monoclonal gammopathies - Biology & translational research

Nayan Yi*[1], Xing Cui[2]

*[1]Shandong University of Traditional Chinese Medicine, Jinan, China;[2]Second Affiliated Hospital of Shandong University of Traditional Chinese Medicine, Center of Oncology and Hematology, Jinan, China;*

### Background

Multiple myeloma (MM) is a prevalent hematological malignancy in the elderly. Carfilzomib (CFZ), an irreversible proteasome inhibitor, is a cornerstone of MM treatment; however, its clinical utility is constrained by significant cardiotoxicity, the highest incidence among proteasome inhibitors. The underlying mechanisms remain incompletely understood.
N6-methyladenosine (m6A) methylation is a prevalent epitranscriptomic modification that regulates RNA metabolism. Its reader proteins, particularly YTHDC2, function as critical molecular hubs linking epigenetic modifications to disease phenotypes, with emerging evidence implicating them in cardiovascular diseases. Nevertheless, whether m6A readers contribute to CFZ-induced cardiotoxicity remains largely unexplored.

### Aims

This study aimed to investigate the mitochondrial mechanism underlying CFZ-induced cardiotoxicity and elucidate how GCBD intervenes via methylation regulation.

### Methods

In vitro and in vivo models of carfilzomib (CFZ)-induced cardiotoxicity were established. MeRIP-qPCR and BNIP3 mRNA half-life assays identified YTHDC2 as the sole downregulated m6A reader following CFZ exposure, which positively regulated the mitophagy receptor BNIP3. In vitro, cardiomyocytes were treated with varying concentrations of Guicaobaidu Decoction (GCBD) to assess cell viability, mitochondrial function, and autophagic flux, alongside Western blotting for LC3, P62, and BNIP3. In vivo, a murine model was employed to evaluate survival rate, myocardial enzyme levels, echocardiography, histopathological changes, and mitophagy-related markers. Finally, UPLC-MS/MS analysis identified paeoniflorin as a key bioactive component of GCBD; molecular docking and in vitro validation confirmed that paeoniflorin alleviates CFZ-induced cardiotoxicity by targeting YTHDC2 to regulate mitophagy.

### Results

1.GCBD attenuates CFZ-induced cytotoxicity and restores cell viability in H9C2 cardiomyocytes.
2.YTHDC2 directly binds and stabilizes BNIP3 mRNA in an m6A-dependent manner under CFZ-induced injury.
3.GCDB restores impaired autophagic flux and promotes complete mitophagy to alleviate CFZ-induced myocardial injury.
4.CFZ exposure disrupts mitochondrial membrane potential and induces abnormal electrophysiological signals in cardiomyocytes.
5. GCBD treatment reverses CFZ-induced mitochondrial dysfunction and restores mitochondrial homeostasis.
6. GCBD administration improves cardiac function, reduces myocardial enzyme release, and attenuates histopathological damage in CFZ-treated mice.
7.GCBD upregulates YTHDC2 expression and restores m6A methylation balance to ameliorate CFZ-induced cardiotoxicity in vivo.
8.Paeoniflorin, the bioactive component of GCBD, directly targets YTHDC2 to recapitulate the cardioprotective effects against CFZ-induced injury.

**Copyright Information:** (Online) ISSN: 2572-9241
©2026 The Author(s). HemaSphere is published by John Wiley & Sons Ltd on behalf of European Hematology Association. This is an open access Abstract Book distributed under the Attribution-NonCommercial-NoDerivs (CC BY-NC-ND), which allows third parties to download the articles and share them with others as long as they credit the author and the Abstract Book, but they cannot change the content in any way or use them commercially.
**Abstract Book Citations:** Authors, Title, HemaSphere, 2026;10:(S1):pages. The abstract book DOIs can be found on the "Author Information" page.
**Disclaimer:** Articles published in the journal HemaSphere exclusively reflect the opinions of the authors. The authors are responsible for all content in their abstracts including accuracy of the facts, statements, citing resources, etc.

### Summary/Conclusion

Carfilzomib induces cardiotoxicity by downregulating BNIP3 expression, disrupting mitochondrial homeostasis. The m⁶A reader YTHDC2 regulates BNIP3 mRNA stability, forming a key pathway in CFZ-induced injury. GCBD protects against cardiotoxicity by upregulating YTHDC2 expression in BNIP3 related mitophagy, restoring mitophagy and improving cardiac function, with its active component paeoniflorin directly binding YTHDC2 as the material basis for this effect.

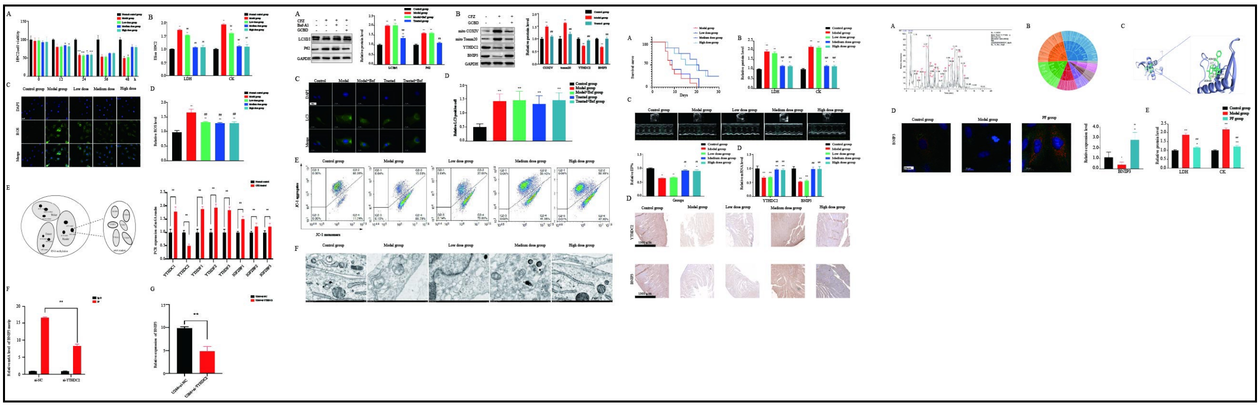

1.GCBD attenuates CFZ-induced cytotoxicity in H9C2 cardiomyocytes through YTHDC2-mediated binding and stabilization of BNIP3 mRNA in an m6A-dependent manner. A: Observation of cell viability in H9C2 cells pretreated with CFZ . B: Elisa of myocardial enzymes LDH and CK in H9C2 cells. C-D: Immunofluorescence and quantification of ROS production in H9C2 cells. E. representative m6A readers PCR expression treated with Carfilzomib, red bar represents cells treated with Carfilzomib, while black represents treated with normal medium, **p < 0.01 compared to normal control group. F. MeRIP-PCR of BNIP3 expression in U266 cells. Red bar represents immunoprecipitation expression, while black represents immunoglobulin. **p < 0.01. G. BNIP3 expression in U266 cells with siRNA. 2. GCBD alleviates CFZ-induced myocardial injury by restoring impaired autophagic flux and promoting complete mitophagy, thereby reversing mitochondrial membrane potential disruption, electrophysiological abnormalities, and mitochondrial dysfunction. A, B. Western blot analysis of representative proteins involved in BNIP3-mediated mitophagy. C, D. LC3 immunofluorescence staining and quantification; scale bar = 20 μm, E. Mitochondrial membrane potential measured by flow cytometry in H9C2 cells subjected to different treatments.F. Mitochondrial ultrastructure observed by transmission electron microscopy 3. GCBD upregulates YTHDC2 expression and restores m6A methylation balance in mice, thereby improving cardiac function and reducing myocardial injury induced by CFZ. A.Kaplan-Meier Survival curve. B. Myocardial enzyme levels of LDH and CK. C. Echocardiography (EF%) of mice. D. YTHDC2 and BNIP3 immunolocalization. 4. Paeoniflorin, the bioactive component of GCBD, directly targets BNIP3 to recapitulate the cardioprotective effects against CFZ-induced injury. A. LC-MS analysis of GCBD in negative ion mode. B. Classification of active components in GCBD. C. Schematic representation of molecular docking between paeoniflorin and YTHDC2. D. Cardiac ultrasound images and expression levels of mitochondrial markers in cardiomyocytes under paeoniflorin treatment, Scale bar: 20 μm. E. Detection of myocardial injury markers in mice following paeoniflorin intervention.

**Copyright Information:** (Online) ISSN: 2572-9241
©2026 The Author(s). HemaSphere is published by John Wiley & Sons Ltd on behalf of European Hematology Association. This is an open access Abstract Book distributed under the Attribution-NonCommercial-NoDerivs (CC BY-NC-ND), which allows third parties to download the articles and share them with others as long as they credit the author and the Abstract Book, but they cannot change the content in any way or use them commercially.
**Abstract Book Citations:** Authors, Title, HemaSphere, 2026;10:(S1):pages. The abstract book DOIs can be found on the "Author Information" page.
**Disclaimer:** Articles published in the journal HemaSphere exclusively reflect the opinions of the authors. The authors are responsible for all content in their abstracts including accuracy of the facts, statements, citing resources, etc.

### (PF749) CHARACTERIZATION OF THE LONG NON-CODING RNA LANDSCAPE IN MULTIPLE MYELOMA REVEALS NOVEL PUTATIVE DRIVER GENES ASSOCIATED WITH POOR OVERALL SURVIVAL

**Topic:** 13. Myeloma and other monoclonal gammopathies - Biology & translational research

Silke Nielsen*[1], Theresa Jakobsen[1], Robin Mjelle[2], Holger Møller[3], Therese Standal[2], Kristine Misund[2], Morten Noergaard Andersen[1, 4, 5], Wencke Walter[6], Lasse Kristensen[1]

*[1]Aarhus University, Department of Biomedicine, Aarhus, Denmark;[2]Norwegian University of Science and Technology, Department of Clinical and Molecular Medicine, Trondheim, Norway; [3]Aarhus University, Department of Clinical Medicine - Department of clinical biochemistry, Aarhus, Denmark; [4]Aarhus University Hospital, Department of Haematology, Aarhus, Denmark; [5]Aarhus University, Department of Clinical Medicine - Dept. of Molecular Medicine (MOMA), Aarhus, Denmark; [6]MLL Munich Leukemia Laboratory, Munich, Germany;*

### Background

Multiple myeloma (MM) is a malignancy of neoplastic plasma cells proliferating in the bone marrow. Despite advances in treatment, MM remains incurable due to treatment resistance and frequent relapses. Long non-coding RNAs (lncRNAs) are non-coding transcripts exceeding 200 nucleotides with diverse functions including transcriptional and translational regulation of genes. Despite being frequently deregulated in cancers, lncRNAs remain largely unexplored and their functions poorly understood.

### Aims

To characterize the lncRNA expression landscape in MM and identify candidates for functional investigation.

### Methods

RNA-seq data from a German cohort (n=191 MM patients, CD138+ plasma cells) and a British cohort (n=28 MM patient samples and n=5 control samples, CD138+ plasma cells) were analyzed for lncRNA expression. Differentially expressed (DE) lncRNAs were identified using DESeq2 with thresholds of |log2 fold-change| > 1 and adjusted p < 0.01. Clinical correlations were evaluated by Kaplan-Meier survival analysis for overall survival (OS) and integrated with cytogenetic risk factors, including translocation t(4;14) and *TP53* mutation data.

### Results

RNA-seq detected 15,085 lncRNAs in the German cohort and 12,824 in the British cohort, with substantial overlap of 12,814 lncRNAs across datasets. Filtering (mean normalized counts >100) identified 110 DE lncRNAs relative to the control samples, nine of which showed prognostic concordance with OS independent of genetic risk factors. Among these were *PURPL* (most significantly deregulated), *POT1-AS1*, and *MIR548XHG*. High *PURPL* expression was associated with poor OS, and this could be validated in an independent Norwegian cohort (n=86 MM patients, CD138+ plasma cells). PURPL-high patients (Gaussian mixture model) exhibited 309 DE mRNAs enriched in membrane potential regulation, inhibitory synapse assembly, and GABA signaling (*GABRG2*, *GABRA3/4* upregulated), all correlating with *PURPL* expression and worse survival.

### Summary/Conclusion

By integrating genome-wide lncRNA studies across multiple MM cohorts with clinical patient data, we characterized lncRNA dysregulation and identified markers of biologically aggressive disease, revealing potential novel therapeutic targets and prognostic biomarkers.

**Copyright Information:** (Online) ISSN: 2572-9241
©2026 The Author(s). HemaSphere is published by John Wiley & Sons Ltd on behalf of European Hematology Association. This is an open access Abstract Book distributed under the Attribution-NonCommercial-NoDerivs (CC BY-NC-ND), which allows third parties to download the articles and share them with others as long as they credit the author and the Abstract Book, but they cannot change the content in any way or use them commercially.
**Abstract Book Citations:** Authors, Title, HemaSphere, 2026;10:(S1):pages. The abstract book DOIs can be found on the "Author Information" page.
**Disclaimer:** Articles published in the journal HemaSphere exclusively reflect the opinions of the authors. The authors are responsible for all content in their abstracts including accuracy of the facts, statements, citing resources, etc.

### (PF750) STAGE-SPECIFIC DISRUPTION OF ERYTHROPOIESIS LEADS TO ANEMIA IN NEWLY DIAGNOSED MULTIPLE MYELOMA PATIENTS.

**Topic:** 13. Myeloma and other monoclonal gammopathies - Biology & translational research

Shihui Wang*[1], Changyu Chen[2], Huizhi Zhao[2], Zunmin Zhu[3]

*[1]Henan Provincial People's Hospital, People's Hospital of Zhengzhou University, Institute of Hematology, ZhengZhou, China; [2]School of Life Sciences, Zhengzhou University, ZhengZhou, China; [3]Henan Provincial People's Hospital, ZhengZhou, China;*

### Background

Multiple myeloma (MM) associated anemia affects over 60% of patients and correlates with poor prognosis. While most studies focus on the impact of disrupted microenvironment on erythropoiesis, the stage-specific erythroid cell defects and their underlying molecular mechanisms remain poorly characterized.

### Aims

To investigate the stage-specific defects and underlying molecular mechanisms of erythropoiesis in newly diagnosed multiple myeloma (NDMM) patients with anemia.

### Methods

Clinical data were collected from 300 patients with newly diagnosed multiple myeloma (NDMM) and 300 healthy controls. Phenotypic and transcriptomic profiles of bone marrow primary erythropoiesis were compared between healthy donors (HDs) and NDMM patients with anemia (NDMM-A). Furthermore, in vitro erythroid culture assays were performed to verify the intrinsic erythropoietic impairments in NDMM-A patients.

### Results

Primary erythropoiesis profiles from bone marrow revealed a global reduction in erythroblasts across all maturation stages, along with significantly impaired colony-forming capacity of erythroid progenitors in NDMM patients. Transcriptomic profiling uncovered stage-specific dysregulation in different stages of erythropoiesis in NDMM-A patients. Erythroid progenitors exhibited downregulation of cell-cycle regulators (e.g., *CDC20*, *AURKB*) and key erythropoietic transcription factors (e.g., *GATA1, GFI1B*), accompanied by upregulation of interferon-response genes. Terminal erythroblasts show upregulation of genes in (oxidative phosphorylation) OXPHOS and p53-mediated apoptosis, which could be responsible for the reduction of terminal erythroblasts. *In vitro* culture assays confirm that CD34+ erythroblasts derived from NDMM patients with anemia exhibit intrinsic defects: decreased proliferation, increased apoptosis, and impaired enucleation capacity.

### Summary/Conclusion

These results demonstrate that impaired erythropoiesis in NDMM involves both extrinsic microenvironmental suppression and intrinsic, stage-specific transcriptional and functional dysregulation within the erythroid lineage.

**Copyright Information:** (Online) ISSN: 2572-9241
©2026 The Author(s). HemaSphere is published by John Wiley & Sons Ltd on behalf of European Hematology Association. This is an open access Abstract Book distributed under the Attribution-NonCommercial-NoDerivs (CC BY-NC-ND), which allows third parties to download the articles and share them with others as long as they credit the author and the Abstract Book, but they cannot change the content in any way or use them commercially.
**Abstract Book Citations:** Authors, Title, HemaSphere, 2026;10:(S1):pages. The abstract book DOIs can be found on the "Author Information" page.
**Disclaimer:** Articles published in the journal HemaSphere exclusively reflect the opinions of the authors. The authors are responsible for all content in their abstracts including accuracy of the facts, statements, citing resources, etc.

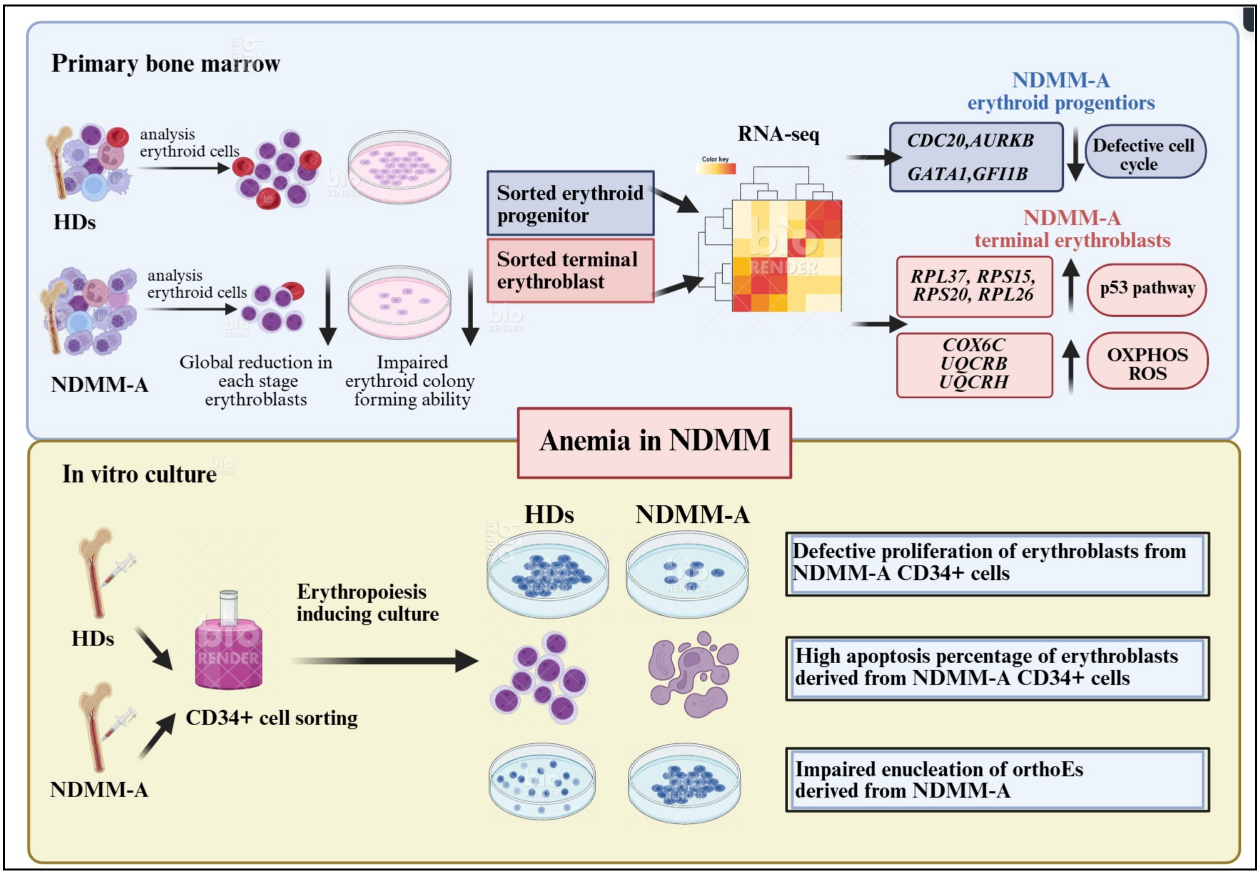

**Copyright Information:** (Online) ISSN: 2572-9241
©2026 The Author(s). HemaSphere is published by John Wiley & Sons Ltd on behalf of European Hematology Association. This is an open access Abstract Book distributed under the Attribution-NonCommercial-NoDerivs (CC BY-NC-ND), which allows third parties to download the articles and share them with others as long as they credit the author and the Abstract Book, but they cannot change the content in any way or use them commercially.
**Abstract Book Citations:** Authors, Title, HemaSphere, 2026;10:(S1):pages. The abstract book DOIs can be found on the "Author Information" page.
**Disclaimer:** Articles published in the journal HemaSphere exclusively reflect the opinions of the authors. The authors are responsible for all content in their abstracts including accuracy of the facts, statements, citing resources, etc.

### (PF751) BONE MARROW MICROENVIRONMENT-DEPENDENT INDUCTION OF MTORC1 CAUSES THERAPY RESISTANCE IN MULTIPLE MYELOMA

**Topic:** 13. Myeloma and other monoclonal gammopathies - Biology & translational research

Maaike Janssens*[1], Guus de Wilde[1], Jennate Elouach[1], Jeroen Guikema[1]

[1]*Amsterdam UMC, Pathology, Amsterdam, The Netherlands;*

### Background

Multiple Myeloma (MM) is a bone marrow (BM) neoplasm characterized by the clonal expansion of terminally differentiated B cells. The BM niche promotes drug resistance and disease progression through soluble factors and cell-cell contact. The PI3K/AKT signaling pathway, central to growth factor receptor signaling, is activated by various microenvironmental cues. We previously showed that the negative regulation of Forkhead box O (FOXO) transcription factors is a key downstream function of AKT in MM, as FOXO activation induces cell death and metabolic shutdown in MM cells. Transcriptome analysis revealed FOXO-dependent upregulation of the Insulin-like Growth Factor-1 Receptor (IGF1R) upon AKT inhibition, suggesting an adaptive pathway enabling MM cells to bypass AKT blockade. Combined AKT and IGF1R inhibition strongly induced cell death in MM cells. However, this was mitigated by co-culture of MM cells with bone marrow stromal cells (BMSC). Further experiments showed that activation of mechanistic Target of Rapamycin Complex 1 (mTORC1) by BMSC-derived soluble factors causes resistance to combined AKT and IGF1R inhibition, which could be overcome by rapamycin treatment. Importantly, mTORC1 inhibition by rapamycin also reversed BMSC-mediated resistance to the proteasome inhibitor Carfilzomib, indicating a more generalized BM microenvironment-induced resistance mechanism involving mTORC1 activation.

### Aims

This project aims to comprehensively characterize pathways involved in BM microenvironment-mediated drug resistance in MM by means of mTORC1-centered whole genome CRISPR screens in MM cell lines.

### Methods

To delineate factors driving mTORC1-dependent drug resistance, we performed whole genome CRISPR screens with MM cells cultured in BMSC-conditioned medium (CM). Parallel functional FACS-based CRISPR screens utilizing the Tor-Signal Indicator (TOSI) system, a PDCD4-degron fluorescent probe serving as mTOR activity sensor, were used to identify genes involved in microenvironment-mediated mTORC1 activation. Candidate genes were validated using pharmacological inhibition, neutralizing antibodies, CRISPR gene-editing and overexpression constructs.

### Results

CM from HS5, but not HS27a, BMSC lines abrogated cytotoxicity of AKT/IGF1R inhibitors. Transcriptomic comparison of these BMSC lines identified factors including IL-6, FGF2 and GM-CSF being secreted by HS5. Next to mTORC1 activation, IL-6 and HS5-CM strongly induced phosphorylation of STAT3. IL-6 supplementation protected MM cells from AKT/IGF1R inhibition, but neutralizing IL-6 in HS5-CM only partially restored drug sensitivity. Inhibition of Janus Kinases (JAK) prevented BMSC-induced resistance and reduced mTORC1 activation by HS5-CM, suggesting a direct role of JAK in mTORC1 activation.

**Copyright Information:** (Online) ISSN: 2572-9241
©2026 The Author(s). HemaSphere is published by John Wiley & Sons Ltd on behalf of European Hematology Association. This is an open access Abstract Book distributed under the Attribution-NonCommercial-NoDerivs (CC BY-NC-ND), which allows third parties to download the articles and share them with others as long as they credit the author and the Abstract Book, but they cannot change the content in any way or use them commercially.
**Abstract Book Citations:** Authors, Title, HemaSphere, 2026;10:(S1):pages. The abstract book DOIs can be found on the "Author Information" page.
**Disclaimer:** Articles published in the journal HemaSphere exclusively reflect the opinions of the authors. The authors are responsible for all content in their abstracts including accuracy of the facts, statements, citing resources, etc.

**Summary/Conclusion**

We have shown that HS5 BMSCs promote resistance of MM cells to AKT/IGF1R inhibition via mTORC1 activation. Neutralizing IL-6 in HS5-CM partially restored sensitivity to AKT/IGF1R inhibition, suggesting involvement of other (combinations of) soluble factors. Besides, JAK inhibition restored sensitivity to AKT/IGF1R inhibition and reduced mTORC1 activity suggesting JAK being involved in BMSC-mediated mTORC1 activation. We are currently investigating the involvement of STAT3 in mTORC1 activation. In addition, whole genome CRISPR screens will identify key regulators of mTORC1 activation, which may offer novel means to alleviate bone microenvironment-mediated therapy resistance in MM.

**Copyright Information:** (Online) ISSN: 2572-9241
©2026 The Author(s). HemaSphere is published by John Wiley & Sons Ltd on behalf of European Hematology Association. This is an open access Abstract Book distributed under the Attribution-NonCommercial-NoDerivs (CC BY-NC-ND), which allows third parties to download the articles and share them with others as long as they credit the author and the Abstract Book, but they cannot change the content in any way or use them commercially.
**Abstract Book Citations:** Authors, Title, HemaSphere, 2026;10:(S1):pages. The abstract book DOIs can be found on the “Author Information” page.
**Disclaimer:** Articles published in the journal HemaSphere exclusively reflect the opinions of the authors. The authors are responsible for all content in their abstracts including accuracy of the facts, statements, citing resources, etc.

### (PF752) PROGNOSTIC BIOMARKERS BEYOND BONE DISEASE IN NEWLY DIAGNOSED MULTIPLE MYELOMA: INTEGRATING PROTEOMIC, GENOMIC, AND BONE METABOLISM SIGNATURES

**Topic:** 13. Myeloma and other monoclonal gammopathies - Biology & translational research

Ioannis Ntanasis-Stathopoulos*[1], Panagiota Efstathia Nikolaou[1, 2], Marios Miliotis[3], Charalampos Filippatos[1], Panagiotis Bakouros[4], Manousos Makridakis[5], Ioannis Kostopoulos[4], Christine Liacos[1], Maria Frantzi[6], Maria Gavriatopoulou[1], Artemis G Hatzigeorgiou[3], Antonia Vlahou[5], Ourania Tsitsiloni[4], Meletios A. Dimopoulos[1], Evangelos Terpos[1]

*[1]Department of Clinical Therapeutics, Medical School, National Kapodistrian University of Athens, Athens, Greece;[2]Laboratory of Pharmacology, Faculty of Pharmacy, National & Kapodistrian University of Athens, Athens, Greece; [3]DIANA-Lab, Department of Computer Science and Biomedical Informatics, University of Thessaly, Lamia, Greece; [4]Section of Animal and Human Physiology, Department of Biology, National and Kapodistrian University of Athens, Athens, Greece; [5]Laboratory of Biotechnology, Academy of Athens Biomedical Research Foundation, Athens, Greece; [6]Department of Biomarker Research, Mosaiques diagnostics GmbH, Hannover, Germany;*

### Background

Myeloma bone disease (MBD) is a common and clinically significant manifestation present in most patients at multiple myeloma (MM) diagnosis.

### Aims

We evaluated the impact of MBD on Overall Survival (OS), Progression-Free Survival (PFS), and Time to Progression (TTP) in newly diagnosed MM (NDMM) patients in the era of modern therapies. As MM is a systemic malignancy originating in bone marrow, we also examined associations between bone-related biomarkers (Sema4D, Activin-A, periostin), markers of bone metabolism, and clinical outcomes. To identify novel circulating biomarkers, plasma proteomics and a pilot genomic analysis of clonal plasma cells and bulk bone marrow cells were performed.

### Methods

Plasma samples from 76 consecutive NDMM patients were analyzed. Sema4D, Activin-A, and periostin were measured by ELISA, and the LEGENDplex™ Human Bone Metabolism Panel (13-plex) quantified 13 additional proteins, including cytokines. To assess these markers at the SMM/MGUS stage, 21 paired samples from the same patients and 25 independent age- and sex-matched SMM/MGUS patients were analyzed. Proteomic profiling for novel biomarker discovery used liquid chromatography–mass spectrometry (LC-MS). Genomic analysis of sorted clonal plasma cells and bulk bone marrow cells from 25 patients employed next-generation sequencing to identify variants associated with MBD or clinical outcomes.

**Copyright Information:** (Online) ISSN: 2572-9241
©2026 The Author(s). HemaSphere is published by John Wiley & Sons Ltd on behalf of European Hematology Association. This is an open access Abstract Book distributed under the Attribution-NonCommercial-NoDerivs (CC BY-NC-ND), which allows third parties to download the articles and share them with others as long as they credit the author and the Abstract Book, but they cannot change the content in any way or use them commercially.
**Abstract Book Citations:** Authors, Title, HemaSphere, 2026;10:(S1):pages. The abstract book DOIs can be found on the “Author Information” page.
**Disclaimer:** Articles published in the journal HemaSphere exclusively reflect the opinions of the authors. The authors are responsible for all content in their abstracts including accuracy of the facts, statements, citing resources, etc.

### Results

NDMM patients had a median age of 59.5 years (range 46–89) and median follow-up of 2.5 years (range 1.7–3.4); 43 (56.6%) were women and 49 (64.5%) had MBD. MBD was not associated with OS (HR: 1.32, 95% CI: 0.35–4.97; p=0.68), PFS (HR: 0.83, 95% CI: 0.39–1.75; p=0.62), or TTP (HR: 0.74, 95% CI: 0.32–1.71; p=0.48), suggesting current treatments mitigate its impact. Sema4D and periostin were elevated in SMM/MGUS patients who progressed to MM (p=0.001; p<0.0001 vs non-progressors). In NDMM, Sema4D predicted PFS and showed a tendency for TTP (Multivariate p=0.03, HR: 3.03, 95% CI: 1.06–8.66; p=0.05, HR: 3.62, 95% CI: 0.98–13.29), highlighting prognostic potential. Multiplate analysis showed that at the MM stage IL-1β predicted PFS (Multivariate p=0.03, HR: 5.55, 95% CI: 1.14–27.03) and osteopontin predicted OS (Multivariate p=0.02, HR: 10.82, 95% CI: 1.29–90.65), underscoring roles of inflammation and bone turnover in relapse.
Proteomic profiling revealed an MBD-associated systemic signature, including upregulation of proteins linked to tumor burden and immune activation (B2M, L1TD1), cytoskeletal remodeling and motility (SHTN1, MYH16, FHOD1), and endothelial, coagulation, and inflammatory pathways (F12, AMBP, ARAP3). A common 13-protein signature (IGKV2-30, CFP, MASP1, F9, CLEC3B, ATRN, FGB, BCHE, ITIH4, C5, CPN1, LRG1, PLG) defined all survival endpoints and was enriched in complement activation, platelet function, and hemostatic pathways. Complement C3, C9, and C7 significantly affected PFS and TTP (C9; PFS multivariate p=0.0075, HR: 4.42, 95% CI:1.49–13.16; TTP multivariate p=0.0044, HR: 8.45, 95% CI:1.95–36.73). Genomic analyses identified variants associated with inferior TTP, including HLA-DRB5 (c.300_306delinsCGGG) and HLA-DQB1 (c.317_319delinsCGG).

### Summary/Conclusion

MBD did not significantly impact OS, PFS, or TTP in NDMM patients. Sema4D emerged as a promising biomarker for early MM progression. The IL-1β–complement cascade axis appears to drive disease progression independently of MBD and represents a potential therapeutic target in NDMM.

**Copyright Information:** (Online) ISSN: 2572-9241
©2026 The Author(s). HemaSphere is published by John Wiley & Sons Ltd on behalf of European Hematology Association. This is an open access Abstract Book distributed under the Attribution-NonCommercial-NoDerivs (CC BY-NC-ND), which allows third parties to download the articles and share them with others as long as they credit the author and the Abstract Book, but they cannot change the content in any way or use them commercially.
**Abstract Book Citations:** Authors, Title, HemaSphere, 2026;10:(S1):pages. The abstract book DOIs can be found on the “Author Information” page.
**Disclaimer:** Articles published in the journal HemaSphere exclusively reflect the opinions of the authors. The authors are responsible for all content in their abstracts including accuracy of the facts, statements, citing resources, etc.

### (PF753) A COMMON GENE EXPRESSION SIGNATURE ACROSS MULTIPLE MYELOMA DISEASE STAGES IDENTIFIES HIGH-RISK OF DISEASE PROGRESSION

**Topic:** 13. Myeloma and other monoclonal gammopathies - Biology & translational research

Claudio Cerchione*[1, 1], Nour Alassi[1], Sarah Tassinari[1], Delia Cangini[1], Irene Azzali[1], Asia Spedaluzzi[1], Sofia Cortesi[1], Giacomo Feliciani[1], Alice Rossi[1], Gerardo Musuraca[1], Nicola Normanno[1], Giorgia Simonetti[1], Emanuela Scarpi[1], Giovanni Martinelli[2], Matteo Marchesini[1]

*[1]Istituto Romagnolo per lo Studio dei Tumori "Dino Amadori" - IRST IRCCS, Meldola (FC), Italy;[2]Alma Mater Studiorum—Università di Bologna, Bologna, Italy, Department of Medical and Surgical Sciences (DIMEC), Bologna, Italy;*

### Background

Early and accurate identification of high-risk (HR) multiple myeloma (MM) is critical to optimize therapeutic strategies. Plasma cell–derived gene expression profiling (GEP) has emerged as a complementary prognostic tool to DNA-based diagnostics, identifying HR patients who may be missed by conventional risk stratification; however, its role in the early asymptomatic phase remains poorly explored.

### Aims

In this first Italian single-center prospective study, we profiled all consecutive patients with smoldering (SMM), newly diagnosed (NDMM), and relapsed/refractory (RRMM) myeloma to determine whether cytogenetic abnormalities and the risk of disease progression could be recapitulated across disease phases using a gene expression profiling (GEP) assay.

### Methods

Thirty-three SMM, 42 NDMM, and 30 RRMM patients were enrolled. Bone marrow samples were analyzed using the MMprofiler assay (Skyline Dx) to assess HR-associated GEP (SKY92 gene signature) and cytogenetics (virtual FISH or vFISH) according to the manufacturer's instructions. Statistical analyses were performed using GraphPad Prism 8 and R (v4.4.2), with two-sided tests, a significance threshold of $P < 0.05$, and Benjamini–Hochberg FDR correction.

**Copyright Information:** (Online) ISSN: 2572-9241
©2026 The Author(s). HemaSphere is published by John Wiley & Sons Ltd on behalf of European Hematology Association. This is an open access Abstract Book distributed under the Attribution-NonCommercial-NoDerivs (CC BY-NC-ND), which allows third parties to download the articles and share them with others as long as they credit the author and the Abstract Book, but they cannot change the content in any way or use them commercially.
**Abstract Book Citations:** Authors, Title, HemaSphere, 2026;10:(S1):pages. The abstract book DOIs can be found on the "Author Information" page.
**Disclaimer:** Articles published in the journal HemaSphere exclusively reflect the opinions of the authors. The authors are responsible for all content in their abstracts including accuracy of the facts, statements, citing resources, etc.

### Results

The SKY92 assay met quality criteria in 81.1% of SMM, 92.8% of NDMM, and 80.0% of RRMM cases, with increasing HR frequency from SMM (18%) to NDMM (43.5%) and RRMM (67%; *P* = 0.0018). vFISH showed high accuracy for t(4;14), t(14;16/20), and gain1q (97.9%, 85.2%, and 87.2%, respectively), but lower performance for del17p (66.7%). NDMM HR SKY92 patients showed a worst overall survival compared to patients with SKY92 standard risk (SR, *P*=0.0079), a trend maintained in RRMM with a nearly significant statistics due to the cohort sample size (*P*=0.093). These findings suggest that HR patients share a common transcriptional signature across disease stages. Accordingly, transcriptional comparison of SKY92 HR and SR NDMM and RRMM revealed *(i)* substantial cross-stage overlap within a SKY92-enriched GEP manifold by principal component analysis (PCA) and *(ii)* shared enrichment of pathways associated with aggressive MM as well as cell proliferation, oncogene-associated targets and DNA repair by gene set enrichment analysis (GSEA). To assess the predictive value of GE-based approaches in asymptomatic myeloma, SMM samples were projected onto the PCA space generated from NDMM and RRMM data, showing partial overlap according to SKY92 risk groups. GSEA comparing SKY92 HR versus SR SMM identified pathways shared with symptomatic HR myeloma (e.g. cell proliferation, oncogene-associated targets and DNA repair), alongside signatures related to early disease biology (e.g. inflammation). Consistently, SKY92 HR SMM patients had a significantly higher risk of progression to symptomatic MM, with 36-month progression rates of 70% versus 8.4% in SR cases (*P* = 0.038); among untreated patients, rates were 70% versus 0% (*P* = 0.173).

### Summary/Conclusion

Our findings support the use of gene expression (GE)–based tools for risk stratification and detection of high-risk (HR) cytogenetic features in symptomatic MM patients, particularly when conventional FISH is unavailable. Moreover, our data indicate that HR MM patients exhibit shared transcriptional programs across asymptomatic and symptomatic phases, consistent with recent evidence of common genomic features between a subset of SMM and overt myeloma, and demonstrate that these patients can be timely identified using clinically applicable GEP-based prognostic assays.

**Copyright Information:** (Online) ISSN: 2572-9241
©2026 The Author(s). HemaSphere is published by John Wiley & Sons Ltd on behalf of European Hematology Association. This is an open access Abstract Book distributed under the Attribution-NonCommercial-NoDerivs (CC BY-NC-ND), which allows third parties to download the articles and share them with others as long as they credit the author and the Abstract Book, but they cannot change the content in any way or use them commercially.
**Abstract Book Citations:** Authors, Title, HemaSphere, 2026;10:(S1):pages. The abstract book DOIs can be found on the “Author Information” page.
**Disclaimer:** Articles published in the journal HemaSphere exclusively reflect the opinions of the authors. The authors are responsible for all content in their abstracts including accuracy of the facts, statements, citing resources, etc.

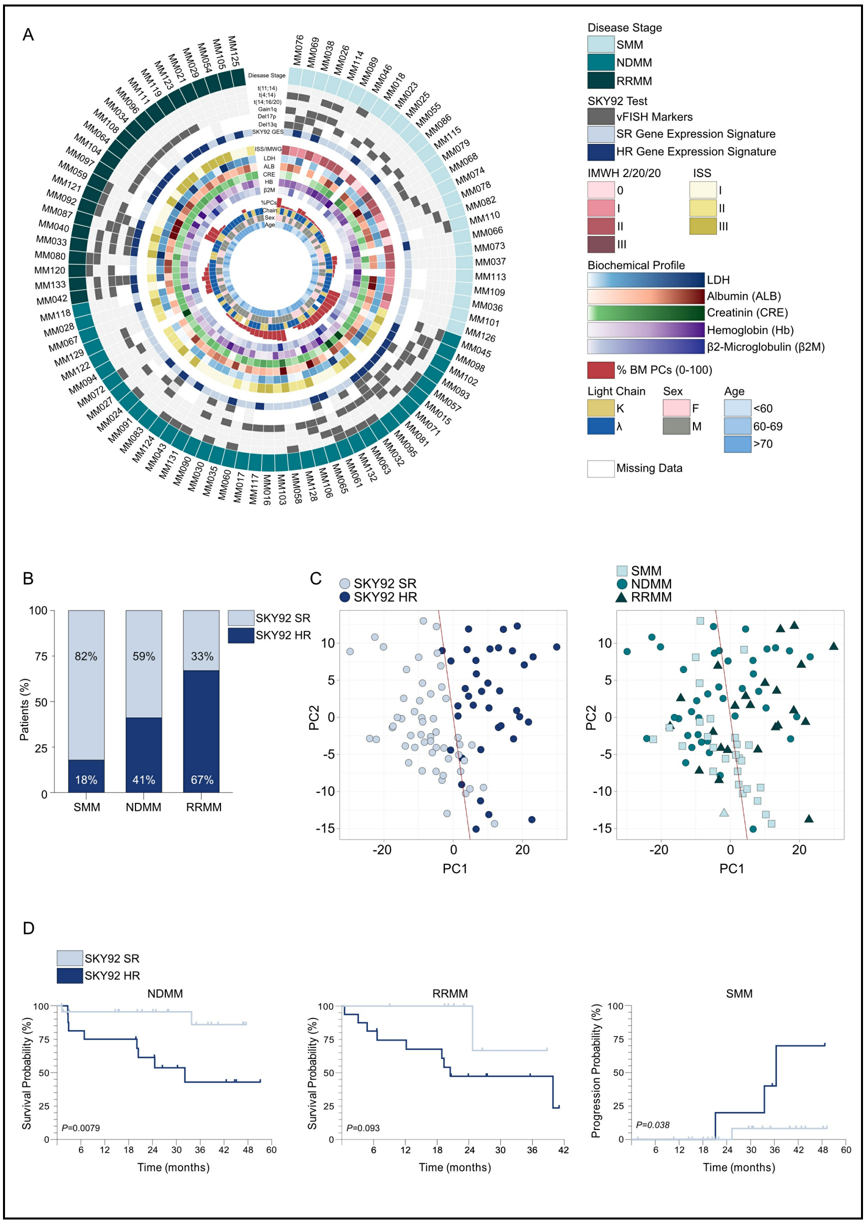

A) Circos plot showing clinical and molecular features of the study cohort (n=90 patients: SMM=27, NDMM=39 and RRMM=24). B) Results of SKY92 test across disease stages calculated as a percentage of SKY92 high risk (HR) detected samples on the total number of patients included in each category. C) PCA score plots of PC1 versus PC2. Left: Samples stratified by SKY92 risk classification primarily along PC1 (dashed red line indicates the main direction of separation). Right: Samples stratified by clinical disease stage. D) Kaplan-Meier overall survival curves of SKY92 HR versus standard risk (SKY92 SR) in NDMM (left, HR=16; SR=23), RRMM (center, HR=16 and SR=8) and SMM (right, HR=5 and SR=22). X-axis, time elapsed since sample collection (months); y-axis probability of survival (NDMM and RRMM) or progression (SMM). Logrank P and number of patients at risk are shown.

**Copyright Information:** (Online) ISSN: 2572-9241
©2026 The Author(s). HemaSphere is published by John Wiley & Sons Ltd on behalf of European Hematology Association. This is an open access Abstract Book distributed under the Attribution-NonCommercial-NoDerivs (CC BY-NC-ND), which allows third parties to download the articles and share them with others as long as they credit the author and the Abstract Book, but they cannot change the content in any way or use them commercially.
**Abstract Book Citations:** Authors, Title, HemaSphere, 2026;10:(S1):pages. The abstract book DOIs can be found on the "Author Information" page.
**Disclaimer:** Articles published in the journal HemaSphere exclusively reflect the opinions of the authors. The authors are responsible for all content in their abstracts including accuracy of the facts, statements, citing resources, etc.

### (PF754) MALONYL-COA–DRIVEN XPO1 MALONYLATION PROMOTES DRUG RESISTANCE THROUGH BECLIN1 NUCLEAR EXPORT–DEPENDENT AUTOPHAGY IN RELAPSED AND REFRACTORY MULTIPLE MYELOMA

**Topic:** 13. Myeloma and other monoclonal gammopathies - Biology & translational research

Guangqi Wang*[1], Yu Hu[1], Chunyan Sun[1], Fengjuan Fan[1], Chi MA[1], Qun Li[1], Linyu Cai[1], Lixin Quan[1], Wanting Yu[1], Dehu Li[1], Meng Wang[1]

*[1]Union Hospital, Tongji Medical College, Huazhong University of Science and Technology, Institute of Hematology, Wuhan, China;*

### Background

Acquired drug resistance is a primary driver of disease relapse and treatment refractoriness in multiple myeloma (MM), yet its underlying molecular mechanisms remain highly complex and incompletely understood. Emerging evidence indicates that metabolic reprogramming and aberrant post-translational modifications (PTMs) enable tumor cells to adapt to therapeutic stress and maintain cellular homeostasis. Malonyl-CoA, a key intermediate of lipid metabolism, has recently been identified as a substrate for lysine malonylation, an underexplored PTM with potential regulatory roles in cancer biology. However, whether malonyl-CoA–driven protein malonylation contributes to mechanisms of therapy resistance in MM remains largely unknown.

### Aims

To determine whether malonyl-CoA–driven XPO1 malonylation contributes to drug resistance in MM and to elucidate the underlying molecular mechanism.

### Methods

Intracellular malonyl-CoA in MM cells was measured by ELISA. Lithium malonyl-CoA was used to modulate intracellular malonyl-CoA level, followed by assessment of drug sensitivity (CCK-8 assay). Global lysine malonylation was analyzed by immunoblotting, and malonyl-lysine–enriched proteomics identified differential modifications in drug-resistant MM cells. To identify the lysine stie(s) involved in XPO1 malonylation, XPO1 mutants that switched from lysine to arginine (K→R) in three lysine sites were generated according to above proteomic results. Drug sensitivity to bortezomib and selinexor were tested in vitro and in vivo comparing XPO1 WT and K455R cells. PTM crosstalk was assessed by examining ubiquitination, phosphorylation, and acetylation of XPO1 WT and K455R. Protein chasing assays were conducted to determine protein stability. Integrated immunoprecipitation–mass spectrometry and nuclear–cytoplasmic fractionation proteomics were performed to elucidate downstream mechanisms of XPO1 malonylation–mediated drug resistance.

**Copyright Information:** (Online) ISSN: 2572-9241
©2026 The Author(s). HemaSphere is published by John Wiley & Sons Ltd on behalf of European Hematology Association. This is an open access Abstract Book distributed under the Attribution-NonCommercial-NoDerivs (CC BY-NC-ND), which allows third parties to download the articles and share them with others as long as they credit the author and the Abstract Book, but they cannot change the content in any way or use them commercially.
**Abstract Book Citations:** Authors, Title, HemaSphere, 2026;10:(S1):pages. The abstract book DOIs can be found on the "Author Information" page.
**Disclaimer:** Articles published in the journal HemaSphere exclusively reflect the opinions of the authors. The authors are responsible for all content in their abstracts including accuracy of the facts, statements, citing resources, etc.

### Results

Malonyl-CoA levels were significantly elevated in primary plasma cells from relapsed and refractory multiple myeloma (RRMM) compared with newly diagnosed MM (NDMM), a finding consistently observed in paired parental and drug-resistant MM cell lines. Pharmacological elevation of malonyl-CoA increased bortezomib resistance in MM cells. Accordingly, global lysine malonylation levels were markedly higher in RRMM samples and resistant MM cell lines. Quantitative malonyl-proteomic profiling identified XPO1 as a resistance-associated protein with significantly increased malonylation in drug-resistant MM cells. Site-directed mutagenesis identified lysine 455 (K455) as the critical malonylation site on XPO1. MM cells expressing a malonylation-deficient XPO1 mutant (K455R) displayed enhanced sensitivity to bortezomib both in vitro and in vivo compared with XPO1 wild-type cells. Loss of XPO1 malonylation did not affect its ubiquitination, phosphorylation, acetylation, or protein stability. Integrated immunoprecipitation–mass spectrometry and nuclear–cytoplasmic proteomic analyses demonstrated that XPO1 malonylation enhanced its interaction with Beclin1, promoted Beclin1 nuclear export, and increased autophagy under therapeutic stress, thereby supporting cellular homeostasis and drug resistance in MM.

### Summary/Conclusion

Malonyl-CoA–triggered XPO1 malonylation facilitates nuclear export of Beclin1, maintaining autophagy and cellular homeostasis in multiple myeloma cells, which supports cell survival under therapeutic stress and contributes to the development of drug resistance.

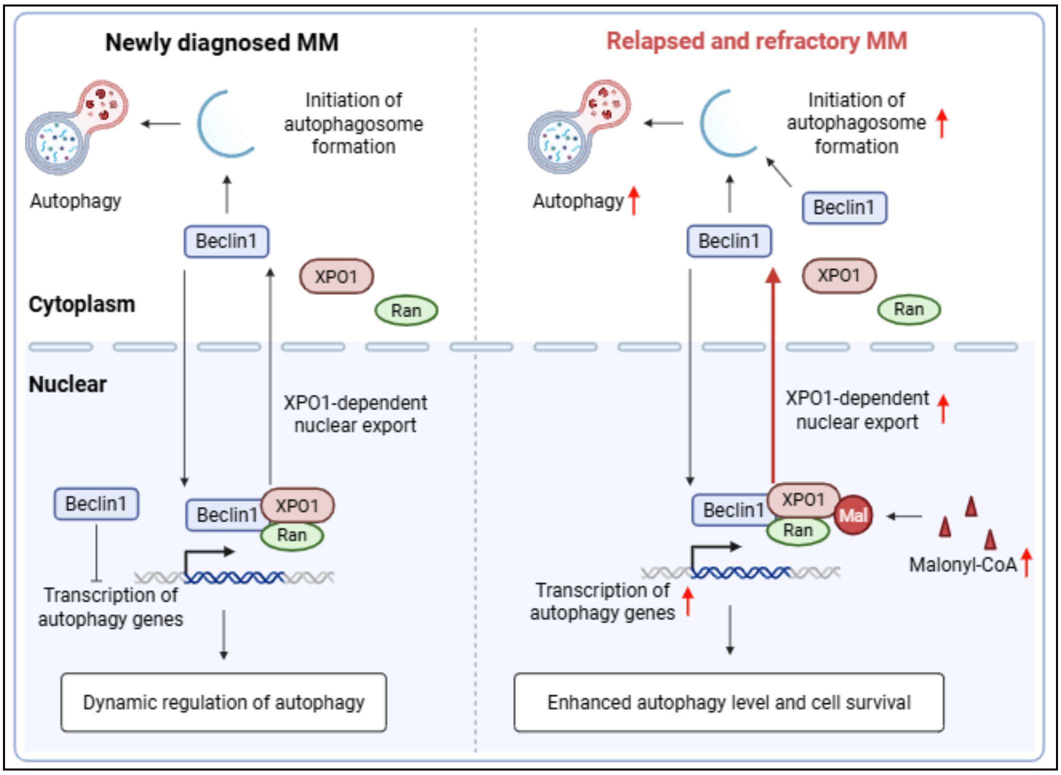

Schematic model depicting the mechanism of XPO1 malonylation-mediated drug resistance in multiple myeloma (MM) . In newly diagnosed MM, Beclin1 dynamically shuttles between nucleus and cytoplasm, maintaining basal autophagy levels. In relapsed/refractory MM, elevated Malonyl-CoA enhances XPO1-dependent nuclear export of Beclin1, promoting autophagy gene transcription and autophagosome formation, thereby increasing autophagy and supporting tumor cell survival. Upward red arrows indicate increased activity or expression.

**Copyright Information:** (Online) ISSN: 2572-9241
©2026 The Author(s). HemaSphere is published by John Wiley & Sons Ltd on behalf of European Hematology Association. This is an open access Abstract Book distributed under the Attribution-NonCommercial-NoDerivs (CC BY-NC-ND), which allows third parties to download the articles and share them with others as long as they credit the author and the Abstract Book, but they cannot change the content in any way or use them commercially.
**Abstract Book Citations:** Authors, Title, HemaSphere, 2026;10:(S1):pages. The abstract book DOIs can be found on the "Author Information" page.
**Disclaimer:** Articles published in the journal HemaSphere exclusively reflect the opinions of the authors. The authors are responsible for all content in their abstracts including accuracy of the facts, statements, citing resources, etc.

### (PF755) IMS-IMWG 2025 CONSENSUS GENOMIC STAGING (CGS) OF MULTIPLE MYELOMA IN COMMPASS IDENTIFIES CLINICAL DIFFERENCES AND SURVIVAL DISPARITIES FOR PATIENTS WITH HIGH AFRICAN GENETIC ANCESTRY

**Topic:** 13. Myeloma and other monoclonal gammopathies - Biology & translational research

Steven Foltz[1], Chaitanya Acharya*[1], Alexander Gout[1], Yuxin Jin[2], David Avigan[3], Ravi Vij[4], Shaji Kumar[5], Sagar Lonial[6], Hearn Cho[1], Katie Wozniak[1], Jonathan Keats[7], Craig Cole[8], John Carpten[2], George Mulligan[1]

*[1]Multiple Myeloma Research Foundation, Norwalk, CT, United States of America;[2]City of Hope National Medical Center, Duarte, CA, United States of America; [3]Beth Israel Deaconess Medical Center, Boston, MA, United States of America; [4]Washington University School of Medicine, St. Louis, MO, United States of America; [5]Mayo Clinic, Rochester, MN, United States of America; [6]Winship Cancer Institute, Emory University School of Medicine, Atlanta, GA, United States of America;[7]Translational Genomics Research Institute, Phoenix, AZ, United States of America; [8]Karmanos Cancer Institute, Detroit, MI, United States of America;*

### Background

Multiple myeloma is the second most common adult blood cancer and remains largely incurable, despite recent advances. The IMS-IMWG Consensus Genomic Staging (CGS) risk criteria combine genomic events with clinical measures to identify patients at high-risk of poor clinical outcome (Avet-Loiseau, et al. *JCO* 2025). The Multiple Myeloma Research Foundation CoMMpass Study (NCT01454297) is a prospective, longitudinal observational trial that enrolled over 1100 patients starting in 2011. Clinical data collection for CoMMpass is now complete, including updated, final progression-free (PFS) and overall survival (OS) outcomes.

### Aims

We evaluated CoMMpass clinical outcomes in the context of both CGS risk criteria and genetic ancestry.

### Methods

Following CGS guidelines, we characterized patients according to the following criteria. Criteria 1: del(17p) with > 20% clonal fraction and/or TP53 mutation. Criteria 2: t(4;14), t(14;16), or t(14;20) along with 1q+ and/or del(1p32). Criteria 3: monoallelic del(1p32) plus 1q+, or biallelic del(1p32). Criteria 4: beta-2 microglobulin >= 5.5 mg/L with normal creatinine (< 1.2 mg/dL, or < 106.082 micromol/L). Genetic ancestry was determined using ADMIXTURE in comparison to 1000 Genomes populations.

**Copyright Information:** (Online) ISSN: 2572-9241
©2026 The Author(s). HemaSphere is published by John Wiley & Sons Ltd on behalf of European Hematology Association. This is an open access Abstract Book distributed under the Attribution-NonCommercial-NoDerivs (CC BY-NC-ND), which allows third parties to download the articles and share them with others as long as they credit the author and the Abstract Book, but they cannot change the content in any way or use them commercially.
**Abstract Book Citations:** Authors, Title, HemaSphere, 2026;10:(S1):pages. The abstract book DOIs can be found on the "Author Information" page.
**Disclaimer:** Articles published in the journal HemaSphere exclusively reflect the opinions of the authors. The authors are responsible for all content in their abstracts including accuracy of the facts, statements, citing resources, etc.

### Results

We evaluated the CGS risk status of 877 CoMMpass patients, finding 69.2% (573/828) to be Standard Risk (SR) and 30.8% (255/828) to be High Risk (HR). 10.6% (91/858) of patients met Criteria 1, 10.7% (93/873) met Criteria 2, 5.6% (49/877) met Criteria 3, and 8.7% (73/835) met Criteria 4.

We found 14.2% (123/867) of patients to have high African-like genetic ancestry (AFR-high) and 85.8% (744/867) to have low African-like genetic ancestry (AFR-low). Genetic ancestry was over 99% concordant with self-reported ethnicity.
We observed significantly fewer HR patients within the AFR-high group (20%, 23/115) than in the AFR-low group (32.4%, 231/712) (Fisher's exact test, p = 0.0065). The AFR-high group met HR criteria less frequently for all categories; however, a statistically significant difference was observed only for Criteria 3 (p-value = 0.03). Higher levels of creatinine in AFR-high patients decreased the likelihood of meeting the normal creatinine requirement for Criteria 4.

HR patients showed worse PFS (median 26.9 months vs. 49.4 months; log-rank p < 0.0001) and OS (median OS 67.7 months vs. median not reached; p < 0.0001). Among patients receiving first line triplet therapy and stem cell transplant, HR patients showed worse PFS (median 42.0 months vs. 76.6 months; p < 0.0001) and OS (median 102.4 months vs. median not reached; p < 0.0001). Using Cox proportional hazards models with covariates for age, sex, genetic ancestry, CGS risk, transplant status, and drug treatment, AFR-high patients showed significantly worse PFS (HR = 1.51, 95% CI 1.19-1.93; p = 0.00081); OS trended worse but was not statistically significant (HR = 1.30, 95% CI 0.95-1.80; p = 0.11).

### Summary/Conclusion

CGS HR patients comprise 30.8% of CoMMpass and show significantly worse PFS and OS than SR patients when treated with CoMMpass-era standard of care therapies. AFR-high patients met HR criteria at a lower rate than AFR-low patients, which may impact patient recruitment and equitable access to clinical trials targeting HR patients unless specific strategies are implemented to address this difference. AFR-high patients demonstrated significantly worse PFS than AFR-low patients after controlling for common clinical covariates.

**Copyright Information:** (Online) ISSN: 2572-9241
©2026 The Author(s). HemaSphere is published by John Wiley & Sons Ltd on behalf of European Hematology Association. This is an open access Abstract Book distributed under the Attribution-NonCommercial-NoDerivs (CC BY-NC-ND), which allows third parties to download the articles and share them with others as long as they credit the author and the Abstract Book, but they cannot change the content in any way or use them commercially.
**Abstract Book Citations:** Authors, Title, HemaSphere, 2026;10:(S1):pages. The abstract book DOIs can be found on the "Author Information" page.
**Disclaimer:** Articles published in the journal HemaSphere exclusively reflect the opinions of the authors. The authors are responsible for all content in their abstracts including accuracy of the facts, statements, citing resources, etc.

### (PF756) LIQUID BIOPSY–BASED THREE-DIMENSIONAL TELOMERE PROFILING DISTINGUISHES AL AMYLOIDOSIS FROM MULTIPLE MYELOMA AT DIAGNOSIS

**Topic:** 13. Myeloma and other monoclonal gammopathies - Biology & translational research

Yulia Shifrin[1], Asieh Alikhah[1], Zahabiya Husain[1], Josiph Pranjeta[2], Jason Valent[2], Shahzad Raza[2], Faiz Anwer[2], Christy Samaras[2], Louis Williams[2], Jack Khouri[2], Diana Basali[2], Claudio Brunstein[2], Willem Vanheeckeren[2], Sabine Mai*[3], Sandra Mazzoni[2]

*[1]Telo Genomics Corp, Toronto, Canada;[2]Taussig Cancer Institute, Cleveland, United States of America;[3]University of Manitoba, WINNIPEG, Canada;*

### Background

Light-chain (AL) amyloidosis and multiple myeloma (MM) are plasma cell dyscrasias characterized by a typical clonal expansion of plasma cells in the bone marrow. AL amyloidosis is defined by tissue deposition of misfolded immunoglobulin light chains, leading to progressive multiorgan dysfunction and poor prognosis. While MM diagnostics are increasingly incorporating liquid biopsy approaches, AL diagnosis remains dependent on detecting a monoclonal immunoglobulin or an abnormal serum free light chain (FLC) ratio, followed by an invasive tissue biopsy to confirm amyloid deposition. To date, no studies have reported the use of peripheral blood–based liquid biopsy for the diagnosis or risk stratification of AL amyloidosis.

### Aims

Spatial three-dimensional (3D) telomere profiling at the single-cell level has been shown to be a strong prognostic tool across the myeloma disease spectrum, from monoclonal gammopathy of undetermined significance (MGUS) to smoldering MM (SMM) to symptomatic MM. Telomere profiles have been used to identify SMM patients at high risk of progression from precursor states and detect emerging resistance to first-line therapy in newly-diagnosed MM (NDMM). This study aims to explore whether single-cell 3D telomere profiling of circulating plasma cells (CTC) from the peripheral blood can differentiate AL amyloidosis from MM.

### Methods

We previously established a workflow for isolation, immunophenotyping, and 3D telomere profiling of circulating MM plasma cells from peripheral blood, enabling characterization of minimal residual disease (MRD) and clonal heterogeneity in NDMM patients (doi.org/10.1182/blood-2025-2217). In this study, our previously established workflow was used to isolate CTCs from peripheral blood samples of a mixed MM/AL cohort. Isolated CTCs were immunophenotyped, and the single-cell 3D telomere profile of the cells was evaluated. Hierarchical cluster analysis was performed based on spatial telomere architecture parameters.

### Results

We used a TeloView, a software specifically designed to quantify key 3D telomeric parameters on a single-cell level, to identify and quantify the number of telomeres (an indicator of aneuploidy), their length (measured by relative fluorescent intensity), number of aggregates, the spatial distribution of telomeres throughout the cell cycle (axial or *a/c* ratio), nuclear volume, and the telomere numbers to the nuclear volume ratio. Hierarchical cluster analysis stratified patients into distinct clusters according to 3D telomere architecture. Notably, AL cases formed two discrete clusters clearly separated from MM profiles, indicating biologically distinct telomere signatures detectable in CTCs.

**Copyright Information:** (Online) ISSN: 2572-9241
©2026 The Author(s). HemaSphere is published by John Wiley & Sons Ltd on behalf of European Hematology Association. This is an open access Abstract Book distributed under the Attribution-NonCommercial-NoDerivs (CC BY-NC-ND), which allows third parties to download the articles and share them with others as long as they credit the author and the Abstract Book, but they cannot change the content in any way or use them commercially.
**Abstract Book Citations:** Authors, Title, HemaSphere, 2026;10:(S1):pages. The abstract book DOIs can be found on the "Author Information" page.
**Disclaimer:** Articles published in the journal HemaSphere exclusively reflect the opinions of the authors. The authors are responsible for all content in their abstracts including accuracy of the facts, statements, citing resources, etc.

**Summary/Conclusion**

This study provides the first indication that liquid biopsy combined with single-cell spatial telomere profiling may enable non-invasive identification of AL amyloidosis at diagnosis. These results may provide a foundation for developing methods for peripheral blood–based disease classification in AL, reducing the need for invasive tissue biopsy.

**Copyright Information:** (Online) ISSN: 2572-9241
©2026 The Author(s). HemaSphere is published by John Wiley & Sons Ltd on behalf of European Hematology Association. This is an open access Abstract Book distributed under the Attribution-NonCommercial-NoDerivs (CC BY-NC-ND), which allows third parties to download the articles and share them with others as long as they credit the author and the Abstract Book, but they cannot change the content in any way or use them commercially.
**Abstract Book Citations:** Authors, Title, HemaSphere, 2026;10:(S1):pages. The abstract book DOIs can be found on the "Author Information" page.
**Disclaimer:** Articles published in the journal HemaSphere exclusively reflect the opinions of the authors. The authors are responsible for all content in their abstracts including accuracy of the facts, statements, citing resources, etc.

### (PF757) UTILISING WHOLE PROTEOME ANALYSIS OF EX-VIVO IMID/CELMOD TREATED PRIMARY MYELOMA PATIENT SAMPLES TO UNDERSTAND DRUG RESISTANCE MECHANISMS

**Topic:** 13. Myeloma and other monoclonal gammopathies - Biology & translational research

Sarah Bird*[1, 2], Fernando Sialana[1], Shannon Martin[1], Marc Leiro[1], Ananya Sukhtankar[1], Salomon Morales[1], Jyoti Choudhary[1], Charlotte Pawlyn[1, 2]

*[1]The Institute of Cancer Research, London, United Kingdom;[2]The Royal Marsden Hospital, London, United Kingdom;*

### Background

Immunomodulatory drugs (IMiDs) are approved for the treatment of myeloma as part of combination regimes and as maintenance. IMiDs function as molecular glue degraders, binding to the CRBN component of the CRL4$^{CRBN}$ E3 ubiquitin ligase and designating neosubstrates for degradation via the proteasome. They have direct action against myeloma cells but also increase the activity of T/NK cells. Next generation agents (CELMoDs) are more potent degraders, with clinical activity in IMiD resistant patients. Agnostic analysis of the degradome across IMiDs and CELMoDs in primary myeloma cells has not previously been undertaken.

### Aims

- To generate patient-specific proteome-wide *ex-vivo* IMiD and CELMoD-induced degradation profiles.
- To compare these profiles in samples from clinically IMiD sensitive and resistant patients, exposing likely mechanisms of resistance.

### Methods

CD138+ myeloma cells and CD8+ T cells were isolated from bone marrow aspirates. Cells, processed <24hr from BMA, were incubated for 4hr with lenalidomide (Len, 10uM), pomalidomide (Pom, 10uM), iberdomide (Iber, 1uM) and mezigdomide (Mezi, 1uM) vs DMSO control. Myeloma cell samples were analysed using TMT-based quantitative mass spectrometry. For each protein log2FCs were generated for each drug compared to DMSO and analysed using two-sided one sample t-tests in Perseus. T cell samples were analysed using the Jess (Simple Western) for Ikaros, Aiolos and Cereblon.

**Copyright Information:** (Online) ISSN: 2572-9241
©2026 The Author(s). HemaSphere is published by John Wiley & Sons Ltd on behalf of European Hematology Association. This is an open access Abstract Book distributed under the Attribution-NonCommercial-NoDerivs (CC BY-NC-ND), which allows third parties to download the articles and share them with others as long as they credit the author and the Abstract Book, but they cannot change the content in any way or use them commercially.
**Abstract Book Citations:** Authors, Title, HemaSphere, 2026;10:(S1):pages. The abstract book DOIs can be found on the "Author Information" page.
**Disclaimer:** Articles published in the journal HemaSphere exclusively reflect the opinions of the authors. The authors are responsible for all content in their abstracts including accuracy of the facts, statements, citing resources, etc.

**Results**

TMT-based whole proteomes (~7000 proteins) were generated from *ex-vivo* samples from 19 patients (9 patients with all drugs and 10 with at least one IMiD and one CELMoD). Six patients were clinically sensitive/unexposed to IMiDs and 13 were resistant. None had been exposed to CELMoDs.

Degradation of Ikaros and Aiolos was demonstrated in all patients classified as clinically sensitive to IMiDs (6/6). In contrast, of the 13 resistant patients only 9/13 (69%) showed evidence of degradation. This suggests loss of CRBN function led to resistance in a subset of patients, whilst in the remainder resistance arose through alternate mechanisms, such as uncoupling of MYC/IRF4 from Ikaros/Aiolos control.

To explore the degradome in the primary sample context, the 15 patients demonstrating degradation were analysed. Degradation of Ikaros/Aiolos was confirmed with all IMiDs/CELMoDs and of ZFP91 with pomalidomide and CELMoDs. No novel neosubstrates across any of the IMiD/CELMoDs were identified and there was no difference in the spectrum of neosubstrates degraded between clinically sensitive and resistant patients.

The expected increase in magnitude of degradation of Ikaros/Aiolos was demonstrated with the more potent CELMoDs compared to IMiDs, despite a lower concentration of CELMoDs. Of the IMiDs Pom was a more potent degrader than Len, and within the CELMoDs Mezi was a more potent degrader than Iber.

To understand how resistance may occur clinically in the subset of resistant patients with preserved degradation, a separate analysis of CD8+ T cells from 11 patients was undertaken. Of these, 4 were from clinically IMiD sensitive patients and Ikaros/Aiolos degradation was seen in all. Of the 7 from resistant patients, 2 showed no evidence of Ikaros/Aiolos degradation, suggesting this may have contributed to the clinical resistance phenotype.

**Summary/Conclusion**

Exploring the degradome with IMiDs and CELMoDs in a large and well annotated collection of *ex-vivo* primary myeloma samples for the first time has provided insights into mechanisms of resistance.

**Copyright Information:** (Online) ISSN: 2572-9241
©2026 The Author(s). HemaSphere is published by John Wiley & Sons Ltd on behalf of European Hematology Association. This is an open access Abstract Book distributed under the Attribution-NonCommercial-NoDerivs (CC BY-NC-ND), which allows third parties to download the articles and share them with others as long as they credit the author and the Abstract Book, but they cannot change the content in any way or use them commercially.
**Abstract Book Citations:** Authors, Title, HemaSphere, 2026;10:(S1):pages. The abstract book DOIs can be found on the “Author Information” page.
**Disclaimer:** Articles published in the journal HemaSphere exclusively reflect the opinions of the authors. The authors are responsible for all content in their abstracts including accuracy of the facts, statements, citing resources, etc.

### (PF758) LNCRNA NEAT1 ORCHESTRATES A CERNA NETWORK GOVERNING CORE PATHWAYS OF PROLIFERATION AND CELLULAR DYNAMICS IN MULTIPLE MYELOMA

**Topic:** 13. Myeloma and other monoclonal gammopathies - Biology & translational research

Domenica Ronchetti[1], Ilaria Silvestris[1], Valentina Traini[1], Giuseppina Fabbiano[2], Andrea Devecchi[3], Noemi Puccio[4], Federica Torricelli[4], Ilaria Craparotta[5], Roberto Piva[6], Antonino Neri[7], Luca Agnelli[3], Francesco Passamonti[1, 2], Niccolò Bolli*[1, 2], Elisa Taiana[2]

*[1]University of Milan, Department of Oncology and Hemato-Oncology, Milan, Italy;[2]IRCCS Fondazione Ca' Granda Ospedale Maggiore Policlinico, Hematology, Milan, Italy; [3]Fondazione IRCCS Istituto Nazionale dei Tumori, Department of Diagnostic Innovation, Milano, Italy; [4]Azienda USL-IRCCS di Reggio Emilia, Laboratory of Translational Research, Reggio Emilia, Italy;[5]Mario Negri IRCCS, Oncology Department, Milano, Italy; [6]University of Turin, Department of Molecular Biotechnology and Health Sciences, Turin, Italy; [7]Azienda USL-IRCCS di Reggio Emilia, Scientific Directorate, Reggio Emilia, Italy;*

### Background

The lncRNA NEAT1 is overexpressed in multiple myeloma (MM) plasma cells, playing a central role in disease pathogenesis by promoting MM progression. The competing endogenous RNA (ceRNA) network, defined by shared miRNAs among different transcripts, contributes to fine regulate several cellular processes. Perturbation of this network may unsettle the transcriptomic balance, potentially leading to pathological condition. While deregulation of NEAT1-driven ceRNA circuits are described in several cancers, evidence in MM are virtually absent.

### Aims

To elucidate NEAT1 involvement in ceRNA network in MM.

### Methods

RNAseq analysis was performed on NEAT1-KD AMO1, LP1, and NCI-H929 MM cell lines, and in NEAT1-transactivated AMO1 cells (AMO1-OVX). Differentially expressed transcripts were identified by LIMMA VOOM R package. StarBase 2.0 and TarBase databases were used to select miRNAs targeting NEAT1 and mRNAs. Statistical analyses were performed using the Wilcoxon rank-sum, Kruskal-Wallis, and Dunn tests. GSEA was performed on pre-ranked differentially expressed genes based on fold change. AMO-1 cells were electroporated with miRNA mimics (Life Technologies) by using the Neon Transfection System.

**Copyright Information:** (Online) ISSN: 2572-9241
©2026 The Author(s). HemaSphere is published by John Wiley & Sons Ltd on behalf of European Hematology Association. This is an open access Abstract Book distributed under the Attribution-NonCommercial-NoDerivs (CC BY-NC-ND), which allows third parties to download the articles and share them with others as long as they credit the author and the Abstract Book, but they cannot change the content in any way or use them commercially.
**Abstract Book Citations:** Authors, Title, HemaSphere, 2026;10:(S1):pages. The abstract book DOIs can be found on the "Author Information" page.
**Disclaimer:** Articles published in the journal HemaSphere exclusively reflect the opinions of the authors. The authors are responsible for all content in their abstracts including accuracy of the facts, statements, citing resources, etc.

### Results

We identified 41 putative target genes in the NEAT1-driven ceRNA network by selecting transcripts that were concurrently downregulated in NEAT1-KD AMO1 cells and upregulated in AMO1-OVX cells. We then defined a list of 97 miRNAs that can be sponged by NEAT1, target at least one of these 41 mRNAs, and exhibit appreciable expression levels in MM plasma cells (relative intensity levels $\log_2 > 4$ in at least 10 samples, GSE87830). Overall, we defined a ceRNA network comprising 1.146 edges and 139 nodes. To validate our results, we assessed the positive correlation between each candidate mRNAs and NEAT1 across available MM datasets (GSE116294, GSE13591, GSE6477, CoMMpass). 13 out of 41 targets showed a significant Spearman's correlation with NEAT1 in at least one dataset. Furthermore, all 41 targets exhibited expression trends similar to NEAT1 in NEAT1-KD LP1 and NCI-H929 cells, as well as in NEAT1-KD KMS27 cells (GSE178868), with 13 targets reaching statistical significance in at least one cell line.
To explore the functional implication of the ceRNA network, we performed functional enrichment analyses (EnrichR) of the 41 targets, identifying 78 significant gene sets related to cell cycle, senescence, and RHO GTPase effectors. Notably, 17 gene sets were significantly enriched ($-1.5 \leq$ NES $\geq 1.5$, padj $< 0.05$) by GSEA in at least one experimental condition, including NEAT1-KD LP1, NCI-H929, and KMS27 cells, AMO1-OVX cells, or the extreme NEAT1 expression quartiles in the CoMMpass dataset. Importantly, gene sets related to cell cycle, senescence, and RHO GTPase effectors were validated in 5 out of 6 experimental conditions. Finally, *in vitro* experimental results obtained in AMO1 cells indicate that perturbations of specific miRNAs (miR-195, miR-106a-5p, and miR-106b-5p) in the ceRNA network lead to reduced expression of genes involved in the cell cycle pathway, including *KIF11*, *NSD2*, *PRKCA*, *ATAD2*, and *TPX2*.

### Summary/Conclusion

Our study strongly suggests NEAT1 involvement in the ceRNA network in MM. Notably, through ceRNA-mediated mechanisms, NEAT1 appears to influence biological processes related to the cell cycle, in agreement with our previous published data showing that NEAT1-KD negatively affects proliferation and viability of MM cells.

**Copyright Information:** (Online) ISSN: 2572-9241
©2026 The Author(s). HemaSphere is published by John Wiley & Sons Ltd on behalf of European Hematology Association. This is an open access Abstract Book distributed under the Attribution-NonCommercial-NoDerivs (CC BY-NC-ND), which allows third parties to download the articles and share them with others as long as they credit the author and the Abstract Book, but they cannot change the content in any way or use them commercially.
**Abstract Book Citations:** Authors, Title, HemaSphere, 2026;10:(S1):pages. The abstract book DOIs can be found on the "Author Information" page.
**Disclaimer:** Articles published in the journal HemaSphere exclusively reflect the opinions of the authors. The authors are responsible for all content in their abstracts including accuracy of the facts, statements, citing resources, etc.

### (PF759) A NEW 3D EX VIVO MODEL OF BONE MARROW FOR PROGNOSIS AND DRUG SCREENING

**Topic:** 13. Myeloma and other monoclonal gammopathies - Biology & translational research

Margarida Domingos[1, 2], Ana C. Queirós[1], Cristina João[1, 3, 4], Emilie Carneiro*[1]

[1]*Champalimaud Foundation, Myeloma Lymphoma research group, Lisbon, Portugal;* [2]*Instituto Superior Técnico, Bioengineering Department, Lisbon, Portugal;* [3]*Champalimaud Foundation, Hemato-Oncology Unit, Lisbon, Portugal;* [4]*NOVA University, NOVA Medical School, Lisbon, Portugal;*

### Background

Multiple Myeloma (MM) is a chronic bone marrow (BM) malignancy characterized by marked interpatient heterogeneity and frequent development of drug resistance. Both tumour-intrinsic features and the BM microenvironment contribute to treatment failure, underscoring the need for physiologically relevant ex vivo platforms capable of supporting personalized drug sensitivity profiling. However, existing drug testing models often lack microenvironmental complexity, require high cell input, and show limited scalability for high-throughput applications.

### Aims

To address these challenges, we developed BioMarrow, a miniaturized 3D ex vivo culture platform designed to recapitulate key features of the BM niche while enabling high-throughput drug screening with low cell input.

### Methods

Culture conditions were systematically optimized by evaluating cell density and Matrigel-based 3D configurations. Assay protocols for viability, cytotoxicity, and drug sensitivity were subsequently refined for miniaturized, high-throughput formats. The platform was first validated using the MM.1S cell line and then applied to patient-derived BM samples. MM.1S cells and primary BM mononuclear cells were seeded in 384-well plates under either 2D conditions or embedded in growth factor-reduced Matrigel (25-100%) and cultured for 48 hours.

### Results

Optimal MM.1S cells growth was achieved at 5,000-10,000 cells per well in 50% Matrigel, yielding significantly higher growth rates compared with 2D culture. Drug sensitivity profiling using the following MM treatment regimens: (1) lenalidomide-dexamethasone, (2) bortezomib-dexamethasone, (3) daratumumab, and (4) daratumumab-bortezomib-lenalidomide-dexamethasone, revealed reproducible, dose-dependent responses with measurable IC50 values and robust drug sensitivity scores.
Translation to primary BM samples demonstrated that BioMarrow supports short-term ex vivo culture with low cell input (100,000 cells per well) while preserving BM cellular complexity. Across patient samples, malignant plasma cells remained highly viable (>95%) after 48 hours of 3D culture. Spectral flow cytometry enabled identification and quantification of aberrant plasma cells (CD38+/CD138+/CD19−/CD56−/+) and confirmed preservation of clinically relevant plasma cell fractions over time.

**Copyright Information:** (Online) ISSN: 2572-9241
©2026 The Author(s). HemaSphere is published by John Wiley & Sons Ltd on behalf of European Hematology Association. This is an open access Abstract Book distributed under the Attribution-NonCommercial-NoDerivs (CC BY-NC-ND), which allows third parties to download the articles and share them with others as long as they credit the author and the Abstract Book, but they cannot change the content in any way or use them commercially.
**Abstract Book Citations:** Authors, Title, HemaSphere, 2026;10:(S1):pages. The abstract book DOIs can be found on the “Author Information” page.
**Disclaimer:** Articles published in the journal HemaSphere exclusively reflect the opinions of the authors. The authors are responsible for all content in their abstracts including accuracy of the facts, statements, citing resources, etc.

**Summary/Conclusion**

BioMarrow is a novel and practical 3D ex vivo culture platform for MM patient-derived bone marrow cells that maintains high malignant plasma cell viability over 48 hours while requiring low cell input. By preserving BM cellular complexity and enabling reproducible, high-throughput drug sensitivity profiling, this system represents a promising functional tool to support personalized therapeutic decision-making in Multiple Myeloma, including the evaluation of combination regimens and immunotherapy-based approaches.

**Copyright Information:** (Online) ISSN: 2572-9241
©2026 The Author(s). HemaSphere is published by John Wiley & Sons Ltd on behalf of European Hematology Association. This is an open access Abstract Book distributed under the Attribution-NonCommercial-NoDerivs (CC BY-NC-ND), which allows third parties to download the articles and share them with others as long as they credit the author and the Abstract Book, but they cannot change the content in any way or use them commercially.
**Abstract Book Citations:** Authors, Title, HemaSphere, 2026;10:(S1):pages. The abstract book DOIs can be found on the "Author Information" page.
**Disclaimer:** Articles published in the journal HemaSphere exclusively reflect the opinions of the authors. The authors are responsible for all content in their abstracts including accuracy of the facts, statements, citing resources, etc.

### (PF760) CXCR4 REWIRES MITOCHONDRIAL FUNCTION TO SUSTAIN METABOLIC PLASTICITY AND SURVIVAL IN MULTIPLE MYELOMA

**Topic:** 13. Myeloma and other monoclonal gammopathies - Biology & translational research

Katia Beider*[1, 2], Olga Ostrovsky[1], Hila Magen[1], Avichai Shimoni[1], Amnon Peled[3], Arnon Nagler[1]

*[1]Division of Hematology, Sheba Medical Center, Ramat Gan, Israel;[2]Jerusalem Multidisciplinary College, Jerusalem, Israel;*
*[3]Goldyne Savad Institute of Gene Therapy, Hadassah-Hebrew University Medical Center, Jerusalem, Israel;*

### Background

Acquired resistance to proteasome inhibitors (PIs) is a major barrier to sustained response in multiple myeloma (MM). While CXCR4 signaling is linked to aggressive disease biology, the metabolic adaptations that contribute to PI resistance are incompletely defined.

### Aims

We compared MM cell lines with low endogenous CXCR4 to matched models with exogenous CXCR4 overexpression, and to MM cells with acquired resistance to Bortezomib (Bort) generated in our laboratory.

### Methods

Label-free proteomics were performed on purified mitochondrial fractions to define resistance-associated mitochondrial programs.

### Results

CXCR4 overexpression established a mitochondrial state with coordinated increases in glucose uptake/phosphorylation capacity (GLUT3, HK1, PDK1), mitochondrial biogenesis machinery (TFAM, TOMM/TIMM components), and a reinforced respiratory backbone including the Complex II/SDH axis, together with strong antioxidant capacity (GST and thioredoxin system components). Despite the glycolytic signature, CXCR4-high and Bort-resistant cells showed reduced functional dependence on glycolysis, with attenuated cytotoxic responses to glycolytic inhibitors and transient growth suppression followed by recovery after drug withdrawal, compared with PI-sensitive cells. In parallel, both CXCR4-high and Bort-resistant MM cells exhibited greater mitochondrial fitness, including increased mitochondrial mass, higher membrane potential, and elevated ATP production, together with increased basal ROS, supporting survival under metabolic and proteotoxic stress.
Acquired PI resistance was associated with a distinct mitochondria-centered adaptation integrating metabolic flexibility, respiratory support, and apoptosis resistance. Mitochondrial proteomics showed upregulation of SQOR, suggesting enhanced electron delivery to the CoQ pool and respiratory support under stress, and increased SUCLG2, consistent with reinforced TCA-linked bioenergetic capacity.
In parallel, resistant cells showed marked elevation of BCL-XL, indicating a higher mitochondrial apoptosis threshold. The focused panel further revealed increased mitochondrial import/assembly and inner membrane biogenesis nodes (TIMM9/10, OXA1L, COX17), increased anti-apoptotic and stress-tolerance factors (BCL-2, HSPB1, CLPP), and reduced stress inducibility upon bortezomib, consistent with a pre-adapted resistant state. Accordingly, PI exposure elicited divergent metabolic responses. In PI-sensitive cells, Bort suppressed AKT/mTOR activity with concomitant downregulation of glycolytic genes and reduced ATP and mitochondrial polarization. In contrast, PI-resistant CXCR4-high cells maintained glycolytic gene expression and exhibited increased ATP levels and mitochondrial polarization upon Bort, indicating an adaptive shift toward oxidative metabolism under PI stress. Importantly, co-treatment with dual BCL-2/BCL-XL inhibitor AZD430 and bortezomib effectively abrogated resistance, restoring PI responsiveness in resistant CXCR4-high MM cells.

**Copyright Information:** (Online) ISSN: 2572-9241
©2026 The Author(s). HemaSphere is published by John Wiley & Sons Ltd on behalf of European Hematology Association. This is an open access Abstract Book distributed under the Attribution-NonCommercial-NoDerivs (CC BY-NC-ND), which allows third parties to download the articles and share them with others as long as they credit the author and the Abstract Book, but they cannot change the content in any way or use them commercially.
**Abstract Book Citations:** Authors, Title, HemaSphere, 2026;10:(S1):pages. The abstract book DOIs can be found on the “Author Information” page.
**Disclaimer:** Articles published in the journal HemaSphere exclusively reflect the opinions of the authors. The authors are responsible for all content in their abstracts including accuracy of the facts, statements, citing resources, etc.

### Summary/Conclusion

CXCR4 programs a mitochondria-centered high-capacity, high-buffering phenotype that promotes metabolic plasticity, supports survival under PI stress, and likely enhances bone marrow niche adaptation and intra-tumor heterogeneity. These findings define mitochondria-centered apoptosis buffering as a key dependency in PI-resistant MM, and provide a strong rationale for dual BCL-2/BCL-XL inhibition as a strategy to restore PI sensitivity.

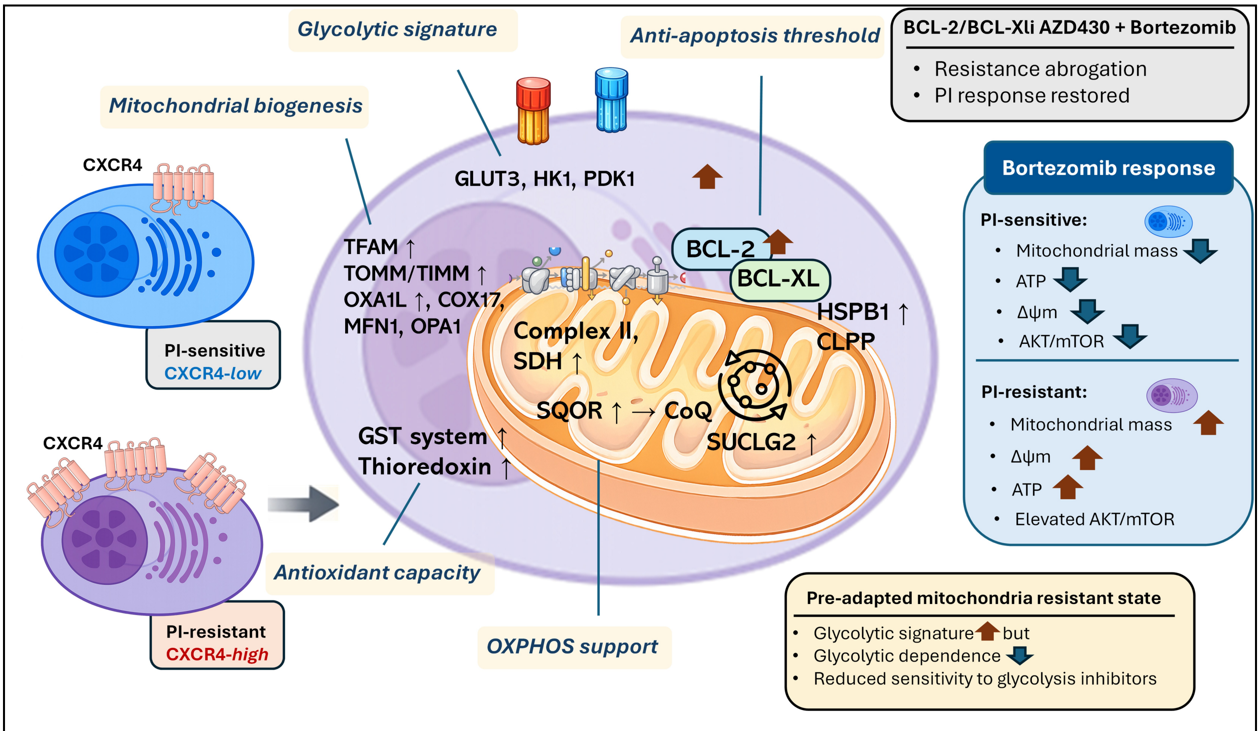

CXCR4 overexpression and acquired proteasome inhibitor (PI) resistance in multiple myeloma are associated with a pre-adapted mitochondria-centered state featuring enhanced mitochondrial biogenesis/import, respiratory support, antioxidant buffering, mitochondrial fitness, and elevated anti-apoptotic threshold (notably BCL-XL). Although glycolytic transcripts are increased, resistant cells show reduced functional dependence on glycolysis and limited mitochondrial depolarization. Upon bortezomib exposure, PI-sensitive cells undergo metabolic collapse, whereas resistant CXCR4-high cells maintain metabolic output and increase mitochondrial polarization, consistent with adaptive oxidative remodeling. Dual BCL-2/BCL-XL inhibition (AZD430) restores bortezomib responsiveness.

**Copyright Information:** (Online) ISSN: 2572-9241
©2026 The Author(s). HemaSphere is published by John Wiley & Sons Ltd on behalf of European Hematology Association. This is an open access Abstract Book distributed under the Attribution-NonCommercial-NoDerivs (CC BY-NC-ND), which allows third parties to download the articles and share them with others as long as they credit the author and the Abstract Book, but they cannot change the content in any way or use them commercially.
**Abstract Book Citations:** Authors, Title, HemaSphere, 2026;10:(S1):pages. The abstract book DOIs can be found on the “Author Information” page.
**Disclaimer:** Articles published in the journal HemaSphere exclusively reflect the opinions of the authors. The authors are responsible for all content in their abstracts including accuracy of the facts, statements, citing resources, etc.

### (PF761) PHARMACOLOGIC BCMA UPREGULATION WITH ATRA ENHANCES BELANTAMAB MAFODOTIN ACTIVITY IN MULTIPLE MYELOMA

**Topic:** 13. Myeloma and other monoclonal gammopathies - Biology & translational research

Amaya Llorente*[1], Laura Álvarez[1], Mónica González[1], Verónica Vidal[1], Raquel García Ruiz[2], Carmen Montes-Gaisán[2], Maria Aranzazu Bermudez Rodriguez[2], Enrique M. Ocio[2], Patricia Maiso[1]

*[1]Instituto de Investigación Valdecilla, IDIVAL, Neoplasias hematológicas, Santander, Spain;[2]Hospital Universitario Marqués de Valdecilla, Hematology, Santander, Spain;*

### Background

B-cell maturation antigen (BCMA, TNFRSF17) is the target of the antibody–drug conjugate Belantamab mafodotin (BLMF), approved for relapsed/refractory Multiple myeloma (MM). ADC efficacy critically depends on target antigen density and internalization. BCMA expression varies across disease stages, with reduced levels in advanced MM, potentially limiting therapeutic benefit. Pharmacologic modulation of antigen density may therefore enhance anti-BCMA strategies.

### Aims

To determine whether pharmacologic upregulation of BCMA using ATRA enhances BLMF binding, internalization, and cytotoxic activity, and to establish antigen-density modulation as a therapeutic strategy to improve BLMF efficacy in MM patients.

### Methods

BCMA expression was analyzed by flow cytometry in bone marrow plasma cells from MGUS (n=10), smoldering MM (n=4), newly diagnosed MM (n=29), and relapsed/refractory MM (n=13) patients.
MM cell lines (MM1S, RPMI8226, H929) were treated with standard anti-myeloma agents and ATRA. BCMA levels were quantified by flow cytometry, western blot, and qPCR. BLMF binding and internalization were assessed by confocal microscopy and flow cytometry. Cell viability and drug interactions were analyzed by MTT and SynergyFinder. Apoptosis and downstream signaling were evaluated by Annexin V/PI staining and caspase/PARP cleavage.

### Results

BCMA expression was significantly higher in newly diagnosed MM compared to MGUS and relapsed/refractory MM (p<0.01). However, reduced antigen density was observed in heavily pretreated patients—the population receiving BLMF (Median BCMA intensity: MGUS = 19,75; NDMM = 62,029; RRMM = 32,070).
Most anti-myeloma agents reduced BCMA expression, with Panobinostat consistently downregulating BCMA. In contrast, ATRA significantly increased BCMA levels (p<0.001 MM1S; p<0.05 H929; p=0.07 RPMI8226) confirmed at mRNA and protein levels, supporting transcriptional regulation.
ATRA pretreatment enhanced BLMF binding and internalization (p<0.05). Functionally, the combination demonstrated consistent synergistic cytotoxicity (synergy scores: 14, 24, 17) and significantly increased apoptosis compared to BLMF alone, with enhanced activation of caspase-3, -7, and -8 and increased PARP cleavage.

**Copyright Information:** (Online) ISSN: 2572-9241
©2026 The Author(s). HemaSphere is published by John Wiley & Sons Ltd on behalf of European Hematology Association. This is an open access Abstract Book distributed under the Attribution-NonCommercial-NoDerivs (CC BY-NC-ND), which allows third parties to download the articles and share them with others as long as they credit the author and the Abstract Book, but they cannot change the content in any way or use them commercially.
**Abstract Book Citations:** Authors, Title, HemaSphere, 2026;10:(S1):pages. The abstract book DOIs can be found on the “Author Information” page.
**Disclaimer:** Articles published in the journal HemaSphere exclusively reflect the opinions of the authors. The authors are responsible for all content in their abstracts including accuracy of the facts, statements, citing resources, etc.

**Summary/Conclusion**

Advanced MM is associated with reduced BCMA expression, potentially limiting anti-BCMA efficacy. Pharmacologic BCMA upregulation with ATRA enhances BLMF binding, internalization, and apoptosis induction, translating into synergistic cytotoxicity. These findings identify antigen-density modulation as a novel and clinically actionable strategy to enhance BCMA-targeted therapies in relapsed/refractory MM.

**Copyright Information:** (Online) ISSN: 2572-9241
©2026 The Author(s). HemaSphere is published by John Wiley & Sons Ltd on behalf of European Hematology Association. This is an open access Abstract Book distributed under the Attribution-NonCommercial-NoDerivs (CC BY-NC-ND), which allows third parties to download the articles and share them with others as long as they credit the author and the Abstract Book, but they cannot change the content in any way or use them commercially.
**Abstract Book Citations:** Authors, Title, HemaSphere, 2026;10:(S1):pages. The abstract book DOIs can be found on the "Author Information" page.
**Disclaimer:** Articles published in the journal HemaSphere exclusively reflect the opinions of the authors. The authors are responsible for all content in their abstracts including accuracy of the facts, statements, citing resources, etc.

### (PF4568) EARLIER CILTACABTAGENE AUTOLEUCEL (CILTA-CEL) USE AND BETTER IMMUNE FITNESS (IF) BASED ON CORRELATIVE ANALYSIS OF PERIPHERAL BLOOD AND BONE MARROW TUMOR MICROENVIRONMENT (TME) FROM CARTITUDE-4

**Topic:** 13. Myeloma and other monoclonal gammopathies - Biology & translational research

Samir Parekh*[1], Katherine Li[2], Niels W C J van de Donk[3], Yi Lin[4], Thomas Martin[5], Siyu Han[2], Rocio Montes de Oca[2], Mahipal Suraneni,[6], Michael Gormley[2], Brandon Hodkinson[2], Jinzhou Yuan[2], Feng Wang[7], Ashwini Kumar[2], Da Yin[2], Denis Smirnov[2], Jaymala Patel[2], Nikoletta Lendvai[8], Deepu Madduri[8], Jieqing Zhu[9], Mythili Koneru[9], Nitin Patel[9], Dong Geng[9], Vicki Plaks[2], Simon Harrison[10, 11, 12, 13]

*[1]Icahn School of Medicine at Mount Sinai, New York, United States of America;[2]Johnson & Johnson, Spring House, United States of America; [3]Amsterdam University Medical Center, Vrije Universiteit Amsterdam, Amsterdam, The Netherlands;[4]Mayo Clinic, Rochester, United States of America; [5]University of California San Francisco, San Francisco, United States of America; [6]Johnson & Johnson, San Diego, United States of America; [7]Johnson & Johnson, Shanghai, China; [8]Johnson & Johnson, Raritan, United States of America; [9]Legend Biotech USA Inc., Somerset, United States of America; [10]Peter MacCallum Cancer Centre, Melbourne, VIC, Australia; [11]Royal Melbourne Hospital, Melbourne, VIC, Australia; [12]Translation Laboratory, Centre of Excellence in Cellular Immunotherapy, Peter MacCallum Cancer Centre, Melbourne, VIC, Australia; [13]Sir Peter MacCallum Department of Oncology, University of Melbourne, Parkville, VIC, Australia;*

### Background

Cilta-cel is approved in the US/EU for patients (pts) with lenalidomide-refractory multiple myeloma (MM) after ≥1 prior line of therapy (pLOT) based on CARTITUDE-1 and -4 (CART-1, CART-4). In CART-1, cilta-cel led to deep, durable responses and significant survival benefit in relapsed/refractory MM (RRMM) after ≥3 pLOT. In CART-4, cilta-cel led to significant overall survival benefit and more pts achieving deep, sustained minimal residual disease negativity vs standard of care in lenalidomide-refractory MM after 1–3 pLOT.

### Aims

Investigate cilta-cel mechanism of action by correlating peripheral blood and bone marrow TME biomarkers with progression-free survival (PFS) and number of pLOT in pts with RRMM from CART-1 and CART-4.

### Methods

Biomarker analyses used peripheral blood and bone marrow aspirates (BMAs). Immunophenotyping by flow cytometry was performed using peripheral blood at baseline/time of apheresis. Baseline IF was assessed by pLOT and in association with PFS. Gene set enrichment scores were derived from RNA sequencing of the TME using BMAs. Mixed-effects models identified signatures/pathways modulated over time (day [D] 28 and 6 months [mo] postinfusion vs screening) and in association with PFS and pLOT.

**Copyright Information:** (Online) ISSN: 2572-9241
©2026 The Author(s). HemaSphere is published by John Wiley & Sons Ltd on behalf of European Hematology Association. This is an open access Abstract Book distributed under the Attribution-NonCommercial-NoDerivs (CC BY-NC-ND), which allows third parties to download the articles and share them with others as long as they credit the author and the Abstract Book, but they cannot change the content in any way or use them commercially.
**Abstract Book Citations:** Authors, Title, HemaSphere, 2026;10:(S1):pages. The abstract book DOIs can be found on the “Author Information” page.
**Disclaimer:** Articles published in the journal HemaSphere exclusively reflect the opinions of the authors. The authors are responsible for all content in their abstracts including accuracy of the facts, statements, citing resources, etc.

**Results**

In total, 176 CART-4 and 97 CART-1 pts received cilta-cel as study treatment. Immunophenotyping data were available from 248 peripheral blood samples (1 pLOT, n=56; 2 pLOT, n=61; ≥3 pLOT, n=131). At apheresis, CD4+ naive T cells (%) were higher in pts with 1 or 2 pLOT vs ≥3 pLOT with no difference in pts with ≥3 pLOT with further breakdown. Higher baseline CD4+ naive T-cell levels correlated with longer PFS, suggesting negative impact on peripheral IF with additional LOT may plateau with ≥3 pLOT.
TME gene expression data were available from 148 CART-4 BMA samples (screening, n=50; D28, n=50; 6 mo, n=48). Gene expression signatures suggest B-cell and antibody depletion at D28 after cilta-cel, with partial recovery at 6 mo and corroborated by flow cytometry and immunoglobulin quantification. Elevated expression of myeloid cell-associated genes, including tumor-associated macrophages (TAM, likely M1), and cytotoxic T-cell associated genes were also observed at D28. Elevated gene expression associated with B-cell receptors, T-cell differentiation and activation, and cytokine signaling pathways were seen at both D28 and 6 mo.
Pts with longer PFS (>18 mo) had higher M1 TAM levels at D28; pts with shorter PFS had higher regulatory T-cell levels and more suppressed interferon pathway genes at 6 mo, suggesting relapse may be associated with more suppressive immune response. In pts with fewer pLOT, more profound B-cell depletion on D28, better recovery at 6 mo, and elevated B-cell receptor signaling were inferred from gene expression data. Pts with 1 pLOT also had higher elevation of M1 TAM expression on D28 from screening vs 3 pLOT, similar to pts with longer PFS.

**Summary/Conclusion**

Correlative biomarker data suggest longer PFS is associated with better baseline IF and stronger postinfusion immune responses as observed in peripheral blood and TME. Peripheral IF was more pronounced in pts with 1–2 pLOT vs ≥3 pLOT where deterioration plateaued, suggesting limited impact of T-cell IF on PFS beyond third LOT. Other covariates, including factors within immune TME and tumor burden, may significantly balance compromised intrinsic T-cell IF at apheresis, contributing to PFS durability after 3 pLOT. Results support increased benefits in treating MM with cilta-cel in earlier LOT.

**Copyright Information:** (Online) ISSN: 2572-9241
©2026 The Author(s). HemaSphere is published by John Wiley & Sons Ltd on behalf of European Hematology Association. This is an open access Abstract Book distributed under the Attribution-NonCommercial-NoDerivs (CC BY-NC-ND), which allows third parties to download the articles and share them with others as long as they credit the author and the Abstract Book, but they cannot change the content in any way or use them commercially.
**Abstract Book Citations:** Authors, Title, HemaSphere, 2026;10:(S1):pages. The abstract book DOIs can be found on the “Author Information” page.
**Disclaimer:** Articles published in the journal HemaSphere exclusively reflect the opinions of the authors. The authors are responsible for all content in their abstracts including accuracy of the facts, statements, citing resources, etc.

### (PF763) UPDATED RESULTS OF A PHASE 1 FIRST-IN-HUMAN STUDY OF CEMSIDOMIDE, A NOVEL MONODAC® DEGRADER, WITH DEXAMETHASONE (DEX) IN PATIENTS WITH (RRMM)

**Topic:** 14. Myeloma and other monoclonal gammopathies - Clinical

Sagar Lonial*[1], Andrew Yee[2], Paul Richardson[3], Sikander Ailawadhi[4], Saurabh Chhabra[5], Wilson Gonsalves[6], Jesus Berdeja[7], Shambavi Richard[8], Jeffrey Matous[9], Urvi A. Shah[10], Mark Schroeder[11], Nisha Joseph[12], Sumedha Javalikar[13], Amro Ali[13], Leah Leahy[13], Uma Chandrasekaran[13], Riadh Lobbardi[13], Rong Chu[13], Eunju Hurh[13], Leonard Reyno[13], Binod Dhakal[13]

*[1]Winship Cancer Institute, Emory University School of Medicine, Atlanta, United States of America;[2]Massachusetts General Hospital Cancer Center, Boston, United States of America; [3]Dana-Farber Cancer Institute, Harvard Medical School, Boston, United States of America; [4]Division of Hematology, Mayo Clinic, Jacksonville, United States of America;[5]Mayo Clinic, Phoenix, United States of America; [6]Mayo Clinic, Rochester, United States of America; [7]Tennessee Oncology, Nashville, United States of America; [8]Mount Sinai Hospital, New York, United States of America;[9]Colorado Blood Cancer Institute, Denver, United States of America; [10]Memorial Sloan Kettering Cancer Center, New York, United States of America; [11]Washington University School of Medicine, St. Louis, United States of America; [12]Winship Cancer Institute, Emory University, Atlanta, United States of America; [13]C4 Therapeutics, Watertown, United States of America;*

### Background

Cemsidomide (CFT7455) is a novel and highly potent Ikaros Family Zinc Finger Protein 1/3 (IKZF1/3) MonoDAC® cereblon-based degrader. Cemsidomide has demonstrated best-in-class activity in MM preclinical models and induces immune activation.

### Aims

To report updated results with additional pts and longer follow-up from the cemsidomide plus dexamethasone (DEX) phase 1 dose escalation and expansion cohorts.

### Methods

CFT7455-1101(NCT04756726) is an open-label, phase 1/2, multi-center, first-in-human study evaluating safety, efficacy, pharmacokinetics (PK), and pharmacodynamics (PD) of cemsidomide in pts with RRMM and R/R NHL. Eligible MM pts must have received lenalidomide, pomalidomide, a proteasome inhibitor, and an anti-CD38 antibody. The primary objective is to characterize the safety and tolerability of cemsidomide in combination with DEX and to determine the MTD and/or RP2D. Secondary objectives include antimyeloma activity per IMWG response criteria, PK, and PD. Dose escalation was guided by a Bayesian logistic regression model with cohorts of 3-6 pts and a provision for additional pts at doses declared safe.

**Copyright Information:** (Online) ISSN: 2572-9241
©2026 The Author(s). HemaSphere is published by John Wiley & Sons Ltd on behalf of European Hematology Association. This is an open access Abstract Book distributed under the Attribution-NonCommercial-NoDerivs (CC BY-NC-ND), which allows third parties to download the articles and share them with others as long as they credit the author and the Abstract Book, but they cannot change the content in any way or use them commercially.
**Abstract Book Citations:** Authors, Title, HemaSphere, 2026;10:(S1):pages. The abstract book DOIs can be found on the "Author Information" page.
**Disclaimer:** Articles published in the journal HemaSphere exclusively reflect the opinions of the authors. The authors are responsible for all content in their abstracts including accuracy of the facts, statements, citing resources, etc.

### Results

As of November 26, 2025, 73 MM pts have been treated with cemsidomide plus DEX. Enrollment is completed and 8 pts remain on treatment. The median age is 67 (range 39-90) and pts received a median of 7 prior lines (range 3-22). 81% of pts were penta-drug exposed and 75% had previous CAR-T therapy or a bispecific antibody. 32% of pts have extramedullary disease. Cemsidomide doses explored included 50 µg MWF (n=6), 37.5 µg QD (n=12), 62.5 µg QD (n=16), 75 µg QD (n=20), and 100 µg QD (n=19), all on a 14 day on/14 day off dosing schedule. All doses were declared safe and 100 µg was the maximum administered dose. Systemic exposure of cemsidomide increased dose proportionally. Across doses, cemsidomide produced greater than 50% and 80% of IKZF1 and IKZF3 degradation respectively, and T-cell activation by flow cytometry and cytokine expression. 4 pts had a dose-limiting toxicity: 1 pt at 62.5 µg had grade 4 neutropenia >7 days; 3 pts at 100 µg had 5 DLT events (grade 4 neutropenia >7 days, grade 3 ALT increase, grade 3 febrile neutropenia, grade 3 pneumonia in 2 subjects). Grade 3-4 treatment emergent adverse events (AEs) occurring in ≥10% of pts included neutropenia (58%), infections (30%), anemia (25%), leukopenia (25%), thrombocytopenia (11%), lymphopenia (11%). The majority of grade ≥3 neutropenia occurred in cycles 1-2. 45% of pts received G-CSF and 6% of pts experienced grade 3-4 febrile neutropenia. No pts experienced grade 3-4 rash, fatigue, nausea, or vomiting. 4 pts had AEs resulting in dose reduction and 4 pts had an AE resulting in discontinuation (none considered related). The median relative dose intensity of cemsidomide is 0.942. The ORR among all pts is 36% (26/72) with a clinical benefit rate (MR or better) of 50% (36/72). The ORR at the 75 µg and 100 µg doses is 40% (8/20) and 53% (10/19).

### Summary/Conclusion

Oral cemsidomide with DEX demonstrates compelling efficacy, tolerability and PK/PD in a heavily pre-treated RRMM population, the majority of whom had received a CAR-T or bispecific antibody. Grade 3-4 toxicities were primarily cycle 1-2 myelosuppression, which were manageable with limited dose reductions and discontinuations. Further development includes a phase 2 MOMENTUM study (NCT07284758) and a phase 1 b study of cemsidomide and elranatamab (NCT07280013). Updated safety and antimyeloma endpoints will be presented at the conference.

**Copyright Information:** (Online) ISSN: 2572-9241
©2026 The Author(s). HemaSphere is published by John Wiley & Sons Ltd on behalf of European Hematology Association. This is an open access Abstract Book distributed under the Attribution-NonCommercial-NoDerivs (CC BY-NC-ND), which allows third parties to download the articles and share them with others as long as they credit the author and the Abstract Book, but they cannot change the content in any way or use them commercially.
**Abstract Book Citations:** Authors, Title, HemaSphere, 2026;10:(S1):pages. The abstract book DOIs can be found on the "Author Information" page.
**Disclaimer:** Articles published in the journal HemaSphere exclusively reflect the opinions of the authors. The authors are responsible for all content in their abstracts including accuracy of the facts, statements, citing resources, etc.

### (PF764) DURABLE CLINICAL BENEFIT WITH B-CELL MATURATION ANTIGEN THERAPY, BELANTAMAB MAFODOTIN, POMALIDOMIDE, AND DEXAMETHASONE, IN RELAPSED/REFRACTORY MULTIPLE MYELOMA: DREAMM-8 LONG-TERM RESPONDER ANALYSIS

**Topic:** 14. Myeloma and other monoclonal gammopathies - Clinical

Meletios A. Dimopoulos*[1, 2], Meral Beksaç[3], Sosana Delimpasi[4], Vladimir Vorobyev[5], Hang Quach[6], Ivan Špička[7], Jakub Radocha[8], Paweł Robak[9], Kihyun Kim[10], Michele Cavo[11], Syed F. Zafar[12], Ainslee Moore[13], Kristin Morris[14], Margaret Polinkovsky[15], Kelly Brock[15], Joanna Grams[16], Elisabet E. Manasanch[15], María-Victoria Mateos[17], Suzanne Trudel[18]

*[1]Department of Clinical Therapeutics, School of Medicine, National and Kapodistrian University of Athens, Athens, Greece; [2]Department of Medicine, Korea University, Seoul, South Korea; [3]Department of Hematology, Ankara Liv Hospital, Istinye University, Ankara, Türkiye; [4]General Hospital Evangelismos, Athens, Greece; [5]Leningrad Regional Clinical Hospital, Saint-Petersburg, Russia; [6]University of Melbourne, St Vincent's Hospital Melbourne, Melbourne, VIC, Australia; [7]First Faculty of Medicine, Charles University and General Hospital, Prague, Czechia; [8]4th Department of Internal Medicine – Hematology, University Hospital Hradec Králové, Charles University, Faculty of Medicine in Hradec Králové, Hradec Králové, Czechia; [9]Medical University of Łódź, Łódź, Poland; [10]Sungkyunkwan University, Samsung Medical Center, Seoul, South Korea; [11]Department of Medical and Surgical Sciences, University of Bologna, Bologna, Italy; [12]Florida Cancer Specialists and Research Institute, Fort Myers, FL, United States of America; [13]GSK, Stevenage, United Kingdom; [14]GSK, Durham, NC, United States of America; [15]GSK, Collegeville, PA, United States of America; [16]GSK, Warsaw, Poland; [17]Hematology Department, University Hospital of Salamanca/IBSAL/Cancer Research Center-IBMCC (USAL-CSIC, Salamanca, Spain; [18]Department of Medical Oncology and Hematology, Princess Margaret Cancer Centre, Toronto, ON, Canada;*

### Background

B-cell maturation antigen (BCMA)–directed therapies have changed the relapsed/refractory multiple myeloma (RRMM) treatment landscape. In the phase 3, open-label, randomized DREAMM-8 trial (NCT04484623), belantamab mafodotin, pomalidomide and dexamethasone (BPd) demonstrated significant progression-free survival (PFS) benefit compared with pomalidomide, bortezomib, and dexamethasone (PVd) in patients with RRMM who received ≥1 prior line of therapy (LOT).

### Aims

Conduct a post hoc analysis to examine the characteristics of and outcomes in patients achieving sustained clinical benefit with BPd (ie, long-term responders [LTRs]).

### Methods

DREAMM-8 is an ongoing, phase 3, open-label study evaluating BPd and PVd in patients with RRMM with ≥1 prior LOT, including lenalidomide. Patients were randomized 1:1 and treatment continued until disease progression, unacceptable toxicity, withdrawal of consent, or death, whichever occurred first. The primary endpoint was PFS; secondary endpoints included minimal residual disease (MRD) negativity ($10^{-5}$), PFS2, response rates, and safety. LTRs were patients with PFS ≥30 months (data cutoff: July 7, 2025; median follow-up, 36 months in both arms).

**Copyright Information:** (Online) ISSN: 2572-9241
©2026 The Author(s). HemaSphere is published by John Wiley & Sons Ltd on behalf of European Hematology Association. This is an open access Abstract Book distributed under the Attribution-NonCommercial-NoDerivs (CC BY-NC-ND), which allows third parties to download the articles and share them with others as long as they credit the author and the Abstract Book, but they cannot change the content in any way or use them commercially.
**Abstract Book Citations:** Authors, Title, HemaSphere, 2026;10:(S1):pages. The abstract book DOIs can be found on the "Author Information" page.
**Disclaimer:** Articles published in the journal HemaSphere exclusively reflect the opinions of the authors. The authors are responsible for all content in their abstracts including accuracy of the facts, statements, citing resources, etc.

**Results**

Of the 302 patients in DREAMM-8 (BPd, n=155; PVd, n=147), 81 were LTRs. The BPd arm had more than twice the proportion of LTRs (n=59 [38%]) as the PVd arm (n=22 [15%]). Baseline characteristics between treatment arms were generally well balanced. In LTRs, median PFS potentially favored BPd (not reached [NR]; 95% CI, NR-NR) vs PVd (NR; 95% CI, 42.7 months-NR), with a hazard ratio (HR) of 0.72 (95% CI, 0.20-2.53). BPd LTRs continued to demonstrate potential benefits after the first subsequent therapy, with median PFS2 (95% CI) of NR (NR-NR) in the BPd LTR arm and NR (44.2 months-NR) in the PVd LTR arm (HR, 0.33; 95% CI, 0.07-1.69). LTRs treated with BPd vs PVd had deeper responses: 76% vs 59% had complete response or better (≥ CR), 56% vs 32% had MRD negativity plus ≥ CR overall (73% vs 54% of patients with ≥ CR), and 39% vs 18% had sustained MRD negativity for ≥ 12 months, respectively. A higher proportion of patients with high-risk cytogenetics (ie, ≥1 of t[4;14], t[14;16], or del[17p13]) treated with BPd vs PVd achieved long-term response (27% vs 11%). The safety profile in LTRs was generally consistent with that previously reported.

**Summary/Conclusion**

More than one-third of patients treated with BPd achieved LTR status, representing a 2.5-fold higher rate than with PVd, with similar rates in patients with high-risk cytogenetics; median PFS with BPd remains NR. These findings suggest that this broadly accessible BCMA-targeted regimen delivers potential durable clinical benefit, characterized by deep and sustained disease-free survival.
© 2026 American Society of Clinical Oncology, Inc. Reused with permission. This abstract was accepted and previously presented at the 2026 ASCO Annual Meeting. All rights reserved.

**Funding Statement:** Sponsored by GSK. Editorial support provided by Nucleus Global, an Inizio company and funded by GSK (study ID 207499). Drug linker technology licensed from Seagen Inc.; monoclonal antibody produced using POTELLIGENT Technology licensed from Biowa.

**Clinical trial registration:** NCT04484623

**Copyright Information:** (Online) ISSN: 2572-9241
©2026 The Author(s). HemaSphere is published by John Wiley & Sons Ltd on behalf of European Hematology Association. This is an open access Abstract Book distributed under the Attribution-NonCommercial-NoDerivs (CC BY-NC-ND), which allows third parties to download the articles and share them with others as long as they credit the author and the Abstract Book, but they cannot change the content in any way or use them commercially.
**Abstract Book Citations:** Authors, Title, HemaSphere, 2026;10:(S1):pages. The abstract book DOIs can be found on the “Author Information” page.
**Disclaimer:** Articles published in the journal HemaSphere exclusively reflect the opinions of the authors. The authors are responsible for all content in their abstracts including accuracy of the facts, statements, citing resources, etc.

### (PF765) IMPACT OF SUSTAINED MRD NEGATIVITY ON LONG-TERM OUTCOME OF TRANSPLANT ELIGIBLE NEWLY DIAGNOSED MULTIPLE MYELOMA PATIENTS ENROLLED IN THE RANDOMIZED PHASE 2 FORTE TRIAL

**Topic:** 14. Myeloma and other monoclonal gammopathies - Clinical

Giuseppe Bertuglia*[1], Mattia D'agostino[1], Delia Rota-Scalabrini[2], Angelo Belotti[3], Laura Paris[4], Paolo Corradini[5], Francesco Vassallo[6], Stelvio Ballanti[7], Sara Aquino[8], Michele Cea[9, 10], Nicola Giuliani[11, 12], Francesca Patriarca[13], Massimo Gentile[14], Mario Luppi[15], Salvatore Palmieri[16], Giuseppe Pietrantuono[17], Renato Bassan[18], Federico Monaco[19], Renato Zambello[20, 21], Maria Teresa Petrucci[22], Massimo Offidani[23], Elena Zamagni[24], Benedetto Bruno[1], Mario Boccadoro[25], Pellegrino Musto[26], Francesca Gay[1]

*[1]Division of Hematology, AOU Città della Salute e della Scienza di Torino, University of Torino and Department of Molecular Biotechnology and Health Sciences, University of Torino, Turin, Italy; [2]Division of Oncology, Candiolo Cancer Institute, FPO, IRCCS, University of Torino Medical School, Candiolo, Italy; [3]Department of Hematology, ASST Spedali Civili di Brescia, Brescia, Italy; [4]Department of Oncology and Hematology, Azienda Socio-Sanitaria Territoriale Papa Giovanni XXIII, Bergamo, Italy; [5]Fondazione IRCCS Istituto Nazionale dei Tumori Milano, University of Milano, Milan, Italy;[6]S.C. Ematologia, Azienda Ospedaliera Santa Croce e Carle, Cuneo, Italy; [7]Sezione di Ematologia e Immunologia Clinica, Ospedale Santa Maria Della Misericordia, Perugia, Italy; [8]Ematologia e Terapie cellulari, IRCCS Ospedale Policlinico San Martino, Genova, Italy; [9]Clinic of Hematology, Department of Internal Medicine and Medical Specialties (DiMI), University of Genoa, Genoa, Italy; [10]IRCCS Azienda Ospedaliera Metropolitana, Genoa, Italy; [11]Department of Medicine and Surgery, University of Parma, Parma, Italy; [12]Hematology, Azienda Ospedaliero-Universitaria di Parma, Parma, Italy;[13]Hematology and Transplant Center Unit, Udine University Hospital, DAME, University of Udine, Udine, Italy; [14]Hematology Unit, Department of Onco-Hematology, AO of Cosenza, Cosenza, Italy; [15]Department of Medical and Surgical Sciences, University of Modena and Reggio Emilia, Hematology Unit, AOU Policlinico, Modena, Italy; [16]Hematology, Hospital "Antonio Cardarelli", Naples, Italy; [17]Hematology and Stem Cell Transplantation Unit, Centro di Riferimento Oncologico della Basilicata (IRCCS-CROB), Rionero in Vulture, Italy; [18]Division of Hematology, Ospedale dell'Angelo, Mestre-Venice, Italy; [19]SC Ematologia, Azienda Ospedaliera SS. Antonio e Biagio e Cesare Arrigo, Alessandria, Italy; [20]University of Padova, Department of Medicine, Hematology Unit, Padova, Italy;[21]Veneto Institute of Molecular Medicine, Padova, Italy; [22]Ematologia - Dipartimento di Medicina Traslazionale e di Precisione - Azienda ospedaliera Universitaria Policlinico Umberto I, Università Sapienza di Roma, Roma, Italy; [23]Hematology Unit, AOU Ospedali Riuniti di Ancona, Ancona, Italy; [24]Seragnoli Institute of Hematology, Bologna University School of Medicine, Bologna, Italy;[25]European Myeloma Network, Torino, Italy, Torino, Italy; [26]Department of Precision and Regenerative Medicine and Ionian Area, Aldo Moro University School of Medicine, Bari, Italy;*

### Background

Novel combinations induce high rates of minimal residual disease (MRD) negativity (neg) in multiple myeloma (MM) patients (pts). However, the optimal duration of MRD negative status to predict long term outcomes remains a matter of debate.

### Aims

We analyzed the impact of different sustained (sust) MRD-neg cut-offs on progression free survival (PFS) and overall survival (OS) in transplant eligible newly diagnosed MM pts treated in the randomized phase 2 FORTE trial (NCT02203643). A subgroup analysis according to a modified Consensus Genomic Staging (mCGS as described in Avet-Loiseau et al JCO 2025 without the use of molecular data), and the presence of an MGUS-like profile at diagnosis (Burgos et al JCO 2023) was performed in pts with SustMRD <3 years (y) vs ≥ 3-y.

**Copyright Information:** (Online) ISSN: 2572-9241
©2026 The Author(s). HemaSphere is published by John Wiley & Sons Ltd on behalf of European Hematology Association. This is an open access Abstract Book distributed under the Attribution-NonCommercial-NoDerivs (CC BY-NC-ND), which allows third parties to download the articles and share them with others as long as they credit the author and the Abstract Book, but they cannot change the content in any way or use them commercially.
**Abstract Book Citations:** Authors, Title, HemaSphere, 2026;10:(S1):pages. The abstract book DOIs can be found on the “Author Information” page.
**Disclaimer:** Articles published in the journal HemaSphere exclusively reflect the opinions of the authors. The authors are responsible for all content in their abstracts including accuracy of the facts, statements, citing resources, etc.

### Methods

We included all pts enrolled in the trial who were randomized to maintenance to receive Carfilzomib for up two years plus lenalidomide until progression or intolerance (KR) or lenalidomide alone (R). MRD was assessed in the bone marrow in pts with ≥ very good partial response by multiparameter flow cytometry (sensitivity of $10^{-5}$) at premaintenance and every 6 months thereafter. MRD sample until 60 months from maintenance were analysed. SustMRD-neg was defined as consecutive MRD-negative evaluations for at least 1, 2, 3, 4 or 5 years without intercurrent MRD-positive results.

### Results

356 pts were randomized to maintenance according to study protocol. At pre-maintenance, 232 patients (65%) were negative, and 124 patients (35%) were positive or not evaluable. Median follow-up from maintenance randomization was of 85.9 months and 80% of pts had at least one MRD-neg sample within 60 months. The rate of at least 1, 2, 3, 4, 5-years sustMRD neg was 53%, 42%, 36%, 28% and 20% respectively, while 24% of pts lost MRD-neg during the first year. Compared to MRD-positive patients (those who never achieved MRD-neg during follow-up; n=83), MRD-neg lasting <1 year (HR 0.72; 95% CI 0.47-1.09), 1 year (HR 0.40; 95% CI 0.22-0.72), 2 years (HR 0.36; 95% CI 0.19-0.66), 3 years (HR 0.17; 95% CI 0.07-0.40), 4 years (HR 0.15; 95% CI 0.06-0.35) and 5 years (HR 0.06; 95% CI 0.03-0.15) significantly and progressively improved pts' PFS. In pts with at least 5-year SustMRD neg, a 7-year PFS rate of 91% and OS rate of 98% was observed.
In pts with ≥ 3-years sustMRD neg vs < 3-year sustMRD, 7-years PFS rates were 84.7% vs 35.4% (HR 0.16, 95% CI 0.10-0.27) and 7-years OS rates were 97% vs 69% (HR 0.08, 95% CI 0.02-0.27).
Among patients with ≥ 3-years sustMRD neg, 7-years PFS rates were 84% for mCGS standard risk and 83% for mCGS high risk (HR 0.8, 95% CI 0.28-2.27); among patients with < 3-y sustMRD, mGCS classification retain its prognostic role (7-years PFS high vs. standard: 23% vs 43%, HR 10.5, 95% CI 5.68 – 19.6; 7-year OS: 51% vs 77%, HR 2.76, 95% CI 1.64–4.63).
Among patients with < 3-years sustMRD neg, the presence of an MGUS-like profile predicted a significantly better PFS and OS compared to non MGUS-like pts (7-years PFS 67% vs 33%, HR 0.37 95% CI 0.17-0.85; 7-years OS 93% vs 66%, HR 0.15 95% CI 0.02-1.08).

### Summary/Conclusion

Duration of MRD negativity, and in particular SustMRD neg for more than 3 years, significantly improved PFS and OS in NDMM pts. Achieving ≥3-y sustMRD neg strongly impact on outcome also in high-risk pts ; MGUS-like pts may have favourable outcomes despite the absence of 3-y sustMRD neg.

**Copyright Information:** (Online) ISSN: 2572-9241
©2026 The Author(s). HemaSphere is published by John Wiley & Sons Ltd on behalf of European Hematology Association. This is an open access Abstract Book distributed under the Attribution-NonCommercial-NoDerivs (CC BY-NC-ND), which allows third parties to download the articles and share them with others as long as they credit the author and the Abstract Book, but they cannot change the content in any way or use them commercially.
**Abstract Book Citations:** Authors, Title, HemaSphere, 2026;10:(S1):pages. The abstract book DOIs can be found on the "Author Information" page.
**Disclaimer:** Articles published in the journal HemaSphere exclusively reflect the opinions of the authors. The authors are responsible for all content in their abstracts including accuracy of the facts, statements, citing resources, etc.

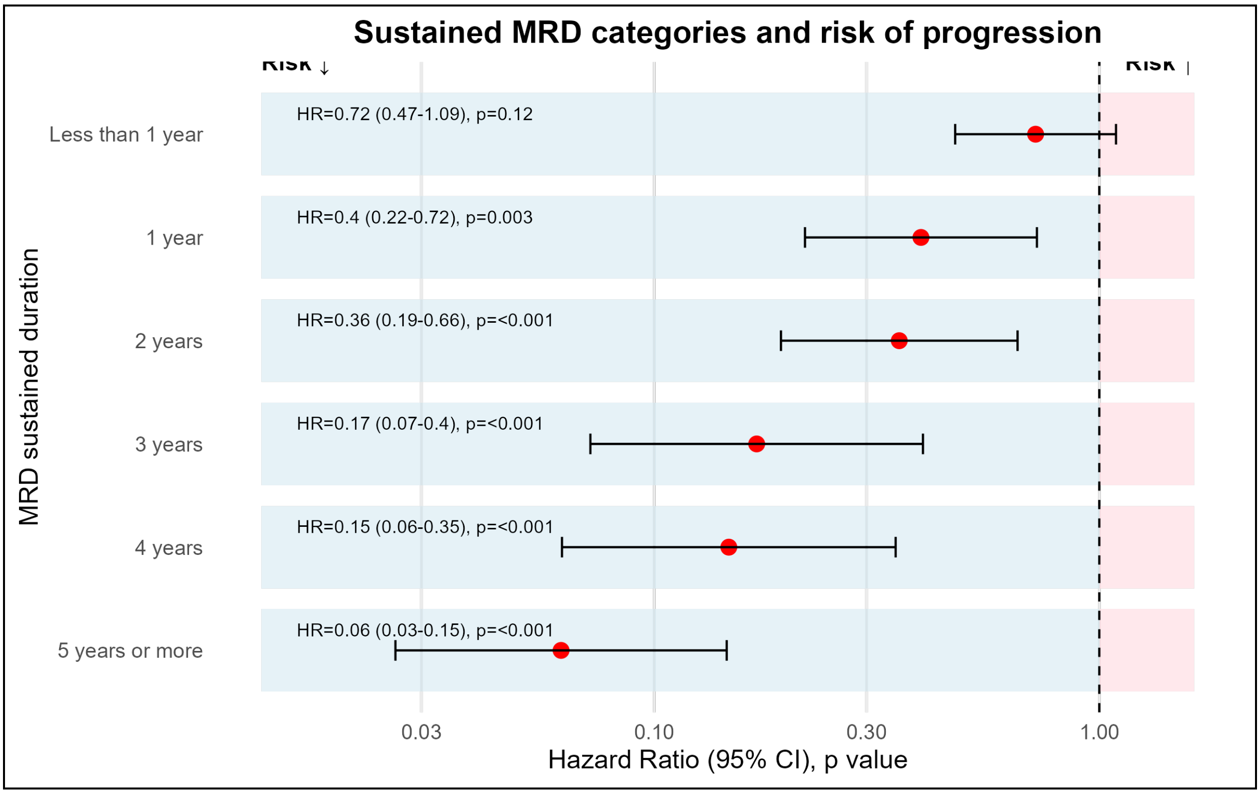

Forest plot showing the impact of the duration of sustained MRD negativity (<1 year, 1 year, 2 years, 3 years, 4 years, and ≥5 years) on progression-free survival (PFS), compared with patients who never achieved MRD negativity during maintenance therapy. Hazard ratios (HRs) and 95% confidence intervals (CIs) are reported for each duration category. The blue shaded area indicates a lower risk of progressive disease (PD), whereas the red shaded area indicates an increased risk of PD.

**Copyright Information:** (Online) ISSN: 2572-9241
©2026 The Author(s). HemaSphere is published by John Wiley & Sons Ltd on behalf of European Hematology Association. This is an open access Abstract Book distributed under the Attribution-NonCommercial-NoDerivs (CC BY-NC-ND), which allows third parties to download the articles and share them with others as long as they credit the author and the Abstract Book, but they cannot change the content in any way or use them commercially.
**Abstract Book Citations:** Authors, Title, HemaSphere, 2026;10:(S1):pages. The abstract book DOIs can be found on the "Author Information" page.
**Disclaimer:** Articles published in the journal HemaSphere exclusively reflect the opinions of the authors. The authors are responsible for all content in their abstracts including accuracy of the facts, statements, citing resources, etc.

### (PF766) ISATUXIMAB–RD INDUCTION FOLLOWED BY ISA-R MAINTENANCE COMPARED TO RD INDUCTION FOLLOWED BY R MAINTENANCE IN ELDERLY PTS WITH NDMM WITH EMPHASIS ON FRAIL AND NON-FRAIL PTS (AGMT MM04 TRIAL)

**Topic:** 14. Myeloma and other monoclonal gammopathies - Clinical

Heinz Ludwig*[1], Thomas Melchardt[2], Evangelos Terpos[3], Jelena Bila[4], Martin Schreder[5], Sarah Bernhard[1], Klaus Podar[6, 7], Eirini Katodritou[8], Thamer Sliwa[9], Predrag Djurdjevic[10], Sosana Delimpasi[11], Michael Leisch[2], Marko Mitrovic[4], Maria Gavriatopoulou[3], Ioannis Ntanasis-Stathopoulos[3], Eva-Maria Autzinger[12], August Zabernigg[13], Michael Fillitz[14], Ewald Woell[15], Petra Pichler[16], Irene Sáenz de Tejada[17], Bruno Paiva[17], Richard Greil[18, 19, 20, 21]

*[1]Wilhelminen Cancer Research Institute, First Department of Medicine, Center for Oncology, Hematology, and Palliative Care, Clinic Ottakring, Vienna, Austria; [2]Paracelsus Medical University, Department of Internal Medicine III With Haematology, Medical Oncology, Haemostaseology, Infectiology and Rheumatology, Salzburg, Austria; [3]National and Kapodistrian University of Athens, Department of Clinical Therapeutics, School of Medicine, Athens, Austria; [4]University Clinical Center of Serbia, Clinic of the Hematology, Belgrade, Serbia; [5]Clinic Ottakring, First Department of Medicine, Center for Oncology, Hematology, and Palliative Care, Vienna, Austria; [6]University Hospital Krems, Division of Internal Medicine 2, Krems an der Donau, Austria;[7]Karl Landsteiner University of Health Sciences, Division of Molecular Oncology and Hematology, Krems an der Donau, Austria; [8]Theagenio Cancer Hospital, Thessaloniki, Greece; [9]LKH Hochsteiermark-Leoben, Department of Hemato-Oncology, Leoben, Austria; [10]University Clinical Center of Kragujevac, Clinic of Hematology, Kragujevac, Serbia;[11]Evangelismos Hospital, Hematology, Athens, Greece; [12]Hospital Barmherzige Schwestern, First Department of Medicine, Hematology and Oncology, Vienna, Austria; [13]County Hospital Kufstein, Department of Internal Medicine, Kufstein, Austria;[14]Hanusch Krankenhaus, Department of Internal Medicine, Vienna, Austria; [15]St. Vinzenz Hospital, Department of Internal Medicine, Zams, Austria; [16]University Hospital of St. Pölten, Department of Internal Medicine, St. Pölten, Austria;[17]University of Navarra, IdiSNA, CIBERONC, Department of Hematology, Center for Applied Medical Research, Cancer Center Clinica Universidad de Navarra, Navarra, Spain; [18]Austrian Group for Medical Tumor Therapy (AGMT) GmbH, Salzburg, Austria;[19]Salzburg Cancer Research Institute-Laboratory for Immunological and Molecular Cancer Research (SCRI-LIMCR), Salzburg, Austria; [20]Salzburg Cancer Research Institute-Center for Clinical Cancer and Immunology Trials (SCRI-CCCIT) GmbH, Salzburg, Austria; [21]Cancer Cluster Salzburg, Salzburg, Austria;*

### Background

Data on isatuximab (Isa) combinations in very elderly pts are not available so far. Here, we compared Isa-lenalidomide-dexamethasone (Isa-Rd) induction followed by Isa-R maintenance with Rd induction followed by R maintenance in elderly, predominately frail pts.

### Aims

We aimed to evaluate potential benefits of combining Isa with Rd induction and R maintenance therapy with special focus on MRD results after induction therapy and on frail pts.

**Copyright Information:** (Online) ISSN: 2572-9241
©2026 The Author(s). HemaSphere is published by John Wiley & Sons Ltd on behalf of European Hematology Association. This is an open access Abstract Book distributed under the Attribution-NonCommercial-NoDerivs (CC BY-NC-ND), which allows third parties to download the articles and share them with others as long as they credit the author and the Abstract Book, but they cannot change the content in any way or use them commercially.
**Abstract Book Citations:** Authors, Title, HemaSphere, 2026;10:(S1):pages. The abstract book DOIs can be found on the “Author Information” page.
**Disclaimer:** Articles published in the journal HemaSphere exclusively reflect the opinions of the authors. The authors are responsible for all content in their abstracts including accuracy of the facts, statements, citing resources, etc.

### Methods

Pts were randomized to either 8 cycles of Isa-Rd or Rd; Isa 10mg/kg on d 1, 8, 15, 22 in cycle 1, subsequently on d 1 and 15; R 25mg, d 1-21 q 28 d, dexamethasone 40mg once weekly (20mg in pts aged ≥75 years). Maintenance therapy was administered for 24 cycles; Isa was given at 10mg/kg q 28 d, and R at 5-10mg, d 1-21 q 28 d. MRD was evaluated at the end of induction therapy using NGF with a sensitivity of $10^{-6}$. Circulating tumor cells (CTC) were assessed at d 1 and 8 of the study. PFS and OS estimates were calculated according to Kaplan-Meier and compared using the log-rank test. This trial is registered on ClinicalTrials.gov (NCT04891809).

This investigator-initiated study received funding from Sanofi. The funder had no influence on the study design, data analysis or interpretation.

### Results

One hundred thirty-seven pts have been enrolled. Median age was 77 yrs (range 70-91 yrs). Frailty assessment by IMWG algorithm categorized 72.2% as frail. ISS stage I/II/III: 41 (29.9%)/47 (34.3%)/46 (33.6%), ECOG status 0/1/2: 36 (26.2%)/70 (51.1%)/31 (22.6%). Cytogenetics: t(4;14) 8 (5.8%), t(14;16) 4 (2.9%), del17p 21 (15.3%), ampl1q21 31 (22.6%) of 110-115 pts with results available. Median follow-up was 21.2 mos.

The efficacy analysis was performed in the ITT population. The median time to best response (≥PR) was 3.0 mos in Isa-Rd and 2.8 in Rd. The overall response was 88.5% in the Isa-Rd and 86.6% in the Rd group. (CR: 25.7% vs. 9%, p=0.013, VGPR: 31.4% vs. 20.9%, p=0.179, PR: 31.4% vs. 56.7%, p=0.003, and ORR: 88.5% vs. 86.6%, p=0.799, respectively). ORR was similar in frail and non-frail pts (85.9% vs. 92.1%, p=0.398).

MRD was evaluated in 92 pts after 8 induction cycles. MRDneg at $10^{-6}$ was achieved in 18/52 (34.6%) in the Isa-Rd and in only 1/40 (2.5%) pt of the Rd group (p<0.001). PFS analysis showed a median PFS of 31.8 mos in the Isa-Rd vs. 22.2 mos in the Rd group, respectively (p=0.017), while OS did not differ between both groups. Frail pts showed a marked tendency for shorter PFS (25.3 mos vs. not reached, p=0.058) and a tendency for shorter OS (48.1 mos vs. not reached, p=0.104). CTC data were available in 64 pts. Those with CTC detectable at d 8 had significantly shorter PFS (p=0.006). The PFS rate at 24 mos was 34.4% in CTC pos. vs. 68.6% in CTC neg. pts, respectively.

There was no significant difference in AEs between both groups with the exception of procedural complications (15.7% vs.1.5%, p=0.005), and weight loss (8.6% vs. 0%, p=0.028) which were more frequent in the Isa-Rd group. Frail pts had more grade ≥3 infections (30.3% vs. 13.2%, p=0.049) and numerically, but not statistically significantly, more frequent treatment discontinuations (25.3% vs. 15.8%, p=0.264).

### Summary/Conclusion

Isa-Rd followed by Isa-R maintenance resulted in higher CR/VGPR and MRDneg rates, and longer PFS. Frail pts had a marked tendency for shorter PFS and OS, and significantly more grade ≥3 infections.

**Copyright Information:** (Online) ISSN: 2572-9241
©2026 The Author(s). HemaSphere is published by John Wiley & Sons Ltd on behalf of European Hematology Association. This is an open access Abstract Book distributed under the Attribution-NonCommercial-NoDerivs (CC BY-NC-ND), which allows third parties to download the articles and share them with others as long as they credit the author and the Abstract Book, but they cannot change the content in any way or use them commercially.
**Abstract Book Citations:** Authors, Title, HemaSphere, 2026;10:(S1):pages. The abstract book DOIs can be found on the “Author Information” page.
**Disclaimer:** Articles published in the journal HemaSphere exclusively reflect the opinions of the authors. The authors are responsible for all content in their abstracts including accuracy of the facts, statements, citing resources, etc.

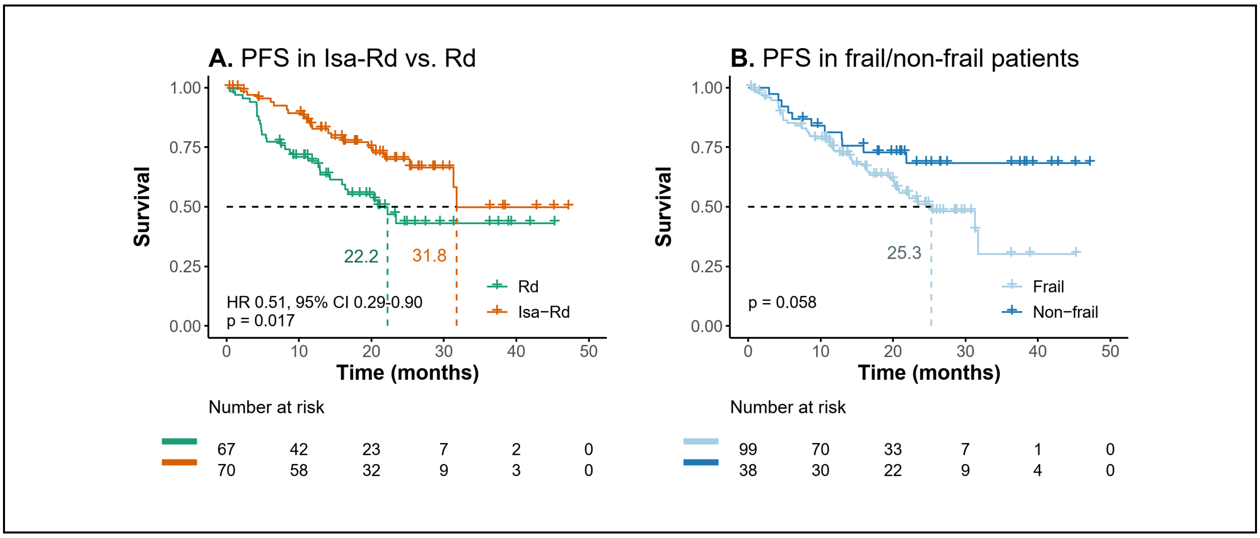

Figure 1. A. PFS in patients treated with Isa-Rd followed by Isa-R and in those receiving Rd followed by R maintenance. B. PFS in frail and non-frail patients.

**Copyright Information:** (Online) ISSN: 2572-9241
©2026 The Author(s). HemaSphere is published by John Wiley & Sons Ltd on behalf of European Hematology Association. This is an open access Abstract Book distributed under the Attribution-NonCommercial-NoDerivs (CC BY-NC-ND), which allows third parties to download the articles and share them with others as long as they credit the author and the Abstract Book, but they cannot change the content in any way or use them commercially.
**Abstract Book Citations:** Authors, Title, HemaSphere, 2026;10:(S1):pages. The abstract book DOIs can be found on the "Author Information" page.
**Disclaimer:** Articles published in the journal HemaSphere exclusively reflect the opinions of the authors. The authors are responsible for all content in their abstracts including accuracy of the facts, statements, citing resources, etc.

### (PF767) CILTACABTAGENE AUTOLEUCEL IN LENALIDOMIDE-REFRACTORY MULTIPLE MYELOMA RESPONDING TO BRIDGING THERAPY: CARTITUDE-4 CYTOGENETIC SUBGROUP ANALYSIS

**Topic:** 14. Myeloma and other monoclonal gammopathies - Clinical

Niels W C J van de Donk*[1], Roberto Mina[2], Cyrille Touzeau[3], Yael Cohen[4], Katja C. Weisel[5], Wilfried Roeloffzen[6], Carlos Fernández de Larrea[7], Tadao Ishida[8], P. Joy Ho[9], Arnab Ghosh[10], Nina Benachour[11], Carolina Lonardi[12], Quanlin Li[13], Agnes Balogh[11], Silva Koskinen[14], Nikoletta Lendvai[10], Nitin Patel[15], Paula Rodríguez-Otero[16], Natalie Callander[17], Salomon Manier[18]

*[1]Amsterdam University Medical Center, Vrije Universiteit Amsterdam, Amsterdam, The Netherlands; [2]Winship Cancer Institute, Emory University, Atlanta, United States of America; [3]Service d'Hématologie, Centre Hospitalier Universitaire (CHU) Hôtel-Dieu,, Nantes, France; [4]Tel Aviv Sourasky (Ichilov) Medical Center, Gray Faculty of Medical and Health Sciences, Tel Aviv University, Tel Aviv, Israel; [5]University Medical Center Hamburg-Eppendorf, Hamburg, Germany; [6]University Medical Center Groningen, Groningen, The Netherlands; [7]Hospital Clinic of Barcelona, Institut d'Investigacions Biomèdiques August Pi i Sunyer (IDIBAPS), University of Barcelona, Barcelona, Spain; [8]Japanese Red Cross Medical Center, Tokyo, Japan; [9]Royal Prince Alfred Hospital, Sydney, Australia; [10]Johnson & Johnson, Raritan, United States of America; [11]Johnson & Johnson, Beerse, Belgium; [12]Johnson & Johnson, Buenos Aires, Argentina; [13]Johnson & Johnson, Apex, United States of America; [14]Johnson & Johnson, Espoo, Finland; [15]Legend Biotech USA Inc., Somerset, United States of America; [16]Cancer Center Clínica Universidad de Navarra, Cima, Pamplona, Spain; [17]Carbone Cancer Center, University of Wisconsin, Madison, United States of America; [18]University of Lille, CHU Lille, Lille, France;*

### Background

CARTITUDE-4 (NCT04181827) showed significant overall survival and progression-free survival benefits of ciltacabtagene autoleucel (cilta-cel) in patients with lenalidomide-refractory multiple myeloma after 1–3 prior lines of therapy. Emerging data underscore the importance of successful bridging therapy; deeper responses (partial response or better, ≥PR) during bridging therapy were associated with better survival and safety outcomes in patients treated with cilta-cel.

### Aims

To present efficacy and safety in patients with high- and standard-risk cytogenetics who received cilta-cel as study treatment and responded to bridging therapy.

### Methods

The CARTITUDE-4 as-treated set comprised patients who received a single cilta-cel infusion as study treatment after apheresis, at least 1 cycle of bridging therapy, and lymphodepletion. High-risk cytogenetics were defined as positivity for del(17)p, t(14;16), t(4;14) or gain/amp(1q); standard-risk patients were negative for these cytogenetic abnormalities. Responses to bridging therapy were assessed based on International Myeloma Working Group criteria. Efficacy and safety from as-treated patients with ≥PR to bridging therapy were analyzed in high- and standard-risk cytogenetics subgroups.

**Copyright Information:** (Online) ISSN: 2572-9241
©2026 The Author(s). HemaSphere is published by John Wiley & Sons Ltd on behalf of European Hematology Association. This is an open access Abstract Book distributed under the Attribution-NonCommercial-NoDerivs (CC BY-NC-ND), which allows third parties to download the articles and share them with others as long as they credit the author and the Abstract Book, but they cannot change the content in any way or use them commercially.
**Abstract Book Citations:** Authors, Title, HemaSphere, 2026;10:(S1):pages. The abstract book DOIs can be found on the "Author Information" page.
**Disclaimer:** Articles published in the journal HemaSphere exclusively reflect the opinions of the authors. The authors are responsible for all content in their abstracts including accuracy of the facts, statements, citing resources, etc.

### Results

Of 176 patients who received cilta-cel as study treatment (median follow-up, 33.6 months), 64 patients had high-risk cytogenetics and achieved ≥PR to bridging therapy. In these patients, median progression-free survival and overall survival were not reached; 30-month progression-free survival and overall survival rates were 65.1% (95% confidence interval [CI], 51.4–75.8) and 87.2% (95% CI, 76.1–93.4), respectively. In 40 patients with standard-risk cytogenetics and ≥PR to bridging therapy, median progression-free survival and overall survival were not reached; 30-month progression-free survival and overall survival rates were 85.0% (95% CI, 69.6–93.0) and 92.5% (95% CI, 78.5–97.5), respectively. Safety analysis included 64 patients with high-risk and 40 with standard-risk cytogenetics. In the high-risk subgroup, cytokine release syndrome was reported in 73.4% of patients, serious nonhematologic adverse events in 64.1%, grade 3/4 infections in 43.8%, and immune effector cell (IEC)–associated neurotoxicity syndrome in 7.8%. Corresponding rates in the standard-risk subgroup were 70.0%, 57.5%, 30.0%, and 0, respectively. Nonrelapse mortality (NRM) occurred in 9 patients (high-risk, 7; standard-risk, 2); there were 4 infection-related deaths in the high-risk population. No cases of IEC–parkinsonism were reported in high- or standard-risk subgroups who responded to bridging therapy.

### Summary/Conclusion

This analysis showed survival and response benefits of cilta-cel in patients with high- and standard-risk cytogenetics who had achieved ≥PR to bridging therapy, with over 85% of patients alive at 30 months. Infections were a key cause of NRM; no patients had IEC–parkinsonism. These data highlight the profound benefit that a single cilta-cel infusion can provide to patients with high- and standard-risk cytogenetics when multiple myeloma is well controlled at time of infusion.

© 2026 American Society of Clinical Oncology, Inc. Reused with permission. This abstract was accepted and previously presented at the 2026 ASCO Annual Meeting. All rights reserved.

**Copyright Information:** (Online) ISSN: 2572-9241
©2026 The Author(s). HemaSphere is published by John Wiley & Sons Ltd on behalf of European Hematology Association. This is an open access Abstract Book distributed under the Attribution-NonCommercial-NoDerivs (CC BY-NC-ND), which allows third parties to download the articles and share them with others as long as they credit the author and the Abstract Book, but they cannot change the content in any way or use them commercially.
**Abstract Book Citations:** Authors, Title, HemaSphere, 2026;10:(S1):pages. The abstract book DOIs can be found on the "Author Information" page.
**Disclaimer:** Articles published in the journal HemaSphere exclusively reflect the opinions of the authors. The authors are responsible for all content in their abstracts including accuracy of the facts, statements, citing resources, etc.

### (PF768) VALIDATION OF A MYELOMA ELDERLY PROGNOSTIC SCORE (MEPS) BASED ON FRAILTY AND HIGH-RISK CYTOGENETICS IN TRANSPLANT INELIGIBLE PATIENTS: A COLLABORATION OF THE GREEK AND PORTUGUESE MYELOMA STUDY GROUPS

**Topic:** 14. Myeloma and other monoclonal gammopathies - Clinical

Eirini Katodritou*[1], Bruna Velosa Ferreira[2], Efstathios Kastritis[3], Dimitra Dalampira[1], Emmanouil Spanoudakis[4], Joana Vieira[5], Sosana Delimpasi[6], Ioannis Ntanasis-Stathopoulos[3], Aikaterini Daioy[1], Helena Martins[5], Elpiniki Rentzeperi[1], Foteini Theodorakakou[3], Elisabeth Karadrakonti[1], Graca Luiza O P Neto Vasconcelos Esteves[5], Vasiliki Tara[1], Vasiliki Douka[7], Dionysios Stoumpos[8], Xanthippi Dimitriadou[1], Catarina Isabel Batista Geraldes Santos[9, 10], Vasiliki Palaska[1], Maria Tsirogianni[11], Maria Kotsopoulou[8], Rui Bergantim[12, 13, 14], Maria Gavriatopoulou[3], Adriana Roque[9, 10], Konstantinos Kostikas[15], Meletios A. Dimopoulos[3], Cristina João[2, 16], Evangelos Terpos[3]

*[1]Theagenio Cancer Hospital, Hematology-Oncology, Thessaloniki, Greece; [2]Champalimaud Foundation, Lisbon, Portugal, Hemato-Oncology Department, Lisbon, Portugal; [3]National and Kapodistrian University, School of Medicine, Athens, Clinical Therapeutics, Athens, Greece; [4]Democritus University, Alexandroupolis, Hematology, Alexandroupolis, Greece; [5]Unidade Local de Saúde de Santa Maria (ULS Santa Maria), Lisbon, Portugal., Hematology and Bone Marrow Transplantation Service, Lisbon, Portugal; [6]Evangelismos General Hospital, Hematology and Bone Marrow transplantation Unit, Athens, Greece; [7]"G. Papanikolaou" General Hospital, Department of Hematology and Bone Marrow transplantation Unit, Thessaloniki, Greece; [8]Metaxa Cancer Hospital, Hematology, Athens, Greece; [9]Unidade Local de Saúde de Coimbra (ULS Coimbra), Clinical Hematology Service, Coimbra, Portugal; [10]Faculty of Medicine, University of Coimbra (FMUC), Coimbra, Portugal; [11]Agios Savvas Cancer Hospital, Hematology, Athens, Greece; [12]Unidade Local de Saúde São João, Porto, Portugal, Hematology, Porto, Portugal; [13]Institute for Research and Innovation in Health, Porto, Portugal, Cancer Drug Resistance Group, i3S, Porto, Portugal; [14]Faculty of Medicine, University of Porto (FMUP), Porto, Portugal; [15]HIPA (Health Information Platform Services), Thessaloniki, Greece; [16]NOVA Medical School, Nova University, Lisbon, Portugal, Lisbon, Portugal;*

### Background

Established prognostic systems, such as the second revision of the International Staging System (R2-ISS), often underperform in elderly, non–transplant-eligible (NTE) patients with multiple myeloma (MM), as they do not incorporate clinically relevant factors such as age, performance status, or renal function.

### Aims

Herein, we aimed to develop and evaluate a Prognostic Score for NTE MM patients integrating frailty and disease biology. To demonstrate the prognostic value of the model, we performed external validation in an independent patient cohort and concurrently applied machine learning (ML) to thoroughly assess its predictive capacity.

### Methods

We analyzed 882 newly diagnosed NTE MM patients (M/F: 448/434) with full R2-ISS data from the Greek registry (2003-2024). Factors with the greatest prognostic impact were used to develop a Myeloma Elderly Prognostic Score (MEPS), which was subsequently validated in an independent Portuguese cohort (n=300); MEPS assigned one point each for the strongest predictors in the multivariate Cox regression analysis, which identified four distinct risk groups. To establish absolute predictive ceilings, we utilized Random Survival Forests (RSF) across 10 repeated seeds (70/30 train/test splits), defining baseline predictive (RSF_10_Pred) and treatment-adjusted explanatory (RSF_12_Exp) models.

**Copyright Information:** (Online) ISSN: 2572-9241
©2026 The Author(s). HemaSphere is published by John Wiley & Sons Ltd on behalf of European Hematology Association. This is an open access Abstract Book distributed under the Attribution-NonCommercial-NoDerivs (CC BY-NC-ND), which allows third parties to download the articles and share them with others as long as they credit the author and the Abstract Book, but they cannot change the content in any way or use them commercially.
**Abstract Book Citations:** Authors, Title, HemaSphere, 2026;10:(S1):pages. The abstract book DOIs can be found on the "Author Information" page.
**Disclaimer:** Articles published in the journal HemaSphere exclusively reflect the opinions of the authors. The authors are responsible for all content in their abstracts including accuracy of the facts, statements, citing resources, etc.

**Results**

In the Greek cohort (median age 73, range 65-91), the median follow up was 64 months (95% CI: 57.5-70.4), and the median OS was 57 months (95% CI: 50-64). In the multivariate analysis, age ≥75 (HR: 1.45, p<0.001), eGFR <40 mL/min/1.73 m² (HR: 1.46, p=0.002), performance status by ECOG scale ≥2 (HR: 1.83; p<0.001) and ultra-high-risk MM (UHR), defined as the occurrence of ≥2 cytogenetic abnormalities, (HR: 1.73, p=0.003), remained independent adverse predictors for OS, whereas R2-ISS lost its significance. Incorporating all four significant parameters, the proposed MEPS stratified patients into four distinct risk groups with a median OS of 96, 65, 39, and 15.3 months, respectively (HR: low vs. high 0.19, low-intermediate vs. high 0.30, intermediate-high vs. high 0.51; p<0.001) (Figure 1); MEPS retained its high significance regardless of upfront treatments or disease response; ML defined the OS predictive ceiling at a C-index of 0.696±0.024 (RSF_12_Exp) and 0.677±0.027 (RSF_10_Pred). The simple 4-point MEPS achieved a C-index of 0.658±0.025, capturing most of the baseline signal and drastically outperforming R2-ISS (0.587±0.022). In the external Portuguese cohort (median age 75, range 49-89) median follow-up was 38.1 months and median OS was 38 months (95% CI: 33.3-41.6); MEPS again demonstrated better prognostic accuracy and discrimination compared with the R2-ISS (C-index 0.785 vs. 0.733; p=0.004).

**Summary/Conclusion**

Validated by advanced ML and an independent external cohort, MEPS provides reliable, frailty-centered and biologically driven risk stratification for real-world NTE MM. By approaching the computational predictive ceiling, MEPS stands as an operational and highly superior alternative to R2-ISS for elderly patients.

**Copyright Information:** (Online) ISSN: 2572-9241
©2026 The Author(s). HemaSphere is published by John Wiley & Sons Ltd on behalf of European Hematology Association. This is an open access Abstract Book distributed under the Attribution-NonCommercial-NoDerivs (CC BY-NC-ND), which allows third parties to download the articles and share them with others as long as they credit the author and the Abstract Book, but they cannot change the content in any way or use them commercially.
**Abstract Book Citations:** Authors, Title, HemaSphere, 2026;10:(S1):pages. The abstract book DOIs can be found on the "Author Information" page.
**Disclaimer:** Articles published in the journal HemaSphere exclusively reflect the opinions of the authors. The authors are responsible for all content in their abstracts including accuracy of the facts, statements, citing resources, etc.

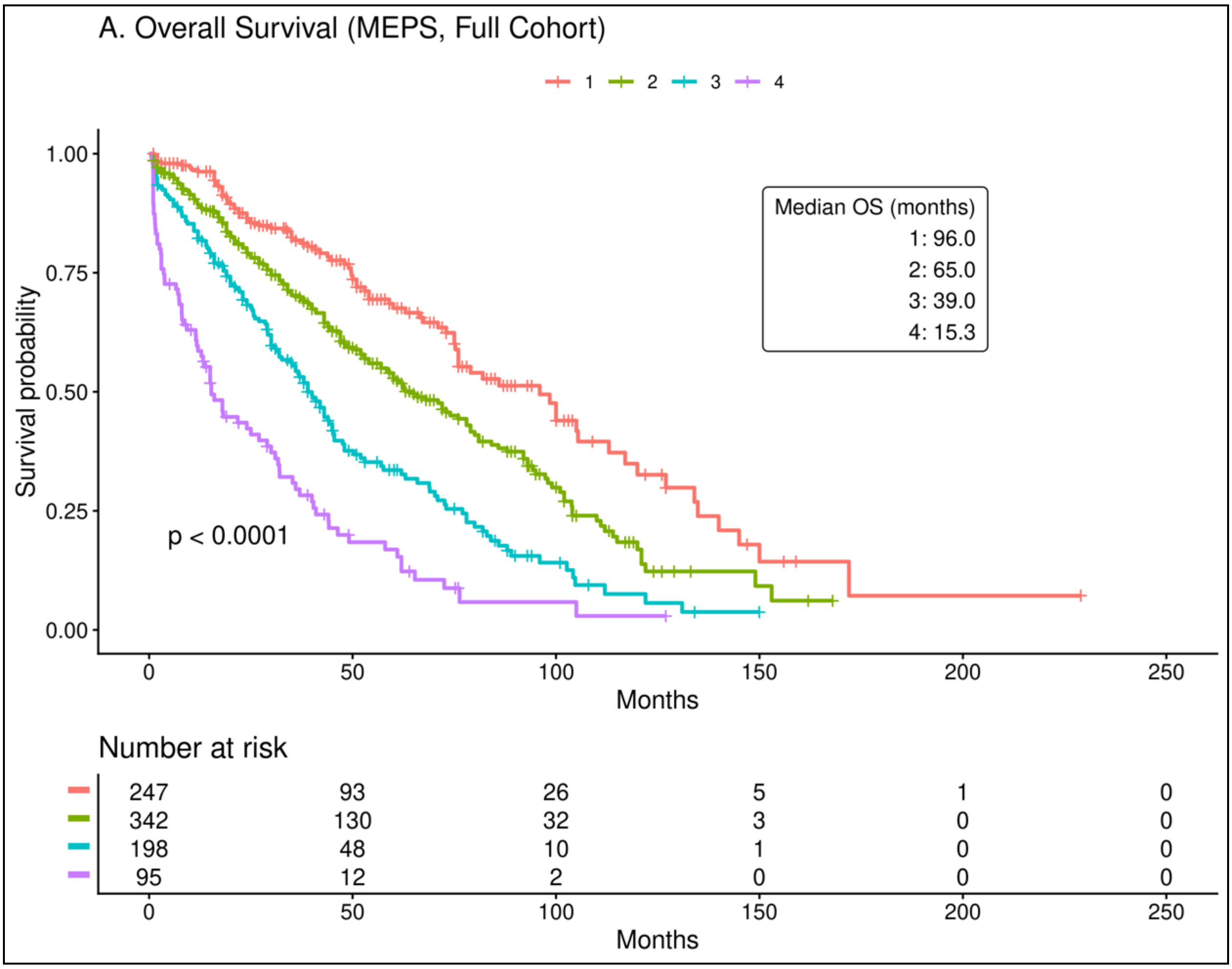

Figure 1. OS was estimated using Kaplan–Meier by MEPS_4 category (age ≥75, ECOG ≥2, eGFR <40 mL/min/1.73m², ultra-high-risk cytogenetics; grouped as 0, 1, 2, ≥3). Median OS was reported per stratum (Greek cohort; n=882)

**Copyright Information:** (Online) ISSN: 2572-9241
©2026 The Author(s). HemaSphere is published by John Wiley & Sons Ltd on behalf of European Hematology Association. This is an open access Abstract Book distributed under the Attribution-NonCommercial-NoDerivs (CC BY-NC-ND), which allows third parties to download the articles and share them with others as long as they credit the author and the Abstract Book, but they cannot change the content in any way or use them commercially.
**Abstract Book Citations:** Authors, Title, HemaSphere, 2026;10:(S1):pages. The abstract book DOIs can be found on the "Author Information" page.
**Disclaimer:** Articles published in the journal HemaSphere exclusively reflect the opinions of the authors. The authors are responsible for all content in their abstracts including accuracy of the facts, statements, citing resources, etc.

### (PF769) RISK OF AL AMYLOIDOSIS IS ASSOCIATED WITH GRADE OF FREE LIGHT CHAIN ELEVATION AND DURATION OF EXPOSURE

**Topic:** 14. Myeloma and other monoclonal gammopathies - Clinical

Maximilian Steinhardt*[1, 2, 3], Eli Muchtar[1, 4], Taxiarchis Kourelis[1], Rahma Warsame[1], Francis Buadi[1], David Dingli[1], Nelson Leung[1, 5], Joselle Cook[1], Ronald Go[1], Suzanne Hayman[1], Wilson Gonsalves[1], Prashant Kapoor[1], Yi Lin[1], Mustaqeem Siddiqui[1], Moritz Binder[1], Nadine Abdallah[1], Saurabh Zanwar[1], Tamer Hellou[1], Amie Fonder[1], Miriam Hobbs[1], Robert Kyle[1], K Martin Kortum[2], Hermann Einsele[2], Vincent Rajkumar[1], Shaji Kumar[1], Morie Gertz[1], Angela Dispenzieri[1]

*[1]Mayo Clinic, Department of Biomedical Informatics, Rochester, United States of America;[2]University Hospital Wuerzburg, Department of Internal Medicine II, Wuerzburg, Germany; [3]University Hospital Wuerzburg, Interdisciplinary Amyloidosis Center, Wuerzburg, Germany; [4]Sheba Medical Center, Department of Hematology, Tel Hashomer, Israel;[5]Mayo Clinic, Department of Nephrology, Rochester, United States of America;*

### Background

Systemic light chain amyloidosis (AL) is caused by organ deposition of misfolded free light chains (FLC) leading to organ dysfunction and failure. A clonal immunoglobulin-producing disorder precedes AL.

### Aims

To investigate risk factors for the development of AL across precursor states.

### Methods

We retrospectively analyzed a single-center cohort of 1,950 systemic AL patients with complete data for precursor classification diagnosed 2010–2024 and compared those who developed AL from a prior diagnosed precursor disease with those where the precursor was found during work-up. Additionally, we studied 4040 MGUS and 459 SMM patients with matched follow-up and available FLC results under prospective surveillance due to their monoclonal protein process. We assessed age, sex, bone marrow plasma cell counts (BMPC), light chain characteristics, M-protein, and cytogenetic abnormalities at the time of AL diagnosis in the AL cohort and at precursor diagnosis in the MGUS/SMM cohort. Associations with time-to-AL were assessed using Kaplan-Meier and Cox models. ROC analysis determined discriminatory thresholds for regression analyses.

**Copyright Information:** (Online) ISSN: 2572-9241
©2026 The Author(s). HemaSphere is published by John Wiley & Sons Ltd on behalf of European Hematology Association. This is an open access Abstract Book distributed under the Attribution-NonCommercial-NoDerivs (CC BY-NC-ND), which allows third parties to download the articles and share them with others as long as they credit the author and the Abstract Book, but they cannot change the content in any way or use them commercially.
**Abstract Book Citations:** Authors, Title, HemaSphere, 2026;10:(S1):pages. The abstract book DOIs can be found on the “Author Information” page.
**Disclaimer:** Articles published in the journal HemaSphere exclusively reflect the opinions of the authors. The authors are responsible for all content in their abstracts including accuracy of the facts, statements, citing resources, etc.

**Results**

In the AL cohort, 258 (13.2%) had a prior diagnosed plasma cell disorder. Comparing those to the group diagnosed with MGUS- or SMM-like phenotypes during AL workup, patients with subsequent AL development were older (median 70 vs 64 years), had lower difference between involved and uninvolved FLCs (dFLC, median 11.0 vs 16.1 mg/dL in MGUS, 28.9 vs 41.3 mg/dL in SMM), higher M-protein concentrations (median 0.62 vs 0.0 g/dL in MGUS, 1.4 vs 0.3 g/dL in SMM), and lower rates of t(11;14) (41.7% vs 59.8% and 38.9 vs 52.6%, respectively). Patients developing AL from SMM had a relatively shorter time to AL (median 34.2 months versus 61.3 months) compared to those from MGUS. Patients developing AL after known MM or LPL commonly lacked deep hematologic response before AL (≤VGPR in 78% of MM, 100% of LPL patients).
In patients followed for MGUS and SMM, 74 (1.8%) and 33 (7.2%) respectively developed AL. Patients who developed AL were more likely to have λ light chain restriction (74.3% vs 26.1% with MGUS, 81.8 vs 30.3% with SMM), had higher baseline dFLC levels (median 21.75 vs 1.4 mg/dL and 31.4 vs 12.9 mg/dL), and were more likely to carry t(11;14) (48.0% vs 24.9% and 50% vs 25.1%, respectively). The time to a 5% cumulative risk of AL was the shortest in patients with dFLC >80 mg/dL (20 months), followed by dFLC 25-80 mg/dL (79 months) and 10-25 mg/dL (95 months), and was not reached in patients with dFLC <10 mg/dL (figure 1). ROC analyses identified dFLC thresholds of 6.4 mg/dL (AUC 0.90) as a major driver of AL; in multivariable analysis, dFLC >6.4 mg/dL was the strongest independent predictor of AL development (HR 19.4), followed by λ light chain type (HR 4.5) and SMM precursor state (HR 1.6). Other risk factors included M-protein >1.5 g/dL, t(11;14), and BMPC >5% (HR 3.8, 3.0, and 2.6, not included in multivariable due to lower numbers).

**Summary/Conclusion**

Risk of AL is associated with sustained exposure to monoclonal free light chains. The likelihood of development is influenced by the grade of light chain elevation as well as their characteristics, particularly λ isotype. In MM and LPL, AL typically occurred in the setting of lack of deep hematologic response. These findings refine our knowledge of AL pathophysiology and support continued clinical surveillance in patients with monoclonal protein disorders and persistently elevated dFLC.

**Copyright Information:** (Online) ISSN: 2572-9241
©2026 The Author(s). HemaSphere is published by John Wiley & Sons Ltd on behalf of European Hematology Association. This is an open access Abstract Book distributed under the Attribution-NonCommercial-NoDerivs (CC BY-NC-ND), which allows third parties to download the articles and share them with others as long as they credit the author and the Abstract Book, but they cannot change the content in any way or use them commercially.
**Abstract Book Citations:** Authors, Title, HemaSphere, 2026;10:(S1):pages. The abstract book DOIs can be found on the "Author Information" page.
**Disclaimer:** Articles published in the journal HemaSphere exclusively reflect the opinions of the authors. The authors are responsible for all content in their abstracts including accuracy of the facts, statements, citing resources, etc.

**Figure 1: Risk of AL development by baseline dFLC levels**

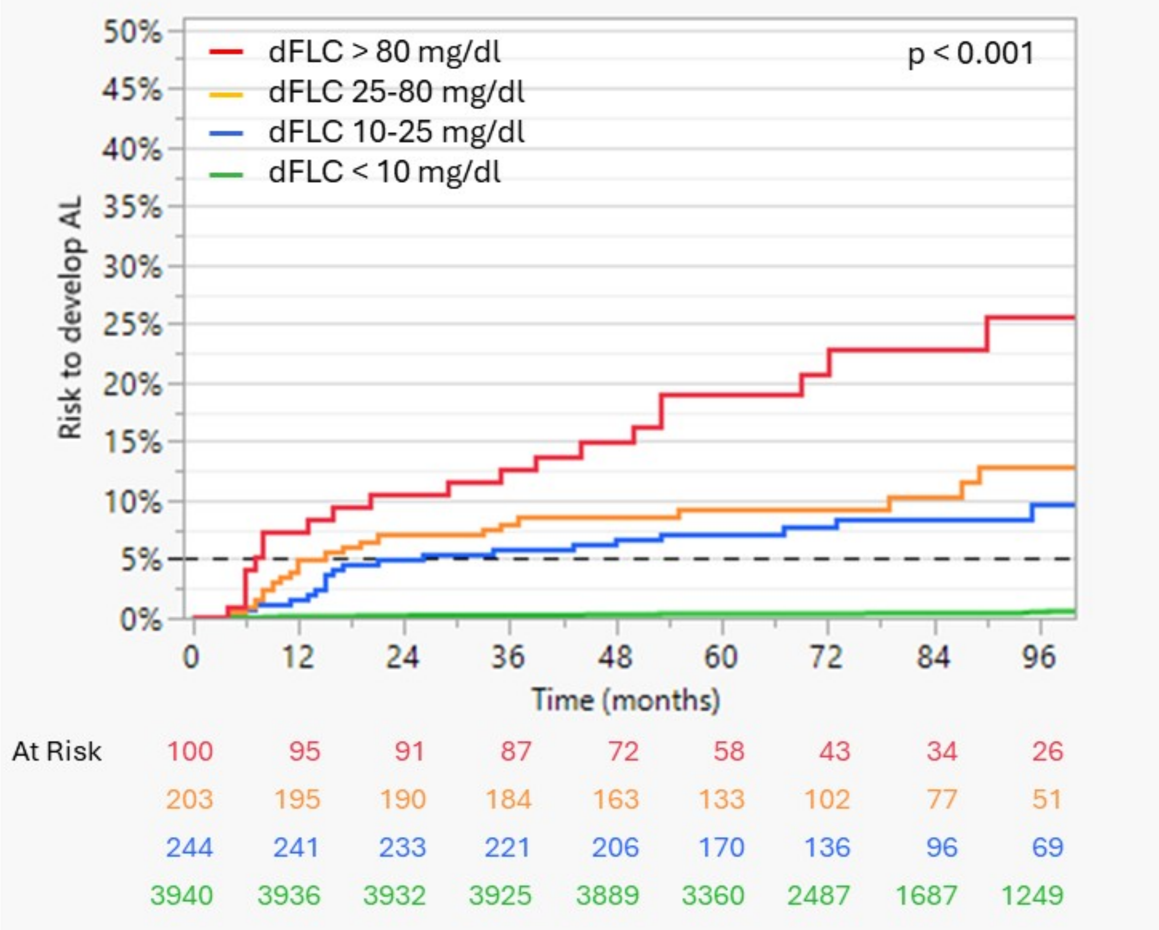

Copyright Information: (Online) ISSN: 2572-9241
©2026 The Author(s). HemaSphere is published by John Wiley & Sons Ltd on behalf of European Hematology Association. This is an open access Abstract Book distributed under the Attribution-NonCommercial-NoDerivs (CC BY-NC-ND), which allows third parties to download the articles and share them with others as long as they credit the author and the Abstract Book, but they cannot change the content in any way or use them commercially.
**Abstract Book Citations:** Authors, Title, HemaSphere, 2026;10:(S1):pages. The abstract book DOIs can be found on the "Author Information" page.
**Disclaimer:** Articles published in the journal HemaSphere exclusively reflect the opinions of the authors. The authors are responsible for all content in their abstracts including accuracy of the facts, statements, citing resources, etc.

### (PF771) REVISED DYNAMIC FRAILTY-TAILORED THERAPY (DYNAFIT-V2) IN ELDERLY PATIENTS WITH NEWLY DIAGNOSED MULTIPLE MYELOMA: A PROSPECTIVE MULTICENTER STUDY

**Topic:** 14. Myeloma and other monoclonal gammopathies - Clinical

Zhang Yingjie[1], Hua Wang[2], Yi Tao[3], Xiaoqi Qin[4], Xinyue Liang[1], Yufeng Zhu[1], Xintian MA[1], Yuhang Su[1], Jianyu Xue[1], Siyao Mu[1], Jian-qing Mi[3], Yun Dai[1], Fengyan Jin*[1]

*[1]First Hospital of Jilin University, Hematology, Changchun, China;[2]Sun Yet-Sen University Cancer Center, Guangzhou, China; [3]Ruijin Hospital Affiliated to Shanghai Jiao Tong University School of Medicine, Shanghai, China;[4]Second Hospital of Shanxi Medical University, Taiyuan, China;*

### Background

Early treatment discontinuation (TD) due to frailty remains a major challenge in the management of elderly patients with newly diagnosed multiple myeloma (NDMM). Our previous study has shown the feasibility and benefit of bortezomib, lenalidomide, and dexamethasone (VRd)-based dynamic frailty-tailored therapy (DynaFiT) in this population. Based on its safety profile, the anti-CD38 monoclonal antibody daratumumab in combination with VRd (D-VRd) has been recommended as first-line therapy for elderly patients with NDMM. Accordingly, we revised the DynaFiT by integrating daratumumab (designated DynaFiT-v2).

### Aims

The study aims to evaluate the efficacy and safety of the D-VRd-based DynaFiT-v2 in NDMM patients aged ≥ 65 years.

### Methods

This is a prospective multicenter study (NCT06099912). After enrollment, participants received eight cycles of D-VRd for induction, with the doses of bortezomib, lenalidomide, and dexamethasone adjusted according to longitudinal changes in the frailty category (defined by IMWG-FI) at each cycle. The primary endpoint was overall response rate (ORR) during induction. Other key endpoints included TD, adverse events (AEs), minimal residual disease (MRD), PFS, and OS.

### Results

At the time of analysis, 132 patients were enrolled, including 54 (41%) fit, 19 (14%) intermediate fit, and 59 (45%) frail as defined at baseline. Of 118 patients who had geriatric assessments for at least twice, 46 (39%) experienced changes at least once in the frailty category: improvement in 30/53 (57%, including 25% fit and 32% intermediate fit) of frail and 6/17 (35%) of intermediate fit patients, but deterioration in 10/48 (21%) of fit patients (**Figure 1a-c**). 51 patients had completed all eight cycles of induction and proceeded to maintenance, whose trajectory of the dynamic changes in the frailty category is illustrated in **Figure 1d**. During induction, 23/132 (17%) patients experienced TD, including 8/54 (15%) fit, 2/19 (11%) intermediate fit patients, and 13/59 (22%) frail patients, with 3/54 (6%), 1/19 (5%), and 7/59 (12%) within the first four cycles, mainly because of non-hematologic toxicity (mostly infections). The competing risk of AE-associated TD was higher in frail than non-frail (fit and intermediate fit) patients (HR 1.75, 95% CI 0.59-2.85, $P = 0.08$). The ORR was 94.8% (61.5% CR or sCR; 15.4% VGPR; 17.9% PR) 100% (43.7%, 37.5%, 18.8%), and 93.2% (40.9%, 22.7%, 29.5%) for fit, intermediate-fit, and frail patients, respectively. Of 78 patients who received at least four cycles of induction, 52.9%, 63.6%, and 51.5% of fit, intermediate-fit, and frail patients achieved undetectable MRD. Cumulative grade ≥3 non-hematologic and hematologic toxicities were reported in 32 (24%) and 48 (36%) patients, with infection (14%) as the most common grade ≥3 non-hematologic AEs. Median PFS of frail and non-frail patients was 26.5 months and not reached (HR 2.44, 95% CI 0.97-6.13, $P = 0.059$); median OS was 26.5 months vs. not reached (HR 4.74, 95% CI 1.33–16.94, $P = 0.017$).

**Copyright Information:** (Online) ISSN: 2572-9241
©2026 The Author(s). HemaSphere is published by John Wiley & Sons Ltd on behalf of European Hematology Association. This is an open access Abstract Book distributed under the Attribution-NonCommercial-NoDerivs (CC BY-NC-ND), which allows third parties to download the articles and share them with others as long as they credit the author and the Abstract Book, but they cannot change the content in any way or use them commercially.
**Abstract Book Citations:** Authors, Title, HemaSphere, 2026;10:(S1):pages. The abstract book DOIs can be found on the "Author Information" page.
**Disclaimer:** Articles published in the journal HemaSphere exclusively reflect the opinions of the authors. The authors are responsible for all content in their abstracts including accuracy of the facts, statements, citing resources, etc.

### Summary/Conclusion

DynaFiT-v2 improves the fitness status and reduces TD, augmenting therapeutic efficacy and safety in elderly patients, particularly frail patients.

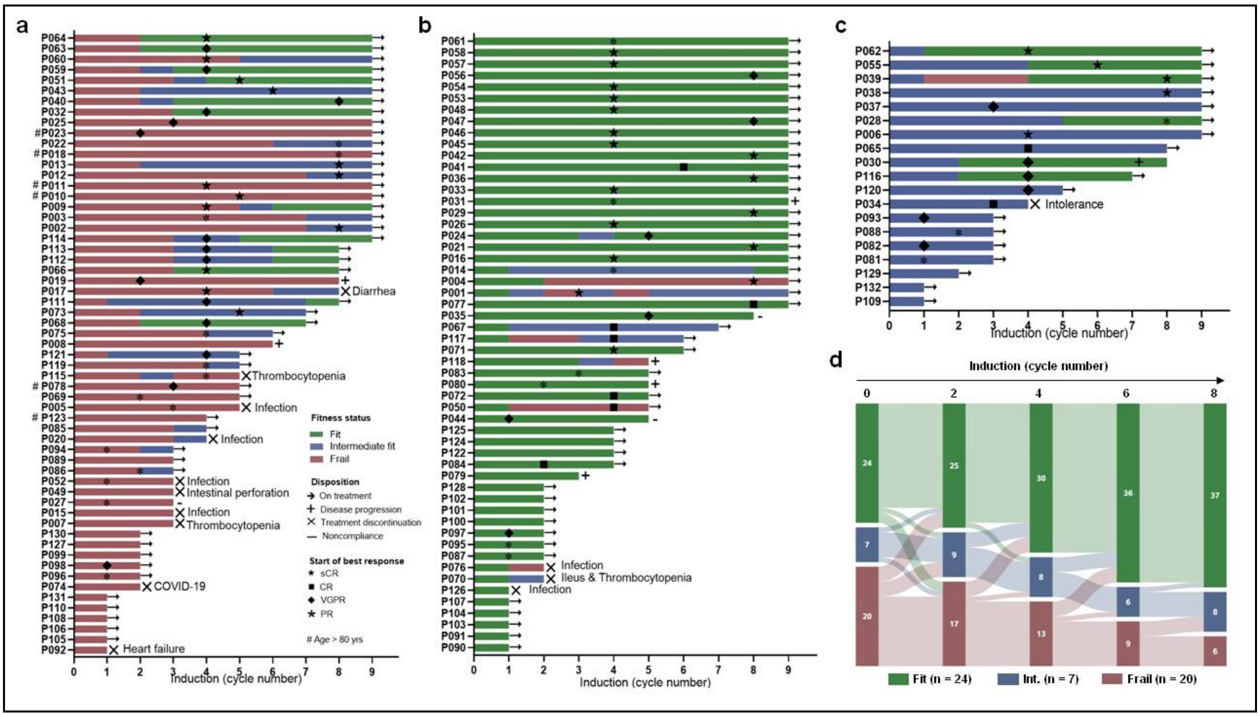

**Copyright Information:** (Online) ISSN: 2572-9241
©2026 The Author(s). HemaSphere is published by John Wiley & Sons Ltd on behalf of European Hematology Association. This is an open access Abstract Book distributed under the Attribution-NonCommercial-NoDerivs (CC BY-NC-ND), which allows third parties to download the articles and share them with others as long as they credit the author and the Abstract Book, but they cannot change the content in any way or use them commercially.
**Abstract Book Citations:** Authors, Title, HemaSphere, 2026;10:(S1):pages. The abstract book DOIs can be found on the "Author Information" page.
**Disclaimer:** Articles published in the journal HemaSphere exclusively reflect the opinions of the authors. The authors are responsible for all content in their abstracts including accuracy of the facts, statements, citing resources, etc.

### (PF772) INTEGRATED RISK STRATIFICATION IN MULTIPLE MYELOMA USING SOLUBLE BCMA AND CIRCULATING TUMOR CELLS

**Topic:** 14. Myeloma and other monoclonal gammopathies - Clinical

Masanori Toho*[1], Rikako Tabata[1], Atsushi Uehara[1], Hajime Sakuma[1], Kosuke Shima[1], Daichi Terunuma[1], Kazuki Nagao[1], Naoya Fukuda[1], Kentaro Narita[1], Masami Takeuchi[1], Kosei Matsue[1]

[1]*Kameda Medical Center, Kamogawa, Japan;*

### Background

Multiple myeloma (MM) is a heterogeneous disease, and accurate risk stratification is essential for treatment decisions. Serum soluble B-cell maturation antigen (sBCMA) is a highly sensitive biomarker reflecting total tumor burden. Circulating tumor cells (CTC) indicate aggressive disease biology and are associated with a poor prognosis. While both are established independent prognostic markers, the clinical utility of combining sBCMA and CTC remains unclear. We hypothesized that this dual liquid biopsy approach would provide better risk stratification than conventional staging systems.

### Aims

To evaluate the prognostic impact of combining sBCMA and CTC in patients with multiple myeloma compared to R-ISS.

### Methods

We retrospectively reviewed 352 patients diagnosed with MM at Kameda Medical Center between 2017 and 2025. After excluding patients with missing data or those who did not receive treatment, 264 patients were included in the final analysis. Serum sBCMA levels were quantified by ELISA. CTC counts were determined using 6- or 8-color flow cytometry ($1.0\times10^{-5}$). Optimal cut-off values for sBCMA and CTC were determined using maximally selected rank statistics to maximize the log-rank statistic for progression-free survival (PFS). The calculated cut-offs were 363.6 ng/mL for sBCMA and approximately $9.24\times10^{-5}$ for CTC. Patients were stratified into four groups (CTC/sBCMA): Hi/Hi (n=109), Hi/Lo (n=93), Lo/Hi (n=9), and Lo/Lo (n=53).

### Results

The Lo/Hi group (n=9) was excluded from survival analysis due to the small sample size. Univariate analysis demonstrated significant differences in both progression-free survival (PFS) and overall survival (OS) among the Hi/Hi, Hi/Lo, and Lo/Lo groups ($p < 0.01$ for all comparisons). The Hi/Hi (high-CTC/high-sBCMA) group showed the most dismal prognosis, while the Lo/Lo group identified a population with excellent outcomes. Although small sample size (n=9), Lo/Hi showed an inferior trend. Multivariate analysis was performed including age (≥65 vs. <65 years), autologous stem cell transplantation (ASCT) status, and R-ISS. In the PFS model, R-ISS stage III (HR 3.06, 95% CI 1.29-7.21, p=0.011) and the sBCMA/CTC subgroups remained independent predictors of poor survival. Strikingly, in the OS model, while all sBCMA/CTC subgroups maintained independent significance (Hi/Hi: HR 4.20, 95% CI 1.78-9.95, p=0.001; Hi/Lo: HR 2.92, 95% CI 1.22-7.00, p=0.016), R-ISS stage III lost its statistical significance (HR 2.54, 95% CI 0.92-7.07, p=0.073). This prognostic superiority was observed even after adjusting for age and ASCT status. The optimized CTC cut-off was approximately 1.0 × 10^-4, consistent with previous large-scale validation studies (Garcés et al., JCO 2020).

**Copyright Information:** (Online) ISSN: 2572-9241
©2026 The Author(s). HemaSphere is published by John Wiley & Sons Ltd on behalf of European Hematology Association. This is an open access Abstract Book distributed under the Attribution-NonCommercial-NoDerivs (CC BY-NC-ND), which allows third parties to download the articles and share them with others as long as they credit the author and the Abstract Book, but they cannot change the content in any way or use them commercially.
**Abstract Book Citations:** Authors, Title, HemaSphere, 2026;10:(S1):pages. The abstract book DOIs can be found on the "Author Information" page.
**Disclaimer:** Articles published in the journal HemaSphere exclusively reflect the opinions of the authors. The authors are responsible for all content in their abstracts including accuracy of the facts, statements, citing resources, etc.

**Summary/Conclusion**

In conclusion, the combination of sBCMA and CTC is useful for identifying high-risk patients more accurately than conventional staging systems. Our results showed that these markers were independent predictors of OS even after adjusting for age and ASCT status, while R-ISS lost its statistical significance. These findings suggest that the combination of sBCMA and CTC directly reflects the aggressive biology of MM. This approach provides a non-invasive and repeatable method for deciding treatment strategies in clinical practice.

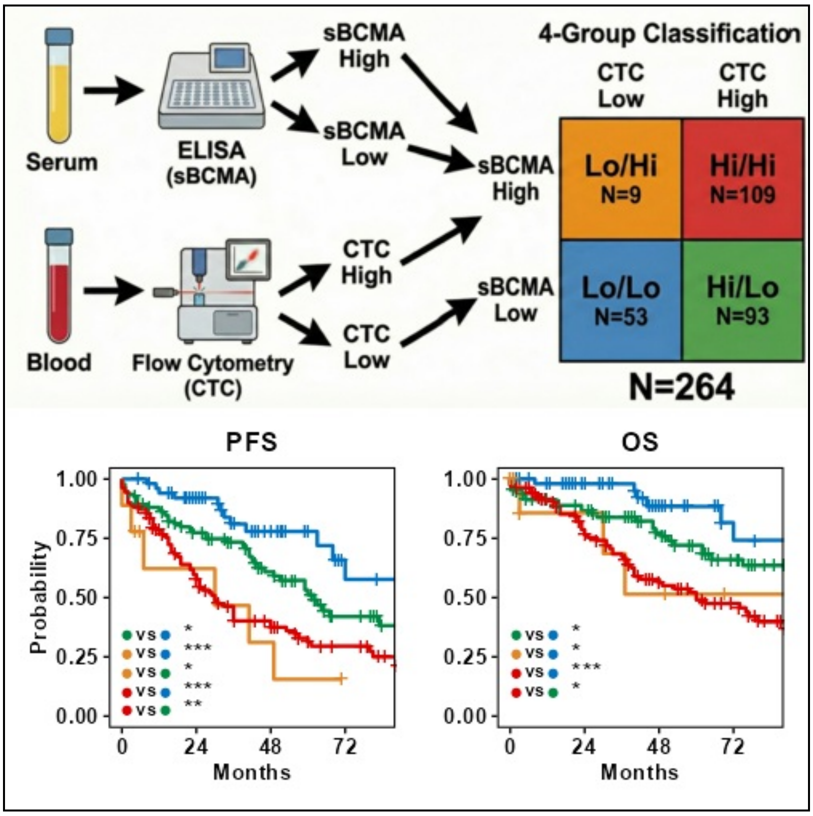

**Copyright Information:** (Online) ISSN: 2572-9241
©2026 The Author(s). HemaSphere is published by John Wiley & Sons Ltd on behalf of European Hematology Association. This is an open access Abstract Book distributed under the Attribution-NonCommercial-NoDerivs (CC BY-NC-ND), which allows third parties to download the articles and share them with others as long as they credit the author and the Abstract Book, but they cannot change the content in any way or use them commercially.
**Abstract Book Citations:** Authors, Title, HemaSphere, 2026;10:(S1):pages. The abstract book DOIs can be found on the "Author Information" page.
**Disclaimer:** Articles published in the journal HemaSphere exclusively reflect the opinions of the authors. The authors are responsible for all content in their abstracts including accuracy of the facts, statements, citing resources, etc.

### (PF773) PROMISING PERFORMANCE OF PET/CT USING [68GA]GA-PENTIXAFOR FOR INITIAL STAGING AND THERAPEUTIC EVALUATION OF MULTIPLE MYELOMA PATIENTS IN FIRST LINE TREATMENT OR RELAPSE: INTERIM ANALYSIS.

**Topic:** 14. Myeloma and other monoclonal gammopathies - Clinical

Chloé François*[1, 2], Cyrille Touzeau[2, 3], Laurent Garderet[4], Charles Mesguich[5], Cyrielle Aveline[6], Clément Bailly[1, 2], Bastien Jamet[1, 2], Francoise Kraeber-Bodere[1, 2], Caroline Milin[1, 2]

*[1]Nuclear Medicine Department, CHU Nantes, Nantes Université, Nantes, France;[2]Nantes Université, Univ Angers, CHU Nantes, INSERM, CNRS, CRCI2NA, Nantes, France; [3]Hematology Department, CHU Nantes, Nantes Université, Nantes, France; [4]Hematology Department, Hôpital Pitié-Salpêtrière, Assistance Publique Hôpitaux de Paris (AP-HP), Sorbonne Université, Paris, France; [5]Nuclear Medicine Department, CHU Bordeaux, Bordeaux, France;[6]Nuclear Medicine Department, Hôpital Tenon, Assistance Publique Hôpitaux de Paris (AP-HP), Sorbonne Université, Paris, France;*

### Background

Molecular imaging targeting CXCR4, highly expressed on malignant plasma cells, is a promising complementary approach to [$^{18}$F]FDG [FDG], with potential to improve detection and characterization of high-risk multiple myeloma (MM).

### Aims

This prospective multicenter phase II study evaluates the performance of [$^{68}$Ga]Ga-PentixaFor PET/CT [PentixaFor] compared with FDG PET/CT for initial staging and therapeutic response assessment in newly diagnosed or relapsed MM (NCT04561492).

### Methods

This study planned to include 60 symptomatic MM patients according to the IMWG criteria. All patients underwent both whole-body PentixaFor and FDG PET/CT at baseline and during response assessment. Images were acquired 60 minutes after injection of 100-200 MBq of PentixaFor and 3 MBq/kg of FDG and were independently reviewed by two experienced nuclear medicine physicians. Focal lesion (FL) was defined as focal bone marrow uptake above background on ≥2 consecutive slices. Diffuse bone marrow involvement (BMI) was defined as an uptake in axial and appendicular skeleton above liver uptake. Paramedullary (PMD) and extra-medullary disease (EMD) were defined as soft tissue involvement with and without contiguous bone involvement, respectively. The gold standard for MM lesion detection was based on functional and conventional imaging at baseline and follow-up.
Whole-body maximal standardized uptake value ($SUV_{max}$) and target-to-blood pool ratio (TBR) defined as $SUVmax/SUVmean_{blood\ pool}$ were calculated for BMI, FL, PMD and EMD. Per-patient comparisons used McNemar tests; per-lesion and semi-quantitative analyses used paired Wilcoxon tests. PET response was assessed using IMPeTUs criteria and biological response using IMWG criteria.

**Copyright Information:** (Online) ISSN: 2572-9241
©2026 The Author(s). HemaSphere is published by John Wiley & Sons Ltd on behalf of European Hematology Association. This is an open access Abstract Book distributed under the Attribution-NonCommercial-NoDerivs (CC BY-NC-ND), which allows third parties to download the articles and share them with others as long as they credit the author and the Abstract Book, but they cannot change the content in any way or use them commercially.
**Abstract Book Citations:** Authors, Title, HemaSphere, 2026;10:(S1):pages. The abstract book DOIs can be found on the "Author Information" page.
**Disclaimer:** Articles published in the journal HemaSphere exclusively reflect the opinions of the authors. The authors are responsible for all content in their abstracts including accuracy of the facts, statements, citing resources, etc.

**Results**

At interim analysis, 59/60 patients had been enrolled; 38 were evaluable for staging including 35 for response (3 awaiting post-ASCT evaluation). At staging, PET positivity tended to be higher with PentixaFor than with FDG (89% vs 74%; p=0.058). FL were more frequently detected with PentixaFor compared to FDG (58% vs 39%; p=0.035). BMI was significantly better detected with PentixaFor than with FDG (82% vs 26%; $p=1.19\times10^{-5}$). Discordances favored PentixaFor-positive/FDG-negative vs FDG-positive/PentixaFor-negative (21% vs 5%), particularly for BMI detection (22 vs 1 patients).
*Per-lesion analysis* identified 712 PentixaFor-positive lesions vs 153 FDG-positive lesions (p=0.017), including 689 vs 133 FL (p=0.004), 18 vs 18 PMD and 5 vs 2 EMD. Overall discordances favored PentixaFor (593 vs 41), mainly for FL (586 vs 36). Four high-burden patients accounted for 528 PentixaFor-positive FL. Semi-quantitative parameters were significantly higher with PentixaFor. Median FL SUVmax was 18.6 [8.7–45.2] with PentixaFor vs 4.9 [4.5–8.7] with FDG (p=0.012), with corresponding TBR of 8.8 [3.8–24.6] vs 2.9 [2.1–5] (p=0.034). BM uptake (L4–L5 SUVmax) was also higher with PentixaFor than with FDG (6.2 [5.1–7.6] vs 3.4 [3.1–4.1]; $p=3.6\times10^{-7}$).
*During therapeutic response assessment*, complete response (CR) rates were more frequent with FDG (81% vs 50%) mainly due to persistent BM uptake above liver background on PentixaFor. PentixaFor PET response was more closely associated with suboptimal biological response, as 67% of patients with <VGPR failed to achieve CR vs 14% with FDG.

**Summary/Conclusion**

These interim results support the clinical relevance of [$^{68}$Ga]Ga-PentixaFor PET/CT for MM staging demonstrating higher lesion detection and improved tumor-to-background contrast compared with FDG.

**Copyright Information:** (Online) ISSN: 2572-9241
©2026 The Author(s). HemaSphere is published by John Wiley & Sons Ltd on behalf of European Hematology Association. This is an open access Abstract Book distributed under the Attribution-NonCommercial-NoDerivs (CC BY-NC-ND), which allows third parties to download the articles and share them with others as long as they credit the author and the Abstract Book, but they cannot change the content in any way or use them commercially.
**Abstract Book Citations:** Authors, Title, HemaSphere, 2026;10:(S1):pages. The abstract book DOIs can be found on the "Author Information" page.
**Disclaimer:** Articles published in the journal HemaSphere exclusively reflect the opinions of the authors. The authors are responsible for all content in their abstracts including accuracy of the facts, statements, citing resources, etc.

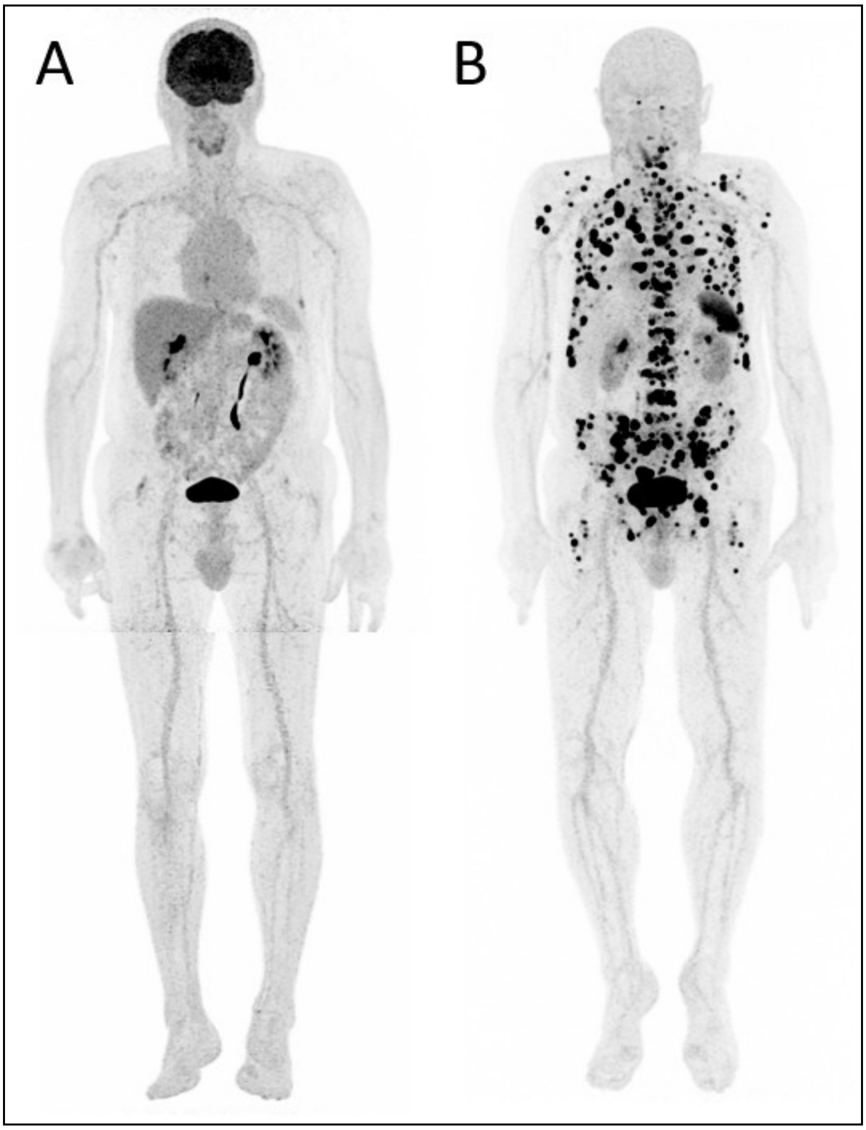

Maximum intensity projection (MIP) PET images of a patient with symptomatic multiple myeloma. (A) [18F]FDG PET showing no focal lesions. (B) [68Ga]Ga-PentixaFor PET demonstrating extensive disease with approximately 250 focal lesions.

**Copyright Information:** (Online) ISSN: 2572-9241
©2026 The Author(s). HemaSphere is published by John Wiley & Sons Ltd on behalf of European Hematology Association. This is an open access Abstract Book distributed under the Attribution-NonCommercial-NoDerivs (CC BY-NC-ND), which allows third parties to download the articles and share them with others as long as they credit the author and the Abstract Book, but they cannot change the content in any way or use them commercially.
**Abstract Book Citations:** Authors, Title, HemaSphere, 2026;10:(S1):pages. The abstract book DOIs can be found on the "Author Information" page.
**Disclaimer:** Articles published in the journal HemaSphere exclusively reflect the opinions of the authors. The authors are responsible for all content in their abstracts including accuracy of the facts, statements, citing resources, etc.

### (PF774) ELRANATAMAB IN COMBINATION WITH DARATUMUMAB AND LENALIDOMIDE IN TRANSPLANT-INELIGIBLE PATIENTS WITH NEWLY DIAGNOSED MULTIPLE MYELOMA: RESULTS FROM MAGNETISMM-6 PART 1

**Topic:** 14. Myeloma and other monoclonal gammopathies - Clinical

Carmine Liberatore*[1], Hang Quach[2], Ja Min Byun[3], Hanlon Sia[4], H. Miles Prince[5], Ignacio Casas-Avilés[6], Alexander Lesokhin[7], Karin Gogat-Marchant[8], Sharon T. Sullivan[9], Eric Leip[9], Alexander Neuhof[10], Meletios A. Dimopoulos[11]

*[1]Hematology Unit, Santo Spirito Hospital, Pescara, Italy; [2]St. Vincent's Hospital, Melbourne, University of Melbourne, VIC, Australia; [3]Department of Internal Medicine, Seoul National University Hospital, Seoul National University College of Medicine, Seoul, South Korea; [4]Pindara Private Hospital, Queensland, Australia; [5]Epworth Healthcare, Melbourne, VIC, Australia; [6]Servicio de Hematología y Hemoterapia, Hospital San Pedro de Alcántara, 10003, Cáceres, Spain; [7]Division of Hematology and Oncology, Memorial Sloan Kettering Cancer Center/Weill Cornell Medical College, New York, United States of America; [8]Pfizer Inc, Paris, France; [9]Pfizer Inc, Cambridge, MA, United States of America; [10]Pfizer Pharma GmbH, Berlin, Germany; [11]National and Kapodistrian University of Athens, Department of Clinical Therapeutics, Athens, Greece;*

### Background

MagnetisMM-6 (NCT05623020) is a phase 3, open-label, 2-arm randomized study evaluating the efficacy and safety of elranatamab (ELRA) in combination with daratumumab and lenalidomide (EDR) versus daratumumab, bortezomib, lenalidomide, and dexamethasone in patients with transplant-ineligible (TI) newly diagnosed multiple myeloma (NDMM). The dose-finding part 1 of the study showed early and promising efficacy of EDR with a predictable safety profile in patients with TI NDMM (Quach et al. J Clin Oncol 2025). At dose level G (DLG), with a median (range) follow-up of 4.6 (1.2-6.2) months, investigator-assessed confirmed objective response rate (ORR) for EDR was 91.9% (95% CI, 78.1-98.3); 81.1% achieved very good partial response or better (≥VGPR).

### Aims

To report updated efficacy and safety data for EDR at the selected recommended phase 3 dose (RP3D)–DLG–in patients with NDMM.

### Methods

In DLG, eligible patients had TI (age ≥65 or <65 years with comorbidities impacting the possibility of transplant) NDMM per IMWG criteria, Eastern Cooperative Oncology Group performance status ≤2, and adequate liver, renal, and bone marrow function. Patients received subcutaneous (SC) ELRA monotherapy (Cycle [C]0) as a priming regimen (12 mg day [D]1 and 32 mg D4), followed by ELRA 76 mg on D8, then ELRA 76 mg SC every 4 weeks (Q4W) starting on C1D1; daratumumab 1800 mg SC weekly (D1, D8, D15, D22 in C1-C2), every 2 weeks (D1, D15 in C3-C6), and Q4W (D1 in C7+); and oral lenalidomide 25 mg daily on D1-D21 in 28-day cycles. Endpoints assessed include safety and preliminary efficacy.

**Copyright Information:** (Online) ISSN: 2572-9241
©2026 The Author(s). HemaSphere is published by John Wiley & Sons Ltd on behalf of European Hematology Association. This is an open access Abstract Book distributed under the Attribution-NonCommercial-NoDerivs (CC BY-NC-ND), which allows third parties to download the articles and share them with others as long as they credit the author and the Abstract Book, but they cannot change the content in any way or use them commercially.
**Abstract Book Citations:** Authors, Title, HemaSphere, 2026;10:(S1):pages. The abstract book DOIs can be found on the "Author Information" page.
**Disclaimer:** Articles published in the journal HemaSphere exclusively reflect the opinions of the authors. The authors are responsible for all content in their abstracts including accuracy of the facts, statements, citing resources, etc.

### Results

In DLG, 37 patients with TI NDMM received ≥1 dose of ELRA. As of September 25, 2025 (data cutoff), the median (range) follow-up was 13.7 (1.2-15.2) months, and treatment was ongoing in 31 patients. Confirmed ORR by investigator was 97.3% (95% CI, 85.8-99.9), with 25 (67.6%) patients achieving complete response or better (≥CR), 35 (94.6%) ≥VGPR, and 1 (2.7%) partial response as best response. Biochemical ≥CR (without bone marrow) was achieved by 83.8% of patients. Median (range) time to response was 1.5 (0.3-4.2) months, median time to ≥CR was 7.5 (3.3-12.5) months. Minimal residual disease (MRD) negativity ($10^{-5}$) rate was 100% in patients with ≥CR who were evaluable for MRD (n=21).

Treatment-emergent adverse events (TEAEs) were reported in 100% (maximum Grade [G]3/4 94.6%) of patients, hematological TEAEs in 83.8% (all G3/4), and infections in 78.4% (G3/4 24.3%). The most frequent TEAEs (any grade ≥20% or G3/4 ≥10%) are shown in Table 1. Cytokine release syndrome occurred in 62.2% (all ≤G2); 1 case of G2 immune effector cell-associated neurotoxicity syndrome was reported. There was one G5 *Candida* pneumonia in a patient who did not receive prophylactic immunoglobulin replacement. Infections of bacterial, viral, fungal, and unspecified origin were reported in 35.1%, 35.1%, 13.5%, and 51.4% of patients, respectively. Thirty-four (91.9%) patients received immunoglobulin replacement at least once during treatment.

### Summary/Conclusion

In patients with TI NDMM, EDR at the RP3D demonstrated rapid and deep responses, with responses deepening with longer follow-up. The safety profile was manageable and consistent with the known toxicities of the individual components, with no new safety signals observed. These findings support further evaluation of EDR in Part 2 of the MagnetisMM-6 study. Results with a longer follow-up will be presented at EHA 2026.

**Table 1. Most frequent (any grade in ≥20% or maximum grade 3/4 in ≥10% of pts) TEAEs[a]**

| TEAEs, % | Any grade | Grade 3/4 |
|---|---|---|
| Hematologic | | |
| Neutropenia | 78.4 | 78.4 |
| Anemia | 35.1 | 18.9 |
| Thrombocytopenia | 18.9 | 13.5 |
| Nonhematologic | | |
| Cytokine release syndrome | 62.2 | 0 |
| Pyrexia | 37.8 | 0 |
| Diarrhea | 35.1 | 5.4 |
| Cough | 32.4 | 0 |
| Nausea | 29.7 | 0 |
| Rash | 29.7 | 8.1 |
| Injection site reaction | 29.7 | 0 |
| Hypogammaglobulinemia | 27.0 | 2.7 |
| Upper respiratory tract infection | 4.3 | 0 |
| Decreased appetite | 21.6 | 5.4 |
| Peripheral sensory neuropathy | 21.6 | 5.4 |
| Constipation | 21.6 | 0 |
| Fatigue | 21.6 | 0 |
| Peripheral edema | 21.6 | 0 |
| Asthenia | 18.9 | 10.8 |

[a] Preferred terms were based on MedDRA v28.0 coding dictionary

Copyright Information: (Online) ISSN: 2572-9241
©2026 The Author(s). HemaSphere is published by John Wiley & Sons Ltd on behalf of European Hematology Association. This is an open access Abstract Book distributed under the Attribution-NonCommercial-NoDerivs (CC BY-NC-ND), which allows third parties to download the articles and share them with others as long as they credit the author and the Abstract Book, but they cannot change the content in any way or use them commercially.
Abstract Book Citations: Authors, Title, HemaSphere, 2026;10:(S1):pages. The abstract book DOIs can be found on the "Author Information" page.
Disclaimer: Articles published in the journal HemaSphere exclusively reflect the opinions of the authors. The authors are responsible for all content in their abstracts including accuracy of the facts, statements, citing resources, etc.

### (PF775) BEYOND BONE MARROW: SOLUBLE BCMA AS A LONGITUDINAL SURROGATE FOR MRD AND ITS DISTINCT KINETICS UNDER BISPECIFIC ANTIBODY THERAPY

**Topic:** 14. Myeloma and other monoclonal gammopathies - Clinical

Masanori Toho*[1], Rikako Tabata[1], Atsushi Uehara[1], Hajime Sakuma[1], Kosuke Shima[1], Daichi Terunuma[1], Kazuki Nagao[1], Naoya Fukuda[1], Kentaro Narita[1], Masami Takeuchi[1], Kosei Matsue[1]

[1]*Kameda Medical Center, Kamogawa, Japan;*

### Background

Soluble B-cell maturation antigen (sBCMA) is a serum biomarker reflecting total plasma cell mass. While sBCMA levels correlate with bone marrow (BM) measurable residual disease (MRD) at single time points, it remains unclear whether sBCMA changes follow longitudinal MRD dynamics. Furthermore, the impact of BCMA-targeted therapies on sBCMA interpretive frameworks has not been fully evaluated.

### Aims

This study aimed to validate the longitudinal utility of sBCMA and define how its interpretation should change depending on the treatment modality.

### Methods

We conducted a retrospective analysis at Kameda Medical Center using two distinct patient cohorts.
**Part 1 (Longitudinal Validation):** From the 369 patients who underwent BM assessments between 2017 and 2025, 117 patients fulfilled predefined eligibility criteria: achievement of ≥VGPR and absence of biochemical progression during follow-up. Patients treated with BCMA-targeted therapies were excluded. These patients underwent 8-color flow-cytometric MRD (MFC-MRD; $1{\times}10^{-5}$) without interim biochemical relapse. Trends were defined as: Increasing (≥ 100% sBCMA rise, ≥ 50% rise from baseline ≥ 30 ng/mL, or ≥ 1-log MRD rise), Declining (≥ 25% sBCMA drop or ≥ 1-log MRD drop), and Stable (not meeting above criteria).
**Part 2 (Modality-Specific Impact):** sBCMA levels at the time of CR with confirmed MRD negativity were compared between patients treated with BCMA-targeted BsAbs (n=35), GPRC5D-targeted BsAbs (n=14), or conventional therapies (anti-CD38 antibodies, n=50).

### Results

**(Part 1)** sBCMA trajectories showed high parallel concordance with MFC-MRD kinetics over a median follow-up of 12 months. The analysis showed 85.7% (12 of 14) concordance for Increasing patterns and 95.1% (39 of 41) for Stable patterns, confirming that sBCMA accurately tracks bone marrow MRD dynamics. With a median follow-up from the second BM assessment, patients with an Increasing sBCMA pattern reached a significantly shorter PFS compared to those with non-increasing patterns ($p < 0.01$, Figure 1). Notably, within the Stable sBCMA group, 30.4% of patients remained MRD-positive. However, these patients achieved a PFS comparable to those who maintained MRD negativity.
**(Part 2)** BCMA-targeted BsAbs reduced sBCMA to extremely low levels (n=25, median 0.5 ng/mL), which were significantly lower than those seen with GPRC5D-targeted BsAbs (n=6, median 3.2 ng/mL) or conventional therapies (n=50, median 9.6 ng/mL) ($p < 0.01$). This profound depletion correlated with the loss of normal plasma cells and peripheral B-cells. Importantly, in patients who relapsed after BCMA-targeted therapy (n=3), sBCMA re-elevation was markedly diminished despite significant BM plasma cell expansion. This subtle rise often overlapped with levels seen during normal hematopoietic recovery, representing a practical difficulty in distinguishing early relapse from physiological marrow restoration.

**Copyright Information:** (Online) ISSN: 2572-9241
©2026 The Author(s). HemaSphere is published by John Wiley & Sons Ltd on behalf of European Hematology Association. This is an open access Abstract Book distributed under the Attribution-NonCommercial-NoDerivs (CC BY-NC-ND), which allows third parties to download the articles and share them with others as long as they credit the author and the Abstract Book, but they cannot change the content in any way or use them commercially.
**Abstract Book Citations:** Authors, Title, HemaSphere, 2026;10:(S1):pages. The abstract book DOIs can be found on the "Author Information" page.
**Disclaimer:** Articles published in the journal HemaSphere exclusively reflect the opinions of the authors. The authors are responsible for all content in their abstracts including accuracy of the facts, statements, citing resources, etc.

**Summary/Conclusion**

sBCMA is a reliable and minimally invasive biomarker that tracks longitudinal MRD changes in multiple myeloma. However, BCMA-targeted therapies cause a severe depletion of normal plasma cells, requiring different interpretations of sBCMA levels depending on the treatment. Our findings suggest that sBCMA levels during MRD negativity reflect a deeper biological response than current bone marrow tests. Conversely, the attenuated rise in sBCMA at relapse after BCMA-targeted therapy means that we must monitor these patients carefully to ensure early detection of disease progression.

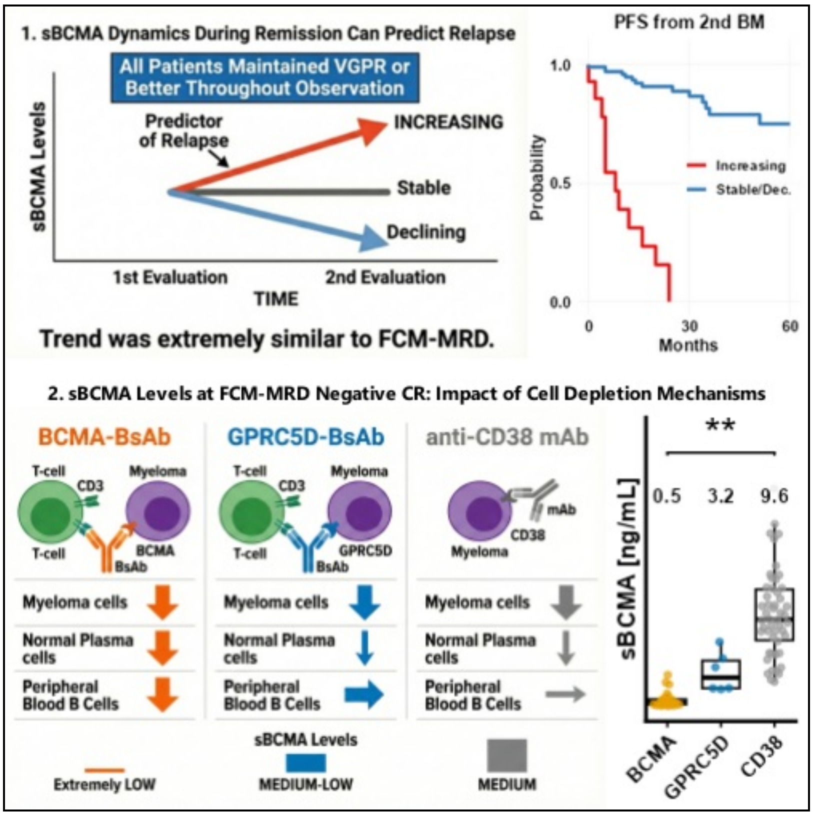

**Copyright Information:** (Online) ISSN: 2572-9241
©2026 The Author(s). HemaSphere is published by John Wiley & Sons Ltd on behalf of European Hematology Association. This is an open access Abstract Book distributed under the Attribution-NonCommercial-NoDerivs (CC BY-NC-ND), which allows third parties to download the articles and share them with others as long as they credit the author and the Abstract Book, but they cannot change the content in any way or use them commercially.
**Abstract Book Citations:** Authors, Title, HemaSphere, 2026;10:(S1):pages. The abstract book DOIs can be found on the "Author Information" page.
**Disclaimer:** Articles published in the journal HemaSphere exclusively reflect the opinions of the authors. The authors are responsible for all content in their abstracts including accuracy of the facts, statements, citing resources, etc.

### (PF776) PFS2 OUTCOMES BY PRIOR THERAPY FROM DREAMM-8: BELANTAMAB MAFODOTIN, POMALIDOMIDE, AND DEXAMETHASONE VS POMALIDOMIDE, BORTEZOMIB, AND DEXAMETHASONE IN PATIENTS WITH RELAPSED/REFRACTORY MULTIPLE MYELOMA

**Topic:** 14. Myeloma and other monoclonal gammopathies - Clinical

Güldane Cengiz Seval*[1], Sosana Delimpasi[2], Vladimir Vorobyev[3], Hang Quach[4], Ivan Špička[5], Jakub Radocha[6], Youngil Koh[7], Tadeusz Robak[8], Felipe de Arriba de la Fuente[9], Esther González García[10], Pelin Aytan[11], Silvia Mangiacavalli[12], Elena Zamagni[13], Margaret Polinkovsky[14], Marie Duggan[15], Joanna Grams[16], Elisabet E. Manasanch[14], María-Victoria Mateos[17], Suzanne Trudel[18], Meletios A. Dimopoulos[19, 20]

*[1]Department of Hematology, Ankara University, Ankara, Türkiye;[2]General Hospital Evangelismos, Athens, Greece;[3]Leningrad Regional Clinical Hospital, Saint- Petersburg, Saint- Petersburg, Russia; [4]University of Melbourne, St Vincent's Hospital Melbourne, Melbourne, VIC, Australia; [5]First Faculty of Medicine, Charles University and General Hospital, Prague, Czechia;[6]4th Department of Internal Medicine – Hematology, University Hospital Hradec Králové, Charles University, Faculty of Medicine in Hradec Králové, Hradec Králové, Czechia; [7]Department of Internal Medicine, Seoul National University Hospital, Seoul, South Korea; [8]Medical University of Łódź, Department of Experimental Hematology, Łódź, Poland; [9]Hematology Department, Hospital General Universitario Morales Meseguer, IMIB-Pascual Parrilla, University of Murcia, Murcia, Spain; [10]Jefa de Hematologia, Hospital Universitario Cabueñes-Gijón, Asturias, Spain; [11]Department of Hematology, Mersin University Hospital, Mersin, Türkiye; [12]Institute of Hematology, I.R.C.C.S. Policlinico San Matteo, University of Pavia, Pavia, Italy; [13]Department of Hematology, University of Bologna, Bologna, Italy; [14]GSK, Collegville, PA, United States of America; [15]GSK, Stevenage, United Kingdom; [16]GSK, Warsaw, Poland; [17]Hematology Department, University Hospital of Salamanca/IBSAL/Cancer Research Center-IBMCC (USAL-CSIC), Salamanca, Spain; [18]Princess Margaret Cancer Centre, Department of Medical Oncology and Hematology, Toronto, ON, Canada; [19]Department of Clinical Therapeutics, School of Medicine, National and Kapodistrian University of Athens, Athens, Greece; [20]Department of Medicine, Korea University, Seoul, South Korea;*

### Background

B-cell maturation antigen (BCMA)–directed therapies have transformed the treatment landscape in relapsed/refractory multiple myeloma (RRMM). Belantamab mafodotin (belamaf), a BCMA-targeting antibody-drug conjugate, in combination with pomalidomide and dexamethasone (BPd) significantly improved progression-free survival (PFS) vs pomalidomide, bortezomib, and dexamethasone (PVd) (hazard ratio [HR], 0.52; 95% CI, 0.37-0.73; *P*<0.001) in patients with RRMM in DREAMM-8 (median follow-up, 22 months). This PFS benefit was maintained with extended follow-up (HR, 0.49; 95% CI, 0.36-0.67; median follow-up 36 months); median PFS2 also favored BPd vs PVd (47.1 vs 21.7 months, respectively; HR, 0.52; 95% CI, 0.38-0.70), indicating sustained clinical benefit over time.

### Aims

Report PFS2 outcomes from DREAMM-8 in subgroups based on prior therapy to further understand the long-term impact of BPd and inform treatment sequencing.

### Methods

DREAMM-8 (NCT04484623) is a phase 3, randomized, open-label study evaluating BPd vs PVd in patients with RRMM with ≥1 prior line of therapy including lenalidomide (LEN). Patients could not have received prior BMCA-targeted therapy. PFS2 was defined as time from randomization to disease progression after initiation of subsequent antimyeloma therapy or death. PFS2 was assessed based on prior-treatment subgroups.

**Copyright Information:** (Online) ISSN: 2572-9241
©2026 The Author(s). HemaSphere is published by John Wiley & Sons Ltd on behalf of European Hematology Association. This is an open access Abstract Book distributed under the Attribution-NonCommercial-NoDerivs (CC BY-NC-ND), which allows third parties to download the articles and share them with others as long as they credit the author and the Abstract Book, but they cannot change the content in any way or use them commercially.
**Abstract Book Citations:** Authors, Title, HemaSphere, 2026;10:(S1):pages. The abstract book DOIs can be found on the "Author Information" page.
**Disclaimer:** Articles published in the journal HemaSphere exclusively reflect the opinions of the authors. The authors are responsible for all content in their abstracts including accuracy of the facts, statements, citing resources, etc.

**Results**

In the BPd arm (data cutoff: July 7, 2025; median follow-up, 36 months), PFS2 benefit was maintained in all subgroups, including patients who were LEN refractory and anti-CD38 exposed/refractory. In LEN-refractory patients, BPd treatment led to almost a 3-fold median PFS2 improvement vs PVd (41.7 vs 15.0 months). A total of 56/155 patients (36%) in the BPd arm and 93/147 (63%) in the PVd arm had received any first subsequent therapy (FST) by data cutoff. Across both arms, 36% of patients received steroids, 28% received anti-CD38 therapies (>50% of patients with any subsequent therapy in each arm), and 23% received proteasome inhibitors as FST. A total of 21 patients (7%) received novel therapies (BPd, n=6 [4%]; PVd, n=15 [10%]) as FST, including BCMA-targeted bispecific antibodies (bsAbs), non-BCMA bsAbs, cereblon E3 ligase modulators, venetoclax, and belamaf. Improved median PFS2 was observed in patients who had novel therapies as FST (n=21) vs non-novel agents (n=128) (26.0 vs 20.1 months; HR, 0.61; 95% CI, 0.34-1.09).

**Summary/Conclusion**

BCMA-directed therapy, BPd, resulted in improved PFS2 outcomes vs PVd, demonstrating sustained clinical benefit beyond initial belamaf therapy. This benefit was seen regardless of prior treatment, with LEN-refractory patients experiencing almost a 3-fold PFS2 benefit, and despite a higher proportion of patients receiving novel therapies as FST in the PVd arm. PFS2 benefit with high use of subsequent anti-CD38 agents supports sequencing of belamaf combinations early in the treatment paradigm prior to anti-CD38 agents.
© 2026 American Society of Clinical Oncology, Inc. Reused with permission. This abstract was accepted and previously presented at the 2026 ASCO Annual Meeting. All rights reserved.

**Funding Statement:** Sponsored by GSK. Editorial support provided by Nucleus Global, an Inizio company and funded by GSK (study ID 207499). Drug linker technology licensed from Seagen Inc.; monoclonal antibody produced using POTELLIGENT Technology licensed from BioWa.

**Copyright Information:** (Online) ISSN: 2572-9241
©2026 The Author(s). HemaSphere is published by John Wiley & Sons Ltd on behalf of European Hematology Association. This is an open access Abstract Book distributed under the Attribution-NonCommercial-NoDerivs (CC BY-NC-ND), which allows third parties to download the articles and share them with others as long as they credit the author and the Abstract Book, but they cannot change the content in any way or use them commercially.
**Abstract Book Citations:** Authors, Title, HemaSphere, 2026;10:(S1):pages. The abstract book DOIs can be found on the "Author Information" page.
**Disclaimer:** Articles published in the journal HemaSphere exclusively reflect the opinions of the authors. The authors are responsible for all content in their abstracts including accuracy of the facts, statements, citing resources, etc.

### (PF777) VALIDATION OF THE IMS–IMWG GENOMIC RISK CLASSIFICATION IN A LARGE COHORT OF PATIENTS WITH TRANSPLANT-ELIGIBLE MULTIPLE MYELOMA

**Topic:** 14. Myeloma and other monoclonal gammopathies - Clinical

Damian Mikulski*[1], Mark R. Tanner[2], Qaiser Bashir[2], Samer A. Srour[2], Neeraj Saini[2], Paul Lin[2], Jeremy L. Ramdial[2], Yago Nieto[2], Guilin Tang[3], Yosra Aljawai[2], Krina Patel[4], Partow Kebriaei[2], Jing Christine Ye[4], Mahmoud R. Gaballa[4], Sheeba K. Thomas[4], Robert Z Orlowski[4], Richard E. Champlin[2], Elizabeth J. Shpall[2], Muzaffar H. Qazilbash[2], Oren Pasvolsky[4]

*[1]Medical University of Lodz, Department of Biostatistics and Translational Medicine, Lodz, Poland;[2]The University of Texas MD Anderson Cancer Center, Department of Stem Cell Transplantation and Cellular Therapy, Houston, United States of America; [3]The University of Texas MD Anderson Cancer Center, Department of Hematopathology, Houston, United States of America; [4]The University of Texas MD Anderson Cancer Center, Department of Lymphoma and Myeloma, Houston, United States of America;*

### Background

The International Myeloma Society–International Myeloma Working Group (IMS–IMWG) classification provides a genomically driven definition of high-risk (HR) multiple myeloma (MM). However, independent validation and a deeper understanding of interactions among its defining HR features are needed.

### Aims

To validate the prognostic performance of the IMS–IMWG classification and to characterize co-occurrence patterns among its constituent HR features in a large transplant-eligible cohort.

### Methods

We conducted a retrospective analysis of patients with newly diagnosed MM treated with triplet-based induction followed by autologous transplantation (autoHCT) between 2010 and 2021. Survival outcomes were evaluated using Kaplan–Meier analyses and multivariable Cox regression. Pairwise enrichment of high-risk features was assessed using observed-to-expected (O/E) ratios with false discovery rate (FDR) correction.

**Copyright Information:** (Online) ISSN: 2572-9241
©2026 The Author(s). HemaSphere is published by John Wiley & Sons Ltd on behalf of European Hematology Association. This is an open access Abstract Book distributed under the Attribution-NonCommercial-NoDerivs (CC BY-NC-ND), which allows third parties to download the articles and share them with others as long as they credit the author and the Abstract Book, but they cannot change the content in any way or use them commercially.
**Abstract Book Citations:** Authors, Title, HemaSphere, 2026;10:(S1):pages. The abstract book DOIs can be found on the “Author Information” page.
**Disclaimer:** Articles published in the journal HemaSphere exclusively reflect the opinions of the authors. The authors are responsible for all content in their abstracts including accuracy of the facts, statements, citing resources, etc.

**Results**

A total of 1,193 patients were included. Induction regimens included VRD (bortezomib, lenalidomide, dexamethasone) in 612 patients (51.3%), KRD (carfilzomib, lenalidomide, dexamethasone) in 341 (28.6%), and VCD (bortezomib, cyclophosphamide, dexamethasone) in 240 (20.1%). Overall, 289 patients (24.2%) met the IMS–IMWG HR criteria. Of these, 239 patients (20.0%) met a single HR criterion, 42 (3.5%) met two criteria, and 8 (0.7%) met three criteria. The prevalence of individual HR features was as follows: del(17p) in 123 patients (10.3%); high-risk IgH translocations [t(4;14), t(14;16), or t(14;20)] co-occurring with 1q gain and/or del(1p32) in 121 patients (10.1%); del(1p32) with concomitant 1q gain or amplification in 36 patients (3.0%); and elevated β2-microglobulin (>5.5 mg/dL) in the setting of preserved renal function in 67 patients (5.6%).
Median PFS was 33.9 months in HR patients compared with 63.3 months in standard-risk (SR) patients (hazard ratio [HR], 1.93; 95% CI, 1.62–2.30), whereas median OS was 81.3 versus 128.9 months, respectively (HR, 2.58; 95% CI, 2.01–3.31). Each individual IMS–IMWG HR features was associated with adverse outcomes, and the risk of progression or death increased in a stepwise fashion with the number of HR criteria met (Figure 1). In multivariable models, IMS–IMWG-defined HR MM remained independently associated with inferior PFS (HR 1.92; 95% CI, 1.54–2.40) and OS (HR 2.23; 95% CI, 1.67–2.96).
Among patients meeting ≥2 IMS–IMWG HR criteria, the most prevalent constellation was del(17p) co-occurring with high-risk IgH translocations and chromosome 1 abnormalities. Pairwise co-occurrence analysis confirmed significant non-random enrichment between del(17p) and high-risk IgH translocations (O/E ratio 2.0; FDR 0.002), as well as between del(17p) and elevated β2-microglobulin in the absence of renal dysfunction (O/E 2.03; FDR 0.020). Among patients harboring ≥2 HR features, those with concomitant del(17p) and high-risk translocations with chromosome 1 aberrations showed numerically inferior PFS and OS compared with other multiple HR criteria, although these differences did not reach statistical significance.

**Summary/Conclusion**

In this large cohort of transplant-eligible MM patients, the IMS–IMWG classification demonstrated robust and independent prognostic value. Risk increased in a graded manner with the accumulation of HR features, and del(17p) emerged as a central abnormality with preferential co-occurrence alongside other HR criteria.

**Copyright Information:** (Online) ISSN: 2572-9241
©2026 The Author(s). HemaSphere is published by John Wiley & Sons Ltd on behalf of European Hematology Association. This is an open access Abstract Book distributed under the Attribution-NonCommercial-NoDerivs (CC BY-NC-ND), which allows third parties to download the articles and share them with others as long as they credit the author and the Abstract Book, but they cannot change the content in any way or use them commercially.
**Abstract Book Citations:** Authors, Title, HemaSphere, 2026;10:(S1):pages. The abstract book DOIs can be found on the “Author Information” page.
**Disclaimer:** Articles published in the journal HemaSphere exclusively reflect the opinions of the authors. The authors are responsible for all content in their abstracts including accuracy of the facts, statements, citing resources, etc.

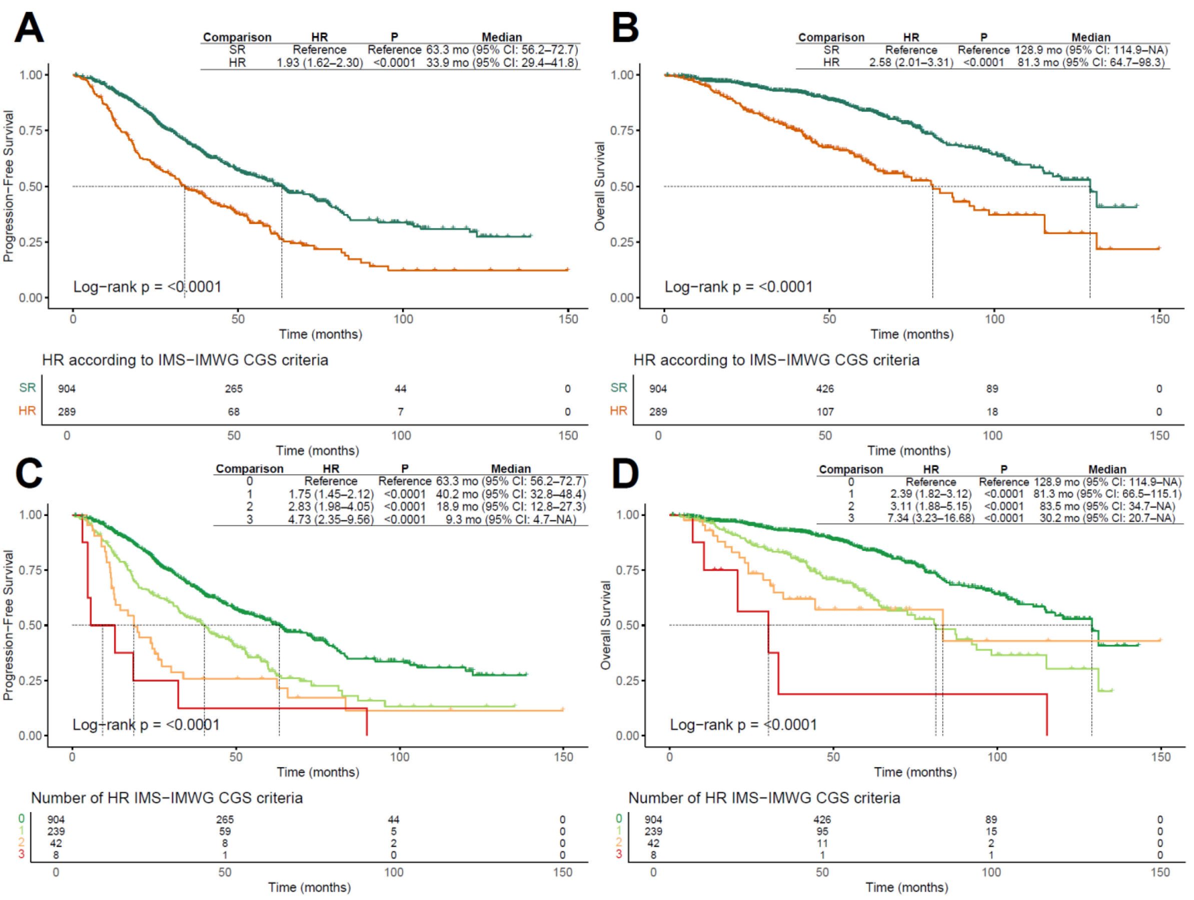

**Figure 1. Prognostic impact of the IMS–IMWG high-risk classification and cumulative high-risk burden.** (A) Progression-free survival (PFS) and (B) overall survival (OS) in patients with multiple myeloma treated with triplet-based induction and autologous transplantation, stratified according to International Myeloma Society–International Myeloma Working Group (IMS–IMWG) high-risk (HR) versus standard-risk (SR) categories. (C) PFS and (D) OS according to the number of fulfilled IMS–IMWG high-risk criteria (0, 1, 2, or 3), demonstrating a stepwise worsening of outcomes with increasing high-risk burden. Survival curves were estimated using the Kaplan–Meier method and compared using the log-rank test. Hazard ratios (HRs) with 95% confidence intervals are shown.

**Copyright Information:** (Online) ISSN: 2572-9241
©2026 The Author(s). HemaSphere is published by John Wiley & Sons Ltd on behalf of European Hematology Association. This is an open access Abstract Book distributed under the Attribution-NonCommercial-NoDerivs (CC BY-NC-ND), which allows third parties to download the articles and share them with others as long as they credit the author and the Abstract Book, but they cannot change the content in any way or use them commercially.
**Abstract Book Citations:** Authors, Title, HemaSphere, 2026;10:(S1):pages. The abstract book DOIs can be found on the "Author Information" page.
**Disclaimer:** Articles published in the journal HemaSphere exclusively reflect the opinions of the authors. The authors are responsible for all content in their abstracts including accuracy of the facts, statements, citing resources, etc.

### (PF778) UNRAVELING 1Q$^+$/T(4;14) DOUBLE-HIT MYELOMA: CLINICAL AGGRESSIVENESS, EXTRAMEDULLARY RELAPSE, AND MOLECULAR TARGETS FROM A MULTICENTER AND MULTI-OMIC STUDY

**Topic:** 14. Myeloma and other monoclonal gammopathies - Clinical

Yi Tao*[1], Niu Qiao[1], Ying Guo[2], Shu-Jun Sun[1], Kun-Ming Liu[3], Wanyan Ouyang[1], Wenyan Yu[1], Bing-Bing Zhao[4], Jie Hao[5], Wei Wei[6], Jian-qing Mi[1]

*[1]Shanghai Institute of Hematology, State Key Laboratory of Medical Genomics, National Research Center for Translational Medicine at Shanghai, Department of Hematology, Shanghai, China; [2]The First People's Hospital of Jiujiang City, Department of Hematology, Jiujiang, China; [3]The First People's Hospital of Suzhou City, Department of Hematology, Suzhou, China;[4]411 Hospital Affiliated to Shanghai University, Department of Hematology, Shanghai, China; [5]Bei Zhan Hospital, Shanghai, Shanghai, China; [6]Zhabei Central Hospital, Shanghai, Shanghai, China;*

### Background

The concurrent presence of 1q gain/amplification (1q$^+$) and t(4;14) identifies a unique double-hit (DH) subset in newly diagnosed multiple myeloma (NDMM), yet its clinical behavior and underlying biological mechanisms remain largely unclear.

### Aims

This study aimed to systematically define the clinical characteristics, treatment efficacy, and molecular landscape of the 1q$^+$/t(4;14) DH subgroup in NDMM.

### Methods

Clinical data from a multi-center cohort and multi-omic profiles from the CoMMpass database were combined for integrative analysis. We retrospectively evaluated 841 NDMM patients, assessing survival outcomes, measurable residual disease (MRD), and secondary extramedullary disease (sEMD) incidence. Clonal evolutionary patterns at relapse were examined, and mutational, transcriptomic, and pathway analyses were performed across DH and non-DH subgroups.

### Results

Of 841 patients, 106 (12.5%) had 1q$^+$/t(4;14), exhibiting highly aggressive features: 53% classified as R2-ISS IV, high 1q$^+$ clonal burden, median PFS 26 months, 3-year OS 67%, 24% MRD negativity, and 26% 3-year sEMD risk. Extraosseous extramedullary disease and del(17p) were independent adverse factors for PFS, while MRD negativity was associated with prolonged PFS. Autologous stem cell transplantation and triplet induction (proteasome inhibitor + immunomodulatory drug + anti-CD38 antibody) strongly predicted achievement of MRD negativity. Four distinct clonal evolution patterns (linear, branching, differentiation, stable) were detected at relapse. Multi-omic analysis revealed significantly elevated mutation frequencies in DIS3 (30%), TP53 (14%), and FGFR3 (14%). Pairwise transcriptomic comparisons uncovered 30 DH-specific upregulated genes, 67% mapping to 1q, including immune-regulatory CD70, proliferation-associated SEMA4A, and HDACi-responsive ATF3, representing candidate therapeutic targets. Gene set enrichment analysis demonstrated activation of MAPK, cell cycle, and EZH2-related stem cell pathways, accompanied by suppressed immune signatures, consistent with the high-risk phenotype and sEMD propensity.

**Copyright Information:** (Online) ISSN: 2572-9241
©2026 The Author(s). HemaSphere is published by John Wiley & Sons Ltd on behalf of European Hematology Association. This is an open access Abstract Book distributed under the Attribution-NonCommercial-NoDerivs (CC BY-NC-ND), which allows third parties to download the articles and share them with others as long as they credit the author and the Abstract Book, but they cannot change the content in any way or use them commercially.
**Abstract Book Citations:** Authors, Title, HemaSphere, 2026;10:(S1):pages. The abstract book DOIs can be found on the "Author Information" page.
**Disclaimer:** Articles published in the journal HemaSphere exclusively reflect the opinions of the authors. The authors are responsible for all content in their abstracts including accuracy of the facts, statements, citing resources, etc.

### Summary/Conclusion

The 1q⁺/t(4;14) double-hit subtype constitutes a biologically distinct and clinically high-risk NDMM subgroup. These results underscore the value of MRD-directed treatment strategies and support the development of consolidative CAR-T therapy and novel DH-specific targeted interventions.

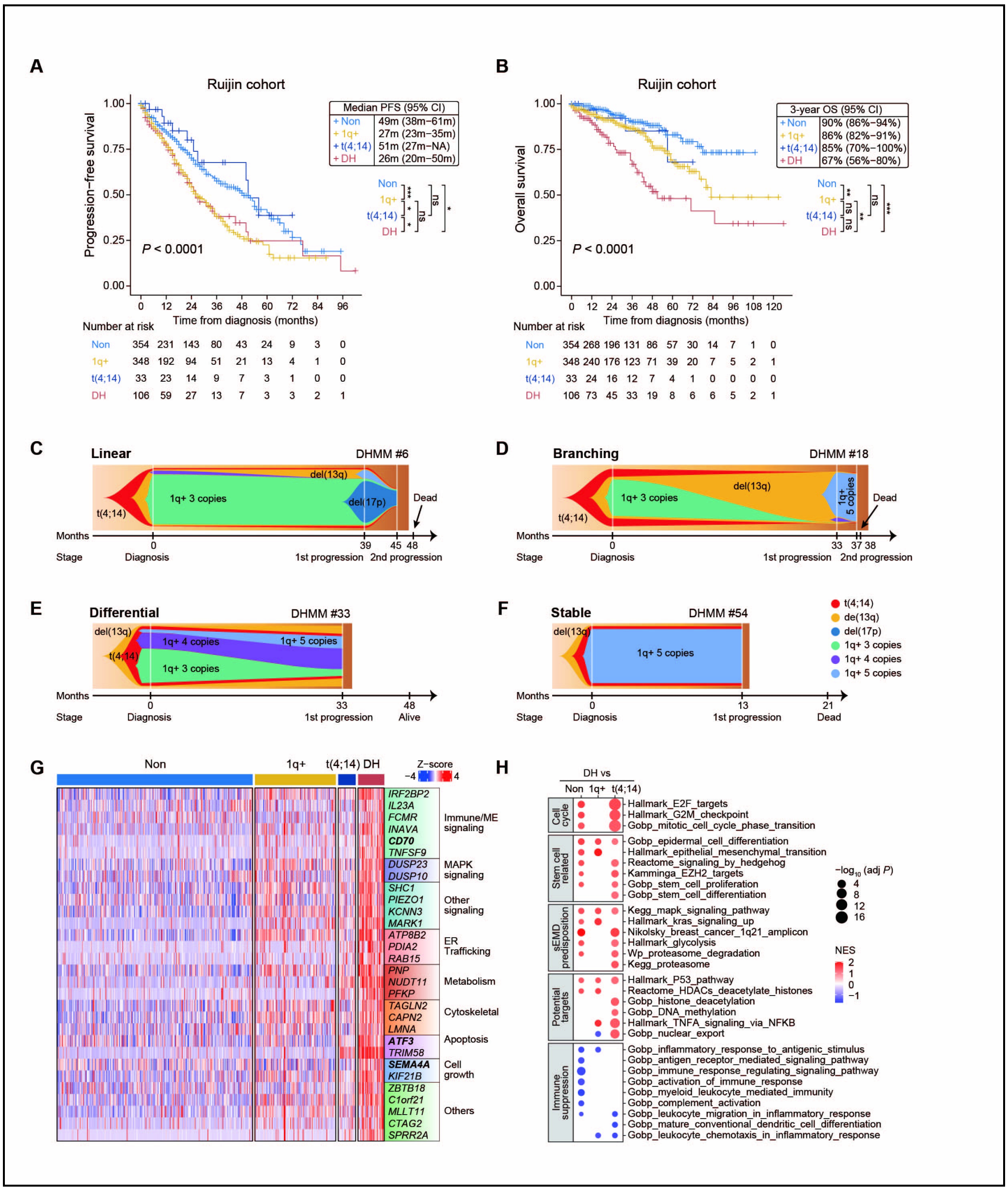

Figure. Clinical and molecular features of the 1q⁺/t(4;14) DHMM A-B. Kaplan-Meier curves depicting PFS (A) and OS (B) in Ruijin cohort, stratified by genetic aberration status; C-F. Fish plots depicting the cytogenetic evolutionary patterns of four representative DHMM patients at relapse: linear (F; Patient #6), branching (G; Patient #18), differential (H; Patient #33), and stable (I; Patient #54) patterns; G. Heatmap of 30 overlapping DEGs across four groups and their functional categorization; H. Dot plot showing enriched GO/Hallmark pathways of DEGs from three DH subgroup comparisons.

**Copyright Information:** (Online) ISSN: 2572-9241
©2026 The Author(s). HemaSphere is published by John Wiley & Sons Ltd on behalf of European Hematology Association. This is an open access Abstract Book distributed under the Attribution-NonCommercial-NoDerivs (CC BY-NC-ND), which allows third parties to download the articles and share them with others as long as they credit the author and the Abstract Book, but they cannot change the content in any way or use them commercially.
**Abstract Book Citations:** Authors, Title, HemaSphere, 2026;10:(S1):pages. The abstract book DOIs can be found on the "Author Information" page.
**Disclaimer:** Articles published in the journal HemaSphere exclusively reflect the opinions of the authors. The authors are responsible for all content in their abstracts including accuracy of the facts, statements, citing resources, etc.

### (PF779) DARATUMUMAB RETREATMENT AFTER CONTINUOUS FRONTLINE DARA-VCD WITH BACKBONE SWITCH IN TRANSPLANT-INELIGIBLE PATIENTS WITH NEWLY DIAGNOSED MULTIPLE MYELOMA: RESULTS FROM THE MULTICENTER GMMG-DADA STUDY

**Topic:** 14. Myeloma and other monoclonal gammopathies - Clinical

Tim Richardson*[1], Christina Thielen[1], Axel Benner[2], Kaya Miah[2], Udo Holtick[1], Mathias Hänel[3], Peter Staib[4], Britta Besemer[5], Yon-Dschun Ko[6], Martin Schmidt-Hieber[7], Florian Kaiser[8], Timon Hansen[9], Maika Klaiber[10], Sebastian Theurich[11], Swen Weßendorf[12], Michael Neise[13], Christian Schmitt[14], Ullrich Graeven[15], Manfred Hensel[16], Cyrus Khadanpour[17], Joana Millo[18], Stefanie Huhn[19], Philipp Reichert[19], Michael Hundemer[20], Niels Weinhold[20], Marc-Steffen Raab[20], Hartmut Goldschmidt[20], Christoph Scheid[1]

*[1]University hospital of cologne, Cologne, Germany;[2]German Cancer Research Center (DKFZ), Heidelberg, Germany, Heidelberg, Germany; [3]Department III of Internal Medicine, Klinikum Chemnitz, Chemnitz, Germany;[4]Department of Haematology and Oncology, St. Antonius-Hospital Eschweiler, Eschweiler, Germany; [5]Department of Internal Medicine II, University of Tübingen, Tübingen, Germany; [6]Department of Haematology and Oncology, Johanniter Hospital Bonn, Bonn, Germany; [7]Department II of Haematology/Oncology, Medizinische Universität Lausitz – Carl Thiem, Cottbus, Germany;[8]ÜBAG-MVZ Dr. Vehling-Kaiser GmbH, Landshut, Germany; [9]Hämatologisch-Onkologische Praxis Altona (HOPA), Hamburg, hamburg, Germany; [10]Department of Oncology and Haematology, Marien-Hospital Düsseldorf, Düsseldorf, Germany; [11]Department of Medicine III, LMU University Hospital, Ludwig-Maximilians-Universität (LMU), Munich, Germany, München, Germany; [12]Department of General Internal Medicine, Oncology/Haematology, Gastroenterology and Infectiology, Klinikum Esslingen GmbH, Esslingen, Germany, Esslingen, Germany; [13]OnkoMed GbR Dr. M. Neise, Dr. A. Lollert, Dr. D. Neise, MVZ West Krefeld, Krefeld, Germany, Krefeld, Germany; [14]Haematology and Oncology, Bethanien Hospital Frankfurt, Frankfurt/Main, Germany, Frankfurt, Germany; [15]Department of Haematology and Oncology, Kliniken Maria Hilf GmbH, Mönchengladbach, Germany, Mönchengladbach, Germany; [16]Mannheimer Onkologie Praxis, Mannheim, Germany, Mannheim, Germany; [17]Universitätsklinik für Innere Medizin - Onkologie und Haematologie, Oldenburg, Dermany, Oldenburg, Germany; [18]Haematology, Oncology and Palliative Care Winnenden, Rems-Murr-Klinikum gGmbH, Winnenden, Germany, Winnenden, Germany; [19]GMMG Central Laboratory and Biobank Multiple Myeloma, Heidelberg University Hospital, Heidelberg, Germany, Heidelberg, Germany; [20]Heidelberg Myeloma Center, Department of Medicine V, Heidelberg University Hospital and Medical Faculty, Heidelberg, Germany, Heidelberg, Germany;*

### Background

Daratumumab is increasingly used in the frontline therapy of transplant-ineligible patients with newly diagnosed multiple myeloma (NDMM) and is commonly continued until progression. The GMMG-DADA trial specifically enrolled a frail, elderly population, including patients with renal impairment and dialysis dependence, for whom lenalidomide-based regimens are often unsuitable, making Dara-VCd a particularly relevant frontline choice. Prospective data on daratumumab retreatment with protocol-defined backbone switching in this setting are lacking.

### Aims

To evaluate feasibility and efficacy of daratumumab re-treatment with an IMiD-based backbone after continuous frontline daratumumab exposure until progression and to explore the impact of duration of frontline response on second-line outcomes in a real-world elderly NDMM population.

**Copyright Information:** (Online) ISSN: 2572-9241
©2026 The Author(s). HemaSphere is published by John Wiley & Sons Ltd on behalf of European Hematology Association. This is an open access Abstract Book distributed under the Attribution-NonCommercial-NoDerivs (CC BY-NC-ND), which allows third parties to download the articles and share them with others as long as they credit the author and the Abstract Book, but they cannot change the content in any way or use them commercially.
**Abstract Book Citations:** Authors, Title, HemaSphere, 2026;10:(S1):pages. The abstract book DOIs can be found on the "Author Information" page.
**Disclaimer:** Articles published in the journal HemaSphere exclusively reflect the opinions of the authors. The authors are responsible for all content in their abstracts including accuracy of the facts, statements, citing resources, etc.

### Methods

GMMG-DADA is a prospective, multicenter phase II study enrolling 67 transplant-ineligible NDMM patients (median age 74 years; including patients with renal failure and dialysis) treated with 8 cycles of Dara-VCd followed by daratumumab-bortezomib-dexamethasone maintenance until progression or intolerance. At first relapse, patients received daratumumab-lenalidomide-dexamethasone (DRd) per protocol. Response was assessed by IMWG criteria. Patients initiating second-line therapy were stratified by time from start of frontline to start of second-line treatment (<12 months vs ≥12 months).

### Results

Frontline Dara-VCd achieved an ORR of 86% and a VGPR-or-better rate of 71%. Of 67 enrolled patients, 26 have progressed so far and were started on second-line DRd. Best responses to second-line therapy according to time to relapse are shown below:

| Time to 2nd line | CR | VGPR | PR | SD | PD |
|---|---|---|---|---|---|
| ≥12 months (n=16) | 2 | 7 | 5 | 1 | 1 |
| <12 months (n=10) | 0 | 2 | 4 | 2 | 2 |

**Best Response to Second-Line Dara-Rd by Time to Relapse for First-Line therapy:** *IMWG best responses to second-line DRd in 26 patients stratified by early (<12 months) versus late (≥12 months) relapse after frontline Dara-VCd.*

The overall 2L ORR was 77%. Patients relapsing ≥12 months after frontline initiation achieved an ORR of 88%, with deep responses (CR/VGPR) in 56% and a very low rate of progressive disease (6%) at a median follow-up of 11.6 months from second-line initiation. Patients relapsing <12 months had an ORR of 60%, fewer deep responses (20%) and higher rates of PD (20%). Median PFS2 and OS were not yet reached. No unexpected safety signals were observed during re-treatment. In the daratumumab-naive POLLUX population, DRd achieved an ORR of 93% (VGPR or better 76%, CR or better 43%) versus 76% for Rd alone (VGPR or better 44%, CR or better 19%).
The responses observed in GMMG-DADA after prior continuous daratumumab exposure without any wash-out phase suggest partial preservation of daratumumab sensitivity when switching to an IMiD-based backbone, in particular in relapses occurring later than 12 months. In contrast early relapse represents functional high-risk patients with a medical need for alternative therapy such as CAR-T cells.

### Summary/Conclusion

GMMG-DADA provides unique prospective data on daratumumab re-treatment after continuous frontline exposure until progression with protocol-defined backbone switching in a frail, elderly NDMM population including patients with renal dysfunction. Daratumumab re-treatment with DRd was feasible and showed clinically meaningful activity, particularly in patients with relapses after 12 months. Functional high-risk patients relapsing within 12 months may not benefit from such a retreatment.

**Copyright Information:** (Online) ISSN: 2572-9241
©2026 The Author(s). HemaSphere is published by John Wiley & Sons Ltd on behalf of European Hematology Association. This is an open access Abstract Book distributed under the Attribution-NonCommercial-NoDerivs (CC BY-NC-ND), which allows third parties to download the articles and share them with others as long as they credit the author and the Abstract Book, but they cannot change the content in any way or use them commercially.
**Abstract Book Citations:** Authors, Title, HemaSphere, 2026;10:(S1):pages. The abstract book DOIs can be found on the "Author Information" page.
**Disclaimer:** Articles published in the journal HemaSphere exclusively reflect the opinions of the authors. The authors are responsible for all content in their abstracts including accuracy of the facts, statements, citing resources, etc.

### (PF780) A PRELIMINARY STUDY ON THE SAFETY AND EFFICACY OF INTRATHECAL COMBINED WITH INTRAVENOUS CAR-T CELL THERAPY FOR B-CELL MALIGNANCIES WITH CENTRAL NERVOUS SYSTEM INVOLVEMENT

**Topic:** 14. Myeloma and other monoclonal gammopathies - Clinical

Yanli Feng*[1], Yi Wang[1]

[1]*Shaanxi Provincial People's Hospital, Department of Hematology, Xi'an, China;*

### Background

CAR-T cell therapy has demonstrated clear efficacy in relapsed/refractory B-cell malignancies, including B-cell acute lymphoblastic leukemia (B-ALL), diffuse large B-cell lymphoma (DLBCL), and multiple myeloma (MM). CAR-T cells can penetrate the blood-brain barrier and are effective in patients with central nervous system (CNS) involvement. Recently, the mainly infusion of CAR-T was through intravenous. The evaluation of the safety and efficacy of combined intrathecal and intravenous CAR-T infusion remain scarce.

### Aims

To evaluate the safety and clinical efficacy of intrathecal combined with intravenous CAR-T therapy in patients with relapsed/refractory B-cell malignancies accompanied by CNS involvement.

### Methods

This study analyzed patients with B-cell malignancies and CNS involvement treated at our center from January 2025 to February 2026 using intrathecal combined with intravenous CAR-T therapy. Flow cytometry was used to detect the CAR-T cell expansion and cytokine levels in cerebrospinal fluid (CSF) and peripheral blood (PB). Efficacy was assessed via CSF minimal residual disease detection and MRI imaging. Patient demographics and adverse events were collected, with follow-up until February 28, 2025.

### Results

A cohort of 8 patients were include: 5 with B-ALL, 2 with DLBCL, and 1 with MM. CNS manifestations included isolated CSF positivity by flow cytometry in 5 B-ALL cases, isolated parenchymal lesions on MRI in 2 relapsed/refractory DLBCL cases, and both CSF and parenchymal positivity in 1 MM case.
All patients received standard fludarabine-cyclophosphamide lymphodepletion before CAR-T therapy. Relapsed/refractory B-ALL and DLBCL were targets by CD19/or CD22, and BCMA for relapsed/refractory MM. 37.5% (3/8) of the patients recieved intravenous CAR-T first, and followed intrathecal CAR-T after 7 days; while 62.5% (5/8) were given concurrently.
Significant CAR-T expansion was observed in CSF post CAR-T, with a median peak of 52.8% (range: 17.7%–88.6%), median time to peak in CSF was 4 days (range: 3–5 days). Cytokine release syndrome (CRS) occurred in 100% of patients (62.5% grade 1–2, 37.5% grade 3). ICANS (grade 1–2) affected 50%, and infections occurred in 12.5%. No grade ≥3 severe adverse events or treatment-related deaths were reported. Common symptoms included fever, hypotension, headache, dizziness, nausea, and vomiting; only 2 patients had minor seizures. All events were transient and manageable.
Median follow-up was 4 months (range: 2–7 months). At 1 month post-CAR-T, the overall objective response rate (ORR) was 100%, including 5 complete responses (CR), 1 very good partial response (VGPR), and 2 partial responses (PR). During 2–7 months of follow-up, 50% (4/8) relapsed; 2 achieved CR again after second intrathecal/intravenous CAR-T with different targets.

**Copyright Information:** (Online) ISSN: 2572-9241
©2026 The Author(s). HemaSphere is published by John Wiley & Sons Ltd on behalf of European Hematology Association. This is an open access Abstract Book distributed under the Attribution-NonCommercial-NoDerivs (CC BY-NC-ND), which allows third parties to download the articles and share them with others as long as they credit the author and the Abstract Book, but they cannot change the content in any way or use them commercially.
**Abstract Book Citations:** Authors, Title, HemaSphere, 2026;10:(S1):pages. The abstract book DOIs can be found on the “Author Information” page.
**Disclaimer:** Articles published in the journal HemaSphere exclusively reflect the opinions of the authors. The authors are responsible for all content in their abstracts including accuracy of the facts, statements, citing resources, etc.

**Summary/Conclusion**

Intrathecal combined with intravenous CAR-T cell therapy enables rapid CNS tumor clearance in B-cell malignancies with CNS involvement, with favorable safety. These findings establish intrathecal CAR-T as a promising strategy for CNS-limited B-cell tumors.

**Copyright Information:** (Online) ISSN: 2572-9241
©2026 The Author(s). HemaSphere is published by John Wiley & Sons Ltd on behalf of European Hematology Association. This is an open access Abstract Book distributed under the Attribution-NonCommercial-NoDerivs (CC BY-NC-ND), which allows third parties to download the articles and share them with others as long as they credit the author and the Abstract Book, but they cannot change the content in any way or use them commercially.
**Abstract Book Citations:** Authors, Title, HemaSphere, 2026;10:(S1):pages. The abstract book DOIs can be found on the "Author Information" page.
**Disclaimer:** Articles published in the journal HemaSphere exclusively reflect the opinions of the authors. The authors are responsible for all content in their abstracts including accuracy of the facts, statements, citing resources, etc.

### (PF781) FUNCTIONAL CURE IN WALDENSTROM'S MACROGLOBULINEMIA: DETERMINANTS OF 10-YEAR PROGRESSION-FREE SURVIVAL

**Topic:** 14. Myeloma and other monoclonal gammopathies - Clinical

Maria Gavriatopoulou*[1], Ioannis Ntanasis-Stathopoulos[1], Charalampos Filippatos[1], Panagiotis Malandrakis[1], Ilias Katsadouros[1], Panagiotis Boutsikos[1], Magdalini Migkou[1], Nikolaos Kanellias[1], Evangelos Eleutherakis-Papaiakovou[1], Foteini Theodorakakou[1], Eirini Solia[1], Evangelos Terpos[1], Efstathios Kastritis[1], Meletios A. Dimopoulos[1]

*[1]National and Kapodistrian University of Athens, Department of Clinical Therapeutics, Athens, Greece;*

### Background

Waldenström Macroglobulinemia (WM) is a low-grade lymphoma characterized by increasingly prolonged survival especially after the integration of Bruton Tyrosine Kinase inhibitors.

### Aims

In this study, we attempt to characterize the clinical and biological determinants that allow a specific subset of patients to achieve functional cure (defined as exceeding 10 years of progression free survival (PFS)).

### Methods

We conducted a retrospective analysis of 326 consecutive, unselected WM newly diagnosed patients who initiated frontline therapy in a tertiary academic center between 2002 and 2016. To evaluate long-term outcomes, a minimum follow-up of 10 years was required for inclusion. Patients treated primarily for cold-agglutinin disease or WM related neuropathy were excluded. Independent predictors of 10-year PFS were identified using multivariate logistic and Cox regression models.

**Copyright Information:** (Online) ISSN: 2572-9241
©2026 The Author(s). HemaSphere is published by John Wiley & Sons Ltd on behalf of European Hematology Association. This is an open access Abstract Book distributed under the Attribution-NonCommercial-NoDerivs (CC BY-NC-ND), which allows third parties to download the articles and share them with others as long as they credit the author and the Abstract Book, but they cannot change the content in any way or use them commercially.
**Abstract Book Citations:** Authors, Title, HemaSphere, 2026;10:(S1):pages. The abstract book DOIs can be found on the "Author Information" page.
**Disclaimer:** Articles published in the journal HemaSphere exclusively reflect the opinions of the authors. The authors are responsible for all content in their abstracts including accuracy of the facts, statements, citing resources, etc.

**Results**

A total of 326 unselected patients were included; median age was 70 (range 35- 92) and 191 patients (58.6%) were males. The 10-year OS and PFS rates for the whole cohort was 52.1% and 25.3%, respectively. Among all patients, 39 (12.0%) achieved a PFS of more than 10 years after frontline treatment. The median follow-up among them was 15.8 years and the 15-year OS and PFS rates were 59.5% and 36.5%, respectively. Long-term progression-free survivors were younger at diagnosis, with a median age of 67 compared to 71 for others (p=0.057). A higher proportion of females was observed among those with ≥10-year PFS (56.4% vs. 39.4%, p=0.056). Several baseline characteristics highlighted a more favorable prognostic profile among long-term survivors. Patients achieving ≥10-year PFS presented with significantly lower rates of severe hypoalbuminemia (23.1% vs. 44.3%, p=0.015) and high serum b2-microglobulin levels (>3 mg/L: 12.9% vs. 35.4%, p=0.014). Increased LDH levels were less frequent in the long-term PFS group (5.6% vs. 19.2%, p=0.060). According to the R-IPSSWM stratification, a significantly higher prevalence of low-risk disease was observed among long-term survivors compared to the others (p<0.001), with 69.2% diagnosed with "Very low" or "Low" risk disease and only 2.6% with "High" risk disease. In contrast, the group of patients with PFS of <10 years consisted of a significantly larger proportion of “High” (19.5%) or “Very high” (10.8%) risk individuals. The distribution of first-line therapy was similar between groups (p=0.738), with dexamethasone-rituximab-cyclophosphamide being the most commonly used regimen in both the ≥10-year PFS (53.8%) and the <10-year (52.3%) groups. Importantly, patients achieving "functional cure" demonstrated a higher major response rate (≥PR) to initial therapy compared to others (76.9% vs. 60.3%, p=0.053). In the multivariate logistic regression for the identification of independent predictors of a ≥10-year PFS, normal serum albumin (≥3.5 g/dL) was the primary clinical determinant of a "functional cure" (OR=2.51, p=0.022). In parsimonious models, the R-IPSSWM score and female gender were also independently associated with prolonged PFS both in logistic (OR=0.54 and OR=2.03, p<0.001 and p=0.046; respectively) and Cox analyses (HR=1.27 and HR=0.69; respectively, p<0.001).

**Summary/Conclusion**

A "functional cure" was observed in approximately 12% of unselected patients treated for newly diagnosed WM. This long-term disease-free status is primarily determined by baseline factors expressed in the R-IPSSWM model, rather than the specific choice of induction regimen.

**Copyright Information:** (Online) ISSN: 2572-9241
©2026 The Author(s). HemaSphere is published by John Wiley & Sons Ltd on behalf of European Hematology Association. This is an open access Abstract Book distributed under the Attribution-NonCommercial-NoDerivs (CC BY-NC-ND), which allows third parties to download the articles and share them with others as long as they credit the author and the Abstract Book, but they cannot change the content in any way or use them commercially.
**Abstract Book Citations:** Authors, Title, HemaSphere, 2026;10:(S1):pages. The abstract book DOIs can be found on the “Author Information” page.
**Disclaimer:** Articles published in the journal HemaSphere exclusively reflect the opinions of the authors. The authors are responsible for all content in their abstracts including accuracy of the facts, statements, citing resources, etc.

### (PF782) WHOLE-BODY [18F]FDG-PET/MRI IN RELAPSED/REFRACTORY MULTIPLE MYELOMA PATIENTS TREATED BY IDECABTAGENE VICLEUCEL FOR DISEASE DETECTION AND PREDICTION OF SURVIVAL.

**Topic:** 14. Myeloma and other monoclonal gammopathies - Clinical

Bastien Jamet*[1], Francoise Kraeber-Bodere[1], Huajun Sun[1], Chloé François[1], Hatem Necib[1], Aurelien Monnet[2], Eric Frampas[2], Nicolas Blin[3], Nicoletta Lilli[3], Caroline Milin[1], Philippe Moreau[3], Cyrille Touzeau[3]

*[1] 1Nantes Université, Univ Angers, CHU Nantes, INSERM, CNRS, CRCI2NA, Médecine Nucléaire, Nantes, France; [2] Nantes Université, Univ Angers, CHU Nantes, INSERM, CNRS, CRCI2NA, Radiologie, Nantes, France; [3] Nantes Université, Univ Angers, CHU Nantes, INSERM, CNRS, CRCI2NA, Hématologie, Nantes, France;*

### Background

Idecabtagene-vicleucel (Ide-cel) chimeric antigen receptor (CAR)-T-cell therapy is approved in patients with relapsed/refractory multiple myeloma (RRMM) but up to date there is no established model able to predict outcome before and after therapy in order to identify patients who may benefit the most from CAR-T. Combining whole-body [$^{18}$F]-fluorodeoxyglucose-positron emission tomography ([$^{18}$F]FDG-PET) and diffusion-weighted MRI (DW-MRI) in a single simultaneous scan could be interesting in this setting.

### Aims

The aims of this study were to assess the diagnostic value (detection rate of MM-related medullary or extra-medullary disease (EMD)) and the prognostic value for progression-free survival (PFS) of whole-body simultaneous [$^{18}$F]FDG-PET/MRI imaging performed before and after Ide-cel therapy in RRMM workup.

### Methods

Patients with triple-class (or more) RRMM who received at least two prior lines of treatment were prospectively enrolled in this monocentric study between May 2021 and August 2025. All patients received a personalized bridging therapy and underwent whole-body simultaneous multi-parametric [$^{18}$F]FDG-PET/MRI imaging on a 3T Biograph mMR device before Ide-cel (after bridging therapy) and one to three months after Ide-cel infusion.

**Copyright Information:** (Online) ISSN: 2572-9241
©2026 The Author(s). HemaSphere is published by John Wiley & Sons Ltd on behalf of European Hematology Association. This is an open access Abstract Book distributed under the Attribution-NonCommercial-NoDerivs (CC BY-NC-ND), which allows third parties to download the articles and share them with others as long as they credit the author and the Abstract Book, but they cannot change the content in any way or use them commercially.
**Abstract Book Citations:** Authors, Title, HemaSphere, 2026;10:(S1):pages. The abstract book DOIs can be found on the “Author Information” page.
**Disclaimer:** Articles published in the journal HemaSphere exclusively reflect the opinions of the authors. The authors are responsible for all content in their abstracts including accuracy of the facts, statements, citing resources, etc.

**Results**

A total of 72 patients underwent [$^{18}$F]FDG-PET/MRI imaging before Ide-cel. Overall, active residual disease was detected in 64/72 patients (89%). By univariate analysis, presence of diffuse bone marrow involvement (BMI) pattern on PET (HR, 2.67; 95%CI, 1.12-6.35, p = 0.03) or MRI (HR, 2.54; 95%CI, 1.17-5.5, p = 0.02) was an adverse prognostic parameter for PFS. Presence of 2 HRCA (HR, 2.58; 95%CI, 1.09-6.12, p = 0.03) was also associated with shorter PFS.

A total of 66 patients underwent [$^{18}$F]FDG-PET/MRI imaging after Ide-cel infusion for response assessment. Biological overall response rate (ORR, partial response or better) was of 97%. MRI detected residual disease (response assessment category (RAC) 2 or higher) in 9/66 (14%) of the patients and PET (no complete metabolic response) in 7/66 (11%). Overall, [$^{18}$F]FDG-PET/MRI allowed to increase the number of patients with residual disease detection to 13/66 (20%). Patients with residual disease on DW-MRI according to MY-RADS (RAC2 or higher) had shorter PFS (HR, 4.11; 95%CI, 1.8-9.4, $p = <10^{-3}$) than those without (RAC1). On the other hand, achieving at least a partial response on PET significantly improved the PFS (p = 0.0046). [$^{18}$F]FDG-PET/MRI combination allowed identifying three different risk groups (double MRI and PET negativity, MRI and/or PET positivity, MRI and PET positivity, Figure) with a very high-risk (MRI and PET positivity) group.

**Summary/Conclusion**

Simultaneous [$^{18}$F]FDG-PET/MRI imaging increased the diagnostic and prognostic value of PET and MRI alone in RRMM patients treated by Ide-cel. Presence of diffuse BMI pattern on PET/MRI before Ide-cel was an adverse prognostic parameter for PFS, as well as presence of residual disease according to standardized criteria after Ide-cel.

**Copyright Information:** (Online) ISSN: 2572-9241
©2026 The Author(s). HemaSphere is published by John Wiley & Sons Ltd on behalf of European Hematology Association. This is an open access Abstract Book distributed under the Attribution-NonCommercial-NoDerivs (CC BY-NC-ND), which allows third parties to download the articles and share them with others as long as they credit the author and the Abstract Book, but they cannot change the content in any way or use them commercially.
**Abstract Book Citations:** Authors, Title, HemaSphere, 2026;10:(S1):pages. The abstract book DOIs can be found on the "Author Information" page.
**Disclaimer:** Articles published in the journal HemaSphere exclusively reflect the opinions of the authors. The authors are responsible for all content in their abstracts including accuracy of the facts, statements, citing resources, etc.

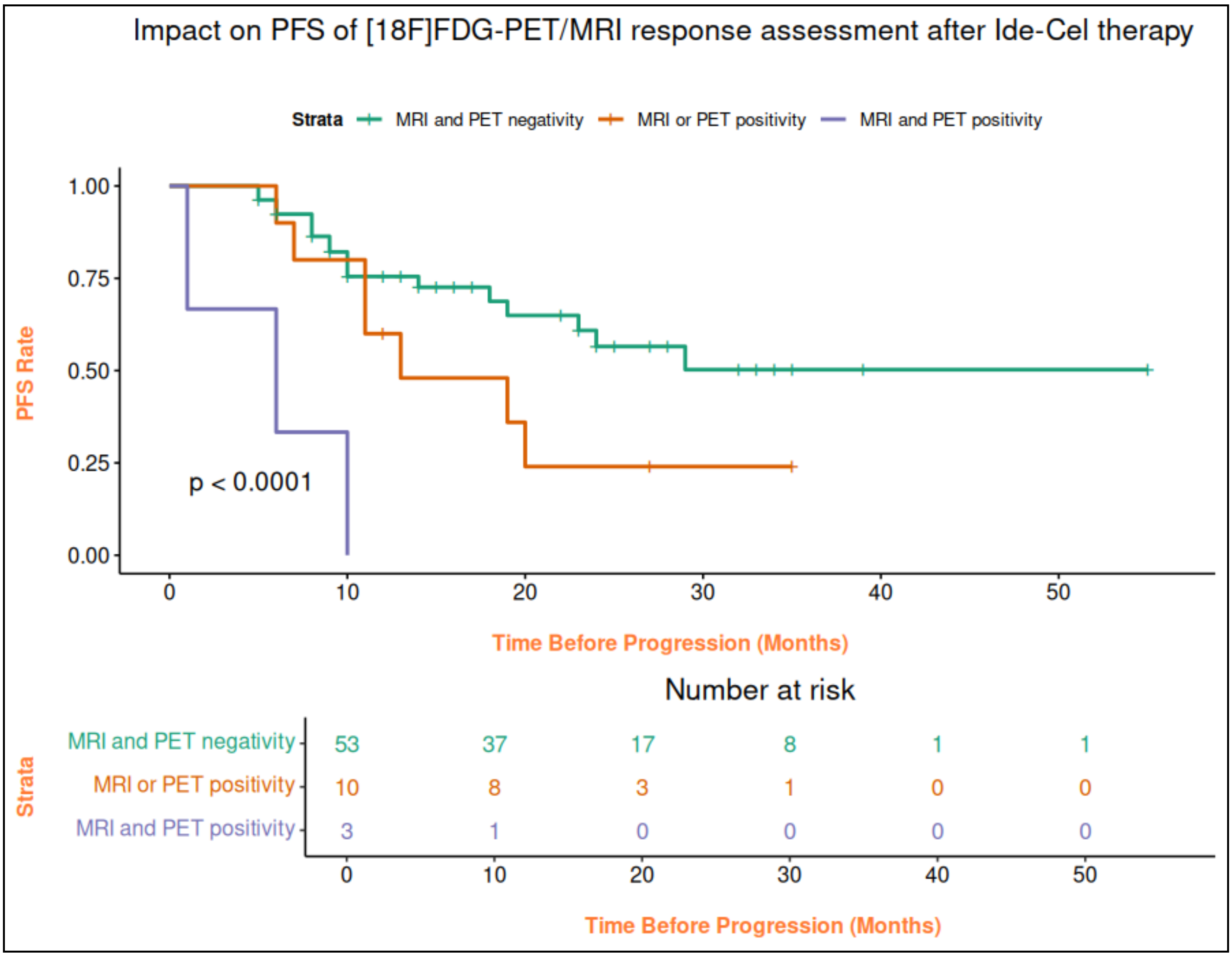

**Copyright Information:** (Online) ISSN: 2572-9241
©2026 The Author(s). HemaSphere is published by John Wiley & Sons Ltd on behalf of European Hematology Association. This is an open access Abstract Book distributed under the Attribution-NonCommercial-NoDerivs (CC BY-NC-ND), which allows third parties to download the articles and share them with others as long as they credit the author and the Abstract Book, but they cannot change the content in any way or use them commercially.
**Abstract Book Citations:** Authors, Title, HemaSphere, 2026;10:(S1):pages. The abstract book DOIs can be found on the “Author Information” page.
**Disclaimer:** Articles published in the journal HemaSphere exclusively reflect the opinions of the authors. The authors are responsible for all content in their abstracts including accuracy of the facts, statements, citing resources, etc.

### (PF783) IMPACT OF AUTOLOGOUS STEM CELL TRANSPLANTATION FOLLOWING QUADRUPLET THERAPY (DARA-VRD) ON SURVIVAL OUTCOMES IN MULTIPLE MYELOMA: A REAL-WORLD DATA ANALYSIS

**Topic:** 14. Myeloma and other monoclonal gammopathies - Clinical

Lanzhu Yue*[1], Bojana Mandic[1], Vladimir Otasevic[1], Ying Li[1], Heidi Cho[1]

[1]*Parexel International, Shanghai, China;*

### Background

Multiple myeloma (MM) remains an incurable hematologic malignancy despite significant therapeutic advances. The quadruplet regimen combining daratumumab, bortezomib, lenalidomide and dexamethasone (Dara-VRd) demonstrates superior efficacy in newly diagnosed MM (NDMM) patients. However, whether autologous stem cell transplantation (ASCT) still brings survival benefits following highly effective quadruplet regimens is not well established.

### Aims

This study aims to evaluate the impact of ASCT on overall survival in NDMM patients receiving Dara-VRd induction therapy using real-world data (RWD).

### Methods

We conducted a retrospective cohort study using the TriNetX USA network on February 26, 2026. We identified patients with MM who were diagnosed after January 1, 2019 and received Dara-VRd regimen as induction therapy and stratified them into two treatment cohorts: patients who underwent ASCT consolidation (ASCT group) and those who did not (non-ASCT group). Risk ratios (RR), hazard ratios (HR), 95% confidence intervals (CI), and Kaplan-Meier analysis with log-rank test were used to investigate the primary outcomes: risk of death and overall survival. Propensity score matching was used to control the impact of potential confounding factors.

### Results

A total of 2060 patients were identified, who received Dara-VRd frontline induction therapy and had data available regarding subsequent ASCT and survival within 5 years of treatment initiation. The patients' mean age at treatment initiation was 63.19 ± 10.27 years and 56.78% were males. 37.38% of the patients (770/2060) underwent ASCT. Patients who underwent ASCT demonstrated a statistically significant lower risk of death compared to patients who did not undergo ASCT (RR 0.67, 95% CI 0.470-0.956). ASCT group demonstrated significant survival benefit and lower risk of death compared to the non-ASCT group (survival probability at 5 years: 88.84% vs. 75.69%, log-rank test p<0.0001; HR0.354, 95% CI 0.236-0.53). Median survival was not reached for both cohorts at the time of analysis, indicating the high efficacy of Dara-VRd in both groups. However, ASCT consolidation provided additional survival benefit, reducing the risk of death by 64.6%. To minimize confounding factors, propensity score matching was performed for baseline covariates, including age, sex, ethnicity, ECOG, extramedullary disease, hypertensive diseases and renal function. In the matched cohorts, ASCT group still showed significantly improved survival rate over non-ASCT group (survival probability at 5 years: 89.88% vs. 78.31%, log-rank test p<0.0001; HR 0.371, 95% CI 0.234-0.588). While median survival time was not reached for both groups, ASCT group showed a lower risk of death compared to non-ASCT group (RR 0.6, 95% CI 0.386-0.932).

**Copyright Information:** (Online) ISSN: 2572-9241
©2026 The Author(s). HemaSphere is published by John Wiley & Sons Ltd on behalf of European Hematology Association. This is an open access Abstract Book distributed under the Attribution-NonCommercial-NoDerivs (CC BY-NC-ND), which allows third parties to download the articles and share them with others as long as they credit the author and the Abstract Book, but they cannot change the content in any way or use them commercially.
**Abstract Book Citations:** Authors, Title, HemaSphere, 2026;10:(S1):pages. The abstract book DOIs can be found on the "Author Information" page.
**Disclaimer:** Articles published in the journal HemaSphere exclusively reflect the opinions of the authors. The authors are responsible for all content in their abstracts including accuracy of the facts, statements, citing resources, etc.

**Summary/Conclusion**

In this real-world analysis, ASCT consolidation following Dara-VRd quadruplet induction therapy was associated with significantly reduced mortality risk and increased survival probability in MM patients. These findings suggest that ASCT continues to provide meaningful clinical benefit in the era of highly effective quadruplet regimens. Our results support the continued use of ASCT as consolidation therapy for transplant-eligible patients following Dara-VRd induction. Prospective randomized trials are warranted to definitively establish the role of ASCT in the evolving treatment landscape.

**Copyright Information:** (Online) ISSN: 2572-9241
©2026 The Author(s). HemaSphere is published by John Wiley & Sons Ltd on behalf of European Hematology Association. This is an open access Abstract Book distributed under the Attribution-NonCommercial-NoDerivs (CC BY-NC-ND), which allows third parties to download the articles and share them with others as long as they credit the author and the Abstract Book, but they cannot change the content in any way or use them commercially.
**Abstract Book Citations:** Authors, Title, HemaSphere, 2026;10:(S1):pages. The abstract book DOIs can be found on the "Author Information" page.
**Disclaimer:** Articles published in the journal HemaSphere exclusively reflect the opinions of the authors. The authors are responsible for all content in their abstracts including accuracy of the facts, statements, citing resources, etc.

### (PF784) FIRST-IN-HUMAN STUDY OF CT0596, AN ALLOGENEIC BCMA CAR T IN RELAPSED/REFRACTORY MULTIPLE MYELOMA AND PRIMARY PLASMA CELL LEUKEMIA: SAFETY, EFFICACY, AND PHARMACOKINETIC AT RECOMMENDED DOSE

**Topic:** 14. Myeloma and other monoclonal gammopathies - Clinical

Juan Du*[1, 2], Wanting Qiang[1], Lina Jin[1], Pei Guo[1], Xi Chen[1], Liying Peng[1], Xinying Zhao[3], Daijing Yuan[3], Yi Luo[3], Zonghai Li[3]

*[1]Shanghai Changzheng Hospital, Department of Hematology, Myeloma & Lymphoma Center, Shanghai, China;[2]Ren Ji Hospital, Shanghai Jiao Tong University School of Medicine, Department of Hematology, Shanghai, China; [3]CARsgen Therapeutics Co. Ltd., Shanghai, China;*

### Background

CT0596 is an allogeneic BCMA CAR T-cell product, which also targets NKG2A to resist host NK cell rejection, and is engineered with triple gene knockout of TRAC, B2M, and NKG2A to mitigate graft-versus-host disease (GVHD), host immune rejection, and increase anti-tumor activities, respectively.

### Aims

Herein, we report preliminary safety, efficacy, and pharmacokinetic (PK) data at recommended dose level, i.e. $4.5\times10^8$ $CAR^+$ T cell.

### Methods

This study was designed to evaluate the safety, preliminary efficacy, and PK profile of CT0596 in patients with relapsed/refractory multiple myeloma (R/RMM, ≥3 prior lines) or primary plasma cell leukemia (R/R pPCL, ≥1 prior lines). An i3+3 dose-escalation design was employed with four planned dose levels:$1.5\times10^8$, $3.0\times10^8$, $4.5\times10^8$, and $6.0\times10^8$ CAR+ T cells. Patients underwent lymphodepletion with fludarabine (30 mg/m²) and cyclophosphamide (500 mg/m²) for 3 days, followed by CT0596 cells infusion.

**Copyright Information:** (Online) ISSN: 2572-9241
©2026 The Author(s). HemaSphere is published by John Wiley & Sons Ltd on behalf of European Hematology Association. This is an open access Abstract Book distributed under the Attribution-NonCommercial-NoDerivs (CC BY-NC-ND), which allows third parties to download the articles and share them with others as long as they credit the author and the Abstract Book, but they cannot change the content in any way or use them commercially.
**Abstract Book Citations:** Authors, Title, HemaSphere, 2026;10:(S1):pages. The abstract book DOIs can be found on the “Author Information” page.
**Disclaimer:** Articles published in the journal HemaSphere exclusively reflect the opinions of the authors. The authors are responsible for all content in their abstracts including accuracy of the facts, statements, citing resources, etc.

**Results**

Eight patients, including 6 R/RMM and 2 R/R pPCL, received CT0596 at the 4.5×$10^8$ $CAR^+$ T cell dose level. Disease subtypes included IgA (n=2), IgG (n=3), and kappa light chain (n=3). The median age was 64.5 years (range 49, 70). All patients were heavily pretreated with a median of 3.5 prior lines of therapy (range 2, 6), and 3 had undergone prior autologous stem cell transplant. Most patients had advanced disease (ISS Stage III: n=5; R-ISS Stage III: n=3), 1 had extramedullary disease, and 5 patients had high-risk cytogenetics.

Hematologic toxicities were the most frequent adverse event (AE), occurring in all patients. Grade 3 infections were observed in 2 patients (25%). Grade 1/2 cytokine release syndrome (CRS) occurred in 7 patients (87.5%) with no grade ≥3 events reported. One patient developed grade 3 haemophagocytic lymphohistiocytosis, which resolved after treatment. No immune effector cell-associated neurotoxicity syndrome, GVHD, treatment-related deaths or study discontinuation due to AE was observed.

As of February 10, 2026, all 8 patients were evaluable for efficacy. Six patients achieved stringent complete response (sCR) (n=4) or very good partial response (VGPR) (n=2) following the initial 4.5×$10^8$ infusion. One R/R MM patient achieved PR with extramedullary disease and ongoing response at Month 8 after retreatment with 4.5×$10^8$, following failure of initial 3.0×$10^8$ infusion. One overweight pPCL patient (102 kg) who received reduced intensity lymphodepletion progressed after the initial 4.5×$10^8$ infusion, but achieved sCR following retreatment with 6.0×$10^8$. Both pPCL patients achieved sCR. Of the 6 MM patients, 3 achieved sCR, 2 achieved VGPR, and 1 achieved PR, as shown in the figure. All patients attained MRD negativity at a sensitivity of $10^{-6}$ at 4 weeks after the effective infusion. With a median follow-up of 5.86 months, 6 patients maintained ongoing responses.

CT0596 CAR-T cells expanded in all patients following infusion, with median $C_{max}$ of 100,078 copies/μg gDNA (range 262–389,586), median $T_{max}$ of 10.5 days (range 5–21), and median $AUC_{0-t}$ of 732,105 day × copies/μg gDNA (range 2,136–4,705,799).

**Summary/Conclusion**

At the recommended dose level, CT0596 demonstrated a manageable safety profile with no unexpected toxicities and promising efficacy in heavily pretreated patients with R/R MM and pPCL. Pharmacokinetic data supported robust in vivo expansion of CT0596. These preliminary results warrant further dose expansion and clinical development of CT0596.

**Copyright Information:** (Online) ISSN: 2572-9241
©2026 The Author(s). HemaSphere is published by John Wiley & Sons Ltd on behalf of European Hematology Association. This is an open access Abstract Book distributed under the Attribution-NonCommercial-NoDerivs (CC BY-NC-ND), which allows third parties to download the articles and share them with others as long as they credit the author and the Abstract Book, but they cannot change the content in any way or use them commercially.
**Abstract Book Citations:** Authors, Title, HemaSphere, 2026;10:(S1):pages. The abstract book DOIs can be found on the "Author Information" page.
**Disclaimer:** Articles published in the journal HemaSphere exclusively reflect the opinions of the authors. The authors are responsible for all content in their abstracts including accuracy of the facts, statements, citing resources, etc.

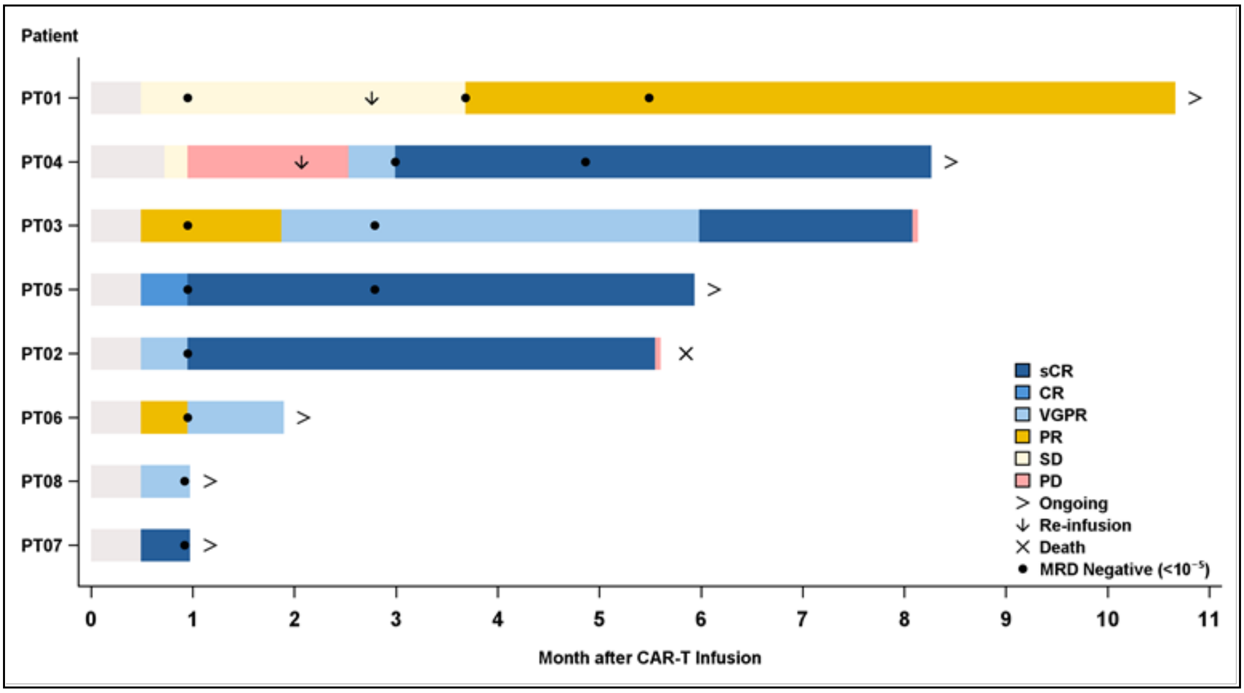

Swimming Plot of Individual Response

**Copyright Information:** (Online) ISSN: 2572-9241
©2026 The Author(s). HemaSphere is published by John Wiley & Sons Ltd on behalf of European Hematology Association. This is an open access Abstract Book distributed under the Attribution-NonCommercial-NoDerivs (CC BY-NC-ND), which allows third parties to download the articles and share them with others as long as they credit the author and the Abstract Book, but they cannot change the content in any way or use them commercially.
**Abstract Book Citations:** Authors, Title, HemaSphere, 2026;10:(S1):pages. The abstract book DOIs can be found on the "Author Information" page.
**Disclaimer:** Articles published in the journal HemaSphere exclusively reflect the opinions of the authors. The authors are responsible for all content in their abstracts including accuracy of the facts, statements, citing resources, etc.

### (PF785) MAGNAZ-TRIAL: A MULTICENTER PHASE II STUDY OF ZANUBRUTINIB TREATMENT IN ANTI-MAG POLYNEUROPATHY

**Topic:** 14. Myeloma and other monoclonal gammopathies - Clinical

Marieke Bruinsma[1, 2], Johannes van de Mortel[1], Josephine Vos[3], Filip Eftimov[2], Frances Verheij[4], Ruben van Eijk[1, 5], Nicolette Notermans[1], Alexander Vrancken[1], Monique Minnema*[4]

*[1]UMC Utrecht, Department of Neurology and Clinical Neurophysiology, Utrecht, The Netherlands;[2]Amsterdam UMC, Department of Neurology and Clinical Neurophysiology, Amsterdam, The Netherlands; [3]University of Amsterdam, Cancer Center Amsterdam, Amsterdam UMC, Department of Hematology, Amsterdam, The Netherlands; [4]UMC Utrecht, Department of Hematology, Utrecht, The Netherlands; [5]School of Medicine, Stanford University, Department of Biomedical Data Science and Center for Innovative Study Design, Stanford, United States of America;*

### Background

Polyneuropathy associated with immunoglobulin M monoclonal gammopathy of unknown significance and myelin associated glycoprotein antibodies (IgM MGUS anti-MAG PNP) is a chronic and progressive polyneuropathy causing significant neurological impairment. The IgM MGUS with anti-MAG reactivity produced by a B-cell clone is believed to be the cause of the neuropathy. Although Rituximab is considered standard therapy, only 30% of patients showed clinical improvement in two prospective trials, and the relationship to the reduction of M-protein levels remains unclear. IgM MGUS anti-MAG PNP frequently harbors the same MYD88 mutation found in Waldenström's Macroglobulinemia (WM), which increases sensitivity to Bruton's Tyrosine Kinase (BTK) inhibitors like Zanubrutinib.

### Aims

We hypothesize that the combination of Zanubrutinib and Rituximab treatment improves neurological and hematological outcomes in patients with IgM MGUS anti-MAG PNP. In addition, we investigate safety and tolerability of this treatment.

### Methods

We conducted a multicenter, single-arm, open-label phase II study in patients with IgM MGUS anti-MAG PNP with a follow-up of 3 years. Inclusion criteria were MAG antibody titers ≥10.000 BTU, bone marrow infiltration ≤10% and demyelinating polyneuropathy per EFNS/PNS criteria. Patients received Rituximab (375 mg/m$^2$) 4 times weekly combined with Zanubrutinib (320mg) daily. Hematologic and neurological responses were assessed at 6 and 12 months. Participants with at least partial hematological response (PR) at 6 months (per modified IWWM-6 criteria), stayed on treatment. The primary endpoint was the proportion of patients achieving ≥2 points improvement on the INCAT disability scale (clinician-rated scale assessing functional disability) at 12 months. Molecular markers were analyzed by Next-Generation Sequencing on B-cell–selected bone marrow aspirates. Anti-MAG titers were monitored throughout the study.

**Copyright Information:** (Online) ISSN: 2572-9241
©2026 The Author(s). HemaSphere is published by John Wiley & Sons Ltd on behalf of European Hematology Association. This is an open access Abstract Book distributed under the Attribution-NonCommercial-NoDerivs (CC BY-NC-ND), which allows third parties to download the articles and share them with others as long as they credit the author and the Abstract Book, but they cannot change the content in any way or use them commercially.
**Abstract Book Citations:** Authors, Title, HemaSphere, 2026;10:(S1):pages. The abstract book DOIs can be found on the "Author Information" page.
**Disclaimer:** Articles published in the journal HemaSphere exclusively reflect the opinions of the authors. The authors are responsible for all content in their abstracts including accuracy of the facts, statements, citing resources, etc.

**Results**

Enrollment has been completed with 49 patients screened of whom 33 (73% men) were included between March 2024 and December 2025. Mean age was 66 years (SD 8.4), 23 (70%) patients had an anti-MAG titer >70.000 BTU. A MYD88 p.L265P mutation was detected in 27 (87%) patients, one patient had an additional CXCR4 mutation and 3 had additional TP53 mutations. Twenty-six (79%) patients had an IgM kappa M protein. Median total IgM level was 3.7 g/L (IQR 2.8 – 6.0). Nineteen (57.6%) patients had prior treatment for polyneuropathy, 16 of whom had received rituximab. Hematological efficacy and safety results are presented for the 26 patients who have reached the 6 months timepoint. Best achieved hematological response within 6 months was VGPR in 31%, MR in 46% and SD in 23%. No safety concerns were observed. Two patients withdrew before 6 months: one due to visit burden and one due to development of metastases of a previously known oncologic disease. Fatigue was the most prevalent adverse event, affecting 18 (69%) patients, followed by bruising in 16 (62%). Ten mild to moderate infections occurred in 10 (38%) patients. No cardiac symptoms were observed, except for palpitations in 2 patients.

**Summary/Conclusion**

The MAGNAZ trial is the first study on efficacy and safety of Zanubrutinib and Rituximab combination treatment in patients with IgM MGUS anti-MAG PNP. The MYD88 mutation was present in 87% of the patients and at 6 months a hematological response was achieved in 77%. Neurological endpoints at 12 months are not yet reached.

**Copyright Information:** (Online) ISSN: 2572-9241
©2026 The Author(s). HemaSphere is published by John Wiley & Sons Ltd on behalf of European Hematology Association. This is an open access Abstract Book distributed under the Attribution-NonCommercial-NoDerivs (CC BY-NC-ND), which allows third parties to download the articles and share them with others as long as they credit the author and the Abstract Book, but they cannot change the content in any way or use them commercially.
**Abstract Book Citations:** Authors, Title, HemaSphere, 2026;10:(S1):pages. The abstract book DOIs can be found on the "Author Information" page.
**Disclaimer:** Articles published in the journal HemaSphere exclusively reflect the opinions of the authors. The authors are responsible for all content in their abstracts including accuracy of the facts, statements, citing resources, etc.

### (PF786) PERIPHERAL BLOOD MASS SPECTROMETRY REVEALS EARLY RELAPSE DYNAMICS AFTER MAINTENANCE DISCONTINUATION IN MULTIPLE MYELOMA

**Topic:** 14. Myeloma and other monoclonal gammopathies - Clinical

Anna Pula*[1], David Barnidge[2], Jennifer Cooperider[1], Frank Wen[1], Ken Jiang[1], Andrzej Jakubowiak[1], Benjamin Derman[1]

[1]*University of Chicago, Section of Hematology/Oncology, Chicago, United States of America;*[2]*The Binding Site, Birmingham, United Kingdom;*

### Background

Discontinuation of maintenance therapy guided by sustained multimodal measurable residual disease (MRD) negativity is feasible in multiple myeloma (MM). However, a subset of patients experiences disease relapse after treatment cessation. Blood-based mass spectrometry may enable earlier detection of resurgence and improve post-discontinuation surveillance and management.

### Aims

To evaluate the incidence, timing, and prognostic significance of peripheral blood mass spectrometry (MS) resurgence after maintenance discontinuation in multiple myeloma.

### Methods

We analyzed samples from patients participating in MRD2STOP (NCT04108624), who discontinued maintenance following confirmation of sustained MRD negativity at the $10^{-6}$ threshold. Patients only restarted treatment upon meeting IMWG progression criteria. Patients had conventional myeloma assessments performed every 3 months and bone marrow (BM) assessments yearly. Peripheral blood (PB) was assessed every six months first by EXENT then LC-MS if negative by EXENT. EXENT and LC-MS were classified as positive, negative, or oligoclonal (indicating multiple small clones of uncertain disease attribution). Cumulative incidence of assay-specific resurgence through three years was estimated among patients negative or oligoclonal at screening. Associations between timepoint–specific assay positivity and MRD-free survival (MRDFS) were evaluated at screening and at six-month intervals using Cox proportional hazards models.

### Results

Among 67 evaluable patients with a median follow-up of 44.7 months from discontinuation, there were 20 MRD $10^{-6}$ resurgences, 9 progressions, and 5 deaths (2 unrelated to progression). Of the 61 patients who had baseline mass spectrometry prior to discontinuation, 13 (21%) were EXENT-positive, 3 (5%) were EXENT negative but LC-MS–positive, and 18 (30%) were negative by both methods. Twenty-three patients were EXENT-negative but oligoclonal by LC-MS. In three cases, only EXENT was performed and was negative, and in one case only LC-MS was performed, showing an oligoclonal pattern. Among 41 patients negative at screening by both assays, MS resurgence was detected in 10 (24%) patients. The cumulative incidence of resurgence by EXENT was 6.1% at 12 months, 14.2% at 24 months, and 14.2% at 36 months. LC-MS demonstrated a cumulative incidence of 4.8% at 12 months, 16.4% at 24 months, and 28.6% at 36 months (figure 1).
Positivity by EXENT and LC-MS at the time of discontinuation (versus negative or oligoclonal) was not associated with MRDFS. However, EXENT positivity at month 6 was associated with inferior MRDFS (HR 4.32, 95% CI 1.32–14.1, p=0.01), and LC-MS positivity at the same time point also predicted shorter MRDFS (HR 3.93, 95% CI 1.38–11.14, p=0.01).
Across months 12–36, EXENT and LC-MS demonstrated 80.6% and 81% concordance with BM MRD at $10^{-6}$, with fair agreement ($\kappa$ = 0.23 and 0.35) and no significant systematic discordance (McNemar p = 0.15 and 0.68).
Of the 9 progression events with MS samples available, 7 (77.8%) had MS positivity prior to progression.

**Copyright Information:** (Online) ISSN: 2572-9241
©2026 The Author(s). HemaSphere is published by John Wiley & Sons Ltd on behalf of European Hematology Association. This is an open access Abstract Book distributed under the Attribution-NonCommercial-NoDerivs (CC BY-NC-ND), which allows third parties to download the articles and share them with others as long as they credit the author and the Abstract Book, but they cannot change the content in any way or use them commercially.
**Abstract Book Citations:** Authors, Title, HemaSphere, 2026;10:(S1):pages. The abstract book DOIs can be found on the “Author Information” page.
**Disclaimer:** Articles published in the journal HemaSphere exclusively reflect the opinions of the authors. The authors are responsible for all content in their abstracts including accuracy of the facts, statements, citing resources, etc.

**Summary/Conclusion**

Peripheral blood mass spectrometry reveals dynamic MS resurgence after maintenance discontinuation in MM in the MRD2STOP trial. Cumulative incidence of EXENT positivity is higher at early timepoints, whereas LC-MS continues to detect disease even at later timepoints. Baseline positivity lacks prognostic value, while month-6 positivity strongly predicts relapse. These findings support risk-adapted surveillance to guide early intervention.

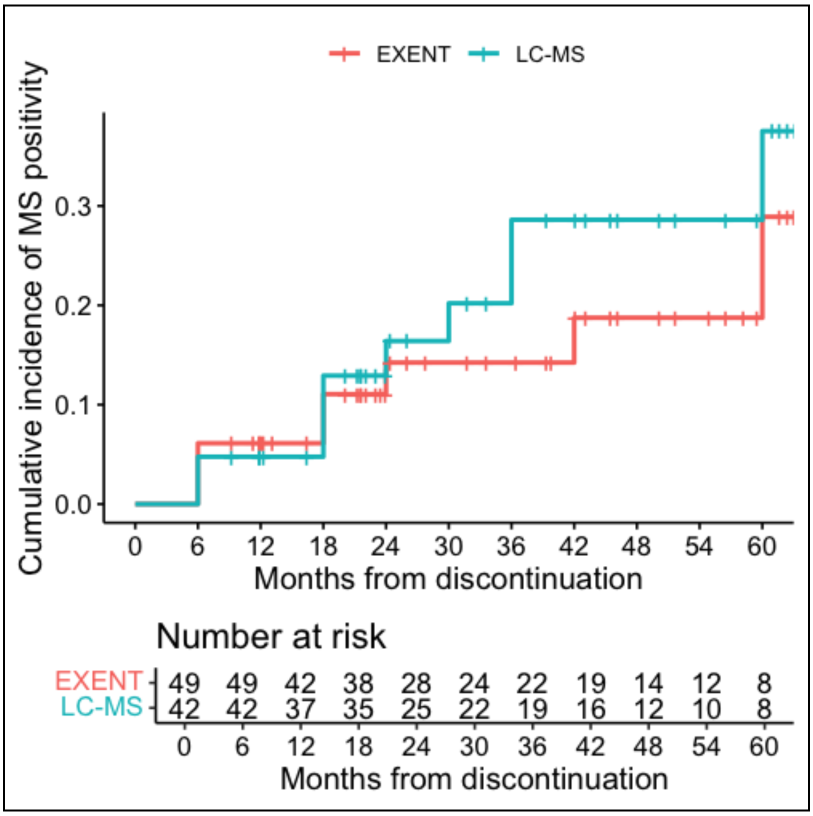

Cumulative incidence of mass spectrometry resurgence detected by EXENT and LC-MS among screening-negative or oligoclonal patients.

**Copyright Information:** (Online) ISSN: 2572-9241
©2026 The Author(s). HemaSphere is published by John Wiley & Sons Ltd on behalf of European Hematology Association. This is an open access Abstract Book distributed under the Attribution-NonCommercial-NoDerivs (CC BY-NC-ND), which allows third parties to download the articles and share them with others as long as they credit the author and the Abstract Book, but they cannot change the content in any way or use them commercially.
**Abstract Book Citations:** Authors, Title, HemaSphere, 2026;10:(S1):pages. The abstract book DOIs can be found on the "Author Information" page.
**Disclaimer:** Articles published in the journal HemaSphere exclusively reflect the opinions of the authors. The authors are responsible for all content in their abstracts including accuracy of the facts, statements, citing resources, etc.

### (PF787) WHOLE GENOME SEQUENCING OF MULTIPLE MYELOMA CELLS WITH A NOVEL CLINICAL ASSAY UNCOVERS DIVERSITY OF GENETIC ALTERATIONS UNDERLYING IMMUNOTHERAPY RESISTANCE

**Topic:** 14. Myeloma and other monoclonal gammopathies - Clinical

Bruno Paiva*[1], Peter Voorhees[2], Patricia Greipp[3], Danielle Sookiasian[4], Julian Hess[4], Marisa Demeo[4], Vicki Pounder[4], Sarah Calkins[4], Reid Meyer[3], Linda Baughn[3], Christine Liacos[5], Konstantina Taouxi[5], Alexandra Papadimou[5], Meletios A. Dimopoulos[5], Efstathios Kastritis[5], Jesus Berdeja[6], Daniel Auclair[4], Valentina Nardi[4], Thomas Mullen[4], Francois Aguet[4], Shaji Kumar[3]

*[1]Cancer Center Clínica Universidad de Navarra, CIMA, IDISNA, CIBERONC, Pamplona, Spain;[2]Levine Cancer Institute, Atrium Health Wake Forest University School of Medicine, Department of Hematologic Oncology and Blood Disorders, Charlotte, United States of America; [3]Mayo Clinic, Division of Laboratory Genetics, Department of Laboratory Medicine and Pathology, Rochester, United States of America; [4]Predicta Biosciences, Cambridge, United States of America; [5]National and Kapodistrian University of Athens, Department of Clinical Therapeutics, Athens, Greece; [6]Greco-Hainsworth Centers for Research at Tennessee Oncology, Nashville, United States of America;*

### Background

Detecting genetic abnormalities is a required component of diagnostic workup and for potential individualization of therapy selection in multiple myeloma (MM) and its precursor conditions. Presently, this requires invasive bone marrow (BM) biopsies, hindering early detection, frequent longitudinal monitoring, and precise therapy selection. The current standard for detecting genetic alterations in MM is fluorescence in situ hybridization (FISH), which cannot detect point mutations and other clinically relevant alterations. Consequently, the IMS-IMWG guidelines were recently updated to require next-generation sequencing for the classification of high-risk MM. To address these needs, we recently launched GenoPredicta, a CLIA-approved LDT that enables routine monitoring, informing diagnosis, and treatment selection by comprehensively characterizing MM genomes with whole genome sequencing (WGS) from as few as 50 circulating tumor cells (CTCs) isolated from peripheral blood (PB) or tumor cells from BM. In addition to genetic alterations relevant for risk stratification and prognosis, identifying alterations in therapeutic targets for guiding MM immunotherapies and detecting potential resistance mechanisms is becoming increasingly important.

### Aims

To characterize the spectrum and prevalence of genetic mechanisms conferring resistance to therapy in clinical samples characterized with the GenoPredicta assay.

### Methods

Tumor cells were isolated from BM or PB samples using fluorescence-activated cell sorting (after immunomagnetic CD138+ enrichment for PB) and subjected to WGS. Copy number alterations, translocations, and single nucleotide variants and indels were identified with a fully automated pipeline that generates physician-ready clinical reports from raw sequencing data in ~6h.

**Copyright Information:** (Online) ISSN: 2572-9241
©2026 The Author(s). HemaSphere is published by John Wiley & Sons Ltd on behalf of European Hematology Association. This is an open access Abstract Book distributed under the Attribution-NonCommercial-NoDerivs (CC BY-NC-ND), which allows third parties to download the articles and share them with others as long as they credit the author and the Abstract Book, but they cannot change the content in any way or use them commercially.
**Abstract Book Citations:** Authors, Title, HemaSphere, 2026;10:(S1):pages. The abstract book DOIs can be found on the "Author Information" page.
**Disclaimer:** Articles published in the journal HemaSphere exclusively reflect the opinions of the authors. The authors are responsible for all content in their abstracts including accuracy of the facts, statements, citing resources, etc.

**Results**

Here, we describe results from a cohort 40 patients representing a mix of newly diagnosed and relapsed/refractory MM. Notably, half of the patients had alterations relevant for IMS-IMWG risk stratification (e.g., TP53 mutations), informing therapy selection, or conferring resistance to therapy that are not detectable by FISH or part of standard FISH panels. 90% of samples had at least 1 event not detectable by FISH. Moreover, ~20% of detected translocations are not captured by standard FISH panels, including potentially high-risk IGL::MYC translocations. 35% of samples had *TP53* mutations and/or deletions, emphasizing the advantage of WGS-based approaches for comprehensively characterizing MM samples. Crucially, 45% of samples had mutations and/or deletions in therapy target genes leading to biallelic inactivation or loss, including targets of T cell engager therapies (e.g., BCMA and GPRC5D) and immunomodulatory therapies (e.g., CRBN, CD38). The detected alterations ranged from large-scale deletions (Mb- to arm-level) to small focal deletions (kb-scale) and point mutations. In all instances, the resistance-conferring alterations were consistent with patients' prior lines of therapy.

**Summary/Conclusion**

We demonstrate that WGS-based characterization of MM from BM or CTCs enables comprehensive profiling of MM genomes, including a large diversity of genetic resistance mechanisms only detectable by WGS. Moreover, CTC-based profiling, which is increasingly recognized for its independent prognostic value, will enable more frequent monitoring, enabling both early detection of such alterations and more precise selection and guidance of therapy.

**Copyright Information:** (Online) ISSN: 2572-9241
©2026 The Author(s). HemaSphere is published by John Wiley & Sons Ltd on behalf of European Hematology Association. This is an open access Abstract Book distributed under the Attribution-NonCommercial-NoDerivs (CC BY-NC-ND), which allows third parties to download the articles and share them with others as long as they credit the author and the Abstract Book, but they cannot change the content in any way or use them commercially.
**Abstract Book Citations:** Authors, Title, HemaSphere, 2026;10:(S1):pages. The abstract book DOIs can be found on the "Author Information" page.
**Disclaimer:** Articles published in the journal HemaSphere exclusively reflect the opinions of the authors. The authors are responsible for all content in their abstracts including accuracy of the facts, statements, citing resources, etc.

### (PF788) MOSAIC CHROMOSOMAL ABERRATIONS PRIOR TO AND AT DIAGNOSIS OF MULTIPLE MYELOMA

**Topic:** 14. Myeloma and other monoclonal gammopathies - Clinical

Cecilie Maeng*[1], Mathias Ærendahl Heldbo[2], Pei Meng[3], Simon Husby[1], Ole Birger Vesterager Pedersen[4], Erik Sørensen[1], Christian Erikstrup[5], Christina Mikkelsen[2], Nanna Brøns[1], Henrik Ullum[6], Bitten Jensen[5], Mie Bruun[7], Estrid Høgdall[1], Bo Porse[3], Sisse Rye Ostrowski[1], Henrik Hjalgrim[8], Sigrún Thorsteinsdóttir[2], Kirsten Grønbæk[1]

*[1]University Hospital of Copenhagen (Rigshospitalet), Department of Immunology, Copenhagen N, Denmark;[2]University Hospital of Copenhagen (Rigshospitalet), Copenhagen N, Denmark; [3]University of Copenhagen, Biotech Research and Innovation Centre, Copenhagen N, Denmark; [4]University of Copenhagen, Copenhagen N, Denmark; [5]Aarhus University Hospital, Aarhus, Denmark; [6]Danish Ministry of Health, Copenhagen, Denmark; [7]Odense University Hospital, Odense, Denmark; [8]Danish Cancer Institute, Copenhagen, Denmark;*

### Background

Chromosomal abnormalities have prognostic impact for individuals with multiple myeloma (MM) and precursor conditions, but current studies are limited to smaller and diseased populations. Mosaic chromosomal aberrations (mCAs) are detectable in peripheral blood and may reflect clonal hematopoiesis, circulating plasma cells, and/or early disease-associated genomic instability, but their occurrence before MM and their association with survival after MM diagnosis remain uncertain.

### Aims

The aim of the study was to 1/ examine mCA occurrence in individuals with blood samples obtained >90 days prior to MM diagnosis (pre-MM) compared with age- and sex-matched controls, and 2/ examine associations between mCAs and overall survival (OS) among MM patients.

### Methods

We analyzed samples from 177,555 individuals from the Copenhagen Hospital Biobank (CHB) and the Danish Blood Donor Study (DBDS), and mCAs were called using the MoChA pipeline. Pre-MM individuals were matched 1:5 to controls without hematological cancer on age and sex. Associations between pre-MM and mCA subgroups were assessed using binomial logistic regression. In MM patients, OS from diagnosis was evaluated with Kaplan–Meier curves and Cox regression analysis, adjusted for age and sex.

**Copyright Information:** (Online) ISSN: 2572-9241
©2026 The Author(s). HemaSphere is published by John Wiley & Sons Ltd on behalf of European Hematology Association. This is an open access Abstract Book distributed under the Attribution-NonCommercial-NoDerivs (CC BY-NC-ND), which allows third parties to download the articles and share them with others as long as they credit the author and the Abstract Book, but they cannot change the content in any way or use them commercially.
**Abstract Book Citations:** Authors, Title, HemaSphere, 2026;10:(S1):pages. The abstract book DOIs can be found on the “Author Information” page.
**Disclaimer:** Articles published in the journal HemaSphere exclusively reflect the opinions of the authors. The authors are responsible for all content in their abstracts including accuracy of the facts, statements, citing resources, etc.

### Results

We included 175,941 controls, 310 pre-MM individuals, and 661 MM patients. In the pre-MM individuals, samples were taken median 2.9 years (IQR 1.1-5.6) before diagnosis, with a maximum of 12 years before diagnosis. In the matched case–control analysis (310 pre-MM; 1,550 controls), pre-MM was associated with increased prevalence of autosomal mCAs (18% vs 12%), any chromosomal gain (7.4% vs 2.6%), any chromosomal loss (7.7% vs 3.8%), and especially known MM-associated mCAs (1 gain, 1q CNLOH, 21 gain, 13q CNLOH, 17p loss) (2.9% vs 0.9%). Loss of X in females was not increased compared to matched controls (table 1)

| mCA subgroups | Odds ratio | 95%CI |
|---|---|---|
| Loss of X | 1.24 | 0.80 - 1.91 |
| Autosomal mCA | 1.61 | 1.16 - 2.22 |
| Any gain | 2.95 | 1.72 - 4.95 |
| Any loss | 2.12 | 1.28 - 3.42 |
| MM-associated mCAs | 3.28 | 1.36 - 7.55 |

A total of 416 MM patients had the sample for mCA testing taken at or <365 days after diagnosis with a median time of 7 days (IQR: 3-37) days after diagnosis. In this subgroup, autosomal mCAs (N=92 (22%)) were associated with poorer OS (adjusted hazard ratio (HR) 1.47, 95% confidence interval (CI) 1.14-1.90). The OS tended especially decreased in patients carrying any chromosomal gain (N=38 (9.1%), (HR 1.50, 95%CI 1.05–2.14)), any chromosomal loss (N=50 (12%), (HR 2.2, 95%CI 1.61-3.03)), and known MM-associated mCAs (N=32 (7.7%), (HR 1.90, 95%CI 1.29-2.80)). Loss of X in females (N=42 (25%)) may impact OS, but was not statistically significantly (HR 1.43, 95%CI 0.96–2.12).

### Summary/Conclusion

Autosomal mCAs were more frequent in individuals who later developed MM compared to age- and sex-matched individuals, especially chromosomal gains and known MM-associated mCAs. Additionally, MM patients carrying mCA had worse overall survival compared to MM patients without mCA. These findings confirm chromosomal aberrations as predictor of MM risk and prognosis and find similar patterns of mCAs in peripheral blood years prior to diagnosis of MM as known from bone marrow testing at diagnosis.  This opens the possibility to create larger studies on mCAs in peripheral blood to achieve a better understanding of myelomagenesis, early detection, and create better and less invasive prognostic tools, in addition to monoclonal protein and free light chain levels.

**Copyright Information:** (Online) ISSN: 2572-9241
©2026 The Author(s). HemaSphere is published by John Wiley & Sons Ltd on behalf of European Hematology Association. This is an open access Abstract Book distributed under the Attribution-NonCommercial-NoDerivs (CC BY-NC-ND), which allows third parties to download the articles and share them with others as long as they credit the author and the Abstract Book, but they cannot change the content in any way or use them commercially.
**Abstract Book Citations:** Authors, Title, HemaSphere, 2026;10:(S1):pages. The abstract book DOIs can be found on the "Author Information" page.
**Disclaimer:** Articles published in the journal HemaSphere exclusively reflect the opinions of the authors. The authors are responsible for all content in their abstracts including accuracy of the facts, statements, citing resources, etc.

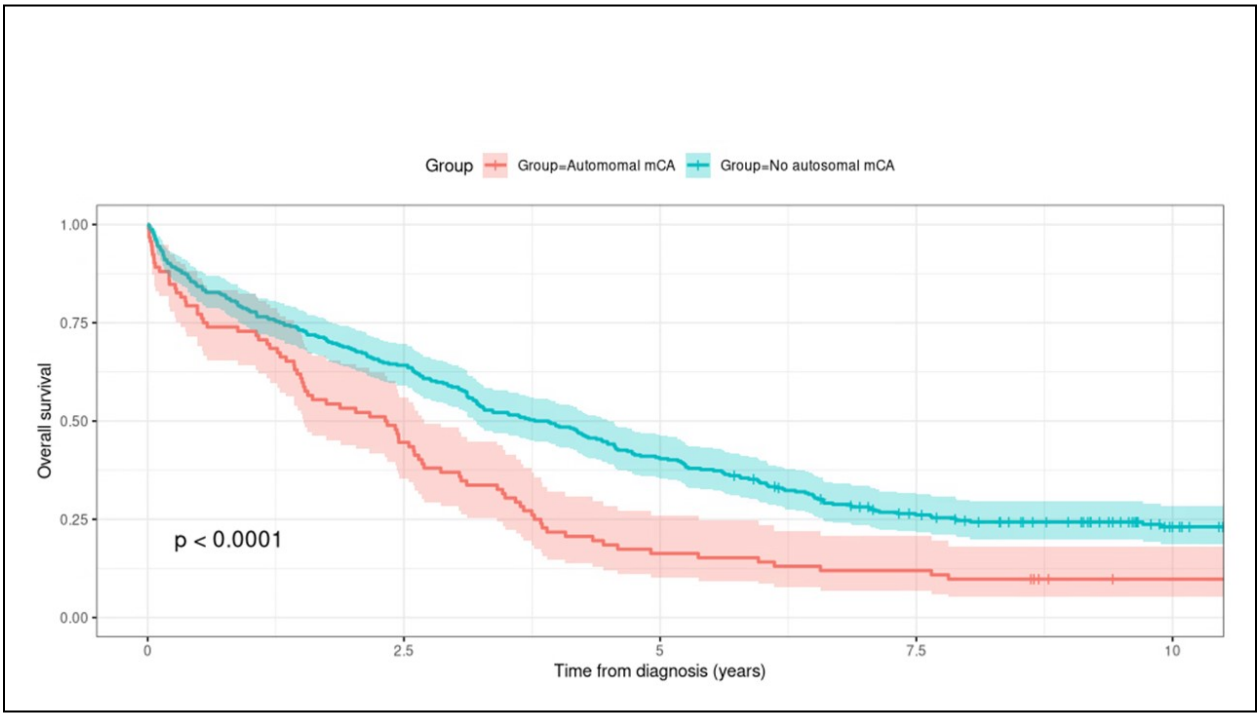

Figure 1: Overall survival from time of diagnosis in multiple myeloma patients with (red line) and without (blue line) autosomal mosaic chromosomal aberrations in peripheral blood

**Copyright Information:** (Online) ISSN: 2572-9241
©2026 The Author(s). HemaSphere is published by John Wiley & Sons Ltd on behalf of European Hematology Association. This is an open access Abstract Book distributed under the Attribution-NonCommercial-NoDerivs (CC BY-NC-ND), which allows third parties to download the articles and share them with others as long as they credit the author and the Abstract Book, but they cannot change the content in any way or use them commercially.
**Abstract Book Citations:** Authors, Title, HemaSphere, 2026;10:(S1):pages. The abstract book DOIs can be found on the "Author Information" page.
**Disclaimer:** Articles published in the journal HemaSphere exclusively reflect the opinions of the authors. The authors are responsible for all content in their abstracts including accuracy of the facts, statements, citing resources, etc.

### (PF789) EXTRAPOLATED PROGRESSION-FREE SURVIVAL WITH BELANTAMAB MAFODOTIN/LENALIDOMIDE/DEXAMETHASONE EXCEEDS 7 YEARS IN INTERMEDIATE-FIT AND FRAIL, TRANSPLANT-INELIGIBLE, NEWLY DIAGNOSED MULTIPLE MYELOMA

**Topic:** 14. Myeloma and other monoclonal gammopathies - Clinical

Evangelos Terpos*[1], Maria Gavriatopoulou[1], Charalampos Filippatos[1], Zeyad Khalaf[2], Ioannis Ntanasis-Stathopoulos[1], Nikolaos Kanellias[1], Eirini Solia[1], Panagiotis Malandrakis[1], Foteini Theodorakakou[1], Magdalini Migkou[1], Evangelos Eleutherakis-Papaiakovou[1], Jorge Mouro[3], Benga Kazeem[4], Vinay Jadhav[5], Ioannis Ninos[6], Efstathios Kastritis[1], Meletios A. Dimopoulos[1, 7]

*[1]Department of Clinical Therapeutics, National and Kapodistrian University of Athens, Athens, Greece;[2]GSK, London, United Kingdom; [3]GSK, Baar, Switzerland; [4]GSK, Stevenage, United Kingdom; [5]GSK, Bengaluru, India;[6]Veeda Lifesciences, Milan, Italy; [7]Department of Medicine, Korea University, Seoul, South Korea;*

### Background

B-cell maturation antigen (BCMA)-targeting agents have demonstrated transformational survival benefits for patients (pts) with multiple myeloma (MM) from first relapse. Belantamab mafodotin (belamaf) is a unique BCMA-targeting modality as an antibody drug conjugate that is approved as part of triplet combinations for relapsed/refractory MM based on robust efficacy shown vs standards of care in the phase 3 DREAMM-7/DREAMM-8 trials. In a phase 1/2 study (NCT04808037), the tolerability and efficacy of belamaf plus lenalidomide and dexamethasone (BRd) in transplant-ineligible newly diagnosed MM (TI-NDMM) was evaluated; pts were required to be intermediate-fit or frail per IMWG criteria, a population requiring tailored management. BRd showed substantial clinical activity with the recommended phase 2 dose of belamaf (1.9 mg/kg every 8 weeks [Q8W]) and overall (2.5/1.9/1.4 mg/kg Q8W) (median progression-free survival [mPFS] not reached [NR] at a median follow-up of 28.8 months; 18-month PFS estimate 82.9%). Anti-CD38-based regimens (daratumumab, lenalidomide, and dexamethasone with or without bortezomib [DRd and DVRd]; isatuximab plus VRd [isaVRd]) are standard regimens for TI-NDMM based on the phase 3 MAIA, CEPHEUS and IMROZ trials. In these trials, mPFS was 61.9 months for DRd (median follow-up 64.5 months), NR at median follow-up 58.7 months for DVRd (54-month PFS estimate 68.1%), and NR at median follow-up of 59.7 months for IsaVRd (60-month PFS estimate 63.2%). Given the transformational efficacy of belamaf-based regimens in the second-line or later setting, including superiority vs DVd, there is interest in understanding the long-term PFS of different dose levels in the NDMM setting.

### Aims

Predict the long-term mPFS of BRd using survival extrapolation.

### Methods

At the March 1, 2025 datacut of NCT04808037, individual pt-level data from 66 pts were used to model predicted mPFS of BRd using the combined study cohorts (median follow-up 28.8 months) and the 1.9 mg/kg-only cohort (median follow-up 24.6 months). Modeling was performed using standard parametric distributions including exponential, Weibull, log-logistic, log-normal, and gamma distributions.

### Results

The combined cohorts included 18 PFS events among 66 pts, 4 with COVID-19-related deaths. Predicted mPFS for the combined cohorts ranged from 86.7–115.5 months (**Table**). In the 1.9 mg/kg-only cohort, there were 11 PFS events among 42 pts, including 1 with COVID-19-related death. Predicted mPFS for the 1.9 mg/kg cohort ranged from 83.7–101.8 months.

**Copyright Information:** (Online) ISSN: 2572-9241
©2026 The Author(s). HemaSphere is published by John Wiley & Sons Ltd on behalf of European Hematology Association. This is an open access Abstract Book distributed under the Attribution-NonCommercial-NoDerivs (CC BY-NC-ND), which allows third parties to download the articles and share them with others as long as they credit the author and the Abstract Book, but they cannot change the content in any way or use them commercially.
**Abstract Book Citations:** Authors, Title, HemaSphere, 2026;10:(S1):pages. The abstract book DOIs can be found on the “Author Information” page.
**Disclaimer:** Articles published in the journal HemaSphere exclusively reflect the opinions of the authors. The authors are responsible for all content in their abstracts including accuracy of the facts, statements, citing resources, etc.

**Summary/Conclusion**

In light of observed and predicted outcomes with standards of care such as anti-CD38-based regimens, model-predicted mPFS of BRd in TI-NDMM (83.7–101.8 months for 1.9 mg/kg-only cohort and 86.7–115.5 months for combined cohorts) was longer than the observed mPFS for the triplet (DRd) and comparable to that expected for the quadruplets (DVRd: 96–118 months; isaVRd: 90 months). These results are particularly important given all pts in our analysis were intermediate-fit or frail, representing a population with unmet need. While acknowledging the limitations of this analysis (e.g., small sample size, small numbers at risk at later timepoints in Kaplan-Meier analyses, and duration of follow-up), the predicted mPFS in the combined cohorts and the 1.9 mg/kg-only cohort were similar, suggesting potential for high efficacy with BRd for TI-NDMM. BRd using a 1.9 mg/kg belamaf dose is being evaluated further for TI-NDMM in the phase 3 DREAMM-10 trial.

Funding: Supported by GSK.

**Table.** Predicted mPFS of BRd

| | Overall (2.5/1.9/1.4 mg/kg) | | | 1.9 mg/kg | | |
|---|---|---|---|---|---|---|
| Distribution | Predicted mPFS, months | 95% LCL | 95% UCL | Predicted mPFS, months | 95% LCL | 95% UCL |
| Exponential | 86.7 | 54.4 | 137.7 | 83.7 | 46.2 | 151.3 |
| Weibull | 96.5 | 48.4 | 183.3 | 87.6 | 38.0 | 189.0 |
| Log-logistic | 105.7 | 50.2 | 224.1 | 94.2 | 38.7 | 232.1 |
| Log-normal | 115.5 | 50.5 | 257.7 | 101.8 | 38.1 | 263.9 |
| Gamma | 93.1 | 48.8 | 165.2 | 84.5 | 38.2 | 167.0 |

LCL, lower confidence level; UCL, upper confidence level

**Copyright Information:** (Online) ISSN: 2572-9241
©2026 The Author(s). HemaSphere is published by John Wiley & Sons Ltd on behalf of European Hematology Association. This is an open access Abstract Book distributed under the Attribution-NonCommercial-NoDerivs (CC BY-NC-ND), which allows third parties to download the articles and share them with others as long as they credit the author and the Abstract Book, but they cannot change the content in any way or use them commercially.
**Abstract Book Citations:** Authors, Title, HemaSphere, 2026;10:(S1):pages. The abstract book DOIs can be found on the “Author Information” page.
**Disclaimer:** Articles published in the journal HemaSphere exclusively reflect the opinions of the authors. The authors are responsible for all content in their abstracts including accuracy of the facts, statements, citing resources, etc.

### (PF790) EARLY RESOLUTION OF TALQUETAMAB ORAL SIDE EFFECTS IN RELAPSED/REFRACTORY MULTIPLE MYELOMA (RRMM): UPDATED WATERLESS EMPIRICAL TASTE TEST (WETT) AND PATIENT REPORTED OUTCOMES (PRO) DATA FROM TALISMAN

**Topic:** 14. Myeloma and other monoclonal gammopathies - Clinical

Niels W C J van de Donk*[1], Rakesh Popat[2], Beth Faiman[3], Junichiro Yuda[4], Kihyun Kim[5], Abel Costa Neto[6], Alexa Laheij[7], Richard Doty[8], James Omel[9], Brea Lipe[10], Larysa Sanchez[11], Ajai Chari[12], Alexander Lin[8], Herve Sroussi[13], Mariana Fernandez[14], Kathleen Gray[15], Svetlana Gavrilov[16], Gloria Aguilar-Soto[17], Kasey Bolyard[16], Jennifer Schultz[18], Keqin Qi[18], Diana Cortes-Selva[19], Jacqueline Fabry[19], Sheri Skerget[19], Jenny Zhang[19], Christoph Heuck[19], Kelly Hawks[19], Mark Wildgust[16], Lori Parisi[16], Leo Rasche[20]

*[1]Amsterdam University Medical Center, Amsterdam, The Netherlands; [2]University College London Hospitals NHS Foundation Trust, London, United Kingdom; [3]Cleveland Clinic, Cleveland, United States of America; [4]National Cancer Center Hospital East, Kashiwa, Japan; [5]Samsung Medical Center, Sungkyunkwan University School of Medicine, Seoul, South Korea; [6]D'or Institute for Research and Education, São Paulo, Brazil; [7]University of Amsterdam and Vrije Universiteit, and Amsterdam University Medical Centers, University of Amsterdam, Amsterdam, The Netherlands; [8]University of Pennsylvania, Philadelphia, United States of America; [9]The Central Nebraska Myeloma Support Group, Grand Island, United States of America; [10]University of Rochester Medical Center, Rochester, United States of America; [11]Icahn School of Medicine at Mount Sinai, New York, United States of America; [12]University of California, San Francisco, San Francisco, United States of America; [13]Brigham and Women's Hospital, and Harvard School of Dental Medicine, Boston, United States of America; [14]Johnson & Johnson, Madrid, Spain; [15]Johnson & Johnson, Bridgewater, United States of America; [16]Johnson & Johnson, Raritan, United States of America; [17]Johnson & Johnson, Leiden, The Netherlands; [18]Johnson & Johnson, Titusville, United States of America; [19]Johnson & Johnson, Spring House, United States of America; [20]University Hospital of Würzburg, Würzburg, Germany;*

### Background

In the phase 1/2 MonumenTAL-1 study, talquetamab (Tal) elicited high response rates; 3-year median overall survival (OS) was not reached, with a 3-year OS rate of 60.8% in patients (pts) with RRMM. Pts treated with Tal often experience taste changes (eg, dysgeusia) and other oral side effects, but assessments for these events are limited. Better characterizing and improving the time course of these events may allow more pts to benefit from long-term durable responses with Tal. The WETT is an innovative tool to objectively detect discrete taste changes; measure onset, severity, and resolution of dysgeusia; and evaluate the effectiveness of prophylactic interventions. Initial TALisman results showed that dysgeusia is reported early (2 weeks after treatment), with resolution beginning at the end of 3 months.

### Aims

We report updated TALisman data with more pts and longer follow-up, and a 3-month futility analysis of Tal with dexamethasone oral rinse prophylaxis (prophy Dex) vs Tal only (control).

### Methods

TALisman (NCT06500884) is an ongoing phase 2 study. Eligible pts have RRMM and prior exposure to a PI, IMiD, and anti-CD38 antibody. Pts are ineligible if they have severe dysgeusia at baseline (prior to Tal) per WETT. Pts are randomized to control or experimental prophylaxis (eg, prophy Dex) for oral side effects starting 7 days before the first step-up dose and are longitudinally assessed. Pts achieving very good partial response or better may reduce dosing from 0.8 mg/kg every 2 weeks to every 4 weeks from cycle 5. Co-primary endpoints are dysgeusia incidence and severity, time to first severe dysgeusia, and dysgeusia improvement/resolution at 3 and 6 months per WETT.

**Copyright Information:** (Online) ISSN: 2572-9241
©2026 The Author(s). HemaSphere is published by John Wiley & Sons Ltd on behalf of European Hematology Association. This is an open access Abstract Book distributed under the Attribution-NonCommercial-NoDerivs (CC BY-NC-ND), which allows third parties to download the articles and share them with others as long as they credit the author and the Abstract Book, but they cannot change the content in any way or use them commercially.
**Abstract Book Citations:** Authors, Title, HemaSphere, 2026;10:(S1):pages. The abstract book DOIs can be found on the "Author Information" page.
**Disclaimer:** Articles published in the journal HemaSphere exclusively reflect the opinions of the authors. The authors are responsible for all content in their abstracts including accuracy of the facts, statements, citing resources, etc.

### Results

As of December 2025, 30 pts were randomized (15 to control, 15 to prophy Dex); median age was 62.5 years; 56.7% were male. 25 pts completed ≥3 cycles and 11 completed ≥6 cycles. Overall, 86.7% had dysgeusia and 76.7% had severe dysgeusia at any time point. The highest proportion of pts with any grade dysgeusia occurred at C3D8 with the control (80.0%) and at C2D1 with prophy Dex (92.3%) (**Figure**). Median time to first dysgeusia was 15 days (control) and 8 days (prophy Dex); median time to first severe dysgeusia was 68 days and 9 days, respectively. Prophy Dex did not result in improved outcomes vs the control in a futility analysis at the end of month 3; improvement/resolution of dysgeusia was observed in 63.6% (control) vs 27.3% (prophy Dex) at the end of month 3, and in 60.0% vs 40.0%, respectively, at the end of month 5. Overall, the highest proportion of pts with severe dysgeusia (~60%) by WETT was detected earlier (C1D15) vs severe/complete loss by the Scale of Subjective Total Taste Acuity (STTA; C4D1). Additional safety, PRO, and salivary data, and initial pregabalin prophylaxis data, will be presented at the congress.

### Summary/Conclusion

TALisman data confirm that dysgeusia measured by WETT begins to improve/resolve after 3 months of Tal, with 60% showing improvement/resolution at the end of 5 months. Severe dysgeusia measured by objective WETT preceded subjective severe/complete loss by STTA. Prophy Dex did not improve rates of dysgeusia resolution, although small numbers limit definitive conclusions. Emerging data from TALisman help to inform management strategies and clarify expectations for pts, caregivers, and healthcare providers to facilitate ongoing treatment and clinical benefit from Tal. TALisman is ongoing and exploring additional interventions to prevent dysgeusia and improve pt experience.

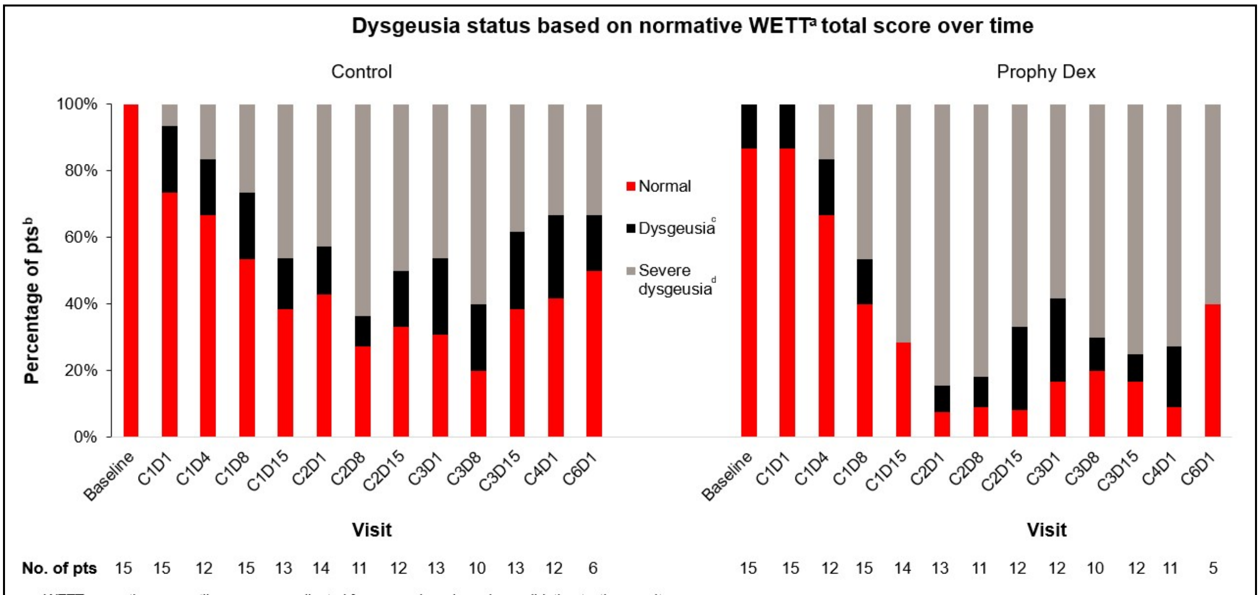

[a]WETT normative percentile scores are adjusted for age and sex based on validation testing results.
[b]Percentages are calculated with the number of pts in the treated analysis set with data available as the denominator.
[c]Defined as scores of 25th percentile or below by normative WETT.
[d]Defined as scores of 10th percentile or below by normative WETT.
C, cycle; D, day.

**Copyright Information:** (Online) ISSN: 2572-9241
©2026 The Author(s). HemaSphere is published by John Wiley & Sons Ltd on behalf of European Hematology Association. This is an open access Abstract Book distributed under the Attribution-NonCommercial-NoDerivs (CC BY-NC-ND), which allows third parties to download the articles and share them with others as long as they credit the author and the Abstract Book, but they cannot change the content in any way or use them commercially.
**Abstract Book Citations:** Authors, Title, HemaSphere, 2026;10:(S1):pages. The abstract book DOIs can be found on the "Author Information" page.
**Disclaimer:** Articles published in the journal HemaSphere exclusively reflect the opinions of the authors. The authors are responsible for all content in their abstracts including accuracy of the facts, statements, citing resources, etc.

### (PF791) ISATUXIMAB PLUS POMALIDOMIDE-DEXAMETHASONE IN RELAPSED/REFRACTORY MULTIPLE MYELOMA: SUBGROUP ANALYSIS BY PRIOR ANTI-CD38 EXPOSURE FROM THE PHASE 3 IRAKLIA TRIAL

**Topic:** 14. Myeloma and other monoclonal gammopathies - Clinical

Albert Oriol*[1], Marek Hus[2], Raimundo Gazitua[3], Aijun Liao[4], Wojciech Janowski[5], Cindy Lee[6], Gabor Mikala[7, 8], Artur Jurczyszyn[9], Alejandro Berkovits[10], Jin Lu[11], Fredrik Schjesvold[12], Silvia Ling[13], Andrew Spencer[14], Sikander Ailawadhi[15], Tondre Buck[16], Umer Khan[17], Florence Suzan[18], Maya Stefanova-Urena[19], Vania Hungria[20], Ivan Špička[21]

*[1]Institut Català d'Oncologia and Josep Carreras Research Institute, Hospital Germans Trias i Pujol, Barcelona, Spain;[2]University of Lublin, Lubin, Poland; [3]Servicio de Hematologia, Instituto Oncologico Fundacion Arturo Lopez Perez, Santiago, Chile; [4]Department of Hematology, Shengjing Hospital of China Medical University, Shenyang, Liaoning, China;[5]Department of Haematology, Calvary Mater Newcastle, Waratah, Australia; [6]Department of Haematology, The Royal Adelaide Hospital, SA Health, Adelaide, Australia; [7]South Pest Central Hospital, National Institute for Hematology and Infectious Diseases, Budapest, Hungary; [8]Semmelweis University, Department of Internal Medicine and Hematology, Budapest, Hungary; [9]Plasma Cell Dyscrasias Center, Department of Hematology, Jagiellonian University Medical College, Kraków, Poland; [10]Inmunocel, Santiago, Chile; [11]Peking University, Beijing, China; [12]Oslo Myeloma Center, Department of Hematology, Oslo University Hospital, Oslo, Norway; [13]Sydney Adventist Hospital, Wahroonga, NSW, Australia; [14]Monash University, Melbourne, Australia; [15]Division of Hematology/Oncology, Mayo Clinic, Jacksonville, FL, United States of America; [16]Spartanburg Medical Center, Center for Hematology/Oncology, Spartanburg, SC, United States of America; [17]Sanofi, Cambridge, MA, United States of America; [18]Sanofi R&D, Vitry-sur-Seine, France; [19]Sanofi, Morristown, NJ, United States of America;[20]Department of Hematology and Oncology, Clínica São Germano, São Paulo, Brazil; [21]1st Department of Medicine – Department of Hematology, First Faculty of Medicine, Charles University and General Hospital, Prague, Czechia;*

### Background

With the growing use of anti-CD38 therapies in early multiple myeloma (MM) management, there is a need for data in patients previously exposed to anti-CD38 therapy to guide decision making. In the Phase 3 IRAKLIA trial (NCT05405166), patients with relapsed/refractory MM (RRMM) received the anti-CD38 monoclonal antibody isatuximab (Isa) subcutaneously (SC) delivered via an innovative on-body injector (OBI) or intravenously (IV), with pomalidomide and dexamethasone (Pd).

### Aims

In this post hoc analysis, we examined outcomes in IRAKLIA patients with vs without prior anti-CD38 exposure.

### Methods

RRMM patients ≥18 years who provided informed consent and had ≥1 prior line of therapy including lenalidomide and a proteasome inhibitor were randomized to receive Isa SC (n=263) or IV (n=268) weekly in Cycle 1, then every 2 weeks, with Pd. Patients with prior anti-CD38 exposure <9 months (mos) before randomization or intolerance to anti-CD38 were excluded. Refractory patients were defined as those who failed to achieve minimal response on treatment or had progression within 60 days after last dose. Patients receiving Isa SC or IV were pooled.

**Copyright Information:** (Online) ISSN: 2572-9241
©2026 The Author(s). HemaSphere is published by John Wiley & Sons Ltd on behalf of European Hematology Association. This is an open access Abstract Book distributed under the Attribution-NonCommercial-NoDerivs (CC BY-NC-ND), which allows third parties to download the articles and share them with others as long as they credit the author and the Abstract Book, but they cannot change the content in any way or use them commercially.
**Abstract Book Citations:** Authors, Title, HemaSphere, 2026;10:(S1):pages. The abstract book DOIs can be found on the "Author Information" page.
**Disclaimer:** Articles published in the journal HemaSphere exclusively reflect the opinions of the authors. The authors are responsible for all content in their abstracts including accuracy of the facts, statements, citing resources, etc.

**Results**

Overall, 67 patients (12.6%) had prior anti-CD38 exposure with a median washout period of 20.2 mos, of which 26 (38.8%) were anti-CD38 refractory. Baseline characteristics were largely consistent between patients with or without prior anti-CD38 exposure; the median follow-up for the overall population was 12 mos. Patients with prior anti-CD38 exposure had an overall response rate (ORR) of 52.2%, 12-mo progression-free survival (PFS) rate of 40.6%, and median PFS of 8.5 mos. In patients without prior exposure, an ORR of 73.5%, 12-mo PFS of 68.8%, and non-estimable (NE) median PFS were reported. Anti-CD38 non-refractory vs refractory patients had an ORR of 48.8% vs 57.7%, 12-mo PFS of 38.9% vs 42.2%, and median PFS of 9.0 vs 7.5 mos. Patients with a longer washout period (>median of 20.2 mos) had an ORR of 63.6%, 12-mo PFS of 61.8% and NE median PFS, whereas patients whose prior anti-CD38 therapy ended more recently (≤median of 20.2 mos) had an ORR of 41.2%, 12-mo PFS of 19.2%, and median PFS of 6.7 mos. The safety profile of Isa + Pd was similar in patients with vs without prior anti-CD38 exposure.

**Summary/Conclusion**

Isa + Pd demonstrated clinical benefit across anti-CD38–naïve, anti-CD38–exposed, and anti-CD38–refractory patients. A washout period >median of 20.2 mos resulted in outcomes comparable to those in anti-CD38–naïve patients, and outcomes were consistent between anti-CD38–exposed and anti-CD38–refractory patients after a >9-mo washout period. Efficacy of Isa + Pd in patients with prior anti-CD38 exposure from the IRAKLIA trial presents an opportunity to address an unmet need in a difficult-to-treat patient population.

© 2026 American Society of Clinical Oncology, Inc. Reused with permission. This abstract was accepted and previously presented at the 2026 ASCO Annual Meeting. All rights reserved.

**Copyright Information:** (Online) ISSN: 2572-9241
©2026 The Author(s). HemaSphere is published by John Wiley & Sons Ltd on behalf of European Hematology Association. This is an open access Abstract Book distributed under the Attribution-NonCommercial-NoDerivs (CC BY-NC-ND), which allows third parties to download the articles and share them with others as long as they credit the author and the Abstract Book, but they cannot change the content in any way or use them commercially.
**Abstract Book Citations:** Authors, Title, HemaSphere, 2026;10:(S1):pages. The abstract book DOIs can be found on the “Author Information” page.
**Disclaimer:** Articles published in the journal HemaSphere exclusively reflect the opinions of the authors. The authors are responsible for all content in their abstracts including accuracy of the facts, statements, citing resources, etc.

### (PF792) LONG-TERM OUTCOMES WITH SUSTAINED MINIMAL RESIDUAL DISEASE NEGATIVITY IN BELANTAMAB MAFODOTIN–TREATED PATIENTS WITH RELAPSED/REFRACTORY MULTIPLE MYELOMA: AN UPDATE FROM DREAMM-8

**Topic:** 14. Myeloma and other monoclonal gammopathies - Clinical

Suzanne Trudel[1], Güldane Cengiz Seval[2], Sosana Delimpasi[3], Vladimir Vorobyev[4], Hang Quach[5], Ivan Špička[6], Jakub Radocha[7], Youngil Koh[8], Paweł Robak[9], Felipe de Arriba de la Fuente[10], Esther González García[11], Silvia Mangiacavalli[12], Margaret Polinkovsky[13], Jie MA[14], Ainslee Moore[15], Kelly Brock[16], Joanna Grams[17], Elisabet E. Manasanch[16], María-Victoria Mateos[18], Meletios A. Dimopoulos*[19, 20]

*[1]Department of Medical Oncology and Hematology, Princess Margaret Cancer Centre, Toronto, ON, Canada;[2]Department of Hematology, Ankara University, Ankara, Türkiye; [3]General Hospital Evangelismos, Athens, Greece; [4]Leningrad Regional Clinical Hospital, Saint-Petersburg, Russia; [5]University of Melbourne, St Vincent's Hospital Melbourne, Melbourne, VIC, Australia; [6]First Faculty of Medicine, Charles University and General Hospital, Prague, Czechia; [7]4th Department of Internal Medicine – Hematology, University Hospital Hradec Králové and Faculty of Medicine in Hradec Králové, Charles University, Hradec Králové, Czechia; [8]Department of Internal Medicine, Seoul National University Hospital, Seoul, South Korea;[9]Medical University of Łódź, Łódź, Poland; [10]Hematology Department, Hospital General Universitario Morales Meseguer, IMIB-Pascual Parrilla, University of Murcia, Murcia, Spain; [11]Jefa de Hematologia, Hospital Universitario Cabueñes-Gijón, Gijon, Spain;[12]Institute of Hematology, I.R.C.C.S. Policlinico San Matteo, Pavia, Italy; [13]GSK, Collegville, PA, United States of America; [14]GSK, Waltham, MA, United States of America; [15]GSK, Stevenage, United Kingdom; [16]GSK, Collegeville, PA, United States of America; [17]GSK, Warsaw, Poland; [18]University Hospital of Salamanca/IBSAL/Cancer Research Center-IBMCC (USAL-CSIC), Hematology Department, Salamanca, Spain; [19]Department of Clinical Therapeutics, School of Medicine, National and Kapodistrian University of Athens, Athens, Greece; [20]Department of Medicine, Korea University, Seoul, South Korea;*

### Background

Targeting B-cell maturation antigen (BCMA) has shifted the relapsed/refractory multiple myeloma (RRMM) landscape, offering new treatment options for these patients (pts). In the phase 3 DREAMM-8 trial (NCT04484623), the BCMA-directed antibody-drug conjugate (ADC) belantamab mafodotin combined with pomalidomide and dexamethasone (BPd) demonstrated significant progression-free survival (PFS) benefit and higher rates of complete response (CR)–based minimal residual disease (MRD) negativity vs the standard-of-care pomalidomide, bortezomib, dexamethasone (PVd) in pts with RRMM and ≥1 prior line of therapy (LOT), including lenalidomide.

### Aims

MRD negativity is associated with improved survival in MM. As such, this long-term, postbaseline update describes the characteristics and clinical outcomes of pts with sustained MRD negativity from DREAMM-8.

### Methods

Pts with ≥1 prior LOT including lenalidomide were randomized 1:1 to BPd or PVd. The primary endpoint was PFS (independent review committee assessed). PFS2 was an investigator-assessed secondary endpoint, and CR-based sustained MRD negativity (≥12 months) was an exploratory endpoint. Pts with ≥ CR were tested for MRD negativity by next-generation sequencing at $10^{-5}$ sensitivity.

**Copyright Information:** (Online) ISSN: 2572-9241
©2026 The Author(s). HemaSphere is published by John Wiley & Sons Ltd on behalf of European Hematology Association. This is an open access Abstract Book distributed under the Attribution-NonCommercial-NoDerivs (CC BY-NC-ND), which allows third parties to download the articles and share them with others as long as they credit the author and the Abstract Book, but they cannot change the content in any way or use them commercially.
**Abstract Book Citations:** Authors, Title, HemaSphere, 2026;10:(S1):pages. The abstract book DOIs can be found on the "Author Information" page.
**Disclaimer:** Articles published in the journal HemaSphere exclusively reflect the opinions of the authors. The authors are responsible for all content in their abstracts including accuracy of the facts, statements, citing resources, etc.

**Results**

With a median follow-up of 35.8 months overall at data cutoff (July 7, 2025), the pts in the intention-to-treat population who received BPd (n=155) vs PVd (n=147) were >4× more likely to achieve ≥ CR + MRD negativity (27.7% vs 6.1%) and sustained MRD negativity (15.5% vs 2.7%). MRD-negativity rates were also higher with BPd vs PVd in pts with ≥ CR (43/67 [64.2%] vs 9/25 [36.0%]), as were rates of sustained MRD negativity (24/43 [55.8%] vs 4/9 [44.4%]) among all ≥ CR + MRD-negative pts.
Pts with sustained MRD negativity had durable PFS in both arms; 1 of 24 pts with BPd and 0 of 4 with PVd experienced PFS events. In pts without sustained MRD negativity, median PFS was 21.1 months with BPd (HR vs sustained MRD negative, 0.04; 95% CI, 0.01-0.32) vs 10.2 months with PVd (HR vs sustained MRD negative, not estimable [NE]). Sustained MRD negativity was also associated with durable PFS2 in both arms; 2 of 25 pts with BPd and 0 of 4 with PVd experienced PFS2 events. Median PFS2 was 20.2 months with BPd (HR vs sustained MRD negative, 0.08; 95% CI, 0.02-0.33) vs 9.3 months with PVd (HR vs sustained MRD negative, NE) in pts without sustained MRD negativity. Results were similar in pts with vs without ≥ CR + MRD negativity.
BPd-treated pts with MRD negativity (n=43) received 1-3 prior LOTs, with most (74.4%) having had 1. MRD-positive pts treated with BPd (n=112) received up to 6 prior LOTs, where 44.6% received 1 and 17.0% received >3. High-risk cytogenetics were present for nearly 40% of pts with CR-based MRD negativity in the BPd arm.

**Summary/Conclusion**

Pts receiving BPd vs PVd were >4× more likely to achieve sustained MRD negativity, which was associated with improved PFS and PFS2. In the BPd arm, MRD-negative vs -positive pts more often had 1 prior LOT. These findings provide additional support for BPd, a BCMA-directed ADC regimen, in earlier LOTs for RRMM, including in pts with high-risk cytogenetics.

© 2026 American Society of Clinical Oncology, Inc. Reused with permission. Abstract accepted & presented at ASCO 2026. All rights reserved

Sponsored by GSK. Editorial support provided by Nucleus Global, Inizio (GSK funded; study ID 207499). Drug linker technology licensed from Seagen Inc.; monoclonal antibody produced using POTELLIGENT Technology licensed from BioWa.

**Copyright Information:** (Online) ISSN: 2572-9241
©2026 The Author(s). HemaSphere is published by John Wiley & Sons Ltd on behalf of European Hematology Association. This is an open access Abstract Book distributed under the Attribution-NonCommercial-NoDerivs (CC BY-NC-ND), which allows third parties to download the articles and share them with others as long as they credit the author and the Abstract Book, but they cannot change the content in any way or use them commercially.
**Abstract Book Citations:** Authors, Title, HemaSphere, 2026;10:(S1):pages. The abstract book DOIs can be found on the “Author Information” page.
**Disclaimer:** Articles published in the journal HemaSphere exclusively reflect the opinions of the authors. The authors are responsible for all content in their abstracts including accuracy of the facts, statements, citing resources, etc.

### (PF793) ELIGIBILITY AND TREATMENT DECISION FACTORS, ALTERNATIVE TREATMENTS AND OUTCOMES OF MULTIPLE MYELOMA PATIENTS CONSIDERED FOR CAR T-CELL THERAPY IN GERMANY. A REAL-WORLD TRINETX DATABASE STUDY.

**Topic:** 14. Myeloma and other monoclonal gammopathies - Clinical

Zuzana Dostalova*[1], Francisco Henriquez[1], Kai Strobel[1], Laurent Garderet[2], Arthur Gerl[3], Hermann Einsele[4], Eilidh Duncan[5], Markus Rückert[1], Solène Clavreul[5]

*[1]TriNetX Oncology GmbH, Freiburg, Germany; [2]Clinical Haematology Department, Sorbonne Université, Pitié-Salpêtrière Hospital, APHP, Paris, France; [3]Praxis für Hämatologie und Onkologie, Munich, Germany; [4]Universitätsklinikum Würzburg, Medizinische Klinik und Poliklinik II, Würzburg, Germany; [5]Myeloma Patients Europe, Brussels, Belgium;*

### Background

CAR T-cell therapies (CAR-T) showed high response rates in multiple myeloma (MM) patients in clinical trials, with the promise of durable responses. Real-world (RW) studies showed comparable outcomes. However, it is unclear if all eligible patients can currently receive CAR-T, why some do not, and how this impacts their outcomes.

### Aims

To decipher the RW CAR-T eligibility of MM patients in Germany since the first approval of a MM CAR-T in the European Union in 08/2021, understand why some eligible patients do not receive CAR-T and their outcomes.

### Methods

We conducted a retrospective analysis using the TriNetX RW database which includes 10,933 MM patients from Germany with a study period from 18/08/2021 to 30/06/2025 (data cut-off). Patients matching at least one approved MM CAR-T indication at the beginning of a new line of therapy (LoT) and/or for whom CAR-T eligibility had been assessed by their physician were included. Patients' characteristics, type of care centre, ineligibility factors, reasons for not receiving CAR-T, alternative treatments and outcomes (overall response rate (ORR), time to next treatment (TTNT) and overall survival (OS)), as reported by the treating physician, were analysed.

**Copyright Information:** (Online) ISSN: 2572-9241
©2026 The Author(s). HemaSphere is published by John Wiley & Sons Ltd on behalf of European Hematology Association. This is an open access Abstract Book distributed under the Attribution-NonCommercial-NoDerivs (CC BY-NC-ND), which allows third parties to download the articles and share them with others as long as they credit the author and the Abstract Book, but they cannot change the content in any way or use them commercially.
**Abstract Book Citations:** Authors, Title, HemaSphere, 2026;10:(S1):pages. The abstract book DOIs can be found on the "Author Information" page.
**Disclaimer:** Articles published in the journal HemaSphere exclusively reflect the opinions of the authors. The authors are responsible for all content in their abstracts including accuracy of the facts, statements, citing resources, etc.

### Results

551 patients matched an approved CAR-T indication at the beginning of a new LoT during the study period. 98.5% (n=543) were assessed by their physician for CAR-T eligibility, 10.3% (n=57) were considered eligible and 1.6% (n=9) received it. A total of 2,763 MM patients from the database had been physician-assessed for CAR-T eligibility during the study period, of which 10.0% (n=275) were considered eligible to CAR-T and 1.6% (n=45) received it while 8.3% (n=230) did not.
Main non-eligibility reasons were high biological age (77% of 2,491 ineligible patients), comorbidities (29%), poor general condition (29%) and no expected benefit (20%). Ineligible patients with high biological age had a median age of 75.4 [27.6-97.9]. Main comorbidities were hypertension (51.9% of ineligible patients), renal disease (32.6%), cardiovascular disease (30.7%), chronic obstructive pulmonary disease (22.4%), arrhythmia (20.5%), diabetes (20.5%) and heart disease (20.8%).
Main reasons for eligible patients not receiving CAR-T were patient's refusal (41.0%), no expected benefit (23.3%), CAR-T not yet administered (12.3%), other/unknown (11.5%), not suitable after further examination (7.5%) and patient did not show up at CAR-T centre (7.5%).
At 4 LoT+, alternative treatments received by eligible patients not receiving CAR-T (n=105) were varied with EPd, Isa-Kd, Isa-Pd combinations most frequently used (in resp. 19%, 12% and 9% of patients) and outcomes of patients treated with CAR-T were better than those treated with alternative treatments with an ORR of 90.0% (n=20) vs 55.1% (n=78), and an estimated median TTNT of 33.4 (95% CI 22.4-NE) vs 15.7 months (95% CI 12.9-NE).

### Summary/Conclusion

This RW analysis shows that only 10.3% of MM patients matching an approved CAR-T indication in Germany were considered eligible to CAR-T in 2021-25 and 1.6% received it. Eligibility assessments were conducted in nearly all patients matching an approved CAR-T indication. Main ineligibility factors were high biological age, comorbidities and poor general condition. 41% of eligible patients refused CAR-T despite its clear benefit. Longer follow up is needed to compare outcomes at 2-3 LoT and analyse OS data. Qualitative analysis of reasons for declining CAR-T and impact of type of care centre will be further described.

**Copyright Information:** (Online) ISSN: 2572-9241
©2026 The Author(s). HemaSphere is published by John Wiley & Sons Ltd on behalf of European Hematology Association. This is an open access Abstract Book distributed under the Attribution-NonCommercial-NoDerivs (CC BY-NC-ND), which allows third parties to download the articles and share them with others as long as they credit the author and the Abstract Book, but they cannot change the content in any way or use them commercially.
**Abstract Book Citations:** Authors, Title, HemaSphere, 2026;10:(S1):pages. The abstract book DOIs can be found on the "Author Information" page.
**Disclaimer:** Articles published in the journal HemaSphere exclusively reflect the opinions of the authors. The authors are responsible for all content in their abstracts including accuracy of the facts, statements, citing resources, etc.

### (PF794) PLASMACYTOMA REDUCED QUICKLY IN PATIENTS(PTS) WITH RELAPSED/REFRACTORY MULTIPLE MYELOMA BY APONERMIN +KT-DECP REGIMEN

**Topic:** 14. Myeloma and other monoclonal gammopathies - Clinical

Menghan Liu[1], Jingrong Wang[2], Yanru Zhang[1], Xiaoqi Qin[3], Weiwei Tian[4], Yuechen Zhang[1], Lefu Huang[1], Jing Jia[1], Wenjing Li[1], Donghai Han[5], Yanping MA[3], Da Gao[5], Guangzhong Yang[1], Wen Gao[1], Yan Yan[6], Aijun Liao[7], Liping Su[2], Honghu Zhu[1], Wenming Chen[1], Aijun Liu*[1]

*[1]Beijing Chaoyang Hospital, affiliated to Capital Medical University, Beijing, China;[2]Shanxi Cancer Hospital (Shanxi Cancer Center, Taiyuan, China; [3]Second hospital of Shanxi Medical University, Taiyuan, China;[4]Shanxi Bethune Hospital, Taiyuan, China; [5]The Affiliated Hospital of Inner Mongolia Medical University, Hohhot, China;[6]Bayannur Hospital, Bayannur, China; [7]ShengJing Hospital of China Medical University, Shenyang, China;*

### Background

Extramedullary Disease (EMD) occurred often in Relapsed/refractory multiple myeloma (RRMM), the survival was still poor (3-year OS: 58%). Aponermin (Apo), the first approved DR4/DR5 agonist, triggers the extrinsic apoptotic pathway through caspase cascade activation.

### Aims

This study was to evaluate the efficacy and safety of Apo+KT-DECP* regimen in pts of RRMM and EMD.

### Methods

This multicenter, single-arm, phase 2 study (NCT05013190) aimed to enroll 31 pts with EMD-confirmed RRMM according to IMWG criteria. Patients received Apo-KT-DECP regimen*at least 4-6 cycles; and adding radiotherapy when efficacy less than complete remission (CR). The primary endpoint was overall response rate (ORR) after two cycles of treatment. Key secondary endpoints include CR, very good partial remission (VGPR), Time to response (TOR), Progression-Free Survival (PFS) and safety.

### Results

31 pts have been enrolled in this study, median age was 60 years, male was 41.9%, median prior lines of therapy was 2(range:1–9), 29.0% of pts with EMD≥3(1-7), 32.3% of pts with bone-independent and paraskeletal mass together, 25.8% of pts with big EMD(diameter≥5cm), high-risk cytogenetics was 29.0%, EMD of 3 pts with blast-type plasma cell, 2 pts with malignant pleural effusion, 3 triple-class refractory (TCR) was 38.7%, and no BCMA exposure pts.
By the data cutoff, all enrolled pts had received a median of 6 cycle of regimen as induction therapy (range: 2–6), 48.4%(15/31) of pts entered to maintenance. Median follow-up of 7 months, median PFS were not reached, 6-month and 12-month PFS rate was 79.3% and 58.1%, respectively. ORR of 2-cycle was 78.6% (22/28), much better than the efficacy of DECP in 20 pts with EMD(ORR 55%) that was published in 2014(National Medical Journal of China). The best response included 32.3% of pts achieving ≥VGPR (25.8% achieving ≥CR, 2 pts with radiotherapy)
In this study, 67.7% (21/31) of patients experienced adverse events (AEs) of any grade. The most common AEs were anemia (74.2%), thrombocytopenia (67.7%), infection (64.5%), and leukopenia (58.1%). The most frequent Grade 3/4 adverse events were leukopenia (35.5%), infection (32.3%), and thrombocytopenia (29.0%).

**Copyright Information:** (Online) ISSN: 2572-9241
©2026 The Author(s). HemaSphere is published by John Wiley & Sons Ltd on behalf of European Hematology Association. This is an open access Abstract Book distributed under the Attribution-NonCommercial-NoDerivs (CC BY-NC-ND), which allows third parties to download the articles and share them with others as long as they credit the author and the Abstract Book, but they cannot change the content in any way or use them commercially.
**Abstract Book Citations:** Authors, Title, HemaSphere, 2026;10:(S1):pages. The abstract book DOIs can be found on the "Author Information" page.
**Disclaimer:** Articles published in the journal HemaSphere exclusively reflect the opinions of the authors. The authors are responsible for all content in their abstracts including accuracy of the facts, statements, citing resources, etc.

**Summary/Conclusion**

Our study demonstrates that EMD decreased quickly, PFS was prolonged obviously with less toxicity in RRMM by the Apo-KT-DECP regimen. An ORR of 78.6% was achieved in pts with EMD in 2months and 58.1% of pts were not progressed in 12 months, a high-risk population with unmet need, supporting further evaluation of this combination.
Clinical trial information: NCT05013190

**Copyright Information:** (Online) ISSN: 2572-9241
©2026 The Author(s). HemaSphere is published by John Wiley & Sons Ltd on behalf of European Hematology Association. This is an open access Abstract Book distributed under the Attribution-NonCommercial-NoDerivs (CC BY-NC-ND), which allows third parties to download the articles and share them with others as long as they credit the author and the Abstract Book, but they cannot change the content in any way or use them commercially.
**Abstract Book Citations:** Authors, Title, HemaSphere, 2026;10:(S1):pages. The abstract book DOIs can be found on the "Author Information" page.
**Disclaimer:** Articles published in the journal HemaSphere exclusively reflect the opinions of the authors. The authors are responsible for all content in their abstracts including accuracy of the facts, statements, citing resources, etc.

### (PF795) BONE MARROW QUALITY INDEX (BMQI) AS A MARKER OF SAMPLE QUALITY IN POST-TREATMENT MRD ASSESSMENT: DATA FROM THE REMNANT TRIAL

**Topic:** 14. Myeloma and other monoclonal gammopathies - Clinical

Jón Þórir Óskarsson*[1, 2], Liv Osnes[3], Mari Evensen[4], Pegah Abdollahi[5], Anne-Marie Rasmussen[5], Frida Bugge Askeland[5, 6], Anna Lysén[5], Einar Haukås[7], Sæmundur Rögnvaldsson[1, 8], Sigrún Thorsteinsdóttir[1, 9], Anette L Eilertsen[10], Magnus Moksnes[11], Galina Tsykunova[12], Nils Morten Leknes[13], Birgitte Dahl Eiken[14], Vidar Stavseth[15], Rolke Jürgen[16], Eivind Samstad[17, 18, 19], Randi Fykse Hallstensen[20], Damian Szatkowski[21], Sigurdur Yngvi Kristinsson[1, 22], Tobias S. Slørdahl[23], Fredrik Schjesvold[5], Mona Høysæter Fenstad[4]

*[1]University of Iceland, Faculty of Medicine, Reykjavík, Iceland;[2]Landspítali University Hospital, Department of Laboratory Hematology, Reykjavík, Iceland; [3]Oslo University Hospital, Department of Immunology, Oslo, Norway; [4]St. Olavs University Hospital, Department of Immunology and Transfusion Medicine, Trondheim, Norway; [5]Oslo University Hospital, Oslo Myeloma Center, Oslo, Norway; [6]University of Oslo, Institute of Clinical Medicine, Oslo, Norway;[7]Stavanger University Hospital, Department of Hematology and Oncology, Stavanger, Norway; [8]Sahlgrenska University Hospital, Gothenburg, Sweden; [9]Rigshospitalet, Department of Hematology, Copenhagen, Denmark; [10]Akershus University Hospital, Department of Hematology, Oslo, Norway; [11]Vestfold Hospital, Cancer and Hematology Center, Tønsberg, Norway; [12]Haukeland University Hospital, Department of Medicine, Bergen, Norway; [13]University Hospital of North Norway, Department of Hematology, Tromsø, Norway; [14]Hospital Østfold, Department of Oncology, Kalnes, Norway; [15]Levanger Hospital, Department of Medicine, Levanger, Norway; [16]Sørlandet Hospital, Kristiansand, Norway; [17]Ålesund Hospital, Department of Medicine, Ålesund, Norway; [18]Møre og Romsdal Hospital Trust, Department of Research and Innovation, Ålesund, Norway; [19]Norwegian University of Science and Technology, Department of Health Sciences in Ålesund, Ålesund, Norway; [20]Nordland Hospital, Department of Hematology, Bodø, Norway; [21]Førde Central Hospital, Department of Hematology and Oncology, Førde, Norway;[22]Landspítali University Hospital, Department of Hematology, Reykjavík, Iceland; [23]St. Olavs University Hospital, Department of Hematology, Trondheim, Norway;*

### Background

Hemodilution of bone marrow (BM) aspirates negatively affects plasma cell enumeration and may lead to false-negative measurable residual disease (MRD) results in multiple myeloma (MM). We recently developed a Bone Marrow Quality Index (BMQI) to quantify hemodilution in diagnostic BM aspirates, based on the distribution of BM-associated cell populations captured by the EuroFlow next-generation flow (NGF) method. BMQI may provide an objective measure of BM sample quality to improve interpretation and prognostic accuracy of MRD assessments. Its performance in the post-treatment MRD setting remains to be evaluated.

### Aims

To assess whether BMQI functions as a marker of sample quality in the post-treatment MRD setting in newly diagnosed MM patients from the REMNANT trial.

**Copyright Information:** (Online) ISSN: 2572-9241
©2026 The Author(s). HemaSphere is published by John Wiley & Sons Ltd on behalf of European Hematology Association. This is an open access Abstract Book distributed under the Attribution-NonCommercial-NoDerivs (CC BY-NC-ND), which allows third parties to download the articles and share them with others as long as they credit the author and the Abstract Book, but they cannot change the content in any way or use them commercially.
**Abstract Book Citations:** Authors, Title, HemaSphere, 2026;10:(S1):pages. The abstract book DOIs can be found on the "Author Information" page.
**Disclaimer:** Articles published in the journal HemaSphere exclusively reflect the opinions of the authors. The authors are responsible for all content in their abstracts including accuracy of the facts, statements, citing resources, etc.

### Methods

This analysis was conducted within the REMNANT study (NCT04513639), a phase II/III trial in transplant-eligible, newly diagnosed MM evaluating MRD-guided management following first-line treatment. Patients were treated with bortezomib-lenalidomide-dexamethasone (VRd) induction, high-dose melphalan with autologous stem cell transplantation, VRd consolidation, and lenalidomide maintenance. Patients who achieved MRD negative CR following first-line therapy were randomized to Arm A (MRD monitoring by NGF every 4 months; second-line therapy at MRD reappearance) or Arm B (second-line therapy at IMWG-defined progressive disease). EuroFlow MM-MRD NGF was used to analyze BM samples obtained after consolidation. Per REMNANT protocol, MRD negativity was defined as $<10^{-5}$ and required BM representativeness QC based on an overall operator assessment of cellularity and marrow-associated cell populations. Separately, for this analysis, BMQI was calculated as: *(%myeloid precursors×0.37) + (%nucleated red cells×0.14)*, with lower values indicating greater hemodilution. Logistic regression was used to assess the association between BMQI and MRD negativity. For analyses of sustained MRD negativity, we included Arm A patients with follow-up MRD assessments. The association between BMQI (continuous) and time to MRD reappearance was evaluated using a Cox proportional hazards model. Kaplan-Meier curves were used to describe sustained MRD negativity, stratified by median BMQI (2.82; BMQI-low vs BMQI-high).

### Results

In total, BM samples from 267 patients were included; 174/267 (65.2%) were MRD negative by NGF. BMQI values were significantly lower in MRD-neg compared to MRD-pos samples (median 2.54 vs 2.95; $p<0.01$). Higher BMQI values were associated with a significantly lower probability of MRD negativity (logistic regression coefficient -0.28; $p<0.05$). Among 76 Arm A participants with follow-up, the median follow-up was 24 months (range 4-44). In this cohort, BMQI was significantly associated with sustained MRD negativity, with a 1.6-fold higher hazard of MRD reappearance per 1-unit lower BMQI (95% CI 1.01-2.44; $p<0.05$). Sustained MRD-negativity was observed in 34/38 (89.5%) in the BMQI-high group compared to 26/38 (68.4%) in the BMQI-low group ($p<0.05$; Figure).

### Summary/Conclusion

In this cohort of newly diagnosed MM patients after initial therapy, lower BMQI values were associated with a higher probability of MRD negativity and a shorter time to MRD reappearance, despite protocol QC aimed at excluding samples not representative of BM. This pattern supports BMQI as a marker of hemodilution in the post-treatment MRD setting, objectively informing the reliability of MRD results and the need for repeat MRD assessment.

**Copyright Information:** (Online) ISSN: 2572-9241
©2026 The Author(s). HemaSphere is published by John Wiley & Sons Ltd on behalf of European Hematology Association. This is an open access Abstract Book distributed under the Attribution-NonCommercial-NoDerivs (CC BY-NC-ND), which allows third parties to download the articles and share them with others as long as they credit the author and the Abstract Book, but they cannot change the content in any way or use them commercially.
**Abstract Book Citations:** Authors, Title, HemaSphere, 2026;10:(S1):pages. The abstract book DOIs can be found on the "Author Information" page.
**Disclaimer:** Articles published in the journal HemaSphere exclusively reflect the opinions of the authors. The authors are responsible for all content in their abstracts including accuracy of the facts, statements, citing resources, etc.

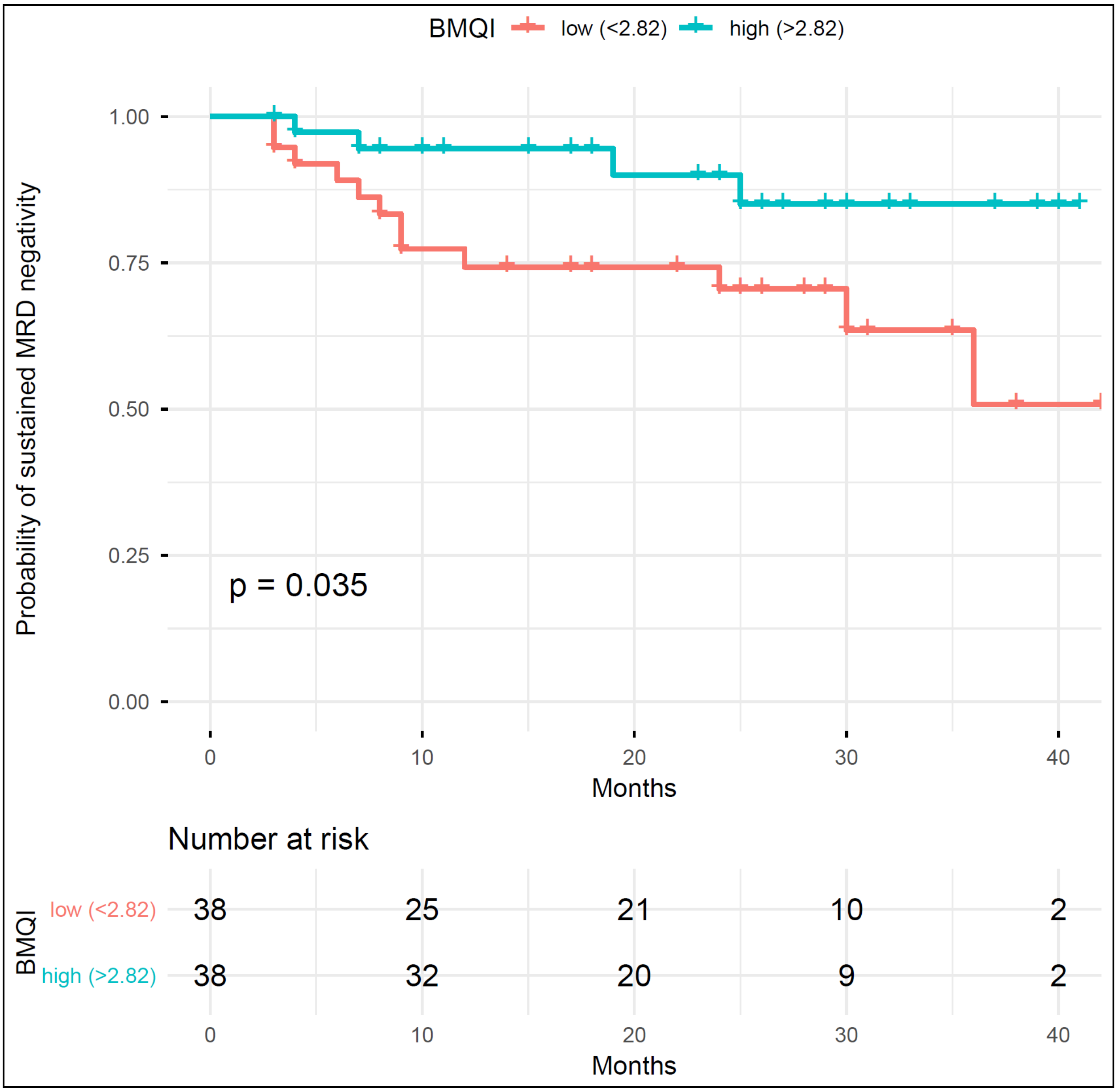

Figure. Kaplan-Meier curves for sustained MRD negativity in REMNANT Arm A patients by BMQI group (low <2.82 vs high ≥2.82). Patients with higher BMQI (less hemodiluted BM samples) showed longer duration of MRD negativity than those with lower BMQI (log-rank p<0.05; n=76).

**Copyright Information:** (Online) ISSN: 2572-9241
©2026 The Author(s). HemaSphere is published by John Wiley & Sons Ltd on behalf of European Hematology Association. This is an open access Abstract Book distributed under the Attribution-NonCommercial-NoDerivs (CC BY-NC-ND), which allows third parties to download the articles and share them with others as long as they credit the author and the Abstract Book, but they cannot change the content in any way or use them commercially.
**Abstract Book Citations:** Authors, Title, HemaSphere, 2026;10:(S1):pages. The abstract book DOIs can be found on the "Author Information" page.
**Disclaimer:** Articles published in the journal HemaSphere exclusively reflect the opinions of the authors. The authors are responsible for all content in their abstracts including accuracy of the facts, statements, citing resources, etc.

### (PF796) CAR-T VERSUS BCMA BISPECIFIC ANTIBODIES AFTER PRIOR BCMA THERAPY IN RELAPSED/REFRACTORY MULTIPLE MYELOMA: A MULTICENTER REAL-WORLD ANALYSIS

**Topic:** 14. Myeloma and other monoclonal gammopathies - Clinical

Osama Younis[1], Sencer Goklemez[2], Barry Paul[3], Hira Shaikh[4], Christopher Strouse[4], Anita Mazloom[5], Prerna Mewawalla[6], Mansi R. Shah[7], Zahra Mahmoudjafar[8], Jordan Snyder[8], Muhammad Umair Mushtaq[8], Jeries Kort[8], Alma Habib[8], Shebli Atrash*[3], Al-Ola Abdallah[8], Omar Alkharabsheh[2], Abdullah M. Khan[9]

*[1]University of Jordan, School of Medicine, Amman, Jordan; [2]University of Cincinnati, Cincinnati, Ohio, United States of America; [3]Levine Cancer Institute, Charlotte, North Carolina, United States of America; [4]University of Iowa, Iowa, United States of America; [5]University of South Alabama, Mobile, Alabama, United States of America; [6]Allegheny Health Network, Pittsburgh, Pennsylvania, United States of America; [7]Rutgers Cancer Institute, New Brunswick, New Jersey, United States of America; [8]University of Kansas Cancer Center, Kansas City, Kansas, United States of America; [9]The Ohio State University Comprehensive Cancer Center, Columbus, Ohio, United States of America;*

**Background**

Several BCMA-directed therapies (BDT) have been approved for relapsed/refractory multiple myeloma (RRMM); yet, optimal sequencing following prior BCMA exposure remains unknown. Prior BDT may impair T-cell fitness and alter BCMA expression, potentially influencing subsequent treatment efficacy. We present a multicenter real-world comparison of chimeric antigen receptor T-cell therapy (CAR-T) versus BCMA bispecific antibodies (BsAbs) in the BDT-exposed setting.

**Methods**

We conducted a multicenter retrospective cohort study across seven U.S. academic centers (May 2021–April 2024) within the U.S. Myeloma Innovations Research Collaborative (USMIRC). Patients with RRMM treated with CAR-T or BCMA BsAbs after prior BDT exposure were included. Progression-free survival (PFS) and overall survival (OS) were estimated using Kaplan–Meier methods and restricted mean survival time (RMST) analysis. Multivariate Cox regression identified survival predictors.

**Results**

Among 81 BDT-exposed patients, 22 (27%) received CAR-T (cilta-cel 45%, ide-cel 55%) and 59 (73%) received teclistamab. Baseline characteristics were comparable, including age (64 vs. 68 years), ECOG 0–1 (80% vs. 82%), R-ISS I–II (78% vs. 72%), EMD (59% vs. 46%), and median prior lines of therapy (7.5 vs. 8). Patients receiving teclistamab had higher rates of deletion 17p (22% vs. 4.5%). Prior BCMA exposure differed significantly (p<0.001) : CAR-T patients more often had prior antibody drug conjugate (ADC) (57%) or BsAbs(38%), while patients who received teclistamab more frequently had prior CAR-T (49%), ADC (20%), or multiple BDT (19%).
Overall response rate was 64% in the CAR-T versus 54% with teclistamab, with comparable ≥VGPR rates(50% vs. 45%). The CR rate for patients receiving cilta-cel was 30% vs 8.3% for ide-cel. VGPR rates were comparable between the two treatments. Median PFS was short in both groups (4.9 vs. 2.7 months; HR 0.96, p=0.9). Despite similar PFS, OS favored CAR-T, with RMST analysis demonstrating that CAR-T patients lived an average of 122 days longer (RMST difference: –122 days, 95% CI: –242 to –2.3, p=0.046). Achieving ≥VGPR was the only significant independent predictor of both PFS (HR=0.20, p<0.001) and OS (HR=0.2, p<0.001) in multivariate analysis.
CAR-T was associated with higher rates of grade 3–4 cytopenias at day 30 (neutropenia: 41% vs. 14%, p<0.001; thrombocytopenia: 52% vs. 10%, p=0.002), whereas teclistamab was associated with a higher acuteinfection rate (49% vs. 14%, p=0.004). CRS and ICANS rates were similar between the groups.

**Copyright Information:** (Online) ISSN: 2572-9241
©2026 The Author(s). HemaSphere is published by John Wiley & Sons Ltd on behalf of European Hematology Association. This is an open access Abstract Book distributed under the Attribution-NonCommercial-NoDerivs (CC BY-NC-ND), which allows third parties to download the articles and share them with others as long as they credit the author and the Abstract Book, but they cannot change the content in any way or use them commercially.
**Abstract Book Citations:** Authors, Title, HemaSphere, 2026;10:(S1):pages. The abstract book DOIs can be found on the "Author Information" page.
**Disclaimer:** Articles published in the journal HemaSphere exclusively reflect the opinions of the authors. The authors are responsible for all content in their abstracts including accuracy of the facts, statements, citing resources, etc.

### Summary/Conclusion

In BDT-exposed RRMM, both CAR-T and BCMA BsAbs produced modest and short-lived responses. CAR-T demonstrated a significant modest OS advantage by RMST analysis, while depth of response was the strongest determinant of survival regardless of modality. These findings support prioritizing the most effective BDT earlier in the disease course, although differences in prior BCMA exposure between cohorts may confound outcomes and warrant prospective validation.

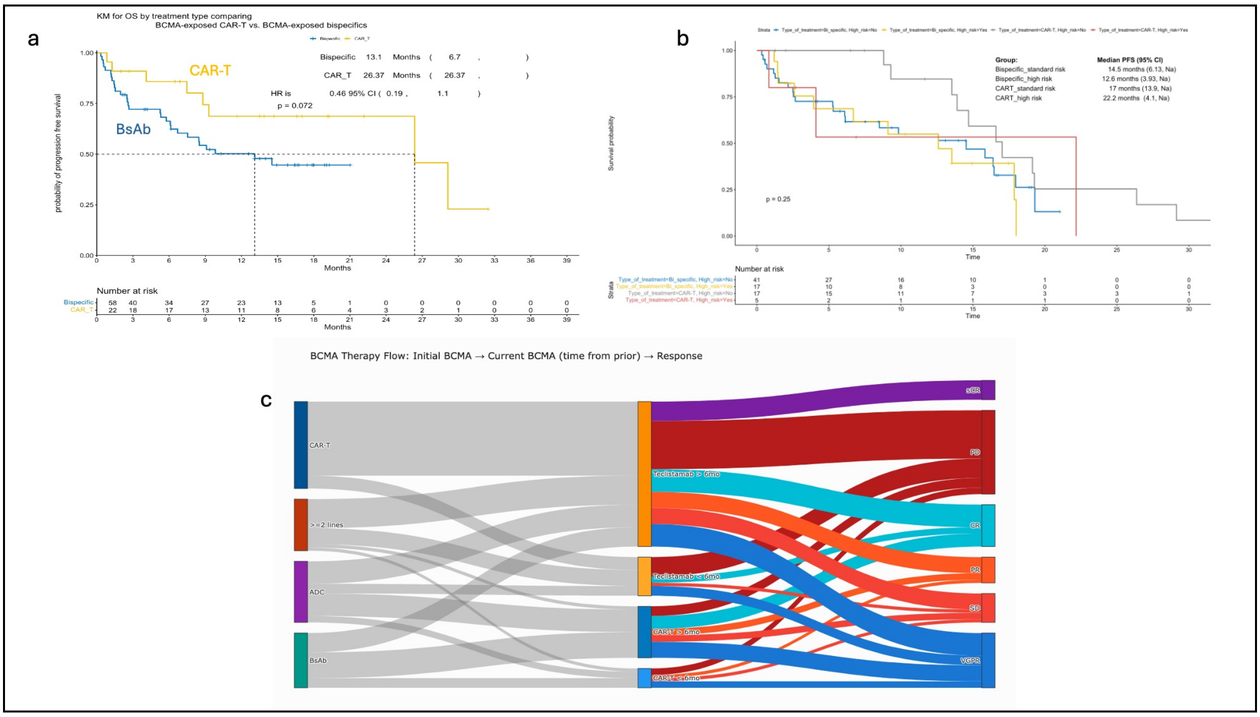

A) Overall survival comparison between BDT-exposed CAR-T and Teclistamab patients. B) KM plot showing the OS difference between BDT-exposed patients stratified by high risk cytogenetics and treatment type. C) Shows the responses of BDT-exposed patients based on the most recent BDT and the time from last BDT.

**Copyright Information:** (Online) ISSN: 2572-9241
©2026 The Author(s). HemaSphere is published by John Wiley & Sons Ltd on behalf of European Hematology Association. This is an open access Abstract Book distributed under the Attribution-NonCommercial-NoDerivs (CC BY-NC-ND), which allows third parties to download the articles and share them with others as long as they credit the author and the Abstract Book, but they cannot change the content in any way or use them commercially.
**Abstract Book Citations:** Authors, Title, HemaSphere, 2026;10:(S1):pages. The abstract book DOIs can be found on the "Author Information" page.
**Disclaimer:** Articles published in the journal HemaSphere exclusively reflect the opinions of the authors. The authors are responsible for all content in their abstracts including accuracy of the facts, statements, citing resources, etc.

### (PF797) CIBMTR REAL-WORLD OUTCOMES OF OLDER PATIENTS TREATED WITH IDECABTAGENE VICLEUCEL

**Topic:** 14. Myeloma and other monoclonal gammopathies - Clinical

Doris Hansen*[1], Othman Salim Akhtar[2, 3], Ajay Nooka[4], Surbhi Sidana[5], Krina Patel[6], Marcello Pasquini[2, 3], Sophie Hello[7], Maryna Levikova-Denzler[7], Romain Piault[7], Devender Dhanda[7], Omar Nadeem[8]

*[1]Moffitt Cancer Center, Tampa, Florida, United States of America;[2]Medical College of Wisconsin, Milwaukee, Wisconsin, United States of America; [3]Center of Internal Blood & Marrow Transplant Research, Milwaukee, Wisconsin, United States of America; [4]Winship Cancer Institute, Atlanta, Georgia, United States of America;[5]Stanford University School of Medicine, Stanford, California, United States of America; [6]The University of Texas MD Anderson Cancer Center, Houston, Texas, United States of America; [7]Bristol Myers Squibb, Princeton, New Jersey, United States of America;[8]Dana-Farber Cancer Institute, Boston, Massachusetts, United States of America;*

### Background

Idecabtagene vicleucel (ide-cel) is approved for patients (pts) with relapsed/refractory multiple myeloma (RRMM) after ≥ 2 prior lines of therapy. RRMM is increasingly prominent in the elderly population, with a median age at diagnosis of 71 yrs (Turresson et al. *Eur J Haematol.* 2018;101:237). A prior real-world study (Akhtar et al. *Blood Adv.* 2025;54:212) reported outcomes by age with a median follow-up of 11.6 mo.

### Aims

Using data with longer follow-up, a higher number of pts, and an age cut-off of 75 yrs, this analysis further evaluates effectiveness and safety of ide-cel in older pts.

### Methods

This retrospective study used real-world data from the CIBMTR registry and included pts who received commercial ide-cel for RRMM between 12 May 2021 and
10 August 2023 and had the 100-day post-infusion form completed. Outcomes included overall response rate (ORR), progression-free survival (PFS), overall survival (OS), and safety. Pts were analyzed by age (> 75 vs ≤ 75 yrs).

**Copyright Information:** (Online) ISSN: 2572-9241
©2026 The Author(s). HemaSphere is published by John Wiley & Sons Ltd on behalf of European Hematology Association. This is an open access Abstract Book distributed under the Attribution-NonCommercial-NoDerivs (CC BY-NC-ND), which allows third parties to download the articles and share them with others as long as they credit the author and the Abstract Book, but they cannot change the content in any way or use them commercially.
**Abstract Book Citations:** Authors, Title, HemaSphere, 2026;10:(S1):pages. The abstract book DOIs can be found on the “Author Information” page.
**Disclaimer:** Articles published in the journal HemaSphere exclusively reflect the opinions of the authors. The authors are responsible for all content in their abstracts including accuracy of the facts, statements, citing resources, etc.

**Results**

Of 996 pts, 149 (15.0%) were aged > 75 yrs. Median follow-up was 24.4 mo. Compared with pts ≤ 75 yrs, pts > 75 yrs had numerically higher prevalence of comorbidities (83.9% vs 77.3%), high-risk cytogenetics (28.2% vs 22.2%), and Eastern Cooperative Oncology Group (ECOG) performance status score ≥ 2 (9.4% vs 4.4%). Median age at diagnosis was 66.3 yrs for all pts. Time from diagnosis to infusion was similar between groups. Time from leukapheresis to infusion was also similar between the > 75 yrs and ≤ 75 yrs pts (46 [20.4] days vs 45 [11.9] days).
There was no detriment in ORR (95% CI) in pts > 75 vs ≤ 75 yrs (84.6% [77.7–90.0] vs 76.7% [73.7–79.5],*P*=0.03), including VGPR+ rates of 67.8% vs 62.2%. Median PFS (95% CI) was comparable (12.4 [10.6–17.2] mo vs 10.2 [9.3–11.8] mo). At 2 yrs, 29.5% (21.3–38.2) of pts > 75 yrs and 25.2% (22.1–28.4) of pts ≤ 75 yrs were alive and progression free. OS (95% CI) at 24 mo was similar (56.4% [46.9–64.9] vs 61.8% [58.2–65.1]).
Grade ≥ 3 cytokine release syndrome occurred in 2.0% of pts > 75 yrs vs 3.7% of pts ≤ 75 yrs, while grade ≥ 3 immune cell–associated neurotoxicity syndrome (ICANS) occurred in 8.1% vs 4.7% (*P*=0.09). Treatment-related mortality (95% CI) was higher in pts > 75 yrs at 12 mo (6.9% [3.5–11.8] vs 3.6% [2.5–5.1]) and 24 mo (10.1% [5.5–16.2] vs 4.9% [3.6–6.6]). Prolonged cytopenia occurred in 22.1% of pts > 75 yrs and 29.9% of pts ≤ 75 yrs. Rates of infections (60.4% vs 63.0%) were similar between groups. The 3 main causes of death of pts > 75 yrs included myeloma progression (28.9%), bacterial and viral infections (4.7%), and > 75 yrs intercranial hemorrhage (1.3%).

**Summary/Conclusion**

With extended follow-up, ide-cel effectiveness (ORR, PFS), as well as safety, was broadly comparable across age groups. Findings are directionally consistent with 6-mo outcomes reported with an earlier data cut and continue to highlight the benefit of ide-cel in older pts who may typically be overlooked for CAR T therapy.

**Copyright Information:** (Online) ISSN: 2572-9241
©2026 The Author(s). HemaSphere is published by John Wiley & Sons Ltd on behalf of European Hematology Association. This is an open access Abstract Book distributed under the Attribution-NonCommercial-NoDerivs (CC BY-NC-ND), which allows third parties to download the articles and share them with others as long as they credit the author and the Abstract Book, but they cannot change the content in any way or use them commercially.
**Abstract Book Citations:** Authors, Title, HemaSphere, 2026;10:(S1):pages. The abstract book DOIs can be found on the "Author Information" page.
**Disclaimer:** Articles published in the journal HemaSphere exclusively reflect the opinions of the authors. The authors are responsible for all content in their abstracts including accuracy of the facts, statements, citing resources, etc.

### (PF798) COMPARATIVE EFFICACY AND SAFETY OF CHIMERIC ANTIGEN RECEPTOR (CAR) T-CELL THERAPY VERSUS BISPECIFIC ANTIBODIES FOR RELAPSED/REFRACTORY MULTIPLE MYELOMA: A SYSTEMATIC REVIEW AND NETWORK META-ANALYSIS

**Topic:** 14. Myeloma and other monoclonal gammopathies - Clinical

Rahul Falodia*[1], Shauna Sequeira[2]

*[1]All India Institute of Medical Sciences, Jodhpur, India;[2]Seth GS Medical College & KEM Hospital, Mumbai, India;*

### Background

The rapid emergence of B-cell maturation antigen (BCMA)- and GPRC5D-directed T-cell redirecting therapies has revolutionized the treatment paradigm for relapsed/refractory multiple myeloma (RRMM). However, head-to-head randomized trials comparing CAR T-cell therapies with bispecific antibodies (BsAbs) are entirely lacking. This creates a critical clinical void for optimal treatment sequencing, particularly in resource-constrained healthcare systems where the logistical intensity of a "one-and-done" cellular infusion must be weighed against the cumulative toxicity of continuous outpatient therapy.

### Aims

To conduct a Bayesian network meta-analysis (NMA) indirectly comparing the aggregate clinical efficacy, survival outcomes, and specific high-grade toxicity profiles of approved CAR-T therapies versus BsAbs in patients with RRMM.

### Methods

A PRISMA-compliant systematic review was conducted across PubMed, Embase, and the Cochrane Library for prospective phase 1-3 trials evaluating CAR-T (cilta-cel, ide-cel) and BsAbs (teclistamab, elranatamab, linvoseltamab, talquetamab). A Bayesian NMA using a random-effects generalized linear mixed model was performed. To bridge the single-arm BsAb trials with Phase 3 CAR-T RCTs (CARTITUDE-4, KarMMa-3) without collapsing the network, matched-adjusted indirect comparisons (MAIC) incorporating standard-of-care (SOC) synthetic control cohorts were utilized as common bridge nodes. Primary endpoints evaluated were Overall Response Rate (ORR), Progression-Free Survival (PFS) via Hazard Ratios (HR), and rates of Grade 3 infections and Cytokine Release Syndrome (CRS).

### Results

The final network comprised 7 pivotal trials encompassing over 1,000 heavily pretreated patients. Bayesian synthesis demonstrated that CAR-T therapies yielded a significantly superior depth of response compared to BsAbs. Cilta-cel ranked highest for the probability of achieving a Complete Response or better (SUCRA: 94%), significantly outperforming continuous BsAbs (Pooled Odds Ratio [OR] vs SOC: 14.2 for CAR-T vs. 6.8 for BsAbs; $p<0.05$). Regarding survival, CAR-T modalities demonstrated a superior PFS profile (HR vs SOC: 0.32 [95% CrI: 0.21-0.45]) compared to BsAbs (HR: 0.52 [95% CrI: 0.38-0.71]). Toxicity profiling revealed a stark clinical dichotomy: while CAR-T therapies exhibited higher initial rates of severe (Grade 3) CRS (3.5% vs. 0.8% for BsAbs) and ICANS, BsAbs were associated with a profoundly higher cumulative incidence of Grade 3 severe infections (40.2% vs. 15.4% for CAR-T). This infectious burden in the BsAb cohort was heavily driven by continuous T-cell exhaustion and chronic hypogammaglobulinemia resulting from ongoing, indefinite dosing schedules.

**Copyright Information:** (Online) ISSN: 2572-9241
©2026 The Author(s). HemaSphere is published by John Wiley & Sons Ltd on behalf of European Hematology Association. This is an open access Abstract Book distributed under the Attribution-NonCommercial-NoDerivs (CC BY-NC-ND), which allows third parties to download the articles and share them with others as long as they credit the author and the Abstract Book, but they cannot change the content in any way or use them commercially.
**Abstract Book Citations:** Authors, Title, HemaSphere, 2026;10:(S1):pages. The abstract book DOIs can be found on the "Author Information" page.
**Disclaimer:** Articles published in the journal HemaSphere exclusively reflect the opinions of the authors. The authors are responsible for all content in their abstracts including accuracy of the facts, statements, citing resources, etc.

**Summary/Conclusion**

CAR T-cell therapy delivers superior progression-free survival and depth of response compared to continuous bispecific antibodies in RRMM, accompanied by a distinct safety profile favoring fewer long-term severe infections. For global health systems weighing therapeutic resource allocation, the acute logistical and intensive care burden of upfront CAR-T administration must be balanced against the cumulative infectious toxicity, need for chronic supportive care, and continuous financial strain associated with indefinite BsAb administration. These quantifiable findings provide a critical evidence-based hierarchy to guide the sequencing of immunotherapies in multiple myeloma.

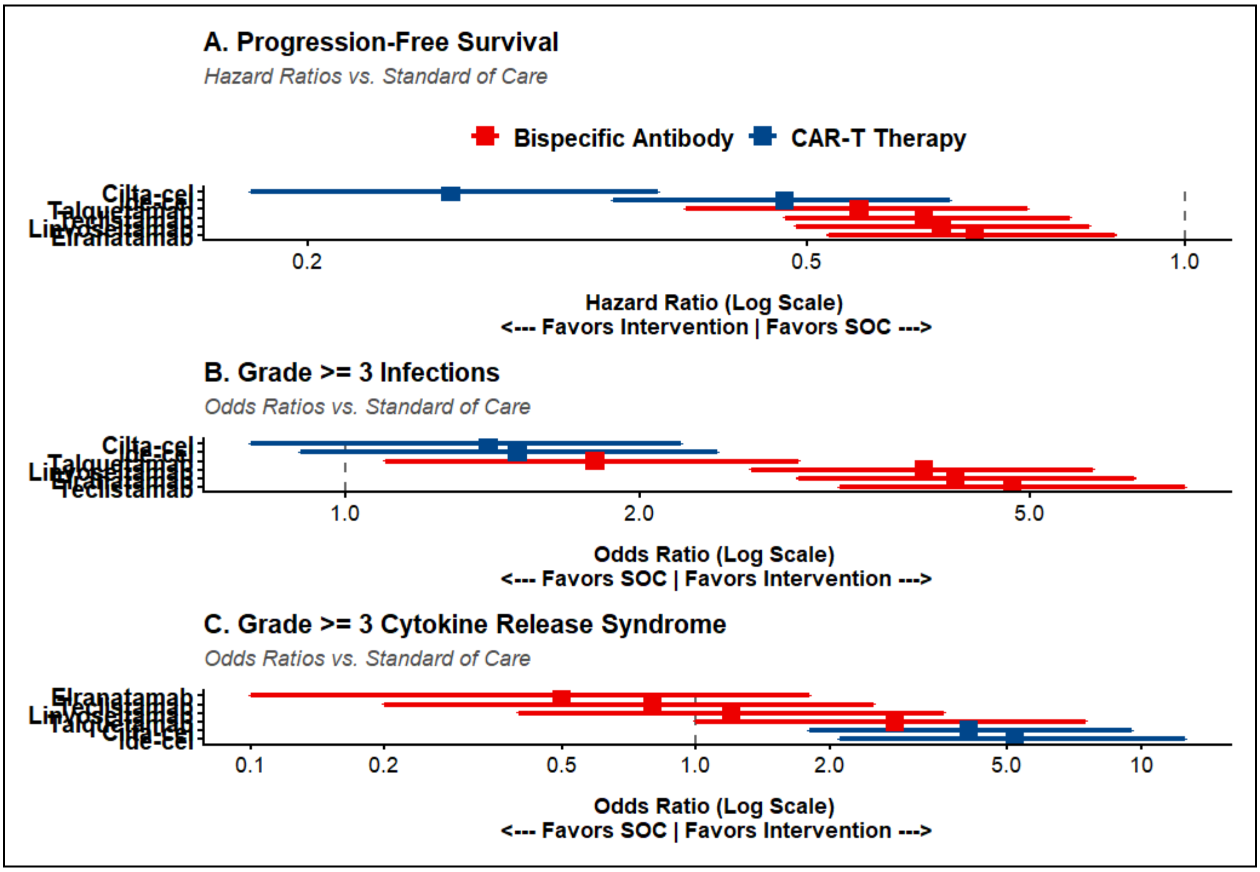

**Copyright Information:** (Online) ISSN: 2572-9241
©2026 The Author(s). HemaSphere is published by John Wiley & Sons Ltd on behalf of European Hematology Association. This is an open access Abstract Book distributed under the Attribution-NonCommercial-NoDerivs (CC BY-NC-ND), which allows third parties to download the articles and share them with others as long as they credit the author and the Abstract Book, but they cannot change the content in any way or use them commercially.
**Abstract Book Citations:** Authors, Title, HemaSphere, 2026;10:(S1):pages. The abstract book DOIs can be found on the "Author Information" page.
**Disclaimer:** Articles published in the journal HemaSphere exclusively reflect the opinions of the authors. The authors are responsible for all content in their abstracts including accuracy of the facts, statements, citing resources, etc.

### (PF799) EFFICACY OF SEQUENCED BCMA-DIRECTED THERAPIES IN RELAPSED/REFRACTORY MULTIPLE MYELOMA – A RETROSPECTIVE, MULTICENTER, INTERNATIONAL COHORT STUDY (SEQ-BCMA STUDY)

**Topic:** 14. Myeloma and other monoclonal gammopathies - Clinical

Christoph Schaefers*[1], Maximilian Steinhardt[2], Jan Frenking[3], Tim Richardson[4], Raphael Teipel[5], Maximilian Alexander Funk[6], Christian Michel[7], Lisa M Sgonina[8], Evgenii Shumilov[8], Markus Maulhardt[9], Thomas Pabst[10], Normann Steiner[11], Wolfgang Willenbacher[11, 12], Michael Leisch[13], Thomas Melchardt[13], Irene Strassl[14, 15], Marie Harzer[1], Lilan Gatti[3], David Böckle[16], Stephan Bohl[16], Igor Wolfgang Blau[16], Jan Vorwerk[17], Annamaria Brioli[18], Sandra Sauer[3], Maximilian Al-Bazaz[1], Jule Artzenroth[1], Leandra Bartke[1], Leon Cords[1], Abdulaziz Kamili[1], Ricardo Kosch[1], Winfried Alsdorf[1], Christine Riedhammer[2], Julia Mersi[2], Florian Eisele[2], K Martin Kortum[2], Kai Rejeski[6], Sebastian Theurich[6], Christof Scheid[4], Marc-Steffen Raab[3], Carsten Bokemeyer[1], Hermann Einsele[2], Katja C. Weisel[1], Leo Rasche[2], Lisa Leypoldt[1, 19]

*[1]University Medical Center Hamburg-Eppendorf, Department of Hematology, Oncology and Bone Marrow Transplantation With Section of Pneumology, Hamburg, Germany; [2]University Hospital Würzburg, Department of Internal Medicine II, Würzburg, Germany; [3]University Hospital Heidelberg, Internal Medicine V and National Center for Tumor Diseases, Heidelberg, Germany; [4]University Hospital Cologne, Department of Internal Medicine I, Cologne, Germany; [5]University Hospital of Dresden, Department of Internal Medicine I, Dresden, Germany; [6]LMU University Hospital, LMU Munich, Department of Medicine III-Hematology/Oncology, Munich, Germany; [7]University Hospital Mainz, Department of Internal Medicine III, Mainz, Germany; [8]University Hospital Münster, Department of Medicine A, Hematology, Oncology and Pneumology, Münster, Germany; [9]University Medical Center Göttingen, Department for Hematology and Medical Oncology, Göttingen, Germany; [10]University Hospital Inselspital and University of Bern, Department of Medical Oncology, Bern, Switzerland; [11]Comprehensive Cancer Center Innsbruck, Medical University of Innsbruck, Department of Internal Medicine V, Innsbruck, Austria; [12]syndena GmbH, connect to cure, Innsbruck, Austria; [13]Paracelsus Medical University, Cancer Cluster Salzburg, Department of Internal Medicine III with Haematology, Medical Oncology, Haemostaseology, Infectiology and Rheumatology, Oncologic Center, Salzburg Cancer Research Institute - Laboratory for Immunological and Molecular Cancer Research (SCRI-LIMCR), Salzburg, Austria; [14]Ordensklinikum Linz, Department of Internal Medicine I: Hematology with Stem Cell Transplantation, Hemostaseology and Medical Oncology, Linz, Austria; [15]Johannes Kepler University Linz, Medical Faculty, Linz, Austria; [16]Charité-University Medicine Berlin, Department of Internal Medicine, Berlin, Germany; [17]University Medical Center Schleswig-Holstein (UKSH) and University Cancer Center Schleswig- Holstein (UCCSH), Campus Lübeck, Department of Hematology and Oncology, Lübeck, Germany; [18]Hannover Medical School Hannover, Department of Hematology, Hemostasis, Oncology, and Stem Cell Transplantation, Hannover, Germany; [19]University Medical Center Hamburg-Eppendorf, Mildred Scheel Early Career Center, Hamburg, Germany;*

### Background

The introduction of BCMA-directed treatments (BCMA-Tx) has revolutionized the treatment of relapsed/refractory multiple myeloma (RRMM). However, it has also created a new group of patients (pts) with unmet needs: those who relapse after receiving BCMA-Tx treatment. Currently, three BCMA-Tx modalities are available: CAR T cells, bispecific antibodies (bsAb), and an antibody drug conjugate (ADC). Yet, limited data exist on 1) the use of BCMA-sequences in the real world, 2) outcomes of subsequent BCMA-Tx depending on the first BCMA-Tx, and 3) whether certain sequencing strategies should be avoided or preferred.

### Aims

We aimed to investigate the outcomes of all 9 conceivable BCMA-after-BCMA-directed treatment strategies in a real-world analysis.

**Copyright Information:** (Online) ISSN: 2572-9241
©2026 The Author(s). HemaSphere is published by John Wiley & Sons Ltd on behalf of European Hematology Association. This is an open access Abstract Book distributed under the Attribution-NonCommercial-NoDerivs (CC BY-NC-ND), which allows third parties to download the articles and share them with others as long as they credit the author and the Abstract Book, but they cannot change the content in any way or use them commercially.
**Abstract Book Citations:** Authors, Title, HemaSphere, 2026;10:(S1):pages. The abstract book DOIs can be found on the “Author Information” page.
**Disclaimer:** Articles published in the journal HemaSphere exclusively reflect the opinions of the authors. The authors are responsible for all content in their abstracts including accuracy of the facts, statements, citing resources, etc.

### Methods

In this retrospective, multicenter, real-world analysis, RRMM pts were included if they had received ≥2 BCMA-Tx, regardless of the number of interim treatment lines. Baseline characteristics were summarized descriptively; PFS and OS were analyzed using Kaplan-Meier estimates with Benjamini-Hochberg-adjusted log-rank tests and median follow-up (mFU) by reverse Kaplan-Meier.

### Results

137 pts from 16 centers in Germany, Austria, and Switzerland were included. Median age was 64 years (range 34-86), median prior lines of therapy were 6 (3-15) at the time of 2nd BCMA-Tx. High-risk cytogenetics (HRCA; according to R-ISS) were present in 50/114 evaluable (ev.) pts. CAR T was the most frequent 1st BCMA-Tx (84 pts, 61%), followed by ADC (37 pts, 27%) and bsAb (16 pts, 12%). Of note, HRCA were more frequent among 1st BCMA-Tx with bsAb pts (60%, 9/15 ev. pts) vs CAR T (39%, 27/69 ev. pts) or ADC (47%, 14/30 ev. pts). Overall, pts had a median of 1 (0-6) line and 365 days (5-1507) between BCMA-Tx, with bsAb as the most frequent 2nd BCMA-Tx (n=78, 57%), followed by CAR T (n=50, 36%) and ADC (n=9, 7%). The four most frequent sequences were CAR T→bsAb (n=60, 44%), ADC→CAR T (n=19, 14%), ADC→bsAb (n=18, 13%), and CAR T→CAR T (ide cel→cilta cel, n=18, 13%). Overall response rate to 2nd BCMA-Tx was 67% (83/123 ev. pts), and 35% achieved ≥ CR or VGPR MRD negativity. Median PFS after 2nd BCMA-Tx of the full cohort was 8.4 months (mFU 13.1 months [mo]) and 2nd BCMA CAR T yielded a numerically superior mPFS of 15.31 mo compared to 4.0 mo for bsAb. With regard to how the 1st BCMA-Tx influences CAR T as 2nd BCMA-Tx, most favorable outcomes were seen with CAR T→CAR T (mPFS not reached), followed by ADC→CAR T (mPFS 16.8 mo) compared to bsAb→CAR T, which showed diminished survival (n=13, mPFS 6.5 mo, p=0.03). Moreover, for pts relapsing after prior CAR T, CAR T showed superior outcomes compared to bsAb (mPFS not reached vs 4 mo; p=0.03). 2nd BCMA-Tx was not significantly affected by the time between 1st and 2nd BCMA-Tx (<3, 3-6, vs. >6 mo; p=0.34). Median OS of the full cohort since 2nd BCMA-Tx was 29 mo, at a mFU of 13.9 months. Five pts received a 3rd BCMA-Tx, of which 2 achieved a response.

### Summary/Conclusion

Repeated BCMA-Tx yields high response rates in real-world practice, but patterns differ: while CAR T provides superior PFS after CAR T or ADC, its outcome appears attenuated after bsAb, indicating that CAR T should be given first. The relatively short PFS in the CAR T→bsAb cohort underscores the unmet need to further improve the effectiveness of post-BCMA regimens. These data also support the feasibility of "CAR T-after-CAR T" strategies and provide a framework for informed clinical sequencing of BCMA-Tx.

Copyright Information: (Online) ISSN: 2572-9241
©2026 The Author(s). HemaSphere is published by John Wiley & Sons Ltd on behalf of European Hematology Association. This is an open access Abstract Book distributed under the Attribution-NonCommercial-NoDerivs (CC BY-NC-ND), which allows third parties to download the articles and share them with others as long as they credit the author and the Abstract Book, but they cannot change the content in any way or use them commercially.
Abstract Book Citations: Authors, Title, HemaSphere, 2026;10:(S1):pages. The abstract book DOIs can be found on the “Author Information” page.
Disclaimer: Articles published in the journal HemaSphere exclusively reflect the opinions of the authors. The authors are responsible for all content in their abstracts including accuracy of the facts, statements, citing resources, etc.

### (PF800) OUTCOMES AND UNMET NEED IN LENALIDOMIDE-EXPOSED AND LENALIDOMIDE-REFRACTORY 2-4L RRMM FLATIRON PATIENTS

**Topic:** 14. Myeloma and other monoclonal gammopathies - Clinical

Omar Alkharabsheh*[1], Katie Gravagna[2], Amol Dhamane[2], Amir Samuel[3], Jessica Katz[2], Jiaqi Fang[2]

*[1]University of Cincinnati College of Medicine, Cincinnati, United States of America; [2]Bristol Myers Squibb, Lawrenceville, United States of America; [3]Bristol Myers Squibb, Boudry, Switzerland;*

### Background

Lenalidomide (LEN) is widely used in early lines of therapy (LOTs) for treatment of newly diagnosed and relapsed/refractory multiple myeloma (RRMM) patients. In the frontline setting, both transplant eligible and ineligible patients often receive prolonged LEN as LEN is incorporated into several triplet or quadruplet regimens. There are ongoing clinical trials in the LEN exposed (LEN-E)/refractory (LEN-R) RRMM patient population; however, there is limited real-world evidence to understand existing unmet need for such population to contextualize these novel treatments.

### Aims

The aim of this study was to assess real-world outcomes of US RRMM patients after LEN exposure by LEN-refractory status.

### Methods

This retrospective observational study used the nationwide Flatiron Health electronic health record database. Eligible adults had RRMM; received 1–3 prior lines of therapy with a 2L start date on or after 01/01/2019 through October 2025; and were LEN-E before their index therapy line. Descriptive statistics were reported for demographic characteristics, disease status, and prior treatments. Progression free survival (PFS) and overall survival (OS) were estimated using Kaplan–Meier methods, and associated factors were evaluated using multivariable Cox models.

### Results

Among 3,910 patients initiating 2L therapy, 2,738 (70.0%) were LEN-E. LEN exposure increased in later lines: N=1,665 (83.3%) in 3L; N=708 (88.9%) in 4L. Demographic and clinical characteristics were consistent across lines. DARA with pomalidomide was the top regimen for all cohorts, at 9.4% for 2L, 12.4% for 3L, and 8.9% for 4L. DARA with LEN was the second most common regimen for both 2L and 3L, at 7.7% and 4.5% respectively. The post-LEN cohorts were also highly LEN-R: 67.7% in 2L, 73.0% in 3L, and 78.1% in 4L. Similarly, DARA-R status increased from 12.2% at 2L to 45.6% at 3L and 67.8% at 4L, reflecting increasing exposure rates (16.0% at 2L, 54.4% at 3L, and 77.4% at 4L). Triple-class exposure and refractoriness (TCR) also increased substantially from 2L to 4L, with TCR increasing from 5.4% in 2L to 49.6% in 4L.

PFS in the LEN-E cohorts was 15.2 months (95% CI: 13.6, 16.6) in 2L, 8.0 months (95% CI: 7.2, 8.8) in 3L, and 7.2 months (95% CI: 6.2, 8.5) in 4L. Similarly, OS was 66.7 months (95% CI: 61.5, 73.9) in 2L post-LEN patients, followed by 44.0 months (95% CI: 36.0, 50.8) in 3L and 30.8 months (95% CI: 27.6, 36.9) in 4L. In comparison, for LEN-R patients, PFS was lower: 14.3 months (95% CI: 12.7, 16.6) in 2L, 7.5 months (95% CI: 6.7,8.4) in 3L, and 6.9 months (95% CI: 6.1,7.9) in 4L. OS in LEN-R patients followed a similar trend, at 66.2 months (95% CI: 60.5,-) in 2L, 41.8 months (95% CI: 33.9,50.8) in 3L, and 29.0 months (95% CI: 23.0,34.4) in 4L.

Multivariable models identified frailty, ISS stage II/III status, high-risk cytogenetics, and triple-class refractoriness as consistently associated with significantly worse OS and PFS across lines.

**Copyright Information:** (Online) ISSN: 2572-9241
©2026 The Author(s). HemaSphere is published by John Wiley & Sons Ltd on behalf of European Hematology Association. This is an open access Abstract Book distributed under the Attribution-NonCommercial-NoDerivs (CC BY-NC-ND), which allows third parties to download the articles and share them with others as long as they credit the author and the Abstract Book, but they cannot change the content in any way or use them commercially.
**Abstract Book Citations:** Authors, Title, HemaSphere, 2026;10:(S1):pages. The abstract book DOIs can be found on the "Author Information" page.
**Disclaimer:** Articles published in the journal HemaSphere exclusively reflect the opinions of the authors. The authors are responsible for all content in their abstracts including accuracy of the facts, statements, citing resources, etc.

### Summary/Conclusion

In this large real-world US cohort of LEN-E patients with RRMM, outcomes worsened with each additional line of therapy. The high frequency of refractoriness, particularly in later lines, demonstrates substantial unmet need and the limited durability of available therapeutic options in LEN-E and LEN-R patients. These findings underscore the importance of novel therapeutic mechanisms and optimized treatment sequencing for patients with RRMM who progress after LEN exposure.

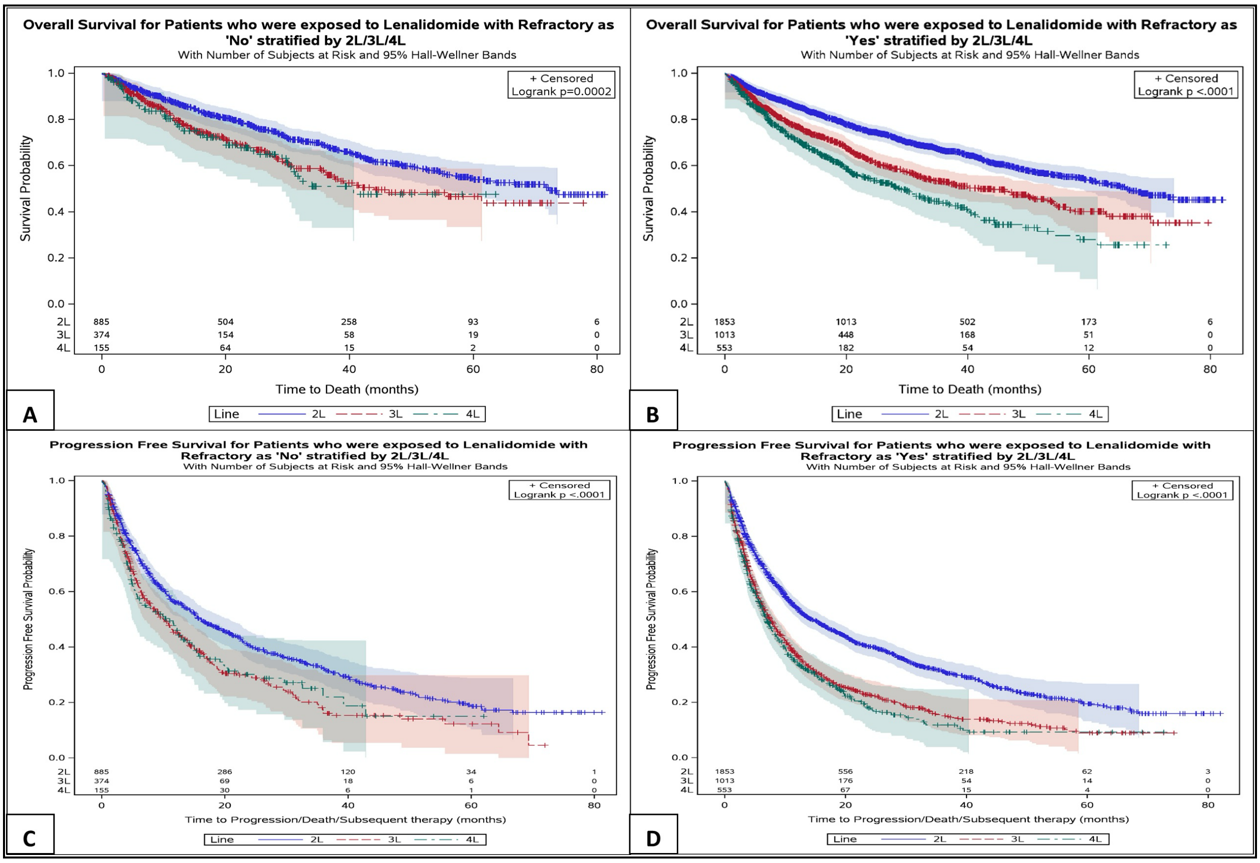

Figure 1. Outcomes by LEN-R status

**Copyright Information:** (Online) ISSN: 2572-9241
©2026 The Author(s). HemaSphere is published by John Wiley & Sons Ltd on behalf of European Hematology Association. This is an open access Abstract Book distributed under the Attribution-NonCommercial-NoDerivs (CC BY-NC-ND), which allows third parties to download the articles and share them with others as long as they credit the author and the Abstract Book, but they cannot change the content in any way or use them commercially.
**Abstract Book Citations:** Authors, Title, HemaSphere, 2026;10:(S1):pages. The abstract book DOIs can be found on the "Author Information" page.
**Disclaimer:** Articles published in the journal HemaSphere exclusively reflect the opinions of the authors. The authors are responsible for all content in their abstracts including accuracy of the facts, statements, citing resources, etc.

### (PF801) SIGNIFICANT IMPROVEMENTS IN SURVIVAL OUTCOMES IN SMOLDERING MULTIPLE MYELOMA: A POPULATION-BASED STUDY

**Topic:** 14. Myeloma and other monoclonal gammopathies - Clinical

Malin Hultcrantz*[1, 2], Baojing Li[3], Therese Andersson[4], Rickard Strandberg[3], Michael Crowther[3], Sigurdur Yngvi Kristinsson[5, 6]

[1]*Memorial Sloan Kettering Cancer Center, Myeloma Service, Department of Medicine, New York, United States of America;* [2]*Karolinska Institute, Department of Medicine, Solna, Stockholm, Sweden;* [3]*Red Door Analytics AB, Stockholm, Sweden;* [4]*Karolinska Institute, Department of Medical Epidemiology and Biostatistics, Stockholm, Sweden;* [5]*University of Iceland, Department of Science, Reykjavík, Iceland;* [6]*Landspitali - The National University Hospital of Iceland, Reykjavik, Iceland;*

### Background

Smoldering multiple myeloma (SMM) is an asymptomatic precursor condition to multiple myeloma (MM) with a heterogenous risk of progression. Over the past 10-15 years, the diagnostic criteria for SMM and MM have evolved and early intervention trials have sought to improve the long-term outcome in SMM. However, contemporary population-based data on long-term survival trends remain limited.

### Aims

To assess overall survival, relative survival ratios, and cause of death in a population-based study using Swedish healthcare registers of individuals with SMM compared to the general population.

### Methods

The National Blood Cancer Register which captures information on SMM and MM, were used to identify all individuals with SMM diagnosed in Sweden between 2008 and 2022 with follow up through 2023. Life tables were obtained through Statistics Sweden and through the Swedish Register for Total Population, up to 10 controls were chosen randomly matched for age, gender, and region using data from Statistics Sweden and the Swedish Register for Total Population. Outcomes were captured using the Swedish Cancer Register and Cause of Death Register. Overall Survival (OS) and progression to MM were assessed using Kaplan-Meier estimates and Cox regression. Relative survival ratios (RSRs) were calculated using flexible parametric models to estimate excess mortality.

### Results

We identified 2,007 individuals with SMM; there were 1,108 men (55%) and 899 women (45%) and median age was 73 years. A total of 19,720 matched controls were identified and included. The median follow up in the SMM cohort was 4.39 years.
Median OS in individuals with SMM was 7.24 years (95% CI: 6.66-7.74). The median OS in the individuals with SMM under 60 years was not reached, it was 6.5 years in individuals aged 70-79 years, and 3.5 years for those aged 80 years or above.
RSRs were significantly reduced in SMM patients, particularly at 10- and 15-year after diagnosis, with the most pronounced reduction observed in the older age groups, 70-79 and over 80 years (Figure 1). The RSR was similar in men and women. The RSR improved markedly over time; the 5-year RSR improved from 0.59 to 0.81, the 10-year RSR from 0.31 to 0.62 and the 15-year RSR from 0.18 to 0.48 in SMM patients diagnosed 2018 compared to those diagnosed in 2008. However, a significant excess mortality persisted in the older age groups in recent calendar periods.
During the study period, 794 individuals with SMM progressed to MM. The leading cause of death was MM, hazard ratio (HR) 499 (95% confidence interval [CI] 297.92-836.75). The risk of death from infections was significantly increased, HR 2.73 (95% CI 2.10-3.55). The risk of death from cardiovascular diseases was 1.66 (95% CI 1.43-1.93) while the risk of death from solid tumors was not significantly increased, HR 1.16 (95% 0.94-1.43).

**Copyright Information:** (Online) ISSN: 2572-9241
©2026 The Author(s). HemaSphere is published by John Wiley & Sons Ltd on behalf of European Hematology Association. This is an open access Abstract Book distributed under the Attribution-NonCommercial-NoDerivs (CC BY-NC-ND), which allows third parties to download the articles and share them with others as long as they credit the author and the Abstract Book, but they cannot change the content in any way or use them commercially.
**Abstract Book Citations:** Authors, Title, HemaSphere, 2026;10:(S1):pages. The abstract book DOIs can be found on the "Author Information" page.
**Disclaimer:** Articles published in the journal HemaSphere exclusively reflect the opinions of the authors. The authors are responsible for all content in their abstracts including accuracy of the facts, statements, citing resources, etc.

### Summary/Conclusion

In this large population-based study, we found that the OS in individual with SMM was significantly reduced compared to the general population. The leading cause of death was multiple myeloma. Importantly, the RSR improved significantly over time across all age groups. These findings highlight the importance of accurate diagnosis and suggest that modern therapeutic strategies and refined risk stratification models, including dynamic and genomic prediction models, are translating into better real-world outcomes. Early identification of high risk SMM remains vital for further reducing excess mortality.

**Figure.** Predicted 1-, 5-, 10-, and 15-year relative survival ratios (RSRs) in patients with SMM by calendar year from 2008 to 2018 stratified by age at diagnosis.

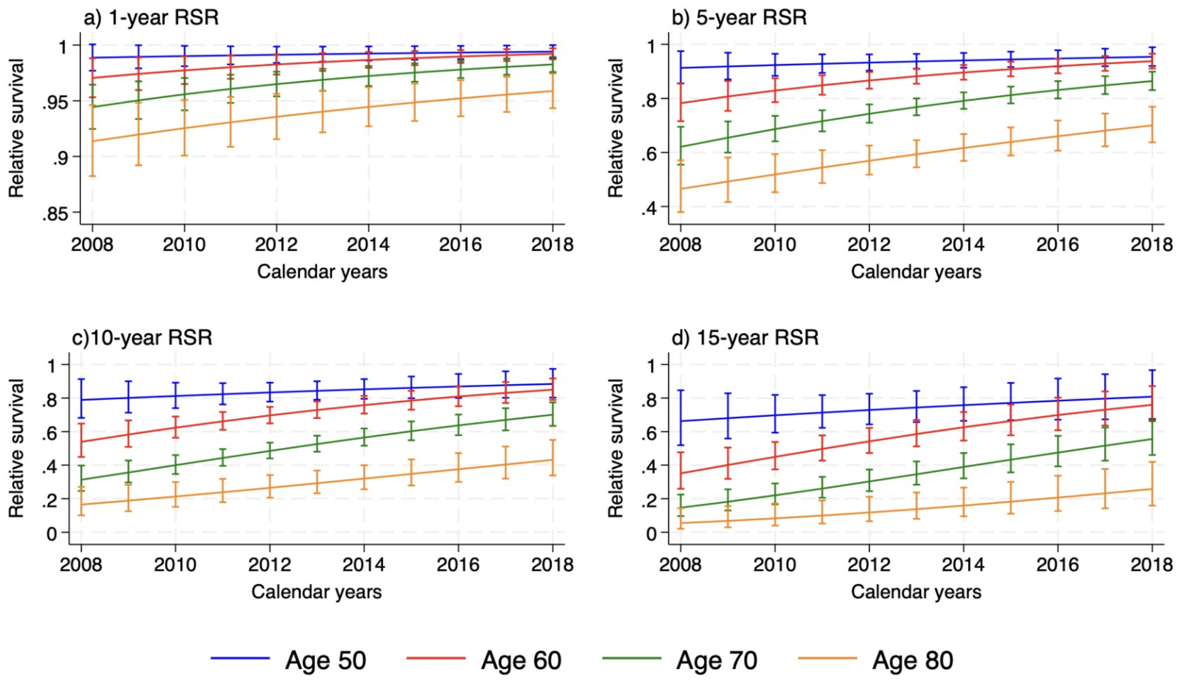

**Copyright Information:** (Online) ISSN: 2572-9241
©2026 The Author(s). HemaSphere is published by John Wiley & Sons Ltd on behalf of European Hematology Association. This is an open access Abstract Book distributed under the Attribution-NonCommercial-NoDerivs (CC BY-NC-ND), which allows third parties to download the articles and share them with others as long as they credit the author and the Abstract Book, but they cannot change the content in any way or use them commercially.
**Abstract Book Citations:** Authors, Title, HemaSphere, 2026;10:(S1):pages. The abstract book DOIs can be found on the “Author Information” page.
**Disclaimer:** Articles published in the journal HemaSphere exclusively reflect the opinions of the authors. The authors are responsible for all content in their abstracts including accuracy of the facts, statements, citing resources, etc.

### (PF802) MORTALITY AND CLINICAL RISK FACTORS IN PATIENTS WITH MULTIPLE MYELOMA.

**Topic:** 14. Myeloma and other monoclonal gammopathies - Clinical

Maria Ordoñez Garcia*[1], Ane Brieva Lopez[1], Rafael Garcia Carretero[2], Juan Sastre Ortega[1], Mariana Cerrato Salas[1], Pavel Manuel Acevedo Pérez[1], Maria Angeles Andreu Costa[1]

*[1]Hospital Universitario de Móstoles, Madrid, Spain;[2]Hospital Universitario Severo Ochoa, Madrid, Spain;*

### Background

Multiple myeloma (MM) is a complex hematologic malignancy characterized by the proliferation of plasma cells. While therapeutic advancements have significantly improved survival rates in recent years, hospitalized patients remain a highly vulnerable population due to the inherent nature of the disease and its associated treatments.

### Aims

The objective of this study was to analyze the trends in hospital admissions and in-hospital mortality among patients with multiple myeloma in Spain between 2020 and 2023, and to identify the clinical and demographic risk factors associated with mortality during these hospitalizations.

### Methods

This retrospective study utilized centralized data from the Spanish National Health System's Minimum Basic Data Set (MBDS-H), which captures approximately 97% of all hospital discharge reports in Spain. Patients were identified using ICD-10-CM codes for multiple myeloma (C90.00, C90.01, and C90.02). Trends in mortality over the four-year period were assessed using Poisson regression models. To identify independent risk factors for in-hospital mortality, a multivariate logistic regression analysis was performed, incorporating variables such as age, sex, comorbidities, and clinical complications.

### Results

The study included a total of 20,249 patients. While annual hospitalized patients rose from 4,677 in 2020 to 5,360 in 2023, the in-hospital mortality rate showed a significant declining trend, decreasing from 11.9% to 9.6% ($p = 0.028$). Multivariate analysis revealed that the highest risks for mortality were associated with SARS-CoV-2 infection (OR = 1.77; 95% CI: 1.54–2.04), relapsed MM (OR = 1.75; 95% CI: 1.5–2.02), and cerebrovascular disease (OR = 1.7; 95% CI: 1.37–2.11). A Charlson Comorbidity Index (CCI) > 4 also significantly increased mortality risk (OR = 1.67). On the contrary, hematopoietic stem cell transplantation (HSCT) was the strongest protective factor (OR = 0.36; 95% CI: 0.3–0.43), followed by being in complete remission (OR = 0.53; 95% CI: 0.44–0.63).

### Summary/Conclusion

In-hospital mortality for MM in Spain has significantly improved between 2020 and 2023. However, the presence of acute infectious complications and severe cardiovascular or cerebrovascular comorbidities remains a critical determinant of survival, outweighing the impact of age alone.

**Copyright Information:** (Online) ISSN: 2572-9241
©2026 The Author(s). HemaSphere is published by John Wiley & Sons Ltd on behalf of European Hematology Association. This is an open access Abstract Book distributed under the Attribution-NonCommercial-NoDerivs (CC BY-NC-ND), which allows third parties to download the articles and share them with others as long as they credit the author and the Abstract Book, but they cannot change the content in any way or use them commercially.
**Abstract Book Citations:** Authors, Title, HemaSphere, 2026;10:(S1):pages. The abstract book DOIs can be found on the "Author Information" page.
**Disclaimer:** Articles published in the journal HemaSphere exclusively reflect the opinions of the authors. The authors are responsible for all content in their abstracts including accuracy of the facts, statements, citing resources, etc.

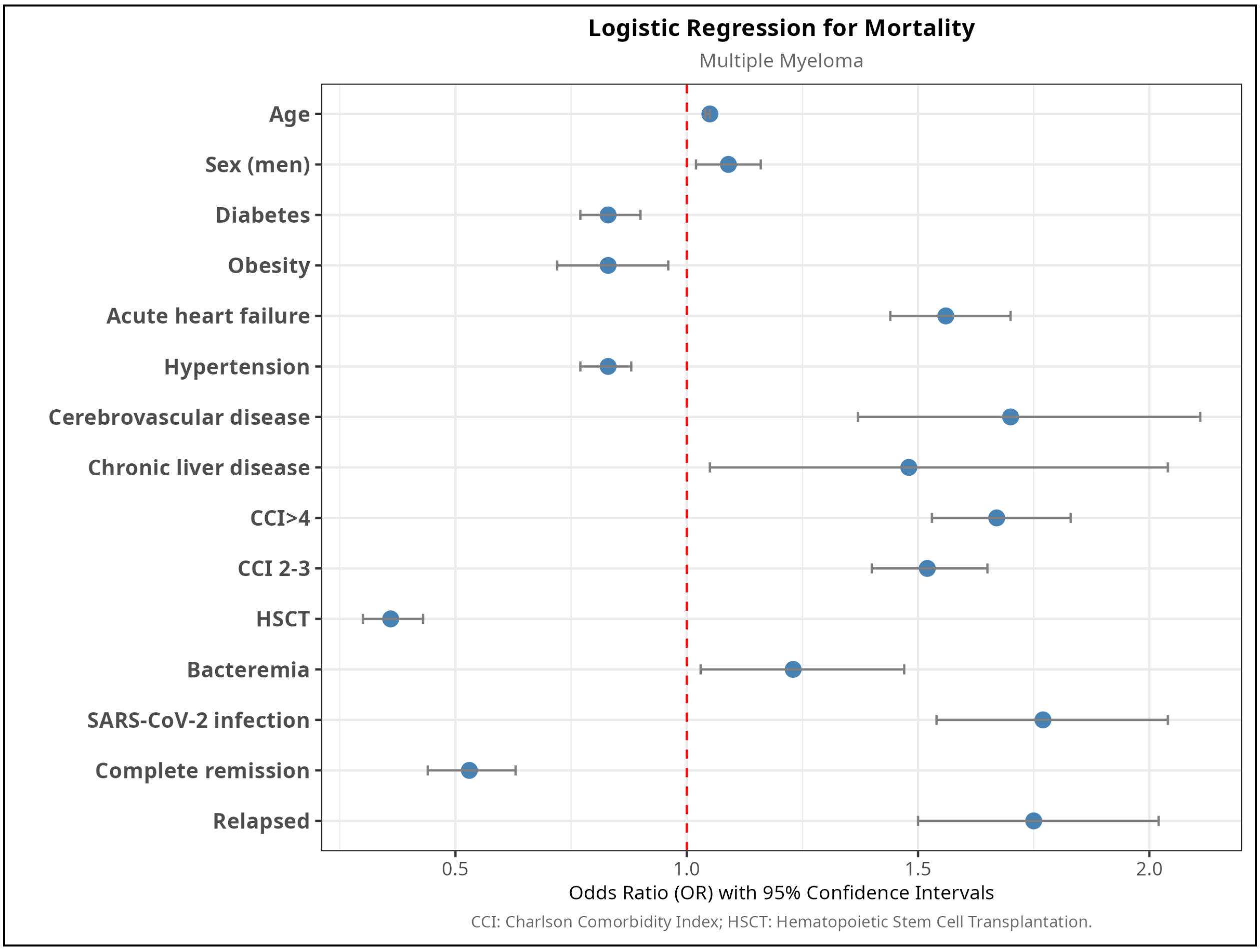

Figure 1. Logistic regression for mortality.

**Copyright Information:** (Online) ISSN: 2572-9241
©2026 The Author(s). HemaSphere is published by John Wiley & Sons Ltd on behalf of European Hematology Association. This is an open access Abstract Book distributed under the Attribution-NonCommercial-NoDerivs (CC BY-NC-ND), which allows third parties to download the articles and share them with others as long as they credit the author and the Abstract Book, but they cannot change the content in any way or use them commercially.
**Abstract Book Citations:** Authors, Title, HemaSphere, 2026;10:(S1):pages. The abstract book DOIs can be found on the "Author Information" page.
**Disclaimer:** Articles published in the journal HemaSphere exclusively reflect the opinions of the authors. The authors are responsible for all content in their abstracts including accuracy of the facts, statements, citing resources, etc.

### (PF803) DEEP PERIPHERAL IMMUNE PHENOTYPING REVEALS PROFOUND T-CELL SENESCENCE, NK-CELL DYSREGULATION, AND MONOCYTE SKEWING IN RELAPSED/REFRACTORY MULTIPLE MYELOMA

**Topic:** 14. Myeloma and other monoclonal gammopathies - Clinical

Xuelin Han[1], Ting Zhang[1], Yajing Zhang*[1]

[1]*Beijing GoBroad Boren Hospital, Beijing, China;*

### Background

T-cell engaging therapies (CAR-T, bispecifics) revolutionized relapsed/refractory multiple myeloma (RRMM) treatment, but resistance remains a hurdle. While bone marrow dysregulation is well-documented, the peripheral blood (PB) immune landscape—vital for T-cell manufacturing and systemic surveillance—remains under-characterized. Defining exact PB quantitative and phenotypic defects is crucial for optimizing immunotherapies and predicting therapy readiness.

### Aims

To comprehensively map the multi-lineage systemic immune landscape of RRMM patients via high-parameter flow cytometry, identifying distinct peripheral immunoparesis signatures and novel biomarkers of profound immune exhaustion.

### Methods

We retrospectively analyzed PB samples from advanced RRMM and plasma cell leukemia (PCL) patients using a 52-parameter flow cytometry panel. Subsets of T cells (Naive, Tcm, Tem, TEMRA, Treg, and PD1+/TIM3+ exhausted), B cells, NK cells, and monocytes were precisely quantified.

### Results

The cohort exhibited a globally compromised, highly senescent immune profile with four distinct hallmarks:1. Severe T-cell Skewing & Senescence: Extreme CD4/CD8 inversion (median 0.55, range 0.37-0.80; normal 0.8-2.7) was driven by expanded CD3+CD8+ T cells (median 52.12%), notably accumulating Effector Memory (Tem, ≤51.27%) and TEMRA (≤37.51%) subsets. Profound exhaustion occurred, with PD1+ and TIM3+ T cells reaching 47.95% and 41.13% respectively. Tregs constituted up to 37.7% of CD4+ T cells.2. Profound B-cell Aplasia: Systemic humoral immunity was severely depleted. CD19+ B cells were <1.5% of lymphocytes (median 0.50%, range 0.00-1.80%), indicating sustained immunoparesis.3. NK-cell Regulatory Shift: Despite frequently decreased total NK cells, a striking shift occurred from cytotoxic CD56dimCD16+ to immature/regulatory CD56hiCD16- subsets (median 10.39%, peaking at 26.06%; normal ≤5.0%), suggesting suppressed innate cytotoxicity.4. Pro-inflammatory Monocyte Expansion: Total monocytes expanded (≤12.35%), with notable skewing towards intermediate and non-classical (CD14lowCD16hi) inflammatory subsets (≤31.75%; normal ≤5.0%).

### Summary/Conclusion

RRMM patients harbor a profoundly dysregulated and senescent systemic immune environment. The unique tetrad of CD4/CD8 inversion with TEMRA accumulation, B-cell aplasia, CD56hi NK skewing, and non-classical monocyte expansion defines a distinct "Immune-Evasive/Senescent" phenotype. These findings provide an immunological rationale for therapy failure, underscoring the urgent need for early immune-resetting interventions or exhaustion-blockades (e.g., anti-PD1/TIM3) alongside T-cell engagers to overcome RRMM systemic barriers.

**Copyright Information:** (Online) ISSN: 2572-9241
©2026 The Author(s). HemaSphere is published by John Wiley & Sons Ltd on behalf of European Hematology Association. This is an open access Abstract Book distributed under the Attribution-NonCommercial-NoDerivs (CC BY-NC-ND), which allows third parties to download the articles and share them with others as long as they credit the author and the Abstract Book, but they cannot change the content in any way or use them commercially.
**Abstract Book Citations:** Authors, Title, HemaSphere, 2026;10:(S1):pages. The abstract book DOIs can be found on the “Author Information” page.
**Disclaimer:** Articles published in the journal HemaSphere exclusively reflect the opinions of the authors. The authors are responsible for all content in their abstracts including accuracy of the facts, statements, citing resources, etc.

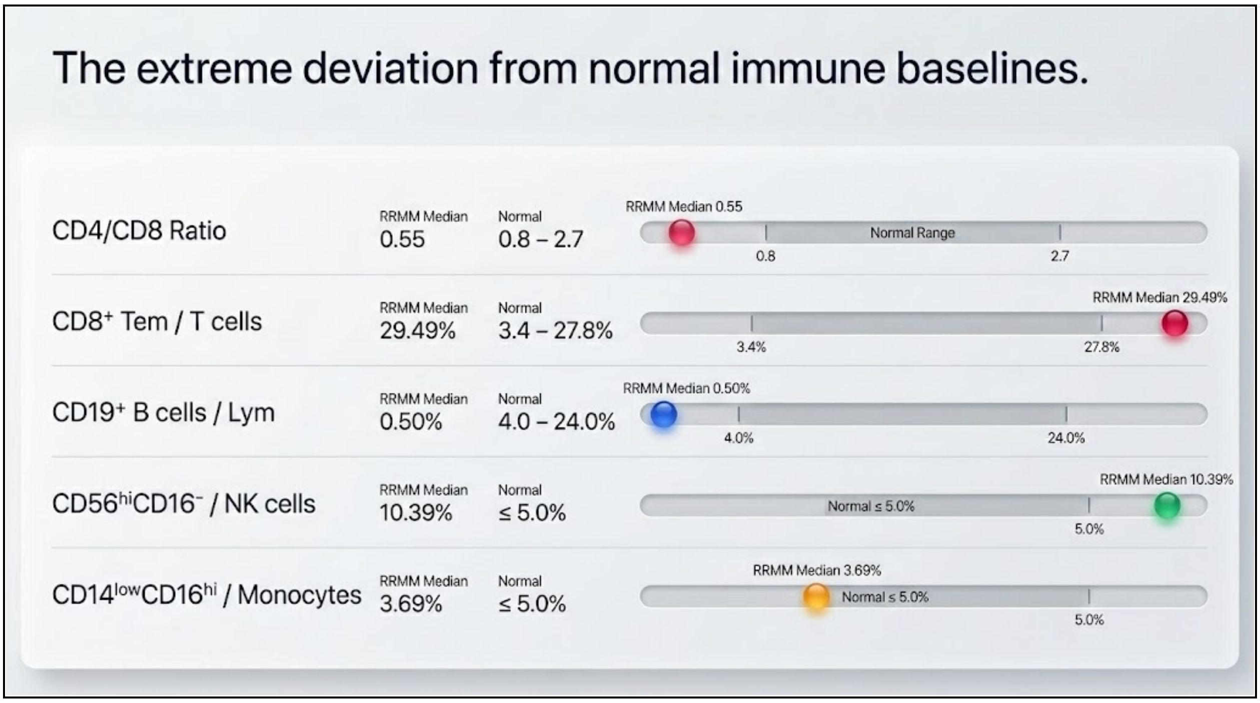

The extreme deviation from normal immune baselines

**Copyright Information:** (Online) ISSN: 2572-9241
©2026 The Author(s). HemaSphere is published by John Wiley & Sons Ltd on behalf of European Hematology Association. This is an open access Abstract Book distributed under the Attribution-NonCommercial-NoDerivs (CC BY-NC-ND), which allows third parties to download the articles and share them with others as long as they credit the author and the Abstract Book, but they cannot change the content in any way or use them commercially.
**Abstract Book Citations:** Authors, Title, HemaSphere, 2026;10:(S1):pages. The abstract book DOIs can be found on the "Author Information" page.
**Disclaimer:** Articles published in the journal HemaSphere exclusively reflect the opinions of the authors. The authors are responsible for all content in their abstracts including accuracy of the facts, statements, citing resources, etc.

### (PF804) REAL-WORLD PATIENT CHARACTERISTICS, GENERAL SAFETY PROFILE, AND TREATMENT PATTERNS OF ELRANATAMAB (ELRA) USE IN RELAPSED REFRACTORY MULTIPLE MYELOMA: INTERIM RESULTS OF THE AMBRELA STUDY

**Topic:** 14. Myeloma and other monoclonal gammopathies - Clinical

Thomas Chalopin*[1], Mohamad Mohty[2], Cyrille Touzeau[3], Camille Villesuzanne[4], Manon Sapet[5], Guido Nador[6], Sikha-M'benard Dabo[7], Pierre-Alexandre Squara[7], Justine Wenta[7], Elodie Priol[8], Margaux Dubois[8], Alexandre Combes[8], Laurent Frenzel[9], Aurore Perrot[10]

*[1]Adult and Pediatric Hematology and Cell Therapy Services at Regional University Hospital Center of Tours, Tours, France; [2]Clinical Hematology and Cellular Therapy Services at Public Assistance Hospital of Paris - Saint-Antoine Hospital, Paris, France; [3]Department of Medicine - Clinical Hematology Services at Nantes University Hospital Centre, Nantes, France;[4]Cancerology Center - Hematology Service at Brive Hospital Center, Brive, France; [5]Hematology and Cell Therapy Service at Saint-Étienne University Hospital Center, Saint-Etienne, France; [6]Pfizer Inc., London, United Kingdom; [7]Pfizer Inc., Paris, France; [8]Pfizer France, Paris, France; [9]Adult Hematology service at Public Assistance Hospital of Paris - Necker–Enfants Malades Hospital, Paris, France; [10]Department of Hematology at Toulouse University Cancer Institute-Oncopole, Toulouse, France;*

### Background

ELRA, a BCMA-CD3 bispecific antibody, was approved for the treatment of relapsed/refractory multiple myeloma based on the phase 2 registrational MagnetisMM-3 study (MM-3, NCT04649359). ELRA administration includes 2 step-up priming doses followed by a full fixed dose of 76 mg weekly until week 24, biweekly (Q2W) when response is achieved until week 48, and monthly (Q4W) thereafter if response is maintained.

### Aims

To compare real-world patient (pt) characteristics between the AMbrELA and MM-3 cohorts and describe the real-world safety profile and treatment patterns of ELRA.

### Methods

AMbrELA (EUPAS1000000074) is an observational ambispective study evaluating the effectiveness and safety of ELRA in pts who initiated ELRA in the early access program in France since May 2023. Pts starting treatment retrospectively were selected for this 2$^{nd}$ interim analysis. Adverse events (AEs) that occurred before pt inclusion were collected if they were treatment-related (TR), while all AEs that occurred after inclusion were collected regardless of cause. Data cut-off was July 31, 2025. A 3$^{rd}$ interim analysis is planned on February 28, 2026, to update these results.

**Copyright Information:** (Online) ISSN: 2572-9241
©2026 The Author(s). HemaSphere is published by John Wiley & Sons Ltd on behalf of European Hematology Association. This is an open access Abstract Book distributed under the Attribution-NonCommercial-NoDerivs (CC BY-NC-ND), which allows third parties to download the articles and share them with others as long as they credit the author and the Abstract Book, but they cannot change the content in any way or use them commercially.
**Abstract Book Citations:** Authors, Title, HemaSphere, 2026;10:(S1):pages. The abstract book DOIs can be found on the "Author Information" page.
**Disclaimer:** Articles published in the journal HemaSphere exclusively reflect the opinions of the authors. The authors are responsible for all content in their abstracts including accuracy of the facts, statements, citing resources, etc.

**Results**

A total of 89 pts from 30 centers were included. Median follow-up was 13.4 months (mo) (95% CI, 11.5-16.6).
Pts from AMbrELA (vs MM-3 BCMA-naive pts) were older (median [range], 72.0 years [50-92] vs 68.0 years [36-89]), more often female (52.8% vs 44.7%) and more likely to have ECOG PS (Eastern Cooperative Oncology Group Performance Status) score ≥2 (38.5% vs 5.7%) or ISS/R-ISS (Revised-International Staging System) ≥II (80.4% vs 70.7%). Presence of extramedullary disease (28.2% vs 31.7%), bone marrow plasma cells ≥50% (28.8% vs 21.1%), and prior lines of therapy (median [range], 4 [1-15] vs 5 [2-22]) were similar between cohorts. Class exposure rates were similar for triple-class (93.3% vs 100%) or penta-drug exposition(67.4% vs 70.7%) but pts in AMbrELA were less likely to have triple-class (37.1% vs 96.7%) or penta-drug refractoriness (19.1% vs 42.3%); 12.4% vs 0% had prior BCMA-directed therapy.
In AMbrELA, high risk myeloma was defined by the presence of del(17p). Among pts with genetic screening available (n=65/89), 10 had the mutation. In MM-3, it was defined by t(4;14), t(14;16) and del(17p); 25.2% were high risk.
Overall, 71.9% of pts had ≥1 AE; 28.1% had ≥1 serious AE. Common AEs (>5%)/TR-AEs were cytokine release syndrome (36.0%/34.8%), asthenia (6.7%/3.4%), pyrexia (6.7%/4.5%), neutropenia (5.6%/2.2%) and injection site reaction (5.6%/5.6%). Most AEs and TR-AEs were not serious.
A total of 23.6% of pts had ≥1 infection (vs 69.9% in MM-3), 9.0% had ≥1 serious infection, and 10.1% had >1 infection. Median (range) time to onset and resolution was 4.1 mo (0-21) and 0.6 mo (0-3), respectively.
Most pts (84.3%) received ELRA weekly; 29.2% switched to Q2W and 15.7% to Q4W. Median (range) time from 1st dose of ELRA to 1st Q2W dose was 5.3 mo (1-8) and time to 1st Q4W dose was 6.6 mo (1-16). ELRA was administered in an outpatient setting in 73.0% of pts.

**Summary/Conclusion**

Overall, pts in the AMbrELA cohort were older and had a poorer general condition compared with BCMA-naïve pts in the MM-3 trial. The real-world safety profile of ELRA was consistent with that observed in MM-3, except for a lower rate of infections, which may reflect improved management in routine clinical practice in France. The dosing regimen appeared to transition to a monthly schedule more rapidly than recommended.

**Copyright Information:** (Online) ISSN: 2572-9241
©2026 The Author(s). HemaSphere is published by John Wiley & Sons Ltd on behalf of European Hematology Association. This is an open access Abstract Book distributed under the Attribution-NonCommercial-NoDerivs (CC BY-NC-ND), which allows third parties to download the articles and share them with others as long as they credit the author and the Abstract Book, but they cannot change the content in any way or use them commercially.
**Abstract Book Citations:** Authors, Title, HemaSphere, 2026;10:(S1):pages. The abstract book DOIs can be found on the “Author Information” page.
**Disclaimer:** Articles published in the journal HemaSphere exclusively reflect the opinions of the authors. The authors are responsible for all content in their abstracts including accuracy of the facts, statements, citing resources, etc.

### (PF805) RISK FACTORS FOR VENOUS THROMBOEMBOLISM IN 8,672 MYELOMA PATIENTS FROM THE SWEDISH MYELOMA REGISTRY

**Topic:** 14. Myeloma and other monoclonal gammopathies - Clinical

Mariana Villegas- Scivetti*[1, 2], Ingigerdur Sverrisdottir[1, 2], Sigrun Einarsdottir[1, 2], Gunnar Larfors[3], Karin Larsson[3], Moshtaak Karma[4], Cecilie Hveding Blimark[1, 2]

*[1]Sahlgrenska University Hospital, Dep. of Hematology and Coagulation, Gothenburg, Sweden;[2]Sahlgrenska Academy, Gothenburg University, Department of Medicine, Gothenburg, Sweden; [3]Uppsala University Hospital, Uppsala, Sweden, Uppsala, Sweden; [4]Södra Älvsborg Hospital,, Borås,, Sweden;*

### Background

Venous thromboembolism (VTE) risk in patients with multiple myeloma (MM) is high, especially within six months of diagnosis when immunomodulatory drugs (IMiDs) are used. Despite prophylactic measures, evidence on how comorbidities, patient characteristics, and treatment influence VTE risk during the IMiDs era is limited.

### Aims

To identify risk factors for VTE and estimate cumulative incidence among newly diagnosed MM patients

### Methods

All patients with newly diagnosed MM recorded in the Swedish Myeloma Registry (2008–2021) were included and linked to national health registries for data on first VTE, clinical characteristics, and 34 comorbidities. Follow-up ended at death or 1 January 2023. Cumulative incidence was estimated with death as a competing risk. Cox regression adjusted for age, sex, and diagnosis year identified independent predictors, reported as hazard ratios (HR) with 95% confidence intervals (CI)

### Results

We included 8,672 NDMM patients, 62% were men, median age: 72 yrs (range 20-101) at diagnosis. Cumulative incidence of VTE was 6% (95% CI 6.0–7.2) at 6 months and 10.1% (95% CI 9.3–10.9) at 12 months. The distribution of VTE across three time periods showed significantly fewer events in the later period (2018- 2021).

We found 1,005 patients with first VTE after MM diagnosis; 62% were men, mean age: 69.2 (±10.1) vs 71.8 (±11.2) yrs for non-VTE patients ($p<0.001$). A univariate analysis identified obesity, thrombophilia, previous VTE, treatment with autologous stem cell transplant (ASCT), male sex, and younger age as risk factors. Comorbidities such as previous cancer, atrial fibrillation, diabetes mellitus, valvular disease, heart failure, ischemic heart disease, chronic kidney disease, hypertension, and dementia were significant factors for not having VTE. VTE patients had higher haemoglobin levels ($p < 0.001$) and ISS stage II disease ($p=0.016$). Calcium and creatinine levels, and bone disease were not significant factors.

In the multivariable Cox regression (Table) obesity, chronic lung disease, thrombophilia, ASCT, and previous VTE all increased the risk of VTE diagnosis. In contrast valvular, disease decreased the risk of VTE. When age groups were examined separately, the risk decreased with age. VTE incidence was comparabel across comorbidity groups, but higher in the patients with none or 1 comorbidity after one year (Figure).

**Copyright Information:** (Online) ISSN: 2572-9241
©2026 The Author(s). HemaSphere is published by John Wiley & Sons Ltd on behalf of European Hematology Association. This is an open access Abstract Book distributed under the Attribution-NonCommercial-NoDerivs (CC BY-NC-ND), which allows third parties to download the articles and share them with others as long as they credit the author and the Abstract Book, but they cannot change the content in any way or use them commercially.
**Abstract Book Citations:** Authors, Title, HemaSphere, 2026;10:(S1):pages. The abstract book DOIs can be found on the "Author Information" page.
**Disclaimer:** Articles published in the journal HemaSphere exclusively reflect the opinions of the authors. The authors are responsible for all content in their abstracts including accuracy of the facts, statements, citing resources, etc.

| Characteristic | HR | 95%CI | p-value |
|---|---|---|---|
| Age groups* | | | |
| <65 | Ref. | Ref. | |
| 65-79 | 0.78 | 0.62-0.99 | 0.040 |
| >80 | 0.75 | 0.50-1.12 | 0.2 |
| Obesity** | 2.90 | 1.39-3.14 | <0.001 |
| Valvular disease** | 0.57 | 0.35-0.91 | 0.017 |
| Chronic lung disease** | 1.92 | 1.29-2.86 | 0.001 |
| Thrombophilia** | 3.50 | 1.74-7.03 | <0.001 |
| Prior Venous thrombosis** | 45.7 | 38.0-54.9 | <0.001 |
| Autolog stem cell stransplant** | 1.24 | 1.03, 1.49 | 0.024 |

Table. Risk factors of venous thromboembolism after multiple myeloma diagnosis. Abbreviations: HR: Hazard ratio, 96%CI: Confidence interval. *Adjusted for year of diagnosis and sex. **Adjusted for year of diagnosis, sex and age

### Summary/Conclusion

In this large population study with 1,005 patients with VTE, the incidence reached 6% at six months, with a lower incidence in the latest time period. Independent predictors included obesity, chronic lung disease, thrombophilia, prior VTE, and ASCT, while valvular disease was protective. Younger patients were at the highest risk, suggesting thromboprophylaxis may be suboptimal in this group. Further analysis of the association between different myeloma treatments, comorbidities and thromboprophylaxis is needed to identify preventive factors for VTE.

**Copyright Information:** (Online) ISSN: 2572-9241
©2026 The Author(s). HemaSphere is published by John Wiley & Sons Ltd on behalf of European Hematology Association. This is an open access Abstract Book distributed under the Attribution-NonCommercial-NoDerivs (CC BY-NC-ND), which allows third parties to download the articles and share them with others as long as they credit the author and the Abstract Book, but they cannot change the content in any way or use them commercially.
**Abstract Book Citations:** Authors, Title, HemaSphere, 2026;10:(S1):pages. The abstract book DOIs can be found on the "Author Information" page.
**Disclaimer:** Articles published in the journal HemaSphere exclusively reflect the opinions of the authors. The authors are responsible for all content in their abstracts including accuracy of the facts, statements, citing resources, etc.

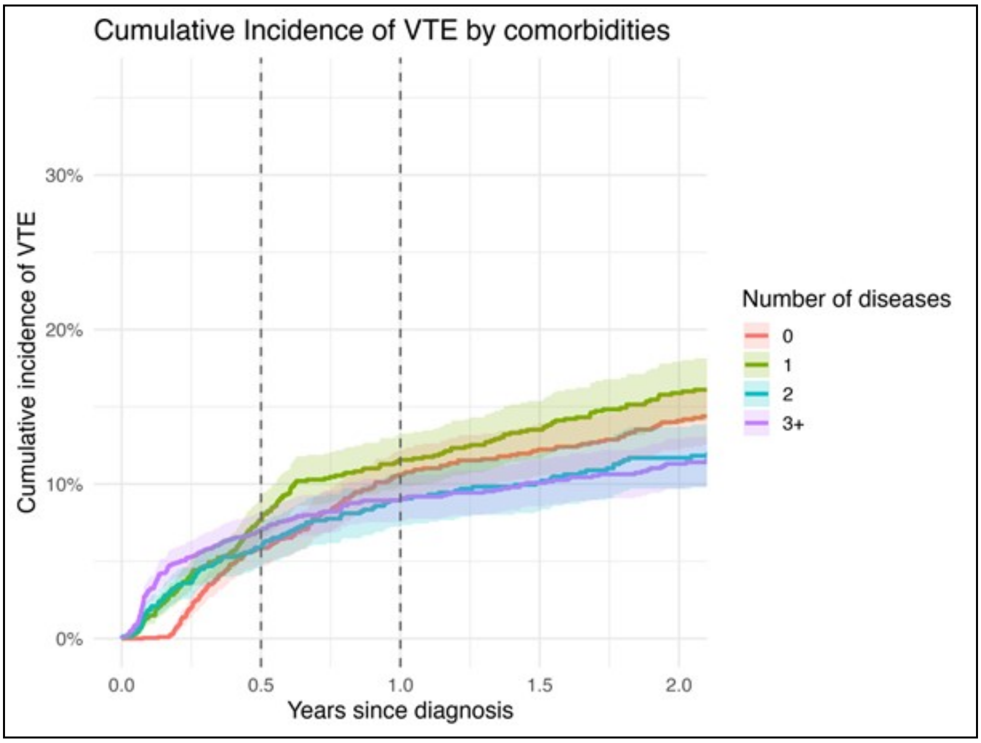

Cumulative incidence of VTE by comorbidities

**Copyright Information:** (Online) ISSN: 2572-9241
©2026 The Author(s). HemaSphere is published by John Wiley & Sons Ltd on behalf of European Hematology Association. This is an open access Abstract Book distributed under the Attribution-NonCommercial-NoDerivs (CC BY-NC-ND), which allows third parties to download the articles and share them with others as long as they credit the author and the Abstract Book, but they cannot change the content in any way or use them commercially.
**Abstract Book Citations:** Authors, Title, HemaSphere, 2026;10:(S1):pages. The abstract book DOIs can be found on the "Author Information" page.
**Disclaimer:** Articles published in the journal HemaSphere exclusively reflect the opinions of the authors. The authors are responsible for all content in their abstracts including accuracy of the facts, statements, citing resources, etc.

### (PF806) THE ROLE OF WHOLE-BODY STIR MRI IN ASSESSING FIRST BIOCHEMICAL RELAPSE IN MULTIPLE MYELOMA AND INFORMING THE INITIATION OF SECOND-LINE THERAPY

**Topic:** 14. Myeloma and other monoclonal gammopathies - Clinical

Tania Kreuzbauer*[1], James Rowland[1, 2, 3], Manil Chouhan[2, 4], Amanda Goh[1, 2, 5], Peter Mollee[1, 2, 3]

*[1]Princess Alexandra Hospital, Department of Haematology, Brisbane, Australia;[2]The University of Queensland, Faculty of Medicine, Brisbane, Australia; [3]Queensland Amyloidosis Centre, Brisbane, Australia; [4]Princess Alexandra Hospital, Department of Diagnostic Radiology, Brisbane, Australia; [5]Mater Pathology, Brisbane, Australia;*

### Background

Multiple myeloma remains incurable, and most patients will relapse. Optimal timing of salvage therapy is important, and patients are commonly retreated at biochemical progression. Whole-body STIR MRI with diffusion weighted imaging (DWI) can detect bone involvement before cortical destruction and may guide treatment initiation in early asymptomatic biochemical progression.

### Aims

To evaluate the role of STIR MRI DWI in guiding second-line therapy initiation in otherwise asymptomatic first biochemical relapse of myeloma.

### Methods

We conducted a single-center retrospective audit of patients with myeloma diagnosed from April 2012 to August 2025 at first relapse from ≥PR to first-line therapy. The preferred restaging imaging modality was whole-body STIR MRI with DWI and dedicated spinal sequences. IMWG response and relapse criteria was used, with monthly serum protein electrophoresis and light chain assessment, excluding regular urine BJP. Rapid biochemical relapse was defined per Sonneveld (ASH Education Book 2017). Second-line therapy initiation at relapse was at the treating haematologist's discretion.

### Results

340 patients with newly diagnosed symptomatic myeloma who achieved ≥PR to first-line therapy were identified. Median age at diagnosis was 66 (range 31-89) and 60% were male. ISS stage was I (18%), II (60%), III (22%), and 29% had high-risk cytogenetics defined by t(4:14), t(14:16), or del(17p) by FISH. Bortezomib-based induction was most common, and 43% received autologous stem cell transplantation. Best IMWG response was CR (43%), VGPR (27%), and PR (30%).
162 patients (48%) have relapsed at a median of 22 months. At IMWG defined progressive disease (PD), 54% had clear treatment indications: meeting CRAB criteria exclusive of MRI findings (19%), high-risk FISH at diagnosis (17%), rapid biochemical PD (10%), or renal failure at diagnosis (8%), leaving 75 patients (46%) in slow biochemical PD without urgent indications for salvage. MRI was performed in 46 of these patients at a median of 44 days from biochemical PD (range: -43 to 468 days): 25 (54%) demonstrated progressive focal bone lesions of whom all had focal lesions at diagnosis, 16 showed stable/treated lesions and 5 had no lesions.
Thus, 21 patients had slow biochemical PD without MRI proven bone progression or other clear indications for salvage. 20 received second-line therapy (median time to treatment 111 days), of which 5 commenced immediately at clinician discretion. 15 patients were initially observed, until 6 (40%) subsequently developed clinically significant symptoms; 5 experienced progressive bone disease (range: 6-16 months), and 1 developed renal failure after 38 days. 9 were retreated without clinical progression after a median of 133 days. 1 was never retreated, with MRI 3 years later showing no further lesions.

**Copyright Information:** (Online) ISSN: 2572-9241
©2026 The Author(s). HemaSphere is published by John Wiley & Sons Ltd on behalf of European Hematology Association. This is an open access Abstract Book distributed under the Attribution-NonCommercial-NoDerivs (CC BY-NC-ND), which allows third parties to download the articles and share them with others as long as they credit the author and the Abstract Book, but they cannot change the content in any way or use them commercially.
**Abstract Book Citations:** Authors, Title, HemaSphere, 2026;10:(S1):pages. The abstract book DOIs can be found on the "Author Information" page.
**Disclaimer:** Articles published in the journal HemaSphere exclusively reflect the opinions of the authors. The authors are responsible for all content in their abstracts including accuracy of the facts, statements, citing resources, etc.

**Summary/Conclusion**

Whole-body MRI identified progressive focal bone lesions in 54% of patients with slow biochemical first relapse and no other indications to treat. 40% of those without new MRI detected lesions subsequently developed new bone disease predominantly within 1 year of observation. This high incidence of bone progression at, or shortly after biochemical PD suggests most patients require therapy at this point. While a small cohort may safely be observed whilst planning second-line therapy, surveillance imaging with STIR MRI DWI at short intervals (e.g. 6 months) is needed to identify asymptomatic bone relapse, enabling early intervention to prevent morbidity from destructive bone disease.

**Copyright Information:** (Online) ISSN: 2572-9241
©2026 The Author(s). HemaSphere is published by John Wiley & Sons Ltd on behalf of European Hematology Association. This is an open access Abstract Book distributed under the Attribution-NonCommercial-NoDerivs (CC BY-NC-ND), which allows third parties to download the articles and share them with others as long as they credit the author and the Abstract Book, but they cannot change the content in any way or use them commercially.
**Abstract Book Citations:** Authors, Title, HemaSphere, 2026;10:(S1):pages. The abstract book DOIs can be found on the "Author Information" page.
**Disclaimer:** Articles published in the journal HemaSphere exclusively reflect the opinions of the authors. The authors are responsible for all content in their abstracts including accuracy of the facts, statements, citing resources, etc.

### (PF807) OVERALL SURVIVAL AND TIME TO NEXT TREATMENT IN GERMAN POST-LEN RRMM PATIENTS: A REAL-WORLD PERSPECTIVE

**Topic:** 14. Myeloma and other monoclonal gammopathies - Clinical

Florian Weissinger*[1], Kai Strobel[2], Katie Gravagna[3], Amol Dhamane[3], Amir Samuel[4], Stefan Schilling[2], Markus Rückert[2]

*[1]Evangelische Klinikum Bethel, Bielefeld, Germany; [2]TriNetX, Freiburg, Germany; [3]Bristol Myers Squibb, Lawrenceville, United States of America; [4]Bristol Myers Squibb, Boudry, Switzerland;*

### Background

Lenalidomide (LEN) has long been a foundational therapy in multiple myeloma (MM). It is frequently used in combination with other agents, including proteasome inhibitors and anti-CD38 monoclonal antibodies, in relapsed and refractory (RR) MM patients. The widespread use of LEN-containing regimens has resulted in a substantial proportion of RRMM patients having early LEN exposure, which poses clinical challenges in the subsequent sequencing of treatments.

### Aims

Our study objective was to characterize and assess the outcomes of a predominantly LEN-exposed RRMM population using real-world data.

### Methods

A retrospective, multicenter, survey-based cohort study was conducted across Germany as part of the TherapyMonitor Multiple Myeloma research project (N=9,477). Characteristics, treatment patterns, and outcomes for patients with a newly initiated 2nd-4th line of therapy [LOT] between 01/01/2019 and 31/12/2024 were assessed. Patient cohorts were mutually inclusive. We reported descriptive statistics for baseline patient characteristics and treatment patterns. Kaplan-Meier curves were created for time to next treatment (TTNT) and overall survival (OS) outcomes. TTNT was defined as time from index to death, start of new LOT, or censor. OS was defined as time from index to death or censor.

### Results

3,552 RRMM LEN-exposed RRMM patients who had initiated 2L therapy were included. LEN-R (refractory) rates increased with later lines of therapy: 40.2% in 2L, 78.5% in 3L, and 92.8% in 4L. Patient characteristics were consistent across LOTs for post-LEN RRMM patients, although the percentage classified as ISS III increased notably after 2L. The median age was 72 years across LOTs, and about 46% of patients were classified as having limited fitness. Only 13.6% received a transplant in 1L. Prior exposure to anti-CD38 treatments increased with increasing LOT: 12.0% in 2L, 40.1% in 3L, and 64.2% in 4L. The most common treatments received by post-LEN patients varied by LOT with no single SoC, but pomalidomide- and daratumumab-containing regimens were frequent in 3L and 4L.
OS among both LEN-exposed and LEN-R patients decreased with advancing lines of therapy. In 2L LEN-R, OS was 61.1 (95% CI: 53.8, not reached (NR)) months, compared to 39.1 (95% CI: 36.6, 42.1) for 3L LEN-R and 25.7 (95% CI: 24.3, 27.2) for 4L LEN-R. LEN-exposed patients had numerically larger OS per LOT compared to LEN-R. TTNT in LEN-R patients was 17.2 months (95% CI: 15.9, 19.2) in 2L, 14.1 (95% CI: 13.7, 14.8) in 3L, and 12.2 (95% CI: 12.0, 12.8) in 4L (**Fig. 1**) versus 18.4 months (95% CI: 17.4, 19.3) in 2L, 14.4 (95% CI: 13.8, 15.0) in 3L, and 12.2 (95% CI: 12.0, 12.7) in 4L for LEN-E.

**Copyright Information:** (Online) ISSN: 2572-9241
©2026 The Author(s). HemaSphere is published by John Wiley & Sons Ltd on behalf of European Hematology Association. This is an open access Abstract Book distributed under the Attribution-NonCommercial-NoDerivs (CC BY-NC-ND), which allows third parties to download the articles and share them with others as long as they credit the author and the Abstract Book, but they cannot change the content in any way or use them commercially.
**Abstract Book Citations:** Authors, Title, HemaSphere, 2026;10:(S1):pages. The abstract book DOIs can be found on the “Author Information” page.
**Disclaimer:** Articles published in the journal HemaSphere exclusively reflect the opinions of the authors. The authors are responsible for all content in their abstracts including accuracy of the facts, statements, citing resources, etc.

### Summary/Conclusion

The OS and TTNT outcomes of LEN-exposed RRMM patients are still suboptimal across 2L-4L, especially for patients in later lines or those who are LEN-refractory. Given the large percentage of LEN-refractory patients in this population, especially as lines advance, this study highlights the opportunity for more effective treatments in the LEN-exposed RRMM patient populations to help address this unmet need.

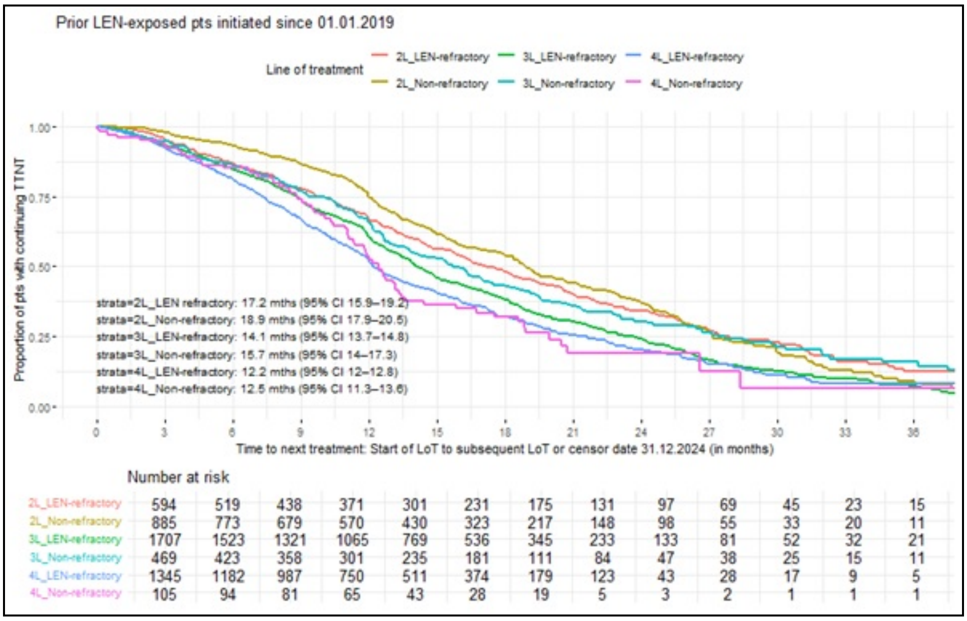

Figure 1. Time to next treatment by LEN-refractory status for each LOT

**Copyright Information:** (Online) ISSN: 2572-9241
©2026 The Author(s). HemaSphere is published by John Wiley & Sons Ltd on behalf of European Hematology Association. This is an open access Abstract Book distributed under the Attribution-NonCommercial-NoDerivs (CC BY-NC-ND), which allows third parties to download the articles and share them with others as long as they credit the author and the Abstract Book, but they cannot change the content in any way or use them commercially.
**Abstract Book Citations:** Authors, Title, HemaSphere, 2026;10:(S1):pages. The abstract book DOIs can be found on the "Author Information" page.
**Disclaimer:** Articles published in the journal HemaSphere exclusively reflect the opinions of the authors. The authors are responsible for all content in their abstracts including accuracy of the facts, statements, citing resources, etc.

### (PF808) THE RELATIONSHIP BETWEEN MINIMUM RESIDUAL DISEASE NEGATIVE COMPLETE RESPONSE RATE AND PROGRESSION-FREE SURVIVAL TREATMENT EFFECTS IN RELAPSED REFRACTORY MULTIPLE MYELOMA: A NOVEL PERSPECTIVE

**Topic:** 14. Myeloma and other monoclonal gammopathies - Clinical

C. Ola Landgren*[1], Harlan Campbell[2], Kevin Towle[2], Jenny Chen[2], Ying Qui[3], Paulo Maclag[3], Gabriela Hernandez Rivera[4], Yu-Hsuan Shih[3], Shannon Cope[2]

*[1]University of Miami, Miami, United States of America;[2]Precision AQ, Vancouver, Canada; [3]Bristol Myers Squibb, Princeton, United States of America; [4]Bristol Myers Squibb, Boudry, Switzerland;*

### Background

Minimum residual disease complete response (MRDneg CR) is a sensitive marker for depth of response in multiple myeloma (MM) and recent Food and Drug Administration (FDA) guidance (January 2026) concluded that MRDneg CR may support accelerated approvals when appropriately justified. To date, no new therapies have been approved based solely on MRDneg CR as a primary endpoint. MRDneg CR is prognostic and pooled analyses have demonstrated an association between MRDneg CR and progression-free survival (PFS) in MM. Accumulating clinical data may further support MRDneg CR as an intermediate endpoint, particularly in earlier lines of therapy in relapsed/refractory multiple myeloma (RRMM). Additionally, the inconsistent reporting of MRDneg CR in randomized controlled trials (RCTs) results in a fragmented evidence base of multiple disconnected networks, precluding standard network meta-analysis (NMA) based on MRDneg CR alone.

### Aims

The first objective of this study was to evaluate the trial-level association between MRDneg CR and PFS within earlier lines of therapy in RRMM. The second objective was to leverage the association between MRDneg CR and PFS to facilitate indirect treatment comparisons (ITCs) across multiple interventions, incorporating studies reporting PFS only to connect the evidence network.

### Methods

A systematic literature review was conducted to identify RCTs reporting MRDneg CR at any time ($10^{-5}$ threshold) and PFS evaluating treatments for RRMM patients in 2-4L setting with who were not required to be refractory to any specific class/agent. For the first objective, a weighted least squares (WLS) model was fit based on reported log-odds ratio for MRDneg CR at any time and log-hazard ratio for PFS. Additional analyses were performed using surrogacy models recommended for health technology assessments. For the second objective, relative treatment effects for interventions across the network were estimated using two bivariate NMA models.

### Results

Eleven RCTs reported treatment effects on MRDneg at any time and PFS for the target population, which provided evidence of a strong association between MRDneg CR and PFS. The WLS model estimated an R-squared of 0.73 (95% confidence interval [CI]: 0.27, 0.92). The relationship between MRDneg CR and PFS enabled ITCs across all interventions of interest with respect to MRDneg CR (including interventions for which no MRD data were reported) and additionally provided more precise estimates with respect to PFS (compared to models considering PFS alone). Finally, for new interventions, the bivariate NMA models allowed for predictions of PFS benefits relative to existing regimens based on early MRDneg CR results.

**Copyright Information:** (Online) ISSN: 2572-9241
©2026 The Author(s). HemaSphere is published by John Wiley & Sons Ltd on behalf of European Hematology Association. This is an open access Abstract Book distributed under the Attribution-NonCommercial-NoDerivs (CC BY-NC-ND), which allows third parties to download the articles and share them with others as long as they credit the author and the Abstract Book, but they cannot change the content in any way or use them commercially.
**Abstract Book Citations:** Authors, Title, HemaSphere, 2026;10:(S1):pages. The abstract book DOIs can be found on the "Author Information" page.
**Disclaimer:** Articles published in the journal HemaSphere exclusively reflect the opinions of the authors. The authors are responsible for all content in their abstracts including accuracy of the facts, statements, citing resources, etc.

**Summary/Conclusion**

There is a strong association between MRDneg CR and PFS within lines of therapy in RRMM which supports MRDneg CR as a potential surrogate endpoint for this population of interest. Furthermore, leveraging the MRDneg CR and PFS surrogacy relationship allows for informative and precise ITCs using bivariate NMA. This approach can support decision-making for accelerated approvals and guideline development.

**Copyright Information:** (Online) ISSN: 2572-9241
©2026 The Author(s). HemaSphere is published by John Wiley & Sons Ltd on behalf of European Hematology Association. This is an open access Abstract Book distributed under the Attribution-NonCommercial-NoDerivs (CC BY-NC-ND), which allows third parties to download the articles and share them with others as long as they credit the author and the Abstract Book, but they cannot change the content in any way or use them commercially.
**Abstract Book Citations:** Authors, Title, HemaSphere, 2026;10:(S1):pages. The abstract book DOIs can be found on the “Author Information” page.
**Disclaimer:** Articles published in the journal HemaSphere exclusively reflect the opinions of the authors. The authors are responsible for all content in their abstracts including accuracy of the facts, statements, citing resources, etc.

### (PF809) PATIENT PREFERENCES FOR ATTRIBUTES OF RELAPSED/REFRACTORY MULTIPLE MYELOMA TREATMENT: FINDINGS FROM A DISCRETE CHOICE EXPERIMENT

**Topic:** 14. Myeloma and other monoclonal gammopathies - Clinical

Thomas W Leblanc*[1], Rakesh Popat[2], Colin Todd Kennedy[3], Josh Coulter[4], Soohyun Hwang[5], Anna Dumas[5], Kathleen Beusterien[5], David Hughes[6], Guido Nador[7], Joseph Cappelleri[8], Aster Meche[4], Richa Shah[4], Marco Dibonaventura[4], Mohsena Sumaya[4], Patrick Hlavacek[4]

*[1]Division of Hematologic Malignancies and Cellular Therapy, Department of Medicine, Duke University School of Medicine, Durham, United States of America; [2]Hematology, University College London Hospitals NHS Foundation Trust, London, United Kingdom; [3]Independent patient and research advocate, Coto de Caza, United States of America; [4]Pfizer Inc, New York, United States of America; [5]Oracle Life Sciences, Austin, United States of America; [6]Pfizer Inc, Cambridge, United States of America; [7]Pfizer Ltd., Surrey, United Kingdom; [8]Pfizer Inc, Groton, United States of America;*

### Background

The treatment landscape for relapsed or refractory multiple myeloma (RRMM) has evolved with novel immunotherapies, including bispecific antibodies (BsAbs). As options increase, incorporating patient preferences into treatment decisions remains critical given the trade-offs in efficacy, safety, and treatment burden. While patients with RRMM value attributes beyond clinical benefit, including mode of administration, side effects, and ability to maintain quality of life [Hydren 2024; Syeed 2025; Fifer 2025], data on how they prioritize attributes of currently available BsAb treatments remain limited.

### Aims

To quantify preferences for BsAb treatment attributes among patients with RRMM.

### Methods

We conducted an online, cross-sectional survey including a discrete choice experiment among adults with self-reported RRMM in the United States and Europe from September to December 2025. Attributes and levels were identified through literature review and qualitative interviews (Fig 1). Preference weights for attribute levels were estimated via a hierarchical Bayes model of responses to a series of tasks involving choices between treatment profiles representing different combinations of attribute levels. Attribute relative importance (RI) was computed at the respondent level for each attribute as the difference between preference weights for the most versus least preferred levels, divided by the sum of the ranges of all attributes and then multiplying this ratio by 100. The attribute RIs, which range from 0-100% and sum to 100%, were averaged across respondents with 95% confidence intervals computed; higher RI values indicate stronger preference.

**Copyright Information:** (Online) ISSN: 2572-9241
©2026 The Author(s). HemaSphere is published by John Wiley & Sons Ltd on behalf of European Hematology Association. This is an open access Abstract Book distributed under the Attribution-NonCommercial-NoDerivs (CC BY-NC-ND), which allows third parties to download the articles and share them with others as long as they credit the author and the Abstract Book, but they cannot change the content in any way or use them commercially.
**Abstract Book Citations:** Authors, Title, HemaSphere, 2026;10:(S1):pages. The abstract book DOIs can be found on the "Author Information" page.
**Disclaimer:** Articles published in the journal HemaSphere exclusively reflect the opinions of the authors. The authors are responsible for all content in their abstracts including accuracy of the facts, statements, citing resources, etc.

### Results

Data from 290 patients were analyzed (mean age: 60.0 years; female: 47.6%). Most identified as White/Caucasian (67.3%) and had attained a university degree or higher (65.3%). Most patients were pretreated, with 26.2% having received three prior lines of therapy (LOTs) and 16.6% with four or more. For all naturally ordered attributes, unambiguously better levels were preferred to worse levels.n average, hospitalization at treatment initiation (from 12 consecutive days to no hospitalization) was the most important attribute (RI=22.8%), followed by improvements in 2-year overall survival from 50% to 70% (RI=18.7%) and duration of response from 9 to 48 months (RI=16.8%) (Fig 1). The next most important attributes were dosing schedule (RI=10.3%) and reducing skin-related side effects from 73% to 0% (RI=9.7%). The least important attributes were reducing risk of serious infections from 55% to 17% (RI=8.7%), followed by short-term inflammation in the brain from 9% to 3% (RI=5.3%). Consistent with these preference estimates, patients would be willing to accept a nearly 10% decrease in 2-year overall survival (e.g., from 70 to 60%) (change in RI=9.3%) to avoid 5 days in the hospital at treatment initiation (e.g., reducing hospital stays from 12 to 7) (change in RI=9.5%), holding all else constant.

### Summary/Conclusion

Hospitalization at treatment initiation was most important attribute to patients, followed by efficacy outcomes, while safety attributes were less influential. Patients placed high value on minimizing prolonged inpatient stays, highlighting the importance of treatment approaches and care-delivery settings that reduce early hospitalization and disruption to daily life. In later LOTs, this study characterizes how patients prioritize attributes of BsAbs beyond efficacy and may help inform discussions around treatment decisions.

**Figure 1. Mean Relative Importance of Treatment Attributes in the Discrete Choice Experiment (Overall Sample)**

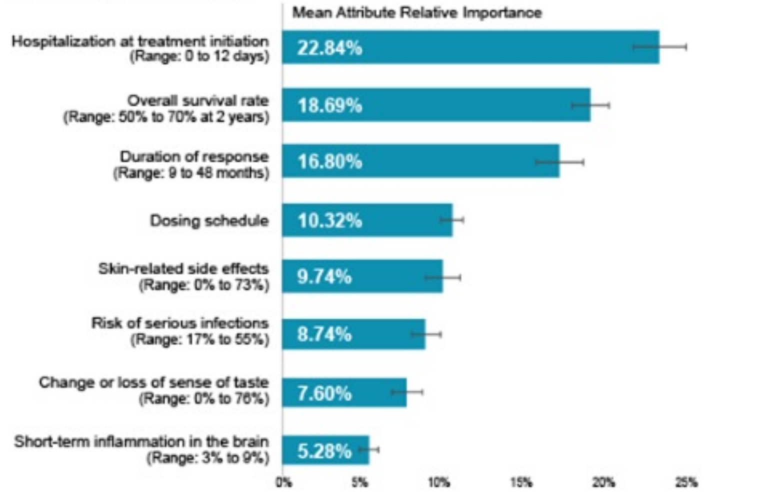

**Note.** Error bars represent 95% confidence intervals. Attribute relative importance estimates sum to 100% across attributes. Hospitalization required at treatment initiation was defined as no hospitalization or hospitalization for 4 days within a 9-day period, or for 5, 9, or 12 consecutive days. Dosing schedule included subcutaneous injection once weekly, once every 2 weeks, or once weekly for 24 weeks followed by dosing every 2 or 4 weeks, as well as intravenous infusion once weekly for 13 weeks followed by dosing every 2 weeks or once weekly for 13 weeks, followed by dosing every 2 weeks for 10 weeks and then every 4 weeks. Overall survival rate was defined as 50, 60, or 70 out of 100 patients alive at least 2 years after treatment initiation. Duration of response was defined as response lasting 9, 19, 29, or 48 months among responders. Short-term inflammation in the brain was defined as occurring in 3, 5, or 9 out of 100 patients, with symptoms including headache, confusion, neurologic impairment, seizures, or drowsiness, lasting an average of 2 days. Change or loss of sense of taste was defined as occurring in 0, 37, or 76 out of 100 patients, lasting an average of 43 days or longer. Risk of serious infections (e.g., lung infections, shingles, tuberculosis) was defined as occurring in 17, 29, 42, or 55 out of 100 patients and requiring medical intervention or hospitalization. Skin-related side effects were defined as occurring in 0, 25, or 73 out of 100 patients and included rash, dry skin, or scaly skin.

**Copyright Information:** (Online) ISSN: 2572-9241
©2026 The Author(s). HemaSphere is published by John Wiley & Sons Ltd on behalf of European Hematology Association. This is an open access Abstract Book distributed under the Attribution-NonCommercial-NoDerivs (CC BY-NC-ND), which allows third parties to download the articles and share them with others as long as they credit the author and the Abstract Book, but they cannot change the content in any way or use them commercially.
**Abstract Book Citations:** Authors, Title, HemaSphere, 2026;10:(S1):pages. The abstract book DOIs can be found on the “Author Information” page.
**Disclaimer:** Articles published in the journal HemaSphere exclusively reflect the opinions of the authors. The authors are responsible for all content in their abstracts including accuracy of the facts, statements, citing resources, etc.

### (PF810) CERTIFIED MEDICAL EDUCATION LEADS TO CLAIMS CONFIRMED CHANGES IN CLINICAL PRACTICE REGARDING MANAGEMENT OF MULTIPLE MYELOMA

**Topic:** 14. Myeloma and other monoclonal gammopathies - Clinical

Sarah Elizabeth Bomba*[1], Andrew Small[1], Megan Whitney[1], Katie Lucero[1]

[1]*Medscape Education, New York, United States of America;*

### Background

Multiple myeloma (MM) is a hematologic malignancy requiring evidence-based treatment strategies across the disease continuum. Despite availability of anti-CD38 monoclonal antibody-based regimens for newly diagnosed MM (NDMM) and novel cellular therapies (bispecific antibodies, CAR T-cell therapies) for relapsed/refractory MM (RRMM), these treatments remain underutilized, particularly in community practice settings. Key barriers include limited knowledge of practice guidelines, insufficient awareness of clinical trial data, challenges in identifying appropriate patients, and difficulty selecting evidence-based treatments.

### Aims

To determine if participation in a curriculum-based CME initiative increased the evidence-directed use of anti-CD38 monoclonal antibody-based regimens in NDMM and novel cellular therapies (bispecific antibodies, CAR T-cell therapies) in RRMM among hematologist/oncologists treating patients with multiple myeloma.

### Methods

A curriculum of 4 self-directed online CME activities was offered between December 2024 and March 2025. This real-world outcome study (RWO) was designed to assess the impact of the CME activities on the number of patients receiving anti-CD38 regimens, number of patients receiving bispecific antibodies, and number of patients referred to CAR T centers, measured 3 months post-participation. A retrospective matched case-control study was conducted using medical and pharmacy claims aggregated with patients and the coding clinicians between December 2024 through June 2025. Hematologist/oncologist learners who saw ≥1 patient with multiple myeloma in the 6 months prior to participation were matched 1:1 with non-learner controls based on exposed month, specialty, geography (state and zip code), and volume of multiple myeloma patients. Data were pulled for 3 months pre and 3 months-post first CME activity participation date ("index date"). Independent samples t-tests assessed statistical significance.

### Results

A total of 20,662 patients with MM were seen by clinicians in the CME cohort during the evaluation period. The majority of learners (72%) practiced in community settings, representing 69% of MM patients treated.
9%* more patients with MM received anti-CD38 regimens in the CME group (n=3,994) compared to the Control group (n=3,675), a difference of 319 additional patients.
54%* more patients with MM received bispecific antibodies in the CME group (n=300) versus the Control group (n=195), a difference of 105 additional patients.
10%* more patients with MM were referred to CAR T centers in the CME group (n=8,550) compared to the Control group (n=7,785), a difference of 765 additional patients.
Supporting these practice changes, learners demonstrated a 42% improvement in knowledge related to clinical trial data for R/R MM treatment (48% to 68% correct), a 6% increase for NDMM treatment (87% to 92% correct), and 65% more learners reported confidence in applying clinical evidence (20% to 33%).

**Copyright Information:** (Online) ISSN: 2572-9241
©2026 The Author(s). HemaSphere is published by John Wiley & Sons Ltd on behalf of European Hematology Association. This is an open access Abstract Book distributed under the Attribution-NonCommercial-NoDerivs (CC BY-NC-ND), which allows third parties to download the articles and share them with others as long as they credit the author and the Abstract Book, but they cannot change the content in any way or use them commercially.
**Abstract Book Citations:** Authors, Title, HemaSphere, 2026;10:(S1):pages. The abstract book DOIs can be found on the "Author Information" page.
**Disclaimer:** Articles published in the journal HemaSphere exclusively reflect the opinions of the authors. The authors are responsible for all content in their abstracts including accuracy of the facts, statements, citing resources, etc.

**Summary/Conclusion**

This 3-month interim analysis demonstrates that targeted medical education effectively translated knowledge improvements into measurable increases in evidence-based utilization of anti-CD38 monoclonal antibodies and utilization of novel cellular therapies for multiple myeloma. The particularly strong impact on bispecific antibody use (+54%) suggests education successfully addressed implementation barriers. Six-month follow-up data, anticipated in February 2026, will determine if these practice improvements are sustained over time.

**Copyright Information:** (Online) ISSN: 2572-9241
©2026 The Author(s). HemaSphere is published by John Wiley & Sons Ltd on behalf of European Hematology Association. This is an open access Abstract Book distributed under the Attribution-NonCommercial-NoDerivs (CC BY-NC-ND), which allows third parties to download the articles and share them with others as long as they credit the author and the Abstract Book, but they cannot change the content in any way or use them commercially.
**Abstract Book Citations:** Authors, Title, HemaSphere, 2026;10:(S1):pages. The abstract book DOIs can be found on the "Author Information" page.
**Disclaimer:** Articles published in the journal HemaSphere exclusively reflect the opinions of the authors. The authors are responsible for all content in their abstracts including accuracy of the facts, statements, citing resources, etc.

### (PF811) REAL-WORLD OUTCOMES OF TECLISTAMAB IN HEAVILY PRETREATED RELAPSED/REFRACTORY MULTIPLE MYELOMA WITH TRUE EXTRAMEDULLARY DISEASE: RESULTS FROM THE DANISH ABCD STUDY

**Topic:** 14. Myeloma and other monoclonal gammopathies - Clinical

Astrid Hundebøll Torpe*[1, 2], Jonathan Thorsen[2, 3, 4], Katrine Fladeland Iversen[3], Sarah Leeth Farmer[3], Thomas Lund[3, 5], Charlotte Toftmann Hansen[6], Lene Kongsgaard Nielsen[6, 7, 8, 9], Nicolai Grønne Dahlager Jørgensen[10], Maja Ølholm Vase[11], Elena Manuela Teodorescu[12], Christina Wennerström[13], Morten Salomo[13], Agoston Szabo[1, 2, 3]

*[1]Department of Hematology, Copenhagen University Hospital, Rigshospitalet, Copenhagen, Denmark;[2]Dept. of Clinical Medicine, University of Copenhagen, Copenhagen, Denmark; [3]Dept. of Hematology, Vejle Hospital, Vejle, Denmark; [4]COPSAC, Herlev and Gentofte Hospital, University of Copenhagen, Copenhagen Prospective Studies on Asthma in Childhood, Copenhagen, Denmark; [5]Centre for Innovative Medical Technology, Odense University Hospital, Odense, Denmark;[6]Dept. of Hematology, Odense University Hospital, Odense, Denmark; [7]Section of Hematology, Dept. of Internal Medicine, Gødstrup Hospital, Herning, Denmark; [8]Department of Clinical Medicine, Aarhus University, Aarhus, Denmark;[9]Department of Clinical Research, University of Southern Denmark, Odense, Denmark; [10]Dept. of Hematology, Zealand University Hospital Roskilde, Roskilde, Denmark; [11]Dept. of Hematology, Aarhus University Hospital, Skejby, Aarhus, Denmark; [12]Dept. of Hematology, Aalborg University Hospital, Aalborg, Denmark; [13]Janssen-Cilag A/S, a Johnson & Johnson Company, Copenhagen, Denmark;*

### Background

Teclistamab (Tec), the first approved bispecific antibody (BsAbs) has shown clinical benefit for relapsed/refractory multiple myeloma (RRMM). Patients with true extramedullary disease (EMD) represent a difficult to treat high-risk subgroup of RRMM. Previously published real-world (RW) studies about BsAbs have used heterogeneous EMD definitions; outcomes in true, non-osseous EMD are poorly described.

### Aims

The aim of this study was to evaluate outcomes in a nationwide cohort of RRMM patients with true non-osseous EMD who were treated with Tec.

### Methods

This retrospective multicenter study included all consecutive Danish patients receiving Tec from reimbursement (Feb. 2024) until data cut-off (Nov. 2025). Data were collected by patient chart reviews and included demographics, prior lines of therapy (pLOT), overall response rates (ORR), progression-free survival (PFS), duration of response (DOR), overall survival (OS) and dosing patterns.
True EMD was defined as soft-tissue plasmacytoma not contiguous with bone, confirmed by PET-CT, MRI or CT. Paraskeletal plasmacytomas were excluded. The study was funded by Johnson & Johnson.

**Copyright Information:** (Online) ISSN: 2572-9241
©2026 The Author(s). HemaSphere is published by John Wiley & Sons Ltd on behalf of European Hematology Association. This is an open access Abstract Book distributed under the Attribution-NonCommercial-NoDerivs (CC BY-NC-ND), which allows third parties to download the articles and share them with others as long as they credit the author and the Abstract Book, but they cannot change the content in any way or use them commercially.
**Abstract Book Citations:** Authors, Title, HemaSphere, 2026;10:(S1):pages. The abstract book DOIs can be found on the "Author Information" page.
**Disclaimer:** Articles published in the journal HemaSphere exclusively reflect the opinions of the authors. The authors are responsible for all content in their abstracts including accuracy of the facts, statements, citing resources, etc.

**Results**

A total of 105 patients received Tec. Median follow-up was 11 months (IQR 5-14). At Tec initiation, 30 patients had true, non-osseous EMD (EMD group); 75 did not (No-EMD group). The method of detection of true, non-osseous EMD was PET-CT scan in 73%, MRI in 13%, CT scan in 13% of cases.
Patients in the EMD vs No-EMD group were similar in terms of median age at Tec initiation (72 vs 71 years), median age at diagnosis (66 vs 63 years), median pLOTs (4 vs 4), prior autologous transplant (73% vs 71%), triple-class exposure (97% vs 99%), and triple-class refractoriness (80% vs 75%). EMD patients had a shorter duration of time from diagnosis to teclistamab initiation (4 vs 6 years), a higher prevalence of adverse FISH defined as t(4;14), t(14;16), or del(17p) (43% vs. 33%), and a lower presence of measurable disease at Tec initiation (80% vs 88%).
Compared with No-EMD patients, EMD patients had lower ORR (30% vs 73%), higher rates of progressive disease as best response (33% vs 8%), shorter median PFS (3.4 months vs not reached; 12-month PFS 38% vs 64%), and shorter median OS (11.7 months vs not reached; 12-month OS 47% vs 77%).
Median DOR was not reached in either group; 12-month DOR was 86% among responding EMD patients (9/30) and 76% in No-EMD patients.
In this RW-cohort, extended Tec dosing intervals was applied at physicians' discretion, deviating from the label recommendations. EMD patients more frequently remained on weekly dosing (Q1W) than No-EMD patients. At 3 months after Tec initiation, the proportion of patients receiving Q1W dosing was 61% (EMD) vs. 52% (No-EMD); at 6 months 30% vs 23%; and at 9 months 19% vs 5%.

**Summary/Conclusion**

In this nationwide RW study, RRMM patients with true non-osseous EMD have poorer outcomes with Tec monotherapy than No-EMD patients. Primary refractoriness was more frequent and median PFS was shorter compared with No-EMD patients, despite higher treatment intensity in the EMD group.
However, in the group of EMD patients who achieved partial response or better, DOR was long and comparable to No-EMD patients and consistent with results from the MajesTEC-1 trial.
These findings suggest that a durable benefit may be achieved by the patients who respond to Tec. Nevertheless, the majority of true EMD patients continue to face a significant unmet need for more effective therapies to achieve comprehensive disease control.

**Copyright Information:** (Online) ISSN: 2572-9241
©2026 The Author(s). HemaSphere is published by John Wiley & Sons Ltd on behalf of European Hematology Association. This is an open access Abstract Book distributed under the Attribution-NonCommercial-NoDerivs (CC BY-NC-ND), which allows third parties to download the articles and share them with others as long as they credit the author and the Abstract Book, but they cannot change the content in any way or use them commercially.
**Abstract Book Citations:** Authors, Title, HemaSphere, 2026;10:(S1):pages. The abstract book DOIs can be found on the "Author Information" page.
**Disclaimer:** Articles published in the journal HemaSphere exclusively reflect the opinions of the authors. The authors are responsible for all content in their abstracts including accuracy of the facts, statements, citing resources, etc.

### (PF812) MIF/MTOR PATHWAY DRIVES INFLAMMATION IN IMCD WITH ANA POSITIVITY PRONE TO SEVERE DISEASE AND WORSE PROGNOSIS

**Topic:** 14. Myeloma and other monoclonal gammopathies - Clinical

Shengnan Ding*[1], Xuejiao Yin[1], Qiumei Yao[1], Yi Liu[1], Jiaying Ge[1], Choon Kiat Ong[2], Haitao Meng[1], Soon Thye Lim[2], Hongyan Tong[1], Liangshun You[1]

*[1]The First Affiliated Hospital, College of Medicine, Zhejiang University, Hematology, Hangzhou, China;[2]Division of Medical Oncology, National Cancer Centre, Singapore, Singapore;*

### Background

Idiopathic multicentric Castleman disease (iMCD) is a heterogeneous spectrum of rare disorders characterized by lymphadenopathy and systemic inflammation, which can often be accompanied by autoantibodies, such as anti-nuclear antibodies (ANA), and may, on occasion, complicated with autoimmune diseases (AID). It has been reported that iMCD and AID may share certain immune inflammatory mechanisms and cross similarities in the clinical-molecular level, but there lacks more irrefutable and systematic evidence, resulting in misdiagnosis or superior treatment.

### Aims

To investigate the characteristics of iMCD with or without ANA positivity and AID with "iMCD-like" lymphadenopathy pathology by integrating clinical cohort analysis and single-cell RNA sequencing of peripheral blood mononuclear cells.

### Methods

We enrolled a cohort of 92 newly diagnosed iMCD patients and and 20 patients accompanied by autoimmune disease and lymph node pathology suggesting iMCD from 2020 to 2025. Additionally, we employed single-cell RNA sequencing from a cohort of 21 individuals, including 12 iMCD patients with ANA positivity or not, 3 AID with "iMCD-like" lymphadenopathy pathology, as well as 3 healthy donors and 3 paried iMCD patients with ANA positivity after treatment with Siltuximab.

### Results

Patients with ANA positivity and AID tended to exhibit severe diseases, however Patients with ANA positivity showed poorest overall survival (OS). Single-cell transcriptomic profiling unveiled that ANA activates PI3K-Akt-mTOR pathway by elevated Macrophage migration inhibitory factor (MIF) from Plasma cell which interacted with pDC. Decreased IL-6 signaling score of Plasma cell in ANA positivity group suggested that the inflammation was driven by MIF but not IL-6 in peripheral blood of ANA positivity group. Additionally, after the treatment of Siltuximab, MIF still existed to activate the PI3K-Akt-mTOR pathway, and mTOR showed higher expression, which underlined the approval of mTOR inhibitor, such as rapamycin for iMCD after Siltuximab treatment.

### Summary/Conclusion

Our findings identify MIF/mTOR pathway as a driver of immune activation and disease severity in iMCD with ANA positivity, and provide new theoretical basis on mTOR inhibitor treating iMCD patients.

**Copyright Information:** (Online) ISSN: 2572-9241
©2026 The Author(s). HemaSphere is published by John Wiley & Sons Ltd on behalf of European Hematology Association. This is an open access Abstract Book distributed under the Attribution-NonCommercial-NoDerivs (CC BY-NC-ND), which allows third parties to download the articles and share them with others as long as they credit the author and the Abstract Book, but they cannot change the content in any way or use them commercially.
**Abstract Book Citations:** Authors, Title, HemaSphere, 2026;10:(S1):pages. The abstract book DOIs can be found on the "Author Information" page.
**Disclaimer:** Articles published in the journal HemaSphere exclusively reflect the opinions of the authors. The authors are responsible for all content in their abstracts including accuracy of the facts, statements, citing resources, etc.

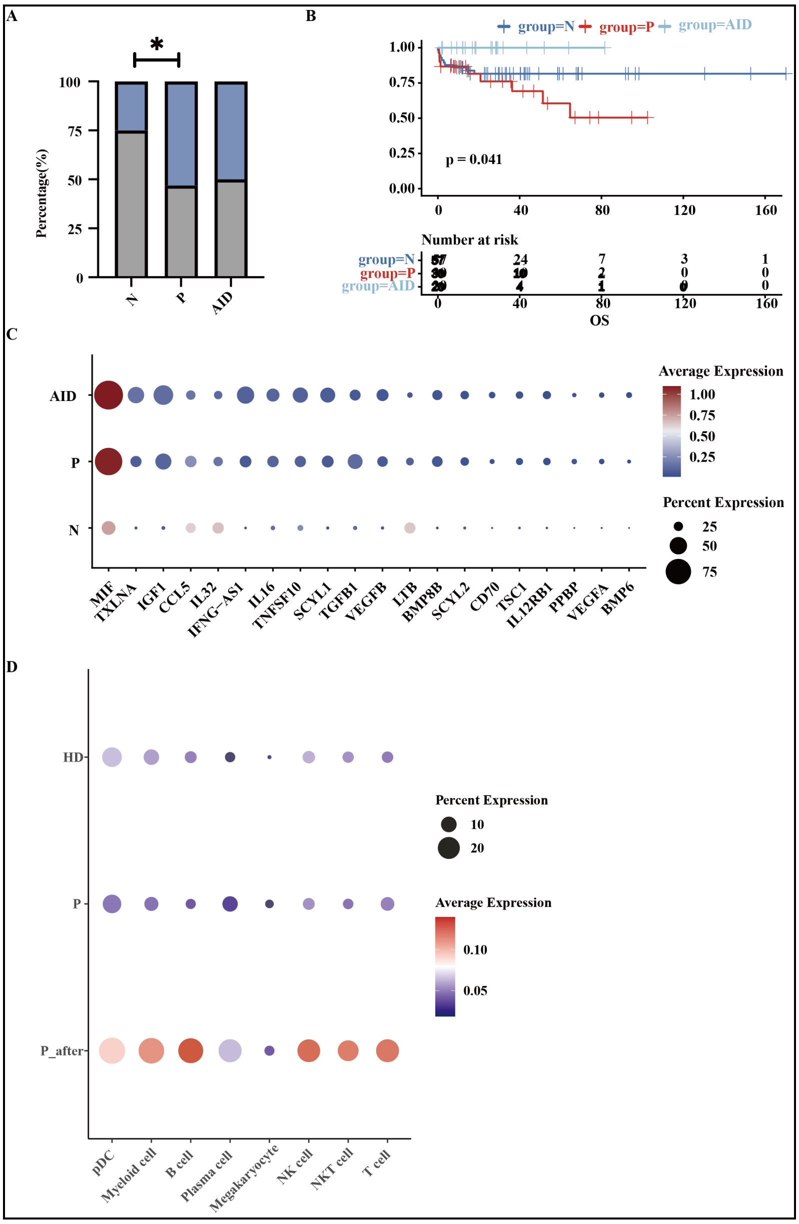

(A) Severe disease in iMCD patients with ANA positivity or not and AID. (B) OS in iMCD patients with ANA positivity or not and AID. (C) Expression level of cytokines in Plasma cell of iMCD patients with ANA positivity or not and AID. (D) mTOR expression of healthy donor (HD) and iMCD patients with ANA positivity before and after treatment.

Copyright Information: (Online) ISSN: 2572-9241
©2026 The Author(s). HemaSphere is published by John Wiley & Sons Ltd on behalf of European Hematology Association. This is an open access Abstract Book distributed under the Attribution-NonCommercial-NoDerivs (CC BY-NC-ND), which allows third parties to download the articles and share them with others as long as they credit the author and the Abstract Book, but they cannot change the content in any way or use them commercially.
Abstract Book Citations: Authors, Title, HemaSphere, 2026;10:(S1):pages. The abstract book DOIs can be found on the “Author Information” page.
Disclaimer: Articles published in the journal HemaSphere exclusively reflect the opinions of the authors. The authors are responsible for all content in their abstracts including accuracy of the facts, statements, citing resources, etc.

### (PF813) SUSTAINED MRD NEGATIVITY FOR 4 YEARS IS A SIGNIFICANT MARKER OF PROGNOSIS IN PATIENTS WITH HIGH-RISK MULTIPLE MYELOMA

**Topic:** 14. Myeloma and other monoclonal gammopathies - Clinical

Huan Liu[1], Juan Li*[1]

[1] *The First Affiliated Hospital of Sun Yat-sen University, Guangzhou, China;*

### Background

Although numerous studies have shown that achieving minimal residual disease (MRD) negative status is an important sign of favorable survival for multiple myeloma (MM) patients, especially patients with high-risk cytogenetic abnormalities, the optimal duration of MRD-negative maintenance remains unclear.

### Aims

The study aimed to explore the prognostic value of the necessary duration of MRD negativity in transplant-eligible newly diagnosed multiple myeloma (TE-NDMM) patients.

### Methods

This research included 223 TE-NDMM patients who underwent initial treatment and conducted at least two consecutive MRD negative assessments. We analyzed the influence of sustained MRD negativity on survival in different risk stratification.

### Results

A total of 147 (65.92%) standard-risk patients and 76 (34.08%) high-risk patients were enrolled. The median time of follow-up was 54.51 months. PFS in high-risk patients with MRD negativity duration ≥ 2 years was similar to patients in standard-risk group (70.77 vs 67.38 months, $P$=0.529). In standard-risk patients, PFS had no significant difference between MRD negativity maintaining 2-3 years and the duration more than 3 years (72.25months vs. 107.50months, $P$=0.103). While in high-risk group, there was no difference between duration for 4-5 years and ≥ 5 years (NR vs. 84.53months, $P$ = 0.136). And the duration over 2 years was associated with superior OS for all patients.

### Summary/Conclusion

Long term maintaining MRD-negative status improved prognosis of MM patients. Sustained 2-year MRD negativity can overcome the adverse influence from high-risk cytogenetics and the duration over 4 years was correlated with longer PFS in high-risk MM patients.

**Copyright Information:** (Online) ISSN: 2572-9241
©2026 The Author(s). HemaSphere is published by John Wiley & Sons Ltd on behalf of European Hematology Association. This is an open access Abstract Book distributed under the Attribution-NonCommercial-NoDerivs (CC BY-NC-ND), which allows third parties to download the articles and share them with others as long as they credit the author and the Abstract Book, but they cannot change the content in any way or use them commercially.
**Abstract Book Citations:** Authors, Title, HemaSphere, 2026;10:(S1):pages. The abstract book DOIs can be found on the “Author Information” page.
**Disclaimer:** Articles published in the journal HemaSphere exclusively reflect the opinions of the authors. The authors are responsible for all content in their abstracts including accuracy of the facts, statements, citing resources, etc.

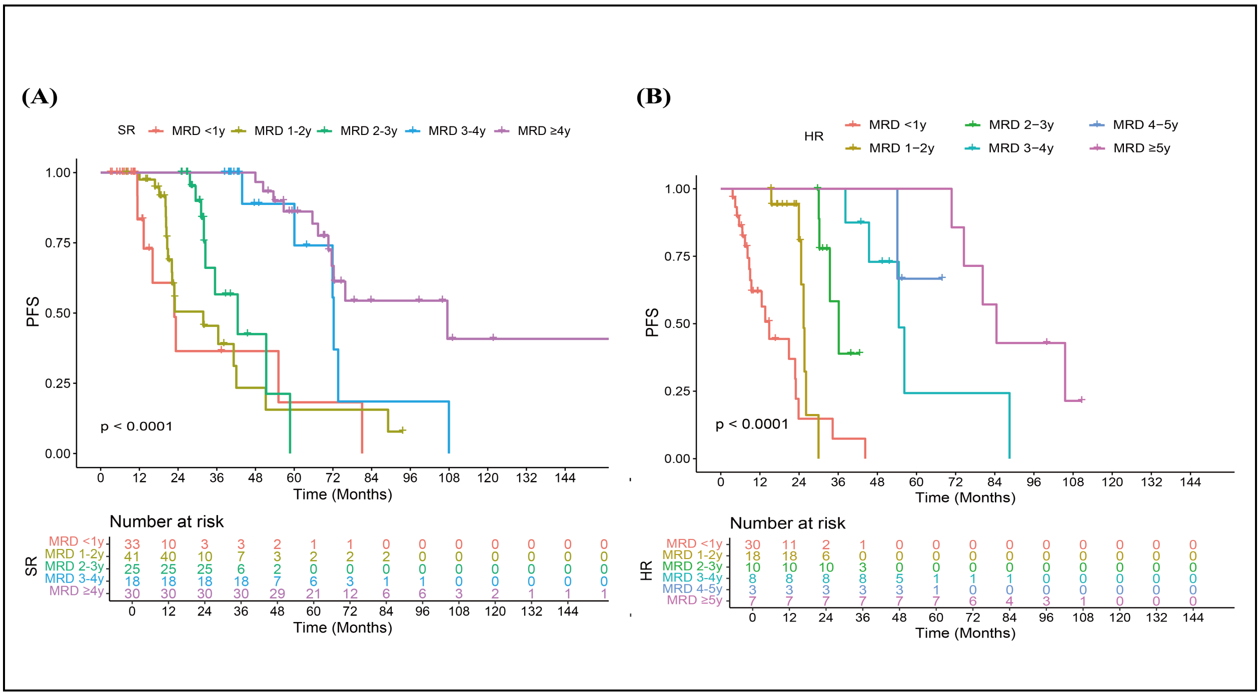

**Copyright Information:** (Online) ISSN: 2572-9241
©2026 The Author(s). HemaSphere is published by John Wiley & Sons Ltd on behalf of European Hematology Association. This is an open access Abstract Book distributed under the Attribution-NonCommercial-NoDerivs (CC BY-NC-ND), which allows third parties to download the articles and share them with others as long as they credit the author and the Abstract Book, but they cannot change the content in any way or use them commercially.
**Abstract Book Citations:** Authors, Title, HemaSphere, 2026;10:(S1):pages. The abstract book DOIs can be found on the "Author Information" page.
**Disclaimer:** Articles published in the journal HemaSphere exclusively reflect the opinions of the authors. The authors are responsible for all content in their abstracts including accuracy of the facts, statements, citing resources, etc.

### (PF814) IMPACT OF BRIDGING THERAPY AND DISEASE RESPONSE ON OUTCOMES AFTER ARI0002H BCMA CAR-T IN RELAPSED/REFRACTORY MULTIPLE MYELOMA

**Topic:** 14. Myeloma and other monoclonal gammopathies - Clinical

Anna de Daniel*[1, 2], Aina Oliver[1, 3], Natalia Tovar[1], Nuria Martinez-Cibrian[1], Nil Albiol[1], M. Teresa Cibeira[1, 3], Europa Azucena Gonzalez[1], Daniel Munarriz[1], Sergio Navarro[1], Paola Charry Espana[1], Joan Mañe[2, 3], David Moreno[1, 3], Valentín Ortiz-Maldonado[1], Beatriz Merchan[1], Alexandra Martínez Roca[1], Jose Miguel Mateos Perez[1, 2], Laura Rosiñol Dachs[1, 3], Sara Varea[1], Julio Delgado[1], Avaro Urbano Ispizua[1, 2, 3], Manel Juan[1, 3], Luis Gerardo Rodriguez-Lobato[1, 3], Carlos Fernández de Larrea[1, 2, 3]

*[1]Hospital Clínic de Barcelona, Immunology, Barcelona, Spain;[2]Universitat de Barcelona, Barcelona, Spain;[3]Institut d'Investigacions Biomèdiques August Pi i Sunyer (IDIBAPS), Barcelona, Spain;*

### Background

ARI0002h (cesni-cel), a BCMA-directed CAR-T developed at Hospital Clínic de Barcelona, demonstrated high efficacy and durable responses in relapsed/refractory multiple myeloma (RRMM), achieving a median progression-free survival (mPFS) of 20 months and a 24-month overall survival (OS) rate of 63% in a phase II trial, which led to a compassionate use program and national approval in Spain under Hospital Exemption. In this setting, optimizing disease control prior to infusion remains relevant, and the impact of bridging therapy (BT) during manufacturing is still under study.

### Aims

To evaluate whether BT, its type, and response influence mPFS and mOS after ARI0002h.

### Methods

We retrospectively analyzed 69 RRMM patients treated with ARI0002h. BT was defined as therapy between leukapheresis and lymphodepletion. Response was defined as >25% reduction in serum M-protein or involved free light chain, progression as >25% increase, and stable disease otherwise. mPFS from infusion was the primary endpoint.

**Copyright Information:** (Online) ISSN: 2572-9241
©2026 The Author(s). HemaSphere is published by John Wiley & Sons Ltd on behalf of European Hematology Association. This is an open access Abstract Book distributed under the Attribution-NonCommercial-NoDerivs (CC BY-NC-ND), which allows third parties to download the articles and share them with others as long as they credit the author and the Abstract Book, but they cannot change the content in any way or use them commercially.
**Abstract Book Citations:** Authors, Title, HemaSphere, 2026;10:(S1):pages. The abstract book DOIs can be found on the "Author Information" page.
**Disclaimer:** Articles published in the journal HemaSphere exclusively reflect the opinions of the authors. The authors are responsible for all content in their abstracts including accuracy of the facts, statements, citing resources, etc.

**Results**

Sixty-nine patients were included with a median follow-up of 21.8 months. mPFS was 22.4 months and 24-month OS was 78.9% (95%CI 68.8–90.6). BT was administered in 46/69 (67%): cyclophosphamide bolus (48%), pomalidomide-based (28%), carfilzomib-based (15%), and chemotherapy (6.5%); all received a single cycle. Among bridged patients, per prespecified definitions, 15 responded (30%), 16 had stable disease (35%), 14 progressed (30%) and one was not evaluable. Only prior treatment lines were significantly associated with non-response (OR 2.56, 95%CI 1.25–6.67; p=0.026). mPFS was 31.0 months without BT vs. 18.2 months with BT. Pomalidomide-based regimens were associated with lower risk of progression (HR 0.29, 95%CI 0.09–0.98; p=0.047), even after adjustment for prior lines of therapy (aHR 0.29; p=0.046). Patients achieving response had mPFS comparable to no BT (23.2 months; HR 1.51, p=0.39), whereas stable disease (12.7 months; HR 2.53, p=0.038) and progression (13.3 months; HR 3.92, p=0.002) were associated with inferior mPFS. mOS did not differ. The vein-to-vein interval was longer with BT (36 vs. 29 days; p=0.009). In multivariate analysis, progression to BT independently predicted inferior mPFS (HR 6.72, p<0.001). CRS was less frequent with BT (61% vs. 87%; p=0.029) with no differences in grade. Three IEC-HS events occurred (1 no-BT, 2 BT) and one grade 1 ICANS without BT. All events resolved.

**Table 1. Baseline Characteristics According to Use of Bridging Therapy**

| Characteristics | No Bridging (n=23) | Bridging (n=46) | p-value |
|---|---|---|---|
| **Age at infusion, years** | 64[39–73] | 61.5[43–74] | 0.76 |
| **Female, n(%)** | 10(43) | 27(59) | 0.31 |
| **Prior lines of therapy** | 4[2–10] | 3.0[2–6] | 0.004 |
| **ISS stage III, n(%)** | 6(29) | 12(33) | 0.58 |
| **Triple-refractory, n(%)** | 13(57) | 18(39) | 0.21 |
| **High-risk cytogenetics†‡, n(%)** | 4(36) | 10(45) | 0.72 |
| **Extramedullary disease, n(%)** | 2(9) | 9(20) | 0.31 |
| **Serum M-protein, g/L** | 11.6(1.5–22.3) | 12.2(7.7–19.6) | 0.81 |
| **Bone marrow plasma cells, %** | 9.5(1–24) | 10(2–20) | 0.69 |

† Defined as t(4;14), t(14;16), or del(17p).
‡ Percentage among evaluable patients.

**Copyright Information:** (Online) ISSN: 2572-9241
©2026 The Author(s). HemaSphere is published by John Wiley & Sons Ltd on behalf of European Hematology Association. This is an open access Abstract Book distributed under the Attribution-NonCommercial-NoDerivs (CC BY-NC-ND), which allows third parties to download the articles and share them with others as long as they credit the author and the Abstract Book, but they cannot change the content in any way or use them commercially.
**Abstract Book Citations:** Authors, Title, HemaSphere, 2026;10:(S1):pages. The abstract book DOIs can be found on the "Author Information" page.
**Disclaimer:** Articles published in the journal HemaSphere exclusively reflect the opinions of the authors. The authors are responsible for all content in their abstracts including accuracy of the facts, statements, citing resources, etc.

### Summary/Conclusion

In this cohort, shorter mPFS among patients receiving BT likely reflects patient selection and disease behavior during manufacturing. Disease control mitigated this risk, whereas progression independently identified a high-risk subgroup. BT in this cohort was significantly associated with lower incidence of CRS, an effect not explained by differences in baseline tumor burden, and warrants further investigation in the context of this CAR-T construct.

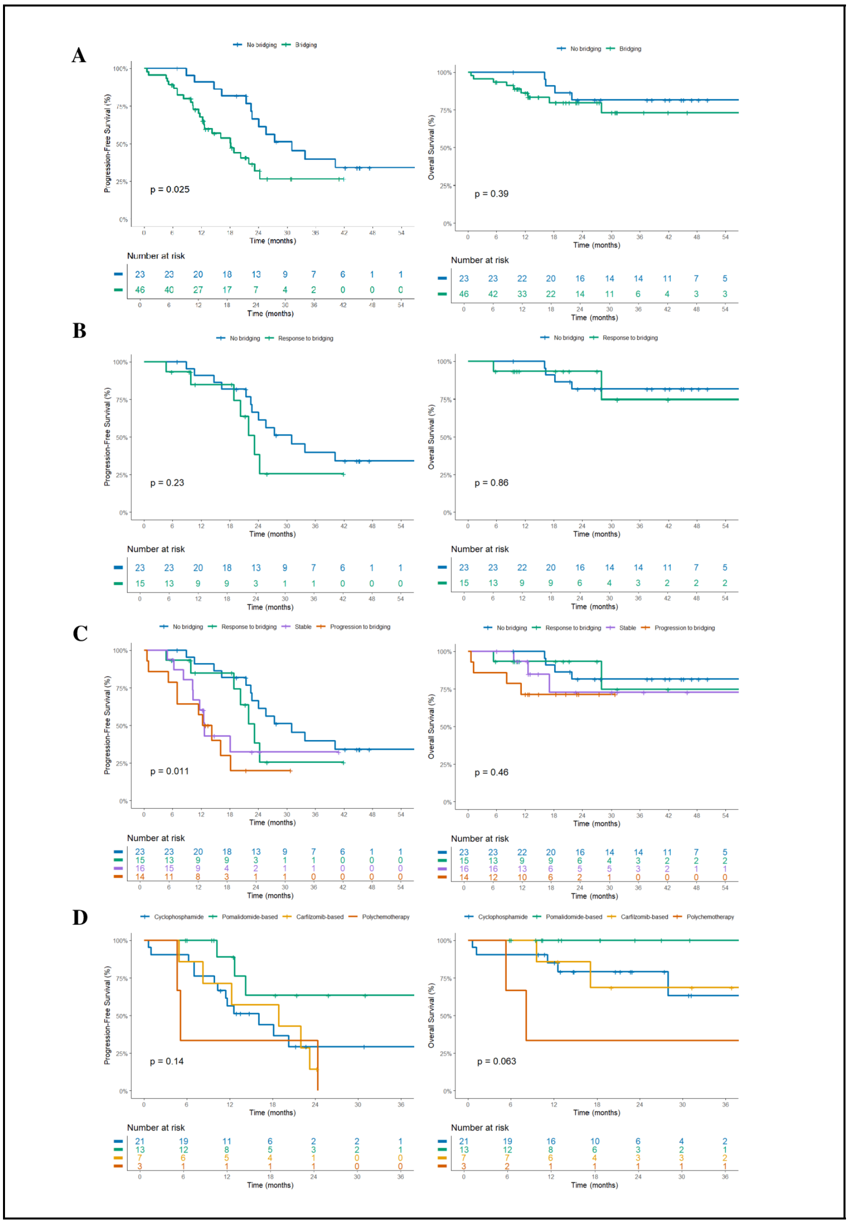

Figure 1. Kaplan–Meier curves for progression-free survival (left) and overall survival (right) according to bridging therapy use (A), response to bridging (B), response category (C), and bridging regimen (D).

**Copyright Information:** (Online) ISSN: 2572-9241
©2026 The Author(s). HemaSphere is published by John Wiley & Sons Ltd on behalf of European Hematology Association. This is an open access Abstract Book distributed under the Attribution-NonCommercial-NoDerivs (CC BY-NC-ND), which allows third parties to download the articles and share them with others as long as they credit the author and the Abstract Book, but they cannot change the content in any way or use them commercially.
**Abstract Book Citations:** Authors, Title, HemaSphere, 2026;10:(S1):pages. The abstract book DOIs can be found on the "Author Information" page.
**Disclaimer:** Articles published in the journal HemaSphere exclusively reflect the opinions of the authors. The authors are responsible for all content in their abstracts including accuracy of the facts, statements, citing resources, etc.

### (PF815) FIRST-LINE DARATUMUMAB–LENALIDOMIDE–DEXAMETHASONE IN NEWLY DIAGNOSED SYSTEMIC AL AMYLOIDOSIS: A UK NATIONAL AMYLOIDOSIS CENTRE EXPERIENCE

**Topic:** 14. Myeloma and other monoclonal gammopathies - Clinical

Sargam Vohra[1, 2], Suraj Vasudevan*[1, 2], Sriram Ravichandran[1, 2], Jahanzaib Khwaja[1, 2], Marianna Fontana[2], Carol Whelan[2], Helen Lachmann[2], Julian Gillmore[2], Ashutosh Wechalekar[1, 2]

*[1]University College London Hospitals NHS Foundation Trust, Amyloidosis, London, United Kingdom;[2]National Amyloidosis Centre, Amyloidosis, London, United Kingdom;*

### Background

Systemic immunoglobulin light chain (AL) amyloidosis is a rare multisystem disorder caused by clonal plasma cell or B-cell production of amyloidogenic free light chains, most commonly affecting the heart and kidneys. Outcomes have improved with daratumumab-based regimens, particularly daratumumab-cyclophosphamide-bortezomib-dexamethasone (Dara-CyBorD), now a licensed first-line. However, a substantial proportion of patients present with peripheral or autonomic neuropathy, limiting exposure to proteasome inhibitors. Daratumumab–lenalidomide–dexamethasone (DRD) has high response rates in first-line for transplant-ineligible multiple myeloma but has not been studied in newly diagnosed AL amyloidosis.

### Aims

To describe the clinical characteristics, efficacy, and tolerability of first-line DRD in newly diagnosed systemic AL amyloidosis.

### Methods

We retrospectively identified 25 patients with newly diagnosed systemic AL amyloidosis treated first-line with DRD from the UK National Amyloidosis Centre database between 2023 and 2025. All patients provided written informed consent in accordance with the Declaration of Helsinki. Haematological responses were assessed at 3, 6, and 12 months on an intention-to-treat basis. Organ responses were evaluated at 12 months. Outcomes included toxicity, overall survival (OS), event-free survival (EFS), progression-free survival (PFS), and time to next treatment (TTNT). OS was calculated from DRD initiation to death from any cause.

### Results

Median age was 75 years (range 60–87). Cardiac involvement was present in 20/25 (80%) and renal involvement in 11/25 (44%); 10/25 (40%) had Mayo stage ≥3 disease. Median baseline dFLC was 363.1 mg/L (range 23.5–14,788.4), NT-proBNP 3,371 ng/L (range 100–20,360), and creatinine 84 µmol/L (range 40–398). Three patients progressed and received subsequent therapy; four died while on DRD. Eighteen remain on treatment.

Haematological response was evaluable in 21, 22, and 20 patients at 3, 6, and 12 months. At 3, 6, and 12 months, overall response was 76.2%, 77.3%, and 70%, with ≥VGPR rates of 61.9%, 72.7%, and 65% respectively. Best responses were CR in 12/25 (48%), VGPR in 5/25 (20%), and PR in 3/25 (12%). Among 17 patients with longitudinal response data, 9 achieved best response by 3 months, while responses deepened in 3 with continued therapy, including conversion to CR at 12 months in 3 patients. Cardiac response was evaluable in 10 patients, with responses observed in 5 (50%).

Median OS and EFS were not reached. Overall survival at 12, 24, and 36 months was 74%, 65%, and 65%. Thirty-four adverse events were recorded; gastrointestinal toxicity, musculoskeletal symptoms, and fatigue occurred in 16%, infections in 12%, and one patient developed grade 3 neutropenia. Eight hospitalisations occurred in five patients, mainly due to infection or cardiac decompensation. No patients discontinued DRD due to toxicity.

**Copyright Information:** (Online) ISSN: 2572-9241
©2026 The Author(s). HemaSphere is published by John Wiley & Sons Ltd on behalf of European Hematology Association. This is an open access Abstract Book distributed under the Attribution-NonCommercial-NoDerivs (CC BY-NC-ND), which allows third parties to download the articles and share them with others as long as they credit the author and the Abstract Book, but they cannot change the content in any way or use them commercially.
**Abstract Book Citations:** Authors, Title, HemaSphere, 2026;10:(S1):pages. The abstract book DOIs can be found on the "Author Information" page.
**Disclaimer:** Articles published in the journal HemaSphere exclusively reflect the opinions of the authors. The authors are responsible for all content in their abstracts including accuracy of the facts, statements, citing resources, etc.

### Summary/Conclusion

First-line DRD is effective and well tolerated in newly diagnosed systemic AL amyloidosis, achieving rapid and deep haematological responses comparable to Dara-VCD with encouraging survival, including in advanced cardiac disease. DRD represents a useful first-line option for patients unsuitable for proteasome inhibitor-based therapy. Prospective studies are warranted to define its role relative to, or following suboptimal response to, Dara-CyBorD.

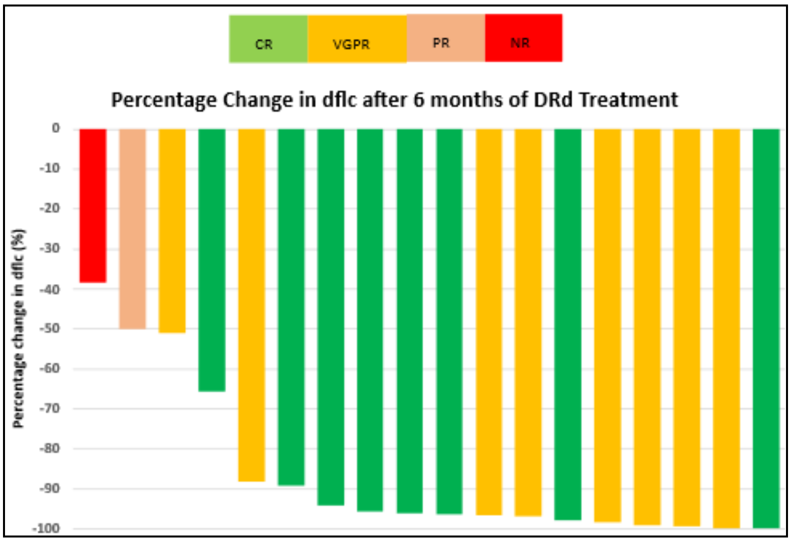

Percentage change in dFLC at 6 months after starting DRd first-line for each patient in Systemic AL Amyloidosis

Copyright Information: (Online) ISSN: 2572-9241
©2026 The Author(s). HemaSphere is published by John Wiley & Sons Ltd on behalf of European Hematology Association. This is an open access Abstract Book distributed under the Attribution-NonCommercial-NoDerivs (CC BY-NC-ND), which allows third parties to download the articles and share them with others as long as they credit the author and the Abstract Book, but they cannot change the content in any way or use them commercially.
**Abstract Book Citations:** Authors, Title, HemaSphere, 2026;10:(S1):pages. The abstract book DOIs can be found on the "Author Information" page.
**Disclaimer:** Articles published in the journal HemaSphere exclusively reflect the opinions of the authors. The authors are responsible for all content in their abstracts including accuracy of the facts, statements, citing resources, etc.

### (PF816) COMPLETE CARDIAC RESPONSE IMPROVES THE SURVIVAL OF PATIENTS WITH AL AMYLOIDOSIS AND IMPAIRED GLOBAL LONGITUDINAL STRAIN

**Topic:** 14. Myeloma and other monoclonal gammopathies - Clinical

Foteini Theodorakakou*[1], Ioannis Petropoulos[1], Christina Zotika[1], Maria-Eirini Tselegkidi[1], Nikolaos Kanellias[1], Eirini Solia[1], Vassiliki Spiliopoulou[1], Magdalini Migkou[1], Evangelos Eleutherakis-Papaiakovou[1], Asimina Papanikolaou[2], Maria Gavriatopoulou[1], Evangelos Terpos[1], Meletios A. Dimopoulos[1], Alexandros Briasoulis[1], Kimon Stamatelopoulos[1], Efstathios Kastritis[1]

*[1]National and Kapodistrian University of Athens, Department of Clinical Therapeutics, Athens, Greece;[2]Evangelismos Hospital, Athens, Greece;*

### Background

The main predictor of survival in AL amyloidosis is the severity of cardiac involvement. Global longitudinal strain (GLS) has integrated in the current staging system and can identify an ultra-high-risk population with very poor outcomes (Khwaja et al J Clin Oncol 2025). Patients who achieve complete cardiac response, tend to have excellent outcomes and data regarding GLS and graded cardiac response not available yet.

### Aims

To evaluate the impact of graded cardiac response and baseline GLS on the outcomes of patients with AL amyloidosis and cardiac involvement.

### Methods

The analysis included 167 previously untreated consecutive patients with AL amyloidosis and cardiac involvement with available GLS measured at baseline, before treatment initiation.

**Copyright Information:** (Online) ISSN: 2572-9241
©2026 The Author(s). HemaSphere is published by John Wiley & Sons Ltd on behalf of European Hematology Association. This is an open access Abstract Book distributed under the Attribution-NonCommercial-NoDerivs (CC BY-NC-ND), which allows third parties to download the articles and share them with others as long as they credit the author and the Abstract Book, but they cannot change the content in any way or use them commercially.
**Abstract Book Citations:** Authors, Title, HemaSphere, 2026;10:(S1):pages. The abstract book DOIs can be found on the "Author Information" page.
**Disclaimer:** Articles published in the journal HemaSphere exclusively reflect the opinions of the authors. The authors are responsible for all content in their abstracts including accuracy of the facts, statements, citing resources, etc.

### Results

Median age was 67 (43-95), 56% were males, 80% had λ light chain type while 51% had also renal, 26% liver and 26% nerve involvement. Mayo stage distribution was 50%, 25% and 25% for stages 2, 3a and 3b. Initial treatment was bortezomib-based without daratumumab in 42% and daratumumab-based in 57%. Median baseline GLS was -13.4 ([-5.7] - [-24]). GLS was correlated with NTproBNP (r=-0.366, p<0.001) and hsTnT (r=-0.5667, p<0.001), and Mayo stage (r=-0.524, p<0.001), but not with clone characteristics such as bone marrow involvement, or free light chain levels. At 6-month landmark, hematologic response rate was 76% for CR/VGPR, 15% PR and 7% NR/PD. Graded cardiac response rates for carCR, carVGPR, carPR and carNR/PD at 6 months were 5.5%, 18%, 35.5% and 50%, at 12 months was 8%, 28%, 35%, 29% and at 24 months 10%, 41%, 28% and 21%, respectively.
Per baseline GLS levels (≥ -9% vs < -9%) graded cardiac response rates at 6 months were 9.8%, 11.5%, 31% and 47.5%, vs 0%, 21%, 39%, 31% and 40% for CR, VGPR, PR, NR/PD (p=0.034), at 12 months was 19%, 14% , 39.5% and 30% vs 4%, 37%, 28% and 31.5% (p=0.012) and at 24 months was 6%, 23%, 29% and 43% vs 7%, 37% 19% and 37% (p=0.508). Baseline GLS was significantly associated with graded cardiac response. In binary logistic regression analysis lower baseline GLS was significantly associated with increased likelihood of achieving carCR at 6 months (OR 1.53, 95% CI 1.139-2.071, p=0.005) and at 12 months (OR 1.345, 95% CI 1.114-1.624, p=0.002). After adjustment for hematologic response at 6 months, baseline GLS remained independent predictor of achieving carCR at 6 months (OR 1.638, 95% CI 1.166-2.303, p=0.004) and at 12 months (OR 1.330, 95% CI 1.107-.599, p=0.002).
Median OS of the whole cohort was 70 months. In univariate analysis GLS < -9% was associated with improved survival (median OS not reached vs 67.7 months; HR 0.561, 95%CI 0.326-0.968, p=0.038). Among patients with GLS >-9, achievement of complete cardiac response at 12 and 24 months was associated with longer OS (Figure 1), while there was no significant difference at 6 months for patients with GLS < -9%. Estimated 2-year survival rate per graded cardiac response at 6 months was 74%, 92% and 100% (p=0.042) for carNR, carPR and carVGPR for patients with impaired GLS (≥-9%) and 73%, 95%, 100% and 100%(p=0.135) for carNR, carPR, carVGPR and carCR.

### Summary/Conclusion

In patients with cardiac AL amyloidosis GLS≥-9% at baseline is associated with lower probability of achieving complete cardiac response independently of hematologic response. However, achievement of carCR is associated with improved outcomes of patients with impaired GLS and this highlights the importance of very effective anti-clonal treatments.

**Copyright Information:** (Online) ISSN: 2572-9241
©2026 The Author(s). HemaSphere is published by John Wiley & Sons Ltd on behalf of European Hematology Association. This is an open access Abstract Book distributed under the Attribution-NonCommercial-NoDerivs (CC BY-NC-ND), which allows third parties to download the articles and share them with others as long as they credit the author and the Abstract Book, but they cannot change the content in any way or use them commercially.
**Abstract Book Citations:** Authors, Title, HemaSphere, 2026;10:(S1):pages. The abstract book DOIs can be found on the "Author Information" page.
**Disclaimer:** Articles published in the journal HemaSphere exclusively reflect the opinions of the authors. The authors are responsible for all content in their abstracts including accuracy of the facts, statements, citing resources, etc.

### (PF817) PROGNOSTIC ROLE OF PROGRESSION IN WALDENSTROM MACROGLOBULINEMIA PATIENTS, A REPORT FROM THE FRENCH INNOVATIVE LYMPHOMA ORGANIZATION (FILO) REGISTRY

**Topic:** 14. Myeloma and other monoclonal gammopathies - Clinical

Lydia Montes*[1], Cecile Tomowiak[2], Daniela Robu[3], Eric Durot[4], Damien Roos Weil[5], Olivier Tournilhac[6], Elise Toussaint[7], Fontanet Bijou[8], Katell Le Du[9], Diane Lara[10], Sophie De Guibert[11], Florian Bouclet[12], Bruno Royer[13], Lea Greub[14], Benedicte Hivert Fournier[15], Adeline Lhermitte[16], Marie Christine Béné[17], Pierre Morel[1]

*[1]CHU Amiens Picardie, Amiens, France; [2]CHU Poitiers, Poitiers, France; [3]Centre Hospitalier de Lens, Lens, France; [4]CHU de Reims, Reims, France; [5]Hopital Pitie Salpetriere, Paris, France; [6]CHU Clermont Ferrand, Clermont Ferrand, France; [7]CHU Strasbourg, Strasbourg, France; [8]Institut Bergonie, Bordeaux, France; [9]Clinique Nantes Confluent, Nantes, France; [10]CH de Libourne, Libourne, France; [11]CHU de Rennes, Rennes, France; [12]Centre Henri Becquerel, Rouen, France; [13]Hopital Saint Louis, Paris, France; [14]CH de Mont de Marsan, Mont de Marsan, France; [15]GHICL, Lille, France; [16]FILO, Tours, France; [17]Nantes University, Nantes, France;*

### Background

In Waldenström macroglobulinemia (WM) the adverse prognostic value of early progression before 24 months (POD24) has been reported, especially in patients who received in first line a bendamustine rituximab combination (BendaR). However, conflicting results have been reported in patients who received other chemoimmunotherapy (CIT) such as the dexamethasone/rituximab/cyclophosphamide (DRC) combination.

### Aims

To address this issue, we took advantage of the availability of the mortality data of the French reference population until 2024/01/01 to review the outcome and assess the relative survival of the 401 patients who initiated first line treatment between 1989/12/10 and 2022/12/31 and who were enrolled in the FILO symptomatic WM registry.

### Methods

In 2018, 11 centers merged their prospectively maintained local databases. Since 2022, they prospectively enroll symptomatic WM patients with 21 other centers, provided that the rate of patients lost to follow-up remains <15% in each center. Thus we could compare the survival of the patients to the survival of a group of subjects with the same demographic structure (relative survival).

**Copyright Information:** (Online) ISSN: 2572-9241
©2026 The Author(s). HemaSphere is published by John Wiley & Sons Ltd on behalf of European Hematology Association. This is an open access Abstract Book distributed under the Attribution-NonCommercial-NoDerivs (CC BY-NC-ND), which allows third parties to download the articles and share them with others as long as they credit the author and the Abstract Book, but they cannot change the content in any way or use them commercially.
**Abstract Book Citations:** Authors, Title, HemaSphere, 2026;10:(S1):pages. The abstract book DOIs can be found on the "Author Information" page.
**Disclaimer:** Articles published in the journal HemaSphere exclusively reflect the opinions of the authors. The authors are responsible for all content in their abstracts including accuracy of the facts, statements, citing resources, etc.

**Results**

Median age was 68 years, (interquartile range: 61; 76), M/F=1.93, 149 patients received DRC (after 2008/06) and 46 bendaR. Median PFS and OS were 44 months (95% confidence interval [95CI]: 36; 52) and 127 months (95CI: 116; 144), respectively. With a median follow-up of alive patients of 102 months, and 14% of patients lost to follow-up at the date of analysis (01/01/2024), 207 deaths were recorded (51%, WM related: 57). With a similar follow-up and 16% of patients lost to follow-up, 57 deaths were recorded (39%, WM related: 8) in the patients treated with DRC. The rate of patients lost to follow-up was 32% in patients treated with BendaR, preventing further analyses.
Landmark analysis set at 24 months showed an adverse prognostic value of POD24 for subsequent OS in the whole series (p=0.02, HR=1.49, 95%CI: 1.06; 2.0.9) but not in the patients treated with DRC (p=0.62). Simlarly time dependent POD24 analysis showed that survival after 2 years without POD24 was better than survival after POD24 in the whole series (p=0.005, with validity of the PH assumption), but not in patients receiving DRC (p=0,8). Survival after progression, the endpoint of clinical interest for the management of progressive patients was not influenced by the previous time to progression (with cut-offs set monthly during the first 6 years).
We assessed the relative survival of patients without POD24, taking into account only their survival after 24 months. Five-years estimates was 89% (95CI: 85; 94) and 94% (95CI: 88; 102) in the whole series and in the patients with DRC (Figure 1) respectively. At 10 years it was 71% (95CI: 64; 79) and 70% (95CI: 53; 93) respectively. Similar results were observed, considering survival after 12 months of patients progression-free (without POD12), and survival after 36 months of patients progression-free (without POD36) at these times points. However, the 5-year relative survival after 36-months, of patients with DRC without POD36 was also different from 1.

**Summary/Conclusion**

Some landmark analyses and other analysis of POD24 showed significant differences. Nevertheless, survival after early progression was not worst than survival after late progression. With an appropriate follow-up, we found that long term relative survival after 24 months remains different from 1 in patients progression-free at 24 months, even after the DRC regimen.

**Copyright Information:** (Online) ISSN: 2572-9241
©2026 The Author(s). HemaSphere is published by John Wiley & Sons Ltd on behalf of European Hematology Association. This is an open access Abstract Book distributed under the Attribution-NonCommercial-NoDerivs (CC BY-NC-ND), which allows third parties to download the articles and share them with others as long as they credit the author and the Abstract Book, but they cannot change the content in any way or use them commercially.
**Abstract Book Citations:** Authors, Title, HemaSphere, 2026;10:(S1):pages. The abstract book DOIs can be found on the "Author Information" page.
**Disclaimer:** Articles published in the journal HemaSphere exclusively reflect the opinions of the authors. The authors are responsible for all content in their abstracts including accuracy of the facts, statements, citing resources, etc.

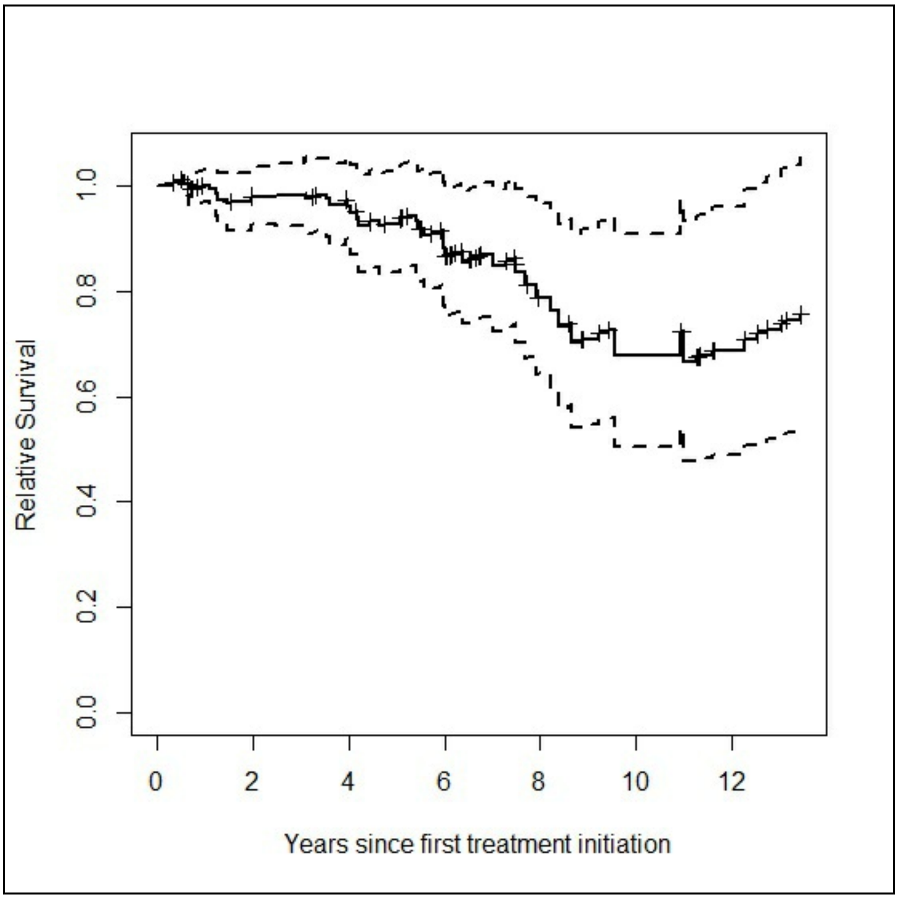

Figure 1: relative survival after 24 months of patients treated with DRC and progression-free at 24 months (without POD24)

**Copyright Information:** (Online) ISSN: 2572-9241
©2026 The Author(s). HemaSphere is published by John Wiley & Sons Ltd on behalf of European Hematology Association. This is an open access Abstract Book distributed under the Attribution-NonCommercial-NoDerivs (CC BY-NC-ND), which allows third parties to download the articles and share them with others as long as they credit the author and the Abstract Book, but they cannot change the content in any way or use them commercially.
**Abstract Book Citations:** Authors, Title, HemaSphere, 2026;10:(S1):pages. The abstract book DOIs can be found on the "Author Information" page.
**Disclaimer:** Articles published in the journal HemaSphere exclusively reflect the opinions of the authors. The authors are responsible for all content in their abstracts including accuracy of the facts, statements, citing resources, etc.

### (PF818) PATIENT-REPORTED OUTCOMES (PROS) AND MINIMAL RESIDUAL DISEASE (MRD) NEGATIVITY IN NEWLY DIAGNOSED MULTIPLE MYELOMA (NDMM) PATIENTS TREATED WITH QUADRUPLET THERAPY: POST-HOC ANALYSIS OF GMMG-HD7 TRIAL

**Topic:** 14. Myeloma and other monoclonal gammopathies - Clinical

Elias K. Mai*[1], Uta Bertsch[1, 2], Roland Fenk[3], Britta Besemer[4], Amelie Boquoi[5], Roland Schroers[6], Ivana von Metzler[7], Mathias Hänel[8], Christoph Mann[9], Lisa Leypoldt[10], Bernhard Heilmeier[11], Stefanie Huhn[1], Christof Scheid[12], Igor Wolfgang Blau[13], Steffen Luntz[14], Tobias Holderried[15], Karolin Trautmann[16], Deniz Nogueira Gezer[17], Maika Klaiber[18], Martin Müller[19], Evgenii Shumilov[20], Wolfgang Knauf[21], Christian Michel[22], Thomas Geer[23], Hendrik Riesenberg[24], Christoph Lutz[25], Marc-Steffen Raab[1], Martin Hoffmann[26], Erin Comerford[27], Maeva Kyheng[28], Feng Lin[29], Jerome Msihid[30], Hans Juergen Dr. Salwender[31], Katja C. Weisel[10], Hartmut Goldschmidt[1, 2]

*[1]Heidelberg University, Heidelberg Myeloma Center, Department of Internal Medicine V, Heidelberg University Hospital and Medical Faculty Heidelberg, Heidelberg, Germany; [2]National Center for Tumor Diseases (NCT) Heidelberg, Heidelberg, Germany; [3]University Hospital Dusseldorf, Department of Hematology, Oncology and Clinical Immunology, Dusseldorf, Germany; [4]University Hospital Tubingen, Department of Internal Medicine II, Tubingen, Germany;[5]University Hospital Essen, Department for Hematology and Stem Cell Transplantation, Essen, Germany; [6]Ruhr-University Bochum, Medical Clinic II—Hematology and Oncology, Bochum, Germany; [7]Goethe-University Frankfurt, University Hospital, Department of Medicine II - Hematology and Oncology, Frankfurt am Main, Germany; [8]Klinikum Chemnitz, Department of Internal Medicine III, Chemnitz, Germany; [9]University Hospital Gießen and Marburg, Department for Hematology, Oncology and Immunology, Marburg, Germany; [10]University Medical Center Hamburg-Eppendorf, Department of Oncology, Hematology and BMT, Hamburg, Germany; [11]Hospital Barmherzige Brueder Regensburg, Clinic for Oncology and Hematology, Regensburg, Germany; [12]University Hospital Cologne, Department of Internal Medicine I, Cologne, Germany; [13]Charite University Medicine Berlin, Charite University Medicine Berlin, Berlin, Germany; [14]Coordination Centre for Clinical Trials (KKS) Heidelberg, Heidelberg, Germany; [15]University Hospital Bonn, Department of Hematology, Oncology, Stem Cell Transplantation, Immune and Cell Therapy, Clinical Immunology and Rheumatology, Bonn, Germany; [16]University Hospital Dresden, Department of Internal Medicine I, Dresden, Germany; [17]RWTH Aachen University, Department of Hematology, Oncology, Hemostaseology, and Stem Cell Transplantation, Faculty of Medicine, Aachen, Germany; [18]Marien Hospital Dusseldorf, Clinic for Hematology, Oncology and Palliative Care, Dusseldorf, Germany; [19]Klinikum Siloah Hannover, Clinic for Hematology, Oncology and Immunology, Hannover, Germany; [20]University Hospital Munster, Department of Medicine A, Hematology, Oncology and Pneumology, Department of Medicine A, Hematology, Oncology and Pneumology, Germany; [21]Center for Hematology and Oncology Bethanien, Frankfurt am Main, Germany; [22]University Hospital Mainz, Department of Internal Medicine III, Mainz, Germany; [23]Diakoneo Clinic Schwabisch-Hall, Department of Internal Medicine III, Schwabisch-Hall, Germany; [24]Hematology/Oncology Center, Bielefeld, Germany; [25]Hematology/Oncology Center, Koblenz, Germany; [26]Clinic Ludwigshafen, Clinic Ludwigshafen, Ludwigshafen, Germany;[27]Sanofi, Sanofi, United States of America; [28]Aixial, Sèvres, France; [29]Sanofi, Morristown, United States of America; [30]Sanofi, Gentilly, France; [31]AK Altona and AK St Georg, Asklepios Tumorzentrum Hamburg, Hamburg, Germany;*

### Background

MRD negativity is becoming a key clinical endpoint in multiple myeloma (MM) due to its association with improved progression-free and overall survival. This was demonstrated in part 1 of the phase 3 GMMG-HD7 trial (NCT03617731), which compared isatuximab plus lenalidomide (Len), bortezomib, and dexamethasone (Isa-RVd) induction therapy with Len, bortezomib, and dexamethasone (RVd) alone in transplant-eligible (TE) NDMM patients. However, there remains limited data on the potential impact of MRD status on health-related quality of life (HRQoL) in patients with TE NDMM.

**Copyright Information:** (Online) ISSN: 2572-9241
©2026 The Author(s). HemaSphere is published by John Wiley & Sons Ltd on behalf of European Hematology Association. This is an open access Abstract Book distributed under the Attribution-NonCommercial-NoDerivs (CC BY-NC-ND), which allows third parties to download the articles and share them with others as long as they credit the author and the Abstract Book, but they cannot change the content in any way or use them commercially.
**Abstract Book Citations:** Authors, Title, HemaSphere, 2026;10:(S1):pages. The abstract book DOIs can be found on the "Author Information" page.
**Disclaimer:** Articles published in the journal HemaSphere exclusively reflect the opinions of the authors. The authors are responsible for all content in their abstracts including accuracy of the facts, statements, citing resources, etc.

### Aims

The aim of this *post-hoc* analysis was to assess the association between achieving MRD negativity vs remaining MRD positive on HRQoL in each treatment arm, and whether the effect differed between treatments.

### Methods

In part 1 of the GMMG-HD7 trial, NDMM patients were randomized (1:1) to Isa-RVd or RVd induction therapy. The induction treatment period consisted of three cycles of Isa-RVd or RVd followed by an intensification phase with autologous stem cell transplantation (ASCT). MRD was measured after induction and after intensification. Patients were grouped as MRD-negative (MRD-neg), MRD-positive (MRD-pos), or discordant when results differed between time points. MRD negativity was defined as $<10^{-5}$ using next-generation flow cytometry. PROs to measure HRQoL included the EORTC-QLQ-C30 and the EORTC QLQ-MY20 at baseline, after induction, and 60–90 days after intensification. The association between MRD status and mean change in PROs between baseline and post-intensification was evaluated using an ANCOVA model adjusted for baseline PRO and R-ISS score within each treatment arm. Increasing scores on the QLQ-C30 General Health Status/QoL (GHS/QoL) and functioning scales indicate improvement of functioning/HRQoL, whereas increasing scores on QLQ-C30 and MY20 symptom scales/items indicate worsening. Treatment-by-subgroup interaction tests were also performed to assess the heterogeneity of MRD status effect between the treatments.

### Results

A total of 662 patients were randomized in the GMMG-HD7 trial to receive Isa-RVd (n=331) or RVd (n=329) induction therapy, followed by single or tandem ASCT. Among patients with available MRD data at both timepoints (n=516), 236 patients (45.7%) achieved MRD negativity and 137 patients (26.6%) remained MRD-pos. For the QLQ-C30 GHS/QoL, the least square (LS) mean difference (95% CI) for MRD-neg vs MRD-pos patients was 2.26 (-4.88 to 9.39) in the Isa-RVd group and -0.02 (-5.77 to 5.74) in the RVd group. The LS mean (95% CI) difference in QLQ-C30 emotional function score (MRD-neg vs MRD-pos) was 4.83 (-3.15 to 12.81) in the Isa-RVd group and 1.45 (-5.27 to 8.17) in the RVd group. The QLQ-C30 pain score LS mean (95% CI) difference for MRD-neg vs MRD-pos patients was -3.56 (-13.74 to 6.60) in the Isa-RVd group and -7.42 (-15.93 to 1.10) in the RVd group. Changes in PRO numerically favored MRD-neg patients compared with MRD-pos but were not statistically significant. Moreover, consistent effects were observed across treatments (interaction $p>0.10$).

### Summary/Conclusion

While MRD status was not significantly associated with HRQoL changes in either treatment arm, patients who achieved MRD negativity demonstrated a trend toward improved PROs in both treatment groups. However, further investigation using longitudinal PRO data and larger sample sizes is warranted.

**Copyright Information:** (Online) ISSN: 2572-9241
©2026 The Author(s). HemaSphere is published by John Wiley & Sons Ltd on behalf of European Hematology Association. This is an open access Abstract Book distributed under the Attribution-NonCommercial-NoDerivs (CC BY-NC-ND), which allows third parties to download the articles and share them with others as long as they credit the author and the Abstract Book, but they cannot change the content in any way or use them commercially.
**Abstract Book Citations:** Authors, Title, HemaSphere, 2026;10:(S1):pages. The abstract book DOIs can be found on the "Author Information" page.
**Disclaimer:** Articles published in the journal HemaSphere exclusively reflect the opinions of the authors. The authors are responsible for all content in their abstracts including accuracy of the facts, statements, citing resources, etc.

### (PF819) INFLAMMATORY MIMICS: MASS SPECTROMETRY RESOLVES FALSE-POSITIVE PARAPROTEINS ON SERUM IMMUNOFIXATION

**Topic:** 14. Myeloma and other monoclonal gammopathies - Clinical

Neela Easton*[1], Tamara Kaneshiro[1], Roger Pearse[1], Victor Jiminez Zepeda[1], Mark Bustoros[1], Ruben Niesvizky[1], Mateo Mejia Saldarriaga[1]

[1] *Weill Cornell Medicine Hematology & Oncology, New York, United States of America;*

### Background

Serum protein electrophoresis (SPEP) and immunofixation electrophoresis (sIFE) are standard screening tools for monoclonal gammopathies. In polyclonal hypergammaglobulinemia—such as that seen with chronic inflammation or infection—increased background noise can produce false-positive monoclonal bands, leading to misdiagnosis of monoclonal gammopathy of undetermined significance (MGUS) and unnecessary downstream workup. Mass spectrometry-based assays, with superior sensitivity and specificity, offer a tool to resolve diagnostic uncertainty.

### Aims

We described clinical and laboratory features of patients with false-positive sIFE—defined as positive sIFE with negative Mass-Fix (Mayo Clinic Laboratories)—referred for MGUS evaluation and characterized their course.

### Methods

We performed a retrospective descriptive analysis of patients who met International Myeloma Working Group (IMWG) criteria for MGUS at a single center from 2012-2025 who were later found to have discordant results (positive SPEP/sIFE, negative Mass-Fix). Progression to multiple myeloma or other plasma cell dyscrasia was defined by IMWG criteria.

### Results

37 patients were identified with median age 73 (IQR 68-80); 70.3% were female. At initial positive sIFE, 13/37 (35%) had autoimmune disease, 4/37 (11%) recent infection, 4/37 (11%) recent monoclonal antibody exposure, and 10/37 (27%) recent vaccination. 12/37 (32%) had no identifiable inflammatory trigger **(Figure 1A).** Median M-protein was 0.05 g/dL (range 0-1.4). Heavy chain and light chain isotypes are shown in **Figure 1B**. 14/37 (37.8%) patients developed a new or changed heavy or light chain isotype on follow-up sIFE, and 11/37 (29.7%) had dense polyclonal background on at least one report, both suggestive of a reactive process. Median kappa/lambda ratio was 1.44 (IQR 1.20-1.58); only 7 patients had an abnormal ratio. Of 28/37 (75.7%) of patients with urine immunofixation available, 8/28 (28.6%) were positive. Median time from first positive sIFE to Mass-Fix testing was 18.8 months (IQR 10.3-34.8). 35/37 (94.6%) had negative Mass-Fix results; 2 had initially positive results that subsequently became negative. Over a median follow-up of 24.8 months (IQR 13.8-44), no patients progressed to smoldering or symptomatic myeloma. At last follow-up, 31/37 (83.8%) had persistently positive sIFE despite negative Mass-Fix**(Figure 1C).** Median number of clinic visits was 2 (range 1-16) (**Figure 1D**).

**Copyright Information:** (Online) ISSN: 2572-9241
©2026 The Author(s). HemaSphere is published by John Wiley & Sons Ltd on behalf of European Hematology Association. This is an open access Abstract Book distributed under the Attribution-NonCommercial-NoDerivs (CC BY-NC-ND), which allows third parties to download the articles and share them with others as long as they credit the author and the Abstract Book, but they cannot change the content in any way or use them commercially.
**Abstract Book Citations:** Authors, Title, HemaSphere, 2026;10:(S1):pages. The abstract book DOIs can be found on the "Author Information" page.
**Disclaimer:** Articles published in the journal HemaSphere exclusively reflect the opinions of the authors. The authors are responsible for all content in their abstracts including accuracy of the facts, statements, citing resources, etc.

### Summary/Conclusion

In this cohort, Mass-Fix clarified false-positive sIFE results meeting IMWG criteria for MGUS in 37 patients. These results were frequently associated with identifiable inflammatory or immunological triggers, including autoimmune disease, infection, monoclonal antibody exposure, and vaccination. Despite persistently positive sIFE in 83.8% of patients at last follow-up, no patients progressed to smoldering or multiple myeloma, supporting the benign nature of these findings. These results underscore a significant downstream healthcare utilization burden associated with false-positive immunofixation, including a median diagnostic delay of 18.8 months before Mass-Fix clarification. Broader adoption of mass spectrometry-based assays in the initial evaluation of suspected monoclonal paraproteinemia may reduce unnecessary workup and patient anxiety, though long-term follow-up remains necessary to confirm the absence of late progression.

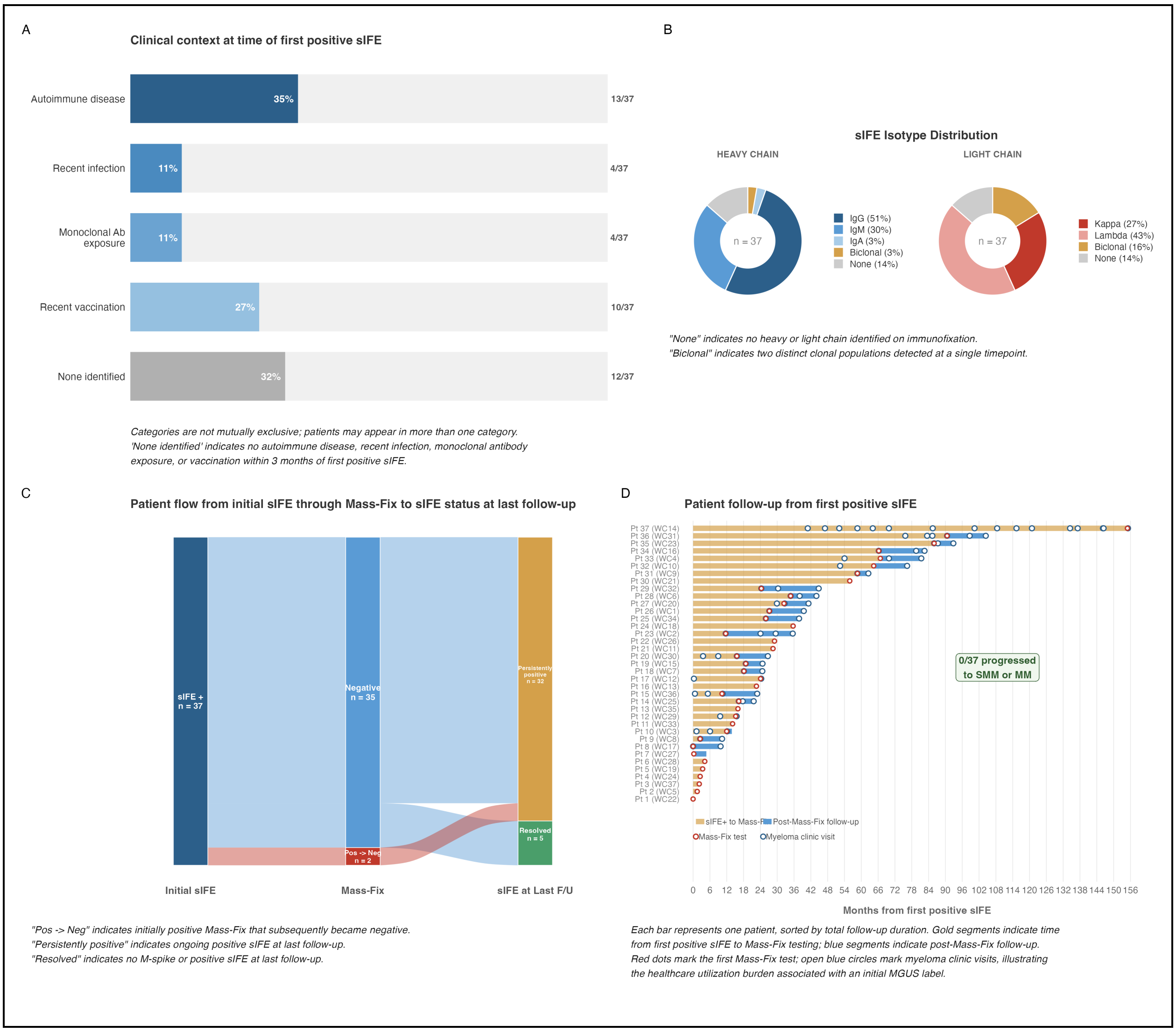

**Copyright Information:** (Online) ISSN: 2572-9241
©2026 The Author(s). HemaSphere is published by John Wiley & Sons Ltd on behalf of European Hematology Association. This is an open access Abstract Book distributed under the Attribution-NonCommercial-NoDerivs (CC BY-NC-ND), which allows third parties to download the articles and share them with others as long as they credit the author and the Abstract Book, but they cannot change the content in any way or use them commercially.
**Abstract Book Citations:** Authors, Title, HemaSphere, 2026;10:(S1):pages. The abstract book DOIs can be found on the "Author Information" page.
**Disclaimer:** Articles published in the journal HemaSphere exclusively reflect the opinions of the authors. The authors are responsible for all content in their abstracts including accuracy of the facts, statements, citing resources, etc.

### (PF820) IMPACT OF SPECIFIC CYTOGENETIC ABNORMALITIES ON MRD KINETICS AND PROGNOSTIC VALUE IN MULTIPLE MYELOMA

**Topic:** 14. Myeloma and other monoclonal gammopathies - Clinical

Alberto Blanco Sánchez*[1], Andrés Arroyo[1], Ajai Chari[2], Thomas Martin[2], Shagun Arora[2], Anupama Kumar[2], Nina Shah[2], Alfred Chung[2], Darren Pan[2], Jodi Lipof[2], Joaquín Martinez-Lopez[1], Jeffrey Wolf[2]

*[1]Hospital Universitario 12 de Octubre, Madrid, Spain;[2]University of California San Francisco, San Francisco, United States of America;*

### Background

Achieving undetectable measurable residual disease (uMRD) is associated with improved progression-free survival (PFS) and overall survival (OS) in patients with multiple myeloma (MM). The presence of high-risk cytogenetic abnormalities (HRCA) has been associated with lower rates of uMRD. However, most studies dichotomize patients into high-risk versus standard-risk groups, and few have explored MRD kinetics and its prognostic impact according to individual cytogenetic alterations.

### Aims

To evaluate the impact of specific cytogenetic abnormalities on the rate and kinetics of MRD clearance, and the prognostic significance of achieving uMRD in each group.

### Methods

Clinical records of consecutive patients with MM and at least one bone marrow MRD assessment at University of California San Francisco (UCSF) between 2013 and May 2024 were reviewed. Among 636 patients, 459 patients were evaluated from the start of first-line therapy, and prior to disease progression. MRD was assessed with the clonoSEQ® assay. A cutoff of $10^{-6}$ was used to define uMRD. High-risk cytogenetics were defined as del(17p), t(4;14), t(14;16) or 1q gain/amplification. Time to first achievement of uMRD and PFS were estimated using the Kaplan–Meier method.

### Results

Baseline characteristics are summarized in Table 1. Median age at treatment initiation was 62 years (interquartile range 56-67). Cytogenetic data were available for 411 patients (90%), of whom 128 (31%) were classified as high-risk. The t(11;14) was present in 64 patients (16%), t(4;14) in 42 (10%), del(17p) in 40 (10%) and 1q gain/amp in 49 (12%). Overall, 189 (41%) patients achieved uMRD at any time during follow-up.

Patients with t(11;14) achieved lower uMRD rates compared to those without this abnormality (25% vs 44%, p=0.006), with a trend toward delayed MRD clearance (p=0.067). However, in this subgroup, achieving uMRD was not associated with improved PFS (p=0.51). Patients with t(4;14) had comparable uMRD rate (52% vs 40%, p=0.176) but achieved uMRD more rapidly (p=0.013). In this subgroup, uMRD was also not associated with improved PFS (p=0.34). Patients with del(17p) or 1q gain/amplification showed similar uMRD rates and comparable MRD kinetics to those lacking these HRCA. In both subgroups, achieving uMRD was significantly associated with improved PFS (Figure 1).

**Copyright Information:** (Online) ISSN: 2572-9241
©2026 The Author(s). HemaSphere is published by John Wiley & Sons Ltd on behalf of European Hematology Association. This is an open access Abstract Book distributed under the Attribution-NonCommercial-NoDerivs (CC BY-NC-ND), which allows third parties to download the articles and share them with others as long as they credit the author and the Abstract Book, but they cannot change the content in any way or use them commercially.
**Abstract Book Citations:** Authors, Title, HemaSphere, 2026;10:(S1):pages. The abstract book DOIs can be found on the "Author Information" page.
**Disclaimer:** Articles published in the journal HemaSphere exclusively reflect the opinions of the authors. The authors are responsible for all content in their abstracts including accuracy of the facts, statements, citing resources, etc.

**Table 1. Main patient characteristics (n=459)**

| | |
|---|---|
| Male, n (%) | 280 (61%) |
| Female, n (%) | 179 (39%) |
| Median age (IQR), y | 62 (56-67) |
| M protein, n (%) | |
| Missing | 29 (6.3%) |
| IgG | 251 (58.4%) |
| IgA | 69 (16%) |
| Light Chains | 96 (22.3%) |
| Other | 14 (3.3%) |
| ISS, n (%) | |
| Missing | 84 (18.3%) |
| I | 140 (37%) |
| II | 124 (33%) |
| III | 111 (30%) |
| Cytogenetics, n (%) | |
| Missing | 48 (10.5%) |
| Standard-risk | 283 (68.9%) |
| t(11;14) | 64 (15.6%) |
| HRCA | 128 (31.1%) |
| Del(17p) | 40 (9.7%) |
| t(4;14) | 42 (10.2%) |
| 1q gain/amplification | 49 (11.9%) |
| t(14;16) | 18 (4.4%) |
| Double HRCA | 19 (4.8%) |
| Induction, n (%) | |
| PI | 411 (89.5%) |
| IMiD | 371 (80.8%) |
| Daratumumab | 87 (19%) |
| Quadruplet | 70 (15.3%) |
| ASCT, n (%) | 368 (80.2%) |
| Maintenance, n (%) | 392 (85.4%) |
| PI | 85 (21.7%) |
| IMiD | 259 (66.1%) |
| Daratumumab | 138 (35.2%) |
| Combination | 148 (37.8%) |

### Summary/Conclusion

Our findings suggest that the underlying cytogenetic landscape influences both the kinetics of MRD clearance and its prognostic significance in multiple myeloma. MRD negativity does not appear to confer uniform prognostic benefit across all cytogenetic subgroups, highlighting biological heterogeneity that may not be captured by conventional high- versus standard-risk classifications.

**Copyright Information:** (Online) ISSN: 2572-9241
©2026 The Author(s). HemaSphere is published by John Wiley & Sons Ltd on behalf of European Hematology Association. This is an open access Abstract Book distributed under the Attribution-NonCommercial-NoDerivs (CC BY-NC-ND), which allows third parties to download the articles and share them with others as long as they credit the author and the Abstract Book, but they cannot change the content in any way or use them commercially.
**Abstract Book Citations:** Authors, Title, HemaSphere, 2026;10:(S1):pages. The abstract book DOIs can be found on the "Author Information" page.
**Disclaimer:** Articles published in the journal HemaSphere exclusively reflect the opinions of the authors. The authors are responsible for all content in their abstracts including accuracy of the facts, statements, citing resources, etc.

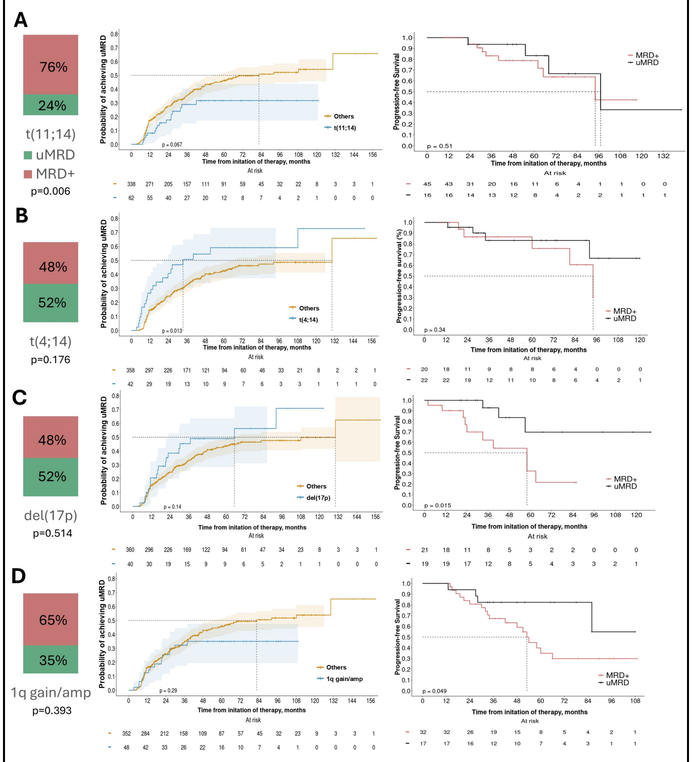

Left: rates of undetectable measurable residual disease (uMRD) according to the main cytogenetic abnormalities. Middle: Kaplan-Meier curves for time to uMRD according to the main cytogenetic abnormalities. Right: Kaplan-Meier curves for progression free survival (PFS) depending on MRD status. A: patients with t(11;14), B: patients with t(4;14), C: patients with del(17p), D: patients with 1q gain or amplification.

**Copyright Information:** (Online) ISSN: 2572-9241
©2026 The Author(s). HemaSphere is published by John Wiley & Sons Ltd on behalf of European Hematology Association. This is an open access Abstract Book distributed under the Attribution-NonCommercial-NoDerivs (CC BY-NC-ND), which allows third parties to download the articles and share them with others as long as they credit the author and the Abstract Book, but they cannot change the content in any way or use them commercially.
**Abstract Book Citations:** Authors, Title, HemaSphere, 2026;10:(S1):pages. The abstract book DOIs can be found on the "Author Information" page.
**Disclaimer:** Articles published in the journal HemaSphere exclusively reflect the opinions of the authors. The authors are responsible for all content in their abstracts including accuracy of the facts, statements, citing resources, etc.

### (PF821) IGH REARRANGEMENTS IN AL AMYLOIDOSIS, CLINICAL CHARACTERISTICS AND OUTCOMES OF A POPULATION BASED STUDY

**Topic:** 14. Myeloma and other monoclonal gammopathies - Clinical

Flores Weverling*[1, 2], Mirian Brink[2], Anne-Marie van der Kevie-Kersemaekers[3], Monique Minnema[1]

*[1]University Medical Center Utrecht, Department of Hematology, Utrecht, The Netherlands;[2]Netherlands Comperhensive Cancer Organisation (IKNL), Department of Research and Development, Utrecht, The Netherlands; [3]University Medical Center Utrecht, Department of Clinical Genetics, Utrecht, The Netherlands;*

### Background

Amyloid light chain (AL) amyloidosis is a plasma cell disease characterized by the deposition of amyloid fibrils, leading to progressive organ damage. The past decade, translocation t(11;14) gained interest as it is the most prevalent cytogenetic aberration in AL amyloidosis, ~40% of the patients harbor this translocation. Shorter event-free survival rates were found in t(11;14)-positive (+) patients treated with bortezomib-based regimens. Currently, treatment strategies have changed with the introduction of daratumumab-containing combination which have prolonged survival especially in t(11;14) positive patients.

### Aims

In this population-based study, we aim to assess the impact of IGH rearrangements (ie. t(11;14), t(4;14), t(14;16) and t(14q32)) on clinical characteristics, hematologic response and overall survival (OS) in treated patients with AL amyloidosis.

### Methods

Patients ≥18 years diagnosed with AL amyloidosis between 2021-2024 were identified in the Netherlands Cancer Registry, survival was available until February 1, 2025. We excluded patients with lymphoplasmacytic lymphoma (n = 61), no treatment (n = 81), no chromosome 14 information (n = 128) or autologous stem cell transplantation (n = 65). Patients were categorized in IGH-negative (IGH-) or IGH-positive (IGH+). IGH+ caeses were further categorized as t(11;14)+; t(14q32)+ or t(4;14)/t(14;16)+.
Best hematological response and OS were assessed by IGH status and by treatment regimen: bortezomib-based without addition of daratumumab (BOR-only) and daratumumab-based upfront regimens (DARA-based). OS was defined as death by any cause.

### Results

Of 287 patient, 131 (33%) were IGH- and 156 IGH+. Of the latter, 44% were t(11;14)+, 40% were t(14q32)+, 6% were t(4;14)+ or t(14;16)+ and 2% with another IGH rearrangement. Patients with t(11;14) had lower levels of paraprotein (0.09 g/L vs. 4.8 g/L, p<0.01), more light chain only disease (53% vs 26%, p<0.01) and were less often accompanied by hyperdiploidy (9% vs. 45%, p<0.01) compared with IGH- patients. To a lesser extent t(14q32)+ patients showed a similar pattern (paraprotein 2.3 g/l, light chain only 44% and hyperdiploidy 17%), suggesting that the majority of patients in this group may carry an underlying t(11;14).
Hematologic responses were evaluable in 243 patients (85%). Complete response (CR) rates were comparable between IGH- and IGH+ patients (IGH-, hem-CR: 45%; [95% confidence interval [CI] 36-55] vs. IGH+ hem-CR 45% [95% CI 37-53]) in the total cohort. In the BOR-based subcohort, IGH- patients achieved higher CR rates (51% [95% CI 38-64]) than IGH+ patients (25% [95% CI 16-37]). In contrast, among DARA-based treated patients, CR rates were lower in IGH- patients (42% [95% CI 30-56]) compared to IGH+ patients (64% [95% CI 51-74]).
One-year OS in IGH- patients was 69% (95% CI 63-74) compared to 61% (95% CI 52-72).

**Copyright Information:** (Online) ISSN: 2572-9241
©2026 The Author(s). HemaSphere is published by John Wiley & Sons Ltd on behalf of European Hematology Association. This is an open access Abstract Book distributed under the Attribution-NonCommercial-NoDerivs (CC BY-NC-ND), which allows third parties to download the articles and share them with others as long as they credit the author and the Abstract Book, but they cannot change the content in any way or use them commercially.
**Abstract Book Citations:** Authors, Title, HemaSphere, 2026;10:(S1):pages. The abstract book DOIs can be found on the “Author Information” page.
**Disclaimer:** Articles published in the journal HemaSphere exclusively reflect the opinions of the authors. The authors are responsible for all content in their abstracts including accuracy of the facts, statements, citing resources, etc.

**Summary/Conclusion**

Translocation t(11;14) was the most common IGH rearrangement and was associated with a distinct clinical profile, including more frequent light-chain-only disease, lower paraprotein levels, and less hyperdiploidy. Hematologic response rates were similar between IGH- and IGH+ patients. However, in the BOR-only cohort IGH+ patients achieved fewer complete responses, whereas in DARA-based regimens they showed higher complete response rates. These findings support the transition from inferior outcomes with BOR-only therapy to superior outcomes with DARA-based regimens in patients with IGH-rearranged disease, most of whom carry t(11;14).

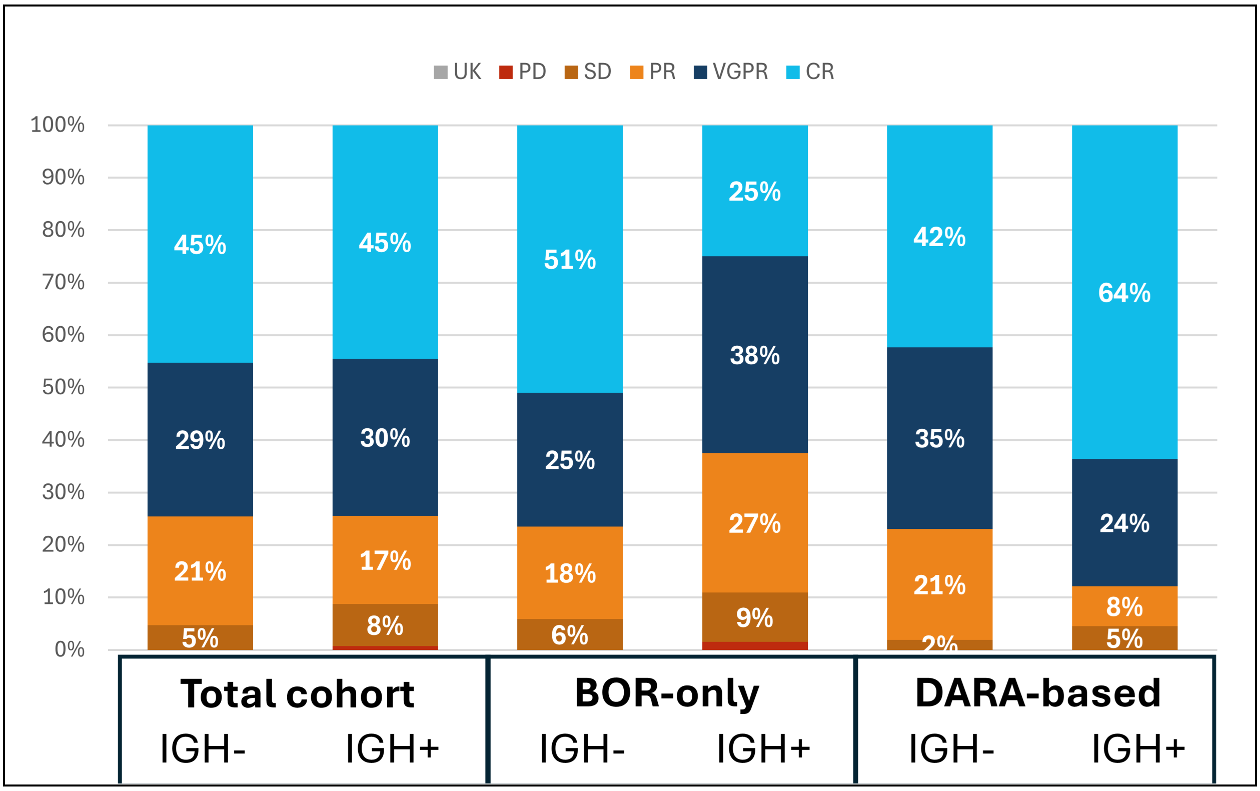

Figure 1 - Best hematologic responses in total cohort, bortezomib-based regimens without daratumumab (BOR-only) and daratumumab-based upfront regimens (DARA-based)

**Copyright Information:** (Online) ISSN: 2572-9241
©2026 The Author(s). HemaSphere is published by John Wiley & Sons Ltd on behalf of European Hematology Association. This is an open access Abstract Book distributed under the Attribution-NonCommercial-NoDerivs (CC BY-NC-ND), which allows third parties to download the articles and share them with others as long as they credit the author and the Abstract Book, but they cannot change the content in any way or use them commercially.
**Abstract Book Citations:** Authors, Title, HemaSphere, 2026;10:(S1):pages. The abstract book DOIs can be found on the "Author Information" page.
**Disclaimer:** Articles published in the journal HemaSphere exclusively reflect the opinions of the authors. The authors are responsible for all content in their abstracts including accuracy of the facts, statements, citing resources, etc.

### (PF822) THE INTERVAL AND RISK FACTORS FROM LOSS OF COMPLETE RESPONSE TO DEFINITE RELAPSE IN NEWLY DIAGNOSED MULTIPLE MYELOMA AFTER AUTOLOGOUS STEM CELL TRANSPLANTATION

**Topic:** 14. Myeloma and other monoclonal gammopathies - Clinical

Yang Liu*[1], Jingyi Bi[1], Jin Lu[1]

*[1]Peking University People's Hospital, Hematology, Beijing, China;*

### Background

Based on IMWG criteria, relapse from complete response (CR) is an endpoint for disease-free survival in multiple myeloma (MM), and clinical treatment is not adjusted for isolated loss of CR (LoCR) unless relapse criteria are met. However, clinical data on the time from LoCR to definite biochemical/clinical relapse and its associated risk factors remain scarce. Clarifying these aspects is meaningful for understanding the prognosis of this subgroup and formulating subsequent treatment strategies, which lacks relevant research support currently.

### Aims

This study retrospectively analyzes newly diagnosed MM patients who achieved CR then experienced LoCR after autologous stem cell transplantation (ASCT) to evaluate the time interval from LoCR to biochemical/clinical relapse and identify the risk factors for rapid disease progression in this population.

### Methods

A retrospective review was conducted on ASCT-treated MM patients at Peking University People's Hospital from 2011 to 2022, with 47 eligible patients enrolled per strict criteria (post-ASCT CR followed by LoCR, unchanged maintenance therapy after LoCR, regular 3-months' follow-up, etc.). LoCR was defined as reappearance of M-protein (or elevated dFLC for non-measurable M-protein cases) without meeting definitive relapse criteria. The study used swimmer plots to show individual disease timelines, Kaplan-Meier analysis for cumulative relapse incidence, and univariate Cox regression to screen risk factors for shorter time to relapse, with subgroup analysis for high-risk vs. standard-risk patients.

### Results

The median interval from LoCR to definite relapse was 8.0 months (range: 2.0–35.0 months); 87.2% (41/47) patients progressed to definitive relapse during follow-up, while 12.8% (6/47) had isolated biochemical recurrence only. Kaplan-Meier analysis showed 3-, 6-, and 12-month relapse-free rates of 19.1%, 36.2%, and 70.2% after LoCR, respectively, with notable heterogeneity in individual disease progression. Univariate Cox regression identified three key factors: shorter time from CR to LoCR (HR=0.961, p[illegible]0.001), R-ISS stage III (HR=2.182, p=0.017) and t(11;14) (HR=0.373, p=0.046) were associated with accelerated relapse.

**Copyright Information:** (Online) ISSN: 2572-9241
©2026 The Author(s). HemaSphere is published by John Wiley & Sons Ltd on behalf of European Hematology Association. This is an open access Abstract Book distributed under the Attribution-NonCommercial-NoDerivs (CC BY-NC-ND), which allows third parties to download the articles and share them with others as long as they credit the author and the Abstract Book, but they cannot change the content in any way or use them commercially.
**Abstract Book Citations:** Authors, Title, HemaSphere, 2026;10:(S1):pages. The abstract book DOIs can be found on the "Author Information" page.
**Disclaimer:** Articles published in the journal HemaSphere exclusively reflect the opinions of the authors. The authors are responsible for all content in their abstracts including accuracy of the facts, statements, citing resources, etc.

| n (%) | All Patients(n=47) |
|---|---|
| Male | 32 (68.1) |
| Age (median,range) | 54 (35,67) |
| ISS (1/2/3) | 12/16/19 (25.6/34.0/40.4) |
| R-ISS (1/2/3/NA) | 7/26/10/4 (14.9/55.3/21.3/8.5) |
| 1q21+ | 26 (55.3) |
| Del(17P)+ | 2 (4.3[illegible] |
| T(4;14)+ | 10 (21.3) |
| T(11;14)+ | 7 (14.9) |
| High LDH | 6 (12.8) |
| Low PLT | 4 (8.5) |
| PC in peripheral (0/1/2/3) | 39/3/3/2 (83.0/6.3/6.3/4.4) |
| EMD | 14(29.8) |
| Induction Therapy (RVd/VCd/Others) | 28/9/10 (59.6/19.1/21.3) |
| Single OR Tandem ASCT | 42/5 (89.4/10.6) |
| Maintenance (R/IR/V/Others) | 20/10/2/15 (42.6/21.3/4.2/31.9) |

**Copyright Information:** (Online) ISSN: 2572-9241
©2026 The Author(s). HemaSphere is published by John Wiley & Sons Ltd on behalf of European Hematology Association. This is an open access Abstract Book distributed under the Attribution-NonCommercial-NoDerivs (CC BY-NC-ND), which allows third parties to download the articles and share them with others as long as they credit the author and the Abstract Book, but they cannot change the content in any way or use them commercially.
**Abstract Book Citations:** Authors, Title, HemaSphere, 2026;10:(S1):pages. The abstract book DOIs can be found on the "Author Information" page.
**Disclaimer:** Articles published in the journal HemaSphere exclusively reflect the opinions of the authors. The authors are responsible for all content in their abstracts including accuracy of the facts, statements, citing resources, etc.

### Summary/Conclusion

This real-world study confirms a median of 8 months' interval from LoCR to definite relapse in ASCT-treated MM patients, with R-ISS stage and time from CR to LoCR as risk factors for rapid progression. Larger prospective studies are needed to validate these findings.

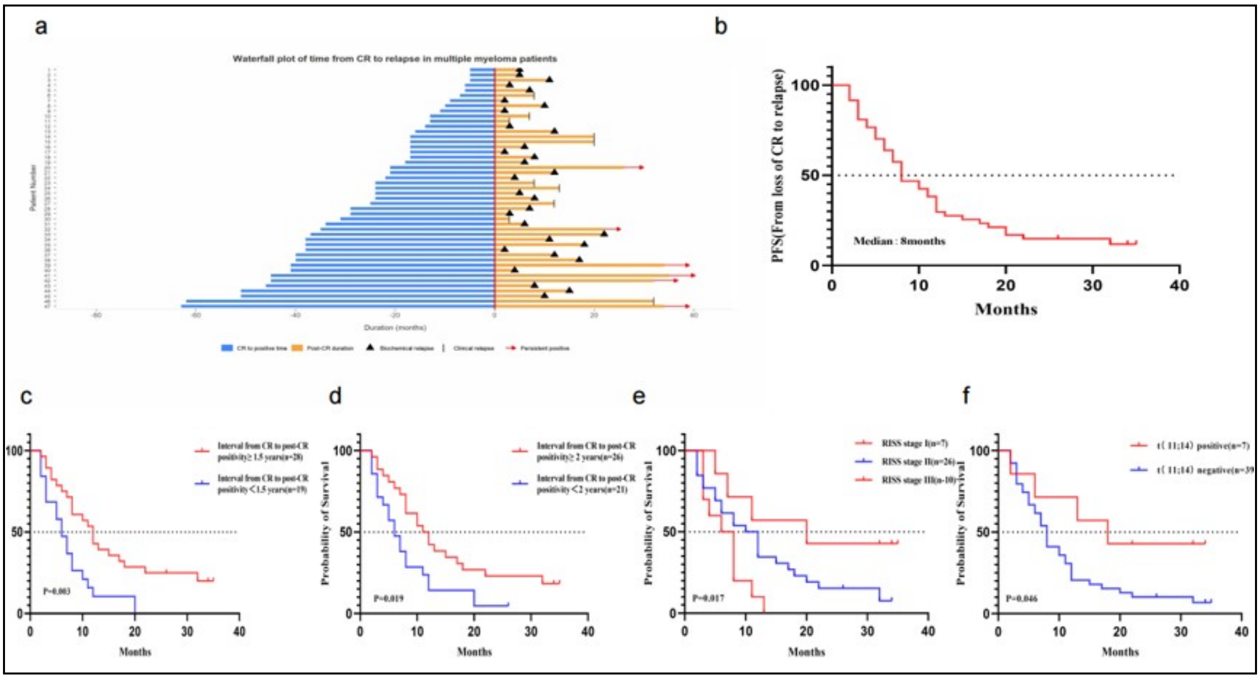

**Copyright Information:** (Online) ISSN: 2572-9241
©2026 The Author(s). HemaSphere is published by John Wiley & Sons Ltd on behalf of European Hematology Association. This is an open access Abstract Book distributed under the Attribution-NonCommercial-NoDerivs (CC BY-NC-ND), which allows third parties to download the articles and share them with others as long as they credit the author and the Abstract Book, but they cannot change the content in any way or use them commercially.
**Abstract Book Citations:** Authors, Title, HemaSphere, 2026;10:(S1):pages. The abstract book DOIs can be found on the "Author Information" page.
**Disclaimer:** Articles published in the journal HemaSphere exclusively reflect the opinions of the authors. The authors are responsible for all content in their abstracts including accuracy of the facts, statements, citing resources, etc.

### (PF823) INFLAMMATION SCORE (I-SCORE) IN ELDERLY PATIENTS WITH MULTIPLE MYELOMA: A NEW EASY TOOL TO ASSESS PROGNOSIS.

**Topic:** 14. Myeloma and other monoclonal gammopathies - Clinical

Velia Bongarzoni*[1], Barbara Anaclerico[1], Gabriella Tomei[2], Sonia More[3], Francesca Di Landro[4], Francesca Fazio[5], Carmine Liberatore[6], Giusy Antolino[7], Silvia Gentili[8], Laura De Padua[9], Luca Cupelli[10], Svitlana Gumenyuk[11], Ombretta Annibali[12], Mariagrazia Garzia[13], Luciano Fiori[14], Angela Rago[15], Paola Arcuri[4], Elena Rossi[4], Maria Teresa Petrucci[5], Massimo Offidani[3], Alessandro Andriani[2], Roberto Latagliata[2]

*[1]UOC Hematology - San Giovanni Hospital, Rome, Italy;[2]UOC Hematology - Santa Rosa Hospital, Viterbo, Italy;[3]Hematology Clinic, AOU delle Marche, Ancona, Italy; [4]Hematology Clinic, Policlinico Agostino Gemelli, Rome, Italy;[5]Hematology, Department of Translational and Precision Medicine, Sapienza University of Rome, Rome, Italy;[6]Clinical Hematology Unit, Department of Oncology and Hematology - Santo Spirito Civil Hospital, Pescara, Italy; [7]Hematology - Sant'Andrea Hospital, Rome, Italy;[8]UOSD Hematology - AST Macerata, Civitanova Marche, Italy;[9]UOC Hematology - Spaziani Hospital, Frosinone, Italy; [10]UOC Hematology - Sant'Eugenio Hospital, Rome, Italy; [11]Hematology - IRCCS Regina Elena National Cancer Institute, Rome, Italy; [12]Hematology - Campus Biomedico University, Rome, Italy; [13]UOC Hematology - San Camillo Hospital, Rome, Italy; [14]UOC Hematology - Santa Maria Goretti Hospital, Latina, Italy; [15]UOSD Hematology - ASL Roma 1, Rome, Italy;*

### Background

In patients with Multiple Myeloma (MM) chronic inflammation promotes malignant progression by altering tumor microenvironment (TME) and proliferation, with development of drug resistance. Aging itself is associated with a chronic low-grade inflammation, the so-called inflammaging, due to imbalance of the immune system. Interleukin-6 (IL-6) increases many inflammatory parameters, like Reactive C Protein (RCP), Fibrinogen (Fb) and Ferritin (Fe).  However, the relationship between these inflammatory biomarkers and the prognosis of MM is still unknown.

### Aims

To provide an easy and cost-effective prognostic model, we evaluated inflammation parameters in newly diagnosed elderly (>75 years) patients with MM and correlated their levels with age, haemoglobin (Hb), ISS and overall survival (OS).

### Methods

A cohort of 109 elderly patients with MM newly diagnosed in 11 hematology departments in central Italy from Jan 2019 to Apr 2025 was collected and analysed. Patients were stratified according to blood levels of RCP, Fb and Fe. An Inflammation score (I-score) was built by assigning 1 point each to elevated RCP (>0.5 mg/L), Fb (>400 mg/dl) and Fe (>275 µg/L) and two different risk groups were defined: low-risk group (I-score 0) with 0-1 point and high-risk group  (I-Score 1) with 2-3 points.

**Copyright Information:** (Online) ISSN: 2572-9241
©2026 The Author(s). HemaSphere is published by John Wiley & Sons Ltd on behalf of European Hematology Association. This is an open access Abstract Book distributed under the Attribution-NonCommercial-NoDerivs (CC BY-NC-ND), which allows third parties to download the articles and share them with others as long as they credit the author and the Abstract Book, but they cannot change the content in any way or use them commercially.
**Abstract Book Citations:** Authors, Title, HemaSphere, 2026;10:(S1):pages. The abstract book DOIs can be found on the “Author Information” page.
**Disclaimer:** Articles published in the journal HemaSphere exclusively reflect the opinions of the authors. The authors are responsible for all content in their abstracts including accuracy of the facts, statements, citing resources, etc.

### Results

Among the 109 patients, 73 (66.9%) had an I-score 0 and 36 (33.1%) an I-score 1.  MM was preceded by MGUS and SMM in 33,9% and 12,8% of the patients in the whole cohort, without differences between the two I-score groups. No difference was also observed between the two I-score groups as to median age  (79.7 versus 80.9 years, p=0.28). Patients in the I-score 1 had a significant higher rate of anemia (Hb ≤10 g/dl), renal impairement (creatinine levels > 2 mg/L) and β2 microglobulin elevation (p<0.0001, p=0.0003 and p= 0.0025, respectively). In addition, patients in the I-score 1 group had a higher rate of ISS 3 compared to patients in the I-score 0 group (p=0.0086).  As to treatment, 34 patients (31.19%) received dara-based triplets, 38 (34.8%) bortezomib-based doublets, 25 (22.9%) lenalidomide-based schemes and 12 (11%) MP and other low dose therapy. At the last follow-up, 67 patients (61,64%) are still alive, 5 (4,6%) were lost to follow-up and 36 (33%) died. Median OS of the whole cohort was 47.0 months (95%CI 32.0 – 65.0). Median OS of patients in the I-score 0 group was 51.9 months (95%CI 38.3 – 72.3) compared to 30.2 months (95%CI 12.6 – 68.8) of patients in the I-score 1 group (p=0.0083) (Figure).

### Summary/Conclusion

I-score, which is very simple to assess,  showed a good correlation with parameters of disease aggressiveness at diagnosis and with OS, implementing ISS staging and providing a cost-effective risk stratification tool. It could be a good method to identify high-risk MM patients, in particular among elderly patients, in which karyotypic and molecular analyses are not routinarily performed.

Figure – Overall survival according to I-score

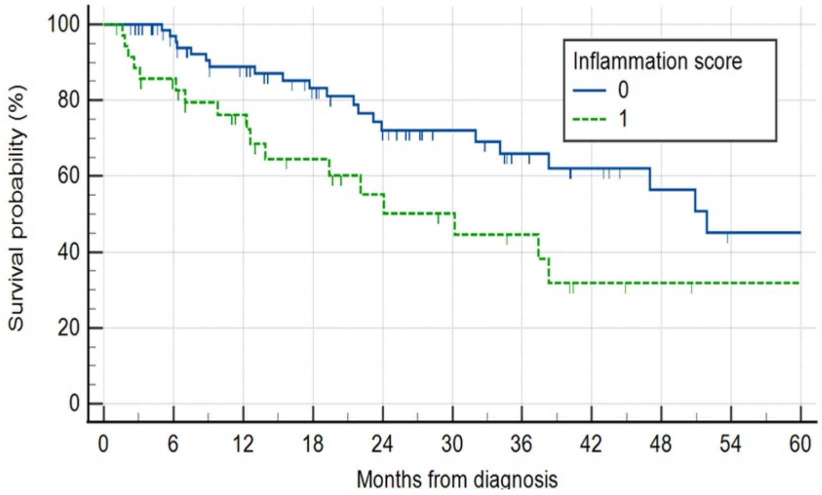

**Copyright Information:** (Online) ISSN: 2572-9241
©2026 The Author(s). HemaSphere is published by John Wiley & Sons Ltd on behalf of European Hematology Association. This is an open access Abstract Book distributed under the Attribution-NonCommercial-NoDerivs (CC BY-NC-ND), which allows third parties to download the articles and share them with others as long as they credit the author and the Abstract Book, but they cannot change the content in any way or use them commercially.
**Abstract Book Citations:** Authors, Title, HemaSphere, 2026;10:(S1):pages. The abstract book DOIs can be found on the "Author Information" page.
**Disclaimer:** Articles published in the journal HemaSphere exclusively reflect the opinions of the authors. The authors are responsible for all content in their abstracts including accuracy of the facts, statements, citing resources, etc.

### (PF824) ROLE OF COMPLETE OR VERY GOOD PARTIAL RESPONSE (CR/VGPR) AND EARLY PROGRESSION AFTER DEXAMETHASONE RITUXIMAB CYCLOPHOSPHAMIDE (DRC) +/- BORTEZOMIB IN WALDENSTRÖM MACROGLOBULINEMIA IN THE ECWM1 TRIAL

**Topic:** 14. Myeloma and other monoclonal gammopathies - Clinical

Pierre Morel*[1], Efstathios Kastritis[2], Caroline Skrzypczak[1], Alexander Grunenberg[3], Lydia Montes[1], Cecile Tomowiak[4], Roman Hájek[5], Andreas Viardot[3], Olivier Tournilhac[6], Therese Aurran[7], Florian Bouclet[8], Hacene Zerazhi[9], Benedicte Hivert Fournier[10], Damien Roos Weil[11], Sophie De Guibert[12], Lena Brandefors[13], Daniela Robu[14], Ramón García Sanz[15], Maria Ramos Lopes Gomes Da Silva[16], Eva Kimby[17], Birgit Schmelzle[3], Dajana Kaszynski[3], Melanie Verlay[14], Jens Dreyhaupt[3], Meletios A. Dimopoulos[2], Christian Buske[3]

*[1]CHU Amiens Picardie, Amiens, France; [2]National and Kapodistrian University of Athens, Alexandra Hospital, Athens, Greece; [3]University Hospital Ulm, Ulm, Germany; [4]CHU Poitiers, Poitiers, France; [5]University Hospital Ostrava and University of Ostrava, Ostrava, Czechia; [6]CHU Clermont Ferrand, Clermont Ferrand, France; [7]Institut Paoli Calmettes, Marseille, France; [8]Centre Henri Becquerel, Rouen, France; [9]Centre Hospitalier d'Avignon, Avignon, France; [10]GHICL, Lille, France; [11]Hopital Pitie Salpetriere, Paris, France; [12]CHU de Rennes, Rennes, France; [13]Sunderbyn hospital, Lulea, Sweden; [14]Centre Hospitalier de Lens, Lens, France; [15]Hospital General Universitario Gregorio Marañón, Madrid, Spain; [16]Instituto Portugues de Oncologia de Lisboa, Lisboa, Portugal; [17]Karolinska Institute, Stockholm, Sweden;*

### Background

It is one of the major goals to develop early surrogate markers for treatment outcome in the management of Waldenström's Macroglobulinemia (WM). We recently reported on the ECWM1 trial, which is one of the largest prospectively randomized clinical trials performed in WM (Blood 2025; 146 Suppl 1: 223). The final analysis of the ECWM1 trial showed that the DRC regimen with or without Bortezomib was associated with an excellent treatment outcome in the majority of patients, with a median progression-free survival (PFS) of 50.1 and 55.9 months, respectively and with 15% overall death in the patient population after a median follow-up of 68.8 months. On the other hand a limited number of patients showed early progression indicating a limited number of high-risk patients identifiable at baseline.

### Aims

We took advantage of the prespecified follow-up monitoring prospectively performed in 195 patients of the study to assess the prognostic role of the depth of response and progression.

### Methods

Response was assessed with PFS and subsequent PFS (SPFS, starting from response assessment) as endpoints. We also fitted a 4 states model of survival (OS, treatment initiation, response, progression, final status). These analyses were repeated with time to 2nd treatment (T2T) and subsequent T2T (ST2T, starting from response assessment) as endpoint and a similar 4 states model (2nd treatment instead of progression). The endpoints for assessing progression were OS and survival after the first progression (SA1P). We also fitted a 3 states model of OS.

**Copyright Information:** (Online) ISSN: 2572-9241
©2026 The Author(s). HemaSphere is published by John Wiley & Sons Ltd on behalf of European Hematology Association. This is an open access Abstract Book distributed under the Attribution-NonCommercial-NoDerivs (CC BY-NC-ND), which allows third parties to download the articles and share them with others as long as they credit the author and the Abstract Book, but they cannot change the content in any way or use them commercially.
**Abstract Book Citations:** Authors, Title, HemaSphere, 2026;10:(S1):pages. The abstract book DOIs can be found on the "Author Information" page.
**Disclaimer:** Articles published in the journal HemaSphere exclusively reflect the opinions of the authors. The authors are responsible for all content in their abstracts including accuracy of the facts, statements, citing resources, etc.

### Results

In Cox models of PFS and T2T with response coded by three time dependent covariates, only attainment of a CR/VGPR retained a significant prognostic value. Dynamic landmarking analysis over the first 36 months, identified a significant favorable effect only for achieving CR/VGPR for SPFS for nearly all time points after 7 months. Similar results were observed for ST2T.
The four states model provided fixed horizon prediction of being progression-free at 5 years after treatment initiation. In agreement with previous results, it showed higher probability of being progression-free only in case of achieving CR/VGPR. Once again, similar results were observed in fixed horizon prediction analyses of T2T at 5 years. Thus, the 36 patients who achieved CR/VGPR at 12 months had a median SPFS of 62 months (95% confidence inyterval [95CI]:56; n.a.) vs. 37 months (95CI: 26; 48, p=0.0022, Figure 1) for the 126 patients that did not achieve CR/VGPR.
There was no significant difference in survival after 24 months between patients being progression-free at this time point or not (p=0.20). SA1P was shorter only for the 38 patients who progressed before 22 months. In agreement with these results, only this limited subset of patients had reduced fixed horizon prediction of being alive at 5 years after treatment initiation.

### Summary/Conclusion

In patients with WM receiving first-line chemoimmunotherapy, failure to achieve CR/VGPR at 12 months, is associated with significantly shorter PFS. This endpoint should be considered as an endpoint of interest for future trials, providing a potential early surrogate for PFS in clinical trials in WM.

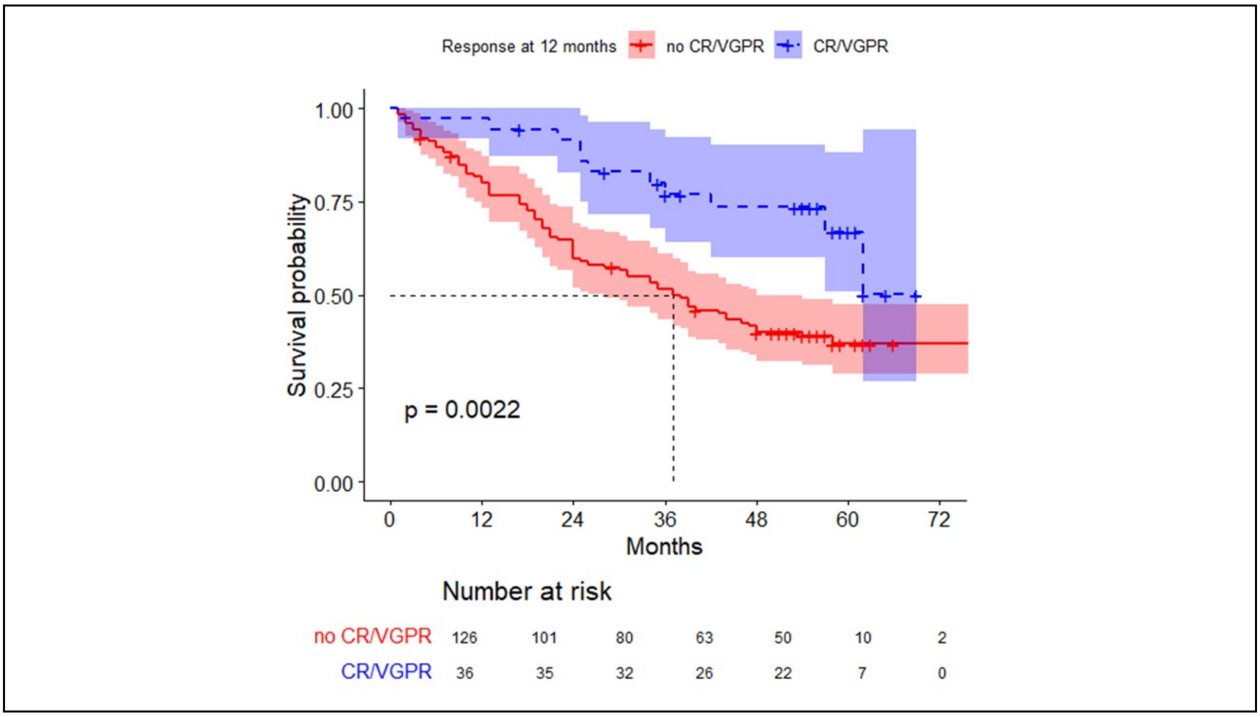

Figure 1: Progression-free survival after 12 months according to CR/VGPR status at this time point

Copyright Information: (Online) ISSN: 2572-9241
©2026 The Author(s). HemaSphere is published by John Wiley & Sons Ltd on behalf of European Hematology Association. This is an open access Abstract Book distributed under the Attribution-NonCommercial-NoDerivs (CC BY-NC-ND), which allows third parties to download the articles and share them with others as long as they credit the author and the Abstract Book, but they cannot change the content in any way or use them commercially.
Abstract Book Citations: Authors, Title, HemaSphere, 2026;10:(S1):pages. The abstract book DOIs can be found on the "Author Information" page.
Disclaimer: Articles published in the journal HemaSphere exclusively reflect the opinions of the authors. The authors are responsible for all content in their abstracts including accuracy of the facts, statements, citing resources, etc.

### (PF825) UPDATED SAFETY OF ISATUXIMAB SUBCUTANEOUS AT-HOME ADMINISTRATION BY ON-BODY INJECTOR IN RELAPSED/REFRACTORY MULTIPLE MYELOMA IN THE PHASE 3 IRAKLIA STUDY

**Topic:** 14. Myeloma and other monoclonal gammopathies - Clinical

Sikander Ailawadhi[1], Fredrik Schjesvold[2], Claudio Cerchione*[3], Péter Rajnics[4], Massimo Offidani[5], Masaki Ri[6], Satoshi Ito[7], Enrique M. Ocio[8], Christine Rojas[9], Florence Suzan[10], Rick Zhang[11], Maya Stefanova-Urena[11], María-Victoria Mateos[12], Silvia Ling[13]

*[1]Division of Hematology/Oncology, Mayo Clinic, Jacksonville, FL, United States of America;[2]Oslo Myeloma Center, Department of Hematology, Oslo University Hospital, Oslo, Norway; [3]Hematology Unit, Istituto Romagnolo per lo Studiodei Tumori "Dino Amadori" - IRST IRCCS, Meldola (FC), Italy; [4]Mór Kaposi Teaching Hospital, Kaposvár, Hungary; [5]Clinica di Ematologica Ospedali Riuniti Ancona, Ancona, Italy; [6]Nagoya City University Institute of Medical and Pharmaceutical Sciences, Department of Hematology and Oncology, Nagoya, Japan; [7]Yamagata University Faculty of Medicine, Yamagata, Japan;[8]Valdecilla Health Research Institute, Universidad de Cantabria, Hematology Department, Santander, Spain; [9]Universidad Católica del Maule, Talca, Chile; [10]Sanofi, Vitry-sur-Seine, France; [11]Sanofi, Morristown, NJ, United States of America; [12]Hospital Universitario de Salamanca-Instituto Biosanitario de Salamanca, Salamanca, Spain; [13]Department of Haematology, Liverpool Hospital, NSW, Australia;*

### Background

At-home administration of oncology therapies may help address health disparities and treatment adherence barriers, while offering greater flexibility and convenience to support long-term disease control. At-home administration was studied in a subset of patients in IRAKLIA (NCT05405166), an ongoing, global, non-inferiority Phase 3 trial investigating isatuximab (Isa) subcutaneous (SC) versus intravenous (IV) administration plus pomalidomide and dexamethasone (Pd) in patients with relapsed/refractory multiple myeloma (RRMM). In IRAKLIA, Isa SC is administered using an on-body injector (OBI)—a wearable, automated injector with a small, retractable needle. Isa SC OBI showed non-inferiority to Isa IV for the co-primary endpoints of efficacy and pharmacokinetics, with higher patient satisfaction and a lower incidence of infusion reactions. Previously, we showed the safety and feasibility of at-home administration in IRAKLIA.

### Aims

Here, we show an update of the results of at-home Isa SC OBI administration by an HCP with data from an additional 93 at-home injections.

### Methods

In IRAKLIA, patients with RRMM aged ≥18 years with ≥1 prior line of therapy were randomly assigned 1:1 to Isa SC OBI (1400 mg; n=263) or IV (10 mg/kg; n=268) weekly in Cycle (C)1, then every 2 weeks, + P (4 mg/day, Day [D]1–21) + d (40 mg [20 mg if aged ≥75 years] weekly) in 4-week cycles. After 5 cycles, at-home administration by an HCP was optional for Isa SC OBI patients on D15 from C6 onward (D1 visits from C6 remained clinic based). At-home eligibility depended on an absence of infusion reactions at C4 and C5, hematology test results at D1 of each cycle, investigator judgment, and patient willingness. During at-home visits, HCPs applied and disposed of Isa SC OBI, evaluated the injection site and vital signs, and completed documentation. For the first isatuximab injection performed at home, an additional assessment was to be performed 30 min after the end of the injection. Thereafter, local tolerability was assessed at end of injection.

**Copyright Information:** (Online) ISSN: 2572-9241
©2026 The Author(s). HemaSphere is published by John Wiley & Sons Ltd on behalf of European Hematology Association. This is an open access Abstract Book distributed under the Attribution-NonCommercial-NoDerivs (CC BY-NC-ND), which allows third parties to download the articles and share them with others as long as they credit the author and the Abstract Book, but they cannot change the content in any way or use them commercially.
**Abstract Book Citations:** Authors, Title, HemaSphere, 2026;10:(S1):pages. The abstract book DOIs can be found on the "Author Information" page.
**Disclaimer:** Articles published in the journal HemaSphere exclusively reflect the opinions of the authors. The authors are responsible for all content in their abstracts including accuracy of the facts, statements, citing resources, etc.

### Results

In the ongoing IRAKLIA trial, 16 (6.1%) out of 263 patients treated with Isa SC OBI participated in at-home administration (cut-off date: August 29, 2025; median follow-up period: 21.75 months), with 148 injections performed (none interrupted). Most patients started at-home administration at C6 (n=7), with the remaining patients starting at C7+ (n=9). More than half (68.8%) switched to at-home administration and have not returned to the hospital for the D15 visit of their next cycle. The median injection duration was similar between clinic (13 min) and at-home administration (12 min). At-home administration was well tolerated with no new safety signals and a 100% completed injection rate. It was also associated with a low rate of injection-site reactions (ISRs; n=1); the single grade 1 ISR consisted of injection-site swelling, which resolved the same day. No other adverse events, systemic infusion reactions, or device events were associated with at-home administration.

### Summary/Conclusion

Updated data from the ongoing IRAKLIA trial demonstrated that at-home administration by an HCP is feasible and well tolerated. Median injection time and success rate are consistent with in-clinic use. These data support the safety of Isa SC OBI administration at home by an HCP in addition to in the clinic, enabling more flexible, accessible, and comfortable care for patients with MM.

**Copyright Information:** (Online) ISSN: 2572-9241
©2026 The Author(s). HemaSphere is published by John Wiley & Sons Ltd on behalf of European Hematology Association. This is an open access Abstract Book distributed under the Attribution-NonCommercial-NoDerivs (CC BY-NC-ND), which allows third parties to download the articles and share them with others as long as they credit the author and the Abstract Book, but they cannot change the content in any way or use them commercially.
**Abstract Book Citations:** Authors, Title, HemaSphere, 2026;10:(S1):pages. The abstract book DOIs can be found on the “Author Information” page.
**Disclaimer:** Articles published in the journal HemaSphere exclusively reflect the opinions of the authors. The authors are responsible for all content in their abstracts including accuracy of the facts, statements, citing resources, etc.

### (PF826) CLONAL PLASMA CELL S-PHASE AFTER AUTOLOGOUS STEM CELL TRANSPLANTATION PREDICTS SURVIVAL OUTCOMES IN MULTIPLE MYELOMA

**Topic:** 14. Myeloma and other monoclonal gammopathies - Clinical

Tamer Hellou[1], Daniel Packard[1], Maximilian Steinhardt*[1], Shaji Kumar[1], Angela Dispenzieri[1], Saurabh Zanwar[1], Dragan Jevremovic[1], Horatiu Olteanu[1], Pedro Horna[1], Gregory Otteson[1], Francis Buadi[1], David Dingli[1], Suzanne Hayman[1], Prashant Kapoor[1], Nelson Leung[1], Joselle Cook[1], Nadine Abdallah[1], Moritz Binder[1], Eli Muchtar[1], Rahma Warsame[1], Taxiarchis Kourelis[1], Ronald Go[1], Yi Lin[1], Vincent Rajkumar[1], Wilson Gonsalves[1], Morie Gertz[1]

[1]*Mayo Clinic, Department of Biomedical Informatics, Rochester, United States of America;*

**Background**

Risk stratification in multiple myeloma (MM) relies on staging systems assessing baseline variables; however, early relapses regularly occur in patients with favorable risk features, suggesting further factors may be relevant for prognosis. An increased proportion of clonal plasma cells in S-phase at diagnosis identifies a high-risk phenotype associated with inferior survival, independent of established prognostic models.

**Aims**

To evaluate the clinical significance of S-phase after ASCT and its association with survival outcomes.

**Methods**

We performed a retrospective analysis of 1136 patients with MM undergoing ASCT at Mayo Clinic Rochester between 2013 and 2024. Patients without available post-ASCT assessment of clonal plasma cell S-phase within one year were excluded, resulting in a final evaluable cohort of 150 patients. The proportion of clonal plasma cells in S-phase was determined by DNA content between G0/G1 and G2/M peaks and reported as a percentage. High-risk cytogenetics by FISH were defined as proof of either del(17p), t(4;14), t(14;16), t(14;20), or 1q21 gain/amp. S-phase was assessed at the first post-ASCT marrow evaluation within predefined windows: day +60 (D45–75) and/or day +100 (D76–130); when both were available, the earlier assessment was used. Patients were categorized as low (<2%) or high (≥2%) post-ASCT S-phase. Patients with paired S-phase assessments before and post-ASCT, were further classified as low-low, high-low, low-high, and high-high by S-phase results. Multivariable Cox proportional hazards models adjusted for cytogenetic risk, maintenance therapy, disease response at transplantation, International Staging System (ISS), and age.

**Copyright Information:** (Online) ISSN: 2572-9241
©2026 The Author(s). HemaSphere is published by John Wiley & Sons Ltd on behalf of European Hematology Association. This is an open access Abstract Book distributed under the Attribution-NonCommercial-NoDerivs (CC BY-NC-ND), which allows third parties to download the articles and share them with others as long as they credit the author and the Abstract Book, but they cannot change the content in any way or use them commercially.
**Abstract Book Citations:** Authors, Title, HemaSphere, 2026;10:(S1):pages. The abstract book DOIs can be found on the "Author Information" page.
**Disclaimer:** Articles published in the journal HemaSphere exclusively reflect the opinions of the authors. The authors are responsible for all content in their abstracts including accuracy of the facts, statements, citing resources, etc.

### Results

Thirty-six patients (24%) had high post-ASCT S-phase (≥2%) and 114 (76%) had low post-ASCT S-phase. Advanced ISS stage was more frequent among patients with high post-ASCT S-phase (ISS II/III: 78% vs 57%; p = 0.02), whereas cytogenetic risk was not associated with S-phase categories. Baseline demographic and treatment characteristics, including age, depth of response prior to ASCT, induction regimen, and maintenance therapy, were comparable
Patients with high post-ASCT S-phase experienced significantly inferior PFS compared with those with low post-ASCT S-phase, with a median PFS of 17.0 versus 39.7 months, respectively (p < 0.001). In multivariable Cox regression analysis adjusting for cytogenetic risk, maintenance therapy, disease response before ASCT, ISS stage, and age, high S-phase post-ASCT remained independently associated with inferior PFS (HR 1.76, 95% CI 1.00–3.09; p = 0.049). In an analysis of 142 patients with available paired S-phase assessments, dynamic S-phase trajectories further stratified outcomes. Median PFS was longest in patients with persistently low S-phase (low→low; 39.7 months), intermediate in those converting from high to low S-phase (high→low; 27.2 months), and shortest in patients with newly emergent or persistently high post-ASCT S-phase (low→high: 15.0 months; high→high: 11.0 months) (p = 0.0019).

### Summary/Conclusion

High proliferation defined by S-phase ≥2% of residual clonal plasma cells after ASCT identifies a high-risk subset of MM patients independent of common risk stratifications and depth of response. Changes in post-ASCT S-phase status may have a role in refining risk stratification during treatment. Post-ASCT clonal plasma cell S-phase represents a flow cytometry-based biomarker that can inform risk-adapted therapeutic strategies.

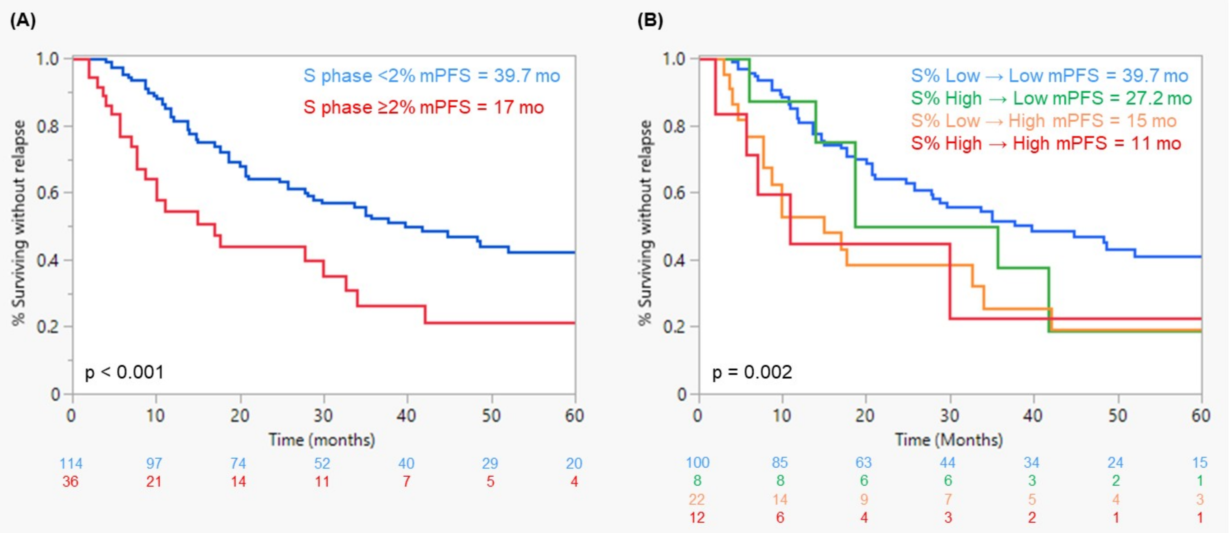

**Figure 1. Progression-free survival according to post-ASCT clonal plasma cell S-phase**
**(A)** Kaplan–Meier plot of PFS stratified by post-ASCT S-phase high/low
**(B)** Kaplan–Meier plot of PFS by longitudinal S-phase results prior/post ASCT

Copyright Information: (Online) ISSN: 2572-9241
©2026 The Author(s). HemaSphere is published by John Wiley & Sons Ltd on behalf of European Hematology Association. This is an open access Abstract Book distributed under the Attribution-NonCommercial-NoDerivs (CC BY-NC-ND), which allows third parties to download the articles and share them with others as long as they credit the author and the Abstract Book, but they cannot change the content in any way or use them commercially.
Abstract Book Citations: Authors, Title, HemaSphere, 2026;10:(S1):pages. The abstract book DOIs can be found on the "Author Information" page.
Disclaimer: Articles published in the journal HemaSphere exclusively reflect the opinions of the authors. The authors are responsible for all content in their abstracts including accuracy of the facts, statements, citing resources, etc.

### (PF827) PROGNOSTIC VALUE OF SERUM FERRITIN KINETICS IN ANTI-BCMA CAR-T THERAPY FOR RELAPSED/REFRACTORY MULTIPLE MYELOMA

**Topic:** 14. Myeloma and other monoclonal gammopathies - Clinical

James Di Palma-Grisi*[1], Egor Volcotrub[1], Yedidyah Weiss[1], Jason Romancik[2], Eli Zolotov[2], Noa Biran[2], David H. Vesole[2], Pooja Phull[2], Joseph Franz[2], David Siegel[2], Harsh Parmar[2]

*[1]Hackensack University Medical Center, Hackensack, United States of America;[2]John Theurer Cancer Center, Hackensack, United States of America;*

### Background

Anti-BCMA CAR-T therapy has become a standard-of-care treatment for patients with relapsed/refractory multiple myeloma (RRMM). Serum ferritin is an inflammatory marker that increases following CAR-T therapy, and the ferritin level prior to CAR-T infusion has been shown to have prognostic value (Liu et. al., 2023). We evaluated the prognostic impact of post-infusion ferritin kinetics in patients with RRMM treated with BCMA directed CAR-T therapy.

### Aims

To assess the prognostic value of ferritin kinetics post-CAR-T infusion in patients with RRMM.

### Methods

We performed a retrospective single center analysis of 241 consecutive patients who received BCMA-directed CAR-T therapy (cilta-cel or ide-cel) between June 2021 and January 2026. Ferritin levels were collected on days 1 through 9 post-infusion. The area under the curve (AUC) of ferritin was calculated using the trapezoidal rule. Patients were stratified by the 75th percentile threshold for AUC (AUC>=5600 ng/ml.days) (H-FA vs L-FA) and separately for peak ferritin levels (>=1231ng/ml) (H-FP vs L-FP). Progression-free survival (PFS) and overall survival (OS) were analyzed using the Kaplan-Meier method. Univariate and multivariate analyses were performed using the Cox proportional hazards model.

**Copyright Information:** (Online) ISSN: 2572-9241
©2026 The Author(s). HemaSphere is published by John Wiley & Sons Ltd on behalf of European Hematology Association. This is an open access Abstract Book distributed under the Attribution-NonCommercial-NoDerivs (CC BY-NC-ND), which allows third parties to download the articles and share them with others as long as they credit the author and the Abstract Book, but they cannot change the content in any way or use them commercially.
**Abstract Book Citations:** Authors, Title, HemaSphere, 2026;10:(S1):pages. The abstract book DOIs can be found on the "Author Information" page.
**Disclaimer:** Articles published in the journal HemaSphere exclusively reflect the opinions of the authors. The authors are responsible for all content in their abstracts including accuracy of the facts, statements, citing resources, etc.

### Results

Demographic characteristics were as follows: the median age was 67.7 years, 57.4% were male, patients had received a median of 5 prior lines of therapy (IQR 4-6) (LoT), and 53.2% of patients had high-risk cytogenetics by fluorescent in-situ hybridisation. The majority of patients (87.6%) were triple-class refractory. 74.3% patients received cilta-cel while 26.1% patients received ide-cel. The ORR was lower in the H-FA group (81.7% vs 92.8%, p=0.018). Patients with H-FA had a significantly shorter median PFS compared to patients with L-FA (12.2 vs 25.1 months, p<0.0001) and shorted median OS (42.2 vs 44.9 months, p=0.01). Univariate analysis is reported in Table 1. On multivariate analysis, H-FA remained an independent predictor of inferior PFS (HR=2.0, p=0.001) and OS (HR 1.87, p=0.03) even after adjusting for CAR-T product and lymphodepletion therapy. In the subgroup analysis, the ferritin AUC was prognostic for PFS in both the cilta-cel cohort (26.8 months vs 16.9 months; p =0.007) and the ide-cel cohort (12.6 vs 5.2 months; p=0.37). Peak ferritin >=1231ng/ml was independently associated with an inferior OS on multivariate analysis (HR 1.9, p=0.03) but did not reach significance for PFS (HR=1.5, p=0.08). Subgroup analysis for peak ferritin was as follows: the median OS for cilta-cel was not reached for the high peak or the low peak group (p=0.3) but the median OS for ide-cel was 44.9 months in the the high peak group and 14.9 months in the low peak group (p=0.038).

| | HR for PFS | HR for OS |
|---|---|---|
| Age > 68 years | 0.98 (p=0.9) | 1.2 (p=0.6) |
| Male gender | 0.79 (p=0.27) | 1.4 (p=0.26) |
| Fludarabine-cycophosphamide vs bendamustine | 0.44 (p=0.0002) | 0.6 (p=0.15) |
| Cilta-cel vs ida-cel | 0.32 (p<0.0001) | 0.46 (p=0.01) |
| ISS > 2 | 1.2 (p=0.47) | 1.9 (p=0.15) |
| Extramedullary disease | 1.1 (p=0.76) | 1.1 (p=0.76) |
| LoT > 4 | 2.2 (p=0.05) | 1.4 (p=0.6) |
| H-FA | 2.0 (p=0.001) | 1.87 (p=0.03) |
| H-FP | 1.5 (p=0.08) | 1.9 (p=0.03) |

Table 1. Univariate analyses for median PFS and OS.

### Summary/Conclusion

Elevated post-infusion serum ferritin measured as both AUC and peak level is associated with inferior survival outcomes in patients treated with CAR-T therapy. AUC is a more robust prognostic marker than peak ferritin alone. Ferritin kinetics may serve as biomarkers to identify patients at risk of early progression following CAR-T.

**Copyright Information:** (Online) ISSN: 2572-9241
©2026 The Author(s). HemaSphere is published by John Wiley & Sons Ltd on behalf of European Hematology Association. This is an open access Abstract Book distributed under the Attribution-NonCommercial-NoDerivs (CC BY-NC-ND), which allows third parties to download the articles and share them with others as long as they credit the author and the Abstract Book, but they cannot change the content in any way or use them commercially.
**Abstract Book Citations:** Authors, Title, HemaSphere, 2026;10:(S1):pages. The abstract book DOIs can be found on the "Author Information" page.
**Disclaimer:** Articles published in the journal HemaSphere exclusively reflect the opinions of the authors. The authors are responsible for all content in their abstracts including accuracy of the facts, statements, citing resources, etc.

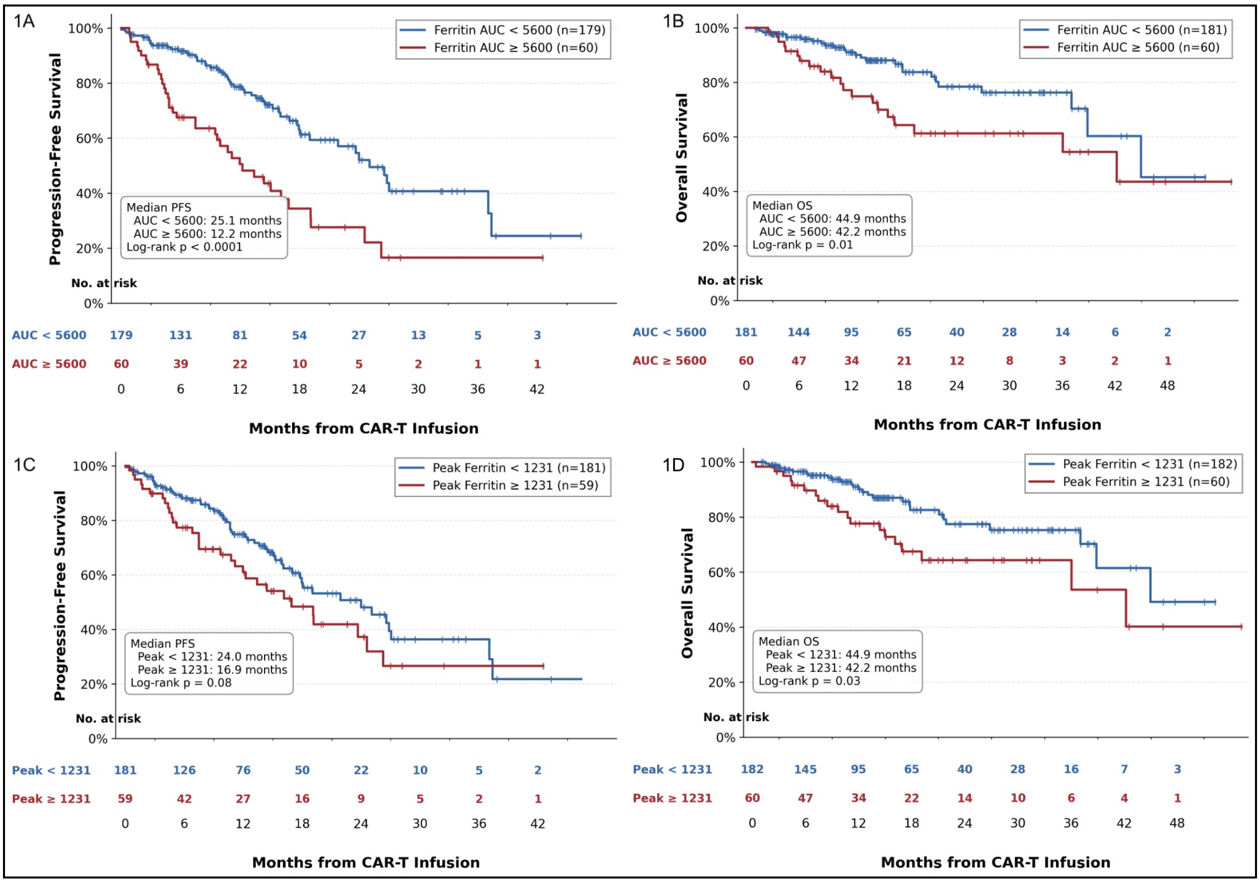

Figure 1. Kaplan-Meier curves for post-CAR-T infusion ferritin AUC PFS (1A) and OS (1B) and peak ferritin PFS (1C) and OS (1D).

**Copyright Information:** (Online) ISSN: 2572-9241
©2026 The Author(s). HemaSphere is published by John Wiley & Sons Ltd on behalf of European Hematology Association. This is an open access Abstract Book distributed under the Attribution-NonCommercial-NoDerivs (CC BY-NC-ND), which allows third parties to download the articles and share them with others as long as they credit the author and the Abstract Book, but they cannot change the content in any way or use them commercially.
**Abstract Book Citations:** Authors, Title, HemaSphere, 2026;10:(S1):pages. The abstract book DOIs can be found on the "Author Information" page.
**Disclaimer:** Articles published in the journal HemaSphere exclusively reflect the opinions of the authors. The authors are responsible for all content in their abstracts including accuracy of the facts, statements, citing resources, etc.

### (PF828) BCMA CAR-T THERAPY WITH RADIOTHERAPY BRIDGING AND MAINTENANCE ACHIEVES UNPRECEDENTED DURABLE RESPONSE IN EXTRAMEDULLARY MYELOMA

**Topic:** 14. Myeloma and other monoclonal gammopathies - Clinical

Lili Zhou*[1], Jian-qing Mi[2], Aibin Liang[3, 4]

*[1]Shanghai Sinounited health, International CAR-T cell therapy center, Hematology department, shanghai, China;[2]Shanghai Institute of Hematology, State Key Laboratory of Medical Genomics, National Research Center for Translational Medicine at Shanghai, Ruijin Hospital Affiliated to Shanghai Jiao Tong University School of Medicine, Shanghai 200025, China., hematology department, shanghai, China; [3]Tongji Hospital, Shanghai, China, Department of Hematology,, shang, China;[4]Tongji Hospital, Shanghai, China, Department of Hematology,, shanghai, China;*

### Background

Extramedullary disease (EMD) in relapsed/refractory multiple myeloma (RRMM) presents a significant clinical challenge due to its aggressive nature and resistance to conventional treatments. Despite promising approaches including B cell maturation antigen (BCMA) targeted chimeric antigen receptor (CAR) T-cell therapy , outcomes for EMD MM remain suboptimal with significantly shorter survival.

### Aims

Given the limited published data focused on this highly challenging subgroup, we provide meaningful real world expeiences with clinical insight , which may inform future therapeutic strategies to improve  long term survival of this poor group of patients with EMM.

### Methods

This multi-centered, retrospective study was to evaluate the clinical efficacy and safety of a novel tri-modal strategy in high-risk RRMM patients with EMD. This strategy integrated bridging radiotherapy, fully humanized BCMA-CAR T-cell therapy (equecabtagene autoleucel), and post-CAR-T maintenance. We retrospectively analyzed twenty consecutive R/R EMD-MM patients from three centers., including 10 consecutive R/R EMD-MM patients who received the fully humanized BCMA-CAR T-cell therapy at the authors' center (6 patients) and two other participating hospitals (Ruijin and Tongji Hospitals, 4 patients) between Januray 2023 and December 2024. Clinical summaries of the 10 cases are provided in Supplementary Table 1. For comparison, ten propensity-matched patients from the same period, who received standard of care (SOC) treatment, were included as a control group. Key inclusion criteria required patients to have: i) high-risk RRMM; ii) EMD confirmed by imaging (e.g., CT, MRI, or PET-CT); iii) adequate organ function to tolerate the procedure; and iv) provide informed consent. Major exclusion criteria included active uncontrolled infections, severe concomitant diseases, pregnancy, or other conditions precluding CAR-T therapy.

**Copyright Information:** (Online) ISSN: 2572-9241
©2026 The Author(s). HemaSphere is published by John Wiley & Sons Ltd on behalf of European Hematology Association. This is an open access Abstract Book distributed under the Attribution-NonCommercial-NoDerivs (CC BY-NC-ND), which allows third parties to download the articles and share them with others as long as they credit the author and the Abstract Book, but they cannot change the content in any way or use them commercially.
**Abstract Book Citations:** Authors, Title, HemaSphere, 2026;10:(S1):pages. The abstract book DOIs can be found on the “Author Information” page.
**Disclaimer:** Articles published in the journal HemaSphere exclusively reflect the opinions of the authors. The authors are responsible for all content in their abstracts including accuracy of the facts, statements, citing resources, etc.

### Results

During median 24 months follow up (range 13–31 months), CAR-T group demonstrated 100% overall response rate（ORR）at three months with 90% complete remission (CR) , significantly higher than the 10.0%(1/10) ORR with partical remission (PR) observed in the SOC group ($p < 0.001$). Kaplan-Meier analysis revealed significantly superior PFS in the CAR-T cohort compared to SOC group (NR vs 3.0 months, 95% CI: 0.01–0.15, $p<0.001$). 90% (9/10) CAR-T patients maintained continuous CR throughout the follow-up time (median 24 months, , whereas 1/10 relapsed at 9m post BCMA-CAR T cell infusion and 10/10 patients in SOC group progressed within 12 months. The 12-month and 24-month PFS rate was 90% for the CAR-T group versus 10% for the SOC group ($p<0.001$ by log-rank test). Median OS was not reached in CAR-T group (0% mortality at median follow-up of 24 months) versus 5.5 months (90% mortality within 24 months).

### Summary/Conclusion

This study demonstrates unprecedented efficacy for the strategy of radiation therapy as bridging with fully humanized BCMA-CAR T cell therapy and maintenance in high-risk RRMM patients with EMD.

**Copyright Information:** (Online) ISSN: 2572-9241
©2026 The Author(s). HemaSphere is published by John Wiley & Sons Ltd on behalf of European Hematology Association. This is an open access Abstract Book distributed under the Attribution-NonCommercial-NoDerivs (CC BY-NC-ND), which allows third parties to download the articles and share them with others as long as they credit the author and the Abstract Book, but they cannot change the content in any way or use them commercially.
**Abstract Book Citations:** Authors, Title, HemaSphere, 2026;10:(S1):pages. The abstract book DOIs can be found on the "Author Information" page.
**Disclaimer:** Articles published in the journal HemaSphere exclusively reflect the opinions of the authors. The authors are responsible for all content in their abstracts including accuracy of the facts, statements, citing resources, etc.

### (PF829) MOLECULAR MARKERS AS A POSSIBLE FACTOR IN PROGRESSION IN MULTIPLE MYELOMA

**Topic:** 14. Myeloma and other monoclonal gammopathies - Clinical

Zhanna Kozich*[1], Natallia Klimkovich[2], Tatiana Rudenkova[2], Janna Pugacheva[1], Volha Bylitskaya[1], Dmitry Blizin[1]

*[1]REPUBLICAN RESEARCH CENTER FOR RADIATION MEDICINE AND HUMAN ECOLOGY, Gomel, Belarus;[2]Belarusian State Medical University, Minsk, Belarus;*

### Background

New methods of treatment are not a radical option for treating multiple myeloma (MM). The disease progresses steadily, but at varying rates. Determining gene changes that influence progression can help develop personalized treatment strategies and approaches.

### Aims

To assess the role of molecular markers in the development of multiple myeloma progression

### Methods

For the study, a group of 102 patients with newly diagnosed MM was formed, who were observed at the RRCRM&HE. Observation period is from 2018 to 2025. The median age was 64 years (58.0 and 70.0 years), with women predominating (56.9%). All patients underwent general clinical examinations, bone marrow aspiration biopsy with immunophenotyping, and molecular genetic testing. PCR was used to detect alterations in the NRAS, KRAS, BRAF, ATM, and DIS3 genes.

### Results

All patients with MM at the time of diagnosis had various types of mutations and polymorphisms of the NRAS, KRAS, BRAF, ATM, DIS3 genes. Disease progression during follow-up developed in 65 (63.7%) patients. Thus, mutations in the NRAS genes were detected in 33.3% of cases, of which progression occurred in 50% of cases (p=0.269), KRAS was detected in 69.2% of which progression occurred in 50% of cases (p=0.198), BRAF was detected in 23.1% of which progression occurred in 28.6% of cases (p=0.681), ATM was detected in 82.1% of which progression occurred in 85.7% of cases (p=0.75), polymorphism in the DIS3 gene were detected in 10.3%, of which no progression was detected in patients. Moreover, the PFS differed depending on the presence of these mutations and polymorphisms. When a change in the NRAS genes (p=0.0027) or a KRAS mutation (p=0.00029) was detected, the PFS was significantly lower compared to the absence of this mutation. Detection of BRAF had no effect on PFS. The lowest PFS was found with the combination of KRAS/NRAS (p=0.00004). Isolated detection of changes in the ATM and DIS3 genes did not affect PFS. However, when KRAS/NRAS is combined with polymorphisms in the ATM gene, or NRAS/ATM, the PFS in these patients was significantly improved (p=0.035, p=0.012, respectively).

### Summary/Conclusion

The identified gene changes may serve as prognostic markers of the risk of MM progression. It is possible that polymorphisms in the ATM and DIS3 genes promote remodeling of damaged DNA and may compensate for the adverse outcomes in patients with a combination of KRAS/NRAS mutations, providing an improvement in progression-free survival.

**Copyright Information:** (Online) ISSN: 2572-9241
©2026 The Author(s). HemaSphere is published by John Wiley & Sons Ltd on behalf of European Hematology Association. This is an open access Abstract Book distributed under the Attribution-NonCommercial-NoDerivs (CC BY-NC-ND), which allows third parties to download the articles and share them with others as long as they credit the author and the Abstract Book, but they cannot change the content in any way or use them commercially.
**Abstract Book Citations:** Authors, Title, HemaSphere, 2026;10:(S1):pages. The abstract book DOIs can be found on the "Author Information" page.
**Disclaimer:** Articles published in the journal HemaSphere exclusively reflect the opinions of the authors. The authors are responsible for all content in their abstracts including accuracy of the facts, statements, citing resources, etc.

### (PF830) COMPARATIVE EFFICACY OF LINVOSELTAMAB VERSUS TECLISTAMAB IN TRIPLE-CLASS EXPOSED RELAPSED/REFRACTORY MULTIPLE MYELOMA: UPDATED MATCHING-ADJUSTED INDIRECT COMPARISON WITH LONGER FOLLOW-UP

**Topic:** 14. Myeloma and other monoclonal gammopathies - Clinical

Hans C. Lee*[1], Naresh Bumma[2], Joshua Richter[3], Jeffrey A. Zonder[4], James E. Hoffman[5], Zheng-Yi Zhou[6], Viviana Garcia-Horton[6], Mirko Fillbrunn[6], Hongjue Wang[6], Matthew Mattera[6], Wenxin MA[6], Timothy J. Inocencio[7], Yingxin Xu[7], James Harnett[7], Tito Roccia[7], Kate Knorr[7], Glenn S. Kroog[7], Karen Rodriguez Lorenc[7], Qiufei MA[7], Sundar Jagannath[3]

*[1]Sarah Cannon Research Institute, Nashville, TN, United States of America;[2]The Ohio State University Comprehensive Cancer Center, Columbus, OH, United States of America; [3]Icahn School of Medicine at Mount Sinai, New York, NY, United States of America; [4]Karmanos Cancer Institute, Detroit, MI, United States of America;[5]University of Miami Health System, Miami, FL, United States of America; [6]Analysis Group, Inc., Boston, MA, United States of America; [7]Regeneron Pharmaceuticals, Inc., Tarrytown, NY, United States of America;*

### Background

Linvoseltamab and teclistamab are anti-B-cell maturation antigen (BCMA)×CD3 bispecific antibodies evaluated in the LINKER-MM1 and MajesTEC-1 studies, respectively, and approved for the treatment of triple-class exposed (TCE) relapsed/refractory multiple myeloma (RRMM). In the absence of head-to-head studies, a previous matching-adjusted indirect comparison (MAIC) was used to assess their relative efficacy at ~14-month median follow-up (mFU). We present updated results with longer follow-up for both studies.

### Aims

To compare the efficacy of linvoseltamab versus teclistamab for the treatment of patients with RRMM after exposure to ≥3 prior therapies, including a proteasome inhibitor, an immunomodulatory drug, and an anti-CD38 monoclonal antibody (i.e., TCE RRMM).

### Methods

After excluding 10 patients with prior BCMA antibody–drug conjugate exposure from LINKER-MM1 to align with MajesTEC-1 eligibility, patient-level data from LINKER-MM1 (linvoseltamab 200 mg, N=107; data cut-off [DCO] July 2024; mFU 21.3 months) and aggregate data from MajesTEC-1 (N=165; DCO August 2023; mFU 30.4 months) were used.
Patients from LINKER-MM1 were weighted to match MajesTEC-1 using prespecified prognostic factors, as described previously (Lee et al. *Clin Lymphoma Myeloma Leuk* 2025). MAICs were rerun for objective response rate (ORR), very good partial response or better (≥VGPR) rate, complete response or better (≥CR) rate, duration of response (DOR), progression-free survival (PFS) and overall survival (OS) duration, and time to next treatment (TTNT). Treatment effects were estimated using odds ratios (ORs) and hazard ratios (HRs) with 95% confidence intervals (CIs). Restricted mean survival time (RMST) was evaluated at 30 months.

### Results

In both unadjusted comparisons and MAICs adjusted for six key prognostic factors (cytogenetic risk, age, refractory status, International Staging System stage, Eastern Cooperative Oncology Group performance status, extramedullary disease/plasmacytoma status; effective sample size 82.5), treatment with linvoseltamab resulted in numerically higher ORR, ≥VGPR, and ≥CR rates, and significantly longer DOR, PFS, OS, and TTNT versus teclistamab (**Table**). Compared with prior MAICs with shorter follow-up, adjusted HRs for DOR and TTNT further decreased in favor of linvoseltamab, whereas other results remained stable.

**Copyright Information:** (Online) ISSN: 2572-9241
©2026 The Author(s). HemaSphere is published by John Wiley & Sons Ltd on behalf of European Hematology Association. This is an open access Abstract Book distributed under the Attribution-NonCommercial-NoDerivs (CC BY-NC-ND), which allows third parties to download the articles and share them with others as long as they credit the author and the Abstract Book, but they cannot change the content in any way or use them commercially.
**Abstract Book Citations:** Authors, Title, HemaSphere, 2026;10:(S1):pages. The abstract book DOIs can be found on the “Author Information” page.
**Disclaimer:** Articles published in the journal HemaSphere exclusively reflect the opinions of the authors. The authors are responsible for all content in their abstracts including accuracy of the facts, statements, citing resources, etc.

### Summary/Conclusion

With longer follow-up, linvoseltamab continued to demonstrate comparative efficacy advantages versus teclistamab, reinforcing its potential as an effective treatment option for TCE RRMM.

**Table. Unadjusted and adjusted outcomes for linvoseltamab versus teclistamab across key efficacy endpoints**

| Outcome, linvoseltamab versus teclistamab | Unadjusted | Adjusted for six key prognostic factors |
|---|---|---|
| | *OR (95% CI)* | *OR (95% CI)* |
| ORR | 1.44 (0.97–2.13) | 1.50 (0.97–2.32) |
| ≥VGPR | 1.19 (0.83–1.72) | 1.17 (0.79–1.74) |
| ≥CR | 1.29 (0.91–1.82) | 1.21 (0.83–1.76) |
| | *HR (95% CI); RMST difference, months (95% CI)* | *HR (95% CI); RMST difference, months (95% CI)* |
| DOR | 0.58 (0.36–0.96);* 3.66 (0.77–6.55)* | 0.54 (0.31–0.92);* 4.06 (1.13–6.98)* |
| PFS | 0.57 (0.40–0.82);* 5.13 (2.01–8.25)* | 0.55 (0.36–0.82);* 5.43 (2.04–8.81)* |
| OS | 0.68 (0.47–0.98);* 2.99 (0.22–5.75)* | 0.64 (0.43–0.97);* 3.06 (0.08–6.04)* |
| TTNT | 0.42 (0.28–0.62);* 7.10 (4.27–9.94)* | 0.41 (0.27–0.64);* 7.12 (4.15–10.09)* |

*p<0.05. OR >1, HR <1, or RMST >0 indicate better efficacy with linvoseltamab versus teclistamab.

© 2026 American Society of Clinical Oncology, Inc. Reused with permission. This abstract was accepted and previously presented at the 2026 ASCO Annual Meeting. All rights reserved.

**Copyright Information:** (Online) ISSN: 2572-9241
©2026 The Author(s). HemaSphere is published by John Wiley & Sons Ltd on behalf of European Hematology Association. This is an open access Abstract Book distributed under the Attribution-NonCommercial-NoDerivs (CC BY-NC-ND), which allows third parties to download the articles and share them with others as long as they credit the author and the Abstract Book, but they cannot change the content in any way or use them commercially.
**Abstract Book Citations:** Authors, Title, HemaSphere, 2026;10:(S1):pages. The abstract book DOIs can be found on the "Author Information" page.
**Disclaimer:** Articles published in the journal HemaSphere exclusively reflect the opinions of the authors. The authors are responsible for all content in their abstracts including accuracy of the facts, statements, citing resources, etc.

### (PF831) PROGNOSTIC VALUE OF TIME-TO-TREATMENT AND REGIMENS IN WALDENSTROM MACROGLOBULINEMIA: A REAL-WORLD MULTICENTER STUDY

**Topic:** 14. Myeloma and other monoclonal gammopathies - Clinical

Kyungju Kim*[1], Ja Min Byun[1], Dong Hyun Kim[1], Sung-Soo Park[2], Hee Jeong Cho[3], Jaewon Hyung[4], Christopher Liam Chin Keong[5], Yang Liang Boo[5]

*[1]Seoul National University Hospital, Internal Medicine, Seul, South Korea;[2]Catholic Hematology Hospital, Seoul St. Mary's Hospital, Catholic University, Hematology/Oncology, Seoul, South Korea; [3]Kyungpook National University Hospital, Hematology/Oncology, Daegu, South Korea; [4]Asan Medical Center, Department of Oncology, Seoul, South Korea;[5]Hospital Sultanah Aminah, Haematology Unit, Medical Department, Jalan Persiaran Abu Bakar Sultan, Malaysia;*

### Background

In Waldenström macroglobulinemia (WM), conventional prognostic models are largely based on static variables and do not account for dynamic course of the disease. Time-to-lymphoma treatment within 12 months (TLT12) and time-to-next treatment within 12 months (TNT12) may offer dynamic assessment of the disease behavior and treatment effectiveness. Their prognostic value, however, has not been systematically evaluated in WM. In parallel, although various frontline options are available in practice, real-world comparative data on treatment outcomes remain limited.

### Aims

The primary endpoint was to explore the prognostic value of TLT12 and TNT12. The secondary endpoint was to compare the treatment outcomes of frontline regimens including BTK inhibitors.

### Methods

This multicenter study included newly diagnosed WM patients from six tertiary hospitals in South Korea and Malaysia, between January 2011 and April 2025. Patients were stratified by TLT12 and TNT12 status, according to treatment exposure and follow-up duration. Survival outcomes, including overall survival (OS) and WM-related survival (WRS), were compared according to TLT12 and TNT12 status.

**Copyright Information:** (Online) ISSN: 2572-9241
©2026 The Author(s). HemaSphere is published by John Wiley & Sons Ltd on behalf of European Hematology Association. This is an open access Abstract Book distributed under the Attribution-NonCommercial-NoDerivs (CC BY-NC-ND), which allows third parties to download the articles and share them with others as long as they credit the author and the Abstract Book, but they cannot change the content in any way or use them commercially.
**Abstract Book Citations:** Authors, Title, HemaSphere, 2026;10:(S1):pages. The abstract book DOIs can be found on the "Author Information" page.
**Disclaimer:** Articles published in the journal HemaSphere exclusively reflect the opinions of the authors. The authors are responsible for all content in their abstracts including accuracy of the facts, statements, citing resources, etc.

**Results**

Among 218 patients, TLT12-positive group (N=168) had significantly lower hemoglobin and albumin levels, higher β2-microglobulin and rIPSS-WM risk categories compared to TLT12-negative patients (N = 50). TLT12-positive group demonstrated inferior overall survival (OS) and WM-related survival. (WRS). The median OS and WRS were 88 months and 112 months respectively in TLT12-positive group, while neither endpoint was reached in the TLT12-negative group. Additionally, TLT12-positivity was independently associated with inferior OS in multivariable analysis with a hazard ratio (HR) of 2.62 (95% CI: 1.04-6.55).
Regarding frontline treatments, bendamustine + rituximab (BR) was the most common first-line regimen (N=81), followed by rituximab + cyclophosphamide-based regimen (N=30), rituximab + proteasome inhibitor-based regimen (N=8), and others, including rituximab monotherapy and BTK inhibitors (N=64). BR achieved the highest response rate (VGPR/CR, 50.6%) and the most favorable progression-free survival, with 4- and 8-year PFS rates of 74.1% and 46.1%, respectively.
Among patients who received first-line treatment, TNT12-positive group (N=34) had a shorter median time to first-line treatment and a higher use of non-BR-containing regimens. Cox analysis for OS did not show a statistically significant hazard ratio in TNT12 status, whereas first-line BR exposure and BTK inhibitor use were associated with improved OS (HR 0.43, 95% CI: 0.22-0.87, HR 0.48, 95% CI 0.22-1.03).
BTK inhibitors (N=37; 3 treatment-naïve, 34 relapsed/refractory) used in any line of treatment showed high response rates, with a VGPR/CR rate of 27.0%, and an overall response rate of 89.2%. The median duration of response was not reached in most patients, consistent with rapid and durable treatment effect.

**Summary/Conclusion**

TLT12 provides prognostic information in WM, with early treatment initiation within 12 months of diagnosis associated with worse survival outcomes. TNT12 did not independently predict OS, but the choice of treatment regimen, particularly first-line BR and BTK inhibitor in any line, appears to influence long-term outcomes.

**Copyright Information:** (Online) ISSN: 2572-9241
©2026 The Author(s). HemaSphere is published by John Wiley & Sons Ltd on behalf of European Hematology Association. This is an open access Abstract Book distributed under the Attribution-NonCommercial-NoDerivs (CC BY-NC-ND), which allows third parties to download the articles and share them with others as long as they credit the author and the Abstract Book, but they cannot change the content in any way or use them commercially.
**Abstract Book Citations:** Authors, Title, HemaSphere, 2026;10:(S1):pages. The abstract book DOIs can be found on the “Author Information” page.
**Disclaimer:** Articles published in the journal HemaSphere exclusively reflect the opinions of the authors. The authors are responsible for all content in their abstracts including accuracy of the facts, statements, citing resources, etc.

**Figure**. Survival outcomes based on TLT12 and TNT12 status (A), (D); Overall survival, (B), (E); Waldenström macroglobulinemia-related survival, (C), (F); Multivariable analysis of overall survival.

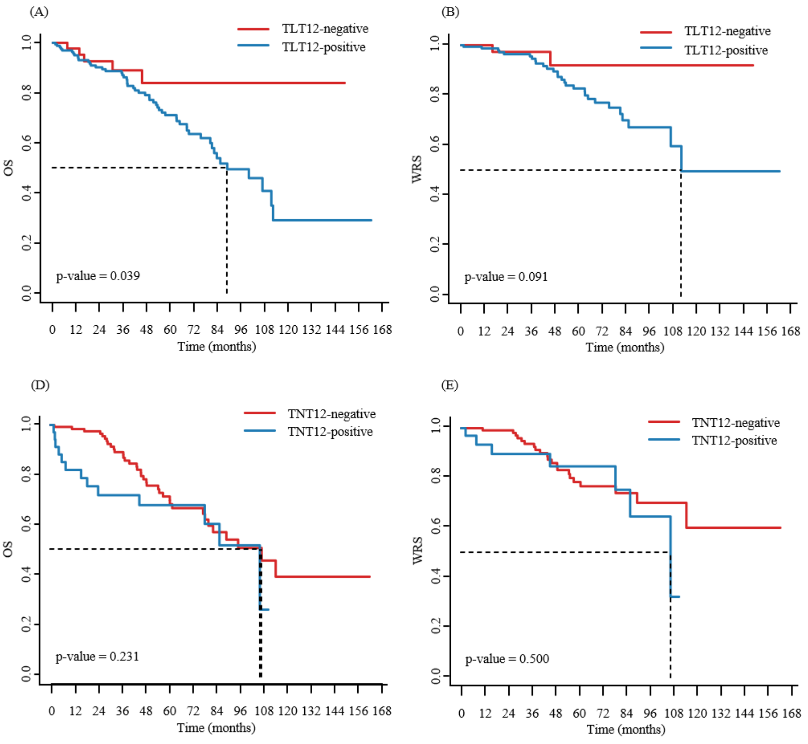

(C)

| Variables | Multivariable | |
|---|---|---|
| | HR (95% CI) | P-value |
| Age | 1.04 (1.01 – 1.07) | 0.016 |
| Sex, male | | |
| ECOG < 2 | | |
| Lymphadenopathy | | |
| Hepatosplenomegaly | | |
| Hb < 10 g/dL | | |
| Platelet < 100×10$^9$/L | | |
| Albumin < 3.5 g/dL | | |
| LDH > ULN | | |
| IgM > 7000 mg/dL | | |
| β2-microglobulin > 3 mg/L | | |
| MYD88 mutation | | |
| CXCR4 mutation | | |
| TLT12-positive | 2.62 (1.04 – 6.55) | 0.040 |

(F)

| Variables | Multivariable | |
|---|---|---|
| | HR (95% CI) | P-value |
| Age | 1.03 (0.99 – 1.06) | 0.126 |
| Sex, male | | |
| ECOG < 2 | | |
| Lymphadenopathy | | |
| Hepatosplenomegaly | | |
| Hb < 10 g/dL | 0.61 (0.33 – 1.13) | 0.115 |
| Platelet < 100×10$^9$/L | | |
| Albumin < 3.5 g/dL | 1.83 (0.93 – 3.60) | 0.079 |
| LDH > ULN | | |
| IgM > 7000 mg/dL | | |
| β2-microglobulin > 3 mg/L | | |
| MYD88 mutation | | |
| CXCR4 mutation | | |
| Treatment lines > 2 | | |
| First-line treatment | | |
| Others | 1.00 | |
| BR | 0.43 (0.22 – 0.87) | 0.018 |
| BTK inhibitor use | 0.48 (0.22 – 1.03) | 0.060 |
| TNT12-positive | | |

**Copyright Information:** (Online) ISSN: 2572-9241
©2026 The Author(s). HemaSphere is published by John Wiley & Sons Ltd on behalf of European Hematology Association. This is an open access Abstract Book distributed under the Attribution-NonCommercial-NoDerivs (CC BY-NC-ND), which allows third parties to download the articles and share them with others as long as they credit the author and the Abstract Book, but they cannot change the content in any way or use them commercially.
**Abstract Book Citations:** Authors, Title, HemaSphere, 2026;10:(S1):pages. The abstract book DOIs can be found on the "Author Information" page.
**Disclaimer:** Articles published in the journal HemaSphere exclusively reflect the opinions of the authors. The authors are responsible for all content in their abstracts including accuracy of the facts, statements, citing resources, etc.

### (PF832) MATCHING-ADJUSTED INDIRECT COMPARISON FOR BELANTAMAB MAFODOTIN WITH POMALIDOMIDE AND DEXAMETHASONE VS DARATUMUMAB WITH POMALIDOMIDE AND DEXAMETHASONE IN RELAPSED/REFRACTORY MULTIPLE MYELOMA

**Topic:** 14. Myeloma and other monoclonal gammopathies - Clinical

Joshua Richter[1], Meral Beksaç*[2], Alessandro Corso[3], Katerina Papadimitropoulou[4], Leanne Cooper[4], Nicholas Meader[5], Molly Purser[6], Alex Bates[5], Sandhya Sapra[6], Nick Ballew[6]

*[1]Mount Sinai Hospital, Division of Hematology and Medical Oncology, New York, NY, United States of America;[2]Ankara Liv Hospital, Istinye University, Department of Hematology, Ankara, Türkiye; [3]Ospedale di Legnano, Division of Hematology, Milan, Italy; [4]FIECON, London, United Kingdom; [5]GSK, London, United Kingdom; [6]GSK, Upper Providence, PA, United States of America;*

### Background

Belantamab mafodotin (belamaf) with pomalidomide and dexamethasone (BPd) is approved for patients (pts) with relapsed/refractory multiple myeloma (RRMM) in multiple countries based on results of the phase 3 DREAMM-8 trial. DREAMM-8 evaluated BPd vs pomalidomide with bortezomib and dexamethasone, allowing enrollment of pts previously treated with daratumumab.

### Aims

To support clinical decision-making in the absence of head-to-head comparison, a matching-adjusted indirect comparison (MAIC) was performed to estimate the relative clinical efficacy of BPd vs daratumumab with pomalidomide and dexamethasone (DPd) in pts with RRMM who received ≥1 prior therapy line (2L+), including lenalidomide (len).

### Methods

A systematic literature review was undertaken and updated as of January 2024 to identify efficacy/safety data in 2L+ RRMM, and studies were assessed for common treatment arms and pt characteristics. MAICs of progression-free survival (PFS) and minimal residual disease negativity (MRD-) were conducted using the len-exposed populations from phase 3 trials of BPd (DREAMM-8) and DPd (APOLLO). As these trials lack a common comparator arm, an unanchored approach was required. Individual pt data (IPD) were available for BPd-treated pts (n=155) from DREAMM-8, while only published summary data were available for DPd-treated pts (n=151) from APOLLO. BPd-treated pts from DREAMM-8 were weighted (using IPD) to match DPd-treated pts from APOLLO in terms of age (<75 , ≥75 years), prior lines of therapy (LOT; 1, 2–3, ≥4), refractoriness to len/immunomodulatory drugs (IMD; yes, no), refractoriness to proteasome inhibitors (PI; yes, no), International Staging System (ISS) stage (1/2, 3), cytogenetic risk (standard, high), Eastern Cooperative Oncology Group performance status (ECOG PS; 0, 1, 2), and creatinine clearance (CrCl; ≤60, >60 mL/min). Outcomes for weighted BPd-treated pts were compared to those for DPd-treated pts. DREAMM-8 interim analysis 1 (MRD-) and 3 (PFS) data were used for analysis. Cox regression and logistic regression were performed for PFS and MRD- comparisons, respectively.

### Results

After matching, equal proportions in both arms were: ≥75 years (17%); had 2–3 prior LOT (75%); were refractory to an IMD (79%) or PI (47%); had ISS stage 3 (22%); ECOG PS of 1 (36%); had CrCl ≤60 mL/min (26%); and had high cytogenetic risk (38%). Effective sample size of weighted BPd-treated pts was 65.6. PFS favored BPd, with medians of 18.1 vs 12.4 months (hazard ratio 0.74 [95% confidence interval 0.50, 1.09]). MRD- also favored BPd, with rates of 25.8% vs 8.6% (odds ratio 2.73 [95% confidence interval 1.26, 5.92]). Only MRD- was statistically significant.

**Copyright Information:** (Online) ISSN: 2572-9241
©2026 The Author(s). HemaSphere is published by John Wiley & Sons Ltd on behalf of European Hematology Association. This is an open access Abstract Book distributed under the Attribution-NonCommercial-NoDerivs (CC BY-NC-ND), which allows third parties to download the articles and share them with others as long as they credit the author and the Abstract Book, but they cannot change the content in any way or use them commercially.
**Abstract Book Citations:** Authors, Title, HemaSphere, 2026;10:(S1):pages. The abstract book DOIs can be found on the “Author Information” page.
**Disclaimer:** Articles published in the journal HemaSphere exclusively reflect the opinions of the authors. The authors are responsible for all content in their abstracts including accuracy of the facts, statements, citing resources, etc.

**Summary/Conclusion**

While MAICs have inherent limitations, BPd demonstrated improved relative clinical efficacy vs DPd when adjusting for differences in key baseline pt characteristics. These findings demonstrate the importance and utility of BPd in the 2L+ RRMM setting with the growing use of len with daratumumab in early LOT.

**Funding:** GSK

This abstract was previously submitted to the American Society of Clinical Oncology (ASCO) Annual Meeting, June 11–14, 2026, and is submitted on behalf of the original authors with their permission. ©2026 American Society of Clinical Oncology, Inc. Reused with permission. All rights reserved.

**Copyright Information:** (Online) ISSN: 2572-9241
©2026 The Author(s). HemaSphere is published by John Wiley & Sons Ltd on behalf of European Hematology Association. This is an open access Abstract Book distributed under the Attribution-NonCommercial-NoDerivs (CC BY-NC-ND), which allows third parties to download the articles and share them with others as long as they credit the author and the Abstract Book, but they cannot change the content in any way or use them commercially.
**Abstract Book Citations:** Authors, Title, HemaSphere, 2026;10:(S1):pages. The abstract book DOIs can be found on the "Author Information" page.
**Disclaimer:** Articles published in the journal HemaSphere exclusively reflect the opinions of the authors. The authors are responsible for all content in their abstracts including accuracy of the facts, statements, citing resources, etc.

### (PF833) COMPARISON OF CLONOTYPIC PEPTIDE (EASYM) AND INTACT IMMUNOGLOBULIN LIGHT CHAIN (EXENT) PERIPHERAL BLOOD-BASED MASS SPECTROMETRY IN MULTIPLE MYELOMA

**Topic:** 14. Myeloma and other monoclonal gammopathies - Clinical

Jessie Zhao*[1, 2], Tiffany Khong[1, 2], Abir Khaled[3], Zac McDonald[3], Que Thanh Lam[4], Nicole Jenkins[4], Sridurga Mithraprabhu[1, 2], Nicholas Bingham[1, 2], Sueh-Li Lim[1, 2], Daniel Wong[1, 2], Liqiang Yang[3], Andrew Spencer[1, 2]

*[1]The Alfred Hospital, Department of Clinical Haematology, Melbourne, Australia;[2]Monash University, Australian Centre for Blood Diseases, Melbourne, Australia; [3]Rapid Novor, Kitchener, Canada; [4]The Alfred Hospital, Department of Pathology, Melbourne, Australia;*

### Background

Peripheral blood-based mass spectrometry (PB-based MS) offers the potential for highly sensitive, non-invasive sequential minimal residual disease (MRD) assessment in multiple myeloma (MM). Two approaches are commonly employed: intact immunoglobulin light chain MS (e.g. EXENT), which offers higher throughput, and clonotypic peptide MS (e.g. EasyM), which is personalized and more sensitive. EasyM has been compared to bone marrow-based MRD assessment but direct comparison with EXENT has not been reported.

### Aims

To evaluate the sensitivity and correlation of EasyM and EXENT in paired longitudinal MM serum samples.

### Methods

We retrospectively identified MM patients from the Australasian Leukaemia and Lymphoma Group MM19 (ACTRN12616000772448) and MM21 (ACTRN12618001490268) trials with baseline monoclonal protein (M-protein) ≥2 g/L by serum protein electrophoresis and/or free light chains (FLC) ≥100 mg/L. MM19 evaluated the addition of ixazomib to thalidomide-dexamethasone consolidation for 12 months in transplant-eligible newly diagnosed MM (TE NDMM) patients undergoing frontline autologous stem cell transplantation (ASCT), whilst MM21 evaluated intensive salvage with daratumumab-lenalidomide-dexamethasone as re-induction for 4 cycles and post-ASCT consolidation for 12 cycles, followed by lenalidomide maintenance until progressive disease (PD) in TE NDMM patients failing (<partial response) frontline bortezomib-based induction. Serum samples from screening, pre-ASCT, post-ASCT, mid-consolidation, end-of-consolidation, complete response (CR), and PD timepoints were analysed by both EasyM and EXENT. Responses were defined by International Myeloma Working Group criteria. Spearman's rank coefficient assessed correlation between assays.

**Copyright Information:** (Online) ISSN: 2572-9241
©2026 The Author(s). HemaSphere is published by John Wiley & Sons Ltd on behalf of European Hematology Association. This is an open access Abstract Book distributed under the Attribution-NonCommercial-NoDerivs (CC BY-NC-ND), which allows third parties to download the articles and share them with others as long as they credit the author and the Abstract Book, but they cannot change the content in any way or use them commercially.
**Abstract Book Citations:** Authors, Title, HemaSphere, 2026;10:(S1):pages. The abstract book DOIs can be found on the "Author Information" page.
**Disclaimer:** Articles published in the journal HemaSphere exclusively reflect the opinions of the authors. The authors are responsible for all content in their abstracts including accuracy of the facts, statements, citing resources, etc.

### Results

53 patients with 215 paired samples were included, comprising IgG (n=42), IgA (n=6), FLC (n=4), and IgM (n=1) isotypes. EasyM detected residual M-proteins at lower levels than EXENT (IgA: 0.001 vs <0.015 g/L; IgG: 0.009 vs 0.053 g/L; FLC: 0.010 vs unquantifiable, respectively). Among samples with measurable EasyM concentrations ≤0.14 g/L, only 30% were detected and quantified by EXENT, further demonstrating EasyM's superior sensitivity. Sequential EasyM identified progressively rising levels in 4 patients, preceding relapse by a median of 9.9 (range 1.8-32.1) months; EXENT declined in two patients despite impending relapse and in one patient at confirmed PD. Of the 215 paired samples, M-protein was undetectable in only 2 (0.9%) by EasyM versus 20 (9.3%) by EXENT, with 197 (91.6%) concordant. All discordant samples were EasyM-positive/EXENT-negative and discordance increased with depth of response (Figure 1): partial response 1/58 (1.7%), very good partial response 4/48 (8.3%), and CR 13/31 (41.9%). Notably, EXENT and EasyM remained positive in 17/31 (54.8%) and 30/31 (96.8%) of CR samples, respectively, underscoring residual disease detected by MS beyond conventional methods. Across 171 paired quantifiable samples, EasyM and EXENT showed strong correlation (rho=0.92, p<0.001).

### Summary/Conclusion

EasyM demonstrates superior sensitivity while maintaining strong correlation with EXENT. EXENT and EasyM detected residual M-protein in 54.8% and 96.8% of CR samples, respectively, highlighting its clinical relevance and potential for sequential PB-based MS MRD monitoring in MM. Prospective studies are warranted to define optimal thresholds, assess clinical utility, and optimise integration of PB-based MS with bone marrow-based MRD assessment in MM.

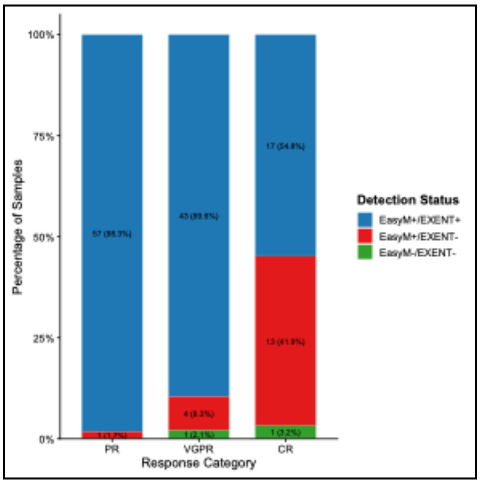

Figure 1. Detection of residual M-protein by EasyM and EXENT across International Myeloma Working Group response categories. PR = partial response; VGPR = very good partial response; CR = complete response; EasyM+ = EasyM-positive; EasyM- = EasyM-negative; EXENT+ = EXENT-positive; EXENT- = EXENT-negative.

**Copyright Information:** (Online) ISSN: 2572-9241
©2026 The Author(s). HemaSphere is published by John Wiley & Sons Ltd on behalf of European Hematology Association. This is an open access Abstract Book distributed under the Attribution-NonCommercial-NoDerivs (CC BY-NC-ND), which allows third parties to download the articles and share them with others as long as they credit the author and the Abstract Book, but they cannot change the content in any way or use them commercially.
**Abstract Book Citations:** Authors, Title, HemaSphere, 2026;10:(S1):pages. The abstract book DOIs can be found on the "Author Information" page.
**Disclaimer:** Articles published in the journal HemaSphere exclusively reflect the opinions of the authors. The authors are responsible for all content in their abstracts including accuracy of the facts, statements, citing resources, etc.

### (PF834) FRAILTY AND QUALITY OF LIFE IN NEWLY DIAGNOSED MULTIPLE MYELOMA PATIENTS

**Topic:** 14. Myeloma and other monoclonal gammopathies - Clinical

Klara Frederikke Koue Rydahl*[1, 2, 3, 4], Trine Allerslev Horsbøl[5], Sören Möller[6], Birgit Bjerre Høyer[7], Bo Amdi Jensen[8], Carsten Helleberg[9], Anja Klostergaard[10], Henrik Gregersen[11], Sarah Leeth Farmer[12], Mikael Frederiksen[13], Anne K. Mylin[9], Morten Saaby Steffensen[14], Per Trøllund Pedersen[15], Niels Abildgaard[1, 2, 16], Lene Kongsgaard Nielsen[1, 2, 4, 14]

*[1]Quality of Life Research Center, Department of Haematology, Odense University Hospital, Odense, Denmark;[2]Department of Clinical Research, University of Southern Denmark, Odense, Denmark; [3]Department of Affective Disorders, Aarhus University Hospital, Aarhus, Denmark; [4]Department of Clinical Medicine, Aarhus University, Aarhus, Denmark;[5]National Institute of Public Health, University of Southern Denmark, Copenhagen, Denmark; [6]Department of Public Health, Epidemiology, Biostatistics, and Biodemography, University of Southern Denmark, Odense, Denmark; [7]OPEN, Open Patient data Explorative Network, Odense University Hospital, Odense, Denmark; [8]Department of Haematology, Zealand University Hospital, Roskilde, Denmark; [9]Department of Haematology, Copenhagen University Hospital, Copenhagen, Denmark; [10]Department of Haematology, Aarhus University Hospital, Aarhus, Denmark; [11]Department of Haematology, Aalborg University Hospital, Aalborg, Denmark; [12]Department of Haematology, Vejle Hospital, Vejle, Denmark; [13]Department of Haematology, Hospital of Southern Jutland, Sønderborg, Denmark; [14]Department of Haematology, Gødstrup Hospital, Herning, Denmark;[15]Department of Haematology, South West Jutland Hospital, Esbjerg, Denmark; [16]Department of Haematology, Odense University Hospital, Odense, Denmark;*

### Background

Multiple myeloma (MM) patients report lower quality of life (QoL) than patients with other cancers. Most MM QoL evidence comes from clinical trials, with younger and fitter populations than real-world MM patients. Therefore, knowledge about QoL in frail MM patients is limited, despite data suggesting that frailty is associated with poorer QoL. Such insights are crucial for guiding treatment decisions, supportive care, and rehabilitation.

### Aims

To investigate whether frail newly diagnosed MM (NDMM) patients report lower global QoL, physical functioning, and more fatigue and pain than fit NDMM patients at inclusion and during two years of follow-up.

### Methods

Data were obtained from "Quality of Life among Danish Multiple Myeloma Patients" survey. NDMM patients starting first-line treatment were enrolled between 2017 and 2021, and classified at inclusion using the International Myeloma Working Group frailty scale. Mean differences in QoL domains between fit and frail patients were analyzed using linear regression adjusted for sex, inclusion year, level of education, and civil status. Mean differences exceeding domain-specific minimal important difference (MID) were considered clinically relevant.

**Copyright Information:** (Online) ISSN: 2572-9241
©2026 The Author(s). HemaSphere is published by John Wiley & Sons Ltd on behalf of European Hematology Association. This is an open access Abstract Book distributed under the Attribution-NonCommercial-NoDerivs (CC BY-NC-ND), which allows third parties to download the articles and share them with others as long as they credit the author and the Abstract Book, but they cannot change the content in any way or use them commercially.
**Abstract Book Citations:** Authors, Title, HemaSphere, 2026;10:(S1):pages. The abstract book DOIs can be found on the "Author Information" page.
**Disclaimer:** Articles published in the journal HemaSphere exclusively reflect the opinions of the authors. The authors are responsible for all content in their abstracts including accuracy of the facts, statements, citing resources, etc.

### Results

Among 403 NDMM patients (median age 71.0 years; interquartile range 62.0–75.0), 193 were classified as fit, 134 as intermediate fit, and 76 as frail. At inclusion, frail patients reported clinically relevant lower global QoL and physical functioning, and higher fatigue compared to fit patients. Physical functioning differences remained clinically relevant throughout follow-up. Fatigue differences were clinically relevant at baseline and two years, but not at one year. Adjusted global QoL differences diminished during follow-up. Pain did not differ clinically relevantly between frail and fit patients at any time. The mean scores and mean score differences are presented in the Table.

**Mean estimates at inclusion, one, and two years for fit and frail NDMM patients. Statistically significant adjusted mean differences are marked in bold if they exceed the minimalimportant difference threshold.**

| Quality of life domains (MID) | Time year (y) | Fit Mean estimates (SD) | Frail Mean estimates (SD) | Adjusted[1] difference between fit and frail (95% confidence interval) |
|---|---|---|---|---|
| Global quality of life (7.66) | Inclusion | 56.95 (25.62) | 49.11 (31.11) | **-8.73 (-16.22; -1.24)** |
| | 1 y | 69.48 (19.69) | 63.24 (23.58) | -5.18 (11.68; 0.94) |
| | 2 y | 66.23 (22.33) | 60.61 (25.25) | -2.97 (-10.50;4.56) |
| Physical functioning (7.44) | Inclusion | 75.01 (24.24) | 58.57 (33.99) | **-17.80 (-25.05; -10.55)** |
| | 1 y | 79.21 (17.40) | 67.80 (24.73) | **-10.40 (-16.43; -4.37)** |
| | 2 y | 78.87 (18.13) | 64.02 (24.58) | **-12.92 (-19.37;-6.46)** |
| Fatigue (8.48) | Inclusion | 38.40 (28.45) | 51.97 (34.17) | **13.81 (5.75; 21.88)** |
| | 1 y | 30.79 (21.07) | 38.78 (22.50) | 7.01 (0.21; 13.81) |
| | 2 y | 31.10 (22.34) | 44.93 (25.76) | **12.38 (4.86;19.89)** |
| Pain (10.29) | Inclusion | 42.31 (34.74) | 48.68 (40.35) | 7.52 (-2.37; 17.42) |
| | 1 y | 22.69 (22.93) | 28.93 (23.83) | 6.15 (-1.55; 13.85) |
| | 2 y | 23.70 (24.31) | 31.88 (31.20) | 8.92 (0.14;17.69) |

[1]*Adjusted for sex, education level, inclusion year, and civil status*
MID; minimal important difference, SD; standard deviation

### Summary/Conclusion

Frailty was associated with lower global QoL, physical functioning, and higher fatigue in NDMM patients. After two years, frail patients maintained clinically relevant lower physical functioning and higher fatigue than fit patients. These findings highlight the need for targeted interventions addressing fatigue and impaired physical functioning in frail MM patients.

**Copyright Information:** (Online) ISSN: 2572-9241
©2026 The Author(s). HemaSphere is published by John Wiley & Sons Ltd on behalf of European Hematology Association. This is an open access Abstract Book distributed under the Attribution-NonCommercial-NoDerivs (CC BY-NC-ND), which allows third parties to download the articles and share them with others as long as they credit the author and the Abstract Book, but they cannot change the content in any way or use them commercially.
**Abstract Book Citations:** Authors, Title, HemaSphere, 2026;10:(S1):pages. The abstract book DOIs can be found on the "Author Information" page.
**Disclaimer:** Articles published in the journal HemaSphere exclusively reflect the opinions of the authors. The authors are responsible for all content in their abstracts including accuracy of the facts, statements, citing resources, etc.

### (PF835) ANTIDIABETIC MEDICATION USE AND MONOCLONAL GAMMOPATHY OF UNDETERMINED SIGNIFICANCE: ASSOCIATIONS WITH PREVALENCE AND M-PROTEIN TRAJECTORIES IN THE ISTOPMM STUDY

**Topic:** 14. Myeloma and other monoclonal gammopathies - Clinical

Kirstine Ostenfeld Larsen*[1], Sæmundur Rögnvaldsson[2, 3], Baojing Li[4], Joshua Entrop[4], Andri Ólafsson[2], Ásdís Rósa Þórðardóttir[2], Brynjar Viðarsson[5], Páll Torfi Önundarson[2], Bjarni Agnar Agnarsson[5], Margrét Sigurðardóttir[5], Ingunn Þorsteinsdóttir[5], Ísleifur Ólafsson[5], Elías Eyþórsson[5], Ásbjörn Jónsson[6], Thorvardur Jon Love[2, 5], Sigurdur Yngvi Kristinsson[2, 5], Sigrún Thorsteinsdóttir[1, 2]

*[1]Rigshospitalet, Department of Hematology, Copenhagen, Denmark; [2]University of Iceland, Faculty of Medicine, Reykjavík, Iceland; [3]Sahlgrenska University Hospital, Gothenburg, Sweden; [4]Red Door Analytics AB, Stockholm, Sweden; [5]Landspítali University Hospital, Reykjavík, Iceland; [6]Akureyri University Hospital, Akureyri, Iceland;*

### Background

Obesity, and possibly type 2 diabetes, are associated with increased incidence of monoclonal gammopathy of undetermined significance (MGUS) and multiple myeloma (MM). Treatment with antidiabetic medication has been proposed to reduce the risk of progression from MGUS to MM; however, their relationship with MGUS prevalence or M-protein dynamics remains unclear. Using the Iceland Screens, Treats, or Prevents Multiple Myeloma (iStopMM) study we examined this more closely.

### Aims

1. To assess the association between prior antidiabetic medication use and MGUS prevalence at screening in individuals with diabetes.
2. To estimate the effect of antidiabetic medication use on longitudinal M-protein levels in individuals with MGUS and diabetes.

### Methods

For the MGUS prevalence analysis all individuals screened for MGUS in the iStopMM study with a prior diabetes diagnosis (N=6,425) were included. Logistic regression models estimated prevalence odds ratios (ORs) of MGUS comparing ever versus never users of different antidiabetic drug classes, adjusted for age and sex.

For M-protein trajectory analyses, 192 individuals with MGUS, diabetes, and repeated M-protein measurements were included. Follow-up began at MGUS screening and exposure to antidiabetic drugs was updated monthly. Linear mixed-effects models estimated the effect of cumulative drug exposure on longitudinal M-protein levels, adjusting for age, sex, BMI and HbA1c at screening and time-varying BMI and HbA1c using inverse probability weighting. Average M-protein concentration were predicted over 0-5 years of cumulative drug exposure.

**Copyright Information:** (Online) ISSN: 2572-9241
©2026 The Author(s). HemaSphere is published by John Wiley & Sons Ltd on behalf of European Hematology Association. This is an open access Abstract Book distributed under the Attribution-NonCommercial-NoDerivs (CC BY-NC-ND), which allows third parties to download the articles and share them with others as long as they credit the author and the Abstract Book, but they cannot change the content in any way or use them commercially.
**Abstract Book Citations:** Authors, Title, HemaSphere, 2026;10:(S1):pages. The abstract book DOIs can be found on the "Author Information" page.
**Disclaimer:** Articles published in the journal HemaSphere exclusively reflect the opinions of the authors. The authors are responsible for all content in their abstracts including accuracy of the facts, statements, citing resources, etc.

### Results

No significant associations were found between the use of any antidiabetic medication and MGUS prevalence overall (Figure 1). GLP-1 agonist use was associated with a higher odds of MGUS among men (OR 1.83, 95% CI 1.10-3.05), but not among women (OR 1.06, 95% CI 0.49-2.33). 94% of all GLP-1 agonist users diagnosed with MGUS were obese.
The M-protein trajectory analyses showed that at a given time point, greater cumulative metformin exposure was associated with lower M-protein levels (expβ 0.992, 95% CI 0.986, 0.996; Table 1), corresponding to approximately 0.8% lower levels per one-month difference in cumulative exposure. Associations for SGLT2 and GLP-1/DPP-4 were directionally similar but not statistically significant.

| Drug groups | Expβ 95% CI |
|---|---|
| **Hypoglycaemic agents** | 1.001 (0.996-1.006) |
| **GLP-1/DPP-4** | 0.997 (0.990-1.003) |
| **SGLT2** | 0.992 (0.984-1.000) |
| **Metformin** | 0.992 (0.986-0.999) |

**Table 1.** Expβ for cumulative time in months on drug groups from linear mixed effect models of longitudinal M-protein-levels.

### Summary/Conclusion

In this large population-based screening cohort, the use of antidiabetic medication was not significantly associated with MGUS prevalence. The association between GLP-1 agonist use and MGUS prevalence found in men is likely due to the high prevalence of obesity among GLP-1 agonist users given the established association between obesity and MGUS prevalence.
Metformin exposure was associated with lower levels of M-protein over time independent of BMI and HbA1c changes. This trend was also seen in patients treated with SGLT2 inhibitors and GLP-1 agonists/DPP-4 inhibitor, although not statistically significant. This suggests a potential physiological effect on plasma cell clone dynamics which warrants further investigation into these agents as modifiers of disease activity in plasma cell dyscrasias.

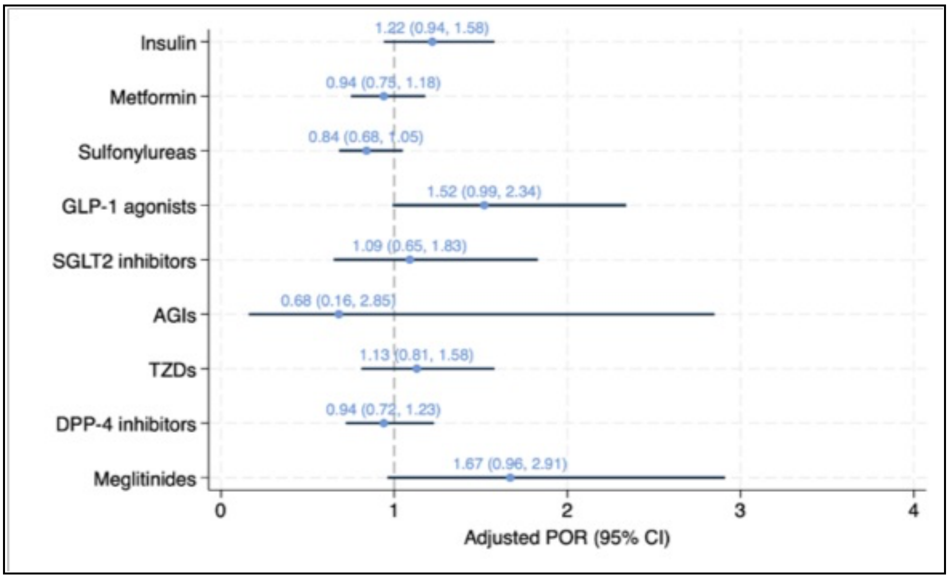

Figure 1. Forest plot of the adjusted prevalence odds ratios (PORs) of MGUS comparing never and ever users of each drug, adjusted for age and sex.

**Copyright Information:** (Online) ISSN: 2572-9241
©2026 The Author(s). HemaSphere is published by John Wiley & Sons Ltd on behalf of European Hematology Association. This is an open access Abstract Book distributed under the Attribution-NonCommercial-NoDerivs (CC BY-NC-ND), which allows third parties to download the articles and share them with others as long as they credit the author and the Abstract Book, but they cannot change the content in any way or use them commercially.
**Abstract Book Citations:** Authors, Title, HemaSphere, 2026;10:(S1):pages. The abstract book DOIs can be found on the "Author Information" page.
**Disclaimer:** Articles published in the journal HemaSphere exclusively reflect the opinions of the authors. The authors are responsible for all content in their abstracts including accuracy of the facts, statements, citing resources, etc.

### (PF836) UNRESTRAINED LYMPHOCYTOSIS AS A PREDICTOR OF A DELAYED NON-ICANS NEUROTOXICITY FOLLOWING CILTACABTAGENE AUTOLEUCEL CAR T-CELL THERAPY IN MULTIPLE MYELOMA: A REAL-WORLD SINGLE-CENTER EXPERIENCE

**Topic:** 14. Myeloma and other monoclonal gammopathies - Clinical

Inna Shaforostova*[1], Marie-Noëlle Kronig[1], Katja Seipel[2], Martina Bertschinger[1], Seok-Yun Lee[1], Marc Wehrli[1], Urban Novak[1], Ulrike Bacher[3], Thomas Pabst[1]

*[1]Inselspital Bern University Hospital, Medical Oncology, Bern, Switzerland; [2]University of Bern, Department for Biomedical Research, Bern, Switzerland; [3]Inselspital Bern University Hospital, Department of Hematology, Bern, Switzerland;*

### Background

Ciltacabtagene autoleucel (cilta-cel), has significantly improved outcomes in relapsed/refractory multiple myeloma (MM), providing deep and durable responses even in heavily pretreated patients. However, cilta-cel is associated with rare, delayed, potentially life-threatening neurotoxicities (non–immune effector cell–associated neurotoxicities (NINTs)), including movement and neurocognitive treatment-emergent events (MNTs). Unrestrained lymphocytosis, defined by the absolute lymphocyte count (ALC) ≥3.0 G/l, was recently identified as a potential risk marker. Prophylactic treatment with corticosteroids may reduce the risk of late neurotoxicities in these patients.

### Aims

The aim of our study was to evaluate the association of lymphocytosis with neurotoxicity profiles and clinical outcomes in MM patients, who received cilta-cel.

### Methods

We retrospectively analyzed 41 MM patients treated with cilta-cel at a single center between September 6, 2024, and January 9, 2026. Demographic and clinical characteristics were collected from day 0 to day +30 following CAR T-cell infusion. An ALC threshold of ≥3.0 G/l was used to define unrestrained lymphocytosis.

**Copyright Information:** (Online) ISSN: 2572-9241
©2026 The Author(s). HemaSphere is published by John Wiley & Sons Ltd on behalf of European Hematology Association. This is an open access Abstract Book distributed under the Attribution-NonCommercial-NoDerivs (CC BY-NC-ND), which allows third parties to download the articles and share them with others as long as they credit the author and the Abstract Book, but they cannot change the content in any way or use them commercially.
**Abstract Book Citations:** Authors, Title, HemaSphere, 2026;10:(S1):pages. The abstract book DOIs can be found on the "Author Information" page.
**Disclaimer:** Articles published in the journal HemaSphere exclusively reflect the opinions of the authors. The authors are responsible for all content in their abstracts including accuracy of the facts, statements, citing resources, etc.

**Results**

Median follow-up was 202 days (range, 17 – 489 days). Peripheral lymphocytosis occurred in 29% (12/41) of patients and developed earlier compared with patients with ALC <3.0 G/l (median 12 vs 19 days; p=0.015). In ALC ≥3.0 G/l group a significant higher ferritin level (3606 vs 534 µg/l, p=0.015) and earlier CART-cell expansion (164 vs 55 copies/µg DNA, p=0.04) was registered.
Neurological symptoms occurred in 22% (9/41) of patients (6/12 in the ALC ≥3.0 G/l group and 3/29 in ALC <3.0 G/l group), with distinct patterns by lymphocytosis status. The onset of neurological symptoms was earlier in the ALC <3.0 G/l group (median 1 vs 14 days, p=0.09).
11/12 patients with unrestrained lymphocytosis received prophylactic dexamethasone ten mg twice daily for three days.
6/12 patients with ALC ≥3.0 G/l developed neurological symptoms: 2/6 developed early immune effector cell–associated neurotoxicity syndrome (ICANS) grade 1 and 3, respectively; 4/6 patients showed NINTs concurrent with lymphocytosis, including intention tremor in all 4 patients and MNTs in 2/4 patients. All 4 patients received corticosteroids, 3/4 improved, while 1/4 progressed with severe lumbosacral plexopathy and death.
In contrast, 3/29 patients with ALC <3.0 G/l developed early ICANS (2/3) or non-CAR T-cell related events (ischemic stroke and paraparesis due to pseudoprogression after CAR T-cell infusion); no NINTs were observed.
Overall response was 83% in both groups; 1-year progression-free survival and overall survival were numerically lower in patients with ALC ≥3.0 G/l (56% vs 82%, p=0.22, and 72% vs 89%, p=0.19, respectively).

**Summary/Conclusion**

Post–cilta-cel lymphocytosis may identify patients at increased risk for delayed neurotoxicity; hyperferritinemia and early CAR T-cell expansion could represent potential risk factors. Additionally, lymphocytosis may be associated with inferior outcomes, warranting validation in prospective studies.

**Copyright Information:** (Online) ISSN: 2572-9241
©2026 The Author(s). HemaSphere is published by John Wiley & Sons Ltd on behalf of European Hematology Association. This is an open access Abstract Book distributed under the Attribution-NonCommercial-NoDerivs (CC BY-NC-ND), which allows third parties to download the articles and share them with others as long as they credit the author and the Abstract Book, but they cannot change the content in any way or use them commercially.
**Abstract Book Citations:** Authors, Title, HemaSphere, 2026;10:(S1):pages. The abstract book DOIs can be found on the "Author Information" page.
**Disclaimer:** Articles published in the journal HemaSphere exclusively reflect the opinions of the authors. The authors are responsible for all content in their abstracts including accuracy of the facts, statements, citing resources, etc.

### (PF837) OUTCOMES AFTER FRONTLINE QUADRUPLET FAILURE IN TRANSPLANT-ELIGIBLE NEWLY DIAGNOSED MULTIPLE MYELOMA

**Topic:** 14. Myeloma and other monoclonal gammopathies - Clinical

Luis Gerardo Rodriguez-Lobato*[1], Itziar Carro Arostegui[2], Anna de Daniel[1], Iago Arribas Castro[2], Cristina Motllo Borrella[3], Marcela Vázquez-Rodríguez[4], Natalia Tovar[1], M. Teresa Cibeira[1], Jose Miguel Mateos Perez[1], Laura Rosiñol Dachs[1], Anna Sureda[2], Carlos Fernández de Larrea[1]

*[1]Hematology Department, Hospital Clínic and IDIBAPS, Universitat de Barcelona, Barcelona, Spain;[2]Institut Català d'Oncologia Hospitalet, IDIBELL, Universitat de Barcelona, Hematology, Barcelona, Spain; [3]Hospital Universitari Parc Taulí, Hematology, Sabadell, Spain; [4]Hospital Universitari Sagrat Cor, Hematology, Barcelona, Spain;*

### Background

Quadruplet (quad) regimens incorporating anti-CD38 MoAb have become a standard frontline approach for transplant-eligible newly diagnosed multiple myeloma (TE-NDMM). However, limited data are available on baseline factors associated with relapse and outcomes after second-line (2L) therapy following quad-based induction.

### Aims

To characterize baseline factors associated with relapse and outcomes after 2L therapy, including progression-free survival after 2L (PFS2), in TE-NDMM patients treated with frontline quad regimens.

### Methods

We performed a retrospective multicenter analysis within the GEMMAC (Grup d'Estudi del Mieloma Múltiple de Catalunya), between 01/2019 and 05/2025. Approximately 40% received first-line therapy within a clinical trial. Responses were assessed according to IMWG criteria, and minimal residual disease (MRD) was analysed when available. Relapse timing was assessed from treatment initiation using 6- and 18-month landmarks.

**Copyright Information:** (Online) ISSN: 2572-9241
©2026 The Author(s). HemaSphere is published by John Wiley & Sons Ltd on behalf of European Hematology Association. This is an open access Abstract Book distributed under the Attribution-NonCommercial-NoDerivs (CC BY-NC-ND), which allows third parties to download the articles and share them with others as long as they credit the author and the Abstract Book, but they cannot change the content in any way or use them commercially.
**Abstract Book Citations:** Authors, Title, HemaSphere, 2026;10:(S1):pages. The abstract book DOIs can be found on the "Author Information" page.
**Disclaimer:** Articles published in the journal HemaSphere exclusively reflect the opinions of the authors. The authors are responsible for all content in their abstracts including accuracy of the facts, statements, citing resources, etc.

### Results

Ninety-eight patients were included in the study. Median age was 61 years; 21% of patients had high-risk cytogenetics (IMWG 2025 criteria). Induction regimens were predominantly daratumumab-based (69% DVRd; 17% DVTD; 10% IsaVRd). Post-induction overall response rate (ORR) was 93%, with 83% achieving ≥VGPR and 42% ≥CR. MRD negativity ($10^{-5}$) was achieved in 55% of evaluable patients after induction. Upfront autologous stem cell transplantation (ASCT) was performed in 82% of patients, further deepening responses, with ≥CR rates increasing to 68% and MRD negativity ($10^{-5}$) increasing to 79% among evaluable patients post-ASCT.
After a median follow-up of 22 months, 14 patients (14%) experienced disease progression. Seven patients progressed early, all within the first 6 months from treatment initiation, while the remaining 7 patients relapsed after 18 months, with a median time to relapse of 45 months (IQR 26–63). Patients who relapsed were more likely to present adverse baseline features, including ECOG-PS≥3, ISS-III, and elevated LDH at diagnosis. The 12- and 24-month PFS rates for the overall cohort were 93% and 91%, respectively; median PFS was not reached. OS data were immature, with 12- and 24-month OS rates of 98% and 95%, respectively. 2L regimens were heterogeneous and predominantly based on standard therapies, most commonly carfilzomib-based combinations (43%); no patients received bispecific antibodies or CAR T-cell therapy in 2L. ORR to 2L therapy was 45%. Among patients relapsing within 18 months from treatment initiation, ORR to 2L therapy was 43% (3/7), compared with 50% (2/4) in those relapsing later with available response data. Median PFS2 was 4.3 months in patients relapsing within 18 months versus 10 months in those relapsing later. Among 7 patients relapsing prior to first ASCT, salvage therapy failed to enable ASCT in 4 cases, whereas 3 patients achieved sufficient response to proceed to ASCT in 2L.

### Summary/Conclusion

In this multicenter cohort of TE-NDMM patients treated with frontline quad regimens, relapse was associated with adverse baseline clinical features, including poor performance status, advanced ISS stage, and elevated LDH. Outcomes after 2L therapy using standard regimens were suboptimal, particularly in patients with early relapse, and frequently failed to rescue patients. These findings highlight an unmet need for improved early risk stratification and more effective salvage strategies, potentially including novel immunotherapies, following frontline quad failure.

**Copyright Information:** (Online) ISSN: 2572-9241
©2026 The Author(s). HemaSphere is published by John Wiley & Sons Ltd on behalf of European Hematology Association. This is an open access Abstract Book distributed under the Attribution-NonCommercial-NoDerivs (CC BY-NC-ND), which allows third parties to download the articles and share them with others as long as they credit the author and the Abstract Book, but they cannot change the content in any way or use them commercially.
**Abstract Book Citations:** Authors, Title, HemaSphere, 2026;10:(S1):pages. The abstract book DOIs can be found on the “Author Information” page.
**Disclaimer:** Articles published in the journal HemaSphere exclusively reflect the opinions of the authors. The authors are responsible for all content in their abstracts including accuracy of the facts, statements, citing resources, etc.

### (PF838) COMPREHENSIVE MGUS IDENTIFICATION OVER 13 YEARS ALLOWS REAL WORLD ANALYSIS OF FOLLOW UP AND OUTCOMES

**Topic:** 14. Myeloma and other monoclonal gammopathies - Clinical

Aidan Haslam*[1, 2], Yiu Wayn Ker[3], Rohina Mufti[4], Suniva Kansakar[5], Andrew Bacon[1], Henry Crosland[6], Chris Bunce[2], Alex Richter[7, 8], Guy Pratt[9, 10]

*[1]University Hospitals Birmingham NHS Foundation Trust, Birmingham, United Kingdom;[2]University of Birmingham, School of Biosciences, Birmingham, United Kingdom; [3]University of Birmingham, School of Medicine, Birmingham, United Kingdom; [4]University Hopsitals Birmingham NHS Foundation Trust, Birmingham, United Kingdom;[5]University Hopsitals Birmingham NHS Foundation Trust, Haematology, Birmingham, United Kingdom; [6]University Hospitals Birmingham NHS Foundation Trust, Haematology, Birmingham, United Kingdom; [7]University Hospitals Birmingham NHS Foundation Trust, Immunology, Birmingham, United Kingdom; [8]University of Birmingham, Clinical Immunology Service, Birmingham, United Kingdom;[9]University Hosptals Birmingham NHS Foundation Trust, Haematology, Birmingham, United Kingdom; [10]University of Birmingham, School of Cancer and Genomic Sciences, Birmingham, United Kingdom;*

### Background

Monoclonal Gammopathy of Undetermined Significance (MGUS) is a common, precursor condition to serious haematological diseases, primarily Multiple Myeloma (MM). MGUS monitoring may improve outcomes by allowing early intervention during disease progression. MGUS however, is often diagnosed incidentally by non-haematologists, which may lead to variation in outcome reporting, clinical management and referrals to haematology. By using a combination of regional laboratory and hospital databases with national cancer registrations to comprehensively identify all cases of MGUS within a trust, we bridge this gap to unbiasedly report on MGUS outcomes over a 13-year period at a single trust.

### Aims

Identify all patients with newly identified MGUS at a large NHS trust from 2006-2019 and audit referral rates, monitoring frequency and outcomes.

### Methods

MGUS cases were identified using a regional laboratory database of serum electrophoresis and free light chain results. Results compatible with a plasma cell disorder were linked with national cancer registrations and hospital records to exclude pre-existing malignancy and to identify cases progressing to MM, Waldenström's Macroglobulinaemia or Solitary Plasmacytoma at least one year after the first MGUS-compatible blood test. Patients with a bone marrow demonstrating smouldering myeloma within 1 year of their first blood test were also removed. Records were then individually reviewed to confirm diagnoses and complete a trust-wide audit.

**Copyright Information:** (Online) ISSN: 2572-9241
©2026 The Author(s). HemaSphere is published by John Wiley & Sons Ltd on behalf of European Hematology Association. This is an open access Abstract Book distributed under the Attribution-NonCommercial-NoDerivs (CC BY-NC-ND), which allows third parties to download the articles and share them with others as long as they credit the author and the Abstract Book, but they cannot change the content in any way or use them commercially.
**Abstract Book Citations:** Authors, Title, HemaSphere, 2026;10:(S1):pages. The abstract book DOIs can be found on the "Author Information" page.
**Disclaimer:** Articles published in the journal HemaSphere exclusively reflect the opinions of the authors. The authors are responsible for all content in their abstracts including accuracy of the facts, statements, citing resources, etc.

### Results

977 patients with a putative diagnosis of MGUS were identified of which 196 were labelled as progressors. 78% of the MGUS cases and 54% of the progressions were confirmed by review of patient records (Table 1). Among patients progressing to MM, prior haematology follow-up was associated with lower rates of severe CRAB features and higher rates of treatment at diagnosis (Table 2). Substantial morbidity persisted however, and all six cases of spinal cord compression occurred in patients previously reviewed by haematology.

Table 1. MGUS management and progression

| | Progression from MGUS | |
|---|---|---|
| | **Stable**<br>N = 659[1] | **Progressive**<br>N = 105[1] |
| **Follow-up duration**[2] | 6.9 (4.3-9.9) | 4.7 (3.0-7.5) |
| **Monoclonal gammopathy of clinical significance** | 37 (5.6%) | 8 (7.6%) |
| **Venous thromboembolic disease** | 16 (2.4%) | 2 (1.9%) |
| **Attended haematology clinic**[3] | 532 (81%) | 74 (70%) |
| **Had a bone marrow**[3] | 101 (16%) | 40 (38%) |
| **Progression type** | | |
| Chronic lymphocytic lymphoma | 0 | 4 (3.8%) |
| Multiple Myeloma | 0 | 67 (64%) |
| Non-Hodgkin's lymphoma | 0 | 23 (22%) |
| Solitary Plasmacytoma[3] | 0 | 3 (2.9%) |
| Waldenstrom's Macroglobulinaemia | 0 | 8 (7.6%) |

[1]n (%)

[2]Years until progression, loss to follow up or death n (IQR)

[3]During follow-up or ≥9 months prior to progression

[4]All subsequently progressed to Multiple Myeloma

Table 2. Complications from Multiple Myeloma at diagnosis

| | Attended Haematology Clinic | |
|---|---|---|
| | **Did not attend haematology clinic**<br>N = 16[1] | **Attended haematology clinic**<br>N = 54[1] |
| **Presence of CRAB features** | 13 (81%) | 39 (72%) |
| **Required dialysis due to plasma cell disorder** | 1 (6.3%) | 0 |
| **Spinal cord compression** | 0 | 6 (11%) |
| **Pathological fracture** | 6 (38%) | 13 (24%) |
| **Transfusion for symptomatic anaemia** | 2 (13%) | 5 (9.3%) |
| **Received treatment for myeloma** | 11 (69%) | 48 (91%) |
| **Hypercalcaemia requiring intravenous fluids** | 3 (19%) | 2 (3.7%) |

[1]n (%)

**Copyright Information:** (Online) ISSN: 2572-9241
©2026 The Author(s). HemaSphere is published by John Wiley & Sons Ltd on behalf of European Hematology Association. This is an open access Abstract Book distributed under the Attribution-NonCommercial-NoDerivs (CC BY-NC-ND), which allows third parties to download the articles and share them with others as long as they credit the author and the Abstract Book, but they cannot change the content in any way or use them commercially.
**Abstract Book Citations:** Authors, Title, HemaSphere, 2026;10:(S1):pages. The abstract book DOIs can be found on the "Author Information" page.
**Disclaimer:** Articles published in the journal HemaSphere exclusively reflect the opinions of the authors. The authors are responsible for all content in their abstracts including accuracy of the facts, statements, citing resources, etc.

**Summary/Conclusion**

Comprehensive identification and audit of MGUS during a 13-year period reveals high morbidity at progression with 9% of MM patients developing spinal cord compression. This is despite 79% of patients being reviewd by a haematologist in clinic. Future work will examine the frequency of monitoring and imaging.

**Copyright Information:** (Online) ISSN: 2572-9241
©2026 The Author(s). HemaSphere is published by John Wiley & Sons Ltd on behalf of European Hematology Association. This is an open access Abstract Book distributed under the Attribution-NonCommercial-NoDerivs (CC BY-NC-ND), which allows third parties to download the articles and share them with others as long as they credit the author and the Abstract Book, but they cannot change the content in any way or use them commercially.
**Abstract Book Citations:** Authors, Title, HemaSphere, 2026;10:(S1):pages. The abstract book DOIs can be found on the "Author Information" page.
**Disclaimer:** Articles published in the journal HemaSphere exclusively reflect the opinions of the authors. The authors are responsible for all content in their abstracts including accuracy of the facts, statements, citing resources, etc.

### (PF839) REAL-WORLD OUTCOMES AND SUBSEQUENT TREATMENT UTILIZATION FOLLOWING ANTI-B-CELL MATURATION ANTIGEN ANTIBODY-DRUG CONJUGATE EXPOSURE IN PATIENTS WITH MULTIPLE MYELOMA

**Topic:** 14. Myeloma and other monoclonal gammopathies - Clinical

Meletios A. Dimopoulos*[1], Jorge Monge[2], Efstathios Kastritis[1], Xavier Leleu[3], David S. Siegel[4], Enrique M. Ocio[5], Madhu Nagaraj[6], Rafla Hassan[6], Sundar Jagannath[7], Katja C. Weisel[8], Nicolle Bonar[9], Mostafa Shokoohi[9], Christian Hampp[10], Jeannette Green[10], Olivier Humblet[10], Alexander Breskin[10], Glenn S. Kroog[10], Qiufei MA[10], Shaji Kumar[6]

*[1]National and Kapodistrian University of Athens, Alexandra Hospital, Athens, Greece; [2]University of Colorado Anschutz, Aurora, CO, United States of America; [3]Centre Hospitalier Universitaire, Poitiers, France; [4]Hackensack University Medical Center, Hackensack, NJ, United States of America; [5]Hospital Universitario Marqués de Valdecilla, Santander, Spain;[6]Mayo Clinic, Rochester, MN, United States of America; [7]The Mount Sinai Hospital, New York, NY, United States of America;[8]University Medical Center Hamburg-Eppendorf, Hamburg, Germany; [9]EVERSANA, Toronto, ON, Canada; [10]Regeneron Pharmaceuticals, Inc., Tarrytown, NY, United States of America;*

### Background

The anti-B–cell maturation antigen (BCMA) antibody-drug conjugate (ADC) belantamab mafodotin is approved in the USA and EU for relapsed/refractory multiple myeloma (RRMM). Understanding treatment options and patient outcomes following ADC failure is clinically important.

### Aims

This study describes real-world post-ADC treatment patterns and clinical outcomes in patients with RRMM.

### Methods

Adult patients with MM who received an ADC and initiated ≥1 subsequent line of therapy (LOT) were included. The index date was the day the first post-ADC LOT was initiated. Data were obtained from chart reviews at International Myeloma Foundation International Myeloma Working Group (IMF IMWG) academic sites, and US electronic health records from primarily community sites (COTA and Guardian Research Network [GRN]). Patient characteristics and index regimens were summarized descriptively. Time to next treatment (TTNT) and overall survival (OS) were estimated with the Kaplan–Meier estimator.

protease inhibitor/immunomodulatory drug/anti-CD38) after ADC. Median (95% CI) TTNT and OS were 4.9 months (2.8–non-estimable [NE]) and 12.0 months (4.9–NE), respectively.

-->

**Copyright Information:** (Online) ISSN: 2572-9241
©2026 The Author(s). HemaSphere is published by John Wiley & Sons Ltd on behalf of European Hematology Association. This is an open access Abstract Book distributed under the Attribution-NonCommercial-NoDerivs (CC BY-NC-ND), which allows third parties to download the articles and share them with others as long as they credit the author and the Abstract Book, but they cannot change the content in any way or use them commercially.
**Abstract Book Citations:** Authors, Title, HemaSphere, 2026;10:(S1):pages. The abstract book DOIs can be found on the "Author Information" page.
**Disclaimer:** Articles published in the journal HemaSphere exclusively reflect the opinions of the authors. The authors are responsible for all content in their abstracts including accuracy of the facts, statements, citing resources, etc.

**Results**

The IMF IMWG cohort (n=40) had a mean age of 70.8 years, a median of 7 prior LOTs, 82.5% of patients had triple-class refractory (TCR) MM, 55% were female, and 95% were White. Overall, 85% received ADC monotherapy (70% belantamab mafodotin) and 15% received combination therapy. Before receiving ADC, without considering corticosteroids, patients were commonly exposed to lenalidomide (100%), bortezomib (92.5%), daratumumab (90%), and pomalidomide (90%). Over a median follow-up of 26 months after the index date, 29 unique index post-ADC regimens were reported; often selinexor-based (27.5%) or bispecific antibodies (15%). Chimeric antigen receptor (CAR) T cell therapy was administered to 5% of patients, with 60% re-treated with a pre-ADC drug class (protease inhibitor/immunomodulatory drug/anti-CD38) after ADC. Median (95% confidence interval [CI]) TTNT and OS were 10.8 months (7.0–20.6) and 19.0 months (13.4–25.1), respectively.
The COTA-GRN cohort (n=39) had a mean age of 67.5 years, a median of 8 prior LOTs, 51.3% of participants had TCR MM, 49% were female, and 76% were White. Overall, 56% received ADC monotherapy (belantamab mafodotin) and 44% received combination therapy. Before receiving ADC, without considering corticosteroids, patients were commonly exposed to daratumumab and lenalidomide (97.4% each), and carfilzomib, bortezomib, and pomalidomide (94.9% each). Over a median follow-up of 9.2 months, 32 unique index post-ADC regimens were reported, often selinexor-based (23.1%) or venetoclax-based (17.9%); none of the patients received CAR T cell therapy, and 54% were re-treated with a pre-ADC drug class (protease inhibitor/immunomodulatory drug/anti-CD38) after ADC. Median (95% CI) TTNT and OS were 4.9 months (2.8–non-estimable [NE]) and 12.0 months (4.9–NE), respectively.

**Summary/Conclusion**

Findings highlight that most patients with heavily pretreated BCMA-ADC-exposed MM were re-treated with similar therapy classes. Academic centers used novel therapies (i.e., CAR T cell therapy and bispecific antibodies) more often, and their patients had longer survival than those treated at community sites. However, clinical outcomes remained suboptimal, emphasizing the unmet need for effective, accessible post-ADC treatments.

© 2026 American Society of Clinical Oncology, Inc. Reused with permission. This abstract was accepted and previously presented at the 2026 ASCO Annual Meeting. All rights reserved.

**Copyright Information:** (Online) ISSN: 2572-9241
©2026 The Author(s). HemaSphere is published by John Wiley & Sons Ltd on behalf of European Hematology Association. This is an open access Abstract Book distributed under the Attribution-NonCommercial-NoDerivs (CC BY-NC-ND), which allows third parties to download the articles and share them with others as long as they credit the author and the Abstract Book, but they cannot change the content in any way or use them commercially.
**Abstract Book Citations:** Authors, Title, HemaSphere, 2026;10:(S1):pages. The abstract book DOIs can be found on the “Author Information” page.
**Disclaimer:** Articles published in the journal HemaSphere exclusively reflect the opinions of the authors. The authors are responsible for all content in their abstracts including accuracy of the facts, statements, citing resources, etc.

### (PF840) REAL-WORLD EFFECTIVENESS AND SAFETY OF DARATUMUMAB, POMALIDOMIDE AND DEXAMETHASONE (DPD) IN RELAPSED/REFRACTORY MULTIPLE MYELOMA: PRELIMINARY RESULTS OF THE MULTICENTER EMN DARAPD STUDY

**Topic:** 14. Myeloma and other monoclonal gammopathies - Clinical

Ombretta Annibali*[1, 2], Francesca Fazio[3], Martina Tinelli[4], Paola Tacchetti[5], Irene Attucci[6], Claudio Cartia[7], Gregorio Barilà[8], Sonia More'[9], Massimo Gentile[10], Francesca Guidotti[11], Martina Tomassi[12], Bernardo Rossini[13], Barbara Anaclerico[14], Francesca Rezzonico[15], Valeria Tomarchio[1], Cristina Clissa[4], Marco Talarico[5, 16], Elisabetta Antonioli[6], Silvia Mangiacavalli[7], Edoardo Scomazzon[8], Monica Di Cecca[1], Marcello Capriata[3], Alfonso Piciocchi[17], Alice Donnini[1], Luigi Rigacci[1], Massimo Offidani[9], Maria Teresa Petrucci[3]

*[1]Fondazione Policlinico Universitario Campus Bio Medico, ROme, Italy; [2]Università Campus Bio Medico, Rome, Italy; [3]Hematology, Department of Translational and Precision Medicine, Sapienza University of Rome, Rome, Italy; [4]azienda ospedaliera universitaria di Verona, Verona, Italy; [5]IRCCS Azienda Ospedaliero-Universitaria di Bologna, Istituto di Ematologia "Seràgnoli", Bologna, Italy, Bologna, Italy; [6]Department of Hematology, Careggi Hospital and University of Florence, Florence, Italy;, FLorence, Italy; [7]Fondazione I.R.C.C.S. Policlinico San Matteo – Pavia, Pavia, Italy; [8]U.O.C. Ematologia, Ospedale san Bortolo, Vicenza, Italy; [9]Azienda Ospedaliero Universitaria delle Marche, Ancona, Italy; [10]A.O. of Cosenza, Cosenza, Italy; [11]UOC Ematologia, Ospedale Valduce, Como, Como, Italy; [12]IRCCS ISTITUTO NAZIONALE TUMORI REGINA ELENA, Roma, Rome, Italy; [13]Hematology and Cell Therapy Unit, IRCCS Istituto Tumori "Giovanni Paolo II", Bari, Italy; [14]OSPEDALE SAN GIOVANNI ADDOLORATA, Rome, Italy; [15]U.O.C. Ematologia, Ospedale di Legnano, ASST Ovest Milanese, Legnano, Italy; [16]Dipartimento di Scienze Mediche e Chirurgiche, Università di Bologna,, Bologna, Italy; [17]Fondazione Gimema, Roma, Italy;*

### Background

The combination of daratumumab, pomalidomide and dexamethasone (DPd) demonstrated superior progression-free survival (PFS) and overall response rate (ORR) compared with pomalidomide–dexamethasone in the phase III APOLLO trial. Nevertheless, patients enrolled in clinical trials are often highly selected, and real-world data in heterogeneous relapsed/refractory multiple myeloma (RRMM) populations are still limited. The Real-Life DaraPD study was designed to evaluate the effectiveness and safety of DPd in routine clinical practice across multiple Italian hematology centers.

### Aims

The primary objective was to assess ORR according to International Myeloma Working Group (IMWG) criteria. Secondary objectives included evaluation of PFS, overall survival (OS), depth of response, and safety in a real-world RRMM population.

### Methods

This is a multicenter, retrospective-prospective observational study including consecutive RRMM patients aged ≥18 years who received DPd from January 2023 onwards after at least one prior line including lenalidomide and a proteasome inhibitor. Data were collected through a centralized electronic REDCap platform. Responses were assessed according to IMWG criteria. PFS was defined as the time from DPd initiation to disease progression or death from any cause; OS was defined as the time from treatment start to death from any cause. Survival curves were estimated using the Kaplan–Meier method.

**Copyright Information:** (Online) ISSN: 2572-9241
©2026 The Author(s). HemaSphere is published by John Wiley & Sons Ltd on behalf of European Hematology Association. This is an open access Abstract Book distributed under the Attribution-NonCommercial-NoDerivs (CC BY-NC-ND), which allows third parties to download the articles and share them with others as long as they credit the author and the Abstract Book, but they cannot change the content in any way or use them commercially.
**Abstract Book Citations:** Authors, Title, HemaSphere, 2026;10:(S1):pages. The abstract book DOIs can be found on the "Author Information" page.
**Disclaimer:** Articles published in the journal HemaSphere exclusively reflect the opinions of the authors. The authors are responsible for all content in their abstracts including accuracy of the facts, statements, citing resources, etc.

**Results**

A total of 93 patients were included in this preliminary analysis. Median age was 63 years (range 39–86), and 53% were male. DPd was administered as second-line therapy in 49% of patients, third-line in 37%, and fourth or later line in 14%. At the start of DPd, 93% of patients were treated at relapse (58% biochemical and 42% clinical relapse), while 6.7% had refractory disease.
Seventy-four percent had previously undergone autologous stem cell transplantation. Regarding prior therapies, 49% had received VTD, 23% Rd, and 18% KRd, reflecting broad prior exposure to immunomodulatory drugs and proteasome inhibitors. No patients had previously received anti-BCMA bispecific antibodies or CAR-T therapy.
High-risk cytogenetic abnormalities were observed in a relevant proportion of patients, including del(17p) (9.7%), t(4;14) (2.2%), t(14;16) (5.4%), and 1q gain/amplification (14%). ISS stage at DPd initiation was I in 56%, II in 26%, and III in 18% of evaluable patients.
The best ORR (≥PR) was 88%, with ≥VGPR achieved in 59% and CR/sCR in 26% of patients. Median time to best response was 4 cycles. After a median follow-up of approximately 23 months, median PFS was 13 months (95% CI 11–22). Patients achieving CR/sCR had significantly longer median PFS compared with those without CR (29 vs 11 months).
Median OS was 23 months (95% CI 16–28), with half of deaths attributable to progressive disease. No unexpected safety signals emerged compared with the known toxicity profile of DPd.

**Summary/Conclusion**

In this real-world multicenter RRMM cohort, DPd demonstrated high response rates and clinically meaningful survival outcomes, consistent with pivotal clinical trial data despite a heterogeneous population and prior transplantation exposure. Depth of response was strongly associated with prolonged PFS. These preliminary results confirm DPd as an effective and feasible therapeutic option in routine clinical practice.

**Copyright Information:** (Online) ISSN: 2572-9241
©2026 The Author(s). HemaSphere is published by John Wiley & Sons Ltd on behalf of European Hematology Association. This is an open access Abstract Book distributed under the Attribution-NonCommercial-NoDerivs (CC BY-NC-ND), which allows third parties to download the articles and share them with others as long as they credit the author and the Abstract Book, but they cannot change the content in any way or use them commercially.
**Abstract Book Citations:** Authors, Title, HemaSphere, 2026;10:(S1):pages. The abstract book DOIs can be found on the "Author Information" page.
**Disclaimer:** Articles published in the journal HemaSphere exclusively reflect the opinions of the authors. The authors are responsible for all content in their abstracts including accuracy of the facts, statements, citing resources, etc.

### (PF841) MINIMAL RESIDUAL DISEASE TRAJECTORIES IN NEWLY DIAGNOSED MULTIPLE MYELOMA AND SURVIVAL OUTCOMES; A RETROSPECTIVE COHORT STUDY

**Topic:** 14. Myeloma and other monoclonal gammopathies - Clinical

Panagiotis Malandrakis*[1], Ioannis Ntanasis-Stathopoulos[1], Ioannis Kostopoulos[2], Charalampos Filippatos[1], Nikolaos Kanellias[1], Sotirios Manganas[1], Magdalini Migkou[1], Vassiliki Spiliopoulou[1], Foteini Theodorakakou[1], Evangelos Eleutherakis-Papaiakovou[1], Efstathios Kastritis[1], Maria Gavriatopoulou[1], Ourania Tsitsiloni[2], Meletios A. Dimopoulos[1], Evangelos Terpos[1]

*[1]National and Kapodistrian University of Athens, Department of Clinical Therapeutics, Athens, Greece;[2]National and Kapodistrian University of Athens, Biology Department, Athens, Greece;*

### Background

Minimal residual disease (MRD) assessment is used to evaluate treatment response in multiple myeloma (MM) and as such is a surrogate marker of progression-free survival (PFS) and overall survival (OS). Consecutive MRD evaluations offer important information regarding the depth and the duration of response to treatment and can be used to guide the treatment decisions.

### Aims

In this single center retrospective study patients with newly diagnosed MM (NDMM) with at least 3 consecutive MRD tests, were evaluated. The aim of the study was to compare PFS between subgroups.

### Methods

Four subgroups of patients were identified: sustained MRD positive (sMRDpos), sustained MRD negative (sMRDneg), converted to MRD negative (conMRDneg) and converted to MRD positive (conMRDpos).  Baseline characteristics were assessed between the four groups.

**Copyright Information:** (Online) ISSN: 2572-9241
©2026 The Author(s). HemaSphere is published by John Wiley & Sons Ltd on behalf of European Hematology Association. This is an open access Abstract Book distributed under the Attribution-NonCommercial-NoDerivs (CC BY-NC-ND), which allows third parties to download the articles and share them with others as long as they credit the author and the Abstract Book, but they cannot change the content in any way or use them commercially.
**Abstract Book Citations:** Authors, Title, HemaSphere, 2026;10:(S1):pages. The abstract book DOIs can be found on the "Author Information" page.
**Disclaimer:** Articles published in the journal HemaSphere exclusively reflect the opinions of the authors. The authors are responsible for all content in their abstracts including accuracy of the facts, statements, citing resources, etc.

### Results

Overall, 335 NDMM patients were included in the analysis. The median age at diagnosis was 59 years (range 35-89), 55.2% were males and the median number of MRD assessments was 5 (range 3-14). One hundred patients (29.9%) patients received quadruplet regimen as induction. Regarding risk stratification, 56 (16.7%) were ISS-3, and 52 (15.5%) were labeled as high-risk per the novel IMS/IMWG HRMM criteria. The median number of MRD evaluations in each patient was 5 (range 3-13).
The median follow-up of the cohort was 6.1 years. Patient distribution according to MRD status was as follows: 191 (57%) sustained negativity, 40 (12%) sustained positivity, 53 (15.8%) converted to negative, whereas 51 (15.2%) converted to positive. Median PFS and OS were not achieved in any of the MRD subgroups.
Sustained MRD negativity was associated with superior PFS compared to all the other subgroups (HR=0.21, 95% CI: 0.10 – 0.42, p<0.001). Moreover, sustained MRD positivity was associated with significantly inferior PFS compared to sMRDneg respectively (HR=4.84; 95% CI: 1.91 – 12.30, p<0.001). The cMRDpos subgroup had also inferior PFS compared to the sMRDneg (HR= 6.04, 95% CI: 2.74 – 13.31, p<0.001). Intrestingly, even one MRD positive result, at any time-point, was associated with inferior PFS compared to the sustained negativity subgroup (HR= 4.29, 95% CI: 2.05 – 8.99, p<0.001).
Among the sustained MRD negative patients, high risk cytogenetics yielded a trend towards worse PFS (HR= 1.42. 95% CI: 0.30 - 6.69, p=0.659), possibly suggesting that sMRDneg overrides high risk cytogenetics.
Among 200 patients that were tested, 20 were found positive for t(11;14). At a median follow—up of 4.2 years, there was no significant difference in the 4year PFS among those bearing the translocation versus the rest of the cohort (88.6 vs 85.6%).
Finally, among the 165 patients who sustained MRD negativity, the median number of consecutive MRD tests were 5 (range 3-14). Among these patients with more than 5 tests had superior PFS, compared to less than 5, with a HR= 0.04, 95% CI: 0.01 – 0.33, p=0.003 signaling no further need of additional consecutive MRD testing in such patients.

### Summary/Conclusion

In patients with NDMM in the real-world setting, sustained MRD negativity is associated with prolonged PFS, even in HR patients, whereas the presence of even one MRD positive result impacted PFS.

**Copyright Information:** (Online) ISSN: 2572-9241
©2026 The Author(s). HemaSphere is published by John Wiley & Sons Ltd on behalf of European Hematology Association. This is an open access Abstract Book distributed under the Attribution-NonCommercial-NoDerivs (CC BY-NC-ND), which allows third parties to download the articles and share them with others as long as they credit the author and the Abstract Book, but they cannot change the content in any way or use them commercially.
**Abstract Book Citations:** Authors, Title, HemaSphere, 2026;10:(S1):pages. The abstract book DOIs can be found on the "Author Information" page.
**Disclaimer:** Articles published in the journal HemaSphere exclusively reflect the opinions of the authors. The authors are responsible for all content in their abstracts including accuracy of the facts, statements, citing resources, etc.

### (PF842) THE HIDDEN COST OF INFECTION, SEPTIC SHOCK AND SURVIVAL IN WALDENSTROM MACROGLOBULINEMIA

**Topic:** 14. Myeloma and other monoclonal gammopathies - Clinical

Abdulhaq Al Jafari*[1], Abdulmoiz Aljafari[2]

[1]*University of Novi Sad, Medical Faculty, Novi Sad, Serbia;*[2]*Hamilton Medical Center, Dalton, United States of America;*

### Background

Waldenström Macroglobulinemia weakens the immune system and makes patients more vulnerable to severe infections. Septic shock is the most dangerous form of infection and often leads to poor outcomes but its impact in patients with Waldenström Macroglobulinemia is not well understood. This study looks at how septic shock affects hospital outcomes in this vulnerable population using national data.

### Aims

The aim of this study is to evaluate how septic shock affects hospitalized patients with Waldenström Macroglobulinemia. We seek to better understand its impact on in hospital mortality length of stay and major clinical complications using national inpatient data.

### Methods

We conducted a retrospective analysis using the National Inpatient Sample (NIS) database from 2016 to 2022. Adult patients hospitalized with a diagnosis of Waldenström Macroglobulinemia who develop septic shock were identified using ICD-10 codes. Baseline characteristics were compared using t-tests. Multivariable logistic regression was used to assess the odds of mortality and developing complications, adjusting for age, sex, race, Charlson Comorbidity Index, insurance type, hospital region, hospital location, teaching status, and median household income by ZIP code. A two-tailed p-value <0.05 was considered statistically significant.

### Results

Among 68,924 patients hospitalized with Waldenström Macroglobulinemia, 2,565 (3.7%) developed septic shock. Patients with septic shock were older (mean age 74.1 vs. 73.1 years) and more likely to be female (56.2% vs. 43.7%). 3,355 (4.8%) patients died from the cohort. Patients with septic shock had 39.3% mortality rate compared to 3.5 % to those without septic shock. aOR of mortality increased by 1738% in septic shock patients with p<0.001. The average length of stay (LOS) was 6.2 days. Patients with septic shock had their LOS increased by 6 days on average compared to those without septic shock (p<0.001). Patients with septic shock had a significant increase in their odds of developing complications, with odds of developing Acute respiratory failure, AKI, DIC, pancytopenia, hepatic failure, being on mechanical ventilation, and blood transfusions were all increased signifcantly (551%, 643%, 1035%, 183%, 888%, 2444%, 124% with p<0.001).

### Summary/Conclusion

This national study explores the impact of septic shock on hospitalized patients with Waldenström Macroglobulinemia. Using inpatient data from across the United States we compared patients who developed septic shock with those who did not. Septic shock was associated with a much higher risk of death longer hospital stays and significantly more organ related complications. Overall the findings show that septic shock has a severe effect on outcomes in patients with Waldenström Macroglobulinemia and highlight the importance of early recognition and timely management in this vulnerable population.

**Copyright Information:** (Online) ISSN: 2572-9241
©2026 The Author(s). HemaSphere is published by John Wiley & Sons Ltd on behalf of European Hematology Association. This is an open access Abstract Book distributed under the Attribution-NonCommercial-NoDerivs (CC BY-NC-ND), which allows third parties to download the articles and share them with others as long as they credit the author and the Abstract Book, but they cannot change the content in any way or use them commercially.
**Abstract Book Citations:** Authors, Title, HemaSphere, 2026;10:(S1):pages. The abstract book DOIs can be found on the “Author Information” page.
**Disclaimer:** Articles published in the journal HemaSphere exclusively reflect the opinions of the authors. The authors are responsible for all content in their abstracts including accuracy of the facts, statements, citing resources, etc.

### (PF843) INCIDENCE AND SURVIVAL OF PLASMA CELL LEUKAEMIA IN THE MODERN ERA: AN ENGLISH COHORT STUDY FROM THE UNCOVER GROUP AND UKMRA FRAILTY GROUP.

**Topic:** 14. Myeloma and other monoclonal gammopathies - Clinical

Thea Chandler*[1, 2], Indrani Karpha[3, 4], Nurunnahar Akter[4], Temitope Erinfolami[4], Mark Bishton[5, 6], Brogan Johnston[3], Yeong Lim[3, 4], Nagesh Kalakonda[3, 4], Gillian Brearton[3], Sally Moore[7], Hira Mian[8], Christopher Parrish[1, 9], Frances Seymour[1, 10], Charlotte Pawlyn[11, 12], Catrin Tudur Smith[4], Andrew Pettitt[3, 4], Gordon Cook[1, 9, 10]

*[1]Leeds Cancer Centre, Leeds Teaching Hospitals Trust, Haematology, Leeds, United Kingdom;[2]National Institute for Health and Care Research, Leeds, United Kingdom; [3]The Clatterbridge Cancer Centre NHS Foundation Trust, Liverpool, United Kingdom; [4]University of Liverpool, Liverpool, United Kingdom; [5]University of Nottingham, Nottingham, United Kingdom; [6]Nottingham University Hospitals NHS Trust, Nottingham, United Kingdom; [7]University Hospitals Bristol and Weston, Bristol, United Kingdom; [8]McMaster University, Ontario, Canada; [9]Cancer Research UK Clinical Trials Unit, Leeds Institute of Clinical Trial Research, Leeds, United Kingdom; [10]National Institute for Health and Care Research, Leeds Biomedical Research Centre (BRC), Leeds, United Kingdom; [11]The Royal Marsden NHS Foundation Trust, London, United Kingdom; [12]The Institute of Cancer Research, London, United Kingdom;*

### Background

Plasma cell leukaemia (PCL) is a rare but extremely aggressive malignancy with a high mortality. There is a paucity of data on PCL epidemiology and survival, with evidence mostly limited to small case series or cohort studies. UNCOVER is a blood cancer health data research programme that utilises the National Cancer Registration Dataset (NCRD). The NCRD records information on all patients diagnosed cancer at NHS institutions in England (Int J Epidemiol 2020; 49(1):16–16h). Here, we report the demographics, incidence and survival of patients with PCL on an England-wide scale.

### Methods

Data was selected for patients diagnosed with PCL (ICD-O-3: 9733/3) between January 2014 and December 2022 with follow-up until July 2024. Crude and adjusted incidence rate ratios (IRRs) were estimated and compared between groups using Poisson regression, and calendar time trends were assessed. All-cause overall survival (OS) was assessed using Kaplan-Meier methods and an adjusted Cox regression model. Cause-specific (Fine-Gray model) and relative (Pohar Perme method) survival were estimated. All models were adjusted for age at diagnosis, gender, index of multiple deprivation (IMD) quintile, year of diagnosis, and government region, while Cox and Fine-Gray models were also adjusted for ethnicity and Charlson co-morbidity index (CCI).

### Results

A total of 306 patients were identified. 52% were female and the median age at diagnosis was 71 (IQR 60–80) years, with 55.2% of cases aged 70 or older. 99.3% of diagnoses were primary PCL, while 0.7% of patients had a prior diagnosis of myeloma. Most patients had no significant comorbidities (CCI 0 = 68.3%). The age standardised incidence rate was 0.71 per million person years. Adjusted incidence rate ratios (IRRs) increased with age (IRR 40.0 for age ≥80 vs <50; p<0.001) and socioeconomic deprivation (IRR 1.78 for IMD1 vs IMD5; p=0.023) and varied with ethnicity (IRR 1.76 for Black vs White ethnicity; p<0.001). Other co-variates including gender and year of diagnosis were not significantly associated with incidence.
245 patients (80.1%) had died after a median follow-up of 6.6 (IQR 5.8–7.5) years, with death attributed to blood cancer in 84.1% of cases. 1- and 5-year OS rates were 45.8% and 21.0%, respectively. Increasing age was a risk factor for shorter OS (HR 4.42 for ≥80 vs <50 years, 95% CI 2.17–9.04; p<0.001) and cause-specific survival (CSS) (HR 3.88 for ≥80 vs <50 years, 95% CI 1.86–8.09; p<0.001). Neither OS nor CSS was associated with gender, ethnicity, deprivation, geographical region, CCI and year of diagnosis.

**Copyright Information:** (Online) ISSN: 2572-9241
©2026 The Author(s). HemaSphere is published by John Wiley & Sons Ltd on behalf of European Hematology Association. This is an open access Abstract Book distributed under the Attribution-NonCommercial-NoDerivs (CC BY-NC-ND), which allows third parties to download the articles and share them with others as long as they credit the author and the Abstract Book, but they cannot change the content in any way or use them commercially.
**Abstract Book Citations:** Authors, Title, HemaSphere, 2026;10:(S1):pages. The abstract book DOIs can be found on the "Author Information" page.
**Disclaimer:** Articles published in the journal HemaSphere exclusively reflect the opinions of the authors. The authors are responsible for all content in their abstracts including accuracy of the facts, statements, citing resources, etc.

### Summary/Conclusion

This is by far the largest European study of PCL epidemiology and survival hitherto reported. In addition to being strongly associated with increasing age, PCL incidence was also significantly higher among people of Black ethnicity and in areas of socioeconomic deprivation. Survival was dismal and strongly associated with age, with only one fifth of patients aged ≥80 surviving 12 months and no improvement in outcome among patients diagnosed in successive calendar years. Together, these findings raise intriguing questions concerning the causal relationship between deprivation and PCL and highlight the unmet need for effective PCL treatment, especially in older people and despite significant advances in myeloma therapy.

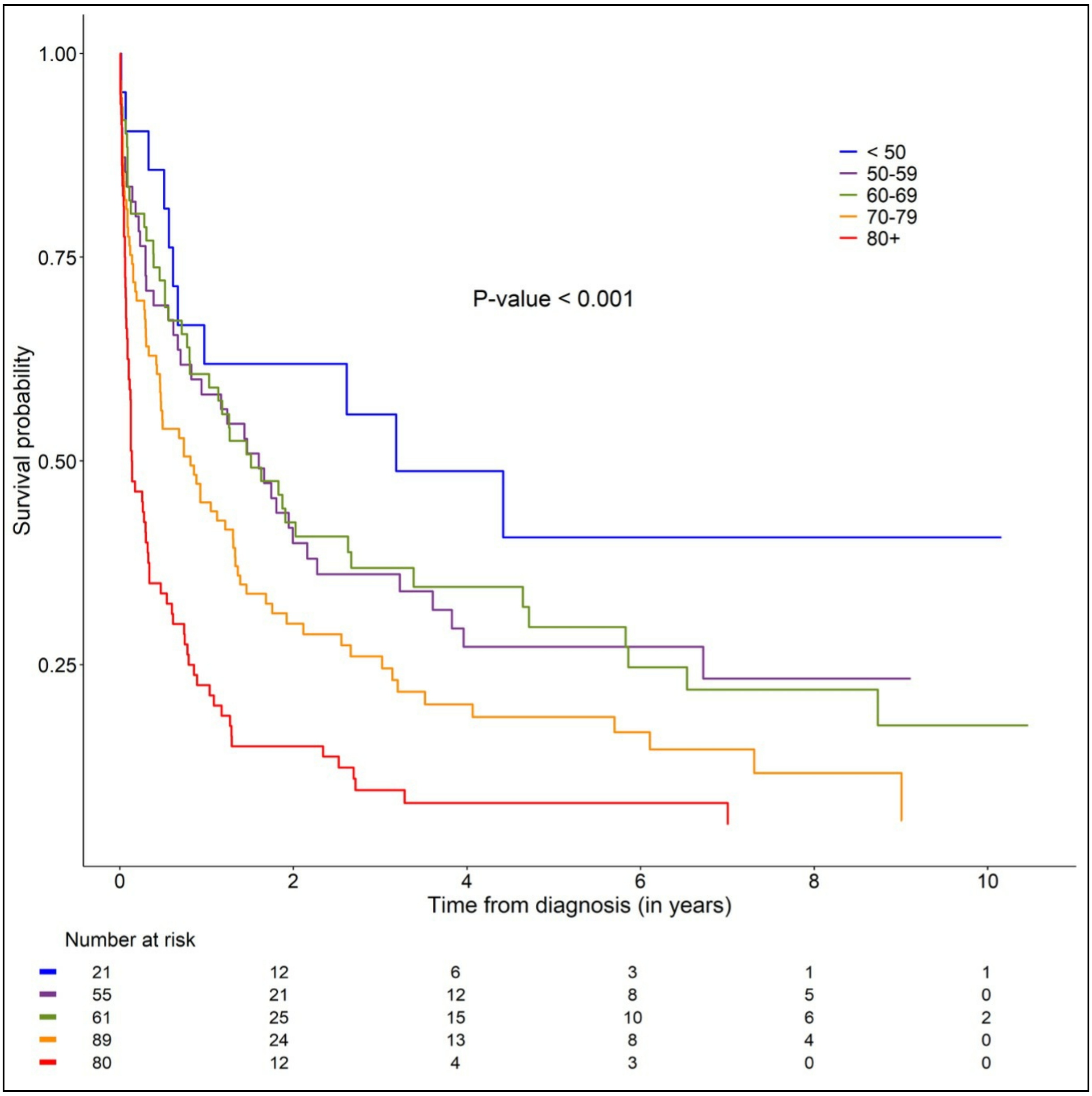

Figure: Kaplan-Meier plots for overall survival by age group

**Copyright Information:** (Online) ISSN: 2572-9241
©2026 The Author(s). HemaSphere is published by John Wiley & Sons Ltd on behalf of European Hematology Association. This is an open access Abstract Book distributed under the Attribution-NonCommercial-NoDerivs (CC BY-NC-ND), which allows third parties to download the articles and share them with others as long as they credit the author and the Abstract Book, but they cannot change the content in any way or use them commercially.
**Abstract Book Citations:** Authors, Title, HemaSphere, 2026;10:(S1):pages. The abstract book DOIs can be found on the “Author Information” page.
**Disclaimer:** Articles published in the journal HemaSphere exclusively reflect the opinions of the authors. The authors are responsible for all content in their abstracts including accuracy of the facts, statements, citing resources, etc.

### (PF844) NORMAL PLASMA CELLS AS A PROGNOSTIC BIOMARKER AND PREDICTOR OF EARLY SEVERE INFECTION IN NEWLY DIAGNOSED MULTIPLE MYELOMA

**Topic:** 14. Myeloma and other monoclonal gammopathies - Clinical

Qun Li*[1], Meng Wang[1], Min Zhang[1], Li Cai[1], Wendi Zhang[1], Dehu Li[1], Jinhua Wang[1], Aoshuang Xu[1], Yu Hu[1], Chunyan Sun[1]

*[1]Union Hospital, Tongji Medical College, Huazhong University of Science and Technology, Institute of Hematology, Wuhan, China;*

### Background

The pathogenesis of multiple myeloma (MM) involves a dynamic interplay between normal and malignant plasma cells within the bone marrow. While malignant plasma cells have been extensively studied, the clinical relevance of residual normal plasma cells (NPCs) remains incompletely understood.

### Aims

To validate the prognostic value of NPCs and investigate their association with the risk of early severe infection in newly diagnosed MM.

### Methods

We retrospectively analyzed 782 newly diagnosed MM patients who underwent baseline bone marrow flow cytometry. The NPCs ratio was defined as the proportion of NPCs among total bone marrow plasma cells. Survival outcomes were evaluated using Kaplan–Meier analysis and multivariable Cox regression. The association between NPCs ratio and early grade ≥3 infections was further assessed in 587 patients with complete medical records. Early severe infection was defined as grade ≥3 infection occurring within 3 months of diagnosis according to CTCAE v5.0. For infection risk model development, variable selection was performed using a combination of the Boruta algorithm and LASSO regression. Intersecting variables were incorporated into multivariable logistic regression to construct an infection prediction model. Model performance was evaluated by area under the receiver operating characteristic curve (AUC), calibration plots, and decision curve analysis.

### Results

Among 782 MM patients, 119 (15.2%) exhibited a high NPCs ratio (≥5%) at diagnosis. These patients presented with a more favorable clinical profile, including lower frequencies of advanced ISS/R-ISS stages, high-risk cytogenetics, anemia, renal dysfunction and lower β2-microglobulin levels (all $P<0.05$). Survival outcomes were markedly superior in the high NPCs group compared with the low-ratio group, with a median PFS of 58.7 versus 27.2 months and a median OS of 90.1 versus 45.6 months (both $P < 0.05$). In multivariable Cox analysis, a high NPCs ratio remained an independent favorable prognostic factor for both PFS and OS. The NPCs ratio was inversely correlated with immunoparesis ($P<0.001$). The incidence of early severe infections was significantly lower in patients with NPCs ≥5% compared with those with NPCs <5% (13.6% vs. 39.5%, $P<0.001$). Five predictors were incorporated to construct a 5-Factor Myeloma Infection Score (5-MIS) including NPCs≥5%, β2-microglobulin >5.5 mg/L, D-dimer >1.29 μg/mL, eGFR <60 mL/min/1.73 m², and hemoglobin <100 g/L. The model demonstrated robust discrimination in the training (AUC=0.733), validation (AUC=0.715), and overall cohorts (AUC=0.728). Calibration plots showed strong agreement between predicted and observed outcomes, and decision curve analysis supported its clinical utility.

**Copyright Information:** (Online) ISSN: 2572-9241
©2026 The Author(s). HemaSphere is published by John Wiley & Sons Ltd on behalf of European Hematology Association. This is an open access Abstract Book distributed under the Attribution-NonCommercial-NoDerivs (CC BY-NC-ND), which allows third parties to download the articles and share them with others as long as they credit the author and the Abstract Book, but they cannot change the content in any way or use them commercially.
**Abstract Book Citations:** Authors, Title, HemaSphere, 2026;10:(S1):pages. The abstract book DOIs can be found on the "Author Information" page.
**Disclaimer:** Articles published in the journal HemaSphere exclusively reflect the opinions of the authors. The authors are responsible for all content in their abstracts including accuracy of the facts, statements, citing resources, etc.

### Summary/Conclusion

NPCs represent an independent favorable prognostic biomarker and a protective factor against early severe infection in newly diagnosed MM.

**Copyright Information:** (Online) ISSN: 2572-9241
©2026 The Author(s). HemaSphere is published by John Wiley & Sons Ltd on behalf of European Hematology Association. This is an open access Abstract Book distributed under the Attribution-NonCommercial-NoDerivs (CC BY-NC-ND), which allows third parties to download the articles and share them with others as long as they credit the author and the Abstract Book, but they cannot change the content in any way or use them commercially.
**Abstract Book Citations:** Authors, Title, HemaSphere, 2026;10:(S1):pages. The abstract book DOIs can be found on the "Author Information" page.
**Disclaimer:** Articles published in the journal HemaSphere exclusively reflect the opinions of the authors. The authors are responsible for all content in their abstracts including accuracy of the facts, statements, citing resources, etc.

### (PF846) CLINICAL FACTORS AT FIRST MOBILIZATION FOR ASSESSING THE NEED TO STORE EXCESS CRYOPRESERVED AUTOLOGOUS STEM CELLS FOR FUTURE SALVAGE TRANSPLANTATION IN MULTIPLE MYELOMA

**Topic:** 14. Myeloma and other monoclonal gammopathies - Clinical

Laura Renners*[1], Anika Bergert[1], Angeliki Giesecke[1], Svenja Gehring[1], Ben-Niklas Bärmann[1], Guido Kobbe[1], Sascha Dietrich[1], Roland Fenk[1], Romans Zukovs[1]

*[1]University Hospital Düsseldorf, Medical Faculty, Heinrich Heine University Düsseldorf, Düsseldorf, Germany;*

### Background

High-dose chemotherapy (HDT) followed by autologous stem cell transplantation (ASCT) remains an integral part of most intensive therapy protocols for multiple myeloma (MM). The amount of stem cells collected prior to transplantation usually exceeds that required for the first line therapy in order to provide an option for possible salvage HDT-ASCT in relapse. However, additional or even prolonged stem cell apheresis involves repeated intensified medical intervention for the patient and the storage of cryopreserved stem cells increases the burden on the healthcare system.

### Aims

We looked for clinical factors that could help assessing the need to store excess stem cells after first-line mobilisation. The second aim was to test the clinical applicability and replicability of a score system previously proposed for accessing the probability of salvage therapy (*A. Vaishnav et al, ASH 2023*).

### Methods

Consecutive MM patients who underwent first-line HDT and ASCT between 1994 and 2024 were included in this single-centre retrospective analysis. The median follow-up time was 64 months and a minimum follow-up period of 12 months was required for inclusion. Clinical parameters assessed at diagnosis and prior to mobilization were evaluated in order to identify predictors. Routine clinical data such as age, renal function, peripheral blood counts, tumorload, type of induction therapy, best response and amount of collected stem cells/ transplants were among the parameters assessed.

### Results

A total of 867 subjects treated with ASCT in first-line were consequently identified and included in the research. The median age was 57 (27 - 75) years with 61% male subjects. 531 (61%) subjects experienced relapse and 88 (10%) received a salvage HDT-ASCT. Age ≤65 years (p= 0,023), age ≤55 years (p= 0,002), serum Creatinine ≤1,2 mg/dl (p= 0,031), CKD-Stage <II (p= 0,019), type of induction regimen (p= 0,039) and number of collected stem cell transplants (p= 0,04) were all univariate statistically significant predictive factors for the utilization of cryopreserved stem cells for salvage therapy in future. The score reported by Vaishnav et al. based on age, comorbidity index and renal function was replicable in our analysis on univariate assessment as well (p= 0,005). In multivariate analysis, age ≤55 remained as only independent predictive factor. Interestingly, the number of salvage therapies performed peaked in subjects treated with proteasome-inhibitor- and immunomodulator-based double and triple induction regimens. In contrast, only two salvage HDT-ASTCs have been performed in subjects treated with modern quadruple therapies in the studied population to date.

**Copyright Information:** (Online) ISSN: 2572-9241
©2026 The Author(s). HemaSphere is published by John Wiley & Sons Ltd on behalf of European Hematology Association. This is an open access Abstract Book distributed under the Attribution-NonCommercial-NoDerivs (CC BY-NC-ND), which allows third parties to download the articles and share them with others as long as they credit the author and the Abstract Book, but they cannot change the content in any way or use them commercially.
**Abstract Book Citations:** Authors, Title, HemaSphere, 2026;10:(S1):pages. The abstract book DOIs can be found on the "Author Information" page.
**Disclaimer:** Articles published in the journal HemaSphere exclusively reflect the opinions of the authors. The authors are responsible for all content in their abstracts including accuracy of the facts, statements, citing resources, etc.

**Summary/Conclusion**

We provide the age of over 55 years as a single factor for clinical decision-making regarding the need for storage of excess cells. This could reduce the financial and technical burden of an excessive long-time transplant storage.

**Copyright Information:** (Online) ISSN: 2572-9241
©2026 The Author(s). HemaSphere is published by John Wiley & Sons Ltd on behalf of European Hematology Association. This is an open access Abstract Book distributed under the Attribution-NonCommercial-NoDerivs (CC BY-NC-ND), which allows third parties to download the articles and share them with others as long as they credit the author and the Abstract Book, but they cannot change the content in any way or use them commercially.
**Abstract Book Citations:** Authors, Title, HemaSphere, 2026;10:(S1):pages. The abstract book DOIs can be found on the "Author Information" page.
**Disclaimer:** Articles published in the journal HemaSphere exclusively reflect the opinions of the authors. The authors are responsible for all content in their abstracts including accuracy of the facts, statements, citing resources, etc.

### (PF847) MULTI-OMICS PROFILING AND MEASURABLE RESIDUAL DISEASE ANALYSIS USING LIQUID BIOPSIES FROM MULTIPLE MYELOMA PATIENTS IN CLINICAL ROUTINE – A TRANSLATIONAL STUDY

**Topic:** 14. Myeloma and other monoclonal gammopathies - Clinical

Jan Vorwerk*[1], Lorena Rudolph[2], Anke Schilhabel[3], Philipp Nakov[3], Sabrina Neumann[1], Mouhamad Khouja[3], Jennifer Wigger[1], Theresa Stadler[1], Alessandra Hartke[1], Mona Merz[1], Lorenz Oelschläger[1, 4], Benedikt Färber[1], Leopold Schawe[1], Theo Leitner[1], Helal Ahmed[1, 5], Marah Hussein[1], Subbaiah Chary Nimmagadda[1], Pradeep Kumar Patnana[1], Eva Dazert[1], Jasmin Ostermann[6], Luna Reifenrath[2, 7], Ulrich Günther[2], Axel Künstner[8], Hauke Busch[8], Felix Sommer[9], Lisa Leypoldt[10, 11], Michaela Kotrova[3], Nikos Darzentas[3], Christiane Pott[3], Niklas Gebauer[1], Nikolaschristiancornelius Bubnoff[1], Alvaro Mallagaray[2], Timo Gemoll[6], Monika Brüggemann[3], Cyrus Khandanpour[1, 12, 13]

*[1]Department of Hematology and Oncology, University Cancer Center Schleswig-Holstein (UCCSH), University Hospital Schleswig-Holstein (UKSH) – Campus Lübeck, Lübeck, Germany; [2]Institute of Chemistry and Metabolomics, University of Lübeck, Lübeck, Germany; [3]Department of Hematology and Oncology, University Cancer Center Schleswig-Holstein (UCCSH), University Hospital Schleswig-Holstein (UKSH) – Campus Kiel, Kiel, Germany; [4]Division of Immunobiology, Cincinnati Children's Hospital, Cincinnati, United States of America; [5]Faculty of Medicine and Health Sciences, Amran University, Amran, Yemen; [6]Section for Translational Surgical Oncology and Biobanking, Department of Surgery, University Hospital Schleswig-Holstein (UKSH) – Campus Lübeck, Lübeck, Germany; [7]Department of Medicine I, University Hospital Schleswig-Holstein (UKSH) – Campus Lübeck, Lübeck, Germany; [8]Lübeck Institute of Experimental Dermatology (LIED), University of Lübeck, Lübeck, Germany; [9]Institute of Clinical Molecular Biology, Kiel University and University Hospital Schleswig-Holstein (UKSH) – Campus Kiel, Kiel, Germany; [10]Department of Oncology, Hematology, and Bone Marrow Transplantation, University Medical Center Hamburg-Eppendorf (UKE), Hamburg, Germany; [11]Mildred Scheel Early Career Center, University Medical Center Hamburg-Eppendorf (UKE), Hamburg, Germany; [12]Department of Hematology and Oncology, Klinikum Oldenburg, Oldenburg, Germany; [13]University Medicine Oldenburg, Carl von Ossietzky University of Oldenburg, Oldenburg, Germany;*

### Background

Bone marrow- (BM-) based molecular profiling and measurable residual disease (MRD) assessment are central to multiple myeloma (MM) management, but focal lesions, spatial heterogeneity, and extramedullary disease can limit the representativeness of single-site BM sampling. Plasma-derived cell-free DNA (cfDNA) and complementary plasma multi-omics may provide a minimally invasive, systemic readout of disease burden and treatment response.

### Aims

This study, therefore, aims to evaluate the complementarity of cfDNA sequencing and plasma multi-omics with BM-based MRD and molecular profiling in MM patients.

### Methods

20 adult MM patients were sampled at screening and up to four follow-ups during therapy. BM aspirates were assessed by next-generation flow cytometry (NGF) at screening. Patient-matched CD138-enriched BM genomic DNA (gDNA) and cfDNA underwent hybrid-capture targeted sequencing to detect clonal immunoglobulin rearrangements, single-nucleotide variants, and structural variants, enabling patient-specific longitudinal cfDNA MRD tracking. In parallel, plasma proteomics – mass spectrometry-based label-free profiling and Olink® technology – as well as NMR-based metabolomic, lipoprotein, and glycoprotein profiling were performed.

**Copyright Information:** (Online) ISSN: 2572-9241
©2026 The Author(s). HemaSphere is published by John Wiley & Sons Ltd on behalf of European Hematology Association. This is an open access Abstract Book distributed under the Attribution-NonCommercial-NoDerivs (CC BY-NC-ND), which allows third parties to download the articles and share them with others as long as they credit the author and the Abstract Book, but they cannot change the content in any way or use them commercially.
**Abstract Book Citations:** Authors, Title, HemaSphere, 2026;10:(S1):pages. The abstract book DOIs can be found on the "Author Information" page.
**Disclaimer:** Articles published in the journal HemaSphere exclusively reflect the opinions of the authors. The authors are responsible for all content in their abstracts including accuracy of the facts, statements, citing resources, etc.

### Results

Paired NGF and baseline molecular profiling in BM and plasma were available for 10 of 20 patients (all progressive disease (PD) at screening). NGF was MRD-positive in 6 of 10 and MRD-negative in 4 of 10. At baseline, myeloma-associated molecular markers were detected in 9 of 10 patients in BM and/or cfDNA. cfDNA recapitulated BM-derived variants while also identifying clinically relevant mutations not captured in paired BM samples, including RAS-pathway alterations. All 3 baseline patients with extramedullary involvement had detectable cfDNA markers, despite BM negativity 2 of these cases. Longitudinal cfDNA MRD remained positive in persistent PD and cleared in responders. In one case, cfDNA detected molecular recurrence before clinical relapse. Plasma proteomics identified a response-associated signature: in discovery profiling, 34 of 334 quantified proteins differed across states and separated PD vs. controlled disease with cross-validated AUROC 0.85 (PD) and 0.95 (very good partial response (VGPR) or complete remission (CR)), while an independent targeted plasma protein panel also distinguished PD and CR (AUROC 0.80 and 0.78, respectively). NMR-based metabolomic/lipoprotein profiling showed a consistent response gradient from PD → VGPR → CR. Integrated NMR-derived glycoprotein/lipoprotein composite features separated response groups in multivariate modeling (cross-validated AUROC ≥0.87).

### Summary/Conclusion cfDNA sequencing complements BM-based MRD by capturing spatially heterogeneous disease and enabling minimally invasive longitudinal monitoring, including early molecular relapse detection. Integrating cfDNA with plasma multi-omics adds response-associated systemic signatures that may support personalized disease surveillance.

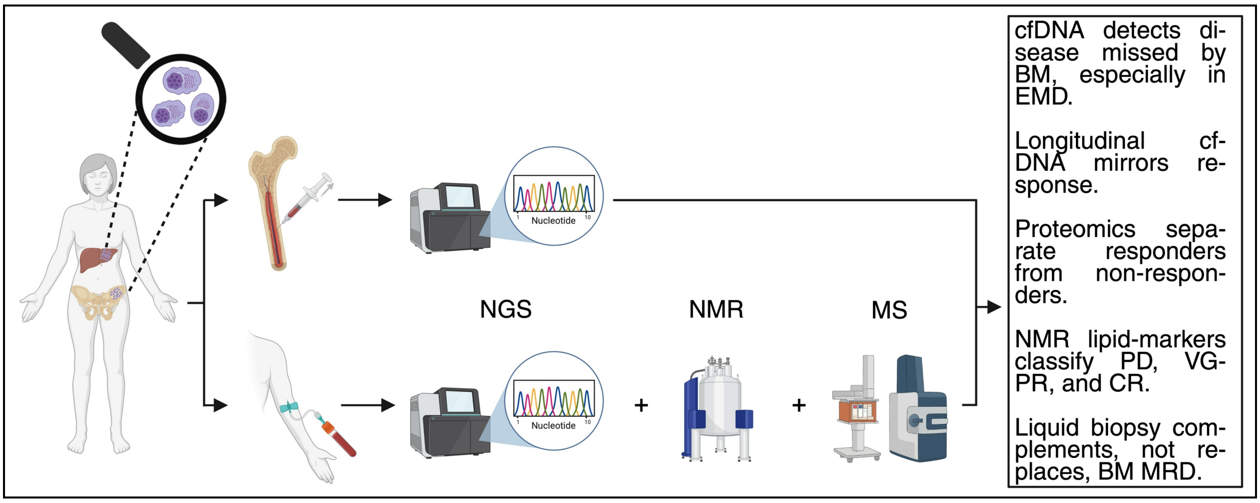

**Copyright Information:** (Online) ISSN: 2572-9241
©2026 The Author(s). HemaSphere is published by John Wiley & Sons Ltd on behalf of European Hematology Association. This is an open access Abstract Book distributed under the Attribution-NonCommercial-NoDerivs (CC BY-NC-ND), which allows third parties to download the articles and share them with others as long as they credit the author and the Abstract Book, but they cannot change the content in any way or use them commercially.
**Abstract Book Citations:** Authors, Title, HemaSphere, 2026;10:(S1):pages. The abstract book DOIs can be found on the “Author Information” page.
**Disclaimer:** Articles published in the journal HemaSphere exclusively reflect the opinions of the authors. The authors are responsible for all content in their abstracts including accuracy of the facts, statements, citing resources, etc.

### (PF848) FACTORS INFLUENCING LONG PROGRESSION-FREE SURVIVAL IN MULTIPLE MYELOMA PATIENTS RECEIVING IXAZOMIB-BASED ALL-ORAL REGIMENS

**Topic:** 14. Myeloma and other monoclonal gammopathies - Clinical

Liang Zhao[1], Xiao He[1], Qin Yang[1], Yan Zhu[1], Shuping Chen[1], Yanjuan He*[1]

*[1]Xiangya hospital, Central south university, Department of hematology, Changsha, China;*

### Background

Treatment of multiple myeloma (MM) has advanced rapidly, leading to a significant extension of patient survival and rendering MM a chronic disease. MM therapies are predominantly administered via injection, which compromises patient convenience. Ixazomib is the only oral proteasome inhibitor, and ixazomib(I)-based all-oral regimens offer remarkable convenience in clinical practice. However, in real-world settings, we observed substantial heterogeneity in treatment outcomes: some patients achieve excellent responses, while others show unsatisfactory efficacy. Thus, it is crucial to identify patient subgroups with extremely favorable outcomes to optimize individualized treatment strategies.

### Aims

This study aimed to evaluate and compare the clinical characteristics between MM patients with long PFS (lPFS) and those with short PFS (sPFS) who received I-based all-oral treatment regimens.

### Methods

A retrospective analysis was performed on 82 MM patients who received I-based all-oral therapy at our center between July 2018 and January 2023. Survival outcomes, demographic characteristics, and clinical features were compared between the lPFS and sPFS groups. Prognostic factor analysis was conducted using multivariate Cox regression.

### Results

**Copyright Information:** (Online) ISSN: 2572-9241
©2026 The Author(s). HemaSphere is published by John Wiley & Sons Ltd on behalf of European Hematology Association. This is an open access Abstract Book distributed under the Attribution-NonCommercial-NoDerivs (CC BY-NC-ND), which allows third parties to download the articles and share them with others as long as they credit the author and the Abstract Book, but they cannot change the content in any way or use them commercially.
**Abstract Book Citations:** Authors, Title, HemaSphere, 2026;10:(S1):pages. The abstract book DOIs can be found on the “Author Information” page.
**Disclaimer:** Articles published in the journal HemaSphere exclusively reflect the opinions of the authors. The authors are responsible for all content in their abstracts including accuracy of the facts, statements, citing resources, etc.

| Predictors | | Hazard Ratio | 95%CI | p |
|---|---|---|---|---|
| Age at diagnosis | | **1.148** | 1.063-1.239 | **<0.001** |
| Sex | Male vs Female | 0.927 | 0.336-2.348 | 0.874 |
| ECOG-PS | 2 | 1.018 | 0.193-5.379 | 0.983 |
| | 3 | 1.351 | 0.22-8.281 | 0.745 |
| | 4 | 5.596 | 0.517-60.52 | 0.156 |
| RISS stage | 2 | 0.807 | 0.182-3.582 | 0.777 |
| | 3 | 0.303 | 0.037-2.501 | 0.268 |
| Bone destruction | | 2.509 | 0.966-6.514 | 0.059 |
| High-risk cytogenetics | | **3.374** | 1.152-9.879 | **0.027** |
| I-based regimens | others vs IRD | 1.381 | 0.529-3.605 | 0.51 |
| ASCT | | 1.954 | 0.49-7.787 | 0.342 |
| Maintenance therapy | | **0.213** | 0.054-0.833 | **0.026** |
| I-based regimens efficacy | ≥VGPR vs <VGPR | 1.095 | 0.322-3.726 | 0.884 |
| I dose | 4mg vs 3mg | 2.258 | 0.883-5.776 | 0.089 |
| Treatment type | | 0.518 | 0.194-1.383 | 0.189 |

All 82 patients received I-based all-oral regimens, predominantly IRD, ITD, and ID regimens. Among them, 29 were treated with ixazomib as initial therapy, while the remaining 53 were classified into the *in-class* transition (iCT) group. Based on PFS duration, 34 patients (41%) were categorized into the lPFS group (PFS ≥ 36 months), and 48 patients (59%) into the sPFS group (PFS < 36 months), with a statistically significant difference in PFS. OS also differed significantly between the two groups: the median OS was not reached in the lPFS group versus 70 months in the sPFS group (Fig. 1A). Compared with the sPFS group, the lPFS group had a significantly younger age, lower RISS stage, lower percentage of bone destruction, and better efficacy of I-based regimens (all P < 0.05, Fig. 1B-E). There was a trend toward lower proportions of ISS stage 1 (P = 0.057) and ixazomib 3mg usage (P = 0.055) in the

**Copyright Information:** (Online) ISSN: 2572-9241
©2026 The Author(s). HemaSphere is published by John Wiley & Sons Ltd on behalf of European Hematology Association. This is an open access Abstract Book distributed under the Attribution-NonCommercial-NoDerivs (CC BY-NC-ND), which allows third parties to download the articles and share them with others as long as they credit the author and the Abstract Book, but they cannot change the content in any way or use them commercially.
**Abstract Book Citations:** Authors, Title, HemaSphere, 2026;10:(S1):pages. The abstract book DOIs can be found on the "Author Information" page.
**Disclaimer:** Articles published in the journal HemaSphere exclusively reflect the opinions of the authors. The authors are responsible for all content in their abstracts including accuracy of the facts, statements, citing resources, etc.

lPFS group. No significant differences were observed in other demographic and clinical features between the two groups. Further multivariate Cox regression analysis revealed that advanced age (HR=1.148) and high-risk cytogenetics (HR=3.374) were significantly negatively associated with prolonged PFS, while maintenance therapy (HR=0.213) was beneficial for extending PFS (Table).

### Summary/Conclusion

In conclusion, the most significant factors affecting prognosis were age and high-risk cytogenetics (both poor prognostic factors), and maintenance therapy (a protective factor). Therefore, patients who derive the greatest benefit from I-based all-oral regimens (either as initial therapy or iCT) are characterized by a younger age, absence of high-risk cytogenetics, and adherence to maintenance therapy.

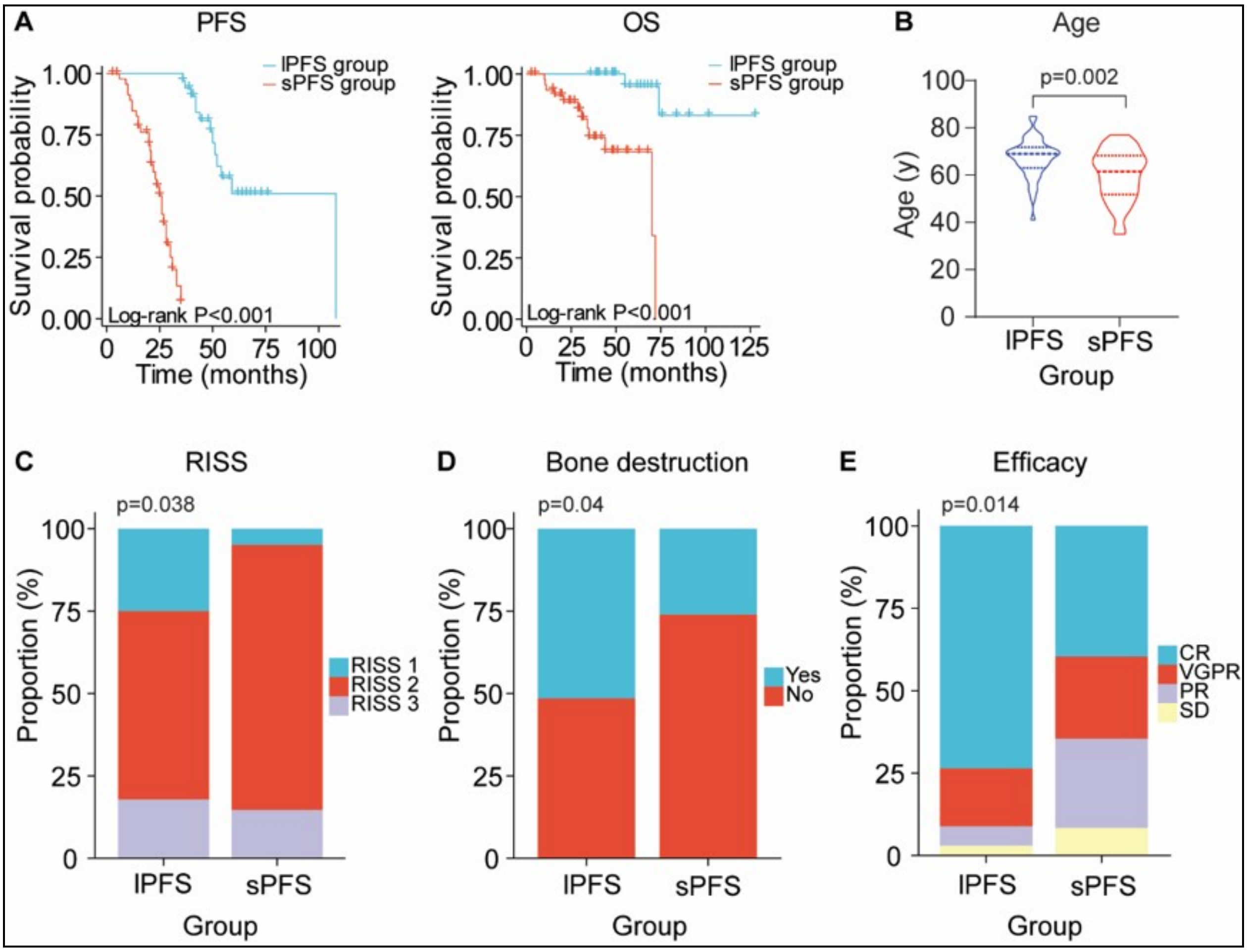

Figure 1. Prognosis and patient characteristics in the lPFS and sPFS groups. A. PFS (left) and OS (right) of patients in the lPFS and sPFS groups. B–E. Comparisons of age (B), RISS stage (C), bone destruction (D), and best response to ixazomib-based regimens (E) between the lPFS and sPFS groups. lPFS, long progression-free survival; sPFS, short progression-free survival. OS, overall survival.

Copyright Information: (Online) ISSN: 2572-9241
©2026 The Author(s). HemaSphere is published by John Wiley & Sons Ltd on behalf of European Hematology Association. This is an open access Abstract Book distributed under the Attribution-NonCommercial-NoDerivs (CC BY-NC-ND), which allows third parties to download the articles and share them with others as long as they credit the author and the Abstract Book, but they cannot change the content in any way or use them commercially.
Abstract Book Citations: Authors, Title, HemaSphere, 2026;10:(S1):pages. The abstract book DOIs can be found on the “Author Information” page.
Disclaimer: Articles published in the journal HemaSphere exclusively reflect the opinions of the authors. The authors are responsible for all content in their abstracts including accuracy of the facts, statements, citing resources, etc.

### (PF849) LOWER INFECTIONS WITH ELRANATAMAB (ELRA) IN MULTIPLE MYELOMA (RRMM) IN REAL-WORLD SETTINGS THAN IN MAGNETISMM-3 (MM-3)

**Topic:** 14. Myeloma and other monoclonal gammopathies - Clinical

Rahul Banerjee*[1], Aaron Rosenberg[2], Chai Kim[3], David Hughes[4], Guido Nador[5], Rickard Sandin[6], Jenna Collins[7], Trong Le[7], Amy Pierre[7], Ayush Kris[7], Yong Chen[3]

*[1]Fred Hutchinson Cancer Center, Seattle, United States of America;[2]UC Davis Comprehensive Cancer Center, Sacramento, United States of America; [3]Pfizer Inc, New York, United States of America;[4]Pfizer Inc, Cambridge, United States of America; [5]Pfizer Ltd, Surrey, United Kingdom;[6]Pfizer AB, Stockholm, Sweden;[7]Flatiron Health, New York, United States of America;*

### Background

Elranatamab (ELRA) induces durable responses in RRMM, but is associated with infectious complications. In the MM-3 trial leading to ELRA's approval (Lesokhin Nat Med 2023), any-grade and Grade ≥3 infections occurred in 70% and 40% of patients, respectively; the trial enrolled patients between 02/2021 and 01/2022. Given evolving coronavirus-related morbidity and increased use of intravenous immunoglobulin (IVIG), real-world (RW) data are needed to better characterize contemporary infection risk and management.

### Aims

To assess incidence, timing, and management of infections in a RW cohort of patients treated with ELRA.

### Methods

This retrospective, observational analysis used data from the Flatiron Health Database. Adult patients (≥18 years) with RRMM and triple-class refractory disease who initiated ELRA treatment between 08/2023 and 07/2025 were included.
Patient demographic and clinical characteristics, and safety outcomes were summarized descriptively, including overall infections incidence, classified as non-respiratory viral (adenovirus, influenza, rhinovirus, hepatitis B), bacterial (sepsis, bacteremia, pneumonia), or unspecified pulmonary infections (pneumonia, pneumonitis, bronchiolitis, tracheitis), as well as time to onset, resolution, and treatment.

**Copyright Information:** (Online) ISSN: 2572-9241
©2026 The Author(s). HemaSphere is published by John Wiley & Sons Ltd on behalf of European Hematology Association. This is an open access Abstract Book distributed under the Attribution-NonCommercial-NoDerivs (CC BY-NC-ND), which allows third parties to download the articles and share them with others as long as they credit the author and the Abstract Book, but they cannot change the content in any way or use them commercially.
**Abstract Book Citations:** Authors, Title, HemaSphere, 2026;10:(S1):pages. The abstract book DOIs can be found on the "Author Information" page.
**Disclaimer:** Articles published in the journal HemaSphere exclusively reflect the opinions of the authors. The authors are responsible for all content in their abstracts including accuracy of the facts, statements, citing resources, etc.

**Results**

Among 115 ELRA patients, 45% were male; median age was 71 years, with 37% aged ≥75 years; the median time from MM diagnosis to ELRA initiation was 7.2 years (IQR, 4.3–10.3). Patients were managed in academic (35%), community (33%), or mixed academic–community (32%) settings. The median follow-up was 11.5 months (IQR, 6.4–15.6).
Overall, 27 (23%) patients experienced ≥ 1 infection during ELRA. Median time to infection was 77 days (IQR, 31–175 days). Among patients with an infection, 13 (48%) had documented resolution, 9 (34%) had either ongoing or resolving resolution at last follow-up, and N=5 had no documented resolution status. The median time to resolution was 11 days (IQR, 6–21 days). By infection subtype, bacterial was most common (N=18, 16%), followed by non-respiratory viral (N=13, 11%) and unspecified pulmonary infections (N=5, 4%).
All patients with bacterial infections (N=18) received treatment; 17 (94%) received antibiotics, 12 (67%) required hospitalization and N=7 (39%) had a therapy hold. Among patients with non-respiratory viral infections, N=9 (69%) received treatment; N=2 (15%) received antiviral therapy, N=5 (38%) required hospitalization and N=5 (38%) had a therapy hold. Among those with unspecified pulmonary infections, N=5 (83%) received treatment; N=4 (67%) required hospitalization and N=3 (50%) had a therapy hold. Across all infection subtypes, no treatment discontinuations or infection-related deaths were documented. Further analyses of IVIG usage before and during ELRA treatment will be presented at the meeting.

**Summary/Conclusion**

Any-grade infections occurred in 23% of RW patients treated with ELRA at a median follow-up of 11.5 months. This proportion appears numerically lower than that reported in MM-3 despite similar median follow-up (MM-3: 14.7 months vs RW cohort: 11.5 months). This difference may reflect incomplete capture of infections in our RW database or, alternatively, evolving trends in coronavirus morbidity and IVIG use in our more contemporary cohort. Notably, no infections resulted in treatment discontinuation or death.

**Copyright Information:** (Online) ISSN: 2572-9241
©2026 The Author(s). HemaSphere is published by John Wiley & Sons Ltd on behalf of European Hematology Association. This is an open access Abstract Book distributed under the Attribution-NonCommercial-NoDerivs (CC BY-NC-ND), which allows third parties to download the articles and share them with others as long as they credit the author and the Abstract Book, but they cannot change the content in any way or use them commercially.
**Abstract Book Citations:** Authors, Title, HemaSphere, 2026;10:(S1):pages. The abstract book DOIs can be found on the "Author Information" page.
**Disclaimer:** Articles published in the journal HemaSphere exclusively reflect the opinions of the authors. The authors are responsible for all content in their abstracts including accuracy of the facts, statements, citing resources, etc.

### (PF850) FIRST-LINE USE OF CARFILZOMIB-BASED REGIMENS IMPROVES SURVIVAL OF NEWLY DIAGNOSED MULTIPLE MYELOMA PATIENTS WITH EXTRAMEDULLARY PLASMACYTOMAS: A REAL-WORLD RETROSPECTIVE STUDY

**Topic:** 14. Myeloma and other monoclonal gammopathies - Clinical

Ziying Jian[1], Yifan Jia[1], Zheng Ge*[1]

*[1]Zhongda Hospital Southeast University, Institute of Hematology Southeast University, Department of Hematology, Nanjing, China;*

### Background

The mSMART 4.0 defines newly diagnosed multiple myeloma (NDMM) with extramedullary diseases as a high-risk subtype, which elaborates the difficulties and urgency of treatment in MM patients with extramedullary plasmacytomas. Carfilzomib is widely used as salvage treatment in refractory and relapsed multiple myeloma (RRMM). In recent years, there have been scattered case reports of its therapeutics in extramedullary lesions of RRMM. but lack of real-world data in NDMM.

### Aims

To explore the efficiency and prognosis of NDMM patients with extramedullary plasmacytomas treated with carfilzomib-based regimen.

### Methods

Our study included 46 MM patients with extramedullary plasmacytomas in our center between January 1, 2020 and January 31, 2025. Clinical and laboratory data were collected. The primary endpoints were median overall survival (mOS) and median progression-free survival (mPFS). Data processing and analysis were performed using Prism 8, version 8.2.1.

### Results

Of all enrolled patients, the median age was 66.0 years (41.0 to 88.0 years). Myeloma type was similarly distributed in IgG (16/46), IgA (15/46) and light chain only (13/46) subtypes. 27 NDMM and 19 RRMM patients were involved. 37.0% (17/46) of patients accompanied with extramedullary (EMD) plasmacytomas, 24.8% (16/46) with paraskeletal (PS) lesions and 38.2% (13/46) with both EMD and PS lesions. Carf group included MM with extramedullary plasmacytomas treated with carfizomib-based regimen. Non Carf group included no carfizomib used patients. The frequencies of clinical characteristics were similar between the two groups (Table 1). First-line use of carfizomib-based regimen prolonged mPFS in NDMM with extramedullary plasmacytomas (Carf v.s. Non Carf: 1134 v.s. 615 days, *P*=0.0295) (Figure 1A). 80% ((4/5) of Carf group patients achieved at least very good partial response (VGPR), while that was 63.0% (26/41) in the Non Carf group. The overall response rate (ORR) was 100% in the Carf group. Meanwhile, carfizomib-based regimen seemed to improve mOS in NDMM with extramedullary plasmacytomas (undefined v.s. 2050 days).(Figure 1B).

After relapse, the carfizomib-based regimen still enabled 25.0% (2/8) of the patients to achieve at least VGPR, 62.5% (5/8) achieved at least PR. But in the Non Carf group, they were only 5.88% (1/17) and 41.18% (7/17). In NDMM and RRMM patients with extramedullary diseases, the median OS was 2631 days in the Carf group, significantly longer than that in the Non Carf group (1213 days, *P*=0.0362) (Figure 1C).

**Copyright Information:** (Online) ISSN: 2572-9241
©2026 The Author(s). HemaSphere is published by John Wiley & Sons Ltd on behalf of European Hematology Association. This is an open access Abstract Book distributed under the Attribution-NonCommercial-NoDerivs (CC BY-NC-ND), which allows third parties to download the articles and share them with others as long as they credit the author and the Abstract Book, but they cannot change the content in any way or use them commercially.
**Abstract Book Citations:** Authors, Title, HemaSphere, 2026;10:(S1):pages. The abstract book DOIs can be found on the "Author Information" page.
**Disclaimer:** Articles published in the journal HemaSphere exclusively reflect the opinions of the authors. The authors are responsible for all content in their abstracts including accuracy of the facts, statements, citing resources, etc.

Table 1 Patient characteristics at baseline

| | All patients (N = 46) | Non Carf (N = 32) | Carf (N = 14) |
|---|---|---|---|
| **Age, years** | 66.0(41.0-88.0) | 66.5(41.0-88.0) | 65.0(42.0-76.0) |
| **Sex** | | | |
| Male | 20 | 15 | 5 |
| Female | 26 | 17 | 9 |
| **Myeloma Type** | | | |
| IgG | 16 | 8 | 8 |
| IgA | 15 | 14 | 1 |
| Light chain only | 13 | 8 | 5 |
| Others | 2 | 2 | 0 |
| **NDMM** | | | |
| Yes | 27 | 22 | 5 |
| No | 19 | 10 | 9 |
| **ISS** | | | |
| I | 4 | 1 | 3 |
| II | 17 | 12 | 5 |
| III | 22 | 16 | 6 |
| **R-ISS** | | | |
| I | 2 | 1 | 1 |
| II | 16 | 13 | 3 |
| III | 22 | 10 | 2 |
| Unknown | 16 | 8 | 8 |
| **High-risk cytogenetics (mSMRT 3.0)** | | | |
| Yes | 23 | 18 | 5 |
| No | 18 | 11 | 7 |
| Not reported | 5 | 3 | 2 |
| **Extramedullary plasmacytomas by type** | | | |
| EMD plasmacytomas | 17 | 11 | 6 |
| PS plasmacytomas | 16 | 12 | 4 |
| EMD and PS plasmacytomas | 8 | 4 | 4 |
| **Bone marrow plasma cell categories** | | | |
| 0.0%–10.0% | 18 | 10 | 8 |
| >10.0% and <60.0% | 25 | 20 | 5 |
| ≥60.0% | 3 | 2 | 1 |

### Summary/Conclusion

Our study indicated that first-line use of carfizomib-based regimen improved outcome in NDMM with extramedullary plasmacytomas by longer mPFS and mOS, higher response rate. Meanwhile, it improved mOS in NDMM and RRMM with extramedullary plasmacytomas. Further prospective studies are needed to validate its long-term therapeutic value.

**Copyright Information:** (Online) ISSN: 2572-9241
©2026 The Author(s). HemaSphere is published by John Wiley & Sons Ltd on behalf of European Hematology Association. This is an open access Abstract Book distributed under the Attribution-NonCommercial-NoDerivs (CC BY-NC-ND), which allows third parties to download the articles and share them with others as long as they credit the author and the Abstract Book, but they cannot change the content in any way or use them commercially.
**Abstract Book Citations:** Authors, Title, HemaSphere, 2026;10:(S1):pages. The abstract book DOIs can be found on the "Author Information" page.
**Disclaimer:** Articles published in the journal HemaSphere exclusively reflect the opinions of the authors. The authors are responsible for all content in their abstracts including accuracy of the facts, statements, citing resources, etc.

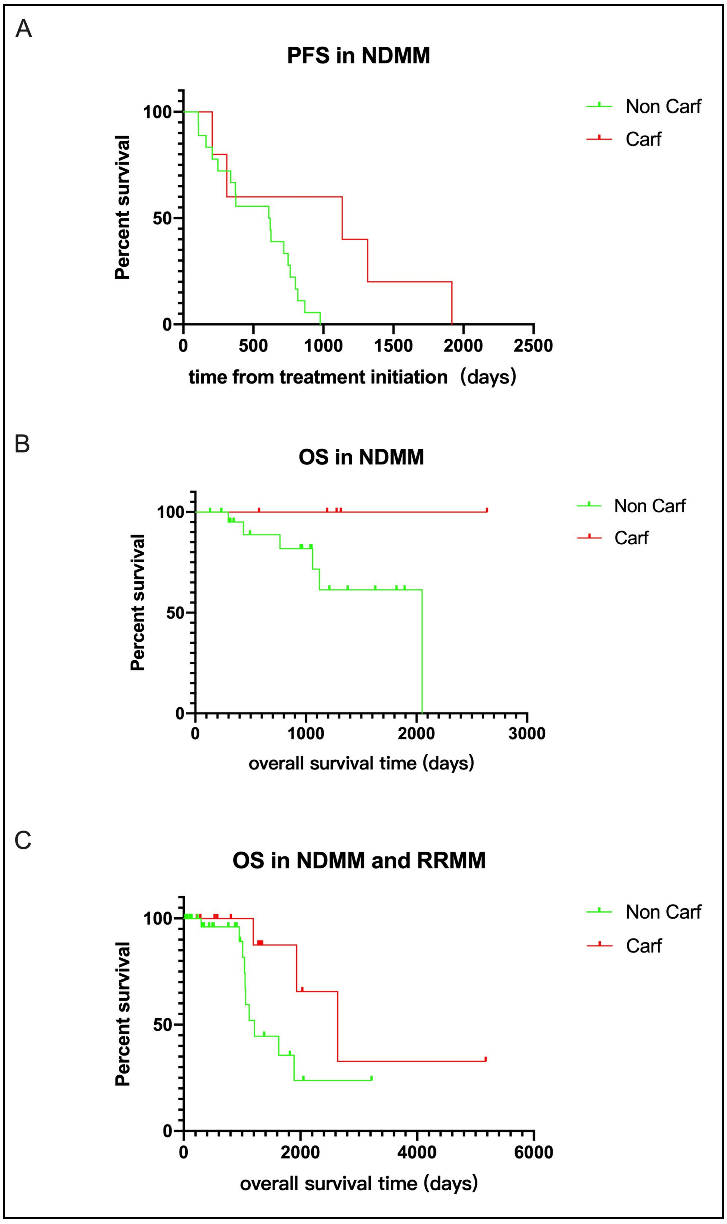

Figure 1:PFS,OS in NDMM and RRMM with extramedullary plasmacytomas.

**Copyright Information:** (Online) ISSN: 2572-9241
©2026 The Author(s). HemaSphere is published by John Wiley & Sons Ltd on behalf of European Hematology Association. This is an open access Abstract Book distributed under the Attribution-NonCommercial-NoDerivs (CC BY-NC-ND), which allows third parties to download the articles and share them with others as long as they credit the author and the Abstract Book, but they cannot change the content in any way or use them commercially.
**Abstract Book Citations:** Authors, Title, HemaSphere, 2026;10:(S1):pages. The abstract book DOIs can be found on the "Author Information" page.
**Disclaimer:** Articles published in the journal HemaSphere exclusively reflect the opinions of the authors. The authors are responsible for all content in their abstracts including accuracy of the facts, statements, citing resources, etc.

### (PF851) EFFICACY AND SAFETY OF CESNICABTAGENE AUTOLEUCEL (ARI0002H), AN ACADEMIC BCMA-TARGETED CAR T-CELL THERAPY, IN RELAPSED/REFRACTORY LIGHT-CHAIN AMYLOIDOSIS

**Topic:** 14. Myeloma and other monoclonal gammopathies - Clinical

Jose Miguel Mateos Perez*[1], Aina Oliver[1], Natalia Tovar[1], Nuria Martinez-Cibrian[2], Nil Albiol[2], Beatriz Merchan[2], Alexandra Martínez Roca[2], Valentín Ortiz-Maldonado[2], Laura Rosiñol Dachs[1], Julio Delgado[2], Avaro Urbano Ispizua[2], Europa Azucena Gonzalez[3], Marta Español-Rego[3], Sergio Navarro[3], Pilar Ayora[2], Paola Charry Espana[2], Mercedes Montoro[2], Luis Gerardo Rodriguez-Lobato[1], Carlos Fernández de Larrea[1], M. Teresa Cibeira[1]

*[1]Hospital Clinic of Barcelona, Amyloidosis and Multiple Myeloma Unit, Hematology Department, IDIBAPS, University of Barcelona, Barcelona, Spain; [2]Hospital Clinic of Barcelona, Oncoimmunotherapy Unit, Hematology Department, IDIBAPS, University of Barcelona, Barcelona, Spain; [3]Hospital Clinic of Barcelona, Inmunology Department, Barcelona, Spain;*

### Background

Systemic light chain (AL) amyloidosis is a rare plasma cell disorder that causes severe and potentially life-threatening organ dysfunction. Therapeutic options at relapse are scarce and mostly come from proven indications in multiple myeloma (MM). *Cesnicabtagene autoleucel* (cesni-cel, or ARI0002h) is a BCMA-directed CAR-T fully developed in Hospital Clinic of Barcelona (HCB) and approved in Spain under the Hospital Exemption pathway for relapsed/refractory (R/R) MM.

### Aims

To evaluate the efficacy and safety of ARI0002h in patients with R/R AL amyloidosis.

### Methods

Retrospective single-center study of 5 patients with R/R AL amyloidosis treated with cesni-cel under compassionate use. Cesni-cel ($3x10^6$CAR+ cells/kg) was administered in three fractions (10%, 30%, 60%), after lymphodepletion with fludarabine (30mg/$m^2$) and cyclophosphamide (300mg/$m^2$) on days –6 to -4. A non-fractionated booster dose ($3x10^6$CAR+ cells/kg) was administered at 3 months in responders with no limiting toxicities. Mayo and Revised Mayo 2012 amyloidosis staging systems were applied. Hematologic and organic response were assessed by ISA criteria.

### Results

Baseline characteristics are depicted in **Table 1**. Median prior lines of therapy were 3 (range 2-5); all patients were triple-exposed and none had received prior BCMA-directed treatment. Four (80%) received cesni-cel for both hematologic and organ progression, while one patient presented with hematologic progression only. One grade 1 CRS occurred on day 8; no specific treatment was needed. No neurologic events were observed. Four patients (80%) presented hypogammaglobulinemia but only one grade 3 infection due to BK virus cystitis occurred with proven bladder amyloidosis. No cardiac or renal events appeared during apheresis, infusion, or hospitalization. A booster dose was administered in 4 patients (80%) with no side effects.
At a median follow-up of 7.7 months (range, 3–66), hematologic overall and very good partial response or better (≥VGPR) rates were 100%. Free light chain (FLC) suppression occurred in 100% at a median time of 29 days (range, 15-62). All patients achieved undetectable bone marrow measurable residual disease. All patients remain alive without progression at 66, 21, 8, 6 and 3 months. During follow-up, one patient (25%) achieved organ complete response, 1 (25%) VGPR and 2 (50%) stable disease.

**Table 1**

**Copyright Information:** (Online) ISSN: 2572-9241
©2026 The Author(s). HemaSphere is published by John Wiley & Sons Ltd on behalf of European Hematology Association. This is an open access Abstract Book distributed under the Attribution-NonCommercial-NoDerivs (CC BY-NC-ND), which allows third parties to download the articles and share them with others as long as they credit the author and the Abstract Book, but they cannot change the content in any way or use them commercially.
**Abstract Book Citations:** Authors, Title, HemaSphere, 2026;10:(S1):pages. The abstract book DOIs can be found on the "Author Information" page.
**Disclaimer:** Articles published in the journal HemaSphere exclusively reflect the opinions of the authors. The authors are responsible for all content in their abstracts including accuracy of the facts, statements, citing resources, etc.

| Variables; median (range) | At diagnosis | At screening |
|---|---|---|
| **Age (years)** | 52 (49–66) | 58 (50–66) |
| **Heavy and light chain isotype, n(%)** | | |
| IgG/IgA/IgD/BJ | 2 (40)/ 1 (20)/ 1 (20)/ 1 (20) | |
| Lambda | 5 (100) | |
| **Serum M protein (g/L)** | 15.5 (13–24) | 2.5 (0–20) |
| **Involved FLC (mg/L)** | 389.5 (147–3471) | 352 (54–967) |
| **Bone marrow plasma cells (%)*** | 29 (8–96) | 14.5 (5–48) |
| **NT-proBNP (pg/mL)** | 647 (211–21186) | 277 (192–949) |
| **Interventricular septum thickness (mm)** | 13 (7–14) | 13 (9–15) |
| **24h proteinuria (g/24h)** | 2.37 (0.2–2.37) | 0.8 (0.18–11.4) |
| **Organic disease** | | |
| Heart, n (%) | 3 (60) | 3 (60) |
| Kidney, n (%) | 1 (20) | 2 (40) |
| Muscular, n (%) | 1 (20) | 1 (20) |
| Gastrointestinal, n (%) | 3 (60) | 3 (60) |
| Total affected organs | 3 (1–4) | 4 (2–4) |
| **Mayo stage, n (%)** | | |
| I | | 3 (60) |
| II | 4 (80) | 2 (40) |
| IIIa | 1 (20) | |

**Copyright Information:** (Online) ISSN: 2572-9241
©2026 The Author(s). HemaSphere is published by John Wiley & Sons Ltd on behalf of European Hematology Association. This is an open access Abstract Book distributed under the Attribution-NonCommercial-NoDerivs (CC BY-NC-ND), which allows third parties to download the articles and share them with others as long as they credit the author and the Abstract Book, but they cannot change the content in any way or use them commercially.
**Abstract Book Citations:** Authors, Title, HemaSphere, 2026;10:(S1):pages. The abstract book DOIs can be found on the "Author Information" page.
**Disclaimer:** Articles published in the journal HemaSphere exclusively reflect the opinions of the authors. The authors are responsible for all content in their abstracts including accuracy of the facts, statements, citing resources, etc.

| | | |
|---|---|---|
| **Revised Mayo stage 2012, n (%)** | | |
| I | | 2 (40) |
| II | 3 (60) | 1 (20) |
| III | 1 (20) | 2 (40) |
| IV | 1 (20) | |

| | |
|---|---|
| **Prior drug exposure/refractoriness (%)** | |
| Bortezomib | 100/20 |
| Lenalidomide | 100/40 |
| Anti-CD38 therapy | 100/60 |
| Carfilzomib | 40/20 |
| Pomalidomide | 20/0 |

| | |
|---|---|
| **Time from diagnosis to infusion (months)** | 61 (3–81) |

*Cytogenetic at diagnosis (n=3) one patient without alterations and two with t(11;14)

### Summary/Conclusion

Cesni-cel is safe in patients with R/R AL amyloidosis and provides deep and sustained hematologic responses.

Copyright Information: (Online) ISSN: 2572-9241
©2026 The Author(s). HemaSphere is published by John Wiley & Sons Ltd on behalf of European Hematology Association. This is an open access Abstract Book distributed under the Attribution-NonCommercial-NoDerivs (CC BY-NC-ND), which allows third parties to download the articles and share them with others as long as they credit the author and the Abstract Book, but they cannot change the content in any way or use them commercially.
**Abstract Book Citations:** Authors, Title, HemaSphere, 2026;10:(S1):pages. The abstract book DOIs can be found on the "Author Information" page.
**Disclaimer:** Articles published in the journal HemaSphere exclusively reflect the opinions of the authors. The authors are responsible for all content in their abstracts including accuracy of the facts, statements, citing resources, etc.

### (PF852) SAFETY AND EFFICACY OF AUTOLOGOUS DUAL TARGETING BCMA/CD19 CAR-T FKC289 IN SUBJECTS WITH RELAPSED / REFRACTORY LIGHT CHAIN AMYLOIDOSIS

**Topic:** 14. Myeloma and other monoclonal gammopathies - Clinical

Jing Li*[1], Shiyang Gu[1], Yuhong Ren[1], Yang Yang[1], Jiadai Xu[1], Yian Zhang[1], Tianhong Xu[1], Xiaochen Wang[2], Gongxian Jia[2], Zisong Zhou[2], Wen Shi[2], Peng Liu[1]

*[1]Zhongshan Hospital Fudan University, Shanghai, China;[2]Shanghai FosunKairos Biotechnology Co.,LTD., Shanghai, China;*

### Background

Although Daratumumab-based regimen has been proven efficacy for newly diagnosed patients, there are still huge unmet medical needs for the relapsed/refractory Light chain amyloidosis (r/r-ALA). BCMA-directed CAR-T therapies can rapidly suppress circulating free light chains in plasma-cell disorders, yet single-target therapies may lead to resistance due to antigen loss. Dual-target CAR-T therapy introduces an innovative treatment strategy for deeper eradication of malignant plasma cells and secreted light chains. Currently, we launched this investigator initiated trial (IIT), first-in-human Phase 1 trial (ChiCTR2500100410) to evaluate the safety and preliminary efficacy of the BCMA/CD19 CAR-T product FKC289 in patients with r/r ALA.

### Aims

To explore the safety, tolerability, response rate, and long-term efficacy of FKC289 in r/r ALA.

### Methods

From May 2025 to Feb 2026, 4 patients with r/r ALA were enrolled and infused with FKC289 CAR-T at a dose of $1 \times 10^5$ cells/kg in 3 subjects and $3 \times 10^5$ cells/kg in 1 subject. Since the fourth subject was infused shortly before database lock, here we report the first 3 subjects who were dosed at $1 \times 10^5$ cells/kg. The primary objective was to evaluate safety and tolerability. CRS and ICANS were graded according to the ASTCT 2019 criteria. The secondary objectives included assessing efficacy and PK profile for r/r ALA patients receiving FKC289 treatment. Response was evaluated based on the NCCN Guidelines for Systemic Light Chain Amyloidosis (2023).

**Copyright Information:** (Online) ISSN: 2572-9241
©2026 The Author(s). HemaSphere is published by John Wiley & Sons Ltd on behalf of European Hematology Association. This is an open access Abstract Book distributed under the Attribution-NonCommercial-NoDerivs (CC BY-NC-ND), which allows third parties to download the articles and share them with others as long as they credit the author and the Abstract Book, but they cannot change the content in any way or use them commercially.
**Abstract Book Citations:** Authors, Title, HemaSphere, 2026;10:(S1):pages. The abstract book DOIs can be found on the "Author Information" page.
**Disclaimer:** Articles published in the journal HemaSphere exclusively reflect the opinions of the authors. The authors are responsible for all content in their abstracts including accuracy of the facts, statements, citing resources, etc.

### Results

The median age of 3 patients was 61 years (range: 45-73). All subjects were male, had ECOG scores 2. All subjects were treated with daratumumab, bortezomib, cyclophosphamide and dexamethasone in the previous line, none of them had received CAR-T therapy or autologous transplantation. The incidence of CRS was 33.3% (1/3), no CRS of grade 3 or higher was observed. No ICANS was reported. The median time to peak concentrations was 14 (range, 10-21) days, with a median peak concentration of $9.19 \times 10^5$ (range, $4.51 \times 10^5$ to $1.13 \times 10^6$) copies/μg DNA by qPCR. All subjects achieved hematological completely response (CR), with a median follow-up of 6.57 months. For subjects diagnosed with multiple organ diseases involvement such as heart or kidney, 66.7% (2/3) subjects achieved organ response, including one patient achieved cardiac partial response (PR) and kidney CR, another patient achieved kidney very good partial response (VGPR).

Table 1 Baseline characteristics

| Characteristics | N=3 |
|---|---|
| Age, median (range), y | 61 (45, 73) |
| Sex (M/F), n | 3/0 |
| Time from diagnosis (months), median (range) | 5.30 (3.60, 17.1) |
| ECOG performance-status score-2, n (%) | 3 (100%) |
| Involved organ, n (%) | |
| Cardiac | 3 (100%) |
| Kidney | 2 (66.7%) |
| Liver | 2 (66.7%) |
| Others (peripheral nervous, digestive system, etc) | 3 (100%) |
| Mayo AL amyloidosis 2004 stage - IIIa, n (%) | 3(100%) |
| Urinary proteinuria, median (rang), g/24 h | 3.77 (0.27, 7.27) |
| Serum creatinine, median (rang), mg/dL | 84.0 (71.0, 99.0) |
| eGFR, median (rang), ml/min/1·73m$^2$ | 79.0 (78.0, 96.0) |
| Serum albumin, median (rang), g/L | 36.0 (31.0, 44.0) |
| dFLC at screening, median (rang), mg/L | 86.6 (80.9, 95.6) |
| Previous treatment, n (%) | |
| Daratumumab | 3 (100%) |
| Bortezomib | 3 (100%) |
| Cyclophosphamide | 3 (100%) |
| Dexamethasone | 3 (100%) |
| Autologous transplantation | 0 |

### Summary/Conclusion

Dual-target BCMA/CD19 CAR-T therapy demonstrated favorable safety and efficacy in patients with r/r-ALA. These findings highlight its potential as a promising therapeutic treatment option for patients with r/r ALA, warranting further investigation.

**Copyright Information:** (Online) ISSN: 2572-9241
©2026 The Author(s). HemaSphere is published by John Wiley & Sons Ltd on behalf of European Hematology Association. This is an open access Abstract Book distributed under the Attribution-NonCommercial-NoDerivs (CC BY-NC-ND), which allows third parties to download the articles and share them with others as long as they credit the author and the Abstract Book, but they cannot change the content in any way or use them commercially.
**Abstract Book Citations:** Authors, Title, HemaSphere, 2026;10:(S1):pages. The abstract book DOIs can be found on the "Author Information" page.
**Disclaimer:** Articles published in the journal HemaSphere exclusively reflect the opinions of the authors. The authors are responsible for all content in their abstracts including accuracy of the facts, statements, citing resources, etc.

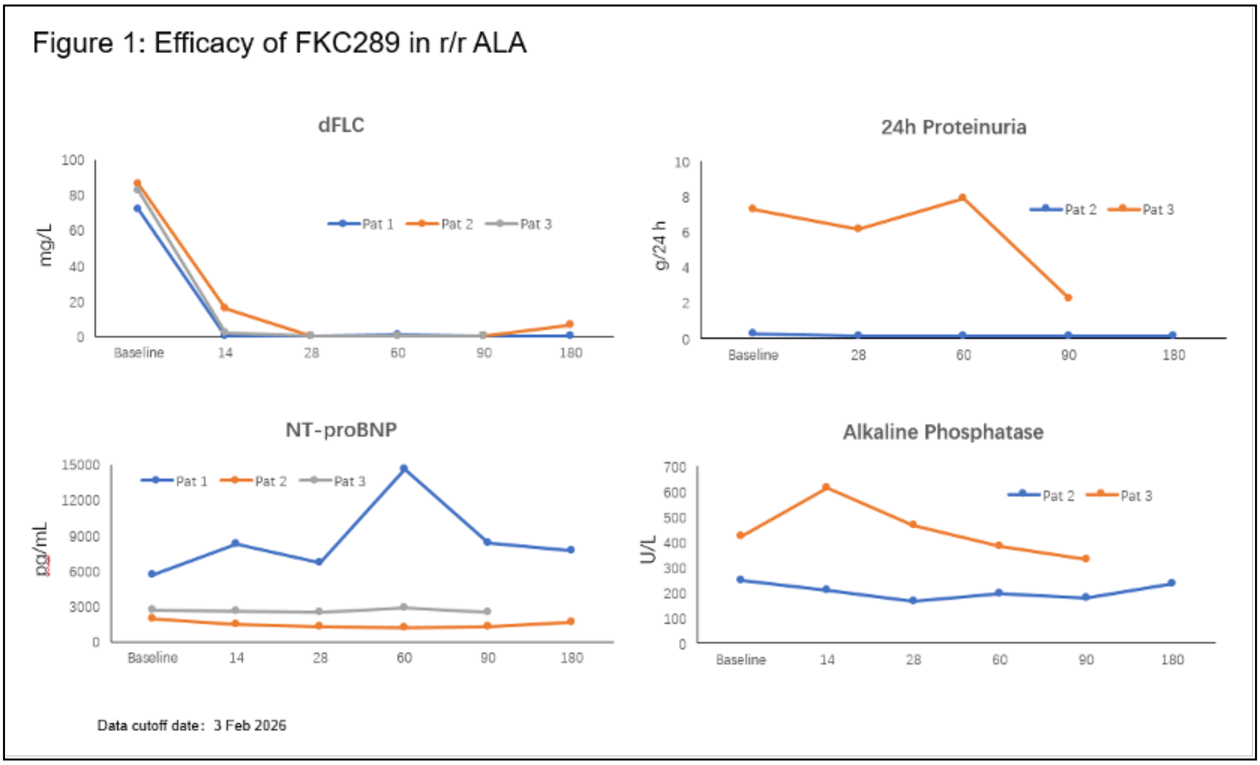

Figure 1. Efficacy of FKC289 in r/r ALA

**Copyright Information:** (Online) ISSN: 2572-9241
©2026 The Author(s). HemaSphere is published by John Wiley & Sons Ltd on behalf of European Hematology Association. This is an open access Abstract Book distributed under the Attribution-NonCommercial-NoDerivs (CC BY-NC-ND), which allows third parties to download the articles and share them with others as long as they credit the author and the Abstract Book, but they cannot change the content in any way or use them commercially.
**Abstract Book Citations:** Authors, Title, HemaSphere, 2026;10:(S1):pages. The abstract book DOIs can be found on the "Author Information" page.
**Disclaimer:** Articles published in the journal HemaSphere exclusively reflect the opinions of the authors. The authors are responsible for all content in their abstracts including accuracy of the facts, statements, citing resources, etc.

### (PF853) PRECLINICAL CHARACTERIZATION OF CGT1145, A NOVEL, WILD-TYPE-SPARING, JAK2 V617F MUTANT-SELECTIVE INHIBITOR

**Topic:** 15. Myeloproliferative neoplasms - Biology & translational research

Mark J Chicarelli*[1], Michelle Crow[1], Kim Alley[1], Tanna Bettendorf[1], Abiezer Blandon[1], Alexandra Born[1], Richard Brizendine[1], Paul Carlson[1], Payal Chatterjee[1], Brad Fell[1], John Fischer[1], Jennifer Fulton[1], Anna Guarnieri[1], Ravi Jalluri[1], Amber Johnson[1], Keith Koch[1], Vijay Kumar[1], Cori Malinky[1], Matt McDonald[1], Colin McHugh[1], Maralee McVean[1], Bradley Newhouse[1], Scott Niman[1], Rob Rieger[1], John Robinson[1], Mareli Rodriquez[1], Leah Salituro[1], Patrick Salvo[1], Francis Sullivan[1], John Trujillo[1], Brooklynn Venteicher[1], Logan Vine[1], Shannon Winski[1], Hannah Work[1], Yeyun Zhou[1]

[1]*Cogent Biosciences, Inc., Boulder, United States of America;*

### Background

JAK2 V617F is the most common mutation in BCR-ABL-negative myeloproliferative neoplasms (MPNs), present in ~95% of patients with polycythemia vera and ~50% of patients with essential thrombocythemia or primary myelofibrosis. This acquired point mutation in the JAK2 pseudokinase (JH2) domain results in the constitutive activation of JAK2 kinase (JH1) signaling leading to cytokine-independent growth of hematopoietic cells. Approved JAK2 inhibitors, such as ruxolitinib, are kinase domain binders that can alleviate symptoms and improve outcomes in patients with MPNs. However, even with long-term treatment, this therapy rarely leads to reductions in variant allele frequency or molecular remission due to dose-limiting toxicities from wild type (WT) JAK family target engagement. A JAK2 V617F mutant-selective inhibitor has the potential to selectively target mutant clones and alter pathological biology, while avoiding hematologic tolerability issues. Herein, we report the preclinical characterization of CGT1145, a JAK2 V617F JH2 mutant-selective inhibitor, which shows potential best-in-class potency and selectivity against JAK2 WT.

### Results

A custom HTRF competition binding assay, using purified JH1 and JH2 domains and custom fluorescent tracers, demonstrated preferential binding to the JH2 domain (6300 nM JH1 / 0.3 nM JH2, 21,000-fold selective). Cellular activity was measured via HTRF phospho-STAT5 in GM-CSF stimulated TF-1 (JAK2 WT) and HEL 92.1.7 (JAK2 V617F homozygous) cells. CGT1145 has an $IC_{50}$ of 91 nM in the mechanistic JAK2 V617F cellular assay and is 89-fold selective over JAK2 WT in this assay. Additional assays have been performed and support superior selectivity in inhibition of pSTAT5. This compound shows high kinome selectivity with advanceable oral bioavailability across species. Target engagement in vivo was studied using athymic nude mice subcutaneously implanted with HEL 92.1.7 cells. At 4-hours post-dose, CGT1145 showed dose dependent inhibition of tumor pSTAT5, reaching >95% inhibition at 50 mg/kg (AlphaLISA). In a mouse model of splenomegaly which mimics human MPN, athymic nude mice were implanted intravenously with Ba/F3-EPOR-JAK2V617F cells. Treatment with CGT1145 led to dose dependent inhibition of splenomegaly, with normalized spleen weights (equivalent to naïve controls and ruxolitinib treatment) at 50 mg/kg BID and 100 mg/kg QD. The efficacy of CGT1145 was further evaluated in a heterozygous JAK2 V617F transgenic mouse model, which recapitulates a classic MPN-like phenotype. CGT1145 treatment normalized spleen weights and hematologic parameters, and decreased erythroid precursor accumulation in the spleen. These findings indicate restoration of bone marrow function and attenuation of extramedullary hematopoiesis, supporting the potential of CGT1145 as a disease modifying therapy. Additionally, CGT1145 was well tolerated over the course of all in vivo efficacy studies.

**Copyright Information:** (Online) ISSN: 2572-9241
©2026 The Author(s). HemaSphere is published by John Wiley & Sons Ltd on behalf of European Hematology Association. This is an open access Abstract Book distributed under the Attribution-NonCommercial-NoDerivs (CC BY-NC-ND), which allows third parties to download the articles and share them with others as long as they credit the author and the Abstract Book, but they cannot change the content in any way or use them commercially.
**Abstract Book Citations:** Authors, Title, HemaSphere, 2026;10:(S1):pages. The abstract book DOIs can be found on the “Author Information” page.
**Disclaimer:** Articles published in the journal HemaSphere exclusively reflect the opinions of the authors. The authors are responsible for all content in their abstracts including accuracy of the facts, statements, citing resources, etc.

**Summary/Conclusion**

CGT1145 is a potential best-in-class, JAK2 WT sparing, JAK2 V617F JH2 mutant-selective inhibitor that has the potential to provide significantly higher target engagement compared to approved JAK inhibitors. By selectively inhibiting JAK2 V617F signaling while preserving JAK2 WT signaling, CGT1145 has the potential to more effectively eradicate JAK2 V617F MPN-propagating cells, induce molecular remission, and minimize toxicities associated with non-selective JAK inhibition.

**Copyright Information:** (Online) ISSN: 2572-9241
©2026 The Author(s). HemaSphere is published by John Wiley & Sons Ltd on behalf of European Hematology Association. This is an open access Abstract Book distributed under the Attribution-NonCommercial-NoDerivs (CC BY-NC-ND), which allows third parties to download the articles and share them with others as long as they credit the author and the Abstract Book, but they cannot change the content in any way or use them commercially.
**Abstract Book Citations:** Authors, Title, HemaSphere, 2026;10:(S1):pages. The abstract book DOIs can be found on the "Author Information" page.
**Disclaimer:** Articles published in the journal HemaSphere exclusively reflect the opinions of the authors. The authors are responsible for all content in their abstracts including accuracy of the facts, statements, citing resources, etc.

### (PF854) MUTANT SHP2 CONDENSATES PROMOTE GENOMIC INSTABILITY THROUGH PAK1-ASSOCIATED MICROTUBULE DYSREGULATION IN JMML

**Topic:** 15. Myeloproliferative neoplasms - Biology & translational research

Chunmou Li[1], Zhiguang Chang[1], Zhiyong Peng[2], Lingling MA[1], Yan Xiao[1], Yao Guo[1], Dengyang Zhang[1], Yuming Zhao[1], Liuting Yu[1], Junbin Huang[1], Hongman Xue[1], Chun Chen[1], Zhizhuang Zhao[3], Yun Chen*[1]

*[1]The Seventh Affiliated Hospital, Sun Yat-sen University, Shenzhen, China;[2]Guangzhou First People's Hospital, Guangzhou, China; [3]The University of Oklahoma Health Sciences Center, Oklahoma City, United States of America;*

### Background

Juvenile myelomonocytic leukemia (JMML) is a rare but aggressive pediatric myeloproliferative neoplasm driven by hyperactivation of the Ras signaling pathway. SHP2, encoded by *PTPN11*, functions as a critical positive regulator of Ras signaling, and pathogenic gain-of-function mutations in *PTPN11* are the leading driver in approximately 35% of JMML patients. In our preliminary study, *PTPN11* copy number gain (CNG) occurred in 68.7% of mutant JMML colonies, with significantly higher levels (6.4±3.2) compared to wild-type controls (2.0±0.2). The high frequency of PTPN11 copy number gain (CNG) suggests underlying genomic instability in mutant JMML cells. While various SHP2 mutant proteins have been found to undergo liquid-liquid phase separation (LLPS), the potential link between this phenomenon, genomic instability and *PTPN11* CNG remains to be elucidated.

### Aims

This study aims to investigate the mechanistic link between mutant SHP2 phase separation, genomic instability, and PTPN11 copy number amplification, and to elucidate the impact of such genomic amplification on the pathogenesis and progression of JMML.

### Methods

Phase-separated droplet formation was evaluated using confocal imaging in stable HCD-57 cell lines with specific SHP2 mutations (D61Y, E76K, A72V, and G503A). Mutant SHP2-interacting proteins were identified through IP-MS and prioritized by bioinformatic analysis for further mechanistic validation. Then, to experimentally evaluate the pathogenic impact of mutant *PTPN11* gene dosage *in vivo*, we established transgenic mice expressing human SHP2-E76K mutant under the hematopoietic-specific *Vav* promoter, with defined transgene copy numbers.

**Copyright Information:** (Online) ISSN: 2572-9241
©2026 The Author(s). HemaSphere is published by John Wiley & Sons Ltd on behalf of European Hematology Association. This is an open access Abstract Book distributed under the Attribution-NonCommercial-NoDerivs (CC BY-NC-ND), which allows third parties to download the articles and share them with others as long as they credit the author and the Abstract Book, but they cannot change the content in any way or use them commercially.
**Abstract Book Citations:** Authors, Title, HemaSphere, 2026;10:(S1):pages. The abstract book DOIs can be found on the "Author Information" page.
**Disclaimer:** Articles published in the journal HemaSphere exclusively reflect the opinions of the authors. The authors are responsible for all content in their abstracts including accuracy of the facts, statements, citing resources, etc.

**Results**

All four stable cell lines exhibited mutant SHP2 LLPS, with SHP2-A72V showing the strongest droplet formation. KEGG analysis of IP-MS–identified proteins revealed significant enrichment in microtubule remodeling. Notably, PAK1ip1 was upregulated whereas TFCB, a key microtubule stabilizer, was reduced, highlighting PAK1 as a central interactor in mutant SHP2 condensates. Confocal microscopy demonstrated clear spatial co-localization between PAK1 and mutant SHP2 condensates. Immunoblotting further showed a significant down-regulation of PAK1 in SHP2-mutant HCD57 cells, accompanied by the up-regulation of genomic instability markers pH2A.X and the p-Chk1/Chk1 ratio. Treatment with the allosteric PAK1 inhibitor NVS-PAK1-1 led to a dose- and time-dependent increase in these genomic instability markers. Conversely, the levels of these proteins gradually declined following inhibitor withdrawal or transient PAK1 overexpression.
To directly assess the in vivo impact of mutant Ptpn11 gene dosage, we generated three independent transgenic mouse lines carrying approximately 1, 2, or 6 copies of the SHP2-E76K transgene. With the increase in *Ptpn11* copy number, the mice exhibited an earlier disease onset and exacerbated symptoms, which more closely recapitulated the clinical features of pediatric JMML patients. Mice from the 6-copy line developed significant thrombocytopenia and anemia as early as 3 weeks of age, consistent with early-onset disease of JMML, and these abnormalities persisted into adulthood.

**Summary/Conclusion**

In summary, mutant SHP2 forms phase-separated condensates that sequester and functionally exhaust PAK1, promoting genomic instability, enabling Ptpn11 copy number gain, and accelerating JMML progression.

**Copyright Information:** (Online) ISSN: 2572-9241
©2026 The Author(s). HemaSphere is published by John Wiley & Sons Ltd on behalf of European Hematology Association. This is an open access Abstract Book distributed under the Attribution-NonCommercial-NoDerivs (CC BY-NC-ND), which allows third parties to download the articles and share them with others as long as they credit the author and the Abstract Book, but they cannot change the content in any way or use them commercially.
**Abstract Book Citations:** Authors, Title, HemaSphere, 2026;10:(S1):pages. The abstract book DOIs can be found on the “Author Information” page.
**Disclaimer:** Articles published in the journal HemaSphere exclusively reflect the opinions of the authors. The authors are responsible for all content in their abstracts including accuracy of the facts, statements, citing resources, etc.

### (PF855) GALECTIN-1 AS A MEDIATOR OF THROMBOTIC RISK IN MYELOPROLIFERATIVE NEOPLASMS?

**Topic:** 15. Myeloproliferative neoplasms - Biology & translational research

Natalie J Jooss*[1], Alejandro J Cagnoni[2, 3], Florian Laforets[1], Lauren Murphy[1, 4], Laura Nunn[5], Kirsty Crozier[5], Ashley Chee[5], Michael Smith[1, 4], Charlotte Lees[1], Rupen Hargreaves[1], Damaris Darby[6], Athanasia Panopoulou[6], Juan M Pérez Sáez[3], Abdullah Khan[1], Martine Jandrot-Perrus[7], Mirta Schattner[8], Johan Heemskerk[9], Adam Mead[1, 5], Gabriel A Rabinovich[3, 10, 11], Bethan Psaila[1, 4, 5]

*[1]Weatherall Institute of Molecular Medicine, University of Oxford, Oxford, United Kingdom;[2]Departamento de Química Orgánica, Facultad de Ciencias Exactas y Naturales, Universidad de Buenos Aires, Buenos Aires, Argentina; [3]Laboratorio de Glicomedicina, IBYME-CONICET, Buenos Aires, Argentina; [4]Ludwig Institute for Cancer Research, University of Oxford, Oxford, United Kingdom; [5]Oxford University Hospitals NHS Trust, Oxford, United Kingdom; [6]Acute Multidisciplinary Imaging and Interventional Centre, University of Oxford,, Oxford, United Kingdom; [7]UMR S1144 Université Paris Cité, INSERM, Paris, France; [8]Institute of Experimental Medicine, CONICET, National Academy of Medicine, Buenos Aires, Argentina;[9]Synapse Research Institute, Maastricht, The Netherlands; [10]CaixaResearch Institute, Barcelona, Spain; [11]Departamento de Química Biológica, Facultad de Ciencias Exactas y Naturales, Universidad de Buenos Aires, Buenos Aires, Argentina;*

### Background

Thrombotic events are a major cause of morbidity and mortality for patients with myeloproliferative neoplasms (MPNs), with an increased incidence of arterial and venous events including heart attacks, strokes and deep vein thrombosis, affecting ~10% of patients with myelofibrosis, ~20% with essential thrombocythemia and ~30% with polycythemia vera (PV). The increased incidence of thromboses is reduced, but not completely ameliorated, by current primary prevention strategies including aspirin and cytoreduction. We recently identified Galectin-1 (Gal1) as a novel driver of progression in MPNs (Li *et al., Sci Trans Med* 2024), showing induction of Gal1 expression by both the MPN clone and the cells in the bone marrow niche. We also observed increased platelet Gal1 mRNA as a sensitive biomarker of disease trajectory. Other groups reported increased platelet Gal1 protein in MPNs (Kelliher *et al., Blood Advances* 2024). Galectins, including Gal1, are β-galactoside binding proteins and have previously been implicated as platelet agonists. Gal1 has been shown to activate platelets through αIIbβ3, however, its impact on platelet function in MPNs has not been studied.

### Aims

In the present study we investigate whether elevated Gal1 levels in patients with MPNs may contribute to the pro-thrombotic phenotype, and if pharmacological blockade of Gal1 might be a viable novel therapeutic strategy.

### Methods

To interrogate platelet - Gal1 interactions we utilised fluorescence microscopy, multi-colour flow cytometry, biolayer interferometry and western blotting as well as a parallel flow chamber to probe for whole blood thrombus formation.

**Copyright Information:** (Online) ISSN: 2572-9241
©2026 The Author(s). HemaSphere is published by John Wiley & Sons Ltd on behalf of European Hematology Association. This is an open access Abstract Book distributed under the Attribution-NonCommercial-NoDerivs (CC BY-NC-ND), which allows third parties to download the articles and share them with others as long as they credit the author and the Abstract Book, but they cannot change the content in any way or use them commercially.
**Abstract Book Citations:** Authors, Title, HemaSphere, 2026;10:(S1):pages. The abstract book DOIs can be found on the "Author Information" page.
**Disclaimer:** Articles published in the journal HemaSphere exclusively reflect the opinions of the authors. The authors are responsible for all content in their abstracts including accuracy of the facts, statements, citing resources, etc.

### Results

We show for the first time that Gal1 induces thrombus formation in a whole blood parallel flow chamber at arterial shear rates, including samples from patients with PV who were receiving aspirin therapy. Further, we demonstrate that Gal1 dose-dependently supports platelet adhesion. When assessed using flow cytometry, Gal1 induces αIIbβ3 activation ($p < 0.01$) in a dose-dependant manner, and exerts additive effects to low-dose thrombin ($p<0.05$), indicating that Gal1 might prime platelets to be more sensitive for activation upon secondary agonist exposure.

Gal1 supports formation of large platelet aggregates and pro-coagulant, phosphatidylserine(PS)-exposing platelets. As GPVI is known to trigger PS-exposure, this observation prompted us to examine whether GPVI may also play a role in Gal1-induced platelet activation, in addition to αIIbβ3. Indeed, biolayer interferometry assays confirmed specific and glycan-dependent binding of Gal1 to GPVI ($K_D$ = 0.24 µM) and western blots of washed platelets stimulated with Gal1 indicated phosphorylation of GPVI downstream signalling molecules. Additionally, inhibition of GPVI signalling with PRT-318 significantly reduced platelet spreading ($p<0.0001$) as well as thrombus formation ($p<0.001$) on Gal1.

Finally, antibody-mediated neutralisation of Gal1 significantly impaired thrombus formation in response to Gal1, in whole blood from healthy donors as well as PV patients on aspirin therapy (for PV samples $p<0.0005$, Fig1).

### Summary/Conclusion

Collectively, these findings indicate that Gal1 mediates platelet activation through αIIbβ3 outside-in signalling as well as via GPVI-dependent mechanisms. Given our prior work identifying a mechanistic role for Gal1 in myelofibrosis and leukaemic transformation in MPNs, targeting Gal1 in MPNs presents a potential multi-pronged strategy to improve patient outcomes in MPNs.

Figure 1: Antibody-mediated neutralisation of Gal1 abolishes thrombus formation in PV patients

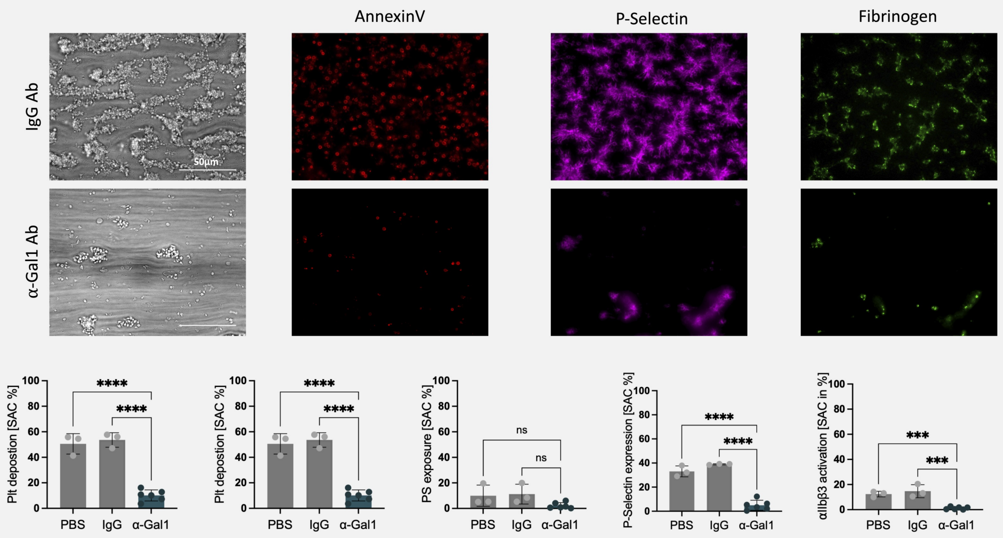

PV patient whole blood perfused through a parallel flow chamber at arterial shear rates over Gal1+vWF-BP+PolyL. Thrombus formation and expression of platelet activation markers is abolished by an anti-Gal1 neutralising antibody, t-test, n=4.

Copyright Information: (Online) ISSN: 2572-9241
©2026 The Author(s). HemaSphere is published by John Wiley & Sons Ltd on behalf of European Hematology Association. This is an open access Abstract Book distributed under the Attribution-NonCommercial-NoDerivs (CC BY-NC-ND), which allows third parties to download the articles and share them with others as long as they credit the author and the Abstract Book, but they cannot change the content in any way or use them commercially.
Abstract Book Citations: Authors, Title, HemaSphere, 2026;10:(S1):pages. The abstract book DOIs can be found on the "Author Information" page.
Disclaimer: Articles published in the journal HemaSphere exclusively reflect the opinions of the authors. The authors are responsible for all content in their abstracts including accuracy of the facts, statements, citing resources, etc.

### (PF856) COMBINED MEK AND PI3KΔ INHIBITION OVERCOMES RASA2-MEDIATED RESISTANCE IN PTPN11-MUTANT JMML

**Topic:** 15. Myeloproliferative neoplasms - Biology & translational research

Yuming Zhao*[1], Yao Guo[1], Zhiyong Peng[2], Tianqi Liang[1], Liuting Yu[1], Dengyang Zhang[1], Lingling MA[1], Chun Chen[1], Zhizhuang Zhao[3], Yun Chen[1]

*[1]The Seventh Affiliated Hospital of Sun Yat-sen University, Shenzhen, China;[2]Guangzhou First People's Hospital, Guangzhou, China; [3]University of Oklahoma, Oklahoma, United States of America;*

### Background

Juvenile myelomonocytic leukemia (JMML) is a rare and aggressive pediatric malignancy driven by Ras pathway mutations. *PTPN11*mutations occur in ~35% of patients and correlate with poor prognosis. A recent clinical trial showed that the MEK inhibitor Trametinib achieves a 50% response rate in JMML; however, patients harboring *PTPN11* mutations exhibit limited response. This resistance likely stems from MEK inhibition triggering a PI3K/Akt bypass via the relief of negative feedback. The molecular details of this JMML-specific loop remain unclear.

### Aims

This study aims to elucidate the molecular mechanisms of feedback-driven resistance to MEK inhibition in*PTPN11*-mutant JMML and to validate dual MEK and PI3K blockade as a rational therapeutic strategy.

### Methods

To model *PTPN11*-driven JMML, we engineered HCD-57 cells to express *PTPN11* mutants and established Trametinib-resistant derivatives. Findings were validated using CDX, *PTPN11-E76K* transgenic and PDX mouse models. Ras activity was quantified via pulldown, with transcriptional changes characterized by RNA-seq and qPCR. In vitro, viability, apoptosis, and colony formation were assessed. In vivo, efficacy was measured by survival, leukemic burden, and organ infiltration.

### Results

Trametinib treatment in *PTPN11*-mutant HCD-57 cells induces significant ERK suppression alongside compensatory AKT activation. In long-term resistant cells, this evolves into sustained ERK inhibition and steady-state AKT activation. Mechanistic studies reveal that MEK inhibition downregulates multiple RAS-GAPs, specifically *RASA2*. This impaired negative feedback is corroborated by pulldown assays showing elevated levels of RAS-GTP. *RASA2* knockdown mimics the resistant phenotype by inducing spontaneous AKT activation, while its overexpression rescues sensitivity by abrogating compensatory phosphorylation. Collectively, *RASA2* downregulation relieves inhibitory constraints on RAS, driving a signaling shift toward the PI3K/AKT bypass.
To counteract this, we combined Trametinib with the PI3Kδ inhibitor Idelalisib, demonstrating robust synergy. In*PTPN11*-mutant HCD-57 cells and primary JMML cells, the combination induced superior G1 arrest and apoptosis via concurrent suppression of p-ERK and p-AKT. In vivo, the combination significantly outperformed single agents, prolonging survival and reducing leukemic burden in BM. Notably, PDX-based limiting dilution assays confirmed superior targeting of leukemia-initiating cell frequency. Dual blockade was well tolerated without overt toxicity.

**Copyright Information:** (Online) ISSN: 2572-9241
©2026 The Author(s). HemaSphere is published by John Wiley & Sons Ltd on behalf of European Hematology Association. This is an open access Abstract Book distributed under the Attribution-NonCommercial-NoDerivs (CC BY-NC-ND), which allows third parties to download the articles and share them with others as long as they credit the author and the Abstract Book, but they cannot change the content in any way or use them commercially.
**Abstract Book Citations:** Authors, Title, HemaSphere, 2026;10:(S1):pages. The abstract book DOIs can be found on the "Author Information" page.
**Disclaimer:** Articles published in the journal HemaSphere exclusively reflect the opinions of the authors. The authors are responsible for all content in their abstracts including accuracy of the facts, statements, citing resources, etc.

**Summary/Conclusion**

Our study uncovers that *PTPN11*-mutant JMML cells bypass MEK inhibition via Ras-driven feedback activation of the PI3K/Akt pathway. We show that Idelalisib effectively suppresses this bypass, synergizing with Trametinib to overcome the inherent resistance. These findings establish a strong rationale for dual MEK/PI3Kδ blockade as a novel therapy for high-risk JMML, supporting its transition into clinical trials.

**Copyright Information:** (Online) ISSN: 2572-9241
©2026 The Author(s). HemaSphere is published by John Wiley & Sons Ltd on behalf of European Hematology Association. This is an open access Abstract Book distributed under the Attribution-NonCommercial-NoDerivs (CC BY-NC-ND), which allows third parties to download the articles and share them with others as long as they credit the author and the Abstract Book, but they cannot change the content in any way or use them commercially.
**Abstract Book Citations:** Authors, Title, HemaSphere, 2026;10:(S1):pages. The abstract book DOIs can be found on the "Author Information" page.
**Disclaimer:** Articles published in the journal HemaSphere exclusively reflect the opinions of the authors. The authors are responsible for all content in their abstracts including accuracy of the facts, statements, citing resources, etc.

### (PF857) SIMULTANEOUS ANTIBODY TARGETING OF MUTANT CALRETICULIN TO PROXIMAL AND DISTAL EPITOPES FOR ENHANCED PRECISION MEDICINE FOR TYPE 1 VS TYPE 2 MYELOFIBROSIS

**Topic:** 15. Myeloproliferative neoplasms - Biology & translational research

Denis Tvorogov*[1], Chloe Thompson-Peach[1], Mara Dottore[1], Kelly Lim[1], Vashe Chandrakanthan[1], Maha Kamel[1], Romana Panagopoulos[1], Sandra Jenker[1], Pramod Nair[1], Frank Stomski[1], Luke Bland[1], Stan Gronthos[1], Sharon Paton[1], Hannah Wardill[1], Stuart Pitson[1], Paul Moretti[1], Timothy Hercus[1], Winnie Kan[1], Johannes Foßelteder[2], Andreas Reinisch[2], Angel Lopez[1], Daniel Thomas[1]

*[1]Adelaide University, School of Medicine, Adelaide, Australia;[2]University of Graz, Graz, Austria;*

### Background

Frameshift mutations in *CALR* give rise to myelofibrosis and are classified as Type 1 (del52) or Type 2 (ins5) according to the degree of wildtype sequence retained adjacent to the neopeptide, with each type conferring different clinical outcomes. Targeting strategies specific for Type 1 vs Type 2 mutations would have enormous clinical utility in the treatment and prevention of myelofibrosis as responses to ruxoltinib are not durable nor mutation-specific.

### Aims

Our aim was to develop humanized antibodies against distal and proximal epitopes of mutant CALR and test them against Type 1, Type 1-like, Type 2 and Type 2-like mutations using primary patient assays and in vivo models.

### Methods

TF-1 cells were obtained from ATCC and cultured in RPMI with 10% (v/v) foetal bovine serum (FBS) supplemented with 2 ng/mL of human GM-CSF and 25 mM HEPES. TF-1 TpoR cells were cultured in RPMI with 10% FBS supplemented with 10 ng/mL of human TPO. SET2 cells (DSMZ # 608) were cultured in RPMI with 10% FBS. For cell survival analysis, cells analysed with Annexin V–APC according to the manufacturer's protocol. Cells containing the human CALR mutations (del52, ins5, del61, Type 5, Type 40) were generated through lentiviral transduction of TF-1 and Ba/F3 cells expressing the TpoR as previously described. Megakaryocyte differentiation assays were performed as previously described (Tvorogov et al *EMBO Reports*). Mesenchymal stromal cells from healthy donors were used to make transplantable ossicles in NSG mice as previous described (Reinish, Thomas et al, *Nature Medicine*).

**Copyright Information:** (Online) ISSN: 2572-9241
©2026 The Author(s). HemaSphere is published by John Wiley & Sons Ltd on behalf of European Hematology Association. This is an open access Abstract Book distributed under the Attribution-NonCommercial-NoDerivs (CC BY-NC-ND), which allows third parties to download the articles and share them with others as long as they credit the author and the Abstract Book, but they cannot change the content in any way or use them commercially.
**Abstract Book Citations:** Authors, Title, HemaSphere, 2026;10:(S1):pages. The abstract book DOIs can be found on the "Author Information" page.
**Disclaimer:** Articles published in the journal HemaSphere exclusively reflect the opinions of the authors. The authors are responsible for all content in their abstracts including accuracy of the facts, statements, citing resources, etc.

### Results

We demonstrate that dual targeting of Type 1 (del52) mutant CALR with two monoclonal antibodies directed against distinct epitopes in CALR have significant advantages compared to single agent treatment in the eradication of primary megakaryocyte progenitors in vitro and in a humanized ossicle microenvironment leading to improved survival in xenograft models. Dual targeting was superior in blocking constitutive STAT5 and ERK phosphorylation induced by del52 and prevented accumulation of JAK2 phosphorylation, overcoming ruxolitinib resistance. In contrast, Type 2 mutations showed increased CALR dimerization and were partially resistant to antibody targeting but could be impacted by ruxolitinib triple combination.

To further test efficacy for Type 1 in a humanized microenvironment, we developed an *ex vivo* assay in which $CALR^{del52}$ CD34+ cells from a PMF patient were exposed to IgG, h9H11, h4D7 or combination for 2 hours prior to engraftment into ossicles derived from healthy stromal cells. Notably, the number of $CALR^{+}vWF^{+}CD45^{low}$ cells/field of view were observed in IgG treated after 10 weeks but were significantly reduced in all three treatment groups, with virtually no $CALR^{+}$ cells engrafting in the combination group (IgG vs h9H11/h4D7, *P*=0.001).

### Summary/Conclusion

Together, our data demonstrate an ultra-precision medicine approach tailored to either Type 1 OR Type 2 mutation classes will be required for maximal efficacy and complete blockade of JAK/STAT signalling, with far-reaching implications for patient management.The development of a therapeutic approach for CALR-driven MPN has enormous clinical potential.

**Copyright Information:** (Online) ISSN: 2572-9241
©2026 The Author(s). HemaSphere is published by John Wiley & Sons Ltd on behalf of European Hematology Association. This is an open access Abstract Book distributed under the Attribution-NonCommercial-NoDerivs (CC BY-NC-ND), which allows third parties to download the articles and share them with others as long as they credit the author and the Abstract Book, but they cannot change the content in any way or use them commercially.
**Abstract Book Citations:** Authors, Title, HemaSphere, 2026;10:(S1):pages. The abstract book DOIs can be found on the "Author Information" page.
**Disclaimer:** Articles published in the journal HemaSphere exclusively reflect the opinions of the authors. The authors are responsible for all content in their abstracts including accuracy of the facts, statements, citing resources, etc.

### (PF858) PRECLINICAL EVALUATION OF INCA035784, A NOVEL T-CELL–REDIRECTING ANTIBODY FOR THE TREATMENT OF MUTCALR-DRIVEN MYELOPROLIFERATIVE NEOPLASMS (MPNS)

**Topic:** 15. Myeloproliferative neoplasms - Biology & translational research

Eleanor Murphy*[1], Veethika Pandey[2], Moritz Reichert[1], Ashwini Kulkarni[2], Yuqi Shen[1], Florian Laforets[1], Anna Godfrey[3], Sophie Michael[1], Obsie Baissa[1], Abdullah Khan[1], Mark Rupar[2], Liang-Chuan Wang[2], Chryssa Kanellopoulou[2], Ricardo MacArron[2], Patrick Mayes[2], Bethan Psaila[1]

*[1]MRC Weatherall Institute of Molecular Medicine and Ludwig Institute for Cancer Research, University of Oxford, Oxford, United Kingdom; [2]Incyte Corporation, Wilmington, DE, United States of America;[3]Cambridge University Hospitals NHS Foundation Trust, Cambridge, United Kingdom;*

### Background

Mutations in exon 9 of the calreticulin gene (mut*CALR*) are the second most common drivers of MPNs, occurring in 25% of essential thrombocythemia and 35% of myelofibrosis (MF) cases. Cell surface-expressed mutCALR in complex with the thrombopoietin receptor (TPOR) is an attractive cancer cell-specific target. Antibodies directed against mutant C-terminus neoepitopes of mutCALR are currently in phase 1 trials. INCA035784 is a novel T-cell–redirecting antibody that binds within the N-domain of mutCALR, which is invariant across known *CALR* mutations. Therefore, INCA035784 is hypothesized to show equipotent efficacy across all C-terminal variants.

### Aims

To evaluate the ability of INCA035784 to target cells from patients with a range of *CALR* mutations, including in assays that mimic the human tumor microenvironment.

### Methods

INCA035784 was evaluated in cells from patients with mutCALR variants (n=9). Cytotoxicity against hematopoietic stem and progenitor cells (HSPC, n=4) and megakaryocyte progenitors (MKP, n=5) was tested using healthy donor-derived and autologous T cells with defined effector:target (E:T) ratios. Activity in physiological contexts was assessed using minimally manipulated total mononuclear cells (n=2) and human bone marrow (BM) organoids. Target cell depletion and T-cell activation were measured by ddPCR and flow cytometry. Binding of INCA035784 was assessed in Ba/F3 cell lines expressing atypical Type 1/2 mutations or Type 2 mutCALR variants expressing different truncations in the C-terminus using flow cytometry. Variant mutCALR protein structures were modeled using AlphaFold3.

**Copyright Information:** (Online) ISSN: 2572-9241
©2026 The Author(s). HemaSphere is published by John Wiley & Sons Ltd on behalf of European Hematology Association. This is an open access Abstract Book distributed under the Attribution-NonCommercial-NoDerivs (CC BY-NC-ND), which allows third parties to download the articles and share them with others as long as they credit the author and the Abstract Book, but they cannot change the content in any way or use them commercially.
**Abstract Book Citations:** Authors, Title, HemaSphere, 2026;10:(S1):pages. The abstract book DOIs can be found on the "Author Information" page.
**Disclaimer:** Articles published in the journal HemaSphere exclusively reflect the opinions of the authors. The authors are responsible for all content in their abstracts including accuracy of the facts, statements, citing resources, etc.

**Results**

INCA035784 showed selective and equipotent binding to mutCALR$^+$ cells. Induction of cytotoxicity and dose-dependent T-cell activation were confirmed. No binding was observed on cells with stress-induced surface exposed wild-type CALR, confirming selectivity for the mutCALR-TPOR complex. Target cell killing was demonstrated in T cell coculture assays with CD34$^+$ cells and CD34-derived MKPs upon treatment with INCA035784, with efficacy ranging from 20%–90% compared to mock×CD3 antibody (**Figure A**; n=5). Cell killing increased with higher E:T ratios (**Figure B**). A 50% reduction in variant allele frequency was observed with INCA035784 treatment in a patient sample with Type 2 *CALR* mutation in coculture with autologous T cells (1:1 E:T ratio). INCA035784-induced killing was also observed in healthy and fibrotic human BM organoids. Flow cytometry binding assessment of INCA035784 on Ba/F3 variants with atypical Type 1/2 mutations and on Type 2 variants harboring truncated C-termini demonstrated that INCA035784 retains binding to mutCALR regardless of modifications in the C-domain. Atypical mut*CALR* variants may produce a structurally distinct C-terminus that can impact the binding of C-terminus–directed antibodies. We identified and modeled 4 such C-termini from patients, demonstrating differences from canonical Type 2 variants (**Figure C–E**). Histologic analysis of BM from one of these patients showed MF with characteristic MK hyperproliferation, but no staining with the mutant C-terminus–specific CAL2 antibody. However, INCA035784 induced cytotoxicity of CD34$^+$ HSPCs with autologous T cells in this patient sample.

**Summary/Conclusion**

These findings suggest that INCA035784, by targeting the conserved mutCALR N-domain, may have advantages over C-terminal–directed therapies, especially in patients harboring atypical mutCALR variants in addition to patients with canonical Type 1/2 mut*CALR*.

**Copyright Information:** (Online) ISSN: 2572-9241
©2026 The Author(s). HemaSphere is published by John Wiley & Sons Ltd on behalf of European Hematology Association. This is an open access Abstract Book distributed under the Attribution-NonCommercial-NoDerivs (CC BY-NC-ND), which allows third parties to download the articles and share them with others as long as they credit the author and the Abstract Book, but they cannot change the content in any way or use them commercially.
**Abstract Book Citations:** Authors, Title, HemaSphere, 2026;10:(S1):pages. The abstract book DOIs can be found on the "Author Information" page.
**Disclaimer:** Articles published in the journal HemaSphere exclusively reflect the opinions of the authors. The authors are responsible for all content in their abstracts including accuracy of the facts, statements, citing resources, etc.

**Figure:** INCA035784 reliably eliminates mutCALR⁺ target cells with canonical Type 1 or Type 2 variants.

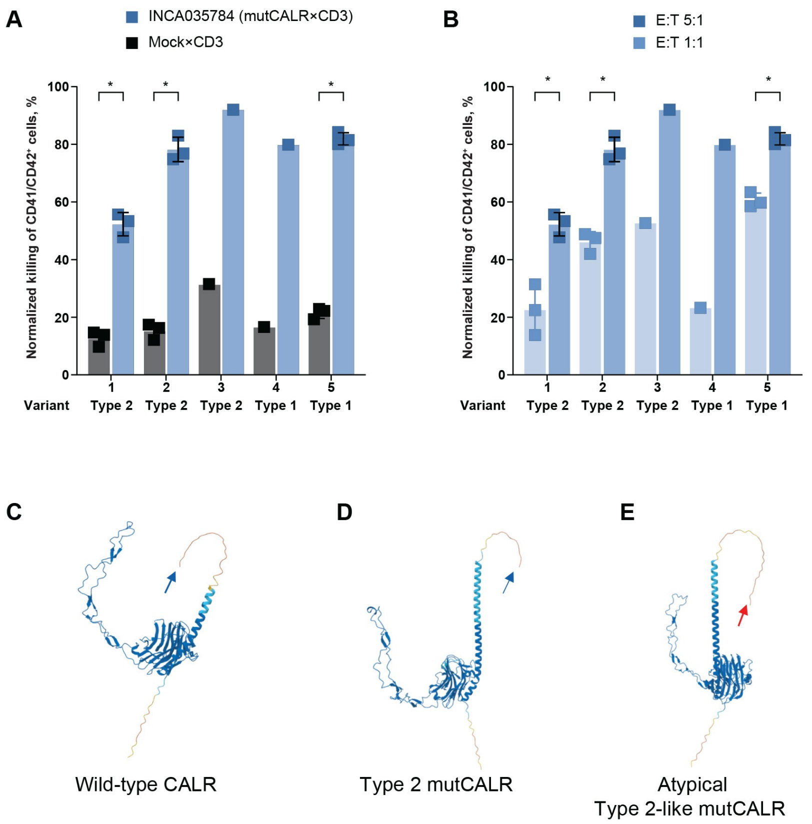

**A-B)** CD34⁺ cells from patients with mutCALR⁺ MPNs were cultured for 6 days with TPO and SCF to initiate megakaryocytic differentiation and co-cultured with T cells. Cytotoxicity of CD41/CD42⁺ cells was measured and normalized to untreated controls (*represents $P$<0.05; unpaired $t$-test).

**A)** Cytotoxicity induced by INCA035784 compared with mock×CD3 control in patients with Type 1/2 mutCALR.

**B)** Comparison of cytotoxicity induced by INCA035784 between 1:1 and 5:1 E:T ratios.

**C-E)** Comparative structural modeling with AlphaFold3 of wild-type, canonical Type 2, and atypical Type 2-like mutations reveal aberrant C-terminal (red arrow) folding.

E, effector; MPN, myeloproliferative neoplasm; mutCALR, mutations in exon 9 of the calreticulin gene; SCF, stem cell factor; T, target; TPO, thrombopoietin.

**Copyright Information:** (Online) ISSN: 2572-9241
©2026 The Author(s). HemaSphere is published by John Wiley & Sons Ltd on behalf of European Hematology Association. This is an open access Abstract Book distributed under the Attribution-NonCommercial-NoDerivs (CC BY-NC-ND), which allows third parties to download the articles and share them with others as long as they credit the author and the Abstract Book, but they cannot change the content in any way or use them commercially.
**Abstract Book Citations:** Authors, Title, HemaSphere, 2026;10:(S1):pages. The abstract book DOIs can be found on the "Author Information" page.
**Disclaimer:** Articles published in the journal HemaSphere exclusively reflect the opinions of the authors. The authors are responsible for all content in their abstracts including accuracy of the facts, statements, citing resources, etc.

### (PF859) A HUMANIZED OSSICLE MODEL OF MYELOFIBROSIS REVEALS THPO-DRIVEN FIBROSIS, OSTEOSCLEROSIS AND SPP1-DEPENDENT MICROENVIRONMENTAL REMODELING

**Topic:** 15. Myeloproliferative neoplasms - Biology & translational research

Hongzhe Li[1], Alba Sierras[1], Rong Fan[1], Katharina Schallmoser[2], Anne Hultquist[3], Stefan Scheding*[1]

*[1]Lund Stem Cell Centre, Department of molecular haematology, Lund, Sweden;[2]Paracelsus Medical University Salzburg, Department of Transfusion Medicine, Salzburg, Austria; [3]Skane University Hospital, Department of Hematology, Lund, Sweden;*

### Background

Myelofibrosis (MF) is the most severe form of the myeloproliferative neoplasms (MPN). Current MF therapies — excluding allogeneic stem cell transplantation — are not curative and have limited effects on fibrosis, highlighting the urgent need for more effective targeted therapies. Although murine models have successfully recapitulated certain aspects of MF, they fall short in reflecting human MF pathophysiology.

### Aims

We therefore aimed to establish a humanized MF model by transplanting thrombopoietin (THPO)-overexpressing human bone marrow (BM) CD34+ cells into humanized BM tissues established in immunodeficient NSG mice.

### Methods

Culture-expanded human BM stromal cells were injected subcutaneously into NSG mice to generate human bone marrow tissues (huOssicles). After 7–8 weeks, CD34$^+$ BM cells transduced with THPO-GFP or GFP control lentivirus were injected intraossically (i.o.) following sublethal irradiation. Ossicles were harvested after 4 – 10 weeks. Fibrosis was graded on reticulin-stained sections according to established criteria (blinded assessment). Human hematopoiesis was analyzed by flow cytometry, histology and immunohistochemistry. Bone remodeling was quantified on H&E and trichrome-stained sections.

### Results

THPO overexpression in CD34 BM cells was first evaluated in vitro, demonstrating enhanced CD41a$^+$CD42b$^+$ megakaryocytic differentiation independent of exogenous THPO (THPO: 1.5±0.13-fold, GFP 0.65±0.07-fold, p=0.019). In vivo, transplantation of THPO-overexpressing CD34$^+$ cells induced progressive reticulin fibrosis in the ossicles, designated as HuMF ossicles. Fibrosis was not detectable at week 4 but became prominent by weeks 5–6 and was stable at week 8 (median grade 2 in THPO group vs grade 0 in controls, p<0.01). Human hematopoiesis in HuMF ossicles showed myeloid skewing and significantly increased megakaryocyte clusters (median 2 in THPO group vs 0 in controls, p<0.01). THPO-huMF ossicles exhibited increased redistribution of human CD45$^+$ cells to femur and spleen, with significantly elevated splenic engraftment despite unchanged spleen size, consistent with extramedullary hematopoiesis. HuMF ossicles showed features of osteosclerosis, including increased bone-to-total area ratio (median 1.3 in THPO group vs 0.9 in controls, p<0.01) and expanded trabecular bone with higher osteoid content (median 1.63-fold in THPO group compared with controls), indicating pathological remodeling.
CD56$^+$ osteoprogenitors and SPP1/OPN expression were markedly upregulated in huMF ossicles, and this was validated in BM biopsies from primary MF patients. Functionally, in-vivo anti-SPP1 treatment attenuated myeloid skewing, reduced megakaryocyte frequency and clustering, and decreased fibrosis in 8-week HuMF ossicles. A trend toward reduced splenic human cell content and bone area was observed.

**Copyright Information:** (Online) ISSN: 2572-9241
©2026 The Author(s). HemaSphere is published by John Wiley & Sons Ltd on behalf of European Hematology Association. This is an open access Abstract Book distributed under the Attribution-NonCommercial-NoDerivs (CC BY-NC-ND), which allows third parties to download the articles and share them with others as long as they credit the author and the Abstract Book, but they cannot change the content in any way or use them commercially.
**Abstract Book Citations:** Authors, Title, HemaSphere, 2026;10:(S1):pages. The abstract book DOIs can be found on the "Author Information" page.
**Disclaimer:** Articles published in the journal HemaSphere exclusively reflect the opinions of the authors. The authors are responsible for all content in their abstracts including accuracy of the facts, statements, citing resources, etc.

**Summary/Conclusion**

We established a humanized model of myelofibrosis based on transplantation of THPO overexpressing CD34+ cells into humanized ossicles in NSG mice. This model recapitulates key hallmarks of myelofibrosis, including fibrosis, megakaryocyte clustering, osteosclerosis and extramedullary hematopoietic redistribution. Activation of $CD56^{+}$ osteoprogenitors and upregulation of SPP1/OPN contributed to pathological niche remodeling, and SPP1 neutralization partially reversed MF features. This humanized platform thus provides a translationally relevant model to dissect microenvironment-driven MF pathogenesis and evaluate targeted therapies.

**Copyright Information:** (Online) ISSN: 2572-9241
©2026 The Author(s). HemaSphere is published by John Wiley & Sons Ltd on behalf of European Hematology Association. This is an open access Abstract Book distributed under the Attribution-NonCommercial-NoDerivs (CC BY-NC-ND), which allows third parties to download the articles and share them with others as long as they credit the author and the Abstract Book, but they cannot change the content in any way or use them commercially.
**Abstract Book Citations:** Authors, Title, HemaSphere, 2026;10:(S1):pages. The abstract book DOIs can be found on the "Author Information" page.
**Disclaimer:** Articles published in the journal HemaSphere exclusively reflect the opinions of the authors. The authors are responsible for all content in their abstracts including accuracy of the facts, statements, citing resources, etc.

### (PF860) BLOOD-BASED SINGLE-CELL PROFILING OF CIRCULATING PROGENITORS RESOLVES DISEASE STATE, PROGRESSION RISK, AND CLONAL IDENTITY ACROSS MYELOPROLIFERATIVE NEOPLASMS

**Topic:** 15. Myeloproliferative neoplasms - Biology & translational research

Liran Shlush[1], Gal Dadi*[1], Oren Milman[1], Amos Tanay[1], Sigal Tavor[1, 2], Adrian Duek[3], Vikas Gupta[4], Mark Minden[4]

*[1]Weizmann Institute of Science, molecular cell biology, Rehovot, Israel;[2]assuta medical center tel aviv, Tel Aviv, Israel;[3]assuta medical center ashdod, ashdod, Israel; [4]Princess Margaret Cancer Centre, Toronto, Canada;*

### Background

Clinical distinction across the polycythemia vera (PV)–essential thrombocythemia (ET)–myelofibrosis (MF) continuum relies on bone marrow pathology, an invasive and variably interpreted procedure that limits longitudinal assessment. Genotype and routine blood indices incompletely resolve disease state, risk of fibrotic transformation, and the clonal nature of triple-negative thrombocytosis.

### Aims

The goal of this study was to establish whether circulating hematopoietic stem and progenitor cell (cHSPC) states can be used as a quantitative, non-invasive substrate for disease classification and risk assessment across myeloproliferative neoplasms.
We aimed to:

1. Construct patient-level representations of circulating progenitor composition and transcriptional program activity using a reference model of adult cHSPC states.
2. Train and evaluate classifiers based on these representations to distinguish PV, ET, and MF and to detect impending fibrotic progression before clinical manifestation.
3. Integrate single-cell transcriptional profiles with targeted DNA sequencing to associate progenitor state with clonal burden and pathway activity.
4. Test whether circulating cHSPC-based models can provide diagnostic classification in clinically ambiguous triple-negative MPN cases.

### Methods

We performed single-cell RNA sequencing of 530,607 cHSPCs from 83 patients with PV, ET, or MF and integrated these data with targeted DNA sequencing of canonical MPN hotspots. Using a reference model of adult cHSPC states, we derived patient-level progenitor compositions and gene-program activities from peripheral blood and trained classifiers to distinguish disease states and predict progression.

### Results

Circulating progenitor architectures differentiated PV, ET, and MF. MF was characterized by expansion of megakaryocyte progenitors, depletion of lymphoid progenitors, and pervasive activation of S- and G2/M-phase programs. Integrative models based on progenitor composition and gene-program activity distinguished MF from PV/ET and identified PV and ET patients who later progressed to MF prior to clinical diagnosis. A SOCS2-centered transcriptional program in early cHSPCs tracked with MPN clonal burden and was selectively inducible by thrombopoietin in vitro, linking circulating progenitor state to JAK-STAT pathway activity. Triple-negative ET cases exhibited elevated SOCS2 program activity and MPN-like progenitor architectures, supporting their classification as clonal ET rather than secondary thrombocytosis despite absence of canonical driver mutations.

**Copyright Information:** (Online) ISSN: 2572-9241
©2026 The Author(s). HemaSphere is published by John Wiley & Sons Ltd on behalf of European Hematology Association. This is an open access Abstract Book distributed under the Attribution-NonCommercial-NoDerivs (CC BY-NC-ND), which allows third parties to download the articles and share them with others as long as they credit the author and the Abstract Book, but they cannot change the content in any way or use them commercially.
**Abstract Book Citations:** Authors, Title, HemaSphere, 2026;10:(S1):pages. The abstract book DOIs can be found on the "Author Information" page.
**Disclaimer:** Articles published in the journal HemaSphere exclusively reflect the opinions of the authors. The authors are responsible for all content in their abstracts including accuracy of the facts, statements, citing resources, etc.

**Summary/Conclusion**

Noninvasive peripheral-blood cHSPC transcriptional signatures provide a quantitative readout of MPN disease state and progression risk.This approach resolves PV, ET, and MF, anticipates fibrotic transformation in PV/ET, and supports molecular classification of triple-negative ET, enabling blood-based diagnosis and longitudinal monitoring across the MPN spectrum.

**Copyright Information:** (Online) ISSN: 2572-9241
©2026 The Author(s). HemaSphere is published by John Wiley & Sons Ltd on behalf of European Hematology Association. This is an open access Abstract Book distributed under the Attribution-NonCommercial-NoDerivs (CC BY-NC-ND), which allows third parties to download the articles and share them with others as long as they credit the author and the Abstract Book, but they cannot change the content in any way or use them commercially.
**Abstract Book Citations:** Authors, Title, HemaSphere, 2026;10:(S1):pages. The abstract book DOIs can be found on the “Author Information” page.
**Disclaimer:** Articles published in the journal HemaSphere exclusively reflect the opinions of the authors. The authors are responsible for all content in their abstracts including accuracy of the facts, statements, citing resources, etc.

### (PF861) LNK/SH2B3 VARIANTS AT THE N-TERMINAL AND PH DOMAIN DEFINE A NOVEL MODULATORY REGION FOR JAK2-MEDIATED BIOLOGICAL ACTIVITY IN MYELOPROLIFERATIVE NEOPLASMS

**Topic:** 15. Myeloproliferative neoplasms - Biology & translational research

Elisbetta Dondi[1], Olivier Schischmanoff[1], Diego Elrio[2], Bruno Cassinat[2], Olli Silvennoinen[3], Nadine Blank[1], Laura Eugenia Velazquez Medina*[2]

*[1]UMR 1349 INSERM, UFR SMBH, Université Sorbonne Paris Nord, Bobigny, France;[2]U1342 INSERM, Hôpital Saint-Louis, Institut de Recherche Saint-Louis, Paris, France; [3]Institute of Biotechnology, HiLIFE, Biocenter 2, University of Helsinki, Helsinki, Finland;*

### Background

LNK/SH2B3 adaptor protein is a pivotal negative regulator of cytokine signaling in the hematopoietic system. Somatic and germline mutations in *SH2B3* have been identified across the spectrum of myeloproliferative neoplasms (MPN) and other hematological malignancies. While the loss of LNK function is known to promote JAK/STAT activation, the specific biological consequences of missense mutations outside the SH2 domain, particularly within the amino (N)-terminal and pleckstrin-homology (PH) regions, in the context of MPN pathogenesis and phenotypic evolution, remain poorly understood.

### Aims

We were interested in assessing the molecular mechanism and defining the biological effect on MPN phenotype of two distinct *SH2B3* missense mutations, the recurrent PH domain mutation E208Q and the N-terminal variant G152R.

### Methods

For this, we conducted a structure-function study of these MPN-associated mutations by characterizing functionally, molecularly and in silico *Sh2b3*$^{-/-}$-derived progenitors and hematopoietic cell lines expressing WT and MPN mutated forms of LNK alone or with JAK2 mutated forms. These analyses allowed to examine their impact on protein localization, JAK2 interaction, and myeloid cell proliferation.

### Results

Clinical screening has identified the E208Q variant as a recurrent mutation in MPN patients, occurring in both somatic and germline contexts alongside various driver mutations. To assess the functional consequences of this *SH2B3* mutation, we retrovirally transduced human erythroleukemia HEL cells (JAK2-V617F+) with either the E208Q or the G152R variant, previously identified in our laboratory. In HEL cells, expression of E208Q mutant failed to suppress proliferation and correlated with hyperactivation of JAK2, STAT5, and MAPK pathways. *In silico* modeling suggested that E208Q alters the PI-binding pocket of the PH domain. This was confirmed by localization studies, via cell fractionation and confocal microscopy, showing that E208Q proteins were primarily sequestered in the cytosol compared to wild-type (WT)-LNK, with significantly reduced plasma membrane recruitment. Conversely, the G152R mutation showed slightly increased membrane localization but higher levels of JAK2 phosphorylation. Importantly, we identified a novel interaction between the LNK N-terminal region plus PH domain (NPH) and the FERM/SH2-like (SH2L) domains of non-phosphorylated JAK2 by performing co-immunoprecipitation studies of truncated LNK and JAK2 constructs. This indicates that the LNK NPH region binds to JAK2 even in its basal state. While LNK mutations did not abolish this binding, they disrupted the spatial orientation and subsequent inhibitory capacity of the LNK-JAK2 complex. Functional studies performed using *Sh2b3*-deficient or heterozygous murine Lin- progenitor cells demonstrated that E208Q exerts a dominant-negative effect over WT-LNK, evidenced by a significant increase in both the number and size of megakaryocyte colonies as evaluated in colony-forming unit assays.

**Copyright Information:** (Online) ISSN: 2572-9241
©2026 The Author(s). HemaSphere is published by John Wiley & Sons Ltd on behalf of European Hematology Association. This is an open access Abstract Book distributed under the Attribution-NonCommercial-NoDerivs (CC BY-NC-ND), which allows third parties to download the articles and share them with others as long as they credit the author and the Abstract Book, but they cannot change the content in any way or use them commercially.
**Abstract Book Citations:** Authors, Title, HemaSphere, 2026;10:(S1):pages. The abstract book DOIs can be found on the "Author Information" page.
**Disclaimer:** Articles published in the journal HemaSphere exclusively reflect the opinions of the authors. The authors are responsible for all content in their abstracts including accuracy of the facts, statements, citing resources, etc.

**Summary/Conclusion**

Our findings reveal that the NPH region of LNK acts as a critical regulatory scaffold that maintains JAK2 activity at basal levels. Mutations in this region act in a dominant-negative fashion by impairing proper subcellular localization and membrane translocation. By disrupting the dynamics of the LNK-JAK2 complex, these variants facilitate JAK2 pre-activation and cooperate with other activating mutations (like V617F) to drive the phenotypic switch and progression of MPNs.

**Copyright Information:** (Online) ISSN: 2572-9241
©2026 The Author(s). HemaSphere is published by John Wiley & Sons Ltd on behalf of European Hematology Association. This is an open access Abstract Book distributed under the Attribution-NonCommercial-NoDerivs (CC BY-NC-ND), which allows third parties to download the articles and share them with others as long as they credit the author and the Abstract Book, but they cannot change the content in any way or use them commercially.
**Abstract Book Citations:** Authors, Title, HemaSphere, 2026;10:(S1):pages. The abstract book DOIs can be found on the "Author Information" page.
**Disclaimer:** Articles published in the journal HemaSphere exclusively reflect the opinions of the authors. The authors are responsible for all content in their abstracts including accuracy of the facts, statements, citing resources, etc.

### (PF862) MICE EXPRESSING KITD814V IN THE MAST CELL LINEAGE SHOW A SYSTEMIC MASTOCYTOSIS PHENOTYPE WITH SKIN LESIONS THAT CAN BE REVERSED BY TREATMENT WITH SPECIFIC TYROSINE KINASE INHIBITORS

**Topic:** 15. Myeloproliferative neoplasms - Biology & translational research

Simona Stivala*[1], Alina Makeeva[1], Tiago Almeida[1], Laetitia Clauss[1], Etnik Sheremeti[1], Elena Ratti[1], Martina Konantz[1], Karin Hartmann[1]

[1] *University Hospital of Basel, Department of Biomedicine, Basel, Switzerland;*

### Background

Mastocytosis is characterized by an abnormal expansion of mast cells (MC) in one or various organs such as bone marrow (BM), skin and/or intestine, driven in >90% of patients by the KIT D816V mutation. KIT D816V-specific inhibitors reduce symptoms and prolong survival in systemic mastocytosis, but not all patients benefit from this therapy; therefore, there is an urgent need for novel treatment options.

### Aims

Aim of the project was the characterization of a novel mouse line expressing KitD814V (homologous to human KIT D816V) specifically in the mast cell lineage as a preclinical model for systemic mastocytosis.

### Methods

To advance treatment development, we generated by CRISPR/Cas9 technology a novel pre-clinical mastocytosis mouse model carrying the Kit D814V mutation. Expression of Kit D814V was restricted to the MC lineage by Mcpt5-Cre-mediated recombination. The phenotype resembling mastocytosis, specifically skin lesions, the number of MC in various organs, serum levels of murine MC protease 1 (mMCP-1) and peripheral blood count, was evaluated over time. Moreover, mice were monitored for disease progression upon treatment with a specific KIT inhibitor.

### Results

Constitutive expression of Kit D814V in the MC lineage resulted in progressive eczematous skin lesions in mice, starting from 7 weeks of age, located on the neck, between the ears and around the eyes. Video recording of single mice showed a scratching behavior (3-17 episodes in a 2-minute time frame) that was absent in control mice expressing Kit WT. MC accumulated in the skin, BM, spleen liver and stomach, while peripheral blood count was unaltered, consistent with a typical phenotype of systemic mastocytosis. Expansion of MC also resulted in elevated serum levels of mMCP-1 and markedly reduced body weight already at 6 weeks of age. Correspondingly, MC progenitors in peripheral blood and spleen were elevated in mice expressing the mutant Kit compared to control mice. Olink analysis revealed a significant upregulation of inflammatory cytokines such as TNFalpha and IL-6, chemotactic factors (IL-16, Ccl2 and Ccl12), and immune checkpoint molecules (PD-L1 and PD-L2). In order to investigate the response to clinically relevant tyrosine kinase inhibitors, mice were randomized to receive either avapritinib 30 mg/kg or vehicle by oral gavage for 16 days. Treatment resulted in a significant reduction in MC accumulation in the skin, BM and liver, paralleled by a five-fold reduction in mMCP-1 serum levels, and was well tolerated as shown by stable body weight. Moreover, avapritinib-treated mice showed no scratching bouts, indicating resolution of chronic itch, and no skin lesions.

**Copyright Information:** (Online) ISSN: 2572-9241
©2026 The Author(s). HemaSphere is published by John Wiley & Sons Ltd on behalf of European Hematology Association. This is an open access Abstract Book distributed under the Attribution-NonCommercial-NoDerivs (CC BY-NC-ND), which allows third parties to download the articles and share them with others as long as they credit the author and the Abstract Book, but they cannot change the content in any way or use them commercially.
**Abstract Book Citations:** Authors, Title, HemaSphere, 2026;10:(S1):pages. The abstract book DOIs can be found on the "Author Information" page.
**Disclaimer:** Articles published in the journal HemaSphere exclusively reflect the opinions of the authors. The authors are responsible for all content in their abstracts including accuracy of the facts, statements, citing resources, etc.

### Summary/Conclusion

We have generated a novel mouse line expressing the oncogenic Kit D814V mutation specifically in the MC lineage. Mutant mice present a phenotype closely resembling systemic mastocytosis, with expansion of MC in skin, BM, spleen and liver, and chronic itch as indicated by frequent scratching and appearance of progressive skin lesions. The phenotype is corrected by treatment with the tyrosine kinase inhibitor avapritinib, suggesting the relevance as a preclinical model. Therefore, this mouse line could represent an invaluable tool for the investigation of the pathogenesis of the disease, and the identification of novel targetable pathways.

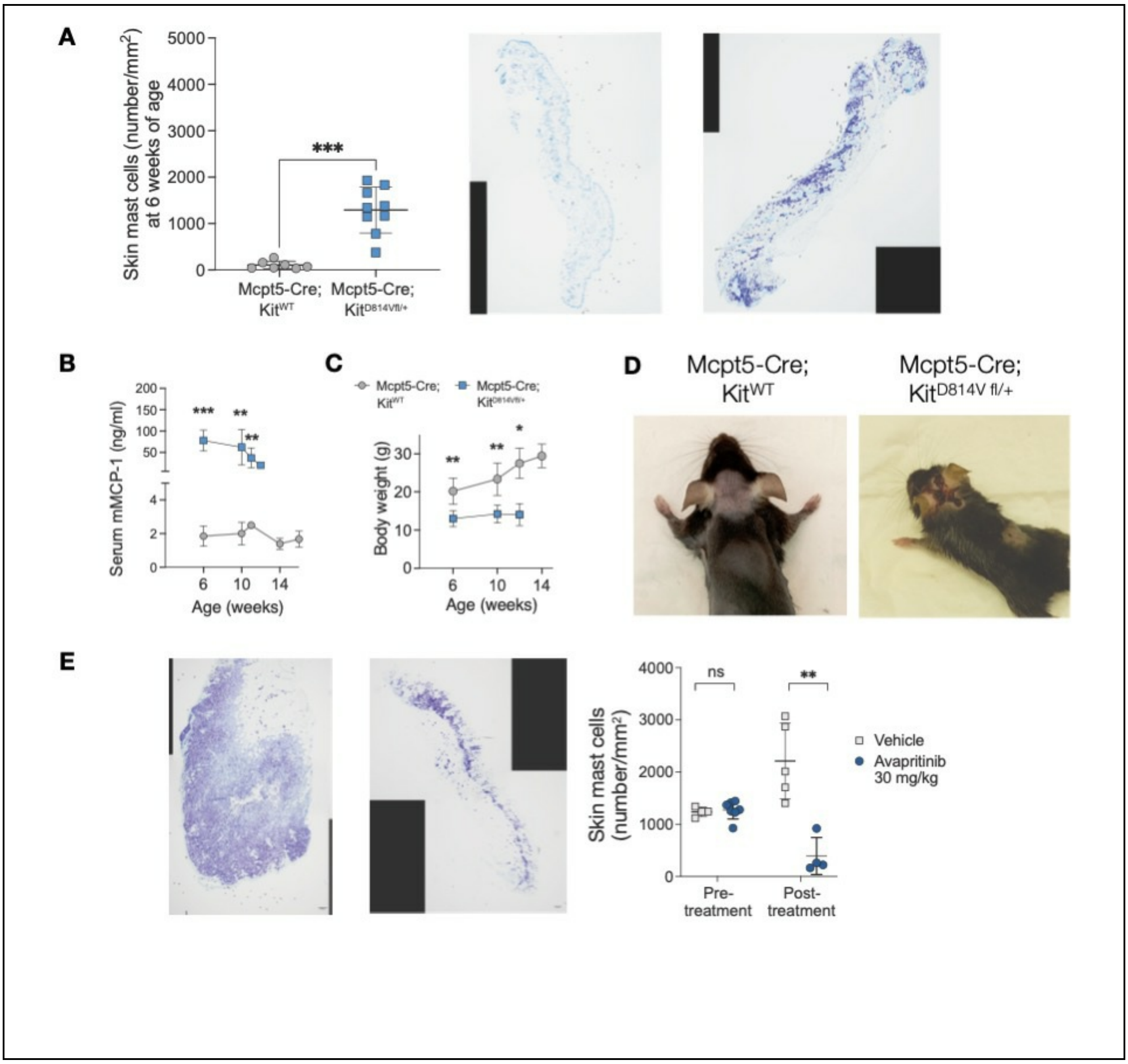

Figure 1 (A) Skin mast cell quantification (left) and representative micrographs (right) from mice expressing KitWT or KitD814V. (B) Serum levels of mMCP-1 are significantly elevated in Mcpt5-Cre; KitD814V fl/+ mice, while BW is reduced (C). (D) Representative pictures showing skin lesions in Mcpt5-Cre; KitD814V fl/+ mice due to scratching. (E) Representative micrographs (left) and quantification (right) of skin mast cells in vehicle- or avapritinib-treated mice.

**Copyright Information:** (Online) ISSN: 2572-9241
©2026 The Author(s). HemaSphere is published by John Wiley & Sons Ltd on behalf of European Hematology Association. This is an open access Abstract Book distributed under the Attribution-NonCommercial-NoDerivs (CC BY-NC-ND), which allows third parties to download the articles and share them with others as long as they credit the author and the Abstract Book, but they cannot change the content in any way or use them commercially.
**Abstract Book Citations:** Authors, Title, HemaSphere, 2026;10:(S1):pages. The abstract book DOIs can be found on the "Author Information" page.
**Disclaimer:** Articles published in the journal HemaSphere exclusively reflect the opinions of the authors. The authors are responsible for all content in their abstracts including accuracy of the facts, statements, citing resources, etc.

### (PF864) JAK2V617F SIGNALLING DRIVES A PATHOLOGICAL PHOSPHORYLATION PROFILE ON HYPOXIA INDUCIBLE FACTOR-1 THAT ALTERS FUNCTION IN HSCS

**Topic:** 15. Myeloproliferative neoplasms - Biology & translational research

Ruth Ellerington*[1, 2], Angelica Varesi[3, 4], David Kealy[1, 2], Catherine Hawley[1, 2], Bryce Drylie[1, 2, 5], Kristin Hope[3, 4], John Dick[3, 4], Katherine Bridge[1, 2]

*[1]Centre for Blood Research, University of York, Biology, York, United Kingdom;[2]York Biomedical Research Institute, University of York, York, United Kingdom; [3]Princess Margaret Cancer Centre, University Health Network, Toronto, Canada;[4]University of Toronto, Toronto, Canada; [5]Barts Cancer Institute, Queen Mary University of London, York, United Kingdom;*

### Background

Hypoxia-inducible factor-1 (HIF-1) is a master regulator of transcription, coordinating a repertoire of target genes regulating essential cell survival processes in hypoxia and is frequently activated within malignancy. Whilst malignant context is known to direct the role of HIFs within oncogenesis, these mechanisms are not well characterised. JAK2V617F signalling stabilises HIF-1 independent of hypoxia and our group has recently demonstrated that the JAK2V617F-HIF-1 regulon correlates with MPN disease severity and worsened patient survival outcomes. Our study identified that JAK2V617F-induced HIF-1α stabilisation is mediated via PIM1 kinase and causes two unique phosphorylation events on HIF-1α: T498 and S500. The role of these novel phosphorylation events in JAK2V617F-mediated HIF-1 stabilisation and activity remains unclear.

### Aims

To characterise the mechanism by which HIF-1α is phosphorylated at the T498 and S500 sites, to understand the mechanism by which the T498 and S500 phosphorylation sites stabilise HIF-1α, and to characterise the role of the T498 and S500 phosphorylation sites in MPN disease pathogenesis.

### Methods

The mechanism of JAK2V617F-specific HIF-1α phosphorylation was investigated by *in vitro* kinase assay using purified recombinant HIF-1a oxygen-dependent domain (ODD) segment peptide in conjunction with flow cytometry and immunoblotting in paired isogenic cell lines. Human CD34+ HSPC were engineered to contain the JAK2V617F-specific HIF-1α phosphorylations through CRISPR/Cas9 + AAV6 homologous directed recombination. The impact of these mutations on lineage skewing and stem cell properties were evaluated through extensive In vitro immunophenotypic assays during liquid culture, colony forming unit experiments and in vivo xenograft assays.

### Results

Despite being both necessary and sufficient for HIF-1α stabilisation in the presence of the JAK2V617F mutation, PIM1 does not directly phosphorylate HIF-1α at T498/ S500. Due to its proximity to canonical hydroxylation sites in the ODD of HIF-1α, phosphorylation at the T498 and S500 sites interferes with canonical normoxic HIF-1α hydroxylation and subsequent targeting for proteasomal degradation. When inserted into huamn human CD34+ HSPC, these JAK2V617F-specific phosphorylation events are sufficient to cause an MPN-recapitulating myeloid bias and retention of stemness in *in vitro* and *in vivo.*

**Copyright Information:** (Online) ISSN: 2572-9241
©2026 The Author(s). HemaSphere is published by John Wiley & Sons Ltd on behalf of European Hematology Association. This is an open access Abstract Book distributed under the Attribution-NonCommercial-NoDerivs (CC BY-NC-ND), which allows third parties to download the articles and share them with others as long as they credit the author and the Abstract Book, but they cannot change the content in any way or use them commercially.
**Abstract Book Citations:** Authors, Title, HemaSphere, 2026;10:(S1):pages. The abstract book DOIs can be found on the "Author Information" page.
**Disclaimer:** Articles published in the journal HemaSphere exclusively reflect the opinions of the authors. The authors are responsible for all content in their abstracts including accuracy of the facts, statements, citing resources, etc.

**Summary/Conclusion**

JAK2V617F-specific phosphorylation at the T498 and S500 sites of HIF-1α drive the contribution of HIF-1 to MPN pathogenesis via retention of stem-like cell qualities and a shift to a myeloid phenotype, via inhibition of HIF-1s normoxic proteasomal degradation.

**Copyright Information:** (Online) ISSN: 2572-9241
©2026 The Author(s). HemaSphere is published by John Wiley & Sons Ltd on behalf of European Hematology Association. This is an open access Abstract Book distributed under the Attribution-NonCommercial-NoDerivs (CC BY-NC-ND), which allows third parties to download the articles and share them with others as long as they credit the author and the Abstract Book, but they cannot change the content in any way or use them commercially.
**Abstract Book Citations:** Authors, Title, HemaSphere, 2026;10:(S1):pages. The abstract book DOIs can be found on the "Author Information" page.
**Disclaimer:** Articles published in the journal HemaSphere exclusively reflect the opinions of the authors. The authors are responsible for all content in their abstracts including accuracy of the facts, statements, citing resources, etc.

### (PF865) SHARED GENETIC ARCHITECTURE OF CLONAL HAEMATOPOIESIS AND LEUKOCYTE TELOMERE LENGTH IMPLICATES VSIG4 ON THE X CHROMOSOME AS A NOVEL RISK LOCUS FOR TET2-MUTANT CLONAL HAEMATOPOIESIS

**Topic:** 15. Myeloproliferative neoplasms - Biology & translational research

Aleksandra Kamizela*[1], Jocelyn Andrews[2], George Vassiliou[1], Siddhartha Kar[2]

*[1]University of Cambridge, Cambridge Stem Cell Institute, Department of Haematology, Cambridge, United Kingdom;[2]University of Cambridge, Early Cancer Institute, Department of Oncology, Cambridge, United Kingdom;*

### Background

Despite being driven by acquired somatic mutations, clonal haematopoiesis (CH) is impacted by inherited genetic variation, highlighting the importance of identifying germline risk loci to uncover biological mechanisms governing clonal emergence and expansion. Most germline genetic determinants of CH identified to date are driven by associations with *DNMT3A*-mutant CH. *TET2* is the second most mutated gene in CH, comprising ~20% of detectable CH. Only a few risk loci have been reported for *TET2*-mutant CH (*TET2*-CH). Among these, *TERT*, which encodes the catalytic core of telomerase and is a central regulator of telomere length, is the most strongly associated locus. Moreover, longer leukocyte telomere length (LTL) is a major causal risk factor for the development of *TET2*-CH.

### Aims

To identify new germline susceptibility loci for *TET2*-mutant clonal haematopoiesis by leveraging its established genetic and causal relationship with leukocyte telomere length.

### Methods

Our analyses were based on 3,918 *TET2*-CH cases and 342,869 controls without detectable CH (as defined by whole-exome sequencing) and LTL measurements (from a combination of quantitative PCR and whole-genome sequencing) in 462,666 individuals – all from the UK Biobank. Array-based and imputed genotype data covering ~8 million common (minor allele frequency >0.5%) variants were used for association analyses. Cross-trait meta-analysis was performed using 'Multi-trait Analysis of GWAS' (MTAG) to boost statistical power for *TET2*-CH germline discovery, followed by linkage disequilibrium-based clumping ($r^2 < 0.05$) using the 1000 Genomes reference panel. For each genome-wide significant lead variant (MTAG $p < 5\times10^{-8}$), the 200 kb surrounding region was subjected to Bayesian colocalisation analysis using HyPrColoc software, with posterior probability >80% considered strong evidence of colocalisation.

**Copyright Information:** (Online) ISSN: 2572-9241
©2026 The Author(s). HemaSphere is published by John Wiley & Sons Ltd on behalf of European Hematology Association. This is an open access Abstract Book distributed under the Attribution-NonCommercial-NoDerivs (CC BY-NC-ND), which allows third parties to download the articles and share them with others as long as they credit the author and the Abstract Book, but they cannot change the content in any way or use them commercially.
**Abstract Book Citations:** Authors, Title, HemaSphere, 2026;10:(S1):pages. The abstract book DOIs can be found on the "Author Information" page.
**Disclaimer:** Articles published in the journal HemaSphere exclusively reflect the opinions of the authors. The authors are responsible for all content in their abstracts including accuracy of the facts, statements, citing resources, etc.

### Results

Cross-trait meta-analysis leveraging LTL genome-wide association data followed by colocalisation identified six risk loci for *TET2*-CH. At each locus, the lead variant reached genome-wide significance (MTAG $p<5\times10^{-8}$), *and* colocalisation demonstrated strong evidence (posterior probability >80%) of a shared causal variant underlying both *TET2*-CH and LTL. Five of these loci have been previously reported at genome-wide significance in single-trait analysis for *TET2*-CH risk (*THRB*, *RPN1*, *TERT*, *ATM*, and *TCL1A*). The sixth risk locus was novel, marked by variant rs585168 (OR=1.11; 95% CI:1.05-1.18; MTAG $p=6.2\times10^{-16}$) 3,900 base pairs upstream of *VSIG4* at Xq12 and predicted to regulate *VSIG4* based on variant-to-gene functional annotations. VSIG4, an immune checkpoint, is known to regulate macrophage activation by reprogramming mitochondrial pyruvate metabolism, and in turn controls inflammatory gene programs involving IL-1β and the NLRP3 inflammasome. Additional analyses of 979 myelodysplastic syndrome (MDS) cases and 378,296 controls in the FinnGen cohort showed that rs585168 (*VSIG4*/Xq12) is also associated with MDS risk ($p=1.6\times10^{-3}$).

### Summary/Conclusion

By exploiting the shared genetic architecture between leukocyte telomere length and *TET2*-mutant clonal hematopoiesis, we identify *VSIG4* on the X chromosome as a novel germline risk locus for *TET2*-CH. These findings highlight immunometabolic regulation of macrophage function as a previously unrecognized pathway influencing CH, with potential relevance to CH-associated inflammatory and malignant outcomes.

**Copyright Information:** (Online) ISSN: 2572-9241
©2026 The Author(s). HemaSphere is published by John Wiley & Sons Ltd on behalf of European Hematology Association. This is an open access Abstract Book distributed under the Attribution-NonCommercial-NoDerivs (CC BY-NC-ND), which allows third parties to download the articles and share them with others as long as they credit the author and the Abstract Book, but they cannot change the content in any way or use them commercially.
**Abstract Book Citations:** Authors, Title, HemaSphere, 2026;10:(S1):pages. The abstract book DOIs can be found on the "Author Information" page.
**Disclaimer:** Articles published in the journal HemaSphere exclusively reflect the opinions of the authors. The authors are responsible for all content in their abstracts including accuracy of the facts, statements, citing resources, etc.

### (PF866) JAK2V617F-POSITIVE MPN MICROENVIRONMENT DRIVES T LYMPHOCYTE EXHAUSTION

**Topic:** 15. Myeloproliferative neoplasms - Biology & translational research

Mariana Medeiros[1, 2], Allana Carvalho[1, 2], Camila Garcia[1, 2], Larissa Binelli[1, 2], Beatriz Adjafre[1, 2], Isabela Arruda[1, 2], Priscila Santos Scheucher[1], Josiane Schiavinato[1], Cleide Lúcia Araújo Silva[2], Rafael Pacca[1, 2], Leonardo Palma[1], Lorena Pontes*[1, 2]

*[1]Ribeirao Preto Medical School - University of Sao Paulo, Hematology Division, Department of Medical Imaging, Hematology, and Clinical Oncology, Ribeirao Preto, Brazil; [2]Ribeirão Preto Blood Center Foundation - University of Sao Paulo, Ribeirao Preto, Brazil;*

### Background

Myelofibrosis (MF), driven by mutations in hematopoietic stem cells that activate JAK/STAT signaling, is an advanced myeloproliferative neoplasm (MPN) with an adverse prognosis. Survival is influenced by the mutational profile, with CALR-mutated MF showing improved outcomes compared to *JAK2V617F*- and *MPL*-mutated patients. Given the central role of JAK/STAT in immune activation and cytotoxicity, we hypothesize that these mutations may differentially impair immune surveillance.

### Aims

This study aimed to determine whether JAK2V617F-driven myelofibrosis differentially impairs T cell immune surveillance and to distinguish the intrinsic effects of mutant hematopoiesis from the extrinsic impact of the MPN microenvironment on T cell differentiation and exhaustion.

### Methods

We immunophenotyped T cells from the peripheral blood of untreated MF patients [10 *JAK2* wild type (8 *CALR*, 2 *MPL*), 18 *JAK2V617F*] and 5 healthy donors (HD). To compare the direct versus microenvironmental effects of JAK2V617F on immune cells, we generated a chimeric murine model in which sub-lethally irradiated CD45.1 recipients were transplanted with total bone marrow from Jak2V617F or *Jak2WT* (CD45.2) donors. Splenic T cells from both graft and host were analyzed.

### Results

*JAK2*-mutated MF patients showed a higher frequency of CD3+ T lymphocytes compared to *JAK2WT* patients and an increased frequency of CD8+ T cells, with a slight but not significant reduction in CD4+ T cells. Regarding the memory phenotype, *JAK2*-mutated MF patients presented a higher frequency of effector memory and terminally differentiated T lymphocytes in the CD4 compartment compared to HD, but similar frequencies compared to *JAK2WT* patients. The same was observed in the CD8 compartment; however, the increase in terminally differentiated T lymphocytes was more pronounced in *JAK2*-mutated MF compared to both HD and *JAK2WT* patients. In addition, *JAK2*-mutated patients showed reduced CD8 activating signaling via NKG2D.

In the chimeric mice, compared to controls (mice transplanted with *JAK2WT* cells), *Jak2V617F* CD45.2 grafts presented reduced T lymphocytes but an increased CD8+ fraction, suggesting that *Jak2V617F*-mutant hematopoiesis intrinsically disrupts T cell development. Conversely, MPN-exposed CD45.1 host cells showed expansion of CD8+ T cells, particularly the effector memory subtype. In addition, MPN exposure led to a CD8 exhausted phenotype, marked by an increase in TIM-3+ and PD-1+ cells, suggesting that soluble factors extrinsically promote immune exhaustion.

**Copyright Information:** (Online) ISSN: 2572-9241
©2026 The Author(s). HemaSphere is published by John Wiley & Sons Ltd on behalf of European Hematology Association. This is an open access Abstract Book distributed under the Attribution-NonCommercial-NoDerivs (CC BY-NC-ND), which allows third parties to download the articles and share them with others as long as they credit the author and the Abstract Book, but they cannot change the content in any way or use them commercially.
**Abstract Book Citations:** Authors, Title, HemaSphere, 2026;10:(S1):pages. The abstract book DOIs can be found on the "Author Information" page.
**Disclaimer:** Articles published in the journal HemaSphere exclusively reflect the opinions of the authors. The authors are responsible for all content in their abstracts including accuracy of the facts, statements, citing resources, etc.

**Summary/Conclusion**

Together, these findings demonstrate that *JAK2V617F*-driven MPN impairs T cell immunity through both intrinsic and extrinsic mechanisms. While mutant hematopoiesis directly disrupts T cell development and skews CD8+ differentiation, the pro-inflammatory microenvironment promotes T cell exhaustion. These combined effects may contribute to defective immune surveillance in MF and help explain differences in clinical behavior according to mutational status.

**Copyright Information:** (Online) ISSN: 2572-9241
©2026 The Author(s). HemaSphere is published by John Wiley & Sons Ltd on behalf of European Hematology Association. This is an open access Abstract Book distributed under the Attribution-NonCommercial-NoDerivs (CC BY-NC-ND), which allows third parties to download the articles and share them with others as long as they credit the author and the Abstract Book, but they cannot change the content in any way or use them commercially.
**Abstract Book Citations:** Authors, Title, HemaSphere, 2026;10:(S1):pages. The abstract book DOIs can be found on the "Author Information" page.
**Disclaimer:** Articles published in the journal HemaSphere exclusively reflect the opinions of the authors. The authors are responsible for all content in their abstracts including accuracy of the facts, statements, citing resources, etc.

### (PF867) PRECLINICAL EVIDENCE OF TARGETING ACVR1/HEPCIDIN BY NUVISERTIB, AN ORAL INVESTIGATIONAL PIM1 KINASE INHIBITOR, SUGGESTS POTENTIAL MECHANISTIC BASIS FOR HEMOGLOBIN BENEFIT IN MYELOFIBROSIS

**Topic:** 15. Myeloproliferative neoplasms - Biology & translational research

Joseph M Scandura[1], Jinny Lee*[2], Ronald Hoffman[3], Jason Foulks[2], John Mascarenhas[3], Adam Siddiqui[2], Firas El Chaer[4], Lindsay Rein[5], Raajit K Rampal[6], Vikas Gupta[7], Claire Harrison[8], Ruben Mesa[9], Florian Heidel[10], Francesco Passamonti[11], Kazuya Shimoda[12], Noriko Fukuhara[13], Alessandra Iurlo[11], Shuichi Shirane[14], Akiyoshi Takami[15], Junichiro Yuda[16], Tamanna Haque[6], Michiko Ichii[17], James McCloskey[18], Samah Alimam[19], Giulia Benevolo[20], Nikki Granacher[21], Alessandro Lucchesi[22], Karen Ansaldo[2], Sujan Kabir[2], Eric I Sbar[2], Masataka Seki[2], Jatin Shah[2], Steven L Warner[2], Prithviraj Bose[23]

*[1]Weill Cornell Medicine, New York, United States of America;[2]Sumitomo Pharma America, Inc., Marlborough, United States of America; [3]Icahn School of Medicine at Mount Sinai, New York, United States of America;[4]Miami Cancer Institute, Baptist Health, Miami, United States of America; [5]Duke University Medical Center, Durham, United States of America;[6]Memorial Sloan Kettering Cancer Center, New York, United States of America; [7]Princess Margaret Cancer Centre, Toronto, Canada;[8]Guy's and St Thomas' NHS Foundation Trust, London, United Kingdom; [9]Atrium Health Levine Cancer Institute, Charlotte, United States of America; [10]Hannover Medical School (MHH), Hematology, Hemostasis, Oncology and Stem Cell Transplantation, Hannover, Germany; [11]Foundation IRCCS Ca' Granda Ospedale Maggiore Policlinico, Milan, Italy; [12]University of Miyazaki, Miyazaki, Japan; [13]Tohoku University Hospital, Sendai, Japan; [14]Juntendo University School of Medicine, Tokyo, Japan; [15]Aichi Medical University School of Medicine, Nagakute, Japan; [16]National Cancer Center Hospital East, Kashiwa, Japan; [17]The University of Osaka Graduate School of Medicine, Osaka, Japan; [18]John Theurer Cancer Center, Hackensack University Medical Center, Hackensack, United States of America; [19]University College London Hospitals, London, United Kingdom; [20]Hematology U, Città della Salute e della Scienza, Turin, Italy; [21]ZAS Cadix, Antwerp, Belgium;[22]IRCCS Istituto Romagnolo per lo Studio e la Cura dei Tumori (IRST) "DinoAmadori", Meldola, Italy; [23]The University of Texas MD Anderson Cancer Center, Houston, United States of America;*

### Background

PIM1 expression is upregulated in CD34+ cells from patients (pts) with myelofibrosis (MF). In preclinical mouse models, PIM1 knockout (KO) was shown to prevent MF progression without affecting platelet counts, whereas pan-PIM KO resulted in thrombocytopenia (Dutta 2022 and An 2013). Cytopenias due to disease pathogenesis and/or treatment-emergent toxicity remain major limitations of MF treatments and are associated with poor prognosis. Nuvisertib (nuvi) is an oral investigational PIM1 inhibitor currently being evaluated in pts with relapsed and refractory (R/R) MF in a Phase 1/2 study. Preliminary clinical data showed that nuvi monotherapy is well tolerated, exhibiting minimal myelosuppression and demonstrating clinical activity through improvements in symptoms, spleen size, and fibrosis. Mean hemoglobin (Hgb) remained stable during the 24-week treatment period. In pts with baseline Hgb <10 g/dL, 6 of 25 (24%) showed Hgb response [mean ≥1.0 g/dL Hgb increase for ≥12 weeks without transfusion, including 3 pts with ≥1.5 g/dL Hgb increase]. As a central regulator of iron homeostasis, hepcidin is elevated in pts with MF and anemia. The goal of this study was to investigate the mechanism by which nuvi may support Hgb stability or improvement through modulation of hepcidin.

### Methods

Plasma hepcidin levels were measured by ELISA in pts (n=75) with R/R MF treated with nuvi monotherapy from a Phase 1/2 clinical study (NCT04176198, Arm 1). Paired C1D1 predose and 6-hour samples were available for 70 pts. HepG2 cells were stimulated with BMP6 following pre-incubation with nuvi, and hepcidin-encoding *Hamp* mRNA expression was quantified by RT-qPCR. Western blot analyses were performed to assess BMP6-induced SMAD1/5/8 phosphorylation and IL-6-induced STAT3 phosphorylation. Biochemical kinase activity assay, structure-based modeling, and co-crystal structure analysis were used to characterize ACVR1 engagement.

**Copyright Information:** (Online) ISSN: 2572-9241
©2026 The Author(s). HemaSphere is published by John Wiley & Sons Ltd on behalf of European Hematology Association. This is an open access Abstract Book distributed under the Attribution-NonCommercial-NoDerivs (CC BY-NC-ND), which allows third parties to download the articles and share them with others as long as they credit the author and the Abstract Book, but they cannot change the content in any way or use them commercially.
**Abstract Book Citations:** Authors, Title, HemaSphere, 2026;10:(S1):pages. The abstract book DOIs can be found on the "Author Information" page.
**Disclaimer:** Articles published in the journal HemaSphere exclusively reflect the opinions of the authors. The authors are responsible for all content in their abstracts including accuracy of the facts, statements, citing resources, etc.

**Results**

In the Phase 1/2 clinical study, reductions in hepcidin levels were observed in 68.6% of pts (48 of 70) as early as 6 hours after the first dose of nuvi monotherapy. An inverse correlation between hepcidin and Hgb levels was observed (Figure 1). Notably, hepcidin reduction and its correlation with Hgb appeared to be primarily driven by pts with baseline Hgb <10 g/dL. In a preclinical *in vitro* study, nuvi treatment resulted in a dose-dependent reduction in *Hamp* mRNA (EC50= 380 nM), comparable to the ACVR1/JAK inhibitor momelotinib (MMB) (EC50= 470 nM). Combined treatment with nuvi and MMB resulted in additive suppression of *Hamp* mRNA expression in preclinical study. Mechanistically, nuvi inhibited BMP6-induced SMAD1/5/8 phosphorylation by ACVR1 but did not affect IL-6-induced STAT3 phosphorylation by JAK, whereas MMB inhibited both pathways. A biochemical kinase activity assay demonstrated that nuvi inhibits ACVR1 with an IC50 of 135 nM. Structure-based modeling and co-crystal structure determination further confirmed direct binding of nuvi to ACVR1.

**Summary/Conclusion**

*In vitro* and biochemical data demonstrated that nuvi may function as a dual PIM1 and ACVR1 inhibitor, providing a potential mechanistic explanation for Hgb improvement observed in pts with MF in an ongoing Phase 1/2 study. In addition, the combinatorial activity of nuvi and MMB in reducing *Hamp* mRNA *in vitro* supports continued clinical evaluation of this combination in the ongoing Phase 1/2 study, including exploration of hepcidin levels and correlations to Hgb improvements.

**Copyright Information:** (Online) ISSN: 2572-9241
©2026 The Author(s). HemaSphere is published by John Wiley & Sons Ltd on behalf of European Hematology Association. This is an open access Abstract Book distributed under the Attribution-NonCommercial-NoDerivs (CC BY-NC-ND), which allows third parties to download the articles and share them with others as long as they credit the author and the Abstract Book, but they cannot change the content in any way or use them commercially.
**Abstract Book Citations:** Authors, Title, HemaSphere, 2026;10:(S1):pages. The abstract book DOIs can be found on the “Author Information” page.
**Disclaimer:** Articles published in the journal HemaSphere exclusively reflect the opinions of the authors. The authors are responsible for all content in their abstracts including accuracy of the facts, statements, citing resources, etc.

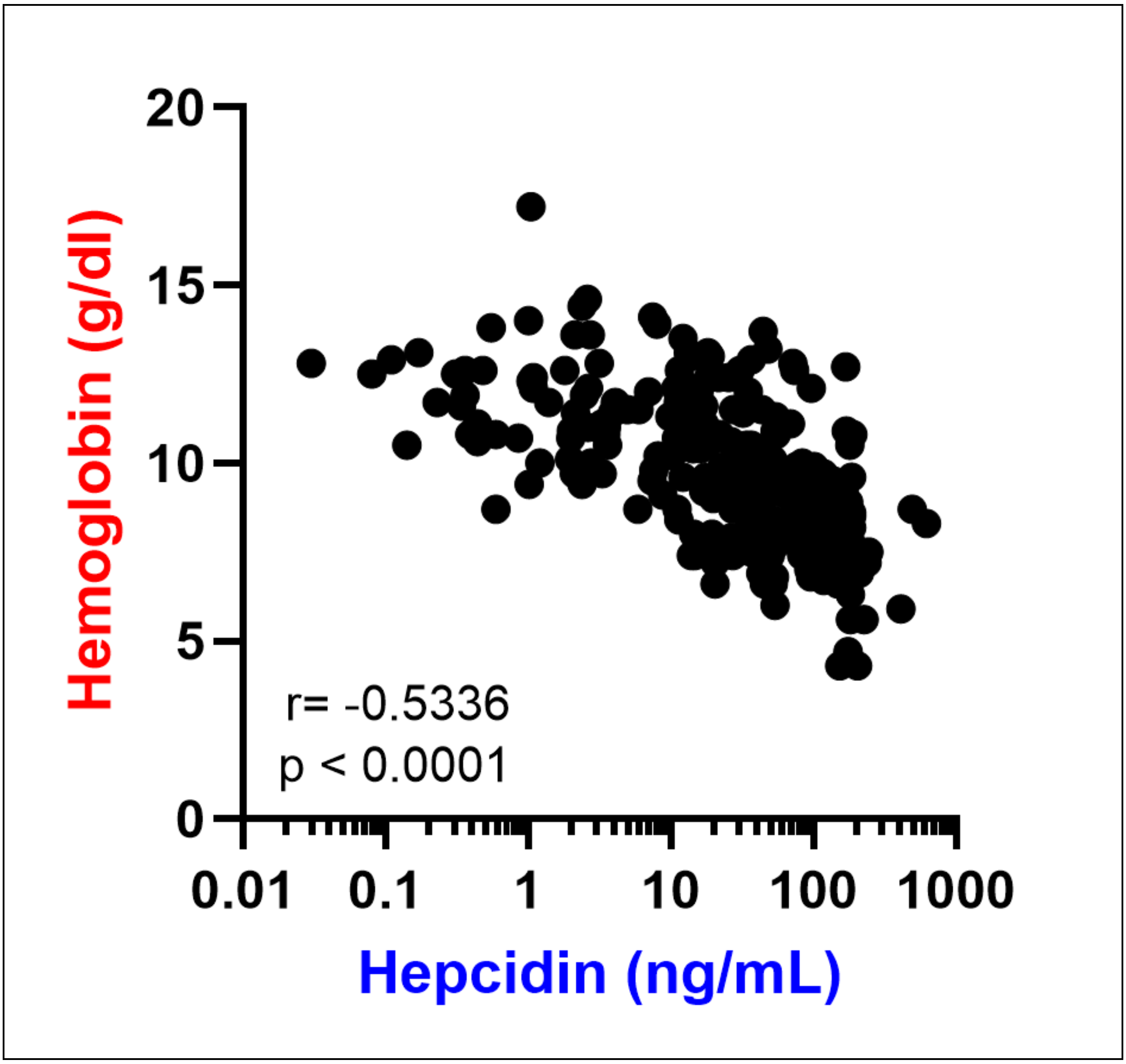

Figure 1. Plasma hepcidin levels are inversely correlated with Hgb. For each patient, multiple time points were included as available, with each dot representing a single time-point measurement.

**Copyright Information:** (Online) ISSN: 2572-9241
©2026 The Author(s). HemaSphere is published by John Wiley & Sons Ltd on behalf of European Hematology Association. This is an open access Abstract Book distributed under the Attribution-NonCommercial-NoDerivs (CC BY-NC-ND), which allows third parties to download the articles and share them with others as long as they credit the author and the Abstract Book, but they cannot change the content in any way or use them commercially.
**Abstract Book Citations:** Authors, Title, HemaSphere, 2026;10:(S1):pages. The abstract book DOIs can be found on the "Author Information" page.
**Disclaimer:** Articles published in the journal HemaSphere exclusively reflect the opinions of the authors. The authors are responsible for all content in their abstracts including accuracy of the facts, statements, citing resources, etc.

### (PF868) FROM CLONAL HEMATOPOIESIS TO MYELOPROLIFERATIVE NEOPLASMS: A THROMBOINFLAMMATORY PERSPECTIVE ON JAK2V617F

**Topic:** 15. Myeloproliferative neoplasms - Biology & translational research

Lea Löffler*[1], Laura Massarenti[2, 3], Mads Larsen[2], Isabella Davidsen[1], Vibe Skov[1], Lasse Kjær[1], Troels Wienecke[4, 5], Christina Ellervik[5, 6, 7], Morten Kranker Larsen[1], Claus Nielsen[2, 3], Hans Hasselbalch[1, 5]

*[1]Zealand University Hospital, Department of Hematology, Roskilde, Denmark; [2]Rigshospitalet, Institute for Inflammation Research (IIR), Department of Rheumatology and Spine Diseases, Copenhagen, Denmark; [3]University of Copenhagen, Department of Odontology, Copenhagen, Denmark; [4]Zealand University Hospital, Department of Neurology, Roskilde, Denmark; [5]University of Copenhagen, Department of Clinical Medicine, Copenhagen, Denmark; [6]Zealand University Hospital, Department of Clinical Biochemistry, Køge, Denmark; [7]Boston Children's Hospital & Harvard Medical School, Department of Laboratory Medicine, Boston, United States of America;*

**Background**

Clonal hematopoiesis (CH) is linked to thromboembolic complications and progression to hematological malignancies, including myeloproliferative neoplasms (MPN). The *JAK2V617F* (*JAK2*) mutation occurs as CH in 3.1% of the Danish population. Progression from *JAK2*-CH to MPN is increasingly viewed as a continuum driven by chronic inflammation, but it is unclear whether thromboinflammatory signatures are present at the CH stage.

**Aims**

To characterize circulating thromboinflammatory markers in individuals with *JAK2*-CH longitudinally and assess associations with *JAK2* variant allele frequency (VAF) and progression to MPN.

**Methods**

This study was based on the Danish General Suburban Population Study (21,205 citizens, 2010-2013). *JAK2* mutation status was determined using digital droplet PCR in 19,985 stored baseline samples, identifying 599 individuals with the *JAK2* mutation. The present study included 44 *JAK2*-positive individuals and 44 age- and sex-matched *JAK2*-negative controls. Among the *JAK2*-positive individuals, 15 developed incident MPN (*JAK2*-MPN) during the follow-up period (2018-January 2026), while 29 remained classified as *JAK2*-CH. Thromboinflammatory biomarker analyses were performed on serum samples obtained at baseline and at first follow-up. For the *JAK2*-MPN group, the follow-up sample preceded clinical diagnosis. Cytokines (IL-1β, IL-6, IL-10 and TNF-α) were measured using the Meso Scale Discovery S-PLEX Pro-Inflammatory panel. Additional markers reflecting endothelial activation, inflammation and platelet activation (VCAM-1, ICAM-1, thrombomodulin, P-selectin, E-selectin, IL-8, sCD40L, and suPAR) were quantified using Luminex assays. Associations between biomarker levels, *JAK2* VAF and disease evolution were evaluated using non-parametric tests and regression analyses with correction for multiple testing.

**Copyright Information:** (Online) ISSN: 2572-9241
©2026 The Author(s). HemaSphere is published by John Wiley & Sons Ltd on behalf of European Hematology Association. This is an open access Abstract Book distributed under the Attribution-NonCommercial-NoDerivs (CC BY-NC-ND), which allows third parties to download the articles and share them with others as long as they credit the author and the Abstract Book, but they cannot change the content in any way or use them commercially.
**Abstract Book Citations:** Authors, Title, HemaSphere, 2026;10:(S1):pages. The abstract book DOIs can be found on the "Author Information" page.
**Disclaimer:** Articles published in the journal HemaSphere exclusively reflect the opinions of the authors. The authors are responsible for all content in their abstracts including accuracy of the facts, statements, citing resources, etc.

**Results**

Baseline characteristics were similar between the three groups (Controls, *JAK2*-CH & *JAK2*-MPN) with respect to age, sex, BMI and smoking, while *JAK2* VAF was higher in the *JAK2*-MPN group compared to those remaining *JAK2*-CH (median 3.3% and 1.1% respectively, p=0.03). Median follow-up time until end of follow-up or date of MPN diagnosis was 14.4 years (interquartile range 13.2-15.0). At baseline, sCD40L levels were significantly higher in the *JAK2*-MPN group compared to *JAK2*-CH (p=0.03) and Controls (p=0.004), while P-selectin levels were higher in *JAK2*-MPN compared to Controls (p=0.04), but did not differ significantly from *JAK2*-CH. In regression analyses adjusted for age and sex, *JAK2* VAF at baseline was positively associated with baseline levels of sCD40L (p=0.003), IL-8 (p=0.04) and TNF-α (p=0.03).
Longitudinally, the concentration of several biomarkers increased more in both *JAK2*-CH and -MPN than in Controls, including sCD40L, P-selectin, IL-1β, IL-8 and TNF-α, with the largest increases in the *JAK2*-MPN group. In contrast, IL-10 serum concentrations were significantly higher in *JAK2*-CH at follow-up but not in *JAK2*-MPN (p=0.001). Changes in the levels of VCAM-1 (p=0.01), IL-8 (p=0.02), suPAR (p=0.04) and sCD40L (p=0.04) were positively associated with *JAK2* VAF over time.

**Summary/Conclusion**

Our findings suggest early and progressive thromboinflammatory dysregulation in *JAK2*-CH, years before MPN diagnosis. Platelet activation, particularly elevated sCD40L, may help identify individuals at higher risk of progression, while increased IL-10 may reflect protective immune regulation in non-progressors.

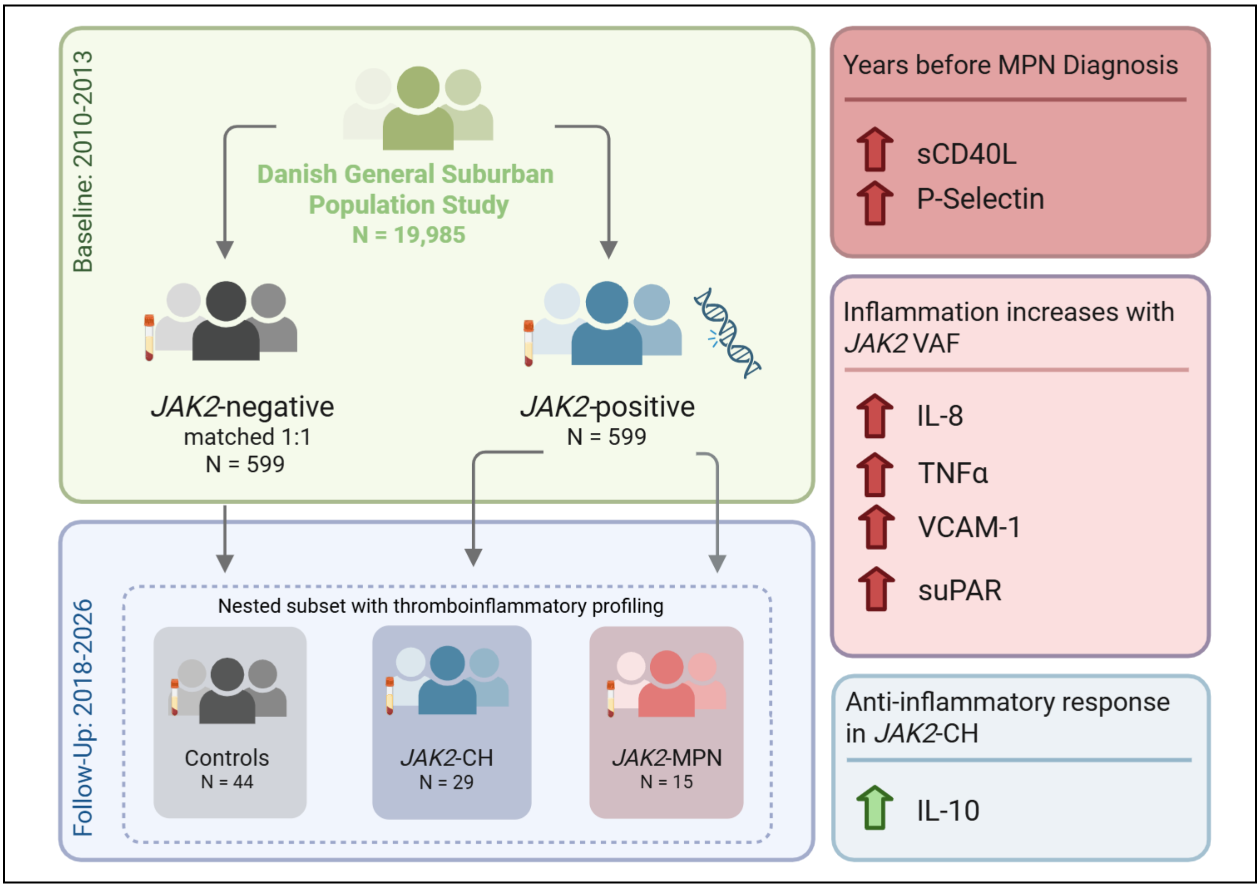

MPN: Myeloproliferative Neoplasm. JAK2: JAK2V617F. CH: Clonal Hematopoiesis. Created in https://BioRender.com.

**Copyright Information:** (Online) ISSN: 2572-9241
©2026 The Author(s). HemaSphere is published by John Wiley & Sons Ltd on behalf of European Hematology Association. This is an open access Abstract Book distributed under the Attribution-NonCommercial-NoDerivs (CC BY-NC-ND), which allows third parties to download the articles and share them with others as long as they credit the author and the Abstract Book, but they cannot change the content in any way or use them commercially.
**Abstract Book Citations:** Authors, Title, HemaSphere, 2026;10:(S1):pages. The abstract book DOIs can be found on the "Author Information" page.
**Disclaimer:** Articles published in the journal HemaSphere exclusively reflect the opinions of the authors. The authors are responsible for all content in their abstracts including accuracy of the facts, statements, citing resources, etc.

### (PF869) AN INDUCIBLE TRANSGENIC SCL-CRE;KITD814V MOUSE MODEL RECAPITULATES ADVANCED SYSTEMIC MASTOCYTOSIS WITH SM-AHN AND VALIDATES AVAPRITINIB RESPONSE

**Topic:** 15. Myeloproliferative neoplasms - Biology & translational research

Martina Konantz*[1], Laetitia Clauss[1], Alina Makeeva[1], Marc Usart[1], Elena Ratti[1], Etnik Sheremeti[1], Simona Stivala[1], Karin Hartmann[1, 2, 3]

*[1]University Hospital Basel and University of Basel, Department of Biomedicine, Basel, Switzerland;[2]University Hospital Basel, Department of Dermatology, Division of Allergy, Basel, Switzerland; [3]University Hospital Basel and University of Basel, Department of Clinical Research, Basel, Switzerland;*

### Background

Systemic mastocytosis (SM) is a heterogeneous disease characterized by the clonal expansion of mast cells (MC), primarily driven by the KIT D816V mutation. SM is classified into cutaneous and systemic subtypes, including chronic and advanced forms (AdvSM) such as SM-AHN (SM with an associated hematological neoplasm). While treatment options have improved, the development of effective therapies remains slow, partly due to the lack of well-established pre-clinical models.

### Aims

We generated an inducible transgenic Scl-CreER$^{T2}$;Kit$^{+/D814V}$ mouse line expressing KitD814V in hematopoietic stem and progenitor cells to better model AdvSM, specifically SM-AHN of myeloid origin.

### Methods

Disease progression after tamoxifen induction was monitored using serial blood analyses, skin biopsies, histology and flow cytometry. Transgene copy number was quantified by digital droplet PCR (ddPCR). IgE- and MRGPRX2-mediated anaphylaxis were performed using DNP-specific IgE and ciprofloxacin, respectively. Symptomatic mice received oral avapritinib (30 mg/kg body weight daily for 16 days).

**Copyright Information:** (Online) ISSN: 2572-9241
©2026 The Author(s). HemaSphere is published by John Wiley & Sons Ltd on behalf of European Hematology Association. This is an open access Abstract Book distributed under the Attribution-NonCommercial-NoDerivs (CC BY-NC-ND), which allows third parties to download the articles and share them with others as long as they credit the author and the Abstract Book, but they cannot change the content in any way or use them commercially.
**Abstract Book Citations:** Authors, Title, HemaSphere, 2026;10:(S1):pages. The abstract book DOIs can be found on the “Author Information” page.
**Disclaimer:** Articles published in the journal HemaSphere exclusively reflect the opinions of the authors. The authors are responsible for all content in their abstracts including accuracy of the facts, statements, citing resources, etc.

**Results**

Mutant mice (n=23) developed a symptomatic disease phenotype with 100% penetrance after a median latency of 139 days and a median survival of 112 days, whereas non-induced (n=5) and control littermates (n=25) remained healthy. ddPCR revealed 3–5 transgene copies without correlation to disease severity. Mutant mice compared to controls exhibited marked MC accumulation in the skin (458 ± 398 vs. 71 ± 31 MC/mm², p<0.001) and stomach (204 ± 229 vs. 43 ± 22 MC/mm², p<0.01), splenomegaly in 80% of animals, and significantly elevated serum Mcpt1 levels. Serum tryptase (Mcpt6) levels were likewise increased and positively correlated with Mcpt1. Mutant mice experienced significantly greater temperature drops following MRGPRX2-mediated anaphylaxis compared to control littermates, while IgE-mediated anaphylaxis remained unaffected.

17 of 23 mutant mice (75%) developed hematological abnormalities consistent with SM-AHN. Anemia and thrombocytopenia were observed in 26% and 42% of mice, respectively. Bone marrow (BM) analysis demonstrated increased LSK and multipotent progenitors, reduced MC progenitors (0.020 ± 0.025% vs. 0.056 ± 0.044% of live cells, p<0.05), and elevated mature FcεRI$^{+}$c-KIT$^{+}$ MC (1.01 ± 0.344% vs. 0.52 ± 0.163%). Sorted LSK (Lin$^{-}$Sca1$^{+}$c-Kit$^{+}$) and progenitor populations expressed KitD814V, confirming involvement of early hematopoietic compartments. Despite increased MC burden, diagnostic criteria for human mast cell leukemia were not met. Avapritinib treatment (n=12/group) significantly improved disease parameters, including reduced skin and gastric MC numbers, decreased serum Mcpt1 levels, normalization of peripheral blood counts and reduced spleen weight. Importantly, avapritinib normalized LSK and mature BM MC frequencies and ameliorated MRGPRX2-mediated anaphylaxis.

**Summary/Conclusion**

We established and characterized a novel inducible transgenic mouse line, Scl-CreER$^{T2}$;Kit$^{+/D814V}$, that faithfully recapitulates key features of AdvSM, specifically SM-AHN of myeloid origin (SM-AMN) arising from early hematopoietic progenitors. Therapeutic response to avapritinib validates this model as a robust and translationally relevant pre-clinical in vivo platform to evaluate novel treatment strategies in AdvSM.

**Copyright Information:** (Online) ISSN: 2572-9241
©2026 The Author(s). HemaSphere is published by John Wiley & Sons Ltd on behalf of European Hematology Association. This is an open access Abstract Book distributed under the Attribution-NonCommercial-NoDerivs (CC BY-NC-ND), which allows third parties to download the articles and share them with others as long as they credit the author and the Abstract Book, but they cannot change the content in any way or use them commercially.
**Abstract Book Citations:** Authors, Title, HemaSphere, 2026;10:(S1):pages. The abstract book DOIs can be found on the “Author Information” page.
**Disclaimer:** Articles published in the journal HemaSphere exclusively reflect the opinions of the authors. The authors are responsible for all content in their abstracts including accuracy of the facts, statements, citing resources, etc.

### (PF870) INVESTIGATING GSK3B SIGNALLING AXIS AS A NOVEL THERAPEUTIC VULNERABILITY IN JAK2V617F MYELOPROLIFERATIVE NEOPLASMS

**Topic:** 15. Myeloproliferative neoplasms - Biology & translational research

Aparna Sinha*[1, 2], Jakub Lukaszonek[1, 2], Catherine Hawley[1, 2], Danielle Powdrill[3], Alyssa Cull[1, 2], Kate Foley[3], David Kent[1, 2], Ines Hahn[2], Katherine Bridge[1, 2]

*[1]University of York, Centre for Blood Research, York, United Kingdom;[2]University of York, York Biomedical Research Institute, York, United Kingdom; [3]York Teaching Hospitals NHS Foundation Trust, York, United Kingdom;*

### Background

Myeloproliferative Neoplasms (MPNs) are characterised by clonal expansion of terminally differentiated myeloid cells encompassing red blood cells (Polycythaemia Vera [PV]), platelets (Essential Thrombocythemia [ET]), and bone marrow stem cells (Primary Myelofibrosis [PMF]). More than 95% of PV and 50-60% of ET/PMF patients carry the JAK2V617F mutation. Our group recently identified HIF-1α as pathologically stabilised downstream of JAK2V617F signalling, activating a non-canonical hypoxia regulon associated with disease progression and transformation to blast phase in MPN patients. We identified GSK3β as one such gene within the JAK2V617F-HIF-1α regulon.

### Aims

Applying a multimodal approach in human, mouse, and *Drosophila* to dissect the core molecular mechanisms of GSK3β expression and activity in JAK2V617F MPNs.

### Methods

JAK2V617F MPN patient peripheral blood mononuclear cells (PBMCs) were isolated and seeded on methylcellulose media with rh SCF, rh GM-CSF, rh IL3, rh G-CSF, and without EPO for 14 days. LiCl was added for GSK3β inhibition, and MK2206 was added for GSK3β activation. Colonies were imaged using the STEMVision Colony Counter and counted manually. JAK2 WT or V617F+/- mice were used to isolate Lin- cKit+ Sca1+ EPCR+ CD34- CD150+ cells (ESLAMs). The cells were expanded for 28 days and treated with LiCl and MK2206 for 7 days. Post treatment, ESLAMs were characterised by Lineage, Sca-1, cKit, CD34, CD16/32, and CD41a expression. JAK2 homolog, hop mutant fly line, $hop^{T42/}$FM7Tb, were maintained at 25 $^{o}$C on standard yeast-sucrose-agar food. For GSK3β inhibition and activation, Formula 4-24® Instant Drosophila Medium was mixed with LiCl and MK2206, respectively, and the flies were treated for 7 days. Next, all 3rd instar larvae were counted, and $hop^{T42}$ mutants were imaged for tumour score calculation.

### Results

Activation of GSK3β resulted in a 2.3-fold reduction in myeloid colony-forming (M-CFUs) in ET and a 1.7-fold reduction in PV patients. Conversely, GSK3β inhibition led to a 1.2-fold increase in M-CFUs in ET patients, and interestingly, no change was observed in PV patients. Similarly, in *ex vivo* expansion cultures of HSC from $JAK^{VF/WT}$ mice, GSK3β activation also decreased the megakaryocyte population, whereas GSK3β inhibition increased megakaryocytes. Furthermore, in *Drosophila*, GSK3β activation in hopT42/FM7Tb decreased tumour formation in a dose-dependent manner, with a 2.9-fold decrease at a high dose of MK2206. Contrarily, inhibition of GSK3β increased tumour formation by 5.4-fold and increased tumour size.

**Copyright Information:** (Online) ISSN: 2572-9241
©2026 The Author(s). HemaSphere is published by John Wiley & Sons Ltd on behalf of European Hematology Association. This is an open access Abstract Book distributed under the Attribution-NonCommercial-NoDerivs (CC BY-NC-ND), which allows third parties to download the articles and share them with others as long as they credit the author and the Abstract Book, but they cannot change the content in any way or use them commercially.
**Abstract Book Citations:** Authors, Title, HemaSphere, 2026;10:(S1):pages. The abstract book DOIs can be found on the "Author Information" page.
**Disclaimer:** Articles published in the journal HemaSphere exclusively reflect the opinions of the authors. The authors are responsible for all content in their abstracts including accuracy of the facts, statements, citing resources, etc.

**Summary/Conclusion**

These data suggest that GSK3β inhibition promotes myeloid cell differentiation, indicating a putative tumour suppressive role in JAK2V617F MPNs. Future studies will determine how HIF-1α regulates GSK3β expression at the chromatin and the role of GSK3β in myeloid cell differentiation *in vitro.*

**Copyright Information:** (Online) ISSN: 2572-9241
©2026 The Author(s). HemaSphere is published by John Wiley & Sons Ltd on behalf of European Hematology Association. This is an open access Abstract Book distributed under the Attribution-NonCommercial-NoDerivs (CC BY-NC-ND), which allows third parties to download the articles and share them with others as long as they credit the author and the Abstract Book, but they cannot change the content in any way or use them commercially.
**Abstract Book Citations:** Authors, Title, HemaSphere, 2026;10:(S1):pages. The abstract book DOIs can be found on the "Author Information" page.
**Disclaimer:** Articles published in the journal HemaSphere exclusively reflect the opinions of the authors. The authors are responsible for all content in their abstracts including accuracy of the facts, statements, citing resources, etc.

### (PF871) LOSS OF CBP DRIVES MYELOID-DERIVED SUPPRESSOR CELL EXPANSION AND PREDICTS POOR PROGNOSIS VIA METTL14-DEPENDENT M6A MODIFICATION IN MYELOPROLIFERATIVE NEOPLASMS

**Topic:** 15. Myeloproliferative neoplasms - Biology & translational research

Rumeng Li[1], Tian Zeng[1], Jian Huang*[1]

[1]*The First Affiliated Hospital, Zhejiang University School of Medicine, Hangzhou, China;*

### Background

Classical Ph-negative myeloproliferative neoplasms (MPNs) comprise a group of rare hematopoietic stem cell disorders characterized by excessive blood cell proliferation. Current treatments demonstrate limited remission rates and fail to prevent transformation to acute myeloid leukemia (AML). Deep exploration of pathogenesis and novel therapeutic targets is urgently needed.

### Aims

This study aims to elucidate how the Cbp/Lyn/METTL14 signaling axis drives myeloproliferative neoplasm progression through epigenetic regulation of MDSC expansion and immune microenvironment remodeling, thereby identifying novel therapeutic targets.

### Methods

We analyzed 300 MPN patients and 20 controls by flow cytometry to quantify MDSCs and immune infiltration, and examined Cbp, Lyn, and METTL14 expression correlating with MDSC abundance and survival. Epigenetic mechanisms of Cbp downregulation were investigated. Using SET2 and HEL cells, we studied Cbp manipulation effects on proliferation, apoptosis, and JAK/STAT/Lyn pathways. Dot-blot and m6A sequencing analyzed Cbp/Lyn regulation of METTL14-mediated methylation. Transcriptome sequencing and GSEA elucidated the Cbp/Lyn-METTL14-m6A axis in MDSC function and immune microenvironment regulation.

### Results

The study found that MDSC numbers were significantly elevated in MPN patient bone marrow, particularly prominent in the myelofibrosis (MF) subtype, and high MDSC levels correlated with poor patient prognosis. MDSC expansion showed significant negative correlation with Cbp gene expression. In an in vitro co-culture system, IL-6 successfully induced MDSC expansion, accompanied by decreased Cbp expression and elevated Lyn kinase activity in MPN cells. Mechanistic studies revealed that Cbp was downregulated in MPNs, negatively correlated with enhanced Lyn kinase activity and increased MDSC numbers, and low Cbp expression was an independent prognostic factor for poor outcomes. This downregulation was associated with abnormal CpG island methylation in the promoter region. Functionally, Cbp specifically bound and inhibited Lyn kinase activity, thereby negatively regulating PI3K/Akt and JAK/STAT5 signaling pathways to promote apoptosis. With prolonged Cbp expression, its function was primarily localized to the nucleus. Further exploration identified methyltransferase METTL14 as the most significantly altered gene in the Cbp differential expression profile. METTL14 was highly expressed in MPNs, negatively correlated with Cbp levels, positively correlated with MDSC numbers, and high expression also indicated poor prognosis. GO and GSEA analyses suggested that the Cbp/Lyn/METTL14 axis may influence MDSC-mediated immunosuppression by regulating downstream m6A immune pathway-related target genes. Preliminary clinical data showed that myelofibrosis patients with high MDSCs responded poorly to treatment.

**Copyright Information:** (Online) ISSN: 2572-9241
©2026 The Author(s). HemaSphere is published by John Wiley & Sons Ltd on behalf of European Hematology Association. This is an open access Abstract Book distributed under the Attribution-NonCommercial-NoDerivs (CC BY-NC-ND), which allows third parties to download the articles and share them with others as long as they credit the author and the Abstract Book, but they cannot change the content in any way or use them commercially.
**Abstract Book Citations:** Authors, Title, HemaSphere, 2026;10:(S1):pages. The abstract book DOIs can be found on the "Author Information" page.
**Disclaimer:** Articles published in the journal HemaSphere exclusively reflect the opinions of the authors. The authors are responsible for all content in their abstracts including accuracy of the facts, statements, citing resources, etc.

**Summary/Conclusion**

In summary, this study elucidates a novel mechanism whereby a signaling axis composed of Cbp deficiency, Lyn kinase activation, and METTL14 upregulation drives MDSC expansion and shapes an immunosuppressive microenvironment through epigenetic modifications and key signaling pathways, thereby promoting MPN development and progression, providing potential strategies for targeted therapy.his research was supported by the National Natural Science Foundation of China (No. 82570191) and the Zhejiang Provincial Health Highlevel Innovative Talent Project (2022-2026).* Correspondence to:Jian Huang, email: househuang@zju.edu.cn

**Copyright Information:** (Online) ISSN: 2572-9241
©2026 The Author(s). HemaSphere is published by John Wiley & Sons Ltd on behalf of European Hematology Association. This is an open access Abstract Book distributed under the Attribution-NonCommercial-NoDerivs (CC BY-NC-ND), which allows third parties to download the articles and share them with others as long as they credit the author and the Abstract Book, but they cannot change the content in any way or use them commercially.
**Abstract Book Citations:** Authors, Title, HemaSphere, 2026;10:(S1):pages. The abstract book DOIs can be found on the "Author Information" page.
**Disclaimer:** Articles published in the journal HemaSphere exclusively reflect the opinions of the authors. The authors are responsible for all content in their abstracts including accuracy of the facts, statements, citing resources, etc.

### (PF872) INCB160058 SELECTIVELY TARGETS JAK2V617F-DRIVEN HEMATOPOIESIS IN DIVERSE AND DRUG-RESISTANT MODELS OF MYELOPROLIFERATIVE NEOPLASMS (MPNS)

**Topic:** 15. Myeloproliferative neoplasms - Biology & translational research

Hamza Celik*[1], Gaurang Trivedi[1], Angelo B. A. Laranjeira[1], Brittney Wass[1], Eddy Yue[1], Yanran Ai[1], Caroline Marty[2, 3], Elodie Rosa[2, 3], Maxime Evrard[2, 3], William Vainchenker[2, 3], Florence Pasquier[2, 3, 4], Cynthia Timmers[1], Sunkyu Kim[1], Ricardo MacArron[1], Isabelle Plo[2, 3], Matthew Stubbs[1]

*[1]Incyte Corporation, Wilmington, DE, United States of America;[2]INSERM U1287, Gustave Roussy, Villejuif, France;[3]Université Paris-Saclay, Gustave Roussy, Villejuif, France; [4]Clinical Hematology Department, Gustave Roussy, Villejuif, France;*

### Background

INCB160058 is a first-in-class, orally bioavailable, Janus kinase 2 (JAK2) V617F mutant-selective inhibitor, studied in phase 1 clinical trials (eg NCT06313593) in patients (pts) with MPNs harboring a *JAK2*V617F mutation. INCB160058 uniquely binds the JAK2 pseudokinase domain (JH2), resulting in a novel mechanism of action. INCB160058 selectively inhibits ligand-independent oncogenic JAK2V617F homodimers associated with class I cytokine receptors (eg thrombopoietin receptor), while preserving physiological, cytokine-dependent signaling. This unique mode of action leads to inhibition of pathogenic JAK2/STAT5 signaling and proliferation in JAK2V617F$^{+}$ cells with minimal impact on wild-type counterparts (Stubbs. *Blood* 2023;142[Suppl 1]:860).

### Aims

To evaluate the activity of INCB160058 using in vitro and ex vivo cellular models, including JAK2V617F$^{+}$ cells harboring secondary mutations or having previously been treated with MPN-directed therapy.

### Methods

We evaluated the effect of INCB160058 on the clonogenic potential of JAK2V617F$^{+}$ CD34$^{+}$ hematopoietic stem and progenitor cells using colony-forming unit (CFU) assays and assessed *JAK2*V617F-dependent erythroid and megakaryocyte differentiation potential through liquid culture assays. We also assessed the in vitro efficacy of INCB160058 on cells exhibiting persistence to ruxolitinib, established by exposing *JAK2*V617F-dependent SET2 cells to increasing drug concentrations, resulting in a population capable of proliferating despite high-dose ruxolitinib treatment.

**Copyright Information:** (Online) ISSN: 2572-9241
©2026 The Author(s). HemaSphere is published by John Wiley & Sons Ltd on behalf of European Hematology Association. This is an open access Abstract Book distributed under the Attribution-NonCommercial-NoDerivs (CC BY-NC-ND), which allows third parties to download the articles and share them with others as long as they credit the author and the Abstract Book, but they cannot change the content in any way or use them commercially.
**Abstract Book Citations:** Authors, Title, HemaSphere, 2026;10:(S1):pages. The abstract book DOIs can be found on the “Author Information” page.
**Disclaimer:** Articles published in the journal HemaSphere exclusively reflect the opinions of the authors. The authors are responsible for all content in their abstracts including accuracy of the facts, statements, citing resources, etc.

**Results**

In CFU assays, INCB160058 inhibited colony formation by JAK2V617F$^+$ CD34$^+$ cells, irrespective of additional mutations, including high-risk variants (eg *ASXL1*, *SF3B1)*, with minimal impact on normal CD34$^+$ cells. These findings show that INCB160058 effectively inhibited aberrant proliferation of MPN CD34$^+$ cells, irrespective of co-mutations, highlighting that oncogenic growth remains primarily dependent on the *JAK2*V617F mutation. In liquid culture assays, INCB160058 selectively impaired cytokine-independent *JAK2*V617F-driven erythroid and megakaryocytic maturation of CD34$^+$ cells from JAK2V617F$^+$ polycythemia vera, essential thrombocythemia, and myelofibrosis pt samples, while sparing erythroid and megakaryocyte maturation of CD34$^+$ cells from healthy donors. Dose-dependent INCB160058 treatment reduced oncogenic signaling pathways (eg, pSTAT5) and JAK2/STAT5 target gene expression (eg, *PIM1)* in JAK2V617F$^+$ CD34$^+$ cells, with minimal effects on normal CD34$^+$ cells. INCB160058 treatment inhibited STAT5 phosphorylation and suppressed proliferation to a similar extent in ruxolitinib-persistent and inhibitor-naive JAK2V617F-dependent SET2 cells, suggesting potential benefit in ruxolitinib-resistant or refractory MPNs. Currently, approved JAK2 inhibitors for MPNs induce paradoxical hyperphosphorylation of the JAK2 activation loop (Y1007/1008) associated with withdrawal syndrome; in contrast, INCB160058 reduced activation loop phosphorylation, consistent with its binding to the JH2 domain of JAK2V617F, supporting the potential of INCB160058 to overcome ruxolitinib resistance and mitigate withdrawal-related signaling effects in clinical settings.

**Summary/Conclusion**

Our findings demonstrate that INCB160058 selectively targets *JAK2*V617F-mediated pathways and inhibits constitutive differentiation of CD34$^+$ cells driven by the mutation, while preserving cytokine-dependent signaling for normal hematopoietic differentiation across numerous clinically relevant in vitro and ex vivo MPN models.

**Copyright Information:** (Online) ISSN: 2572-9241
©2026 The Author(s). HemaSphere is published by John Wiley & Sons Ltd on behalf of European Hematology Association. This is an open access Abstract Book distributed under the Attribution-NonCommercial-NoDerivs (CC BY-NC-ND), which allows third parties to download the articles and share them with others as long as they credit the author and the Abstract Book, but they cannot change the content in any way or use them commercially.
**Abstract Book Citations:** Authors, Title, HemaSphere, 2026;10:(S1):pages. The abstract book DOIs can be found on the "Author Information" page.
**Disclaimer:** Articles published in the journal HemaSphere exclusively reflect the opinions of the authors. The authors are responsible for all content in their abstracts including accuracy of the facts, statements, citing resources, etc.

### (PF873) DISCOVERY AND CHARACTERIZATION OF DMR-001, AN FC-NULL, HALF-LIFE OPTIMIZED MONOCLONAL ANTIBODY SELECTIVELY TARGETING MUTANT CALRETICULIN

**Topic:** 15. Myeloproliferative neoplasms - Biology & translational research

Byron H. Kwan[1], Shannon E. Elf*[2], Kevin J. Kramer[1], Mohammad Murshid Alam[1], Ines Castro[1], Ninad Varkhede[1], Ghassan N. Fayad[1], Michael Meehl[1], Jason Oh[1], Hussam Shaheen[1]

*[1]Paragon Therapeutics, Waltham, United States of America; [2]Huntsman Cancer Institute, Internal Medicine, Salt Lake City, United States of America;*

### Background

Essential thrombocythemia (ET) and myelofibrosis (MF) are myeloproliferative neoplasms (MPNs) driven by frameshift mutations in calreticulin (CALR), which are found in approximately 25% and 35% of cases, respectively. These mutations produce a neomorphic mutant C-terminus that permits binding to and causes constitutive activation of the thrombopoietin receptor (TpoR), which drive a range of disease symptoms from increased risk of thrombosis to severe, life-threatening bone marrow fibrosis. Until the development of monoclonal antibodies (mAbs) selectively targeting mutant CALR (mutCALR), disease modifying treatments were not available. Here we describe the discovery and preclinical activity of a novel, selective, anti-mutCALR mAb with high affinity and anti-proliferative potency against both Type 1 and Type 2 mutations.

### Aims

To engineer and characterize an Fc-null, half-life extended mutCALR-targeted mAb with activity against both major mutCALR variants and drug-like properties enabling convenient clinical dosing.

### Methods

Antibody discovery campaigns utilized engineered phage and yeast display libraries to identify clones specific to the mutCALR epitope. Target specificity was increased through iterative rounds of negative selection against wild-type (wt) CALR and positive selection against Type 1 and Type 2 mutCALR antigens. Select antibody candidates were further optimized using *in silico* engineering, which included Fc mutations to prolong half-life. Resulting clones were next evaluated for binding affinity to mutCALR by surface plasmon resonance (SPR) and for selectivity towards mutCALR over wtCALR using ELISA and cell-binding assays. *In vitro* functional profiling further informed clinical candidate selection, focusing on the ability to reduce proliferation and attenuate STAT3/5 phosphorylation in Ba/F3 cells co-expressing TpoR and mutant CALR. Pharmacokinetic properties were assessed in non-human primates (NHPs). To facilitate comparison, a reference mAb was generated based on publicly disclosed structures and evaluated in parallel.

### Results

The development candidate (DMR-001) has high affinity for both Type 1 and Type 2 mutCALR ($K_D$ = 0.074 nM and 0.31 nM, respectively). Enhanced affinity compared to the reference mAb translated into increased cell-surface binding and improvements in functional potency. DMR-001 achieved up to approximately 3-fold greater inhibition of Type 1 mutCALR–dependent proliferation and ≥10-fold greater inhibition of Type 2 mutCALR–dependent proliferation relative to the reference mAb. Additionally, suppression of mutCALR/TpoR-driven STAT3 and STAT5 phosphorylation was observed in both Type 1 and Type 2 mutCALR reporter assays. DMR-001 showed no detectable binding to wtCALR in cell-based or biochemical binding assays. Fc engineering resulted in a markedly prolonged terminal half-life in NHPs relative to the reference mAb ($t_{1/2}$ ≈ 15 d vs. 3 d).

**Copyright Information:** (Online) ISSN: 2572-9241
©2026 The Author(s). HemaSphere is published by John Wiley & Sons Ltd on behalf of European Hematology Association. This is an open access Abstract Book distributed under the Attribution-NonCommercial-NoDerivs (CC BY-NC-ND), which allows third parties to download the articles and share them with others as long as they credit the author and the Abstract Book, but they cannot change the content in any way or use them commercially.
**Abstract Book Citations:** Authors, Title, HemaSphere, 2026;10:(S1):pages. The abstract book DOIs can be found on the “Author Information” page.
**Disclaimer:** Articles published in the journal HemaSphere exclusively reflect the opinions of the authors. The authors are responsible for all content in their abstracts including accuracy of the facts, statements, citing resources, etc.

**Summary/Conclusion**

DMR-001 is a potent, selective mutCALR-targeted mAb with activity across both major CALR mutation subtypes and demonstrates the potential to treat patients with MPNs using infrequent subcutaneous dosing. These data support the initiation of clinical trials with DMR-001 as a differentiated therapeutic candidate for patients with ET and MF.

**Copyright Information:** (Online) ISSN: 2572-9241
©2026 The Author(s). HemaSphere is published by John Wiley & Sons Ltd on behalf of European Hematology Association. This is an open access Abstract Book distributed under the Attribution-NonCommercial-NoDerivs (CC BY-NC-ND), which allows third parties to download the articles and share them with others as long as they credit the author and the Abstract Book, but they cannot change the content in any way or use them commercially.
**Abstract Book Citations:** Authors, Title, HemaSphere, 2026;10:(S1):pages. The abstract book DOIs can be found on the “Author Information” page.
**Disclaimer:** Articles published in the journal HemaSphere exclusively reflect the opinions of the authors. The authors are responsible for all content in their abstracts including accuracy of the facts, statements, citing resources, etc.

### (PF874) SINGLE-CELL TRANSCRIPTOMICS REVEALS SHARED DISEASE-SPECIFIC HEMATOPOIETIC STEM CELL PROGRAMS IN JMML AND RALD AND JMML-SPECIFIC ZEB1 HYPERACTIVATION

**Topic:** 15. Myeloproliferative neoplasms - Biology & translational research

Alexandr Ikonnikov*[1], Egor Volchkov[2, 3, 4], Medina Gurzhihanova[3], Vlada Tuliavko[2], Victor Matveev[3], Dmitry Evseev[5], Elena Osipova[3], Alexandra Semchenkova[3], Evgeny Denisov[2, 6], Michail Maschan[3]

*[1]Peoples' Friendship University of Russia (RUDN University),, Research Institute of Molecular and Cellular Medicine, Moscow, Russia; [2]Research Institute Of Molecular And Cellular Medicine Peoples' Friendship University Of Russia (RUDN University), Moscow, Russia; [3]Dmitry Rogachev National Medical Research Center of Pediatric Hematology, Oncology and Immunology, Moscow, Russia; [4]Botkin Hospital, Moscow Healthcare Department, Moscow, Russia; [5]Johannes Kepler University Linz, Departament of Pediatrics and Adolescent Medicine, Linz, Austria; [6]Tomsk National Research Medical Center, Moscow, Russia;*

### Background

Juvenile myelomonocytic leukemia (JMML) and autoimmune RAS-associated leukoproliferative disorder (RALD) are rare hematologic diseases characterized by overlapping clinical features driven by mutations in the RAS signaling pathway. JMML is a malignant clonal disorder with high risk of progression, while RALD is a non-malignant disorder associated with immune dysregulation. The absence of definitive molecular markers complicates accurate diagnosis and treatment.

### Aims

We aimed to characterize the transcriptional and metabolic profiles of JMML and RALD at single-cell resolution, identify disease-specific regulatory networks, and discover potential diagnostic biomarkers that distinguish JMML from healthy hematopoiesis.

### Methods

Bone marrow cells were obtained from eight JMML patients carrying mutations in PTPN11, NF1, NRAS, KRAS, and CBL and one patient with RALD. Healthy bone marrow scRNA-seq datasets from seven donors were used as controls. After quality filtering, 59,651 cells were included in the analysis. Single-cell libraries were prepared using the 10x Genomics Chromium platform and Single Cell 3′ Reagent Kit, with sequencing on Genolab M (GeneMind, China). Bioinformatic analysis included preprocessing, cell-type annotation, and integration using Scanpy v1.9.5, batch-effect correction with Harmony, metabolic pathway analysis with GSEApy and DecoupleR, and regulatory network inference using CellOracle.

### Results

Analysis revealed elevated Ras-MAPK signaling in hematopoietic stem cells (HSCs) and megakaryocyte-erythroid progenitors in JMML and RALD, alongside enhanced myelomonocytic proliferation. In an aggressive JMML case with a PTPN11 mutation, HSCs, early progenitors, and erythroid lineage cells were markedly expanded. Granulocyte-monocyte progenitors showed increased GTPase activity, histone deacetylation, and decreased mRNA splicing pathway activity (FDR $< 0.05$). Principal component analysis showed strong similarity between JMML and RALD HSCs (Pearson correlation = 0.88) and negative correlation with healthy controls (Pearson correlation = −0.67 and −0.65). Regulatory network analysis revealed significant differences between JMML and normal bone marrow. The transcription factor ZEB1 emerged as a central regulatory hub with increased connectivity to hematopoietic differentiation and survival-associated genes, consistent with acquisition of aberrant stem cell persistence programs. Notably, the long non-coding RNA AC016735.1 was selectively expressed in JMML HSCs and absent in healthy controls, identifying a candidate disease-specific biomarker.

**Copyright Information:** (Online) ISSN: 2572-9241
©2026 The Author(s). HemaSphere is published by John Wiley & Sons Ltd on behalf of European Hematology Association. This is an open access Abstract Book distributed under the Attribution-NonCommercial-NoDerivs (CC BY-NC-ND), which allows third parties to download the articles and share them with others as long as they credit the author and the Abstract Book, but they cannot change the content in any way or use them commercially.
**Abstract Book Citations:** Authors, Title, HemaSphere, 2026;10:(S1):pages. The abstract book DOIs can be found on the "Author Information" page.
**Disclaimer:** Articles published in the journal HemaSphere exclusively reflect the opinions of the authors. The authors are responsible for all content in their abstracts including accuracy of the facts, statements, citing resources, etc.

**Summary/Conclusion**

Our findings raise the possibility that JMML and RALD may arise from a shared abnormal RAS-driven hematopoietic stem cell state. Rather than being completely distinct diseases, they may represent biologically related conditions along a spectrum of disrupted hematopoiesis. In JMML, malignant progression may be linked to stabilization of regulatory networks and activation of stem cell persistence programs, including ZEB1-centered gene regulatory network rewiring and disease-specific lncRNA expression. These findings may contribute to the development of improved diagnostic approaches and potential therapeutic strategies for pediatric RAS-associated disorders.

**Copyright Information:** (Online) ISSN: 2572-9241
©2026 The Author(s). HemaSphere is published by John Wiley & Sons Ltd on behalf of European Hematology Association. This is an open access Abstract Book distributed under the Attribution-NonCommercial-NoDerivs (CC BY-NC-ND), which allows third parties to download the articles and share them with others as long as they credit the author and the Abstract Book, but they cannot change the content in any way or use them commercially.
**Abstract Book Citations:** Authors, Title, HemaSphere, 2026;10:(S1):pages. The abstract book DOIs can be found on the "Author Information" page.
**Disclaimer:** Articles published in the journal HemaSphere exclusively reflect the opinions of the authors. The authors are responsible for all content in their abstracts including accuracy of the facts, statements, citing resources, etc.

### (PF875) MEGACALR IHC: AI-DRIVEN QUANTIFICATION OF MUTANT CALR EXPRESSION IN MEGAKARYOCYTES

**Topic:** 15. Myeloproliferative neoplasms - Biology & translational research

Thomas Fletcher*[1], Anna Kotanska[1], Xuezi Hu[1], Sam Maxwell[1], Len Brandes[1], Ruoyu Wang[1], Sanam Loghavi[2], David Ross[3], Fatima Zahra Jelloul[2], Ken Young[4], Florian Heidel[5], Jens Rittscher[1, 6], Alan Aberdeen[1], Sharon Ruane[1], Daniel Royston[7], Korsuk Sirinukunwattana[1]

*[1]Ground Truth Labs, Oxford, United Kingdom; [2]The University of Texas MD Anderson Cancer Center, Department of Hematopathology, Houston, United States of America; [3]Royal Adelaide Hospital and SA Pathology, Department of Hematology, Adelaide, Australia; [4]Duke University Medical Center, Durham, United States of America; [5]Hannover Medical School, Hematology, Hemostasis, Oncology and Stem Cell Transplantation, Hannover, Germany; [6]University of Oxford, Department of Engineering Science, Oxford, United Kingdom; [7]University of Oxford, Radcliffe Department of Medicine, Oxford, United Kingdom;*

### Background

Calreticulin (CALR) mutations are the second most common driver in myeloproliferative neoplasms (MPNs). With recent development of CALR-targeted therapies, scalable and quantitative tools are needed to assess treatment response. Current assessment of mutant CALR using immunohistochemistry (IHC) relies on binary slide-level grading, which provides no information on mutant megakaryocyte (Mk) quantity or expression levels, while exhaustive manual annotation of all Mks in a sample is impractical. We developed MegaCALR IHC, a deep learning pipeline that detects Mks in whole-slide images (WSIs) of mutant CALR IHC-stained bone marrow and identifies mutant CALR expression.

### Aims

Development and preliminary validation of the MegaCALR IHC pipeline:
(1) Segmentation and classification accuracy against a multi-pathologist consensus,
(2) Robustness to variation in cell size, stain intensity, and diagnosis,
(3) Inter-scanner variation for both Mk detection and classification.

### Methods

The MegaCALR pipeline consists of two AI models. Mks are first segmented and then classified as one of three IHC-classes: CALR '-Negative', '-Low Positive', or '-High Positive'. The pipeline was trained on WSIs of 133 mutant CALR-IHC-stained samples. Validation was performed on a held-out set of 23 WSIs, from which subsets were drawn for different analyses: segmentation accuracy (n=10), classification accuracy (n=10), inter-scanner precision (n=10 pairs, scanners: Hamamatsu, Philips). Diagnoses represented were ET, pre-PMF, PMF, Normal. Model evaluation was performed on the consensus labels of five board-certified hematopathologists. Segmentation consensus was derived from 20 FoVs (2 per WSI, 512 × 512 $\mu m^2$) exhaustively annotated. Classification consensus was derived from pathologist class labels on 492 candidate Mks from the same WSIs.

**Copyright Information:** (Online) ISSN: 2572-9241
©2026 The Author(s). HemaSphere is published by John Wiley & Sons Ltd on behalf of European Hematology Association. This is an open access Abstract Book distributed under the Attribution-NonCommercial-NoDerivs (CC BY-NC-ND), which allows third parties to download the articles and share them with others as long as they credit the author and the Abstract Book, but they cannot change the content in any way or use them commercially.
**Abstract Book Citations:** Authors, Title, HemaSphere, 2026;10:(S1):pages. The abstract book DOIs can be found on the "Author Information" page.
**Disclaimer:** Articles published in the journal HemaSphere exclusively reflect the opinions of the authors. The authors are responsible for all content in their abstracts including accuracy of the facts, statements, citing resources, etc.

### Results

Segmentation: MegaCALR detection F1 was 0.78 (recall: 0.81, precision: 0.75) and mean per-cell IoU was within the range of pathologists (pipeline: 0.80 vs. pathologists 0.61-0.84 range). Detection fell within the range of individual pathologist-vs-consensus agreement (pipeline: 0.78 vs pathologists: 95%CI=0.59-0.90). F1 was consistently ≥0.76 across all stain-intensity bins and ≥0.86 for cell diameter bins > 20 µm (0.42–0.70 for smaller cells) and was 0.76-0.92 across diagnoses, comparable to results seen for pathologists.

Classification: Per-IHC-class F1 scores were 0.96 for 'Negative', 0.82 for 'Low Positive', and 0.83 for 'High Positive'. Quadratic Weighted Kappa (QWK) (pipeline vs consensus) was 0.89, within the range of pathologist QWK (95%CI = 0.88-0.96). Prediction and consensus distributions were consistent across diagnosis groups (Fisher p > 0.06) and bins of cell diameters (Fisher p > 0.4).

Inter-scanner consistency: Mk count Intraclass Correlation Coefficient (ICC) was 0.99 (95% CI: 0.95–1.00). Per-IHC-class proportion ICCs were 'Negative': 0.99, 'Low Positive': 0.94, and 'High Positive': 0.96. Bland-Altman analysis showed no systematic bias in either detected Mk counts (bias 6.2%, p = 0.084) or IHC class proportions (bias within 1.0-3.1%, p>0.76) between scanners.

### Summary/Conclusion

Performance of both Mk segmentation and CALR expression classification was within the range of individual pathologists, with robust performance across cell size, stain intensity, diagnostic subtype, and scanner. MegaCALR IHC enables scalable, quantitative assessment of mutant CALR expression in clinical trials and translational research.

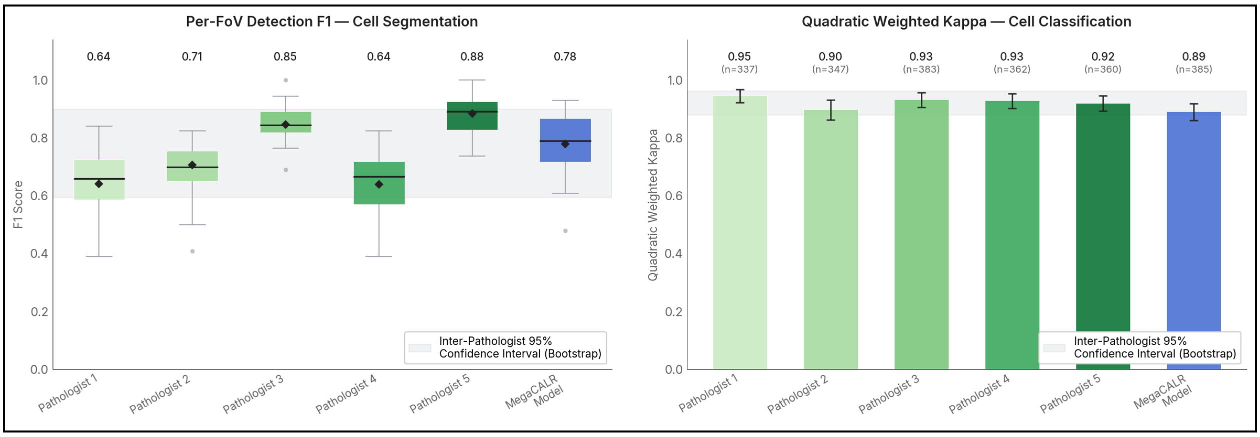

Figure 1: MegaCALR pipeline segmentation and classification accuracy versus five board-certified hematopathologists. Detection F1 (left; 20 FoVs, 10 WSIs) and three-class quadratic weighted kappa (right; up to n = 385 Mks) both fell within the inter-pathologist 95% bootstrap confidence intervals (grey bands), with pipeline F1 = 0.78 (pathologist 95% CI: 0.59–0.90) and QWK = 0.89 (pathologist 95% CI: 0.88–0.96). All metrics are computed against the multi-pathologist consensus reference standard.

**Copyright Information:** (Online) ISSN: 2572-9241
©2026 The Author(s). HemaSphere is published by John Wiley & Sons Ltd on behalf of European Hematology Association. This is an open access Abstract Book distributed under the Attribution-NonCommercial-NoDerivs (CC BY-NC-ND), which allows third parties to download the articles and share them with others as long as they credit the author and the Abstract Book, but they cannot change the content in any way or use them commercially.
**Abstract Book Citations:** Authors, Title, HemaSphere, 2026;10:(S1):pages. The abstract book DOIs can be found on the "Author Information" page.
**Disclaimer:** Articles published in the journal HemaSphere exclusively reflect the opinions of the authors. The authors are responsible for all content in their abstracts including accuracy of the facts, statements, citing resources, etc.

### (PF876) DISTINCT CYTOKINE EXPRESSION SIGNATURES IN ADOLESCENT AND YOUNG ADULTS (AYA) AND NON-AYA PATIENTS WITH BCR::ABL NEGATIVE MYELOPROLIFERATIVE NEOPLASM (MPN)

**Topic:** 15. Myeloproliferative neoplasms - Biology & translational research

Alice Diepers*[1], Danny Misiak[1], Susann Schulze[1], Christina Zahn[1], Nadja Jäkel[1], Dietrich Kämpfe[2], Regina Moeller[3], Maik Schwarz[4], Haifa Kathrin Al-Ali[1]

*[1]Martin Luther University Halle-Wittenberg, Germany, Halle (Saale), Germany; [2]Praxis Rathausplatz, Luedenscheid, Germany; [3]Haematologisch-onkologischen Gemeinschaftspraxis, Halle (Saale), Halle (Saale), Germany; [4]MVZ Adorf - Schwerpunktpraxis für Haematologie und Onkologie, Adorf, Germany;*

### Background

Inflammation is a hallmark of MPN and can be modulated by therapeutics such as Interferon (IFN) or JAK inhibitors. The inflammatory milieu caused by the malignant clone is compounded by age and studies indicate that MPN patients (pts) show signs of accelerated immune aging. It is known that pts aged 15-39 years (y) (AYA) exhibit clinical and genetic differences compared to older pts. Little is known about the impact of age at diagnosis and duration of disease on the level of inflammation.

### Aims

Characterize expression (exp) signatures of 50 cytokines in pts with MPN based on age at diagnosis within the multicenter MARY project of the East German Study Group for Hematology and Oncology.

### Methods

Phenotype and genetic data were collected from 122 consecutive adult pts [45 AYA/77 non-AYA, (60% female)] after ethical approval and signed informed consent. MARY was supported by a fund from Incyte Bioscience International Sàrl, Morges, Switzerland. Per protocol, stratification to AYA or non-AYA was based on age at diagnosis and not age at enrolment. Whole transcriptome sequencing from blood derived RNA was done on an Illumina NovaSeq 6000 resulting in 150 bp strand-specific paired-end reads. Raw reads were quality checked, aligned and annotated to the human genome (HG38). After batch-correction using ComBat_Seq, reads were normalized utilizing trimmed mean of M-values (TMM) normalization resulting in normalized fragments per kilobase of transcript per million fragments mapped (FPKM). The FPKM values were then log2-transformed.

**Copyright Information:** (Online) ISSN: 2572-9241
©2026 The Author(s). HemaSphere is published by John Wiley & Sons Ltd on behalf of European Hematology Association. This is an open access Abstract Book distributed under the Attribution-NonCommercial-NoDerivs (CC BY-NC-ND), which allows third parties to download the articles and share them with others as long as they credit the author and the Abstract Book, but they cannot change the content in any way or use them commercially.
**Abstract Book Citations:** Authors, Title, HemaSphere, 2026;10:(S1):pages. The abstract book DOIs can be found on the “Author Information” page.
**Disclaimer:** Articles published in the journal HemaSphere exclusively reflect the opinions of the authors. The authors are responsible for all content in their abstracts including accuracy of the facts, statements, citing resources, etc.

### Results

Median age at diagnosis of AYA and non-AYA was 33 and 52y respectively. Within the AYA cohort, 15 pts were >40y at enrolment (OldAYA) with a median MPN duration of 143 versus 14 months for the remaining AYA pts (p<0.001). Only one progression to post-ET-MF occurred in OldAYA pts. ET (p<0.001), *CALR* mutations and a triple negative genotype (*p*=0.004) were more common in the entire AYA cohort, while *JAK2*V617F and a higher allele burden were associated with older age (*p*<0.001). Irrespective of MPN type, driver mutations, or therapy, AYA including OldAYA exhibited a predominant pro-inflammatory (Th1) cytokine exp profile [IFN-gamma, IL-2, IL-24 (an IL-10 family cytokine regulating inflammation), and Interleukin-1 receptor antagonist (an anti-inflammatory competitive inhibitor of IL-1alpha and IL-1beta)] while in non-AYA, immunosuppressive (Th2) cytokine exp signatures were leading [IFN-beta, TGF-beta (a primary driver of fibrosis), IL-4 (*p*<0.001), IL-19 (an IL-10 family cytokine promoting Th2 response), IL-33 (an IL-1 family cytokine driving Th2 response), and IL-37 (a master suppressor of Th1 and Th2 responses)] (figure). In AYA, IL-6 exp was the dominant Th2 response (p=0.05). Although TNF-alpha exp was similar across cohorts, TNFR1, the receptor responsible for most of the TNF inflammatory effects, was highly exp in non-AYA. Exp of cellular senescence markers as INHBA (a critical marker for tumor-associated fibroblasts) and GDF15 (a proxy for epigenetic aging) was significantly higher in non-AYA (p=0.004).

### Summary/Conclusion

Inflammation in young MPN pts is characterized by a Th1-dominant response that involves IFN-gamma, persists over years, and promotes anti-tumor immunity while the Th2 bias of elderly pts fosters an immunosuppressive environment through cytokines like IL-4 and is linked to progression. To maximize therapeutic effects of a targeted modification of the immune system, defining an MPN Th1/Th2 ratio based on age at diagnosis is crucial.

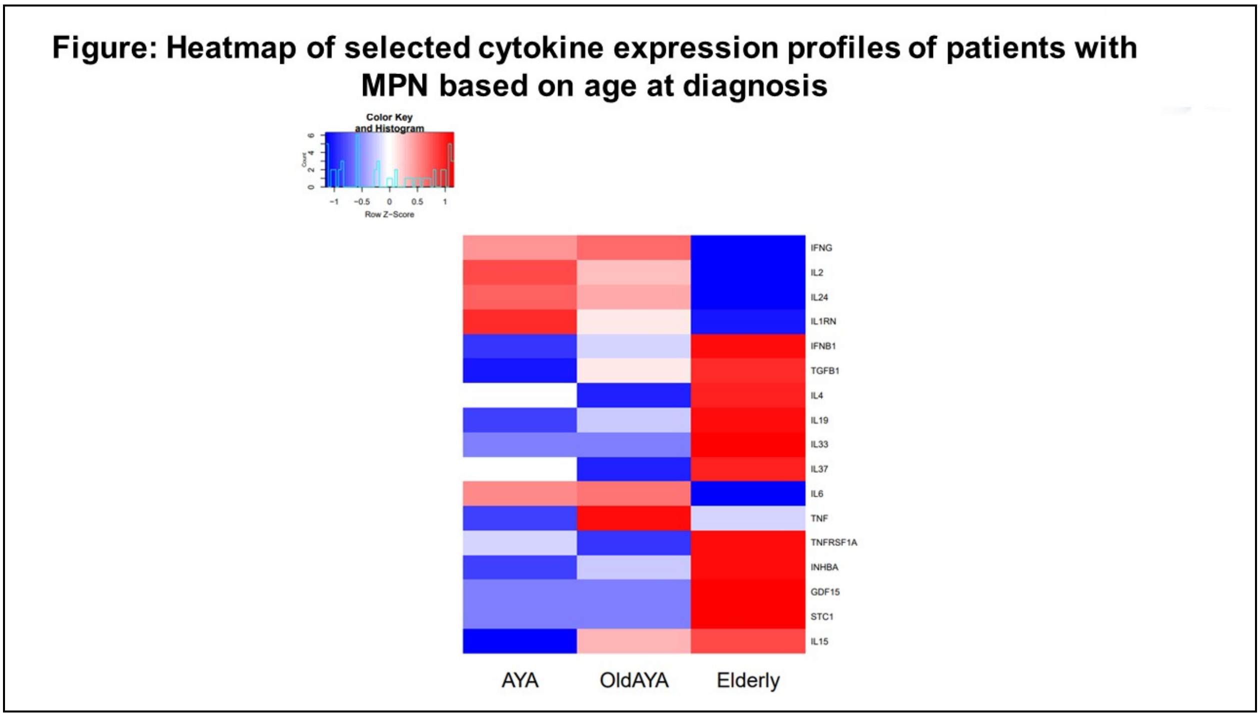

Cytokine expression profiles in AYA and Non-AYA patients with MPN

Copyright Information: (Online) ISSN: 2572-9241
©2026 The Author(s). HemaSphere is published by John Wiley & Sons Ltd on behalf of European Hematology Association. This is an open access Abstract Book distributed under the Attribution-NonCommercial-NoDerivs (CC BY-NC-ND), which allows third parties to download the articles and share them with others as long as they credit the author and the Abstract Book, but they cannot change the content in any way or use them commercially.
Abstract Book Citations: Authors, Title, HemaSphere, 2026;10:(S1):pages. The abstract book DOIs can be found on the “Author Information” page.
Disclaimer: Articles published in the journal HemaSphere exclusively reflect the opinions of the authors. The authors are responsible for all content in their abstracts including accuracy of the facts, statements, citing resources, etc.

### (PF877) JAK2 VAF IN POLYCYTHEMIA VERA PATIENTS TREATED WITH ROPEGINTERFERON AND RUXOLITINIB IN A REAL LIFE COHORT: RESULTS FROM A SINGLE CENTER RETROSPECTIVE STUDY.

**Topic:** 15. Myeloproliferative neoplasms - Biology & translational research

Giulia Berutto[1], Andrea Cavallaro[2], Bianca Patarnello[1], Scaldaferri Matilde[3], Paola Francia di Celle[4], Ludovica Riera[4], Lorenzo Celona[1], Eleonora Boscaro[1], Chiara Frairia[1], Marco Cerrano[1], Elisa Santambrogio[1], Giuseppe Lanzarone[2], Stefano D'ardia[1], Ernesta Audisio[1], Giulia Benevolo[2], Eloise Beggiato[2], Roberto Freilone[1], Valentina Giai*[1]

*[1]Division of Hematology, Città della Salute e della Scienza, Torino, Italy;[2]Hematology, University of Torino, Città della Salute e della Scienza, Torino, Italy; [3]Pharmacy, Città della Salute e della Scienza, Torino, Italy;[4]Città della Salute e della Scienza, Division of Pathology, Torino, Italy;*

### Background

In the last decades, polycythemia vera (PV) patients showed an increase of clinical and prognostic benefits due to newest treatments as JAK2 inhibitors and interferons. It is known that the variant allele frequency (VAF) of JAK2 is associated with a more severe and symptomatic disease. Nowadays, scientific data agree that some drugs, such as Ruxolitinib and Interferon, are able to reduce JAK2 VAF; currently, real life data on influence of different treatments on JAK2 VAF and its response implications are missing and real clinical benefit of JAK2 VAF reduction is yet to be demonstrated.

### Aims

The aim of the study is to evaluate through real world data JAK2 VAF in PV patients and its correlation with haematological responses and treatment.

### Methods

A single-center retrospective real life study was conducted at “Città della salute e della Scienza” in Turin, Italy. Clinical responses were evaluated according to Barosi G et al, Blood 2013. The V617F mutation quantification was performed using the Ipsogen JAK2 MutaQuant kit. Statistic tests were run through GraphPad Prism Software.

**Copyright Information:** (Online) ISSN: 2572-9241
©2026 The Author(s). HemaSphere is published by John Wiley & Sons Ltd on behalf of European Hematology Association. This is an open access Abstract Book distributed under the Attribution-NonCommercial-NoDerivs (CC BY-NC-ND), which allows third parties to download the articles and share them with others as long as they credit the author and the Abstract Book, but they cannot change the content in any way or use them commercially.
**Abstract Book Citations:** Authors, Title, HemaSphere, 2026;10:(S1):pages. The abstract book DOIs can be found on the “Author Information” page.
**Disclaimer:** Articles published in the journal HemaSphere exclusively reflect the opinions of the authors. The authors are responsible for all content in their abstracts including accuracy of the facts, statements, citing resources, etc.

**Results**

Ninety-two patients were enrolled (56 males and 36 females) with a median age at diagnosis of 58 years (range 18-82). Nineteen patients (21%) were treated with Hydroxyurea (HU), 27 patients (29%) with Ropeginterferon (Ropeg), 40 patients (43%) with Ruxolitinib (Ruxo) and 3 patients with Ruxo and Ropeg (3%) (Table 1).
Median follow up was 10 years. The median treatment duration was 2 years for Ropeg and HU, and 3 years for Ruxo.
Complete remissions (CR) at 12, 24 and 36 months were 7/13 (54%), 18/31 (58%), 16/22 (73%) for Ruxo patients, 7/20 (35%), 7/13 (54%), 4/7 (57%) for Ropeg patients and 7/16 (44%), 4/12 (33%) and 5/10 (50%) for HU patients (Fig.1).
The quantification of JAK2 VAF was performed at diagnosis, pre and post therapy. At the diagnosis, mean JAK2 VAF was 45.4 (±22.9); a significant difference was spotted among pre and post therapy JAK2 VAF: 45.1 vs 33.2 (p: 0.023) (Fig.2). Median time between pre and post JAK2 measurements was 44 months and median time between therapy start and JAK2 test post treatment was 15 months. No significant differences in JAK2 VAF were detected between pre and post samples of Ropeg patients (38.8 vs 31.9, p: 0.3), with the median time from the two test of 40 months, and HU patients; a significant decrease of JAK2 VAF in pre and post treatment was displayed among Ruxo patients (64 vs 41.7, p: 0.034) with a median time between the 2 samples of 69.8 months (Fig.3). Then we looked at each patients JAK2 VAF post treatment and the respective hematological response: interestingly, in the Ropeg group, 3 out of 4 patients with No Response (NR) showed an increase of JAK2 VAF post treatment; in Ruxo group, patients with no reduction of JAK2 VAF, showed only a Partial Response, while those with a significant reduction of JAK2 VAF displayed a CR (Fig.4).
Median overall survival was not reached and the probability of survival of the population was 78% at 16 years (Fig.5). Myelofibrosis (MF) evolution was detected in 13/92 (14%) patients and the median time from the diagnosis to MF evolution was 12,7 years.

**Summary/Conclusion**

According to our single center retrospective study, we showed that therapies influence JAK2 VAF and hematologic response in PV patients. In this scenario, modeling JAK2 VAF could be the key to induce long term remissions and improve prognosis and quality of life of PV patients.

**Copyright Information:** (Online) ISSN: 2572-9241
©2026 The Author(s). HemaSphere is published by John Wiley & Sons Ltd on behalf of European Hematology Association. This is an open access Abstract Book distributed under the Attribution-NonCommercial-NoDerivs (CC BY-NC-ND), which allows third parties to download the articles and share them with others as long as they credit the author and the Abstract Book, but they cannot change the content in any way or use them commercially.
**Abstract Book Citations:** Authors, Title, HemaSphere, 2026;10:(S1):pages. The abstract book DOIs can be found on the "Author Information" page.
**Disclaimer:** Articles published in the journal HemaSphere exclusively reflect the opinions of the authors. The authors are responsible for all content in their abstracts including accuracy of the facts, statements, citing resources, etc.

*Table 1*

| | Median Age (range) | High risk at diagnosis | Therapy | Median Age at therapy start (range) | Median time of therapy in years (range) |
|---|---|---|---|---|---|
| All | 58 (18-82) | 49/92 (53%) | 92 | 67,5 (31-88) | 2 (<1-13) |
| Ropeginterferon | 51 (29-76) | 8/49 (16%) | 27 (29%) | 64 (31-84) | 2 (<1-13) |
| Ruxolitinib | 58,5 (18-82) | 23/49 (47%) | 40 (43%) | 70 (52-88) | 3 (1-10) |
| Hydroxyurea | 72 (48-80) | 18/49 (37%) | 19 (21%) | 72 (48-80) | 2 (<1-10) |
| 2 Therapies (Ropeg→Ruxo) | 58 (38-60) | 0 | 3 (3%) | 53 (42-60) | 8 (7-10) |
| No therapy | 50 (40-56) | 0 | 3 (3%) | | |

*Figure 1*

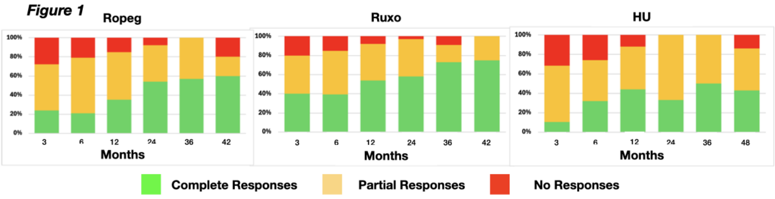

*Figure 2*

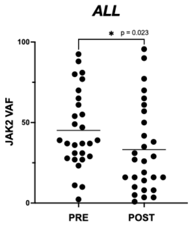

*Figure 3*

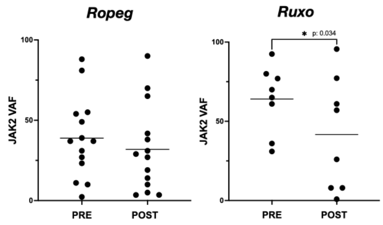

*Figure 4*

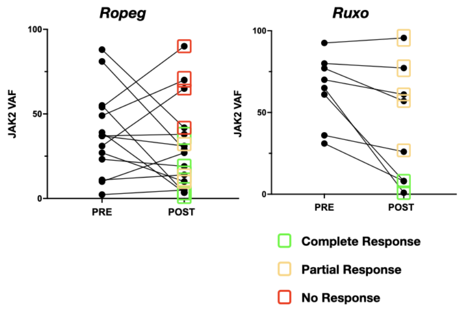

*Figure 5*

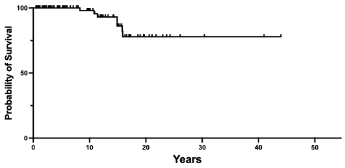

**Copyright Information:** (Online) ISSN: 2572-9241
©2026 The Author(s). HemaSphere is published by John Wiley & Sons Ltd on behalf of European Hematology Association. This is an open access Abstract Book distributed under the Attribution-NonCommercial-NoDerivs (CC BY-NC-ND), which allows third parties to download the articles and share them with others as long as they credit the author and the Abstract Book, but they cannot change the content in any way or use them commercially.
**Abstract Book Citations:** Authors, Title, HemaSphere, 2026;10:(S1):pages. The abstract book DOIs can be found on the "Author Information" page.
**Disclaimer:** Articles published in the journal HemaSphere exclusively reflect the opinions of the authors. The authors are responsible for all content in their abstracts including accuracy of the facts, statements, citing resources, etc.

### (PF878) DNA METHYLATION PROFILING IDENTIFIES MYC AND MAPK PATHWAY DEREGULATION IN TRIPLE NEGATIVE MDS/MPN WITH SF3B1 MUTATION AND THROMBOCYTOSIS

**Topic:** 15. Myeloproliferative neoplasms - Biology & translational research

Marion Divoux[1, 2, 3], Ghislain Fievet[1], Natacha Dreumont[1], Laurence Schenone[1], Ramia Safar[1], Maud D Aveni[3], Marc Muller[4], Eric Solary[5], Pierre Feugier[1, 3], François Girodon[6, 7], Sébastien Hergalant[1], Julien Broseus*[1, 2]

*[1]Université de Lorraine, Inserm, NGERE, Nancy, France;[2]CHRU-Nancy, Hematology Laboratory, Laboratory department, Vandoeuvre-lès-nancy, France; [3]CHRU-Nancy, Clinical Hematology Department, Vandoeuvre-lès-nancy, France;[4]CHRU-Nancy, Genetics Laboratory, Laboratory department, Vandoeuvre-lès-nancy, France; [5]Villejuif-Gustave Roussy, Inserm U1287, Villejuif, France; [6]CHU de Dijon, Dijon, France;[7]CHRU Dijon, Haematology Laboratory, Dijon, France;*

### Background

Myelodysplastic/myeloproliferative neoplasm with *SF3B1* mutation and thrombocytosis (MDS/MPN-*SF3B1*-T) is a clonal disorder of hematopoietic stem cells, displaying overlapping characteristics of both myelodysplastic neoplasms with *SF3B1* mutation (MDS-*SF3B1*) and essential thrombocythemia (ET). While a somatic mutation in *JAK2, CALR or MPL* accounts for thrombocytosis in about 50% of patients with MDS/MPN-*SF3B1*-T (1), megakaryocyte proliferation remains unexplained in so-called "triple-negative" MDS/MPN-*SF3B*1-T cases.

### Aims

To investigate whether epigenetic deregulation might contribute to thrombocytosis in "triple-negative" cases.

### Methods

We conducted a comparative analysis of DNA methylation using reduced representation bisulfite sequencing (RRBS) in peripheral blood or bone marrow cells collected from 9 patients with triple-negative MDS/MPN-*SF3B1*-T. The same analysis was performed simultaneously in peripheral blood or bone marrow cells collected from 6 *JAK2V617F* MDS/MPN-*SF3B1*-T samples, 8 *JAK2V617F* ET samples, 8 MDS-*SF3B1* samples, and 3 normal bone marrows. Analysis was performed by using a custom *R* software package, *MethylKit* (2), which integrates CpG positions within differentially methylated regions (DMRs).

### Results

RRBS analysis identified 174 hypomethylated DMRs and 99 hypermethylated DMRs between *JAK2V617F* MDS/MPN-*SF3B1*-T and triple-negative MDS/MPN-*SF3B1*-T. By using a relevance score to pinpoint DMRs potentially involved in thrombocytosis unrelated to *JAK2V617F*, we identified 59 relevant DMRs (score ≥ 4/5) linked to 44 genes. Most of these genes were connected to the mitogen-activated protein kinase (MAPK) and related pathways. Using quantitative polymerase chain reaction (qPCR), we measured transcript levels for eight MAPK-related candidate genes across the studied cohort, validating overexpression of five negative and three positive MAPK regulator genes. MYC likewise appeared hypomethylated in triple negative compared with $JAK2^{V617F}$ MDS/MPN-*SF3B1*-T. Such an epigenetic deregulation of MYC expression was previously depicted in trisomy 8-associated MPN and associated with myelofibrosis (3), highlighting the importance of MYC methylation in disease progression.

**Copyright Information:** (Online) ISSN: 2572-9241
©2026 The Author(s). HemaSphere is published by John Wiley & Sons Ltd on behalf of European Hematology Association. This is an open access Abstract Book distributed under the Attribution-NonCommercial-NoDerivs (CC BY-NC-ND), which allows third parties to download the articles and share them with others as long as they credit the author and the Abstract Book, but they cannot change the content in any way or use them commercially.
**Abstract Book Citations:** Authors, Title, HemaSphere, 2026;10:(S1):pages. The abstract book DOIs can be found on the "Author Information" page.
**Disclaimer:** Articles published in the journal HemaSphere exclusively reflect the opinions of the authors. The authors are responsible for all content in their abstracts including accuracy of the facts, statements, citing resources, etc.

### Summary/Conclusion

In conclusion, a profiling method using RRBS data associates megakaryocyte proliferation in triple negative MDS/MPN-*SF3B1*-T with epigenetic deregulation of MAPK and MYC pathways through altered DNA methylation, arguing for a contribution of epigenetics to the pathophysiology of this chronic myeloid malignancy.

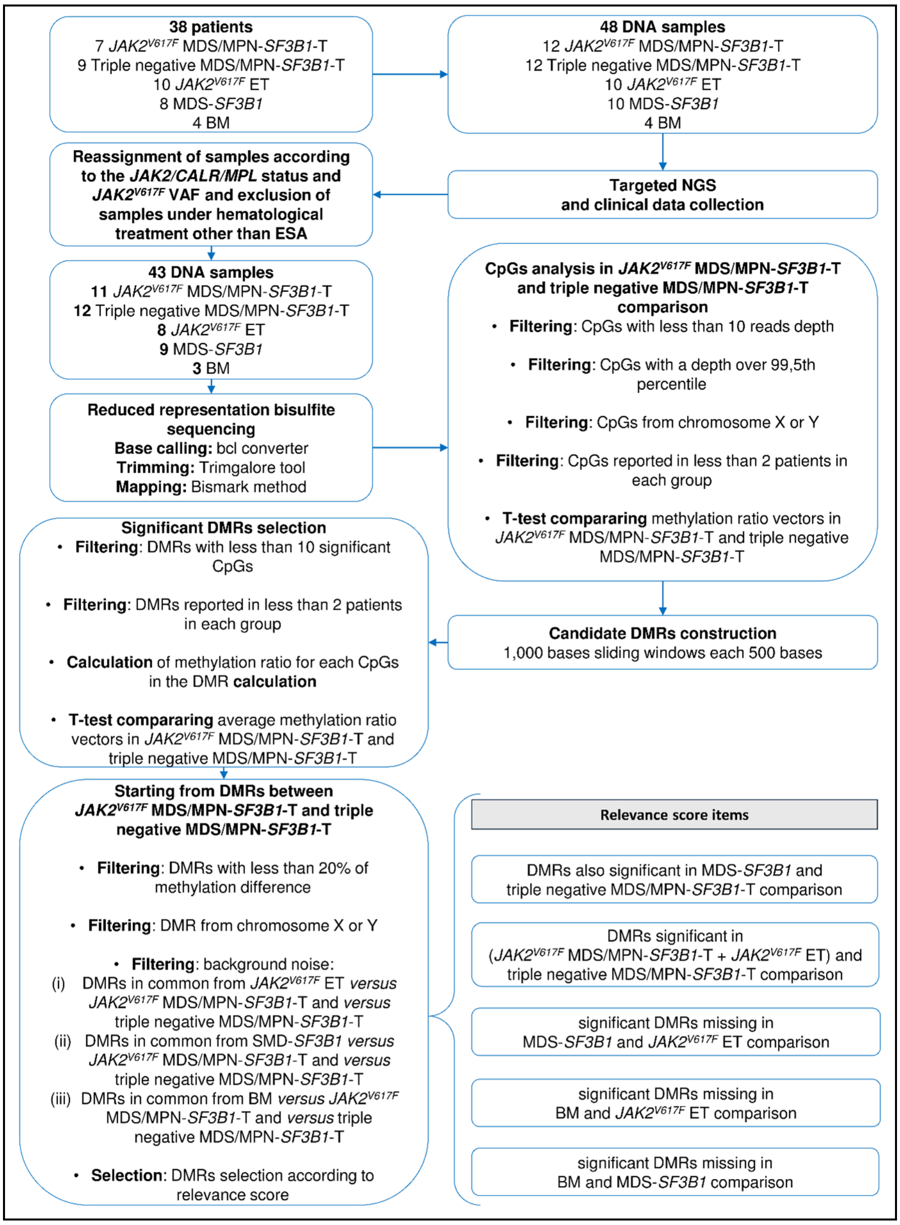

Figure 1. Reduced representation bisulfite sequencing analysis pipeline. JAK2V617F MDS/MPN-SF3B1-T = JAK2V617F mutated myelodysplastic/myeloproliferative neoplasm with SF3B1 mutation and thrombocytosis with mutated JAK2V617F; triple negative MDS/MPN-SF3B1-T = triple negative myelodysplastic/myeloproliferative neoplasm with SF3B1 mutation and thrombocytosis; JAK2V617F ET = JAK2V617F mutated essential thrombocythemia; MDS-SF3B1 = myelodysplastic neoplasm with mutated SF3B1; BM = healthy bone marrows; NGS = Next generation sequencing; DMR = Differentially methylated region; VAF = variant allele frequency.

**Copyright Information:** (Online) ISSN: 2572-9241
©2026 The Author(s). HemaSphere is published by John Wiley & Sons Ltd on behalf of European Hematology Association. This is an open access Abstract Book distributed under the Attribution-NonCommercial-NoDerivs (CC BY-NC-ND), which allows third parties to download the articles and share them with others as long as they credit the author and the Abstract Book, but they cannot change the content in any way or use them commercially.
**Abstract Book Citations:** Authors, Title, HemaSphere, 2026;10:(S1):pages. The abstract book DOIs can be found on the "Author Information" page.
**Disclaimer:** Articles published in the journal HemaSphere exclusively reflect the opinions of the authors. The authors are responsible for all content in their abstracts including accuracy of the facts, statements, citing resources, etc.

### (PF879) USP22 STABILIZES NAP1L1 TO ACTIVATE PI3K/AKT SIGNALING AND PROMOTE CLONAL EXPANSION OF CD34+ CELLS IN MYELODYSPLASTIC SYNDROMES

**Topic:** 15. Myeloproliferative neoplasms - Biology & translational research

Lijuan Li*[1], Jiayi Xie[1]

[1] *Tianjin Medical University General Hospital, Tianjin, China;*

### Background

Myelodysplastic syndromes (MDS) are clonal hematopoietic stem/progenitor cell disorders with ineffective hematopoiesis and variable risk of progression. Our prior single-cell RNA sequencing (scRNA-seq) suggested nucleosome assembly protein 1-like 1 (NAP1L1) is upregulated in MDS CD34+ cells.

### Aims

To define the functional role of NAP1L1 in MDS and determine whether the deubiquitinase USP22 stabilizes NAP1L1 to enhance PI3K/AKT signaling and clonal proliferation.

### Methods

Public/previous scRNA-seq data of unselected bone marrow cells from MDS and healthy controls were analyzed for NAP1L1 expression. CD34+ cells from newly diagnosed MDS patients (IPSS-R–stratified) and healthy donors were validated by qRT-PCR and immunoblotting, with correlation to clinical indices. NAP1L1 was silenced in SKM-1 and MDS-L cells (siRNA); proliferation (EdU), apoptosis (Annexin V/PI), and cell cycle (PI) were assessed at 48 h. RNA-seq with GO/KEGG enrichment was performed after NAP1L1 knockdown. NAP1L1 interactors were identified by affinity purification–MS; interaction with USP22 was confirmed by co-immunoprecipitation. Proteasome dependence and half-life were tested by MG132 and cycloheximide chase; ubiquitination linkage was assessed. Rescue assays used siUSP22 ± NAP1L1 overexpression. The USP22 inhibitor USP22-IN-1 was tested in MDS-L/SKM-1 and primary MDS CD34+ cells.

### Results scRNA-seq showed increased NAP1L1 in the HSPC compartment of MDS versus controls ($P<0.0001$). In CD34+ cells, NAP1L1 mRNA was higher in higher-risk MDS than healthy donors ($P<0.0001$) and lower-risk MDS ($P<0.001$), with no difference between lower-risk MDS and controls ($P=0.1586$). NAP1L1 correlated positively with IPSS-R score ($r=0.422$, $P<0.05$) . NAP1L1 knockdown reduced proliferation (both $P<0.0001$), increased apoptosis ($P<0.01$), and induced G0/G1 arrest ($P<0.001$) with reduced S and G2/M fractions (both $P<0.05$). RNA-seq identified 997 differentially expressed transcripts ($P<0.05$) and highlighted PI3K/AKT pathway enrichment; immunoblotting confirmed decreased p-PI3K and p-AKT with minimal change in total PI3K/AKT. USP22 was identified as a NAP1L1 interactor; USP22 depletion reduced NAP1L1 protein but not mRNA, was reversed by MG132, shortened NAP1L1 half-life, and increased K48-linked polyubiquitination. USP22 knockdown inhibited proliferation and increased apoptosis ($P<0.05$) with reduced NAP1L1 and PI3K/AKT signaling; NAP1L1 overexpression partially rescued these effects ($P<0.05$). USP22-IN-1 decreased proliferation and increased apoptosis in cell lines and primary MDS CD34+ cells (all $P<0.05$), accompanied by reduced NAP1L1, p-PI3K and p-AKT.

**Copyright Information:** (Online) ISSN: 2572-9241
©2026 The Author(s). HemaSphere is published by John Wiley & Sons Ltd on behalf of European Hematology Association. This is an open access Abstract Book distributed under the Attribution-NonCommercial-NoDerivs (CC BY-NC-ND), which allows third parties to download the articles and share them with others as long as they credit the author and the Abstract Book, but they cannot change the content in any way or use them commercially.
**Abstract Book Citations:** Authors, Title, HemaSphere, 2026;10:(S1):pages. The abstract book DOIs can be found on the "Author Information" page.
**Disclaimer:** Articles published in the journal HemaSphere exclusively reflect the opinions of the authors. The authors are responsible for all content in their abstracts including accuracy of the facts, statements, citing resources, etc.

**Summary/Conclusion**

NAP1L1 is overexpressed in MDS CD34⁺ cells and associates with higher disease risk. USP22 stabilizes NAP1L1 by limiting K48-linked ubiquitination, thereby sustaining PI3K/AKT activation and promoting clonal proliferation. Targeting the USP22–NAP1L1 axis may represent a therapeutic vulnerability in MDS.

**Copyright Information:** (Online) ISSN: 2572-9241
©2026 The Author(s). HemaSphere is published by John Wiley & Sons Ltd on behalf of European Hematology Association. This is an open access Abstract Book distributed under the Attribution-NonCommercial-NoDerivs (CC BY-NC-ND), which allows third parties to download the articles and share them with others as long as they credit the author and the Abstract Book, but they cannot change the content in any way or use them commercially.
**Abstract Book Citations:** Authors, Title, HemaSphere, 2026;10:(S1):pages. The abstract book DOIs can be found on the "Author Information" page.
**Disclaimer:** Articles published in the journal HemaSphere exclusively reflect the opinions of the authors. The authors are responsible for all content in their abstracts including accuracy of the facts, statements, citing resources, etc.

### (PF880) DECIPHERING THE IMPACT OF CBL DOUBLE-HIT ALTERATIONS ACROSS MYELOID MALIGNANCIES

**Topic:** 15. Myeloproliferative neoplasms - Biology & translational research

Anna Stengel*[1], Torsten Haferlach[1], Wencke Walter[1]

[1]*MLL Munich Leukemia Laboratory, Munich, Germany;*

### Background

*CBL* encodes an E3 ubiquitin ligase that negatively regulates tyrosine kinase signaling, and its mutations drive RAS/MAPK pathway activation in myeloid neoplasms. Biallelic *CBL* alterations typically arise through missense mutations (mut) accompanied by 11q copy-neutral loss of heterozygosity (CN-LOH), and are enriched in CMML and MDS/MPN, where they associate with proliferative features, resistance to *JAK* inhibition, and adverse outcome.

### Aims

To assess the prevalence and genomic context of *CBL* double-hit (dh) and to evaluate their molecular and clinical correlates across myeloid malignancies.

### Methods

We analyzed samples received 2022 - 2025 and identified 212 patients with *CBL* mut with a variant allele frequency (VAF) >50% accompanied by 11q deletion (del) or 11q CN-LOH (discovery cohort). All samples underwent targeted panel sequencing of 40 myeloid genes and copy-number profiling using the xGen Human Copy Number Variant Backbone Hybridization Panel. Findings were validated in an independent cohort of 4,928 patients across 31 hematologic neoplasms analyzed by WGTS and available clinical data (validation cohort).

### Results

Among 212 patients with *CBL* dh, 201 (95%) showed 11q CN-LOH and 11 (5%) had 11q del. The cohort included CMML (35%), AML (18%), MDS/MPN (16%), MDS (15%) and MPN (15%) with a median age of 71 years (range 19–89, 60% male) and mainly normal karyotypes (59%). Recurrent cytogenetic alterations comprised 7q del/monosomy 7 (8%), trisomy 8 (7%), trisomy 21 (3%), 4q/*TET2* del (2%), and additional CN-LOH on 7q (19 cases) and 4q (13 cases), which strongly associated with *EZH2* and *TET2* mut, respectively. Complex karyotypes were infrequent (2%). Cases had a median of three co-muts, with frequent muts in *ASXL1* (64%), *TET2* (45%), *SRSF2* (39%), *RUNX1* (16%), *EZH2* (15%), *SETBP1* (14%), *U2AF1* (11%), and *JAK2* (11%), while *NPM1* and *TP53* muts were rare (3% and 2%, respectively). Of note, 62/377 cases with a VAF <50% were dh, thus a 50% VAF threshold would miss ~15% of cases. In the validation cohort, 129 *CBL*-mut patients were identified, including n=26 with a VAF >50% (all dh). Clinically, *CBL* dh patients displayed a more proliferative phenotype than single-hit (sh) patients, with higher white blood cell counts (median 17 810/µl vs. 11 400/µl, p=0.035), lower platelets (57 500/µl vs. 106 500/µl, p=0.011), higher bone marrow blast percentages (9.5% vs. 4.5%, p<0.001), and significantly inferior overall survival (13 vs. 40 months, p=0.005), while co-mut burden was similar (median n=3). Analysis of mut topology showed that 83% of dh cases harbored *CBL* muts in the RING domain compared to 64% in sh cases. In the discovery cohort, follow-up data were available for 110 patients, of which 17 progressed to AML or had AML arising from CMML/MDS/MPN, with a median time to progression of 12 months (range 3–31 months).

**Copyright Information:** (Online) ISSN: 2572-9241
©2026 The Author(s). HemaSphere is published by John Wiley & Sons Ltd on behalf of European Hematology Association. This is an open access Abstract Book distributed under the Attribution-NonCommercial-NoDerivs (CC BY-NC-ND), which allows third parties to download the articles and share them with others as long as they credit the author and the Abstract Book, but they cannot change the content in any way or use them commercially.
**Abstract Book Citations:** Authors, Title, HemaSphere, 2026;10:(S1):pages. The abstract book DOIs can be found on the "Author Information" page.
**Disclaimer:** Articles published in the journal HemaSphere exclusively reflect the opinions of the authors. The authors are responsible for all content in their abstracts including accuracy of the facts, statements, citing resources, etc.

**Summary/Conclusion**

*CBL* dh in myeloid neoplasms are predominantly generated by 11q CN-LOH, are enriched for canonical RING-domain muts, and define a distinct molecular subset characterized by proliferative disease features and inferior survival. The co-mut pattern indicates that *CBL* muts arise on a background of epigenetic, splicing, and transcription factor alterations. These findings support systematic assessment of 11q CN-LOH and *CBL* del in routine diagnostics (not all *CBL* dh cases are identified by VAF cut-off) to refine molecular risk stratification and identify candidates for potential RAS/MAPK-targeted therapeutic strategies.

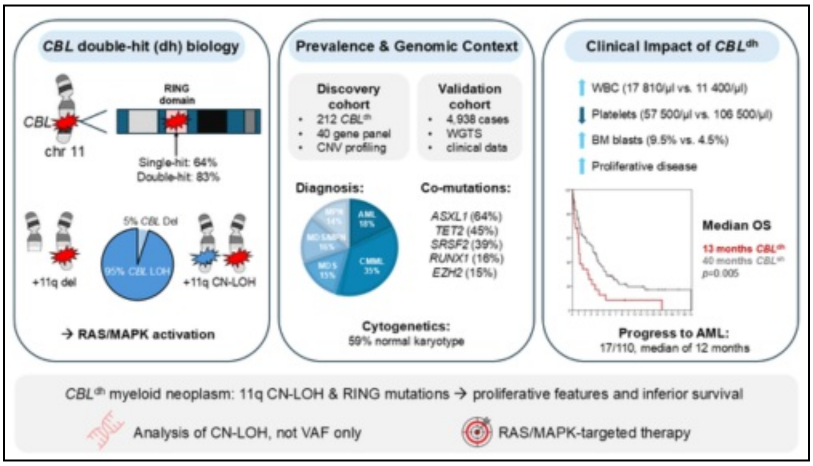

**Copyright Information:** (Online) ISSN: 2572-9241
©2026 The Author(s). HemaSphere is published by John Wiley & Sons Ltd on behalf of European Hematology Association. This is an open access Abstract Book distributed under the Attribution-NonCommercial-NoDerivs (CC BY-NC-ND), which allows third parties to download the articles and share them with others as long as they credit the author and the Abstract Book, but they cannot change the content in any way or use them commercially.
**Abstract Book Citations:** Authors, Title, HemaSphere, 2026;10:(S1):pages. The abstract book DOIs can be found on the "Author Information" page.
**Disclaimer:** Articles published in the journal HemaSphere exclusively reflect the opinions of the authors. The authors are responsible for all content in their abstracts including accuracy of the facts, statements, citing resources, etc.

### (PF881) HDAC INHIBITORS SUPPRESS SURVIVAL OF NEOPLASTIC MAST CELLS AND COUNTERACT CYTOKINE-INDUCED UPREGULATION OF IMMUNE CHECKPOINT MOLECULES PD-L1, CD47 AND TIM-3

**Topic:** 15. Myeloproliferative neoplasms - Biology & translational research

Yüksel Filik Schüppler*[1], Barbara Peter[1], Gabriele Stephanzl[2], Lina Degenfeld-Schonburg[1, 2], Daniela Berger[2], Stefan Kubicek[3], Michel Arock[4], Gregor Hörmann[5], Peter Valent[1, 2]

*[1]Ludwig Boltzmann Institute for Hematology and Oncology, Medical University of Vienna, Vienna, Austria;[2]Department of Internal Medicine I, Division of Hematology & Hemostaseology, Medical University of Vienna, Department of Internal Medicine I, Vienna, Austria; [3]CeMM Research Center for Molecular Medicine of the Austrian Academy of Sciences, Vienna, Austria;[4]French Center of Reference for Mastocytosis (CEREMAST), Pitié-Salpêtrière Hospital, Paris, France; [5]MLL Munich Leukemia Laboratory, Munich, Germany;*

### Background

Systemic mastocytosis (SM) is a clonal hematopoietic stem cell disorder characterized by the expansion and accumulation of neoplastic mast cells (MC) in one or more organs. In most patients, SM is associated with the *KIT* D816V mutation, which confers resistance to multiple tyrosine kinase inhibitors (TKIs), including imatinib. Midostaurin and avapritinib are FDA and EMA approved TKIs that target KIT D816V. However, long-lasting remissions occur only in a subset of patients and many relapse during therapy.

### Aims

The aim of this study was to develop novel therapeutic strategies capable of overcoming drug resistance in neoplastic mast cells in advanced systemic mastocytosis.

### Methods

A robot-based high-throughput multidrug cell viability screen using eight different MC lines, including the *KIT* D816V+ cell lines HMC-1.2 and ROSA$^{\text{KIT D816V}}$ was performed. Selected candidates were tested using proliferation assays, apoptosis by flow cytometry assays, qPCR, immunocytochemistry, and Western blot analysis.

### Results

We identified several compounds that effectively inhibit survival of *KIT* D816V+ MC lines. Among these, HDAC inhibitors were the most potent agents. The HDAC inhibitors panobinostat and quisinostat were selected for further validation experiments and showed strong anti-proliferative effects in HMC-1.1, HMC-1.2, ROSA$^{\text{KIT WT}}$, and ROSA$^{\text{KIT D816V}}$ cell lines, as well as in the multidrug-resistant cell lines MCPV-1.1, MCPV-1.2, MCPV-1.3 and MCPV-1.4. Panobinostat and quisinostat also suppressed proliferation of primary neoplastic MC obtained from patients with advanced SM. Both HDAC inhibitors profoundly reduced cell survival by inducing apoptosis across all tested cell lines, including the multidrug-resistant MCPV-1 subclones. We next assessed HDAC expression in neoplastic mast cells and found that all MC lines as well as primary neoplastic MC from patients with various subtypes of SM express HDAC1 and HDAC2 mRNA. Immunocytochemical analysis confirmed HDAC1 and HDAC2 protein expression in all cell lines. In Western blot experiments, panobinostat and quisinostat induced dose-dependent acetylation of histones H3 and H4 in HMC-1 and ROSA cells, whereas no effects on KIT phosphorylation were observed. In addition, both inhibitors downregulated MYC mRNA expression in these cells. Moreover, combinations of HDAC inhibitors (panobinostat or quisinostat) and KIT-targeting drugs (midostaurin or avapritinib) produced cooperative growth-inhibitory effects in all HMC-1 and ROSA subclones. Finally, we found that panobinostat and quisinostat downregulate cytokine-induced expression and/or constitutive expression of PD-L1, CD47 and TIM-3 in HMC-1 and ROSA cells in a dose-dependent manner.

**Copyright Information:** (Online) ISSN: 2572-9241
©2026 The Author(s). HemaSphere is published by John Wiley & Sons Ltd on behalf of European Hematology Association. This is an open access Abstract Book distributed under the Attribution-NonCommercial-NoDerivs (CC BY-NC-ND), which allows third parties to download the articles and share them with others as long as they credit the author and the Abstract Book, but they cannot change the content in any way or use them commercially.
**Abstract Book Citations:** Authors, Title, HemaSphere, 2026;10:(S1):pages. The abstract book DOIs can be found on the “Author Information” page.
**Disclaimer:** Articles published in the journal HemaSphere exclusively reflect the opinions of the authors. The authors are responsible for all content in their abstracts including accuracy of the facts, statements, citing resources, etc.

**Summary/Conclusion**

HDAC inhibitors are novel potent inhibitors of growth and survival of neoplastic mast cells including multidrug-resistant subclones. Both agents induce histone acetylation and downregulate MYC mRNA expression. Both drugs also exert cooperative growth-inhibitory effects when combined with midostaurin or avapritinib. Furthermore, HDAC inhibitors counteract cytokine-driven upregulation of immune checkpoint molecules. These findings identify HDAC inhibition as a promising therapeutic strategy in advanced SM. Whether these drugs can be successfully applied *in vivo* in patients with advanced SM needs to be determined in clinical studies.

**Copyright Information:** (Online) ISSN: 2572-9241
©2026 The Author(s). HemaSphere is published by John Wiley & Sons Ltd on behalf of European Hematology Association. This is an open access Abstract Book distributed under the Attribution-NonCommercial-NoDerivs (CC BY-NC-ND), which allows third parties to download the articles and share them with others as long as they credit the author and the Abstract Book, but they cannot change the content in any way or use them commercially.
**Abstract Book Citations:** Authors, Title, HemaSphere, 2026;10:(S1):pages. The abstract book DOIs can be found on the "Author Information" page.
**Disclaimer:** Articles published in the journal HemaSphere exclusively reflect the opinions of the authors. The authors are responsible for all content in their abstracts including accuracy of the facts, statements, citing resources, etc.

### (PF883) ANAGRELIDE INDUCED GRP78 PRODUCTION IN MPN CELLS LEADING TO LYSYL OXIDASE EXPRESSION IN BONE MARROW STROMAL CELLS

**Topic:** 15. Myeloproliferative neoplasms - Biology & translational research

Yuma Sakamoto*[1], Kei Nakajima[1], Keita Kirito[1]

[1]*University of Yamanashi, Hematology and Oncology, CHUO-SHI, YAMANASHI, Japan;*

### Background

Anagrelide is widely used as cytoreductive therapy for essential thrombocythemia (ET), especially for younger age patients, dependent on the notion that anagrelide is not a teratogenic agent. However, retrospective observational studies pointed out the increased odds of developing to myelofibrosis in anagrelide treated patients. In our institution, among 27 ET patients who received anagrelide, 7 patients developed myelofibrosis during or after treatment with anagrelide.
In our previous study, we reported that *JAK2*V617F positive myeloproliferative neoplasms (MPN) derived cell lines produced chaperon protein Glucose-Regulated Protein 78 kDa (GRP78) into the culture media (Nakajima et al. Leukemia Rec 2024) and these processes were regulated by transcription factor ATF4 dependent on aberrant JAK2 activation. We also confirmed that GRP78 produced into culture media activated JNK pathway in human bone marrow stromal cell line , HS-5 and enhanced expression of Lysyl oxidase (LOX) , one of the most important enzymes for the homeostasis of extracellular matrix. Interestingly, several groups reported that anagrelide activate ATF4 in normal human megakaryocytes.

### Aims

Based on these notions ,we speculated that anagrelide might enhanced production of GRP78 from MPN cells and induced LOX expression in bone marrow stromal cells leading to development of myelofibrosis.

### Methods

To confirm the hypothesis, we treated cell lines with MPN-driver mutations (JAK2V617F or mutant forms of calreticulin) with anagrelide and analyzed changes of ATF4 activation and GRP78 production. We also evaluated whether addition of anagrelide in co-culture of MPN cells and HS-5 enhanced LOX levels in HS-5 cells. Expression of LOX levles were evaluated with bone marrow samples from anagrelide associated MF patients. This study was approved by IRB in University of Yamanashi.

### Results

We found that anagrelide induced activation of ATF4 and elevation of GRP78 in these cells. Co-culture of HS-5 cells with MPN cells induced LOX expression as we have previously reported and addition of anagrelide further enhanced LOX levels. Finally, we compared expression levels of LOX with bone marrow biopsy samples from 3 ET patients who developed myelofibrosis under anagrelide treatment. We confirmed the increase of LOX levels after progression to myelofibrosis in all three patients.

### Summary/Conclusion

Our present findings suggested that enhanced LOX expression in bone marrow stromal cells  induced by MPN-cell derived GRP78 might be involved in anagrelide associated development of myelofibrosis.

**Copyright Information:** (Online) ISSN: 2572-9241
©2026 The Author(s). HemaSphere is published by John Wiley & Sons Ltd on behalf of European Hematology Association. This is an open access Abstract Book distributed under the Attribution-NonCommercial-NoDerivs (CC BY-NC-ND), which allows third parties to download the articles and share them with others as long as they credit the author and the Abstract Book, but they cannot change the content in any way or use them commercially.
**Abstract Book Citations:** Authors, Title, HemaSphere, 2026;10:(S1):pages. The abstract book DOIs can be found on the “Author Information” page.
**Disclaimer:** Articles published in the journal HemaSphere exclusively reflect the opinions of the authors. The authors are responsible for all content in their abstracts including accuracy of the facts, statements, citing resources, etc.

### (PF884) MUTANT CALRETICULIN-SPECIFIC MONOCLONAL ANTIBODY, INCA033989, PRODUCES CLONAL MOLECULAR RESPONSES THAT CORRELATE WITH CLINICAL RESPONSES IN PATIENTS WITH MYELOFIBROSIS

**Topic:** 16. Myeloproliferative neoplasms - Clinical

Jyoti Nangalia*[1], Claire Harrison[2], Jason Gotlib[3], John Mascarenhas[4], Chenwei Tian[5], Betty Lamothe[5], Tatiana Zinger[5], Erin Crowgey[5], Evan Braunstein[5], Steffen Koschmieder[6]

*[1]Wellcome Sanger Institute, Hinxton, United Kingdom; [2]Guy's and St Thomas' NHS Foundation Trust, London, United Kingdom; [3]Stanford Cancer Institute/Stanford University School of Medicine, Stanford, CA, United States of America; [4]Tisch Cancer Institute, Icahn School of Medicine at Mount Sinai, New York, NY, United States of America; [5]Incyte Corporation, Wilmington, DE, United States of America; [6]RWTH Aachen University, Faculty of Medicine, and Center for Integrated Oncology (CIO-ABCD), Aachen, Germany;*

### Background

Frameshift mutations in exon 9 of the calreticulin gene (mut*CALR*) are found in 25-30% of patients (pts) with myelofibrosis (MF). However, complex clonal architectures may impact treatment response. INCA033989, a novel, fully human, Fc-silenced, IgG1 monoclonal antibody, selectively targets mut*CALR* in complex with thrombopoietin receptor to inhibit oncogenic signaling and cell proliferation. INCA033989 is under investigation in 2 ongoing, phase 1, first-in-human trials enrolling pts with essential thrombocythemia or MF (NCT05936359; NCT06034002).

### Aims

To evaluate the impact of INCA033989 treatment on mut*CALR* clones and co-occurring mutations, and to correlate with clinical outcomes.

### Methods

Next-generation sequencing (NGS) using a targeted 37-gene NGS panel (Archer® VariantPlex® Core Myeloid panel) was performed on peripheral blood samples from 76 pts with MF at multiple time points (n>400 samples). Single-cell sequencing (Tapestri$^{TM}$) on peripheral blood mononuclear cells was performed on a subset with available samples (n=33 pts) using custom-made 37-gene DNA and 46-protein panels for genotyping and immunophenotyping at baseline and posttreatment time points. Efficacy assessments included spleen volume reduction of ≥25% (SVR25) and ≥35% (SVR35) at any time, as well as anemia response.

**Copyright Information:** (Online) ISSN: 2572-9241
©2026 The Author(s). HemaSphere is published by John Wiley & Sons Ltd on behalf of European Hematology Association. This is an open access Abstract Book distributed under the Attribution-NonCommercial-NoDerivs (CC BY-NC-ND), which allows third parties to download the articles and share them with others as long as they credit the author and the Abstract Book, but they cannot change the content in any way or use them commercially.
**Abstract Book Citations:** Authors, Title, HemaSphere, 2026;10:(S1):pages. The abstract book DOIs can be found on the "Author Information" page.
**Disclaimer:** Articles published in the journal HemaSphere exclusively reflect the opinions of the authors. The authors are responsible for all content in their abstracts including accuracy of the facts, statements, citing resources, etc.

### Results

Among 76 pts with MF analyzed, 57% (43/76) had Type 1 and 43% (33/76) had non-Type 1 mut*CALR*. Two pts harbored ≥1 *CALR* mutation with a distinct variant allele frequency (VAF). In addition, 2 pts had a co-occurring *MPL* p.W515 mutation (VAF, 3% and 9%). At baseline, mean mut*CALR* VAF was 38%. Loss of heterozygosity at *CALR* loci was inferred in 4 pts, notably all had Type 2 mut*CALR*. At least 1 co-occurring somatic mutation occurred in 86% of pts, most frequently in *ASXL1* (47%), *TET2* (32%), and *EZH2* (12%).

Among pts with co-occurring mutations and spleen assessments, SVR25 and SVR35 was achieved in 56% (35/63) and 40% (25/63) of pts, respectively (compared with 57% and 42%, respectively, in all pts irrespective of co-occurring mutations). Of 34 pts with an *ASXL1* mutation, 59% (20/34) had SVR25 and 47% (16/34) had SVR35. Anemia response was achieved in 51% (23/45) of all pts, 54% (21/39) of pts with a co-occurring mutation, and 46% of pts with an *ASXL1* mutation.

Reduction in mut*CALR* VAF via NGS occurred in 92% of pts (69/75), with 7 achieving ≥ 25% reduction. Tapestri analysis identified a reduction in mut*CALR* hematopoietic stem and progenitor cells (HSPCs) in all pts with SVR25 (n=24) (**Figure**). Intrapatient mut*CALR* clones displayed similar response to INCA033989 treatment regardless of the presence of co-occurring mutations.

### Summary/Conclusion

In pts with MF, comparable spleen and anemia responses were observed regardless of the presence of co-occurring somatic mutations, indicating that clonal complexity does not limit clinical response to INCA03989 treatment. In addition, single-cell immunophenotyping and genotyping data demonstrated a rapid reduction of mut*CALR* HSPCs in the majority of pts, despite modest mut*CALR* VAF reduction via bulk NGS, supporting the disease-modifying activity of INCA033989.

**Figure. Treatment with INCA033989 results in reduction of the percentage of mut*CALR*-positive hematopoietic stem and progenitor cells (of total peripheral blood mononuclear cells) in a majority of patients and correlates with spleen volume response**

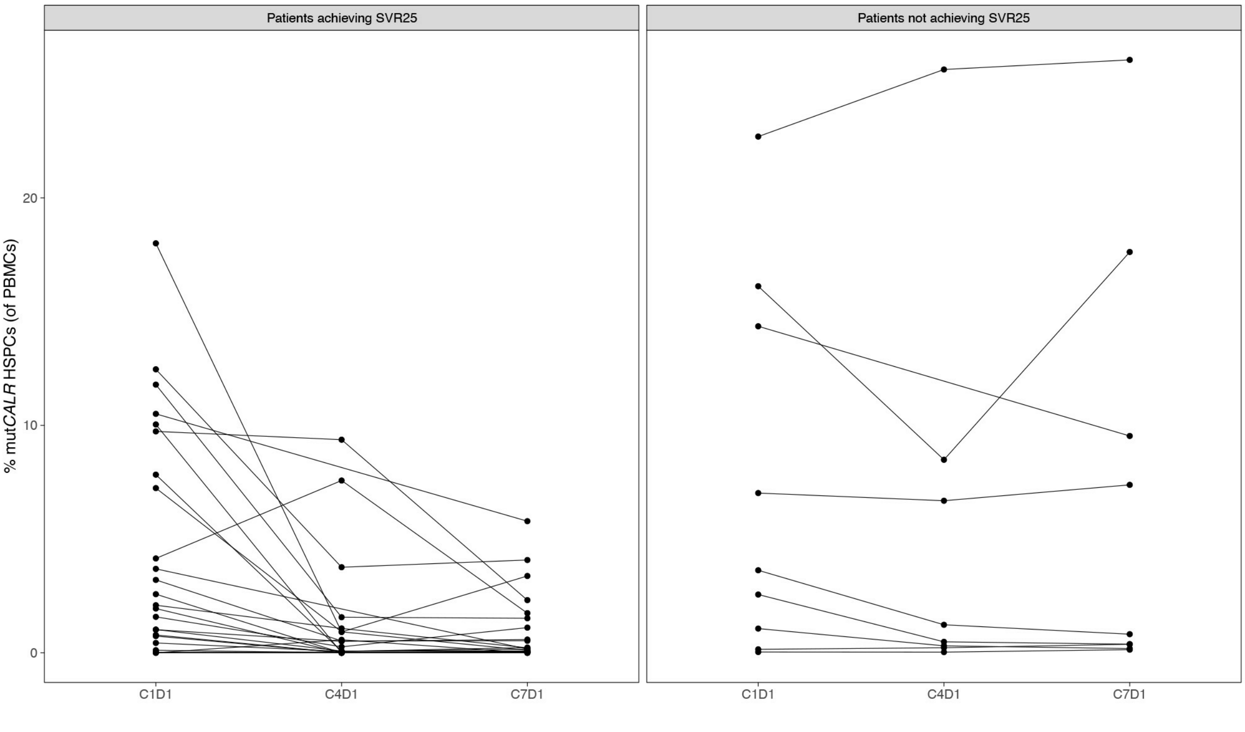

C, cycle; D, day; HSPC, hematopoietic stem and progenitor cell; mut*CALR*, mutation in exon 9 of the calreticulin gene; PBMC, peripheral blood mononuclear cell; SVR, spleen volume reduction.

**Copyright Information:** (Online) ISSN: 2572-9241
©2026 The Author(s). HemaSphere is published by John Wiley & Sons Ltd on behalf of European Hematology Association. This is an open access Abstract Book distributed under the Attribution-NonCommercial-NoDerivs (CC BY-NC-ND), which allows third parties to download the articles and share them with others as long as they credit the author and the Abstract Book, but they cannot change the content in any way or use them commercially.
**Abstract Book Citations:** Authors, Title, HemaSphere, 2026;10:(S1):pages. The abstract book DOIs can be found on the "Author Information" page.
**Disclaimer:** Articles published in the journal HemaSphere exclusively reflect the opinions of the authors. The authors are responsible for all content in their abstracts including accuracy of the facts, statements, citing resources, etc.

### (PF885) THE EFFECT OF BEZUCLASTINIB ON THE PATHOBIOLOGY OF ADVANCED SYSTEMIC MASTOCYTOSIS: RESULTS FROM THE PIVOTAL APEX TRIAL

**Topic:** 16. Myeloproliferative neoplasms - Clinical

Tracy I. George*[1], Daniel J. Deangelo[2], Khalid Shoumariyeh[3], Tania Jain[4], Cristina Papayannidis[5], Jay L. Patel[1], Jonathan Lambert[6], Karin Hartmann[7], Jens Panse[8], Miguel Piris-Villaespesa[9], Cristina Bulai Livideanu[10], Anton Rets[1], Karen Moser[1], Madhu Menon[1], Amanda Pilla[11], Kimberly Dishman[11], Jenna Zhang[11], Catherine Wang[11], Rachael Easton[11], Deepti H. Radia[12]

*[1]University of Utah, ARUP Laboratories, Salt Lake City, United States of America;[2]Dana-Farber Cancer Institute, Boston, United States of America; [3]University of Freiburg, Department of Hematology, Freiburg, Germany; [4]Johns Hopkins University, Sidney Kimmel Comprehensive Cancer Center, Baltimore, United States of America; [5]IRCCS Azienda Ospedaliero-Universitaria di Bologna, Bolgna, Italy; [6]University College Hospital, University College of London Hospitals NHS Foundation Trust, Department of Haematology, London, United Kingdom; [7]University of Basel, Basel, Switzerland; [8]RWTH Aachen University, Department of Hematology, Oncology, Hemostaseology and Stem Cell Transplantation, Aachen, Germany; [9]Hospital Universitario Ramón y Cajal, Madrid, Spain; [10]Toulouse University Hospital, Dermatology Department, Toulouse, France; [11]Cogent Biosciences, Inc., Waltham, United States of America; [12]Guy's and St. Thomas' NHS Foundation Trust, London, United Kingdom;*

### Background

Systemic mastocytosis (SM) is characterized by abnormal mast cell (MC) accumulation, driven by the gain-of-function *KIT* p.D816V mutation in ~95% of patients. Advanced SM (AdvSM) is a life-threatening form that includes aggressive SM, SM with associated hematologic neoplasm, and mast cell leukemia. Pathological and molecular markers of disease burden, such as bone marrow (BM) MC burden and characterization, serum tryptase, and *KIT* p.D816V variant allele frequency (VAF), are used to determine AdvSM subtype and disease severity, monitor disease modification, and predict prognosis and survival.
Bezuclastinib is an investigational, oral, potent, selective tyrosine kinase inhibitor (TKI) with activity against KIT D816V. Primary results from the Apex trial of bezuclastinib demonstrated favorable efficacy and safety in AdvSM. Herein we report the effect of bezuclastinib on pathological markers of SM from the pivotal portion of Apex.

### Methods

Apex (NCT04996875) is a Phase 2, open-label, multipart trial evaluating bezuclastinib in AdvSM. The pivotal Part 2 portion evaluated bezuclastinib at the selected dose of 150 mg once daily in adults with and without measurable C-findings. Primary endpoint was overall response rate (ORR) per mIWG-MRT-ECNM criteria, reported separately. Secondary endpoints included ORR per pure pathologic response (PPR) criteria and reductions in disease markers.
BM biopsy and aspirate, serum tryptase, and *KIT* p.D816V VAF were collected and tested centrally at baseline, every three 28-day cycles for the first 12 months, and then every six cycles. MC burden and aggregation, immunophenotype, and morphology were assessed; immunohistochemical staining was performed on BM sections.

**Copyright Information:** (Online) ISSN: 2572-9241
©2026 The Author(s). HemaSphere is published by John Wiley & Sons Ltd on behalf of European Hematology Association. This is an open access Abstract Book distributed under the Attribution-NonCommercial-NoDerivs (CC BY-NC-ND), which allows third parties to download the articles and share them with others as long as they credit the author and the Abstract Book, but they cannot change the content in any way or use them commercially.
**Abstract Book Citations:** Authors, Title, HemaSphere, 2026;10:(S1):pages. The abstract book DOIs can be found on the "Author Information" page.
**Disclaimer:** Articles published in the journal HemaSphere exclusively reflect the opinions of the authors. The authors are responsible for all content in their abstracts including accuracy of the facts, statements, citing resources, etc.

### Results

In Part 2, 81 patients with AdvSM received bezuclastinib: median age (range), 70 (43-87) years; 28% female; 93% with MC aggregates at baseline. Median (range) baseline BM MC burden was 35% (3-95), serum tryptase was 191 ng/mL (23.3-1987.0), and *KIT* p.816V VAF in blood was 6.7% (0-91.4).

As of the September 19, 2025 primary analysis, ORR was 80.2% per PPR criteria (median treatment duration 9.4 months). Of patients with pertinent baseline and ≥1 postbaseline BM assessments, 76% had clearance of MC aggregates, 69% had serum tryptase reduction to <20 ng/mL, and 28% had undetectable *KIT* p.D816V VAF, each representing a key threshold to assess treatment response in SM.

At Cycle 3, bezuclastinib treatment led to reductions in BM MCs, CD25 and CD30 expression, and spindle-shaped MCs; early Cycle 3 reductions observed in these markers continued to deepen at Cycle 6 (Table 1). Mean percentage reduction in BM MCs in biopsy was 79% at Cycle 6.

**Table 1: Reductions in BM Disease Markers Following Bezuclastinib Treatment**

| Marker | Baseline | Bezuclastinib Cycle 3 Day 1 | Bezuclastinib Cycle 6 Day 1 |
|---|---|---|---|
| | | Mean [N] | |
| BM MCs (biopsy), % | 41 [81] | 19 [74] | 8 [61] |
| BM MCs (aspirate), % | 9 [67] | 5 [46] | 2 [32] |
| CD25 Expression, % | 88 [81] | 53 [75] | 31 [60] |
| CD30 Expression, % | 25 [80] | 10 [75] | 2 [60] |
| Spindle-Shaped MCs (biopsy), % | 59 [77] | 34 [71] | 30 [57] |
| Spindle-Shaped MCs (aspirate), % | 60 [43] | 36 [24] | 40 [17] |

**Copyright Information:** (Online) ISSN: 2572-9241
©2026 The Author(s). HemaSphere is published by John Wiley & Sons Ltd on behalf of European Hematology Association. This is an open access Abstract Book distributed under the Attribution-NonCommercial-NoDerivs (CC BY-NC-ND), which allows third parties to download the articles and share them with others as long as they credit the author and the Abstract Book, but they cannot change the content in any way or use them commercially.
**Abstract Book Citations:** Authors, Title, HemaSphere, 2026;10:(S1):pages. The abstract book DOIs can be found on the "Author Information" page.
**Disclaimer:** Articles published in the journal HemaSphere exclusively reflect the opinions of the authors. The authors are responsible for all content in their abstracts including accuracy of the facts, statements, citing resources, etc.

**Summary/Conclusion**

In patients with AdvSM from Apex, bezuclastinib demonstrated high response rates and significant changes in BM MC burden, aggregation, immunophenotype, and morphology, as well as in serum tryptase and *KIT* p.D816V VAF. Substantial reductions in objective markers of disease burden, to the extent of normalization/elimination in many cases, signifies modification of underlying AdvSM pathobiology with bezuclastinib treatment.

**Copyright Information:** (Online) ISSN: 2572-9241
©2026 The Author(s). HemaSphere is published by John Wiley & Sons Ltd on behalf of European Hematology Association. This is an open access Abstract Book distributed under the Attribution-NonCommercial-NoDerivs (CC BY-NC-ND), which allows third parties to download the articles and share them with others as long as they credit the author and the Abstract Book, but they cannot change the content in any way or use them commercially.
**Abstract Book Citations:** Authors, Title, HemaSphere, 2026;10:(S1):pages. The abstract book DOIs can be found on the "Author Information" page.
**Disclaimer:** Articles published in the journal HemaSphere exclusively reflect the opinions of the authors. The authors are responsible for all content in their abstracts including accuracy of the facts, statements, citing resources, etc.

### (PF886) DIVESIRAN, A NOVEL GALNAC CONJUGATED SIRNA, REDUCES PHLEBOTOMIES, IMPROVES IRON STORES AND SYMPTOMS IN POLYCYTHEMIA VERA PATIENTS IN SANRECO PHASE 1 STUDY.

**Topic:** 16. Myeloproliferative neoplasms - Clinical

Marina Kremyanskaya*[1], Ronald Hoffman[1], Lee Ping Chew[2], Azizan Sharif[3], Boyan Semov[4], Ahlam Naila Kori[5], Hanlon Sia[6], Carolyn Grove[7], Ashley Ng[8], Akash Kalro[9], Indu Raman[10], Ganesh Kasinathan[11], Andrew Perkins[12], Michael Szarek[13], Nick Allison[14], Olubunmi Oduwole[14], Monica Gomez-Palou[14], Henry Fok[14], Steven Romano[14], Curtis Rambaran[14], Alberto Martinez[14]

*[1]Icahn School of Medicine at Mount Sinai, New York, United States of America;[2]Hospital Umum Sarawak, Kurching, Malaysia; [3]Hospital Saltanah Aminah, Johor Bahru, Malaysia;[4]Medical Centre Leo Clinic EOOD, Plovdiv, Bulgaria;[5]Hospital Tengku Ampuan Afzan, Kuantan, Malaysia; [6]Pindara Private Hospital, Benowa, Australia; [7]Linear Clinical Research, Nedlands, Australia; [8]Royal Melbourne Hospital, Peter MacCallum Cancer Center, Melbourne, Australia;[9]Ashford Cancer Centre Research, Tennyson Centre, Kurralta Park, Australia; [10]Epworth Healthcare, Richmond, Australia; [11]Hospital Sultanah Nur Zahirah, Kuala Terengganu, Malaysia; [12]The Alfred Hospital, Melbourne, Australia; [13]University of Colorado School of Medicine, Divison of Cardiology, Aurora, United States of America; [14]Silence Therapeutics, London, United Kingdom;*

### Background

Polycythemia Vera (PV) is a myeloproliferative neoplasm characterized by excessive erythrocytosis and increased risk of thromboembolic events which are linked to elevated hematocrit (HCT) levels (>45%) (Marchioli et al 2013). Pts experience fatigue, brain-fog and other debilitating symptoms. HCT levels are routinely controlled with phlebotomies (PHL), often in combination with cytoreductive therapies (CRT). Hepcidin is the central regulator of iron homeostasis and PV patients (pts) have reduced levels (Ginzburg et al 2018). We employed a novel approach to alter iron distribution in PV pts using a small interfering RNA (siRNA). siRNAs are precision medicines that engage their mRNA targets and silence gene expression. Divesiran (SLN124) is a GalNAc conjugated siRNA that increases hepcidin synthesis by silencing *TMPRSS6 (a negative regulator of hepcidin production),* restricting iron availability for erythropoiesis. We present data from the completed phase 1 (Ph1) part of the ongoing Ph1/2 SANRECO trial (NCT05499013) of divesiran in PV pts.

### Aims

The aim is to evaluate the safety and tolerability of divesiran and its effects on the number of PHL and symptoms.

### Methods

Ph1 is an open label dose finding study enrolling PHL dependent PV pts. Eligibility criteria included: i) PV diagnosis as per 2016 WHO criteria with ≥3 PHL in the 6 months (m) or ≥5 PHL in 12m prior to screening; ii) treatment with PHL alone or on stable doses of CRT. 3 dosing cohorts Ct1, Ct2 and Ct3 (3, 6 or 9 mg/kg of divesiran respectively) enrolling up to 8 pts each, given up to 4 doses by subcutaneous injection every 6 weeks (w). After the last dose (w18), pts were followed up for 16w (w34). Symptom scores using MPN-SAF-TSS (MPN-10), iron and erythroid biomarkers were assessed throughout the study.

**Copyright Information:** (Online) ISSN: 2572-9241
©2026 The Author(s). HemaSphere is published by John Wiley & Sons Ltd on behalf of European Hematology Association. This is an open access Abstract Book distributed under the Attribution-NonCommercial-NoDerivs (CC BY-NC-ND), which allows third parties to download the articles and share them with others as long as they credit the author and the Abstract Book, but they cannot change the content in any way or use them commercially.
**Abstract Book Citations:** Authors, Title, HemaSphere, 2026;10:(S1):pages. The abstract book DOIs can be found on the “Author Information” page.
**Disclaimer:** Articles published in the journal HemaSphere exclusively reflect the opinions of the authors. The authors are responsible for all content in their abstracts including accuracy of the facts, statements, citing resources, etc.

**Results**

21 pts were enrolled. Mean age 56.3 years (range 32 to 71), 16/21 male, 11/21 white, 10/21 Asian, 12/21 high-risk PV (age ≥60) and 14/21 on CRT. The baseline (BL) HCT was 47.0% (1.2), mean (SEM), (range 39% to 59%). Divesiran was well tolerated without dose limiting toxicities, most common treatment emergent adverse events (TEAE) were injection site reactions (ISRs) (44/138) in 67% pts. No treatment-related serious adverse events or TEAEs leading to discontinuation were observed. In the 6m prior to dosing, 21 pts had a total of 80 PHL. There were a total of 5 PHL in the dosing interval (14% pts), and 4 in follow up (FU) of 16 weeks (19% pts). HCT (% points) decreased at w24 by -3.6 (1.7) Ct1, -4.8 (1.2) Ct2 and -4.6 (3.5) Ct3, mean (SEM). Serum iron (6.0umol (0.9)) and TSAT (8.6% (1.7)) were low at BL and were further reduced at w34 (serum iron w34, 3.9umol (0.2); TSAT 5.6%(0.4)) mean (SEM) signalling iron restriction while ferritin (ug/L) levels were increased from BL 28.1 (15.2) to 48.0 (16.2) at w34 showing improved iron stores. The majority of pts reported significantly improved MPN-10 total symptom scores between BL and w34 (Figure 1) p= 0.0350. In 14 pts with FU data after the last dose received in Ph1 (w18), the median time to first PHL was 287 days.

**Summary/Conclusion**

Divesiran is a first in class siRNA targeting the hepcidin pathway being developed for treatment of PV. It is well-tolerated (doses up to 9 mg/kg), decreases the need for PHL, improves iron distribution and PV symptoms with a (Q6W) dosing regimen. It has a prolonged duration of effect that persists after the final dose suggesting it may be an effective and convenient treatment option for PV.

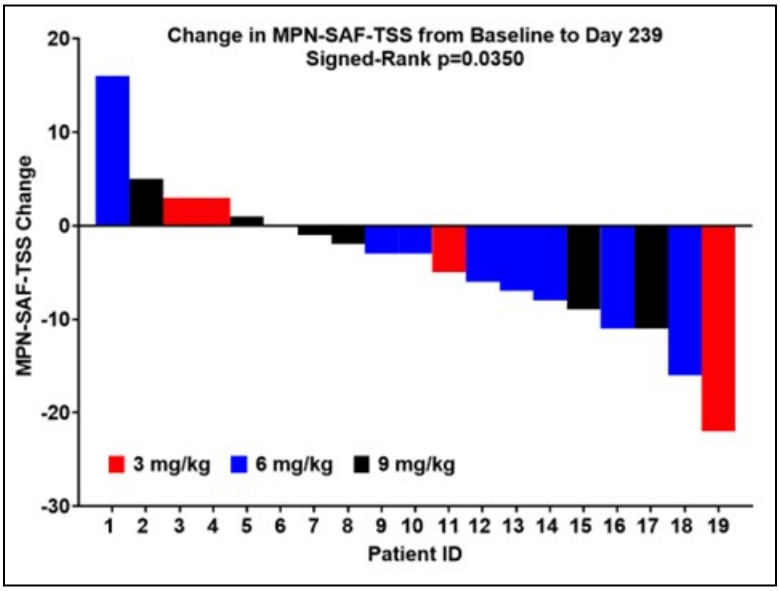

**Copyright Information:** (Online) ISSN: 2572-9241
©2026 The Author(s). HemaSphere is published by John Wiley & Sons Ltd on behalf of European Hematology Association. This is an open access Abstract Book distributed under the Attribution-NonCommercial-NoDerivs (CC BY-NC-ND), which allows third parties to download the articles and share them with others as long as they credit the author and the Abstract Book, but they cannot change the content in any way or use them commercially.
**Abstract Book Citations:** Authors, Title, HemaSphere, 2026;10:(S1):pages. The abstract book DOIs can be found on the "Author Information" page.
**Disclaimer:** Articles published in the journal HemaSphere exclusively reflect the opinions of the authors. The authors are responsible for all content in their abstracts including accuracy of the facts, statements, citing resources, etc.

### (PF887) A NOVEL MACHINE LEARNING-BASED APPROACH IN 1206 PATIENTS RELIABLY DIFFERENTIATES CLONAL FROM NON-CLONAL EOSINOPHILIA: THE EOSINOPHILIA DIFFERENTIATION SCORE (EDS)

**Topic:** 16. Myeloproliferative neoplasms - Clinical

Johannes Lübke*[1], Vito Dangelo[1], Guillaume Lefèvre[2, 3], Julien Rohmer[4, 5], Julien Rossignol[6, 7], Leonard Krenz[1], Nicole Naumann[1], Alice Fabarius[1], Wolf-Karsten Hofmann[1], Olivier Hermine[6, 7], Georgia Metzgeroth[1], Jean-Emmanuel Kahn[8], Juliana Schwaab[1], Andreas Reiter[1]

*[1]University Hospital Mannheim, Department of Hematology and Oncology, Manheim, Germany; [2]National Referral Center for Hypereosinophilic Syndromes (CEREO), Lille, France; [3]Université de Lille, U1286-INFINITE-Institute for Translational Research in Inflammation, Lille, France; [4]Foch Hospital, Department of Internal Medicine, Suresnes, France; [5]National Referral Center for Hypereosinophilic Syndromes (CEREO), Suresnes, France; [6]Necker-Enfants Malades University Hospital, Paris, France; [7]French National Reference Center for Mastocytoses (CEREMAST), Paris, France; [8]Hôpital Foch, Université Versailles-Saint-Quentin-en-Yvelines, Service de Médecine Interne, Centre de Référence des Syndromes Hyperéosinophiliques-CEREO, Suresnes, France;*

### Background

The diagnostic work-up of persistent eosinophilia with organ involvement remains challenging. The clinically most relevant causes include clonal (e.g., myeloid/lymphoid neoplasms with eosinophilia and tyrosine kinase gene fusions, MLN-TK; systemic mastocytosis, SM) and non-clonal disorders (e.g., idiopathic hypereosinophilic syndrome, iHES; eosinophilic granulomatosis with polyangiitis, EGPA). Early distinction between clonal and non-clonal eosinophilia is essential to guide a potentially complex and costly genetic work-up and the substantially different treatment options aimed at preventing eosinophil-mediated end-organ damage through tissue infiltration and irreversible fibrosis.

### Aims

To develop and validate the Eosinophilia Differentiation Score (EDS) for rapid and reliable differentiation between clonal and non-clonal eosinophilia.

### Methods

Among 1206 patients with persistent eosinophilia >0.5 $x10^9$/L for ≥4 weeks, the discovery cohort included 461 patients from the German Registry on Disorders of Eosinophils and Mast Cells (GREM): clonal eosinophilia (n=257, 56%; MLN-*PDGFRA/PDGFRB* fusions, n=102, 22%; MLN-TK with other fusion genes, n=24, 5%; SM, n=131, 28%) and non-clonal eosinophilia (n=204, 44%; idiopathic HES, n=153, 33%; EGPA, n=19, 4%; other disorders, n=32, 7%). Missing data were handled by random forest-based imputation. Clinical and laboratory candidate variables (n=23) were selected using a random forest-informed, two-step logistic regression. The EDS was computed as a weighted linear combination of selected variables. Organ involvement was captured both as organ-specific predictors and as the total number of involved organ sites (based on clinical/laboratory assessment, imaging, and/or biopsy). Discrimination was assessed by receiver operating characteristic (ROC) analysis and quantified by the area under the ROC curve (AUC). For interpretability, the ROC-derived optimal cut-off was centered at 0. For the external validation cohort, we used pooled data from 745 patients from the prospective multicenter COHESion study and the National Referral Center for Hypereosinophilic Syndromes (CEREO): clonal eosinophilia (n=182, 24%; MLN-*PDGFRA/PDGFRB* fusions, n=153, 21%; SM, n=5, 1%; other disorders, n=24, 3%) and non-clonal eosinophilia (n=563, 76%; idiopathic HES, n=377, 51%; EGPA, n=56, 8%; other disorders, n=130, 17%). Clinical utility was evaluated by decision curve analysis (DCA).

**Copyright Information:** (Online) ISSN: 2572-9241
©2026 The Author(s). HemaSphere is published by John Wiley & Sons Ltd on behalf of European Hematology Association. This is an open access Abstract Book distributed under the Attribution-NonCommercial-NoDerivs (CC BY-NC-ND), which allows third parties to download the articles and share them with others as long as they credit the author and the Abstract Book, but they cannot change the content in any way or use them commercially.
**Abstract Book Citations:** Authors, Title, HemaSphere, 2026;10:(S1):pages. The abstract book DOIs can be found on the "Author Information" page.
**Disclaimer:** Articles published in the journal HemaSphere exclusively reflect the opinions of the authors. The authors are responsible for all content in their abstracts including accuracy of the facts, statements, citing resources, etc.

### Results

Seven variables were identified as predictors for clonal eosinophilia, including the continuous variables: (i) higher serum tryptase, (ii) higher vitamin B12, (iii) lower immunoglobin E, (iv) lower lactate dehydrogenase, (v) lower number of involved organ sites, and the categorical variables: (vi) absence of the composite variable "asthma or ear-nose-throat [ENT] involvement," and (vii) presence of splenomegaly. A diagnostic model using a weighted linear combination of these parameters demonstrated excellent discrimination with sensitivity 95% (AUC 0.984) in the discovery cohort and 96% (AUC 0.959) in the validation cohort. DCA demonstrated the highest net benefit for EDS across a broad range of threshold probabilities compared with alternative strategies, including individual features (e.g., splenomegaly, asthma/ENT involvement).

### Summary/Conclusion

The EDS reliably differentiates clonal from non-clonal eosinophilia, and facilitates early, individualized decisions regarding genetic testing and treatment options.

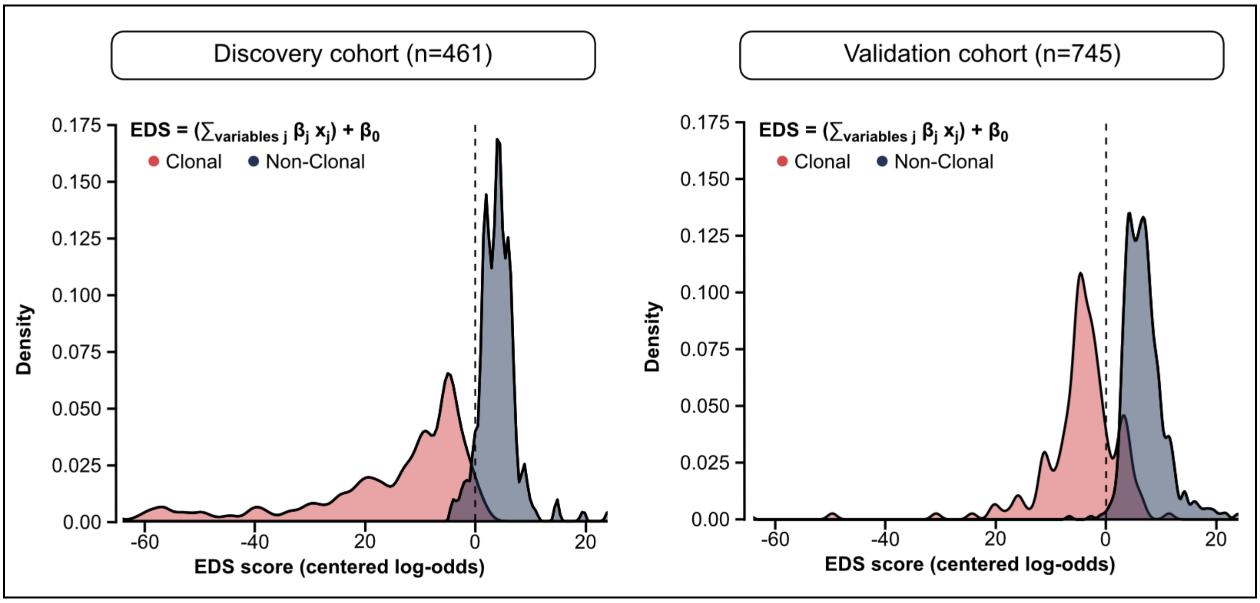

Density plots showing discrimination of clonal versus non-clonal eosinophilia in the discovery and training cohorts using the Eosinophilia Differentiation Score (EDS). βj, coefficient for predictor j; xj, weight of predictor j; β0, intercept used to center the EDS at 0.

**Copyright Information:** (Online) ISSN: 2572-9241
©2026 The Author(s). HemaSphere is published by John Wiley & Sons Ltd on behalf of European Hematology Association. This is an open access Abstract Book distributed under the Attribution-NonCommercial-NoDerivs (CC BY-NC-ND), which allows third parties to download the articles and share them with others as long as they credit the author and the Abstract Book, but they cannot change the content in any way or use them commercially.
**Abstract Book Citations:** Authors, Title, HemaSphere, 2026;10:(S1):pages. The abstract book DOIs can be found on the "Author Information" page.
**Disclaimer:** Articles published in the journal HemaSphere exclusively reflect the opinions of the authors. The authors are responsible for all content in their abstracts including accuracy of the facts, statements, citing resources, etc.

### (PF888) NOVEL PROGNOSTIC RISK SCORING SYSTEM FOR PATIENTS WITH ADVANCED SYSTEMIC MASTOCYTOSIS TREATED WITH MIDOSTAURIN OR AVAPRITINIB: DEVELOPMENT AND VALIDATION OF THE REVISED MUTATION-ADJUSTED RISK SCORE

**Topic:** 16. Myeloproliferative neoplasms - Clinical

Johannes Lübke*[1], Aaron Zakharyan[2], Jason Gotlib[3], Julien Rossignol[4], Daniel J. Deangelo[5], Deepti H. Radia[6], Tracy I. George[7], Michael Deininger[8], Orianne Debeaupuis[9], Nicole Naumann[1], Georgia Metzgeroth[1], Alice Fabarius[1], Wolf-Karsten Hofmann[1], Saša Dimitrijević[10], Olivier Hermine[4], Juliana Schwaab[1], Andreas Reiter[1]

*[1]University Hospital Mannheim, Department of Hematology and Oncology, Manheim, Germany; [2]Blueprint Medicines Corporation, Cambridge, MA, United States of America; [3]Stanford Cancer Institute/Stanford University School of Medicine, Division of Hematology, Stanford, CA, United States of America; [4]Imagine Institute, INSERM U1163, Necker-Enfants Malades University Hospital, Paris University, Paris, France; [5]Dana-Farber Cancer Institute, Department of Medical Oncology, Boston, MA, United States of America; [6]Guy's and St Thomas' NHS Foundation Trust, London, United Kingdom; [7]University of Utah, ARUP Laboratories, Salt Lake City, UT, United States of America; [8]Michael Deininger Laboratory, University of Michigan, Division of Hematology/Oncology, Ann Arbor, MI, United States of America; [9]Université Paris Cité, Institut Imagine, Laboratoire d'Immunogénétique des Maladies Autoimmunes Pédiatriques, INSERM UMR1163, Paris, France; [10]Blueprint Medicines GmbH, Zug, Switzerland;*

### Background

Overall survival (OS) of patients with advanced systemic mastocytosis (AdvSM) has improved since the introduction of the multikinase inhibitor midostaurin and the more selective *KIT* D816V inhibitor avapritinib. The Revised Mutation-Adjusted Risk Score (MARS-R), a continuous-scale OS risk scoring system, was recently developed to better predict OS in the era of AdvSM patients being treated with midostaurin and/or avapritinib.

### Aims

Further development and validation of the final MARS-R model and its application in the EXPLORER (NCT02561988) and PATHFINDER (NCT03580655) studies to account for baseline OS risk when evaluating survival in different treatment lines.

### Methods

The training cohort (n=287) combined (i) midostaurin real-world data (n=120) from the German Registry on Disorders of Eosinophils and Mast Cells and (ii) avapritinib clinical trial data (n=167) from the Phase 1 EXPLORER and Phase 2 PATHFINDER studies. Clinical and genetic candidate risk variables at treatment start were selected via stepwise Cox multivariable modeling. MARS-R was derived as a weighted sum of the selected predictors. External validation used 103 *KIT* D816V-positive AdvSM patients treated with midostaurin at the French National Reference Center for Mastocytosis (CEREMAST), including 28 who later received avapritinib. Avapritinib given ≥3 months after the last midostaurin and/or cladribine dose was considered as second-line (2L+) treatment.

**Copyright Information:** (Online) ISSN: 2572-9241
©2026 The Author(s). HemaSphere is published by John Wiley & Sons Ltd on behalf of European Hematology Association. This is an open access Abstract Book distributed under the Attribution-NonCommercial-NoDerivs (CC BY-NC-ND), which allows third parties to download the articles and share them with others as long as they credit the author and the Abstract Book, but they cannot change the content in any way or use them commercially.
**Abstract Book Citations:** Authors, Title, HemaSphere, 2026;10:(S1):pages. The abstract book DOIs can be found on the "Author Information" page.
**Disclaimer:** Articles published in the journal HemaSphere exclusively reflect the opinions of the authors. The authors are responsible for all content in their abstracts including accuracy of the facts, statements, citing resources, etc.

### Results

The continuous MARS-R comprises 8 adverse risk variables: the 3 continuous predictors older age (years), lower platelets (x$10^9$/L), and higher monocytes (x$10^9$/L), and the 5 categorical predictors male sex, absence of skin involvement, and mutations in *ASXL1*, *RUNX1*, and/or *SETBP1*. Using equal percentile-based cutoffs of the continuous MARS-R, 3 risk groups were defined: low risk (LR, n=95, 33%; median OS [mOS] not reached [NR]), intermediate risk (IR, n=96, 33%; mOS 5.7 years), and high risk (HiR, n=96, 33%; mOS 1.9 years) (P<0.001). Discrimination was confirmed in the validation cohort (LR, n=34, 33%, mOS NR; IR, n=26, 25%, 2.7 years; HiR, n=43, 42%, 1.4 years) (P<0.001). MARS-R retained prognostic performance when applied separately to midostaurin and avapritinib subgroups within both the training and validation cohorts. In a multivariable Cox model, pooling training and validation cohorts and adjusting for relevant confounders, a higher MARS-R score (hazard ratio [HR] 2.56; 95% CI 2.05–3.19) and avapritinib treatment (HR 0.52; 95% CI 0.35–0.76) were each independently associated with better OS. Across all risk categories, avapritinib-treated patients had improved OS compared with those treated with midostaurin (LR, mOS NR vs. 7.4 years, P=0.012; IR, mOS NR vs. 3.8 years, P=0.079; HiR, mOS 4.1 vs. 1.7 years, P=0.079). Among avapritinib-treated patients in the EXPLORER and PATHFINDER studies, we compared OS for first line (1L) vs. 2L+ treatment. Among patients with a starting dose of 100 and 200 mg, and after adjustment for baseline OS risk (by MARS-R), 1L avapritinib was associated with superior OS (HR 0.36; 95% CI 0.18–0.70) in both studies.

### Summary/Conclusion

MARS-R robustly stratifies OS risk in AdvSM across real-world as well as clinical study patients and enables risk-adjusted OS comparisons across treatment lines. Avapritinib use is associated with improved OS, particularly in the 1L setting. A web-based MARS-R calculator is available at www.mars-r.com.

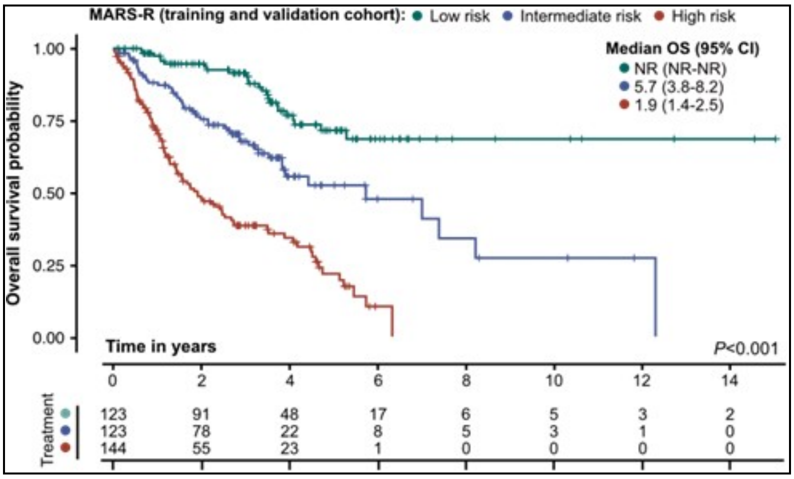

The figure shows Kaplan–Meier estimates of overall survival (OS) stratified by MARS-R risk categories in the pooled training and validation cohorts (N=390 patients). CI, confidence interval; MARS-R, Revised Mutation-Adjusted Risk Score; NR, not reached.

**Copyright Information:** (Online) ISSN: 2572-9241
©2026 The Author(s). HemaSphere is published by John Wiley & Sons Ltd on behalf of European Hematology Association. This is an open access Abstract Book distributed under the Attribution-NonCommercial-NoDerivs (CC BY-NC-ND), which allows third parties to download the articles and share them with others as long as they credit the author and the Abstract Book, but they cannot change the content in any way or use them commercially.
**Abstract Book Citations:** Authors, Title, HemaSphere, 2026;10:(S1):pages. The abstract book DOIs can be found on the "Author Information" page.
**Disclaimer:** Articles published in the journal HemaSphere exclusively reflect the opinions of the authors. The authors are responsible for all content in their abstracts including accuracy of the facts, statements, citing resources, etc.

### (PF889) TREATMENT STRATEGIES AND LIVE BIRTH RATES IN MPN PREGNANCIES: A SINGLE-CENTER ANALYSIS OF 165 PREGNANCIES

**Topic:** 16. Myeloproliferative neoplasms - Clinical

Kristine Poghosyan*[1], Anait Melikyan[1], Sofia Starchenko[1], Elena Gilyazitdinova[1], Irina Soubortseva[1], Sergey Kulikov[1]

[1]*National Medical Research Center for Hematology, Moscow, Russia;*

**Background**

Pregnancy in women with Ph-negative myeloproliferative neoplasms (MPNs) is associated with an increased risk of miscarriage and stillbirth. The use of acetylsalicylic acid (ASA), low-molecular-weight heparin (LMWH), and/or interferon-alpha (IFN-α) during pregnancy has been shown to improve live birth rates.

**Aims**

Impact of disease subtype and treatment on pregnancy course and outcomes in Ph-negative MPNs.

**Methods**

This retrospective study analyzed pregnancy outcomes in women with Ph-negative MPNs treated at the National Medical Research Center of Hematology between 2014 and 2024, with a focus on treatment regimens administered during pregnancy.

**Results**

The study included 165 pregnancies in 122 patients with Ph-negative MPNs: essential thrombocythemia (ET), n=63 (78 pregnancies), polycythemia vera (PV), n=40 (57 pregnancies), and primary myelofibrosis (PMF), n=19 (30 pregnancies). Live birth rates by MPN subtype were 80.7% (63/78) in ET, 52.6% (30/57) in PV, and 63.3% (19/30) in PMF, yielding an overall rate of 67.8% in the cohort. Of the 165 pregnancies, 137 received treatment with the following regimens: triple therapy (IFN-α + LMWH + ASA) in 33.3%, IFN-α + ASA in 29%, LMWH + ASA in 14.5%, and ASA monotherapy in 15.5% of cases.

**Pregnancy outcome depending on treatment regimens**

In the general group of MPN patient with pregnancies live birth rates varied significantly by regimen: 77.4% (41/53) with triple therapy, 73% (27/37) with IFN-α+ASA, 91.7% (22/24) with ASA+LMWH, and 56.5% (13/23) with ASA monotherapy (p=0.045). The ASA+LMWH combination was associated with the highest live birth rate (OR 5; 95% CI 1.1–25; p=0.022), while ASA monotherapy yielded the lowest (56.5%; OR 0.42; 95% CI 0.16–1.05; p=0.05).

**The effect of IFN and LMWH on pregnancy outcome in MPN**

The role of cytoreductive and anticoagulant therapy during pregnancy was assessed by comparing outcomes in patients who received IFN-α and/or LMWH during pregnancy versus those who did not. Overall, 58.9% (93/158) of pregnancies received IFN-α. Live birth rates were 76.3% (71/93) with IFN-α versus 63.0% (41/65) without (OR 1.92; 95% CI 0.94–3.85; p=0.07). Among pregnancies receiving LMWH (n=84), the live birth rate was 78.5% (66/84) compared to 62.0% (46/74) in those without LMWH (OR 2.22; 95% CI 1.1–4.55; p=0.02)

**Copyright Information:** (Online) ISSN: 2572-9241
©2026 The Author(s). HemaSphere is published by John Wiley & Sons Ltd on behalf of European Hematology Association. This is an open access Abstract Book distributed under the Attribution-NonCommercial-NoDerivs (CC BY-NC-ND), which allows third parties to download the articles and share them with others as long as they credit the author and the Abstract Book, but they cannot change the content in any way or use them commercially.
**Abstract Book Citations:** Authors, Title, HemaSphere, 2026;10:(S1):pages. The abstract book DOIs can be found on the "Author Information" page.
**Disclaimer:** Articles published in the journal HemaSphere exclusively reflect the opinions of the authors. The authors are responsible for all content in their abstracts including accuracy of the facts, statements, citing resources, etc.

**Summary/Conclusion**

This study demonstrates that in pregnant women with Ph-negative MPNs, treatment significantly impacts live birth rates. The overall live birth rate was 67.8%, varying by MPN subtype. Among treatment regimens, ASA+LMWH was associated with the highest live birth rate (91.7%), while ASA monotherapy yielded the poorest outcome (56.5%). IFN-α showed a trend toward higher live birth rates (p=0.07), but statistical significance was observed only for LMWH (p=0.02). These findings support the use of risk-adapted thromboprophylaxis and suggest that ASA monotherapy may be insufficient in this setting.

**Copyright Information:** (Online) ISSN: 2572-9241
©2026 The Author(s). HemaSphere is published by John Wiley & Sons Ltd on behalf of European Hematology Association. This is an open access Abstract Book distributed under the Attribution-NonCommercial-NoDerivs (CC BY-NC-ND), which allows third parties to download the articles and share them with others as long as they credit the author and the Abstract Book, but they cannot change the content in any way or use them commercially.
**Abstract Book Citations:** Authors, Title, HemaSphere, 2026;10:(S1):pages. The abstract book DOIs can be found on the "Author Information" page.
**Disclaimer:** Articles published in the journal HemaSphere exclusively reflect the opinions of the authors. The authors are responsible for all content in their abstracts including accuracy of the facts, statements, citing resources, etc.

### (PF890) THROMBOEMBOLIC AND PROGRESSION EVENTS IN PHLEBOTOMY-DEPENDENT PATIENTS WITH POLYCYTHEMIA VERA (PV): LONG-TERM RESULTS FROM THE PHASE 2 REVIVE AND THRIVE OPEN-LABEL EXTENSION RUSFERTIDE STUDIES

**Topic:** 16. Myeloproliferative neoplasms - Clinical

Naveen Pemmaraju*[1], Andrew Kuykendall[2], Aaron T. Gerds[3], Jason Gotlib[4], Ellen Ritchie[5], Kristen Pettit[6], Uttam Kumar Nath[7], Victor Priego[8], Jennifer Lucas[9], David Oubre[10], Greg Vosganian[11], Antonieta Sosa[11], Pedro Oyuela[11], Elizabeth Lindemulder[11], Jie Xiao[11], Philipp Dinh[11], Arturo Molina[11], Marina Kremyanskaya[12]

*[1]The University of Texas MD Anderson Cancer Center, Houston, TX, United States of America;[2]H. Lee Moffitt Cancer Center and Research Institute, Tampa, FL, United States of America; [3]Hematology and Medical Oncology, Cleveland Clinic - Taussig Cancer Institute, Cleveland, OH, United States of America; [4]Department of Medicine, Division of Hematology, Stanford Cancer Institute/Stanford University School of Medicine, Stanford, CA, United States of America; [5]Hematology, Weill Cornell Medical College, New York Presbyterian Hospital, New York, NY, United States of America; [6]Rogel Cancer Center, University of Michigan Health, Ann Arbor, MI, United States of America; [7]Department of Medical Oncology Haematology, All India Institute of Medical Sciences, Rishikesh, India; [8]The Center for Cancer & Blood Disorders (CCBD), Bethesda, MD, United States of America;[9]Marin Cancer Center, Greenbrae, CA, United States of America; [10]Pontchartrain Cancer Center, Covington, LA, United States of America; [11]Protagonist Therapeutics, Inc., Newark, CA, United States of America; [12]Tisch Cancer Institute, Icahn School of Medicine at Mount Sinai, New York, NY, United States of America;*

### Background

Patients (pts) with polycythemia vera (PV) have an elevated risk of morbidity and mortality due to disease transformation, cardiovascular (CV) events, and thromboembolic events (TEs). In the phase 2 REVIVE study (NCT04057040), the hepcidin mimetic rusfertide was superior to placebo in controlling hematocrit (Hct) to <45% while reducing the need for therapeutic phlebotomy (PHL) in PHL-dependent pts with PV (Kremyanskaya M, et al. *N Engl J Med.* 2024;390:723-35). Pts in REVIVE who enrolled in the open-label extension portion of the study were eligible to continue receiving rusfertide in the open-label phase 2 THRIVE extension study (NCT06033586).

### Aims

To present long-term results from pts who enrolled in REVIVE and THRIVE, including data on the occurrence of TEs and other clinical events relevant to pts with PV.

### Methods

This analysis included all pts who enrolled in REVIVE (intention-to-treat [ITT] population) and pts who transitioned to THRIVE. TEs (arterial and venous) and hemorrhage terms were defined according to the US FDA's Office of New Drugs (OND) Custom Medical Queries (OCMQs) version 4.0 standards. Thrombosis-free survival (TFS) is defined as absence of death or arterial thrombosis (eg, ischemic or hemorrhagic stroke, myocardial infarction [MI], transient ischemic attack [TIA]) or venous thrombosis (eg, pulmonary embolism [PE], deep vein thrombosis [DVT], abdominal/splanchnic thrombosis) events. Event-free survival (EFS) is defined as TFS and absence of grade ≥3 hemorrhage or post-PV progression event (eg, acute myeloid leukemia [AML], myelodysplastic syndromes, myelofibrosis [MF]). Median (95% confidence interval [CI]) times to TFS and EFS were calculated overall and in PV risk and prior and concurrent cytoreductive therapy subgroups.

**Copyright Information:** (Online) ISSN: 2572-9241
©2026 The Author(s). HemaSphere is published by John Wiley & Sons Ltd on behalf of European Hematology Association. This is an open access Abstract Book distributed under the Attribution-NonCommercial-NoDerivs (CC BY-NC-ND), which allows third parties to download the articles and share them with others as long as they credit the author and the Abstract Book, but they cannot change the content in any way or use them commercially.
**Abstract Book Citations:** Authors, Title, HemaSphere, 2026;10:(S1):pages. The abstract book DOIs can be found on the "Author Information" page.
**Disclaimer:** Articles published in the journal HemaSphere exclusively reflect the opinions of the authors. The authors are responsible for all content in their abstracts including accuracy of the facts, statements, citing resources, etc.

### Results

The data cutoff date was 10 December 2025. Seventy pts enrolled in REVIVE (median age, 57.5 years [yrs]; male, 70%; high-risk PV, 55.7%; use of concurrent cytoreductive therapy (CRT), 47.1%). Of these 70 pts, 12 (17.1%) had a history of TE(s) prior to study entry. Forty-six of 70 pts (65.7%) who enrolled in REVIVE transitioned to THRIVE. After a median (95% CI) follow up of 4.4 (3.6-4.6) yrs, 7 of 70 pts (10.0%) had ≥1 TE(s) (9 events: 7 arterial, 2 venous). All 7 pts with on-study TE(s) had prior risk factors (eg, hypertension) and high-risk PV (4 had TE[s] prior to study entry, including 3 venous, and 2 arterial events), and 4 received hydroxyurea at study entry. Six of 7 pts with on-study TEs continued receiving rusfertide post-TE; 3 remain ongoing. Median (95% CI) TFS was not reached in the ITT population or in the following subgroups: low- or high-risk PV, pts who received CRT prior to study entry, and pts who received concurrent CRT. After 5 yrs, there was an 88% (95% CI: 0.76-0.94) probability that a patient would not have a TE over this period. Nine pts had ≥1 event evaluable for EFS, including two grade 3 bleeding events of grade 3 hemorrhage and 2 events of post-PV progression (AML and MF). No pts died on either study. After a median (95% CI) follow up of 4.4 (2.7-4.6) yrs, median EFS was also not reached in the ITT population or in the subgroups stated previously.

### Summary/Conclusion

In the phase 2 REVIVE and THRIVE studies, after >4 yrs of follow-up, 10% of the patient population had a TE (all had risk factors and high-risk PV prior to study entry). Two pts (2.8%) had a post-PV progression event. Long-term follow-up will continue to further investigate TEs and other events following rusfertide exposure.

**Copyright Information:** (Online) ISSN: 2572-9241
©2026 The Author(s). HemaSphere is published by John Wiley & Sons Ltd on behalf of European Hematology Association. This is an open access Abstract Book distributed under the Attribution-NonCommercial-NoDerivs (CC BY-NC-ND), which allows third parties to download the articles and share them with others as long as they credit the author and the Abstract Book, but they cannot change the content in any way or use them commercially.
**Abstract Book Citations:** Authors, Title, HemaSphere, 2026;10:(S1):pages. The abstract book DOIs can be found on the "Author Information" page.
**Disclaimer:** Articles published in the journal HemaSphere exclusively reflect the opinions of the authors. The authors are responsible for all content in their abstracts including accuracy of the facts, statements, citing resources, etc.

### (PF891) EFFECT OF RUSFERTIDE ON CYTOREDUCTIVE THERAPY USE IN PATIENTS WITH PHLEBOTOMY DEPENDENT POLYCYTHEMIA VERA: POST HOC ANALYSIS FROM THE RANDOMIZED CONTROLLED PHASE 3 VERIFY STUDY

**Topic:** 16. Myeloproliferative neoplasms - Clinical

Francesca Palandri*[1], Greg Vosganian[2], Andrew Kuykendall[3], Aniket Bankar[4], Naveen Pemmaraju[5], Valentin Garcia Gutierrez[6], Alessandro Lucchesi[7], Jiří Mayer[8], Harry Gill[9], Antonín Hluší[10], Daniel Sasca[11], Joseph M Scandura[12], David Ross[13], Maria Laura Fox[14], Alessandro Maria Vannucchi[15], Steffen Koschmieder[16], Jean-Jacques Kiladjian[17], Philipp Dinh[2], Jie Xiao[2], Vidhyavathi Venkataraman[2], Elizabeth Lindemulder[2], Arturo Molina[2], Joseph Shatzel[18]

*[1]IRCCS Azienda Ospedaliero-Universitaria di Bologna - Institute of Hematology "L. and A. Seràgnoli", Bologna, Italy;[2]Protagonist Therapeutics, Inc., Newark, CA, United States of America; [3]Moffitt Cancer Center, Tampa, FL, United States of America; [4]Princess Margaret Malignancy Centre, Toronto, Canada;[5]MD Anderson Cancer Center, Houston, TX, United States of America; [6]Hospital Universitario Ramón y Cajal Instituto Ramón y Cajal de Investigación Sanitaria, Universidad de Alcalá, Madrid, Spain; [7]IRCCS Istituto Romagnolo per lo Studio dei Tumori (IRST) "Dino Amadori", Meldola, Italy; [8]University Hospital and Masaryk University, Brno, Czechia; [9]Department of Medicine, School of Clinical Medicine, LKS Faculty of Medicine, the University of Hong Kong, Hong Kong, Hong Kong; [10]Palacky University and University Hospital Olomouc, Olomouc, Czechia; [11]Universitaetsmedizin der Johannes Gutenberg - Universitaet Mainz, Mainz, Germany; [12]Silver MPN Center/Weill Cornell Medicine, New York, NY, United States of America; [13]Royal Adelaide Hospital, Adelaide, Australia; [14]Vall d'Hebron Institute of Oncology (VHIO), Hospital Universitario Vall d'Hebron, Barcelona, Spain; [15]Azienda Ospedaliero-Universitaria Careggi, Firenze, Italy; [16]RWTH Aachen University, Faculty of Medicine, and Center for Integrated Oncology (CIO-ABCD), Aachen, Germany; [17]Hôpital Saint-Louis, Université Paris Diderot, Paris, France; [18]Oregon Health & Science University, Portland, OR, United States of America;*

### Background

Rusfertide is a first-in-class, subcutaneously injected peptide mimetic of hepcidin. The phase 3 VERIFY study (NCT05210790) investigating rusfertide with current standard of care (SOC) therapy in patients (pts) with polycythemia vera (PV) met its prespecified primary endpoint of clinical response (absence of phlebotomy [PHL] eligibility) and all four key secondary endpoints vs. placebo (PBO) with SOC (Kuykendall AT, et al *Blood*. 2025;146(S1): abstract 81). The safety profile of rusfertide in VERIFY was also consistent with prior phase 2 studies.

### Aims

To describe the effect of rusfertide on CRT use in VERIFY.

### Methods

VERIFY is an ongoing phase 3 study that randomized pts (1:1) with PV to rusfertide or PBO with concurrent SOC therapy (ie, PHL with or without cytoreductive therapy [CRT]). After completing the 32-week double blind period, pts could receive rusfertide during the open-label period for 3+ years. Data from the 285 pts exposed to rusfertide in VERIFY were reviewed in a post-hoc analysis to identify pts who (1) discontinued CRT without restarting CRT; (2) reduced their dose of CRT (ie, reduced weekly CRT dose [any magnitude] used at randomization lasting ≥12 wks without returning to the baseline weekly CRT dose); or (3) started a new CRT or increased their weekly CRT dose that was used at baseline. Data are presented descriptively.

**Copyright Information:** (Online) ISSN: 2572-9241
©2026 The Author(s). HemaSphere is published by John Wiley & Sons Ltd on behalf of European Hematology Association. This is an open access Abstract Book distributed under the Attribution-NonCommercial-NoDerivs (CC BY-NC-ND), which allows third parties to download the articles and share them with others as long as they credit the author and the Abstract Book, but they cannot change the content in any way or use them commercially.
**Abstract Book Citations:** Authors, Title, HemaSphere, 2026;10:(S1):pages. The abstract book DOIs can be found on the "Author Information" page.
**Disclaimer:** Articles published in the journal HemaSphere exclusively reflect the opinions of the authors. The authors are responsible for all content in their abstracts including accuracy of the facts, statements, citing resources, etc.

**Results**

As of 10 December 2025, median (range) duration of exposure for the 285 rusfertide-treated pts was 88.1 (2–158) wks. At the cutoff date, >80% of pts (n=242) remain ongoing in the study and continue to receive rusfertide with or without CRT. More than half of pts (156/285; 54.7%) were receiving CRT at baseline. Throughout the course of the trial, for pts receiving PHL with CRT, 12.2% of pts discontinued CRT, 41.7% of pts reduced and/or discontinued CRT (29.5% reduced without discontinuing CRT), and 12.2% started a new CRT or increased their weekly CRT dose from baseline (**Table**). In pts who discontinued CRT without restarting any new CRT, median (range) time from first dose of rusfertide to CRT discontinuation was 28.1 (5.4–125.9) wks. In pts who reduced their dose of CRT, median (range) time from first dose of rusfertide to first dose reduction of CRT was 20.1 (2.0–96.1) wks. Median hematocrit (Hct) remained <45% in pts who reduced or discontinued their CRT dose up to 48 wks prior to and 48 wks following the change in CRT. During rusfertide exposure for the year following dose reduction or discontinuation of CRT, the median PHL rate was 0 PHL/year.

**Summary/Conclusion**

In this post-hoc analysis of the phase 3 VERIFY study, 41.7% of pts with PHL-dependent PV who were treated with CRT at baseline discontinued or reduced their dose of CRT following exposure to rusfertide. In these pts, Hct levels remained under 45%, suggesting that rusfertide may reduce PHL and/or CRT burden in pts with PV who have poorly controlled Hct.

**Table.**

| | **PHL+CRT n=156** | | | | | **PHL Only** | **PHL+CRT Or PHL Only*** |
|---|---|---|---|---|---|---|---|
| | HU n=109 | Interferon n=38 | Ruxolitinib n=7 | Other n=2 | Any CRT n=156 | n=129 | n=285 |
| Discontinued CRT, n (%) | 15 (13.8) | 4 (10.5) | 0 | 0 | 19 (12.2) | N/A | 19 (6.7) |
| Reduced and/or discontinued CRT, n (%) | 47 (43.1) | 16 (42.1) | 1 (14.3) | 1 (50.0) | 65 (41.7) | N/A | 65 (22.8) |
| Increased dose/ New CRT, n (%) | 14 (12.8) | 4 (10.5) | 1 (14.3) | 0 | 19 (12.2) | 20 (15.5) | 39 (13.7) |
| No CRT change, n (%) | 48 (44.0) | 18 (47.4) | 5 (71.4) | 1 (50.0) | 72 (46.2) | 109 (84.5) | 181 (63.5) |

HU, hydroxyurea; N/A, not applicable.
*All pts treated with rusfertide (n=285). In VERIFY (N=293 pts), 2 pts were randomized but not dosed; 6 pts (PBO group) discontinued VERIFY before receiving rusfertide.

**Copyright Information:** (Online) ISSN: 2572-9241
©2026 The Author(s). HemaSphere is published by John Wiley & Sons Ltd on behalf of European Hematology Association. This is an open access Abstract Book distributed under the Attribution-NonCommercial-NoDerivs (CC BY-NC-ND), which allows third parties to download the articles and share them with others as long as they credit the author and the Abstract Book, but they cannot change the content in any way or use them commercially.
**Abstract Book Citations:** Authors, Title, HemaSphere, 2026;10:(S1):pages. The abstract book DOIs can be found on the "Author Information" page.
**Disclaimer:** Articles published in the journal HemaSphere exclusively reflect the opinions of the authors. The authors are responsible for all content in their abstracts including accuracy of the facts, statements, citing resources, etc.

### (PF892) BENEFIT OF RUSFERTIDE MAINTAINED IN PATIENTS (PTS) WITH LOW-RISK OR HIGH-RISK POLYCYTHEMIA VERA (PV): EFFICACY AND SAFETY SUBGROUP ANALYSIS FROM THE RANDOMIZED CONTROLLED PHASE 3 VERIFY STUDY

**Topic:** 16. Myeloproliferative neoplasms - Clinical

Valentin Garcia Gutierrez*[1], Aniket Bankar[2], Naveen Pemmaraju[3], Kristen Pettit[4], Joseph Shatzel[5], Alessandro Lucchesi[6], Jiří Mayer[7], Abdulraheem Yacoub[8], Harry Gill[9], Antonín Hluší[10], Daniel Sasca[11], Joseph M Scandura[12], David Ross[13], Francesca Palandri[14], Maria Laura Fox[15], Alessandro Maria Vannucchi[16], Steffen Koschmieder[17], Jean-Jacques Kiladjian[18], A. Peter Morello[19], Philipp Dinh[19], Harinath Reddy Goli[19], Greg Vosganian[19], Sarita Khanna[19], Arturo Molina[19], Andrew Kuykendall[20]

*[1]Hospital Universitario Ramón y Cajal Instituto Ramón y Cajal de Investigación Sanitaria, Universidad de Alcalá, Madrid, Spain; [2]Princess Margaret Malignancy Centre, Toronto, Canada;[3]The University of Texas MD Anderson Cancer Center, Houston, TX, United States of America; [4]Rogel Cancer Center, University of Michigan Health, Ann Arbor, MI, United States of America; [5]Oregon Health & Science University, Portland, OR, United States of America;[6]IRCCS Istituto Romagnolo per lo Studio dei Tumori (IRST) "Dino Amadori", Meldola, Italy; [7]University Hospital and Masaryk University, Brno, Czechia; [8]University of Kansas Malignancy Center, Westwood, KS, United States of America; [9]Department of Medicine, School of Clinical Medicine, LKS Faculty of Medicine, the University of Hong Kong, Hong Kong, Hong Kong; [10]Palacky University and University Hospital Olomouc, Olomouc, Czechia; [11]Universitaetsmedizin der Johannes Gutenberg - Universitaet Mainz, Mainz, Germany;[12]Silver MPN Center/Weill Cornell Medicine, New York, NY, United States of America; [13]Royal Adelaide Hospital, Adelaide, Australia;[14]IRCCS Azienda Ospedaliero-Universitaria di Bologna - Institute of Hematology "L. and A. Seràgnoli", Bologna, Italy; [15]Vall d'Hebron Institute of Oncology (VHIO), Hospital Universitario Vall d'Hebron, Barcelona, Spain; [16]Azienda Ospedaliero-Universitaria Careggi, Firenze, Italy; [17]RWTH Aachen University, Faculty of Medicine, and Center for Integrated Oncology (CIO-ABCD), Aachen, Germany; [18]Hôpital Saint-Louis, Université Paris Diderot, Paris, France;[19]Protagonist Therapeutics, Inc., Newark, CA, United States of America; [20]Moffitt Cancer Center, Tampa, FL, United States of America;*

### Background

The phase 3 VERIFY study (NCT05210790) assessed standard-of-care with rusfertide or placebo (PBO) in phlebotomy (PHL)-dependent pts with PV. VERIFY met its primary endpoint (p<0.0001) and all four key secondary endpoints (p<0.05 for each endpoint; Kuykendall AT, et al. *J Clin Oncol.* 2025;43(17_suppl.):LBA3).

### Aims

The objective of this post-hoc analysis is to investigate differences between pts with low-risk PV (ie, <60 years [yrs] old and no thromboembolic event [TE] prior to study entry) and high-risk PV (≥60 yrs old and/or occurrence of a TE prior to study entry) in the rusfertide and PBO arms.

### Methods

In VERIFY, pts requiring frequent PHL with or without cytoreductive therapy (CRT) to achieve and maintain hematocrit (Hct) <45% were randomized (1:1) to receive once-weekly rusfertide or PBO from baseline to Week 32. After 32 wks, pts were eligible to continue receiving rusfertide during the open-label parts of the study. The primary endpoint was the proportion of pts achieving a clinical response (ie, absence of PHL eligibility) from Wks 20-32. Four key secondary endpoints were evaluated from baseline to Week 32: the mean number of PHLs, proportion of pts with Hct <45%, and mean change in scores of the PROMIS Fatigue SF-8a and MFSAF version 4.0 patient reported outcome (PRO) questionnaires.

**Copyright Information:** (Online) ISSN: 2572-9241
©2026 The Author(s). HemaSphere is published by John Wiley & Sons Ltd on behalf of European Hematology Association. This is an open access Abstract Book distributed under the Attribution-NonCommercial-NoDerivs (CC BY-NC-ND), which allows third parties to download the articles and share them with others as long as they credit the author and the Abstract Book, but they cannot change the content in any way or use them commercially.
**Abstract Book Citations:** Authors, Title, HemaSphere, 2026;10:(S1):pages. The abstract book DOIs can be found on the "Author Information" page.
**Disclaimer:** Articles published in the journal HemaSphere exclusively reflect the opinions of the authors. The authors are responsible for all content in their abstracts including accuracy of the facts, statements, citing resources, etc.

### Results

As of 10 December 2025, this analysis included 293 pts (median age overall, 57 yrs; 156 pts had low-risk PV [53.2%; median age, 52.0 yrs]; 137 pts had high-risk PV [46.8%; median age, 66.0 yrs]). In the high-risk group, 117 pts (39.9%) received prior CRT; 34% of high-risk pts in the rusfertide arm received rusfertide only. During Wks 20-32, significantly more pts achieved a clinical response in the rusfertide vs PBO arm regardless of risk group (nominal p<0.0001) and concurrent CRT status (**Table**). Irrespective of risk group, pts in the rusfertide arm had statistically significant improvements in the mean number of PHLs received (nominal p<0.0001) and the percentage of pts with Hct <45% (nominal p<0.0001) vs PBO. Numeric improvements were also observed in PROs at Week 32 for both risk groups with rusfertide vs PBO. Overall, treatment-emergent adverse events (AEs) in rusfertide-treated pts were similar in the high-risk (96.1%) and low-risk PV (94.2%) groups. Overall, serious AEs were more common in pts with high-risk PV (14.7%) vs low-risk PV (6.4%). In the high-risk group, 45 pts (32.8%) had a history of TE(s) prior to study entry; 20 pts (14.6%) were <60 yrs old. From baseline to Week 32, TEs were similar in the rusfertide and PBO arms irrespective of risk group (0 TEs in pts with high-risk PV in each arm; 1 TE [rusfertide arm] in a patient with low-risk PV). In pts treated with rusfertide during the open-label period, TEs were observed in 3 pts with low-risk PV and 3 pts with high-risk PV (5 of these 6 pts remain on treatment).

### Summary/Conclusion

In this post hoc analysis of the randomized VERIFY trial in PV, the benefit of rusfertide was maintained for response and all four key secondary endpoints vs PBO irrespective of risk group at baseline. As expected, there were more serious AEs in older pts with high-risk vs younger pts with low-risk PV throughout the study.

**Table.**

| | Randomized N=293 | | | |
|---|---|---|---|---|
| | High Risk n=137 | | Low-Risk n=156 | |
| | **Rusfertide** | **PBO** | **Rusfertide** | **PBO** |
| **With Or Without Concurrent CRT, n** | 66 | 71 | 81 | 75 |
| Responders, n (%) | 47 (71.2) | 22 (31.0) | 65 (80.2) | 26 (34.7) |
| **Concurrent CRT, n** | 50 | 52 | 32 | 28 |
| Responders, n (%) | 36 (72.0) | 17 (32.7) | 23 (71.9) | 9 (32.1) |
| **No concurrent CRT, n** | 16 | 19 | 49 | 47 |
| Responders, n (%) | 11 (68.8) | 5 (26.3) | 42 (85.7) | 17 (36.2) |

CRT, cytoreductive therapy; PBO, placebo

**Copyright Information:** (Online) ISSN: 2572-9241
©2026 The Author(s). HemaSphere is published by John Wiley & Sons Ltd on behalf of European Hematology Association. This is an open access Abstract Book distributed under the Attribution-NonCommercial-NoDerivs (CC BY-NC-ND), which allows third parties to download the articles and share them with others as long as they credit the author and the Abstract Book, but they cannot change the content in any way or use them commercially.
**Abstract Book Citations:** Authors, Title, HemaSphere, 2026;10:(S1):pages. The abstract book DOIs can be found on the “Author Information” page.
**Disclaimer:** Articles published in the journal HemaSphere exclusively reflect the opinions of the authors. The authors are responsible for all content in their abstracts including accuracy of the facts, statements, citing resources, etc.

### (PF893) LONG-TERM EFFICACY OF GECACITINIB ON MYELOFIBROSIS GRADE AND GENE MUTATION LEVEL: A PHASE IIIB EXTENSION STUDY IN PREVIOUSLY ENROLLED PATIENTS

**Topic:** 16. Myeloproliferative neoplasms - Clinical

Yi Zhang*[1], Jian Huang[1], Min Yang[1], Wenjuan Yu[1], Hongyan Tong[1], Jie Jin[1]

*[1]The First Affiliated Hospital, Zhejiang University School of Medicine, Hematology, Hangzhou, China, Hematology, Hangzhou, China;*

### Background

Myelofibrosis (MF) is characterized by ineffective hematopoiesis, splenomegaly, and progressive bone marrow fibrosis (BMF). Improvement in BMF grade and reduction in driver mutation burden represent key efficacy endpoints in myelofibrosis therapy. Longitudinal monitoring of these parameters during long-term treatment enables timely assessment of therapeutic response and disease progression. Gecacitinib, an oral novel JAK and ACVR1 inhibitor, has been shown to improve splenomegaly, symptom burden, and overall survival in patients with intermediate or high-risk myelofibrosis.

### Aims

We conducted a phase 3b study (NCT07342712) to evaluate the changes in BMF grade and mutant allele burden from baseline in patients with MF who had previously participated in Gecacitinib clinical trials and were still receiving treatment.

### Methods

Patients with MF who had participated in prior Gecacitinib clinical trials (ZGJAK016: phase 3, JAK inhibitor naïve MF; ZGJAK002: phase 2, Optimal dosing frequency exploration; ZGJAK006: phase 2, Ruxolitinib-intolerant MF; ZGJAK017: phase 2, Ruxolitinib-refractory/relapsed MF) and were eligible for long-term treatment continuation were recruited into this phase 3b study. The primary endpoint was the proportion of patients with worsening/stable/improving myelofibrosis grade after treatment. The secondary endpoint was the change in mutant allele burden. Successfully enrolled patients underwent bone marrow biopsy and mutant allele burden evaluation as indicated by follow-up, and then once per year until Gecacitinib was permanently discontinued.

### Results

A total of 18 patients were enrolled. At baseline, 94.5% (17/18) of the patients had overt BMF (Grade 2 or Grade 3). Genetic mutation analysis revealed *JAK2*$^{V617F}$ mutations in 77.8% (14/18) of patients, calreticulin (*CALR*) mutations in 16.7% (3/18), and triple-negative in 5.6% (1/18). The *JAK2*$^{V617F}$ mutant allele burden was 90.7 % (range, 44-94.9). Hemoglobin was 116 g/L (range, 74-181), and platelet count was $260\times10^9$/L (range, 107-904). Patient baseline characteristics are shown in Table 1.
The median duration of Gecacitinib treatment was 56 months (range, 45-78). Among the evaluable patients, 33.3% (6/18) achieved improvement in BMF grade, and 16.7% (3/18) demonstrated ≥2-grade improvement. Strikingly, two patients achieved BMF regression to Grade 0, representing complete reversal from baseline. BMF grade stabilization was achieved in 38.9% (7/18). Progression of BMF grade was noted in 27.8% (5/18) of patients. At the molecular level, among evaluable patients, 85.7% (12/14) exhibited a reduction in *JAK2*$^{V617F}$ allele burden posttreatment. The median percentage decrease in *JAK2*$^{V617F}$ allele burden was −38.5% (range, −100% to 34.4%). Notably, 78.6% (11/14) of patients achieved ≥10% reduction and 35.7% (5/14) achieved ≥50% reduction in mutant allele burden. Furthermore, one patient converted from *JAK2* $^{V617F}$-positive at baseline to negativity, indicating deep molecular response induced by Gecacitinib therapy.

**Copyright Information:** (Online) ISSN: 2572-9241
©2026 The Author(s). HemaSphere is published by John Wiley & Sons Ltd on behalf of European Hematology Association. This is an open access Abstract Book distributed under the Attribution-NonCommercial-NoDerivs (CC BY-NC-ND), which allows third parties to download the articles and share them with others as long as they credit the author and the Abstract Book, but they cannot change the content in any way or use them commercially.
**Abstract Book Citations:** Authors, Title, HemaSphere, 2026;10:(S1):pages. The abstract book DOIs can be found on the "Author Information" page.
**Disclaimer:** Articles published in the journal HemaSphere exclusively reflect the opinions of the authors. The authors are responsible for all content in their abstracts including accuracy of the facts, statements, citing resources, etc.

### Summary/Conclusion

These clinical observations are consistent with the proposed mechanism of action of JAK inhibition, and also align with the prominent role of Gecacitinib in myelofibrosis. Gecacitinib not only substantially improves BMF grade, but also reduces driver mutant allele burden.

**Table 1. Demographics and Baseline Characteristics**

| Variables | Patients (n=18) |
|---|---|
| Age, years | 60.5 (37, 72) |
| Gender | |
| Male | 7 (38.9%) |
| Female | 11 (61.1%) |
| Myelofibrosis subtype | |
| PMF | 7 (38.9%) |
| Post–PV MF | 8 (44.4%) |
| Post–ET MF | 3 (16.7%) |
| Myelofibrosis grading | |
| MF-1 | 1 (5.6%) |
| MF-2 | 10 (55.6%) |
| MF-3 | 7 (38.9%) |
| Mutational profile | |
| $JAK2^{V617F}$ | 14 (77.8%) |
| CALR | 3 (16.7%) |
| Triple-negative | 1 (5.6%) |
| DIPSS risk status | |
| Int-1 | 4 (22.2%) |
| Int-2 | 14 (77.8%) |
| Status at screening | |
| JAKI naïve | 13 (72.2%) |
| Ruxolitinib intolerant | 3 (16.7%) |
| Ruxolitinib refractory/relapsed | 2 (11.1%) |
| MPN-SAF TSS | 15 (2, 63) |
| Spleen size,$cm^3$ | 1152.2 (601.2, 3094.3) |
| Hemoglobin, g/L | 116 (74, 181) |
| WBC count, $\times 10^9$/L | 14.3 (2.1, 40.7) |
| Platelet count, $\times 10^9$/L | 260 (107, 904) |
| Peripheral blasts,% | 1 (0, 2) |
| $JAK2^{V617F}$ VAF, % | 90.7 (44, 94.9) |
| Duration of the disease, months | 76.5 (52, 198) |
| Duration of previous ruxolitinib treatment, months | 21.6(7.7, 37.3) |

Note: Data are median (range) or n (%).
Abbreviations: DIPSS, Dynamic International Prognostic Scoring System; PMF, primary myelofibrosis; Post–PV MF: post-polycythemia vera myelofibrosis; Post–ET MF: post-essential thrombocythemia myelofibrosis; TSS, total symptom score; VAF: variant allele frequency.

Copyright Information: (Online) ISSN: 2572-9241
©2026 The Author(s). HemaSphere is published by John Wiley & Sons Ltd on behalf of European Hematology Association. This is an open access Abstract Book distributed under the Attribution-NonCommercial-NoDerivs (CC BY-NC-ND), which allows third parties to download the articles and share them with others as long as they credit the author and the Abstract Book, but they cannot change the content in any way or use them commercially.
Abstract Book Citations: Authors, Title, HemaSphere, 2026;10:(S1):pages. The abstract book DOIs can be found on the "Author Information" page.
Disclaimer: Articles published in the journal HemaSphere exclusively reflect the opinions of the authors. The authors are responsible for all content in their abstracts including accuracy of the facts, statements, citing resources, etc.

### (PF894) EVALUATION OF PELABRESIB (PELA) AS ADD-ON THERAPY TO JANUS KINASE INHIBITOR (JAKI) RUXOLITINIB (RUX) IN MYELOFIBROSIS (MF) PATIENTS: RESULTS FROM ARM 2 OF THE OPEN-LABEL, PHASE 2 MANIFEST STUDY

**Topic:** 16. Myeloproliferative neoplasms - Clinical

Marina Kremyanskaya*[1], Claire Harrison[2], Vikas Gupta[3], Raajit K Rampal[4], Moshe Talpaz[5], Alessandro Maria Vannucchi[6], Andrew Kuykendall[7], Ruben Mesa[8], Prithviraj Bose[9], Jonathan Lambert[10], Jean-Jacques Kiladjian[11], Francesca Palandri[12], Monika Wroclawska[13], Grace Cleary[14], Claudia Gorcea-Carson[15], John Mascarenhas[1]

*[1]Tisch Cancer Institute, New York, NY, United States of America;[2]Guy's and St. Thomas' NHS Foundation Trust, London, United Kingdom; [3]Princess Margaret Cancer Centre, Toronto, Canada;[4]Memorial Sloan Kettering Cancer Center, New York, NY, United States of America; [5]University of Michigan, Ann Arbor, MI, United States of America; [6]Azienda Ospedaliero-Universitaria Careggi, University of Florence, Florence, Italy; [7]Moffitt Cancer Center, Tampa, FL, United States of America; [8]Wake Forest University School of Medicine, Winston-Salem, NC, United States of America; [9]University of Texas MD Anderson Cancer Center, Houston, TX, United States of America; [10]University College London Hospitals, NHS Foundation Trust, London, United Kingdom; [11]Hôpital Saint-Louis, Université de Paris, Paris, France;[12]IRCCS Azienda Ospedaliero-Universitaria di Bologna, Istituto di Ematologia "Seràgnoli", Bologna, Italy; [13]Novartis Pharma AG, Basel, Switzerland; [14]Novartis Ireland Limited, Dublin, Ireland; [15]Novartis Pharmaceuticals UK Limited, London, United Kingdom;*

### Background

MF is a clonal myeloproliferative neoplasm associated with splenomegaly, cytopenias and progressive bone marrow fibrosis (BMF) (Mughal. *Int J Gen Med.* 2014). Although RUX, a JAKi, is a standard therapy for patients with splenomegaly and/or symptoms, responses may be suboptimal/transient; and features, such as anemia, may worsen, presenting an unmet need in MF patients (Harrison. *Cancer.* 2024). PELA (DAK539), an investigational bromodomain and extra-terminal inhibitor, modulates pro-inflammatory and pro-fibrotic pathways in MF pathogenesis (Rampal. *Nat Med.* 2025).

### Aims

The MANIFEST study (NCT02158858) assessed PELA across various clinical settings; we report results from Arm 2, which evaluated PELA added to ongoing RUX in suboptimal responders.

### Methods

Arm 2 enrolled adults with MF receiving RUX for ≥6 months at a stable dose for ≥8 weeks, with persistent splenomegaly or transfusion dependence. Patients were assigned to Cohort 2B (non-transfusion dependent [non-TD]) or Cohort 2A (transfusion dependent [TD]). PELA 125 mg was administered orally once daily on Days 1–14 of 21-day cycles; RUX was continued twice daily at pre-study dose. Primary endpoints were spleen volume reduction ≥35% (SVR35) at Week 24 (W24) (Cohort 2B) and conversion from TD to transfusion independence (TI; Cohort 2A). Secondary and exploratory endpoints included total symptom score reduction ≥50% (TSS50), BMF, haemoglobin improvement and safety.

**Copyright Information:** (Online) ISSN: 2572-9241
©2026 The Author(s). HemaSphere is published by John Wiley & Sons Ltd on behalf of European Hematology Association. This is an open access Abstract Book distributed under the Attribution-NonCommercial-NoDerivs (CC BY-NC-ND), which allows third parties to download the articles and share them with others as long as they credit the author and the Abstract Book, but they cannot change the content in any way or use them commercially.
**Abstract Book Citations:** Authors, Title, HemaSphere, 2026;10:(S1):pages. The abstract book DOIs can be found on the "Author Information" page.
**Disclaimer:** Articles published in the journal HemaSphere exclusively reflect the opinions of the authors. The authors are responsible for all content in their abstracts including accuracy of the facts, statements, citing resources, etc.

**Results**

Eighty-seven patients were treated (TD cohort = 59, non-TD cohort = 28). Median age was 69 years (41, 83); patients were Dynamic International Prognostic Scoring System category Intermediate-1 (8%), Intermediate-2 (63%) or high risk (29%). Pre-study RUX dose ranged 5–25 mg twice daily. Prior RUX median treatment duration was 30.2 months (3, 117), median spleen volume was 1860 $cm^3$ (121, 7673), and median TSS was 19.7 (1, 61.6). W24 SVR35 was achieved in 15/83 (18.1%) evaluable patients, 9/55 (16.4%) TD patients and 6/28 (21.4%) non-TD patients. Overall SVR35 at any time was 28.8% and durable SVRs were observed with a median spleen response duration of 180.9 weeks (95% CL: 73.0, NE [not evaluable]). Among TD patients, TI was achieved by 12/46 (26.1%), with median time to TI being 12.4 weeks (0.1,45.1) and median TI duration being 68.0 weeks (95% CL: 34.7, NE). In non-TD patients, 6/28 (21.4%) achieved a hemoglobin response. W24 TSS50 was achieved by 32/85 (37.6%) evaluable patients, 21/58 (36.2%) TD patients and 11/27 (40.7%) non-TD patients; 47/82 (57%) evaluable patients had TSS50 at any time. W24 TSS mean absolute change (SD) was -7.39 (10.269). Among evaluable patients, BMF improved by ≥1 grade in 15/48 (31.3%) at W24 and 13/35 (37.1%) at W48. Most common grade ≥3 treatment-emergent adverse events were thrombocytopenia (25.3%) and anemia (23%). No new safety signals were identified; overall safety profile was consistent with known PELA and RUX effects.

**Summary/Conclusion**

PELA added to RUX in MF patients with suboptimal response demonstrated meaningful clinical benefit, with improvements in SVR, TSS, haemoglobin, TD and BMF, alongside a generally manageable safety profile. These findings reinforce the therapeutic value of PELA+RUX across diverse MF settings including suboptimal responders to RUX.

**Copyright Information:** (Online) ISSN: 2572-9241
©2026 The Author(s). HemaSphere is published by John Wiley & Sons Ltd on behalf of European Hematology Association. This is an open access Abstract Book distributed under the Attribution-NonCommercial-NoDerivs (CC BY-NC-ND), which allows third parties to download the articles and share them with others as long as they credit the author and the Abstract Book, but they cannot change the content in any way or use them commercially.
**Abstract Book Citations:** Authors, Title, HemaSphere, 2026;10:(S1):pages. The abstract book DOIs can be found on the “Author Information” page.
**Disclaimer:** Articles published in the journal HemaSphere exclusively reflect the opinions of the authors. The authors are responsible for all content in their abstracts including accuracy of the facts, statements, citing resources, etc.

### (PF895) BIOMETRIC DATA SHOWS GREATER ALIGNMENT WITH HAEMATOLOGIC RESULTS THAN SYMPTOM BURDEN IN PATIENTS WITH MYELOPROLIFERATIVE NEOPLASMS – UPDATES FROM THE MYMPNVOICE PATIENT APP

**Topic:** 16. Myeloproliferative neoplasms - Clinical

Patrick Harrington*[1], Kim Summers[2], Jay Corr[2], Muna Yusuf[2], Andrea Preston[2], Alfonso Portabales[2], Debbie Street[3], Mark Taylor[3], Amna Sheikh[1], Rebecca Frewin[4], Alesia Khan[5], Samah Alimam[6], Claire Woodley[1], Catherine Loughran[7], Priya Sriskandarajah[1], Ali Mahdi[8], Andrea Duminuco[9], Shahram Kordasti[10], Orlando Agrippa[2], Claire Harrison[1]

*[1]Guy's and St Thomas' NHS Foundation Trust, London, United Kingdom;[2]Sanius Health, London, United Kingdom;[3]MPN Voice, London, United Kingdom; [4]Gloucestershire Hospitals NHS Trust, Gloucester, United Kingdom;[5]Leeds Hospital NHS Trust, Leeds, United Kingdom; [6]University College London Hospitals, London, United Kingdom; [7]University Hospital Leicester, Leicester, United Kingdom; [8]Aneurin Bevan University Health Board, Newport, United Kingdom; [9]University of Catania, Catania, Italy; [10]King's College London, London, United Kingdom;*

### Background

Wearable technology and mobile applications provide the opportunity for continuous patient monitoring. Here we provide an update on the myMPNVoice app, launched in April 2024.

### Methods

Participants with an MPN diagnosis enrolled by self-referral through an online platform. The app allows patients to record MPN-10 total symptom score (TSS) and other patient reported outcomes (PRO). Biometric data are collected from a Withings watch that feeds into the app. A Linear Mixed Model was used to account for repeated measures, with age and sex as covariates.

Electronic health record (EHR) data were received from primary and tertiary care settings. Files were read with an optical character recognition (OCR) software (Amazon Textract or PyPDF2). An augmented Large Language Model (LLM, Llama) was used to identify a concept of interest, and of their negation, before extracting relevant values. Retrieval Augmented Generation (RAG) improved the LLM's medical domain knowledge. Paired mean values for haematologic laboratory results (haemoglobin [Hb], white cell count [WCC], platelets) and biometric and PRO data, over a weekly period, were assessed.

**Copyright Information:** (Online) ISSN: 2572-9241
©2026 The Author(s). HemaSphere is published by John Wiley & Sons Ltd on behalf of European Hematology Association. This is an open access Abstract Book distributed under the Attribution-NonCommercial-NoDerivs (CC BY-NC-ND), which allows third parties to download the articles and share them with others as long as they credit the author and the Abstract Book, but they cannot change the content in any way or use them commercially.
**Abstract Book Citations:** Authors, Title, HemaSphere, 2026;10:(S1):pages. The abstract book DOIs can be found on the "Author Information" page.
**Disclaimer:** Articles published in the journal HemaSphere exclusively reflect the opinions of the authors. The authors are responsible for all content in their abstracts including accuracy of the facts, statements, citing resources, etc.

**Results**

487 patients were enrolled in the study with data available from 449 patients. Of these 238 (49%) had ET, 157 (32%) had PV, 78 (16%) had MF and 14 (3%) MPN not otherwise specified (NOS). Patients had a median follow up of 300 days. Median age was 58 years (range 24-85), and 64% were female.
In ET, the highest MPN-10 TSS and lowest EQ-5D-5L scores were observed in patients receiving hydroxycarbamide (HC), while the lowest MPN-10 TSS and highest EQ-5D-5L were in those undergoing active surveillance (AS) (Figure 1a). Patients taking HC had lowest activity levels, whereas those receiving IFN had poorest overall sleep quality.
In PV, the highest MPN-10 TSS and lowest EQ-5D-5L were recorded in patients treated with ruxolitinib, while the lowest MPN-10 TSS and highest EQ-5D-5L were again observed in the AS group. Comparing typical first line therapies, a higher symptom burden and lower QoL was observed for IFN compared with HC.
Significant inverse correlations were observed between multiple PRO metrics and biometric parameters. Increasing MPN-10 TSS was associated with reduced active duration, steps, and calorie expenditure amongst other metrics (Figure 1b).
Regarding EHR laboratory results, data was received in 330 patients with laboratory values available in 223. There was greater alignment with biometric data than with patient reported symptom data. In ET, there was significant inverse correlation between platelet count and step count and distance (r=-0.22/p=0.001, r=-0.18/p=0.007), whereas no correlation with symptoms was observed (MPN10 TSS r=0.03/p=0.66, Figure 1c). Similarly, for WCC in ET there was inverse correlation with active time, distance, steps and calorie expenditure (r=-0.26/p<0.001, r=-0.35/p<0.001, r=-0.38/p<0.001, r=-0.18/p=0.01) but no association with symptoms (MPN-10 TSS r=0.02/p=0.7). Finally, for Hb, there was again, inverse correlation with steps and distance in ET patients (r=-0.4/p<0.001, r=-0.39/p<0.001) but no correlation with MPN-10 TSS (r=0.06/p=0.35).

**Summary/Conclusion**

In this study we present novel data describing correlation between TSS and various biometric data in MPN. We also show, for the first time, that biometrics are more aligned with laboratory results than symptoms, suggesting these may be more relevant as an endpoint in future clinical trials.

**Copyright Information:** (Online) ISSN: 2572-9241
©2026 The Author(s). HemaSphere is published by John Wiley & Sons Ltd on behalf of European Hematology Association. This is an open access Abstract Book distributed under the Attribution-NonCommercial-NoDerivs (CC BY-NC-ND), which allows third parties to download the articles and share them with others as long as they credit the author and the Abstract Book, but they cannot change the content in any way or use them commercially.
**Abstract Book Citations:** Authors, Title, HemaSphere, 2026;10:(S1):pages. The abstract book DOIs can be found on the "Author Information" page.
**Disclaimer:** Articles published in the journal HemaSphere exclusively reflect the opinions of the authors. The authors are responsible for all content in their abstracts including accuracy of the facts, statements, citing resources, etc.

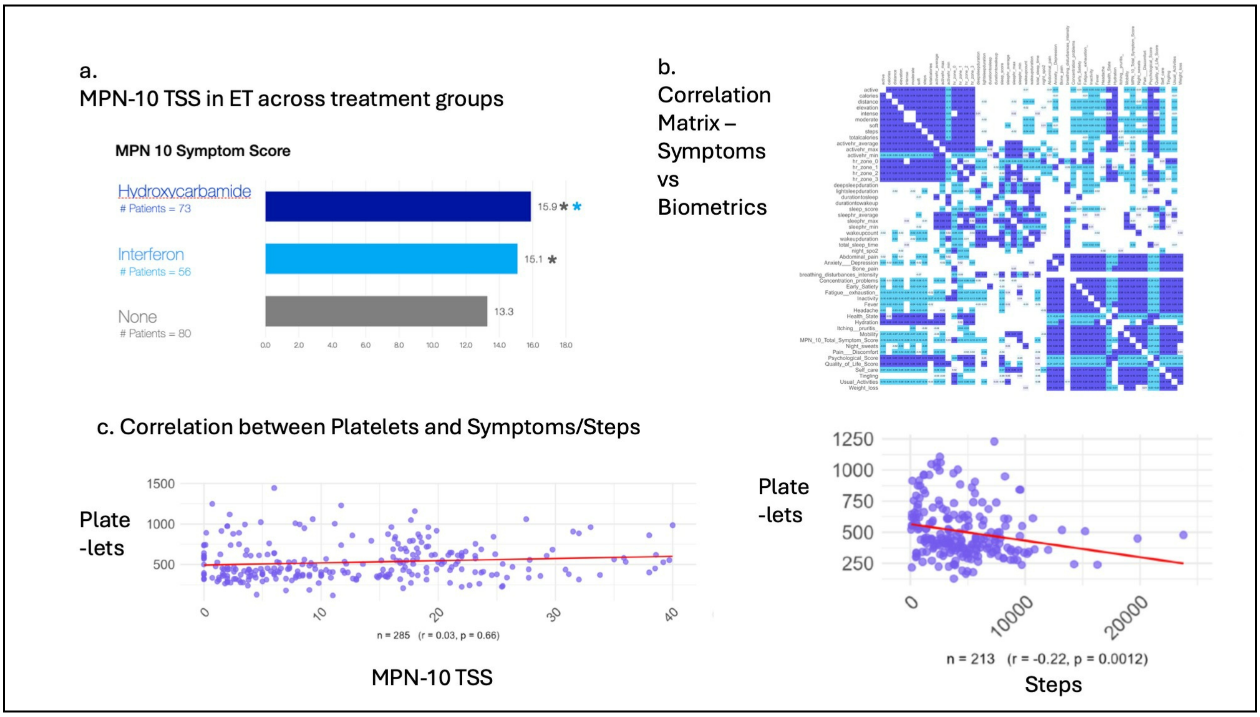

**Copyright Information:** (Online) ISSN: 2572-9241
©2026 The Author(s). HemaSphere is published by John Wiley & Sons Ltd on behalf of European Hematology Association. This is an open access Abstract Book distributed under the Attribution-NonCommercial-NoDerivs (CC BY-NC-ND), which allows third parties to download the articles and share them with others as long as they credit the author and the Abstract Book, but they cannot change the content in any way or use them commercially.
**Abstract Book Citations:** Authors, Title, HemaSphere, 2026;10:(S1):pages. The abstract book DOIs can be found on the "Author Information" page.
**Disclaimer:** Articles published in the journal HemaSphere exclusively reflect the opinions of the authors. The authors are responsible for all content in their abstracts including accuracy of the facts, statements, citing resources, etc.

### (PF896) SAFETY RESULTS OF THE T2020-004 PHASE I/II TRIAL OF TRAMETINIB AND RISK-STRATIFIED CHEMOTHERAPY FOR CHILDREN WITH NEWLY DIAGNOSED JUVENILE MYELOMONOCYTIC LEUKEMIA: A REPORT FROM THE TACL CONSORTIUM

**Topic:** 16. Myeloproliferative neoplasms - Clinical

Elliot Stieglitz*[1], Lauren Pommert[2], Reuven Schore[3], Dana Salzberg[4], Jamie Stokke[5], Jennifer Agrusa[6], Ziedulla Abdullaev[7], Janel Long-Boyle[1], Bill Chang[8], Yueh-Yun Chi[5], Christopher Dvorak[1], Ellynore Florendo[5], Omkar Singh[7], Erica Southworth[1], Sarah Tasian[9], Marielle Yohe[7], Deepa Bhojwani[5], Alan S Wayne[5], Mignon Loh[10]

*[1]University of California, San Francisco, San Francisco, United States of America;[2]Cincinnati Children's Hospital, Cincinnati, United States of America; [3]Children's National Hospital, Washington, United States of America;[4]Phoenix Children's Hospital, Phoenix, United States of America; [5]Children's Hospital Los Angeles, Los Angeles, United States of America;[6]C.S. Mott Children's Hospital, Ann Arbor, United States of America; [7]National Cancer Institute, National Institutes of Health, Bethesda, United States of America; [8]Oregon Health & Science University, Portland, United States of America;[9]Children's Hospital of Philadelphia, Philadelphia, United States of America; [10]Seattle Children's Hospital, Seattle, United States of America;*

### Background

Clinical trials and practice guidelines for children with juvenile myelomonocytic leukemia (JMML) have historically mandated hematopoietic stem cell transplantation (HSCT) for all patients. DNA methylation and mutational burden have been shown in retrospective studies to predict transplant-free survival. Separately, the MEK inhibitor trametinib demonstrated clinical activity in children with relapsed/refractory JMML (Stieglitz *Cancer Discovery* 2024). We therefore designed T2020-004, the first prospective risk-stratified clinical trial in JMML, in which patients receive trametinib-based regimens with or without consolidative HSCT based on DNA methylation testing and mutational burden.

### Aims

The primary objectives are to determine the safety of trametinib and azacitidine in patients with lower-risk (LR) JMML and the safety of trametinib in combination with intensive chemotherapy in patients with high-risk (HR) JMML. Secondary objectives are to determine event-free survival in LR patients treated without HSCT and to determine the rate of molecular remission (variant allele frequency <1%) in HR patients prior to HSCT.

### Methods

T2020-004 is a phase I/II clinical trial sponsored by the Therapeutic Advances in Childhood Leukemia and Lymphoma Consortium (TACL). Phase I uses a Rolling 6 design in which children with LR JMML (low DNA methylation and 1 somatic RAS pathway gene mutation) receive trametinib (dose level 1 [DL1]=0.032 mg/kg/day for patients ≤6 years and 0.025 mg/kg/day for patients >6 years; days 1–28) in combination with azacitidine (75 mg/m²/day; days 1–5) for up to 12 cycles. LR patients only proceed to HSCT in the setting of progressive disease. Children with HR JMML (intermediate/high DNA methylation and >1 somatic mutation, including monosomy 7) receive the same trametinib and azacitidine backbone with fludarabine (30 mg/m²/day, or 1 mg/kg if <12 kg; days 6–10) and cytarabine (2,000 mg/m²/day, or 67 mg/kg if <12 kg; days 6–10) for two cycles. Consolidative HSCT following trametinib-based chemotherapy was recommended for all HR patients.

**Copyright Information:** (Online) ISSN: 2572-9241
©2026 The Author(s). HemaSphere is published by John Wiley & Sons Ltd on behalf of European Hematology Association. This is an open access Abstract Book distributed under the Attribution-NonCommercial-NoDerivs (CC BY-NC-ND), which allows third parties to download the articles and share them with others as long as they credit the author and the Abstract Book, but they cannot change the content in any way or use them commercially.
**Abstract Book Citations:** Authors, Title, HemaSphere, 2026;10:(S1):pages. The abstract book DOIs can be found on the "Author Information" page.
**Disclaimer:** Articles published in the journal HemaSphere exclusively reflect the opinions of the authors. The authors are responsible for all content in their abstracts including accuracy of the facts, statements, citing resources, etc.

**Results**

Phase I enrollment began in October 2024. Five patients were enrolled in the LR cohort with n=1 each for mutations in *CBL*, *NRAS*, *KRAS*, *NF1*, and *PTPN11*. No dose-limiting toxicities (DLTs) were observed, and DL1 was declared the recommended phase 2 dose (RP2D). One patient with clinical NF1 proceeded to HSCT per provider preference. The remaining four LR patients have completed 4 to 12 cycles of therapy (data cut-off 1 February 2026) and continue to undergo molecular monitoring. Six patients were enrolled in the HR cohort with mutations in *KRAS* (n=1), *NF1* (n=2), *NRAS* (n=1), and *PTPN11* (n=2). All patients harbored secondary mutations and/or monosomy 7 and had intermediate or high DNA methylation signatures. One patient experienced a DLT of grade 4 *Clostridioides difficile* gastrointestinal infection. DL1 was declared the RP2D for this cohort. Both patients with *NF1*-mutant JMML achieved molecular remission after two cycles of therapy, and all 6 HR patients proceeded to HSCT. No disease- or treatment-related deaths have been reported to date.

**Summary/Conclusion**

T2020-004 is the first prospective JMML precision medicine trial incorporating clinical-grade DNA methylation and mutation testing for risk stratification. Trametinib-based therapy was well tolerated in both LR and HR patients with successful RP2D identification. The phase 2 efficacy component of the trial is now enrolling in both LR and HR cohorts.

**Copyright Information:** (Online) ISSN: 2572-9241
©2026 The Author(s). HemaSphere is published by John Wiley & Sons Ltd on behalf of European Hematology Association. This is an open access Abstract Book distributed under the Attribution-NonCommercial-NoDerivs (CC BY-NC-ND), which allows third parties to download the articles and share them with others as long as they credit the author and the Abstract Book, but they cannot change the content in any way or use them commercially.
**Abstract Book Citations:** Authors, Title, HemaSphere, 2026;10:(S1):pages. The abstract book DOIs can be found on the "Author Information" page.
**Disclaimer:** Articles published in the journal HemaSphere exclusively reflect the opinions of the authors. The authors are responsible for all content in their abstracts including accuracy of the facts, statements, citing resources, etc.

### (PF897) STRATIFYING AUTOIMMUNE RISK IN MPN THERAPY: INDEPENDENT ROLES OF INTERFERON-Α AND RUXOLITINIB WITH PREDICTIVE BIOMARKERS

**Topic:** 16. Myeloproliferative neoplasms - Clinical

Guangshuai Teng*[1], Yuhui Zhang[1], Ke Shang[1], Anna Li[1], Chenxiao Du[1], Yan Wang[1], Yanqi Li[1], Huiqin Zhang[1], Lan Peng[1], Yifan Duan[1], Weiping Yuan[1, 2], Jie Bai[1]

*[1]The Second Hospital of Tianjin Medical University, Department of Hematology, Tianjin, China;[2]Institute of Hematology & Blood Diseases Hospital, Chinese Academy of Medical Sciences & Peking Union Medical College, State Key Laboratory of Experimental Hematology, National Clinical Research Center for Blood Diseases, Tianjin, China;*

### Background

Myeloproliferative neoplasms (MPNs) are intrinsically linked to immune dysregulation, with a high prevalence of autoimmune diseases (AID). Interferon-α (IFN-α), a cornerstone therapy, exerts immune activation that may paradoxically precipitate AID-a critical safety concern that remains poorly characterized. Ruxolitinib (RUX), a JAK1/2 inhibitor, possesses immunomodulatory properties, but whether it influences the risk of IFN-α-associated AID is unknown in combined therapy.

### Aims

To evaluate the impact of IFN-α and RUX therapy on AID risk in MPN patients and to identify predictive biomarkers for AID susceptibility.

### Methods

We conducted a retrospective cohort study of MPN patients. A Fine-Gray competing risk model (with death as competing event) identified risk factors for AID. Propensity score matching (PSM) balanced baseline characteristics; AID-free survival was compared by Kaplan-Meier. Pre- and post-treatment serum cytokines were measured. Treatment-biomarker interaction analyses explored modifiers of RUX's protective effect.

### Results

A total of 473 MPN patients were analyzed. Multivariable analysis identified IFN-α therapy as an independent risk factor for AID (HR=401, 95%CI:154–10.93, P=0.005). Other independent predictors included prior AID history (HR=2.62, P=0.04), serum globulin ≥35 g/L (HR=2.94, P=0.024), peripheral blood $CD19^+$ B cells >18% (HR=3.03, P=0.018), and anti-Ro52 positivity (HR=4.05, P=0.004). In contrast, RUX treatment was protective (HR=0.16, P=0.005). After PSM, IFN-α treated patients had significantly shorter AID-free survival, while RUX treated patients had significantly longer AID-free survival (both P<0.05). Development of AID was associated with inferior fibrosis-free and overall survival (P<0.05). Mechanistically, IFN-α monotherapy increased IL-6 and IFN-γ (both P<0.05), whereas RUX monotherapy suppressed pro-inflammatory cytokines (IL-1β, IL-6, IL-17A, TNF-α; all P<0.05). Interaction analysis showed that RUX's protective effect was independent of baseline immunological markers.

### Summary/Conclusion

IFN-α is an independent risk factor for new-onset AID in MPN, while RUX exerts a protective effect. Baseline $CD19^+$ B-cell expansion (>18%) and anti-Ro52 antibodies are novel predictive biomarkers for AID susceptibility. The association of AID with poorer survival supports risk stratification for IFN-α therapy, suggesting that RUX co-treatment may mitigate autoimmune toxicity in high-risk patients. Prospective validation of these biomarkers is warranted.

**Copyright Information:** (Online) ISSN: 2572-9241
©2026 The Author(s). HemaSphere is published by John Wiley & Sons Ltd on behalf of European Hematology Association. This is an open access Abstract Book distributed under the Attribution-NonCommercial-NoDerivs (CC BY-NC-ND), which allows third parties to download the articles and share them with others as long as they credit the author and the Abstract Book, but they cannot change the content in any way or use them commercially.
**Abstract Book Citations:** Authors, Title, HemaSphere, 2026;10:(S1):pages. The abstract book DOIs can be found on the "Author Information" page.
**Disclaimer:** Articles published in the journal HemaSphere exclusively reflect the opinions of the authors. The authors are responsible for all content in their abstracts including accuracy of the facts, statements, citing resources, etc.

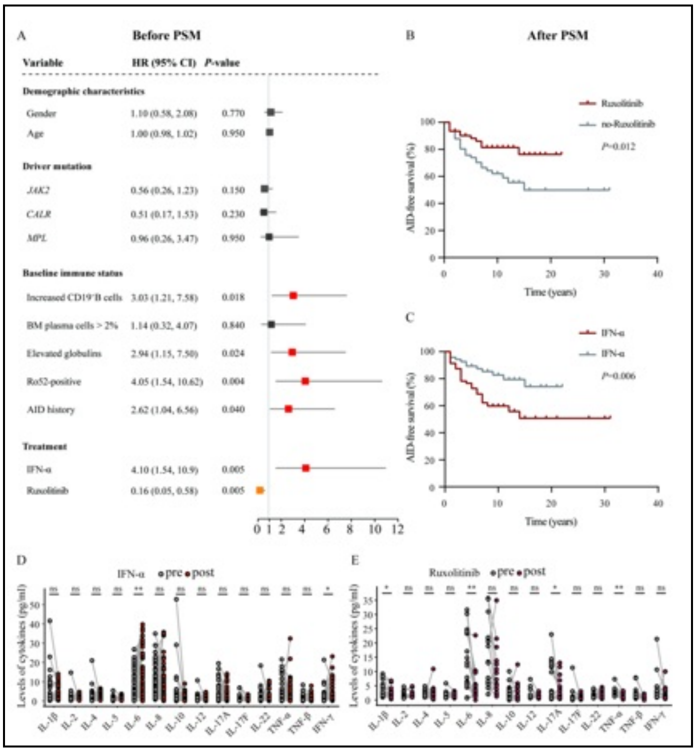

Figure. Independent and Opposing Roles of IFN-α and RUX in Modulating AID Risk in MPN. (A) Forest plot of multivariable Fine-Gray competing risk analysis (with death as competing event) identifying independent risk factors for AID. (B-C) AID-free survival after propensity score matching (n=76/group). PSM was performed on gender, age, elevated CD19+ B cells, elevated bone marrow plasma cells, Ro52 positivity, elevated globulin, and prior AID history to balance groups. (D-E) Longitudinal changes in serum cytokine levels before and after IFN-α or ruxolitinib treatment. * P<0.05, ** P<0.01, *** P<0.001 by paired t-test.

**Copyright Information:** (Online) ISSN: 2572-9241
©2026 The Author(s). HemaSphere is published by John Wiley & Sons Ltd on behalf of European Hematology Association. This is an open access Abstract Book distributed under the Attribution-NonCommercial-NoDerivs (CC BY-NC-ND), which allows third parties to download the articles and share them with others as long as they credit the author and the Abstract Book, but they cannot change the content in any way or use them commercially.
**Abstract Book Citations:** Authors, Title, HemaSphere, 2026;10:(S1):pages. The abstract book DOIs can be found on the "Author Information" page.
**Disclaimer:** Articles published in the journal HemaSphere exclusively reflect the opinions of the authors. The authors are responsible for all content in their abstracts including accuracy of the facts, statements, citing resources, etc.

### (PF898) IMPACT OF RUSFERTIDE ON POLYCYTHEMIA VERA (PV)-RELATED SYMPTOMS AND PATIENT-REPORTED OUTCOME-RELATED ITEMS IN THE RANDOMIZED, DOUBLE-BLIND PHASE 3 VERIFY STUDY

**Topic:** 16. Myeloproliferative neoplasms - Clinical

Alessandro Lucchesi*[1], Aniket Bankar[2], Kristen Pettit[3], Naveen Pemmaraju[4], Joseph Shatzel[5], Valentin Garcia Gutierrez[6], Jiří Mayer[7], Abdulraheem Yacoub[8], Harry Gill[9], Antonín Hluší[10], Daniel Sasca[11], Joseph M Scandura[12], David Ross[13], Francesca Palandri[14], Maria Laura Fox[15], Alessandro Maria Vannucchi[16], Steffen Koschmieder[17], David Cella[18], Philipp Dinh[19], Jie Xiao[19], A. Peter Morello[19], Vidhyavathi Venkataraman[19], Sarita Khanna[19], Arturo Molina[19], Andrew Kuykendall[20]

*[1]IRCCS Istituto Romagnolo per lo Studio dei Tumori (IRST) "Dino Amadori", Meldola, Italy; [2]Princess Margaret Malignancy Centre, Toronto, Canada; [3]Rogel Cancer Center, University of Michigan Health, Ann Arbor, MI, United States of America; [4]The University of Texas MD Anderson Cancer Center, Houston, TX, United States of America; [5]Oregon Health & Science University, Portland, OR, United States of America; [6]Hospital Universitario Ramón y Cajal Instituto Ramón y Cajal de Investigación Sanitaria, Universidad de Alcalá, Madrid, Spain; [7]University Hospital and Masaryk University, Brno, Czechia; [8]University of Kansas Malignancy Center, Westwood, KS, United States of America; [9]Department of Medicine, School of Clinical Medicine, LKS Faculty of Medicine, the University of Hong Kong, Hong Kong, Hong Kong; [10]Palacky University and University Hospital Olomouc, Olomouc, Czechia; [11]Universitaetsmedizin der Johannes Gutenberg - Universitaet Mainz, Mainz, Germany; [12]Silver MPN Center/Weill Cornell Medicine, New York, NY, United States of America; [13]Royal Adelaide Hospital, Adelaide, Australia; [14]IRCCS Azienda Ospedaliero-Universitaria di Bologna - Institute of Hematology "L. and A. Seràgnoli", Bologna, Italy; [15]Vall d'Hebron Institute of Oncology (VHIO), Hospital Universitario Vall d'Hebron, Barcelona, Spain; [16]Azienda Ospedaliero-Universitaria Careggi, Firenze, Italy; [17]RWTH Aachen University, Faculty of Medicine, and Center for Integrated Oncology (CIO-ABCD), Aachen, Germany; [18]Northwestern University, Chicago, IL, United States of America; [19]Protagonist Therapeutics, Inc., Newark, CA, United States of America; [20]Moffitt Cancer Center, Tampa, FL, United States of America;*

### Background

Polycythemia vera (PV) is a chronic myeloproliferative neoplasm associated with erythrocytosis and symptom burden. Rusfertide is a first-in-class hepcidin mimetic peptide administered subcutaneously that controls erythrocytosis. In the phase 3 VERIFY study (NCT05210790) in patients (pts) with PV, rusfertide added to standard-of-care (SOC) treatment vs placebo (PBO) with SOC met the primary endpoint of clinical response and all four key secondary endpoints at Week 32 (Kuykendall AT et al. *J Clin Oncol* 2025;43(17_suppl):LBA3). Two of the key secondary endpoints focused on the Patient Reported Outcomes Measurement Information System (PROMIS) Fatigue Short Form (SF)-8a and the Myeloproliferative Neoplasm Symptom Assessment Form (MFSAF) version 4.0.

### Aims

This analysis reports on the change from baseline at Week 32 in total scores and between-group differences in the single items used in the PROMIS Fatigue SF-8a and MFSAF questionnaires.

### Methods

In VERIFY, pts entered their responses to the PROMIS Fatigue SF-8a and MFSAF questionnaires in electronic diaries prior to dosing at baseline and scheduled time points. Pts recorded responses to the PROMIS Fatigue SF-8a over the prior 7 days using a five-point scale (ie, "Not at all" to "Very much") and to the MFSAF over the prior 24 hours using an 11-point scale (ie, "0 [Absent]" to "10 [Worst Imaginable]"). Mean (standard deviation [SD]) change from baseline at Week 32 in the PROMIS Fatigue SF-8a T score and MFSAF total symptom score 7 (TSS7) were calculated. Individual item responses to both questionnaires were also examined. A negative change from baseline at Week 32 indicates improvement; a positive number indicates worsening.

**Copyright Information:** (Online) ISSN: 2572-9241
©2026 The Author(s). HemaSphere is published by John Wiley & Sons Ltd on behalf of European Hematology Association. This is an open access Abstract Book distributed under the Attribution-NonCommercial-NoDerivs (CC BY-NC-ND), which allows third parties to download the articles and share them with others as long as they credit the author and the Abstract Book, but they cannot change the content in any way or use them commercially.
**Abstract Book Citations:** Authors, Title, HemaSphere, 2026;10:(S1):pages. The abstract book DOIs can be found on the "Author Information" page.
**Disclaimer:** Articles published in the journal HemaSphere exclusively reflect the opinions of the authors. The authors are responsible for all content in their abstracts including accuracy of the facts, statements, citing resources, etc.

### Results

At randomization, 293 pts (median age, 57 years; 46.8% with high-risk PV) were enrolled in the rusfertide (n=147) or PBO (n=146) arms. A total of 275 pts (134 [91.2%] and 141 [96.6%] in each respective arm) completed 32 weeks of treatment. Mean (SD) change from baseline at Week 32 in the PROMIS Fatigue SF-8a T-score was -3.87 (8.22) and -1.06 (8.14) in the rusfertide and PBO arms, respectively (LS means [standard error of the mean (SEM)] difference [rusfertide–PBO], -1.98 [0.88]; p=0.0252) (**Figure**). Although mean change from baseline at Week 32 improved in both arms for each of the eight fatigue-related items in the PROMIS Fatigue SF-8a, numeric improvements in each item were greater in the rusfertide arm. Mean (SD) change from baseline at Week 32 in the MFSAF TSS7 was -2.96 (8.04) and -0.06 (5.72) in the rusfertide and PBO arms, respectively (LS means [SEM] difference [rusfertide–PBO], -1.87 [0.82]; p=0.0240). Mean change from baseline at Week 32 improved in the rusfertide arm for all seven items in the MFSAF (**Figure**). In contrast, two items (abdominal discomfort and worst bone pain) worsened in the PBO arm. Numerically, improvements in all items were more favorable in the rusfertide arm, including those items where improvements were also recorded in the PBO arm.

### Summary/Conclusion

In VERIFY, rusfertide-treated pts had a significant improvement in the PROMIS Fatigue SF-8a T-score and MFSAF TSS7 at Week 32 vs PBO (p<0.03). All items in the PROMIS Fatigue-SF-8a and MFSAF improved in the rusfertide arm vs. PBO. Although items improved in the PBO arm, numerically greater improvements in all items were observed in the rusfertide arm. These findings support the key PRO-focused secondary endpoints in VERIFY, further demonstrating that rusfertide broadly improved symptomatology-related items in pts with PV.

**Copyright Information:** (Online) ISSN: 2572-9241
©2026 The Author(s). HemaSphere is published by John Wiley & Sons Ltd on behalf of European Hematology Association. This is an open access Abstract Book distributed under the Attribution-NonCommercial-NoDerivs (CC BY-NC-ND), which allows third parties to download the articles and share them with others as long as they credit the author and the Abstract Book, but they cannot change the content in any way or use them commercially.
**Abstract Book Citations:** Authors, Title, HemaSphere, 2026;10:(S1):pages. The abstract book DOIs can be found on the "Author Information" page.
**Disclaimer:** Articles published in the journal HemaSphere exclusively reflect the opinions of the authors. The authors are responsible for all content in their abstracts including accuracy of the facts, statements, citing resources, etc.

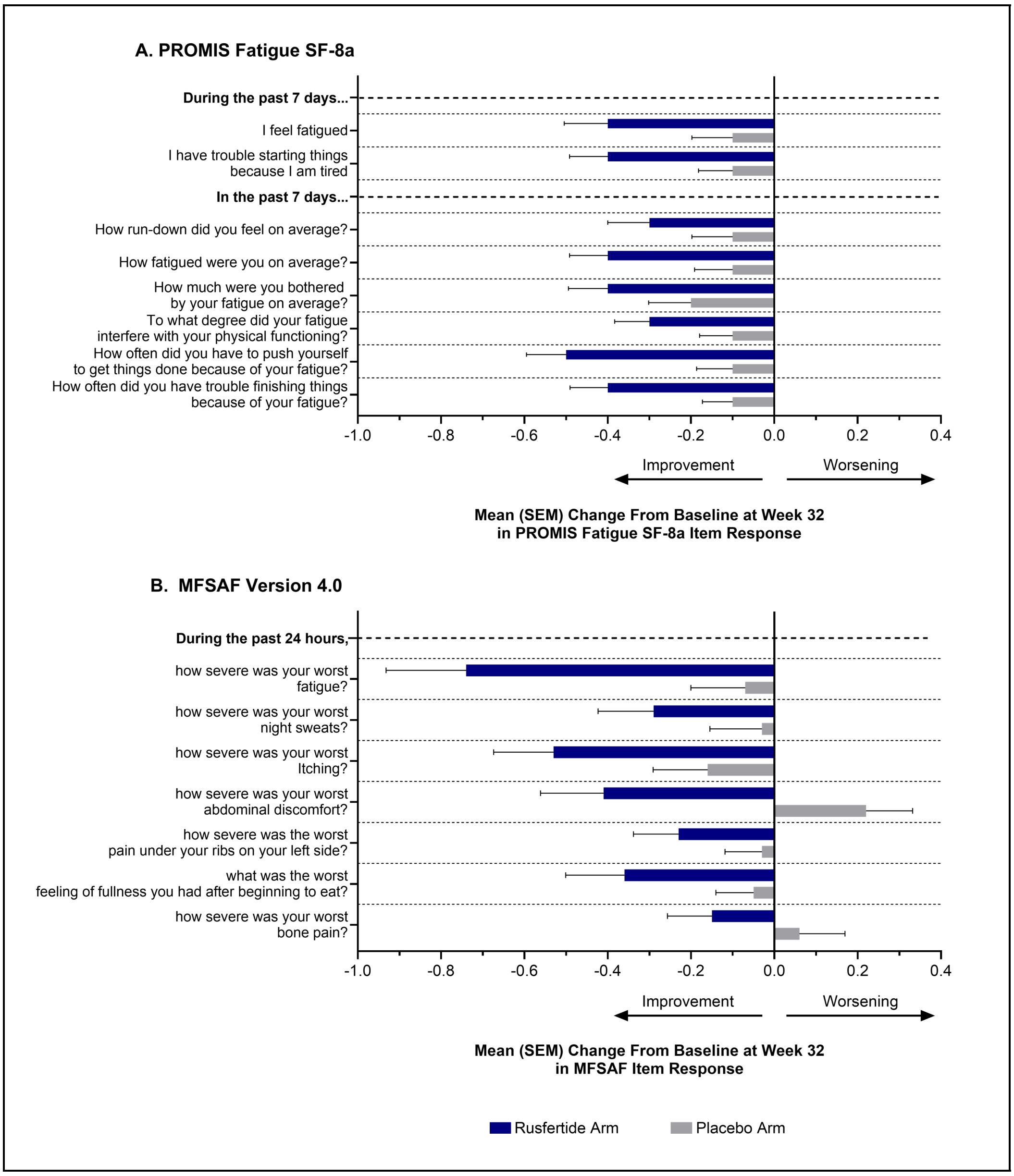

**Copyright Information:** (Online) ISSN: 2572-9241
©2026 The Author(s). HemaSphere is published by John Wiley & Sons Ltd on behalf of European Hematology Association. This is an open access Abstract Book distributed under the Attribution-NonCommercial-NoDerivs (CC BY-NC-ND), which allows third parties to download the articles and share them with others as long as they credit the author and the Abstract Book, but they cannot change the content in any way or use them commercially.
**Abstract Book Citations:** Authors, Title, HemaSphere, 2026;10:(S1):pages. The abstract book DOIs can be found on the "Author Information" page.
**Disclaimer:** Articles published in the journal HemaSphere exclusively reflect the opinions of the authors. The authors are responsible for all content in their abstracts including accuracy of the facts, statements, citing resources, etc.

### (PF899) IMPACT OF METFORMIN (MET) ON THE INCIDENCE OF SECOND MALIGNANCIES (SMS) IN OLDER PATIENTS WITH POLYCYTHEMIA VERA (PV) OR ESSENTIAL THROMBOCYTHEMIA (ET) IN THE UNITED STATES

**Topic:** 16. Myeloproliferative neoplasms - Clinical

Jessica Stempel*[1, 2], Rong Wang[2, 3], Lourdes Mendez[1], Jan Bewersdorf[1], Maximilian Stahl[1], Luis E. Aguirre[1], Alain Mina[1], Amer M. Zeidan[1, 2], Nikolai Podoltsev[1, 2], Xiaomei MA[2, 3]

*[1]Yale School of Medicine, New Haven, United States of America;[2]Yale University, Cancer Outcomes, Public Policy, and Effectiveness Research (COPPER) Center, New Haven, United States of America; [3]Yale School of Public Health, Chronic Disease Epidemiology, New Haven, United States of America;*

### Background

Patients with myeloproliferative neoplasms (MPNs) have an increased risk of SMs including solid and hematologic malignancies (*Landtblom et al. 2018*). MET has been associated with a reduced incidence of MPNs (*Kristensen et al. 2024),* generating interest in its potential antineoplastic effects. However, no study has examined MET use in relation to the risk of SMs in patients with MPNs.

### Aims

To evaluate the association between MET exposure and SM risk in patients diagnosed with PV or ET.

### Methods

We conducted a retrospective cohort study using the Surveillance, Epidemiology, and End Results-Medicare linked database to evaluate MET exposure and SMs risk in patients diagnosed with PV or ET during 2008-2019. Eligible patients were aged 66-99 years, continuously enrolled in Medicare Parts A/B/D and followed for ≥6 months after MPN diagnosis. MET users were defined as patients receiving MET ≥6 months before MPN diagnosis and continuing for ≥180 days. SM events counted if diagnosed ≥30 days after MPN diagnosis. Patients were followed from diagnosis until 12/31/2020, death, or loss of coverage, whichever occurred first. Multivariable Fine-Gray proportional subdistribution hazards models, with death as a competing risk, were used to estimate the association between MET exposure and SM risk, adjusting for sociodemographic and clinical covariates.

**Copyright Information:** (Online) ISSN: 2572-9241
©2026 The Author(s). HemaSphere is published by John Wiley & Sons Ltd on behalf of European Hematology Association. This is an open access Abstract Book distributed under the Attribution-NonCommercial-NoDerivs (CC BY-NC-ND), which allows third parties to download the articles and share them with others as long as they credit the author and the Abstract Book, but they cannot change the content in any way or use them commercially.
**Abstract Book Citations:** Authors, Title, HemaSphere, 2026;10:(S1):pages. The abstract book DOIs can be found on the "Author Information" page.
**Disclaimer:** Articles published in the journal HemaSphere exclusively reflect the opinions of the authors. The authors are responsible for all content in their abstracts including accuracy of the facts, statements, citing resources, etc.

**Results**

Of 6576 eligible patients (PV, n=2937; ET, n=3639), 944 (14.4%) had diabetes mellitus (DM). A total of 546 were MET users, and 6030 were non-users. Most MPN patients were female (62.7%), had ≤2 comorbid conditions (77.3%), and received influenza immunization (61.9%) (indicative of healthcare access). MET users were frailer and had higher comorbidity scores than non-users ($p<0.01$). A total of 1453 patients (22.1%) had a prior history of cancer, and the percentage was similar between MET users and non-users ($p=0.41$).
The median follow-up for the cohort was 3.25 years (interquartile range [IQR], 1.89-5.33). Among MET users, the median proportion of days covered (PDC) was 82.4% (IQR 57.3%–95.2%), indicating sustained drug exposure throughout follow-up. Overall, 715 patients (10.9%) developed a SM: 526 solid, 81 lymphoid, and 108 myeloid. Patients with DM had a higher unadjusted SM incidence rate than those without DM (31.2 vs 27.0 per 1,000 person-years). Among patients with DM, MET use was associated with a lower incidence of SM compared to non-users (23.9 vs 35.7 per 1,000 person-years).
In multivariable competing-risk models, MET use was associated with a significantly lower risk of any SM (hazard ratio [HR]=0.70, 95% confidence interval [CI] 0.50–0.97; $p=0.03$), while DM was independently associated with increased risk (HR=1.27, 95% CI 1.04–1.56, $p=0.02$) (**Figure**). Compared with those without DM, patients with DM not receiving MET had a higher risk (HR=1.27, 95% CI 1.04–1.56). MET users with DM had a risk comparable to those without DM (HR=0.88, 95% CI 0.66–1.19) and a reduced risk compared to MET non-users with DM (HR=0.70, 95% CI 0.50–0.97). When considering the duration of MET exposure, increasing MET PDC was associated with a lower risk of SMs (per 10% increase: HR=0.93, 95% CI 0.89–0.97). Similar exposure-response associations were observed for solid (HR=0.93, 95% CI 0.88–0.98) and lymphoid (HR = 0.83, 95% CI 0.69–0.99) SM subgroups.

**Summary/Conclusion**

Our study suggests that MET exposure is associated with a lower risk of SM in older patients with PV or ET, with a significant MET exposure-response effect.

**Copyright Information:** (Online) ISSN: 2572-9241
©2026 The Author(s). HemaSphere is published by John Wiley & Sons Ltd on behalf of European Hematology Association. This is an open access Abstract Book distributed under the Attribution-NonCommercial-NoDerivs (CC BY-NC-ND), which allows third parties to download the articles and share them with others as long as they credit the author and the Abstract Book, but they cannot change the content in any way or use them commercially.
**Abstract Book Citations:** Authors, Title, HemaSphere, 2026;10:(S1):pages. The abstract book DOIs can be found on the "Author Information" page.
**Disclaimer:** Articles published in the journal HemaSphere exclusively reflect the opinions of the authors. The authors are responsible for all content in their abstracts including accuracy of the facts, statements, citing resources, etc.

**Figure. Multivariable analysis of the second malignancy (SM) risk by metformin (MET) exposure and diabetes mellitus (DM) status (n=6576)**

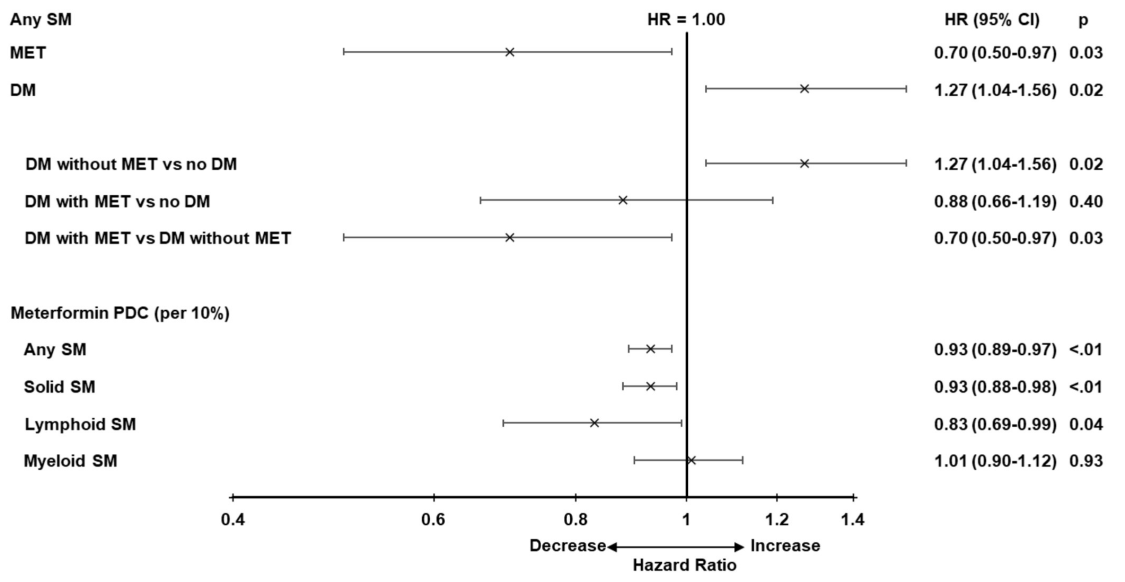

CI: confidence interval, DM: diabetes mellitus, HR: hazard ratio, MET: metformin, PDC: proportion of days covered, SM: second malignancy

**Copyright Information:** (Online) ISSN: 2572-9241
©2026 The Author(s). HemaSphere is published by John Wiley & Sons Ltd on behalf of European Hematology Association. This is an open access Abstract Book distributed under the Attribution-NonCommercial-NoDerivs (CC BY-NC-ND), which allows third parties to download the articles and share them with others as long as they credit the author and the Abstract Book, but they cannot change the content in any way or use them commercially.
**Abstract Book Citations:** Authors, Title, HemaSphere, 2026;10:(S1):pages. The abstract book DOIs can be found on the “Author Information” page.
**Disclaimer:** Articles published in the journal HemaSphere exclusively reflect the opinions of the authors. The authors are responsible for all content in their abstracts including accuracy of the facts, statements, citing resources, etc.

### (PF900) MODEST INCREASES IN JAK2V617F AND CALR MUTATION ALLELE BURDEN PREDICT ADVERSE OUTCOMES AND ARE ASSOCIATED WITH ANAGRELIDE IN MYELOPROLIFERATIVE NEOPLASMS

**Topic:** 16. Myeloproliferative neoplasms - Clinical

Zi Yun Ng[1, 2, 3], Laurane Cottin[4], Marine Demoy[4], Damien Luque-Paz[4], Corentin Orvain[4], Wayne-Corentin Lambert[5], Jean Christophe Ianotto[6], Eric Lippert[5], David Ross[7], Diana Iarossi[7], Alesia Khan[8], Daniel Lock[8], Catherine Truong[9], Kamel Laribi[9], Yunfan Yang[10], Laura Li Gagnon[1, 11], Valerie Ugo[4], Claire Harrison[1], Jennifer O'sullivan*[1, 12]

*[1]Guy's and St. Thomas' NHS Foundation Trust, London, United Kingdom;[2]Royal Perth Hospital, Perth, Australia;[3]Rockingham General Hospital, Perth, Australia; [4]Univ Angers, Nantes Université, CHU Angers, Inserm, CNRS, CRCI2NA, F-49000, Angers, France; [5]Service d'Hématologie biologique, Hôpital de la Cavale Blanche, CHU de Brest, Brest, France;[6]Service d'Hématologie et d'Hémostase clinique, Institut de Cancérologie et Hématologie, CHU de Brest, Brest, France; [7]Dept of Haematology, Royal Adelaide Hospital and Flinders Medical Centre, Adelaide, Australia; [8]Haematological Malignancy Diagnostic Service, Leeds Teaching Hospital NHS Trust, Leeds, United Kingdom; [9]Hématologie Clinique, CH Le Mans, Le Mans, France; [10]Department of Haematology, West China Hospital, Sichuan University, Chengdu, Sichuan, China; [11]Department of Haematology, CHU de Québec-Université Laval, Quebec, Canada; [12]Medical Research Council (MRC) Weatherall Institute of Molecular Medicine, University of Oxford, Oxford, United Kingdom;*

### Background

*JAK2*$^{V617F}$ variant allele frequency (VAF) reductions correlate with clinical benefit in myeloproliferative neoplasms (MPN), but less is known about *CALR* VAF dynamics.

### Aims

We evaluated dynamics of *CALR* & *JAK2*$^{V617F}$ VAF in MPN patients treated with cytoreduction & correlate with clinical characteristics/outcomes.

### Methods

Sequential cases collated from international centres & MAJIC-ET study. *CALR* & *JAK2*$^{V617F}$ VAF were quantified using various assays at respective centres.
MR defined as per IWG-MRT/ELN 2013 criteria; MRs only calculated if baseline VAF ≥20%. "PMR20", defined as 20-49% decrease in VAF was chosen *a priori* as it has been shown to correlate with clinical/histological response & overall survival (OS). VAF changes 0-1% were categorised as "no change", a 2-19% decrease in VAF as "non-MR decrease" & ≥2% increase as "increase".

**Copyright Information:** (Online) ISSN: 2572-9241
©2026 The Author(s). HemaSphere is published by John Wiley & Sons Ltd on behalf of European Hematology Association. This is an open access Abstract Book distributed under the Attribution-NonCommercial-NoDerivs (CC BY-NC-ND), which allows third parties to download the articles and share them with others as long as they credit the author and the Abstract Book, but they cannot change the content in any way or use them commercially.
**Abstract Book Citations:** Authors, Title, HemaSphere, 2026;10:(S1):pages. The abstract book DOIs can be found on the "Author Information" page.
**Disclaimer:** Articles published in the journal HemaSphere exclusively reflect the opinions of the authors. The authors are responsible for all content in their abstracts including accuracy of the facts, statements, citing resources, etc.

### Results

347 patients with *CALR* (*n*=190) & *JAK2*$^{V617F}$ (*n*=157) mutations with VAF measurements ≥6 months apart were included. Essential Thrombocythaemia was the most common (*n*=235, 67.7%), followed by primary/secondary myelofibrosis (MF; *n*=73, 21.1%), pre-fibrotic MF (*n*=17, 4.9%), polycythaemia vera (*n*=15, 4.3%), MPN-unclassified (*n*=6, 1.7%) & blast-phase MPN (*n*=1, 0.3%). Treatments included interferon (*n*=132, 38.0%), ruxolitinib (*n*=70, 20.2%), anagrelide (*n*=29, 8.4%), hydroxycarbamide (*n*=88, 25.4%) & other therapies (*n*=28, 8.0%).
Median change in VAF (ΔVAF) was +0.4% (range -65.95 to +64%). Median VAF measurement intervals were 66 months (range 6-246). Median follow-up was 123 months (range 8-469) & median treatment duration was 55 months (range 4-289).
Non-MPN driver mutations (NDM) were present in 45.3% (*n*=120/265) with ≥2 NDMs in 49.2% (*n*=59/120) of these patients. Most prevalent NDMs were *TET2* & *ASXL1* (12.7%, *n*=44, & 7.7%, *n*=27, of patients respectively). High molecular risk (defined by MIPSS70+), *RAS* pathway & *TP53* mutations were identified in 10.1% (*n*=35), 2.0% (*n*=7) & 2.8% (*n*=10) of patients respectively.
MR responses by treatment are described in Figure 1A with no differences by driver mutation. 86.2% of anagrelide-treated patients (*n*=25/29) had an increase in driver VAF compared with 37.9% (*n*=50/132), 45.7% (32/70) & 59% (52/88) interferon-, ruxolitinib- & hydroxycarbamide-treated patients respectively, $p<0.0001$ (Figure 1A). No association was noted between MR & haematological response, age at diagnosis, MPN subtype, disease duration or prior treatment lines.
OS & progression-free survival (PFS) did not differ by MPN subtype. Overall, 18.2% (*n*=63) had a progression event. At the 18-month landmark (*n*=100; 19 events), patients with a ΔVAF ≥10% were more likely to have a subsequent inferior PFS compared with those achieving MR or stable VAF ($p$=0.0011, Figure 1B), which remained significant after adjusting for driver mutation, *TP53/RAS* mutation status & centre ($p$=0.027).

### Summary/Conclusion

Anagrelide was associated with VAF increase in most patients irrespective of driver mutation. This finding aligns with prior reports linking anagrelide exposure to fibrotic transformation & supports its positioning below alternative cytoreductive therapies with greater disease-modifying potential. Furthermore, our data show that even modest allele burden expansions (ΔVAF ≥10%) are independently associated with progression and may serve as a predictive marker of disease evolution. In the era of emerging mutation-directed therapies that may achieve rapid reductions in allele burden, these findings underscore the prognostic relevance of serial VAF monitoring.

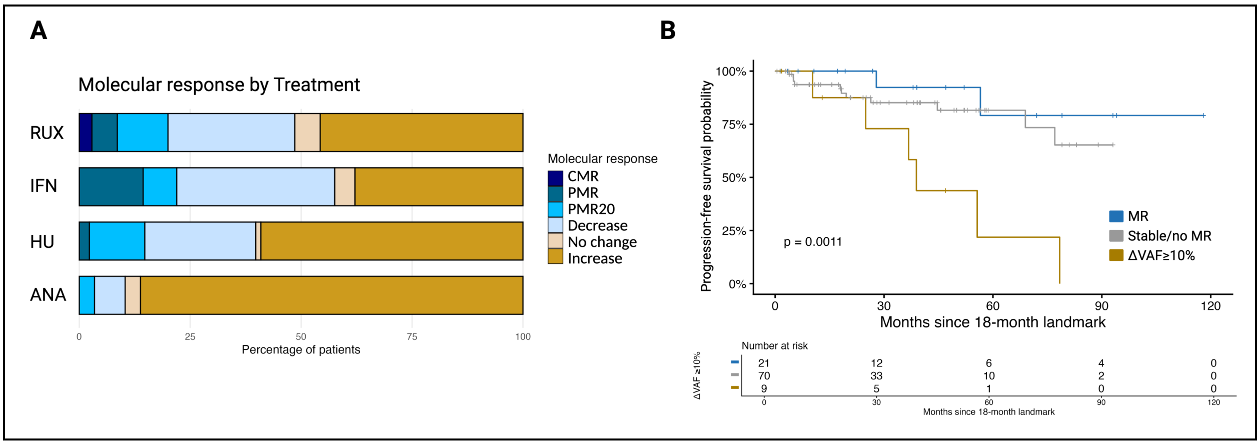

Figure 1. (A) Stacked barchart showing distribution of molecular response categories by treatment type. (B) Kaplan-Meier curve from 18-month landmark analysis showing progression-free survival (PFS) stratified by molecular response (MR), stable/no MR and VAF increase ΔVAF ≥10%. CMR=complete molecular response; PMR=partial molecular response; PMR20=20-49% reduction in allele burden; RUX=ruxolitinib; IFN=interferon; HC=hydroxycarbamide; ANA=anagrelide.

**Copyright Information:** (Online) ISSN: 2572-9241
©2026 The Author(s). HemaSphere is published by John Wiley & Sons Ltd on behalf of European Hematology Association. This is an open access Abstract Book distributed under the Attribution-NonCommercial-NoDerivs (CC BY-NC-ND), which allows third parties to download the articles and share them with others as long as they credit the author and the Abstract Book, but they cannot change the content in any way or use them commercially.
**Abstract Book Citations:** Authors, Title, HemaSphere, 2026;10:(S1):pages. The abstract book DOIs can be found on the "Author Information" page.
**Disclaimer:** Articles published in the journal HemaSphere exclusively reflect the opinions of the authors. The authors are responsible for all content in their abstracts including accuracy of the facts, statements, citing resources, etc.

### (PF901) EFFICACY AND SAFETY OF ROPEGINTERFERON ALFA-2B IN JAPANESE PATIENTS WITH ESSENTIAL THROMBOCYTHEMIA: SUBGROUP ANALYSIS OF THE SURPASS ET TRIAL

**Topic:** 16. Myeloproliferative neoplasms - Clinical

Yuka Sugimoto*[1], Keita Kirito[2], Shuichi Shirane[3, 4], Katsuto Takenaka[5], Michiko Ichii[6], Yoko Kubuki[7], Tomoki Ito[8], Michiaki Koike[9], Akihiko Gotoh[10], Hiroki Yamaguchi[11], Sheena Lin[12], Oleh Zagrijtschuk[13], Albert Qin[12], Hiroaki Kawase[14], Mai Sato[14], Norio Komatsu[3, 4, 14], Toshiaki Sato[14], Kazuya Shimoda[7]

*[1]Mie University Graduate School of Medicine, Department of Hematology and Oncology, Mie, Japan;[2]University of Yamanashi, Department of Hematology and Oncology, Yamanashi, Japan; [3]Juntendo University Graduate School of Medicine, Department of Hematology, Tokyo, Japan; [4]Juntendo University Graduate School of Medicine, Department of Advanced Hematology, Tokyo, Japan; [5]Ehime University Graduate School of Medicine, Department of Hematology, Clinical Immunology and Infectious Diseases, Ehime, Japan; [6]Osaka University Graduate School of Medicine, Department of Hematology and Oncology, Osaka, Japan; [7]University of Miyazaki, Division of Hematology, Diabetes, and Endocrinology, Department of Internal Medicine, Miyazaki, Japan; [8]Kansai Medical University, First Department of Internal Medicine, Osaka, Japan;[9]Juntendo University Shizuoka Hospital, Department of Hematology, Shizuoka, Japan; [10]Tokyo Medical University, Department of Hematology, Tokyo, Japan; [11]Nippon Medical School, Department of Hematology, Tokyo, Japan; [12]PharmaEssentia Corporation, Taipei, Taiwan; [13]PharmaEssentia USA Corporation, Burlington, Massachusetts, United States of America; [14]PharmaEssentia Japan KK, Tokyo, Japan;*

### Background

Essential thrombocythemia (ET) is a myeloproliferative neoplasm characterized by elevated blood counts, thrombosis symptoms and risks, and progression to myelofibrosis or acute leukemia. According to the Japanese Society of Hematology's Guidelines for the Treatment of Hematologic Malignancies, the first-line treatment is observation or low-dose aspirin for low-risk ET patients and hydroxyurea (HU) or anagrelide(ANA) for high-risk. Ropeginterferon alfa-2b (ropegIFN) is a new-generation interferon therapy approved for polycythemia vera. Here, we present a study assessing ropegIFN in Japanese ET patients.

### Aims

This study aimed to evaluate the efficacy and safety of ropeIFN in Japanese ET patients.

### Methods

This was a subgroup analysis of the global SURPASS-ET trial, a randomized, open-label, multicenter phase III study comparing ropegIFN with ANA in ET patients resistant or intolerant to HU. A total of 174 patients, including 39 Japanese, received ropegIFN (n=91) or ANA (n=83). The starting dose of ropegIFN was 250 mcg. The dose was titrated to 350 mcg at week2 and to 500 mcg from week 4, every 2 weeks if tolerated. The primary endpoint was the rate of patients demonstrating a response at months9 and 12, according to the modified European LeukemiaNet (ELN) criteria. Secondary endpoints included symptoms, thromboembolic events, spleen size, driver mutation variant allele frequency (VAF), and safety.

**Copyright Information:** (Online) ISSN: 2572-9241
©2026 The Author(s). HemaSphere is published by John Wiley & Sons Ltd on behalf of European Hematology Association. This is an open access Abstract Book distributed under the Attribution-NonCommercial-NoDerivs (CC BY-NC-ND), which allows third parties to download the articles and share them with others as long as they credit the author and the Abstract Book, but they cannot change the content in any way or use them commercially.
**Abstract Book Citations:** Authors, Title, HemaSphere, 2026;10:(S1):pages. The abstract book DOIs can be found on the “Author Information” page.
**Disclaimer:** Articles published in the journal HemaSphere exclusively reflect the opinions of the authors. The authors are responsible for all content in their abstracts including accuracy of the facts, statements, citing resources, etc.

**Results**

49 Japanese patients were screened and 39 were randomized (22 to ropegIFN group, 17 to ANA group). All randomized Japanese patients received an investigational drug. Of the 39, 34 (87.2%) completed the trial: 20 (90.9%) in the ropegIFN group and 14 (82.4%) in the ANA group. RopegIFN showed superiority over ANA for the primary endpoint (50.0% vs. 5.9%; p=0.0047) in Japanese patients. RopegIFN consistently showed higher rates across hematologic response and symptom improvement. Thrombotic events occurred in no patients in the ropegIFN group and 2 patients (13.3%) in the ANA group. For ropegIFN, the mean change in *JAK2* V617F VAF from baseline was -8.67% at 6 months (n=16) and -11.11% at 12 months (n=16), showing a statistically significant decrease at both time points (p = 0.0018 and p = 0.0116). For ANA, the mean change from baseline was +3.60% at 6 months (n=12) and +3.90% at 12 months (n=12), showing a significant increase at both time points (p=0.0024 and p=0.0185). At baseline, 4 out of 22 patients were *CALR* mutation-positive in the ropegIFN group versus 2 out of 17 in the ANA group. One patient in the ropegIFN group showed a decrease in *CALR* VAF, while an increase was observed in the ANA group. No Japanese patient was positive for the *MPL* mutation.
RopegIFN showed a better safety profile than ANA, with fewer Grade ≥3 treatment emergent adverse events (TEAEs) (13.6% vs. 29.4%), serious AEs (9.1% vs. 11.8%), TEAEs leading to interruption of the investigational drug (18.2% vs. 35.3%). For ropegIFN, most frequently observed TEAEs were increased urinary β2-microglobulin (59.1%), pyrexia (54.5%), anemia (50.0%), fatigue (40.9%), and liver function abnormalities (22.7%). For ANA, most frequently observed TEAEs were headache (47.1%), palpitation (35.3%), anemia (29.4%), and peripheral edema (29.4%).

**Summary/Conclusion**

RopegIFN outperformed ANA for the primary and secondary efficacy endpoints and demonstrated a favourable safety profilein Japanese patients. The findings indicate ropegIFN has superior efficacy and safety over ANA in ET patients resistant or intolerant to HU.

**Copyright Information:** (Online) ISSN: 2572-9241
©2026 The Author(s). HemaSphere is published by John Wiley & Sons Ltd on behalf of European Hematology Association. This is an open access Abstract Book distributed under the Attribution-NonCommercial-NoDerivs (CC BY-NC-ND), which allows third parties to download the articles and share them with others as long as they credit the author and the Abstract Book, but they cannot change the content in any way or use them commercially.
**Abstract Book Citations:** Authors, Title, HemaSphere, 2026;10:(S1):pages. The abstract book DOIs can be found on the "Author Information" page.
**Disclaimer:** Articles published in the journal HemaSphere exclusively reflect the opinions of the authors. The authors are responsible for all content in their abstracts including accuracy of the facts, statements, citing resources, etc.

### (PF902) POST-MARKETING STUDY OF ROPEGINTERFERON ALFA-2B IN PATIENTS INTOLERANT TO HYDROXYUREA OR WITH A HISTORY OF SKIN CANCER

**Topic:** 16. Myeloproliferative neoplasms - Clinical

Tiziano Barbui*[1], Arianna Ghirardi[1], Francesca Fenili[1], Patryk Sobieralski[2], Novella Pugliese[3], Giuseppe Palumbo[4], Valerio De Stefano[5], Alessandra Iurlo[6], Marco Santoro[7], Annalisa Condorelli[8, 9], Daniele Sannipoli[10], Elena Elli[11], Giuseppe Gaetano Loscocco[12], Massimiliano Bonifacio[13], Francesca Palandri[14], Elisa Rumi[15], Alessandro Allegra[16], Erika Morsia[17], Davide Rapezzi[18], Matteo Bellia[19], Monia Marchetti[20], Gessica Marchesini[21], Massimo Breccia[22], Rossella Stella[23], Mario Tiribelli[24], Rosa Daffini[25], Elisabetta Abruzzese[26], Giulia Benevolo[27], Francesco Cavazzini[28], Amedeo Votto[29], Margherita Maffioli[30], Angelo Michele Carella[31], Alessandra Ricco[32], Marianna Caramella[33], Irene Bertozzi[34], Roberta Rocconi[35], Paola Guglielmelli[12], Alessandro Rambaldi[1], Alessandro Maria Vannucchi[12]

*[1]FROM - Research Foundation Bergamo Hospital - ETS, Bergamo, Italy; [2]Department of Hematology and Transplantology, Medical University of Gdańsk, University Clinical Center, Gdańsk, Poland; [3]Hematology and Stem Cell Trasplant unit, University of Naples, "Federico II", Naples, Italy; [4]Department of Medical, Surgical and Advanced Technologies Sciences "G.F. Ingrassia", University of Catania, Catania, Italy; [5]Catholic University and Policlinico "A. Gemelli" IRCCS Foundation, Rome, Italy; [6]Hematology Division, Foundation IRCCS Cà Granda Ospedale Maggiore Policlinico, Milan, Italy; [7]Hematology Unit, AOP "Paolo Giaccone", Palermo, Italy; [8]Hematology Division, ASST Papa Giovanni XXIII, Bergamo, Italy; [9]University of Milano-Bicocca, Milan, Italy; [10]Hematology and Bone Marrow Trasplantation Unit, IRCCS Ospedale San Raffaele, Milan, Italy; [11]Hematology Division, Fondazione IRCCS "San Gerardo dei Tintori", Monza, Italy; [12]Department of Experimental and Clinical Medicine, Center for Research and Innovation of Myeloproliferative Neoplasms, AOU Careggi, University of Florence, Florence, Italy; [13]Department of Engineering for Innovation Medicine, Section of Innovation Biomedicine, University of Verona, Verona, Italy; [14]Institute of Hematology "L. e A. Seràgnoli" IRCCS S. Orsola-Malpighi Alma Mater Studiorum - University of Bologna, Bologna, Italy; [15]Department of Molecular Medicine, University of Pavia; Precision Medicine and Molecular Hematology, Fondazione IRCCS Policlinico San Matteo, Pavia, Italy; [16]University of Messina, Messina, Italy; [17]Hematology Unit, AOU delle Marche, Department of Clinical and Molecular Sciences, DISCLIMO, Università Politecnica delle Marche, Ancona, Italy; [18]Hematology Division, ASO "Santa Croce e Carle", Cuneo, Italy; [19]Division of Hematology, Department of Translational Medicine, Università del Piemonte Orientale and Azienda Ospedaliero-Universitaria Maggiore della Carità, Novara, Italy; [20]Hematology Unit, University Hospital "SS Antonio e Biagio e Cesare Arrigo", Alessandria, Italy; [21]Hematology Unit, San Bortolo Hospital, ULSS8 Berica, Vicenza, Italy; [22]Policlinico Umberto I and Sapienza University, Rome, Italy;[23]University Hospital Giuliano Isontina Ospedale Maggiore, Trieste, Italy; [24]Division of Hematology and BMT, Department of Medicine, ASUFC and University of Udine, Udine, Italy;[25]ASST Spedali Civili, Brescia, Italy; [26]S. Eugenio Hospital, Tor Vergata University, Rome, Italy;[27]Hematology Unit, Città della Salute e della Scienza, Turin, Italy; [28]University of Ferrara, Department of Medical Sciences, Hematology Unit, Ferrara, Italy;[29]Department of Clinical and Experimental Medicine, Hematology Unit, University of Pisa & AOUP, Pisa, Italy; [30]ASST Sette Laghi, Ospedale di Circolo, Varese, Italy; [31]"Casa Sollievo della Sofferenza" Foundation, San Giovanni Rotondo, Italy; [32]Unit of Hematology and Stem Cell Transplantation, AOUC Policlinico, Bari, Italy; [33]ASST GOM Niguarda Department of Hematology, Niguarda Cancer Center, Milan, Italy; [34]Clinica Medica 1, Department of Medicine – DIME, Università degli Studi di Padova, Padova, Italy;[35]Hospital dell'Angelo, Mestre, Italy;*

### Background

Ropeginterferon alfa-2b (ropeg) is approved for polycythemia vera (PV) irrespective of risk, but real-world access in Europe is shaped by national reimbursement policies. In Italy and Poland, reimbursement is limited to hydroxyurea-intolerant patients (HU-I), women planning pregnancy, and patients with history of skin cancer (H-SC) leaving outcomes in these underrepresented subgroups in clinical trials and poorly characterized in clinical practice.

**Copyright Information:** (Online) ISSN: 2572-9241
©2026 The Author(s). HemaSphere is published by John Wiley & Sons Ltd on behalf of European Hematology Association. This is an open access Abstract Book distributed under the Attribution-NonCommercial-NoDerivs (CC BY-NC-ND), which allows third parties to download the articles and share them with others as long as they credit the author and the Abstract Book, but they cannot change the content in any way or use them commercially.
**Abstract Book Citations:** Authors, Title, HemaSphere, 2026;10:(S1):pages. The abstract book DOIs can be found on the "Author Information" page.
**Disclaimer:** Articles published in the journal HemaSphere exclusively reflect the opinions of the authors. The authors are responsible for all content in their abstracts including accuracy of the facts, statements, citing resources, etc.

### Aims

To assess the effectiveness and safety of ropeg in HU-I and H-SC patients. Complete hematologic response (CHR) was the primary endpoint and clinical outcomes, treatment exposure, vascular events, disease progression, secondary malignancies, and adverse events were secondary endpoints.

### Methods

This international, prospective, multicenter, non-interventional study (NCT06506084) enrolled patients with WHO-defined PV patients who were eligible for ropeg treatment according to reimbursement criteria in Italian and Polish centers. Data were collected via eCRF during routine clinical practice per SmPC. The study is sponsored by "FROM, Research Foundation Bergamo Hospital – ETS (Italy)".

### Results

- *Baseline characteristics*: Among HU-I patients (n=211), hematological tolerance included blood cytopenias (15.6%) and non-hematologic intolerance (83.4%): mucocutaneous, gastrointestinal systemic symptoms. The H-SC group (n=51) was older (median 73 yrs) with longer HU exposure and included basalioma, squamaous cell carcinomas and cutaneous melanoma.
- *Dosing and discontinuation*: Median doses were 125-150 µg every 2 weeks. Only 18.8% of intolerant patients required escalation to 500 µg. Discontinuation, regardless of causality, occurred in 19.4% and 25.5% in HU-I and H-SC patients, respectively.
- *Major events:* Among these two groups, after a median follow-up of 15 months, 13 (6.2%) and 3 (5.9%) patients experienced at least one major event (thrombosis, bleeding, myelofibrosis, solid tumors, death).
- *Hematological, molecular and symptoms response:* CHR in HU-I rise steadily from 14.3% at screening to 29.2% at 12 months and 41.0% at 24 months. In the H-SC cohort CHR increases from 18.0% at screening to 33.3% at 12 months, then remains stable at 18 and 24 months. Overall, JAK2 VAF declined from 45-50% to 15-20% at 24 months, with 57.1% of patients achieving partial molecular response and 14.3% complete/deep molecular response (VAF<2%) by 24 months. Ropeg treatment maintained stable symptom control over 12 months without significant improving or worsening, though fatigue and itching remained major unresolved burdens.
- *Neutrophil-to-Lymphocyte ratio* (NLR): In a proportion of HU-I patients, NLR at baseline was elevated and declined progressively over time after ropeg therapy, showing that HU does not control inflammation and may have a role in the genesis of intolerance.
- *Safety:* Adverse events occurred in 14.7% of HU-I and 29.4% of H-SC (most mild-to-moderate). Notably, no new cutaneous malignancies occurred in the H-SC cohort despite heavy prior HU exposure.

### Summary/Conclusion

This study supports ropeg as a valuable therapeutic option for HU-intolerant patients and those with prior skin cancer. Despite some challenges such as discontinuation and disease complications, the study shows encouraging results, with substantial hematologic and molecular improvement even in difficult-to-treat subgroups. The study is ongoing and future analyses will be crucial to confirm these benefits and to refine patient selection for optimized long-term care.

**Copyright Information:** (Online) ISSN: 2572-9241
©2026 The Author(s). HemaSphere is published by John Wiley & Sons Ltd on behalf of European Hematology Association. This is an open access Abstract Book distributed under the Attribution-NonCommercial-NoDerivs (CC BY-NC-ND), which allows third parties to download the articles and share them with others as long as they credit the author and the Abstract Book, but they cannot change the content in any way or use them commercially.
**Abstract Book Citations:** Authors, Title, HemaSphere, 2026;10:(S1):pages. The abstract book DOIs can be found on the "Author Information" page.
**Disclaimer:** Articles published in the journal HemaSphere exclusively reflect the opinions of the authors. The authors are responsible for all content in their abstracts including accuracy of the facts, statements, citing resources, etc.

### (PF903) EARLY-ONSET PHILADELPHIA-NEGATIVE MPN: AGE-DEPENDENT MUTATION DISTRIBUTION AND AGE-INDEPENDENT THROMBOTIC RISK

**Topic:** 16. Myeloproliferative neoplasms - Clinical

Olga Demska*[1], Magdalena Karasek[1], Marta Sobas[1], Yan Beauverd[2], Natalia Curto-Garcia[3], Parvis Sadjadian[4], Lee-Yung Shih[5], Timothy Devos[6], Serena Galli[7], Mary Frances McMullin[8], Anna Armatys[9], Joanna Wącław-Koziara[10], Zdrenghea Mihnea Tudor[11], Laurence Legros[12], François Girodon[13], Krzysztof Lewandowski[14], Anna Angona Figueras[15], Jan Samuelsson[16], Aitor Abuin Blanco[17], Pascale Cony-Makhoul[18], Angela Collins[19], Chloe James[20], Rajko Kusec[21], Marie Lauermannova[22], Maria Soledad Noya Pereira[23], Marielle Wondergem[24], Iryna Dudchenko[25], Joanna Góra-Tybor[26], Tomasz Sacha[10], Weronika Lebowa[10], Patryk Sobieralski[27], Danijela Lekovic[28], Kamel Laribi[29], Anna Kulikowska de Nałęcz[30], Jean-Loup Demory[31], Katell Le Du[32], Sonja Zweegman[33], Carlos Besses Raebel[15], Radek Skoda[34], Stephane Giraudier[35], Martin Griesshammer[4], Jean-Jacques Kiladjian[36], Jean Christophe Ianotto[37], Claire Harrison[3]

*[1]Collegium Medicum in Bydgoszcz, Nicolaus Copernicus University in Torun, Department of Hematology, Bydgoszcz, Poland; [2]Geneva University Hospitals, Division of Hematology, Department of Oncology,, Geneva, Switzerland;[3]Guy's and St Thomas' National Health Service Foundation Trust, Department of Hematology, London, United Kingdom; [4]University Clinic for Hematology, Oncology, Hemostasis, and Palliative Care, Johannes Wesling Medical Center, University of Bochum, Minden, Germany; [5]Division of Hematology-Oncology, Chang Gung Memorial Hospital, Taipei, Taiwan;[6]University Hospitals Leuven and Department of Microbiology and Immunology, Laboratory of Molecular Immunology (Rega Institute), Katholieke Universiteit Leuven, Department of Hematology, Leuven, Belgium; [7]Department of Biomedicine, Experimental Hematology, University Hospital and University, Basel, Switzerland; [8]Hematology, Belfast City Hospital, Queen's University Belfast, Belfast, United Kingdom; [9]University of Silesia, Department of Hematology and Bone Marrow Transplantation, Katowice, Poland;[10]Collegium Medicum, Jagiellonian University, Department of Hematology,, Krakow, Poland; [11]Iuliu Hatieganu University of Medicine and Pharmacy, Department of Hematology, Cluj-Napoca, Romania; [12]Paul Brousse Hospital, Department of Hematology, Paris, France; [13]Laboratory of Biological Hematology, University Hospital, Dijon, France; [14]University of Medical Sciences, Department of Hematology and Bone Marrow Transplantation, Poznan, Poland; [15]Hospital del Mar, Department of Hematology, Barcelona, Spain; [16]University Hospital, Department of Hematology, Linkoping, Sweden; [17]University Hospital, Department of Hematology, Santiago de Compostela, Spain; [18]Annecy-Genevois Hospital Centre, Department of Hematology, Pringy, France; [19]Norfolk and Norwich University Hospitals National Health Service Trust, Department of Hematology, Norwich, United Kingdom; [20]Biology of Cardiovascular Diseases, University of Bordeaux, INSERM, UMR1034, Pessac, France; [21]University Hospital Dubrava, School of Medicine, University of Zagreb, Zagreb, Croatia; [22]Institute of Hematology and Blood Transfusion, Prague, Czechia;[23]University Hospital Juan Canalejo-Complejo Hospitalario Universitario a Coruña, Department of Hematology, La Coruña, Spain; [24]Amsterdam University Medical Centers, Universiteit Amsterdam, Cancer Center, Department of Hematology, Amsterdam, The Netherlands; [25]Sumy State University, Medical Institute, Respiratory Medicine Center, Department of Internal Medicine,, Sumy, Ukraine; [26]Medical University of Lodz, Department of Hematology, Lodz, Poland;[27]Medical University of Gdańsk, Gdańsk, Poland; [28]Clinic of Hematology, University Clinical Center Serbia, Belgrade, Serbia Faculty of Medicine, University of Belgrade, Belgrade, Serbia; [29]Le Mans Hospital, Department of Hematology, Le Mans, France; [30]State Hospital, Opole, Poland; [31]St. Vincent De Paul Hospital, Department of Hematology, Lille, France; [32]The Confluent, Private Hospital, Nantes, France; [33]University Medical Center, Department of Hematology, Amsterdam, The Netherlands;[34]Baylor College of Medicine, Dan L. Duncan Comprehensive Cancer Center, Houston, United States of America; [35]INSERM UMRS 1131, St Louis Hospital, AP-HP, Department of Cellular Biology,, Paris, France; [36]Université de Paris, Assistance Publique des Hôpitaux de Paris (AP-HP), Hôpital Saint-Louis, Centre d'Investigations Cliniques 1427, INSERM, Paris, France; [37]Institute of Oncology and Hematology, University Hospital, Department of Hematology, Brest, France;*

**Copyright Information:** (Online) ISSN: 2572-9241
©2026 The Author(s). HemaSphere is published by John Wiley & Sons Ltd on behalf of European Hematology Association. This is an open access Abstract Book distributed under the Attribution-NonCommercial-NoDerivs (CC BY-NC-ND), which allows third parties to download the articles and share them with others as long as they credit the author and the Abstract Book, but they cannot change the content in any way or use them commercially.
**Abstract Book Citations:** Authors, Title, HemaSphere, 2026;10:(S1):pages. The abstract book DOIs can be found on the "Author Information" page.
**Disclaimer:** Articles published in the journal HemaSphere exclusively reflect the opinions of the authors. The authors are responsible for all content in their abstracts including accuracy of the facts, statements, citing resources, etc.

### Background

Age is incorporated into thrombotic risk stratification in Philadelphia-negative myeloproliferative neoplasms (MPN Ph-neg). However, the independent impact of age at diagnosis within exclusively young patients remains unclear.

### Aims

To compare molecular distribution and vascular outcomes between patients diagnosed ≤25 and 26–40 years and to identify independent predictors of thrombosis during follow-up.

### Methods

We analyzed a multicenter retrospective cohort of 726 patients aged ≤40 years at diagnosis with essential thrombocythemia (ET), polycythemia vera (PV), or myelofibrosis (MF). Patients were stratified into two age groups (≤25 vs 26–40 years). Categorical variables were compared using chi-square or Fisher's exact test. Multivariable logistic regression was performed to identify independent predictors of thrombosis during follow-up. Age at diagnosis was entered as a continuous variable. Analyses were restricted to patients with available data for each variable.

### Results

Overall driver mutation distribution was: JAK2 434/726 (59.8%), CALR 103/726 (14.2%), MPL 8/726 (1.1%), and triple-negative 162/726 (22.3%). An age-dependent shift in driver mutation distribution was observed, reaching statistical significance in ET (p=0.019), where patients ≤25 years showed a higher relative proportion of CALR-mutated cases, whereas JAK2 mutations predominated in those aged 26–40 years. Across disease subtypes, history of thrombosis occurred in 38/170 (22.4%) PV, 12/61 (19.7%) MF, and 46/464 (9.9%) ET patients (p=0.00012). Within PV, no significant age-related differences were observed for history of thrombosis (21.1% vs 26.2%; p=0.49) or thrombosis during follow-up (17.1% vs 15.7%; p=0.82). In multivariable analysis adjusting for age, disease subtype, interferon exposure, JAK2 mutation, and prior thrombosis, prior thrombosis independently predicted thrombosis during follow-up (OR 3.99; 95% CI 2.23–7.14; p<0.001), as did JAK2 mutation (OR 2.85; 95% CI 1.39–5.82; p=0.004). Age at diagnosis (p=0.81), disease subtype (p=0.45), and interferon exposure (p=0.82) were not independent predictors. Cytoreductive treatment patterns did not differ significantly between age groups across ET (p=0.27), PV (p=0.86), or MF (p=0.97).

### Summary/Conclusion

In this young MPN cohort (≤40 years), future thrombotic risk was independently driven by biological factors—JAK2 mutation and prior thrombosis— rather than by chronological age, while interferon exposure was not independently associated with risk in this analysis. These findings suggest that within early-onset MPN, molecular background and clinical history of thrombosis outweigh incremental age differences in determining vascular risk, supporting mutation-oriented rather than age-oriented risk assessment in young patients.

**Copyright Information:** (Online) ISSN: 2572-9241
©2026 The Author(s). HemaSphere is published by John Wiley & Sons Ltd on behalf of European Hematology Association. This is an open access Abstract Book distributed under the Attribution-NonCommercial-NoDerivs (CC BY-NC-ND), which allows third parties to download the articles and share them with others as long as they credit the author and the Abstract Book, but they cannot change the content in any way or use them commercially.
**Abstract Book Citations:** Authors, Title, HemaSphere, 2026;10:(S1):pages. The abstract book DOIs can be found on the "Author Information" page.
**Disclaimer:** Articles published in the journal HemaSphere exclusively reflect the opinions of the authors. The authors are responsible for all content in their abstracts including accuracy of the facts, statements, citing resources, etc.

### (PF904) SYMPTOM DISTRIBUTION IN PATIENTS WITH HEREDITARY ALPHA-TRYPTASEMIA (HαT) AND SYSTEMIC MASTOCYTOSIS (SM): DATA FROM A LARGE GERMAN REGISTRY COHORT

**Topic:** 16. Myeloproliferative neoplasms - Clinical

Pia Platthoff*[1], Daniel Koch[1], Juliana Schwaab[2], Debroha Christen[3], Nikolas Von Bubnoff[1], Dagmar von Bubnoff[4], Friederike Wortmann[1]

*[1]University Hospital Schleswig-Holstein, Hematology and Oncology, Lübeck, Germany; [2]University Hospital Mannheim, Hematology/Oncology, Mannheim, Germany; [3]University Hospital RWTH Aachen, Hematology/Oncology, Aachen, Germany; [4]University Hospital Schleswig-Holstein, Dermatology/Allergology, Lübeck, Germany;*

### Background

Hereditary alpha-tryptasemia (HαT) is an autosomal dominant genetic trait characterized by high-normal (8–11.4 µg/L) to elevated (>11.4 µg/L) baseline serum tryptase (BST) levels due to monoallelic alpha-tryptase gene amplifications on the TPSAB1 locus. HαT is associated with multisystem symptoms. An important differential diagnosis is systemic mastocytosis (SM), a clonal mast cell disorder characterized by pathologic mast cell accumulation in various organs. SM is also associated with elevated BST levels, and approximately 90% of patients harbor the KIT-D816V mutation. Given clinical and biochemical overlap, distinguishing HαT from SM may be challenging.

### Aims

The aim of this study was to describe the symptom distribution and labaratory characteristics in a large registry cohort stratified according to HαT and SM status.

### Methods

Submissions to a nationwide registry were obtained from individuals with high-normal or elevated basal serum tryptase (BST) levels. Clinical and laboratory data, structured symptom questionnaires, and information on systemic mastocytosis (SM) status and bone marrow biopsy (BMB) were collected. Testing for HαT and cKIT-D816V mutation analysis were performed centrally at the University Hospital Schleswig-Holstein, Campus Lübeck.

### Results

A total of 1196 individuals were included in the registry (median age 52 years, range 8-91; 67.2% female). HαT genotyping was performed in 1120 individuals, of whom 42.4% tested positive. Symptom questionnaires were available for 570 participants. Participants were stratified by HAT and SM status into four groups: HAT+/SM+ (n=16; median age 53 years), HAT-/SM+ (n=69; median age 55 years), HAT+/SM- (n=264; median age 52 years) and HAT-/SM- (n=160; median age 60 years). BST levels differed significantly between groups (p<0.001). The highest median BST was observed in individuals with HAT+/SM+ (43.2 µg/L; range 26.5-102), followed by HAT+/SM- (14.1 µg/L; range 4.6-100), whereas the lowest median BST was found in the HAT-/SM- group (9.6 µg/L; range 1.8-200). Across all four groups, the median total symptom score was 26 (range 0–54), corresponding to an overall moderate symptom burden; 43.5% participants scored within the predefined moderate range (19-36 points). Symptom scores differed significantly between groups (p=0.004). The highest median symptom score was observed in HAT+/SM- individuals (median 27.5 points; range 0-54). HAT+/SM+ individuals had a median score of 23.5 (range 7-45), whereas the lowest median score was observed in the HAT-/SM- group (median 21 points; range 1-51). Pain-related and neurological symptoms were numerically more frequent in HAT-positive individuals, particularly in the HAT+/SM– group, compared with HAT-/SM– individuals.

**Copyright Information:** (Online) ISSN: 2572-9241
©2026 The Author(s). HemaSphere is published by John Wiley & Sons Ltd on behalf of European Hematology Association. This is an open access Abstract Book distributed under the Attribution-NonCommercial-NoDerivs (CC BY-NC-ND), which allows third parties to download the articles and share them with others as long as they credit the author and the Abstract Book, but they cannot change the content in any way or use them commercially.
**Abstract Book Citations:** Authors, Title, HemaSphere, 2026;10:(S1):pages. The abstract book DOIs can be found on the "Author Information" page.
**Disclaimer:** Articles published in the journal HemaSphere exclusively reflect the opinions of the authors. The authors are responsible for all content in their abstracts including accuracy of the facts, statements, citing resources, etc.

**Summary/Conclusion**

BST levels varied across groups, with the highest values observed in HAT+/SM+ individuals. Although SM was associated with higher BST concentrations, overall symptom burden did not parallel BST levels.
Both HAT and SM were associated with higher total symptom scores compared with HAT-/SM-. Notably, HAT+/SM- exhibited the highest overall symptom burden within this cohort, suggesting a potential contribution of HAT to systemic symptom severity. Pain-related and neurological symptoms were more prominent in HAT-positive individuals, indicating a distinct symptom profile in this subgroup.The small size of the HAT+/SM+ subgroup (n=16) limits the interpretability of comparisons involving this group.

**Copyright Information:** (Online) ISSN: 2572-9241
©2026 The Author(s). HemaSphere is published by John Wiley & Sons Ltd on behalf of European Hematology Association. This is an open access Abstract Book distributed under the Attribution-NonCommercial-NoDerivs (CC BY-NC-ND), which allows third parties to download the articles and share them with others as long as they credit the author and the Abstract Book, but they cannot change the content in any way or use them commercially.
**Abstract Book Citations:** Authors, Title, HemaSphere, 2026;10:(S1):pages. The abstract book DOIs can be found on the "Author Information" page.
**Disclaimer:** Articles published in the journal HemaSphere exclusively reflect the opinions of the authors. The authors are responsible for all content in their abstracts including accuracy of the facts, statements, citing resources, etc.

### (PF905) TREATMENT PATTERNS AND COMPLICATIONS IN ESSENTIAL THROMBOCYTHEMIA – A POPULATION-BASED STUDY OF 3,234 PATIENTS IN SWEDEN

**Topic:** 16. Myeloproliferative neoplasms - Clinical

Anna Ravn Landtblom*[1, 2], Yuliya Leontyeva[3], Francesca Coutinho[4], Xiaoqin Yang[4], Therese Andersson[3]

*[1]Center for Hematology and Regenerative Medicine, Department of Medicine, Huddinge, Karolinska Institutet, Stockholm, Sweden; [2]Department of Hematology, Karolinska University Hospital, Stockholm, Sweden; [3]Department of Medical Epidemiology and Biostatistics, Karolinska Institutet, Stockholm, Sweden; [4]Merck Sharp and Dohme LLC, Rahway, United States of America;*

### Background

Essential thrombocythemia (ET) has a long expected clinical course, thus complications occurring during the disease course and treatment choices are important for health, survival and well-being of patients. Real world data that quantifies complications and treatment patterns in a comprehensive way are lacking.

### Aims

The aim of this work is to describe treatment patterns and complications in patients with ET in a population-based register study with real-world treatment.

### Methods

We identified adult patients with a diagnosis of ET from the Swedish Blood Cancer Quality register, diagnosed 2008-2021. Information of prescribed drugs were retrieved from the Prescribed Drug Register. Complications of interest were thrombosis, bleeding, disease progression to myelofibrosis (MF), acute myeloid leukemia (AML) or myelodysplastic syndrome (MDS), and skin cancers. Skin cancers were considered a relevant complication as hydroxyurea (HU) is associated with an increased risk of skin cancers. Complications were identified from Patient Register and the Cause of death Register, thus defined as diagnoses mentioned while requiring health care or causing death. Cumulative incidence functions were estimated, treating death due to other causes as a competing risk.

### Results

Among 3,234 patients with ET, the median age at diagnosis was 69 years, 63.1% were JAK2-mutated. Within the 10 years preceding the diagnosis, 16% had a bleeding event and 21% had a thrombotic event. As per revised-IPSET thrombosis, 71.3% of patients were intermediate or high risk at diagnosis. The median follow-up from diagnosis was 6.5 years (95% CI 6.2-6.7). Estimated median time from diagnosis to first cytoreductive treatment was 3 months, and 90% of patients were estimated to have received cytoreductive treatment within 12 years from diagnosis. HU was used as first cytoreductive medication in 90.1%, while 8.1% received interferon, 1.5% anagrelide and 0.3% busulphan. A second treatment was initiated in 16.0%; among which HU was given in 19.1%, interferon 26.3%, anagrelide 37.5%, and busulphan 19.6%. Among the 793 ET patients of very low and low-risk at diagnosis, 39.9% were treated with cytoreductive treatment prior to changing into high/intermediate risk group. Estimated median time from diagnosis to any complication was 8.3 years. Estimated cumulative incidence proportion during 12 years was for thrombosis 29% (95% CI 27-32), bleeding 36% (95% CI 33-38), MF 5% (95% CI 4-7), AML 4% (95% CI 3-5), MDS 4% (95% CI 3-6) and skin cancer 10% (95% CI 9-12). In Figure 1, estimated stacked cumulative incidence functions of first complication are demonstrating that 60% of patients are expected to experience at least one complication within 12 years. Estimated median survival was 12.1 years.

**Copyright Information:** (Online) ISSN: 2572-9241
©2026 The Author(s). HemaSphere is published by John Wiley & Sons Ltd on behalf of European Hematology Association. This is an open access Abstract Book distributed under the Attribution-NonCommercial-NoDerivs (CC BY-NC-ND), which allows third parties to download the articles and share them with others as long as they credit the author and the Abstract Book, but they cannot change the content in any way or use them commercially.
**Abstract Book Citations:** Authors, Title, HemaSphere, 2026;10:(S1):pages. The abstract book DOIs can be found on the "Author Information" page.
**Disclaimer:** Articles published in the journal HemaSphere exclusively reflect the opinions of the authors. The authors are responsible for all content in their abstracts including accuracy of the facts, statements, citing resources, etc.

**Summary/Conclusion**

In this large population-based study of 3,234 ET patients treated in clinical practice in Sweden, a high cumulative incidence of complications of 60% is demonstrated. Treatment was dominated by HU, in both first and later treatments suggesting the occurrence of HU recycling in later treatment lines. A large proportion of very low and low risk patients received treatment, highlighting the long duration of treatment, and the need to have several treatment options available to find what is suitable for each patient. The results of this study elucidate a need for innovative treatments, as treatment options today are limited, and complications are common.

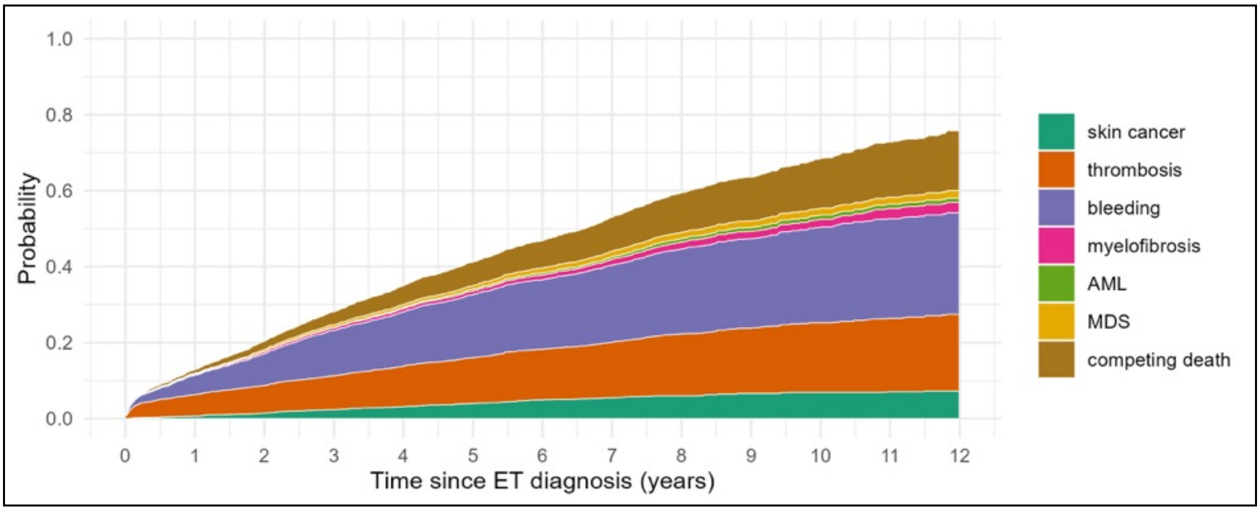

Figure 1. Cumulative incidence of first complication among individuals with ET in Sweden.

**Copyright Information:** (Online) ISSN: 2572-9241
©2026 The Author(s). HemaSphere is published by John Wiley & Sons Ltd on behalf of European Hematology Association. This is an open access Abstract Book distributed under the Attribution-NonCommercial-NoDerivs (CC BY-NC-ND), which allows third parties to download the articles and share them with others as long as they credit the author and the Abstract Book, but they cannot change the content in any way or use them commercially.
**Abstract Book Citations:** Authors, Title, HemaSphere, 2026;10:(S1):pages. The abstract book DOIs can be found on the "Author Information" page.
**Disclaimer:** Articles published in the journal HemaSphere exclusively reflect the opinions of the authors. The authors are responsible for all content in their abstracts including accuracy of the facts, statements, citing resources, etc.

### (PF906) NEUTROPHIL-LYMPHOCYTE RATIO AND JAK2 V617F ALLELE BURDEN AS PROGNOSTIC INDICATORS OF BIOLOGICAL ACTIVITY AND THROMBOTIC RISK IN POLYCYTHEMIA VERA.

**Topic:** 16. Myeloproliferative neoplasms - Clinical

Andrea Bender*[1], Ana Ines Varela[2], Nehuen Gasparini[1], Eugenia Bender[1], MacArena Giuliani[1], Federico Torreguitart[1], Cecilia Lang[1], María Paula Iommi[1], Castello Celia[3], Lisa Lopez Ares[4], Fabian Meriño Rojel[4], Rosana Venchi[4], Oscar Gomez[5], Juan Carlos Herrera[6], Juan Luis Maradei[7], Gustavo Taborda[8], Marita Rios Part[9], Yesica Nuñez[10], Lorena Vargas Garcia[11], Marisa Sandoval[12], Evangelina Agriello[1]

*[1]LEB, Bahía Blanca, Buenos Aires, Argentina; [2]Srio. Las Lomas, Buenos Aires, Argentina; [3]Consultorios de Especialidades Médicas Morales (CEMM), Bariloche, Rio Negro, Argentina; [4]Srio. Juan XXIII, Gral. Roca, Rio Negro, Argentina; [5]Centro médico hematológico, Trelew, Chubut, Argentina; [6]Hematología Herrera, Comodoro Rivadavia, Chubut, Argentina; [7]Hosp. Emilio Ferreyra, Necochea, Buenos Aires, Argentina; [8]Policlínico Modelo de Cipolletti, Cipolletti, Rio Negro, Argentina; [9]Clínica del Valle, Comodoro Rivadavia, Chubut, Argentina; [10]Clínica Chacabuco, Tandil, Buenos Aires, Argentina; [11]Srio. San Crlos, Bariloche, Rio Negro, Argentina; [12]Universidad Nacional del Sur, Catedra de Hematología Clínica, Bahía Blanca, Argentina;*

### Background

Polycythemia vera (PV) is a clonal myeloid neoplasm characterized by myeloid proliferation with absolute erythrocytosis, driven by JAK2V617F mutation that amplifies hematopoietic signaling, promoting uncontrolled clonal expansion and myeloid differentiation. Patients (pts) with PV are at increased risk of developing thrombotic events (TE)

The increase of JAK2 allele burden (JAB) is associated with thrombosis, clonal expansion, and progression to myelofibrosis. The neutrophil-to-lymphocyte ratio (NLR) is hypothesized to correlate with JAB as an easily accessible indicator of disease activity that could be incorporated into decision-making in patients with PV.

Monitoring the biological activity of PV through these markers could allow identification of patients who could benefit from the initiation of disease-modifying therapies with a widely available method.

### Aims

To evaluate the correlation between NLR and JAB and to describe the distribution of these variables among classical thrombotic risk categories in patients with PV.

### Methods

Pts with PV (2022 WHO/ICC) were retrospectively evaluated (2020–2025). Clinical and complete blood count data were recorded at diagnosis. The presence of the JAB was assessed by RT-PCR (TaqMan probe SNP assay. Kruskal–Wallis test and Pearson correlation was used.Continuous variables were expressed as median (IQR) and compared using the Mann–Whitney U test. JAB, NLR and leukocyte count was evaluated using Spearman's correlation (p value <0.05 as significant).

**Copyright Information:** (Online) ISSN: 2572-9241
©2026 The Author(s). HemaSphere is published by John Wiley & Sons Ltd on behalf of European Hematology Association. This is an open access Abstract Book distributed under the Attribution-NonCommercial-NoDerivs (CC BY-NC-ND), which allows third parties to download the articles and share them with others as long as they credit the author and the Abstract Book, but they cannot change the content in any way or use them commercially.
**Abstract Book Citations:** Authors, Title, HemaSphere, 2026;10:(S1):pages. The abstract book DOIs can be found on the "Author Information" page.
**Disclaimer:** Articles published in the journal HemaSphere exclusively reflect the opinions of the authors. The authors are responsible for all content in their abstracts including accuracy of the facts, statements, citing resources, etc.

### Results

The study cohort (n=129) had a median age of 71 yrs (65–79; F/M: 1.3). Median values at diagnosis were: hg 17.8 g/dL (16.5–19.5), leu 13.8 ×10⁹/L (8.9–17.2), and pts 557 ×10⁹/L (347–746). Leukocytosis was present in 61%, 32% had palpable splenomegaly, and 14.5% had prior TE. The median JAB was 48% (35–58). Overall, 51.2% (63/129) of pts had high NLR (cut-off 3.5). This group showed significantly higher leu counts compared to those with low NLR. Likewise, JAB was significantly higher in the high NLR group. A significant positive correlation was observed between JAB and NLR (r=0.412, p<0.05) and between JAB and leu count (r=0.562, p<0.05).
The conventional prognostic model, based on age and prior TE, categorized 82% of pts (n=106) as high risk (HR). Within thisgroup, 48.1% (n=51) had NLR <3.5 with a median JAB of 46% (27–55). The remaining 51.9% (n=55) had high NLR with a median JAB of 52% (49–68). 18% (n=23) were low-risk (LR) pts. In this subgroup, 15/23 (65%) had low NLR with a median JAB of 30.0% (20–47), and 8/23 (35%) had high NLR with a median AB of 46.5% (41–67).

### Summary/Conclusion

NLR ≥3.5 constitutes an accessible marker to identify a PV phenotype with higher proliferative disease. The direct correlation between NLR and JAB, and the association between the latter and leukocyte count, confirms that systemic inflammatory status reflects the magnitude of the malignant clone.
These findings question the sufficiency of conventional stratification models. NLR allows identification, within the HR group, of a subgroup with lower JAB and a more favorable inflammatory profile, in whom clinical risk may be overestimated. Conversely, risk may be underestimated in pts classified as LR who present elevated NLR and higher JAB, representing a subgroup requiring closer clinical monitoring.
If validates in larger cohorts, these results would justify the integration of this markers into future prognostic models to refine evaluation at diagnosis and follow up to optimize therapeutic decision-making.

| | Total Cohort (n=129) | NRL < 3.5 (n=66) | NRL ≥ 3.5 (n=63) | P value |
|---|---|---|---|---|
| **Age** | 72 (65-79) | 72 (63–80) | 73 (69–79) | NS |
| **Hemoglobin (g/dL)** | 17.8 (16.5-19.5) | 17.8 (16.2–19.1) | 18.2 (16.6–20.0) | NS |
| **Leukocytes (x10⁹/L)** | 13.8 (8.9-17.2) | 10.3 (7.7–13.7) | 15.6 (10.8–18.7) | 0.000 |
| ***Neutrophils*(x10⁹/L)** | 8.3 (4.9-10.8) | 5.0 (3.4–7.2) | 10.2 (7.8–13.0) | 0.000 |
| ***lymphocytes* (x10⁹/L)** | 2.5 (1.7-3.0) | 2.6 (2.1–3.7) | 1.8 (1.4–2.3) | 0.000 |
| **Alelic burden *JAK2V617F (%)*** | 48 (35-58) | 42 (27–55) | 51 (45–68) | 0.000 |
| **Alelic burden *JAK2V617F-HR patients (%)*** | - | 46 (27.5-55) | 52 (49-68) | 0.003 |
| **Alelic burden *JAK2V617F-LR patients (%)*** | - | 30(20.5-47.5) | 46.5(41-67) | 0.066 |

Table 1.

Copyright Information: (Online) ISSN: 2572-9241
©2026 The Author(s). HemaSphere is published by John Wiley & Sons Ltd on behalf of European Hematology Association. This is an open access Abstract Book distributed under the Attribution-NonCommercial-NoDerivs (CC BY-NC-ND), which allows third parties to download the articles and share them with others as long as they credit the author and the Abstract Book, but they cannot change the content in any way or use them commercially.
Abstract Book Citations: Authors, Title, HemaSphere, 2026;10:(S1):pages. The abstract book DOIs can be found on the "Author Information" page.
Disclaimer: Articles published in the journal HemaSphere exclusively reflect the opinions of the authors. The authors are responsible for all content in their abstracts including accuracy of the facts, statements, citing resources, etc.

### (PF907) MULTIPLE TET2 MUTATIONS CONFER INCREASED RISK OF LEUKEMIC TRANSFORMATION IN PATIENTS WITH MYELOFIBROSIS

**Topic:** 16. Myeloproliferative neoplasms - Clinical

Tian Zeng[1], Rumeng Li[1], Qinmei Han[1, 1], Bingbing Wen[1], Zhikang Zheng[1], Xiaojing Bao[1], Jian Huang*[1]

[1]*The First Affiliated Hospital, Zhejiang University School of Medicine, Hangzhou, China;*

### Background

TET2 is a recurrently mutated gene in myeloid malignancies and is commonly identified in clonal hematopoiesis of indeterminate potential (CHIP). In Chronic Myelomonocytic Leukemia, biallelic TET2 mutations have been associated with distinct clinical behavior and outcomes. In Myelofibrosis (MF), although TET2 mutations are frequently detected, they are not categorized as high molecular risk (HMR) lesions.

### Aims

In this study, we aimed to evaluate the clinical impact of multiple TET2 mutations on leukemic transformation in MF patients.

### Methods

Molecular profiling was performed using next-generation sequencing with a custom panel targeting 155 myeloid-associated genes. We retrospectively analyzed 185 MF patients with available NGS data. Multi-TET2 was defined as the presence of two or more distinct TET2 mutations detected by NGS, regardless of variant allele frequency (VAF). Categorical variables were compared using the Chi-square test, and continuous variables using the Kruskal-Wallis test. Leukemia-free survival (LFS) was estimated using the Kaplan–Meier method and compared by log-rank testing. Hazard ratios (HRs) were calculated using Cox regression analysis.

### Results

A total of 185 patients with MF and available NGS data were included in the analysis. The median age of the cohort was 57.0 years (IQR 48.0–67.0), with a nearly equal distribution between males and females (50.8% male, 49.2% female). Among them, 36 patients (19.46%) harbored multiple TET2 mutations (multi-TET2), 17 (9.19%) had a single TET2 mutation (mono-TET2), and 132 (71.35%) were TET2 wild-type (wt). Age differed significantly among the three groups ($P < 0.05$), whereas no statistically significant differences were observed in white blood cell count, hemoglobin level, platelet count, lactate dehydrogenase, peripheral blood blasts, diagnosis subtype, bone marrow fibrosis grade, sex, leukemic transformation rate at baseline, constitutional symptoms, transfusion dependence, or cytogenetic risk category (all $P > 0.05$). When survival outcomes were examined, no significant difference in leukemia-free survival (LFS) was observed between overall TET2-mutated patients and wild-type patients (log-rank $P = 0.500$). Similarly, mono-TET2 mutations were not associated with inferior LFS compared with wild-type cases (log-rank $P = 0.656$). In contrast, patients harboring multiple TET2 mutations exhibited significantly shorter LFS compared with wild-type patients (log-rank $P = 0.048$; HR 3.09, 95% CI: 1.01–9.46), with a median LFS of 60 months, while the median was not reached in the wild-type group. Moreover, multi-TET2 patients also had significantly inferior LFS compared with mono-TET2 patients (log-rank $P = 0.029$; HR 4.04, 95% CI: 1.16–14.12). Patients harboring multi-TET2 mutations exhibited a significantly higher number of mutated genes compared with mono-TET2 cases (median 8.0 vs 3.5, $P < 0.001$), indicating a markedly more complex molecular profile in the multi-hit subgroup.

**Copyright Information:** (Online) ISSN: 2572-9241
©2026 The Author(s). HemaSphere is published by John Wiley & Sons Ltd on behalf of European Hematology Association. This is an open access Abstract Book distributed under the Attribution-NonCommercial-NoDerivs (CC BY-NC-ND), which allows third parties to download the articles and share them with others as long as they credit the author and the Abstract Book, but they cannot change the content in any way or use them commercially.
**Abstract Book Citations:** Authors, Title, HemaSphere, 2026;10:(S1):pages. The abstract book DOIs can be found on the “Author Information” page.
**Disclaimer:** Articles published in the journal HemaSphere exclusively reflect the opinions of the authors. The authors are responsible for all content in their abstracts including accuracy of the facts, statements, citing resources, etc.

**Summary/Conclusion**

Patients harboring multiple TET2 mutations exhibit a significantly increased risk of leukemic transformation compared with both mono-TET2 and wild-type patients. Multi-hit TET2 may therefore define a biologically distinct and higher-risk subgroup that warrants separate clinical consideration.
This research was supported by the National Natural Science Foundation of China (No. 82570191) and the Zhejiang Provincial Health High level Innovative Talent Project (2022-2026).
†Correspondence to: Prof Jian Huang, E-mail: househuang@zju.edu.cn

**Copyright Information:** (Online) ISSN: 2572-9241
©2026 The Author(s). HemaSphere is published by John Wiley & Sons Ltd on behalf of European Hematology Association. This is an open access Abstract Book distributed under the Attribution-NonCommercial-NoDerivs (CC BY-NC-ND), which allows third parties to download the articles and share them with others as long as they credit the author and the Abstract Book, but they cannot change the content in any way or use them commercially.
**Abstract Book Citations:** Authors, Title, HemaSphere, 2026;10:(S1):pages. The abstract book DOIs can be found on the "Author Information" page.
**Disclaimer:** Articles published in the journal HemaSphere exclusively reflect the opinions of the authors. The authors are responsible for all content in their abstracts including accuracy of the facts, statements, citing resources, etc.

### (PF908) A PHASE IB STUDY OF 9MW3011, A FIRST-IN-CLASS ANTI-TMPRSS6 ANTIBODY, IN RELAPSED/REFRACTORY PATIENTS WITH POLYCYTHEMIA VERA

**Topic:** 16. Myeloproliferative neoplasms - Clinical

Bing Li*[1], Min Zhang[2], Jie Bai[3], Jian Huang[4], Hu Zhou[5], Jieyu Wang[6, 7], Zhitian Hu[8], Ning Wang[8], Fang Tian[8], Mengxiao Li[8], Jianchi MA[8], Zhijian Xiao[1]

*[1]State Key Laboratory of Experimental Hematology, National Clinical Research Center for Blood Diseases, Haihe Laboratory of Cell Ecosystem, Institute of Hematology & Blood Diseases Hospital, Chinese Academy of Medical Sciences & Peking Union Medical College, Tianjin, China; [2]Institute of Hematology, Union Hospital, Tongji Medical College, HUST, Wuhan, China;[3]The Second Hospital of Tianjin Medical University, Tianjin, China; [4]The First Affiliated Hospital ,Zhejiang University School of Medicine, Hangzhou, China; [5]The Affiliated Cancer Hospital of Zhengzhou University & Henan Cancer Hospital, Zhengzhou 450008, China, Henan, China; [6]Jiangxi Provincial Key Laboratory of Hematological Diseases, Department of Hematology, The First Affiliated Hospital, Jiangxi Medical College, Nanchang University, Jiangxi, China; [7]Jiangxi Clinical Research Center for Hematologic Disease, Nanchang 330006, Jiangxi, China, Jiangxi, China; [8]Mabwell (Shanghai) Bioscience Co., Ltd, Shanghai, China;*

### Background

Polycythemia vera (PV) is a chronic myeloproliferative neoplasm characterized by erythrocytosis. Current first-line medications are limited by resistance and intolerance, resulting in unmet needs in cytoreductive therapy burden. 9MW3011 is a novel humanized monoclonal antibody targeting transmembrane serine protease 6 (TMPRSS6), modulates the BMP/SMAD–hepcidin pathway to inhibit erythropoiesis and may offer a novel therapeutic option, with potential to improve patient-reported outcomes, as evaluated in a phase Ib clinical study (NCT06752746).

### Aims

To evaluate the safety and tolerability of multiple intravenous (IV) administrations of 9MW3011 injection in Relapsed/Refractory Chinese patients with PV, and to further assess its pharmacokinetic (PK) and pharmacodynamic (PD) characteristics and immunogenicity, while preliminarily exploring its therapeutic effects.

### Methods

Eligible participants were randomized into three dose groups (150 mg IV Q4W, 150 mg IV Q2W, and 300 mg IV Q4W), with evaluations of safety, tolerability, and pharmacological parameters. The treatment period consisted of five dosing cycles, and participants who withdrew within five dosing cycles were classified as early withdrawals.

Preliminary efficacy was evaluated based on cytoreductive-therapy-free (CTF) status in terms of duration and rates. CTF is considered as the absence of cytoreductive intervention such as hydroxyurea and interferon-α. The need for intervention was determined according to baseline hematocrit (Hct) levels: for participants with baseline Hct <45%, post-treatment Hct ≥45% (with ≥3% increase) or ≥48%; for patients with baseline Hct ≥45%, an Hct increase ≥3%.

**Copyright Information:** (Online) ISSN: 2572-9241
©2026 The Author(s). HemaSphere is published by John Wiley & Sons Ltd on behalf of European Hematology Association. This is an open access Abstract Book distributed under the Attribution-NonCommercial-NoDerivs (CC BY-NC-ND), which allows third parties to download the articles and share them with others as long as they credit the author and the Abstract Book, but they cannot change the content in any way or use them commercially.
**Abstract Book Citations:** Authors, Title, HemaSphere, 2026;10:(S1):pages. The abstract book DOIs can be found on the "Author Information" page.
**Disclaimer:** Articles published in the journal HemaSphere exclusively reflect the opinions of the authors. The authors are responsible for all content in their abstracts including accuracy of the facts, statements, citing resources, etc.

**Results**

25 participants were enrolled and assigned to three dose groups (Figure 1a). As of July 31, 2024, all participants had completed scheduled study visits.
Overall, 9MW3011 was well tolerated with a favorable safety profile across all dose levels (Figure 1b). The most common adverse events were laboratory test abnormalities. No deaths or serious adverse reactions were reported. Adverse reaction rates were consistent across dose groups, with no dose-dependent trends. No clinically significant safety risk signals were identified.
Preliminary efficacy was also observed (Figure 1c and 1d). The median CTF duration increased with dose, reaching 120, 139, and 197 days in the 150 mg Q4W, 150 mg Q2W, and 300 mg Q4W groups, respectively. Rates of participants remaining CTF were 43% in the 150 mg Q4W group, 57% in the 150 mg Q2W group, and 83% in the 300 mg Q4W group, showing an increasing trend with higher drug exposure of 9MW3011.

**Summary/Conclusion**

These findings indicate that 9MW3011 demonstrated favorable safety and tolerability in Chinese patients with PV and showed potential to prolong time free from cytoreductive therapy, thereby reducing cytoreductive treatment-related adverse reaction, suggesting a potential improvement in quality of life. Above all support further clinical investigations.
**Corresponding Author:** Zhijian Xiao, zjxiao@ihcams.ac.cn

**Copyright Information:** (Online) ISSN: 2572-9241
©2026 The Author(s). HemaSphere is published by John Wiley & Sons Ltd on behalf of European Hematology Association. This is an open access Abstract Book distributed under the Attribution-NonCommercial-NoDerivs (CC BY-NC-ND), which allows third parties to download the articles and share them with others as long as they credit the author and the Abstract Book, but they cannot change the content in any way or use them commercially.
**Abstract Book Citations:** Authors, Title, HemaSphere, 2026;10:(S1):pages. The abstract book DOIs can be found on the "Author Information" page.
**Disclaimer:** Articles published in the journal HemaSphere exclusively reflect the opinions of the authors. The authors are responsible for all content in their abstracts including accuracy of the facts, statements, citing resources, etc.

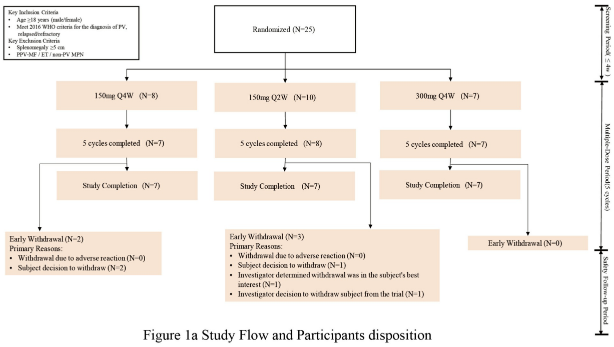

Figure 1a Study Flow and Participants disposition

| Adverse Event Category | 150mg Q4W | 150mg Q2W | 300mg Q4W | Total |
|---|---|---|---|---|
| | (N=8) | (N=10) | (N=7) | (N=25) |
| | n (%) | n (%) | n (%) | n (%) |
| Treatment-Emergent Adverse Events (TEAE) | 7(87.5) | 9(90.0) | 6(85.7) | 22(88.0) |
| Drug-related TEAE | 4(50.0) | 5(50.0) | 3(42.9) | 12(48.0) |
| Grade ≥3 TEAE | 2(25.0) | 2(20.0) | 1(14.3) | 5(20.0) |
| Drug-related Grade ≥3 TEAE | 0 | 0 | 0 | 0 |
| Serious Adverse Events (SAE) | 1(12.5) | 2(20.0) | 0 | 3(12.0) |
| Drug-related SAE | 0 | 0 | 0 | 0 |
| Discontinuations due to AEs | 0 | 0 | 0 | 0 |
| Deaths | 0 | 0 | 0 | 0 |

Figure 1b Safety

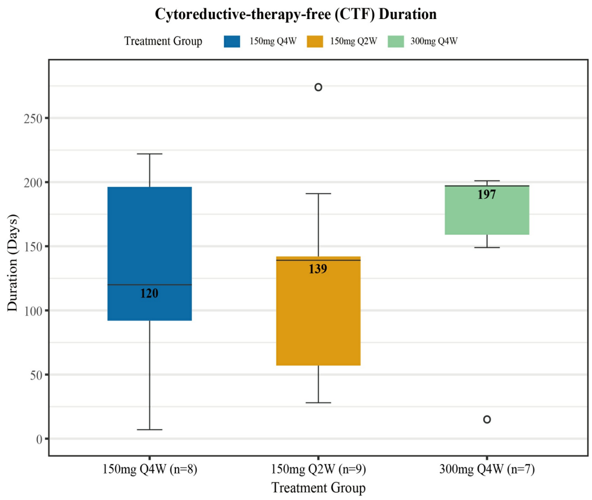

Figure 1c Preliminary Efficacy

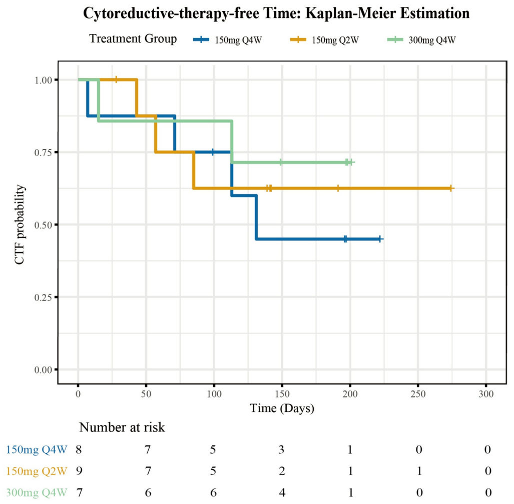

Figure 1d KM Curve

Favorable Safety, Tolerability, and Preliminary Efficacy of 9MW3011

**Copyright Information:** (Online) ISSN: 2572-9241
©2026 The Author(s). HemaSphere is published by John Wiley & Sons Ltd on behalf of European Hematology Association. This is an open access Abstract Book distributed under the Attribution-NonCommercial-NoDerivs (CC BY-NC-ND), which allows third parties to download the articles and share them with others as long as they credit the author and the Abstract Book, but they cannot change the content in any way or use them commercially.
**Abstract Book Citations:** Authors, Title, HemaSphere, 2026;10:(S1):pages. The abstract book DOIs can be found on the "Author Information" page.
**Disclaimer:** Articles published in the journal HemaSphere exclusively reflect the opinions of the authors. The authors are responsible for all content in their abstracts including accuracy of the facts, statements, citing resources, etc.

### (PF909) RESTORE-PV – A PHASE 2 OPEN-LABEL STUDY EVALUATING THE SAFETY AND EFFICACY OF THE ANTI-TMPRSS6 MONOCLONAL ANTIBODY DISC-3405 IN PARTICIPANTS WITH POLYCYTHEMIA VERA

**Topic:** 16. Myeloproliferative neoplasms - Clinical

Naseema Gangat*[1], Ayalew Tefferi[1], Aaron T. Gerds[2], Michael Grunwald[3], Anna B. Halpern[4, 5], Stephen Oh[6], Caitlin O'neill[7], Jeanne Palmer[8], Lindsay Rein[9], Candido Rivera[10], Gary Schiller[11], Joseph Shatzel[12], Lynn Guiducci[12], Jennifer Vaughn[13], Anne Wofford[14], Alison Kracunas[15], Marcus Carden[15], Will Savage[15], Prithviraj Bose[16], Naveen Pemmaraju[16]

*[1]Mayo Clinic, Rochester, United States of America; [2]Cleveland Clinic, Cleaveland, United States of America; [3]Atrium Health Levine Cancer Institute, Charlotte, United States of America; [4]Fred Hutchinson Cancer Center, Seattle, United States of America; [5]University of Washington, Seattle, United States of America; [6]Washington University, St. Louis, United States of America; [7]USC Norris Comprehensive Cancer Center, Los Angeles, United States of America; [8]Mayo Clinic, Pheonix, United States of America; [9]Duke University School of Medicine, Durham, United States of America; [10]Mayo Clinic, Jacksonville, United States of America; [11]David Geffen School of Medicine at UCLA, Los Angeles, United States of America; [12]Oregon Health & Science University, Portland, United States of America; [13]Ohio State University Comprehensive Cancer Center, Columbus, United States of America; [14]Atrium Health Wake Forest Baptist, Winston Salem, United States of America; [15]Disc Medicine, Watertown, United States of America; [16]MD Anderson Cancer Center, Houston, United States of America;*

### Background

Polycythemia vera (PV) is a myeloproliferative neoplasm associated with pathologic erythrocytosis driven by somatic mutations in the Janus kinase (JAK) 2 gene. A key treatment goal is tomaintain hematocrit (HCT) below 45% to reduce thromboembolic complications. Current standard of care, including phlebotomy (PHL), can be burdensome, may lead to intolerable adverse effects, and can be associated with symptoms of iron deficiency. DISC-3405 is an investigational, novel, humanized immunoglobulin (Ig)G1 monoclonal antibody (mAb) that targets transmembrane serine protease 6 (TMPRSS6, also known as matriptase-2) to stimulate endogenous production of hepcidin to restrict iron availability for erythropoiesis. DISC-3405 is being developed as a potential treatment for people living with PV requiring HCT reduction by PHL regardless of baseline risk or concurrent cytoreductive therapy (CRT).

### Aims

To assess the safety and efficacy of DISC-3405in people with PV requiring PHL.

**Copyright Information:** (Online) ISSN: 2572-9241
©2026 The Author(s). HemaSphere is published by John Wiley & Sons Ltd on behalf of European Hematology Association. This is an open access Abstract Book distributed under the Attribution-NonCommercial-NoDerivs (CC BY-NC-ND), which allows third parties to download the articles and share them with others as long as they credit the author and the Abstract Book, but they cannot change the content in any way or use them commercially.
**Abstract Book Citations:** Authors, Title, HemaSphere, 2026;10:(S1):pages. The abstract book DOIs can be found on the "Author Information" page.
**Disclaimer:** Articles published in the journal HemaSphere exclusively reflect the opinions of the authors. The authors are responsible for all content in their abstracts including accuracy of the facts, statements, citing resources, etc.

**Methods**

RESTORE-PV (NCT06985147) is a multicenter, open-label, 52-week Phase 2 study enrolling up to 60 people living with PV across 14 sites in the United States into 3 cohorts (20 per cohort) to assess the safety, tolerability, pharmacokinetics (PK), pharmacodynamics (PD), and therapeutic effect of DISC-3405 on PV disease control as evidenced by HCT reduction, PHL requirements, and measures of quality of life. Participants must meet 2022 WHO diagnostic criteria, need at least 3 PHLs in 26 weeks or 5 PHLs in the 52 weeks prior to screening, and at least 1 PHL must be within 12 weeks prior to screening. Participants receiving CRT (ie, hydroxyurea [HU], interferon, ruxolitinib) must have been on the therapy for at least 6 months and on a stable PV treatment regimen for at least 2 months. Participants requiring PHL at HCT levels <45% and clinically significant thrombosis within 2 months prior to study treatment were excluded.

After an observation period of 4 to12 weeks, participants are treated in a 12-week dose escalation period and then with 300 mg every 2 weeks (Cohort A) or 300 mg every 4 weeks (Cohort B) for 20 weeks for the primary efficacy period, followed by another 20 weeks of dosing up to 300 mg every 2 weeks. Cohort C is optional with a regimen to be informed by initial results. After 52 weeks, participants can continue in an optional 2-year continuation phase. The main efficacy endpoints of the study include the proportion of participants who are PHL free, as well as the number of PHLs during these treatment periods and proportion of participants maintaining HCT values <45%. A safety review committee monitors accumulating data to ensure safety of study participants.

**Results**

Sixteen adults (mean age 57.7 years ; SD 13.4) were enrolled into the study at the time of the data cut. Thirteen (81.3%) participants were male, all 16 were White, and 7 (43.8%) were receiving concurrent CRT: 4 (25%) HU and 3 (18.8%) interferon. Mean HCT at screening was 44.5% (SD 1.8), and the mean number of PHLs 52 weeks before screening was 6.8 (SD 1.8).

**Summary/Conclusion**

DISC-3405 is the first anti-TMPRSS6 mAb tested to reduce PHL burden in patients with PV. Additional baseline and observation period details will be presented.

**Copyright Information:** (Online) ISSN: 2572-9241
©2026 The Author(s). HemaSphere is published by John Wiley & Sons Ltd on behalf of European Hematology Association. This is an open access Abstract Book distributed under the Attribution-NonCommercial-NoDerivs (CC BY-NC-ND), which allows third parties to download the articles and share them with others as long as they credit the author and the Abstract Book, but they cannot change the content in any way or use them commercially.
**Abstract Book Citations:** Authors, Title, HemaSphere, 2026;10:(S1):pages. The abstract book DOIs can be found on the “Author Information” page.
**Disclaimer:** Articles published in the journal HemaSphere exclusively reflect the opinions of the authors. The authors are responsible for all content in their abstracts including accuracy of the facts, statements, citing resources, etc.

### (PF910) LONGITUDINAL ANALYSIS OF IRON DEFICIENCY MARKERS IN PATIENTS WITH POLYCYTHEMIA VERA ENROLLED IN THE PROSPECTIVE OBSERVATIONAL REVEAL STUDY

**Topic:** 16. Myeloproliferative neoplasms - Clinical

Jamile Shammo[1], Salman Fazal[2], Jennifer Hamer-Maansson[3], Valkal Bhatt[3], Amber Essenmacher[3], Michael Grunwald*[4]

*[1]Northwestern University, Division of Hematology & Oncology, Chicago, IL, United States of America;[2]Allegheny Health Cancer Institute, Division of Hematology and Cellular Therapy, Pittsburgh, PA, United States of America; [3]Incyte Corporation, Wilmington, DE, United States of America; [4]Levine Cancer Institute, Atrium Health, Department of Hematologic Oncology and Blood Disorders, Charlotte, NC, United States of America;*

### Background

Patients with polycythemia vera (PV) frequently require phlebotomies to lower hematocrit, leading to an iron deficiency-state over time. This further impairs quality of life (QoL) with worsening fatigue, headache, and cognitive impairment. Iron deficiency can contribute to cardiovascular and thrombotic events that elevate the overall morbidity and mortality risk associated with PV.

### Aims

This post hoc analysis of prospective data from the REVEAL study assessed the time-dependence of iron deficiency markers and their association with treatment in patients with PV.

### Methods

REVEAL (NCT02252159) was a US-based, prospective, non-interventional, multicenter study to longitudinally inform disease and clinical characteristics, patient journey, and treatment patterns of adults (≥18 years) with PV. Patients were enrolled across 227 academic/community practices and followed for ≥36 months during their usual PV care. The study included optional quarterly measurements of iron deficiency, defined as abnormal values for any of total iron, ferritin, transferrin saturation (TSAT), mean corpuscular volume (MCV), and total iron-binding capacity (TIBC). Phlebotomy burden on QoL was assessed using the Phlebotomy Burden Questionnaire (PBQ-21).

### Results

Among 2510 patients enrolled, 2411 (96%) had data for ≥1 iron marker available during the study, of whom data were available for: MCV, n=2073 (86%); ferritin, n=530 (22%); total iron, n=428 (18%); TIBC, n=402 (17%); and TSAT, n=163 (7%). Overall, 1364 (57%) patients had evidence of iron deficiency at any time; median age was 66 years (range 22-95); and most were treated in community practice (82%). Almost one-third (757/2411 [31%]) had iron deficiency at baseline (low total iron, 197/757 [26%]; low ferritin, 248/757 [33%]; low TSAT, 104/757 [14%]; low MCV, 544/757 [72%]; TIBC >ULN, 81/757 [11%]). Of 1654 patients with iron marker data available but without iron deficiency at baseline, 607 (37%) developed iron deficiency during the study. Time dependent changes in iron deficiency markers are shown (**Figure**). Over 48 months of visits, there was a trend towards increased total iron and ferritin and decreased TIBC.
During the study, 1509 patients (63%) received ≥1 phlebotomy annually. More patients with vs without iron deficiency received ≥1 phlebotomy during the study (1045/1364 [77%] vs 464/1047 [44%]), and the median (range) total number of phlebotomies was higher in patients with vs without iron deficiency (7 [1-61] vs 4 [1-53]).
Of patients who completed the PBQ-21 at baseline, in the past 3 months, mean travel time to phlebotomy procedure was 1.8 h (standard deviation [SD], 2.9; n=1127); mean procedure time was 2.3 h (SD, 3.1; n=1123); mean missed work time was 4.9 h (SD, 13.2; n=502); and mean total treatment time was 4.1 h (SD, 5.1; n=1123).

**Copyright Information:** (Online) ISSN: 2572-9241
©2026 The Author(s). HemaSphere is published by John Wiley & Sons Ltd on behalf of European Hematology Association. This is an open access Abstract Book distributed under the Attribution-NonCommercial-NoDerivs (CC BY-NC-ND), which allows third parties to download the articles and share them with others as long as they credit the author and the Abstract Book, but they cannot change the content in any way or use them commercially.
**Abstract Book Citations:** Authors, Title, HemaSphere, 2026;10:(S1):pages. The abstract book DOIs can be found on the "Author Information" page.
**Disclaimer:** Articles published in the journal HemaSphere exclusively reflect the opinions of the authors. The authors are responsible for all content in their abstracts including accuracy of the facts, statements, citing resources, etc.

### Summary/Conclusion

The REVEAL study has provided the largest dataset describing longitudinal changes in iron deficiency in patients with PV. Over half of the patients (57%) were iron deficient at some point during the study. The phlebotomy rate was higher in patients with iron deficiency during the study compared with those without, suggesting that the state of iron deficiency does not always prevent the requirement for additional phlebotomy. Phlebotomy burden was notable; patients spent around half a workday for each phlebotomy procedure. These real-world data provide information that may be used to inform and complement randomized controlled clinical trials.

**Figure. Changes Over Time in Iron Deficiency Markers in Enrolled Patients With Evaluable Iron Marker Data.**

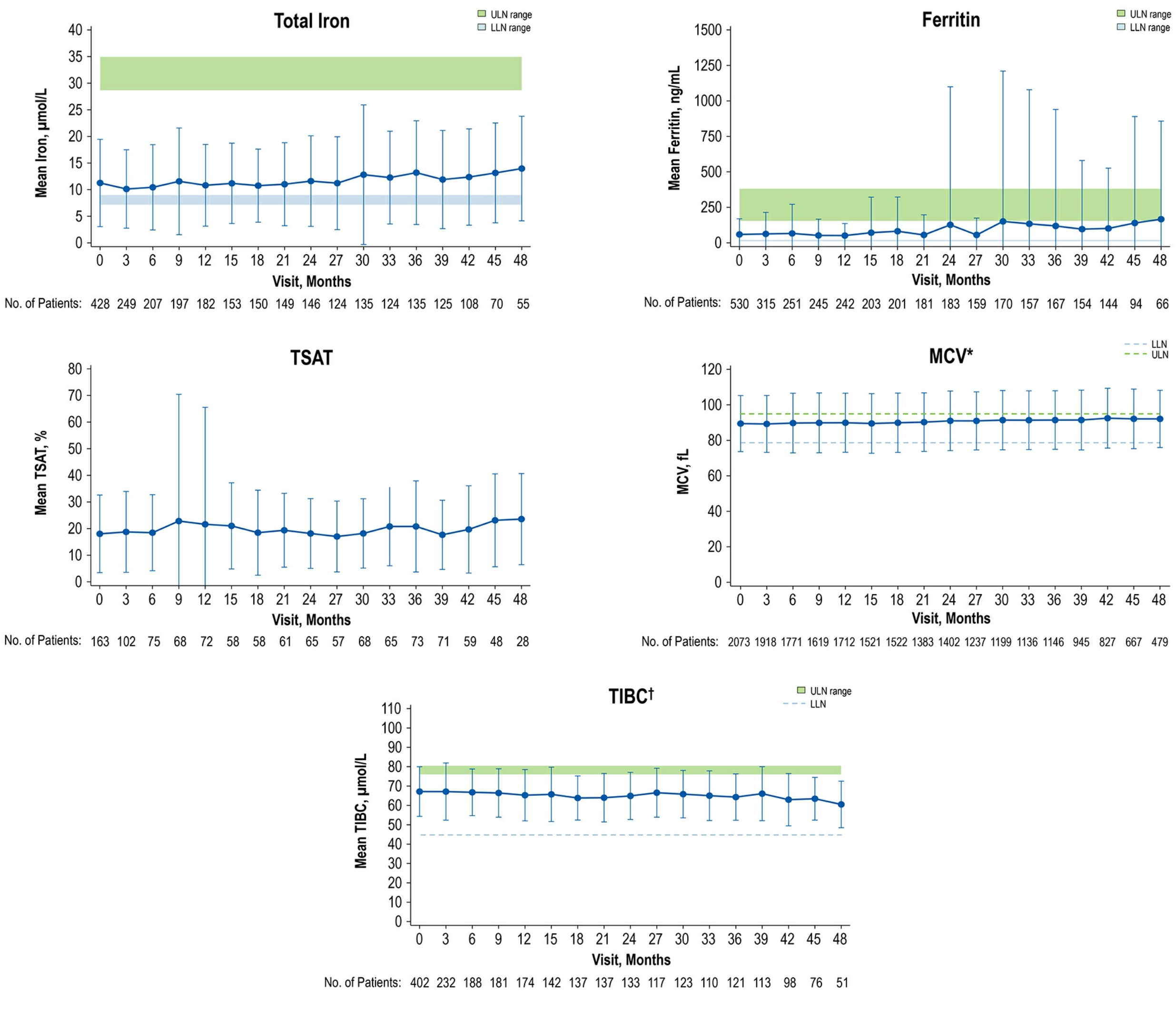

Data are mean (±SD). *One outlier at month 45 has been removed from the figure. †One outlier at month 18 has been removed from the figure. LLN, lower limit of normal; MCV, mean corpuscular volume; SD, standard deviation; TIBC, total iron-binding capacity; TSAT, transferrin saturation; ULN, upper limit of normal.

**Copyright Information:** (Online) ISSN: 2572-9241
©2026 The Author(s). HemaSphere is published by John Wiley & Sons Ltd on behalf of European Hematology Association. This is an open access Abstract Book distributed under the Attribution-NonCommercial-NoDerivs (CC BY-NC-ND), which allows third parties to download the articles and share them with others as long as they credit the author and the Abstract Book, but they cannot change the content in any way or use them commercially.
**Abstract Book Citations:** Authors, Title, HemaSphere, 2026;10:(S1):pages. The abstract book DOIs can be found on the "Author Information" page.
**Disclaimer:** Articles published in the journal HemaSphere exclusively reflect the opinions of the authors. The authors are responsible for all content in their abstracts including accuracy of the facts, statements, citing resources, etc.

### (PF911) AGE AS A CORE DISEASE MODIFIER: DISTINCT CLINICAL, MOLECULAR, AND PROGNOSTIC LANDSCAPES OF ESSENTIAL THROMBOCYTHEMIA IN ADOLESCENTS AND YOUNG ADULTS

**Topic:** 16. Myeloproliferative neoplasms - Clinical

Jia Li[1], Xiaojing Bao[1], Xiudi Yang[1], Honglan Qian[2], Binbin Lai[3], Xiao Yan[3], Jie Jin[1], Hongyan Tong[1], Jian Huang*[1]

*[1]The First Affiliated Hospital, Zhejiang University School of Medicine, Hangzhou, China;[2]The First Affiliated Hospital of Wenzhou Medical University, Wenzhou, China; [3]Department of Hematology, The First Affiliated Hospital of Ningbo University, Ningbo, China;*

### Background

Essential thrombocythemia (ET) in adolescents and young adults (AYA, aged 15-39 years) represents a distinct clinical entity with a not fully defined biological basis and prognosis compared to older patients.

### Aims

To delineate the clinical and molecular characteristics, long-term outcomes, and risk factors for post-ET myelofibrosis (MF) progression in AYA patients with ET.

### Methods

In this multicenter retrospective study, 1728 ET patients from 29 hospitals across China were enrolled and stratified into an AYA group (n=328) and a non-AYA group (age ≥40 years, n=1400). Clinical profiles, genomic landscapes, long-term outcomes, and risk factors for post-ET MF progression were compared between groups.

### Results

The AYA group had fewer cardiovascular risks and lower thrombosis rates, but more frequently had platelet counts >1000×$10^9$/L. Molecular profiling revealed that AYA patients were enriched for *CALR* mutations and triple-negative status, whereas *JAK2* predominated in non-AYA patients. Non-driver mutations (*TET2*, *DNMT3A*, *ASXL1*, *SH2B3*) were less frequent in AYA patients. Consequently, AYA patients achieved superior long-term survival (15-year OS: 98.2% vs. 90.9%, *P*=0.008; MFS: 77.7% vs. 60.9%, *P*=0.017; LFS: 100% vs. 93.7%, *P*=0.030). Analysis of post-ET MF progression risks identified age-specific patterns: baseline bone marrow fibrosis and smoking were shared independent risk factors; *CALR* mutation was specific to AYA patients, while splenomegaly, hemoglobin <125 g/L, and RDW >15% were specific to non-AYA patients.

### Summary/Conclusion

AYA-ET represents a unique clinical and molecular subtype with a favorable prognosis, which argues for age-stratified management. Young patients are enriched for *CALR* mutations, which independently heighten their risk of progressing to post-ET MF. These findings underscore the specific need for targeted therapies against *CALR*-mutant clones in this population.

This research was supported by the National Natural Science Foundation of China (No. 82570191) and the Zhejiang Provincial Health High level Innovative Talent Project (2022-2026).

Correspondence to: Prof Jian Huang, E-mail: househuang@zju.edu.cn.

**Copyright Information:** (Online) ISSN: 2572-9241
©2026 The Author(s). HemaSphere is published by John Wiley & Sons Ltd on behalf of European Hematology Association. This is an open access Abstract Book distributed under the Attribution-NonCommercial-NoDerivs (CC BY-NC-ND), which allows third parties to download the articles and share them with others as long as they credit the author and the Abstract Book, but they cannot change the content in any way or use them commercially.
**Abstract Book Citations:** Authors, Title, HemaSphere, 2026;10:(S1):pages. The abstract book DOIs can be found on the "Author Information" page.
**Disclaimer:** Articles published in the journal HemaSphere exclusively reflect the opinions of the authors. The authors are responsible for all content in their abstracts including accuracy of the facts, statements, citing resources, etc.

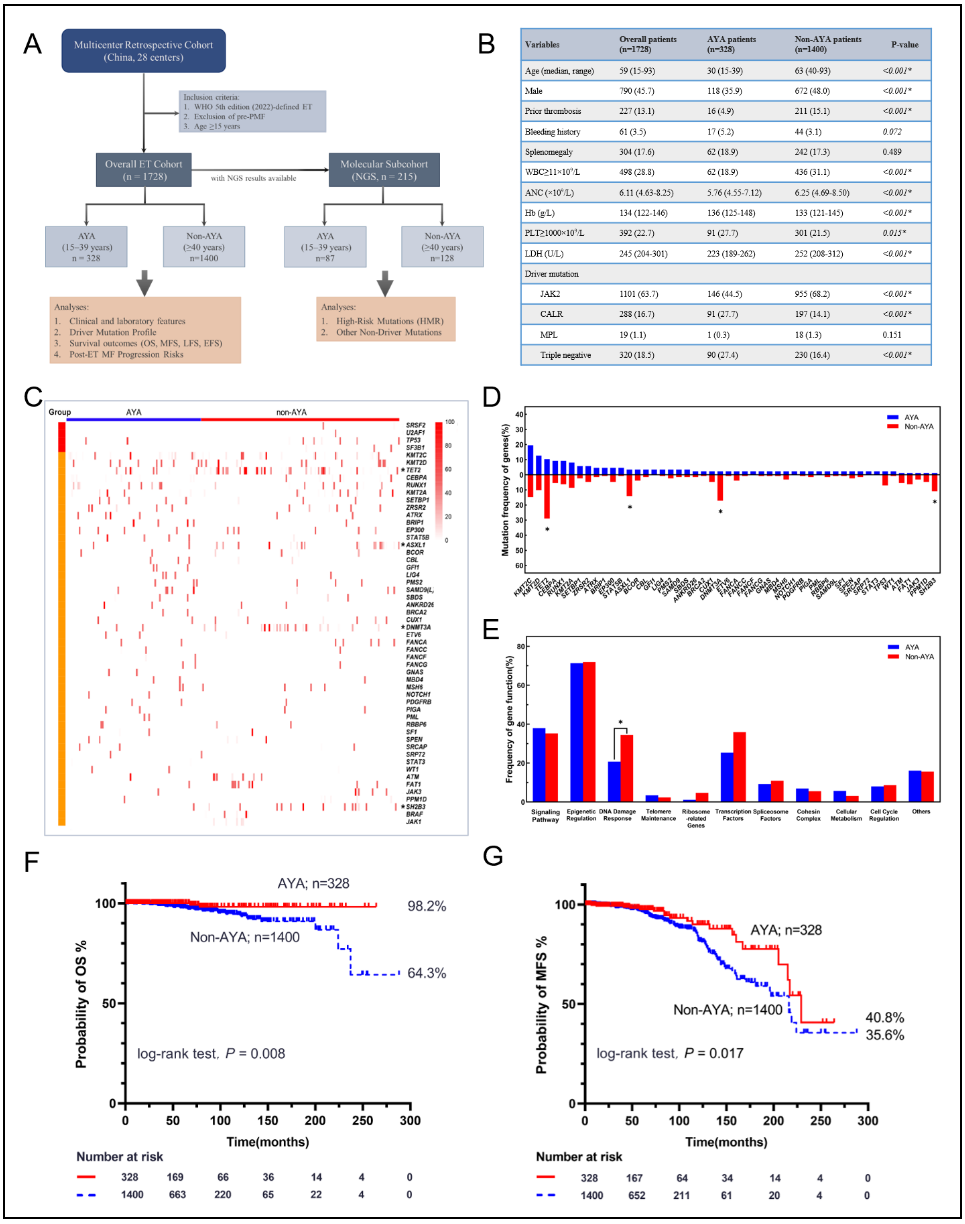

| Variables | Overall patients (n=1728) | AYA patients (n=328) | Non-AYA patients (n=1400) | P-value |
|---|---|---|---|---|
| Age (median, range) | 59 (15-93) | 30 (15-39) | 63 (40-93) | <0.001* |
| Male | 790 (45.7) | 118 (35.9) | 672 (48.0) | <0.001* |
| Prior thrombosis | 227 (13.1) | 16 (4.9) | 211 (15.1) | <0.001* |
| Bleeding history | 61 (3.5) | 17 (5.2) | 44 (3.1) | 0.072 |
| Splenomegaly | 304 (17.6) | 62 (18.9) | 242 (17.3) | 0.489 |
| WBC≥11×10⁹/L | 498 (28.8) | 62 (18.9) | 436 (31.1) | <0.001* |
| ANC (×10⁹/L) | 6.11 (4.63-8.25) | 5.76 (4.55-7.12) | 6.25 (4.69-8.50) | <0.001* |
| Hb (g/L) | 134 (122-146) | 136 (125-148) | 133 (121-145) | <0.001* |
| PLT≥1000×10⁹/L | 392 (22.7) | 91 (27.7) | 301 (21.5) | 0.015* |
| LDH (U/L) | 245 (204-301) | 223 (189-262) | 252 (208-312) | <0.001* |
| Driver mutation | | | | |
| JAK2 | 1101 (63.7) | 146 (44.5) | 955 (68.2) | <0.001* |
| CALR | 288 (16.7) | 91 (27.7) | 197 (14.1) | <0.001* |
| MPL | 19 (1.1) | 1 (0.3) | 18 (1.3) | 0.151 |
| Triple negative | 320 (18.5) | 90 (27.4) | 230 (16.4) | <0.001* |

Figure 1. (A) Flow diagram of study subject selection. (B) Comparison of clinical characteristics and driver mutations between AYA (n=328) and non-AYA (n=1400) patients. (C) Heatmap comparing non-driver gene mutations and their variant allele frequencies (VAF) between AYA and non-AYA patients. (D) Mutation landscape aggregated by functional pathways in AYA versus non-AYA patients. (E) Mutation spectrum of the 50 most frequently mutated non-driver genes in AYA versus non-AYA patients. (F) Overall survival (OS) and (G) myelofibrosis-free survival (MFS) in AYA versus non-AYA patients.

**Copyright Information:** (Online) ISSN: 2572-9241
©2026 The Author(s). HemaSphere is published by John Wiley & Sons Ltd on behalf of European Hematology Association. This is an open access Abstract Book distributed under the Attribution-NonCommercial-NoDerivs (CC BY-NC-ND), which allows third parties to download the articles and share them with others as long as they credit the author and the Abstract Book, but they cannot change the content in any way or use them commercially.
**Abstract Book Citations:** Authors, Title, HemaSphere, 2026;10:(S1):pages. The abstract book DOIs can be found on the "Author Information" page.
**Disclaimer:** Articles published in the journal HemaSphere exclusively reflect the opinions of the authors. The authors are responsible for all content in their abstracts including accuracy of the facts, statements, citing resources, etc.

### (PF912) HEMATOLOGIC CONTROL AND IMPROVED SAFETY FOLLOWING CROSSOVER FROM ANAGRELIDE TO ROPEGINTERFERON ALFA-2B IN PATIENTS WITH ESSENTIAL THROMBOCYTHEMIA: DATA FROM THE SURPASS-ET STUDY

**Topic:** 16. Myeloproliferative neoplasms - Clinical

Lucia Masarova*[1], Harry Gill[2], Zhijian Xiao[3], Jie Jin[4], Norio Komatsu[5], Keita Kirito[6], Toshiaki Sato[5], Oleh Zagrijtschuk[7], Albert Qin[8], Ruben Mesa[9]

*[1]MD Anderson Cancer Center, Houston, United States of America;[2]LKS Faculty of Medicine, the University of Hong Kong, Hong Kong, Hong Kong; [3]National Clinical Research Centre for Blood Diseases, Institute of Haematology and Blood Diseases Hospital, Chinese Academy of Medical Sciences & Peking Union Medical College, State Key Laboratory of Experimental Haematology, Tianjin, China; [4]The First Affiliated Hospital, Zhejiang University School of Medicine, Hangzhou, China;[5]PharmaEssentia Japan KK, Tokyo, Japan; [6]University of Yamanashi, Department of Haematology and Oncology, Yamanashi, Japan;[7]PharmaEssentia Corporation, Burlington, United States of America; [8]PharmaEssentia Corporation, Taipei, Taiwan; [9]Atrium Health Wake Forest Baptist Comprehensive Cancer Center, Winston-Salem, NC, United States of America;*

### Background

In patients with essential thrombocythemia (ET), optimal cytoreduction aims to control platelet (PLT) and leukocyte (WBC) counts while maintaining tolerability. Patients receiving anagrelide in the core phase of the phase 3 SURPASS-ET study were allowed to crossover to ropeginterferon alfa-2b in the extension phase regardless of their response status, safety and tolerability to anagrelide in the core 12 month period of the study. We assessed hematologic control and safety before and after treatment transition.

### Aims

We assessed hematologic control and safety before and after treatment transition from anagrelide to ropeginterferon alfa-2b in the SURPASS-ET study.

### Methods

This analysis included 39 patients with ET who received anagrelide during the core study and transitioned to ropeginterferon alfa-2b in the extension phase. Hematologic parameters were evaluated over two predefined time windows:
(1) 12 weeks prior to crossover (anagrelide period) and
(2) 12 weeks following crossover (ropeginterferon alfa-2b period).
Endpoints included mean platelet counts ($\times10^9$/L), mean leukocyte counts ($\times10^9$/L) during the biweekly visits 12 weeks prior to the crossover as also 12 weeks following the crossover, proportion of patients achieving platelet and WBC control (<400 $\times10^9$/L; <9.5 $\times10^9$/L, respectively) at the last visit before the transition and at the end of the 12 weeks period post-crossover, and incidence of treatment-emergent adverse events (AEs) during each 12 weeks period (anagrelide, before crossover, and ropeginterferon alfa-2b, after crossover).
Descriptive statistics were used to summarize changes between periods.

**Copyright Information:** (Online) ISSN: 2572-9241
©2026 The Author(s). HemaSphere is published by John Wiley & Sons Ltd on behalf of European Hematology Association. This is an open access Abstract Book distributed under the Attribution-NonCommercial-NoDerivs (CC BY-NC-ND), which allows third parties to download the articles and share them with others as long as they credit the author and the Abstract Book, but they cannot change the content in any way or use them commercially.
**Abstract Book Citations:** Authors, Title, HemaSphere, 2026;10:(S1):pages. The abstract book DOIs can be found on the "Author Information" page.
**Disclaimer:** Articles published in the journal HemaSphere exclusively reflect the opinions of the authors. The authors are responsible for all content in their abstracts including accuracy of the facts, statements, citing resources, etc.

**Results**

Thirty-nine patients were evaluable for hematologic outcomes. During the 12 weeks prior to switch, mean platelet count was 624.5 $\times 10^9$/L (SD 480.8, 95% CI 561.6-687.4), compared with 428.7 $\times 10^9$/L (SD 168.8, 95% CI 381.2-476.1) in the 12 weeks following initiation of ropeginterferon alfa-2b ($p=0.0098$). The proportion of patients achieving platelet control (<400 $\times 10^9$/L) increased from 28.2% to 54.8% post-switch.
Mean leukocyte counts were 12.6 $\times 10^9$/L (SD 6.0, 95% CI 11.8-13.4) before switch and 7.2 $\times 10^9$/L (SD 4.1, 95% CI 6.1-8.4) after switch ($p<0.0001$), with leukocyte normalization (<9.5 $\times 10^9$/L) observed in 41.0% vs 83.9%, respectively.
Adverse events occurred in 61.5% of patients during the anagrelide period and 56.4% during the ropeginterferon alfa-2b period. The most frequent AEs in the pre-crossover period were headache and palpitations, while post-switch AEs included, among others, flu-like symptoms and injection-site reactions.

**Summary/Conclusion**

In this short-term pre-/post-crossover analysis, transition from anagrelide to ropeginterferon alfa-2b was associated with improved/stable platelet and leukocyte control over 12 weeks, with a manageable safety profile, supporting feasibility of transitioning patients from anagrelide to ropeginterferon alfa-2b.

**Copyright Information:** (Online) ISSN: 2572-9241
©2026 The Author(s). HemaSphere is published by John Wiley & Sons Ltd on behalf of European Hematology Association. This is an open access Abstract Book distributed under the Attribution-NonCommercial-NoDerivs (CC BY-NC-ND), which allows third parties to download the articles and share them with others as long as they credit the author and the Abstract Book, but they cannot change the content in any way or use them commercially.
**Abstract Book Citations:** Authors, Title, HemaSphere, 2026;10:(S1):pages. The abstract book DOIs can be found on the "Author Information" page.
**Disclaimer:** Articles published in the journal HemaSphere exclusively reflect the opinions of the authors. The authors are responsible for all content in their abstracts including accuracy of the facts, statements, citing resources, etc.

### (PF913) MEASURABLE RESIDUAL DISEASE IN MYELOFIBROSIS: CLONAL CORRELATES FROM A PROSPECTIVE PHASE II STUDY

**Topic:** 16. Myeloproliferative neoplasms - Clinical

Nico Gagelmann*[1], Noha Awais[2], Richard Hao[2], Zachariah Defilipp[2], Peter van Galen[2], Colman Lindsley[1], Gabriela Hobbs[3]

*[1]Dana-Farber Cancer Institute, Boston, United States of America; [2]Division of Hematology, Brigham and Women's Hospital, Boston, Massachusetts, Boston, United States of America; [3]Leukemia Service, Massachusetts General Hospital, Boston, MA, Boston, United States of America;*

### Background

Measurable residual disease (MRD) monitoring guides management in several myeloid diseases, but remains yet undefined in myelofibrosis. Allogeneic hematopoietic cell transplantation (HCT) is the only clone-erasing treatment option to date and therefore the optimal environment to investigate clonal dynamics.

### Aims

We performed targeted, error-corrected next-generation sequencing (ec-NGS) on serial samples from patients enrolled in a prospective phase II trial of peri- and post-HCT ruxolitinib to characterize MRD and correlates of relapse.

### Methods

We quantified somatic variants in 43 genes across pre-HCT screening and serial post-HCT samples (Day+100 [D100], month 6, month 12, and end-of-treatment). Molecular remission was defined per current consensus as driver mutation not detected by an assay with minimum 1% VAF sensitivity (Salit et al. *Leukemia* 2025). We further quantified persistence of pre-HCT mutations (VAF≥0.0001) and incident/new mutations (VAF≥0.001). High-molecular-risk (HMR) mutations included *ASXL1, SRSF2, EZH2, IDH1/2, U2AF1, TP53, KRAS*, and *NRAS*. Donor chimerism was quantified from the same NGS data using a SNP fingerprint panel via maximum-likelihood, mixture-model, and regression-based methods.
Thirty-six patients had screening samples, 27 had paired screen+D100, and 23 had month 6/12 or end of treatment. The median age was 64 years, 83% had DIPSS+ intermediate-2 or high-risk disease, and 89% had splenomegaly.

**Copyright Information:** (Online) ISSN: 2572-9241
©2026 The Author(s). HemaSphere is published by John Wiley & Sons Ltd on behalf of European Hematology Association. This is an open access Abstract Book distributed under the Attribution-NonCommercial-NoDerivs (CC BY-NC-ND), which allows third parties to download the articles and share them with others as long as they credit the author and the Abstract Book, but they cannot change the content in any way or use them commercially.
**Abstract Book Citations:** Authors, Title, HemaSphere, 2026;10:(S1):pages. The abstract book DOIs can be found on the “Author Information” page.
**Disclaimer:** Articles published in the journal HemaSphere exclusively reflect the opinions of the authors. The authors are responsible for all content in their abstracts including accuracy of the facts, statements, citing resources, etc.

**Results**

Prior to HCT, MPN phenotypic drivers included *JAK2* (67%) *CALR* (17%), and *MPL* (11%). Most patients (86%) had additional myeloid driver mutations (range 1-4; median VAF 37%), including 67% with ≥1 HMR mutation.
At D100, 28% of *JAK2* mutations remained detectable, all below the 1% consensus remission threshold (VAF 0.07-0.4%). Pre-HCT HMR mutation status was not associated with molecular persistence. No *CALR* or *MPL* persistence was found at any VAF. Five of 27 (19%) had chimerism <95% at D100, of whom 4 (80%) were NGS-negative and subsequently achieved full chimerism. Both triple-negative patients achieved full chimerism (>95%) and were NGS-negative.
Among 6 patients with clinical relapse, no distinct baseline clinical pattern was observed. Both patients with pre-HCT *TP53* mutations relapsed (2/2, 100%), compared with 4/34 (12%) without *TP53*, relapses were enriched for *SRSF2* (33% vs 10%) and *U2AF1* (33% vs 13%) mutations. Despite 5 of 6 achieving full chimerism at Day100, detectable mutations were derived from the pre-HCT MPN clone in 4 patients (defined by phenotypic driver: 3 with *JAK2* and 1 with *CALR*) while 1 patient had a newly acquired *PTPN11*. Of the 6 relapses, 4 had relapse-persistent HMR mutation (*ASXL1*, *U2AF1*, *TP53*), and 5 of 6 showed evidence of subclonal evolution, most commonly involving RAS/MAPK pathway genes (*KRAS*, *NRAS*, *PTPN11*), and *TP53*.
New post-HCT *DNMT3A* and/or *TET2* mutations not detected pre-HCT were found in 70%, consistent with donor-derived clonal hematopoiesis (CH). Although recipient and donor age did not differ between those with and without donor CH, all recipients of matched sibling donors had donor CH.

**Summary/Conclusion**

Residual MPN clones frequently persist after HCT below consensus remission thresholds. *TP53* mutations at baseline, persistent HMR mutations, and emergent RAS/MAPK subclonal evolution were the dominant molecular features of relapse. Integrating high-sensitivity molecular monitoring into post-HCT surveillance could optimize MRD definition and prompt preemptive intervention in larger prospective trials.

**Copyright Information:** (Online) ISSN: 2572-9241
©2026 The Author(s). HemaSphere is published by John Wiley & Sons Ltd on behalf of European Hematology Association. This is an open access Abstract Book distributed under the Attribution-NonCommercial-NoDerivs (CC BY-NC-ND), which allows third parties to download the articles and share them with others as long as they credit the author and the Abstract Book, but they cannot change the content in any way or use them commercially.
**Abstract Book Citations:** Authors, Title, HemaSphere, 2026;10:(S1):pages. The abstract book DOIs can be found on the “Author Information” page.
**Disclaimer:** Articles published in the journal HemaSphere exclusively reflect the opinions of the authors. The authors are responsible for all content in their abstracts including accuracy of the facts, statements, citing resources, etc.

### (PF914) RISK FACTORS FOR PROGRESSIVE KIDNEY IMPAIRMENT AMONG PATIENTS WITH POLYCYTHEMIA VERA (PV) ARE RECAPITULATED AND TREATABLE IN MOUSE MODELS OF PV

**Topic:** 16. Myeloproliferative neoplasms - Clinical

Brandi Reeves*[1], Marcelo A. Szymanski de Toledo[2], Fatima Trebak[1], Malgorzata Kasztan[1], Vimal Derebail[3], Rafal Pawlinski[1], Steffen Koschmieder[2], Jennifer Hamer-Maansson[4], Valkal Bhatt[4]

*[1]University of North Carolina at Chapel Hill, Department of Medicine, Division of Hematology and Oncology, Chapel Hill, NC, United States of America; [2]RWTH Aachen University, Department of Hematology, Oncology, Hemostaseology, and Stem Cell Transplantation, Faculty of Medicine, Aachen, Germany; [3]University of North Carolina, Department of Medicine, Division of Nephrology and Hypertension, Chapel Hill, NC, United States of America; [4]Incyte Corporation, Wilmington, DE, United States of America;*

### Background

Chronic kidney disease (CKD) prevalence is 2-3 fold higher in patients (pts) with myeloproliferative neoplasms (MPN) vs the general population; 30% of pts with MPN have moderate-severe CKD and 20% have rapid estimated glomerular filtration rate (eGFR) decline (Christensen AS. *Leukemia Res*. 2014; Gecht J. *Cancers (Basel)*. 2021). Limited data exist on CKD progression in MPN, particularly in US pts.

### Aims

We report a post hoc analysis of real-world data to identify potential associations between rapid kidney function decline and PV progression in pts enrolled in the P**r**ospective Obs**e**rvational Study of patients with Polycythemia **Ve**ra in US Clinic**al** Practices (REVEAL; NCT02252159). Kidney impairment and treatment response in *JAK2*V617F-driven mouse models were also assessed.

### Methods eGFR decline from baseline during the 4-y study was assessed in pts with baseline and ≥2 post-baseline measurements of serum creatinine and *JAK2*V617F status in 2 subgroups: rapid (>3 mL/min/1.73 $m^2$/y); non-rapid (≤3) eGFR decline. PV progression on study was defined by meeting any of the criteria outlined in **Table 1**. GFR was measured transcutaneously in 14-week-old vavCre-driven *JAK2*V617F$^+$ transgenic mice and wild-type (WT) controls. Competitive hematopoietic stem cell transplantation was performed in 8-week-old C57BL/6 mice receiving a 1:1 mix of SCL-Cre-driven *JAK2*V617F$^+$ transgenic and WT cells. After engraftment, mice received interferon (IFN)-α or vehicle for 8 weeks and kidneys were collected for blinded histologic scoring.

**Copyright Information:** (Online) ISSN: 2572-9241
©2026 The Author(s). HemaSphere is published by John Wiley & Sons Ltd on behalf of European Hematology Association. This is an open access Abstract Book distributed under the Attribution-NonCommercial-NoDerivs (CC BY-NC-ND), which allows third parties to download the articles and share them with others as long as they credit the author and the Abstract Book, but they cannot change the content in any way or use them commercially.
**Abstract Book Citations:** Authors, Title, HemaSphere, 2026;10:(S1):pages. The abstract book DOIs can be found on the "Author Information" page.
**Disclaimer:** Articles published in the journal HemaSphere exclusively reflect the opinions of the authors. The authors are responsible for all content in their abstracts including accuracy of the facts, statements, citing resources, etc.

**Results**

Of 2510 pts enrolled in REVEAL, 624 were included in this analysis (rapid eGFR decline, n=153 [24.5%]; non-rapid eGFR decline, n=471 [75.5%]); 20% had evidence of moderate-severe kidney dysfunction at enrollment. Baseline characteristics were similar between subgroups. In both subgroups, kidney function deteriorated significantly over time.
Significantly more pts with rapid eGFR decline had history of thromboembolism (TE; 30.7% vs 20.0%, *P*=0.0057) and high-risk PV (age ≥60 y or history of TE, 87.6% vs 79.8%, *P*=0.0311; age ≥60 y and history of TE, 22.9% vs 15.1%, *P*=0.0256). Baseline eGFR and LDH were abnormal in both subgroups and significantly higher in the rapid vs non-rapid eGFR decline subgroup (median eGFR, 84.7 vs 76.7, *P*=0.0012; median LDH, 252.5 vs 209.0 U/L, *P*=0.0007). Incidence of PV progression was higher in patients with vs without rapid eGFR decline, reflecting increased bone marrow (BM) progression of myelofibrosis (MF; 8.5% vs 2.8%, *P*=0.0020).
Mean (±SD) GFR was reduced in *JAK2*V617F$^+$ transgenic mice (n=12) vs WT controls (n=5) (235.8±12.8 vs 261.1±4.5 mL/min, *P*=0.0006). Mesangial expansion was prominent in glomeruli of 1-yr-old *JAK2*V617F$^+$ transgenic mice (n=7) vs controls (n=3) (2.8±0.5 vs 1.5±0.8, *P*=0.0417). Tubular dilation was prominent in *JAK2*V617F$^+$ BM chimeras (n=3) vs controls (n=3) (1.6±0.3 vs 0.7±0.4, *P*=0.0306) and reduced following 8 weeks of IFNα therapy (0.5±0.2, *P*=0.0085).

**Summary/Conclusion**

In this large, prospective study of pts with PV, rapid eGFR decline was associated with novel early markers (history of TE, high-risk PV, elevated LDH, and BM progression of MF). Observed effects of *JAK2*V617F$^+$ and age on kidney dysfunction in mice were consistent with effects of PV progression on kidney dysfunction in pts. These findings support regular monitoring of kidney function and LDH in pts with PV. Further studies are warranted to determine the impact of rapid eGFR decline on PV outcomes and if PV-directed therapy may confer renal protection.

**Table 1. PV Progression Criteria**

| |
|---|
| **Criterion 1:** Death owing to MF/MDS/AML |
| **Criterion 2:** New/worsening splenomegaly and ≥2 of: WBC count >11×10$^9$/L, Hb <10 g/dL, and platelet count <100×10$^9$/L |
| **Criterion 3:** Bone marrow biopsy with fibrosis grade ≥2 or pathologic diagnosis of MF |
| **Criterion 4:** Circulating blasts >1% and new/worsening splenomegaly |

AML, acute myeloid leukemia; Hb, hemoglobin; MDS, myelodysplastic syndrome; MF, myelofibrosis; PV, polycythemia vera; WBC, white blood cell.

**Copyright Information:** (Online) ISSN: 2572-9241
©2026 The Author(s). HemaSphere is published by John Wiley & Sons Ltd on behalf of European Hematology Association. This is an open access Abstract Book distributed under the Attribution-NonCommercial-NoDerivs (CC BY-NC-ND), which allows third parties to download the articles and share them with others as long as they credit the author and the Abstract Book, but they cannot change the content in any way or use them commercially.
**Abstract Book Citations:** Authors, Title, HemaSphere, 2026;10:(S1):pages. The abstract book DOIs can be found on the "Author Information" page.
**Disclaimer:** Articles published in the journal HemaSphere exclusively reflect the opinions of the authors. The authors are responsible for all content in their abstracts including accuracy of the facts, statements, citing resources, etc.

### (PF4569) PREVALENCE AND UTILITY OF JAK2V617F TESTING IN PATIENTS WITH CRYPTOGENIC STROKE

**Topic:** 16. Myeloproliferative neoplasms - Clinical

Maria Alicia Senin Magan*[1, 2], Patricia Vélez[1, 2], Elisa Cuadrado[2, 3], Angel Ois[2, 3], Marta Vallverdú[2], Raquel Longarón[2, 4], Concepción Fernández-Rodriguez[2, 4], Barbara Ymaine[2, 4], Lierni Fernández Ibarrondo[2], Irene Botella Pérez[2], Laura Camacho[2, 4], Joan Gibert[2], Lucía Gómez-Pérez[1], Sara García Ávila[1], Christelle Ferrà[1, 2], Carles Besses[2], Beatriz Bellosillo[2, 4]

*[1]Hospital del Mar, Hematology, Barcelona, Spain;[2]IMIM Hospital del Mar Medical Research Institute, Barcelona, Spain; [3]Hospital del Mar, Neurology, Barcelona, Spain;[4]Hospital del Mar, Pathology, Barcelona, Spain;*

### Background

Cryptogenic stroke (CS) accounts for up to 20% of all strokes and require exclusion of an attributable cardioembolic, atherosclerotic, lacunar or thrombophilic cause. Chronic myeloproliferative neoplasms (MPNs) are anuncommon cause of stroke and are not included in the comprehensive diagnostic workup of CS.
The *JAK2*V617F mutation defines most MPNs, but it can also be detected in up to 3% of the healthy population and as clonal hematopoiesis of indeterminate potential (CHIP) with a low variant allele frequency, being associated with cardiovascular risk.
It is unknown whether testing for *JAK2* in this group of patients could contribute to the etiological and prognostical workup of CS.

### Aims

To assess the prevalence of the *JAK2*V617F mutation in patients with CS without a prior diagnosis of MPN.
To compare patients with low VAF (0.1-≤1%), *JAK2*-mutated (>1%,) and wild-type status and evaluate their risk of stroke recurrence.

### Methods

A retrospective study of an evolutionary cohort of 7,550 stroke patients was conducted. Patients were treated at Hospital del Mar (Barcelona) between 2004 and 2024, with DNA samples stored at the time of the event.
A total of 550 patients diagnosed with CS without a prior diagnosis of MPN were selected.
Clinical variables, vascular risk factors, and laboratory parameters were collected.
The *JAK2*V617F mutation was assessed using allele-specific PCR (sensitivity 0.01%). The positive cutoff was established at a VAF>0.1%.
The group of LowVAF was defined as VAF between 0.1-≤1%. The group of *JAK2*-mutated status was defined as VAF >1%.
A comparative analysis was performed between *JAK2*-mutated, LowVAF and wild-type groups using Fisher's exact test and logistic regression with the R statistical software package.
Early stroke recurrence (ESR) was defined as a new cerebrovascular event within 3 months.

**Copyright Information:** (Online) ISSN: 2572-9241
©2026 The Author(s). HemaSphere is published by John Wiley & Sons Ltd on behalf of European Hematology Association. This is an open access Abstract Book distributed under the Attribution-NonCommercial-NoDerivs (CC BY-NC-ND), which allows third parties to download the articles and share them with others as long as they credit the author and the Abstract Book, but they cannot change the content in any way or use them commercially.
**Abstract Book Citations:** Authors, Title, HemaSphere, 2026;10:(S1):pages. The abstract book DOIs can be found on the "Author Information" page.
**Disclaimer:** Articles published in the journal HemaSphere exclusively reflect the opinions of the authors. The authors are responsible for all content in their abstracts including accuracy of the facts, statements, citing resources, etc.

**Results**

The 3 groups of patients were well balanced in clinical-biological characteristics.
About 8.5% of patients with CS were carriers of *JAK2*V617F: 3% (17/550) were *JAK2*mutated (VAF >1%) and 5.5% (30/550) had lowVAF (VAF 0.1–≤1%). The frequency of *JAK2* was higher in patients > 75 years (13.3% versus 7.9% in those between 55-75 and 4.5% in those <55 years, p = 0.0145).
Six patients were diagnosed with MPN during the follow-up, 4 of them in the group of *JAK2*mutated patients (2PV, 2ET), 1 in *JAK2* wild-type group (*MPL* mutated) and the other in LowVAF group (unclassificable MPN).
ESR occurred in 13% (71/550), with rates of 12% (60/503) in wild type, 20% (6/30) in CHIP, and 35.2% (6/17) in *JAK2*mutated patients (p=0.0118).
In multivariable logistic regression adjusted for age, sex, and baseline stroke severity, *JAK2*V617F mutated status was associated with ESR (OR 3.45; 95% CI 1.12–11.85; p=0.032), Figure 1.

**Summary/Conclusion**

The prevalence of *JAK2* mutations in patients with CS and no prior history of laboratory abnormalities was, in our cohort, 3 to 4 times higher than that reported in the general population. *JAK2*-mutated patients with CS showed up to a three-fold higher probability of recurrent stroke in our series, demonstrating the importance of *JAK2* testing in identifying patients at high risk of recurrence.
The study expansion is underway to assess the prevalence of the *JAK2* mutation in 1000 patients with a determined cause of stroke, and the data will be updated at the meeting.

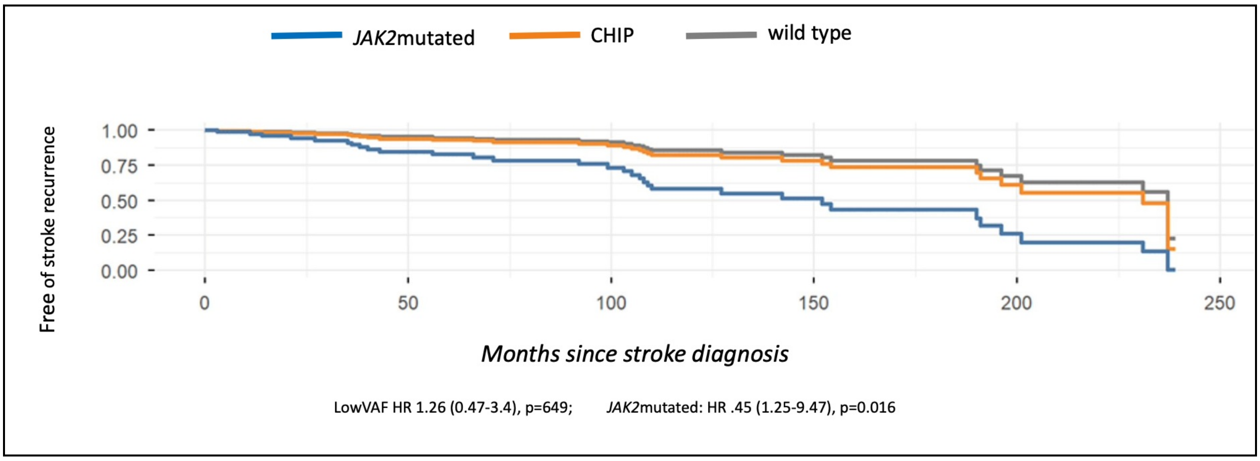

Figure 1. Risk of Recurrence of stroke within 3 months from diagnosis in 550 patients with Cryotogenic stroke.

**Copyright Information:** (Online) ISSN: 2572-9241
©2026 The Author(s). HemaSphere is published by John Wiley & Sons Ltd on behalf of European Hematology Association. This is an open access Abstract Book distributed under the Attribution-NonCommercial-NoDerivs (CC BY-NC-ND), which allows third parties to download the articles and share them with others as long as they credit the author and the Abstract Book, but they cannot change the content in any way or use them commercially.
**Abstract Book Citations:** Authors, Title, HemaSphere, 2026;10:(S1):pages. The abstract book DOIs can be found on the "Author Information" page.
**Disclaimer:** Articles published in the journal HemaSphere exclusively reflect the opinions of the authors. The authors are responsible for all content in their abstracts including accuracy of the facts, statements, citing resources, etc.

### (PF915) A PHASE 1 STUDY OF THE PRMT5 INHIBITOR AZD3470 IN PATIENTS WITH RELAPSED/REFRACTORY CLASSIC HODGKIN LYMPHOMA (PRIMAVERA)

**Topic:** 17. Hodgkin lymphoma - Clinical

Pier Luigi Zinzani*[1], Franck Morschhauser[2], Vincent Ribrag[3], Elizabeth H. Phillips[4], Herve Ghesquieres[5], Hun Lee[6], Peter Borchmann[7], Paul J. Bröckelmann[7], Tae Min Kim[8], Katharine Lewis[9], Jakub Svoboda[10], Antonia Rodriguez Izquierdo[11], Wonseog Kim[12], Anna Sureda[13], Graham Collins[14], Kaitlyn Beyfuss[15], Cedric Dos Santos[16], Richard F. Olsson[17], Enrico Derenzini[18]

*[1] 1. Policlinico Sant'Orsola-Malpighi, University of Bologna, Bologna, Italy;[2]Centre Hospitalier Universitaire de Lille, Lille, France; [3]Institut Gustave Roussy, Villejuif, France; [4]Christie NHS Foundation Trust and Division of Cancer Sciences, University of Manchester, Manchester, United Kingdom; [5]Hospital Lyon Sud, Lyon, France; [6]MD Anderson Cancer Center, Houston, TX, United States of America; [7]Universitätsklinikum Köln, Cologne, Germany; [8]Seoul National University Hospital, Seoul, South Korea; [9]Linear Clinical Research, Sir Charles Gairdner Hospital, and University of Western Australia, Perth, Australia;[10]Lymphoma Program, Abramson Cancer Center, University of Pennsylvania, Philadelphia, PA, United States of America; [11]Hospital Universitario 12 de Octubre, Madrid, Spain; [12]Division of Hematology and Oncology, Department of Medicine, Samsung Medical Center, Sungkyunkwan University School of Medicine, Seoul, South Korea; [13]Instituto Catalán de Oncología - Hospital Duran i Reynals, Barcelona, Spain; [14]14. Oxford Cancer and Haematology Centre, Oxford, United Kingdom; [15]Hematology R&D, AstraZeneca, Mississauga, Canada; [16]Hematology/Oncology R&D, AstraZeneca, South San Francisco, CA, United States of America; [17]Hematology R&D, AstraZeneca, Gothenburg, Sweden; [18]IEO European Institute of Oncology IRCCS, Milan, Italy;*

### Background

The epigenetic enzyme protein arginine methyltransferase 5 (PRMT5) plays a critical role in cell proliferation and differentiation; PRMT5 dysregulation is associated with cancer development. Methylthioadenosine (MTA) is an endogenous partial inhibitor of PRMT5 that accumulates in cancer cells deficient in MTA phosphorylase (MTAP). In classic Hodgkin lymphoma (cHL), we previously reported that >80% of primary patient (pt) tumor samples are MTAP deficient (Urosevic J, ASH 2023). The MTA-cooperative PRMT5 inhibitor AZD3470 preferentially binds to the MTA-bound state of PRMT5, increasing its target engagement to MTAP-deficient cancer cells.

### Aims

To determine the safety and preliminary efficacy of AZD3470 in a first-in-human Phase 1 study in patients with relapsed/refractory (r/r) cHL (NCT06137144).

### Methods

Eligible pts were ≥18 years with r/r cHL after ≥3 prior lines of therapy (including brentuximab vedotin (BV) and anti-PD-1). All patients provided written informed consent. In dose escalation, pts received oral AZD3470 monotherapy QD in ascending dose levels (DLs) using an mTPI-2 design. Primary endpoints were incidence and severity of treatment-emergent adverse events (TEAEs) and dose-limiting toxicity (DLT). Secondary endpoints were overall response rate (ORR) and complete response rate (CRR) per Lugano 2014 criteria.

**Copyright Information:** (Online) ISSN: 2572-9241
©2026 The Author(s). HemaSphere is published by John Wiley & Sons Ltd on behalf of European Hematology Association. This is an open access Abstract Book distributed under the Attribution-NonCommercial-NoDerivs (CC BY-NC-ND), which allows third parties to download the articles and share them with others as long as they credit the author and the Abstract Book, but they cannot change the content in any way or use them commercially.
**Abstract Book Citations:** Authors, Title, HemaSphere, 2026;10:(S1):pages. The abstract book DOIs can be found on the "Author Information" page.
**Disclaimer:** Articles published in the journal HemaSphere exclusively reflect the opinions of the authors. The authors are responsible for all content in their abstracts including accuracy of the facts, statements, citing resources, etc.

**Results**

As of Nov 25, 2025, 39 pts had received AZD3470 at DL ranging from 1 to 8. Most pts were male (62%) with stage IV disease (77%) and a median age of 42 (range 25–78) years. Pts had received a median of 6 prior lines of anticancer therapy (range 3–14); all had had prior BV and anti-PD1 treatments, and 20 (51%) and 5 (13%) pts had had prior autologous and allogeneic hematopoietic stem cell transplantation, respectively. All patients evaluable for MTAP protein expression were MTAP deficient. Median duration of exposure was 15 weeks (range 0.1–45.1), with treatment ongoing in 16 pts (41%). TEAEs occurred in 85% of pts, predominantly grades 1 or 2. The most common TEAEs (any grade) were anemia (28%) and nausea (15%), followed by asthenia, constipation, fatigue, and neutropenia (13% each). Grade ≥3 TEAEs occurred in 28% of pts, most commonly neutropenia (related) and hypokalemia (n=2 each). Serious TEAEs occurred in 5 (13%) pts. Two pts had dose reductions due to TEAEs (grade 4 hypertriglyceridemia and grade 3 esophagitis). No DLTs, treatment discontinuations nor deaths due to TEAEs were reported. Of the 31 pts evaluable for efficacy, 14 had an objective response, with responses at doses ≥DL4. The highest response rate was observed at doses ≥DL7 (n=10) with an ORR of 80% and CRR of 50%.

**Summary/Conclusion**

AZD3470 monotherapy was well tolerated up to DL8, with no DLTs and mainly low-grade AEs. Incidences of grade ≥3 AEs and serious TEAEs were low. Importantly, both the ORR and CRR were dose-dependent and notably high in this heavily pretreated cHL population. Dose optimization is ongoing and further safety and efficacy will be reported.

© 2026 American Society of Clinical Oncology, Inc. Reused with permission. This abstract was accepted and previously presented at the 2026 ASCO Annual Meeting. All rights reserved.

**Copyright Information:** (Online) ISSN: 2572-9241
©2026 The Author(s). HemaSphere is published by John Wiley & Sons Ltd on behalf of European Hematology Association. This is an open access Abstract Book distributed under the Attribution-NonCommercial-NoDerivs (CC BY-NC-ND), which allows third parties to download the articles and share them with others as long as they credit the author and the Abstract Book, but they cannot change the content in any way or use them commercially.
**Abstract Book Citations:** Authors, Title, HemaSphere, 2026;10:(S1):pages. The abstract book DOIs can be found on the "Author Information" page.
**Disclaimer:** Articles published in the journal HemaSphere exclusively reflect the opinions of the authors. The authors are responsible for all content in their abstracts including accuracy of the facts, statements, citing resources, etc.

### (PF916) PEMBROLIZUMAB IN CHILDREN AND YOUNG ADULTS WITH LOW-RISK CLASSIC HODGKIN LYMPHOMA WITH SLOW EARLY RESPONSE TO FRONT-LINE CHEMOTHERAPY: UPDATED RESULTS FROM KEYNOTE-667

**Topic:** 17. Hodgkin lymphoma - Clinical

Kara Kelly*[1], Melgar Toledo Mario[2], Bradford S. Hoppe[3], Christopher J. Forlenza[4], Sharon Castellino[5], Maitane Andion Catalan[6], Julie Krystal[7], Adam Lamble[8], Flavio Luisi[9], Fabio Molina Morales[10], Aarati Rao[11], Stacy Cooper[12], Oscar González-Llano[13], Luis Juarez Villegas[14], Christine Mauz-Koerholz[15, 16], Juan Shen[17], Pallavi Pillai[17], Rushdia Yusuf[17], Lisa Giulino-Roth[18]

*[1]Roswell Park Comprehensive Cancer Center, Buffalo, United States of America; [2]Unidad Nacional de Oncologia Pediatrica, Guatemala City, Guatemala; [3]Mayo Clinic, Jacksonville, United States of America; [4]Memorial Sloan Kettering Cancer Center, New York, United States of America; [5]Emory University, Aflac Cancer and Blood Disorders Center, Children's Healthcare of Atlanta, Atlanta, United States of America; [6]Hospital Infantil Universitario Nino Jesus, Madrid, Spain; [7]Cohen Children's Medical Center of New York, Queens, United States of America; [8]Seattle Children's Hospital, Seattle, United States of America; [9]Instituto de Oncologia Pediatrica—GRAACC—UNIFESP, São Paulo, Brazil; [10]Oncomédica S.A.S, Monteria, Colombia; [11]The Permanente Medical Group, Kaiser Permanente Oakland, Oakland, United States of America; [12]Johns Hopkins University, Baltimore, United States of America; [13]Hospital Universitario "Dr. Jose Eleuterio Gonzalez", Monterrey, Mexico; [14]Hospital Infantil de Mexico Federico Gomez, Mexico City, Mexico; [15]Justus-Liebig University of Giessen, Giessen, United States of America; [16]Medical Faculty of the Martin-Luther-University of Halle-Wittenberg, Halle, Germany; [17]Merck & Co., Inc., Rahway, United States of America; [18]Weill Cornell Medicine, New York, United States of America;*

### Background

Children and young adults with classic Hodgkin lymphoma (cHL) and slow early response (SER) to front-line chemotherapy are at risk of relapse. The open-label, phase 2 KEYNOTE-667 study (NCT03407144) evaluated consolidation with pembrolizumab plus chemotherapy with or without radiotherapy (RT) followed by maintenance pembrolizumab in children and young adults with cHL and SER to front-line chemotherapy. Prior analysis of participants (pts) with low-risk cHL and SER to front-line ABVD showed that consolidation with pembrolizumab plus AVD followed by RT and pembrolizumab maintenance had promising antitumor activity and manageable safety.

### Aims

To present updated results with an additional 13 months of follow-up for pts with low-risk cHL.

### Methods

Eligible pts were aged 3-25 years; had newly diagnosed stage IA, IB, or IIA cHL without bulky disease; had measurable disease per investigator; and had a performance status score of ≥50. Pts received induction with 2 cycles of ABVD followed by early response assessment. Pts with rapid early response (RER; Deauville score [DS] 1-3) received nonstudy therapy at investigator's discretion. Pts with SER (DS 4 or 5) received consolidation with 3 cycles of pembrolizumab 2 mg/kg up to 200 mg (aged 3-17 years) or 200 mg (aged 18-25 years) IV Q3W plus 2 cycles of AVD (days 1 and 15; Q4W) followed by late response assessment (LRA; between C2D22 and C2D28 of AVD). After LRA, all pts with low-risk cHL and SER received involved-site RT: 21.6 Gy for complete PET response (DS 1-3) or 30.6-36.0 Gy for partial PET response (DS 4 or 5). Pts received pembrolizumab for up to 17 cycles; pts with a complete response (CR) for ≥2 cycles could stop after 8 cycles. Primary end point was objective response rate (ORR) per Cheson 2007 IWG criteria by BICR. Secondary end points were PET negativity after consolidation, 2-year event-free survival (EFS) after enrollment, overall survival (OS), and safety in pts with SER.

**Copyright Information:** (Online) ISSN: 2572-9241
©2026 The Author(s). HemaSphere is published by John Wiley & Sons Ltd on behalf of European Hematology Association. This is an open access Abstract Book distributed under the Attribution-NonCommercial-NoDerivs (CC BY-NC-ND), which allows third parties to download the articles and share them with others as long as they credit the author and the Abstract Book, but they cannot change the content in any way or use them commercially.
**Abstract Book Citations:** Authors, Title, HemaSphere, 2026;10:(S1):pages. The abstract book DOIs can be found on the "Author Information" page.
**Disclaimer:** Articles published in the journal HemaSphere exclusively reflect the opinions of the authors. The authors are responsible for all content in their abstracts including accuracy of the facts, statements, citing resources, etc.

**Results**

A total of 78 pts with low-risk cHL were enrolled; 10 had SER to front-line ABVD and received consolidation with pembrolizumab plus AVD and then RT after LRA. At data cutoff (October 1, 2025), 6 pts with SER had completed consolidation and pembrolizumab maintenance and 4 discontinued due to CR. Pts received a median of 11.5 cycles of pembrolizumab (range, 8-17) in the consolidation and maintenance phase. Median time from enrollment to database cutoff for pts with SER was 39.0 months (range, 24.6-63.9). All 10 pts (100%) with SER who received consolidation had an LRA. At LRA, 6 pts (60%) were PET-negative by BICR; 7 (70%) were PET-negative by investigator, of whom 6 received a reduced dose of RT. After end of pembrolizumab, ORR was 100% (95% CI, 69-100; 8 CR, 2 partial response). Median EFS was not reached (NR; 95% CI, 21-NR); 2-year EFS was 90% and 3-year EFS was 90%. Median OS was NR (95% CI, NR-NR); 3-years OS was 100%. Treatment-related adverse events (AEs) occurred in 8 pts (80%; grade 3 or 4, n=4 [40%]). No pts discontinued treatment or died due to treatment-related AEs. Immune-mediated AEs occurred in 3 pts (30%).

**Summary/Conclusion**

With additional follow-up, consolidation with pembrolizumab plus AVD followed by RT and pembrolizumab maintenance continued to show promising antitumor activity (ORR, 100%; 2-year EFS rate, 90%) and manageable safety in pts with low-risk cHL and SER to front-line ABVD. Seven pts (70%) were PET negative by investigator, of whom 6 received a reduced dose of RT.

**Copyright Information:** (Online) ISSN: 2572-9241
©2026 The Author(s). HemaSphere is published by John Wiley & Sons Ltd on behalf of European Hematology Association. This is an open access Abstract Book distributed under the Attribution-NonCommercial-NoDerivs (CC BY-NC-ND), which allows third parties to download the articles and share them with others as long as they credit the author and the Abstract Book, but they cannot change the content in any way or use them commercially.
**Abstract Book Citations:** Authors, Title, HemaSphere, 2026;10:(S1):pages. The abstract book DOIs can be found on the "Author Information" page.
**Disclaimer:** Articles published in the journal HemaSphere exclusively reflect the opinions of the authors. The authors are responsible for all content in their abstracts including accuracy of the facts, statements, citing resources, etc.

### (PF917) VALIDATION OF THE ADVANCED-STAGE HODGKIN LYMPHOMA INTERNATIONAL PROGNOSTIC INDEX (A-HIPI) IN THE GHSG HD21 TRIAL

**Topic:** 17. Hodgkin lymphoma - Clinical

Hishan Tharmaseelan*[1], Janina Jablonski[1], Susan Parsons[2], Angie Rodday[2], Andrew M. Evens[3], Josée Zijlstra[4], Alexander Fosså[5], Daniel Molin[6], Mark Hertzberg[7], Urban Novak[8, 9], Martin Fehr[8, 10], Richard Greil[11], Thomas Melchardt[11, 12], Michael Fuchs[1], Helen Kaul[1], Peter Borchmann[1], Justin Ferdinandus[1]

*[1]University of Cologne, Faculty of Medicine and University Hospital of Cologne, Department I of Internal Medicine, and Center for Integrated Oncology Aachen Bonn Cologne Düsseldorf (CIO ABCD), and German Hodgkin Study Group (GHSG), Cologne, Germany; [2]Institute for Clinical Research and Health Policy Studies, Tufts Medical Center, Boston, MA, United States of America; [3]Division of Blood Disorders, Rutgers Cancer Institute New Jersey, New Brunswick, NJ, United States of America,[4]Department of Hematology, Amsterdam UMC, Vrije Universiteit, Cancer Center, Amsterdam, The Netherlands; [5]Department of Oncology, Oslo University Hospital, Oslo, Norway; [6]Department of Immunology, Genetics and Pathology, Cancer Immunotherapy, Uppsala University, Uppsala, Sweden; [7]Prince of Wales Hospital Department of Haematology Sydney and Australasian Leukaemia Lymphoma Group (ALLG), Melbourne, Australia; [8]Swiss Cancer Institute (SCI), Bern, Switzerland;[9]Medical Oncology, Inselspital, Bern University Hospital and University of Bern, Bern, Switzerland; [10]Cantonal Hospital St. Gallen, St. Gallen, Switzerland; [11]Arbeitsgemeinschaft Medikamentöse Tumortherapie (AGMT), Salzburg, Austria; [12]Department of Internal Medicine III with Haematology, Medical Oncology, Haemostaseology, Infectiology and Rheumatology, Paracelsus Medical University, Salzburg, Austria;*

### Background

The Advanced-Stage Hodgkin Lymphoma International Prognostic Index (A-HIPI) was developed to predict 5-year progression-free survival (PFS) and overall survival (OS) in advanced-stage classical Hodgkin lymphoma patients, using individual patient data from modern clinical trials and validated in prominent registries. This study aimed to validate the A-HIPI in patients treated within the GHSG HD21 trial (NCT02661503), comparing BrECADD (brentuximab vedotin, etoposide, cyclophosphamide, doxorubicin, dacarbazine, and dexamethasone) versus eBEACOPP (bleomycin, etoposide, doxorubicin, cyclophosphamide, vincristine, procarbazine, and prednisone) regimens.

### Aims

To validate the A-HIPI model for predicting 5-year OS and PFS in HD21 patients by assessing discrimination ability and calibration accuracy, and to compare its performance with the original International Prognostic Score (IPS-7).

### Methods

The analysis included 1,446 patients from the HD21 intention-to-treat population (n=1,473) with available A-HIPI variables and laboratory values within pre-defined ranges. The A-HIPI models respectively incorporate multiple variables (e.g. age, sex, stage, bulk disease, lymphocyte count, hemoglobin, and albumin levels) to calculate individual 5-year PFS and OS probabilities. Calibration was assessed by comparing observed and predicted probabilities of 5-year events within quintiles of predicted probabilities and the estimation of calibration-in-the-large and calibration intercepts and slopes. Kaplan-Meier curves by quintiles were generated. Discrimination was assessed using Harrell's C statistic for both, A-HIPI and IPS-7, and bootstrap confidence intervals were calculated. Separate analyses were performed for the total cohort and by treatment arm (BrECADD: 729 patients; eBEACOPP: 717 patients)

**Copyright Information:** (Online) ISSN: 2572-9241
©2026 The Author(s). HemaSphere is published by John Wiley & Sons Ltd on behalf of European Hematology Association. This is an open access Abstract Book distributed under the Attribution-NonCommercial-NoDerivs (CC BY-NC-ND), which allows third parties to download the articles and share them with others as long as they credit the author and the Abstract Book, but they cannot change the content in any way or use them commercially.
**Abstract Book Citations:** Authors, Title, HemaSphere, 2026;10:(S1):pages. The abstract book DOIs can be found on the "Author Information" page.
**Disclaimer:** Articles published in the journal HemaSphere exclusively reflect the opinions of the authors. The authors are responsible for all content in their abstracts including accuracy of the facts, statements, citing resources, etc.

### Results

In the HD21 validation cohort, A-HIPI distribution for both OS and PFS was comparable to the development cohort, but only 27 OS and 112 PFS events occurred. For PFS, the C-statistic of A-HIPI was 0.57 (95% CI 0.52-0.63), compared to 0.61 (95% CI 0.56-0.66) for IPS-7. Calibration-in-the-large was 16.2% (predicted: 23.9%, observed: 7.7%) and the calibration slope was 0.32 (95% CI 0.07-0.57) with differences between predicted and observed event rates in quintiles increasing from Q0 (11.7%) to Q4 (22.7%). In the BrECADD arm, the lowest PFS risk quintile showed a discernibly better outcome, compared to other quintiles. In this group, the calibration slope was 0.33 (95% CI -0.03-0.68) and the C-statistic for A-HIPI was 0.61 (95% CI 0.52-0.69) versus 0.64 (95% CI 0.55-0.73) for IPS-7. In the eBEACOPP arm, the highest PFS risk quintile showed a discernibly worse outcome compared to other quintiles, the calibration slope was 0.33 (95% CI -0.17-0.83) and the C-statistic for A-HIPI was 0.55 (95% CI 0.49-0.63), compared to 0.59 (95% CI 0.52-0.65) for IPS-7. Limited OS events precluded survival model validation.

### Summary/Conclusion

While A-HIPI distribution in HD21 mirrored the development cohort, the model overestimates the PFS event rate, especially in patients with high predicted risk by A-HIPI. Therefore, recalibration of the A-HIPI may be necessary to reflect cure rates with modern treatments.

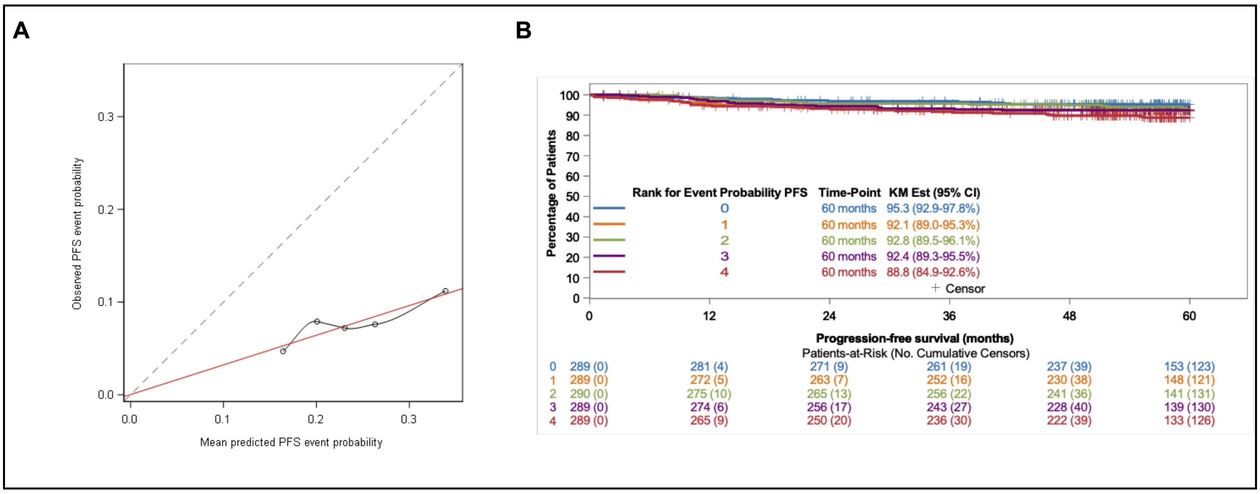

A: Calibration plots for the A-HIPI in the HD21 trial; B: PFS split by A-HIPI risk quintiles (HD21 full cohort)

**Copyright Information:** (Online) ISSN: 2572-9241
©2026 The Author(s). HemaSphere is published by John Wiley & Sons Ltd on behalf of European Hematology Association. This is an open access Abstract Book distributed under the Attribution-NonCommercial-NoDerivs (CC BY-NC-ND), which allows third parties to download the articles and share them with others as long as they credit the author and the Abstract Book, but they cannot change the content in any way or use them commercially.
**Abstract Book Citations:** Authors, Title, HemaSphere, 2026;10:(S1):pages. The abstract book DOIs can be found on the “Author Information” page.
**Disclaimer:** Articles published in the journal HemaSphere exclusively reflect the opinions of the authors. The authors are responsible for all content in their abstracts including accuracy of the facts, statements, citing resources, etc.

### (PF918) CHOP/MINICHOP AND TISLELIZUMAB IN ELDERLY PATIENTS WITH CLASSICAL HODGKIN LYMPHOMA (CHL). FIRST REPORT OF THE PHASE II CATER-HL TRIAL (HD12) FROM THE AUSTRALASIAN LYMPHOMA AND LEUKAEMIA GROUP (ALLG).

**Topic:** 17. Hodgkin lymphoma - Clinical

Tara Cochrane*[1, 2], Sze Ting Lee[3], Robin Gasiorowski[4, 5], Shane Gangatharan[6, 7], Fernando Roncolato[8], William Renwick[9, 10], Jock Simpson[11, 12], Amanda Johnston[13], Hans Hahn[14, 15], Philip Wong[16, 17], Masa Lasica[18], Ann Solterbeck[19], Robert Traficante[19], Sally Mapp[20, 21], Bianca Devitt[22, 23], Dhvani Pandya[24], Julia Carlson[24], Tracey Gerber[24], Mark Hertzberg[25], Eliza Hawkes[3, 26], Colm Keane[20, 27]

*[1]Gold Coast University Hospital, Haematology, Southport, Australia;[2]Griffith University, School of Medicine and Dentistry, Griffith Health, Nathan, Australia; [3]Olivia Newton John Cancer Research Institute at Austin Health, Melbourne, Australia;[4]Concord Hospital, Sydney, Australia; [5]University of Sydney, Sydney, Australia;[6]Fiona Stanley Hospital, Murdoch, Australia; [7]University of Western Australia, Crawley, Australia; [8]St George Hospital, Sydney, Australia;[9]Western Health, Footscray, Australia; [10]University of Melbourne, Melbourne, Australia; [11]Mid North Coast Cancer Institute, Port Macquarie, Australia; [12]University of UNSW, Sydney, Australia; [13]Westmead Hospital, Sydney, Australia; [14]Royal Adelaide Hospital and The Queen Elizabeth Hospital, Adelaide, Australia; [15]SA Pathology, Adelaide, Australia; [16]Toowoomba Hospital, Toowoomba, Australia; [17]University of Queensland, Rural Clinical School, Brisbane, Australia; [18]St Vincent's Hospital, Melbourne, Australia; [19]Statistical Revelations Pty Ltd, Ocean Grove, Australia; [20]Princess Alexandra Hospital, Brisbane, Australia;[21]University of Queensland, Faculty of Medicine, Brisbane, Australia; [22]Eastern Health, Oncology, Box Hill, Australia;[23]Monash University, Eastern Health Clinical School, Melbourne, Australia; [24]Australasian Leukaemia and Lymphoma Group, Melbourne, Australia;[25]Prince of Wales Hospital, Randwick, Australia; [26]Monash University, School of Public Health and Preventive Medicine, Melbourne, Australia;[27]University of Queensland, Frazer Institute, Brisbane, Australia;*

### Background

Single agent anti-PD-1 therapy is active in some older patients (pts) with cHL, however responses are not sustained. The combination of anti-PD-1 with Adriamycin, vinblastine, dacarbazine (AVD) is effective in older pts with cHL however data are limited in pts >70. CHOP (cyclophosphamide, doxorubicin, vincristine, prednisone) is active and deliverable in older cHL pts, but has not been combined with anti-PD-1.

### Aims

CATER-HL (ALLG-HD12, ACTRN12622000207718p) is a single arm phase II study of anti-PD-1 agent, tislelizumab (T), with CHOP/miniCHOP in older (≥61 years) pts with untreated cHL. Primary end point is complete remission (CR) rate (Deauville score 1-3 on PET-CT) and deliverability of T monotherapy (absence of grade 3/4 toxicity and absence of symptomatic progressive disease [PD] requiring cessation of T monotherapy). Key secondary end points include overall response (ORR)/CR post therapy, PFS, OS and quality of life (QOL).

### Methods

Eligible pts had stage 2 unfavourable to stage 4 disease and no anthracycline or immunotherapy contraindications. Patients received 3 cycles of T monotherapy (200mg q3W), followed by T in combination with 4-6 cycles of CHOP (miniCHOP if aged >80 or frail) (T-CHOP[mini]) q3W. Consolidation radiotherapy (RT) or 4 extra doses of T was allowed for those with residual PET-CT avidity following T-CHOP[mini] or if stage 2 (RT). PET-CT scans were performed at screening, after T monotherapy, after 2 cycles of T-CHOP[mini], post final cycle of T-CHOP[mini] and after any extra T/RT.

**Copyright Information:** (Online) ISSN: 2572-9241
©2026 The Author(s). HemaSphere is published by John Wiley & Sons Ltd on behalf of European Hematology Association. This is an open access Abstract Book distributed under the Attribution-NonCommercial-NoDerivs (CC BY-NC-ND), which allows third parties to download the articles and share them with others as long as they credit the author and the Abstract Book, but they cannot change the content in any way or use them commercially.
**Abstract Book Citations:** Authors, Title, HemaSphere, 2026;10:(S1):pages. The abstract book DOIs can be found on the "Author Information" page.
**Disclaimer:** Articles published in the journal HemaSphere exclusively reflect the opinions of the authors. The authors are responsible for all content in their abstracts including accuracy of the facts, statements, citing resources, etc.

### Results

35 pts from 11 Australian sites were enrolled from May 2023-2025. Baseline characteristics: median age 74 (range 63-90), ≥70 years 69%, male 66%, ECOG 0-1 86%, white/European ethnicity 77%, advanced stage 89% with median international prognostic score 4 (range 2-6), EBER-ish positive 46%, median Charlson's comorbidity score 4 (range 3–12) and frailty score ≥4 26%. The CR rate after T monotherapy was 17% (ORR 69%); deliverability was 86%. Five pts ceased T monotherapy prematurely; 2 from immune related adverse event (IRAE) of grade 4 hepatitis and 3 for other reasons: PD (1), EBV+ mouth ulcer and increasing frailty (1), declined further therapy (1). Thirty pts commenced T-CHOP[mini] (CHOP:70%, miniCHOP:30%). The ORR after 2 cycles of T-CHOP[mini] was 87% (26/30; CR 50%/PR 37%). At the end of T-CHOP[mini], the ORR was 77% (23/30; CR 63%/PR17%, PD 7%, indeterminate response 7%, data pending 2 pts). Median follow up is 384 days (range 24–735). Estimated 2 year PFS/OS is 77%/94% (see figure). Grade 3/4 treatment emergent AEs affected 46% of pts and comprised: infection 14%, fever 9%, hepatitis 11%, peripheral neuropathy 6%, confusion 6%. IRAEs affected 31% pts and comprised: thyroid dysfunction 14%, hepatitis 11%, pancreatitis 3%, raised creatinine kinase 3%, pruritis 3%. Two pts died, both from PD. No patient died from treatment related toxicity. QOL improved on treatment; there was median improvement of 10% after T monotherapy, 10% after 2 cycles of T-CHOP[mini] and 14% at end of therapy.

### Summary/Conclusion

A lead in T monotherapy strategy is deliverable and demonstrated early metabolic response. Sequential treatment with T-CHOP[mini] is well tolerated and results in promising PFS, OS and improvements in QOL in this predominant over 70 year old cohort. Given the challenges of delivering AVD-based therapy in this age group, this sequential approach represents a pragmatic curative-intent alternative for older/ frail pts unsuitable for standard regimens.

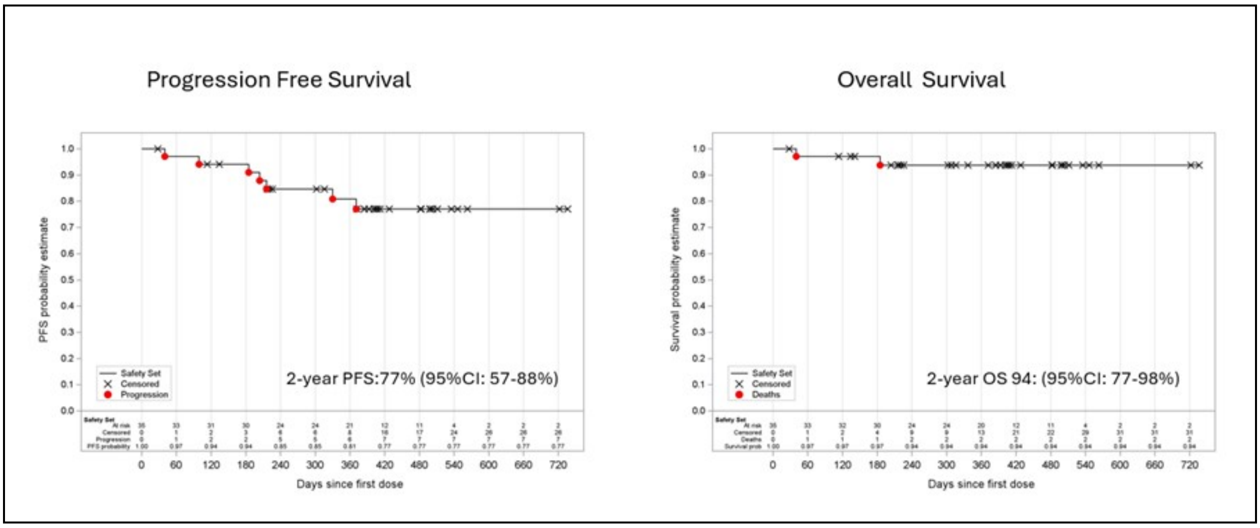

**Copyright Information:** (Online) ISSN: 2572-9241
©2026 The Author(s). HemaSphere is published by John Wiley & Sons Ltd on behalf of European Hematology Association. This is an open access Abstract Book distributed under the Attribution-NonCommercial-NoDerivs (CC BY-NC-ND), which allows third parties to download the articles and share them with others as long as they credit the author and the Abstract Book, but they cannot change the content in any way or use them commercially.
**Abstract Book Citations:** Authors, Title, HemaSphere, 2026;10:(S1):pages. The abstract book DOIs can be found on the "Author Information" page.
**Disclaimer:** Articles published in the journal HemaSphere exclusively reflect the opinions of the authors. The authors are responsible for all content in their abstracts including accuracy of the facts, statements, citing resources, etc.

### (PF919) SEX-SPECIFIC CHARACTERISTICS AND OUTCOMES IN NODULAR LYMPHOCYTE-PREDOMINANT HODGKIN LYMPHOMA: AN ANALYSIS FROM THE GERMAN HODGKIN STUDY GROUP

**Topic:** 17. Hodgkin lymphoma - Clinical

Dennis A. Eichenauer*[1], Ina Bühnen[1], Michael Fuchs[1], Aylin Basaran[1], Karolin Behringer[1], Anne Sophie Robertz[1], Bastian von Tresckow[2], Sylvia Hartmann[3], Peter Borchmann[1]

*[1]University Hospital Cologne, First Department of Internal Medicine, Cologne, Germany; [2]University Hospital Essen, Department for Hematology and Stem Cell Transplantation, Essen, Germany; [3]University Hospital Essen, Institute for Pathology, Essen, Germany;*

### Background

Nodular lymphocyte-predominant Hodgkin lymphoma (NLPHL) accounts for ≈5% of all Hodgkin lymphoma cases. Roughly 75% of NLPHL patients are male. According to some analyses, women appear to have a higher median age at NLPHL diagnosis than men. Larger analyses on sex-specific pathologic and clinical characteristics as well as outcomes have not been conducted until now.

### Aims

To investigate sex-specific characteristics and outcomes in NLPHL.

### Methods

Patients treated for newly diagnosed NLPHL within prospective German Hodgkin Study Group (GHSG) clinical trials (HD10-HD18, RIPL, LP, LPHD) with available information on the histopathological growth pattern (GP) were included in this analysis. Baseline and outcome characteristics were analyzed according to the patient´s sex.

### Results

Overall, 583 patients were eligible for the analysis. Of these, the majority (74%) were male. The median age was 39 years (men: 39 years; women: 40.5 years). Patients presented with stage IA disease without risk factors, other early favorable stages, early unfavorable stages and advanced stages according to the GHSG criteria in 29% (men: 28%; women: 30%), 35% (men: 33%, women: 42%), 12% (men: 12%; women: 13%) and 24% (men: 27%; women: 15%) of cases, respectively. They had GP ABCF in 83% (men: 82%; women: 85%) and a low-risk LP-IPS (0-1 points) in 84% (men: 82%; women: 91%). The bone marrow was involved in 3% (men: 3%; women: 1%), the liver in 2% (men: 2%; women: 1%) and the spleen in 10% (men: 12%; women: 3%). Median follow-up was 6.5 years for progression-free survival (PFS) and 7.2 years for overall survival (OS). The 5-year and 10-year PFS estimates were 83.9% and 73.8% for men and 91.7% and 84.2% for women (HR: 0.67; 95%-CI: 0.42-1.09), the respective OS rates were 95.1% and 93.3% for men and 97.9% each for women (HR: 0.35; 95-CI: 0.1-1.15). In the GP ABCF group, PFS did not differ between the sexes (HR: 0.85; 95%-CI: 0.51-1.42) whereas there was a PFS difference in favor of women in the GP DE group (HR: 0.13; 95%-CI: 0.02-0.94). There were no OS differences between men and women in both patients with GP ABCF and individuals with GP DE. In the case of disease recurrence (men: 65 cases; women: 19 cases), the median time to relapse was 2.7 years (range: 0.2-12 years) for men and 5.1 years (range: 0.9-11 years) for women. Salvage treatment consisted of high-dose chemotherapy and autologous stem cell transplantation in 38% (men: 46%; women: 12%), conventional chemotherapy in 28% (men: 31%; women: 18%) and an anti-CD20 antibody +/- radiotherapy in 32% (men: 24%; women: 59%) of cases. Seven of 24 deaths (29.2%) (GP ABCF: 1/15 (6.7%); GP DE 6/9 (66.7%)) in men were considered NLPHL-related whereas none of the 5 deaths in women was due to NLPHL.

**Copyright Information:** (Online) ISSN: 2572-9241
©2026 The Author(s). HemaSphere is published by John Wiley & Sons Ltd on behalf of European Hematology Association. This is an open access Abstract Book distributed under the Attribution-NonCommercial-NoDerivs (CC BY-NC-ND), which allows third parties to download the articles and share them with others as long as they credit the author and the Abstract Book, but they cannot change the content in any way or use them commercially.
**Abstract Book Citations:** Authors, Title, HemaSphere, 2026;10:(S1):pages. The abstract book DOIs can be found on the "Author Information" page.
**Disclaimer:** Articles published in the journal HemaSphere exclusively reflect the opinions of the authors. The authors are responsible for all content in their abstracts including accuracy of the facts, statements, citing resources, etc.

**Summary/Conclusion**

This analysis comprising NLPHL patients from 12 GHSG studies revealed several sex-specific differences in terms of baseline characteristics including a higher proportion of female patients diagnosed in early stages and a more common involvement of the spleen in men. Regarding outcomes, women tended to have an improved overall PFS and had a significantly better PFS in the subgroup of GP DE patients. The median time to relapse was longer for women and they more often had low-intensity salvage treatment. Unlike in men, no death in women was NLPHL-related. These findings underscore that sex plays a role concerning presentation and outcomes in NLPHL.

**Copyright Information:** (Online) ISSN: 2572-9241
©2026 The Author(s). HemaSphere is published by John Wiley & Sons Ltd on behalf of European Hematology Association. This is an open access Abstract Book distributed under the Attribution-NonCommercial-NoDerivs (CC BY-NC-ND), which allows third parties to download the articles and share them with others as long as they credit the author and the Abstract Book, but they cannot change the content in any way or use them commercially.
**Abstract Book Citations:** Authors, Title, HemaSphere, 2026;10:(S1):pages. The abstract book DOIs can be found on the "Author Information" page.
**Disclaimer:** Articles published in the journal HemaSphere exclusively reflect the opinions of the authors. The authors are responsible for all content in their abstracts including accuracy of the facts, statements, citing resources, etc.

### (PF920) OUTCOME AND PROGNOSTIC VALUE OF PET-2 IN STAGE IV CLASSICAL HODGKIN LYMPHOMA TREATED WITH FRONTLINE BRENTUXIMAB VEDOTIN PLUS AVD: A REAL-WORLD MULTINATIONAL GELTAMO-GATLA STUDY

**Topic:** 17. Hodgkin lymphoma - Clinical

Jose Ignacio Trucco*[1], Marta Pena[2], Maria Encuentra Marti[2], Laura Korin[3], Rocio Figueroa Mora[4], Carmen Martínez[5], Guadalupe Antelo Perez[6], Marta Elisa Zerga[7], María Belén Navarro[8], Carolina Mahuad[7], Carmen Maria Alonso Prieto[9], Lorena Fiad[10], Maria Jimena Cannata Ortiz[11], Malena Rocca[12], Nicholas Kellenher[13], Mariana Rosenhain[14], Blanca Sanchez Gonzales[15], Luciano Salvano[16], Lucia Villalon Blanco[17], Javier Romano[18], Veronica Musso[19], Alessandra Comai[20], Fernando Warley[21], Carlos Santorum[22], Florencia Negri Aranguren[23], Maria Orlova[24], Amalia Cerutti[25], Valeria Drouet[26], Lautaro Sardu[27], Julia Valerga Couste[28], Trinidad Viviani[28], Eva Domingo[2], Astrid Pavlovsky[29], Anna Sureda[2]

*[1]Hospital Universitario Austral, Hematology, Pilar, Argentina; [2]ICO Hospital Duran i Reinals, L'Hospitalet de Llobregat, Spain; [3]Instituto Alexander Fleming, Hematology, Ciudad Autonoma de Buenos Aires, Argentina; [4]Clinica Universitaria de Navarra, Hematology, Pamplona, Spain; [5]Hospital Clínic De Barcelona, Hematology, Barcelona, Spain; [6]CEMIC, Hematology, Ciudad Autonoma de Buenos Aires, Argentina; [7]Hospital Alemán, Hematology, Ciudad Autonoma de Buenos Aires, Argentina; [8]Hospital Universitario Puerta De Hierro Majadahonda, Majadahonda, Spain; [9]Hospital Arnau de Vilanova, Valencia, Spain; [10]Hospital Italiano La Plata, La Plata, Argentina; [11]Hospital Universitario De La Princesa, Madrid, Spain; [12]Sanatorio Allende, Córdoba, Argentina; [13]Hospital Josep Trueta, Girona, Spain; [14]Hospital General de Agudos Enrique Tornu, Buenos Aires, Argentina; [15]Hospital del Mar, Barcelona, Spain; [16]Clínica Universitaria Reina Fabiola, Córdoba, Argentina; [17]Hospital Universitario Fundación Alcorcón, Alcorcón, Spain; [18]Hospital Nacional Alejandro Posadas, Buenos Aires, Argentina; [19]Centro Privado de Oncologia Milstein, Cordoba, Argentina; [20]Hospital General De Catalunya, Sant Cugat del Vallès, Spain; [21]Universidad del Hospital Italiano de Buenos Aires, Ciudad Autonoma de Buenos Aires, Argentina; [22]Hospital Cesar Milstein, Ciudad Autonoma de Buenos Aires, Argentina; [23]Instituto Privado de Hematologia y Hemoterapia, Paraná, Argentina; [24]Hospital Italiano de Buenos Aires, Ciudad Autonoma de Buenos Aires, Argentina; [25]Sanatorio Británico, Rosario, Argentina; [26]Hospital General de Agudos Carlos Durand, Ciudad Autonoma de Buenos Aires, Argentina; [27]Hospital El Cruce, Florencio Varela, Argentina; [28]GATLA, Ciudad Autonoma de Buenos Aires, Argentina; [29]Centro de Hematologia Pavlovsky /FUNDALEU, Ciudad Autonoma de Buenos Aires, Argentina;*

### Background

The ECHELON-1 trial demonstrated improved modified progression-free survival and overall survival (OS) with brentuximab vedotin plus AVD (Bv-AVD) compared with ABVD in advanced-stage classical Hodgkin lymphoma (cHL). Real-world evidence in stage IV cHL remains limited, and the prognostic value of interim PET (PET-2) in this setting has not been clearly defined.

### Aims

To evaluate metabolic complete remission (mCR) rate, 2-year event-free survival (EFS) and overall survival (OS) and the prognostic value of PET-2 in patients (pts) with stage IV cHL treated with frontline Bv-AVD in routine clinical practice within a joint GELTAMO (Spain) and GATLA (Argentina) collaboration.

**Copyright Information:** (Online) ISSN: 2572-9241
©2026 The Author(s). HemaSphere is published by John Wiley & Sons Ltd on behalf of European Hematology Association. This is an open access Abstract Book distributed under the Attribution-NonCommercial-NoDerivs (CC BY-NC-ND), which allows third parties to download the articles and share them with others as long as they credit the author and the Abstract Book, but they cannot change the content in any way or use them commercially.
**Abstract Book Citations:** Authors, Title, HemaSphere, 2026;10:(S1):pages. The abstract book DOIs can be found on the "Author Information" page.
**Disclaimer:** Articles published in the journal HemaSphere exclusively reflect the opinions of the authors. The authors are responsible for all content in their abstracts including accuracy of the facts, statements, citing resources, etc.

### Methods

This retrospective, multicentre cohort study included consecutive pts (≥16 years) with newly diagnosed stage IV cHL treated with frontline Bv-AVD between 2/2019 and 3/2025 in 10 centres in Spain and 18 in Argentina. EFS was defined as time from diagnosis to progression, relapse, death, change of treatment due to toxicity or progression, or failure to achieve complete response at end of treatment (EOT). OS was defined as time from diagnosis to death from any cause or last follow-up. PET-2 was considered evaluable if performed after ≥2 cycles. Metabolic complete remission (mCR) was defined as Deauville Score (DS) 1-3. OS was estimated using Kaplan–Meier methodology. Variables analysed for both EFS and OS were age (<60 vs. ≥ 60 yrs), IPS (1-3 vs. 4-7), B symptoms, bulky disease, PET-2 status, and extranodal disease. Multivariate analyses were performed using Cox regression models, including the same variables.

### Results

A total of 109 pts were included. Median age was 42.9 yrs (range 15–82), 79.4% had ECOG 0–1, 69.7% had B symptoms and 53.2% ≥2 extranodal sites. Bulky disease was documented in 27.5%, IPS ≥4 in 37.6%. The median number of Bv doses was 12 (IQR 10–12). PET-2 was performed in 100/109 pts (91.7%); 80.8% achieved mCR. In 7/20 PET-2–positive pts, treatment was modified to a new line of therapy at physician discretion. EOT PET was available in 98/109 (89.9%), with an mCR rate of 88%. Consolidative radiotherapy was administered in 3.8%. Median follow-up for the entire cohort was 33.8 months (m) (95% CI 29.5-38.1). With a median follow-up of 27.1 m for EFS, the estimated 2-year EFS was 80.9% (95% CI 73.5–89.1) (Fig 1A). With a median follow-up of 33.7 m for OS, the 2-year OS was 96.1% (95% CI 92.4–99.9) (Fig. 1B). mCR at PET-2 was associated with improved EFS (83.0% vs. 57.4%; p= 0.002) but not OS (Fig 1C). IPS ≥ 4 was associated with shorter OS (98.4% vs. 85.2%; p= 0.04). In multivariable analysis, PET-2 positivity remained independently associated with shorter EFS (HR 3.8; p=0.004), with no significant impact on OS.

### Summary/Conclusion

This large binational real-world study, restricted to pts with stage IV classical Hodgkin lymphoma, demonstrates that frontline Bv-AVD achieves favorable outcomes, with 2-year EFS and OS rates of 80.9% and 96.1%, respectively, consistent with the results of the ECHELON-1 trial.

To our knowledge, this represents the largest real-world evidence series published exclusively in stage IV patients and the first to show that PET-2 positivity independently predicts inferior EFS with Bv-AVD, although the implications for treatment adaptation require prospective validation.

This collaborative GELTAMO–GATLA analysis provides a relevant benchmark for the development of future PET-adapted strategies in this high-risk population.

**Copyright Information:** (Online) ISSN: 2572-9241
©2026 The Author(s). HemaSphere is published by John Wiley & Sons Ltd on behalf of European Hematology Association. This is an open access Abstract Book distributed under the Attribution-NonCommercial-NoDerivs (CC BY-NC-ND), which allows third parties to download the articles and share them with others as long as they credit the author and the Abstract Book, but they cannot change the content in any way or use them commercially.
**Abstract Book Citations:** Authors, Title, HemaSphere, 2026;10:(S1):pages. The abstract book DOIs can be found on the "Author Information" page.
**Disclaimer:** Articles published in the journal HemaSphere exclusively reflect the opinions of the authors. The authors are responsible for all content in their abstracts including accuracy of the facts, statements, citing resources, etc.

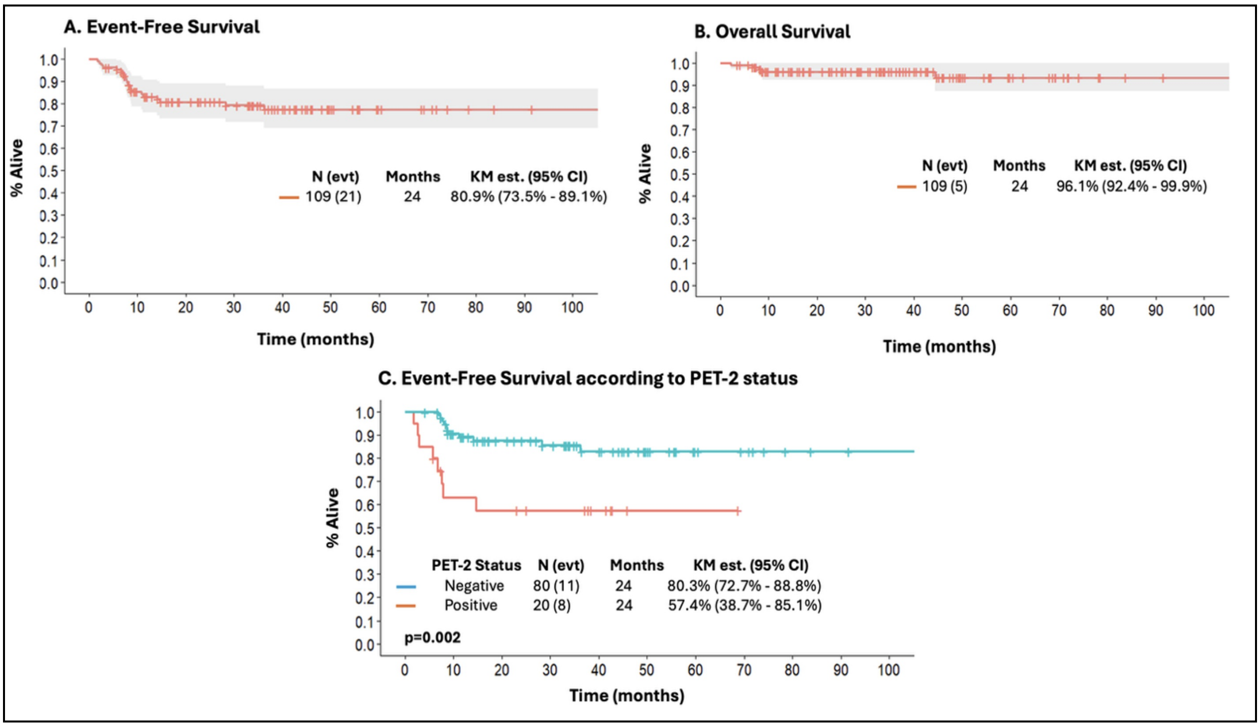

Figure 1. A. Event-free Survival; B. Overall Survival; C. Event-Free Survival according to PET-2 status

**Copyright Information:** (Online) ISSN: 2572-9241
©2026 The Author(s). HemaSphere is published by John Wiley & Sons Ltd on behalf of European Hematology Association. This is an open access Abstract Book distributed under the Attribution-NonCommercial-NoDerivs (CC BY-NC-ND), which allows third parties to download the articles and share them with others as long as they credit the author and the Abstract Book, but they cannot change the content in any way or use them commercially.
**Abstract Book Citations:** Authors, Title, HemaSphere, 2026;10:(S1):pages. The abstract book DOIs can be found on the "Author Information" page.
**Disclaimer:** Articles published in the journal HemaSphere exclusively reflect the opinions of the authors. The authors are responsible for all content in their abstracts including accuracy of the facts, statements, citing resources, etc.

### (PF921) A GERIATRIC RISK SCORE INTEGRATING METABOLIC TUMOR BURDEN AND HOST VULNERABILITY OUTPERFORMS CONVENTIONAL TOOLS AND GUIDES TREATMENT INTENSIFICATION IN OLDER HODGKIN LYMPHOMA PATIENTS

**Topic:** 17. Hodgkin lymphoma - Clinical

Peiqi Zhao*[1], Yihan Wang[2], Min Bai[3], Chen Huang[4], Jiesong Wang[5], Yajun Li[6], Yanan Jiang[7], Ganggang Wang[8], Feng Zhu[9], Shu Zhao[10], Wei Guo[11], Shukai Qiao[12], Jianxia He[13], Ji MA[14], Lei Zhu[15], Li Li[16], Wenyue Yu[17], Shaozhen Chen[5, 18], Junqing Xu[19], Ou Bai[11], Hui Zhou[6], Qingyuan Zhang[10], Liping Su[20], Yuanlin Xu[2], Hui-Lai Zhang[1]

*[1]Department of Lymphoma, Tianjin Medical University Cancer Institute & Hospital, National Clinical Research Center for Cancer, Tianjin's Clinical Research Center for Cancer, State Key Laboratory of Druggability Evaluation and Systematic Translational Medicine, Tianjin Medical University, Tianjin, 300060, China, Tianjin, China; [2]Department of Internal Medicine, Affiliated Cancer Hospital of Zhengzhou University & Henan Cancer Hospital, Zhengzhou, 450008, China, Zhengzhou, China; [3]Department of Hematology, Shanxi Province Cancer Hospital/ Shanxi Hospital Affiliated to Cancer Hospital, Chinese Academy of Medical Sciences/Cancer Hospital Affiliated to Shanxi Medical University, Taiyuan, 030013, China, Taiyuan, China; [4]Department of Hematology, The Fourth Hospital of Hebei Medical University, 169 Tianshan Road, Shijiazhuang, 050035, China, Shijiazhuang, China; [5]Department of Lymphoma & Head and Neck Oncology, College of Clinical Medicine for Oncology, Fujian Medical University, Fuzhou, 350014China, Fuzhou, China; [6]Department of Lymphoma and Hematology, The Affiliated Cancer Hospital of Xiangya School of Medicine, Central South University/Hunan Cancer Hospital, Changsha, 410013, China, Changsha, China; [7]Department of Hematology, Yantai Yuhuangding Hospital Affiliated to Qingdao University, Yantai, 264000China, Yantai, China; [8]Cancer Center, Shanxi Bethune Hospital, Shanxi Academy of Medical Sciences, Third Hospital of Shanxi Medical University, Tongji Shanxi Hospital, Taiyuan, 030032, China, Taiyuan, China; [9]Department of Hematology, the Affiliated Hospital of Xuzhou Medical University, Xuzhou, 221006, China, Xuzhou, China; [10]Department of Medical Oncology, Harbin Medical University Cancer Hospital, Harbin Medical University, Harbin, 150081, China, Haerbin, China; [11]Department of Hematology, The First Hospital of Jilin University, Changchun, 130033, China, Changchun, China; [12]Department of Hematology, the Second Hospital of Hebei Medical University, Shijiazhuang, 050000China, Shijiazhuang, China; [13]Department of Hematology, Shanxi Provincial People's Hospital, Taiyuan 030012, China, Taiyuan, China; [14]Department of Hematology, Shandong Cancer Hospital and Institute, Shandong First Medical University and Shandong Academy of Medical Sciences, Jinan, 250117, China, Jinan, China; [15]Department of Molecular Imaging and Nuclear Medicine, Tianjin's Clinical Research Center for Cancer, Key Laboratory of Cancer Prevention and Therapy, National Clinical Research Center for Cancer, Tianjin Medical University Cancer Institute and Hospital, Tianjin Medical University, Tianjin, 300060, China, Tianjin, China; [16]Clinical Pathology Department, Shanxi Bethune Hospital, Shanxi Academy of Medical Sciences, Third Hospital of Shanxi Medical University, Tongji Shanxi Hospital, Taiyuan, 030032, China, Taiyuan, China; [17]Cancer Research Center, The First Affiliated Hospital of USTC, Division of Life Sciences and Medicine, University of Science and Technology of China, Hefei, 230036, China, Hefei, China; [18]Department of Lymphoma & Head and Neck Oncology, College of Clinical Medicine for Oncology, Fujian Medical University, Fuzhou, 350014, China, Fuzhou, China; [19]Department of Hematology, Yantai Yuhuangding Hospital Affiliated to Qingdao University, Yantai, 264000, China, Yantai, China; [20]Department of HematologyShanxi Province Cancer Hospital/ Shanxi Hospital Affiliated to Cancer Hospital, Chinese Academy of Medical Sciences/Cancer Hospital Affiliated to Shanxi Medical University, Taiyuan, 030013, China, Taiyuan, China;*

### Background

Outcomes for older patients (≥60 years) with Hodgkin lymphoma (HL) remain poor due to therapeutic under-treatment and overtreatment. The International Prognostic Score (IPS) is suboptimal, and the Comprehensive Geriatric Assessment (CGA), while valuable, fails to fully capture tumor biology. A tool integrating host vulnerability with quantitative disease burden is urgently needed to guide personalized therapy.

**Copyright Information:** (Online) ISSN: 2572-9241
©2026 The Author(s). HemaSphere is published by John Wiley & Sons Ltd on behalf of European Hematology Association. This is an open access Abstract Book distributed under the Attribution-NonCommercial-NoDerivs (CC BY-NC-ND), which allows third parties to download the articles and share them with others as long as they credit the author and the Abstract Book, but they cannot change the content in any way or use them commercially.
**Abstract Book Citations:** Authors, Title, HemaSphere, 2026;10:(S1):pages. The abstract book DOIs can be found on the "Author Information" page.
**Disclaimer:** Articles published in the journal HemaSphere exclusively reflect the opinions of the authors. The authors are responsible for all content in their abstracts including accuracy of the facts, statements, citing resources, etc.

### Aims

To develop and validate a multidimensional Geriatric Risk Score (GRS) that synthesizes metabolic tumor burden and geriatric parameters for precise risk stratification and to evaluate its utility in guiding treatment intensity decisions.

### Methods

In this multicenter, retrospective study, 306 newly diagnosed older HL patients (median age 66, range 60-88) from 14 Chinese centers (2006-2024) were analyzed. All had baseline 18F-FDG PET/CT (for Total Lesion Glycolysis, TLG) and CGA. Patients were randomly split 8:2 into training (n=250) and validation (n=56) cohorts. A prognostic model for overall survival (OS) was developed using LASSO and multivariable Cox regression. Its discrimination (C-index), calibration, and clinical utility (Decision Curve Analysis, DCA) were compared to IPS and CGA. Treatment outcomes and toxicity profiles were analyzed within GRS risk strata. Targeted sequencing (n=76) explored molecular correlates.

### Results

Four independent predictors for OS were identified: age >68 years, TLG >192.5 mL, hemoglobin <105 g/L, and lymphocyte-depleted histology. The derived continuous GRS (range 0-7) stratified patients into low- (score 0-1), intermediate- (score 2-3), and high-risk (score 4-7) groups with markedly distinct 5-year OS (91.2%, 63.5%, 23.6%; p<0.0001). The GRS's predictive accuracy (validation C-index: 0.786) was superior to both CGA (0.671) and IPS (0.451). DCA confirmed the superior net clinical benefit of the GRS across a wide range of risk thresholds. Critically, the GRS reclassified 30.1% (78/259) of CGA-"fit" patients into the GRS-high-risk category, a group with a molecular profile (e.g., enrichment of *PPM1D*and *TET2*mutations) and outcomes distinct from GRS-low-risk "fit" patients.

Among GRS-high-risk patients, those receiving standard-intensity (ABVD/BEACOPP) or novel agent-based (BV/PD-1) regimens had significantly superior OS compared to those receiving reduced-intensity therapy (HR 0.22, 95% CI 0.08-0.58, p=0.002), despite a higher incidence of grade 3-4 hematologic toxicity (65% vs. 40%, p=0.03), which was manageable. Conversely, in the GRS-low-risk group, outcomes were excellent regardless of regimen intensity, supporting the feasibility of de-escalation strategies in this subset. The GRS also more accurately identified patients at risk for early progression (<12 months) than CGA alone (AUC 0.76 vs. 0.61).

### Summary/Conclusion

We developed and validated a novel Geriatric Risk Score that seamlessly integrates metabolic tumor burden and host vulnerability. It significantly outperforms existing tools, uncovers a high-risk, biologically distinct population within ostensibly "fit" patients, and provides actionable evidence: GRS-high-risk patients derive a substantial survival benefit from full-intensity or novel therapies, supporting treatment intensification with proactive support. The GRS-low-risk group represents an ideal population for future de-escalation trials. This tool enables a shift from age-based to biologically- and vulnerability-informed personalized therapy for older HL patients.

**Copyright Information:** (Online) ISSN: 2572-9241
©2026 The Author(s). HemaSphere is published by John Wiley & Sons Ltd on behalf of European Hematology Association. This is an open access Abstract Book distributed under the Attribution-NonCommercial-NoDerivs (CC BY-NC-ND), which allows third parties to download the articles and share them with others as long as they credit the author and the Abstract Book, but they cannot change the content in any way or use them commercially.
**Abstract Book Citations:** Authors, Title, HemaSphere, 2026;10:(S1):pages. The abstract book DOIs can be found on the "Author Information" page.
**Disclaimer:** Articles published in the journal HemaSphere exclusively reflect the opinions of the authors. The authors are responsible for all content in their abstracts including accuracy of the facts, statements, citing resources, etc.

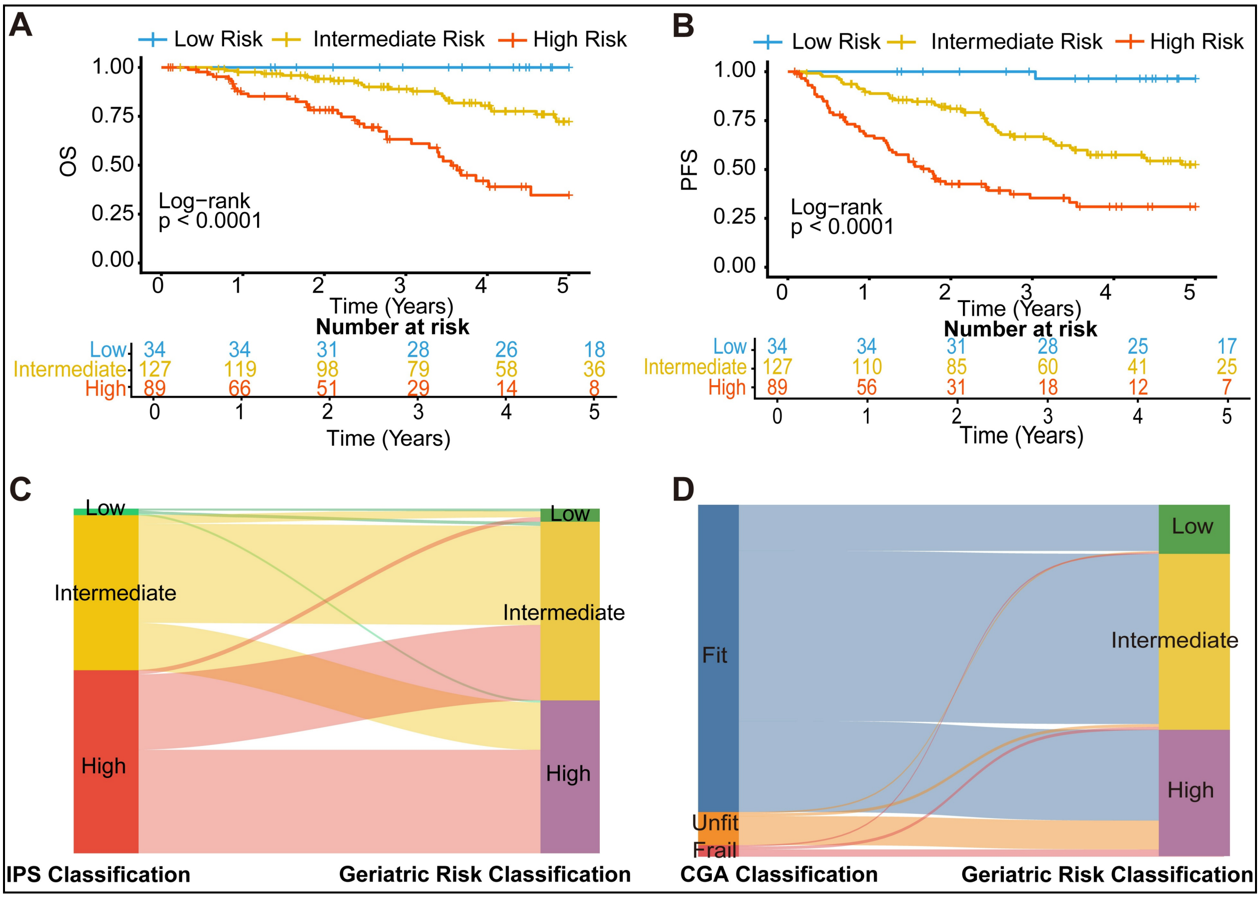

Risk stratification and reclassification dynamics. The three-category risk model defined for the composite Geriatric Risk Score. Kaplan–Meier survival curves for overall survival (A) and progression-free survival (B) based on the Geriatric Risk Classification, along with Sankey plots showing the comparison of the new Geriatric Risk Classification with the IPS (Low risk: score 0–1; Intermediate risk: score 2–3; High risk: score 4–7) (C) and the CGA (Fit, Unfit, Frail) (D).

**Copyright Information:** (Online) ISSN: 2572-9241
©2026 The Author(s). HemaSphere is published by John Wiley & Sons Ltd on behalf of European Hematology Association. This is an open access Abstract Book distributed under the Attribution-NonCommercial-NoDerivs (CC BY-NC-ND), which allows third parties to download the articles and share them with others as long as they credit the author and the Abstract Book, but they cannot change the content in any way or use them commercially.
**Abstract Book Citations:** Authors, Title, HemaSphere, 2026;10:(S1):pages. The abstract book DOIs can be found on the "Author Information" page.
**Disclaimer:** Articles published in the journal HemaSphere exclusively reflect the opinions of the authors. The authors are responsible for all content in their abstracts including accuracy of the facts, statements, citing resources, etc.

### (PF922) REAL WORLD EFFICACY AND SAFETY COMPARISON OF SALVAGE REGIMENS COMBINING BRENTUXIMAB VEDOTIN AND CHEMOTHERAPY IN SECOND-LINE HODGKIN LYMPHOMA: A LYSA MULTICENTER RETROSPECTIVE STUDY.

**Topic:** 17. Hodgkin lymphoma - Clinical

Juliette Wencel*[1], Arnaud Pages[2], Florian Chevillon[3], Pilar Dutari[4], Lucie Laemmel[5], Maxime Vignac[1], Emilie Corvilain[6], Pamela Sfeir[2], Milena Kohn[7], Guillemette Fouquet[8, 9, 10], Maxence Lohez[8], Baptiste Delapierre (Moisdon)[11], Gaétan Basile[5], Gaelle Laboure[12], Amina Cherait[13], Carole Soussain[14], Adrien Grenier[15], Thorsten Braun[16], Laure Ricard[17], Sophie Bernard[18], Axel Andre[19], Deau Bénédicte[20], Morgane Cheminant[1], Jean Galtier[5], Robin Noel[4], Loïc Renaud[2]

*[1]Necker-Enfants Malades University Hospital, Department of Hematology, Paris, France;[2]Gustave Roussy Cancer Campus, Department of Hematology, Villejuif, France; [3]Hôpitaux Universitaires Paris Nord, Department of AJA, Paris, France;[4]Institut Paoli-Calmettes, Department of Hematology, Marseille, France; [5]CHU de Bordeaux, Department of Hematology-Transplantation, Pessac, France; [6]Hôpital Saint-Louis, Department of Immunopathology, Paris, France;[7]Centre Hospitalier de Versailles, Department of Hematology, Versailles, France; [8]CH Sud Francilien, Department of Hematology, Corbeil-Essonne, France; [9]CH Sud Francilien, Paris Saclay University, Corbeil-Essonne, France; [10]Gustave Roussy Institute, UMR 1287, Villejuif, France; [11]CHU de Caen, Department of Hematology, Caen, France; [12]Centre hospitalier de Libourne, Department of Hematology, Libourne, France; [13]Argenteuil Hospital, Department of Hematology, Argenteuil, France; [14]Institut Curie, Department of Hematology, St Cloud, France; [15]Pitié-Salpêtrière Hospital, Department of Hematology and cellular therapies, Paris, France; [16]Hôpital Avicenne, Department of Hematology, Paris, France; [17]Hôpital Universitaire St. Antoine, Paris, France;[18]Centre Hospitalier de la Cote Basque, Department of Hematology, Bayonne, France; [19]Centre Henri Mondor, Department of Lymphoid Hematology, Créteil, France; [20]Hôpital Cochin, Department of Hematology, Paris, France;*

### Background

The treatment of relapsed or refractory (R/R) Hodgkin lymphoma (HL) involves polychemotherapy usually combined with Brentuximab vedotin (Bv) or anti-PD1 followed by autologous stem cell transplantation (ASCT) for eligible patients. Various polychemotherapy combinations are commonly used, based on phase II studies. No direct comparison of these associations allows for an evidence-based choice and treatment selection often reflects institutional habits.

### Aims

To compare the efficacy and toxicity of Bv-chemotherapy regimens in patients with R/R HL who have been treated in a second line setting.

### Methods

This retrospective multicenter study included patients with R/R Hodgkin lymphoma treated with Bv-chemotherapy with intent to proceed to ASCT across 19 LYSA-affiliated centers in France between 2014 and 2025. Data collection was performed with a standardized electronic case report form. Efficacy outcomes included overall survival (OS), complete response (CR) rate and progression-free survival (PFS) and were assessed using Log-rank tests or adjusted Cox models. Safety was graded according to CTCAE v4.0.

**Copyright Information:** (Online) ISSN: 2572-9241
©2026 The Author(s). HemaSphere is published by John Wiley & Sons Ltd on behalf of European Hematology Association. This is an open access Abstract Book distributed under the Attribution-NonCommercial-NoDerivs (CC BY-NC-ND), which allows third parties to download the articles and share them with others as long as they credit the author and the Abstract Book, but they cannot change the content in any way or use them commercially.
**Abstract Book Citations:** Authors, Title, HemaSphere, 2026;10:(S1):pages. The abstract book DOIs can be found on the “Author Information” page.
**Disclaimer:** Articles published in the journal HemaSphere exclusively reflect the opinions of the authors. The authors are responsible for all content in their abstracts including accuracy of the facts, statements, citing resources, etc.

**Results**

Among 404 patients with R/R HL eligible to ASCT included in the study, 288 received one of the three most commonly used Bv-chemotherapy combinations. 81 patients (28.1%) were treated with Bv-bendamustine (Bv-benda), 110 (38.2%) with Bv-ICE and 97 (33.7%) with Bv-DHAC/X/P (Bv-DHA). The median age was 33 years (range 18-69), and median follow-up was 41.3 months. ABVD first line treatment was more frequent in patients receiving Bv-benda compared to Bv-ICE or Bv-DHA ($p<0.001$). Age at relapse, Ann Arbor stage at relapse and time to relapse were also significantly different ($p<0.001$, $p<0.001$ and $p=0.005$ respectively). 97 patients (33.7%) had a refractory disease (< 3 months after the end of treatment (EOT)) and 118 patients (41.0%) relapsed more than 1 year after EOT. 215 patients (74.7%) underwent ASCT after second line salvage therapy, 24 (8.3%) after third line.
CR was observed in 72.4% of patients without difference between treatment groups (77.5% for Bv-benda, 76.1% for Bv-ICE and 67.0% for Bv-DHA; $p=0.208$). Similarly, 24-months PFS rate was 63.9% (71.5% (IC95% 60.9-80.2) for Bv-benda, 60.2% (IC95% 50.5-69.1) with Bv-ICE, 60.4% (IC95% 50.2-69.8) with Bv-DHA), with no significant difference in PFS between the treatment groups (log rank $p=0.480$). In multivariable Cox analysis adjusted for first-line treatment, age, time to relapse, and Ann Arbor stage at relapse, PFS was not significantly different between the three groups ($p=0.202$) : using Bv-DHA as the comparator, the HR for the Bv-benda group was 0.60 (95% CI, 0.33-1.07) and 0.84 (95% CI, 0.53-1.35) for Bv-ICE group.
Hematological toxicity was higher with Bv-ICE exposure in terms of both red blood cell ($p<0.001$) and platelet transfusions ($p<0.001$). Hospitalization median duration was significantly shorter in patients treated with Bv-benda (1.7 days/cycle) compared to those treated with Bv-ICE (4.3 days/cycle) or Bv-DHA (3.2 days/cycle). Severe infections rates were similar between the groups ($p=0.811$). Allergic reactions were reported in 34% of Bv-benda-treated patients, including 6.3% of grade 3-4.

**Summary/Conclusion**

Bv-bendamustine, Bv-ICE and Bv-DHA showed similar efficacy as second-line Bv based salvage treatments for HL, with Bv-ICE being associated with significantly increased toxicity. These results provide real-world comparative data that may guide treatment selection among Bv-based salvage regimens.

**Copyright Information:** (Online) ISSN: 2572-9241
©2026 The Author(s). HemaSphere is published by John Wiley & Sons Ltd on behalf of European Hematology Association. This is an open access Abstract Book distributed under the Attribution-NonCommercial-NoDerivs (CC BY-NC-ND), which allows third parties to download the articles and share them with others as long as they credit the author and the Abstract Book, but they cannot change the content in any way or use them commercially.
**Abstract Book Citations:** Authors, Title, HemaSphere, 2026;10:(S1):pages. The abstract book DOIs can be found on the “Author Information” page.
**Disclaimer:** Articles published in the journal HemaSphere exclusively reflect the opinions of the authors. The authors are responsible for all content in their abstracts including accuracy of the facts, statements, citing resources, etc.

### (PF923) DURABLE COMPLETE RESPONSES AFTER NIVOLUMAB DISCONTINUATION IN RELAPSED/REFRACTORY CLASSIC HODGKIN LYMPHOMA: SEVEN-YEAR FOLLOW-UP AND RETREATMENT OUTCOMES

**Topic:** 17. Hodgkin lymphoma - Clinical

Liudmila Fedorova*[1], Lepik Kirill[1], Elena Kondakova[1], Andrey Chekalov[1], Polina Kotselyabina[1], Evgeniya Borzenkova[1], Natalya Mikhaylova[1], Alexander Kulagin[1]

*[1]RM Gorbacheva Research Institute, Pavlov University, Saint-Petersburg, Russia;*

### Background

Previously published data from a limited patient cohort demonstrated the possibility of achieving durable remission in patients with relapsed/refractory classic Hodgkin lymphoma (r/r cHL) following discontinuation of checkpoint inhibitors (CPI) in complete response (CR). However, long-term follow-up data for this patient population remain lacking. In addition, outcomes after CPI re-treatment in these patients have not yet been sufficiently characterized. Given the steadily increasing number of patients receiving CPI, refining prognostic assessment for those who discontinue therapy for various reasons is crucial for optimizing subsequent treatment strategies.

### Aims

To analyze long-term outcomes in an expanded cohort of patients with r/r cHL whose nivolumab (nivo) therapy was discontinued in CR, and to evaluate the efficacy of CPI re-treatment in this population.

### Methods

The retrospective analysis included 47 patients with r/r cHL who were treated with nivo 3 mg/kg (n=28) or 40 mg (n=19). Therapy with nivo was discontinued in CR for various reasons. Overall survival (OS) and progression-free survival (PFS) were analyzed in the entire cohort.
In 19 out of 22 patients with disease relapse, CPI monotherapy was re-initiated. In CPI retreatment cohort, overall response rate (ORR), PFS, OS and adverse events (AEs) were analyzed. Response was evaluated by PET-CT using LYRIC criteria. PFS was censored by the time of additional therapy initiation in group of nivo retreatment. AEs were analyzed by NCI CTCAE v5.0.
Univariate Cox regression analysis was performed to identify prognostic factors for PFS in the nivolumab discontinuation cohort (sex, r/r status, prior autologous HCT, prior brentuximab vedotin, B symptoms, ECOG status at nivo initiation, number of prior therapy lines, nivo dosage, severe AEs, therapy duration, early response to nivo).

### Results

Patient characteristics are presented in Table 1. In only 5 patients (11%), nivo was discontinued due to grade 3-4 AEs. Median follow-up after nivo discontinuation was 84 months (4-109). Relapse occurred in 24 patients (51%). Median PFS was 25.8 months (95%CI: 13.9-NA), 7-year PFS was 44.9% (95%CI: 32.1-62.8), Four patients (9%) died due to progressive multifocal leukoencephalopathy after allogeneic hematopoietic cell transplantation (HCT) (n=1), disease progression (n=1), and unknown reasons (n=2). Median OS was not reached, 7-y OS was 88.4% (95%CI: 78.3-99.8) (Fig.1).
Retreatment with mono-nivo was initiated in 19 pts. Median follow-up in this cohort was 68 (25-96) mo. ORR was 74%: CR n=9 (47%), partial response n=5 (26%), and indeterminate response n=5 (26%). Median PFS was 22.7 months (95%CI: 16.5-NA), 5-y PFS 34.7% (95%CI: 18.4-65.5). Eight patients (42%) developed AEs during nivo retreatment, while only 3 (16%) experienced grade 3-4 AEs.
Univariate Cox regression analysis did not identify any significant prognostic factors for PFS.

**Copyright Information:** (Online) ISSN: 2572-9241
©2026 The Author(s). HemaSphere is published by John Wiley & Sons Ltd on behalf of European Hematology Association. This is an open access Abstract Book distributed under the Attribution-NonCommercial-NoDerivs (CC BY-NC-ND), which allows third parties to download the articles and share them with others as long as they credit the author and the Abstract Book, but they cannot change the content in any way or use them commercially.
**Abstract Book Citations:** Authors, Title, HemaSphere, 2026;10:(S1):pages. The abstract book DOIs can be found on the "Author Information" page.
**Disclaimer:** Articles published in the journal HemaSphere exclusively reflect the opinions of the authors. The authors are responsible for all content in their abstracts including accuracy of the facts, statements, citing resources, etc.

### Summary/Conclusion

Patients who achieve CR to mono-nivo demonstrate durable remissions with long-term follow-up, raising the question of the potential curative role of CPI in a subset of patients. Re-treatment with CPI monotherapy appears to be an effective and safe therapeutic strategy in this population.

Table 1. Patient characteristics

| Variable | N=47 |
|---|---|
| Median age, years (range) | 29 (17-50) |
| Male/female, n (%) | 15/32 (32/68) |
| Primary refractory, n (%)<br>Early relapse, n (%) | 27 (57)<br>8 (43) |
| Prior autologous stem cell transplantation, n (%) | 17 (36) |
| Prior brentuximab vedotin, n (%) | 19 (40) |
| Therapy lines before Nivo therapy, n (range) | 5 (1-10) |
| B symptoms at Nivo therapy initiation, n (%) | 22 (47) |
| ECOG status at Nivo therapy initiation, n (%)<br>0-1<br>≥2 | <br>27 (57)<br>18 (38) |
| Nivo dose, n (%)<br>40 mg<br>3 mg/kg | <br>28 (60)<br>19 (40) |
| Number of Nivo cycles, n (range) | 24 (3-38) |
| Number of Nivo cycles before the best response achievement, n (range) | 6 (6-29) |

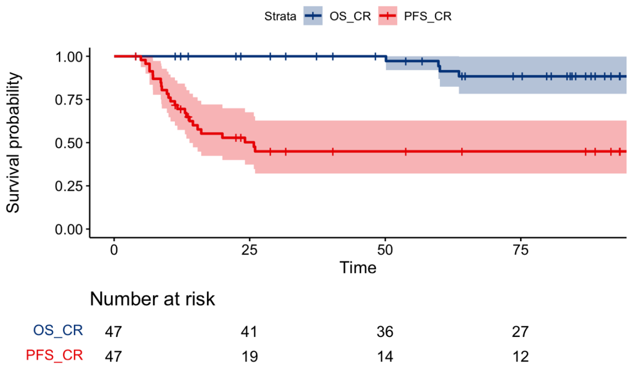

Figure 1. Overall and progression-free survival

**Copyright Information:** (Online) ISSN: 2572-9241
©2026 The Author(s). HemaSphere is published by John Wiley & Sons Ltd on behalf of European Hematology Association. This is an open access Abstract Book distributed under the Attribution-NonCommercial-NoDerivs (CC BY-NC-ND), which allows third parties to download the articles and share them with others as long as they credit the author and the Abstract Book, but they cannot change the content in any way or use them commercially.
**Abstract Book Citations:** Authors, Title, HemaSphere, 2026;10:(S1):pages. The abstract book DOIs can be found on the "Author Information" page.
**Disclaimer:** Articles published in the journal HemaSphere exclusively reflect the opinions of the authors. The authors are responsible for all content in their abstracts including accuracy of the facts, statements, citing resources, etc.

### (PF924) INTERIM RESULTS OF A PET-GUIDED IMMUNE CHECKPOINT INHIBITOR–BASED FIRST-LINE STRATEGY IN ADVANCED-STAGE CLASSICAL HODGKIN LYMPHOMA

**Topic:** 17. Hodgkin lymphoma - Clinical

Anna Kravtsova*[1, 1], Daria Koroleva[1], Olga Shchetsova[1], Madina Bagova[1], Aminat Magomedova[1], Oleg Margolin[1], Ekaterina Gitelzon[1], Alexey Subbotin[2], Evgeniy Zvonkov[1, 3], Yana Mangasarova[1, 3]

*[1]National Medical Research Center for Hematology, Moscow, Russia;[2]N.N. Blokhin National Medical Research Center of Oncology, Moscow, Russia; [3]The Russian University of Medicine, Moscow, Russia;*

### Background

Advanced-stage classical Hodgkin lymphoma (AS-cHL) has historically been treated with 6–8 cycles of BEACOPP-like regimens followed by consolidative radiotherapy (RT) to residual masses. While effective, this approach is associated with substantial acute and long-term toxicity. The integration of immunotherapy and PET-guided treatment adaptation offers the potential to reduce treatment intensity while maintaining efficacy.
We conducted a single-center prospective study of a PET-adapted nivolumab-based first-line strategy in AS-cHL (NCT07209059) with chemotherapy de-escalation and RT omission in patients achieving early complete metabolic response (CMR).

### Aims

To evaluate the efficacy and safety of this PET-adapted immune checkpoint inhibitor–based first-line strategy (scheme 1) in previously untreated AS-cHL.

### Methods

Thirty-seven patients with newly diagnosed AS-cHL were treated between October 2024 and February 2026. Median age was 28 years (range, 18–43); 54% were female. Stage III–IV disease was present in 57%, and 81% were classified as AS according to GHSG criteria. Median IPS was 1 (range, 0–4; ≥3 in 16%). B symptoms and bulky disease were observed in 57% each; ECOG ≥2 in 19%. Two patients had a history of autoimmune thyroiditis without active disease or need for treatment at study entry.
Nivolumab was administered at a fixed dose of 40 mg every 2 weeks (N40). The protocol included two induction cycles of N40 plus EACOPD-14. Patients achieving CMR (Deauville score [DS] 1–3) received two consolidation cycles of N40-AVD followed by two maintenance N40 administrations. Patients with partial metabolic response (PMR, DS4–5) after induction continued N40-EACOPD-14 up to six cycles.
Response was assessed according to LYRIC criteria (PET2/4/6). Adverse events were graded per CTCAE v5.0.

**Copyright Information:** (Online) ISSN: 2572-9241
©2026 The Author(s). HemaSphere is published by John Wiley & Sons Ltd on behalf of European Hematology Association. This is an open access Abstract Book distributed under the Attribution-NonCommercial-NoDerivs (CC BY-NC-ND), which allows third parties to download the articles and share them with others as long as they credit the author and the Abstract Book, but they cannot change the content in any way or use them commercially.
**Abstract Book Citations:** Authors, Title, HemaSphere, 2026;10:(S1):pages. The abstract book DOIs can be found on the “Author Information” page.
**Disclaimer:** Articles published in the journal HemaSphere exclusively reflect the opinions of the authors. The authors are responsible for all content in their abstracts including accuracy of the facts, statements, citing resources, etc.

**Results**

At the time of analysis 30 patients completed treatment, 6 were receiving consolidation, 1 remained on induction. After two induction cycles (PET2, n=36 evaluable) CMR was achieved in 30/36 patients (83%), PMR (DS4) was observed in 6/36 (17%). One case (3%) of pseudoprogression was recorded. At end-of-treatment assessment CMR rate was 100% (30/30). No patient received radiotherapy. As of February 2026, no disease progression or relapse has been observed. All patients who completed therapy remain in remission. Median follow-up was 9.1 months (range, 2.2–18.0).
Treatment was generally well tolerated: grade 3-4 neutropenia: 17%, febrile neutropenia: 10%, no grade ≥3 infectious complications were observed, no transfusion support was required
Grade 3 immune-related AEs occurred in 3 patients (8%): thyroiditis (n=2; one with concomitant immune-mediated polyneuropathy), periarthritis (n=1). All events resolved with prednisone (0.5 mg/kg) without treatment discontinuation. Importantly, in the two patients with pre-existing autoimmune thyroiditis, no clinical or biochemical reactivation was observed during nivolumab therapy.

**Summary/Conclusion**

The PET-adapted N40-based strategy resulted in high early metabolic response rates with manageable toxicity in patients with AS-cHL. Chemotherapy de-escalation and omission of RT in AS-cHL patients achieving early CMR appear feasible. Longer follow-up is required to assess durability of response.

**Copyright Information:** (Online) ISSN: 2572-9241
©2026 The Author(s). HemaSphere is published by John Wiley & Sons Ltd on behalf of European Hematology Association. This is an open access Abstract Book distributed under the Attribution-NonCommercial-NoDerivs (CC BY-NC-ND), which allows third parties to download the articles and share them with others as long as they credit the author and the Abstract Book, but they cannot change the content in any way or use them commercially.
**Abstract Book Citations:** Authors, Title, HemaSphere, 2026;10:(S1):pages. The abstract book DOIs can be found on the "Author Information" page.
**Disclaimer:** Articles published in the journal HemaSphere exclusively reflect the opinions of the authors. The authors are responsible for all content in their abstracts including accuracy of the facts, statements, citing resources, etc.

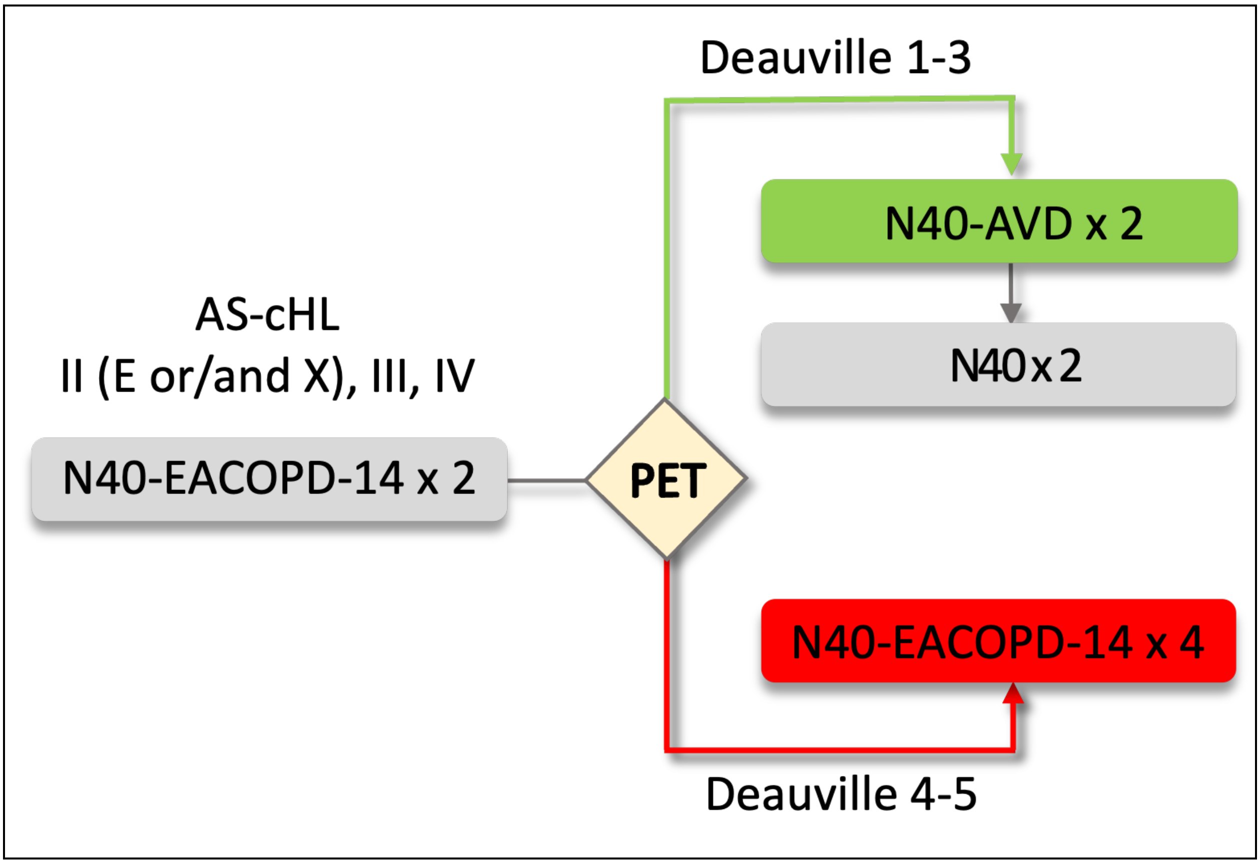

Scheme 1. AS-cHL – advanced-stage classical Hodgkin Lymphoma; N40-EACOPD-14: nivolumab 40 mg on day 0, etoposide 100 mg/m² on days 1–3, doxorubicin 25 mg/m² on day 1, cyclophosphamide 650 mg/m² on day 1, vincristine 1,4 mg/m² on day 8, prednisolone 40 mg/m² on days 1–7, and dacarbazine 375 mg/m² on day 1, administered intravenously in a 14-day cycle; N40-AVD: nivolumab 40 mg, doxorubicin 25 mg/m², vinblastine 6 mg/m², and dacarbazine 375 mg/m², administered intravenously on days 1 and 15 of a 28-day cycle

**Copyright Information:** (Online) ISSN: 2572-9241
©2026 The Author(s). HemaSphere is published by John Wiley & Sons Ltd on behalf of European Hematology Association. This is an open access Abstract Book distributed under the Attribution-NonCommercial-NoDerivs (CC BY-NC-ND), which allows third parties to download the articles and share them with others as long as they credit the author and the Abstract Book, but they cannot change the content in any way or use them commercially.
**Abstract Book Citations:** Authors, Title, HemaSphere, 2026;10:(S1):pages. The abstract book DOIs can be found on the "Author Information" page.
**Disclaimer:** Articles published in the journal HemaSphere exclusively reflect the opinions of the authors. The authors are responsible for all content in their abstracts including accuracy of the facts, statements, citing resources, etc.

### (PF925) PROGNOSTIC VALUE OF PET METRICS IN CHILDREN AND YOUNG ADULTS WITH RELAPSED/REFRACTORY CLASSIC HODGKIN LYMPHOMA TREATED WITH NIVOLUMAB AND BRENTUXIMAB VEDOTIN ON CHECKMATE 744

**Topic:** 17. Hodgkin lymphoma - Clinical

Sarah Milgrom*[1], Stephen Daw[2], Kara Kelly[3], Peter Cole[4], Paul Harker-Murray[5], Thierry Leblanc[6], Diederik Grootendorst[7], Jennifer Krajewski[7], Naveen Boora[7], Jenny Zhang[7], Bradford S. Hoppe[8]

*[1]University of Colorado, Aurora, United States of America;[2]University College Hospital, London, United Kingdom; [3]Roswell Park Comprehensive Cancer Center and University at Buffalo Jacobs School of Medicine and Biomedical Sciences, Buffalo, United States of America; [4]Rutgers Cancer Institute of New Jersey, New Brunswick, United States of America;[5]Children's Wisconsin, Milwaukee, United States of America; [6]Hôpital Robert-Debré Assistance Publique - Hôpitaux de Paris, Paris, France;[7]Bristol Myers Squibb, Princeton, United States of America; [8]Mayo Clinic, Jacksonville, United States of America;*

### Background

In classic Hodgkin lymphoma (cHL) treated with conventional chemotherapy, PET-derived baseline total metabolic tumor volume (TMTV) and interim disease response are independently associated with progression-free survival (PFS). Whether these metrics retain prognostic relevance in relapsed/refractory (R/R) cHL treated with novel immunotherapy-based combinations is unknown. CheckMate 744 (NCT02927769) was a Phase II, risk- and response-adapted trial of nivolumab plus brentuximab vedotin (N+Bv) in children and young adults with R/R cHL.

### Aims

This secondary analysis of CheckMate 744 explored the association of 1) interim PET response with PFS, 2) baseline TMTV with interim PET response, and 3) baseline TMTV with PFS.

### Methods

Patients were stratified into a low-risk (R1, n=27) and standard-risk (R2, n=43) cohort. All patients received N+Bv as induction; those without a complete metabolic response (CMR) after 4 cycles received intensification to bendamustine+Bv. Subsequent consolidation comprised involved-site radiotherapy (R1) or high-dose chemotherapy & autologous stem cell transplantation (R2). Interim PET response was assigned centrally and defined using the Lugano 2014 criteria, with Deauville 1-3 defined as a CMR. Subjects were classified as having a rapid CMR (CMR after 2 cycles of N+Bv), non-rapid CMR (CMR later during protocol therapy), or non-CMR (no CMR documented during protocol therapy). TMTV, defined as the total body lymphoma volume (cc) with an SUV > 4, was assessed centrally in a blinded fashion.

**Copyright Information:** (Online) ISSN: 2572-9241
©2026 The Author(s). HemaSphere is published by John Wiley & Sons Ltd on behalf of European Hematology Association. This is an open access Abstract Book distributed under the Attribution-NonCommercial-NoDerivs (CC BY-NC-ND), which allows third parties to download the articles and share them with others as long as they credit the author and the Abstract Book, but they cannot change the content in any way or use them commercially.
**Abstract Book Citations:** Authors, Title, HemaSphere, 2026;10:(S1):pages. The abstract book DOIs can be found on the "Author Information" page.
**Disclaimer:** Articles published in the journal HemaSphere exclusively reflect the opinions of the authors. The authors are responsible for all content in their abstracts including accuracy of the facts, statements, citing resources, etc.

### Results

Across 70 patients (27 R1, 43 R2), the median baseline TMTV was 18.95 (range: 1.1-540.8). Forty-eight patients (69%) had a rapid CMR, 6 (9%) had a non-rapid CMR, and 16 (23%) had a non-CMR. Median baseline TMTV was 19.02 (range: 2.2-540.8) for the rapid CMR subset, 6.8 (range: 1.1-52.1) for non-rapid CMR, and 25.18 (range: 1.7-441.9) for non-CMR. Median change in TMTV from baseline to after cycle 4 was -100% (range: +100% - -100%). In the R1 cohort (median follow-up 65.0 months [range 40.7-79.8]), there was 1 PFS event. In the R2 cohort (median follow-up 78.0 months [range 72.2-85.6]), there were 3 PFS events. Characteristics of the 4 subjects with PFS events are summarized in the Table. The relationship between baseline TMTV, interim PET response, and PFS is summarized in the Figure. All 4 PFS events occurred in patients with a rapid CMR. In the R1 cohort, 3-year PFS was 100% after a non-rapid CMR and non-CMR and was 95% after a rapid CMR. In the R2 cohort, 3-year PFS was 100% after a non-rapid CMR and non-CMR and was 89% after a rapid CMR.

Table: Patients with PFS Events

| Patient | Arm | TMTV Baseline (cc) | TMTV PET4 (cc) | Deauville PET2 | Deauville PET4 |
|---|---|---|---|---|---|
| *1* | R1 | 20.3 | 0 | 3 | 3 |
| *2* | R2 | 198.3 | 2.2 | 2 | 2 |
| *3* | R2 | 5.55 | 1.3 | 1 | 2 |
| *4* | R2 | 37.8 | 1.3 | 2 | 5 |

### Summary/Conclusion

Checkmate 744 yielded exceptional PFS rates in R/R pediatric and young adult cHL, with very few events over long follow-up. The small number of PFS events limits the assessment for associations. Nonetheless, baseline TMTV showed no apparent correlation with interim PET response or PFS, suggesting that the prognostic weight of tumor bulk, established in conventionally treated cHL, may not translate to novel immunotherapy-based regimens. Conversely, there was a correlation between interim PET response and PFS, although the event number was small; this association was in an unexpected direction with all PFS events observed following a rapid CMR, most plausibly reflecting de-escalation, as patients with a rapid CMR received less intensive therapy.

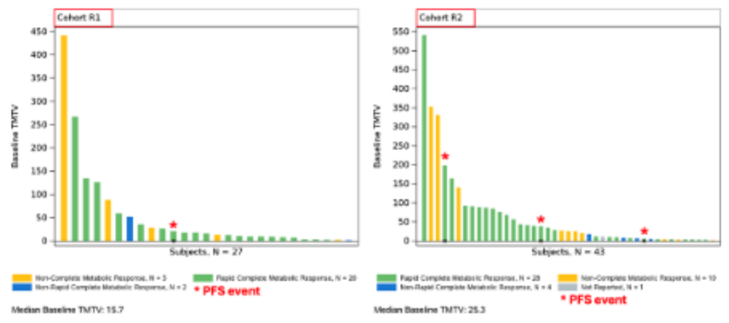

Relationship Between Baseline TMTV, Interim PET Response, and PFS

**Copyright Information:** (Online) ISSN: 2572-9241
©2026 The Author(s). HemaSphere is published by John Wiley & Sons Ltd on behalf of European Hematology Association. This is an open access Abstract Book distributed under the Attribution-NonCommercial-NoDerivs (CC BY-NC-ND), which allows third parties to download the articles and share them with others as long as they credit the author and the Abstract Book, but they cannot change the content in any way or use them commercially.
**Abstract Book Citations:** Authors, Title, HemaSphere, 2026;10:(S1):pages. The abstract book DOIs can be found on the "Author Information" page.
**Disclaimer:** Articles published in the journal HemaSphere exclusively reflect the opinions of the authors. The authors are responsible for all content in their abstracts including accuracy of the facts, statements, citing resources, etc.

### (PF926) PEMBROLIZUMAB (PEMBRO) IN PEDIATRIC PARTICIPANTS WITH HIGH-RISK CLASSIC HODGKIN LYMPHOMA (CHL): AN EXPLORATORY ANALYSIS OF CIRCULATING TUMOR DNA (CTDNA) FROM THE PHASE 2 KEYNOTE-667 STUDY

**Topic:** 17. Hodgkin lymphoma - Clinical

Christine Mauz-Koerholz*[1, 2], Luciana Vinti[3], Gerard Michel[4], Auke Beishuizen[5], Franca Fagioli[6, 7], Lorena Valero-Arrese[8], Salvatore Buffardi[9], Michaela Cepelova[10], Stephen Daw[11], Antony Ceraulo[12], Thierry Leblanc[13], Anne Thorwarth[14], Charlotte Rigaud[15], Theresa Jox[16], Maximilian Dörrbecker[17], Stefan Gattenlöhner[16], Marek Bartkuhn[17], MacKenzie Edmondson[18], Yuehui Zhao[18], Kevin Pham[18], Pallavi Pillai[18], Razvan Cristeau[18], Carol Pena[18], Andreas Bräuninger[16]

*[1]Justus-Liebig University of Giessen, Giessen, Germany; [2]Medical Faculty of the Martin-Luther-University of Halle-Wittenberg, Halle, Germany; [3]IRCCS Ospedale Pediatrico Bambino Gesù, Rome, Italy; [4]CHU de Marseille Hôpital de la Timone Enfants, Marseille, France; [5]Princess Máxima Centre, Utrecht, The Netherlands; [6]Ospedale Infantile Regina Margherita, Turin, Italy; [7]University of Turin, Turin, Italy; [8]Hospital Universitari Vall d'Hebron, Barcelona, Spain; [9]Azienda Ospedaliera di Rilievo Nazionale Santobono Pausilipon, Naples, Italy; [10]University Hospital Motol and 2nd Medical Faculty Charles University, Prague, Czechia; [11]University College London Hospitals NHS Foundation Trust, London, United Kingdom; [12]Institut d'Hématologie et d'Oncologie Pédiatrique (IHOPe), Lyon, France; [13]Hôpital Universitaire Robert-Debré, Paris, France; [14]Charité – Universitätsmedizin Berlin Campus Virchow-Klinikum, Berlin, Germany; [15]Gustave Roussy, Villejuif, France; [16]Institute of Pathology, Justus-Liebig University of Giessen, Giessen, Germany; [17]Biomedical Informatics and System Medicine, University of Giessen, Giessen, Germany; [18]Merck & Co., Inc., New Jersey, United States of America;*

### Background ctDNA is a promising biomarker in cHL. KEYNOTE-667 (NCT03407144) is evaluating pembro + chemotherapy consolidation with or without radiotherapy (RT) followed by pembro maintenance in children and young adults with cHL and a slow early response (SER) to front-line chemotherapy.

### Aims

To perform an exploratory analysis of ctDNA among participants (pts) with high-risk cHL in KEYNOTE-667.

### Methods

Pts aged 3-17 y with newly diagnosed stage IIEB-IVB cHL received 2 cycles of OEPA followed by early response assessment. Pts with rapid early response (RER; Deauville score [DS] 1-3) received nonstudy therapy. Pts with SER (DS 4 or 5) received consolidation with 4 cycles of COPDAC-28 + 6 cycles of pembro 2 mg/kg up to 200 mg IV Q3W followed by late response assessment (LRA). Pts with PET-positive disease at LRA (DS 4 or 5) received RT (28.8 Gy); pts with PET-negative disease received no RT. Pts with SER continued to receive pembro for a total of 17 cycles. Blood samples for ctDNA were collected from 150 pts at screening, during induction (cycle [C] 1, day [D] 28), and after induction (C2D28); from all pts with SER (n≈40) during consolidation (C1D28, C2D28, C4D28) and at pembro discontinuation; and from the first 40 pts with RER during consolidation (end of C1, C2, and C4) and ~8 mo after treatment end. Plasma ctDNA was analyzed using a custom, hybrid capture-based, targeted next-generation sequencing assay (Institute of Pathology, University of Giessen).

**Copyright Information:** (Online) ISSN: 2572-9241
©2026 The Author(s). HemaSphere is published by John Wiley & Sons Ltd on behalf of European Hematology Association. This is an open access Abstract Book distributed under the Attribution-NonCommercial-NoDerivs (CC BY-NC-ND), which allows third parties to download the articles and share them with others as long as they credit the author and the Abstract Book, but they cannot change the content in any way or use them commercially.
**Abstract Book Citations:** Authors, Title, HemaSphere, 2026;10:(S1):pages. The abstract book DOIs can be found on the "Author Information" page.
**Disclaimer:** Articles published in the journal HemaSphere exclusively reflect the opinions of the authors. The authors are responsible for all content in their abstracts including accuracy of the facts, statements, citing resources, etc.

**Results**

ctDNA was analyzed for 43 pts with SER and 38 with RER. Most commonly mutated genes (≥20% pts) were *SOCS1*, *STAT6*, *TNFAIP3*, *NFKBIE*, and *IGLL5*. Median maximum variant allele frequency (mVAF) at baseline was higher in pts with SER (10.6% [range, 0.0-34.0]) vs RER (6.2% [range, 0.0-34.2]) (AUROC, 0.61 [95% CI, 0.49-0.74]). All pts with SER achieved an objective response per Cheson 2007 IWG criteria by BICR; baseline ctDNA did not differentiate type of response (CR vs PR: AUROC, 0.45 [95% CI, 0.25-0.65]) or LRA PET status (negative vs positive: AUROC, 0.39 [95% CI, 0.20-0.58]). ctDNA data were analyzed for 34 pts with RER and 37 with SER during (C1D28) and after (C2D28) induction. Of 26 pts with RER who were ctDNA positive at baseline, 24 (92%) had ctDNA clearance at C1D28; 26 (100%) had clearance at C2D28. Of 32 pts with SER who were ctDNA positive at baseline, 16 (50%) had ctDNA clearance at C1D28; 21 (66%) had clearance at C2D28. At C2D28, there was a trend toward higher mVAF in pts with SER vs RER (AUROC, 0.66 [95% CI, 0.59-0.74]). In pts with SER, there was no association between C2D28 ctDNA and type of response (CR vs PR: AUROC, 0.45 [95% CI, 0.26-0.65]) or LRA PET status (negative vs positive: AUROC, 0.42 [95% CI, 0.25-0.59]). ctDNA data were available for 11 pts with RER and 27 with SER during consolidation and maintenance. No pts with RER were ctDNA positive at start of consolidation, C1, C2, or C4; 1 pt with RER became ctDNA positive after 8 mo. In pts with SER, 11 were ctDNA positive at start of consolidation, 2 at C1, 2 at C2, 1 at C4, and 1 after 8 mo.

**Summary/Conclusion**

A trend toward higher ctDNA levels was observed in pts with SER vs RER at baseline and end of induction. For pts with SER, there was no association between baseline or postinduction ctDNA level and type of response or LRA PET status. During induction, there was rapid clearance of ctDNA, with more pts with RER than SER having complete ctDNA clearance. These results support ctDNA as a potential biomarker in cHL.

**Copyright Information:** (Online) ISSN: 2572-9241
©2026 The Author(s). HemaSphere is published by John Wiley & Sons Ltd on behalf of European Hematology Association. This is an open access Abstract Book distributed under the Attribution-NonCommercial-NoDerivs (CC BY-NC-ND), which allows third parties to download the articles and share them with others as long as they credit the author and the Abstract Book, but they cannot change the content in any way or use them commercially.
**Abstract Book Citations:** Authors, Title, HemaSphere, 2026;10:(S1):pages. The abstract book DOIs can be found on the “Author Information” page.
**Disclaimer:** Articles published in the journal HemaSphere exclusively reflect the opinions of the authors. The authors are responsible for all content in their abstracts including accuracy of the facts, statements, citing resources, etc.

### (PF927) REAL-WORLD OUTCOMES OF PEMBROLIZUMAB PLUS GVD AS SALVAGE THERAPY AND BRIDGE TO AUTOLOGOUS STEM CELL TRANSPLANTATION IN RELAPSED/REFRACTORY CLASSICAL HODGKIN LYMPHOMA: A MULTICENTER GATLA STUDY

**Topic:** 17. Hodgkin lymphoma - Clinical

Astrid Pavlovsky*[1], Fernando Warley[2], Luciano Salvano[3], Maria Orlova[2], Luciana Guanchiale[4], Laura Korin[5], Carolina Mahuad[6], Jose Trucco[7], Amalia Cerutti[8], Florencia Negri Aranguren[9], Guadalupe Antelo Perez[10], Bruno Wannesson[1], Javier Romano[11], Lautaro Sardu[12], Veronica Musso[13], Etelvina Lucia MacChiavello[14], Nadia Scebba[15], Trinidad Viviani[16], Lorena Fiad[15]

*[1]Centro de Hematología Pavlovsky /FUNDALEU, Ciudad Autónoma de Buenos Aires, Argentina;[2]Hospital Italiano de Buenos Aires, Ciudad Autónoma de Buenos Aires, Argentina; [3]Clínica Universitaria Reina Fabiola, Córdoba, Argentina; [4]Hospital Privado Universitario de Córdoba, Córdoba, Argentina; [5]Instituto Alexander Fleming, Ciudad Autónoma de Buenos Aires, Argentina; [6]Hospital Alemán, Ciudad Autónoma de Buenos Aires, Argentina; [7]Hospital Austral, Buenos Aires, Argentina; [8]Sanatorio Británico, Rosario, Argentina; [9]Instituto Privado de Hematología y Hemoterapia, Paraná, Argentina; [10]CEMIC, Ciudad Autónoma de Buenos Aires, Argentina; [11]Hospital Posadas, Buenos Aires, Argentina; [12]Hospital El Cruce, Buenos Aires, Argentina; [13]Instituto de Trasplante y Alta Complejidad, Buenos Aires, Argentina; [14]Sanatorio La Pequeña Familia, Buenos Aires, Argentina; [15]Hospital Italiano de La Plata, Buenos Aires, Argentina; [16]GATLA, Buenos Aires, Argentina;*

### Background

In a phase 2 study including 39 patients (pts.) with relapsed/refractory classical Hodgkin lymphoma (R/R cHL) who were candidates for autologous stem cell transplantation (ASCT), pembrolizumab, gemcitabine, vinorelbine, and liposomal doxorubicin (P-GVD) achieved a complete metabolic response (CMR) rate of 95%, with 95% of patients proceeding to ASCT. This findings, support P-GVD as a highly effective bridge to transplant in this population.

### Aims

To report real-world outcomes in a broader, unselected population of pts. with R/R cHL eligible for ASCT who receive P-GVD.

### Methods

We conducted a retrospective multicenter study within the GATLA cooperative group, including consecutive pts. with R/R cHL eligible for ASCT who were treated with P-GVD. Pts. received P-GVD followed by PET with the intention to consolidate with ASCT. Baseline characteristics, PET response, immune-related adverse events (irAEs), transplant rates, were analyzed.

**Copyright Information:** (Online) ISSN: 2572-9241
©2026 The Author(s). HemaSphere is published by John Wiley & Sons Ltd on behalf of European Hematology Association. This is an open access Abstract Book distributed under the Attribution-NonCommercial-NoDerivs (CC BY-NC-ND), which allows third parties to download the articles and share them with others as long as they credit the author and the Abstract Book, but they cannot change the content in any way or use them commercially.
**Abstract Book Citations:** Authors, Title, HemaSphere, 2026;10:(S1):pages. The abstract book DOIs can be found on the "Author Information" page.
**Disclaimer:** Articles published in the journal HemaSphere exclusively reflect the opinions of the authors. The authors are responsible for all content in their abstracts including accuracy of the facts, statements, citing resources, etc.

### Results

Fifty-two pts. initiated treatment with P-GVD. The median age at diagnosis was 32 years (IQR, 17–69). Forty-six pts (88.5%) received ABVD as first-line therapy, and 6 (11.5%) BV-AVD. AETHERA high-risk features were present in 46 pts (88.5%): 69.2% had primary refractory disease, 11.5% relapsed within the first year, and 36.5% had extranodal relapse. B symptoms were present in 65.4%, and bulky disease was observed in 32.7%. P-GVD was administered as first salvage therapy in 35 pts (67.3%) and as second salvage in the remainder. PET response assessment was available in 49 pts (94.2%); 3 pts. discontinued therapy after 1 cycle due to toxicity.
After 2 cycles of PGVD, 38 patients underwent PET/CT evaluation: 29 (76%) achieved CR, 5 (13%) PR, and 4 (11%) PD. Among these, 25 patients did not receive further PGVD beyond cycle 2 (CR 21, PR 2, PD 2). 13/38 received additional cycles and were re-evaluated after 3–4 cycles (CR 8, PR 3, PD 2). 24 patients had a PET/CT assessment after 3–4 cycles, including those continuing beyond cycle 2 and those first evaluated at this time point.
Overall, 36/49 pts (73.5%) achieved CMR after planned treatment. Thirty-six (73.5%) proceeded to ASCT directly after P-GVD, (30 in CMR and 6 in PR), 6 pts in CMR did not undergo ASCT and 1 pt received allogeneic transplantation as consolidation, 4/36 pts relapsed after ASCT. (Fig.1)
With a median follow up of 14.7 months (IQR 10.3-26.2 months) 3/52 pts died: 1 due to COVID-19 during P-GVD, 1 due to graft-versus-host disease (GVHD) following allogeneic transplantation after relapsed post-ASCT and 1 due of sudden unexplained death. IrAR occurred in 8/52 pts (15.4%) and were manageable with standard interventions.

### Summary/Conclusion

In this real-world multicenter cohort, P-GVD produced a CMR rate of 73.5%, lower than the 95% reported by Moskowitz et al., and enabled 77.6% of patients to proceed to ASCT, compared with 95% in the phase 2 trial. These differences likely reflect a less selected population, including patients who received PGVD as $2^{nd}$salvage outside of a clinical trial setting and a higher proportion of primary refractory pts. Nonetheless, P-GVD remained an effective bridge to transplantation in routine practice, including in a cohort enriched for primary refractory disease. These findings provide important real-world context for PD-1–based chemo-immunotherapy strategies as a bridge to ASCT. Our cooperative group is now conducting a prospective trial in this setting to more clearly define its safety profile and efficacy.

**Copyright Information:** (Online) ISSN: 2572-9241
©2026 The Author(s). HemaSphere is published by John Wiley & Sons Ltd on behalf of European Hematology Association. This is an open access Abstract Book distributed under the Attribution-NonCommercial-NoDerivs (CC BY-NC-ND), which allows third parties to download the articles and share them with others as long as they credit the author and the Abstract Book, but they cannot change the content in any way or use them commercially.
**Abstract Book Citations:** Authors, Title, HemaSphere, 2026;10:(S1):pages. The abstract book DOIs can be found on the "Author Information" page.
**Disclaimer:** Articles published in the journal HemaSphere exclusively reflect the opinions of the authors. The authors are responsible for all content in their abstracts including accuracy of the facts, statements, citing resources, etc.

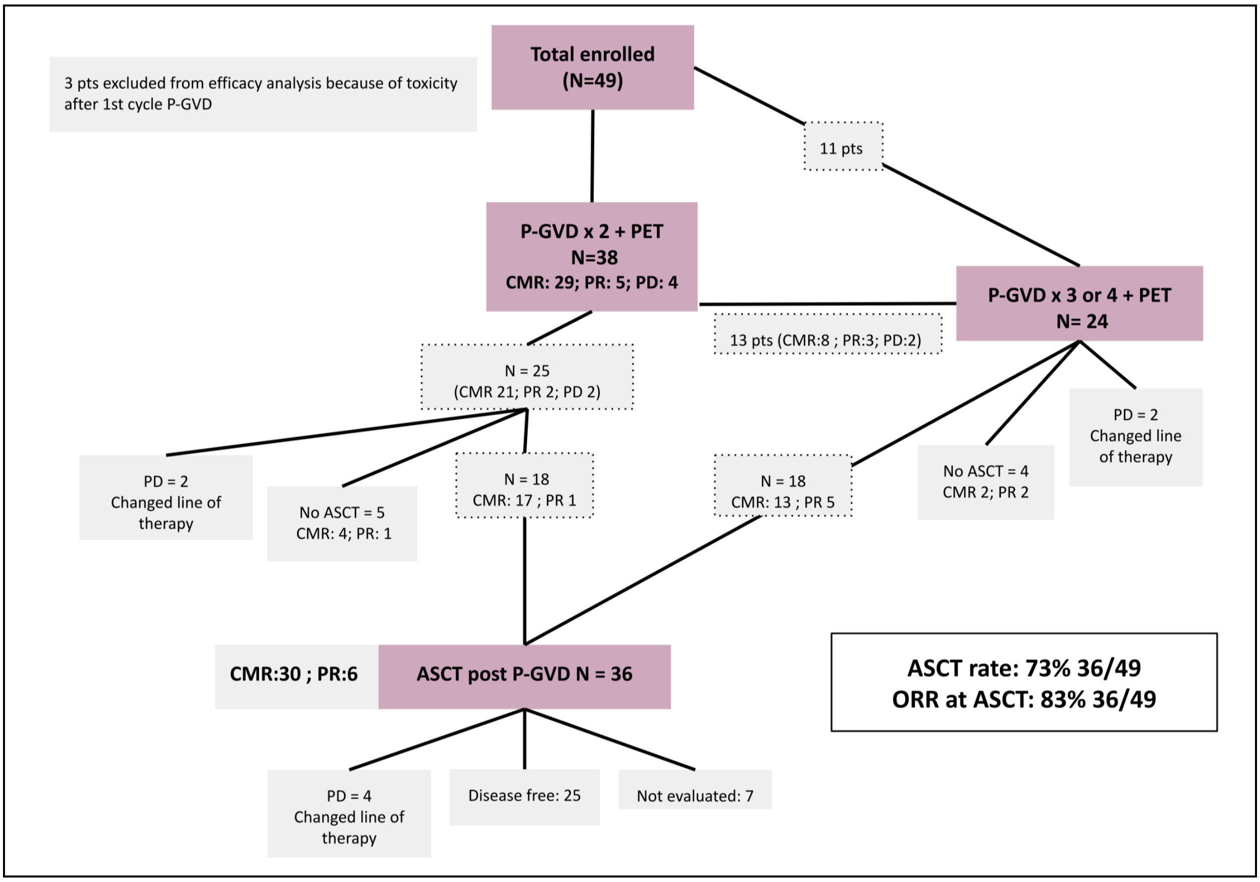

Figure 1. Flow chart. CMR: complete metabolic response, PR: partial response, PD: progressive disease, ASCT: autologous stem cell transplant, P-GVD: Pembrolizumab, Gemcitabine, Vinorelbine, Liposomal Doxurubicine, ORR: overall response rate.

**Copyright Information:** (Online) ISSN: 2572-9241
©2026 The Author(s). HemaSphere is published by John Wiley & Sons Ltd on behalf of European Hematology Association. This is an open access Abstract Book distributed under the Attribution-NonCommercial-NoDerivs (CC BY-NC-ND), which allows third parties to download the articles and share them with others as long as they credit the author and the Abstract Book, but they cannot change the content in any way or use them commercially.
**Abstract Book Citations:** Authors, Title, HemaSphere, 2026;10:(S1):pages. The abstract book DOIs can be found on the "Author Information" page.
**Disclaimer:** Articles published in the journal HemaSphere exclusively reflect the opinions of the authors. The authors are responsible for all content in their abstracts including accuracy of the facts, statements, citing resources, etc.

### (PF928) EXTERNAL VALIDATION OF THE EARLY-STAGE CHL INTERNATIONAL PROGNOSTIC INDEX (E-HIPI) IN A MULTICENTRIC ITALIAN COHORT

**Topic:** 17. Hodgkin lymphoma - Clinical

Alessandro Cellini*[1], Martina De Ferrari[1], Giovanni Manfredi Assanto[2], Alessandra Romano[3], Gabriella Santuccio[3], Candida Vitale[4], Maria Chiara Montalbano[4], Francesco Angotzi[1], Andrea Serafin[1], Nicolò Danesin[1], Arianna Bevilacqua[1], Mattia D'antiga[1], Giovanni Leone[1], Marta Coscia[5], Francesco Di Raimondo[3], Ilaria Del Giudice[2], Francesco Piazza[1], Livio Trentin[1], Andrea Visentin[1]

*[1]Università di Padova, Department of Medicine, Padova, Italy; [2]Università Sapienza, Department of Translational and Precision Medicine, Roma, Italy; [3]A.O.U. "G. Rodolico-S. Marco", Hematology and BMT Unit, Catania, Italy; [4]Università di Torino, Department of Molecular Biotechnology and Health Sciences,, Torino, Italy; [5]Università of Insubria, Department of Medicine and Surgery, Varese, Italy;*

### Background

Early-stage classical Hodgkin lymphoma (cHL) is highly curable, yet a relevant minority of affected patients experience relapse or have a primary refractory disease. The Early-stage cHL International Prognostic Index (E-HIPI) is a recently proposed prognostic model that predicts 2-year progression-free survival (PFS) using sex, maximum tumor diameter (MTD), hemoglobin (Hb) and albumin. Independent evaluation in contemporary PET-adapted cohorts is warranted.

### Aims

To externally validate E-HIPI in a multicenter Italian cohort of early-stage cHL treated with PET-adapted combined-modality therapy.

### Methods

Patients diagnosed with early-stage cHL between 2007 and 2025 were retrospectively enrolled across 4 Italian centers. All subjects were treated with PET/CT-adapted ABVD or BEACOPP, with PFS being defined as time from diagnosis to progression/relapse or death, whichever occurred first. E-HIPI 2-year PFS was computed using published coefficients. Missing predictors were handled using multiple imputation by chained equations. Discrimination was assessed by the means of Harrel's C-index of concordance and calibration at 2 years (calibration-in-the-large, and calibration slope). Two-year PFS and overall survival (OS) was estimated by the Kaplan–Meier method.

### Results

A total of 271 patients were included; median age was 31 years (range 18–65), 134 (49%) were female, and 251 (93%) had stage II disease. Bulky disease was present in 86 (32%) individuals, whereas B symptoms were reported by 66 (25%) patients. First-line treatment was ABVD-based in 261 (96%); 7 (3%) received an HD17-like regimen (2 cycles escalated BEACOPP followed by 2 ABVD), and 3 (1%) received an unspecified regimen. Albumin values were missing in 16 patients and MTD values were missing in 37 subjects. Baseline characteristics of interest were as follows: median Hb 13 g/dL (8.5-16.5), median albumin 4 g/dL (2.6-5.3), mean MTD 5.6 cm (1-22.3). After a median follow-up of 63 months (4-178), 38 patients experienced disease relapse/progression, and 8 subjects died, with a 2-year PFS was 87.4% (95% CI 83.6–91.7) and a 2-year OS of 100% (CI not estimable). After the implementation of the E-HIPI prognostic model discrimination for 2-year PFS was moderate, with a C-index of 0.654, 95% CI 0.55–0.75). Predicted risk was systematically lower than observed at 24 months (observed–predicted +6.3%, 95% CI 2.6–10.4%), while the calibration slope was 1.162 (95% CI 0.444–1.941).

**Copyright Information:** (Online) ISSN: 2572-9241
©2026 The Author(s). HemaSphere is published by John Wiley & Sons Ltd on behalf of European Hematology Association. This is an open access Abstract Book distributed under the Attribution-NonCommercial-NoDerivs (CC BY-NC-ND), which allows third parties to download the articles and share them with others as long as they credit the author and the Abstract Book, but they cannot change the content in any way or use them commercially.
**Abstract Book Citations:** Authors, Title, HemaSphere, 2026;10:(S1):pages. The abstract book DOIs can be found on the "Author Information" page.
**Disclaimer:** Articles published in the journal HemaSphere exclusively reflect the opinions of the authors. The authors are responsible for all content in their abstracts including accuracy of the facts, statements, citing resources, etc.

### Summary/Conclusion

E-HIPI was successfully validated in this multicenter cohort, yielding C-index values close to the ones reported in the training cohort and in the external validation cohort included in the original publication. While these findings support the usage of the model for risk stratification, E-HIPI however, systematically underestimated 2-year progression risk, indicating that recalibration of baseline risk may be required before clinical implementation.

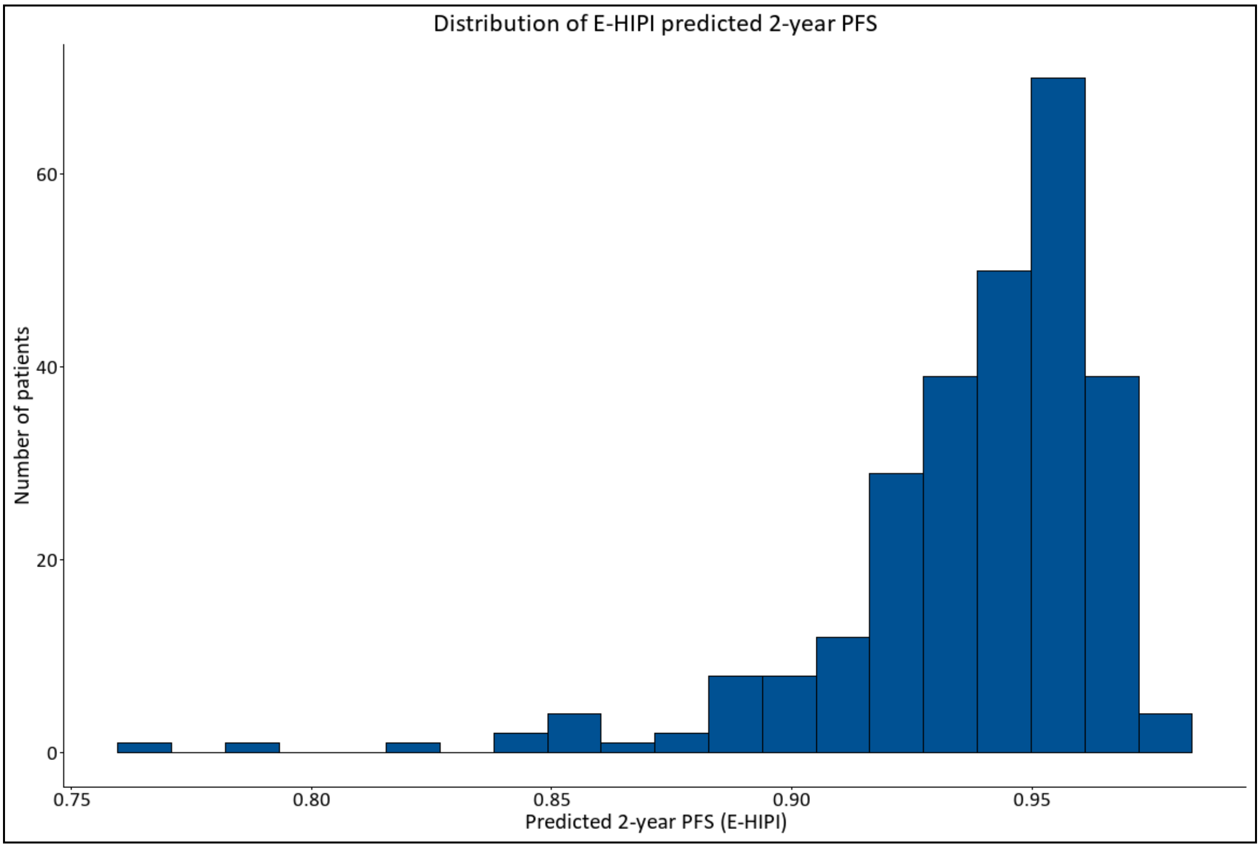

Distribution of the E-HIPI estimated 2-year progression-free survival

**Copyright Information:** (Online) ISSN: 2572-9241
©2026 The Author(s). HemaSphere is published by John Wiley & Sons Ltd on behalf of European Hematology Association. This is an open access Abstract Book distributed under the Attribution-NonCommercial-NoDerivs (CC BY-NC-ND), which allows third parties to download the articles and share them with others as long as they credit the author and the Abstract Book, but they cannot change the content in any way or use them commercially.
**Abstract Book Citations:** Authors, Title, HemaSphere, 2026;10:(S1):pages. The abstract book DOIs can be found on the "Author Information" page.
**Disclaimer:** Articles published in the journal HemaSphere exclusively reflect the opinions of the authors. The authors are responsible for all content in their abstracts including accuracy of the facts, statements, citing resources, etc.

### (PF929) SUPERIOR EFFICACY OF BRECADD VS PET-GUIDED ABVD AND BEACOPP IN ADVANCED-STAGE FRONTLINE HODGKIN LYMPHOMA: A MATCHING-ADJUSTED INDIRECT TREATMENT COMPARISON OF LONG-TERM FOLLOW-UP DATA OF HD21 STUDY

**Topic:** 17. Hodgkin lymphoma - Clinical

Fjoralba Kristo*[1], Alexa Molinari[2], Mingxin Liu[3], Thomas Melchardt[4], Hishan Tharmaseelan[5], Christian Jaworek[5], Hoora Moradian[3], Athanasios Zomas[6], Vanessa Stache[6], Victoria Federico Paly[2], Ajibade Ashaye[1]

*[1]Takeda Development Center Americas, Inc. (TDCA), Cambridge, United States of America;[2]Takeda Pharmaceuticals America, Inc., Lexington, United States of America; [3]Cytel Inc., Cambridge, United States of America; [4]Department of Internal Medicine III, Paracelsus Medical University, Salzburg, Austria; [5]Department I of Internal Medicine, Center for Integrated Oncology Aachen Bonn Cologne Duesseldorf (CIO ABCD), German Hodgkin Study Group (GHSG), Faculty of Medicine and University Hospital Cologne, University of Cologne, Cologne, Germany; [6]Takeda Pharmaceuticals International AG, Zurich, Switzerland;*

### Background

Brentuximab vedotin, etoposide, cyclophosphamide, doxorubicin, dacarbazine, dexamethasone (BrECADD) is a preferred frontline treatment for advanced-stage Hodgkin lymphoma (aHL) in global guidelines. Positron emission tomography (PET)-guided doxorubicin, bleomycin, vinblastine, and dacarbazine (ABVD) and escalated bleomycin, etoposide, doxorubicin, cyclophosphamide, vincristine, procarbazine and prednisone (BEACOPP) are also recommended regimens.

### Aims

A systematic literature review (SLR) and indirect treatment comparison of clinical trials was conducted to assess long-term comparative efficacy for BrECADD vs PET-guided ABVD and BEACOPP among various age groups with aHL.

### Methods

The SLR identified the phase 3 HD21 trial for BrECADD, RATHL and SWOG S0816 for PET-guided ABVD, and HD9 for BEACOPP. Patient-level data from HD21 (randomized cohorts [18–60 years, median follow-up [FU]: 61.6 months], non-randomized cohort [61–75 years, median FU: 34.3 months], and pooled data from both cohorts applying censoring rules) were weighted for comparison with aggregate data from the comparators using an unanchored matching-adjusted indirect comparison (MAIC). Hazard ratios (HR) with 95% confidence intervals (CI) for progression-free survival (PFS) and overall survival (OS) were generated using weighted Cox regression. For the base case, analyses were adjusted for baseline age, sex, International Prognostic Score, Eastern Cooperative Oncology Group performance status, stage, and B symptoms and censored at 36 months. Additional scenario and sensitivity analyses were conducted to explore inclusion/exclusion of model covariates and censoring cutoff timepoints.

### Results

Patient characteristics were well balanced between trials after MAIC reweighting. In the base case, BrECADD (HD21, 18–60 years, stage III and IV subgroup) significantly improved PFS and OS vs PET-guided ABVD in the SWOG S0816 intent-to-treat population (PFS HR: 0.23 [95% CI: 0.16, 0.33], p<0.001; OS HR: 0.31 [95% CI: 0.15, 0.64], p=0.005). Among patients 18–60 years, BrECADD (HD21, stage IIB, III, IV subgroup) also significantly improved PFS and OS vs PET-guided ABVD in RATHL (PFS HR: 0.25 [95% CI: 0.17, 0.38], p<0.001); OS HR: 0.37 [95% CI; 0.17, 0.79], p=0.002). Scenario analysis results of the MAICs between HD21 and RATHL (e.g., PET +/-, older adults, and pooled HD21 population including the HD21 61–75 cohort) were generally consistent with those in the 18–60 group. Among patients 66–75 years, BrECADD significantly improved OS vs BEACOPP in the HD9 trial (HR: 0.28 [95% CI: 0.10, 0.82], p=0.008).

**Copyright Information:** (Online) ISSN: 2572-9241
©2026 The Author(s). HemaSphere is published by John Wiley & Sons Ltd on behalf of European Hematology Association. This is an open access Abstract Book distributed under the Attribution-NonCommercial-NoDerivs (CC BY-NC-ND), which allows third parties to download the articles and share them with others as long as they credit the author and the Abstract Book, but they cannot change the content in any way or use them commercially.
**Abstract Book Citations:** Authors, Title, HemaSphere, 2026;10:(S1):pages. The abstract book DOIs can be found on the "Author Information" page.
**Disclaimer:** Articles published in the journal HemaSphere exclusively reflect the opinions of the authors. The authors are responsible for all content in their abstracts including accuracy of the facts, statements, citing resources, etc.

### Summary/Conclusion

BrECADD is robustly associated with significantly improved PFS and OS vs PET-guided ABVD across the 18–60 and 18–75 year-old populations in MAIC analyses of long-term FU data. A similar trend in OS was observed against BEACOPP in older adults. These results of this MAIC further support the use of BrECADD as a preferred frontline regimen for adults with aHL.

**Figure 1. Full-adjusted Cox regression results for progression-free survival and overall survival**

| Comparison | Scenario | Age | Stage | ESS/N | Index arm | Comparator arm | HR (95% CI) | p-value |
|---|---|---|---|---|---|---|---|---|
| Progression-free survival | | | | | | | | |
| BrECADD vs PET-guided ABVD | BC | 18-60 | III,IV | 619.64/631 | HD21 randomized (stage III & IV) | SWOG S0816 ITT | 0.23 (0.16, 0.33) | <0.001 |
| BrECADD vs PET-guided ABVD | BC | 18-60 | IIB,III,IV | 440.93/748 | HD21 randomized ITT | RATHL (age ≤ 60) | 0.25 (0.17, 0.38) | <0.001 |
| BrECADD vs PET-guided ABVD* | Naive | 61-75 | IIB,III,IV | 45.86/85 | HD21 nonrandomized ITT | RATHL (age > 60) | 0.45 (0.23, 0.92) | 0.024 |
| BrECADD vs PET-guided ABVD | BC | 18-75 | IIB,III,IV | 438.54/833 | HD21 pooled ITT | RATHL ITT | 0.25 (0.17, 0.37) | <0.001 |
| BrECADD vs PET-guided ABVD | BC | 18-75 | III,IV | 632.20/713 | HD21 pooled (stage III & IV) | RATHL (stage III & IV) | 0.25 (0.17, 0.36) | <0.001 |
| BrECADD vs PET-guided ABVD | BC | 18-75 | IIB,III,IV | 160.75/269 | HD21 pooled ITT, PET2+ | RATHL (PET2+) | 0.18 (0.10, 0.33) | <0.001 |
| BrECADD vs PET-guided ABVD | BC | 18-75 | IIB,III,IV | 237.84/485 | HD21 pooled ITT, PET2- | RATHL (PET2-) | 0.22 (0.12, 0.40) | <0.001 |
| Overall survival | | | | | | | | |
| BrECADD vs PET-guided ABVD | BC | 18-60 | III,IV | 619.64/631 | HD21 randomized (stage III & IV) | SWOG S0816 ITT | 0.31 (0.15, 0.64) | 0.005 |
| BrECADD vs PET-guided ABVD | BC | 18-60 | IIB,III,IV | 440.93/748 | HD21 randomized ITT | RATHL (age≤60) | 0.37 (0.17, 0.79) | 0.002 |
| BrECADD vs PET-guided ABVD* | Naive | 61-75 | IIB,III,IV | 45.86/85 | HD21 nonrandomized ITT | RATHL (age>60) | 0.73 (0.34, 1.57) | 0.416 |
| BrECADD vs PET-guided ABVD | BC | 18-75 | IIB,III,IV | 438.54/833 | HD21 pooled ITT | RATHL ITT | 0.45 (0.25, 0.83) | 0.004 |
| BrECADD vs PET-guided ABVD | BC | 18-75 | III,IV | 632.20/713 | HD21 pooled (stage III & IV) | RATHL (stage III&IV) | 0.42 (0.23, 0.75) | 0.003 |
| BrECADD vs PET-guided ABVD | BC | 18-75 | IIB,III,IV | 160.75/269 | HD21 pooled ITT, PET2+ | RATHL (PET2+) | 0.17 (0.06, 0.47) | <0.001 |
| BrECADD vs PET-guided ABVD | BC | 18-75 | IIB,III,IV | 237.84/485 | HD21 pooled ITT, PET2- | RATHL (PET2-) | 0.59 (0.26, 1.36) | 0.177 |
| BrECADD vs BEACOPP | BC | 66-75 | III,IV | 25.84/47 | HD21 nonrandomized (stage III & IV, age >65) | HD9 ITT (age 66-75) | 0.28 (0.10, 0.82) | 0.008 |

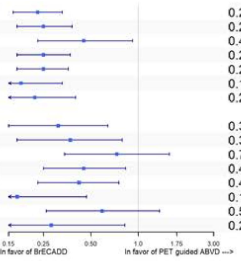

Notes: *Naïve (unweighted) results presented as patient characteristics were not available for the RATHL 61–75 subgroup. Abbreviations: ABVD, doxorubicin, bleomycin, vinblastine, and dacarbazine; BC, base case; BEACOPP, bleomycin, etoposide, doxorubicin, cyclophosphamide, vincristine, procarbazine, and prednisone; BrECADD, brentuximab vedotin, etoposide, cyclophosphamide, doxorubicin, dacarbazine, and dexamethasone; CI, confidence interval; ESS, effective sample size; HR, hazard ratio; ITT, intent to treat; PET, positron emission tomography.

Copyright Information: (Online) ISSN: 2572-9241
©2026 The Author(s). HemaSphere is published by John Wiley & Sons Ltd on behalf of European Hematology Association. This is an open access Abstract Book distributed under the Attribution-NonCommercial-NoDerivs (CC BY-NC-ND), which allows third parties to download the articles and share them with others as long as they credit the author and the Abstract Book, but they cannot change the content in any way or use them commercially.
Abstract Book Citations: Authors, Title, HemaSphere, 2026;10:(S1):pages. The abstract book DOIs can be found on the "Author Information" page.
Disclaimer: Articles published in the journal HemaSphere exclusively reflect the opinions of the authors. The authors are responsible for all content in their abstracts including accuracy of the facts, statements, citing resources, etc.

### (PF930) ASSOCIATION OF CIRCULATING TUMOUR DNA AND EBV DNA CHARACTERISTICS WITH SURVIVAL OUTCOMES OF CHILDREN AND YOUNG ADULTS WITH HODGKIN LYMPHOMA IN EAST AFRICA

**Topic:** 17. Hodgkin lymphoma - Clinical

Clara Chamba*[1], Heavenlight Christopher[1], Ismail Legason[2], Alex Mremi[3], Paul Ntemi[4], Emmanuel Balandya[1], Hadija Mwamtemi[5], Edrick Elias[6], Carol Achola[7], Anthony Cutts[8], Eli Mkwizu[3], Anna Schuh[8]

*[1]Muhimbili University of Health and Allied Sciences, Haematology, Dar-es-Salaam, Tanzania;[2]St. Mary's Hospital, Lacor, Uganda; [3]KCMC University, Moshi, Tanzania; [4]Bugando Medical Centre, Mwanza, Tanzania; [5]Muhimbili National Hospital, Dar-es-Salaam, Tanzania; [6]Catholic University of Health and Allied Sciences, Mwanza, Tanzania;[7]Central Public Health Laboratories, Kampala, Uganda; [8]University of Oxford, Oxford, United Kingdom;*

### Background

Hodgkin lymphoma (HL) is frequently associated with Epstein–Barr virus (EBV), particularly in low- and middle-income settings. Circulating tumour DNA (ctDNA) and EBV-derived plasma DNA have emerged as minimally invasive biomarkers for disease monitoring. Plasma-based mutation profiling enables characterisation of recurrent HL driver alterations, particularly within JAK–STAT and NF-κB pathways. However, the prognostic relevance of circulating EBV characteristics, mutation profiles, and fragmentomic metrics remains incompletely defined

### Aims

To evaluate ctDNA, EBV DNA characteristics, mutation profiles, and their association with survival outcomes in patients with newly diagnosed HL treated with ABVD.

### Methods

Plasma cfDNA underwent targeted next-generation sequencing. Somatic variants were identified using a validated bioinformatic pipeline with depth and variant allele frequency (VAF) thresholds to generate high-confidence calls. Mutation-derived measures included total variants, genes mutated, and hotspot analysis. EBV-derived markers (EBER1, EBER2, EBNA2, maximum EBV load, EBV proportion, and EBV entropy) and fragmentomic metrics (size ratio and fragment entropy) were quantified. ctDNA was estimated from cfDNA concentration and median VAF. Overall survival (OS) and event-free survival (EFS) were assessed using Kaplan–Meier methods, with landmark estimates at 6 and 12 months. An event was defined as death, progression, relapse, or initiation of second-line therapy. Associations were evaluated using univariate Cox models. Median follow-up was 74.7 weeks.

**Copyright Information:** (Online) ISSN: 2572-9241
©2026 The Author(s). HemaSphere is published by John Wiley & Sons Ltd on behalf of European Hematology Association. This is an open access Abstract Book distributed under the Attribution-NonCommercial-NoDerivs (CC BY-NC-ND), which allows third parties to download the articles and share them with others as long as they credit the author and the Abstract Book, but they cannot change the content in any way or use them commercially.
**Abstract Book Citations:** Authors, Title, HemaSphere, 2026;10:(S1):pages. The abstract book DOIs can be found on the “Author Information” page.
**Disclaimer:** Articles published in the journal HemaSphere exclusively reflect the opinions of the authors. The authors are responsible for all content in their abstracts including accuracy of the facts, statements, citing resources, etc.

**Results**

Among 50 patients, 30 (60%) harboured at least one somatic alteration. Across these cases, 104 variants were identified across 96 unique loci (median 2.5 per sample, IQR 1.25–4). Median VAF was 0.06 (IQR 0.034–0.077) with median sequencing depth 1,047× (IQR 681–1,422). Variants were predominantly missense, with additional frameshift, splice-site, and truncating mutations. The mutational landscape was dominated by genes regulating cytokine signalling and immune pathways. SOCS1 was the most frequently altered gene (25/50, 50%). NF-κB pathway regulators were also recurrently mutated, including NFKBIE (14%) and TNFAIP3 (12%). Mutations in B2M and ITPKB (each 10%) suggest immune evasion and B-cell signalling perturbation, while EP300 and STAT6 were infrequent (2% each). Hotspot analysis identified recurrent loci in NFKBIE and SOCS1, with a dominant NFKBIE locus mutated in five samples and dispersed recurrent SOCS1 positions consistent with classical HL mutational patterns.
Six- and 12-month OS were 93.8% (95% CI 87.3–100) and 86.1% (76.2–97.2). Corresponding EFS rates were 81.6% (71.4–93.2) and 64.0% (50.4–81.3). Elevated lactate dehydrogenase (LDH) was significantly associated with inferior EFS (HR 1.82, 95% CI 1.18–2.80; p=0.007). A higher EBV fragment size ratio (proportion of 180–200 bp EBV DNA fragments) showed a modest association with inferior EFS (HR 1.48, 95% CI 1.01–2.16; p=0.043). Plasma EBV-derived biomarkers, ctDNA levels, mutation burden, and individual gene alterations were not associated with survival.

**Summary/Conclusion**

Plasma mutation profiling demonstrated recurrent disruption of JAK–STAT and NF-κB pathways consistent with classical HL biology; however, these alterations were not prognostic. Clinical factors, particularly LDH, remained the dominant predictors of outcome, while EBV fragment size ratio showed a modest exploratory association with EFS.

**Copyright Information:** (Online) ISSN: 2572-9241
©2026 The Author(s). HemaSphere is published by John Wiley & Sons Ltd on behalf of European Hematology Association. This is an open access Abstract Book distributed under the Attribution-NonCommercial-NoDerivs (CC BY-NC-ND), which allows third parties to download the articles and share them with others as long as they credit the author and the Abstract Book, but they cannot change the content in any way or use them commercially.
**Abstract Book Citations:** Authors, Title, HemaSphere, 2026;10:(S1):pages. The abstract book DOIs can be found on the "Author Information" page.
**Disclaimer:** Articles published in the journal HemaSphere exclusively reflect the opinions of the authors. The authors are responsible for all content in their abstracts including accuracy of the facts, statements, citing resources, etc.

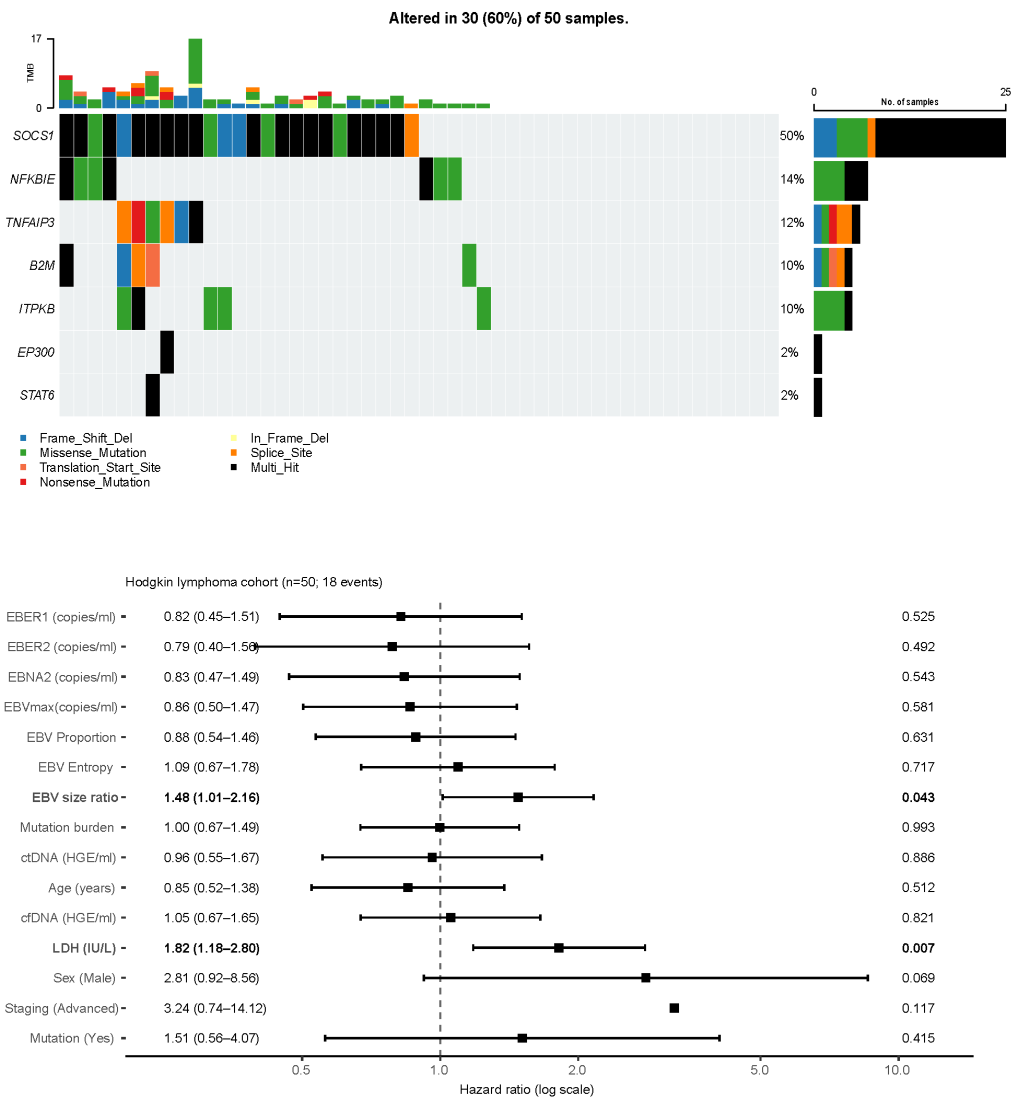

**Plasma Mutation Landscape and Association with Event-Free Survival in Classical Hodgkin Lymphoma**

1. Somatic variants were identified from plasma cell-free DNA using targeted next-generation sequencing and a validated bioinformatic pipeline with predefined depth and variant allele frequency thresholds. Only high-confidence variants are shown. 2. Percentages in the oncoplot represent the proportion of the full cohort (n=50) harbouring alterations in each gene. 3. Mutation burden refers to the total number of detected somatic variants per sample. 4. Hazard ratios (HRs) were derived from univariate Cox proportional hazards models for event-free survival (EFS). 5. An event was defined as death, disease progression, relapse, or initiation of second-line therapy. 6. EBV fragment size ratio represents the proportion of EBV-derived DNA fragments measuring 180–200 base pairs. 7. The vertical dashed line in the forest plot indicates HR = 1. Confidence intervals represent 95% CIs. 8. Bold values indicate statistically significant associations ($p < 0.05$).

**Copyright Information:** (Online) ISSN: 2572-9241
©2026 The Author(s). HemaSphere is published by John Wiley & Sons Ltd on behalf of European Hematology Association. This is an open access Abstract Book distributed under the Attribution-NonCommercial-NoDerivs (CC BY-NC-ND), which allows third parties to download the articles and share them with others as long as they credit the author and the Abstract Book, but they cannot change the content in any way or use them commercially.
**Abstract Book Citations:** Authors, Title, HemaSphere, 2026;10:(S1):pages. The abstract book DOIs can be found on the “Author Information” page.
**Disclaimer:** Articles published in the journal HemaSphere exclusively reflect the opinions of the authors. The authors are responsible for all content in their abstracts including accuracy of the facts, statements, citing resources, etc.

### (PF931) SEPARATING THE IMPACT OF POD24 AND TRANSFORMATION IN FOLLICULAR LYMPHOMA: A PROSPECTIVE BIOPSY-CONFIRMED STUDY

**Topic:** 18. Indolent and mantle-cell non-Hodgkin lymphoma - Clinical

Diana Malarikova*[1, 2], Katerina Benesova[1], Josef Karban[1], Andrea Janikova[3], David Salek[3], Alice Sykorova[4], David Belada[4], Heidi Mociková[5], Vit Prochazka[6], Juraj Duras[7], Petra Blahovcova[1], Vit Campr[8], Zuzana Prouzova[9], Jan Galko[9], Marek Trneny#[1]

*[1]First Department of Internal Medicine – Hematology, General University Hospital and First Faculty of Medicine, Charles University, Prague, Czechia; [2]Department of pathological physiology, First Faculty of Medicine, Charles University, Prague, Czechia; [3]Department of Internal Medicine, Hematology and Oncology, University Hospital Brno, Brno, Czechia; [4]4th Department of Internal Medicine – Haematology, University Hospital Hradec Kralove, Hradec Kralove, Czechia; [5]Clinic of Hematology, University Hospital Královské Vinohrady and Third Faculty of Medicine, Charles University, Prague, Czechia; [6]Hemato-Oncology Clinic, University Hospital Olomouc, Olomouc, Czechia; [7]Department of Hemato-Oncology, University Hospital Ostrava, Ostrava, Czechia; [8]Department of Pathology and Molecular Medicine, Second Faculty of Medicine, Charles University, and University Hospital Motol, Prague, Czechia; [9]Institute of Pathology, First Faculty of Medicine, Charles University and General Hospital, Prague, Czechia;*

### Background

Both early relapse or progression of follicular lymphoma (FL) within 24 months of first-line treatment initiation (POD24) and histologic transformation to diffuse large B-cell lymphoma (DLBCL) are predictors of inferior survival. It is sometimes hypothesized that the poor prognosis associated with POD24 reflects undetected transformation. However, data derived from systematically biopsy-confirmed cohorts remain scarce.

### Aims

To evaluate and compare the impact of POD24 and histologic transformation on survival following first relapse/progression (R/P) of FL.

### Methods

This analysis was conducted within the framework of the prospective NiHiL study (GovTrial No. NCT03199066). Eligible patients had histologically confirmed FL at diagnosis, received first-line therapy, and had biopsy-confirmed first relapse/progression. All histologic specimens were centrally reviewed. POD24 was defined as disease recurrence within 24 months from initiation of first-line treatment. Overall survival (OS) and progression-free survival (PFS) were calculated from initiation of second-line therapy using the Kaplan–Meier method. Median follow-up was 9.2 years.

**Copyright Information:** (Online) ISSN: 2572-9241
©2026 The Author(s). HemaSphere is published by John Wiley & Sons Ltd on behalf of European Hematology Association. This is an open access Abstract Book distributed under the Attribution-NonCommercial-NoDerivs (CC BY-NC-ND), which allows third parties to download the articles and share them with others as long as they credit the author and the Abstract Book, but they cannot change the content in any way or use them commercially.
**Abstract Book Citations:** Authors, Title, HemaSphere, 2026;10:(S1):pages. The abstract book DOIs can be found on the "Author Information" page.
**Disclaimer:** Articles published in the journal HemaSphere exclusively reflect the opinions of the authors. The authors are responsible for all content in their abstracts including accuracy of the facts, statements, citing resources, etc.

**Results**

A total of 445 patients with biopsy-confirmed first R/P were identified: 364 (82%) had relapsed FL and 81 (18%) had transformed DLBCL. Among patients with relapsed FL, 114 (31%) experienced POD24 (POD24-FL) and 250 (69%) relapsed later (non-POD24-FL). Among the 81 transformation events, 41 (51%) occurred within 24 months (POD24-T) and 40 (49%) beyond 24 months (non-POD24-T). Overall, among 155 POD24 events, 114 (74%) were FL and 41 (26%) represented transformation. In contrast, among 290 late relapses, 250 (86%) were FL and only 40 (14%) were transformed lymphomas. At relapse, patients with transformation were significantly older (66 vs 62 years; p<0.001), had worse performance status (≥2: 40% vs 8%; p<0.001), more frequent LDH elevation (72% vs 46%; p=0.001), higher β2-microglobulin (54% vs 32%; p<0.005), and more B symptoms (30% vs 16%; p<0.01) compared with those with relapsed FL. Within the transformation cohort, no significant clinical differences were observed between POD24-T and non-POD24-T. Among relapsed FL, only borderline differences were found between POD24-FL and non-POD24-FL (higher LDH and younger age in POD24-FL). Patients with POD24-FL had significantly inferior OS compared to non-POD24-FL (HR 1.5; p<0.01; median OS 6.8 vs 14.1 years). Patients with POD24-T had markedly worse OS compared with non-POD24-T (HR 2.0; p<0.01; median OS 0.9 vs 5.4 years) and with POD24-FL (HR 3.0; median OS 0.9 vs 6.8 years). The only groups without significant survival difference were non-POD24-T and POD24-FL (HR 1.3; p=NS; median OS 5.4 vs 6.8 years). In multivariable analysis, independent predictors of inferior OS were transformation (HR 2.0; p<0.0001), POD24 (HR 1.7; p<0.001), age (continuous; HR 1.05 per year; p<0.0001) and clinical stage III–IV (HR 2.1; p<0.05).

**Summary/Conclusion**

In this prospective biopsy-confirmed cohort, histologic transformation was identified in 18% of first relapses. Among POD24 events, transformation accounted for 26% of cases, compared with 14% among late relapses. Median OS progressively declined across relapse categories:14.1 years (non-POD24-FL), 6.8 years (POD24-FL), 5.4 years (non-POD24-T) and 0.9 years (POD24-T). Importantly, the adverse prognosis of POD24 in FL cannot be explained by transformation alone, because both early progression and transformation independently predict survival after first relapse.

**Copyright Information:** (Online) ISSN: 2572-9241
©2026 The Author(s). HemaSphere is published by John Wiley & Sons Ltd on behalf of European Hematology Association. This is an open access Abstract Book distributed under the Attribution-NonCommercial-NoDerivs (CC BY-NC-ND), which allows third parties to download the articles and share them with others as long as they credit the author and the Abstract Book, but they cannot change the content in any way or use them commercially.
**Abstract Book Citations:** Authors, Title, HemaSphere, 2026;10:(S1):pages. The abstract book DOIs can be found on the “Author Information” page.
**Disclaimer:** Articles published in the journal HemaSphere exclusively reflect the opinions of the authors. The authors are responsible for all content in their abstracts including accuracy of the facts, statements, citing resources, etc.

### (PF932) RECLASS : REFINEMENT OF MARGINAL ZONE LYMPHOMA CLASSIFICATION AND STAGING IMPROVES PROGNOSTIC DISCRIMINATION IN THE PROSPECTIVE REALYSA COHORT.

**Topic:** 18. Indolent and mantle-cell non-Hodgkin lymphoma - Clinical

Côme Bommier*[1], Marie Donzel[2], Cedric Rossi[3], Luc Matthieu Fornecker[4], Fontanet Bijou[5], Adrien Chauchet[6], Laure Lebras[7], Loic Ysebaert[8], Corinne Haioun[9], Kamal Bouabdallah[10], Thomas Gastinne[11], Nadine Morineau[12], Sandy Amorim[13], Fabrice Jardin[14], Julie Abraham[15], Thierry Lamy De La Chapelle[16], Remy Gressin[17], Ludovic Fouillet[18], Christophe Fruchart[19], Gaelle Olivier[20], Franck Morschhauser[21], Fanny Cherblanc[22], Aurelien Belot[22], Sandra Le Guyader[5], Alain Monnereau[5], Herve Ghesquieres[23], Catherine Thieblemont[24]

*[1]APHP, Hôpital Saint Louis, Paris, France;[2]Hospices Civils de Lyon, Service de Pathologie, Centre Hospitalier Lyon-Sud, Pierre Bénite, France; [3]CHU de Dijon Bourgogne, Dijon, France;[4]Institut de Cancérologie Strasbourg Europe, Strasbourg, France; [5]Institut Bergonié, Bordeaux, France; [6]CHU Besançon, Besançon, France; [7]Centre Léon Bérard, Lyon, France; [8]Institut universitaire du cancer de Toulouse Oncopole, Toulouse, France; [9]Hôpital Henri Mondor, Créteil, France; [10]CHU Bordeaux, Pessac, France; [11]CHU Nantes, Nantes, France; [12]Centre Hospitalier Départemental Vendée, La-Roche-sur-Yon, France; [13]Hôpital Saint Vincent de Paul, Lille, France;[14]Centre Henri Becquerel, Rouen, France; [15]CHU Limoges, Limoges, France; [16]CHU Rennes Pontchaillou, Rennes, France; [17]CHU Grenoble, Grenoble, France; [18]CHU Saint-Etienne, Saint-Etienne, France; [19]CH Dunkerque, Dunkerque, France; [20]CH Niort, Niort, France; [21]CHU Lille, Lille, France; [22]Lymphoma Academic Research Organisation, Pierre Bénite, France; [23]Hôpital Lyon Sud, Pierre Bénite, France;[24]Hopital Saint-Louis, Paris, France;*

### Background

Marginal zone lymphomas (MZL) traditionally comprise three entities: extranodal (EMZL), splenic (SMZL), and nodal (NMZL). Histological classifications do not provide a clinically operational framework integrating the full anatomical extent of disease. The concept of a "disseminated" entity (DissMZL), initially introduced in 2003, was subsequently adopted in the FIL NF10 study, grouping biologically heterogeneous entities and disregarding the prognostic relevance of multiple mucosal site involvement. While blood involvement in MZL may be observed across all subtypes, it has been shown to lack prognostic impact, reflecting disease biology rather than true anatomical dissemination.

### Aims

To redefine MZL classification by developing a hierarchical algorithm (RECLASS) integrating histological entities and anatomical dissemination, and to assess its clinical relevance compared with WHO2016 and FIL classifications.

### Methods

All MZL cases prospectively enrolled in the French REALYSA cohort were analyzed (2018–2023). We developed RECLASS, an algorithm-based system harmonizing histological and anatomical criteria that disregards blood and bone marrow involvement. Entity distribution and dissemination were compared across WHO2016, FIL, and RECLASS. RECLASS entity assignment follows a hierarchical approach: MBL-MZ and primary cutaneous MZL are identified first; mucosal involvement defines EMZL; splenic involvement without mucosal disease defines SMZL; remaining cases are classified as NMZL. Dissemination was defined as stage III–IV (per Lugano 2014 revised staging system) for WHO2016, as DissMZL for FIL, and in RECLASS according to entity-specific rules: multiple mucosal sites or stage III–IV for EMZL, stage III–IV for NMZL, and extension beyond splenic hilar nodes for SMZL. PFS by stage was evaluated using Cox models and Harrell's C-index.

**Copyright Information:** (Online) ISSN: 2572-9241
©2026 The Author(s). HemaSphere is published by John Wiley & Sons Ltd on behalf of European Hematology Association. This is an open access Abstract Book distributed under the Attribution-NonCommercial-NoDerivs (CC BY-NC-ND), which allows third parties to download the articles and share them with others as long as they credit the author and the Abstract Book, but they cannot change the content in any way or use them commercially.
**Abstract Book Citations:** Authors, Title, HemaSphere, 2026;10:(S1):pages. The abstract book DOIs can be found on the "Author Information" page.
**Disclaimer:** Articles published in the journal HemaSphere exclusively reflect the opinions of the authors. The authors are responsible for all content in their abstracts including accuracy of the facts, statements, citing resources, etc.

**Results**

In total, 389 MZL patients were prospectively included in REALYSA, and the median follow-up was 38.4 months (95%CI 36.4-44.0). Among the 136 patients classified as DissMZL by FIL, RECLASS reassigned 63% (n=86) to EMZL, 28% (n=38) to SMZL, and 9% (n=12) to NMZL. Notably, these former DissMZL cases accounted for 38% of all EMZL cases under RECLASS. Compared with WHO2016, RECLASS modified dissemination status in 26% (101/389) of cases, exclusively through downstaging of patients classified as Lugano stage III–IV patients, with no cases upstaged. Disseminated disease was identified in 71% of patients by WHO2016, 35% by FIL, and 45% by RECLASS. RECLASS staging demonstrated stronger prognostic discrimination for PFS (HR 1.90, 95%CI 1.30–2.78; C-index 0.586) than WHO2016 (HR 1.72, 95%CI 1.09-2.73; C-index 0.553) or FIL (HR 1.36; 95%CI 0.93-1.99; C-index 0.544). At 24 months, the absolute difference in PFS between localized and disseminated disease was 11.1% with WHO2016 (88.9% vs 77.8%), 7.5% with FIL (83.7% vs 76.2%), and 13.5% with RECLASS (87.1% vs 73.6%), comforting improved risk separation with RECLASS staging.

**Summary/Conclusion**

By incorporating recent biological and clinical insights while preserving established histological entities, RECLASS provides a coherent clinico-anatomical framework. It reduces conceptual ambiguity in entity attribution, and improves prognostic stratification compared with WHO2016 and FIL. External validation in independent cohorts is warranted before implementation in routine practice.

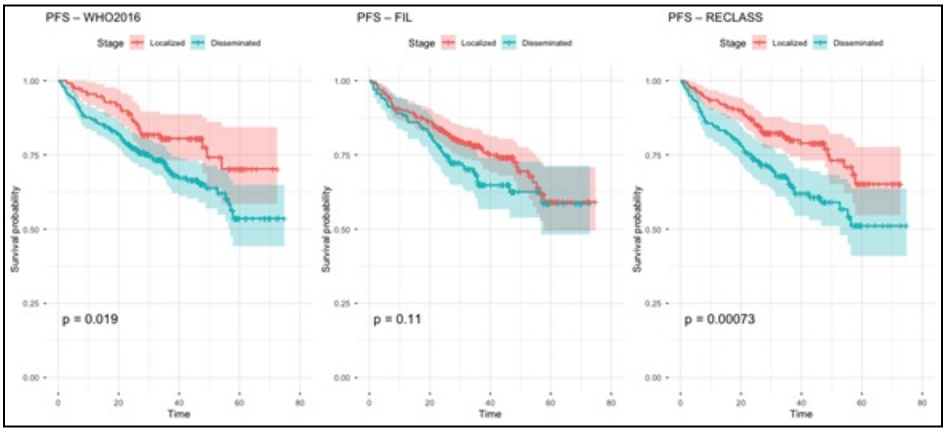

Progression-free survival from diagnosis date, by dissemination status, across the three classifying & staging systems : WHO2016/Lugano2014, FIL (NF10 study), RECLASS (REALYSA study).

**Copyright Information:** (Online) ISSN: 2572-9241
©2026 The Author(s). HemaSphere is published by John Wiley & Sons Ltd on behalf of European Hematology Association. This is an open access Abstract Book distributed under the Attribution-NonCommercial-NoDerivs (CC BY-NC-ND), which allows third parties to download the articles and share them with others as long as they credit the author and the Abstract Book, but they cannot change the content in any way or use them commercially.
**Abstract Book Citations:** Authors, Title, HemaSphere, 2026;10:(S1):pages. The abstract book DOIs can be found on the "Author Information" page.
**Disclaimer:** Articles published in the journal HemaSphere exclusively reflect the opinions of the authors. The authors are responsible for all content in their abstracts including accuracy of the facts, statements, citing resources, etc.

### (PF933) COMBINATION TREATMENT WITH NOVEL BCL2 INHIBITOR SONROTOCLAX (BGB-11417) AND ZANUBRUTINIB IN PATIENTS WITH RELAPSED/REFRACTORY MANTLE CELL LYMPHOMA (R/R MCL): RESULTS FROM A PHASE 1/1B STUDY

**Topic:** 18. Indolent and mantle-cell non-Hodgkin lymphoma - Clinical

Jacob Soumerai*[1], Constantine S. Tam[2], Masa Lasica[3], Marc Hoffmann[4], Ramón García Sanz[5], Johannes Schetelig[6], Talha Munir[7], Robert Weinkove[8, 9], Peter Browett[10], Eva Gonzalez Barca[11], Laura Magnano Mayer[12], Andrea Bernardelli[13], Jing-Zhou Hou[14], Raul Cordoba[15], Binghao Wu[16], Gabriel Man[17], Yiqian Fang[16], Sheel Patel[17], Remus Vezan[17], Stephen Opat[18]

*[1]Mass General Brigham Cancer Institute and Harvard Medical School, Boston, MA, United States of America;[2]Alfred Hospital and Monash University, Melbourne, VIC, Australia; [3]St Vincent's Hospital, Melbourne, Melbourne, VIC, Australia;[4]University of Kansas Cancer Center, Kansas City, KS, United States of America; [5]Gregorio Marañón University Hospital, CIBERONC, Madrid, Spain; [6]Universitatsklinikum Carl Gustav Carus, Technische Universitat Dresden, Dresden, Germany;[7]Leeds Teaching Hospitals NHS Trust, Leeds, United Kingdom; [8]Te Rerenga Ora Blood and Cancer Centre, Te Whatu Ora Health New Zealand Capital Coast & Hutt Valley, Wellington, New Zealand; [9]Cancer Immunotherapy Programme, Malaghan Institute of Medical Research, Wellington, New Zealand; [10]Auckland City Hospital, Grafton, Auckland, New Zealand; [11]Institut Català d'Oncologia Hospitalet, Universitat de Barcelona, IDIBELL, Barcelona, Spain; [12]Hospital Clínic de Barcelona, Barcelona, Spain;[13]Department of Engineering for Innovation Medicine, Section of Hematology, Azienda Ospedaliera Universitaria Integrata di Verona, Verona, Italy; [14]University of Pittsburgh Medical Center, Pittsburgh, PA, United States of America;[15]Hospital Universitario Fundación Jiménez Díaz, Madrid, Spain; [16]BeOne Medicines, Ltd, Shanghai, China; [17]BeOne Medicines, Ltd, San Carlos, CA, United States of America; [18]Lymphoma Research Group, School of Clinical Sciences at Monash Health, Monash University, Clayton, VIC, Australia;*

### Background

MCL is characterized by recurrent relapse after short remission periods. Venetoclax + ibrutinib demonstrated efficacy in R/R MCL but treatments (tx) with improved tolerability and efficacy are needed. Sonrotoclax (sonro), a next-generation BCL2 inhibitor, is a more selective and pharmacologically potent BCL2 inhibitor vs venetoclax, with a shorter half-life and no drug accumulation. Zanubrutinib (zanu) is a next-generation covalent BTK inhibitor with fewer off-target adverse events vs other BTK inhibitors and is FDA approved for R/R MCL.

### Aims

To report safety and efficacy of sonro + zanu (SZ) combination therapy in patients (pts) with R/R MCL with median follow-up of ~2 y.

### Methods

BGB-11417-101 (NCT04277637) is an ongoing open-label, phase 1/1b dose escalation and expansion study. Eligible adults have R/R MCL and received ≥1 prior therapy. Tx started with zanu lead-in (320mg once daily [QD] or 160mg twice daily) for 8-12 wk; then sonro was added. Ramp-up to sonro target dose (80, 160, 320, or 640mg QD) was used to mitigate risk of tumor lysis syndrome (TLS). Combination therapy continued until progressive disease (PD), unacceptable toxicity, or elective discontinuation after 96 wk. The primary endpoint was safety (eg, TLS; Howard criteria); secondary endpoints included overall response rate (ORR; partial response [PR] or better; Lugano 2014 criteria).

**Copyright Information:** (Online) ISSN: 2572-9241
©2026 The Author(s). HemaSphere is published by John Wiley & Sons Ltd on behalf of European Hematology Association. This is an open access Abstract Book distributed under the Attribution-NonCommercial-NoDerivs (CC BY-NC-ND), which allows third parties to download the articles and share them with others as long as they credit the author and the Abstract Book, but they cannot change the content in any way or use them commercially.
**Abstract Book Citations:** Authors, Title, HemaSphere, 2026;10:(S1):pages. The abstract book DOIs can be found on the "Author Information" page.
**Disclaimer:** Articles published in the journal HemaSphere exclusively reflect the opinions of the authors. The authors are responsible for all content in their abstracts including accuracy of the facts, statements, citing resources, etc.

**Results**

As of Dec 6, 2025, 51 pts were enrolled in sonro dose cohorts: 80mg (n=6), 160mg (n=13), 320mg (n=27), and 640mg (n=5). Forty-six pts (90.2%) initiated SZ; as of data cutoff, 25 (49.0%) remained on tx. Median age was 68 y and 70.6% of pts were male. Pts had a median of 1 prior tx (range, 1-4; median duration of last tx, 6.0 mo [range, 0.1-65.6]); prior tx included stem cell transplant (n=15; 1 allogeneic, 14 autologous), CAR-T (n=1), and BTK inhibitor (n=4). Maximum tolerated dose was not reached; RP2D was 320mg QD.
Twenty-one pts (41.2%) discontinued SZ and 6 (11.8%) discontinued zanu only. TEAEs led to discontinuation of SZ in 4 pts (7.8%) and zanu only in 2 (3.9%; diarrhea and cryptococcal meningoencephalitis); none led to discontinuation of sonro only. TEAEs led to death in 2 pts (3.9%; pneumonia; abdominal sepsis). The most common any-grade TEAEs were diarrhea (37.3%), neutropenia (33.3%), and COVID-19 (33.3%). Grade ≥3 TEAEs occurred in 66.7% of pts; neutropenia (21.6%) was the most common. Serious TEAEs occurred in 41.2% of all pts; pneumonia (15.7%) was the most common. In sonro 320mg + zanu pts, the most common any-grade TEAE was diarrhea (51.9%; most grade 1-2) and grade ≥3 TEAE was neutropenia (22.2%). No laboratory or clinical TLS occurred.
In 51 pts across doses, ORR was 80.4%; complete response (CR) rate was 60.8%. Median time to CR was 6.4 mo (range, 1.5-32.5). With a median study follow-up of 25.5 mo (range, 0.7-51.6), 80.6% of pts who achieved CR (25/31) remain in CR. In sonro 320mg + zanu pts (median follow-up, 21.3 mo), ORR was 81.5% (22/27); CR rate was 59.3% (16/27). Median duration of response (DOR) was not reached; 30-mo DOR rate was 77.7% (95% CI, 50.2%-91.2%). Of 4 pts with prior BTK inhibitor therapy, 2 achieved PR. All 6 pts who elected to discontinue tx achieved CR and remain in complete remission (median time off tx, 9.6 mo; range, 2.9-12.1).

**Summary/Conclusion**

SZ is well tolerated with promising antitumor activity. A phase 3 registrational trial assessing sonro 320mg + zanu is ongoing (NCT06742996).

**Copyright Information:** (Online) ISSN: 2572-9241
©2026 The Author(s). HemaSphere is published by John Wiley & Sons Ltd on behalf of European Hematology Association. This is an open access Abstract Book distributed under the Attribution-NonCommercial-NoDerivs (CC BY-NC-ND), which allows third parties to download the articles and share them with others as long as they credit the author and the Abstract Book, but they cannot change the content in any way or use them commercially.
**Abstract Book Citations:** Authors, Title, HemaSphere, 2026;10:(S1):pages. The abstract book DOIs can be found on the “Author Information” page.
**Disclaimer:** Articles published in the journal HemaSphere exclusively reflect the opinions of the authors. The authors are responsible for all content in their abstracts including accuracy of the facts, statements, citing resources, etc.

### (PF935) OUTCOMES OF LISOCABTAGENE MARALEUCEL (LISO-CEL) IN PATIENTS WITH RELAPSED OR REFRACTORY (R/R) MANTLE CELL LYMPHOMA (MCL): FIRST REAL-WORLD DATA FROM THE CIBMTR

**Topic:** 18. Indolent and mantle-cell non-Hodgkin lymphoma - Clinical

Jennifer Huang*[1], Taylor Brooks[2], Jason Romancik[3], Michael L. Wang[4], Elise Chong[5], Uttam Rao[6], Lizamarie Bachier-Rodriguez[7], Amitkumar Mehta[8], Matthew Lunning[9], David Bernasconi[10], Lin Wang[11], Kartik Patel[11], Debasmita Roy[11], Samantha Jaglowski[12], Bradley D. Hunter[13], Iris Isufi[14]

*[1]Fred Hutchinson Cancer Center, University of Washington, Seattle, United States of America;[2]Cleveland Clinic, Cleveland, United States of America; [3]Hackensack University Medical Center, Hackensack, United States of America;[4]The University of Texas MD Anderson Cancer Center, Houston, United States of America; [5]Abramson Cancer Center, University of Pennsylvania, Philadelphia, United States of America; [6]St. David's South Austin Medical Center, Austin, United States of America;[7]Northside Hospital Cancer Institute, Atlanta, United States of America; [8]University of Alabama at Birmingham, Birmingham, United States of America; [9]University of Nebraska Medical Center, Omaha, United States of America;[10]Bristol Myers Squibb, Boudry, Switzerland; [11]Bristol Myers Squibb, Princeton, United States of America; [12]Center for International Blood and Marrow Transplant Research (CIBMTR), Medical College of Wisconsin, Milwaukee, United States of America; [13]Intermountain LDS Hospital, Salt Lake City, United States of America; [14]Yale University School of Medicine, New Haven, United States of America;*

### Background

Liso-cel, an autologous, CD19-directed CAR T cell product, demonstrated deep, durable responses with manageable safety in patients with R/R MCL in the MCL cohort of TRANSCEND NHL 001 (NCT02631044).

### Aims

To present the first report of real-world outcomes for patients with R/R MCL who received liso-cel, using data from Center for International Blood and Marrow Transplant Research (CIBMTR).

### Methods

This retrospective study included patients in the United States with R/R MCL who received commercial liso-cel from May 2024 to September 2025 with ≥ 1 postinfusion assessment reported to CIBMTR. Informed consent for inclusion in the CIBMTR Registry was obtained by treating physicians or site investigators. Effectiveness outcomes included ORR, CR rate, duration of response (DOR), PFS, and OS. Safety outcomes included AEs of special interest, nonrelapse mortality (NRM), and death. Results are descriptive.

**Copyright Information:** (Online) ISSN: 2572-9241
©2026 The Author(s). HemaSphere is published by John Wiley & Sons Ltd on behalf of European Hematology Association. This is an open access Abstract Book distributed under the Attribution-NonCommercial-NoDerivs (CC BY-NC-ND), which allows third parties to download the articles and share them with others as long as they credit the author and the Abstract Book, but they cannot change the content in any way or use them commercially.
**Abstract Book Citations:** Authors, Title, HemaSphere, 2026;10:(S1):pages. The abstract book DOIs can be found on the "Author Information" page.
**Disclaimer:** Articles published in the journal HemaSphere exclusively reflect the opinions of the authors. The authors are responsible for all content in their abstracts including accuracy of the facts, statements, citing resources, etc.

### Results

A total of 94 patients were eligible. Median age was 73 years (range, 46–85), 74% were male, 4% (4/92) had ECOG PS ≥ 2, and 65% (53/81) had ≥ 1 clinically significant comorbidity, with cardiac (31%), pulmonary (31%), and obesity (17%) being most common (> 10%). Largest nodal mass > 10 cm, indicative of bulky disease, was observed in 15% of patients (9/60), 31% had ≥ 2 extranodal involvement, and 5% had CNS involvement. Data on *TP53* mutation status and Ki-67 score were limited. Median number of prior lines of therapy (including HSCT/cell therapy) was 4 (range, 2–12); 69% of patients (62/90) had refractory disease and 72% (67/93) received bridging therapy.
At a median follow-up of 6.0 months (95% CI, 5.9–6.2), ORR was 88% (95% CI, 80–94) with CR rate of 80% (95% CI, 70–87). Median DOR, PFS, and OS were not reached; 6-month DOR, PFS, and OS rates were 79% (95% CI, 55–91), 79% (95% CI, 68–87), and 92% (95% CI, 83–96.5), respectively.
Any-grade cytokine release syndrome and immune effector cell–associated neurotoxicity syndrome were reported in 65% (grade ≥ 3, 2%) and 37% (grade ≥ 3, 12%) of patients, respectively, with no grade 5 events. Clinically significant infections were reported in 23% of patients, grade 4 thrombocytopenia and/or neutropenia persistent at 30 days after infusion in 18%, and second primary malignancies in 5%, with skin malignancies being the most common. Grade 3/4 organ toxicity occurred in 1 (1%) patient, and none had tumor lysis syndrome. Nine (10%) patients died, including 6 (6%) due to PD. The NRM rate at 6 months was 1% (95% CI, 0.1–5.4). Of the 36% of patients planned for outpatient infusion, 59% (20/34) were reported as being hospitalized postinfusion with a median (range) time to hospitalization and stay of 5.5 days (1–35) and 4.5 days (2–32), respectively.

### Summary/Conclusion

These results show that liso-cel is associated with high response rates in a broad population of heavily pretreated patients with R/R MCL with early signs of durable benefit and safety profile consistent with TRANSCEND NHL 001. While longer follow-up is needed, this supports the potential of liso-cel to address critical gaps for patients with limited treatment options, providing strong effectiveness and manageable safety.
©2026 American Society of Clinical Oncology, Inc. Reused with permission. This abstract was submitted to ASCO 2026. All rights reserved.

**Copyright Information:** (Online) ISSN: 2572-9241
©2026 The Author(s). HemaSphere is published by John Wiley & Sons Ltd on behalf of European Hematology Association. This is an open access Abstract Book distributed under the Attribution-NonCommercial-NoDerivs (CC BY-NC-ND), which allows third parties to download the articles and share them with others as long as they credit the author and the Abstract Book, but they cannot change the content in any way or use them commercially.
**Abstract Book Citations:** Authors, Title, HemaSphere, 2026;10:(S1):pages. The abstract book DOIs can be found on the "Author Information" page.
**Disclaimer:** Articles published in the journal HemaSphere exclusively reflect the opinions of the authors. The authors are responsible for all content in their abstracts including accuracy of the facts, statements, citing resources, etc.

### (PF936) PROGNOSTIC IMPACT OF FDG-PET–BASED BONE MARROW ASSESSMENT COMPARED WITH CONVENTIONAL HISTOLOGY IN FOLLICULAR LYMPHOMA: A SUBANALYSIS OF THE FIL FOLL12 TRIAL

**Topic:** 18. Indolent and mantle-cell non-Hodgkin lymphoma - Clinical

Giovanni Manfredi Assanto*[1], Stefano Luminari[2], Luigi Marcheselli[3], Elizabeth Anna Katherine Triumbari[4, 5], Durmo Rexhep[6], Elisa Genuardi[7], Maria Elena Nizzoli[2], Carola Boccomini[8], Benedetta Puccini[9], Alessandro Pulsoni[10], Vincenzo Pavone[11], Annalisa Arcari[12], Annarita Conconi[13], Marco Ladetto[14], Sara Galimberti[15], Ilaria Del Giudice[1], Annibale Versari[6], Stephane Chauvie[16], Massimo Federico[17], Simone Ferrero[7], Luca Guerra[18, 19]

*[1]Sapienza University Of Rome -AOU Policlinico Umberto I, Department of Translational and Precision Medicine, Rome, Italy; [2]Azienda USL-IRCCS di Reggio Emilia, Hematology Unit, Reggio emilia, Italy;[3]Uffici Studi Fondazione Italiana Linfomi, Fondazione Italiana Linfomi Onlus, Alessandria, Italy; [4]Fondazione Policlinico Universitario Campus Biomedico, Nuclear Medicine Unit - Medipass Ergéa Group, Rome, Italy; [5]University of Rome Tor Vergata, Department of Biomedicine and Prevention, Rome, Italy; [6]Azienda USL-IRCCS of Reggio Emilia, Nuclear medicine Unit, Reggio Emilia, Italy;[7]University of Turin, Department of Molecular Biotechnologies and Health Sciences, Turin, Italy; [8]A.O.U. Città della Salute e della Scienza di Torino, Hematology Division, Turin, Italy; [9]Careggi University Hospital, Hematology Division, Florence, Italy; [10]Sapienza University Polo-Pontino, Department of Hematology, SM Goretti Hospital, Latina, Italy; [11]Card. Panico Hospital, Tricase, Italy; [12]Ospedale Guglielmo da Saliceto, Hematology, Piacenza, Italy; [13]Ospedale degli Infermi, Hematology Unit, Department of Internal Medicine, Biella, Italy; [14]SCDU Ematologia AOU SS Antonio e Biagio E Cesare Arrigo, Università del Piemonte Orientale, Department of Translational Medicine, Alessandria, Italy; [15]Pisa University Hospital, University of Pisa, Department of Clinical and Experimental Medicine,, Pisa, Italy; [16]Santa Croce e Carle Hospital, Medical Physics Division, Cuneo, Italy; [17]CHIMOMO department, University of Modena and Reggio Emilia, Modena, Italy; [18]Fondazione IRCCS San Gerardo dei Tintori, Nuclear Medicine, Monza, Italy; [19]University of Milano Bicocca, School of Medicine, Milan, Italy;*

### Background

Bone marrow biopsy (BMB) is an invasive staging procedure for follicular lymphoma (FL) and its results combined with other disease features are employed to calculate FLIPI2 and PRIMA-PI scores. Beyond conventional biopsy bone marrow can also be assessed through FDG-PET bone staging (PETb). We report preliminary data on a comparison of BMB and PETb in assessing BM involvement among FL patients enrolled in the FIL-FOLL12 trial.

### Aims

To compare prognostic value of bone marrow involvement on progression-free survival (PFS) assessed through histologic evaluation and centralized PET review.

### Methods

Untreated stage II–IV FL patients with intermediate–high FLIPI2 risk requiring therapy enrolled in the FIL FOLL12 trial were included. Patients were balanced according to treatment received and arm. BMBs were assessed by morphology and immunohistochemistry at recruiting centers. PET/CT scans were reviewed by two expert nuclear medicine physicians. Pathological bone uptake (PETb+) was defined as mono- or multifocal, or diffuse FDG uptake in bone segments higher than liver activity (SUVmax).

**Copyright Information:** (Online) ISSN: 2572-9241
©2026 The Author(s). HemaSphere is published by John Wiley & Sons Ltd on behalf of European Hematology Association. This is an open access Abstract Book distributed under the Attribution-NonCommercial-NoDerivs (CC BY-NC-ND), which allows third parties to download the articles and share them with others as long as they credit the author and the Abstract Book, but they cannot change the content in any way or use them commercially.
**Abstract Book Citations:** Authors, Title, HemaSphere, 2026;10:(S1):pages. The abstract book DOIs can be found on the "Author Information" page.
**Disclaimer:** Articles published in the journal HemaSphere exclusively reflect the opinions of the authors. The authors are responsible for all content in their abstracts including accuracy of the facts, statements, citing resources, etc.

### Results

This report includes 179 patients; no significant differences in disease characteristics were observed between the revised cohort and the remaining patients enrolled in the trial. High-risk FLIPI2 was noted in 35.4%, and β2-microglobulin was increased in 53% of patients. BMB involvement was present in 58.5% (median infiltration 30%, range 5–95%). PETb was positive in 38 cases (21%), including 14% with focal or multifocal involvement and 7% with diffuse increased uptake (mean SUVMax L4 2.92 vs Mean SUV Liver 2.13). Concordant results between BMB and PETb were observed in 53.6%: among 103 BMB+ patients, 28 were PETb+ (29%); 18 with focal uptake and 10 with diffuse uptake. PETb identified BM involvement in 8 cases missed by BMB, 3 with diffuse uptake and 7 with multifocal uptake,. While BMB+ was not associated with a different risk of PFS compared to BMB-, we observed a significant impact of PETb+ on PFS: PETb+ had 41% 5-year PFS (CI 25-72, p=0.003), while BMB+/PETb- showed 74% (CI 62-83, p=0.27) and BMB-/PETb- 66%(CI 52-72, Figure 1). Focal/multifocal or diffuse increased uptake pattern retained equally prognostic relevance.

We then calculated FLIPI2 and PRIMA-PI using either conventional BMB or PETb to define BM involvement. The agreement between BMB and PETb scores was 87% for FLIPI2 and 67% for PRIMA-PI and the main disagreement was due to reallocation to lower risk groups, FLIPI2 0-2 patients raised from 65% to 74.3% according to FLIPI2-PET. FLIPI2 was confirmed prognostic for PFS and its c-Harrel increased from 0.582 to 0.602 when BMB was substituted with PETb (Table 1), with a 52% (CI 39-64) 5-Year PFS for conventional high risk FLIPI2 shifting to 41% (CI26-56) for high risk FLIPI2-PET. PETb improved performance also for PRIMA-PI (c-Harrell 0.55 vs 0.53) but still inferior to FLIPI2-PET (Table 1).

### Summary/Conclusion

Although BMB detects marrow involvement in most patients, this does not uniformly translate into adverse prognosis. PETb, while less sensitive than BMB, identifies metabolically active and prognostically relevant bone disease. A FDG-PET-based assessment of bone involvement could refine risk stratification in FL and spare BMB in most patients. Extended cohort results will be presented.

**Table 1**

| Score | HR for High risk | CI | c-Harrell |
|---|---|---|---|
| FLIPI2 | 2.03 | 1.25-3.31 | 0.582 |
| FLIPI2-PET | 2.58 | 1.57-4.26 | 0.602 |
| PRIMA-PI | 1.23 | 0.67-2.23 | 0.537 |
| PRIMA-PI-PET | 1.47 | 0.85-2.54 | 0.550 |

**Copyright Information:** (Online) ISSN: 2572-9241
©2026 The Author(s). HemaSphere is published by John Wiley & Sons Ltd on behalf of European Hematology Association. This is an open access Abstract Book distributed under the Attribution-NonCommercial-NoDerivs (CC BY-NC-ND), which allows third parties to download the articles and share them with others as long as they credit the author and the Abstract Book, but they cannot change the content in any way or use them commercially.
**Abstract Book Citations:** Authors, Title, HemaSphere, 2026;10:(S1):pages. The abstract book DOIs can be found on the "Author Information" page.
**Disclaimer:** Articles published in the journal HemaSphere exclusively reflect the opinions of the authors. The authors are responsible for all content in their abstracts including accuracy of the facts, statements, citing resources, etc.

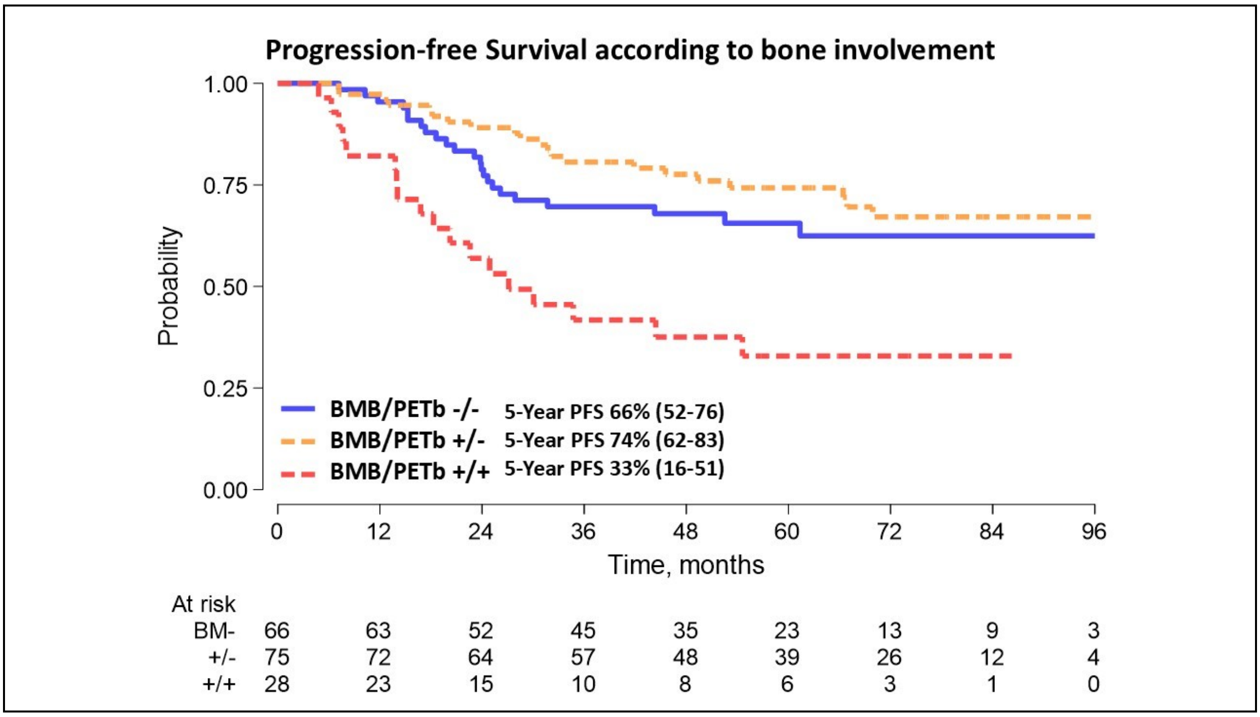

Progression-free survival according to Bone Marrow involvement. Patients which are PETbone+ (BMB+/PETb+) had inferior PFS, while no difference was observed compared to BMB- for BMB+ with no bone FDG pathological uptake.

**Copyright Information:** (Online) ISSN: 2572-9241
©2026 The Author(s). HemaSphere is published by John Wiley & Sons Ltd on behalf of European Hematology Association. This is an open access Abstract Book distributed under the Attribution-NonCommercial-NoDerivs (CC BY-NC-ND), which allows third parties to download the articles and share them with others as long as they credit the author and the Abstract Book, but they cannot change the content in any way or use them commercially.
**Abstract Book Citations:** Authors, Title, HemaSphere, 2026;10:(S1):pages. The abstract book DOIs can be found on the “Author Information” page.
**Disclaimer:** Articles published in the journal HemaSphere exclusively reflect the opinions of the authors. The authors are responsible for all content in their abstracts including accuracy of the facts, statements, citing resources, etc.

### (PF937) RITUXIMAB (R), GOLCADOMIDE (GOLCA) +/- NIVOLUMAB (N) IN TREATMENT-NAIVE FOLLICULAR LYMPHOMA (FL). PRIMARY RESULTS OF THE INVESTIGATOR-LED RANDOMISED PHASE II TOP-FLOR STUDY.

**Topic:** 18. Indolent and mantle-cell non-Hodgkin lymphoma - Clinical

Allison Barraclough[1], Geoff Chong[2, 3], Arina Martynchyk[2, 4], Mridula Mokoonlall[5], Tamara Marconi[6], Jodie B. Palmer[4, 7], Colm Keane[8], Sze Ting Lee[2, 4, 7, 9], Andrew Scott[2, 4, 7, 9], Alexandra Romano[4, 7], Leonid Churilov[9], Denise Lee[2], Melinda Burgess[8], Rebecca Nightingale[4, 7], Andrea Henden[10], Eliza Hawkes*[2, 4, 7, 11]

*[1]Fiona Stanley Hospital, Perth, Australia; [2]Austin Health, Heidelberg, Australia; [3]Grampians Health, Ballarat, Australia; [4]Olivia Newton-John Cancer Research Institute, Heidelberg, Australia; [5]Barwon Health, Geelong, Australia; [6]Eastern Health, Box Hill, Australia; [7]La Trobe University, Bundoora, Australia; [8]University of Queensland, Brisbane, Australia; [9]University of Melbourne, Melbourne, Australia; [10]QIMR Berghofer, Brisbane, Australia; [11]Monash University, Melbourne, Australia;*

### Background

Standard R-lenalidomide (R2) or chemoimmunotherapy is effective in treatment-naïve FL but is associated with Grade 3-4 Adverse Event (AE) rates >65%.  Golca is a potent first-in-class oral Cereblon E3 Ligase Modulator (CELMoD) with dual immunomodulatory and anti-tumor activity 10- to 100-fold enhanced compared to lenalidomide, preferential lymphoid organ distribution and a favourable toxicity profile (Barraclough 2025, Chavez 2024). R-Golca yields responses in 93% of relapsed FL.  We have demonstrated PD1/PDL1i efficacy with R-nivo, and atezolizumab-obinutuzumab-radiotherapy in prior FL studies (Chong 2024; Barraclough 2025).
We report the primary endpoint analysis of our randomised phase II study of R-Golca +/- N in stage II-IV, grade 1-3A treatment-naive FL.

### Aims

To assess efficacy and safety of R-Golca +/-N in treatment-naive FL. Primary endpoint: complete metabolic response rate on FDG PET in the absence of prohibitive toxicity requiring cessation of induction therapy.

### Methods

TOP-FLOR (NCT05788081) is an umbrella randomised Bayesian optimal phase II open label in 1L FL. All patients provided written informed consent prior to commening on study. 40pts were randomised 1:1 to receive 8 cycles of induction R (375 mg/m2 IV,  D1), Golca (0.4mg p.o daily,D1-14) +/- N (480mg IV, D1) Q4W and 2 yrs of R maintenance (Q12W). The primary endpoint was Complete metabolic response rate (CRR) in the absence prohibitive toxicity (CTCAE V5.0 [Grade 3 or 4]) causing cessation of induction, with a predefined futility threshold of 12/20 CRR (60%) in each arm. Arms were not formally compared but randomised for patient equity.

### Results

40 pts were enrolled Median age was 67.7y (range 23-81). 61% were male. CRR was 82.5% (33/40; 95%CI: 68%, 91.3%); 17/20 (85%) for R-Golca and 16/20 (80%) for R-Golca+N; 2 patients permanently ceased induction therapy due to infection (1 Gr4 sepsis cycle 1, [unrelated-commenced prior to D1], 1 Gr 4 COVID-19 in cycle 7);  3 due to progressive disease (**Figure 1**). Most frequent treatment-related AEs were infection (75%, G3+ 20%), neutropenia (58%; all G3+; 73% received G-CSF), maculo-papular rash (25%; G3<1%) and constipation (35%, G3+ <1%). Dose reductions to 0.3mg occurred in 11 patients (6 R-Golca+N; 5 R-Golca) . With a median follow up of 21 months at data cut-off (range 0.7-28.7), median progression free and overall survival have not been reached. To date there have been no deaths on study.

**Copyright Information:** (Online) ISSN: 2572-9241
©2026 The Author(s). HemaSphere is published by John Wiley & Sons Ltd on behalf of European Hematology Association. This is an open access Abstract Book distributed under the Attribution-NonCommercial-NoDerivs (CC BY-NC-ND), which allows third parties to download the articles and share them with others as long as they credit the author and the Abstract Book, but they cannot change the content in any way or use them commercially.
**Abstract Book Citations:** Authors, Title, HemaSphere, 2026;10:(S1):pages. The abstract book DOIs can be found on the "Author Information" page.
**Disclaimer:** Articles published in the journal HemaSphere exclusively reflect the opinions of the authors. The authors are responsible for all content in their abstracts including accuracy of the facts, statements, citing resources, etc.

**Summary/Conclusion**

Rituximab-golcadomide +/-nivolumab is highly effective as 1L therapy for FL with the primary endpoint of this study met in both arms. Toxicity was manageable, most commonly neutropenia that responds to GCSF and low-grade infection. The study continues follow up with a phase III study planned.

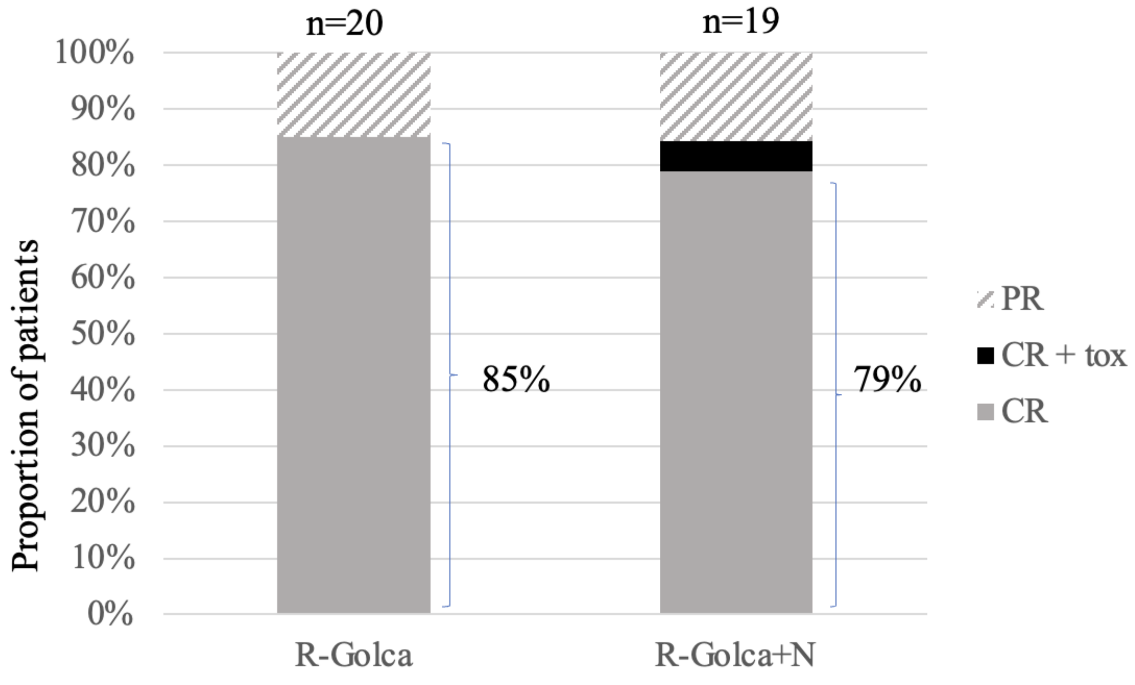

**Figure 1. Best overall response according to lugano in PET/CT evaluable patients during induction treatment with R-Golca+/- Nivo.** PR – partial response, CR-complete response, CR + tox – complete response, toxicity resulting in discontinuation.

**Copyright Information:** (Online) ISSN: 2572-9241
©2026 The Author(s). HemaSphere is published by John Wiley & Sons Ltd on behalf of European Hematology Association. This is an open access Abstract Book distributed under the Attribution-NonCommercial-NoDerivs (CC BY-NC-ND), which allows third parties to download the articles and share them with others as long as they credit the author and the Abstract Book, but they cannot change the content in any way or use them commercially.
**Abstract Book Citations:** Authors, Title, HemaSphere, 2026;10:(S1):pages. The abstract book DOIs can be found on the "Author Information" page.
**Disclaimer:** Articles published in the journal HemaSphere exclusively reflect the opinions of the authors. The authors are responsible for all content in their abstracts including accuracy of the facts, statements, citing resources, etc.

### (PF938) EO2463 (EO) AN OFF-THE-SHELF MULTI-TARGET PEPTIDE IMMUNOTHERAPY: IN VIVO CD8 T CELL EXPANSION KINETICS CORRELATES WITH EFFICACY IN PATIENTS (PTS) WITH FOLLICULAR (FL) AND MARGINAL ZONE (MZL) LYMPHOMA

**Topic:** 18. Indolent and mantle-cell non-Hodgkin lymphoma - Clinical

Jose C. Villasboas*[1], Danielle Wallace[2], Stephen Smith[3], Reid Merryman[4], Francesc Bosch[5], Carlos Grande García[6], Antonio Pinto[7], Luca Arcaini[8, 9], Jean Pierre Marolleau[10], Norma Gutierrez Gutierrez[11], Carla Casulo[2], Brian Till[3], Cristina García Hercé[5], Maria Rocio Figueroa Mora[6], Sara Rattotti[9], Pierre Morel[10], Philippe Rinaudo[12], Jan Fagerberg[12], Jean-Marie Carpier[12], Pier Luigi Zinzani[13], Philippe Armand[4], Stephen Ansell[1]

*[1]Mayo Clinic, Rochester, MN, United States of America;[2]University of Rochester, Rochester, NY, United States of America; [3]Fred Hutchinson Cancer Center, University of Washington, Seattle, WA, United States of America;[4]Dana-Farber Cancer Institute, Boston, MA, United States of America; [5]University Hospital Vall d'Hebron, Barcelona, Spain; [6]Universidad de Navarra, Pamplona, Spain; [7]Fondazione 'G. Pascale', IRCCS, Naples, Italy; [8]Department of Molecular Medicine, University of Pavia, Pavia, Italy; [9]Division of Hematology, Fondazione IRCCS Policlinico San Matteo, Pavia, Italy; [10]Centre Hospitalier Universitaire Amiens-Picardie, Amiens, France; [11]Hospital Universitario De Salamanca, IBSAL, Salamanca, Spain; [12]Enterome, Paris, France; [13]University of Bologna, Bologna, Italy;*

### Background

EO was designed to expand in vivo pre-existing memory CD8 T cells recognizing non-self-proteins from gut bacteria cross-reacting with 4 B cell antigens (BCA). EO includes 4 synthetic HLA-A2 CD8-epitopes with mimicry to CD20, CD22, CD37, and BAFF-receptor, and a CD4 epitope UCP2.

### Aims

Assess immune data and correlations with clinical efficacy in cohorts (C) of trial EONHL1-20/SIDNEY.

### Methods

EO [induction 4xq2 weekly (w), boost q4w] was given SC with Montanide adjuvant. Pts with relapsed FL/MZL in C1 (safety lead-in, n=9) and C4 (extension, n=14) received EO max 15 doses + lenalidomide (from w7 C1/ w1 C4) + rituximab (R) (from w19). Untreated pts with no indication for treatment in C2 (watch-and-wait; n=25) received EO mono max 12 doses. Pts with low tumor burden in C3 (n=6) received EO max 12 doses + R (from w7). Expansion of specific CD8 T cells was assessed on thawed PBMC via flow cytometry using tetramers (EO-mimic or BCA specific) and by IFN-γ ELISPOT, either ex vivo or after in vitro expansion.

**Copyright Information:** (Online) ISSN: 2572-9241
©2026 The Author(s). HemaSphere is published by John Wiley & Sons Ltd on behalf of European Hematology Association. This is an open access Abstract Book distributed under the Attribution-NonCommercial-NoDerivs (CC BY-NC-ND), which allows third parties to download the articles and share them with others as long as they credit the author and the Abstract Book, but they cannot change the content in any way or use them commercially.
**Abstract Book Citations:** Authors, Title, HemaSphere, 2026;10:(S1):pages. The abstract book DOIs can be found on the "Author Information" page.
**Disclaimer:** Articles published in the journal HemaSphere exclusively reflect the opinions of the authors. The authors are responsible for all content in their abstracts including accuracy of the facts, statements, citing resources, etc.

**Results**

Across all cohorts (48/54 pts tested), EO induced EO-mimic and BCA-specific CD8 T cell expansion in 90% and 81% of pts. EO-responses plateaued early (w7-12) in 76% EO- and 73% BCA-responders. Relative frequencies (median max % of total CD8 T cells at any time) were 0.52% for EO-mimic and 0.35% for BCA-specific CD8 T cells. Durable EO-mimic and BCA-specific responses are detectable up to 34 months (mo) after last EO-admin. At response-peak, EO-specific CD8 T cells displayed predominantly an effector-memory phenotype suggesting rapid pro-inflammatory features and cytotoxic potential which was confirmed by in-vitro killing of loaded T2 cells and B cell lines. Anti-CD3 and pathogen-derived epitope (CEFSX peptide pool) responses showed no correlation with EO-responses, indicating EO-expansion of specific CD8 T cells rather than global T cell activation. Max expansion % (at EO-induction) of BCA-specific CD8 T cells in C1+4 was higher for pts with Lugano complete response (CR) (n=11) than for patients without CR (n=9) (Mann-Whitney p=0.04). Likewise, in C2 max expansion was higher for pts with Lugano CR/partial response (PR) (n=9) than for pts without CR/PR (n=12) (p=0.04). No correlation was found between CEFSX response vs clinical response (n=12). A binary biomarker (pos/neg EO-induction expansion of EO-specific CD8 T cells) was tested in C1+4 (n=18). After median follow-up 15.7 mo, median progression-free survival (PFS) for C1+4 (n=23) was 18.5 mo. PFS for biomarker pos pts (19.3 mo) was longer than for biomarker neg pts (14.7 mo) [log-rank p=0.03; HR=0.16 (95% CIs 0.03-1.03)] (Fig). Data on total CD8 T cell expansion at week 7 (compared to baseline) assessed via local clinical lab was available in 15 pts from C1+4. Pts with early CD8 T cell expansion (n=9; 18-237% increase; median PFS 36.6 mo) had longer PFS [log-rank p=0.03; HR=0.13 (95% CIs 0.01-1.16)] vs pts with no expansion (n=6; median PFS 14.7 mo). Biomarker pos pts were numerically enriched in the group with early CD8 expansion (8/9) vs without expansion (2/6), suggesting a potential association between biomarker pos and early CD8 expansion. Individual pts responses to CEFSX were stable over time. PFS is currently too immature in C2 for analyses.

**Summary/Conclusion**

EO2463 rapidly induces extensive in vivo expansion of target-specific CD8 T cells that are both long-lasting and functional. EO2463 induced expansion is associated with clinical outcomes and has the potential to be developed into a predictive biomarker.

**Copyright Information:** (Online) ISSN: 2572-9241
©2026 The Author(s). HemaSphere is published by John Wiley & Sons Ltd on behalf of European Hematology Association. This is an open access Abstract Book distributed under the Attribution-NonCommercial-NoDerivs (CC BY-NC-ND), which allows third parties to download the articles and share them with others as long as they credit the author and the Abstract Book, but they cannot change the content in any way or use them commercially.
**Abstract Book Citations:** Authors, Title, HemaSphere, 2026;10:(S1):pages. The abstract book DOIs can be found on the “Author Information” page.
**Disclaimer:** Articles published in the journal HemaSphere exclusively reflect the opinions of the authors. The authors are responsible for all content in their abstracts including accuracy of the facts, statements, citing resources, etc.

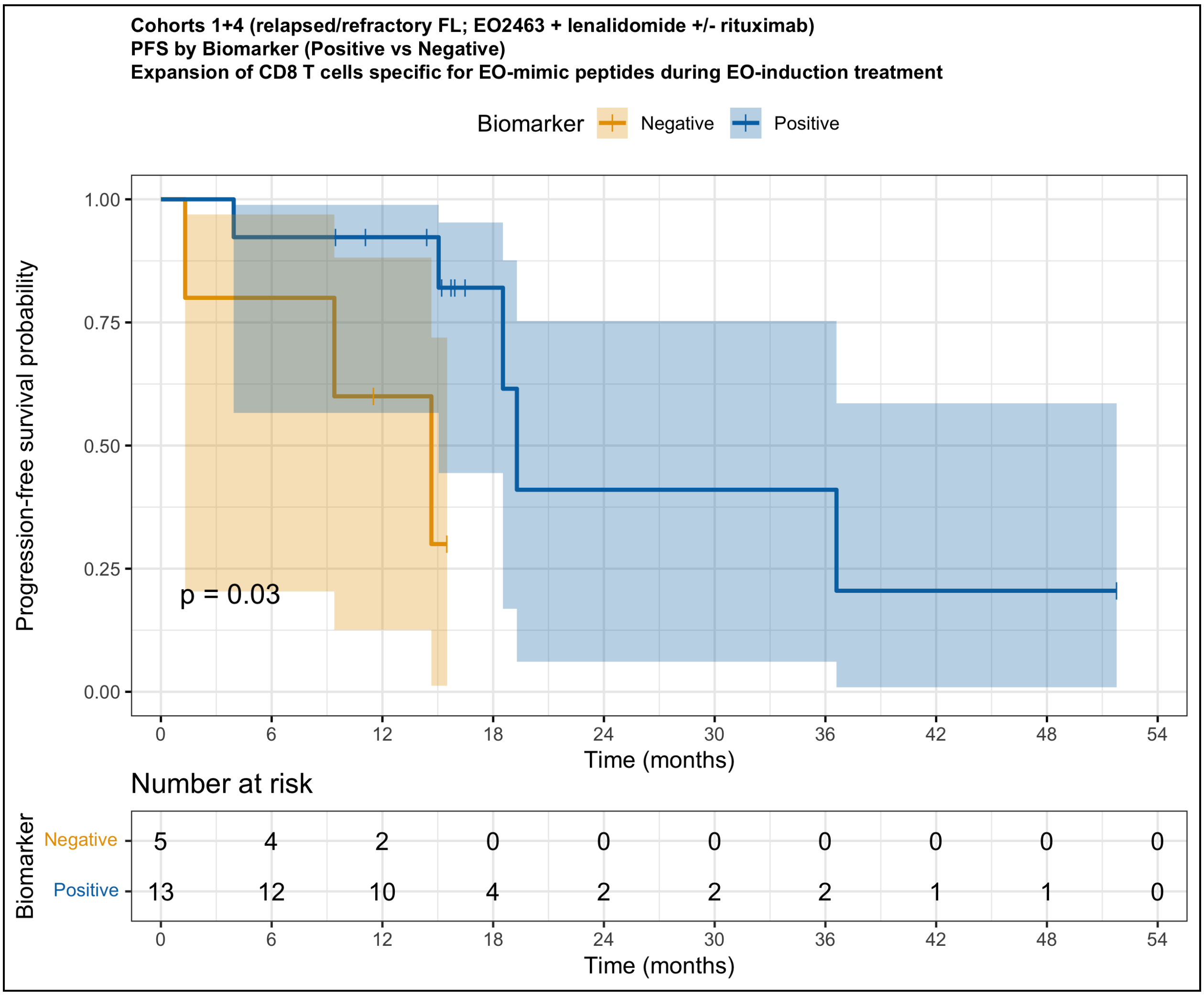

**Copyright Information:** (Online) ISSN: 2572-9241
©2026 The Author(s). HemaSphere is published by John Wiley & Sons Ltd on behalf of European Hematology Association. This is an open access Abstract Book distributed under the Attribution-NonCommercial-NoDerivs (CC BY-NC-ND), which allows third parties to download the articles and share them with others as long as they credit the author and the Abstract Book, but they cannot change the content in any way or use them commercially.
**Abstract Book Citations:** Authors, Title, HemaSphere, 2026;10:(S1):pages. The abstract book DOIs can be found on the "Author Information" page.
**Disclaimer:** Articles published in the journal HemaSphere exclusively reflect the opinions of the authors. The authors are responsible for all content in their abstracts including accuracy of the facts, statements, citing resources, etc.

### (PF939) A MULTICENTER, PHASE II STUDY OF ORELABRUTINIB-LENALIDOMIDE-RITUXIMAB IN PATIENTS WITH UNTREATED MANTLE CELL LYMPHOMA (POLARIS): EFFICACY, SAFETY, AND BIOMARKER ANALYSES

**Topic:** 18. Indolent and mantle-cell non-Hodgkin lymphoma - Clinical

Xiaohui Wang*[1], Zheng Song[1], Huilai Zhang[1]

[1] *Tianjin Medical University Cancer Institute and Hospital, Tianjin, China;*

### Background

The management of mantle cell lymphoma (MCL) is evolving toward chemotherapy-free, targeted combination regimens. We evaluated the safety and efficacy of orelabrutinib combined with lenalidomide and rituximab (OLR) as frontline therapy for previously untreated MCL (ClinicalTrials.gov identifier: NCT05076097).

### Aims

The objective of this study was to evaluate the safety, efficacy, and depth of response of the chemotherapy-free combination of orelabrutinib, lenalidomide, and rituximab (OLR) as frontline therapy in patients with previously untreated mantle cell lymphoma. In addition, this study aimed to characterize longitudinal minimal residual disease dynamics and to explore genomic determinants of treatment response and resistance using high-sensitivity next-generation sequencing, thereby informing the potential of MRD-guided therapeutic strategies in this setting.

### Methods

This multicenter, phase II study evaluated six cycles of OLR induction followed by fixed-duration maintenance for up to 18 cycles. The primary endpoint was complete response rate (CRR) after induction. Secondary endpoints included safety, response outcomes, and survival. Longitudinal minimal residual disease (MRD) assessment and genomic profiling were performed using next-generation sequencing with a sensitivity of $10^{-6}$.

### Results

Twenty-nine patients were enrolled. The CRR after induction was 69.0%, and the best CRR was 89.7%. At a median follow-up of 29.7 months, the median duration of response (DoR), progression-free survival (PFS), and overall survival (OS) were not reached; the 24-month DoR and PFS rates were both 86.1%, with a 100% OS rate. Grade 3–4 hematologic toxicities included neutropenia (62.1%), lymphopenia (17.2%), thrombocytopenia (6.9%), and anemia (10.3%). Nonhematologic toxicities were infrequent and included rash (6.9%), COVID-19 infection (6.9%), pulmonary infection (6.9%), and upper respiratory tract infection (6.9%). Genomic analyses demonstrated enrichment of chromatin remodeling alterations in nonresponders compared with responders (100% vs 47.1%; P = 0.009), including *SETD2* (33.3% vs 0%; P = 0.032) and *SMARCA4* (44.4% vs 5.9%; P = 0.034). Rates of undetectable MRD increased from 64.3% at cycle 3 to 95.5% at the end of treatment.

### Summary/Conclusion

The chemotherapy-free OLR triplet demonstrates high activity and favorable tolerability as frontline therapy for MCL, achieving durable clinical responses and deep molecular remissions. Integrated genomic and ctDNA profiling provides insights into resistance mechanisms and response dynamics, supporting further development of MRD-guided therapeutic strategies.

**Copyright Information:** (Online) ISSN: 2572-9241
©2026 The Author(s). HemaSphere is published by John Wiley & Sons Ltd on behalf of European Hematology Association. This is an open access Abstract Book distributed under the Attribution-NonCommercial-NoDerivs (CC BY-NC-ND), which allows third parties to download the articles and share them with others as long as they credit the author and the Abstract Book, but they cannot change the content in any way or use them commercially.
**Abstract Book Citations:** Authors, Title, HemaSphere, 2026;10:(S1):pages. The abstract book DOIs can be found on the "Author Information" page.
**Disclaimer:** Articles published in the journal HemaSphere exclusively reflect the opinions of the authors. The authors are responsible for all content in their abstracts including accuracy of the facts, statements, citing resources, etc.

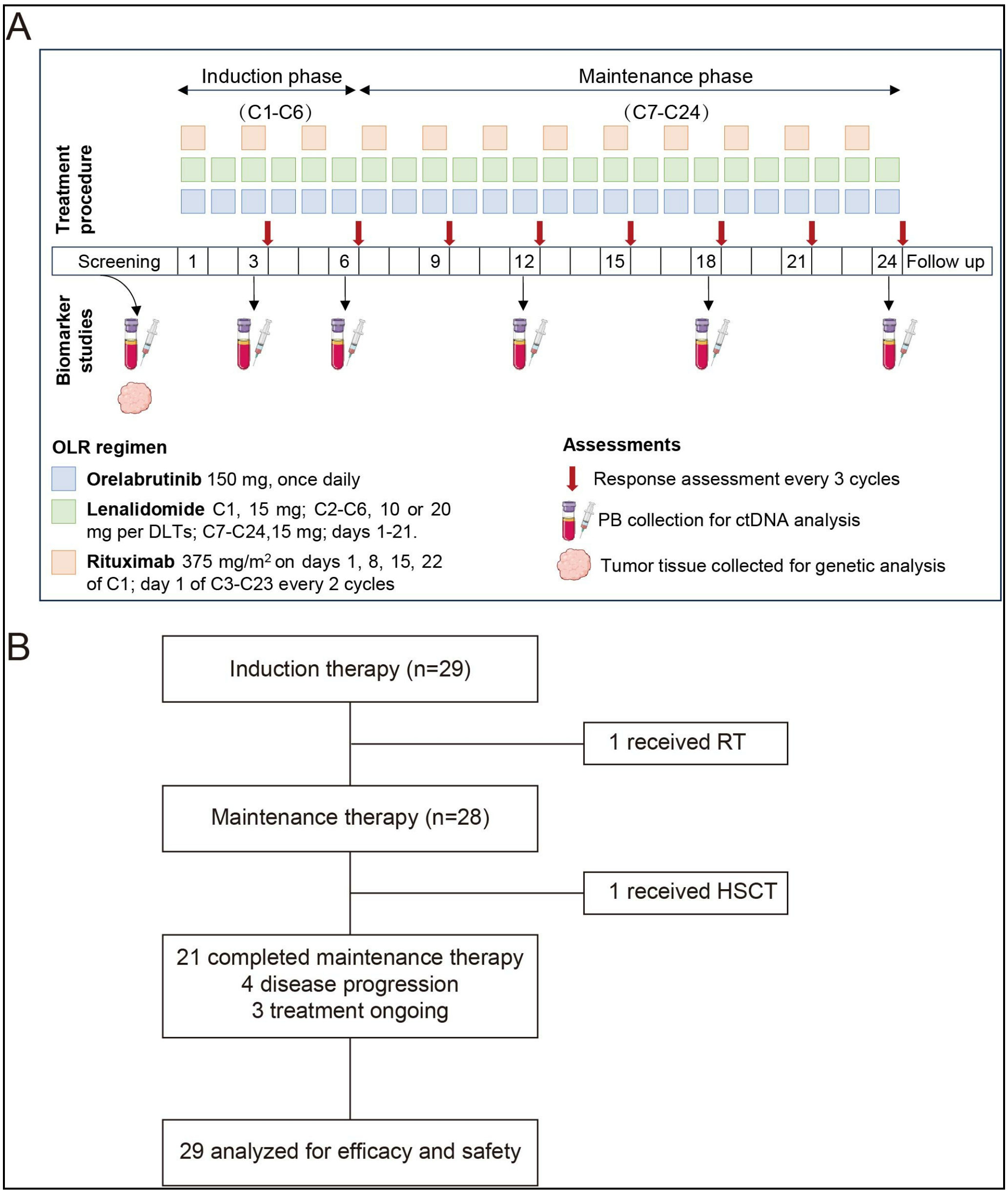

Copyright Information: (Online) ISSN: 2572-9241
©2026 The Author(s). HemaSphere is published by John Wiley & Sons Ltd on behalf of European Hematology Association. This is an open access Abstract Book distributed under the Attribution-NonCommercial-NoDerivs (CC BY-NC-ND), which allows third parties to download the articles and share them with others as long as they credit the author and the Abstract Book, but they cannot change the content in any way or use them commercially.
Abstract Book Citations: Authors, Title, HemaSphere, 2026;10:(S1):pages. The abstract book DOIs can be found on the "Author Information" page.
Disclaimer: Articles published in the journal HemaSphere exclusively reflect the opinions of the authors. The authors are responsible for all content in their abstracts including accuracy of the facts, statements, citing resources, etc.

### (PF940) CLONAL FEATURES AND MOLECULAR PREDICTORS OF MRD NEGATIVITY ATTAINMENT IN WALDENSTROM'S MACROGLOBULINEMIA: CLINICAL IMPLICATIONS FOR THERAPEUTIC DE-ESCALATION

**Topic:** 18. Indolent and mantle-cell non-Hodgkin lymphoma - Clinical

Anastasia Vinnikova*[1], Irina Galtseva[1], Alexander Grachev[1], Kseniya Nikiforova[1], Yulia Tsoy[1], Kulikov Sergey[1], Sofia Starchenko[1], Elena Nikulina[1], Maryina Saliya[1], Valentina Dvirnik[1], Elena Gribanova[1], Andrey Sudarikov[1], Evgeniy Zvonkov[1], Vera Troitskaya[1], Elena Parovichnikova[1]

[1]*National Medical Research Center for Hematology, Moscow, Russia;*

### Background

Waldenström's macroglobulinemia (WM) is characterized by a distinctive bone marrow infiltration of a lymphoplasmacytic clone. Despite improved outcomes with chemoimmunotherapy and BTK inhibitors, the role of Rituximab maintenance remains under discussion. There is a clinical need to identify patients who achieve rapid, deep clonal eradication and might benefit from treatment de-escalation, thereby avoiding the toxicities and costs associated with prolonged maintenance therapy. There is a lack of clear data on the clinical significance of MRD monitoring in Waldenström macroglobulinemia and of the prognostic impact of the *MYD88 (L265P)* mutation on it.

### Aims

To characterize the depletion kinetics of aberrant B-cell and PC populations using multicolor flow cytometry (MFC) and to evaluate their correlation with *MYD88* status via Allele-Specific Polymerase Chain Reaction (AS-PCR) and laboratory parameters,progression rate.

### Methods

A cohort of 70 patients (median age 57; treated 2018–2025) was analyzed via multicolor MFC and AS-PCR for*MYD88*. MRD status was categorized based on the persistence of B-and PC populations (MRD -/-, -/+, +/-, +/+) and integrated with *MYD88* positive/negative status.

### Results

Induction therapy led to a rapid elimination of the aberrant B-cell compartment within the first month.The reduction of aberrant PC was significantly more gradual. Baseline PC counts strongly correlated with initial monoclonal IgM levels. The most significant decrease in IgM concentration was observed one month post-induction. Patients MRD -/- exhibited significantly lower baseline tumor burden (B-cell infiltration, serum IgM, β2-microglobulin) and higher hemoglobin/platelet levels compared to MRD+ groups. At one month, patients with persistent B- and PC populations maintained higher IgM levels than those with isolated PC persistence.
All *MYD88* wild-type patients achieved B-cell MRD negativity (MRD -/-, -/+) post-induction, whereas the mutation was associated with higher residual B-cell counts. The median MYD88 variant allele frequency (VAF) was higher in patients reaching MRD negativity 20.5% and 5.6% in MRD positivity. Moderate concordance was observed between MFC-based MRD monitoring and *MYD88* AS-PCR results. Among 14 patients reaching MRD negativity by MFC, 12 also demonstrated molecular negativity via AS-PCR. Conversely, in 37 patients with any persistent MRD by MFC, 20 achieved molecular negativity by AS-PCR. The *MYD88* mutation frequently persisted in the MRD -/+ subgroup (PCs only), suggesting either varying sensitivity between diagnostic methods or distinct elimination rates of B-cell and PC compartments.
The 3-year progression rate was 10% for *MYD88* wild-type vs. 25% for mutation carriers. The MRD +/+ group reached one-fifth of patients by the fourth year, while no progression was observed in the MRD -/- group.

**Copyright Information:** (Online) ISSN: 2572-9241
©2026 The Author(s). HemaSphere is published by John Wiley & Sons Ltd on behalf of European Hematology Association. This is an open access Abstract Book distributed under the Attribution-NonCommercial-NoDerivs (CC BY-NC-ND), which allows third parties to download the articles and share them with others as long as they credit the author and the Abstract Book, but they cannot change the content in any way or use them commercially.
**Abstract Book Citations:** Authors, Title, HemaSphere, 2026;10:(S1):pages. The abstract book DOIs can be found on the “Author Information” page.
**Disclaimer:** Articles published in the journal HemaSphere exclusively reflect the opinions of the authors. The authors are responsible for all content in their abstracts including accuracy of the facts, statements, citing resources, etc.

**Summary/Conclusion**

The near-total clearance of the B-cell clone following induction therapy, coupled with the absence of progression in MRD-negative patients, suggests that prolonged Rituximab maintenance may be unnecessary for this subgroup. Integrated monitoring of both B-cell and PC compartments provides a superior framework for personalized treatment de-escalation. Furthermore, the persistence of *MYD88* mutations specifically in residual PC population highlights the complexity of clonal populations and supports a robust MRD-driven approach to post-induction management in WM.

*This work builds upon and extends preliminary observations previously reported by our group.*

**Copyright Information:** (Online) ISSN: 2572-9241
©2026 The Author(s). HemaSphere is published by John Wiley & Sons Ltd on behalf of European Hematology Association. This is an open access Abstract Book distributed under the Attribution-NonCommercial-NoDerivs (CC BY-NC-ND), which allows third parties to download the articles and share them with others as long as they credit the author and the Abstract Book, but they cannot change the content in any way or use them commercially.
**Abstract Book Citations:** Authors, Title, HemaSphere, 2026;10:(S1):pages. The abstract book DOIs can be found on the "Author Information" page.
**Disclaimer:** Articles published in the journal HemaSphere exclusively reflect the opinions of the authors. The authors are responsible for all content in their abstracts including accuracy of the facts, statements, citing resources, etc.

### (PF941) IMPROVED CAUSE-SPECIFIC SURVIVAL FOR WALDENSTROM MACROGLOBULINEMIA IN THE ERA OF NOVEL THERAPIES

**Topic:** 18. Indolent and mantle-cell non-Hodgkin lymphoma - Clinical

Muhammad Azeem Khan*[1], Gunjan Gaur[1], Jithma Abeykoon[1], Morie Gertz[1], Shaji Kumar[1], Rebecca King[1], Jonas Paludo[1], Thomas Habermann[1], Ronald Go[1], Joselle Cook[1], Nadine Abdallah[1], Gita Thanarajasingam[1], Francis Buadi[1], Angela Dispenzieri[1], Thompson Thompson[1], Thomas Witzig[1], Wilson Gonsalves[1], Grzegorz Nowakowski[1], David Dingli[1], Vincent Rajkumar[1], Eli Muchtar[1], Surendra Dasari[1], Joseph Novak[1], Patricia Greipp[1], Rahma Warsame[1], Stephen Ansell[1], Prashant Kapoor[1], Saurabh Zanwar[1]

[1]*Mayo Clinic, Rochester, United States of America;*

### Background

Waldenström macroglobulinemia (WM) is a lymphoplasmacytic lymphoma that often presents asymptomatically, with treatment initiated upon symptom development. Treatment options have expanded with the advent of chemoimmunotherapy and Bruton tyrosine kinase inhibitors (BTKi).

### Aims

We aimed to assess whether treatment advances have improved survival in those with active WM (AD).

### Methods

We conducted a single center retrospective study of patients diagnosed with WM at Mayo Clinic, Rochester, MN, between 01/01/1996 and 12/31/2018. AD was defined by symptoms or organ dysfunction attributable to WM. Patients were grouped into time periods in which AD was diagnosed. An analysis comparing characteristics and survival between time periods between 1996-2005 and post-2006 as well as pre-2001, 2001–2007, 2008–2013, and post-2013 was done. Categorical variables were compared using the Chi-square test or Fisher's exact test. Overall survival (OS) and cause specific survival (CSS) was evaluated using the Kaplan–Meier method with group comparisons by log-rank test. Competing risk analysis was performed to account for non–disease-related deaths.

### Results

A total of 1020 patients were included with baseline characteristics outlined in Table 1. Median follow-up was 20.5 and 11.3 years for the 1996–2005 and post-2006 cohorts, respectively ($p < 0.0001$), and 21.3, 20.0, 13.1, and 9.2 years for the pre-2001, 2001–2007, 2008–2013, and post-2013 cohorts ($p < 0.0001$).
Patients treated in more recent eras were older, with a higher proportion aged ≥65 years ($p<0.01$), while other baseline characteristics were comparable. Frontline therapy evolved, with rituximab use increasing from 15% pre-2001 to >70% after 2008 ($p<0.01$), and BTKi used exclusively post-2013 ($p<0.01$).
OS was comparable between the earlier and later time frames, whether divided as 1996–2005 (10.9y, 95% CI 9.8-12.1) and post-2006 (11.9y, CI 10.6-13.5, p=0.64), or as pre-2001 (9.9y, CI 8.9-11.6), 2001–2007 (12y, CI 10.8-13.6) 2008–2013 (11.7y, CI 10.2-13.6) and post-2013 (survival not reached, p=0.86).
CSS improved significantly over successive time periods. In the 2006–2017 cohort versus 1996–2005, CSS was 18.9 versus 15.6 years ($p< 0.0001$). Similarly, CSS increased across the pre-2001, 2001–2007, 2008–2013, and post-2013 cohorts (13.7, 18.9, survival not reached for latter 2 groups, $p < 0.0001$, figure1a). In the four-era cohort, 10-year CSS improved over time: 61% (95% CI 52–70%) pre-2001, 69% (64–76%) in 2001–2007, 74% (69–79%) in 2008–2013, and 82% (77–88%) post-2013. A competing risk analysis

**Copyright Information:** (Online) ISSN: 2572-9241
©2026 The Author(s). HemaSphere is published by John Wiley & Sons Ltd on behalf of European Hematology Association. This is an open access Abstract Book distributed under the Attribution-NonCommercial-NoDerivs (CC BY-NC-ND), which allows third parties to download the articles and share them with others as long as they credit the author and the Abstract Book, but they cannot change the content in any way or use them commercially.
**Abstract Book Citations:** Authors, Title, HemaSphere, 2026;10:(S1):pages. The abstract book DOIs can be found on the "Author Information" page.
**Disclaimer:** Articles published in the journal HemaSphere exclusively reflect the opinions of the authors. The authors are responsible for all content in their abstracts including accuracy of the facts, statements, citing resources, etc.

of deaths from WM versus other causes confirmed a significant improvement in WM-related survival across both split-time cohorts (p < 0.001, figure1b).

Table1.

| | Pre-2001 | 2001 – 2007 | 2008-2013 | Post-2013 | P-Value |
|---|---|---|---|---|---|
| Age ≥ 65 years (%) | 44 | 48 | 57 | 56 | **<0.01** |
| Median age | 64.3 | 64.1 | 66.5 | 66.7 | **0.005** |
| Beta-2-microglobulin > 3μg/dL (%) | 49 | 55 | 57 | 53 | 0.77 |
| Lactate dehydrogenase > ULN (%) | 7 | 8 | 12 | 20 | 0.05 |
| Hemoglobin ≤11.5g/dL (%) | 77 | 69 | 69 | 71 | 0.52 |
| *MYD88*$^{L265P}$ (%) | 76 | 70 | 73 | 78 | 0.63 |
| IPSS-WM High Risk (%) | 57 | 65 | 56 | 50 | 0.41 |
| Amyloidosis at any point (%) | 5 | 8 | 7 | 8 | 0.85 |
| Transformed disease (%) | 6 | 8 | 4 | 4 | 0.25 |
| Rituximab front line treatment (%) | 15 | 59 | 73 | 72 | **<0.01** |
| BTK front line treatment (%) | 0 | 0 | 0 | 26 | **<0.01** |

### Summary/Conclusion

Over the past two decades, with the advent of novel therapies CSS for patients with WM has improved significantly, while OS has remained unchanged. This improvement in disease-specific outcomes appears to be driven by reductions in WM-attributed mortality, as confirmed by competing risk analysis.

**Copyright Information:** (Online) ISSN: 2572-9241
©2026 The Author(s). HemaSphere is published by John Wiley & Sons Ltd on behalf of European Hematology Association. This is an open access Abstract Book distributed under the Attribution-NonCommercial-NoDerivs (CC BY-NC-ND), which allows third parties to download the articles and share them with others as long as they credit the author and the Abstract Book, but they cannot change the content in any way or use them commercially.
**Abstract Book Citations:** Authors, Title, HemaSphere, 2026;10:(S1):pages. The abstract book DOIs can be found on the "Author Information" page.
**Disclaimer:** Articles published in the journal HemaSphere exclusively reflect the opinions of the authors. The authors are responsible for all content in their abstracts including accuracy of the facts, statements, citing resources, etc.

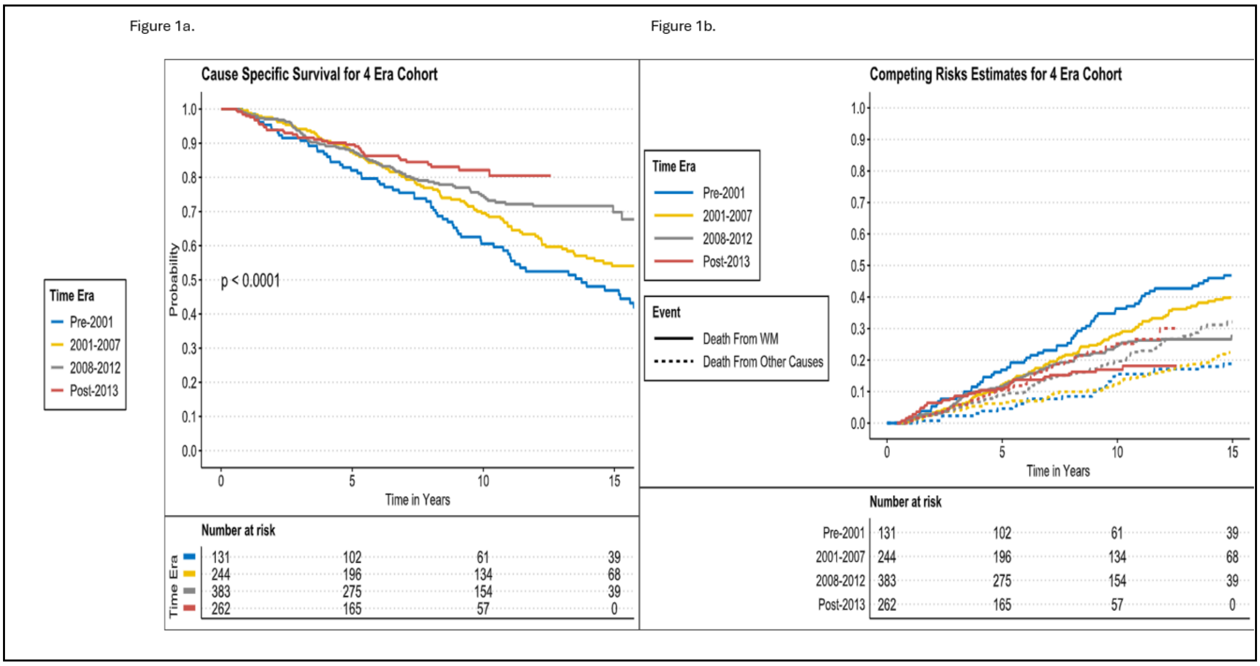

Figure1a. Cause specific survival curve of 4 era cohort. Figure1b. Competing risk analysis of 4 era cohort.

**Copyright Information:** (Online) ISSN: 2572-9241
©2026 The Author(s). HemaSphere is published by John Wiley & Sons Ltd on behalf of European Hematology Association. This is an open access Abstract Book distributed under the Attribution-NonCommercial-NoDerivs (CC BY-NC-ND), which allows third parties to download the articles and share them with others as long as they credit the author and the Abstract Book, but they cannot change the content in any way or use them commercially.
**Abstract Book Citations:** Authors, Title, HemaSphere, 2026;10:(S1):pages. The abstract book DOIs can be found on the “Author Information” page.
**Disclaimer:** Articles published in the journal HemaSphere exclusively reflect the opinions of the authors. The authors are responsible for all content in their abstracts including accuracy of the facts, statements, citing resources, etc.

### (PF942) FLP-R PROGNOSTIC INDEX IN RELAPSED/REFRACTORY MARGINAL ZONE LYMPHOMA: A COMPARATIVE ANALYSIS WITH MZL-IPI, MALT-IPI AND POD24

**Topic:** 18. Indolent and mantle-cell non-Hodgkin lymphoma - Clinical

Prokop Vodicka*[1], Andrea Janíková[2], David Belada[3], Juraj Ďuraš[4], Heidi Mociková[5], Ales Obr[6], Katerina Steinerova[7], Josef Karban[1], David Salek[2], Alice Sykorova[3], Michal Kascak[4], Pavel Klener[1], Samuel Hricko[2], Michal Masár[1], Jozef Michalka[2], Magdalena Klanova[1], Vít Campr[8], Zuzana Prouzova[9], Jitka Dlouhá[10], Petra Blahovcova[10], Marek Trněný[1]

*[1]First Faculty of Medicine, Charles University and General Hospital, First Department of Medicine, Prague, Czechia;[2]Faculty of Medicine, Masaryk University and University Hospital Brno, Department of Internal Medicine, Hematology and Oncology, Brno, Czechia; [3]Faculty of Medicine, Charles University and University Hospital Hradec Kralove, Fourth Department of Internal Medicine – Hematology, Hradec Kralove, Czechia; [4]Medical Faculty of the Ostrava University and University Hospital, Department of Hematology, Ostrava, Czechia; [5]Fakultni nemocnice Kralovske Vinohrady and Third Faculty of Medicine, Charles University, Department of Hematology, Prague, Czechia; [6]Faculty of Medicine and Dentistry, Palacky University and University Hospital, Department of Haemato-Oncology, Olomouc, Czechia; [7]Faculty of Medicine, Charles University and University Hospital Pilsen, Department of Haematology and Oncology, Pilsen, Czechia; [8]Second Faculty of Medicine, Charles University and Motol University Hospital, Department of Pathology and Molecular Medicine, Prague, Czechia; [9]First Faculty of Medicine, Charles University and General Hospital, Institute of Pathology, Prague, Czechia; [10]Czech Lymphoma Study Group, Datacenter, Prague, Czechia;*

### Background

Several prognostic tools have been established to predict outcomes in newly diagnosed marginal zone lymphomas (MZL), including subtype-specific (MALT-IPI) or non-specific (MZL-IPI, POD24) models. However, no dedicated prognostic index exists for relapsed/refractory (r/r) MZL. This is increasingly important as therapeutic landscape evolves, including recent approval of CAR-T for 3+ line (L). The FLP-R index, recently developed for r/r follicular lymphoma (Maurer, ASH 2025; available at: https://rtools.mayo.edu/flp_r_calculator/), identifies a high-risk group (25%) with inferior outcomes, higher risk of lymphoma-related death (LRD), and/or histologic transformation (HT).

### Aims

To evaluate the prognostic performance of FLP-R index for overall survival (OS), event-free survival (EFS), and LRD in r/r MZL, and to compare its prognostic accuracy with MZL-IPI, POD24, and MALT-IPI at evaluated line.

### Methods

We identified r/r MZL cases prospectively enrolled in the NiHiL registry with available FLP-R variables at evaluated line (age, sex, number/type of prior treatment, time from dg., LDH, HGB, and WBC) who initiated L2+ between 2010–2025. Observation was considered as L1 management only. Cases with HT at L1 or evaluated line were excluded; HT was not considered a separate endpoint due to subtype-specific differences in incidence. We used Cox regression, Fine-Gray models, and optimism-corrected C-statistics to assess outcomes by FLP-R and compare performance with other indices.

**Copyright Information:** (Online) ISSN: 2572-9241
©2026 The Author(s). HemaSphere is published by John Wiley & Sons Ltd on behalf of European Hematology Association. This is an open access Abstract Book distributed under the Attribution-NonCommercial-NoDerivs (CC BY-NC-ND), which allows third parties to download the articles and share them with others as long as they credit the author and the Abstract Book, but they cannot change the content in any way or use them commercially.
**Abstract Book Citations:** Authors, Title, HemaSphere, 2026;10:(S1):pages. The abstract book DOIs can be found on the "Author Information" page.
**Disclaimer:** Articles published in the journal HemaSphere exclusively reflect the opinions of the authors. The authors are responsible for all content in their abstracts including accuracy of the facts, statements, citing resources, etc.

**Results**

A total of 485 treatment lines (L2 n=366, L3 n=86, L4 n=33) among 340 r/r MZL patients were analyzed. Subtypes included extranodal MZL (EMZL, n=220; MALT n=181), splenic MZL (SMZL, n=85), nodal MZL (NMZL, n=50), and disseminated MZL (dissMZL, n=130). Median age at start of the evaluated therapy was 68 years; median time from dg. was 47 months. Prior treatments included: R-CVP (28%), R-CHOP (28%), and B-R (5%).
FLP-R stratified patients into low (62%), intermed. (22%), and high (16%) risk groups. High-risk cases were associated with higher age, male sex, elevated LDH and WBC, decreased HGB, worse ECOG PS, shorter time from dg, and prior use of CVP regimens (all P≤0.01).
With a median follow-up of 4.7 years, 5-year OS was 75% (low), 54% (intermed.), and 33% (high; P<0.01; Fig. A). 5-year EFS was 54%, 30%, and 28%, resp. (P<0.01). The 5-year cumulative incidence of LRD was 35.2% (high-risk) vs 4.1% (low) and 11.6% (intermed.; P<0.01; Fig. B). In multivariate analysis of FLP-R categories and MZL subtypes, high FLP-R independently predicted inferior OS (HR=2.89, 95% CI 1.96–4.26), EFS (HR=2.11, 95% CI 1.52–2.94), and LRD (HR=4.85, 95% CI 2.45–9.57); SMZL and dissMZL also predicted outcomes.
Based on C-statistics, FLP-R (C-index: OS 0.63, Fig. C; EFS 0.61, Fig. D; LRD 0.75) outperformed MZL-IPI, POD24, and MALT-IPI in the entire cohort, in SMZL, NMZL, and dissMZL (Fig. C–D). In EMZL, MALT-IPI showed higher accuracy for OS (C-index 0.65 vs 0.60), and MZL-IPI (0.79 vs 0.59) yielded the best discrimination for LRD.

**Summary/Conclusion**

The FLP-R index is a promising prognostic tool for r/r MZL, identifying a high-risk subset (16%) with inferior survival and high LRD. It outperforms MZL-IPI and POD24 in the overall r/r MZL population and may improve stratification in clinical trials. However, its utility is subtype-dependent (highest performance in dissMZL), yet established tools (MALT-IPI) remain superior for EMZL.

**Support:** NU23-03-00127, CU Hem-Onc Cooperatio, BBMRI_CZ LM2023033.

**Copyright Information:** (Online) ISSN: 2572-9241
©2026 The Author(s). HemaSphere is published by John Wiley & Sons Ltd on behalf of European Hematology Association. This is an open access Abstract Book distributed under the Attribution-NonCommercial-NoDerivs (CC BY-NC-ND), which allows third parties to download the articles and share them with others as long as they credit the author and the Abstract Book, but they cannot change the content in any way or use them commercially.
**Abstract Book Citations:** Authors, Title, HemaSphere, 2026;10:(S1):pages. The abstract book DOIs can be found on the "Author Information" page.
**Disclaimer:** Articles published in the journal HemaSphere exclusively reflect the opinions of the authors. The authors are responsible for all content in their abstracts including accuracy of the facts, statements, citing resources, etc.

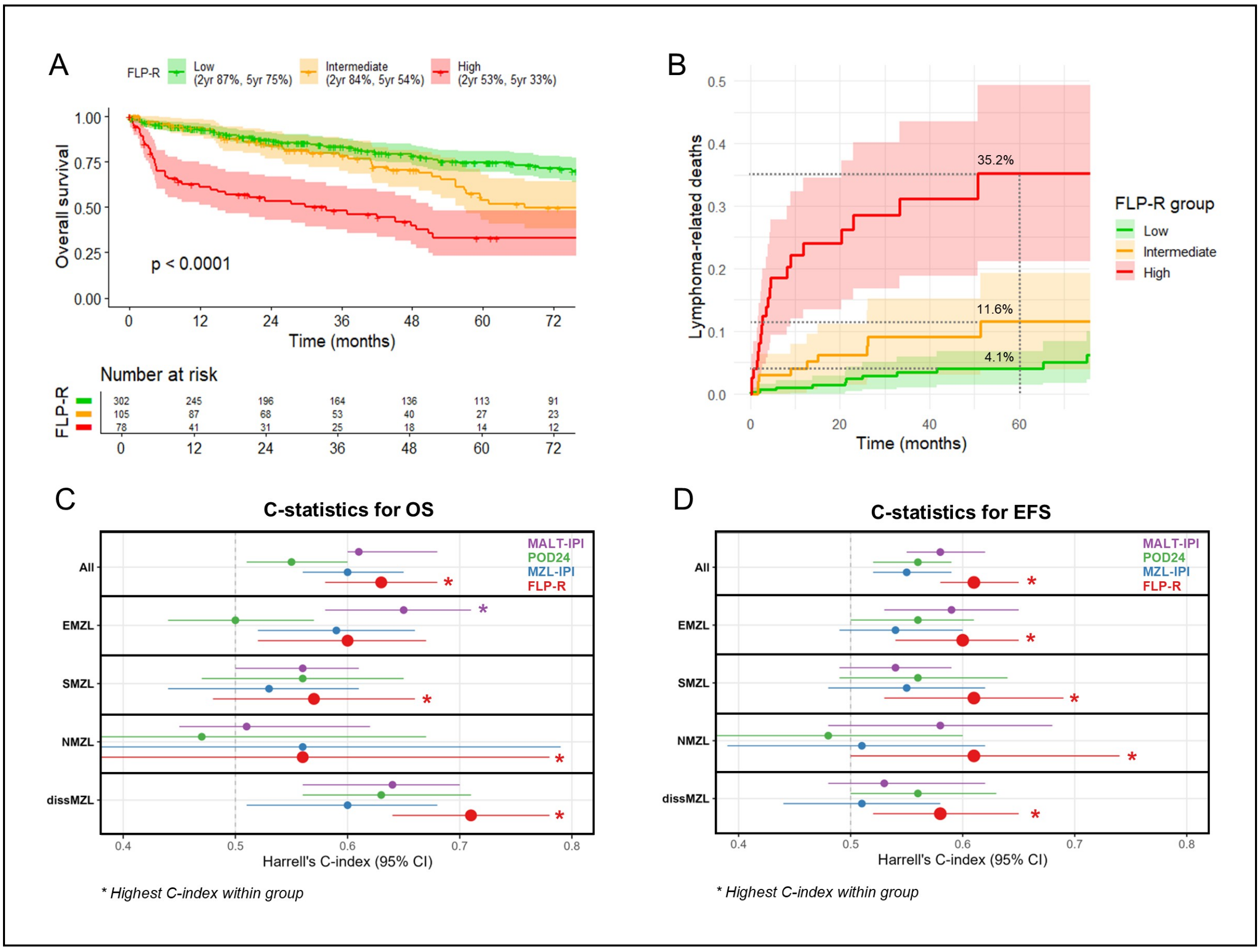

Survival outcomes in r/r MZL stratified by FLP-R risk group: (A) overall survival (OS) from index line, (B) cumulative incidence of lymphoma-related deaths (accounting for non-lymphoma death as a competing risk); comparison of predictive performance of FLP-R with other indices using optimism-corrected C-statistics (C. OS; D. event-free survival).

**Copyright Information:** (Online) ISSN: 2572-9241
©2026 The Author(s). HemaSphere is published by John Wiley & Sons Ltd on behalf of European Hematology Association. This is an open access Abstract Book distributed under the Attribution-NonCommercial-NoDerivs (CC BY-NC-ND), which allows third parties to download the articles and share them with others as long as they credit the author and the Abstract Book, but they cannot change the content in any way or use them commercially.
**Abstract Book Citations:** Authors, Title, HemaSphere, 2026;10:(S1):pages. The abstract book DOIs can be found on the "Author Information" page.
**Disclaimer:** Articles published in the journal HemaSphere exclusively reflect the opinions of the authors. The authors are responsible for all content in their abstracts including accuracy of the facts, statements, citing resources, etc.

### (PF943) PROGNOSTIC VALUE OF PET PARAMETERS IN PATIENTS WITH RELAPSED OR REFRACTORY (R/R) FOLLICULAR LYMPHOMA (FL) TREATED WITH LISOCABTAGENE MARALEUCEL (LISO-CEL): RESULTS FROM THE TRANSCEND FL TRIAL

**Topic:** 18. Indolent and mantle-cell non-Hodgkin lymphoma - Clinical

Anne-Segolene Cottereau*[1], Thibaut Reichert[2], Samuel Griolet[3], Emelie Van Zele[4], Merav Bar[5], David Bernasconi[5], Luciana Moro Bueno[5], Diederik Grootendorst[6], Maria Strocchia[5], Alberto Vasconcelos[5], Joe Xi[6], Franck Morschhauser[7]

*[1]Cochin Hospital, APHP, University Paris Cité, Paris, France;[2]Institut Paoli-Calmettes, Nuclear Medicine, Marseille, France; [3]LYSARC, Centre Hospitalier Lyon-Sud, Pierre-Bénite, Lyon, France; [4]LYSARC, Hôpital Henri Mondor, Créteil, France; [5]Bristol Myers Squibb, Boudry, Switzerland; [6]Bristol Myers Squibb, Princeton, NJ, United States of America;[7]Centre Hospitalier Universitaire de Lille, Groupe de Recherche sur les formes Injectables et les Technologies Associées, Hematolgy, Lille, France;*

### Background

Anti-CD19 chimeric antigen receptor (CAR) T-cell therapy is effective in R/R FL, but evidence on the prognostic relevance of baseline (BL) FDG-PET parameters in this setting remains limited.

### Aims

We evaluated the prognostic value of BL PET parameters and early response in patients with R/R FL who received liso-cel as third-line or later (3L+) treatment in TRANSCEND FL (NCT04245839).

### Methods

Patients with 3L+ FL from the efficacy-evaluable set in TRANSCEND FL with available PET (at baseline and Day 29 after liso-cel infusion) and survival data were included. BL PET parameters included total metabolic tumor volume using an SUV≥4 threshold (TMTV4), standardized maximum inter-lesion distance normalized by body surface area (SDmax), and the presence of focal bone involvement. CAR T cell expansion was characterized by peak transgene expansion (Cmax).Early metabolic response was assessed at Day 29 using the Deauville score. PFS and OS were analyzed using Kaplan–Meier and Cox models, with cutoffs for continuous variables derived using maximally selected rank statistics.

**Copyright Information:** (Online) ISSN: 2572-9241
©2026 The Author(s). HemaSphere is published by John Wiley & Sons Ltd on behalf of European Hematology Association. This is an open access Abstract Book distributed under the Attribution-NonCommercial-NoDerivs (CC BY-NC-ND), which allows third parties to download the articles and share them with others as long as they credit the author and the Abstract Book, but they cannot change the content in any way or use them commercially.
**Abstract Book Citations:** Authors, Title, HemaSphere, 2026;10:(S1):pages. The abstract book DOIs can be found on the "Author Information" page.
**Disclaimer:** Articles published in the journal HemaSphere exclusively reflect the opinions of the authors. The authors are responsible for all content in their abstracts including accuracy of the facts, statements, citing resources, etc.

**Results**

A total of 96 patients were included, with a median age of 61.5 years; 87.5% had advanced-stage disease, 53.1% had BL high-risk FLIPI scores (3–5), 50% met mGELF criteria, and 35.4% received bridging therapy.
A high TMTV (>480 mL) was associated with shorter PFS (HR: 2.68; p=0.010) and OS (HR: 3.00; p=0.036). Median PFS was 31.9 months for patients with high BL TMTV (n=16) versus not reached for patients with low BL TMTV (n=80). In contrast, the TMTV cutoff of >240 mL (n=31) used in the ELARA trial was significantly associated with OS (p=0.043) but not PFS (p=0.081).
High SDmax (>388; n=20) was associated with inferior PFS (HR: 2.99; p=0.007) and OS (HR: 4.03; p=0.009). Similarly, the presence of at least one bone focal lesion (n=13) identified patients with poorer PFS (HR: 3.80; p=0.001) and OS (HR: 6.66; p<0.001). Conversely, high Cmax (>18442 copies per µg; n=61) was associated with superior PFS (HR: 0.25 p<0.001) and OS (HR: 0.13; p=0.0001).
In multivariable analysis, TMTV and Cmax were independent significant prognostic factors for PFS (p=0.009 HR=2.91 and p=0.0002 HR=4.06) and OS (p=0.036 HR=3.28 and p=0.002 HR=7.61). When combining TMTV and Cmax, outcomes were similar for patients with TMTV high/Cmax high (n=9) and those with TMTV low/Cmax high (n=52), suggesting that higher CAR T-cell expansion partially mitigated the poor prognosis associated with high TMTV (Figure 1).
At Day 29 post-infusion, Deauville score was prognostic for survival outcomes: scores of 4–5 were associated with shorter PFS (HR: 6.67; p=0.002) and OS (HR: 10.05; p=0.001) compared with scores of 1–3, while no significant difference was observed between scores 1–2 and 3.

**Summary/Conclusion**

Baseline PET parameters (TMTV, SDmax and focal bone lesions) were robust predictors of survival outcomes in patients with 3L+ R/R FL treated with liso-cel, even after adjusting for CAR T cell expansion (Cmax). Most patients belonged to more favorable prognostic groups across these parameters, reinforcing the clinical benefits of liso-cel in 3L+ FL. These findings support the clinical utility of BL metabolic profiling (alone or combined with expansion kinetics, when feasible) to identify high-risk patient subsets who may benefit from closer disease monitoring or additional therapeutic interventions before progression following CAR T infusion.

**Copyright Information:** (Online) ISSN: 2572-9241
©2026 The Author(s). HemaSphere is published by John Wiley & Sons Ltd on behalf of European Hematology Association. This is an open access Abstract Book distributed under the Attribution-NonCommercial-NoDerivs (CC BY-NC-ND), which allows third parties to download the articles and share them with others as long as they credit the author and the Abstract Book, but they cannot change the content in any way or use them commercially.
**Abstract Book Citations:** Authors, Title, HemaSphere, 2026;10:(S1):pages. The abstract book DOIs can be found on the "Author Information" page.
**Disclaimer:** Articles published in the journal HemaSphere exclusively reflect the opinions of the authors. The authors are responsible for all content in their abstracts including accuracy of the facts, statements, citing resources, etc.

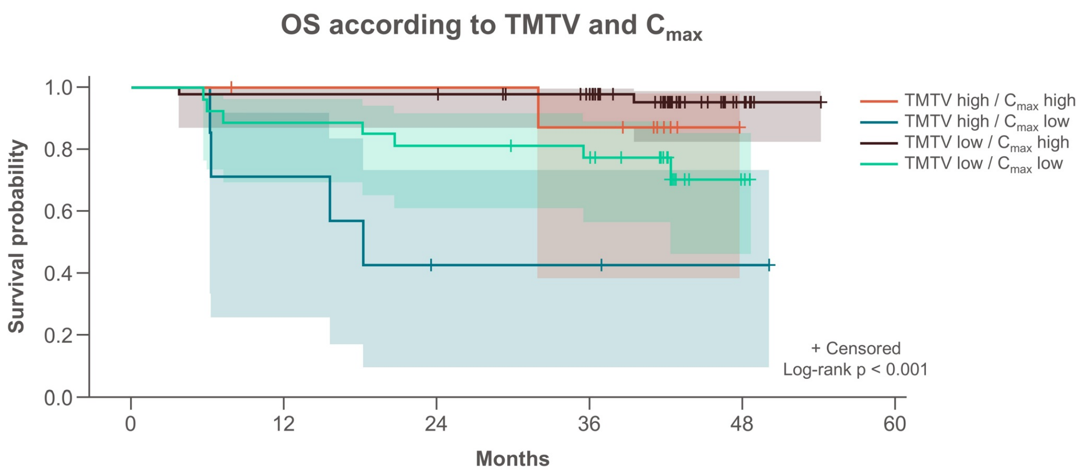

No. at risk

| | 0 | 12 | 24 | 36 | 48 | 60 |
|---|---|---|---|---|---|---|
| TMTV high / $C_{max}$ high | 9 | 8 | 8 | 7 | 0 | |
| TMTV high / $C_{max}$ low | 7 | 5 | 2 | 2 | 1 | 0 |
| TMTV low / $C_{max}$ high | 52 | 51 | 51 | 44 | 9 | 0 |
| TMTV low / $C_{max}$ low | 27 | 24 | 22 | 19 | 2 | 0 |

| | No. of patients | Event | Censored | Median survival (95% CI) |
|---|---|---|---|---|
| TMTV high / $C_{max}$ high | 9 | 11.1% (1) | 88.9% (8) | Not reached (31.9; NA) |
| TMTV high / $C_{max}$ low | 7 | 57.1% (4) | 42.9% (3) | 18.2 (6.2; NA) |
| TMTV low / $C_{max}$ high | 52 | 3.8% (2) | 96.2% (50) | Not reached |
| TMTV low / $C_{max}$ low | 27 | 25.9% (7) | 74.1% (20) | Not reached (42.3; NA) |

Figure 1 :OS according to TMTV and Cmax combination

**Copyright Information:** (Online) ISSN: 2572-9241
©2026 The Author(s). HemaSphere is published by John Wiley & Sons Ltd on behalf of European Hematology Association. This is an open access Abstract Book distributed under the Attribution-NonCommercial-NoDerivs (CC BY-NC-ND), which allows third parties to download the articles and share them with others as long as they credit the author and the Abstract Book, but they cannot change the content in any way or use them commercially.
**Abstract Book Citations:** Authors, Title, HemaSphere, 2026;10:(S1):pages. The abstract book DOIs can be found on the "Author Information" page.
**Disclaimer:** Articles published in the journal HemaSphere exclusively reflect the opinions of the authors. The authors are responsible for all content in their abstracts including accuracy of the facts, statements, citing resources, etc.

### (PF944) THE ZAP TRIAL: FIRST PROSPECTIVE VALIDATION OF AN MRD-ADAPTED, CHEMOTHERAPY-SPARING REGIMEN WITH ZANUBRUTINIB PLUS G-CHOP IN HIGH-RISK FOLLICULAR LYMPHOMA

**Topic:** 18. Indolent and mantle-cell non-Hodgkin lymphoma - Clinical

Peiqi Zhao*[1], Ge Hu[1], Xia Liu[1], Lanfang Li[1], Zhengzi Qian[1], Wei Li[1], Shiyong Zhou[1], Lihua Qiu[1], Xianming Liu[1], Hui-Lai Zhang[1]

*[1]Department of Lymphoma, Tianjin's Clinical Research Center for Cancer, Key Laboratory of Cancer Prevention and Therapy, National Clinical Research Center for Cancer, Tianjin Medical University Cancer Institute and Hospital, Tianjin Medical University, Tianjin, 300060, China, Tianjin, China;*

### Background

Patients with untreated follicular lymphoma (FL) and high-risk FLIPI scores (≥2) experience suboptimal outcomes (5-year PFS ~50%) with standard immunochemotherapy followed by prolonged anti-CD20 maintenance, a strategy burdened by cumulative toxicity. The ROSEWOOD study established the synergy of obinutuzumab and the BTK inhibitor zanubrutinib. We hypothesized that incorporating zanubrutinib into first-line G-CHOP could induce deep, rapid molecular remissions, enabling a response-adapted paradigm to safely minimize both chemotherapy exposure and maintenance duration.

### Aims

The primary aim was to assess the complete metabolic response (CMR) rate after 4 cycles of zanubrutinib + G-CHOP induction. Secondary aims included evaluating the safety and feasibility of MRD-guided therapy de-escalation, and the 12-month progression-free survival (PFS) in this molecularly-defined responder population.

### Methods

ZAP is an ongoing, single-arm, phase II trial (NCT06474481). Eligible patients had untreated grade 1-3A FL, FLIPI 2-5, and required therapy per GELF criteria. All received 4 cycles of zanubrutinib (160mg BID) + standard G-CHOP with pegfilgrastim. Interim restaging included PET-CT (Deauville 1-3 = CMR) and MRD assessment in plasma (tumor-informed ctDNA, PhasED-seq) and bone marrow (ClonoSEQ® NGS; both $10^{-6}$ sensitivity). Patients achieving CMR and dual-modality MRD negativity stopped chemotherapy and proceeded to 12 months of response-adapted maintenance (zanubrutinib + obinutuzumab). All others received 2 additional induction cycles before starting the same maintenance. Correlative studies included serial immune profiling.

### Results

Between Apr 2024-Dec 2024, 32 patients were enrolled (FLIPI 3-5: 78%; bulky disease ≥7cm: 44%). The primary endpoint was met: CMR rate after 4 cycles was 84% (27/32; 95% CI: 68.3-93.1%). MRD negativity rates were 91% (29/32) in ctDNA and 88% (28/32) in BM. A landmark 84% (27/32) of patients achieved the stringent triple-negative endpoint (CMR + ctDNA- + BM-), qualifying for immediate chemotherapy de-escalation. Early ctDNA clearance by C2 (achieved in 75% of patients) was a powerful predictor, with 100% of these patients reaching the triple-negative endpoint at C4 vs. 38% of delayed clearers ($p<0.001$).
**With a median follow-up of 15 months (data cut-off: Jan 2026), the 12-month PFS for the total cohort is 97% (95% CI: 91-100%), and 100% for the triple-negative subgroup.** Patient-reported outcomes showed significant improvement in Global Health Status (Δ +25.4 points, $p<0.001$). Grade ≥3 adverse events were primarily hematologic (neutropenia: 22%; febrile neutropenia: 6%), with no new safety signals for the zanubrutinib combination. Only 1 patient (3%) discontinued due to a BTKi-related AE (atrial fibrillation).

**Copyright Information:** (Online) ISSN: 2572-9241
©2026 The Author(s). HemaSphere is published by John Wiley & Sons Ltd on behalf of European Hematology Association. This is an open access Abstract Book distributed under the Attribution-NonCommercial-NoDerivs (CC BY-NC-ND), which allows third parties to download the articles and share them with others as long as they credit the author and the Abstract Book, but they cannot change the content in any way or use them commercially.
**Abstract Book Citations:** Authors, Title, HemaSphere, 2026;10:(S1):pages. The abstract book DOIs can be found on the “Author Information” page.
**Disclaimer:** Articles published in the journal HemaSphere exclusively reflect the opinions of the authors. The authors are responsible for all content in their abstracts including accuracy of the facts, statements, citing resources, etc.

**Summary/Conclusion**

The ZAP trial is the first to prospectively validate an MRD-guided, response-adapted strategy in frontline FL. Adding zanubrutinib to G-CHOP yielded exceptional early CMR and MRD negativity rates, enabling the safe omission of 33% of planned chemotherapy cycles and halving of maintenance duration in ~85% of high-risk patients, without compromising short-term efficacy. Early ctDNA dynamics robustly identified candidates for de-escalation. This precision approach successfully uncouples treatment intensity from initial risk score, tailoring therapy to individual tumor biology and minimizing patient burden.

**Copyright Information:** (Online) ISSN: 2572-9241
©2026 The Author(s). HemaSphere is published by John Wiley & Sons Ltd on behalf of European Hematology Association. This is an open access Abstract Book distributed under the Attribution-NonCommercial-NoDerivs (CC BY-NC-ND), which allows third parties to download the articles and share them with others as long as they credit the author and the Abstract Book, but they cannot change the content in any way or use them commercially.
**Abstract Book Citations:** Authors, Title, HemaSphere, 2026;10:(S1):pages. The abstract book DOIs can be found on the "Author Information" page.
**Disclaimer:** Articles published in the journal HemaSphere exclusively reflect the opinions of the authors. The authors are responsible for all content in their abstracts including accuracy of the facts, statements, citing resources, etc.

### (PF945) UPDATED EFFICACY AND SAFETY RESULTS OF ROCBRUTINIB FROM THE PHASE 2 ROCK-1 STUDY IN PATIENTS WITH RELAPSED OR REFRACTORY MANTLE CELL LYMPHOMA AND PREVIOUSLY TREATED WITH BTK INHIBITOR

**Topic:** 18. Indolent and mantle-cell non-Hodgkin lymphoma - Clinical

Yuqin Song*[1], Qingqing Cai[2], Xiaoqiong Tang[3], Keshu Zhou[4], Lihong Liu[5], Zhigang Peng[6], Xiaojing Yan[7], Kaiyang Ding[8], Jianzhen Shen[9], Huijing Wu[10], Jie Cui[11], Hui Zhou[12], Jianqiu Wu[13], Hongmei Jing[14], Ming Jiang[15], Xiuhua Sun[16], Yu Yang[17], Kang Yu[18], Wei Yang[19], Zhifeng Li[20], Zhengming Jin[21], Shuhua Yi[22], Fei Li[23], Guowu Wu[24], Xiaolei Wei[25], Gaoxiang Wang[26], Yudan Wu[27], Ningjing Lin[1], Fenlai Tan[28], Yue Shen[28], Yejiang Lou[28], Xiang Xiao[28], Jun Zhu[1]

*[1]Peking University Cancer Hospital, Beijing, China; [2]Sun Yat-sen University Cancer Center, Guangzhou, China; [3]The First Affiliated Hospital of Chongqing Medical University, Chongqing, China; [4]Henan Cancer Hospital, Zhengzhou, China; [5]The Fourth Hospital of Hebei Medical University, Shijiazhuang, China; [6]The First Affiliated Hospital of Guangxi Medical University, Nanning, China; [7]The First Affiliated Hospital of China Medical University, Shenyang, China; [8]Anhui Provincial Cancer Hospital, Hefei, China; [9]Fujian Medical University Union Hospital, Fuzhou, China; [10]Hubei Cancer Hospital, Wuhan, China; [11]Gansu Cancer Hospital, Lanzhou, China; [12]Hunan Cancer Hospital, Changsha, China; [13]Jiangsu Cancer Hospital, Nanjing, China; [14]Peking University Third Hospital, Beijing, China; [15]West China Hospital of Sichuan University, Chengdu, China; [16]The Second Affiliated Hospital of Dalian Medical University, Dalian, China; [17]Fujian Cancer Hospital, Fuzhou, China; [18]The First Affiliated Hospital of Wenzhou Medical University, Wenzhou, China; [19]Shengjing Hospital of China Medical University, Shenyang, China; [20]The First Affiliated Hospital of Xiamen University, Xiamen, China; [21]The First Affiliated Hospital of Soochow University, Suzhou, China; [22]Institute of Hematology & Blood Diseases Hospital, Chinese Academy of Medical Sciences, Tianjin, China; [23]The First Affiliated Hospital of Nanchang University, Nanchang, China; [24]Meizhou People's Hospital, Meizhou, China; [25]Nanfang Hospital of Southern Medical University, Guangzhou, China; [26]Tongji Hospital, Tongji Medical College, Huazhong University of Science and Technology, Wuhan, China; [27]Sun Yat-sen Memorial Hospital, Sun Yat-sen University, Guangzhou, China; [28]Guangzhou Lupeng Pharmaceutical Co., Ltd., Guangzhou, China;*

### Background

Rocbrutinib is a novel, fourth generation BTK inhibitor that incorporates both covalent (non-reversible) and non-covalent (reversible) binding mechanisms. In the primary analysis of the Phase 2 ROCK-1 Trial (NCT05716087), rocbrutinib demonstrated clinically meaningful and durable efficacy with manageable safety profile in heavily pretreated, cBTKi-exposed Chinese patients with relapsed or refractory mantle cell lymphoma (R/R MCL) (Song *et al.* ASH 2025, abs25-7939).

### Aims

Here we present updated efficacy and safety results from the ROCK-1 trial after prolonged follow-up (median, 19.49 months; range, 0.62-28.65).

### Methods

Eligible patients received oral rocbrutinib (150 mg once daily) until disease progression or unacceptable toxicity. The primary endpoint was IRC-assessed overall response rate (ORR) per Lugano 2014 criteria. Key secondary endpoints included duration of response (DOR), progression-free survival (PFS), overall survival (OS), and safety. Adverse events (AEs) were graded according to CTCAE v5.0.

**Copyright Information:** (Online) ISSN: 2572-9241
©2026 The Author(s). HemaSphere is published by John Wiley & Sons Ltd on behalf of European Hematology Association. This is an open access Abstract Book distributed under the Attribution-NonCommercial-NoDerivs (CC BY-NC-ND), which allows third parties to download the articles and share them with others as long as they credit the author and the Abstract Book, but they cannot change the content in any way or use them commercially.
**Abstract Book Citations:** Authors, Title, HemaSphere, 2026;10:(S1):pages. The abstract book DOIs can be found on the "Author Information" page.
**Disclaimer:** Articles published in the journal HemaSphere exclusively reflect the opinions of the authors. The authors are responsible for all content in their abstracts including accuracy of the facts, statements, citing resources, etc.

### Results

As of Jan 05, 2026, among the 61 R/R MCL patients, the median treatment duration was 168 days (range, 9-813), the maximum number of treatment cycles received was 29 (28 days per cycle). Per IRC's assessment, ORR was 63.9% (95% CI, 50.6-75.8), including 23.0% complete response (CR). Median PFS and DOR were 7.39 months (95% CI, 3.71–18.30) and 16.46 months (95% CI, 8.25–not reached), respectively. The efficacy results per investigator were consistent with IRC's results, showing an ORR of 62.3% (95% CI, 49.0–74.4) with CR rate of 26.2%, a median PFS of 5.52 months (95% CI, 3.71–12.68), and a median DOR of 16.46 months (95% CI, 5.55–22.14). After a median follow-up for OS of 23.36 months (range, 0.62-28.65), the median OS was not reached (95% CI, 15.97-not reached), corresponding to an estimated 24-month OS rate of 61.4%. Treatment emergent adverse events (TEAEs) of any grade occurred in more than 15% of patients were thrombocytopenia (43.5%), anemia (32.3%), neutropenia (30.6%), white blood cell count increased (24.2%), lymphocytosis (24.2%), blood creatinine increased (21.0%), white blood cell count decreased (21.0%), hyperuricemia (21.0%), leukopenia (21.0%), pneumonia (19.4%), hypoalbuminemia (17.7%), weight loss (16.1%), hypokalemia (16.1%), constipation (16.1%), and predominantly Grade 1 or 2. Grade ≥3 TEAEs with an incidence of 5% or higher included lymphocytosis (12.9%), anemia (12.9%), pneumonia (12.9%), neutropenia (9.7%), and thrombocytopenia (6.5%). BTKi associated AEs of special interest were infrequent, with major bleeding (Grade ≥3 hemorrhage) of 3.2%, and no atrial fibrillation or flutter was reported. 2 cases of benign neoplasms (one papilloma and one uterine leiomyoma) were reported and deemed unrelated to rocbrutinib. Treatment was interrupted due to TEAEs in 35.5% of patients, mainly were pneumonia (11.3%) and pyrexia (6.5%), and most interruptions did not necessitate dose modification (reduction rate: 3.2%). No TEAEs leading to permanent treatment discontinuation or death.

### Summary/Conclusion

With extended follow-up, rocbrutinib still showed durable efficacy and favorable safety in patients with R/R MCL. A randomized phase 3 confirmatory trial of rocbrutinib versus investigator's choice of BTK inhibitors in patients with R/R MCL is ongoing (NCT07377578).

**Copyright Information:** (Online) ISSN: 2572-9241
©2026 The Author(s). HemaSphere is published by John Wiley & Sons Ltd on behalf of European Hematology Association. This is an open access Abstract Book distributed under the Attribution-NonCommercial-NoDerivs (CC BY-NC-ND), which allows third parties to download the articles and share them with others as long as they credit the author and the Abstract Book, but they cannot change the content in any way or use them commercially.
**Abstract Book Citations:** Authors, Title, HemaSphere, 2026;10:(S1):pages. The abstract book DOIs can be found on the "Author Information" page.
**Disclaimer:** Articles published in the journal HemaSphere exclusively reflect the opinions of the authors. The authors are responsible for all content in their abstracts including accuracy of the facts, statements, citing resources, etc.

### (PF946) THE EFFICACY AND SAFETY OF OBINUTUZUMAB WITH ZANUBRUTINIB AS FIRST-LINE TREATMENT IN OLDER PATIENTS WITH MANTLE CELL LYMPHOMA

**Topic:** 18. Indolent and mantle-cell non-Hodgkin lymphoma - Clinical

Yanan Zhu*[1], Yunfei Lv[1], Chunmei Yang[1], Xingnong Ye[1], Xiang Zhang[1], Xuewu Zhang[1], Juying Wei[1], Ying Lin[2], Lixia Sheng[3], Hongyan Tong[1], Jie Jin[1], Wenjuan Yu[1]

*[1]The First Affiliated Hospital, Zhejiang University School of Medicine, Hangzhou, China;[2]The Second Affiliated Hospital and Yuying Children's Hospital of Wenzhou Medical University, Wenzhou, China; [3]The First Affiliated Hospital of Ningbo University, Ningbo, China;*

### Background

Mantle cell lymphoma (MCL) is a biologically heterogeneous malignancy with increasing incidence in the elderly population. Older patients often present with impaired performance status and limited tolerance to intensive chemotherapy. Therefore, chemo-free therapeutic strategies are urgently needed for newly diagnosed elderly patients with MCL.

### Aims

This study aimed to evaluate the efficacy and safety of obinutuzumab combined with zanubrutinib (GZ regimen) in previously untreated elderly patients with MCL.

### Methods

This is an open-label, multicenter, single-arm, phase II clinical trial (ChiCTR2300071433). Elderly patients (≥65 years) with newly diagnosed MCL were enrolled. Patients with blastoid or pleomorphic histology were excluded. Obinutuzumab was administered at 1000 mg intravenously on days 1, 8, and 15 of cycle 1 and on day 1 of cycles 2–4 (28-day cycles). Zanubrutinib (160 mg orally twice daily) was initiated from cycle 2 as part of induction therapy. Patients who achieved complete remission (CR) received zanubrutinib maintenance. Those achieving partial remission (PR) received two additional cycles of obinutuzumab followed by zanubrutinib maintenance. Zanubrutinib was continued until disease progression, unacceptable toxicity, or treatment discontinuation for any reason. The primary endpoint was complete response rate (CRR). Secondary endpoints included overall response rate (ORR), progression-free survival (PFS), overall survival (OS), and safety profiles.

### Results

A total of 49 patients were enrolled, with a median age of 73 years. Bone marrow involvement was observed in 34 patients. 14 patients were classified as high-risk according to the Simplified Mantle Cell Lymphoma International Prognostic Index (sMIPI), and 23 patients had Ki-67 ≥30%. One patient had a prior history of atrial fibrillation. Next-generation sequencing was performed in 32 patients, identifying TP53 mutations in 7 patients (22%). Among 45 evaluable patients assessed by PET-CT, 36 (80%) achieved CR and 5 achieved PR, resulting in an ORR of 91.1%. With a median follow-up of 12 months, one patient died due to disease progression and one due to COVID-19. Median PFS and OS were not reached. Most adverse events (AEs) were grade 1–2. 3 patients experienced grade 2 infusion-related reactions during the first obinutuzumab infusion. 6 patients developed grade 3–4 thrombocytopenia during the first obinutuzumab cycle. One patient developed new-onset paroxysmal atrial fibrillation, and one experienced grade 1 gastrointestinal bleeding.

**Copyright Information:** (Online) ISSN: 2572-9241
©2026 The Author(s). HemaSphere is published by John Wiley & Sons Ltd on behalf of European Hematology Association. This is an open access Abstract Book distributed under the Attribution-NonCommercial-NoDerivs (CC BY-NC-ND), which allows third parties to download the articles and share them with others as long as they credit the author and the Abstract Book, but they cannot change the content in any way or use them commercially.
**Abstract Book Citations:** Authors, Title, HemaSphere, 2026;10:(S1):pages. The abstract book DOIs can be found on the “Author Information” page.
**Disclaimer:** Articles published in the journal HemaSphere exclusively reflect the opinions of the authors. The authors are responsible for all content in their abstracts including accuracy of the facts, statements, citing resources, etc.

**Summary/Conclusion**

The combination of obinutuzumab and zanubrutinib demonstrated promising efficacy and a manageable safety profile in elderly patients with previously untreated MCL, supporting a chemo-free therapeutic approach in this population.

**Copyright Information:** (Online) ISSN: 2572-9241
©2026 The Author(s). HemaSphere is published by John Wiley & Sons Ltd on behalf of European Hematology Association. This is an open access Abstract Book distributed under the Attribution-NonCommercial-NoDerivs (CC BY-NC-ND), which allows third parties to download the articles and share them with others as long as they credit the author and the Abstract Book, but they cannot change the content in any way or use them commercially.
**Abstract Book Citations:** Authors, Title, HemaSphere, 2026;10:(S1):pages. The abstract book DOIs can be found on the "Author Information" page.
**Disclaimer:** Articles published in the journal HemaSphere exclusively reflect the opinions of the authors. The authors are responsible for all content in their abstracts including accuracy of the facts, statements, citing resources, etc.

### (PF947) CLINICAL OUTCOME AFTER BREXU-CEL THERAPY FAILURE IN MANTLE CELL LYMPHOMAS: AN ANALYSIS OF THE ITALIAN CART-SIE STUDY

**Topic:** 18. Indolent and mantle-cell non-Hodgkin lymphoma - Clinical

Annalisa Chiappella*[1], Anna Dodero[1], Beatrice Casadei[2], Stefania Bramanti[3], Silva Ljevar[4], Maurizio Musso[5], Patrizia Chiusolo[6], Ilaria Cutini[7], Mirko Farina[8], Anna Maria Barbui[9], Jacopo Olivieri[10], Mauro Krampera[11], Angelica Barone[1, 12], Massimo Martino[13], Maria Chiara Tisi[14], Luca Arcaini[15], Alessia Castellino[16], Giovanni Grillo[17], Federica Cavallo[18], Piera Angelillo[19], Mattia Novo[20], Alice Di Rocco[21], Lucia Brunello[22], Federico Stella[1], Rosalba Miceli[4], Pier Luigi Zinzani[2, 23], Paolo Corradini[1, 12]

*[1]Hematology, Fondazione IRCCS Istituto Nazionale dei Tumori, Milano, Italy; [2]IRCCS Azienda Ospedaliero-Universitaria di Bologna, Istituto di Ematologia “Seràgnoli”, Bologna, Italy; [3]IRCCS Humanitas Research Hospital, Transplantation Unit Department of Oncology and Hematology, Rozzano, Italy; [4]Unit of Biostatistics for Clinical Research, Department of Data Science, Fondazione IRCCS Istituto Nazionale dei Tumori, Milano, Italy; [5]UOC di Oncoematologia e TMO, Dipartimento Oncologico "La Maddalena", Palermo, Italy; [6]Ematologia e Trapianto, Dipartimento di Scienze Ematologiche e di laboratorio, Fondazione Policlinico Universitario Agostino Gemelli IRCCS, Sezione di Ematologia, Università Cattolica del Sacro Cuore, Roma, Italy; [7]SOD Ematologia, AAD Trapianto di midollo osseo, Ospedale Careggi, Firenze, Italy;[8]Unit of Blood Disease and Bone Marrow Transplantation, and Unit of Hematology, University of Brescia, ASST Spedali Civili di Brescia, Brescia, Italy; [9]Azienda Socio-Sanitaria Territoriale Papa Giovanni XXIII, Bergamo, Italy; [10]Clinica Ematologica, Centro Trapianti e Terapie Cellulari "Carlo Melzi", Azienda Sanitaria Universitaria Friuli Centrale, Udine, Italy; [11]Hematology and Bone Marrow Transplant Unit, Section of Biomedicine of Innovation, Department of Engineering for Innovative Medicine (DIMI), University of Verona, Verona, Italy; [12]Chair of Hematology, University of Milano, Milano, Italy; [13]Hematology and Stem Cell Transplantation and Cellular Therapies Unit (CTMO), Department of Hemato-Oncology and Radiotherapy, Grande Ospedale Metropolitano "Bianchi-Melacrino-Morelli", Reggio Calabria, Italy; [14]Hematology Unit, San Bortolo Hospital, AULSS 8 Berica, Vicenza, Italy;[15]Department of Molecular Medicine, University of Pavia & Division of Hematology, Fondazione IRCCS Policlinico San Matteo, Pavia, Italy; [16]Division of Hematology - AO S. Croce e Carle, Cuneo, Italy; [17]Dipartimento di Ematologia e Trapianto di midollo, ASST Grande Ospedale Metropolitano Niguarda, Milano, Italy; [18]Division of Hematology, Department of Molecular Biotechnologies and Health Sciences, University of Torino, Torino, Italy; [19]IRCCS San Raffaele Scientific Institute, Milano, Italy; [20]Division of Hematology, AOU “Città della Salute e della Scienza di Torino”, Torino, Italy; [21]Hematology, Department of Translational and Precision Medicine Università La Sapienza di Roma, Roma, Italy; [22]SCDU Ematologia, AOU SS Antonio e Biagio e Cesare Arrigo, Alessandria, Italy; [23]Dipartimento di Scienze Mediche e Chirurgiche, Università di Bologna, Bologna, Italy;*

### Background

The prognosis of relapsed or refractory mantle cell lymphoma (R/R MCL) after chemoimmunotherapy and Bruton tyrosine kinase inhibitor (BTKi) improved with the approval of Brexucabtagene autoleucel (brexu-cel), an autologous CD19-directed chimeric antigen receptor (CAR) T-cell. In the registrative trial ZUMA-2 (Wang, NEJM 2020), brexu-cel demonstrated high activity in heavily pretreated patients; however, disease recurrence after CAR T-cell remains frequent and long-term disease control is not consistently achieved.

### Aims

Aim of the present analysis was to investigate the pattern of treatments and the outcome following progression post CAR T-cell therapy in R/R MCL patients enrolled in the CART-SIE study.

**Copyright Information:** (Online) ISSN: 2572-9241
©2026 The Author(s). HemaSphere is published by John Wiley & Sons Ltd on behalf of European Hematology Association. This is an open access Abstract Book distributed under the Attribution-NonCommercial-NoDerivs (CC BY-NC-ND), which allows third parties to download the articles and share them with others as long as they credit the author and the Abstract Book, but they cannot change the content in any way or use them commercially.
**Abstract Book Citations:** Authors, Title, HemaSphere, 2026;10:(S1):pages. The abstract book DOIs can be found on the “Author Information” page.
**Disclaimer:** Articles published in the journal HemaSphere exclusively reflect the opinions of the authors. The authors are responsible for all content in their abstracts including accuracy of the facts, statements, citing resources, etc.

### Methods

The CART-SIE is an ongoing multicenter observational study collecting data on all consecutive lymphoma patients treated with commercial CAR T-cells in 22 Italian centers. Patients infused with response at day30 available were analyzed. Progression-free survival (PFS), overall survival (OS) and post-relapse OS (pr-OS) were estimated using the Kaplan–Meier method, and group comparisons were done using Cox proportional hazards regression.

### Results

From 2019 to January 2026, 1522 lymphoma patients were enrolled, including 197 MCLs; 172 were infused and evaluable for the analysis. Median age was 64 years (IQR: 56,70), 91% (157) had Ann Arbor stage III or IV; median number of prior lines of therapy was 3 (IQR: 2,3), with 53% (92) refractory to last treatment; 56% (97) failed a prior autologous stem cell transplantation (SCT), 100% were exposed to BTKi. Histological subtypes were 74% (127) classic MCL, 17% (29) blastoid and 9% (16) pleomorphic variant. Bridging therapy was performed in 75% (130) patients. At day 30 after infusion, ORR was 78% (135), with 67% (116) CR; 7 patients died due to treatment related toxicities or progressive disease. At a median follow-up of 18.5 months (IQR: 6.97, 24.67), median PFS was 30.4 months (CI 95% 16.02-NA), with 1-year PFS of 63% (95% CI: 55.5%-71.4%), and median OS was not reached (CI 95% 30.39-NA), with 1-year OS of 79% (95% CI: 72.4%-85.9%). After Brexu-cel infusion, failures were 51. The median time to progression post CAR T was 5.9 months (IQR: 2.99, 11.35). Forty-one out of 51 received a salvage therapy (some patients received multiple therapies): chemotherapy in 31% (13), bispecific antibodies in 19.5% (8), BTKi in 48% (20), and others therapies in 13% (5). Ten patients achieved a CR after salvage, and four were consolidated with allogeneic-SCT. The median post relapse OS was 7.3 months, with 1-year pr-OS 40.3% (95% CI: 27.3%-59.5%). In univariable Cox models, the use of bispecific antibodies was associated with improved pr-OS, while a inferior pr-OS was found in case of use of chemotherapy as salvage post-CAR T, inefficacy of the bridging therapy and a high CAR-HEMATOTOX at baseline.

### Summary/Conclusion

The outcome of R/R MCL patients failing CAR T-cells is very poor. Our data suggest that bispecific antibodies may represent an effective salvage backbone. Future effors should focus in integrating these agents into a treatment algorithm and clarifying the optimal patient selection for allogeneic SCT consolidation.The role of bispecific antibodies and consolidation with allo-SCT requires further investigation.

**Copyright Information:** (Online) ISSN: 2572-9241
©2026 The Author(s). HemaSphere is published by John Wiley & Sons Ltd on behalf of European Hematology Association. This is an open access Abstract Book distributed under the Attribution-NonCommercial-NoDerivs (CC BY-NC-ND), which allows third parties to download the articles and share them with others as long as they credit the author and the Abstract Book, but they cannot change the content in any way or use them commercially.
**Abstract Book Citations:** Authors, Title, HemaSphere, 2026;10:(S1):pages. The abstract book DOIs can be found on the “Author Information” page.
**Disclaimer:** Articles published in the journal HemaSphere exclusively reflect the opinions of the authors. The authors are responsible for all content in their abstracts including accuracy of the facts, statements, citing resources, etc.

### (PF948) PHASE 3 INMIND STUDY OF TAFASITAMAB PLUS LENALIDOMIDE AND RITUXIMAB FOR RELAPSED OR REFRACTORY FOLLICULAR LYMPHOMA: CLINICAL CHARACTERISTICS AND OUTCOMES OF PATIENTS RECEIVING SECOND-LINE TREATMENT

**Topic:** 18. Indolent and mantle-cell non-Hodgkin lymphoma - Clinical

Stefano Luminari*[1, 2], Marek Trněný[3], Laurie H. Sehn[4], Julie Wu[5], Michael Arbushites[5], Oscar Bortolami[6], Ajay Gopal[7]

*[1]Azienda USL-IRCCS di Reggio Emilia, Reggio Emilia, Italy;[2]Surgical, Medical and Dental Department of Morphological Sciences related to Transplant, Oncology and Regenerative Medicine, University of Modena and Reggio Emilia, Reggio Emilia, Italy; [3]Charles University, General Hospital, Prague, Czechia;[4]BC Cancer Centre for Lymphoid Cancer and The University of British Columbia, Vancouver, BC, Canada; [5]Incyte Corporation, Wilmington, DE, United States of America;[6]Incyte Biosciences International Sàrl, Morges, Switzerland; [7]University of Washington, Division of Medical Oncology/Fred Hutchinson Cancer Center, Seattle, WA, United States of America;*

### Background

In patients (pts) with follicular lymphoma (FL), durability of response in second-line (2L) therapy is important, as prognosis worsens with subsequent treatment. Chemoimmunotherapy is often used 1L with immunotherapy preferred for relapsed or refractory (R/R) FL. Tafasitamab (tafa) is a humanized CD19-targeting monoclonal antibody (mAb) approved with lenalidomide (Len) + rituximab (R) for pts with R/R FL. Primary analysis of inMIND (NCT04680052) showed significant improvement in PFS by investigator (INV) in pts with R/R FL, representing a 57% reduction in the risk of progression or death, with a safety profile as expected (Sehn LH, *Lancet* 2026).

### Aims

To evaluate efficacy and safety outcomes in pts with R/R FL who received 2L therapy in inMIND.

### Methods

Pts aged ≥18 y with R/R CD19$^{+}$ and CD20$^{+}$ FL (grade 1-3A), ECOG PS ≤2, and who had received ≥1 prior systemic therapy including an anti-CD20 mAb were eligible. Pts were randomized 1:1 to receive tafa 12 mg/kg IV or placebo (pbo) with standard dosing of Len+R for up to 12x28-day cycles. Analyses included PFS by INV (primary endpoint), PFS by IRC, PET-CR rate, ORR, TTNT, and safety.

**Copyright Information:** (Online) ISSN: 2572-9241
©2026 The Author(s). HemaSphere is published by John Wiley & Sons Ltd on behalf of European Hematology Association. This is an open access Abstract Book distributed under the Attribution-NonCommercial-NoDerivs (CC BY-NC-ND), which allows third parties to download the articles and share them with others as long as they credit the author and the Abstract Book, but they cannot change the content in any way or use them commercially.
**Abstract Book Citations:** Authors, Title, HemaSphere, 2026;10:(S1):pages. The abstract book DOIs can be found on the "Author Information" page.
**Disclaimer:** Articles published in the journal HemaSphere exclusively reflect the opinions of the authors. The authors are responsible for all content in their abstracts including accuracy of the facts, statements, citing resources, etc.

**Results**

Of 548 pts randomized to tafa (n=273) or pbo (n=275), 300 (55%) had 1 prior line of therapy (tafa, n=147; pbo, n=153). Baseline characteristics for 2L pts were generally consistent with the overall FL population and balanced between arms: median age 65 y (range, 31-88); 56% male; 77% FLIPI ≥2; 81% ≥1 GELF criterion; 43% POD24 (progression within 24 months of start of initial treatment). In 2L pts, median PFS by INV was improved with addition of tafa vs pbo (24.0 months vs 15.4 months; hazard ratio [HR] [95% CI] 0.40 [0.26, 0.62]). Median PFS by IRC (not reached [NR] vs 20.7 months; HR [95% CI] 0.44 [0.28, 0.70]), PET-CR rate (50.7% vs 43.4%; odds ratio [OR] [95% CI] 1.3 [0.84, 2.15]), ORR (86.4% vs 73.9%; OR [95% CI] 2.3 [1.26, 4.17]), and median TTNT (NR vs 28.8 months; HR [95% CI] 0.52 [0.32, 0.84]) were also improved with addition of tafa vs pbo in 2L pts. In 2L pts with POD24, improvement was observed in median PFS by INV (19.2 months vs 11.7 months; HR [95% CI] 0.46 [0.26, 0.82]), median PFS by IRC (19.2 months vs 13.6 months; HR [95% CI] 0.51 [0.27, 0.95]), PET-CR rate (45.6% vs 31.8%; OR [95% CI] 2.0 [0.93, 4.13]), ORR (83.6% vs 62.3%; OR [95% CI] 3.5 [1.46, 8.35]), and median TTNT (NR vs 17.0 months; HR [95% CI] 0.62 [0.34, 1.13]). Benefit was also observed with addition of tafa vs pbo in 2L pts who received 1L anti-CD20+chemotherapy (chemo), including those with POD24 (**Table**). In the overall 2L pt subgroup, similar rates of TEAEs (100% vs 99%) were observed in the tafa vs pbo arm. Most common TEAEs (≥20%) in the tafa and pbo arms were neutropenia (47% vs 39%), diarrhea (37% vs 31%), constipation (31% vs 30%), COVID-19 (30% vs 20%), rash (22% vs 24%), fatigue (20% vs 18%), cough (20% vs 17%), nausea (20% vs 14%), and muscle spasm (18% vs 20%). Grade 3 or 4 TEAEs were reported in 69% vs 61% of pts and serious TEAEs in 31% vs 25% of pts in the tafa vs pbo arm, respectively. Three (2%) pts in each arm experienced fatal AEs.

**Summary/Conclusion**

This analysis of R/R FL pts receiving 2L treatment in the inMIND study confirms that addition of tafa to Len+R improved PFS, representing a 60% reduction in risk of progression or death, including in pts receiving prior anti-CD20+chemo or with POD24. Improvements were also observed in PET-CR, ORR, and TTNT. The safety profile was manageable in pts receiving 2L treatment with toxicities as expected.

**Copyright Information:** (Online) ISSN: 2572-9241
©2026 The Author(s). HemaSphere is published by John Wiley & Sons Ltd on behalf of European Hematology Association. This is an open access Abstract Book distributed under the Attribution-NonCommercial-NoDerivs (CC BY-NC-ND), which allows third parties to download the articles and share them with others as long as they credit the author and the Abstract Book, but they cannot change the content in any way or use them commercially.
**Abstract Book Citations:** Authors, Title, HemaSphere, 2026;10:(S1):pages. The abstract book DOIs can be found on the "Author Information" page.
**Disclaimer:** Articles published in the journal HemaSphere exclusively reflect the opinions of the authors. The authors are responsible for all content in their abstracts including accuracy of the facts, statements, citing resources, etc.

| Variable | 1L anti-CD20 + chemo | | 1L anti-CD20 + chemo with POD24 | |
|---|---|---|---|---|
| | Tafa+Len+R (n=122) | Pbo+Len+R (n=132) | Tafa+Len+R (n=46) | Pbo+Len+R (n=59) |
| ***Primary endpoint*** | | | | |
| Median PFS by INV, months | 24.0 | 14.4 | 19.2 | 11.7 |
| HR (95% CI) | 0.45 (0.29, 0.71) | | 0.46 (0.24, 0.88) | |
| ***Secondary and exploratory endpoints*** | | | | |
| Median PFS by IRC, months | NR | 20.1 | 19.1 | 13.3 |
| HR (95% CI) | 0.51 (0.31, 0.82) | | 0.50 (0.25, 0.99) | |
| PET-CR rate, %* | 52.2 | 41.6 | 46.5 | 28.6 |
| OR (95% CI) | 1.6 (0.95, 2.66) | | 2.6 (1.10, 6.16) | |
| ORR, % | 86.9 | 71.2 | 82.6 | 57.6 |
| OR (95% CI) | 2.8 (1.40, 5.45) | | 3.9 (1.46, 10.18) | |
| Median TTNT, months | NR | 28.8 | NR | 15.5 |
| HR (95% CI) | 0.52 (0.31, 0.86) | | 0.58 (0.30, 1.13) | |

**Table. Efficacy outcomes from inMIND in 2L patients who received 1L anti-CD20 + chemo**

*FDG-avid population (1L anti-CD20 + chemo: tafa n=113, pbo n=125; 1L anti-CD20 + chemo with POD24: tafa n=43, pbo n=56). PET-CR rate was defined as the proportion of patients who achieved complete metabolic response at any time after start of treatment as per Lugano classification among the patients with a positive PET scan at baseline, by PET-CR results only.

1L, first line; 2L, second line; chemo, chemotherapy; CI, confidence interval; FDG, fluorodeoxyglucose; HR, hazard ratio; INV, investigator assessment; IRC, independent review committee; Len, lenalidomide; NR, not reached; OR, odds ratio; ORR, overall response rate; pbo, placebo; PET-CR, positron emission tomography complete response; PFS, progression-free survival; POD24, progression of disease within 24 months of start of initial treatment; R, rituximab; tafa, tafasitamab; TTNT, time to next treatment.

Copyright Information: (Online) ISSN: 2572-9241
©2026 The Author(s). HemaSphere is published by John Wiley & Sons Ltd on behalf of European Hematology Association. This is an open access Abstract Book distributed under the Attribution-NonCommercial-NoDerivs (CC BY-NC-ND), which allows third parties to download the articles and share them with others as long as they credit the author and the Abstract Book, but they cannot change the content in any way or use them commercially.
Abstract Book Citations: Authors, Title, HemaSphere, 2026;10:(S1):pages. The abstract book DOIs can be found on the “Author Information” page.
Disclaimer: Articles published in the journal HemaSphere exclusively reflect the opinions of the authors. The authors are responsible for all content in their abstracts including accuracy of the facts, statements, citing resources, etc.

### (PF949) LONG-TERM FOLLOW-UP OF ORELABRUTINIB IN PATIENTS WITH RELAPSED OR REFRACTORY MARGINAL ZONE LYMPHOMA

**Topic:** 18. Indolent and mantle-cell non-Hodgkin lymphoma - Clinical

Lijuan Deng*[1], Zhiming Li[2], Hui-Lai Zhang[3], Haiwen Huang[4], Jianda Hu[5], Lihong Liu[6], Ting Liu[7], Jie Jin[8], Zunmin Zhu[9], Wenyu Li[10], Zhenqian Huang[11], Wen-Rong Huang[12], Keshu Zhou[13], Hai-Yan Yang[14], Mingzhi Zhang[15], Kaiyang Ding[16], Hui Zhou[17], Yu Hu[18], Yuerong Shuang[19], Junning Cao[20], Sujun Gao[21], Dengju Li[22], Zimin Sun[23], Qingyuan Zhang[24], Shuhua Yi[25], Chunyan Ji[26], Liansheng Zhang[27], Yue Du[28], Weige Wang[28], Renbin Zhao[28], Jun Zhu[1], Yuqin Song[1]

*[1]Peking University Cancer Hospital & Institute, Department of Lymphoma, Beijing, China;[2]Sun Yat-sen University Cancer Center, Department of Medical Oncology, Guangzhou, China; [3]Tianjin Medical University Cancer Institute and Hospital, Department of Lymphoma, Tianjin, China; [4]The First Affiliated Hospital of Soochow University, Department of Hematology, Suzhou, China; [5]Fujian Medical University Union Hospital, Department of Hematology, Fuzhou, China;[6]The Fourth Hospital of Hebei Medical University, Department of Hematology, Shijiazhuang, China; [7]West China Hospital, Sichuan University, Department of Hematology, Chengdu, China; [8]The First Affiliated Hospital, Zhejiang University School of Medicine, Department of Hematology, Hangzhou, China; [9]Henan Provincial People's Hospital, Department of Hematology, Zhengzhou, China; [10]Guangdong Provincial People's Hospital, Department of Lymphoma, Guangzhou, China; [11]The First Affiliated Hospital of Guangzhou Medical University, Department of Hematology, Guangzhou, China; [12]The Fifth Medical Center of the Chinese People's Liberation Army General Hospital, Department of Hematology, Beijing, China; [13]Affiliated Cancer Hospital of Zhengzhou University, Henan Cancer Hospital, Department of Hematology, Zhengzhou, China; [14]Zhejiang Cancer Hospital, Department of Oncology, Hangzhou, China; [15]The First Affiliated Hospital of Zhengzhou University, Department of Oncology, Zhengzhou, China;[16]Anhui Province Cancer Hospital, Department of Hematological Oncology, Hefei, China; [17]Hunan Cancer Hospital, Department of Lymphoma and Hematology, Changsha, China; [18]Union Hospital Tongji Medical College Huazhong University of Science and Technology, Department of Hematology, Wuhan, China; [19]Jiangxi Cancer Hospital, Department of Hematology and Lymphoma, Nanchang, China; [20]Fudan University Shanghai Cancer Center, Department of Medical Oncology, Shanghai, China;[21]The First Hospital of Jilin University, Department of Hematology, Changchun, China; [22]Tongji Hospital Tongji Medical College Huazhong University of Science and Technology, Department of Hematology, Wuhan, China; [23]Anhui Provincial Hospital, Department of Hematology, Hefei, China; [24]Cancer Hospital of Harbin Medical University, Department of Internal Medicine-Oncology, Harbin, China;[25]State Key Laboratory of Experimental Hematology, National Clinical Research Center for Blood Diseases, Haihe Laboratory of Cell Ecosystem, Institute of Hematology & Blood Diseases Hospital, Chinese Academy of Medical Sciences and Peking Union Medical College, Tianjin, China; [26]Qilu Hospital of Shandong University, Department of Oncology Medicine, Jinan, China; [27]Lanzhou University Second Hospital, Department of Oncology Medicine, Lanzhou, China;[28]Beijing InnoCare Pharma Tech Co., Ltd., Beijing, China;*

### Background

Marginal zone lymphoma (MZL) is a prevalent indolent B-cell non-Hodgkin lymphoma that develops through pathological B-cell receptor signaling. There is currently no established standard of care for relapsed or refractory (r/r) MZL, and clinical outcomes progressively decline with repeated lines of therapy, underscoring a persistent unmet need for more effective treatment options. Orelabrutinib is a second-generation, highly selective, irreversible Bruton tyrosine kinase (BTK) inhibitor with improved kinase selectivity and an enhanced safety profile compared with first-generation BTK inhibitors. In a multicenter, prospective phase II study (NCT03797456) enrolling predominantly patients previously treated with anti-CD20 monoclonal antibody-based regimens, orelabrutinib demonstrated significant clinical activity in r/r MZL and a well-tolerated safety profile.

### Aims

To report long-term data on the efficacy and safety of orelabrutinib in patients with r/r MZL.

**Copyright Information:** (Online) ISSN: 2572-9241
©2026 The Author(s). HemaSphere is published by John Wiley & Sons Ltd on behalf of European Hematology Association. This is an open access Abstract Book distributed under the Attribution-NonCommercial-NoDerivs (CC BY-NC-ND), which allows third parties to download the articles and share them with others as long as they credit the author and the Abstract Book, but they cannot change the content in any way or use them commercially.
**Abstract Book Citations:** Authors, Title, HemaSphere, 2026;10:(S1):pages. The abstract book DOIs can be found on the "Author Information" page.
**Disclaimer:** Articles published in the journal HemaSphere exclusively reflect the opinions of the authors. The authors are responsible for all content in their abstracts including accuracy of the facts, statements, citing resources, etc.

### Methods

Eligible patients were adults aged ≥18 years with histologically confirmed MZL who had relapsed after or were refractory to prior therapy. Orelabrutinib was administered orally at a dose of 150 mg once daily for 28-day cycles until disease progression, intolerable toxicity, death, or withdrawal of consent. The primary endpoint was overall response rate (ORR) as assessed by the independent review committee (IRC). Secondary endpoints included investigator-assessed ORR, complete response rate (CRR), time to response (TTR), progression-free survival (PFS), overall survival (OS), and safety.

### Results

Between April 25, 2019, and November 6, 2021, 90 patients with r/r MZL were enrolled and treated with orelabrutinib. The median age was 62 years (range, 23-77), and 83 patients (92.2%) had received prior anti-CD20 monoclonal antibody-based therapy. All patients had an ECOG performance status of 0-1, and the majority presented with staged IV disease (68/90, 75.6%). Disease subtypes included splenic MZL in 5 patients (5.6%), nodal MZL in 32 patients (35.6%), and mucosa-associated lymphoid tissue lymphoma in 42 patients (46.7%). Fifty-three patients (58.9%) achieved overall response as assessed by the IRC, with a median TTR of 3.6 months. At a median follow-up of 36.8 (range, 26.1-41.2), the IRC-assessed median PFS was not reached, with a 36-month PFS rate of 75.8%. Investigator-assessed ORR was 58.9% (95% confidence interval [CI], 48.0-69.2), CRR was 12.2%, and median PFS was 44.4 months (95% CI, 27.8-not evaluable). Median OS was not reached, and the 36-month OS rate was 84.7%. Treatment-related adverse events (TRAEs) were reported in 92.8% patients. Grade ≥3 TRAEs occurred in 31.5% patients, with the most common being neutrophil count decreased (14.4%) and white blood cell decreased (8.1%).

### Summary/Conclusion

With long-term follow-up, orelabrutinib demonstrated rapid and durable responses, indicating sustained therapeutic benefit in patients with r/r MZL. Importantly, no new safety signals were observed with extended follow-up.

**Copyright Information:** (Online) ISSN: 2572-9241
©2026 The Author(s). HemaSphere is published by John Wiley & Sons Ltd on behalf of European Hematology Association. This is an open access Abstract Book distributed under the Attribution-NonCommercial-NoDerivs (CC BY-NC-ND), which allows third parties to download the articles and share them with others as long as they credit the author and the Abstract Book, but they cannot change the content in any way or use them commercially.
**Abstract Book Citations:** Authors, Title, HemaSphere, 2026;10:(S1):pages. The abstract book DOIs can be found on the "Author Information" page.
**Disclaimer:** Articles published in the journal HemaSphere exclusively reflect the opinions of the authors. The authors are responsible for all content in their abstracts including accuracy of the facts, statements, citing resources, etc.

### (PF950) IMPACT OF HIV STATUS ON CLINICAL FEATURES AND OUTCOMES OF HHV8-ASSOCIATED MULTICENTRIC CASTLEMAN DISEASE: INSIGHTS FROM A NATIONWIDE GELTAMO STUDY

**Topic:** 18. Indolent and mantle-cell non-Hodgkin lymphoma - Clinical

Gianfranco Lapietra*[1], Aleix Mendez-Lopez[2], Xavier Cintas[1], Eva Gonzalez Barca[3], Mariana Bastos-Oreiro[4], Pau Abrisqueta[5], Sergio Ramos Cillan[6], Noemí Fernández[7], Ana Isabel Jimenez Ubieto[8], Sergio Felipe Pinzón[9], Antonio Gutierrez Garcia[10], Elena Pérez Ceballos[11], Andrea Rivero Arango[12], Carmen Maria Alonso Prieto[13], Alejandro Martín García-Sancho[14], María Belén Navarro[15], Jordina Rovira Solé[16], Araceli Rubio Martínez[17], Santiago Montes Moreno[18], Jose Maria Ribera[19], Gustavo Tapia Melendo[20], Jose Tomás Navarro Ferrando[19]

*[1]Josep Carreras Leukaemia Research Institute, Lymphoid Neoplasms Group, Badalona, Spain; [2]Josep Carreras Leukaemia Research Institute; Servei Hematologia, Institut Català d'Oncologia (ICO)-Badalona, Hospital Germans Trias i Pjuol, Lymphoid Neoplasms Group; Hematology Department, Badalona, Spain; [3]Institut Català d'Oncologia (ICO)-Hospitalet, Universidad de Barcelona, Instituto de Investigación Biomédica de Bellvitge IDIBELL, Hematology Department, Barcelona, Spain; [4]Hospital Universitario Gregorio Marañón, Hematology Department, Madrid, Spain; [5]Vall d'Hebron Hospital Universitari, Vall d'Hebron Institute of Oncology (VHIO), Hematology Department, Barcelona, Spain; [6]Hospital Universitario Fundación Jiménez Díaz, Hematology Department, Madrid, Spain; [7]Hospital Universitario Marqués de Valdecilla (IDIVAL), Hematology Department, Santander, Spain; [8]Hospital Universitario 12 de Octubre, Hematology Department, Madrid, Spain; [9]Hospital del Mar, Hematology Department, Barcelona, Spain; [10]Hospital Universitario Son Espases (IdISBa), Hematology Department, Palma de Mallorca, Spain; [11]H.J.M. Morales Meseguer, IMIB-Pascual Parrilla, Hematology and Oncology Department, Murcia, Spain; [12]Hospital Clinic of Barcelona, August Pi i Sunyer Biomedical Research Institute-IDIBAPS, Hematology Department, Barcelona, Spain; [13]Hospital Arnau de Vilanova, Hematology Department, Valencia, Spain; [14]Hospital Universitario de Salamanca, IBSAL, CIBERONC, Universidad de Salamanca, Hematology Department, Salamanca, Spain; [15]Hospital Universitario Puerta de Hierro, Hematology Department, Majadahonda, Madrid, Spain; [16]Hospital Universitari de Tarragona Joan XXIII, Institut Català d`Oncologia (ICO)-Tarragona, Hematology Department, Tarragona, Spain; [17]Hospital Universitario Miguel Servet, Hematology Department, Zaragoza, Spain; [18]Valdecilla/IDIVAL University Hospital, Anatomic Pathology Department and Translational Hematopathology Lab, Santander, Spain; [19]Josep Carreras Leukaemia Research Institute; Servei Hematologia, Institut Català d'Oncologia (ICO)-Badalona, Hospital Germans Trias i Pjuol; Universitat Autònoma de Barcelona, Lymphoid Neoplasms Group; Hematology Department, Badalona, Spain; [20]Josep Carreras Leukaemia Research Institute; Hospital Germans Trias i Pjuol; Universitat Autònoma de Barcelona, Lymphoid Neoplasms Group; Department of Pathology, Badalona, Spain;*

### Background

Human herpesvirus 8-associated multicentric Castleman disease (HHV8-MCD) is a rare lymphoproliferative disorder characterized by ≥2 lymphadenopathies with typical histology and positivity for HHV8 latency-associated nuclear antigen-1 (LANA-1), systemic symptoms, and inflammatory syndrome. Although it is predominantly described in people living with HIV (HIV+), it has also been reported in HIV-negative (HIV-) patients (pts). In both settings, regimens including rituximab (RTX) are considered the gold standard for first-line treatment. Due to its rarity, HHV8-MCD remains poorly characterized, with limited and controversial data on differences regarding HIV status.

### Aims

To characterize the clinical presentation and prognosis of HHV8-MCD in a real-life setting, stratifying pts according to HIV status, in order to identify differences between the two cohorts.

**Copyright Information:** (Online) ISSN: 2572-9241
©2026 The Author(s). HemaSphere is published by John Wiley & Sons Ltd on behalf of European Hematology Association. This is an open access Abstract Book distributed under the Attribution-NonCommercial-NoDerivs (CC BY-NC-ND), which allows third parties to download the articles and share them with others as long as they credit the author and the Abstract Book, but they cannot change the content in any way or use them commercially.
**Abstract Book Citations:** Authors, Title, HemaSphere, 2026;10:(S1):pages. The abstract book DOIs can be found on the "Author Information" page.
**Disclaimer:** Articles published in the journal HemaSphere exclusively reflect the opinions of the authors. The authors are responsible for all content in their abstracts including accuracy of the facts, statements, citing resources, etc.

### Methods

We conducted a multicenter retrospective analysis across 21 centers in Spain, using data from the national registry GELTAMO-CAST-2020-018. To be included in the study, patients were required to have a histologically confirmed diagnosis of HHV8-MCD and a known HIV virologic status at the time of diagnosis. Comparisons between HIV+ and HIV- pts were conducted using non-parametric tests, and survival was analyzed by Kaplan–Meier curves with the Log-rank test.

### Results

Out of 176 overall Castleman disease cases recorded in the registry, 57 met the inclusion criteria for the study. Fifty-one pts (89%) were male; median age was 47 (range, 25–88). Forty-one cases (72%) were HIV+, all of them on treatment with antiretroviral therapy (ART); 16 (28%) were HIV-. HIV+ pts were significantly younger at diagnosis compared with HIV- (median age, 42 vs. 68.5; p< 0.001). They also more frequently presented with concomitant Kaposi sarcoma (KS) (56% vs. 19%; p = 0.017) and tended to have a higher prevalence of EBV positivity, as assessed by EBER hybridization on lymph node tissue (46% vs. 10%; p = 0.059). Furthermore, 7 HIV+ pts (12%) had an aggressive B-cell non-Hodgkin lymphoma (B-NHL) concomitant with or subsequent to the diagnosis of HHV8-MCD (3 plasmablastic, 2 primary effusion, 1 HHV8+ diffuse large B-cell, 1 diffuse large B-cell, NOS). No cases of lymphoma were observed in the HIV- cohort. At data cut-off, 50 pts had received first-line therapy, with RTX administered in 41/50 cases (82%). For the purpose of the study, only pts treated with the gold-standard were considered for response and survival analyses. The overall response rate (ORR) was significantly higher in HIV+ pts than in HIV- pts (85% vs. 50%; p = 0.032). After a median follow-up of 38 months (range, 1–179), no statistically significant differences were observed according to HIV status in terms of overall survival (OS) or progression-free survival (PFS) (p=0.243 and p=0.459, respectively). However, HIV+ pts with B-NHL had significantly worse outcomes compared with those without B-NHL (median OS, 13 months vs. not reached; p = 0.004; median PFS, 7 months vs. 58 months; p = 0.018). Stratification by both HIV status and B-NHL confirmed the adverse prognostic impact of B-NHL, while HIV+ pts without B-NHL tended to have better outcomes than HIV- pts (Figure 1).

### Summary/Conclusion

HHV8-MCD in HIV+ pts, compared to HIV-, is characterized by younger age at diagnosis and higher frequency of KS, B-NHL and EBV. HIV infection per se does not appear to adversely affect prognosis. However, the presence of B-NHL in this setting remains a negative prognostic factor.

**Copyright Information:** (Online) ISSN: 2572-9241
©2026 The Author(s). HemaSphere is published by John Wiley & Sons Ltd on behalf of European Hematology Association. This is an open access Abstract Book distributed under the Attribution-NonCommercial-NoDerivs (CC BY-NC-ND), which allows third parties to download the articles and share them with others as long as they credit the author and the Abstract Book, but they cannot change the content in any way or use them commercially.
**Abstract Book Citations:** Authors, Title, HemaSphere, 2026;10:(S1):pages. The abstract book DOIs can be found on the “Author Information” page.
**Disclaimer:** Articles published in the journal HemaSphere exclusively reflect the opinions of the authors. The authors are responsible for all content in their abstracts including accuracy of the facts, statements, citing resources, etc.

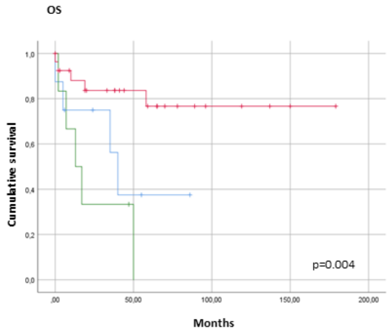

| | HIV- cohort | HIV+ cohort without B-NHL | HIV+ cohort with B-NHL |
|---|---|---|---|
| **Median OS (months)** (95% CI) | 40 (29-50) | Not reached | 13 (1-25) |

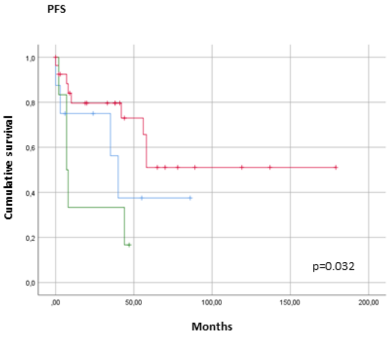

| | HIV- cohort | HIV+ cohort without B-NHL | HIV+ cohort with B-NHL |
|---|---|---|---|
| **Median PFS (months)** (95% CI) | 40 (29-50) | Not reached | 7 (2-11) |

Legend:

HIV+ without B-NHL

HIV-

HIV+ with B-NHL

Kaplan-Meier curves showing overall survival (top) and progression-free survival (bottom) stratified by HIV status and B-NHL in HHV8-MCD patients treated with rituximab.

**Copyright Information:** (Online) ISSN: 2572-9241
©2026 The Author(s). HemaSphere is published by John Wiley & Sons Ltd on behalf of European Hematology Association. This is an open access Abstract Book distributed under the Attribution-NonCommercial-NoDerivs (CC BY-NC-ND), which allows third parties to download the articles and share them with others as long as they credit the author and the Abstract Book, but they cannot change the content in any way or use them commercially.
**Abstract Book Citations:** Authors, Title, HemaSphere, 2026;10:(S1):pages. The abstract book DOIs can be found on the “Author Information” page.
**Disclaimer:** Articles published in the journal HemaSphere exclusively reflect the opinions of the authors. The authors are responsible for all content in their abstracts including accuracy of the facts, statements, citing resources, etc.

### (PF951) EFFICACY, SAFETY, AND GENETIC ANALYSIS OF ORELABRUTINIB COMBINED WITH RITUXIMAB AS FIRST-LINE SYSTEMIC TREATMENT FOR MARGINAL ZONE LYMPHOMA

**Topic:** 18. Indolent and mantle-cell non-Hodgkin lymphoma - Clinical

Xian Li[1], Yun Liang*[1], Wenbin Qian[1], Yaping Xie[2], Wenping Wu[3], Ying Lu[4]

*[1]The Second Affiliated Hospital, College of Medicine, Zhejiang University, Department of Hematology, Hangzhou, China; [2]Affiliated Hangzhou First People's Hospital, Zhejiang University School of Medicine, Department of Hematology, Hangzhou, China; [3]People's Hospital of Quzhou, Department of Hematology, Quzhou, China; [4]The Affiliated People's Hospital of Ningbo University, Department of Hematology, Ningbo, China;*

### Background

Marginal zone lymphoma (MZL) is a heterogeneous B-cell malignancy that is often managed with chemoimmunotherapy in the first-line setting, but chemotherapy entails considerable toxicity. Orelabrutinib, a novel oral BTK inhibitor which offers improved target selectivity and fewer off-target effects. This study evaluates the chemo-free combination of orelabrutinib plus rituximab in untreated MZL.

### Methods

This is a prospective, single-arm, multicenter phase II clinical trial (NCT07022223). Patients with untreated MZL who met ECOG criteria of 0–2 and were in need of treatment, or who had failed local, were eligible. All participants will receive induction therapy with the orelabrutinib and rituximab regimen. The treatment cycle is 28 days, with a total of 4~6 cycles. The induction treatment period is as follows: Cycle 1: Rituximab, 375 mg/m², on day 1. Cycles 2-6: Orelabrutinib, 150 mg, once daily orally, from day 1-28, plus rituximab 375 mg/m² on day 1. Patients who achieve a partial response (PR) or better will enter a 2-year maintenance period with orelabrutinib. The primary endpoint is the overall response rate (ORR). Secondary endpoints include complete response (CR) rate, PR rate, progression-free survival (PFS), and overall survival (OS). Safety is assessed by monitoring adverse events (AEs).

### Results

From March 2025 to December 2025, a total of 40 patients with MZL (32 MALT, 6 NMZL, 2 SMZL) were enrolled. The median age at diagnosis was 68 years (range 44–84), and 82.5 % of patients were older than 60 years. 21 (52.5%) were female. Most patients had an ECOG score of 0-1 (28/40), Ann Arbor Stage Ⅲ-IV disease (35/40). Twelve patients had an MZL-International Prognostic Index score of ≥2. Thirteen patients had failed prior local therapy, the majority of whom had undergone surgical resection. After three cycles, 25 patients were evaluable, with an ORR of 80 % (20/25) and a CRR of 24 %. Following six cycles, 13 patients were evaluable, achieving an ORR of 100 % (13/13) and a CRR of 62 % (8/13). The median time to response was 72 days. With a median follow-up of 5.7 months (range, 5.1-6.8), the median PFS and OS were not reached, the 1-year PFS rates were 97.2%(95% CI: 92.0-100.0%), and the 1-year OS rates were 100%. Next-generation sequencing (NGS) was performed on 16 patients, a total of 158 mutated genes were identified, with missense mutations predominating (62.7%). The most frequently mutated genes were *NOTCH2, FAT4, LRP1B, PCLO*, and *SYNE1*, all at 31.2 %. Treatment was generally well tolerated, only 13 patients experienced adverse events, the most common adverse events were anemia (15 %), infection (12.5 %) and neutropenia (10 %). Notably, BTK inhibitor–associated atrial fibrillation (0 %) and bradycardia (0 %) were absent.

### Summary/Conclusion

The combination of orelaburtinib and rituximab shows significant activity in MZL, with a manageable toxicity profile.

**Copyright Information:** (Online) ISSN: 2572-9241
©2026 The Author(s). HemaSphere is published by John Wiley & Sons Ltd on behalf of European Hematology Association. This is an open access Abstract Book distributed under the Attribution-NonCommercial-NoDerivs (CC BY-NC-ND), which allows third parties to download the articles and share them with others as long as they credit the author and the Abstract Book, but they cannot change the content in any way or use them commercially.
**Abstract Book Citations:** Authors, Title, HemaSphere, 2026;10:(S1):pages. The abstract book DOIs can be found on the "Author Information" page.
**Disclaimer:** Articles published in the journal HemaSphere exclusively reflect the opinions of the authors. The authors are responsible for all content in their abstracts including accuracy of the facts, statements, citing resources, etc.

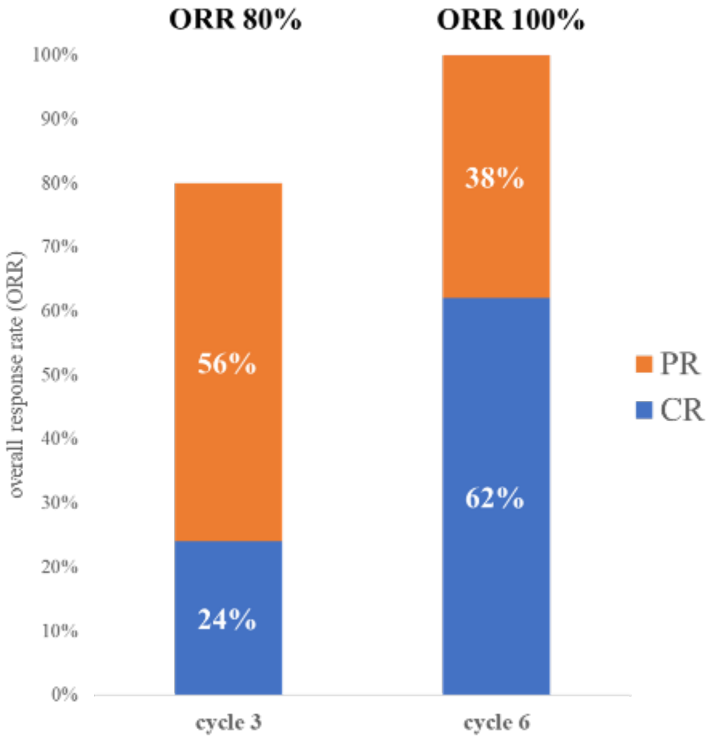

Figure 1. ORR Assessed at Cycle 3 and Cycle 6

**Copyright Information:** (Online) ISSN: 2572-9241
©2026 The Author(s). HemaSphere is published by John Wiley & Sons Ltd on behalf of European Hematology Association. This is an open access Abstract Book distributed under the Attribution-NonCommercial-NoDerivs (CC BY-NC-ND), which allows third parties to download the articles and share them with others as long as they credit the author and the Abstract Book, but they cannot change the content in any way or use them commercially.
**Abstract Book Citations:** Authors, Title, HemaSphere, 2026;10:(S1):pages. The abstract book DOIs can be found on the "Author Information" page.
**Disclaimer:** Articles published in the journal HemaSphere exclusively reflect the opinions of the authors. The authors are responsible for all content in their abstracts including accuracy of the facts, statements, citing resources, etc.

### (PF952) INCIDENCE OF INFECTIONS IN PATIENTS TREATED WITH RITUXIMAB-BENDAMUSTINE WITH OR WITHOUT ACALABRUTINIB FOR UNTREATED MANTLE CELL LYMPHOMA: ANALYSIS OF THE PHASE 3 ECHO TRIAL

**Topic:** 18. Indolent and mantle-cell non-Hodgkin lymphoma - Clinical

Martin Dreyling*[1], Ian W. Flinn[2], Melina Boutin[3], Eva Gonzalez Barca[4], Jiefen Munley[5], Moana Hodari[6], Victoria Otero[7], Andre Goy[8]

*[1]Medizinische Klinik III, LMU Hospital, Munchen, Germany; [2]Tennessee Oncology and OneOncology, Nashville, TN, United States of America; [3]Centre Intégré de Cancérologie de la Montérégie, Hôpital Charles-Lemoyne, Greenfield Park, Canada; [4]Institut Català dÒncolgia Hospitalet, Universidad de Barcelona, IDIBELL, Barcelona, Spain Microbiology Department, Hospital Universitari i Politècnic La Fe, València, Spain; [5]AstraZeneca, Wilmington, DE, United States of America;[6]AstraZeneca, Cambridge, United Kingdom; [7]AstraZeneca, Gaithersburg, MD, United States of America;[8]John Theurer Cancer Center, Hackensack Meridian Health, Hackensack, NJ, United States of America;*

### Background

Bendamustine-rituximab (BR) has been shown to be efficacious and well tolerated, but infections are a known risk. Adding acalabrutinib (A) to BR (ABR) significantly improved progression-free survival over BR in older patients (pts) with treatment-naive (TN) mantle cell lymphoma (MCL) in the phase 3 ECHO trial (NCT02972840), with an overall favorable safety profile; nevertheless, infections were more frequent, warranting detailed characterization.

### Aims

To analyze infections that occurred during the ECHO trial, including impact by phase of treatment and COVID-19 pandemic.

### Methods

Pts ≥65 y of age with TN MCL were randomized 1:1 to receive ABR or placebo (PBR). Treatment phases consisted of induction (ABR or PBR for 6 cycles), maintenance 1 (M1; AR or PR for 24 cycles), and maintenance 2 (M2; A or P until progressive disease or unacceptable toxicity). Crossover from PBR to A was permitted at disease progression.

**Copyright Information:** (Online) ISSN: 2572-9241
©2026 The Author(s). HemaSphere is published by John Wiley & Sons Ltd on behalf of European Hematology Association. This is an open access Abstract Book distributed under the Attribution-NonCommercial-NoDerivs (CC BY-NC-ND), which allows third parties to download the articles and share them with others as long as they credit the author and the Abstract Book, but they cannot change the content in any way or use them commercially.
**Abstract Book Citations:** Authors, Title, HemaSphere, 2026;10:(S1):pages. The abstract book DOIs can be found on the “Author Information” page.
**Disclaimer:** Articles published in the journal HemaSphere exclusively reflect the opinions of the authors. The authors are responsible for all content in their abstracts including accuracy of the facts, statements, citing resources, etc.

### Results

At the data cutoff (15 Feb 2024; median time on study 44.9 mo [range <0.1–81.3]), the safety analysis set included 297 pts in each arm. In total, 232 pts (78.1%) in the ABR arm vs 214 (72.1%) in the PBR arm had an infection. Median age of those who had an infection was 71 y in both arms. The most common (≥15%) infections (preferred terms; ABR vs PBR) were COVID-19 (30.6% vs 24.6%), upper respiratory tract infection (18.2% vs 15.5%), pneumonia (16.2% vs 14.1%), and COVID-19 pneumonia (15.8% vs 14.1%). Grade 5 infections occurred in 10.4% (ABR) vs 6.7% (PBR) of pts, grade 5 non-COVID-19 infections occurred in 2.4% vs 1.0%, and grade 5 opportunistic infections occurred in 9.1% vs 6.7%. Infections required hospitalization in 39.1% (ABR) vs 34.7% (PBR) and ICU admission in 7.7% vs 8.1%. Induction was complete for most pts (72.7% [ABR] vs 74.4% [PBR]) before the COVID-19 pandemic. Most pts were in the M1/2 phases after the start of the pandemic (M1: 69.0%, n=205 [ABR] vs 61.6%, n=183 [PBR]; M2: 76.8%, n=228 vs 70.4%, n=209). Rates and severity of infection were similar between arms during induction (**Table**). Despite having fewer drugs in the regimen, the rate and severity of infection increased in the M1 and M2 periods, coinciding with the COVID-19 pandemic, during which most patients had completed induction and entered the M1 and M2 periods. Fatal COVID-19 events were highest during M2. Overall, COVID-19 infections occurred in 38.4% (ABR) vs 30.3% (PBR). Most COVID-19 infections occurred during the Omicron wave and beyond (26 Nov 2021 to 14 Feb 2024) (infection: 34.0% [ABR] vs 23.6% [PBR]; death: 6.1% vs 3.0%), overlapping with M1/2. COVID-19 vaccination was received by 44.4% (ABR) vs 40.7% (PBR); 33.0% vs 31.6% received an mRNA vaccine and 8.1% (each arm) received a non-mRNA vaccine. Among total COVID-19 deaths (including non–treatment emergent), 17 of 28 (ABR) and 15 of 23 (PBR) were unvaccinated. COVID-19 AEs leading to discontinuation of ≥1 study treatment were reported in 11.1% (n=33; ABR) vs 7.4% (n=22; PBR). Overall, most pts with infections did not have concomitant neutropenia (71.1% vs 70.6%). G-CSF use and infection prophylaxis rates were similar between arms.

### Summary/Conclusion

This analysis contextualizes infections in the ECHO trial, showing that infection rates were highly impacted by COVID-19 infections during the pandemic. Most pts had COVID-19 infection during the M1/2 periods, coinciding with later pandemic phases, with unvaccinated pts having worse outcomes.

**Copyright Information:** (Online) ISSN: 2572-9241
©2026 The Author(s). HemaSphere is published by John Wiley & Sons Ltd on behalf of European Hematology Association. This is an open access Abstract Book distributed under the Attribution-NonCommercial-NoDerivs (CC BY-NC-ND), which allows third parties to download the articles and share them with others as long as they credit the author and the Abstract Book, but they cannot change the content in any way or use them commercially.
**Abstract Book Citations:** Authors, Title, HemaSphere, 2026;10:(S1):pages. The abstract book DOIs can be found on the "Author Information" page.
**Disclaimer:** Articles published in the journal HemaSphere exclusively reflect the opinions of the authors. The authors are responsible for all content in their abstracts including accuracy of the facts, statements, citing resources, etc.

**Table. Most Common Infections by Treatment Period.**
ABR, acalabrutinib + bendamustine + rituximab; M1, maintenance 1; M2, maintenance 2; PBR, placebo + bendamustine + rituximab.

| | ABR N=297 | | PBR N=297 | |
|---|---|---|---|---|
| n (%) | Any grade | Grade ≥3 | Any grade | Grade ≥3 |
| **Induction** | 107 (36.0) | 23 (7.7) | 109 (36.7) | 28 (9.4) |
| Upper respiratory tract infection | 23 (7.7) | 1 (0.3) | 23 (7.7) | 0 |
| Pneumonia | 10 (3.4) | 5 (1.7) | 13 (4.4) | 9 (3.0) |
| Urinary tract infection | 8 (2.7) | 1 (0.3) | 13 (4.4) | 2 (0.7) |
| **M1 period** | 154 (51.9) | 41 (13.8) | 108 (36.4) | 26 (8.8) |
| Pneumonia | 28 (9.4) | 13 (4.4) | 15 (5.1) | 4 (1.3) |
| Upper respiratory tract infection | 27 (9.1) | 0 | 12 (4.0) | 0 |
| COVID-19 | 19 (6.4) | 6 (2.0) | 16 (5.4) | 4 (1.3) |
| **M2 period** | 111 (37.4) | 46 (15.5) | 83 (27.9) | 31 (10.4) |
| COVID-19 | 57 (19.2) | 9 (3.0) | 41 (13.8) | 10 (3.4) |
| COVID-19 pneumonia | 29 (9.8) | 26 (8.8) | 20 (6.7) | 15 (5.1) |
| Upper respiratory tract infection | 12 (4.0) | 0 | 18 (6.1) | 0 |

Copyright Information: (Online) ISSN: 2572-9241
©2026 The Author(s). HemaSphere is published by John Wiley & Sons Ltd on behalf of European Hematology Association. This is an open access Abstract Book distributed under the Attribution-NonCommercial-NoDerivs (CC BY-NC-ND), which allows third parties to download the articles and share them with others as long as they credit the author and the Abstract Book, but they cannot change the content in any way or use them commercially.
Abstract Book Citations: Authors, Title, HemaSphere, 2026;10:(S1):pages. The abstract book DOIs can be found on the "Author Information" page.
Disclaimer: Articles published in the journal HemaSphere exclusively reflect the opinions of the authors. The authors are responsible for all content in their abstracts including accuracy of the facts, statements, citing resources, etc.

### (PF953) TARGETING RESISTANCE TO RITUXIMAB THROUGH FCGRIIB (CD32B) BLOCKADE: BI-1206 + RITUXIMAB + ACALABRUTINIB SHOWS POWERFUL ACTIVITY IN R/R NHL

**Topic:** 18. Indolent and mantle-cell non-Hodgkin lymphoma - Clinical

Laura Fogliatto*[1], Eduardo Munhoz[2], Ana Costa Cordeiro[3], Guilherme Fleury Perini[4], Don Stevens[5], Mariana Bastos-Oreiro[6], Eva Domingo[7], Daniel Morillo[8], Agustin Penedo Coello[9], Erika Bågeman[10], Danijela Lindahl[10], Ingrid Karlsson[10], Linda Mårtensson[10], Zinnia Parra-Guillen[10], Dmytro Piliuhin[10], Karianne Risberg Handeland[10], Anette Sundstedt[10], Ingrid Teige[10], Johan Wallin[10], Björn Frendeus[10], Andres McAllister[10]

*[1]Hospital de Clínicas de Porto Alegre, Porto Alegre, Brazil;[2]Hospital Erasto Gaertner - Liga Paranaense de Combate ao Câncer, Curitiba, Brazil; [3]A.C. Camargo Cancer Center, São Paulo, Brazil; [4]Hospital Israelita Albert Einstein, São Paulo, Brazil; [5]Norton Cancer Institute - Saint Matthews, Louisville, United States of America; [6]Hospital General Universitario Gregorio Marañón, Madrid, Spain; [7]Hospital Duran i Reynals- Institut Catala d'Oncologia, Barcelona, Spain;[8]Hospital Universitario Fundación Jiménez Díaz, Madrid, Spain; [9]University Hospital HM Sanchinarro. START Madrid CIOCC, Madrid, Spain; [10]BioInvent International AB, Lund, Sweden;*

### Background

Anti-CD20 antibodies remain central to the management of NHL, yet a substantial proportion of patients exhibit primary resistance or early relapse. FcγRIIb-mediated rituximab internalization is a known resistance-mechanism, and higher tumor FcγRIIb levels correlate with poor responses. BI-1206, an anti-FcγRIIb IgG1 antibody, blocks this internalization and restores rituximab activity. In relapsed/refractory FL patients, BI-1206 plus rituximab achieved a 59% ORR  with good tolerability.
The ROSEWOOD trial showed that adding the BTKi zanubrutinib (Z) to the Fc-engineered anti-CD20 antibody obinutuzumab (O) increased ORR from 45% to 69%. In contrast, adding the BTKi acalabrutinib to rituximab resulted in only 31% ORR, despite similar kinase profiles for acalabrutinib and zanubrutinib. These findings suggest that the greater activity of the OZ regimen resulted from improved CD20-targeting, highlighting the need to overcome rituximab resistance. Tumor FcγRIIB-mediated internalization is a principal driver of rituximab resistance. Because BI-1206 directly targets this mechanism, and obinutuzumab shows reduced but not blunted FcγRIIB-mediated internalization, combining BI-1206 with rituximab plus acalabrutinib has the potential to further improve treatment responses.

### Aims

Assess safety and efficacy of BI-1206 in combination with rituximab and acalabrutinib in R/R NHL.

### Methods

This single-arm study evaluates BI-1206 with rituximab and acalabrutinib in up to 30 patients with relapsed or refractory NHL. The design compares two subcutaneous (SC) BI-1206 doses of 150 and 225 mg. BI-1206 plus rituximab is administered QW during a 4 week induction. Patients with disease control at week 6 may receive a 2nd induction, then maintenance every eight weeks for up to six cycles. Acalabrutinib is given at 100 mg twice daily for 1 year.

**Copyright Information:** (Online) ISSN: 2572-9241
©2026 The Author(s). HemaSphere is published by John Wiley & Sons Ltd on behalf of European Hematology Association. This is an open access Abstract Book distributed under the Attribution-NonCommercial-NoDerivs (CC BY-NC-ND), which allows third parties to download the articles and share them with others as long as they credit the author and the Abstract Book, but they cannot change the content in any way or use them commercially.
**Abstract Book Citations:** Authors, Title, HemaSphere, 2026;10:(S1):pages. The abstract book DOIs can be found on the "Author Information" page.
**Disclaimer:** Articles published in the journal HemaSphere exclusively reflect the opinions of the authors. The authors are responsible for all content in their abstracts including accuracy of the facts, statements, citing resources, etc.

### Results

As of Jan 26, 2026, 23 patients were treated with BI-1206 in combination with rituximab and acalabrutinib. Nineteen patients experienced treatment-related AEs, the majority of which were mild or moderate (86%). Nine patients had Gr 3 events considered related or possibly related to study treatment: three patients with neutropenia, two with lymphopenia, two patients with IRR related to rituximab. The following Gr 3 events were recorded once: thrombocytopenia, headache, increased transaminases, urticaria and maculo-papular rash.
No treatment-related serious AEs and no Gr 4/5 events were observed.
The overall response rate observed was 80%. Among 20 evaluable patients, 7 achieved complete responses, 9 partial responses, and the remaining patients all exhibited stable disease as best response. In the FL subset (n=13), ORR was 77%. At cut-off date, average treatment duration reached was 4 months. Five patients completed the study duration of one year. Study is still ongoing and data is expected to mature.

### Summary/Conclusion

The combination treatment was well tolerated and demonstrates a highly promising therapeutic effect in R/R FL, achieving an ORR of 77%. Taken together with the approximately 80% ORR usually observed with SoC R2 therapy, these data suggest that the BI-1206 triplet may offer at least comparable efficacy with a highly manageable safety profile.
In this heavily pretreated R/R NHL population with limited therapeutic options, the BI-1206 triplet delivers robust, durable responses and a beneficial, manageable safety profile, making it a compelling future treatment option for patients with R/R FL.
We acknowledge AstraZeneca for providing acalabrutinib.

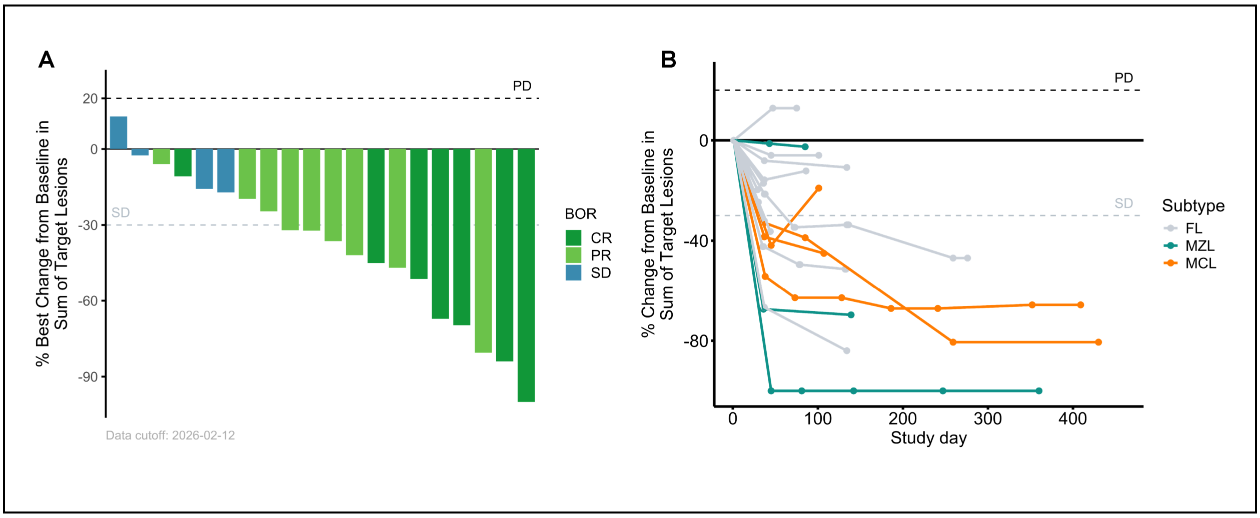

Figure 1 A) Waterfall plot depicting best response as assessed by Lugano criteria (FDG-PET if available, else CT) and largest decrease in tumor burden as assessed by CT. B) Spider plot depicting tumor burden (sum of perpendicular diameter) versus time from treatment start. Color indicate NHL subtype.

**Copyright Information:** (Online) ISSN: 2572-9241
©2026 The Author(s). HemaSphere is published by John Wiley & Sons Ltd on behalf of European Hematology Association. This is an open access Abstract Book distributed under the Attribution-NonCommercial-NoDerivs (CC BY-NC-ND), which allows third parties to download the articles and share them with others as long as they credit the author and the Abstract Book, but they cannot change the content in any way or use them commercially.
**Abstract Book Citations:** Authors, Title, HemaSphere, 2026;10:(S1):pages. The abstract book DOIs can be found on the “Author Information” page.
**Disclaimer:** Articles published in the journal HemaSphere exclusively reflect the opinions of the authors. The authors are responsible for all content in their abstracts including accuracy of the facts, statements, citing resources, etc.

### (PF954) FLP-R: A CLINICAL PREDICTION MODEL FOR FOLLICULAR LYMPHOMA PROGNOSIS IN THE RELAPSED/REFRACTORY SETTING

**Topic:** 18. Indolent and mantle-cell non-Hodgkin lymphoma - Clinical

Jennifer Bøgh Jørgensen*[1, 2], Silje Post Strøm[1], Anders Asmussen[3], Iman Chanchiri[3], Inger Lise Gade[4, 5], Mette Dahl[6], Ruth Salim Raaschou[6], Elliot Cahn[7], Matthew Maurer[7], Tarec El-Galaly[1, 2]

*[1]Aarhus University, Department of clinical medicine, Aarhus, Denmark; [2]Aarhus University Hospital, Department of Hematology, Aarhus, Denmark; [3]Odense University Hospital, Odense, Denmark; [4]Aalborg University Hospital, Aalborg, Denmark; [5]Aalborg University, Aalborg, Denmark; [6]Copenhagen University Hospital, Copenhagen, Denmark; [7]Mayo Clinic, Rochester, United States of America;*

### Background

Prognostic models for patients with non-transformed relapsed/refractory (R/R) follicular lymphoma (FL) are limited. Progression of disease within 24 months (POD24) and number of prior therapy lines, as indicators of high-risk disease, are commonly used to benchmark clinical trial outcomes, but do not sufficiently capture the heterogeneity of outcomes. The Mayo Clinic, Rochester developed a novel risk prediction model for R/R FL, the FLP-R model.

### Aims

We validated the model in a Danish population-based setting.

### Methods

The FLP-R model was developed in the Lymphoma Epidemiology of Outcomes (LEO) and the University of Iowa/Mayo Clinic Lymphoma SPORE Molecular Epidemiology Resource (MER) cohort. The model incorporates age, sex, LDH, HGB, WBC, prior immunochemotherapy, and time since diagnosis to calculate an individual FLP-R score at initiation of therapy lines 2-4. The score reflects the risk of histologic transformation (HT) and/or lymphoma-related death (LD) and stratifies patients into low (0-15), intermediate (16-30), or high (31-100) risk category.
Patients diagnosed with FL from 2010-2022 were identified from Danish National Lymphoma Registry. 640 patients were registered with relapse following initial management with radiotherapy, systemic therapy or watch-and-wait (WAW). Medical records were reviewed and clinical data on all management strategies were extracted. WAW was considered a first-line therapy. Therapy lines administered after HT were excluded.

**Copyright Information:** (Online) ISSN: 2572-9241
©2026 The Author(s). HemaSphere is published by John Wiley & Sons Ltd on behalf of European Hematology Association. This is an open access Abstract Book distributed under the Attribution-NonCommercial-NoDerivs (CC BY-NC-ND), which allows third parties to download the articles and share them with others as long as they credit the author and the Abstract Book, but they cannot change the content in any way or use them commercially.
**Abstract Book Citations:** Authors, Title, HemaSphere, 2026;10:(S1):pages. The abstract book DOIs can be found on the “Author Information” page.
**Disclaimer:** Articles published in the journal HemaSphere exclusively reflect the opinions of the authors. The authors are responsible for all content in their abstracts including accuracy of the facts, statements, citing resources, etc.

### Results

A total of 397 patients with FL grade 1–3A received at least two lines of therapy. Overall, 243 patients were excluded due to histological transformation in first- or second-line therapy (n=172), diagnostic misclassification (n=24), grade 3B FL at diagnosis (n=11), receipt of only one line of therapy (n=17), mixed first-line immunochemotherapy exposure (n=10), or missing records (n=9). Of the patients included, 129 progressed to third line and 46 to fourth line therapy. Initial management was watch-and-wait (WAW) in 63%, while 37% initiated treatment within 90 days of diagnosis; first-line therapy consisted of immunochemotherapy (69%), rituximab monotherapy (12%), or radiotherapy (16%). Median follow-up in the WAW cohort was 17.2 months (IQR 9.9–37.9). Median age at initiation of lines 2–4 was 67, 69, and 68 years, respectively. Median follow-up from initiation of lines 2–4, using reverse Kaplan–Meier, was 6.4, 4.5, and 3.9 years. Crude 5-year overall survival from initiation of lines 2-4 was 71.9% (95% CI 66.2-78.1), 59.4% (95% CI 51.4-68.7), and 56.9% (95% CI 48.9–66.1) respectively.
The distribution of FLP-R risk groups showed a clear shift toward higher risk in later treatment lines. In line 2, 116 patients (29.2%) were classified as low risk, compared with 6 patients (13%) in line 4. Conversely, the proportion of high-risk FLP-R patients increased from 30% in line 2 to 65.2% in line 4. 5-year observed rates of HT/LD in high-risk group were 38.5%, 43%, and 50% in lines 2–4, compared to 18%, 21%, and 36% in low-risk group. The FLPR model across all lines of therapy demonstrated superior discrimination (c-statistic 0.65) compared with FLIPI at diagnosis (c=0.58), line of therapy (c=0.58), POD24 status (c=0.54), or all three (c=0.59).

### Summary/Conclusion

In this population-based cohort, the FLP-R model improved risk discrimination beyond established clinical markers by identifying patients initiating 2L+ therapy at increased risk of transformation and/or lymphoma-related death.

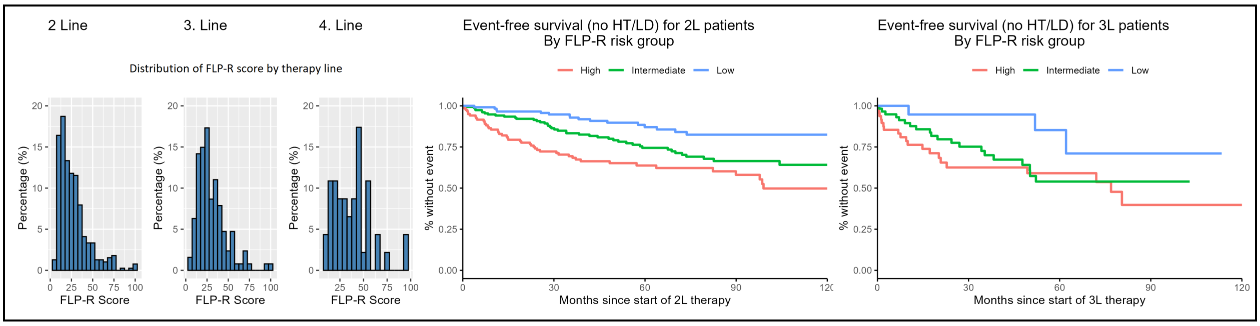

Left: Distribution of continuos FLP-R score by therapy line. Middel: Event-free survival for 2 L patients, by FLP-R risk groups. Right: Event-free survival for 3 L patients, by FLP-R risk groups.

**Copyright Information:** (Online) ISSN: 2572-9241
©2026 The Author(s). HemaSphere is published by John Wiley & Sons Ltd on behalf of European Hematology Association. This is an open access Abstract Book distributed under the Attribution-NonCommercial-NoDerivs (CC BY-NC-ND), which allows third parties to download the articles and share them with others as long as they credit the author and the Abstract Book, but they cannot change the content in any way or use them commercially.
**Abstract Book Citations:** Authors, Title, HemaSphere, 2026;10:(S1):pages. The abstract book DOIs can be found on the "Author Information" page.
**Disclaimer:** Articles published in the journal HemaSphere exclusively reflect the opinions of the authors. The authors are responsible for all content in their abstracts including accuracy of the facts, statements, citing resources, etc.

### (PF955) LTB-TREAT: A VALIDATED INTERNATIONAL PROGNOSTIC MODEL FOR TIME TO TREATMENT IN LOW TUMOUR BURDEN FL.

**Topic:** 18. Indolent and mantle-cell non-Hodgkin lymphoma - Clinical

Luani Barge[1, 2], Lachlan Webb[3], Anita Pelecanos[3], Niamh Waters[4], Ross Salvaris[5], Sam Lai[6], Tessa Potezny[7], Helen Cashman[8], Caitlyn Nguyen-Ngo[9], Denise Lee[10], Costas K. Yannakou[9, 11], Nada Hamad[8], Adrien Chauchet[12], Sylvain Choquet[13], Cedric Rossi[14], Lukshe Kanagaratnam[15], Adelie Herbin[14], Cyrielle Rodier[16, 17], Constantine Tam[7], Abir Bhattacharyya[6], Chan Y. Cheah[5], Eliza Hawkes[4], Kirk Morris[18], Eric Durot[16, 17], Joshua Tobin[1, 2], Greg Hapgood*[1, 2]

*[1]Princess Alexandra Hospital, Brisbane, Australia;[2]School of Medicine, University of Queensland, Brisbane, Australia;[3]QIMR Berghofer Medical Research Institute, Brisbane, Australia; [4]Austin Hospital, Melbourne, Australia; [5]Sir Charles Gairdner Hospital, Perth, Australia; [6]Westmead Hospital, Sydney, Australia; [7]Alfred Hospital and Monash University, Melbourne, Australia; [8]St Vincent's Hospital, Sydney, Australia; [9]Epworth HealthCare, Melbourne, Australia; [10]Eastern Health, Melbourne, Australia; [11]Department of Clinical Pathology, Faculty of Medicine, Dentistry and Health Sciences, University of Melbourne, Melbourne, Australia; [12]Department of Hematology, University Hospital of Besançon, Besançon, Australia; [13]Department of Hematology, APHP, Groupe Hospitalier Pitié-Salpêtrière, Paris, France; [14]Department of Hematology, University Hospital F. Mitterrand and Inserm UMR 1231, Dijon, France; [15]Department of Research and Innovation, University Hospital of Reims, Hôpital Robert Debré, Reims, France; [16]Department of Hematology, University Hospital of Reims, Hôpital Robert Debré, Reims, France; [17]UFR Médecine, Reims, France; [18]Royal Brisbane and Women's Hospital, Brisbane, Australia;*

### Background

Watchful waiting (WW) in low tumour burden follicular lymphoma (LTB FL) defers treatment and has been considered safe. The GELF criteria were developed using overall survival (OS) as the endpoint. With improvements in OS, there is a need to develop a prognostic model to better stratify LTB FL patients. At present, there is no validated prognostic model for time to treatment (TTT) in widespread clinical use.

### Aims

We aimed to develop and externally validate a prognostic model for TTT in patients with LTB FL in the rituximab era.

### Methods

We analysed newly diagnosed patients with FL managed with a WW approach at 8 centres from the Australasian Lymphoma Alliance (ALA). Patients were included if they were ≥18 years with grade 1-3A FL and managed with a WW approach (not treated for ≥3 months from diagnosis). Clinical, imaging and laboratory values, treatment details and outcomes were collected. TTT was defined as time from diagnosis to treatment initiation. The cumulative incidence of TTT was calculated using a competing risk approach with death as a competing risk. Variables with an association with TTT were included in a multivariable regression model to identify prognostic factors to develop a risk score model. A serious complication was defined as organ compression/dysfunction, thrombosis or serous effusion.

**Copyright Information:** (Online) ISSN: 2572-9241
©2026 The Author(s). HemaSphere is published by John Wiley & Sons Ltd on behalf of European Hematology Association. This is an open access Abstract Book distributed under the Attribution-NonCommercial-NoDerivs (CC BY-NC-ND), which allows third parties to download the articles and share them with others as long as they credit the author and the Abstract Book, but they cannot change the content in any way or use them commercially.
**Abstract Book Citations:** Authors, Title, HemaSphere, 2026;10:(S1):pages. The abstract book DOIs can be found on the "Author Information" page.
**Disclaimer:** Articles published in the journal HemaSphere exclusively reflect the opinions of the authors. The authors are responsible for all content in their abstracts including accuracy of the facts, statements, citing resources, etc.

**Results**

266 patients were included with a median follow-up of 5.0 years (range 0.5-17.8 y). The median age was 64 years, 79% had advanced stage and 89% were GELF negative. The median TTT was 5.0 years (95% CI 4.1-6.3y). Serious complications occurred in 10% and transformation in 13% of patients during WW. Elevated lactate dehydrogenase and > 4 nodal groups were associated with shorter TTT in GELF negative patients (n=229) and formed a prognostic model identifying three groups: low 59% (median TTT 6.34 years), intermediate 36% (median TTT 4.08 years), high 5% (median TTT 1.49 years)(*P*<0.001) (Harrell C score 0.607, 95% CI 0.56-0.66)(Figure). There was a higher incidence of serious complications among risk groups (low 7.5% vs intermediate 13.3% vs high 25%; *P*=0.1).
A cohort of 178 patients from four French centres fulfilled the inclusion criteria and represented an external validation cohort (Rodier et al. Hemasphere 2023). Baseline characteristics were similar between the two cohorts. The median follow up was 4.8 years (95% CI 3.6-5.9 years). Elevated LDH (HR 1.99, 95% CI 1.02-3.88, *P*=0.04) and >4 nodal sites (HR 1.86, 95% CI 1.18-2.93, *P*=0.007) were independent predictors of shorter TTT in the validation cohort. Application of the model demonstrated the distributions and outcomes of risk groups were almost superimposable: low (71%, risk score=0, median TTT 5.5 years), intermediate (27% risk score=1, median TTT 2.3 years) and high (2% risk score=2, median TTT 1.3 years)(P=0.0011)(Harrell C score 0.56, 95% CI 0.51-0.61).

**Summary/Conclusion**

LTB-TREAT is a validated prognostic model for TTT in low tumour burden FL. The model is parsimonious, easily-applied, clinically-relevant and was developed using real-world data, enhancing its applicability. It stratifies the cohort into three groups with distinct rates of progression and a trend towards serious complications. The model identifies a low-risk group predicted to have a prolonged TTT with WW and a high-risk group predicted to experience early progression in whom treatment should be considered. These data have implications for treatment decisions and patient counselling regarding management strategies in LTB FL.

**Copyright Information:** (Online) ISSN: 2572-9241
©2026 The Author(s). HemaSphere is published by John Wiley & Sons Ltd on behalf of European Hematology Association. This is an open access Abstract Book distributed under the Attribution-NonCommercial-NoDerivs (CC BY-NC-ND), which allows third parties to download the articles and share them with others as long as they credit the author and the Abstract Book, but they cannot change the content in any way or use them commercially.
**Abstract Book Citations:** Authors, Title, HemaSphere, 2026;10:(S1):pages. The abstract book DOIs can be found on the "Author Information" page.
**Disclaimer:** Articles published in the journal HemaSphere exclusively reflect the opinions of the authors. The authors are responsible for all content in their abstracts including accuracy of the facts, statements, citing resources, etc.

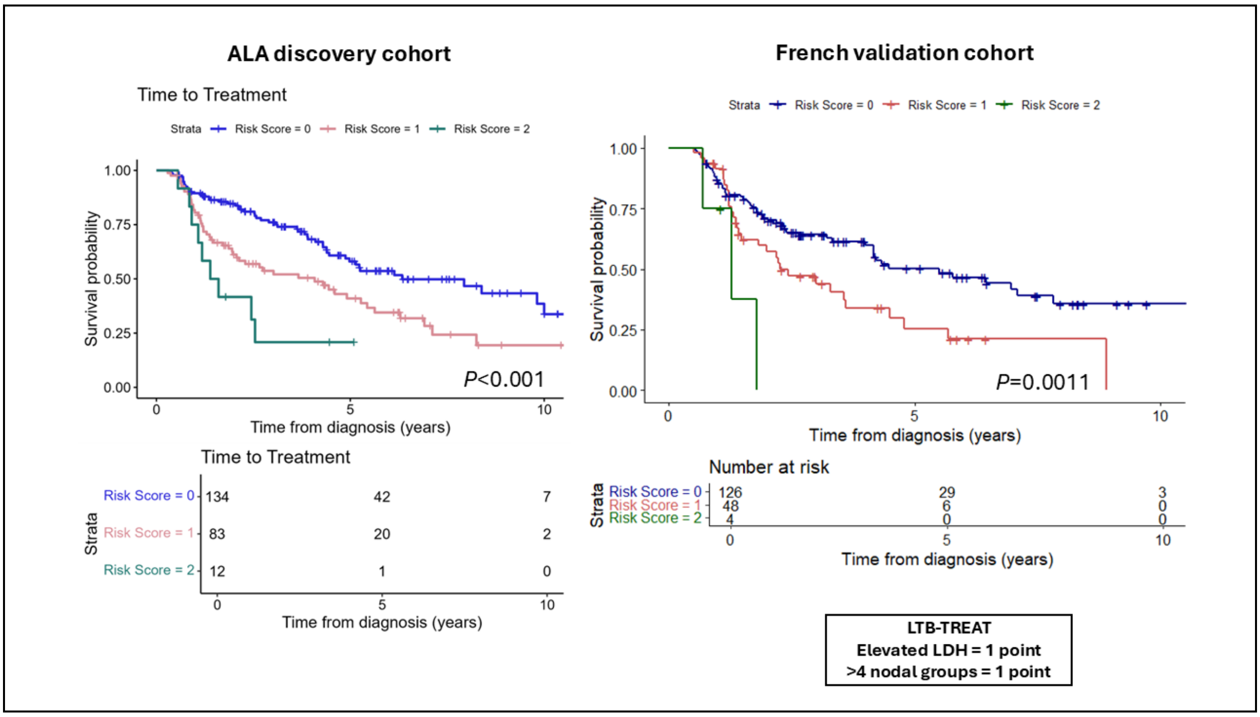

LTB-TREAT: A prognostic model for time to treatment in low tumour burden FL

**Copyright Information:** (Online) ISSN: 2572-9241
©2026 The Author(s). HemaSphere is published by John Wiley & Sons Ltd on behalf of European Hematology Association. This is an open access Abstract Book distributed under the Attribution-NonCommercial-NoDerivs (CC BY-NC-ND), which allows third parties to download the articles and share them with others as long as they credit the author and the Abstract Book, but they cannot change the content in any way or use them commercially.
**Abstract Book Citations:** Authors, Title, HemaSphere, 2026;10:(S1):pages. The abstract book DOIs can be found on the "Author Information" page.
**Disclaimer:** Articles published in the journal HemaSphere exclusively reflect the opinions of the authors. The authors are responsible for all content in their abstracts including accuracy of the facts, statements, citing resources, etc.

### (PF956) ZANUBRUTINIB PLUS R-BAP (RITUXIMAB, BENDAMUSTINE, CYTARABINE, AND PREDNISONE) IN PATIENTS WITH PREVIOUSLY UNTREATED MANTLE CELL LYMPHOMA: A PROSPECTIVE, MULTICENTER, INVESTIGATOR-INITIATED STUDY

**Topic:** 18. Indolent and mantle-cell non-Hodgkin lymphoma - Clinical

Jieming Zhang*[1], Yu Chang[1], Ling Li[1], Xin Li[1], Xinhua Wang[1], Xiaorui Fu[1], Zhenchang Sun[1], Xudong Zhang[1, 1], Linan Zhu[1], Songtao Niu[1], Feifei Nan[1], Shuhao Mei[2], Qiuhong Wang[3], Lei Zhang[1], Mingzhi Zhang[1]

*[1]First Affiliated Hospital of Zhengzhou University, Zhengzhou, China;[2]Xuchang Central Hospital, Xuchang, China;[3]Jiyuan People's Hospital, Jiyuan, China;*

### Background

Mantle cell lymphoma (MCL) is an aggressive B-cell malignancy. While current frontline chemoimmunotherapies achieve high response rates, relapse remains inevitable. Zanubrutinib, a potent next-generation BTKi, has demonstrated favorable efficacy and a superior safety profile. Here, we report preliminary efficacy and safety of zanubrutinib plus R-BAP (rituximab, bendamustine, cytarabine, and prednisone) followed by zanubrutinib maintenance in patients with treatment-naïve (TN) MCL from an ongoing, prospective, multicenter study (NCT05506410).

### Aims

To evaluate the efficacy and safety of zanubrutinib-containing regimens in patients with previously untreated MCL.

### Methods

This prospective, multicenter trial enrolled adult patients with TN MCL. Participants were stratified by age into two cohorts: a younger cohort (<65 years) and an elderly cohort (65–75 years). The younger cohort received 6 cycles of combination therapy every 28 days, consisting of zanubrutinib (160 mg, twice daily), rituximab (375 mg/m², day 0), bendamustine (70 mg/m², days 1–2), cytarabine (400 mg/m², days 1–3), and prednisone (100 mg, days 1–5), followed by 12 months of zanubrutinib maintenance (160 mg, twice daily). The elderly cohort received 4 cycles of the same R-BAP regimen (with prednisone reduced to 60 mg, days 1–5) every 28 days, followed by 4 cycles of rituximab (375 mg/m², day 1) every 21 days and 12 months of zanubrutinib maintenance (160 mg, twice daily). The trial is registered at ClinicalTrials.gov (NCT05506410).

**Copyright Information:** (Online) ISSN: 2572-9241
©2026 The Author(s). HemaSphere is published by John Wiley & Sons Ltd on behalf of European Hematology Association. This is an open access Abstract Book distributed under the Attribution-NonCommercial-NoDerivs (CC BY-NC-ND), which allows third parties to download the articles and share them with others as long as they credit the author and the Abstract Book, but they cannot change the content in any way or use them commercially.
**Abstract Book Citations:** Authors, Title, HemaSphere, 2026;10:(S1):pages. The abstract book DOIs can be found on the “Author Information” page.
**Disclaimer:** Articles published in the journal HemaSphere exclusively reflect the opinions of the authors. The authors are responsible for all content in their abstracts including accuracy of the facts, statements, citing resources, etc.

### Results

From August 2022 to January 2026, 25 eligible patients with TN MCL were enrolled across 3 centers, including 19 in the younger cohort (median age 57 years) and 6 in the elderly cohort (median age 70 years). The data cutoff date was February 22, 2026.
In the younger cohort, 12 patients completed 6 cycles of chemoimmunotherapy and were evaluable for efficacy. The overall response rate (ORR) and complete response (CR) rate were both 91.7% (11/12), including 3 patients with blastoid variant. At a median follow-up of 20 months (range, 6–41), median progression-free survival (PFS) was not reached; estimated PFS rates at 1 and 2 years were 100% and 87.5%, respectively. Median overall survival (OS) was also not reached, with all patients alive. The most frequent grade ≥3 treatment-related adverse events (TRAEs) were leukopenia (50%), neutropenia (50%), and thrombocytopenia (41.7%). Other grade ≥3 adverse events included two cases of pneumonia.
In the elderly cohort, all 6 patients were evaluable for efficacy. The ORR and CR rate were both 83.3% (5/6), with one patient achieving CR during zanubrutinib maintenance. With a median follow-up of 14 months (range, 5–33), disease progression occurred in one patient (with blastoid variant) at 5 months. Median PFS was not reached, and estimated PFS rate at 1 year was 83.3%. 1-year OS rate was 100%. The most frequent grade ≥3 TRAEs were leukopenia (50%), neutropenia (50%), and pneumonia (33.3%).

| Characteristic | Younger Cohort(N=12) | Elderly Cohort(N=6) |
|---|---|---|
| Sex,n(%)<br>Male<br>Female | <br>8 (66.7%)<br>4 (33.3%) | <br>3 (50%)<br>3 (50%) |
| Age,median(range) | 57 (39-64) | 70 (66-72) |
| Intermediate-high MIPI-c,n(%) | 2 (16.7%) | 3 (50%) |
| Ki-67≥30%,n(%) | 9 (75%) | 5 (83.3%) |
| MCL, Blastoid Variant n(%) | 3 (25%) | 2 (33.3%) |

### Summary/Conclusion

In patients with TN MCL, zanubrutinib combined with R-BAP followed by zanubrutinib maintenance demonstrated encouraging efficacy and a manageable safety profile.

**Copyright Information:** (Online) ISSN: 2572-9241
©2026 The Author(s). HemaSphere is published by John Wiley & Sons Ltd on behalf of European Hematology Association. This is an open access Abstract Book distributed under the Attribution-NonCommercial-NoDerivs (CC BY-NC-ND), which allows third parties to download the articles and share them with others as long as they credit the author and the Abstract Book, but they cannot change the content in any way or use them commercially.
**Abstract Book Citations:** Authors, Title, HemaSphere, 2026;10:(S1):pages. The abstract book DOIs can be found on the “Author Information” page.
**Disclaimer:** Articles published in the journal HemaSphere exclusively reflect the opinions of the authors. The authors are responsible for all content in their abstracts including accuracy of the facts, statements, citing resources, etc.

### (PF957) PREDICTIVE ROLE OF POSITRON EMISSION TOMOGRAPHY – COMPUTED TOMOGRAPHY (PET-CT) IN TREATMENT OF MANTLE CELL LYMPHOMA (MCL) WITH CHIMERIC ANTIGEN RECEPTOR (CAR) T-CELLS

**Topic:** 18. Indolent and mantle-cell non-Hodgkin lymphoma - Clinical

Lars Kurch[1, 2], Paul J. Bröckelmann[3, 4], David Reher[5], Beatrice Berneck[5], Carmen Fernandez-Perez[6], Marcel Teichert[7], Georg-Nikolaus Franke[5], Madlen Jentzsch[5], Maximilian Merz[5, 8], Daniel Fürst[9], Sandra Hoffmann[5], Klaus Metzeler[5], Timm Denecke[5, 6], Markus Dietlein[10], Solveig Tiepolt[1], Alexander Drzezga[10], Marco Herling[5], Carmen Diana Herling[5], Bastian von Tresckow[11], Osama Sabri[1], Uwe Platzbecker[5, 12], Conrad-Amadeus Voltin[3, 10], Ron Jachimowicz[3, 4], Vladan Vucinic*[5, 13]

*[1]University Leipzig, Department of Nuclear Medicine, Leipzig, Germany;[2]University Halle, Department of Anaesthesiology and Operative Intensive Care, Halle (Saale), Germany; [3]University of Cologne, Department I of Internal Medicine, Center for Integrated Oncology (CIO), Cologne, Germany; [4]Max Planck Institute for Biology of Ageing, Cologne, Germany;[5]University Leipzig, Hematology, Cellular Therapy, Hemostaseology and Infectious Diseases, Comprehensive Cancer Center Central Germany, Leipzig, Germany; [6]University Leipzig, Department of Radiology, Leipzig, Germany; [7]University of Duisburg Essen, Department of Haematology and Stem Cell Transplantation, West German Cancer Center and German Cancer Consortium, Essen, Germany; [8]Memorial Slone Kettering Cancer Center, New York, United States of America;[9]German Red Cross, Institute for Clinical Transfusion Medicine and Immunogenetics Ulm, Ulm, Germany; [10]University of Cologne, Department of Nuclear Medicine, Cologne, Germany; [11]University of Duisburg Essen, Essen, Germany; [12]University Dresden, Dresden, Germany; [13]Fraunhofer Institute for Celltherapy and Immunology, Leipzig, Germany;*

### Background

Brexucabtagene autoleucel (brexu-cel) is an anti-CD19 chimeric antigen receptor (CAR) T-cell therapy with high initial response rates in relapsed/refractory (r/r) mantle cell lymphoma (MCL). However, durable remissions remain limited, with >50% of patients eventually relapsing. Robust early predictors of long-term outcome after CAR T-cell therapy in MCL are lacking. While positron emission tomography-computed tomography (PET-CT) is established for response assessment and prognostication in aggressive B-cell lymphomas, its role in brexu-cel–treated MCL in real-world practice remains insufficiently defined.

### Aims

We aimed to evaluate the prognostic relevance of PET-CT performed at lymphodepletion (PET-0) and one month after infusion (PET-1) in r/r MCL patients treated with brexu-cel in a multicenter real-world setting.

### Methods

We retrospectively analyzed 28 adults with r/r MCL treated with brexu-cel at three German academic centers (10/2021–12/2024). PET-CT was assessed according to Lugano 2014 criteria. Responses were categorized as complete metabolic remission (CMR), partial metabolic remission (PMR), stable disease (SD), or progressive disease (PD). Quantitative PET parameters (SUVmean/max, metabolic tumor volume [MTV], total lesion glycolysis [TLG]) were analyzed. CAR T-cell expansion measured by PCR was available in a subset (n=14). Progression-free survival (PFS) and overall survival (OS) were estimated using Kaplan–Meier analysis.

**Copyright Information:** (Online) ISSN: 2572-9241
©2026 The Author(s). HemaSphere is published by John Wiley & Sons Ltd on behalf of European Hematology Association. This is an open access Abstract Book distributed under the Attribution-NonCommercial-NoDerivs (CC BY-NC-ND), which allows third parties to download the articles and share them with others as long as they credit the author and the Abstract Book, but they cannot change the content in any way or use them commercially.
**Abstract Book Citations:** Authors, Title, HemaSphere, 2026;10:(S1):pages. The abstract book DOIs can be found on the "Author Information" page.
**Disclaimer:** Articles published in the journal HemaSphere exclusively reflect the opinions of the authors. The authors are responsible for all content in their abstracts including accuracy of the facts, statements, citing resources, etc.

**Results**

Median age was 68.5 years (50–82); 86% were male. Patients had received a median of 4 prior lines (1–6); all were BTK inhibitor–exposed (75% refractory). Twelve patients had prior autologous transplantation (ASCT); 10 had prior bendamustine exposure. Median follow-up from brexu-cel infusion by reverse Kaplan-Meier was 18.9 months (95% CI: 15.1-31.0). Median PFS and OS were 5.3 and 16.9 months, respectively. Cytokine release syndrome (CRS) and immune-effector-cell associated neurotoxicity syndrome (ICANS) occurred in 79% and 54% (median grade 2). CMR was observed in 10 patients at PET-0 and 22 at PET-1. We found no evidence for differing PFS according to PET-0 CMR (log-rank p=0.95, median PFS of CMR and non-CMR estimated to 24.1 and 29.3 months, respectively). In contrast, PET-1 CMR was associated with superior PFS (log-rank p=0.005). Median PFS for pts with PET-1 CMR was not reached and PFS for pts without CMR was estimated to 2.7 months. CMR at PET-1 was not associated with prior therapies (e.g. bendamustine exposure, ASCT), CRS, or ICANS. At 5.3 months, cumulative relapse incidence was 15% vs 50% for PET-1 CMR vs non-CMR (Gray's p=0.197). Day-7 CAR T expansion was higher in responders (Wilcoxon-Mann-Whitney p=0.023; r=0.78 [95% CI: 0.23-0.84]). We found no evidence for further differences in quantitative PET metrics (SUV, MTV, TLG) and laboratory parameters (LDH, platelets, ferritin, hemoglobin) between responders and non-responders.

**Summary/Conclusion**

Our multicenter real-world analysis highlights the challenges regarding prognostication of pts undergoing treatment with brexu-cel for r/r MCL. We could show that metabolic response at one month—but not at lymphodepletion—was associated with improved PFS after brexu-cel in r/r MCL. Early CMR on PET-CT may serve as a clinically accessible risk stratification tool in the absence of validated biomarkers. Prospective validation in larger cohorts is warranted.

**Copyright Information:** (Online) ISSN: 2572-9241
©2026 The Author(s). HemaSphere is published by John Wiley & Sons Ltd on behalf of European Hematology Association. This is an open access Abstract Book distributed under the Attribution-NonCommercial-NoDerivs (CC BY-NC-ND), which allows third parties to download the articles and share them with others as long as they credit the author and the Abstract Book, but they cannot change the content in any way or use them commercially.
**Abstract Book Citations:** Authors, Title, HemaSphere, 2026;10:(S1):pages. The abstract book DOIs can be found on the "Author Information" page.
**Disclaimer:** Articles published in the journal HemaSphere exclusively reflect the opinions of the authors. The authors are responsible for all content in their abstracts including accuracy of the facts, statements, citing resources, etc.

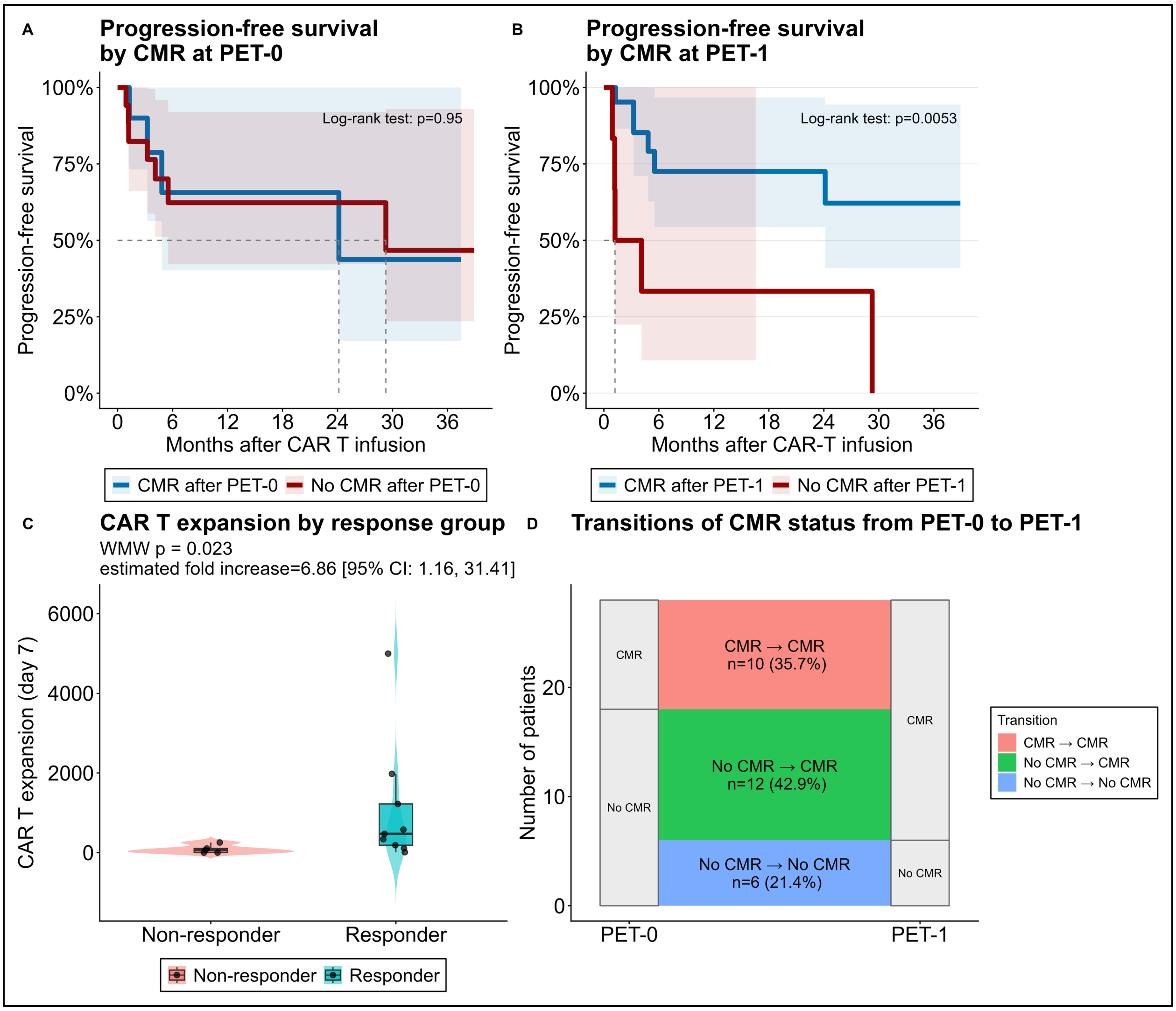

Figure 1: A. Progression-free survival (PFS) in patients achieving Complete metabolic remission (CMR) at PET-0. B. PFS in patients achieving CMR at PET-1. C. Association of CAR T-cell expansion in vivo by response. D. Transition diagram of CMR status from PET-0 to PET-1.

**Copyright Information:** (Online) ISSN: 2572-9241
©2026 The Author(s). HemaSphere is published by John Wiley & Sons Ltd on behalf of European Hematology Association. This is an open access Abstract Book distributed under the Attribution-NonCommercial-NoDerivs (CC BY-NC-ND), which allows third parties to download the articles and share them with others as long as they credit the author and the Abstract Book, but they cannot change the content in any way or use them commercially.
**Abstract Book Citations:** Authors, Title, HemaSphere, 2026;10:(S1):pages. The abstract book DOIs can be found on the "Author Information" page.
**Disclaimer:** Articles published in the journal HemaSphere exclusively reflect the opinions of the authors. The authors are responsible for all content in their abstracts including accuracy of the facts, statements, citing resources, etc.

### (PF958) CLINICAL OUTCOMES AMONG PATIENTS WITH RELAPSED/REFRACTORY MANTLE CELL LYMPHOMA RECEIVING ZANUBRUTINIB OR ACALABRUTINIB IN REAL-WORLD PRACTICE IN THE UNITED STATES

**Topic:** 18. Indolent and mantle-cell non-Hodgkin lymphoma - Clinical

Jing-Zhou Hou[1], Javier Munoz*[2], Gregory Maglinte[3], Manasi Suryavanshi[3], John Li[4], Beverly Chigarira[4], Lindsay Aton[4], Lisa Morere[4], Melissa Hagan[3], G. Rhys Williams[3], Rushir Choksi[1], Yucai Wang[5]

*[1]UPMC, Pittsburgh, United States of America;[2]Mayo Clinic, Phoenix, United States of America;[3]BeOne Medicines, Ltd., San Carlos, United States of America; [4]Integra Connect, West Palm Beach, United States of America;[5]Mayo Clinic, Rochester, United States of America;*

### Background

The second-generation Bruton tyrosine kinase inhibitors (BTKis) zanubrutinib and acalabrutinib are established therapies for relapsed or refractory mantle cell lymphoma (R/R MCL). However, head-to-head trials are lacking.

### Aims

To describe baseline characteristics and compare treatment outcomes among patients with R/R MCL receiving zanubrutinib or acalabrutinib in real-world practice.

### Methods

This is a retrospective, observational study of adult patients with R/R MCL initiating second-line or later (2L+) zanubrutinib or acalabrutinib treatment (Jan 1, 2018 – Jul 31, 2025) in the US community oncology setting. Patients were identified using the Integra Connect PrecisionQ de-identified real-world database and were followed until Oct 31, 2025. Index date was the initiation of a 2L+ BTKi. Outcome measures included time to treatment discontinuation (TTD), time to next treatment (TTNT), and overall survival (OS). Event date was the date of discontinuing a BTKi or death for TTD, and the date of starting a subsequent line of treatment (LOT) or death for TTNT. The probabilities of not discontinuing treatment, not advancing to next LOT from zanubrutinib or acalabrutinib initiation, and OS were estimated using the Kaplan–Meier method.

### Results

Overall, 93 patients received zanubrutinib and 91 received acalabrutinib. Median age was 68 years; most patients were male (76.1%), non-Hispanic (77.2%), and White (84.2%). Median (range) follow-up was 17.3 (3.0-53.3) months for zanubrutinib and 20.2 (0.9-89.0) months for acalabrutinib. Median TTD was 17.8 months (95% confidence interval [CI] 14.7, not reached [NR]) for zanubrutinib and 9.6 months (95% CI 6.5, 15.4) for acalabrutinib (log rank $P$<.01). Primary reasons for discontinuation of zanubrutinib or acalabrutinib were toxicity (11.8% vs 11.0%), disease progression (6.5% vs 5.5%), and death (2.2% vs 4.4%). Median TTNT was 18.2 months (95% CI 15.4, NR) for zanubrutinib and 15.4 months (95% CI 9.6, 26.6) for acalabrutinib (log rank $P$=.15). Median OS was NR (95% CI 30.0, NR) for zanubrutinib and 51.5 months (95% CI 41.7, NR) for acalabrutinib (log rank $P$=.37). Patients on zanubrutinib had a higher probability of not discontinuing treatment, not advancing to next LOT, and survival at 6, 12, and 18 months than those on acalabrutinib (Table).

**Copyright Information:** (Online) ISSN: 2572-9241
©2026 The Author(s). HemaSphere is published by John Wiley & Sons Ltd on behalf of European Hematology Association. This is an open access Abstract Book distributed under the Attribution-NonCommercial-NoDerivs (CC BY-NC-ND), which allows third parties to download the articles and share them with others as long as they credit the author and the Abstract Book, but they cannot change the content in any way or use them commercially.
**Abstract Book Citations:** Authors, Title, HemaSphere, 2026;10:(S1):pages. The abstract book DOIs can be found on the "Author Information" page.
**Disclaimer:** Articles published in the journal HemaSphere exclusively reflect the opinions of the authors. The authors are responsible for all content in their abstracts including accuracy of the facts, statements, citing resources, etc.

### Summary/Conclusion

In this real-world, comparative effectiveness analysis in R/R MCL, patients treated with zanubrutinib were more likely to remain on treatment, less likely to require subsequent LOT, and had better survival probability compared with those who received acalabrutinib. These findings are consistent with other real-world evidence suggesting improved outcomes for zanubrutinib over acalabrutinib. These data underscore the need for future studies evaluating long-term outcomes and optimization of sequencing strategies in real-world settings.

| | TTD | | | TTNT | | | OS | | |
|---|---|---|---|---|---|---|---|---|---|
| | **6 m %** | **12 m %** | **18 m %** | **6 m %** | **12 m %** | **18 m %** | **6 m %** | **12 m %** | **18 m %** |
| Zanu | 80 (72, 89) | 64 (54, 76) | 49 (38, 63) | 83 (75, 91) | 66 (56, 77) | 51 (40, 65) | 93 (88, 99) | 85 (77, 93) | 75 (66, 87) |
| Acala (ref) | 62 (52, 73) | 44 (35, 56) | 34 (25, 46) | 67 (58, 78) | 55 (45, 67) | 45 (35, 57) | 83 (75, 91) | 77 (68, 86) | 69 (59, 80) |
| ***Acala*** acalabrutinib, ***m*** months, ***ref*** reference, ***Zanu*** zanubrutinib | | | | | | | | | |

Table: Real-World Effectiveness Outcomes

**Copyright Information:** (Online) ISSN: 2572-9241
©2026 The Author(s). HemaSphere is published by John Wiley & Sons Ltd on behalf of European Hematology Association. This is an open access Abstract Book distributed under the Attribution-NonCommercial-NoDerivs (CC BY-NC-ND), which allows third parties to download the articles and share them with others as long as they credit the author and the Abstract Book, but they cannot change the content in any way or use them commercially.
**Abstract Book Citations:** Authors, Title, HemaSphere, 2026;10:(S1):pages. The abstract book DOIs can be found on the "Author Information" page.
**Disclaimer:** Articles published in the journal HemaSphere exclusively reflect the opinions of the authors. The authors are responsible for all content in their abstracts including accuracy of the facts, statements, citing resources, etc.

### (PF959) ORELABRUTINIB COMBINED WITH BENDAMUSTINE-RITUXIMAB OR OBINUTUZUMAB FOLLOWED BY ORELABRUTINIB MAINTENANCE IN UNTREATED MARGINAL ZONE LYMPHOMA (OPTIMIZE): A MULTICENTER, SINGLE-ARM, PHASE II STUDY

**Topic:** 18. Indolent and mantle-cell non-Hodgkin lymphoma - Clinical

Xuewu Zhang*[1], De Zhou[1], Yunfei Lv[1], Xiang Zhang[1], Xingnong Ye[1], Chunmei Yang[1], Juying Wei[1], Wanzhuo Xie[1], Haitao Meng[1], Hongyan Tong[1], Jie Jin[1], Wenjuan Yu[1]

[1]*First Affiliated Hospital, Zhejiang University School of Medicine, Department of Haematology, Hangzhou, China;*

### Background

The lack of a standard first-line treatment for marginal zone lymphoma (MZL) and the toxicity of existing systemic therapies have driven the exploration of novel regimens. In recent years, Bruton's tyrosine kinase inhibitors (BTKis) have demonstrated durable responses and a favorable benefit-risk profile across all MZL subtypes in the relapsed/refractory setting. Orelabrutinib is a novel BTKi with higher selectivity and fewer off-target effects. It has been approved by the National Medical Products Administration for the treatment of MZL patients who have received at least one prior line of therapy.

### Aims

To evaluate the efficacy and safety of orelabrutinib plus bendamustine-rituximab or obinutuzumab followed by orelabrutinib maintenance in untreated MZL.

### Methods

Optimize was a multicenter, open-label, phase II study (NCT06504940) that recruited treatment-naïve pts with MZL from 7 centers in China. Fit pts aged 18-70 years (group A) received 3 cycles of induction treatment with orelabrutinib (150 mg/day) plus bendamustine (70 mg/m$^2$ on days 1-2) and rituximab (375 mg/m$^2$ on day 0), while pts aged ≥70 years or unfit pts aged <70 years (group B) received 6 cycles of orelabrutinib (150 mg/day) plus obinutuzumab (1000 mg on days 1, 8, and 15 of cycle 1 and day 1 of cycles 2-6) every 4 weeks. Subsequently, pts without disease progression in both groups continued orelabrutinib maintenance at the same dose and scheduled for up to 21 (group A) or 18 (group B) cycles. The primary endpoint was complete response rate (CRR) at the end of induction treatment for both groups.  Key secondary endpoints in both groups were ORR, PFS, OS, and AEs.

### Results

As of January 2026, a total of 39 pts were enrolled (group A, n=30; group B, n=9). The median age of overall population was 62.0 years (group A, 60.0 years; group B, 72.0 years). The majority of pts presented with MALT lymphoma (overall, 61.5%; group A, 56.7%; group B, 77.8%%) and had Ann Arbor stage II-IV disease (overall, 76.9%; group A, 80%; group B, 66.7%). International Prognostic Index (IPI) score of 2/3 and Ki-67 expression of >20% were reported in 53.8% (group A, 53.3%; group B, 55.6%) and 23.1% (group A, 20.0%; group B, 33.3%) of pts, respectively. At the end of induction treatment, tumor evaluation was conducted in 21 pts in group A and 4 pts in group B. The CRR was 85.7% (18/21) in group A and 75.0% (3/4) in group B, with an ORR of 100.0% in both groups (Table 1). At the data cutoff, the median PFS and OS remained immature. Grade 3-4 adverse events (AEs) were occurred in 40.0% of pts overall (group A, 42.9%; group B, 25%), including thrombocytopenia (overall, 20.0%; group A, 23.8%; group B, 0.0%), leukopenia (overall,20.0%; group A, 23.8%; group B, 0.0%), and neutropenia (overall, 8.0%; group A, 4.8%; group B, 25%). No BTKi-related AEs, such as atrial fibrillation or bleeding, were observed.

**Copyright Information:** (Online) ISSN: 2572-9241
©2026 The Author(s). HemaSphere is published by John Wiley & Sons Ltd on behalf of European Hematology Association. This is an open access Abstract Book distributed under the Attribution-NonCommercial-NoDerivs (CC BY-NC-ND), which allows third parties to download the articles and share them with others as long as they credit the author and the Abstract Book, but they cannot change the content in any way or use them commercially.
**Abstract Book Citations:** Authors, Title, HemaSphere, 2026;10:(S1):pages. The abstract book DOIs can be found on the "Author Information" page.
**Disclaimer:** Articles published in the journal HemaSphere exclusively reflect the opinions of the authors. The authors are responsible for all content in their abstracts including accuracy of the facts, statements, citing resources, etc.

### Summary/Conclusion

Orelabrutinib combined with bendamustine-rituximab or obinutuzumab followed by orelabrutinib maintenance was effective and well-tolerated in untreated pts with MZL. The study is ongoing, with updated efficacy and safety data to be released in the future.

**Table 1. Tumor response at the end of induction treatment**

| | Overall (n=25) | Group A (n=21) | Group B (n=4) |
|---|---|---|---|
| Overall response rate, n (% [95% CI]) | 25 (100.0 [86.3-100.0]) | 21 (100.0 [83.9-100.0]) | 4 (100.0 [39.8-100.0]) |
| Response category, n (%) | | | |
| Complete response | 21 (84.0) | 18 (85.7) | 3 (75.0) |
| Partial response | 4 (16.0) | 3 (14.3) | 1 (25.0) |
| Stable disease | 0 | 0 | 0 |
| Progressive disease | 0 | 0 | 0 |

**Copyright Information:** (Online) ISSN: 2572-9241
©2026 The Author(s). HemaSphere is published by John Wiley & Sons Ltd on behalf of European Hematology Association. This is an open access Abstract Book distributed under the Attribution-NonCommercial-NoDerivs (CC BY-NC-ND), which allows third parties to download the articles and share them with others as long as they credit the author and the Abstract Book, but they cannot change the content in any way or use them commercially.
**Abstract Book Citations:** Authors, Title, HemaSphere, 2026;10:(S1):pages. The abstract book DOIs can be found on the "Author Information" page.
**Disclaimer:** Articles published in the journal HemaSphere exclusively reflect the opinions of the authors. The authors are responsible for all content in their abstracts including accuracy of the facts, statements, citing resources, etc.

### (PF960) INTERPRETABLE MULTIMODAL PET/CT-EHR FUSION VIA MIXTURE-OF-EXPERTS FOR PROGNOSTIC STRATIFICATION IN MANTLE CELL LYMPHOMA: A MULTICENTER STUDY

**Topic:** 18. Indolent and mantle-cell non-Hodgkin lymphoma - Clinical

Chong Jiang*[1], Ming Jiang[2], Chongyang Ding[3], Jing-Yan Xu[4]

*[1]West China Hospital, Sichuan University, Department of Nuclear Medicine, Chengdu, China;[2]West China Hospital, Sichuan University, Department of Oncology, Chengdu, China; [3]Jiangsu Province Hospital, The First Affiliated Hospital of Nanjing Medical University, Nanjing, China; [4]Nanjing Drum Tower Hospital, Nanjing University Medical School, Nanjing, China;*

### Background

Mantle cell lymphoma (MCL) is a rare B-cell malignancy characterized by high biological heterogeneity and variable clinical outcomes. While clinical indices like the Mantle Cell Lymphoma International Prognostic Index (MIPI) and metabolic parameters (e.g., TMTV, TLG) are used for risk stratification, their predictive accuracy remains suboptimal. Traditional deep learning models often process imaging and electronic health record (EHR) data independently, potentially overlooking critical cross-modal associations and lacking the transparency required for clinical translation.

.

### Aims

To develop and validate an interpretable deep learning framework (R-signature) that integrates baseline [18F]FDG PET/CT imaging and EHR data through a Mixture-of-Experts (MoE) fusion network for individualized prognostic stratification in MCL.

### Methods

In this multicenter study, 187 treatment-naïve MCL patients were analyzed (Training: n=125; Validation: n=62). A fusion network was developed using multiple "experts," including vision encoders (MedCLIP, MedSAM), radiomics extractors, and a medical language model (Med-BERT) for clinical text. An attention-based gating mechanism integrated these representations to construct multimodal radiomic signatures (R-signatures) predictive of progression-free survival (PFS) and overall survival (OS). Performance was evaluated using AUC and C-indices, and interpretability was assessed via attention heatmaps, gating weights, and pathologic correlation.

### Results

The R-signatures demonstrated strong discriminatory power, with AUCs of 0.893 (training) and 0.755 (validation) for PFS, and 0.804 and 0.844 for OS. High-risk patients stratified by R-signature had significantly shorter survival (PFS: HR=27.70, P<0.001; OS: HR=6.86, P=0.001). Multiparametric models integrating R-signatures with clinical (WBC, Ki-67, β2-MG) and metabolic (TLG) factors outperformed conventional indices, achieving C-indices of 0.781 for PFS and 0.862 for OS in the validation cohort. Interpretability analysis showed that attention maps focused on hypermetabolic tumor regions, and R-signature values were significantly higher in aggressive blastoid/pleomorphic variants compared to classic histology (P=0.028 and P=0.010).

### Summary/Conclusion

This interpretable PET/CT–EHR fusion framework significantly improves prognostic precision in MCL. By bridging the gap between non-invasive imaging features and underlying biological behavior, this tool provides a robust, clinically translatable method for risk-adapted management and personalized therapy in MCL.

**Copyright Information:** (Online) ISSN: 2572-9241
©2026 The Author(s). HemaSphere is published by John Wiley & Sons Ltd on behalf of European Hematology Association. This is an open access Abstract Book distributed under the Attribution-NonCommercial-NoDerivs (CC BY-NC-ND), which allows third parties to download the articles and share them with others as long as they credit the author and the Abstract Book, but they cannot change the content in any way or use them commercially.
**Abstract Book Citations:** Authors, Title, HemaSphere, 2026;10:(S1):pages. The abstract book DOIs can be found on the "Author Information" page.
**Disclaimer:** Articles published in the journal HemaSphere exclusively reflect the opinions of the authors. The authors are responsible for all content in their abstracts including accuracy of the facts, statements, citing resources, etc.

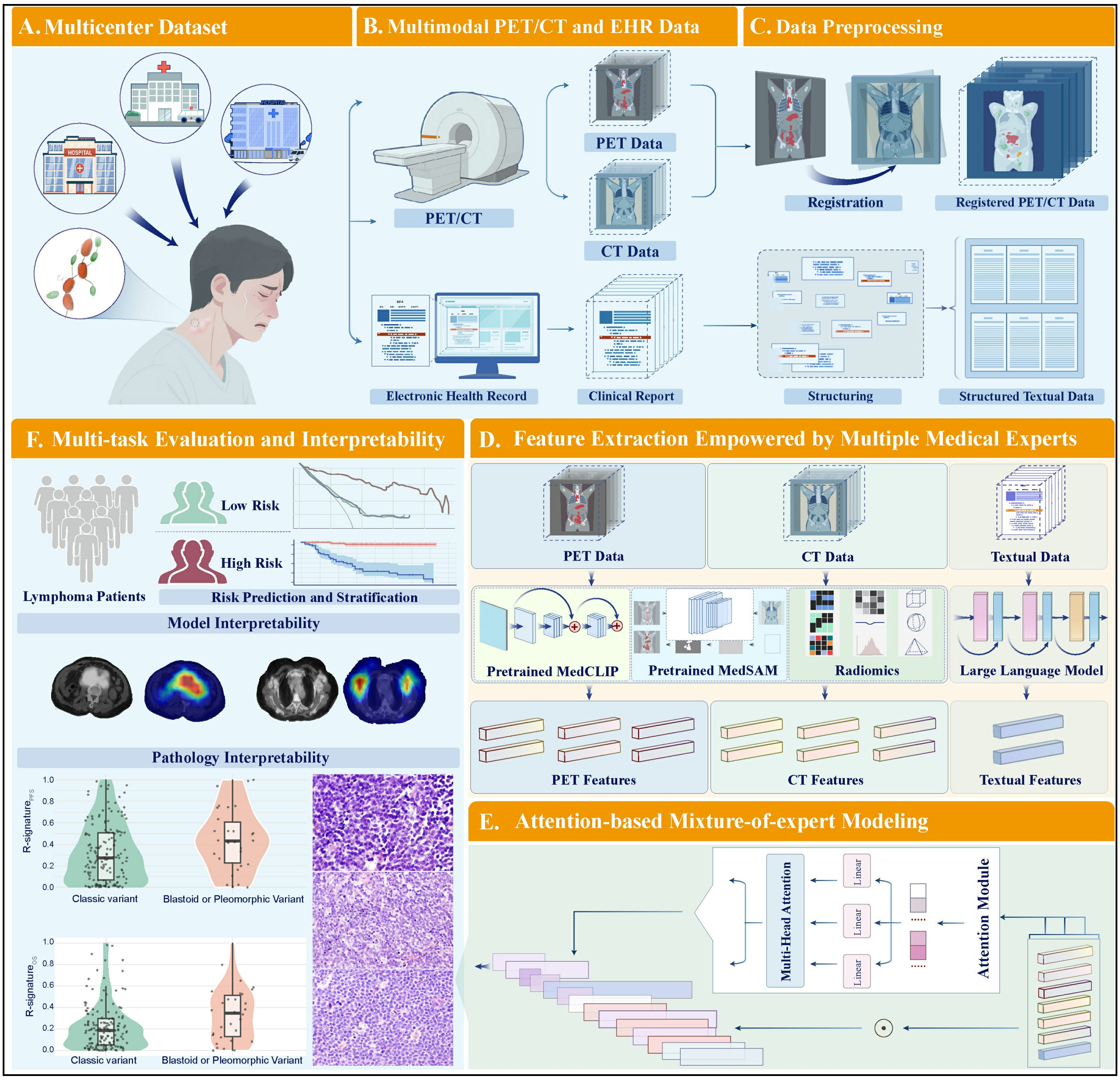

**Copyright Information:** (Online) ISSN: 2572-9241
©2026 The Author(s). HemaSphere is published by John Wiley & Sons Ltd on behalf of European Hematology Association. This is an open access Abstract Book distributed under the Attribution-NonCommercial-NoDerivs (CC BY-NC-ND), which allows third parties to download the articles and share them with others as long as they credit the author and the Abstract Book, but they cannot change the content in any way or use them commercially.
**Abstract Book Citations:** Authors, Title, HemaSphere, 2026;10:(S1):pages. The abstract book DOIs can be found on the "Author Information" page.
**Disclaimer:** Articles published in the journal HemaSphere exclusively reflect the opinions of the authors. The authors are responsible for all content in their abstracts including accuracy of the facts, statements, citing resources, etc.

### (PF961) PHASE 1/2 STUDY OF SONROTOCLAX (BGB-11417) MONOTHERAPY IN BRUTON TYROSINE KINASE (BTK) INHIBITOR-PRETREATED RELAPSED/REFRACTORY MANTLE CELL LYMPHOMA (R/R MCL): A CHINESE SUBPOPULATION ANALYSIS

**Topic:** 18. Indolent and mantle-cell non-Hodgkin lymphoma - Clinical

Yuqin Song*[1], Yuerong Shuang[2], Kaiyang Ding[3], Yu Yang[4], Liqun Zou[5], Xiaojing Xing[6], Zengjun Li[7], Hongwei Xue[8], Tianlei Chen[9], Xiaotong Li[10], Elena Ivanova[11], Gabriel Man[9], Priscille Bourquelot[9], Remus Vezan[9], Olivier Hermine[12], Toby A. Eyre[13], Michael L. Wang[14]

*[1]Peking University Cancer Hospital and Institute, Beijing, China; [2]Jiangxi Province Hospital, Nanchang, China; [3]Anhui Provincial Cancer Hospital, Hefei, China; [4]Fujian Cancer Hospital, Fuzhou, China; [5]West China Hospital, Sichuan University, Chengdu, China; [6]Liaoning Cancer Hospital & Institute, Shenyang, China; [7]Shandong Cancer Hospital, Jinan, China; [8]The Affiliated Hospital of Qingdao University Branch West Coast, Qingdao, China; [9]BeOne Medicines, Ltd, San Carlos, CA, United States of America; [10]BeOne Medicines, Ltd, Shanghai, China; [11]BeOne Medicines, Ltd, Sydney, NSW, Australia; [12]Necker Hospital Assistance Publique Hôpitaux de Paris, Paris Cité University, Imagine Institute, INSERM U 1163, Paris, France; [13]Oxford Cancer and Haematology Centre, Churchill Hospital, Headington, Oxford, United Kingdom; [14]The University of Texas MD Anderson Cancer Center, Houston, TX, United States of America;*

### Background

MCL is a rare, incurable subtype of B-cell non-Hodgkin lymphoma. Sonrotoclax (sonro), a next-generation B-cell lymphoma 2 (BCL2) inhibitor, is a more selective and pharmacologically potent BCL2 inhibitor than venetoclax, with a shorter half-life and no drug accumulation. BGB-11417-201 (NCT05471843) is an ongoing global, open-label, phase 1/2 study. In the global population, sonro was well tolerated, and patients (pts) receiving sonro (n=103) had a significantly higher overall response rate (ORR; 52.4%) vs the historic control (30%; *P*<.0001), meeting the primary endpoint.

### Aims

To report phase 1/2 data for sonro monotherapy in patients in China with BTK inhibitor–pretreated R/R MCL from BGB-11417-201.

### Methods

Eligible adults had R/R MCL and previous exposure to ≥1 anti-CD20–based therapy and ≥1 BTK inhibitor. Pts received once daily oral sonro in the part 1 dose-escalation and safety expansion (160 mg or 320 mg) and the part 2 efficacy expansion (320 mg) via gradual ≈4-wk ramp-up. This subgroup analysis in pts in China evaluated safety (parts 1 and 2) and efficacy endpoints (part 2) including ORR assessed by independent review committee (IRC) or investigator (INV), time to response (TTR), duration of response (DOR), progression-free survival (PFS), and overall survival (OS).

**Copyright Information:** (Online) ISSN: 2572-9241
©2026 The Author(s). HemaSphere is published by John Wiley & Sons Ltd on behalf of European Hematology Association. This is an open access Abstract Book distributed under the Attribution-NonCommercial-NoDerivs (CC BY-NC-ND), which allows third parties to download the articles and share them with others as long as they credit the author and the Abstract Book, but they cannot change the content in any way or use them commercially.
**Abstract Book Citations:** Authors, Title, HemaSphere, 2026;10:(S1):pages. The abstract book DOIs can be found on the “Author Information” page.
**Disclaimer:** Articles published in the journal HemaSphere exclusively reflect the opinions of the authors. The authors are responsible for all content in their abstracts including accuracy of the facts, statements, citing resources, etc.

**Results**

As of July 18, 2025, 55 pts in China were enrolled and assigned to sonro 160-mg (n=10) or 320-mg (part 1, n=11; part 2, n=34) cohorts. Results in the 320-mg cohort (n=45) are reported. Median study follow-up was 14.1 (range, 0.1-28.7) mo. Median age was 66 y and 73% were male. At study entry, 89% of pts had stage III/IV MCL, 27% had bulky disease (longest diameter ≥5 cm), 64% had an intermediate/high simplified MCL International Prognostic Index, and 24% had *TP53* mutation. The median number of prior lines of therapy was 2 (range, 1-8); 47% of pts received ≥3 prior therapies, 20% had ≥2 prior BTK inhibitors, and 80% discontinued their last therapy due to progression. Pt characteristics were generally similar in the global and China population.

In part 2 (n=34), efficacy outcomes by IRC and INV were similar (**Table**). The ORR-IRC was 62%. Median TTR-IRC was 1.8 mo; median DOR-IRC was 15.8 mo. Median PFS-IRC was 11.9 mo; median OS was not reached. As in the global population, clinically meaningful ORR, DOR, and TTR results were observed.

For pts in China who received sonro 320 mg in parts 1 and 2 (n=45), neutrophil count decreased was the most common any-grade (53%) and grade ≥3 TEAE (20%). At cutoff, 27% of pts continued on treatment and 58% discontinued treatment due to disease progression. Serious TEAEs were observed in 27%, most commonly pneumonia and platelet count decreased (each 7%). TEAEs led to treatment discontinuation in 4 pts (9%) and death in 2 (4%; multi-organ disorder and respiratory failure; both assessed as related to disease under study and study treatment). Tumor lysis syndrome (TLS) occurred in 3 pts (7%; 2 laboratory and 1 clinical; all transient and resolved without sequelae). No TLS led to treatment discontinuation. The incidences of grade ≥3 and serious TEAEs and TEAEs leading to death or treatment discontinuation/modification were generally similar in pts in China vs the global population.

**Summary/Conclusion**

As in the global population, sonro monotherapy demonstrated meaningful clinical activity and a manageable safety profile in pts in China with R/R MCL. A global phase 3 trial evaluating sonro + zanubrutinib in pts with R/R MCL (NCT06742996) is ongoing.

**Copyright Information:** (Online) ISSN: 2572-9241
©2026 The Author(s). HemaSphere is published by John Wiley & Sons Ltd on behalf of European Hematology Association. This is an open access Abstract Book distributed under the Attribution-NonCommercial-NoDerivs (CC BY-NC-ND), which allows third parties to download the articles and share them with others as long as they credit the author and the Abstract Book, but they cannot change the content in any way or use them commercially.
**Abstract Book Citations:** Authors, Title, HemaSphere, 2026;10:(S1):pages. The abstract book DOIs can be found on the "Author Information" page.
**Disclaimer:** Articles published in the journal HemaSphere exclusively reflect the opinions of the authors. The authors are responsible for all content in their abstracts including accuracy of the facts, statements, citing resources, etc.

**Table.** Part 2 Efficacy Endpoints in a Subpopulation of Patients in China With R/R MCL Receiving Sonrotoclax Monotherapy

| | **Sonrotoclax 320 mg total n=34** | |
|---|---|---|
| | **Assessed by IRC** | **Assessed by INV** |
| **ORR, n (%)**[a] | 21 (61.8) | 19 (55.9) |
| 95% CI, %[b] | 43.6-77.8 | 37.9-72.8 |
| **CRR, n (%)**[c] | 9 (26.5) | 12 (35.3) |
| 95% CI, %[b] | 12.9-44.4 | 19.7-53.5 |
| **TTR, median (range), months** | 1.8 (1.7-3.5) | 1.8 (1.7-3.7) |
| **DOR, median (95% CI), months** | 15.8 (7.4-NE) | 17.2 (7.4-NE) |
| Follow-up, median (95% CI), months | 15.6 (10.2-20.6) | 15.6 (10.2-20.6) |
| **PFS, median (95% CI), months** | 11.9 (6.3-19.8) | 9.0 (1.7-19.8) |
| Follow-up, median (95% CI), months | 17.4 (12.0-22.8) | 17.4 (12.1-22.8) |
| **OS, median (95% CI), months** | NE (7.7-NE) | |
| Follow-up, median (95% CI), months | 18.7 (16.2-21.0) | |

Medians estimated by the Kaplan-Meier method with 95% CIs estimated using the Brookmeyer and Crowley method with log-log transformation unless otherwise specified. [a]Overall response included patients who achieved a best overall response of CR or PR per the Lugano classification. [b]The 95% CI was estimated using the Clopper-Pearson method. [c]Complete response includes patients who achieved a best overall response of CR.

CR, complete response; CRR, complete response rate; DOR, duration of response; INV, investigator; IRC, independent review committee; NE, not evaluable; ORR, overall response rate; OS, overall survival; PFS, progression-free survival; PR, partial response; TTR, time to response.

**Copyright Information:** (Online) ISSN: 2572-9241
©2026 The Author(s). HemaSphere is published by John Wiley & Sons Ltd on behalf of European Hematology Association. This is an open access Abstract Book distributed under the Attribution-NonCommercial-NoDerivs (CC BY-NC-ND), which allows third parties to download the articles and share them with others as long as they credit the author and the Abstract Book, but they cannot change the content in any way or use them commercially.
**Abstract Book Citations:** Authors, Title, HemaSphere, 2026;10:(S1):pages. The abstract book DOIs can be found on the “Author Information” page.
**Disclaimer:** Articles published in the journal HemaSphere exclusively reflect the opinions of the authors. The authors are responsible for all content in their abstracts including accuracy of the facts, statements, citing resources, etc.

### (PF962) LOW-GRADE CRS AND ICANS WITH RONDECABTAGENE AUTOLEUCEL, A DUAL-TARGETING CD19/CD20 CAR T-CELL PRODUCT CANDIDATE, IN PATIENTS WITH LARGE B-CELL LYMPHOMA: UPDATED SAFETY ANALYSIS

**Topic:** 19. Large B cell lymphomas - Clinical

Sarah M. Larson*[1], Tahir Latif[2], Umar Farooq[3], Locke Bryan[4], Stefan Ciurea[5], Nebu Koshy[6], Boyu Hu[7], Bradley D. Hunter[8], Felix Mensah[9], Mehdi Hamadani[10], Hassaan Yasin[11], William Clark[12], Shashank Cingam[13], Matthew Fero[14], Sami Brake[15], Yeonhee Kim[16], Dave Shook[16], Akil Merchant[17]

*[1]UCLA David Geffen School of Medicine, Hematology and Oncology, Los Angeles, United States of America;[2]University of Cincinnati Medical Center, Cincinnati, United States of America; [3]University of Iowa Health Care, Iowa City, United States of America; [4]Georgia Cancer Center Medical College of Georgia, Augusta, United States of America;[5]University of California, Irvine, UCI Health, Orange, United States of America; [6]Baylor Scott & White Health, Dallas, United States of America;[7]Huntsman Cancer Institute at The University of Utah, Salt Lake City, United States of America; [8]Intermountain Healthcare, Salt Lake City, United States of America; [9]Indiana Blood & Marrow Transplantation, Franciscan Health, Indianapolis, United States of America; [10]Medical College of Wisconsin, Milwaukee, United States of America;[11]University of Louisville, Louisville, United States of America; [12]Virginia Commonwealth University, Richmond, United States of America; [13]LSU Health Shreveport, Shreveport, United States of America; [14]University of New Mexico, Albuquerque, United States of America; [15]Corewell Health, Grand Rapids, United States of America; [16]Lyell Immunopharma, South San Francisco, United States of America; [17]Cedars-Sinai Medical Center, Los Angeles, United States of America;*

### Background

Single-antigen CD19-targeted chimeric antigen receptor (CAR) T-cell therapies are part of routine care for patients with relapsed or refractory (R/R) large B-cell lymphoma (LBCL), yet opportunity remains to improve outcomes. Emerging dual-targeting CD19/CD20 CAR T-cell product candidates represent a next-generation approach to address antigen escape and improve durability of response. Rondecabtagene autoleucel (ronde-cel) is an autologous, dual-targeting CD19/CD20 CAR T-cell product candidate manufactured from CD62L-enriched T cells, which are associated with lower adverse cytokine production, such as IL-6 (Arcangeli 2022). Recent results from a Phase 1/2 trial (NCT05826535) evaluating ronde-cel in LBCL showed durable efficacy and an encouraging safety profile (Larson 2025).

### Aims

Here we report updated safety data from an expanded cohort with extended follow-up. We characterize the incidence, severity, time to onset, and resolution of cytokine release syndrome (CRS) and immune effector cell-associated neurotoxicity syndrome (ICANS), as well as infections and cytopenias.

### Methods

Patients with R/R LBCL (N = 87) received ronde-cel following lymphodepletion with fludarabine and cyclophosphamide. Eligible patients had received at least one prior line of therapy (second-line [2L] or third- or later-line [3L+]). There was no upper age limit. Safety assessments included CRS and ICANS grading per ASTCT consensus criteria, infection monitoring, and hematologic laboratory evaluations. Low-dose dexamethasone (10 mg/day on Days 0, 1, and 2) was introduced for ICANS prophylaxis (N = 37) and is now standard for all patients in the ongoing clinical trial.

**Copyright Information:** (Online) ISSN: 2572-9241
©2026 The Author(s). HemaSphere is published by John Wiley & Sons Ltd on behalf of European Hematology Association. This is an open access Abstract Book distributed under the Attribution-NonCommercial-NoDerivs (CC BY-NC-ND), which allows third parties to download the articles and share them with others as long as they credit the author and the Abstract Book, but they cannot change the content in any way or use them commercially.
**Abstract Book Citations:** Authors, Title, HemaSphere, 2026;10:(S1):pages. The abstract book DOIs can be found on the “Author Information” page.
**Disclaimer:** Articles published in the journal HemaSphere exclusively reflect the opinions of the authors. The authors are responsible for all content in their abstracts including accuracy of the facts, statements, citing resources, etc.

### Results

As of January 9, 2026, 84 patients had received ronde-cel (35 2L, 49 3L+) at the Phase 2 dose of $100 \times 10^6$ CAR T cells. Median age was 64 years (range, 20 to 87), 67% (56/84) had primary refractory disease, 61% (51/84) had ECOG performance status of 1, 30% (25/84) had an International Prognostic Index $^3$ 3, 56% (47/84) had Stage IV disease, 42% (35/84) had elevated lactate dehydrogenase, and median baseline sum of the product of diameters (SPD) was 19 $cm^2$ (range, 1 to 180). Median follow-up was 8 months (range, 0.2 to 26).
Grade 1 or 2 CRS occurred in 60% (50/84) of patients (Grade 1, 36%; Grade 2, 24%), with no Grade ≥ 3 CRS events. Median time to CRS onset was 5 days (range, 1 to 18), and median time to resolution was 3 days (range, 1 to 21). Tocilizumab was administered in 35% (29/84) of patients.
Grade 1 or 2 ICANS occurred in 11% (9/84) of patients (Grade 1, 8%; Grade 2, 2%). Grade ≥ 3 occurred in 3% (1/37) of patients who received dexamethasone prophylaxis versus 13% (6/47) who did not. Median time to ICANS onset in those patients who received dexamethasone prophylaxis was 7 days (range, 4 to 14), and median time to resolution was 3 days (range, 1 to 9).
Grade ≥ 3 infections occurred in 10% (8/84) of patients. Prolonged cytopenias (Grade ≥ 3 not resolved by Day 28) were observed in 23% (19/84) of patients. No deaths attributable to ronde-cel were reported.

### Summary/Conclusion

Ronde-cel continues to show a consistent and manageable safety profile with low rates of CAR T-cell associated toxicities. No Grade ≥ 3 CRS was observed, and <5% of patients reported with Grade ≥ 3 ICANS after the introduction of dexamethasone prophylaxis. These findings support the feasibility of clinical management of ronde-cel across a broad range of treatment settings for LBCL, including outpatient administration.

**Copyright Information:** (Online) ISSN: 2572-9241
©2026 The Author(s). HemaSphere is published by John Wiley & Sons Ltd on behalf of European Hematology Association. This is an open access Abstract Book distributed under the Attribution-NonCommercial-NoDerivs (CC BY-NC-ND), which allows third parties to download the articles and share them with others as long as they credit the author and the Abstract Book, but they cannot change the content in any way or use them commercially.
**Abstract Book Citations:** Authors, Title, HemaSphere, 2026;10:(S1):pages. The abstract book DOIs can be found on the “Author Information” page.
**Disclaimer:** Articles published in the journal HemaSphere exclusively reflect the opinions of the authors. The authors are responsible for all content in their abstracts including accuracy of the facts, statements, citing resources, etc.

### (PF963) FIVE-YEAR FOLLOW-UP OF PATIENTS WITH RELAPSED/REFRACTORY LARGE B-CELL LYMPHOMA WHO ACHIEVED A COMPLETE RESPONSE WITH GLOFITAMAB MONOTHERAPY

**Topic:** 19. Large B cell lymphomas - Clinical

Krish Patel*[1], Carmelo Carlo-Stella[2], Martin Hutchings[3], Franck Morschhauser[4], Gloria Iacoboni[5], Paolo Corradini[6], Fritz C. Offner[7], Lorenzo Falchi[8], Estefania Mulvihill[9], Vanessa Breton[10], Alessia Bottos[9], James Relf[11], Linda Lundberg[9], Michael Dickinson[12], Tycel Phillips[13]

*[1]Sarah Cannon Research Institute, Nashville, TN, United States of America;[2]Humanitas University and IRCCS Humanitas Research Hospital, Milan, Italy; [3]Rigshospitalet and University of Copenhagen, Copenhagen, Denmark; [4]Hôpital Claude Huriez and CHU de Lille, Lille, France; [5]Vall d'Hebron University Hospital, Barcelona, Spain;[6]University of Milan and Fondazione IRCCS Istituto Nazionale dei Tumori, Milan, Italy; [7]Department of Hematology Universitair Ziekenhuis, Gent, Belgium; [8]Lymphoma Service, Memorial Sloan Kettering Cancer Center, New York, NY, United States of America; [9]F. Hoffmann-La Roche Ltd, Basel, Switzerland; [10]Hoffmann-La Roche Ltd, Mississauga, ON, Canada; [11]Roche Products Ltd, Welwyn Garden City, United Kingdom; [12]Peter MacCallum Cancer Centre, Royal Melbourne Hospital and The University of Melbourne, Melbourne, Australia; [13]City of Hope National Medical Center, Duarte, CA, United States of America;*

### Background

Long-term follow-up data demonstrate that chimeric antigen receptor (CAR) T-cell therapy is a curative option for patients with relapsed/refractory (R/R) large B-cell lymphoma (LBCL), particularly those who achieve a complete response (CR). CD20xCD3 bispecific antibodies, such as glofitamab, have demonstrated durable responses with a favorable safety profile in R/R LBCL. However, to date the curative potential of CD20xCD3 bispecific antibodies has not been well defined.

### Aims

To evaluate the long-term outcomes, with ~5 years of follow-up, of glofitamab monotherapy in patients with R/R LBCL who achieved a CR in the Phase 1/2 NP30179 (NCT03075696) trial.

### Methods

Patients with ≥1 prior line of therapy for LBCL received obinutuzumab pre-treatment (1000mg) 7 days prior to the first glofitamab dose. Glofitamab was given intravenously at a fixed dose (0.6–25mg) or with step-up dosing (2.5/10/16 or 30mg) for up to 12 cycles. This exploratory analysis evaluated investigator-assessed duration of CR (DOCR), progression-free survival (PFS) and overall survival (OS) in patients with a CR, as well as the safety and tolerability of glofitamab monotherapy. All patients provided written informed consent.

**Copyright Information:** (Online) ISSN: 2572-9241
©2026 The Author(s). HemaSphere is published by John Wiley & Sons Ltd on behalf of European Hematology Association. This is an open access Abstract Book distributed under the Attribution-NonCommercial-NoDerivs (CC BY-NC-ND), which allows third parties to download the articles and share them with others as long as they credit the author and the Abstract Book, but they cannot change the content in any way or use them commercially.
**Abstract Book Citations:** Authors, Title, HemaSphere, 2026;10:(S1):pages. The abstract book DOIs can be found on the "Author Information" page.
**Disclaimer:** Articles published in the journal HemaSphere exclusively reflect the opinions of the authors. The authors are responsible for all content in their abstracts including accuracy of the facts, statements, citing resources, etc.

### Results

As of September 8, 2025, 107 patients had received ≥0.6mg glofitamab monotherapy and had a CR per investigator assessment. All data herein report for patients who achieved a CR. The median age was 66 years (range: 22–86); 56.1% were male. The median number of prior lines of therapy was 3 (range: 1–9); 18.7% had received prior CAR T-cell therapy; and 47.7% were refractory to their first line of therapy and 77.6% to their last line of therapy.
The median OS follow-up was 58.0 months (~5 years). The majority of patients reached CR at the first tumor assessment; median time to CR was 43.0 days (95% confidence interval [CI]: 42.0–48.0). The median DOCR was 60.0 months (95% CI: 24.9–not estimable [NE]); 57.0% of patients had an ongoing CR. The median PFS was 61.4 months (95% CI: 26.3–NE) (Figure) and the 5-year PFS rate was 51.5% (95% CI: 41.2–61.8). The median OS was NE (95% CI: NE) and the 5-year OS rate was 67.5% (95% CI: 57.8–77.1). Disease-specific survival (censoring deaths unrelated to disease progression) estimated at 5 years was 86.0% (95% CI: 78.6–93.4).
The safety profile of glofitamab in patients with a CR was in line with the pivotal cohort (Dickinson, et al. N Engl J Med 2022). Grade ≥3 adverse events (AEs) were reported in 68 (63.6%) patients, the most common of which was neutropenia (37.4%). Grade 5 AEs occurred in 4 (3.7%) patients (acute myeloid leukemia [n=2], recurrent pancreatic carcinoma [n=1] and COVID-19 [n=1]; none were deemed treatment related). Of patients with a CR who had received full treatment (n=69 [64.5%]), 35 (50.7%) reported ≥1 AE after the end of treatment (EOT). Post EOT, infections were the most common AE (17.4%; Grade 1/2: 11.6%; Grade 3/4: 5.8%), and hypogammaglobulinemia was reported in one (1.4%; Grade 2) patient. Immune recovery was observed, with median B-cell and immunoglobulin G and immunoglobulin M counts above the lower limit of normal 18–24 months after EOT.

### Summary/Conclusion

After a ~5-year follow-up, fixed-duration glofitamab monotherapy demonstrated continued benefit with durable long-term remissions and manageable safety in patients with R/R LBCL who achieved a CR. Overall, the durable remissions observed with glofitamab treatment in patients who achieved a CR suggest a curative potential in a proportion of patients.

**Copyright Information:** (Online) ISSN: 2572-9241
©2026 The Author(s). HemaSphere is published by John Wiley & Sons Ltd on behalf of European Hematology Association. This is an open access Abstract Book distributed under the Attribution-NonCommercial-NoDerivs (CC BY-NC-ND), which allows third parties to download the articles and share them with others as long as they credit the author and the Abstract Book, but they cannot change the content in any way or use them commercially.
**Abstract Book Citations:** Authors, Title, HemaSphere, 2026;10:(S1):pages. The abstract book DOIs can be found on the “Author Information” page.
**Disclaimer:** Articles published in the journal HemaSphere exclusively reflect the opinions of the authors. The authors are responsible for all content in their abstracts including accuracy of the facts, statements, citing resources, etc.

**Figure.** Kaplan–Meier curve of PFS in patients who received ≥0.6mg glofitamab monotherapy and achieved a CR.

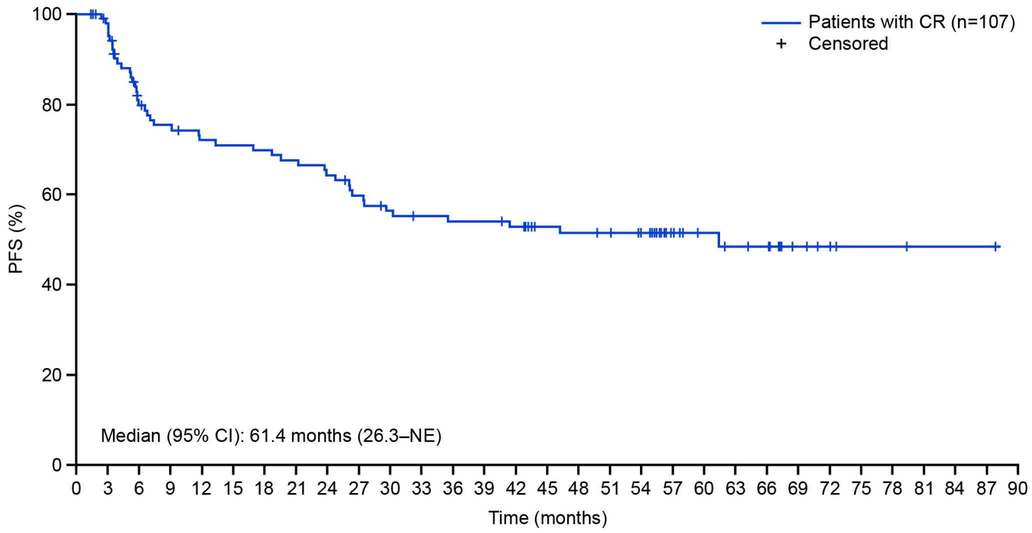

| | | | | | | | | | | | | | | | | | | | | | | | | | | | | | | | |
|---|---|---|---|---|---|---|---|---|---|---|---|---|---|---|---|---|---|---|---|---|---|---|---|---|---|---|---|---|---|---|---|
| Patients with CR (n=107) | 107 | 101 | 75 | 69 | 65 | 64 | 63 | 61 | 58 | 53 | 49 | 47 | 46 | 46 | 44 | 38 | 37 | 36 | 33 | 21 | 17 | 15 | 14 | 6 | 4 | 2 | 2 | 1 | 1 | 1 | NE |

CI, confidence interval; CR, complete response; NE, not estimable; PFS, progression-free survival.

**Copyright Information:** (Online) ISSN: 2572-9241
©2026 The Author(s). HemaSphere is published by John Wiley & Sons Ltd on behalf of European Hematology Association. This is an open access Abstract Book distributed under the Attribution-NonCommercial-NoDerivs (CC BY-NC-ND), which allows third parties to download the articles and share them with others as long as they credit the author and the Abstract Book, but they cannot change the content in any way or use them commercially.
**Abstract Book Citations:** Authors, Title, HemaSphere, 2026;10:(S1):pages. The abstract book DOIs can be found on the “Author Information” page.
**Disclaimer:** Articles published in the journal HemaSphere exclusively reflect the opinions of the authors. The authors are responsible for all content in their abstracts including accuracy of the facts, statements, citing resources, etc.

### (PF964) ZANUBRUTINIB PLUS R-CHOP AS FRONTLINE THERAPY FOR MYD88 L265P /CD79B-MUTATED (MCD) DIFFUSE LARGE B-CELL LYMPHOMA: A PHASE II STUDY.

**Topic:** 19. Large B cell lymphomas - Clinical

Chunmei Yang*[1], Ying Lu[2], Juying Wei[1], Lihong Shou[3], Hongyan Tong[1], Jie Jin[1], Wenjuan Yu[1]

*[1]The First Affiliated Hospital, Zhejiang University School of Medicine, Department of Hematology, Hangzhou, China;[2]Yinzhou People's Hospital, Department of Hematology, Ningbo, China; [3]Huzhou Central Hospital, Huzhou, China;*

### Background

MCD-subtype DLBCL characterized by concurrent MYD88(L265P) and CD79B mutations is driven by chronic active B-cell receptor/NF-κB signaling and is associated with suboptimal outcomes with standard R-CHOP. BTK inhibition represents a rational, biomarker-enriched strategy for this population.

### Aims

The present study aimed to investigate the efficacy and safety of Zanubrutinib in combination with R-CHOP in previously untreated DLBCL patients harboring both MYD88(L265P) and CD79B mutations.

### Methods

Eligible patients with previously untreated DLBCL harboring concurrent MYD88L265P and CD79B mutations (MCD subtype), identified by next-generation sequencing (NGS), and an ECOG performance status of 0–3 received Zanubrutinib 160 mg orally twice daily plus R-CHOP every 21 days. Treatment was response-adapted after four cycles based on interim response: patients achieving complete remission (CR) received two additional cycles of ZR-CHOP followed by rituximab consolidation, whereas patients with non-CR could receive up to four additional cycles of ZR-CHOP. The primary endpoint was progression-free survival (PFS). Secondary endpoints included overall response rate (ORR), overall survival (OS), and safety. Responses were investigator-assessed based on best overall response. Time-to-event endpoints were estimated using the Kaplan–Meier method.

### Results

As of January 18, 2026, 40 patients were enrolled. Median age was 60 years (range, 33–75). Ann Arbor stage III–IV disease was present in 55% (n=22), extranodal involvement in 67.5% (n=27), and IPI ≥2 in 50% (n=20); 85% (n=34) were non-GCB by immunohistochemistry. All patients received ≥1 cycle of ZR-CHOP; 34 (80%) completed protocol therapy. Six patients discontinued treatment early, including three due to documented disease progression (corresponding to PFS events) and three for other reasons. In the intent-to-treat population (N=40), ORR was 95% and the CR rate was 87.5% (35/40). In the non-GCB subgroup (n=34), responses included 30 CR (88.2%) and 2 PR. After a median follow-up of 20 months (range, 6–41), median PFS and OS were not reached; estimated 3-year PFS and OS were 88.5% (95% CI, 71.4–95.7) and 97.5% (95% CI, 83.5–99.6), respectively. Only three PFS events (progressions) were observed at data cut-off; one patient withdrew from the study (censored at withdrawal). No CNS relapse was observed. In patients with IPI 3–5 (n=11), ORR was 90.9% and CR rate 72.7%; the estimated 2-year PFS rate was 90.9% (median follow-up 15 months; range, 6–33), with one case of primary progressive disease (PFS 3 months). ZR-CHOP was generally well tolerated. The most common adverse events (all grades) were neutropenia (95%), pulmonary infection (20%), anemia (10%), and thrombocytopenia (8%). Grade 3–4 adverse events occurred in 62.5%, predominantly neutropenia.

**Copyright Information:** (Online) ISSN: 2572-9241
©2026 The Author(s). HemaSphere is published by John Wiley & Sons Ltd on behalf of European Hematology Association. This is an open access Abstract Book distributed under the Attribution-NonCommercial-NoDerivs (CC BY-NC-ND), which allows third parties to download the articles and share them with others as long as they credit the author and the Abstract Book, but they cannot change the content in any way or use them commercially.
**Abstract Book Citations:** Authors, Title, HemaSphere, 2026;10:(S1):pages. The abstract book DOIs can be found on the "Author Information" page.
**Disclaimer:** Articles published in the journal HemaSphere exclusively reflect the opinions of the authors. The authors are responsible for all content in their abstracts including accuracy of the facts, statements, citing resources, etc.

### Summary/Conclusion

Frontline ZR-CHOP demonstrated high CR rates and encouraging long-term PFS/OS with few progression events and no CNS relapse in previously untreated MYD88(L265P)/CD79B-mutated (MCD) DLBCL, including durable disease control in IPI 3–5 high-risk patients, with a manageable safety profile. These findings support a biomarker-driven BTK inhibitor–based frontline approach for this molecularly high-risk DLBCL subtype and warrant validation in a larger prospective study.

Table Baseline Characteristics

| Characteristic (N=40) | |
|---|---|
| **Median age, years (range)** | 60(33-75) |
| **Sex** | |
| Male | 26(65%) |
| Female | 14(35%) |
| **Ann Arbor stage** | |
| I–II | 18(45%) |
| III–IV | 22(55%) |
| **International prognostic index score** | |
| 0-1 | 20(50%) |
| 2-3 | 14(35%) |
| 4-5 | 6(15%) |
| **B-Symptoms** | |
| A | 33(82.5%) |
| B | 7 (17.5%) |
| **Extranodal disease** | 27 (67.5%) |
| **Cell of origin** | |
| Germinal centre B-cell-like | 6 (15%) |
| Non-germinal centre B-cell-like | 34 (85%) |

Figure Progression-free survival and overall survival

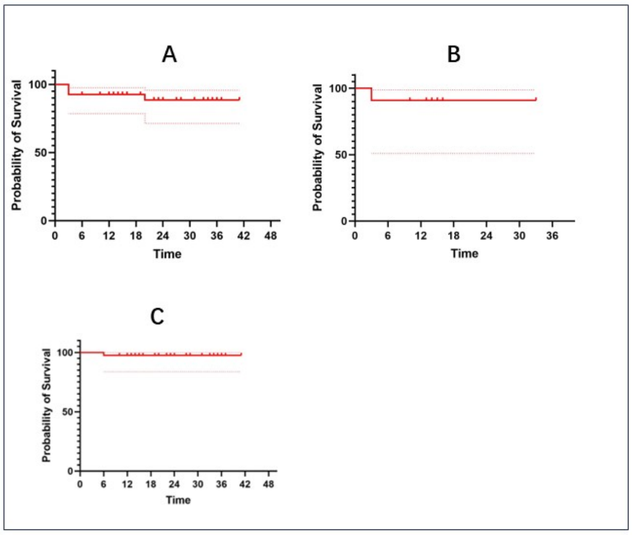

Investigator-assessed PFS in overall population (A) and IPI 3 to 5 (B),OS in overall population (C)

**Copyright Information:** (Online) ISSN: 2572-9241
©2026 The Author(s). HemaSphere is published by John Wiley & Sons Ltd on behalf of European Hematology Association. This is an open access Abstract Book distributed under the Attribution-NonCommercial-NoDerivs (CC BY-NC-ND), which allows third parties to download the articles and share them with others as long as they credit the author and the Abstract Book, but they cannot change the content in any way or use them commercially.
**Abstract Book Citations:** Authors, Title, HemaSphere, 2026;10:(S1):pages. The abstract book DOIs can be found on the "Author Information" page.
**Disclaimer:** Articles published in the journal HemaSphere exclusively reflect the opinions of the authors. The authors are responsible for all content in their abstracts including accuracy of the facts, statements, citing resources, etc.

### (PF965) THE OPTIMATE-TRIAL: AN INVESTIGATOR-INITIATED RANDOMIZED PHASE III STUDY TO OPTIMIZE MATRIX INDUCTION IN NEWLY DIAGNOSED PRIMARY CNS LYMPHOMA (TRIAL IN PROGRESS)

**Topic:** 19. Large B cell lymphomas - Clinical

Gerald Illerhaus*[1], Julia Wendler[1], Andrés José María Ferreri[2], Andreas Viardot[3], Frank Kroschinsky[4], Sebastian Wirtz[5], Louise Stanton[6], Uta Brunnberg[7], Stephan Mathas[8], Jan-Michel Heger[9], Robert Möhle[10], Alexander Röth[11], Lorenz Thurner[12], Florian Scherer[13], Jessica Okosun[14], Elke Valk[1], Jeff Smith[15], Kate Cwynarski[16], Christopher Fox[17], Elisabeth Schorb[13]

*[1]Klinikum Stuttgart, Stuttgart, Germany; [2]IRCCS San Raffaele Scientific Institute, Milano, Italy; [3]Department of Internal Medicine III, University Hospital of Ulm, Ulm, Germany; [4]Carl Gustav Carus University Hospital Dresden, Dresden, Germany; [5]Clinical Trials Unit, Medical Centre University of Freiburg, Freiburg, Germany; [6]Southampton Clinical Trials Unit, University of Southampton, Southampton, United Kingdom; [7]Goethe University Frankfurt, University Hospital, Frankfurt am Main, Germany; [8]Charité University of Medicine Berlin, Berlin, Germany;[9]University Hospital Cologne, Department I of Internal Medicine, Cologne, Germany; [10]Medical University Hospital Tübingen, Medizinische Klinik II, Tübingen, Germany;[11]University Hospital Essen, Department of Hematology and Stem Cell Transplantation, Essen, Germany; [12]Saarland University, Homburg, Germany; [13]Medical Centre, University of Freiburg, Department of Medicine I, Freiburg, Germany;[14]Barts Cancer Institute, Queen Mary University Of London, London, United Kingdom; [15]Clatterbridge Cancer Centre, Liverpool, United Kingdom; [16]University College London Hospitals NHS Foundation Trust, London, United Kingdom; [17]University of Nottingham, Nottingham, United Kingdom;*

### Background

Primary CNS lymphoma (PCNSL) is an aggressive extranodal diffuse large B-cell lymphoma with a dismal prognosis. Curative-intend treatments are associated with substantial toxicity. Induction with MATRix followed by high-dose chemotherapy and autologous stem cell transplantation (HDT-ASCT) is widely used in fit patients ≤70 years. The OptiMATe trial (NCT04931368) evaluates whether a de-escalation of induction can reduce treatment associated morbidity while maintaining efficacy and increasing the proportion of patients who proceed to consolidation HDT-ASCT.

### Aims

To compare event-free survival (EFS) between standard induction therapy and a de-escalated induction strategy in patients with newly diagnosed PCNSL.

### Methods

OptiMATe is a multicentre, open-label, randomized phase III trial across 68 European centres enrolling immunocompetent patients ≤70 years with newly diagnosed PCNSL. Patients are randomized 1:1 to Arm A (four MATRix cycles) or Arm B (R-MTX pre-treatment followed by two MATRix cycles). Patients achieving at least partial remission proceed to thiotepa-conditioned high-dose chemotherapy and HDT-ASCT. The primary endpoint is EFS; key secondary endpoints include overall and progression-free survival, safety, neurocognition, and quality of life, with a minimum follow-up of 24 months.

**Copyright Information:** (Online) ISSN: 2572-9241
©2026 The Author(s). HemaSphere is published by John Wiley & Sons Ltd on behalf of European Hematology Association. This is an open access Abstract Book distributed under the Attribution-NonCommercial-NoDerivs (CC BY-NC-ND), which allows third parties to download the articles and share them with others as long as they credit the author and the Abstract Book, but they cannot change the content in any way or use them commercially.
**Abstract Book Citations:** Authors, Title, HemaSphere, 2026;10:(S1):pages. The abstract book DOIs can be found on the "Author Information" page.
**Disclaimer:** Articles published in the journal HemaSphere exclusively reflect the opinions of the authors. The authors are responsible for all content in their abstracts including accuracy of the facts, statements, citing resources, etc.

**Results**

Enrollment is complete. From June 2021 to February 2026, 331 patients were enrolled and randomized. Patients remain in follow-up and the primary analysis is planned after completion of the prespecified 24-month follow-up. The primary analysis will occur after completion of the planned follow-up. Four Data Monitoring Committee (DMC) meetings have been held, most recently on December 1st, 2025 (data cut off November 12th, 2025). Based on the current safety dataset (n = 298), grade 3–5 adverse events (AEs) were analyzed. A total of 298 patients (147 Arm A; 151 Arm B) were assessable for safety analysis. At least one grade 3–5 AE occurred in 90.5% of Arm A and 86.8% of Arm B. The most frequent grade 3–5 AEs by system organ class were blood and lymphatic system disorders (78.9% vs. 68.9%), infections (53.7% vs. 49.0%) and gastrointestinal disorders (15.6% vs. 21.9%).
By treatment phase, grade 3–5 AEs occurred during induction in 89.8% (132/147) of Arm A and 83.4% (126/151) of Arm B, and during/after consolidation in 72.6% (61/84) of Arm A and 73.0% (84/115) of Arm B.
Fatal AEs (grade 5) occurred during induction in 3.0% (9/298; Arm A: 7, Arm B: 2) and during/after consolidation in 3.5% (7/199; all Arm B). No new or unexpected safety signals were identified at the time of this interim safety analysis.
We implemented a comprehensive biobanking program collecting blood, CSF, and bone marrow at diagnosis, on-treatment, and post-treatment time points (>1,450 samples to date). Samples support translational biomarker, immuno-oncology, and multi-omics studies.

**Summary/Conclusion**

This is the largest randomized phase III de-escalation trial in newly diagnosed primary CNS lymphoma. The study has shown excellent feasibility, outstanding recruitment, and no unexpected toxicity signals. The most recent follow-up and safety data will be presented.
The study is supported by the German Federal Ministry for Research, Technology, and Space (BMFTR) and Cancer Research UK. The sponsor of the trial is Klinikum Stuttgart, UK sites are coordinated by Cancer Research UK Southampton Clinical Trials Unit and Italian sites by the International Extranodal Lymphoma Study Group.

**Copyright Information:** (Online) ISSN: 2572-9241
©2026 The Author(s). HemaSphere is published by John Wiley & Sons Ltd on behalf of European Hematology Association. This is an open access Abstract Book distributed under the Attribution-NonCommercial-NoDerivs (CC BY-NC-ND), which allows third parties to download the articles and share them with others as long as they credit the author and the Abstract Book, but they cannot change the content in any way or use them commercially.
**Abstract Book Citations:** Authors, Title, HemaSphere, 2026;10:(S1):pages. The abstract book DOIs can be found on the “Author Information” page.
**Disclaimer:** Articles published in the journal HemaSphere exclusively reflect the opinions of the authors. The authors are responsible for all content in their abstracts including accuracy of the facts, statements, citing resources, etc.

### (PF966) IMPACT OF PRIOR LINES OF THERAPY ON PROGRESSION RISK AND POST-FAILURE OVERALL SURVIVAL FOLLOWING AXICABTAGENE CILOLEUCEL OR LISOCABTAGENE MARALEUCEL CAR T-CELLS THERAPY.

**Topic:** 19. Large B cell lymphomas - Clinical

Anna Dodero*[1], Annalisa Chiappella[1], Beatrice Casadei[2], Maria Chiara Tisi[3], Ilaria Cutini[4], Silva Ljevar[5], Stefania Bramanti[6], Patrizia Chiusolo[7], Alice Di Rocco[8], Piera Angelillo[9], Massimo Martino[10], Mirko Farina[11], Anna Maria Barbui[12], Barbara Botto[13], Francesca Patriarca[14], Giovanni Grillo[15], Mauro Krampera[16], Marco Ladetto[17], Alessia Castellino[18], Simone Ragaini[19], Luca Arcaini[20], Federico Stella[1], Angelica Barone[1], Anna Guidetti[21], Pier Luigi Zinzani[22], Paolo Corradini[23]

*[1]Fondazione IRCCS Istituto Nazionale dei Tumori, Hematology, Milano, Italy; [2]IRCCS Azienda Ospedaliero-Universitaria di Bologna,, Istituto di Ematologia "Seràgnoli", Bologna, Italy; [3]San Bortolo Hospital, AULSS 8 Berica, Hematology, Vicenza, Italy; [4]Ospedale Careggi, SOD Ematologia, AAD Trapianto di midollo osseo, Firenze, Italy; [5]Fondazione IRCCS Istituto Nazionale dei Tumori, Unit of Biostatistics for Clinical Research, Department of Data Science, Milano, Italy; [6]IRCCS Humanitas Research Hospital,, Transplantation Unit Department of Oncology and Hematology, Rozzano, Italy; [7]Fondazione Policlinico Universitario Agostino Gemelli IRCCS, Università Cattolica del Sacro Cuore, Dipartimento di Scienze Ematologiche e di laboratorio, Roma, Italy; [8]Universita' Sapienza Roma, Hematology, Department of Translational and Precision Medicine, Roma, Italy; [9]IRCCS San Raffaele Scientific Institute, Lymphoma Unit, Milano, Italy; [10]Ospedale Metropolitano "Bianchi-Melacrino-Morelli", Hematology and Stem Cell Transplantation and Cellular Therapies Unit (CTMO), Department of Hemato-Oncology and Radiotherapy,, Reggio Calabria, Italy; [11]University of Brescia, ASST Spedali Civili di Brescia, Unit of Blood Disease and Bone Marrow Transplantation, and Unit of Hematology,, Brescia, Italy; [12]Azienda Socio-Sanitaria Territoriale Papa Giovanni XXIII, Hematology Unit, Bergamo, Italy; [13]AOU "Città della Salute e della Scienza di Torino", Division of Hematology, Torino, Italy; [14]Azienda Sanitaria Universitaria Integrata di Udine, Centro Trapianti e Terapie Cellulari "Carlo Melzi", Udine, Italy; [15]ASST Grande Ospedale Metropolitano Niguarda, Dipartimento di Ematologia e Trapianto di midollo, Milano, Italy; [16]University of Verona, Hematology and Bone Marrow Transplant Unit, Section of Biomedicine of Innovation, Department of Engineering for Innovative Medicine (DIMI), Verona, Italy; [17]AOU SS Antonio e Biagio e Cesare Arrigo, SCDU Ematologia, Alessandria, Italy; [18]AO S. Croce e Carle, Cuneo and Laboratory of Blood Tumor Immunology, Molecular Biotechnology Center "Guido Tarone",, Hematology, Cuneo, Italy; [19]University Hospital A.O.U. "Città della Salute e della Scienza", Hematology, Torino, Italy; [20]University of Pavia, Molecular Medicine, Pavia, Italy; [21]Universita' di MIlano, Hematology, Milano, Italy; [22]Università di Bologna, Dipartimento di Scienze Mediche e Chirurgiche, Bologna, Italy; [23]Fondazione IRCCS Istituto Tumori, Universita' di Milano, Hematology, Milano, Italy;*

### Background

CAR T-cells have improved outcomes for relapsed/refractory aggressive large B-cell lymphomas. Over the last decade, Axicabtagene ciloleucel (Axicel) and Lisocabtagene maraleucel (Lisocel) were approved for patients who had failed two or more therapies, with 5-year OS of 38%- 42%. Recently, based on the Zuma-7 and Transform trials, their use expanded to 2$^{nd}$ (2L), with a 3-year OS of 50%.

### Aims

Our study evaluates how prior therapies influence the risk of progression and post-failure OS (OS2) in real-world settings.

**Copyright Information:** (Online) ISSN: 2572-9241
©2026 The Author(s). HemaSphere is published by John Wiley & Sons Ltd on behalf of European Hematology Association. This is an open access Abstract Book distributed under the Attribution-NonCommercial-NoDerivs (CC BY-NC-ND), which allows third parties to download the articles and share them with others as long as they credit the author and the Abstract Book, but they cannot change the content in any way or use them commercially.
**Abstract Book Citations:** Authors, Title, HemaSphere, 2026;10:(S1):pages. The abstract book DOIs can be found on the "Author Information" page.
**Disclaimer:** Articles published in the journal HemaSphere exclusively reflect the opinions of the authors. The authors are responsible for all content in their abstracts including accuracy of the facts, statements, citing resources, etc.

**Methods**

CART-SIE is a prospective observational study of patients (pts) treated with anti-CD19 CAR T-cells at 22 Italian centers. A total of 627 pts were treated: 604 received Axicel, and 23 Lisocel. Time to progression was calculated from infusion to first documented progression and analyzed using a competing-risks framework, with death as the competing event. Cumulative incidence functions were estimated, and between-group comparisons were performed using univariable and multivariable Fine and Gray models. Six-month progression estimates were derived from the final multivariable model across covariate combinations to identify factors associated with early relapse/progression. Post-failure overall survival (OS2) was measured from relapse/progression to death and estimated using the Kaplan–Meier method

**Results**

Of 627 pts, 221 (35%) received CAR T-cells in the 2L, 282 (45%) in 3$^{rd}$ (3L), and 124 (20%) in 4$^{th}$ or later line (4L+). Patient characteristics differed significantly across groups for the following factors: 1) High-grade B-cell lymphomas were more common in 4L+ and 3L than in 2L (20% vs 17% vs 9%, p<0.001); 2) transformed DLBCL was more frequent in 4L+ and 3L than in 2L (35% vs 17% vs 6.8%, p<0.001); 3) as expected, a greater proportion of pts with primary refractory disease received CAR T-cells in 2L than in 3L or 4L+ (67% vs 21% vs 16%, p<0.001); 4) CAR-Hematotox was more frequently high in 4L+ and 3L than in 2L (70% vs 66% vs 48%, p<0.001). No significant differences were found in stage, IPI, CRP, ferritin, LDH levels, extranodal sites, use of bridging therapy, or time from apheresis to infusion. At a median follow-up of 12.5 months (IQR: 6-23), 260 of 627 (41%) progressed. The estimated cumulative 6- and 24-month progression risk was 35% and 47% in 2L, 38% and 50% in 3L, and 43% and 56% in 4L+. Predicted cumulative incidence of PD within 6 months, extracted from a multivariable model, identified late line of therapy, advanced stage, no response to bridging, and bulky disease as significant factors. The difference in the probability of PD within 6 months was more pronounced between 4L+ and 3L (13.9%) and between 4L and 2L (20.7%). The OS2 was 29.5% (27.5% in 2L, 33% in 3L, and 23.5% in 4L+). Among progressing pts, there was no difference in the use of salvage therapy between those that failed CAR T-cells in 2L and those in 3L or 4L+ (p=ns). The use of bispecific antibodies, alone or in combination, was higher in pts treated with CAR T-cells in 2L (p=0.0009), but they have not yet affected survival.

**Summary/Conclusion**

Despite limitations inherent to our population and the high number of primary refractory pts treated in 2L, this study showed that the probability of PD within 6 months following CAR T-cells was 20% lower in 2L+ than in 4L+. Despite a significant increase in bispecific therapy after 2L CAR T-cells failure, 1-year OS2 remained poor, underscoring a critical unmet clinical need.

**Copyright Information:** (Online) ISSN: 2572-9241
©2026 The Author(s). HemaSphere is published by John Wiley & Sons Ltd on behalf of European Hematology Association. This is an open access Abstract Book distributed under the Attribution-NonCommercial-NoDerivs (CC BY-NC-ND), which allows third parties to download the articles and share them with others as long as they credit the author and the Abstract Book, but they cannot change the content in any way or use them commercially.
**Abstract Book Citations:** Authors, Title, HemaSphere, 2026;10:(S1):pages. The abstract book DOIs can be found on the "Author Information" page.
**Disclaimer:** Articles published in the journal HemaSphere exclusively reflect the opinions of the authors. The authors are responsible for all content in their abstracts including accuracy of the facts, statements, citing resources, etc.

### (PF967) A DISEASE SPECIFIC PROGNOSTIC SCORE FOR IMMUNODEFICIENCY-ASSOCIATED PRIMARY CNS LYMPHOMA: AN INTERNATIONAL PRIMARY CNS LYMPHOMA COLLABORATIVE GROUP (IPCG) STUDY

**Topic:** 19. Large B cell lymphomas - Clinical

Leon D. Kaulen*[1, 2, 3], Lakshmi Nayak[4], Philipp Karschnia[5], Imke Kraai[6], Daniela Galluzzo[7], Fleur de Groot[8], Matthew Witterholt[9], Laura Donovan[10], Sita Bhella[11], Luis Kuschel[12], Christopher P. Fox[13], Sabine Seidel[14], James Paterson[15], Benjamin Richter[16, 17], Karan Dixit[18], Thomas Zeyen[19], Juan Alderuccio[20], Silvia Montoto[21], Outi Kuittinen[22], Benjamin-Leon Traub[1, 2], Sofia Doubrovinskaia[1, 2], Hannah Rohdjess[1, 2], Katharina J. Müller[23], Roelien Enting[24], Jeroen de Bresser[25], Hanne Kuitunen[26], Fabio M. Iwamoto[10], Alvaro J. Alencar[20], Ulrich Herrlinger[19], Michael Platten[16, 17], Kate Cwynarski[15], Joe Mendez[27], Patrick Roth[28], Louisa von Baumgarten[12, 23], Anca Prica[11], Govind Bhagat[29], Kathryn Lurain[9, 30], David Schiff[31], Joost Vermaat[8], Joachim M. Baehring[7], Marcel Nijland[6], Jorg Dietrich[3], Tracy Batchelor[3, 32], Wolfgang Wick[1, 2]

*[1]University Hospital Heidelberg, Department of Neurology, Heidelberg, Germany;[2]German Cancer Research Center (DKFZ) and German Consortium for Translational Cancer Research (DKTK), Clinical Cooperation Unit Neuro-Oncology, Heidelberg, Germany; [3]Massachusetts General Hospital Cancer Center, Harvard Medical School, Department of Neurology, Division of Neuro-Oncology, Boston, United States of America; [4]Dana Farber Cancer Institute, Center for Neuro Oncology, Department of Medical Oncology, Boston, United States of America; [5]Friedrich-Alexander-University Erlangen, Department of Neurosurgery, Erlangen, Germany; [6]University of Groningen, University Medical Center Groningen, Department of Hematology, Groningen, The Netherlands; [7]Yale School of Medicine, Department of Neurology, New Haven, United States of America;[8]Leiden University Medical Center, Leiden University, Department of Hematology, Leiden, The Netherlands; [9]National Cancer Institute, National Institutes of Health, HIV and AIDS Malignancy Branch, Center for Cancer Research, Bethesda, United States of America; [10]Columbia University Irving Medical Center, Department of Neuro-Oncology, New York, United States of America;[11]Princess Margaret Cancer Centre, Division of Medical Oncology and Hematology, Toronto, Canada; [12]Ludwig-Maximilians-University, Department of Neurosurgery, Munich, Germany; [13]School of Medicine, University of Nottingham, Department of Haematology, Nottingham, United Kingdom; [14]Knappschaft Kliniken University Hospital Bochum, Ruhr University Bochum, Department of Neurology, Bochum, Germany; [15]University College London, Department of Haematology, London, United Kingdom; [16]Medical Faculty Mannheim, Mannheim Center for Translation Neuroscience (MCTN), Heidelberg University, Department of Neurology, Mannheim, Germany; [17]German Cancer Research Center (DKFZ), Clinical Cooperation Unit (CCU) Neuroimmunology and Brain Tumor Immunology, Heidelberg, Germany; [18]Feinberg School of Medicine Northwestern University, Division of Neuro-Oncology, Department of Neurology, Chicago, United States of America; [19]University Hospital Bonn, Department of Neurooncology, Center for Neurology and Center for Integrated Oncology (CIO), Bonn, Germany; [20]Sylvester Comprehensive Cancer Center, University of Miami Miller School of Medicine, Department of Medicine, Miami, United States of America; [21]St. Bartholomew's Hospital, Barts Health NHS Trust, Department of Haemato-Oncology, London, United Kingdom; [22]Kuopio University Hospital, University of Eastern Finland, Institute of Clinical Medicine Oncology,, Kuopio, Finland; [23]Ludwig-Maximilians-University, Department of Neurology, Munich, Germany; [24]University Medical Center Groningen, University of Groningen, Department of Neurology, Groningen, The Netherlands; [25]Leiden University Medical Center,, Department of Radiology, Leiden, The Netherlands;[26]Cancer Centre, Oulu University Hospital, Department of Oncology, Oulu, Finland; [27]University of Utah, Department of Neurosurgery, Salt Lake City, United States of America; [28]University Hospital Zurich, University of Zurich, Department of Neurology and Brain Tumor Center, Zürich, Switzerland; [29]Saint Louis University School of Medicine, Department of Pathology, Saint Louis, United States of America; [30]Ohio State University, Division of Hematology, Columbus, United States of America;[31]University of Virginia, Department of Neurology, Division of Neuro-Oncology,, Charlottesville, United States of America; [32]Brigham and Women's Hospital and Massachusetts General Hospital, Harvard Medical School, Department of Neurology, Boston, United States of America;*

**Copyright Information:** (Online) ISSN: 2572-9241
©2026 The Author(s). HemaSphere is published by John Wiley & Sons Ltd on behalf of European Hematology Association. This is an open access Abstract Book distributed under the Attribution-NonCommercial-NoDerivs (CC BY-NC-ND), which allows third parties to download the articles and share them with others as long as they credit the author and the Abstract Book, but they cannot change the content in any way or use them commercially.
**Abstract Book Citations:** Authors, Title, HemaSphere, 2026;10:(S1):pages. The abstract book DOIs can be found on the "Author Information" page.
**Disclaimer:** Articles published in the journal HemaSphere exclusively reflect the opinions of the authors. The authors are responsible for all content in their abstracts including accuracy of the facts, statements, citing resources, etc.

### Background

Immunodeficiency-associated primary central nervous system lymphoma (ID-PCNSL) constitutes a clinicopathologically distinct PCNSL variant. Large clinical studies are lacking and existing prognostic models, developed using immunocompetent cohorts, may not be applicable to this patient population.

### Aims

To characterize clinical and radiological features, treatment outcomes, and prognostic factors in ID-PCNSL and to develop a disease-specific prognostic score.

### Methods

The International Primary CNS Lymphoma Collaborative Group (IPCG) conducted a retrospective multicenter study of ID-PCNSL, diagnosed between 2003-2024, at 23 centers across 7 countries. Clinical, radiological, and pathological data were collected. Survival outcomes were assessed using Kaplan–Meier estimates and multivariable Cox regression. Independent predictors of survival were combined to derive a prognostic score and compared with established MSKCC and IELSG models.

### Results

A total of 308 patients with ID-PCNSL were identified. Underlying immunodeficiency resulted from immunosuppressive therapy for organ transplantation (41.2%) or autoimmune disease (36.7%), and from HIV infection (21.7%). All tumors were classified as diffuse large B-cell lymphomas, with Epstein–Barr virus (EBV) detected in 79.2%.
Most patients (91.4%) received curative-intent induction therapy, predominantly systemic chemotherapy (97.5%), while radiation alone was uncommon (2.1%). Rituximab–methotrexate (RM)–based regimens were most frequently used (69.8%), followed by rituximab-based (15.6%) and methotrexate-based therapy alone (12.7%). Best overall response rate (ORR) to first-line therapy was 77% (complete response 50%; partial response 27%). RM-based chemotherapy achieved higher ORR (85%) compared with rituximab-based (68%, p=0.01) or methotrexate-based regimens alone (52%, p<0.001). Immune reconstitution together with RM-based chemotherapy was associated with prolonged progression-free survival irrespective of immunodeficiency subtype and EBV status.
Outcomes were heterogeneous, with a median overall survival (OS) of 54 months (**Fig. 1A**). Multivariable analysis identified increasing age (HR 1.05 per year, 95% CI 1.02–1.07; p<0.001; **Fig. 1B**), Karnofsky Performance Status (KPS) <70 (HR 3.10, 95% CI 1.67–5.87; p<0.001; **Fig. 1C**), and EBV positivity (HR 3.26, 95% CI 1.47–7.33; p=0.004;**Fig. 1D**) as independent predictors of inferior OS. A prognostic score incorporating these factors (age >60 years, KPS <70, EBV positivity; **Fig. 1E**) stratified patients into three risk groups with median OS of 135, 29, and 3 months for patients with ≤1, 2, and 3 risk factors, respectively (p<0.0001; **Fig. 1F**). The score retained prognostic significance in patients treated with RM-based regimens. Compared with MSKCC and IELSG scores, the new model demonstrated improved performance with lower AIC and higher concordance index and time-dependent AUC.

### Summary/Conclusion

This large international cohort defines the clinical spectrum of ID-PCNSL and demonstrates substantial heterogeneity in outcomes. Immune reconstitution combined with RM-based therapy is associated with high response rates across immunodeficiency subtypes. A simple prognostic score based on age, performance status, and EBV status provides robust risk stratification, remains informative in RM-treated patients, and outperforms established models developed for immunocompetent PCNSL.

**Copyright Information:** (Online) ISSN: 2572-9241
©2026 The Author(s). HemaSphere is published by John Wiley & Sons Ltd on behalf of European Hematology Association. This is an open access Abstract Book distributed under the Attribution-NonCommercial-NoDerivs (CC BY-NC-ND), which allows third parties to download the articles and share them with others as long as they credit the author and the Abstract Book, but they cannot change the content in any way or use them commercially.
**Abstract Book Citations:** Authors, Title, HemaSphere, 2026;10:(S1):pages. The abstract book DOIs can be found on the "Author Information" page.
**Disclaimer:** Articles published in the journal HemaSphere exclusively reflect the opinions of the authors. The authors are responsible for all content in their abstracts including accuracy of the facts, statements, citing resources, etc.

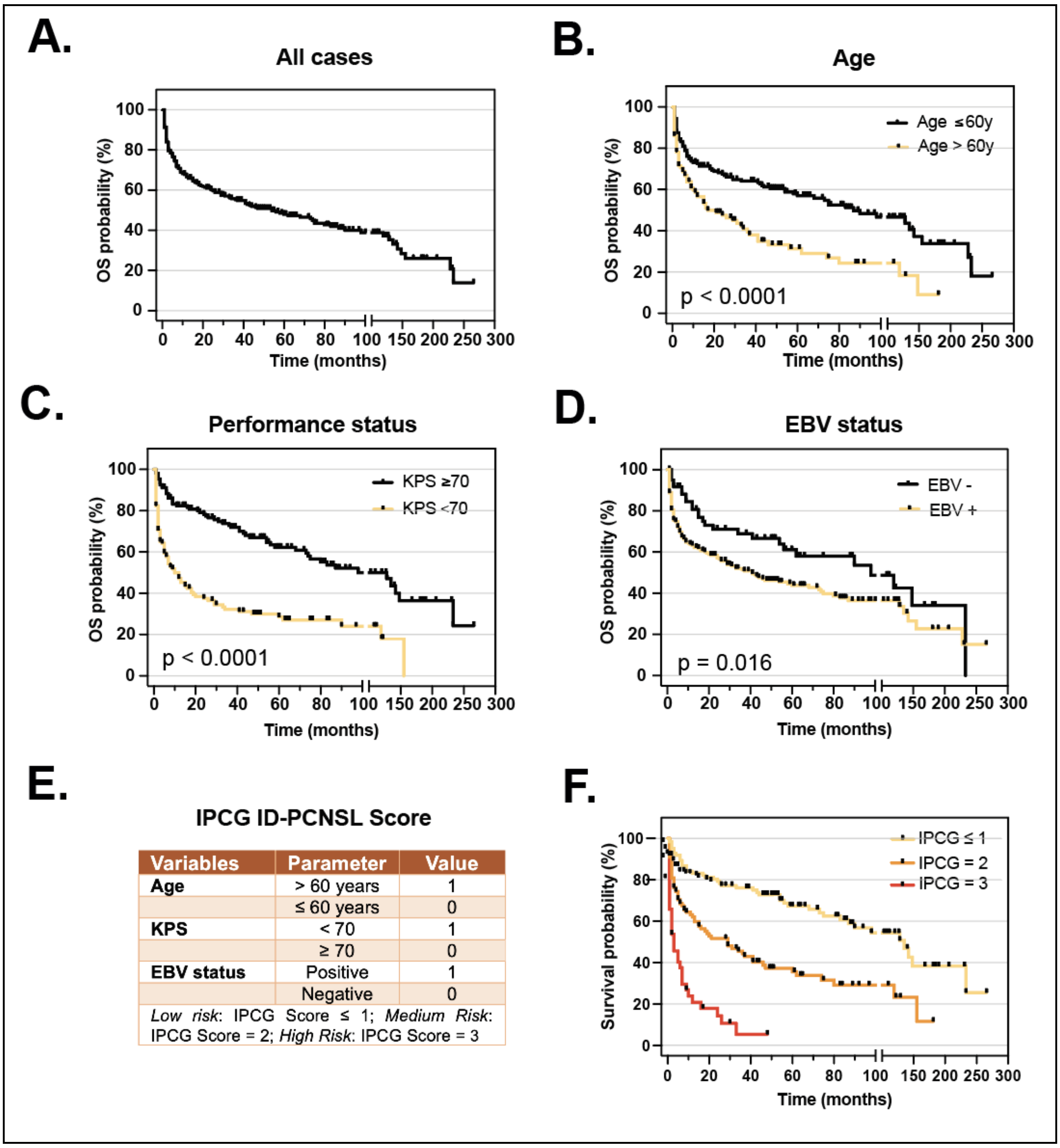

**Copyright Information:** (Online) ISSN: 2572-9241
©2026 The Author(s). HemaSphere is published by John Wiley & Sons Ltd on behalf of European Hematology Association. This is an open access Abstract Book distributed under the Attribution-NonCommercial-NoDerivs (CC BY-NC-ND), which allows third parties to download the articles and share them with others as long as they credit the author and the Abstract Book, but they cannot change the content in any way or use them commercially.
**Abstract Book Citations:** Authors, Title, HemaSphere, 2026;10:(S1):pages. The abstract book DOIs can be found on the "Author Information" page.
**Disclaimer:** Articles published in the journal HemaSphere exclusively reflect the opinions of the authors. The authors are responsible for all content in their abstracts including accuracy of the facts, statements, citing resources, etc.

### (PF968) MOSUNETUZUMAB PLUS POLATUZUMAB VEDOTIN VERSUS RITUXIMAB, GEMCITABINE AND OXALIPLATIN IN LARGE B-CELL LYMPHOMA: UPDATED EFFICACY AND SAFETY FROM THE PHASE 3 SUNMO STUDY INCLUDING IN PATIENT SUBGROUPS

**Topic:** 19. Large B cell lymphomas - Clinical

L. Elizabeth Budde*[1], Jason Westin[2], Dai Maruyama[3], Hui-Lai Zhang[4], Hideki Goto[5], Huangming Hong[6], Mark Fesler[7], Boone Goodgame[8], Zhiming Li[9], Eduardo M. Rego[10], Adam J. Olszewski[11], Nikesh Shah[12], Bei Hu[13], Shen Yin[14], Hao Wu[14], Martin Janousek[15], Song Pham[16], Connie Lee Batlevi[14], Michael C. Wei[14], Wonseog Kim[17]

*[1]City of Hope National Medical Center, Duarte, CA, United States of America;[2]MD Anderson Cancer Center, Houston, TX, United States of America; [3]Cancer Institute Hospital, Japanese Foundation for Cancer Research, Tokyo, Japan;[4]Tianjin Medical University Cancer Institute, Tianjin, China; [5]Hokkaido University Hospital, Sapporo, Japan; [6]Si Chuan Cancer Hospital, Chengdu, China; [7]St. Luke's Hospital, Chesterfield, MO, United States of America;[8]Dell Medical School, University of Texas at Austin, Austin, TX, United States of America; [9]Sun Yat-sen University Cancer Center, Guangzhou, China; [10]Instituto D'Or de Pesquisa e Ensino, São Paulo, Brazil; [11]Brown University, Providence, RI, United States of America; [12]Tampa General Hospital Cancer Institute, Tampa, FL, United States of America; [13]Atrium Health Levine Cancer Institute and Wake Forest School of Medicine, Charlotte, NC, United States of America; [14]Genentech, Inc., South San Francisco, CA, United States of America; [15]F. Hoffmann-La Roche Ltd, Basel, Switzerland; [16]Hoffmann-La Roche Ltd, Mississauga, ON, Canada; [17]Samsung Medical Center, Seoul, South Korea;*

### Background

At the primary analysis, mosunetuzumab plus polatuzumab vedotin (Mosun-Pola) demonstrated superior efficacy versus rituximab, gemcitabine and oxaliplatin (R-GemOx), with infrequent cytokine release syndrome (CRS) events and manageable safety in patients with relapsed/refractory (R/R) large B-cell lymphoma (LBCL) in the Phase 3 SUNMO trial (NCT05171647; Budde, et al. JCO 2025).

### Aims

To report updated efficacy and safety, including in the second-line setting.

### Methods

Patients with R/R LBCL ineligible for autologous stem-cell transplant (ASCT) were randomized 2:1 to Mosun (subcutaneous)-Pola (investigational regimen) or R-GemOx. Dual primary endpoints were independent review committee-assessed progression-free survival (PFS) and objective response rate (ORR); secondary endpoints included complete response (CR) rate, duration of response (DOR), duration of CR (DOCR), and safety. Overall survival (OS) was not assessed as the data cut-off was prior to the pre-defined final OS analysis. All patients provided written informed consent.

**Copyright Information:** (Online) ISSN: 2572-9241
©2026 The Author(s). HemaSphere is published by John Wiley & Sons Ltd on behalf of European Hematology Association. This is an open access Abstract Book distributed under the Attribution-NonCommercial-NoDerivs (CC BY-NC-ND), which allows third parties to download the articles and share them with others as long as they credit the author and the Abstract Book, but they cannot change the content in any way or use them commercially.
**Abstract Book Citations:** Authors, Title, HemaSphere, 2026;10:(S1):pages. The abstract book DOIs can be found on the "Author Information" page.
**Disclaimer:** Articles published in the journal HemaSphere exclusively reflect the opinions of the authors. The authors are responsible for all content in their abstracts including accuracy of the facts, statements, citing resources, etc.

**Results**

At data cut-off (August 8, 2025), 138 and 70 patients were assigned to Mosun-Pola or R-GemOx, respectively. Overall, 91 patients had 1 prior line of therapy (2L; Mosun-Pola, n=61; R-GemOx, n=30) and 117 had ≥2 prior lines of therapy (3L+; Mosun-Pola, n=77; R-GemOx, n=40). With a median follow-up of 28.3 months, Mosun-Pola continued to demonstrate superior PFS benefits over R-GemOx (**Table**; hazard ratio [HR], 0.41; 95% confidence interval [CI]: 0.28–0.60). ORRs with Mosun-Pola were 70.3% vs 40.0% with R-GemOx.
The observed PFS benefit of Mosun-Pola over R-GemOx was similar in 2L (HR, 0.38; 95% CI: 0.22–0.67) and 3L+ (HR, 0.48; 95% CI: 0.29–0.81) subgroups. With Mosun-Pola vs R-GemOx in 2L, the 2-year PFS rates were 40.3% vs 20.1%, and the 2-year DOCR estimates were 60.8% vs 37.5%, respectively. Median DOCR was not reached with Mosun-Pola in 2L and 3L+ subgroups. ORRs with Mosun-Pola vs R-GemOx were 75.4% vs 36.7% in 2L patients, and 66.2% vs 42.5% in 3L+ patients, respectively.
The safety profile was unchanged since the primary analysis. Grade ≥2 CRS occurred in 4% of Mosun-Pola-treated patients and no immune effector cell-associated neurotoxicity syndrome events occurred. The safety profile was consistent in 2L patients: Grade 2 CRS occurred in 3% of Mosun-Pola-treated patients, with no Grade ≥3 CRS events.

**Summary/Conclusion**

Mosun-Pola continues to show notable efficacy with manageable safety in patients with ASCT-ineligible R/R LBCL, particularly in the 2L setting, and represents an effective off-the-shelf alternative for those patients without access to chimeric antigen receptor T-cell therapy.

© 2026 American Society of Clinical Oncology, Inc. Reused with permission. This abstract was accepted and previously presented at the 2026 ASCO Annual Meeting. All rights reserved.

**Table:**

| | Mosun-Pola (n=138) | R-GemOx (n=70) | 2L Mosun-Pola (n=61) | 2L R-GemOx (n=30) | 3L+ Mosun-Pola (n=77) | 3L+ R-GemOx (n=40) |
|---|---|---|---|---|---|---|
| Median PFS, months (95% CI) | 11.6 (5.6–17.6) | 3.8 (2.9–4.1) | 17.6 (5.6–NE) | 3.6 (2.1–5.3) | 8.6 (4.0–16.2) | 3.9 (2.1–5.6) |
| Median DOR, months (95% CI) | 18.8 (11.5–NE) | 6.0 (3.7–23.9) | 18.8 (11.3–NE) | 6.0 (3.9–NE) | 21.5 (9.5–NE) | 5.4 (2.3–18.3) |
| CR, % (95% CI) | 51.4 (42.8–60.0) | 24.3 (14.8–36.0) | 60.7 (47.3–72.9) | 20.0 (7.7–38.6) | 44.2 (32.8–55.9) | 27.5 (14.6–43.9) |
| Median DOCR, months (95% CI) | NE | 18.3 (3.9–NE) | NE (15.6–NE) | 21.4 (3.9–NE) | NE (21.5–NE) | 7.5 (2.3–NE) |

2L, 1 prior line of therapy; 3L+, ≥2 prior lines of therapy; CI, confidence interval; CR, complete response; DOCR, duration of complete response; DOR, duration of response; Mosun-Pola, mosunetuzumab plus polatuzumab vedotin; NE, not estimable; PFS, progression-free survival; R-GemOx, rituximab, gemcitabine and oxaliplatin.

**Copyright Information:** (Online) ISSN: 2572-9241
©2026 The Author(s). HemaSphere is published by John Wiley & Sons Ltd on behalf of European Hematology Association. This is an open access Abstract Book distributed under the Attribution-NonCommercial-NoDerivs (CC BY-NC-ND), which allows third parties to download the articles and share them with others as long as they credit the author and the Abstract Book, but they cannot change the content in any way or use them commercially.
**Abstract Book Citations:** Authors, Title, HemaSphere, 2026;10:(S1):pages. The abstract book DOIs can be found on the “Author Information” page.
**Disclaimer:** Articles published in the journal HemaSphere exclusively reflect the opinions of the authors. The authors are responsible for all content in their abstracts including accuracy of the facts, statements, citing resources, etc.

### (PF969) TIME-DEFINED PD-1 BLOCKADE DURING EARLY CAR-T CONTRACTION OF IL-7/CCL19–ARMORED CAR-T CELLS IS ASSOCIATED WITH RESPONSE DEEPENING IN HIGH-RISK RELAPSED/REFRACTORY LBCL LOGO

**Topic:** 19. Large B cell lymphomas - Clinical

Lixia Sheng*[1], Wen Lei[2], Duo Wu[3], Yi Zhang[4], Laiyan Li[1], Suyan Wang[5], Duobing Zou[4], Ping Zhang[1], Kaihong Xu[1], Shanhao Tang[1], Huijie Hu[6], Tongyu Li[1], Qitian Mu[4], Xiaofei Ye[3], Hui Liu[2], Wenbin Qian[2], Guifang Ouyang[1]

*[1]The First Affiliated Hospital of Ningbo University, Department of Hematology, Ningbo, China;[2]The Second Affiliated Hospital, Zhejiang University School of Medicine, Hematology, Hangzhou, China; [3]Kindstar Biotech and Kindstar Global Precision Medicine Institute, Wuhan, China; [4]The First Affiliated Hospital of Ningbo University, Stem Cell Laboratory, Ningbo, China; [5]Ningbo Clinical Pathology Diagnosis Center, Department of Histopathology, Ningbo, China;[6]School of Medicine, Ningbo University, Ningbo, China;*

### Background

CD19-directed CAR-T therapy induces high response rates in relapsed/refractory large B-cell lymphoma (R/R LBCL), yet durable remission remains suboptimal in patients with bulky disease or PD-L1–enriched tumor microenvironments. CAR-T functional states evolve dynamically after peak expansion, but whether the early contraction phase represents a clinically actionable window for checkpoint modulation remains uncertain.

### Aims

To assess the safety and preliminary efficacy of PD-1 blockade administered during early CAR-T contraction and to explore its association with response deepening in biologically high-risk R/R LBCL.

### Methods

In this investigator-initiated phase I study (NCT04381741), 20 patients with R/R LBCL received lymphodepletion followed by IL-7/CCL19–armored CD19 CAR-T cells ($2\times10^6$/kg). Based on longitudinal CAR-T kinetics, PD-1 blockade (tislelizumab 200 mg) was initiated on day +30 (±2), corresponding to early contraction.Primary endpoints were safety and 3-month objective response rate (ORR). Secondary endpoints included complete response (CR), progression-free survival (PFS), overall survival (OS), and duration of response. Immune profiling included CAR transgene monitoring, multiparameter flow cytometry, single-cell RNA sequencing, and TCR clonotype analysis.

**Copyright Information:** (Online) ISSN: 2572-9241
©2026 The Author(s). HemaSphere is published by John Wiley & Sons Ltd on behalf of European Hematology Association. This is an open access Abstract Book distributed under the Attribution-NonCommercial-NoDerivs (CC BY-NC-ND), which allows third parties to download the articles and share them with others as long as they credit the author and the Abstract Book, but they cannot change the content in any way or use them commercially.
**Abstract Book Citations:** Authors, Title, HemaSphere, 2026;10:(S1):pages. The abstract book DOIs can be found on the “Author Information” page.
**Disclaimer:** Articles published in the journal HemaSphere exclusively reflect the opinions of the authors. The authors are responsible for all content in their abstracts including accuracy of the facts, statements, citing resources, etc.

**Results**

CAR-T expansion peaked at days 14–21 and transitioned to contraction by days 21–30, accompanied by upregulation of PD-1, TIGIT, LAG-3, and TOX, and reduced memory-like subsets. Despite transcriptional exhaustion, day-30 CAR-T cells retained functional plasticity. Ex vivo PD-1 blockade enhanced proliferation (Ki-67 18.8% vs 31.7%, P=0.002), IFN-γ production (14.4% vs 30.6%, P=0.028), and degranulation (30.9% vs 47.9%, P=0.016). suggesting preserved functional reversibility during early contraction. Sequential PD-1 blockade was feasible and was not associated with new CRS or ICANS.At 1 month (prior to PD-1 blockade), CR rate was 30% and PR 60%. Among PR patients, 8/12 (66.7%) achieved CR after PD-1 initiation, resulting in a cumulative CR rate of 70% and a 3-month ORR of 85%. With a median follow-up of 21.8 months, estimated 2-year PFS and OS were 60.0% and 71.1%, respectively. In PD-L1$^{high}$ tumors, CR rates were initially lower (10% vs 90% at 3 months, P=0.001) but increased to 50% following PD-1 blockade, yielding PFS and OS comparable to PD-L1$^{low}$ patients. In patients with bulky disease (n=11), CR at 1 month was 18.2% versus 44.4% in non-bulky disease. After PD-1 blockade, 6/9 bulky patients with residual disease experienced further response improvement. Best CR rates and 2-year PFS/OS were comparable between bulky and non-bulky subgroups .

**Summary/Conclusion**

CAR-T cells undergo an early contraction phase marked by checkpoint-sensitive functional restraint rather than terminal exhaustion. PD-1 blockade timed to this window reprogrammed dysfunctional CAR-T cells, deepened responses, and overcame the negative prognostic impact of PD-L1^high expression and bulky disease—without increasing immune-related toxicity. These results highlight timing as a key therapeutic variable in CAR-T therapy and support a dual-axis strategy combining intrinsic IL-7/CCL19 armoring with phase-specific checkpoint inhibition to enhance durability in immune-resistant LBCL. Given the single-arm design and small sample size, findings are hypothesis-generating and warrant validation in prospective controlled trials.

**Copyright Information:** (Online) ISSN: 2572-9241
©2026 The Author(s). HemaSphere is published by John Wiley & Sons Ltd on behalf of European Hematology Association. This is an open access Abstract Book distributed under the Attribution-NonCommercial-NoDerivs (CC BY-NC-ND), which allows third parties to download the articles and share them with others as long as they credit the author and the Abstract Book, but they cannot change the content in any way or use them commercially.
**Abstract Book Citations:** Authors, Title, HemaSphere, 2026;10:(S1):pages. The abstract book DOIs can be found on the “Author Information” page.
**Disclaimer:** Articles published in the journal HemaSphere exclusively reflect the opinions of the authors. The authors are responsible for all content in their abstracts including accuracy of the facts, statements, citing resources, etc.

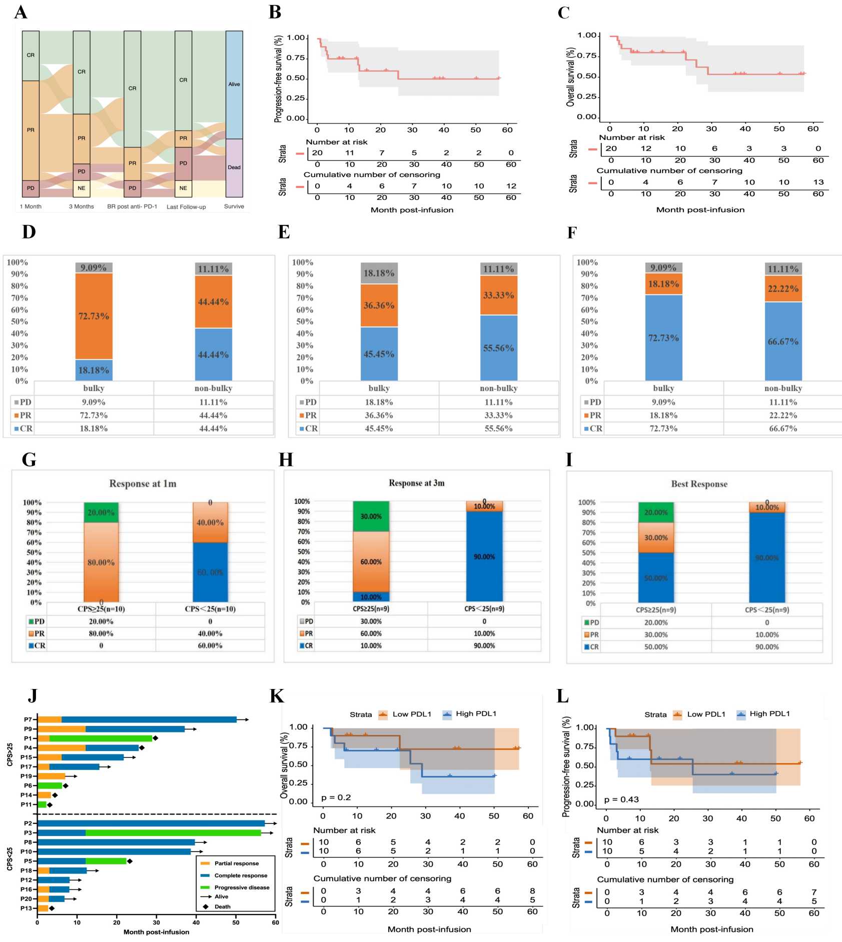

Figure 1. Time-defined PD-1 Blockade Deepens Responses and Improves Survival Following IL-7/CCL19–Armored CAR-T Therapy in High-Risk R/R LBCL. (A) Sankey diagram showing response transitions (e.g., partial response (PR) to complete response (CR)) and clinical outcomes before and after PD-1 blockade. (B, C) Kaplan-Meier curves for overall survival (OS) and progression-free survival (PFS). (D-F) Response rates in bulky vs. non-bulky disease subgroups at 1 month, 3 months, and best response. (G-I) Response rates stratified by PD-L1 expression (CPS ≥ 25 vs. < 25). (J) Swimmer plot illustrating individual response kinetics and remission duration in CPS-high vs. CPS-low patients treated with IL-7/CCL19-armored CAR-T cells followed by tislelizumab. (K, L) Survival analysis stratified by PD-L1 expression (High vs. Low). PR: partial response; FR: full response; CR: complete response; CI: confidence interval.

**Copyright Information:** (Online) ISSN: 2572-9241
©2026 The Author(s). HemaSphere is published by John Wiley & Sons Ltd on behalf of European Hematology Association. This is an open access Abstract Book distributed under the Attribution-NonCommercial-NoDerivs (CC BY-NC-ND), which allows third parties to download the articles and share them with others as long as they credit the author and the Abstract Book, but they cannot change the content in any way or use them commercially.
**Abstract Book Citations:** Authors, Title, HemaSphere, 2026;10:(S1):pages. The abstract book DOIs can be found on the "Author Information" page.
**Disclaimer:** Articles published in the journal HemaSphere exclusively reflect the opinions of the authors. The authors are responsible for all content in their abstracts including accuracy of the facts, statements, citing resources, etc.

### (PF970) MINIMAL HINGE OPTIMIZATION MARKEDLY ENHANCES DUAL CD19/CD20 CAR T-CELL POTENCY AND YIELDS DURABLE 3-YEAR SURVIVAL IN RELAPSED/REFRACTORY B-CELL LYMPHOMA

**Topic:** 19. Large B cell lymphomas - Clinical

Zekai Mao*[1], Juan Peng[1], Ang Zhang[2], Jiaqi Guo[3], Xiao Zhao[4], Yang Cao[1], Fankai Meng[1], Yicheng Zhang[1], Weiwei Tian[3, 5], Xiaoxi Zhou[1], Jia Wei[1, 6]

*[1]Tongji Hospital, Tongji Medical College, Huazhong University of Science and Technology, Hematology, Wuhan, Hubei, China; [2]Department of Research, The Ninth Medical Center, Chinese PLA General Hospital, Beijing, People's Republic of China, Beijing, China; [3]Shanxi Bethune Hospital, Shanxi Academy of Medical Sciences, Third Hospital of Shanxi Medical University, Tongji Shanxi Hospital, Taiyuan, China; [4]Chinese PLA Medical School, Chinese PLA General Hospital, No. 28, Fuxing Road, Haidian District, Beijing 100039, China, Beijing, China; [5]Sino-German Joint Oncological Research Laboratory, Shanxi Bethune Hospital, Shanxi Academy of Medical Sciences, Taiyuan, Shanxi, 030032, China., Taiyuan, China; [6]Immunotherapy Research Center for Hematologic Diseases of Hubei Province, Wuhan, 430030, China., Wuhan, China;*

### Background

Dual CD19/CD20 CAR T-cell strategies aim to prevent antigen escape in relapsed/refractory B-cell lymphoma (r/r BCL). However, subtle structural elements within CAR architecture may critically influence functionality. Moreover, long-term follow-up data for bispecific CAR constructs are limited, and the safety and potential benefit of integrating CAR T-cell therapy with sequential autologous stem cell transplantation (ASCT) have not been fully established.

### Aims

To evaluate whether hinge optimization enhances the functional potency of a tandem CD19/CD20 CAR, and to assess the feasibility of sequential CAR T followed by ASCT consolidation in patients with r/r BCL.

### Methods

A tandem CD19/CD20 CAR incorporating a short "AS" motif between the scFv and hinge domain was engineered. Functional activity was compared with established dual CAR constructs *in vitro* and *in vivo*. A phase Ia/Ib study (NCT05388695) enrolled 50 patients with r/r BCL receiving HinTCAR2019 alone (n=29) or sequential ASCT consolidation (n=21). The primary endpoint was safety, with efficacy and survival evaluated as secondary outcomes.

### Results

Hinge optimization significantly increased CAR surface expression (64.7% vs 39.3% in TanCAR7) and enhanced cytotoxicity against $CD19^+CD20^+$ targets. In high tumor burden xenografts, HinTCAR2019 significantly prolonged survival compared with TanCAR7 ($p<0.0001$).
Clinically, no dose-limiting toxicities occurred. CRS was observed in 68% of patients, with only 2% grade ≥3 events; no grade ≥3 ICANS occurred. The best overall response rate was 88%, including 56% complete responses. At a median follow-up of 25.2 months, median PFS and OS were not reached. Estimated 3-year PFS and OS were 65.3% (95% CI 53.2–80.2) and 74.5% (95% CI 62.7–88.4), respectively. Survival analyses according to consolidation strategy demonstrated higher 3-year PFS and OS rates in the sequential ASCT cohort compared with CAR T therapy alone (85.7% vs 51.2% for PFS; 95.2% vs 59.6% for OS). Sequential ASCT was independently associated with improved overall survival in multivariable analysis (HR 0.12, 95% CI 0.02–0.97; p=0.047) without increased treatment-related toxicity.

**Copyright Information:** (Online) ISSN: 2572-9241
©2026 The Author(s). HemaSphere is published by John Wiley & Sons Ltd on behalf of European Hematology Association. This is an open access Abstract Book distributed under the Attribution-NonCommercial-NoDerivs (CC BY-NC-ND), which allows third parties to download the articles and share them with others as long as they credit the author and the Abstract Book, but they cannot change the content in any way or use them commercially.
**Abstract Book Citations:** Authors, Title, HemaSphere, 2026;10:(S1):pages. The abstract book DOIs can be found on the "Author Information" page.
**Disclaimer:** Articles published in the journal HemaSphere exclusively reflect the opinions of the authors. The authors are responsible for all content in their abstracts including accuracy of the facts, statements, citing resources, etc.

### Summary/Conclusion

Hinge-optimized tandem CD19/CD20 CAR T-cell therapy demonstrates a favorable safety profile and durable clinical activity in r/r BCL. Sequential ASCT consolidation is associated with improved long-term survival without additional toxicity. These findings support further prospective evaluation of this strategy.

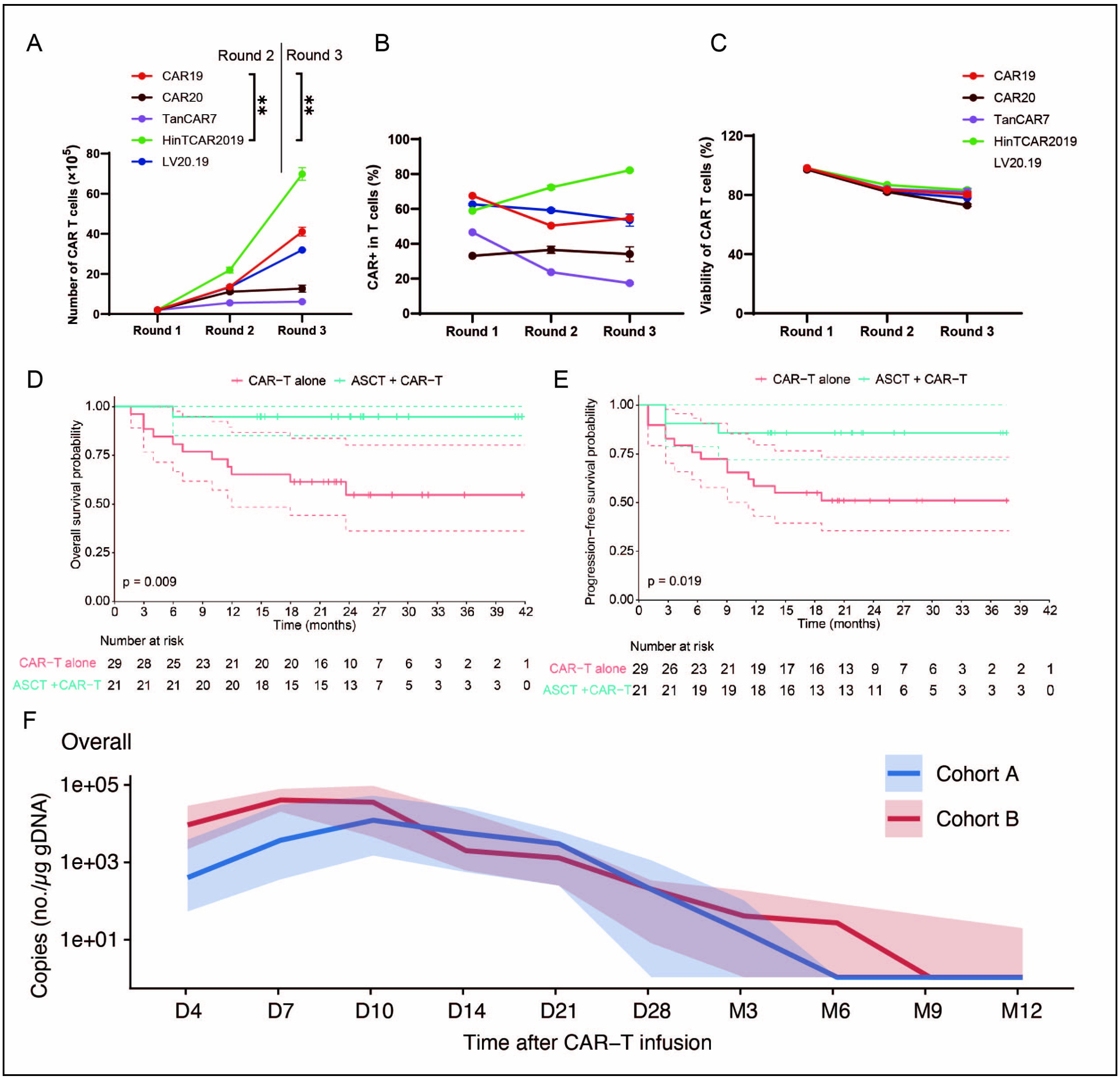

Figure 1. Therapeutic efficacy and in vivo kinetics of HinTCAR2019 in a high–tumor burden model. (A–C) A high–tumor burden model was established by intravenously inoculating NCG mice with $5 \times 10^5$ Raji cells per mouse. Seven days post-engraftment, mice were treated with Mock T cells, CAR19, TanCAR7, or HinTCAR2019. (D–E) Kaplan–Meier survival curves showing overall survival (OS) (D) and progression-free survival (PFS) (E) according to treatment groups, comparing CAR-T therapy alone versus ASCT followed by HinTCAR2019. (F) In vivo expansion and persistence of HinTCAR2019 T cells in peripheral blood following infusion, stratified by treatment cohort (cohort A: CAR-T alone; cohort B: ASCT followed by CAR-T).

**Copyright Information:** (Online) ISSN: 2572-9241
©2026 The Author(s). HemaSphere is published by John Wiley & Sons Ltd on behalf of European Hematology Association. This is an open access Abstract Book distributed under the Attribution-NonCommercial-NoDerivs (CC BY-NC-ND), which allows third parties to download the articles and share them with others as long as they credit the author and the Abstract Book, but they cannot change the content in any way or use them commercially.
**Abstract Book Citations:** Authors, Title, HemaSphere, 2026;10:(S1):pages. The abstract book DOIs can be found on the "Author Information" page.
**Disclaimer:** Articles published in the journal HemaSphere exclusively reflect the opinions of the authors. The authors are responsible for all content in their abstracts including accuracy of the facts, statements, citing resources, etc.

### (PF971) FIRST-LINE ODRONEXTAMAB PLUS CHOP IN DIFFUSE LARGE B-CELL LYMPHOMA: OLYMPIA-3 PART 1B RESULTS

**Topic:** 19. Large B cell lymphomas - Clinical

Bastian von Tresckow*[1], Matthew Matasar[2], Thomas Jandl[3], Rita Assi[4], Philip Kuriakose[5], Chaitanya Iragavarapu[6], Thomas Illmer[7], Neta Goldschmidt[8], Abraham Avigdor[9], Youngwoo Jeon[10], Shin Yeu Ong[11], Costas K. Yannakou[12, 13], Hanlon Sia[14], Ho-Jin Shin[15], Lily Wong Lee Lee[16], Sonia González de Villambrosia Pellón[17], Ahlam Naila Kori[18], Ciel De Vriendt[19], Gottfried von Keudell[20, 21], Sandy Amorim[22], Gerardo Musuraca[23], Monica Tani[24], Eva Donato Martin[25], Antonio Gutierrez Garcia[26], Jurriaan Brouwer-Visser[27], Katherine Kargus[27], Hesham Mohamed[27], Alice Cao[27], Nazia Iqbal[27], Jean-Marie Michot[28]

*[1]West German Cancer Center and German Cancer Consortium (DKTK partner site Essen), University Hospital Essen, University of Duisburg-Essen, Essen, Germany; [2]Rutgers Cancer Institute, New Brunswick, NJ, United States of America;[3]Stony Brook University Cancer Center, Stony Brook University, Stony Brook, NY, United States of America; [4]Indiana University School of Medicine, Indianapolis, IN, United States of America; [5]Henry Ford Health, Detroit, MI, United States of America;[6]Hematology & Cellular Therapy, University of Kentucky College of Medicine, Markey Cancer Center, Lexington, KY, United States of America; [7]Berufsausübungsgemeinschaft, Dresden, Germany; [8]Hadassah Medical Center and Faculty of Medicine, Hebrew University of Jerusalem, Jerusalem, Israel; [9]Chaim Sheba Medical Center, Ramat Gan, Israel and Gray School of Medicine, Tel Aviv University, Tel Aviv, Israel; [10]Yeouido St. Mary's Hospital, The Catholic University of Korea, Seoul, South Korea;[11]Singapore General Hospital, Singapore, Singapore; [12]Epworth HealthCare, Melbourne, Australia; [13]The University of Melbourne, Melbourne, Australia; [14]Pindara Private Hospital, Gold Coast, Australia;[15]Research Institute of Medical Science, Pusan National University Hospital, Pusan National University School of Medicine, Busan, South Korea; [16]Queen Elizabeth Hospital, Kota Kinabalu, Malaysia; [17]Hospital Universitario Marqués de Valdecilla (IDIVAL), Santander, Spain;[18]Hospital Tengku Ampuan Afzan, Kuantan, Malaysia; [19]Ghent University Hospital, Ghent, Belgium;[20]Harvard Medical School, Boston, MA, United States of America;[21]Beth Israel Deaconess Medical Center, Boston, MA, United States of America; [22]Hôpital Saint Vincent de Paul, Lille, France;[23]IRCCS Istituto Romagnolo per lo Studio dei Tumori (IRST) "Dino Amadori" Hematology Unit, Meldola, Italy; [24]Ospedale 'Santa Maria delle Croci' di Ravenna, Ravenna, Italy; [25]Hospital Universitario Doctor Peset, Valencia, Spain;[26]Son Espases University Hospital, Palma, Spain; [27]Regeneron Pharmaceuticals, Inc., Tarrytown, NY, United States of America;[28]Gustave Roussy, Villejuif, France;*

### Background

Approximately 25% of patients (pts) with diffuse large B-cell lymphoma (DLBCL) treated with rituximab + CHOP do not have a complete response (CR), and those with refractory/relapsed disease or high-risk features have poor outcomes. To address this unmet need, bispecific antibody combinations are being evaluated. In Part 1A (dose escalation) of the Phase 3 OLYMPIA-3 study (NCT06091865), odronextamab (Odro)-CHOP demonstrated generally manageable safety (most common treatment-emergent adverse events [TEAEs] were neutropenia [81.8%] and cytokine release syndrome [CRS; 54.5%]) and encouraging preliminary efficacy in pts with previously untreated DLBCL, with CR rates of 100% on positron emission tomography/computed tomography (160 mg dose level, selected for Part 1B dose optimization).

### Aims

To evaluate the safety and efficacy of 2 dosing regimens of Odro-CHOP in Part 1B.

**Copyright Information:** (Online) ISSN: 2572-9241
©2026 The Author(s). HemaSphere is published by John Wiley & Sons Ltd on behalf of European Hematology Association. This is an open access Abstract Book distributed under the Attribution-NonCommercial-NoDerivs (CC BY-NC-ND), which allows third parties to download the articles and share them with others as long as they credit the author and the Abstract Book, but they cannot change the content in any way or use them commercially.
**Abstract Book Citations:** Authors, Title, HemaSphere, 2026;10:(S1):pages. The abstract book DOIs can be found on the "Author Information" page.
**Disclaimer:** Articles published in the journal HemaSphere exclusively reflect the opinions of the authors. The authors are responsible for all content in their abstracts including accuracy of the facts, statements, citing resources, etc.

### Methods

Part 1B included pts aged ≥18 years with untreated CD20+ DLBCL not otherwise specified with at least 1 high-risk feature or high-grade B-cell lymphoma with *MYC*, *BCL2*, and/or *BCL6* rearrangement. Odro-CHOP was administered in 6 × 21-day cycles, with Odro 0.7/4/20 mg step-up dosing from Cycle (C) 1 Day (D) 8. Pts were randomized 1:1 to receive Odro 160 mg QW on C2D8–C5D1 then 320 mg Q2W on C5D8–C6D15 (Regimen [R] 1), or Odro 160 mg QW on C2D8 and C2D15 then 160 mg Q3W on C3D1–C6D1 (R2), plus CHOP. The primary endpoint was safety. Secondary endpoints included investigator-assessed objective response rate (ORR), CR rate, and duration of response (DOR) per 2014 Lugano criteria. Exploratory endpoints included biomarker analysis.

### Results

At data cut-off (August 19, 2025), 40 pts were enrolled in Part 1B (20 per regimen). Median age was 67.5 years, 60.0% of pts were male, and 75.0% had an IPI score of 3–5. Median duration of treatment exposure was 18.1 weeks (R1) and 16.6 weeks (R2); 67.5% of pts completed 6 treatment cycles. Median relative dose intensity was 90.1% in R1 and 100% in R2 for Odro, and 98 to 100% for cyclophosphamide, doxorubicin, and vincristine (CHO) in both regimens. The safety profile of Odro-CHOP was similar in both regimens. TEAEs led to dose interruption/delay in 70.0% vs 50.0% of pts and to CHOP dose reduction in 10.0% vs 5.0% of pts with R1 vs R2, respectively. TEAEs led to treatment discontinuation in 1 pt (Grade 4 neutropenic sepsis, R2). The most common TEAEs were neutropenia (57.5%), CRS (55.0%; predominantly Grade 1 [42.5% of pts]), and anemia (42.5%). ICANS occurred in 3 pts (all Grade 1) and resolved completely. As mandated by the protocol, cytomegalovirus (CMV) monitoring was performed via blood PCR. CMV infections (high-level term) occurred in 20 pts; 7 were symptomatic infections. Efficacy was analyzed as ITT, including 3 pts who discontinued treatment prior to tumor imaging assessment. With a median follow-up of 2.3 months in both regimens, ORRs were 95.0% (R1) and 90.0% (R2), and CR rates were 85.0% (both regimens). Median DOR and duration of CR: not reached. Analyses of pts who reached end of treatment showed 8/12 pts in R1 and 7/13 pts in R2 had undetectable ctDNA (**Figure**).

### Summary/Conclusion

In Part 1B of OLYMPIA-3, the safety profile ofOdro-CHOP was generally manageable, preliminary efficacy was encouraging with no meaningful differences between regimens, and combination with Odro did not impact the delivery of CHO. Given these results, the less frequent Odro dosing regimen (R2) with CHOP was selected as the recommended Phase 3 dose for Part 2 (randomized controlled trial).

© 2026 American Society of Clinical Oncology, Inc. Reused with permission. This abstract was accepted and previously presented at the 2026 ASCO Annual Meeting. All rights reserved.

**Copyright Information:** (Online) ISSN: 2572-9241
©2026 The Author(s). HemaSphere is published by John Wiley & Sons Ltd on behalf of European Hematology Association. This is an open access Abstract Book distributed under the Attribution-NonCommercial-NoDerivs (CC BY-NC-ND), which allows third parties to download the articles and share them with others as long as they credit the author and the Abstract Book, but they cannot change the content in any way or use them commercially.
**Abstract Book Citations:** Authors, Title, HemaSphere, 2026;10:(S1):pages. The abstract book DOIs can be found on the "Author Information" page.
**Disclaimer:** Articles published in the journal HemaSphere exclusively reflect the opinions of the authors. The authors are responsible for all content in their abstracts including accuracy of the facts, statements, citing resources, etc.

**Figure. Absolute ctDNA count in R1 and R2**

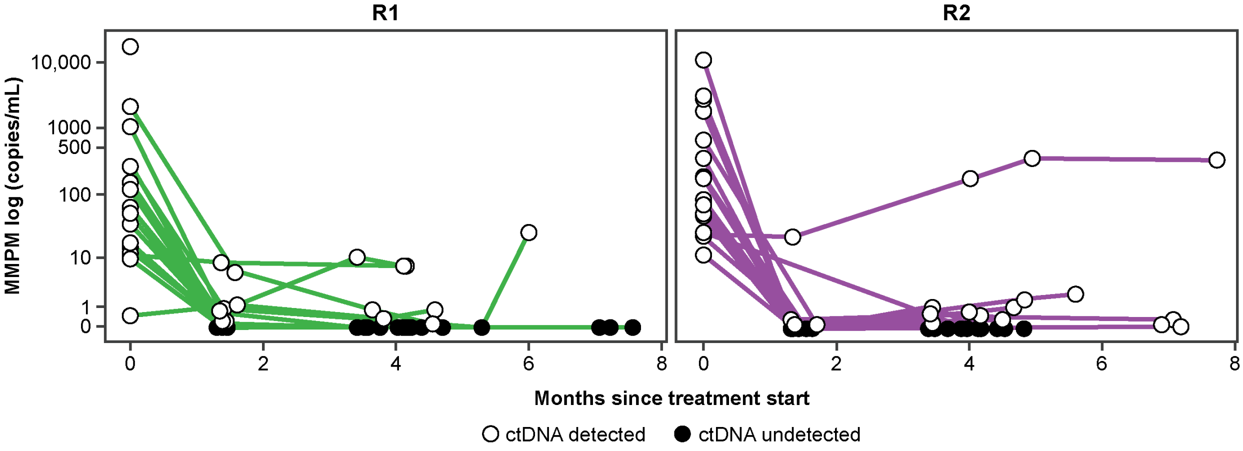

ctDNA, circulating tumor DNA; MMPM, mutant molecules per milliliter.

**Copyright Information:** (Online) ISSN: 2572-9241
©2026 The Author(s). HemaSphere is published by John Wiley & Sons Ltd on behalf of European Hematology Association. This is an open access Abstract Book distributed under the Attribution-NonCommercial-NoDerivs (CC BY-NC-ND), which allows third parties to download the articles and share them with others as long as they credit the author and the Abstract Book, but they cannot change the content in any way or use them commercially.
**Abstract Book Citations:** Authors, Title, HemaSphere, 2026;10:(S1):pages. The abstract book DOIs can be found on the "Author Information" page.
**Disclaimer:** Articles published in the journal HemaSphere exclusively reflect the opinions of the authors. The authors are responsible for all content in their abstracts including accuracy of the facts, statements, citing resources, etc.

### (PF972) PHASE I/II STUDY OF ZANUBRUTINIB IN COMBINATION WITH RITUXIMAB, IFOSFAMIDE, CARBOPLATIN, AND ETOPOSIDE IN RELAPSED/REFRACTORY DIFFUSE LARGE B-CELL LYMPHOMA: INTERIM RESULTS OF THE PHASE II PART

**Topic:** 19. Large B cell lymphomas - Clinical

Hyungwoo Cho*[1], Jaewon Hyung[2], Dong Won Baek[3], Ho Sup Lee[4], Hye Jin Kang[5], Gyeong-Won Lee[6], Jeong-Ok Lee[7], Myung-Won Lee[8], Sungnam Lim[9], Sung Yong Oh[10], Ho-Jin Shin[11], Kwai Han Yoo[12], Young Rok Do[13], Jae-Yong Kwak[14], Yong-Pyo Lee[15], Deok Hwan Yang[16], Dok Hyun Yoon[1]

*[1]Asan Medical Center, University of Ulsan College of Medicine, Seoul, Korea, Seoul, South Korea;[2]Asan Medical Center, University of Ulsan College of Medicine, Seoul, KoreaS, Seoul, South Korea; [3]Department of Hematology/Oncology, Kyungpook National University Hospital, School of Medicine, Kyungpook National University, Daegu, South Korea., Daegu, South Korea; [4]Department of Internal Medicine, Kosin University College of Medicine, Busan, South Korea, Busan, South Korea;[5]"Division of Hematology/Oncology, Department of Internal Medicine, Korea Cancer Center Hospital, Korea Institute of Radiological and Medical Sciences, Seoul, Republic of Korea", Seoul, South Korea; [6]Division of Hematology and Oncology, Department of Internal Medicine, Gyeongsang National University Hospital, Institute of Medical Science, Gyeongsang National University College of Medicine, 79 Gangnam-ro, Jinju, 52727, Korea., Jinju, South Korea; [7]Department of Internal Medicine, Seoul National University Bundang Hospital, Seoul National University College of Medicine, Seongnam, South Korea, Seoul, South Korea; [8]Division of Hematology/Oncology, Department of Internal Medicine,Chungnam National University Hospital, Daejeon, South Korea; [9]Department of Internal Medicine, Inje University College of Medicine, Haeundae Paik Hospital, Busan, South Korea; [10]Department of Internal Medicine, Dong-A University Hospital, Busan, South Korea; [11]"Hematology-oncology Division, Department of Internal Medicine, Pusan National University Hospital, 179 Gudeok-ro, Seo-gu, Busan, 602-739, Korea", Busan, South Korea; [12]Division of Hematology, Department of Internal Medicine Gachon University Gil Medical Center, Gachon University College of Medicine, Incheon, South Korea; [13]"Division of Hematology-Oncology, Department of Internal Medicine, Dongsan Medical Center, Keimyung University School of Medicine, Daegu, Korea", Daegu, South Korea; [14]"Division of Hematology Department of Internal Medicine Jeonbuk National University Medical School Republic of Korea", Jeonju, South Korea; [15]Division of Medical Hematology Chungbuk National University Hospital, Cheongju, South Korea;[16]Department of Hematology-Oncology, Chonnam National University Medical School, Chonnam National University Hwasun Hospital, Jeollanam-do, South Korea;*

### Background

The prognosis of relapsed or refractory diffuse large B-cell lymphoma (R/R DLBCL) is poor, and outcomes are particularly dismal for R/R CNS lymphomas (primary [PCNSL] or secondary [SCNSL]). R-ICE (rituximab, ifosfamide, carboplatin, and etoposide) chemotherapy has shown efficacy in these patients (pts) but the durability of response is limited. Recently, BTK inhibitors have demonstrated encouraging efficacy outcomes in systemic R/R non-GCB DLBCL and R/R CNS lymphomas.

### Aims

This phase I/II study aims to evaluate the efficacy, safety, and feasibility of combining zanubrutinib, a second-generation BTK inhibitor, with R-ICE (ZR-ICE) in pts with R/R systemic non-GCB DLBCL or R/R CNS lymphomas. Here we report the interim efficacy and safety results from the phase II part.

**Copyright Information:** (Online) ISSN: 2572-9241
©2026 The Author(s). HemaSphere is published by John Wiley & Sons Ltd on behalf of European Hematology Association. This is an open access Abstract Book distributed under the Attribution-NonCommercial-NoDerivs (CC BY-NC-ND), which allows third parties to download the articles and share them with others as long as they credit the author and the Abstract Book, but they cannot change the content in any way or use them commercially.
**Abstract Book Citations:** Authors, Title, HemaSphere, 2026;10:(S1):pages. The abstract book DOIs can be found on the "Author Information" page.
**Disclaimer:** Articles published in the journal HemaSphere exclusively reflect the opinions of the authors. The authors are responsible for all content in their abstracts including accuracy of the facts, statements, citing resources, etc.

### Methods

In the phase I part, zanubrutinib 160 mg daily was established as the recommended phase 2 dose based on a standard 3+3 dose escalation design. In the phase II part, pts with R/R systemic non-GCB DLBCL (cohort A) or R/R CNS lymphomas (PCNSL or SCNSL) (cohort B) received zanubrutinib 160 mg daily in combination with R-ICE. The R-ICE regimen consisted of rituximab 375 mg/m² IV (D1), ifosfamide 1670 mg/m² IV (D1–3), etoposide 100 mg/m²/day IV (D1–3), and carboplatin AUC 5 IV (D2). Pts who were candidates for upfront autologous stem cell transplantation (ASCT) and achieved CR or PR after 3 cycles proceeded to ASCT. Pts who were not candidates for upfront ASCT received up to 6 cycles of ZR-ICE. Cohort B pts achieving CR or PR after 6 cycles of ZR-ICE or 3 cycles of ZR-ICE followed by ASCT received zanubrutinib 320 mg daily as maintenance for up to 24 months.

### Results

A total of 52 pts were enrolled in the phase II part (cohort A: n = 38; cohort B: n = 14). The median age was 63 years (range, 33–80), and 34 pts were male (65.4%). The median number of prior lines of therapy was 1 (range, 1–5), and 30 pts (57.7%) had refractory disease to the last line of therapy. Response was evaluable in 44 of the 52 pts. The ORR was 90.9%, with a CR rate of 72.7% (cohort A: ORR 90.3%, CR 74.2%; cohort B: ORR 92.3%, CR 69.2%) (Figure 1A). With a median follow-up of 5.8 months (95% CI, 4.1–8.3), the 6-month PFS rate was 76.3% and the 6-month OS rate was 90.0% for the entire cohort (Figure 1B and 1C). The 6-month PFS and OS rates were 83.2% and 100.0% in cohort A, and 62.3% and 71.6% in cohort B. Eleven pts (all from cohort A) proceeded to upfront ASCT, with no PFS or OS events observed as of data cut-off (Jan 31, 2026). The most frequent grade ≥3 adverse events (AEs) were neutropenia (46.0%), thrombocytopenia (46.0%), and anemia (29.0%). Febrile neutropenia was observed in 5 pts (10.0%). One patient died of treatment-related sepsis. No patient discontinued treatment due to AEs.

### Summary/Conclusion

ZR-ICE demonstrated promising efficacy with a manageable safety profile in R/R systemic non-GCB DLBCL and R/R CNS lymphomas. These interim results support continued enrollment of this ongoing phase II study.

**Copyright Information:** (Online) ISSN: 2572-9241
©2026 The Author(s). HemaSphere is published by John Wiley & Sons Ltd on behalf of European Hematology Association. This is an open access Abstract Book distributed under the Attribution-NonCommercial-NoDerivs (CC BY-NC-ND), which allows third parties to download the articles and share them with others as long as they credit the author and the Abstract Book, but they cannot change the content in any way or use them commercially.
**Abstract Book Citations:** Authors, Title, HemaSphere, 2026;10:(S1):pages. The abstract book DOIs can be found on the "Author Information" page.
**Disclaimer:** Articles published in the journal HemaSphere exclusively reflect the opinions of the authors. The authors are responsible for all content in their abstracts including accuracy of the facts, statements, citing resources, etc.

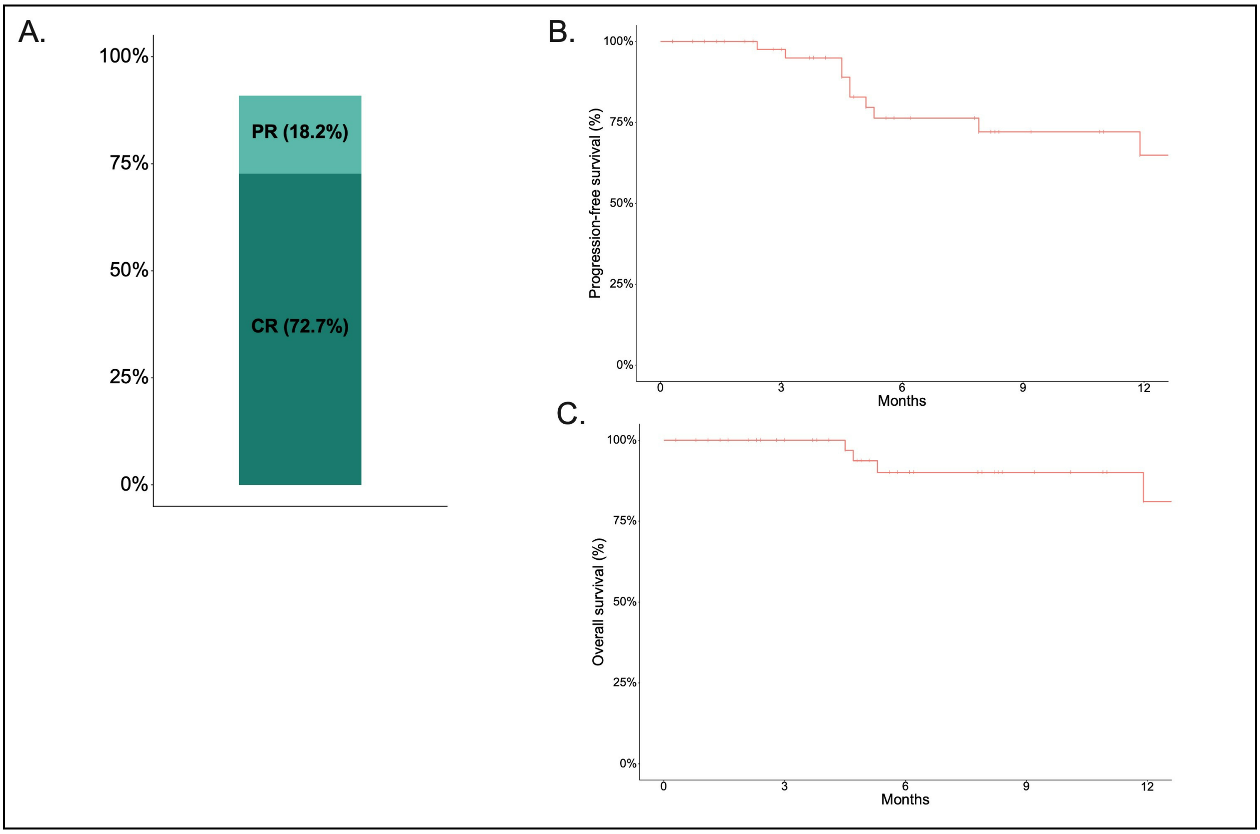

**Copyright Information:** (Online) ISSN: 2572-9241
©2026 The Author(s). HemaSphere is published by John Wiley & Sons Ltd on behalf of European Hematology Association. This is an open access Abstract Book distributed under the Attribution-NonCommercial-NoDerivs (CC BY-NC-ND), which allows third parties to download the articles and share them with others as long as they credit the author and the Abstract Book, but they cannot change the content in any way or use them commercially.
**Abstract Book Citations:** Authors, Title, HemaSphere, 2026;10:(S1):pages. The abstract book DOIs can be found on the "Author Information" page.
**Disclaimer:** Articles published in the journal HemaSphere exclusively reflect the opinions of the authors. The authors are responsible for all content in their abstracts including accuracy of the facts, statements, citing resources, etc.

### (PF973) BIRELENTINIB DEMONSTRATES MANAGEABLE SAFETY PROFILE AND UNIVERSAL ANTITUMOR ACTIVITY ACROSS MOLECULAR SUBTYPES IN DIFFUSE LARGE B-CELL LYMPHOMA

**Topic:** 19. Large B cell lymphomas - Clinical

Lugui Qiu*[1], Yajun Li[2], Keshu Zhou[3], Wenbin Qian[4], Ming Jiang[5], Meifang Zheng[6], Wei Liu[1], Zhiming Li[7], Zengjun Li[8], Pengcheng He[9], Mei Lan[10], Hongmei Jing[11, 11], Kaiyang Ding[12], Fang Zhu[13], Ziyi Liu[14], Lihua Qiu[15]

*[1]State Key Laboratory of Experimental Hematology, National Clinical Medical Research Center for Blood Diseases, Institute of Hematology and Blood Diseases Hospital, Chinese Academy of Medical Sciences and Peking Union Medical College, Tianjin, Tianjin, China; [2]the Affiliated Cancer Hospital of Xiangya School of Medicine, Central South University, Hunan Cancer Hospital, Changsha, China; [3]Henan Cancer Hospital, Affiliated Cancer Hospital of Zhengzhou University, Zhengzhou, China, Zhengzhou, China; [4]The Second Affiliated Hospital Zhejiang University School of Medicine, Hangzhou, China;[5]West China Hospital of Sichuan University, Chengdu, China; [6]Linyi Cancer Hospital, Linyi, China; [7]Sun Yat-sen University Cancer Center, Guangzhou, China; [8]Shandong Cancer Hospital and Institute, Shandong First Medical University and Shandong Academy of Medical Sciences, Jinan, China; [9]The First Affiliated Hospital of Xi'an Jiaotong University, Xian, China;[10]The People's Hospital of Guangxi Zhuang Autonomous Region,, Nanning, China; [11]Department of Hematology, Peking University Third Hospital., Beijing, China; [12]Department of Hematology, The First Ailiated Hospital of USTC, Division of Life Sciences and Medicine, University of Science and Technology of China, Hefei, China; [13]Huazhong University of Science and Technology,, Wuhan, China; [14]Dizal Pharmaceutical Co., Ltd., Shanghai, China; [15]Tianjin Medical University Cancer Institute and Hospital, Tianjin, China;*

### Background

A major hurdle in treating DLBCL is its high molecular heterogeneity. BTK inhibitors show efficacy primarily in the non-GCB (ABC) subtype and certain genomic clusters. T-cell-directed therapies expand the treatment landscape but are constrained by a challenging toxicity profile, including severe adverse events like CRS and ICANS. Therefore, there is a critical need for next-generation targeted agents that provide broad-spectrum activity across molecular subtypes and coupled with a favorable safety profile. LYN and BTK are critical mediators of B-cell receptor (BCR) signalling, driving lymphomagenesis across diverse molecular profiles. Birelentinib (DZD8586), a novel LYN/BTK dual inhibitor with excellent blood-brain barrier (BBB) penetration, is designed to overcome these barriers by targeting multiple components of the BCR signalling pathway. Here, we report updated results from the ongoing Phase 2 TAI-SHAN9 study (NCT06539195) for birelentinib in r/r DLBCL, including an analysis of activity across genetic subtypes.

### Methods

Adult patients aged ≥18 years with histologically confirmed DLBCL and prior standard chemoimmunotherapy were enrolled. Birelentinib was administered orally as monotherapy until disease progression, unacceptable toxicity, or patient withdrawal. Investigators assessed antitumor efficacy per Lugano 2014 criteria using PET and contrast-enhanced CT scans. Adverse events (AEs) were graded using CTCAE v5.0. Based on targeted sequencing, LymphPlex algorithm was applied to assign genetic subtypes, and clinical efficacy was analyzed across genetic subtypes.

**Copyright Information:** (Online) ISSN: 2572-9241
©2026 The Author(s). HemaSphere is published by John Wiley & Sons Ltd on behalf of European Hematology Association. This is an open access Abstract Book distributed under the Attribution-NonCommercial-NoDerivs (CC BY-NC-ND), which allows third parties to download the articles and share them with others as long as they credit the author and the Abstract Book, but they cannot change the content in any way or use them commercially.
**Abstract Book Citations:** Authors, Title, HemaSphere, 2026;10:(S1):pages. The abstract book DOIs can be found on the “Author Information” page.
**Disclaimer:** Articles published in the journal HemaSphere exclusively reflect the opinions of the authors. The authors are responsible for all content in their abstracts including accuracy of the facts, statements, citing resources, etc.

### Results

As of October 20, 2025, 58 patients received DZD8586 at doses of 25 mg (n=4), 50 mg (n=18), and 75 mg (n=36) once daily (QD). The median age was 61.5 years; 35 patients (60.3%) were male, and 27 (46.6%) had International Prognostic Index (IPI) score ≥3. Patients had received a median of 1 (range,1-5) prior lines of therapy, and 45 (77.6%) were refractory to their last regimen. Based on cell-of-origin classification (Han's algorithm), 16 patients (27.6%) were GCB, and 40 (69.0%) were non-GCB. Among 42 efficacy-evaluable patients treated at 50 mg or 75 mg QD, overall objective response rate (ORR) was 47.6% (20/42) and complete response rate (CRR) was 31.0% (13/42). Response was observed across COO subtypes: in GCB DLBCL, the ORR was 58.3% (7/12) and the CRR was 41.7% (5/12); in non-GCB DLBCL, the ORR was 44.8% (13/29) and the CRR was 27.6% (8/29). At a median follow-up of 9.2 months for complete responders, the median duration of response (DoR) was not reached. The longest DoR of ongoing CR exceeded 9.3 months. Birelentinib showed response across LymphPlex genetic subtypes: MCD-like (ORR: 50.0% [6/12], CRR: 33.3% [4/12]); TP53-mut (ORR 50.0% [5/10], CRR: 30.0% [3/10]), and NOS (ORR: 50.0% [7/14], CRR: 35.7% [5/14]). Limited patient numbers in the BN2-like (n=2), ST2-like (n=1), and EZB-like (n=1) subtypes prevented a meaningful evaluation of activity. Common grade 3/4 drug-related AEs included: thrombocytopenia (20.7%), neutropenia (12.1%), and pneumonia (5.2%). No significant drug-related bleeding or arrhythmia was reported. No drug-related AEs led to death.

### Summary/Conclusion

Birelentinib demonstrated universal and potentially durable activity across molecular subtypes, with a manageable safety profile. These findings suggest broad therapeutic potential for birelentinib in DLBCL.

**Copyright Information:** (Online) ISSN: 2572-9241
©2026 The Author(s). HemaSphere is published by John Wiley & Sons Ltd on behalf of European Hematology Association. This is an open access Abstract Book distributed under the Attribution-NonCommercial-NoDerivs (CC BY-NC-ND), which allows third parties to download the articles and share them with others as long as they credit the author and the Abstract Book, but they cannot change the content in any way or use them commercially.
**Abstract Book Citations:** Authors, Title, HemaSphere, 2026;10:(S1):pages. The abstract book DOIs can be found on the "Author Information" page.
**Disclaimer:** Articles published in the journal HemaSphere exclusively reflect the opinions of the authors. The authors are responsible for all content in their abstracts including accuracy of the facts, statements, citing resources, etc.

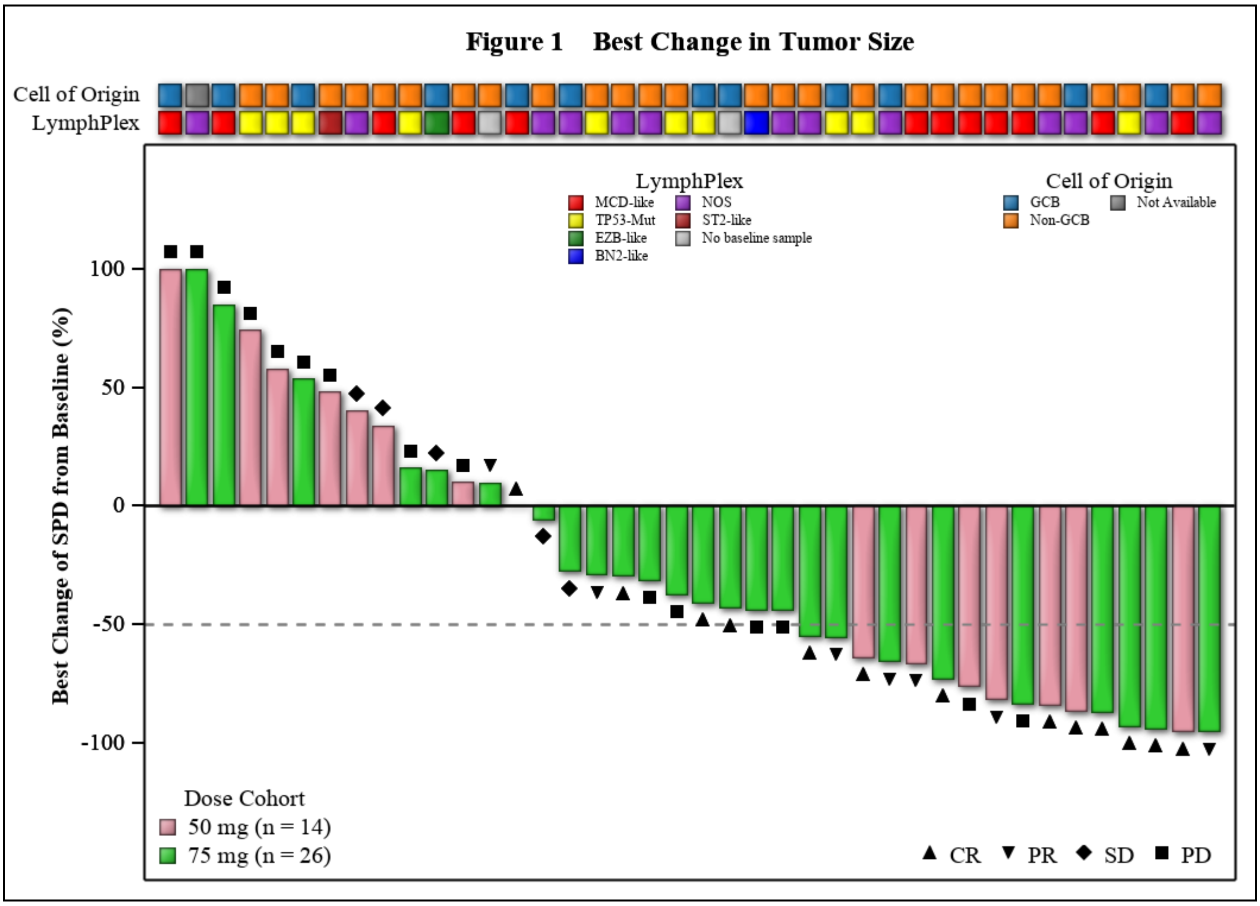

CR: Complete Response; PR: Partial Response; SD: Stable Disease; PD: Progressive Disease; SPD: Sum of the Product of the Perpendicular Diameters. n: Number of participants with at least one change of SPD in the analysis set for each treatment group.

**Copyright Information:** (Online) ISSN: 2572-9241
©2026 The Author(s). HemaSphere is published by John Wiley & Sons Ltd on behalf of European Hematology Association. This is an open access Abstract Book distributed under the Attribution-NonCommercial-NoDerivs (CC BY-NC-ND), which allows third parties to download the articles and share them with others as long as they credit the author and the Abstract Book, but they cannot change the content in any way or use them commercially.
**Abstract Book Citations:** Authors, Title, HemaSphere, 2026;10:(S1):pages. The abstract book DOIs can be found on the "Author Information" page.
**Disclaimer:** Articles published in the journal HemaSphere exclusively reflect the opinions of the authors. The authors are responsible for all content in their abstracts including accuracy of the facts, statements, citing resources, etc.

### (PF974) HUMANIZED PDX MODELS REVEAL DRUG-REVERSIBLE EPIGENETIC IMMUNE EXCLUSION GOVERNING THERAPY OUTCOME IN DLBCL, ALLOWING PATIENTS STRATIFICATION.

**Topic:** 19. Large B cell lymphomas - Clinical

Giovanni Medico*[1], Giorgio Inghirami[1]

[1]*Weill Cornell Medical College, Pathology and Laboratory Medicine, New York, United States of America;*

### Background

Diffuse large B-cell lymphoma (DLBCL) is a heterogenous disease, with a relatively high response rate to first line chemoimmunotherapy; T cell based therapy have played a major part in enhancing previously dismal patient outcomes in second line, but still only ~50% of the patients responds, and no predictive tool is currently available in the clinic.
To elucidate and dissect DLBCL complex tumor-host immune interactions, we established the first fully humanized DLBCL PDX models (HuMice PDX) and performed integrated multi-omics profiling of patient samples.

### Aims in this study we envision a strategy to deliver a clinically applicable predictive patients stratifier, to inform clinicians therapeutic choice. We sought to reproduce immune depleted (IR) and immune rich (IR) DLBCL pathophysiology in vivo, clarify correlation between the ID phenotype and resistance to CAR-T cell therapy. And finally we screened for compounds that could resensitize CAR resistant DLBCL to T cell mediated killing.

### Methods

We analyzed 132 DLBCL (112 R-CHOP, 20 CAR-T patients), 48 matched PDX, 9 HuMice PDX and CAR-19/CAR-BAFFR treated PDX, using bulk RNA-seq, single-cell RNA/TCR-seq, WES/CAPP-seq and CosMx SMI profiling (6000 RNA, 64 proteins). Large DLBCL datasets were used to build a prediction classifier. heterologous/allogeneic CD34+ Human Stem Progenitor Cell (HSPC) were implanted in NSG-SGM3xNBSGW mice, to generate HuMice.

**Copyright Information:** (Online) ISSN: 2572-9241
©2026 The Author(s). HemaSphere is published by John Wiley & Sons Ltd on behalf of European Hematology Association. This is an open access Abstract Book distributed under the Attribution-NonCommercial-NoDerivs (CC BY-NC-ND), which allows third parties to download the articles and share them with others as long as they credit the author and the Abstract Book, but they cannot change the content in any way or use them commercially.
**Abstract Book Citations:** Authors, Title, HemaSphere, 2026;10:(S1):pages. The abstract book DOIs can be found on the "Author Information" page.
**Disclaimer:** Articles published in the journal HemaSphere exclusively reflect the opinions of the authors. The authors are responsible for all content in their abstracts including accuracy of the facts, statements, citing resources, etc.

### Results

We identified REPIX, a 12-gene signature, able to predict whether DLBCL displays an immune-depleted (ID) versus an immune-rich (IR) tumor microenvironment, and able to accurately predict therapy outcome. Deconvolution algorithms applied to large RNAseq datasets showed significantly lower immune infiltration in REPIX-high DLBCL. ID/REPIX-high patients displayed significantly worse outcomes following both R-CHOP-(p<0.0001) and CAR-19 (p=0.002) treatment, compared to IR/REPIX-low patients.
To elucidate the complex host–lymphoma interactions at play, we generated HuMice and implanted them with ID/REPIX-high and IR/REPIX-low PDX models.
Mimicking patients' outcomes, only IR models displayed reduced tumor growth in HuMice, while ID-DLBCL showed unimpeded growth. Flow cytometry, mIHC, and RNAseq/scRNAseq analyses revealed that ID-DLBCL-HuPDX were devoid of Tumor-Infiltrating Lymphocytes (TILs), compared to IR-DLBCL-HuPDX (p<2e-04).
We then tested two different CART (CD19, BAFFR) in-vivo in nine ID- and IR-PDX-HuMice models. IR/REPIX-low models were eradicated, while ID/REPIX-high were refractory to both CAR.
To enhance ID-DLBCL responses, we performed drug screening of clinical phase epigenetic targeting drugs shown to improve immune responses.
Valemetostat and Lenalidomide treatments were associated with improved CAR-T cell killing, both in-vitro and in-vivo. exposure to drug led to a reduction of REPIX score, and enrichment of inflammatory signaling pathways (NFkb, IFNg/a, IFN-dependent chemokines), demonstrating the ability of this therapeutic regimens to convert ID-DLBCL into IR-DLBCL (p<0.05–0.0005)

### Summary/Conclusion

REPIX, a novel classifier, predicts DLBCL T-cell infiltration and therapy outcomes allowing patients stratification.
Humanized PDX accurately reflect DLBCL physiopathology and immune-host interactions, providing a platform for validating next-generation immunotherapies.
Epigenetic therapies reprogram REPIX-high/ID- into REPIX-low/IR-DLBCL, overcoming immune refractoriness and enhancing T-cell infiltration and tumor immunorecognition.

**Copyright Information:** (Online) ISSN: 2572-9241
©2026 The Author(s). HemaSphere is published by John Wiley & Sons Ltd on behalf of European Hematology Association. This is an open access Abstract Book distributed under the Attribution-NonCommercial-NoDerivs (CC BY-NC-ND), which allows third parties to download the articles and share them with others as long as they credit the author and the Abstract Book, but they cannot change the content in any way or use them commercially.
**Abstract Book Citations:** Authors, Title, HemaSphere, 2026;10:(S1):pages. The abstract book DOIs can be found on the "Author Information" page.
**Disclaimer:** Articles published in the journal HemaSphere exclusively reflect the opinions of the authors. The authors are responsible for all content in their abstracts including accuracy of the facts, statements, citing resources, etc.

### (PF975) ZAMTOCABTAGENE AUTOLEUCEL, A TANDEM CD20-CD19 DIRECTED CAR-T CELL THERAPY IN RELAPSED/REFRACTORY LARGE B-CELL LYMPHOMA: CROSSOVER ANALYSIS (THIRD-LINE) FROM THE RANDOMIZED, PIVOTAL DALY 2-EU TRIAL

**Topic:** 19. Large B cell lymphomas - Clinical

Peter Vandenberghe*[1], Tom Van Meerten[2], Pierre Bories[3], Peter Dreger[4], László Gopcsa[5], Pim G. N. J. Mutsaers[6], Matthias Edinger[7], Lajos Gergely[8], Bastian von Tresckow[9, 10], Peter Neumeister[11], Normann Steiner[12], Ulrich Jäger[13], Ana Triguero[14], Laimonas Griškevičius[15], Gloria Iacoboni[16], Francois Lemonnier[17], Jaap Van Doesum[2], Marie José Kersten[18], Nirav N Shah[19], Rimas Orentas[20], Stefan Miltenyi[21], Toon Overstijns[22], Iris Bürger[21], Gregor Zadoyan[22], Corinne Brillant[22], Birte Friedrich[23], Pearl van Heteren[22], Silke Holtkamp[22], Peter Borchmann[24, 25]

*[1]University Hospital Leuven, Leuven, Belgium; [2]University Medical Center Groningen, Groningen, The Netherlands; [3]University Cancer Institute Toulouse Oncopole, Toulouse, France; [4]University Hospital Heidelberg, Heidelberg, Germany; [5]South-Pest Central Hospital, National Institute of Hematology and Infectious Diseases, Budapest, Hungary; [6]Erasmus University Rotterdam, Rotterdam, The Netherlands; [7]University Hospital Regensburg, Regensburg, Germany; [8]University of Debrecen, Debrecen, Hungary; [9]Department of Hematology and Stem Cell Transplantation, West German Cancer Center, Essen, Germany; [10]German Cancer consortium (DKTK partner site Essen), University Hospital Essen, University of Duisburg-Essen, Essen, Germany; [11]Medical University Graz, Graz, Austria; [12]Medical University Innsbruck, Innsbruck, Austria; [13]Medical University of Vienna, Vienna, Austria; [14]Hospital Clinic Barcelona, Barcelona, Spain; [15]Vilnius University Hospital Santaros Klinikos, Vilnius, Lithuania; [16]Vall d'Hebron University Hospital, Barcelona, Spain; [17]CHU Mondor, Créteil, France; [18]Amsterdam University Medical Center, Amsterdam, The Netherlands; [19]Medical College of Wisconsin, Milwaukee, United States of America; [20]Lentigen Technology, Inc., Gaithersburg, United States of America; [21]Miltenyi Biotec B.V. & Co. KG, Bergisch Gladbach, Germany; [22]Miltenyi Biomedicine, Bergisch Gladbach, Germany; [23]Uniklinik Düsseldorf, Düsseldorf, Germany; [24]University of Cologne, Faculty of Medicine, Cologne, Germany; [25]University Hospital Cologne, Cologne Lymphoma Working Group, Department of Internal Medicine I, Center for Integrated Oncology Aachen Bonn Cologne Duesseldorf, Cologne, Germany;*

### Background

Zamtocabtagene autoleucel (zamto-cel) is an autologous, non-cryopreserved tandem CD20-CD19 directed CAR-T cell therapy. In the primary analysis of the DALY 2-EU trial (NCT04844866) in transplant ineligible (TI) adults with relapsed/refractory (r/r) large B-cell lymphoma (LBCL), zamto-cel demonstrated superior event-free survival (EFS; median 6.2 vs 2.5 months), improved best overall response (BOR; 72.0 vs 44.9%) and complete response (CR; 53.7 vs 14.1%) vs R-GemOx (standard of care [SoC]) as assessed by an independent review committee. Zamto-cel had a manageable safety profile, with low rates of grade ≥3 immune cell-associated neurotoxicity syndrome (ICANS; 1.3%) and cytokine release syndrome (CRS; 5.3%).

### Aims

To evaluate the efficacy and safety of zamto-cel for crossover patients (pts) from SoC in DALY 2-EU.

**Copyright Information:** (Online) ISSN: 2572-9241
©2026 The Author(s). HemaSphere is published by John Wiley & Sons Ltd on behalf of European Hematology Association. This is an open access Abstract Book distributed under the Attribution-NonCommercial-NoDerivs (CC BY-NC-ND), which allows third parties to download the articles and share them with others as long as they credit the author and the Abstract Book, but they cannot change the content in any way or use them commercially.
**Abstract Book Citations:** Authors, Title, HemaSphere, 2026;10:(S1):pages. The abstract book DOIs can be found on the "Author Information" page.
**Disclaimer:** Articles published in the journal HemaSphere exclusively reflect the opinions of the authors. The authors are responsible for all content in their abstracts including accuracy of the facts, statements, citing resources, etc.

### Methods

DALY 2-EU is a randomized, European, open-label trial. Informed consent was obtained from adult (≥18 years [y]) pts with r/r (≤24 months after start of first-line therapy) LBCL with Eastern Cooperative Oncology Group performance status ≤2 who met one TI criterion (age ≥65y with impaired organ function or age ≥70y).
Between Oct 2021 and Jul 2024, 168 pts were randomized to zamto-cel or SoC (R-GemOx or Pola-BR). Zamto-cel pts started lymphodepletion (fludarabine and cyclophosphamide) during manufacturing and received zamto-cel (target dose of $2.5 \times 10^6$ CAR-T cells/kg body weight). A protocol amendment (Feb 2023) allowed one-sided crossover from SoC to zamto-cel as third-line (3L) therapy, no crossover occurred before the amendment. Eligible pts had relapsed or progressed ≤1y after randomization, or failed to achieve partial response (PR) or CR in ≥8 weeks. Leukapheresis product was collected after randomization, cryopreserved and used after manufacturing following crossover approval. Bridging therapy was allowed prior to zamto-cel administration. Exploratory efficacy endpoints in the crossover population were investigator assessed and included EFS, progression-free survival (PFS), and BOR. Safety was also evaluated; treatment-emergent adverse events (TEAEs) were defined as any AE occurring from zamto-cel infusion to 90 days post-infusion, and before new anti-lymphoma therapy.

### Results

Following the protocol amendment, 31/54 SoC pts were approved for crossover; 29 pts received zamto-cel, one pt withdrew informed consent and one pt died due to progressive disease (PD). The main reason for crossover was PD during SoC (21 [67.7%] pts). **Figure** shows key patient characteristics. Bridging chemoimmunotherapy was administered to 8 (26.7%) pts; 5 (16.7%) pts received radiotherapy. At data cut-off (15 Jan 2025) and a median follow-up of 7.9 (range: 1.5–8.3) months, BOR was 79.3%, with 65.5% of pts achieving CR. 6-month EFS and PFS rates were both 79% (95% CI 53%–92%). Neutropenia was the most common hematologic TEAE (16 [55.2%] pts). TEAEs leading to death occurred in 4 (13.8%) pts: malignant neoplasm progression (n=2), bronchitis and septic shock (both n=1). Reported rates of any grade CRS and ICANS were 48.3% and 17.2%, respectively; grade ≥3 CRS and ICANS were 6.9% and 3.4%, respectively. No grade 5 CRS or ICANS were observed.

### Summary/Conclusion

In this 3L setting, most intended pts received zamto-cel. Analysis of this mainly refractory 3L pt population revealed clinically meaningful efficacy of zamto-cel with low rates of severe CRS and ICANS, consistent with second-line treatment with zamto-cel.

**Copyright Information:** (Online) ISSN: 2572-9241
©2026 The Author(s). HemaSphere is published by John Wiley & Sons Ltd on behalf of European Hematology Association. This is an open access Abstract Book distributed under the Attribution-NonCommercial-NoDerivs (CC BY-NC-ND), which allows third parties to download the articles and share them with others as long as they credit the author and the Abstract Book, but they cannot change the content in any way or use them commercially.
**Abstract Book Citations:** Authors, Title, HemaSphere, 2026;10:(S1):pages. The abstract book DOIs can be found on the “Author Information” page.
**Disclaimer:** Articles published in the journal HemaSphere exclusively reflect the opinions of the authors. The authors are responsible for all content in their abstracts including accuracy of the facts, statements, citing resources, etc.

| Patient characteristics | Total crossover population[a] (N=30) |
|---|---|
| Median age, years | 74.0 |
| ≥75 years, n (%) | 12 (41.4) |
| Male sex, n (%) | 17 (58.6) |
| Histological diagnosis by investigator assessment, n (%) | |
| DLBCL (NOS) | 20 (66.7) |
| HGBL | 2 (6.7) |
| Other[b] | 8 (26.7) |
| Status after 1L therapy, n (%) | |
| Relapsed | 14 (46.7) |
| Relapsed after ≤12 months | 10 (71.4) |
| Refractory | 16 (53.3) |
| Modified Ann Arbor stage at Screening, n (%) | |
| Stage III + IV | 20 (66.7) |
| IPI score between 3–5 at screening, n (%) | 15 (50.0) |
| ECOG PS at crossover baseline, n (%) | |
| Grade 0 | 11 (36.7) |
| Grade 1 | 13 (43.3) |
| Grade 2 | 5 (16.7) |
| Grade 3 | 1 (3.3) |

[a]Data are from the crossover full analysis set; [b]Other included high-grade BCL NOS (n=5, 16.7%), and disease transformed from an earlier diagnosis of low-grade lymphoma into DLBCL with disease progression after systemic treatment (n=3, 10.0%).
1L, first-line; DLBCL NOS, diffuse large B-cell lymphoma not otherwise specified; ECOG PS, Eastern Cooperative Oncology Group performance status; HGBCL, high-grade B-cell lymphoma; IPI, International Prognostic Index.

Copyright Information: (Online) ISSN: 2572-9241
©2026 The Author(s). HemaSphere is published by John Wiley & Sons Ltd on behalf of European Hematology Association. This is an open access Abstract Book distributed under the Attribution-NonCommercial-NoDerivs (CC BY-NC-ND), which allows third parties to download the articles and share them with others as long as they credit the author and the Abstract Book, but they cannot change the content in any way or use them commercially.
Abstract Book Citations: Authors, Title, HemaSphere, 2026;10:(S1):pages. The abstract book DOIs can be found on the "Author Information" page.
Disclaimer: Articles published in the journal HemaSphere exclusively reflect the opinions of the authors. The authors are responsible for all content in their abstracts including accuracy of the facts, statements, citing resources, etc.

### (PF976) REAL-WORLD OUTCOMES OF AXICABTAGENE CILOLEUCEL VERSUS NON-CAR-T APPROACHES IN SECOND-LINE FOR PATIENTS WITH LARGE B CELL LYMPHOMA: A SPANISH MULTICENTER GELTAMO-GETH STUDY

**Topic:** 19. Large B cell lymphomas - Clinical

Mariana Bastos-Oreiro*[1], Leyre Bento[2], Ana Isabel Jimenez Ubieto[3], Jose María Sánchez-Pina[3], Gloria Iacoboni[4], Cristina García Hercé[4], Rafael Hernani[5], Ana Saus Carreres[5], Paula Fernández-Caldas[1], Mi Kwon[1], Rebeca Bailen[1], Javier Delgado-Serrano[6], Almudena Cabero[7], Lucía López Corral[7], Carola Diaz Aizpun[8], Marta Pena[9], Juan-Manuel Sancho Cia[10], Antonio Gutierrez Garcia[2], Inmaculada Concepcion Herrera Arroyo[11], Hugo Luzardo[12], Judith Vázquez-Álvarez[13], Anabelle Chinea Rodriguez[14], Víctor Noriega Concepción[15], Andrés Sanchez Salinas[16], Miriam Sánchez Escamilla[17], Sonia González de Villambrosia Pellón[17], Jaime Sanz[18], Karen Humala[19], Oriana Jimena Lopez Godino[20], Beatriz Aguado Bueno[21], Santiago F. Browne Arthur[22], Blanca Sanchez-Gonzalez[23], María Teresa Villalobos Prego[24], María Belén Navarro[25], Paola Villafuerte Gutierrez[26], Eva Maria Donato Martin[27], Daniel García-Belmonte[28], Jordina Rovira Sole[29], Claudia Salazar[30], Concepción Nicolás[31], Ana Muntañola Prat[32], Carlos Grande[22], Pascual Balsalobre[33], Auxiliadora Moreno[34], Victor Navarro[4], Pere Barba[4], Pau Abrisqueta Costa[4], Alejandro Martín García-Sancho[7]

*[1]Hospital General Gregorio Marañón. Instituto de investigación sanitaria Gregorio Marañón, Hematology, Madrid, Spain; [2]Hospital Universitario Son Espases, Palma, Spain; [3]Hospital Universitario 12 De Octubre, Madrid, Spain; [4]Hospital Vall d'Hebron, Barcelona, Spain; [5]Hospital Clínico universitario de Valencia, Valencia, Spain; [6]Hospital Universitario Virgen del Rocío, Sevilla, Spain; [7]Antiguo Hospital Clínico de Salamanca, Salamanca, Spain; [8]Hospital Regional Universitario de Málaga, Málaga, Spain; [9]Institut Català d'Oncologia L'Hospitalet, L'Hospitalet de Llobregat, Spain; [10]Hospital Germans Trias I Pujol, Badalona, Spain; [11]Hospital Universitario Reina Sofia, Córdoba, Spain; [12]Hospital Universitario de Gran Canaria Doctor Negrín, Las Palmas de Gran Canaria, Spain; [13]Urgencias Hospital Álvaro Cunqueiro, Vigo, Spain; [14]Hospital Universitario Ramón Y Cajal, Madrid, Spain; [15]Hospital Universitario de A Coruña, A Coruña, Spain; [16]Hospital Clínico Universitario Virgen De La Arrixaca, Murcia, Spain; [17]Hospital Universitario Marqués De Valdecilla, Santander, Spain; [18]Hospital Universitario La Fe, Valencia, Spain; [19]Hospital Universitario La Paz, Madrid, Spain; [20]Hospital General Universitario Morales Meseguer, Murcia, Spain; [21]Hospital Universitario De La Princesa, Madrid, Spain; [22]Clínica Universidad De Navarra, Madrid, Spain; [23]Hospital del Mar, Barcelona, Spain; [24]Hospital Universitario Mútua Terrassa, Terrassa, Spain; [25]Hospital Universitario Puerta De Hierro Majadahonda, Majadahonda, Spain; [26]Urgencias Hospital Universitario Príncipe de Asturias, Alcalá de Henares, Spain; [27]Hospital Universitario Doctor Peset, Valencia, Spain; [28]Hospital Universitario La Zarzuela, Madrid, Spain; [29]Hospital Joan XXIII, Tarragona, Spain; [30]Ivo-Instituto Valenciano de Oncología, Valencia, Spain; [31]Hospital Universitario Central De Asturias, Oviedo, Spain; [32]Hospital de Sant Pau, Barcelona, Spain; [33]Grupo Español de Trasplante de Progenitores Hematopoyéticos y Terapia Celular, Madrid, Spain; [34]Grupo Español de Linfomas GELTAMO, Madrid, Spain;*

### Background

Clinical trials have demonstrated the superiority of anti-CD19 CAR T-cell therapy over standard chemoimmunotherapy (CIT) as second-line (2L) treatment for patients with refractory or early-relapsed large B-cell lymphoma (LBCL). However, real-world (RW) comparative data remain limited, particularly according to transplant eligibility.

### Aims

We aimed to describe RW outcomes of axicabtagene ciloleucel (axi-cel) as 2L treatment for LBCL in Spain and to compare progression-free survival (PFS) and overall survival (OS) between these patients and a RW cohort (non-CAR-T) treated with 2L CIT, stratified by transplant eligibility.

**Copyright Information:** (Online) ISSN: 2572-9241
©2026 The Author(s). HemaSphere is published by John Wiley & Sons Ltd on behalf of European Hematology Association. This is an open access Abstract Book distributed under the Attribution-NonCommercial-NoDerivs (CC BY-NC-ND), which allows third parties to download the articles and share them with others as long as they credit the author and the Abstract Book, but they cannot change the content in any way or use them commercially.
**Abstract Book Citations:** Authors, Title, HemaSphere, 2026;10:(S1):pages. The abstract book DOIs can be found on the "Author Information" page.
**Disclaimer:** Articles published in the journal HemaSphere exclusively reflect the opinions of the authors. The authors are responsible for all content in their abstracts including accuracy of the facts, statements, citing resources, etc.

### Methods

We conducted a retrospective, multicenter, RW study including patients with LBCL receiving 2L treatment in Spain. The CAR-T cohort comprised patients treated with axi-cel (03/2024 to 12/2025), while the non-CAR-T cohort included patients treated with CIT from a large retrospective GELTAMO dataset (01/2013 to 12/2023), encompassing both transplant-eligible (T-E) and transplant-ineligible (T-I) patients. Propensity score matching was applied to balance baseline clinical characteristics between cohorts (gender, age, refractoriness, histology, IPI and stage).

### Results

A total of 311 patients with LBCL treated with 2L axi-cel were identified across 21 participating centers and constituted the global CAR-T cohort. With a median follow-up (mFU) of 25 months (95% CI 21–36), 12-month OS rate was 66.3% (95% CI 59.0–74.4), median not reached. Median PFS was 12.7 months (95% CI 10.4–NR) and 12-month PFS rate was 53.8% (95% CI 47.0–61.7). Overall and complete response rates were 81.3% and 71.1%, respectively. Cytokine release syndrome occurred in 95% of patients (grade 2–4: 37%; grade 3–4: 4%), and immune effector cell–associated neurotoxicity syndrome in 47% (grade 2–4: 26%; grade 3–4: 12.5%).
Comparative analyses were performed against a RW non-CAR-T cohort of 530 patients, including 315 T-E and 213 T-I patients (mFU 50 and 56 months, respectively). After propensity score matching, two well-balanced matched cohorts were obtained: axi-cel versus T-E non-CAR cohort (n=96 vs n=97) and axi-cel versus T-I non-CAR cohort (n=96 vs n=76). Among T-E patients, median PFS was not reached with axi-cel versus 4.60 months (95% CI 3.15–9.46) with CIT (HR 2.06, 95% CI 1.34–3.18; p=0.001), with 12-month PFS rates of 63.7% and 27.0%, respectively. In this subgroup, OS did not differ significantly between axi-cel and non-CAR cohorts (median not reached vs 11.24 months, 95% CI 9.56–18.30; HR 1.22, 95% CI 0.76–1.97; p=0.406) (Figure 1A, B). Among T-I patients, axi-cel was associated with superior PFS compared with CIT (median not reached vs 2.50 months, 95% CI 1.22–11.04; HR 2.84, 95% CI 1.60–5.04; p<0.001), with 12-month PFS of 63.7% and 22.0%, respectively. In this subgroup, axi-cel was also associated with significantly improved OS (median not reached vs 7.69 months, 95% CI 4.67–30.95; HR 2.00, 95% CI 1.10–3.67; p=0.024) (Figure 1C, D). One hundred T-E patients and 23 T-I patients received CAR-T therapy in ≥3L.

### Summary/Conclusion

In this large RW Spanish cohort, axi-cel as 2L showed high response rates, favourable survival, and manageable safety. After adjustment, axi-cel provided a clinically meaningful PFS advantage over CIT in both T-E and T-I patients. An OS benefit was seen in T-I, while in T-E it may have been mitigated by subsequent CAR-T use in ≥3L. These findings support CAR-T as an effective 2L strategy for LBCL in routine practice.

**Copyright Information:** (Online) ISSN: 2572-9241
©2026 The Author(s). HemaSphere is published by John Wiley & Sons Ltd on behalf of European Hematology Association. This is an open access Abstract Book distributed under the Attribution-NonCommercial-NoDerivs (CC BY-NC-ND), which allows third parties to download the articles and share them with others as long as they credit the author and the Abstract Book, but they cannot change the content in any way or use them commercially.
**Abstract Book Citations:** Authors, Title, HemaSphere, 2026;10:(S1):pages. The abstract book DOIs can be found on the “Author Information” page.
**Disclaimer:** Articles published in the journal HemaSphere exclusively reflect the opinions of the authors. The authors are responsible for all content in their abstracts including accuracy of the facts, statements, citing resources, etc.

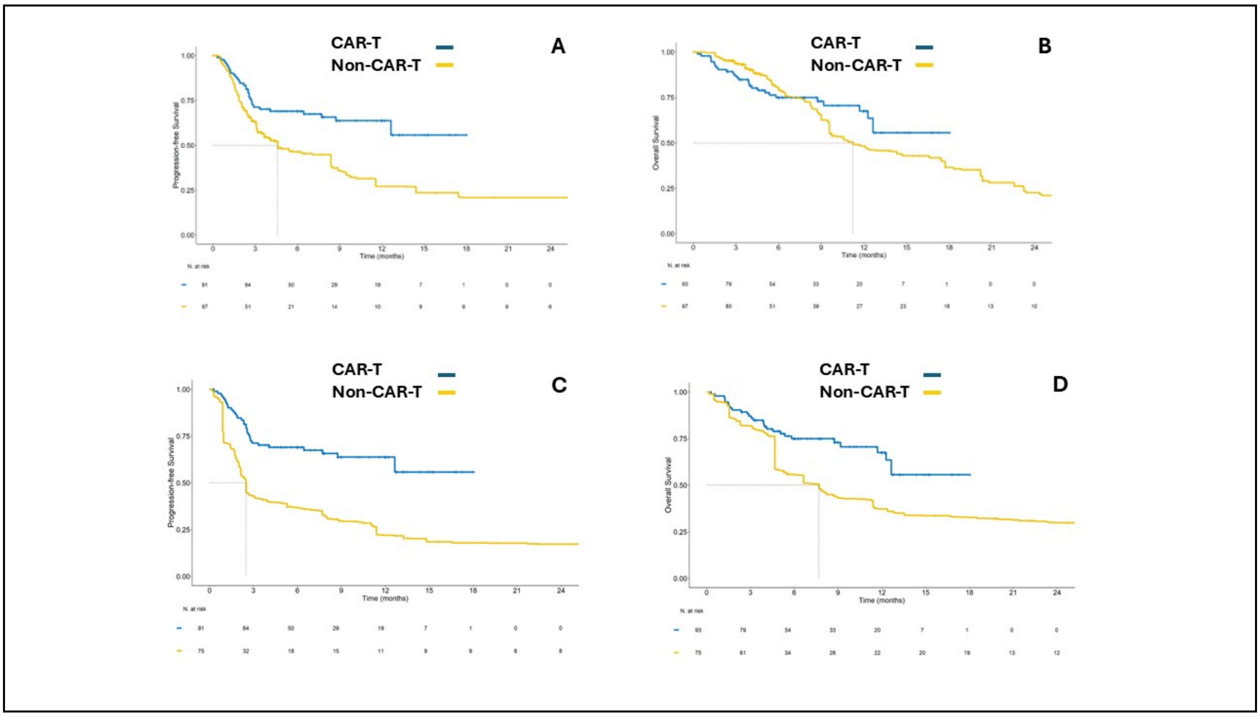

**Copyright Information:** (Online) ISSN: 2572-9241
©2026 The Author(s). HemaSphere is published by John Wiley & Sons Ltd on behalf of European Hematology Association. This is an open access Abstract Book distributed under the Attribution-NonCommercial-NoDerivs (CC BY-NC-ND), which allows third parties to download the articles and share them with others as long as they credit the author and the Abstract Book, but they cannot change the content in any way or use them commercially.
**Abstract Book Citations:** Authors, Title, HemaSphere, 2026;10:(S1):pages. The abstract book DOIs can be found on the "Author Information" page.
**Disclaimer:** Articles published in the journal HemaSphere exclusively reflect the opinions of the authors. The authors are responsible for all content in their abstracts including accuracy of the facts, statements, citing resources, etc.

### (PF977) SUSTAINED REMISSIONS BEYOND 4 YEARS WITH EPCORITAMAB MONOTHERAPY: LONG-TERM FOLLOW-UP RESULTS FROM THE PIVOTAL EPCORE NHL-1 TRIAL IN PATIENTS WITH RELAPSED OR REFRACTORY LARGE B-CELL LYMPHOMA

**Topic:** 19. Large B cell lymphomas - Clinical

Catherine Thieblemont*[1], Chan Y. Cheah[2], Julie Vose[3], Michael Roost Clausen[4], David Cunningham[5], Umar Farooq[6], Tatyana Feldman[7], Herve Ghesquieres[8], Wojciech Jurczak[9], Kim Linton[10], Tycel Phillips[11], Won Seog Kim[12], Pegah Jafarinasabian[13], Barbara D'angelo Månsson[14], Milan Geybels[14], Christian W. Eskelund[14], Martin Hutchings[15], Yasmin Karimi[16]

*[1]Assistance Publique Hôpitaux de Paris - Hôpital Saint-Louis, University of Paris, Department of Hematology, Paris, France;[2]Sir Charles Gairdner Hospital and the University of Western Australia, Perth, WA, Australia; [3]University of Nebraska Medical Center, Omaha, NE, United States of America; [4]Vejle Hospital, Vejle, Denmark; [5]The Royal Marsden NHS Foundation Trust, Sutton, United Kingdom; [6]University of Iowa, Iowa City, IA, United States of America; [7]John Theurer Cancer Center, Hackensack Meridian School of Medicine, Lymphoma Division, Hackensack, NJ, United States of America; [8]Hospices Civils de Lyon, Centre Hospitalier Lyon Sud, Pierre-Bénite, France; [9]Maria Skłodowska-Curie National Research Institute of Oncology, Kraków, Poland; [10]The Christie NHS Foundation Trust, Manchester Cancer Research Centre, and Division of Cancer Sciences, University of Manchester, Manchester, United Kingdom; [11]University of Michigan Comprehensive Cancer Center (Present Affiliation: City of Hope, Duarte, CA, United States), Ann Arbor, MI, United States of America; [12]Samsung Medical Center, Seoul, South Korea; [13]AbbVie, North Chicago, IL, United States of America; [14]Genmab, Copenhagen, Denmark; [15]Rigshospitalet and University of Copenhagen, Copenhagen, Denmark; [16]University of Michigan, Division of Hematology/Oncology, Ann Arbor, MI, United States of America;*

### Background

R/R LBCL is challenging to treat; there remains a need for more effective treatment (tx) options for patients (pts) with LBCL in third-line or later (3L+). Epcoritamab (epcor) is a subcutaneous (SC) CD3×CD20 bispecific antibody approved for the tx of adults with R/R LBCL or R/R FL after ≥ 2 lines of systemic therapy, based on outcomes from the pivotal phase 2 EPCORE® NHL-1 trial (NCT03625037), in which epcor monotherapy led to high CR rates and deep, durable responses with manageable safety in pts with R/R LBCL. Pivotal results have been reported previously (Vose JM, et al. *Blood* 2024;144[Suppl 1]:4480).

### Aims

Here, we report updated data with an extended follow-up of over 4 years.

### Methods

Adults with R/R CD20$^+$ LBCL (including diffuse LBCL, high-grade B-cell lymphoma, primary mediastinal LBCL, and FL grade 3B) and ≥ 2 prior lines of systemic therapy were treated with SC epcor (0.16-mg and 0.8-mg step-up doses followed by 48-mg full doses) in 28-day cycles according to the approved dosing schedule: QW, cycles 1–3; Q2W, cycles 4–9; Q4W, cycles ≥ 10, until progressive disease or unacceptable toxicity. The primary endpoint was ORR per Lugano criteria. Efficacy results presented here are based on investigators' assessment. The data cutoff date was May 2, 2025.

**Copyright Information:** (Online) ISSN: 2572-9241
©2026 The Author(s). HemaSphere is published by John Wiley & Sons Ltd on behalf of European Hematology Association. This is an open access Abstract Book distributed under the Attribution-NonCommercial-NoDerivs (CC BY-NC-ND), which allows third parties to download the articles and share them with others as long as they credit the author and the Abstract Book, but they cannot change the content in any way or use them commercially.
**Abstract Book Citations:** Authors, Title, HemaSphere, 2026;10:(S1):pages. The abstract book DOIs can be found on the "Author Information" page.
**Disclaimer:** Articles published in the journal HemaSphere exclusively reflect the opinions of the authors. The authors are responsible for all content in their abstracts including accuracy of the facts, statements, citing resources, etc.

**Results**

Overall, 157 pts with R/R LBCL received epcor monotherapy in the study. Median age was 64 years and 31% had bulky disease (> 7 cm). Pts were heavily pretreated, with a median number of 3 prior lines of therapy (range, 2–11); 61% had primary refractory disease and 39% had received prior CAR T therapy.

Median follow-up was 49.2 months (range, 0.3–57.0). In total, 59% (92/157) of pts had a response with epcor monotherapy, including 41% (65/157) of pts who had a CR. At data cutoff, 42% (27/65) of CRs were ongoing. The overall median duration of CR was 37.7 months (95% CI, 25.1 to not reached), and the longest ongoing CR at the time of data cutoff was 54.5 months. Only 7 pts with a CR progressed after the first year, 5 progressed in years 1–2, and 2 progressed in years 2–3. Median OS in all pts was 18.5 months (95% CI, 11.7–27.7). At the 4-year landmark, an estimated 32% of all pts and 57% of pts with a CR remained alive. Pts remained on tx for a mean of 11.8 months. The longest duration of therapy at data cutoff was 54.8 months.

Between years 3–4, few pts had an AE; 18 pts were on tx during this time period. Since the last data cutoff, which had a median follow-up of 37.1 months (Vose JM, et al. *Blood* 2024;144[Suppl 1]:4480), 5 pts experienced a serious AE (grade 1 back pain, grade 3 urinary tract infection, grade 3 pneumonia, grade 3 syncope, and grade 5 COVID-19) and 1 pt discontinued tx due to grade 2 bronchitis.

**Summary/Conclusion**

With more than 4 years of follow-up, epcor monotherapy continues to demonstrate deep and durable remissions. Nearly half (42%) of all complete responders remained in remission after more than 4 years of median follow-up and 57% remained alive, demonstrating the potential for long-term disease-free survival with epcor in this population with difficult-to-treat LBCL. The consistent safety profile and prolonged survival benefit highlight epcor as a favorable chemotherapy-free, readily available tx option in the 3L+ LBCL tx setting. © American Society of Hematology (2025). Reused with permission.

**Copyright Information:** (Online) ISSN: 2572-9241
©2026 The Author(s). HemaSphere is published by John Wiley & Sons Ltd on behalf of European Hematology Association. This is an open access Abstract Book distributed under the Attribution-NonCommercial-NoDerivs (CC BY-NC-ND), which allows third parties to download the articles and share them with others as long as they credit the author and the Abstract Book, but they cannot change the content in any way or use them commercially.
**Abstract Book Citations:** Authors, Title, HemaSphere, 2026;10:(S1):pages. The abstract book DOIs can be found on the "Author Information" page.
**Disclaimer:** Articles published in the journal HemaSphere exclusively reflect the opinions of the authors. The authors are responsible for all content in their abstracts including accuracy of the facts, statements, citing resources, etc.

### (PF978) DEVELOPMENT AND VALIDATION OF CAR-IPI, A MACHINE LEARNING–DERIVED PROGNOSTIC SCORE FOR RELAPSED/REFRACTORY LARGE B-CELL LYMPHOMA TREATED WITH ANTI-CD19 CAR T CELLS

**Topic:** 19. Large B cell lymphomas - Clinical

Estelle Bourbon[1], Mad-Helenie Elsensohn[2], Anna Géraud[2], Catherine Thieblemont[3, 3], Roch Houot[4], Guillaume Cartron[5], Ibrahim Yakoubagha[6], Gabriel Brisou[7], Benoit Tessoulin[8], Francois Xavier Gros[9], Pierre Bories[10], Fabien Le Bras[11], Cristina Castilla Llorente[12], Mohamad Mohty[11], Rémy Duléry[11], Marie-Thérèse Rubio[13], Stephanie Guidez[14], Amandine Durand[15], Sylvain Choquet[11], Jacques Olivier Bay[16], Laurianne Drieu la Rochelle[17], Vincent Camus[18], Michael Loschi[19], Emmanuel Bachy*[1]

*[1]Hopital Lyon Sud, oullins pierre benite, France;[2]LYSARC, Oullins-Pierre Bénite, France; [3]Hopital Saint Louis, Paris, France; [4]CHU Rennes, Rennes, France; [5]CHU Montpellier, Montpellier, France; [6]CHU Lille, Lilles, France; [7]Institut Paoli Calmette, Marseille, France; [8]CHU Nantes, Nantes, France; [9]CHU Bordeaux, Bordeaux, France; [10]Oncopole Toulouse, Toulouse, France; [11]APHP, Paris, France; [12]Institut Gustave Roussy, Villejuif, France; [13]CHU Nancy, Nancy, France; [14]CHU Poitiers, Poitiers, France; [15]CHU Dijon, Dijon, France; [16]CHU Clermont Ferrand, Clermont Ferrand, France; [17]CHU Tours, Tours, France; [18]Centre Henri Bequerel, Rouen, France; [19]CHU Nice, Nice, France;*

### Background

Anti-CD19 chimeric antigen receptor (19CAR) T-cell therapy has transformed the prognosis of large B-cell lymphoma (LBCL), yet nearly half of treated patients relapse. Response to and survival after 19CAR T cells is influenced by patient-, tumor- and treatment-related factors. However, no validated prognostic score currently identifies patients at high risk of progression or relapse.

### Aims

To develop a robust international prognostic score, named CAR-IPI, predicting progression-free survival (PFS) after 19CAR T cell therapy for LBCL, independently of CAR product or line of therapy, using machine-learning (ML) approaches and routinely available clinical and biological variables.

### Methods

Patients from the French DESCAR-T registry receiving 19CAR T cells for LBCL in 2$^{nd}$ line or more were selected (n=2259). The cohort was randomly split into training (80%, n=1807) and testing (20%, n=452) sets.

Eighteen clinically relevant variables were screened. The treatment line (2$^{nd}$ vs 3$^{rd}$ or more) and CAR product were combined into a single variable and added to the models as an additional variable or as strata. Missing data were imputed 10 times independently in training and testing sets.

Three ML approaches were applied: elastic net (EN) penalized Cox model, random survival forests (RSF), and support vector machine (SVM). Predictive performances were assessed in each imputed test set using the C-index and time-dependent AUC at 6 and 12 months. A simplified version of the score including four variables (sCAR-IPI) was derived from the 10-variable score CAR-IPI.

**Copyright Information:** (Online) ISSN: 2572-9241
©2026 The Author(s). HemaSphere is published by John Wiley & Sons Ltd on behalf of European Hematology Association. This is an open access Abstract Book distributed under the Attribution-NonCommercial-NoDerivs (CC BY-NC-ND), which allows third parties to download the articles and share them with others as long as they credit the author and the Abstract Book, but they cannot change the content in any way or use them commercially.
**Abstract Book Citations:** Authors, Title, HemaSphere, 2026;10:(S1):pages. The abstract book DOIs can be found on the “Author Information” page.
**Disclaimer:** Articles published in the journal HemaSphere exclusively reflect the opinions of the authors. The authors are responsible for all content in their abstracts including accuracy of the facts, statements, citing resources, etc.

### Results

The EN model demonstrated the best discrimination in the internal validation set and retained 10 variables: sex, histology (de novo vs transformed), Ann Arbor stage, bridging and response to bridging, time from last treatment to infusion, LDH ratio, ferritin, CRP, hemoglobin and neutrophil count (Figure).
Median (range) C-index was 67.9% (67.1–68.2) for EN, 67.3% (65.5–68.3) for RSF, and 61.4% (52.4–65.6) for SVM (Table). Median (range) AUC at 6 months were 74.3% (73.4–75.4), 72.7% (70.2–74.9) and 65.7% (54.0–70.8), respectively (Table). Both C-index and AUC at 6 and 12 months were consistently lower for IPI and CAR-HEMATOTOX compared with the EN model (Table). A simplified sCAR-IPI including LDH ratio, CRP, ferritin and hemoglobin maintained satisfactory predictive performance (Table). External validation in international cohorts is ongoing and will be presented at the meeting.

**Table. Comparative performances of the different prognostic scores.**

| | | C-index (%) | | AUC at 6 months (%) | | AUC at 12 months (%) | |
|---|---|---|---|---|---|---|---|
| | Set | Median | Range | Median | Range | Median | Range |
| CAR-IPI | Testing | 67.9 | 67.1 – 68.2 | 74.3 | 73.4 – 75.4 | 74.5 | 73.7 – 75.2 |
| sCAR-IPI | Testing | 66.1 | 65.4 – 66.7 | 71.2 | 70.2 – 73.1 | 71.4 | 70.2 – 72.0 |
| RSF | Testing | 67.3 | 65.5 – 68.3 | 72.7 | 70.2 – 74.9 | 73.3 | 70.6 – 75.1 |
| SVM | Testing | 61.4 | 52.4 – 65.6 | 65.7 | 54.0 – 70.8 | 64.6 | 53.1 – 70.4 |
| IPI-like | Testing | 61.0 | 60.4 – 61.7 | 64.3 | 63.5 – 65.6 | 64.3 | 63.2 – 65.5 |
| CAR-HEMATOTOX | Testing | 58.8 | 57.4 – 59.6 | 59.5 | 57.6 – 60.9 | 60.1 | 57.5 – 61.3 |

### Summary/Conclusion

Using ML approaches, we developed CAR-IPI, a robust 10-variable prognostic score based on parameters routinely available at lymphodepletion. Both CAR-IPI and its simplified 4-variable version accurately predicted PFS after 19CAR T-cell therapy in LBCL, independently of CAR product and treatment line. The model outperformed existing scores (IPI, CAR-HEMATOTOX) and may improve risk stratification, patient selection and individualized therapeutic strategies.

**Copyright Information:** (Online) ISSN: 2572-9241
©2026 The Author(s). HemaSphere is published by John Wiley & Sons Ltd on behalf of European Hematology Association. This is an open access Abstract Book distributed under the Attribution-NonCommercial-NoDerivs (CC BY-NC-ND), which allows third parties to download the articles and share them with others as long as they credit the author and the Abstract Book, but they cannot change the content in any way or use them commercially.
**Abstract Book Citations:** Authors, Title, HemaSphere, 2026;10:(S1):pages. The abstract book DOIs can be found on the "Author Information" page.
**Disclaimer:** Articles published in the journal HemaSphere exclusively reflect the opinions of the authors. The authors are responsible for all content in their abstracts including accuracy of the facts, statements, citing resources, etc.

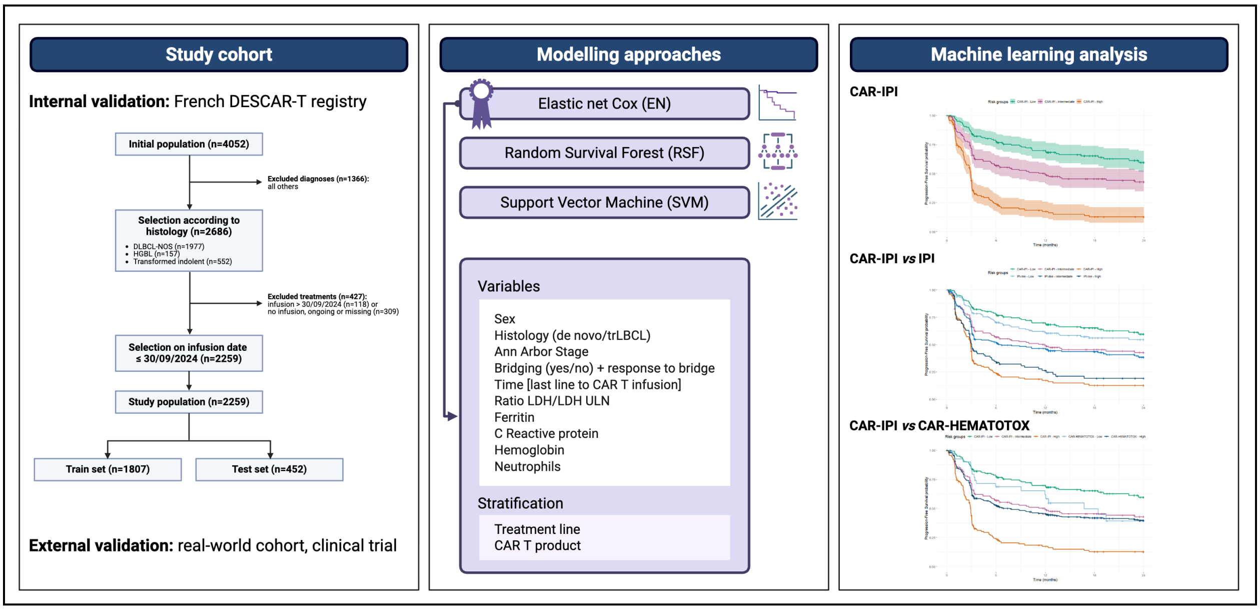

**Copyright Information:** (Online) ISSN: 2572-9241
©2026 The Author(s). HemaSphere is published by John Wiley & Sons Ltd on behalf of European Hematology Association. This is an open access Abstract Book distributed under the Attribution-NonCommercial-NoDerivs (CC BY-NC-ND), which allows third parties to download the articles and share them with others as long as they credit the author and the Abstract Book, but they cannot change the content in any way or use them commercially.
**Abstract Book Citations:** Authors, Title, HemaSphere, 2026;10:(S1):pages. The abstract book DOIs can be found on the "Author Information" page.
**Disclaimer:** Articles published in the journal HemaSphere exclusively reflect the opinions of the authors. The authors are responsible for all content in their abstracts including accuracy of the facts, statements, citing resources, etc.

### (PF979) EARLY EXPERIENCE WITH GLOFITAMAB PLUS POLATUZUMAB VEDOTIN AS BRIDGING THERAPY PRIOR TO CD19-DIRECTED CAR T-CELL THERAPY IN R/R B-CELL LYMPHOMA

**Topic:** 19. Large B cell lymphomas - Clinical

Thomas Eipe*[1], Manju Sengar[1], Alok Shetty[1], Sahitya Rao[1], Lingaraj Nayak[1], Bhausaheb Bagal[1], Anupa John[1], Devanshi Kalra[2], Smrithi Ravikumar[2], Neha Sharma[1], Girish Bs[1], Meghana Cs[1], Anjali Jaiswal[2], Yuktam Yadav[2], Atharva Karulkar[2], Rahul Purwar[3], Hasmukh Jain[1]

*[1]Tata Memorial Centre, Mumbai, India;[2]Immunoadoptive Cell Therapy Private Limited (ImmunoACT), Navi Mumbai, India; [3]Indian Institute of Technology (IIT) Bombay, Mumbai, India;*

### Background

Bridging therapy (BT) prior to CAR T-cell therapy is essential considering high disease burden and prolonged manufacturing timelines. However, BT is primarily guided by physician preference and its impact is poorly characterized. Initial results demonstrated high efficacy in combining Glofitamab (Glofit) with Polatuzumab Vedotin (Pola) in relapsed/refractory (R/R) large B-cell lymphoma (LBCL) (Hutchings M et al. JCO 2025). We evaluated the safety and efficacy of this combination as BT prior to talicabtagene autoleucel (Tali-cel) in patients (pts) with r/r BCL.

### Aims

To evaluate the impact of using glofitamab plus polatuzumab vedotin as bridging therapy in R/R BCL patients undergoing CD19-directed CAR-T cell therapy.

### Methods

This was a single-center, retrospective, real-world study of patients aged ≥15 years with R/R BCL who received Glofit plus Pola as BT prior to Tali-cel between 15 November 2023 and 30 January 2025. During cycle 1, patients received obinutuzumab (1000 mg) or rituximab (375 mg/m²) on day 1, pola (1.8 mg/kg) on day 2, and glofit on days 8 (2.5 mg) and 15 (10 mg) of a 21-day cycle. In subsequent cycles, pola (1.8 mg/kg) and glofit (10 mg) were administered on day 1 of each 21-day cycle. Following Flu-Cy conditioning, patients received a single infusion of Tali-cel at a target dose of ≥5 × $10^6$ CAR-T cells/kg. Post–CAR-T maintenance therapy was permitted at the discretion of the treating physician.

**Copyright Information:** (Online) ISSN: 2572-9241
©2026 The Author(s). HemaSphere is published by John Wiley & Sons Ltd on behalf of European Hematology Association. This is an open access Abstract Book distributed under the Attribution-NonCommercial-NoDerivs (CC BY-NC-ND), which allows third parties to download the articles and share them with others as long as they credit the author and the Abstract Book, but they cannot change the content in any way or use them commercially.
**Abstract Book Citations:** Authors, Title, HemaSphere, 2026;10:(S1):pages. The abstract book DOIs can be found on the "Author Information" page.
**Disclaimer:** Articles published in the journal HemaSphere exclusively reflect the opinions of the authors. The authors are responsible for all content in their abstracts including accuracy of the facts, statements, citing resources, etc.

### Results

As of February 2026, 84 patients underwent leukapheresis for R/R BCL, of whom 27 received Glofit-Pola as BT. In this cohort, the median age was 52 years (range 21–72), and 13 (48%) were females. Patients had received a median of 2 prior lines of therapy (range 1–5); 48% had bulky disease and 22% had bone marrow involvement. The median LDH was 303 (range 164–2027). The median number of BT cycles administered was 1 (range 1–3).
All 27 patients underwent response assessment post-BT, with an ORR of 78% (21/27) and CR in 37% (10/27). One patient had SD and 11 had PD, of whom 2 died due to disease progression prior to CAR-T infusion. The most common adverse event during BT was CRS (30%), including grade 1 in 19%, grade 2 in 7%, and grade 3 in 4%; no neurological events were observed. All CRS events were managed with tocilizumab. There were no ICU care or treatment-related deaths.
Tali-cel was infused in 25 of 27 patients, of whom 3 have not yet reached the day-28 assessment. Among the remaining 22 evaluable patients, 3 died prior to response assessment, all of whom had a poor response to BT. Among the 22 evaluable patients, the ORR at day 28 was 73% (16/22), with CR in 55% (12/22). No patient with PD post-BT achieved response following CAR-T infusion. Following Tali-cel infusion, all patients developed cytopenias. CRS occurred in 59% (13/22), including grade 1–2 in 55% and grade 3–4 in 4%. Grade 2 ICANS was observed in 1 patient. IEC-HS occurred in 27% (6/22), and hypogammaglobulinemia in 45% (10/22). At a median follow-up of 10 months, median PFS was 7.3 months (95% CI, 2.6–12) and median OS was 14.8 months (95% CI, 2.6–27). At data cutoff, 11 responders remain disease-free with ongoing B-cell aplasia.

### Summary/Conclusion

Glofit-Pola as BT in R/R BCL demonstrated high response rates, even with a single cycle. 93% were successfully bridged to CAR T-cell therapy and responses achieved post-BT were mostly maintained following Tali-cel infusion.

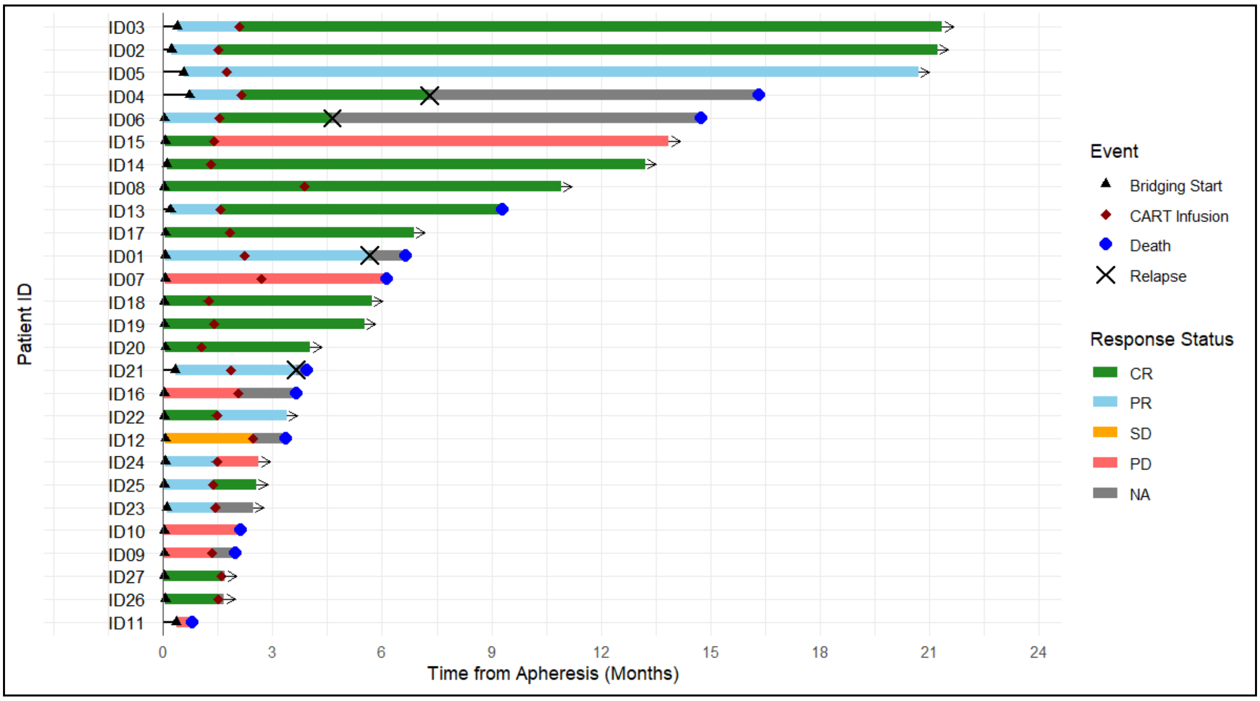

Swimmer plot showing longitudinal responses after Glofit-Pola and Tali-cel

**Copyright Information:** (Online) ISSN: 2572-9241
©2026 The Author(s). HemaSphere is published by John Wiley & Sons Ltd on behalf of European Hematology Association. This is an open access Abstract Book distributed under the Attribution-NonCommercial-NoDerivs (CC BY-NC-ND), which allows third parties to download the articles and share them with others as long as they credit the author and the Abstract Book, but they cannot change the content in any way or use them commercially.
**Abstract Book Citations:** Authors, Title, HemaSphere, 2026;10:(S1):pages. The abstract book DOIs can be found on the "Author Information" page.
**Disclaimer:** Articles published in the journal HemaSphere exclusively reflect the opinions of the authors. The authors are responsible for all content in their abstracts including accuracy of the facts, statements, citing resources, etc.

### (PF980) LOW EXPRESSION OF ZFP36L1 PREDICTS POOR OUTCOMES AND ASSOCIATES WITH MITOCHONDRIAL TRANSLATION PROGRAMS IN DLBCL

**Topic:** 19. Large B cell lymphomas - Clinical

Yingyu Dong*[1], Jiaying Liu[1], Qing Shi[1], Muchen Zhang[1], Weili Zhao[1], Li Wang[1]

[1]*Ruijin Hospital, Shanghai Jiao Tong University School of Medicine., Shanghai Institute of Hematology, Shanghai, China;*

### Background

ZFP36L1 is an AU-rich element (ARE)–binding RNA-binding protein of the TTP family that promotes ARE-dependent mRNA decay and post-transcriptional gene regulation. Although ZFP36L1 has been implicated in multiple malignancies, its clinical and biological relevance to the progression of diffuse large B-cell lymphoma (DLBCL) remains to be fully elucidated.

### Aims

To define the prognostic significance of ZFP36L1 in DLBCL and to characterize associated transcriptional programs, immune contexture, and mitochondrial translation–related features with functional validation.

### Methods

RNA-seq and clinical data from 600 de novo DLBCL patients treated with R-CHOP were analyzed. An optimal cutoff for ZFP36L1 expression was determined using time-dependent ROC analysis, which stratified patients into ZFP36L1-high (n = 280) and ZFP36L1-low (n = 320) groups. Overall survival (OS), progression-free survival (PFS), and baseline clinicopathologic variables were compared between groups. Differential expression and pathway enrichment analyses were conducted using RNA-seq data.

### Results

Low ZFP36L1 expression was associated with inferior OS and PFS (both $p<0.05$), as shown in Kaplan-Meier survival curves. ZFP36L1-low tumors exhibited enrichment of mitochondrial translation and oxidative phosphorylation pathways (Figure A), along with increased tumor-associated macrophage infiltration, indicating a more immunosuppressive tumor microenvironment. In vitro, ZFP36L1 knockdown increased cell proliferation (CCK-8) and mitochondrial activity (ATP levels, HPG incorporation, Figure B), whereas ZFP36L1 overexpression suppressed these effects, supporting a suppressive role for ZFP36L1 in DLBCL aggressiveness.

### Summary/Conclusion

ZFP36L1 downregulation identifies a high-risk DLBCL subset and is linked to enhanced mitochondrial translation programs and an immunosuppressive microenvironment. ZFP36L1 may serve as a prognostic biomarker and inform risk stratification, providing a rationale for therapeutic targeting in DLBCL patients.

**Copyright Information:** (Online) ISSN: 2572-9241
©2026 The Author(s). HemaSphere is published by John Wiley & Sons Ltd on behalf of European Hematology Association. This is an open access Abstract Book distributed under the Attribution-NonCommercial-NoDerivs (CC BY-NC-ND), which allows third parties to download the articles and share them with others as long as they credit the author and the Abstract Book, but they cannot change the content in any way or use them commercially.
**Abstract Book Citations:** Authors, Title, HemaSphere, 2026;10:(S1):pages. The abstract book DOIs can be found on the “Author Information” page.
**Disclaimer:** Articles published in the journal HemaSphere exclusively reflect the opinions of the authors. The authors are responsible for all content in their abstracts including accuracy of the facts, statements, citing resources, etc.

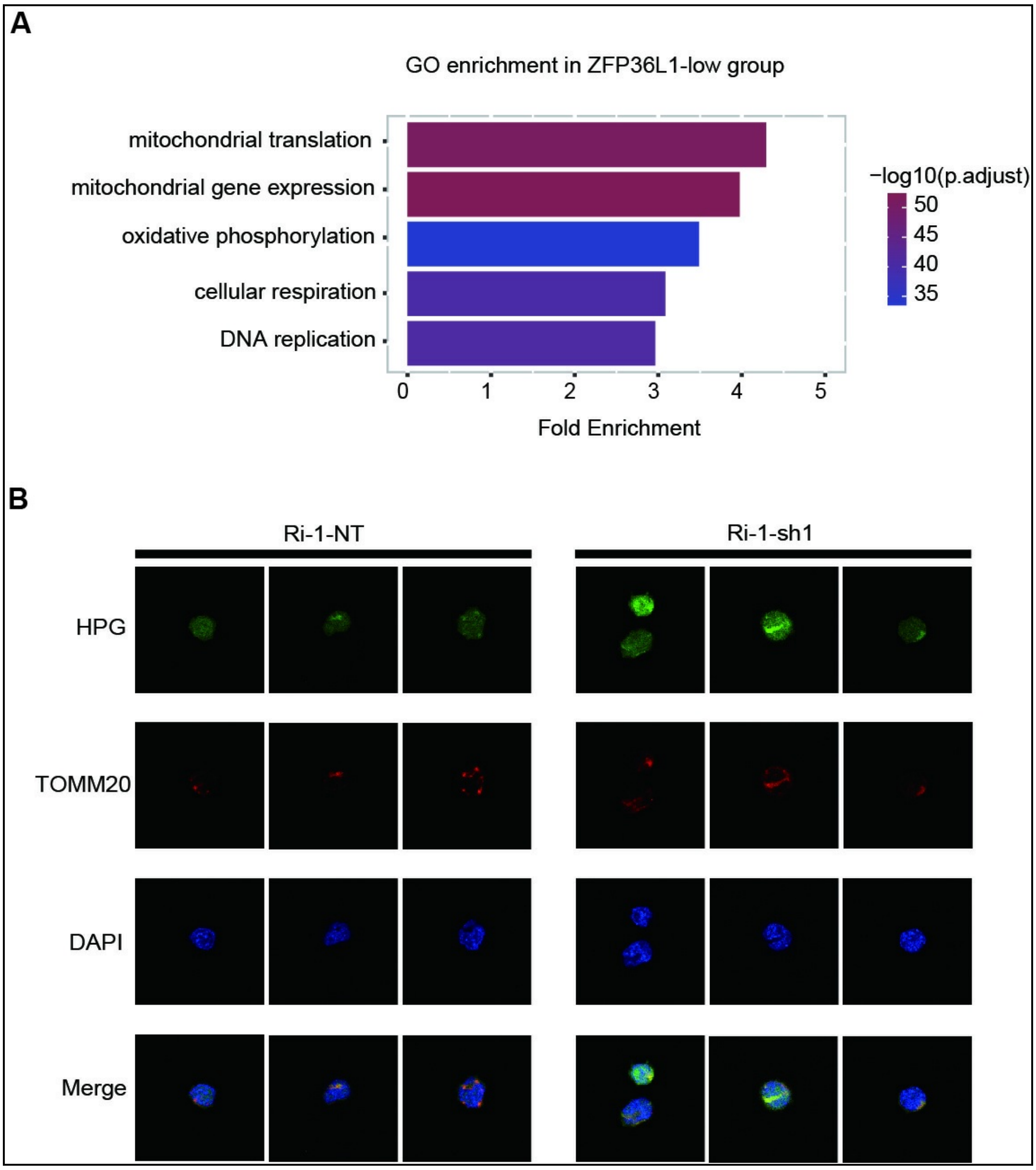

Figure A. GO enrichment analysis of the ZFP36L1-low group. Figure B. Representative confocal images showing HPG incorporation and TOMM20 staining with DAPI and merged channels in Ri-1 NT vs Ri-1 sh1 cells.

**Copyright Information:** (Online) ISSN: 2572-9241
©2026 The Author(s). HemaSphere is published by John Wiley & Sons Ltd on behalf of European Hematology Association. This is an open access Abstract Book distributed under the Attribution-NonCommercial-NoDerivs (CC BY-NC-ND), which allows third parties to download the articles and share them with others as long as they credit the author and the Abstract Book, but they cannot change the content in any way or use them commercially.
**Abstract Book Citations:** Authors, Title, HemaSphere, 2026;10:(S1):pages. The abstract book DOIs can be found on the “Author Information” page.
**Disclaimer:** Articles published in the journal HemaSphere exclusively reflect the opinions of the authors. The authors are responsible for all content in their abstracts including accuracy of the facts, statements, citing resources, etc.

### (PF981) AXICABTAGENE CILOLEUCEL AS SECOND-LINE TREATMENT IN PATIENTS WITH LATE-RELAPSED LARGE B-CELL LYMPHOMA: INTERIM ANALYSIS OF THE LATE-R CLINICAL TRIAL FROM THE SPANISH LYMPHOMA GROUP GELTAMO

**Topic:** 19. Large B cell lymphomas - Clinical

Mariana Bastos-Oreiro*[1], Armando Lopez Guillermo[2], Javier Delgado[3], Izaskun Zeberio Etxetxipia[4], Eva Gonzalez Barca[5], Hugo Daniel Luzardo Henríquez[6], Antonio Gutierrez Garcia[7], Maria Jose Terol Castera[8], Sonia González de Villambrosia Pellón[9], Gloria Iacoboni[10], Ana Isabel Jimenez Ubieto[11], Oriana Jimena Lopez Godino[12], Victor Noriega Concepción[13], Miguel Ángel Canales Albendea[14], Auxiliadora Moreno[15], Madison Davis[16], Davide Bedognetti[16], Margarida Palla Garcia[16], Regina Quiroga[16], Alejandro Martín García-Sancho[17]

*[1]Hospital General Gregorio Marañón. Instituto de investigación sanitaria Gregorio Marañón, Hematology, Madrid, Spain; [2]Hospital Clínic De Barcelona, Hematology, Barcelona, Spain;[3]Hospital Universitario Virgen del Rocío, Sevilla, Spain;[4]Hospital Universitario de Donostia, Hematology, San Sebastian, Spain; [5]Institut Català d'Oncologia L'Hospitalet, L'Hospitalet de Llobregat, Spain; [6]Hospital Universitario de Gran Canaria Doctor Negrín, Las Palmas de Gran Canaria, Spain;[7]Hospital Universitario Son Espases, Palma, Spain; [8]Hospital Clínico universitario de Valencia, Valencia, Spain; [9]Hospital Universitario Marqués De Valdecilla, Santander, Spain; [10]Hospital Vall d'Hebron, Barcelona, Spain; [11]Hospital Universitario 12 De Octubre, Madrid, Spain; [12]Hospital Morales Meseguer, Murcia, Spain; [13]Hospital Universitario de A Coruña, A Coruña, Spain;[14]Clinica Universidad de Navarra, Pamplona, Spain; [15]Grupo Español de Linfomas GELTAMO, Madrid, Spain; [16]Kite, a Gilead Company, Santa Mónica, United States of America; [17]Antiguo Hospital Clínico de Salamanca, Salamanca, Spain;*

### Background

CAR T-cell therapy is the standard of care for patients with diffuse large B-cell lymphoma (DLBCL) experiencing early relapse or primary refractory disease. In contrast, patients who relapse 12 months after first-line chemoimmunotherapy (CIT) represent a clinically heterogeneous population for whom prospective clinical trials specifically evaluating the role of CAR T-cell therapy are lacking.

### Aims

The LATE-R trial was designed to evaluate the efficacy and safety of axicabtagene ciloleucel (axi-cel) in patients with late-relapsing aggressive B-cell lymphomas.

### Methods

This is an open-label, multicenter, single-arm, phase II study sponsored by GELTAMO in collaboration with Kite, a Gilead Company, Inc. Eligible patients were ≥18 years with either DLBCL, transformed follicular lymphoma (tFL), or high-grade B-cell lymphoma (HGBCL), relapsing 12–60 months after completion of first-line CIT. Bridging therapy was allowed at investigator discretion. The primary endpoint was complete response (CR) rate at 3 months after axi-cel infusion, assessed locally by PET-CT according to standard criteria. Secondary endpoints included overall response rate (ORR), progression-free survival (PFS), overall survival (OS) and safety. Herein we present an interim analysis pre-specified in the protocol.

**Copyright Information:** (Online) ISSN: 2572-9241
©2026 The Author(s). HemaSphere is published by John Wiley & Sons Ltd on behalf of European Hematology Association. This is an open access Abstract Book distributed under the Attribution-NonCommercial-NoDerivs (CC BY-NC-ND), which allows third parties to download the articles and share them with others as long as they credit the author and the Abstract Book, but they cannot change the content in any way or use them commercially.
**Abstract Book Citations:** Authors, Title, HemaSphere, 2026;10:(S1):pages. The abstract book DOIs can be found on the “Author Information” page.
**Disclaimer:** Articles published in the journal HemaSphere exclusively reflect the opinions of the authors. The authors are responsible for all content in their abstracts including accuracy of the facts, statements, citing resources, etc.

### Results

As of December 31st 2025 (cut-off), 35 patients underwent leukapheresis and 31 had received axi-cel and were included in the present analysis. Median age was 63 years (range 28–79) and 19 (54%) patients were male. At study entry, 49% had relapsed within 12–24 months and 51% within 24–60 months from first-line CIT; 26% of patients had HBCL double hit, 83% showed Ann Arbor stage III–IV and 74% had an IPI ≥2.
The CR rate and ORR at 3 months among the 21 patients who had reached this time point at the time of data cutoff were 90% and 95%, respectively. With a median follow-up of 5.9 months (Q1-Q3: 2,4-8.7), the median progression-free survival (PFS) and overall survival (OS) had not been reached. The estimated 6-month PFS and OS were 87.0% (95% CI 68.0-100) and 95.7% (95% CI 86.7-100), respectively (Figure).
Regarding safety, a total of 81 adverse events classified as serious adverse events (SAEs) or adverse events of special interest (AESIs) and occurring on or before the cut-off time, were reported. Cytokine release syndrome (CRS) occurred in 74% of patients, predominantly grade 1 (37%) and 2 (28.6%); grade ≥3 CRS was infrequent (8.6%), with no grade 4–5 events. Immune effector cell–associated neurotoxicity syndrome (ICANS) was reported in 45.7% of patients. Most events were low to moderate in severity, with grade 3 and 4 events occurring in 14.6% and 8.3% of patients, respectively; no grade 5 neurotoxicity was observed. Hematologic toxicities were common, with thrombocytopenia (20%) and neutropenia (17.1%) being the most frequently reported grade ≥3 cytopenias. Severe infections were reported in 5 cases (14%), including one patient who died due to a septic shock at 34 days from axi-cel infusion.

### Summary/Conclusion

In this interim analysis of the LATE-R trial, axi-cel demonstrated remarkably high CR rates in patients with late-relapsing aggressive B-cell lymphomas treated in the second-line setting, with a manageable toxicity profile consistent with the known risks of axi-cel. These findings suggest that patients with late CIT relapses could benefit from axi-cel and support its use in this clinical setting.

**Copyright Information:** (Online) ISSN: 2572-9241
©2026 The Author(s). HemaSphere is published by John Wiley & Sons Ltd on behalf of European Hematology Association. This is an open access Abstract Book distributed under the Attribution-NonCommercial-NoDerivs (CC BY-NC-ND), which allows third parties to download the articles and share them with others as long as they credit the author and the Abstract Book, but they cannot change the content in any way or use them commercially.
**Abstract Book Citations:** Authors, Title, HemaSphere, 2026;10:(S1):pages. The abstract book DOIs can be found on the “Author Information” page.
**Disclaimer:** Articles published in the journal HemaSphere exclusively reflect the opinions of the authors. The authors are responsible for all content in their abstracts including accuracy of the facts, statements, citing resources, etc.

### (PF982) PRELIMINARY RESULTS FROM SHORTEN-CTDNA: MEASURABLE RESIDUAL DISEASE-ADAPTED DE-ESCALATION OF FRONTLINE THERAPY FOR DIFFUSE LARGE B-CELL LYMPHOMA

**Topic:** 19. Large B cell lymphomas - Clinical

Hua-Jay Cherng*[1, 2], Beatriz Raposo Corradini[2], Stephanie Meek[3, 4], Cheng-Shiun Leu[1], Sandra Close[3, 4], Jacob Chabon[3, 4], Seda Tolu[1, 2], Jennifer Amengual[1, 2], David Kurtz[3, 4], Barbara Pro[1, 2]

*[1]Columbia University Irving Medical Center, Division of Hematology & Oncology, New York, NY, United States of America; [2]Herbert Irving Comprehensive Cancer Center, New York, NY, United States of America;[3]Foresight Diagnostics, Inc., Boulder, CO, United States of America; [4]Natera, Inc., Austin, TX, United States of America;*

### Background

Detection of circulating tumor DNA (ctDNA)-based measurable/molecular residual disease (MRD) with phased variant (PV) enrichment and detection sequencing (PhasED-seq) is a minimally invasive, clinically validated method of response assessment in diffuse large B-cell lymphoma (DLBCL). Patients (pts) with undetected ctDNA-MRD by PhasED-seq after 2 cycles of frontline therapy experienced a 2-year progression-free survival rate of 96% (Roschewski *JCO* 2025). Real-time ctDNA-MRD testing with the PhasED-seq-based Foresight CLARITY assay to guide de-escalation of frontline DLBCL therapy has not previously been performed.

### Aims

The co-primary aims were to 1) evaluate the feasibility of real-time ctDNA-MRD testing during frontline DLBCL treatment and 2) determine the outcomes of pts who became undetectable for ctDNA-MRD mid-treatment and discontinued chemotherapy early.

### Methods

We performed a single-center investigator-initiated feasibility study (NCT06693830) for pts with newly diagnosed stage II-IV DLBCL. Pts received 4 cycles of standard rituximab, cyclophosphamide, doxorubicin, prednisone, and vincristine (R-CHOP) or polatuzumab vedotin (R-pola-CHP). Whole blood samples were drawn on cycle 4 day 1 (C4D1) for real-time ctDNA-MRD testing. These samples, alongside biopsy tissue to identify patient-specific PVs, were shipped to and analyzed at Foresight Diagnostics, Inc. (Boulder, CO). PET scans were performed on C4D15 (iPET4). Pts with undetected ctDNA-MRD on C4D1 and complete metabolic response (CMR) on iPET4 received rituximab alone for C5-6. All other pts continued standard therapy. Equivocal iPET4 response could be confirmed with biopsy. An end-of-treatment (EOT) PET was performed 10-14 weeks after C6D1. ctDNA-MRD was also evaluated in a batched manner from other timepoints.

**Copyright Information:** (Online) ISSN: 2572-9241
©2026 The Author(s). HemaSphere is published by John Wiley & Sons Ltd on behalf of European Hematology Association. This is an open access Abstract Book distributed under the Attribution-NonCommercial-NoDerivs (CC BY-NC-ND), which allows third parties to download the articles and share them with others as long as they credit the author and the Abstract Book, but they cannot change the content in any way or use them commercially.
**Abstract Book Citations:** Authors, Title, HemaSphere, 2026;10:(S1):pages. The abstract book DOIs can be found on the “Author Information” page.
**Disclaimer:** Articles published in the journal HemaSphere exclusively reflect the opinions of the authors. The authors are responsible for all content in their abstracts including accuracy of the facts, statements, citing resources, etc.

### Results

We report on the first 14 evaluable pts. Median age was 70 years (40-92), 11 (79%) were stage IV, 8 (57%) had elevated lactate dehydrogenase (LDH), 8 (57%) had ≥2 extranodal sites, and 5 (36%) had bulky tumor ≥7.5 cm. The international prognostic index (IPI) was ≥3 for 8 (57%). Four pts received R-CHOP (29%) and 10 R-pola-CHP (71%).
Of the 14 pts, 13/14 (93%) had patient-specific PVs identified, and those 13 had successful real-time ctDNA-MRD testing. Median time from sample collection to report was 8 days (7-11). A median 10 mL plasma was used from each sample, enabling a median empirical sensitivity of 1.38 parts per million. The rate of undetected ctDNA-MRD on C4D1 was 9/13 (69%); of these 9 pts, 6 (67%) were stage IV, 5 (56%) had elevated LDH, 3 (33%) had bulky tumor, and 5 (56%) had IPI ≥3. iPET4 was reported definitively as CMR for 5/9 but equivocal for 4/9 (SUVmax 4.4-7.8, Deauville score 4 [2] or X [2]). These 4 pts had negative biopsies (2 upper endoscopy, 1 colonoscopy, 1 upper endoscopy and thyroid biopsy) which confirmed CMR before de-escalation. Eight pts with undetected ctDNA-MRD de-escalated to rituximab for C5-6 (the other was awaiting C5); to date, the first 6 de-escalated pts assessed with EOT PET all experienced CMR and remain ctDNA-MRD undetected on subsequent timepoints (Figure). Median follow-up after de-escalation was 6.4 months (0.7-7.7) and all remain in remission.

### Summary/Conclusion

Preliminary results from SHORTEN-ctDNA suggest that real-time ctDNA-MRD assessment with Foresight CLARITY to de-escalate frontline therapy in DLBCL is feasible. Short-term clinical outcomes and molecular remissions for pts with undetected mid-treatment ctDNA-MRD who stop chemotherapy early are excellent.

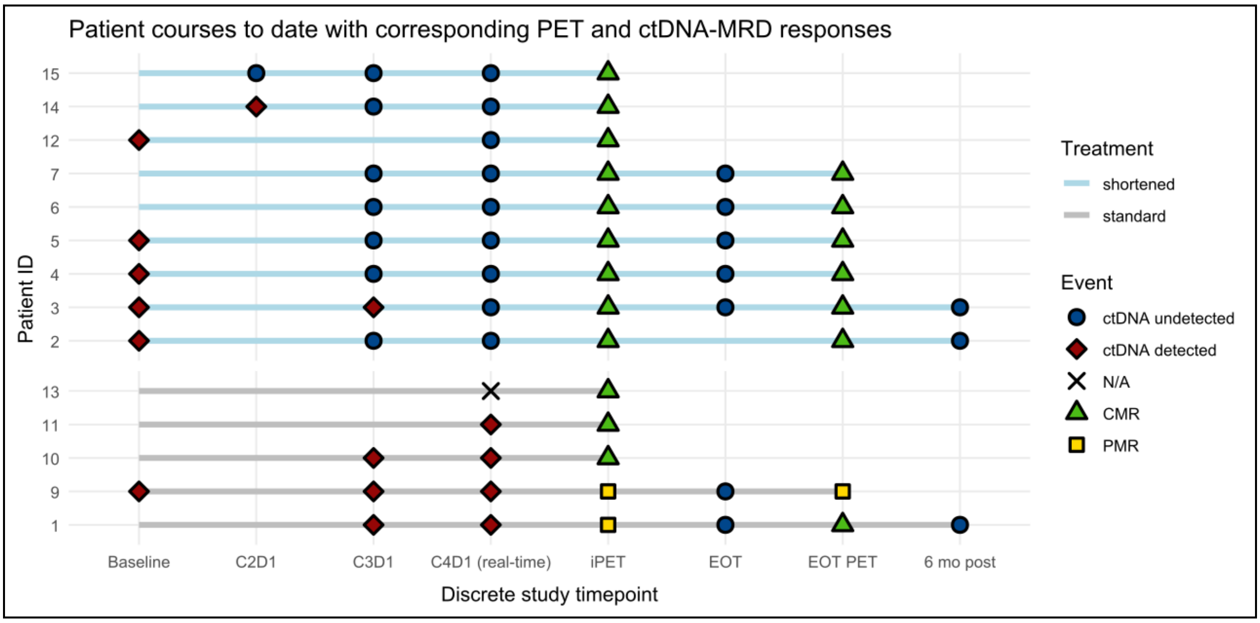

Figure. Swimmer's plot depicting individual patient treatment courses across discrete timepoints with corresponding PET and ctDNA-MRD (when available) responses annotated. Blue and grey bars denote patients who de-escalated treatment or continued standard duration treatment, respectively. Bars are capped at last study assessment available to date.

Copyright Information: (Online) ISSN: 2572-9241
©2026 The Author(s). HemaSphere is published by John Wiley & Sons Ltd on behalf of European Hematology Association. This is an open access Abstract Book distributed under the Attribution-NonCommercial-NoDerivs (CC BY-NC-ND), which allows third parties to download the articles and share them with others as long as they credit the author and the Abstract Book, but they cannot change the content in any way or use them commercially.
Abstract Book Citations: Authors, Title, HemaSphere, 2026;10:(S1):pages. The abstract book DOIs can be found on the "Author Information" page.
Disclaimer: Articles published in the journal HemaSphere exclusively reflect the opinions of the authors. The authors are responsible for all content in their abstracts including accuracy of the facts, statements, citing resources, etc.

### (PF983) POST–CAR-T RELAPSE IN CNS-INVOLVED LYMPHOMA: TREATMENT PATTERNS AND CLINICAL OUTCOMES WITH A FOCUS ON SECOND CAR-T THERAPY

**Topic:** 19. Large B cell lymphomas - Clinical

Rui Liu[1], Zhonghua Fu[1], Fan Yang*[1], Miaomiao Cao[1], Yuelu Guo[1], Lixia MA[1], Biping Deng[2], Alex Chang[3], Xiaoyan Ke[1], Kai Hu[1]

*[1]Beijing GoBroad Hospital, Department of Lymphoma and Myeloma Research Center, Beijing, China; [2]Beijing GoBroad Boren Hospital, Cytology Laboratory, Beijing, China; [3]Shanghai YaKe Biotechnology Ltd., Engineering Research Center of Gene Technology, Ministry of Education, Institute of Genetics, School of Life Sciences, Shanghai, China;*

### Background

Patients with central nervous system (CNS)-involved lymphoma are often excluded from CAR-T clinical trials. Treatment strategies and outcomes after CAR-T failure in this population remain unclear.

### Aims

This study aimed to characterize relapse patterns and post-relapse treatment outcomes in CNS-involved lymphoma patients, with a focus on the efficacy and safety of second CAR-T therapy.

### Methods

We retrospectively analyzed CNS-involved lymphoma patients who relapsed after first CAR-T therapy. Patients were grouped according to post-relapse treatment: second CAR-T, non-CAR-T therapy, or no further treatment. Overall survival (OS) and progression-free survival (PFS) were evaluated, and efficacy and safety of second CAR-T therapy were assessed.

### Results

Among 166 CNS-involved lymphoma patients receiving CAR-T therapy, 77 (46%) experienced relapse. Median age was 54 years (range, 19–79), with 41 (53%) males. Histology included primary CNS lymphoma (PCNSL, 32 [41%]), diffuse large B-cell lymphoma (DLBCL, 36 [47%]), and other aggressive B-cell lymphomas (9 [12%]). CNS involvement was parenchymal in 46 (60%), cerebrospinal fluid in 12 (15%), and both in 19 (25%) patients. Patients received a median of 3 prior lines of therapy (range, 2–6). 3-month response to first CAR-T was complete remission (CR) in 32 (41%), partial remission (PR) in 7 (10%), stable disease (SD) in 3 (4%), and progressive disease (PD) in 35 (45%). Relapse occurred in the CNS only in 51 (66%) patients and systemically ± CNS in 26 (34%), with a median time to relapse of 6.05 months (range, 1.43–49.02).

Post-relapse treatment was heterogeneous: 28 patients received second CAR-T therapy, 36 received other non-CAR-T therapies (including chemotherapy, targeted therapy, and radiotherapy), 2 received CD3×CD20 bispecific antibodies, and 11 patients progressed rapidly without further treatment. Among second CAR-T recipients, the overall response rate (ORR) was 57.1% (16/28), with a CR rate of 46.4% (13/28). Median PFS was 6.28 months, and median OS was 15.81 months, compared with 8.55 months OS in the non-CAR-T group ($p \approx 0.068$). All second CAR-T patients received a different target than their first CAR-T, with a median interval of 9.5 months (range, 0.85–54.87). Twenty-three patients received bridging therapy prior to second CAR-T (median 2 cycles, range 0–6). Safety was manageable: CRS occurred in 23 (82.1%) patients, all grade 1–2; ICANS occurred in 9 (32.1%) patients, with only one grade ≥3 event.

**Copyright Information:** (Online) ISSN: 2572-9241
©2026 The Author(s). HemaSphere is published by John Wiley & Sons Ltd on behalf of European Hematology Association. This is an open access Abstract Book distributed under the Attribution-NonCommercial-NoDerivs (CC BY-NC-ND), which allows third parties to download the articles and share them with others as long as they credit the author and the Abstract Book, but they cannot change the content in any way or use them commercially.
**Abstract Book Citations:** Authors, Title, HemaSphere, 2026;10:(S1):pages. The abstract book DOIs can be found on the "Author Information" page.
**Disclaimer:** Articles published in the journal HemaSphere exclusively reflect the opinions of the authors. The authors are responsible for all content in their abstracts including accuracy of the facts, statements, citing resources, etc.

**Summary/Conclusion**

CNS-involved lymphoma patients have poor prognosis after CAR-T failure, and post-relapse treatment patterns are highly heterogeneous. Second CAR-T therapy can achieve high initial response rates and shows a trend toward improved survival in selected patients, though mortality remains substantial. Further studies with larger cohorts and prospective design are warranted to clarify its true benefit.

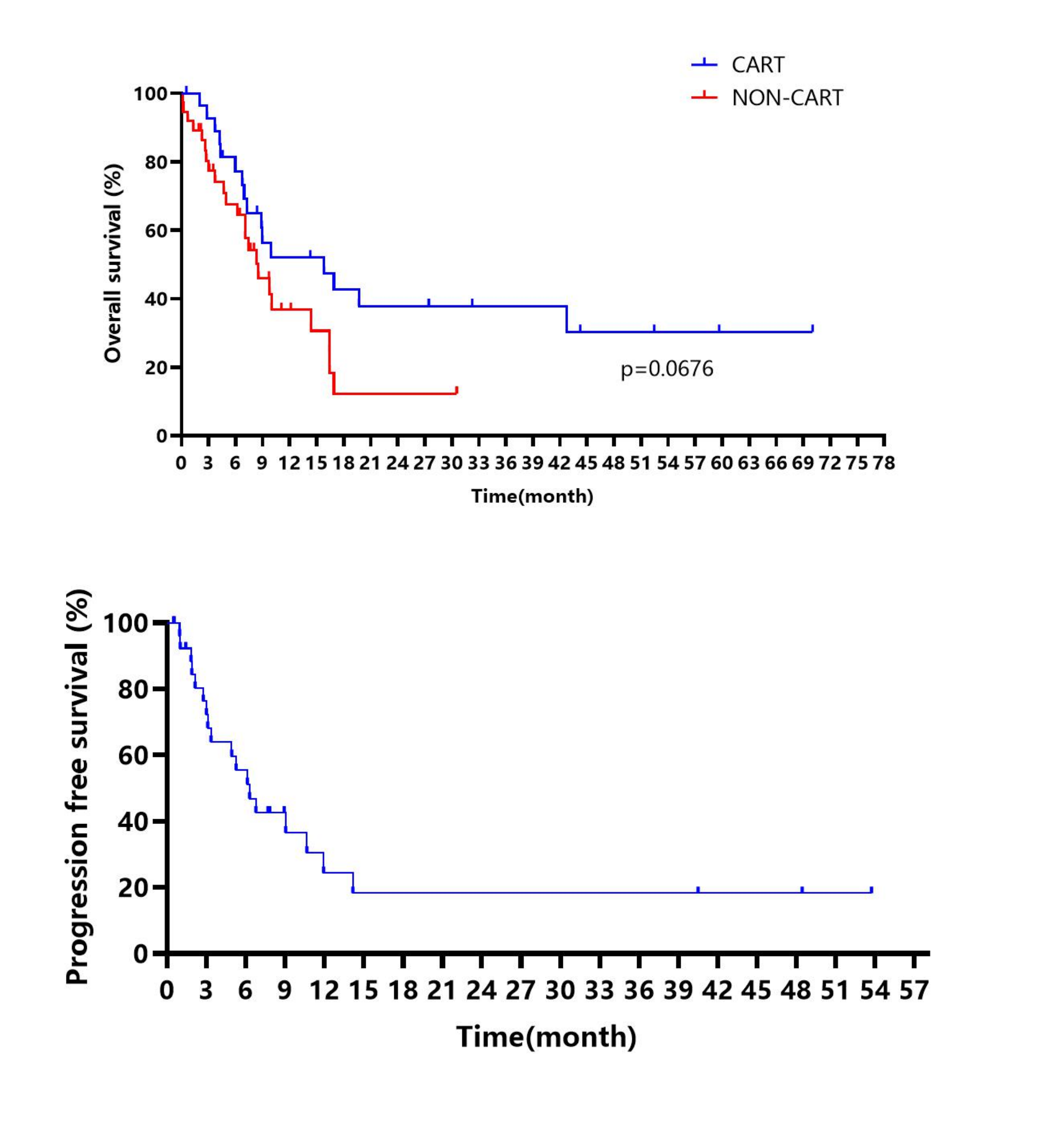

**Copyright Information:** (Online) ISSN: 2572-9241
©2026 The Author(s). HemaSphere is published by John Wiley & Sons Ltd on behalf of European Hematology Association. This is an open access Abstract Book distributed under the Attribution-NonCommercial-NoDerivs (CC BY-NC-ND), which allows third parties to download the articles and share them with others as long as they credit the author and the Abstract Book, but they cannot change the content in any way or use them commercially.
**Abstract Book Citations:** Authors, Title, HemaSphere, 2026;10:(S1):pages. The abstract book DOIs can be found on the "Author Information" page.
**Disclaimer:** Articles published in the journal HemaSphere exclusively reflect the opinions of the authors. The authors are responsible for all content in their abstracts including accuracy of the facts, statements, citing resources, etc.

### (PF984) FEASIBILITY OF PROSPECTIVE REAL-TIME CTDNA MONITORING IN ROUTINE CLINICAL CARE OF DIFFUSE LARGE B-CELL LYMPHOMA PATIENTS

**Topic:** 19. Large B cell lymphomas - Clinical

Halaleh Shakeri[1], Kurt Yakimovich[2], Gregory Cormack[1], Jean-Francois Larouche[3], Tanya Skamene[4], David MacDonald[5], Stephane Doucet[6], Ryan Morin[2], Nathalie Johnson*[1]

*[1]Jewish General Hospital, Montreal, Canada;[2]BC Cancer, Vancouver, Canada;[3]CHU de Quebec, Quebec city, Canada;[4]St-Mary's Hospital, Montreal, Canada; [5]The Ottawa Hospital, Ottawa, Canada;[6]Centre Hospitalier Universitaire de Montreal, Montreal, Canada;*

### Background

Over 25% of patients with diffuse large B cell lymphoma (DLBCL) do not respond to frontline chemotherapy or relapse within 12 months of completing therapy. Half of these patients are eligible for second-line chimeric antigen receptor T cell (CART) therapy, but high tumor burden and low performance status decrease efficacy and increase toxicity. Circulating tumor DNA (ctDNA) is a promising biomarker for monitoring treatment response in DLBCL, but its feasibility in routine clinical practice has not been established. Understanding logistical factors affecting turnaround time and data quality is critical for clinical implementation. We developed a 195-gene panel to detect treatment failure as early as cycle 2, day 1 (C2D1) of first-line therapy in a retrospective cohort of 245 patients.

### Aims

We evaluated the feasibility and clinical utility of our panel to detect early relapsed DLBCL in a prospective cohort of 75 patients. We hypothesized the assay could be performed in real time within 3 weeks of collection. The primary endpoint was the turnaround time from blood collection to ctDNA reporting at C2D1. The secondary objective was to determine the optimal timing for ctDNA testing at C2D1, C3D1, and end of treatment (EOT).

### Methods

Patients with de novo or transformed DLBCL undergoing curative-intent chemotherapy were enrolled from five Canadian hospitals. Plasma samples were collected in Streck tubes at diagnosis (DX), C2D1, C3D1, EOT, and follow-up. ctDNA was extracted from 5mL plasma and hybrid-capture libraries were prepared using a dual-UMI approach, then sequenced on a NextSeq 2000 (1500× coverage). Finally, somatic variants were called using germline-filtered and error-suppressed data. Results did not change clinical management.

**Copyright Information:** (Online) ISSN: 2572-9241
©2026 The Author(s). HemaSphere is published by John Wiley & Sons Ltd on behalf of European Hematology Association. This is an open access Abstract Book distributed under the Attribution-NonCommercial-NoDerivs (CC BY-NC-ND), which allows third parties to download the articles and share them with others as long as they credit the author and the Abstract Book, but they cannot change the content in any way or use them commercially.
**Abstract Book Citations:** Authors, Title, HemaSphere, 2026;10:(S1):pages. The abstract book DOIs can be found on the “Author Information” page.
**Disclaimer:** Articles published in the journal HemaSphere exclusively reflect the opinions of the authors. The authors are responsible for all content in their abstracts including accuracy of the facts, statements, citing resources, etc.

### Results

Between March 2024 and December 2025, 75 patients enrolled, provided 425 samples; 68 completed treatment (study ongoing). The mean age was 63; 44 patients had IPI>3, 10 had prior indolent lymphoma, and 13 experienced disease progression (PD). The mean turnaround time was 18 days, with distant sites (>200 km) requiring 21 days versus 16 days for local sites ($p<0.01$). Delays were due to limited staffing during holidays (n=6) and transport issues (n=7). Sequencing quality and ctDNA detection were consistent across sites. At DX, ctDNA was detectable in 85% of patients, correlated with high LDH ($p<0.001$), advanced stage ($p<0.05$), and IPI>3 ($p<0.001$), but not progression-free survival (PFS) or overall survival (OS). In contrast, detectable ctDNA at C2D1 was predictive of shorter PFS ($p<0.001$) and worse OS ($p<0.01$). PD patients had persistent ctDNA, whereas those achieving complete response (CR) showed rapid ctDNA clearance at C2D1/C3D1 (median ctDNA: CR vs PD, $p<0.01$). The most common baseline mutations were in IGLL5 (42%) and KMT2D (27%). Of the 13 patients with PD, 12 had detectable ctDNA after C2D1; the ctDNA-negative patient died of an isolated central nervous system relapse. 12 received second-line therapy, but only 2 received CART.Barriers to CART included poor performance status and high disease burden (n=6) and age/frailty (n=4). Mortality was 70% among PD patients.

### Summary/Conclusion

Real-time ctDNA profiling is feasible, but logistical challenges for clinical implementation remain. There is significant attrition among refractory DLBCL patients eligible for CART in real-world settings. Our panel can identify high-risk patients as early as C2D1, supporting early intervention trials.

**Copyright Information:** (Online) ISSN: 2572-9241
©2026 The Author(s). HemaSphere is published by John Wiley & Sons Ltd on behalf of European Hematology Association. This is an open access Abstract Book distributed under the Attribution-NonCommercial-NoDerivs (CC BY-NC-ND), which allows third parties to download the articles and share them with others as long as they credit the author and the Abstract Book, but they cannot change the content in any way or use them commercially.
**Abstract Book Citations:** Authors, Title, HemaSphere, 2026;10:(S1):pages. The abstract book DOIs can be found on the “Author Information” page.
**Disclaimer:** Articles published in the journal HemaSphere exclusively reflect the opinions of the authors. The authors are responsible for all content in their abstracts including accuracy of the facts, statements, citing resources, etc.

### (PF985) REAL-WORLD COMPARATIVE STUDY OF THE EFFECTIVENESS, SAFETY, AND HEALTH CARE RESOURCE UTILIZATION (HCRU) OF LISOCABTAGENE MARALEUCEL AND BISPECIFICS IN THIRD-LINE OR LATER LARGE B-CELL LYMPHOMA (LBCL)

**Topic:** 19. Large B cell lymphomas - Clinical

Narendranath Epperla*[1], Paolo F Caimi[2], Jennifer Huang[3, 4], Farah Toron[5], David Bernasconi[6], Robert Braun[6], Fei Fei Liu[7], Sagar Patel[1], Nathan Denlinger[8]

*[1]Huntsman Cancer Institute, University of Utah, Salt Lake City, United States of America;[2]CCF Taussig Cancer Center, Cleveland, United States of America; [3]University of Washington, Seattle, United States of America;[4]Fred Hutchinson Cancer Center, Seattle, United States of America; [5]Bristol Myers Squibb, Uxbridge, United Kingdom; [6]Bristol Myers Squibb, Boudry, Switzerland; [7]Bristol Myers Squibb, Princeton, United States of America;[8]The Ohio State University Comprehensive Cancer Center, Columbus, United States of America;*

### Background

Lisocabtagene maraleucel (liso-cel) and CD3×CD20 T-cell engaging bispecific antibodies (BsAb) are approved LBCL therapies; liso-cel in second line or later and BsAb in third-line (3L) or later (3L+) in the United States (US). Understanding their comparative effectiveness, safety, and HCRU in the real-world (RW) setting can inform optimal treatment selection.

### Aims

To compare RW effectiveness, safety, and HCRU among patients with R/R LBCL treated with liso-cel or BsAb in the 3L+ setting.

### Methods

Patients with LBCL who were newly treated with liso-cel or BsAb (epcoritamab, glofitamab) in the 3L+ setting (April 2022–May 2025) were identified from the US Flatiron Health Research Database, which collects longitudinal electronic health records from the routine clinical setting. Baseline differences were adjusted using inverse probability of treatment weighting (IPTW) for efficacy, safety, and HCRU. Sensitivity analysis was conducted in 3L-only patients.

### Results

A total of 273 patients were included, with 161 receiving liso-cel and 112 receiving BsAb (median [IQR] age at index, 72 y [62–77] vs 75 y [65–81]; DLBCL, not otherwise specified, 87% vs 76%; high-grade B-cell lymphoma, 2% vs 8%; elevated LDH, 28% vs 54%; bulky disease, 66% vs 66%; 3L, 70% vs 54%; and median follow-up time of 12.4 mo vs 9.1 mo).

In both unadjusted and adjusted analyses, liso-cel was associated with a statistically significant improvement in OS (adjusted HR, 0.62; *P*=0.023) and PFS (adjusted HR, 0.53; *P*<0.001) vs BsAb and had significantly higher adjusted ORR (81% vs 64%; *P*=0.004) and CRR (61% vs 28%; *P*<0.001) (**Table 1**).
In a sensitivity analysis of 3L-only patients (liso-cel, n=113; BsAb, n=60), liso-cel had a statistically significant benefit for OS (HR, 0.35; *P*<0.001) and PFS (HR, 0.37; *P*<0.001) with higher ORR (83% vs 64%) and CRR (65% vs 27%) vs BsAb.
Any-grade cytokine release syndrome (CRS) and immune effector cell–associated neurotoxicity syndrome (ICANS) incidence was higher with liso-cel, mostly due to grade 1–2 events (**Table 2**). There was no grade ≥3 CRS with liso-cel and low rates of grade ≥3 ICANS overall. Rates of prolonged cytopenia (liso-cel, 32%; BsAb, 29%) and infections (liso-cel, 8%; BsAb, 14%) were comparable between cohorts. There were lower hospitalization and ICU admission rates with liso-cel vs BsAb across most time periods (**Figure 1**).

**Copyright Information:** (Online) ISSN: 2572-9241
©2026 The Author(s). HemaSphere is published by John Wiley & Sons Ltd on behalf of European Hematology Association. This is an open access Abstract Book distributed under the Attribution-NonCommercial-NoDerivs (CC BY-NC-ND), which allows third parties to download the articles and share them with others as long as they credit the author and the Abstract Book, but they cannot change the content in any way or use them commercially.
**Abstract Book Citations:** Authors, Title, HemaSphere, 2026;10:(S1):pages. The abstract book DOIs can be found on the "Author Information" page.
**Disclaimer:** Articles published in the journal HemaSphere exclusively reflect the opinions of the authors. The authors are responsible for all content in their abstracts including accuracy of the facts, statements, citing resources, etc.

### Summary/Conclusion

In this retrospective, RW analysis, liso-cel demonstrated significantly better efficacy and similar HCRU and safety vs BsAb as a 3L+ therapy for LBCL; further data are needed to compare these treatments in earlier lines of therapy.

**Table 1. Efficacy outcomes**

| Outcome | Liso-cel (n=161) | BsAb (n=112) | *P* value |
|---|---|---|---|
| **OS, (95% CI)** | | | |
| Unadjusted median, mo | NE (20–NE) | 11 (8–NE) | |
| Unadjusted HR | 0.50 (0.33–0.75) | | <0.001 |
| Adjusted HR[a] | n=154 | n=105 | 0.023 |
| | 0.62 (0.41–0.94) | | |
| **PFS, (95% CI)** | | | |
| Unadjusted median, mo | 12 (10–22) | 5 (3–7) | |
| Unadjusted HR | 0.48 (0.34–0.67) | | <0.001 |
| Adjusted HR[a] | n=154 | n=105 | <0.001 |
| | 0.53 (0.37–0.74) | | |
| **ORR, % (95% CI)[b]** | | | |
| Unadjusted | n=138<br>80 (73–87) | n=87<br>66 (55–75) | 0.012 |
| Adjusted[a] | n=133<br>81 (74–87.5) | n=81<br>64 (52–74) | 0.004 |
| **CRR, % (95% CI)[b]** | | | |
| Unadjusted | n=138<br>60 (51.5–68) | n=87<br>28 (18.5–38) | <0.001 |
| Adjusted[a] | n=133<br>61 (52.5–70) | n=81<br>28 (19–39) | <0.001 |

[a]Patients with rare histologies removed after adjustment; [b]Excluding patients with ORR/CRR not documented.
NE, nonevaluable.

**Table 2. Adjusted safety outcomes**

**Copyright Information:** (Online) ISSN: 2572-9241
©2026 The Author(s). HemaSphere is published by John Wiley & Sons Ltd on behalf of European Hematology Association. This is an open access Abstract Book distributed under the Attribution-NonCommercial-NoDerivs (CC BY-NC-ND), which allows third parties to download the articles and share them with others as long as they credit the author and the Abstract Book, but they cannot change the content in any way or use them commercially.
**Abstract Book Citations:** Authors, Title, HemaSphere, 2026;10:(S1):pages. The abstract book DOIs can be found on the “Author Information” page.
**Disclaimer:** Articles published in the journal HemaSphere exclusively reflect the opinions of the authors. The authors are responsible for all content in their abstracts including accuracy of the facts, statements, citing resources, etc.

| Outcome | Liso-cel (n=154) | BsAb (n=105) | *P* value |
|---|---|---|---|
| **CRS, n (%)** | | | |
| Yes | 61 (40) | 27 (26) | 0.02 |
| Grade <3 | 60 (39) | 20 (19) | |
| Grade ≥3 | 0 | 3 (3) | <0.001 |
| Unknown | 1 (1) | 4 (4) | |
| **ICANS, n (%)** | | | |
| Yes | 31 (20) | 7 (7) | 0.003 |
| Grade <3 | 22 (14) | 3 (3) | |
| Grade ≥3 | 8 (5) | 3 (3) | 0.555 |
| Unknown | 1 (1) | 1 (1) | |

Figure 1. Adjusted HCRU across periods. (A) Hospitalizations, (B) ICU admissions, and (C) median duration of hospitalizations

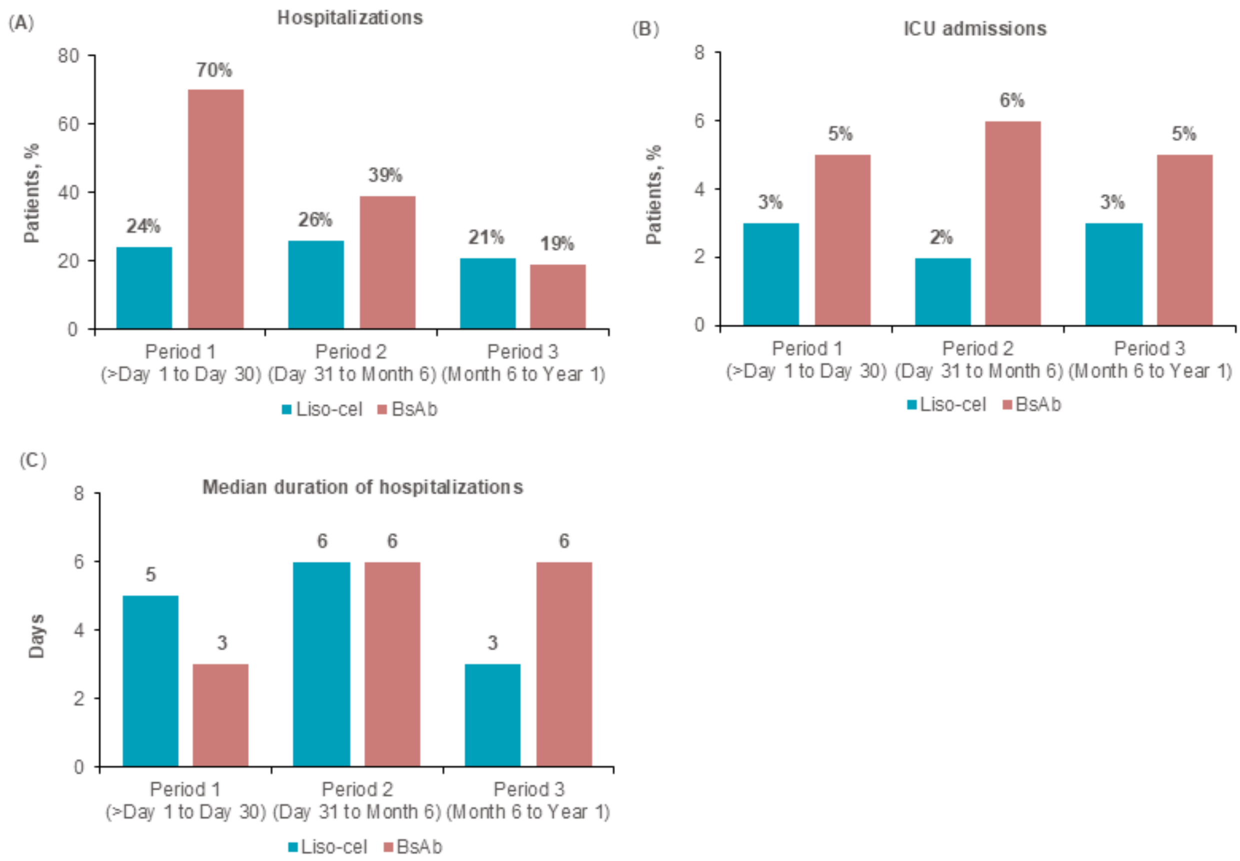

**Copyright Information:** (Online) ISSN: 2572-9241
©2026 The Author(s). HemaSphere is published by John Wiley & Sons Ltd on behalf of European Hematology Association. This is an open access Abstract Book distributed under the Attribution-NonCommercial-NoDerivs (CC BY-NC-ND), which allows third parties to download the articles and share them with others as long as they credit the author and the Abstract Book, but they cannot change the content in any way or use them commercially.
**Abstract Book Citations:** Authors, Title, HemaSphere, 2026;10:(S1):pages. The abstract book DOIs can be found on the "Author Information" page.
**Disclaimer:** Articles published in the journal HemaSphere exclusively reflect the opinions of the authors. The authors are responsible for all content in their abstracts including accuracy of the facts, statements, citing resources, etc.

### (PF986) ALLOGENEIC HEMATOPOIETIC STEM CELL TRANSPLANT AS CONSOLIDATION AFTER CD3XCD20 BISPECIFIC ANTIBODIES IN RELAPSED/REFRACTORY LARGE B-CELL LYMPHOMAS: AN LWP-CTIWP STUDY ON 52 CASES FROM THE EBMT REGISTRY

**Topic:** 19. Large B cell lymphomas - Clinical

Federico Erbella*[1], Jarl Mooyarart[2], Jacques-Emmanuel. Galimard[3], Peter Dreger[4], Francis Ayuk[5], Anoek Broers[6], Amandine Charbonnier[7], Arian Laurence[8], Georg-Nikolaus Franke[9], Ain Kaare[10], Tessa Kerre[11], Jakob Passweg[12], Christof Scheid[13], Maria Aranzazu Bermudez Rodriguez[14], Adrian Bloor[15], Luca Castagna[16], Yves Chalandon[17], Patrice Chevallier[18], Johannes Clausen[19], Rafael Duarte[20], Virginie Gandemer[21], Anna Grassi[22], Arnon Nagler[23], Emma Nicholson[24], Jorinde Hoogenboom[25], Annelot van Amerongen[26], Nicole Santoro[27, 28], Florent Malard[29], Jurgen Kuball[30], Ali Bazarbachi[31], Andrés José María Ferreri[1], Annalisa Ruggeri[32]

*[1]San Raffaele Scientific Institute, Strategic Program on Lymhomas, Milan, Italy; [2]EBMT Leiden Statistical Unit, Leiden, The Netherlands; [3]EBMT Paris Study Office, Paris, France; [4]University of Heidelberg, Heidelberg, Germany; [5]University Hospital Eppendorf, Hamburg, Germany; [6]Erasmus MC Cancer Institute, Rotterdam, The Netherlands; [7]University hospital of Amiens, Amiens, France; [8]University College London Hospital, London, United Kingdom; [9]University Of Leipzig Medical Center, Clinic and Policlinic for Haematology, Cell Therapy, Hemostaseology and Infectious Diseases, Leipzig, Germany; [10]Tartu University Hospital, Tartu, Estonia; [11]Ghent University Hospital, Gent, Belgium; [12]University Hospital | Basel, Basel, Switzerland; [13]University of Cologne, Cologne, Germany; [14]Hospital Universitario Marqués de Valdecilla, Santander, Spain; [15]Christie Hospital Manchester, Manchester, United Kingdom; [16]Centro Trapianto di Midollo - A.O. Ospedali Riuniti Villa Sofia Cervello, Palermo, Italy; [17]University Hospitals of Geneva, Genève, Switzerland; [18]Hôtel-Dieu, Centre Hospitalier Universitaire de Nantes, Nantes, France; [19]Elisabethinen Hospital, Linz, Austria; [20]Clinica Puerta de Hierro, Madrid, Spain; [21]Centre Hospitalier Universitaire de Rennes, Rennes, France; [22]ASST Ospedale Papa Giovanni XXIII, Bergamo, Italy; [23]Chaim Sheba Medical Center, Tel-Hashomer, Israel; [24]Royal Marsden Hospital, Leeds, United Kingdom; [25]EBMT Registry Unit, Leiden, The Netherlands; [26]EBMT Registry, Leiden, The Netherlands; [27]Hematology Unit, Department of Oncology and Hematology, Pescara Hospital, Pescara, Italy; [28]Department of Medicine and Aging Sciences, University of Chieti-Pescara, Chieti, Italy; [29]Hôpital Saint Antoine, AP-HP, Sorbonne Université, Paris, France; [30]University Medical Centre Utrecht, Utrecht, The Netherlands; [31]Bone Marrow Transplantation Program, Department of Internal Medicine, American University of Beirut, Beirut, Lebanon; [32]San Raffaele Scientific Institute, Hematology and Bone Marrow Transplantation Unit, Milan, Italy;*

### Background

Relapsed or refractory large B-cell lymphoma (rrLBCL) is associated with poor outcomes. Chimeric antigen receptor T-cell (CAR-T) therapy cures 30-50% of rrLBCLs, but many still relapse or progress. CD3xCD20 bispecific antibodies (BsAb) show 50-60% overall response rates, with complete responses (CR) in 23-40%, in both CAR-T-naïve and -exposed pts. Nevertheless, over half of responders relapse with 1-year progression-free survival (1y-PFS) of ~80% and ~30% after CR or partial response (PR), respectively. Allogeneic hematopoietic stem cell transplant (HSCT) may be curative in selected cases, possibly enhanced by graft-versus-lymphoma effects in BsAb-sensitive pts. Consolidation with HSCT in BsAb responders is an important mater of investigation, but non-relapse mortality (NRM) and uncertain survival benefit remain major concerns.

### Aims

This is a retrospective EBMT study aimed to address the efficacy and toxicity of consolidative HSCT in rrLBCL patients responding to BsAbs.

**Copyright Information:** (Online) ISSN: 2572-9241
©2026 The Author(s). HemaSphere is published by John Wiley & Sons Ltd on behalf of European Hematology Association. This is an open access Abstract Book distributed under the Attribution-NonCommercial-NoDerivs (CC BY-NC-ND), which allows third parties to download the articles and share them with others as long as they credit the author and the Abstract Book, but they cannot change the content in any way or use them commercially.
**Abstract Book Citations:** Authors, Title, HemaSphere, 2026;10:(S1):pages. The abstract book DOIs can be found on the "Author Information" page.
**Disclaimer:** Articles published in the journal HemaSphere exclusively reflect the opinions of the authors. The authors are responsible for all content in their abstracts including accuracy of the facts, statements, citing resources, etc.

### Methods

Adults with rrLBCL who achieved CR or PR after BsAb therapy and received consolidation HSCT between January 2018 and February 2025 were included. Primary end-points were PFS and NRM. PFS was analyzed according to Kaplan-Meier method and estimated from the date of HSCT until PD, relapse, death, or last visit of follow-up, whichever occur first. NRM was analyzed using cumulative incidence function with relapse as competing event.

### Results

Fifty-two [median age 53 (37-61); 34 males] pts were included. Of these, 44 (84%) were diffuse LBCL, 4 (8%) T-cell/histiocyte-rich LBCLs, 2 (4%) high-grade B-cell lymphomas, and 2 (4%) primary mediastinal LBCL. Nine (17%) had received previous autologous transplant and 40 (77%) CAR-T. Used BsAbs were glofitamab (n=35), epcoritamab (n=14), odronextamab (n=2), and mosunetuzumab (n=1). Karnofsky performance status at HSCT was available in 49 pts and was >70 in all of them. Median time from the start of BsAb therapy to alloHSCT was 175 (IQR 126-255) days. At HSCT, 26 (50%) pts were in CR and 26 in PR. Eleven pts received a matched related donor transplant, 27 unrelated including (17 matched, 7 mismatched and 3 unrelated without HLA reported), and 10 haploidentical; donor type not reported in 4. Conditioning regimens delivered were myeloablative in 15 (29%) and reduced intensity in 36 (71%) pts; missing in 1. With a median follow-up of 13 (95%CI: 12-21.2) months, 20 patients died: 10 (50%) pts relapsed and died of lymphoma and 10 died due to infections (n=4), graft-versus-host disease ± infection (n=3), cardiotoxicity (n=1), unknown (n=2). Two (4%) pts relapsed but were alive at last follow-up. One-year PFS was 57% (95%CI:43-72%) and overall survival (OS) 62% (95%CI:48-76%). According to response status at the time of HSCT, 1y-PFS was 78% (95%CI: 62-95) and 35% (95%CI:15-55), respectively for CR and PR groups (p=0.006) (Fig. A), with a 1y-OS of 78% (95%CI, 61-95) and 47% (95%CI: 27-67), respectively (p=0.01); 1y-NRM was 17% (95%CI: 6-27%) (Fig. B). The 1-year cumulative incidence of relapse was 26% (13-39%).

### Summary/Conclusion

With the limitations inherent to the absence of a suitable comparator and to its retrospective nature, this study suggests that the NRM of consolidative HSCT following BsAb is not higher than that reported with the same approach in pts with rrLBCL. The 1y-PFS observed is similar to those reported with BsAb alone in both CR and PR cohorts. A longer follow-up and further investigation in larger cohorts may clarify the potential benefits of this strategy.

**Copyright Information:** (Online) ISSN: 2572-9241
©2026 The Author(s). HemaSphere is published by John Wiley & Sons Ltd on behalf of European Hematology Association. This is an open access Abstract Book distributed under the Attribution-NonCommercial-NoDerivs (CC BY-NC-ND), which allows third parties to download the articles and share them with others as long as they credit the author and the Abstract Book, but they cannot change the content in any way or use them commercially.
**Abstract Book Citations:** Authors, Title, HemaSphere, 2026;10:(S1):pages. The abstract book DOIs can be found on the “Author Information” page.
**Disclaimer:** Articles published in the journal HemaSphere exclusively reflect the opinions of the authors. The authors are responsible for all content in their abstracts including accuracy of the facts, statements, citing resources, etc.

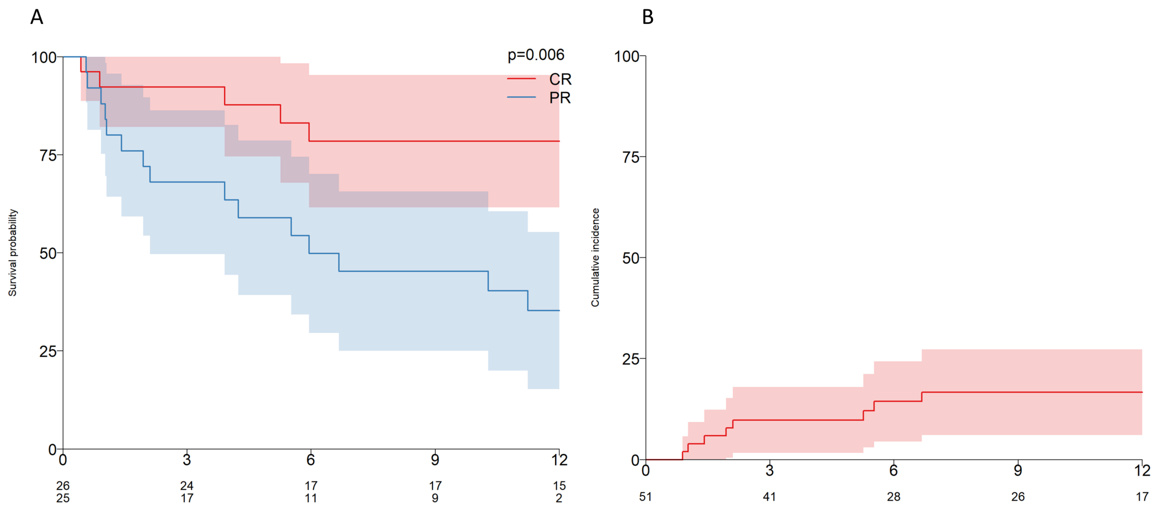

**Figure A.** Kaplan-Meier curves of PFS according to disease response at the time of transplant. **B** Kaplan-Meier curves of NRM of the entire cohort.

**Copyright Information:** (Online) ISSN: 2572-9241
©2026 The Author(s). HemaSphere is published by John Wiley & Sons Ltd on behalf of European Hematology Association. This is an open access Abstract Book distributed under the Attribution-NonCommercial-NoDerivs (CC BY-NC-ND), which allows third parties to download the articles and share them with others as long as they credit the author and the Abstract Book, but they cannot change the content in any way or use them commercially.
**Abstract Book Citations:** Authors, Title, HemaSphere, 2026;10:(S1):pages. The abstract book DOIs can be found on the "Author Information" page.
**Disclaimer:** Articles published in the journal HemaSphere exclusively reflect the opinions of the authors. The authors are responsible for all content in their abstracts including accuracy of the facts, statements, citing resources, etc.

### (PF987) PRIMARY ANALYSIS OF THE POLATUZUMAB VEDOTIN PLUS R-CHP (POLA-R-CHP) COHORT OF FIL-MAB, A PROSPECTIVE, MULTICENTER ITALIAN MULTICOHORT REAL-LIFE STUDY

**Topic:** 19. Large B cell lymphomas - Clinical

Rita Tavarozzi[1, 2], Andrea Evangelista[3], Carlo Visco[4], Umberto Vitolo[5], Stefano Luminari[6], Luca Nassi[7], Sonya De Lorenzo[8], Claudia Castellino[9], Maura Nicolosi[10], Alessandra Tucci[11], Federica Cavallo[12], Vittorio Ruggero Zilioli[13], Riccardo Bruna[14], Alessandro Rambaldi[15], Ivana Rita Casaroli[16], Monica Tani[17], Elisa Lucchini[18], Ugo Consoli[19], Elisabetta Scarpa[20], Elsa Pennese[21], Dario Marino[22], Caterina Bocci[23], Roberta Giachetti[24], Annarita Conconi[25], Alessandra Dondi[26], Mariella Lo Schirico[27], Enrico Carazzai[4], Maria Elena Nizzoli[6], Pio Manlio Mirko Frascione[5], Giovannino Ciccone[3], Alice Di Rocco[28], Andrés José María Ferreri[29], Marco Ladetto*[1, 2]

*[1]University of Eastern Piedmont, Department of Translational Medicine, Novara, Italy;[2]AOU SS Antonio e Biagio e Cesare Arrigo, SCDU of Hematology, Alessandria, Italy; [3]Unit of Clinical Epidemiology, Città della Salute e della Scienza di Torino, Turin, Italy; [4]Section of Hematology, Department of Medicine, Verona, Italy; [5]Candiolo Cancer Institute, FPO-IRCCS, Candiolo (TO), Italy; [6]Surgical, Medical and Dental Department of Morphological Sciences Related to Transplant, Oncology and Regenerative Medicine, University of Modena and Reggio Emilia, Reggio Emilia, Italy; [7]Azienda Ospedaliera Universitaria Careggi, Florence, Italy; [8]U.O.C. Ematologia e T.M.O., AORN "S.G. Moscati", Avellino, Italy;[9]Division of Hematology, Santa Croce e Carle Hospital, Cuneo, Italy; [10]Hematology Division, A.O.U. Città della Salute e della Scienza di Torino, Turin, Italy;[11]Hematology Azienda Socio Sanitaria Territoriale Spedali Civili Brescia, Brescia, Italy; [12]Division of Hematology, Department of Molecular Biotechnologies and Health Sciences, University of Torino/AOU "Città della Salute e della Scienza di Torino", Turin, Italy; [13]Division of Hematology, ASST Grande Ospedale Metropolitano Niguarda, Milan, Italy; [14]SCDU Ematologia, Department of Translational Medicine, University of Eastern Piedmont and AOU Maggiore della Carità, Novara, Italy; [15]Asst Papa Giovanni XXIII, Bergamo, Italy; [16]Fondazione IRCCS San Gerardo dei Tintori, Monza, Italy; [17]Ospedale Santa Maria delle Croci, Ravenna, Italy;[18]SC Ematologia, Azienda Sanitaria Universitaria Integrata, Trieste, Italy; [19]ARNAS Garibaldi di Catania, Catania, Italy; [20]UOC Ematologia, Ospedale Cà Foncello, Treviso, Italy; [21]ASL Pescara, Presidio Ospedaliero Santo Spirito, Pescara, Italy;[22]Medical Oncology 1, Veneto Institute of Oncology IOV-IRCSS, Padova, Italy; [23]U.O.S.D Ematologia Con Autotrapianto Cellule Staminali Ospedale di Civitanova Marche, Civitanova Marche, Italy; [24]SOC Oncohematology, Oncology Department, Azienda USL Toscana Centro, Hospital SS Cosma e Damiano, Pescia, Italy; [25]Ospedale degli Infermi, Biella, Italy; [26]Fondazione Italiana Linfomi ETS, sede di Modena, Modena, Italy; [27]Ospedale San Giacomo, Istituto Oncologico Veneto, Castelfranco Veneto, Italy; [28]Dipartimento di Medicina Traslazionale e di Precisione, Roma, Italy; [29]Lymphoma Unit, IRCCS San Raffaele Scientific Institute, Milano, Italy;*

### Background

Pola-R-CHP is a new standard of care for previously untreated DLBCL(Tilly, NEJM 2022)and is reimbursed in Italy for IPI 3–5. However, real-world (RW) data are still limited. FIL-MAB (NCT06008691) is an independent,academic,multicenter,prospective RW observational study evaluating effectiveness and safety of novel mAb-based therapies across 65 Italian centers and 8 cohorts.

### Aims

The Pola-R-CHP cohort has met the prespecified sample size for primary analysis,enabling assessment of its clinical value in routine practice in a large European country.

**Copyright Information:** (Online) ISSN: 2572-9241
©2026 The Author(s). HemaSphere is published by John Wiley & Sons Ltd on behalf of European Hematology Association. This is an open access Abstract Book distributed under the Attribution-NonCommercial-NoDerivs (CC BY-NC-ND), which allows third parties to download the articles and share them with others as long as they credit the author and the Abstract Book, but they cannot change the content in any way or use them commercially.
**Abstract Book Citations:** Authors, Title, HemaSphere, 2026;10:(S1):pages. The abstract book DOIs can be found on the "Author Information" page.
**Disclaimer:** Articles published in the journal HemaSphere exclusively reflect the opinions of the authors. The authors are responsible for all content in their abstracts including accuracy of the facts, statements, citing resources, etc.

**Methods**

Patients (pts) deemed candidates for Pola-R-CHP at participating centers, were prospectively enrolled and treated per routine practice. Baseline characteristics, feasibility, discontinuation rates, G3–5 AEs, ORR/CRR (PET-CT per Lugano 2014), PFS and OS were estimated by Kaplan–Meier. Outcomes were analyzed overall and benchmarked against the R-CHOP arm of the POLARIX trial using a matching-adjusted indirect comparison (MAIC) approach. Individual patient data were reweighted to match aggregate baseline characteristics(age,sex,stage,ECOG PS,LDH,IPI) of the POLARIX control arm.

**Results**

At data cut-off (Jan 23, 2026), 308 pts were enrolled across 45 FIL centers. For this analysis, only 177 pts enrolled before 30 Jun 2025 were analyzed. Compared to POLARIX arms, our cohort was enriched for high-risk features. Median age 67 yrs (IQR 61–74); stage III–IV 96.6%; elevated LDH 75.7%; extranodal involvement (≥2 sites) 71.4%; ECOG ≥2 22.2%; high-risk IPI (3–5) 93.3%; CNS-IPI high-risk 45.2%. According to histology: de novo DLBCL 72.3% (GCB 30%, ABC/non-GCB 46%, NOS 18% and unknown 6%), transformed from indolent lymphoma 6.8%, HGBL 8.5%, other aggressive forms 6.3%.
155 (89%) completed the planned therapy. Twenty-six patients (9%) discontinued treatment (6% due to PD, 1% to toxicity, 1% to other causes; 1% experienced treatment-related death) and 3 (2%) had missing data. Among evaluable pts who completed treatment (155/174), response rates were: CR 74%, PR 11%, and SD/PD 4%. The current median follow-up is 7.4 months.
At 12 months from enrollment, PFS was 78.2% (95% CI 68.5–85.3, Fig 1A) and OS was 91.2% (95% CI 83.1–95.5, Fig 1B). In the de novo DLBCL subgroup, 12-month PFS and OS were 75.1% (95% CI 62.5–83.9) and 90.6% (95% CI 79.5–95.8), respectively.
After reweighting using a MAIC to align with the baseline characteristics of the POLARIX control group (age >60:70.2%; female:46.7%; stage III–IV:88.2%; ECOG 0–1:82.7%; elevated LDH:64.7%; high IPI:62.0%; HGBL:11.4%; other LBCL:5.0%), adequate covariate balance was achieved with negligible residual differences. In the weighted analysis, estimated 12-mPFS was 87.8% (bootstrap 95% CI 78.3–94.4).
Two toxic deaths(1%, 1 candida septic shock and 1 ischemic stroke) during treatment were reported.Non-fatal toxicities were G3–4 neutropenia 15% (G3 10%, G4 5%); anemia 3%; thrombocytopenia 3%.Non-hematologic: G3-4 infections 9%; other G3 events were 9%(GI, hepatobiliary, cardiac, musculoskeletal, vascular, respiratory, renal, any of them with an incidence ≤2%).An updated analysis will be available by May 30, 2026.

**Summary/Conclusion**

To our knowledge, the primary analysis of the prospective FIL-MAB Pola-R-CHP cohort is the first European RW experience assessing this combination. With the limitation of a short follow-up, our data, including reweighted analyses aligned to the baseline characteristics demonstrate the good applicability and effectiveness of Pola-R-CHP in a broad RW multi-institutional setting.

**Copyright Information:** (Online) ISSN: 2572-9241
©2026 The Author(s). HemaSphere is published by John Wiley & Sons Ltd on behalf of European Hematology Association. This is an open access Abstract Book distributed under the Attribution-NonCommercial-NoDerivs (CC BY-NC-ND), which allows third parties to download the articles and share them with others as long as they credit the author and the Abstract Book, but they cannot change the content in any way or use them commercially.
**Abstract Book Citations:** Authors, Title, HemaSphere, 2026;10:(S1):pages. The abstract book DOIs can be found on the "Author Information" page.
**Disclaimer:** Articles published in the journal HemaSphere exclusively reflect the opinions of the authors. The authors are responsible for all content in their abstracts including accuracy of the facts, statements, citing resources, etc.

### (PF988) DENOSUMAB VERSUS ALENDRONATE FOR PREVENTING STEROID-INDUCED OSTEOPOROSIS IN MALIGNANT LYMPHOMA: A MULTICENTER PHASE III TRIAL

**Topic:** 19. Large B cell lymphomas - Clinical

Shohei Kikuchi*[1], Eiju Negoro[2], Yusuke Kamihara[1], Akinori Wada[1], Yasufumi Masaki[3], Toshihiro Miyamoto[4], Takahiro Yamauchi[2], Tsutomu Sato[1]

*[1]Toyama University Hospital, Hematology, Toyama, Japan; [2]University of Fukui, Hematology and Oncology, Fukui, Japan; [3]Kanazawa Medicai University, Hematology and Immunology, Uchinada, Japan; [4]Kanazawa University, Hematology, Kanazawa, Japan;*

-->

### Background

Since the introduction of R-CHOP and subsequent development of novel immunotherapies, outcomes for lymphomas including DLBCL have improved. With long-term survival now achievable, preventing and managing treatment-related adverse events have become increasingly important. R-CHOP and related regimens use repeated short courses of high-dose glucocorticoids, which can induce glucocorticoid-induced osteoporosis (GIOP) and fractures. However, conventional GIOP guidelines are largely based on chronic low-dose steroid exposure and may not address this distinct setting, termed lymphoma therapy–related GIOP (LTR-GIOP). Although bisphosphonates have some evidence for prophylaxis, the available data are insufficient for LTR-GIOP, and no clinical trials evaluating denosumab in this setting have been reported.

-->

### Aims

To compare the efficacy and safety of denosumab versus alendronate for preventing bone mineral density (BMD) loss in older patients with newly diagnosed lymphoma receiving steroid-containing chemotherapy.

-->

### Methods

The study was named DENOSULY, a prospective, multicenter, open-label, phase III randomized controlled trial that enrolled patients aged 65 years or older with newly diagnosed malignant lymphoma who were scheduled to receive steroid-containing chemotherapy. Participants were stratified by age (≥80 years) and sex and then randomized to receive either denosumab or alendronate. In the denosumab arm, patients received denosumab 60 mg subcutaneously at the initiation of chemotherapy and again at 6 months (two total doses). In the alendronate arm, patients received oral alendronate 35 mg once weekly for 12 months. The primary endpoint was the percent change in lumbar spine BMD at 12 months. Secondary endpoints included percent change in BMD at additional skeletal sites (lumbar spine, total hip, and femoral neck) at 6 and 12 months, longitudinal changes in bone turnover markers at 6 months (TRACP-5b and total P1NP), and safety outcomes, including serious adverse events and adverse events of special interest.

-->

**Copyright Information:** (Online) ISSN: 2572-9241
©2026 The Author(s). HemaSphere is published by John Wiley & Sons Ltd on behalf of European Hematology Association. This is an open access Abstract Book distributed under the Attribution-NonCommercial-NoDerivs (CC BY-NC-ND), which allows third parties to download the articles and share them with others as long as they credit the author and the Abstract Book, but they cannot change the content in any way or use them commercially.
**Abstract Book Citations:** Authors, Title, HemaSphere, 2026;10:(S1):pages. The abstract book DOIs can be found on the "Author Information" page.
**Disclaimer:** Articles published in the journal HemaSphere exclusively reflect the opinions of the authors. The authors are responsible for all content in their abstracts including accuracy of the facts, statements, citing resources, etc.

**Results**

A total of 100 patients were randomized (alendronate, n=48; denosumab, n=52). Baseline characteristics, BMD, and bone turnover markers were comparable between groups. At 12 months, denosumab was superior to alendronate for the primary endpoint—percent change in lumbar spine (L2–L4) BMD (+2.8 ± 4.4% vs −1.3 ± 5.6%, p=0.0010) and (L1–L4) BMD (+2.7 ± 4.5% vs −1.0 ± 4.8%, p=0.0054). Denosumab also showed superiority at femoral neck (+2.8 ± 5.8% vs −3.6 ± 10.3%, p=0.0020), though no significant difference was observed at the total hip. At 6 months, denosumab produced stronger suppression of the bone resorption marker TRACP-5b than alendronate (alendronate 0.2% vs denosumab -36.5%, p=0.0003), while the bone formation marker total P1NP did not differ significantly between groups (alendronate 38.6% vs denosumab 7.5%, p=0.1929). Safety was generally similar across groups. No Grade ≥3 hypocalcemia or osteonecrosis of the jaw occurred, and serious adverse events were not considered causally related to either study drug.

**Summary/Conclusion**

In older patients with newly diagnosed lymphoma receiving R-CHOP and related regimens, denosumab was superior to alendronate for preventing BMD loss at 12 months. Significant benefits were observed at both the lumbar spine and femoral neck, with a favorable safety profile. These findings suggest denosumab may be the preferred prophylactic option for LTR-GIOP.

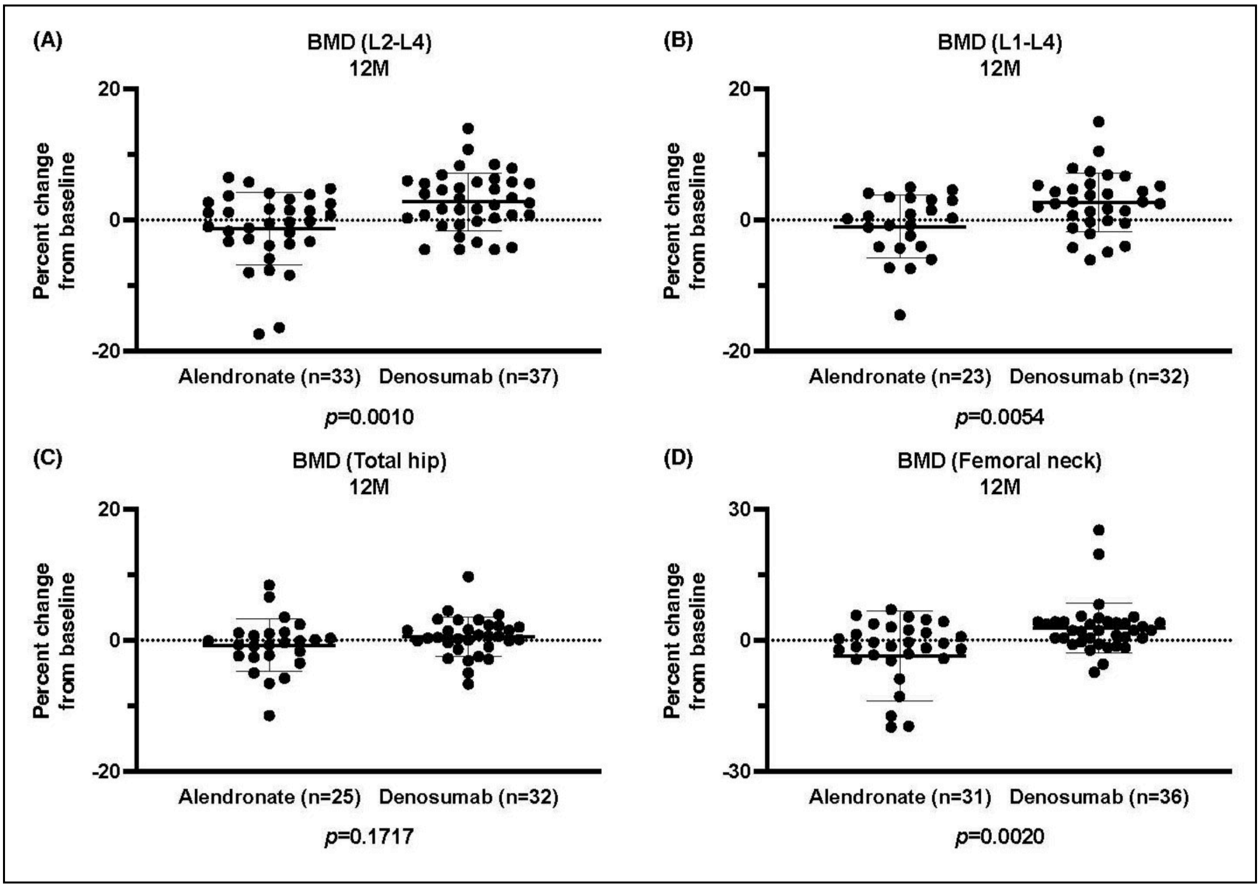

**Copyright Information:** (Online) ISSN: 2572-9241
©2026 The Author(s). HemaSphere is published by John Wiley & Sons Ltd on behalf of European Hematology Association. This is an open access Abstract Book distributed under the Attribution-NonCommercial-NoDerivs (CC BY-NC-ND), which allows third parties to download the articles and share them with others as long as they credit the author and the Abstract Book, but they cannot change the content in any way or use them commercially.
**Abstract Book Citations:** Authors, Title, HemaSphere, 2026;10:(S1):pages. The abstract book DOIs can be found on the "Author Information" page.
**Disclaimer:** Articles published in the journal HemaSphere exclusively reflect the opinions of the authors. The authors are responsible for all content in their abstracts including accuracy of the facts, statements, citing resources, etc.

### (PF989) A FULLY OBJECTIVE 3-VARIABLE PROGNOSTIC SCORE FOR DIFFUSE LARGE B-CELL LYMPHOMA: DEVELOPMENT AND EXTERNAL VALIDATION IN TWO INDEPENDENT COHORTS TOTALING 776 PATIENTS

**Topic:** 19. Large B cell lymphomas - Clinical

Yoshimasa Kamoda*[1], Futoshi Iioka[2], Masako Hirao[1, 1], Hiromitsu Iizuka[1], Michiko Kida[1], Hitoshi Ohno[2], Takashi Akasaka[2], Motoshi Ichikawa[1]

*[1]Department of Hematology, NTT Medical Center Tokyo, Tokyo, Japan;[2]Department of Hematology, Tenri Hospital, Tenri, Japan;*

### Background

The International Prognostic Index (IPI) and NCCN-IPI remain widely used in diffuse large B-cell lymphoma (DLBCL), but they include evaluator-dependent variables such as performance status, extranodal involvement, and Ann Arbor stage, all of which are subject to interobserver variability. Serum albumin has repeatedly been reported as a strong prognostic factor but is not incorporated into existing indices. A simplified and fully objective prognostic score based solely on routine laboratory and demographic variables could improve reproducibility and clinical applicability.

### Aims

To identify robust prognostic factors using data-driven methods and to construct an objective prognostic score that minimizes subjectivity while preserving discrimination.

### Methods

Patients were drawn from two independent Japanese institutions: 402 patients formed the derivation cohort and 374 the external validation cohort. Candidate predictors included age, albumin, LDH, performance status, extranodal involvement, and Ann Arbor stage. Albumin was dichotomized at 3.5 g/dL and LDH at the upper limit of normal based on widely accepted clinical thresholds, ensuring consistency with routine practice. For age, multiple dichotomization schemes (55–85 years) were evaluated using LASSO Cox regression with 1,000 bootstrap resamples, and restricted cubic spline modeling was used to assess the continuous effect of age and identify structural changes in risk. Models incorporating different predictor combinations were compared using C-index and interpretability, and the final model was externally validated.

### Results

Across alternative age thresholds, LASSO consistently selected age and albumin with the highest frequencies. Age >75 years showed the highest selection frequency (95%), and spline analysis demonstrated a noticeable change in slope around this cutoff, supporting its data-driven selection. To evaluate whether a third variable improved discrimination, LDH, performance status, Ann Arbor stage, and extranodal involvement were assessed. Extranodal involvement did not improve discrimination (ΔC-index −0.004). Ann Arbor stage (+0.024), LDH (+0.017), and performance status (+0.014) each provided modest improvements; however, the differences among these ΔC-index values were negligible. Given this similarity and our emphasis on objectivity and reproducibility, LDH was selected as the third variable.

A multivariable Cox model including age >75, albumin <3.5 g/dL, and elevated LDH showed comparable hazard ratios (age HR 2.86; albumin HR 2.78; LDH HR 1.90), supporting a simple additive score (0–3 points). In the derivation cohort, the score stratified overall survival into four groups (5-year OS: 93.6%, 83.3%, 66.7%, 37.4%; C-index 0.749). In the validation cohort, separation was reproduced (5-year OS: 93.2%, 79.9%, 58.8%, 34.2%; C-index 0.745). The score showed clearer separation than the IPI (5-year OS: 90.9%, 74.8%, 70.6%, 50.7%; C-index 0.701) and performance comparable to the NCCN-IPI (5-year OS: 100%, 87.5%, 69.3%, 45.8%; C-index 0.734).

**Copyright Information:** (Online) ISSN: 2572-9241
©2026 The Author(s). HemaSphere is published by John Wiley & Sons Ltd on behalf of European Hematology Association. This is an open access Abstract Book distributed under the Attribution-NonCommercial-NoDerivs (CC BY-NC-ND), which allows third parties to download the articles and share them with others as long as they credit the author and the Abstract Book, but they cannot change the content in any way or use them commercially.
**Abstract Book Citations:** Authors, Title, HemaSphere, 2026;10:(S1):pages. The abstract book DOIs can be found on the "Author Information" page.
**Disclaimer:** Articles published in the journal HemaSphere exclusively reflect the opinions of the authors. The authors are responsible for all content in their abstracts including accuracy of the facts, statements, citing resources, etc.

### Summary/Conclusion

We developed and externally validated a simplified and fully objective 3-variable prognostic score for DLBCL based on age, albumin, and LDH. The score demonstrated robust risk stratification across two independent cohorts totaling 776 patients and offers a reproducible and clinically practical alternative to existing prognostic indices. Because it relies solely on universally available variables, this score can be readily implemented in routine clinical practice.

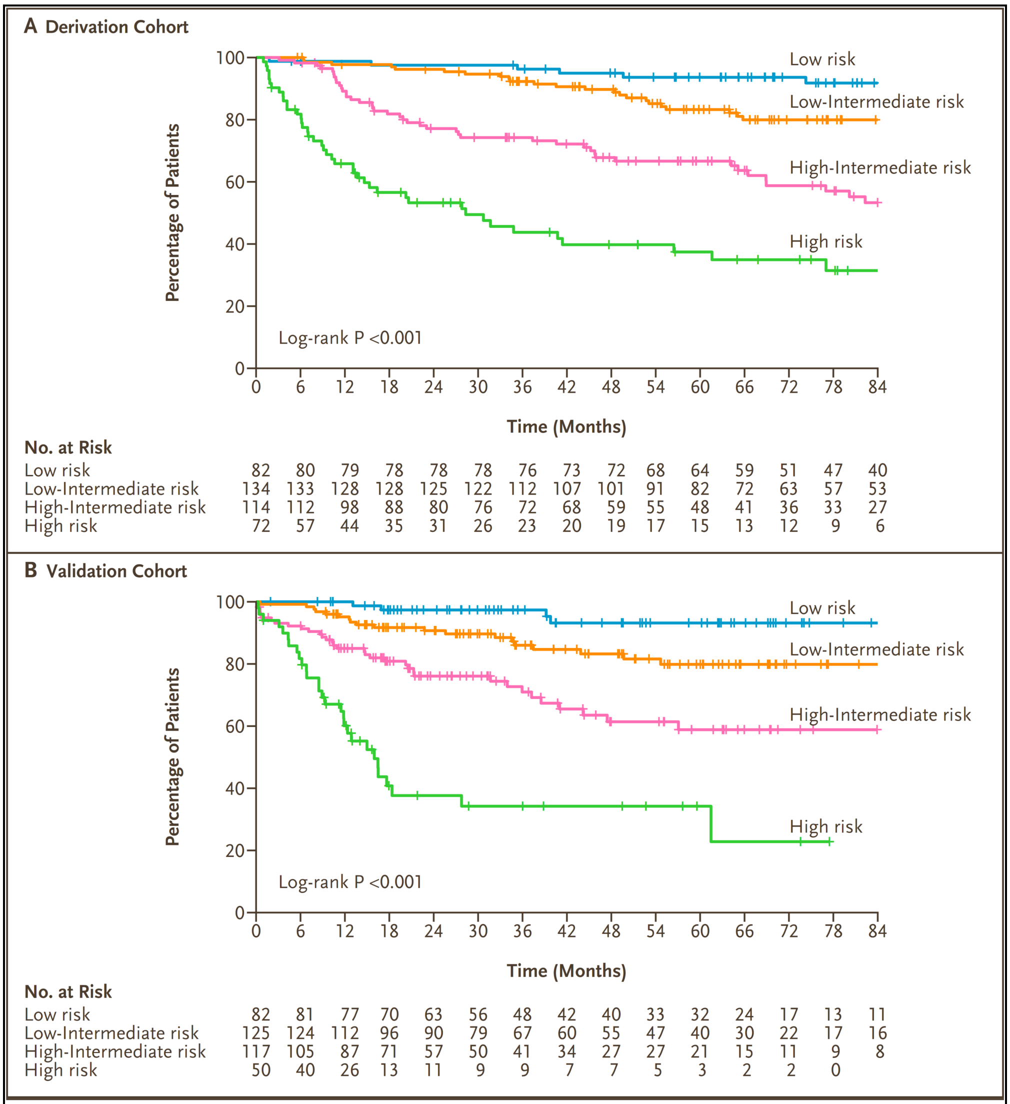

**Figure. Overall survival curves stratified by the 3 variable prognostic score in the derivation (A) and validation (B) cohorts**
(A)The score (0–3 points; age >75 years, albumin <3.5 g/dL, elevated LDH) demonstrated clear separation of four risk groups, with 5 year overall survival rates of 93.6%, 83.3%, 66.7%, and 37.4%. (B)Risk separation observed in the derivation cohort was reproduced, with 5 year overall survival rates of 93.2%, 79.9%, 58.8%, and 34.2%, confirming the external validity and robustness of the scoring system.

**Copyright Information:** (Online) ISSN: 2572-9241
©2026 The Author(s). HemaSphere is published by John Wiley & Sons Ltd on behalf of European Hematology Association. This is an open access Abstract Book distributed under the Attribution-NonCommercial-NoDerivs (CC BY-NC-ND), which allows third parties to download the articles and share them with others as long as they credit the author and the Abstract Book, but they cannot change the content in any way or use them commercially.
**Abstract Book Citations:** Authors, Title, HemaSphere, 2026;10:(S1):pages. The abstract book DOIs can be found on the “Author Information” page.
**Disclaimer:** Articles published in the journal HemaSphere exclusively reflect the opinions of the authors. The authors are responsible for all content in their abstracts including accuracy of the facts, statements, citing resources, etc.

### (PF990) INTRODUCTION OF CAR-T CELL THERAPY DRAMATICALLY IMPROVED OUTCOMES OF PATIENTS WITH RELAPSED/REFRACTORY LARGE B-CELL LYMPHOMA RECEIVING SECOND-LINE TREATMENT AT A POPULATION LEVEL

**Topic:** 19. Large B cell lymphomas - Clinical

Prokop Vodicka*[1], Andrea Janíková[2], David Belada[3], Katerina Steinerova[4], Juraj Ďuraš[5], Heidi Mociková[6], Vít Procházka[7], Kamila Polgarova[1], David Salek[2], Alice Sykorova[3], Diana Malarikova[1], Michal Kascak[5], Katerina Benesova[1], Samuel Hricko[2], Vít Campr[8], Jan Galko[9], Jitka Dlouhá[10], Petra Blahovcova[10], Marek Trněný[1]

*[1]First Faculty of Medicine, Charles University and General Hospital, First Department of Medicine, Prague, Czechia;[2]Faculty of Medicine, Masaryk University and University Hospital Brno, Department of Internal Medicine, Hematology and Oncology, Brno, Czechia; [3]Faculty of Medicine, Charles University and University Hospital Hradec Kralove, Fourth Department of Internal Medicine – Hematology, Hradec Kralove, Czechia; [4]Faculty of Medicine, Charles University and University Hospital Pilsen, Department of Haematology and Oncology, Pilsen, Czechia; [5]Medical Faculty of the Ostrava University and University Hospital, Department of Hematology, Ostrava, Czechia; [6]Fakultni nemocnice Kralovske Vinohrady and Third Faculty of Medicine, Charles University, Department of Hematology, Prague, Czechia; [7]Faculty of Medicine and Dentistry, Palacky University and University Hospital, Department of Haemato-Oncology, Olomouc, Czechia; [8]Second Faculty of Medicine, Charles University and Motol University Hospital, Department of Pathology and Molecular Medicine, Prague, Czechia; [9]First Faculty of Medicine, Charles University and General Hospital, Institute of Pathology, Prague, Czechia; [10]Czech Lymphoma Study Group, Datacenter, Prague, Czechia;*

### Background

The introduction of CAR-T and other innovative therapies has changed the treatment landscape for patients with relapsed/refractory (r/r) large B-cell lymphomas (LBCL). We have previously demonstrated that these novel treatments improved outcomes for r/r LBCL patients in the third-line setting in recent years (EHA 2025, PF939). Subsequently, CAR-T was approved in the second line (L2) in 2022 in our region, creating the potential for similar survival improvements.

### Aims

We aimed to evaluate evolving treatment patterns in the L2 for r/r LBCL and to assess the impact of novel therapies on overall survival (OS) within a real-world patient population on a national-based level.

### Methods

We analyzed data from 524 consecutive patients with r/r LBCL from the prospective NiHiL registry (NCT03199066) who received L2 therapy between 2020–2024. Patients were stratified into two eras based on CAR-T access: Era 1 (2020–2021; n=239) and Era 2 (2023–2024; n=285), with 2022 excluded (transitional year). Patients were included regardless of whether they ultimately received CAR-T. The primary endpoint was OS; secondary endpoints included cumulative incidence (CIF) of lymphoma-related mortality (LRM) and shifts in treatment patterns. Subgroup analysis was conducted for "early-r/r" patients, defined as progressive (PD), refractory disease (stable [SD], partial remission [PR]), or relapse ≤12 months after the end of L1 (n=393). We used Cox regression for time-to-event analysis (OS) and Fine-Gray models for CIF of LRM (non-lymphoma death as a competing risk).

**Copyright Information:** (Online) ISSN: 2572-9241
©2026 The Author(s). HemaSphere is published by John Wiley & Sons Ltd on behalf of European Hematology Association. This is an open access Abstract Book distributed under the Attribution-NonCommercial-NoDerivs (CC BY-NC-ND), which allows third parties to download the articles and share them with others as long as they credit the author and the Abstract Book, but they cannot change the content in any way or use them commercially.
**Abstract Book Citations:** Authors, Title, HemaSphere, 2026;10:(S1):pages. The abstract book DOIs can be found on the "Author Information" page.
**Disclaimer:** Articles published in the journal HemaSphere exclusively reflect the opinions of the authors. The authors are responsible for all content in their abstracts including accuracy of the facts, statements, citing resources, etc.

### Results

The analysis included 524 patients (median age at L2: 69.5 years; ECOG performance status [PS] ≥2: 34%; median time from L1 end to L2: 3 months, primary PD/SD: 33%); baseline characteristics were balanced between eras.
From Era 1 to Era 2, treatment patterns shifted significantly, characterized by increased use of CAR-T (0% vs. 18%) and innovative treatments (1% vs. 15%; incl. BiAbs, ADCs, lenalidomide-based therapy), alongside a decrease in salvage therapy (56% vs. 20%) and autologous SCT (ASCT; 16% vs. 3%; all P<0.01; Fig. A).
After median follow-up of 4.9 (Era 1) and 1.9 years (Era 2), OS improved in Era 2 compared to Era 1 (HR=0.71, 95% CI 0.56–0.91, P=0.01; 2-year OS 56% vs 41%; Fig. B). Although not significant, CIF of LRM was lower in Era 2 (33% vs 41% at 2 years; P=0.09; Fig. C). In multivariate model, the following variables were independent predictors of OS: Era 2 (vs Era 1; HR=0.62), age (per year; HR=1.02) ECOG PS (per point; HR=1.65), primary PD/SD (HR=3.69; all P<0.01).
Among the early-r/r subgroup (n=393), similar shifts were observed: CAR-T and innovative treatment increased (0% to 20% and 1% to 15%, resp.), salvage and ASCT decreased (all P<0.01; Fig. D). OS improved in Era 2 (HR=0.76, P=0.04; 2-year OS 51% vs 37%; Fig. E), and the CIF of LRM significantly decreased (37% vs. 49%, P=0.04; Fig. F). In multivariate analysis, Era 2 (HR=0.63), age at L2 (HR=1.02), ECOG PS (HR=1.62), and primary PD/SD (HR=3.58) were independent predictors of OS (all P<0.01).

### Summary/Conclusion

The adoption of CAR-T and innovative therapeutic strategies has significantly improved the OS of L2-treated r/r LBCL patients in recent years, by reducing the risk of death by 29% in the overall population and by 24% in the early-r/r subgroup. These real-world data confirm that shifting novel therapies to earlier lines of treatment translates into substantial clinical benefits and a reduction in LRM.
**Support:** NU23-03-00127, CU Hem-Onc Cooper, BBMRI_CZ LM2023033.

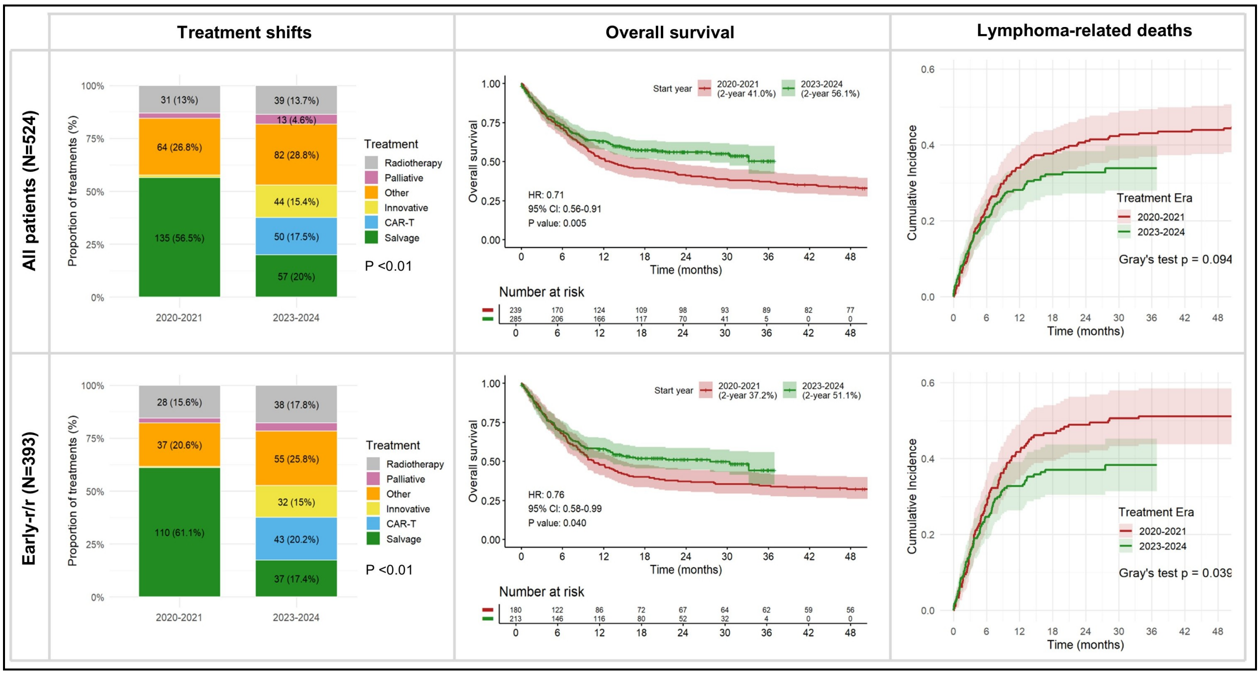

Treatment patterns and outcomes of r/r LBCL patients treated with second line (top row: all patients, N=524; bottom row: "early-r/r" subgroup, N=393): shifts from salvage treatment toward CAR-T and innovative therapies (A, D), improved overall survival in Era 2 (2023–2024) vs. Era 1 (2020–2021; B, E), decrease in lymphoma-related mortality between the two Eras (C, F).

**Copyright Information:** (Online) ISSN: 2572-9241
©2026 The Author(s). HemaSphere is published by John Wiley & Sons Ltd on behalf of European Hematology Association. This is an open access Abstract Book distributed under the Attribution-NonCommercial-NoDerivs (CC BY-NC-ND), which allows third parties to download the articles and share them with others as long as they credit the author and the Abstract Book, but they cannot change the content in any way or use them commercially.
**Abstract Book Citations:** Authors, Title, HemaSphere, 2026;10:(S1):pages. The abstract book DOIs can be found on the "Author Information" page.
**Disclaimer:** Articles published in the journal HemaSphere exclusively reflect the opinions of the authors. The authors are responsible for all content in their abstracts including accuracy of the facts, statements, citing resources, etc.

### (PF991) LARGE-SCALE REAL-WORLD CLINICAL CHARACTERISTICS AND EFFICACY OF DIFFUSE LARGE B-CELL LYMPHOMA ACROSS DISTINCT MOLECULAR SUBTYPES: INTERIM DATA FROM THE BELIEVE STUDY

**Topic:** 19. Large B cell lymphomas - Clinical

Pengpeng Xu*[1], Keshu Zhou[2], Hongmei Jing[3], Songfu Jiang[4], Wenrong Huang[5], Ou Bai[6], Xiaoxi Zhou[7], Jianzhen Shen[8], Xiuhua Sun[9], Xiaobo Wang[9], Xiaojing Yan[10], Ting Niu[11], Haisheng Liu[12], Chunling Wang[13], Wei Sang[14], Yanli Yang[15], Bing Xiu[16], Yingyu Nan[17], Yao Liu[17], Zhimin Zhai[18], Sujiang Zhang[19], Wuping Li[20], Guohui Cui[21], Meiyun Fang[22], Hongling Peng[23], Xiaolei Wei[24], Jing-Yan Xu[25], Li-Jie Xing[26], Xiangxiang Zhou[27], Ying Lu[28], Xudong Zhang[29], Qianqian Cai[30], Jinhai Ren[31], Ying Zhao[32], Zhengzi Qian[33], Zhenling Li[34], Ai-li He[35], Weili Zhao[1]

*[1]Shanghai Institute of Hematology, Shanghai, China;[2]Henan Cancer Hospital, Affiliated Cancer Hospital of Zhengzhou University, Zhengzhou, China; [3]Peking University Third Hospital, Beijing, China; [4]The First Affiliated Hospital of Wenzhou Medical University, Wenzhou, China; [5]The fifth Medical Center,Chinese PLA General Hospital, Beijing, China;[6]The First Hospital of Jilin University, Changchun, China; [7]Tongji Hospital Affiliated to Tongji Medical College, Huazhong University of Science and Technology, Wuhan, China; [8]Fujian Medical University Union Hospita, Fuzhou, China;[9]The Second Hospital of Dalian Medical University, Dalian, China; [10]The First Hospital of China Medial University, Shenyang, China; [11]West China Hospital of Sichuan University, Chengdu, China; [12]The Fourth Hospital of Hebei Medical University, Shijiazhuang, China;[13]Huai'an First People's Hospital, Huai'an, China; [14]The Affiliated Hospital of Xuzhou Medical University, Xuzhou, China;[15]The First Affiliated Hospital of Bengbu Medical College, Bengbu, China; [16]Tongji Hospital, Shanghai, China; [17]Chongqing Cancer Hospital, Chongqing, China; [18]The Second Affiliated Hospital of Anhui Medical University, Hefei, China;[19]Ruijin North Hospital, Shanghai Jiao Tong University School of Medicine, Shanghai, China; [20]Jiangxi Cancer Hospital, Nanchang, China;[21]Union Hospital, Tongji Medical College, Huazhong University of Science and Technology, Wuhan, China; [22]Affiliated Zhongshan Hospital Of Dalian University, Dalian, China; [23]The Second Xiangya Hospital of Central South University, Changsha, China;[24]Nanfang Hospital, Southern Medical University, Guangzhou, China; [25]Nanjing Drum Tower Hospital, Nanjing, China; [26]Shandong Cancer Hospital and Institute, Shandong University, Jinan, China; [27]Shandong Provincial Hospital, Jinan, China;[28]The Affiliated People's Hospital of Ningbo University, Ningbo, China; [29]The First Affliated Hospital of Zhengzhou University, Zhengzhou, China;[30]Ningbo Medical Treatment Center Lihuili Hospital, Ningbo, China; [31]The Second Hospital of Hebei Medical University, Shijiazhuang, China;[32]The First People's Hospital of Foshan, Foshan, China; [33]Tianjin Medical University Cancer Institute and Hospital, Tianjin, China;[34]China-Japan Friendship Hospital, Beijing, China; [35]The Second Affiliated Hospital of Xi'an Jiaotong University, Xi'an, China;*

### Background

Diffuse large B-cell lymphoma (DLBCL) shows extensive molecular heterogeneity. Molecular subtypes can be identified by LymphGen (MCD, BN2, N1, EZB, A53, ST2) and the simplified 38-gene LymphPlex (*TP53*$^{\mathrm{Mut}}$, MCD-like, BN2-like, N1-like, EZB-like, ST2-like). LymphPlex showed high concordance with LymphGen *(Sig Transduct Target Ther 2023:8:145)*. These subtypes differ greatly in clinical features, treatment response and prognosis, but large-sample real-world evidence to guide subtype-tailored management is still limited. The BELIEVE study (NCT 06026488) is a large Chinese real-world registry.

### Aims

To clarify clinical characteristics, treatment patterns and efficacy differences across DLBCL molecular subtypes, and to generate high-quality real-world evidence for personalized therapy.

**Copyright Information:** (Online) ISSN: 2572-9241
©2026 The Author(s). HemaSphere is published by John Wiley & Sons Ltd on behalf of European Hematology Association. This is an open access Abstract Book distributed under the Attribution-NonCommercial-NoDerivs (CC BY-NC-ND), which allows third parties to download the articles and share them with others as long as they credit the author and the Abstract Book, but they cannot change the content in any way or use them commercially.
**Abstract Book Citations:** Authors, Title, HemaSphere, 2026;10:(S1):pages. The abstract book DOIs can be found on the "Author Information" page.
**Disclaimer:** Articles published in the journal HemaSphere exclusively reflect the opinions of the authors. The authors are responsible for all content in their abstracts including accuracy of the facts, statements, citing resources, etc.

### Methods

A total of 3773 DLBCL patients from 35 centers were enrolled. All patients were classified into seven molecular subtypes (BN2-like, EZB-like, MCD-like, N1-like, ST2-like, *TP53*$^{Mut}$ and Others) by NGS. Baseline characteristics, treatment regimens and efficacy were collected. Endpoints included progression-free survival (PFS), complete response rate (CRR), objective response rate (ORR), and overall survival (OS). Herein, efficacy was analyzed across molecular subtypes and first-line regimens.

### Results

Of 3773 patients, 52.2% were male, median age 60 years (IQR 50–68). Key baseline features: non-GCB subtype (58.5%), Ann Arbor stage Ⅲ–Ⅳ disease (58.3%), IPI score 3–5 (38.0%), ≥2 extranodal sites (31.6%), MYC/BCL-2 double expression (28.8%), high-grade B-cell lymphomas with MYC and BCL-2 and/or BCL-6 rearrangements (3.9%). Molecular subtype distribution: Others (31.2%), *TP53*$^{Mut}$ (21.1%), MCD-like (19.9%), BN2-like (10.0%), EZB-like (7.0%), ST2-like (6.3%), N1-like (4.4%). Common first-line treatments: R-CHOP-like (60.0%), R-CHOP plus target agents (11.9%). Among 3599 efficacy-evaluable patients, ORR was 81.3% and CRR 64.1%. BN2-like and N1-like subtypes had favorable responses, while *TP53*$^{Mut}$ had poorest efficacy. R-CHOP plus target agents achieved higher ORR (88.4%) and CRR (74.6%) than the overall cohort. Chemo-free and R-miniCHOP-like showed inferior efficacy.

### Summary/Conclusion

In Chinese DLBCL patients, the most prevalent LymphPlex subtypes are Others, MCD-like and *TP53*$^{Mut}$ subtypes, with marked efficacy heterogeneity. R-CHOP plus target agents significantly improves CRR across multiple subtypes. These data support molecular subtype-tailored personalized treatment for DLBCL. Updated survival and safety data will be presented.

**Copyright Information:** (Online) ISSN: 2572-9241
©2026 The Author(s). HemaSphere is published by John Wiley & Sons Ltd on behalf of European Hematology Association. This is an open access Abstract Book distributed under the Attribution-NonCommercial-NoDerivs (CC BY-NC-ND), which allows third parties to download the articles and share them with others as long as they credit the author and the Abstract Book, but they cannot change the content in any way or use them commercially.
**Abstract Book Citations:** Authors, Title, HemaSphere, 2026;10:(S1):pages. The abstract book DOIs can be found on the "Author Information" page.
**Disclaimer:** Articles published in the journal HemaSphere exclusively reflect the opinions of the authors. The authors are responsible for all content in their abstracts including accuracy of the facts, statements, citing resources, etc.

### (PF992) RE-EVALUATING THE ROLE OF AUTOLOGOUS STEM CELL TRANSPLANTATION IN PRIMARY CNS DIFFUSE LARGE B-CELL LYMPHOMA IN THE TARGETED-THERAPY ERA: A PROPENSITY SCORE–MATCHED MULTICENTER REAL-WORLD STUDY

**Topic:** 19. Large B cell lymphomas - Clinical

Liqun Zou[1], Mengyuan Liu*[1], Kexing Ren[1], Ming Jiang[1], Wei Zhang[2], Hui-Lai Zhang[3], Bingyi Wang[3], Xue Meng[1], Li Xie[1], Na Li[1], Chunli Yang[1], Xi Chen[1], You Feng[1], Tao Hai[1]

*[1]West China Hospital of Sichuan Uniersity, Cheng Du, China;[2]Peking Union Medical College Hospital, Chinese Academy of Medical Sciences & Peking Union Medical College, Beijing, China; [3]Tianjin Medical University Cancer Institute and Hospital, Tianjin, China;*

### Background

The role of autologous stem cell transplantation (ASCT) in primary central nervous system diffuse large B-cell lymphoma (PCNS-DLBCL) is being re-evaluated, particularly in newly diagnosed patients, as BTK inhibitors (BTKi) and lenalidomide are increasingly used in induction, salvage, and maintenance therapy. In real-world clinical practice, ASCT allocation is influenced by baseline fitness and disease characteristics, highlighting the need for confounding control to assess outcomes and clarify whether post-induction consolidation strategies are key to long-term results in the Targeted Therapy era.

### Aims

This study aimed to evaluate the association between ASCT and progression-free survival (PFS) and overall survival (OS) in patients with PCNS-DLBCL treated in the BTKi/lenalidomide era, using propensity score matching (PSM) to adjust for confounding factors. We also examined whether post-induction consolidation strategies (ASCT and/or maintenance) and BTKi/lenalidomide treatment pathways stratify outcomes, including among treatment responders.

### Methods

We conducted a retrospective, three-centre cohort study of PCNS-DLBCL patients treated in routine practice. The primary exposure was ASCT (yes vs no). PSM was performed using nearest-neighbour matching with a target ratio of 1:2 and caliper width of 0.1, adjusting for covariates listed in Table 1. Balance was assessed using standardized mean differences. Endpoints were PFS and OS, analyzed by Kaplan–Meier methods with log-rank comparisons. Prespecified exploratory analyses evaluated six subgroups based on regimen category (BTKi, lenalidomide, or neither) and ASCT status; four subgroups based on ASCT and maintenance therapy; and responder subsets (CR/PR and CR). All analyses were hypothesis-generating.

### Results

Among 291 eligible patients (ASCT group: n=63, non-ASCT group: n=228), PSM yielded a balanced cohort of 133 patients (ASCT: n=53, non-ASCT: n=80). In the matched cohort, ASCT was associated with improved PFS (p=0.023), while OS showed a favorable but non-significant trend (p=0.085), suggesting ASCT may be more consistent impact on disease control than with overall survival. Post-induction consolidation pathways strongly stratified outcomes: four groups defined by ASCT status and maintenance use exhibited clear separation for both PFS (p<0.0001) and OS (p=0.0013). Maintenance therapy was particularly associated with improved outcomes among non-ASCT patients (PFS p=0.00026; OS p=0.0014).
In the Targeted Therapy era, PFS differed across six groups defined by regimen category and ASCT status (p=0.0038), indicating that the effect is not simply additive from “novel agent + ASCT.” This pathway-based stratification remained significant among treatment responders (PFS p=0.025, OS p=0.044).

**Copyright Information:** (Online) ISSN: 2572-9241
©2026 The Author(s). HemaSphere is published by John Wiley & Sons Ltd on behalf of European Hematology Association. This is an open access Abstract Book distributed under the Attribution-NonCommercial-NoDerivs (CC BY-NC-ND), which allows third parties to download the articles and share them with others as long as they credit the author and the Abstract Book, but they cannot change the content in any way or use them commercially.
**Abstract Book Citations:** Authors, Title, HemaSphere, 2026;10:(S1):pages. The abstract book DOIs can be found on the “Author Information” page.
**Disclaimer:** Articles published in the journal HemaSphere exclusively reflect the opinions of the authors. The authors are responsible for all content in their abstracts including accuracy of the facts, statements, citing resources, etc.

### Summary/Conclusion

In patients with PCNS-DLBCL treated in the BTKi/lenalidomide era, ASCT was associated with improved PFS after PSM, while OS showed a favorable trend. Post-induction consolidation strategies most strongly differentiated long-term outcomes, with maintenance therapy being particularly beneficial for patients not receiving ASCT. Outcomes also varied by regimen type (BTKi-based, lenalidomide-based, or neither) and ASCT status. These findings support a risk-adapted approach for selecting ASCT and/or maintenance therapy, highlighting the need for prospective validation.
Key survival curves are presented in the submitted figure.

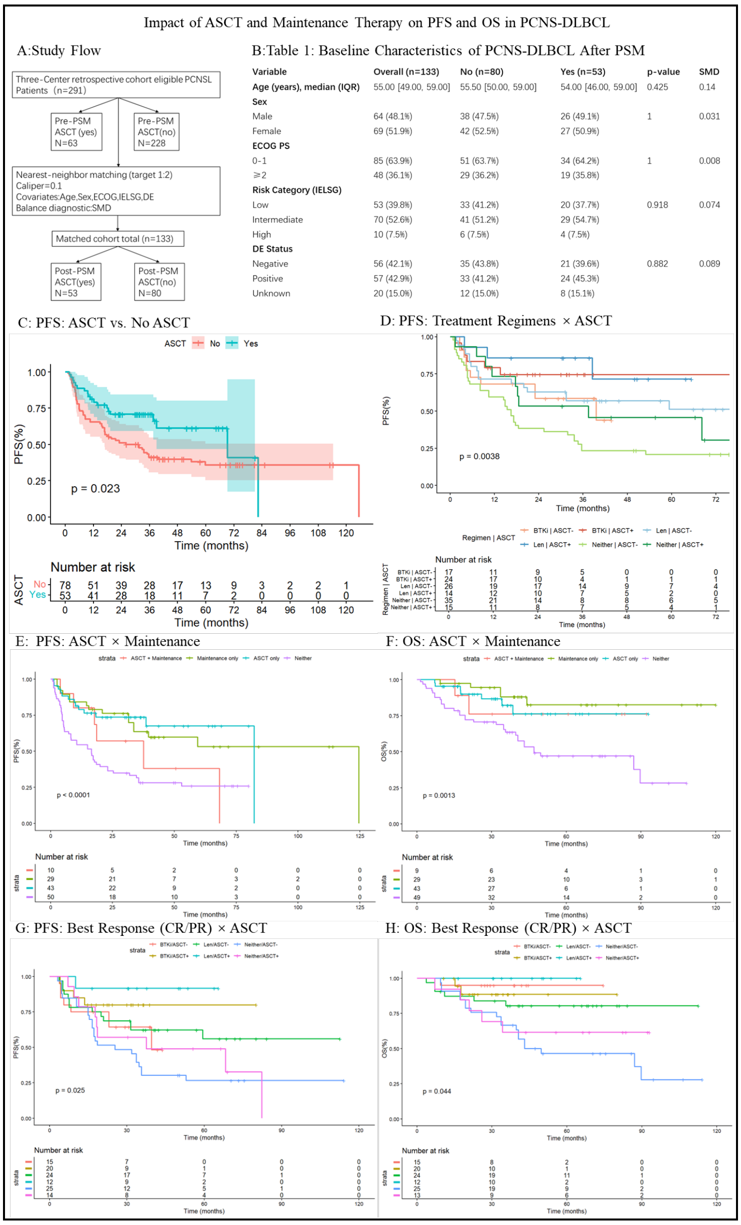

| Variable | Overall (n=133) | No (n=80) | Yes (n=53) | p-value | SMD |
|---|---|---|---|---|---|
| **Age (years), median (IQR)** | 55.00 [49.00, 59.00] | 55.50 [50.00, 59.00] | 54.00 [46.00, 59.00] | 0.425 | 0.14 |
| **Sex** | | | | | |
| Male | 64 (48.1%) | 38 (47.5%) | 26 (49.1%) | 1 | 0.031 |
| Female | 69 (51.9%) | 42 (52.5%) | 27 (50.9%) | | |
| **ECOG PS** | | | | | |
| 0-1 | 85 (63.9%) | 51 (63.7%) | 34 (64.2%) | 1 | 0.008 |
| ⩾2 | 48 (36.1%) | 29 (36.2%) | 19 (35.8%) | | |
| **Risk Category (IELSG)** | | | | | |
| Low | 53 (39.8%) | 33 (41.2%) | 20 (37.7%) | 0.918 | 0.074 |
| Intermediate | 70 (52.6%) | 41 (51.2%) | 29 (54.7%) | | |
| High | 10 (7.5%) | 6 (7.5%) | 4 (7.5%) | | |
| **DE Status** | | | | | |
| Negative | 56 (42.1%) | 35 (43.8%) | 21 (39.6%) | 0.882 | 0.089 |
| Positive | 57 (42.9%) | 33 (41.2%) | 24 (45.3%) | | |
| Unknown | 20 (15.0%) | 12 (15.0%) | 8 (15.1%) | | |

**Copyright Information:** (Online) ISSN: 2572-9241
©2026 The Author(s). HemaSphere is published by John Wiley & Sons Ltd on behalf of European Hematology Association. This is an open access Abstract Book distributed under the Attribution-NonCommercial-NoDerivs (CC BY-NC-ND), which allows third parties to download the articles and share them with others as long as they credit the author and the Abstract Book, but they cannot change the content in any way or use them commercially.
**Abstract Book Citations:** Authors, Title, HemaSphere, 2026;10:(S1):pages. The abstract book DOIs can be found on the "Author Information" page.
**Disclaimer:** Articles published in the journal HemaSphere exclusively reflect the opinions of the authors. The authors are responsible for all content in their abstracts including accuracy of the facts, statements, citing resources, etc.

### (PF993) R-GEMOX+NIVOLUMAB VS R-GEMOX AS 2ND-LINE THERAPY FOR LARGE B-CELL LYMPHOMA IN TRANSPLANT-INELIGIBLE PATIENTS: FINAL ANALYSIS OF THE NIVEAU TRIAL, A PHASE 3 STUDY OF THE AGMT, GLA, HOVON, LYSA AND PLRG

**Topic:** 19. Large B cell lymphomas - Clinical

Gerhard Held*[1], Bettina Altmann[2, 2], Joerg Hoffmann[3], Hervé Tilly[4], Andrea Kerkhoff[5], Thomas Weber[6], Olivier Casasnovas[7], Maria Ramos Lopes Gomes Da Silva[8], Thomas Gastinne[9], Sanne Tonino[10], Stéphane Vigouroux[11], Pierre Feugier[12], Violaine Safar[13], Stephanie Mayer[14], Ulrich Langenkamp[15], Franck Morschhauser[16], Christian W. Scholz[17], Laurence de Leval[18], Philippe Gaulard[19], Thierry Molina[20], Andreas Rosenwald[21], Richard Greil[22], Ulrich Jäger[23], Marc Andre[24], Marie José Kersten[10], Abraham Avigdor[25], Anna Dabrowska-Iwanicka[26], Lorenz Thurner[27], Markus Löffler[28], Marita Ziepert[2], Corinne Haioun[29], Viola Pöschel[30], Roch Houot[31]

*[1]Westpfalz-Klinikum Kaiserslautern, Internal Medicine I, Kaiserslautern, Germany; [2]Institute for Medical Informatics, Statistics, and Epidemiology, University of Leipzig, Leipzig, Germany; [3]University Hospital Marburg, Department of Internal Medicine, Hematology, Oncology and Immunology, Marburg, Germany; [4]Centre Henri-Becquerel, Service d'Hématologie, Rouen, France; [5]University Hospital Muenster, Department for Medicine A, Hematology, Oncology, Hemostaseology and Pneumology, Muenster, Germany; [6]University Hospital Halle (Saale), Martin-Luther-University Halle-Wittenberg, Department of Internal Medicine IV, Oncology and Hematology, Halle (Saale), Germany; [7]CHU François Mitterrand, Dijon, France;[8]Portuguese Institute of Oncology, Lisbon, Hematology, Lisbon, Portugal; [9]CHU Hôtel Dieu - Service d'Hématologie Clinique, Nantes, France; [10]AMC Academisch Medisch Centrum, Hematology, Amsterdam, The Netherlands; [11]Centre Hospitalier Départemental, Service d'Onco-Hématologie, La Roche sur Yon, France; [12]CHU Brabois, Service d'Hématologie, Nancy, France; [13]CHU Lyon Sud, Lyon-Pierre Benite, France; [14]University Hospital Regensburg, Department of Internal Medicine III, Hematology an Oncology, Regensburg, Germany; [15]Rostock University Hospital, Internal Medicine, Rostock, Germany; [16]CHRU-Hôpital Claude Huriez, Service des Maladies du Sang, Lille, France; [17]Vivantes Klinikum am Urban, Department of Internal Medicine, Hematology and Oncology, Berlin, Germany; [18]Institut universitaire de pathologie, Médecine de laboratoire et Pathologie, Lausanne, Switzerland; [19]Hopital Henri Mondor, Département de Pathologie & Inserm U955, Créteil, France; [20]Hôpitaux Universitaires Robert Debré et Necker Enfants Malades, Anatomie et Cytologie Pathologiques, Paris, France; [21]University of Wuerzburg, Institute of Pathology, Wuerzburg, Germany; [22]Paracelsus Medical University, Third Medical Department with Hematology, Medical Oncology, Rheumatology and Infectiology, Salzburg, Austria; [23]Medical University Of Vienna, Department of Hematology and Haemostaseology, Vienna, Austria; [24]Clinique de Mont-Godinne, CHU UCL Namur, Yvoir, Belgium;[25]The Chaim Sheba Medical Center, Division of Hematology and Bone-Marrow Transplantation, Ramat Gan, Israel; [26]Maria Sklodowska-curie National Research Institute Of Oncology, Department of lymphoid malignancies, Warsaw, Poland; [27]Saarland University Medical School, Department of Hematology, Oncology and Rheumatology, Homburg, Germany; [28]Institute for Medical Informatics, Statistics and Epidemiology, University of Leipzig, Leipzig, Germany; [29]Hôpital Henri Mondor, Service d'Hématologie, Créteil, France; [30]Saarland University Medical School, Department of Internal Medicine, Hematology, Oncology and Rheumatology, Homburg, Germany; [31]CHU Pontchaillou, Rennes, Hématologie clinique, Rennes, France;*

### Background

Patients (pts) with relapsed or refractory (*R*/*R*) large B-cell lymphoma (LBCL) after first-line treatment, who are ineligible for high-dose chemotherapy (HDCT) and hematopoietic stem cell transplantation (HSCT) have poor outcomes. Rituximab, gemcitabine and oxaliplatin (R-GemOx) is a common regimen used in this setting.

### Aims

The objective of the open-label, randomized, phase 3 NIVEAU study (NCT03366272) was to evaluate efficacy and safety of Nivolumab added to R-GemOx.

**Copyright Information:** (Online) ISSN: 2572-9241
©2026 The Author(s). HemaSphere is published by John Wiley & Sons Ltd on behalf of European Hematology Association. This is an open access Abstract Book distributed under the Attribution-NonCommercial-NoDerivs (CC BY-NC-ND), which allows third parties to download the articles and share them with others as long as they credit the author and the Abstract Book, but they cannot change the content in any way or use them commercially.
**Abstract Book Citations:** Authors, Title, HemaSphere, 2026;10:(S1):pages. The abstract book DOIs can be found on the "Author Information" page.
**Disclaimer:** Articles published in the journal HemaSphere exclusively reflect the opinions of the authors. The authors are responsible for all content in their abstracts including accuracy of the facts, statements, citing resources, etc.

### Methods

Adult pts with R/R LBCL after 1st-line therapy and not eligible for HDCT/HSCT based on of one of the following criteria: age ≥65 years; age ≥18 years and Hematopoietic Cell Transplantation-specific Comorbidity Index (HCT-CI) score ≥3; or age ≥18 years and prior ASCT (as 1st-line consolidation). Therapy consisted 8 cycles R-GemOx (arm A) or 8 cycles of R-GemOx + nivolumab followed by nivolumab as maintenance for a maximum of 1 year or until progression or unacceptable toxicity (arm B). Primary endpoint was progression-free survival (PFS) after 1 year. Major secondary endpoints were response rates, event-free-survival (EFS) (defined as progression, relapse, death or unplanned lymphoma treatment), overall survival (OS) and toxicity.
Recruitment was stopped in March 2023, because a planned interim analysis revealed futility to demonstrate superiority for the primary endpoint. However, a trend for a better OS was observed in arm B (*Blood* (2023)142(Suppl. 1):435). In total, 270 of 310 initially intended pts have been recruited. Here, we present the final analysis of the NIVEAU trial after a median follow-up of 42 months.

### Results

Three pts did not receive any treatment (1 withdrew informed consent, 1 death, 1 intercurrent disease) resulting in a full analysis set of n=134 (arm A) and n=133 (arm B). Median age was 76 years (range 44-89), 52% of pts were >75 years of age, 33% were primary progressive after first-line therapy, 62% had IPI 3-5. Overall response (OR) and complete response (CR) rates were similar with 37% and 22% vs. 34% and 22% (arm A vs. arm B, throughout the abstract), respectively. Rates of progressive disease (PD) were 53% vs. 45%. 1-year PFS was 27% (95%-CI: 19%-34%) vs. 23% (95%-CI: 16%-30%), (p=0.686). 1-year EFS was 22% (15-29) vs. 22% (14-29), (p=0.897). One-year OS was 51% (43-60) vs. 58% (49-66), (p=0.884). At 2 years, PFS, EFS and OS were 19% (12-26), 17% (10-23) and 39% (30-47) vs. 18% (11-24), 17% (10-23) and 42% (33-50). There was no difference in number of pts with grade 3-5 AEs except gastrointestinal 4% vs. 8% (p=0,179) and hepatobiliary disorders 19% vs. 23% (p=0,431), respectively. No difference in number of pts with immune-related grade 3-5 AEs was observed between both arms. Most common grade 3-5 AEs during nivolumab consolidation in arm B were hepatobiliary disorders 18%, lymphopenia 18%, leukopenia 9%, peripheral neuropathy 9%, neutropenia 9%. In total, 18 treatment-related deaths occurred, 6 (4%) vs. 12 (9%), including 15 caused by infections, 6 vs. 9, respectively.

### Summary/Conclusion

In pts with R/R LBCL, the addition of nivolumab to R-GemOx followed by nivolumab maintenance is feasible without relevant additive toxicity. The addition of Nivolumab did not improve PFS, EFS nor OS. The trend towards improved survival observed in the interim analysis was not confirmed in the final analysis. These results demonstrate that addition of checkpoint inhibition to immunochemotherapy does not improve the outcome in these patients.

**Copyright Information:** (Online) ISSN: 2572-9241
©2026 The Author(s). HemaSphere is published by John Wiley & Sons Ltd on behalf of European Hematology Association. This is an open access Abstract Book distributed under the Attribution-NonCommercial-NoDerivs (CC BY-NC-ND), which allows third parties to download the articles and share them with others as long as they credit the author and the Abstract Book, but they cannot change the content in any way or use them commercially.
**Abstract Book Citations:** Authors, Title, HemaSphere, 2026;10:(S1):pages. The abstract book DOIs can be found on the “Author Information” page.
**Disclaimer:** Articles published in the journal HemaSphere exclusively reflect the opinions of the authors. The authors are responsible for all content in their abstracts including accuracy of the facts, statements, citing resources, etc.

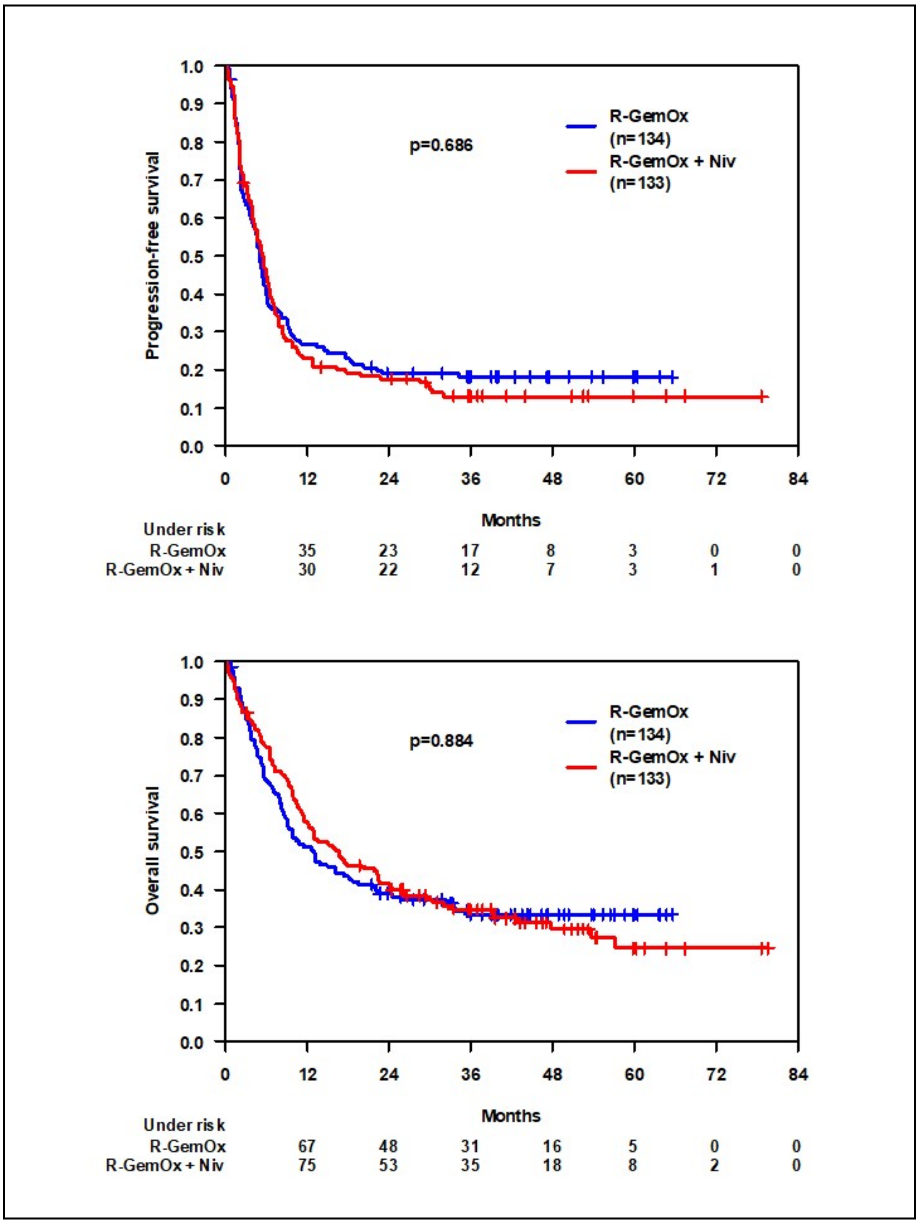

**Copyright Information:** (Online) ISSN: 2572-9241
©2026 The Author(s). HemaSphere is published by John Wiley & Sons Ltd on behalf of European Hematology Association. This is an open access Abstract Book distributed under the Attribution-NonCommercial-NoDerivs (CC BY-NC-ND), which allows third parties to download the articles and share them with others as long as they credit the author and the Abstract Book, but they cannot change the content in any way or use them commercially.
**Abstract Book Citations:** Authors, Title, HemaSphere, 2026;10:(S1):pages. The abstract book DOIs can be found on the "Author Information" page.
**Disclaimer:** Articles published in the journal HemaSphere exclusively reflect the opinions of the authors. The authors are responsible for all content in their abstracts including accuracy of the facts, statements, citing resources, etc.

### (PF994) POLATUZUMAB-BR VS TAFASITAMAB-LENALIDOMIDE IN RELAPSED/REFRACTORY DLBCL: A PROPENSITY SCORE–MATCHED ANALYSIS FROM THE POLISH LYMPHOMA RESEARCH GROUP (PLRG)

**Topic:** 19. Large B cell lymphomas - Clinical

Anna Czyż[1], Magdalena Olszewska-Szopa*[2], Magdalena Malicka[3], Anastazja Szlauer-Stefańska[4], Joanna Sciesinska[5], Joanna Drozd-Sokolowska[6], Anna Kopinska[7], Ewa Paszkiewicz-Kozik[8], Edyta Subocz[9], Mirosław Markiewicz[10], Ewa Wasik-Szczepanek[11], Agnieszka Giza[12], Agnieszka Kołkowska-Leśniak[13], Waldemar Sawicki[14], Artur Matyszewski[15], Monika Długosz-Danecka[16], Grzegorz Helbig[7], Grzegorz Basak[17], Tomasz Wróbel[18], Ewa Lech-Maranda[19]

*[1]Wroclaw Medical University, Wroclaw, Poland, Department and Clinic of Hematology, Cellular Therapies and Internal Medicine, Wrocław, Poland; [2]Wroclaw Medical University, Wroclaw, Poland, Department and Clinic of Hematology, Cellular Therapies and Internal Medicine,, Wrocław, Poland; [3]Department of Hematology, Medical University of Lodz, Lodz, Poland, Łódź, Poland; [4]Bone Marrow Transplantation and Oncohematology Department, Maria Sklodowska-Curie National Research Institute of Oncology (branch in Gliwice), Gliwice, Poland; [5]Department of Hematology and Transplantology, Medical University of Gdansk, Gdańsk, Poland; [6]Department of Hematology, Oncology and Internal Medicine, Medical University of Warsaw, Warsaw, Poland; [7]Department of Hematology and Bone Marrow Transplantation, Faculty of Medicine in Katowice, Medical University of Silesia, Katowice, Poland; [8]Maria Sklodowska-Curie National Research Institute of Oncology, Warsaw, Poland;[9]Maria Sklodowska-Curie National Research Institute of Oncology, Warsaw, Poland 8. Department of Hematology, Warmian-Masurian Cancer Center of The Ministry Of The Interior And Administration's Hospital, Olsztyn, Poland; [10]Department of Hematology, Institute of Medical Sciences, College of Medical Sciences, University of Rzeszow, Rzeszów, Poland; [11]Department of Haematooncology and Bone Marrow Transplantation, Medical University in Lublin, Lublin, Poland; [12]Department of Hematology, Jagiellonian University Medical College, Kraków, Kraków, Poland; [13]Institute of Hematology and Transfusion Medicine, Warsaw, Poland, Warsaw, Poland; [14]Department of Internal Medicine and Hematology, Military Institute of Medicine-National Research Institute, Warsaw, Warsaw, Poland; [15]Department of Internal Medicine, Cardiology and Hypertensionr, L. Rydygier Province Hospital, Suwałki, Suwałki, Poland; [16]. Maria Sklodowska-Curie National Research Institute of Oncology, Krakow, Kraków, Poland;[17]Department of Hematology and Transplantology, Medical University of Gdansk, Poland 5. Department of Hematology, Oncology and Internal Medicine, Medical University of Warsaw, Warsaw, Poland; [18]Department and Clinic of Hematology, Cellular Therapies and Internal Medicine, Wroclaw Medical University, Wrocław, Poland; [19]Institute of Hematology and Transfusion Medicine, Warsaw, Warsaw, Poland;*

### Background

This multicenter study conducted by the Polish Lymphoma Research Group (PLRG) compared real-world outcomes of two salvage regimens—polatuzumab vedotin with bendamustine and rituximab (Pola-BR) and tafasitamab plus lenalidomide (Tafa-Len)—in patients with relapsed/refractory diffuse large B-cell lymphoma (R/R DLBCL). Direct comparative data remain limited.

### Aims

To verify and compare real-world outcomes for Pola-BR and Tafa-Len regimens in R/R DLBCL patients in a routine clinical setting.

**Copyright Information:** (Online) ISSN: 2572-9241
©2026 The Author(s). HemaSphere is published by John Wiley & Sons Ltd on behalf of European Hematology Association. This is an open access Abstract Book distributed under the Attribution-NonCommercial-NoDerivs (CC BY-NC-ND), which allows third parties to download the articles and share them with others as long as they credit the author and the Abstract Book, but they cannot change the content in any way or use them commercially.
**Abstract Book Citations:** Authors, Title, HemaSphere, 2026;10:(S1):pages. The abstract book DOIs can be found on the "Author Information" page.
**Disclaimer:** Articles published in the journal HemaSphere exclusively reflect the opinions of the authors. The authors are responsible for all content in their abstracts including accuracy of the facts, statements, citing resources, etc.

### Methods

In this retrospective analysis, 206 patients with R/R DLBCL treated with Pola-BR (n=152) or Tafa-Len (n=54) in the second or subsequent lines were identified. Propensity score matching (PSM) was performed based on 14 baseline clinical and disease-related variables, including age, sex, ECOG performance status, DLBCL cell-of-origin subtype, clinical stage, IPI at diagnosis and at salvage, type of first-line failure (primary refractory disease vs early relapse vs late relapse), number of prior treatment lines, refractoriness to the last therapy, disease status, bulky disease, and B symptoms at salvage. After PSM, 36 matched patients in each group were included in the comparative analysis.

### Results

The median age was 70 years (IQR 65–73) in the Pola-BR group and 71 years (IQR 65–77) in the Tafa-Len group (p=0.11). Patients had received a median of one prior line of therapy in both groups (range 1–3 vs 1–3; p=1.0). Primary refractory disease was present in 81% of patients in both cohorts; 50% in each group received Pola-BR or Tafa-Len as second-line treatment due to primary refractory disease. Refractoriness to the last therapy was observed in 64% of patients in both cohorts (p=0.80). High-risk salvage IPI was present in 64% of patients treated with Pola-BR and 70% of those treated with Tafa-Len (p=0.87), while bulky disease at salvage was reported in 28% and 19%, respectively (p=0.41).
Overall response rates were significantly higher in the Pola-BR cohort: CR, PR, and <PR were observed in 39%, 28%, and 33% of patients treated with Pola-BR, compared with 14%, 14%, and 72% in the Tafa-Len group (p=0.005). After a median follow-up of 15 months (range, 2-35), the median progression-free survival (PFS) was 7.2 months (95% CI, 4.7–NE) with Pola-BR versus 3.6 months (95% CI, 2.8–NE) with Tafa-Len (p=0.001). PFS curves are presented in *Figure 1*. The one-year overall survival rates (OS) were 50% and 44%, respectively (p=0.20).

### Summary/Conclusion

In this PSM analysis of real-world PLRG data, Pola-BR achieved higher response rates and longer PFS than Tafa-Len in high-risk R/R DLBCL, while OS was comparable. Tafa-Len was developed for transplant-ineligible patients, and primary refractory disease was excluded from L-MIND. However, our cohort was largely composed of primary refractory cases, which may not represent the optimal setting for this regimen. At that time, novel T-cell–engaging (TCE) therapies were not available in the second line. With expanding access to CAR T-cell therapy and bispecific antibodies, TCE approaches should be incorporated early for primary refractory disease and early relapse, consistent with benefits shown in second-line CAR-T trials and the STARGLO study. The optimal positioning of Pola-BR and Tafa-Len in R/R DLBCL requires further clarification given their meaningful efficacy and manageable safety profiles.

**Copyright Information:** (Online) ISSN: 2572-9241
©2026 The Author(s). HemaSphere is published by John Wiley & Sons Ltd on behalf of European Hematology Association. This is an open access Abstract Book distributed under the Attribution-NonCommercial-NoDerivs (CC BY-NC-ND), which allows third parties to download the articles and share them with others as long as they credit the author and the Abstract Book, but they cannot change the content in any way or use them commercially.
**Abstract Book Citations:** Authors, Title, HemaSphere, 2026;10:(S1):pages. The abstract book DOIs can be found on the "Author Information" page.
**Disclaimer:** Articles published in the journal HemaSphere exclusively reflect the opinions of the authors. The authors are responsible for all content in their abstracts including accuracy of the facts, statements, citing resources, etc.

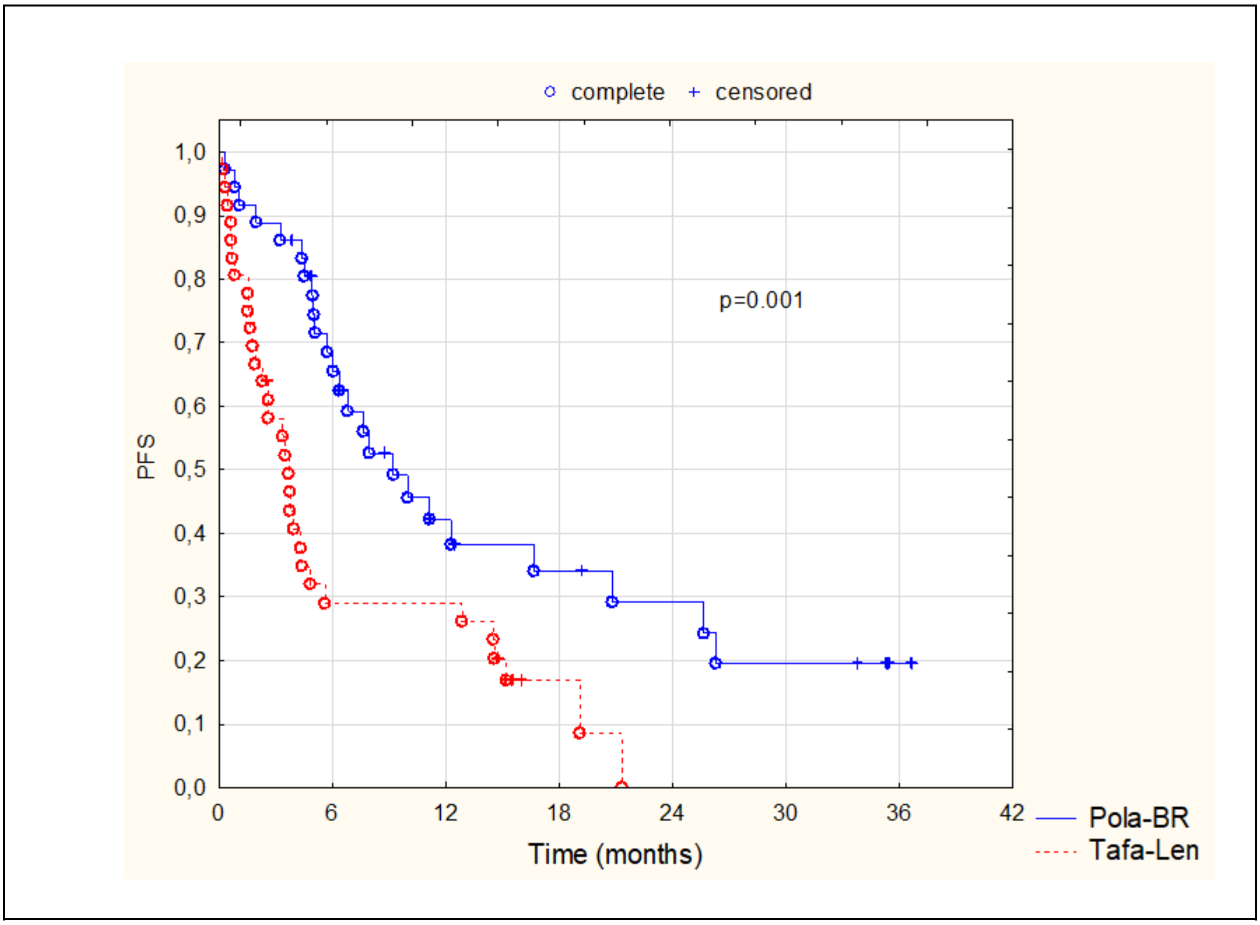

**Copyright Information:** (Online) ISSN: 2572-9241
©2026 The Author(s). HemaSphere is published by John Wiley & Sons Ltd on behalf of European Hematology Association. This is an open access Abstract Book distributed under the Attribution-NonCommercial-NoDerivs (CC BY-NC-ND), which allows third parties to download the articles and share them with others as long as they credit the author and the Abstract Book, but they cannot change the content in any way or use them commercially.
**Abstract Book Citations:** Authors, Title, HemaSphere, 2026;10:(S1):pages. The abstract book DOIs can be found on the "Author Information" page.
**Disclaimer:** Articles published in the journal HemaSphere exclusively reflect the opinions of the authors. The authors are responsible for all content in their abstracts including accuracy of the facts, statements, citing resources, etc.

### (PF995) COMPARATIVE REAL-WORLD OVERALL SURVIVAL IN DIFFUSE LARGE B-CELL LYMPHOMA: CAR T-CELL THERAPIES VERSUS BISPECIFIC ANTIBODIES

**Topic:** 19. Large B cell lymphomas - Clinical

Vladimir Otasevic*[1], Darko Antic[2, 3], Orlando Esparza[1], Kausik Maiti[1], Nancy Lunney[1], Darko Dabic[1], Chris Learn[1], Heidi Cho[1]

*[1]Parexel International, Raleigh, NC, United States of America; [2]Clinic for Hematology, University Clinical Center of Serbia, Belgrade, Serbia; [3]Faculty of Medicine, University of Belgrade, Belgrade, Serbia;*

### Background

Treatment options for diffuse large B-cell lymphoma (DLBCL) have expanded, especially in relapsed/refractory (R/R) cases, with CAR T-cell therapies and CD20×CD3 bispecific antibodies (BsAbs) being notable advances. While trials suggest CAR T-cells may be more effective and improve survival, these findings come from controlled populations that may not mirror clinical practice.

### Aims

This study aims to evaluate overall survival (OS) in DLBCL patients treated with CAR T-cells or BsAbs using real-world data (RWD).

### Methods

A retrospective analysis of RWD was conducted using TriNetX Dataworks Global database on February 21, 2026. Two cohorts were analyzed: (1) DLBCL patients ≤60 years old treated solely with BsAbs (epcoritamab/glofitamab), and (2) DLBCL patients ≤60 years old treated solely with CAR T-therapies (tisacel/axicel/lisocel). Patients treated with both therapeutic options were excluded from analysis to control for “crossover” between cohorts and impact on OS. Standardized mean differences were used to ascertain the distribution balance among baseline variables. Kaplan-Meier analysis, log-rank test, hazard ratios (HR), risk ratios (RR), and 95% confidence intervals (CI) were used to assess the primary outcomes, which were risk of death and OS. Propensity score matching (PSM) was utilized to account for the effects of specific known confounders.

### Results

A total of 561 patients with DLBCL were enrolled, including 123 treated with BsAbs and 438 treated with CAR T-cell therapies. The mean age at the index event (initiation of BsAbs for Cohort 1; initiation of CAR T-cell therapy for Cohort 2) was 47.2±9.2 for Cohort 1 and 44.5±11.3 for Cohort 2 (p=0.015). There were 63% and 66% male patients, respectively (p=0.664). Over a 2-year follow-up period from the index event, Cohort 1 had a significantly higher risk of death compared to Cohort 2 (RR 1.468, 95%CI 1.116-1.932). Cohort 1 had a significantly shorter two-year OS probability and a higher risk of death at two years (47.5% vs. 67.3% survival probability at 2 years from the index event, log-rank test, $\chi 2$=20.034, p<0.001; HR 2.153, 95%CI 1.527-3.037). After PSM for covariates (age at index event, sex, ECOG performance status, arterial hypertension, atherosclerotic heart disease, chronic obstructive pulmonary disease, asthma), Cohort 1 still had a significantly higher risk of death compared to Cohort 2 (RR 1.731, 95%CI 1.150-2.606). In addition, Cohort 1 still had a significantly shorter two-year OS probability and a higher risk of death at two years of follow-up compared to Cohort 2 (47% vs. 72.5% survival probability at 2 years from the index event, log-rank test, $\chi 2$=14.092, p<0.001; HR 2.482, 95%CI 1.520-4.053). A key limitation of this study is the aggregate nature of the data on which the analysis was based.

**Copyright Information:** (Online) ISSN: 2572-9241
©2026 The Author(s). HemaSphere is published by John Wiley & Sons Ltd on behalf of European Hematology Association. This is an open access Abstract Book distributed under the Attribution-NonCommercial-NoDerivs (CC BY-NC-ND), which allows third parties to download the articles and share them with others as long as they credit the author and the Abstract Book, but they cannot change the content in any way or use them commercially.
**Abstract Book Citations:** Authors, Title, HemaSphere, 2026;10:(S1):pages. The abstract book DOIs can be found on the “Author Information” page.
**Disclaimer:** Articles published in the journal HemaSphere exclusively reflect the opinions of the authors. The authors are responsible for all content in their abstracts including accuracy of the facts, statements, citing resources, etc.

### Summary/Conclusion

In this real-world analysis of DLBCL patients ≤60 years old, treatment with BsAbs was linked to worse OS and higher death risk compared to CAR T-cell therapies. These findings remained consistent before and after PSM, highlighting the robustness of the results. While CAR T-cell therapies showed better survival over 2 years, the data's aggregate nature limits detailed clinical interpretation, including disease and treatment factors affecting outcomes. Still, the results support using CAR T-cell therapies over BsAbs for eligible younger patients with R/R DLBCL and emphasize the need for more research with patient-level data to improve comparative assessments.

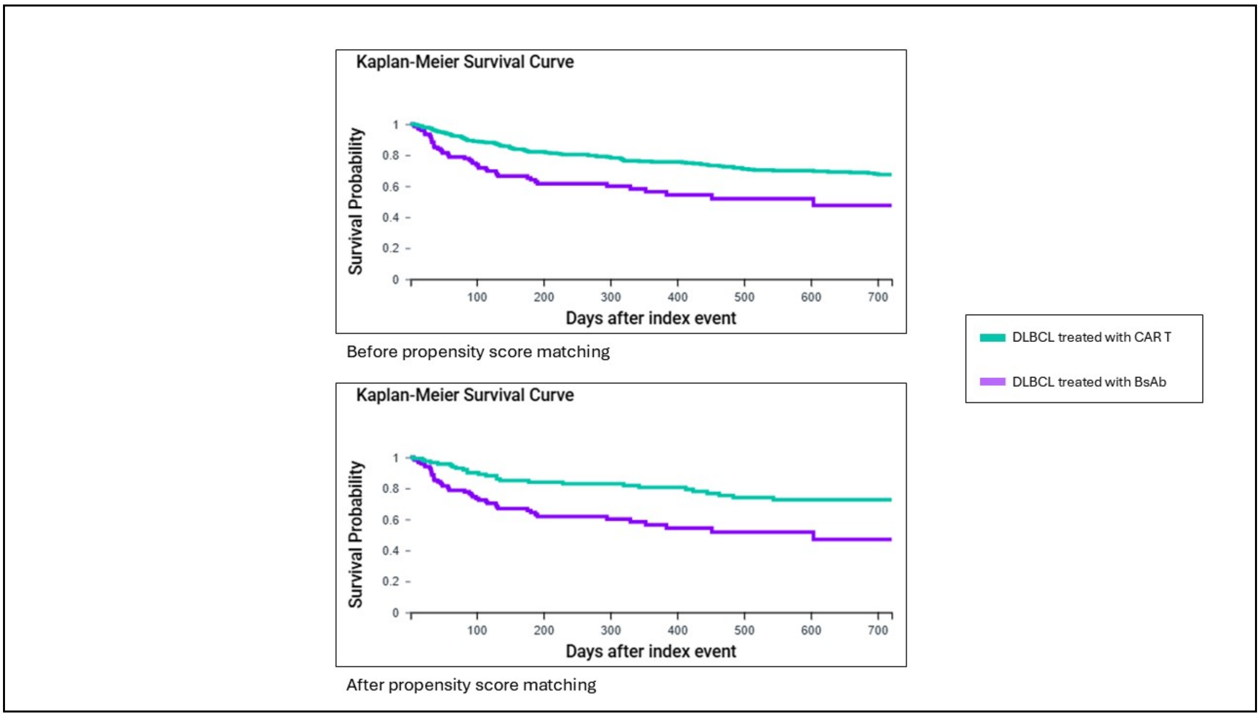

**Copyright Information:** (Online) ISSN: 2572-9241
©2026 The Author(s). HemaSphere is published by John Wiley & Sons Ltd on behalf of European Hematology Association. This is an open access Abstract Book distributed under the Attribution-NonCommercial-NoDerivs (CC BY-NC-ND), which allows third parties to download the articles and share them with others as long as they credit the author and the Abstract Book, but they cannot change the content in any way or use them commercially.
**Abstract Book Citations:** Authors, Title, HemaSphere, 2026;10:(S1):pages. The abstract book DOIs can be found on the "Author Information" page.
**Disclaimer:** Articles published in the journal HemaSphere exclusively reflect the opinions of the authors. The authors are responsible for all content in their abstracts including accuracy of the facts, statements, citing resources, etc.

### (PF996) FEASIBILITY AND SAFETY OF RAPID GLOFITAMAB RAMP-UP FOR THE TREATMENT OF RELAPSED AND REFRACTORY LARGE B-CELL LYMPHOMA

**Topic:** 19. Large B cell lymphomas - Clinical

Celine Liem*[1], Rebecca Wurm-Kuczera *[1], David Böckle[1], Marie Anne-Catherine Neumann[2], Marcel Teichert[3], Urban Novak[4], Merle Klein Helmkamp[3], Evgenii Shumilov[5], Nina Malburg[5], Tim Versteegen[6], Fabian Görke[7], Giulia Magno[8], Susanne Ghandili[9], Joseph Kauer[10], Konstantinos Christofyllakis[11], Thomas Melchardt[12], Peter Dreger[10], Francis Ayuk[9], Marion Subklewe[8], Vladan Vucinic[7], Christiane Pott[6], Hans C. Reinhardt[3], Georg Lenz[5], Bastian von Tresckow[3], Thomas Pabst[4], Peter Borchmann[2], Antonia Busse[1], Ulrich Keller[1], Björn Chapuy[1]

*[1]Charité Universtitätsmedizin Berlin, Berlin, Germany; [2]University Hospital of Cologne, Cologne, Germany; [3]University Hospital Essen, Essen, Germany; [4]University Hospital Bern, Bern, Switzerland; [5]University Hospital Münster, Münster, Germany; [6]University Hospital Schleswig-Holstein, Kiel, Germany; [7]University Hospital Leipzig, Leipzig, Germany; [8]LMU Munich, Munich, Germany; [9]University Medical Center Hamburg-Eppendorf, Hamburg, Germany; [10]Heidelberg University Hospital, Heidelberg, Germany; [11]Saarland University Medical Center, Homburg, Germany; [12]University Clinic Salzburg, Salzburg, Austria;*

### Background

Large B-cell lymphomas (LBCL) are biologically and clinically heterogeneous. Immunochemotherapy cures 75% of patients, but high proliferation rates and clinical need for fast-acting treatments often characterize chemotherapy-refractory or relapsed disease (r/rLBCL). Prognosis in r/rLBCL is substantially diminished despite the use of CD19-directed chimeric antigen receptor T-cells (CAR-T), and approximately 50% of patients relapse after or are refractory to CAR-T, highlighting the medical need for further treatment optimization following CAR-T.
Glofitamab is a CD20xCD3 bispecific, T-cell-engaging monoclonal antibody that is approved for the treatment of r/rLBCL. Standard dosing protocol for glofitamab requires an obinutuzumab-pretreatment to mitigate CRS and a step-up dosing of glofitamab (2.5mg, 10mg) before reaching the full therapeutic dose three-weeks after treatment start.The delayed disease control during the ramp-up phase of glofitamab is contrasted by high proliferation and often high tumor burden at diagnosis of r/rLBCL, limiting the use of single-agent glofitamab and resulting in the development of the BiCAR trial and other off-label accelerated step-up protocols in clinical practice.

### Aims

To assess whether the full dose of glofitamab can be administered more rapidly without compromising patient safety and thereby accelerating disease control in patients with r/rLBCL.

### Methods

In this retrospective multicenter study, we analyzed 83 patients from 11 centers across Germany, Austria and Switzerland who had received glofitamab between December 2020 and November 2025 in an accelerated step-up dosing schedule. These patients (Accelerated Glo group) were further divided into the BiCAR-like group if glofitamab was administered within 1-2 weeks, and the rapid glofitamab group in which glofitamab was administered within less than 1 week. Efficacy including response rates and survival as well as adverse events were analyzed.

**Copyright Information:** (Online) ISSN: 2572-9241
©2026 The Author(s). HemaSphere is published by John Wiley & Sons Ltd on behalf of European Hematology Association. This is an open access Abstract Book distributed under the Attribution-NonCommercial-NoDerivs (CC BY-NC-ND), which allows third parties to download the articles and share them with others as long as they credit the author and the Abstract Book, but they cannot change the content in any way or use them commercially.
**Abstract Book Citations:** Authors, Title, HemaSphere, 2026;10:(S1):pages. The abstract book DOIs can be found on the "Author Information" page.
**Disclaimer:** Articles published in the journal HemaSphere exclusively reflect the opinions of the authors. The authors are responsible for all content in their abstracts including accuracy of the facts, statements, citing resources, etc.

**Results**

We enrolled 83 patients with a median age of 63 years (range: 28-81). Most patients had advanced-stage disease (AnnArbor III+IV: 74%) and a intermediate or high clinical risk (IPI ≥ 3: 44%). About half (48%[40/83]) of the patients had CAR-T-cell therapy prior to glofitamab treatment. 64 patients had received obinutuzumab and glofitamab within 8-14 days (BiCAR-like group), 19 patients had received obinutuzumab and glofitamab within 4-7 days (Rapid Glo group).
The most common adverse events were CRS (47%[39/83]) and infections (18%[15/83]). CRS were mostly grade I (36%[30/39]) and the CRS-rate was lower than in the pivotal trial (63%) and similar to standard glofitamab real world analyses (27-40%), but higher than in the BiCAR-trial (13%). The rate of ICANS was persistently low across studies. Notably, CRS and ICANS of grade 3 or higher did not occur at all in the patients with an accelerated dosing schedule.
The overall response rate for patients who received accelerated glofitamab was 53% (44/83, CR/CMR: 12%; PR: 41%) and similar to prior studies. The median PFS was 6.3 months and the median OS was 21.5 months. Both PFS and OS were significantly higher in the accelerated group compared to the PFS and OS of prior studies with standard dosing (RWA: PFS 3.6 months, OS 5.7 months; pivotal trial: PFS 4.9 months, OS 11.5 months).

**Summary/Conclusion**

Accelerated administration of glofitamab for patients with r/rLBCL is feasible without compromising patients' safety or response, supporting its use in clinical practice for patients with r/rLBCL.

**Copyright Information:** (Online) ISSN: 2572-9241
©2026 The Author(s). HemaSphere is published by John Wiley & Sons Ltd on behalf of European Hematology Association. This is an open access Abstract Book distributed under the Attribution-NonCommercial-NoDerivs (CC BY-NC-ND), which allows third parties to download the articles and share them with others as long as they credit the author and the Abstract Book, but they cannot change the content in any way or use them commercially.
**Abstract Book Citations:** Authors, Title, HemaSphere, 2026;10:(S1):pages. The abstract book DOIs can be found on the "Author Information" page.
**Disclaimer:** Articles published in the journal HemaSphere exclusively reflect the opinions of the authors. The authors are responsible for all content in their abstracts including accuracy of the facts, statements, citing resources, etc.

### (PF997) OUTCOME OF NEWLY DIAGNOSED AGGRESSIVE B-CELL LYMPHOMA WITH CONCURRENT INDOLENT LYMPHOMA: A POOLED POST-HOC ANALYSIS OF FOUR PROSPECTIVE PHASE III DSHNHL/GLA TRIALS

**Topic:** 19. Large B cell lymphomas - Clinical

Dominic Kaddu-Mulindwa[1], German Ott[2], Konstantinos Christofyllakis*[1], Viola Poeschel[3], Moritz Bewarder[4], Annette Staiger[2], Gerhard Held[5], Lorenz Thurner[1], Philipp Staber[1], Norbert Schmitz[6], Fabian Frontzek[7], Lorenz Trümper[8], Markus Löffler[9], Bettina Altmann[9], Marita Ziepert[9]

*[1]University of Saarland, Department of Hematology, Oncology, Clinical Immunology, Rheumatology, Medical School, Homburg, Germany; [2]Robert Bosch Krankenhaus, Stuttgart, Germany; [3]Universität Basel, Basel, Switzerland; [4]Centrum für Hämatologie und Onkologie Bethanien, Frankfurt, Germany; [5]Westpfalzklinikum, Klinik für Innere Medizin 1, Kaiserslautern, Germany; [6]Universitätsklinikum Münster, Medizinische Klinik A, Münster, Germany; [7]BC Cancer Research Centre, Vancouver, Canada; [8]Universitätsmedizin Göttingen, Göttingen, Germany; [9]Universität Leipzig, INSTITUT FÜR MEDIZINISCHE INFORMATIK, STATISTIK UND EPIDEMIOLOGIE, Leipzig, Germany;*

### Background

At diagnosis, a subset of patients with aggressive B-cell lymphoma presents with a concurrent indolent non-Hodgkin lymphoma (iNHL), most commonly follicular lymphoma (FL). As these patients are frequently excluded from prospective trials, outcome data in the rituximab era remain limited.

### Aims

We analysed prevalence, clinico-pathological characteristics, and outcome of patients with aggressive B-cell lymphomas and concurrent iNHL treated within four clinical trials of the DSHNHL/GLA (German High-grade Non-Hodgkin's Lymphoma Study Group/German Lymphoma Alliance).

### Methods

We did a pooled post-hoc analysis of four prospective randomized phase III DSHNHL/GLA trials (RICOVER-60, UNFOLDER, FLYER, MEGA-CHOEP) enrolling patients with newly diagnosed aggressive B-cell lymphoma and treated with R-CHOP-based immunochemotherapy. All cases with concurrent iNHL at diagnosis were identified and confirmed by central reference pathology review according to WHO criteria. Outcomes were compared with patients with aggressive B-cell lymphoma alone. Progression-free survival (PFS) and overall survival (OS) were estimated using Kaplan–Meier methods. Multivariable Cox regression models were adjusted for International Prognostic Index (IPI) factors.

**Copyright Information:** (Online) ISSN: 2572-9241
©2026 The Author(s). HemaSphere is published by John Wiley & Sons Ltd on behalf of European Hematology Association. This is an open access Abstract Book distributed under the Attribution-NonCommercial-NoDerivs (CC BY-NC-ND), which allows third parties to download the articles and share them with others as long as they credit the author and the Abstract Book, but they cannot change the content in any way or use them commercially.
**Abstract Book Citations:** Authors, Title, HemaSphere, 2026;10:(S1):pages. The abstract book DOIs can be found on the "Author Information" page.
**Disclaimer:** Articles published in the journal HemaSphere exclusively reflect the opinions of the authors. The authors are responsible for all content in their abstracts including accuracy of the facts, statements, citing resources, etc.

**Results**

Among 2,155 patients, 898 (42%) were evaluable for further analysis. A concurrent iNHL component was identified in 117 patients (13%) by reference pathology review. Major clinical characteristics were largely comparable between patients with (n=117) and without (n=781) concurrent iNHL (46% female vs. 41%, median age 54 years vs. 56), with a trend towards lower IPI (p= 0.071) in the concurrent group. With a median follow-up of 78 months for PFS and 79 months for OS, patients with concurrent iNHL showed no significant difference in PFS ( 5-years PFS aggressive B-NHL 77% (95% CI: 74-80) vs concurrent iNHL 79% (95% CI: 71-87), p= 0.223 ) nor OS (5-years OS aggressive B-NHL 82% (95% CI: 79-84) vs concurrent iNHL 87% (95% CI: 81-94), p= 0.088) compared with patients with aggressive B-cell lymphoma alone across the pooled cohort and within individual trials. Also, after adjustment for IPI factors the results remained unchanged (PFS: HR=1.0 (95% CI: 0.6-1.6), p=0.978; OS: HR=0.9 (95% CI: 0.5-1.5), p=0.689.

**Summary/Conclusion**

In this large, pooled analysis of prospective phase III trials with centralized pathology review, patients with newly diagnosed aggressive B-cell lymphoma and concurrent indolent B-cell lymphoma had outcomes comparable to those with aggressive B-cell lymphoma alone. With the largest analysis available so far, our findings support using R-CHOP-based treatment strategies for aggressive B-cell lymphoma for patients with concurrent indolent B-cell lymphoma and their inclusion in clinical trials.

**Copyright Information:** (Online) ISSN: 2572-9241
©2026 The Author(s). HemaSphere is published by John Wiley & Sons Ltd on behalf of European Hematology Association. This is an open access Abstract Book distributed under the Attribution-NonCommercial-NoDerivs (CC BY-NC-ND), which allows third parties to download the articles and share them with others as long as they credit the author and the Abstract Book, but they cannot change the content in any way or use them commercially.
**Abstract Book Citations:** Authors, Title, HemaSphere, 2026;10:(S1):pages. The abstract book DOIs can be found on the "Author Information" page.
**Disclaimer:** Articles published in the journal HemaSphere exclusively reflect the opinions of the authors. The authors are responsible for all content in their abstracts including accuracy of the facts, statements, citing resources, etc.

### (PF998) DEVELOPMENT AND EXTERNAL VALIDATION OF A SIMPLE PROGNOSTIC INDEX FOR PRIMARY MEDIASTINAL B-CELL LYMPHOMA: PMBCL-PI

**Topic:** 19. Large B cell lymphomas - Clinical

Oguzhan Koca*[1, 2], Selin Küçükyurt[3], Serkan Akın[4], Isik Atagunduz[5], Figen Atalay[6], Elif Birtaş Ateşoğlu[6], Mesut Ayer[7], İbrahim Barışta[4], Mehmet Baysal[8], Peter Brown[9, 10], Rafiye Ciftciler[11], Ahmet Muzaffer Demir[12], Esra Terzi Demirsoy[13], İmdat Dilek[14], Ali Doğan[15], Tarec El-Galaly[16, 17], Deniz Goren[18], Ahmet Kursad Gunes[19], Serkan Güven[20], Utku İltar[21], Yildiz Ipek[22], Paw Jensen[23, 24], Nesibe Taser Kanat[25], Fatma Keklik Karadag[26], Özge Soyer[14], Thomas Stauffer Larsen[27, 28], Özgür Mehtap[13], Meral Ulukoylu Menguc[5], Mads Munk Nielsen[29], Anne Krogh Nøhr[29], Yasemin Özgür[7], Hacer Berna Afacan Öztürk[19], Işıl Erdoğan Özünal[30], Ibrahim Ethem Pinar[31], Ozan Salim[21], Güray Saydam[32], Cem Selim[11], Marianne Severinsen[24, 33], Nur Soyer[32], Fahri Sahin[32], Osman Sahin[34], Pınar Tığlıoğlu[30], Mehmet Can Ugur[35], Ayşe Uysal[36], Ali Ünal[25], Mahmut Yeral[34], Abdulkerim Yildiz[37], Ahmet Yigitbasi[12], Meltem Kurt Yuksel[38], Merve Yüksel[38], Ahmet Emre Eskazan[3, 39], Rasmus Rask Kragh Jørgensen[23, 29, 39]

*[1]Department of Internal Medicine, Cerrahpasa Faculty of Medicine, Istanbul University-Cerrahpasa, Istanbul, Türkiye; [2]Department of Immunology and Inflammation, Faculty of Medicine, Imperial College London, London, United Kingdom; [3]Department of Internal Medicine, Division of Hematology, Cerrahpasa Faculty of Medicine, Istanbul University-Cerrahpasa, Istanbul, Türkiye; [4]Department of Internal Medicine, Division of Medical Oncology, Hacettepe University, Ankara, Türkiye; [5]Department of Hematology, Marmara University, Istanbul, Türkiye;[6]Department of Internal Medicine, Division of Hematology, Yeditepe University, Istanbul, Türkiye; [7]Department of Hematology, Başakşehir Çam and Sakura City Hospital, Istanbul, Türkiye; [8]Division of Hematology, Department of Internal Medicine, Tekirdağ Namık Kemal University, Tekirdağ, Türkiye;[9]Department of Clinical Medicine, University of Copenhagen, Copenhagen, Denmark; [10]Department of Hematology, Copenhagen University Hospital, Rigshospitalet, Copenhagen, Denmark; [11]Department of Internal Medicine, Division of Hematology, Selçuk University, Konya, Türkiye; [12]Department of Internal Medicine, Division of Hematology, Trakya University, Edirne, Türkiye;[13]Department of Internal Medicine, Division of Hematology, Kocaeli University, Kocaeli, Türkiye; [14]Department of Hematology, Ankara Bilkent City Hospital, Ankara, Türkiye; [15]Department of Internal Medicine, Division of Hematology, Van Yüzüncü Yıl University, Van, Türkiye; [16]Department of Clinical Epidemiology, Molecular Medicine and Hematology, Aarhus University Hospital, Aarhus, Denmark; [17]Department of Clinical Medicine, Aarhus University, Aarhus, Denmark; [18]Department of Internal Medicine, Division of Hematology, Haliç University, Istanbul, Türkiye; [19]Department of Hematology, Ankara Etlik City Hospital, Ankara, Türkiye; [20]Department of Hematology, Çanakkale Mehmet Akif Ersoy State Hospital, Çanakkale, Türkiye;[21]Department of Internal Medicine, Division of Hematology, Akdeniz University, Antalya, Türkiye; [22]Department of Hematology, Kartal Dr. Lütfi Kırdar City Hospital, Istanbul, Türkiye; [23]Department of Hematology, Aalborg University Hospital, Aalborg, Denmark;[24]Department of Clinical Medicine, Aalborg University, Aalborg, Denmark; [25]Department of Internal Medicine, Division of Hematology, Erciyes University, Kayseri, Türkiye; [26]Department of Hematology, İzmir City Hospital, İzmir, Türkiye;[27]Department of Hematology, Odense University Hospital, Odense, Denmark; [28]Department of Clinical Research, University of Southern Denmark, Odense, Denmark; [29]Center for Clinical Data Science, Aalborg University and Aalborg University Hospital, Aalborg, Denmark; [30]Department of Hematology, Göztepe Prof. Dr. Süleyman Yalçın City Hospital, Istanbul, Türkiye;[31]Department of Hematology, Isparta City Hospital, Isparta, Türkiye; [32]Department of Internal Medicine, Division of Hematology, Ege University, Izmir, Türkiye; [33]Clinical Cancer Research Center, Department of Hematology, Aalborg University Hospital, Aalborg, Denmark;[34]Department of Internal Medicine, Division of Hematology, Adana Başkent University, Adana, Türkiye; [35]Department of Hematology, Health Science University, Tepecik Training and Research Hospital, Izmir, Türkiye; [36]Department of Internal Medicine, Division of Hematology, Fırat University, Elazığ, Türkiye; [37]Department of Internal Medicine, Division of Hematology, Hitit University, Çorum, Türkiye; [38]Department of Internal Medicine, Division of Hematology, Ankara University, Ankara, Türkiye;[39]These authors contributed equally as senior authors, Türkiye, Denmark;*

**Copyright Information:** (Online) ISSN: 2572-9241
©2026 The Author(s). HemaSphere is published by John Wiley & Sons Ltd on behalf of European Hematology Association. This is an open access Abstract Book distributed under the Attribution-NonCommercial-NoDerivs (CC BY-NC-ND), which allows third parties to download the articles and share them with others as long as they credit the author and the Abstract Book, but they cannot change the content in any way or use them commercially.
**Abstract Book Citations:** Authors, Title, HemaSphere, 2026;10:(S1):pages. The abstract book DOIs can be found on the "Author Information" page.
**Disclaimer:** Articles published in the journal HemaSphere exclusively reflect the opinions of the authors. The authors are responsible for all content in their abstracts including accuracy of the facts, statements, citing resources, etc.

### Background

Primary mediastinal B-cell lymphoma (PMBCL) is a distinct subtype of aggressive B-cell lymphoma, predominantly affecting young adults. While overall outcomes are favorable, a clinically relevant subset of patients experience early progression or death. To date, no widely accepted, disease-specific prognostic index exists for PMBCL, and risk stratification largely relies on tools developed for diffuse large B-cell lymphoma. Accurate prognostic stratification in PMBCL is clinically relevant for identifying patients at higher risk, informing treatment selection strategies, and optimizing patient selection and stratification in prospective clinical trials.

### Aims

To develop and externally validate a simple, point-based prognostic index for overall survival (OS) and progression-free survival (PFS) in patients with PMBCL, using routinely available baseline clinical variables.

### Methods

A retrospective multicenter cohort of 155 patients with PMBCL diagnosed between 2006–2024 in Turkey was used for model development. The prognostic index incorporated age >45 years, ECOG performance status ≥2, Ann Arbor stage ≥III, and hemoglobin <10.5 g/dL for OS, while a simplified model excluding age was used for PFS. Each variable contributed one point, generating categorical scores, and patients were stratified into low- (score 0), intermediate- (score 1), and high-risk (score ≥2) groups.
External validation used data from the Danish Lymphoma Registry, including 228 patients diagnosed during 2000–2025. Model performance was evaluated using Kaplan–Meier survival analysis with log-rank tests, Cox proportional hazards regression, and discrimination assessed by Harrell's concordance index (C-index).

### Results

Baseline characteristics are summarized in Table 1; both cohorts were young (median age 31 vs 38 years), with greater use of intensified regimens in the validation cohort. In the development cohort, 5-year OS rates were 90%, 77%, and 52%, and corresponding PFS rates were 79%, 56%, and 27% across low-, intermediate-, and high-risk groups ($p$<0.001 for both; Figure 1). In Cox proportional hazards models, compared with the low-risk group, intermediate-risk patients showed a trend toward increased risk for OS (HR 2.36, $p$=0.099), while high-risk patients had a significantly increased risk (HR 6.34, $p$<0.001). For PFS, both intermediate- (HR 2.12, $p$=0.037) and high-risk groups (HR 4.58, $p$<0.001) demonstrated significantly higher hazards. Despite borderline statistical significance for OS in the intermediate-risk group, hazard ratios indicated a consistent risk gradient across groups. Discriminatory performance was good, with C-indices of 0.704 for OS and 0.663 for PFS.
External validation in the Danish cohort confirmed preserved prognostic discrimination. Five-year OS rates across low-, intermediate-, and high-risk groups were 100%, 92%, and 81%, respectively, while corresponding 5-year PFS rates were 90%, 81%, and 63% (p<0.001 for both endpoints; Figure 2). Discrimination remained robust, with C-indices of 0.740 for OS and 0.649 for PFS.

### Summary/Conclusion

The PMBCL-PI is a simple, disease-specific, point-based prognostic index that reliably stratifies patients with PMBCL according to relative risk using routinely available clinical variables. The model demonstrates robust discrimination in both development and external validation cohorts. To enhance clinical applicability, the PMBCL-PI has been implemented as an online decision-support tool, available at https://lymphomapredictor.rn.dk/.

**Copyright Information:** (Online) ISSN: 2572-9241
©2026 The Author(s). HemaSphere is published by John Wiley & Sons Ltd on behalf of European Hematology Association. This is an open access Abstract Book distributed under the Attribution-NonCommercial-NoDerivs (CC BY-NC-ND), which allows third parties to download the articles and share them with others as long as they credit the author and the Abstract Book, but they cannot change the content in any way or use them commercially.
**Abstract Book Citations:** Authors, Title, HemaSphere, 2026;10:(S1):pages. The abstract book DOIs can be found on the "Author Information" page.
**Disclaimer:** Articles published in the journal HemaSphere exclusively reflect the opinions of the authors. The authors are responsible for all content in their abstracts including accuracy of the facts, statements, citing resources, etc.

| Variables | Development cohort | Validation cohort |
|---|---|---|
| n | 155 | 228 |
| Age, years, median (IQR) | 31 (24-40) | 38 (29-47) |
| Age, years, n (%) | | |
| ≤ 45 | 130 (83.9) | 164 (71.9) |
| > 45 | 25 (16.1) | 64 (28.1) |
| Sex, n (%) | | |
| Female | 105 (67.7) | 115 (50.4) |
| Male | 50 (32.3) | 113 (49.6) |
| Stages, n (%) | | |
| Early (I-II) | 113 (72.9) | 97 (42.5) |
| Advanced (III-IV) | 42 (27.1) | 131 (57.5) |
| ECOG, n (%) | | |
| 0-1 | 141 (91.0) | 204 (89.5) |
| 2-4 | 14 (9.0) | 24 (10.5) |
| Hemoglobin, g/dL, n (%) | | |
| < 10.5 | 20 (12.9) | 34 (14.9) |
| ≥ 10.5 | 135 (87.1) | 194 (85.1) |
| First line therapy, n (%) | | |
| R-CHOP | 79 (51.0) | 77 (33.8) |
| Intensified Regimen | 76 (49.0) | 151 (66.2) |
| DA-EPOCH-R | 76 (100) | 10 (6.6) |
| R-CHOEP | 0 (0) | 141 (93.4) |
| OS score, n (%) | | |
| 0 | 86 (55.5) | 69 (30.3) |
| 1 | 45 (29.0) | 94 (41.2) |
| ≥2 | 24 (15.5) | 65 (28.5) |
| PFS score, n (%) | | |
| 0 | 101 (65.2) | 85 (37.3) |
| 1 | 35 (22.6) | 105 (46.0) |
| ≥2 | 19 (12.2) | 38 (16.7) |

**Table 1.** Baseline clinical characteristics of patients in the development (Turkish) and external validation (Danish LYFO) cohorts. Abbreviations: IQR, interquartile range; ECOG, Eastern Cooperative Oncology Group; OS, overall survival; PFS, progression-free survival.

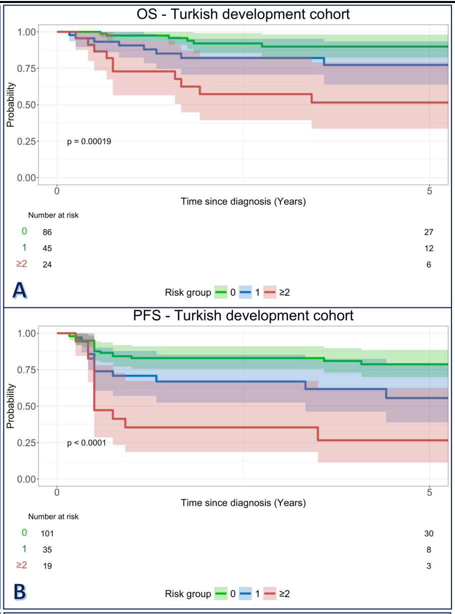

**Figure 1.** Kaplan–Meier survival curves stratified by PMBCL-PI risk groups in the Turkish development cohort. **(A)** Overall survival (OS) and **(B)** progression-free survival (PFS). Patients were categorized into low- (score 0), intermediate- (score 1), and high-risk (score ≥2) groups based on the PMBCL-PI. Log-rank *p*-values are shown in each panel.

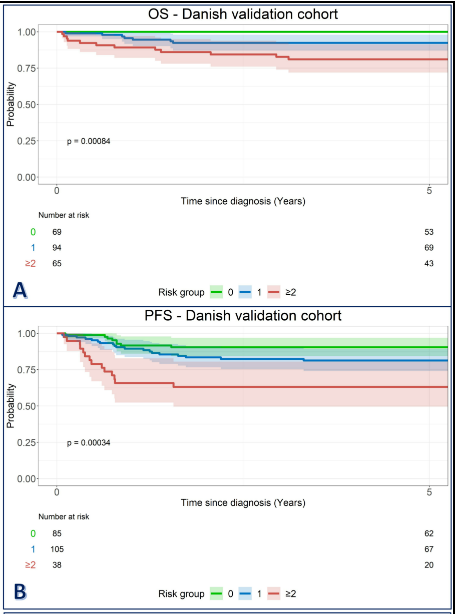

**Figure 2.** Kaplan–Meier survival curves stratified by PMBCL-PI risk groups in the Danish validation cohort. **(A)** Overall survival (OS) and **(B)** progression-free survival (PFS). Patients were categorized into low- (score 0), intermediate- (score 1), and high-risk (score ≥2) groups based on the PMBCL-PI. Log-rank *p*-values are shown in each panel.

**Copyright Information:** (Online) ISSN: 2572-9241
©2026 The Author(s). HemaSphere is published by John Wiley & Sons Ltd on behalf of European Hematology Association. This is an open access Abstract Book distributed under the Attribution-NonCommercial-NoDerivs (CC BY-NC-ND), which allows third parties to download the articles and share them with others as long as they credit the author and the Abstract Book, but they cannot change the content in any way or use them commercially.
**Abstract Book Citations:** Authors, Title, HemaSphere, 2026;10:(S1):pages. The abstract book DOIs can be found on the “Author Information” page.
**Disclaimer:** Articles published in the journal HemaSphere exclusively reflect the opinions of the authors. The authors are responsible for all content in their abstracts including accuracy of the facts, statements, citing resources, etc.

### (PF999) RISK FACTORS FOR EARLY DEATH AND POOR SURVIVAL IN HEMOPHAGOCYTIC LYMPHOHISTIOCYTOSIS AS AN ONSET OF DIFFUSE LARGE B-CELL LYMPHOMA

**Topic:** 19. Large B cell lymphomas - Clinical

Jianai Sun[1], De Zhou[1], Hongyan Tong*[1]

[1] *The First Affiliated Hospital, Zhejiang University School of Medicine, Department of Hematology, Hangzhou, China;*

### Background

Diffuse large B-cell lymphoma with hemophagocytic lymphohistiocytosis as the initial presentation (HLH-onset DLBCL) is a highly aggressive clinical subtype characterized by a "tumor-inflammation self-driving loop". This population exhibits unique clinical features and an extremely poor prognosis; however, robust risk-stratification models and systematic evaluations of treatment strategies remain scarce.

### Aims

This study aimed to: 1) identify independent risk factors for early death (ED, ≤30 days) and develop a bedside scoring model (ED-SSM); 2) establish a novel long-term prognostic system (OS-PSS); 3) evaluate the impact of treatment intensity using inverse probability of treatment weighting (IPTW); and 4) characterize the genomic landscape using targeted next-generation sequencing (NGS).

### Methods

A total of 130 treatment-naïve patients with HLH-onset DLBCL admitted between January 1, 2019, and January 1, 2025, were retrospectively analyzed. Multivariable logistic and Cox proportional hazards models identified prognostic determinants. IPTW was implemented to balance six core baseline parameters (age, platelets, APTT, LDH, albumin, and ferritin) to minimize selection bias. Targeted NGS (475-gene panel) was performed on 38 patients to identify mutational clusters and molecular subtypes.

### Results

The median age was 67 years; 92.3% were in stage III–IV and 86.2% had bone marrow involvement. The ED rate was 19.2%. Multivariable analysis identified age >75 years (OR=25.0, P<0.001), ferritin >5000 μg/L (OR=25.9, P=0.008), and PCT >0.5 ng/mL (OR=5.52, P=0.007) as independent ED predictors. The ED-SSM (0–5 points) achieved an AUC of 0.848. Traditional IPI failed to provide effective risk stratification, as 95.4% of patients clustered in the high-risk category. In contrast, the OS-PSS (incorporating age, platelets, ferritin, and PCT) demonstrated superior discriminative power (C-index 0.746). After IPTW adjustment, standard-intensity chemoimmunotherapy significantly reduced the risk of death compared to no treatment (HR=0.012, 95% CI: 0.005–0.032, P<0.001). Subgroup analysis confirmed that even in fragile patients (age >65 or ECOG >2), standard intensity remained the key driver of survival (HR=0.017, P<0.001). Genomic profiling revealed a high prevalence of the MCD subtype (44.7%), which was significantly associated with inferior progression-free survival (HR=3.87, P=0.024).

### Summary/Conclusion

HLH-onset DLBCL is characterized by high early mortality and unique molecular features. Our ED-SSM and OS-PSS models enable precise bedside risk stratification. Crucially, concurrent standard-intensity lymphoma-directed therapy is essential for improving survival, even in fragile patients. The enrichment of the MCD subtype suggests that integrating targeted agents, such as BTK inhibitors, warrants further investigation.

**Copyright Information:** (Online) ISSN: 2572-9241
©2026 The Author(s). HemaSphere is published by John Wiley & Sons Ltd on behalf of European Hematology Association. This is an open access Abstract Book distributed under the Attribution-NonCommercial-NoDerivs (CC BY-NC-ND), which allows third parties to download the articles and share them with others as long as they credit the author and the Abstract Book, but they cannot change the content in any way or use them commercially.
**Abstract Book Citations:** Authors, Title, HemaSphere, 2026;10:(S1):pages. The abstract book DOIs can be found on the "Author Information" page.
**Disclaimer:** Articles published in the journal HemaSphere exclusively reflect the opinions of the authors. The authors are responsible for all content in their abstracts including accuracy of the facts, statements, citing resources, etc.

### (PF1000) OUTPATIENT TREATMENT WITH GLOFITAMAB PLUS GEMCITABINE AND OXALIPLATIN (GEMOX) IN RELAPSED/REFRACTORY (R/R) DIFFUSE LARGE B-CELL LYMPHOMA (DLBCL): PRELIMINARY SAFETY RESULTS FROM A PHASE 2 STUDY

**Topic:** 19. Large B cell lymphomas - Clinical

Dok Hyun Yoon*[1], David Berz[2], Carmelo Carlo-Stella[3], Rami Kotb[4], Jeong-Ok Lee[5], Charles Herbaux[6], Robert Puckrin[7], Vanja Zeremski[8], Lucile Bussot[9], Gerardo Musuraca[10], Ritika Jagtiani[11], Juana Hernandez[12], Grace Smithen[13], Isabel Prieto Gonzalez-Albo[13], Linda Lundberg[12], James Relf[13], John M. Burke[14]

*[1]Asan Medical Center, University of Ulsan College of Medicine, Seoul, South Korea;[2]Valkyrie Clinical Trials, Los Angeles, CA, United States of America; [3]Humanitas University and Department of Oncology and Hematology, Humanitas Research Hospital, Rozzano-Milano, Italy; [4]CancerCare Manitoba and University of Manitoba, Winnipeg, MB, Canada;[5]Seoul National University Bundang Hospital, Seoul National University College of MedicinE, Seongnam, South Korea; [6]University Hospital of Montpellier, Montpellier, France; [7]Arthur Child Cancer and University of Calgary, Calgary, AB, Canada;[8]Otto von Guericke University Magdeburg, Magdeburg, Germany; [9]Daniel-Hollard Institute, Grenoble, France; [10]IRCCS-Istituto Scientifico Romagnolo per lo Studio e la Cura dei Tumori (IRST) SRL, Meldola, Italy; [11]Genentech, Inc., South San Francisco, CA, United States of America; [12]F. Hoffmann-La Roche Ltd, Basel, Switzerland; [13]Roche Products Ltd, Welwyn Garden City, United Kingdom; [14]SCRI at Rocky Mountain Cancer Centers, Aurora, CO, United States of America;*

### Background

Glofitamab, a CD20xCD3 bispecific antibody, plus GemOx (Glofit-GemOx) has shown significant improvements versus rituximab-GemOx in overall survival and progression-free survival in the randomized Phase 3 STARGLO trial (NCT04408638) in autologous stem-cell transplant (ASCT)-ineligible R/R DLBCL. The observed cytokine release syndrome (CRS) profile with Glofit-GemOx in STARGLO allows for treatment without mandatory hospitalization. Implementing outpatient strategies can further improve patient convenience and optimize healthcare resource utilization.

### Aims

Present preliminary safety results from an ongoing Phase 2 trial (GO45434; NCT06806033), evaluating CRS profile optimization for outpatient Glofit-GemOx treatment via an enhanced steroid premedication, and a revised monitoring regimen for treated patients (pts) with R/R DLBCL.

### Methods

Pts had R/R DLBCL; those with 1 prior line of therapy (LOT) had to be ASCT-ineligible. After 1000mg obinutuzumab pretreatment, pts received Glofit-GemOx (8 cycles plus 4 cycles glofitamab monotherapy), with glofitamab given in step-up doses (SUD; 2.5/10mg) in Cycle (C)1, then 30mg target dose every 21 days from C2. Pts received oral dexamethasone (Dex) 20mg one day prior to and after glofitamab SUD in C1, in addition to the premedication used in STARGLO. Pts were monitored in the outpatient setting for 4 hours during SUD and 90 minutes after the first target dose, in both academic and community centers. Primary endpoint was CRS incidence. Other safety parameters and efficacy were secondary endpoints. All pts provided informed consent. In this ongoing study, 100 pts in 7 countries will be recruited.

**Copyright Information:** (Online) ISSN: 2572-9241
©2026 The Author(s). HemaSphere is published by John Wiley & Sons Ltd on behalf of European Hematology Association. This is an open access Abstract Book distributed under the Attribution-NonCommercial-NoDerivs (CC BY-NC-ND), which allows third parties to download the articles and share them with others as long as they credit the author and the Abstract Book, but they cannot change the content in any way or use them commercially.
**Abstract Book Citations:** Authors, Title, HemaSphere, 2026;10:(S1):pages. The abstract book DOIs can be found on the "Author Information" page.
**Disclaimer:** Articles published in the journal HemaSphere exclusively reflect the opinions of the authors. The authors are responsible for all content in their abstracts including accuracy of the facts, statements, citing resources, etc.

**Results**

At the data cut-off (September 24, 2025), 25 pts with R/R DLBCL had received ≥1 dose of study treatment: 23 completed the SUD, and 15 had received ≥2 glofitamab target doses. Overall, median patient age was 66.4 years; 64% of pts had 1 prior LOT, 36% had ≥2 prior LOT, 64% were refractory to any LOT, and 60% were refractory to their last LOT. Five pts had prior chimeric antigen receptor T-cell therapy and one had prior stem-cell transplant. Most pts received glofitamab in the outpatient setting.
CRS was reported in 44% of pts (Grade [Gr] 1: 32%; Gr 2: 12%; no Gr ≥3) and mainly occurred after the 2.5mg (7/11 pts) and 10mg (3/11 pts) glofitamab doses. Two pts had their first CRS event (both Gr 1) in later cycles (C2 and C4). Serious CRS events were reported in 16% of pts. All CRS events resolved within a median of 2 days; no CRS led to treatment discontinuation. The most common Gr 3/4 adverse events (AEs) were anemia (24%), neutropenia (20%), febrile neutropenia (12%), and thrombocytopenia (12%). Infections occurred in 16% of pts and were mostly Gr 1/2; none led to treatment discontinuation. One patient not exposed to glofitamab had a Gr 5 AE of COVID-19 pneumonia. Immune effector cell-associated neurotoxicity syndrome (ICANS) events were reported in 12% of pts (Gr 1, Gr 2, Gr 3: 4% each); all resolved within 2 days and none led to treatment discontinuation. Two pts had ICANS concurrent with CRS (after the 2.5mg glofitamab dose).

**Summary/Conclusion**

In this preliminary analysis, pts with R/R DLBCL receiving Glofit-GemOx were monitored in the outpatient setting after receiving additional oral Dex one day prior to and after glofitamab infusion in C1. The rate and severity of CRS events were similar to those reported in STARGLO. Data are encouraging and support further optimization of outpatient Glofit-GemOx treatment. Updated data will be presented.

**Copyright Information:** (Online) ISSN: 2572-9241
©2026 The Author(s). HemaSphere is published by John Wiley & Sons Ltd on behalf of European Hematology Association. This is an open access Abstract Book distributed under the Attribution-NonCommercial-NoDerivs (CC BY-NC-ND), which allows third parties to download the articles and share them with others as long as they credit the author and the Abstract Book, but they cannot change the content in any way or use them commercially.
**Abstract Book Citations:** Authors, Title, HemaSphere, 2026;10:(S1):pages. The abstract book DOIs can be found on the “Author Information” page.
**Disclaimer:** Articles published in the journal HemaSphere exclusively reflect the opinions of the authors. The authors are responsible for all content in their abstracts including accuracy of the facts, statements, citing resources, etc.

### (PF1001) REAL-WORLD EFFICACY AND SAFETY OF THE MEDZ-R REGIMEN AS INDUCTION THERAPY IN NEWLY DIAGNOSED PRIMARY CENTRAL NERVOUS SYSTEM LYMPHOMA

**Topic:** 19. Large B cell lymphomas - Clinical

Ming Jiang[1], Xue Meng[2], Yongqian Jia[3], Jing Tan[4], Fangyi Fan[5], Hui Li[6], Kexing Ren[1], Na Li[7], You Feng[7], Liqun Zou*[1]

*[1]West China Hospital, Sichuan University, Department of Medical Oncology of Cancer Center, Chengdu, China;[2]Chengdu Shang Jin Nan Fu Hospital, Shang Jin Hospital of West China Hospital, Sichuan University, Department of Oncology, Chengdu, China; [3]West China Hospital, Sichuan University, Department of Hematology and Institute of Hematology, Chengdu, China;[4]The Third People's Hospital of Chengdu, Chengdu, China; [5]People's Liberation Army The General Hospital of Western Theater Command, Chengdu, China; [6]Sichuan Provincial People's Hospital, Department of Hematology, Chengdu, China;[7]West China School of Public Health and West China Fourth Hospital, Sichuan University,, Department of Oncology, Chengdu, China;*

### Background

Primary central nervous system lymphoma (PCNSL) is a rare and aggressive non-Hodgkin lymphoma with a poorer prognosis compared to systemic diffuse large B-cell lymphoma. High-dose methotrexate-based combinations remain the cornerstone of induction therapy, but outcomes are still suboptimal. Our previous phase II trial reported a complete response (CR) rate exceeding 90% with the MEDZ-R regimen in newly diagnosed PCNSL patients under 70.

### Aims

To evaluate the real-world efficacy and safety of the MEDZ-R regimen as induction therapy in newly diagnosed PCNSL.

### Methods

This multicenter, retrospective real-world study enrolled newly diagnosed PCNSL patients from five institutions. Patients received 5-6 cycles of the MEDZ-R regimen: rituximab (375 mg/m2, day 0), methotrexate (3.5 g/m2, day 1), etoposide (50 mg/m2, days 3–5), liposomal doxorubicin (30 mg/m2, day 3), and zanubrutinib (160mg BID, days 3–21) every 3 weeks. Consolidation (reduced-dose radiotherapy or autologous stem cell transplantation) or maintenance was recommended for patients achieving CR. Radiological assessment was performed every two cycles during induction. Primary endpoint was CR rate. Secondary endpoints included objective response rate (ORR), PFS, OS, and safety (CTCAE v5.0).

### Results

Between May 2023 and Nov 2025, 59 patients were enrolled. Median age was 55 years (range, 27-68); 55.9% were male. Most had non-GCB subtype (72.9%), multiple lesions (59.3%), deep brain involvement (55.9%), and ECOG PS 0-1 (59.3%). Of 57 evaluable patients, ORR was 93.0%, with 52 (91.2%) achieving CR. One patient discontinued due to toxicity after PR at cycle 2; two died from severe infections at diagnosis (not evaluable). Among 52 CR patients, 38 received consolidation (ASCT, n=20; WBRT, n=18), 4 received BTKi maintenance, and 10 were observed. After a median follow-up of 22.0 months (95%CI, 19.0-26.0), median PFS and OS were not reached. The 1-year PFS and OS rates were 84.7% (95%CI, 75.9-94.4%) and 87.5% (95%CI, 79.2-96.6%), respectively. Patients who received consolidation/maintenance had superior PFS compared to those who did not (1-year PFS: 100% vs. 80.0%, p=0.0033), with no significant difference in OS (1-year OS: 97.4% vs. 90.0%, p=0.22). TRAEs occurred in 77.9% (mostly grade 1-2). Grade 3-4 toxicities were predominantly hematological: leukopenia (23.7%) and thrombocytopenia (15.3%), both reversible. Hepatotoxicity (23.7%) and nephrotoxicity (23.7%) were both Grade 1-2. Infectious complications were uncommon, with Grade 3-4 infections occurring in 5.1% of patients; additionally, two patients died due to severe infection at initial diagnosis. Bleeding events were rare (3.4%) and mild.

**Copyright Information:** (Online) ISSN: 2572-9241
©2026 The Author(s). HemaSphere is published by John Wiley & Sons Ltd on behalf of European Hematology Association. This is an open access Abstract Book distributed under the Attribution-NonCommercial-NoDerivs (CC BY-NC-ND), which allows third parties to download the articles and share them with others as long as they credit the author and the Abstract Book, but they cannot change the content in any way or use them commercially.
**Abstract Book Citations:** Authors, Title, HemaSphere, 2026;10:(S1):pages. The abstract book DOIs can be found on the "Author Information" page.
**Disclaimer:** Articles published in the journal HemaSphere exclusively reflect the opinions of the authors. The authors are responsible for all content in their abstracts including accuracy of the facts, statements, citing resources, etc.

### Summary/Conclusion

In this real-world study, the MEDZ-R regimen demonstrated high efficacy (CR >90%) and a manageable safety profile as induction for newly diagnosed PCNSL. These findings support its potential as a promising treatment option.

Table 1. Baseline characteristics

| Variables | Total (n = 59) |
|---|---|
| AGE, M (range) | 55.00 (27-68) |
| Sex, n(%) | |
| Male | 33 (55.93) |
| Female | 26 (44.07) |
| ECOG, n(%) | |
| 0-1 | 35 (59.32) |
| 2-4 | 24 (40.68) |
| IELSG, n(%) | |
| High | 6 (10.17) |
| Intermediate | 31 (52.54) |
| Low | 15 (25.42) |
| NA | 7 (11.86) |
| MSKCC, n(%) | |
| High | 15 (25.42) |
| Intermediate | 25 (42.37) |
| Low | 19 (32.20) |
| COO, n(%) | |
| 0 | 43 (72.88) |
| 1 | 14 (23.73) |
| NA | 2 (3.39) |
| DEL, n(%) | |
| 0 | 24 (40.68) |
| 1 | 33 (55.93) |
| unknow | 2 (3.39) |
| Lesion at first registration, n(%) | |
| Single | 24 (40.68) |
| Multiple | 35 (59.32) |
| Deep Lesions, n(%) | |
| No | 26 (44.07) |
| Yes | 33 (55.93) |
| LDH, n(%) | |
| Normal | 43 (72.88) |
| Elevated | 16 (27.12) |
| CSF Protein, n(%) | |
| Normal | 10 (16.95) |
| Elevated | 26 (44.07) |
| NA | 23 (38.98) |

Table 2. Adverse events

| Type of toxicity | Grade 0 | Grade 1 | Grade 2 | Grade 3 | Grade 4 | Grade 5 |
|---|---|---|---|---|---|---|
| Neutropenia, n(%) | 35 (59.3%) | 3 (5.1%) | 7 (11.9%) | 3 (5.1%) | 11 (18.6%) | 0 (0.0) |
| Anemia, n(%) | 30 (50.8%) | 19 (32.2%) | 7 (11.9%) | 3 (5.1%) | 0 (0.0) | 0 (0.0) |
| Thrombocytopenia, n(%) | 43(72.9%) | 3 (5.1%) | 4 (6.7%) | 2 (3.4%) | 7 (11.9%) | 0 (0.0) |
| Hepatotoxicity, n(%) | 45 (76.3%) | 11 (18.6%) | 3 (5.1%) | 0 (0.0) | 0 (0.0) | 0 (0.0) |
| Nephrotoxicity, n(%) | 45 (76.3%) | 8 (13.6%) | 6 (10.1%) | 0 (0.0%) | 0 (0.0) | 0 (0.0) |
| Infection, n(%) | 47 (79.7%) | 1 (1.7%) | 6 (10.1%) | 3 (5.1%) | 0 (0.0) | 2 (3.4%) |
| Bleeding event, n(%) | 57 (96.6%) | 1 (1.7%) | 1 (1.7%) | 0 (0.0) | 0 (0.0) | 0 (0.0) |

Figuer. Survival outcomes. (A) PFS and (B) OS in all patients. (C) PFS and (D) OS in patients achieving CR after induction, stratified by receipt of maintenance or consolidation .

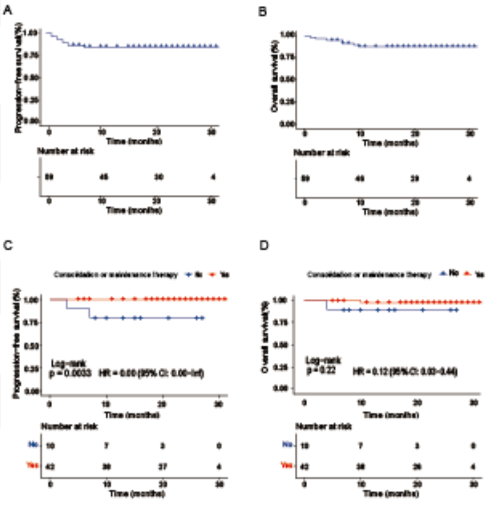

**Copyright Information:** (Online) ISSN: 2572-9241
©2026 The Author(s). HemaSphere is published by John Wiley & Sons Ltd on behalf of European Hematology Association. This is an open access Abstract Book distributed under the Attribution-NonCommercial-NoDerivs (CC BY-NC-ND), which allows third parties to download the articles and share them with others as long as they credit the author and the Abstract Book, but they cannot change the content in any way or use them commercially.
**Abstract Book Citations:** Authors, Title, HemaSphere, 2026;10:(S1):pages. The abstract book DOIs can be found on the "Author Information" page.
**Disclaimer:** Articles published in the journal HemaSphere exclusively reflect the opinions of the authors. The authors are responsible for all content in their abstracts including accuracy of the facts, statements, citing resources, etc.

### (PF1002) THE PREDIC-TO STUDY: AN INTERNATIONAL, REAL-WORLD COHORT STUDY OF VENOUS THROMBOEMBOLISM RISK MODEL STRATIFICATION IN DLBCL PATIENTS ACROSS ASIA, EUROPE, AND UNITED STATES OF AMERICA

**Topic:** 19. Large B cell lymphomas - Clinical

Giulia Bassi*[1, 2], Arianna Zappaterra[1, 2, 3], Andrea Aroldi[1], Silvia Bolis[1], Ivana Rita Casaroli[1], Luisa Verga[1], Lorenza Maria Borin[1], Andrea Carrer[1], Fabrizio Cavalca[1], Giovanni Rindone[1], Elisa Bossi[1], Anna Mochi[1, 2], Stefano Crippa[1, 2], Bianca Maria Granelli[1, 2, 3], Daniele Ramazzotti[2], Fabrizio Marino[4], Carolina Steidl[4], Giulia Pata[4], Elena Rela[4], Laura Leoni[4], Abeer Yaseen[5], Mohammad Makoseh[5], Hira Bani Hani[5], Taylor Brooks[6], Kenji Yokoyama[7], Hikmat Abdel-Razeq[5], Federica Cocito[1], Andrés José María Ferreri[4], Paolo F. Caimi[6], Carlo Gambacorti-Passerini[1, 2], Ivan Civettini[1, 2, 8]

*[1]Hematology Division and Bone Marrow Unit, Fondazione IRCCS San Gerardo dei Tintori, Monza, Italy; [2]Department of Medicine and Surgery, University of Milan-Bicocca, Monza, Italy; [3]ASST Grande Ospedale Metropolitano Niguarda, Niguarda Cancer Center, Department of Haematology,, Milano, Italy; [4]Lymphoma Unit, IRCCS San Raffaele Scientific Institute, Milano, Italy; [5]Department of Medicine, King Hussein Cancer Center, Amman, Jordan; [6]Cleveland Clinic Cancer Institute, Cleveland, Ohio, United States of America; [7]Department of Hematology/Oncology, Tokai University, Hachioji, Tokyo, Japan; [8]Experimental Immunology Unit, IRCCS San Raffaele Scientific Institute, Milano, Italy;*

### Background

Venous thromboembolism (VTE) represents a major cause of morbidity and mortality in diffuse large B-cell lymphoma (DLBCL). Several predictive models, Khorana, ThroLy, and Model IX were developed to stratify thrombotic risk, yet their comparative performance in a DLBCL-exclusive population remains unclear.

### Aims

To comparatively assess the discriminative performance of Khorana, ThroLy, Model IX, and R-IPI for VTE occurrence and overall survival (OS) in a large, real-world, international DLBCL cohort.

### Methods

We retrospectively analyzed 1,504 consecutive DLBCL patients diagnosed between 2004 and 2024 at two European, two Asian and one United States center (ClinicalTrials ID NCT06694779). VTE was defined as any objectively confirmed deep vein thrombosis or pulmonary embolism occurring after diagnosis. Model performance was evaluated using Cox regression and Harrell's concordance index (c-index) with bootstrap-based estimation of 95% confidence intervals.

**Copyright Information:** (Online) ISSN: 2572-9241
©2026 The Author(s). HemaSphere is published by John Wiley & Sons Ltd on behalf of European Hematology Association. This is an open access Abstract Book distributed under the Attribution-NonCommercial-NoDerivs (CC BY-NC-ND), which allows third parties to download the articles and share them with others as long as they credit the author and the Abstract Book, but they cannot change the content in any way or use them commercially.
**Abstract Book Citations:** Authors, Title, HemaSphere, 2026;10:(S1):pages. The abstract book DOIs can be found on the "Author Information" page.
**Disclaimer:** Articles published in the journal HemaSphere exclusively reflect the opinions of the authors. The authors are responsible for all content in their abstracts including accuracy of the facts, statements, citing resources, etc.

### Results

VTE occurred in 219/1504 patients (14.6%), with a 1-year cumulative incidence of 3.3% (95% CI 2.4–4.2). Median time to VTE was 72 days, confirming the early clustering of thrombotic events. VTE independently predicts inferior OS (38.5 vs 172.3 months). In univariate analysis, hemoglobin (hb) level, leukocytosis, hypoalbuminemia, body mass index (BMI) > 30, bulky disease, and deep vein compression, were significantly associated with increased VTE risk ($p<0.05$). Central venous catheter (CVC) placement was not associated with higher VTE incidence (HR 1.00, 95% CI 0.71–1.42). Patients not receiving anthracycline-based chemotherapy showed increased thrombotic risk (HR 2.53, 95% CI 1.68–3.83), likely reflecting baseline frailty and adverse disease features rather than a direct treatment effect. All thrombotic scores significantly stratified VTE risk. High-risk groups showed increased VTE incidence for Model IX (HR 2.73, 95% CI 1.88–3.95), Khorana (HR 2.54, 95% CI 1.91–3.37), and ThroLy (HR 2.54, 95% CI 1.80–3.58). In comparative analysis, Model IX demonstrated the highest discriminative ability for VTE (c-index 0.63, 95% CI 0.59-0.66), followed by Khorana, ThroLy, and R-IPI (see Tab. 1). Bootstrap-based LASSO Cox regression identified additional stable predictors beyond Model IX, including deep vein compression (99.3% selection frequency), BMI>30 (96.6%), hb level (92.8%), neutrophil count (77.9%), and R-IPI (65.8%), while higher albumin levels showed a consistent protective association (96.1% negative selection). These findings suggest residual multidimensional risk information in our cohort, not fully captured by the composite Model IX weighting. Regarding OS prediction, Model IX again showed superior performance (c-index 0.67, 95% CI 0.65–0.69), outperforming ThroLy, R-IPI, and Khorana. While R-IPI retained strong prognostic value for survival, its discriminatory ability for VTE was limited (see Tab. 1). Conversely, VTE-oriented models, particularly Model IX, captured prognostic information relevant to both thrombotic risk and survival, highlighting a partial biological overlap between disease aggressiveness and thrombotic propensity.

### Summary/Conclusion

VTE is a frequent early complication in DLBCL and is associated with inferior survival. Model IX showed the best discrimination for VTE and outperformed the other models for OS, supporting its role as the most informative risk stratification tool in this setting.

*PC, CGP and IC contributed equally

| Models | Variables included | OS (c-index, 95% CI) | OS (ΔAIC) | VTE (c-index, 95% CI) | VTE (ΔAIC) |
|---|---|---|---|---|---|
| Model IX | Lymphoma subtype (FL or DLBCL), albumin concentration (g/L), WBC count>11x10⁹/L, bulky disease | **0,6749** (0.6516 - 0.6982) | -120 | **0.6304** (0.5949 - 0.6659) | - 34 |
| Khorana | Cancer type, PLT count ≥350x10⁹/L, Hb <10 g/dL or use of red-cell growth factors, WBC count >11x10⁹/L, BMI ≥35 | 0,5998 (0.5757 - 0.6240) | - 86 | 0,6276 (0.5916 - 0.6636) | - 49 |
| Throly | Previous VTE/IMA/stroke, ECOG 2-4, BMI > 30, Extranodal localization, Mediastinal involvement, ANC <1 × 109/L, Hb <10 g/dL | 0,6652 (0.6419 - 0.6885) | - 141 | 0.6083 (0.5698 -0.6469) | -35 |
| R-IPI | Age >60y, Serum LDH > URL, ECOG ≥2, Ann Arbor Stage (III-IV), Extranodal Involvement | 0,6553 (0.6355 - 0.6751) | **- 180** | 0,5938 (0.5604 - 0.6273) | **- 76** |

Tab. 1: Risk models discriminative performance for OS and VTE. AIC, Akaike information criterion; c-index, concordance index; OS, overall survival; VTE, venous thromboembolism; IMA, acute myocardial infraction; ANC, absolute neutrophils count; PLT, platelets; WBC, white blood cells; FL, follicular lymphoma; DLBCL, Diffuse large B cell lymphoma, ECOG, eastern cooperative oncology group. ΔAIC: empty model AIC – selected model AIC

Copyright Information: (Online) ISSN: 2572-9241
©2026 The Author(s). HemaSphere is published by John Wiley & Sons Ltd on behalf of European Hematology Association. This is an open access Abstract Book distributed under the Attribution-NonCommercial-NoDerivs (CC BY-NC-ND), which allows third parties to download the articles and share them with others as long as they credit the author and the Abstract Book, but they cannot change the content in any way or use them commercially.
Abstract Book Citations: Authors, Title, HemaSphere, 2026;10:(S1):pages. The abstract book DOIs can be found on the “Author Information” page.
Disclaimer: Articles published in the journal HemaSphere exclusively reflect the opinions of the authors. The authors are responsible for all content in their abstracts including accuracy of the facts, statements, citing resources, etc.

### (PF1003) TEMPORAL TRENDS IN HEPATITIS B RISK FOLLOWING LYMPHOMA: A POPULATION-BASED ANALYSIS USING SWEDISH LYMPHOMA REGISTRY DATA (2002–2024)

**Topic:** 19. Large B cell lymphomas - Clinical

Torsten Dahlén*[1], Karin Ekstrom Smedby[1]

[1]*Karolinska Institutet, Solna, Sweden;*

### Background

Hepatitis B virus (HBV) acquisition remains a significant concern in lymphoma patients. While screening and prophylaxis protocols have improved, long-term population-based data regarding late-onset HBV risk—occurring well after diagnosis—remain scarce in the modern era.

### Aims

To evaluate the risk of late-onset HBV (defined as >9 months post-diagnosis) in lymphoma patients compared to matched comparators, and to assess temporal trends across 5-year diagnosis periods between 2002 and 2024.

### Methods

Using Swedish national registry data, we identified lymphoma patients and matched general population comparators by using the Lymphoma register. A 9-month landmark analysis was performed to isolate late-onset events and mitigate immortal time bias. Cumulative incidence functions (CIF) were estimated using competing risk analysis, with death treated as a competing event. We analysed the full population against comparators and performed a subgroup analysis of treated patients stratified by 5-year periods. Risk estimates were adjusted for age at entry, sex, and region using a Cox regression.

### Results

The study cohort included 59,115 lymphoma cases. Baseline characteristics and 2-year survival rates across 5-year periods are detailed in Table 1. Notably, 2-year survival improved steadily over the study period, increasing from 70.9% in 2000–2004 to 81.3% in 2020–2024. The overall CIF demonstrated a significantly higher HBV burden in lymphoma patients compared to matched comparators throughout the follow-up period (Figure 1A). Among treated patients, the CIF stratified by diagnosis period revealed the evolution of late-onset HBV acquisition over time (Figure 1B). Adjusting for age at entry, sex, and region, the hazard ratio (HR) of late HBV acquisition in the competing risk model was HR 2.13 (95% CI 1.80-2.51), p<0.001.

**Table 1: Patient Characteristics and 2-Year Survival by Period**

| Period | N | Age, Mean (SD) | Females, n (%) | 2-Year Survival |
|---|---|---|---|---|
| 2000–2004 | 6,672 | 66.4 (15.5) | 2,914 (43.7%) | 70.90% |
| 2005–2009 | 12,002 | 66.8 (15.4) | 5,234 (43.6%) | 73.90% |
| 2010–2014 | 13,855 | 67.1 (15.4) | 5,933 (42.8%) | 76.90% |
| 2015–2019 | 15,380 | 68.2 (15.0) | 6,326 (41.1%) | 79.00% |
| 2020–2024 | 11,206 | 68.2 (15.4) | 4,671 (41.7%) | 81.30% |

**Copyright Information:** (Online) ISSN: 2572-9241
©2026 The Author(s). HemaSphere is published by John Wiley & Sons Ltd on behalf of European Hematology Association. This is an open access Abstract Book distributed under the Attribution-NonCommercial-NoDerivs (CC BY-NC-ND), which allows third parties to download the articles and share them with others as long as they credit the author and the Abstract Book, but they cannot change the content in any way or use them commercially.
**Abstract Book Citations:** Authors, Title, HemaSphere, 2026;10:(S1):pages. The abstract book DOIs can be found on the “Author Information” page.
**Disclaimer:** Articles published in the journal HemaSphere exclusively reflect the opinions of the authors. The authors are responsible for all content in their abstracts including accuracy of the facts, statements, citing resources, etc.

### Summary/Conclusion

This nationwide study demonstrates that lymphoma patients face a two-fold increase in the risk of acquiring late-onset Hepatitis B compared to the general population. While the steady improvement in 2-year survival reflects advances in lymphoma management, the persistent and significant gap in HBV risk highlights the necessity for long-term clinical vigilance and potentially extended HBV monitoring or prophylaxis well beyond the initial treatment window especially as HBV incidence in the general population is declining.

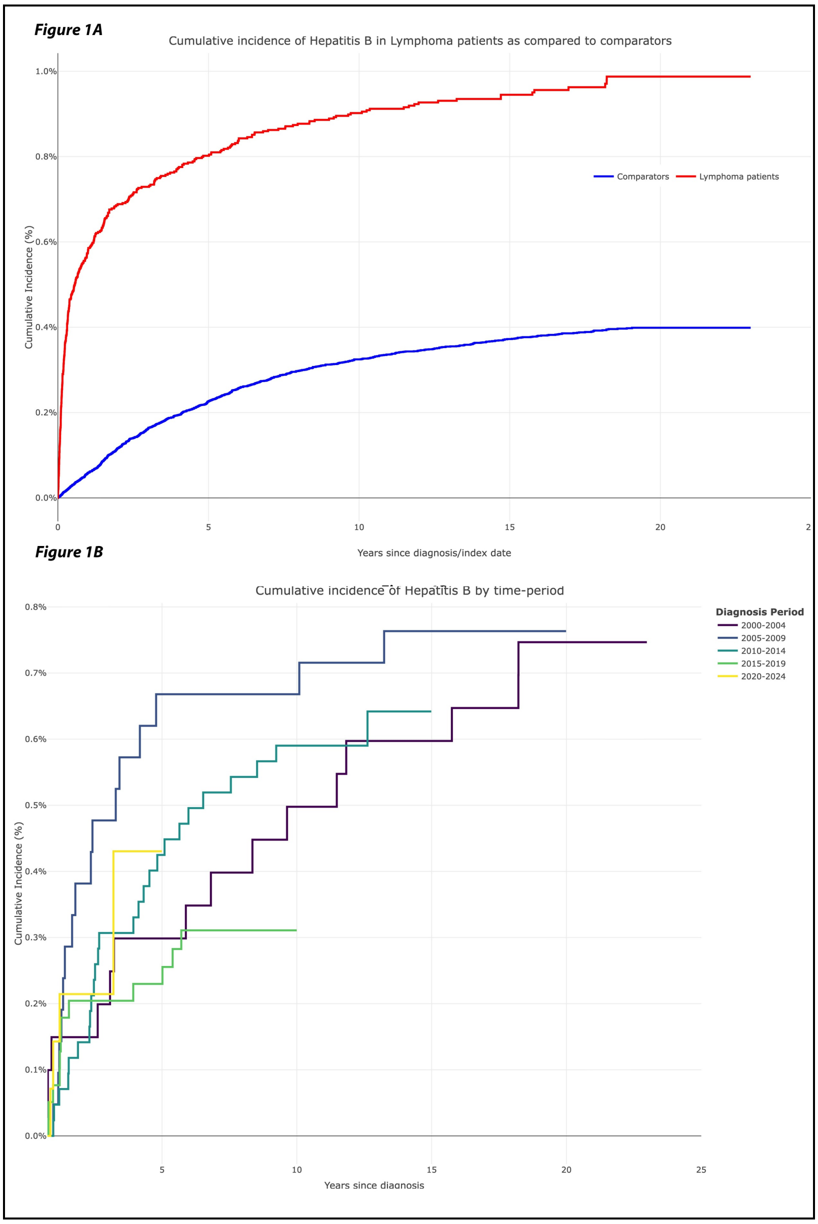

Cumulative-incidence of Hepatits B in Lymphoma patients (a) as compared to comparators (b) by time-period of treated patients

**Copyright Information:** (Online) ISSN: 2572-9241
©2026 The Author(s). HemaSphere is published by John Wiley & Sons Ltd on behalf of European Hematology Association. This is an open access Abstract Book distributed under the Attribution-NonCommercial-NoDerivs (CC BY-NC-ND), which allows third parties to download the articles and share them with others as long as they credit the author and the Abstract Book, but they cannot change the content in any way or use them commercially.
**Abstract Book Citations:** Authors, Title, HemaSphere, 2026;10:(S1):pages. The abstract book DOIs can be found on the "Author Information" page.
**Disclaimer:** Articles published in the journal HemaSphere exclusively reflect the opinions of the authors. The authors are responsible for all content in their abstracts including accuracy of the facts, statements, citing resources, etc.

### (PF1004) REAL-WORLD ASIAN MULTI-CENTRE COHORT STUDY ON OUTCOMES OF POLA-R-CHP IN UNTREATED DIFFUSE LARGE B-CELL LYMPHOMA

**Topic:** 19. Large B cell lymphomas - Clinical

Nicole-Ann Lim*[1], Jing Yuan Tan[2], Yi Chye Law[1], Ya Hwee Tan[1, 3], Jianbang Chiang[1, 3], Esther Wei Yin Chang[1, 3], Eileen Yi Ling Poon[1, 3], Nagavalli Somasundaram[1, 3], Cheng Kiat Lawrence Ng[2], Keith Wong[4], Michelle Limei Poon[5], Soon Thye Lim[1, 3], Jason Yongsheng Chan[1, 3], Raymond Wong[6]

*[1]National Cancer Centre Singapore, Division of Medical Oncology, Singapore, Singapore;[2]Singapore General Hospital, Department of Haematology, Singapore, Singapore; [3]Duke-NUS Medical School, Oncology Academic Clinical Program, Singapore, Singapore; [4]Pok Oi/Tin Shu Wai Hospital, Department of Medicine and Geriatrics, Hong Kong, China;[5]National University Health System, Department of Haematology-Oncology, Singapore, Singapore; [6]Prince of Wales Hospital, The Chinese University of Hong Kong, Sir Y.K. Pao Centre for Cancer and Department of Medicine & Therapeutics, Hong Kong, China;*

### Background

Pola-R-CHP (polatuzumab vedotin with rituximab, cyclophosphamide, doxorubicin, and prednisone) has been approved for first-line treatment of diffuse large B-cell lymphoma (DLBCL). However, real-world outcomes data compared against R-CHOP (rituximab, cyclophosphamide, doxorubicin, and prednisone) remain limited, especially in Asian cohorts where POLARIX subset analysis has suggested overall survival (OS) benefit.

### Aims

We aim to evaluate the efficacy of Pola-R-CHP in a real-world Asian setting for first-line treatment of DLBCL.

### Methods

We conducted a retrospective observational study (APOLLO: Asian POLA-R-CHP reaL-world Outcomes) in an international, multi-centre cohort from Singapore and Hong Kong, involving patients with newly diagnosed DLBCL between January 2015 to 2026. Physician assessed best response rates after treatment completion were documented. Analyses were conducted using Kaplan-Meier models and Cox proportional-hazards analysis. Given differences in follow-up duration between cohorts, analyses were restricted to the first 24 months, during which adequate follow-up was observed in both groups. Restricted mean survival time (RMST) at 24 months was also calculated to compare survival within the fixed time horizon.

**Copyright Information:** (Online) ISSN: 2572-9241
©2026 The Author(s). HemaSphere is published by John Wiley & Sons Ltd on behalf of European Hematology Association. This is an open access Abstract Book distributed under the Attribution-NonCommercial-NoDerivs (CC BY-NC-ND), which allows third parties to download the articles and share them with others as long as they credit the author and the Abstract Book, but they cannot change the content in any way or use them commercially.
**Abstract Book Citations:** Authors, Title, HemaSphere, 2026;10:(S1):pages. The abstract book DOIs can be found on the “Author Information” page.
**Disclaimer:** Articles published in the journal HemaSphere exclusively reflect the opinions of the authors. The authors are responsible for all content in their abstracts including accuracy of the facts, statements, citing resources, etc.

### Results

145 patients treated with Pola-R-CHP and 592 patients treated with R-CHOP were identified. Further 1:1 propensity-matching generated a cohort with 137 patients in both Pola-R-CHP and R-CHOP arms, with median follow-up of 13.9 and 77.4 months, respectively. Median age was 68 years (range, 21-87), with the majority with IPI 3-5 (83.9%). Based on Hans criteria, 84 (30.7%) were germinal centre B-cell-like (GCB), 183 (66.8%) were non-GCB, with remainder unknown. Baseline covariates were balanced, with all standardized mean differences below 0.1. No significant difference was observed in post-relapse treatments between both groups. Complete response (CR) rates were 92.0% for Pola-R-CHP and 84.3% for R-CHOP. CR rates to Pola-R-CHP were similar at 92.3% and 92.0% in DLBCL or transformed DLBCL, respectively. The 2-year OS was 92.5% for Pola-R-CHP versus 77.2% for R-CHOP (HR 0.30, 95% CI 0.13-0.70, p = 0.003); 2-year PFS was 79% for Pola-R-CHP versus 66.2% for R-CHOP (HR 0.57, 95% CI 0.34-0.98, p = 0.038). RMST at 24 months for PFS was 21.0 months for Pola-R-CHP and 19.0 months for R-CHOP (difference 1.99 months, 95% CI 0.23-3.75, p = 0.027); RMST for OS was 23.0 months and 20.7 months, respectively (difference 2.29 months, 95% CI 0.92-3.67, p = 0.001). The survival benefits remain observed for patients with IPI 3-5 (2-year OS 91.4% vs 73.4%, p = 0.0023; 2-year PFS 76.7% vs 62.9%, p = 0.037). Significant PFS and OS benefit to Pola-RCHP over R-CHOP was observed in non-GCB DLBCL but not in the GCB subtype (Figure 1).

### Summary/Conclusion

Our early real-world data suggest that Pola-R-CHP may provide significant OS and PFS benefit over R-CHOP, including in patients with IPI scores 3-5 and in non-GCB DLBCL, highlighting its potential role in higher-risk disease.

**Table 1. Patient characteristics in propensity-matched R-CHOP and Pola-R-CHP groups**

| Characteristic | R-CHOP N = 137 | Pola-R-CHP N = 137 | p-value[1] |
|---|---|---|---|
| Age, years (range) | 69 (21 to 87) | 68 (22 to 85) | 0.2 |
| ECOG ≥ 2 | 20 (15%) | 21 (15%) | >0.9 |
| ≥1 extranodal site | 86 (63%) | 80 (58%) | 0.5 |
| Low-grade component | 23 (62%) | 14 (38%) | 0.2 |
| Stage | | | >0.9 |
| • I-II | 22 (16%) | 22 (16%) | |
| • III-IV | 115 (84%) | 115 (84%) | |
| IPI group | | | >0.9 |
| • Low | 4 (2.9%) | 4 (2.9%) | |
| • Low-intermediate | 19 (14%) | 17 (12%) | |
| • High-intermediate | 55 (40%) | 56 (41%) | |
| • High | 59 (43%) | 60 (44%) | |
| Presence of B symptoms | 43 (31%) | 35 (26%) | 0.3 |
| Male | 72 (53%) | 79 (58%) | 0.5 |
| LDH elevated | 119 (87%) | 124 (91%) | 0.4 |
| Hans Criteria | | | 0.07 |
| • Germinal centre B-cell-like | 50 (36%) | 34 (25%) | |
| • Non-Germinal centre B-cell-like | 85 (62%) | 98 (72%) | |
| • Unknown | 2 (2%) | 5 (4%) | |

[1]Wilcoxon rank sum test; Pearson's Chi-squared test

**Figure 1: Kaplan-Meier curves truncated at 24 months in propensity-matched R-CHOP and Pola-R-CHP groups (A) OS (B) PFS**

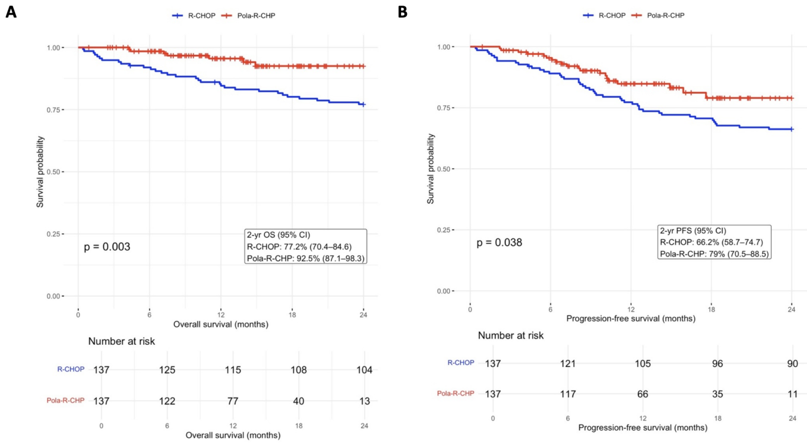

**Copyright Information:** (Online) ISSN: 2572-9241
©2026 The Author(s). HemaSphere is published by John Wiley & Sons Ltd on behalf of European Hematology Association. This is an open access Abstract Book distributed under the Attribution-NonCommercial-NoDerivs (CC BY-NC-ND), which allows third parties to download the articles and share them with others as long as they credit the author and the Abstract Book, but they cannot change the content in any way or use them commercially.
**Abstract Book Citations:** Authors, Title, HemaSphere, 2026;10:(S1):pages. The abstract book DOIs can be found on the "Author Information" page.
**Disclaimer:** Articles published in the journal HemaSphere exclusively reflect the opinions of the authors. The authors are responsible for all content in their abstracts including accuracy of the facts, statements, citing resources, etc.

### (PF1005) PROSPECTIVE VALIDATION OF A WEIGHTED GAH SCORE IN PATIENTS ≥65 YEARS WITH LYMPHOPROLIFERATIVE NEOPLASMS: TOXICITY PREDICTION AND PROGNOSTIC IMPACT

**Topic:** 19. Large B cell lymphomas - Clinical

Josefa Marco Buades*[1], Eva Donato Martin[2], Miguel Fernández Zarzoso[2], Mª José Lis[1], Carmen Amorós[1], Mª Dolores Torregrosa[2], Guillermo Moret[3], Javier De La Rubia Comos[4]

*[1]Consorcio Hospital General Universitario, Valencia, Spain;[2]Hospital Universitario Dr. Peset, Valencia, Spain;[3]Hospital Clinico Universitario, Valencia, Spain;[4]Hospital Universitario y Politecnico La Fe, Valencia, Spain;*

### Background

Lymphoproliferative neoplasms are common in people over 65 years, whose clinical and functional heterogeneity complicates therapeutic decision-making. Conducting a Comprehensive Geriatric Assessment (CGA) is essential for differentiating between "fit" and frail patients. The GAH scale provides a brief instrument that evaluates 30 items across eight key dimensions: nutritional, cognitive, functional, and emotional status, as well as comorbidities, polypharmacy, and health perception.

### Aims

To prospectively validate the GAH scale as a predictor of treatment tolerability and to analyze its impact on therapeutic response, progression-free survival (PFS), and overall survival (OS) in patients with newly diagnosed lymphoproliferative neoplasms.

### Methods

Prospective observational study in patients aged ≥65 years with a first diagnosis of a lymphoproliferative neoplasm. A baseline assessment using the weighted GAH scale was performed before starting first-line active treatment in all patients. Toxicity was defined as the need for therapeutic adjustment (dose reduction, delay, or interruption) or hospital admission. Hematologic and non-hematologic toxicities (CTCAE v5), treatment response, and survival were recorded. To identify the threshold that best distinguishes patients' outcomes, ROC curves were applied, and the value with the best balance of sensitivity and specificity was selected using the Youden index. Prognostic value was assessed using Cox regression models, including toxicity and response as covariates.

**Copyright Information:** (Online) ISSN: 2572-9241
©2026 The Author(s). HemaSphere is published by John Wiley & Sons Ltd on behalf of European Hematology Association. This is an open access Abstract Book distributed under the Attribution-NonCommercial-NoDerivs (CC BY-NC-ND), which allows third parties to download the articles and share them with others as long as they credit the author and the Abstract Book, but they cannot change the content in any way or use them commercially.
**Abstract Book Citations:** Authors, Title, HemaSphere, 2026;10:(S1):pages. The abstract book DOIs can be found on the "Author Information" page.
**Disclaimer:** Articles published in the journal HemaSphere exclusively reflect the opinions of the authors. The authors are responsible for all content in their abstracts including accuracy of the facts, statements, citing resources, etc.

**Results**

106 patients with lymphoproliferative neoplasms were enrolled. Median age was 76 years. The most common diagnoses were chronic lymphocytic leukemia (30.2%), diffuse large B-cell lymphoma (24.5%), and follicular lymphoma (17.9%); an unfavorable baseline prognostic profile predominated. Overall, 68.9% patients experienced some treatment-related toxicity leading to therapy modification, and 52.8% required hospitalization. Patients with toxicity presented significantly higher GAH scale scores (median 50 vs. 40; p=0.018). Using ROC curve analysis, a cut-off point of 55 was identified as the most discriminative (area under the curve, 0.644; p=0.018), with a specificity of 81.8%. Patients with GAH >55 showed a higher frequency of toxicity (85.4% vs. 58.5%; p=0.005) and a higher risk of hospital admission (70.7% vs. 41.5%; p=0.005). Frailty assessed by GAH, was associated with poorer treatment response, with a significantly lower overall response rate was observed in patients with GAH ≤55 versus >55 (86.2% vs 65.9%), with a complete response of 61.5% vs 36.6%, respectively. Regarding survival, patients with a GAH had a median overall survival of 23 months versus not reached in those with GAH ≤55 (p<0.001) (Figure 1), and the median progression-free survival was 14 months versus not reached, respectively. Early mortality (≤8 weeks) occurred exclusively in patients with a GAH >55 (14.6% vs 0%). In the multivariate analysis, a GAH score >55 was established as an independent predictor of toxicity (odds ratio, 4.8) and lower overall survival (hazard ratio, 2.1).

**Summary/Conclusion**

The GAH scale is a valid and specific tool for identifying frailty in older patients with lymphoproliferative neoplasms. A threshold of 55 points is robust for predicting severe toxicity, hospitalizations, and unfavorable clinical outcomes. Its implementation facilitates treatment individualization and safer, more tailored decision-making based on the patient's actual profile.

**Copyright Information:** (Online) ISSN: 2572-9241
©2026 The Author(s). HemaSphere is published by John Wiley & Sons Ltd on behalf of European Hematology Association. This is an open access Abstract Book distributed under the Attribution-NonCommercial-NoDerivs (CC BY-NC-ND), which allows third parties to download the articles and share them with others as long as they credit the author and the Abstract Book, but they cannot change the content in any way or use them commercially.
**Abstract Book Citations:** Authors, Title, HemaSphere, 2026;10:(S1):pages. The abstract book DOIs can be found on the "Author Information" page.
**Disclaimer:** Articles published in the journal HemaSphere exclusively reflect the opinions of the authors. The authors are responsible for all content in their abstracts including accuracy of the facts, statements, citing resources, etc.

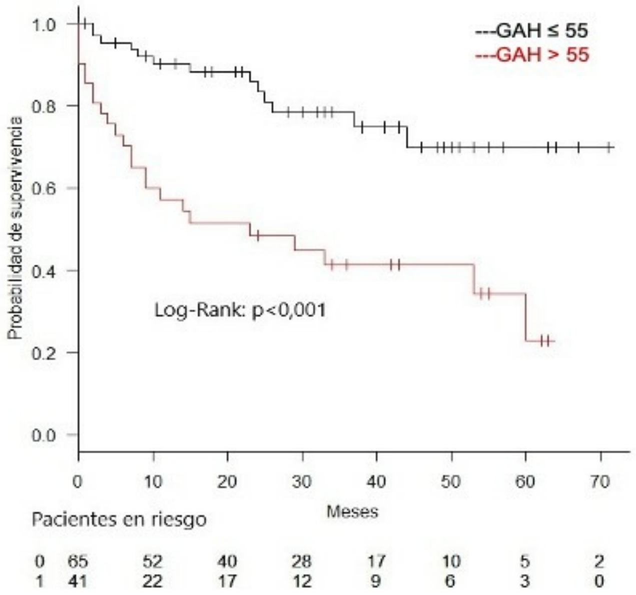

Figure 1. Overall survival curve of the series according to the GAH scale score with a cutoff point at 55.

Copyright Information: (Online) ISSN: 2572-9241
©2026 The Author(s). HemaSphere is published by John Wiley & Sons Ltd on behalf of European Hematology Association. This is an open access Abstract Book distributed under the Attribution-NonCommercial-NoDerivs (CC BY-NC-ND), which allows third parties to download the articles and share them with others as long as they credit the author and the Abstract Book, but they cannot change the content in any way or use them commercially.
Abstract Book Citations: Authors, Title, HemaSphere, 2026;10:(S1):pages. The abstract book DOIs can be found on the "Author Information" page.
Disclaimer: Articles published in the journal HemaSphere exclusively reflect the opinions of the authors. The authors are responsible for all content in their abstracts including accuracy of the facts, statements, citing resources, etc.

### (PF1006) TREATMENT ACCESS ELIMINATES RACIAL DISPARITIES BUT CANNOT OVERCOME STRUCTURAL POVERTY: A NATIONAL ANALYSIS OF SURVIVAL EQUITY IN CURABLE LYMPHOMA

**Topic:** 19. Large B cell lymphomas - Clinical

Shivam Patel*[1], Ansy Patel[2], Pragya Jain[1], Vedant Shah[3], Nency Ganatra[1], Abdullah Jamal[1], Tahir Naqvi[1]

*[1]Baptist hospital of southeast texas, Internal medicine, Beaumont, United States of America;[2]SUNY Upstate Medical University, Syracuse, NY, new york, United States of America; [3]NYMC at St. Mary's General Hospital, Passaic and Saint Clare's Health, Denville, NJ, new jersy, United States of America;*

### Background

Diffuse large B cell lymphoma is one of the few aggressive cancers we can cure, yet who receives that chance depends heavily on zip code, insurance status, and where a patient seeks care. Guideline discordance and socioeconomic disadvantage are known to worsen survival in DLBCL, yet prior studies rely on relative risk estimates and do not separate treatment-attributable from structural contributions to survival inequity.

### Aims

We aimed to quantify absolute survival penalties across insurance, deprivation, geography, race, and facility type, and to decompose each penalty into its preventable and structural components using a national cohort of 91,246 patients.

### Methods

Using the National Cancer Database, we identified patients with DLBCL diagnosed between 2014 and 2020. Guideline-concordant therapy was defined as multiagent chemotherapy plus rituximab. Inverse probability of treatment weighting balanced all 40 covariates before analysis. Survival penalties were quantified as restricted mean survival time differences at 36 months, expressed as life years lost per 1,000 patients. Socioeconomic deprivation was quantified using a composite score derived from five NCDB provided variables: zip code level income quartile, educational attainment quartile, insurance status, urban-rural classification, and distance to the treating facility. Each variable was dichotomized and summed to generate a score ranging from 0 to 5, then categorized as none (0), low (1), Moderate (2), High (3-5). Analyses were repeated within the 55,618 R-CHOP recipients to separate preventable from structural penalties.

### Results

Only 61% received guideline-concordant R-CHOP. Patients who did not were associated with 4.5 fewer months of survival, 373 life-years lost per 1,000, compared to those who received concordant therapy. Uninsured and Medicaid patients lost 3.6 and 3.7 months respectively versus privately insured patients (303 and 307 LYL/1,000; HR 1.72 and 1.65; both $p<0.001$). Socioeconomic deprivation predicted inferior survival in a dose response fashion; the most deprived carried a 36% higher risk of death and lost 1.8 months versus the least deprived (HR 1.36; $p<0.001$). Rural residence and community facility care each contributed 1.1 months of survival loss. Among R-CHOP recipients, the racial survival gap disappeared entirely. Black patients achieved equivalent survival to White patients, confirming that racial disparities in DLBCL reflect unequal access, not biology. Insurance and deprivation penalties told a different story: uninsured patients retained a 2.1-month penalty and Medicaid patients 2.5 months even with R-CHOP, with 57 to 94% of socioeconomic penalties persisting independent of treatment.

**Copyright Information:** (Online) ISSN: 2572-9241
©2026 The Author(s). HemaSphere is published by John Wiley & Sons Ltd on behalf of European Hematology Association. This is an open access Abstract Book distributed under the Attribution-NonCommercial-NoDerivs (CC BY-NC-ND), which allows third parties to download the articles and share them with others as long as they credit the author and the Abstract Book, but they cannot change the content in any way or use them commercially.
**Abstract Book Citations:** Authors, Title, HemaSphere, 2026;10:(S1):pages. The abstract book DOIs can be found on the "Author Information" page.
**Disclaimer:** Articles published in the journal HemaSphere exclusively reflect the opinions of the authors. The authors are responsible for all content in their abstracts including accuracy of the facts, statements, citing resources, etc.

### Summary/Conclusion

Racial survival disparities in DLBCL are entirely preventable through equitable R-CHOP delivery. Socioeconomic penalties are harder to close. Poverty follows patients through the clinic door, and the majority of the survival gap driven by insurance inadequacy and deprivation persists regardless of treatment received. Eliminating survival inequity in curable lymphoma demands both equitable care delivery and the structural interventions that extend well beyond it.

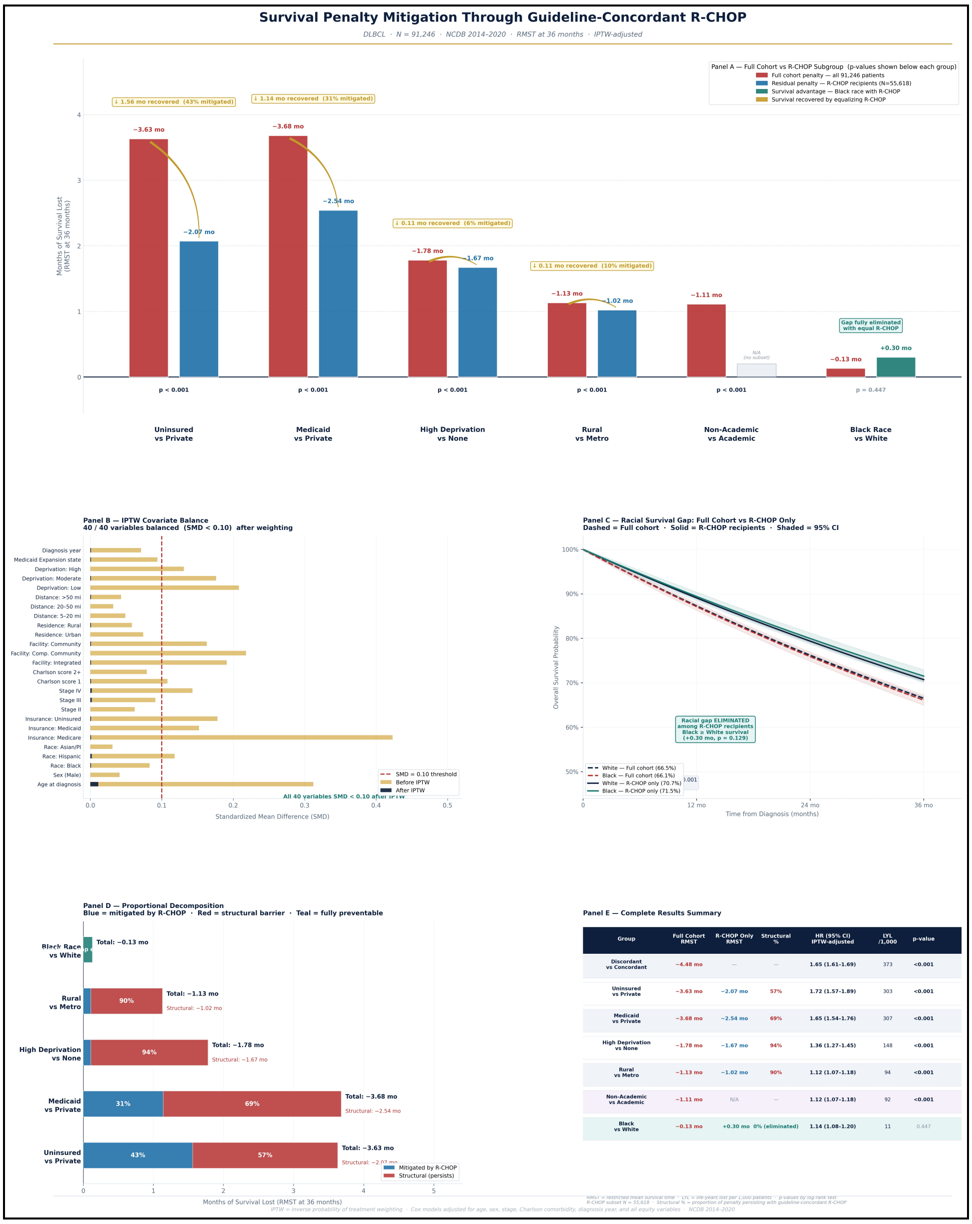

**Copyright Information:** (Online) ISSN: 2572-9241
©2026 The Author(s). HemaSphere is published by John Wiley & Sons Ltd on behalf of European Hematology Association. This is an open access Abstract Book distributed under the Attribution-NonCommercial-NoDerivs (CC BY-NC-ND), which allows third parties to download the articles and share them with others as long as they credit the author and the Abstract Book, but they cannot change the content in any way or use them commercially.
**Abstract Book Citations:** Authors, Title, HemaSphere, 2026;10:(S1):pages. The abstract book DOIs can be found on the "Author Information" page.
**Disclaimer:** Articles published in the journal HemaSphere exclusively reflect the opinions of the authors. The authors are responsible for all content in their abstracts including accuracy of the facts, statements, citing resources, etc.

### (PF1007) EPCORITAMAB + R-MINI-CHOP RESULTS IN 2-YEAR REMISSIONS AND HIGH MRD-NEGATIVITY RATES IN ELDERLY PATIENTS WITH NEWLY DIAGNOSED DLBCL: RESULTS FROM THE EPCORE NHL-2 TRIAL

**Topic:** 19. Large B cell lymphomas - Clinical

David Belada*[1], Juraj Ďuraš[2], Joshua Brody[3], Justin Darrah[4], Yasmin Karimi[5], Franck Morschhauser[6], Gerardo Musuraca[7], Mats Hellström[8], Katerina Kopeckova[9], Lori Leslie[10], Anna Sureda[11], Catherine Thieblemont[12], Francesca Bonello[13], Kojo Osei-Bonsu[14], Mina Khoshdeli[15], Addison Li[15], Aidan Reilly[15], Malene Risum[16], Joost Vermaat[17], Chan Y. Cheah[18]

*[1]Charles University, Hospital and Faculty of Medicine, 4th Department of Internal Medicine – Haematology, Hradec Králové, Czechia; [2]Medical Faculty of the Ostrava University and University Hospital, Department of Hematology, Ostrava, Czechia; [3]Icahn School of Medicine at Mount Sinai, New York, NY, United States of America;[4]Cedars-Sinai Medical Center, Division of Hematology and Cellular Therapy, Department of Medicine, Los Angeles, CA, United States of America; [5]University of Michigan, Division of Hematology/Oncology, Ann Arbor, MI, United States of America; [6]University of Lille, CHU Lille, Department of Hematology, Lille, France; [7]IRCCS Istituto Romagnolo per lo Studio dei Tumori (IRST) "Dino Amadori", Hematology Unit, Meldola, Italy; [8]Uppsala University Hospital, Department of Immunology, Genetics and Pathology, Uppsala, Sweden; [9]Charles University and General University Hospital, First Department of Medicine, First Faculty of Medicine, Prague, Czechia; [10]John Theurer Cancer Center, Hackensack Meridian School of Medicine, Lymphoma Division, Hackensack, NJ, United States of America; [11]Institut Català d'Oncologia-Hospitalet, IDIBELL, Clinical Hematology Department, Barcelona, Spain; [12]Assistance Publique Hôpitaux de Paris - Hôpital Saint-Louis, University of Paris, Department of Hematology, Paris, France; [13]Candiolo Cancer Institute, FPO-IRCCS, Candiolo (Turin), Italy; [14]AbbVie, North Chicago, IL, United States of America; [15]Genmab, Plainsboro, NJ, United States of America; [16]Genmab, Copenhagen, Denmark; [17]Leiden University Medical Center, Department of Hematology, Leiden, The Netherlands; [18]Sir Charles Gairdner Hospital and the University of Western Australia, Perth, WA, Australia;*

### Background

Dose-attenuated rituximab plus cyclophosphamide, doxorubicin, vincristine, and prednisone (R-mini-CHOP) is a standard of care (SOC) for patients (pts) with newly diagnosed (1L) DLBCL unable to receive full-dose R-CHOP due to age or comorbidities. Outcomes with R-mini-CHOP remain suboptimal, with 2-y PFS and OS rates of 47% and 59%, respectively.

### Aims

To report > 2-y follow-up results of epcoritamab, a CD3×CD20 bispecific antibody, plus R-mini-CHOP in elderly pts with 1L DLBCL ineligible for full-dose R-CHOP from the phase 1b/2 EPCORE® NHL-2 trial (NCT04663347).

### Methods

Adults with 1L CD20+ DLBCL, ineligible for full dose R-CHOP due to age ≥ 75 y or ≥ 65 y with comorbidities, received fixed-duration SC epcoritamab 48 mg (QW, cycle(s) [C] 1–2 [21 d each]; Q3W, C3–6 [21 d each]; Q4W, C7–8 [28 d each]; with step-up dosing in C1 [0.16 mg on C1 day 1 (D1) and 0.8 mg on C1D8]) + R-mini-CHOP (Q3W, C1–6). The primary endpoint was ORR per Lugano criteria. MRD negativity, defined as < 1 mutant molecule per mL, was assessed using the exploratory AVENIO Oncology circulating tumor DNA (ctDNA) method.

**Copyright Information:** (Online) ISSN: 2572-9241
©2026 The Author(s). HemaSphere is published by John Wiley & Sons Ltd on behalf of European Hematology Association. This is an open access Abstract Book distributed under the Attribution-NonCommercial-NoDerivs (CC BY-NC-ND), which allows third parties to download the articles and share them with others as long as they credit the author and the Abstract Book, but they cannot change the content in any way or use them commercially.
**Abstract Book Citations:** Authors, Title, HemaSphere, 2026;10:(S1):pages. The abstract book DOIs can be found on the "Author Information" page.
**Disclaimer:** Articles published in the journal HemaSphere exclusively reflect the opinions of the authors. The authors are responsible for all content in their abstracts including accuracy of the facts, statements, citing resources, etc.

**Results**

As of September 21, 2025, 28 pts had received epcoritamab + R-mini-CHOP. Median age was 81 y (range, 74–90), 89% of pts had de novo DLBCL, 46% had germinal center B-cell (GCB) and 39% had non-GCB, 43% had IPI scores 4–5, 64% had elevated LDH, and 39% had bulky disease (≥ 7 cm). Most pts (22/28; 79%) completed treatment (tx) as planned; median relative dose intensity of R-mini-CHOP was 94%.

With a median follow-up of 33.4 mo (range, 2.8–41.1), the ORR was 93% and 86% of pts had a complete response (CR) as best response. Median DOR and DOCR were not reached (NR) at data cutoff. Estimated 2-y DOR and DOCR rates were 79%. Median PFS and median OS were NR. The estimated 2-y PFS and OS rates were 76% and 82%, respectively. 20/22 (91%) pts who completed tx had a CR at end of tx; with a median follow-up for DOCR of 22.6 mo after tx end, 18/20 (90%) remained in CR.

MRD negativity was reported for 20/21 (95%) pts evaluable for MRD at any time; 16/20 (80%) were MRD negative by C3D1. At second assessment (C6D1), 12/16 maintained MRD negativity, 2/16 became MRD positive, and 2/16 were not tested (no sample/off study). High MRD-negativity rates were observed among high-risk subgroups, such as pts with bulky disease (8/9, 89%) and IPI score 3–5 (14/15, 93%).

Most common grade (G) ≥ 3 TEAEs were neutropenia (43%), serious infections (32%), and anemia (14%). Majority (7/9) of G ≥ 3 serious infections occurred within the first 6 cycles of tx during R-mini-CHOP coadministration. TEAEs led to epcoritamab discontinuation in 3 (11%) pts (G2 persistent rhinitis, G2 cytokine release syndrome, and G5 confusional state and cytomegalovirus infection reactivation in a pt aged 90 y also diagnosed with acute cerebrovascular accident).

**Summary/Conclusion**

Fixed-duration epcoritamab + R-mini-CHOP led to high ORR and CR rates, rapid and sustained MRD negativity, and durable remissions in elderly pts with 1L DLBCL ineligible for full-dose R-CHOP. These outcomes compare favorably with historical outcomes of R-mini-CHOP alone. Safety was consistent with prior reports and the known safety profiles of epcoritamab and R-mini-CHOP. These results, along with those from other arms of the trial, support combinations of epcoritamab with SOC across a range of disease settings and pt populations. © American Society of Hematology (2025). Reused with permission.

**Copyright Information:** (Online) ISSN: 2572-9241
©2026 The Author(s). HemaSphere is published by John Wiley & Sons Ltd on behalf of European Hematology Association. This is an open access Abstract Book distributed under the Attribution-NonCommercial-NoDerivs (CC BY-NC-ND), which allows third parties to download the articles and share them with others as long as they credit the author and the Abstract Book, but they cannot change the content in any way or use them commercially.
**Abstract Book Citations:** Authors, Title, HemaSphere, 2026;10:(S1):pages. The abstract book DOIs can be found on the "Author Information" page.
**Disclaimer:** Articles published in the journal HemaSphere exclusively reflect the opinions of the authors. The authors are responsible for all content in their abstracts including accuracy of the facts, statements, citing resources, etc.

### (PF1008) ADDITION OF HIGH-DOSE METHOTREXATE TO STANDARD FIRST-LINE IMMUNOCHEMOTHERAPY IN PATIENTS WITH DIFFUSE LARGE B-CELL LYMPHOMA IS ASSOCIATED WITH ACUTE BUT NOT CHRONIC KIDNEY DISEASE

**Topic:** 19. Large B cell lymphomas - Clinical

Silje Post Strøm*[1, 2], Ahmed Ludvigsen Al-Mashhadi[3, 4, 5], Paw Jensen[4, 6], Thomas Stauffer Larsen[7, 8], Peter Brown[9], Lars Pedersen[1, 2], Christian Fynbo Christiansen[1, 2], Tarec El-Galaly[1, 2, 3, 5]

*[1]Aarhus University and Aarhus University Hospital, Department of Clinical Epidemiology, Aarhus, Denmark;[2]Aarhus University, Department of Clinical Medicine, Aarhus, Denmark; [3]Aarhus University Hospital, Department of Hematology, Aarhus, Denmark; [4]Aalborg University Hospital, Department of Clinical Medicine, Aalborg, Denmark;[5]Aarhus University Hospital, Department of Molecular Medicine, Aarhus, Denmark; [6]Aalborg University Hospital, Department of Haematology, Clinical Cancer Research Center, Aalborg, Denmark; [7]Odense University Hospital, Department of Hematology, Odense, Denmark;[8]University of Southern Denmark, Department of Clinical Research, Odense, Denmark; [9]Rigshospitalet, Department of Hematology, Copenhagen, Denmark;*

### Background

High-dose methotrexate (HD-MTX) remains widely used as CNS prophylaxis in diffuse large B-cell lymphoma (DLBCL), despite a lack of confirmatory randomized clinical trials. Nephrotoxicity, an important HD-MTX-related adverse event, can delay the delivery of curative immunochemotherapy but its occurrence has not been extensively studied in this setting.

### Aims

To explore the association between HD-MTX in first-line treatment of DLBCL and nephrotoxicity.

### Methods

Adults aged 18-79 years with first diagnosis of DLBCL between 01/07-2015 and 31/12-2023 were identified from the Danish Lymphoma Registry. Patients without CNS involvement and with no prior history of kidney disease, according to national laboratory registries and/or diagnosis registries, were included if they were treated with R-CHOP(-like) immunochemotherapy.
Eligible patients were grouped by use of HD-MTX in first line (HD-MTX group vs no-HD-MTX group). The primary endpoint was the 5-year cumulative incidence of stage ≥G3a chronic kidney disease (CKD). Secondary endpoints were 5-year cumulative incidence of stage G5 CKD and 1-year cumulative incidences of any-stage acute kidney injury (AKI) and stage ≥2 AKI. CKD (stage ≥G3a) was defined as renal replacement therapy or two measurements of eGFR<60 at least 90 days apart without eGFR measures of ≥60 in between. Using the same rules, stage G5 was defined by eGFR cutoff of <15. Any-stage AKI was defined as a relative increase in pCr of 1.5x or an absolute increase of 26.5 μmol/L from baseline. Stage ≥2 AKI was defined as a relative increase in pCr of ≥2x from baseline.
Death and re-treatment/relapse were treated as competing risks. Inverse probability of treatment weighting (IPTW) was used to adjust for differences in age, sex, hypertension, diabetes, bulk, Ann Arbor stage, number of extranodal sites, LDH, kidney involvement, B-symptoms, and Charlson Comorbidity Index.

**Copyright Information:** (Online) ISSN: 2572-9241
©2026 The Author(s). HemaSphere is published by John Wiley & Sons Ltd on behalf of European Hematology Association. This is an open access Abstract Book distributed under the Attribution-NonCommercial-NoDerivs (CC BY-NC-ND), which allows third parties to download the articles and share them with others as long as they credit the author and the Abstract Book, but they cannot change the content in any way or use them commercially.
**Abstract Book Citations:** Authors, Title, HemaSphere, 2026;10:(S1):pages. The abstract book DOIs can be found on the “Author Information” page.
**Disclaimer:** Articles published in the journal HemaSphere exclusively reflect the opinions of the authors. The authors are responsible for all content in their abstracts including accuracy of the facts, statements, citing resources, etc.

**Results**

Of 3,919 patients diagnosed with DLBCL in the surveyed period, 1,558 met the inclusion criteria and 1,537 had complete data. Among complete cases, 25% received HD-MTX. The median follow-up was 5.2 years, with 5-year OS estimates of 91% (95%CI: 88-94) for the HD-MTX group vs. 93% (95%CI: 91-94) for the no HD-MTX group.

There were no differences in the IPTW-adjusted 5-year cumulative incidence of CKD (stage ≥G3a) of 5.3% (95%CI: 3.2-7.5) for the HD-MTX group vs. 6.5% (95%CI: 5.0-8.2) with a risk-ratio (RR) of 0.81 (95%CI: 0.5-1.3). The 5-year cumulative incidence of CKD stage G5 was <1%, and similar between the groups. The IPTW-adjusted 1-year cumulative incidence of any-stage AKI was higher for patients in the HD-MTX group 43% (95%CI: 36-50) vs. 15% (95%CI: 13-17) and a RR of 2.8 (95%CI: 2.2-3.6). For stage ≥2 AKI, the IPTW-adjusted 1-year cumulative incidence was 11% (95%CI: 6.3-17) vs. 3.5% (95%CI: 2.5-4.7) with a RR of 3.2 (95%CI: 1.5-5.6) (Figure 1b). For the patients that experienced AKI in the HD-MTX group the median time from initiation of first-line treatment to any-stage AKI was 36 days (IQR: 17-80). Crude comparisons between the groups gave similar results (data not shown).

**Summary/Conclusion**

The use of HD-MTX as CNS prophylaxis was not associated with CKD in patients with DLBCL. However, HD-MTX did lead to a substantially higher cumulative incidence of AKI, which could lead to untoward delays in immunochemotherapy or dose reductions. Without firmly documented efficacy of early intercalated HD-MTX, its use should be deferred till after completion of standard immunochemotherapy.

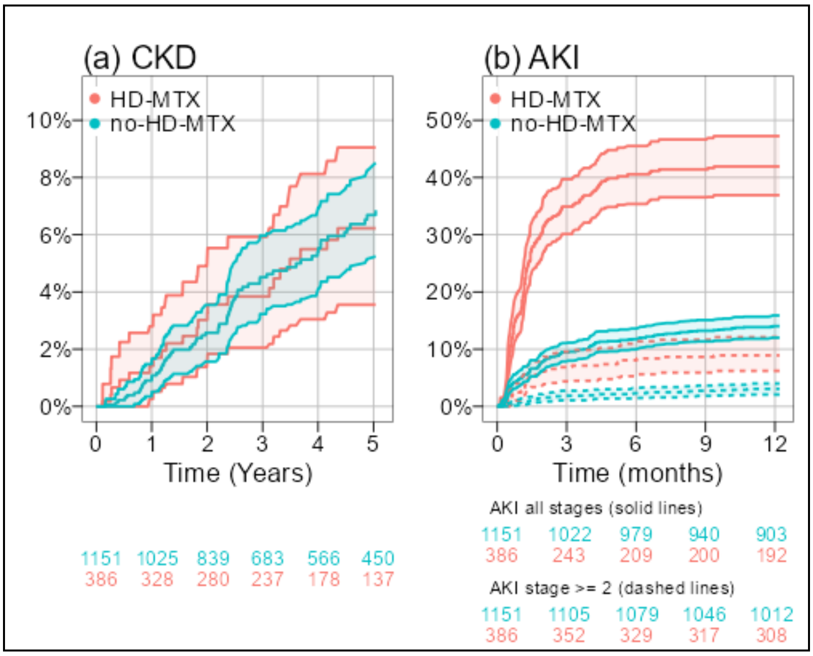

Figure 1: Cumulative incidence

**Copyright Information:** (Online) ISSN: 2572-9241
©2026 The Author(s). HemaSphere is published by John Wiley & Sons Ltd on behalf of European Hematology Association. This is an open access Abstract Book distributed under the Attribution-NonCommercial-NoDerivs (CC BY-NC-ND), which allows third parties to download the articles and share them with others as long as they credit the author and the Abstract Book, but they cannot change the content in any way or use them commercially.
**Abstract Book Citations:** Authors, Title, HemaSphere, 2026;10:(S1):pages. The abstract book DOIs can be found on the "Author Information" page.
**Disclaimer:** Articles published in the journal HemaSphere exclusively reflect the opinions of the authors. The authors are responsible for all content in their abstracts including accuracy of the facts, statements, citing resources, etc.

### (PF1009) NOVA-BCL6-1: A FIRST-IN-HUMAN PHASE 1 TRIAL OF LY4584180, A NOVEL BCL6 MOLECULAR GLUE, IN PATIENTS WITH RELAPSED/REFRACTORY B-CELL MALIGNANCIES (TRIAL IN PROGRESS)

**Topic:** 19. Large B cell lymphomas - Clinical

Philippe Armand[1], Jonathan Lockhart[2], Yair Levy*[3], Vishalkumar Patel[4], Shyamli Singla[4], Jonathan Boss[4], James Pauff[4], Manish Patel[5]

*[1]Dana-Farber Cancer Institute, Boston, MA, United States of America; [2]HCA HealthONE, Denver, CO, United States of America; [3]Texas Oncology, Dallas, TX, United States of America; [4]Eli Lilly and Company, Indianapolis, IN, United States of America; [5]Florida Cancer Specialists/Sarah Cannon Research Institute, Sarasota, FL, United States of America;*

### Background

BCL6 is a protooncogene and key molecular driver of many B-cell malignancies, including diffuse large B-cell lymphoma (DLBCL) and follicular lymphoma (FL). Given its central role in the pathobiology of these diseases, BCL6 represents an attractive therapeutic target. LY4584180 is a novel oral, highly selective molecular glue that induces BCL6 oligomerization, resulting in sequestering of BCL6 polymers into cellular aggregates, leading to polyubiquitination by multiple E3 ligases, and subsequent degradation by the proteasome. LY4584180 has a unique non-cereblon-dependent degradation mechanism that allows combination with IMiDs and CELMoDs thus increasing combinability and therapeutic options. In preclinical studies, LY4584180 demonstrated rapid, potent, and concentration-dependent degradation of BCL6, was antiproliferative across a panel of malignant human B-cell lines and exhibited antitumor activity *in vivo*.[1]

### Aims

The NOVA-BCL6-1 trial aims to determine the PK, optimal dose, safety, and antitumor activity of LY4584180 in relapsed/refractory (R/R) B-cell malignancies.

**Copyright Information:** (Online) ISSN: 2572-9241
©2026 The Author(s). HemaSphere is published by John Wiley & Sons Ltd on behalf of European Hematology Association. This is an open access Abstract Book distributed under the Attribution-NonCommercial-NoDerivs (CC BY-NC-ND), which allows third parties to download the articles and share them with others as long as they credit the author and the Abstract Book, but they cannot change the content in any way or use them commercially.
**Abstract Book Citations:** Authors, Title, HemaSphere, 2026;10:(S1):pages. The abstract book DOIs can be found on the "Author Information" page.
**Disclaimer:** Articles published in the journal HemaSphere exclusively reflect the opinions of the authors. The authors are responsible for all content in their abstracts including accuracy of the facts, statements, citing resources, etc.

### Methods

NOVA-BCL6-1 is a first-in-human, open-label, multicenter phase 1a/b trial of LY4584180 in patients (pts) with R/R B-cell malignancies (NCT07226843). LY4584180 will be administered orally twice daily in 28-day cycles. The trial will be conducted in 2 parts: phase 1a dose escalation/dose optimization and phase 1b dose expansion. Dose escalation will follow the mTPI-2 method and will enroll pts with R/R DLBCL not otherwise specified (NOS), high-grade B-cell lymphoma (HGBCL), and FL (grades 1-3b). Pts with R/R DLBCL transformed from indolent lymphoma, marginal zone lymphoma (MZL), mantle cell lymphoma (MCL), Waldenström macroglobulinemia/lymphoplasmacytic lymphoma (WM/LPL), or chronic lymphocytic leukemia/small lymphocytic lymphoma (CLL/SLL) may be enrolled in backfill. The dose-limiting toxicity evaluation period will be 28 days. Dose optimization will enroll pts with DLBCL-NOS, HGBCL, or FL (grades 1-3b), who will be randomized 1:1 to 2 or more dose levels. Pts with FL grade 1-3a (classic FL) who achieve complete response may stop therapy after 1 year; those with partial response may stop after 2 years; pts with FL grade 3b, DLBCL-NOS, or HGBCL may continue until disease progression or discontinuation. Dose expansion will enroll pts with select tumor types across 5 cohorts to evaluate LY4584180 alone or in combination with other agents (**Table**). Eligible pts (≥18 years) must have confirmed R/R B-cell malignancies with measurable disease, an ECOG PS of 0-1, and ≥2 prior lines of systemic therapy.  Dose expansion eligibility requirements are outlined in the **Table**. Key exclusion criteria include known CNS involvement and significant cardiovascular disease.

**Table**

| Cohort (Treatment) | Tumor Type | Key Eligibility |
|---|---|---|
| • B1 (LY4584180) | • DLBCL-NOS/HGBCL/FL (grade 3b) | • B1a: ≥2 prior lines of therapy including CAR T-  cell therapy<br>• B1b: ≥2 prior lines of therapy; no prior CAR T-cell therapy |
| • B2 (LY4584180) | • FL (grades 1-3a) | • ≥2 prior lines of therapy |
| • B3 (LY4584180) | • MZL, MCL, WM/LPL, CLL/SLL | • ≥2 prior lines of therapy<br>• CLL/SLL: prior BTK inhibitor + BCL2 inhibitor required<br>• WM/LPL: prior BTK inhibitor required |
| • B4 (LY4584180) | • DLBCL transformed from indolent lymphoma | • ≥2 prior lines of therapy |
| • B5 (LY4584180 + rituximab) | • DLBCL-NOS/HGBCL/FL (grade 3b) | • ≥2 prior lines including CAR T-cell therapy unless not a candidate |
| [1]Langan C, et al. Abstract 4594. Poster presented at AACR, April 17-22, 2026 | | |

**Copyright Information:** (Online) ISSN: 2572-9241
©2026 The Author(s). HemaSphere is published by John Wiley & Sons Ltd on behalf of European Hematology Association. This is an open access Abstract Book distributed under the Attribution-NonCommercial-NoDerivs (CC BY-NC-ND), which allows third parties to download the articles and share them with others as long as they credit the author and the Abstract Book, but they cannot change the content in any way or use them commercially.
**Abstract Book Citations:** Authors, Title, HemaSphere, 2026;10:(S1):pages. The abstract book DOIs can be found on the "Author Information" page.
**Disclaimer:** Articles published in the journal HemaSphere exclusively reflect the opinions of the authors. The authors are responsible for all content in their abstracts including accuracy of the facts, statements, citing resources, etc.

### (PF1011) DEVELOPMENT AND VALIDATION OF A CLINICOPATHOLOGICAL PROGNOSTIC MODEL FOR PRIMARY CENTRAL NERVOUS SYSTEM LYMPHOMA

**Topic:** 19. Large B cell lymphomas - Clinical

Yupiao Feng*[1], Xinting Hu[2], Yutang Li[3], Dai Yuan[4], Huiting Qu[2], Xiangxiang Zhou[2]

*[1]Shandong Provincial Hospital, Shandong University, Jinan, China, Jinan, China;[2]Shandong Provincial Hospital Affiliated to Shandong First Medical University, Jinan, China, Department of Hematology, Jinan, China; [3]Shandong Provincial Hospital, Shandong University, Jinan, China, Department of Hematology, Jinan, China; [4]Shandong Provincial Hospital Affiliated to Shandong First Medical University, Jinan, China, Jinan, China;*

### Background

The IELSG and MSKCC prognostic scoring systems, while commonly used for primary central nervous system lymphoma (PCNSL), present certain limitations in clinical practice. The IELSG model is constrained by the inconsistent availability of LDH and CSF protein measurements, whereas the MSKCC system relies solely on age and Karnofsky performance status, potentially limiting its prognostic scope.

### Aims

This study seeks to establish and validate a novel prognostic model leveraging real-world clinical data to enhance predictive accuracy and practical applicability.

### Methods

A retrospective analysis was conducted on 180 newly diagnosed PCNSL patients (≥ 18 years) treated at our center between January 2012 and January 2025. Overall survival (OS) served as the primary endpoint, with progression-free survival (PFS) as the secondary endpoint. Patients were randomly divided into training (70%) and validation (30%) cohorts. Univariable and multivariable Cox regression analyses were performed to identify factors associated with OS. A novel prognostic model was constructed based on multivariable findings and subsequently assessed for calibration, discriminative ability, and clinical utility in both cohorts.

### Results

The median age at diagnosis of the enrolled 180 patients was 60 years (range: 18–82 years), with 56.67% (102/180) being male. Diffuse large B-cell lymphoma (DLBCL) constituted the predominant pathological subtype (95%, 171/180). Treatment modalities included subtotal/total tumor resection in 135 cases (75%), biopsy alone in 45 cases (25%), high-dose methotrexate (HD-MTX)-based chemotherapy in 67 cases (37.22%), and combination therapies (including radiotherapy or hematopoietic cell transplantation) in 30 cases (16.7%). Multivariable Cox regression identified age ≥ 60 years (HR: 2.58, $p < 0.05$), ECOG-PS ≥ 2 (HR: 4.28, $p < 0.05$), and positive MYC expression by immunohistochemistry (HR: 1.85, $p < 0.05$) as independent predictors of OS. Based on independent prognostic factors identified by multivariable Cox regression analysis, we developed a novel prognostic prediction model and visualized it as a nomogram **(Figure 1A)**. The model exhibited good discrimination, with area under the curve values of 0.74 and 0.89 in the training and validation cohorts, respectively **(Figure 1B-C)**. Calibration curves demonstrated high consistency between the predicted probabilities and actual observations. Furthermore, risk stratification based on this model revealed significant differences in survival status among patients in different risk groups, indicating that this risk stratification strategy can effectively differentiate patient prognosis **(Figure 1D-E)**.

**Copyright Information:** (Online) ISSN: 2572-9241
©2026 The Author(s). HemaSphere is published by John Wiley & Sons Ltd on behalf of European Hematology Association. This is an open access Abstract Book distributed under the Attribution-NonCommercial-NoDerivs (CC BY-NC-ND), which allows third parties to download the articles and share them with others as long as they credit the author and the Abstract Book, but they cannot change the content in any way or use them commercially.
**Abstract Book Citations:** Authors, Title, HemaSphere, 2026;10:(S1):pages. The abstract book DOIs can be found on the "Author Information" page.
**Disclaimer:** Articles published in the journal HemaSphere exclusively reflect the opinions of the authors. The authors are responsible for all content in their abstracts including accuracy of the facts, statements, citing resources, etc.

### Summary/Conclusion

This study presents a novel prognostic model integrating age, ECOG-PS, and MYC expression to predict OS in newly diagnosed PCNSL patients. By incorporating high-risk clinicopathological features associated with tumor aggressiveness, this model may facilitate more individualized treatment planning.

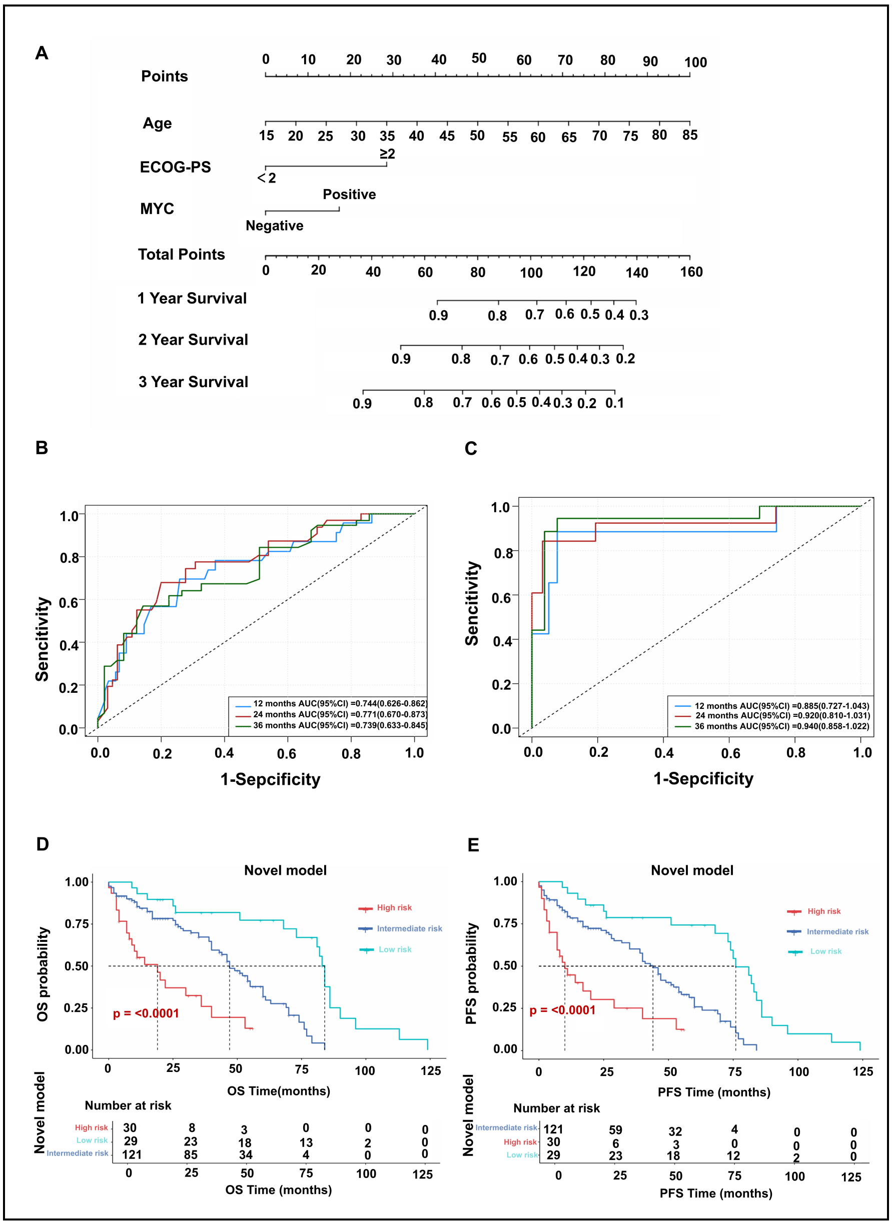

**Copyright Information:** (Online) ISSN: 2572-9241
©2026 The Author(s). HemaSphere is published by John Wiley & Sons Ltd on behalf of European Hematology Association. This is an open access Abstract Book distributed under the Attribution-NonCommercial-NoDerivs (CC BY-NC-ND), which allows third parties to download the articles and share them with others as long as they credit the author and the Abstract Book, but they cannot change the content in any way or use them commercially.
**Abstract Book Citations:** Authors, Title, HemaSphere, 2026;10:(S1):pages. The abstract book DOIs can be found on the "Author Information" page.
**Disclaimer:** Articles published in the journal HemaSphere exclusively reflect the opinions of the authors. The authors are responsible for all content in their abstracts including accuracy of the facts, statements, citing resources, etc.

### (PF1012) EXTRANODAL METABOLIC TUMOR VOLUME IDENTIFIES LARGE B-CELL LYMPHOMA SUBGROUPS THAT BENEFIT FROM POLA-R-CHP

**Topic:** 19. Large B cell lymphomas - Clinical

Hajime Sakuma*[1], Naoya Fukuda[1], Kazuki Nagao[1], Kosuke Shima[1], Masanori Toho[1], Masami Takeuchi[1], Kosei Matsue[1]

[1]*Kameda Medical Center, Department of Hematology/Oncology, Kamogawa, Chiba, Japan;*

### Background

R-CHOP has been the standard first-line regimen for large B-cell lymphoma (LBCL) for over two decades, until the POLARIX trial demonstrated improved progression-free survival (PFS) with polatuzumab vedotin (PV) in Pola-R-CHP. However, overall survival (OS) did not differ, and there remains no validated strategy to identify patients who benefit most from PV-based therapy.

### Aims

To determine whether the composition of total metabolic tumor volume (TMTV), particularly extranodal burden, improves frontline treatment stratification between Pola-R-CHP and R-CHOP in LBCL.

### Methods

We retrospectively analyzed patients with newly diagnosed LBCL treated with R-CHOP or Pola-R-CHP from 2007 to 2025. Baseline positron emission tomography-computed tomography was used to quantify TMTV and extranodal metabolic tumor volume. The proportion of extranodal disease (EN/TMTV) was derived. Associations with 3-year PFS and treatment interactions were evaluated using multivariable Cox models with interaction terms. Survival was assessed using Kaplan Meier analysis.

Extranodal site count greater than one was associated with worse PFS (HR: 1.89 [95% CI: 1.20–2.96];$p < 0.01$), but showed inferior discrimination (C-index: 0.67) and did not identify a subgroup with differential treatment benefit. Sensitivity analyses showed that the advantage of Pola-R-CHP in patients with low TMTV and high EN/TMTV persisted across TMTV thresholds up to 2000 mL.

-->

**Copyright Information:** (Online) ISSN: 2572-9241
©2026 The Author(s). HemaSphere is published by John Wiley & Sons Ltd on behalf of European Hematology Association. This is an open access Abstract Book distributed under the Attribution-NonCommercial-NoDerivs (CC BY-NC-ND), which allows third parties to download the articles and share them with others as long as they credit the author and the Abstract Book, but they cannot change the content in any way or use them commercially.
**Abstract Book Citations:** Authors, Title, HemaSphere, 2026;10:(S1):pages. The abstract book DOIs can be found on the "Author Information" page.
**Disclaimer:** Articles published in the journal HemaSphere exclusively reflect the opinions of the authors. The authors are responsible for all content in their abstracts including accuracy of the facts, statements, citing resources, etc.

**Results**

Among 625 patients (494 R-CHOP and 131 Pola-R-CHP), those receiving Pola-R-CHP were (75.9 years [interquartile range (IQR): 70.5–81.7] vs 73.9 years [IQR: 66.1–81.3]) older and more likely to have advanced disease (72.5% vs 61.3%). Median TMTV was 206 mL. With a median follow up of 36.6 months, PFS and OS were similar between regimens. High TMTV (≥ 350 mL) was associated with inferior survival (hazard ratio [HR]: 2.72 [95% confidence interval (CI): 2.02–3.67]; $p < 0.01$). EN/TMTV alone did not stratify outcomes and was minimally correlated with TMTV ($r = -0.026$).
Adding EN/TMTV improved 3-year PFS prediction beyond TMTV and regimen (C-index: 0.72 vs 0.66; $p = 0.03$). A significant treatment interaction was observed with Pola-R-CHP conferring significantly better PFS only in patients with low TMTV and high EN/TMTV (HR 0.11 [95% CI: 0.02–0.81]; $p = 0.03$; Figure A). This subgroup showed higher rates of sinus (10.1% vs 2.1%) and breast involvement (7.5 % vs 1.2%). Three-year PFS (96.3% vs 74.0%; Figure B) and OS (100% vs 80.2%) in this subgroup was higher for Pola-R-CHP.
Extranodal site count greater than one was associated with worse PFS (HR: 1.89 [95% CI: 1.20–2.96]; $p < 0.01$), but showed inferior discrimination (C-index: 0.67) and did not identify a subgroup with differential treatment benefit. Sensitivity analyses showed that the advantage of Pola-R-CHP in patients with low TMTV and high EN/TMTV persisted across TMTV thresholds up to 2000 mL.

**Summary/Conclusion**

High EN/TMTV identifies a subgroup with low tumor burden who appear to derive particular benefit from Pola-R-CHP. Imaging-derived extranodal burden may provide a rapid, non-invasive biomarker to guide frontline regimen selection and could complement emerging molecular classifiers. External validation integrating molecular and imaging data is warranted.

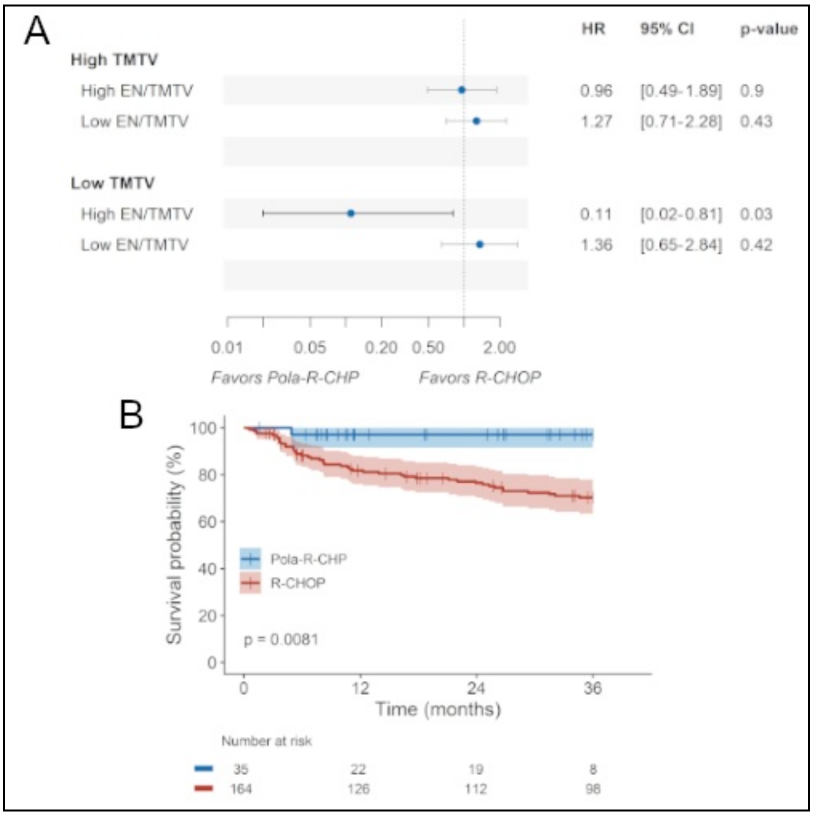

**Copyright Information:** (Online) ISSN: 2572-9241
©2026 The Author(s). HemaSphere is published by John Wiley & Sons Ltd on behalf of European Hematology Association. This is an open access Abstract Book distributed under the Attribution-NonCommercial-NoDerivs (CC BY-NC-ND), which allows third parties to download the articles and share them with others as long as they credit the author and the Abstract Book, but they cannot change the content in any way or use them commercially.
**Abstract Book Citations:** Authors, Title, HemaSphere, 2026;10:(S1):pages. The abstract book DOIs can be found on the “Author Information” page.
**Disclaimer:** Articles published in the journal HemaSphere exclusively reflect the opinions of the authors. The authors are responsible for all content in their abstracts including accuracy of the facts, statements, citing resources, etc.

### (PF1013) DEVELOPMENT OF A PROGNOSTIC MODEL INCORPORATING PLASMA EBV-DNA LEVEL FOR EBV-POSITIVE DIFFUSE LARGE B-CELL LYMPHOMA: A RETROSPECTIVE, SINGLE-CENTER STUDY

**Topic:** 19. Large B cell lymphomas - Clinical

Xinzhu Tang*[1, 2, 3], He Li[1], Ailin Zhao[1], Yuhui Chen[1, 2], Yutong Wang[1, 2], Lang Min[1, 2], Xinai Gan[1, 2], Laduona Wang[1, 2, 3], Jiang Yu[1, 2, 4], Ting Niu[1, 4, 5, 6]

[1]*West China Hospital, Sichuan University, Hematology, Chengdu, China;*[2]*Sichuan University, West China School of Medicine, Chengdu, China;* [3]*West China Lecheng Hospital of Sichuan University, Hematology, Qionghai, China;*[4]*West China Hospital, Sichuan University, Department of Hematology, Institute of Hematology, and Center for High Altitude Medicine, Chengdu, China;* [5]*West China Hospital, Sichuan University, State Key Laboratory of Biotherapy, Collaborative Innovation Center of Biotherapy, Chengdu, China;* [6]*West China Hospital, Sichuan University, National Facility for Translational Medicine (Sichuan), Chengdu, China;*

### Background

Epstein-Barr virus-positive diffuse large B-cell lymphoma (EBV+ DLBCL) is an aggressive subtype accounting for 10%–15% of DLBCL cases worldwide. Despite advances in chemoimmunotherapy, patients with EBV+ DLBCL continue to experience inferior outcomes compared to EBV-negative counterparts. The International Prognostic Index (IPI) is widely used for DLBCL risk stratification, but no dedicated prognostic model exists for EBV+ DLBCL. Emerging evidence suggests that pretreatment plasma EBV-DNA level may serve as a prognostic biomarker. However, models incorporating this parameter are lacking. We therefore conducted a retrospective study to develop a prognostic model integrating plasma EBV-DNA level for EBV+ DLBCL.

### Aims

To evaluate the prognostic impact of plasma EBV-DNA level on overall survival (OS) and progression-free survival (PFS), and to develop a predictive model for OS in patients with EBV+ DLBCL.

### Methods

This single-center retrospective study enrolled 58 patients diagnosed with EBV+ DLBCL at West China Hospital, Sichuan University between January 2010 and January 2025. OS and PFS were analyzed using the Kaplan-Meier method and multivariable Cox proportional hazards regression, and plasma EBV-DNA level was log10-transformed for the regression model. A nomogram was constructed based on the Cox model to visualize probability estimates for OS. The model's discriminatory performance was quantified by Harrell's C-index, after verifying the proportional hazards assumption with the Schoenfeld residuals test.

**Copyright Information:** (Online) ISSN: 2572-9241
©2026 The Author(s). HemaSphere is published by John Wiley & Sons Ltd on behalf of European Hematology Association. This is an open access Abstract Book distributed under the Attribution-NonCommercial-NoDerivs (CC BY-NC-ND), which allows third parties to download the articles and share them with others as long as they credit the author and the Abstract Book, but they cannot change the content in any way or use them commercially.
**Abstract Book Citations:** Authors, Title, HemaSphere, 2026;10:(S1):pages. The abstract book DOIs can be found on the "Author Information" page.
**Disclaimer:** Articles published in the journal HemaSphere exclusively reflect the opinions of the authors. The authors are responsible for all content in their abstracts including accuracy of the facts, statements, citing resources, etc.

### Results

Median age was 57.7 years (range 15.0–83.0). Advanced stage (III/IV) was present in 75.8% of patients, extranodal involvement in 79.3%, B symptoms in 46.6%, and hemophagocytic lymphohistiocytosis (HLH) in 8%. Ki67 index was ≥20% in all 53 patients assessed, and CD30 positivity was 76.6% (36/47). After a median follow-up of 39 months (range 0.7–137.4), the 1-year, 3-year, and 5-year OS rates were 62.1%, 48.3%, and 36.2%, respectively, and the corresponding PFS rates were 60.3%, 44.8%, and 34.5%. Univariate analysis revealed that plasma EBV-DNA level, presence of HLH, ECOG-PS ≥2, and age ≥60 years are independent prognostic factors for both OS and PFS. Multivariate Cox proportional hazards model identified plasma EBV-DNA level (log10-transformed) (HR 1.75; 95% CI, 1.28-2.38; P < 0.001), presence of HLH, and ECOG-PS ≥2 as significant predictors of prognosis in EBV+ DLBCL. The proportional hazards assumption was satisfied (global Schoenfeld test P = 0.797). A prognostic nomogram was constructed to visualize 1-year, 3-year, and 5-year OS probabilities incorporating these three factors, and the model demonstrated good discrimination with a C-index of 0.815 (SE = 0.048).

### Summary/Conclusion

The prognostic model incorporating plasma EBV-DNA level, presence of HLH, and ECOG-PS ≥2 demonstrated robust predictive performance for outcomes in patients with EBV+ DLBCL, and may facilitate risk stratification and individualized treatment decisions.

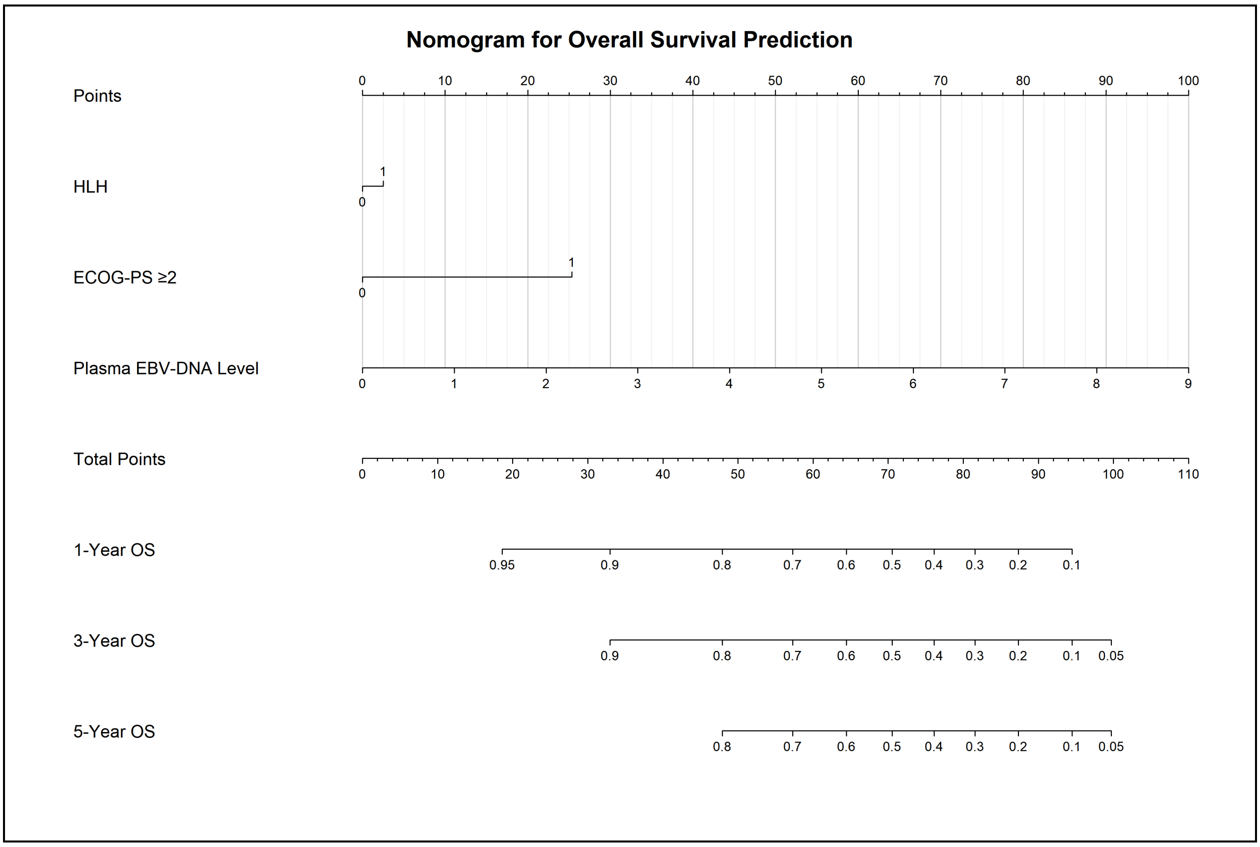

Figure 1. Nomogram for predicting OS in EBV+ DLBCL.

Copyright Information: (Online) ISSN: 2572-9241
©2026 The Author(s). HemaSphere is published by John Wiley & Sons Ltd on behalf of European Hematology Association. This is an open access Abstract Book distributed under the Attribution-NonCommercial-NoDerivs (CC BY-NC-ND), which allows third parties to download the articles and share them with others as long as they credit the author and the Abstract Book, but they cannot change the content in any way or use them commercially.
Abstract Book Citations: Authors, Title, HemaSphere, 2026;10:(S1):pages. The abstract book DOIs can be found on the "Author Information" page.
Disclaimer: Articles published in the journal HemaSphere exclusively reflect the opinions of the authors. The authors are responsible for all content in their abstracts including accuracy of the facts, statements, citing resources, etc.

### (PF1014) REAL-WORLD (RW) TREATMENT PATTERNS AND SURVIVAL OUTCOMES IN PATIENTS (PTS) WITH NEWLY DIAGNOSED HIGH-RISK (HR) LARGE B-CELL LYMPHOMA (LBCL)

**Topic:** 19. Large B cell lymphomas - Clinical

Ran Reshef[1], Javier Munoz*[2], Babatunde Adedokun[3], Qian Huang[3], Hil Hsu[3], Nan Liu[3], Madhu Palivela[3], Zhen-Huan Hu[3], Liana Nikolaenko[3], Richard Lee[3], Julio Chavez[4]

*[1]Columbia University Irving Medical Center, New York, NY, United States of America;[2]Mayo Clinic, Phoenix, AZ, United States of America; [3]Kite, a Gilead Company, Santa Monica, CA, United States of America;[4]Mayo Clinic, Jacksonville, FL, United States of America;*

### Background

LBCL is curable in ~60% of pts treated with first-line (1L) chemoimmunotherapy (CIT); however, outcomes remain poor for pts with HR disease, defined as International Prognostic Index (IPI) 4-5. RW data on contemporary outcomes and subsequent therapy pathways in this population are limited.

### Aims

To evaluate RW treatment patterns, time to next treatment (rwTTNT), overall survival (rwOS), and utilization of second-line (2L) therapies in pts with HR LBCL treated with 1L CIT.

### Methods

This retrospective study used electronic health records from the US-based Flatiron Health database. Pts had HR LBCL, initiated 1L therapy Jan 1, 2011-Feb 28, 2025 (all pts), or Jan 1, 2019-Feb 28, 2025 (recent cohort, a subset of all pts), and had ≥6 mo follow-up opportunity through Aug 31, 2025. Outcomes were rwTTNT, rwOS, 1L therapy utilization, and pathway to 2L therapy and were analyzed descriptively.

**Copyright Information:** (Online) ISSN: 2572-9241
©2026 The Author(s). HemaSphere is published by John Wiley & Sons Ltd on behalf of European Hematology Association. This is an open access Abstract Book distributed under the Attribution-NonCommercial-NoDerivs (CC BY-NC-ND), which allows third parties to download the articles and share them with others as long as they credit the author and the Abstract Book, but they cannot change the content in any way or use them commercially.
**Abstract Book Citations:** Authors, Title, HemaSphere, 2026;10:(S1):pages. The abstract book DOIs can be found on the “Author Information” page.
**Disclaimer:** Articles published in the journal HemaSphere exclusively reflect the opinions of the authors. The authors are responsible for all content in their abstracts including accuracy of the facts, statements, citing resources, etc.

**Results**

In total, 666 pts were included, with 337 in the recent cohort. Median follow-up from start of 1L therapy was 62 and 38 mo, respectively. In the recent cohort, median age was 74 y (IQR, 68-81); 47% were female. Most pts (86%) had DLBCL not otherwise specified; 87% had IPI 4 and 13% had IPI 5. The most common 1L regimens were R-CHOP-based (64%), R-EPOCH-based (12%), and pola-R-CHP-based (6%). Median duration of 1L therapy was 106 d (IQR, 63-133). There were no significant differences in baseline characteristics or 1L regimens between all pts and the recent cohort. Median rwOS (95% CI) from start of 1L therapy was 35 mo (29-47) for all pts and 39 mo (26-56) in the recent cohort. At 2 y, rwOS was 57% for all pts and 58% in the recent cohort. Median rwTTNT was 11 mo for both groups (Figure).
To evaluate pathways to 2L therapy, pts were placed in 3 groups (grp): Grp A received 2L therapy, Grp B was alive without 2L, and Grp C died before receiving 2L therapy. Among all pts, grp proportions were 38% (n=256), 27% (n=179), and 35% (n=231), respectively. In Grp A, the most common 2L regimens were R-ICE (10%), pola-based regimens (8%), R-Benda (8%), and R-CHOP (7%); 1% received CAR T-cell therapy. Overall, 60% (n=401) died; 33% (n=222; Grp A, n=67; Grp C, n=155) died ≤12 mo of completing 1L therapy. In Grp C, median time to death from diagnosis was 6.9 mo; median times to death from start and end of 1L therapy were 6.1 and 1.3 mo, respectively.
In the recent cohort, the proportions in Grps A, B, and C were 32% (n=108), 36% (n=122), and 32% (n=107), respectively. In Grp A, the most common 2L regimens were pola-based regimens (19%), R-CHOP (7%), R-ICE (6%), and R-Gem/Ox (6%); 3% received CAR T-cell therapy. Overall, 48% (n=161) died; 32% (n=108; Grp A, n=27; Grp C, n=81) died ≤12 mo of completing 1L therapy. In Grp C, median time to death from diagnosis was 6.3 mo; median times to death from start and end of 1L therapy were 5.4 and 1.1 mo, respectively.

**Summary/Conclusion**

Outcomes remain poor among pts with HR LBCL despite newer 1L CIT regimens. A significant proportion of pts (73%) progressed to 2L or died even before receiving 2L, highlighting a critical unmet need for improved 1L therapies. Differences in rwOS, but not rwTTNT, between all pts and the recent cohort suggest evolution of 2L+ therapies over time. Study limitations include unknown rationales for and possible misclassification of specific therapy/drug combinations as well as different duration of follow-up and censoring between the recent cohort and all pts.
RR and JM contributed equally.

**Copyright Information:** (Online) ISSN: 2572-9241
©2026 The Author(s). HemaSphere is published by John Wiley & Sons Ltd on behalf of European Hematology Association. This is an open access Abstract Book distributed under the Attribution-NonCommercial-NoDerivs (CC BY-NC-ND), which allows third parties to download the articles and share them with others as long as they credit the author and the Abstract Book, but they cannot change the content in any way or use them commercially.
**Abstract Book Citations:** Authors, Title, HemaSphere, 2026;10:(S1):pages. The abstract book DOIs can be found on the “Author Information” page.
**Disclaimer:** Articles published in the journal HemaSphere exclusively reflect the opinions of the authors. The authors are responsible for all content in their abstracts including accuracy of the facts, statements, citing resources, etc.

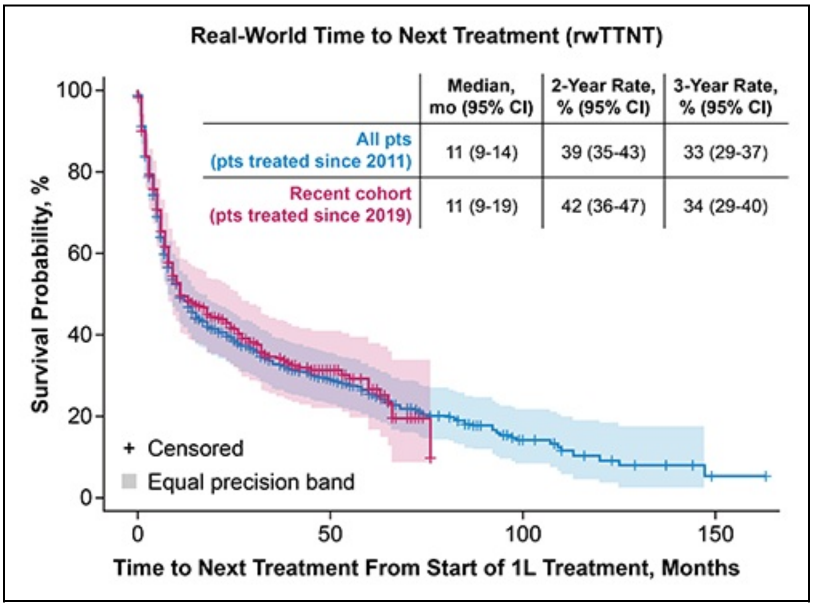

| | Median, mo (95% CI) | 2-Year Rate, % (95% CI) | 3-Year Rate, % (95% CI) |
|---|---|---|---|
| All pts (pts treated since 2011) | 11 (9-14) | 39 (35-43) | 33 (29-37) |
| Recent cohort (pts treated since 2019) | 11 (9-19) | 42 (36-47) | 34 (29-40) |

**Copyright Information:** (Online) ISSN: 2572-9241
©2026 The Author(s). HemaSphere is published by John Wiley & Sons Ltd on behalf of European Hematology Association. This is an open access Abstract Book distributed under the Attribution-NonCommercial-NoDerivs (CC BY-NC-ND), which allows third parties to download the articles and share them with others as long as they credit the author and the Abstract Book, but they cannot change the content in any way or use them commercially.
**Abstract Book Citations:** Authors, Title, HemaSphere, 2026;10:(S1):pages. The abstract book DOIs can be found on the "Author Information" page.
**Disclaimer:** Articles published in the journal HemaSphere exclusively reflect the opinions of the authors. The authors are responsible for all content in their abstracts including accuracy of the facts, statements, citing resources, etc.

### (PF1015) RECEIPT OF HOLDING THERAPY IS ASSOCIATED WITH INFERIOR OUTCOMES ACROSS THE CAR T TREATMENT PATHWAY IN RELAPSED/REFRACTORY LARGE B CELL LYMPHOMA

**Topic:** 19. Large B cell lymphomas - Clinical

Inna Gong*[1, 2], Meenakshi Jeeva[3], Mahmood Aminilari[3], Massimiliano Marinoni[1], Anca Prica[1, 2], Danielle Rodin[3, 4], Sita Bhella[1, 2], Tomohiro Aoki[1, 2], Maria Azab[5], Christine Chen[1, 2], Robert Kridel[1, 2], Vishal Kukreti[1, 2], Abi Vijenthira[1, 2], Chloe Yang[1, 2], Nauman Malik[3, 4], Woodrow Wells[3, 4], Ur Metser[6], Michael Crump[1, 2], David Hodgson[3, 4], John Kuruvilla[1, 2]

*[1]Princess Margaret Cancer Centre, Medical Oncology and Hematology, Toronto, Canada;[2]University of Toronto, Department of Medicine, Toronto, Canada; [3]Princess Margaret Cancer Centre, Radiation Medicine Program, Toronto, Canada;[4]University of Toronto, Department of Radiation Oncology, Toronto, Canada; [5]University of Toronto, Temerty Faculty of Medicine, Toronto, Canada; [6]Princess Margaret Cancer Centre, Joint Department of Medical Imaging, Toronto, Canada;*

### Background

Although CD19-directed chimeric antigen receptor T cell (CAR T) therapy has improved outcomes for relapsed/refractory large B cell lymphoma (R/R LBCL), up to a third of referred pts don't reach infusion. While outcomes associated with bridging therapy (BT) have been described, there is a lack of data reporting on the association of holding therapy (HT) administered between referral and apheresis across the full intention-to-treat (ITT) pathway.

### Aims

To evaluate the association of receipt of HT across the complete CAR T treatment journey from referral through infusion.

### Methods

We retrospectively analyzed 219 patients (pts) with R/R LBCL referred for axicel or tisacel at Princess Margaret Cancer Centre (2017-2024), with follow-up through August 2025. Outcomes were assessed across sequential treatment phases: referral to apheresis (HT), apheresis to infusion (BT), and post-CAR T infusion. HT and BT were selected at the discretion of the treating physician. Cox models evaluated overall survival (OS) in the ITT, apheresed, and infused cohorts. Kaplan-Meier curves estimated OS, with multivariable Cox models assessing the association of HT and BT with outcomes.

**Copyright Information:** (Online) ISSN: 2572-9241
©2026 The Author(s). HemaSphere is published by John Wiley & Sons Ltd on behalf of European Hematology Association. This is an open access Abstract Book distributed under the Attribution-NonCommercial-NoDerivs (CC BY-NC-ND), which allows third parties to download the articles and share them with others as long as they credit the author and the Abstract Book, but they cannot change the content in any way or use them commercially.
**Abstract Book Citations:** Authors, Title, HemaSphere, 2026;10:(S1):pages. The abstract book DOIs can be found on the "Author Information" page.
**Disclaimer:** Articles published in the journal HemaSphere exclusively reflect the opinions of the authors. The authors are responsible for all content in their abstracts including accuracy of the facts, statements, citing resources, etc.

### Results

Of 219 referred pts, 36 (16%) received HT (chemotherapy±radiotherapy [RT] 53%, RT 25%, polatuzumab±RT19%, corticosteroids 3%) and had higher LDH ≥500 U/L (50% vs 27%, p=0.021) while no differences in other factors were observed. Attrition prior to apheresis was significantly higher among pts receiving HT (27.8% vs 11.5%, p=0.015) (Figure A). The most common reason for non-infusion among pts who received HT was disease progression (67%). In ITT analysis (median follow-up 30 months), HT was independently associated with inferior OS (adjusted hazard ratio [aHR] 2.03, 95% CI 1.29-3.17, p=0.002; Figure B); median OS was 5.7 vs 26.6 months for HT vs no HT.

Among 188 apheresed pts, 149 (79%) received BT (chemotherapy±RT 16%, RT 41%, polatuzumab±RT 26%, corticosteroids 17%) and these pts had more refractory disease, elevated LDH, extranodal involvement, and bulky disease defined as ≥7.5 cm (all p<0.05). Attrition between apheresis and infusion was not associated with BT (14.1% vs 23.1%, p=0.17), and receipt of BT was not associated with OS in apheresed pts (aHR 0.93, 95% CI 0.55-1.58, p=0.78).

Overall, 72% received CAR T infusion; median OS in infused pts was 47.9 months. HT remained independently associated with inferior OS after infusion (aHR 2.12, 95% CI 1.11-4.04, p=0.023; Figure C), whereas BT was not (aHR 1.05, 95% CI 0.54–2.05, p=0.88; Figure D) (model adjusted for IPI, ECOG, histologic diagnosis, and CAR T product). No significant interaction was observed between HT and BT (p=0.63), indicating that the association of HT with survival did not differ according to receipt of BT. Median OS was 12.3 months (95% CI 4.8-not reached [NR]) among pts who required HT vs NR among those without HT (95% CI 24.7-NR). Median OS was 47.9 months (95% CI 13.5-NR) for pts who received BT compared to 37 months (95% CI 23.2-NR) for pts who did not receive BT.

### Summary/Conclusion

In this cohort of pts referred for CAR T evaluation, receipt of HT but not BT was independently associated with inferior OS across the CAR T treatment journey through to infusion. This distinction is clinically important as it identifies the pre-apheresis interval as a critical prognostic window, suggesting that pts requiring disease control prior to apheresis represent a high-risk population whose inferior outcomes persist despite eventual CAR T infusion.

**Copyright Information:** (Online) ISSN: 2572-9241
©2026 The Author(s). HemaSphere is published by John Wiley & Sons Ltd on behalf of European Hematology Association. This is an open access Abstract Book distributed under the Attribution-NonCommercial-NoDerivs (CC BY-NC-ND), which allows third parties to download the articles and share them with others as long as they credit the author and the Abstract Book, but they cannot change the content in any way or use them commercially.
**Abstract Book Citations:** Authors, Title, HemaSphere, 2026;10:(S1):pages. The abstract book DOIs can be found on the “Author Information” page.
**Disclaimer:** Articles published in the journal HemaSphere exclusively reflect the opinions of the authors. The authors are responsible for all content in their abstracts including accuracy of the facts, statements, citing resources, etc.

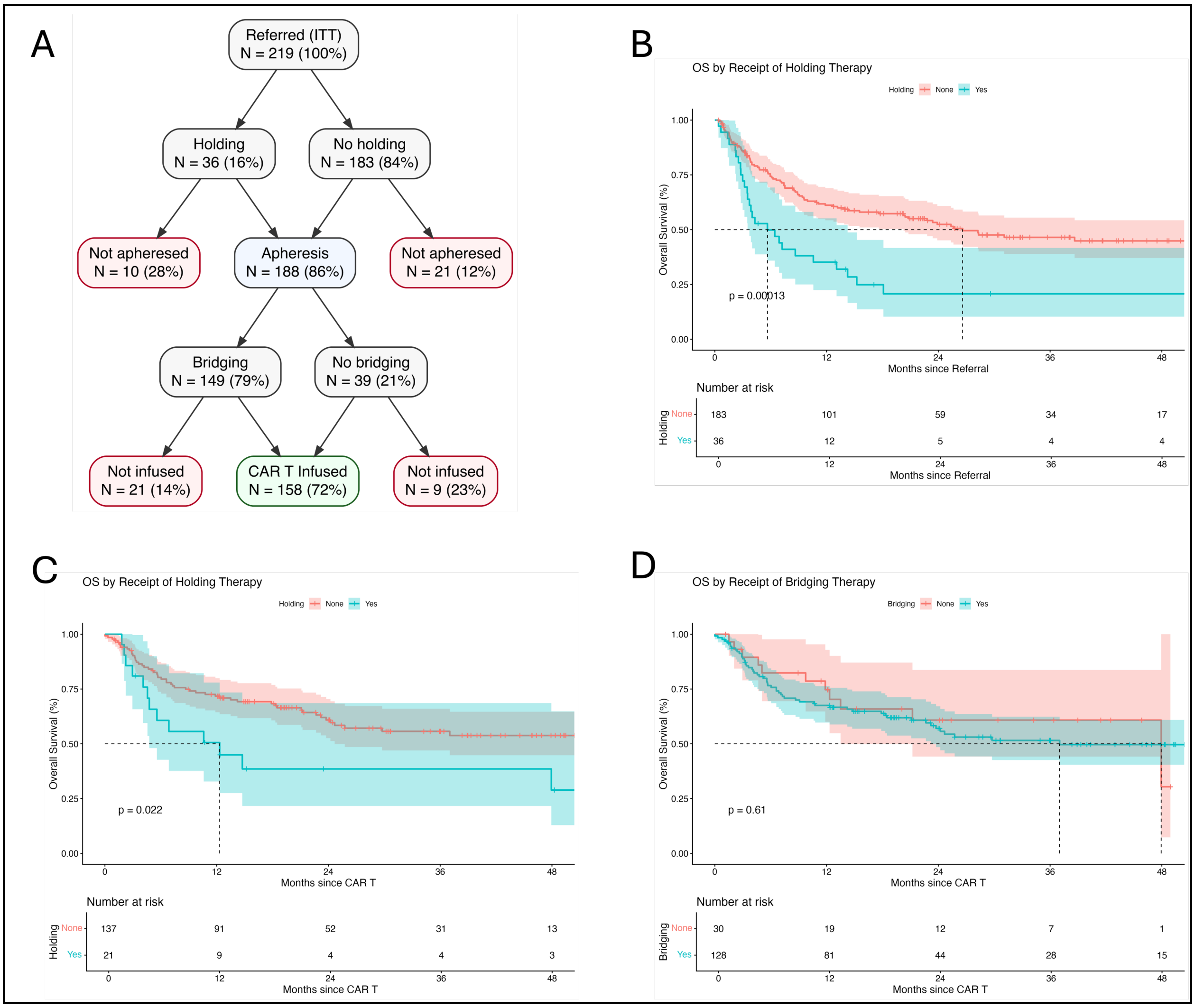

**Copyright Information:** (Online) ISSN: 2572-9241
©2026 The Author(s). HemaSphere is published by John Wiley & Sons Ltd on behalf of European Hematology Association. This is an open access Abstract Book distributed under the Attribution-NonCommercial-NoDerivs (CC BY-NC-ND), which allows third parties to download the articles and share them with others as long as they credit the author and the Abstract Book, but they cannot change the content in any way or use them commercially.
**Abstract Book Citations:** Authors, Title, HemaSphere, 2026;10:(S1):pages. The abstract book DOIs can be found on the "Author Information" page.
**Disclaimer:** Articles published in the journal HemaSphere exclusively reflect the opinions of the authors. The authors are responsible for all content in their abstracts including accuracy of the facts, statements, citing resources, etc.

### (PF1016) THE CLINICAL SIGNIFICANCE OF MYC::BCL6 FUSION IN MYC/BCL6 DOUBLE HIT LYMPHOMA PATIENTS

**Topic:** 19. Large B cell lymphomas - Clinical

Shaoying Li*[1], Jie Xu[1], Lianqun Qiu[1]

[1]*UT MD Anderson Cancer Center, Hematopathology, Houston, United States of America;*

### Background

A small proportion of large B cell lymphoma carry *MYC* and *BCL6* rearrangements, the so called *MYC/BCL6* double hit lymphoma (DHL), of which there are conflicting reports regarding the prognosis. Occasional studies suggested this is likely due to the presence of pseudo *MYC/BCL6* DHL, that is, those carried a single t(3;8) or *MYC::BCL6* fusion, which likely had a better prognosis. However, the number of such studies is limited, and more studies are needed to confirm it.

### Aims

To explore the prognostic significance of *MYC::BCL6* fusion in *MYC/BCL6* DHL patients.

### Methods

FISH analysis was performed on unstained formalin-fixed, paraffin-embedded 4µm tissue sections or bone marrow aspirate smears using *IGH::BCL2* dual-color dual-fusion probes; *MYC* dual-color break-apart probe, *BCL6* dual-color break-apart probe, and *MYC::BCL6* dual-color dual-fusion probes. Patient OS was analyzed using the Kaplan-Meier method and compared using the log-rank test (GraphPad Prism).

### Results

Thirty-six patients were confirmed as *MYC/BCL6* DHL by FISH, including 22 men and 14 women with a median age of 66.5 years (range, 31–99). Thirty (83%) patients had de novo disease, and 6 patients had a history of low-grade non-Hodgkin lymphoma, most commonly follicular lymphoma. 47% (17/36) patients had at least two extranodal sites of disease at diagnosis, 13% (4/31) patients who underwent staging bone marrow biopsy showed involvement by BCL6-DHL, and 14% (3/22) patients tested for CSF had CNS involvement. Approximately 89% and 82% of patients had high-stage disease and a high-intermediate to high IPI score respectively. Morphologically, 44% of *MYC/BCL6* DHL cases had HGBL morphology (including 4 with blastoid features) and 56% had DLBCL morphology. All cases expressed one or more pan B-cell antigens. CD10 was positive in approximately 47% (16/34) of cases assessed, BCL6 was positive in 91% (31/34) and MUM1/IRF4 and BCL2 were each positive in about 70-80% of cases. MYC was positive in 69% (24/35) of cases assessed and concurrent MYC and BCL2 expressions were observed in 44% (16/36) of cases evaluated. According to the Hans algorithm, 50% (17/34) of cases had a germinal center B-cell (GCB) immunophenotype. Ki67 was overall high, with a median of 85% and a range from 50% to virtually 100%. By definition, all cases carried *MYC* and *BCL6* rearrangements but no evidence of *BCL2* rearrangement as assessed by FISH. Further testing for *MYC::BCL6* fusion showed 19% (7/36) cases carried *MYC::BCL6* fusion and 29 cases were negative. The clinicopathologic features of those with *MYC::BCL6* fusion were compared with those without *MYC::BCL6* fusion and most were similar, except high stage disease was more frequently seen in patients without *MYC::BCL6 fusion* (57% vs 93%, p=0.04), while CD10 expression is more frequently seen in *MYC::BCL6* fusion subgroup (86% vs 37%, p=0.03), although the frequency of GCB phenotype did not reach statistical significance (p=0.09). Patients with or without the *MYC::BCL6* fusion received similar induction regiments. Survival analysis showed patients with or without *MYC::BCL6* fusion had no significant difference in overall survival (OS), either all patients or only de novo patients were included (p=0.35 and 0.57 respectively). There was also no significant difference in complete remission rate (40% vs 35%, p=1.0).

**Copyright Information:** (Online) ISSN: 2572-9241
©2026 The Author(s). HemaSphere is published by John Wiley & Sons Ltd on behalf of European Hematology Association. This is an open access Abstract Book distributed under the Attribution-NonCommercial-NoDerivs (CC BY-NC-ND), which allows third parties to download the articles and share them with others as long as they credit the author and the Abstract Book, but they cannot change the content in any way or use them commercially.
**Abstract Book Citations:** Authors, Title, HemaSphere, 2026;10:(S1):pages. The abstract book DOIs can be found on the "Author Information" page.
**Disclaimer:** Articles published in the journal HemaSphere exclusively reflect the opinions of the authors. The authors are responsible for all content in their abstracts including accuracy of the facts, statements, citing resources, etc.

**Summary/Conclusion**

The clinicopathologic features were largely similar betwen MYC/BCL6 DHL patients with or without *MYC::BCL6* fusion. *MYC::BCL6* fusion is not a prognostic factor in *MYC/BCL6* DHL patients.

**Copyright Information:** (Online) ISSN: 2572-9241
©2026 The Author(s). HemaSphere is published by John Wiley & Sons Ltd on behalf of European Hematology Association. This is an open access Abstract Book distributed under the Attribution-NonCommercial-NoDerivs (CC BY-NC-ND), which allows third parties to download the articles and share them with others as long as they credit the author and the Abstract Book, but they cannot change the content in any way or use them commercially.
**Abstract Book Citations:** Authors, Title, HemaSphere, 2026;10:(S1):pages. The abstract book DOIs can be found on the "Author Information" page.
**Disclaimer:** Articles published in the journal HemaSphere exclusively reflect the opinions of the authors. The authors are responsible for all content in their abstracts including accuracy of the facts, statements, citing resources, etc.

### (PF1017) POLA-R-CHP VS R-EPOCH FOR FRONTLINE THERAPY IN DLBCL WITH EXTRANODAL INVOLVEMENT

**Topic:** 19. Large B cell lymphomas - Clinical

Lei Shi[1], Mengzhen Wang[1], Bin Chu[1], Minqiu Lu[1], Shan Gao[1], Yuan Chen[1], Lijuan Fang[1], Qiuqing Xiang[1], Li Bao*[1]

[1]*Beijing Jishuitan Hospital, Capital Medical University, Beijing, China;*

### Background

The newly diagnosed diffuse large B-cell lymphoma (DLBCL) patients with extra nodal organ involvement (ENI) occur in approximately 30-40% and have limited efficacy and poor survival in the era of rituximab. Therefore, the R-EPOCH regimen was generally adopted for such patients in our center. Pola-R-CHP has been proven to improve the progression-free survival (PFS) of some patients in first-line treatment. However, there is still insufficient data available for ENI-DLBCL. This study conducted a retrospective analysis of the clinical data of 83 newly diagnosed patients with ENI-DLBCL. The efficacy and safety of the Pola-R-CHP and R-EPOCH regimens were compared, aiming to provide reasonable treatment for such patients.

### Aims

To compare the efficacy and safety of the Pola-R-CHP and R-EPOCH regimens in patients with newly diagnosed ENI-DLBCL.

### Methods

This is a real-world, single-center, retrospective, observational study. 83 patients who adopted the two regimens were enrolled. Propensity score matching was conducted based on age, sex, COO, stage, IPI, and Bulky ≥7.5cm.

### Results

After six cycles, the Pola-R-CHP group had a higher ORR of 90% (18/22) compared to 63% in the other group. There was no significant difference in the CR rates between the two groups. The Pola-R-CHP group had fewer cases of grade 3 or higher hematological toxicities. The non-hematological toxicities of the two groups were similar, as shown in Table 1. The median follow-up was 7.6 months and 33.6 months, respectively. The median PFS in the Pola-R-CHP group was significantly longer than the R-EPOCH group before matching. But the median OS and PFS showed no statistical difference between the two groups after matching, see figure1.
Table 1. Patient characteristics, efficacy, and safety

**Copyright Information:** (Online) ISSN: 2572-9241
©2026 The Author(s). HemaSphere is published by John Wiley & Sons Ltd on behalf of European Hematology Association. This is an open access Abstract Book distributed under the Attribution-NonCommercial-NoDerivs (CC BY-NC-ND), which allows third parties to download the articles and share them with others as long as they credit the author and the Abstract Book, but they cannot change the content in any way or use them commercially.
**Abstract Book Citations:** Authors, Title, HemaSphere, 2026;10:(S1):pages. The abstract book DOIs can be found on the "Author Information" page.
**Disclaimer:** Articles published in the journal HemaSphere exclusively reflect the opinions of the authors. The authors are responsible for all content in their abstracts including accuracy of the facts, statements, citing resources, etc.

| Characteristics | Unmatched | | | Matched | | |
|---|---|---|---|---|---|---|
| | R-EPOCH N = 56 | Pola-R-CHP N = 27 | P | R-EPOCH N = 32 | Pola-R-CHP N = 22 | P |
| **Age** | | | 0.002 | | | 0.374 |
| Mean (Min, Max) | 53 (20,70) | 61 (20,70) | | 59 (31,70) | 59 (18,79) | |
| **Sex** | | | 0.161 | | | 0.821 |
| Female | 24 (43%) | 16 (59%) | | 17 (53%) | 11 (50%) | |
| Male | 32 (57%) | 11 (41%) | | 15 (47%) | 11 (50%) | |
| **COO** | | | 0.212 | | | 0.496 |
| GCB | 19 (34%) | 13 (48%) | | 13 (41%) | 11 (50%) | |
| nonGCB | 37 (66%) | 14 (52%) | | 19 (59%) | 11 (50%) | |
| **Lugano** | | | 0.658 | | | >0.999 |
| I-II | 5 (9%) | 1 (4%) | | 2 (6%) | 1 (5%) | |
| III-IV | 51 (91%) | 26 (96%) | | 30 (94%) | 21 (95%) | |
| **IPI** | | | 0.7343 | | | 0.361 |
| 1-2 | 9 (16.1%) | 6 (22.2%) | | 6 (18.8%) | 5 (22.7%) | |
| 3 | 9 (16.1%) | 5 (18.5%) | | 3 (9.4%) | 5 (22.7%) | |
| 4-5 | 38 (67.9%) | 16 (59.3%) | | 23 (71.9%) | 12(54.5%) | |
| **Bulky >7.5cm** | 28(50%) | 11(41%) | 0.428 | 15(47%) | 9(41%) | 0.665 |
| **After 4 cycles efficacy** | | | | | | |
| ORR(CR+PR) | 22 (69%) | 21 (95%) | 0.019* | 39 (70%) | 26 (96%) | 0.006* |
| CR | 15 (47%) | 11 (50%) | 0.82 | 29 (52%) | 14 (52%) | >0.99 |
| **After 6 cycles efficacy** | | | | | | |
| ORR(CR+PR) | 20 (63%) | 18 (90%) | 0.030* | 34 (61%) | 22 (88%) | 0.014* |
| CR | 15 (47%) | 14 (70%) | 0.10 | 29 (52%) | 17 (68%) | 0.17 |
| **AEs ≥3 Grade** | | | | | | |
| Neutropenia | 28(50.0%) | 5 (18.5%) | 0.006* | 16 (50.0%) | 4 (18.2%) | 0.017* |
| Thrombocytopenia | 18 (32.1%) | 0 (0.0%) | <0.001* | 9 (28.1%) | 0 (0.0%) | 0.007* |
| Anemia requiring Blood transfusion | 17 (30.4%) | 2 (7.4%) | 0.020* | 9 (28.1%) | 1 (4.6%) | 0.036* |
| Serious infections | 16 (28.6%) | 3 (11.1%) | 0.076 | 9 (28.1%) | 2 (9.1%) | 0.17 |
| Cardiac Toxicity | 5 (8.9%) | 3 (11.1%) | 0.711 | 3 (9.4%) | 3 (13.6%) | 0.68 |

### Summary/Conclusion

Pola-R-CHP regimen provided promising efficacy and lower toxicity for patients with ENI-DLBCL, especially bone involvement.

**Copyright Information:** (Online) ISSN: 2572-9241
©2026 The Author(s). HemaSphere is published by John Wiley & Sons Ltd on behalf of European Hematology Association. This is an open access Abstract Book distributed under the Attribution-NonCommercial-NoDerivs (CC BY-NC-ND), which allows third parties to download the articles and share them with others as long as they credit the author and the Abstract Book, but they cannot change the content in any way or use them commercially.
**Abstract Book Citations:** Authors, Title, HemaSphere, 2026;10:(S1):pages. The abstract book DOIs can be found on the "Author Information" page.
**Disclaimer:** Articles published in the journal HemaSphere exclusively reflect the opinions of the authors. The authors are responsible for all content in their abstracts including accuracy of the facts, statements, citing resources, etc.

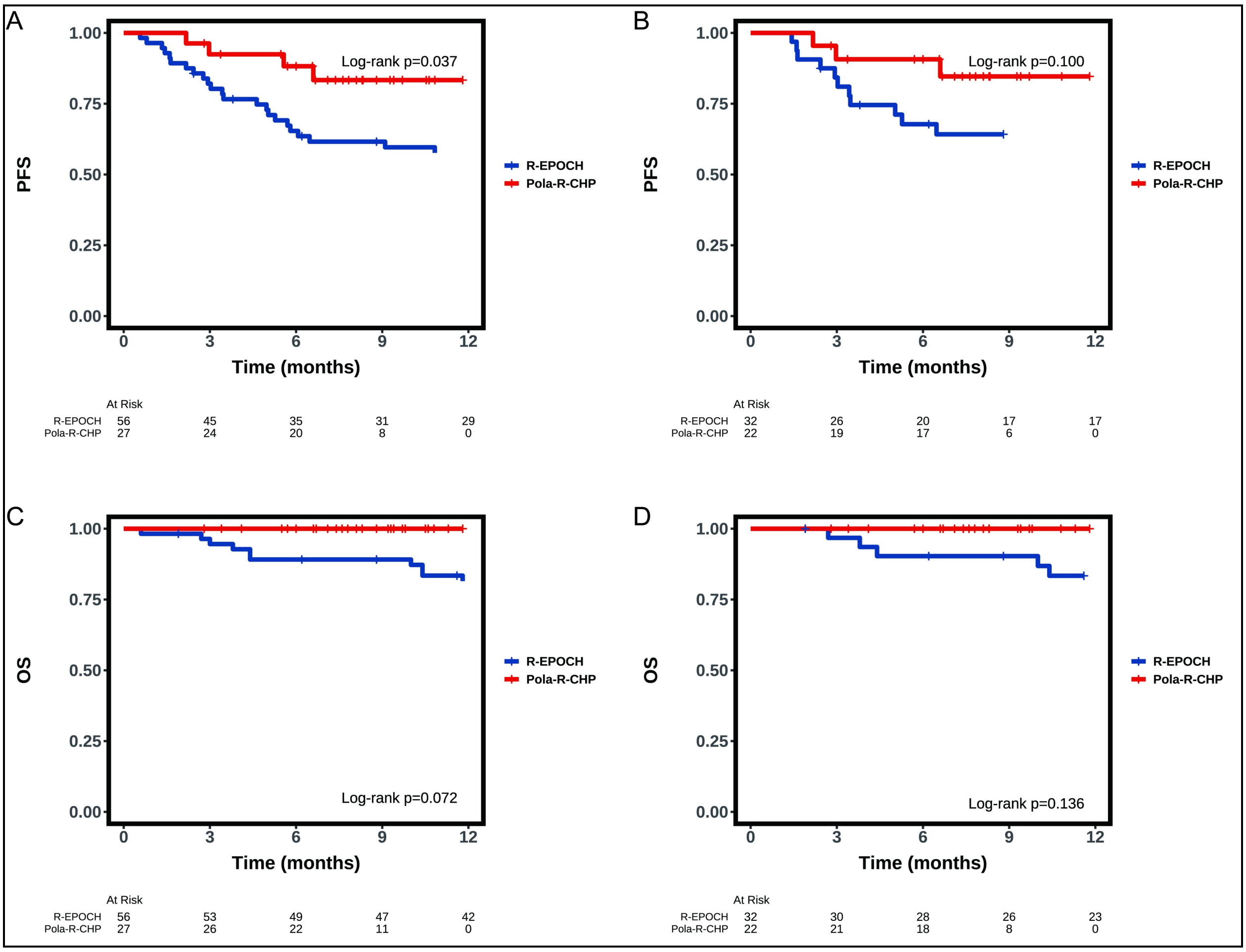

Figure 1. PFS and OS of patients in the R-EPOCH and Polar-R-CHP groups(A and C: before matching; B and D: matching)

**Copyright Information:** (Online) ISSN: 2572-9241
©2026 The Author(s). HemaSphere is published by John Wiley & Sons Ltd on behalf of European Hematology Association. This is an open access Abstract Book distributed under the Attribution-NonCommercial-NoDerivs (CC BY-NC-ND), which allows third parties to download the articles and share them with others as long as they credit the author and the Abstract Book, but they cannot change the content in any way or use them commercially.
**Abstract Book Citations:** Authors, Title, HemaSphere, 2026;10:(S1):pages. The abstract book DOIs can be found on the "Author Information" page.
**Disclaimer:** Articles published in the journal HemaSphere exclusively reflect the opinions of the authors. The authors are responsible for all content in their abstracts including accuracy of the facts, statements, citing resources, etc.

### (PF1018) MULTIVARIABLE ANALYSES OF OVERALL SURVIVAL IN THE PHASE 3 SUNMO, STARGLO AND POLARGO TRIALS IN RELAPSED/REFRACTORY LARGE B-CELL LYMPHOMA

**Topic:** 19. Large B cell lymphomas - Clinical

Matthew Matasar*[1], Jason Westin[2], Jeremy S. Abramson[3], Adam J. Olszewski[4], Song Pham[5], Jue Wang[6], Steven Barrett[6], Iris To[6], Shen Yin[6], Connie Lee Batlevi[6], Lisa Musick[6], Linda Lundberg[7], Michael C. Wei[6], L. Elizabeth Budde[8]

*[1]Rutgers Cancer Institute, New Brunswick, NJ, United States of America;[2]MD Anderson Cancer Center, Houston, TX, United States of America; [3]Massachusetts General Hospital Cancer Center, Boston, MA, United States of America;[4]Brown University, Providence, RI, United States of America; [5]Hoffmann-La Roche Ltd, Mississauga, ON, Canada; [6]Genentech, Inc., South San Francisco, CA, United States of America; [7]F. Hoffmann-La Roche Ltd, Basel, Switzerland; [8]City of Hope National Medical Center, Duarte, CA, United States of America;*

### Background

Phase 3 trials, notably SUNMO (NCT05171647), STARGLO (NCT04408638) and POLARGO (NCT04182204), have shown superior efficacy of combination treatments over rituximab (R) + gemcitabine and oxaliplatin (GemOx) in second line or later large B-cell lymphoma (LBCL). However, baseline imbalances may impact the robustness of efficacy results, even with stratified randomization.

### Aims

To conduct multivariable analyses (MVAs) of SUNMO, STARGLO and POLARGO to assess overall survival (OS). As the target number of OS events was not reached at the SUNMO interim OS analysis, to increase the sample size we also conducted a pooled analysis of mosunetuzumab (Mosun) + polatuzumab vedotin (Pola; data from SUNMO and GO40516 [Phase 2; NCT03671018]) vs R-GemOx (comparators from SUNMO, STARGLO and POLARGO).

### Methods

A multivariable Cox regression model was used to estimate treatment effect while adjusting for potential confounding factors selected by a systematic process involving: 1) univariable analysis of prespecified baseline factors; 2) multicollinearity assessment of pairwise correlation among selected factors; 3) MVA of the experimental arm (SUNMO: Mosun-Pola [an investigational regimen]; STARGLO: glofitamab [Glofit]-GemOx; POLARGO: Pola-R-GemOx [an investigational regimen]) vs the control arm (R-GemOx) and selected non-collinear prognostic baseline factors from steps 1 and 2.

### Results

In SUNMO, patients treated with Mosun-Pola had more high-risk features, such as a higher Eastern Cooperative Oncology Group performance status and greater bulky disease, vs those treated with R-GemOx. The MVA-adjusted hazard ratio (HR, 0.65 [95% confidence interval (CI): 0.41–1.02]) was more favorable than the unadjusted interim OS HR (0.80 [95% CI: 0.54–1.20]) for Mosun-Pola vs R-GemOx. The MVA-adjusted HR for POLARGO was also more favorable than the unadjusted HR; the HR for STARGLO remained largely unchanged (Table).
A pooled MVA was used to increase the sample size for the Mosun-Pola regimen, noting baseline differences between trials (e.g. patients with ≥2 prior lines of therapy: SUNMO, 56%; STARGLO, 37%; POLARGO, 35%). In this pooled analysis, OS HRs favored Mosun-Pola vs R-GemOx in the unadjusted (HR, 0.62 [95% CI: 0.48–0.81]) and MVA-adjusted (HR, 0.59 [95% CI: 0.44–0.81]) models, with lower HRs and narrower 95% CIs vs the MVA of SUNMO alone.

**Copyright Information:** (Online) ISSN: 2572-9241
©2026 The Author(s). HemaSphere is published by John Wiley & Sons Ltd on behalf of European Hematology Association. This is an open access Abstract Book distributed under the Attribution-NonCommercial-NoDerivs (CC BY-NC-ND), which allows third parties to download the articles and share them with others as long as they credit the author and the Abstract Book, but they cannot change the content in any way or use them commercially.
**Abstract Book Citations:** Authors, Title, HemaSphere, 2026;10:(S1):pages. The abstract book DOIs can be found on the "Author Information" page.
**Disclaimer:** Articles published in the journal HemaSphere exclusively reflect the opinions of the authors. The authors are responsible for all content in their abstracts including accuracy of the facts, statements, citing resources, etc.

**Summary/Conclusion**

The MVAs support favorable OS benefits in SUNMO, STARGLO and POLARGO, all with an MVA-adjusted OS HR ≤0.65 vs R-GemOx. The pooled MVA further supports the OS benefit of Mosun-Pola vs R-GemOx. Overall, the MVAs show that baseline imbalances can influence OS estimates, thus multivariable adjustment is important when interpreting pooled or cross-trial comparisons.

© 2026 American Society of Clinical Oncology, Inc. Reused with permission. This abstract was accepted and previously presented at the 2026 ASCO Annual Meeting. All rights reserved.

**Copyright Information:** (Online) ISSN: 2572-9241
©2026 The Author(s). HemaSphere is published by John Wiley & Sons Ltd on behalf of European Hematology Association. This is an open access Abstract Book distributed under the Attribution-NonCommercial-NoDerivs (CC BY-NC-ND), which allows third parties to download the articles and share them with others as long as they credit the author and the Abstract Book, but they cannot change the content in any way or use them commercially.
**Abstract Book Citations:** Authors, Title, HemaSphere, 2026;10:(S1):pages. The abstract book DOIs can be found on the "Author Information" page.
**Disclaimer:** Articles published in the journal HemaSphere exclusively reflect the opinions of the authors. The authors are responsible for all content in their abstracts including accuracy of the facts, statements, citing resources, etc.

**Table. Multivariable analyses of overall survival**

| | SUNMO n=208 | STARGLO n=274 | POLARGO n=255 | Pooled Mosun-Pola vs R-GemOx n=465 |
|---|---|---|---|---|
| **Median follow-up, months (range)** | 23.2 (0–32) | 20.7 (0–36) | 24.6 (0–34) | – |
| **MVA factors** | Treatment, sex, region, baseline ECOG PS, IPI score (study entry), NHL subtype, Ann Arbor stage (study entry), bulky disease (>7.5cm), COO (local), baseline LDH, prior ASCT, trFL | Treatment, sex, region, baseline BMI, IPI score (study entry), bulky disease (≥10 cm), prior lines of systemic therapy, refractory status to last systemic therapy | Treatment, baseline ECOG PS, IPI score (study entry), bulky disease (≥7.5cm), extranodal involvement (study entry), baseline MYC by IHC, China subgroup | Treatment, sex, region, baseline BMI, baseline ECOG PS, NHL subtype, bulky disease (≥7.5cm), baseline LDH, prior lines of systemic therapy (1 vs ≥2), R/R to first-line therapy, prior ASCT, trFL |
| **MVA-adjusted OS, HR (95% CI)** | 0.65 (0.41–1.02) | 0.63 (0.44–0.90) | 0.52 (0.36–0.74) | 0.59 (0.43–0.80) |
| **Unadjusted OS, HR (95% CI)** | 0.80 (0.54–1.20) | 0.62 (0.43–0.88) | 0.60 (0.43–0.83) | 0.62 (0.48–0.81) |

ASCT, autologous stem cell transplant; BMI, body-mass index; CI, confidence interval; COO, cell of origin; ECOG PS, Eastern Cooperative Oncology Group Performance Status; HR, hazard ratio; IHC, immunohistochemistry; IPI, International Prognostic Index; LDH, lactate dehydrogenase; Mosun-Pola, mosunetuzumab plus polatuzumab vedotin; MVA, multivariable analysis; NHL, non-Hodgkin lymphoma; OS, overall survival; R-GemOx, rituximab plus gemcitabine and oxaliplatin; R/R, relapsed/refractory; trFL, transformed folicular lymphoma.

**Copyright Information:** (Online) ISSN: 2572-9241
©2026 The Author(s). HemaSphere is published by John Wiley & Sons Ltd on behalf of European Hematology Association. This is an open access Abstract Book distributed under the Attribution-NonCommercial-NoDerivs (CC BY-NC-ND), which allows third parties to download the articles and share them with others as long as they credit the author and the Abstract Book, but they cannot change the content in any way or use them commercially.
**Abstract Book Citations:** Authors, Title, HemaSphere, 2026;10:(S1):pages. The abstract book DOIs can be found on the "Author Information" page.
**Disclaimer:** Articles published in the journal HemaSphere exclusively reflect the opinions of the authors. The authors are responsible for all content in their abstracts including accuracy of the facts, statements, citing resources, etc.

### (PF1019) IMMUNE DEFICIENCY AND INFECTION RISK FOLLOWING SECOND LINE CAR-T CELLS THERAPY IN RELAPSED REFRACTORY LARGE B CELLS LYMPHOMA

**Topic:** 19. Large B cell lymphomas - Clinical

Mohamad Sabbah*[1, 2, 3], Sofia Brand[1, 4], Agathe Vely[2], Ouedrani Amani[1, 5], Vincent Allain[5], Fabrizia Campana[2], Matteo Bisio[2], Manu Juneja[2], Laetitia Vercellino[1, 6], Maxime Berquier[2], Côme Bommier[1, 2], Roberta Di Blasi[2], Caillat-Zucman Sophie[1, 5], Jérôme Lambert[1, 4], Catherine Thieblemont[1, 2]

*[1]Université Paris Cité, Paris, France; [2]Hopital Saint Louis - Hemato-Oncologie Department, Hemato-Oncology, Paris, France; [3]Institut de Recherche Saint Louis - INSERM UMR 1342, Paris, France;[4]Biostatistics Department, Hôpital Saint Louis, APHP, Paris, France; [5]Immunologie, Hopital Saint Louis, Paris, France;[6]Hopital Saint Louis - Médécine Nucléaire, Paris, France;*

### Background

Anti-CD19 CAR T-cell is approved in second line (L2) for patients with refractory or early relapsed large B-cell lymphoma (R/R LBCL). In L3+, prolonged immune defects associated with infections and non-relapse mortality. However, immune reconstitution (IR) and infectious risk after L2 CAR T-cell therapy remain insufficiently characterized.

### Aims

To describe immune deficiency after L2 anti-CD19 CAR T-cell in R/R LBCL, including: (1) lymphocyte B and T subsets recovery and immunoglobulin levels, (2) incidence, timing and type of infections after month 3 (M3), and (3) association with survival outcomes. Secondary aims were to explore factors associated with immune deficiency and infectious complications, and the impact of immunoglobulin (Ig) supplementation.

### Methods

We performed a retrospective analysis including consecutive patients with R/R LBCL treated in L2 with commercial anti-CD19 CAR T cells between 01/04/2022 and 31/12/2024. Immune monitoring included serum Ig and lymphocyte subsets (including CD4+ T cells and B cells) at M1, M3, M6, M9, M12, and beyond when available. Hypogammaglobulinemia (HypoGG) was defined as hypoGG <4 g/L and severe <2 g/L; Groups were defined according to IR at M3: 1/CD4+ T cells >200/mL, 2/detectable B cells, and 3/IgG >4 g/L) as: no IR, 1, or 2 criteria. Landmark comparison of occurrence of infection post-M3 was performed, and factors associated with reconstitution were explored.

**Copyright Information:** (Online) ISSN: 2572-9241
©2026 The Author(s). HemaSphere is published by John Wiley & Sons Ltd on behalf of European Hematology Association. This is an open access Abstract Book distributed under the Attribution-NonCommercial-NoDerivs (CC BY-NC-ND), which allows third parties to download the articles and share them with others as long as they credit the author and the Abstract Book, but they cannot change the content in any way or use them commercially.
**Abstract Book Citations:** Authors, Title, HemaSphere, 2026;10:(S1):pages. The abstract book DOIs can be found on the “Author Information” page.
**Disclaimer:** Articles published in the journal HemaSphere exclusively reflect the opinions of the authors. The authors are responsible for all content in their abstracts including accuracy of the facts, statements, citing resources, etc.

### Results

A total of 57 patients were included (median age 63 years, 56% male); 53% received axi-cel and 47% liso-cel. Evaluation of the criteria for IR is described in fig 1. At M3 post-CAR T infusion, hypoGG occurred in 11% with no patient experiencing severe hypoGG; all patients had B-cell aplasia, and CD4+ T-cell count was <200/mL in 51% of patients; 8 (16%) patients had not recovered any IR criterion, 23 (45%) had recovered 1 criterion and 20 (39%) had recovered 2 criteria. At M6 and M12, IR meeting all 3 criteria was achieved in 3/34 (9%), 11/42 (26 %), of the patients, respectively. Ig supplementation was administered in 12/57 (21% of patients)

At M3, 56% of patients had already experienced at least one infection; 25% patients experienced at least one severe (grade >=3) infection during follow-up. In the cohort, 49 infections occurred after M3 in 23 patients, including 53 % bacterial, 45 % viral, and 2 % fungal infections.

Mean cumulative count of infections post M3 was not significantly different between groups (MCC at M12 of 0,55 (IC95%: 0, 1,3), 0,96 (IC95%: 0,41, 1,5), 1,2 (IC95%: 0,56, 1,8) for patients with 0, 1 and 2 criteria, respectively)

One-year PFS and OS post CART were 54 % and 77 %, respectively. When starting at M3 landmark post CART infusion, PFS was 83 %, 56 % and 87 % and OS was 88 %, 63 % and 90 % after 1 year for patients with 0, 1 and 2 criteria respectively (p-value 0,2 et 0,07 respectively).

Factors associated with immune deficiency and infections will be described during the presentation.

### Summary/Conclusion

In this real-world L2 R/R LBCL population treated with anti-CD19 CAR T cells, immune deficiency remained frequent, with persistent hypoGG, B-cell aplasia and delayed CD4+ T-cell recovery in a substantial proportion of patients. Infectious complications were common and extended beyond the early post-infusion period. These findings support systematic immune monitoring after L2 CAR T-cell and may help refine prophylactic and Ig replacement strategies.

**Copyright Information:** (Online) ISSN: 2572-9241
©2026 The Author(s). HemaSphere is published by John Wiley & Sons Ltd on behalf of European Hematology Association. This is an open access Abstract Book distributed under the Attribution-NonCommercial-NoDerivs (CC BY-NC-ND), which allows third parties to download the articles and share them with others as long as they credit the author and the Abstract Book, but they cannot change the content in any way or use them commercially.
**Abstract Book Citations:** Authors, Title, HemaSphere, 2026;10:(S1):pages. The abstract book DOIs can be found on the "Author Information" page.
**Disclaimer:** Articles published in the journal HemaSphere exclusively reflect the opinions of the authors. The authors are responsible for all content in their abstracts including accuracy of the facts, statements, citing resources, etc.

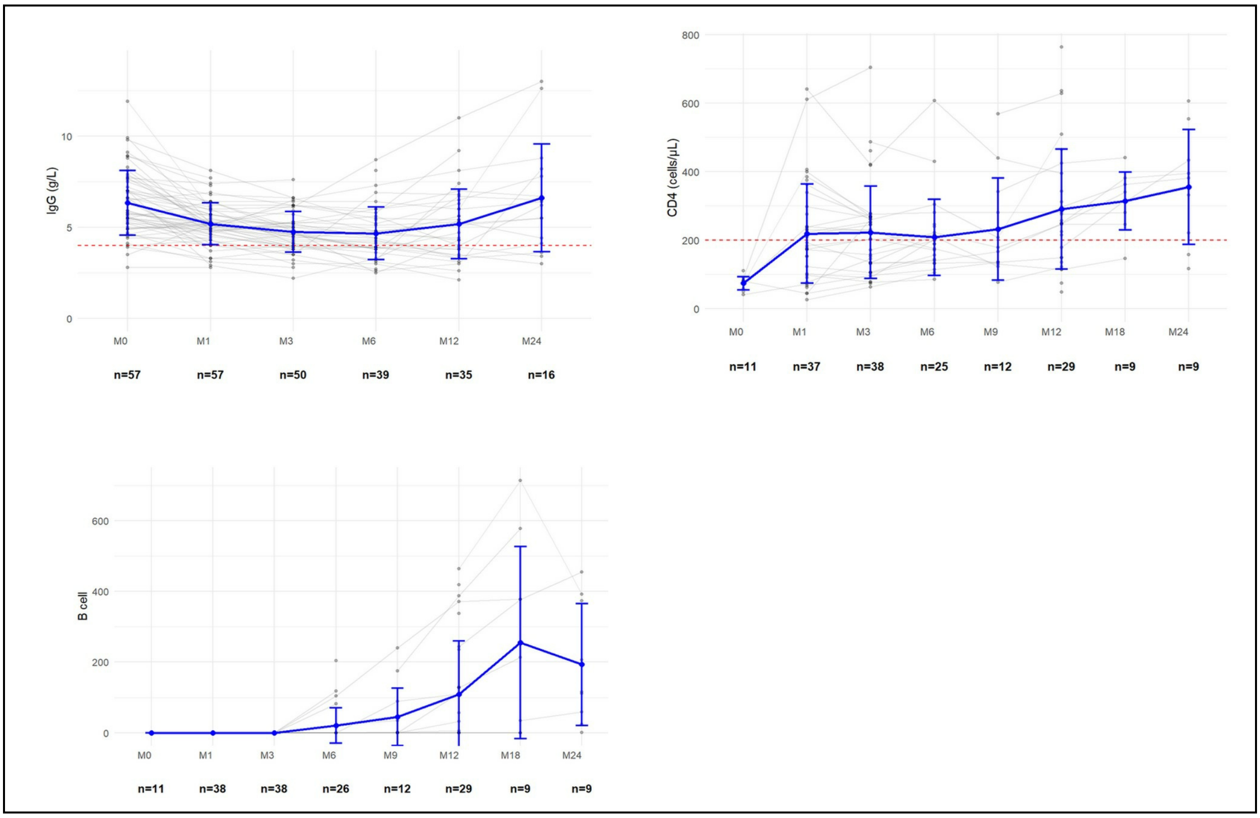

Longitudinal monitoring of CD4 T cells, B cells and immunoglobulin levels following second line CD19 CAR T cell therapy

**Copyright Information:** (Online) ISSN: 2572-9241
©2026 The Author(s). HemaSphere is published by John Wiley & Sons Ltd on behalf of European Hematology Association. This is an open access Abstract Book distributed under the Attribution-NonCommercial-NoDerivs (CC BY-NC-ND), which allows third parties to download the articles and share them with others as long as they credit the author and the Abstract Book, but they cannot change the content in any way or use them commercially.
**Abstract Book Citations:** Authors, Title, HemaSphere, 2026;10:(S1):pages. The abstract book DOIs can be found on the "Author Information" page.
**Disclaimer:** Articles published in the journal HemaSphere exclusively reflect the opinions of the authors. The authors are responsible for all content in their abstracts including accuracy of the facts, statements, citing resources, etc.

### (PF1020) FLOW CYTOMETRY OF VITRECTOMY SAMPLES AS A DIAGNOSTIC TOOL IN PRIMARY VITREORETINAL LYMPHOMA

**Topic:** 19. Large B cell lymphomas - Clinical

Lena-Marie Poczka*[1, 2, 3], Frank Ziemann[1, 2, 4, 5, 6], Gerulf Hänel[1, 2, 7], Johannes Hellmuth[1, 3, 4], Veit Bücklein[1, 2, 7], Louisa von Baumgarten[1, 5], Stephan Thurau[1], Marion Subklewe[1, 2, 3, 5, 6, 7]

*[1]LMU University Hospital, MED III (Hematology/Oncology), Munich, Germany;[2]Laboratory for Leukemia Diagnostics (LfL), LMU Munich, Munich, Germany; [3]German Lymphoma Alliance, Munich, Germany;[4]Laboratory for Experimental Leukemia and Lymphoma Research (ELLF), LMU Munich, Munich, Germany; [5]The Bavarian Cancer Reasearch Center (BZKF), Munich, Germany; [6]German Cancer Consortium (DKTK) and German Research Center (DKFZ), Heidelberg, Germany;[7]Laboratory for Translational Cancer Immunology, LMU Gene Center, Munich, Germany;*

### Background

Primary Vitreoretinal Lymphoma (PVRL) is a rare subtype of predominantly aggressive B-cell lymphoma that manifests in the vitreus and/or retina and is associated with poor clinical outcomes mostly due to frequent involvement of the central nervous system (CNS). PVRL remains a diagnostic challenge for ophthalmologists, pathologists and hematologists, as standardized diagnostic guidelines are lacking.

### Aims

In this retrospective single-center analysis, we aimed to evaluate the utility of multiparametric flow cytometry analysis (MPFC) for the diagnosis of PVRL in vitrectomy samples. Furthermore, we wanted to assess the sensitivity, specificity and predictive values of MPFC in identifying patients at risk for CNS lesions after identification of monoclonal B-cells in the vitreus.

### Methods

A total of 49 *pars plana* vitrectomy (PPV) samples were analyzed for monoclonal B-cells (CD45, CD19, CD5, CD10 and immunoglobulin light chains κ/λ) using MPFC. Statistical comparisons between positive and negative samples were conducted using the Mann–Whitney U test.
To determine the diagnostic accuracy of MPFC for predicting CNS involvement following identification of a monoclonal B-cell population in the vitrectomy sample, 34 patients were followed for a median of 31 months, and MPFC findings were validated against subsequent definitive evidence of lymphoma, including CSF analysis, imaging by MRI or CT and clinical evolution. Sensitivity, specificity, positive predictive value (PPV), and negative predictive value (NPV) of MPFC were calculated.

### Results

While $CD45^+$ cell counts did not differ significantly between groups ($p = 0.18$), the proportion of $CD19^+$ B-cells within the $CD45^+$ population was significantly higher in the PVRL cohort ($p < 0.0001$). The median percentage of $CD19^+$ cells among $CD45^+$ cells in positive samples was 34.3%. In the flow-negative group, the median $CD19^+$ fraction accounted for 2.2% of $CD45^+$ cells. Additionally, we evaluated whether positive MPFC results were associated with increased risk of CNS involvement. MPFC demonstrated a sensitivity of 100% (95% CI: 77.1–100%) and a specificity of 94.4% (95% CI: 77.3–99.1%) for predicting concomitant or subsequent CNS lymphoma in patients with suspected PVRL (PVRL cohort). The median time to diagnosis of CNS lymphoma in the cohort was 13 months (range: 5–19 months). Of note, MPFC analysis provided a high NPV of 100% (95% CI: 83.9–100%) for the development of CNS lymphoma in our cohort since no patient in the flow-negative group developed CNS lesions during follow-up of at least 13 months.

**Copyright Information:** (Online) ISSN: 2572-9241
©2026 The Author(s). HemaSphere is published by John Wiley & Sons Ltd on behalf of European Hematology Association. This is an open access Abstract Book distributed under the Attribution-NonCommercial-NoDerivs (CC BY-NC-ND), which allows third parties to download the articles and share them with others as long as they credit the author and the Abstract Book, but they cannot change the content in any way or use them commercially.
**Abstract Book Citations:** Authors, Title, HemaSphere, 2026;10:(S1):pages. The abstract book DOIs can be found on the "Author Information" page.
**Disclaimer:** Articles published in the journal HemaSphere exclusively reflect the opinions of the authors. The authors are responsible for all content in their abstracts including accuracy of the facts, statements, citing resources, etc.

**Summary/Conclusion**

MPFC analysis of vitrectomy samples represents a valuable diagnostic tool in suspected PVRL. Detection of intravitreal monoclonal B-cells was associated with increased risk of concomitant or subsequent CNS involvement, supporting the need for systematic CNS staging upon initial diagnosis as well as close surveillance during the first year after diagnosis.

**Copyright Information:** (Online) ISSN: 2572-9241
©2026 The Author(s). HemaSphere is published by John Wiley & Sons Ltd on behalf of European Hematology Association. This is an open access Abstract Book distributed under the Attribution-NonCommercial-NoDerivs (CC BY-NC-ND), which allows third parties to download the articles and share them with others as long as they credit the author and the Abstract Book, but they cannot change the content in any way or use them commercially.
**Abstract Book Citations:** Authors, Title, HemaSphere, 2026;10:(S1):pages. The abstract book DOIs can be found on the "Author Information" page.
**Disclaimer:** Articles published in the journal HemaSphere exclusively reflect the opinions of the authors. The authors are responsible for all content in their abstracts including accuracy of the facts, statements, citing resources, etc.

### (PF1021) OUTCOMES OF CONSOLIDATIVE RADIOTHERAPY AND SURVIVAL IN LIMITED-STAGE PRIMARY GASTRIC DIFFUSE LARGE B-CELL LYMPHOMA ACHIEVING COMPLETE RESPONSE: A MULTICENTER STUDY FROM CHINA

**Topic:** 19. Large B cell lymphomas - Clinical

Natalia Sunarto*[1], Ximei Zhang[2]

*[1]National Cancer Center/National Clinical Research Center for Cancer/Cancer Hospital, Chinese Academy of Medical Sciences and Peking Union Medical College, Department of Radiation Oncology, Beijing, China; [2]Tianjin Medical University Cancer Institute and Hospital, Key Laboratory of Cancer Prevention and Therapy, National Clinical Research Center for Cancer, Department of Radiotherapy, Tianjin, China;*

### Background

Primary gastric diffuse large B-cell lymphoma (PG-DLBCL) accounts for approximately 30% of all gastric lymphomas.While R-CHOP has been the standard first-line treatment for over two decades,achieving long-term disease control in a majority of PG-DLBCL patients,the necessity of consolidative radiotherapy (RT) for those reaching complete remission (CR) remains controversial in the rituximab era.

### Aims

This study aims to evaluate the role of consolidative radiotherapy and survival outcomes in patients with limited-stage PG-DLBCL who achieved CR after first-line chemotherapy(CT).

### Methods

Adult patients with limited-stage PG-DLBCL who achieved CR after first-line chemotherapy were retrospectively reviewed from May 2008 to September 2024 across two Chinese institutions. Survival outcomes were estimated using the Kaplan-Meier method and compared with the log-rank test. Standardized mortality ratios (SMRs) were calculated to assess curability by comparing observed deaths with expected deaths in the age- and sex-matched general Chinese population (UN World Population Prospects 2024). A competing risk framework differentiated lymphoma-specific mortality (LSM) from non-lymphoma mortality (NLM), with cumulative incidence functions (CIFs) compared using Gray's test.

### Results

A total of 202 patients were included, with a median age of 55 years (range from 18 to 81). 96 (47.5%) patients received consolidative RT after chemotherapy , while 106 (52.5%) received CT alone. R-CHOP-like regimens were used in 83.2% of cases, with 48% and 48% completing baseline and post-treatment PET/CT assessments, respectively. With a median follow-up of 80.8 months, the 5-year OS, PFS, and LRC rates were 98.5% , 92.2% and 97.6% in the CT+RT group , compared to 86.4% (P=0.06), 83.5(P=0.04)and 95.8% (P=0.5) for the CT-alone group, respectively. Although a statistical difference in PFS was observed, consolidative RT did not significantly improve OS or LRC, consistent with the exceptionally low local recurrence rate (only 6 cases) across the entire cohort. A total of 14 deaths were recorded during follow-up. Notably, only 3 patients (21.4%) died of lymphoma-specific causes, 11 patients (78.6%) died of non-lymphoma-related causes, including second primary malignancies (n=3), hepatic disease (n=1), and other unknown causes (n=7). Survival outcomes were favorable and equivalent to the age- and sex-matched general population in both groups, with SMR of 0.64 (95% CI: 0.17–1.64; P = 0.54) for the CT+RT group and 1.41 (95% CI: 0.68–2.60; P = 0.25) for the CT-alone group, indicating that patients achieved life expectancy comparable to the general population. Competing risk analysis revealed that NLM was a prominent contributor to late mortality events, whereas the cumulative incidence of LSM remained low and comparable between the CT-alone and CT+RT groups (2.3% vs 1.6%, p = 0.53).

**Copyright Information:** (Online) ISSN: 2572-9241
©2026 The Author(s). HemaSphere is published by John Wiley & Sons Ltd on behalf of European Hematology Association. This is an open access Abstract Book distributed under the Attribution-NonCommercial-NoDerivs (CC BY-NC-ND), which allows third parties to download the articles and share them with others as long as they credit the author and the Abstract Book, but they cannot change the content in any way or use them commercially.
**Abstract Book Citations:** Authors, Title, HemaSphere, 2026;10:(S1):pages. The abstract book DOIs can be found on the "Author Information" page.
**Disclaimer:** Articles published in the journal HemaSphere exclusively reflect the opinions of the authors. The authors are responsible for all content in their abstracts including accuracy of the facts, statements, citing resources, etc.

**Summary/Conclusion**

For limited-stage PG-DLBCL achieving CR after first-line chemotherapy, consolidative RT provides limited survival benefit due to exceptional local control and survival normalization. Competing risk analysis identifies NLM as the primary late mortality driver. While RT may not be mandatory for all, it may serve as a strategic enabler for chemotherapy de-escalation. The next step is to further investigate whether RT can serve as a key enabler for chemotherapy de-escalation.

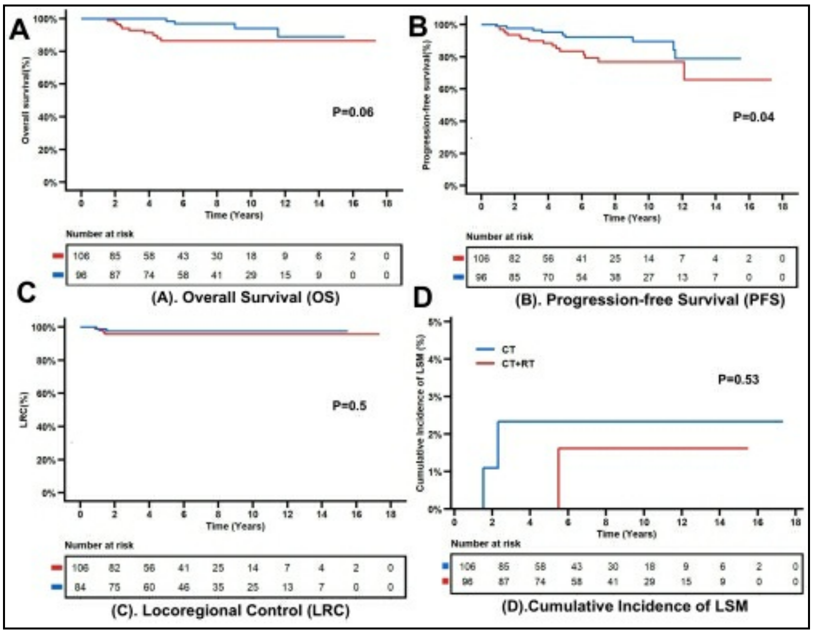

**Copyright Information:** (Online) ISSN: 2572-9241
©2026 The Author(s). HemaSphere is published by John Wiley & Sons Ltd on behalf of European Hematology Association. This is an open access Abstract Book distributed under the Attribution-NonCommercial-NoDerivs (CC BY-NC-ND), which allows third parties to download the articles and share them with others as long as they credit the author and the Abstract Book, but they cannot change the content in any way or use them commercially.
**Abstract Book Citations:** Authors, Title, HemaSphere, 2026;10:(S1):pages. The abstract book DOIs can be found on the "Author Information" page.
**Disclaimer:** Articles published in the journal HemaSphere exclusively reflect the opinions of the authors. The authors are responsible for all content in their abstracts including accuracy of the facts, statements, citing resources, etc.

### (PF1022) POLA-RCHP FOR DLBCL PATIENTS WITH EXTRANODAL INVOLVEMENT: REAL-WORLD EVIDENCE IN AN UNDERREPRESENTED POPULATION

**Topic:** 19. Large B cell lymphomas - Clinical

Xi Chen*[1], Tao Lei[1], Shuiyun Han[1], Haifeng Yu[1], Li Cong[1], Haiyan Yang[1]

[1]*Zhejiang Cancer Hospital, Lymphoma department, Hangzhou, China;*

### Background

The POLARIX trial established polatuzumab vedotin in combination with rituximab, cyclophosphamide, doxorubicin, and prednisone (pola-R-CHP) as a new standard for first-line treatment of diffuse large B-cell lymphoma (DLBCL). However, patients with extranodal involvement—a well-recognized adverse prognostic factor—were underrepresented in that study, and data on the efficacy and safety of this regimen in the real-world population remain scarce. To address this gap, we conducted a single-center retrospective analysis of DLBCL patients with extranodal involvement treated with pola-R-CHP.

### Aims

The primary objectives were to assess overall response rate (ORR), complete response (CR) rate, and short-term survival outcomes; secondary objectives included safety evaluation and exploration of whether the pattern of extranodal involvement (single-site versus multi-site) influenced treatment outcomes.

### Methods

Eligible patients had pathologically confirmed DLBCL with at least one extranodal site, completed ≥2 cycles of pola-R-CHP followed by response assessment, and had no prior anti-lymphoma therapy. All patients were scheduled to receive six cycles of pola-R-CHP and two additional cycles of rituximab monotherapy. Efficacy and safety were assessed per Lugano 2014 criteria and CTCAE version 5.0, respectively. Survival was estimated using the Kaplan-Meier method, with data censored as of January 31, 2026.

### Results

Between January 2023 and November 2025, 103 patients were enrolled, with a median age of 63 years (range 25–84) and a male predominance (58%). All had stage III–IV disease, and 84% were IPI 3–5, reflecting a high-risk population. By Hans algorithm, cell-of-origin was GCB in 38 patients (37%), non-GCB in 61 (59%), and unclassified in 4 (4%). Bulky disease (≥7 cm) was seen in 16 (16%), DEL in 20 (20%), and DHL in 3 (3%). Extranodal involvement was single-site in 45 patients (44%) and multi-sites (≥2) in 58 (56%). The most common sites were bone/bone marrow (43, 42%), gastrointestinal tract (35, 34%), and liver (19, 18%). Among single-site cases, gastrointestinal tract (13, 29%) and bone/bone marrow (7, 16%) predominated.
After a median follow-up of 7.1 months, the ORR was 86%, with 81% of patients achieving CR. Response rates stratified by number of involved sites and by specific extranodal sites are illustrated in Figure 1. The 6-month progression-free survival rate was 85.9%, and the 12-month rate was 77.3%; corresponding overall survival rates were 100% at 6 months and 97.4% at 12 months. With regard to safety, the most frequently reported any-grade adverse events were neutropenia (32%), nausea (25%), and peripheral neuropathy (20%). Grade ≥3 events were observed in 20% of patients, most commonly neutropenia (24%), infections (10%), and anemia (9%). Notably, no patient required treatment discontinuation due to adverse events, and no treatment-related deaths occurred.

**Copyright Information:** (Online) ISSN: 2572-9241
©2026 The Author(s). HemaSphere is published by John Wiley & Sons Ltd on behalf of European Hematology Association. This is an open access Abstract Book distributed under the Attribution-NonCommercial-NoDerivs (CC BY-NC-ND), which allows third parties to download the articles and share them with others as long as they credit the author and the Abstract Book, but they cannot change the content in any way or use them commercially.
**Abstract Book Citations:** Authors, Title, HemaSphere, 2026;10:(S1):pages. The abstract book DOIs can be found on the “Author Information” page.
**Disclaimer:** Articles published in the journal HemaSphere exclusively reflect the opinions of the authors. The authors are responsible for all content in their abstracts including accuracy of the facts, statements, citing resources, etc.

### Summary/Conclusion

To our knowledge, this represents the first real-world evidence demonstrating that pola-R-CHP is both highly active and well tolerated in DLBCL patients with extranodal involvement. Despite the uniformly high-risk features of this cohort, the observed CRR of 81% compares favorably with the 78% CRR reported in the overall POLARIX population. These findings support the use of pola-R-CHP in this challenging subgroup and underscore the need for larger prospective studies to further delineate outcomes according to specific extranodal sites.

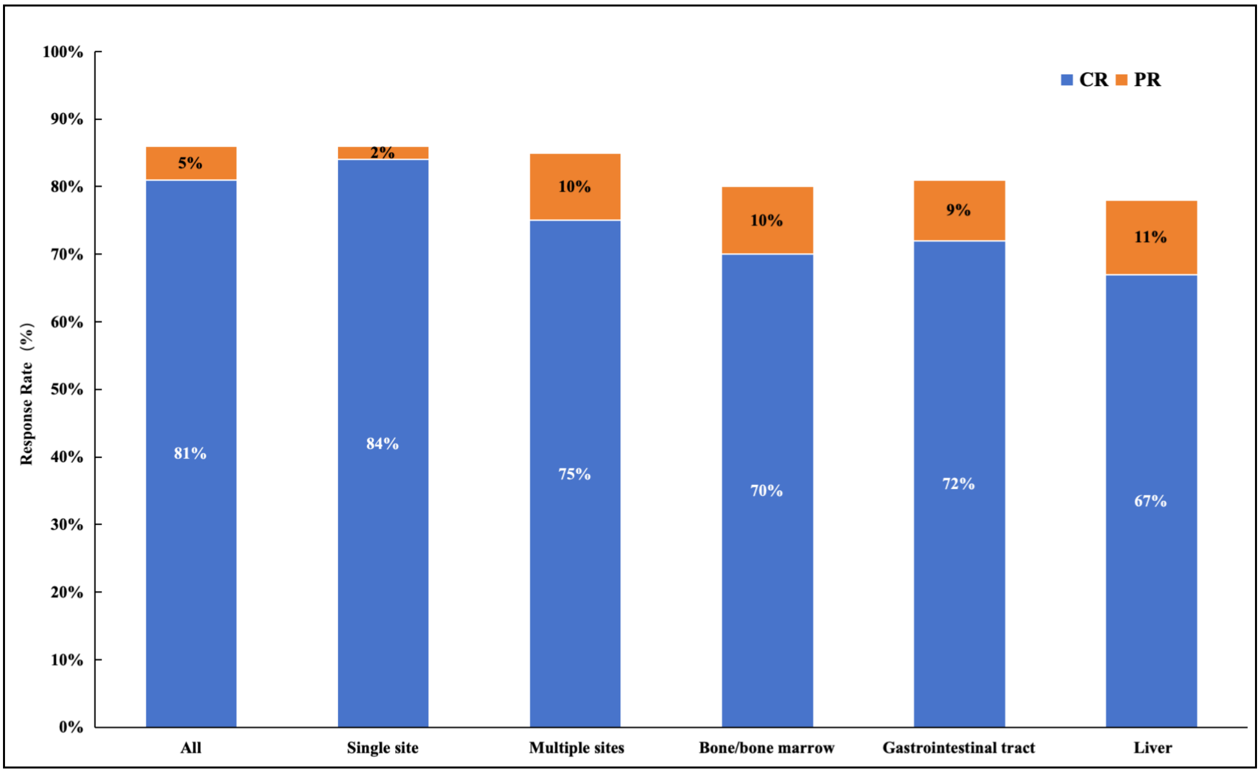

Figure 1. Response rates stratified by number of involved sites and by specific extranodal sites

**Copyright Information:** (Online) ISSN: 2572-9241
©2026 The Author(s). HemaSphere is published by John Wiley & Sons Ltd on behalf of European Hematology Association. This is an open access Abstract Book distributed under the Attribution-NonCommercial-NoDerivs (CC BY-NC-ND), which allows third parties to download the articles and share them with others as long as they credit the author and the Abstract Book, but they cannot change the content in any way or use them commercially.
**Abstract Book Citations:** Authors, Title, HemaSphere, 2026;10:(S1):pages. The abstract book DOIs can be found on the "Author Information" page.
**Disclaimer:** Articles published in the journal HemaSphere exclusively reflect the opinions of the authors. The authors are responsible for all content in their abstracts including accuracy of the facts, statements, citing resources, etc.

### (PF1023) INTERNATIONAL EXPERT CONSENSUS ON REAL-WORLD CAR T-CELL ELIGIBILITY IN LARGE B-CELL LYMPHOMAS: AN E-DELPHI STUDY

**Topic:** 19. Large B cell lymphomas - Clinical

Catherine Thieblemont*[1], Isabelle Fleury[2], Mazyar Shadman[3], Tobias A.W. Holderried[4, 5], Graham Collins[6]

*[1]Université Paris Cité & Assistance Publique des Hôpitaux de Paris (APHP), Hôpital Saint-Louis, Paris, France;[2]CHU de Quebec-Université Laval, Québec, Canada; [3]Fred Hutch Cancer Center, Cellular Immunotherapy, Seattle, United States of America; [4]Universitätsklinikum Bonn, Medizinische Klinik und Poliklinik III, Bonn, Germany;[5]Medizinische Universität Graz, Division of Hematology, Graz, Austria; [6]Oxford University Hospitals NHS Foundation Trust, Haematology, Oxford, United Kingdom;*

### Background

Despite robust clinical evidence, regulatory approval and guideline recommendations in second- and third-line settings, disparities persist for referring patients with relapsed or refractory diffuse large B-cell lymphoma (r/r DLBCL) for chimeric antigen receptor T-cell therapy (CAR T). Recent studies estimate only one-third of CAR T-eligible patients are ultimately treated, partly due to uncertainty regarding referral eligibility criteria. Consensus-driven guidance is needed to support equitable, timely referral practices.

### Aims

To identify and characterize consensus on core clinical eligibility criteria to guide referral of patients with r/r DLBCL by community-based physicians to specialized centers for evaluation for CAR T.

### Methods

An e-Delphi study was conducted in accordance with established consensus reporting standards. All study materials, designed in alignment with ACCORD (ACcurate COnsensus Reporting Document) guidelines, received approval from an independent steering committee of clinical experts, and independent institutional review boards. A panel of 23 expert hematology and/or oncology clinicians in USA, Canada, UK, France, Germany, and Italy, anonymously responded to statements via a web-based questionnaire (≥75% consensus threshold).

**Copyright Information:** (Online) ISSN: 2572-9241
©2026 The Author(s). HemaSphere is published by John Wiley & Sons Ltd on behalf of European Hematology Association. This is an open access Abstract Book distributed under the Attribution-NonCommercial-NoDerivs (CC BY-NC-ND), which allows third parties to download the articles and share them with others as long as they credit the author and the Abstract Book, but they cannot change the content in any way or use them commercially.
**Abstract Book Citations:** Authors, Title, HemaSphere, 2026;10:(S1):pages. The abstract book DOIs can be found on the "Author Information" page.
**Disclaimer:** Articles published in the journal HemaSphere exclusively reflect the opinions of the authors. The authors are responsible for all content in their abstracts including accuracy of the facts, statements, citing resources, etc.

**Results**

All (100%, N=23) reported academic research leadership, including guideline development (74.0%) and board memberships (61%), managing a mean (SD) of 63.7 (57.3) patients with LBCL and referring/prescribing CAR T to 22.2 (16.9), in the last 12 months.
All agreed that CAR T-eligibility is not the same as autologous stem-cell transplant (ASCT)- eligibility, and ASCT-ineligible patients can be considered CAR T eligible; 95.7% agreed that eligibility for CAR T is broader than ASCT.
Panelists agreed (82.6%) on immediate referral for relapse within 12 months post- or, refractoriness to, first-line treatment. Panelists agreed that response to first-line therapy should be evaluated in all patients after 2-4 cycles using interim PET (82.6%) and that patients with stable disease at these timepoints should be referred (87.0%). All agreed to refer patients with progressive disease after 2-4 cycles, and patients with r/r DLBCL after ≥2 lines of systemic therapy.
All agreed that age should neither be an absolute contraindication for referral nor, alone, be an absolute exclusion criterion. For patients aged ≥70 years, 87.0% agreed a formal frailty/comorbidity assessment should be prioritized over age alone when determining referral eligibility.
Performance status (PS) was identified as a core clinical criterion for referral by 91.3%, with automatic referral for an ECOG PS of 0, 1, or 2.
82.6% agreed that patients with creatinine clearance above 30 mL/min should automatically be referred, and 78.2% that cardiovascular function is a core criterion.
Statements not reaching consensus will be re-presented in subsequent Delphi rounds along with the aggregated and anonymized responses from the previous round.

**Summary/Conclusion**

Round 1 analysis demonstrates robust international expert consensus on referral eligibility for CAR T in patients with r/r DLBCL. The panel strongly endorsed broad eligibility for referral independent of ASCT eligibility, and provided clarity regarding disease status, age, performance, and organ function as core referral criteria. These consensus recommendations have the potential to reduce unwarranted variation in referral practices and support more equitable access to CAR T. Final recommendations will be presented at congress.

**Copyright Information:** (Online) ISSN: 2572-9241
©2026 The Author(s). HemaSphere is published by John Wiley & Sons Ltd on behalf of European Hematology Association. This is an open access Abstract Book distributed under the Attribution-NonCommercial-NoDerivs (CC BY-NC-ND), which allows third parties to download the articles and share them with others as long as they credit the author and the Abstract Book, but they cannot change the content in any way or use them commercially.
**Abstract Book Citations:** Authors, Title, HemaSphere, 2026;10:(S1):pages. The abstract book DOIs can be found on the "Author Information" page.
**Disclaimer:** Articles published in the journal HemaSphere exclusively reflect the opinions of the authors. The authors are responsible for all content in their abstracts including accuracy of the facts, statements, citing resources, etc.

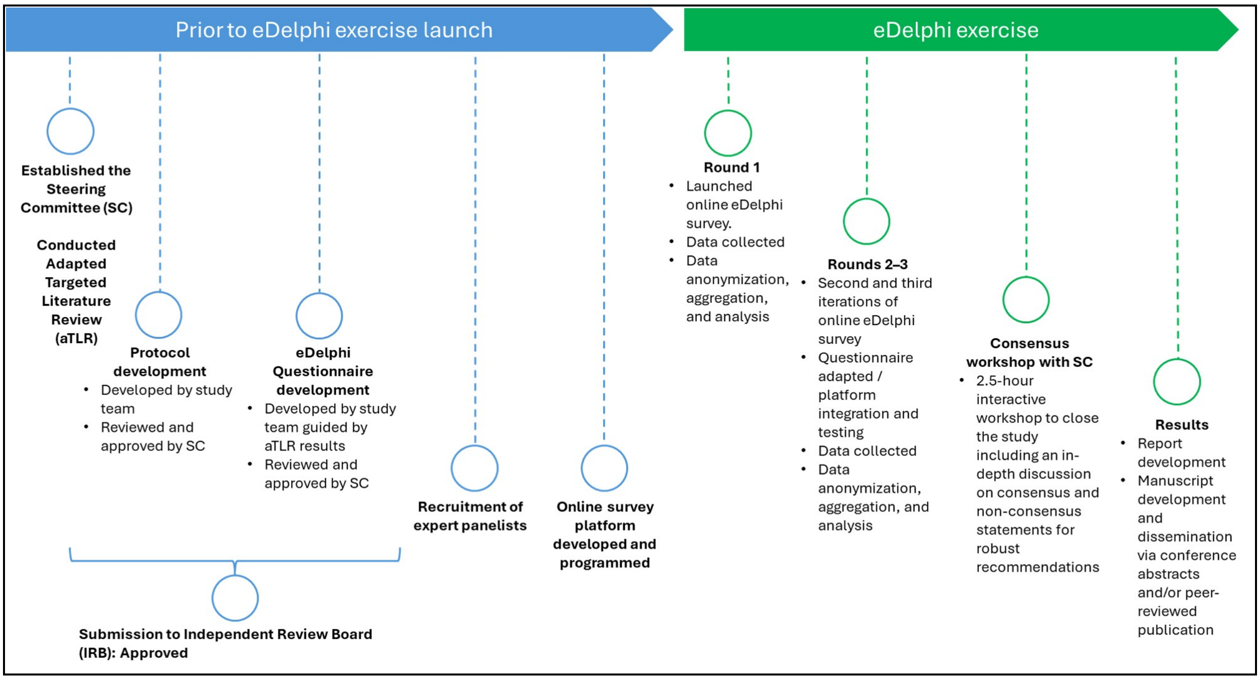

Figure 1: eDelphi Study Design

**Copyright Information:** (Online) ISSN: 2572-9241
©2026 The Author(s). HemaSphere is published by John Wiley & Sons Ltd on behalf of European Hematology Association. This is an open access Abstract Book distributed under the Attribution-NonCommercial-NoDerivs (CC BY-NC-ND), which allows third parties to download the articles and share them with others as long as they credit the author and the Abstract Book, but they cannot change the content in any way or use them commercially.
**Abstract Book Citations:** Authors, Title, HemaSphere, 2026;10:(S1):pages. The abstract book DOIs can be found on the "Author Information" page.
**Disclaimer:** Articles published in the journal HemaSphere exclusively reflect the opinions of the authors. The authors are responsible for all content in their abstracts including accuracy of the facts, statements, citing resources, etc.

### (PF1024) LARGE B-CELL LYMPHOMA TREATMENT WITH LONCASTUXIMAB TESIRINE IN AN ITALIAN REAL-LIFE EXPERIENCE

**Topic:** 19. Large B cell lymphomas - Clinical

Alessandro Broccoli*[1, 2], Lisa Argnani[2], Manuela Zanni[3], Sonya De Lorenzo[4], Lorena Appio[5], Pio Manlio Mirko Frascione[6], Martina Di Palma[7], Natalia Cenfra[8], Francesco Malaspina[9], Anna Vanazzi[10], Anna Dodero[11], Francesca Gaia Rossi[12], Luisa Verga[13], Bruna Riccardo[14], Annalisa Arcari[15], Martina Cantelli[16], Fabiana Esposito[17], Flaminia Bellisario[18], Mattia Novo[19], Elisa Lucchini[20], Silvia Corazza[21], Pier Luigi Zinzani[1, 2]

*[1]IRCCS Azienda Ospedaliero-Universitaria di Bologna, Policlinico di Sant'Orsola, Dipartimento di Scienze Mediche e Chirurgiche, Università di Bologna, Bologna, Italy; [2]Dipartimento di Scienze Mediche e Chirurgiche, Università di Bologna, Bologna, Italy; [3]SCDU Ematologia Azienda Ospedaliera Santi Antonio e Biagio e Cesare Arrigo, Alessandria, Italy;[4]UOC Ematologia e Terapie Cellulari Avanzate - AORN "S. G. Moscati", Avellino, Italy; [5]SC Ematologia, PO Busto Arsizio, ASST Valle Olona, Busto Arsizio, Italy; [6]Istituto di Candiolo, FPO-IRCCS, Candiolo, Italy;[7]Hematology Unit, Onco-hematology Department, AORN Sant'Anna e San Sebastiano, Caserta, Italy; [8]UOC Ematologia con Trapianto, Ospedale "S.M. Goretti", Latina, Italy;[9]IRCCS Istituto Romagnolo per lo studio dei Tumori "Dino Amadori", Meldola, Italy; [10]Divisione di Oncoematologia, Istituto Europeo di Oncologia, Milano, Italy; [11]Dipartimento Ematologia, Fondazione IRCCS INT, Milano, Italy; [12]Fondazione IRCCS Ca' Granda, Ospedale Maggiore Policlinico, S.C. Ematologia, Milano, Italy; [13]IRCCS San Gerardo dei Tintori, SC Ematologia, Monza, Italy; [14]Division of Hematology, Department of Translational Medicine, "Amedeo Avogadro" University of Eastern Piedmont, Novara, Italy; [15]Hematology Unit, Ospedale "Guglielmo da Saliceto", AUSL Piacenza, Piacenza, Italy; [16]U.O.C Ematologia, Ospedale "Santa Maria delle Croci", Ravenna, Italy; [17]Dipartimento di Biomedicina e Prevenzione, Università "Tor Vergata", Roma, Italy; [18]Fondazione Policlinico Universitario "A. Gemelli" IRCCS, Dipartimento Scienze di Laborartorio ed Ematologiche, UOSD Malattie Linfoproliferative Extramidollari, Roma, Italy; [19]Division of Hematology and Allogeneic Stem Cell Transplant Unit, A.O.U. Città della Salute e della Scienza di Torino, Torino, Italy; [20]UCO Ematologia, Azienda Sanitaria Universitaria Giuliano-Isontina, Trieste, Italy; [21]IRCCS Azienda Ospedaliero-Universitaria di Bologna, Policlinico di Sant'Orsola, Bologna, Italy;*

### Background

Loncastuximab tesirine (LT), an anti-CD19 pyrrolobenzodiazepine-conjugated monoclonal antibody, is now approved for the treatment of large B-cell lymphoma (LBCL) patients from their third line onwards. According to the results of the phase 2 registration trial, responses occurred quickly and lasted for over a year in patients receiving at least 2 previous treatments, including chimeric antigen receptor T (CART) cells.

### Aims

An observational study reporting the Italian real-life experience with LT as compassionate use, with the purpose of assessing its effectiveness, in terms of response rate and time to response. Secondary endpoints were progression-free, overall survival, response duration and safety in a cohort of patients with LBCL relapsing after or refractory to at least 2 previous lines.

### Methods

LT was administered in accordance with its prescribing indications until disease progression or unacceptable toxicity, whichever came first. Patients in the study were not subjected to any procedures outside normal practice and the variables collected were retrieved from patients' medical records.

**Copyright Information:** (Online) ISSN: 2572-9241
©2026 The Author(s). HemaSphere is published by John Wiley & Sons Ltd on behalf of European Hematology Association. This is an open access Abstract Book distributed under the Attribution-NonCommercial-NoDerivs (CC BY-NC-ND), which allows third parties to download the articles and share them with others as long as they credit the author and the Abstract Book, but they cannot change the content in any way or use them commercially.
**Abstract Book Citations:** Authors, Title, HemaSphere, 2026;10:(S1):pages. The abstract book DOIs can be found on the "Author Information" page.
**Disclaimer:** Articles published in the journal HemaSphere exclusively reflect the opinions of the authors. The authors are responsible for all content in their abstracts including accuracy of the facts, statements, citing resources, etc.

**Results**

Thirty-seven patients (25 males and 12 females) with LBCL (including 4 cases of double-hit diffuse LBCL) received a median number of 3 cycles (range: 1-16) of LT after a median of 3 (range: 2-12) previous lines of therapy and at a median age of 69 years (range: 26-90). Three patients already received autologous transplantation and 12 (32.4%) CART cells. Eighteen (48.6%) showed refractoriness to their latest treatment immediately before LT. Disease stage was III-IV in 89.2% of the cases, with extranodal disease in 78.4% and bulky masses in 21.6%. ECOG performance status ≥ 2 was reported in 43.2% of the cases. A complete response was obtained in 32.4% of treated patients and a partial response in 10.8% at a median time to response of 3.3 months. The remaining 51.4% of the patients progressed with no response, while 5.4% remained in stable disease. No patient was still receiving LT at the time of present analysis. At a median follow-up of 17.1 months, median progression-free and overall survival were 2.7 and 5.5 months, respectively (Figure). Twenty-nine patients died during treatment period (78.4%), in 72.4% of the cases due to disease progression. Median duration of complete response was 5.4 months. Seven patients developed 17 hematologic adverse events (AEs), including grade 3 anemia, neutropenia and thrombocytopenia in 3, 1 and 3 cases, respectively, and grade 4 neutropenia and thrombocytopenia in 1 and 3 cases, respectively, all considered drug correlated. Twenty-five extra-hematologic AEs were reported in 19 patients, 13 were considered drug-related (occurring in 11 patients), of which 4 were grade ≥ 3 (in 3 patients), including grade 3 skin toxicity and edema in one case each, and 2 grade 3 infections. No treatment-related deaths occurred.

**Summary/Conclusion**

LT displays rapid disease control in a population of highly pretreated LBCL patients with severe presentation. More disappointing survival results have been documented in our population compared to registration study due to much more unfavorable disease characteristics. Extra-hematologic toxicity remains a concern.

**Copyright Information:** (Online) ISSN: 2572-9241
©2026 The Author(s). HemaSphere is published by John Wiley & Sons Ltd on behalf of European Hematology Association. This is an open access Abstract Book distributed under the Attribution-NonCommercial-NoDerivs (CC BY-NC-ND), which allows third parties to download the articles and share them with others as long as they credit the author and the Abstract Book, but they cannot change the content in any way or use them commercially.
**Abstract Book Citations:** Authors, Title, HemaSphere, 2026;10:(S1):pages. The abstract book DOIs can be found on the "Author Information" page.
**Disclaimer:** Articles published in the journal HemaSphere exclusively reflect the opinions of the authors. The authors are responsible for all content in their abstracts including accuracy of the facts, statements, citing resources, etc.

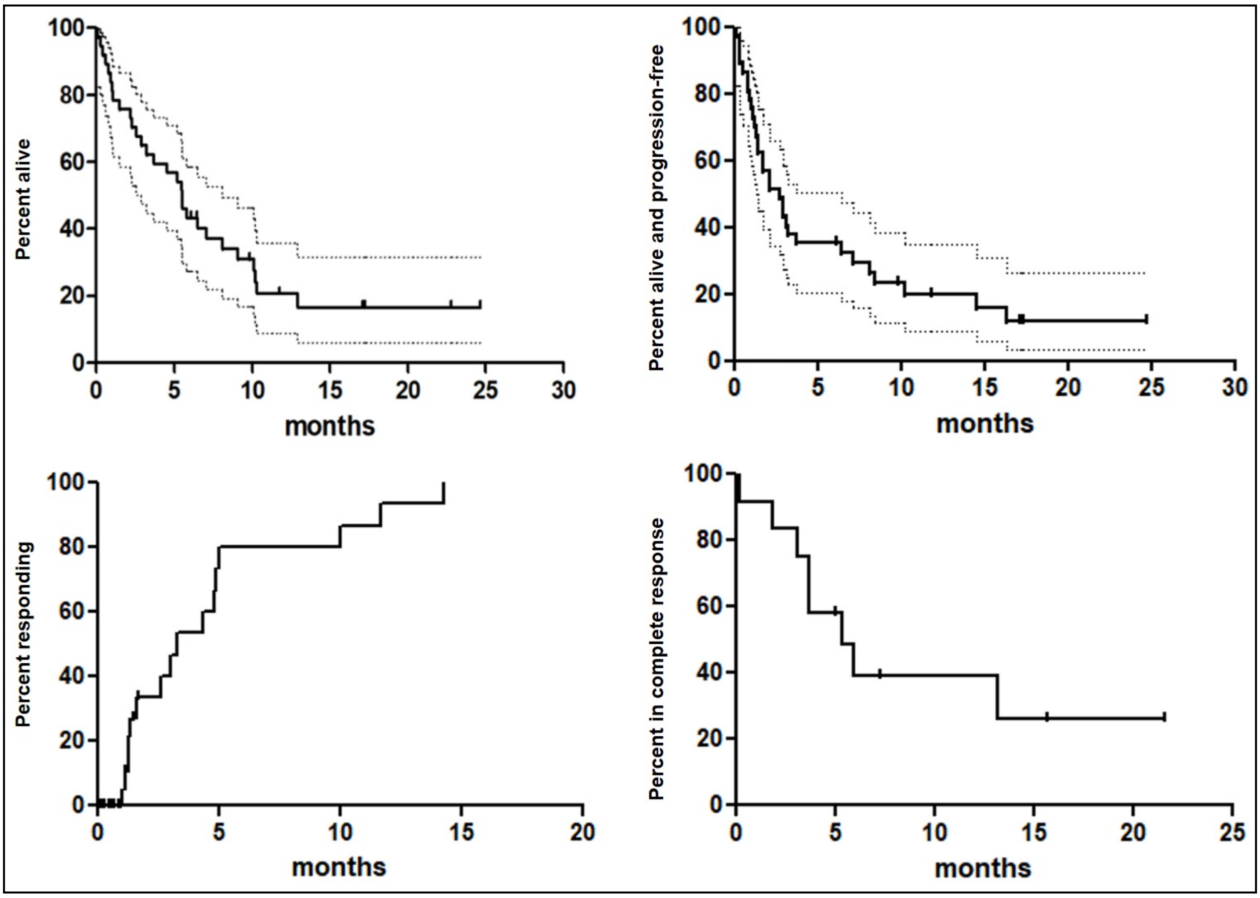

Copyright Information: (Online) ISSN: 2572-9241
©2026 The Author(s). HemaSphere is published by John Wiley & Sons Ltd on behalf of European Hematology Association. This is an open access Abstract Book distributed under the Attribution-NonCommercial-NoDerivs (CC BY-NC-ND), which allows third parties to download the articles and share them with others as long as they credit the author and the Abstract Book, but they cannot change the content in any way or use them commercially.
Abstract Book Citations: Authors, Title, HemaSphere, 2026;10:(S1):pages. The abstract book DOIs can be found on the "Author Information" page.
Disclaimer: Articles published in the journal HemaSphere exclusively reflect the opinions of the authors. The authors are responsible for all content in their abstracts including accuracy of the facts, statements, citing resources, etc.

### (PF1025) METAGENOMIC STUDY ON THE DYNAMIC CHANGES OF GUT MICROBIOME-METABOLOME IN DIFFUSE LARGE B-CELL LYMPHOMA: A COHORT ANALYSIS BASED ON PATIENTS' HEALTHY COHABITANTS AS CONTROL

**Topic:** 19. Large B cell lymphomas - Clinical

Qiling Liu[1], Qingyang Liu[1], Ping Chen[1], Jingwen Tang[1], Lili Dong[1], Cong Feng[1], Sai Huang*[1]

[1]*Chinese PLA General Hospital, Beijing, China;*

### Background

Diffuse large B-cell lymphoma (DLBCL) is the most common type of non-Hodgkin lymphoma. Despite numerous studies focusing on its genomic characteristics and molecular pathogenesis, systematic elucidation of the dynamic changes of gut microbiota during the course of DLBCL remains scarce.

### Aims

This study aims to investigate the dynamic changes of gut microbiome-metabolome in diffuse large B-cell lymphoma.

### Methods

This study compared fecal gut microbiota samples from 9 patients with DLBCL at initial diagnosis, samples from these patients after achieving complete remission (CR) through standard first-line chemotherapy, and samples from their healthy cohabitants living in the same environment as the patients. Differences in microbial composition and metabolic pathways among the 3 groups were investigated through metagenomic and metabolomic analysis.

### Results

Compared with HC, ND patients showed significantly reduced gut microbial diversity (fewer unique genes, impaired robustness) and core microbial dysbiosis: Bacteroidetes relative abundance was elevated (46.3%), while Firmicutes (56.6% in HC, 58.4% in CR) and its derived taxa (Clostridia, Clostridiales, Lachnospiraceae, Eubacterioceae) were reduced. Beneficial SCFA-producing genera (Blautia, Roseburia, Ruminococcus) were depleted, and the Firmicutes/Bacteroidetes (F/B) ratio was decreased—all restored in CR. Macrogenomic evidence suggests that the gut microbiota of patients is associated with multiple metabolic pathways, including reduced function K06147 (ATP-binding cassette, subfamily B, bacterial), elevated function K03088 (RNA polymerase sigma-70 factor, ECF subfamily) and K21572(starch-binding outer membrane protein, SusD/RagB family) in DLBCL patients.

### Summary/Conclusion

This study demonstrates the dynamic remodeling of gut microbiota mediated by disease and treatment in DLBCL patients, supporting the characteristic dysbiosis and metabolic disorders of gut microbiota in untreated newly diagnosed DLBCL patients. After achieving complete remission through standard first-line chemotherapy, the gut microbiota structure of patients approximates the state of their healthy cohabitants. These findings characterize the changes in gut microbiota during the course of DLBCL, suggesting potential insights for the study of the pathogenesis of DLBCL and the development of new therapies.

**Copyright Information:** (Online) ISSN: 2572-9241
©2026 The Author(s). HemaSphere is published by John Wiley & Sons Ltd on behalf of European Hematology Association. This is an open access Abstract Book distributed under the Attribution-NonCommercial-NoDerivs (CC BY-NC-ND), which allows third parties to download the articles and share them with others as long as they credit the author and the Abstract Book, but they cannot change the content in any way or use them commercially.
**Abstract Book Citations:** Authors, Title, HemaSphere, 2026;10:(S1):pages. The abstract book DOIs can be found on the “Author Information” page.
**Disclaimer:** Articles published in the journal HemaSphere exclusively reflect the opinions of the authors. The authors are responsible for all content in their abstracts including accuracy of the facts, statements, citing resources, etc.

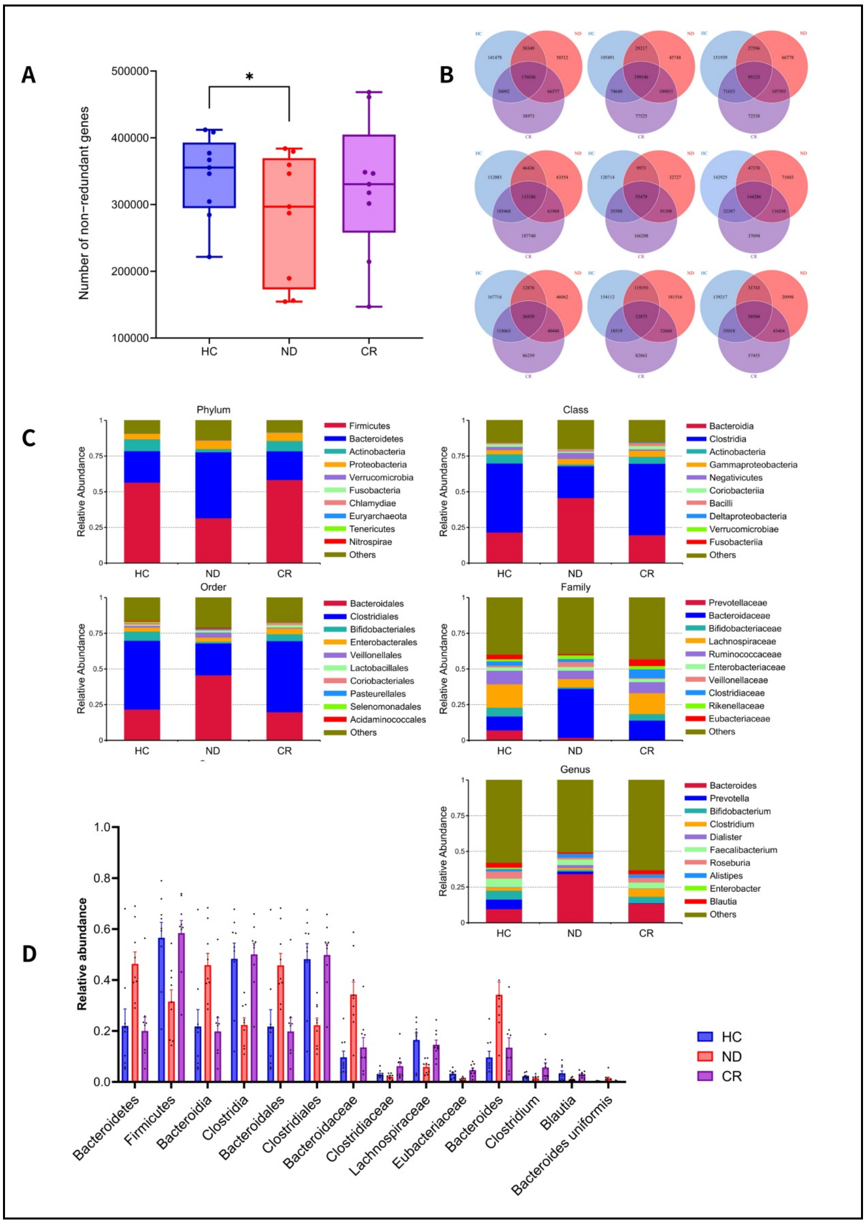

**Copyright Information:** (Online) ISSN: 2572-9241
©2026 The Author(s). HemaSphere is published by John Wiley & Sons Ltd on behalf of European Hematology Association. This is an open access Abstract Book distributed under the Attribution-NonCommercial-NoDerivs (CC BY-NC-ND), which allows third parties to download the articles and share them with others as long as they credit the author and the Abstract Book, but they cannot change the content in any way or use them commercially.
**Abstract Book Citations:** Authors, Title, HemaSphere, 2026;10:(S1):pages. The abstract book DOIs can be found on the "Author Information" page.
**Disclaimer:** Articles published in the journal HemaSphere exclusively reflect the opinions of the authors. The authors are responsible for all content in their abstracts including accuracy of the facts, statements, citing resources, etc.

### (PF1026) EARLY TOXICITY OF AXICABTAGENE CILOLEUCEL BY LINE OF THERAPY IN DLBCL: A REAL-WORLD COMPARISON OF 2L VS ≥3L TREATMENT

**Topic:** 19. Large B cell lymphomas - Clinical

Rebeca Bailen*[1], José María Sánchez-Pina[2], Javier Delgado-Serrano[3], Gloria Iacoboni[4], Almudena Cabero[5], Carola Diaz Aizpun[6], Alberto Mussetti[7], Judith Vázquez-Álvarez[8], Víctor Noriega Concepción[9], Jaime Sanz Caballer[10], Anabelle Chinea Rodriguez[11], Rafael Hernani[12], Pilar Gómez Prieto[13], Miriam Sánchez Escamilla[14], Valentín Ortiz-Maldonado[15], Ana Carolina Caballero Gonzalez[16], Luisa Guerra Domínguez[17], Mariana Bastos-Oreiro[1], Maria Calbacho Robles[2], Juan Luis Reguera Ortega[3], Pere Barba[4], Lucía López Corral[5], Pascual Balsalobre[18], Mi Kwon[1, 19]

*[1]Hospital General Universitario Gregorio Marañón. Instituto de Investigación Sanitaria Gregorio Marañón., Madrid, Spain; [2]Hospital Doce de Octubre., Madrid, Spain;[3]Hospital Universitario Virgen del Rocío, Sevilla, Spain;[4]Hospital Vall d'Hebron. Vall d'Hebron Institute of Oncology (VHIO), Barcelona, Spain; [5]Hospital Clínico Universitario de Salamanca, IBSAL, Salamanca, Spain; [6]Hospital Regional de Málaga, Málaga, Spain;[7]Hospital Duran i Reynals, Instituto Catalán de Oncología, IDIBELL, Barcelona, Spain; [8]Hospital Universitario Álvaro Cunqueiro, Vigo, Spain;[9]Complejo Hospitalario Universitario de A Coruña, A Coruña, Spain; [10]Hospital Universitario y Politécnico La Fe, Valencia, Spain; [11]Hospital Universitario Ramón y Cajal, Madrid, Spain; [12]Hospital Clínico Universitario de Valencia, Instituto de Investigación Sanitaria INCLIVA, Valencia, Spain;[13]Hospital Universitario La Paz, Madrid, Spain; [14]Hospital Universitario de Valdecilla, Santander, Spain;[15]Hospital Clinic, Barcelona, Spain; [16]Hospital de la Santa Creu i Sant Pau, Barcelona, Spain;[17]Hospital Universitario de Gran Canaria Doctor Negrín, Las Palmas de Gran Canaria, Spain; [18]Grupo Español de Trasplante de Progenitores Hematopoyéticos y Terapia Celular, Madrid, Spain; [19]Universidad Complutense de Madrid, Madrid, Spain;*

### Background

Axicabtagene ciloleucel (axi-cel) is approved for relapsed/refractory large B-cell lymphoma, with early toxicities driven by cytokine release syndrome (CRS) and immune effector cell–associated neurotoxicity syndrome (ICANS). Pivotal studies reported typical early onset within the first week and a considerable burden of grade ≥3 events, prompting interest in whether toxicity differs when axi-cel is administered in second line (2L) versus later lines (≥3L).

### Aims

To compare early toxicity patterns (CRS and ICANS) of axi-cel in diffuse large B-cell lymphoma (DLBCL) treated in 2L versus ≥3L, including timing, severity, duration, and toxicity-directed interventions.

### Methods

This retrospective, multicenter study on behalf of the GETH-TC (Spanish Group of Stem Transplantation and Cell Therapy) group compared consecutive DLBCL patients receiving axi-cel in second line from April 2024 (following regulatory approval in Spain) with a previously published historical cohort from our group treated between 2018 and 2021 in third or later line (Bailén et al. *Transpl Cell Ther* 2022). Patients were grouped by line of therapy at infusion: 2L vs ≥3L. CRS and ICANS were graded per ASTCT consensus criteria. Endpoints included incidence (any grade; grade ≥3), time-to-onset (days from infusion), duration (days), ICU admission, and treatment (tocilizumab, corticosteroids, anakinra if applicable). Comparative analyses used $\chi^2$/Fisher for categorical and Mann-Withney/t-test for continuous variables.

**Copyright Information:** (Online) ISSN: 2572-9241
©2026 The Author(s). HemaSphere is published by John Wiley & Sons Ltd on behalf of European Hematology Association. This is an open access Abstract Book distributed under the Attribution-NonCommercial-NoDerivs (CC BY-NC-ND), which allows third parties to download the articles and share them with others as long as they credit the author and the Abstract Book, but they cannot change the content in any way or use them commercially.
**Abstract Book Citations:** Authors, Title, HemaSphere, 2026;10:(S1):pages. The abstract book DOIs can be found on the "Author Information" page.
**Disclaimer:** Articles published in the journal HemaSphere exclusively reflect the opinions of the authors. The authors are responsible for all content in their abstracts including accuracy of the facts, statements, citing resources, etc.

**Results**

A total of N=413 patients were included (2L n=184, ≥3L n=229). Baseline characteristics are summarized in Table 1. Briefly, the 2L cohort showed a better ECOG performance status at infusion, lower R-IPI, lower prior ASCT exposure, lower rates of bulky disease and elevated LDH, and more frequent use of bridging therapy. No differences were observed in the median time from apheresis to infusion between groups.
Comparing 2L vs ≥3L, any-grade CRS occurred in 95% vs 92%, with grade ≥3 CRS in 4% vs 8% ($p=0.002$). Median time-to-onset was 2 days in both groups and median duration 4 vs 5 days ($p=0.02$). Tocilizumab was administered in 61% vs 62% patients (median doses 1.5 vs 2, $p=0.03$), and corticosteroids were administered in 48% vs 32% ($p<0.001$). Any-grade ICANS occurred in 47% vs 52%, with grade ≥3 ICANS in 12.5% vs 21% ($p<0.001$). Median time-to-onset was 6 days in both groups and median duration 3 vs 4 days. Corticosteroids for ICANS were used in 35% vs 43% ($p<0.001$).
ICU admission related to toxicity occurred in 21% vs 24%. No differences were observed in non-relapse mortality (NRM).

**Summary/Conclusion**

In this real-world DLBCL axi-cel cohort, second line of therapy was associated with lower rates of grade ≥3 early toxicities (CRS and ICANS) as compared to ≥3L. Despite this reduced incidence of high-grade toxicity, corticosteroid use for CRS was more frequent in the 2L cohort, likely reflecting a temporal shift toward earlier and more proactive toxicity management in the more recent treatment period. Further analysis are needed to discard an impact of disease characteristics on these findings.

**Copyright Information:** (Online) ISSN: 2572-9241
©2026 The Author(s). HemaSphere is published by John Wiley & Sons Ltd on behalf of European Hematology Association. This is an open access Abstract Book distributed under the Attribution-NonCommercial-NoDerivs (CC BY-NC-ND), which allows third parties to download the articles and share them with others as long as they credit the author and the Abstract Book, but they cannot change the content in any way or use them commercially.
**Abstract Book Citations:** Authors, Title, HemaSphere, 2026;10:(S1):pages. The abstract book DOIs can be found on the “Author Information” page.
**Disclaimer:** Articles published in the journal HemaSphere exclusively reflect the opinions of the authors. The authors are responsible for all content in their abstracts including accuracy of the facts, statements, citing resources, etc.

**TABLE 1. Baseline characteristics and early toxicity outcomes (axi-cel) by line of therapy.** Second-line data are reported as fractions for selected variables according to the number of patients with available data for each variable.

| | 2L (n=184) | ≥3L (n= 229) | P |
|---|---|---|---|
| Age, median (IQR) | 61 (50-69) | 59 (48-67) | NS |
| Male sex, n (%) | 113 (61%) | 140 (61%) | NS |
| ECOG ≥2, n (%) | 7/134 (5%) | 21 (9%) | <0.001 |
| Stage III–IV at diagnosis, n (%) | 81/101(81%) | 184 (80%) | NS |
| IPI ≥3 at apheresis, n (%) | 42/94 (47%) | 124 (59%) | <0.001 |
| Prior ASCT, n (%) | 5 (3%) | 60 (26%) | <0.001 |
| Bulky disease | 18/85 (21%) | 80 (35%) | <0.001 |
| LDH > ULN, n (%) | 46/105 (44%) | 154 (67%) | <0.001 |
| Bridging therapy, n (%) | 127/139 (92%) | 180 (79%) | <0.001 |
| Time leukapheresis to infusion, days median (IQR) | 36 (32-43) | 39 (34-47) | NS |
| **CRS (ASTCT)** | | | |
| Any-grade CRS, n (%) | 176 (95%) | 210 (92%) | NS |
| Grade ≥3 CRS, n (%) | 7/166 (4%) | 18 (7.8%) | 0.002 |
| Time to CRS onset, days median (IQR/range) | 1 (1-3) (0-10) | 2 (1-4) (0-11) | NS |
| CRS duration, days median (IQR/range) | 4 (3-5) (1-15) | 5 (3-7) (1-68) | 0.02 |
| Tocilizumab, n (%) | 113 (61%) | 142 (62%) | NS |
| Tocilizumab doses, median (IQR) | 1.5 (1-2) | 2 (1-2) | 0.03 |
| Steroids for CRS, n (%) | 88 (48%) | 74 (32%) | <0.001 |
| HLH/MAS, n (%) | 2 (1%) | 1 (0.5%) | <0.001 |
| **ICANS (ASTCT)** | | | |
| Any-grade ICANS, n (%) | 86 (47%) | 118 (52%) | NS |
| Grade ≥3 ICANS, n (%) | 23 (12.5%) | 49 (21%) | <0.001 |
| Time to ICANS onset, days median (IQR/range) | 6 (5-7) (0-19) | 6 (5-8) (2-34) | NS |
| ICANS duration, days median (IQR/range) | 3 (1-7) (0-31) | 4 (2-10) (1-56) | NS |
| Steroids for ICANS, n (%) | 64 (35%) | 98 (43%) | <0.001 |
| Anakinra for ICANS, n (%) | 19 (10%) | 28 (12%) | 0.015 |
| **Outcomes** | | | |
| ICU admission, n (%) | 38 (21%) | 56 (24%) | NS |
| Treatment-related mortality, n (%) (day 30/day 100) | 12 (6%) (1 / 4) | 14 (6%) (3 / 3) | NS |
| Length of stay, days median (IQR) | Not available | 21 (18-28) | NA |

Copyright Information: (Online) ISSN: 2572-9241
©2026 The Author(s). HemaSphere is published by John Wiley & Sons Ltd on behalf of European Hematology Association. This is an open access Abstract Book distributed under the Attribution-NonCommercial-NoDerivs (CC BY-NC-ND), which allows third parties to download the articles and share them with others as long as they credit the author and the Abstract Book, but they cannot change the content in any way or use them commercially.
Abstract Book Citations: Authors, Title, HemaSphere, 2026;10:(S1):pages. The abstract book DOIs can be found on the "Author Information" page.
Disclaimer: Articles published in the journal HemaSphere exclusively reflect the opinions of the authors. The authors are responsible for all content in their abstracts including accuracy of the facts, statements, citing resources, etc.

### (PF1027) ZANUBRUTINIB PLUS R-CHOP (ZR-CHOP) IN NON-GERMINAL CENTER B-CELL-LIKE (NON-GCB) DIFFUSE LARGE B-CELL LYMPHOMA PATIENTS WHO WERE POSITRON EMISSION TOMOGRAPHY-POSITIVE AFTER TWO CYCLES OF R-CHOP

**Topic:** 19. Large B cell lymphomas - Clinical

Jie Wang*[1], He Li[1], Chenlu Yang[1], Di Wu[2], Xingli Zou[3], Tao Yang[4], Liangliang MA[5], Fang Xu[6], Jie Wang[2], Minggang Su[7], Sha Zhao[8], Lin Ji[9], Pu Kuang[1], Jie Huang[1], Yao Liu[4], Pengcheng He[2], Ting Niu[1]

*[1]West China Hospital, Sichuan University, Department of Hematology,Institute of Hematology, Chengdu, China;[2]The First Affiliated Hospital of Xi'an Jiaotong University, Department of Hematology, Xi'an, China; [3]Affiliated Hospital of North Sichuan Medical College, Department of Hematology, Nanchong, China; [4]Chongqing University Cancer Hospital, Department of Hematology and Oncology, Chongqing, China; [5]Meishan People's Hospital, Department of Hematology, Meishan, China; [6]Mianyang Central Hospital, School of Medicine, University of Electronic Science and Technology of China, Department of Hematology, Mianyang, China; [7]West China Hospital, Sichuan University, Department of Nuclear Medicine, Chengdu, China; [8]West China Hospital, Sichuan University, Department of Pathology, Chengdu, China;[9]West China Hospital, Sichuan University, Department of Radiology, Chengdu, China;*

### Background

R-CHOP is a standard treatment for previously untreated diffuse large B-cell lymphoma (DLBCL), however, more than one-third of patients are not cured by R-CHOP. There is a clear need to improve the prognosis of patients, especially for those with factors associated with poor prognosis, such as non-GCB subtype and positive interim positron emission tomography (PET). Bruton's tyrosine kinase inhibitors (BTKi) have established therapeutic activity in B-cell malignancies, with modest activity in DLBCL. Zanubrutinib, a potent and selective BTKi, was evaluated in patients with relapsed or refractory (R/R) non-GCB DLBCL. Zanubrutinib showed minimized off-target activities against interleukin-2-inducible T cell kinase (ITK) and reduced interference of ITK-mediated, rituximab-induced, antibody-dependent cellular cytotoxicity. Therefore, ZR-CHOP is a potentially effective regimen.

### Aims

We designed a treatment escalation regimen with ZR-CHOP, followed by zanubrutinib maintenance, for non-GCB DLBCL patients who were PET positive after two cycles of R-CHOP.

### Methods

This was a single arm, multicenter, phase II study. Patients with non-GCB DLBCL who were PET positive (Deauville scores 4 or 5) after two cycles of R-CHOP were enrolled. Patients with PET negative or PD were excluded. Patients received ZR-CHOP in a 21-day cycle for 6 cycles (induction), followed by zanubrutinib monotherapy as maintenance up to 6 months for patients who achieved PR or CR. Zanubrutinib was administered orally twice daily at 160 mg. The primary endpoint was 2-year event-free survival (EFS) from start of RCHOP treatment, defined as disease progression, relapse from CR, initiation of subsequent systemic antilymphoma therapy for either PET-positive or biopsy-proven residual disease upon completion of at least 6 cycles of ZR-CHOP therapy, or death, whichever occurred first. Secondary endpoints included overall remission rate (ORR), complete remission rate, response improvement rate (defined as the proportion of patients whose response was improved from PR to CR or from SD to CR or PR during ZR-CHOP induction), and safety.

**Copyright Information:** (Online) ISSN: 2572-9241
©2026 The Author(s). HemaSphere is published by John Wiley & Sons Ltd on behalf of European Hematology Association. This is an open access Abstract Book distributed under the Attribution-NonCommercial-NoDerivs (CC BY-NC-ND), which allows third parties to download the articles and share them with others as long as they credit the author and the Abstract Book, but they cannot change the content in any way or use them commercially.
**Abstract Book Citations:** Authors, Title, HemaSphere, 2026;10:(S1):pages. The abstract book DOIs can be found on the “Author Information” page.
**Disclaimer:** Articles published in the journal HemaSphere exclusively reflect the opinions of the authors. The authors are responsible for all content in their abstracts including accuracy of the facts, statements, citing resources, etc.

### Results

From May 2022 to June 2024, a total of 28 patients were enrolled. The median age of patients was 59.0 (range, 40-79) years, 53.6 % were male, and 32.1% had ECOG PS of ≥2. A total of 78.6% of patients were stage III-IV; 32.1% had double-expressor lymphoma; most had extra-nodal involvement (82.2%). Eight (28.6 %) patients had an international prognostic index score of ≥3.
As of January 13, 2026, with a median follow-up of 28.3 months (95% CI: 25.3-32.0), the 2-year EFS rate was 38.2% (95% CI: 18.4-57.9), and the median EFS was 17.6 (8.3-NE) months. Patients who achieved CR after 2 cycles of ZR-CHOP had favorable EFS versus those with PR (median EFS: NE vs. 15.0 months). 25 (89.3%) patients achieved best response of CR (42.9%) or PR (46.4%). The response improvement rate was 54.2% (13/24; 95% CI, 32.8-74.5). 27 (96.4%) patients experienced any grade treatment emergent adverse events (TEAE). Grade ≥3 TEAE occurred in 21 (77.8%) patients. Serious adverse events were observed in 5 (18.5%) patients.

### Summary/Conclusion

Our results showed that ZR-CHOP was a safe and effective scheme for non-GCB DLBCL patients who were PET positive after two-cycle R-CHOP. It indicates that adding zanubrutinib to escalate the treatment under the guidance of dynamic PET-CT can improve the remission and median EFS of these patients without significantly increasing toxicity.

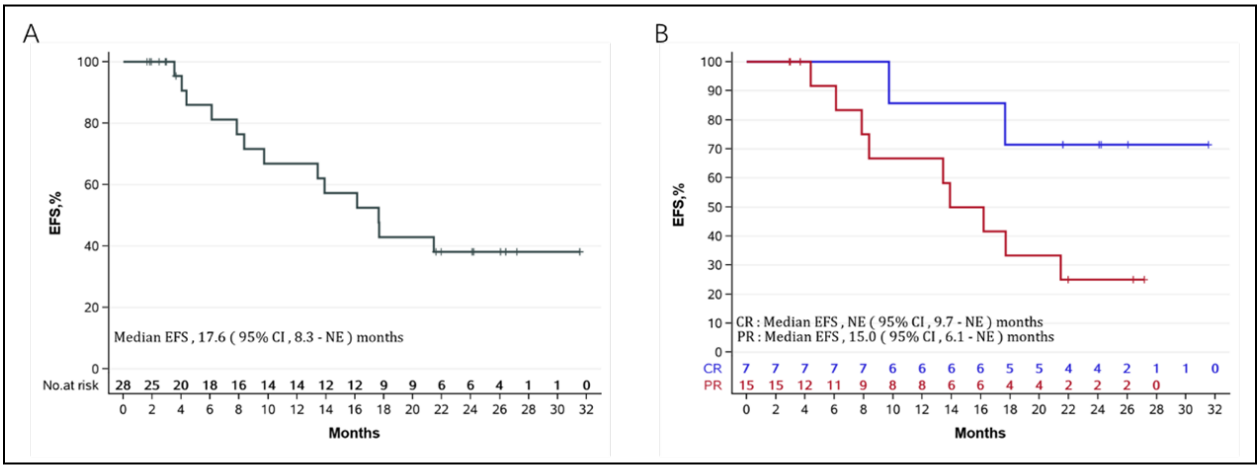

Figure 1 EFS in (A) overall patients and (B) patients achieving CR or PR after 2 cycles of ZR-CHOP

**Copyright Information:** (Online) ISSN: 2572-9241
©2026 The Author(s). HemaSphere is published by John Wiley & Sons Ltd on behalf of European Hematology Association. This is an open access Abstract Book distributed under the Attribution-NonCommercial-NoDerivs (CC BY-NC-ND), which allows third parties to download the articles and share them with others as long as they credit the author and the Abstract Book, but they cannot change the content in any way or use them commercially.
**Abstract Book Citations:** Authors, Title, HemaSphere, 2026;10:(S1):pages. The abstract book DOIs can be found on the "Author Information" page.
**Disclaimer:** Articles published in the journal HemaSphere exclusively reflect the opinions of the authors. The authors are responsible for all content in their abstracts including accuracy of the facts, statements, citing resources, etc.

### (PF1028) EFFICACY OF CAR-T CELL THERAPY IN DOUBLE-EXPRESSOR TP53-POSITIVE RELAPSED/REFRACTORY DIFFUSE LARGE B-CELL LYMPHOMA: A MULTICENTER REAL-WORLD STUDY

**Topic:** 19. Large B cell lymphomas - Clinical

Shihui Ren*[1], Ling Qiu[1], Dan Chen[1], Jiajia Li[1, 2], Yanling Li[1, 2], Mengjiao Li[1, 2], Sihan Lai[1], Yi Su[1], Alex Chang[3, 4], Mingqiang Ren[5], Linhui Wang[6], Feng Zhu[7], Jia Wei[8], Hao Yao[1, 9, 10, 11], Fangyi Fan[1, 2]

*[1]Department of Hematology, The General Hospital of Western Theater Command, Chengdu, China;[2]Department of Clinical Medicine, North Sichuan Medical College, Nanchong, China; [3]Engineering Research Center of Gene Technology, Ministry of Education, Institute of Genetics, School of Life Sciences, Fudan University, Shanghai, China; [4]Shanghai YaKe Biotechnology Ltd., Shanghai, China; [5]Department of Hematology, Affiliated Hospital of Zunyi Medical Univercity, Zunyi, China;[6]Department of Hematology, The People's Hospital of Guizhou Province, Guiyang, China; [7]Department of Hematology, The Affiliated Hospital of Xuzhou Medical University, Xuzhou, China; [8]Department of Hematology, Tongji Hospital, Tongji Medical College, Huazhong University of Science and Technology, Wuhan, China; [9]Institute of Basic Medicine, North Sichuan Medical College, Nanchong, China; [10]College of Medicine, Southwest Jiaotong University, Chengdu, China; [11]Tissue Stress Injury and Functional Repair Key Laboratory of Sichuan Province, Chengdu, China;*

### Background

Double-expressor TP53-positive diffuse large B-cell lymphoma (DLBCL) represents a highly aggressive subtype associated with poor prognosis. Outcomes are particularly dismal in relapsed/refractory (r/r) patients. Chimeric antigen receptor T-cell (CAR-T) therapy has emerged as an effective treatment option for r/r DLBCL; however, its efficacy in this high-risk biological subgroup remains unclear.

### Aims

To evaluate the clinical efficacy, overall survival (OS), and progression-free survival (PFS) of CAR-T therapy in patients with double-expressor TP53-positive r/r DLBCL.

### Methods

This multicenter retrospective study included patients with double-expressor TP53-positive r/r DLBCL who received CAR-T therapy between February 1, 2017, and December 31, 2025, at three tertiary centers. Clinical data were collected and analyzed using SPSS software. Overall response rate (ORR) at 1 and 3 months, median OS, and median PFS were assessed.

### Results

A total of 53 patients were included, with a median follow-up of 8–2,939 days. Thirteen patients were lost to follow-up (24.53%). Fifty-one patients received CAR-T therapy alone (CD19: n=28; CD19/CD22: n=22; CD20/CD22: n=1), and two patients received autologous hematopoietic stem cell transplantation combined with CD19 CAR-T therapy. The median age was 52 years (range 28–74), and 92.45% of patients had stage III–IV disease. The median number of prior treatment lines was 3 (range 1–6). The mortality rate was 50.94%. All 53 patients were evaluable for OS. The median OS was 270 days (95% CI 108.15–431.85). Among 46 evaluable patients, the median PFS was 108 days (95% CI 62.23–146.77). At 1 month post-infusion (n=47), the ORR was 70.21%, including 29.79% complete responses (CR) and 40.42% partial responses (PR). At 3 months (n=45), the ORR declined to 53.33%, with a CR rate of 31.11% and PR rate of 22.22%.

**Copyright Information:** (Online) ISSN: 2572-9241
©2026 The Author(s). HemaSphere is published by John Wiley & Sons Ltd on behalf of European Hematology Association. This is an open access Abstract Book distributed under the Attribution-NonCommercial-NoDerivs (CC BY-NC-ND), which allows third parties to download the articles and share them with others as long as they credit the author and the Abstract Book, but they cannot change the content in any way or use them commercially.
**Abstract Book Citations:** Authors, Title, HemaSphere, 2026;10:(S1):pages. The abstract book DOIs can be found on the "Author Information" page.
**Disclaimer:** Articles published in the journal HemaSphere exclusively reflect the opinions of the authors. The authors are responsible for all content in their abstracts including accuracy of the facts, statements, citing resources, etc.

**Summary/Conclusion**

In patients with double-expressor TP53-positive r/r DLBCL, CAR-T therapy induces initial responses; however, durability remains limited, with short median PFS and OS observed in this high-risk population. These findings suggest that additional therapeutic strategies, including combination or consolidation approaches, may be required to improve long-term outcomes.

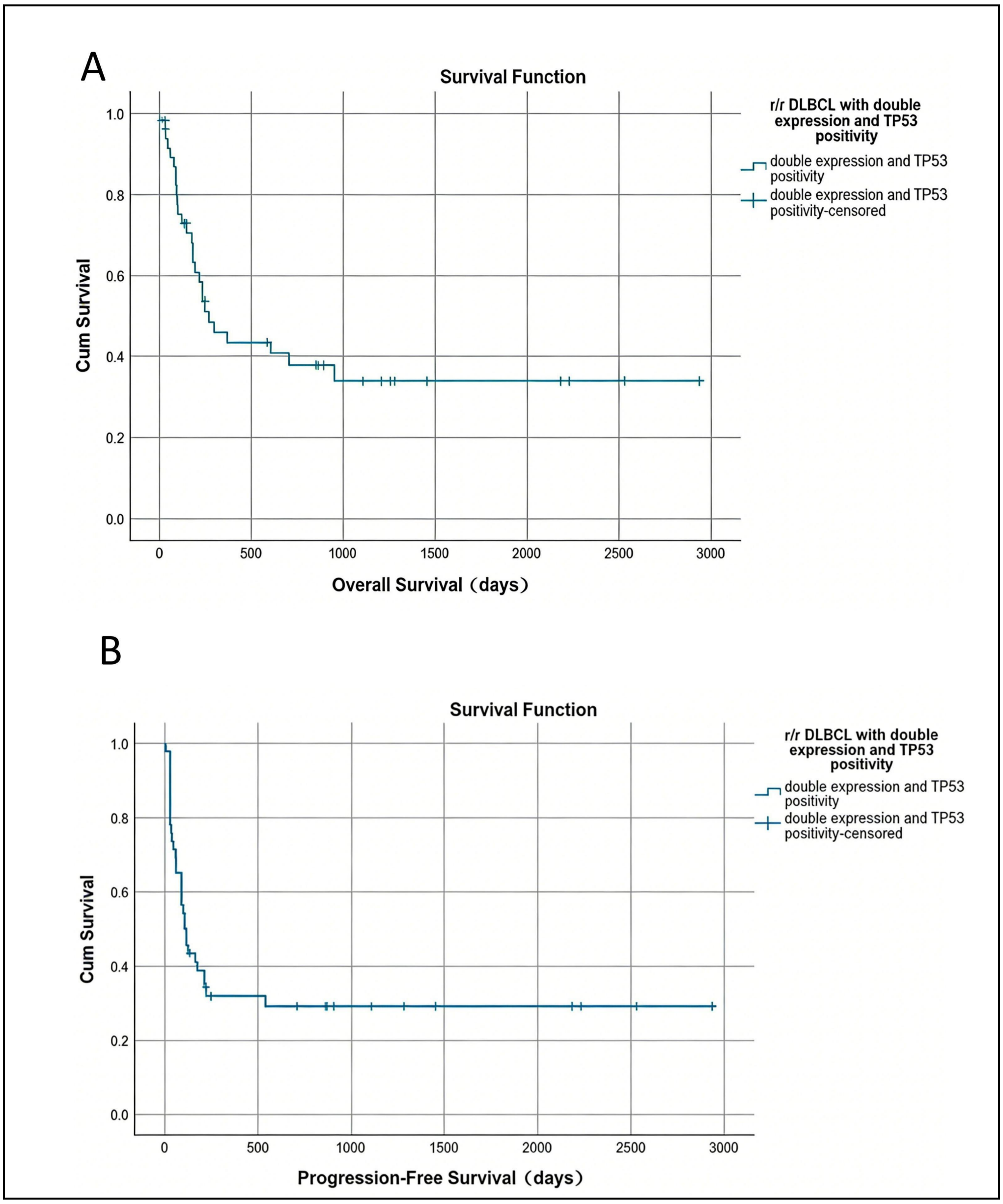

Figure 1. Kaplan–Meier estimates of overall survival (OS) and progression-free survival (PFS) in patients with double-expressor TP53-positive relapsed/refractory diffuse large B-cell lymphoma following CAR-T therapy. (A) Overall survival. (B) Progression-free survival. Tick marks represent censored cases.

**Copyright Information:** (Online) ISSN: 2572-9241
©2026 The Author(s). HemaSphere is published by John Wiley & Sons Ltd on behalf of European Hematology Association. This is an open access Abstract Book distributed under the Attribution-NonCommercial-NoDerivs (CC BY-NC-ND), which allows third parties to download the articles and share them with others as long as they credit the author and the Abstract Book, but they cannot change the content in any way or use them commercially.
**Abstract Book Citations:** Authors, Title, HemaSphere, 2026;10:(S1):pages. The abstract book DOIs can be found on the "Author Information" page.
**Disclaimer:** Articles published in the journal HemaSphere exclusively reflect the opinions of the authors. The authors are responsible for all content in their abstracts including accuracy of the facts, statements, citing resources, etc.

### (PF1029) OVERALL AND PROGRESSION-FREE SURVIVAL IN PATIENTS WITH EPSTEIN-BARR VIRUS-DRIVEN POST-TRANSPLANT LYMPHOPROLIFERATIVE DISEASE (EBV+ PTLD) TREATED WITH TABELECLEUCEL IN THE PHASE 3 ALLELE TRIAL

**Topic:** 20. Other aggressive lymphomas

Sylvain Choquet*[1], Armin Ghobadi[2], Sunita Nasta[3], Sridhar Chaganti[4], Sarah Nikiforow[5], Amer Beitinjaneh[6], Sonali Chaudhury[7], Rabi Hanna[8], Robert Baiocchi[9], Glen Lew[10], Ashley Swanson[10], Sandrine Roye[11], Daan Dierickx[12], Susan Prockop[13]

*[1]University Hospital Pitié Salpêtrière, Paris, France; [2]Washington University, St Louis, MO, United States of America; [3]University of Pennsylvania, Philadelphia, PA, United States of America; [4]University Hospitals Birmingham, Centre for Clinical Haematology, Birmingham, United Kingdom; [5]Dana-Farber Cancer Institute, Boston, United States of America; [6]University of Miami, Jackson Memorial Hospital, Miami, FL, United States of America; [7]Lurie Children's Hospital, Chicago, IL, United States of America; [8]Cleveland Clinic, Cleveland, OH, United States of America; [9]The Ohio State University, James Comprehensive Cancer Center, Columbus, OH, United States of America; [10]Pierre Fabre Pharmaceuticals, Secaucus, NJ, United States of America; [11]Pierre Fabre Laboratories, Medical Care R&D, Toulouse, France; [12]University Hospitals Leuven, Leuven, Belgium; [13]Dana Farber/Boston Children's Hospital Cancer and Blood Disorders Center, Boston, MA, United States of America;*

### Background

Median overall survival (OS) in patients with relapsed/refractory Epstein-Barr virus-associated post-transplant lymphoproliferative disease (R/R EBV+ PTLD) is 0.7 months after hematopoietic cell transplant (HCT) and 4.1 months after solid organ transplant (SOT), representing an unmet therapeutic need (Socié et al. Bone Marrow Transplant, 2024; Dharnidharka et al. Blood, 2021). Tabelecleucel is an off-the-shelf, allogeneic EBV-specific cytotoxic T cell therapy being investigated in patients with R/R EBV+ PTLD after HCT or SOT in the ongoing ALLELE trial (NCT03394365). Results from 86 patients have been reported (Nikiforow et al. ASH 2025).

### Aims

To present updated results from ALLELE with 89 patients and 12.7 months of follow-up, focusing on OS and progression-free survival (PFS).

### Methods

ALLELE is a multicenter, open-label, phase 3 trial. Patients received intravenous tabelecleucel at 2 x $10^6$ cells/kg on days 1, 8, and 15 in 35-day cycles. Disease assessments were conducted at the end of each cycle and treatment response evaluated by independent oncologic response adjudication (IORA). Survival status was assessed for up to 5 years. All eligible patients received tabelecleucel, and efficacy and safety were evaluated in all who received ≥1 dose. The primary endpoint was objective response rate (ORR). Other endpoints included OS and PFS.

**Copyright Information:** (Online) ISSN: 2572-9241
©2026 The Author(s). HemaSphere is published by John Wiley & Sons Ltd on behalf of European Hematology Association. This is an open access Abstract Book distributed under the Attribution-NonCommercial-NoDerivs (CC BY-NC-ND), which allows third parties to download the articles and share them with others as long as they credit the author and the Abstract Book, but they cannot change the content in any way or use them commercially.
**Abstract Book Citations:** Authors, Title, HemaSphere, 2026;10:(S1):pages. The abstract book DOIs can be found on the "Author Information" page.
**Disclaimer:** Articles published in the journal HemaSphere exclusively reflect the opinions of the authors. The authors are responsible for all content in their abstracts including accuracy of the facts, statements, citing resources, etc.

**Results**

As of 1 September 2025, 90 patients were enrolled and 89 received tabelecleucel (32 HCT, 57 SOT). The median (range) age was 42.8 (2.7–81.5) years and 52/89 (58.4%) were male. The overall ORR was 49.4% (44/89, 95% CI 38.7, 60.2). ORR for HCT was 53.1% (17/32, 95% CI 34.7, 70.9) and for SOT was 47.4% (27/57, 95% CI 34.0, 61.0). After a median follow-up time of 12.7 months, the overall median OS (95% CI) was 26.6 months (12.1, not estimable [NE]; Figure 1A). Median OS (95% CI) in HCT was 25.6 months (5.7, NE) and in SOT was NE (9.0, NE). In patients responding to treatment, median OS (95% CI) was NE (NE, NE) overall (Figure 1A), NE (2.9, NE) for HCT, and NE (NE, NE) for SOT. In all patients, the 1-year OS rate (95% CI) was 61.8% (50.4, 71.3) overall, 59.1% (39.2, 74.5) for HCT, and 63.2% (48.9, 74.5) for SOT. In responders, the 1-year OS rate (95% CI) was 83.6% (68.7, 91.8) overall, 76.5% (48.8, 90.4) for HCT, and 88.1% (67.5, 96.0) for SOT. The median duration of response (95% CI) in responders was 23.0 months (15.9, NE). Median PFS (95% CI) in responders was 25.6 months (16.9, NE), with a 1-year PFS rate (95% CI) of 75.0% (58.2, 85.8; Figure 1B). Treatment-related treatment emergent adverse events (TEAEs) of grade ≥3 were reported in 15 patients (16.9%) and treatment-related serious TEAEs in 8 (9.0%). One patient was reported with grade 1 fever deemed a sign of cytokine release syndrome possibly related to treatment. No treatment-related deaths were reported. Cases of graft vs. host disease were reported in 3 HCT patients (9.4%), and transplant rejection in 4 SOT patients (7.0%), none of which were treatment-related. There were no reports of infusion-related reactions, immune effector cell-associated neurotoxicity syndrome, tumor flare, immunogenicity events, or HCT rejection.

**Summary/Conclusion**

Results from ALLELE show OS and PFS benefit with tabelecleucel in patients with R/R EBV+ PTLD, who otherwise have a poor probability of survival. ORR and safety data are consistent with previous reports, supporting tabelecleucel as a novel treatment option for R/R EBV+ PTLD.

**Copyright Information:** (Online) ISSN: 2572-9241
©2026 The Author(s). HemaSphere is published by John Wiley & Sons Ltd on behalf of European Hematology Association. This is an open access Abstract Book distributed under the Attribution-NonCommercial-NoDerivs (CC BY-NC-ND), which allows third parties to download the articles and share them with others as long as they credit the author and the Abstract Book, but they cannot change the content in any way or use them commercially.
**Abstract Book Citations:** Authors, Title, HemaSphere, 2026;10:(S1):pages. The abstract book DOIs can be found on the "Author Information" page.
**Disclaimer:** Articles published in the journal HemaSphere exclusively reflect the opinions of the authors. The authors are responsible for all content in their abstracts including accuracy of the facts, statements, citing resources, etc.

**Figure 1.** Kaplan-Meier plots of (A) overall survival in the full analysis set, responders, and non-responders, and (B) progression-free survival in responders.

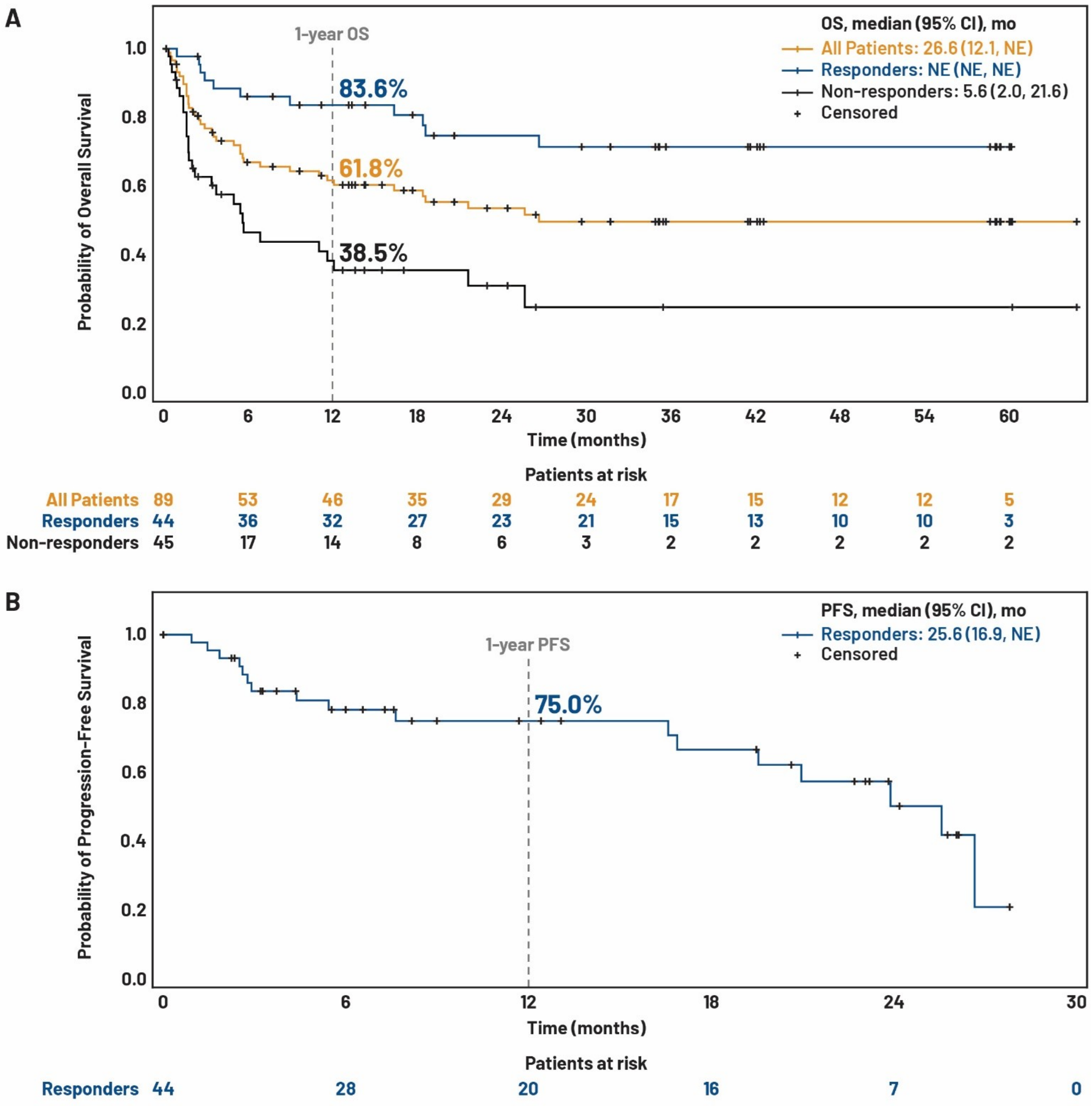

CI: confidence interval; NE: not estimable; OS: overall survival; PFS: progression-free survival.

**Copyright Information:** (Online) ISSN: 2572-9241
©2026 The Author(s). HemaSphere is published by John Wiley & Sons Ltd on behalf of European Hematology Association. This is an open access Abstract Book distributed under the Attribution-NonCommercial-NoDerivs (CC BY-NC-ND), which allows third parties to download the articles and share them with others as long as they credit the author and the Abstract Book, but they cannot change the content in any way or use them commercially.
**Abstract Book Citations:** Authors, Title, HemaSphere, 2026;10:(S1):pages. The abstract book DOIs can be found on the "Author Information" page.
**Disclaimer:** Articles published in the journal HemaSphere exclusively reflect the opinions of the authors. The authors are responsible for all content in their abstracts including accuracy of the facts, statements, citing resources, etc.

### (PF1030) UPDATED CLINICAL DATA FROM THE PHASE 1/2 STUDY OF DIBOTATUG (DR-01), A NON-FUCOSYLATED ANTI-CD94 ANTIBODY, IN PATIENTS WITH RELAPSED/REFRACTORY CYTOTOXIC T/NK CELL LYMPHOMAS

**Topic:** 20. Other aggressive lymphomas

Swaminathan Iyer*[1], Christina Poh[2], Jasmine Zain[3], Christiane Querfeld[2], John C. Reneau[4], Jonathan Brammer[4], Costas K. Yannakou[5, 6], Mengyang Di[7], Cinzia Pellegrini[8], Pier Luigi Zinzani[8], Oleg Akilov[9], Youn H. Kim[10], Michael Khodadoust[10], Enrica Marchi[11], Yok Lam Kwong[12], Ji Hyun Lee[13], Youngil Koh[14], Hyeon-Seok Eom[15], Wonseog Kim[16], Jake Shortt[17], Alejandro Gru[18], Alissa Keegan[19], Wan-Jen Hong[19], Ruoshi Shi[19], Jennifer Richardson[19], Nenad Tomasevic[19], H. Miles Prince[5], Steven Horwitz[3]

*[1]MD Anderson Cancer Center, Houston, United States of America;[2]City of Hope, Duarte, United States of America;[3]Memorial Sloan Kettering Cancer Center, New York, United States of America; [4]The Ohio State University Comprehensive Cancer Center, Columbus, United States of America; [5]Epworth HealthCare, Melbourne, Australia; [6]University of Melbourne, Melbourne, Australia; [7]Fred Hutchinson Cancer Center, Seattle, United States of America; [8]IRCCS Azienda Ospedaliero-Universitaria di Bologna, Bologna, Italy; [9]University of Pittsburgh Medical Center, Pittsburgh, United States of America; [10]Stanford Cancer Center, Stanford, United States of America; [11]University of Virginia Comprehensive Cancer Center, Charlottesville, United States of America; [12]University of Hong Kong, Hong Kong, Hong Kong; [13]Dong-A University, Busan, South Korea; [14]Seoul National University Hospital, Seoul, South Korea; [15]National Cancer Center, Seoul, South Korea; [16]Samsung Medical Center, Seoul, South Korea; [17]Monash University, Victoria, Australia; [18]Columbia University Irving Medical Center, New York, United States of America; [19]Dren Bio, San Carlos, United States of America;*

### Background

Dibotatug (DR-01) is a non-fucosylated human IgG antibody targeting CD94, which is selectively expressed on NK cells, γδ T cells, and terminal effector CD8$^+$T cells. Cytotoxic T/NK cell lymphomas (CTLs) are a group of lymphoma histologies that are characterized by CD94-expressing cytotoxic cells and presence of cytotoxic markers, such as granzyme B, TIA1 or perforin. Dibotatug leads to depletion of CD94$^+$ malignant cells by antibody-dependent cellular cytotoxicity and represents a potential new targeted therapy for patients with relapsed/refractory (R/R) CTL who have no effective therapies.

### Aims

Assess safety, pharmacodynamics, and efficacy of dibotatug in R/R CTL patients.

### Methods

This Phase 1/2 open-label study (NCT05475925) enrolled R/R CTL patients who failed ≥1 prior line of therapy. All patients provided informed consent. Patients received intravenous dibotatug (0.3–10 mg/kg) in one of three induction regimens followed by monthly dosing during dose escalation, followed by dose optimization at 1 or 6 mg/kg and dose expansion at 1 mg/kg with induction regimen of C1D1/D8/D15. Efficacy was assessed using Lugano criteria (Cheson 2014), Olsen criteria (Olsen 2021) for cutaneous histologies or modified TPLL criteria (Staber 2019) for hepatosplenic or leukemic disease. The study also assessed correlations between baseline CD94 expression and responses and CD94$^+$ cell depletion after dibotatug treatment.

**Copyright Information:** (Online) ISSN: 2572-9241
©2026 The Author(s). HemaSphere is published by John Wiley & Sons Ltd on behalf of European Hematology Association. This is an open access Abstract Book distributed under the Attribution-NonCommercial-NoDerivs (CC BY-NC-ND), which allows third parties to download the articles and share them with others as long as they credit the author and the Abstract Book, but they cannot change the content in any way or use them commercially.
**Abstract Book Citations:** Authors, Title, HemaSphere, 2026;10:(S1):pages. The abstract book DOIs can be found on the "Author Information" page.
**Disclaimer:** Articles published in the journal HemaSphere exclusively reflect the opinions of the authors. The authors are responsible for all content in their abstracts including accuracy of the facts, statements, citing resources, etc.

### Results

As of October 2025, 81 patients with CTL were enrolled. Forty-five (56%) were male, median age was 53 (18–82 years) and ECOG performance status was approximately evenly distributed between 0 and 1. The most common histologies enrolled (>5%) were extranodal NK/T-cell lymphoma (ENKTL) (n=19, 23%), primary cutaneous γδ T-cell lymphoma (PCγδTCL) (n=16, 20%), cytotoxic peripheral T-cell lymphoma, not otherwise specified (PTCL-NOS) (n=12, 15%), monomorphic epitheliotropic intestinal T-cell lymphoma (MEITL) (n=8, 10%), subcutaneous panniculitis-like T-cell lymphoma (SPTCL) (n=7, 9%), primary cutaneous CD8+ aggressive epidermotropic cytotoxic T-cell lymphoma (PCAETCL) (n=7, 9%), and hepatosplenic T-cell lymphoma (HSTCL) (n=6, 7%). Median lines of prior therapy were 3 (range 1–14) and 16% (n=13) were post autologous/allogeneic stem cell transplant. Disease was resistant or intolerant to the most recent therapy in 48% (n=39) of patients. Infusion-related reactions were the predominant treatment-related AEs, occurring in 27 (33%) patients primarily during initial administration, and were managed effectively with standard mitigation measures.
In the 47 response-evaluable patients at the selected dose (1 mg/kg), the ORR was 53% (14 CR, 11 PR) with responses across histologic subtypes. Highest ORRs were observed in the following histologies; SPTCL (n=5) with ORR 80% (3 CR, 1 PR), PCγδTCL (n=8) with ORR 75% (1 CR, 5 PR), HSTCL (n=4) with ORR 75% (2 CR, 1 PR), MEITL (n=3) with ORR 67% (1 CR, 1 PR), and ENKTL (n=14) with ORR 50% (5 CR, 2 PR). The longest duration of response is ongoing at 22 months in a patient with PCγδTCL. Biomarker analyses showed near universal expression (95%) of CD94 across histologies and responses were not correlated with CD94 expression level. By C2D1, dibotatug treatment had markedly depleted $CD94^{+}$ cells in patient samples.

### Summary/Conclusion

Dibotatug continues to demonstrate promising safety and efficacy with responses across CTL histologies, supporting development as a targeted therapy in patients with R/R CTL.

**Copyright Information:** (Online) ISSN: 2572-9241
©2026 The Author(s). HemaSphere is published by John Wiley & Sons Ltd on behalf of European Hematology Association. This is an open access Abstract Book distributed under the Attribution-NonCommercial-NoDerivs (CC BY-NC-ND), which allows third parties to download the articles and share them with others as long as they credit the author and the Abstract Book, but they cannot change the content in any way or use them commercially.
**Abstract Book Citations:** Authors, Title, HemaSphere, 2026;10:(S1):pages. The abstract book DOIs can be found on the “Author Information” page.
**Disclaimer:** Articles published in the journal HemaSphere exclusively reflect the opinions of the authors. The authors are responsible for all content in their abstracts including accuracy of the facts, statements, citing resources, etc.

### (PF1031) PRELIMINARY EFFICACY AND SAFETY OF GOLIDOCITINIB IN RELAPSED/REFRACTORY T CELL AND NK CELL LARGE GRANULAR LYMPHOCYTE LEUKEMIA

**Topic:** 20. Other aggressive lymphomas

Ying Yu*[1], Yuxi Li[1], Yuting Yan[1], Tingyu Wang[1], Cui Rui[2], Wenjie Xiong[1], Shuhui Deng[1], Gang An[1], Lugui Qiu[1], Yin Wu[3], Huanling Zhu[4], Yuqing Chen[5], Chenglu Yuan[6], Wanling Wang[7], Wenyong Li[8], Jing Li[9], Huili Xu[10], Jianling Chen[11], Baixin Ye[12], Xiangxiang Zhou[13], Xiaoqing Li[14], Jie Bai[15], Yanliang Bai[16], Ying Lu[17], Ling Pan[4], Yirong Jiang[18], Ling Ye[19], Shuhua Yi[1]

*[1]The State Key Laboratory of Experimental Hematology, National Clinical Medical Research Center for Blood Diseases, Institute of Hematology and Blood Diseases Hospital, Chinese Academy of Medical Sciences and Peking Union Medical College, Tianjin, China; [2]Tianjin First Central Hospital, School of Medicine, Nankai University, Tianjin, China;[3]Department of Hematology, Beijing Chaoyang Hospital, Capital Medical University, Beijing, China; [4]Department of Hematology, West China Hospital, Sichuan University, Chengdu, China; [5]Department of Hematology, Henan Provincial People's Hospital, Zhengzhou, China;[6]Qingdao Campus of Qilu Hospital of Shandong University, Qingdao, China; [7]Department of Hematology, First Affiliated Hospital of Henan University of Chinese Medicine, Zhengzhou, China, Zhengzhou, China; [8]Department of Lymphoma, Jiaozuo People's Hospital, Jiaozuo, China; [9]Department of Hematology, First Affiliated Hospital of Guangxi Medical University, Nanning, China; [10]Department of Hematology and Oncology, The First People's Hospital of Pingdingshan, Pingdingshan, China;[11]Department of Hematology, The Second People's Hospital of Pingdingshan, Pingdingshan, China; [12]Department of Hematology, People's Hospital of Wuhan University, Wuhan, China; [13]Shandong Provincial Hospital Affiliated to Shandong University, Jinan, China; [14]The First Affiliated Hospital of Shenzhen University, Shenzhen, China; [15]The Second Hospital of Tianjin Medical University, Tianjin, China; [16]Department of Hematology, Henan Provincial People's Hospital, Zhengzhou University, Zhengzhou, China; [17]Department of Hematology, Affiliated People's Hospital of Ningbo University, Ningbo, China;[18]Department of Hematology,Affiliated Dongguan People's Hospital, Southern Medical University, Dongguan, China; [19]Department of Hematology, Jining No.1 People's Hospital, Jining, China;*

### Background

Large granular lymphocyte leukemia (LGLL) is a rare and incurable hematologic malignancy with minimal therapeutic advances in recent decades. Immunosuppressive therapy remains the standard first line treatment, including methotrexate, cyclosporine A, and cyclophosphamide. No standard of care is available for refractory or relapsed disease. Golidocitinib, a potent selective JAK1 inhibitor, has shown promising antitumor activity in relapsed/refractory peripheral T cell lymphoma.

### Aims

To evaluate efficacy and safety of golidocitinib monotherapy in patients with relapsed/refractory T/NK-LGLL.

### Methods

This open-label, prospective clinical study evaluates the feasibility, efficacy, and safety of golidocitinib in relapsed/refractory (R/R) indolent T/NK-cell lymphoma, including cutaneous T-cell lymphoma (CTCL), gastrointestinal indolent T-cell lymphoma, and LGLL (NCT06716658). Patients received oral golidocitinib 150 mg once daily in 28-day cycles. Treatment continued until disease progression, intolerable toxicity, or consent withdrawal, up to 24 cycles. The primary endpoint is objective response rate (ORR). Preliminary efficacy and safety data for the LGLL cohort are presented herein.

**Copyright Information:** (Online) ISSN: 2572-9241
©2026 The Author(s). HemaSphere is published by John Wiley & Sons Ltd on behalf of European Hematology Association. This is an open access Abstract Book distributed under the Attribution-NonCommercial-NoDerivs (CC BY-NC-ND), which allows third parties to download the articles and share them with others as long as they credit the author and the Abstract Book, but they cannot change the content in any way or use them commercially.
**Abstract Book Citations:** Authors, Title, HemaSphere, 2026;10:(S1):pages. The abstract book DOIs can be found on the "Author Information" page.
**Disclaimer:** Articles published in the journal HemaSphere exclusively reflect the opinions of the authors. The authors are responsible for all content in their abstracts including accuracy of the facts, statements, citing resources, etc.

**Results**

As of the database cutoff on February 2, 2026, 45 r/r T or NK LGLL patients were enrolled and received at least one cycle of golidocitinib treatment (T-LGLL, n=41; NK-LGLL, n=4). A summary of baseline characteristics is presented as follows: median age 60 years (range 42 to 83), 36 male patients (80.0%), 8 with B symptoms (17.8%), and 11 with splenomegaly (24.4%). Forty-two patients (93.3%) required intervention for cytopenia including transfusion dependent anemia, anemia, severe neutropenia, severe thrombocytopenia, and autoimmune hemolytic anemia. STAT3 mutations were detected in 11 patients (24.4%). All patients had relapsed or refractory disease after prior immunosuppressive therapy. The median number of prior systemic therapy lines was 2 (range 1 to 5). Sixteen patients had failed three or more prior lines including PI3Kδ inhibitor linperlisib and JAK1/2 inhibitor ruxolitinib. Among 36 efficacy evaluable patients, the ORR was 86.1%, the CRR was 61.1%. Among patients with baseline hematologic abnormalities, neutrophil counts normalized in 88.0% (22/25) of those with neutropenia, and hemoglobin levels improved in 79.5% (31/39) of anemic patients. Notably, responses were observed in all 3 evaluable patients lacking STAT3 mutations, including 2 complete responses and 1 partial response. After median follow up of 4.9 (0.46-17.6) months, median progression free survival was 11.7 months (5.8-NE, 95% CI) and median overall survival was not reached. Golidocitinib showed a favorable safety profile, with treatment-related adverse events (TRAEs) mainly grade 1-2 hematologic events. TRAEs occurred in 12 patients (26.7%), and grade 3-4 TRAEs occurred in 4 patients (8.9%). The most frequent TRAEs were neutropenia (6.7%), leukopenia (6.7%), thrombocytopenia (6.7%), and pulmonary infection (6.7%). The most common grade 3-4 TRAEs were neutropenia (4.4%), pulmonary infection (4.4%), and leukopenia (2.2%). Only one grade 5 fatal event of pulmonary infection was reported.

**Summary/Conclusion**

Golidocitinib monotherapy has potent antitumor activity in heavily pretreated R/R T/NK-LGLL patients, with 86.1% ORR and 61.1% CRR. Most patients with baseline hematologic abnormalities had marked hematologic improvements, and its favorable safety profile supports further study in this unmet-need population.

**Copyright Information:** (Online) ISSN: 2572-9241
©2026 The Author(s). HemaSphere is published by John Wiley & Sons Ltd on behalf of European Hematology Association. This is an open access Abstract Book distributed under the Attribution-NonCommercial-NoDerivs (CC BY-NC-ND), which allows third parties to download the articles and share them with others as long as they credit the author and the Abstract Book, but they cannot change the content in any way or use them commercially.
**Abstract Book Citations:** Authors, Title, HemaSphere, 2026;10:(S1):pages. The abstract book DOIs can be found on the “Author Information” page.
**Disclaimer:** Articles published in the journal HemaSphere exclusively reflect the opinions of the authors. The authors are responsible for all content in their abstracts including accuracy of the facts, statements, citing resources, etc.

### (PF1032) SINGLE-CELL ANALYSES IDENTIFY A DISTINCT MYELOID CELL ECOSYSTEM ASSOCIATED WITH TREATMENT RESISTANCE IN ANAPLASTIC LARGE CELL LYMPHOMA

**Topic:** 20. Other aggressive lymphomas

Putri Ramadani*[1, 2], Shinichiro Oshima[2], Yifan Yin[3], Nawar Dakhallah[4], Davidson Zhao[2], Sally Zhang[2], Yusuke Toda[2], Robert Kridel[2], Federico Gaiti[2, 5, 6], Ali Sakhdari[7], Katsuyoshi Takata[8], Rose Chami[4], Bo Ngan[4], Angela Punnett[4], Tomohiro Aoki[2, 9]

*[1]University of Toronto, Molecular Genetics, Toronto, Canada;[2]Princess Margaret Cancer Centre, University Health Network, Toronto, Canada; [3]British Columbia Cancer, Centre for Lymphoid Cancer, Vancouver, Canada;[4]Hospital for Sick Children, Toronto, Canada; [5]University of Toronto, Department of Medical Biophysics, Toronto, Canada;[6]Ontario Institute for Cancer Research, Toronto, Canada; [7]University Health Network, Laboratory Medicine and Pathobiology, Toronto, Canada;[8]University of Toyama, Department of Pathology, Faculty of Medicine, Academic Research Division, Toyama, Japan; [9]University of Toronto, Department of Immunology, Toronto, Canada;*

### Background

Anaplastic large cell lymphoma (ALCL) is an aggressive T-cell lymphoma and further classified into ALK-positive ($ALK^+$) and ALK-negative ($ALK^-$) ALCL. ALCL exhibits heterogeneous clinical presentations, with a predominance in younger patients for $ALK^+$ ALCL, in contrast to an adult-age predominance in $ALK^-$ ALCL. Despite recent therapeutic advances, approximately 25–50% of ALCL patients still experience disease progression and ultimately succumb to their disease. Thus, further studies are required to identify novel therapeutic targets that may improve patient outcomes. To date, prior studies have largely focused on the characterization of lymphoma cells, leading, for example, to the development of ALK-targeted therapies. In contrast, the role of the tumor microenvironment (TME) in ALCL has not been extensively investigated. Notably, one of the characteristic morphologic variants of ALCL, the small cell or lymphohistiocytic pattern, is associated with abundant reactive immune cell infiltrates and poor clinical outcomes, suggesting TME may play a crucial role in the pathogenesis of ALCL.

### Aims

We aim to identify novel therapeutically targetable interactions by comprehensively characterizing TME in ALCL.

### Methods

We performed snRNA-seq from a total of 49 patients across all age groups, including 12 $ALK^+$ ALCL (1–55 years), 14 $ALK^-$ ALCL (14–70 years), 17 reactive lymph nodes (RLN; 0–83 years), and 6 thymic samples (0–15 years). Following low-quality cell filtering and data normalization, expression profiles from all cells were integrated, and the Louvain clustering algorithm was applied to identify major cell types and functional cellular subsets. Tumor cell clusters were distinguished from normal T cells using copy number variation analysis implemented with inferCNV.

**Copyright Information:** (Online) ISSN: 2572-9241
©2026 The Author(s). HemaSphere is published by John Wiley & Sons Ltd on behalf of European Hematology Association. This is an open access Abstract Book distributed under the Attribution-NonCommercial-NoDerivs (CC BY-NC-ND), which allows third parties to download the articles and share them with others as long as they credit the author and the Abstract Book, but they cannot change the content in any way or use them commercially.
**Abstract Book Citations:** Authors, Title, HemaSphere, 2026;10:(S1):pages. The abstract book DOIs can be found on the "Author Information" page.
**Disclaimer:** Articles published in the journal HemaSphere exclusively reflect the opinions of the authors. The authors are responsible for all content in their abstracts including accuracy of the facts, statements, citing resources, etc.

**Results**

By analyzing 277,029 single-nucleus transcriptomes of the snRNA-seq data, unsupervised clustering identified 21 phenotypically distinct clusters, comprising ALCL cells, T cells, myeloid cells, stromal cells, and B cells. Among them, macrophages (mac) were one of the most enriched immune populations within the ALCL TME when compared with RLN ($P = 0.003$), and we focused subsequent analyses on this subset. By applying subclustering, we identified 10 distinct subclusters, including HAVCR2$^+$ mac, C1QA$^+$APOE$^+$ mac, SIGLEC1$^+$PD-L1$^+$ mac, ADGRE1$^+$CXCL9$^+$ mac, SPP1$^+$ mac, cDC1, cDC2, pDCs, and mast cells. Notably, SIGLEC1$^+$PD-L1$^+$ mac and ADGRE1$^+$CXCL9$^+$ mac were predominantly derived from ALCL samples, suggesting disease-specific enrichment and a distinctive role in ALCL pathobiology. Both mac subsets exhibited upregulation of inflammatory transcriptional programs, including IFNγ signaling pathways. SIGLEC1$^+$PD-L1$^+$ mac subcluster was significantly enriched in paediatric ALCL. Furthermore, cell–cell interaction analyses using CellChat revealed significant CXCL9–CXCR3 and SIGLEC1–SPN interactions between mac and ALCL cells. Notably, the CXCL9 (mac)–CXCR3 (ALCL) interaction was uniquely enriched in ALCL patients who experienced relapse, and IL-18 was identified by CytoSig, a cytokine-specific signaling inference framework, as an upstream activator of CXCR3 in ALCL. Together, these findings indicate complex macrophage–ALCL cell interactions that contribute to treatment resistance in ALCL.

**Summary/Conclusion**

SnRNA-seq reveals a distinct inflammatory, mac-driven signalling program that emerges as a key contributor to distinct TME interactions in ALCL. Our insights aid in the development of novel therapeutic strategies for ALCL.

**Copyright Information:** (Online) ISSN: 2572-9241
©2026 The Author(s). HemaSphere is published by John Wiley & Sons Ltd on behalf of European Hematology Association. This is an open access Abstract Book distributed under the Attribution-NonCommercial-NoDerivs (CC BY-NC-ND), which allows third parties to download the articles and share them with others as long as they credit the author and the Abstract Book, but they cannot change the content in any way or use them commercially.
**Abstract Book Citations:** Authors, Title, HemaSphere, 2026;10:(S1):pages. The abstract book DOIs can be found on the “Author Information” page.
**Disclaimer:** Articles published in the journal HemaSphere exclusively reflect the opinions of the authors. The authors are responsible for all content in their abstracts including accuracy of the facts, statements, citing resources, etc.

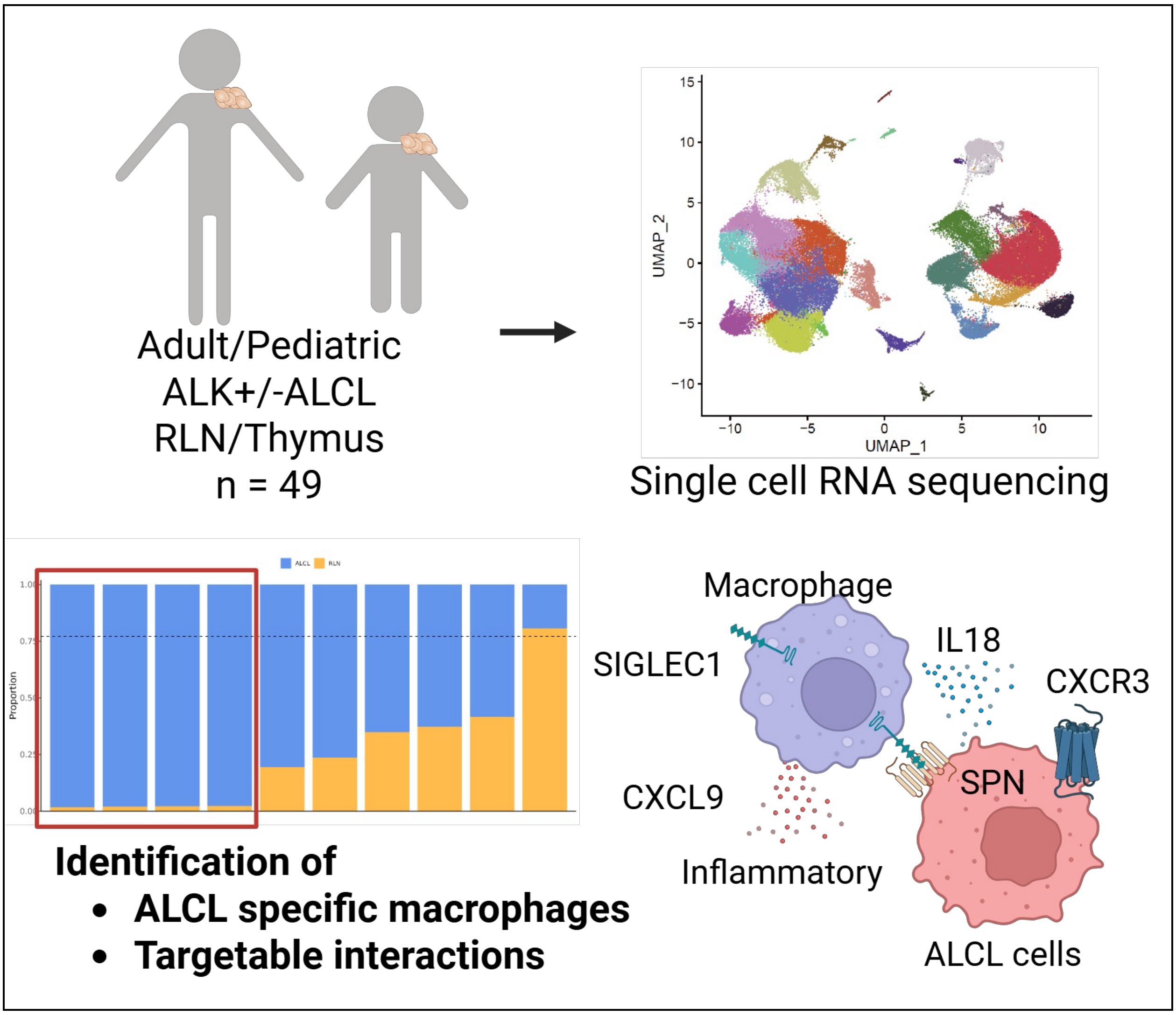

**Copyright Information:** (Online) ISSN: 2572-9241
©2026 The Author(s). HemaSphere is published by John Wiley & Sons Ltd on behalf of European Hematology Association. This is an open access Abstract Book distributed under the Attribution-NonCommercial-NoDerivs (CC BY-NC-ND), which allows third parties to download the articles and share them with others as long as they credit the author and the Abstract Book, but they cannot change the content in any way or use them commercially.
**Abstract Book Citations:** Authors, Title, HemaSphere, 2026;10:(S1):pages. The abstract book DOIs can be found on the "Author Information" page.
**Disclaimer:** Articles published in the journal HemaSphere exclusively reflect the opinions of the authors. The authors are responsible for all content in their abstracts including accuracy of the facts, statements, citing resources, etc.

### (PF1033) FIRST REPORT OF ROCBRUTINIB FOR THE TREATMENT OF PATIENTS WITH RELAPSED OR REFRACTORY PRIMARY CENTRAL NERVOUS SYSTEM LYMPHOMA

**Topic:** 20. Other aggressive lymphomas

Lin Fu[1], Keshu Zhou[2], Xuefei Sun[1], Qingqing Cai[3], Xiuhua Sun[4], Liang Zhong[5], Zheng Wang[5], Nawei Liu[5], Yejiang Lou[5], Yue Shen[5], Yi Chen[6], Fenlai Tan[5], Yuqin Song*[7]

*[1]Beijing Tiantan Hospital, Capital Medical University, Beijing, China;[2]Affiliated Cancer Hospital of Zhengzhou University, Henan Cancer Hospital, Zhengzhou, China; [3]State Key Laboratory of Oncology in South China, Sun Yat-sen University Cancer Center, Guangzhou, China; [4]The Second Hospital of Dalian Medical University, Dalian, China;[5]Guangzhou Lupeng Pharmaceutical Co., Ltd., Guangzhou, China; [6]Newave Pharmaceutical Inc., Pleasanton, CA, United States of America;[7]Peking University Cancer Hospital & Institute (Beijing Cancer Hospital), Beijing, China;*

### Background

Primary central nervous system lymphoma (PCNSL) is a lymphoma arising in the brain, spinal cord, cerebrospinal fluid, or eyes. It represents 4%–6% of all extranodal lymphomas, with approximately 90% classified histopathologically as diffuse large B-cell lymphoma (DLBCL). This disease is highly aggressive, with limited treatment options and poor prognosis, particularly in relapsed or refractory (R/R) cases. Although Bruton's tyrosine kinase inhibitors (BTKi) have demonstrated clinical activity, outcomes remain suboptimal. Rocbrutinib, a highly selective fourth-generation covalent and non-covalent BTKi, has shown promising efficacy in non-GCB DLBCL and exhibits good blood-brain barrier penetration in mice. This report presents the preliminary clinical data from rocbrutinib-treated patients with R/R PCNSL.

### Aims

We report the PCNSL subgroup results from a rocbrutinib phase 1 study (NCT04993690) conducted in China.

### Methods

In this open-label phase 1 study, eligible subjects had a PCNSL diagnosis and had received at least one prior therapy, including a high-dose methotrexate-based regimen; prior BTKi exposure was permitted, including patients who were refractory to covalent BTKi. Subjects received rocbrutinib tablets at 200 mg once daily as monotherapy until disease progression or unacceptable toxicity. Adverse events (AEs) were graded according to CTCAE v5.0. Cerebrospinal fluid (CSF) and peripheral blood were collected for pharmacokinetic analysis. Efficacy was assessed via gadolinium-enhanced magnetic resonance imaging (MRI) using the International PCNSL Collaborative Group Response Criteria.

**Copyright Information:** (Online) ISSN: 2572-9241
©2026 The Author(s). HemaSphere is published by John Wiley & Sons Ltd on behalf of European Hematology Association. This is an open access Abstract Book distributed under the Attribution-NonCommercial-NoDerivs (CC BY-NC-ND), which allows third parties to download the articles and share them with others as long as they credit the author and the Abstract Book, but they cannot change the content in any way or use them commercially.
**Abstract Book Citations:** Authors, Title, HemaSphere, 2026;10:(S1):pages. The abstract book DOIs can be found on the "Author Information" page.
**Disclaimer:** Articles published in the journal HemaSphere exclusively reflect the opinions of the authors. The authors are responsible for all content in their abstracts including accuracy of the facts, statements, citing resources, etc.

### Results

As of 31 December 2025, nine subjects were enrolled, all with a pathological diagnosis of non-GCB DLBCL. The median age was 63 years (range, 49-71). The median number of prior lines of therapy was 2 (range, 1-4); eight (88.9%) subjects had refractory disease. Three subjects had prior covalent BTKi exposure, all of whom discontinued treatment due to disease progression. The median International Extranodal Lymphoma Study Group (IELSG) score was 3 (range, 1-4).
CSF samples were collected 1 to 5 hours post-dose at steady-state. The median rocbrutinib concentration was 5.71 ng/mL (range, 2.89–8.21 ng/mL), which is approximately 40- to 161-fold higher than its *in vitro* potency ($IC_{50}$), confirming effective blood-brain barrier penetration by rocbrutinib.
After a median treatment duration of 3.0 months (range, 1.9-4.7), the overall response rate (ORR) was 88.9%, with a complete response (CR)/unconfirmed complete response (CRu) rate of 22.2%. One subject achieved stable disease, with a 34.8% reduction in target lesions after one month of treatment. Six patients are still on treatment.
The most common treatment-related AEs (TRAEs) (any grade; ≥grade 3) with an incidence ≥20% were neutrophil count decreased (33.3%; 22.2%), white blood cell count decreased (33.3%; 0), hypertriglyceridemia (22.2%; 0), and platelet count decreased (22.2%; 0); most events were grade 1. All TRAEs were grade 1 or 2 except for two cases of grade 3 neutrophil count decreased. No serious AEs (SAEs) or AEs leading to dose reduction, interruption, discontinuation, or death were reported.

### Summary/Conclusion

Rocbrutinib demonstrated effective blood-brain barrier penetration and encouraging single agent activity in patients with PCNSL, along with a manageable safety profile. These findings support its potential for treating R/R PCNSL.

| Characteristics | N=9 |
|---|---|
| **Age, median (range), years** | 63 (49-71) |
| **Gender, N (%)** | |
| Male | 4 (44.4) |
| Female | 5 (55.6) |
| **ECOG performance status, N (%)** | |
| 1 | 7 (77.8) |
| 2 | 2 (22.2) |
| **Pathologic diagnosis-non-GCB DLBCL** | 9 (100) |
| **IELSG, median (range)** | 3 (1-4) |
| **Refractory disease, N (%)** | 8 (88.9) |
| **No. of prior lines of therapy, median (range)** | 2 (1-4) |
| Prior high-dose MTX-based therapy, N (%) | 9 (100) |
| Prior BTKi exposure, N (%) | 3 (33.3) |
| Discontinuation due to PD | 3/3 (100) |
| BTKi refractory | 3/3 (100) |

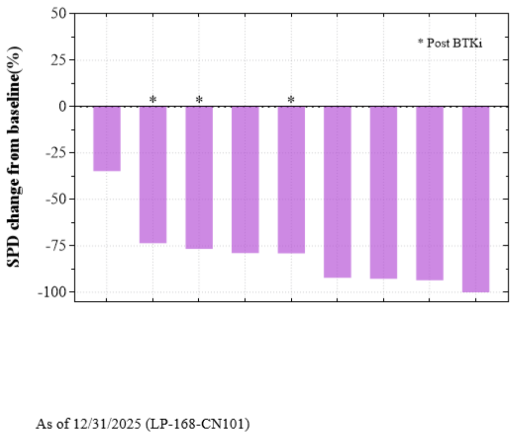

Baseline Characteristics and Waterfall Plot of Subjects with R/R PCNSL

**Copyright Information:** (Online) ISSN: 2572-9241
©2026 The Author(s). HemaSphere is published by John Wiley & Sons Ltd on behalf of European Hematology Association. This is an open access Abstract Book distributed under the Attribution-NonCommercial-NoDerivs (CC BY-NC-ND), which allows third parties to download the articles and share them with others as long as they credit the author and the Abstract Book, but they cannot change the content in any way or use them commercially.
**Abstract Book Citations:** Authors, Title, HemaSphere, 2026;10:(S1):pages. The abstract book DOIs can be found on the "Author Information" page.
**Disclaimer:** Articles published in the journal HemaSphere exclusively reflect the opinions of the authors. The authors are responsible for all content in their abstracts including accuracy of the facts, statements, citing resources, etc.

### (PF1034) REAL-WORLD MANAGEMENT AND OUTCOMES OF PTS WITH PRIMARY MEDIASTINAL B-CELL LYMPHOMA: INSIGHTS FROM THE FRENCH REALYSA COHORT.

**Topic:** 20. Other aggressive lymphomas

Loic Renaud*[1], Cedric Rossi[2], Luc-Matthieu Fornecker[3], Côme Bommier[4], Jean Galtier[5], Francois Lemonnier[6], Loic Ysebaert[7, 7], Romain Guieze[8], Florence Lachenal[9], Bernard Drenou[10], Sandy Amorim[11], Olivier Fitoussi[12], Remy Gressin[13], Thomas Gastinne[14], Adrien Chauchet[15], Julie Abraham[16], Launay Vincent[17], Christophe Fruchart[18], Gaelle Laboure[19], Roch Houot[20], Sophie Dennetiere[21], Laure Lebras[22], Gaelle Olivier[23], Gian Matteo Pica[24], Bastien Lesne[25], Aurelien Belot[25], Alice Pauc[25], Fanny Cherblanc[25], Gandhi Laurent Damaj[26], Ludovic Fouillet[27], Adrien Gilbert[28], Franck Morschhauser[29], Herve Ghesquieres[30], Vincent Camus[31]

*[1]APHP - hôpital Cochin, Hématologie clinique, Paris, France; [2]CHU Dijon, Service d'hématologie, Dijon, France; [3]chru de Strasbourg, Hématologie, Strasbourg, France; [4]APHP - Hôpital St Louis, Service d'onco-hématologie, Paris, France; [5]CHU Bordeaux, Hématologie, Bordeaux, France; [6]APHP - CHU Henri Mondor, Service d'hématologie lymphoïde, Créteil, France; [7]institut universitaire de cancérologie de Toulouse, Hématologie, Toulouse, France; [8]CHU Clermont Ferrand, Hématologie, Clermont Ferrand, France; [9]CH pierre Oudot, Hématologie, Bourgoin-Jallieu, France; [10]CH Mulhouse, Hématologie, Mulhouse, France; [11]GHICL- Hopital St Vincent de Paul, Hématologie, lille, France; [12]Polyclinique Bordeaux Nord Aquitaine, Hématologie, Bordeaux, France; [13]CHU Grenoble, Hématologie, Grenoble, France; [14]chu nantes, hématologie, Nantes, France; [15]CHU Jean Minjoz, hématologie, Besançon, France; [16]CHU limoges, Hématologie, Limoges, France; [17]CH Yves le Foll, Service d'hématologie clinique, Saint-Brieuc, France; [18]CH de Dunkerque, Hématologie, Dunkerque, France; [19]CH de Libourne, Hématologie, Libourne, France; [20]chu Rennes, Hématologie, Rennes, France; [21]CH de Roubaix, Hématologie, Roubaix, France; [22]CENTRE LEON BERARD, Hématologie, Lyon, France; [23]CH de niort, Hématologie, Niort, France; [24]Centre Hospitalier Métropole Savoie, hématologie, Chambéry, France; [25]LYSARC, The Lymphoma Academic Research Organisation, Hôpital Lyon Sud - HCL, biostatistics, Pierre-Bénite, France; [26]CHU Caen, Hématologie, Caen, France; [27]CHU St Etiennes, Hématologie, St Etiennes, France; [28]HEMATOLOGIE, CLCC RENE HUGUENIN INSTITUT CURIE SAINT CLOUD, Hématologie, Saint Cloud, France; [29]Hôpital Claude Huriez - CHU de Lille, Service des maladies du sang, Lille, France;[30]Hôpital Lyon Sud, hématologie, Pierre-Bénite, France; [31]CHU Rouen, Hématologie, Rouen, France;*

### Background

**Primary mediastinal B-cell lymphoma (PMBL) is a rare and aggressive subtype of lymphoma that typically affects young patients (pts). Excellent outcomes following first-line immunochemotherapy have been reported in clinical trials; however, prospective real-world data in this setting remain limited.**

### Methods

REal world dAta in LYmphoma and Survival in Adults (REALYSA) is a prospective, multicenter, non-interventional cohort study that included pts newly diagnosed with lymphoma in France. We analyzed the PMBL population enrolled between November 2018 and August 2023. The primary endpoint was progression-free survival (PFS).

**Copyright Information:** (Online) ISSN: 2572-9241
©2026 The Author(s). HemaSphere is published by John Wiley & Sons Ltd on behalf of European Hematology Association. This is an open access Abstract Book distributed under the Attribution-NonCommercial-NoDerivs (CC BY-NC-ND), which allows third parties to download the articles and share them with others as long as they credit the author and the Abstract Book, but they cannot change the content in any way or use them commercially.
**Abstract Book Citations:** Authors, Title, HemaSphere, 2026;10:(S1):pages. The abstract book DOIs can be found on the "Author Information" page.
**Disclaimer:** Articles published in the journal HemaSphere exclusively reflect the opinions of the authors. The authors are responsible for all content in their abstracts including accuracy of the facts, statements, citing resources, etc.

### Results

Of the 5,823 pts included in the REALYSA cohort, 150 (2.6%) had PMBL. The median age at diagnosis was 35.5 years (range 19–75) and 60% were female. Overall, 51% had bulky disease and 53% had localized disease (stage I–II). An ECOG of 0–1 was observed in 90% of pts, 73% had an IPI score of 0–2, 6.8% had a CNS-IPI ≥4, and 3.3% had kidney or adrenal involvement. Response assessment was mainly performed using PET-CT (98% at C2, 98% at C4 and 93% at end of treatment (EoT)).
First-line treatment consisted of R-ACVBP in 43% of pts (n=64), R-CHOP14 in 34% (n=51), R-CHOP21 in 21.3% (n=32) and R-EPOCH in 2% (n=3). Consolidative radiotherapy was delivered to 4% of patients (n=6). Autologous stem cell transplantation (ASCT) was performed in 4.7% of patients (n=7), mainly following R-CHOP14 (n=4). After four cycles, the CR rate was higher with R-ACVBP (71.9%) compared with R-CHOP14 (55%), R-CHOP21 (60%), and R-EPOCH (66.7%).
After a median follow-up of 35.5 months (IQR, 33.8–37.5), median PFS and overall survival (OS) were not reached. The estimated 3-year PFS and OS rates were 87.7% (95% CI, 81.1–92.1) and 99.3% (95% CI, 95.2–99.9), respectively. The progression rate was 25% with R-CHOP21 (n=8) and <10% with R-CHOP14 (7.8%, n=4) and R-ACVBP (7.8%, n=5) (Figure 1). When considering PFS from the EoT, calculated from the EoT date in patients alive and progression-free at EoT (regardless of response status), 3-year PFS was 97.8% (95% CI, 85.3–99.7) with R-CHOP14 , 95.2% (95% CI, 85.7–98.4) with R-ACVBP, and 85% with R-CHOP21 (p = 0.0859). The use of R-CHOP14 increased over time relative to R-CHOP21, rising from 50% in 2019 to 66.7% in 2023.

Nineteen pts (12.7%) progressed, with a median time to progression of 6 months from initiation of 1st-line treatment, including 11 pts with progressive disease at the Eot. Second-line treatment consisted of DHAX/C (n=14 pts), ICE (n=2 pts), combined with rituximab (n=14pts) or brentuximab-vedotin (bv) (n=2 pts). Two pts received bv-nivolumab. One was not treated. In addition, some pts underwent radiotherapy (n=2), CAR T-cells (n=4) or ASCT (n=5). Seven pts achieved a CR, two achieved a partial response while nine (50%) progressed under salvage therapy. Ten patients subsequently relapsed and received third-line treatment consisting of CAR T-cells (n=7), bv plus anti–PD-1 (n=2), or chemotherapy (n=1). Three patients experienced disease progression, and one died without documented progression. Overall, two deaths were reported; one related to lymphoma and one due to COVID19.

### Summary/Conclusion

In this prospective real-world cohort, PMBL patients achieved excellent survival outcomes. R-CHOP21 was associated with inferior PFS compared with R-CHOP14 and R-ACVBP, which showed similar efficacy, confirming the relevance of dose-dense regimens in routine practice. (REALYSA, NCT03869619)

**Copyright Information:** (Online) ISSN: 2572-9241
©2026 The Author(s). HemaSphere is published by John Wiley & Sons Ltd on behalf of European Hematology Association. This is an open access Abstract Book distributed under the Attribution-NonCommercial-NoDerivs (CC BY-NC-ND), which allows third parties to download the articles and share them with others as long as they credit the author and the Abstract Book, but they cannot change the content in any way or use them commercially.
**Abstract Book Citations:** Authors, Title, HemaSphere, 2026;10:(S1):pages. The abstract book DOIs can be found on the "Author Information" page.
**Disclaimer:** Articles published in the journal HemaSphere exclusively reflect the opinions of the authors. The authors are responsible for all content in their abstracts including accuracy of the facts, statements, citing resources, etc.

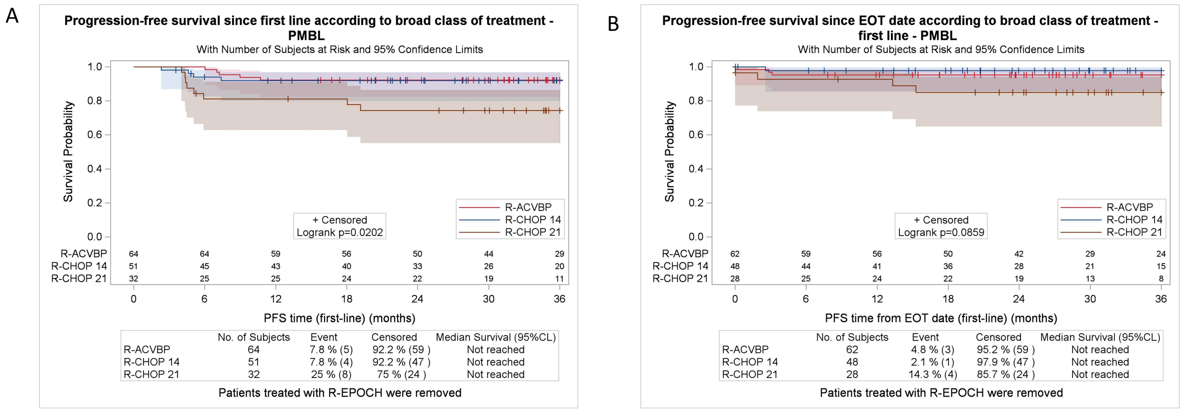

**Copyright Information:** (Online) ISSN: 2572-9241
©2026 The Author(s). HemaSphere is published by John Wiley & Sons Ltd on behalf of European Hematology Association. This is an open access Abstract Book distributed under the Attribution-NonCommercial-NoDerivs (CC BY-NC-ND), which allows third parties to download the articles and share them with others as long as they credit the author and the Abstract Book, but they cannot change the content in any way or use them commercially.
**Abstract Book Citations:** Authors, Title, HemaSphere, 2026;10:(S1):pages. The abstract book DOIs can be found on the "Author Information" page.
**Disclaimer:** Articles published in the journal HemaSphere exclusively reflect the opinions of the authors. The authors are responsible for all content in their abstracts including accuracy of the facts, statements, citing resources, etc.

### (PF1035) THE REAL-WORLD EFFICACY OF BRUTON'S TYROSINE KINASE INHIBITORS PLUS HIGH-DOSE METHOTREXATE BASED INDUTION TREATMENT FOR UNTREATED PRIMARY CNS LYMPHOMA: A SINGLE-CENTER RETROSPECTIVE ANALYSIS.

**Topic:** 20. Other aggressive lymphomas

He Huang*[1], Peng Zhang[1], Lulu Zhang[1], Zhao Wang[1], Yu Wang[1], Xiaojie Fang[1], Chengcheng Guo[2], Jiahao Tian[1], Haimei Ye[1]

*[1]Sun Yat-sen University Cancer Center, State Key Laboratory of Oncology in South China, Guangdong Provincial Clinical Research Center for Cancer, Sun Yat-sen University, Department of Medical Oncology, Guangzhou, China; [2]Sun Yat-sen University Cancer Center, State Key Laboratory of Oncology in South China, Guangdong Provincial Clinical Research Center for Cancer, Sun Yat-sen University, Department of Neurosurgery, Guangzhou, China;*

### Background

Primary central nervous system lymphoma (PCNSL) is a rare, aggressive malignant tumor with poor prognosis, and the optimal standard treatment remains undefined. High-dose methotrexate (HD-MTX) based regimens continue to be the first-line treatment for newly diagnosed PCNSL. In PCNSL, alterations in B-cell receptor (BCR) signaling pathway-related genes, such as *MYD88* and *CD79B*, have been shown to mediate responses to Bruton's tyrosine kinase inhibitors (BTKi).

### Aims

This single-center retrospective analysis aims to evaluate the efficacy of BTKi plus HD-MTX based chemotherapy regimen as indution treatment for newly diagnosed PCNSL patients in a real-world cohort.

### Methods

We enrolled adult patients with histologically confirmed primary CNS large B cell lymphoma who had received induction treatment with the HD-MTX based chemotherapy regimen, and the combination of BTKi (included ibrutinib, zanubrutinib and orelabrutinib) between January 2020 and June 2025. HD-MTX is defined as ≥3.0 g/m2. When patients achieved complete response (CR) or partial response (PR), autologous stem cell transplantation (ASCT) or whole brain radiation treatment (WBRT) was recommended as consolidation treatment. Treatment responses were assessed by MRI/PET. Categorical variables were analyzed using chi-square or Fisher's exact tests. Survival was estimated with the Kaplan-Meier method. R software, version 4.3.2 were used for data analysis. A two-sided $P < 0.05$ indicated a significant difference.

**Copyright Information:** (Online) ISSN: 2572-9241
©2026 The Author(s). HemaSphere is published by John Wiley & Sons Ltd on behalf of European Hematology Association. This is an open access Abstract Book distributed under the Attribution-NonCommercial-NoDerivs (CC BY-NC-ND), which allows third parties to download the articles and share them with others as long as they credit the author and the Abstract Book, but they cannot change the content in any way or use them commercially.
**Abstract Book Citations:** Authors, Title, HemaSphere, 2026;10:(S1):pages. The abstract book DOIs can be found on the "Author Information" page.
**Disclaimer:** Articles published in the journal HemaSphere exclusively reflect the opinions of the authors. The authors are responsible for all content in their abstracts including accuracy of the facts, statements, citing resources, etc.

### Results

Between January 2020 and June 2025, 132 patients were enrolled, with a median age of 58 years (range, 18-83). 69 of 132 (52.3%) patients were male, 63 (47.7%) were female. Based on cell of origin (COO) classification, the majority of patients exhibited the non-germinal center B-cell (non-GCB) subtype (n=97, 73.5%). Baseline imaging analysis indicated that most patients presented with deep structural involvement (n=87, 65.9%) and multiple lesions (n=75, 56.8%). In the choice of BTKi, 48 patients used ibrutinib (36.4%), 58 used orelabrutinib (43.9%) and 26 used zanubrutinib (19.7%). As of the cut-off date (December 31, 2025), the median follow-up time was 23.6 (range, 2.2-64.3) month. The objective response rate (ORR) after induction treatment was 88.6%. Specifically, 107 patients (81.1%) achieved CR and 10 patients (7.5%) achieved PR. The CRR for the ibrutinib, orelabrutinib, and zanubrutinib groups were 79.2%, 82.8%, and 80.8%, respectively, with no statistically significant difference ($\chi^2$=0.223, $P$= 0.895). 55 patients received ASCT or WBRT as consolidation treatment. The median progression free survival (PFS) and overall survival (OS) was not reached (NR) (95% CI NR-NR). The 2- year PFS and OS rates were 76.0% (95%CI 69.0%-85.0%) and 83.1% (95%CI 77.3%-91.5%), respectively. There was no significant difference in PFS among the three BTKi ($P$= 0.283), but a significant improvement in OS was observed in the orelabrutinib group (HR 0.26, 95% CI 0.09-0.78, $P$= 0.016). In univariate and multivariate analyses, baseline characteristics such as age, gender, LDH, ECOG PS, number of lesions, deep structural involvement were not significantly associated with PFS and OS.

### Summary/Conclusion

This single-center retrospective analysis confirms the efficacy of adding BTKi especially orelabrutinib as indution treatment in a real-world Chinese patient population. These results support the use of BTKi-containing treatment regimens as a first-line therapy for PCNSL in clinical practice.

**Copyright Information:** (Online) ISSN: 2572-9241
©2026 The Author(s). HemaSphere is published by John Wiley & Sons Ltd on behalf of European Hematology Association. This is an open access Abstract Book distributed under the Attribution-NonCommercial-NoDerivs (CC BY-NC-ND), which allows third parties to download the articles and share them with others as long as they credit the author and the Abstract Book, but they cannot change the content in any way or use them commercially.
**Abstract Book Citations:** Authors, Title, HemaSphere, 2026;10:(S1):pages. The abstract book DOIs can be found on the "Author Information" page.
**Disclaimer:** Articles published in the journal HemaSphere exclusively reflect the opinions of the authors. The authors are responsible for all content in their abstracts including accuracy of the facts, statements, citing resources, etc.

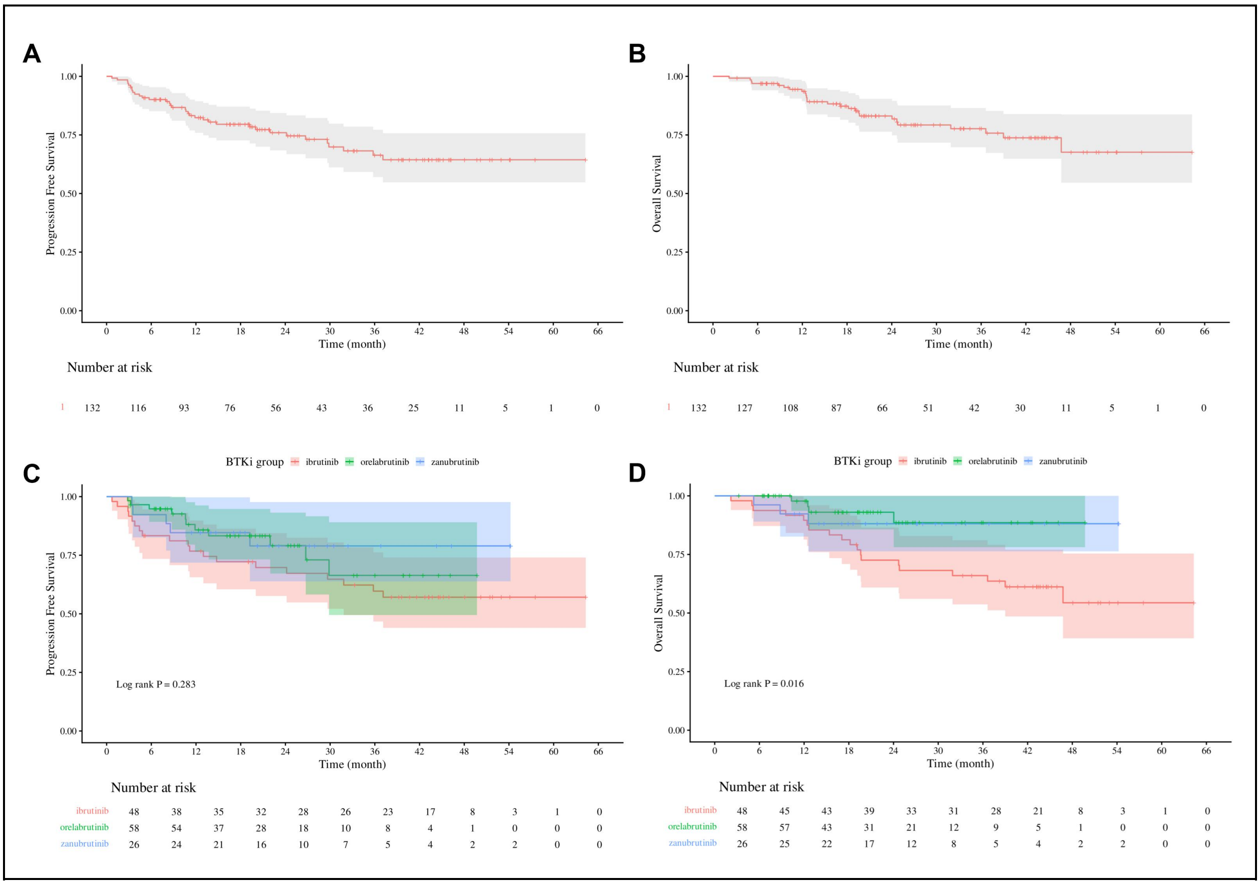

Kaplan–Meier plots for survival outcomes. (A) PFS of all patients, (B) OS of all patients, (C) PFS of different BTKi treatment group and (D) OS of different BTKi treatment group

**Copyright Information:** (Online) ISSN: 2572-9241
©2026 The Author(s). HemaSphere is published by John Wiley & Sons Ltd on behalf of European Hematology Association. This is an open access Abstract Book distributed under the Attribution-NonCommercial-NoDerivs (CC BY-NC-ND), which allows third parties to download the articles and share them with others as long as they credit the author and the Abstract Book, but they cannot change the content in any way or use them commercially.
**Abstract Book Citations:** Authors, Title, HemaSphere, 2026;10:(S1):pages. The abstract book DOIs can be found on the “Author Information” page.
**Disclaimer:** Articles published in the journal HemaSphere exclusively reflect the opinions of the authors. The authors are responsible for all content in their abstracts including accuracy of the facts, statements, citing resources, etc.

### (PF1036) CHIDAMIDE IN COMBINATION WITH CHOP IN PREVIOUSLY UNTREATED PERIPHERAL T-CELL LYMPHOMA WITH T FOLLICULAR HELPER PHENOTYPE–RESULTS OF PHASE II SWIFT TRIAL

**Topic:** 20. Other aggressive lymphomas

Qingqing Cai*[1], Yi Xia[1], Huiqiang Huang[1], Rong Tao[2], Jianbo Liu[3], Runhui Zheng[3], Qike Zhang[4], Dongfeng Zeng[5], Xiaojun Xu[6], Qingsong Yin[7], Xiaojie Fang[1], Bing Bai[1], Man Nie[1], Yuchen Zhang[1], Shenrui Bai[1]

*[1]Sun Yat-sen University Cancer Center, Guangzhou, China;[2]Fudan University Shanghai Cancer Center, Shanghai, China;[3]The Fifth Affiliated Hospital of Guangzhou Medical University, Guangzhou, China; [4]Gansu Provincial Hospital, Department of Hematology, Lanzhou, China; [5]Daping Hospital, Third Military Medical University (Army Medical University),, Chongqing, China; [6]The Seventh Affiliated Hospital, Sun Yat-Sen University, Shenzhen, China; [7]The Affiliated Cancer Hospital of Zhengzhou University and Henan Cancer Hospital, Zhengzhou, China;*

### Background

Peripheral T-Cell Lymphoma with T-follicular helper phenotype (PTCL-TFH), which mainly includes angioimmunoblastic T-cell lymphoma (AITL), is a biologically distinct subtype characterized by frequent epigenetic alterations. Chidamide is an oral, histone deacetylase (HDAC) inhibitor that targets HDAC subtypes 1, 2, 3, and 10, has shown encouraging activity in R/R PTCL-TFH.

### Aims

This study aimed to evaluate the efficacy and safety of chidamide in combination with CHOP (cyclophosphamide, doxorubicin, vincristine, and prednisone) in previously untreated PTCL-TFH patients.

### Methods

This is a multicenter, open-label, single-arm phase II study (NCT05572983). Patients with previously untreated PTCL-TFH were enrolled and received chidamide (20 mg, oral, biweekly) combined with CHOP regimen for up to six induction cycles. Patients achieving complete response (CR) after induction entered a maintenance phase with chidamide for up to two years. The primary endpoint was CR rate. Secondary endpoints included overall response rate (ORR), progression-free survival (PFS), overall survival (OS), and safety.

### Results

Between May 2023 and July 2025, a total of 47 patients were enrolled, and enrollment was completed. All patients had the AITL subtype. The median age was 62 years (IQR, 54–67), and 25.5% were female. Advanced-stage disease (stage III–IV) was present in 85.0% of patients, and 48.9% had extranodal involvement. The median number of induction cycles was 5 (range, 1–6), with 33 patients completing six cycles of induction therapy. Among 35 patients who underwent post-treatment PET/CT evaluation, the best CR rate during the induction phase was 85.2% (30/35), with an ORR of 100%. At the end of induction therapy, the CR rate was 87.9% (29/33), and the ORR remained 100%. As of October 2025, disease progression was observed in 10 patients. The most common grade 3–4 adverse events were neutropenia (42.6%), thrombocytopenia (19.1%), infections (17.0%), anemia (6.4%), and elevated transaminases (2.1%).

### Summary/Conclusion

Chidamide in combination with CHOP demonstrated promising efficacy with a manageable safety profile in previously untreated PTCL-TFH patients. A randomized controlled trial is currently ongoing to further validate these findings (NCT07139353).

**Copyright Information:** (Online) ISSN: 2572-9241
©2026 The Author(s). HemaSphere is published by John Wiley & Sons Ltd on behalf of European Hematology Association. This is an open access Abstract Book distributed under the Attribution-NonCommercial-NoDerivs (CC BY-NC-ND), which allows third parties to download the articles and share them with others as long as they credit the author and the Abstract Book, but they cannot change the content in any way or use them commercially.
**Abstract Book Citations:** Authors, Title, HemaSphere, 2026;10:(S1):pages. The abstract book DOIs can be found on the "Author Information" page.
**Disclaimer:** Articles published in the journal HemaSphere exclusively reflect the opinions of the authors. The authors are responsible for all content in their abstracts including accuracy of the facts, statements, citing resources, etc.

### (PF1037) THE AITL-HZ SCORE INCORPORATING EBV AND COMPLEMENT C3 OUTPERFORMS EXISTING PROGNOSTIC MODELS FOR CHINESE AITL PATIENTS

**Topic:** 20. Other aggressive lymphomas

Mengmeng Dong*[1], Yi Zhong[2], Fang Yu[2], Yi Zhao[2], Lixia Zhu[2], Fengping Zhou[3], Linlin Huang[4], Xiujin Ye[5], Zhen Cai[5], Jin Zhang[3], Ye Baodong[4], Qingming Wang[6], He Jingsong[5]

*[1]The First Affiliated Hospital, Zhejiang University, School of Medicine, Bone Marrow Transplantation Center, Hangzhou, China; [2]The First Affiliated Hospital, Zhejiang University, School of Medicine, Hangzhou, China;[3]Sir Run Run Shaw Hospital, affiliated with the Zhejiang University School of Medicine, Hangzhou, China; [4]The First Affiliated Hospital of Zhejiang Chinese Medical University, Hangzhou, China; [5]The First Affiliated Hospital, School of Medicine, Zhejiang University, Hangzhou, China;[6]The Second Affiliated Hospital of Nanchang University, Hangzhou, China;*

### Background

Angioimmunoblastic T-cell lymphoma (AITL) is an aggressive malignancy with a dismal and heterogeneous prognosis, predominantly affecting the elderly. While several prognostic models exist, their predictive accuracy in diverse populations, particularly within the Chinese cohort, remains suboptimal. Identifying novel, robust biomarkers is critical for refining risk stratification and guiding personalized treatment strategies in the era of immunochemotherapy.

### Aims

This multicenter study aimed to comprehensively characterize the clinical landscape of AITL in China, identify novel prognostic factors, and develop a superior prognostic model that integrates these factors to outperform current international scoring systems.

### Methods

We conducted a retrospective analysis of 350 patients diagnosed with AITL across four major Chinese medical centers from August 2015 to November 2023. LASSO regression was employed to identify risk factors for progression-free survival (PFS) and overall survival (OS). ROC analysis was used to develop predictive models and compare prognostic value of various international scoring systems in our cohort.

**Copyright Information:** (Online) ISSN: 2572-9241
©2026 The Author(s). HemaSphere is published by John Wiley & Sons Ltd on behalf of European Hematology Association. This is an open access Abstract Book distributed under the Attribution-NonCommercial-NoDerivs (CC BY-NC-ND), which allows third parties to download the articles and share them with others as long as they credit the author and the Abstract Book, but they cannot change the content in any way or use them commercially.
**Abstract Book Citations:** Authors, Title, HemaSphere, 2026;10:(S1):pages. The abstract book DOIs can be found on the "Author Information" page.
**Disclaimer:** Articles published in the journal HemaSphere exclusively reflect the opinions of the authors. The authors are responsible for all content in their abstracts including accuracy of the facts, statements, citing resources, etc.

### Results

In multicenter study involving 350 AITL patients with an average age of 65.36 years (59.14% male), the median progression-free survival (PFS) was 14.5 months and median overall survival (OS) 24.8 months during follow-up. Multivariate analysis identified multiple baseline clinical indicators associated with PFS and OS. We incorporated both PFS-and OS-associated factors into LASSO regression analysis. Based on overlapping factors identified through LASSO regression, we established prognostic correlates: age ≥65 years, ECOG score ≥2, platelet count ≤150×10^9/L, LDH> upper limit of normal (ULN), β2-MG> ULN, log(EBV copy number) ≥3.0, C3 ≥40 mg/dl, and extranodal involvement ≥2. ROC analysis validated the predictive model using 75% of samples for prediction and 25% for verification. Results showed Internal validation C indices (95% CI) for PFS and OS at 0.7018 (0.6638,0.7398) and 0.7588 (0.7243,0.7934), respectively. Building upon previous research, we developed a new AITL score system named AITL-HZ (Low Risk: 0 or 1 risk factors; Low-Mid Risk: 2 or 3 risk factors; Mid-High Risk: 4 or 5 risk factors; High Risk: ≥6 risk factors). The study introduced EBV copy number and C3 complement as novel risk factors compared to existing prognostic scoring systems. The K-M curve analysis further demonstrated that patients with different AITL-HZ scores exhibited distinct PFS and OS outcomes (p values were <0.001).

Furthermore, we compared AITL-HZ scores with existing prognostic scoring systems (including IPI, PIT, ATPI, AITL score (2021 Blood), and AITL score (2022 Chinese)). ROC analysis of multivariate predictors demonstrated that the AITL-HZ score showed AUC values of 0.699 for PFS and 0.780 for OS, both significantly outperforming previous prognostic evaluation systems.

### Summary/Conclusion

We have developed and internally validated the AITL-HZ score, a novel and powerful prognostic model for AITL. By uniquely integrating EBV DNA load and complement C3 with established clinical factors, it provides superior risk stratification compared to all current standard models in this large Chinese cohort. The AITL-HZ score holds significant promise for enhancing clinical decision-making, guiding therapeutic intensity, and refining patient stratification in future clinical trials. External validation is warranted to confirm its generalizability.

**Copyright Information:** (Online) ISSN: 2572-9241
©2026 The Author(s). HemaSphere is published by John Wiley & Sons Ltd on behalf of European Hematology Association. This is an open access Abstract Book distributed under the Attribution-NonCommercial-NoDerivs (CC BY-NC-ND), which allows third parties to download the articles and share them with others as long as they credit the author and the Abstract Book, but they cannot change the content in any way or use them commercially.
**Abstract Book Citations:** Authors, Title, HemaSphere, 2026;10:(S1):pages. The abstract book DOIs can be found on the "Author Information" page.
**Disclaimer:** Articles published in the journal HemaSphere exclusively reflect the opinions of the authors. The authors are responsible for all content in their abstracts including accuracy of the facts, statements, citing resources, etc.

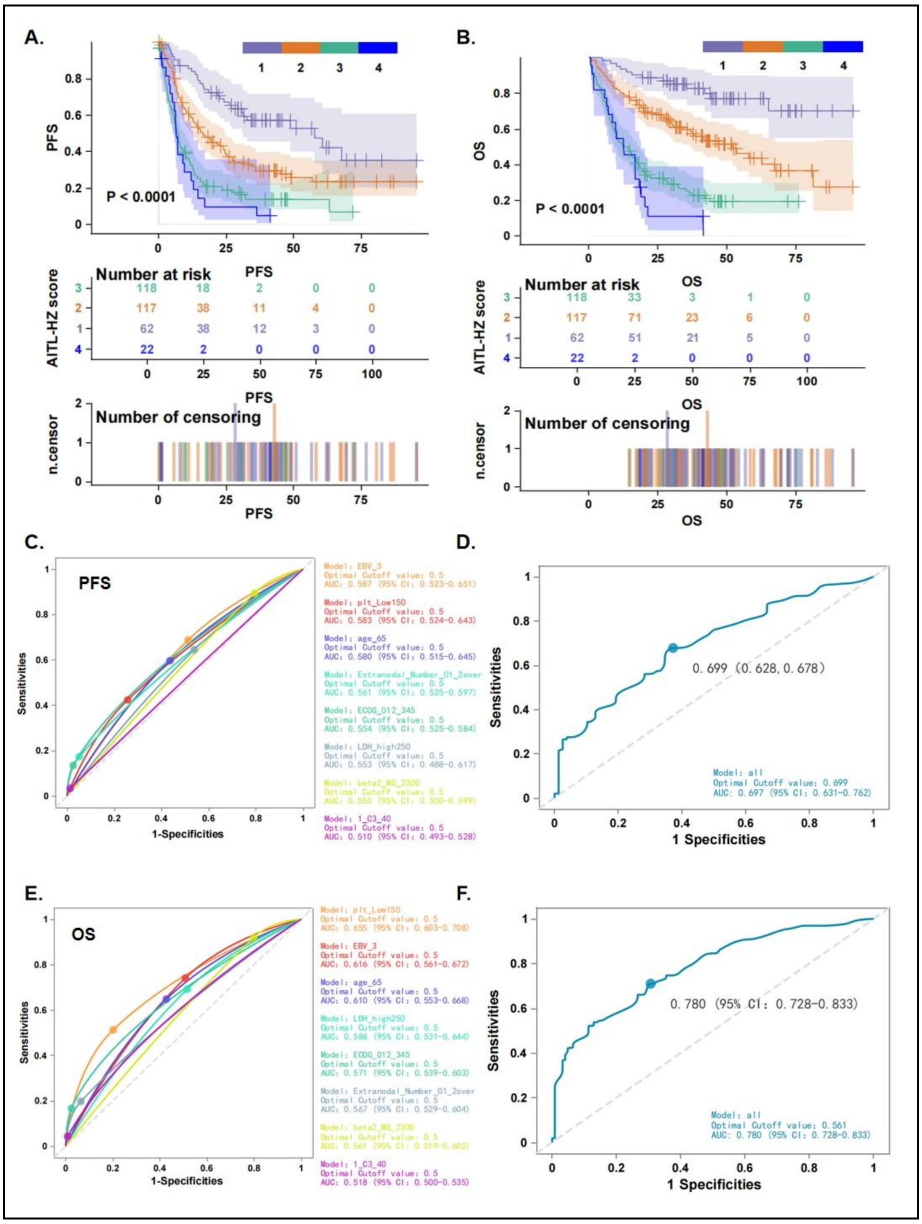

**Copyright Information:** (Online) ISSN: 2572-9241
©2026 The Author(s). HemaSphere is published by John Wiley & Sons Ltd on behalf of European Hematology Association. This is an open access Abstract Book distributed under the Attribution-NonCommercial-NoDerivs (CC BY-NC-ND), which allows third parties to download the articles and share them with others as long as they credit the author and the Abstract Book, but they cannot change the content in any way or use them commercially.
**Abstract Book Citations:** Authors, Title, HemaSphere, 2026;10:(S1):pages. The abstract book DOIs can be found on the "Author Information" page.
**Disclaimer:** Articles published in the journal HemaSphere exclusively reflect the opinions of the authors. The authors are responsible for all content in their abstracts including accuracy of the facts, statements, citing resources, etc.

### (PF1038) BLOOD CANCER ASSOCIATED HAEMOPHAGOCYTIC LYMPHOHISTIOCYTOSIS IS MORE LIKELY IN YOUNGER PATIENTS, AND ONLY AGE AND T-, NKT- AND NK-CELL LYMPHOMA DIAGNOSIS IMPACT SURVIVAL.

**Topic:** 20. Other aggressive lymphomas

Nurunnahar Akter[1], Harry Wilson[2], Rodothea Amerikanou[3], Sneha Tandon[4], Satyen Gohil[3], Callum Wright[5], Trung Nguyen[6], Vasanta Nanduri[7], Kristian Bowles[8, 9], Indrani Karpha[10], Nikesh Chavda[11], Matthew Collin[2, 5], Yeong Jer Lim[1, 10], Nagesh Kalakonda[1, 10], Andrew Pettitt[1, 10], Catrin Tudur Smith[1], Mark Bishton*[12, 13]

*[1]University of Liverpool, Liverpool, United Kingdom; [2]University of Newcastle, Newcastle, United Kingdom; [3]University College London Hospitals NHS Foundation Trust, London, United Kingdom; [4]Bart's Health NHS Trust, London, United Kingdom; [5]Newcastle upon Tyne Hospitals NHS Foundation Trust, Newcastle, United Kingdom; [6]Great Ormond Street Hospital for Children, London, United Kingdom; [7]West Hertfordshire Teaching Hospitals NHS Trust, Watford, United Kingdom; [8]Norfolk and Norwich University Hospitals NHS Foundation Trust, Norwich, United Kingdom; [9]Norwich Medical School, Norwich, United Kingdom; [10]The Clatterbridge Cancer Centre NHS Foundation Trust, Liverpool, United Kingdom; [11]UNIVERSITY HOSPITALS BRISTOL AND WESTON NHS FOUNDATION TRUST, Bristol, United Kingdom; [12]University of Nottingham, Nottingham, United Kingdom; [13]Nottingham University Hospitals NHS Trust, Nottingham, United Kingdom;*

### Background

Haemophagocytic Lymphohistiocytosis (HLH) is a life-threatening syndrome of hyperinflammation frequently triggered by blood cancer.

### Aims

The UNCOVER project utilised the National Cancer Registration Dataset in England with linked data on hospital admissions to assess the incidence and survival of HLH in the context of blood cancer and the impact of patient characteristics and disease.

### Methods

Patients (pts) in England with blood cancer diagnosed 2014-2022 were considered. Pts with HLH were identified by admission ICD-10 codes D76.1 and D76.2, or death certificate using D76.1, D76.2 or D76.3. Crude incidence rates within the blood cancer population were calculated and incidence rate ratios (IRR) estimated by Poisson regression adjusting for selected patient characteristics. Interactions between age and disease subtype were assessed in a separate Poisson model. Overall survival (OS) was calculated from date of HLH admission by Kaplan–Meier, and Cox regression modelling examined risk factors.

**Copyright Information:** (Online) ISSN: 2572-9241
©2026 The Author(s). HemaSphere is published by John Wiley & Sons Ltd on behalf of European Hematology Association. This is an open access Abstract Book distributed under the Attribution-NonCommercial-NoDerivs (CC BY-NC-ND), which allows third parties to download the articles and share them with others as long as they credit the author and the Abstract Book, but they cannot change the content in any way or use them commercially.
**Abstract Book Citations:** Authors, Title, HemaSphere, 2026;10:(S1):pages. The abstract book DOIs can be found on the "Author Information" page.
**Disclaimer:** Articles published in the journal HemaSphere exclusively reflect the opinions of the authors. The authors are responsible for all content in their abstracts including accuracy of the facts, statements, citing resources, etc.

**Results**

We identified 541 pts with blood cancer and HLH, 362 (67%) via hospital admission, 293 (54.2%) by death certificate and 114 (21%) in both. Median age at HLH diagnosis was 58 years (IQR 36-68), 351 (64.9%) were male. The commonest blood cancer subtypes were aggressive B-cell lymphoma (n=127 [23.5%]), T-/NKT-/NK-cell lymphoma (n=119 [22.0%]), acute leukaemia/high-grade MDS (n=87 [16.1%]), and Hodgkin lymphoma (n=63 [11.6%]). The overall crude incidence rate of HLH in blood cancer was 1.82/1000 cases (95%CI 1.66-1.97) although rates doubled over the study period.
The adjusted IRR of HLH in blood cancer was higher in males (vs females IRR 1.28 95%CI 1.08-1.54; p-value 0.005), younger pts <18 years (vs ref >75 years IRR 10.78 95%CI 7.33-15.86; global p-value <0.001), later years 2022 (vs ref 2014 IRR 2.11 95%CI 1.44-3.15; global p-value <0.001) and T-/NKT-/NK-cell lymphoma (vs ref acute leukaemia/high grade MDS IRR 4.43 95%CI 3.33-5.91). There was a significant difference in adjusted incidence by geographical regions (global p-value 0.003) due to high rates in London (unadjusted incidence rate 3.09/1000 95%CI 2.55-3.72) and low rates in the North-East (unadjusted incidence rate 0.65/1000 95%CI 0.31-1.19).
The liklehood of a diagnosis of HLH progressively declined with increasing age for all blood cancer subtypes, except Hodgkin lymphoma which increased in ages over 45 years, and the interaction of age and disease was significant (global p<0.001).
Overall crude 1-year OS was 34% (95%CI 30.2-38.2) with no change over time. Pts <18 years had 1-year OS 57.5% (95%CI 46.1-71.6), although OS progressively declined with age (global p-value <0.001; figure 1). 1-year OS was better in acute leukaemia/high-grade MDS, driven by higher numbers in young pts, with no difference in OS for other diseases (p=0.025). In the multivariable regression model, blood cancer subtype was not significantly associated with OS (global p=0.053), however, compared with acute leukaemia, pts with T-/NKT-/NK-cell lymphoma had worse OS (HR 1.50 95%CI 1.06–2.12; p=0.024). Calendar year, gender, ethnicity, deprivation, region or co-morbidities did not impact OS.

**Summary/Conclusion**

The reported incidence of HLH within blood cancer doubled between 2014-2022 but with significant geographical variation. Risk factors included T-/NTK-/NK-lymphoma, male gender and younger age except for Hodgkin lymphoma where HLH was more likely in older pts. Survival remains dismal, static over time and only age and blood cancer subtype predict OS.

**Copyright Information:** (Online) ISSN: 2572-9241
©2026 The Author(s). HemaSphere is published by John Wiley & Sons Ltd on behalf of European Hematology Association. This is an open access Abstract Book distributed under the Attribution-NonCommercial-NoDerivs (CC BY-NC-ND), which allows third parties to download the articles and share them with others as long as they credit the author and the Abstract Book, but they cannot change the content in any way or use them commercially.
**Abstract Book Citations:** Authors, Title, HemaSphere, 2026;10:(S1):pages. The abstract book DOIs can be found on the “Author Information” page.
**Disclaimer:** Articles published in the journal HemaSphere exclusively reflect the opinions of the authors. The authors are responsible for all content in their abstracts including accuracy of the facts, statements, citing resources, etc.

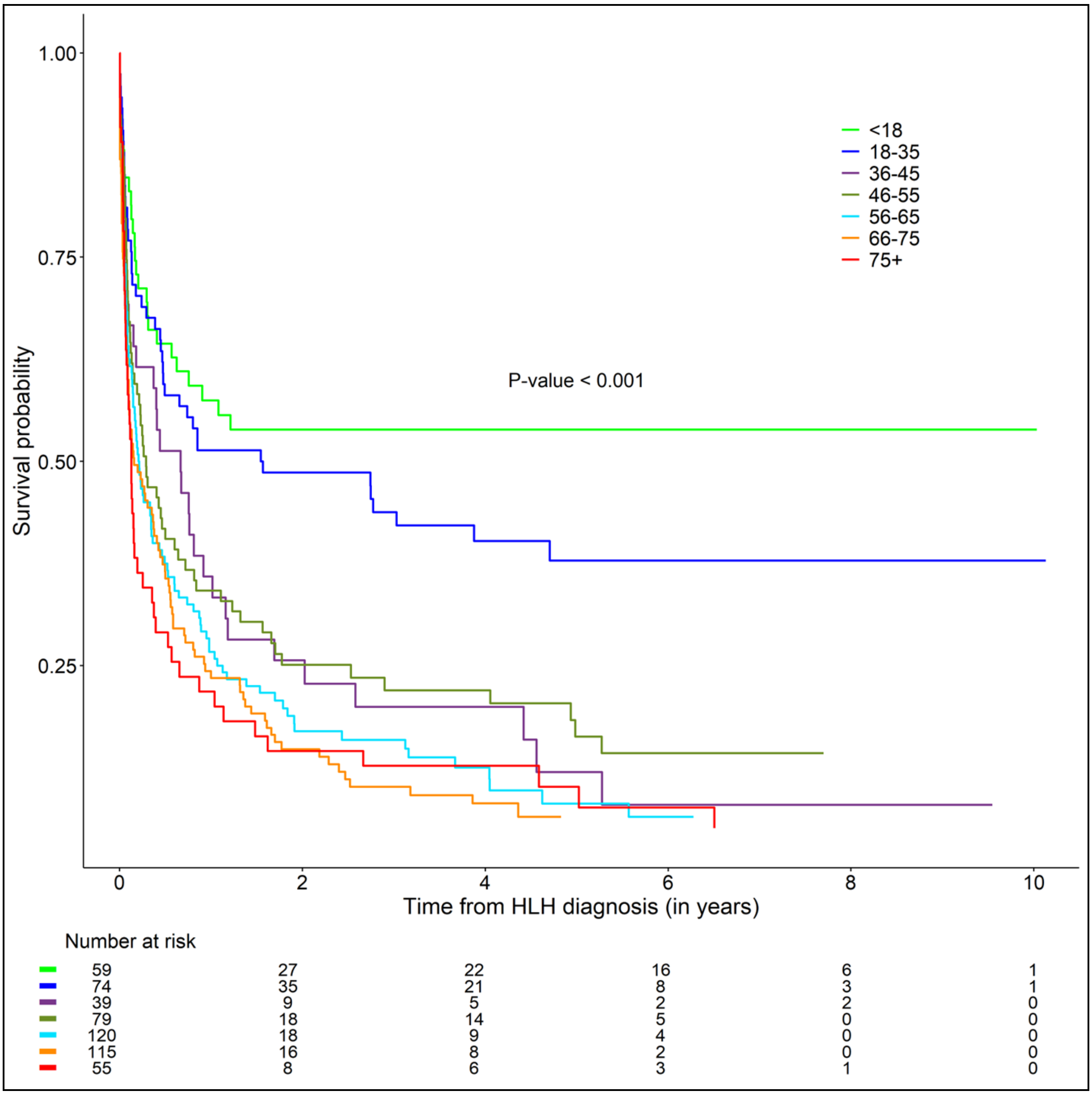

**Copyright Information:** (Online) ISSN: 2572-9241
©2026 The Author(s). HemaSphere is published by John Wiley & Sons Ltd on behalf of European Hematology Association. This is an open access Abstract Book distributed under the Attribution-NonCommercial-NoDerivs (CC BY-NC-ND), which allows third parties to download the articles and share them with others as long as they credit the author and the Abstract Book, but they cannot change the content in any way or use them commercially.
**Abstract Book Citations:** Authors, Title, HemaSphere, 2026;10:(S1):pages. The abstract book DOIs can be found on the "Author Information" page.
**Disclaimer:** Articles published in the journal HemaSphere exclusively reflect the opinions of the authors. The authors are responsible for all content in their abstracts including accuracy of the facts, statements, citing resources, etc.

### (PF1039) COMPARABLE EFFICACY AND SAFETY OF BRENTUXIMAB VEDOTIN -BASED THERAPY TO THE ECHELON TRIALS: REAL-WORLD EVIDENCE FROM THE BRAVE STUDY

**Topic:** 20. Other aggressive lymphomas

Mingci Cai*[1], Ou Bai[2], Hui-Lai Zhang[3], Chuanxu Liu[4], Jianli Wang[5], Ziheng Guo[5], Weili Zhao[1]

*[1]Shanghai Institute of Hematology, State Key Laboratory of Medical Genomics, National Research Center for Translational Medicine at Shanghai, Ruijin Hospital affiliated to Shanghai Jiao Tong University School of Medicine, Shanghai, China; [2]The First Bethune Hospital of Jilin University, Changchun, China; [3]Tianjin Medical University Cancer Institute and Hospital, Tianjin, China; [4]Fudan University Shanghai Cancer Center, Shanghai, China;[5]Takeda (China) International Trading Co., Ltd, Shanghai, China;*

### Background

Brentuximab vedotin (BV) is a pivotal therapy for CD30-positive lymphomas, with efficacy and safety established in ECHELON-1, -2, and -3 for cHL, PTCL and BCL. Real-world evidence in Chinese patients remains limited. The BRAVE study is the first large-scale, multicenter, observational real-world study to evaluate BV-based regimens across lymphoma subtypes in China.

### Aims

To characterize baseline demographics, treatment patterns, and outcomes of BV-treated patients in China, and to compare these findings descriptively with the ECHELON trials.

### Methods

BRAVE (NCT04837222) prospectively enrolled adult patients with CD30+ lymphoma treated with BV-based regimens from 30 centers in China. Demographics, disease features, treatment exposure, and outcomes were collected. The analysis focused on safety (AEs, ADRs), dose intensity, efficacy (ORR, CR, PFS), and response kinetics (TTCR). Baseline characteristics, treatment exposure, response kinetics, and safety were compared at the disease-population level and efficacy comparisons were further restricted to regimen-matched subgroups: BV+AVD for cHL, BV+CHP for PTCL.

### Results

*Baseline:* ND cHL patients in BRAVE were older (median age≥60 years: 24.9% vs 13%) with poorer performance status (ECOG≥2: 7.8% vs 4%) than ECHELON-1. ND PTCL baseline characteristics were comparable, though ECHELON-2 had more stage III-IV disease (81% vs 73.3%). The R/R DLBCL cohort in BRAVE was younger (median age 51.5 vs 74 years) with more ECOG ≥2 (30.0% vs 11%), while ECHELON-3 had higher IPI scores (≥3: 60% vs 35.0%).
*Safety:* The overall safety profile of BV in BRAVE was manageable and generally consistent with the ECHELON trials. Serious AEs and Serious ADRs were reported for 14.6% and 4.1% of patients, with infections being the most common SAEs.
*Efficacy:* Efficacy was evaluated in the full analysis set. BRAVE patients received fewer BV cycles and, in some cohorts, lower dose intensity than RCTs, yet achieved rapid and clinically meaningful responses. In the ND cHL cohort, a median of 7 cycles at a dose intensity of 1.2 mg/kg yielded an ORR of 91.9% (CR 59.3%) with a TTCR of 2.17 months, compared to 12 cycles at a predefined 1.2 mg/kg in ECHELON-1 (ORR 86%, CR 73%). In the ND PTCL cohort, 4 median cycles at 1.63 mg/kg produced an ORR of 71.0% (CR 49.5%) with a median TTCR of 2.79 months, versus 6 cycles in ECHELON-2 (ORR 83%, CR 68%). For R/R DLBCL, only 2.5 median cycles at 1.69 mg/kg achieved an ORR of 50.0% (CR 25.0%) with a median TTCR of 1.58 months, identical to that reported in ECHELON-3 (1.58 months; 5 cycles at 1.133 mg/kg; ORR 64%, CR 40%). At a median follow-up of 8.84 months, median PFS was not reached in ND cHL, was 7.4 months for ND PTCL (41.9% censoring at cutoff), and 4.7 months for R/R DLBCL, in line with ECHELON-3.

**Copyright Information:** (Online) ISSN: 2572-9241
©2026 The Author(s). HemaSphere is published by John Wiley & Sons Ltd on behalf of European Hematology Association. This is an open access Abstract Book distributed under the Attribution-NonCommercial-NoDerivs (CC BY-NC-ND), which allows third parties to download the articles and share them with others as long as they credit the author and the Abstract Book, but they cannot change the content in any way or use them commercially.
**Abstract Book Citations:** Authors, Title, HemaSphere, 2026;10:(S1):pages. The abstract book DOIs can be found on the "Author Information" page.
**Disclaimer:** Articles published in the journal HemaSphere exclusively reflect the opinions of the authors. The authors are responsible for all content in their abstracts including accuracy of the facts, statements, citing resources, etc.

### Summary/Conclusion

To our knowledge, this is the first large-scale real-world analysis of BV across CD30+lymphoma subtypes in Chinese patients. Even with shorter treatment courses and certain unfavorable baseline features, BV produced efficacy that closely paralleled the ECHELON benchmarks, particularly in cHL. However, CR rates were consistently lower, which may reflect the reduced dose intensity and shorter treatment duration in real-world practice, suggesting that optimizing BV exposure could further improve depth of response. The convergent TTCR across trial and real-world settings points to BV's capacity to drive deep remissions early. These data support the real-world effectiveness of BV.

**Table 1. Baseline characteristics**

| | BRAVE ND stage III/IV cHL（N=205） | ECHELON-1 ND stage III/IV cHL BV+AVD （N=664） | BRAVE ND PTCL (N=258) | ECHELON-2 ND PTCL BV+CHP(N=226) | BRAVE R/R DLBCL (N=20) | ECHELON-3 R/R DLBCL BV+Len+R（N=112） |
|---|---|---|---|---|---|---|
| **Age (years)** | | | | | | |
| **Median** | 35.0 (IQR 25.0, 59.0) | 35(range 18-82) | 55.0 (IQR 38.0, 67.0) | 58.0 (IQR 45-67) | 51.5 (IQR 30.0, 69.0) | 74 (range 29-87) |
| **≥60 years, n (%)** | 51 (24.9) | 84(13) | 110 (42.6) | - | 9 (45.0) | 79 (71)[a] |
| **Gender, n (%)** | | | | | | |
| **Male** | 118 (57.6) | 378(57) | 159 (61.6) | 133 (59) | 11 (55.0) | 60 (54) |
| **Female** | 87 (42.4) | 286(43) | 99 (38.4) | 93 (41) | 9 (45.0) | 52 (46) |
| **ECOG, n (%)** | | | | | | |
| **0-1** | 186(90.7) | 636(96) | 188 (72.9) | 174(77) | 13 (65.0) | 100(89) |
| **≥2** | 16(7.8) | 28(4) | 63 (24.4) | 51(23) | 6 (30.0) | 12 (11) |
| **Unknown** | 3(1.5) | 0 | 7 (2.7) | 1(<1) | 1 (5.0) | 0 |
| **IPS/IPI score[b], n (%)** | | | | | | |
| **0-3/0-2** | 144(70.2) | 495(75) | 137(53.1) | 127(56) | 10 (50.0) | 45 (40) |
| **≥4/≥3** | 51(24.9) | 169(25) | 106(41.1) | 99(44) | 7 (35.0) | 67 (60) |
| **Unknown** | 10(4.9) | 0 | 15(5.8) | 0 | 3 (15.0) | 0 |
| **Ann Arbor staging, n (%)** | | | | | | |
| **I-II** | 0 | 1(<1) | 51 (19.8) | 42(19) | 5(25.0) | 29(26) |
| **III-IV** | 205(100.0) | 662(99.7) | 189 (73.3) | 184(81) | 13 (65.0) | 83(74) |
| **Unknown** | 0 | 1(<1) | 18 (7.0) | 0 | 2 (10.0) | 0 |
| | **a：patients≥65 years**<br>**b：IPS score (0-3 or ≥4) for cHL patients while IPI score (0-2 or ≥3) for both PTCL and DLBCL patients** | | | | | |

**Table 2. Treatment outcomes**

| | BRAVE ND stage III/IV cHL (N=155) | BRAVE ND stage III/IV cHL BV+AVD(N=135) | ECHELON-1 ND stage III/IV cHL BV+AVD (N=664) | BRAVE ND PTCL (N=210) | BRAVE ND PTCL BV+CHP (N=164) | ECHELON-2 ND PTCL BV+CHP(N=226) | BRAVE R/R DLBCL (N=16) | ECHELON-3 R/R DLBCL BV+Len+R（N=112） |
|---|---|---|---|---|---|---|---|---|
| **Best Overall response, n(%)** | | | | | | | | |
| **ORR(≥PR)** | 136 (87.7) | 124(91.9) | 571(86) | 149 (71.0) | 124(75.6) | 188 (83) | 8 (50.0) | 72 (64) |
| **CR** | 85 (54.8) | 80 (59.3) | 485(73) | 104 (49.5) | 86(52.4) | 153 (68) | 4 (25.0) | 45 (40) |
| **PR** | 51 (32.9) | 44 (32.6) | 86(13) | 45 (21.4) | 38(23.2) | 35 (15) | 4 (25.0) | 27 (24) |
| **SD** | 10 (6.5) | 6 (4.4) | - | 15 (7.1) | 9(5.5) | 5 (2) | 2 (12.5) | 7 (6) |
| **PD** | 6 (3.9) | 4 (3.0) | 90(14) | 17 (8.1) | 11(6.7) | 15 (7) | 2 (12.5) | 23 (21) |
| **Unknown** | 3(1.9) | 1(0.7) | - | 29(13.8) | 20(12.2) | 18 (8) | 4 (25.0) | 10(9) |
| **mTTR, months** | 2.14 (IQR 1.87, 2.73) | - | - | 2.46 (IQR 1.91, 3.02) | - | - | 1.91 (IQR 1.58, 2.23) | - |
| **mTTCR, months** | 2.17 (IQR 1.94, 3.35) | - | - | 2.79 (IQR 2.20, 3.19) | - | - | 1.58 (IQR 1.10, 1.89) | 1.58 (range 1.2,7.3) |
| **Treatment exposure[c]** | | | | | | | | |
| **Median BV dose intensity, mg/kg** | 1.200 (range 0.6, 2.33) | - | ≤1.2[d] | 1.630 (range 0.77, 2.87) | - | 1.786 (IQR 1.685,1.800) | 1.690 (range 1, 2.08) | 1.133 (range 0.612,1.260) |
| **Median cycles of BV received(range)** | 7.0 (1,22) | - | 12.0(1, 12) | 4.0(1.13) | - | 6.0(1,8) | 2.5 (1.7) | 5.0 (1,34) |
| | **c：The patient population of BRAVE assessed for treatment exposure corresponds to the safety set, aligning with the cohort described in the baseline characteristics.**<br>**d：BV dose was predefined as 1.2mg/kg in the regimen of ECHELON-1 trial-BV+AVD arm.** | | | | | | | |

Copyright Information: (Online) ISSN: 2572-9241
©2026 The Author(s). HemaSphere is published by John Wiley & Sons Ltd on behalf of European Hematology Association. This is an open access Abstract Book distributed under the Attribution-NonCommercial-NoDerivs (CC BY-NC-ND), which allows third parties to download the articles and share them with others as long as they credit the author and the Abstract Book, but they cannot change the content in any way or use them commercially.
Abstract Book Citations: Authors, Title, HemaSphere, 2026;10:(S1):pages. The abstract book DOIs can be found on the “Author Information” page.
Disclaimer: Articles published in the journal HemaSphere exclusively reflect the opinions of the authors. The authors are responsible for all content in their abstracts including accuracy of the facts, statements, citing resources, etc.

### (PF1040) MANAGEMENT AND OUTCOME OF SECONDARY CENTRAL NERVOUS SYSTEM (CNS) INVOLVEMENT IN AN INTERNATIONAL COHORT OF 62 PATIENTS WITH PRIMARY MEDIASTINAL LARGE B-CELL LYMPHOMA (PMLBCL)

**Topic:** 20. Other aggressive lymphomas

Andrés José María Ferreri*[1], Vittoria Tarantino[2], Piera Angelillo[2], Izel Okcu[3], Anna Dabrowska-Iwanicka[4], Sabrina Pelliccia[5], Maria Dimou[6], Felicetto Ferrara[7], Agnieszka Giza[8], Sara Veronica Usai[9], Pier Luigi Zinzani[10], Paolo Fiore[2], Yucai Wang[3], Alessia Castellino[11], Papageorgiou Sotirios[12], Alessandra Tucci[13], Luigi Petrucci[14], Luca Arcaini[15], Federica Cocito[16], Kate Cwynarski[17], Andrew Davies[18], Andrea Ferrario[19], Frederico Lisboa Nogueira[20], Cristina Muzi[21], Daniela Onofrillo[22], Praveen Ramakrishnan Geethkumar[23], Mariana Salvador Chalup[20], Potito Rosario Scalzulli[24], Monica Tani[25], Maria Chiara Tisi[26], Federico Erbella[2], Fabrizio Marino[2], Elena Flospergher[2], Fabiola De Mattia[2], Maurilio Ponzoni[2], Emilio Iannitto[27], Teresa Calimeri[2], Theodoros Vassilakopoulos[6]

*[1]Università Vita-Salute San Raffaele, Milano, Italy; [2]IRCCS San Raffaele Scientific Institute, Strategic Program on Lymphomas, Milano, Italy; [3]Mayo Clinic, Division of Hematology, Rochester, MN, United States of America;[4]Maria Sklodowska-Curie National Research Institute of Oncology, Dept. of Lymphoid Malignancies, Warsaw, Poland; [5]A.O.U. Sant'Andrea, Dipartimento Scienze Oncologiche, U.O.C. Ematologia, Rome, Italy; [6]Laikon General Hospital, National and Kapodistrian University of Athens, Department of Haematology and Bone Marrow Transplantation, Athens, Greece; [7]AORN Cardarelli Hospital, Division of Hematology and Stem Cell Transplantation Program, Naples, Italy; [8]Jagiellonian University Medical College, Department of Haematology, Krakow, Poland; [9]Ospedale Oncologico, Struttura Complessa di Ematologia e CTMO, Cagliari, Italy; [10]Institute of Hematology "L. e A. Seràgnoli", Lymphoma and Chronic Lymphoproliferative Syndromes Unit, Bologna, Italy; [11]AO Santa Croce e Carle, Division of Hematology, Cuneo, Italy; [12]University General Hospital "Attikon", National and Kapodistrian University of Athens, Second Department of Internal Medicine, Propaedeutic, Hematology Unit, Athens, Greece; [13]ASST Spedali Civili, UO Ematologia, Brescia, Italy; [14]University Sapienza, Hematology Department of Translation and Precision Medicine, Rome, Italy; [15]Fondazione IRCCS Policlinico San Matteo & Department of Molecular Medicine, University of Pavia, Division of Hematology, Pavia, Italy; [16]ASST Monza Ospedale San Gerardo, UO Ematologia, Monza, Italy; [17]University College London Hospitals, Department of Haematology, London, United Kingdom; [18]CRUK Centre, University of Southampton, Faculty of Medicine, Southampton Experimental Cancer Medicines Centre, Southampton, United Kingdom; [19]ASST Sette Laghi, Division of Hematology, Varese, Italy; [20]Grupo Oncoclínicas, Hematological Malignancies and Stem Cell Transplantation, Clinica Hematológica, Belo Horizonte, Brazil; [21]ASST Grande Ospedale Metropolitano Niguarda, SC Ematologia, Milano, Italy; [22]Spirito Santo Hospital, Pediatric Hematology and Oncology Unit, Department of Hematology, Pescara, Italy; [23]Harold C. Simmons Comprehensive Cancer Center, UT Southwestern Medical Center, Division of Hematologic Malignancies and Cellular Therapy, Dallas, United States of America; [24]Fondazione IRCCS "Casa Sollievo della Sofferenza", Division of Hematology, San Giovanni Rotondo, Italy; [25]Santa Maria delle Croci Hospital, Hematology Unit, Ravenna, Italy;[26]San Bortolo Hospital, Cell Therapy and Hematology, Vicenza, Italy; [27]Casa di Cura "La Maddalena", Hematology and BMT, Department of Oncology, Palermo, Italy;*

### Background

CNS involvement in PMLBCL is rare and associated with poor outcomes. The clinical features, prognosis and efficacy of modern CNS-directed therapies, including autologous transplantation (ASCT), and anti-CD19 CAR T-cell therapy (CAR-T), remain poorly defined.

### Aims

To provide management recommendations for CNS involvement in PMLBCL in routine practice.

**Copyright Information:** (Online) ISSN: 2572-9241
©2026 The Author(s). HemaSphere is published by John Wiley & Sons Ltd on behalf of European Hematology Association. This is an open access Abstract Book distributed under the Attribution-NonCommercial-NoDerivs (CC BY-NC-ND), which allows third parties to download the articles and share them with others as long as they credit the author and the Abstract Book, but they cannot change the content in any way or use them commercially.
**Abstract Book Citations:** Authors, Title, HemaSphere, 2026;10:(S1):pages. The abstract book DOIs can be found on the "Author Information" page.
**Disclaimer:** Articles published in the journal HemaSphere exclusively reflect the opinions of the authors. The authors are responsible for all content in their abstracts including accuracy of the facts, statements, citing resources, etc.

### Methods

Data on pts with PMLBCL and CNS involvement at diagnosis or relapse treated between 2001 and 2024 in 27 centers across 7 countries were analyzed (NCT06024694).

### Results

62 pts (median age 30, range 14-52; 30 males) were included. At initial PMLBCL diagnosis, most pts had bulky disease (90%), elevated LDH (82%), advanced stage (63%), extranodal disease (58%), B symptoms (47%), and aa-IPI ≥2 (66%). First-line therapy consisted of R-CHOP14/21 (n=36), R-M/VACOP-B (n=15), or R-DA-EPOCH (n=11); 16 received mediastinal irradiation. CNS prophylaxis included high-dose methotrexate (HD-MTX) in 4 pts, intrathecal chemotherapy (ITC) in 8, both in 3.
CNS involvement occurred at diagnosis in 1 (2%) pt, at first (early) relapse in 53 (85%), and at third (late) relapse in 8 (13%), with median time to CNS relapse of 7 (0-27) months. The CNS was the sole relapse site in 36 (58%) pts, all at first relapse. Lymphoma involved the brain in 51 pts, brain + meninges in 6, spinal cord in 1, meninges alone in 4.
Late CNS relapse (n=8) was uniformly terminal and associated with uncontrolled systemic disease. Among pts with early CNS relapse (n=53), 46 (87%) received curative-intent chemotherapy (chemo), including HD-MTX-based regimens in 43. Sixteen (33%) achieved complete remission (CR), while 30 had progressive disease (PD). Responders received consolidation with ASCT (n=10), brain irradiation (n=2), or both (n=4); all but two remain alive and disease-free at 23-218 months (median 90). The 30 pts with PD received brain irradiation-based therapy (n=12), ITC (n=3), high-dose ifosfamide/cytarabine-based chemo (n=8), checkpoint inhibitors (n=2), or BSC (n=5); 13 (9 after brain irradiation, 3 after ITC, 1 after chemo) achieved CR and proceeded with CAR-T (n=8), ASCT (n=4), or ibrutinib (n=1); all remain alive and disease-free at 10-132 months (median 45).
At a median follow-up of 65 (10-218) months, 12 pts with early CNS involvement remain relapse-free and 28 are alive and disease-free, with 5-year PFS and OS of 24% (95%CI=6-48) and 51% (95%CI=45-57) (Fig. A). Thirty pts treated with HD-MTX-based therapy followed by ASCT/WBRT if responsive, or by brain irradiation or ITC as bridging to CAR-T or ASCT if refractory, had a 5-year OS of 83% (95%CI=63-92), with 25 alive and disease-free.
OS improved in the last decade (5-year: 67% vs. 38%; p=0.04) (Fig. B), coinciding with increased use of MATRix, ASCT and CAR-T. Multivariable analysis confirmed improved OS with treatment in 2015-2024 (HR=0.42), early CNS relapse (HR=0.21), and upfront HD-MTX use (HR=0.13), while meningeal or systemic disease did no impact outcome. Results were unchanged when multivariable analysis was limited to pts with early CNS relapse.

### Summary/Conclusion

In the largest series reported to date, durable remissions were achieved in PMLBCL pts with early CNS relapse treated with HD-MTX–based chemo followed by ASCT and/or brain irradiation. CAR-T, preceded by brain irradiation or ITC as bridging, provides long-term disease control even in HD-MTX-refractory pts. In contrast, late CNS involvement in multiply relapsed PMLBCL has a dismal prognosis.

**Copyright Information:** (Online) ISSN: 2572-9241
©2026 The Author(s). HemaSphere is published by John Wiley & Sons Ltd on behalf of European Hematology Association. This is an open access Abstract Book distributed under the Attribution-NonCommercial-NoDerivs (CC BY-NC-ND), which allows third parties to download the articles and share them with others as long as they credit the author and the Abstract Book, but they cannot change the content in any way or use them commercially.
**Abstract Book Citations:** Authors, Title, HemaSphere, 2026;10:(S1):pages. The abstract book DOIs can be found on the “Author Information” page.
**Disclaimer:** Articles published in the journal HemaSphere exclusively reflect the opinions of the authors. The authors are responsible for all content in their abstracts including accuracy of the facts, statements, citing resources, etc.

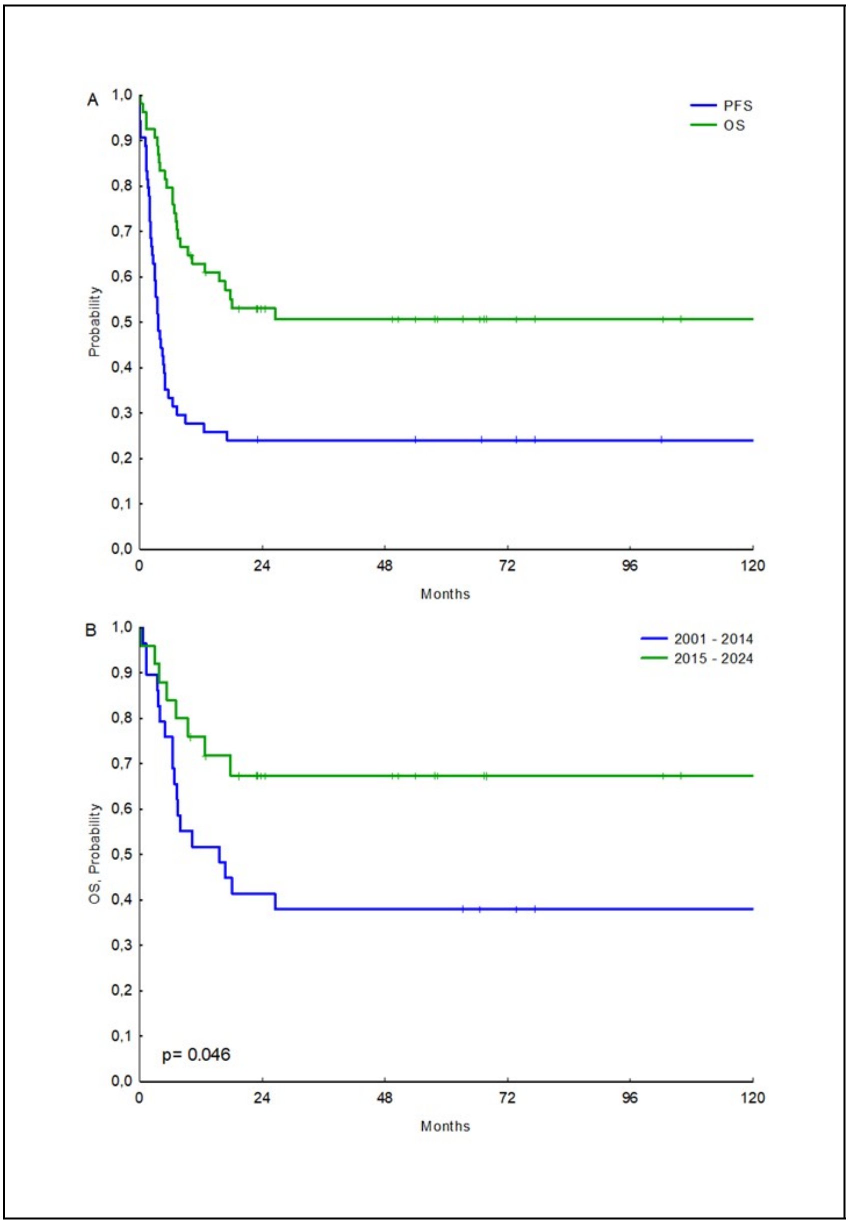

**Copyright Information:** (Online) ISSN: 2572-9241
©2026 The Author(s). HemaSphere is published by John Wiley & Sons Ltd on behalf of European Hematology Association. This is an open access Abstract Book distributed under the Attribution-NonCommercial-NoDerivs (CC BY-NC-ND), which allows third parties to download the articles and share them with others as long as they credit the author and the Abstract Book, but they cannot change the content in any way or use them commercially.
**Abstract Book Citations:** Authors, Title, HemaSphere, 2026;10:(S1):pages. The abstract book DOIs can be found on the "Author Information" page.
**Disclaimer:** Articles published in the journal HemaSphere exclusively reflect the opinions of the authors. The authors are responsible for all content in their abstracts including accuracy of the facts, statements, citing resources, etc.

### (PF1041) TABELECLEUCEL FOR ALLOGENEIC OR SOLID ORGAN TRANSPLANT RECIPIENTS WITH EBV+ PTLD AFTER FAILURE OF RITUXIMAB OR RITUXIMAB PLUS CHEMOTHERAPY: PAEDIATRIC SUBGROUP ANALYSIS OF THE PHASE 3 ALLELE STUDY

**Topic:** 20. Other aggressive lymphomas

Sonali Chaudhury[1], Ran Reshef[2], Sarah Nikiforow[3], Diane George[2], Neena Kapoor[4], Joerg Krueger[5], Michael Kent[6], Theresa Cole[7], Pere Barba*[8, 9], Eduardo Olavarria[10], Roberta Valenti[11], Anke Friedetzky[12], Sandrine Roye[12], Susan Prockop[13, 14]

*[1]Lurie Children's Hospital, Chicago, IL, United States of America;[2]Columbia University Irving Medical Center, New-York, United States of America; [3]Dana-Farber Cancer Institute, Boston, United States of America; [4]Cancer and Blood Disease Institute, Children's Hospital Los Angeles, Los Angeles, United States of America; [5]The Hospital for Sick Children, Toronto, Canada; [6]Atrium Health Levine Childrens, Charlotte, United States of America;[7]Children's Cancer Centre, Royal Children's Hospital, Melbourne, Australia; [8]Hospital Universitari Vall d'Hebron-Universitat Autónoma de Barcelona, Barcelona, Spain;[9]Cell Engineering and Hematologic Immunotherapy lab. Vall Hebron Institute of Oncology, Barcelona, Spain; [10]Imperial College Healthcare NHS Trust, London, United Kingdom; [11]Pierre Fabre Médicament, Boulogne-Billancourt, France; [12]Institut de Recherche Pierre Fabre, Toulouse, France; [13]Dana Farber/Boston Children's Hospital Cancer and Blood Disorders Center, Boston, United States of America; [14]Harvard Medical School, Boston, United States of America;*

### Background

Epstein–Barr virus-associated post-transplant lymphoproliferative disease (EBV+ PTLD) is an ultra-rare complication with low survival if initial treatment fails. Tabelecleucel is an off-the-shelf allogenic EBV-specific cytotoxic T cell therapy. Results in the overall population have been presented previously (Ghobadi et al. ASH 2024).

### Aims

We report results from 12 paediatric patients who have participated in the ALLELE trial.

### Methods

ALLELE is a multicentre, open-label Phase 3 trial including patients of any age. Patients received intravenous tabelecleucel at 2 x $10^6$ cells/kg on days 1, 8, and 15 in 35-day cycles. Response was evaluated by independent oncologic response adjudication based on clinical and radiographic evidence. After treatment, disease status was evaluated for up to 2 years and survival status up to 5 years. The primary objective was objective response rate (ORR). Secondary objectives included rates of complete response (CR), partial response (PR), time to response (TTR), duration of response (DOR), and overall survival (OS). Safety analyses were conducted in all patients who received ≥1 dose of tabelecleucel.

**Copyright Information:** (Online) ISSN: 2572-9241
©2026 The Author(s). HemaSphere is published by John Wiley & Sons Ltd on behalf of European Hematology Association. This is an open access Abstract Book distributed under the Attribution-NonCommercial-NoDerivs (CC BY-NC-ND), which allows third parties to download the articles and share them with others as long as they credit the author and the Abstract Book, but they cannot change the content in any way or use them commercially.
**Abstract Book Citations:** Authors, Title, HemaSphere, 2026;10:(S1):pages. The abstract book DOIs can be found on the "Author Information" page.
**Disclaimer:** Articles published in the journal HemaSphere exclusively reflect the opinions of the authors. The authors are responsible for all content in their abstracts including accuracy of the facts, statements, citing resources, etc.

### Results

At data cutoff on 10 September 2024, 86 patients ranging in age from 2.7–81.5 years were enrolled and treated with tabelecleucel. Among patients, 12 were <17 years of age, 3 had hematopoietic cell transplantation (HCT) and 9 solid organ transplant (SOT). The median (range) age of paediatric patients was 8.8 (2.7–16.8) years. Two patients were aged 2–<6 years, 5 were 6–<12 years and 5 were 12–<17 years. At the time of initial PTLD diagnosis, there were 9 patients with stage III (n=4) or stage IV (n=5) disease (Lugano classification, LYRIC modification), and 3 with unknown stage. All 12 patients had extranodal disease and 8 had lymph node disease at screening. The median (range) time from PTLD diagnosis to first tabelecleucel dose was 3.0 (0.6–43.8) months. The ORR in paediatric patients was 50.0% (6/12 patients): CR was reported in 4/12 patients (1 HCT and 3 SOT), and PR was observed in 2/12 patients (1 HCT and 1 SOT). For the 6 responders, the median (range) TTR was 1.6 (0.9–4.7) months. Two of the responders had a durable response of >6 months. At the time of data cutoff, 3 of the responders were censored with no disease progression or death. After a median (range) follow-up time of 5.2 (1.1–52.7) months, at the time of data cutoff, 50% (6/12) of patients were still alive, including 5 responders and 1 non-responder. Six patients developed treatment-emergent serious adverse events (TESAEs). Fatal SAEs were reported in 2 patients, none were related to treatment. 4 TESAEs (in 2 patients) were considered treatment-related. One SOT patient reported grade 1 pyrexia, considered a sign of possible treatment-related cytokine release syndrome. There were no reports of tumour flare reactions, infusion reactions, immune effector cell associated neurotoxicity, transmission of infectious agent, graft vs host disease, or transplant rejection. Tabelecleucel showed a favourable safety profile in the paediatric population.

### Summary/Conclusion

This analysis of 12 paediatric patients from the ALLELE trial suggests that the risk/benefit profile in the paediatric subgroup is consistent with that reported in the overall population. These findings support the use of tabelecleucel in paediatric patients with refractory/relapsed EBV+ PTLD who have poor survival and limited treatment options.

**Copyright Information:** (Online) ISSN: 2572-9241
©2026 The Author(s). HemaSphere is published by John Wiley & Sons Ltd on behalf of European Hematology Association. This is an open access Abstract Book distributed under the Attribution-NonCommercial-NoDerivs (CC BY-NC-ND), which allows third parties to download the articles and share them with others as long as they credit the author and the Abstract Book, but they cannot change the content in any way or use them commercially.
**Abstract Book Citations:** Authors, Title, HemaSphere, 2026;10:(S1):pages. The abstract book DOIs can be found on the "Author Information" page.
**Disclaimer:** Articles published in the journal HemaSphere exclusively reflect the opinions of the authors. The authors are responsible for all content in their abstracts including accuracy of the facts, statements, citing resources, etc.

### (PF1042) MITOXANTRONE HYDROCHLORIDE LIPOSOME-BASED CMOP±R REGIMEN FOR PREVIOUSLY UNTREATED NON-HODGKIN LYMPHOMA: RESULTS FROM A PHASE 3 MULTICENTER STUDY

**Topic:** 20. Other aggressive lymphomas

Tao You[1], Zhou Jin[2], Qian Wang[2], Xiangping Zong[2], Yun Wang[2], Xiao MA[2], Depei Wu[2], Haiwen Huang*[2]

*[1]The First Affiliated Hospital of Soochow University, Department of Hematology, Suzhou, China;[2]The First Affiliated Hospital Of Soochow University, Hematology, Suzhou, China;*

### Background

Non-Hodgkin lymphoma (NHL) is one of the most common hematologic malignancies in China. Conventional anthracycline-based regimens (e.g., R-CHOP) are associated with cumulative cardiotoxicity. Mitoxantrone hydrochloride liposome (Lipo-MIT), a novel liposomal formulation, offers improved cardiac safety. The CMOP±R regimen (cyclophosphamide, Lipo-MIT, vincristine, prednisone ± rituximab) showed promising results in a phase 2 study (ORR 84.6% in FAS; 97.1% in EAS).

### Aims

To evaluate the efficacy and safety of CMOP±R as first-line treatment for previously untreated NHL in a larger phase 3 cohort.

### Methods

This multicenter, single-arm, prospective phase 3 study enrolled patients aged 18–75 years with previously untreated NHL and ECOG PS 0–2. Patients received CMOP±R every 21 days for up to 6 cycles. The primary endpoint was ORR in the efficacy analysis set (EAS), assessed per Lugano 2014 criteria. Secondary endpoints included CR rate, PFS, OS, and safety. ORR/CR rates were calculated using the Clopper-Pearson method; PFS/OS were estimated by Kaplan-Meier analysis. A matching-adjusted indirect comparison (MAIC) was performed versus R-CHOP (DLBCL) and CHOP (PTCL).

### Results

Of 92 screened patients, 82 were enrolled (FAS/SS, n=82; data cutoff: January 2026). The median age was 59 years (range, 24–75), and 59.8% were female. Histologic subtypes included DLBCL (56.1%), FL (15.9%), and PTCL-NOS (13.4%). Stage III–IV disease was present in 53.7%, 32.9% had IPI ≥3, and 70.7% were CD20-positive. In the efficacy analysis set (EAS, n=48), ORR was 89.6% (95% CI, 77.3–96.5), with a CR rate of 50.0% (95% CI, 35.2–64.8). After a median follow-up of 8.7 months, median PFS and OS were not reached. The 12-month PFS and OS rates were 87.6% (95% CI, 76.8–93.6) and 94.5% (95% CI, 85.8–97.9), respectively. Grade ≥3 TEAEs included neutropenia (35.4%), leukopenia (30.5%), thrombocytopenia (12.2%), and anemia (11.0%). No clinically meaningful changes in LVEF were observed (66% at baseline and post-treatment; p=0.275), and no treatment-related cardiac deaths occurred. In the MAIC analysis, after reweighting, CMOP+R showed comparable ORR to R-CHOP in DLBCL (87.0% vs 82.5%), while CMOP demonstrated higher ORR than CHOP in PTCL (76.9% vs 64.0%).

### Summary/Conclusion

CMOP±R demonstrates high efficacy (ORR 89.6%, CR 50.0%) and a manageable safety profile in previously untreated NHL, with excellent cardiac safety. These phase 3 results confirm and extend the phase 2 findings, supporting CMOP±R as an effective and cardio-safe first-line treatment option for NHL patients, particularly those with cardiac risk factors.

**Copyright Information:** (Online) ISSN: 2572-9241
©2026 The Author(s). HemaSphere is published by John Wiley & Sons Ltd on behalf of European Hematology Association. This is an open access Abstract Book distributed under the Attribution-NonCommercial-NoDerivs (CC BY-NC-ND), which allows third parties to download the articles and share them with others as long as they credit the author and the Abstract Book, but they cannot change the content in any way or use them commercially.
**Abstract Book Citations:** Authors, Title, HemaSphere, 2026;10:(S1):pages. The abstract book DOIs can be found on the “Author Information” page.
**Disclaimer:** Articles published in the journal HemaSphere exclusively reflect the opinions of the authors. The authors are responsible for all content in their abstracts including accuracy of the facts, statements, citing resources, etc.

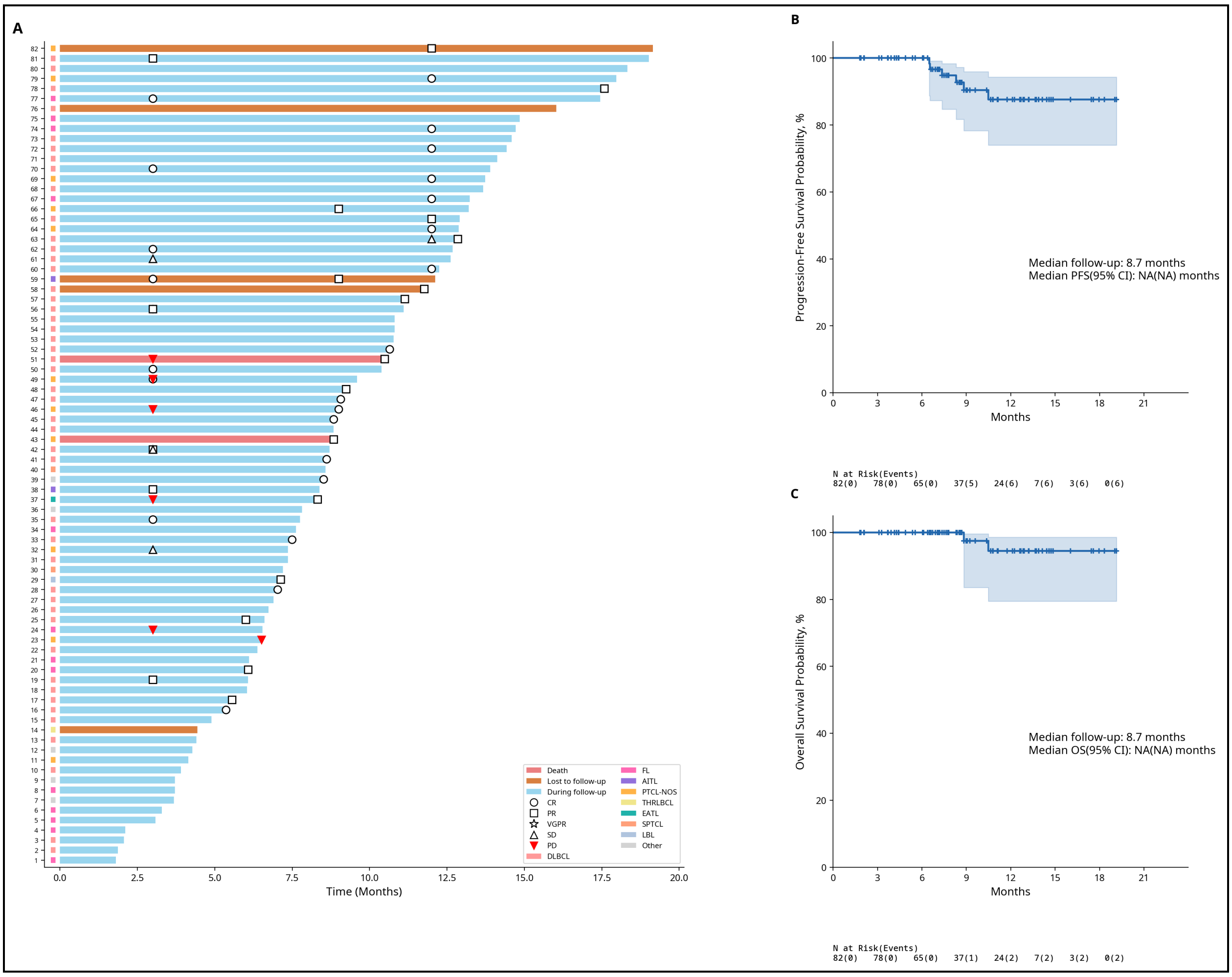

Figure. Swimmer plot and Kaplan-Meier survival curves for patients treated with CMOP regimen.

**Copyright Information:** (Online) ISSN: 2572-9241
©2026 The Author(s). HemaSphere is published by John Wiley & Sons Ltd on behalf of European Hematology Association. This is an open access Abstract Book distributed under the Attribution-NonCommercial-NoDerivs (CC BY-NC-ND), which allows third parties to download the articles and share them with others as long as they credit the author and the Abstract Book, but they cannot change the content in any way or use them commercially.
**Abstract Book Citations:** Authors, Title, HemaSphere, 2026;10:(S1):pages. The abstract book DOIs can be found on the "Author Information" page.
**Disclaimer:** Articles published in the journal HemaSphere exclusively reflect the opinions of the authors. The authors are responsible for all content in their abstracts including accuracy of the facts, statements, citing resources, etc.

### (PF1043) FINAL RESULTS OF THE PHASE II S-IDE STUDY: ADDING SELINEXOR TO IFOSFAMIDE, ETOPOSIDE, AND DEXAMETHASONE DID NOT IMPROVE OUTCOMES IN RELAPSED/REFRACTORY PERIPHERAL T-CELL LYMPHOMAS

**Topic:** 20. Other aggressive lymphomas

Annalisa Chiappella*[1], Anna Dodero[1], Chiara Pagani[2], Silva Ljevar[3], Claudia Castellino[4], Michele Clerico[5], Silvia Gambara[6], Luca Arcaini[7], Pellegrino Musto[8], Jacopo Olivieri[9], Anna Guidetti[1, 10], Rosalba Miceli[3], Anisa Bermema[1], Cristiana Carniti[1], Paolo Corradini[1, 10]

*[1]Hematology, Fondazione IRCCS Istituto Nazionale dei Tumori, Milano, Italy; [2]Hematology Division, ASST Spedali Civili di Brescia, Brescia, Italy; [3]Unit of Biostatistics for Clinical Research, Department of Data Science, Fondazione IRCCS Istituto Nazionale dei Tumori, Milano, Italy; [4]Division of Hematology - AO S. Croce e Carle, Cuneo, Italy;[5]Division of Hematology and Stem Cell Transplant Unit, University of Torino, AOU Città della Salute e della Scienza, Torino, Italy; [6]Hematology, Azienda USL-IRCCS, Reggio Emilia, Italy; [7]Department of Molecular Medicine, University of Pavia & Division of Hematology, Fondazione IRCCS Policlinico San Matteo, Pavia, Italy; [8]Department of Precision and Regenerative Medicine and Ionian Area, "Aldo Moro" University School of Medicine, and Unit of Hematology and Stem Cell Transplantation, AOUC Policlinico, Bari, Italy; [9]Clinica Ematologica, Centro Trapianti e Terapie Cellulari "Carlo Melzi", Azienda Sanitaria Universitaria Friuli Centrale, Udine, Italy; [10]Chair of Hematology, University of Milano, Milano, Italy;*

### Background

Relapsed/refractory (R/R) peripheral T-cell lymphomas (PTCLs), excluding CD30+ ALK positive, have a poor prognosis, with overall response rate [ORR, complete (CR), plus partial (PR)] of 30-40% and a median progression-free survival (PFS) and overall survival (OS) of 3 and 5.5 months. Selinexor, a selective inhibitor of nuclear exportin that is overexpressed in PTCLs, showed promising results (ORR 91%) when combined with dexamethasone, ifosfamide, carboplatin and etoposide, in a small phase I trial (Tang, Haematol 2021). Based on these data, we conducted the investigator-initiated, non-profit multicenter pilot phase II study PTCL S-IDE (selinexor, ifosfamide, etoposide, dexamethasone; carboplatin omitted to reduce hematologic toxicity); EudraCT 2021-006229-23.

### Aims

To evaluate ORR after 4 cycles of S-IDE in R/R PTCLs [PTCL-NOS, angioimmunoblastic T cell (AITL), anaplastic ALK negative (ALK neg), T follicular helper (TFH)] after at least one anthracycline containing regimen, with or without prior high dose chemotherapy and stem cell transplantation (SCT).

### Methods

R/R PTCLs aged 18-75 years were enrolled to receive 4 cycles of 21-day S-IDE: selinexor (40 mg on day 3, 5 and 7); ifosfamide 5g/mq on day 2; etoposide 100 mg/mq on days 1-3; dexamethasone 20 mg/day on days 3-7. Patients achieving CR after 4 S-IDE proceeded to allo-SCT or auto-SCT based on donor availability, prior SCT, age and comorbidities; patients ineligible for SCT could receive selinexor maintenance (60 mg weekly) until progression or unacceptable toxicity. Primary endpoint was ORR at the end of 4 courses of S-IDE; secondary endpoints were 18-months PFS and OS. A sample size of 30 patients provided 94% power (two-sided one-sample binomial test, $\alpha$=0.05) to detect an ORR increase from 50% (null) to 80% (alternative).

**Copyright Information:** (Online) ISSN: 2572-9241
©2026 The Author(s). HemaSphere is published by John Wiley & Sons Ltd on behalf of European Hematology Association. This is an open access Abstract Book distributed under the Attribution-NonCommercial-NoDerivs (CC BY-NC-ND), which allows third parties to download the articles and share them with others as long as they credit the author and the Abstract Book, but they cannot change the content in any way or use them commercially.
**Abstract Book Citations:** Authors, Title, HemaSphere, 2026;10:(S1):pages. The abstract book DOIs can be found on the "Author Information" page.
**Disclaimer:** Articles published in the journal HemaSphere exclusively reflect the opinions of the authors. The authors are responsible for all content in their abstracts including accuracy of the facts, statements, citing resources, etc.

**Results**

From August 2022 to April 2025, 30 patients were enrolled in 9 Italian centers, and were evaluable for response. Median age was 66 years (IQR 60;71); 83% (25) had stage III-IV; 47% (14) were PTCL-NOS, 27% (8) ALK neg, 10% (3) AITL and 16% (5) TFH. All patients received one prior line of therapy, 30% (9) had failed prior auto-SCT, and 46% (14) were primary refractory. After induction, ORR was 43% (13), with CR 33% (10). Among the 10 patients in CR, 6 underwent allo-SCT consolidation; 1 elderly patient started selinexor maintenance and remains on therapy at cycle 22; 3 did not proceed to transplant (donor unavailability, n=1; comorbidities, n=2). Of the patients not in CR at the end of S-IDE, 3 subsequently underwent allo-SCT after additional salvage therapy. At the time of analysis, all transplanted patients were alive and in ongoing CR. At a median follow-up of 13 months (IQR: 6.81, 16.61), the median PFS was 4.13 months, with 12-months PFS 30.3% (95% CI: 16.9%-54.3%) and the median OS was 7.27 months, with 12-months OS 41.8% (95% CI: 26.4%-66.1%). The most frequent toxicities were hematological, in 57% (17) of the patients. Treatment related mortality was 10% (3) patients: one due to West Nile infection not related to the treatment, and 2 due to pneumonia.

**Summary/Conclusion**

In this pilot phase II study S-IDE, the primary end-point was not met, with ORR of 43% at the end of induction. Overall, S-IDE did not improve outcomes in R/R PTCLs compared with currently available salvage approaches.

**Copyright Information:** (Online) ISSN: 2572-9241
©2026 The Author(s). HemaSphere is published by John Wiley & Sons Ltd on behalf of European Hematology Association. This is an open access Abstract Book distributed under the Attribution-NonCommercial-NoDerivs (CC BY-NC-ND), which allows third parties to download the articles and share them with others as long as they credit the author and the Abstract Book, but they cannot change the content in any way or use them commercially.
**Abstract Book Citations:** Authors, Title, HemaSphere, 2026;10:(S1):pages. The abstract book DOIs can be found on the "Author Information" page.
**Disclaimer:** Articles published in the journal HemaSphere exclusively reflect the opinions of the authors. The authors are responsible for all content in their abstracts including accuracy of the facts, statements, citing resources, etc.

### (PF1044) MOLECULAR LANDSCAPE OF PEDIATRIC B-CELL LYMPHOBLASTIC LYMPHOMA

**Topic:** 20. Other aggressive lymphomas

Ekaterina Romanenko*[1], Alexey Kulikov[2], Egor Volchkov[1, 3, 4], Dmitry Abramov[1], Maria Gridina[5], Veniamin Fishman[5], Nikita Torgunakov[5], Natalia Miakova[1]

*[1]Dmitry Rogachev National Medical Research Center Of Pediatric Hematology, Oncology and Immunology, Moscow, Russia; [2]Lomonosov Moscow State University, Moscow, Russia; [3]Research Institute of Molecular and Cellular Medicine, RUDN University, Moscow, Russia; [4]Botkin Hospital, Moscow, Russia; [5]Institute of Cytology and Genetics, Novosibirsk, Russia;*

### Background

Pediatric B-cell lymphoblastic lymphoma (B-LBL) is a rare and aggressive subtype of non-Hodgkin lymphoma that typically presents as an extramedullary tumor with limited bone marrow involvement (<25% blasts). Despite strong biological similarities between B-LBL and B-cell acute lymphoblastic leukemia (B-ALL), treatment stratification in B-LBL remains largely based on clinical stage rather than molecular features. In contrast, risk assessment in B-ALL incorporates recurrent chromosomal abnormalities and gene deletions, including alterations of *IKZF1*, *ERG*, and other leukemia-associated genes with established prognostic value. Whether similar molecular markers may improve prognostic stratification in pediatric B-LBL remains insufficiently studied.

### Aims

To characterize the genetic landscape of pediatric B-LBL and evaluate the potential prognostic relevance of molecular alterations.

### Methods

Sixty-five pediatric patients with a median age of 7.9 years (range 0.4-17.1), diagnosed with B-LBL between 2002 and 2025 and with available tumor samples were included in the analysis. The male-to-female ratio was 1.1:1. Copy number alterations were analyzed using multiplex ligation-dependent probe amplification (MLPA) with SALSA Probemix P335-C2 ALL-IKZF1 and P202-C1 IKZF1-ERG kits (MRC-Holland, The Netherlands). Genome-wide structural variant analysis using modified Hi-C whole-genome sequencing (Gridina et al., 2024) was additionally performed in 20 patients. Genetic risk groups were assigned according to the Moorman et al. (2014) classification. The median follow-up duration was 1.6 years.

### Results

The 5-year event-free survival (EFS) and overall survival (OS) were 65% (95% CI: 50–84%) and 87% (95% CI: 75–100%), respectively. No significant association with outcome was observed for hyperdiploidy, *IKZF1* deletion (isolated or combined with *ERG* deletion), other recurrent copy number alterations, or Moorman genetic risk groups. Hi-C–based analysis identified structural rearrangements in the majority of evaluated cases, including *ETV6* rearrangements, *PAX5::TLE5*, *TCF3::PBX1*, *MYC::IGH*, *BCR::ABL1*, *RTF1::NUTM1*, *MLLT10::PICALM*, and *IGH* rearrangements with unidentified partners. Among them, patients harboring *MYC::IGH*, *BCR::ABL1* experienced disease relapse. The *MYC::IGH* fusion, classically associated with Burkitt lymphoma, reflects highly aggressive tumor biology and may contribute to unfavorable outcomes in B-LBL. The presence of *BCR::ABL1* represents a therapeutically actionable alteration requiring incorporation of tyrosine kinase inhibitors, as outcomes without targeted therapy are historically inferior. These findings emphasize the clinical relevance of comprehensive genomic profiling for identification of biologically high-risk subtypes

**Copyright Information:** (Online) ISSN: 2572-9241
©2026 The Author(s). HemaSphere is published by John Wiley & Sons Ltd on behalf of European Hematology Association. This is an open access Abstract Book distributed under the Attribution-NonCommercial-NoDerivs (CC BY-NC-ND), which allows third parties to download the articles and share them with others as long as they credit the author and the Abstract Book, but they cannot change the content in any way or use them commercially.
**Abstract Book Citations:** Authors, Title, HemaSphere, 2026;10:(S1):pages. The abstract book DOIs can be found on the "Author Information" page.
**Disclaimer:** Articles published in the journal HemaSphere exclusively reflect the opinions of the authors. The authors are responsible for all content in their abstracts including accuracy of the facts, statements, citing resources, etc.

**Summary/Conclusion**

Prognostic genetic markers established in pediatric B-ALL were not confirmed to be predictive in pediatric B-LBL, suggesting that direct transfer of leukemia-based molecular risk models may be inappropriate. At the same time, genome-wide analysis revealed clinically important structural alterations, including potentially high-risk and targetable rearrangements. Comprehensive molecular characterization of pediatric B-LBL is therefore essential for defining biological disease subtypes and developing risk-adapted therapeutic approaches. Further studies in larger cohorts are required to refine molecular stratification in this rare disease.

**Copyright Information:** (Online) ISSN: 2572-9241
©2026 The Author(s). HemaSphere is published by John Wiley & Sons Ltd on behalf of European Hematology Association. This is an open access Abstract Book distributed under the Attribution-NonCommercial-NoDerivs (CC BY-NC-ND), which allows third parties to download the articles and share them with others as long as they credit the author and the Abstract Book, but they cannot change the content in any way or use them commercially.
**Abstract Book Citations:** Authors, Title, HemaSphere, 2026;10:(S1):pages. The abstract book DOIs can be found on the "Author Information" page.
**Disclaimer:** Articles published in the journal HemaSphere exclusively reflect the opinions of the authors. The authors are responsible for all content in their abstracts including accuracy of the facts, statements, citing resources, etc.

### (PF1045) AN OPEN LABEL, PHASE 2 TRIAL EVALUATING DARATUMUMAB-BORTEZOMIB-DEXAMETHASONE COMBINATION IN RELAPSED OR REFRACTORY PLASMABLASTIC LYMPHOMA: THE DALYA TRIAL ON BEHALF OF THE FONDAZIONE ITALIANA LINFOMI

**Topic:** 20. Other aggressive lymphomas

Andrés José María Ferreri*[1, 2], Emanuele Ravano[3], Federico Erbella[1], Luisa Verga[4], Angela Ferrari[5], Roberta Battistini[6], Guido Gini[7], Francesco Piazza[8], Luca Nassi[9], Francesca Maria Quaglia[10], Agostino La Porta[11], Alessandro Nonis[1, 12], Fabio Facchetti[13, 14], Maurilio Ponzoni[1, 15], Michele Spina[16], Alessandro Re[17]

*[1]IRCCS San Raffaele Scientific Institute, Strategic Program on Lymphomas, Milano, Italy; [2]Università Vita-Salute San Raffaele, Milano, Italy; [3]ASST Grande Ospedale Metropolitano Niguarda, SC Ematologia, Milano, Italy; [4]ASST Monza Ospedale San Gerardo, UO Ematologia, Monza, Italy; [5]Azienda Unità Sanitaria Locale-IRCCS, Arcispedale Santa Maria Nuova, UO Ematologia, Reggio Emilia, Italy; [6]Azienda Ospedaliera San Camillo Forlanini, UO Ematologia, Roma, Italy; [7]AOU delle Marche, Università Politecnica delle Marche, Clinic of Hematology, Ancona, Italy; [8]Azienda Ospedale - Università di Padova, Dipartimento di Medicina, UOC di Ematologia, Padova, Italy; [9]Azienda Ospedaliera Universitaria Careggi, UOC di Ematologia, Firenze, Italy; [10]Azienda Ospedaliero Universitaria Integrata di Verona, UO di Ematologia, Verona, Italy; [11]Fondazione Italiana Linfomi ETS, sede di Modena, Area Gestione Studi, Alessandria, Italy; [12]Centro Universitario di Statistica nelle Scienze Biomediche, Università Vita-Salute San Raffaele, Milano, Italy; [13]Università di Brescia, Dipartimento di medicina Molecolare e Traslazionale, I Servizio di Anatomia Patologica, Brescia, Italy; [14]Dipartimento di Medicina Molecolare e Traslazionale, I Servizio di Anatomia Patologica, Brescia, Italy; [15]Unità Operativa di Anatomia e Istologia Patologica, Milano, Italy; [16]Centro di Riferimento Oncologico di Aviano, Aviano, Italy; [17]ASST Spedali Civili, UO Ematologia, Brescia, Italy;*

### Background

Plasmablastic lymphoma (PBL) is a rare, aggressive B-cell malignancy often associated with HIV infection. International guidelines recommend intensified chemotherapy (i.e., EPOCH, CODOX-M/IVAC, HyperCVAD) and consideration of autologous stem cell transplantation (ASCT) in CR1; however, these recommendations lack prospective evidence. Prognosis is poor in both HIV-negative and HIV-positive patients, with frequent relapses and a median post-relapse survival of ~3 months. The molecular overlap between PBL and multiple myeloma, particularly high expression of plasma cell markers (i.e., CD38, CD138, MUM1), suggests that anti-myeloma agents may be effective in PBL. On this basis, we conducted a phase 2 trial (DALYA) addressing daratumumab, bortezomib, and dexamethasone as salvage therapy for relapsed/refractory (R/R) PBL.

### Aims

To assess the activity and tolerability of daratumumab, bortezomib, and dexamethasone in patients with R/R PBL. Here, we report the first-step analysis.

**Copyright Information:** (Online) ISSN: 2572-9241
©2026 The Author(s). HemaSphere is published by John Wiley & Sons Ltd on behalf of European Hematology Association. This is an open access Abstract Book distributed under the Attribution-NonCommercial-NoDerivs (CC BY-NC-ND), which allows third parties to download the articles and share them with others as long as they credit the author and the Abstract Book, but they cannot change the content in any way or use them commercially.
**Abstract Book Citations:** Authors, Title, HemaSphere, 2026;10:(S1):pages. The abstract book DOIs can be found on the “Author Information” page.
**Disclaimer:** Articles published in the journal HemaSphere exclusively reflect the opinions of the authors. The authors are responsible for all content in their abstracts including accuracy of the facts, statements, citing resources, etc.

**Methods**

DALYA is a single-arm phase II trial. Eligible patients had histologically confirmed PBL per WHO 2017 criteria (CD38 expression in ≥5% tumor cells), received ≥1 prior line of chemotherapy, and had undergone ASCT or were not eligible for transplantation. Additional criteria were ECOG PS ≤3, age ≥18 ys, and ≥1 measurable lesion. Both HIV-negative and HIV-positive patients were eligible, provided HIV infection was controlled with cART. Treatment was administered as detailed in the Table. The primary endpoint was overall response rate (ORR; CR + PR), assessed after the first course and every three courses thereafter. Best response per Lugano 2014 criteria was analysed. Toxicity was graded per CTCAE. The null hypothesis assumed an ORR of 15%, tested against a one-sided alternative hypothesis of 35%. The planned sample size was 28 patients (type I error 0.05; power 0.80), using Simon's two-stage design. In the first stage, 15 patients were enrolled; if >2 responses were observed, 13 additional patients would be accrued. The regimen would be considered active with ≥8 responses overall.

**Results**

Fifteen patients (median age 62, range 33-80; 14 males) were included in the first-step analysis; 3 had HIV infection. Most patients had advanced stage (n=13), increased LDH (n=11), extranodal disease (n=12), and IPI ≥2 (n=15). Median number of prior lines was 1 (range 1-3); one pt had prior ASCT.
The primary endpoint was met: 2 patients achieved CR and 4 had PR, yielding an ORR of 40% (95%CI=15-65). Notably, 4 responses were achieved after the first course, which consisted of daratumumab single agent.
Treatment was generally well tolerated. Eighty-one adverse events (AEs) were reported: 68 (83%) were grade 1-2 and 13 (16%) were grade ≥3. Among grade ≥3 AEs, 9 were hematologic and 4 non-hematologic (infections). All but one pt recovered; one pt died of septic shock. No treatment interruptions or clinically relevant delays occurred.

**Summary/Conclusion**

Daratumumab-bortezomib-dexamethasone met predefined efficacy criteria in the first step of the DALYA trial, showed acceptable toxicity, and supports further investigation of these agents in PBL, including future incorporation of these drugs into frontline therapy. Accrual is ongoing.

Table. Regimen assessed in the DALYA trial

| Phase | Cycle # | Interval (days) | Daratumumab (subcutaneous) | Bortezomib (subcutaneous) | Dexamethasone (oral route) |
|---|---|---|---|---|---|
| Induction | 1 | 21 | 1800 mg days 1,8, & 15 | - | - |
| | 2-3 | 21 | 1800 mg days 1,8, & 15 | 1,3 mg/m$^2$ days 1,4,8, &11 | 20 mg ds 1,2,4,5,8,9,11,12 |
| | 4-9 | 28 | 1800 mg on day 1 | 1,3 mg/m$^2$ days 1,4,8, &11 | 20 mg days 1,2,4,8,11 |
| Maintenance | 10-15 | 28 | 1800 mg on day 1 | - | - |

**Copyright Information:** (Online) ISSN: 2572-9241
©2026 The Author(s). HemaSphere is published by John Wiley & Sons Ltd on behalf of European Hematology Association. This is an open access Abstract Book distributed under the Attribution-NonCommercial-NoDerivs (CC BY-NC-ND), which allows third parties to download the articles and share them with others as long as they credit the author and the Abstract Book, but they cannot change the content in any way or use them commercially.
**Abstract Book Citations:** Authors, Title, HemaSphere, 2026;10:(S1):pages. The abstract book DOIs can be found on the "Author Information" page.
**Disclaimer:** Articles published in the journal HemaSphere exclusively reflect the opinions of the authors. The authors are responsible for all content in their abstracts including accuracy of the facts, statements, citing resources, etc.

### (PF1046) REAL-WORLD CHARACTERISTICS AND SURVIVAL OUTCOMES OF AGGRESSIVE NATURAL KILLER CELL LEUKEMIA IN THE UNITED STATES

**Topic:** 20. Other aggressive lymphomas

Cher Ying Foo*[1], Ojasav Sehrawat[1], Waqar Haque[2], Dua Azim[1], Bin S Teh[2]

*[1]Rochester General Hospital, Internal Medicine, Rochester, United States of America;[2]Houston Methodist Hospital, Radiation Oncology, Houston, United States of America;*

### Background

Aggressive natural killer cell leukemia (ANKL) is a rare and highly aggressive hematologic malignancy characterized by systemic proliferation of mature NK cells, and is frequently associated with Epstein-Barr virus. Patients with ANKL exhibit a fulminant clinical course with rapidly progressive multiorgan involvement, leading to a poor prognosis and median overall survival (OS) of a few months after diagnosis. Due to the rarity of this disease, contemporary clinical data are mostly limited to small institutional cohorts, predominantly from Asia. Real-world treatment patterns and survival outcomes of patients with ANKL in the United States remain poorly characterized.

### Aims

We conducted the first national population-level study to characterize the clinical characteristics, treatment patterns, and outcomes for patients with ANKL in the United States.

### Methods

The National Cancer Database (NCDB) was queried for patients with ANKL diagnosed between 2004 to 2023. Only cases with pathologic and/or cytologic confirmation were included to reduce diagnostic misclassification. Baseline demographic, socioeconomic, and treatment characteristics were described. Overall survival was assessed using Kaplan-Meier analysis and compared with log-rank tests. Univariable and multivariable Cox proportional hazards models were used to evaluate associations with survival, incorporating age, sex, race/ethnicity, insurance status, Charlson-Deyo comorbidity score, chemotherapy, and hematopoietic stem cell transplantation (HSCT).

### Results

A total of 339 patients with ANKL were identified. The median age at diagnosis was 60 years (IQR 43–72). 67.3% of patients were male. The cohort was predominantly White (79.4%), with 10.8% Asian/Pacific Islander and 9.9% Black patients. Most patients resided in metropolitan areas (81.3%), and the majority had Charlson-Deyo comorbidity score of 0 (78.2%). The median time from diagnosis to treatment was 9 days (IQR 3–26). Chemotherapy was administered in 63.3%, immunotherapy in 10.1%, and HSCT in 3.9% of patients. The median OS was 4.01 months (IQR 0.97–41.94). Male sex was associated with significantly worse survival (HR 1.33, 95% CI 1.03–1.72, p=0.028). Uninsured patients had markedly higher mortality risk compared to patients with health insurance (HR 1.90, 95% CI 1.15–3.12, p=0.049). Race, ethnicity, income, education, urbanization, and comorbidity score were not significantly associated with survival. HSCT was the strongest predictor of improved OS in both univariable (HR 0.36, 95% CI 0.17–0.77, p=0.0017) and multivariable analysis (HR 0.35, 95% CI 0.16–0.76, p=0.0021), adjusting for age, sex, comorbidity, and chemotherapy. Chemotherapy alone was not independently associated with survival benefit on multivariable analysis (HR 1.11, 95% CI 0.85–1.45, p=0.43).

**Copyright Information:** (Online) ISSN: 2572-9241
©2026 The Author(s). HemaSphere is published by John Wiley & Sons Ltd on behalf of European Hematology Association. This is an open access Abstract Book distributed under the Attribution-NonCommercial-NoDerivs (CC BY-NC-ND), which allows third parties to download the articles and share them with others as long as they credit the author and the Abstract Book, but they cannot change the content in any way or use them commercially.
**Abstract Book Citations:** Authors, Title, HemaSphere, 2026;10:(S1):pages. The abstract book DOIs can be found on the "Author Information" page.
**Disclaimer:** Articles published in the journal HemaSphere exclusively reflect the opinions of the authors. The authors are responsible for all content in their abstracts including accuracy of the facts, statements, citing resources, etc.

**Summary/Conclusion**

In this largest national cohort to date, ANKL demonstrated poor survival, with median OS of only four months. Chemotherapy remained the primary treatment modality, however it was not associated with survival benefit. HSCT was the only treatment modality independently associated with improved survival, underscoring the need for early transplant evaluation in eligible patients. Insurance-based disparities in outcomes highlight access-to-care barriers in this rare disease. These findings emphasize an urgent need for improved therapeutic strategies and equitable access to specialized care for patients with ANKL.

**Copyright Information:** (Online) ISSN: 2572-9241
©2026 The Author(s). HemaSphere is published by John Wiley & Sons Ltd on behalf of European Hematology Association. This is an open access Abstract Book distributed under the Attribution-NonCommercial-NoDerivs (CC BY-NC-ND), which allows third parties to download the articles and share them with others as long as they credit the author and the Abstract Book, but they cannot change the content in any way or use them commercially.
**Abstract Book Citations:** Authors, Title, HemaSphere, 2026;10:(S1):pages. The abstract book DOIs can be found on the "Author Information" page.
**Disclaimer:** Articles published in the journal HemaSphere exclusively reflect the opinions of the authors. The authors are responsible for all content in their abstracts including accuracy of the facts, statements, citing resources, etc.

### (PF1047) FACILITY-LEVEL DIFFERENCES TRANSLATE INTO LIVES LOST: ELDERLY PRIMARY CNS LYMPHOMA IN THE NATIONAL CANCER DATABASE

**Topic:** 20. Other aggressive lymphomas

Rosa Rios*[1], Guilherme Perini[2], Ysaith Orellana Ascencio[3], Joel Michalek[4], Qianqian Liu[4], Adolfo Diaz Duque[4]

[1]*Hospital Luis Díaz Soto, Hematology, La Habana, Cuba;* [2]*Hospital Israelita Albert Einstein, Sao Paulo, Brazil;* [3]*UT Health Houston, Houston, United States of America;* [4]*UT Health San Antonio / Mays Cancer Center, San Antonio, United States of America;*

### Background

Primary Central Nervous System Lymphoma (PCNSL) is a rare, aggressive subtype of non-Hodgkin lymphoma with increasing incidence in the U.S (ACSPMID:19273630). Outcomes in older adults remain poor; prior cohorts of patients aged >70 years reported a 2-year overall survival of 50% (NCBIPMID:32241841). Facility-level differences in care have been reported, but their impact on older adults is unclear. We compared academic vs community cancer programs in elderly PCNSL to evaluate demographic, socioeconomic, and survival differences.

### Aims

Primary Aim:
To evaluate whether treatment facility type (Academic Cancer Programs vs Community Cancer Programs) is associated with differences in overall survival (OS) among patients aged ≥75 years with primary CNS lymphoma (PCNSL) using a national database.

### Methods

A retrospective cohort study of patients diagnosed with PCNSL between 2004-2022 using the National Cancer Database (NCDB) for patients ≥75 years old. Patients were classified by treatment facility: ACP (academic/research programs including NCI-designated centers) vs. CCP (community, comprehensive community, and integrated network cancer programs). Demographic, clinical, and treatment characteristics were compared using chi-square and Wilcoxon tests. Kaplan-Meier and Cox proportional hazards models were used to compare overall survival (OS), adjusting for age, race/ethnicity, insurance status, Charlson-Deyo comorbidity index, and distance traveled for care.

### Results

A total of 5,013 patients aged ≥75 years with PCNSL were identified, including 3,204 treated at ACP and 1,809 at CCP. Median age (79 years), sex distribution, comorbidity burden, and stage were similar across facility types. CCP treated a higher proportion of patients from lower-income areas and non–Medicaid expansion states (p<0.001), while ACP patients traveled farther for care (median 14.9 vs 9.2 miles, p<0.001).
Treatment delivery differed significantly. ACP patients were less likely to receive no treatment (6.2% vs 11.6%, p<0.001) and more likely to receive systemic therapy (70.2% vs 63.2%, p<0.001). Time to systemic therapy was shorter at ACP (median 15 vs 18 days, p=0.001), including chemotherapy and immunotherapy initiation (both p<0.001). Radiation was more frequently used at CCP (24% vs 20%, p=0.002).
Early mortality was higher at CCP (30-day: 3.9% vs 3.1%, p=0.013; 90-day: 13.0% vs 10.3%, p=0.011). Overall survival favored ACP, with higher 2-year (30.8% vs 23.2%), 5-year (18.9% vs 12.8%), and 10-year survival (8.8% vs 5.2%). Adjusted median survival was longer at ACP (0.48 vs 0.32 years).

**Copyright Information:** (Online) ISSN: 2572-9241
©2026 The Author(s). HemaSphere is published by John Wiley & Sons Ltd on behalf of European Hematology Association. This is an open access Abstract Book distributed under the Attribution-NonCommercial-NoDerivs (CC BY-NC-ND), which allows third parties to download the articles and share them with others as long as they credit the author and the Abstract Book, but they cannot change the content in any way or use them commercially.
**Abstract Book Citations:** Authors, Title, HemaSphere, 2026;10:(S1):pages. The abstract book DOIs can be found on the “Author Information” page.
**Disclaimer:** Articles published in the journal HemaSphere exclusively reflect the opinions of the authors. The authors are responsible for all content in their abstracts including accuracy of the facts, statements, citing resources, etc.

**Summary/Conclusion**

Among patients aged ≥75 years with PCNS, OS differed significantly by treatment facility type, with a statistically and clinically meaningful survival advantage observed among patients treated at ACP compared with CCP. OS difference was consistent across short- and long-term follow-up with lower early mortality, higher rates of treatment delivery, and shorter time to initiation of therapy at ACP. These results point to system-level gaps in care for older adults with PCNSL and underscore the need to strengthen referral pathways to ACP and build structured academic–community partnerships. Doing so could expand timely access to specialized multidisciplinary care and clinical trials for vulnerable patients more often treated in lower-resource settings.

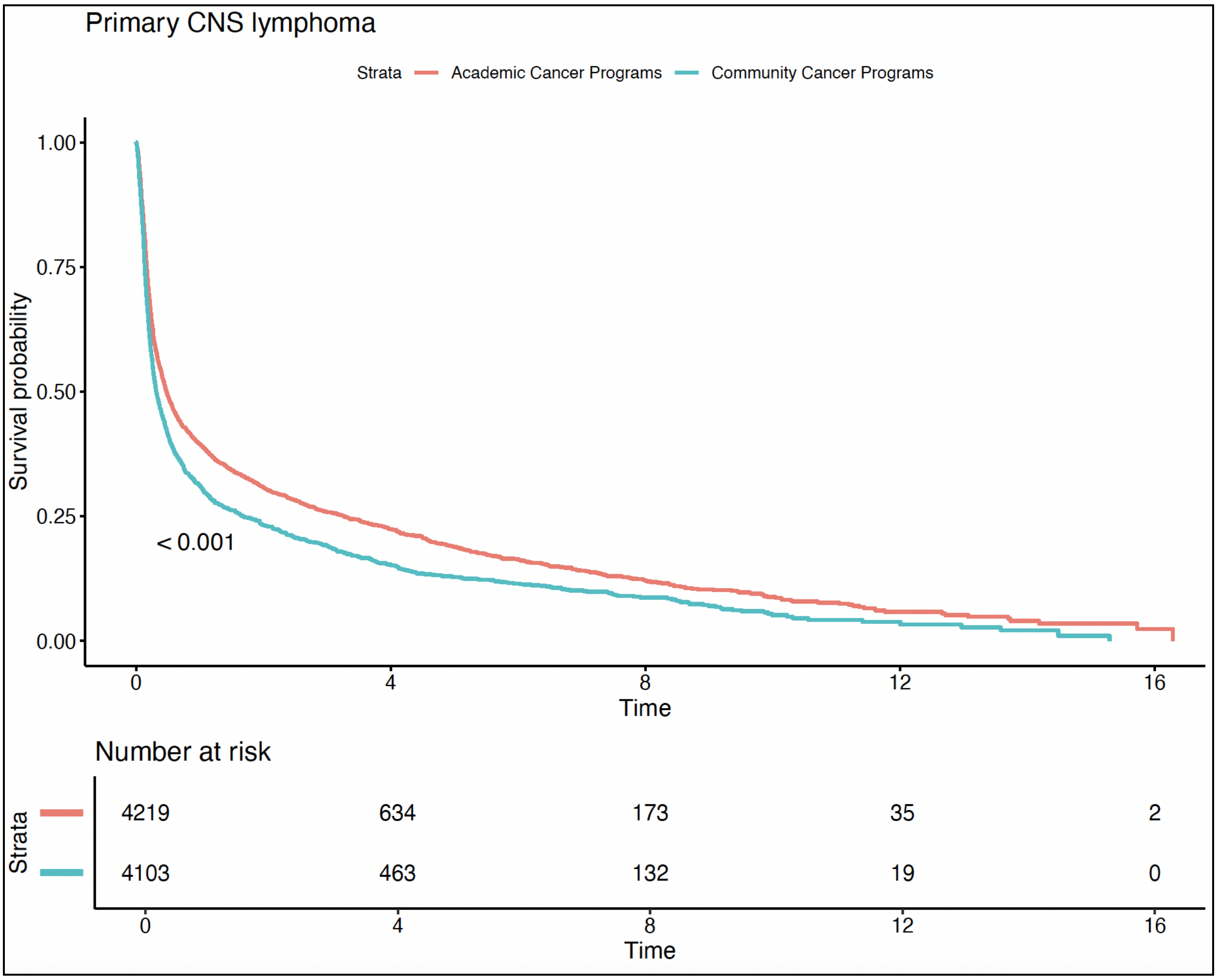

**Copyright Information:** (Online) ISSN: 2572-9241
©2026 The Author(s). HemaSphere is published by John Wiley & Sons Ltd on behalf of European Hematology Association. This is an open access Abstract Book distributed under the Attribution-NonCommercial-NoDerivs (CC BY-NC-ND), which allows third parties to download the articles and share them with others as long as they credit the author and the Abstract Book, but they cannot change the content in any way or use them commercially.
**Abstract Book Citations:** Authors, Title, HemaSphere, 2026;10:(S1):pages. The abstract book DOIs can be found on the "Author Information" page.
**Disclaimer:** Articles published in the journal HemaSphere exclusively reflect the opinions of the authors. The authors are responsible for all content in their abstracts including accuracy of the facts, statements, citing resources, etc.

### (PF1048) AMPLIFICATION OF STAT3 AND TP53 MAY CONFER POOR PROGNOSIS IN PERIPHERAL T-CELL LYMPHOMA: ANALYSIS OF A SINGLE-CENTER COHORT

**Topic:** 20. Other aggressive lymphomas

Alessandro Putelli*[1], Nicola Bianchi[1], Anna Maria Barbui[1], Silvia Ferrari[1], Monica Rondi[1], Giulia Ghirardi[1], Emanuela Carlotti[1], Silvia Salmoiraghi[1], Denise Morotti[1], Giuseppe Gritti[1]

[1]*ASST Papa Giovanni XXIII, Bergamo, Italy;*

### Background

Peripheral T-cell lymphomas (PTCLs) are a heterogeneous group of T-cell neoplasms with variable clinical-biological presentation and, overall, poor prognosis. The increasing integration of next-generation sequencing (NGS) into clinical practice is rapidly refining the molecular landscape of PTCL, defining potential prognostic markers and therapeutic targets. While *STAT3* mutations and *TP53* mutations and deletion are increasingly recognized as poor prognostic factors, the role of copy gains of specific genes is still largely unexplored.

### Aims

Aim of the study was to provide a comprehensive molecular characterization of a cohort of patients with nodal PTCL and to correlate genetic alterations to clinical outcome.

### Methods

We conducted a single-center retrospective study of patients with nodal PTCL treated from 2011 to 2025. Genomic DNA was extracted from Formalin-Fixed Paraffin-Embedded (FFPE) diagnostic biopsy. A custom panel for mutations, insertion-deletions and copy number variations (CNVs), involving 88 genes frequently altered in PTCLs was used (Agilent Technologies). CNVs were annotated only with 6 or more gene copies.

### Results

Fifty-six patients were analyzed, 23 (41%) had a diagnosis of PTCL-NOS, 16 (29%) of angioimmunoblastic T-cell lymphoma (AITL), 15 (27%) of anaplastic large cell lymphoma (ALCL) ALK-, and 2 (4%) of ALCL ALK+. Median age was 60 years, 17 patients (30%) were female. Most of the patients had stage IV disease (n=38, 68%) and an IPI score 3-5 (n=34, 61%) Median number of lines of treatment was 2 (range 1-5). The most common first-line regimens used were CHOEP or CHOP-14 in 24 patients (42.9%), CHOP-21 in 13 (23.2%) and brentuximab-CHP in 12 (21.4%). The median follow-up was 16 months (range 0-117 months). Twenty-eight patients (50%) obtained a CR after first line, the estimated 24-months PFS and OS were 35% and 60%, respectively. At least one genomic alteration was found in all the samples tested. As expected, *TET2, DNMT3A, FAT1, IDH2, RHOA* were the most mutated genes in AITL population. PTCL-NOS mutational landscape was more heterogeneous as only *TET2, SETD1B, TP53* were mutated in more than 20% of patients. *KMT2C, KMT2D, PDPK1, TET2, TP53, CREBBP* were the most frequently mutated genes among ALCL ALK-. The genes harboring a CNV in more than 20% of patients were *MYC, STAT5B, IRF4, RB1, PTEN, STAT3, ASXL3,* and *TP53.* Eight patients had a copy gain of *STAT3,* one with a diagnosis of AITL, 4 of ALCL ALK-, 3 of PTCL-NOS. These patients showed inferior PFS (HR 6.42 95%CI 2.52-16.35; p < 0.001) and OS (HR 4.86 95%CI 1.95-12.14; p < 0.001). A worse PFS was also observed in the 6 patients with *TP53* amplification (HR 2.93, 95%IC 1.02-8.39; p = 0.045), although no difference in OS was observed.

**Copyright Information:** (Online) ISSN: 2572-9241
©2026 The Author(s). HemaSphere is published by John Wiley & Sons Ltd on behalf of European Hematology Association. This is an open access Abstract Book distributed under the Attribution-NonCommercial-NoDerivs (CC BY-NC-ND), which allows third parties to download the articles and share them with others as long as they credit the author and the Abstract Book, but they cannot change the content in any way or use them commercially.
**Abstract Book Citations:** Authors, Title, HemaSphere, 2026;10:(S1):pages. The abstract book DOIs can be found on the "Author Information" page.
**Disclaimer:** Articles published in the journal HemaSphere exclusively reflect the opinions of the authors. The authors are responsible for all content in their abstracts including accuracy of the facts, statements, citing resources, etc.

**Summary/Conclusion**

This study provides the molecular characterization of a cohort of nodal PTCL. The custom 88-gene panel effectively identified molecular alterations, showing robust performance. The mutations found were consistent with existing literature. With the limitations of the small number of patients, our findings shows that *STAT3* and *TP53* amplifications are associated with a detrimental impact on outcome. These results warrant further validation in larger cohort of patients.

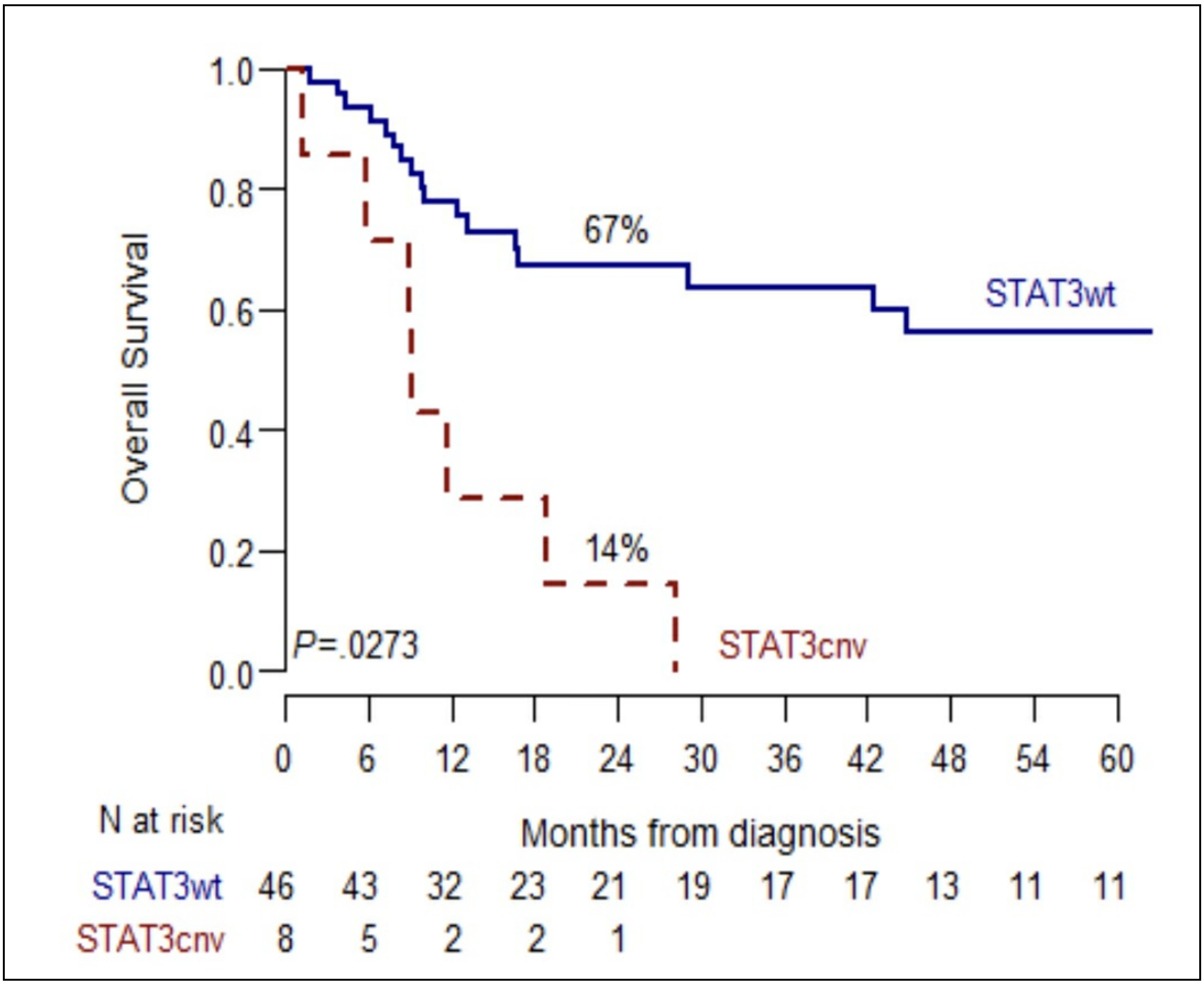

OS STAT3 CNV vs wt Kaplan-Meier

**Copyright Information:** (Online) ISSN: 2572-9241
©2026 The Author(s). HemaSphere is published by John Wiley & Sons Ltd on behalf of European Hematology Association. This is an open access Abstract Book distributed under the Attribution-NonCommercial-NoDerivs (CC BY-NC-ND), which allows third parties to download the articles and share them with others as long as they credit the author and the Abstract Book, but they cannot change the content in any way or use them commercially.
**Abstract Book Citations:** Authors, Title, HemaSphere, 2026;10:(S1):pages. The abstract book DOIs can be found on the "Author Information" page.
**Disclaimer:** Articles published in the journal HemaSphere exclusively reflect the opinions of the authors. The authors are responsible for all content in their abstracts including accuracy of the facts, statements, citing resources, etc.

### (PF1049) FINAL RESULTS OF A PHASE II STUDY OF THE ANTI-PROGRAMMED CELL DEATH LIGAND-1 (PD-L1) ANTIBODY ATEZOLIZUMAB IN RELAPSED/REFRACTORY EXTRANODAL NK/T-CELL LYMPHOMA: THE NCCH1903/ATTACK STUDY

**Topic:** 20. Other aggressive lymphomas

Shinichi Makita*[1], Junji Koya[2], Yasuhiro Suzuki[3], Momoko Nishikori[4], Noriko Fukuhara[5], Akiko Maeshima[6], Akihiro Hirakawa[7], Satoshi Kawashima[8], Yukari Nagasaka[8], Yuta Ito[2], Yasunori Kogure[2], Ayumi Fujimoto[9, 10], Hirokazu Nagai[3], Kengo Takeuchi[9, 10, 11], Keisuke Kataoka[2, 12], Koji Izutsu[1]

*[1]National Cancer Center Hospital, Department of Hematology, Tokyo, Japan;[2]National Cancer Center Research Institute, Division of Molecular Oncology, Tokyo, Japan; [3]NHO Nagoya Medical Center, Department of Hematology, Nagoya, Japan; [4]Kyoto University, Department of Hematology, Kyoto, Japan;[5]Tohoku University, Department of Hematology, Sendai, Japan; [6]National Cancer Center Hospital, Department of Pathology, Tokyo, Japan;[7]Institute of Science Tokyo, Department of Clinical Biostatistics, Tokyo, Japan; [8]National Cancer Center Hospital, Clinical Research Support Office, Tokyo, Japan;[9]The Cancer Institute, Japanese Foundation for Cancer Research, Division of Pathology, Tokyo, Japan; [10]The Cancer Institute, Japanese Foundation for Cancer Research, Pathology Project for Molecular Targets, Tokyo, Japan; [11]Cancer Institute Hospital, Japanese Foundation for Cancer Research, Department of Pathology, Tokyo, Japan; [12]Keio University School of Medicine, Division of Hematology, Department of Medicine, Tokyo, Japan;*

### Background

Patients with relapsed/refractory (R/R) extranodal natural killer/T-cell lymphoma (ENKTL) have limited treatment options, particularly those who fail or are ineligible for asparaginase-containing regimens. Previous studies have suggested the efficacy of immune checkpoint inhibitors for R/R ENKTL. We conducted a pivotal phase II study of atezolizumab, a widely approved anti-PD-L1 antibody, in patients with R/R ENKTL (NCCH1903/ATTACK study). Based on the results of the primary analysis, atezolizumab was approved for the treatment of R/R ENKTL in Japan in September 2025. Here, we report the final results of the pivotal ATTACK study with longer follow-up data.

### Aims

To evaluate the long-term efficacy and safety of atezolizumab in patients with R/R ENKTL.

### Methods

The NCCH1903/ATTACK study was a multi-center single-arm phase II study of single-agent atezolizumab (1200 mg, day1, 21-day cycle, up to 2 years) in patients with R/R ENKTL (jRCT2031190177). Key eligibility criteria included histologically confirmed ENKTL, age ≥12 years, ECOG performance status 0-2, ≥1 prior line of systemic therapy, and the presence of measurable disease. All patients were required to have received prior SMILE therapy (dexamethasone, methotrexate, ifosfamide, asparaginase, and etoposide) or were ineligible due to age or comorbidities. Written informed consent was obtained before enrollment. The primary endpoint was the independent review committee (IRC)-assessed objective response rate (ORR) based on CT criteria of Lugano classification. Secondary endpoints included progression-free survival (PFS), overall survival (OS), duration of response (DoR), and safety. The target accrual was between 12 and 20 patients, and ≥3 responders were required to declare the efficacy of atezolizumab based on our Bayesian design. The final analysis was conducted after completion of follow-up for all enrolled patients (data cutoff: 2 September 2024).

Copyright Information: (Online) ISSN: 2572-9241
©2026 The Author(s). HemaSphere is published by John Wiley & Sons Ltd on behalf of European Hematology Association. This is an open access Abstract Book distributed under the Attribution-NonCommercial-NoDerivs (CC BY-NC-ND), which allows third parties to download the articles and share them with others as long as they credit the author and the Abstract Book, but they cannot change the content in any way or use them commercially.
Abstract Book Citations: Authors, Title, HemaSphere, 2026;10:(S1):pages. The abstract book DOIs can be found on the “Author Information” page.
Disclaimer: Articles published in the journal HemaSphere exclusively reflect the opinions of the authors. The authors are responsible for all content in their abstracts including accuracy of the facts, statements, citing resources, etc.

### Results

A total of 14 patients were enrolled between July 2020 and September 2022, and 13 patients were evaluable for efficacy. The median age was 72 years (IQR 66-77), and 10 patients (77%) had stage IV disease. At the final analysis (data cutoff: September 2024), an 18-month extension from the primary analysis cutoff in March 2023, the IRC-assessed ORR was 53.8% (7/13; 95% CI, 25.1–80.8), including 4 complete responses (CR, 30.8%), meeting the prespecified Bayesian efficacy criterion. The median PFS was 2.4 months, and the median OS was 10.3 months. Notably, the median DoR was 26.5 months and the 1-year DoR rate was 80.0% (95% CI, 20.4–96.9). All four patients who achieved CR experienced durable responses. Two patients completed the 2-year protocol treatment and remained in CR at the data cutoff. The remaining two CR patients discontinued atezolizumab due to adverse events during Cycle 10 and Cycle 18; however, their responses were maintained for 20 months and 16 months, respectively, after the last dose of atezolizumab. PD-L1 status was evaluable in three of the four patients with CR; all harbored PD-L1 structural variants and/or demonstrated PD-L1 expression by immunohistochemistry. Treatment-related adverse events were manageable and no new safety signals were observed after the primary analysis.

### Summary/Conclusion

Atezolizumab demonstrated substantial and durable efficacy in patients with R/R ENKTL. Patients who achieved CR experienced durable responses.

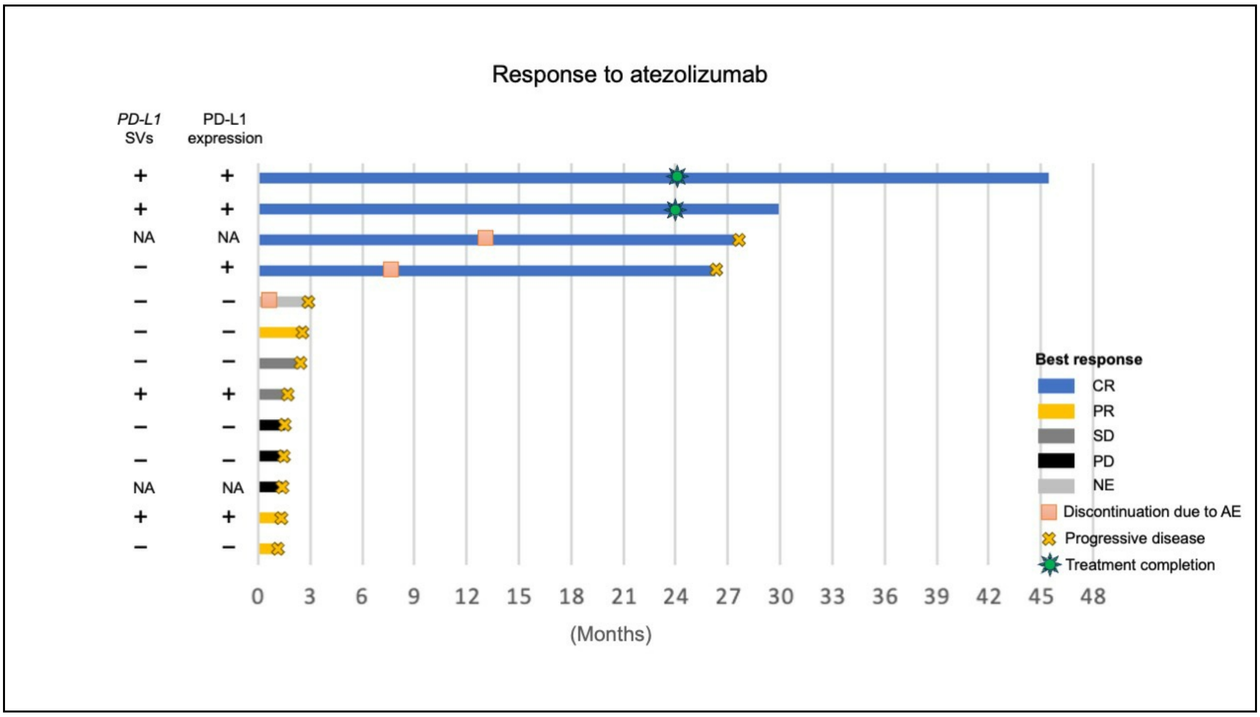

Copyright Information: (Online) ISSN: 2572-9241
©2026 The Author(s). HemaSphere is published by John Wiley & Sons Ltd on behalf of European Hematology Association. This is an open access Abstract Book distributed under the Attribution-NonCommercial-NoDerivs (CC BY-NC-ND), which allows third parties to download the articles and share them with others as long as they credit the author and the Abstract Book, but they cannot change the content in any way or use them commercially.
Abstract Book Citations: Authors, Title, HemaSphere, 2026;10:(S1):pages. The abstract book DOIs can be found on the “Author Information” page.
Disclaimer: Articles published in the journal HemaSphere exclusively reflect the opinions of the authors. The authors are responsible for all content in their abstracts including accuracy of the facts, statements, citing resources, etc.

### (PF1050) PROPOSED DEFINITION FOR 'PRIMARY REFRACTORY' DISEASE IN NODAL PERIPHERAL T-CELL LYMPHOMA: AN AUSTRALASIAN-CANADIAN COLLABORATIVE STUDY

**Topic:** 20. Other aggressive lymphomas

Chathuri Abeyakoon[1, 2], Cameron Wellard[3], Eliza Chung[3], David Hodgson[4], Danielle Rodin[4], Anca Prica[5], Tomohiro Aoki[5], Abi Vijenthira[5], Stephen Opat[1, 2], Erica Wood[1, 3], Zoe McQuilten[3, 6], Eliza Hawkes[3, 7], Michael Crump[5], Gareth Gregory[2, 8], John Kuruvilla*[5]

*[1]Monash Health, Monash Haematology, Melbourne, Australia;[2]Monash University, Department of Medicine, School of Clinical Sciences at Monash Health, Melbourne, Australia; [3]Monash University, School of Public Health and Preventive Medicine, Melbourne, Australia; [4]Princess Margaret Cancer Centre, Division of Radiation Oncology, Toronto, Canada;[5]Princess Margaret Cancer Centre, Division of Medical Oncology and Haematology, Toronto, Canada; [6]Alfred Health, Haematology, Melbourne, Australia; [7]Austin Health, Olivia Newton-John Cancer Research Institute, Melbourne, Australia;[8]Fiona Stanley Hospital, Haematology Department, Perth, Australia;*

### Background

There is no universally accepted definition of 'primary refractory' disease in nodal peripheral T-cell lymphoma (nPTCL), with heterogeneous criteria applied across registry analyses and clinical studies. Pivotal phase II trials of novel agents in relapsed/refractory nPTCL have not reported outcomes specific to this subgroup, limiting interpretation and cross-study comparisons.

### Aims

To determine the optimal time-based definition of 'primary refractory' disease in nPTCL by evaluating survival outcomes according to time-to-relapse (TTR) post primary treatment (1L).

### Methods

We performed a retrospective analysis of the Australasian Lymphoma and Related Diseases Registry (LaRDR; Jan 2016–Dec 2023) and the Princess Margaret Cancer Centre lymphoma database (PMH; Nov 2011–Mar 2024). Adults (≥18 years) treated with 1L therapy for nPTCL were included, comprising PTCL-not otherwise specified, T follicular helper (TFH)–type lymphomas (angioimmunoblastic T-cell lymphoma, PTCL with TFH phenotype, follicular T-helper lymphoma), and anaplastic large cell lymphoma. Patients (pts) were stratified by TTR from end of 1L therapy: stable/progressive disease (SD/PD) after 1L; relapse ≤3months (m), 3–6m, 6–12m, and >12m. Progression-free survival-2 (PFS2) and overall survival-2 (OS2) were calculated from first progression to second progression or death, and to death from any cause, respectively.

### Results

A total of 451 pts were included (LaRDR 285; PMH 166). 1L therapy was typically CHOP-based. Imaging responses to 1L were available in 319 pts (PET/CT 282, CT 37). 358/451 experienced SD/PD to or relapse after 1L: SD/PD (n=100), ≤3m (n=49), 3–6m (n=26), 6–12m (n=50), and >12m (n=133). Median follow-up was 43m (95% CI 39–48).
Baseline characteristics, median PFS and OS by TTR are shown in Table 1.
Pts with SD/PD or relapse ≤6m from 1L had significantly inferior outcomes compared with those relapsing >6m, clearly separating two prognostic groups (Table 1).
In multivariate analysis, pts with SD/PD or relapse ≤6m from 1L had significantly inferior PFS (adjusted hazard ratio [aHR] 2.1 (95%CI 1.5-3), p<0.001) and OS (aHR 2.8 (95%CI 1.8-4), p<0.001) when adjusted for age, nodal subtype, ECOG and gender.

**Copyright Information:** (Online) ISSN: 2572-9241
©2026 The Author(s). HemaSphere is published by John Wiley & Sons Ltd on behalf of European Hematology Association. This is an open access Abstract Book distributed under the Attribution-NonCommercial-NoDerivs (CC BY-NC-ND), which allows third parties to download the articles and share them with others as long as they credit the author and the Abstract Book, but they cannot change the content in any way or use them commercially.
**Abstract Book Citations:** Authors, Title, HemaSphere, 2026;10:(S1):pages. The abstract book DOIs can be found on the "Author Information" page.
**Disclaimer:** Articles published in the journal HemaSphere exclusively reflect the opinions of the authors. The authors are responsible for all content in their abstracts including accuracy of the facts, statements, citing resources, etc.

**Table 1**

| | **SD/PD** | **≤3m** | **3-6m** | **6-12m** | **>12m** | **P value** |
|---|---|---|---|---|---|---|
| **N** | 100 | 49 | 26 | 50 | 133 | |
| **Median age, years (IQR)** | 62 (55-72) | 66 (55-77) | 63 (56-71) | 66 (58-75) | 58 (44-68) | <0.001 |
| **Female** | 33/100 (33%) | 12/49 (25%) | 8/26 (31%) | 19/50 (38%) | 49/133 (37%) | 0.56 |
| **nPTCL subtype**<br>PTCL-NOS<br>TFH<br>ALCL | <br>42/100 (42%)<br>36/100 (36%)<br>22/100 (22%) | <br>26/49 (53%)<br>19/49 (39%)<br>4/49 (8%) | <br>11/26 (42%)<br>8/26 (31%)<br>7/26 (27%) | <br>24/50 (48%)<br>18/50 (36%)<br>8/50 (16%) | <br>37/133 (28%)<br>40/133 (30%)<br>56/133 (42%) | <0.001 |
| **ECOG**<br>2-4 | <br>28/82 (34%) | <br>16/39 (41%) | <br>8/18 (44%) | <br>11/39 (28%) | <br>12/111 (11%) | <0.001 |
| **B symptoms** | 45/89 (51%) | 23/44 (52%) | 11/21 (52%) | 17/43 (40%) | 51/121 (42%) | 0.52 |
| **Survival by TTR** | | | | | | |
| **Median PFS2** | 3.1m<br>(95%CI 2.1-4.8) | 1.8m<br>(95%CI 1.1–5.2) | 3.7m<br>(95%CI 1.8–8) | 11.1m<br>(95%CI 4–17) | 10m<br>(95%CI 5.7–14) | <0.001 |
| **Median OS2** | 8.5m<br>(95%CI 5-12) | 16.9m<br>(95%CI 1.8–34) | 7.9m<br>(95%CI 3.1–NR) | 43.6m<br>(95%CI 16–54) | 28.3m<br>(95%CI 17.4–NR) | <0.001 |
| **36m PFS** | 6.3% (95%CI 2.4-12.7) | | | 16.3% (95%CI 8.1-27) | | **<0.001** |
| **36m OS** | 20% (95%CI 12-30) | | | 49% (95%CI 36-61) | | **<0.001** |

**Copyright Information:** (Online) ISSN: 2572-9241
©2026 The Author(s). HemaSphere is published by John Wiley & Sons Ltd on behalf of European Hematology Association. This is an open access Abstract Book distributed under the Attribution-NonCommercial-NoDerivs (CC BY-NC-ND), which allows third parties to download the articles and share them with others as long as they credit the author and the Abstract Book, but they cannot change the content in any way or use them commercially.
**Abstract Book Citations:** Authors, Title, HemaSphere, 2026;10:(S1):pages. The abstract book DOIs can be found on the "Author Information" page.
**Disclaimer:** Articles published in the journal HemaSphere exclusively reflect the opinions of the authors. The authors are responsible for all content in their abstracts including accuracy of the facts, statements, citing resources, etc.

### Summary/Conclusion

Pts with SD/PD or relapse ≤6m following 1L represent a distinct high-risk group in nPTCL. We propose defining this cohort as ‘primary refractory’ disease. Although our pts received global standard care, external validation in international cohorts is planned. A unified definition would enable accurate risk stratification, consistent outcome reporting and improved trial design for high-risk pts.

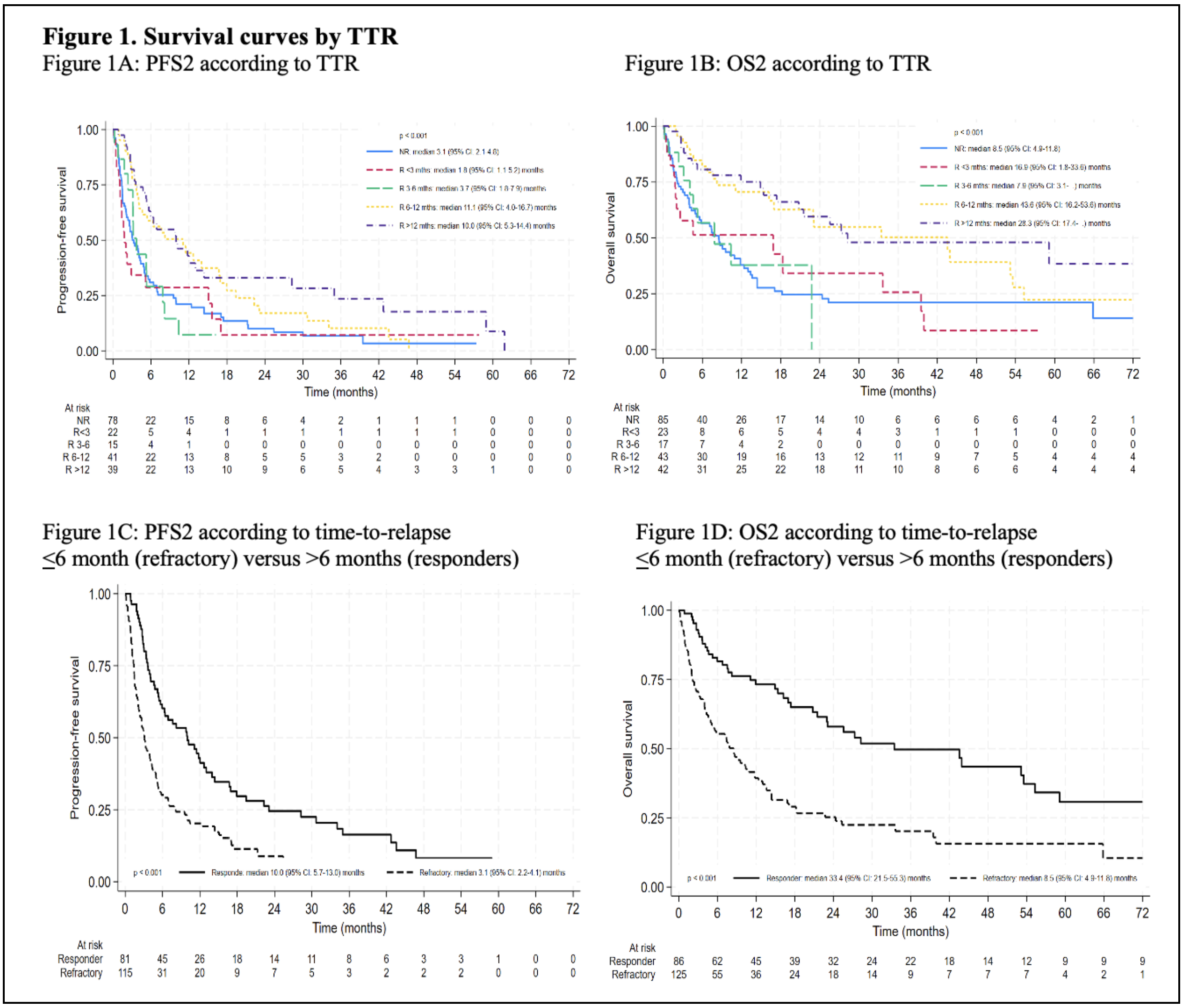

TTR, time-to-relapse; PFS2, progression free survival-2; OS, overall survival-2; NR, non responders with stable disease/progressive disease following frontline therapy; R, responders; mths, months

Copyright Information: (Online) ISSN: 2572-9241
©2026 The Author(s). HemaSphere is published by John Wiley & Sons Ltd on behalf of European Hematology Association. This is an open access Abstract Book distributed under the Attribution-NonCommercial-NoDerivs (CC BY-NC-ND), which allows third parties to download the articles and share them with others as long as they credit the author and the Abstract Book, but they cannot change the content in any way or use them commercially.
Abstract Book Citations: Authors, Title, HemaSphere, 2026;10:(S1):pages. The abstract book DOIs can be found on the “Author Information” page.
Disclaimer: Articles published in the journal HemaSphere exclusively reflect the opinions of the authors. The authors are responsible for all content in their abstracts including accuracy of the facts, statements, citing resources, etc.

### (PF1051) BRENTUXIMAB VEDOTIN PLUS CHP-LIKE VERSUS CHOEP REGIMEN FOR THE FIRST-LINE TREATMENT OF PTCL PATIENTS: A MULTI-CENTER PROPENSITY-MATCHED POPULATION-BASED STUDY

**Topic:** 20. Other aggressive lymphomas

Jing Chen[1], Fang Shu Guan[2], Zhipeng Cheng[1], Yingying Wu[1], Lin Liu[1], Lu Zhang[1], Fang Liu[1], Guohui Cui[1], Heng Mei[1], Fanjun Cheng[1], Yi Zhao[2], Jun Fang*[1]

*[1]Union Hospital, Tongji Medical College, Huazhong University of Science and Technology, Department of Hematology, Wuhan, China; [2]The First Affiliated Hospital, School of Medicine, Zhejiang University, Bone Transplantation Center, Hangzhou, China;*

### Background

Peripheral T-cell lymphomas (PTCLs) constitute a heterogeneous subgroup of non-Hodgkin lymphomas (NHLs) that arise from mature T lymphocytes and natural killer (NK) cells. The treatment strategy for newly diagnosed PTCL is undergoing continuous refinement and therapeutic approaches. Commonly recommended regimens include cyclophosphamide, doxorubicin, vincristine, and prednisone (CHOP), cyclophosphamide, doxorubicin, vincristine, etoposide, and prednisone (CHOEP), dose-adjusted etoposide, prednisone, vincristine, cyclophosphamide, doxorubicin (DA-EPOCH), and brentuximab vedotin (BV) plus cyclophosphamide, doxorubicin, and prednisone (CHP). However, the optimal frontline treatment option is not completely defined.

### Aims

To compare the efficacy and safety of BV plus CHP-like regimen and CHOEP regimen as first-line treatment for newly diagnosed PTCLs.

### Methods

This retrospective multicenter study enrolled 113 eligible patients. Using 1:1 optimal propensity score-matching (PSM), we matched patients by gender, age, disease stage, bone marrow infiltration, extra-nodal involvement, Eastern Cooperative Oncology Group (ECOG) performance status, and PTCL subtype, resulting in two balanced cohorts of 45 patients each. The primary study endpoint was progression-free survival (PFS). The key secondary endpoints were overall survival (OS), objective response rate (ORR), complete remission (CR), duration of response (DOR) and safety.

### Results

The baseline characteristics were well balanced between the BV plus CHP-like and CHOEP groups after PSM. With a median follow-up of 27.2 months, the 2-year PFS rates were 70.7% (95% confidence intervals [CI]: 58.5%-85.5%) and 45.3% (95% CI: 32.7%-62.7%) for the BV plus CHP-like and CHOEP groups, respectively (P = 0.012). The 2-year OS rate was 81.0% (95% CI: 68.8%-95.3%) in the BV plus CHP-like group and 75.1% (95% CI: 62.7%-90.1%) in the CHOEP group (P = 0.550). The CR rate was 50.0% for patients treated with CHOEP and 56.8% for those administered BV plus CHP-like regimens, while the corresponding ORR was 61.4% and 68.2%, respectively. No statistically significant differences were observed between the two groups (CR, P = 0.521; ORR, P = 0.503). The DOR in the BV plus CHP-like group was significantly prolonged compared to the CHOEP group (81.9% vs. 63.0%, P = 0.035). The BV plus CHP-like group had a significant lower any-grade leukopenia (P = 0.048), neutropenia (P = 0.038), anemia (P = 0.026), and thrombocytopenia (P = 0.008) rates compared to the CHOEP group. However, there were no significant differences in the incidence of non-hematologic toxicities between the two groups.

**Copyright Information:** (Online) ISSN: 2572-9241
©2026 The Author(s). HemaSphere is published by John Wiley & Sons Ltd on behalf of European Hematology Association. This is an open access Abstract Book distributed under the Attribution-NonCommercial-NoDerivs (CC BY-NC-ND), which allows third parties to download the articles and share them with others as long as they credit the author and the Abstract Book, but they cannot change the content in any way or use them commercially.
**Abstract Book Citations:** Authors, Title, HemaSphere, 2026;10:(S1):pages. The abstract book DOIs can be found on the "Author Information" page.
**Disclaimer:** Articles published in the journal HemaSphere exclusively reflect the opinions of the authors. The authors are responsible for all content in their abstracts including accuracy of the facts, statements, citing resources, etc.

**Summary/Conclusion**

The results of our real-world data and PSM analysis demonstrated that BV plus CHP-like regimen presented superior PFS and DOR compared with CHOEP, suggesting a potent ability to effectively delay disease progression. Furthermore, this analysis revealed that the BV plus CHP-like regimen was associated with a lower incidence of hematological toxicities than the CHOEP regimen. BV plus CHP-like regimen could be a promising first-line PTCLs regimen with improved disease control and favorable safety, which deserves further study.

**Copyright Information:** (Online) ISSN: 2572-9241
©2026 The Author(s). HemaSphere is published by John Wiley & Sons Ltd on behalf of European Hematology Association. This is an open access Abstract Book distributed under the Attribution-NonCommercial-NoDerivs (CC BY-NC-ND), which allows third parties to download the articles and share them with others as long as they credit the author and the Abstract Book, but they cannot change the content in any way or use them commercially.
**Abstract Book Citations:** Authors, Title, HemaSphere, 2026;10:(S1):pages. The abstract book DOIs can be found on the “Author Information” page.
**Disclaimer:** Articles published in the journal HemaSphere exclusively reflect the opinions of the authors. The authors are responsible for all content in their abstracts including accuracy of the facts, statements, citing resources, etc.

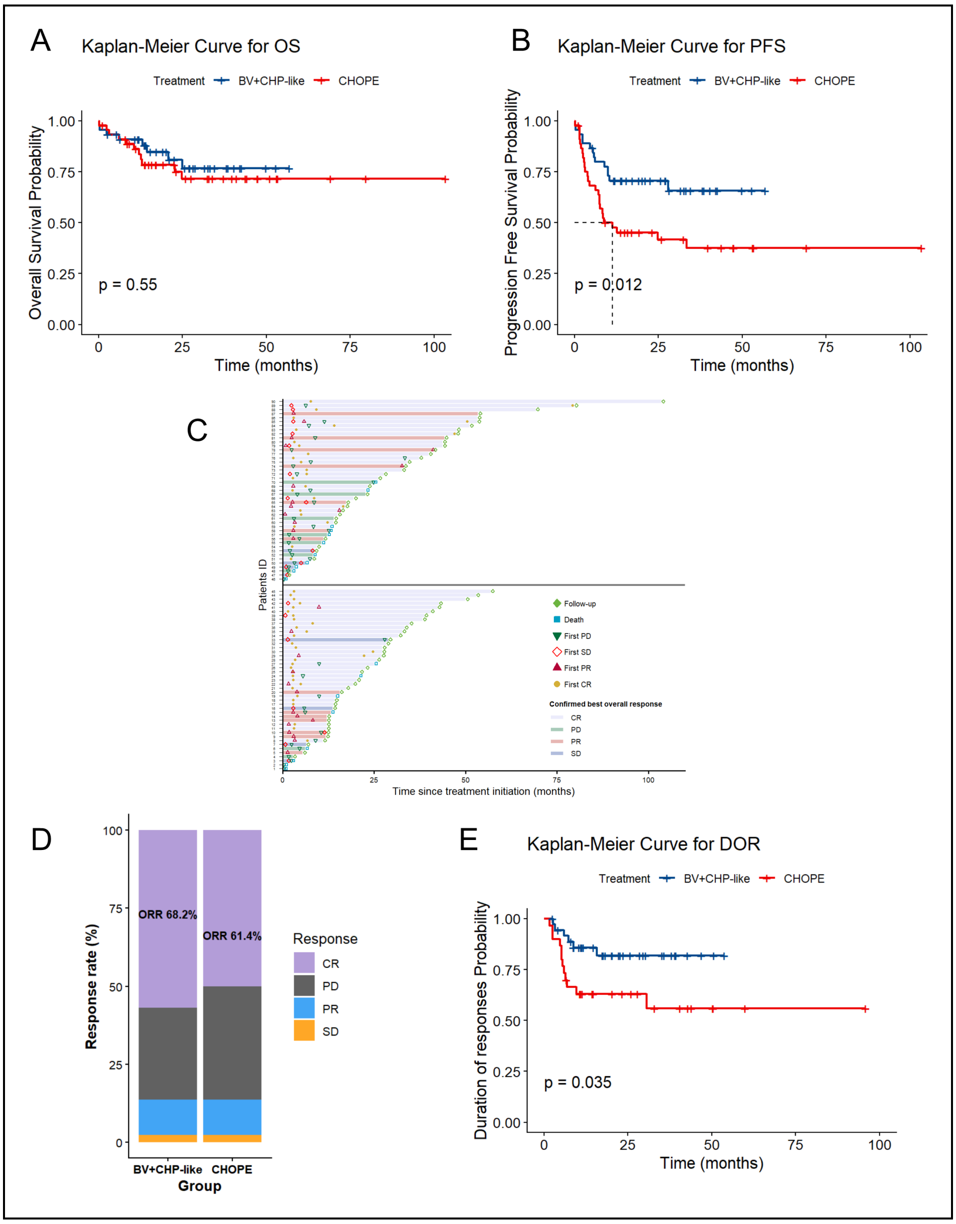

Survival analysis and clinical response profiles of BV plus CHP-like (IDs 1-45) vs. CHOPE (IDs 46-90) regimens in newly diagnosed peripheral T-cell lymphomas. (A) Kaplan-Meier curve for OS; (B) Kaplan-Meier curve for PFS ; (C) swim plot of individual patient response trajectories over time; (D) Response rate distribution; (E) Kaplan-Meier curve for DOR. Abbreviations: OS, overall survival; PFS, progression-free survival; CR, complete remission; PR, partial remission; SD, stable disease; PD, progressive disease; DOR, duration of response.

**Copyright Information:** (Online) ISSN: 2572-9241
©2026 The Author(s). HemaSphere is published by John Wiley & Sons Ltd on behalf of European Hematology Association. This is an open access Abstract Book distributed under the Attribution-NonCommercial-NoDerivs (CC BY-NC-ND), which allows third parties to download the articles and share them with others as long as they credit the author and the Abstract Book, but they cannot change the content in any way or use them commercially.
**Abstract Book Citations:** Authors, Title, HemaSphere, 2026;10:(S1):pages. The abstract book DOIs can be found on the "Author Information" page.
**Disclaimer:** Articles published in the journal HemaSphere exclusively reflect the opinions of the authors. The authors are responsible for all content in their abstracts including accuracy of the facts, statements, citing resources, etc.

### (PF1052) RARE GERMLINE VARIANTS SHAPE SUSCEPTIBILITY AND PROGNOSIS IN B-CELL LYMPHOMAS

**Topic:** 20. Other aggressive lymphomas

Weicheng Ren*[1], Mingyu Yang[1], Hassan Abolhassani[1], Man Nie[2], Xianhuo Wang[3], Hui Wan[1], Zhiming Li[2], Hui-Lai Zhang[3], Qiang Pan-Hammarström[1]

*[1]Karolinska Institutet, Stockholm, Sweden; [2]Sun Yat-Sen University Cancer Center, Guangzhou, China; [3]Tianjin Medical University Cancer Institute and Hospital, TIanjin, China;*

**Background**

Non-Hodgkin lymphoma (NHL) is the most common hematologic malignancy and comprises over 60 entities, including diffuse large B-cell lymphoma (DLBCL), follicular lymphoma (FL), and mantle cell lymphoma (MCL). These subtypes exhibit marked genetic and clinical heterogeneity, and current molecular classification systems predominantly rely on somatic genomic alterations. However, accumulating evidence indicates that inherited genetic variation also contributes to lymphoma susceptibility and clinical behavior. Epidemiological and twin studies support a heritable component to NHL risk, and genome-wide association studies have identified common susceptibility loci with modest effect sizes. Moreover, several genes recurrently mutated somatically in lymphomas have also been implicated in germline predisposition syndromes. Despite these observations, the landscape and clinical impact of rare damaging germline variants in B-cell lymphoma remain incompletely characterized.

**Aims**

We sought to define the contribution of rare damaging germline variants in genes related to inborn errors of immunity (IEI), DNA damage response and repair (DDR), and lymphoma driver pathways to lymphoma development, molecular heterogeneity, and prognosis.

**Methods**

Whole-exome and whole-genome sequencing data from our institutional lymphoma cohort and NHL cases from the UK Biobank were analyzed to systematically identify rare, potentially pathogenic germline variants. Analyses focused on genes involved in immune regulation (IEI), genome stability and DNA repair (DDR), and established lymphoma-associated driver pathways. Germline mutation burden, inheritance patterns, and associations with clinical outcomes were evaluated across subtypes.

**Results**

Patients with DLBCL, FL, and MCL harbored substantially more rare germline coding variants than somatic mutations within the same gene categories, particularly in IEI and DDR genes, supporting a role for inherited susceptibility in lymphomagenesis. Increased germline mutation burden—especially within IEI genes—was associated with inferior survival in DLBCL. Germline IEI variants showed minimal overlap with somatic driver mutations, indicating distinct biological roles in cancer predisposition. Notably, nearly half of patients exhibited genotypes consistent with Mendelian inheritance patterns, including confirmed biallelic defects (~9%) in IEI genes such as *XIAP, IRAK1, G6PD, BRCA2,* and *FANCB.* Patients harboring biallelic defects in IEI genes exhibited significantly worse overall survival in DLBCL and FL.

**Copyright Information:** (Online) ISSN: 2572-9241
©2026 The Author(s). HemaSphere is published by John Wiley & Sons Ltd on behalf of European Hematology Association. This is an open access Abstract Book distributed under the Attribution-NonCommercial-NoDerivs (CC BY-NC-ND), which allows third parties to download the articles and share them with others as long as they credit the author and the Abstract Book, but they cannot change the content in any way or use them commercially.
**Abstract Book Citations:** Authors, Title, HemaSphere, 2026;10:(S1):pages. The abstract book DOIs can be found on the “Author Information” page.
**Disclaimer:** Articles published in the journal HemaSphere exclusively reflect the opinions of the authors. The authors are responsible for all content in their abstracts including accuracy of the facts, statements, citing resources, etc.

**Summary/Conclusion**

Rare damaging germline variants substantially contribute to B-cell lymphoma susceptibility, molecular heterogeneity, and clinical outcome. Integrating germline genetic information with somatic profiling may refine risk stratification, improve prognostic assessment, and provide a foundation for precision management strategies in B-cell lymphomas.

**Copyright Information:** (Online) ISSN: 2572-9241
©2026 The Author(s). HemaSphere is published by John Wiley & Sons Ltd on behalf of European Hematology Association. This is an open access Abstract Book distributed under the Attribution-NonCommercial-NoDerivs (CC BY-NC-ND), which allows third parties to download the articles and share them with others as long as they credit the author and the Abstract Book, but they cannot change the content in any way or use them commercially.
**Abstract Book Citations:** Authors, Title, HemaSphere, 2026;10:(S1):pages. The abstract book DOIs can be found on the "Author Information" page.
**Disclaimer:** Articles published in the journal HemaSphere exclusively reflect the opinions of the authors. The authors are responsible for all content in their abstracts including accuracy of the facts, statements, citing resources, etc.

### (PF1053) EFFICACY AND SAFETY OF GOLIDOCITINIB-CONTAINING REGIMENS IN RELAPSED/REFRACTORY PERIPHERAL T CELL LYMPHOMA: A REAL WORLD STUDY

**Topic:** 20. Other aggressive lymphomas

Shan Meng[1], Hui Zhang[1], Yilin Zhang[1], Bai-yan Wang[1], Liufang Gu*[1]

[1]*the Second Affiliated Hospital of Xi'an Jiaotong University, Hematology, Xi'an, China;*

### Background

The prognosis of relapsed/refractory peripheral T-cell lymphoma (R/R PTCL) is poor, particularly when complicated by hemophagocytic lymphohistiocytosis (HLH). Golidocitinib, a novel JAK1 inhibitor, offers a new therapeutic option. However, its real-world efficacy, especially in patients with HLH, and the associated risk of severe infections require urgent evaluation.

### Aims

In this single-center, retrospective, real world study, we aimed to evaluate the efficacy and infection risks of golidocitinib in R/R PTCL.

### Methods

From November 2023 to October 2025, totally 25 R/R PTCL patients treated with golidocitinib-containing regimens (150 mg orally daily) were included. Efficacy and safety were assessed in the overall population and in the HLH subgroup. Response was evaluated per the Lugano 2014 criteria, with endpoints including objective response rate (ORR), complete response (CR) rate, progression-free survival (PFS), and overall survival (OS). Adverse events (AEs) were graded by CTCAE v5.0, with special focus on infectious complications. The informed consents have been obtained from all the patients.

### Results

The median age was 55 years, and 76% were male. At baseline, Lugano staging was I-II in 24% (6/25) of patients and III-IV in 76% (19/25), and 28% (7/25) of patients had HLH. After a median follow-up of 4.0 months, the investigator-assessed ORR was 44% (11/25) and the CR rate was 8% (2/25) in the overall population. Notably, in the HLH subgroup (n=7), the ORR was 57% (4/7), suggesting therapeutic potential in this high-risk population. Median PFS and OS were 3.2 months and 4.0 months for the entire cohort, respectively. The estimated 6-month PFS and OS rates were 64.9% (95% CI: 39.4%-81.8%) and 79.0% (95% CI: 51.1%-92.0%), respectively. Infection was the most prominent safety concern. The most common grade ≥3 non-hematologic AEs were lung infection (20%, 5/25) and sepsis (8%, 2/25). Of particular note, two patients developed Pneumocystis jirovecii pneumonia (PJP) despite standard prophylaxis, one of whom died; one patient developed cytomegalovirus (CMV) infection. Hematologic toxicity was significant, with grade ≥3 lymphocytopenia, neutropenia, and leukopenia occurring in 60%, 52%, and 48% of patients, respectively. Among the four fatal cases, two were patients with baseline HLH who died from respiratory failure due to fungal pneumonia (involving Aspergillus, Candida, and PJP); the other two died from intestinal hemorrhage related to disease progression.

**Copyright Information:** (Online) ISSN: 2572-9241
©2026 The Author(s). HemaSphere is published by John Wiley & Sons Ltd on behalf of European Hematology Association. This is an open access Abstract Book distributed under the Attribution-NonCommercial-NoDerivs (CC BY-NC-ND), which allows third parties to download the articles and share them with others as long as they credit the author and the Abstract Book, but they cannot change the content in any way or use them commercially.
**Abstract Book Citations:** Authors, Title, HemaSphere, 2026;10:(S1):pages. The abstract book DOIs can be found on the “Author Information” page.
**Disclaimer:** Articles published in the journal HemaSphere exclusively reflect the opinions of the authors. The authors are responsible for all content in their abstracts including accuracy of the facts, statements, citing resources, etc.

**Summary/Conclusion**

This real-world study indicates that golidocitinib-containing regimens show preliminary efficacy in R/R PTCL, including in high-risk patients with HLH. However, treatment is associated with significant immunosuppression and a high risk of severe infections, particularly opportunistic infections (e.g., PJP, fungal, CMV), which may occur despite prophylaxis. Therefore, rigorous infection monitoring, aggressive prophylaxis, and prompt intervention are mandatory. Larger prospective studies are needed to optimize combination strategies and infection management protocols for golidocitinib-based therapy.

**Copyright Information:** (Online) ISSN: 2572-9241
©2026 The Author(s). HemaSphere is published by John Wiley & Sons Ltd on behalf of European Hematology Association. This is an open access Abstract Book distributed under the Attribution-NonCommercial-NoDerivs (CC BY-NC-ND), which allows third parties to download the articles and share them with others as long as they credit the author and the Abstract Book, but they cannot change the content in any way or use them commercially.
**Abstract Book Citations:** Authors, Title, HemaSphere, 2026;10:(S1):pages. The abstract book DOIs can be found on the "Author Information" page.
**Disclaimer:** Articles published in the journal HemaSphere exclusively reflect the opinions of the authors. The authors are responsible for all content in their abstracts including accuracy of the facts, statements, citing resources, etc.

### (PF1054) REAL-WORLD OUTCOMES OF PERIPHERAL AND CUTANEOUS T-CELL LYMPHOMAS IN KAZAKHSTAN: A NATIONWIDE MULTICENTER STUDY

**Topic:** 20. Other aggressive lymphomas

Ayazhan Umutbayeva*[1], Nazarii Shokun[2], Raigul Ramazanova[1], Aidana Shalabay[3], Aigerim Koshkarbayeva[3], Madina Zhumabay[3], Saule Gabbassova[3], Mariyam Akhmetova[1], Nurken Zhappargaly[1], Anastasiia Maliar[4], Madina Valiyeva[1], Massimo Federico[5], Akmaral Jazyltayeva[3]

*[1]Kazakh National Medical University, Almaty, Kazakhstan;[2]University of Salento, Lecce, Italy; [3]Kazakh National Institute of Oncology and Radiology, Almaty, Kazakhstan; [4]National cancer institute, Kyiv, Ukraine; [5]University of Modena and Reggio Emilia, Modena, Italy;*

### Background

Peripheral T-cell lymphomas (PTCL) and cutaneous T-cell lymphomas (CTCL) are rare, biologically heterogeneous entities that account for approximately 5–15% of all lymphomas worldwide. Despite therapeutic advances, outcomes remain poor compared with B-cell lymphomas, and robust real-world data from Central Asia are largely lacking. Such data are essential to benchmark outcomes, understand treatment patterns, and identify unmet clinical needs in routine practice.

### Aims

To analyze the clinical characteristics, treatment patterns, and outcomes of patients with PTCL and CTCL treated in Kazakhstan in a nationwide real-world setting.

### Methods

We conducted a retrospective multicenter cohort study including patients diagnosed with PTCL or CTCL between 2014 and 2024 across 12 hematology centers in Kazakhstan. Data on disease presentation, histological subtype, frontline treatment, response, and survival outcomes were collected. Progression-free survival (PFS) and overall survival (OS) were estimated using standard time-to-event methods. Survival comparisons were performed across patterns of disease involvement. To reduce heterogeneity, a predefined subgroup analysis focused on patients with nodal disease.

### Results

A total of 466 cases were collected over 10 years, with involvement types including nodal 285 (61%), cutaneous 85 (18%), extranodal 66 (14%), and leukemic 30 (6.4%). The corresponding 3-year PFS and OS rates were 43.3%/47.3%, 60.2%/64.8%, 28.5%/29.7%, and 30%/28.1%, respectively (p-value <0.005 cutaneous vs other and no difference between nodal, extranodal, and leukemic), with a median follow-up of 39.5 mos (1–120 mos). For more homogeneous analysis, only cases belonging to the nodal and extranodal groups were selected (n=351, 75%). The five most frequent subtypes were PTCL, NOS (n=181, 52%), ALCL, ALK- (n=45, 13%), TFHL (n=35, 10%), intestinal T-cell lymphoma (n=27, 7.7%), and ENKTL (n=25, 7.1%).
The most frequent regimen for this group was CHOP, CHOEP, and BV-CHP in 121(36%), 75 (23%), and 72 (22%) cases, respectively. The overall response rate was 58%, where complete response (CR) or partial response (PR) was achieved in 110 (38%) and 59 (20%) cases, respectively, and 124 (42%) encountered progressive disease (PD). For patients with CR or PR, 3-year PFS was 74% and 34%, respectively, vs PD in 27%, with differences between CR vs PR or PD and a p-value >0.05 for PR vs PD. Thirty-two (9.3%) patients were consolidated with autologous stem cell transplantation (auto-HSCT) after first-line therapy, with 3-year PFS of 76%.

**Copyright Information:** (Online) ISSN: 2572-9241
©2026 The Author(s). HemaSphere is published by John Wiley & Sons Ltd on behalf of European Hematology Association. This is an open access Abstract Book distributed under the Attribution-NonCommercial-NoDerivs (CC BY-NC-ND), which allows third parties to download the articles and share them with others as long as they credit the author and the Abstract Book, but they cannot change the content in any way or use them commercially.
**Abstract Book Citations:** Authors, Title, HemaSphere, 2026;10:(S1):pages. The abstract book DOIs can be found on the "Author Information" page.
**Disclaimer:** Articles published in the journal HemaSphere exclusively reflect the opinions of the authors. The authors are responsible for all content in their abstracts including accuracy of the facts, statements, citing resources, etc.

**Summary/Conclusion**

In this largest real-world PTCL/CTCL cohort reported from Kazakhstan, survival outcomes varied markedly according to disease involvement, with cutaneous lymphomas demonstrating significantly superior PFS and OS compared with nodal, extranodal, and leukemic presentations. Depth of response was strongly associated with long-term disease control, and consolidation with auto-HSCT was associated with favorable PFS in selected patients. These findings provide an important regional benchmark and underscore the need for improved risk-adapted approaches, wider access to novel agents, and prospective collaborative studies to improve outcomes in PTCL across Central Asia.

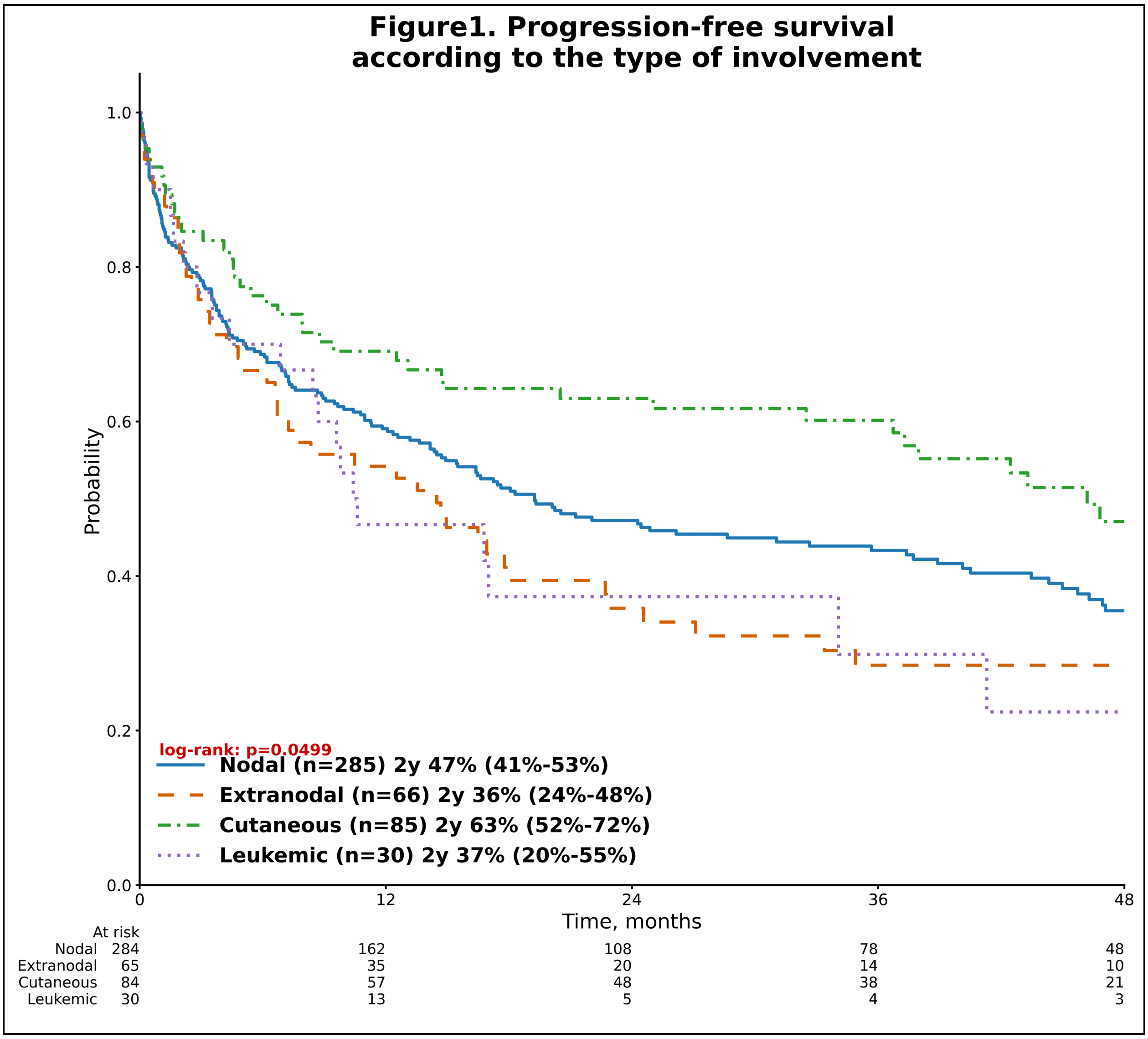

**Copyright Information:** (Online) ISSN: 2572-9241
©2026 The Author(s). HemaSphere is published by John Wiley & Sons Ltd on behalf of European Hematology Association. This is an open access Abstract Book distributed under the Attribution-NonCommercial-NoDerivs (CC BY-NC-ND), which allows third parties to download the articles and share them with others as long as they credit the author and the Abstract Book, but they cannot change the content in any way or use them commercially.
**Abstract Book Citations:** Authors, Title, HemaSphere, 2026;10:(S1):pages. The abstract book DOIs can be found on the “Author Information” page.
**Disclaimer:** Articles published in the journal HemaSphere exclusively reflect the opinions of the authors. The authors are responsible for all content in their abstracts including accuracy of the facts, statements, citing resources, etc.

### (PF1055) INCIDENCE AND PREDICTORS OF CARDIOVASCULAR EVENTS AFTER ANTHRACYCLINE-CONTAINING CHEMOTHERAPY IN LYMPHOMA: A RETROSPECTIVE LONGITUDINAL COHORT

**Topic:** 20. Other aggressive lymphomas

Dewan Zhao*[1], Boliang Li[2], Guirong Song[2], Xiuli Sun[1]

*[1]The First Affiliated Hospital of Dalian Medical University, Dalian, China;[2]Dalian Medical University, Dalian, China;*

### Background

Anthracyclines are central to lymphoma chemotherapy but carry a broad risk of cardiovascular adverse events (CVEs). Evidence is mainly from diffuse large B-cell lymphoma (DLBCL) cohorts receiving CHOP-based regimens, with limited data on subclinical endpoints and longitudinal trajectories of cardiac biomarkers—particularly B-type natriuretic peptide (BNP) and high-sensitivity troponin I (hsTnI).

### Aims

To establish a single-center retrospective longitudinal cohort to quantify CVE incidence and spectrum after anthracycline-containing chemotherapy in lymphoma, identify key risk factors, and characterize longitudinal BNP and hsTnI trajectories to inform risk stratification and monitoring.

### Methods

Hospitalized adults (≥18 years) with lymphoma treated at the Department of Hematology, The First Affiliated Hospital of Dalian Medical University (January 2011–October 2024) who received guideline-based anthracycline-containing regimens for ≥4 cycles were included. The median number of chemotherapy cycles was six, indicating substantial cumulative exposure. Exclusions were baseline cardiovascular disease requiring intervention, prior cardiotoxic therapy, or severe active infection. Cumulative CVE incidence (2022 ESC cardio-oncology) was estimated using Kaplan–Meier. Potential predictors were screened by univariable Cox; variables with P<0.10 entered multivariable Cox with likelihood ratio–based backward selection; proportional hazards and multicollinearity were assessed. Restricted cubic splines (RCS) evaluated dose–response relationships between baseline BNP/hsTnI and CVE risk. Longitudinal BNP and hsTnI were analyzed using linear mixed-effects models(LMMs) (fixed: time, CVE status, time×status; adjusted for centered age and cardiovascular history; random intercept/slope). Multiple imputation was applied for baseline creatine kinase-MB (CK-MB), hsTnI, and BNP when missingness was <25%. Sensitivity analyses repeated Cox and RCS in complete cases (n=220) and used an Andersen–Gill model for recurrent CVEs.

### Results

Among 279 patients (median follow-up, 223 days), 70 (25.1%) developed CVEs. Cumulative incidence was 0.7% at 30 days, 7.2% at 90 days, 21.0% at 180 days, and 26.9% at 360 days after chemotherapy initiation. Of CVE cases, 52.86% occurred during chemotherapy cycles, 25.72% experienced ≥2 distinct CVEs, and median time to first CVE was 129.5 days. Arrhythmias were most frequent (n=31; atrial fibrillation n=12; QTc prolongation n=11), followed by thrombotic/embolic events (n=26; venous thrombosis n=14; ischemic stroke n=10; pulmonary embolism n=2). Independent risk factors were age (HR 1.042, 95% CI 1.021–1.063; P<0.001), hypertension history (HR 1.838, 95% CI 1.092–3.095; P=0.022), baseline BNP (HR 1.001, 95% CI 1.000–1.002; P=0.010), and baseline hsTnI (HR 1.015, 95% CI 1.004–1.026; P=0.007). In LMMs, CVE patients had higher baseline BNP (β 0.456, 95% CI 0.217–0.695; P<0.001) without a significant temporal trend, whereas baseline hsTnI was similar but increased faster over time in CVE patients (β 0.072, 95% CI 0.017–0.127; P=0.010). Sensitivity analyses were consistent.

**Copyright Information:** (Online) ISSN: 2572-9241
©2026 The Author(s). HemaSphere is published by John Wiley & Sons Ltd on behalf of European Hematology Association. This is an open access Abstract Book distributed under the Attribution-NonCommercial-NoDerivs (CC BY-NC-ND), which allows third parties to download the articles and share them with others as long as they credit the author and the Abstract Book, but they cannot change the content in any way or use them commercially.
**Abstract Book Citations:** Authors, Title, HemaSphere, 2026;10:(S1):pages. The abstract book DOIs can be found on the "Author Information" page.
**Disclaimer:** Articles published in the journal HemaSphere exclusively reflect the opinions of the authors. The authors are responsible for all content in their abstracts including accuracy of the facts, statements, citing resources, etc.

### Summary/Conclusion

CVEs were common and occurred early after anthracycline-based therapy in lymphoma. Age, hypertension, and baseline BNP/hsTnI independently predicted risk; BNP reflected baseline vulnerability, while rising hsTnI indicated ongoing myocardial injury. Integrating baseline stratification with on-treatment biomarker monitoring may improve early detection and prevention.

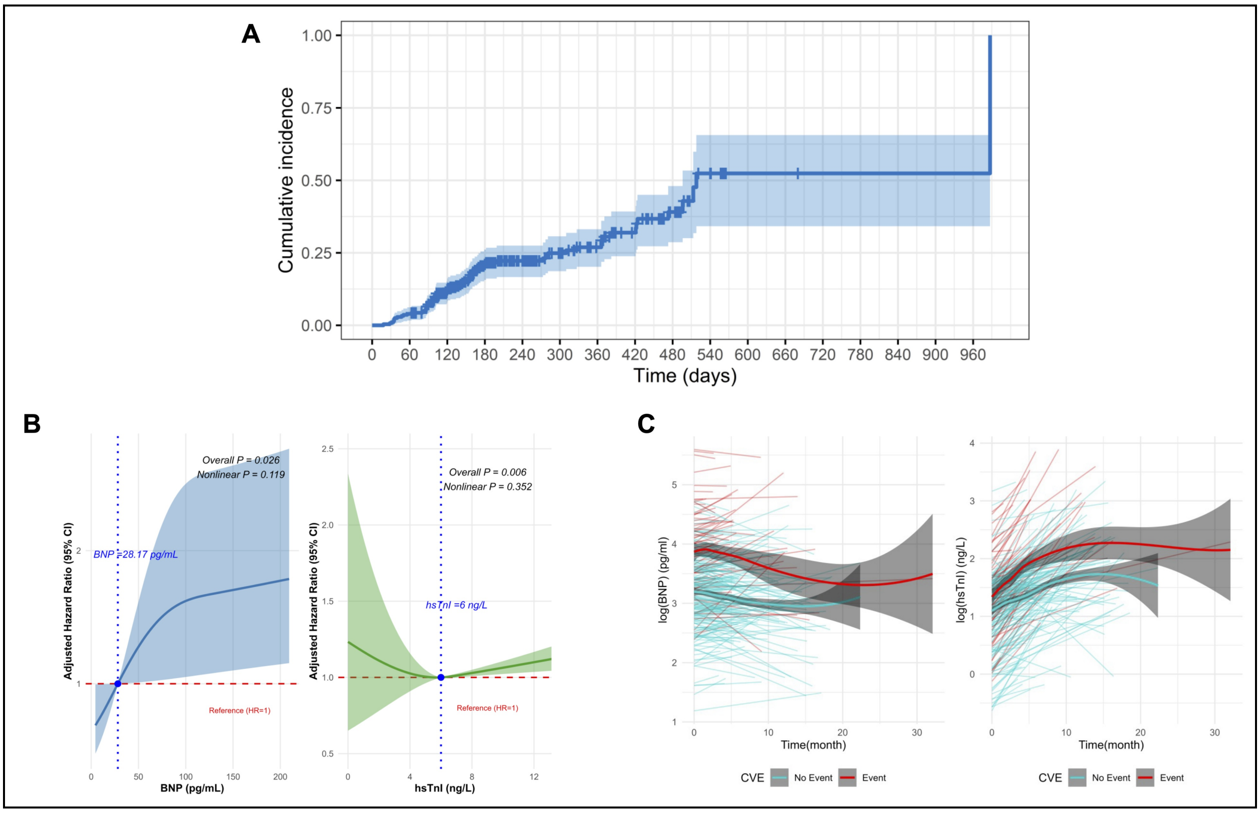

Figure 1. Cumulative incidence of cardiovascular adverse events (CVEs) and biomarker-based risk/trajectory patterns in lymphoma patients receiving anthracycline-containing chemotherapy. (A) Kaplan–Meier curve showing the cumulative incidence of first CVE during follow-up. (B) Restricted cubic spline (RCS) plots illustrating the dose–response relationships between baseline B-type natriuretic peptide (BNP) and high-sensitivity troponin I (hsTnI) with CVE risk. After adjustment for age, hypertension, and the other biomarker, CVE risk increased with higher BNP and hsTnI, with no evidence of nonlinearity (P for nonlinearity: BNP=0.119; hsTnI=0.285). hsTnI was referenced to 6 ng/L; 95% confidence intervals (CIs) were wider below this level and risk increased steadily above it. (C) Longitudinal trajectories of cardiac biomarkers by CVE status. Locally estimated scatterplot smoothing (LOESS) curves depict changes in BNP (left) and hsTnI (right) during follow-up according to CVE occurrence. BNP was higher at baseline in patients who developed CVEs and remained consistently higher, with no clear temporal trend in either group. Baseline hsTnI was comparable between groups, but hsTnI increased more rapidly over time in patients who developed CVEs, leading to progressively widening differences between the groups.

**Copyright Information:** (Online) ISSN: 2572-9241
©2026 The Author(s). HemaSphere is published by John Wiley & Sons Ltd on behalf of European Hematology Association. This is an open access Abstract Book distributed under the Attribution-NonCommercial-NoDerivs (CC BY-NC-ND), which allows third parties to download the articles and share them with others as long as they credit the author and the Abstract Book, but they cannot change the content in any way or use them commercially.
**Abstract Book Citations:** Authors, Title, HemaSphere, 2026;10:(S1):pages. The abstract book DOIs can be found on the "Author Information" page.
**Disclaimer:** Articles published in the journal HemaSphere exclusively reflect the opinions of the authors. The authors are responsible for all content in their abstracts including accuracy of the facts, statements, citing resources, etc.

### (PF1056) SURVIVAL BENEFIT ASSOCIATED WITH TREATMENT AT ACADEMIC CANCER PROGRAMS IN OLDER ADULTS WITH PTCL: A NATIONAL CANCER DATABASE ANALYSIS

**Topic:** 20. Other aggressive lymphomas

Henry Idrobo*[1], Laura Reyes Uribe[2], Joel Michalek[3], Qianqian Liu[3], Adolfo Diaz Duque[3]

*[1]Universidad Tecnológica de Pereira, Clínica Central del Eje, Pereira, Colombia;[2]DHR Health, Edinburg, United States of America; [3]UT Health San Antonio / Mays Cancer Center, San Antonio, United States of America;*

### Background

Peripheral T cell lymphoma (PTCL) is an aggressive mature T cell neoplasm associated with poor prognosis and limited therapeutic options. It predominantly affects older adults and is frequently diagnosed at advanced stage. Outcomes for this disease remain inferior compared with many B cell lymphomas despite advances in contemporary lymphoma care. Evidence guiding optimal management in older adults with PTCL remains limited, in part due to the low representation of this population in prospective clinical studies. Population based and real world analyses have shown that older age, comorbidity burden, and differences in access to specialized lymphoma care are independently associated with inferior outcomes beyond underlying disease characteristics. The impact of treatment facility type on outcomes among older adults with PTCL remains insufficiently defined.

### Aims

Primary Aim

To evaluate whether treatment facility type (Academic Cancer Programs vs Community Cancer Programs) is associated with differences in overall survival (OS) among patients aged ≥75 years with peripheral T-cell lymphoma (PTCL) in a large national cohort.

### Methods

We conducted a retrospective analysis of patients aged 75 years (y) and older diagnosed with PTCL in the US between 2004 and 2022 using the National Cancer Database. Demographic, socioeconomic, clinical, treatment, and survival characteristics were compared between patients treated at Academic Cancer Programs (ACP) and Community Cancer Programs (CCP). Kaplan-Meier and Cox regression analyses were used to compare overall survival (OS) between the two cohorts. Variables used for adjustment included age, ethnicity, insurance status, distance from hospital, and Charlson-Deyo comorbidity score.

### Results

Of 4,533 patients aged 75 y and older with PTCL, 2,402 (53%) were treated at ACP and 2,131 (47%) at CCP. Patients treated at ACP were slightly younger, with a median age of 80 y compared to 81 y at CCP, p value 0.002. Most patients in both groups presented with advanced stage disease. Most patients had Medicare coverage, and comorbidity burden did not differ meaningfully between cohorts. Treatment patterns differed by facility type. Patients treated at ACP were more likely to receive treatment, whereas a substantial proportion of patients treated at CCP had no documented treatment or had missing or unknown treatment status. Time from diagnosis to initiation of systemic therapy was shorter at ACP compared with CCP. On survival analysis, two y survival was 29% at ACP vs. 26% at CCP, five y survival was 17% vs. 14%, and ten y survival was 8% vs. 6%. OS was significantly higher among patients treated at ACP compared with CCP, p value 0.047.

**Copyright Information:** (Online) ISSN: 2572-9241
©2026 The Author(s). HemaSphere is published by John Wiley & Sons Ltd on behalf of European Hematology Association. This is an open access Abstract Book distributed under the Attribution-NonCommercial-NoDerivs (CC BY-NC-ND), which allows third parties to download the articles and share them with others as long as they credit the author and the Abstract Book, but they cannot change the content in any way or use them commercially.
**Abstract Book Citations:** Authors, Title, HemaSphere, 2026;10:(S1):pages. The abstract book DOIs can be found on the "Author Information" page.
**Disclaimer:** Articles published in the journal HemaSphere exclusively reflect the opinions of the authors. The authors are responsible for all content in their abstracts including accuracy of the facts, statements, citing resources, etc.

### Summary/Conclusion

Among patients aged 75 y and older with PTCL in this national cohort, treatment at ACP was associated with superior OS compared with CCP. Differences in outcomes by facility type suggest that access to specialized lymphoma expertise may influence survival in this population. These findings support the need for timely referral to ACP, consideration of transfer of care when appropriate, and greater use of expert consultation, access to clinical trials and guideline based management for older adults with PTCL.

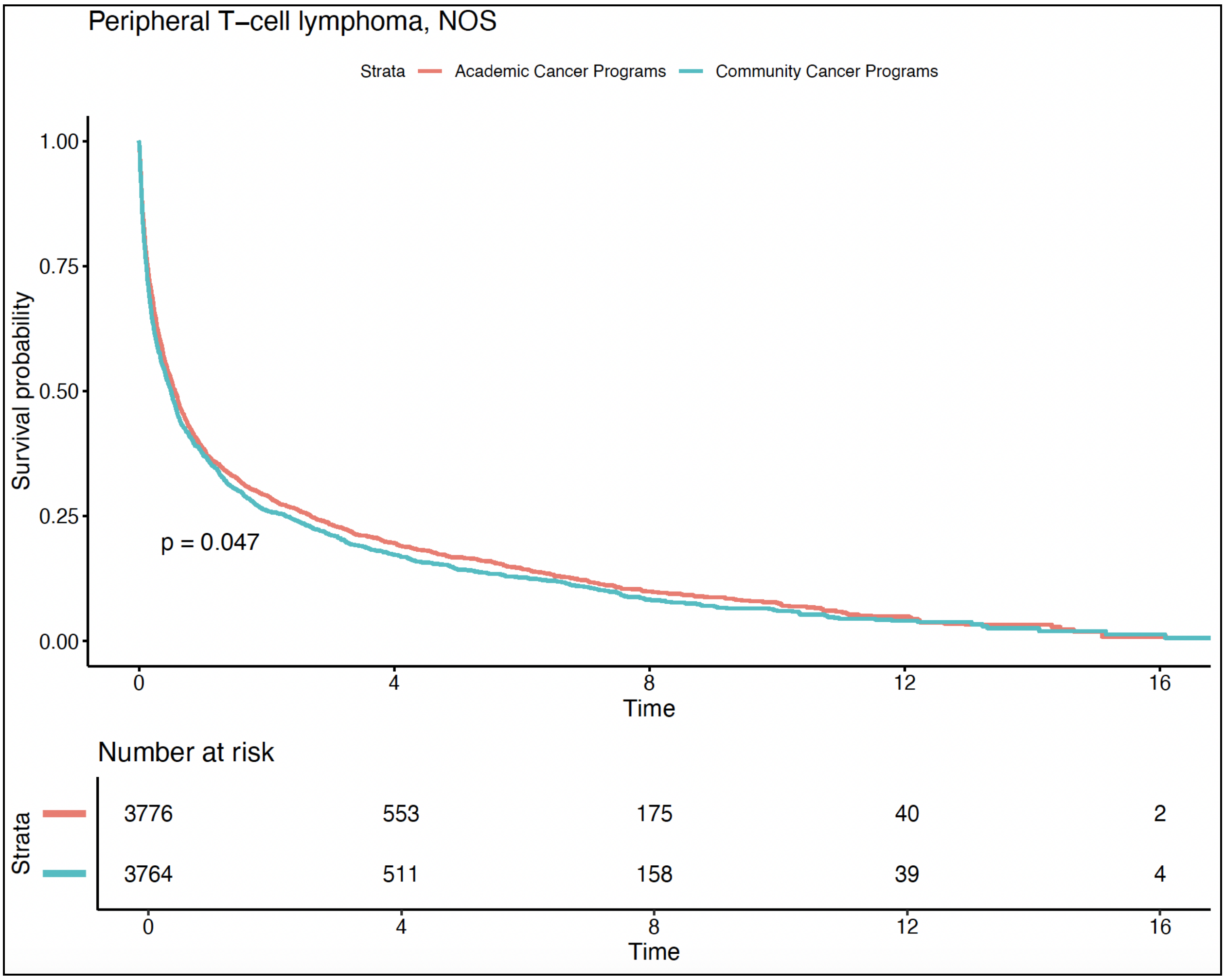

**Copyright Information:** (Online) ISSN: 2572-9241
©2026 The Author(s). HemaSphere is published by John Wiley & Sons Ltd on behalf of European Hematology Association. This is an open access Abstract Book distributed under the Attribution-NonCommercial-NoDerivs (CC BY-NC-ND), which allows third parties to download the articles and share them with others as long as they credit the author and the Abstract Book, but they cannot change the content in any way or use them commercially.
**Abstract Book Citations:** Authors, Title, HemaSphere, 2026;10:(S1):pages. The abstract book DOIs can be found on the "Author Information" page.
**Disclaimer:** Articles published in the journal HemaSphere exclusively reflect the opinions of the authors. The authors are responsible for all content in their abstracts including accuracy of the facts, statements, citing resources, etc.

### (PF1057) PHASE IB STUDY OF SHR0302 PLUS SHR2554 IN RELAPSED/REFRACTORY PERIPHERAL T-CELL LYMPHOMA: SAFETY RUN-IN AND INITIAL EFFICACY

**Topic:** 20. Other aggressive lymphomas

Rong Tao*[1, 2], Chuanxu Liu[1, 2], Da-Hui Li[1, 2], Wen-Hao Zhang[1, 2], Shiyu Jiang[1, 2], Changhong Zhao[3], Miao Zhang[3]

*[1]Department of Lymphoma and Medical Oncology, Fudan University Shanghai Cancer Center, Shanghai, China;[2]Research Center for Lymphoma, Fudan University, Shanghai, China; [3]Department of Clinical Research & Development, Jiangsu Hengrui Pharmaceuticals Co., Ltd., Nanjing, China;*

### Background

Peripheral T-cell lymphoma (PTCL) remains difficult to treat after relapse, with limited effective options. Aberrant JAK-STAT signaling and epigenetic dysregulation are implicated in PTCL pathogenesis, providing a rationale for dual JAK1 and EZH2 inhibition. SHR0302 is a highly selective JAK1 inhibitor; SHR2554 is an oral EZH2 inhibitor that has shown activity in relapsed/refractory (R/R) PTCL and has received conditional approval by China's National Medical Products Administration (NMPA) for adults with R/R PTCL after ≥1 prior systemic therapy.

### Aims

To evaluate safety/tolerability and preliminary antitumor activity of SHR0302 plus SHR2554 and to inform selection of the recommended phase II dose (RP2D) within an overall phase Ib/II study (NCT06519526). We report the phase Ib 3+3 safety run-in and an initial efficacy readout per the prespecified two-stage design.

### Methods

This investigator-initiated, open-label phase Ib/II study enrolled adults (18–70 years) with histologically confirmed R/R PTCL. SHR0302 was administered orally once daily at 4 mg or 8 mg with fixed-dose SHR2554 300 mg orally twice daily in 28-day cycles. Dose-limiting toxicities (DLTs) were assessed during Cycle 1 using a standard 3+3 design (CTCAE v5.0). Efficacy was assessed by dose level using a Simon two-stage design: stage 1 expands to 6 response-evaluable patients and proceeds if ≥2 achieve CR/PR; stage 2 expands to a total of 13 response-evaluable patients, with ≥5 CR/PR in 13 as the prespecified threshold. Responses were assessed per Lugano 2014 by CT/MRI every two cycles (±7 days); PET-CT was performed at baseline and end of Cycle 4. The study was approved by the institutional ethics committee and all patients provided written informed consent.

### Results

As of Jan 28, 2026, 14 patients were treated (SHR0302 4 mg n=9; 8 mg n=5): PTCL-NOS (n=4), angioimmunoblastic T-cell lymphoma (AITL, n=8), and anaplastic large cell lymphoma (ALCL, n=2); median prior lines of therapy was 2. No DLTs were observed during Cycle 1 at either dose level. Grade ≥3 adverse events occurring in ≥10% included neutrophil decrease (28.6%), white blood cell decrease (21.4%), and platelet decrease (14.3%). Ten patients were response-evaluable at data cut-off (4 mg n=6; 8 mg n=4); four were not evaluable due to patient-related withdrawal/discontinuation prior to the first scheduled assessment. Among response-evaluable patients (n=10), best overall responses were CR 2 (20.0%), PR 2 (20.0%), SD 4 (40.0%), and PD 2 (20.0), yielding an ORR of 40.0% (4/10) and DCR of 80.0% (8/10). At 4 mg (stage 1; n=6), ORR was 50.0% (3/6) with CR 33.3% (2/6) and DCR 83.3% (5/6), meeting the prespecified criterion to proceed. At 8 mg, preliminary outcomes to date among evaluable patients (n=4) showed ORR 25.0% and DCR 75.0%, with stage-1 enrollment ongoing. RP2D has not yet been selected and will be determined based on the integrated safety and activity profile across dose levels.

**Copyright Information:** (Online) ISSN: 2572-9241
©2026 The Author(s). HemaSphere is published by John Wiley & Sons Ltd on behalf of European Hematology Association. This is an open access Abstract Book distributed under the Attribution-NonCommercial-NoDerivs (CC BY-NC-ND), which allows third parties to download the articles and share them with others as long as they credit the author and the Abstract Book, but they cannot change the content in any way or use them commercially.
**Abstract Book Citations:** Authors, Title, HemaSphere, 2026;10:(S1):pages. The abstract book DOIs can be found on the "Author Information" page.
**Disclaimer:** Articles published in the journal HemaSphere exclusively reflect the opinions of the authors. The authors are responsible for all content in their abstracts including accuracy of the facts, statements, citing resources, etc.

**Summary/Conclusion**

SHR0302 plus SHR2554 demonstrated manageable safety without observed DLTs and showed initial antitumor activity in R/R PTCL. Completion of stage-1 evaluations across dose levels and further follow-up are ongoing to inform RP2D selection and subsequent study stages.

**Copyright Information:** (Online) ISSN: 2572-9241
©2026 The Author(s). HemaSphere is published by John Wiley & Sons Ltd on behalf of European Hematology Association. This is an open access Abstract Book distributed under the Attribution-NonCommercial-NoDerivs (CC BY-NC-ND), which allows third parties to download the articles and share them with others as long as they credit the author and the Abstract Book, but they cannot change the content in any way or use them commercially.
**Abstract Book Citations:** Authors, Title, HemaSphere, 2026;10:(S1):pages. The abstract book DOIs can be found on the "Author Information" page.
**Disclaimer:** Articles published in the journal HemaSphere exclusively reflect the opinions of the authors. The authors are responsible for all content in their abstracts including accuracy of the facts, statements, citing resources, etc.

### (PF1058) TARGETING TNFR2 WITH BI-1808 WITH OR WITHOUT PEMBROLIZUMAB: IMMUNE ACTIVATION AND PROMISING RESPONSES IN ADVANCED CUTANEOUS T-CELL LYMPHOMAS

**Topic:** 20. Other aggressive lymphomas

Christiane Querfeld[1], Stefan Barta*[2], Stephen Morris[3], Daniel Morillo[4], Richard Cowan[5], Pablo Ortiz[6], Sean Lim[7], Susanne Gertsson[8], Petra Holmkvist[8], Ingrid Karlsson[8], Linda Mårtensson[8], Danijela Lindahl[8], Dmytro Piliuhin[8], Anette Sundstedt[8], Ingrid Teige[8], Johan Wallin[8], Michael Chisamore[9], Björn Frendeus[8, 10], Andres McAllister[8]

*[1]City of Hope Comprehensive Cancer Center, Duarte, CA, United States of America;[2]University of Pennsylvania Hospital, Philadelphia, United States of America; [3]Guy's and St Thomas' NHS Foundation Trust, London, United Kingdom;[4]Hospital Universitario Fundación Jiménez Díaz, Madrid, Spain; [5]The Christie Foundation Trust, Manchester, United Kingdom;[6]Catedrático de Dermatología, Hospital Universitario 12 de Octubre,Universidad Complutense, Madrid, Spain; [7]University Hospital Southampton NHS Foundation Trust, Southampton, United Kingdom; [8]BioInvent International AB, Lund, Sweden;[9]Merck & Co., Inc, Rahway, NJ, United States of America; [10]Centre for Cancer Immunology, University of Southampton, Southampton, United Kingdom;*

### Background

Cutaneous T cell lymphomas (CTCL) are rare nonHodgkin lymphomas arising from malignant skinhoming T cells. The most common subtypes are mycosis fungoides (MF), typically an indolent skin-limited disease, and Sézary syndrome (SS), an aggressive leukemic variant characterized by circulating malignant T cells and erythroderma.Outcomes for advanced CTCL subtypes remain poor, with limited 20-60% 5-year survival.
TNFR2 has emerged as a pathogenic driver in CTCL through gain-of-function alterations that increase expression in malignant $CD4^{+}CD26^{-}$ cells. BI-1808, an IgG1 monoclonal antibody directed against TNFR2, blocks ligand engagement and induces FcγR-mediated depletion of regulatory T cells, leading to enhanced intratumoral $CD8^{+}$ T-cell activity. Clinical studies have demonstrated antitumor activity in CTCL and solid tumors, supporting TNFR2 targeting as a promising broad immunotherapeutic approach.
Preclinical data indicates a positive combinatorial effect of BI-1808 and pembrolizumab. Pembrolizumab has shown promising results in clinical trials in CTCL.

### Aims

Safety and preliminary efficacy are currently investigated in CTCL patients, in a sub-cohort of the ongoing Phase 2a clinical trial 19-BI-1808-01.

### Methods

The study is designed to enroll 20 patients at 1000 mg BI-1808 Q3W, followed by 10 patients to receive 1000 mg BI-1808 + 200 mg Q3W pembrolizumab. The study also includes a dose optimization phase enrolling 10+10 patients evaluating BI-1808 at 250 mg vs 1000 mg Q3W currently ongoing.

**Copyright Information:** (Online) ISSN: 2572-9241
©2026 The Author(s). HemaSphere is published by John Wiley & Sons Ltd on behalf of European Hematology Association. This is an open access Abstract Book distributed under the Attribution-NonCommercial-NoDerivs (CC BY-NC-ND), which allows third parties to download the articles and share them with others as long as they credit the author and the Abstract Book, but they cannot change the content in any way or use them commercially.
**Abstract Book Citations:** Authors, Title, HemaSphere, 2026;10:(S1):pages. The abstract book DOIs can be found on the "Author Information" page.
**Disclaimer:** Articles published in the journal HemaSphere exclusively reflect the opinions of the authors. The authors are responsible for all content in their abstracts including accuracy of the facts, statements, citing resources, etc.

**Results**

As of February 19, 2026, the signal-seeking portion of the study has been fully enrolled. 20 patients with advanced stage CTCL received BI-1808 as single-agent Q3W (13 MF, 8 SS), and 9 patients received BI-1808 in combination with pembrolizumab (6 MF, 3 SS). MF patients had previously received a median of 5 (2-13) prior systemic treatments, and SS patients 3 (1-14).
Treatment related AEs were classified as mild or moderate. G3 TEAEs included one skin reaction in the monotherapy arm, and in the combination arm one instance of pulmonary embolism, and one case of colitis were observed. Disease "flares" (characterized by increased skin peeling, erythema, and pruritus) were observed early during treatment in several cases, and were shown to be related to immune activation, depletion of T regs and influx of CD8+ T cells.
Immunofluorescence multiplex staining of skin biopsies showed a significant increase in CD8+ infiltration and accompanying granzyme B elevation in responding patients at 5 weeks after start of treatment. Serum analysis showed rapid and sustained induction of IL-12. IL-12 is crucial for counteracting the protumor Th2 cytokine profile and switching the balance in favor of Th1 $CD8^{+}$ T cells and NK cells.

Out of 15 CTCL mSWAT evaluable cases in the single agent arm, 1 SS patient exhibited complete response (CR) lasting approximately 2 years to date and ongoing, 5 participants (3 MF, 2 SS) exhibited confirmed partial response (PR) as best clinical response, and 8 participants showed stable disease (SD). Out of 8 evaluable patients in the combination arm, 4 patients exhibited PR at first assessment (3 MF, 1 SS), and 2 patients SD.
More complete data will be disclosed in the poster.

**Summary/Conclusion**

Current data from the signal seeking cohort of BI-1808 in CTCL show promising efficacy associated with strong immune activation in patients with advanced CTCL, leading to an impressive objective response rate of 40% in BI-1808 single agent, and 50% in combination with pembrolizumab.

**Copyright Information:** (Online) ISSN: 2572-9241
©2026 The Author(s). HemaSphere is published by John Wiley & Sons Ltd on behalf of European Hematology Association. This is an open access Abstract Book distributed under the Attribution-NonCommercial-NoDerivs (CC BY-NC-ND), which allows third parties to download the articles and share them with others as long as they credit the author and the Abstract Book, but they cannot change the content in any way or use them commercially.
**Abstract Book Citations:** Authors, Title, HemaSphere, 2026;10:(S1):pages. The abstract book DOIs can be found on the "Author Information" page.
**Disclaimer:** Articles published in the journal HemaSphere exclusively reflect the opinions of the authors. The authors are responsible for all content in their abstracts including accuracy of the facts, statements, citing resources, etc.

### (PF1059) CHANGES IN LEAN MUSCLE MASS, MUSCLE STRENGTH, AND PHYSICAL PERFORMANCE FOLLOWING FIRST-LINE CHEMOTHERAPY IN PATIENTS WITH MALIGNANT LYMPHOMA

**Topic:** 20. Other aggressive lymphomas

Charlotte Grønset*[1], Mary Jarden[1], Jan Christensen[2], Casper Simonsen[3], Anders Tolver[4], Charlotte Suetta[5], Martin Hutchings[1]

*[1]Copenhagen University Hospital, Rigshospitalet, Denmark, Department of Hematology, Copenhagen, Denmark;[2]Copenhagen University Hospital, Rigshospitalet, Denmark, Department of Occupational Therapy and Physiotherapy, Copenhagen, Denmark; [3]Copenhagen University Hospital, Rigshospitalet, Denmark, Centre for Physical Activity Research, Copenhagen, Denmark; [4]Danish Cancer Society, Statistics and Data Analysis, Copenhagen, Denmark; [5]Copenhagen University Hospital, Bispebjerg and Frederiksberg Hospital, Geriatric Research Unit, Copenhagen, Denmark;*

**Background**

Sarcopenia is a strong predictor of treatment toxicity, cancer-related mortality, and reduced survival in patients with cancer, yet it remains underrecognized in clinical practice. In malignant lymphoma, muscle function and sarcopenia classification are rarely assessed as part of routine care, despite their prognostic relevance for treatment tolerance and outcomes during intensive chemotherapy.

**Aims**

To our knowledge, this is the first prospective cohort study to evaluate changes in muscle mass, strength, and physical performance during chemotherapy in patients with lymphoma and to examine their association with treatment tolerability.

**Methods**

A single-center prospective cohort study of adult patients (≥18 years) recently diagnosed with aggressive lymphoma and planned for first-line anthracycline-based chemotherapy. Assessments were conducted before treatment initiation and at six months, including body composition (DEXA), handgrip strength, 30-sec Sit-to-Stand test and 10-meter gait speed. Low muscle parameters and sarcopenia classification were defined according to the European Working Group on Sarcopenia in Older People2 (EWGSOP2) guidelines. Furthermore, results of muscle parameters were compared to age- and sex-matched healthy Danish individuals to explore muscle dysfunction levels.

**Copyright Information:** (Online) ISSN: 2572-9241
©2026 The Author(s). HemaSphere is published by John Wiley & Sons Ltd on behalf of European Hematology Association. This is an open access Abstract Book distributed under the Attribution-NonCommercial-NoDerivs (CC BY-NC-ND), which allows third parties to download the articles and share them with others as long as they credit the author and the Abstract Book, but they cannot change the content in any way or use them commercially.
**Abstract Book Citations:** Authors, Title, HemaSphere, 2026;10:(S1):pages. The abstract book DOIs can be found on the “Author Information” page.
**Disclaimer:** Articles published in the journal HemaSphere exclusively reflect the opinions of the authors. The authors are responsible for all content in their abstracts including accuracy of the facts, statements, citing resources, etc.

**Results**

Both assessments were completed by 58 patients (median age 62, range 18–87; 28% female). Significant changes were observed from baseline to six months follow-up in lean body mass, with a decrease by 1.7 kg (95% CI −2.55 to −0.85, p = 0.0002). Conflicting significant results were seen in relation to upper and lower muscle strength. Upper muscle strength (handgrip) decreased by 1.9 kg (95% CI −3.15 to −0.71, p = 0.002), whereas lower muscle strength (Sit-To-Stand) increased by 1.4 repetitions (95% CI 0.15 to 2.69, p = 0.03). No changes in physical performance were observed.
At baseline, 34% of patients (median age 69) had impaired muscle strength, muscle mass, or physical performance according to EWGSOP2 criteria, with 3% classified as sarcopenic and 12% as possible sarcopenia. At follow-up, the prevalence increased to 5% with severe sarcopenia, 10% with sarcopenia, and 17% with possible sarcopenia.
Compared with healthy, age- and sex-matched adults, 49 patients (84%; 14 females) with a median age of 53 years (range 18–83) had low or severely low muscle strength, muscle mass, or physical performance at baseline. At six-month follow-up, 51 patients (88%; 14 females) with a mean age of 54 years (range 18–83) exhibit low or severely low muscle parameters.
No association was found between muscle parameters and treatment tolerability (dose delay, hospital admissions or treatment response).

**Summary/Conclusion**

Patients with lymphoma experience marked declines in lean body mass and muscle strength from chemotherapy initiation to six-month follow-up. Routine assessment of muscle function can identify those at risk of functional deterioration. Early, targeted rehabilitation integrated into clinical care may help preserve function and support patients throughout treatment.

**Copyright Information:** (Online) ISSN: 2572-9241
©2026 The Author(s). HemaSphere is published by John Wiley & Sons Ltd on behalf of European Hematology Association. This is an open access Abstract Book distributed under the Attribution-NonCommercial-NoDerivs (CC BY-NC-ND), which allows third parties to download the articles and share them with others as long as they credit the author and the Abstract Book, but they cannot change the content in any way or use them commercially.
**Abstract Book Citations:** Authors, Title, HemaSphere, 2026;10:(S1):pages. The abstract book DOIs can be found on the “Author Information” page.
**Disclaimer:** Articles published in the journal HemaSphere exclusively reflect the opinions of the authors. The authors are responsible for all content in their abstracts including accuracy of the facts, statements, citing resources, etc.

### (PF1060) MENDELIAN RANDOMIZATION REVEALS THE CAUSATIVE ROLE OF GENETICALLY PREDICTED VIRAL INFECTION IN PAN-LYMPHOMA RISK

**Topic:** 20. Other aggressive lymphomas

Wanying Chen[1, 2], Zhou Jin[1, 2], Qian Wang[1, 2], Depei Wu[1, 2], Tao You[1, 2], Haiwen Huang*[1, 2]

*[1]The First Affiliated Hospital Of Soochow University, Hematology, Suzhou, China;[2]National Clinical Research Center for Hematologic Diseases, The First Affiliated Hospital of Soochow University., SUZHOU, China;*

### Background

While observational studies have suggested associations between viral infections and lymphoma risk, causal relationships remain unclear. This pan-lymphoma analysis aims to comprehensively explore the role of common viral infections, including HBV, HCV, EBV, CMV, HIV, HSV, HTLV and VZV, in lymphoma development.

### Methods

We conducted a two-sample bidirectional MR analysis using GWAS Catalog and FinnGen R11 data to examine associations between genetically predicted viral infections and multiple lymphoma subtypes (HL, DLBCL, FL, MZL, MCL, WM, NFL, NHL, TNK, and CLL). Causal effects were evaluated via inverse variance-weighted (IVW) methods and validated through sensitivity analyses (MR-PRESSO, MR-Egger, leave-one-out). External validation was performed using chi-square and survival analyses in the UK Biobank.

### Results

Longitudinal analysis in the UK Biobank identified 26 significant associations. Kaplan-Meier (K-M) and Cumulative Incidence Function (CIF) analyses, adjusting for competing mortality risks, revealed significant associations for EBV with TNK (CIF P=0.0414, K-M P=0.0047) and WM (CIF P=0.0067, K-M P=0.00054). HBV predicted WM development (CIF P=0.040, K-M P=0.011). Notably, HCV showed a profound impact on NAS (CIF P=0.0095, K-M P=0.00064) and WM (CIF/K-M P<0.0001). HIV significantly accelerated cumulative incidence of NAS and WM (both P<0.05). HTLV was linked to HL and WM predisposition (P<0.05), while VZV predicted TNK incidence (CIF P<0.05, K-M P=0.023). Across all pairs, CIF substantiated that viral exposures modulate long-term lymphoma risk post-mortality adjustment.Bi-directional MR analysis revealed 10 significant causal associations. Negative associations were found between CMV IgG and HL (OR 0.86, 95% CI 0.76-0.98, P<0.05) and CMV pp52 and non-FL (OR 0.88, 95% CI 0.79-0.99, P=0.026). EBV IgG showed protective effects against FL (OR 0.91, 95% CI 0.85-0.99, P<0.05) and WM (OR 0.75, 95% CI 0.59-0.95, P<0.05); EBV EBNA-1 was negatively associated with HL (OR 0.72, 95% CI 0.53-0.97, P<0.05). Conversely, elevated EBV EA-D increased WM risk (OR 2.23, 95% CI 1.09-4.54, P<0.05), while EBV VCA p18 showed an inverse relationship with WM (OR 0.97, 95% CI 0.94-0.99, P=0.039). HIV increased MCL risk (OR 1.01, 95% CI 1.00-1.02, P<0.05).HSV-1 mgG-1 (OR 1.72, 95% CI 1.05-2.82, P<0.05), HSV-2 mgG-1 (OR 1.38, 95% CI 1.01-1.88, P<0.05), and HSV-2 IgG (OR 1.45, 95% CI 1.05-2.01, P<0.05) were associated with higher WM risk. HSV-1 mgG-1 was linked to increased MZL risk (OR 1.52, 95% CI 1.08-2.14, P<0.05) but decreased TNK risk (OR 0.75, 95% CI 0.58-0.99, P<0.05). HSV-2 IgG increased HL risk (OR 1.14, 95% CI 1.03-1.27, P<0.05), while HSV-1 IgG was protective against non-specific NHL (OR 0.93, 95% CI 0.87-0.99, P<0.05).

### Summary/Conclusion

Our findings provide evidence for the causative role of various viral infections in the development of different lymphoma types. These results offer new insights into the pathogenesis of lymphoma and suggest potential viral prophylactic targets for lymphoma prevention.

**Copyright Information:** (Online) ISSN: 2572-9241
©2026 The Author(s). HemaSphere is published by John Wiley & Sons Ltd on behalf of European Hematology Association. This is an open access Abstract Book distributed under the Attribution-NonCommercial-NoDerivs (CC BY-NC-ND), which allows third parties to download the articles and share them with others as long as they credit the author and the Abstract Book, but they cannot change the content in any way or use them commercially.
**Abstract Book Citations:** Authors, Title, HemaSphere, 2026;10:(S1):pages. The abstract book DOIs can be found on the "Author Information" page.
**Disclaimer:** Articles published in the journal HemaSphere exclusively reflect the opinions of the authors. The authors are responsible for all content in their abstracts including accuracy of the facts, statements, citing resources, etc.

### (PF1061) TOTAL METABOLIC TUMOR VOLUME AND CIRCULATING TUMOR DNA AS EARLY PROGNOSTIC FACTORS IN RELAPSED/REFRACTORY DIFFUSE LARGE B-CELL LYMPHOMA: RESULTS FROM THE PHASE 3 STARGLO TRIAL

**Topic:** 21. Lymphoma biology & translational research

William Townsend*[1], Anton Belousov[2], Jeremy S. Abramson[3], Matthew Ku[4], Huiqiang Huang[5], Christopher P. Fox[6], Hui-Lai Zhang[7], Dok Hyun Yoon[8], Wonseog Kim[9], Herve Ghesquieres[10], Charles Herbaux[11], Qing-Yuan Zhang[12], Haifaa Abdulhaq[13], Skander Jemaa[14], Estefania Mulvihill[2], Martine Joanna Kallemeijn[2], Murali Kesavan[15], Linda Lundberg[2], Alessia Bottos[2], Gareth P. Gregory[16]

*[1]University College London Hospitals, London, United Kingdom; [2]F. Hoffmann-La Roche Ltd, Basel, Switzerland; [3]Massachusetts General Hospital Cancer Center, Boston, MA, United States of America; [4]St Vincent's Hospital, University of Melbourne, Melbourne, VIC, Australia; [5]Sun Yat-sen University Cancer Center, Guangzhou, China;[6]School of Medicine, University of Nottingham, Nottingham, United Kingdom; [7]Tianjin Medical University Cancer Institute, Tianjin, China;[8]Asan Medical Center, University of Ulsan College of Medicine, Seoul, South Korea; [9]Samsung Medical Center, Sungkyunkwan University School of Medicine, Seoul, South Korea; [10]Hôpital Lyon Sud – Hospices Civils de Lyon, Lyon, France; [11]University Hospital of Montpellier, Montpellier, France; [12]Harbin Medical University Cancer Hospital, Harbin, China; [13]University of California San Francisco, Fresno Campus, CA, United States of America; [14]Genentech, Inc., South San Francisco, CA, United States of America; [15]Roche Products Ltd, Welwyn Garden City, United Kingdom; [16]Fiona Stanley Hospital and School of Clinical Sciences at Monash Health, Monash University, Melbourne, VIC, Australia;*

### Background

The Phase 3 STARGLO trial (NCT04408638) demonstrated significant 3-year overall survival and progression-free survival (PFS) benefits with fixed-duration glofitamab in combination with gemcitabine and oxaliplatin (GemOx; Glofit-GemOx), supporting its curative potential in autologous stem cell transplant (ASCT)-ineligible relapsed/refractory (R/R) diffuse large B-cell lymphoma (DLBCL). Nevertheless, identifying robust baseline and on-treatment biomarkers for the prediction of long-term outcomes remains a critical need.

### Aims

To evaluate the prognostic value of baseline total metabolic tumor volume (TMTV) alongside baseline and longitudinal circulating tumor DNA (ctDNA) for the prediction of long-term outcomes in the STARGLO trial.

### Methods

Patients (pts) with ASCT-ineligible R/R DLBCL were randomized 2:1 to Glofit-GemOx or rituximab-GemOx (R-GemOx). ctDNA was analyzed using the AVENIO Oncology Assay NHL test and a customized linked somatic variants method, where co-occurring somatic variants 175 base pairs apart on single DNA fragments were identified. TMTV was assessed by fluorine-18 fluorodeoxyglucose positron emission tomography-computed tomography (PET-CT). Efficacy was assessed by PET-CT using Lugano criteria. Multivariate Kaplan–Meier and Cox-regression analyses were inverse probability weighting-adjusted to account for potential sampling bias in the biomarker-evaluable population. All pts provided written informed consent.

**Copyright Information:** (Online) ISSN: 2572-9241
©2026 The Author(s). HemaSphere is published by John Wiley & Sons Ltd on behalf of European Hematology Association. This is an open access Abstract Book distributed under the Attribution-NonCommercial-NoDerivs (CC BY-NC-ND), which allows third parties to download the articles and share them with others as long as they credit the author and the Abstract Book, but they cannot change the content in any way or use them commercially.
**Abstract Book Citations:** Authors, Title, HemaSphere, 2026;10:(S1):pages. The abstract book DOIs can be found on the "Author Information" page.
**Disclaimer:** Articles published in the journal HemaSphere exclusively reflect the opinions of the authors. The authors are responsible for all content in their abstracts including accuracy of the facts, statements, citing resources, etc.

**Results**

As of May 1, 2025, among TMTV-evaluable pts (n=246), high baseline TMTV (≥125cm$^3$, median) was prognostic for inferior PFS (hazard ratio [HR], 1.49, p=0.004). Glofit-GemOx showed improved PFS over R-GemOx irrespective of TMTV burden (high TMTV: HR, 0.37, p<0.001; low TMTV: HR, 0.30, p<0.001). Among ctDNA-evaluable pts (n=161), higher baseline ctDNA (≥200 mutant molecules/mL, median) was associated with inferior PFS (HR, 1.68, p=0.001). Glofit-GemOx demonstrated improved PFS over R-GemOx across subgroups with both high (HR, 0.51, p=0.024) and low (HR, 0.28, p=0.002) ctDNA. Among 144 pts with both biomarkers, a combined TMTV/ctDNA analysis yielded superior prognostic stratification compared with either biomarker alone. Longitudinal ctDNA analyses showed higher rates of undetected ctDNA with Glofit-GemOx vs R-GemOx across all timepoints: 47.7% vs 9.5% at Days 25–65 (early treatment), 65.6% vs 32.1% at Days 65–110 (mid-treatment), and 70.0% vs 31.6% at Days 110–220 (**Figure**). Among pts treated with Glofit-GemOx, undetected ctDNA status at Cycle 3 was strongly associated with prolonged PFS (p<0.001) and showed greater prognostic value vs PET-CT-assessed complete response (CR) at Cycle 4 (24-month PFS difference: 60% by ctDNA status [detected vs undetected]; 25% by PET-CT [CR vs non-CR]). Similarly, at the end of treatment, undetected ctDNA status was a prognostic indicator of long-term outcomes, with a 24-month PFS of 81% vs 30% for pts with undetected vs detected ctDNA, respectively.

**Summary/Conclusion**

These results demonstrate the potential for TMTV and ctDNA as powerful prognostic tools in R/R DLBCL. Glofit-GemOx demonstrated superior efficacy over R-GemOx regardless of tumor burden (TMTV or ctDNA). In addition, longitudinal ctDNA monitoring supports early response assessment, with undetected ctDNA status by Cycle 3 emerging as a strong predictor of long-term PFS. Higher rates of undetectable ctDNA were observed with Glofit-GemOx vs R-GemOx, validating its clinical benefit and supporting the superior efficacy observed in the primary analysis of STARGLO.

**Copyright Information:** (Online) ISSN: 2572-9241
©2026 The Author(s). HemaSphere is published by John Wiley & Sons Ltd on behalf of European Hematology Association. This is an open access Abstract Book distributed under the Attribution-NonCommercial-NoDerivs (CC BY-NC-ND), which allows third parties to download the articles and share them with others as long as they credit the author and the Abstract Book, but they cannot change the content in any way or use them commercially.
**Abstract Book Citations:** Authors, Title, HemaSphere, 2026;10:(S1):pages. The abstract book DOIs can be found on the "Author Information" page.
**Disclaimer:** Articles published in the journal HemaSphere exclusively reflect the opinions of the authors. The authors are responsible for all content in their abstracts including accuracy of the facts, statements, citing resources, etc.

**Figure.** Undetected ctDNA rate by treatment arm and timepoint.

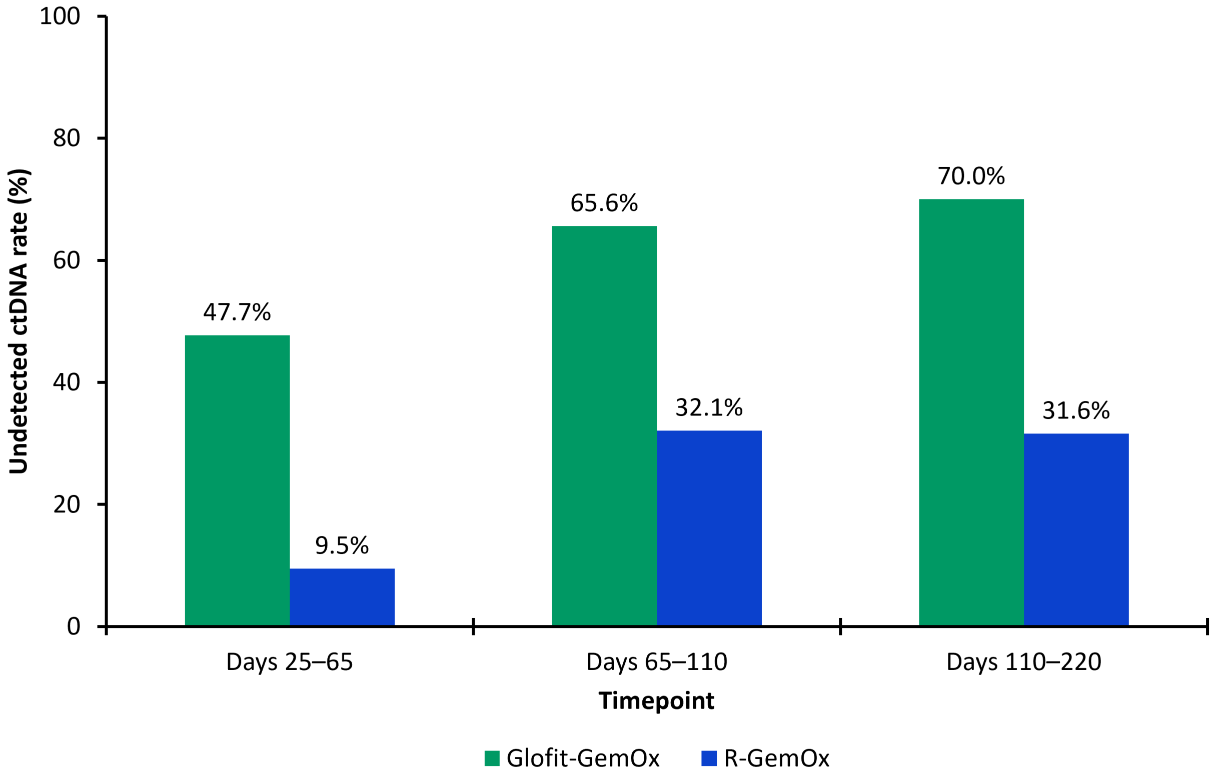

ctDNA, circulating tumor DNA; GemOx, gemcitabine and oxaliplatin; glofit, glofitamab; R, rituximab.

**Copyright Information:** (Online) ISSN: 2572-9241
©2026 The Author(s). HemaSphere is published by John Wiley & Sons Ltd on behalf of European Hematology Association. This is an open access Abstract Book distributed under the Attribution-NonCommercial-NoDerivs (CC BY-NC-ND), which allows third parties to download the articles and share them with others as long as they credit the author and the Abstract Book, but they cannot change the content in any way or use them commercially.
**Abstract Book Citations:** Authors, Title, HemaSphere, 2026;10:(S1):pages. The abstract book DOIs can be found on the "Author Information" page.
**Disclaimer:** Articles published in the journal HemaSphere exclusively reflect the opinions of the authors. The authors are responsible for all content in their abstracts including accuracy of the facts, statements, citing resources, etc.

### (PF1062) SPATIAL TRANSCRIPTOMICS REVEALS STEPWISE FORMATION OF DISTINCT CUTANEOUS NICHES IN NODAL T FOLLICULAR HELPER CELL LYMPHOMA

**Topic:** 21. Lymphoma biology & translational research

Yuri Tsuboi*[1], Sakurako Suma[2], Yuki Shimizu[1], Kenichi Makishima[2], Manabu Fujisawa[3, 4], Yasuhito Suehara[3], Atsushi Uehara[2, 5], Daisuke Kaji[6], Kyohei Sano[7], Ayumu Tsubosaka[7], Yoshiyuki Nakamura[8], Kosei Matsue[5], Yasunori Ota[9], Miwako Kakiuchi[7], Shumpei Ishikawa[7], Mamiko Sakata-Yanagimoto[2, 3, 10]

*[1]Graduate School of Comprehensive Human Sciences, University of Tsukuba, Department of Hematology, Tsukuba, Japan; [2]University of Tsukuba Hospital, Department of Hematology, Tsukuba, Japan; [3]Institute of Medicine, University of Tsukuba, Department of Hematology, Tsukuba, Japan; [4]BC Cancer, Center for Lymphoid Cancer, Vancouver, Canada; [5]Kameda Medical Center, Division of Hematology/Oncology, Department of Hematology, Kamogawa, Japan; [6]Toranomon Hospital, Department of Hematology, Minato-ku, Japan; [7]Graduate School of Medicine, The University of Tokyo, Department of Preventive Medicine, Bunkyo-ku, Japan; [8]Institute of Medicine, University of Tsukuba, Department of Dermatology, Tsukuba, Japan; [9]The Institute of Medical Science, The University of Tokyo, Department of Pathology, Minato-ku, Japan; [10]Transborder Medical Research Center, University of Tsukuba, Division of Advanced Hemato-Oncology, Tsukuba, Japan;*

### Background

Nodal T follicular helper cell lymphoma (TFHL) is an aggressive mature T-cell lymphoma with frequent extranodal involvement. Cutaneous lesions occur in approximately 50% of patients and are associated with poor prognosis. Despite their high frequency, the pathological features of these cutaneous lesions remain poorly defined. We previously demonstrated that TFHL tumor cells form a niche with germinal center B cells via the CD40LG-CD40 axis in lymph nodes (*Blood*, 2022). However, normal skin contains very few B cells, and the mechanisms supporting tumor survival and proliferation in this environment remain unclear.

### Aims

To characterize the tumor microenvironment of TFHL cutaneous lesions and to elucidate the mechanisms of extranodal infiltration.

### Methods

We performed single-nucleus RNA sequencing (snRNA-seq) on four TFHL skin samples using the 10X Chromium platform. Subsequently, spatial transcriptomics was conducted using the 6,000-gene panel CosMx platform on 42 formalin-fixed paraffin-embedded samples (28 TFHL skin samples and 14 normal skin controls) to validate the spatial distribution of cell populations identified by snRNA-seq and to analyze tumor niche formation.

**Copyright Information:** (Online) ISSN: 2572-9241
©2026 The Author(s). HemaSphere is published by John Wiley & Sons Ltd on behalf of European Hematology Association. This is an open access Abstract Book distributed under the Attribution-NonCommercial-NoDerivs (CC BY-NC-ND), which allows third parties to download the articles and share them with others as long as they credit the author and the Abstract Book, but they cannot change the content in any way or use them commercially.
**Abstract Book Citations:** Authors, Title, HemaSphere, 2026;10:(S1):pages. The abstract book DOIs can be found on the “Author Information” page.
**Disclaimer:** Articles published in the journal HemaSphere exclusively reflect the opinions of the authors. The authors are responsible for all content in their abstracts including accuracy of the facts, statements, citing resources, etc.

**Results**

snRNA-seq yielded 114,095 high-quality cells, classified into 51 subclusters. Tumor cells were identified by TFH markers, including *PDCD1*, *CXCR5*, and *CXCL13*. Comprehensive annotation revealed subtypes of T cells, myeloid cells, B cells, fibroblasts, endothelial cells, and keratinocytes, identifying specialized populations such as lipid-associated macrophages (LAMs), fibroblastic reticular cell-like (FRC-like) fibroblasts, and high endothelial venules (HEVs).
Projection of these annotations onto the spatial transcriptomics dataset (792,467 cells) confirmed significant increases in myeloid cells ($p = 0.000002$) and B cells ($p = 0.00075$) in TFHL skin lesions compared with normal skin. Spatial density analysis showed that HEVs, fibroblasts, LAMs, and plasma cells increased with tumor proportion and co-localized with tumor cells.
Spatial niche analysis identified two tumor-enriched niches with distinct compositions. Niche 1 was characterized by three main cell types: vasculature-associated cells such as HEVs, various types of fibroblasts, and macrophages including LAMs, while Niche 2 comprised FRC-like fibroblasts, B cells, and plasma cells. Notably, Niche 2 expanded in correlation with tumor proportion, suggesting a more established tumor microenvironment.
Cell-cell interaction analysis revealed that the CXCL12-CXCR4 as the dominant signaling axis across both niches. Additionally, tumor cells in Niche 1 primarily signal to LAMs via CD40LG-CD40, with supporting signals from HEVs and fibroblasts. Niche 2 exhibited more complex bidirectional interactions between tumor cells and FRC-like fibroblasts, B cells, and plasma cells.

**Summary/Conclusion**

Our findings indicate that TFHL establishes distinct cutaneous tumor niches through a stepwise process. Initially, a limited population of tumor cells infiltrates the skin and reshapes the stromal microenvironment, triggering CXCL12 upregulation in HEVs and fibroblasts to generate Niche 1, thereby facilitating the recruitment of CXCR4-expressing immune cells. As tumor proportion increases, Niche 2 emerges with expansion of FRC-like fibroblasts and recruitment of B cells and plasma cells, creating a lymph node-like microenvironment. These insights shed light on the progressive evolution of extranodal tumor niches.

**Copyright Information:** (Online) ISSN: 2572-9241
©2026 The Author(s). HemaSphere is published by John Wiley & Sons Ltd on behalf of European Hematology Association. This is an open access Abstract Book distributed under the Attribution-NonCommercial-NoDerivs (CC BY-NC-ND), which allows third parties to download the articles and share them with others as long as they credit the author and the Abstract Book, but they cannot change the content in any way or use them commercially.
**Abstract Book Citations:** Authors, Title, HemaSphere, 2026;10:(S1):pages. The abstract book DOIs can be found on the “Author Information” page.
**Disclaimer:** Articles published in the journal HemaSphere exclusively reflect the opinions of the authors. The authors are responsible for all content in their abstracts including accuracy of the facts, statements, citing resources, etc.

### (PF1063) EARLY OUTCOME PREDICTION OF DIFFUSE LARGE B-CELL LYMPHOMA PATIENTS USING ON-TREATMENT OMICS DATA

**Topic:** 21. Lymphoma biology & translational research

Tom van den Bosch*[1, 2], Saba Ghasemi[3], Steven Wang[3], Nils Groenewegen[3], Monique van Eijndhoven[3], Cristina A. Gómez Martín[3], Florent Mouliere[3, 4], Marcel Nijland[5], D. Michiel Pegtel[3], Renee de Menezes[2], Martine Chamuleau[1]

*[1]Amsterdam UMC, Department of Hematology, Amsterdam, The Netherlands;[2]Netherlands Cancer Institute, Department of Biostatistics, Amsterdam, The Netherlands; [3]Amsterdam UMC, Department of Pathology, Amsterdam, The Netherlands; [4]University of Manchester, Cancer Research UK National Biomarker Centre, Manchester, United Kingdom;[5]University Medical Center Groningen, Department of Hematology, Groningen, The Netherlands;*

### Background

Currently, standard-of-care treatment for patients with Diffuse Large B-Cell Lymphoma (DLBCL) consists of 6 cycles of R-CHOP facing two significant issues:

1. Ineffective treatment: 30-40% of patients either relapse or do not respond. An early switch to alternative treatments could improve outcome of these patients.
2. Overtreatment: fewer cycles of therapy might be sufficient for some patients with low-risk disease, allowing de-escalation to 4 cycles reducing unnecessary toxicities.

Early outcome prediction could tackle both issues. Currently available tools (International Prognostic Index (IPI), interim PET-scans and other biomarkers) have prognostic significance but their low predictive power does not allow use in clinical practice yet.

### Aims

We aimed to combine pre-treatment data with on-treatment liquid-biopsy derived data to predict patient outcomes. Specifically, we used both circulating tumor DNA (ctDNA) and extracellular vesicle (EV) derived omics. EVs are small lipid nanoparticles that are secreted by both healthy and tumor cells. As such, data from EVs are informative of tumor cells, the microenvironment and the immune response. We hypothesized that better predictions are possible earlier on by combining all the above mentioned data. Our main goal was to stratify patients into three groups with pre-determined clinically relevant 2yr progression-free survival (PFS) rates (Low-risk > 90%, Intermediate-risk 20-90%, High-risk < 20%).

**Copyright Information:** (Online) ISSN: 2572-9241
©2026 The Author(s). HemaSphere is published by John Wiley & Sons Ltd on behalf of European Hematology Association. This is an open access Abstract Book distributed under the Attribution-NonCommercial-NoDerivs (CC BY-NC-ND), which allows third parties to download the articles and share them with others as long as they credit the author and the Abstract Book, but they cannot change the content in any way or use them commercially.
**Abstract Book Citations:** Authors, Title, HemaSphere, 2026;10:(S1):pages. The abstract book DOIs can be found on the “Author Information” page.
**Disclaimer:** Articles published in the journal HemaSphere exclusively reflect the opinions of the authors. The authors are responsible for all content in their abstracts including accuracy of the facts, statements, citing resources, etc.

### Methods

We used data from 2 clinical studies (HOVON-152 and HOVON-902), involving a unique dataset from 115 DLBCL patients treated with R-CHOP and R-CHOP-like regimens, containing four different data modalities:
Pre-treatment baseline characteristics

1. Age, IPI score, sex, *BCL2* and *BCL6* translocation status

On-treatment (after 1 cycle of R-CHOP) liquid biopsy characteristics:

2. CtDNA-derived copy numbers
3. EV-derived whole transcriptomics mRNA sequencing
4. EV-derived miRNA sequencing

All sequencing was performed on the Novaseq 6000 using paired-end sequencing. We explored all possible combinations of modalities to predict 2yr PFS using a logistic-ridge regression model. Models were trained by using 10x nested cross validation and performance was compared by the AUC on all test sets. Patient stratification was compared to the current gold standard method, interim PET-CT scan (iPET).

### Results

A training set of 72 patients showed that, while single data categories have low predictive accuracy for 2yr PFS (AUC 0.53-0.67, median 0.63), combining different data modalities significantly improves the AUC. The best combination involved on-treatment copy numbers and mRNA (AUC 0.74). The model created by these data predicted a group of patients (n=9/9) as high risk (median predicted probability > 0.5, PPV=100%, Figure 1). The model's ability to predict low-risk patients was lower, while still outperforming iPET assessment (n=6/27 patients with an event, NPV=78%, 9/27 iPET positive, Figure 1). In an independent validation set (n=43), the model also predicted high-risk patients (n=3/3, PPV=100%).

### Summary/Conclusion

The combination of ctDNA copy numbers and EV-derived mRNA expression after 1 cycle of therapy was able to identify high-risk patients with a predictive value of 100%. Combined information on both dying and living cells might reflect tumor and immune response on treatment and is independent of baseline patient and tumor characteristics, holding promise for clinical implementation in the future. For further validation, we will expand our dataset data with 95 more patients.

**Copyright Information:** (Online) ISSN: 2572-9241
©2026 The Author(s). HemaSphere is published by John Wiley & Sons Ltd on behalf of European Hematology Association. This is an open access Abstract Book distributed under the Attribution-NonCommercial-NoDerivs (CC BY-NC-ND), which allows third parties to download the articles and share them with others as long as they credit the author and the Abstract Book, but they cannot change the content in any way or use them commercially.
**Abstract Book Citations:** Authors, Title, HemaSphere, 2026;10:(S1):pages. The abstract book DOIs can be found on the "Author Information" page.
**Disclaimer:** Articles published in the journal HemaSphere exclusively reflect the opinions of the authors. The authors are responsible for all content in their abstracts including accuracy of the facts, statements, citing resources, etc.

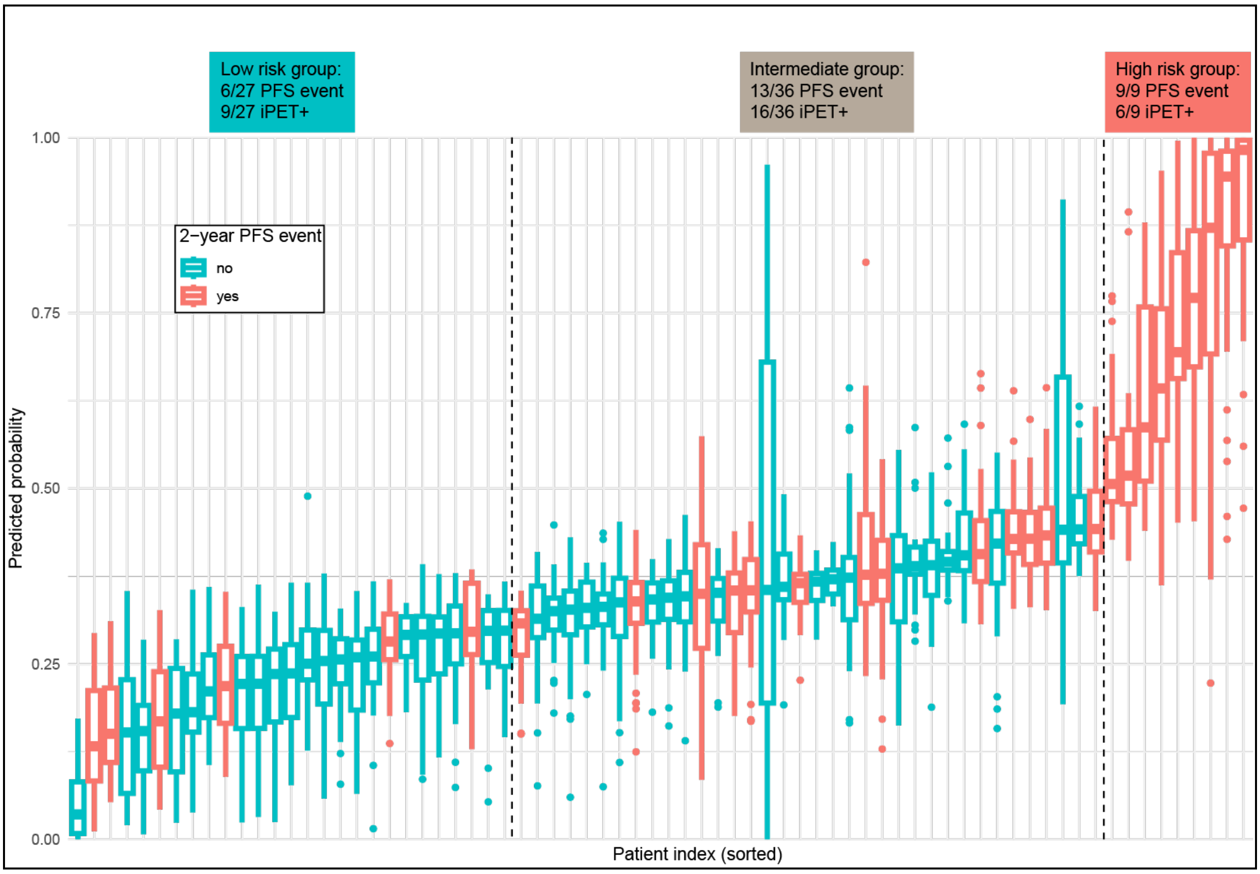

Figure 1: Predicted probability of 2-year progression free survival for 72 DLBCL patients. Each box shows the distribution of test set predictions for one patient (box: 1st to 3rd quantile and median values, outer lines: 1.5x interquartile distance from box, points: outliers). Patients were sorted by their median prediction and colored by survival status(2-year PFS event: red, no event: teal).

**Copyright Information:** (Online) ISSN: 2572-9241
©2026 The Author(s). HemaSphere is published by John Wiley & Sons Ltd on behalf of European Hematology Association. This is an open access Abstract Book distributed under the Attribution-NonCommercial-NoDerivs (CC BY-NC-ND), which allows third parties to download the articles and share them with others as long as they credit the author and the Abstract Book, but they cannot change the content in any way or use them commercially.
**Abstract Book Citations:** Authors, Title, HemaSphere, 2026;10:(S1):pages. The abstract book DOIs can be found on the "Author Information" page.
**Disclaimer:** Articles published in the journal HemaSphere exclusively reflect the opinions of the authors. The authors are responsible for all content in their abstracts including accuracy of the facts, statements, citing resources, etc.

### (PF1064) PLASMA PROTEOMIC SIGNATURES DISTINGUISH LYMPHOMA FROM OTHER CANCERS AND NON-MALIGNANT CONDITIONS IN PATIENTS PRESENTING WITH NON-SPECIFIC SYMPTOMS

**Topic:** 21. Lymphoma biology & translational research

Fredrika Wannberg*[1], María Bueno Álvez[2], Viktoria Hjalmar[1, 3], Tamás Pongrácz[1], Karin Ekstrom Smedby[4, 5], Katherina Aguilera[1], Mikael Åberg[6], Charlotte Thålin[1], Axel Rosell[1, 7]

*[1]Karolinska Institutet, Department of Clinical Sciences, Danderyd Hospital, Stockholm, Sweden;[2]Science for Life Laboratory, Department of Protein Science, KTH Royal Institute of Technology, Stockholm, Sweden; [3]Division of Specialist Medical Care, Diagnostic center, Danderyd Hospital, Stockholm, Sweden; [4]Clinical Epidemiology Division, Department of Medicine Solna, Karolinska Institutet, Stockholm, Sweden; [5]Department of Hematology, Karolinska University Hospital, Stockholm, Sweden; [6]Department of Medical Sciences, Clinical Chemistry and SciLifeLab Affinity Proteomics, Uppsala University, Uppsala, Sweden; [7]Center for Hematology and Regenerative Medicine (HERM), NEO, Department of Medicine, Huddinge, Karolinska Institutet, Stockholm, Sweden;*

### Background

Early discrimination of lymphoma from non-malignant conditions and other cancers remains clinically challenging, particularly in patients presenting with unspecific symptoms. Blood-based proteomic profiling combined with machine learning has the potential to improve diagnostic stratification in this challenging and heterogeneous patient population. Here we developed and evaluated a lymphoma-specific classification model in patients with unspecific symptoms.

### Aims

To identify plasma protein signatures specific for lymphoma and assess their diagnostic performance against a heterogeneous symptomatic control population.

### Methods

A total of 456 patients referred to the Diagnostic Center, Danderyd Hospital, Stockholm, Sweden, due to non-specific symptoms and signs of cancer that can reflect an underlying cancer were included and sampled prior to diagnostic work-up. Plasma levels of 1463 proteins were measured using Olink Explore 1536. Lymphoma cases were compared to controls diagnosed with other malignancies, non-malignant conditions or no definite diagnosis. Differential protein expression analysis was performed, and *P*-values were derived from two-sided moderated *t*-tests from limma linear models and adjusted for multiple testing with the Benjamini–Hochberg method, followed by penalized logistic regression to develop a lymphoma classification model. Performance was evaluated using receiver operating characteristic (ROC) analysis and was further assessed across control subgroups.

### Results

Fifty-three proteins were upregulated in lymphoma, and 1 was downregulated compared to controls (Figure A). The final multivariable model included 29 proteins (Figure C-D) and demonstrated strong discrimination between lymphoma and controls, achieving an area under the ROC curve (AUC) of 0.98. Lymphoma cases (diffuse large B cell lymphoma [*n*=7], Hodgkin lymphoma [*n*=5], follicular lymphoma [*n*=4], mantle cell lymphoma [*n*=2], T-cell lymphoma [*n*=2], chronic lymphocytic leukemia [*n*=2], Waldenstrom macroglobulinemia [*n*=1], bronchial-associated lymphoid tissue lymphoma [*n*=1], *n*=24 in total) remained separable when compared to all controls combined (*n*=432), as well as across stratified control subgroups, including autoimmune (*n*=55, AUC=0.98), infectious (*n*=32, AUC=0.98), inflammatory (*n*=19, AUC=1), other cancers (*n*=136, AUC=0.97), other diseases (*n*=49, AUC=1) and symptomatic patients without a definite diagnosis (*n*=141, AUC=0.99) (Figure B).

**Copyright Information:** (Online) ISSN: 2572-9241
©2026 The Author(s). HemaSphere is published by John Wiley & Sons Ltd on behalf of European Hematology Association. This is an open access Abstract Book distributed under the Attribution-NonCommercial-NoDerivs (CC BY-NC-ND), which allows third parties to download the articles and share them with others as long as they credit the author and the Abstract Book, but they cannot change the content in any way or use them commercially.
**Abstract Book Citations:** Authors, Title, HemaSphere, 2026;10:(S1):pages. The abstract book DOIs can be found on the "Author Information" page.
**Disclaimer:** Articles published in the journal HemaSphere exclusively reflect the opinions of the authors. The authors are responsible for all content in their abstracts including accuracy of the facts, statements, citing resources, etc.

**Summary/Conclusion**

Targeted plasma proteomics combined with machine learning enables accurate discrimination of lymphoma from both non-malignant conditions and other cancers. Several of the identified proteins are well established in hematological malignancies. The TNF receptor superfamily member 13C (TNFRSF13C), also known as the B-cell activating factor receptor (BAFF-R), is a pro-survival receptor expressed in many B-cell malignancies. C–C chemokine ligand 17 (CCL17) is closely related to hematological malignancies, particularly Hodgkin lymphoma, where it is used clinically. T-cell leukemia/lymphoma 1A (TCL1A) is a known oncogenic protein that is overexpressed in various T- and B-cell malignancies. The HER3 receptor (ERBB3) showed lower levels in lymphoma compared to controls, likely reflecting its involvement in solid tumors. These findings support the potential clinical utility of blood-based proteomic signatures for detection of lymphoma in diagnostically challenging settings, as well as the potential of proteomic profiling to transform clinical medicine.

The last three authors of this abstract contributed equally to it.

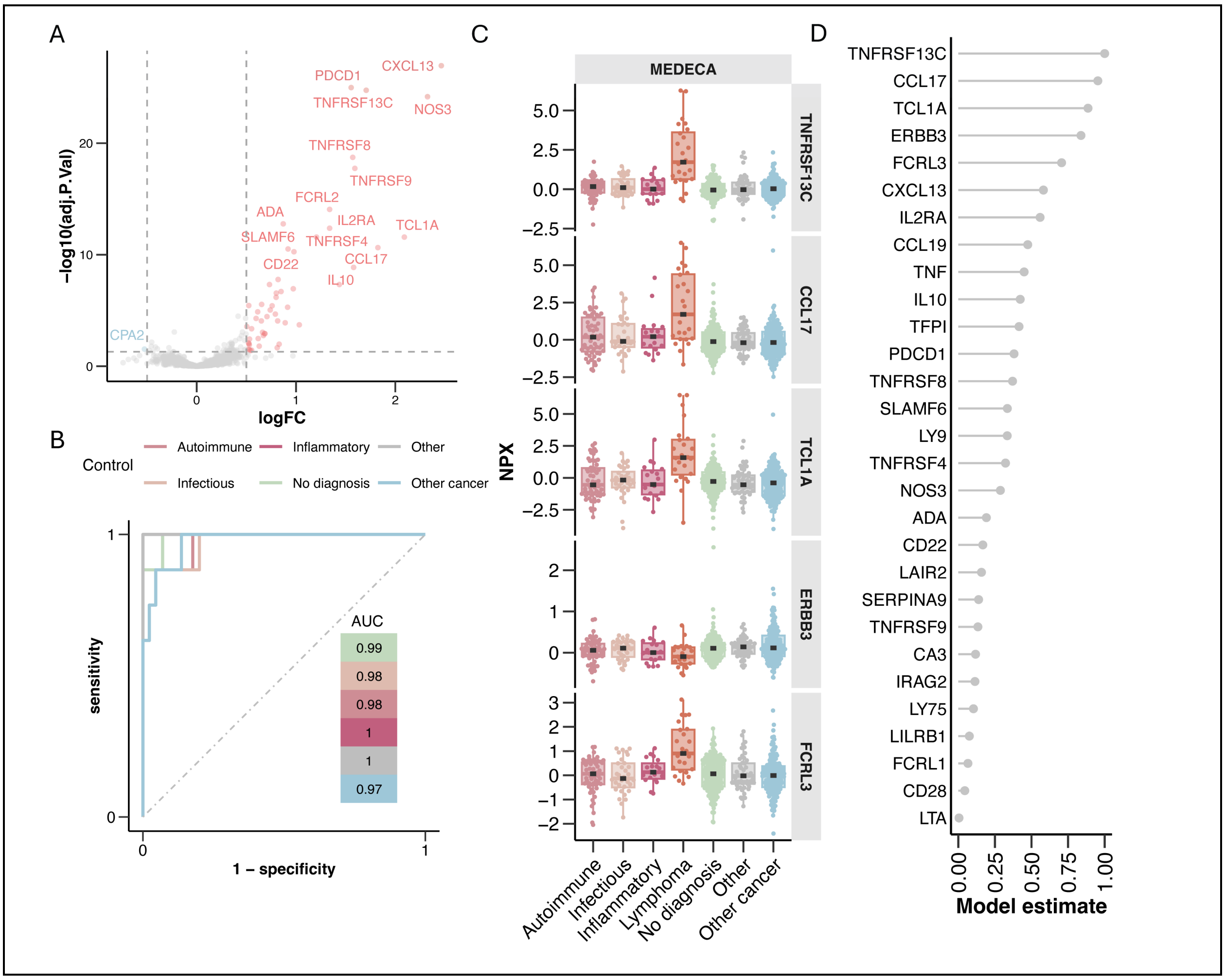

**Copyright Information:** (Online) ISSN: 2572-9241
©2026 The Author(s). HemaSphere is published by John Wiley & Sons Ltd on behalf of European Hematology Association. This is an open access Abstract Book distributed under the Attribution-NonCommercial-NoDerivs (CC BY-NC-ND), which allows third parties to download the articles and share them with others as long as they credit the author and the Abstract Book, but they cannot change the content in any way or use them commercially.
**Abstract Book Citations:** Authors, Title, HemaSphere, 2026;10:(S1):pages. The abstract book DOIs can be found on the "Author Information" page.
**Disclaimer:** Articles published in the journal HemaSphere exclusively reflect the opinions of the authors. The authors are responsible for all content in their abstracts including accuracy of the facts, statements, citing resources, etc.

### (PF1066) SPATIAL PROFILING OF T CELL SUBSETS REVEALS DISTINCT IMMUNE MICROENVIRONMENT PATTERNS ASSOCIATED WITH SURVIVAL IN DIFFUSE LARGE B CELL LYMPHOMA

**Topic:** 21. Lymphoma biology & translational research

Filippa Wikström*[1, 2], Angelica Johansson[1, 2, 3], Ida Hammer[1], Anna Porwit[4], Mats Jerkeman[5], Sara Ek[1, 2, 3]

*[1]Lund University, Department of Immunotechnology, Lund, Sweden;[2]Lund University, Lund Cancer Center (LUCC), Lund, Sweden; [3]Lund University, CREATE Health Translational Cancer Center, Lund, Sweden;[4]Lund University, Department of Clinical Sciences, Lund, Sweden; [5]Lund University, Department of Medical Oncology, Lund, Sweden;*

**Background**

Diffuse large B cell lymphoma (DLBCL) is a heterogeneous disease with variable clinical outcomes. Success of standard treatment with immunochemotherapy (R-CHOP) is still limited, with a 5-year overall survival of 75%. Current clinical risk stratification relies primarily on the International Prognostic Index (IPI), based on clinical parameters.
The tumor immune microenvironment (TIME) is increasingly recognized as a critical determinant of tumor progression, yet its heterogeneity and spatial organization remain largely uncharacterized. While the PD1/PD-L1 axis is central to immune regulation, immune checkpoint blockade has demonstrated limited efficacy in DLBCL, underscoring the need for deeper insight into tumor-immune interactions. Incorporating spatial immune metrics with genetic information and clinical parameters may refine biological understanding and prognostic modeling.

**Aims**

This study aimed to (i) in depth investigate the role of infiltrating T-lymphocyte subsets in relation to clinical outcomes and to (ii) identify novel biomarkers for better patient stratification by linking T cell infiltration and spatial organization of the tumor immune microenvironment to genetic factors and outcome.

**Methods**

The study included 650 patients diagnosed with DLBCL between 2000-2014 in southern Sweden. 365 patients diagnosed with DLBCL-NOS were selected for analysis. To investigate T cell infiltration, a 6-plex panel targeting DAPI, CD20, CD3, CD8, FoxP3 and PD1 was applied to TMA slides. Transcriptional analysis was performed using GeoMx DSP. DNA samples from 74 of 365 patients were sequenced with a targeted panel (GMS lymphoid panel, TWIST Bioscience).

**Copyright Information:** (Online) ISSN: 2572-9241
©2026 The Author(s). HemaSphere is published by John Wiley & Sons Ltd on behalf of European Hematology Association. This is an open access Abstract Book distributed under the Attribution-NonCommercial-NoDerivs (CC BY-NC-ND), which allows third parties to download the articles and share them with others as long as they credit the author and the Abstract Book, but they cannot change the content in any way or use them commercially.
**Abstract Book Citations:** Authors, Title, HemaSphere, 2026;10:(S1):pages. The abstract book DOIs can be found on the “Author Information” page.
**Disclaimer:** Articles published in the journal HemaSphere exclusively reflect the opinions of the authors. The authors are responsible for all content in their abstracts including accuracy of the facts, statements, citing resources, etc.

**Results**

Multiplex immunofluorescence using a 6-plex panel showed that T cell infiltration exhibited great variability between patients. Unsupervised clustering of immune cell frequencies identified six TIME subtypes, each associated with distinct overall survival outcomes. The B cell-rich, immune-poor tumors were associated with poorer outcomes, while the B cell-poor, CD8+ T cell-rich tumors were associated with superior outcomes, outperforming other immune-rich B cell-poor TIME subtypes. Differential gene expression analysis revealed upregulated genes linked to the B cells of the B cell-rich, immune-poor TIME, including BRCA1, NASP, MYBL2, HELLS, BIRC5 and HMGA1.
Spatial analysis further characterized the TIMES and revealed differences in cell colocalization. Significantly more attraction between PD1$^{+}$ T cells and tumor cells was observed in the TIME linked to inferior survival compared to the TIME linked to superior survival, independent of B cell frequency. Greater attraction was seen between CD8$^{+}$ T cells, CD4$^{+}$ T cells and Tregs in the TIME linked to worse prognosis, suggesting limited engagement with tumor cells.
Neighborhood analysis identified five recurrent cellular neighborhoods across the cohort, reflecting important spatial structures within the TIME.

**Summary/Conclusion**

Across patients we identified substantial heterogeneity in the tumor immune microenvironment, revealing six distinct TIME subtypes with different clinical outcomes. Spatial, transcriptional, and neighborhood-level analyses demonstrated that poor-prognosis TIMEs were characterized by dysfunctional T cell organization, altered T cell-tumor interactions, and unique gene expression patterns, highlighting spatial immune architecture as a promising biomarker to improve patient stratification beyond current models.

**Copyright Information:** (Online) ISSN: 2572-9241
©2026 The Author(s). HemaSphere is published by John Wiley & Sons Ltd on behalf of European Hematology Association. This is an open access Abstract Book distributed under the Attribution-NonCommercial-NoDerivs (CC BY-NC-ND), which allows third parties to download the articles and share them with others as long as they credit the author and the Abstract Book, but they cannot change the content in any way or use them commercially.
**Abstract Book Citations:** Authors, Title, HemaSphere, 2026;10:(S1):pages. The abstract book DOIs can be found on the "Author Information" page.
**Disclaimer:** Articles published in the journal HemaSphere exclusively reflect the opinions of the authors. The authors are responsible for all content in their abstracts including accuracy of the facts, statements, citing resources, etc.

### (PF1067) GENOMIC AND TRANSCRIPTOMIC LANDSCAPE OF FOLLICULAR LYMPHOMA

**Topic:** 21. Lymphoma biology & translational research

Matin Salehi*[1, 2, 3], Wei Wu[1, 4], Alessio Bruscaggin[1], Deborah Piffaretti[1], Riccardo Moia[5], Maria Elena Nizzoli[6], Benedetta Donati[6], Janine Schmidt[7], Johannes Hellmuth[8], Katia Pini[1], Federico Jauk[1, 3, 9], Lodovico Terzi Di Bergamo[1, 2], Riccardo Castiglioni[1], Alexandra Valera[9], Luciano Cascione[2, 3, 10], Georgia A. Galimberti[1], Gabriela Forestieri[1], Simone Bocchetta[1], Maria Pirosa[1, 3, 11], Ilaria Romano[1, 3, 11], Renzo Boldorini[12], Emanuele Zucca[3, 11], Francesco Bertoni[3, 10], Francisco Vega[13], Leticia Quintanilla-Fend[7], Oliver Weigert[8], Luca Mazzucchelli[9], Stefano Luminari[6], Gianluca Gaidano[5], Davide Rossi[1, 3, 11], Adalgisa Condoluci[1, 3, 11]

*[1]Institute of Oncology Research, Laboratory of Experimental Hematology, Bellinzona, Switzerland;[2]Swiss Institute of Bioinformatics, Bioinformatics Core Unit, Bellinzona, Switzerland; [3]Università della Svizzera Italiana, Faculty of Biomedical Sciences, Lugano, Switzerland; [4]The First Affiliated Hospital of Nanjing Medical University, Nanjing, China;[5]Università del Piemonte Orientale, 5Division of Hematology, Department of Translational Medicine, Novara, Italy; [6]Hematology AUSL IRCCS Reggio Emilia, Reggio Emilia, Italy; [7]Institute of Pathology and Neuropathology, Eberhard Karls University of Tübingen and Comprehensive Cancer Center, University Hospital Tübingen, Tübingen, Germany; [8]Department of Medicine III, University Hospital, Ludwig-Maximilians-University, Munich, Germany; [9]Division of Pathology, Ente Ospedaliero Cantonale, Locarno, Switzerland; [10]Institute of Oncology Research, Lymphoma Genomics, Bellinzona, Switzerland;[11]Clinic of Hematology, Oncology Institute of Southern Switzerland, Ente Ospedaliero Cantonale, Bellinzona, Switzerland; [12]Pathology Department, University Hospital "Maggiore della Carità", University of Eastern Piedmont, Novara, Italy; [13]Division of Pathology, Department of Hematopathology, The University of Texas M.D. Anderson Cancer Center, Houston, TX, United States of America;*

### Background

Follicular lymphoma (FL) is a biologically and clinically heterogeneous B-cell malignancy historically defined by the *BCL2* rearrangement (*BCL2*-R). FL also includes cases lacking *BCL2*-R (*BCL2*-R$^{neg}$/CD23$^{pos}$ FL, G3B FL), duodenal-type FL (DTFL), pediatric-type FL (PTFL), and primary cutaneous follicle center lymphoma (PCFCL), which differ clinically, pathologically, or molecularly from classic FL (cFL). Despite growing recognition of this diversity, molecular boundaries between FL entities and determinants of adverse prognosis (i.e., POD24) remain incompletely defined.

### Aims

To characterize the molecular boundaries of the different FL entities and to improve the understanding of the biological basis of adverse prognosis of FL.

### Methods

We analyzed a large cohort (n=415) of clinico-pathologically annotated FL cases for mutations, copy number abnormalities (CNAs), *BCL2*-R, and transcriptomics. The series included cFL (n=304), *BCL2*-R$^{neg}$/CD23$^{pos}$ FL (n=50), and G3B FL (n=8) from IOSI-EMA004 (NCT03436602) and IOSI-EMA003 (NCT03280394); PTFL (n=20) and DTFL (n=21) from international consortia; and PCFCL (n=12) from MDACC.

**Copyright Information:** (Online) ISSN: 2572-9241
©2026 The Author(s). HemaSphere is published by John Wiley & Sons Ltd on behalf of European Hematology Association. This is an open access Abstract Book distributed under the Attribution-NonCommercial-NoDerivs (CC BY-NC-ND), which allows third parties to download the articles and share them with others as long as they credit the author and the Abstract Book, but they cannot change the content in any way or use them commercially.
**Abstract Book Citations:** Authors, Title, HemaSphere, 2026;10:(S1):pages. The abstract book DOIs can be found on the "Author Information" page.
**Disclaimer:** Articles published in the journal HemaSphere exclusively reflect the opinions of the authors. The authors are responsible for all content in their abstracts including accuracy of the facts, statements, citing resources, etc.

### Results

Despite subtype-specific enrichments, FL entities exhibit an overlapping spectrum of mutations and CNAs, with no single pattern defining the disease. Unsupervised clustering identified three genetic subtypes: *BCL2*-R$^{neg}$-like FL (29%), enriched for *STAT6*, *CREBBP* (KAT), *FOXO1*, *SOCS1*, and *TNFRSF14*, encompassing nearly all *BCL2*-R$^{neg}$/CD23$^{pos}$ FL and G3B FL, with a fraction of PTFL and PCFCL; chromosomally unstable FL (30%), with *TP53* mutations, 9p (*CDKN2A/B*) loss, and widespread CNAs, distributed across FL; and *BCL2*-R$^{pos}$-like FL (39%), enriched for *BCL2*-R, mutations in *CREBBP* (non-KAT), *EZH2*, and SHM-targeted genes, including cFL and DTFL. These results indicate that coordinated genetic profiles alone cannot fully recapitulate clinico-pathological FL subtypes. FL lacking *BCL2*-R may enter the differential diagnosis with marginal zone lymphoma (MZL). Using a classification tree, genetic profiling identified *BCL2*-R$^{neg}$ FL (n=111) from MZL (n=253) with a positive predictive value (PPV) of 92.6% and negative predictive value (NPV) of 91.4%. Key discriminators included mutations in *CREBBP* (KAT), *STAT6*, *BCL2*, *EZH2*, *GNA13* and *FOXO1*.
Chromosomally unstable FL more frequently presented with anemia, bulky disease, and elevated β2-microglobulin levels. These patients also exhibited higher-risk scores across multiple prognostic models and were enriched for high-risk immune-response gene expression profile. Consistently, they showed inferior overall and relative survival. The NPV for POD24 was high (82%) but comparable to other models, including FLIPI, PRIMA-PI, m7-FLIPI, POD24-PI, and immune-response or cell-of-origin gene expression signatures. The PPV for POD24 in chromosomally unstable FL was modest (30%), similar to that of other models, highlighting that current tools have limited accuracy for positive risk stratification and only moderate discriminative ability.

### Summary/Conclusion

Our analyses show that: i) neither individual genetic alterations nor specific constellations of recurrent lesions provide sufficient discriminatory power to reliably distinguish among FL entities; ii) genetic profiling can help differentiate *BCL2*-R$^{neg}$ FL from MZL; iii) the limited accuracy for POD24 of existing tools likely reflects their inability to capture the combined effects of genomic instability, tumor differentiation state, and microenvironmental context.

**Copyright Information:** (Online) ISSN: 2572-9241
©2026 The Author(s). HemaSphere is published by John Wiley & Sons Ltd on behalf of European Hematology Association. This is an open access Abstract Book distributed under the Attribution-NonCommercial-NoDerivs (CC BY-NC-ND), which allows third parties to download the articles and share them with others as long as they credit the author and the Abstract Book, but they cannot change the content in any way or use them commercially.
**Abstract Book Citations:** Authors, Title, HemaSphere, 2026;10:(S1):pages. The abstract book DOIs can be found on the "Author Information" page.
**Disclaimer:** Articles published in the journal HemaSphere exclusively reflect the opinions of the authors. The authors are responsible for all content in their abstracts including accuracy of the facts, statements, citing resources, etc.

### (PF1068) INTEGRATION OF END-OF-TREATMENT CIRCULATING TUMOR DNA WITH PET-CT IMPROVES RESPONSE ASSESSMENT IN CLASSICAL HODGKIN LYMPHOMA

**Topic:** 21. Lymphoma biology & translational research

Vincent Camus*[1], Mathieu Viennot[2], Oumaima Perrin[3], Emilie Lévêque[3], Elodie Bohers[2], Philippe Ruminy[2], Vinciane Rainville[2], Marie-Delphine Lanic[2], Mélody Caillot[2], Stéphanie Becker[4], Pierre Decazes[4], David Tonnelet[4], Dominique Penther[5], Benoit Berby[5], Antoine Ittel[5], Julien Masliah-Planchon[5], Elena-Liana Veresezan[6], Fanny Drieux[6], Justine Loret[7], Lucie Burel[7], Julie Libraire[7], Hervé Tilly[1], Aspasia Stamatoullas Bastard[1], Sydney Dubois[1], Pierre-Julien Viailly[2], Fabrice Jardin[1]

*[1]Centre Henri Becquerel, Department of Hematology and INSERM U1245 "Cancer & Brain Genomics", Rouen, France;[2]Centre Henri Becquerel, INSERM U1245 "Cancer & Brain Genomics", Rouen, France; [3]Centre Henri Becquerel, Clinical Research Unit / Biostatistics, Rouen, France; [4]Centre Henri Becquerel, Department of Nuclear Medicine and AIMS, Rouen, France;[5]Centre Henri Becquerel, Biopathology Department, Laboratory of Genetic Oncology, Rouen, France; [6]Centre Henri Becquerel, Biopathology Department, Laboratory of Pathology, Rouen, France; [7]Centre Henri Becquerel, Clinical Research Unit, Rouen, France;*

### Background

End-of-treatment (EoT) PET-CT is central for response assessment in classical Hodgkin lymphoma (cHL) but imperfectly predicts relapse risk. Circulating tumor DNA (ctDNA) minimal residual disease (MRD) may complement imaging to refine post-therapy risk stratification.

### Aims

We evaluated whether combining EoT MRD and EoT PET-CT improves prediction of progression or death within 2 years (yrs) compared with either modality alone.

### Methods

We analyzed stored plasma from a single-center prospective cohort (NCT02815137) of 69 newly diagnosed cHL patients (pts) treated with ABVD or BEACOPP ± consolidative radiotherapy (RT). Plasma was collected at diagnosis and EoT. ctDNA was assessed using UMI-based targeted NGS; baseline alterations were used for patient-specific tracking. MRD was defined as positive when ≥2 mutated phased-variant (PV) doublets remained detectable at EoT. EoT PET-CT was dichotomized using Deauville score (1–3 negative; 4–5 positive). To avoid immortal-time bias, prognostic performance for 2-year events was assessed using a landmark analysis including pts alive and progression-free at EoT with both tests available.

**Copyright Information:** (Online) ISSN: 2572-9241
©2026 The Author(s). HemaSphere is published by John Wiley & Sons Ltd on behalf of European Hematology Association. This is an open access Abstract Book distributed under the Attribution-NonCommercial-NoDerivs (CC BY-NC-ND), which allows third parties to download the articles and share them with others as long as they credit the author and the Abstract Book, but they cannot change the content in any way or use them commercially.
**Abstract Book Citations:** Authors, Title, HemaSphere, 2026;10:(S1):pages. The abstract book DOIs can be found on the "Author Information" page.
**Disclaimer:** Articles published in the journal HemaSphere exclusively reflect the opinions of the authors. The authors are responsible for all content in their abstracts including accuracy of the facts, statements, citing resources, etc.

### Results

Overall, 69 cHL pts were included; median age was 36 yrs (range 18–82), 57% were female, 52% had stage III–IV disease, and 52% had B symptoms. Baseline ctDNA was informative in 63/68 (93%, missing plasma: 1) with a median (Q1–Q3) of 212.5 (110–282) evaluable PV doublets; median ctDNA concentration was 62 (24.4–312.9) tHGE/mL. Frequent baseline alterations included *IGH (*92.7%), *IGL* (91%), *BCL6* (85%), *BCL7A* (84%), *SOCS1* (81%); *LPP* (78%), *IGK* (75%) and *CIITA* (72%).

Among 68 pts with treatment data available, frontline therapy was ABVD/AVD-based in 30 (44%)—most with consolidative RT (26/30, 87%)—and BEACOPP-based (often de-escalated to ABVD/AVD after 2 cycles when interim PET-CT was negative) in 38 (56%). At EoT, PET-CT was negative in 58/65 (89%). EoT MRD (PV) was negative in 49/56 (88%) evaluable pts (with 5 baseline non-informative and 7 missing EoT samples). After a median follow-up of 3 yrs, 11 progressions occurred: 10/11 (91%) were biopsied. Overall, 2-year PFS and OS from treatment start were 83% and 100%, respectively.

The primary landmark analysis included 56/69 pts (missing plasmas: 6; MRD non-informative: 5; early progression before EoT: 1; missing PET-CT: 1) corresponding to 8 PFS events within 2 yrs. PET alone showed sensitivity 0.5 (95%CI 0.15–0.85), specificity 0.98 (0.94–1), positive predictive value (PPV) 0.8 (0.45–1) and negative predictive value (NPV) 0.92 (0.85–1). MRD alone showed good sensitivity 0.75 (0.45–1), specificity 0.98 (0.94–1), PPV 0.86 (0.60–1) and NPV 0.96 (0.91–1). Using the combined rule PET-CT(+) or MRD(+), sensitivity increased to 0.88 (0.47–1) while specificity remained high at 0.96 (0.86–0.99); PPV was 0.78 (0.4–0.97) and NPV 0.98 (0.89–1). Only 1/47 pts with both tests negative experienced an event within 2 yrs, resulting in 2-year PFS rate of 98% (95%CI 0.94–1) for pts with both PET and MRD negative versus 22% (95%CI 0.07–0.75) when either test was positive ($p<0.001$, Figure 1).

### Summary/Conclusion

Integrating EoT MRD with PET-CT significantly improves early risk stratification in cHL, substantially increasing sensitivity for 2-year PFS prediction while preserving high specificity and an excellent negative predictive value. This pragmatic combined approach may help refine post-treatment surveillance strategies and warrants validation in larger multicenter cohorts.

**Copyright Information:** (Online) ISSN: 2572-9241
©2026 The Author(s). HemaSphere is published by John Wiley & Sons Ltd on behalf of European Hematology Association. This is an open access Abstract Book distributed under the Attribution-NonCommercial-NoDerivs (CC BY-NC-ND), which allows third parties to download the articles and share them with others as long as they credit the author and the Abstract Book, but they cannot change the content in any way or use them commercially.
**Abstract Book Citations:** Authors, Title, HemaSphere, 2026;10:(S1):pages. The abstract book DOIs can be found on the "Author Information" page.
**Disclaimer:** Articles published in the journal HemaSphere exclusively reflect the opinions of the authors. The authors are responsible for all content in their abstracts including accuracy of the facts, statements, citing resources, etc.

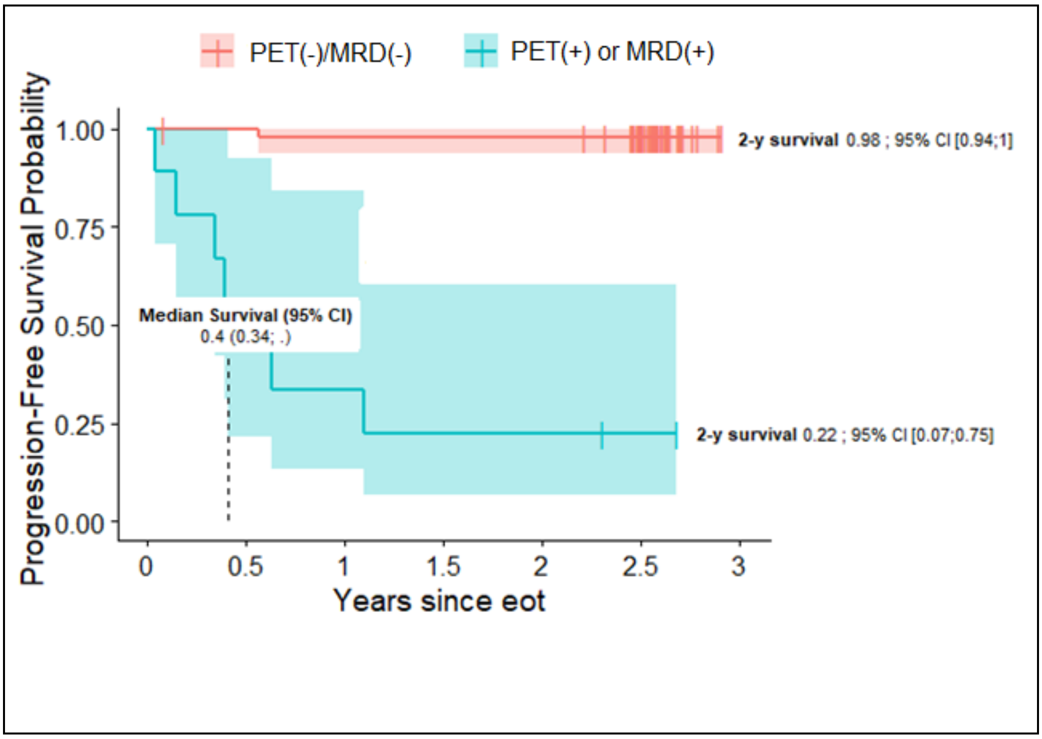

Figure 1: Progression-free survival according to end of treatment (EoT) PET-CT and ctDNA MRD results.

**Copyright Information:** (Online) ISSN: 2572-9241
©2026 The Author(s). HemaSphere is published by John Wiley & Sons Ltd on behalf of European Hematology Association. This is an open access Abstract Book distributed under the Attribution-NonCommercial-NoDerivs (CC BY-NC-ND), which allows third parties to download the articles and share them with others as long as they credit the author and the Abstract Book, but they cannot change the content in any way or use them commercially.
**Abstract Book Citations:** Authors, Title, HemaSphere, 2026;10:(S1):pages. The abstract book DOIs can be found on the "Author Information" page.
**Disclaimer:** Articles published in the journal HemaSphere exclusively reflect the opinions of the authors. The authors are responsible for all content in their abstracts including accuracy of the facts, statements, citing resources, etc.

### (PF1069) REDUCED CD20 EXPRESSION AND INTRATUMORAL CD3+ T CELLS FOLLOWING EPCORITAMAB TREATMENT ARE ASSOCIATED WITH PROGRESSIVE DISEASE IN A SUBSET OF DIFFUSE LARGE B-CELL LYMPHOMA AND FOLLICULAR LYMPHOMA

**Topic:** 21. Lymphoma biology & translational research

Monica Wielgos-Bonvallet*[1], Mahesh Bachu[1], Isil Altintas[2], Han Si[1], Kubra Karagoz[1], Matthew Loya[1], Prianka Chohan[1], Scott Milos[1], Chan Y. Cheah[3], Eliza Hawkes[4], Michael Roost Clausen[5], Christopher Morehouse[1], Melih Acar[6], David Soong[1], Andrew J. Steele[1]

*[1]Genmab, Plainsboro, NJ, United States of America;[2]Genmab, Utrecht, The Netherlands;[3]Sir Charles Gairdner Hospital and the University of Western Australia, Perth, WA, Australia; [4]Olivia Newton John Cancer Research Institute at Austin Health, Melbourne, VIC, Australia; [5]Vejle Hospital, Vejle, Denmark; [6]AbbVie Inc., South San Francisco, CA, United States of America;*

### Background

Epcoritamab (epcor), a subcutaneous CD3×CD20 bispecific antibody, has demonstrated significant clinical benefit in DLBCL and FL as a monotherapy and in combinations. Modulation in CD20 expression has been reported with other CD3×CD20 bispecific antibodies, most often at disease progression, although the exact timing of these changes remains unclear.

### Aims

To describe modulation in CD20 expression in patients pre- and post- treatment (tx) with epcor, evaluate the impact of mutations and transcriptional downregulation on CD20 expression, and assess the association between intratumoral CD3+ T cells and progressive disease in the EPCORE NHL-1 trial (NCT03625037).

### Methods

Tumor biopsies from patients with R/R DLBCL or FL treated with 48-mg epcor in NHL-1 were collected. CD20 protein expression with and without PAX5 and T cells (CD3+) was quantified by immunohistochemistry at baseline, on tx (cycle 2 day 15 [C2D15]), or at the end of tx (EOT). Whole exome sequencing and EdgeSeq were performed on available patient samples to investigate mechanisms underlying reduced CD20 expression. Clinical responses at week 6 and EOT were assessed by investigator per Lugano criteria.

### Results

High baseline CD20 expression (> 80% positive tumor cells) was observed in 89% (319/357) of biomarker-evaluable biopsies from patients with DLBCL and FL; CD20 expression < 10% was infrequent. Clinical responses were observed in some patients with CD20 expression < 10%, suggesting epcor may retain clinical benefit at all levels of CD20 expression. Paired baseline and on-tx/EOT biopsies were analyzed in a biomarker-evaluable subset of 23 patients with baseline CD20 expression > 10% (PR, n = 3; SD, n = 2; PD, n = 18); patients with CR were excluded due to no detectable tumor at C2D15, limiting generalizability. CD20 expression was maintained on tx in 4 of 5 patients with PR/SD. CD20 expression < 10% post baseline was observed in 11 of 18 patients with PD, including those who had biopsies at C2D15 (2 of 6), or at EOT (9 of 12). One patient who initially had a PR demonstrated CD20 expression < 10% on tx and later progressed. Of the 12 patients with CD20 expression < 10% post baseline, 7 (58%) harbored *MS4A1* mutations in the extracellular and transmembrane domains; transcriptional downregulation was observed in 2 (17%) patients. Notably, 1 patient with SD who maintained CD20 expression at C2D15 harbored a pre-existing missense mutation in extracellular loop 2 that may have interfered with epcor binding. Of the 18 pts with PD, 7 maintained CD20 expression, of whom 4 showed on-tx and EOT reductions in intratumoral CD3 T cells.

**Copyright Information:** (Online) ISSN: 2572-9241
©2026 The Author(s). HemaSphere is published by John Wiley & Sons Ltd on behalf of European Hematology Association. This is an open access Abstract Book distributed under the Attribution-NonCommercial-NoDerivs (CC BY-NC-ND), which allows third parties to download the articles and share them with others as long as they credit the author and the Abstract Book, but they cannot change the content in any way or use them commercially.
**Abstract Book Citations:** Authors, Title, HemaSphere, 2026;10:(S1):pages. The abstract book DOIs can be found on the "Author Information" page.
**Disclaimer:** Articles published in the journal HemaSphere exclusively reflect the opinions of the authors. The authors are responsible for all content in their abstracts including accuracy of the facts, statements, citing resources, etc.

### Summary/Conclusion

Whilst most patients with DLBCL and FL have high baseline CD20 expression and high response rates to epcor, clinical responses were also observed in some patients with baseline CD20 expression < 10%. Reduced CD20 expression on tx or at EOT was observed in patients with PD, and this was linked to *MS4A1* mutations and transcriptional downregulation in a subset of patients. Decreased intratumoral T-cell numbers were also identified as a potential mechanism of resistance. Similar changes in CD20 expression at progression have been reported with other CD3xCD20 bispecific antibodies, suggesting antigen modulation may contribute to resistance in some patients. These findings support further exploration of strategies to overcome antigen escape and restore therapeutic sensitivity.

**Copyright Information:** (Online) ISSN: 2572-9241
©2026 The Author(s). HemaSphere is published by John Wiley & Sons Ltd on behalf of European Hematology Association. This is an open access Abstract Book distributed under the Attribution-NonCommercial-NoDerivs (CC BY-NC-ND), which allows third parties to download the articles and share them with others as long as they credit the author and the Abstract Book, but they cannot change the content in any way or use them commercially.
**Abstract Book Citations:** Authors, Title, HemaSphere, 2026;10:(S1):pages. The abstract book DOIs can be found on the "Author Information" page.
**Disclaimer:** Articles published in the journal HemaSphere exclusively reflect the opinions of the authors. The authors are responsible for all content in their abstracts including accuracy of the facts, statements, citing resources, etc.

### (PF1070) OUTCOMES BY LYMPHOMAP ARCHETYPES IN UNTREATED DIFFUSE LARGE B-CELL LYMPHOMA FROM THE POLARIX TRIAL

**Topic:** 21. Lymphoma biology & translational research

R. Andrew Harkins*[1], Will Harris[2], Michael Green[1], Alex F. Herrera[3], Fabrice Jardin[4], Georg Lenz[5], Franck Morschhauser[6], Matthew Sugidono[2], Mark Yan[7], Connie Lee Batlevi[2], Christopher Flowers[1], Yanwen Jiang[2]

*[1]M.D. Anderson Cancer Center, Houston, TX, United States of America; [2]Genentech, Inc., South San Francisco, CA, United States of America; [3]City of Hope, Duarte, CA, United States of America; [4]University of Rouen, Rouen, United States of America; [5]University Hospital Münster, Münster, United States of America; [6]Lille University Hospital Center, Lille Cedex, France; [7]Roche, Inc., Basel, Switzerland;*

### Background

Single-cell multiomics helped identify three tumor microenvironment archetypes in large B-cell lymphoma (LBCL): FMAC, high tumor-associated fibroblasts and macrophages; LN, high naive and memory T cells; TEX, high exhausted CD8+ T cells (Li X, et al. Cancer Cell 2025) with FMAC associated with inferior outcomes with first-line rituximab with cyclophosphamide, doxorubicin, vincristine, and prednisone (R-CHOP). In the POLARIX study, sustained and significant 5-year progression-free survival (PFS)/disease-free survival benefits favoring polatuzumab vedotin with rituximab, cyclophosphamide, doxorubicin, and prednisone (Pola-R-CHP) were observed (Morschhauser F, et al. J Clin Oncol 2025) supporting its use as a standard of care for patients with untreated intermediate/high-risk LBCL.

### Aims

A post hoc exploratory analysis was performed to investigate the clinical outcomes of LymphoMAP subtypes in POLARIX.

### Methods

LymphoMAP subtypes were determined from global gene expression patterns (GEPs) using the LymphoMapR classifier. Cell of origin (COO) status was determined by NanoString. Hazard ratios (HRs) were estimated by an unstratified Cox proportional hazard model and adjusted for International Prognostic Index (IPI) score (2 vs 3–5), age (≤60 vs >60 years) and COO. All statistics are descriptive.

**Copyright Information:** (Online) ISSN: 2572-9241
©2026 The Author(s). HemaSphere is published by John Wiley & Sons Ltd on behalf of European Hematology Association. This is an open access Abstract Book distributed under the Attribution-NonCommercial-NoDerivs (CC BY-NC-ND), which allows third parties to download the articles and share them with others as long as they credit the author and the Abstract Book, but they cannot change the content in any way or use them commercially.
**Abstract Book Citations:** Authors, Title, HemaSphere, 2026;10:(S1):pages. The abstract book DOIs can be found on the "Author Information" page.
**Disclaimer:** Articles published in the journal HemaSphere exclusively reflect the opinions of the authors. The authors are responsible for all content in their abstracts including accuracy of the facts, statements, citing resources, etc.

**Results**

GEP data were available for 669 patients (Pola-R-CHP, n=331; R-CHOP, n=338; baseline characteristics were comparable with the intention-to-treat population); among which 244 (33.5%), 229 (34.2%), and 196 (29.3%) were FMAC, LN, and TEX, respectively (**Table**). Balanced baseline clinical characteristics were seen across subtypes, except for a lower proportion of LN with high IPI (3–5) and a higher proportion of TEX as activated B-cell (ABC) diffuse LBCL (DLBCL; **Table**).
With a median follow-up 61 months, the adjusted HRs for PFS favored Pola-R-CHP compared with R-CHOP in all subtypes and were 0.66 (95% confidence interval [CI]: 0.43–1.01) for FMAC, 0.79 (95% CI: 0.51–1.23) for LN, and 0.81 (95% CI: 0.50–1.32) for TEX. The 5-year PFS estimates were 66.0% (95% CI: 57.1–76.2), 64.6% (95% CI: 55.4–75.4), 68.8% (95% CI: 59.7–79.4) for FMAC, LN, and TEX, respectively in the Pola-R-CHP arm, and 56.9% (95% CI: 47.6-68.0), 61.2% (95% CI: 52.8–70.9), 56.6% (95% CI: 45.8–69.9) for FMAC, LN, and TEX, respectively in the R-CHOP arm.
A similar trend for overall survival (OS) improvement was observed in the FMAC subtype with 5-year OS estimates of 82.5% (95% CI: 76.3–89.3) versus 72.0% (95% CI: 63.8–81.2) with Pola-R-CHP versus R-CHOP (HR 0.64; 95% CI: 0.37–1.10).
Trends of PFS and OS improvements with Pola-R-CHP over R-CHOP were observed in patients with ABC-DLBCL across all LymphoMAP subtypes. For patients with germinal center B-cell DLBCL, no benefit was observed with Pola-R-CHP versus R-CHOP in the TEX subtype.

**Summary/Conclusion**

In this exploratory analysis, Pola-R-CHP demonstrated PFS and OS improvement trends over R-CHOP in all three LymphoMAP subtypes, especially for the FMAC subtype. Future prospective validation is required.

© 2026 American Society of Clinical Oncology, Inc. Reused with permission. This abstract was accepted and previously presented at the 2026 ASCO Annual Meeting. All rights reserved.

**Copyright Information:** (Online) ISSN: 2572-9241
©2026 The Author(s). HemaSphere is published by John Wiley & Sons Ltd on behalf of European Hematology Association. This is an open access Abstract Book distributed under the Attribution-NonCommercial-NoDerivs (CC BY-NC-ND), which allows third parties to download the articles and share them with others as long as they credit the author and the Abstract Book, but they cannot change the content in any way or use them commercially.
**Abstract Book Citations:** Authors, Title, HemaSphere, 2026;10:(S1):pages. The abstract book DOIs can be found on the "Author Information" page.
**Disclaimer:** Articles published in the journal HemaSphere exclusively reflect the opinions of the authors. The authors are responsible for all content in their abstracts including accuracy of the facts, statements, citing resources, etc.

**Table:**

| n (%), unless stated | FMAC (n=244) | LN (n=229) | TEX (n=196) | BEP (n=669) |
|---|---|---|---|---|
| Pola-R-CHP<br>R-CHOP | 134 (55)<br>110 (45) | 105 (46)<br>124 (54) | 92 (47)<br>104 (53) | 331 (50)<br>338 (50) |
| Median age, years [min–max] | 65<br>[22–80] | 66<br>[19–80] | 64<br>[22–80] | 65<br>[19–80] |
| IPI 3–5 | 157 (64) | 125 (55) | 133 (68) | 415 (62) |
| Bulky | 109 (45) | 99 (43) | 87 (44) | 295 (44) |
| Extranodal | 174 (71) | 146 (64) | 143 (73) | 463 (69) |
| Cell of origin<br>ABC<br>GCB<br>Unclassified<br>Unknown | <br>68 (28)<br>138 (57)<br>20 (8)<br>18 (7) | <br>56 (24)<br>135 (59)<br>34 (15)<br>4 (2) | <br>90 (46)<br>65 (33)<br>38 (19)<br>3 (2) | <br>214 (32)<br>338 (50)<br>92 (14)<br>25 (4) |

ABC, activated B-cell; BEP, biomarker evaluable population; FMAC, high tumor-associated fibroblasts and macrophages; GCB, germinal center B-cell; IPI, International Prognostic Index; LN, high naive and memory T cells; Pola-R-CHP, polatuzumab vedotin with rituximab, cyclophosphamide, doxorubicin, and prednisone; R-CHOP, rituximab with cyclophosphamide, doxorubicin, vincristine, and prednisone; TEX, high exhausted CD8+ T cells.

Copyright Information: (Online) ISSN: 2572-9241
©2026 The Author(s). HemaSphere is published by John Wiley & Sons Ltd on behalf of European Hematology Association. This is an open access Abstract Book distributed under the Attribution-NonCommercial-NoDerivs (CC BY-NC-ND), which allows third parties to download the articles and share them with others as long as they credit the author and the Abstract Book, but they cannot change the content in any way or use them commercially.
Abstract Book Citations: Authors, Title, HemaSphere, 2026;10:(S1):pages. The abstract book DOIs can be found on the "Author Information" page.
Disclaimer: Articles published in the journal HemaSphere exclusively reflect the opinions of the authors. The authors are responsible for all content in their abstracts including accuracy of the facts, statements, citing resources, etc.

### (PF1071) CHROMOSOME 8Q COPY NUMBER GAIN PREDICTS EARLY RELAPSE IN HIGH-GRADE DIFFUSE LARGE B-CELL LYMPHOMA WITH MYC AND BCL2 REARRANGEMENTS

**Topic:** 21. Lymphoma biology & translational research

Jurriaan Janssen[1], Daphne de Jong[2], Tom van den Bosch*[3], Erik van Dijk[1], Matias Mendeville[1], Tjitske Los-de Vries[1], Lianne Koens[1], Phylicia Stathi[1], Jacqueline Egthuijsen[1], Paul van Eijk[1], Pieternella Lugtenburg[4], Wolfram Klapper[5], Ulrich Dührsen[6], Andreas Huettmann[6], Julia Richter[5], Bauke Ylstra[1], Martine Chamuleau[1]

*[1]Amsterdam UMC, Department of Pathology, Amsterdam, The Netherlands;[2]Netherlands Cancer Institute, Department of Biostatistics, Amsterdam, The Netherlands; [3]Amsterdam UMC, Department of Hematology, Amsterdam, The Netherlands; [4]University Medical Center Rotterdam, Erasmus MC Cancer Institute, Rotterdam, The Netherlands;[5]University Hospital Schleswig-Holstein, Department of Pathology, Kiel, Germany; [6]University Hospital Essen, Department of Hematology, Essen, Germany;*

### Background

Large B-cell lymphoma (LBCL) comprises biologically heterogeneous entities with variable clinical outcome. Diffuse large B-cell lymphoma/high-grade B-cell lymphoma with concurrent *MYC* and *BCL2* rearrangements (DLBCL/HGBL-*MYC/BCL2, hereafter HGBL-DH (double-hit))* represents a distinct entity characterized by frequent early refractory or relapsed disease. These patients are treated with more intensified treatment regimens than patients with DLBLC without MYC and BCL2 rearrangements (DLBCL-NOS (not otherwise specified)), however outcomes for *HGBL-DH* patients remain substantially inferior to those of DLBCL-NOS patients. Additional biomarkers to identify high-risk patients within the HGBL-DH subgroup are lacking. While *MYC* rearrangements (*MYC*-R) in relation to its partner gene (Ig or non-Ig) and copy numbers at the MYC chromosome 8q locus have been studied in great detail, the relation of the various architectures with outcome has remained underexplored.

### Aims

We aimed to stratify HGBL-DH patients based on their complete genomic architecture, and study the association with patient outcome in a large set of LBCL patients enriched for MYC translocations.

### Methods

We obtained comprehensive next-generation sequencing of 76 HGBL-DH and 660 DLBCL-NOS samples derived from four clinical trials and one multicenter cohort. From this sequencing, we derived genomic mutations, translocations, and genome-wide copy number alterations. *MYC-R* and large-scale chromosome 8 gains were integrated to resolve their complete genomic architecture.

**Copyright Information:** (Online) ISSN: 2572-9241
©2026 The Author(s). HemaSphere is published by John Wiley & Sons Ltd on behalf of European Hematology Association. This is an open access Abstract Book distributed under the Attribution-NonCommercial-NoDerivs (CC BY-NC-ND), which allows third parties to download the articles and share them with others as long as they credit the author and the Abstract Book, but they cannot change the content in any way or use them commercially.
**Abstract Book Citations:** Authors, Title, HemaSphere, 2026;10:(S1):pages. The abstract book DOIs can be found on the “Author Information” page.
**Disclaimer:** Articles published in the journal HemaSphere exclusively reflect the opinions of the authors. The authors are responsible for all content in their abstracts including accuracy of the facts, statements, citing resources, etc.

### Results

Chromosome 8q gains were significantly more frequent in HGBL-DH(47%, 38/76) compared to DLBCL-NOS (23%, 125/546, Fisher's exact p = 0.002) and were associated with unbalanced *MYC::non-IGH* rearrangements. Four distinct configurations of 8q architecture in relation to MYC in HGBL-DH were observed (Figure 1A):

(I) 8q Gains restricted to the region centromeric to and ending at the 8q21.4 *MYC* locus. – 20% 15/76 patients.
(II) 8q Gains restricted to the region telomeric to and ending at the 8q21.4 *MYC* locus. – 7% 5/76 patients.
(III) Complete or partial 8q gains encompassing the 8q21.4 *MYC* locus, and with copy-number breakpoints outside 8q21.4. – 24% 18/76 patients.
(IV) No 8q24.21 *MYC* locus gain – 50%, 38/76 patients.

Patients with configuration I-III had significantly fewer MYC::IGH translocations (3/38) compared to configuration IV (15/38, Fisher's exact p = 0.001). Across all configuration, patients were treated with intensified treatment in similar proportions (conf I: 73%, 11/15 intensified, conf II: 40%, 2/5, conf III 83%, 15/18, conf IV 81%, 29/36, chi-square p = 0.22). In multivariable analysis of 73 HGBL-DH patients, with cohort and IPI score as covariables, 8q gains with a breakpoint outside of MYC (configuration III) were associated with inferior 1 year PFS (Figure 1B). Kaplan Meier analysis showed that HGBL-DH patients without 8q gains (configuration IV) showed favorable outcomes compared to HGBL-DH patients with 8q gains (configuration I-III) and comparable outcomes to DLBCL-NOS patients (Figure 1C).

### Summary/Conclusion

In conclusion, HGBL-DH patients with chromosome 8q copy number gain defines a subgroup of HGBL-DH patients with high risk of refractory/relapsed disease, while HGBL-DH patients without chromosome 8q gain have comparable outcomes to DLBCL-NOS patients. This allows upfront risk stratification and guiding of tailored treatment strategies based on chromosome 8q status.

**Copyright Information:** (Online) ISSN: 2572-9241
©2026 The Author(s). HemaSphere is published by John Wiley & Sons Ltd on behalf of European Hematology Association. This is an open access Abstract Book distributed under the Attribution-NonCommercial-NoDerivs (CC BY-NC-ND), which allows third parties to download the articles and share them with others as long as they credit the author and the Abstract Book, but they cannot change the content in any way or use them commercially.
**Abstract Book Citations:** Authors, Title, HemaSphere, 2026;10:(S1):pages. The abstract book DOIs can be found on the "Author Information" page.
**Disclaimer:** Articles published in the journal HemaSphere exclusively reflect the opinions of the authors. The authors are responsible for all content in their abstracts including accuracy of the facts, statements, citing resources, etc.

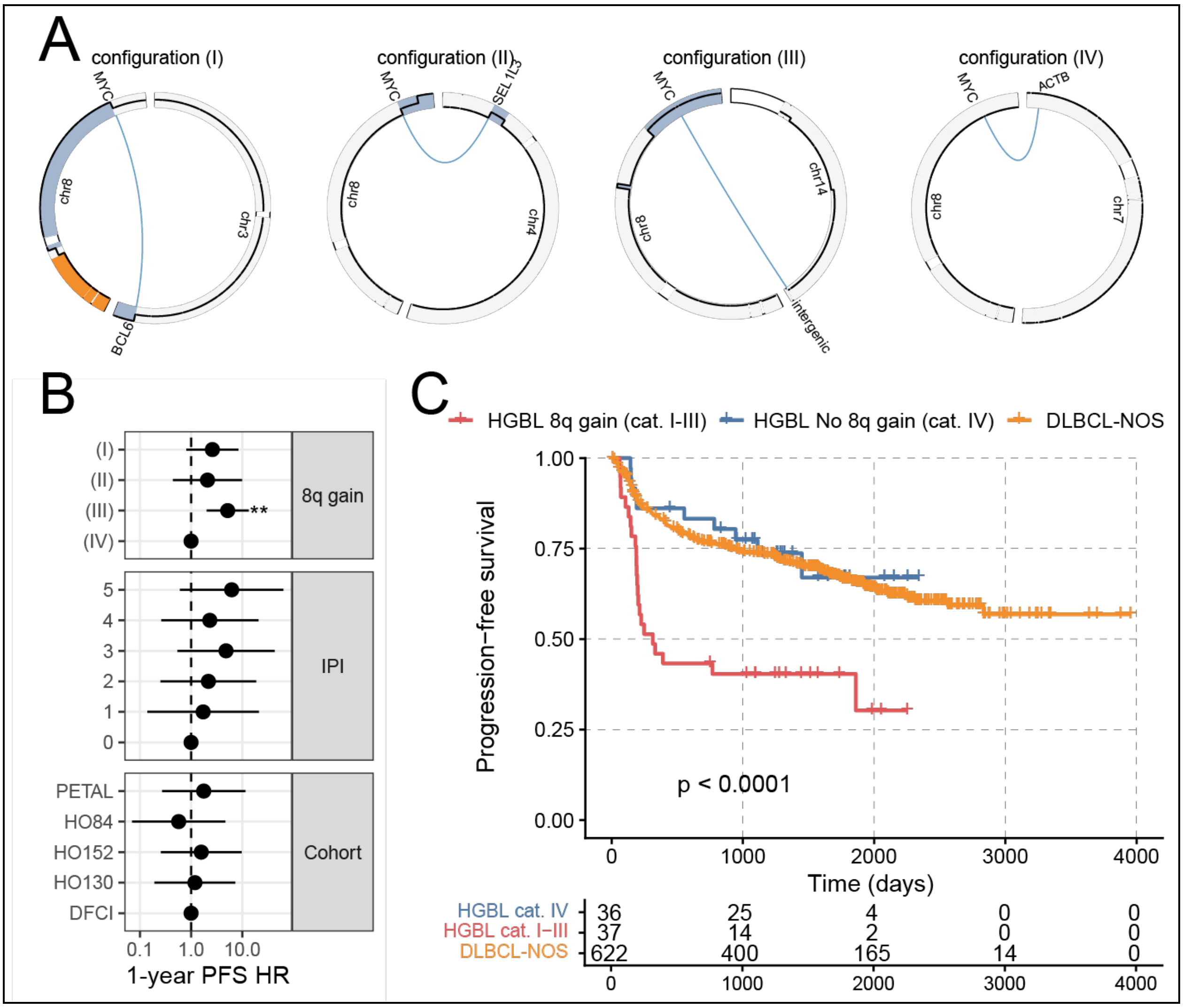

Figure 1: Different configurations of chromosome 8q copy number gain are predictive of HGBL-DH patient survival. (A) Representative examples of the four identified chromosome 8q gain configurations. (B) Hazard ratios (points) and confidence intervals (lines) for 1-year progression-free survival of the four chromosome 8q configurations in HGBL-DH from a multivariable Cox regression model including corrections for IPI and dataset. (C) Survival of HGBL-DH patients with chromosome 8q gain (configuration I-III) versus no gain (configuration IV) compared to DLBCL-NOS patients with reported log-rank p-value.

**Copyright Information:** (Online) ISSN: 2572-9241
©2026 The Author(s). HemaSphere is published by John Wiley & Sons Ltd on behalf of European Hematology Association. This is an open access Abstract Book distributed under the Attribution-NonCommercial-NoDerivs (CC BY-NC-ND), which allows third parties to download the articles and share them with others as long as they credit the author and the Abstract Book, but they cannot change the content in any way or use them commercially.
**Abstract Book Citations:** Authors, Title, HemaSphere, 2026;10:(S1):pages. The abstract book DOIs can be found on the "Author Information" page.
**Disclaimer:** Articles published in the journal HemaSphere exclusively reflect the opinions of the authors. The authors are responsible for all content in their abstracts including accuracy of the facts, statements, citing resources, etc.

### (PF1072) BRIGATINIB RESISTANCE IN PEDIATRIC ALK-POSITIVE ANAPLASTIC LARGE CELL LYMPHOMA IS DRIVEN BY GPX4-MEDIATED FERROPTOSIS SUPPRESSION

**Topic:** 21. Lymphoma biology & translational research

Gerardo Enrique Abarca Ríos*[1, 2], Lucy Hare[3, 4], Veronika Navrkalová[1, 2], Suzanne Turner[1, 3], Sarka Pospisilova[1, 2]

*[1]Institute of Medical Genetics and Genomics and Department of Internal Medicine, Hematology and Oncology, Faculty of Medicine, Masaryk University and University Hospital Brno, Brno, Czechia; [2]Central European Institute of Technology, Masaryk University, Brno, Czechia; [3]Division of Cellular and Molecular Pathology, Department of Pathology, University of Cambridge, Cambridge, United Kingdom; [4]Department of Paediatric Haematology, Oncology and Palliative Care, Addenbrooke's Hospital, Cambridge, United Kingdom;*

### Background

Anaplastic large cell lymphoma (ALCL) is a mature T-cell malignancy frequently driven by *ALK* gene rearrangements. Despite the efficacy of targeted therapy, resistance to ALK tyrosine kinase inhibitors can emerge through secondary *ALK* mutations/amplifications or bypass signaling mechanisms. Clinically, this manifests as treatment resistance or relapse on cessation of therapy. These challenges highlight a critical need for combinatorial therapeutic strategies designed to target and inhibit these bypass resistance pathways.

### Aims

This study aimed to identify novel bypass signaling pathways that promote resistance to brigatinib in ALK-positive ALCL and to evaluate rational combinatorial treatment strategies to target these escape mechanisms.

### Methods

Whole-genome CRISPR activation (CRISPRa) screens were performed in ALK-positive ALCL cell lines under ALK-inhibitor pressure to identify resistance drivers. The candidate gene *GPX4* was validated using single-gene CRISPRa-mediated overexpression and doxycycline-inducible shRNA knockdown. Cellular fitness and ferroptotic markers, including production of reactive oxygen species (ROS), lipid peroxidation, labile iron ($Fe^{2+}$), and glutathione levels, were quantified. Finally, synergy between brigatinib and the GPX4 inhibitor RSL3 or the upstream mevalonate pathway inhibitor rosuvastatin was assessed using the Zero Interaction Potency (ZIP) synergy modeling.

**Copyright Information:** (Online) ISSN: 2572-9241
©2026 The Author(s). HemaSphere is published by John Wiley & Sons Ltd on behalf of European Hematology Association. This is an open access Abstract Book distributed under the Attribution-NonCommercial-NoDerivs (CC BY-NC-ND), which allows third parties to download the articles and share them with others as long as they credit the author and the Abstract Book, but they cannot change the content in any way or use them commercially.
**Abstract Book Citations:** Authors, Title, HemaSphere, 2026;10:(S1):pages. The abstract book DOIs can be found on the "Author Information" page.
**Disclaimer:** Articles published in the journal HemaSphere exclusively reflect the opinions of the authors. The authors are responsible for all content in their abstracts including accuracy of the facts, statements, citing resources, etc.

**Results**

CRISPRa screens identified the selenoprotein GPX4, a key regulator of ferroptosis, as a mediator of brigatinib resistance in ALK-positive ALCL models. Functional validation confirmed that GPX4 overexpression drives resistance by preventing drug-induced lipid peroxidation and preserving redox homeostasis. We demonstrate that after 48 hours of treatment with brigatinib, GPX4-overexpressing cells exhibited significantly lower levels of total ROS, lipid peroxides, and $Fe^{2+}$ and increased glutathione levels, compared with non-targeting controls. Conversely, GPX4 knockdown significantly reduced basal proliferation by inducing ferroptotic cell death, which was evidenced by increased levels of total ROS, lipid peroxides, and $Fe^{2+}$, accompanied by glutathione depletion. This intrinsic dependency on GPX4 for survival sensitized knockdown models to brigatinib, resulting in an enhanced treatment response *in vitro*. Therapeutically, direct GPX4 inhibition with RSL3 synergized with brigatinib to overcome resistance in GPX4-overexpressing ALK-positive ALCL models. In addition, targeting the upstream mevalonate pathway with rosuvastatin, which depletes precursor proteins required for GPX4 translation, synergistically enhanced brigatinib response in wild-type *ex vivo* patient-derived xenograft ALCL models. These synergistic combinations are currently being evaluated in ongoing *in vivo* preclinical studies to confirm their therapeutic potential.

**Summary/Conclusion**

We have established the GPX4-ferroptosis axis as a non-mutational mechanism of resistance to brigatinib in pediatric ALCL. Combining ALK inhibitors with ferroptosis inducers, such as GPX4 inhibitors or mevalonate pathway inhibitors like rosuvastatin, represents a promising strategy to achieve more durable outcomes for children with ALK-positive ALCL.
Funded by the European Union's Horizon Europe MSCA Doctoral Network FANTOM under grant agreement No: 101072735 and Masaryk University grant MUNI/A/1733/2025.

**Copyright Information:** (Online) ISSN: 2572-9241
©2026 The Author(s). HemaSphere is published by John Wiley & Sons Ltd on behalf of European Hematology Association. This is an open access Abstract Book distributed under the Attribution-NonCommercial-NoDerivs (CC BY-NC-ND), which allows third parties to download the articles and share them with others as long as they credit the author and the Abstract Book, but they cannot change the content in any way or use them commercially.
**Abstract Book Citations:** Authors, Title, HemaSphere, 2026;10:(S1):pages. The abstract book DOIs can be found on the “Author Information” page.
**Disclaimer:** Articles published in the journal HemaSphere exclusively reflect the opinions of the authors. The authors are responsible for all content in their abstracts including accuracy of the facts, statements, citing resources, etc.

### (PF1073) THE STRESS-SENSING MARCH5 AXIS GOVERNS BLOOD CANCER APOPTOTIC RESILIENCE VIA TRACTABLE PROTEIN–PROTEIN INTERACTIONS

**Topic:** 21. Lymphoma biology & translational research

Thomas Lew*[1, 2], Mark van Delft[1], Luuk Heitink[1], Simon Cobbold[1], Christine White[1], Jason Bong[1], Philip Hittmeyer[3], Christopher Riffkin[1], Angela Georgiou[1], Adrienne Hilton[1], Allan Huang[1], Yin Yuan[1], Miles Horton[1], Mark Dawson[2], Andrew Roberts[1, 2], David Huang[1]

*[1]Walter and Eliza Hall Institute of Medical Research,, Melbourne, Australia;[2]Peter MacCallum Cancer Centre & the Royal Melbourne Hospital, Melbourne, Australia; [3]University of Vienna, Vienna, Austria;*

### Background

Blood cancer cells must endure organellar stress from oncogenesis and treatment, including DNA damage, ER stress and mitochondrial dysfunction. Pro-survival BCL-2 family proteins mitigate apoptosis, yet alternative survival mechanisms frequently prevail, limiting the curative potential of BH3-mimetics such as venetoclax (VEN). How stress signals are integrated to enforce apoptotic restraint — and how these mechanisms can be therapeutically exploited — remains incompletely defined.

### Aims

To identify core mediators of stress tolerance in hematologic malignancies and define therapeutically tractable vulnerabilities that enforce BCL-2 dependence.

### Methods

We developed an augmented genome-wide CRISPR/Cas9 “death” screen to positively select sensitizing knockout events in BH3-mimetic–resistant mantle cell lymphoma (MCL). Hits were validated across AML, DLBCL and myeloma models. Mechanistic studies employed CRISPR editing, dTag degradation, co-immunoprecipitation, diGly proteomics, split-nanoluciferase assays, subcellular fractionation and xenograft models.

**Copyright Information:** (Online) ISSN: 2572-9241
©2026 The Author(s). HemaSphere is published by John Wiley & Sons Ltd on behalf of European Hematology Association. This is an open access Abstract Book distributed under the Attribution-NonCommercial-NoDerivs (CC BY-NC-ND), which allows third parties to download the articles and share them with others as long as they credit the author and the Abstract Book, but they cannot change the content in any way or use them commercially.
**Abstract Book Citations:** Authors, Title, HemaSphere, 2026;10:(S1):pages. The abstract book DOIs can be found on the “Author Information” page.
**Disclaimer:** Articles published in the journal HemaSphere exclusively reflect the opinions of the authors. The authors are responsible for all content in their abstracts including accuracy of the facts, statements, citing resources, etc.

**Results**

The mitochondrial E3 ligase MARCH5 and E2 enzyme UBE2J2 emerged as consistent synthetic lethal sensitizers to BCL-2 inhibition. MARCH5 loss induced multi-log sensitization to VEN and BCL-XL inhibition across diverse blood cancers, including TP53-mutant lines, and conferred marked intolerance to DNA damage, ER stress and mitochondrial insults. By contrast, solid tumor cells were sensitized only to BCL-XL inhibition, and MCL-1 dependence was largely unaffected across contexts.
UBE2J2 was required for MARCH5-mediated apoptotic restraint in blood cancers but dispensable in solid tumors. MARCH5 variants unable to bind UBE2J2 failed to suppress apoptosis, demonstrating that disrupting this interface is sufficient to enforce BCL-2/XL dependence.
Genome-wide reversal screening and diGly proteomics excluded a single dominant ubiquitin substrate. Instead, mitofusin-2 (MFN2) emerged as an essential co-factor. Co-immunoprecipitation confirmed a MARCH5–UBE2J2–MFN2 tripartite complex at mitochondria–ER contact sites (MERCS). BCL-2, BCL-XL, BAX and BAK co-localized to MERCS, whereas MCL-1 was notably absent, providing a spatial explanation for selective synthetic lethality. Targeting the MARCH5 c-terminus disrupted MFN2 engagement and enforced BCL-2/XL dependence and stress intolerance.
Acute organellar stress induced rapid dissociation of MFN2 and UBE2J2 from MARCH5 upstream of apoptosis, confirmed by co-IP and split-nanoluciferase assays. These stresses increased BCL-2 dependence, indicating that loss of complex integrity—by stress or genetic disruption—resets the apoptotic threshold. Disruption of the MARCH5:MFN2 interaction in vivo reverted VEN-refractory lymphoma to treatment-sensitive with prolonged survival.

**Summary/Conclusion**

MARCH5–UBE2J2–MFN2 form a stress-sensing complex at MERCS that integrates convergent organellar damage signals to reinforce local BCL-2/XL function and restrain apoptosis. Its deterioration under stress or targeted disruption collapses stress tolerance and enforces selective BCL-2/XL addiction in blood cancers. Targeting its protein–protein interfaces provides a rational, lineage-selective strategy to enhance venetoclax and related therapies.

**Copyright Information:** (Online) ISSN: 2572-9241
©2026 The Author(s). HemaSphere is published by John Wiley & Sons Ltd on behalf of European Hematology Association. This is an open access Abstract Book distributed under the Attribution-NonCommercial-NoDerivs (CC BY-NC-ND), which allows third parties to download the articles and share them with others as long as they credit the author and the Abstract Book, but they cannot change the content in any way or use them commercially.
**Abstract Book Citations:** Authors, Title, HemaSphere, 2026;10:(S1):pages. The abstract book DOIs can be found on the "Author Information" page.
**Disclaimer:** Articles published in the journal HemaSphere exclusively reflect the opinions of the authors. The authors are responsible for all content in their abstracts including accuracy of the facts, statements, citing resources, etc.

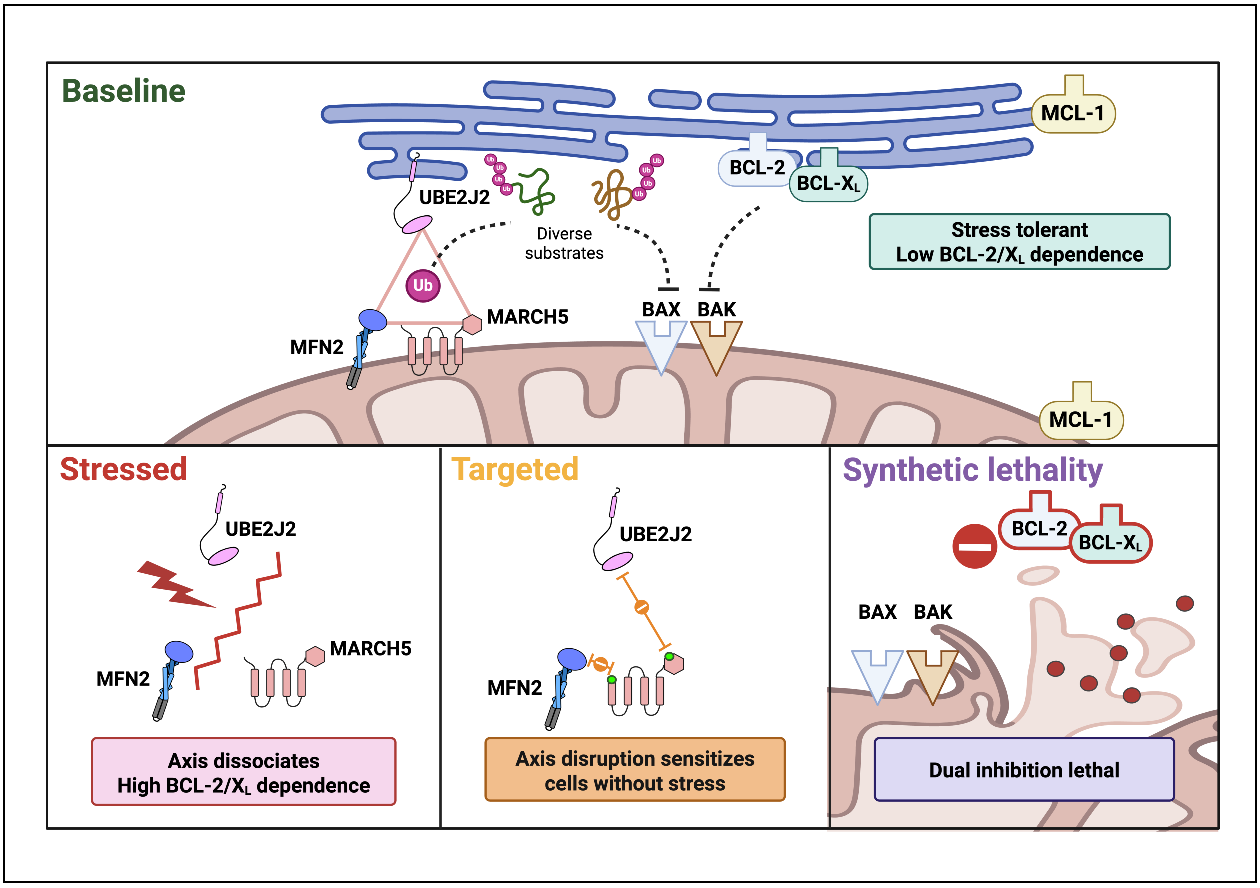

**Copyright Information:** (Online) ISSN: 2572-9241
©2026 The Author(s). HemaSphere is published by John Wiley & Sons Ltd on behalf of European Hematology Association. This is an open access Abstract Book distributed under the Attribution-NonCommercial-NoDerivs (CC BY-NC-ND), which allows third parties to download the articles and share them with others as long as they credit the author and the Abstract Book, but they cannot change the content in any way or use them commercially.
**Abstract Book Citations:** Authors, Title, HemaSphere, 2026;10:(S1):pages. The abstract book DOIs can be found on the "Author Information" page.
**Disclaimer:** Articles published in the journal HemaSphere exclusively reflect the opinions of the authors. The authors are responsible for all content in their abstracts including accuracy of the facts, statements, citing resources, etc.

### (PF1074) GENOMIC ARCHITECTURE OF IRF4 REARRANGEMENTS IN CHILDREN AND ADULT LARGE B-CELL LYMPHOMAS

**Topic:** 21. Lymphoma biology & translational research

Ariadna Colmenero*[1], Leonie Frauenfeld[1], Sara Mato[1], Julia Salmerón[1], Vicente Arredondo Prats[1], Juan Enric Ramis-Zaldivar[1], Anna Montaner[1], Natalia Castrejón-de-Anta[2], Blanca González Farré[2], Noelia García[1], Jaume Verdu[3], Mara Andrés[4], Maitane Andion Catalan[5], Daniel Azorin[6], Verónica Celis[7], María Socorro Rodriguez-Pinilla[8], Carlos Aliste Santos[9], Eric Hsi[10], Gabriela Gheorghe[11], Rachel Mariani[12], Kristian Schafernak[12], Anna Shestakova[13], Madhu Menon[13], Francisco Llamas[14], Konnie Hebeda[15], Tapan M Bhavsar[16], Armando Lopez Guillermo[2], Elaine Jaffe[17], Leticia Quintanilla-Fend[18], Elias Campo[1], Ferran Nadeu[1], Olga Balagué[2], Itziar Salaverria[1]

*[1]Institut d'Investigacions Biomèdiques August Pi i Sunyer (IDIBAPS), Barcelona, Spain;[2]Hospital Clinic de Barcelona, Hematopathology Section, Barcelona, Spain; [3]Hospital Clínico Universitario, Biomedical Research Institute INCLIVA de Valencia, Pediatric Oncology Department, Valencia, Spain; [4]Hospital La Fe, Pediatric Oncology Department, Valencia, Spain; [5]Hospital Niño Jesús, Pediatric Oncology Depart, Barcelona, Spain; [6]Hospital Niño Jesús, Pathology Department, Madrid, Spain; [7]Hospital Sant Joan de Déu, Pediatric Oncology Department, Esplugues de Llobregat, Spain; [8]Health Research Institute-Fundación Jiménez Diaz University Hospital, Universidad Autónoma de Madrid, Pathology Department, Madrid, Spain; [9]Hospital Clínico Universitario de Santiago de Compostela, Pathology Department, Santiago de Compostela, Spain; [10]Mayo Clinic, Department of Laboratory Medicine and Pathology, Rochester MN, United States of America; [11]St. Jude Children's Research Hospital, Department of Pathology, Memphis, TN, United States of America; [12]Phoenix Children's Hospital, Division of Pathology and Laboratory Medicine, Phoenix, AZ, United States of America; [13]University of Utah/ARUP Laboratories, Department of Pathology, Salt Lake City, UT, United States of America; [14]CHU de Rennes, Department of Pathology, Rennes, France; [15]Radboud University Medical Center, Department of Pathology, Nijmegen, The Netherlands; [16]The George Washington University School of Medicine and Health Sciences, Department of Pathology and Laboratory Medicine, Washington DC, United States of America; [17]National Cancer Institute, National Institutes of Health, Bethesda, Maryland, United States of America; [18]Institute of Pathology and Neuropathology, University Hospital and Comprehensive Cancer Center Tübingen, Tübingen, Germany;*

### Background

Large B-cell lymphoma with *IRF4* rearrangement (LBCL-*IRF4*) predominantly affects children and young adults (CAYA) and typically presents as localized disease with an excellent outcome. Although *IRF4*::*IGH* translocations are detected in most cases, cryptic rearrangements have been proposed, and it remains unclear whether all *IRF4* rearrangement (*IRF4*-R) share a common genomic architecture, involve non-IG partners, or evade detection by FISH. NGS has revealed a distinct mutational profile including *IRF4* and NF-κB pathway mutations. Whether diffuse large B-cell lymphomas (DLBCL) with *IRF4*-R in adults represents the same biological entity is uncertain.

### Aims

To define *IRF4*-R architecture, characterize the mutational profile, and determine whether CAYA and adult *IRF4*-R tumors share similar genomic features, supporting more precise diagnosis.

**Copyright Information:** (Online) ISSN: 2572-9241
©2026 The Author(s). HemaSphere is published by John Wiley & Sons Ltd on behalf of European Hematology Association. This is an open access Abstract Book distributed under the Attribution-NonCommercial-NoDerivs (CC BY-NC-ND), which allows third parties to download the articles and share them with others as long as they credit the author and the Abstract Book, but they cannot change the content in any way or use them commercially.
**Abstract Book Citations:** Authors, Title, HemaSphere, 2026;10:(S1):pages. The abstract book DOIs can be found on the "Author Information" page.
**Disclaimer:** Articles published in the journal HemaSphere exclusively reflect the opinions of the authors. The authors are responsible for all content in their abstracts including accuracy of the facts, statements, citing resources, etc.

### Methods

We assembled a retrospective cohort of 42 patients including 28 pediatric (median age: 11y) and 7 young adult (median age: 29.5y) LBCL-*IRF4* as well as 7 adult DLBCL with *IRF4*-R (median age: 67.5y). All cases were classified according to current classification of Lymphoid Neoplasms. FISH analyses for *BCL2*, *BCL6*, *MYC*, IGH, and *IRF4* were performed using commercial and custom break-apart probes, with additional custom probes to validate novel *IRF4* partners. Copy-number alterations were assessed by Oncoscan/Cytoscan arrays (n=42), and cell-of-origin was determined using the *Lymph2Cx* assay. Structural variants (SV) and mutations were profiled in 37 tumors using a custom targeted NGS panel (SV-NGS) covering 168 lymphoma-related genes and key SV loci. Whole-exome sequencing (WES) was performed on 38 cases. Genetic DLBCL subtypes were assigned using the *LymphGen* algorithm.

### Results

Primary involvement in pediatric cases predominantly affected the head and neck region, including tonsils, whereas adult patients more frequently presented with advanced-stage (III/IV) disease at onset ($P<0.05$) and showed a higher rate of extranodal involvement outside the head and neck and gastrointestinal tract. *IRF4*-R were identified by FISH in 38/42 tumors, mostly with balanced patterns. *BCL2*-R were restricted to two adult tumors, one of which also harbored *BCL6*-R. A commercial *IRF4* probe showed discordant results compared with the custom probe, raising concerns about misinterpretation of unbalanced FISH patterns. SV-NGS confirmed *IRF4*-R in 85% of cases, and single-base resolution analysis revealed that 81% of translocations in CAYA LBCL-*IRF4* involved IGH, with 77% of breakpoints located 3′ to *IRF4* (*EXOC2*), whereas 3 out of 5 adult DLBCL with *IRF4*-R showed breakpoints 5′ of or within *IRF4* (**Figure 1A**). Novel translocations, including *IRF4*::*MIR142* in two CAYA cases and *IRF4*::*BATF* in one adult, were identified. WES uncovered previously unreported mutations in *YY1* and *ZC3H12A* and confirmed frequent alterations in B-cell receptor/NF-κB pathway genes (**Figure 1B**). No CAYA tumors were classified by *LymphGen*, while adult *IRF4*-R DLBCL exhibited greater genetic complexity, enriched for *KMT2D*, *DTX1*, and *MYD88*-L265P mutations, with four cases assigned to EZB or MCD subtypes. Stratification by a 40-year threshold revealed significantly inferior EFS in older patients ($P<0.05$), whereas *TP53* mutations and 17p deletions did not significantly impact outcome, with only one relapsed patient.

### Summary/Conclusion

In conclusion, *IRF4*-R architecture and mutational profile diverge according to age, supporting that not all adult cases should be classified as LBCL-*IRF4*.

**Copyright Information:** (Online) ISSN: 2572-9241
©2026 The Author(s). HemaSphere is published by John Wiley & Sons Ltd on behalf of European Hematology Association. This is an open access Abstract Book distributed under the Attribution-NonCommercial-NoDerivs (CC BY-NC-ND), which allows third parties to download the articles and share them with others as long as they credit the author and the Abstract Book, but they cannot change the content in any way or use them commercially.
**Abstract Book Citations:** Authors, Title, HemaSphere, 2026;10:(S1):pages. The abstract book DOIs can be found on the “Author Information” page.
**Disclaimer:** Articles published in the journal HemaSphere exclusively reflect the opinions of the authors. The authors are responsible for all content in their abstracts including accuracy of the facts, statements, citing resources, etc.

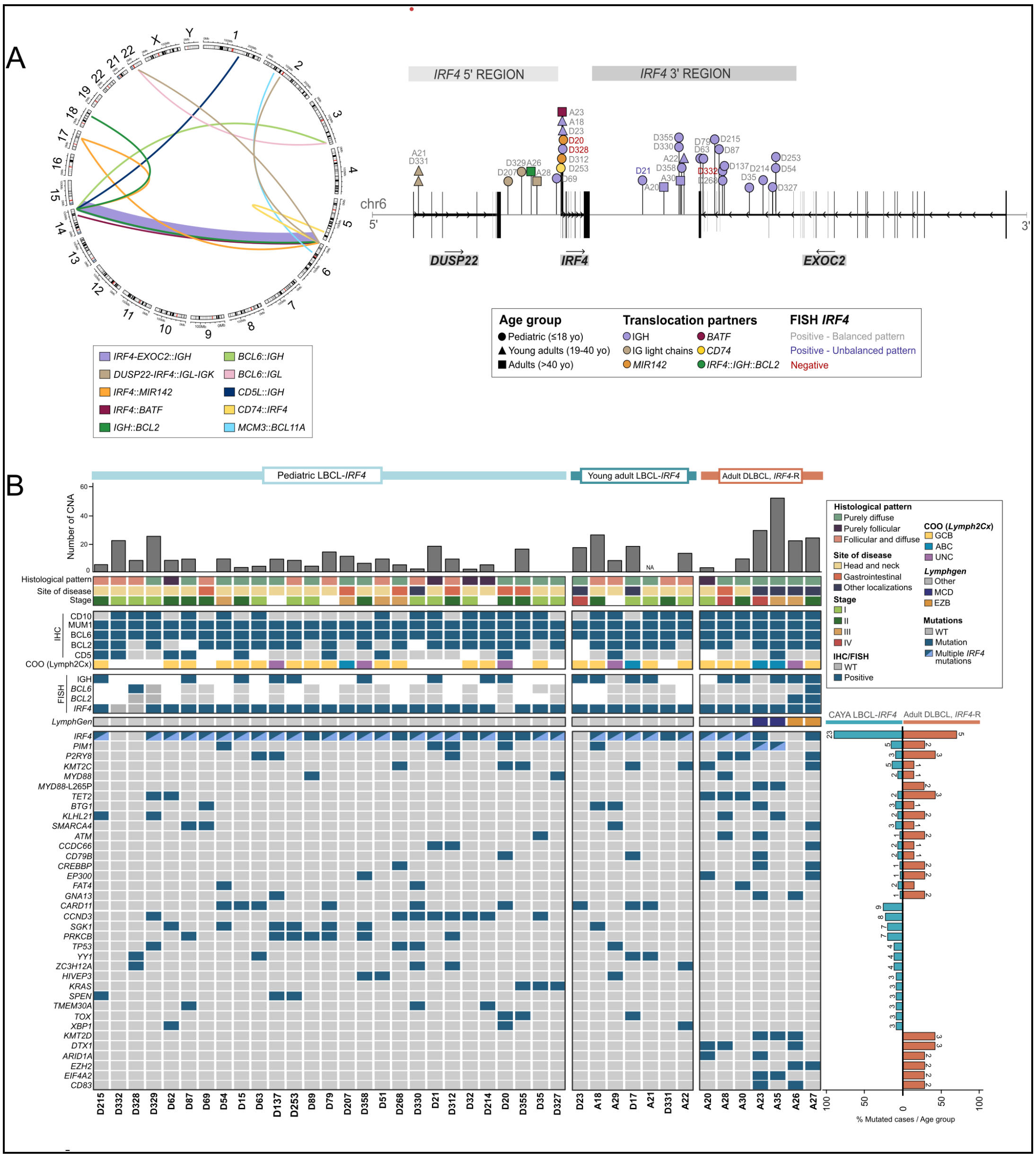

Figure 1. Genomic characterization of CAYA LBCL-IRF4 and adult DLBCL, IRF4-R. (A) Global landscape of rearrangements detected by SV-NGS in the 37 analyzed cases by the SV-NGS approach and characterization of IRF4 rearrangements at base-pair resolution in 32 IRF4-R LBCL cases. (B) Oncoprint summarizing key features of 41 IRF4-R cases stratified into three age groups. Each column represents an individual case; each row shows a specific feature category. Clinical parameters, morphology, immunohistochemistry (IHC), and fluorescence in situ hybridization (FISH) results are depicted. Molecular classification includes LymphGen-based subgroup prediction, cell-of-origin (COO) assignment by the Lymph2Cx assay, and mutation profiles of recurrently altered genes. The top bar plot indicates the total number of copy number alterations (CNA) per case. The right bar plot displays the proportion of cases carrying mutations in the CAYA (<40 years) LBCL-IRF4 cohort compared with adult DLBCL, IRF4-R.

**Copyright Information:** (Online) ISSN: 2572-9241
©2026 The Author(s). HemaSphere is published by John Wiley & Sons Ltd on behalf of European Hematology Association. This is an open access Abstract Book distributed under the Attribution-NonCommercial-NoDerivs (CC BY-NC-ND), which allows third parties to download the articles and share them with others as long as they credit the author and the Abstract Book, but they cannot change the content in any way or use them commercially.
**Abstract Book Citations:** Authors, Title, HemaSphere, 2026;10:(S1):pages. The abstract book DOIs can be found on the "Author Information" page.
**Disclaimer:** Articles published in the journal HemaSphere exclusively reflect the opinions of the authors. The authors are responsible for all content in their abstracts including accuracy of the facts, statements, citing resources, etc.

### (PF1075) PHASE 3 INMIND TRIAL OF TAFASITAMAB (TAFA) PLUS LENALIDOMIDE (LEN) AND RITUXIMAB (R) IN RELAPSED/REFRACTORY (R/R) FOLLICULAR LYMPHOMA (FL): ANALYSES OF BIOMARKERS PREDICTIVE OF PATIENT (PT) RESPONSE

**Topic:** 21. Lymphoma biology & translational research

Sarah Reinke[1], Wolfram Klapper[1], Taehyung (Simon) Kim[2], Michael Arbushites[2], Lucila Sackmann[3], Haobo Ren[2], Diana Alvarez Arias*[2]

[1]*Department of Pathology, Hematopathology Section and Lymph Node Registry, University Hospital Schleswig-Holstein, Campus Kiel, Kiel, Germany;* [2]*Incyte Corporation, Wilmington, DE, United States of America;*[3]*Incyte Biosciences International Sàrl, Morges, Switzerland;*

### Background

Tafa, a humanized CD19-targeting monoclonal antibody (mAb) that has an enhanced Fc domain vs R, is approved in combination with Len for R/R diffuse large B-cell lymphoma (DLBCL) and with Len+R (R2) for R/R FL. Approval for R/R FL was based on the phase 3, placebo-controlled inMIND study (NCT04680052) (Sehn LH. *Lancet* 2026). Studies have shown that natural killer (NK) cell counts (Duell J. *Haematologica* 2024), neutrophil-to-lymphocyte ratio (NLR) (Wang J. *Medicine* 2016), and proportions of Ki67$^{+}$ cells (Han J. *Leukemia & Lymphoma* 2025) at baseline were predictive of response in pts with DLBCL or FL.

### Aims

To identify and evaluate biomarkers predictive of response to tafa+R2 vs R2 in inMIND.

### Methods

Pts with R/R CD19$^{+}$ and CD20$^{+}$ FL (grade 1-3A) who had received ≥1 prior systemic therapy, including an anti-CD20 mAb, were randomized 1:1 to tafa 12 mg/kg IV or placebo with standard dosing of R2. Baseline NK cell counts and NLR were measured by flow cytometry in peripheral blood (PB). Baseline CD19, CD20, and Ki67 expression in biopsies were assessed centrally by immunohistochemistry using anti-CD19 antibody clone BT51E, anti-CD20 antibody clone L26, and anti-Ki67 antibody clone MM1.

### Results

Analyses included 528 pts with FL from inMIND; the number of samples included in each analysis was dependent on data availability. Higher NK cell counts were associated with response to tafa+R2 (p=0.03) but not R2 (p=0.77) (**Figure A-B**). Counts of other immune cell types, such as T cells (p=0.92) or monocytes (p=0.23), were not associated with response to tafa+R2. In the tafa arm, ≥50 NK cells/µl (approximately half of the lowest normal NK range) was associated with longer progression-free survival (PFS) vs <50 NK cells/µl (hazard ratio [HR] [95% CI] 0.53 [0.30-0.93], p=0.03) (**Figure C**). Consistent with prior reports, pts with NLR-high scores (≥7.2) had shorter PFS vs pts with NLR-low scores (<7.2) (HR [95% CI] 1.86 [1.23-2.79], p=0.003). The benefit of tafa+R2 vs R2 was observed in both NLR-high and NLR-low subgroups. The prognostic value of the proportion of Ki67$^{+}$ cells was not confirmed (p=0.14), even when analyzed by POD24 status (defined as progression within 24 months of initial diagnosis) or FLIPI score. Pts with both high (Ki67 ≥30%) and low proliferative (Ki67 <30%) FL had longer PFS with tafa+R2 vs R2. Neither CD19 nor CD20 baseline expression was predictive of response. Even pts with ≤50% CD19$^{+}$ lymphoma cells achieved response with tafa+R2. All pts with CD20$^{-}$/low (<75% positivity) biopsies responded to tafa+R2 (n=6, ORR=100%); however, only a proportion responded to R2 (n=5, ORR=60%). Expression of both CD19 and CD20 was generally retained at the end of tafa+R2 treatment.

**Copyright Information:** (Online) ISSN: 2572-9241
©2026 The Author(s). HemaSphere is published by John Wiley & Sons Ltd on behalf of European Hematology Association. This is an open access Abstract Book distributed under the Attribution-NonCommercial-NoDerivs (CC BY-NC-ND), which allows third parties to download the articles and share them with others as long as they credit the author and the Abstract Book, but they cannot change the content in any way or use them commercially.
**Abstract Book Citations:** Authors, Title, HemaSphere, 2026;10:(S1):pages. The abstract book DOIs can be found on the “Author Information” page.
**Disclaimer:** Articles published in the journal HemaSphere exclusively reflect the opinions of the authors. The authors are responsible for all content in their abstracts including accuracy of the facts, statements, citing resources, etc.

### Summary/Conclusion

Higher NK counts at baseline in PB were significantly associated with responses to tafa+R2. The enhanced Fc region of tafa may allow more robust engagement with FcR-expressing effector cells, such as NK cells, vs R, contributing to the enhanced efficacy of tafa+R2 vs R2 in inMIND. These data suggest that pts with better immune fitness were more likely to achieve response with tafa+R2; however, responses in pts with low NK counts were also observed with tafa+R2. Our data confirmed NLR >7.2 as a prognostic biomarker for shorter PFS in pts with FL; however, benefit of tafa+R2 vs R2 was also observed in this group. Importantly, response to tafa+R2 was independent of baseline CD19 or CD20 expression levels, with all CD20$^{-}$/low pts achieving a response.

**Figure. High NK counts at baseline were significantly associated with responses to tafa+R2.**
High NK cell counts were associated with response to tafa+R2 (A) but not R2 (B). PFS was also longer for patients with high NK counts versus those with low NK counts (C).

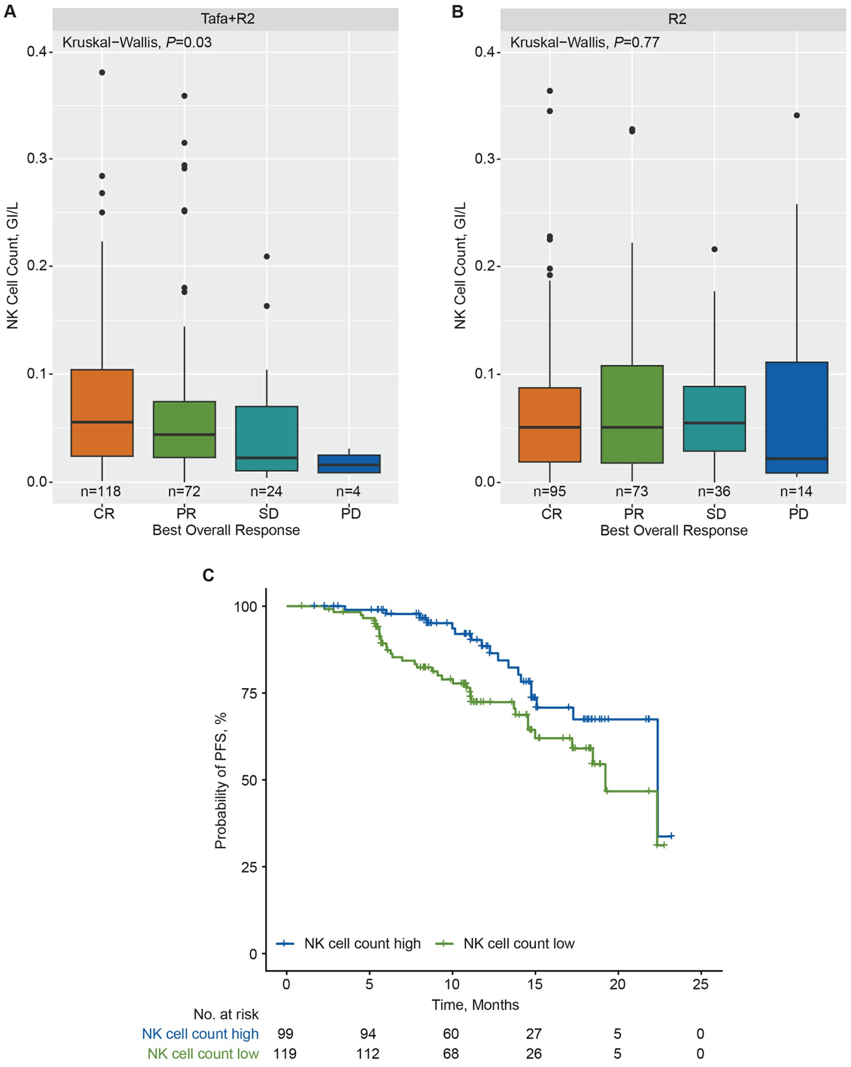

CR, complete response; NK, natural killer; PD, progressive disease; PFS, progression-free survival; PR, partial response; R2, lenalidomide and rituximab; SD, stable disease; tafa, tafasitamab.

**Copyright Information:** (Online) ISSN: 2572-9241
©2026 The Author(s). HemaSphere is published by John Wiley & Sons Ltd on behalf of European Hematology Association. This is an open access Abstract Book distributed under the Attribution-NonCommercial-NoDerivs (CC BY-NC-ND), which allows third parties to download the articles and share them with others as long as they credit the author and the Abstract Book, but they cannot change the content in any way or use them commercially.
**Abstract Book Citations:** Authors, Title, HemaSphere, 2026;10:(S1):pages. The abstract book DOIs can be found on the “Author Information” page.
**Disclaimer:** Articles published in the journal HemaSphere exclusively reflect the opinions of the authors. The authors are responsible for all content in their abstracts including accuracy of the facts, statements, citing resources, etc.

### (PF1076) INTEGRATED SNV/CNV PROFILING AT DIAGNOSIS IN MANTLE CELL LYMPHOMA IDENTIFIES GENOMIC CORRELATES OF CLINICAL HETEROGENEITY AND OUTCOME

**Topic:** 21. Lymphoma biology & translational research

Jana Karolová*[1, 2], Dmitry Manakov[2], Kristina Forsterová[1], Lucie Winkowska[3], Ales Obr[4], Andrea Janíková[5], Marek Trneny#[1], Pavel Klener[1, 2]

*[1]General University Hospital, Charles University, First Dept. of Internal Medicine- Hematology, Prague, Czechia;[2]First Faculty of Medicine, Charles University, Institute of Pathological Physiology, Prague, Czechia; [3]CLIP, Second Faculty of Medicine and University Hospital Motol and Homolka, Department of Pediatric Hematology/Oncology, Prague, Czechia; [4]Palacký University and University Hospital, Olomouc, Department of Hemato-Oncology, Olomouc, Czechia; [5]University Hospital Brno, Faculty of Medicine, Masaryk University, Brno, Department of Internal Medicine - Hematooncology, Brno, Czechia;*

### Background

Mantle cell lymphoma (MCL) is clinically and genetically heterogeneous. Most studies describe diagnostic mutational profiles in unselected MCL cohorts. Here, we took a different approach and enriched for extremes of clinical course by comparing patients (pts) with early relapse to those with long-term remission.

### Aims

To identify diagnostic mutational patterns associated with poor versus favorable prognosis.

### Methods

Whole-exome sequencing was conducted on diagnostic tumors from 89 MCL pts, grouped as Favorable (F, n=28) and Unfavorable (UF, n=61). F included patients without relapse at analysis (NR, n=15) and late relapsers (LR, relapse ≥7 years; n=13). UF comprised early relapsers (ER, relapse within 2 years of diagnosis; n=35) and relapsed-refractory patients (R/R, n=26). All patients received standard immunochemotherapy.

**Copyright Information:** (Online) ISSN: 2572-9241
©2026 The Author(s). HemaSphere is published by John Wiley & Sons Ltd on behalf of European Hematology Association. This is an open access Abstract Book distributed under the Attribution-NonCommercial-NoDerivs (CC BY-NC-ND), which allows third parties to download the articles and share them with others as long as they credit the author and the Abstract Book, but they cannot change the content in any way or use them commercially.
**Abstract Book Citations:** Authors, Title, HemaSphere, 2026;10:(S1):pages. The abstract book DOIs can be found on the "Author Information" page.
**Disclaimer:** Articles published in the journal HemaSphere exclusively reflect the opinions of the authors. The authors are responsible for all content in their abstracts including accuracy of the facts, statements, citing resources, etc.

### Results

UF showed higher coding (*p*=0.029) and overall (*p*=0.011) mutation burdens than F, with more coding SNVs (median 50 vs 37; *p*=0.03). The most aggressive group (R/R) had the highest coding SNV burden (median 58.5) compared with NR (median 31; *p*<0.01). In the full cohort, the most frequently mutated genes were *TP53* (27%), *ATM* (27%), *CCND1* (20%), and *KMT2D* (18%). Among recurrent coding alterations (mutated in ≥3 cases), UF was enriched for *ATM* (34% vs 11%) and *TP53* (33% vs 14%) and harbored UF-exclusive alterations in *NOTCH1* and the NF-κB pathway regulator *NFKBIE* (~10–11% in UF vs 0% in F). In contrast, F more often carried *NSD2* alterations (18% vs 8%) and showed few, if any, of the secondary destabilizing hits observed in UF. The prevalence of *TP53* variants increased with clinical aggressiveness, from LR 1/13 (7.7%) and NR 3/15 (20%) to ER 11/35 (31.4%) and R/R 9/26 (34.6%). A similar trend was observed for *ATM* (NR/LR 6.7% and 15.4% vs ER 14/35 [40%; 5 cases with two ATM variants] and R/R 7/26 [26.9%; 2 cases with two variants]). *NOTCH1*/*NOTCH2* alterations were restricted to ER and R/R (1/28 in NR/LR vs 8/35 in ER and 3/26 in R/R; 18%), supporting an association with aggressive disease. CNV burden was also higher in UF than F, with more gains (median 18 vs 10; p=0.06) and significantly more losses (median 28 vs 20; p=0.03). Recurrent UF deletions involved 9p21.3 (*CDKN2A*/*B*), 11q23.2 (*ATM*/*BIRC3*), 13q14.2 (*MIR15A*/*MIR16-1*), and 17p13.1 (*TP53*); *CDKN2A*/*B* deletion was notably enriched in UF versus F (52.5% vs 10.7%). UF also showed broader losses, including *RB1* deletion (45.9% vs 17.9%) and higher deletion frequencies affecting *TP53* (49.2% vs 21.4%), *ATM* (34.4% vs 21.4%), and *MIR15A*/*MIR16-1* (47.5% vs 25.0%), along with additional losses in *FOXO3*, *PRDM1*, and *TNFAIP3*. Amplifications of *BCL2* and *CDK4* were more common in UF than F (27.9% vs 10.7% and 18% vs 3.6%, respectively). Biallelic *TP53* inactivation increased with aggressiveness (NR 6.7%, LR 15.4%, ER 22.9%, R/R 50%), whereas *TP53*-WT was most common in F (67.9%), specifically in NR (73.3%). In *TP53*-WT patients (n=45), *ATM* mutations were enriched in UF versus F (50.0% vs 10.5%; p<0.01) and correlated with worse outcomes (see Figure 1).

### Summary/Conclusion

Integrated diagnostic SNV/CNV profiling highlights genomic correlates of clinical heterogeneity and outcome in MCL. Adverse course (UF) was associated with a higher frequency of *TP53* alterations, *CDKN2A/B* deletion, *NOTCH1/2*, and *ATM* (including within *TP53*-WT), together with UF-exclusive *NFKBIE*, broader CNV loss (e.g., *RB1*), and more frequent gains of *BCL2* and *CDK4*. A favorable course was characterized by predominance of *TP53*-WT and higher *NSD2* alterations.

**Copyright Information:** (Online) ISSN: 2572-9241
©2026 The Author(s). HemaSphere is published by John Wiley & Sons Ltd on behalf of European Hematology Association. This is an open access Abstract Book distributed under the Attribution-NonCommercial-NoDerivs (CC BY-NC-ND), which allows third parties to download the articles and share them with others as long as they credit the author and the Abstract Book, but they cannot change the content in any way or use them commercially.
**Abstract Book Citations:** Authors, Title, HemaSphere, 2026;10:(S1):pages. The abstract book DOIs can be found on the “Author Information” page.
**Disclaimer:** Articles published in the journal HemaSphere exclusively reflect the opinions of the authors. The authors are responsible for all content in their abstracts including accuracy of the facts, statements, citing resources, etc.

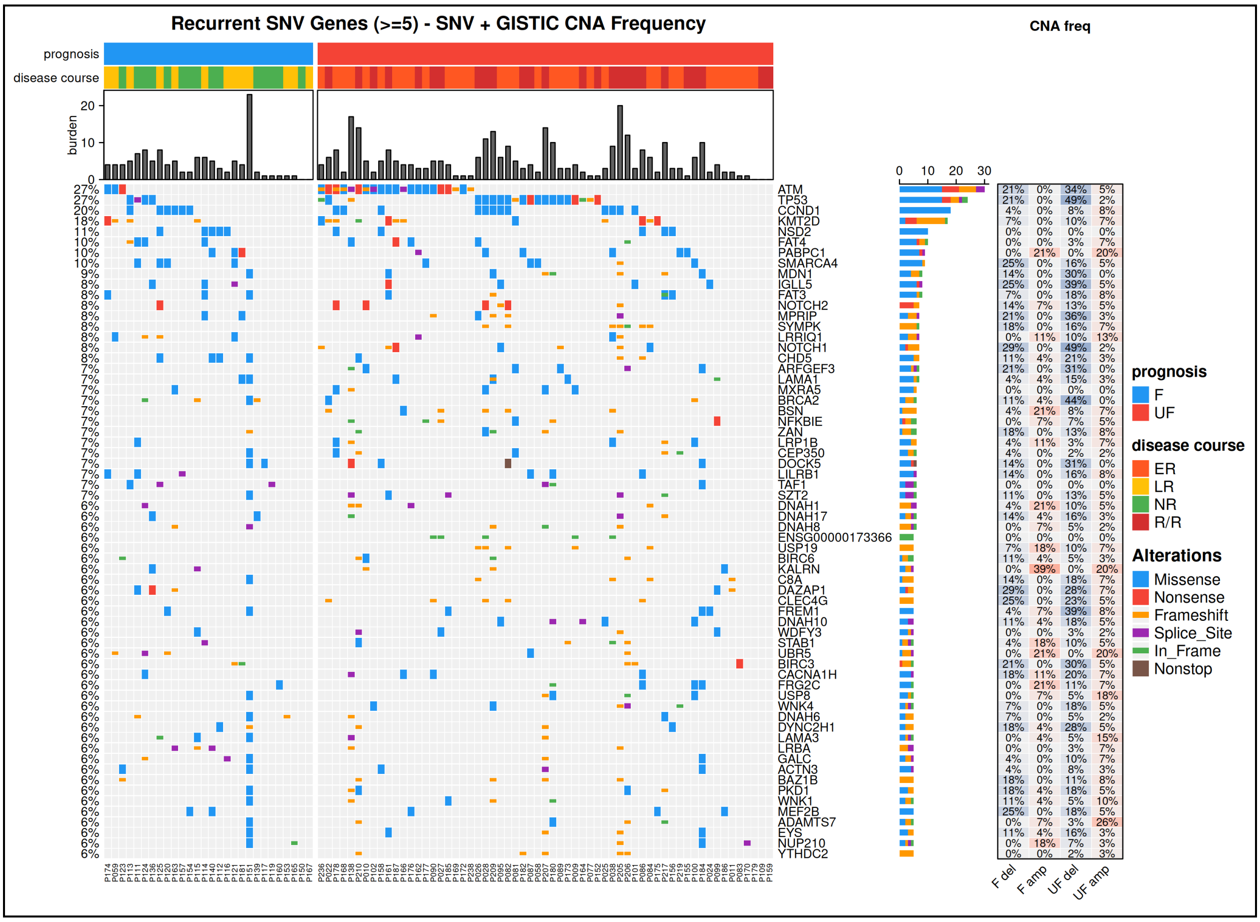

Figure 1: Genomic landscape of recurrently mutated genes in MCL Favorable (F) and Unfavorable (UF) cohorts. Oncoprint displaying coding somatic mutations in 64 genes mutated in ≥5 of 89 MCL patients at diagnosis, split by prognosis group (F, n=28; UF, n=61). Mutation types are color-coded: missense (blue), nonsense (red), frameshift (orange), splice site (purple), in-frame indel (green), and nonstop (brown). Top annotations show the prognosis group and disease course subgroup: no relapse at time of analysis (NR), late relapse (LR), early relapse (ER), relapsed-refractory (R/R), with a bar plot indicating per-patient mutation burden (number of affected genes). Left-side percentages denote the proportion of patients carrying a mutation in each gene. The right-side panel displays GISTIC2.0-derived copy number alteration (CNA) frequencies for each gene's genomic locus across the F and UF cohorts independently, with amplification frequency shown in red and deletion frequency in blue.

**Copyright Information:** (Online) ISSN: 2572-9241
©2026 The Author(s). HemaSphere is published by John Wiley & Sons Ltd on behalf of European Hematology Association. This is an open access Abstract Book distributed under the Attribution-NonCommercial-NoDerivs (CC BY-NC-ND), which allows third parties to download the articles and share them with others as long as they credit the author and the Abstract Book, but they cannot change the content in any way or use them commercially.
**Abstract Book Citations:** Authors, Title, HemaSphere, 2026;10:(S1):pages. The abstract book DOIs can be found on the “Author Information” page.
**Disclaimer:** Articles published in the journal HemaSphere exclusively reflect the opinions of the authors. The authors are responsible for all content in their abstracts including accuracy of the facts, statements, citing resources, etc.

### (PF1077) RHOA IS ESSENTIAL FOR NPM-ALK-DRIVEN LYMPHOMAGENESIS AND REPRESENTS A PROMISING THERAPEUTIC TARGET IN ALK+ ALCL

**Topic:** 21. Lymphoma biology & translational research

Vera Mugoni[1], Giulia Mura[1], Monica MacCagno[1], Roberta Tagliero[1], Ilaria Negri[1], Marta Rubin[1], Francesca Pancioni[1], Chiara Pighi[1], Mariapia Russo[1], Maria Vittoria Di Marco[1, 2], Rocco Giovanni Piazza[3], Roberto Chiarle[1, 4, 5], Claudia Voena*[1]

*[1]University of Turin, Department of Molecular Biotechnology and Health Sciences, Turin, Italy;[2]Krantz Family Center for Cancer Research, Mass General Brigham Cancer Institute, Departments of Medicine, Massachusetts General Hospital and Harvard Medical School, Boston, United States of America; [3]University of Milan-Bicocca, Department of Medicine and Surgery, Monza, Italy; [4]Boston Children's Hospital, Harvard Medical School, Department of Pathology, Boston, United States of America; [5]European Institute of Oncology IRCCS, Division of Haematopathology, Milan, Italy;*

### Background

Rho GTPases are critical players in the biology of T cell lymphoma. In ALK+ anaplastic large cell lymphoma (ALCL), the oncogenic NPM-ALK modulates the activation status of the RhoGTPAses family members Cdc42, Rac1 and RhoA. Importantly, NPM-ALK induced hyperactivation of Cdc42 and the downstream MAPK pathway has already been exploited as a therapeutic vulnerability in ALK+ ALCL. We previously demonstrated that, in contrast, NPM-ALK suppresses RHOA activity in ALCL, indicating a potential critical role for RHOA in ALK-driven lymphomagenesis

### Aims

We aim to investigate how RhoA activation levels fine-tune ALK-driven lymphomagenesis and assess the therapeutic potential of targeting the RhoA-axis.

### Methods

We generated the NPM-ALK/CD4Cre/RhoA$^{fl/fl}$ Tg mouse models from which we derived primary murine cell lines to dissect cell-autonomous alterations *in vitro*. *In vivo* profiling of NPM-ALK or NPM-ALK/RhoA$^{fl/fl}$ cell expansion and spreading was characterized by combined fluorescence-activated cell sorting (FACS) and Immunohistochemical (IHC) analysis of primary and secondary lymphoid organs or by IVIS bioluminescent imaging.
In human ALK+ lymphoma cell lines (TS, JB6, and COST), we abrogated RhoA expression using CRISPR/Cas technology. To dissect fractions of active small RhoGTPase family members, we utilized G-LISA and pull-down assays. We assessed cellular rewiring in the absence of RhoA using omics approaches, including RNA-Seq and phospho-kinase arrays. We also explored altered biological processes using luminescent cell viability assays, immunofluorescence and FACS analysis. We also assessed the effect of RHOA deficiency on the downstream effectors ROCK1/2 and pCofilin/pMLC. We then compared ALK tyrosine kinase inhibitor (TKI) sensitivity in RHOA$^{WT}$ and RHOA$^{KO}$ ALK+ ALCL cells by assessing cell viability using CellTiterGLO.

**Copyright Information:** (Online) ISSN: 2572-9241
©2026 The Author(s). HemaSphere is published by John Wiley & Sons Ltd on behalf of European Hematology Association. This is an open access Abstract Book distributed under the Attribution-NonCommercial-NoDerivs (CC BY-NC-ND), which allows third parties to download the articles and share them with others as long as they credit the author and the Abstract Book, but they cannot change the content in any way or use them commercially.
**Abstract Book Citations:** Authors, Title, HemaSphere, 2026;10:(S1):pages. The abstract book DOIs can be found on the "Author Information" page.
**Disclaimer:** Articles published in the journal HemaSphere exclusively reflect the opinions of the authors. The authors are responsible for all content in their abstracts including accuracy of the facts, statements, citing resources, etc.

**Results**

*In vivo* RhoA deletion impaired lymphoma development resulting a in significant extension of survival in NPM-ALK/RhoA$^{fl/fl}$ mice. While thymic development was not affected in RhoA-deficient mice, a significant reduction of CD3+ T cells was observed in lymph nodes and peripheral blood. *In vitro,* in both primary murine and human cell lines, RhoA deletion affected lymphoma cell proliferation and migration, and altered cell morphology and actin organization. *In vivo* dissemination experiments using intravenous injection of primary murine cells derived from NPM-ALK and NPM-ALK/RhoA$^{fl/fl}$ mice demonstrated a markedly reduced migratory and homing capacity in RhoA-deficient cells compared to their respective controls. RhoA knock-out ALK+ human lymphoma cells underwent transcriptional rewiring, characterized by repression of genes in mitotic spindle organization, cell cycle checkpoint regulation, alongside activation of transcripts associated with oxidative phosphorylation. In addition, loss of RhoA resulted in downregulated phosphorylation of several key signaling molecules, including STAT1, CHK2, and GSK-3a/b. Functionally, RhoA knock-out ALK+ lymphoma cells displayed increased sensitivity to the ALK-TKI crizotinib, and the pharmacological inhibition of the RhoA downstream effector ROCK further enhanced the effects of crizotinib.

**Summary/Conclusion**

RhoA deletion impairs ALK-mediated lymphomagenesis, affects lymphoma cell growth, and increases sensitivity to ALK-targeted therapies. Loss of RhoA markedly alters T cell fitness, suggesting its potential as a therapeutic target in ALK+ lymphomas.

**Copyright Information:** (Online) ISSN: 2572-9241
©2026 The Author(s). HemaSphere is published by John Wiley & Sons Ltd on behalf of European Hematology Association. This is an open access Abstract Book distributed under the Attribution-NonCommercial-NoDerivs (CC BY-NC-ND), which allows third parties to download the articles and share them with others as long as they credit the author and the Abstract Book, but they cannot change the content in any way or use them commercially.
**Abstract Book Citations:** Authors, Title, HemaSphere, 2026;10:(S1):pages. The abstract book DOIs can be found on the "Author Information" page.
**Disclaimer:** Articles published in the journal HemaSphere exclusively reflect the opinions of the authors. The authors are responsible for all content in their abstracts including accuracy of the facts, statements, citing resources, etc.

### (PF1078) PD-L1 AMPLIFICATION BUILDS AN IMMUNE FORTRESS IN EBV+ DLBCL: SPATIAL MULTI-OMICS REVEALS PHYSICAL T-CELL EXCLUSION ALONGSIDE FUNCTIONAL SUPPRESSION

**Topic:** 21. Lymphoma biology & translational research

Axel Künstner*[1], Matthias Kümmel[2], Anke Fähnrich[1], Hanno Witte[2, 3], Annika Raschdorf[4], Yamil Maluje[1], Wolfram Bernd[5], Veronica Bernard[5], Stephanie Stölting[5], Hartmut Merz[5], Nikolaschristiancornelius Bubnoff[2], Stefanie Derer-Petersen[4], Hauke Busch[1], Philipp Lohneis[5], Niklas Gebauer[2]

*[1]University of Lübeck, Medical Systems Biology Group, Lübeck, Germany; [2]University Hospital of Schleswig-Holstein, Department of Hematology and Oncology, Lübeck, Germany; [3]Federal Armed Forces Hospital Ulm, Department of Hematology and Oncology, Ulm, Germany; [4]University Hospital Schleswig-Holstein, Institute of Nutritional Medicine, Lübeck, Germany; [5]Hämatopathologie Lübeck, Lübeck, Germany;*

### Background

In EBV-associated diffuse large B-cell lymphoma (EBV$^+$ DLBCL NOS), copy number gain of the PD-L1 locus (9p24.1) is a recurrent genomic event, observed in 24% of the cases in our cohort. While PD-L1 overexpression is recognized as a mechanism of immune evasion, the spatial architecture through which PD-L1 amplification remodels the tumor microenvironment (TME) remains poorly characterized. Whether PD-L1 gain drives physical exclusion of immune effectors, functional suppression, or both, has not been resolved at the tissue level.

### Aims

To characterize the spatial immune architecture of PD-L1 gain versus normal copy number EBV$^+$ DLBCL at both mesoscale and single-cell resolution, and to determine whether PD-L1 amplification establishes a distinct immune exclusion phenotype.

### Methods

We applied a multi-platform strategy to a retrospective cohort of archival FFPE EBV$^+$ DLBCL cases. Genomic profiling was performed by whole-exome sequencing (WES, n=34) and OncoScan copy number array (n=32, partially overlapping) to define PD-L1 copy number status and broader genomic context. Spatial analyses were conducted on a subset of cases using Visium spatial transcriptomics (13 PD-L1 Normal, 5 PD-L1 Gain) and CosMx spatial proteomics (8 Normal, 3 Gain), with single-cell RNA sequencing providing a reference atlas for cell type deconvolution (DWLS). T-cell exhaustion was scored using a curated gene signature (*PDCD1*, *HAVCR2*, *LAG3*, *TOX*). Statistical comparisons used mixed-effects models with patient-level random intercepts and non-parametric effect size estimation (Cliff's $\delta$).

### Results

CosMx analysis revealed that despite comparable overall T-cell proximity to tumor cells (84% vs 69% within 50 µm, gain *vs* normal), direct tumor-immune cell contact was virtually abolished in PD-L1 Gain tumors (0.0% *vs* 0.6%, p=0.02), defining a physical contact blockade rather than immune desert formation. Structural barrier evidence corroborated this finding: cancer-associated fibroblasts (CAFs) were enriched 1.18-fold at tumor boundaries in PD-L1 gain cases (p=0.030), with cycling immune cells simultaneously excluded from the tumor interface, which additionally displayed transcriptional hallmarks of metabolic immune suppression. Orthogonally, Visium transcriptomics demonstrated significantly elevated T-cell exhaustion signatures (*PDCD1*, *HAVCR2*, *LAG3*, *TOX*) in tumor-proximal regions of PD-L1 gain tumors, revealing a co-existing layer of functional suppression alongside the structural barrier.

**Copyright Information:** (Online) ISSN: 2572-9241
©2026 The Author(s). HemaSphere is published by John Wiley & Sons Ltd on behalf of European Hematology Association. This is an open access Abstract Book distributed under the Attribution-NonCommercial-NoDerivs (CC BY-NC-ND), which allows third parties to download the articles and share them with others as long as they credit the author and the Abstract Book, but they cannot change the content in any way or use them commercially.
**Abstract Book Citations:** Authors, Title, HemaSphere, 2026;10:(S1):pages. The abstract book DOIs can be found on the "Author Information" page.
**Disclaimer:** Articles published in the journal HemaSphere exclusively reflect the opinions of the authors. The authors are responsible for all content in their abstracts including accuracy of the facts, statements, citing resources, etc.

**Summary/Conclusion**

PD-L1 amplification in EBV⁺ DLBCL establishes a dual-layer immune fortress: a CAF-reinforced structural barrier preventing direct cytotoxic engagement, and a transcriptional exhaustion program impairing T-cell function within the TME. This architecture - replacing the adaptive Treg-mediated immunosuppression seen in PD-L1 normal tumors with structural immune exclusion - represents a distinct evasion strategy with potential implications for understanding resistance to immune checkpoint blockade in this disease.

**Copyright Information:** (Online) ISSN: 2572-9241
©2026 The Author(s). HemaSphere is published by John Wiley & Sons Ltd on behalf of European Hematology Association. This is an open access Abstract Book distributed under the Attribution-NonCommercial-NoDerivs (CC BY-NC-ND), which allows third parties to download the articles and share them with others as long as they credit the author and the Abstract Book, but they cannot change the content in any way or use them commercially.
**Abstract Book Citations:** Authors, Title, HemaSphere, 2026;10:(S1):pages. The abstract book DOIs can be found on the "Author Information" page.
**Disclaimer:** Articles published in the journal HemaSphere exclusively reflect the opinions of the authors. The authors are responsible for all content in their abstracts including accuracy of the facts, statements, citing resources, etc.

### (PF1079) ASSOCIATION OF GERMLINE IEI GENE MUTATIONS WITH DISEASE SUBTYPES AND PROGNOSIS IN PEDIATRIC LYMPHOMA

**Topic:** 21. Lymphoma biology & translational research

Keyan Yang*[1], Yang Liu[2], Ying Liu[2], Jing Yang[3], Yanlong Duan[3], Yueping Jia[4], Yunpeng Dai[5], Guotao Guan[5], Caiyan Zhang[1], Qinlong Zheng[1], Yonghong Zhang[2]

*[1]Beijing GoBroad Boren Hospital, Molecular Laboratory Diagnostics, Beijing, China;[2]Beijing GoBroad Boren Hospital, Department of Pediatric Lymphoma, Beijing, China; [3]National Center for Children's Health, Beijing Children's Hospital, Capital Medical University, Department of Pediatric Oncology, Beijing, China; [4]Peking University People's Hospital, Department of Pediatrics, Beijing, China; [5]Shandong Provincial Hospital Affiliated to Shandong University, Department of Pediatrics Hematology and Endocrinology, Jinan, China;*

**Background**

Germline inborn errors of immunity (IEI) are increasingly recognized as predisposing factors for pediatric malignancies, yet their role in pediatric lymphoma has not been well established.

**Aims**

To retrospectively analyze pediatric lymphoma patients with germline susceptibility genes sequencing at Beijing GoBroad Boren Hospital, exploring IEI gene mutations associations with disease subtypes and prognosis.

**Methods**

We included 348 pediatric lymphoma patients (December 2017-May 2025) who underwent sequencing for germline susceptibility genes. Only pathogenic and likely pathogenic (P/LP) variants were included in the analysis. Categorical variables were compared by Chi-square or Fisher's exact test. Survival was analyzed by Kaplan-Meier and Cox regression with log-rank test, using R 3.6.2.

**Copyright Information:** (Online) ISSN: 2572-9241
©2026 The Author(s). HemaSphere is published by John Wiley & Sons Ltd on behalf of European Hematology Association. This is an open access Abstract Book distributed under the Attribution-NonCommercial-NoDerivs (CC BY-NC-ND), which allows third parties to download the articles and share them with others as long as they credit the author and the Abstract Book, but they cannot change the content in any way or use them commercially.
**Abstract Book Citations:** Authors, Title, HemaSphere, 2026;10:(S1):pages. The abstract book DOIs can be found on the "Author Information" page.
**Disclaimer:** Articles published in the journal HemaSphere exclusively reflect the opinions of the authors. The authors are responsible for all content in their abstracts including accuracy of the facts, statements, citing resources, etc.

**Results**

Our cohort included 348 patients, of whom 260 were males (74.7%). The median age was 10 years (IQR: 6-13). The most common disease subtypes were T-LBL (115, 33.0%), followed by Burkitt lymphoma (57, 16.4%), ALK+ ALCL (50, 14.4%), B-LBL (37, 10.6%), HL (31, 8.9%), DLBCL (20, 5.7%), HGBL (10, 2.9%), NKTCL (7, 2.0%) and PMBCL (4, 1.1%). The median follow-up was 38.2 months (95% CI: 33.1-48.0), and the 5-year OS was 76.5% (95% CI: 71.4-82.1%).
Germline variants in IEI-related genes were identified in 270 patients (77.6%), with a mean of 1.55 variants per patient (SD=1.30). The top five variants: *MPEG1* p.P316S (59,17.0%), *TRIM38* p.R164X (30,8.6%), *NCF2* p.R395W (29,8.3%), *TNFAIP3* p.F127C (21, 6.0%), *FANCA* p.S1088F (17,4.9%), and *HAVCR2* p.Y82C (17,4.9%). The variant *POLD1* p.R19H was enriched in B-LBL (OR=13.41, p=0.009). *FANCA* p.S1088F and *THBD* p.D486Y were enriched in NKTCL (*FANCA* p.S1088F: OR=8.56, p=0.041; *THBD* p.D486Y: OR=11.74, p=0.024). *IFNA2* p.A120T was enriched in ALK+ ALCL (OR=4.21, p=0.041). *TIRAP* p.E132K was enriched in PMBCL (OR=35.48, p=0.045). *IRF7* p.Q412R was enriched in HGBL (OR=7.33, p=0.049)
Further classification of the detected germline variants according to the nine IEI categories revealed that the most frequently involved categories were: Congenital defects of phagocyte number or function (61, 17.5%), Autoinflammatory Disorders (58, 16.7%), Bone marrow failure (50, 14.4%), Combined immunodeficiencies with associated or syndromic features (47, 13.5%), Defects in intrinsic and innate immunity (38, 10.9%), Complement Deficiencies (31, 8.9%), Diseases of Immune Dysregulation (29, 8.3%), Immunodeficiencies affecting cellular and humoral immunity (14, 4.0%), and Predominantly Antibody Deficiencies (4, 1.1%). Notably, genes related to Complement Deficiencies were significantly enriched in NKTCL patients (OR=8.28, p=0.018), while genes associated with Immunodeficiencies affecting cellular and humoral immunity (IACHI) were significantly enriched in DLBCL patients (OR=5.04, p=0.039). We did not observe a significant association between these enrichments and prognosis. In Burkitt lymphoma, IACHI mutations were associated with significantly worse OS (HR=8.81, 95%CI:2.18-35.5, p=0.00022).

**Summary/Conclusion**

Approximately 80% of pediatric lymphoma patients carry germline IEI mutations, with *MPEG1* p.P316S being the most frequent. Burkitt lymphoma patients with IACHI mutations have significantly poorer survival, suggesting that genetic background-informed individualized therapy warrants consideration.
(Keyan Yang and Yang Liu contributed equally, Qinlong Zheng and Yonghong Zhang contributed equally).

**Copyright Information:** (Online) ISSN: 2572-9241
©2026 The Author(s). HemaSphere is published by John Wiley & Sons Ltd on behalf of European Hematology Association. This is an open access Abstract Book distributed under the Attribution-NonCommercial-NoDerivs (CC BY-NC-ND), which allows third parties to download the articles and share them with others as long as they credit the author and the Abstract Book, but they cannot change the content in any way or use them commercially.
**Abstract Book Citations:** Authors, Title, HemaSphere, 2026;10:(S1):pages. The abstract book DOIs can be found on the “Author Information” page.
**Disclaimer:** Articles published in the journal HemaSphere exclusively reflect the opinions of the authors. The authors are responsible for all content in their abstracts including accuracy of the facts, statements, citing resources, etc.

### (PF1080) GENETIC ALTERATIONS IN PEDIATRIC B-CELL NON-HODGKIN LYMPHOMA IN PATIENTS WITH INBORN ERRORS OF IMMUNITY

**Topic:** 21. Lymphoma biology & translational research

Sara Mato*[1, 2], Leonie Frauenfeld[1, 2], Julia Richter[3], Ariadna Colmenero[1, 2], Julia Salmerón[1], Angela Deya-Martínez[4], Natalia Castrejón-de-Anta[5], Eugenia García-Fernández[6], Konnie Hebeda[7], Ingrid Simonitsch-Klupp[8, 8], Jaume Verdu[9, 10], Mara Andrés[11], Andishe Attarbaschi[12], Birgit Burkhardt[13], Kenneth Scholten[14], Noelia García[1], María José Ortega[15], Pilar Gurrera-Garcia[16], Berta González Martínez[17], Margarita Llavador[18], Luis Ignacio González[19], Eduardo Quiroga[20], Itziar Astigarraga[21, 22], Idoia Martin-Guerrero[23, 24], Elias Campo[1, 2, 5], Wolfram Klapper[3], Olga Balagué[5], Itziar Salaverria[1]

*[1]Fundació de Recerca Clínic Barcelona-Institut d'Investigacions Biomèdiques August Pi i Sunyer (FRCB-IDIBAPS), Molecular Genetics of Pediatric Lymphoma, Barcelona, Spain; [2]Universitat de Barcelona, Barcelona, Spain;[3]UKSH, Pathology Department,, Kiel, Germany; [4]Clinical Immunology and Primary Immunodeficiencies Unit, Hospital Sant Joan de Déu, Barcelona, Spain; [5]Hospital Clinic de Barcelona, Pathology Department, Barcelona, Spain;[6]Hospital Univesitario La PAz, Pathology department, Madrid, Spain; [7]Radboud UMC, Pathology department, Nijmegen, The Netherlands; [8]AKH Wien, Pathology department, Wien, Austria; [9]Hospital Clínico de Valencia, Pediatric Oncology, Valencia, Spain; [10]Biomedical Research institute, INCLIVA, Valencia, Spain; [11]Hospital La Fe, Pediatric Oncology, Valencia, Spain; [12]St. Anna Children's Cancer Research Institute (CCRI),, Wien, Austria; [13]Department of Pediatric Hematology and Oncology and NHL-BFM Study Center, University Hospital Muenster, Münster, Germany; [14]University Hospital Münster, Department of Pediatric Hematology and Oncology and NHL-BFM Study Center, Münster, Germany; [15]Hospital Virgen de las Nieves, Pediatric Oncology, Granada, Spain;[16]Hospital La Paz, Pediatric Oncology, Madrid, Spain; [17]Hospital Universitario La Paz, Pediatric Oncology, Madrid, Spain; [18]Hospital La Fe, Pathology department, Valencia, Spain; [19]Hospital 12 Octubre, Immunodeficiencies unit, Madrid, Spain;[20]Hospital Virgen del Rocío, Pediatric Oncology, Sevilla, Spain; [21]BioBizkaia Health Research Institute, Pediatric Oncology Group, Barakaldo, Spain; [22]Hospital Universitario Cruces, Pediatrics department, Barakaldo, Spain;[23]University of the Basque Country (UPV/EHU), Department of Genetics, Physical Anthropology & Animal Physiology, Science and Technology Faculty, Leioa, Spain; [24]BioBizkaia Health Research Institute, Barakaldo, Pediatric Oncology group, Barakaldo, Spain;*

### Background

Inborn errors of immunity (IEI), also known as primary immunodeficiencies, are rare monogenic disorders characterized by impaired immune function. Children with IEI have an increased risk of developing non-Hodgkin lymphomas (NHL). The clinical management of these patients is challenging, due to its poor prognosis and the frequent therapy-related toxicity observed in IEI patients.

### Aims

This study aims to unravel the biological factors driving the development of B-NHL in the IEI setting.

### Methods

Fifty-four samples from 49 patients with known IEI or suspected pre-existing condition were recruited from Spanish Pediatric Hospitals and European centers from the European Inter-Group for Childhood NHL (EICNHL). According to the International Union of Immunological Societies (IUIS) classification, we refined the initial series ending up with a cohort of 44 samples from 34 *bona-fide* IEI patients (20M/14F; mean age at lymphoma diagnosis 8 years). Samples were mainly recruited at initial diagnosis, with sequential samples available for 5 patients. The presence of *MYC*, *BCL2*, *BCL6*, *IRF4* breaks, and the 11q aberration was investigated by FISH. Twenty-four samples were analyzed by OncoScan/CytoScan copy number (CN)-arrays. Whole Exome Sequencing was performed on 21 tumors, and gene expression profile (GEP) on 24 B-NHL (PanCancer Immune profiling, NanoString).

**Copyright Information:** (Online) ISSN: 2572-9241
©2026 The Author(s). HemaSphere is published by John Wiley & Sons Ltd on behalf of European Hematology Association. This is an open access Abstract Book distributed under the Attribution-NonCommercial-NoDerivs (CC BY-NC-ND), which allows third parties to download the articles and share them with others as long as they credit the author and the Abstract Book, but they cannot change the content in any way or use them commercially.
**Abstract Book Citations:** Authors, Title, HemaSphere, 2026;10:(S1):pages. The abstract book DOIs can be found on the "Author Information" page.
**Disclaimer:** Articles published in the journal HemaSphere exclusively reflect the opinions of the authors. The authors are responsible for all content in their abstracts including accuracy of the facts, statements, citing resources, etc.

### Results

Combined immunodeficiencies with syndromic features (synd-IEI) affected 19/34 patients. Seven patients had diseases of immune dysregulation (dys-IEI), 3 presented combined immunodeficiencies and 3 antibody disorders. DLBCL was the most frequent diagnosis (31/44) (**Fig.1A**). Most patients (16/20, 80%) had advanced stage of disease at diagnosis. Five patients relapsed and 3 had clonally unrelated second lymphoid malignancies. Eleven patients died (42%), mainly due to lymphoma (4/11) or IEI-related complications (3/11).

Most tumors (14/28) were GCB, 7/28 ABC and 7/28 intermediate/unclassified. *MYC*-R was found in 3/30 tumors and *BCL6*-R in 1/22. Fifteen tumors (44%) were EBV-positive. Unsupervised analysis on GEP revealed two differentiated clusters, mainly defined by EBV status. Cluster1 (C1) included 9 tumors, all but one EBV-positive, whereas C2 consisted of 15 tumors, 12 of them EBV-negative. Differential GE analysis showed an upregulation on IFN-γ pathway and T-cell signaling in C1 tumors, whereas C2 lymphomas presented upregulation of B-cell-related genes, such as *POU2F2, MS4A1* and *AICDA* (**Fig.1B**). C1 tumors also had upregulation of immune checkpoint genes *PDCD1LG2* and *CD274*/*PD-L1.* Accordingly, EBV-positive B-NHL showed recurrent (4/16, 25%) gains/amplifications in 9p24.1/*PD-L1*, virtually absent in EBV-negative tumors. CN analysis revealed an increased genetic complexity in B-NHL from synd-IEI patients in comparison to other IEI subgroups (**Fig1.C**) and B-NHL in pediatric immunocompetent patients. The mutational landscape was highly heterogenous, with no significant differences in mutational burden according to EBV status, IEI or B-NHL (**Fig1.D**). However, EBV-negative tumors showed a set of recurrent mutations targeting chromatin modifiers among others, virtually absent in EBV-positive, which had a more heterogeneous mutational profile.

### Summary/Conclusion

B-NHL in the context of IEI represents a heterogeneous subset of tumors with mutational and gene expression profiles probably influenced by EBV infection. Synd-IEI seem to predispose to increased genetic complexity in pediatric B-NHL.

**Copyright Information:** (Online) ISSN: 2572-9241
©2026 The Author(s). HemaSphere is published by John Wiley & Sons Ltd on behalf of European Hematology Association. This is an open access Abstract Book distributed under the Attribution-NonCommercial-NoDerivs (CC BY-NC-ND), which allows third parties to download the articles and share them with others as long as they credit the author and the Abstract Book, but they cannot change the content in any way or use them commercially.
**Abstract Book Citations:** Authors, Title, HemaSphere, 2026;10:(S1):pages. The abstract book DOIs can be found on the “Author Information” page.
**Disclaimer:** Articles published in the journal HemaSphere exclusively reflect the opinions of the authors. The authors are responsible for all content in their abstracts including accuracy of the facts, statements, citing resources, etc.

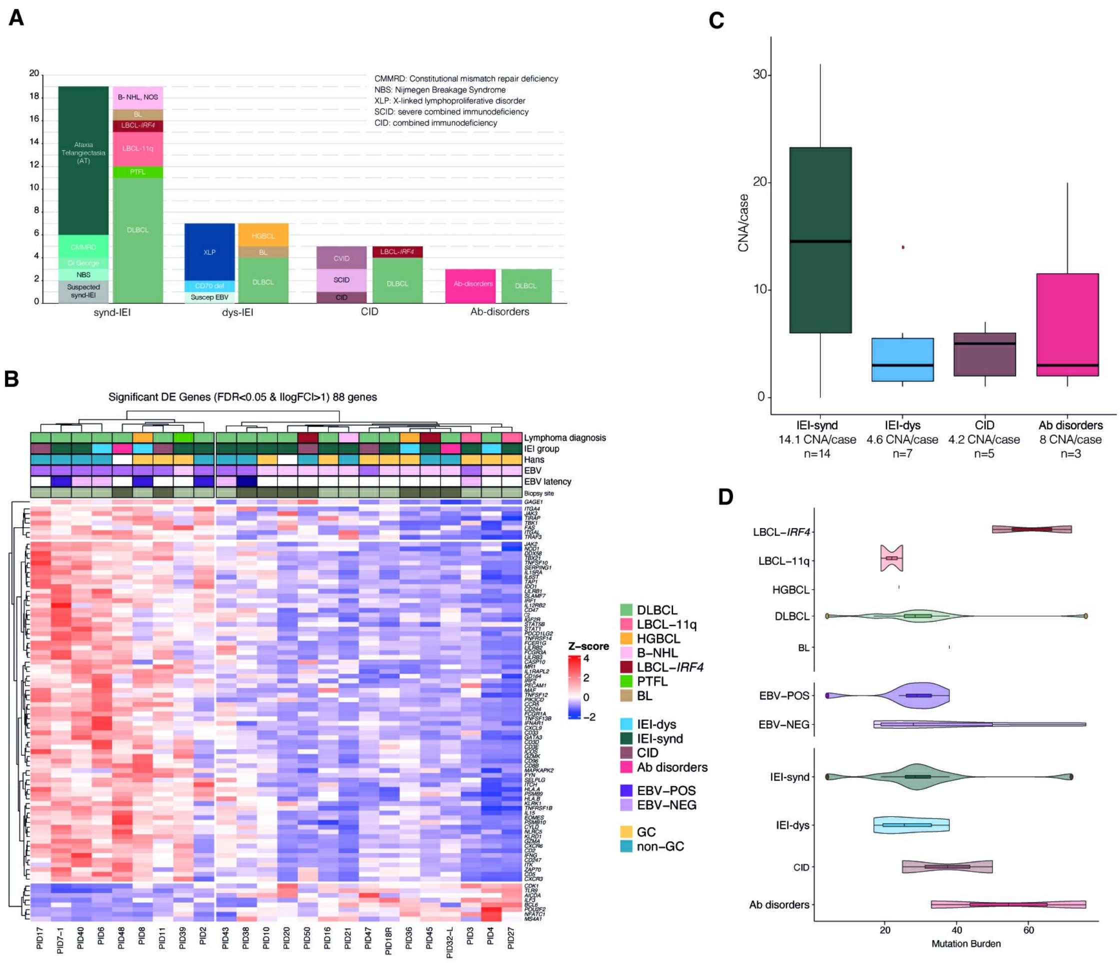

**Figure 1. The genetic landscape of B-cell lymphomas in pediatric patients with inborn errors of immunity (IEI).** Distribution and correspondence of IEI and lymphoma diagnosis in the refined cohort (**A**). Differentially expressed genes between cluster 1 and cluster 2 (**B**). Comparison of total copy number alterations according to IEI diagnosis (**C**). Comparison of mutational burden according to IEI subset, lymphoma diagnosis and EBV status on the tumor (**D**).

Figure 1. The genetic landscape of B-cell lymphomas in pediatric patients with inborn errors of immunity (IEI). Distribution and correspondence of IEI and lymphoma diagnosis in the refined cohort (A). Differentially expressed genes between cluster 1 and cluster 2 (B). Comparison of total copy number alterations according to IEI diagnosis (C). Comparison of mutational burden according to IEI subset, lymphoma diagnosis and EBV status on the tumor (D).

**Copyright Information:** (Online) ISSN: 2572-9241
©2026 The Author(s). HemaSphere is published by John Wiley & Sons Ltd on behalf of European Hematology Association. This is an open access Abstract Book distributed under the Attribution-NonCommercial-NoDerivs (CC BY-NC-ND), which allows third parties to download the articles and share them with others as long as they credit the author and the Abstract Book, but they cannot change the content in any way or use them commercially.
**Abstract Book Citations:** Authors, Title, HemaSphere, 2026;10:(S1):pages. The abstract book DOIs can be found on the "Author Information" page.
**Disclaimer:** Articles published in the journal HemaSphere exclusively reflect the opinions of the authors. The authors are responsible for all content in their abstracts including accuracy of the facts, statements, citing resources, etc.

### (PF1081) PHARMACODYNAMIC BIOMARKERS SUPPORT THE CLINICAL BENEFIT OF EPCORITAMAB PLUS RITUXIMAB AND LENALIDOMIDE (R2) IN PATIENTS WITH RELAPSED/REFRACTORY FOLLICULAR LYMPHOMA (R/R FL): ANALYSES FROM EPCORE FL-1

**Topic:** 21. Lymphoma biology & translational research

Neha Dixit[1], Eunchan Bae[1], Binhuan Wang[1], Melissa Loh[1], Yekyung Seong[1], Kevin Zhao[1], Abel Costa Neto[2], Marcel Nijland[3], Panagiotis Tsirigotis[4], Alessandra Tucci[5], Su Peng Yeh[6], Gauri Sunkersett[1], Rastislav Moskal[7], Isil Altintas[8], Lorenzo Falchi*[9], Franck Morschhauser[10], Edith Szafer-Glusman[1]

*[1]AbbVie Inc., North Chicago, United States of America;[2]D'Or Institute for Research and Education, São Paulo, Brazil;[3]University of Groningen, University Medical Center Groningen, Groningen, The Netherlands;[4]Attikon University Hospital, National and Kapodistrian University of Athens, 2nd Department of Internal Medicine and Research Institute, Hematology Unit, Athens, Greece;[5]ASST Spedali Civili di Brescia, Brescia, Italy;[6]China Medical University, Division of Hematology and Oncology, Department of Internal Medicine, Taichung, Taiwan;[7]AbbVie Inc., Prague, Czechia;[8]Genmab, Utrecht, The Netherlands;[9]Memorial Sloan Kettering Cancer Center, Lymphoma Service, New York, United States of America;[10]Hôpital Claude Huriez, Lille, France;*

### Background

In the global, phase 3 EPCORE FL-1 trial (NCT05409066), fixed-duration epcoritamab (epcor), a CD3xCD20 T cell engaging bispecific antibody, plus $R^2$ (E+$R^2$) showed superior responses and progression-free survival (PFS) compared with $R^2$ alone and a manageable safety profile in patients (pts) with 2L+ FL.

### Aims

Investigate biomarkers associated with the MOA and clinical response to E+$R^2$

### Methods

Pts with CD20+ R/R FL were randomized to E+$R^2$ or $R^2$ for up to 12 cycles. Epcor was initiated via a 2 or 3 step-up dose (SUD) regimen followed by full doses. Dual primary endpoints were best overall response (assessed by independent review committee per Lugano criteria) and PFS. Baseline tumor samples were tested for CD20 and PAX5 expression by immunohistochemistry. Peripheral blood immune phenotypes and B cell recovery were assessed by flow cytometry. Plasma cytokine levels were analyzed by multiplex immunoassay.

**Copyright Information:** (Online) ISSN: 2572-9241
©2026 The Author(s). HemaSphere is published by John Wiley & Sons Ltd on behalf of European Hematology Association. This is an open access Abstract Book distributed under the Attribution-NonCommercial-NoDerivs (CC BY-NC-ND), which allows third parties to download the articles and share them with others as long as they credit the author and the Abstract Book, but they cannot change the content in any way or use them commercially.
**Abstract Book Citations:** Authors, Title, HemaSphere, 2026;10:(S1):pages. The abstract book DOIs can be found on the "Author Information" page.
**Disclaimer:** Articles published in the journal HemaSphere exclusively reflect the opinions of the authors. The authors are responsible for all content in their abstracts including accuracy of the facts, statements, citing resources, etc.

**Results**

Baseline tumor samples had high CD20/PAX5 expression (>80%) in 97.3% and 97.1% of pts in E+$R^2$ and $R^2$ arms, respectively, with no correlation to clinical response (data cut: 24 May 2025). In the E+$R^2$ arm, pro-inflammatory cytokines peaked 24 h after first full epcor dose, with lower median peak levels in pts who received 3SUD vs 2SUD (IL-6, 0.63 vs 1.58 pg/mL; IFNγ, 6.77 vs 27.5 pg/mL) or 2SUD epcor monotherapy (IL-6, 18 pg/mL; IFNγ, 53 pg/mL; Linton *Lancet Haematol* 2024). With E+$R^2$, peak IL-6 and IFNγ levels were higher in pts with vs without CRS events (3SUD, median: IL-6, 1.89 vs 0.63 pg/mL; IFNγ, 22.6 vs 6.48 pg/mL). Reduced peak cytokine levels with 3SUD vs 2SUD correlated with decreased CRS incidence (23% vs 43%, respectively), which was lower than reported with epcor monotherapy (66%).
T cells exhibited transient margination after first full epcor dose in C1, followed by recovery and expansion of CD4+ and CD8+ T cell subsets, with greater CD4 and CD8 increases in the E+$R^2$ arm (fold change [FC] 1.7 and 2.2) compared with $R^2$ alone (FC 1.4 and 1.2). HLA-DR+CD38+CD8+ effector memory (EM) T-cells expanded more with E+$R^2$ compared with $R^2$ (peak FC 2.3 vs 1.8). CD56+ NK cells in E+$R^2$ showed 1.5 FC peak expansion and elevated functional and proliferation markers (GZB, HLA-DR, CD38, Ki67). In the E+$R^2$ arm, pts with complete response (CR) had 3-fold higher baseline CD8+ EM T-cells compared with the limited pts with progressive disease (PD) and 50% lower Treg levels compared with pts with PD or partial response (PR). NK cells from pts with PD had 2.6-fold higher baseline expression of NKG2A (inhibitory marker) compared with pts with PR or CR.
Absolute counts of circulating CD19+ B cells were rapidly depleted in pts in the E+$R^2$ arm, reaching undetectable levels (<10 cells/µL) within the first 2 weeks of treatment (tx) in 99% of pts. Peripheral B cell levels recovered to baseline by 12 months after completing E+$R^2$ tx and to healthy donor levels by 16 months, similar to $R^2$ arm.

**Summary/Conclusion**

Pharmacodynamic analyses from the EPCORE FL-1 study support a biologic rationale for the improved clinical outcomes with E+$R^2$ by linking controlled cytokine dynamics with decreased CRS events and enhanced effector T cell expansion and activation compared with $R^2$ alone. Higher baseline levels of effector CD8+ T cells, fewer Tregs, and active NK cells are linked to a better response to E+$R^2$, underscoring the importance of these cell subsets. In line with the manageable safety profile, reversible B cell depletion following E+$R^2$ is consistent with transient immunosuppression and B cell reconstitution.

**Copyright Information:** (Online) ISSN: 2572-9241
©2026 The Author(s). HemaSphere is published by John Wiley & Sons Ltd on behalf of European Hematology Association. This is an open access Abstract Book distributed under the Attribution-NonCommercial-NoDerivs (CC BY-NC-ND), which allows third parties to download the articles and share them with others as long as they credit the author and the Abstract Book, but they cannot change the content in any way or use them commercially.
**Abstract Book Citations:** Authors, Title, HemaSphere, 2026;10:(S1):pages. The abstract book DOIs can be found on the "Author Information" page.
**Disclaimer:** Articles published in the journal HemaSphere exclusively reflect the opinions of the authors. The authors are responsible for all content in their abstracts including accuracy of the facts, statements, citing resources, etc.

### (PF1082) LONGITUDINAL SINGLE-CELL ANALYSIS REVEALS CLONAL EVOLUTION AND THERAPEUTIC RESISTANCE IN LYMPHOMA ASSOCIATED WITH INBORN ERROR OF IMMUNITY

**Topic:** 21. Lymphoma biology & translational research

Hui Wan*[1], Likun Du[1], Rui Sun[1], Weicheng Ren[1], Klaus Warnatz[2], Qiang Pan-Hammarstrom[1]

*[1]Karolinska Institutet, Department of Medical Biochemistry and Biophysics, Stockholm, Sweden;[2]Medical Center - University of Freiburg, Department of Rheumatology and Clinical Immunology, Freiburg, Germany;*

### Background

Patients with inborn error of immunity or autoimmune diseases have a significantly increased risk of developing B-cell lymphomas; however, the molecular mechanisms linking chronic immune dysregulation to malignant transformation remain poorly defined. Persistent immune activation and defective immune tolerance are thought to create a permissive environment for lymphomagenesis, but direct longitudinal evidence in humans is limited. Activated PI3Kδ syndrome (APDS), caused by germline *PIK3CD* or *PIK3R1* mutations and characterized by immune hyperactivation and lymphoproliferation, represents a unique human model to dissect how immune dysregulation evolves into lymphoma.

### Aims

To dissect the clonal and molecular evolution of tumor B cells from pre-malignant to malignant transformation and therapeutic resistance.

### Methods

We analyzed longitudinal peripheral blood and tumor samples collected before lymphoma diagnosis, at diagnosis, and at relapse from a uniquely informative APDS case progressing from systemic lupus erythematosus to marginal zone lymphoma (MZL). Single-cell RNA sequencing (scRNA-seq), single-cell B-cell receptor sequencing (scBCR-seq), bulk genomic profiling, and functional antibody assays were integrated to reconstruct clonal evolution across disease stages (**Fig. A**), and dissect the molecular features of pre-tumor and tumor B cells.

### Results

Malignant B-cell clones were identified not only in lymphoid tissue but also in peripheral blood, and notebly, the malignant B cells already detectable and expanded prior to clinical diagnosis (**Fig. B**). Single-cell analyses revealed pronounced intratumoral heterogeneity, with tumor B cells spanning at least four phenotypic states: proliferating B-cell–like, germinal center–like, memory B-cell–like, and plasma-like populations, and they were prefer in different tissues (**Fig. C**). These distinct subsets showed preferential distribution across different tissues (**Fig. D**). Notably, Three of four malignant subsets exhibited low or absent *CD20* expression, providing a potential explanation for rituximab treatment failure (**Fig. E**). Functional characterization of tumor-derived BCRs demonstrated marked autoreactivity against nucleosomes, double-stranded DNA, and single-stranded DNA (**Fig. F**). Genomic analyses identified cooperating mutations, including *TET2* and *BRWD3*, suggesting stepwise acquisition of oncogenic events (**Fig. G**).

**Copyright Information:** (Online) ISSN: 2572-9241
©2026 The Author(s). HemaSphere is published by John Wiley & Sons Ltd on behalf of European Hematology Association. This is an open access Abstract Book distributed under the Attribution-NonCommercial-NoDerivs (CC BY-NC-ND), which allows third parties to download the articles and share them with others as long as they credit the author and the Abstract Book, but they cannot change the content in any way or use them commercially.
**Abstract Book Citations:** Authors, Title, HemaSphere, 2026;10:(S1):pages. The abstract book DOIs can be found on the "Author Information" page.
**Disclaimer:** Articles published in the journal HemaSphere exclusively reflect the opinions of the authors. The authors are responsible for all content in their abstracts including accuracy of the facts, statements, citing resources, etc.

**Summary/Conclusion**

Together, these findings support a model in which germline *PIK3CD* mutation impairs B-cell tolerance, enabling autoreactive clones to escape immune checkpoints, expand under chronic self-antigen stimulation, and accumulate additional genetic alterations that drive malignant transformation. These results offer mechanistic insight into primary immunodeficieny-associated and autoimmune-associated lymphoma and support the development of tailored therapeutic strategies for lymphoma patients with complex immune disorders.

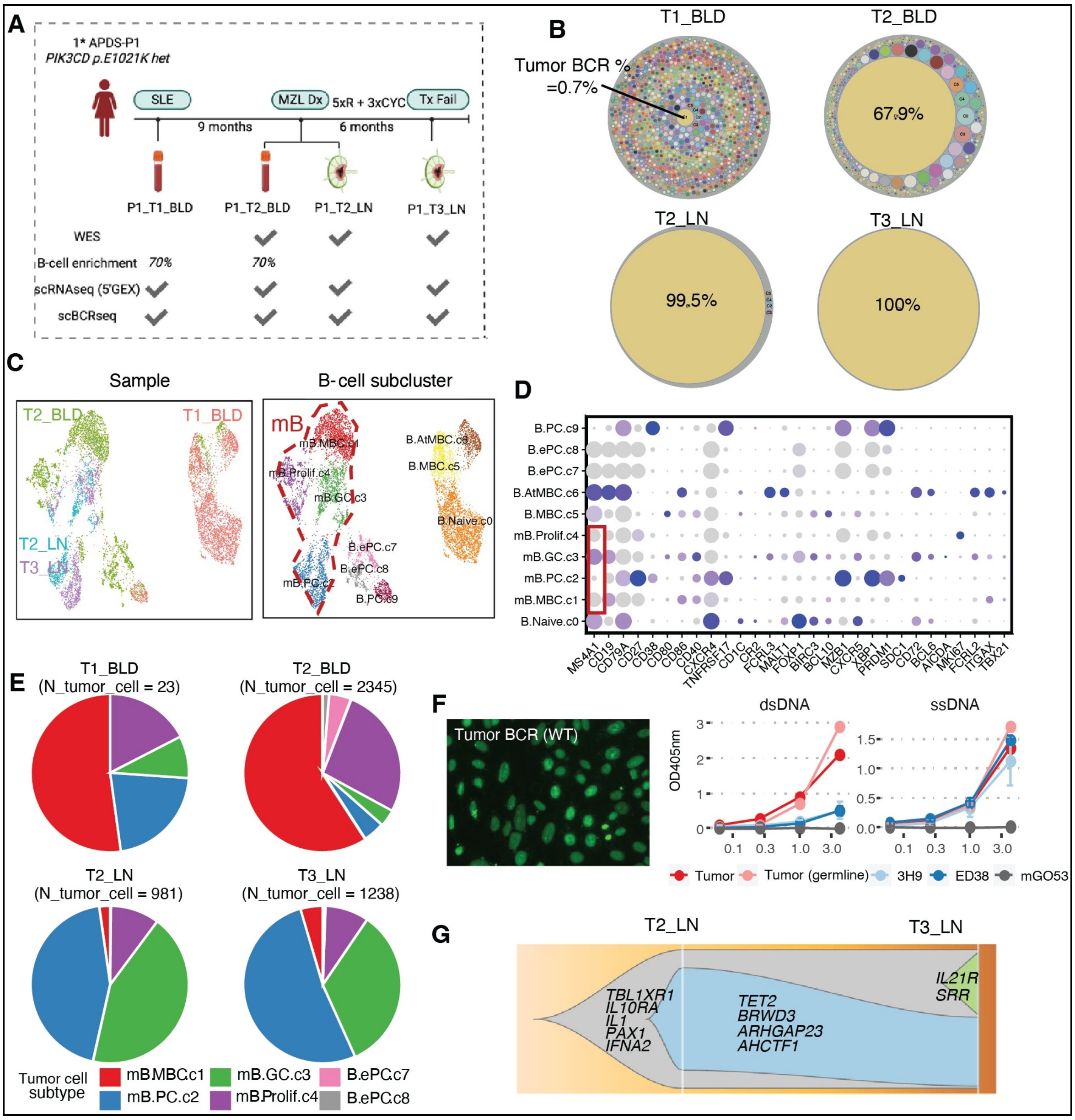

**Copyright Information:** (Online) ISSN: 2572-9241
©2026 The Author(s). HemaSphere is published by John Wiley & Sons Ltd on behalf of European Hematology Association. This is an open access Abstract Book distributed under the Attribution-NonCommercial-NoDerivs (CC BY-NC-ND), which allows third parties to download the articles and share them with others as long as they credit the author and the Abstract Book, but they cannot change the content in any way or use them commercially.
**Abstract Book Citations:** Authors, Title, HemaSphere, 2026;10:(S1):pages. The abstract book DOIs can be found on the "Author Information" page.
**Disclaimer:** Articles published in the journal HemaSphere exclusively reflect the opinions of the authors. The authors are responsible for all content in their abstracts including accuracy of the facts, statements, citing resources, etc.

### (PF1083) SINGLE-CELL RNA SEQUENCING REVEALS THE TUMOR MICROENVIRONMENT AND MOLECULAR FEATURES OF MALIGNANT T CELLS IN ADVANCED CUTANEOUS T-CELL LYMPHOMA

**Topic:** 21. Lymphoma biology & translational research

Jieting Wang*[1], Yingjun Wang[1], Lai Wei[1], Xiaohe Yang[1], Mingzhi Zhang[1], Xinhua Wang[1]

[1] *The First Affiliated Hospital of Zhengzhou University, Department of oncology, Zhengzhou, China;*

### Background

Cutaneous T-cell lymphoma (CTCL) is a heterogeneous group of non-Hodgkin lymphomas characterized by malignant T-cell proliferation in the skin. Advanced stages (IIB–IV) confer a dismal prognosis, with a 5-year overall survival rate below 30%, underscoring an urgent unmet need for novel therapeutic targets. The tumor microenvironment (TME) and unique molecular features of malignant T cells drive CTCL progression and immune evasion. Single-cell RNA sequencing (scRNA-seq) facilitates high-resolution dissection of these complex cellular ecosystems, providing an unprecedented perspective for exploring disease pathogenesis.

### Aims

To construct a comprehensive single-cell atlas of the TME in advanced CTCL, delineate the specific molecular signatures of malignant T cells, and identify and functionally validate the key oncogenic drivers underlying CTCL pathogenesis.

### Methods

We performed scRNA-seq on lesional skin biopsies from 6 advanced CTCL patients and normal skin tissues from 3 healthy controls using the 10x Genomics platform. Following rigorous quality control and unsupervised clustering, malignant T cell populations in tumor tissues were precisely identified by integrating single-cell copy number variation analysis (InferCNV), aneuploidy inference (CopyKAT), and malignancy-associated gene expression profiling. Differential expression analysis identified candidate genes, which were further validated by immunohistochemistry (IHC) in an independent cohort of 40 formalin-fixed paraffin-embedded (FFPE) CTCL samples, and their expression levels correlated with clinicopathological characteristics and patient survival outcomes. Functional assays were conducted in two CTCL cell lines (Hut78, HH) via lentiviral-mediated shRNA knockdown, with proliferation assessed by CCK-8 assay and apoptosis by flow cytometry.

### Results

A total of 73,789 single cells were successfully profiled, identifying 9 major cellular subsets in the CTCL TME. Malignant T cells exhibited a unique mixed phenotypic profile, co-expressing naive T-cell signature genes (TCF7, LEF1) and T-cell exhaustion markers (TIGIT, PDCD1). PAGE5(CT16) was identified as the most markedly upregulated gene in malignant T cells relative to non-malignant T cells. IHC validation confirmed robust PAGE5 protein expression in CTCL tumor tissues, with no detectable expression in normal skin tissues. In the independent 40-patient CTCL cohort, high PAGE5 expression correlated significantly with advanced clinical stage ($P=0.008$) and markedly shorter progression-free survival (PFS) (median: 10 vs. 20 months, $P<0.001$). In vitro functional assays showed that targeted knockdown of PAGE5 in Hut78 and HH cells significantly reduced proliferation ($P<0.01$) and notably increased apoptotic rates ($P<0.05$).

**Copyright Information:** (Online) ISSN: 2572-9241
©2026 The Author(s). HemaSphere is published by John Wiley & Sons Ltd on behalf of European Hematology Association. This is an open access Abstract Book distributed under the Attribution-NonCommercial-NoDerivs (CC BY-NC-ND), which allows third parties to download the articles and share them with others as long as they credit the author and the Abstract Book, but they cannot change the content in any way or use them commercially.
**Abstract Book Citations:** Authors, Title, HemaSphere, 2026;10:(S1):pages. The abstract book DOIs can be found on the "Author Information" page.
**Disclaimer:** Articles published in the journal HemaSphere exclusively reflect the opinions of the authors. The authors are responsible for all content in their abstracts including accuracy of the facts, statements, citing resources, etc.

**Summary/Conclusion**

This study provides a high-resolution single-cell map of advanced CTCL, revealing a unique malignant T-cell subset with a distinct mixed naive/exhausted phenotypic signature. We identified PAGE5 as a novel oncogenic driver in CTCL, with high expression correlating significantly with aggressive disease and poor clinical prognosis; its role in promoting malignant T-cell proliferation and inhibiting apoptosis is functionally validated. Collectively, PAGE5 represents a potential diagnostic biomarker and therapeutic target for advanced CTCL, providing a new direction for the clinical management of this recalcitrant disease.

**Copyright Information:** (Online) ISSN: 2572-9241
©2026 The Author(s). HemaSphere is published by John Wiley & Sons Ltd on behalf of European Hematology Association. This is an open access Abstract Book distributed under the Attribution-NonCommercial-NoDerivs (CC BY-NC-ND), which allows third parties to download the articles and share them with others as long as they credit the author and the Abstract Book, but they cannot change the content in any way or use them commercially.
**Abstract Book Citations:** Authors, Title, HemaSphere, 2026;10:(S1):pages. The abstract book DOIs can be found on the “Author Information” page.
**Disclaimer:** Articles published in the journal HemaSphere exclusively reflect the opinions of the authors. The authors are responsible for all content in their abstracts including accuracy of the facts, statements, citing resources, etc.

### (PF1084) RELAPSE IN PEDIATRIC ALK⁺ ALCL IS ASSOCIATED WITH EARLY INFLAMMATORY SIGNALING AND TUMOR MICROENVIRONMENT REMODELING

**Topic:** 21. Lymphoma biology & translational research

Rebekka Salzmann*[1], Anna Tosi[2], Carlo Zanon[1], Alice Cani[1], Anna Corrà[1], Marta Pillon[3], Elisa Carraro[3], Lara Mussolin[1, 3]

*[1]Istituto Ricerca Pediatrica, Padova, Italy; [2]Veneto Institute of Oncology, Padova, Italy; [3]Maternal and Child Health Department, University of Padova, Padova, Italy;*

### Background

About 30% of pediatric ALK⁺ ALCL cases relapse, yet the underlying mechanisms remain unclear. We previously showed that miR-146a-5p is enriched in small extracellular vesicles at diagnosis in patients who later relapse. Mainly secreted by T-cells and alternatively activated macrophages (M2), it might reflect a pre-activated tumor microenvironment (TME) and may drive a self-reinforcing inflammatory niche, as miR-146a-5p can promote macrophage polarization toward the M2 phenotype. Although high macrophage infiltration predicts poor prognosis in adult ALCL, their role in pediatric disease remains poorly defined. Moreover, Macrophages can supply cholesterol, while ALK⁺ ALCL cells are cholesterol auxotrophic, suggesting a potential metabolic interaction.

### Aims

Our aim is to identify early TME features associated with relapse in pediatric ALK⁺ ALCL and to investigate mechanisms driving these programs.

### Methods

Plasma cytokines/chemokines from 27 patients were quantified at diagnosis. Spatial transcriptomics (GeoMX DSP) was performed on nine diagnostic biopsies to characterize TME signatures. Transwell co-culture systems were used to investigate secretome-mediated tumor-macrophage crosstalk using the ALK⁺ ALCL cell lines Karpas299 and SupM2. THP-1 cell line was used as a macrophage model, and findings were validated in primary macrophages. THP-1–derived and primary macrophages were polarized toward an M2 phenotype. Tumor cells were sequenced before and 24-72 h into co-culture. Secretome profiling of THP-1 macrophages was performed prior to co-culture. Migration, invasion, proliferation, cell-cycle and clonogenic capacity were assessed.

### Results

At diagnosis, patients who later relapsed displayed elevated plasma cytokines (IL-6, IL-8, IL-15, TNFα, CXCL9) and in lymph nodes a TME characterized by strong CD8⁺ T-cell signatures, enhanced cytotoxic programs, and high CXCL9 and CCL5 expression. Tumor cells exhibited reduced antigen presentation and features of partial immune escape, while macrophages were moderately activated with increased CXCL9, CCL5, ICAM1. *In vitro* M2 predominantly secreted immunomodulatory cytokines (CCL8, CCL2, CXCL10), extracellular matrix-remodeling factors (CCL5, TIMP2, IL-8), and induced tumor cells to adopt pro-inflammatory, ECM-remodeling, and TME-adaptive transcriptional programs with reduced metabolism and cell-cycle activity. Notably, cholesterol homeostasis pathways were significantly enriched. Functional assays confirmed increased migration and invasion, with reduced proliferation, clonogenicity, and shortened S-phase. Conversely, ALK+ ALCL further promoted M2 polarization.

**Copyright Information:** (Online) ISSN: 2572-9241
©2026 The Author(s). HemaSphere is published by John Wiley & Sons Ltd on behalf of European Hematology Association. This is an open access Abstract Book distributed under the Attribution-NonCommercial-NoDerivs (CC BY-NC-ND), which allows third parties to download the articles and share them with others as long as they credit the author and the Abstract Book, but they cannot change the content in any way or use them commercially.
**Abstract Book Citations:** Authors, Title, HemaSphere, 2026;10:(S1):pages. The abstract book DOIs can be found on the “Author Information” page.
**Disclaimer:** Articles published in the journal HemaSphere exclusively reflect the opinions of the authors. The authors are responsible for all content in their abstracts including accuracy of the facts, statements, citing resources, etc.

### Summary/Conclusion

Relapse in pediatric ALK$^+$ ALCL is linked to an early chronically inflamed, partially immunosuppressive TME. Elevated plasma markers parallel strong intratumoral CD8$^+$ T-cell and cytotoxic programs, indicating dysregulated sustained immune activation. Macrophages may support this niche via CXCL9/CCL5 signaling, promoting tumor infiltration and TME remodeling. Tumor–macrophage interactions drive a migratory, low-proliferative TME-adaptive phenotype while reinforcing M2 polarization, sustaining inflammatory and ECM-remodeling programs. CXCL9 pathways emerge as central mediators and potential relapse-prevention targets. Upregulation of cholesterol homeostasis in ALK$^+$ ALCL cells suggests metabolic support by macrophages, to be further studied via lipidomics.

**Copyright Information:** (Online) ISSN: 2572-9241
©2026 The Author(s). HemaSphere is published by John Wiley & Sons Ltd on behalf of European Hematology Association. This is an open access Abstract Book distributed under the Attribution-NonCommercial-NoDerivs (CC BY-NC-ND), which allows third parties to download the articles and share them with others as long as they credit the author and the Abstract Book, but they cannot change the content in any way or use them commercially.
**Abstract Book Citations:** Authors, Title, HemaSphere, 2026;10:(S1):pages. The abstract book DOIs can be found on the “Author Information” page.
**Disclaimer:** Articles published in the journal HemaSphere exclusively reflect the opinions of the authors. The authors are responsible for all content in their abstracts including accuracy of the facts, statements, citing resources, etc.

### (PF1085) PATIENT-DERIVED LYMPHOMA SPHEROIDS AS PRECLINICAL MODELS FOR PREDICTING PATIENT RESPONSES TO ADVANCED THERAPIES IN B-NHL: EXAMPLE OF GLOFITAMAB IN FOLLICULAR LYMPHOMA

**Topic:** 21. Lymphoma biology & translational research

Paul Marcoux[1], Fabien Gava[1], Marie Tosolini[1], Pauline Gravelle[1], Christina Schniederjohann[2], Sonia Quertinmont[1], Neus Serrat[3], Fanny Bouquet[4], Sylvia Herter[5], Karin Tarte[6], Mikael Roussel[6], Pierre Sesques[7, 7], Caroline Bret[8], Cedric Rossi[9], Pierre Aubert[10], Franck Morschhauser[11], Guillaume Cartron[12], Wolfgang Huber[13], Sascha Dietrich[14], Loic Ysebaert[15, 16], Pierre Brousset[17], Peter-Martin Bruch[14, 14], Patricia Perez Galan[3], Christine Bezombes*[1], Laurent Camille[17]

*[1]Research Center in Cancer de Toulouse, Toulouse, France; [2]Medical Faculty and University Hospital Düsseldorf, Department of Hematology, Heidelberg, Germany; [3]Institut d'Investigacions Biomèdiques August Pi i Sunyer, Barcelona, Spain;[4]F.Hoffmann-La Roche, Basel, Switzerland; [5]Roche Innovation Center Zürich, Schlieren, Schlieren, Switzerland; [6]Unité Mixte de Recherche U1236, Rennes, France; [7]University Hospital of Lyon, Lyon, France; [8]Institut de Génétique Humaine, Montpellier, France; [9]Clinical Haematology Dijon University Hospital, Dijon, France; [10]Univ. Grenoble-Alpes, CHU Grenoble Alpes, Department of Hematology, Grenoble, France; [11]Department of Hematology, Lille, France; [12]Department of Clinical Hematology, Montpellier, France; [13]European Molecular Biology Laboratory, Heidelberg, Germany; [14]Department of Hematology, Oncology and Clinical Immunology, Medical Faculty and University Hospital Düsseldorf, Düsseldorf, Germany; [15]Department of Hematology, IUCT-O, Toulouse, France; [16]IUCT-O, Dit of hematology, Toulouse, France; [17]Institut Universitaire du Cancer de Toulouse - Oncopole, Dpt of pathology, Toulouse, France;*

### Background

Immunotherapy with anti-CD20 monoclonal antibodies combined with chemotherapy has improved treatment in follicular lymphoma (FL), but about 30% of patients experience relapse or refractory (R/R) disease, leading to poor outcomes. Second-generation bispecific antibodies (BsAbs), like glofitamab, have shown durable complete responses even in CAR-T refractory patients. However, some patients fail to respond or develop resistance, underscoring the need for predictive factors.

### Aims

We aimed to identify predictive biomarkers of glofitamab response and uncover actionable resistance mechanisms.

### Methods

The efficacy of glofitamab was evaluated in patient-derived lymphoma spheroids (PDLS) from FL R/R. PDLS were characterized for cell viability, immune cell composition, and immune escape using flow cytometry and single-cell RNA sequencing. Tumoral B-cell depletion, T-cell proliferation, and activation markers were assessed after 3 days of treatment. Additional analysis was conducted with CODEX.

### Results

FL-PDLS maintained immune cell composition similar to their original samples, with CD19 (~80%), CD4 (~10%), CD8 (~2.5%), NK (~0.8%), and γδ T (~0.1%) cells. Exhaustion markers on FL samples and PDLS showed CD4+ T cells expressing CD28>PD1>TIGIT>TIM3>LAG3>41BB, and CD8+ T cells with PD1>CD28>TIGIT>TIM3>LAG3>41BB. Glofitamab treatment caused two distinct response profiles based B-cell depletion, with different immune microenvironment compositions. High responders had fewer LAG3+ or TIGIT+ CD4+ cells and lower IL-6 levels than low responders. Glofitamab response correlated positively with IFNγ, Granzyme B (validated by CODEX), and CD20 expression. ScRNAseq analysis showed high responders had more activated CD8+ cells whereas low responders had more CD4+ Tfh cells.

**Copyright Information:** (Online) ISSN: 2572-9241
©2026 The Author(s). HemaSphere is published by John Wiley & Sons Ltd on behalf of European Hematology Association. This is an open access Abstract Book distributed under the Attribution-NonCommercial-NoDerivs (CC BY-NC-ND), which allows third parties to download the articles and share them with others as long as they credit the author and the Abstract Book, but they cannot change the content in any way or use them commercially.
**Abstract Book Citations:** Authors, Title, HemaSphere, 2026;10:(S1):pages. The abstract book DOIs can be found on the “Author Information” page.
**Disclaimer:** Articles published in the journal HemaSphere exclusively reflect the opinions of the authors. The authors are responsible for all content in their abstracts including accuracy of the facts, statements, citing resources, etc.

**Summary/Conclusion**

Using a multimodal patient-derived 3D platform (PDLS), we identify two major predictive biomarkers of glofitamab resistance in relapsed/refractory B-NHL: exhausted $CD8^+$
T cells and high levels of functionally active $CD4^+$
Tfh cells that promote tumor support. Our findings indicate that elevated functional Tfh cells in FL patients may reduce response to glofitamab, highlighting their potential role in therapeutic resistance. These results support combinatorial treatment strategies integrating bispecific antibodies (BsAbs) with checkpoint inhibition or approaches disrupting Tfh–B-cell interactions. Moreover, this study validates PDLS as a relevant preclinical model for investigating immune escape mechanisms and identifying biomarkers predictive of immunotherapy response.

**Copyright Information:** (Online) ISSN: 2572-9241
©2026 The Author(s). HemaSphere is published by John Wiley & Sons Ltd on behalf of European Hematology Association. This is an open access Abstract Book distributed under the Attribution-NonCommercial-NoDerivs (CC BY-NC-ND), which allows third parties to download the articles and share them with others as long as they credit the author and the Abstract Book, but they cannot change the content in any way or use them commercially.
**Abstract Book Citations:** Authors, Title, HemaSphere, 2026;10:(S1):pages. The abstract book DOIs can be found on the "Author Information" page.
**Disclaimer:** Articles published in the journal HemaSphere exclusively reflect the opinions of the authors. The authors are responsible for all content in their abstracts including accuracy of the facts, statements, citing resources, etc.

### (PF1086) CK2 INHIBITION REPROGRAMS THE EPIGENETIC LANDSCAPE AND ENHANCES SENSITIVITY TO TARGETED THERAPY IN GERMINAL CENTER DIFFUSE LARGE B CELL LYMPHOMA

**Topic:** 21. Lymphoma biology & translational research

Amir Yami*[1, 2], Laura Quotti Tubi[1, 2], Iris Hxhiu[1, 2], Juairia Uddin Dekha[1, 2], Jessica Ceccato[1, 2], Nicolò Danesin[1], Mattia D'antiga[1], Marco Carraro[1], Greta Scapinello[1], Andrea Visentin[1], Fabrizio Vianello[1, 2], Livio Trentin[1], Suzanne Cory[3, 4], Sabrina Manni[1, 2], Francesco Piazza[1, 2]

*[1]Department of Medicine, Hematology Unit, University of Padova, Italy., Padova, Italy;[2]Veneto Institute of Molecular Medicine (VIMM), Padova, Italy;[3]Walter and Eliza Hall Institute of Medical Research, Melbourne, VIC, Australia, Melbourne, Australia;[4]Department of Medical Biology, The University of Melbourne, Melbourne, VIC, Australia, Melbourne, Australia;*

### Background

Diffuse large B-cell lymphoma (DLBCL), particularly the germinal center B-cell like (GCB) subtype, is characterized by extensive epigenetic dysregulation, including aberrant histone methylation (e.g., H3K27me3 via EZH2 mutations) and disrupted histone acetylation due to frequent alterations in CREBBP/EP300. While these chromatin abnormalities are recognized drivers of lymphomagenesis, upstream regulators coordinating both methylation and acetylation programs remain incompletely defined. Protein kinase CK2 is overexpressed and constitutively active in DLBCL and has been linked to survival signaling; however, its role as a potential central and overlooked regulator of chromatin architecture in DLBCL has not been systematically explored.

### Aims

To determine whether CK2 functions as a master regulator of epigenetic programs in GCB-DLBCL and to evaluate the therapeutic consequences of CK2 inhibition alone or in rational combinations.

### Methods

Multiple DLBCL models were investigated, including germinal center derived cell lines (OCI-LY1, OCI-LY19, Pfeiffer), *CK2α* and *CK2β* gene silencing systems, and the Vav-BCL2 murine model characterized by enlarged germinal centers and lymphoma phenotype. Notably, the Vav-BCL2 model demonstrated overexpression of CK2α and CK2β, mirroring finding in the human GCB-DLBCL context. Models were analyzed for viability, mechanistic signaling alterations, and histone modification changes using purified histone fractions after treatment with two distinct CK2 inhibitors, CX-4945 and the highly selective SGC-CK2-1.

**Copyright Information:** (Online) ISSN: 2572-9241
©2026 The Author(s). HemaSphere is published by John Wiley & Sons Ltd on behalf of European Hematology Association. This is an open access Abstract Book distributed under the Attribution-NonCommercial-NoDerivs (CC BY-NC-ND), which allows third parties to download the articles and share them with others as long as they credit the author and the Abstract Book, but they cannot change the content in any way or use them commercially.
**Abstract Book Citations:** Authors, Title, HemaSphere, 2026;10:(S1):pages. The abstract book DOIs can be found on the “Author Information” page.
**Disclaimer:** Articles published in the journal HemaSphere exclusively reflect the opinions of the authors. The authors are responsible for all content in their abstracts including accuracy of the facts, statements, citing resources, etc.

**Results**

CK2 inhibition reduced viability and induced apoptotic signaling across DLBCL models. Pharmacologic and genetic targeting of CK2 increased repressive histone methylation, particularly H3K27me3, alongside modulation of DNMT1, indicating coordinated regulation of histone and DNA methylation pathways. CK2 inhibition was also associated with reduced ATP citrate lysate activity at specific phosphorylation site (Serine 455) and histone deacetylase 2 (HDAC2), suggesting impaired acetyl-CoA dependent acetylation dynamics and disruption of deacetylase mediated chromatin regulation. Acetylation marks, including H3K27ac and H3K9ac, displayed dynamic remodeling patterns consistent with altered enhancer and promoter activity. Notably, CK2 inhibition reduced expression of BCL6, a central germinal center oncogenic driver, supporting disruption of the germinal center transcriptional program. RNA sequencing analyses are currently underway to define the transcriptional consequences of CK2 inhibition on promoter regions of germinal center related genes and to elucidate how CK2 dependent alterations in epigenetic reshape the chromatin landscape. Findings were recapitulated in CK2 knockdown systems and extended to the Vav-BCL2 murine model, where CK2 targeting modulated chromatin states linked to lymphoma maintenance. Importantly, combination treatment of CK2 inhibitors with the EZH2 inhibitor tazemetostat demonstrated synergistic anti lymphoma effects compared to single agent exposure, highlighting functional cooperation between CK2 signaling and PRC2 dependent chromatin regulation.

**Summary/Conclusion**

CK2 emerges as a central regulator of epigenetic balance in GCB-DLBCL, sustaining lymphoma survival through coordinated control of chromatin remodeling and BCL6 expression. Therapeutic targeting of CK2, especially in combination with tazemetostat, may provide a novel strategy to exploit epigenetic vulnerabilities in DLBCL.

**Copyright Information:** (Online) ISSN: 2572-9241
©2026 The Author(s). HemaSphere is published by John Wiley & Sons Ltd on behalf of European Hematology Association. This is an open access Abstract Book distributed under the Attribution-NonCommercial-NoDerivs (CC BY-NC-ND), which allows third parties to download the articles and share them with others as long as they credit the author and the Abstract Book, but they cannot change the content in any way or use them commercially.
**Abstract Book Citations:** Authors, Title, HemaSphere, 2026;10:(S1):pages. The abstract book DOIs can be found on the “Author Information” page.
**Disclaimer:** Articles published in the journal HemaSphere exclusively reflect the opinions of the authors. The authors are responsible for all content in their abstracts including accuracy of the facts, statements, citing resources, etc.

### (PF1087) INTEGRATIVE MULTI-OMICS FRAMEWORK IDENTIFIES TGFB1–CXCL12 SIGNALING AS A CONSERVED IMMUNOREGULATORY NETWORK IN HIGH-GRADE B-CELL LYMPHOMA AND REACTIVE LYMPHOID TISSUES

**Topic:** 21. Lymphoma biology & translational research

Keisuke Kidoguchi*[1], Ryo Yanagiya[1], Hiroshi Ureshino[1], Shinya Kimura[1]

[1]*Saga University, Division of Hematology, Respiratory Medicine and Oncology, Saga, Japan;*

### Background

High-grade B-cell lymphoma (HGBCL), including double-hit lymphoma (DHL), is characterized by aggressive clinical behavior. While we recently identified intrinsic mitotic perturbations in DHL cells (Blood Neoplasia 2025), the extrinsic microenvironmental interactions in these lymphomas remain incompletely understood.

### Methods

Publicly available single-cell RNA-seq data of Diffuse large B-cell lymphoma (DLBCL) and DHL (EGAD50000001491; Cancer Cell 2025) was obtained. *In silico* cell-cell-communication inference was calculated using LIANA. The validation for reactive lymph node was analyzed with *in situ* single-cell spatial platform; Xenium. Publicly available spatial transcriptomic datasets of DLBCL and DHL (GSE289194; Nature Genetics, 2025) were obtained for analysis.

### Results

We developed an integrative analytical framework leveraging a publicly available single-cell RNA-seq dataset of the DLBCL microenvironment. Lymphoma microenvironment (LME) cells were more abundant in DHL than in DLBCL. Moreover, M1-like macrophage signatures were less enriched in DHL compared with DLBCL, whereas M2-like macrophage signatures were more prominent in DHL. *In silico* analysis revealed recurrent activation of TGFB1-, SPP1-, LGALS3 and CXCL12-associated signaling pathways, suggesting the presence of stable immunomodulatory axes. LIANA analysis highlighted monocyte-derived macrophages and fibroblast-like stromal cells as central hubs transmitting TGFB1–TGFBR1/2, SPP1–CD44, and CXCL12–CXCR4 signals. To evaluate these findings within non-tumorous tissue organization, we performed spatial validation using publicly available reactive lymph node demonstration data. We hypothesized that HGBCL utilize physiological immune-regulatory circuits; therefore, we examined whether these signaling modules align with the tumor microenvironment. Spatial mapping revealed that TGFB1 and CXCL12 module scores localized to distinct immune niches. Spatial transcriptomic analysis using CosMx revealed that perivascular SPP1–CXCL12 double-positive cells are enriched in DHL compared with DLBCL, suggesting that an immunosuppressive M2 macrophage signature represents a key hallmark of DHL. Finally, the clinical significance of *TGFB1* and *CXCL12* was evaluated using an external validation dataset (GSE31312). Upregulation of *TGFB1* and its receptor *TGFBR2* was associated with poorer prognosis. Although *CXCL12* mRNA expression itself was not significantly associated with event-free survival in DLBCL, higher expression of *CXCR4*, the receptor for *CXCL12*, was associated with inferior outcomes in patients with DLBCL.

**Copyright Information:** (Online) ISSN: 2572-9241
©2026 The Author(s). HemaSphere is published by John Wiley & Sons Ltd on behalf of European Hematology Association. This is an open access Abstract Book distributed under the Attribution-NonCommercial-NoDerivs (CC BY-NC-ND), which allows third parties to download the articles and share them with others as long as they credit the author and the Abstract Book, but they cannot change the content in any way or use them commercially.
**Abstract Book Citations:** Authors, Title, HemaSphere, 2026;10:(S1):pages. The abstract book DOIs can be found on the "Author Information" page.
**Disclaimer:** Articles published in the journal HemaSphere exclusively reflect the opinions of the authors. The authors are responsible for all content in their abstracts including accuracy of the facts, statements, citing resources, etc.

### Summary/Conclusion

This spatial concordance supports the hypothesis that the immunosuppressive network in HGBCL might be a "hijacked" conservation of physiological immune-regulatory mechanisms (Cell Metab. 2019). Although the sample of Xenium was non-tumorous lymph node, our results indicate that common immune suppressive gene signature was underlie between HGBCL and reactive lymph node. By integrating transcriptional and spatial signals, our approach will shed a light on the tumor ecosystems and generating hypotheses for future studies aimed at understanding immune suppression in HGBCL. Further investigation is warranted to unveil characterizing immunoregulatory networks in HGBCL.

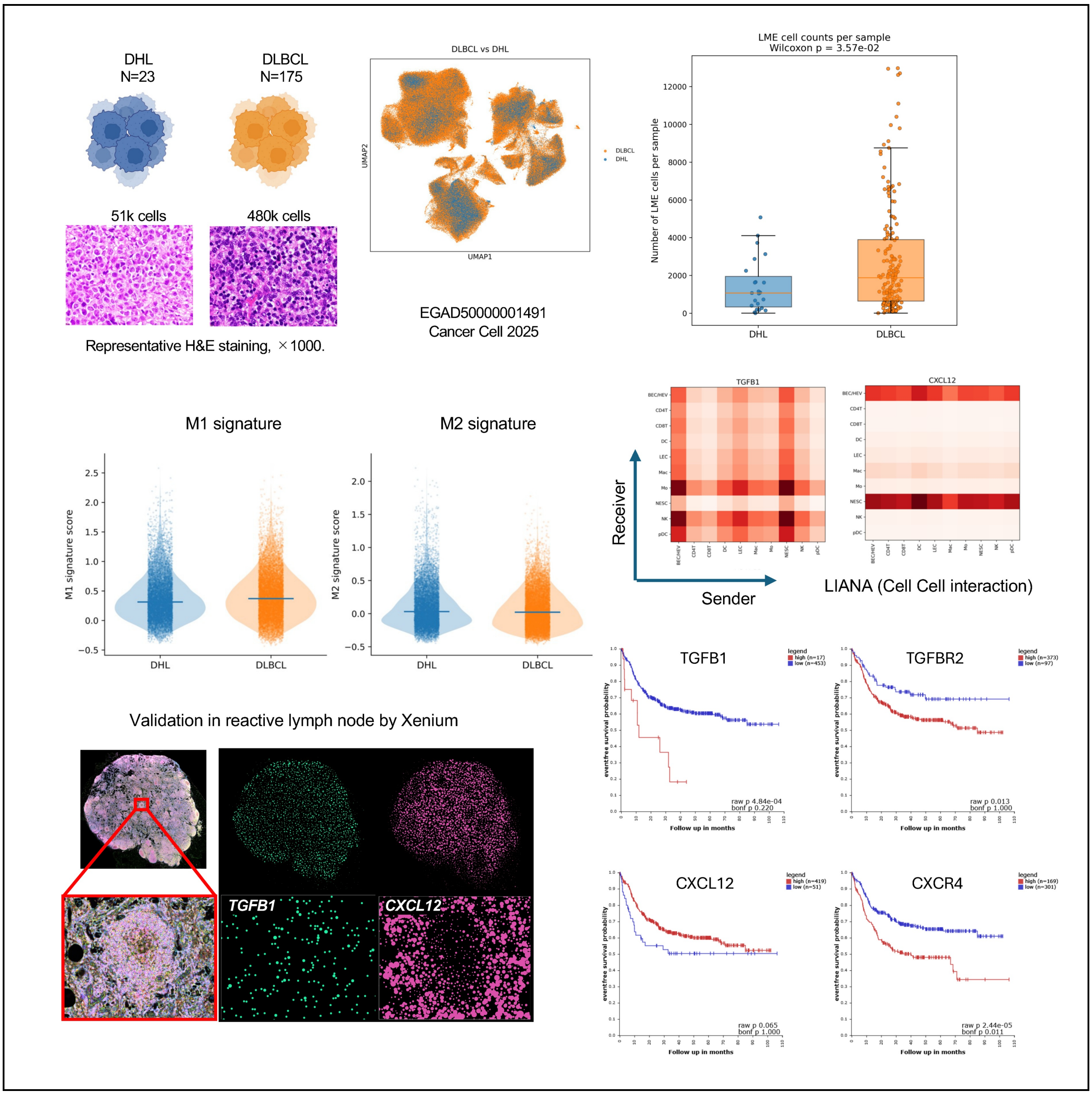

**Copyright Information:** (Online) ISSN: 2572-9241
©2026 The Author(s). HemaSphere is published by John Wiley & Sons Ltd on behalf of European Hematology Association. This is an open access Abstract Book distributed under the Attribution-NonCommercial-NoDerivs (CC BY-NC-ND), which allows third parties to download the articles and share them with others as long as they credit the author and the Abstract Book, but they cannot change the content in any way or use them commercially.
**Abstract Book Citations:** Authors, Title, HemaSphere, 2026;10:(S1):pages. The abstract book DOIs can be found on the "Author Information" page.
**Disclaimer:** Articles published in the journal HemaSphere exclusively reflect the opinions of the authors. The authors are responsible for all content in their abstracts including accuracy of the facts, statements, citing resources, etc.

### (PF1088) PERIPHERAL PROTEOMIC SIGNATURES ASSOCIATED WITH CYTOKINE RELEASE SYNDROME (CRS) IN PATIENTS (PTS) WITH DIFFUSE LARGE B-CELL LYMPHOMA (DLBCL) AND FOLLICULAR LYMPHOMA (FL) TREATED WITH ODRONEXTAMAB

**Topic:** 21. Lymphoma biology & translational research

Stefano Luminari*[1, 2], Geoff Chong[3], Francesca Lorraine Wei Inng Lim[4], Jennifer L. Crombie[5], Tahmineh Tabrizian[6], Yuan Zhu[6], Weiwei Tao[6], Erica Chio[6], Hesham Mohamed[6], Jurriaan Brouwer-Visser[6]

*[1]Azienda Unità Sanitaria Locale – IRCCS of Reggio Emilia, Reggio Emilia, Italy;[2]University of Modena and Reggio Emilia, Modena, Italy; [3]Olivia Newton-John Cancer Wellness & Research Centre, Heidelberg, VIC, Australia;[4]Singapore General Hospital, Singapore, Singapore; [5]Dana-Farber Cancer Institute, Boston, MA, United States of America; [6]Regeneron Pharmaceuticals, Inc., Tarrytown, NY, United States of America;*

### Background

CRS is a common adverse event associated with bispecific antibody (bsAb) therapy. The step-up regimen for odronextamab (Odro; CD20×CD3 bsAb), was modified during the ELM-2 study (NCT03888105) to include steroid pre-treatment (Tx) and an intermediate dose (0.7/4/20 mg), helping to mitigate risk of Grade (Gr)≥2 CRS (Kim et al. *Blood* 2022).

### Aims

To explore serum analytes in pts with relapsed/refractory DLBCL or FL treated with Odro in ELM-2, to better characterize pathways/proteomic signatures associated with CRS, and to elucidate the biological mechanisms driving CRS.

### Methods

A high-throughput proteomics approach (Olink Explore HT assay) was used to analyze serum proteins at baseline (BL) and on Tx. CRS severity (Gr≥2 vs Gr1/no CRS) was analyzed by disease type, step-up regimen, and CAR-HEMATOTOX score (CAR-HT$^{low}$ [0–1] vs CAR-HT$^{high}$ [≥2]; DLBCL only). Proteins associated with CRS were investigated using differential expression analyses. Cytokine data from a standard multiplex assay were used for comparison. Proteins were considered differentially abundant if they met minimal fold change (<0.67 or >1.5) and false discovery rate (FDR)-adjusted p value criteria (p<0.05=significant difference). Gene set enrichment analysis was conducted using fgsea in R to identify enriched MSigDB hallmark pathways associated with comparisons of interest.

**Copyright Information:** (Online) ISSN: 2572-9241
©2026 The Author(s). HemaSphere is published by John Wiley & Sons Ltd on behalf of European Hematology Association. This is an open access Abstract Book distributed under the Attribution-NonCommercial-NoDerivs (CC BY-NC-ND), which allows third parties to download the articles and share them with others as long as they credit the author and the Abstract Book, but they cannot change the content in any way or use them commercially.
**Abstract Book Citations:** Authors, Title, HemaSphere, 2026;10:(S1):pages. The abstract book DOIs can be found on the “Author Information” page.
**Disclaimer:** Articles published in the journal HemaSphere exclusively reflect the opinions of the authors. The authors are responsible for all content in their abstracts including accuracy of the facts, statements, citing resources, etc.

### Results

Serum samples were analyzed from 50 DLBCL and 28 FL pts. At BL (after steroid pre-Tx; before Odro Tx), protein expression associated with cell proliferation and metabolic activation was significantly higher in DLBCL pts, whereas FL pts had higher expression of serum CD21 (FL diagnostic marker), ADGRG2 (an adhesion GPCR), and CD1C (expressed by conventional dendritic cells).

In all pts receiving the modified vs original regimen, the allograft rejection pathway was significantly downregulated and the coagulation pathway was enriched at BL, likely due to steroid pre-Tx. Lower serum IFNγ and IL6 levels (multiplex cytokine assay) were also observed.

Comparing Gr≥2 vs Gr1/no CRS BL samples, several inflammatory pathways (allograft rejection, IFNγ, inflammatory response, IL6/JAK/STAT3) were enriched in FL pts with Gr≥2 CRS but not in DLBCL pts with Gr≥2 CRS. The allograft rejection pathway (and other inflammatory pathways) was enriched in CAR-HT$^{high}$ vs CAR-HT$^{low}$ BL DLBCL samples.

On Tx, inflammatory pathways (allograft rejection, IL6/JAK/STAT3, IL2/STAT5) were enriched in DLBCL Gr≥2 vs Gr1/no CRS pts, but more notably in FL. Unlike DLBCL, FL pts with Gr≥2 CRS also had a sustained enrichment of cellular/metabolic stress response pathways (oxidative phosphorylation, fatty acid metabolism, heme metabolism; **Figure**).

To distinguish a specific "CRS signature" from an Odro signature (proteins altered by Odro in pts with Gr1/no CRS), the differential expression profile at BL vs end of infusion each week in Gr1/no CRS pts was compared with the profile of Gr≥2 CRS. These analyses identified a CRS signature comprising a sustained (Week 1–4) elevation of immune/inflammatory markers including significant elevation of IL6, IFNγ, IL17A, and IL2 in pts with Gr≥2 CRS.

### Summary/Conclusion

Our analysis suggests that steroid pre-Tx attenuates the allograft rejection pathway and other inflammatory markers, potentially contributing to a reduced rate of Gr≥2 CRS in FL and DLBCL. FL pts with an inflamed systemic BL proteome may have a higher risk of Gr≥2 CRS. Further studies are needed to determine if proteomic profiling can identify pts at higher risk of severe CRS.

**Copyright Information:** (Online) ISSN: 2572-9241
©2026 The Author(s). HemaSphere is published by John Wiley & Sons Ltd on behalf of European Hematology Association. This is an open access Abstract Book distributed under the Attribution-NonCommercial-NoDerivs (CC BY-NC-ND), which allows third parties to download the articles and share them with others as long as they credit the author and the Abstract Book, but they cannot change the content in any way or use them commercially.
**Abstract Book Citations:** Authors, Title, HemaSphere, 2026;10:(S1):pages. The abstract book DOIs can be found on the "Author Information" page.
**Disclaimer:** Articles published in the journal HemaSphere exclusively reflect the opinions of the authors. The authors are responsible for all content in their abstracts including accuracy of the facts, statements, citing resources, etc.

**Figure. Pathway analysis over time comparing Grade ≥2 CRS vs Grade 1/no CRS in patients with FL and DLBCL.** Immune/inflammatory pathways (red box) and cellular/metabolic stress response-related pathways (purple box) are more enriched in FL patients with Grade ≥2 CRS compared with DLBCL patients at all timepoints

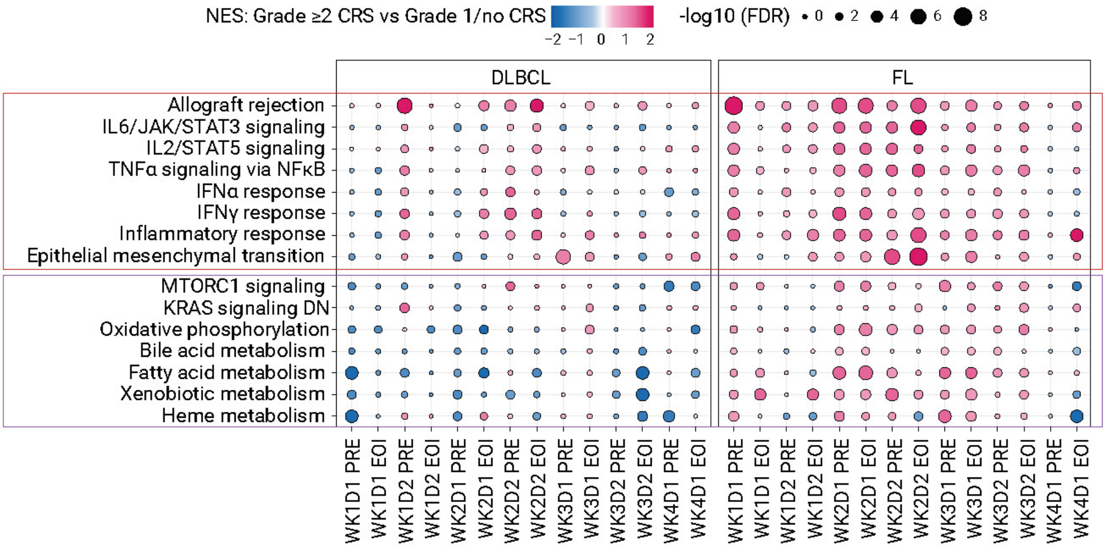

D, day; EOI, end of infusion; NES, normalized enrichment score; PRE, pre-dose; WK, week.

**Copyright Information:** (Online) ISSN: 2572-9241
©2026 The Author(s). HemaSphere is published by John Wiley & Sons Ltd on behalf of European Hematology Association. This is an open access Abstract Book distributed under the Attribution-NonCommercial-NoDerivs (CC BY-NC-ND), which allows third parties to download the articles and share them with others as long as they credit the author and the Abstract Book, but they cannot change the content in any way or use them commercially.
**Abstract Book Citations:** Authors, Title, HemaSphere, 2026;10:(S1):pages. The abstract book DOIs can be found on the "Author Information" page.
**Disclaimer:** Articles published in the journal HemaSphere exclusively reflect the opinions of the authors. The authors are responsible for all content in their abstracts including accuracy of the facts, statements, citing resources, etc.

### (PF1089) PRETREATMENT CTDNA-BASED RISK PREDICTION IN DLBCL: PANEL-INDEPENDENT COPY-NUMBER ANALYSIS OVERCOMES INTER-ASSAY THRESHOLD VARIABILITY

**Topic:** 21. Lymphoma biology & translational research

Rebecca Goodhew*[1, 2, 3], Joanna Krupka[1, 2], Ilias Moutsopoulos[1, 2], Natasha Cutmore[1, 2], Christopher Trethewey[4], Alimu Dayimu[5], Livia Raso-Barnett[6], Jane Price[2, 7], Kay Elston[2, 7], Sateesh Nagumantry[8], Mamatha Karanth[9], Matthew Ahearne[10, 11], Nimish Shah[12], Christopher Fox[13, 14], Shubha Anand[4], Dan Hodson[1, 2, 3]

*[1]Cambridge Stem Cell Institute, University of Cambridge, Cambridge, United Kingdom; [2]Department of Haematology, University of Cambridge, Cambridge, United Kingdom; [3]Department of Haematology, Cambridge University Hospitals Foundation Trust, Cambridge, United Kingdom; [4]Cancer Molecular Diagnostics Laboratory, Department of Oncology, University of Cambridge, Cambridge, United Kingdom; [5]Cambridge Clinical Trials Unit—Cancer Theme, University of Cambridge, Cambridge, United Kingdom; [6]Haemato-Oncology Diagnostic Service, Cambridge University Hospitals NHS Trust, Cambridge, United Kingdom; [7]Cambridge Blood and Stem Cell Biobank, University of Cambridge, Cambridge, United Kingdom; [8]North-West Anglia Foundation Trust, Peterborough, United Kingdom; [9]West Suffolk Hospital, Bury St Edmunds, United Kingdom; [10]University Hospitals of Leicester NHS Trust, Leicester, United Kingdom; [11]University of Leicester, Leicester, United Kingdom; [12]Norfolk and Norwich University Hospitals NHS Trust, Norwich, United Kingdom; [13]Nottingham University Hospitals NHS Trust, Nottingham, United Kingdom; [14]School of Medicine, University of Nottingham, Nottingham, United Kingdom;*

### Background

Large B-cell lymphoma (LBCL), is the commonest aggressive non-Hodgkin lymphoma. A plethora of emerging novel therapeutic modalities creates exciting opportunities to intensify first-line therapy. Such escalation would ideally be reserved for patients at highest risk to avoid unnecessary additional toxicity to standard risk patients who already achieve excellent outcomes with conventional immunochemotherapy. Circulating tumour DNA (ctDNA) is a promising pre-treatment risk stratification tool; however, implementation is limited by poor standardisation and substantial inter-assay variability in ctDNA thresholds.

### Methods

We benchmarked pretreatment ctDNA-based risk prediction in 184 prospectively recruited patients receiving first-line therapy for LBCL. We compared the predictive performance of pretreatment ctDNA burden estimated using single-nucleotide variants (SNVs) identified by multiple variant-calling workflows, or genome-wide copy-number aberrations (CNAs) inferred using ichorCNA. Clinical outcomes were evaluated for time to tumour progression (TTP) and progression-free survival (PFS).

**Copyright Information:** (Online) ISSN: 2572-9241
©2026 The Author(s). HemaSphere is published by John Wiley & Sons Ltd on behalf of European Hematology Association. This is an open access Abstract Book distributed under the Attribution-NonCommercial-NoDerivs (CC BY-NC-ND), which allows third parties to download the articles and share them with others as long as they credit the author and the Abstract Book, but they cannot change the content in any way or use them commercially.
**Abstract Book Citations:** Authors, Title, HemaSphere, 2026;10:(S1):pages. The abstract book DOIs can be found on the "Author Information" page.
**Disclaimer:** Articles published in the journal HemaSphere exclusively reflect the opinions of the authors. The authors are responsible for all content in their abstracts including accuracy of the facts, statements, citing resources, etc.

**Results**

All SNV-based approaches predicted clinical risk, but absolute ctDNA quantification varied markedly. Even within the same cohort, thresholds defining high-risk disease required separate optimisation for each SNV calling strategy and differed by >10-fold. This variation arose partly from different detection limits for low-allele-fraction variants leading to substantial differences in average mutant allele fraction across different variant calling approaches (matched-germline, unmatched-germline and tumour-only strategies employing different variant calling tools). This variability presents a practical barrier to deploying SNV-based risk stratification across centres, sequencing pipelines and panel designs.
As an alternative, we derived tumour fraction from genome-wide CNAs. Panel-based sequencing profiles including off-target reads were normalised to cfDNA from 51 healthy individuals. A tumour fraction >0.1 was identified as optimal to define high risk. In univariate analyses, CNA-based stratification strongly predicted TTP (HR 4.97, 95% CI 2.52–9.83, p<0.0001) and PFS (HR 3.02, 95% CI 1.80-5.05, p<0.0001), with greater prognostic impact than any SNV-based approach. CNA-derived tumour fraction remained independently predictive after adjustment for the International Prognostic Index and total metabolic tumour volume.
We then assessed technical robustness to variables that typically differ across ctDNA platforms, including genomic regions analysed and reference panel size. Tumour fraction from panel-based sequencing showed strong concordance with low-pass whole-genome sequencing (Pearson's r >0.9). Tumour fraction remained stable when chromosome arms were iteratively excluded to simulate alternative panel design, suggesting that risk prediction was driven by global tumour-burden signal rather than individual copy number aberrations. Quantification was also stable when at least 20 healthy cfDNA samples were used as reference.

**Summary/Conclusion**

In conclusion, CNA-based estimation of ctDNA tumour fraction provides a technically robust, panel-independent approach to pretreatment risk stratification in LBCL. Application of this computationally simple approach facilitates harmonisation of risk prediction without the requirement for individual centres to standardise sequencing panels and variant calling pipelines.

**Copyright Information:** (Online) ISSN: 2572-9241
©2026 The Author(s). HemaSphere is published by John Wiley & Sons Ltd on behalf of European Hematology Association. This is an open access Abstract Book distributed under the Attribution-NonCommercial-NoDerivs (CC BY-NC-ND), which allows third parties to download the articles and share them with others as long as they credit the author and the Abstract Book, but they cannot change the content in any way or use them commercially.
**Abstract Book Citations:** Authors, Title, HemaSphere, 2026;10:(S1):pages. The abstract book DOIs can be found on the "Author Information" page.
**Disclaimer:** Articles published in the journal HemaSphere exclusively reflect the opinions of the authors. The authors are responsible for all content in their abstracts including accuracy of the facts, statements, citing resources, etc.

### (PF1090) AID INACTIVATION PROMOTES APOBEC-MEDIATED MUTAGENESIS IN GERMINAL CENTER LYMPHOMA

**Topic:** 21. Lymphoma biology & translational research

Meiqi Ren[1], Eirini Sidiropoulou[1], Rafail Tasaki[1], Irene Gonzalez-Menendez[2], Seunghoon Kim[3, 4], Carolin Brombach[1], Leticia Quintanilla-Fend[2], Jana Ihlow[4], Björn Chapuy[5], Reiner Siebert[6], Fotini Papavasiliou[1], Sandrine Sander*[1, 3]

*[1]German Cancer Research Center (DKFZ), Heidelberg, Germany;[2]Tübingen University Hospital, Tübingen, Germany; [3]University Medicine Greifswald (UMG), Greifswald, Germany; [4]Charite Berlin, Berlin, Germany;[5]Charité, University Medical Center Berlin, Campus Benjamin Franklin, Berlin, Germany; [6]Institute of Human Genetics, Ulm University and Ulm University Medical Center, Ulm, Germany, Ulm, Germany;*

### Background

Most human lymphomas have their origin in the germinal center (GC) reaction where the cytidine deaminase AID (encoded by the *AICDA* gene) targets the B cell genome. Although AID's activity at non-immunoglobulin (Ig) genes is reported, this off-target effect is not the only source of tumorigenic mutations in lymphoma patients.

### Aims

Thus, we aimed for a better understanding of AID independent mechanisms of mutagenesis in GC B cell derived lymphomagenesis.

### Methods

Genetic *Aicda* knockout was combined in a pre-clinical mouse lymphoma model characterized by GC B cell specific MYC and PI3K co-activation. In-depth characterization of pre-malignant and malignant GC B cells was achieved by flow cytometry, immunohistology and next generation sequencing technologies. The murine findings were confirmed using data derived from human B cells and lymphoma cells provided by the ICGC MMML-seq consortium.

### Results

In young mice, AID deficiency promoted GC formation, although GC B cell proliferation, survival and differentiation were similar in AID proficient and deficient animals. Upon aging, GC B cell derived lymphomagenesis was unperturbed in the absence of AID. In AID deficient tumors the lack of mutations in Ig genes was in contrast to the increased mutation load in non-Ig genes compared to AID proficient lymphoma. Gene expression profiling and the detection of distinct mutational signatures pointed to the function of the cytidine deaminase APOBEC1 in murine AID deficient lymphoma.

The mining of human data uncovered the expression of APOBEC3 family members in human B cells, including *APOBEC3B* induction in GC B cells, thereby mimicking murine *Apobec1* expression kinetics. Although *APOBEC3B* expression levels were variable across different GC B cell derived lymphoma entities, the positive correlation of its expression and presence of APOBEC related DNA signatures indicates its relevance in human lymphomagenesis.

### Summary/Conclusion

Our research provides experimental evidence of APOBEC mediated DNA mutagenesis in an autochthonous mouse lymphoma model and supports these findings in lymphoma patient data sets. Thus, we unveiled AID independent transformation in lymphoma prone GC B cells and highlight APOBEC enzymes as mutation driver in GC B cell derived lymphomagenesis.

**Copyright Information:** (Online) ISSN: 2572-9241
©2026 The Author(s). HemaSphere is published by John Wiley & Sons Ltd on behalf of European Hematology Association. This is an open access Abstract Book distributed under the Attribution-NonCommercial-NoDerivs (CC BY-NC-ND), which allows third parties to download the articles and share them with others as long as they credit the author and the Abstract Book, but they cannot change the content in any way or use them commercially.
**Abstract Book Citations:** Authors, Title, HemaSphere, 2026;10:(S1):pages. The abstract book DOIs can be found on the "Author Information" page.
**Disclaimer:** Articles published in the journal HemaSphere exclusively reflect the opinions of the authors. The authors are responsible for all content in their abstracts including accuracy of the facts, statements, citing resources, etc.

### (PF1091) RITUXIMAB-MODIFIED MNO₂-COATED ZIF-8 NANOPLATFORM LOADED WITH DOXORUBICIN FOR TARGETED THERAPY OF B-CELL LYMPHOMA

**Topic:** 21. Lymphoma biology & translational research

Ling Qiu[1], Shihui Ren[1], Mengjiao Li[1, 2], Yanling Li[1, 2], Jiajia Li[1, 2], Caiyang Li[1, 3], Hao Yao*[1, 3, 4, 5], Fangyi Fan[1, 2]

*[1]Department of Hematology, The General Hospital of Western Theater Command, Chengdu, China;[2]Department of Clinical Medicine, North Sichuan Medical College, Nanchong, China; [3]Institute of Basic Medicine, North Sichuan Medical College, Nanchong, China; [4]College of Medicine, Southwest Jiaotong University, Chengdu, China;[5]Tissue Stress Injury and Functional Repair Key Laboratory of Sichuan Province, Chengdu, China;*

### Background

B-cell lymphoma remains a prevalent hematologic malignancy requiring more effective and targeted therapeutic strategies. Although rituximab (RTX) and doxorubicin (DOX) are standard agents, their clinical efficacy may be limited by suboptimal tumor targeting and systemic toxicity.

### Aims

To construct a $MnO_2$-coated ZIF-8 nanoplatform loaded with DOX and surface-modified with RTX (ZIF-8@DOX@$MnO_2$@RTX), and to evaluate its antitumor efficacy and molecular mechanisms in B-cell lymphoma.

### Methods

ZIF-8 nanoparticles were coated with $MnO_2$, loaded with DOX, and conjugated with RTX. Nanoparticle morphology, hydrodynamic size, surface potential, drug encapsulation efficiency, and antibody coupling rate were characterized. In vitro assays in DAUDI B-cell lymphoma cells assessed proliferation, apoptosis, invasion, and cell cycle distribution. In vivo antitumor efficacy was evaluated using a xenograft mouse model. Transcriptome sequencing was performed to identify potential molecular targets, and key proteins were validated by western blotting.

### Results

The RTX + DOX combination demonstrated stronger tumor growth inhibition and increased apoptosis compared with either agent alone. ZIF-8@DOX@$MnO_2$@RTX nanoparticles exhibited a core size of approximately 100 nm, a hydrodynamic diameter of 198.6 nm, a surface potential of 3.94 mV, a DOX encapsulation efficiency of 68.5%, and an RTX conjugation efficiency of 71.5%. The nanoplatform demonstrated favorable stability and serum compatibility. In vitro, ZIF-8@DOX@$MnO_2$@RTX significantly inhibited proliferation and invasion of DAUDI cells, induced apoptosis, and promoted cell cycle arrest. In vivo, the nanoplatform markedly suppressed tumor growth in transplanted mice without observable systemic toxicity. Transcriptomic analysis identified early growth response 2 (EGR2) as a key differentially expressed target. Western blot confirmed that ZIF-8@DOX@$MnO_2$@RTX downregulated EGR2 and the anti-apoptotic protein BCL2.

### Summary/Conclusion

ZIF-8@DOX@$MnO_2$@RTX effectively inhibits tumor growth and cellular invasion in B-cell lymphoma while promoting apoptosis through regulation of EGR2 signaling. This targeted nanoplatform represents a promising strategy for precision chemoimmunotherapy in B-cell lymphoma.

**Copyright Information:** (Online) ISSN: 2572-9241
©2026 The Author(s). HemaSphere is published by John Wiley & Sons Ltd on behalf of European Hematology Association. This is an open access Abstract Book distributed under the Attribution-NonCommercial-NoDerivs (CC BY-NC-ND), which allows third parties to download the articles and share them with others as long as they credit the author and the Abstract Book, but they cannot change the content in any way or use them commercially.
**Abstract Book Citations:** Authors, Title, HemaSphere, 2026;10:(S1):pages. The abstract book DOIs can be found on the "Author Information" page.
**Disclaimer:** Articles published in the journal HemaSphere exclusively reflect the opinions of the authors. The authors are responsible for all content in their abstracts including accuracy of the facts, statements, citing resources, etc.

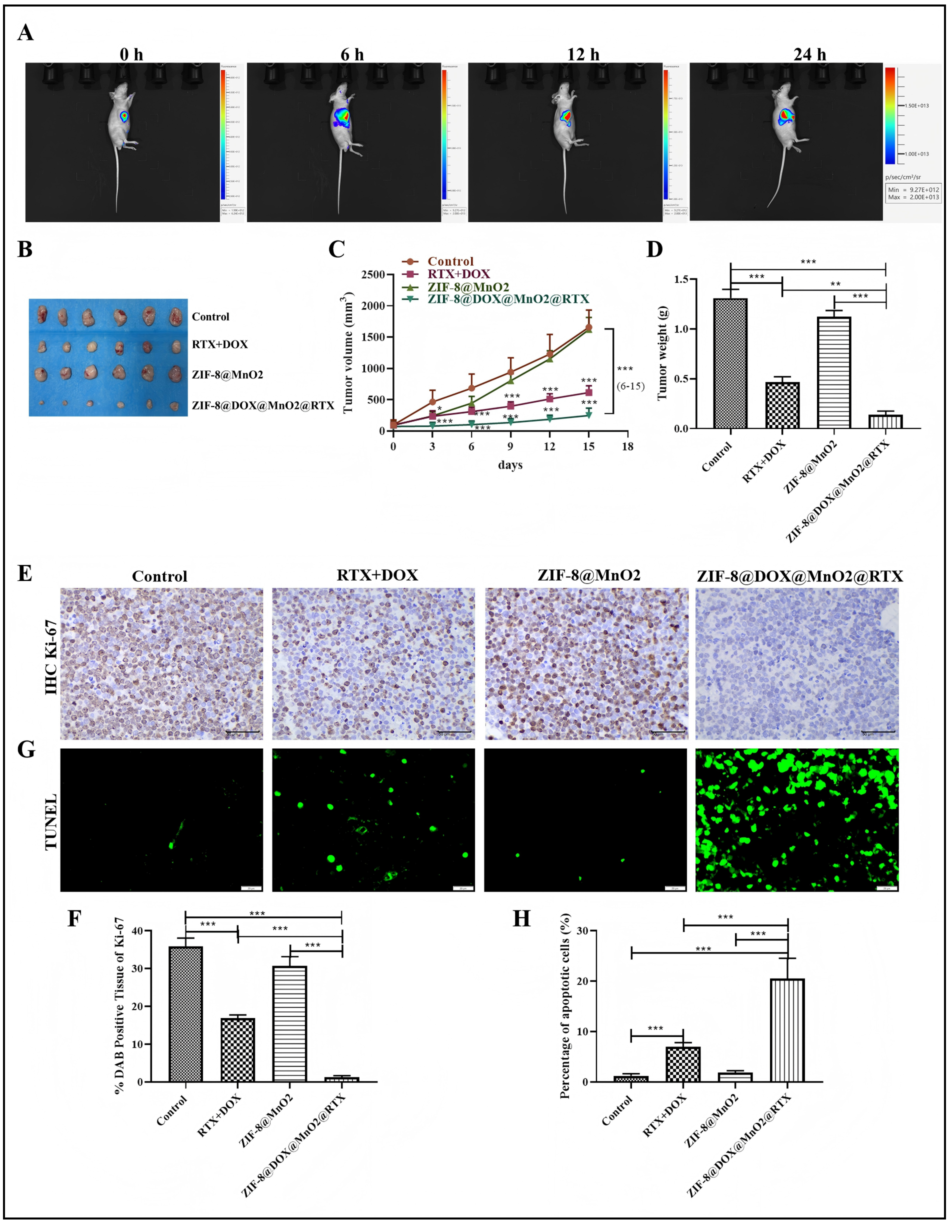

Figure. Superior in vivo antitumor efficacy of ZIF-8@DOX@MnO₂@RTX in a B-cell lymphoma xenograft model. (A) Tumor growth curves demonstrating that ZIF-8@DOX@MnO₂@RTX profoundly suppressed tumor progression, significantly outperforming RTX monotherapy, DOX monotherapy, and even the RTX + DOX combination regimen. The nanoplatform achieved the most sustained and pronounced tumor growth inhibition throughout the treatment course. (B) Representative images of excised tumors at endpoint, illustrating a striking reduction in tumor burden in the ZIF-8@DOX@MnO₂@RTX group compared with all comparator arms. (C) Quantitative tumor weight analysis confirming the superior antitumor potency of ZIF-8@DOX@MnO₂@RTX, with statistically significant reductions versus conventional treatment strategies. (D) Immunohistochemical analysis (Ki-67 and TUNEL staining) demonstrating robust suppression of tumor proliferation and marked induction of apoptosis following ZIF-8@DOX@MnO₂@RTX treatment. Collectively, these findings establish the enhanced therapeutic efficacy of the RTX-modified nanoplatform in vivo. Data are presented as mean ± SD. Statistical significance was determined by one-way ANOVA with appropriate post hoc testing. *P < 0.05, **P < 0.01.

Copyright Information: (Online) ISSN: 2572-9241
©2026 The Author(s). HemaSphere is published by John Wiley & Sons Ltd on behalf of European Hematology Association. This is an open access Abstract Book distributed under the Attribution-NonCommercial-NoDerivs (CC BY-NC-ND), which allows third parties to download the articles and share them with others as long as they credit the author and the Abstract Book, but they cannot change the content in any way or use them commercially.
Abstract Book Citations: Authors, Title, HemaSphere, 2026;10:(S1):pages. The abstract book DOIs can be found on the “Author Information” page.
Disclaimer: Articles published in the journal HemaSphere exclusively reflect the opinions of the authors. The authors are responsible for all content in their abstracts including accuracy of the facts, statements, citing resources, etc.

### (PF1092) PATIENTS WITH T CELLS MAINTAINING AN IMMUNE SIGNATURE FEATURING EARLY DIFFERENTIATION STAGE EXHIBIT A SUPERIOR CLINICAL RESPONSE TO PEMBROLIZUMAB IN RELAPSED LOW-GRADE LYMPHOMA

**Topic:** 21. Lymphoma biology & translational research

Zhi-Zhang Yang*[1], Xinyi Tang[1], Yucai Wang[1], Vaishali Bhardwaj[1], Prithviraj Mukherjee[1], Qiang Pan-Hammarstrom[2], Patrizia Mondello[1], Stephen Ansell[1]

[1]*Mayo Clinic, Rochester, United States of America;* [2]*Karolinska Institutet, Stockholm, Sweden;*

### Background

While overall clinical efficacy of PD-1 blockade therapy with pembrolizumab in relapsed or refractory low-grade lymphoma is limited, some patients show partial or complete response. The underlying cellular and molecular mechanisms that account for the response are largely unknown.

### Aims

This study is to explore transcriptomic and proteomic changes in T cells in patients with relapsed low-grade lymphoma treated with pembrolizumab and to identify the gene signature that differentiates patients who respond from those who don't.

### Methods

Peripheral blood nuclear cells (PBMCs) from 23 patients with relapsed low-grade lymphoma (FL, n=18; MZL, n=2; LPL/WM, n=3) who received single agent pembrolizumab were analyzed using single cell RNA sequencing (scRNA-seq, n=11) and mass cytometry (CyTOF, n=21). PBMCs from healthy donors (n=8) and patients with newly diagnosed untreated low-grade lymphoma (n=5) served as controls. Computationally isolated T cells from PBMCs were analyzed using Seurat algorithm.

### Results

Among 11 patients with scRNA-seq, 3 patients were responders (RESP, 1 complete and 2 partial responses), 4 had progressive disease (PD) and 4 had stable disease (SD). Blood samples from each patient were collected at various time points of treatment including baseline (BL, n=11), cycle 2 (Cyc2, n=5), cycle 3 (Cyc3, n=8), 3 months (3m, n=5), 6 months (6m, n=3), 12 months (12m, n=1) and at progression (Prog, n=7). At BL, peripheral blood from patients with low-grade lymphoma contained a T cell profile featuring significantly increased numbers of effector ($T_{EM}$) and terminally differentiated ($T_{EMRA}$) memory T cells, associated with loss of CD27 and CD28, when compared to untreated patients and healthy donors. Prior to pembrolizumab, we observed the presence of $CD4^+$ and $CD8^+$ naïve T ($T_N$) cells in RESP patients, but their presence was almost absent in PD patients. In contrast, $T_{EM}$ and $T_{EMRA}$ were significantly less abundant in RESP patients compared to PD patients. Supporting this, GSEA analysis using the human immunologic signature gene sets (C7 set) from the Molecular Signatures Database revealed that gene sets that are more highly expressed in $T_N$ than $T_{EM}$ and exhausted memory T cells were markedly upregulated in RESP compared to PD patients. Based on analysis for differentially expressed genes, we identified a panel of 11 genes (*CCR7*, *SELL*, *TCF7*, *LEF1*, *MAL*, *IL7R, LTB, THEM4, CD27*, *CD28* and *FLT3LG)* as a gene signature. This gene signature was significantly more abundant in RESP than both PD and SD patients at BL. Furthermore, RESP patients maintained the gene signature over the course of treatment while PD patients showed a substantial reduction of the score at Prog, which was associated with an expansion of $T_{EM}$ and $T_{EMRA}$ cells and a reduction of $T_N$ and $T_{CM}$ cells.

**Copyright Information:** (Online) ISSN: 2572-9241
©2026 The Author(s). HemaSphere is published by John Wiley & Sons Ltd on behalf of European Hematology Association. This is an open access Abstract Book distributed under the Attribution-NonCommercial-NoDerivs (CC BY-NC-ND), which allows third parties to download the articles and share them with others as long as they credit the author and the Abstract Book, but they cannot change the content in any way or use them commercially.
**Abstract Book Citations:** Authors, Title, HemaSphere, 2026;10:(S1):pages. The abstract book DOIs can be found on the "Author Information" page.
**Disclaimer:** Articles published in the journal HemaSphere exclusively reflect the opinions of the authors. The authors are responsible for all content in their abstracts including accuracy of the facts, statements, citing resources, etc.

**Summary/Conclusion**

Our results demonstrate that patients with relapsed low-grade lymphoma started with a T cell profile highly distinct from patients with untreated low-grade lymphoma and healthy donors. Patients who have increased circulating T cells with early differentiation stage tend to have a better clinical response to pembrolizumab. Identification of a T-cell gene signature may help stratify cancer patients who are likely to respond to immunotherapy with PD-1 blockade.

**Copyright Information:** (Online) ISSN: 2572-9241
©2026 The Author(s). HemaSphere is published by John Wiley & Sons Ltd on behalf of European Hematology Association. This is an open access Abstract Book distributed under the Attribution-NonCommercial-NoDerivs (CC BY-NC-ND), which allows third parties to download the articles and share them with others as long as they credit the author and the Abstract Book, but they cannot change the content in any way or use them commercially.
**Abstract Book Citations:** Authors, Title, HemaSphere, 2026;10:(S1):pages. The abstract book DOIs can be found on the "Author Information" page.
**Disclaimer:** Articles published in the journal HemaSphere exclusively reflect the opinions of the authors. The authors are responsible for all content in their abstracts including accuracy of the facts, statements, citing resources, etc.

### (PF1094) TARGETING HELLS-MCM5 AXIS TO EXPLOIT TRANSCRIPTION-REPLICATION CONFLICTS AS NOVEL THERAPEUTIC STRATEGY IN ANAPLASTIC LARGE CELL LYMPHOMA

**Topic:** 21. Lymphoma biology & translational research

Valentina Fragliasso*[1], Selene Mallia[1], Giulia Gambarelli[1], Emanuele Vitale[1], Federica Torricelli[1], Pierluigi Di Chiaro[1], Rebecca Vezzani[1], Elisa Salviato[1], Veronica Manicardi[1], Benedetta Donati[1], Noemi Puccio[1], Antonino Neri[1], Alessia Ciarrocchi[1]

[1]*AUSL-IRCCS di Reggio Emilia, Reggio Emilia, Italy;*

### Background

Transcription and replication are the primary drivers of chromatin architecture remodeling; however, the spatiotemporal coordination of these processes at individual loci remains a fundamental question in cancer biology. The chromatin remodeler HELLS is aberrantly expressed in multiple tumors and has been implicated as a transcriptional sentinel that safeguards oncogenic programs in T-cell lymphoma. However, its role in coordinating transcription-replication dynamics to maintain lymphoma cell fitness remains largely uncharacterized.

### Aims

We aim to characterize the role of HELLS in coordinating transcription and replication dynamics to preserve genome integrity in aggressive Anaplastic Large Cell Lymphoma.

### Methods

We employed a multi-omics approach (ChIP-seq, ATAC-seq, and CUT&Tag-sequencing) to map chromatin features and R-loop distribution in Anaplastic Large Cell Lymphoma (ALCL) cell models (MAC2A, TLBR-2). Transcription-replication conflicts (TRCs) were quantified via proximity ligation assays (PLA) for PCNA and initiating RNAPII (Ser5), while DNA fiber analysis tracked replication fork dynamics. Translational impact was assessed through proliferation assays using the selective CDK9 inhibitor AZD4573 and the replicative stress-inducing agent etoposide.

### Results

HELLS depletion led to a genome-wide boost in R-loop signals, particularly at promoters, and a marked increase in replication fork stalling assessed by DNA fiber analysis. PLA and ChIP-qPCR revealed that HELLS loss enhances collisions between the replisome and the transcriptional machinery at highly transcribed sites. Importantly, RNaseH1 overexpression in HELLS depleted-cells efficiently suppressed these conflicts, establishing R-loops as the primary drivers of HELLS-dependent replication stress. Starting from functional screening of a list of direct HELLS-target genes potentially implicated in genomic stability, we identified the replicative helicase subunit MCM5 as the primary downstream mediator of HELLS-driven stability. Inducible MCM5-knockdown phenocopies HELLS loss, resulting in impaired proliferation, elevated R-loop levels, increased TRCs involving RNAPII-ser5 and PCNA collisions and enhanced replication fork stalling in lymphoma cells. Conversely, restoring MCM5 expression rescues replication dynamics and suppresses R-loop accumulation in HELLS-deficient cells. Mechanistically, HELLS ensures adequate MCM5 levels to maintain proper origin licensing. In its absence, the activation of dormant origins within gene bodies leads to catastrophic head-on TRCs. Importantly, the HELLS-MCM5 module defines targetable vulnerability. HELLS or MCM5 knockdown significantly sensitized lymphoma cells to CDK9 inhibition. Furthermore, the concurrent pharmacological targeting of transcription (by using AZD4573) and replication (by using etoposide) resulted in a synergistic suppression of cell growth, exceeding the efficacy of single-agent treatments.

**Copyright Information:** (Online) ISSN: 2572-9241
©2026 The Author(s). HemaSphere is published by John Wiley & Sons Ltd on behalf of European Hematology Association. This is an open access Abstract Book distributed under the Attribution-NonCommercial-NoDerivs (CC BY-NC-ND), which allows third parties to download the articles and share them with others as long as they credit the author and the Abstract Book, but they cannot change the content in any way or use them commercially.
**Abstract Book Citations:** Authors, Title, HemaSphere, 2026;10:(S1):pages. The abstract book DOIs can be found on the "Author Information" page.
**Disclaimer:** Articles published in the journal HemaSphere exclusively reflect the opinions of the authors. The authors are responsible for all content in their abstracts including accuracy of the facts, statements, citing resources, etc.

**Summary/Conclusion**

Our findings identify the HELLS-MCM5 axis as a master coordinator of transcription-replication dynamics, essential for the survival of aggressive ALCL. By uncoupling these processes, we established a strategic therapeutic window wherein the pharmacological exacerbation of TRCs overwhelms the genome maintenance capacity of cancer cells. These data define a novel therapeutic paradigm for aggressive, "orphan" malignancies like ALCL through the precision targeting of transcriptional and replicative stress.

**Copyright Information:** (Online) ISSN: 2572-9241
©2026 The Author(s). HemaSphere is published by John Wiley & Sons Ltd on behalf of European Hematology Association. This is an open access Abstract Book distributed under the Attribution-NonCommercial-NoDerivs (CC BY-NC-ND), which allows third parties to download the articles and share them with others as long as they credit the author and the Abstract Book, but they cannot change the content in any way or use them commercially.
**Abstract Book Citations:** Authors, Title, HemaSphere, 2026;10:(S1):pages. The abstract book DOIs can be found on the “Author Information” page.
**Disclaimer:** Articles published in the journal HemaSphere exclusively reflect the opinions of the authors. The authors are responsible for all content in their abstracts including accuracy of the facts, statements, citing resources, etc.

### (PF1095) DIOSCIN INHIBITS DIFFUSE LARGE B-CELL LYMPHOMA BY MODULATING ACACA-DEPENDENT HISTONE H3K27 ACETYLATION AND INDUCING MITOPHAGY

**Topic:** 21. Lymphoma biology & translational research

Qianqian Guo*[1], Xiaoli Zhou[1], Xiangxiang Zhou[1], Shunfeng Hu[1]

[1]*Shandong Provincial Hospital Affiliated to Shandong First Medical University, Jinan, China;*

### Background

Diffuse large B-cell lymphoma (DLBCL) is characterized by high heterogeneity and poor prognosis. Dioscin is a natural steroidal saponin from traditional Chinese medicine with known antitumor properties and clinical applications in liver and cardiovascular disorders.

### Aims

This study aims to elucidate the effects of dioscin in DLBCL development and its regulatory mechanism on downstream targets and histone acetylation in DLBCL.

### Methods

The FDA anti-tumor drug library (n=1746) was used to screen effective anti-DLBCL compounds and potential synergistic agents. In-vitro assays, including cell proliferation, cell cycle, and apoptosis, were performed to evaluate anti-lymphoma effects, and xenograft DLBCL mouse models were established to assess in-vivo efficacy. Drug affinity responsive target stability (DARTS)-based mass spectrum, molecular docking, RNA-seq, confocal immunofluorescence, and ChIP-seq were used to explore the potential mechanism.

**Copyright Information:** (Online) ISSN: 2572-9241
©2026 The Author(s). HemaSphere is published by John Wiley & Sons Ltd on behalf of European Hematology Association. This is an open access Abstract Book distributed under the Attribution-NonCommercial-NoDerivs (CC BY-NC-ND), which allows third parties to download the articles and share them with others as long as they credit the author and the Abstract Book, but they cannot change the content in any way or use them commercially.
**Abstract Book Citations:** Authors, Title, HemaSphere, 2026;10:(S1):pages. The abstract book DOIs can be found on the "Author Information" page.
**Disclaimer:** Articles published in the journal HemaSphere exclusively reflect the opinions of the authors. The authors are responsible for all content in their abstracts including accuracy of the facts, statements, citing resources, etc.

### Results

To explore novel therapeutic strategies for DLBCL, drug screening was performed based on the FDA anti-tumor drug library (n=1746), and dioscin was identified as an effective anti-DLBCL compound. Subsequently, dioscin treatment was found to significantly inhibit cell proliferation in DLBCL cells in a time- and dose-dependent manner, increase the proportion of cells in the G0/G1 phase, and promote cell apoptosis. Moreover, in DLBCL xenograft models, dioscin markedly suppressed tumor growth rates, weight, and tumor volume compared with the control group.

To further elucidate the direct target of dioscin in DLBCL, we combined DARTS-based mass spectrometry and molecular docking, and identified acetyl-CoA carboxylase α (ACACA), a key enzyme involved in fatty acid synthesis, as a potential dioscin-binding protein. Further DARTS validation confirmed the interaction between dioscin and ACACA. Importantly, ACACA was highly expressed in DLBCL tissues, which was significantly associated with poor prognosis in DLBCL patients. Functional experiments showed that targeting ACACA inhibited cell proliferation and induced cell cycle arrest and apoptosis in DLBCL.

To investigate the downstream mechanism, RNA-seq analysis revealed that both dioscin treatment and ACACA targeting were associated with activation of the mitophagy pathway. Further experiments showed that dioscin and ACACA knockdown increased mitochondrial dysfunction and promoted PINK1-Parkin-dependent mitophagy in DLBCL cells. Mechanistically, dioscin and ACACA regulated intracellular acetyl-CoA levels and enhanced histone acetylation, particularly H3K27ac. Integrative analysis of RNA-seq and ChIP-seq data identified HK2, a known PINK1-interacting protein, as a key downstream target of ACACA. ACACA-mediated H3K27ac enrichment at the HK2 promoter promoted its transcription. Consistently, treatment with the HK2 inhibitor BNBZ partially reversed the pro-mitophagy and anti-proliferative effects induced by ACACA knockdown and dioscin treatment. Furthermore, in-vitro and in-vivo experiments revealed that dioscin exhibited synergistic anti-lymphoma effects when combined with chidamide, a selective histone deacetylase inhibitor, and combination treatment markedly enhanced H3K27ac levels.

### Summary/Conclusion

In summary, our findings showed that dioscin suppressed DLBCL progression by targeting ACACA and inducing mitophagy via H3K27ac, highlighting its potential as a novel therapeutic strategy for DLBCL treatment

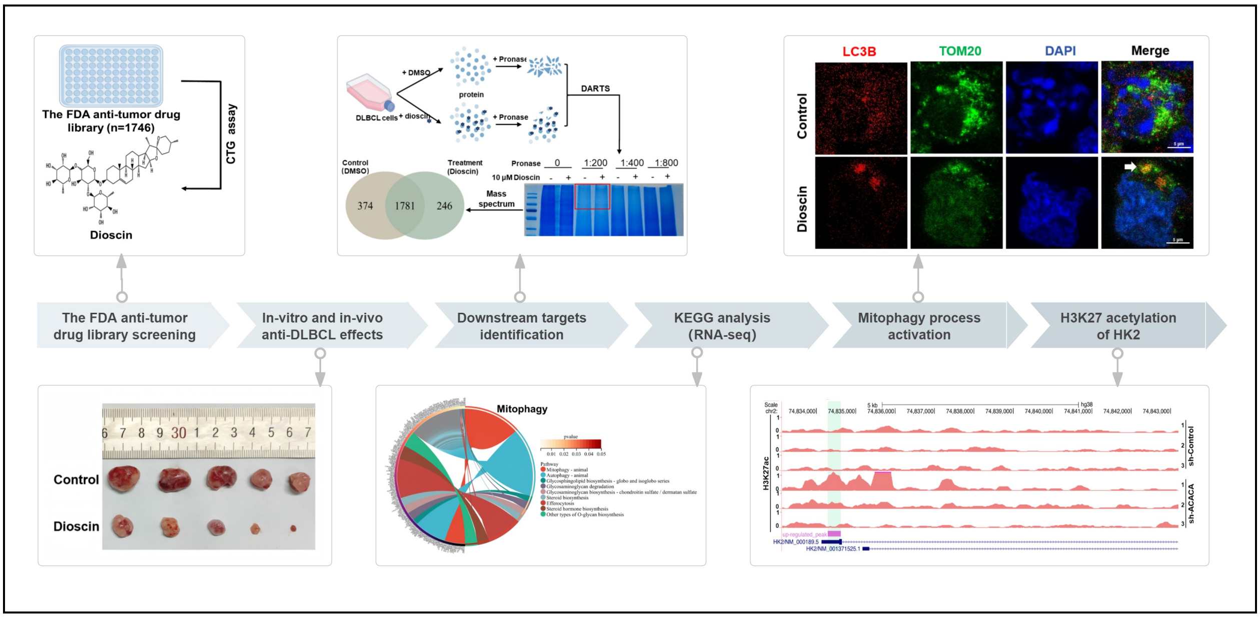

**Copyright Information:** (Online) ISSN: 2572-9241
©2026 The Author(s). HemaSphere is published by John Wiley & Sons Ltd on behalf of European Hematology Association. This is an open access Abstract Book distributed under the Attribution-NonCommercial-NoDerivs (CC BY-NC-ND), which allows third parties to download the articles and share them with others as long as they credit the author and the Abstract Book, but they cannot change the content in any way or use them commercially.
**Abstract Book Citations:** Authors, Title, HemaSphere, 2026;10:(S1):pages. The abstract book DOIs can be found on the "Author Information" page.
**Disclaimer:** Articles published in the journal HemaSphere exclusively reflect the opinions of the authors. The authors are responsible for all content in their abstracts including accuracy of the facts, statements, citing resources, etc.

### (PF1096) INTEGRATED MOLECULAR AND METABOLIC PROFILING IDENTIFIES DETERMINANTS OF OUTCOME WITH LONCASTUXIMAB TESIRINE IN RELAPSED/REFRACTORY LARGE B-CELL LYMPHOMA

**Topic:** 21. Lymphoma biology & translational research

Ciro Maria Improta*[1, 2], Martina di Trani[2], Chiara Signori[1, 2], Jacopo Calabrese De Feo[1, 2], Francesco Corrado[1, 2], Alessandro Davini[3], Marcello Rodari[4], Davide Rossi[5, 6, 7], Enrico Derenzini[3, 8], Carmelo Carlo-Stella[1, 2]

*[1]Humanitas University, Department of Biomedical Sciences, Pieve Emanuele, Milan, Italy; [2]IRCCS Humanitas Research Hospital, Hematology, Rozzano, Milan, Italy; [3]European Institute of Oncology IRCCS, Oncohematology Division, Milan, Italy; [4]IRCCS Humanitas Research Hospital, Nuclear Medicine Unit, Rozzano, Milan, Italy; [5]Institute of Oncology Research, Laboratory of Experimental Hematology, Bellinzona, Switzerland; [6]Oncology Institute of Southern Switzerland, Ente Ospedaliero Cantonale, Clinic of Hematology, Bellinzona, Switzerland; [7]Università della Svizzera Italiana, Faculty of Biomedical Sciences, Lugano, Switzerland; [8]University of Milan, Department of Health Sciences, Milan, Italy;*

### Background

Relapsed or refractory large B-cell lymphoma (LBCL) remains associated with poor prognosis. Loncastuximab Tesirine is a CD19 directed antibody drug conjugate with demonstrated clinical activity in heavily pretreated disease. However, treatment benefit is heterogeneous and no validated biomarkers currently enable biological stratification or prediction of outcome, underscoring the need to define determinants of response.

### Aims

To evaluate the prognostic and predictive role of circulating tumor DNA (ctDNA) and total metabolic tumor volume (TMTV), alone and in combination, in patients with relapsed or refractory LBCL treated with Loncastuximab Tesirine.

### Methods

This single-center retrospective analysis included 41 patients with relapsed or refractory LBCL treated at Humanitas Research Hospital within the LOTIS 1 and LOTIS 2 trials. ctDNA and TMTV were analyzed prior to therapy initiation (Baseline, BL), at cycle 3 (C3) and at end of treatment (EoT). ctDNA was assessed using Cancer Personalized Profiling by Deep Sequencing (CAPP-Seq) and the tumor burden was expressed as haploid genome equivalents per mL (hGE/mL) of plasma. Copy number variation (CNV) analysis from ctDNA was performed using CNVkit to detect focal and broad chromosomal alterations. 3 patients with undetectable BL ctDNA were excluded from subsequent analyses. TMTV was calculated using an SUV threshold of 4.0. Optimal biomarker cutoffs were defined by ROC analysis. Progression-free survival (PFS) was estimated using the Kaplan-Meier method, censoring patients at the time of stem cell transplantation when applicable.

**Copyright Information:** (Online) ISSN: 2572-9241
©2026 The Author(s). HemaSphere is published by John Wiley & Sons Ltd on behalf of European Hematology Association. This is an open access Abstract Book distributed under the Attribution-NonCommercial-NoDerivs (CC BY-NC-ND), which allows third parties to download the articles and share them with others as long as they credit the author and the Abstract Book, but they cannot change the content in any way or use them commercially.
**Abstract Book Citations:** Authors, Title, HemaSphere, 2026;10:(S1):pages. The abstract book DOIs can be found on the "Author Information" page.
**Disclaimer:** Articles published in the journal HemaSphere exclusively reflect the opinions of the authors. The authors are responsible for all content in their abstracts including accuracy of the facts, statements, citing resources, etc.

### Results

Overall response rate (ORR) was 53.7%, including 17.1% complete responses (CR) and 36.6% partial responses. BL ctDNA and TMTV were not significantly associated with ORR or CR. ORR did not differ according to TP53 status (TP53 wild-type:11/18; TP53-mutated:10/20; odds ratio 1.55, p=0.53). Complete responses were numerically more frequent in TP53 wild-type patients (5/18 vs 2/20), although not statistically significant (odds ratio 3.35, p=0.22). CNV analysis identified 4 patients with biallelic TP53 inactivation (TP53 mutation with concurrent 17p deletion), with no isolated 17p loss; none achieved CR. In the overall population, median PFS was 3.6 months. BL ctDNA and TMTV were significantly associated with PFS. A ROC-derived ctDNA cut-off of 245 hGE/mL (AUC 0.667) identified inferior outcomes (median PFS 2.7 vs 7.8 months). High BL TMTV (>135 mL; AUC 0.745) was similarly associated with shorter PFS (2.6 vs 7.8 months). Early molecular response strongly predicted outcome, with ctDNA negativity at C3 associated with longer PFS (8.6 vs 3.7 months, p=0.004). Combined ctDNA-TMTV stratification identified progressively inferior outcomes with increasing risk factors. No differences in BL ctDNA or TMTV were observed between TP53 groups, and TP53 status did not independently impact PFS. No de novo CD19 mutations emerged during treatment.

### Summary/Conclusion

In this cohort treated with Loncastuximab Tesirine, baseline tumor burden captured by ctDNA and TMTV appears to be the main determinant of outcome in this polyrefractory setting. Baseline biomarkers do not predict depth of response but influence durability of benefit, and early ctDNA clearance identifies patients with improved disease control. Lack of prognostic impact of TP53 alterations and CD19 loss suggests alternative resistance mechanisms. Integrated molecular and metabolic profiling enables meaningful risk stratification in relapsed or refractory LBCL.

**Copyright Information:** (Online) ISSN: 2572-9241
©2026 The Author(s). HemaSphere is published by John Wiley & Sons Ltd on behalf of European Hematology Association. This is an open access Abstract Book distributed under the Attribution-NonCommercial-NoDerivs (CC BY-NC-ND), which allows third parties to download the articles and share them with others as long as they credit the author and the Abstract Book, but they cannot change the content in any way or use them commercially.
**Abstract Book Citations:** Authors, Title, HemaSphere, 2026;10:(S1):pages. The abstract book DOIs can be found on the “Author Information” page.
**Disclaimer:** Articles published in the journal HemaSphere exclusively reflect the opinions of the authors. The authors are responsible for all content in their abstracts including accuracy of the facts, statements, citing resources, etc.

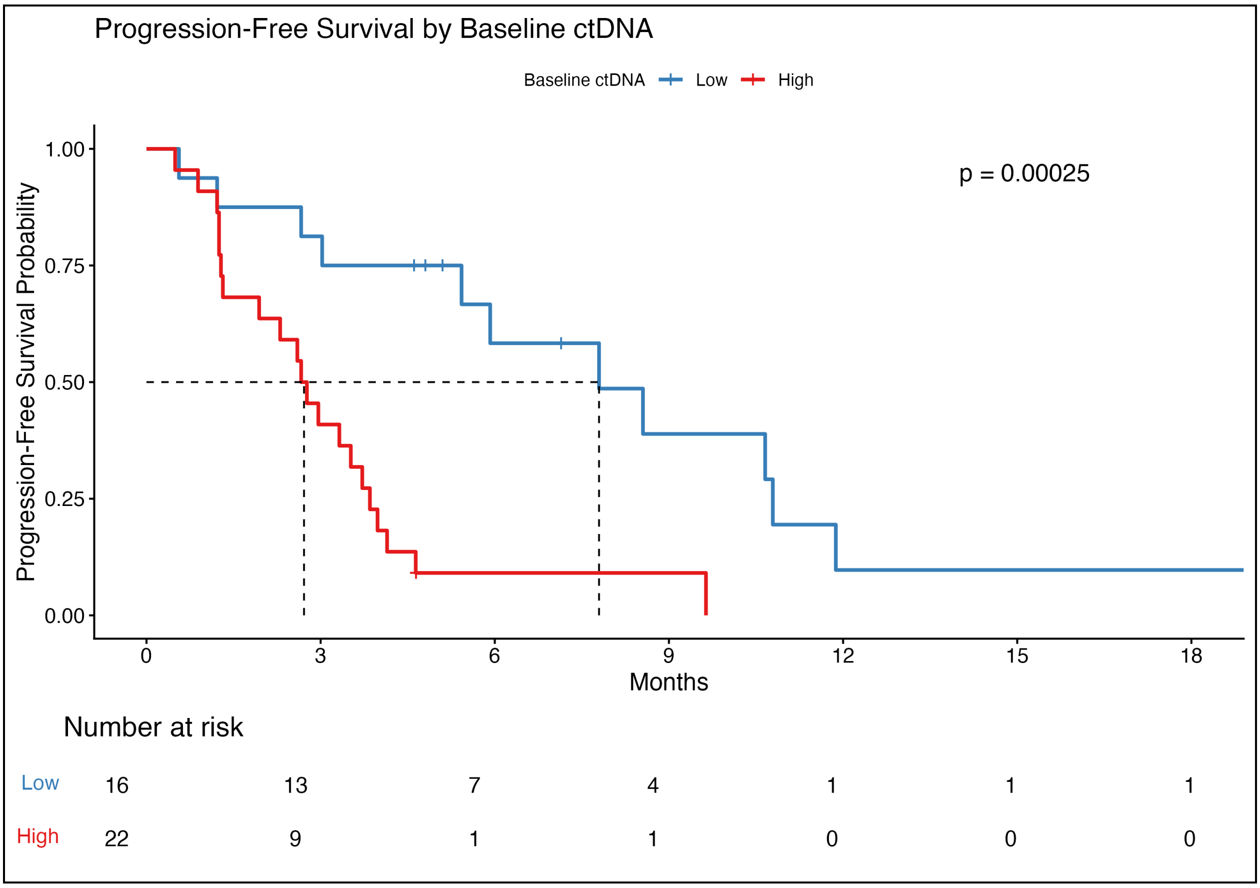

Kaplan–Meier estimates of progression-free survival (PFS) according to baseline ctDNA levels dichotomized using the optimal cutoff identified by Youden index (245 hGE/mL). Statistical comparisons were performed using the log-rank test. Patients with non-detectable baseline ctDNA (<0.0001 hGE/mL) were excluded.

**Copyright Information:** (Online) ISSN: 2572-9241
©2026 The Author(s). HemaSphere is published by John Wiley & Sons Ltd on behalf of European Hematology Association. This is an open access Abstract Book distributed under the Attribution-NonCommercial-NoDerivs (CC BY-NC-ND), which allows third parties to download the articles and share them with others as long as they credit the author and the Abstract Book, but they cannot change the content in any way or use them commercially.
**Abstract Book Citations:** Authors, Title, HemaSphere, 2026;10:(S1):pages. The abstract book DOIs can be found on the "Author Information" page.
**Disclaimer:** Articles published in the journal HemaSphere exclusively reflect the opinions of the authors. The authors are responsible for all content in their abstracts including accuracy of the facts, statements, citing resources, etc.

### (PF1097) DURABLE RESPONSES WITH RONDECABTAGENE AUTOLEUCEL (DUAL-TARGETING CD19/CD20 CAR T-CELLS) ARE ASSOCIATED WITH HIGHER PROPORTION OF CYTOTOXIC T CELLS WITH MEMORY POTENTIAL IN INFUSION PRODUCTS

**Topic:** 21. Lymphoma biology & translational research

Akil Merchant*[1], Ben Harris[2], Tahir Latiff[3], Umar Farooq[4], Locke Bryan[5], Stefan Ciurea[6], Nebu Koshy[7], Viola Lam[8], Shobha Potluri[8], Sarah M. Larson[9]

*[1]Cedars-Sinai Medical Center, Division of Hematology and Cellular Therapy, Los Angeles, United States of America;[2]Lyell Immunopharma, South San Francisco, United States of America; [3]University of Cincinnati Medical Center, Cincinnati, United States of America; [4]University of Iowa Health Care, Iowa City, United States of America; [5]Medical College of Georgia, Hematology Oncology Blood & Marrow Transplantation and Cellular Therapy, Augusta, United States of America; [6]University of California Irvine Health, Chao Family Comprehensive Cancer Center, Hematology Oncology, Department of Medicine, Orange, United States of America; [7]Baylor Scott & White Health, Dallas, United States of America;[8]Lyell Immunopharma, Translational Sciences, South San Francisco, United States of America; [9]UCLA David Geffen School of Medicine, Hematology and Oncology, Los Angeles, United States of America;*

### Background

Achieving durable complete responses (CR) remains a key goal of CAR T-cell therapy for large B-cell lymphoma (LBCL). Higher proportion of cells with memory phenotype in CAR T-cell products has been associated with higher expansion and favorable outcomes (Deng 2020), and CD62L is an established marker of memory T cells (Gattinoni 2017). Rondecabtagene autoleucel (ronde-cel) is an autologous, dual-targeting CD19/CD20 CAR T-cell product candidate manufactured from CD62L-enriched cells, a process designed to preserve higher proportion of cells with memory phenotype and improve durable responses without extending manufacturing time. High rates (76%) of CR with 18-month median progression-free survival were reported from the Phase 1/2 clinical trial of ronde-cel in third- or later-line (3L+) patients (Larson 2025).

### Aims

Evaluate the association between ronde-cel's durability of response in patients treated in the Phase 1/2 trial and ronde-cel product characteristics conferred by CD62L enrichment.

### Methods

Ronde-cel drug product phenotype was assessed by single-cell RNAseq (scRNAseq). Peripheral blood (PB) was collected post-infusion at multiple time-points, and phenotype (scRNAseq) and ex vivo functionality (co-culture) were assessed. Publicly available scRNAseq data from FDA-approved CAR T-cell therapies (Deng 2020, Haradhvala 2022, Li 2023, Yu 2025) were used for comparison.

**Copyright Information:** (Online) ISSN: 2572-9241
©2026 The Author(s). HemaSphere is published by John Wiley & Sons Ltd on behalf of European Hematology Association. This is an open access Abstract Book distributed under the Attribution-NonCommercial-NoDerivs (CC BY-NC-ND), which allows third parties to download the articles and share them with others as long as they credit the author and the Abstract Book, but they cannot change the content in any way or use them commercially.
**Abstract Book Citations:** Authors, Title, HemaSphere, 2026;10:(S1):pages. The abstract book DOIs can be found on the "Author Information" page.
**Disclaimer:** Articles published in the journal HemaSphere exclusively reflect the opinions of the authors. The authors are responsible for all content in their abstracts including accuracy of the facts, statements, citing resources, etc.

**Results**

Transcriptomic analysis on CD8+CAR+ compartment of ronde-cel products (N = 42) shows higher expression of memory-related genes such as CD62L, IL7R, and LEF1 compared to approved CD19 CAR T-cell therapies (N = 120). Deeper phenotypic analysis of ronde-cel products using cluster analysis on scRNAseq data found a cytotoxic cluster of cells (defined by high GZMB, IFNG, CCL3, and CCL4 gene expression). Differential expression of ronde-cel's cytotoxic cluster to an analogous cytotoxic cluster from an FDA-approved therapy showed higher expression of memory markers (LEF1, IL7R, BACH2) in ronde-cel. These cells co-expressing cytotoxic genes (GZMB, IFNG, CCL3, CCL4) and memory-associated genes (LEF1, IL7R, BACH2) are referred to as Cytotoxic T cells with Memory Potential ($T_{cmp}$). Cells of similar phenotype were previously reported in pre-clinical studies (Deak 2022). Cells with memory phenotype in ronde-cel persisted as late as Day 28 and Day 56 after ronde-cel infusion with a median of 83% at D28 (N = 10) and 73% at D56 (N = 4). Ronde-cel CAR+ cells from D28 and D56 PB post-infusion can proliferate, secrete cytokines, and kill tumor cells ex-vivo, confirming the capacity of ronde-cel's memory cells to recall cytotoxic function. Patients with ³ 12 months of sustained CR had higher proportions of $T_{cmp}$ cells in CD8+CAR+ cells of ronde-cel products (N = 8, median = 23, IQR [10 - 28]) compared to patients experiencing progressive disease by Month 3 (N = 6, median = 7, IQR [6 - 9]); p-value = 0.029 Wilcoxon test).

**Summary/Conclusion**

Ronde-cel manufactured with CD62L enrichment produced infusion products with higher expression of memory-related genes compared with FDA-approved CD19 CAR T-cell therapies for LBCL. This memory potential also existed in the cytotoxic cells of ronde-cel products, persisted post-infusion, and allowed for functionality two-months post-infusion. Patients with a higher proportion of $T_{cmp}$ cells were more likely to achieve durable responses (≥12 months CR). These findings support the hypothesis of an association between ronde-cel's durability of response and CD62L enrichment during manufacturing.

**Copyright Information:** (Online) ISSN: 2572-9241
©2026 The Author(s). HemaSphere is published by John Wiley & Sons Ltd on behalf of European Hematology Association. This is an open access Abstract Book distributed under the Attribution-NonCommercial-NoDerivs (CC BY-NC-ND), which allows third parties to download the articles and share them with others as long as they credit the author and the Abstract Book, but they cannot change the content in any way or use them commercially.
**Abstract Book Citations:** Authors, Title, HemaSphere, 2026;10:(S1):pages. The abstract book DOIs can be found on the “Author Information” page.
**Disclaimer:** Articles published in the journal HemaSphere exclusively reflect the opinions of the authors. The authors are responsible for all content in their abstracts including accuracy of the facts, statements, citing resources, etc.

### (PF1098) TAXONOMIC AND FUNCTIONAL INTESTINAL MICROBIOME PROFILES DISTINGUISH MANTLE CELL LYMPHOMA PATIENTS FROM CONTROLS AND PERSIST ACROSS DISEASE STATES

**Topic:** 21. Lymphoma biology & translational research

Justin Desroches*[1], Keith Lau[2], Pedro Dimitriu[3], Alina Gerrie[1], David Scott[1], Andrew J Cowan[1], Christopher Venner[1], Laurie H. Sehn[1], Kerry Savage[1], Diego Villa[1]

*[1]BC Cancer Centre for Lymphoid Cancer and Division of Medical Oncology, Vancouver, Canada;[2]University of British Columbia, Department of Internal Medicine, Vancouver, Canada; [3]Microbiome Insights Inc., Vancouver, Canada;*

### Background

Mantle cell lymphoma (MCL) is a heterogeneous B-cell lymphoma with frequent gastrointestinal (GI) involvement and incompletely defined biological drivers. Given its near-universal GI tract involvement, the GI microbiome may influence disease biology and treatment response. While the microbiome composition has been linked to toxicity and outcomes in other non-Hodgkin lymphomas, it has never been characterized in MCL.

### Aims

To define taxonomic and functional GI microbiome profiles in MCL versus controls, and to evaluate variation across disease states and therapies.

### Methods

We analyzed 228 stool samples: 136 from MCL patients and 92 from close contact controls. Patients with other GI diseases or antibiotic exposure within one month were excluded. Sampling reflected the spectrum of MCL management: observation (OBS), immediately prior to first-line therapy (1L-PRE), in remission after first-line therapy (1L-POST), and relapsed/refractory (RR). Shotgun metagenomic sequencing (NovaSeq X) was performed. After quality control and host depletion, taxonomic and functional profiles were generated. Differential abundance was assessed using multivariable models with FDR correction ($q \leq 0.10$). Alpha diversity (Shannon) and beta diversity (Bray–Curtis) were evaluated using ANOVA and PERMANOVA.

**Copyright Information:** (Online) ISSN: 2572-9241
©2026 The Author(s). HemaSphere is published by John Wiley & Sons Ltd on behalf of European Hematology Association. This is an open access Abstract Book distributed under the Attribution-NonCommercial-NoDerivs (CC BY-NC-ND), which allows third parties to download the articles and share them with others as long as they credit the author and the Abstract Book, but they cannot change the content in any way or use them commercially.
**Abstract Book Citations:** Authors, Title, HemaSphere, 2026;10:(S1):pages. The abstract book DOIs can be found on the “Author Information” page.
**Disclaimer:** Articles published in the journal HemaSphere exclusively reflect the opinions of the authors. The authors are responsible for all content in their abstracts including accuracy of the facts, statements, citing resources, etc.

### Results

Among 136 MCL patients (median age 69 years; 67% male), samples were derived from 15 (11%) on OBS, 18 (13%) 1L-PRE, 52 (38%) 1L-POST, and 51 (38%) RR. Compared with controls, MCL patients had reduced alpha diversity (Shannon difference 0.3, $p<0.001$) and distinct taxonomic and functional composition (PERMANOVA $p<0.001$) (Figure 1A). Across five groups (controls, OBS, 1L-PRE, 1L-POST, RR), alpha diversity differed overall (ANOVA F=2.7, p=0.01), with trends toward lower diversity in active disease states (Figure 1B). Within patients, multivariable models did not show significant overall diversity differences across disease states.

Beta diversity differed between patients and controls for taxonomy (F=2.0, $p<0.001$; $R^2\approx0.03$) and function (F=2.1, $p<0.001$; $R^2\approx0.03$). Global PERMANOVA across all groups remained significant (both $p<0.001$), though pairwise contrasts between disease states were non-significant.

Taxonomically, disease-associated differences centered on Enterocloster-related species, with additional shifts in Flavonifractor plautii and multiple Blautia taxa (FDR≤0.10). Functionally, broader alterations were observed, including branched-chain amino acid biosynthesis pathways, histidine metabolism, membrane-associated zinc metalloproteases, and undecaprenyl diphosphate synthase (Figure 1C).

Alpha diversity was lower in 1L-PRE versus OBS (−0.5, SE 0.2, $p_{adj}<0.05$) and higher in 1L-POST versus 1L-PRE (0.4, SE 0.2, $p_{adj}<0.05$), though PERMANOVA across these states was non-significant. BTK inhibitor exposure (p=0.03) and cellular therapy (ASCT, CAR-T) (p=0.03) were independently associated with differences in alpha diversity, modest taxonomic variation, and limited functional effects.

### Summary/Conclusion

MCL is associated with GI microbiome alterations characterized by reduced diversity, distinct community structure, and marked functional pathway shifts. Functional changes, particularly in amino acid metabolism, provide a stronger disease-associated signal than taxonomy alone. These findings establish the first microbiome reference framework in MCL, support incorporation of microbiome endpoints into prospective studies, and identify biologically plausible pathways for therapeutic intervention.

**Copyright Information:** (Online) ISSN: 2572-9241
©2026 The Author(s). HemaSphere is published by John Wiley & Sons Ltd on behalf of European Hematology Association. This is an open access Abstract Book distributed under the Attribution-NonCommercial-NoDerivs (CC BY-NC-ND), which allows third parties to download the articles and share them with others as long as they credit the author and the Abstract Book, but they cannot change the content in any way or use them commercially.
**Abstract Book Citations:** Authors, Title, HemaSphere, 2026;10:(S1):pages. The abstract book DOIs can be found on the “Author Information” page.
**Disclaimer:** Articles published in the journal HemaSphere exclusively reflect the opinions of the authors. The authors are responsible for all content in their abstracts including accuracy of the facts, statements, citing resources, etc.

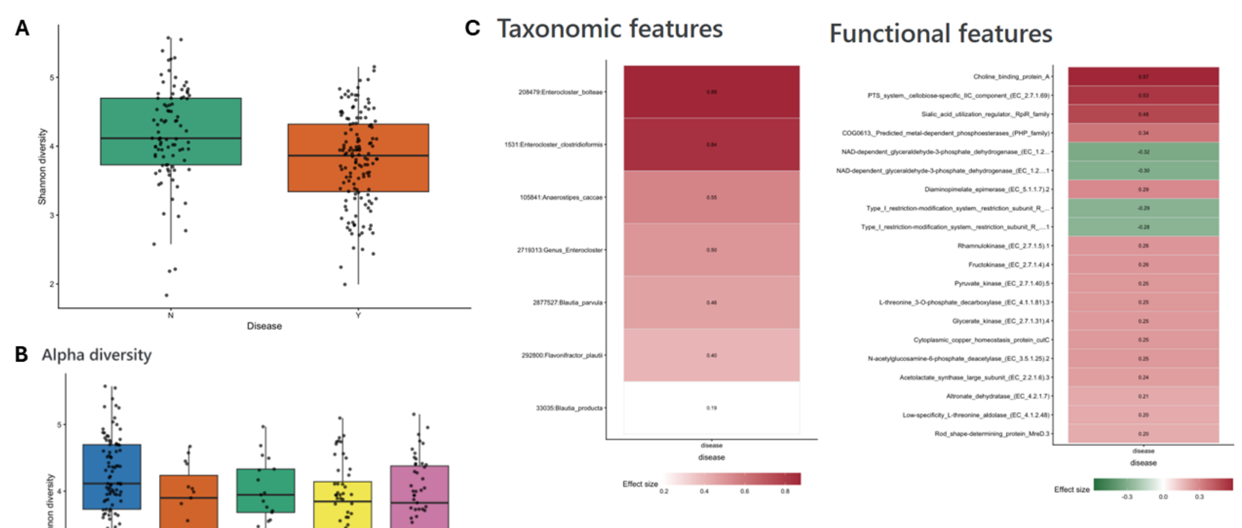

**Figure 1**

A. Alpha diversity in healthy controls vs MCL patients
B. Alpha diversity across disease states and controls
C. Effect sizes of taxonomic and functional features in MCL patients vs healthy controls

**Copyright Information:** (Online) ISSN: 2572-9241
©2026 The Author(s). HemaSphere is published by John Wiley & Sons Ltd on behalf of European Hematology Association. This is an open access Abstract Book distributed under the Attribution-NonCommercial-NoDerivs (CC BY-NC-ND), which allows third parties to download the articles and share them with others as long as they credit the author and the Abstract Book, but they cannot change the content in any way or use them commercially.
**Abstract Book Citations:** Authors, Title, HemaSphere, 2026;10:(S1):pages. The abstract book DOIs can be found on the "Author Information" page.
**Disclaimer:** Articles published in the journal HemaSphere exclusively reflect the opinions of the authors. The authors are responsible for all content in their abstracts including accuracy of the facts, statements, citing resources, etc.

### (PF1099) INTEGRATIVE TRANSCRIPTOMIC PROFILING REVEAL THE MOLECULAR LANDSCAPE AND REGULATORY DRIVERS OF BLASTOID MANTLE CELL LYMPHOMA

**Topic:** 21. Lymphoma biology & translational research

Lu Jiang[1], Xiaozhong Shen[1], Yaohui Huang[1], Yan Yu[1], Weili Zhao[1], Li Wang*[1]

*[1]Shanghai Institute of Hematology, State Key Laboratory of Medical Genomics, National Research Center for Translational Medicine at Shanghai, Ruijin Hospital, Shanghai Jiao Tong University School of Medicine, Shanghai, China;*

### Background

Mantle cell lymphoma (MCL) is a heterogeneous B-cell malignancy. The blastoid variant (bMCL) is characterized by intense proliferative activity and an aggressive clinical course compared to classical MCL (cMCL). However, the molecular boundaries between these subtypes remain partially defined, and morphological diagnosis can be subjective, necessitating a more robust and objective molecular classification framework.

### Aims

To develop a high-performance molecular classifier for MCL subtypes and identify the core transcriptomic signatures and transcription factor (TF) networks that drive the aggressive phenotype of bMCL.

### Methods

We performed bulk RNA-seq on a cohort of MCL patients clinically stratified into Blastoid and Classical subtypes. A Random Forest (RF) classifier was developed using Leave-One-Out Cross-Validation (LOOCV) to identify diagnostic markers. To provide cross-platform validation, a 100-gene "Blastoid Signature" was derived from our single-cell RNA-seq (scRNA-seq) dataset of aggressive MCL clusters (MCL-6, -7, -8), with BCR/TCR and ribosomal signals excluded. Enrichment scores were calculated using the latest GSVA (ssGSEA) framework. Regulatory networks were inferred using decoupleR with the CollecTRI database, and functional pathways were assessed via GSEA.

### Results

The Random Forest classifier achieved excellent performance with an AUC of 0.899 (LOOCV), identifying 30 key features. These top markers were predominantly involved in cell cycle progression and DNA damage repair. The scRNA-seq derived signature was significantly enriched in the bulk bMCL group compared to cMCL (Wilcoxon $P = 0.0022$), confirming the consistency of aggressive features across biological scales. GSEA revealed significant upregulation of E2F targets, MYC targets, and G2/M checkpoint pathways in bMCL, while immune effector processes were downregulated. TF activity analysis via decoupleR identified ELK4, HMGA2, and E2F family members as top regulatory drivers ($p < 0.05$) of the blastoid transition, suggesting a profound rewiring of the proliferative transcriptional program.

### Summary/Conclusion

Our study establishes a robust molecular classifier that accurately distinguishes bMCL from cMCL. By integrating bulk and single-cell transcriptomics, we demonstrate that the bMCL phenotype is driven by a coordinated hijacking of cell cycle regulatory networks and TF-mediated metabolic rewiring. These findings provide a molecular basis for refined risk stratification and highlight specific TF-driven proliferative hubs as potential therapeutic targets for aggressive MCL.

**Copyright Information:** (Online) ISSN: 2572-9241
©2026 The Author(s). HemaSphere is published by John Wiley & Sons Ltd on behalf of European Hematology Association. This is an open access Abstract Book distributed under the Attribution-NonCommercial-NoDerivs (CC BY-NC-ND), which allows third parties to download the articles and share them with others as long as they credit the author and the Abstract Book, but they cannot change the content in any way or use them commercially.
**Abstract Book Citations:** Authors, Title, HemaSphere, 2026;10:(S1):pages. The abstract book DOIs can be found on the "Author Information" page.
**Disclaimer:** Articles published in the journal HemaSphere exclusively reflect the opinions of the authors. The authors are responsible for all content in their abstracts including accuracy of the facts, statements, citing resources, etc.

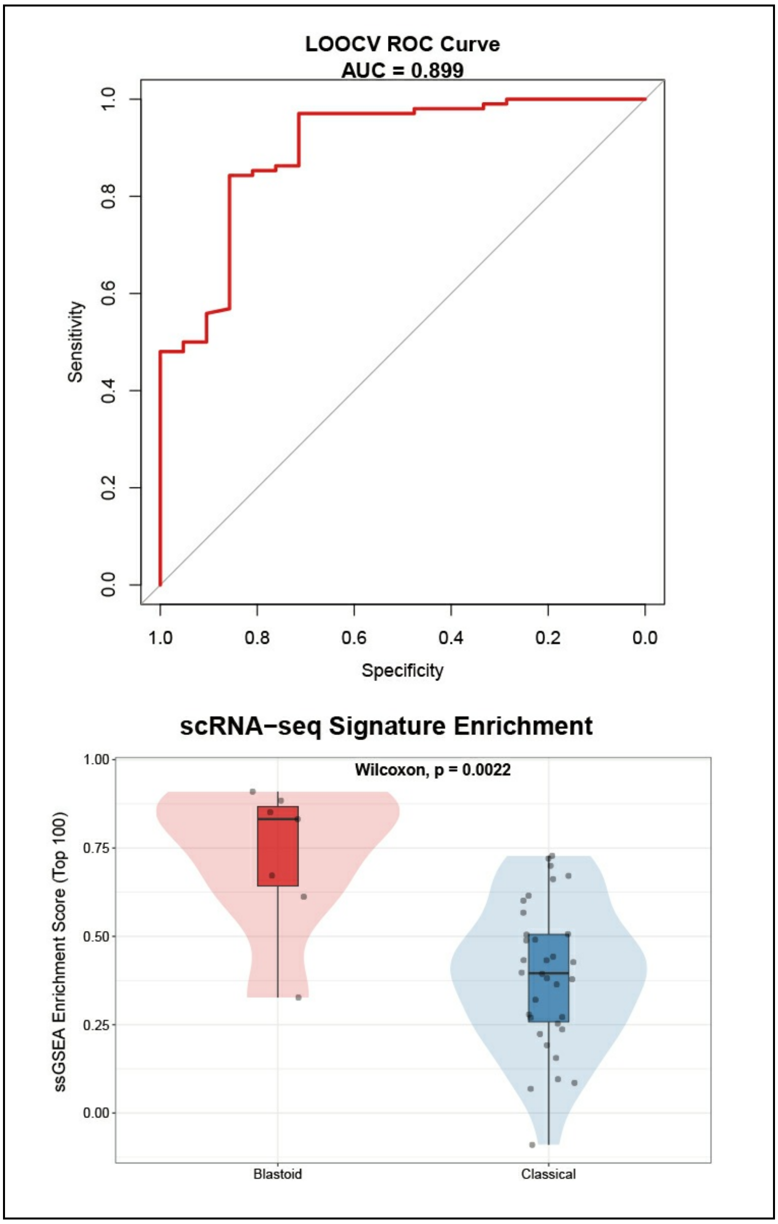

Copyright Information: (Online) ISSN: 2572-9241
©2026 The Author(s). HemaSphere is published by John Wiley & Sons Ltd on behalf of European Hematology Association. This is an open access Abstract Book distributed under the Attribution-NonCommercial-NoDerivs (CC BY-NC-ND), which allows third parties to download the articles and share them with others as long as they credit the author and the Abstract Book, but they cannot change the content in any way or use them commercially.
Abstract Book Citations: Authors, Title, HemaSphere, 2026;10:(S1):pages. The abstract book DOIs can be found on the "Author Information" page.
Disclaimer: Articles published in the journal HemaSphere exclusively reflect the opinions of the authors. The authors are responsible for all content in their abstracts including accuracy of the facts, statements, citing resources, etc.

### (PF1100) CLINICAL AND MOLECULAR CHARACTERISTICS AND THEIR IMPACT ON PROGNOSIS IN CHINESE PEDIATRIC MATURE B-CELL NON-HODGKIN LYMPHOMA

**Topic:** 21. Lymphoma biology & translational research

Yang Li[1], Ling Jin[2], Ying Liu[3], Keyan Yang[1], Yanlong Duan[2], Xiaowen Zhai[4], Meng Zhang[2], Pan Wu[5], Jianwen Zhou[6], Ansheng Liu[7], Xiaomei Yang[8], Xiaojun Yuan[9], Yunpeng Dai[10], Yueping Jia[11], Jian Jiang[12], Jian Wang[13], Rong Liu[14], Baoxi Zhang[15], Qinlong Zheng*[1], Yonghong Zhang[3]

*[1]Beijing GoBroad Boren Hospital, Molecular Laboratory Diagnostics, Beijing, China;[2]National Center for Children's Health, Beijing Children's Hospital, Capital Medical University, Department of Hematology/Oncology, Beijing, China; [3]Beijing GoBroad Boren Hospital, Department of Pediatric Lymphoma, Beijing, China; [4]Children's Hospital of Fudan University, National Center for Children's Health, Department of Hematology &Oncology, Shanghai, China; [5]Hunan Children's Hospital, Department of Hematology, Changsha, China; [6]Zhengzhou Children's Hospital, Department of Hematology & Oncology, Zhengzhou, China; [7]Xi'an Children's Hospital, Department of Hematologic Oncology, Xi'an, China;[8]Shandong University Affiliated Hospital (Jinan Children's Hospital), Department of Pediatric Hematology/Oncology, Jinan, China; [9]Xinhua Hospital, Shanghai Jiao Tong University School of Medicine, Department of Pediatric Hematology/Oncology, Shanghai, China; [10]Shandong Provincial Hospital Affiliated to Shandong University, Department of Pediatrics Hematology and Endocrinology, Jinan, China; [11]Peking University People's Hospital, Department of Pediatrics, Beijing, China; [12]Affiliated Hospital of Qingdao University, Department of Pediatric Hematology and Oncology, Qingdao, China; [13]Anhui Provincial Children's Hospital, Department of Hematology and Oncology, Hefei, China; [14]Capital institute of pediatric, Department of Hematology/Oncology, Beijing, China;[15]The Second Hospital of Hebei Medical University, Department of Pediatrics, Shijiazhuang, China;*

### Background

Mature B-cell non-Hodgkin lymphoma (MB-NHL) is an important subtype of pediatric non-Hodgkin lymphoma. A deeper understanding of the genetic profiles of these patients may help optimize clinical management. However, large-cohort studies focusing on Chinese pediatric populations remain limited.

### Aims

This study aimed to investigate the impact of clinical characteristics and genetic mutations on clinical outcomes in Chinese pediatric patients with MB-NHL, to inform individualized treatment strategies.

### Methods

This retrospective study included 1,368 patients with MB-NHL (1116 male/252 female; aged ≤ 18 years, median age 7 years), who were treated at multiple centers of the China Network of Childhood Lymphoma (CNCL) from 2017 to 2025. Targeted next-generation sequencing (NGS) using a panel covering 262 lymphoma-associated genes was performed on tumor samples from 177 patients. Survival analyses (Kaplan-Meier method and log-rank tests) were used to evaluate the relationships between clinical and molecular indicators and prognosis.

**Copyright Information:** (Online) ISSN: 2572-9241
©2026 The Author(s). HemaSphere is published by John Wiley & Sons Ltd on behalf of European Hematology Association. This is an open access Abstract Book distributed under the Attribution-NonCommercial-NoDerivs (CC BY-NC-ND), which allows third parties to download the articles and share them with others as long as they credit the author and the Abstract Book, but they cannot change the content in any way or use them commercially.
**Abstract Book Citations:** Authors, Title, HemaSphere, 2026;10:(S1):pages. The abstract book DOIs can be found on the "Author Information" page.
**Disclaimer:** Articles published in the journal HemaSphere exclusively reflect the opinions of the authors. The authors are responsible for all content in their abstracts including accuracy of the facts, statements, citing resources, etc.

**Results**

Clinical data and outcomes were available for 1,286 of the 1,368 patients in this cohort. Patients with bone marrow (BM) involvement (EFS: $p < 0.0001$; OS: $p < 0.0001$) or central nervous system (CNS) involvement (EFS: $p < 0.0001$; OS: $p < 0.0001$) demonstrated significantly poorer prognosis than those without these features. Furthermore, patients with CNS3 stage disease had a significantly worse prognosis than those with CNS1 or CNS2 disease (EFS: $p < 0.0001$; OS: $p < 0.0001$, Fig.1a).
Targeted NGS results from 177 patients were analyzed to characterize the genetic profile of Chinese pediatric MB-NHL. Mutations were identified in 170 patients (96%), involving a total of 147 genes. A total of 769 somatic mutations were identified, with an average of 4.3 mutations per patient (range: 1-13). Of these, 131 (74%) harbored three or more mutations. The most frequently mutated genes were *TP53* (46%), *ID3* (42%), *ARID1A* (26%), *CCND3* (24%) and *DDX3X* (21%). Comparative analysis of genetic mutations based on distinct clinical features revealed that mutations in *TP53*, *ID3*, and *ARID1A* were significantly enriched in BL, while *SOCS1* and *STAT6* mutations were significantly enriched in DLBCL. Additionally, *TP53* and *ID3* showed significant enrichment in patients with CNS3 stage disease, and *ID3* mutations were significantly enriched in patients with BM involvement.
Prognostic analysis indicated that patients with mutations in *TP53* (EFS: $p < 0.0001$; OS: $p = 0.0019$, Fig.1b), *ID3* (EFS: $p = 0.0095$; OS: $p = 0.038$, Fig.1c), *ARID1A* (EFS: $p = 0.023$; OS: $p = 0.037$, Fig.1d), and *KMT2D* (EFS: $p = 0.013$; OS: $p = 0.027$, Fig.1e) had a significantly poorer prognosis compared to those without these mutations. Furthermore, we assessed the cumulative prognostic impact of these four genes. Patients with mutations in more than two of these four genes had significantly worse outcomes than those with two or fewer mutations (EFS: $p < 0.0001$; OS: $p = 0.0024$, Fig.1f).

**Summary/Conclusion**

This study demonstrates that BM involvement and CNS involvement are significant adverse prognostic factors in Chinese pediatric MB-NHL. Mutations in *TP53*, *ID3*, *ARID1A*, and *KMT2D* were associated with poor outcomes. The cumulative number of mutations in these four genes effectively stratified patients into distinct prognostic groups, highlighting its potential for refining risk stratification. (Yang Li and Ling Jin contributed equally, Qinlong Zheng and Yonghong Zhang contributed equally).

**Copyright Information:** (Online) ISSN: 2572-9241
©2026 The Author(s). HemaSphere is published by John Wiley & Sons Ltd on behalf of European Hematology Association. This is an open access Abstract Book distributed under the Attribution-NonCommercial-NoDerivs (CC BY-NC-ND), which allows third parties to download the articles and share them with others as long as they credit the author and the Abstract Book, but they cannot change the content in any way or use them commercially.
**Abstract Book Citations:** Authors, Title, HemaSphere, 2026;10:(S1):pages. The abstract book DOIs can be found on the "Author Information" page.
**Disclaimer:** Articles published in the journal HemaSphere exclusively reflect the opinions of the authors. The authors are responsible for all content in their abstracts including accuracy of the facts, statements, citing resources, etc.

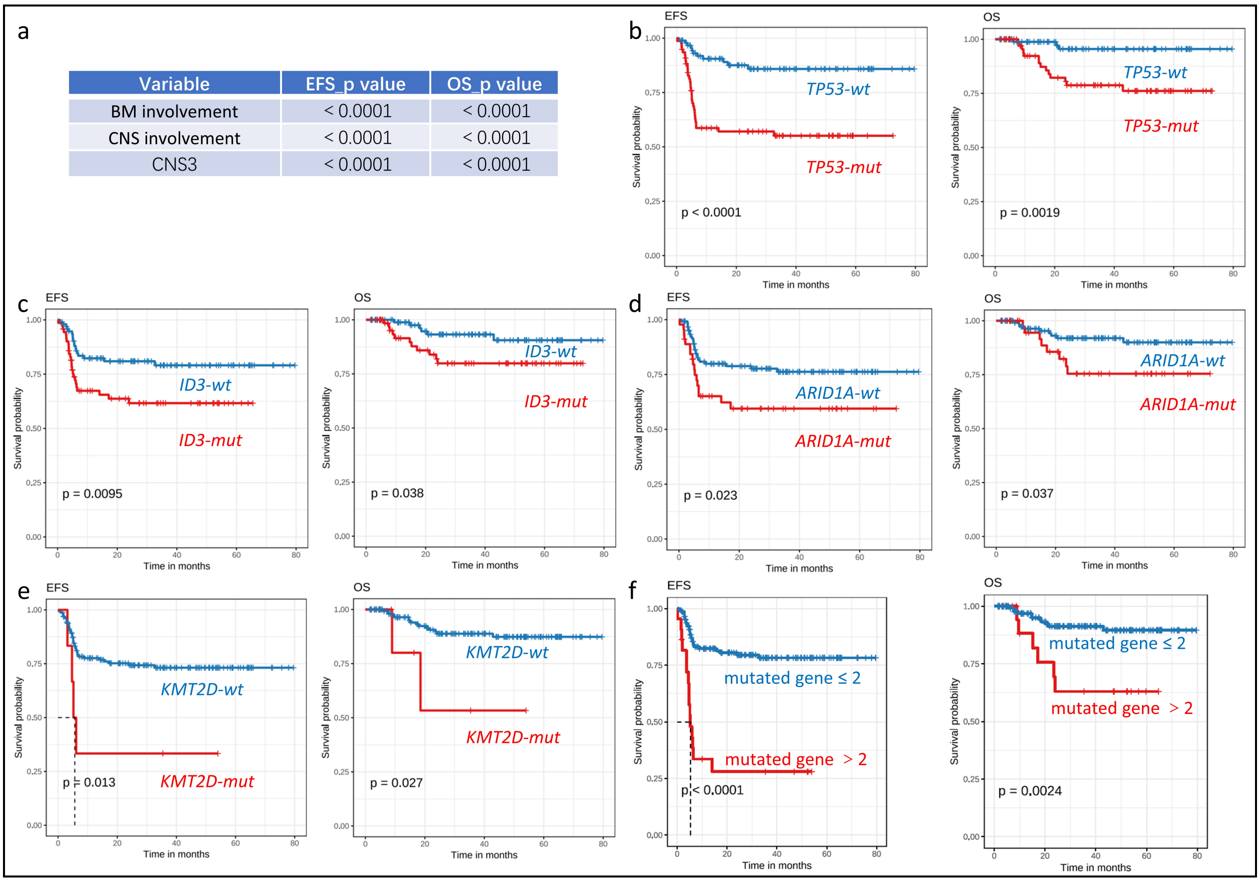

| Variable | EFS_p value | OS_p value |
|---|---|---|
| BM involvement | < 0.0001 | < 0.0001 |
| CNS involvement | < 0.0001 | < 0.0001 |
| CNS3 | < 0.0001 | < 0.0001 |

Figure 1. Clinical and Molecular Characteristics and Their Impact on Prognostic in Chinese Pediatric Patients with MB-NHL.

**Copyright Information:** (Online) ISSN: 2572-9241
©2026 The Author(s). HemaSphere is published by John Wiley & Sons Ltd on behalf of European Hematology Association. This is an open access Abstract Book distributed under the Attribution-NonCommercial-NoDerivs (CC BY-NC-ND), which allows third parties to download the articles and share them with others as long as they credit the author and the Abstract Book, but they cannot change the content in any way or use them commercially.
**Abstract Book Citations:** Authors, Title, HemaSphere, 2026;10:(S1):pages. The abstract book DOIs can be found on the "Author Information" page.
**Disclaimer:** Articles published in the journal HemaSphere exclusively reflect the opinions of the authors. The authors are responsible for all content in their abstracts including accuracy of the facts, statements, citing resources, etc.

### (PF1101) WHOLE-GENOME SEQUENCING REVEALS DISTINCT MOLECULAR CLASSIFICATION PATTERNS IN DIFFUSE LARGE B-CELL LYMPHOMA IN CHINESE PATIENTS

**Topic:** 21. Lymphoma biology & translational research

Mingyu Yang*[1, 2, 3], Weicheng Ren[1], Man Nie[4], Xianhuo Wang[5], Hui Wan[1], Xilong Tang[2], Dongbing Liu[2, 3], Xiaofei Ye[6], Zhiming Li[4], Hui-Lai Zhang[5], Kui Wu[2, 3], Qiang Pan-Hammarstrom[1, 5]

*[1]Karolinska institutet, Division of Immunology, Department of Medical Biochemistry and Biophysics, Stockholm, Sweden;[2]BGI-research, State Key Laboratory of Genome and Multi-omics Technologies, Shenzhen, China; [3]BGI-Genomics, Shenzhen, China; [4]Sun Yat-Sen University Cancer Center, Department of Medical Oncology, State Key Laboratory of Oncology in South China, Collaborative Innovation Center for Cancer Medicine, Guangzhou, China; [5]Tianjin Medical University Cancer Institute and Hospital, Department of Lymphoma, Tianjing, China; [6]Kindstar Global Precision Medicine Institute, Wuhan, China;*

### Background

Diffuse large B-cell lymphoma (DLBCL) is the most common aggressive B-cell non-Hodgkin's lymphoma and exhibits genetic and clinical heterogeneity. Traditionally, DLBCL has been classified into germinal center B-cell–like (GCB) and activated B-cell–like (ABC) subtypes based on cell-of-origin. More recently, whole-exome sequencing (WES)-based molecular classification systems, including DLBCL-Classifier (DLBclass) and LymphGen, have stratified DLBCL into five to seven genetic subtypes, respectively. These frameworks define genetic entities such as MCD (C5), BN2 (C1), N1, EZB-MYC+ (C3), EZB-MYC− (C3), ST2 (C4), and A53 (C2), each characterized by recurrent genetic alterations affecting key oncogenic pathways, including NF-κB signaling, B-cell receptor signaling, chromatin modification, and TP53 dysfunction. However, most of these classification frameworks were largely derived from Western cohorts, and potential differences in the prevalence, genomic architecture, and clinical relevance of genetic subtypes in Chinese patients remain insufficiently characterized.

### Aims

To investigate population-specific molecular heterogeneity in DLBCL and assess whether existing genomic classification frameworks accurately capture the genetic architecture and clinical implications of DLBCL in Chinese patients.

### Methods

We performed non-negative matrix factorization (NMF)-based clustering on 385 DLBCL Whole-genome sequencing (WGS) samples, integrating recurrent driver mutations, focal copy number alterations, and structural variants to define molecular subtypes.

### Results

Seven molecular subtypes were identified in Chinese DLBCL, each characterized by distinct genomic alteration patterns and clinical outcomes: CH-C1 (CNV-deletion); CH-C2 (*KMT2D, IGLL5, CREBBP* mutations); CH-C3 (*KLF2, BTG2, SGK1, TNFAIP3, CD70, ZFP36L1*, and *SOCS1* mutations); CH-C4 (defined by *BCL6*-associated translocations and *BCL6*/*UBE2A* mutations); CH-C5 (characterized by recurrent copy number gains and *TP53* mutations); CH-C6 (*PIM1, MYD88, CD79B* mutations) and CH-C7 (*BCL2*/*EZH2* mutations and BCL2/*MYC* translocations). Most subtypes corresponded to established LymphGen/DLBclass categories, whereas the CNV-deletion cluster may represent a novel molecular subgroup. Compared with Western cohorts, the distribution of molecular subtypes exhibited population-specific differences. In the Chinese cohort, frequencies were CH-C1 (~6%), CH-C2 (C3/EZB-like, ~24%), CH-C3 (C1/4/BN2/ST2-like, ~24%), CH-C4 (C1/BN2-like, ~6%), CH-C5 (C2/A53-like, ~21%), CH-C6 (C5/MCD-like, ~13%), and CH-C7 (C3/EZB-like, ~5%). In the Western cohort (DLBclass), corresponding subtype frequencies were C3/EZB-like (18%), C4/ST2-like (17%), C1/BN2-like (18%), C2/A53-like (22%), and C5/MCD-like (21%).

**Copyright Information:** (Online) ISSN: 2572-9241
©2026 The Author(s). HemaSphere is published by John Wiley & Sons Ltd on behalf of European Hematology Association. This is an open access Abstract Book distributed under the Attribution-NonCommercial-NoDerivs (CC BY-NC-ND), which allows third parties to download the articles and share them with others as long as they credit the author and the Abstract Book, but they cannot change the content in any way or use them commercially.
**Abstract Book Citations:** Authors, Title, HemaSphere, 2026;10:(S1):pages. The abstract book DOIs can be found on the "Author Information" page.
**Disclaimer:** Articles published in the journal HemaSphere exclusively reflect the opinions of the authors. The authors are responsible for all content in their abstracts including accuracy of the facts, statements, citing resources, etc.

### Summary/Conclusion

WGS identified an additional CNV-deletion–dominant molecular subtype in Chinese DLBCL that is not captured by current classification frameworks. Moreover, the distribution of molecular subtypes differed between Chinese and Western cohorts, highlighting population-specific genomic architecture and the need for regionally informed classification strategies in DLBCL.

**Copyright Information:** (Online) ISSN: 2572-9241
©2026 The Author(s). HemaSphere is published by John Wiley & Sons Ltd on behalf of European Hematology Association. This is an open access Abstract Book distributed under the Attribution-NonCommercial-NoDerivs (CC BY-NC-ND), which allows third parties to download the articles and share them with others as long as they credit the author and the Abstract Book, but they cannot change the content in any way or use them commercially.
**Abstract Book Citations:** Authors, Title, HemaSphere, 2026;10:(S1):pages. The abstract book DOIs can be found on the “Author Information” page.
**Disclaimer:** Articles published in the journal HemaSphere exclusively reflect the opinions of the authors. The authors are responsible for all content in their abstracts including accuracy of the facts, statements, citing resources, etc.

### (PF1102) THE NEUROENDOCRINE-METABOLIC-IMMUNE AXIS IN B-CELL LYMPHOMA: ROLE OF ACBP/DBI IN GLUCOCORTICOID RESISTANCE AND IMMUNOSUPPRESSION

**Topic:** 21. Lymphoma biology & translational research

Hui Pan*[1], Shengyan Zhao[2], Ting Niu[1]

*[1]Department of Hematology, Institute of Hematology,and Center for High Altitude Medicine, West China Hospital, Sichuan University, Chengdu, China; [2]Department of Biotherapy, Cancer Center and State Key Laboratory of Biotherapy/Collaborative Innovation Center for Biotherapy, West China Hospital, Sichuan University, Chengdu, China;*

### Background

B-cell lymphoma (BCL) is a highly heterogeneous hematologic malignancy with 30-40% of patients exhibiting resistance to current treatments, involving synergistic effects of tumor metabolic reprogramming and immunosuppressive microenvironment. Stress hormones glucocorticoids (GCs) construct a complex "neuroendocrine-metabolic-immune" regulatory network by inducing acyl-CoA binding protein (ACBP/DBI) release, which acts on GABA_A receptor to upregulate TSC22D3 in dendritic cells (DCs), thereby inhibiting antigen presentation. However, the role of this axis in BCL, particularly in acquired GCs resistance and immune escape, remains unexplored.

### Aims

This study aims to elucidate how ACBP functions as a central hub mediating metabolic adaptation in BCL cells and microenvironment immunosuppression, and to explore dual-function therapeutic strategies targeting ACBP.

### Methods

Integrating multi-omics, single-cell sequencing, preclinical models, and clinical samples: 1) Lipidomics and metabolic flux analysis of ACBP knockout in GCs-resistant/sensitive lymphoma cells; CRISPR/Cas9 validation of ACBP in GCs-induced apoptosis resistance. 2) Eμ-Myc transgenic lymphoma models and CD11c-Cre; ACBP^(fl/fl) conditional knockout mice, combined with scRNA-seq and CUT&Tag to dissect GCs-ACBP regulation on DC differentiation (cDC1), lipid reprogramming (PPARγ-FABP4), and epigenetic modifications (TSC22D3-mediated H3K27me3). 3) Human/mouse cross-reactive anti-ACBP monoclonal antibodies (αACBP mAb) via phage display, evaluated with R-CHOP or CD19 CAR-T in GCs-resistant BCL PDX models.

### Results

1) ACBP/DBI expression was highest in lymphoma cell lines among all tested. In DLBCL patients, ACBP positively correlated with NR3C1; plasma ACBP decreased significantly post-treatment and was pre-elevated in cancer-prone individuals. 2) GCs upregulated Acbp/Dbi, Nr3c1, and Tsc22d3 in DCs while inhibiting cDC1 marker Irf8 and type I IFN signaling; αACBP mAb reversed these effects, restoring DC antigen presentation. 3) Genetic epistasis confirmed that DC Tsc22d3 knockout and ACBP inhibition act on the same pathway to alleviate GCs-mediated immunosuppression. 4) αACBP mAb restored cDC1 proportion in BCL-loaded DCs and enhanced CD8+ T cell activation.

**Copyright Information:** (Online) ISSN: 2572-9241
©2026 The Author(s). HemaSphere is published by John Wiley & Sons Ltd on behalf of European Hematology Association. This is an open access Abstract Book distributed under the Attribution-NonCommercial-NoDerivs (CC BY-NC-ND), which allows third parties to download the articles and share them with others as long as they credit the author and the Abstract Book, but they cannot change the content in any way or use them commercially.
**Abstract Book Citations:** Authors, Title, HemaSphere, 2026;10:(S1):pages. The abstract book DOIs can be found on the "Author Information" page.
**Disclaimer:** Articles published in the journal HemaSphere exclusively reflect the opinions of the authors. The authors are responsible for all content in their abstracts including accuracy of the facts, statements, citing resources, etc.

**Summary/Conclusion**

ACBP/DBI is a key molecule connecting neuroendocrine signals (GCs), tumor metabolic reprogramming, and DC dysfunction. GCs induce ACBP release, promoting tumor lipid metabolic adaptation and GCs resistance while driving DCs toward tolerogenic phenotype, constructing an immunosuppressive microenvironment. Dual-function ACBP targeting (blocking intracellular metabolism and extracellular immunosuppression) may concurrently reverse GCs resistance and restore antitumor immunity, providing a foundation for precision therapy in relapsed/refractory BCL. The proposed "ACBP-metabolism-immunity" score offers a potential biomarker for personalized treatment.

**Copyright Information:** (Online) ISSN: 2572-9241
©2026 The Author(s). HemaSphere is published by John Wiley & Sons Ltd on behalf of European Hematology Association. This is an open access Abstract Book distributed under the Attribution-NonCommercial-NoDerivs (CC BY-NC-ND), which allows third parties to download the articles and share them with others as long as they credit the author and the Abstract Book, but they cannot change the content in any way or use them commercially.
**Abstract Book Citations:** Authors, Title, HemaSphere, 2026;10:(S1):pages. The abstract book DOIs can be found on the "Author Information" page.
**Disclaimer:** Articles published in the journal HemaSphere exclusively reflect the opinions of the authors. The authors are responsible for all content in their abstracts including accuracy of the facts, statements, citing resources, etc.

### (PF1104) CLONAL COMPLEXITY DEFINES DISTINCT TUMOR-INTRINSIC BIOLOGY AND PROGNOSIS IN DIFFUSE LARGE B-CELL LYMPHOMA

**Topic:** 21. Lymphoma biology & translational research

Takahiro Haeno*[1, 2], Kazuko Sakai[1, 3], Shuji Minamoto[1, 2], Daiki Nakatsu[1], Marco De Velasco[1], Shinya Rai[2], Hirokazu Tanaka[2], Itaru Matsumura[2], Kazuto Nishio[1, 3]

*[1]Kindai University Faculty of Medicine, Department of Genome Biology, Osaka, Japan;[2]Kindai University Faculty of Medicine, Department of Hematology and Rheumatology, Osaka, Japan; [3]Kindai University Faculty of Medicine, Center for Genomics, Life Science Research Institute, Osaka, Japan;*

**Background**

Intratumor heterogeneity (ITH) is increasingly recognized as a determinant of clinical outcome across cancers, yet its practical assessment remains challenging, particularly in routine biopsy specimens. We developed a simple method to quantify ITH from copy number variation (CNVs) data derived from formalin-fixed, paraffin-embedded (FFPE) samples, generating a Clonal Composition (CC) number that estimates the number of coexisting subclones. This approach was previously validated in ovarian cancer and hepatocellular carcinoma, where higher CC numbers were associated with inferior outcomes. Here, we apply this framework to diffuse large B-cell lymphoma (DLBCL), a disease with established inter-patient genetic heterogeneity but unresolved intratumor evolutionary complexity.

**Aims**

We aimed to determine whether ITH can be quantitatively assessed in DLBCL, and to evaluate its association with the gene expression profiles, mutational landscape, tumor micro environment, and clinical outcomes, and to elucidate its impact on disease biology.

**Methods**

Gene expression, copy number analyses and gene mutation analysis were performed in patients with newly diagnosed advanced-stage DLBCL using FFPE tumor specimens. CC number was calculated using a clustering approach that clusters all segments with same the B-allele frequency (BAF) and log2 ratio (log2R) values obtained from whole-genome copy number assay and then combines clusters corresponding the same percentage of aberrant cells (%AC). The correlations between CC number and biological, clinical characteristics, and prognosis were analyzed (Kindai University Faculty of Medicine Ethics Committee: Approval number R03-283).

**Copyright Information:** (Online) ISSN: 2572-9241
©2026 The Author(s). HemaSphere is published by John Wiley & Sons Ltd on behalf of European Hematology Association. This is an open access Abstract Book distributed under the Attribution-NonCommercial-NoDerivs (CC BY-NC-ND), which allows third parties to download the articles and share them with others as long as they credit the author and the Abstract Book, but they cannot change the content in any way or use them commercially.
**Abstract Book Citations:** Authors, Title, HemaSphere, 2026;10:(S1):pages. The abstract book DOIs can be found on the “Author Information” page.
**Disclaimer:** Articles published in the journal HemaSphere exclusively reflect the opinions of the authors. The authors are responsible for all content in their abstracts including accuracy of the facts, statements, citing resources, etc.

**Results**

Using our approach, we could estimate the tumor CC number in 74 of the 86 patients (86.0%) initially enrolled in our study. Of the 74 cases, 15 were classified as Mono-CC (CC= 0), and 59 were classified as Poly-CC (CC=1,2,3). At a median follow-up time of 47.5 months (range, 0-116, months), patients with Poly-CC tumors (CC≥1) had a 5-year event-free survival rate of 41.5%, compared with 71.1% in those with Mono-CC tumors (CC = 0) (Log-rank p=0.079, Figure 1A). This association reached statistical significance in the activated B-cell (ABC) subtype (n=35, p=0.0450; 83.3% vs 31.5%, Figure 1 B) but not in the germinal center B-cell (GCB) subtype (n=30, p=0.66; 55.5% vs 50.0%). Gene set enrichment analysis revealed upregulation of cell cycle–related pathways in Poly-CC tumors, consistent with the significantly higher Ki-67 positivity rate than in Mono-CC tumors, as confirmed by immunohistochemistry (p=0.00227). Within the ABC subtype, Poly-CC (Poly-ABC) tumors exhibited more differentiated transcriptional states and enrichment of IRF4-associated gene signatures as compared with Mono-CC (Mono-ABC) tumors. Conversely, IFN-γ and IFN-α response pathways and IL-6/JAK-STAT3 signaling pathway, were markedly suppressed in the Poly-ABC tumors. Furthermore, Poly-ABC tumors carried a significantly higher number of pathogenic mutations as compared with Mono-ABC tumors (p=0.0147).

**Summary/Conclusion**

These results suggest that clonal complexity captures tumor-intrinsic features and biological diversity in DLBCL, especially in ABC subtype, offering novel insights into the disease pathogenesis.

**Copyright Information:** (Online) ISSN: 2572-9241
©2026 The Author(s). HemaSphere is published by John Wiley & Sons Ltd on behalf of European Hematology Association. This is an open access Abstract Book distributed under the Attribution-NonCommercial-NoDerivs (CC BY-NC-ND), which allows third parties to download the articles and share them with others as long as they credit the author and the Abstract Book, but they cannot change the content in any way or use them commercially.
**Abstract Book Citations:** Authors, Title, HemaSphere, 2026;10:(S1):pages. The abstract book DOIs can be found on the “Author Information” page.
**Disclaimer:** Articles published in the journal HemaSphere exclusively reflect the opinions of the authors. The authors are responsible for all content in their abstracts including accuracy of the facts, statements, citing resources, etc.

Figure 1

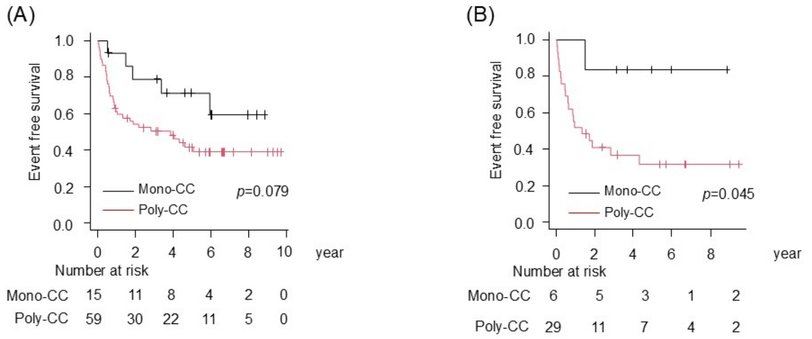

(A) Kaplan-Meier curves for event-free survival (EFS) based on the clonal composition. (B) Kaplan-Meier curves for EFS based on the clonal composition in patient with ABC-DLBCL; the p values listed on each graph were calculated by the log-rank test.

**Copyright Information:** (Online) ISSN: 2572-9241
©2026 The Author(s). HemaSphere is published by John Wiley & Sons Ltd on behalf of European Hematology Association. This is an open access Abstract Book distributed under the Attribution-NonCommercial-NoDerivs (CC BY-NC-ND), which allows third parties to download the articles and share them with others as long as they credit the author and the Abstract Book, but they cannot change the content in any way or use them commercially.
**Abstract Book Citations:** Authors, Title, HemaSphere, 2026;10:(S1):pages. The abstract book DOIs can be found on the "Author Information" page.
**Disclaimer:** Articles published in the journal HemaSphere exclusively reflect the opinions of the authors. The authors are responsible for all content in their abstracts including accuracy of the facts, statements, citing resources, etc.

### (PF1105) TARGETING RNF20 INHIBITS THE PROGRESSION OF DIFFUSE LARGE B-CELL LYMPHOMA THROUGH REGULATING UBIQUITINATION-DEPENDENT PHASE SEPARATION OF FUS

**Topic:** 21. Lymphoma biology & translational research

Qianqian Guo*[1], Xiaoli Zhou[1], Xiangxiang Zhou[1], Shunfeng Hu[1]

[1]*Shandong Provincial Hospital Affiliated to Shandong First Medical University, Jinan, China;*

### Background

RNF20, an E3 ubiquitin ligase, plays a critical role in transcriptional regulation, chromatin remodeling, and tumorigenesis. Liquid-liquid phase separation (LLPS) is a biophysical process that drives the formation of membraneless condensates, thereby organizing intracellular biochemical reactions in space and time.

### Aims

This study aims to explore the prognostic value and functional significance of RNF20 in diffuse large B-cell lymphoma (DLBCL) and its mechanism on LLPS.

### Methods

Lymph node biopsies from 140 DLBCL patients and 41 cases of reactive lymphoid hyperplasia were collected with informed consent. Co-immunoprecipitation (CO-IP), mass spectrometry, and ChIP sequencing were performed. LLPS was assessed by confocal microscopy and fluorescence recovery after photobleaching (FRAP). Full-length and domain-truncated constructs of RNF20 and FUS were generated, and ubiquitination-deficient FUS mutants (K316R, K334R, and K365R) were created by site mutagenesis for functional analyses. The xenograft DLBCL mouse model was established.

**Copyright Information:** (Online) ISSN: 2572-9241
©2026 The Author(s). HemaSphere is published by John Wiley & Sons Ltd on behalf of European Hematology Association. This is an open access Abstract Book distributed under the Attribution-NonCommercial-NoDerivs (CC BY-NC-ND), which allows third parties to download the articles and share them with others as long as they credit the author and the Abstract Book, but they cannot change the content in any way or use them commercially.
**Abstract Book Citations:** Authors, Title, HemaSphere, 2026;10:(S1):pages. The abstract book DOIs can be found on the "Author Information" page.
**Disclaimer:** Articles published in the journal HemaSphere exclusively reflect the opinions of the authors. The authors are responsible for all content in their abstracts including accuracy of the facts, statements, citing resources, etc.

### Results

Firstly, RNF20 expression was significantly elevated in DLBCL tissues (n = 140) compared with reactive lymphoid hyperplasia controls (n = 41), and high RNF20 expression was associated with poor prognosis in DLBCL patients (n = 134, $p$ = 0.0001). Functionally, stable RNF20 overexpression and RNF20 knockdown cell lines were established via lentiviral transduction. RNF20 knockdown inhibited cell proliferation, induced apoptosis and G2/M phase arrest, whereas RNF20 overexpression promoted cell growth. In vivo, RNF20 deficiency suppressed tumor growth in xenograft models. Notably, RNF20 knockdown significantly enhanced the sensitivity of DLBCL cells to ibrutinib both in vitro and in vivo.

Mechanistically, Co-IP combined with mass spectrometry identified FUS as a key RNF20-interacting protein, which was validated by Co-IP and confocal microscopy. Domain truncation plasmid of RNF20 and FUS were constructed and RNF20 was found to interact with FUS through its CC1 domain, while the RRM domain of FUS was responsible for this interaction. Ubiquitination assays further revealed that RNF20 promoted K63-linked ubiquitination of FUS at the K334 site in a RING domain-dependent manner. Rescue experiments showed that RING domain truncation of RNF20 abolished the ubiquitination of FUS, whereas mutation of K334 of FUS significantly reduced RNF20-mediated ubiquitination.

Importantly, RNF20 overexpression enhanced FUS condensate formation and accelerated FRAP recovery. Both the K334 mutation of FUS and the RING-domain truncation of RNF20 impaired FUS LLPS and reduced fluorescence recovery dynamics in DLBCL cells, indicating that RNF20 promoted FUS phase separation through RING-dependent ubiquitination at K334. ChIP-seq analyses further showed that RNF20 overexpression enhanced the binding of phase-separated FUS to chromatin, particularly at genes involved in cell cycle regulation.

### Summary/Conclusion

In summary, we firstly identified the prognostic value of RNF20 and uncovered that RNF20 promoted DLBCL progression by inducing ubiquitination-dependent phase separation of FUS.

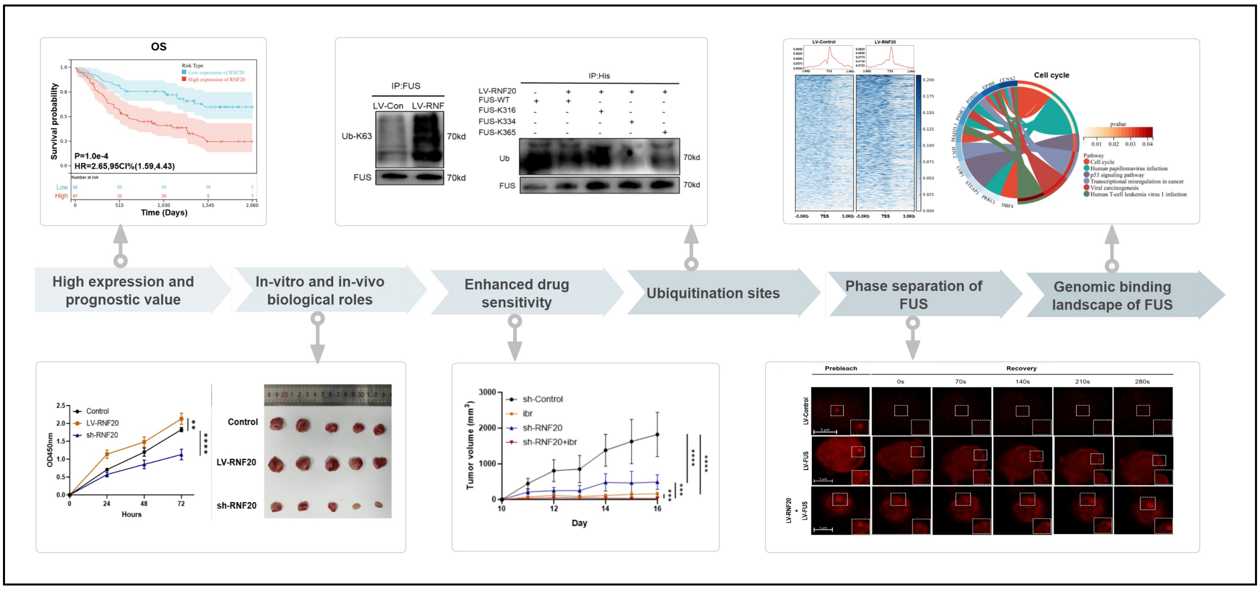

**Copyright Information:** (Online) ISSN: 2572-9241
©2026 The Author(s). HemaSphere is published by John Wiley & Sons Ltd on behalf of European Hematology Association. This is an open access Abstract Book distributed under the Attribution-NonCommercial-NoDerivs (CC BY-NC-ND), which allows third parties to download the articles and share them with others as long as they credit the author and the Abstract Book, but they cannot change the content in any way or use them commercially.
**Abstract Book Citations:** Authors, Title, HemaSphere, 2026;10:(S1):pages. The abstract book DOIs can be found on the "Author Information" page.
**Disclaimer:** Articles published in the journal HemaSphere exclusively reflect the opinions of the authors. The authors are responsible for all content in their abstracts including accuracy of the facts, statements, citing resources, etc.

### (PF1106) PHASED VARIANTS REVEAL PROTO-ONCOGENE PROMOTER HYPERMUTATION AND CFDNA FRAGMENTATION PATTERNS IN DLBCL

**Topic:** 21. Lymphoma biology & translational research

Luca Varga*[1, 2], Péter Király[3], Márk Plander[4], Réka Ráhel Bicskó[5], Miklós Egyed[6], Tímea Gurbity Pálfi[7], Zsófia Zsuzsanna Simon[8], Gabriella Szepesi[1, 2], Nóra Szoboszlai[1, 2], Nikolett Szeifer[1, 2], Richárd Hanza[1, 2], Róbert Horváth[9], Tamás László[1, 2], Tamás Schneider[3], Szilamér Kovács[4], Tamás Szendrei[4], Hermina Sánta[10], Arpad Batai[11], Árpád Szomor[10], Lajos Gergely[5], Laszlo Rejto[12], Zita Borbenyi[7], Zsolt Nagy[13], Masszi Tamás Pál[13], Erika Tóth[3], Réka Mózes[9], Judit Csomor[9], András Matolcsy[9], Donát Alpár[1, 2], Botond Timár[1, 2, 9], Andras Masszi[3], Bence Bátai[1, 2, 13], Csaba Bödör[1, 2]

*[1]Semmelweis University, HCEMM-SE Molecular Oncohematology Research Group, Budapest, Hungary; [2]Semmelweis University, MTA-SE Momentum Molecular Oncohematology Research Group, Budapest, Hungary; [3]National Institute of Oncology, Budapest, Hungary; [4]Vas Vármegyei Markusovszky Egyetemi Oktató Kórház, Szombathely, Hungary; [5]University of Debrecen, Division of Hematology, Department of Internal Medicine, Debrecen, Hungary; [6]Kaposi Mór University Teaching Hospital of County Somogy, Kaposvár, Hungary; [7]University of Szeged, Szeged, Hungary; [8]St. Borbála Hospital, Tatabánya, Hungary; [9]Semmelweis University, Department of Pathology and Experimental Cancer Research, Budapest, Hungary; [10]Fejér County Szent György University Teaching Hospital, Székesfehérvár, Hungary; [11]Central Hospital of Southern Pest, Budapest, Hungary; [12]Hospitals of County Szabolcs-Szatmár-Bereg and University Teaching Hospital, Nyíregyháza, Hungary; [13]Semmelweis University, Department of Internal Medicine and Haematology, Budapest, Hungary;*

### Background

Diffuse large B-cell lymphoma (DLBCL) is a clinically and molecularly heterogeneous disease for which robust, widely applicable patient-stratification tools are still lacking. Analysis of circulating tumor DNA (ctDNA) has emerged as a promising approach for disease monitoring, yet its utility for molecular characterization and stratification remains incompletely understood. Phased variants (PVs) – closely spaced somatic mutations occurring in cis – represent stable, region-specific genomic features that may provide additional insight into DLBCL biology and improve ctDNA-based stratification.

### Aims

Our goal was to characterise a nationwide real-world DLBCL cohort to improve our understanding of the disease's molecular landscape and investigate the applicability of cfDNA for molecular profiling and patient stratification.

### Methods

In Hungarian oncohematological centres 138 newly diagnosed DLBCL patients were prospectively enrolled in liquid biopsy collection between 2021-2023, of which 116 had corresponding diagnostic FFPE samples available. We performed targeted next generation DNA sequencing using a hybrid-capture Agilent XT HS2 panel spanning 251 genes, 4 translocation breakpoints and genome-wide CNA calling using off-target reads on FFPE samples and a smaller, 98 gene panel for ctDNA profiling involving highly-mutated regions in >5% of lymphoma genomes (Kurtz et al., 2021) for phased variant identification. Variant calling and analysis were performed using a custom pipeline.

**Copyright Information:** (Online) ISSN: 2572-9241
©2026 The Author(s). HemaSphere is published by John Wiley & Sons Ltd on behalf of European Hematology Association. This is an open access Abstract Book distributed under the Attribution-NonCommercial-NoDerivs (CC BY-NC-ND), which allows third parties to download the articles and share them with others as long as they credit the author and the Abstract Book, but they cannot change the content in any way or use them commercially.
**Abstract Book Citations:** Authors, Title, HemaSphere, 2026;10:(S1):pages. The abstract book DOIs can be found on the "Author Information" page.
**Disclaimer:** Articles published in the journal HemaSphere exclusively reflect the opinions of the authors. The authors are responsible for all content in their abstracts including accuracy of the facts, statements, citing resources, etc.

### Results

Of the 116 patients with FFPE available, 50.8% was successfully classified into LymphGen subtypes, which increased to 62.9% with the inclusion of variants detected in cfDNA. Notably, 7/57 unclassified (*Other*) cases were reclassified as MCD and 4/57 as EZB subtype, highlighting potential therapeutic targets. Median 111.5 (range: 17-286) phased variants were identified in tumor tissue samples. In 86 of the 116 cases, PVs identified from tumor tissue DNA were present in cfDNA as well, with a median of 58.4% of PVs recalled from the cfDNA compartment. PVs were most commonly found in *IGK*, *IGHM* regions, and the *BCL6*, *BCL2*, *PIM1*, *MYC*, *BCL7A*, *CXCR4*, *TMSB4X* genes. With the exception of *MYC*, the majority of PVs affected 5'UTR or promoter regions. We determined ctDNA fragmentation levels using normalised Shannon entropy on fragment lengths, which revealed ctDNA to be significantly more fragmented to wild-type DNA and total cfDNA. Gene-level analysis of cfDNA fragmentation revealed that *BCL2* and *MYC*, which showed low PV recall, are characterized by low expression in whole blood and high expression in lymphocytes based on GTEx data. Consequently, high background cfDNA abundance masks ctDNA-derived fragmentation signals, impairing phased variant detection in these key driver genes.

### Summary/Conclusion

CfDNA has been shown to be a viable and robust tool to help patient care in several cancers. CtDNA carries potentially relevant information on the disease; in our case, LymphGen classification improved with the use of ctDNA variants and parallel analysis of ctDNA and tumor tissue DNA allocated cases to therapeutically relevant LymphGen subtypes. Aberrant hypermutation in proto-oncogene promoter sites have been described in DLBCL and utilised as PVs for disease monitoring. We demonstrated that regions with lower cfDNA fragmentation and higher cfDNA amounts have worse PV recall, highlighting caveats to improve further in the non-invasive genotyping of DLBCL.

**Copyright Information:** (Online) ISSN: 2572-9241
©2026 The Author(s). HemaSphere is published by John Wiley & Sons Ltd on behalf of European Hematology Association. This is an open access Abstract Book distributed under the Attribution-NonCommercial-NoDerivs (CC BY-NC-ND), which allows third parties to download the articles and share them with others as long as they credit the author and the Abstract Book, but they cannot change the content in any way or use them commercially.
**Abstract Book Citations:** Authors, Title, HemaSphere, 2026;10:(S1):pages. The abstract book DOIs can be found on the "Author Information" page.
**Disclaimer:** Articles published in the journal HemaSphere exclusively reflect the opinions of the authors. The authors are responsible for all content in their abstracts including accuracy of the facts, statements, citing resources, etc.

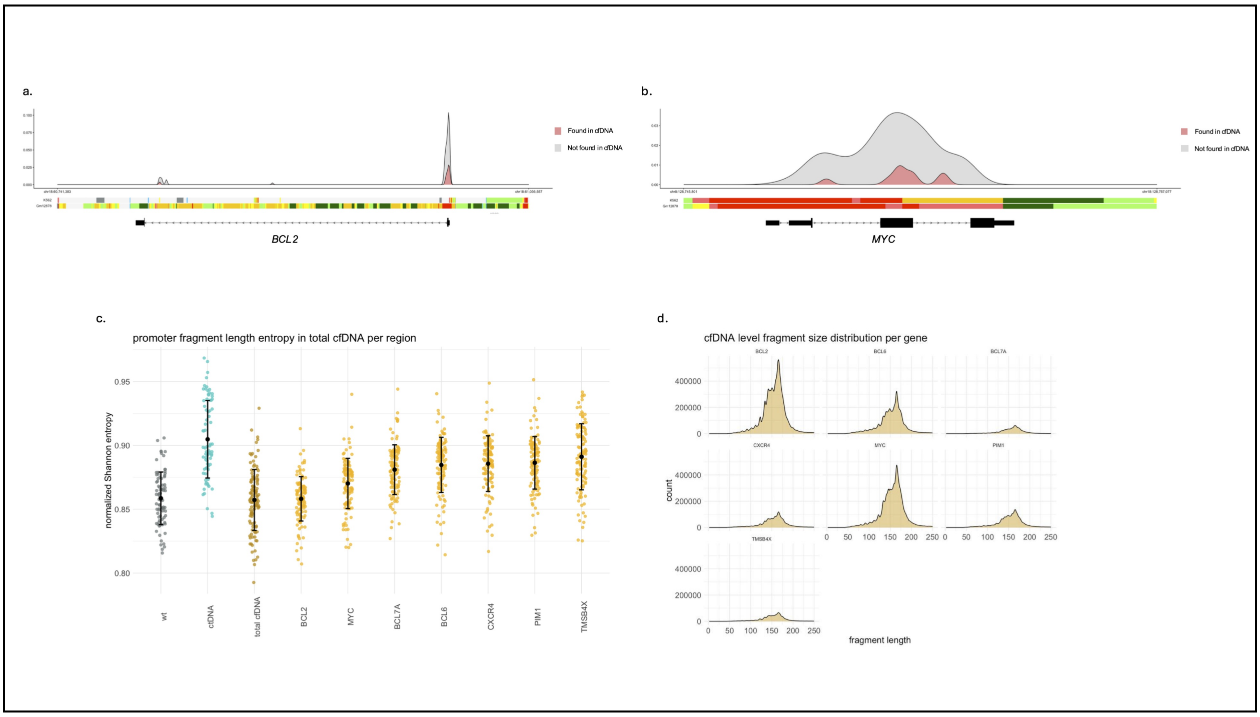

a: PV distribution in the BCL2 gene. The majority of PVs were detected in the promoter region. The red peaks represent PVs recalled from cfDNA, the overall recall percentage was 19.20%. b: PV distribution in the MYC gene, red peaks represent PVs recalled from cfDNA. PVs were detected across the entire gene, with 8.74% overall recall from cfDNA. c: total cfDNA fragment length entropy of frequently hypermutated genes compared to wild-type cfDNA (grey), PV containing ctDNA fragments (blue), total cfDNA (light brown). d: cfDNA fragment sizes and cfDNA amounts in frequently hypermutated regions. MYC and BCL2 show reduced fragmentation compared to other genes and elevated cfDNA amounts.

**Copyright Information:** (Online) ISSN: 2572-9241
©2026 The Author(s). HemaSphere is published by John Wiley & Sons Ltd on behalf of European Hematology Association. This is an open access Abstract Book distributed under the Attribution-NonCommercial-NoDerivs (CC BY-NC-ND), which allows third parties to download the articles and share them with others as long as they credit the author and the Abstract Book, but they cannot change the content in any way or use them commercially.
**Abstract Book Citations:** Authors, Title, HemaSphere, 2026;10:(S1):pages. The abstract book DOIs can be found on the "Author Information" page.
**Disclaimer:** Articles published in the journal HemaSphere exclusively reflect the opinions of the authors. The authors are responsible for all content in their abstracts including accuracy of the facts, statements, citing resources, etc.

### (PF1107) PROTEOLYSIS-TARGETING CHIMERA (PROTAC) TARGETING BRUTON TYROSINE KINASE (BTK) DEGRADATION IN MANTLE CELL LYMPHOMA

**Topic:** 21. Lymphoma biology & translational research

Xiaohui Wang*[1], Huilai Zhang[1]

[1] *Tianjin Medical University Cancer Institute and Hospital, Tianjin, China;*

### Background

Mantle cell lymphoma (MCL) is characterized by Bruton tyrosine kinase (BTK)-signaling which activates the B-cell receptor (BCR) signaling cascade. Although BTK-inhibitors are effective therapy, acquired resistance is common. Proteolysis-targeting chimera molecules (PROTACs) facilitating BTK-degradation (BTK-PROTAC) are a potential therapy.

### Aims

The objective of this study was to develop and characterize novel BTK-directed proteolysis-targeting chimeras and to evaluate their antitumor activity in mantle cell lymphoma. Specifically, we aimed to identify a potent BTK-PROTAC capable of efficiently degrading BTK, suppressing BCR-signaling, and overcoming acquired resistance to BTK inhibitors driven by BTK variants, and to validate its therapeutic efficacy and underlying mechanisms in both in vitro and in vivo MCL models.

### Methods

We designed 24 BTK-PROTACs and screened their activity against MCL cell lines through half maximal inhibitory concentration (IC50) assays and BTK-degradation efficiency. Effects of BTK-PROTAC on differentially expressed genes and on activation of the BCR-signaling cascade were studied by RNA-seq and molecular experiments. BTK variant cells were developed to assess the ability of BTK-PROTAC to reverse acquired BTK-inhibitor resistance *in vitro* assays and in mouse models.

### Results

Amongst BTK-PROTACs we synthesized, C23 had the strongest ability to degrade BTK and inhibit proliferation of several MCL cell lines. C23 induced cell apoptosis and suppressed cancer growth by activating the ubiquitin–proteasome pathway, thereby inhibiting the BCR-signaling cascade including NF-κB and PI3K–AKT–mTOR signaling. Notably, compared with BGB-16673 and NX-2127, C23 not only efficiently degraded BTK but also induced the degradation of IKZF1 and IKZF3. C23 degraded BTK and inhibited the growth of BTK^C481S and BTK^L528W variant cells, and suppressed tumor growth in mouse xenograft models harboring BTK^C481S and BTK^L528W variants.

### Summary/Conclusion

The C23 BTK-PROTAC inhibits MCL cells with BTK variants resistant to BTK-inhibitors *in vitro* and *in vivo* models. BTK-PROTAC C23 is a potential therapy of BTK-inhibitor resistant MCL.

**Copyright Information:** (Online) ISSN: 2572-9241
©2026 The Author(s). HemaSphere is published by John Wiley & Sons Ltd on behalf of European Hematology Association. This is an open access Abstract Book distributed under the Attribution-NonCommercial-NoDerivs (CC BY-NC-ND), which allows third parties to download the articles and share them with others as long as they credit the author and the Abstract Book, but they cannot change the content in any way or use them commercially.
**Abstract Book Citations:** Authors, Title, HemaSphere, 2026;10:(S1):pages. The abstract book DOIs can be found on the "Author Information" page.
**Disclaimer:** Articles published in the journal HemaSphere exclusively reflect the opinions of the authors. The authors are responsible for all content in their abstracts including accuracy of the facts, statements, citing resources, etc.

### (PF264) A LIVING THERAPEUTIC PLATFORM FOR LOCALIZED IN SITU MODULATION OF MACROPHAGE PYROPTOSIS AMELIORATES GVHD WHILE PRESERVING GVL

**Topic:** 22. Stem cell transplantation - Experimental

Zhuo Wan*[1], Yueru Ji[1], Xueqian Yan[1], Li Liu[1], Weiwei Qin[1]

[1] *Tangdu hospital of Fourth military medical university, Hematology, Xi'an, China;*

### Background

Acute graft-versus-host disease (aGVHD) is a major complication of allogeneic hematopoietic stem cell transplantation, with the gastrointestinal tract as a primary target organ. Current management relies on systemic immunosuppression, which may impair graft-versus-leukemia (GVL) activity.Macrophage NLRP3 inflammasome activation and pyroptosis are increasingly associated with intestinal GVHD pathology through amplification of local inflammatory signaling. However, approaches enabling localized modulation of intestinal inflammasome activity while preserving systemic anti-tumor immunity remain lacking.

### Aims

To characterize macrophage NLRP3 inflammasome activation and pyroptosis-related signaling in intestinal GVHD and assess the potential of a gut-targeted therapeutic platform for localized inflammasome modulation to attenuate GVHD without impairing graft-versus-leukemia (GVL) activity.

### Methods

Single-cell RNA sequencing data from intestinal biopsies of GVHD patients were analyzed to characterize macrophage-associated inflammatory programs.A pharmacophore-guided virtual screening approach was applied to an in-house peptide library to identify candidate inhibitors targeting the NLRP3 NACHT domain. Binding affinity was assessed by surface plasmon resonance (SPR), and functional effects were evaluated in LPS/nigericin-stimulated murine bone marrow–derived macrophages.For localized delivery, Escherichia coli Nissle 1917 was genetically engineered to enhance outer membrane vesicle (OMV) production and reduce endotoxin activity. An arabinose-inducible construct expressing ClyA-fused BigLEN (EcNSmart) was generated for gut-restricted delivery.Therapeutic efficacy was evaluated in murine allo-HSCT models of acute GVHD, including a graft-versus-leukemia model using luciferase-expressing A20 lymphoma cells.

### Results

Single-cell transcriptomic analysis demonstrated enrichment of inflammasome and pyroptosis-associated gene signatures in intestinal macrophages from GVHD patients, with increased expression of NLRP3 and IL1B correlating with disease severity.BigLEN was identified as a high-affinity NLRP3 NACHT-binding peptide (KD $1.7\times10^{-7}$ M). In vitro, BigLEN inhibited ASC oligomerization and macrophage pyroptosis following LPS/nigericin stimulation. Intravenous administration reduced early clinical scores but did not improve survival, suggesting insufficient tissue-targeted delivery.The engineered strain EcNSmart preferentially localized to the colon after oral administration, and OMVs were internalized by lamina propria macrophages. In murine allo-HSCT models, EcNSmart treatment significantly improved survival and reduced intestinal histopathological injury compared with free peptide or wild-type EcN. Colonic ASC oligomerization, caspase-1 activation, GSDMD cleavage, LDH release, and macrophage membrane permeability were markedly reduced.EcNSmart-treated mice exhibited decreased intestinal and splenic Th1/Th17 frequencies without alteration of global CD69 expression. In a leukemia co-transplant model, anti-tumor control was maintained, indicating preservation of GVL activity.

**Copyright Information:** (Online) ISSN: 2572-9241
©2026 The Author(s). HemaSphere is published by John Wiley & Sons Ltd on behalf of European Hematology Association. This is an open access Abstract Book distributed under the Attribution-NonCommercial-NoDerivs (CC BY-NC-ND), which allows third parties to download the articles and share them with others as long as they credit the author and the Abstract Book, but they cannot change the content in any way or use them commercially.
**Abstract Book Citations:** Authors, Title, HemaSphere, 2026;10:(S1):pages. The abstract book DOIs can be found on the “Author Information” page.
**Disclaimer:** Articles published in the journal HemaSphere exclusively reflect the opinions of the authors. The authors are responsible for all content in their abstracts including accuracy of the facts, statements, citing resources, etc.

**Summary/Conclusion**

A programmable, OMV-based probiotic platform enables localized modulation of NLRP3 inflammasome activity in the gut, significantly alleviating GVHD without impairing GVL activity. This approach offers a promising strategy for targeted immunomodulation in GVHD, with potential applications in other mucosal inflammatory diseases.

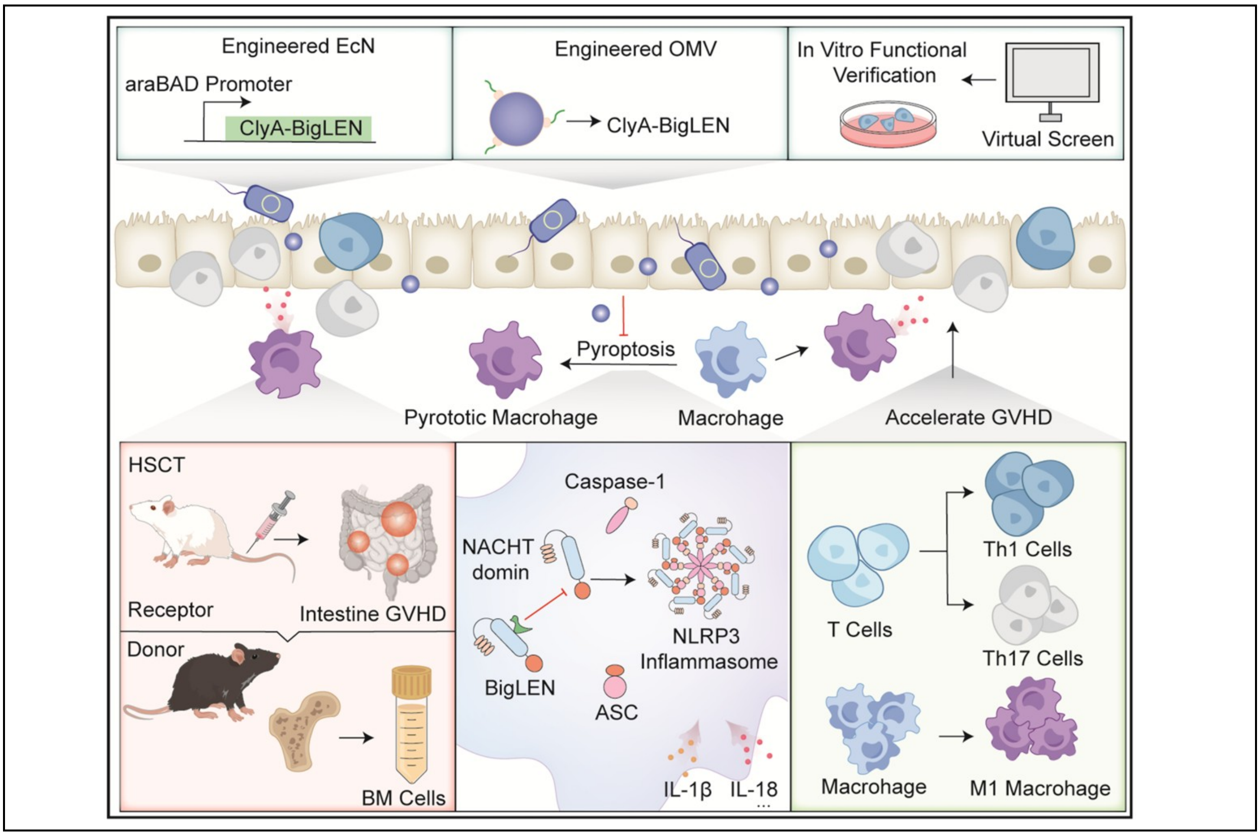

**Copyright Information:** (Online) ISSN: 2572-9241
©2026 The Author(s). HemaSphere is published by John Wiley & Sons Ltd on behalf of European Hematology Association. This is an open access Abstract Book distributed under the Attribution-NonCommercial-NoDerivs (CC BY-NC-ND), which allows third parties to download the articles and share them with others as long as they credit the author and the Abstract Book, but they cannot change the content in any way or use them commercially.
**Abstract Book Citations:** Authors, Title, HemaSphere, 2026;10:(S1):pages. The abstract book DOIs can be found on the "Author Information" page.
**Disclaimer:** Articles published in the journal HemaSphere exclusively reflect the opinions of the authors. The authors are responsible for all content in their abstracts including accuracy of the facts, statements, citing resources, etc.

### (PF1108) ALLOGENEIC TRANSPLANTATION: DISEASE RESPONSE AND COMPARATIVE TREATMENT STUDIES

**Topic:** 22. Stem cell transplantation - Experimental

Zhe Zou[1], Yangwei Li[1], Xiaodong Lyu*[1]

[1]*Henan Cancer Hospital, Central Laboratory, Zhengzhou, China;*

### Background

Chimerism (CHIM) analysis following allogeneic hematopoietic stem cell transplantation (aHSCT) is critical for assessing engraftment and predicting relapse risk in hematological malignancies. The current gold standard, short tandem repeat (STR) analysis, has limited sensitivity, with a detection limit of 1–5%, rendering low-level CHIM undetectable and potentially overlooking prognostic indicators. To address this limitation, we developed a novel next-generation sequencing (NGS)-based method, termed Multiple Single Nucleotide Polymorphism sequencing (MSNPseq). MSNPseq targets 100 DNA fragments (~200 bp each), each containing 4–8 single nucleotide polymorphism (SNP) loci.

### Aims

Our results demonstrate that MSNPseq offers superior sensitivity and specificity compared to STR, enabling the reclassification of patients with undetectable CHIM by STR and refining their CHIM status for improved prognostic accuracy.

### Methods

MSNPseq is based on Ion AmpliSeq technology to amplify 100 fragments for sequencing on the Ion Torrent PGM platform, achieving a sequencing depth >2000× for 96.8% of loci. The sensitivity and accuracy of MSNPseq were validated using simulated chimeric samples and post-transplant clinical samples, benchmarked against STR analysis. Calibration models were developed using pre-transplant sequencing data from donors and recipients to derive correction coefficients tailored for clinical applications. Assessments were conducted at three time points (TPs), spaced 30 days apart, following aHSCT, with ethical approval from participating hospitals and informed consent from all patients. Progression-free survival (PFS) and overall survival (OS) were analyzed across patient groups based on CHIM status to evaluate transplant outcomes, predict prognosis, and detect relapse at later stages.

### Results

In a cohort of 741 patients post-aHSCT, MSNPseq detected complete donor chimerism (cCHIM, 100% donor) in 201, 238, and 239 patients at TP 1, 2, and 3 (30-day intervals), respectively. Subthreshold donor chimerism (sCHIM, 95% to <100% donor), a range where STR may lack sufficient detection accuracy, was observed in 339, 301, and 288 patients, microchimerism (mCHIM, 99% to <100% donor) in 200, 191, and 180 patients, and incomplete donor chimerism (iCHIM, ≤95% donor) in 17, 18, and 30 patients at TP 1, 2, and 3, respectively. Patients with iCHIM exhibited significantly worse PFS and OS compared to those with cCHIM or sCHIM at all TPs. OS was also significantly worse in the sCHIM group versus the cCHIM group at TP 2 ($p = 0.001$) and TP 3 ($p = 0.013$), with a trend at TP 1 ($p = 0.06$). Similarly, patients with mCHIM showed significantly worse OS than those with cCHIM at TP 2 ($p < 0.001$), with a trend at TP 3 ($p = 0.11$). Interestingly, among patients with sCHIM, those with mCHIM exhibited distinct prognostic differences compared to other patients, underscoring the need for further classification within this group. These findings highlight the critical importance of MSNPseq, as sCHIM and mCHIM are frequently misclassified as cCHIM by STR, potentially impacting risk stratification and therapeutic outcomes.

**Copyright Information:** (Online) ISSN: 2572-9241
©2026 The Author(s). HemaSphere is published by John Wiley & Sons Ltd on behalf of European Hematology Association. This is an open access Abstract Book distributed under the Attribution-NonCommercial-NoDerivs (CC BY-NC-ND), which allows third parties to download the articles and share them with others as long as they credit the author and the Abstract Book, but they cannot change the content in any way or use them commercially.
**Abstract Book Citations:** Authors, Title, HemaSphere, 2026;10:(S1):pages. The abstract book DOIs can be found on the “Author Information” page.
**Disclaimer:** Articles published in the journal HemaSphere exclusively reflect the opinions of the authors. The authors are responsible for all content in their abstracts including accuracy of the facts, statements, citing resources, etc.

**Summary/Conclusion**

MSNPseq's superior sensitivity over STR enables precise chimerism stratification, revealing prognostic differences in sCHIM and mCHIM that are often missed by traditional methods. This enhanced detection capability can significantly improve risk assessment and guide therapeutic decisions post-aHSCT, potentially improving patient outcomes.

**Copyright Information:** (Online) ISSN: 2572-9241
©2026 The Author(s). HemaSphere is published by John Wiley & Sons Ltd on behalf of European Hematology Association. This is an open access Abstract Book distributed under the Attribution-NonCommercial-NoDerivs (CC BY-NC-ND), which allows third parties to download the articles and share them with others as long as they credit the author and the Abstract Book, but they cannot change the content in any way or use them commercially.
**Abstract Book Citations:** Authors, Title, HemaSphere, 2026;10:(S1):pages. The abstract book DOIs can be found on the "Author Information" page.
**Disclaimer:** Articles published in the journal HemaSphere exclusively reflect the opinions of the authors. The authors are responsible for all content in their abstracts including accuracy of the facts, statements, citing resources, etc.

### (PF1109) ALLOGENEIC HSC TRANSPLANTATION INCREASES ACETYLCHOLINE PRODUCTION BY DONOR-DERIVED LYMPHOCYTES, WHICH RESTRAINS T CELL ACTIVATION AND EXPANSION VIA ALPHA7 NICOTINIC RECEPTOR - IMPLICATIONS FOR GVHD

**Topic:** 22. Stem cell transplantation - Experimental

Jose Antonio Bejarano Garcia*[1, 2, 3], Michal Remen[4], Massimo Andreis[4], Lam Khuat[1, 3], Claudia Mosquera Rubal[5], Luis Espinosa-Hevia[5], Gabriel Jiménez[2], Thomas Henderson[2, 6], José Quesada-Salas[2, 6], Guillermo Oliva-Ariza[2], Johannes Tossounidis[4, 7], Jakob Passweg[7], Joerg Halter[7], Perez Simon Josè Antonio[2, 8], Rafael Duarte[5], Petya Apostolova[4, 7], Simón Méndez-Ferrer[1, 2, 3, 6]

*[1]Department of Haematology, University of Cambridge, Cambridge, United Kingdom;[2]University Hospital Virgen del Rocío, Instituto de Biomedicina de Sevilla (IBIS)/CSIC, Seville, Spain; [3]NHS Blood and Transplant, Cambridge, United Kingdom; [4]Department of Biomedicine, University and University Hospital, Basel, Switzerland; [5]Department of Hematology, Hospital Universitario Puerta de Hierro Majadahonda, Madrid, Spain; [6]Department of Medical Physiology and Biophysics, University of Seville, Seville, Spain; [7]Division of Hematology, University Hospital Basel, Basel, Switzerland; [8]Department of Hematology, University of Seville, Seville, Spain;*

### Background

Acetylcholine (ACh) produced by T cells curbs inflammation (PMID:40307453). ACh regulates the migration of hematopoietic stem cell (HSC) and leukocytes (PMID:30361261) through the a7 nicotinic ACh receptor (a7nAChR). Cholinergic signals are induced by conditioning regimens required for HSC transplantation (HSCT) and subsequently regulate HSC proliferation after syngeneic transplantation (PMID:35087060). However, the role of ACh or a7nAChR in allogeneic HSCT (allo-HSCT) is unknown. An incomplete duplication of the gene encoding α7nAChR has generated during evolution the human-specific gene *CHRFAM7A* (PMID:11829490), which functions as a dominant negative isoform that inhibits α7nAChR signaling (PMID:21047781). Functional *CHRFAM7A* variants are present in 50-75% humans and include a 2 base-pair deletion ($CHRFAM7A^{\Delta 2bp}$) associated with inflammation after injury (PMID:33956875).

### Aims

To investigate the role of cholinergic signals, and particularly α7nAChR, on the outcome of allo-HSCT.

### Methods

Splenocytes from C57BL/6-background were transplanted into lethally-irradiated BALB/c recipients to induce graft-vs-host disease (GVHD). Choline acetyltransferase (*ChAT)*-GFP transgenic mice were used to track ACh-producing cells. T cells were activated *in vitro* using Concanavalin A (ConA). Cellular phenotyping, activation and proliferation were measured through flow cytometry after treatment with calcineurin inhibitor (cyclosporine A, CsA) or a7nAChR positive allosteric modulator (PNU-120596). Extracellular flux assays were used for metabolic studies. Digital droplet PCR (ddPCR) was used to genotype a7nAChR variants in human PBMNC DNA.

Copyright Information: (Online) ISSN: 2572-9241
©2026 The Author(s). HemaSphere is published by John Wiley & Sons Ltd on behalf of European Hematology Association. This is an open access Abstract Book distributed under the Attribution-NonCommercial-NoDerivs (CC BY-NC-ND), which allows third parties to download the articles and share them with others as long as they credit the author and the Abstract Book, but they cannot change the content in any way or use them commercially.
Abstract Book Citations: Authors, Title, HemaSphere, 2026;10:(S1):pages. The abstract book DOIs can be found on the "Author Information" page.
Disclaimer: Articles published in the journal HemaSphere exclusively reflect the opinions of the authors. The authors are responsible for all content in their abstracts including accuracy of the facts, statements, citing resources, etc.

**Results**

*In vivo* T cell activation was associated with cholinergic induction (A-B) and 3 to 45-fold increased frequency of *ChAT*-GFP$^+$ CD4$^+$/CD8$^+$ T cells in mesenteric lymph nodes (LNs) and spleen (C-D), suggesting cholinergic T cell expansion during GVHD. Strikingly, despite their increased ConA-induced activation (E), *ChAT*-GFP$^+$ T cells proliferated less than *ChAT*-GFP$^-$ T cells (F). Importantly, enrichment of sorted *ChAT*-GFP$^+$ T cells within *ChAT*-GFP$^{+/-}$ mixed cultures significantly restrained *ChAT*-GFP$^-$ T cell proliferation (G). These results suggest a homeostatic role of ACh production by activated T cells, which constrains overall T cell expansion.

Enhanced a7nAChR signaling with PNU-120596 reduced T cell proliferation and activation (H-I). We compared these effects with clinically standard GVHD prophylaxis (CysA). Overall, PNU-120596 showed similar effects and synergised with CysA reducing T cell activation (J) and proliferation (K). The latter was explained by reduced nutrient uptake and energy production: combinatorial treatment reduced the expression of the amino acid transporter CD98 (L) as well as T-cell glycolysis and oxidative phosphorylation by 50%, compared with monotherapy (M-N).

To investigate the role of a7nAChR variants in clinical allo-HSCT, we genotyped donors of allo-HSCT for blood cancer treatment. A preliminary analysis (149 patients) showed a trend towards increased GVHD in recipients of cells homozygous for *CHRFAM7A*$^{\Delta 2bp}$ (O). A larger validation cohort (249 patients) confirmed reduced GVHD-free survival (p=0.007) in recipients of *CHRFAM7A*$^{\Delta 2bp}$/*CHRFAM7A*$^{\Delta 2bp}$ grafts, compared with other genotypes (P).

**Summary/Conclusion**

Allo-HSCT increases ACh production by donor-derived lymphocytes, which restrains T cell activation and expansion via α7nAChR. Cholinergic modulation and/or donor selection based on *CHRFAM7A*$^{\Delta 2bp}$ genotype might impact the risk of GVHD.

**Copyright Information:** (Online) ISSN: 2572-9241
©2026 The Author(s). HemaSphere is published by John Wiley & Sons Ltd on behalf of European Hematology Association. This is an open access Abstract Book distributed under the Attribution-NonCommercial-NoDerivs (CC BY-NC-ND), which allows third parties to download the articles and share them with others as long as they credit the author and the Abstract Book, but they cannot change the content in any way or use them commercially.
**Abstract Book Citations:** Authors, Title, HemaSphere, 2026;10:(S1):pages. The abstract book DOIs can be found on the "Author Information" page.
**Disclaimer:** Articles published in the journal HemaSphere exclusively reflect the opinions of the authors. The authors are responsible for all content in their abstracts including accuracy of the facts, statements, citing resources, etc.

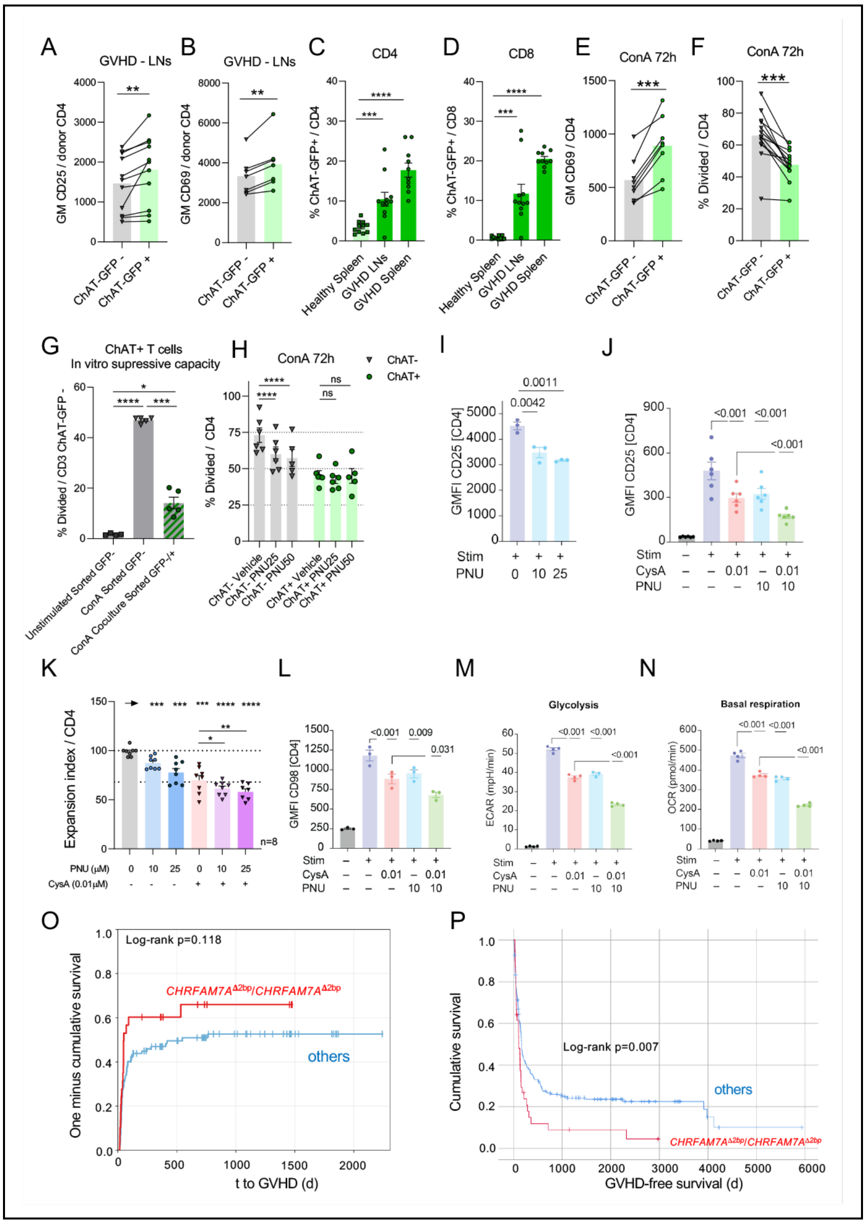

Figure legend: A) Flow cytometry geometric mean (GM) fluorescence intensity of CD25 activation marker in donor CD4+ T cells from host mesenteric lymph nodes (LNs) 6d after transplantation. B) Flow cytometry GM fluorescence intensity of CD69 activation marker in donor CD4+ T cells harvested from the host mesenteric lymph nodes (LNs) 6d after transplantation. C-D) Frequency of cholinergic ChAT-GFP- T cells (ChAT) CD4+/CD8+ T cells harvested from the spleen or the host mesenteric lymph nodes (LNs) at baseline or 6d after transplantation. E) Flow cytometry GM fluorescence intensity of CD69 activation marker in ChAT-GFP+/- T cells after 72h in vitro stimulation with Concanavalin A (ConA). F) Frequency of ChAT-GFP+/- proliferative T cells after 72h treatment with PNU-120596 upon in vitro stimulation with Concanavalin A (ConA). G) Frequency of ChAT-GFP- dividing T cells cultured alone or in co-culture with ChAT-GFP+ T cells (1:1), upon in vitro stimulation with Concanavalin A (ConA). H) Frequency of ChAT-GFP+/- proliferative T cells after 72h treatment with PNU-120596 upon in vitro stimulation with Concanavalin A (ConA). I-J) GM fluorescence intensity for CD25 on CD4+ T cells. CD3+ splenocytes from C57BL/6 mice were activated with ConA in the presence of the indicated pharmacological treatments. K) Expansion index of CD4+ T cells. CD3+ splenocytes from C57BL/6 mice were activated with ConA in the presence of the indicated pharmacological treatments. L) GM fluorescence intensity for CD98 on CD4+ T cells. CD3+ splenocytes from C57BL/6 mice were activated with ConA in the presence of the indicated pharmacological treatments. M-N) Extracellular acidification rate (M) and oxygen consumption rate (N) upon supplementation of the indicated metabolic compounds. CD3+ splenocytes from C57BL/6 mice were activated with ConA in the presence of the indicated pharmacological treatments. O) One minus cumulative survival showing time to development of acute GVHD in a cohort of recipients of grafts from donors

**Copyright Information:** (Online) ISSN: 2572-9241
©2026 The Author(s). HemaSphere is published by John Wiley & Sons Ltd on behalf of European Hematology Association. This is an open access Abstract Book distributed under the Attribution-NonCommercial-NoDerivs (CC BY-NC-ND), which allows third parties to download the articles and share them with others as long as they credit the author and the Abstract Book, but they cannot change the content in any way or use them commercially.
**Abstract Book Citations:** Authors, Title, HemaSphere, 2026;10:(S1):pages. The abstract book DOIs can be found on the "Author Information" page.
**Disclaimer:** Articles published in the journal HemaSphere exclusively reflect the opinions of the authors. The authors are responsible for all content in their abstracts including accuracy of the facts, statements, citing resources, etc.

homozygous for CHRFAM7AΔ2bp vs. other genotypes (n=149). P) Time to development of acute GVHD in recipients of grafts from donors homozygous for CHRFAM7AΔ2bp vs. other genotypes. Validation cohort, n=249.

**Copyright Information:** (Online) ISSN: 2572-9241
©2026 The Author(s). HemaSphere is published by John Wiley & Sons Ltd on behalf of European Hematology Association. This is an open access Abstract Book distributed under the Attribution-NonCommercial-NoDerivs (CC BY-NC-ND), which allows third parties to download the articles and share them with others as long as they credit the author and the Abstract Book, but they cannot change the content in any way or use them commercially.
**Abstract Book Citations:** Authors, Title, HemaSphere, 2026;10:(S1):pages. The abstract book DOIs can be found on the "Author Information" page.
**Disclaimer:** Articles published in the journal HemaSphere exclusively reflect the opinions of the authors. The authors are responsible for all content in their abstracts including accuracy of the facts, statements, citing resources, etc.

### (PF1110) CLONAL DYNAMICS OF THE TCRB REPERTOIRE REVEAL PREDICTIVE IMMUNE SIGNATURES OF POST-TRANSPLANT COMPLICATIONS

**Topic:** 22. Stem cell transplantation - Experimental

Zhe Zou[1], Shixin Lu[2], Yangwei Li[1], Yunfeng Song[1], Xiao Liu[3], Xiaodong Lyu*[1]

[1]*Henan Cancer Hospital, Central Laboratory, Zhengzhou, China;* [2]*NeoImmune Co., LTD, Shenzhen, China;* [3]*Tsinghua University Shenzhen International Graduate School, Shenzhen, China;*

### Background

Early complications following allogeneic hematopoietic stem cell transplantation (allo-HSCT)-including acute graft-versus-host disease (aGVHD), cytomegalovirus (CMV) reactivation, and Epstein–Barr virus (EBV) reactivation-remain major causes of post-transplant morbidity. Although perturbations of the T-cell receptor (TCR) repertoire accompany these events, existing studies typically analyze single complications, limited time windows, or coarse repertoire indices, and few have explored clonal origins or applied deep learning for early prediction.

### Aims

This study integrates pre-transplant baseline profiling, serial post-transplant monitoring, clonal source attribution, donor–recipient similarity analysis, and reinforcement learning-based predictive modeling. It provides a detailed, mechanistically grounded map of early T-cell reconstitution, identifies preclinical indicators of GVHD, CMV, and EBV, and establishes a framework for precision immune monitoring. This framework combines longitudinal repertoire profiling with deep learning-based prediction. These insights have the potential to inform early intervention strategies, optimize patient management, and advance both the mechanistic understanding and clinical application of T-cell repertoire monitoring in allo-HSCT recipients.

### Methods

We performed longitudinal TRB repertoire profiling of donor-recipient pairs across three early post-transplant time points, incorporating diversity metrics, donor-recipient clonal relationships, clonal-origin decomposition (residual recipient clones, donor-like mature clones, and novel clones from donor-derived stem cells), and repertoire similarity analyses. Event-free and event-positive groups were compared across aggregated and time-specific samples. Finally, we developed a reinforcement-learning-based deep neural network trained on CDR3 features to predict aGVHD, CMV, and EBV at pre-event time points.

### Results

Baseline repertoire diversity differed between future event-positive and event-free groups, with the strongest separation observed for EBV reactivation. Before clinical onset, patients who subsequently developed any complication exhibited significantly reduced diversity, with consistent findings across aGVHD, CMV, and EBV. Longitudinal modeling revealed impaired immune reconstitution in event-positive patients, marked by accelerated diversity decline during the first three months after transplant. Clonal-origin analysis showed that CMV reactivation involved substantial reductions in both donor-like mature clones and newly generated clones, indicating pervasive antigen-driven remodeling. Donor-recipient repertoire similarity was significantly reduced in CMV cases, particularly at the second post-transplant month, preceding reactivation. The deep-learning model demonstrated progressively improved predictive performance as event onset approached, achieving strong discrimination for all three complications at the final pre-event time point (AUC up to 0.897).

**Copyright Information:** (Online) ISSN: 2572-9241
©2026 The Author(s). HemaSphere is published by John Wiley & Sons Ltd on behalf of European Hematology Association. This is an open access Abstract Book distributed under the Attribution-NonCommercial-NoDerivs (CC BY-NC-ND), which allows third parties to download the articles and share them with others as long as they credit the author and the Abstract Book, but they cannot change the content in any way or use them commercially.
**Abstract Book Citations:** Authors, Title, HemaSphere, 2026;10:(S1):pages. The abstract book DOIs can be found on the "Author Information" page.
**Disclaimer:** Articles published in the journal HemaSphere exclusively reflect the opinions of the authors. The authors are responsible for all content in their abstracts including accuracy of the facts, statements, citing resources, etc.

**Summary/Conclusion**

Integrated repertoire profiling reveals early immunologic disturbances preceding major allo-HSCT complications, identifies event-specific immune trajectories-most notably CMV-driven repertoire divergence-and supports the feasibility of sequence-based deep learning for pre-event prediction. These findings establish a multi-dimensional immunogenomic framework that may inform future risk-adapted monitoring and preemptive intervention strategies.

**Copyright Information:** (Online) ISSN: 2572-9241
©2026 The Author(s). HemaSphere is published by John Wiley & Sons Ltd on behalf of European Hematology Association. This is an open access Abstract Book distributed under the Attribution-NonCommercial-NoDerivs (CC BY-NC-ND), which allows third parties to download the articles and share them with others as long as they credit the author and the Abstract Book, but they cannot change the content in any way or use them commercially.
**Abstract Book Citations:** Authors, Title, HemaSphere, 2026;10:(S1):pages. The abstract book DOIs can be found on the "Author Information" page.
**Disclaimer:** Articles published in the journal HemaSphere exclusively reflect the opinions of the authors. The authors are responsible for all content in their abstracts including accuracy of the facts, statements, citing resources, etc.

### (PF1111) CIRCULATING MICRORNA SEQUENCING REVEALS CONSISTENT EXPRESSION DYNAMICS ACROSS MOBILIZATION STRATEGIES AND PREDICTIVE MARKERS OF EFFICACY IN MULTIPLE MYELOMA PATIENTS

**Topic:** 22. Stem cell transplantation - Experimental

Damian Mikulski*[1], Jacek Burzyński[1], Izabela Dróżdż[2], Urszula Smyczyńska[1], Małgorzata Misiewicz[3], Jan Wojdal[3], Julia Piotrowska[1], Agnieszka Pluta[3], Agnieszka Wierzbowska[3], Wojciech Fendler[1]

*[1]Medical University of Lodz, Department of Biostatistics and Translational Medicine, Lodz, Poland;[2]Medical University of Lodz, Department of Clinical Genetics, Lodz, Poland; [3]Medical University of Lodz, Department of Hematology, Lodz, Poland;*

### Background

Efficient hematopoietic stem cell (HSC) mobilization is essential for successful autologous stem cell transplantation (auto-SCT) in multiple myeloma (MM). Mobilization failure and the need for rescue plerixafor or chemomobilization have become more frequent in the era of quadruplet induction therapy. Reliable predictors of mobilization failure are lacking in routine practice. Circulating microRNAs (miRNAs) may reflect systemic regulatory processes involved in hematopoietic and inflammatory responses.

### Aims

To characterize circulating miRNA dynamics during mobilization, to identify molecular markers associated with mobilization efficacy in MM patients undergoing auto-SCT and to assess the impact of chemomobilization on the miRNA profile.

### Methods

Patients were prospectively recruited between October 2019 and September 2025. Serum was collected before mobilization and on the day of planned first apheresis. Poor mobilizers were defined according to Gruppo Italiano Trapianto di Midollo Osseo (GITMO) criteria and/or need for rescue plerixafor. Total RNA was isolated from serum, and small RNA libraries were prepared using the QIAseq miRNA Library Kit and sequenced on the Illumina NextSeq 550 platform. Differential expression analysis was conducted using limma-voom models, adjusting for induction regimen. Predictive modeling was performed using elastic net variable selection followed by multivariable logistic regression with five-fold cross-validation. Pathway enrichment analysis was conducted using miRPath v4 and Reactome.

### Results

A total of 49 serum samples from 33 patients were analyzed; 19 (57.6%) received daratumumab-bortezomib-thalidomide-dexamethasone (Dara-VTd) and 14 (42.4%) triplet regimens (VTd or VCd: bortezomib-cyclophosphamide-dexamethasone). Comparison of poor versus good mobilizers identified 39 nominally significant miRNAs with consistent directionality across induction cohorts (Figure 1A). Enrichment analysis highlighted pathways involved in cell cycle regulation and mitosis, as well as p53 signaling, PI3K–AKT signaling, and RHO GTPase–mediated processes.
Dynamic analysis revealed a largely concordant direction of miRNA regulation after granulocyte colony-stimulating factor (G-CSF) in both groups; however, poor mobilizers exhibited markedly reduced effect sizes. Chemomobilization induced broader and stronger expression changes, resembling the pattern observed in good mobilizers. Seven miRNAs showed consistent direction of regulation across chemo-, good-, and poor-mobilization cohorts, with the greatest magnitude observed in good mobilizers.
Finally, predictive modeling identified a two-miRNA signature (hsa-miR-192-5p, hsa-miR-574-3p) with an AUC of 0.917 (95% CI 0.796–1.00) (Figure 1B) for discriminating between poor and good mobilizers in 5-fold cross-validation.

**Copyright Information:** (Online) ISSN: 2572-9241
©2026 The Author(s). HemaSphere is published by John Wiley & Sons Ltd on behalf of European Hematology Association. This is an open access Abstract Book distributed under the Attribution-NonCommercial-NoDerivs (CC BY-NC-ND), which allows third parties to download the articles and share them with others as long as they credit the author and the Abstract Book, but they cannot change the content in any way or use them commercially.
**Abstract Book Citations:** Authors, Title, HemaSphere, 2026;10:(S1):pages. The abstract book DOIs can be found on the "Author Information" page.
**Disclaimer:** Articles published in the journal HemaSphere exclusively reflect the opinions of the authors. The authors are responsible for all content in their abstracts including accuracy of the facts, statements, citing resources, etc.

### Summary/Conclusion

Poor mobilizers exhibit a quantitatively attenuated miRNA response, indicating incomplete activation rather than opposing regulation. Seven miRNAs were consistently deregulated across all cohorts in the same direction, defining a shared mobilization-associated expression pattern. Chemomobilization shifts the circulating miRNA profile toward that observed in good mobilizers, reflecting quantitative enhancement of this pattern. Baseline circulating miRNA profiling identifies a predictive signature of mobilization failure with high discriminative performance and potential to guide personalized mobilization strategies.

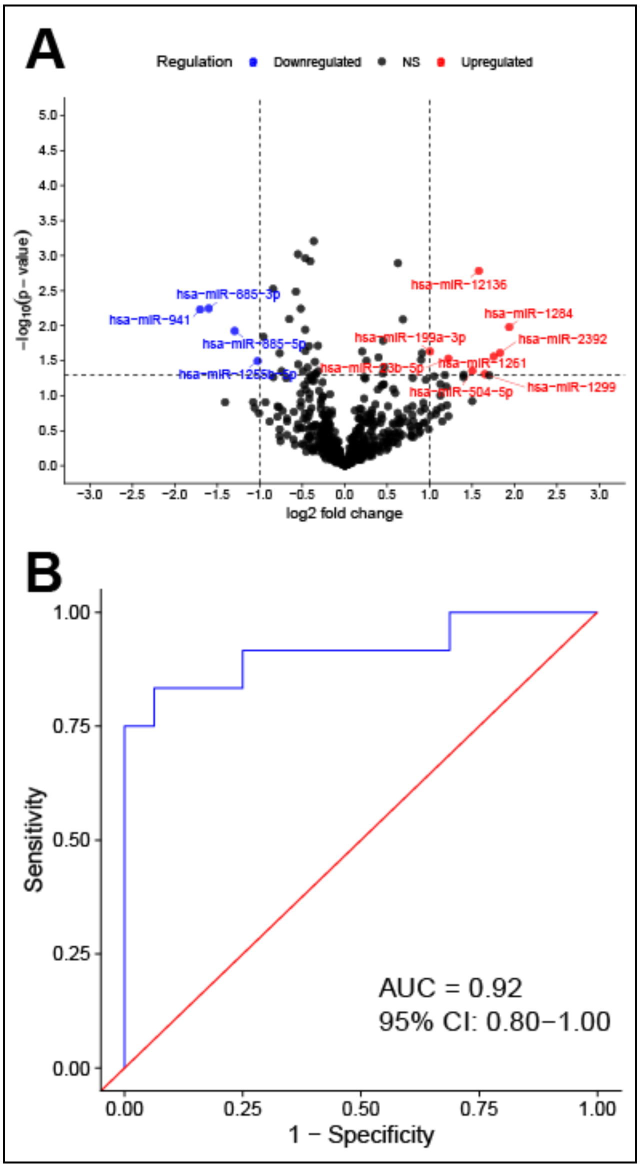

Figure 1. (A) Volcano plot showing differential expression of circulating miRNAs between poor and good mobilizers prior to stem cell mobilization. Only selected miRNAs with larger effect sizes are annotated for clarity. (B) Receiver operating characteristic (ROC) curve demonstrating the performance of the miRNA-based predictive model distinguishing poor from good mobilizers. The final two-miRNA signature (hsa-miR-192-5p and hsa-miR-574-3p) achieved an area under the curve (AUC) of 0.92 (95% CI: 0.80–1.00) in five-fold cross-validation.

**Copyright Information:** (Online) ISSN: 2572-9241
©2026 The Author(s). HemaSphere is published by John Wiley & Sons Ltd on behalf of European Hematology Association. This is an open access Abstract Book distributed under the Attribution-NonCommercial-NoDerivs (CC BY-NC-ND), which allows third parties to download the articles and share them with others as long as they credit the author and the Abstract Book, but they cannot change the content in any way or use them commercially.
**Abstract Book Citations:** Authors, Title, HemaSphere, 2026;10:(S1):pages. The abstract book DOIs can be found on the "Author Information" page.
**Disclaimer:** Articles published in the journal HemaSphere exclusively reflect the opinions of the authors. The authors are responsible for all content in their abstracts including accuracy of the facts, statements, citing resources, etc.

### (PF1112) EPIGENETIC MODULATION ENABLES EXPANSION OF FUNCTIONAL HUMAN HSPCS IN A CHEMICALLY DEFINED SYSTEM FOR CELL AND GENE CLINICAL APPLICATIONS

**Topic:** 22. Stem cell transplantation - Experimental

Nobuchika Yamamoto*[1], Naoki Otsu[1], Maho Okuda[1], Tomonori Tawara[1], Kyoko Kikuchi[1], Motoo Watanabe[1], Nobuyuki Arakawa[1]

[1]*Celaid Therapeutics Inc., Research & Development Division, Tokyo, Japan;*

### Background

Ex vivo expansion of functional human hematopoietic stem progenitor cells (HSPCs) remains a major bottleneck in hematopoietic transplantation and gene-modified HSPC therapies. Cord blood provides a clinically valuable source of HSPCs; however, limited stem cell numbers constrain engraftment kinetics and broader clinical application. Scalable, chemically defined expansion systems that preserve long-term repopulating capacity are critically needed to enable next-generation gene and cell therapy manufacturing.

### Aims

To develop a chemically defined platform enabling large-scale expansion of functional human HSPCs through targeted epigenetic modulation.

### Methods

Building on our previously established chemically defined, we screened small-molecule epigenetic modulators to identify compounds that selectively promote self-renewal and expansion of human HSPCs. Human cord blood $CD34^+$ cells were cultured under defined conditions, and phenotypic expansion of $CD201^+CD34^+CD45RA^-Lineage^-$ cells was assessed. Functional capacity was evaluated by colony-forming unit (CFU) assays and long-term hematopoietic reconstitution in immunodeficient NOG mice.

### Results

Screening identified a novel modulator of histone lysine demethylases that enabled approximately 400-fold expansion of $CD201^+CD34^+CD45RA^-Lineage^-$ HSPCs under chemically defined conditions. Notably, $CD34^+$ cells were maintained at >70% after 14 days of culture. Expanded cells demonstrated significantly enhanced multilineage hematopoietic reconstitution in NOG mice compared with uncultured cord blood derived $CD34^+$ cells. CFU assays revealed a significant increase in CFU-GEMM colonies, supporting preservation of multilineage potential.

### Summary/Conclusion

Targeted epigenetic modulation enables robust, large-scale expansion of functional human HSPCs while preserving in vivo engraftment capacity under chemically defined conditions. In addition to supporting the ongoing U.S. clinical development of CLD-001, our cord blood–derived expanded HSPC product, this scalable platform may be broadly applicable to peripheral blood–derived HSPC gene therapy manufacturing and future iPSC-derived hematopoietic stem cell production, providing a unified framework for next-generation gene and cell therapy applications.

**Copyright Information:** (Online) ISSN: 2572-9241
©2026 The Author(s). HemaSphere is published by John Wiley & Sons Ltd on behalf of European Hematology Association. This is an open access Abstract Book distributed under the Attribution-NonCommercial-NoDerivs (CC BY-NC-ND), which allows third parties to download the articles and share them with others as long as they credit the author and the Abstract Book, but they cannot change the content in any way or use them commercially.
**Abstract Book Citations:** Authors, Title, HemaSphere, 2026;10:(S1):pages. The abstract book DOIs can be found on the “Author Information” page.
**Disclaimer:** Articles published in the journal HemaSphere exclusively reflect the opinions of the authors. The authors are responsible for all content in their abstracts including accuracy of the facts, statements, citing resources, etc.

### (PF1113) EARLIER EMERGENCE OF PROLIFERATIVE CD8+ T CELLS IS ASSOCIATED WITH IMPROVED PROGNOSIS IN R/R DLBCL PATIENTS TREATED WITH AUTO-HSCT PLUS UMBILICAL CORD BLOOD INFUSION

**Topic:** 22. Stem cell transplantation - Experimental

Zhengchang He*[1], Xi Zhang[1], Yongqian Jia[2]

*[1]The Second Affiliated Hospital of Army Medical University, Hematology, Chongqing, China;[2]West China Hospital, Sichuan University, Hematology, Chengdu, China;*

### Background

Autologous hematopoietic stem cell transplantation (Auto-HSCT) remains a potentially curative treatment for patients with relapsed/refractory diffuse large B-cell lymphoma (R/R DLBCL); however, disease relapse still occurs in a subset of patients following transplantation. A critical vulnerability period exists after Auto-HSCT between the decline of chemotherapy-induced cytotoxic effects and the reconstitution of host antitumor immunity, creating a window for tumor recurrence. Human umbilical cord blood stem cells (hUCBSCs) represent a potential cellular source capable of mediating a graft-versus-tumor (GVT) effect. Based on this rationale, we conducted a clinical study evaluating a novel strategy combining Auto-HSCT with unrelated hUCBSC infusion in patients with R/R DLBCL. In our cohort of 44 patients, the 2-year overall survival (OS) and progression-free survival (PFS) rates were 90.9% and 86.3%, respectively. Although these outcomes compare favorably with historical data, the biological mechanisms by which Auto-HSCT combined with hUCBSC infusion may prevent relapse remain unclear.

### Aims

To investigate the immune mechanisms associated with improved survival in R/R DLBCL patients undergoing auto-HSCT plus unrelated hUCBSC infusion (the combined strategy).

### Methods

We enrolled 24 R/R DLBCL patients (14 relapsed and 10 refractory; median age 35 years). Among them, 12 patients underwent the combined strategy, and 12 patients received auto-HSCT strategy alone. Both strategy utilized the BEAM conditioning. The combined strategy protocol was conducted as follows: after conditioning, patients received Auto-HSCT followed by a single infusion of unrelated hUCBSCs on day +2. The unrelated hUCBSCs units must satisfy the requirement of HLA matching ≥ 5/10 loci and blood type-compatible units will be prioritized for selection. Unrelated hUCBSCs was infused as a single dose, with the total nucleated cell (TNC) count ranging from 5 - 15 × $10^8$ and $CD34^+$ cell count from 1.5 - 3.5 × $10^6$. Peripheral blood samples were collected from patients on transplantation Day 0, Day 7, Day 21, and Day 28 for flow cytometric analysis and single-cell transcriptomic sequencing.

### Results

In the combined strategy, the median time to neutrophil and platelet engraftment was 11 days (range, 10–21) and 14 days (range, 12–31), respectively. The percentage of cells derived from unrelated hUCBSCs in peripheral blood remained below 0.01% after 90 days. In the control cohort (Auto-HSCT alone), median engraftment occurred at 11 days (range, 10–19) for neutrophils and 12 days (range, 10–20) for platelets. Despite similar hematologic recovery, the combined regimen demonstrated superior immune reconstitution kinetics. Proliferative CD8+ T cells (CD8+Tprolif) emerged 2-5 days earlier (median occurred at 4 days), and stem cell-like memory CD8+ T cells (CD8+Tscm) emerged 8-10 days earlier (median occurred at 9 days) in the combined group compared to the control group. Furthermore, earlier activation of the CD8+ T-cell compartment was consistently observed in patients receiving the combined hUCBSC infusion.

**Copyright Information:** (Online) ISSN: 2572-9241
©2026 The Author(s). HemaSphere is published by John Wiley & Sons Ltd on behalf of European Hematology Association. This is an open access Abstract Book distributed under the Attribution-NonCommercial-NoDerivs (CC BY-NC-ND), which allows third parties to download the articles and share them with others as long as they credit the author and the Abstract Book, but they cannot change the content in any way or use them commercially.
**Abstract Book Citations:** Authors, Title, HemaSphere, 2026;10:(S1):pages. The abstract book DOIs can be found on the "Author Information" page.
**Disclaimer:** Articles published in the journal HemaSphere exclusively reflect the opinions of the authors. The authors are responsible for all content in their abstracts including accuracy of the facts, statements, citing resources, etc.

### Summary/Conclusion

Infusion of unrelated hUCBSCs may compensate for the lack of a graft-versus-tumor (GVT) effect in Auto-HSCT. The GVT observed in the combined strategy appears to be associated with the earlier emergence of CD8⁺ Tprolif cells, CD8⁺ Tscm cells, and the accelerated activation of CD8⁺ T cells following unrelated hUCBSC infusion.

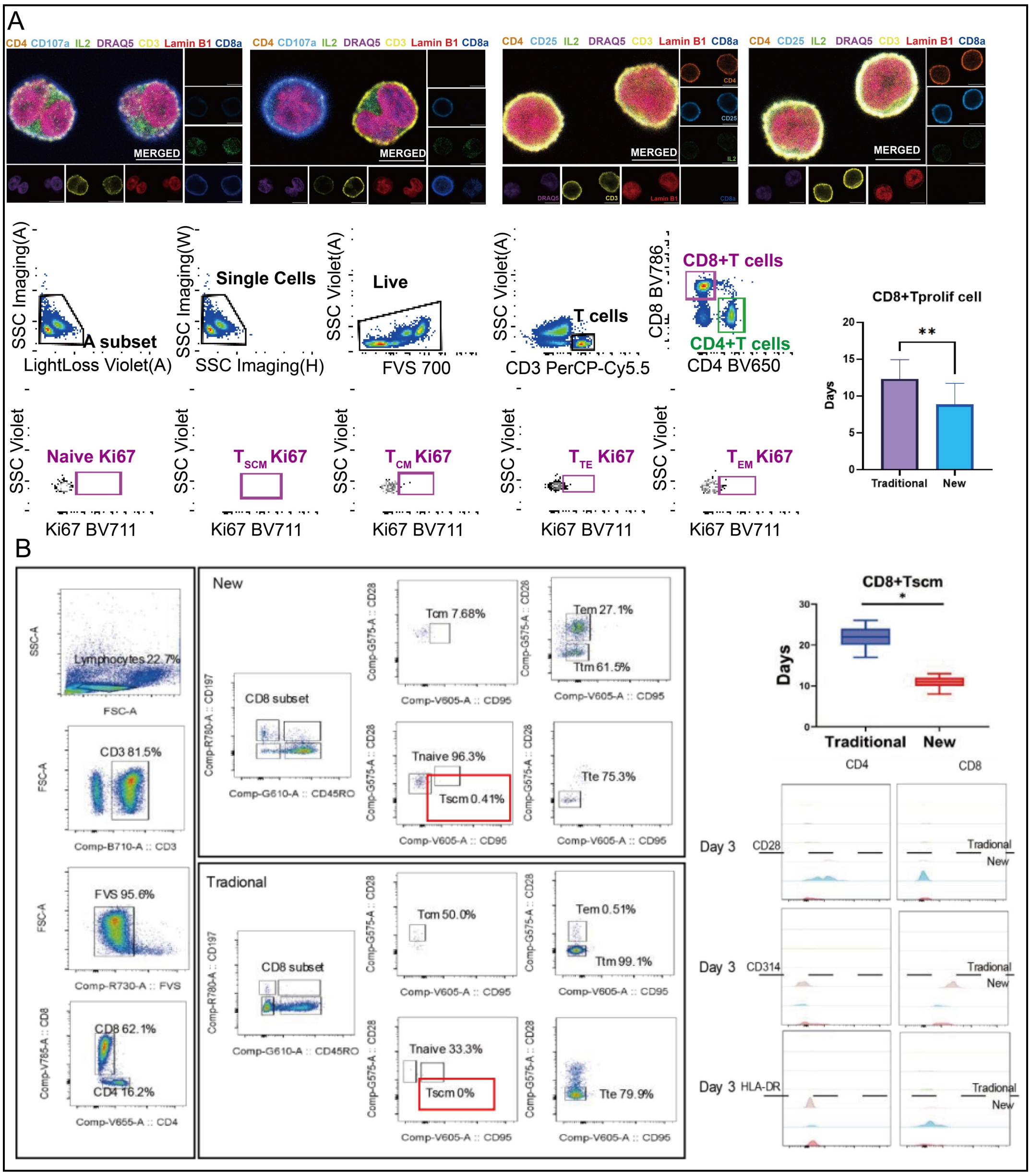

**Copyright Information:** (Online) ISSN: 2572-9241
©2026 The Author(s). HemaSphere is published by John Wiley & Sons Ltd on behalf of European Hematology Association. This is an open access Abstract Book distributed under the Attribution-NonCommercial-NoDerivs (CC BY-NC-ND), which allows third parties to download the articles and share them with others as long as they credit the author and the Abstract Book, but they cannot change the content in any way or use them commercially.
**Abstract Book Citations:** Authors, Title, HemaSphere, 2026;10:(S1):pages. The abstract book DOIs can be found on the "Author Information" page.
**Disclaimer:** Articles published in the journal HemaSphere exclusively reflect the opinions of the authors. The authors are responsible for all content in their abstracts including accuracy of the facts, statements, citing resources, etc.

### (PF1114) PROLIFERATING TCF1LOW PD-1HIGH POPULATION IN HUMAN CD8+ T-CELLS IS ASSOCIATED WITH THE ONSET OF AGVHD AND SUPPRESSED BY PTCY

**Topic:** 22. Stem cell transplantation - Experimental

Kazuya Asano*[1], Shugo Kowata[1], Yuki Seki[1], Kentaro Kobayashi[1], Rino Nozaki[1], Sawako Kitamura[1], Takahiro Maeta[1], Maki Nishiya[1], Yoshiaki Okano[1], Tatsuo Oyake[1], Shigeki Ito[1]

[1]*Iwate Medical University, Hematology and Oncology, 2-1-1 Idaidori, Shiwa-gun, Yahaba-cho, Iwate, Japan, Japan;*

### Background

Graft-versus-host disease (GVHD) is a major complication following allogeneic hematopoietic stem cell transplantation (allo-HSCT). Donor T-cells can be exposed to persistent antigenic stimulation during this process, which may induce exhaustion-associated phenotypes in alloreactive T-cells. Transitory exhausted T-cells have been reported to retain alloreactivity and may contribute to the pathogenesis of GVHD. However, longitudinal data in human peripheral blood are limited, and the clinical relevance of these phenotypes remains unclear.

### Aims

We investigated the relationship between TCF1/PD-1 expression on $CD8^+$ T-cells in peripheral blood and (i) immune reconstitution after auto-HSCT and allo-HSCT, (ii) GVHD development and severity, and (iii) GVHD prophylaxis regimens.

### Methods

We retrospectively analyzed 36 patients who underwent transplantation between 2023 and 2025 at a single institution (auto-HSCT, n=13; allo-HSCT, n=23). Peripheral-blood $CD8^+$ T-cell subsets were defined by TCF1 and PD-1 expression intensity, and subset proportions were analyzed from day 0 to day 105 post-transplant (weeks 1–15). Weekly group comparisons were performed using two-sided Mann–Whitney U tests, with $P<0.05$ considered significant.

### Results

The allo-HSCT group showed a higher proportion of the $TCF1^{low}$ $PD\text{-}1^{high}$ $CD8^+$ T-cell subset compared to the auto-HSCT group at multiple weeks (W4–6, W10, W12–15; $P < 0.05$). The proportion of this subset was also higher in the allo-HSCT group within the $Ki67^+$ $CD8^+$ T-cell population (W2–3, W10, W12; $P < 0.05$). In the allo-HSCT group, the $TCF1^{low}$ $PD\text{-}1^{high}$ subset within the $Ki67^+$ $CD8^+$ T-cell population was higher in severe aGVHD cases (W5–9, W10–11, W13–14; $P < 0.05$). In contrast, patients receiving post-transplant cyclophosphamide (PTCy)-based prophylaxis showed a lower proportion of this subset (W2–3, W9–12, W14; $P < 0.05$). In contrast, no significant differences in this subset were observed regarding the occurrence of all-grade aGVHD or the use of ATG-based prophylaxis ($P > 0.05$).

**Copyright Information:** (Online) ISSN: 2572-9241
©2026 The Author(s). HemaSphere is published by John Wiley & Sons Ltd on behalf of European Hematology Association. This is an open access Abstract Book distributed under the Attribution-NonCommercial-NoDerivs (CC BY-NC-ND), which allows third parties to download the articles and share them with others as long as they credit the author and the Abstract Book, but they cannot change the content in any way or use them commercially.
**Abstract Book Citations:** Authors, Title, HemaSphere, 2026;10:(S1):pages. The abstract book DOIs can be found on the “Author Information” page.
**Disclaimer:** Articles published in the journal HemaSphere exclusively reflect the opinions of the authors. The authors are responsible for all content in their abstracts including accuracy of the facts, statements, citing resources, etc.

### Summary/Conclusion

The TCF1$^{low}$ PD-1$^{high}$ CD8$^{+}$ T-cell subset increased after allo-HSCT compared with auto-HSCT. Within the allo-HSCT patients, proliferation of this subset was associated with severe aGVHD, and was suppressed by PTCy-based prophylaxis. These findings suggest that this proliferating subset may be a potential indicator of severe aGVHD risk and that PTCy may mitigate early GVHD by suppressing the proliferation of this subset.

Figure 1. (A) Gating strategy for identifying proliferating (Ki-67$^{+}$) TCF1$^{low}$ PD-1$^{high}$ CD8$^{+}$ T-cells. (B) Longitudinal proportions of these cells according to severe acute GVHD (●, Grade III-IV; ■, Others) and (C) according to PTCy status (●, PTCy; ■, Others). *$P$ < 0.05.

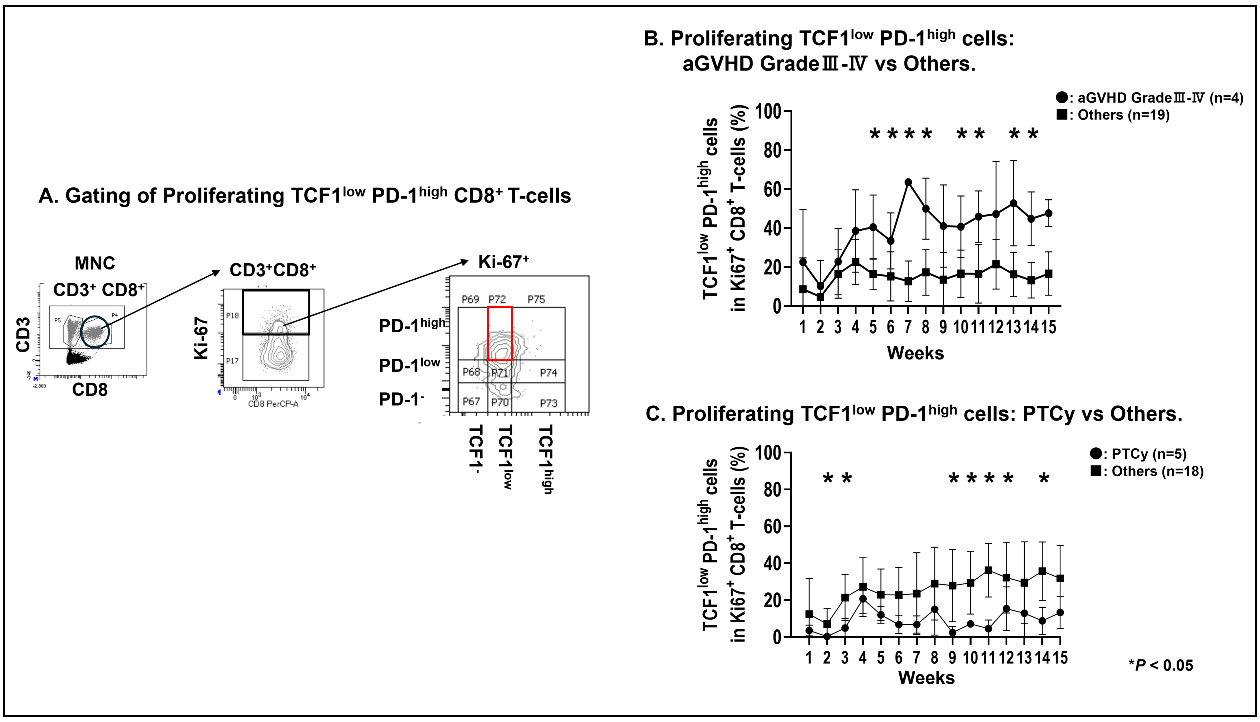

**Copyright Information:** (Online) ISSN: 2572-9241
©2026 The Author(s). HemaSphere is published by John Wiley & Sons Ltd on behalf of European Hematology Association. This is an open access Abstract Book distributed under the Attribution-NonCommercial-NoDerivs (CC BY-NC-ND), which allows third parties to download the articles and share them with others as long as they credit the author and the Abstract Book, but they cannot change the content in any way or use them commercially.
**Abstract Book Citations:** Authors, Title, HemaSphere, 2026;10:(S1):pages. The abstract book DOIs can be found on the "Author Information" page.
**Disclaimer:** Articles published in the journal HemaSphere exclusively reflect the opinions of the authors. The authors are responsible for all content in their abstracts including accuracy of the facts, statements, citing resources, etc.

### (PF1115) IMPACT OF EARLY POST-TRANSPLANT MEASURABLE RESIDUAL DISEASE ON OUTCOMES OF PATIENTS WITH RELAPSED/ REFRACTORY AML: A POST-HOC ANALYSIS OF THE PROSPECTIVE ASAP TRIAL

**Topic:** 23. Stem cell transplantation - Clinical

Katharina Egger-Heidrich*[1, 2], Christian Reicherts[3], Gesine Bug[4], Eva Wagner-Drouet[5], Lutz Peter Müller[6], Christoph Schmid[7], Stefan Walter Krause[8], Wolfgang Bethge[9], Edgar Jost[10], Georg-Nikolaus Franke[11], Stefan A. Klein[12], Judith Niederland[13], Martin Kaufmann[14], Kerstin Schäfer-Eckart[15], Henning Baldauf[2], Falk Heidenreich[1, 2], Ulf-Peter Guenther[16], Ilaria Visco[16], Gerhard Schoefl[16], Markus Fuhrmann[16], Friedrich Stölzel[17], Sarah Trost[2], Christoph Röllig[1], Malte von Bonin[1], Désirée Kunadt[1], Björn Steffen[4], Beate Hauptrock[5], Christoph Schliemann[3], Katja Sockel[1], Fabian Lang[4], Oliver Kriege[5], Judith Schaffrath[6], Hubert Serve[4], Jan-Henrik Mikesch[3], Jan Moritz Middeke[1], Martin Bornhäuser[1, 18], Johannes Schetelig[1, 2], Matthias Stelljes[3]

*[1]University Hospital Carl Gustav Carus, Technische Universität Dresden, Department of Internal Medicine I, Dresden, Germany; [2]DKMS Group, Clinical Trials Unit, Dresden, Germany; [3]University Hospital Münster, Department of Medicine A, Hematology, Oncology, and Pneumology, Münster, Germany; [4]Goethe University Frankfurt, University Hospital, Department of Medicine 2, Frankfurt, Germany; [5]University Medical Center, Mainz, Germany; [6]University Hospital, Martin Luther University Halle-Wittenberg, Department of Internal Medicine IV, Halle (Saale), Germany; [7]Augsburg University Hospital and Medical Faculty,, Department of Hematology and Oncology, Augsburg, Germany; [8]University Hospital Erlangen, Department of Internal Medicine 5, Erlangen, Germany; [9]University Hospital Tübingen, Department of Hematology, Oncology, Clinical Immunology, and Rheumatology, Tübingen, Germany; [10]Rheinisch-Westfälische Technische Hochschule Aachen University and Center for Integrated Oncology Aachen Bonn Cologne Düsseldorf, Department of Hematology, Oncology, Hemostaseology, and Stem Cell Transplantation, Aachen, Germany; [11]University of Leipzig Medical Center, Department for Hematology, Cellular Therapy, Hemostaseology and Infectious Diseases, Leipzig, Germany; [12]Universitätsmedizin Mannheim, Department of Hematology and Oncology, Mannheim, Germany; [13]Helios Hospital Berlin-Buch, Department of Hematology and Cell Therapy, Berlin, Germany; [14]Robert-Bosch-Krankenhaus, Department for Hematology, Oncology and Palliative Medicine, Stuttgart, Germany; [15]Paracelsus Medizinische Privat-Universität, Klinikum Nürnberg, Klinik für Innere Medizin 5, Nuremberg, Germany; [16]DKMS Life Science Lab GmbH, Dresden, Germany; [17]University Hospital Schleswig-Holstein, Kiel University, Division of Stem Cell Transplantation and Cellular Immunotherapy, Kiel, Germany; [18]National Center for Tumor Diseases, Dresden, Germany;*

### Background

The ASAP trial (#NCT02461537) was a randomized, multi-center trial that investigated the effects of remission induction chemotherapy (RIST) followed by allogeneic (allo) HCT versus immediate alloHCT (disease control - DISC) in relapsed/ refractory AML after induction chemotherapy. No survival advantage was observed for patients who had received RIST prior to alloHCT. However, biological disease risk (classified according to ELN 2022) was a major risk factor for survival. As part of ancillary research, peripheral blood or bone marrow aspirate samples were collected on day +56 post alloHCT to assess the prognostic power of measurable residual disease (MRD).

### Aims

The purpose of this analysis was to evaluate the impact of MRD status on the risk of relapse and survival endpoints in patients with (morphological) complete remission on day +56 after alloHCT.

**Copyright Information:** (Online) ISSN: 2572-9241
©2026 The Author(s). HemaSphere is published by John Wiley & Sons Ltd on behalf of European Hematology Association. This is an open access Abstract Book distributed under the Attribution-NonCommercial-NoDerivs (CC BY-NC-ND), which allows third parties to download the articles and share them with others as long as they credit the author and the Abstract Book, but they cannot change the content in any way or use them commercially.
**Abstract Book Citations:** Authors, Title, HemaSphere, 2026;10:(S1):pages. The abstract book DOIs can be found on the "Author Information" page.
**Disclaimer:** Articles published in the journal HemaSphere exclusively reflect the opinions of the authors. The authors are responsible for all content in their abstracts including accuracy of the facts, statements, citing resources, etc.

### Methods

Peripheral blood samples were collected at study inclusion and genomic DNA was extracted. Mutational profiles were determined with high sensitivity using a custom panel (TWIST Biosciences) in combination with Duplex-Sequencing. Based on these results, up to 5 markers were selected for MRD tracking, whereby well-established markers were always included. On day +56 (+/- 30 days) after alloHCT, bone marrow aspirates were collected and genomic DNA was isolated. The frequencies of patient-specific markers were quantified using UMI-based, highly sensitive amplicon sequencing. MRD positivity was assumed if the variant allele frequency of at least one of the five analyzed target mutations exceeded 0.1%.
We evaluated the prognostic significance of MRD status for relapse and survival endpoints by means of Kaplan-Meier estimates, cumulative incidence curves and multivariable Cox models, adjusted for patient age, sex, ELN 2022 risk, ECOG, HCT-CI and donor type.

### Results

In the ASAP trial, 225 patients achieved (morphological) complete remission (CR) on day +56. Samples for MRD analysis were available from 131 CR patients. Of these, 28 (21%) were classified as MRD positive and 88 (67%) MRD negative. In 15 (11%) patients, analysis failed due to insufficient material. There were no differences in these proportions between DISC (15 $MRD^{pos}$ and 44 $MRD^{neg}$ ) and RIST (13 $MRD^{pos}$ and 44 $MRD^{neg}$) patients. Patients who were $MRD^{neg}$ at this early time point after alloHCT had significantly longer relapse-free survival (RFS) and long-term overall survival (OS) ($MRD^{neg}$: 2-year RFS 64% [95% CI: 53-73] versus $MRD^{pos}$: 2-year RFS 29% [95% CI: 14-46]; $MRD^{neg}$ 2-year OS 75% [95% CI: 65-83] versus $MRD^{pos}$: 2-year OS 50% [95% CI: 31-67]), respectively. Multivariate hazard ratios ($MRD^{pos}$ versus $MRD^{neg}$) were 3.1 ([95%-CI: 1.8-5.4], p<0.001) and 2.8 ([95%-CI: 1.5-5.2], p=0.001) for RFS and OS, respectively. These findings were consistent across ELN2022 subgroups.

### Summary/Conclusion

Early MRD negativity after alloHCT is predictive for favorable OS and reduced risk of relapse. Early MRD monitoring may optimize post alloHCT follow-up and indicate patients who could benefit from preemptive therapy (e.g., rapid tapering of immunosuppression, DLIs, targeted therapies). Optimal timing and frequency of MRD testing, preferred material (bone marrow versus peripheral blood samples) and most informative marker panels require further investigations.

**Copyright Information:** (Online) ISSN: 2572-9241
©2026 The Author(s). HemaSphere is published by John Wiley & Sons Ltd on behalf of European Hematology Association. This is an open access Abstract Book distributed under the Attribution-NonCommercial-NoDerivs (CC BY-NC-ND), which allows third parties to download the articles and share them with others as long as they credit the author and the Abstract Book, but they cannot change the content in any way or use them commercially.
**Abstract Book Citations:** Authors, Title, HemaSphere, 2026;10:(S1):pages. The abstract book DOIs can be found on the “Author Information” page.
**Disclaimer:** Articles published in the journal HemaSphere exclusively reflect the opinions of the authors. The authors are responsible for all content in their abstracts including accuracy of the facts, statements, citing resources, etc.

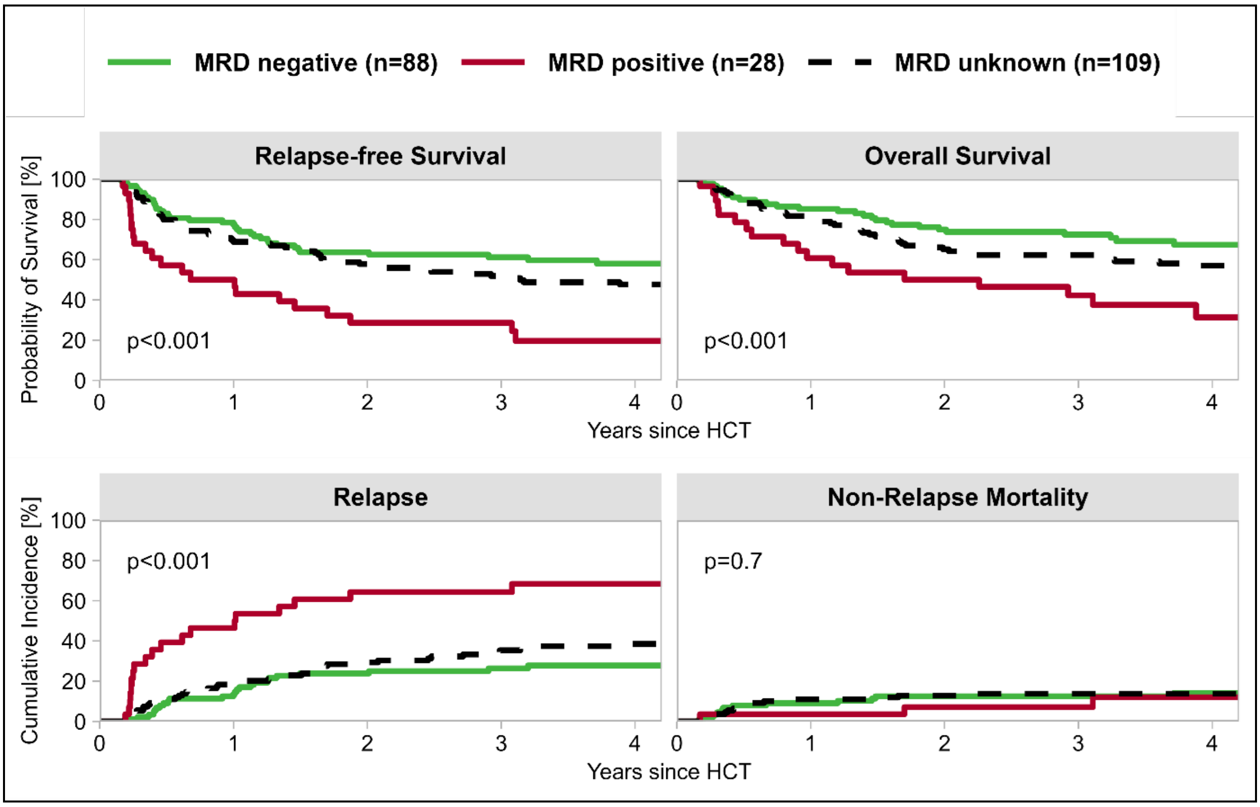

**Copyright Information:** (Online) ISSN: 2572-9241
©2026 The Author(s). HemaSphere is published by John Wiley & Sons Ltd on behalf of European Hematology Association. This is an open access Abstract Book distributed under the Attribution-NonCommercial-NoDerivs (CC BY-NC-ND), which allows third parties to download the articles and share them with others as long as they credit the author and the Abstract Book, but they cannot change the content in any way or use them commercially.
**Abstract Book Citations:** Authors, Title, HemaSphere, 2026;10:(S1):pages. The abstract book DOIs can be found on the "Author Information" page.
**Disclaimer:** Articles published in the journal HemaSphere exclusively reflect the opinions of the authors. The authors are responsible for all content in their abstracts including accuracy of the facts, statements, citing resources, etc.

### (PF1116) OUTCOME OF PATIENTS TREATED FOR T-ALL WITH HSCT IN THE FORUM COHORT

**Topic:** 23. Stem cell transplantation - Clinical

Jeanne Martin*[1], Paulina Kurzmann[2], Peter Bader[3], Jean-Hugues Dalle[1], Akif Yesilipek[4], Franco Locatelli[5], Adriana Balduzzi[6], Julia Palma[7], Herbert Pichler[2], Marc Bierings[8], Jacek Wachowiak[9], Cristina Diaz-de-Heredia[10], Marianne Ifversen[11], Raquel Noemí Staciuk[12], Petr Sedlacek[13], Eugenios Goussetis[14], Jochen Buechner[15], Christina Peters[16], Marie-Emilie Dourthe[17]

*[1]Robert Debré hospital, Pediatric Hematology and Immunology Department, Paris, France; [2]Children's Cancer Research Institute, Biostatistical core unit, Vienna, Austria; [3]Goethe University Frankfurt, University Hospital, Department of Pediatrics, Division for Stem Cell Transplantation and Immunology, Frankfurt, Germany; [4]Medical Park Antalya Hospital, Antalya, Türkiye; [5]IRCCS Bambino Gesù Children's Hospital, Catholic University of the Sacred Heart, Pediatric hematology and oncology, Rome, Italy; [6]Fondazione IRCCS San Gerardo dei Tintori, Pediatric Stem Cell Transplant Unit, Monza, Italy; [7]Hospital Luis Calvo Mackenna, Santiago, Chile; [8]Princess Máxima Center for Pediatric Oncology, Utrecht, The Netherlands;[9]Poznań University of Medical Sciences, Department of Pediatric Oncology, Hematology and Transplantology, Poznan, Poland; [10]Hospital Universitari Vall d'Hebron.Vall d'Hebron Resarch, Division of Pediatric Hematology and Oncology, Barcelona, Spain; [11]Copenhagen University Hospital Rigshospitalet, Copenhagen, Denmark; [12]Hospital de Pediatria "Juan P. Garrahan, buenos aires, Argentina; [13]University Hospital Motol, Department of Pediatric Hematology and Oncology,, prague, Czechia;[14]Agia Sofia Children's Hospital, Stem Cell Transplant Unit, Athens, Greece; [15]Oslo University Hospital, Department of Pediatric Hematology and Oncology, oslo, Norway; [16]St. Anna Children's Hospital, Medical University of Vienna, Department of Pediatrics and Adolescent Medicine, Vienna, Austria; [17]GHU APHP Nord - Université Paris Cité, Robert Debré Hospital, Pediatric Hematology and Immunology Department, Paris, France;*

### Background

The prognosis of relapsed T-cell acute lymphoblastic leukemia (T-ALL) remains poor compared with B-ALL, with limited access to innovative targeted therapies or immunotherapies. Consequently, indications for allogeneic hematopoietic stem cell transplantation (allo-HSCT) are broader in T-ALL, including high-risk patients in first complete remission (CR1) and most patients in CR2.

### Aims

The aim of this study was to assess the toxicity and efficacy of alloHSCT in pediatric T-ALL patients comparing with B-ALL and to evaluate conditioning regimens according to age and disease status at transplant.

### Methods

We analyzed data from the prospective international FORUM cohort (NCT01949129) including children and adolescents with B-ALL or T-ALL undergoing allo-HSCT with either TBI–etoposide or myeloablative busulfan- or treosulfan-based conditioning, according to age (≤4 vs >4 years) and disease status (CR1 vs CR2 or beyond).

**Copyright Information:** (Online) ISSN: 2572-9241
©2026 The Author(s). HemaSphere is published by John Wiley & Sons Ltd on behalf of European Hematology Association. This is an open access Abstract Book distributed under the Attribution-NonCommercial-NoDerivs (CC BY-NC-ND), which allows third parties to download the articles and share them with others as long as they credit the author and the Abstract Book, but they cannot change the content in any way or use them commercially.
**Abstract Book Citations:** Authors, Title, HemaSphere, 2026;10:(S1):pages. The abstract book DOIs can be found on the "Author Information" page.
**Disclaimer:** Articles published in the journal HemaSphere exclusively reflect the opinions of the authors. The authors are responsible for all content in their abstracts including accuracy of the facts, statements, citing resources, etc.

**Results**

Between 2013 and october 2025, 1685 patients underwent HSCT from an HLA-identical sibling donor (MSD) or 9-10/10 matched donor, across 88 international centers.
The cohort included 392 T-ALL and 1,293 B-ALL patients. Three-year overall survival (OS) was 74% in T-ALL and 77% in B-ALL, with corresponding Event-Free-Survival (EFS) of 70% and 64%. The Cumulative Incidence of Relapse (CIR) was higher in B-ALL (29%) than in T-ALL (20%, p=0.019), while cumulative incidence of non relapse mortality (CI-NRM) was similar (10% vs 8%, p=0.125).
Among children ≤4 years old, no significant differences in OS, EFS, CIR or CI-NRM were observed between phenotypes (3y EFS 65% vs 52% and OS 73% vs 70%).
Among patients >4 years old, outcomes were comparable between T-ALL and B-ALL (3y EFS 72% vs 68% and OS 74% vs 80%).
Among 392 T-ALL patients, 38 patients were aged **≤4 years old** and 315 patients were aged **> 4 years old.**
Among the patients **≤4 years old,** only 5 (13%) received TBI-etoposide (all >2 years old). At 3 years, the EFS and OS were **66%** and **73%** respectively with a CI-NRM of 5% and a CIR of 29%.
Among the 231 patients **> 4 years old with T-ALL in CR1,** (median age of 10.2 years (4.1-18.6)), 157 (68%) received TBI-etoposide. At 3 years, the EFS and OS were **76% and 78**% respectively with a CI NRM of 7% and CIR of 16%. There was no difference in EFS, OS, GRFS, CI-NRM or CIR according to type of donor nor conditioning (3y EFS **76% vs 74%** and **OS 77% vs 77%** respectively in TBI-etoposide vs chemo-conditioning group).
Among the 84 patients **> 4 years old with T-ALL in CR2** or later, (median age of 10.3 years (4-19.5)), 67 (80%) received TBI-etoposide. The timing of relapse was distributed as follows: very early 38%, early 27%, late 30%. At 3 years, the EFS and OS were **60% and 65%** respectively with a GRFS of 52%, a CI-NRM of 13% and CIR of 26%. There was no significative impact of donor type or site or timing of the previous relapse in EFS, OS, GRFS, CI NRM or CIR. In contrast, there was a significant superiority of TBI-etoposide over chemo-conditioning for 3 year-EFS (**67% vs 31%,** p-value=0.01), OS (**72% vs 38%,** p-value=0.005), GRFS (**58% vs 25%,** p-value=0.014) and CIR (**19% vs 56%,** p-value=0.002) but no difference in CI NRM (14% vs 13%).

**Summary/Conclusion**

Allo-HSCT allows cure of many patients with T-ALL, especially when transplanted in CR1. Use of TBI- etoposide must be preferred in patients with ≥CR2. In patients transplanted in CR1, chemo-based conditioning achieved similar survival outcome as TBI-etoposide.

**Copyright Information:** (Online) ISSN: 2572-9241
©2026 The Author(s). HemaSphere is published by John Wiley & Sons Ltd on behalf of European Hematology Association. This is an open access Abstract Book distributed under the Attribution-NonCommercial-NoDerivs (CC BY-NC-ND), which allows third parties to download the articles and share them with others as long as they credit the author and the Abstract Book, but they cannot change the content in any way or use them commercially.
**Abstract Book Citations:** Authors, Title, HemaSphere, 2026;10:(S1):pages. The abstract book DOIs can be found on the “Author Information” page.
**Disclaimer:** Articles published in the journal HemaSphere exclusively reflect the opinions of the authors. The authors are responsible for all content in their abstracts including accuracy of the facts, statements, citing resources, etc.

| conditioning | Patients | Events | 2-yrs GRFS | 3-yrs. GRFS | 5-yrs. GRFS | p-value |
|---|---|---|---|---|---|---|
| TBI | 63 | 27 | 0.58±0.06 | 0.58±0.06 | 0.58±0.06 | 0.014 |
| CHEMO | 16 | 12 | 0.25±0.11 | 0.25±0.11 | 0.25±0.11 | |

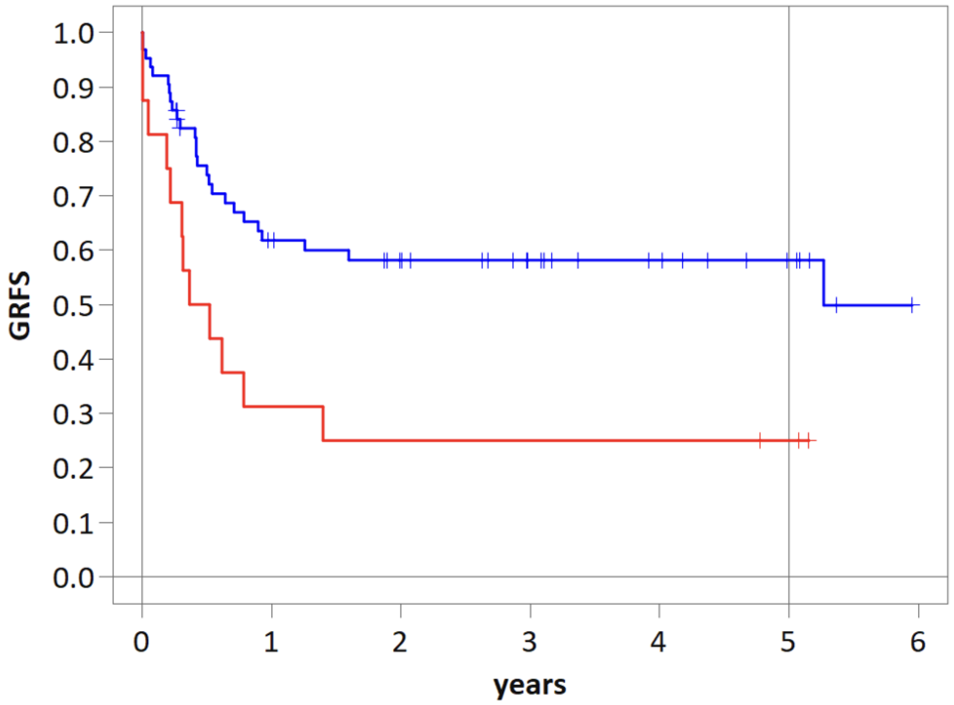

Figure : GRFS with respect to conditioning, T-ALL MSD/MD > 4 with alloHSCT in CR2

**Copyright Information:** (Online) ISSN: 2572-9241
©2026 The Author(s). HemaSphere is published by John Wiley & Sons Ltd on behalf of European Hematology Association. This is an open access Abstract Book distributed under the Attribution-NonCommercial-NoDerivs (CC BY-NC-ND), which allows third parties to download the articles and share them with others as long as they credit the author and the Abstract Book, but they cannot change the content in any way or use them commercially.
**Abstract Book Citations:** Authors, Title, HemaSphere, 2026;10:(S1):pages. The abstract book DOIs can be found on the "Author Information" page.
**Disclaimer:** Articles published in the journal HemaSphere exclusively reflect the opinions of the authors. The authors are responsible for all content in their abstracts including accuracy of the facts, statements, citing resources, etc.

### (PF1117) ROMIPLOSTIM N01 FOR PLATELET RECOVERY AFTER UNRELATED UMBILICAL CORD BLOOD TRANSPLANTATION IN PATIENTS WITH HEMATOLOGICAL MALIGNANCIES: A PHASE II, SINGLE-ARM STUDY

**Topic:** 23. Stem cell transplantation - Clinical

Wanjie Wang*[1, 2, 3], Aijie Huang[1, 2, 3], Wen Yao[1], Kaidi Song[1], Ping Qiang[1], Yongsheng Han[1], Meijuan Tu[1], Qing Cheng[1], Baolin Tang[1, 2, 3], Xiaoyu Zhu[1, 2, 3]

*[1]Department of Hematology, The First Affiliated Hospital of University of Science and Technology of China, Division of Life Sciences and Medicine, University of Science and Technology of China, Hefei, China; [2]Anhui Provincial Key Laboratory of Blood Research and Applications, Hefei, China; [3]Blood and Cell Therapy Institute, Division of Life Sciences and Medicine, University of Science and Technology of China, Hefei, China;*

### Background

Delayed hematopoietic recovery, particularly platelet recovery, remains a major limitation of unrelated umbilical cord blood transplantation (UCBT) due to the limited graft cell dose. Romiplostim N01 may accelerate platelet reconstitution after UCBT, but prospective clinical data are limited.

### Aims

To evaluate the efficacy and safety of romiplostim N01 for promoting platelet recovery after UCBT in adults with hematologic malignancies.

### Methods

This single-center, single-arm phase II trial (Dec 2024-Dec 2026) targeted enrollment of 34 adults with hematologic malignancies after UCBT. Romiplostim N01 was administered for 4 doses at 5ug/kg, initiated between day 1 and day 28 post-UCBT. Primary endpoints were time to platelet engraftment and the cumulative platelet engraftment rate by day 28 (platelets ≥ $20\times10^9$/L for 7 consecutive days without transfusion). Secondary endpoints included time to platelet recovery (≥ $50\times10^9$/L for 7 consecutive days without transfusion), achievement of platelets ≥ $100\times10^9$/L after stopping transfusions, total platelet transfusion volume within 4 weeks, cumulative neutrophil engraftment by day 28 (ANC ≥ $0.5\times10^9$/L for 3 consecutive days), bone marrow megakaryocyte counts at week 4, and safety.

**Copyright Information:** (Online) ISSN: 2572-9241
©2026 The Author(s). HemaSphere is published by John Wiley & Sons Ltd on behalf of European Hematology Association. This is an open access Abstract Book distributed under the Attribution-NonCommercial-NoDerivs (CC BY-NC-ND), which allows third parties to download the articles and share them with others as long as they credit the author and the Abstract Book, but they cannot change the content in any way or use them commercially.
**Abstract Book Citations:** Authors, Title, HemaSphere, 2026;10:(S1):pages. The abstract book DOIs can be found on the "Author Information" page.
**Disclaimer:** Articles published in the journal HemaSphere exclusively reflect the opinions of the authors. The authors are responsible for all content in their abstracts including accuracy of the facts, statements, citing resources, etc.

**Results**

As of 25 Feb 2026, 30 patients were enrolled. One withdrew after the first dose due to liver function impairment. Among 29 evaluable patients, the median age was 37 years (range 18-57 years), and 48% were female. Diagnoses included acute myeloid leukemia (AML, n=14), acute lymphoblastic leukemia (ALL, n=10), mixed phenotype acute leukemia (MPAL, n=1), myelodysplastic syndromes (MDS, n=3), and extranodal NK/T-cell lymphoma (ENKTL, n=1). All received myeloablative conditioning and ≥ 6/10 HLA-matched UCB grafts. Median infused doses were TNC $2.04 \times 10^7$/kg (range 0.61-9.40 $\times 10^7$/kg) and $CD34^+$ $1.78 \times 10^5$/kg (range 0.65-3.90$\times 10^5$/kg). Neutrophil engraftment occurred at a median of 16 days (range 12-25 days) with a day-28 cumulative rate of 100%. Platelet engraftment was achieved in 27/29 at a median of 35 days (range 25-74 days); cumulative platelet engraftment was 24.1% at day 28 (95% CI 12.3-44.1) and 89.7% by day 60 (95% CI 75.7-97.4). Platelet recovery (PLT ≥ $50 \times 10^9$/L) occurred in 24 patients at a median of 42.5 days (range 18-141 days); 21 reached PLT ≥ $100 \times 10^9$/L at a median of 52 days (range 33-314 days). Median platelet transfusion requirement was 8 units (range 4-19 units). Grade II-IV aGVHD occurred in 7/29 (24.1%), CMV infection in 13/29 (44.8%), and hemorrhagic cystitis in 11/29 (37.9%). No treatment-related SAEs were observed.

**Summary/Conclusion**

In this interim analysis, romiplostim N01 may promote platelet engraftment and recovery after unrelated UCBT, with no treatment-related SAEs. Final analyses are ongoing.

**Copyright Information:** (Online) ISSN: 2572-9241
©2026 The Author(s). HemaSphere is published by John Wiley & Sons Ltd on behalf of European Hematology Association. This is an open access Abstract Book distributed under the Attribution-NonCommercial-NoDerivs (CC BY-NC-ND), which allows third parties to download the articles and share them with others as long as they credit the author and the Abstract Book, but they cannot change the content in any way or use them commercially.
**Abstract Book Citations:** Authors, Title, HemaSphere, 2026;10:(S1):pages. The abstract book DOIs can be found on the "Author Information" page.
**Disclaimer:** Articles published in the journal HemaSphere exclusively reflect the opinions of the authors. The authors are responsible for all content in their abstracts including accuracy of the facts, statements, citing resources, etc.

### (PF1118) IMPACT OF DONOR AGE ON OUTCOMES OF ALTERNATIVE DONOR HEMATOPOIETIC STEM CELL TRANSPLANTATION IN PEDIATRIC SEVERE APLASTIC ANEMIA

**Topic:** 23. Stem cell transplantation - Clinical

Jingyu Gao*[1]

[1]*Shanghai Children's Medical Center (SCMC) affiliated to Shanghai Jiao Tong University School of Medcine, Hematopoietic Stem Cell Transplantation Department, Shanghai, China;*

### Background

Allogeneic hematopoietic stem cell transplantation (allo-HSCT) is a definitive therapeutic intervention for pediatric patients with severe aplastic anemia (SAA). In the absence of a matched sibling donor (MSD), identifying the optimal donor source remains a clinical challenge.

### Aims

The primary objective was to evaluate clinical outcomes across different donors to identify key factors that optimize donor selection for pediatric SAA.

### Methods

We conducted a retrospective analysis of pediatric SAA patients who underwent HSCT from matched unrelated donors (MUD), mismatched unrelated donors (MMUD), and mismatched related donors (MMRD) at a single center between 2001 and 2024.

### Results

The study cohort comprised 488 patients (MUD: n=194; MMUD: n=97; MMRD: n=197). The median recipient and donor ages were 7.6 years (range: 0.8–17.8) and 31 years (range: 2–50), respectively. No statistically significant differences were observed between MUD, MMUD, and MMRD regarding the incidence of primary graft failure (1.6%, 2.0%, and 4.4%, respectively; p=0.37) or non-relapse mortality (11.5%, 15.2%, and 12.9%, respectively; p=0.60). MUD recipients experienced significantly lower rates of Grade III-IV acute GVHD (4.0%) than MMUD (20.4%) and MMRD (12.6%) ($p < 0.001$). Overall survival (OS) were 88.7% for MUD, 84.8% for MMUD, and 76.7% for MMRD (p=0.30). Multivariable modeling identified advanced donor age, rather than donor type, as a significant independent predictor of inferior OS (Hazard Ratio [HR], 1.38; 95% CI, 1.02–1.86; p=0.04).

### Summary/Conclusion

Our findings demonstrate that MUD, MMUD, and MMRD are viable alternative donors for pediatric SAA patients lacking an MSD. Additionally, donor age is a more significant determinant, and a younger donor is preferable in donor selection.

**Copyright Information:** (Online) ISSN: 2572-9241
©2026 The Author(s). HemaSphere is published by John Wiley & Sons Ltd on behalf of European Hematology Association. This is an open access Abstract Book distributed under the Attribution-NonCommercial-NoDerivs (CC BY-NC-ND), which allows third parties to download the articles and share them with others as long as they credit the author and the Abstract Book, but they cannot change the content in any way or use them commercially.
**Abstract Book Citations:** Authors, Title, HemaSphere, 2026;10:(S1):pages. The abstract book DOIs can be found on the “Author Information” page.
**Disclaimer:** Articles published in the journal HemaSphere exclusively reflect the opinions of the authors. The authors are responsible for all content in their abstracts including accuracy of the facts, statements, citing resources, etc.

### (PF1119) PHASE II STUDY OF AUTOLOGOUS HEMATOPOIETIC STEM CELL TRANSPLANTATION COMBINED WITH AUTOLOGOUS CD19-CAR T CELL INFUSION IN ADULTS WITH HIGH-RISK B-CELL ACUTE LYMPHOBLASTIC LEUKEMIA: CLINICAL OUTCOMES

**Topic:** 23. Stem cell transplantation - Clinical

Erlie Jiang[1], Rongli Zhang*[1], Aiming Pang[1], Donglin Yang[1], Yi He[1], Jialin Wei[1], Yigeng Cao[1], Xin Chen[1], Wenbin Cao[1], Chen Liang[1], Qiaoling MA[1], Weihua Zhai[1], Qingzhen Liu[1], Tingting Zhang[1], Yuanyuan Shi[1], Xiaofei Ni[1], Mengnan Lv[1], Jiali Wang[1], Fengjiao Wang[1], Mingyang Wang[1], Xiaohui Zheng[1], Dan Feng[1]

[1]*Institute of Hematology & Blood Diseases Hospital, Tianjin, China;*

### Background

Autologous hematopoietic stem cell transplantation (auto-HSCT) is effective for adult B-cell acute lymphoblastic leukemia (B-ALL) patients achieving minimal residual disease (MRD)-negative status after first-line therapy. High-risk patients remain prone to relapse. Inaticabtagene autoleucel (Inati-cel), a CD19-targeted CAR-T derived from the HI19a clone with a 4-1BB/CD3-ζ costimulatory domain, has demonstrated potent activity in relapsed/refractory (r/r) B-ALL. Combining auto-HSCT with autologous CD19-CAR T cells may eliminate residual leukemia and reduce relapse.

### Aims

To evaluate the feasibility, safety, and efficacy of auto-HSCT combined with autologous CD19-CAR T therapy in high-risk adult B-ALL.

### Methods

This single-arm phase II study (NCT06608342) enrolled high-risk adult B-ALL patients who achieved CR and MRD negativity after induction and consolidation therapy, from April 2024. Patients received myeloablative conditioning followed by infusion of autologous stem cells. Inati-cel was given on day +7 post-transplant. Follow-up was continued for 24 months. Key assessments included CAR-T expansion, cytokines, hematopoietic reconstitution, and MRD (qPCR, $10^{-6}$). The primary endpoint was two-year relapse incidence. Secondary endpoints included: non-relapse mortality, PFS, OS, infection incidence, and the Karnofsky Performance Status score.

**Copyright Information:** (Online) ISSN: 2572-9241
©2026 The Author(s). HemaSphere is published by John Wiley & Sons Ltd on behalf of European Hematology Association. This is an open access Abstract Book distributed under the Attribution-NonCommercial-NoDerivs (CC BY-NC-ND), which allows third parties to download the articles and share them with others as long as they credit the author and the Abstract Book, but they cannot change the content in any way or use them commercially.
**Abstract Book Citations:** Authors, Title, HemaSphere, 2026;10:(S1):pages. The abstract book DOIs can be found on the "Author Information" page.
**Disclaimer:** Articles published in the journal HemaSphere exclusively reflect the opinions of the authors. The authors are responsible for all content in their abstracts including accuracy of the facts, statements, citing resources, etc.

**Results**

As of February 20, 2026, 15 patients (median 37 years; 33.3% male) were included in this analysis. Genetic abnormalities: BCR-ABL1 40%, MLL rearrangement 13.3%, IKZF1 6.7%, MEF2D::BCL9 rearrangement 6.7%, IGH rearrangement 6.7%. All achieved CR with MRD negativity pre-HSCT; Median 2 (range: 1–4) consolidation cycles. Conditioning: 13 TBI-based, 2 non-TBI. Median $CD34^{+}$ dose: 2.66 (range: 1.68–10.28)×$10^{6}$/kg; Median Inati-cel dose: 0.58(range: 0.41–0.68)×$10^{8}$ cells. At a median follow-up of 13.4 months, all remained MRD-negative; Neither median PFS nor OS had been reached; Estimated 1-year PFS and OS rates were both 100%. Neutrophil and platelet engraftment occurred at median 13 (range: 9–17) days and 20.5 (range: 10–159) days post-transplant, respectively. Inati-cel infusion had no impact on hematopoietic recovery.
CAR-T cells expanded in vivo with peak levels around day 14 and remained detectable for up to 12 months in one patient. In most patients, IL-5, IL-6 and IL-8 levels peaked within 7 days after CAR-T cell infusion and subsequently declined. Increases in IL-10, IL-17, TNF-γ were also observed. Following CAR-T infusion, one patient developed grade 2 cytokine release syndrome (CRS); no cases of immune effector cell-associated neurotoxicity syndrome (ICANS) were reported. One patient developed immune-mediated cystitis. Two patients developed bacteremia caused by Klebsiella pneumoniae and Escherichia coli. Nine patients experienced intestinal infections, one had a pulmonary infection, and five developed perianal infections. All infections occurred during the neutropenic phase and were successfully managed, with no infection-related fatalities.

**Summary/Conclusion**

Auto-HSCT combined with autologous CD19-CAR T cell infusion is feasible and safe in high-risk adult B-ALL. Longer follow-up is needed to assess long-term efficacy.

**Copyright Information:** (Online) ISSN: 2572-9241
©2026 The Author(s). HemaSphere is published by John Wiley & Sons Ltd on behalf of European Hematology Association. This is an open access Abstract Book distributed under the Attribution-NonCommercial-NoDerivs (CC BY-NC-ND), which allows third parties to download the articles and share them with others as long as they credit the author and the Abstract Book, but they cannot change the content in any way or use them commercially.
**Abstract Book Citations:** Authors, Title, HemaSphere, 2026;10:(S1):pages. The abstract book DOIs can be found on the "Author Information" page.
**Disclaimer:** Articles published in the journal HemaSphere exclusively reflect the opinions of the authors. The authors are responsible for all content in their abstracts including accuracy of the facts, statements, citing resources, etc.

### (PF1120) LONG-TERM EFFICACY AND SAFETY OF DONOR-DERIVED CAR-T CELLS INTEGRATED INTO ALLOGENEIC HSCT CONDITIONING FOR ULTRA-HIGH-RISK R/R B-NHL, T-LBL, AND MULTIPLE MYELOMA: A 5-YEAR FOLLOW-UP STUDY

**Topic:** 23. Stem cell transplantation - Clinical

Fan Yang*[1], Rui Liu[1], Zhonghua Fu[1], Yuelu Guo[1], Lixia MA[1], Miaomiao Cao[1], Biping Deng[2], Xiaoyan Ke[1], Kai Hu[1]

*[1]Beijing Gobroad Hospital, Department of Lymphoma and Myeloma Research Center, Beijing, China; [2]Beijing Gobroad Boren Hospital, Cytology Laboratory, Beijing, China;*

### Background

The prognosis for patients with refractory/relapsed (r/r) aggressive non-Hodgkin lymphoma (NHL) and multiple myeloma (MM) who have failed both autologous chimeric antigen receptor (CAR)-T cell therapy and autologous hematopoietic stem cell transplantation (ASCT) is dismal. We previously reported the feasibility and early efficacy of allogeneic HSCT (allo-HSCT) using a conditioning regimen that incorporates donor-derived CAR-T cells. However, long-term outcomes remain to be established.

### Aims

To evaluate the long-term clinical efficacy, durability of response, and 5-year safety profile of donor-derived CAR-T cells integrated into allo-HSCT conditioning for patients with r/r NHL and MM.

### Methods

Between September 2020 and October 2023, 25 heavily pre-treated patients were enrolled, including B-cell NHL (n=12), Burkitt lymphoma (n=4), T-lymphoblastic lymphoma (T-LBL, n=2), and MM (n=7). All patients had progressive disease (PD) after multiple lines of therapy, including chemotherapy (n=8), ASCT (n=11), and autologous CAR-T therapy (n=22). Donors included haploidentical (n=19), matched sibling (n=5), and matched unrelated (n=1). Target antigens for donor CAR-T cells were CD19/CD22 (B-NHL), CD7 (T-LBL), and BCMA (MM). Conditioning regimens were Bu/Flu-based for NHL and Mel/Flu-based for MM, both incorporating donor CAR-T cells. Graft-versus-host disease (GVHD) prophylaxis consisted of tacrolimus, MMF, short-term MTX, and ATG.

### Results

The median dose of donor-derived CAR-T cells infused was 2.5(range,1.87-4.0)×$10^6$/kg. All patients achieved successful engraftment, with median neutrophil and platelet recovery on days 13 and 16, respectively. Full donor chimerism was confirmed in all cases by day 28. After a median follow-up of 52.4 months (range, 21.6–83.2), the overall response rate (ORR) and complete response rate (CRR) were 83.3% (20/24) and 70.8% (17/24), respectively (one MM patient died early of myocardial infarction and was not evaluable). Among the 20 responders, the median duration of response (DOR) was 22 months (range, 4–72). The 48-month progression-free survival (PFS) and overall survival (OS) rates for these responders were 30.4% (95% CI: 13.8–53.8%) and 39.1% (95% CI: 18.8–58.8%), respectively. Median PFS and OS were 6.8 months and 16.0 months, respectively (Figure 1). Disease relapse occurred in 6/20 responders (30%) at a median of 3.7 months; notably, 2/2 patients with relapsed Burkitt lymphoma achieved sustained CR after subsequent donor CAR-T cell infusions. Non-relapse mortality (NRM) was primarily driven by severe infections (n=7) (Figure 2). Grade 3–4 cytokine release syndrome (CRS) occurred in 16.4%, and grade 1–2 acute GVHD in 27.5%. Late-onset adverse events (>30 days) included five cases of grade 3 extensive chronic GVHD.

**Copyright Information:** (Online) ISSN: 2572-9241
©2026 The Author(s). HemaSphere is published by John Wiley & Sons Ltd on behalf of European Hematology Association. This is an open access Abstract Book distributed under the Attribution-NonCommercial-NoDerivs (CC BY-NC-ND), which allows third parties to download the articles and share them with others as long as they credit the author and the Abstract Book, but they cannot change the content in any way or use them commercially.
**Abstract Book Citations:** Authors, Title, HemaSphere, 2026;10:(S1):pages. The abstract book DOIs can be found on the “Author Information” page.
**Disclaimer:** Articles published in the journal HemaSphere exclusively reflect the opinions of the authors. The authors are responsible for all content in their abstracts including accuracy of the facts, statements, citing resources, etc.

### Summary/Conclusion

Allo-HSCT conditioning incorporating donor-derived CAR-T cells demonstrates durable efficacy and a manageable safety profile in a subset of ultra-high-risk r/r NHL and MM patients who failed prior ASCT and autologous CAR-T. While high pre-transplant tumor burden remains a challenge, this strategy provides a potential curative option. Severe late-onset infection remains a critical concern requiring vigilant management.

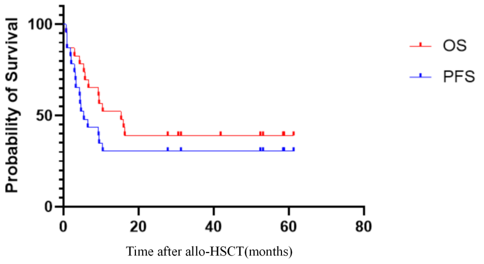

Figure 1 Kaplan–Meier estimates of progression-free survival and overall survival of responded patients

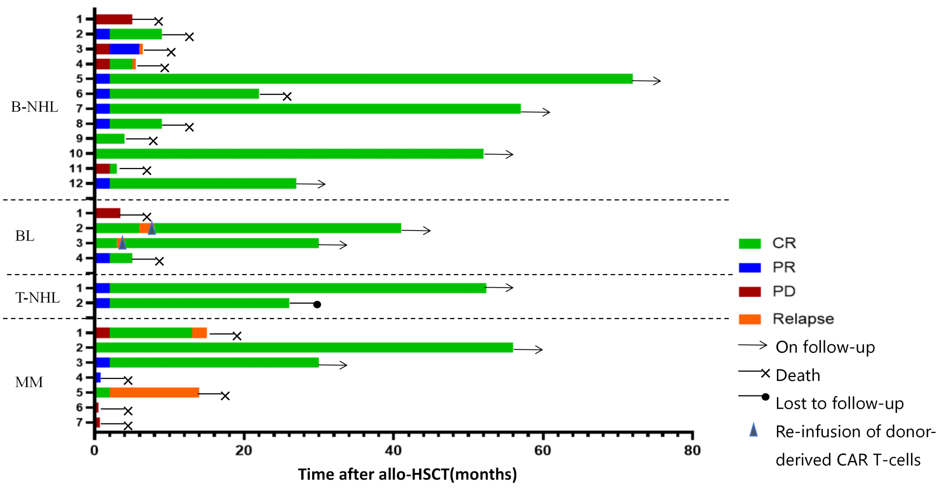

Figure 2 Swimmer plot showing the clinical response and follow-up of individual patients treated with allo-HSCT combined with donor-derived CAR-T cells.

Copyright Information: (Online) ISSN: 2572-9241
©2026 The Author(s). HemaSphere is published by John Wiley & Sons Ltd on behalf of European Hematology Association. This is an open access Abstract Book distributed under the Attribution-NonCommercial-NoDerivs (CC BY-NC-ND), which allows third parties to download the articles and share them with others as long as they credit the author and the Abstract Book, but they cannot change the content in any way or use them commercially.
Abstract Book Citations: Authors, Title, HemaSphere, 2026;10:(S1):pages. The abstract book DOIs can be found on the "Author Information" page.
Disclaimer: Articles published in the journal HemaSphere exclusively reflect the opinions of the authors. The authors are responsible for all content in their abstracts including accuracy of the facts, statements, citing resources, etc.

### (PF1121) SUPERIOR OUTCOMES WITH B-LEADER-MATCHED HAPLOIDENTICAL VERSUS MATCHED SIBLING DONORS IN AL/MDS PATIENTS AGED ≥40 YEARS

**Topic:** 23. Stem cell transplantation - Clinical

Yujun Wei*[1, 1, 2], Jingwen Tang[1, 3], Ruoling Yang[1, 2, 3], Yangliu Shao[1, 3], Songhua Luan[1], Lu Wang[3], Lili Wang[1], Fei Li[3], Yongli Wu[1], Kun Qian[3], Bo Peng[3], Haoyang Zhang[1, 3], Dongxue Ge[1, 2, 3], Yu Fang[1, 3], Liuqing Huang[1, 3], Qingyang Liu[1, 3], Liping Dou[1, 3], Dai-Hong Liu[1, 2, 3]

[1]*Department of Hematology, the First Medical Center of Chinese PLA General Hospital, Beijing, 100853, China, Beijing, China;* [2]*School of Medicine, Nankai University, Tianjin, 300071, China, Tianjin, China;* [3]*State Key Laboratory of Experimental Hematology, Senior Department of Hematology, the Fifth Medical Center of PLA General Hospital, Beijing, 100039, China, Beijing, China;*

### Background

Hematopoietic stem-cell transplantation (HSCT) is a curative option for and myelodysplastic syndrome (MDS). HLA matching is a pivotal determinant of outcomes in HSCT, with an HLA-matched sibling donors (MSDs) representing the optimal donor choice, primarily because of favorable engraftment and lower rates of severe graft-versus-host disease (GVHD). Extensive research has indicated that donor age is a pivotal determinant of transplant outcomes. Studies have indicated that hematopoietic stem cells from older donors may exhibit potential clonal hematopoiesis. With advancing safety profiles in allogeneic hematopoietic stem cell transplantation (allo-HSCT), determining whether HLA-matched siblings remain the optimal donor choice for older adults (≥40 years) and whether haploidentical-HSCT demonstrates superior survival outcomes have become pivotal focuses in transplantation research.

### Aims

The goal of this study was to explore the efficacy of haploidentical or matched sibling allo-HSCT in patients over the age of 40 years with acute leukemia (AL) and myelodysplastic syndrome (MDS).

### Methods

In this retrospective study, 195 consecutive AL/MDS patients (≥40 years) who received allo-HSCT between December 2014 and December 2023 were included and analyzed using a 1:2 matched-pair design. The cohort comprised 130 patients with haploidentical donors (HIDs) and 65 with HLA-matched sibling donors (MSDs). Each patient in the MSDs-HSCT group was matched to two patients who received transplants from young haploidentical donors (offspring). Matching was based on patient age using the nearest-neighbor algorithm. For HLA-matching analysis, “DRB1 mismatch” was defined as the presence of at least a single allele-level mismatch between the donor and recipient at the HLA-DRB1 locus. Similarly, “B-leader match” status was determined on the basis of amino acid sequence identity in the leader peptide region of HLA-B. HLA-B leader matching was performed according to the HLA-B leader mismatch calculator (https://www.ebi.ac.uk/ipd/imgt/hla/dpb.html).

**Copyright Information:** (Online) ISSN: 2572-9241
©2026 The Author(s). HemaSphere is published by John Wiley & Sons Ltd on behalf of European Hematology Association. This is an open access Abstract Book distributed under the Attribution-NonCommercial-NoDerivs (CC BY-NC-ND), which allows third parties to download the articles and share them with others as long as they credit the author and the Abstract Book, but they cannot change the content in any way or use them commercially.
**Abstract Book Citations:** Authors, Title, HemaSphere, 2026;10:(S1):pages. The abstract book DOIs can be found on the “Author Information” page.
**Disclaimer:** Articles published in the journal HemaSphere exclusively reflect the opinions of the authors. The authors are responsible for all content in their abstracts including accuracy of the facts, statements, citing resources, etc.

### Results

Patients who received transplants from younger haploidentical donors with mismatched DRB1 exhibited a significantly lower 5-year cumulative incidence of relapse (15.4%) than those who received MSD-HSCT (45.3%) or transplants from younger haploidentical DRB1-matched donors (26.7%; *p* < 0.001). However, the 5-year nonrelapse mortality (NRM) was highest in the younger haploidentical B-leader-mismatched group (43.8%) compared with that in the B-leader-matched group 1 (5.4%) and MSD group (6.3%). Accordingly, compared with recipients of older MSD donors or younger B-leader-mismatched donors, recipients of younger haploidentical B-leader-matched donors exhibited superior overall survival (OS) and disease-free survival (DFS). In the multivariate analysis, having a younger haploidentical B-leader-mismatched donor was identified as an independent adverse prognostic factor for OS (hazard ratio = 2.03; 95% confidence interval: 1.03–3.98; *p* = 0.040).

### Summary/Conclusion

In AL/MDS patients aged ≥40 years, compared with MSD donors, younger haploidentical B-leader-matched and DRB1-mismatched donors provided superior survival outcomes.

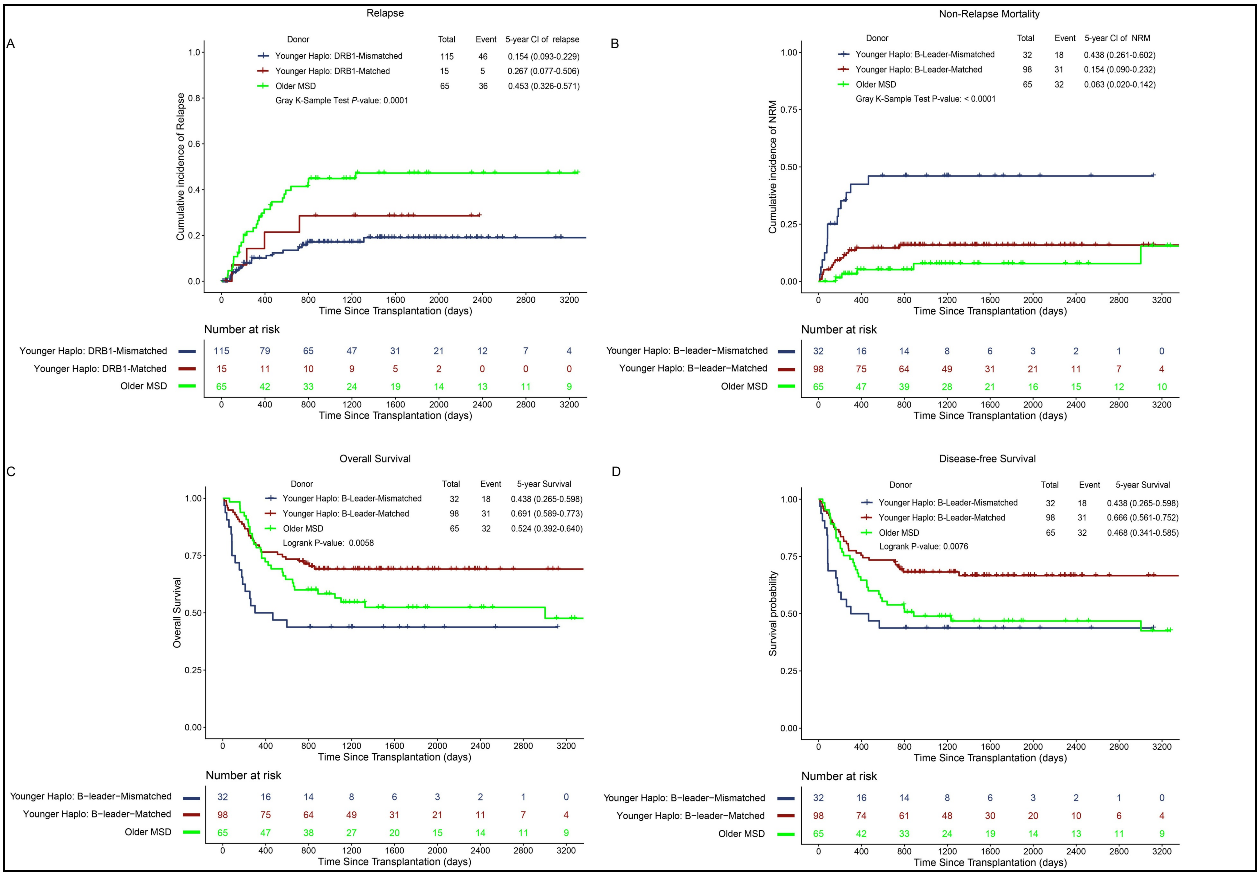

**Copyright Information:** (Online) ISSN: 2572-9241
©2026 The Author(s). HemaSphere is published by John Wiley & Sons Ltd on behalf of European Hematology Association. This is an open access Abstract Book distributed under the Attribution-NonCommercial-NoDerivs (CC BY-NC-ND), which allows third parties to download the articles and share them with others as long as they credit the author and the Abstract Book, but they cannot change the content in any way or use them commercially.
**Abstract Book Citations:** Authors, Title, HemaSphere, 2026;10:(S1):pages. The abstract book DOIs can be found on the "Author Information" page.
**Disclaimer:** Articles published in the journal HemaSphere exclusively reflect the opinions of the authors. The authors are responsible for all content in their abstracts including accuracy of the facts, statements, citing resources, etc.

### (PF1122) INTEGRATING IMS–IMWG GENOMIC RISK WITH R2-ISS IMPROVES RISK STRATIFICATION IN TRANSPLANT-ELIGIBLE MULTIPLE MYELOMA

**Topic:** 23. Stem cell transplantation - Clinical

Damian Mikulski*[1], Mark R. Tanner[2], Qaiser Bashir[2], Samer A. Srour[2], Neeraj Saini[2], Paul Lin[2], Jeremy L. Ramdial[2], Yago Nieto[2], Guilin Tang[3], Yosra Aljawai[2], Krina Patel[4], Partow Kebriaei[2], Jing Christine Ye[4], Mahmoud R. Gaballa[4], Sheeba K. Thomas[4], Robert Z Orlowski[4], Richard E. Champlin[2], Elizabeth J. Shpall[2], Muzaffar H. Qazilbash[2], Oren Pasvolsky[4]

[1]*Medical University of Lodz, Department of Biostatistics and Translational Medicine, Lodz, Poland;*[2]*The University of Texas MD Anderson Cancer Center, Department of Stem Cell Transplantation and Cellular Therapy, Houston, United States of America;* [3]*The University of Texas MD Anderson Cancer Center, Department of Hematopathology, Houston, United States of America;* [4]*The University of Texas MD Anderson Cancer Center, Department of Lymphoma and Myeloma, Houston, United States of America;*

### Background

The recently proposed International Myeloma Society–International Myeloma Working Group (IMS-IMWG) classification provides a genomically driven definition of high-risk (HR) multiple myeloma (MM), yet its relationship to established clinical staging systems is not clearly defined.

### Aims

To assess concordance between IMS–IMWG risk classification and Revised International Staging System (R-ISS)/Second Revision of the International Staging System (R2-ISS), and to determine whether combining IMS–IMWG with R2-ISS improves risk stratification in transplant-eligible patients.

### Methods

We retrospectively analyzed transplant-eligible patients treated with triplet-based induction followed by autologous transplantation (autoHCT) at our institution. Patients were classified according to IMS–IMWG, R-ISS, and R2-ISS criteria. Progression-free survival (PFS) and overall survival (OS) were assessed using Kaplan–Meier methods and multivariable Cox proportional hazards models. Model discrimination was evaluated using concordance indices.

**Copyright Information:** (Online) ISSN: 2572-9241
©2026 The Author(s). HemaSphere is published by John Wiley & Sons Ltd on behalf of European Hematology Association. This is an open access Abstract Book distributed under the Attribution-NonCommercial-NoDerivs (CC BY-NC-ND), which allows third parties to download the articles and share them with others as long as they credit the author and the Abstract Book, but they cannot change the content in any way or use them commercially.
**Abstract Book Citations:** Authors, Title, HemaSphere, 2026;10:(S1):pages. The abstract book DOIs can be found on the “Author Information” page.
**Disclaimer:** Articles published in the journal HemaSphere exclusively reflect the opinions of the authors. The authors are responsible for all content in their abstracts including accuracy of the facts, statements, citing resources, etc.

**Results**

A total of 914 patients were included. Induction regimens consisted of VRD (bortezomib, lenalidomide, dexamethasone) in 426 patients (46.6%), KRD (carfilzomib, lenalidomide, dexamethasone) in 325 (35.6%), and VCD (bortezomib, cyclophosphamide, dexamethasone) in 163 (17.8%). Based on IMS–IMWG criteria, 676 patients (74.0%) were classified as standard risk (SR) and 238 (26.0%) as HR. Using R-ISS, most patients were classified as stage II (60.8%), followed by stage I (21.9%) and stage III (17.3%). Application of the IMS–IMWG criteria resulted in a stepwise enrichment of HR disease across R-ISS stages, with 1.5%, 27.9%, and 50.6% of patients in stages I, II, and III, respectively, reclassified as HR. According to the R2-ISS, patients were distributed across stages I (10.8%), II (36.0%), III (42.0%), and IV (11.2%). No patients classified as R2-ISS stage I met IMS–IMWG HR criteria, whereas HR disease was observed in 7.0% of stage II, 33.6% of stage III, and 84.3% of stage IV patients.
Despite classification as IMS–IMWG SR, substantial outcome heterogeneity persisted. Stratification using R2-ISS identified a distinct intermediate-risk subgroup, in which SR patients classified as R2-ISS stage III or IV experienced significantly inferior PFS (HR 1.63, 95%CI: 1.28–2.07) and OS (1.89, 95%CI: 1.30–2.74) compared with SR patients in R2-ISS stages I–II, while maintaining outcomes superior to those observed in IMS–IMWG HR disease (Figure 1). In contrast, stratification of SR patients by R-ISS demonstrated less pronounced discrimination between stages I–II and stage III. Concordance index analyses consistently favored the combined R2-ISS plus IMS–IMWG model over R-ISS–based or single-system approaches, reflecting improved separation of risk groups across survival endpoints.
Notably, the distinct outcomes observed across groups defined by the combined IMS–IMWG and R2-ISS stratification remained significant in multivariable analyses. Compared with SR patients classified as R2-ISS stages I–II, those with SR disease and R2-ISS stage III or IV had a higher risk of progression (HR 1.61, 95% CI 1.26–2.07; P<0.001) and death (HR 1.65, 95% CI 1.13–2.40; P<0.001).

**Summary/Conclusion**

Combining IMS–IMWG genomic risk classification with R2-ISS enhances risk stratification in transplant-eligible MM, identifying an intermediate-risk subgroup and improving prognostic discrimination beyond established staging systems.

**Copyright Information:** (Online) ISSN: 2572-9241
©2026 The Author(s). HemaSphere is published by John Wiley & Sons Ltd on behalf of European Hematology Association. This is an open access Abstract Book distributed under the Attribution-NonCommercial-NoDerivs (CC BY-NC-ND), which allows third parties to download the articles and share them with others as long as they credit the author and the Abstract Book, but they cannot change the content in any way or use them commercially.
**Abstract Book Citations:** Authors, Title, HemaSphere, 2026;10:(S1):pages. The abstract book DOIs can be found on the “Author Information” page.
**Disclaimer:** Articles published in the journal HemaSphere exclusively reflect the opinions of the authors. The authors are responsible for all content in their abstracts including accuracy of the facts, statements, citing resources, etc.

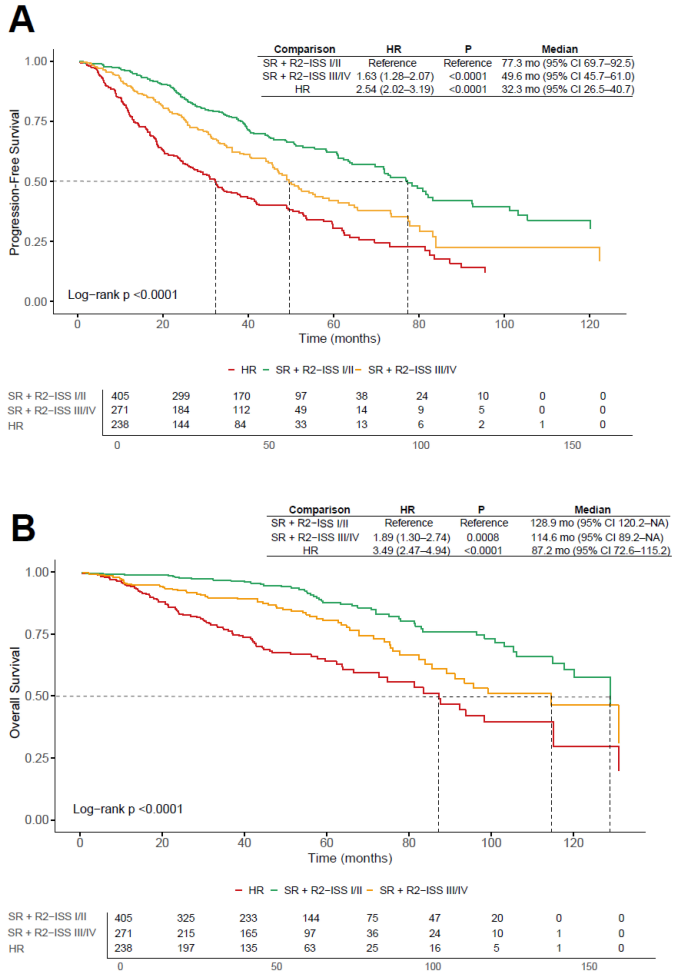

**Figure 1. Identification of an intermediate-risk subgroup using combined IMS–IMWG and R2-ISS stratification.** Kaplan–Meier estimates of (A) progression-free survival (PFS) and (B) overall survival (OS) stratified by combined International Myeloma Society–International Myeloma Working Group (IMS–IMWG) risk status and second revision of the International Staging System (R2-ISS). Patients classified as IMS–IMWG high–risk (HR) are compared with IMS–IMWG standard-risk (SR) patients, further stratified by R2-ISS stage (SR + R2-ISS I/II vs SR + R2-ISS III/IV). Survival curves were compared using the log-rank test; hazard ratios with 95% confidence intervals are shown.

**Copyright Information:** (Online) ISSN: 2572-9241
©2026 The Author(s). HemaSphere is published by John Wiley & Sons Ltd on behalf of European Hematology Association. This is an open access Abstract Book distributed under the Attribution-NonCommercial-NoDerivs (CC BY-NC-ND), which allows third parties to download the articles and share them with others as long as they credit the author and the Abstract Book, but they cannot change the content in any way or use them commercially.
**Abstract Book Citations:** Authors, Title, HemaSphere, 2026;10:(S1):pages. The abstract book DOIs can be found on the "Author Information" page.
**Disclaimer:** Articles published in the journal HemaSphere exclusively reflect the opinions of the authors. The authors are responsible for all content in their abstracts including accuracy of the facts, statements, citing resources, etc.

### (PF1123) GENETIC ABNORMALITIES INFLUENCE THE PROGNOSTIC VALUE OF PRE-TRANSPLANT MEASURABLE RESIDUAL DISEASE IN PATIENTS WITH RELAPSED/REFRACTORY ACUTE MYELOID LEUKAEMIA: A MULTICENTRE STUDY

**Topic:** 23. Stem cell transplantation - Clinical

Yue Jin*[1], Peng Zhao[1], Haixia Fu[1], Qiusha Huang[1], Ying Lu[2], Dingming Wan[3], Xi Zhang[4], Fang Zhou[5], Nainong Li[6], Li Liu[7], Pengcheng He[8], Shifeng Lou[9], Fei Li[10], Han Zhu[11], Yunya Luo[12], Jin Wu[1], Zhuo-Yu An[1], Li-Ping Yang[1], Chencong Wang[1], Fengxue Zhu[1], Xiaodong Mo[1], Xiang-Yu Zhao[1], Yu Wang[1], Yingjun Chang[1], Lanping Xu[1], Xiaojun Huang[1], Xiaohui Zhang[1]

*[1]Peking University People's Hospital, Peking University Institute of Hematology, National Clinical Research Center for Hematologic Disease, Beijing Key Laboratory of Cell and Gene Therapy for Hematologic Malignancies, Peking University, Beijing, China., Beijing, China; [2]Department of Hematology, The Affiliated People's Hospital of Ningbo University., ningbo, China; [3]The First Affiliated Hospital of Zhengzhou University, Zhengzhou, China;[4]Medical Center of Hematology, Xinqiao Hospital of Army Medical University State Key Laboratory of Trauma and Chemical Poisoning Chongqing Key Laboratory of Hematology and Microenvironment., Chongqing, China; [5]Hematology Department, The 960th Hospital of The People's Liberation Army (PLA) Joint Logistics Support Force, Jinan, China; [6]Fujian Institute of Hematology,Fujian Provincial Key Laboratory on Hematology,Fujian Medical University Union Hospital, Fuzhou, China; [7]The Second Affiliated Hospital of Air Force Medical University, Xi'an, China; [8]Department of hematology, the first affiliated hospital of xi'an jiaotong university, Xi'an, China; [9]Department of Hematology, The Second Affiliated Hospital of Chongqing Medical University; Chongqing Medical University., Chongqing, China; [10]Senior Department of Hematology, The Fifth Medical Center of Chinese People's Liberation Army(PLA) General Hospital., Beijing, China; [11]Department of Hematology, Jiangsu Province Hospital, The First Affiliated Hospital of Nanjing Medical University., Nanjing, China; [12]The second xiangya hospital of central south university, Changsha, China;*

### Background

Allogeneic haematopoietic stem cell transplantation (allo-HSCT) is a curative treatment for patients with relapsed/refractory acute myeloid leukaemia (R/R AML). Achieving complete remission (CR) before HSCT is associated with improved prognosis, but the prognostic value of pre-HSCT measurable residual disease (MRD) remains controversial. Genetic abnormalities are a cornerstone of AML prognostic stratification. In addition, variability exists in the methods used for MRD assessment. Systematic comparative studies evaluating whether the prognostic stratification role of MRD assessment methods differs across AML patients with distinct genetic backgrounds are lacking.

### Aims

This study aimed to investigate the differential prognostic value of pre-transplant multiparameter flow cytometry-based MRD (MFC-MRD), Wilms' tumour 1 (WT1) overexpression, and molecular MRD across AML subtypes with distinct genetic backgrounds and to determine whether these MRD methods provide complementary prognostic information.

### Methods

A multicentre study involving contemporaneous cohorts of R/R AML patients who achieved CR or CR with incomplete haematologic recovery (CRi) before HSCT in 12 HSCT centres in China was conducted. The probabilities of overall survival (OS) were estimated using the Kaplan–Meier method and compared using the log-rank test. For prognostic analyses, a least absolute shrinkage and selection operator (LASSO)-penalized Cox proportional hazards model was applied for variable selection. The selected variables were then entered into a multivariable Cox proportional hazards model. A backward stepwise elimination procedure was applied to derive the final model.

**Copyright Information:** (Online) ISSN: 2572-9241
©2026 The Author(s). HemaSphere is published by John Wiley & Sons Ltd on behalf of European Hematology Association. This is an open access Abstract Book distributed under the Attribution-NonCommercial-NoDerivs (CC BY-NC-ND), which allows third parties to download the articles and share them with others as long as they credit the author and the Abstract Book, but they cannot change the content in any way or use them commercially.
**Abstract Book Citations:** Authors, Title, HemaSphere, 2026;10:(S1):pages. The abstract book DOIs can be found on the "Author Information" page.
**Disclaimer:** Articles published in the journal HemaSphere exclusively reflect the opinions of the authors. The authors are responsible for all content in their abstracts including accuracy of the facts, statements, citing resources, etc.

### Results

Among the 403 patients included, the independent prognostic factors for overall survival (OS) included MFC-MRD positivity (HR 1.59), WT1 overexpression (HR 1.59), FLT3-ITD mutation (HR 1.70), TP53 mutation (HR 1.91), KMT2A rearrangement (KMT2Ar) (HR 2.14), and complex karyotypes (HR 2.06; all P<0.05). According to the ELN 2022 genetic risk classification, pre-HSCT MFC-MRD positivity was associated with poor OS in patients classified as having adverse risk (HR 1.95, P=0.009). By contrast, patients in the favourable- and intermediate-risk groups had comparable OS values, regardless of their MRD status (P=0.1 and 0.2, respectively). Similarly, WT1 overexpression before HSCT was associated with poor OS in patients in the adverse risk group (HR 1.91; P=0.01). For patients in the favourable- and intermediate-risk groups, OS was comparable regardless of the WT1 expression status (P=0.5 and 0.1, respectively). According to subgroup analyses of specific genetic abnormalities, pre-HSCT MFC-MRD positivity was associated with poor OS in patients harbouring FLT3-ITD (HR 2.64), TP53 mutation (HR 3.53), and complex karyotypes (HR 2.05; all P<0.05). WT1 overexpression before HSCT was associated with poor OS in patients with TP53 mutation (HR 2.97), myelodysplasia-related gene mutation (HR 3.73), and DNMT3A mutation (HR 3.03; all P<0.05). In patients with NPM1 mutations or KMT2Ar, PCR-detected MRD positivity predicted significantly poor OS (NPM1 mutation: HR 4.68, P=0.0007; KMT2Ar: HR 3.11, P=0.007).

### Summary/Conclusion

This study emphasizes the importance of combining MRD assessments with genetic profiling to refine pre-HSCT risk stratification, potentially leading to more personalized treatment and post-transplant surveillance strategies.

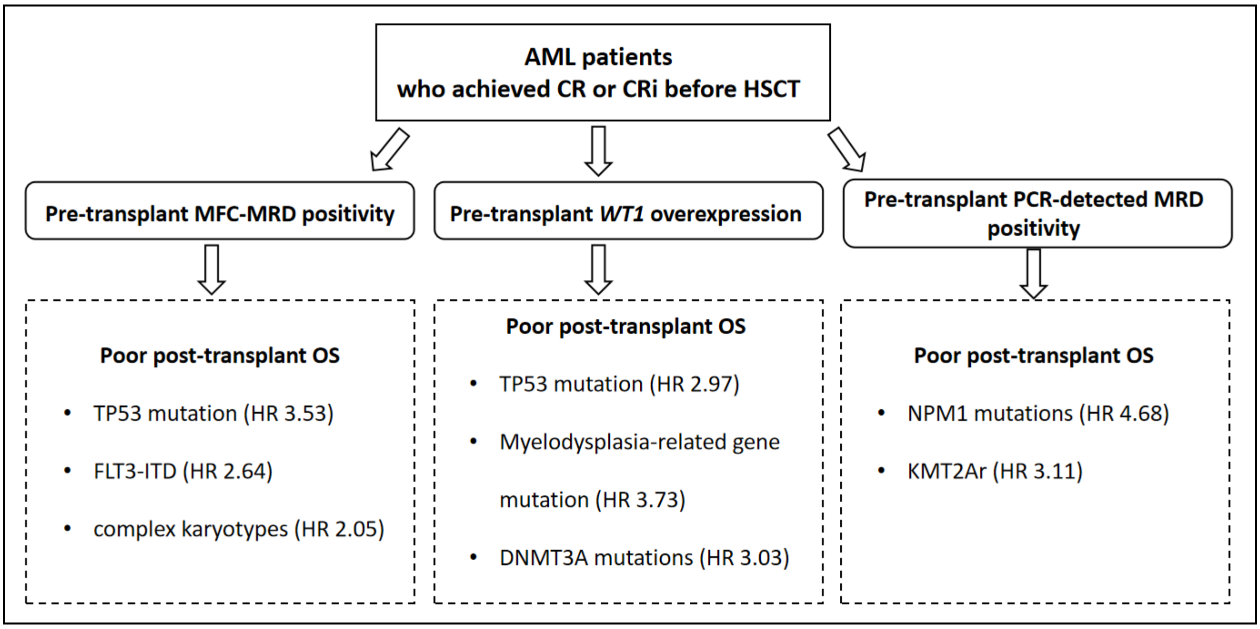

**Copyright Information:** (Online) ISSN: 2572-9241
©2026 The Author(s). HemaSphere is published by John Wiley & Sons Ltd on behalf of European Hematology Association. This is an open access Abstract Book distributed under the Attribution-NonCommercial-NoDerivs (CC BY-NC-ND), which allows third parties to download the articles and share them with others as long as they credit the author and the Abstract Book, but they cannot change the content in any way or use them commercially.
**Abstract Book Citations:** Authors, Title, HemaSphere, 2026;10:(S1):pages. The abstract book DOIs can be found on the "Author Information" page.
**Disclaimer:** Articles published in the journal HemaSphere exclusively reflect the opinions of the authors. The authors are responsible for all content in their abstracts including accuracy of the facts, statements, citing resources, etc.

### (PF1125) THE SAFETY AND EFFICACY OF BELUMOSUDIL AND RUXOLITINIB COMBINATION IN RUXOLITINIB REFRACTORY CGVHD

**Topic:** 23. Stem cell transplantation - Clinical

Yibo Wu*[1, 2, 3, 4], Lizhen Liu[1, 2, 3, 4], Yi Chen[5], Xiaoyu Lai[1, 2, 3, 4], Yan Chen[6], Ying Lu[7], Luxin Yang[1, 2, 3, 4], Lan Sun[5], Jiayi He[5], Fenfang Tong[6], Junjie Cao[7], Lin Li[1, 2, 3, 4], Ji-Min Shi[1, 2, 3, 4], Yanmin Zhao[1, 2, 3, 4], Xiaolin Yuan[8], Panpan Zhu[1, 2, 3, 4], He Huang[1, 2, 3, 4], Yajing Xu[6], Yi Luo[1, 2, 3, 4]

*[1]Bone Marrow Transplantation Center, the First Affiliated Hospital, Zhejiang University School of Medicine, Hangzhou, China; [2]Liangzhu Laboratory, Hangzhou, China; [3]Institute of Hematology, Zhejiang University, Hangzhou, China;[4]Zhejiang Province Engineering Research Center for Stem Cell and Immunity Therapy,, Hangzhou, China; [5]the First Affiliated Hospital of Wenzhou Medical University, Department of hematology, Wenzhou, China; [6]Xiangya Hospital, Central South University,, Department of hematology, Changsha, China; [7]the Affiliated People's Hospital of Ningbo University, Department of hematology, Ningbo, China; [8]Zhejiang Cancer Hospital, Hangzhou Institute of Medicine (HIM), Chinese Academy of Sciences, Department of Hematology,, Hangzhou, China;*

### Background

Despite therapeutic advances with JAK and ROCK inhibitors in chronic GVHD (cGVHD), durable responses remain limited. Patients with advanced cGVHD after multiple prior therapies require simultaneous targeting of multiple pathways. Preclinical data support synergistic effects through dual JAK/ROCK pathway inhibition, potentially overcoming resistance mechanisms.

### Aims

To evaluate the safety and efficacy of combination therapy with belumosudil and ruxolitinib in patients with cGVHD refractory to ruxolitinib.

### Methods

We conducted a multicenter study across four centers, enrolling 57 patients with ruxolitinib-refractory cGVHD (63.2% severe NIH-grade; 66.7% with ≥5 prior therapies). Eligible patients had progression or suboptimal response after ≥3 months of ruxolitinib. The primary endpoint was 6-month overall response rate (ORR), with secondary endpoints including best overall response (BOR), failure-free survival (FFS), adverse events, symptom improvement, and corticosteroid reduction.

### Results

At median follow-up of 364 days, 3 month-ORR was 59.6% (95%CI 47.7-70.5) , with a BOR of 75.4% (63.2–84.7). The 6-month ORR was 50.9% (38.1–63.7), with a BOR of 68.4% (56.4–80.5). Patients with lung involvement (63.2%) showed 83.3% symptom-based response (including 29.2% complete responses) versus 3.7% FEV1 improvement. Twelve-month FFS was 94.5% (87.1-100). Corticosteroid doses were successfully reduced in 86.4% of patients. Following three months of combination therapy, 23 patients successfully discontinued ruxolitinib; nonetheless, 22.8% (13.8–35.3) of patients ultimately required next-line immunosuppressive therapy. The combination demonstrated a favorable safety profile, with grade ≥3 pneumonia occurring in only 5.3% of patients and no treatment-related discontinuations or severe hematologic toxicity observed.

| Variables | N= 57 |
|---|---|

**Copyright Information:** (Online) ISSN: 2572-9241
©2026 The Author(s). HemaSphere is published by John Wiley & Sons Ltd on behalf of European Hematology Association. This is an open access Abstract Book distributed under the Attribution-NonCommercial-NoDerivs (CC BY-NC-ND), which allows third parties to download the articles and share them with others as long as they credit the author and the Abstract Book, but they cannot change the content in any way or use them commercially.
**Abstract Book Citations:** Authors, Title, HemaSphere, 2026;10:(S1):pages. The abstract book DOIs can be found on the "Author Information" page.
**Disclaimer:** Articles published in the journal HemaSphere exclusively reflect the opinions of the authors. The authors are responsible for all content in their abstracts including accuracy of the facts, statements, citing resources, etc.

| | |
|---|---|
| Age, median(range),y | 42(17-68) |
| male | 29(50.9%) |
| Disease, n(%) | |
| AML | 26 (45.6%) |
| ALL | 21 (36.8%) |
| MDS | 5 (8.8%) |
| Others | 5 (8.8%) |
| Donor type, n(%) | |
| MSD | 12 (21.1%) |
| HRD | 35 (61.4%) |
| URD | 10 (17.5%) |
| Conditioning intensity, n(%) | |
| MAC | 51 (89.5%) |
| RIC | 6 (10.5%) |
| Median time of cGVHD diagnosis from transplantation | 215 (68-1512) days |
| Median time from cGVHD diagnosis to initiating ruxolitinib | 138 (0-3636) days |
| Median time from initiating ruxolitinib to adding belumosudil | 287 (69-2471) days |

**Copyright Information:** (Online) ISSN: 2572-9241
©2026 The Author(s). HemaSphere is published by John Wiley & Sons Ltd on behalf of European Hematology Association. This is an open access Abstract Book distributed under the Attribution-NonCommercial-NoDerivs (CC BY-NC-ND), which allows third parties to download the articles and share them with others as long as they credit the author and the Abstract Book, but they cannot change the content in any way or use them commercially.
**Abstract Book Citations:** Authors, Title, HemaSphere, 2026;10:(S1):pages. The abstract book DOIs can be found on the "Author Information" page.
**Disclaimer:** Articles published in the journal HemaSphere exclusively reflect the opinions of the authors. The authors are responsible for all content in their abstracts including accuracy of the facts, statements, citing resources, etc.

| | |
|---|---|
| Median time from cGVHD diagnosis to ruxolitinib & belumosudil combination | 574 (82-4001) days |
| Median follow up from ruxolitinib & belumosudil combination | 364 (162-777) days |
| NIH cGVHD severity at the strat of R+B, n(%) | |
| Severe | n=36 (63.2%) |
| Moderate | n=21 (36.8%) |
| Median systemic LOTs before R+B | 5 (3-8) |
| ≥5 prior LOTs | 40 (70.2%) |
| Median number of organs involved with cGVHD at the start of R + B | 4(2-7) |
| ≥4 organs involved | 37 (64.9%) |
| Organ involvement, n(%) | |
| skin | 30 (52.6%) |
| eyes | 46 (80.7%) |
| mouth | 40 (70.2%) |
| esophagus | 17 (29.8%) |
| Upper gastrointestinal | 6 (10.5%) |
| Lower gastrointestinal | 2 (3.5%) |
| liver | 6 (10.5%) |

**Copyright Information:** (Online) ISSN: 2572-9241
©2026 The Author(s). HemaSphere is published by John Wiley & Sons Ltd on behalf of European Hematology Association. This is an open access Abstract Book distributed under the Attribution-NonCommercial-NoDerivs (CC BY-NC-ND), which allows third parties to download the articles and share them with others as long as they credit the author and the Abstract Book, but they cannot change the content in any way or use them commercially.
**Abstract Book Citations:** Authors, Title, HemaSphere, 2026;10:(S1):pages. The abstract book DOIs can be found on the "Author Information" page.
**Disclaimer:** Articles published in the journal HemaSphere exclusively reflect the opinions of the authors. The authors are responsible for all content in their abstracts including accuracy of the facts, statements, citing resources, etc.

| | |
|---|---|
| lung | 36 (63.2%) |
| Joints/fascia | 29 (50.9%) |
| Vulvo/vaginal | 22 (38.6%) |
| Prior systemic cGVHD therapy type | |
| Corticosteroids | 57 (100.0%) |
| Cyclosporin | 55 (96.5%) |
| Tacrolimus | 36(63.2%) |
| Mycophenolate mofetil | 33 (57.9%) |
| Ruxolitinib | 57 (100.0%) |
| Methotrexate | 24 (42.1%) |
| Imatinib | 5 (8.8%) |
| Mesenchymal stem cells | 18 (31.6%) |
| Others | 7 (12.3%) |

Table1 Baseline patient demographics and clinical characteristics

**Summary/Conclusion**

These results confirm the clinically meaningful efficacy and safety of dual JAK/ROCK inhibition in this heavily pretreated population. These findings support the mechanistic rationale for concurrent pathway inhibition and warrant prospective validation in randomized controlled trials.

**Copyright Information:** (Online) ISSN: 2572-9241
©2026 The Author(s). HemaSphere is published by John Wiley & Sons Ltd on behalf of European Hematology Association. This is an open access Abstract Book distributed under the Attribution-NonCommercial-NoDerivs (CC BY-NC-ND), which allows third parties to download the articles and share them with others as long as they credit the author and the Abstract Book, but they cannot change the content in any way or use them commercially.
**Abstract Book Citations:** Authors, Title, HemaSphere, 2026;10:(S1):pages. The abstract book DOIs can be found on the "Author Information" page.
**Disclaimer:** Articles published in the journal HemaSphere exclusively reflect the opinions of the authors. The authors are responsible for all content in their abstracts including accuracy of the facts, statements, citing resources, etc.

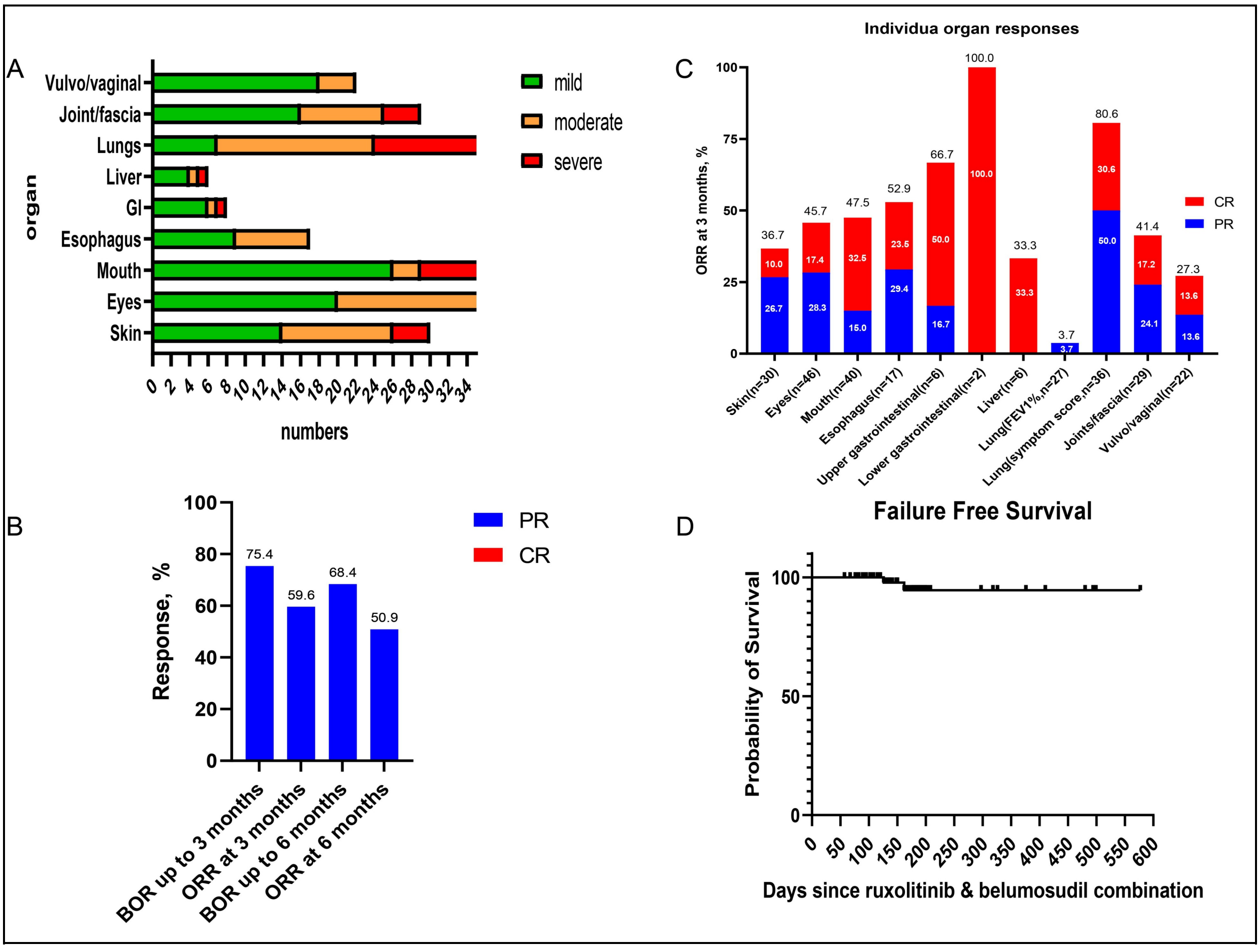

Figure 1 The efficacy of ruxolitinib and belumosudil combination for cGVHD (A) Grade by organ system at start before combination. (B) BOR and ORR to combination therapy. (C) Response to combination ruxolitinib and belumosudil by organ system. (D) Estimated failure free survival.

**Copyright Information:** (Online) ISSN: 2572-9241
©2026 The Author(s). HemaSphere is published by John Wiley & Sons Ltd on behalf of European Hematology Association. This is an open access Abstract Book distributed under the Attribution-NonCommercial-NoDerivs (CC BY-NC-ND), which allows third parties to download the articles and share them with others as long as they credit the author and the Abstract Book, but they cannot change the content in any way or use them commercially.
**Abstract Book Citations:** Authors, Title, HemaSphere, 2026;10:(S1):pages. The abstract book DOIs can be found on the “Author Information” page.
**Disclaimer:** Articles published in the journal HemaSphere exclusively reflect the opinions of the authors. The authors are responsible for all content in their abstracts including accuracy of the facts, statements, citing resources, etc.

### (PF1126) THIOTEPA-FLUDARABINE-TREOSULFAN VS THIOTEPA-FLUDARABINE-BUSULFAN IN ALLOGENEIC HEMATOPOIETIC CELL TRANSPLANTATION FOR ADULT AML PATIENTS IN CR. A STUDY FROM THE ACUTE LEUKEMIA WORKING PARTY OF EBMT.

**Topic:** 23. Stem cell transplantation - Clinical

Enrico Maffini*[1], Maud Ngoya[2], Raffaella Cerretti[3], Anna Maria Raiola[4], Peter Pal Remenyi[5], Jaime Sanz[6], Stella Santarone[7], Simona Sica[8], Giorgio La Nasa[9], Angelo Michele Carella[10], Stefania Bramanti[11], Chiara Nozzoli[12], Anna Paola Iori[13], Didier Blaise[14], Matteo Parma[15], Alexandros Spyridonidis[16], Bipin Savani[17], Arnon Nagler[18], Fabio Ciceri[19], Mohamad Mohty[20]

*[1]IRCCS Azienda Ospedaliero Universitaria di Bologna, Trapianto e terapie cellulari, Bologna, Italy;[2]EBMT ALWP Statistical Unit, Paris, France; [3]Rome Transplant Network, Department of Hematology, Stem Cell Transplant Unit, Fondazione Policlinico Tor Vergata, Roma, Italy; [4]IRCCS Azienda Ospedaliera Metropolitana, Ematologia e Terapie cellulari, Genova, Italy; [5]Del-pesti Centrumkorhaz, Budapest, Hungary; [6]University Hospital La Fe, Valencia, Spain; [7]Hematology Unit, Santo Spirito Hospital, Pescara, Italy; [8]Fondazione Policlinico Universitario Agostino Gemelli IRCCS, Roma, Italy;[9]Hematology Unit, Businco Hospital, ARNAS Brotzu, Department of Medical Sciences and Public Health, University of Cagliari, Cagliari, Italy; [10]IRCCS, Hematology and Stem Cell Transplantation Unit "Casa Sollievo della Sofferenza" Hospital, San Giovanni Rotondo, Italy; [11]IRCCS Humanitas Research Hospital, Haematology, Rozzano, Italy; [12]Cellular Therapies and Transfusion Medicine and Bone Marrow Transplant, AOU Careggi Hospital, Firenze, Italy; [13]Department of Translational and Precision Medicine, Policlinico Umberto I Hospital, Sapienza University of Rome, Roma, Italy; [14]Institut Paoli Calmettes, Marseille, France; [15]IRCCS Ospedale San Gerardo, S.S Trapianti di midollo Osseo, Monza, Italy; [16]University Hospital of Patras, Department of Internal Medicine, Bone Marrow Transplantation Unit, patras, Greece; [17]Vanderbilt University Medical Center, Department of Haematology-Oncology, Nashville, United States of America; [18]Chaim Sheba Medical Center and Tel Aviv University, Hematology Division, Tel-Hashomer, Ramat-Gan, Israel; [19]San Raffaele Scientific Institute, Milan, Italy; [20]Sorbonne University, Clinical Hematology and Cellular Therapy Department, Saint Antoine Hospital, INSERM UMRs 938, Paris, France;*

### Background

The combination of two alkylating agents in thiotepa-busulfan-fludarabine (TBF) conditioning is frequently used in patients with acute myeloid leukemia treated with allogeneic hematopoietic cell transplantation (HCT), however its toxicity is significant. To overcome this problem, one possible solution is to replace busulfan with treosulfan.

### Methods

This study analysed the outcomes of allografted patients treated with TBF or thiotepa-treosulfan-fludarabine (TTF) for acute myeloid leukemia in first or second complete remission between 2010 and 2023 and reported to the Acute Leukemia Working Party of the European Society for Blood and Marrow Transplantation. Donors were human leukocyte antigen-matched related or matched unrelated donors, and haploidentical relatives. A total of 3623 patients received TBF, while 265 received TTF. Median age at HCT was 57.9 (range 18-79) years. Graft-versus-host disease (GvHD) prophylaxis was post-transplant cyclophosphamide in 54.7%.

**Copyright Information:** (Online) ISSN: 2572-9241
©2026 The Author(s). HemaSphere is published by John Wiley & Sons Ltd on behalf of European Hematology Association. This is an open access Abstract Book distributed under the Attribution-NonCommercial-NoDerivs (CC BY-NC-ND), which allows third parties to download the articles and share them with others as long as they credit the author and the Abstract Book, but they cannot change the content in any way or use them commercially.
**Abstract Book Citations:** Authors, Title, HemaSphere, 2026;10:(S1):pages. The abstract book DOIs can be found on the "Author Information" page.
**Disclaimer:** Articles published in the journal HemaSphere exclusively reflect the opinions of the authors. The authors are responsible for all content in their abstracts including accuracy of the facts, statements, citing resources, etc.

**Results**

Median follow-up for the entire population was 3.1 (95% confidence interval [CI] 3-3.2) years. Two-year overall survival and progression-free survival were superimposable between the two groups, with: 69.8% (95% CI 68.1-71.3) and 69.8% (95% CI 63.1-75.5) for TBF and TTF, respectively; 64.3% (95% CI 62.6-65.9) and 64.7% (95% CI 57.8-70.7) for TBF and TTF, respectively. Two-year cumulative incidence of relapse and non-relapse mortality were: 17.4% (95% CI 16.1 - 18.8) and 14.5% (95% CI 10-19.8) for TBF and TTF, respectively; 18.3% (95% CI 17 - 19.6) and 20.8% (95% CI 15.8 - 26.3) for TBF and TTF, respectively (Figure 1). Multivariate analysis confirmed prolonged overall and progression-free survival for patients with non-adverse cytogenetics (hazard ratio [HR] 0.69, 95% CI 0.59-0.79, p<0.001; HR 0.7, CI 95% 0.61-0.80, p<0.001, respectively), and with Karnofsky performance status score ≥90 (HR 0.75, CI 95% 0.64-0.87, p<0.001; HR 0.75, 95% CI 0.66-0.86, p<0.001, respectively), but inferior for those with older patient age at HCT - stratified by 2 years (HR 1.05, CI 95% 1.04-1.06, p<0.001; 1.04, CI 95% 1.03-1.05, p<0.001, respectively). Non-adverse cytogenetics was also associated with lower risk of relapse (HR 0.55, 95% CI 0.46-0.67, p<0.001). Non-relapse mortality was negatively influenced by increasing patient age at HCT (HR 1.08, 95% CI 1.06-1.09, p<0.001) and positively influenced by Karnofsky performance status score ≥90 (HR 0.71, 95% CI 0.58-0.86, p<0.001) and use of a sibling (HR 0.56, 95% CI 0.42-0.74, p<0.001). Grade II/IV and III/IV acute GvHD were: 27% (95% CI 25.5 - 28.6) and 9.8% (95% CI 8.8 - 10.9) for TBF, respectively; 20.7% (95% CI 15.8-26.1) and 8.6% (95% CI 5.4-12.6) for TTF, respectively. Overall and extensive chronic GvHD were: 28.6% (95% CI 27-30.3) and 4.7% (95% CI 4-5.6) for TBF, respectively; 27.3% (95% CI 21.2-33.8) and 5.4% (95% CI 2.8-9.4) for TTF, respectively. The female to male transplant combination was associated with an increased risk of chronic GvHD (HR 1.33, 95% CI 1.12-1.57, p<0.001), while more recent year of HCT – stratified by 5 years – was associated with lower risk of developing both overall chronic GvHD (HR 0.73, 95% CI 0.64-0.84, p<0.001), and extensive chronic GvHD (HR 0.58, 95% CI 0.42-0.79, p<0.001).

**Summary/Conclusion**

In conclusion, this study demonstrates that patients prepared for transplant according to the TTF regimen have outcomes comparable to those receiving TBF. Prospective studies will be needed to confirm these results.

**Copyright Information:** (Online) ISSN: 2572-9241
©2026 The Author(s). HemaSphere is published by John Wiley & Sons Ltd on behalf of European Hematology Association. This is an open access Abstract Book distributed under the Attribution-NonCommercial-NoDerivs (CC BY-NC-ND), which allows third parties to download the articles and share them with others as long as they credit the author and the Abstract Book, but they cannot change the content in any way or use them commercially.
**Abstract Book Citations:** Authors, Title, HemaSphere, 2026;10:(S1):pages. The abstract book DOIs can be found on the "Author Information" page.
**Disclaimer:** Articles published in the journal HemaSphere exclusively reflect the opinions of the authors. The authors are responsible for all content in their abstracts including accuracy of the facts, statements, citing resources, etc.

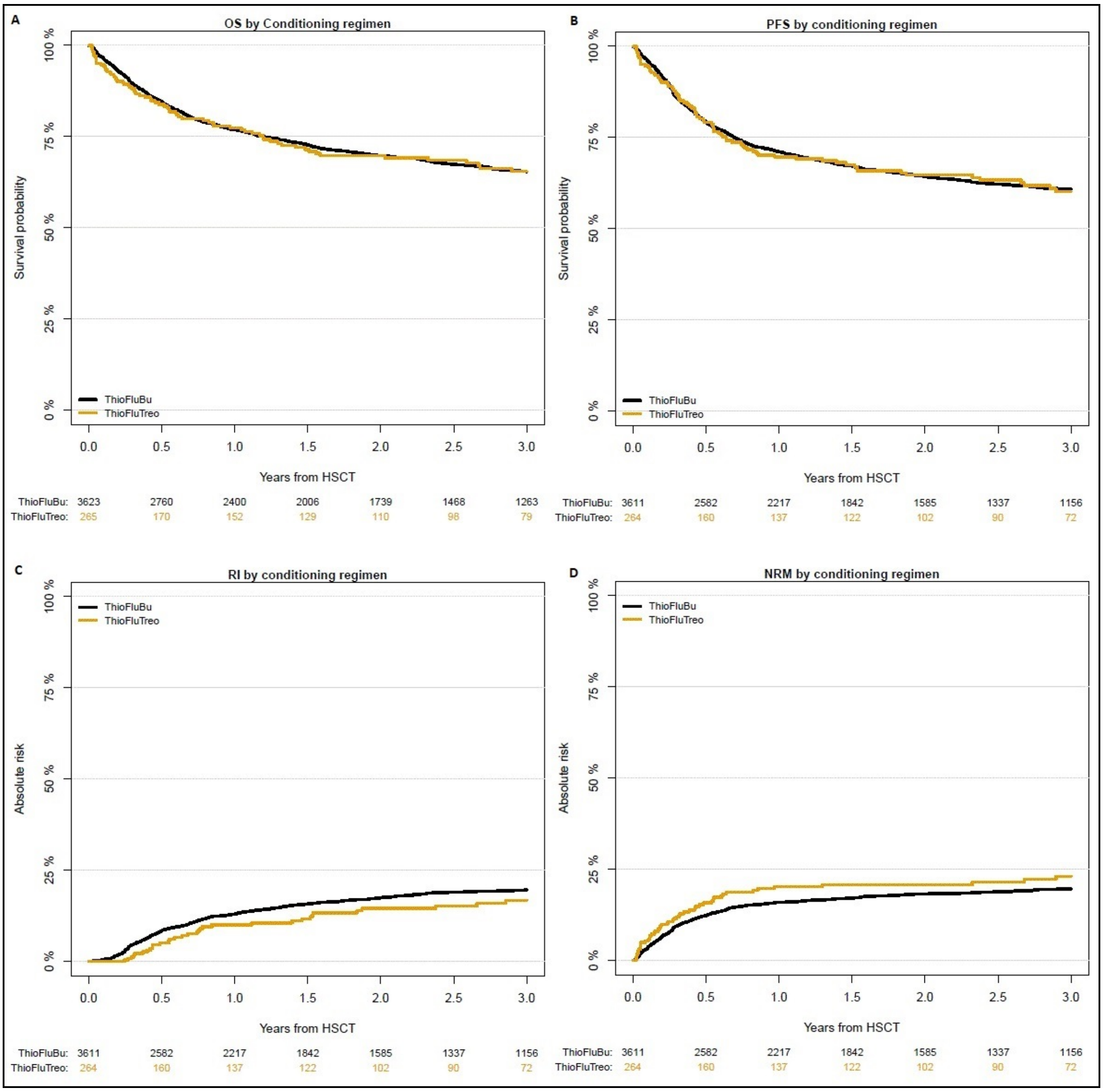

**Copyright Information:** (Online) ISSN: 2572-9241
©2026 The Author(s). HemaSphere is published by John Wiley & Sons Ltd on behalf of European Hematology Association. This is an open access Abstract Book distributed under the Attribution-NonCommercial-NoDerivs (CC BY-NC-ND), which allows third parties to download the articles and share them with others as long as they credit the author and the Abstract Book, but they cannot change the content in any way or use them commercially.
**Abstract Book Citations:** Authors, Title, HemaSphere, 2026;10:(S1):pages. The abstract book DOIs can be found on the “Author Information” page.
**Disclaimer:** Articles published in the journal HemaSphere exclusively reflect the opinions of the authors. The authors are responsible for all content in their abstracts including accuracy of the facts, statements, citing resources, etc.

### (PF1127) HOSPITAL BASED OUTPATIENT DAILY DOSING MYELOABLATIVE INTRAVENOUS AND PHARMACOKINETICALLY GUIDED BUSULFAN BASED CONDITIONING FOR ALLOGENEIC HEMATOPOIETIC STEM CELL TRANSPLANT – SINGLE CENTER EXPERIENCE

**Topic:** 23. Stem cell transplantation - Clinical

Mathias Palmer*[1], Jade Braun[1], Gabe Bartoo[1], Robert Wolf[1], Michel Benz[1], Aasiya Matin[1], Mehrdad Hefazi[1], Abhishek Mangaonkar[1], Mithun Shah[1], David Dingli[1], William Hogan[1], Hassan Alkhateeb[1]

[1]*Mayo Clinic, Hematology, Rochester, United States of America;*

### Background

Busulfan (BU) is the cornerstone for radiation-free myeloablative conditioning (MAC) in preparation for allogeneic hematopoietic cell transplant (alloHCT). At Mayo Clinic Rochester, transplant care is performed in the setting of Hospital-based outpatient care (HBO) with daily evaluation, laboratory assessment and intervention as needed. BU once daily dosing (OD) has been shown to be safe based on Cilburn et al[1] (ASH 2023 Poster 721) and Pasquini et al[2] (2017).

### Aims

Here we present our single-institution experience of HBO MAC OD BU in patients undergoing alloHCT.

### Methods

Patients who underwent alloHCT and received OD MAC Bu from 2024-2025 at Mayo Clinic Rochester were identified. Overall survival (OS) and progression free survival (PFS) were assessed using Kaplan Meier methodology.

Blood sampling to calculate BuPK occurred at 15 minutes, 1,2,3 and 5 hours from finishing Bu infusion on 1$^{st}$ Day of conditioning, while AUC was calculated on 2$^{nd}$ day of Bu targeting 5000 (mcmol/L)/(min), and dose adjustment was implemented as indicated by the 2$^{nd}$ dose of Bu.

**Copyright Information:** (Online) ISSN: 2572-9241
©2026 The Author(s). HemaSphere is published by John Wiley & Sons Ltd on behalf of European Hematology Association. This is an open access Abstract Book distributed under the Attribution-NonCommercial-NoDerivs (CC BY-NC-ND), which allows third parties to download the articles and share them with others as long as they credit the author and the Abstract Book, but they cannot change the content in any way or use them commercially.
**Abstract Book Citations:** Authors, Title, HemaSphere, 2026;10:(S1):pages. The abstract book DOIs can be found on the "Author Information" page.
**Disclaimer:** Articles published in the journal HemaSphere exclusively reflect the opinions of the authors. The authors are responsible for all content in their abstracts including accuracy of the facts, statements, citing resources, etc.

**Results**

A total of 36 consecutive patients received MAC OD BU; 25 (69.4%) received BU with Fludarabine and 10 (27.8%) received BU with Cyclophosphamide. Administration was mostly in HBO care (n=33, 92%); the first three patients were hospitalized for the conditioning. The median age at transplant was 47 years (19-63), with a median HCT-CI of 2 (0-8). AML (n=18, 50%) and MDS (n=10 patients, 27.8%) were the most common indications for alloHCT. High risk disease was present in 7 (38.9%) patients with AML and 6 (60%) with MDS. Donors were primarily matched unrelated donor (n=28, 77.8%) and mismatched unrelated donor (n=6, 16.7%). All but one patient received peripheral blood stem cell product (n=35, 97.2%). GVHD prophylaxis regimen was mainly post-transplant cyclophosphamide based (n=35, 97.2%)
BU was dosed as 130 mg/m2/day in 20 patients (55.6%) and 3.2 mg/kg/day in 15 patients (41.7%) and 3.4 mg/kg/day in 1 patient. The median calculated BU AUC was 5120 (mcmol/L)/(min) (3960 – 7560). Dose adjustment was indicated in 22 patients (61.1%): (9 (41%) required dose increase, 13 (59%) required dose reduction). Median time to neutrophil and platelet engraftment was 15 and 18 days, respectively; none of the patients experienced graft failure. Median Day 90 CD3 and CD33 chimerism were 80% and 100%, respectively. SOS occurred in 2 patients (5.6%) and was treated successfully with Defibrotide; there were no major adverse cardiopulmonary events, otherwise thrombotic microangiopathy occurred in 1 patient (2.8%). At day 100, there were at total of 7 relapses (19.4 %) with one death (2.8%) due to relapse. The day 100 non-relapse mortality was 0%. After a median follow up of 365 days, there were 6 (16.7%) deaths and 13 (36.1%) relapses; both median OS and PFS were not reached. The estimated 1-year PFS was 61.6% (46.9 – 80.8) and 1-year OS was 80.9% (68.2 – 96). All deaths occurred following relapse except one patient who developed severe GI GVHD without relapse.

**Summary/Conclusion**

Hospital-based outpatient once daily PK-guided Busulfan dosing is a safe, feasible, and effective myeloablative conditioning. This is a practical strategy limiting inpatient utilization in large volume transplant centers.

**Copyright Information:** (Online) ISSN: 2572-9241
©2026 The Author(s). HemaSphere is published by John Wiley & Sons Ltd on behalf of European Hematology Association. This is an open access Abstract Book distributed under the Attribution-NonCommercial-NoDerivs (CC BY-NC-ND), which allows third parties to download the articles and share them with others as long as they credit the author and the Abstract Book, but they cannot change the content in any way or use them commercially.
**Abstract Book Citations:** Authors, Title, HemaSphere, 2026;10:(S1):pages. The abstract book DOIs can be found on the “Author Information” page.
**Disclaimer:** Articles published in the journal HemaSphere exclusively reflect the opinions of the authors. The authors are responsible for all content in their abstracts including accuracy of the facts, statements, citing resources, etc.

### (PF1128) RESPIRATORY TRACT INVASIVE FUNGAL INFECTIONS AFTER ALLOGENEIC HEMATOPOIETIC CELL TRANSPLANTATION WITH POST-TRANSPLANT CYCLOPHOSPHAMIDE IN AML AND MDS: A STUDY CONDUCTED ON BEHALF OF GETH-TC

**Topic:** 23. Stem cell transplantation - Clinical

Maria Queralt Salas*[1], Pedro Chorao[2], Pablo Granados[3], Ana Bataller Alfonso[3], María Suárez-Lledó[4], Estefanía Perez-López[5], Lucía López Corral[5], Clara Aparicio Pérez[6], Adolfo Jesus Saez Marin[7], Laura López-Molero[2], Sara Fernandez-Luis[8], Ana María Pilar González Rodríguez[9], Sara Villar-Fernandez[10], Slivia Filaferro[11], Irene Garcia Cadenas[12], José Luis Piñana[2]

*[1]Hospital Clínic de Barcelona, Hematology, Barcelona, Spain;[2]Hospital Clínico de Valencia, Valencia, Spain; [3]Hospital Universitari i Politècnic La Fe, Valencia, Spain; [4]Hospital Clínic de Barcelona, Barcelona, Spain;[5]Hospital Universitario de Salamanca, Salamanca, Spain; [6]Hospital Universitario Reina Sofía, Córdoba, Spain;[7]Hospital Universitario 12 de Octubre, Madrid, Spain; [8]Hospital Universitario Marqués de Valdecilla, Santander, Spain;[9]Hospital Universitario Central de Asturias, Oviedo, Spain; [10]Clinica Universidad Navarra, Pamplona, Spain; [11]Grupo Español de Trasplante Hematopoyético y Terapia Celular (GETH-TC), Madrid, Spain; [12]Hospital de Sant Pau, Barcelona, Spain;*

### Background

Allogeneic hematopoietic cell transplantation (allo-HCT) is a potentially curative therapy for patients with acute myeloid leukemia (AML) and myelodysplastic syndromes (MDS). However, prolonged neutropenia and sustained immunosuppression increase the risk of life-threatening infectious complications, particularly invasive fungal infections (IFI). Post-transplant cyclophosphamide (PTCy) has become a standard graft-versus-host disease (GVHD) prophylaxis across donor types, yet contemporary data on respiratory IFI in this setting are limited.

### Aims

To evaluate the incidence, risk factors, and outcomes of respiratory tract IFI in adult AML and MDS patients undergoing allo-HCT with PTCy.

### Methods

We conducted a retrospective multicenter study including consecutive adult AML or MDS patients who underwent a first peripheral blood allo-HCT with PTCy between 2017 and 2024 across nine centers. IFI episodes (possible, probable, or proven) were identified from medical records. Only the first IFI episode was analyzed, and infections diagnosed after relapse were excluded. The primary endpoint was cumulative incidence (CI) of IFI, considering relapse and death as competing risks.

**Copyright Information:** (Online) ISSN: 2572-9241
©2026 The Author(s). HemaSphere is published by John Wiley & Sons Ltd on behalf of European Hematology Association. This is an open access Abstract Book distributed under the Attribution-NonCommercial-NoDerivs (CC BY-NC-ND), which allows third parties to download the articles and share them with others as long as they credit the author and the Abstract Book, but they cannot change the content in any way or use them commercially.
**Abstract Book Citations:** Authors, Title, HemaSphere, 2026;10:(S1):pages. The abstract book DOIs can be found on the "Author Information" page.
**Disclaimer:** Articles published in the journal HemaSphere exclusively reflect the opinions of the authors. The authors are responsible for all content in their abstracts including accuracy of the facts, statements, citing resources, etc.

### Results

795 patients were included (median age 57 years); 577 had AML and 218 MDS. Reduced-intensity conditioning was used in 40%. Donors were matched related (24.9%), matched unrelated (28.1%), mismatched unrelated (10.1%), or haploidentical (37.1%); 93.7% received PTCy 50 mg/kg for two days. Forty-five patients (5.7%) had IFI before transplant.
Median neutrophil and platelet engraftment occurred at 17 and 23 days. Graft failure (GF) was diagnosed in 19 patients (2.4%). Day +100 and 2-year CI of grade 3–4 aGVHD and mod/severe cGVHD were 10.5% and 21.8%. With a median follow-up of 2.5 years, 215 patients (27.0%) relapsed and 313 (39.4%) died.
Fifty-seven IFI episodes were identified. Median time to diagnosis was 69 days (IQR 22–179). CI at 180 days, 1 year, and 2 years was 5.4%, 6.4%, and 6.7%. Five infections involved the paranasal sinuses; the remainder were pulmonary. Microbiological documentation (culture or PCR) was available in 31 cases (54%): Aspergillus spp. in 25 (69.4%), Mucorales in 5 (13.9%), and other fungi in 1 (2.8%). At diagnosis, 28 patients had breakthrough infection. All-cause mortality was 66.6%; with day +30 and +100 mortality rates 21.1% and 36.8%.
In univariate analysis, baseline risk factors for IFI included prior IFI (HR 2.35, p=0.031), HCT-CI >3 (HR 2.03, p=0.009), and high/very high DRI (HR 1.90, p=0.026). Time-dependent events associated with increased IFI risk were GF (HR 16.5, p<0.001), grade 3–4 aGVHD (HR 3.35, p<0.001), mod/severe cGVHD (HR 2.95, p=0.008), and CMV reactivation (HR 2.03, p=0.023).
Patients with IFI had inferior outcomes. Two-year overall survival was 39.6% versus 68.7% in those without IFI (p<0.001), and two-year non-relapse mortality was 29.8% versus 15.0% (p=0.001). In multivariable time-dependent analysis, IFI remained independently associated with worse survival (HR 2.60, p<0.001) after adjustment for age >60 years, HCT-CI >3, high/very high DRI, haploidentical donor, GF, grade 3–4 aGVHD, and mod/severe cGVHD.

### Summary/Conclusion

Respiratory IFI incidence in the PTCy setting was relatively low (6–7%) but associated with early post-transplant complications, particularly GF and GVHD, and with markedly inferior survival. These findings support risk-adapted surveillance and preventive strategies in in the PTCy era.

**Copyright Information:** (Online) ISSN: 2572-9241
©2026 The Author(s). HemaSphere is published by John Wiley & Sons Ltd on behalf of European Hematology Association. This is an open access Abstract Book distributed under the Attribution-NonCommercial-NoDerivs (CC BY-NC-ND), which allows third parties to download the articles and share them with others as long as they credit the author and the Abstract Book, but they cannot change the content in any way or use them commercially.
**Abstract Book Citations:** Authors, Title, HemaSphere, 2026;10:(S1):pages. The abstract book DOIs can be found on the “Author Information” page.
**Disclaimer:** Articles published in the journal HemaSphere exclusively reflect the opinions of the authors. The authors are responsible for all content in their abstracts including accuracy of the facts, statements, citing resources, etc.

### (PF1129) RISK FACTORS AND PROGNOSTIC INDICATORS OF ACUTE RESPIRATORY FAILURE FOLLOWING ALLOGENEIC HEMATOPOIETIC STEM CELL TRANSPLANTATION: A RETROSPECTIVE COHORT STUDY

**Topic:** 23. Stem cell transplantation - Clinical

Shijia Li[1], Depei Wu[1], Xiaojin Wu*[1]

[1]*the First Affiliated Hospital of Soochow University, Suzhou, China;*

### Background

Allogeneic hematopoietic stem cell transplantation (allo-HSCT) has become a major curative treatment for hematologic diseases, and the number of transplants has increased steadily in recent years. Despite continuous improvements in transplant techniques and peri-transplant management, transplant-related non-relapse mortality (NRM) remains one of the principal barriers to long-term survival. Post-transplant pulmonary complications account for the largest proportion of NRM, and acute respiratory failure (ARF) represents a leading cause of intensive care unit (ICU) admission and death among HSCT recipients.

### Aims

To investigate the clinical characteristics of acute respiratory failure following allogeneic hematopoietic stem cell transplantation and to identify risk and prognostic factors associated with the development of ARF and patient outcomes.

### Methods

A retrospective study was conducted, including patients who underwent allo-HSCT at the First Affiliated Hospital of Soochow University between 2015 and 2022. Clinical data were collected, and follow-up continued until December 30, 2025. The clinical characteristics of patients who developed ARF after allo-HSCT were analyzed. Logistic regression analysis was performed to identify risk factors for the occurrence of ARF. Kaplan-Meier survival curves were plotted, and Cox proportional hazards models were used to analyze prognostic factors affecting survival outcomes.

### Results

A total of 844 allo-HSCT patients were included in the study, of whom 112 developed acute respiratory failure (ARF) during the first year post-transplant, resulting in an overall incidence rate of 13.27%. Pulmonary infection was the most common trigger for ARF across all stages of immune reconstitution. Multivariate logistic regression analysis identified several independent risk factors for ARF, including non-remission of the underlying disease, pre-transplant pulmonary infection, higher infused nucleated cell dose, and pre-transplant $FEV_1$ <80% predicted.Among patients with ARF, the median post-transplant survival was 7.8 months (95% CI, 4.67–13.87). Compared to the non-ARF group, the ARF group had a significantly lower 3-year overall survival (OS) rate (36.6% vs 85.1%, P<0.05) and a significantly higher 3-year NRM (52.7% vs 9.3%, P<0.05). In multivariable Cox regression among ARF patients, disease relapse, thrombocytopenia, lower oxygenation index, acidosis, elevated total bilirubin, and $FEV_1$ <80% predicted were independent predictors of mortality.

**Copyright Information:** (Online) ISSN: 2572-9241
©2026 The Author(s). HemaSphere is published by John Wiley & Sons Ltd on behalf of European Hematology Association. This is an open access Abstract Book distributed under the Attribution-NonCommercial-NoDerivs (CC BY-NC-ND), which allows third parties to download the articles and share them with others as long as they credit the author and the Abstract Book, but they cannot change the content in any way or use them commercially.
**Abstract Book Citations:** Authors, Title, HemaSphere, 2026;10:(S1):pages. The abstract book DOIs can be found on the "Author Information" page.
**Disclaimer:** Articles published in the journal HemaSphere exclusively reflect the opinions of the authors. The authors are responsible for all content in their abstracts including accuracy of the facts, statements, citing resources, etc.

### Summary/Conclusion

ARF after allo-HSCT is common and is associated with poor outcomes. Pre-transplant assessment of pulmonary function, pulmonary infection status, and disease control, together with attention to graft cell dose during infusion, may facilitate risk stratification. After ARF onset, disease status, platelet count, oxygenation index, acid–base status, total bilirubin, and pulmonary function may help inform prognostic assessment.

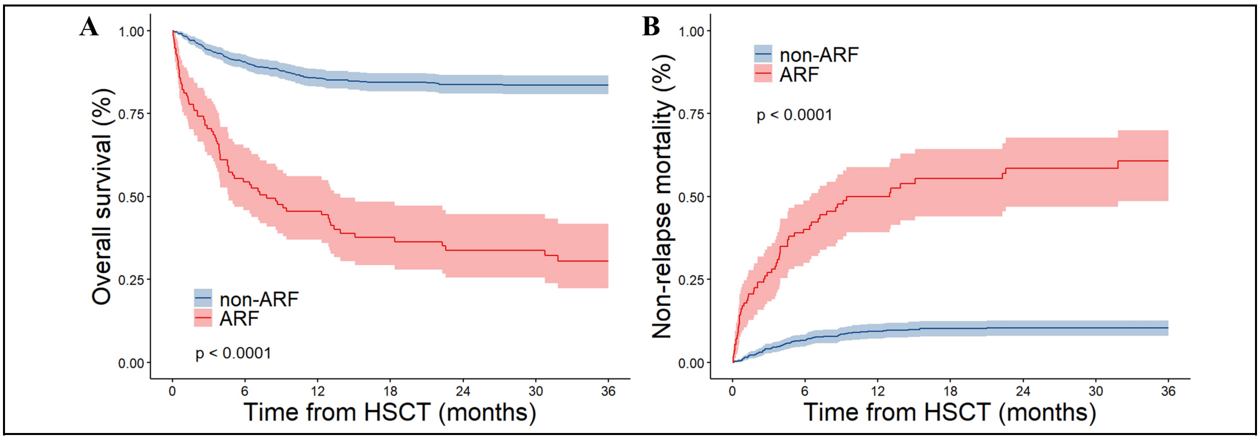

Figure Impact of acute respiratory failure (ARF) after HSCT on survival outcomes. (A) Kaplan–Meier estimates of overall survival (OS) from the time of hematopoietic stem cell transplantation (HSCT) comparing patients with ARF versus those without ARF (non-ARF). (B) Cumulative incidence of non-relapse mortality (NRM) from HSCT in the ARF and non-ARF groups. Shaded areas represent 95% confidence intervals. P values were calculated using the log-rank test for OS (A) and Gray's test for NRM (B); $P < 0.0001$ for both comparisons.

**Copyright Information:** (Online) ISSN: 2572-9241
©2026 The Author(s). HemaSphere is published by John Wiley & Sons Ltd on behalf of European Hematology Association. This is an open access Abstract Book distributed under the Attribution-NonCommercial-NoDerivs (CC BY-NC-ND), which allows third parties to download the articles and share them with others as long as they credit the author and the Abstract Book, but they cannot change the content in any way or use them commercially.
**Abstract Book Citations:** Authors, Title, HemaSphere, 2026;10:(S1):pages. The abstract book DOIs can be found on the "Author Information" page.
**Disclaimer:** Articles published in the journal HemaSphere exclusively reflect the opinions of the authors. The authors are responsible for all content in their abstracts including accuracy of the facts, statements, citing resources, etc.

### (PF1130) CPX-351 BEFORE ALLOGENEIC STEM CELL TRANSPLANTATION, A REAL-WOLRD FRENCH EXPERIENCE

**Topic:** 23. Stem cell transplantation - Clinical

Eléonore Kaphan*[1], Hélène Kemp[2], Nicolas Vallet[3], Flore Sicre de Fontbrune[1], Marie Robin[1], Alienor Xhaard[1], Lionel Ades[4, 5], Lin Pierre Zhao[4, 5], Pierre Walczak[4], Régis Peffault de Latour[1, 6], David Michonneau[1, 6]

*[1]Hematology and Transplantation, Saint-Louis Hospital, Assistance Publique des Hôpitaux de Paris, Paris, France;[2]Department of Hematology, University Hospital of Amiens, Amiens, France; [3]Hematology and Cell Therapy Department, Tours University Hospital, Inserm U1069, Tours, France; [4]Hematologie Séniors, Hôpital Saint-Louis, Paris, France;[5]Paris Leukemia Institute and Université Paris Cité, Paris, France; [6]INSERM UMR 1342, Translational Immunology in Immunotherapy and Hematology, IHU Leukemia, Paris, France;*

### Background

CPX-351, a liposomal fixed-ratio formulation of daunorubicin and cytarabine, has demonstrated superior overall survival (OS) compared with standard 7+3 in therapy-related acute myeloid leukemia (t-AML) and AML with myelodysplasia-related changes (AML-MRC). However, its impact on post-allogeneic stem cell transplantation (allo-SCT) outcomes remains unclear.

### Aims

It was hypothesized in the pivotal trial that this survival benefit might be attributable to deeper remissions prior to allo-SCT and/or improved pre-transplant clinical status in patients treated with CPX-351. However, as the study was not specifically designed to assess post-transplant outcomes, it did not allow for a formal evaluation of whether the survival benefit was driven by differences in pre-transplant disease status or by improved general condition and transplant-related outcomes associated with CPX-351.

### Methods

Patients with t-AML or AML-MRC undergoing allo-SCT between 2017 and 2023 at three French centers were included. Matching criteria included age, sex, underlying disease, conditioning intensity, disease status at transplant, donor type, and stem cell source. Data were extracted from the EBMT registry. Primary endpoint was post-transplant OS; secondary endpoints included relapse-free survival (RFS), non-relapse mortality (NRM), acute and chronic graft-versus-host disease (GVHD), and GVHD-relapse-free survival (GRFS). Survival outcomes were estimated using Kaplan-Meier methods and competing-risk analyses.

**Copyright Information:** (Online) ISSN: 2572-9241
©2026 The Author(s). HemaSphere is published by John Wiley & Sons Ltd on behalf of European Hematology Association. This is an open access Abstract Book distributed under the Attribution-NonCommercial-NoDerivs (CC BY-NC-ND), which allows third parties to download the articles and share them with others as long as they credit the author and the Abstract Book, but they cannot change the content in any way or use them commercially.
**Abstract Book Citations:** Authors, Title, HemaSphere, 2026;10:(S1):pages. The abstract book DOIs can be found on the “Author Information” page.
**Disclaimer:** Articles published in the journal HemaSphere exclusively reflect the opinions of the authors. The authors are responsible for all content in their abstracts including accuracy of the facts, statements, citing resources, etc.

### Results

A total of 75 patients were analyzed (25 CPX-351; 50 controls). Baseline characteristics were well balanced. Median follow-up was longer in controls (33.9 months, IQR 9.2–47.8) than in the CPX-351 group (18.3 months, IQR 6.5–40.8; p=0.016), reflecting a later median transplant year in the CPX-351 group (2021 vs. 2019; p<0.001). The median time to neutrophil engraftment (19.0 days [IQR 16–25] vs. 22.8 days [IQR 15–25]; p=0.1) and platelet recovery (14.0 days [IQR 11–23] vs. 35.2 days [IQR 14–36]; p=0.08) were similar within both groups (control vs CPX-351). Median hospital stay duration (31.9 days vs. 34.4 days, p=0.2) and rehospitalization rates within three months (27% vs. 36%, p=0.6) were similar within both groups. At 12 months post-transplant, OS was similar between CPX-351 and controls (63.5% [95% CI 47.1–85.7] for CPX-351 vs. 63.6% [95%CI 51.5–78.6] for controls; p=0.51). RFS did not differ significantly (59.4% [95%CI 42.8–82.5] vs. 62.7% [95%CI 50.4–78.0]; p=0.37). NRM at 12 months was comparable (18.8%, [95%CI, 0–36.1]  vs. 16.6%, [95% CI, 3.9–17.7], p=0.7). Incidence of acute GVHD and chronic GVHD were similar between groups. GRFS at 12 months was 49.8% (95% CI, 33.3–74.7) in the CPX-351 group versus 37.5% (95% CI, 26.1–53.8) in the control group, without significant differences (p=0.86). Relapse remained the leading cause of death in both cohorts.

### Summary/Conclusion

This study represents the first real-world multicenter matched analysis of post-allo-SCT outcomes following CPX-351. Contrary to pivotal trial findings suggesting improved post-transplant survival, we observed no significant survival advantage after allo-SCT compared with conventional induction. These results suggest that the clinical benefit of CPX-351 may primarily occur before transplantation through deeper remissions rather than improved post-transplant survival. Prospective studies specifically designed to assess transplant outcomes are warranted to optimize the positioning of CPX-351 in high-risk AML.

**Copyright Information:** (Online) ISSN: 2572-9241
©2026 The Author(s). HemaSphere is published by John Wiley & Sons Ltd on behalf of European Hematology Association. This is an open access Abstract Book distributed under the Attribution-NonCommercial-NoDerivs (CC BY-NC-ND), which allows third parties to download the articles and share them with others as long as they credit the author and the Abstract Book, but they cannot change the content in any way or use them commercially.
**Abstract Book Citations:** Authors, Title, HemaSphere, 2026;10:(S1):pages. The abstract book DOIs can be found on the “Author Information” page.
**Disclaimer:** Articles published in the journal HemaSphere exclusively reflect the opinions of the authors. The authors are responsible for all content in their abstracts including accuracy of the facts, statements, citing resources, etc.

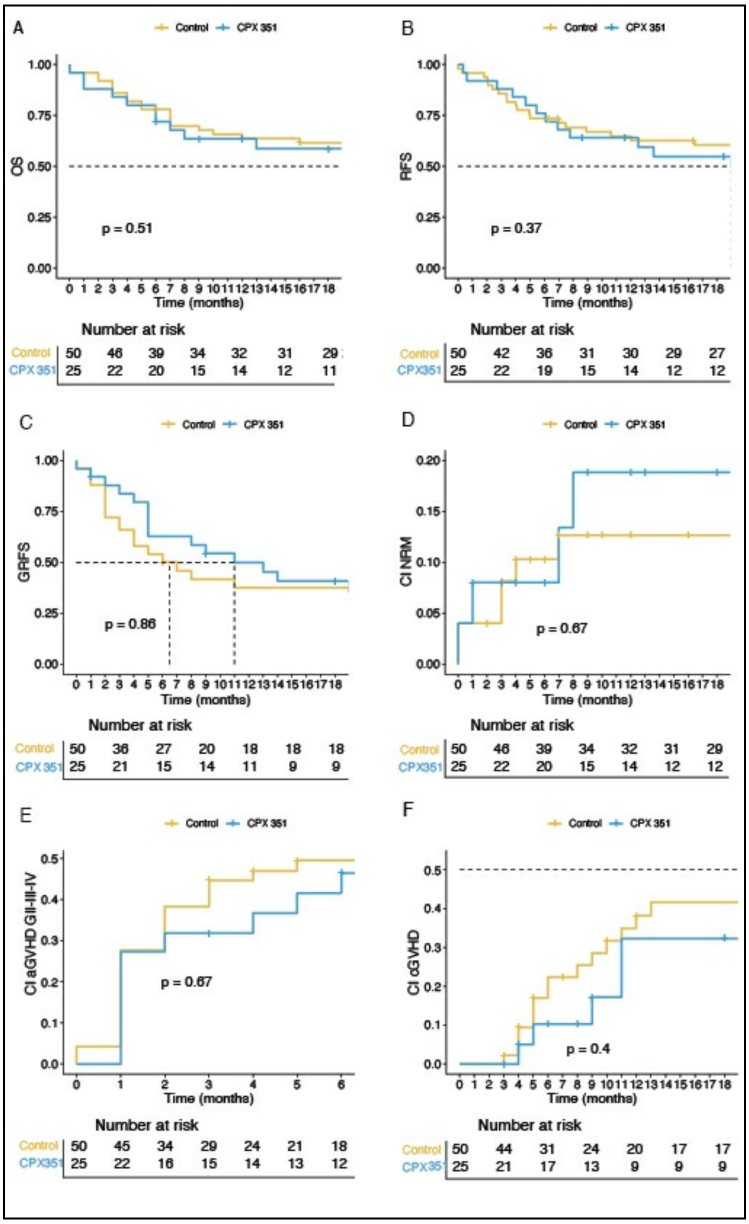

Main outcomes after allogeneic stem cell transplantation, comparison between CPX-351 and control groups. (A) Kaplan Meier (KM) curve describing overall survival, (B) KM curve showing relapse free survival, (C) KM curve reporting graft versus host disease relapse free survival (GRFS), (D) cumulative incidence (CI) curve of non-relapse mortality (NRM), (E) CI of all grade of acute graft versus host disease (aGVHD) and (F) for chronic graft versus host disease (cGVHD).

**Copyright Information:** (Online) ISSN: 2572-9241
©2026 The Author(s). HemaSphere is published by John Wiley & Sons Ltd on behalf of European Hematology Association. This is an open access Abstract Book distributed under the Attribution-NonCommercial-NoDerivs (CC BY-NC-ND), which allows third parties to download the articles and share them with others as long as they credit the author and the Abstract Book, but they cannot change the content in any way or use them commercially.
**Abstract Book Citations:** Authors, Title, HemaSphere, 2026;10:(S1):pages. The abstract book DOIs can be found on the "Author Information" page.
**Disclaimer:** Articles published in the journal HemaSphere exclusively reflect the opinions of the authors. The authors are responsible for all content in their abstracts including accuracy of the facts, statements, citing resources, etc.

### (PF1131) IMMUNOSUPPRESSIVE STRATEGY USING LOW-DOSE ATG COMBINED WITH ANTI-CD25 ANTIBODY FOR GVHD PROPHYLAXIS IN HAPLOIDENTICAL HEMATOPOIETIC STEM CELL TRANSPLANTATION

**Topic:** 23. Stem cell transplantation - Clinical

Peipei Li[1], Xianghua Wang[1], Yujie Jiang[1], Xiaohui Sui[1], Ying Li[1], Dai Yuan[1], Xiaosheng Fang*[1]

*[1]Shandong Provincial Hospital Affiliated to Shandong First Medical University, Jinan, China;*

### Background

Graft-versus-host disease (GVHD) is a major complication of haploidentical hematopoietic stem cell transplantation (haplo-HSCT). Although anti-thymocyte globulin (ATG)-based prophylaxis is widely used, the incidence of severe acute and chronic GVHD remains high. Our previous studies showed that low-dose ATG (7.5 mg/kg) could lower the risk of viral reactivation without compromising GVHD prevention, but it does not significantly improve the incidence of severe GVHD. Humanized anti-CD25 monoclonal antibody (mAb) enhances GVHD prevention by selectively eliminating activated T cells, exhibiting synergistic effects with ATG.

### Aims

To evaluate the efficacy and safety of adding humanized anti-CD25 mAb to low-dose ATG for GVHD prophylaxis in haplo-HSCT.

### Methods

This study prospectively enrolled 39 patients with malignant hematologic diseases undergoing first haplo-HSCT at Shandong Provincial Hospital between September 2022 and August 2025. Diagnoses included acute myeloid leukemia (AML) (46.2%, n=18), myelodysplastic syndrome (MDS) (15.4%, n=6), and T-lymphoblastic lymphoma (T-LBL) (2.5%, n=1). Most patients were in complete remission (69.2%, n=27) or minimal residual disease-positive (MRD+) status (15.4%, n=6) prior to transplantation. All patients underwent conditioning with BU/CY or modified BU/CY regimens, with the baseline GVHD prophylaxis consisting of mycophenolate mofetil + cyclosporine + short-course methotrexate + low-dose ATG (2.5 mg/kg on days -4, -3, and -2). Based on clinical decision-making, patients were divided into the CD25+ group (anti-CD25 mAb 1 mg/kg on days +4 and +7, n=21) and the control group (n=18).

### Results

Median follow-up was 13.14 months (range: 0.85-34.56), with similar baseline characteristics. Regarding the primary endpoint, the CD25+ group showed a trend toward reduction in overall aGVHD incidence (33.3% [7/21] vs 61.1% [11/18], P=0.083), with an absolute risk reduction of 27.8%; the incidence of grade II-IV aGVHD decreased from 38.9% (7/18) in the control group to 14.3% (3/21) (P=0.141). The cGVHD incidence was similar (19.7% [4/21] vs 11.1% [2/18], P=0.667). In terms of hematopoietic reconstitution, neutrophil engraftment was significantly accelerated in the CD25+ group (+11 days [10-19 days] vs +13.5 days [10-24 days], P=0.011), and the median time to platelet engraftment was also shortened (+12 days [11-22 days] vs +14 days [11-51 days], P=0.066). For safety, CMV and EBV were comparable between groups, with no increased risk of viral infection. Infection-related mortality rate was lower in the CD25+ group (4.8% [1/21] vs 27.8% [5/18], P=0.077), and one patient in the control group died from EBV-PTLD complicated by sepsis. Owing to the short follow-up, survival data are still immature, and further follow-up is being conducted to mature the endpoints.

**Copyright Information:** (Online) ISSN: 2572-9241
©2026 The Author(s). HemaSphere is published by John Wiley & Sons Ltd on behalf of European Hematology Association. This is an open access Abstract Book distributed under the Attribution-NonCommercial-NoDerivs (CC BY-NC-ND), which allows third parties to download the articles and share them with others as long as they credit the author and the Abstract Book, but they cannot change the content in any way or use them commercially.
**Abstract Book Citations:** Authors, Title, HemaSphere, 2026;10:(S1):pages. The abstract book DOIs can be found on the “Author Information” page.
**Disclaimer:** Articles published in the journal HemaSphere exclusively reflect the opinions of the authors. The authors are responsible for all content in their abstracts including accuracy of the facts, statements, citing resources, etc.

**Summary/Conclusion**

Our preliminary study showed that the low-dose ATG combined with anti-CD25 mAb for GVHD prophylaxis in haplo-HSCT demonstrates favorable tolerability, reducing the incidence of aGVHD without increasing infection or relapse rates. Importantly, hematopoietic recovery was accelerated, with significantly faster neutrophil engraftment and a trend toward earlier platelet recovery. This promising strategy warrants further validation in larger prospective studies.

**Copyright Information:** (Online) ISSN: 2572-9241
©2026 The Author(s). HemaSphere is published by John Wiley & Sons Ltd on behalf of European Hematology Association. This is an open access Abstract Book distributed under the Attribution-NonCommercial-NoDerivs (CC BY-NC-ND), which allows third parties to download the articles and share them with others as long as they credit the author and the Abstract Book, but they cannot change the content in any way or use them commercially.
**Abstract Book Citations:** Authors, Title, HemaSphere, 2026;10:(S1):pages. The abstract book DOIs can be found on the "Author Information" page.
**Disclaimer:** Articles published in the journal HemaSphere exclusively reflect the opinions of the authors. The authors are responsible for all content in their abstracts including accuracy of the facts, statements, citing resources, etc.

### (PF1132) IMPACT OF CONCURRENT GRAFT-VERSUS-HOST DISEASE ON LONG-TERM SURVIVAL IN CRITICALLY ILL PATIENTS FOLLOWING ALLOGENEIC HEMATOPOIETIC STEM CELL TRANSPLANTATION FOR HEMATOLOGICAL MALIGNANCIES

**Topic:** 23. Stem cell transplantation - Clinical

Zhixue Li*[1], Peng Zhao[1], Haixia Fu[1], Yuanyuan Zhang[1], Chenhua Yan[1], Yao Chen[1], Yuhong Chen[1], Meng Lv[1], Yu-Qian Sun[1], Xiaodong Mo[1], Xiang-Yu Zhao[1], Huiying Zhao[2], Fengxue Zhu[2], Yu Wang[1], Yingjun Chang[1], Lanping Xu[1], Xiaojun Huang[1], Xiaohui Zhang[1]

*[1]Peking University People's Hospital, Peking University Institute of Haematology, Beijing, China;[2]Peking University People's Hospital, Department of Critical Care Medicine, Beijing, China;*

### Background

After allogeneic haematopoietic stem cell transplantation (allo-HSCT) for haematologic malignancies, 10%–20% of patients develop critical illness, which is associated with a poor prognosis. Graft-versus-host disease (GVHD) is a common but potentially life-threatening complication following allo-HSCT and may substantially affect survival among critically ill patients. However, few studies have examined the impact of GVHD on the long-term survival of critically ill patients after transplantation.

### Aims

To investigate the impact of GVHD on the long-term outcomes of critically ill patients who have undergone allo-HSCT for haematologic malignancies, with a particular emphasis on ICU survivors.

### Methods

This cohort included all critically ill patients who underwent allo-HSCT for haematological malignancies at our centre from January 2010 to June 2025. Patients were excluded from the study if they were admitted to the ICU for temporary intensive care. Only the first ICU admission was considered.

**Copyright Information:** (Online) ISSN: 2572-9241
©2026 The Author(s). HemaSphere is published by John Wiley & Sons Ltd on behalf of European Hematology Association. This is an open access Abstract Book distributed under the Attribution-NonCommercial-NoDerivs (CC BY-NC-ND), which allows third parties to download the articles and share them with others as long as they credit the author and the Abstract Book, but they cannot change the content in any way or use them commercially.
**Abstract Book Citations:** Authors, Title, HemaSphere, 2026;10:(S1):pages. The abstract book DOIs can be found on the "Author Information" page.
**Disclaimer:** Articles published in the journal HemaSphere exclusively reflect the opinions of the authors. The authors are responsible for all content in their abstracts including accuracy of the facts, statements, citing resources, etc.

**Results**

A cohort of 726 critically ill patients who underwent allo-HSCT for haematological malignancies was included (470 males and 256 females). Of them, 198 (27.3%) patients had concurrent GVHD and 528 (72.7%) patients had no concurrent GVHD. Patients with concurrent GVHD were admitted to the ICU later after transplantation (256.0 days vs. 167.5 days, $p < 0.001$) and had a shorter ICU length of stay. Long-term survival in the overall cohort was poor, with a 12-month overall survival rate of 27.7% (Figure A). The 12-month overall survival was significantly lower in patients with concurrent GVHD than in those without concurrent GVHD (16.2% vs. 32.8%, log-rank $p < 0.001$, Figure B/C). Concurrent GVHD ($p = 0.002$, HR 1.342, 95% CI 1.117 to 1.613), relapse of haematological malignancies ($p = 0.015$, HR 1.510, 95% CI 1.083 to 2.105), sinusoidal obstruction syndrome (SOS) ($p = 0.048$, HR 1.632, 95% CI 1.004 to 2.651), and ≥ 2 organ failures during ICU stay ($p < 0.001$, HR 1.864, 95% CI 1.559 to 2.228) were identified as independent prognostic factors for overall survival. Patients with controlled GVHD (55/198, 27.8%) had favourable outcomes, with a 12-month overall survival rate of 41.8%, which was comparable with those without concurrent GVHD; By contrast, almost all patients with uncontrolled GVHD died, with a 12-month overall survival rate of 4.9% (Figure D). In patients with uncontrolled GVHD, age ≥ 40 years at HSCT ($p = 0.011$, OR 2.852, 95% CI 1.267 to 6.418) and ≥ 2 organ failures during ICU stay ($p = 0.006$, OR 3.645, 95% CI 1.444 to 9.200) were identified as independent prognostic factors for in-ICU mortality. ICU survivors (288/726, 39.7%), defined as those who survived to 7 days after ICU discharge, demonstrated rather favourable long-term outcomes, with a 12-month overall survival rate of 69.1%. Patients with concurrent GVHD had a significantly lower 12-month overall survival rate than those without concurrent GVHD (47.6% vs. 75.0%, log-rank $p < 0.001$).

**Summary/Conclusion**

In conclusion, in this critically ill cohort with a poor long-term prognosis after transplantation for haematologic malignancies, concurrent GVHD significantly reduced long-term survival. Relapse of haematological malignancies, SOS, ≥ 2 organ failures during ICU stay, and the need for invasive mechanical ventilation were identified as independent risk factors for overall survival. Notably, among ICU survivors who achieved short-term survival, the presence of GVHD was associated with a further reduction in long-term survival.

**Copyright Information:** (Online) ISSN: 2572-9241
©2026 The Author(s). HemaSphere is published by John Wiley & Sons Ltd on behalf of European Hematology Association. This is an open access Abstract Book distributed under the Attribution-NonCommercial-NoDerivs (CC BY-NC-ND), which allows third parties to download the articles and share them with others as long as they credit the author and the Abstract Book, but they cannot change the content in any way or use them commercially.
**Abstract Book Citations:** Authors, Title, HemaSphere, 2026;10:(S1):pages. The abstract book DOIs can be found on the “Author Information” page.
**Disclaimer:** Articles published in the journal HemaSphere exclusively reflect the opinions of the authors. The authors are responsible for all content in their abstracts including accuracy of the facts, statements, citing resources, etc.

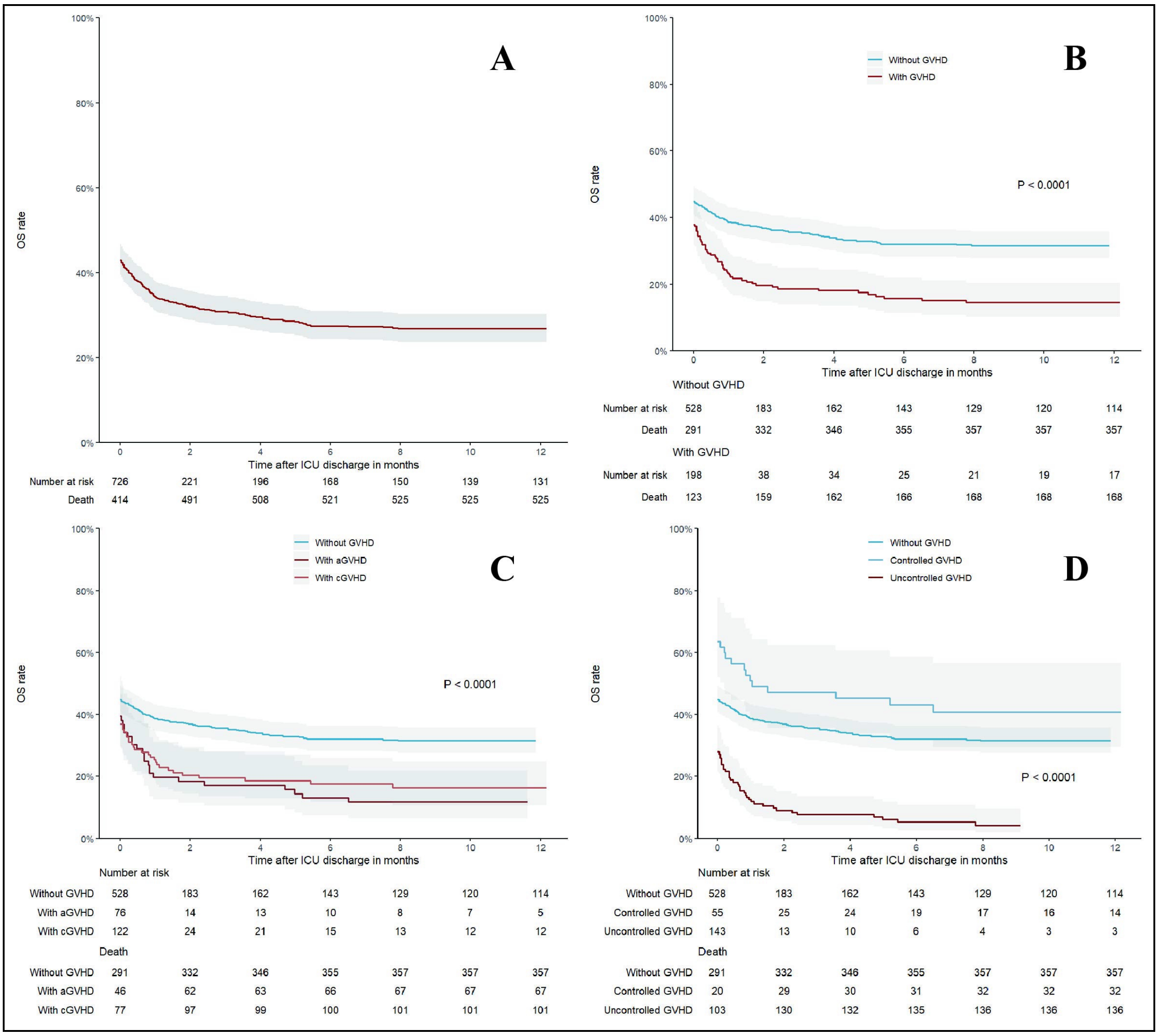

**Copyright Information:** (Online) ISSN: 2572-9241
©2026 The Author(s). HemaSphere is published by John Wiley & Sons Ltd on behalf of European Hematology Association. This is an open access Abstract Book distributed under the Attribution-NonCommercial-NoDerivs (CC BY-NC-ND), which allows third parties to download the articles and share them with others as long as they credit the author and the Abstract Book, but they cannot change the content in any way or use them commercially.
**Abstract Book Citations:** Authors, Title, HemaSphere, 2026;10:(S1):pages. The abstract book DOIs can be found on the "Author Information" page.
**Disclaimer:** Articles published in the journal HemaSphere exclusively reflect the opinions of the authors. The authors are responsible for all content in their abstracts including accuracy of the facts, statements, citing resources, etc.

### (PF1133) BURDEN, SPECTRUM, AND PROGNOSTIC IMPACT OF INFECTIONS AFTER ALLOHSCT IN PATIENTS ≥65 YEARS: A GETH-TC STUDY

**Topic:** 23. Stem cell transplantation - Clinical

Sara Fernandez-Luis*[1], Mónica Baile-González[2], Alejandro Avendaño[2], Cristina Alburquerque-Prieto[1], Lucrecia Yáñez[1], Maria Queralt Salas[3], Paula Sánchez[4], Alberto Doblas Marquez[5], Marta Luque Garrido[5], Candela Ceballos Bolaños[6], Nerea Arratibel[6], María José Jiménez[7], María Huguet[7], Clara Aparicio Pérez[8], Carmen Martin[8], Ana María Pilar González Rodríguez[9], Lucia Garcia[10], Leyre Bento[10], Andrés Sanchez Salinas[11], Lucia Garcia Tomas[12], Inmaculada Heras Fernando[12], Sara Villar-Fernandez[13], Jose Juan Rifon Roca[13], Irene Garcia Cadenas[4]

*[1]Hospital Universitario Marqués de Valdecilla - IDIVAL, Santander, Spain;[2]Complejo Asistencial Universitario de Salamanca-IBSAL, Hematology Department, Salamanca, Spain; [3]Hospital Clinic de Barcelona, ICHMO, Hematopoietic Cell Transplantation Unit, Barcelona, Spain; [4]Hospital de Sant Pau, Hematology Department, Barcelona, Spain;[5]Hospital Regional de Málaga, Málaga, Spain; [6]Hospital Universitario de Donostia, Hematology Department, Donostia, Spain;[7]Institut Catalá d'Oncologia de Badalona, Hospital Universitari Germans Trias i Pujol; Institut d'Investigació contra la Leucemia Josep Carreras, Hematology Department, Badalona, Spain; [8]Hospital Universitario Reina Sofía, Córdoba, Spain;[9]Hospital Universitario Central de Asturias, Oviedo, Spain; [10]Hospital Universitario Son Espases, IdISBa,, Palma de Mallorca, Spain; [11]Hospital Virgen de la Arrixaca-IMIB Pascual Parrilla, Murcia, Spain; [12]Hospital Morales Meseguer, Murcia, Spain; [13]Clínica Universidad de Navarra, Pamplona, Spain;*

### Background

Infectious complications remain a leading cause of morbidity and mortality after allogeneic hematopoietic stem cell transplantation (alloHSCT). As the use of alloHSCT expands in older adults, the impact of age-related immune dysfunction on infectious burden and outcomes remains unclear.

### Aims

To characterise the incidence, risk factors, and clinical impact of infections -focusing on bacterial and fungal events- in alloHSCT recipients aged ≥65 years.

### Methods

This retrospective multicentre study included consecutive patients aged ≥65 years undergoing a first alloHSCT at 13 Spanish centres between 2011 and 2023. All microbiologically documented infections were analysed during the first year post-transplant, or until relapse or death, whichever occurred first. Multivariable models were used to assess risk factors and survival impact.

**Copyright Information:** (Online) ISSN: 2572-9241
©2026 The Author(s). HemaSphere is published by John Wiley & Sons Ltd on behalf of European Hematology Association. This is an open access Abstract Book distributed under the Attribution-NonCommercial-NoDerivs (CC BY-NC-ND), which allows third parties to download the articles and share them with others as long as they credit the author and the Abstract Book, but they cannot change the content in any way or use them commercially.
**Abstract Book Citations:** Authors, Title, HemaSphere, 2026;10:(S1):pages. The abstract book DOIs can be found on the "Author Information" page.
**Disclaimer:** Articles published in the journal HemaSphere exclusively reflect the opinions of the authors. The authors are responsible for all content in their abstracts including accuracy of the facts, statements, citing resources, etc.

**Results**

We analysed 726 patients (median age 67 years, range 65-82). Donors were HLA-identical siblings (28.0%), haploidentical (31.7%), matched unrelated (30.9%), and mismatched unrelated (9.5%). Myeloablative conditioning was used in 21.5% of patients, and 53.9% received post-transplant cyclophosphamide-based GvHD prophylaxis.
Overall, 78.2% of patients developed ≥1 infectious episode (median 2, range 1-22). The cumulative incidence (CI) of any infection at days +30, +100, and +365 was 47.3%, 69.1%, and 77.9%, respectively.
Bacterial infections were the most common, 54.8% of patients experienced ≥1 episode (812 isolates) and the CI at days +30, +100, and +365 was 33.6%, 44.4%, and 53.6%, respectively. Gram-negative pathogens predominated (55.7%) and 21.2% of isolates were multidrug-resistant. The overall bacterial infection density was 4.31 per 1,000 patient-days. ECOG ≥1 (IRR 1.4, $p$=0.005) and haploidentical donor (IRR 2.7, $p$<0.001) were independently associated with higher infection density; whereas antibiotic prophylaxis was protective (IRR 0.6, $p$<0.001).
Fungal infections were identified in 117 patients (16.1%); including 89 cases of invasive fungal disease (IFD), mainly mould infections. The 1-year CI of IFD was 11.7%, with most cases occurring before day +100 (8.4%). The IFD density was 0.47 per 1,000 patient-days. ECOG ≥1 (IRR 1.6, $p$=0.023), haploidentical donor (IRR 2.0, $p$=0.011), and echinocandin prophylaxis (IRR 2.6, $p$<0.001) were associated with higher density; while the 2018-2023 period (vs. 2011-2017) was associated with lower density (IRR 0.5, $p$=0.001).
At 1-year, overall survival (OS) and non-relapse mortality (NRM) were 62.9% and 23.9%. Infection-related mortality (IRM) at days +30, +100, and +365 was 2.5%, 6.3%, and 12.4%, respectively, with *Aspergillus* spp. and *Escherichia coli* as the leading pathogens. Both acute (HR 3.3, $p$<0.001) and chronic (HR 2.9, $p$<0.001) GvHD were associated with higher IRM.
In univariate analyses, the occurrence of any infection was associated with inferior OS (HR 1.6, $p$<0.001) and increased NRM (HR 3.4, $p$<0.001). Moreover, this negative association was observed when the impact of bacterial infections (HR 1.7, $p$<0.001 for OS; HR 3.2, $p$<0.001 for NRM) and IFD (HR 1.8, $p$<0.001 for OS; HR 3.7, $p$<0.001 for NRM) was individually evaluated.

**Summary/Conclusion**

In older alloHSCT recipients, infections -particularly bacterial and fungal- are highly prevalent and strongly compromise survival by increasing NRM. Functional status, haploidentical transplants, and GvHD development are key determinants of infectious burden, highlighting the need for tailored, risk-adapted preventive strategies in this expanding patient population.

**Copyright Information:** (Online) ISSN: 2572-9241
©2026 The Author(s). HemaSphere is published by John Wiley & Sons Ltd on behalf of European Hematology Association. This is an open access Abstract Book distributed under the Attribution-NonCommercial-NoDerivs (CC BY-NC-ND), which allows third parties to download the articles and share them with others as long as they credit the author and the Abstract Book, but they cannot change the content in any way or use them commercially.
**Abstract Book Citations:** Authors, Title, HemaSphere, 2026;10:(S1):pages. The abstract book DOIs can be found on the "Author Information" page.
**Disclaimer:** Articles published in the journal HemaSphere exclusively reflect the opinions of the authors. The authors are responsible for all content in their abstracts including accuracy of the facts, statements, citing resources, etc.

### (PF1134) ASSOCIATIONS OF AGE AND PRE-TRANSPLANT EMOTIONAL STATUS WITH EARLY IMMUNE RECONSTITUTION AFTER PEDIATRIC HEMATOPOIETIC STEM CELL TRANSPLANTATION

**Topic:** 23. Stem cell transplantation - Clinical

Jiali MA*[1], Uet Yu[1], Yi Chen[1], Binghua Zhang[1], Xiaodong Wang[1]

*[1]Blood and Marrow Transplantation Center, Shanghai Children's Medical Center, Shanghai Jiao Tong University School of Medicine, Shanghai, China;*

### Background

Early immune reconstitution after hematopoietic stem cell transplantation (HSCT) is essential for infection control and early clinical outcomes in pediatric patients. The first month following transplantation represents a critical phase of immune recovery. Although biological and transplant-related factors influencing early immune recovery have been widely studied, the impact of recipient age and pre-transplant emotional status on early immune reconstitution remains insufficiently characterized in pediatric HSCT.

### Aims

This prospective study aimed to investigate the associations between recipient age and pre-transplant emotional status and early immune reconstitution in pediatric HSCT recipients.

### Methods

A prospective observational study was conducted from May 2025 to December 2025. A total of 208 pediatric patients undergoing first HSCT were enrolled, including 58 females and 150 males, with a mean age of 8.53 ± 4.21 years. Underlying diseases included acute lymphoblastic leukemia(n = 69), aplastic anemia(n = 33), acute myeloid leukemia(n = 33), as well as other benign(n = 81) and malignant(n = 127) disorders. Fully matched donors were used in 86 cases. Conditioning regimens included T-cell depletion(n = 10), myeloablative conditioning(n = 186), and non-myeloablative conditioning(n = 12). Children aged 8 years and older completed Screen for Child Anxiety Related Emotional Disorders and the Children's Depression Inventory prior to transplantation. Early transplant-related complications, including graft-versus-host disease, sinusoidal obstruction syndrome / veno-occlusive disease, hemorrhagic cystitis, and viral reactivation, were prospectively recorded. Immune reconstitution parameters, including $CD3^+$, $CD4^+$, $CD8^+$ T cells, natural killer(NK) cells, and regulatory T cells(Treg), were assessed at one month after HSCT. Written informed consent was obtained. Univariate analyses were initially performed to screen potential variables. Variables with $P < 0.20$ in univariate analyses were subsequently entered into multivariable linear regression models to identify independent factors associated with early immune recovery.

### Results

Multivariable regression analyses demonstrated that donor type, recipient age, and pre-transplant depressive symptoms were independently associated with early T-cell reconstitution. Specifically, fully matched donor transplantation, younger age, and lower pre-transplant depression levels were associated with better recovery of $CD3^+$ T cells (donor type: β=−1.409, p=0.011; age: β=−0.160, p=0.022; depression: β=−0.082, p=0.030), $CD4^+$ T cells (donor type: β=−1.081, p=0.012; age: β=−0.169, p=0.020; depression: β=−0.086, p=0.031), and $CD8^+$ T cells (donor type: β=−0.952, p=0.020; age: β=−0.170, p=0.020; depression: β=−0.081, p=0.032). In addition, NK cell reconstitution was independently associated with recipient age (β=0.081, p=0.021) and $CD34^+$ cell dose (β=0.091, p=0.027). Higher pre-transplant depressive symptom scores were independently associated with lower Treg levels (β=−0.105, p=0.033).

**Copyright Information:** (Online) ISSN: 2572-9241
©2026 The Author(s). HemaSphere is published by John Wiley & Sons Ltd on behalf of European Hematology Association. This is an open access Abstract Book distributed under the Attribution-NonCommercial-NoDerivs (CC BY-NC-ND), which allows third parties to download the articles and share them with others as long as they credit the author and the Abstract Book, but they cannot change the content in any way or use them commercially.
**Abstract Book Citations:** Authors, Title, HemaSphere, 2026;10:(S1):pages. The abstract book DOIs can be found on the "Author Information" page.
**Disclaimer:** Articles published in the journal HemaSphere exclusively reflect the opinions of the authors. The authors are responsible for all content in their abstracts including accuracy of the facts, statements, citing resources, etc.

**Summary/Conclusion**

Recipient age and pre-transplant depressive symptoms are independently associated with early immune reconstitution after pediatric HSCT, particularly affecting T-cell subsets and regulatory T cells. These findings suggest that emotional vulnerability before transplantation may contribute to early immune dysregulation and highlight the potential clinical value of integrating psychological assessment into pre-transplant evaluation in pediatric HSCT.

**Copyright Information:** (Online) ISSN: 2572-9241
©2026 The Author(s). HemaSphere is published by John Wiley & Sons Ltd on behalf of European Hematology Association. This is an open access Abstract Book distributed under the Attribution-NonCommercial-NoDerivs (CC BY-NC-ND), which allows third parties to download the articles and share them with others as long as they credit the author and the Abstract Book, but they cannot change the content in any way or use them commercially.
**Abstract Book Citations:** Authors, Title, HemaSphere, 2026;10:(S1):pages. The abstract book DOIs can be found on the "Author Information" page.
**Disclaimer:** Articles published in the journal HemaSphere exclusively reflect the opinions of the authors. The authors are responsible for all content in their abstracts including accuracy of the facts, statements, citing resources, etc.

### (PF1135) IMMUNE RECONSTITUTION AFTER HAPLO-IDENTICAL PERIPHERAL BLOOD STEM CELL TRANSPLANT: A PROSPECTIVE REAL-WORLD STUDY

**Topic:** 23. Stem cell transplantation - Clinical

Himani Gupta*[1], Swapnil Tripathi[1], Syed Gafoor[1], Mayank Soni[1], Disha Kakkar[1], Jegan Kr[1], Reshmi Harikumar Pillai[1], Saravana Kumar[1], Priya Kunwar[1], Pooja Srivastava[1], Mayur Dakh[1], Sameer Shaikh[1], Rohan Halder[1], Archit Pandharipande[1], Aakanksha Singh[1], Sakshi Bhatnagar[1], Rajat Jain[1], Vipul Sheth[1], Dinesh Bhurani[1], Narendra Agrawal[1]

*[1]RAJIV GANDHI CANCER INSTITUTE AND RESEARCH CENTRE, DEPARTMENT OF CLINICAL HEMATOLOGY, New Delhi, India;*

### Background

Haplo-identical heamatopoetic stem cell transplant (Haplo-HSCT) with post transplant cyclophosphamide (PTCy) has expanded donor availability worldwide. However, detailed immune reconstitution (IR) kinetics particularly following peripheral blood stem cell (PBSC) grafts in high CMV seroprevalence, resource limited setting remain poorly characterised. Understanding IR patterns and their clinical correlations is essential for optimising post transplant outcomes.

### Aims

To evaluate IR kinetics post haplo-HSCT with PTCy and correlate immune recovery with pre, peri and post transplant factors.

### Methods

This prospective observational study included 20 adult patients with heterogenous hematological disorder who underwent haplo HSCT with PTCy based GVHD prophylaxis using PBSC graft conducted between October 2024 and December 2025. IR of absolute lymphocyte count (ALC), innate immune cells including NK cells, monocytes (classical and non classical), granulocytes and adaptive immune cells including T cell subsets(CD3+ CD4+, CD3+ CD8+), T regulatory cells (Tregs), B cells were assessed via flowcytometry at three time points post transplant (day+30, day+100 and day+180). Immune cell recovery was categorised based on increasing trend and normalisation of values at day+180 time point.IR was correlated with pre (recipient, donor, and transplant related factors), peri (neutrophils engraftment) and post transplant factors (viral infections, acute and chronic GVHD, relapse and mortality). Median follow up was 7 months.

### Results

The median age was 33 years. ALC showed progressive recovery at day+30, +100 and +180 (with median of 250–> 755–> 1270 cells/mcl). Among innate immune cells, monocytes demonstrated early expansion followed by stabilisation while NK cells showed gradual recovery. Adaptive immune cells recovery was delayed with T cells gradual expansion and persistent CD4+: CD8+ inversion throughout follow up period. B cells recovery was slow but progressive. Younger recipient showed superior ALC recovery (p value-0.02). Early neutrophil engraftment was associated with improved CD4+ T cell recovery p value-0.02. At follow up, viral infections occurred in 45% patients, acute GVHD in 35%, chronic GVHD in 40%, relapse in 15% and overall survival was 77%. Though not statistically significant, a hypothetical trend was observed wherein patients with no viral infections showed better monocyte and granulocyte recovery (p value 0.09, 0.07 respectively). Recovery of Tregs and Tregs: CD4+ T cells ratio showed lesser incidence of acute GVHD with p value of 0.05 and 0.02 respectively. ALC and CD8+ T cells recovery were observed in 64.7% and 70.6% non relapsed and survivors vs 0% relapsed and non survivors (p value-0.07) (p value-0.049) respectively.

**Copyright Information:** (Online) ISSN: 2572-9241
©2026 The Author(s). HemaSphere is published by John Wiley & Sons Ltd on behalf of European Hematology Association. This is an open access Abstract Book distributed under the Attribution-NonCommercial-NoDerivs (CC BY-NC-ND), which allows third parties to download the articles and share them with others as long as they credit the author and the Abstract Book, but they cannot change the content in any way or use them commercially.
**Abstract Book Citations:** Authors, Title, HemaSphere, 2026;10:(S1):pages. The abstract book DOIs can be found on the "Author Information" page.
**Disclaimer:** Articles published in the journal HemaSphere exclusively reflect the opinions of the authors. The authors are responsible for all content in their abstracts including accuracy of the facts, statements, citing resources, etc.

**Summary/Conclusion**

Haplo-HSCT with PTCy results in early innate and delayed adaptive immune reconstitution. Recipient age significantly influences immune recovery. CD4+ T cell recovery may serve as surrogate marker of graft potency. Delayed innate immune recovery may contribute to high viral infection burden. Tregs recovery strongly correlated with reduced acute GVHD risk, highlighting its potential role in guiding immunosuppression modulation. In a resource constraint setting, ALC may function as surrogate marker for graft versus leukaemia effect.

**Copyright Information:** (Online) ISSN: 2572-9241
©2026 The Author(s). HemaSphere is published by John Wiley & Sons Ltd on behalf of European Hematology Association. This is an open access Abstract Book distributed under the Attribution-NonCommercial-NoDerivs (CC BY-NC-ND), which allows third parties to download the articles and share them with others as long as they credit the author and the Abstract Book, but they cannot change the content in any way or use them commercially.
**Abstract Book Citations:** Authors, Title, HemaSphere, 2026;10:(S1):pages. The abstract book DOIs can be found on the "Author Information" page.
**Disclaimer:** Articles published in the journal HemaSphere exclusively reflect the opinions of the authors. The authors are responsible for all content in their abstracts including accuracy of the facts, statements, citing resources, etc.

### (PF1136) ROMIPLOSTIM N01 FOR PROMOTING PLATELET ENGRAFTMENT AFTER ALLO-HSCT: INTERIM RESULTS OF A PHASE II EXPLORATORY TRIAL FROM NICHE COHORT

**Topic:** 23. Stem cell transplantation - Clinical

Shan Jiang*[1], Erlie Jiang[1], Aiming Pang[1]

[1] *Institute of Hematology and Blood Diseases Hospital, Chinese Academy of Medical Sciences and Peking Union Medical College, Tianjin, China, Tianjin, China, stem cell transplant center, Tianjin, China;*

### Background

Delayed platelet engraftment occurs in 5–25% of patients after allogeneic hematopoietic stem cell transplantation (allo-HSCT) and has been identified as an independent risk factor for transplant-related mortality. Accelerating platelet engraftment facilitates hematopoietic reconstitution, reduces bleeding risk and transfusion requirements, and may improve patients' quality of life and prognosis. Romiplostim, a long-acting thrombopoietin receptor agonist, has shown preliminary efficacy in chemotherapy-related thrombocytopenia and autologous HSCT, but evidence for its use to promote platelet engraftment after allo-HSCT is lacking.

### Aims

To evaluate the efficacy and safety of romiplostim N01 in promoting platelet engraftment and improving early platelet recovery following allo-HSCT in patients with malignant hematologic diseases.

### Methods

Leveraging a NICHE cohort, we conducted a single-arm, single-center clinical trial (IIT2021011-EC-1) and designed to enroll 21 adult patients (ECOG ≤2, expected survival ≥3 months) undergoing first allo-HSCT for malignant hematologic disease. Treatment with romiplostim N01 started on day +1 after transplantation with fixed dose 5μg/kg administered subcutaneously once weekly until platelet complete response (defined as no platelet transfusion for 7 consecutive days with platelet count $\geq 50\times10^9$/L) or for a maximum of 28 days. The primary endpoint was platelet engraftment by day +21; secondary endpoints included times to platelet engraftment, platelet counts $\geq 50\times10^9$/L and $\geq 100\times10^9$/L, and platelet transfusion volumes at weeks 2 and 4 post-transplant.

### Results

As of December 2025, 17 patients had been enrolled; all received myeloablative conditioning and completed romiplostim N01 treatment. The median infused total nucleated cell dose was $10.6\times10^8$/kg, and the median $CD34^+$ cell dose was $5.16\times10^6$/kg. The median time to platelet engraftment was 13 days, with all patients achieving engraftment by day +21 and a platelet count of $65\times10^9$/L, and the time to reach $\geq 50\times10^9$/L was 19 days. Median platelet transfusion volumes at weeks 2 and 4 were 4 U, and the duration of transfusion requirement or grade 4 thrombocytopenia was 10 days. No treatment-interrupting or treatment-related adverse events were observed through last follow-up.

### Summary/Conclusion

In summary, this interim analysis indicates that romiplostim N01 may accelerate platelet engraftment after allo-HSCT and demonstrated favorable tolerability and safety during the observation period; however, its clinical value requires confirmation in larger studies.

**Copyright Information:** (Online) ISSN: 2572-9241
©2026 The Author(s). HemaSphere is published by John Wiley & Sons Ltd on behalf of European Hematology Association. This is an open access Abstract Book distributed under the Attribution-NonCommercial-NoDerivs (CC BY-NC-ND), which allows third parties to download the articles and share them with others as long as they credit the author and the Abstract Book, but they cannot change the content in any way or use them commercially.
**Abstract Book Citations:** Authors, Title, HemaSphere, 2026;10:(S1):pages. The abstract book DOIs can be found on the "Author Information" page.
**Disclaimer:** Articles published in the journal HemaSphere exclusively reflect the opinions of the authors. The authors are responsible for all content in their abstracts including accuracy of the facts, statements, citing resources, etc.

### (PF1137) THE COMBINATION OF RUXOLITINIB AND EXTRACORPOREAL PHOTOPHERESIS IS A HIGHLY EFFECTIVE SECOND LINE TREATMENT FOR CHRONIC GRAFT-VERSUS-HOST DISEASE

**Topic:** 23. Stem cell transplantation - Clinical

Andrea Varkonyi*[1], Marienn Reti[1], Ágnes Király[1], Krisztina Jeszenszky[1], Melinda Paksi[1], Viktor Lakatos[1], Alexandra Raska[1], Éva Torbágyi[1], János Fábián[1], László Gopcsa[1], Peter Pal Remenyi[1], Nikolett Wohner[1]

*[1]Central Hospital of Southern-Pest, National Institute of Hematology and Infectious Diseases, Department of Hematology and Stem Cell Transplantation, Innovative Cell Therapy Center, Budapest, Hungary;*

### Background

Allogeneic hematopoietic stem cell transplantation (allo-HSCT) is potentially curative for hematologic malignancies, but its success is limited by graft-versus-host disease (GVHD). Chronic GVHD (cGVHD) affects one third to half of long-term survivors and is a major cause of late non-relapse morbidity and mortality. Standard first-line therapy with prolonged glucocorticoids frequently leads to steroid-refractory (SR) or steroid-dependent (SD) disease and long-tem toxicity. Ruxolitinib is an established second-line agent in SR cGVHD, while extracorporeal photopheresis (ECP) is an effective immunomodulatory option with a relative lack of toxicity and immunosuppression. Both are recommended as preferred second-line therapies, but data on their combined use in larger, well-characterized cGVHD cohorts remain limited.

### Aims

We evaluated the efficacy and safety of combined ECP and ruxolitinib (RUX+ECP) treatment and explored clinical predictors of response.

### Methods

We performed a single-center retrospective study of 80 patients with SR or SD cGVHD, treated between 2017 and 2025 at the Department of Stem Cell Transplantation and Innovative Cell Therapy, South-Pest Central Hospital, Budapest, Hungary. SR cGVHD was defined as progression despite ≥1mg/kg/day prednisone for 1–2 weeks or lack of improvement on ≥0.5mg/kg/day for 1–2 months, SD cGVHD as flare when tapering prednisone below 0.25mg/kg/day. All patients received ruxolitinib 5–10 mg twice daily combined with ECP, initiated twice weekly and then tapered individually. Responses were assessed by NIH consensus criteria as complete response (CR), partial response (PR), or no response (NR). Statistical analyses were performed using GraphPad Prism 10.6.1.

### Results

Median age at start of treatment was 45.5 years (IQR 31.25–53). Underlying diseases were predominantly AML (38/80,47.5%) and B/T-ALL (19/80,23.8%). Myeloablative conditioning was used in 59 patients (73.8%) and reduced-intensity conditioning in 21 (26.2%). Donor types included matched unrelated donors in 32 patients (40.0%), HLA-identical related donors in 27 (33.8%), and haploidentical related donors in 21 (26.2%). Overall, 32 patients (40.0%) achieved CR and 36 (45.0%) PR, yielding an overall response rate of 68/80 (85.0%), 12 patients (15.0%) were non-responders. Median time to response was 30 days (IQR 21–60). Neither baseline cGVHD severity ($\chi^2$=0.62, df=2, p=0.73) nor donor type ($\chi^2$=1.7, df=2, p=0.42) significantly influenced response to RUX+ECP. A prior history of acute GVHD (aGvHD) likewise had no statistically significant impact on response (92.3% vs 81.4% responders without vs with prior aGVHD; $\chi^2$=1.61, df=1, p=0.20; Fisher's exact test p=0.31).

**Copyright Information:** (Online) ISSN: 2572-9241
©2026 The Author(s). HemaSphere is published by John Wiley & Sons Ltd on behalf of European Hematology Association. This is an open access Abstract Book distributed under the Attribution-NonCommercial-NoDerivs (CC BY-NC-ND), which allows third parties to download the articles and share them with others as long as they credit the author and the Abstract Book, but they cannot change the content in any way or use them commercially.
**Abstract Book Citations:** Authors, Title, HemaSphere, 2026;10:(S1):pages. The abstract book DOIs can be found on the "Author Information" page.
**Disclaimer:** Articles published in the journal HemaSphere exclusively reflect the opinions of the authors. The authors are responsible for all content in their abstracts including accuracy of the facts, statements, citing resources, etc.

**Summary/Conclusion**

Here we report the largest cohort to date to evaluate the efficacy and safety of RUX+ECP in cGVHD. In this SR or SD cGVHD population, we observed an overall response rate of 85%, which compares favorably with historical cohorts treated with ruxolitinib monotherapy (60–75%). Response rates were consistently high across the patient population, independent of donor types, baseline cGVHD severity or the history of aGVHD, suggesting that the benefit of this combination extends across clinically diverse risk groups. No relevant safety signal emerged. These findings support RUX+ECP as a highly active and broadly applicable second-line strategy in cGVHD and warrant confirmation in prospective, controlled studies.

**Copyright Information:** (Online) ISSN: 2572-9241
©2026 The Author(s). HemaSphere is published by John Wiley & Sons Ltd on behalf of European Hematology Association. This is an open access Abstract Book distributed under the Attribution-NonCommercial-NoDerivs (CC BY-NC-ND), which allows third parties to download the articles and share them with others as long as they credit the author and the Abstract Book, but they cannot change the content in any way or use them commercially.
**Abstract Book Citations:** Authors, Title, HemaSphere, 2026;10:(S1):pages. The abstract book DOIs can be found on the "Author Information" page.
**Disclaimer:** Articles published in the journal HemaSphere exclusively reflect the opinions of the authors. The authors are responsible for all content in their abstracts including accuracy of the facts, statements, citing resources, etc.

### (PF1138) EFFICACY OF LOW-DOSE ATG COMBINED WITH PTCY IN CHILDREN WITH CHRONIC GRANULOMATOUS DISEASE UNDERGOING HAPLOIDENTICAL HEMATOPOIETIC STEM CELL TRANSPLANTATION

**Topic:** 23. Stem cell transplantation - Clinical

Yingjian Si*[1], Wei Lu[1], Wei Chen[1], Xiangfeng Tang[1], Haifei Zhou[2]

*[1]National Engineering Laboratory for Birth Defects Prevention and Control of Key Technology, Department of Pediatrics, the Seventh Medical Center of PLA General Hospital, Beijing, China; [2]Beijing BFR Gene Diagnostics, Beijing, China;*

### Background

Chronic granulomatous disease (CGD) is a primary immunodeficiency (PID) caused by defects of the NADPH oxidase complex in phagocytes, and is characterized by life-threatening infections, immune dysregulation and granulomatous inflammation. Allogeneic hematopoietic stem cell transplantation (allo-HSCT) is the only curative treatment of CGD, but rejection and graft-versus-host disease (GVHD) remain major challenges.

### Aims

The retrospective study aims to explore efficacy of GVHD prophylaxis regimens utilizing low-dose anti-thymocyte globulin (ATG) combined with post-transplant cyclophosphamide (PTCy) (the Combined group) compared to standard-dose ATG alone ( the ATG group) during haplo-HSCT for pediatric patients with CGD, as well as differences of post-tranplant immune reconstitution.

### Methods

A cohort of 70 genetically confirmed pediatric CGD patients undergoing haplo-HSCT at our institution was analyzed. Patients were stratified into two groups according to prophylaxis regimens and comparative analyses were performed for hematologic recovery, engraftment kinetics, incidence of GVHD, overall survival (OS), event-free survival (EFS), and GVHD/event-free survival (GEFS). Moreover, high-throughput immune repertoire profiling assessed T-cell receptor (TCR) diversity, B-cell receptor (BCR) diversity and quantitative lymphocyte subsets following haplo-HSCT.

### Results

Results showed that the Combined group exhibited significantly faster neutrophil engraftment (median: 12.5 vs. 14.0 days; P = 0.007), accelerated platelet engraftment (median days: 12.0 [9.2-16.3] vs. 21.1 [15.3–29.1]; P < 0.001) and comparable 6-month donor chimerism rates (93.8% vs 100.0%, P = 0.205). Cumulative incidence of mild acute GVHD (21.9% vs. 23.7%; P = 0.446) and chronic GVHD (3.1% vs. 5.3%; P > 0.05) were comparable either. At 6 months following transplant, the Combined group had significantly higher CD3+, CD4+, and CD8+ T-cell counts (all P < 0.05), and faster immune recovery of TCR and BCR diversity evidenced by greater top-10 clone diversity (P = 0.001 for both). No significant differences were observed in 2-year OS, EFS, or GEFS.

### Summary/Conclusion

In conclusion, the combination of low-dose ATG with PTCy effectively reduces the cumulative incidence of both acute and cGVHD while promoting accelerated hematopoietic and immune reconstitution post-haplo-HSCT, without adversely affecting long-term survival outcomes. This regimen may constitute a promising therapeutic strategy for managing CGD in the context of haplo-HSCT.

**Copyright Information:** (Online) ISSN: 2572-9241
©2026 The Author(s). HemaSphere is published by John Wiley & Sons Ltd on behalf of European Hematology Association. This is an open access Abstract Book distributed under the Attribution-NonCommercial-NoDerivs (CC BY-NC-ND), which allows third parties to download the articles and share them with others as long as they credit the author and the Abstract Book, but they cannot change the content in any way or use them commercially.
**Abstract Book Citations:** Authors, Title, HemaSphere, 2026;10:(S1):pages. The abstract book DOIs can be found on the “Author Information” page.
**Disclaimer:** Articles published in the journal HemaSphere exclusively reflect the opinions of the authors. The authors are responsible for all content in their abstracts including accuracy of the facts, statements, citing resources, etc.

### (PF1139) MYELOABLATIVE FRACTIONATED BUSULFAN, FLUDARABINE, CLADRIBINE, THIOTEPA, AND VENETOCLAX (CLADILLAC) CONDITIONING FOR HIGH-RISK AML: A PHASE 2 TRIAL

**Topic:** 23. Stem cell transplantation - Clinical

Uday R. Popat*[1], Roland Bassett[1], Amin Majid Alousi[1], Gheath Al-Atrash[1], Yosra Aljawai[1], Qaiser Bashir[1], George Chen[1], Warren Fingrut[1], Chitra Hosing[1], Jin Im[1], Partow Kebriaei[1], David Marin[1], Yago Nieto[1], Amanda Olson[1], Betul Oran[1], Muzaffar H. Qazilbash[1], Jeremy L. Ramdial[1], Neeraj Saini[1], Portia Smallbone[1], Samer A. Srour[1], Richard E. Champlin[1], Elizabeth J. Shpall[1]

[1]*The University of Texas MD Anderson Cancer Center, Houston, United States of America;*

### Background

Patients with high-risk acute myeloid leukemia (AML) have poor outcomes despite undergoing stem cell transplantation (SCT). Non-relapse mortality (NRM) post SCT is often driven by conditioning regimen toxicity and graft-versus-host disease (GVHD). To mitigate conditioning toxicity, we extended the schedule of myeloablative busulfan (Bu) to 3 weeks. To address GVHD, we incorporated post-transplant cyclophosphamide (PTCy). To reduce relapse, we added cladribine, thiotepa, and venetoclax—agents with known activity in AML. We evaluated this regimen in a prospective phase 2 trial in high-risk AML.

### Aims

To evaluate a myeloablative busulfan conditioning regimen containing cladribine, thiotepa, and venetoclax in patients with high-risk AML undergoing allogeneic SCT.

### Methods

High-risk AML was defined by primary refractory/relapsed disease, measurable residual disease (MRD) positivity, ELN22 adverse-risk, or secondary AML. Eligible patients were 18–70 years old and had a matched or haploidentical related donor, or a 7/8 or 8/8 HLA-matched unrelated donor. The conditioning regimen included outpatient Bu 100 mg/m² on days -20 and -13, fludarabine 10 mg/m², cladribine 10 mg/m², and Bu pharmacokinetically dosed to reach a total systemic exposure of 20,000 ±12% µmol/min on days -6 to -3. Venetoclax 400 mg was given daily from days -22 to -3. GVHD prophylaxis was PTCy 50 mg/kg on days 3 and 4, tacrolimus ± MMF. The primary endpoint was progression-free survival (PFS). A sample size of 50 provided >80% power to detect an increase in 1-year PFS from 30% (historical) to 47%, with a 5% Type I error rate (ClinicalTrials.gov: NCT04708054).

### Results

Fifty patients (22 female, 28 male) with a median age of 55 years (range: 18–70) were enrolled from December 2021 to August 2023. Disease characteristics included: primary refractory/relapsed AML (32%), MRD+ (74%), ELN22 adverse risk (66%), and secondary AML (22%). TP53 and FLT3 mutations were each present in 24% of patients. Donors were matched unrelated (52%), matched sibling (26%), haploidentical (14%), and mismatched unrelated (8%). Median HCT-CI score was 2 (range: 0–7); 38% had a Karnofsky performance score ≤80%.
At a median follow up of 28 months, median PFS was not reached (95% CI: 11 months–NR). One-year and 3-year PFS were 60% (95% CI: 48–75) and 58% (46–73), respectively. Three-year overall survival (OS), NRM, and relapse rates were 64% (52–79), 16% (6–26), and 26% (14–38), respectively. TP53 wildtype patients had better outcomes, with 3-year OS of 74%, PFS of 68%, NRM of 18%, and relapse rate of 13%.
Median time to neutrophil and platelet engraftment was 15 (range: 12–31) and 21 days (range: 5–50), respectively. No graft failures occurred. At day 30, T cell and myeloid donor chimerism were 100%. Grade 2–4 acute GVHD occurred in 20% (8.8–31), grade 3–4 in 6% (0–13), chronic GVHD in 13% (2–19), and moderate-to-severe chronic GVHD in 11% (0–16).

**Copyright Information:** (Online) ISSN: 2572-9241
©2026 The Author(s). HemaSphere is published by John Wiley & Sons Ltd on behalf of European Hematology Association. This is an open access Abstract Book distributed under the Attribution-NonCommercial-NoDerivs (CC BY-NC-ND), which allows third parties to download the articles and share them with others as long as they credit the author and the Abstract Book, but they cannot change the content in any way or use them commercially.
**Abstract Book Citations:** Authors, Title, HemaSphere, 2026;10:(S1):pages. The abstract book DOIs can be found on the "Author Information" page.
**Disclaimer:** Articles published in the journal HemaSphere exclusively reflect the opinions of the authors. The authors are responsible for all content in their abstracts including accuracy of the facts, statements, citing resources, etc.

**Summary/Conclusion**

This study met its primary endpoint, demonstrating a promising 3-year PFS in patients with high-risk AML. These findings support further investigation of this regimen.

**Table 1. Summary of outcomes (N=50).**

| | At 1 year | At 2 years | At 3 years |
|---|---|---|---|
| **Overall survival** | 74% | 64% | 64% |
| **Progression-free survival** | 60% | 58% | 58% |
| **Relapse** | 24% | 26% | 26% |
| **Non-relapse mortality** | 16% | 16% | 16% |
| **Median follow-up: 28 months** | | | |
| **Acute GVHD grades 2-4, day 100** | 20% | | |
| **Acute GVHD grades 3-4, day 100** | 6% | | |
| **Moderate/severe chronic GVHD, 3 years** | 11% | | |
| | | | |
| **Neutrophil engraftment: Median (range) days** | 15 (12-31) | | |
| **Platelet engraftment: Median (range) days** | 21 (5-50) | | |
| **T cell chimerism at day 30: Median (range) days** | 100 (100-100) | | |
| **Myeloid chimerism at day 30: Median (range) days** | 100 (100-100) | | |
| | | | |
| **Grade 3-5 toxicity at day 100 (≥10%)** | **N (%)** | | |
| Febrile neutropenia | 27 (54) | | |
| Bacterial infection | 21 (42) | | |
| Diarrhea | 6 (12) | | |

Copyright Information: (Online) ISSN: 2572-9241
©2026 The Author(s). HemaSphere is published by John Wiley & Sons Ltd on behalf of European Hematology Association. This is an open access Abstract Book distributed under the Attribution-NonCommercial-NoDerivs (CC BY-NC-ND), which allows third parties to download the articles and share them with others as long as they credit the author and the Abstract Book, but they cannot change the content in any way or use them commercially.
Abstract Book Citations: Authors, Title, HemaSphere, 2026;10:(S1):pages. The abstract book DOIs can be found on the "Author Information" page.
Disclaimer: Articles published in the journal HemaSphere exclusively reflect the opinions of the authors. The authors are responsible for all content in their abstracts including accuracy of the facts, statements, citing resources, etc.

### (PF1140) EXPLORATION OF A NOVEL AGVHD PROPHYLAXIS REGIMEN BASED ON GECACITINIB IN THE PERITRANSPLANT PERIOD OF PATIENTS WITH MYELOFIBROSIS: A PILOT OPEN-LABEL STUDY

**Topic:** 23. Stem cell transplantation - Clinical

Na Xu*[1], Junwei Huang[1], Yangpeng Chen[1], Yan Zhang[1], Fen Huang[1], Zhiping Fan[1], Hua Jin[1], Min Dai[1], Jing Sun[1], Li Xuan[1], Qifa Liu[1]

*[1]Nanfang Hospital, Southern Medical University CN, Hematology, Guangzhou, China;*

### Background

Myelofibrosis (MF) is a Philadelphia chromosome-negative myeloproliferative neoplasms. Allogeneic hematopoietic stem cell transplantation (allo-HCT) remains the only curative option for MF, despite the development of JAK inhibitors and other non-transplant therapies. Poor graft function (PGF) and graft-versus-host disease (GVHD) are major life-threatening complications of allo-HCT. Previous studies showed that ruxolitinib combined with standard GVHD prophylaxis reduces GVHD incidence in MF without increasing transplant-related mortality, but is associated with haematological toxicity and higher infection risk. Gecacitinib, a JAK/ACVR1 inhibitor, exerts efficacy in MF including splenic volume reduction and haematopoietic recovery. This is the first study to explore a novel acute GVHD (aGVHD) prophylaxis regimen with gecacitinib during the peritransplant period in MF patients undergoing allo-HCT.

### Aims

To evaluate the efficacy and safety of gecacitinib-based aGVHD prophylaxis in MF patients receiving allo-HCT.

### Methods

This pilot study with an expansion cohort enrolled adult MF patients to receive gecacitinib (day - 3 to + 30) peri-transplant, combined with standard myeloablative conditioning (MAC) regimen of decitabine (DAC) + thiotepa/busulfan/fludarabine (TBF), and standard aGVHD prophylaxis including cyclosporine A (CsA), methotrexate (MTX), mycophenolate mofetil (MMF), and anti-thymocyte globulin (ATG). Primary endpoints were grade 2 - 4 aGVHD incidence and donor cell engraftment (granulopoietic/megakaryopoietic recovery). Secondary endpoints included splenic response rate, treatment-related adverse events (TRAEs) graded by CTCAE v5.0, treatment discontinuation, chronic GVHD (cGVHD), grade 3 - 4 infection, overall survival (OS), progression-free survival (PFS), cumulative incidence of disease relapse/progression and nonrelapse mortality.

### Results

A total of 10 MF patients (median age 45 years, range 33 - 61; 7 males, 3 females) undergoing allo-HCT were enrolled from June 2025 to February 2026, with a follow-up of ~ 100 days. Among them, 6 had primary myelofibrosis (PMF) and 4 had post-polycythemia vera/essential thrombocythemia MF. No aGVHD was observed in any patients during follow-up. The median time to neutrophil engraftment was 13 days (range 8 - 18 days) and the median time to platelet engraftment was 20 days (range 8 - 25 days). All patients achieved full donor chimerism at day 28 post-transplant. Mean hemoglobin showed a continuous upward trend (range 55 - 107 g/L). Mean platelet counts recovered early to normal and were maintained at 108 - 168 × $10^9$/L. Mean leukocyte and neutrophil counts normalised at day +30 and +18 post-transplant respectively, and remained normal thereafter. All patients completed gecacitinib treatment; the splenic response rate was 89% (median splenic volume reduction of 27%, range -17% to 42%), with 22% achieving ≥ 30% reduction. Two patients had CMV reactivation, which was rapidly resolved with antiviral therapy. Four patients had grade 1 TRAEs (diarrhoea (n=3), urinary tract infection (n=1)). No grade ≥ 3 TRAEs or treatment discontinuation occurred. All 10 patients were alive without relapse at the last follow-up.

**Copyright Information:** (Online) ISSN: 2572-9241
©2026 The Author(s). HemaSphere is published by John Wiley & Sons Ltd on behalf of European Hematology Association. This is an open access Abstract Book distributed under the Attribution-NonCommercial-NoDerivs (CC BY-NC-ND), which allows third parties to download the articles and share them with others as long as they credit the author and the Abstract Book, but they cannot change the content in any way or use them commercially.
**Abstract Book Citations:** Authors, Title, HemaSphere, 2026;10:(S1):pages. The abstract book DOIs can be found on the “Author Information” page.
**Disclaimer:** Articles published in the journal HemaSphere exclusively reflect the opinions of the authors. The authors are responsible for all content in their abstracts including accuracy of the facts, statements, citing resources, etc.

**Summary/Conclusion**

Peritransplant gecacitinib is safe and well-tolerated in MF patients undergoing allo-HCT, with highly promising early efficacy in aGVHD prophylaxis and potential to promote post-transplant haematopoietic recovery. Larger cohort studies with longer follow-up are warranted to confirm these preliminary findings.

**Copyright Information:** (Online) ISSN: 2572-9241
©2026 The Author(s). HemaSphere is published by John Wiley & Sons Ltd on behalf of European Hematology Association. This is an open access Abstract Book distributed under the Attribution-NonCommercial-NoDerivs (CC BY-NC-ND), which allows third parties to download the articles and share them with others as long as they credit the author and the Abstract Book, but they cannot change the content in any way or use them commercially.
**Abstract Book Citations:** Authors, Title, HemaSphere, 2026;10:(S1):pages. The abstract book DOIs can be found on the "Author Information" page.
**Disclaimer:** Articles published in the journal HemaSphere exclusively reflect the opinions of the authors. The authors are responsible for all content in their abstracts including accuracy of the facts, statements, citing resources, etc.

### (PF1141) LONGITUDINAL TCR SEQUENCING REVEALS DONOR-DEPENDENT IMMUNE RECONSTITUTION PATTERNS AFTER PEDIATRIC ALLOGENEIC HSCT FOR PRIMARY IMMUNODEFICIENCIES

**Topic:** 23. Stem cell transplantation - Clinical

Guanghua Zhu*[1], Wei Yang[1], Jie Zheng[1], Bin Wang[1], Yanhui Luo[1], Jun Yang[1], Chenguang Jia[1], Yuanfang Jing[1], Wei Chen[1], Shunjin Zhang[2], Xiaoxia Wang[2], Jinghua Wu[2], Xiao Liu[3], Maoquan Qin[1]

*[1]Beijing Children's Hospital, Capital Medical University, Beijing, China;[2]NeoImmune Co., LTD, Shenzhen, China; [3]Tsinghua University Shenzhen International Graduate School, Shenzhen, China;*

### Background

Allogeneic hematopoietic stem cell transplantation (allo-HSCT) is a curative treatment for severe primary immunodeficiencies (PIDs). However, the factors influencing T-cell receptor (TCR) repertoire reconstitution post-transplant in children are not well understood, particularly regarding the interplay between donor immune diversity and recipient immune recovery.

### Aims

To longitudinally characterize TCR repertoire recovery in pediatric PID patients undergoing allo-HSCT and evaluate the impact of donor factors, ATG prophylaxis, and viral reactivation.

### Methods

Thirty-two pediatric PID patients and their corresponding donors (123 longitudinal samples) were analyzed pre-transplant and at 3, 6, and 12 months post-HSCT. Patients were stratified as severe combined immunodeficiency(SCID, 25%) or non-SCID (75%). TCR β-chain sequencing was performed using high-throughput methods. Repertoire diversity was assessed by Shannon index and top clonotype frequencies. Associations with donor age, donor relatedness, ATG use, and post-transplant complications were evaluated using mixed-effects models and nonparametric testing.

### Results

SCID patients had significantly lower baseline TCR diversity compared with non-SCID patients ($P<0.05$). Post-transplant, SCID patients demonstrated progressive increases in TCR diversity over 12 months, whereas non-SCID patients showed an early decline at 3 months followed by gradual, but incomplete, recovery over 12 months.

Recipients of pediatric donors and related donors (including haploidentical family donors) exhibited significantly higher TCR diversity at 6 and 12 months compared with adult or unrelated donors (all $P<0.05$). ATG prophylaxis was associated with delayed repertoire diversification, particularly in non-SCID patients. In contrast, letermovir administration did not significantly affect global TCR diversity at any time point.

Lower pre-transplant recipient TCR diversity was associated with subsequent acute GVHD ($P<0.05$), while chronic GVHD and CMV or EBV reactivation were not significantly correlated with overall diversity. Clonal tracking demonstrated predominant replacement by newly generated donor-derived clonotypes. Viral reactivation was associated with enrichment of CMV- and EBV-related clonotypes without sustained contraction of global diversity.

**Copyright Information:** (Online) ISSN: 2572-9241
©2026 The Author(s). HemaSphere is published by John Wiley & Sons Ltd on behalf of European Hematology Association. This is an open access Abstract Book distributed under the Attribution-NonCommercial-NoDerivs (CC BY-NC-ND), which allows third parties to download the articles and share them with others as long as they credit the author and the Abstract Book, but they cannot change the content in any way or use them commercially.
**Abstract Book Citations:** Authors, Title, HemaSphere, 2026;10:(S1):pages. The abstract book DOIs can be found on the "Author Information" page.
**Disclaimer:** Articles published in the journal HemaSphere exclusively reflect the opinions of the authors. The authors are responsible for all content in their abstracts including accuracy of the facts, statements, citing resources, etc.

**Summary/Conclusion**

In pediatric PID patients undergoing allo-HSCT, TCR repertoire reconstitution is influenced by baseline immune status, donor age and relatedness, and ATG exposure. Newly generated donor-derived clones dominate long-term recovery, while virus-associated expansions reflect adaptive immune remodeling. TCR sequencing may support immune monitoring after pediatric transplantation.

**Copyright Information:** (Online) ISSN: 2572-9241
©2026 The Author(s). HemaSphere is published by John Wiley & Sons Ltd on behalf of European Hematology Association. This is an open access Abstract Book distributed under the Attribution-NonCommercial-NoDerivs (CC BY-NC-ND), which allows third parties to download the articles and share them with others as long as they credit the author and the Abstract Book, but they cannot change the content in any way or use them commercially.
**Abstract Book Citations:** Authors, Title, HemaSphere, 2026;10:(S1):pages. The abstract book DOIs can be found on the "Author Information" page.
**Disclaimer:** Articles published in the journal HemaSphere exclusively reflect the opinions of the authors. The authors are responsible for all content in their abstracts including accuracy of the facts, statements, citing resources, etc.

### (PF1142) DONOR-DERIVED CYTOGENETIC ALTERATIONS AFTER ALLOGENEIC HEMATOPOIETIC STEM CELL TRANSPLANTATION: A RETROSPECTIVE SINGLE-CENTER COHORT STUDY

**Topic:** 23. Stem cell transplantation - Clinical

Ragavi Rasiah[1], Sarusan Kathirchelvan[1], Jakob Passweg[1], Joerg Halter[1], Maria Stoikou[2], Brigitta Glaeser[2], Sven Cichon[2], Darius Juskevicius[1], Friedel Wenzel[2], Corinne Widmer*[1]

*[1]University Hospital Basel and University of Basel, Division of Hematology, Basel, Switzerland;[2]University Hospital Basel and University of Basel, Institute of Medical Genetics and Pathology, Basel, Switzerland;*

### Background

Cytogenetic analysis is a cornerstone in the diagnostic and prognostic evaluation of hematologic malignancies and is recommended by the WHO for myeloid neoplasms. While karyotyping at diagnosis is essential to establish a cytogenetic baseline, the clinical value of routine post-transplant cytogenetic assessment after allogeneic hematopoietic stem cell transplantation (allo HCT) remains insufficiently defined. In particular, the frequency and clinical relevance of donor-derived cytogenetic alterations are poorly characterized.

### Aims

We aimed to investigate the occurrence, characteristics, and clinical relevance of post-transplant cytogenetic abnormalities, with a specific focus on donor-associated karyotypic alterations in a well-defined allo HCT cohort.

### Methods

We performed a retrospective single-center study including adult patients (≥18 years) who underwent allo HCT between January 2020 and December 2024 at our institution. Cytogenetic data at initial diagnosis, prior to transplantation, and after transplantation (mainly within three months after allo HCT) were extracted from diagnostic reports. Only patients with complete cytogenetic datasets at diagnosis and post-transplant evaluation were included in the final analysis. Conventional karyotyping was performed according to standard protocols and described using ISCN criteria. In sex-mismatched transplants, donor versus recipient origin was determined by sex chromosome configuration; in sex-matched cases, additional molecular data were considered.

### Results

Of 557 patients with available data, 324 complete datasets were eligible for analysis. Prior to transplantation, 63 patients (19.4%) showed pathological cytogenetics. After allo HCT, 290 patients (89.5%) demonstrated a normal donor-derived karyotype. Post-transplant cytogenetic abnormalities were detected in 34 karyograms from 33 patients. Among these, 28 cases were classified as patient disease-related abnormalities confirmed in the presence of concomitant molecular relapse and decreasing chimerism. In 6 cases (1.8% of the cohort) donor-derived alterations in the karyogram were demonstrated. Four of the donor-associated cases showed isolated loss of the Y chromosome, consistent with an age-related phenomenon in the respective donors. Two patients exhibited donor-derived chromosomal translocations with potential clinical relevance. One patient showed a clonal t(10;17)(q26;q21) three months after transplantation without evidence of neoplasia during three years of follow-up, suggestive of a constitutional origin of this translocation. In contrast, the second patient developed donor-derived AML within three years after allo HCT, presenting with a pathological karyotype despite persistent 100% donor chimerism. However, karyotyping at 3 months after transplantation showed at this timepoint a normal karyotype.

**Copyright Information:** (Online) ISSN: 2572-9241
©2026 The Author(s). HemaSphere is published by John Wiley & Sons Ltd on behalf of European Hematology Association. This is an open access Abstract Book distributed under the Attribution-NonCommercial-NoDerivs (CC BY-NC-ND), which allows third parties to download the articles and share them with others as long as they credit the author and the Abstract Book, but they cannot change the content in any way or use them commercially.
**Abstract Book Citations:** Authors, Title, HemaSphere, 2026;10:(S1):pages. The abstract book DOIs can be found on the “Author Information” page.
**Disclaimer:** Articles published in the journal HemaSphere exclusively reflect the opinions of the authors. The authors are responsible for all content in their abstracts including accuracy of the facts, statements, citing resources, etc.

**Summary/Conclusion**

Clinically relevant cytogenetic abnormalities after allo HCT are uncommon, and donor-derived karyotypic alterations occur rarely. Most donor-associated findings, particularly loss of the Y chromosome, likely reflect age-related phenomena without immediate clinical consequences. The routine use of post-transplant cytogenetic surveillance to detect donor-derived abnormalities appears to have limited diagnostic yield and should be interpreted within the broader clinical context.

**Copyright Information:** (Online) ISSN: 2572-9241
©2026 The Author(s). HemaSphere is published by John Wiley & Sons Ltd on behalf of European Hematology Association. This is an open access Abstract Book distributed under the Attribution-NonCommercial-NoDerivs (CC BY-NC-ND), which allows third parties to download the articles and share them with others as long as they credit the author and the Abstract Book, but they cannot change the content in any way or use them commercially.
**Abstract Book Citations:** Authors, Title, HemaSphere, 2026;10:(S1):pages. The abstract book DOIs can be found on the "Author Information" page.
**Disclaimer:** Articles published in the journal HemaSphere exclusively reflect the opinions of the authors. The authors are responsible for all content in their abstracts including accuracy of the facts, statements, citing resources, etc.

### (PF1143) A MOLECULAR-BASED RISK SCORE FOR PREDICTING LEUKEMIA-FREE SURVIVAL IN ADULT AML PATIENTS UNDERGOING ALLO-HSCT

**Topic:** 23. Stem cell transplantation - Clinical

Shuang Li*[1], Huixian Wu[1], Xiaoxia Hu[2], Fang Zhou[3], Xiong Ni[4], Yi Ding[5], Jiangbo Wan[6], Xiaorui Wang[1], Yu Cai[1], Juan Yang[1], Yin Tong[1], Huiying Qiu[1], Chongmei Huang[1], Kun Zhou[1], Liping Wan[1], Xianmin Song[1]

*[1]Shanghai General Hospital, Shanghai Jiao Tong University, Shanghai, China;[2]Shanghai Rui Jin Hospital, Shanghai Jiao Tong University, Shanghai, China; [3]The 960th Hospital of The People's Liberation Army (PLA) Joint Logistics Support Force, Shanghai, China; [4]Changhai Hospital, Naval Medical University, Shanghai, China;[5]Tongji Hospital of Tongji University, Shanghai, China; [6]Xinhua Hospital, Shanghai Jiao Tong University, Shanghai, China;*

### Background

Allogeneic hematopoietic stem cell transplantation (allo-HSCT) remains to be the cornerstone of curative therapies for acute myeloid leukemia (AML). However, a precise and comprehensive prognostic model for AML patients undergoing allo-HSCT, particularly at the molecular level, is still lacking.

### Aims

To develop a precise and comprehensive prognostic risk scoring system (PRSS) which integrates genomic and clinical factors in adult AML patients undergoing allo-HSCT.

### Methods

A total of 389 adult AML patients undergoing their first allo-HSCT between 2013 and 2021 were enrolled in the training cohort. An independent multicenter cohort of 266 adult AML patients who underwent their first allo-HSCT between September 2018 and August 2022 was used to validate the PRSS. Univariate and multivariate Cox regression models were used to identify the independent predictive variables, with weighted points assigned proportional to their hazard ratios. The model's performance was evaluated using Harrell's C-statistics and time-dependent receiver operating characteristic curves (AUCs). Kaplan- Meier curves were plotted for risk stratification visualization.

### Results

Seven key genetic mutations linked to LFS were identified and classified as favorable (DNMT3A, CEBPA bZIP domain, NPM1 without FLT3-ITD or with FLT3-ITD plus tyrosine kinase inhibitors), unfavorable (NRAS, GATA2), and high-risk (TP53, U2AF1). Multivariate analysis identified five independent predictors for LFS: molecular risk, cytogenetic risk, pre-transplant disease status, age, and HCT-CI score. Based on the total weighted score of these factors, AML-PRSS stratified patients into four risk groups: low (3-year LFS, 90%), intermediate (71%, vs. low-risk, HR=3.28, P<0.001), high (49%, HR=6.98, P<0.001), and very high (26%, HR=12.42, P<0.001). The AML-PRSS also effectively predicted overall survival (OS), the risk of relapse, and non-relapse mortality (NRM). Its predictive value was validated in a multicenter validation cohort demonstrating superior discriminative ability compared to existing models.

**Copyright Information:** (Online) ISSN: 2572-9241
©2026 The Author(s). HemaSphere is published by John Wiley & Sons Ltd on behalf of European Hematology Association. This is an open access Abstract Book distributed under the Attribution-NonCommercial-NoDerivs (CC BY-NC-ND), which allows third parties to download the articles and share them with others as long as they credit the author and the Abstract Book, but they cannot change the content in any way or use them commercially.
**Abstract Book Citations:** Authors, Title, HemaSphere, 2026;10:(S1):pages. The abstract book DOIs can be found on the "Author Information" page.
**Disclaimer:** Articles published in the journal HemaSphere exclusively reflect the opinions of the authors. The authors are responsible for all content in their abstracts including accuracy of the facts, statements, citing resources, etc.

**Summary/Conclusion**

The AML-PRSS integrates genetic, clinical, and transplant-related factors to stratify AML patients into four risk groups. It offers clinicians a reliable tool for individualized risk assessment in AML patients undergoing allo-HSCT, aiding in precise treatment planning and decision-making to improve patient survival.

**Copyright Information:** (Online) ISSN: 2572-9241
©2026 The Author(s). HemaSphere is published by John Wiley & Sons Ltd on behalf of European Hematology Association. This is an open access Abstract Book distributed under the Attribution-NonCommercial-NoDerivs (CC BY-NC-ND), which allows third parties to download the articles and share them with others as long as they credit the author and the Abstract Book, but they cannot change the content in any way or use them commercially.
**Abstract Book Citations:** Authors, Title, HemaSphere, 2026;10:(S1):pages. The abstract book DOIs can be found on the "Author Information" page.
**Disclaimer:** Articles published in the journal HemaSphere exclusively reflect the opinions of the authors. The authors are responsible for all content in their abstracts including accuracy of the facts, statements, citing resources, etc.

### (PF1144) POST-TRANSPLANT ASCIMINIB TREATMENT AS MAINTENANCE, PRE-EMPTIVE OR RELAPSE THERAPY IN PHILADELPHIA-POSITIVE ADVANCED LEUKEMIAS: MULTICENTER REAL-WORLD DATA ANALYSIS

**Topic:** 23. Stem cell transplantation - Clinical

Mohamed Isam Sharif*[1], Yulia Vlasova[2], Daniel Wolff[3], Kathrin Reuthner[3], Francesca Bonifazi[4], Ahmet Emre Eşkazan[5], Osman Sahin[6], Mehmet Sezgin Pepeler[7], Leylagul Kaynar[8], Mohsen Alzahrani[9], Mohamed Abu Haleeqa[10], Acy Quixada[11], Ivan Moiseev[2], Elena Morozova[2], Dennis Kim[1]

*[1]University of Toronto; Princess Margaret Cancer Centre, University Health Network, Division of Hematology, Department of Medicine, Toronto, Canada; [2]Raisa Gorbacheva Memorial Institute of Children's Oncology, Hematology and Transplantation, Pavlov First Saint Petersburg State Medical University, Saint Petersburg, Russia; [3]University Hospital Regensburg, University of Regensburg, Regensburg, Germany, Department of Internal Medicine III, Regensburg, Germany; [4]IRCCS Azienda Ospedaliero-Universitaria di Bologna, Institute of Hematology "L. e A. Seràgnoli", University of Bologna, Bologna, Italy; [5]Cerrahpaşa Faculty of Medicine, Istanbul University-Cerrahpaşa, Division of Hematology, Department of Internal Medicine, Istanbul, Türkiye; [6]Faculty of Medicine, Baskent University Adana Bone Marrow Transplantation Center, Division of Hematology, Department of Internal Medicine,, Adana, Türkiye; [7]Ankara Bilkent City Hospital, Department of Hematology,, Ankara, Türkiye;[8]Faculty of Medicine, Istanbul Medipol University, Department of Hematology,, Istanbul, Türkiye; [9]King Abdulaziz Medical City, Ministry of National Guard Health Affairs, Department of Oncology and Hematology, Riyadh, Saudi Arabia; [10]Abu Dhabi Stem Cells Center (ADSCC), Abu Dhabi, United Arab Emirates; [11]Hematology and Bone Marrow Transplant Unit, Hospital Português da Bahia / Federal University of Bahia (UFBA), Hematology and Bone Marrow Transplant Unit, Salvador, Brazil;*

### Background

Post-transplant relapse remains a major cause of treatment failure after allogeneic hematopoietic stem cell transplantation (allo-HCT) in Philadelphia chromosome–positive (Ph+) leukemias, including advanced CML and Ph+ ALL/AML. Tyrosine kinase inhibitors (TKIs) are frequently used after allo-HCT as maintenance or pre-emptive therapy to reduce relapse risk and improve relapse-free survival (RFS), although no clear recommendation exists regarding optimal agent selection. Asciminib is a first-in-class allosteric inhibitor targeting the myristoyl pocket of ABL1, with activity against multiple ATP-site resistant mutations and a favorable toxicity profile. While it has demonstrated superior tolerability compared with other TKIs in CML-CP, data on its use in the post-transplant setting are limited.

### Methods

We performed a multicenter retrospective analysis to evaluate the efficacy and safety of asciminib used as maintenance, pre-emptive, or relapse therapy following allo-HCT in patients with advanced Ph+ CML, Ph+ ALL, and Ph+ AML. Outcomes included cumulative incidence of relapse (CIR), non-relapse mortality (NRM), relapse-free survival (RFS), overall survival (OS), failure-free survival (FFS), and event-free survival (EFS). Incidence of graft-versus-host disease (GVHD) and outcomes were compared among the three treatment strategies.

**Copyright Information:** (Online) ISSN: 2572-9241
©2026 The Author(s). HemaSphere is published by John Wiley & Sons Ltd on behalf of European Hematology Association. This is an open access Abstract Book distributed under the Attribution-NonCommercial-NoDerivs (CC BY-NC-ND), which allows third parties to download the articles and share them with others as long as they credit the author and the Abstract Book, but they cannot change the content in any way or use them commercially.
**Abstract Book Citations:** Authors, Title, HemaSphere, 2026;10:(S1):pages. The abstract book DOIs can be found on the "Author Information" page.
**Disclaimer:** Articles published in the journal HemaSphere exclusively reflect the opinions of the authors. The authors are responsible for all content in their abstracts including accuracy of the facts, statements, citing resources, etc.

### Results

Thirty-four patients from seven countries (Canada, Germany, Italy, Russia, Türkiye, Saudi Arabia, UAE) were included. Nineteen were male. Diagnoses were CML in chronic phase (n=21), accelerated phase (n=8), blastic phase (n=2), Ph+ AML (n=1), and Ph+ ALL (n=1). ABL1 mutations were detected in 14 patients, including T315I (n=10) and M244V (n=2).
Asciminib was initiated for maintenance (n=7), pre-emptive therapy (n=20), or relapse treatment (n=5) at a median of 209 days post-HCT (range, 21 days–5.5 years). Treatment was generally well tolerated; dose reduction occurred in 2 patients and discontinuation due to adverse events in 1, with 2 additional discontinuations due to severe infection. Reported adverse events included anemia (n=9), neutropenia (n=4), and thrombocytopenia (n=10).
With a median follow-up of 24 months, 9 patients (26%) completed planned therapy (median duration 9.7 months). Thirteen remained on asciminib (12 in remission, 1 with active disease), while 12 discontinued due to relapse/progression (n=4), intolerance/resistance (n=2), or non-relapse mortality in remission (n=6). Seven relapse events occurred on therapy: loss of molecular response (n=2) and resistance/progression (n=5).
At 2 years, the overall CIR was 21.8%, differing significantly across groups: maintenance (0%), pre-emptive (18.4%), and relapse therapy (60%; p=0.05). The 2-year RFS was 64.7% overall, favoring maintenance (71.4%) and pre-emptive therapy (72.2%) compared with relapse treatment (40%; p=0.488). No significant difference in OS was observed among groups (p=0.807).

### Summary/Conclusion

In this multicenter real-world analysis, post-transplant asciminib was feasible and well tolerated, with a low discontinuation rate due to toxicity. Maintenance and pre-emptive strategies were associated with encouraging disease control and lower CIR, whereas outcomes were inferior when used for overt relapse. Prospective studies are warranted to further define the role of asciminib as post-transplant therapy in Ph+ leukemias.

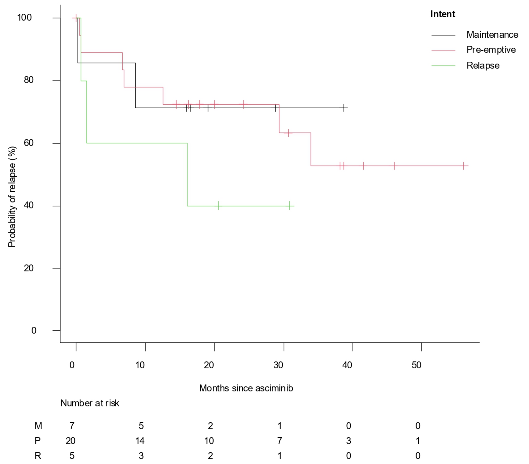

**RFS: median 34.1 months (95% CI 12.6 – NA);** maintenance NR (0.26 – NA), pre-emptive NR (12.6 – NA), relapse 16.1 (0.7 – NA); p=0.488.

RFS: median 34.1 months (95% CI 12.6 – NA); maintenance NR (0.26 – NA), pre-emptive NR (12.6 – NA), relapse 16.1 (0.7 – NA); p=0.488

**Copyright Information:** (Online) ISSN: 2572-9241
©2026 The Author(s). HemaSphere is published by John Wiley & Sons Ltd on behalf of European Hematology Association. This is an open access Abstract Book distributed under the Attribution-NonCommercial-NoDerivs (CC BY-NC-ND), which allows third parties to download the articles and share them with others as long as they credit the author and the Abstract Book, but they cannot change the content in any way or use them commercially.
**Abstract Book Citations:** Authors, Title, HemaSphere, 2026;10:(S1):pages. The abstract book DOIs can be found on the "Author Information" page.
**Disclaimer:** Articles published in the journal HemaSphere exclusively reflect the opinions of the authors. The authors are responsible for all content in their abstracts including accuracy of the facts, statements, citing resources, etc.

### (PF1145) TREATED WITH EMAPALUMAB AS A BRIDGE TO HEMATOPOIETIC STEM CELL TRANSPLANTATION FOR REFRACTORY/RECURRENT EPSTEIN-BARR VIRUS-ASSOCIATED HEMOPHAGOCYTIC LYMPHOHISTIOCYTOSIS

**Topic:** 23. Stem cell transplantation - Clinical

Na Wei*[1], Jingshi Wang[1], Zhao Wang[1]

*[1]Beijing Friendship Hospital、Capital Medical University, Beijing, China;*

### Background

Hemophagocytic lymphohistiocytosis (HLH) is a life-threatening hyperinflammatory disorder. Epstein-Barr virus (EBV) is the most common pathogen in infection-associated HLH. EBV-HLH who EBV infects T/NK cells had poor prognosis, especially in refractory or relapsed (r/r) patients. Emapalumab is a fully human monoclonal antibody targeting interferon-gamma (IFNγ), had demonstrated favorable efficacy in patients with primary HLH and part of secondary HLH. However, the exact role of emapalumab in EBV-HLH remains unclear.

### Aims

To evaluate the therapeutic potential of emapalumab in the management of EBV-HLH.

### Methods

Collect and analyze clinical data from patients diagnosed with r/r EBV-HLH who received at least one dose of emapalumab at the Department of Hematology, Beijing Friendship Hospital, Capital Medical University, between April 2023 and August 2025. Evaluate the efficacy and safety of emapalumab-based regimens in patients with r/r EBV-HLH.

### Results

23 patients with r/r EBV-HLH were enrolled in this study, including 17 males and 6 females. The median age was 32 years (range, 3–46 years). All patients showed T/NK lymphocytes infection or multilineage involvement by EBV. The median time from HLH diagnosis to the first administration of emapalumab was 68 days (range, 26–128 days). All patients had received prior therapy with ruxolitinib and etoposide-containing regimens. During emapalumab treatment, all 23 patients received JAK inhibitor, 19 patients received corticosteroids. The median number of emapalumab administrations was 2 (range, 1–5), and 7 patients received ≥3 doses. The median initial dose was 1.3 mg/kg (range, 0.6–3.75 mg/kg), 2 adult patients received dose escalation during subsequent treatment, whereas no dose modifications were performed in others. Following treatment with emapalumab, the most remarkable change was observed in temperature. Of the 18 patients with fever, 15 became afebrile. Reduction in ferritin was observed in 17 of 22 patients. Decreased transaminase and/or bilirubin levels were documented in half of the patients. Two patients died within one week after the first administration. Among 21 patients who could evaluate treatment response, the ORR was 43.5%, including 10 cases with PR and 11 cases with NR. The median time to best treatment response was 7 days. Patients without complex chromosomal karyotypes and those receiving ≥3 doses of emapalumab achieved superior treatment efficacy. Administration of emapalumab did not result in an elevation of EBV-DNA levels. No infusion reactions or emapalumab-related hepatic or renal toxicity was observed during treatment. Four patients developed new-onset pulmonary infections. No infection-related deaths were reported. Follow-up to December 15, 2025, among 23 patients, 15 had died and 8 were alive, with a 1-year OS rate of 34%. The median survival time after the first dose of emapalumab was 32.5 days (range, 2–760 days). Patients who achieved PR had superior survival compared with those with NR, 1-year OS was 50.9% vs 20% ($p$=0.051). Patients who underwent allo-HSCT after emapalumab therapy had significantly better survival, the 1-year OS was 68.6% in transplant group versus 0% in non-transplant group ($p < 0.001$).

**Copyright Information:** (Online) ISSN: 2572-9241
©2026 The Author(s). HemaSphere is published by John Wiley & Sons Ltd on behalf of European Hematology Association. This is an open access Abstract Book distributed under the Attribution-NonCommercial-NoDerivs (CC BY-NC-ND), which allows third parties to download the articles and share them with others as long as they credit the author and the Abstract Book, but they cannot change the content in any way or use them commercially.
**Abstract Book Citations:** Authors, Title, HemaSphere, 2026;10:(S1):pages. The abstract book DOIs can be found on the "Author Information" page.
**Disclaimer:** Articles published in the journal HemaSphere exclusively reflect the opinions of the authors. The authors are responsible for all content in their abstracts including accuracy of the facts, statements, citing resources, etc.

**Summary/Conclusion**

Emapalumab is effective and safe in the treatment of patients with r/r EBV-HLH. Patients who are able to receive sequential allo-HSCT achieve significant survival benefit. Emapalumab may act as a bridge to allo-HSCT for these patients.

**Copyright Information:** (Online) ISSN: 2572-9241
©2026 The Author(s). HemaSphere is published by John Wiley & Sons Ltd on behalf of European Hematology Association. This is an open access Abstract Book distributed under the Attribution-NonCommercial-NoDerivs (CC BY-NC-ND), which allows third parties to download the articles and share them with others as long as they credit the author and the Abstract Book, but they cannot change the content in any way or use them commercially.
**Abstract Book Citations:** Authors, Title, HemaSphere, 2026;10:(S1):pages. The abstract book DOIs can be found on the "Author Information" page.
**Disclaimer:** Articles published in the journal HemaSphere exclusively reflect the opinions of the authors. The authors are responsible for all content in their abstracts including accuracy of the facts, statements, citing resources, etc.

### (PF1146) A PHASE II STUDY OF A BUSULFAN IN COMBINATION WITH FLAG CONDITIONING REGIMEN FOR ALLOGENEIC HEMATOPOIETC STEM CELL TRANSPLANTATION

**Topic:** 23. Stem cell transplantation - Clinical

Xiawei Zhang*[1], Liping Dou[2], Chun-Ji Gao[2]

[1]*Medical School of Chinese PLA, Beijing, China;* [2]*PLA General Hospital, BEIJING, China;*

### Background

Allogeneic hematopoietic stem cell transplantation (allo-HSCT) is the optimal approach to cure acute myeloid leukemia (AML) and myelodysplastic syndrome (MDS). Busulfan/Cyclophosphamide (BuCy, Cytarabine, Busulfan, Cyclophosphamide) has been a classical conditioning regimen for allo-HSCT. Busulfan/Cyclophosphamide (BuCy, Cytarabine, Busulfan, Cyclophosphamide) has been a classical conditioning regimen for allo-SCT. Substitution of Cyclophosphamide in the BuCy regimen with Fludarabine (BuF) resulted in reduced toxicity and similar efficacy. FLAG (fludarabine, cytarabine and granulocyte colony-stimulating factor) is a chemotherapy regimen that has been proved to be effective for AML/MDS as salvage therapy. To reduce the relapse and improve the survival of those patients with AML/MDS who receive allo-HSCT, the novel conditioning regimen consisting of Busulfan/FLAG was developed.

### Aims

The purpose of this study is to determine the safety and efficacy of Busulfan/FLAG (fludarabine, cytarabine and granulocyte colony-stimulating factor) conditioning regimen in patients with acute myeloid leukemia and myelodysplastic syndrome undergoing allogeneic hematopoietic stem cell transplantation.

### Methods

We conducted a prospective, phase 2, single-arm trial of a busulfan in combination with FLAG conditioning regimen for allogenetic hematopoietic stem cell transplantation. (NCT02784561). Eligible patients were 12 to 65 years of age and had a diagnosis of AML or MDS. The conditioning regimen for peripheral blood stem cell transplantation consisted of busulfan (3.2 mg/kg/day intravenously on days -10 to -8), fludarabine (30 mg/m²/day on days -7 to -3), cytarabine (1.6 g/m²/day on days -7 to -3), and granulocyte colony-stimulating factor (G-CSF, 5 µg/kg/day on days -8 to -3). In addition, patients with haploidentical or matched unrelated donors received rabbit anti-thymocyte globulin (ATG, Thymoglobuline) at 2.5 mg/kg/day on days -5 to -2. The primary outcome was the cumulative incidence of relapse at 2 years after HSCT. Efficacy was assessed in all patients who received treatment and completed transplantation, and safety in all patients who received treatment.

### Results

Between July 18, 2016, and July 22, 2025, 109 patients were enrolled, including 82 acute myeloid leukemia (AML) and 27 with myelodysplastic syndromes (MDS) . Median follow-up was 15·4 months (IQR 8·8-23·6) for patients treated with treosulfan and 17·4 months (6·3-23·4) for those treated with busulfan. With a data cutoff of January 22, 2026, median follow-up was 23.97 months (IQR 5.83-32.96) for patients treated Busulfan/FLAG conditioning regimen. 2-year cumulative incidence of relapse was 25.7% (95% CI: 17.3%-34.9%) in all patients, 26.8% (95% CI: 17.1$–37.4%) for patients with AML, and 21.5% (95% CI: 7.2%–40.7%) for those with MDS. 2-year disease-free survival was 52.3% (95% CI: 41.9%-61.8%) all patients, 50.7% (95% CI: 38.8%–61.4%) for patients with AML, and 58.4% (95% CI: 35.7%–75.6%) for those with MDS. The 2-year overall survival rates were 63.9% (95% CI: 53.3–72.6%) in all patients, 63.9% (95% CI:52.0%-73.6%) with AML and 64.4% (95% CI: 40.0%-80.9%) for those with MDS.The 100-day cumulative incidence of acute graft-versus-host disease was 31.2% (95% CI: 22.7%–40.0%).

**Copyright Information:** (Online) ISSN: 2572-9241
©2026 The Author(s). HemaSphere is published by John Wiley & Sons Ltd on behalf of European Hematology Association. This is an open access Abstract Book distributed under the Attribution-NonCommercial-NoDerivs (CC BY-NC-ND), which allows third parties to download the articles and share them with others as long as they credit the author and the Abstract Book, but they cannot change the content in any way or use them commercially.
**Abstract Book Citations:** Authors, Title, HemaSphere, 2026;10:(S1):pages. The abstract book DOIs can be found on the "Author Information" page.
**Disclaimer:** Articles published in the journal HemaSphere exclusively reflect the opinions of the authors. The authors are responsible for all content in their abstracts including accuracy of the facts, statements, citing resources, etc.

**Summary/Conclusion**

This study provides evidence that busulfan/FLAG conditioning regimen may serve as a promising dimension in patients with AML and MDS undergoing HSCT.

**Copyright Information:** (Online) ISSN: 2572-9241
©2026 The Author(s). HemaSphere is published by John Wiley & Sons Ltd on behalf of European Hematology Association. This is an open access Abstract Book distributed under the Attribution-NonCommercial-NoDerivs (CC BY-NC-ND), which allows third parties to download the articles and share them with others as long as they credit the author and the Abstract Book, but they cannot change the content in any way or use them commercially.
**Abstract Book Citations:** Authors, Title, HemaSphere, 2026;10:(S1):pages. The abstract book DOIs can be found on the "Author Information" page.
**Disclaimer:** Articles published in the journal HemaSphere exclusively reflect the opinions of the authors. The authors are responsible for all content in their abstracts including accuracy of the facts, statements, citing resources, etc.

### (PF1147) ALLOGENEIC STEM CELL TRANSPLANTATION FOR SECONDARY HEMATOLOGIC MALIGNANCIES ARISING FROM PLASMA CELL NEOPLASM

**Topic:** 23. Stem cell transplantation - Clinical

Masaya Maegaki*[1], Masatomo Kuno[2], Fumisato Takagi[3], Shohei Mizuno[4], Kazuhito Suzuki[5], Nobuhiro Tsukada[6], Kimikazu Yakushijin[7], Naoyuki Uchida[8], Takahiro Fukuda[9], Noriko Doki[10], Yuta Katayama[11], Masatsugu Tanaka[12], Junya Kanda[13], Masashi Sawa[14], Noboru Asada[15], Makoto Onizuka[16], Yoshinobu Kanda[17], Noriyoshi Yoshinaga[13], Yoshiko Atsuta[18], Koji Kawamura[1]

*[1]Tottori University, Yonago, Japan; [2]Osaka Metropolitan University Graduate School of Medicine, Department of Hematology, Osaka, Japan; [3]Hamamatsu University School of Medicine, Division of Hematology, Internal Medicine III, Hamamatus, Japan; [4]Aichi Medical University, Division of Hematology, Department of Internal Medicine, Nagakute, Japan;[5]The Jikei University School of Medicine, Division of Clinical Oncology and Hematology, Department of Internal Medicine, Tokyo, Japan; [6]Japanese Red Cross Medical Center, Department of Hematology, Tokyo, Japan; [7]Kobe University Hospital, Department of Medical Oncology and Hematology, Kobe, Japan; [8]TORANOMON HOSPITAL, Federation of National Public Service Personnel Mutual Aid Associations, Tokyo, Japan; [9]National Cancer Center Hospital, Tokyo, Japan; [10]Tokyo Metropolitan Cancer and Infectious Diseases Center, Komagome Hospital, Tokyo, Japan; [11]Hiroshima Red Cross Hospital & Atomic-bomb Survivors Hospital, Hiroshima, Japan; [12]Kanagawa Cancer Center, Yokohama, Japan; [13]Kyoto University Hospital, Kyoto, Japan; [14]Anjo Kosei Hospital, Anjo, Japan; [15]Okayama University Hospital, Okayama, Japan; [16]Tokai University School of Medicine, Isehara, Japan; [17]Jichi Medical University, Division of Hematology, Shimotsuke, Japan; [18]Japanese Data Center for Hematopoietic Cell Transplantation, Nagakute, Japan;*

### Background

The prognosis of plasma cell neoplasms (PCNs), including multiple myeloma, has improved due to advances in treatment. However, PCNs remain incurable and often require continuous, long-term chemotherapy. The incidence of secondary hematologic malignancies arising from PCNs is considered to be increasing. Nevertheless, the efficacy and safety of allogeneic hematopoietic stem cell transplantation (allo-HSCT) in such cases have not been fully evaluated.

### Aims

This study aimed to evaluate the impact of underlying PCNs on treatment outcomes after allo-HSCT.

### Methods

We retrospectively analyzed cases of secondary acute myeloid leukemia (AML), myelodysplastic syndrome (MDS), and acute lymphoblastic leukemia (ALL) that developed following PCN or other malignancy. This study was approved by the Japanese Data Center for Hematopoietic Cell Transplantation and the Institutional Review Board of Tottori University. Eligible patients had received their first allo-HSCT from 2000 to 2023, and were aged 16 years or older at transplantation. Patients with secondary hematologic malignancies, such as AML originated from MDS, and AML/ALL arising from chronic myeloid leukemia, were excluded.

**Copyright Information:** (Online) ISSN: 2572-9241
©2026 The Author(s). HemaSphere is published by John Wiley & Sons Ltd on behalf of European Hematology Association. This is an open access Abstract Book distributed under the Attribution-NonCommercial-NoDerivs (CC BY-NC-ND), which allows third parties to download the articles and share them with others as long as they credit the author and the Abstract Book, but they cannot change the content in any way or use them commercially.
**Abstract Book Citations:** Authors, Title, HemaSphere, 2026;10:(S1):pages. The abstract book DOIs can be found on the “Author Information” page.
**Disclaimer:** Articles published in the journal HemaSphere exclusively reflect the opinions of the authors. The authors are responsible for all content in their abstracts including accuracy of the facts, statements, citing resources, etc.

**Results**

A total of 91 patients with a history of PCN (PCN group) and 1,779 patients with other malignancies (Control group) were included. AML, MDS, and ALL accounted for 35.2%, 52.7%, and 12.1% in PCN group, and 57.6%, 36.0%, and 6.4% in Control group, respectively. In PCN group, 78 patients (85.7%) had underlying multiple myeloma, while the remaining patients had plasmacytoma, light-chain or heavy-chain amyloidosis, light-chain deposition syndrome, and POEMS syndrome. Patients in PCN group were older, had a higher proportion of males, and more frequently received reduced-intensity conditioning compared with Control group. However, performance status (PS), frequency of comorbidities and disease risk at transplantation did not differ between the groups. The median duration of follow-up for censored patients were 837 days (range 30–4668 days) in PCN group and 1435 days (range 27–7588) in Control group, respectively (P = 0.004). in Control group. There was no significant difference in overall survival (OS) between the groups, with median OS of 12.5 months (95% CI, 7.0–20.2 months) in PCN group and 14.4 months (95% CI, 12.8–16.9 months, P = 0.27) in Control group, respectively. In multivariate analysis, older age, male sex, performance status, presence of comorbidities, higher disease risk at transplantation, and receiving transplantation before 2011 had a significantly negative impact on survival. On the other hand, the presence of underlying PCN was not associated with increased mortality. The 5-year cumulative incidence of relapse was 43.8% (95% CI, 31.8–55.2%) in PCN group and 36.6% (95% CI, 34.3–38.9%, P = 0.35) in Control group, and the 5-year non-relapse mortality (NRM) was 31.0% (95% CI, 20.7–41.8%) and 32.1% (95% CI, 29.7–34.4%, P = 1.0), respectively. The major causes of death in both groups were disease progression, followed by infection, non-infectious respiratory failure, and acute graft versus host disease.

**Summary/Conclusion**

Compared with a history of other malignancies, underlying PCNs did not significantly increase the risk of mortality after allo-HSCT. Careful patient selection based on factors such as age, PS, comorbidities, and the nature of the secondary hematologic malignancy is essential.

**Copyright Information:** (Online) ISSN: 2572-9241
©2026 The Author(s). HemaSphere is published by John Wiley & Sons Ltd on behalf of European Hematology Association. This is an open access Abstract Book distributed under the Attribution-NonCommercial-NoDerivs (CC BY-NC-ND), which allows third parties to download the articles and share them with others as long as they credit the author and the Abstract Book, but they cannot change the content in any way or use them commercially.
**Abstract Book Citations:** Authors, Title, HemaSphere, 2026;10:(S1):pages. The abstract book DOIs can be found on the "Author Information" page.
**Disclaimer:** Articles published in the journal HemaSphere exclusively reflect the opinions of the authors. The authors are responsible for all content in their abstracts including accuracy of the facts, statements, citing resources, etc.

### (PF1148) LONGITUDINAL ASSESSMENT OF COMPLEMENT FACTOR BA AND CLINICAL RESPONSE IN ADULT TRANSPLANT-ASSOCIATED THROMBOTIC MICROANGIOPATHY

**Topic:** 23. Stem cell transplantation - Clinical

Min Wu*[1], Jiayu Huang[1], Su Li[2], Chuanhe Jiang[1], Luxiang Wang[1], Zilu Zhang[1], Huijin Zhao[1], Xiaoxia Hu[1]

*[1]Shanghai Institute of Hematology, State Key Laboratory of Medical Genomics, National Research Center for Translational Medicine at Shanghai, Ruijin Hospital Affiliated to Shanghai Jiao Tong University School of Medicine, Shanghai, China;*
*[2]Department of Hematology, Liquan Hospital, GoBroad Medical Institute of Hematology (Shanghai Center), Shanghai, China;*

### Background

Transplant-associated thrombotic microangiopathy (TA-TMA) is a severe endothelial injury syndrome following allogeneic hematopoietic stem cell transplantation (allo-HSCT). Complement dysregulation has been implicated in its pathogenesis; however, the pattern and temporal dynamics of complement activation in adult patients remain incompletely defined.

### Aims

To characterize the longitudinal kinetics of complements, especially for complement factor Ba and sC5b-9, in adult patients with TA-TMA treated with the factor B inhibitor iptacopan.

### Methods

Between July 11, 2025 and February 15, 2026, fourteen consecutive adult patients with TA-TMA and treated with iptacopan were retrospectively analyzed. Response was assessed according to Jodele criteria.

**Copyright Information:** (Online) ISSN: 2572-9241
©2026 The Author(s). HemaSphere is published by John Wiley & Sons Ltd on behalf of European Hematology Association. This is an open access Abstract Book distributed under the Attribution-NonCommercial-NoDerivs (CC BY-NC-ND), which allows third parties to download the articles and share them with others as long as they credit the author and the Abstract Book, but they cannot change the content in any way or use them commercially.
**Abstract Book Citations:** Authors, Title, HemaSphere, 2026;10:(S1):pages. The abstract book DOIs can be found on the "Author Information" page.
**Disclaimer:** Articles published in the journal HemaSphere exclusively reflect the opinions of the authors. The authors are responsible for all content in their abstracts including accuracy of the facts, statements, citing resources, etc.

**Results**

TA-TMA developed a median of 39 days post-transplant (IQR: 22–114), predominantly triggered by infection (n=9) and acute graft versus host disease (n=5). The most common symptom was hypertension (n = 7, 50%), followed by confusion (n=2, 14.3%) and renal dysfunction (n = 1, 7.1%). Baseline laboratory abnormalities included elevated LDH (median 347 IU/L) and clinically significant proteinuria (approaching 1 g/24 h in individual cases).
According to institutional protocol, calcineurin inhibitors were discontinued and converted to endothelial-sparing immunosuppression once the TA-TMA diagnosis was established. Iptacopan was initiated a median of 3.5 days (IQR 0.25–9.75) after TA-TMA diagnosis.
Based on historical clinical observations, systemic terminal sC5b-9 amplification typically occurs at a median of 7 days following clinical TA-TMA diagnosis. Consistent with this kinetic model, terminal sC5b-9 elevation was uncommon at diagnosis in the present cohort.
At initiation of TA-TMA related therapy, Ba elevation was observed in 88.9% (8/9) of evaluable patients, whereas sC5b-9 elevation was present in 11.1% (1/9). Median baseline Ba was 1096.61 ng/mL(reference 226–740 ng/mL), while median sC5b-9 was 134.8 ng/mL(reference 15–345 ng/mL) and largely within the reference range. These findings suggest that factor Ba may capture upstream alternative pathway activation not uniformly reflected by terminal sC5b-9 levels, supporting its role as a sensitive indicator of early complement dysregulation.
Longitudinal evaluation showed a decline in Ba following treatment initiation, with median levels decreasing to 620.51 ng/mL by week 4 (−43.4%). This reduction was temporally aligned with improvement in platelet counts (median 21.0 to 57.0 $\times 10^9$/L) and decline in LDH (median 347 to 251 IU/L).
Among the 14 treated patients, 7 were evaluable at week 4 (3 had not yet reached follow-up, 1 died early due to infection, and 3 had incomplete laboratory data). In this evaluable cohort, 1 achieved CR and 5 achieved PR (overall response rate 85.7%). Overall survival for the entire cohort was 92.8% (13/14) at last follow-up.

**Summary/Conclusion**

In this single-center adult cohort, TA-TMA was associated with elevated upstream factor Ba despite frequently non-elevated terminal sC5b-9. Early initiation of iptacopan enabled longitudinal evaluation of complement kinetics. The serial decline in factor Ba paralleled hematologic improvement, supporting further evaluation of Ba as a potential dynamic biomarker in adult TA-TMA.

**Copyright Information:** (Online) ISSN: 2572-9241
©2026 The Author(s). HemaSphere is published by John Wiley & Sons Ltd on behalf of European Hematology Association. This is an open access Abstract Book distributed under the Attribution-NonCommercial-NoDerivs (CC BY-NC-ND), which allows third parties to download the articles and share them with others as long as they credit the author and the Abstract Book, but they cannot change the content in any way or use them commercially.
**Abstract Book Citations:** Authors, Title, HemaSphere, 2026;10:(S1):pages. The abstract book DOIs can be found on the "Author Information" page.
**Disclaimer:** Articles published in the journal HemaSphere exclusively reflect the opinions of the authors. The authors are responsible for all content in their abstracts including accuracy of the facts, statements, citing resources, etc.

### (PF1149) TRANSPLANTATION STRATEGIES FOR LYMPHOMA ASSOCIATED HEMOPHAGOCYTIC LYMPHOHISTIOCYTOSIS-A MULTICENTER, REALWORLD STUDY

**Topic:** 23. Stem cell transplantation - Clinical

Jiexian MA*[1], Xinyuan MA[1]

[1]*Huadong Hospital, Fudan University, Shanghai, China;*

### Background

Optimal transplant modality for lymphoma-associated hemophagocytic lymphohistiocytosis (LAHS) remains less defined.

### Aims

To assess the efficacy and safety of different HSCT in different subtypes LAHS.

### Methods

In this multi-center, real-world study, we accessed the efficacy and safety of different hematopoietic stem cell transplantation (HSCT) strategy in each subtype lymphoma.

### Results

177 LAHS (96 with B-cell LAHS and 81 with T-cell LAHS) patients diagnosed between July 2020 and December 2025 were enrolled in this study. Among 96 B-LAHS and 24 T-LAHS patients achieved remission after induction chemotherapy, those received auto-HSCT(n=58) had a prolonged overall survival (OS) and progression-free survival(PFS) compared to those(n=62) without auto-HSCT consolidation (not reached vs. 35m, p=0.003 for OS; not reached vs. 17m, p=0.0006 for PFS) in B-LAHS and (not reached vs. 23.5m, p=0.001 for OS; not reached vs. 20m, p<0.001 for PFS) in T-LAHS. Notably, for 57 chemo-refractory T-LAHS patients, most died within 6 months. Those who underwent allo-HSCT had a significant prolonged OS and PFS compared to those without HSCT (not reached vs. 5m, p=0.003 for OS; 45m vs. 4m, p=0.003 for PFS). Auto-HSCT and allo-HSCT became independent prognosis factor in chemo-sensitive and refractory LAHS respectively. Even in unfavorable LAHS, HSCT still significantly improves the prognosis.

### Summary/Conclusion

These findings indicate chemo-sensitive LAHS could benefit from auto-HSCT, while chemo-refractory LAHS should consider allo-HSCT early after HLH control.

**Copyright Information:** (Online) ISSN: 2572-9241
©2026 The Author(s). HemaSphere is published by John Wiley & Sons Ltd on behalf of European Hematology Association. This is an open access Abstract Book distributed under the Attribution-NonCommercial-NoDerivs (CC BY-NC-ND), which allows third parties to download the articles and share them with others as long as they credit the author and the Abstract Book, but they cannot change the content in any way or use them commercially.
**Abstract Book Citations:** Authors, Title, HemaSphere, 2026;10:(S1):pages. The abstract book DOIs can be found on the "Author Information" page.
**Disclaimer:** Articles published in the journal HemaSphere exclusively reflect the opinions of the authors. The authors are responsible for all content in their abstracts including accuracy of the facts, statements, citing resources, etc.

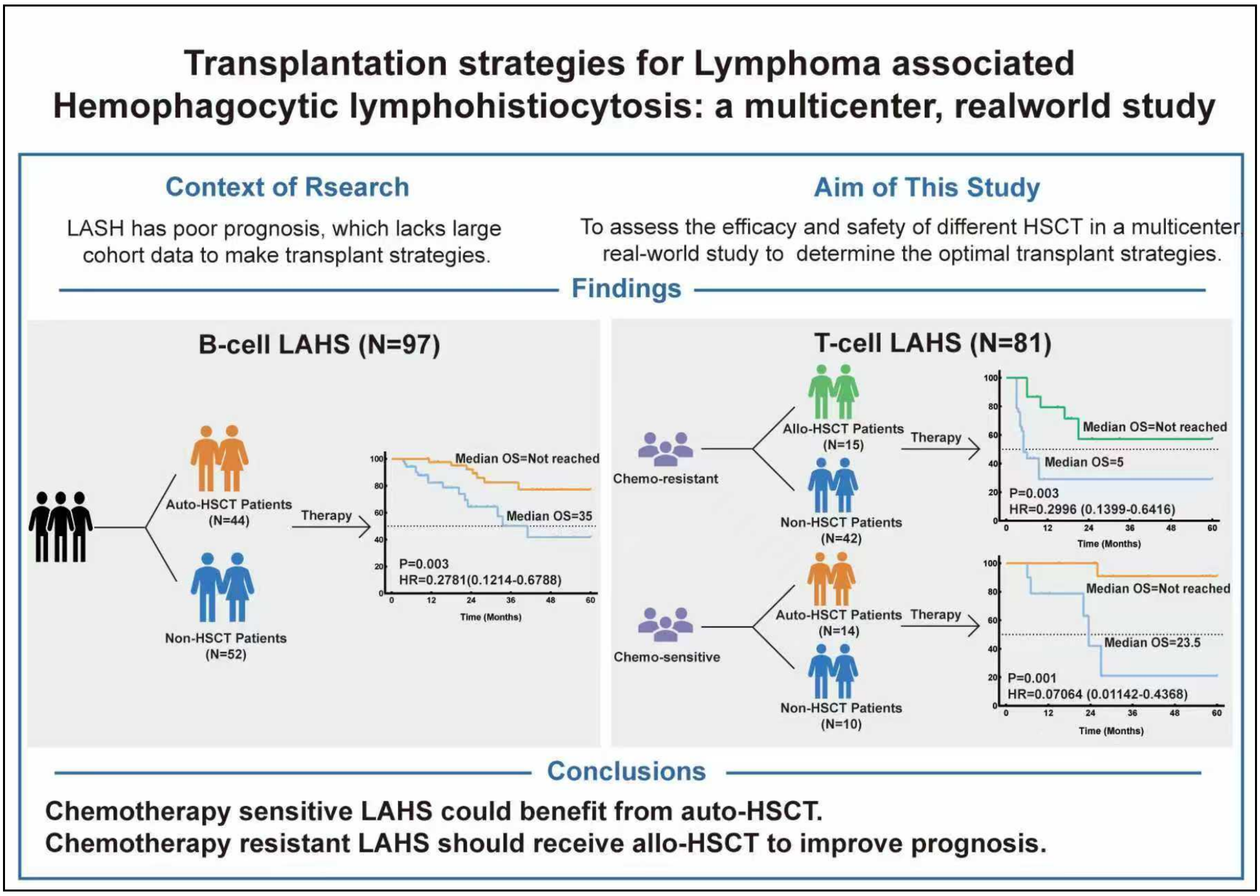

**Copyright Information:** (Online) ISSN: 2572-9241
©2026 The Author(s). HemaSphere is published by John Wiley & Sons Ltd on behalf of European Hematology Association. This is an open access Abstract Book distributed under the Attribution-NonCommercial-NoDerivs (CC BY-NC-ND), which allows third parties to download the articles and share them with others as long as they credit the author and the Abstract Book, but they cannot change the content in any way or use them commercially.
**Abstract Book Citations:** Authors, Title, HemaSphere, 2026;10:(S1):pages. The abstract book DOIs can be found on the "Author Information" page.
**Disclaimer:** Articles published in the journal HemaSphere exclusively reflect the opinions of the authors. The authors are responsible for all content in their abstracts including accuracy of the facts, statements, citing resources, etc.

### (PF1150) IMPACT OF RECIPIENT AGE ON OUTCOMES OF ALLOGENEIC HEMATOPOIETIC STEM CELL TRANSPLANTATION: AN ANALYSIS FROM THE CHINESE BONE MARROW TRANSPLANT REGISTRY GROUP (CBMTRG)

**Topic:** 23. Stem cell transplantation - Clinical

Xiaoli Liang*[1], Jing Liu[2], Li Xuan[3], Yao Sun[4], Jia Chen[5], Jia Liu[1], Haixia Fu[2], Congcong Wang[3], Shengye Lu[2], Lanping Xu[2], Xiaoqi Wang[1], Ruihao Huang[1], Depei Wu[5], Dai-Hong Liu[4], Qifa Liu[3], Xi Zhang[1], Xiao-Jun Huang[2]

*[1]Xinqiao Hospital of Army Medical University, Medical Center of Hematology, chongqing, China;[2]ChinaPeking University People′s Hospital, Peking University Institute of Hematology, National Clinical Research Center for Hematologic Disease, Beijing Key Laboratory of Hematopoietic Stem Cell Transplantation, Beijing, China; [3]Institute of Hematology, Southern Medical University, Guangzhou, China; [4]Chinese PLA General Hospital, Department of Hematology in the Fifth Medical Center of PLA General Hospital, Beijing, China; [5]Department of Hematology, the First Affiliated Hospital of Soochow University, Suzhou, China;*

### Background

With advances in allogeneic hematopoietic stem cell transplantation (allo-HSCT), the proportion of elderly recipients is increasing. However, whether older age independently affects outcomes beyond disease characteristics, and whether the predominant failure mode is relapse or non-relapse mortality (NRM), remains controversial. This study aimed to determine the independent effect of recipient age on transplant outcomes and its dominant pathway using propensity score matching (PSM) to balance confounders.

### Aims

This study aimed to: (1) evaluate whether older recipient age independently influences outcomes after allo-HSCT after rigorously balancing disease-related and clinical confounding factors using propensity score matching; and (2) determine whether the inferior prognosis associated with advanced age is primarily driven by increased relapse risk or non-relapse mortality, thereby informing targeted strategies to improve outcomes in elderly transplant recipients.

### Methods

We retrospectively enrolled 1619 patients with hematologic malignancies undergoing first allo-HSCT between January 2018 and December 2023 at five Chinese centers. Recipients were divided into young (≤35 years) and elderly (≥50 years) groups. PSM was performed to balance 11 covariates (including disease type, pre-transplant disease status, HCT-CI score, donor type, etc.) in a 1:1 ratio, yielding 694 patients (347 per group) for analysis. Overall survival (OS), NRM, cumulative incidence of relapse (CIR), and acute/chronic graft-versus-host disease (GVHD) were compared. Statistical analyses included Kaplan-Meier method, Cox regression, and Fine-Gray competing risk models.

### Results

With a median follow-up of 26.9 months, the elderly group had significantly lower 3-year OS (75.5% vs. 83.1%, HR=1.59, 95%CI: 1.13–2.24, P=0.007) and higher 3-year NRM (13.9% vs. 9.0%, SHR=1.61, 95%CI: 1.01–2.57, P=0.044) compared to the young group. No significant differences were observed in 3-year CIR (20.6% vs. 20.3%, P=0.640), grade II-IV acute GVHD (24.6% vs. 21.9%, P=0.350), or chronic GVHD (45.3% vs. 43.9%, P=0.360). After multivariable adjustment, older age remained an independent risk factor for OS (aHR=1.55, 95%CI: 1.10–2.18, P=0.013), with a borderline significant increase in NRM (aSHR=1.59, 95%CI: 0.99–2.54, P=0.053). Cause-of-death analysis showed infection as the leading cause of NRM in both groups, with a trend toward higher proportions of organ dysfunction and hemorrhage in the elderly.

**Copyright Information:** (Online) ISSN: 2572-9241
©2026 The Author(s). HemaSphere is published by John Wiley & Sons Ltd on behalf of European Hematology Association. This is an open access Abstract Book distributed under the Attribution-NonCommercial-NoDerivs (CC BY-NC-ND), which allows third parties to download the articles and share them with others as long as they credit the author and the Abstract Book, but they cannot change the content in any way or use them commercially.
**Abstract Book Citations:** Authors, Title, HemaSphere, 2026;10:(S1):pages. The abstract book DOIs can be found on the “Author Information” page.
**Disclaimer:** Articles published in the journal HemaSphere exclusively reflect the opinions of the authors. The authors are responsible for all content in their abstracts including accuracy of the facts, statements, citing resources, etc.

**Summary/Conclusion**

After balancing confounders, older recipient age is an independent risk factor for inferior outcomes after allo-HSCT, primarily driven by increased NRM rather than relapse or GVHD. Improving transplant tolerance through optimized conditioning regimens, enhanced organ protection, and infection control is key to improving prognosis in elderly patients.

**Copyright Information:** (Online) ISSN: 2572-9241
©2026 The Author(s). HemaSphere is published by John Wiley & Sons Ltd on behalf of European Hematology Association. This is an open access Abstract Book distributed under the Attribution-NonCommercial-NoDerivs (CC BY-NC-ND), which allows third parties to download the articles and share them with others as long as they credit the author and the Abstract Book, but they cannot change the content in any way or use them commercially.
**Abstract Book Citations:** Authors, Title, HemaSphere, 2026;10:(S1):pages. The abstract book DOIs can be found on the "Author Information" page.
**Disclaimer:** Articles published in the journal HemaSphere exclusively reflect the opinions of the authors. The authors are responsible for all content in their abstracts including accuracy of the facts, statements, citing resources, etc.

### (PF1151) ASSESSMENTS OF BONE HEALTH AMONG PATIENTS WITH CHRONIC GRAFT-VERSUS-HOST DISEASE RECEIVING AXATILIMAB IN THE AGAVE-201 TRIAL

**Topic:** 23. Stem cell transplantation - Clinical

Amandeep Salhotra*[1], Gabriela Thoennissen[2], Jordan Fortunato[3], Zhenyi Xue[3], Doris Ponce[4]

*[1]City of Hope National Medical Center, Hematology, Duarte, United States of America;[2]Incyte Biosciences International Sàrl, Morges, Switzerland; [3]Incyte Corporation, Wilmington, United States of America; [4]Memorial Sloan Kettering Cancer Center, New York, United States of America;*

### Background

Bone mineral density (BMD) loss is a common complication of allogeneic hematopoietic stem cell transplantation, often related to pretransplantation chemotherapy, radiation exposure, and immunosuppressive therapy for chronic graft-versus-host disease (cGVHD). BMD loss results from gonadal hormonal deficiencies, disturbances in calcium and vitamin D homeostasis, and osteoblast and osteoclast dysfunction. Colony-stimulating factor 1 (CSF-1), a CSF-1 receptor (CSF-1R) ligand, is a key regulator in bone-remodeling pathways, as shown in CSF-1−knockout and CSF-1−deficient mice, which present with severe skeletal phenotypes. Axatilimab (AXA) is a high-affinity anti−CSF-1R monoclonal antibody that depletes monocytes and macrophages, key drivers of inflammation and fibrosis in cGVHD. Thus, the potential effects of AXA on bone health are of clinical interest.

### Aims

To evaluate bone-health parameters in patients with refractory cGVHD who received AXA in the pivotal AGAVE-201 trial (NCT04710576).

### Methods

Patients were randomized to intravenous AXA 0.3 mg/kg every 2 weeks (Q2W), 1 mg/kg Q2W, or 3 mg/kg every 4 weeks (Q4W). Bone turnover markers, bone-specific alkaline phosphatase (BAP) and C-telopeptide of cross-linked type 1 collagen (CTX), were assessed at baseline, throughout treatment, and at treatment end. The data cutoff was March 30, 2025.

**Copyright Information:** (Online) ISSN: 2572-9241
©2026 The Author(s). HemaSphere is published by John Wiley & Sons Ltd on behalf of European Hematology Association. This is an open access Abstract Book distributed under the Attribution-NonCommercial-NoDerivs (CC BY-NC-ND), which allows third parties to download the articles and share them with others as long as they credit the author and the Abstract Book, but they cannot change the content in any way or use them commercially.
**Abstract Book Citations:** Authors, Title, HemaSphere, 2026;10:(S1):pages. The abstract book DOIs can be found on the “Author Information” page.
**Disclaimer:** Articles published in the journal HemaSphere exclusively reflect the opinions of the authors. The authors are responsible for all content in their abstracts including accuracy of the facts, statements, citing resources, etc.

### Results

Of the 241 patients enrolled, 239 received AXA (0.3 mg/kg Q2W, n=79; 1 mg/kg Q2W, n=81; 3 mg/kg Q4W, n=79). Among all 241 patients, the median (range) baseline age was 53 (7–81) years; 151 patients (62.7%) were male, and median (range) follow-up was 31.2 (0–46.5) months. Prior bone-related medications included vitamin D (50.6%), calcium combinations (14.9%), calcium (13.7%), bisphosphonates (11.6%), and calcium carbonate (1.7%). Concomitant bone-related medications included vitamin D (56.8%), calcium combinations (19.1%), bisphosphonates (18.3%), calcium (17.8%), and calcium carbonate (2.9%). Baseline concomitant corticosteroid use was reported in 65.3% of patients, with a mean daily dose of 19.4 mg. Common (>5%) bone-related medical conditions in patients' medical histories included osteoporosis (19.5%), osteopenia (16.2%), and osteonecrosis (7.9%). Changes from baseline in BAP and CTX at Cycle 6 Day 1 and treatment end were minimal (**Table**). Bone-related treatment-emergent adverse events (TEAEs) were grade 1/2 and included osteopenia (n=6 [2.5%]), osteoarthritis (n=5 [2.1%]), bone pain, osteonecrosis (both n=4 [1.7%]), musculoskeletal pain (n=2 [0.8%]), bone infarction, coccydynia, increased vitamin D, and osteochondroma (each n=1 [0.4%]). Fractures (any grade) occurred in 17 patients (7.1%), and ≥2 fractures occurred in 3 patients (1.3%).

### Table. Baseline and Changes in Bone Biomarkers in Treated Patients

| | | Axatilimab all doses (N=239) | |
|---|---|---|---|
| **Parameter** | **Visit** | **n** | **Mean (SE)** |
| | Baseline | 233 | 26.2 (1.47) |
| BAP, U/L | CFB to C6D1 | 135 | –0.2 (1.52) |
| | CFB to EOT | 110 | –0.4 (0.88) |
| | Baseline | 233 | 0.4 (0.02) |
| CTX, µg/L | CFB to C6D1 | 136 | –0.1 (0.02) |
| | CFB to EOT | 112 | –0.1 (0.02) |

BAP, bone-specific alkaline phosphatase; C6D1, Cycle 6 Day 1; CFB, change from baseline; CTX, C-telopeptide of cross-linked type 1 collagen; EOT, end of treatment.

### Summary/Conclusion

In AGAVE-201, AXA was associated with minimal changes in bone turnover markers. Bone-related TEAEs were rare, with few patients having multiple fractures; this is notable since 65.3% of patients received concomitant corticosteroids. These results may indicate that AXA had minimal effects on bone metabolic markers, although additional analyses are needed.

**Copyright Information:** (Online) ISSN: 2572-9241
©2026 The Author(s). HemaSphere is published by John Wiley & Sons Ltd on behalf of European Hematology Association. This is an open access Abstract Book distributed under the Attribution-NonCommercial-NoDerivs (CC BY-NC-ND), which allows third parties to download the articles and share them with others as long as they credit the author and the Abstract Book, but they cannot change the content in any way or use them commercially.
**Abstract Book Citations:** Authors, Title, HemaSphere, 2026;10:(S1):pages. The abstract book DOIs can be found on the "Author Information" page.
**Disclaimer:** Articles published in the journal HemaSphere exclusively reflect the opinions of the authors. The authors are responsible for all content in their abstracts including accuracy of the facts, statements, citing resources, etc.

### (PF1152) A PROSPECTIVE COHORT STUDY: THE ASSOCIATION BETWEEN NUTRITIONAL STATUS OF HSCT PATIENTS BEFORE TRANSPLANTATION AND EARLY INFECTION AND NON-RELAPSE MORTALITY

**Topic:** 23. Stem cell transplantation - Clinical

Qian Zhou[1], Xueqian Yan[1], Zhuo Wan[1], Dan Fan[1], Yueru Ji[1], Jing Yan[1], Weiwei Qin*[1]

[1] *Tangdu Hospital, Fourth Military Medical University,, Xi'an, China;*

### Background

Infection and non-relapse mortality are the main causes of death in the early stage after Hematopoietic Stem Cell Transplantation（HSCT）. Current clinical prevention and control mainly focus on pathogen monitoring and adjustment of immunosuppressants, but the systematic assessment of the host's basic condition-nutritional status is often marginalized. Malnutrition can impair immune function and tissue repair, and may increase the risk of the aforementioned complications. However, the association between multidimensional nutritional status before transplantation and clinical outcomes remains to be clarified, and there is currently a lack of sufficient evidence from prospective studies for this.

### Aims

To investigate the association between pre-transplant nutritional status and early post-transplant infection as well as non-relapse mortality (NRM) in patients undergoing hematopoietic stem cell transplantation (HSCT), and to provide evidence-based guidance for clinical nutritional intervention.

### Methods

This prospective cohort study enrolled 109 patients undergoing HSCT at the Department of Hematology, Tangdu Hospital, Fourth Military Medical University, from July 2024 to November 2025. Inclusion criteria were: confirmed hematologic diseases (AML/ALL/AA/MM/NHL), scheduled HSCT, age 18-70 years, adequate communication ability, and written informed consent. Exclusion criteria included history of gastrointestinal surgery or therapy affecting nutrient absorption, other malignancies within 5 years (except cured in situ tumors), psychiatric disorders, participation in other clinical trials within 4 weeks, or poor compliance. Pre-transplant nutritional status was assessed using PG-SGA, BMI, serum albumin, fat-free mass index (FFMI), and phase angle (PhA). Primary endpoints were incidence of infections (bacteremia, pneumonia, viral infections) and non-relapse mortality within 100 days post-transplant. Logistic regression and Cox proportional hazards models were used to analyze associations between nutritional parameters and outcomes, and to evaluate their predictive value.

### Results

Among the 109 patients, the prevalence of severe malnutrition was 59.60%. Early post-transplant infections occurred in 30 patients (27.52%), and non-relapse mortality occurred in 9 patients (8.26%). Multivariate logistic regression identified pre-transplant hypoalbuminemia (OR=3.191, 95% CI: 1.303-7.816, p=0.011) and low phase angle (OR=0.608, 95% CI: 0.376-0.984, p=0.043) as independent risk factors for early infection. Multivariate Cox regression showed that hypoalbuminemia (HR=3.537, 95% CI: 1.232-10.154, p=0.019), low phase angle (HR=0.556, 95% CI: 0.324-0.954, p=0.033), and BMI <18.5 (HR=2.912, 95% CI: 1.057-8.021, p=0.039) were independent risk factors for NRM.

**Copyright Information:** (Online) ISSN: 2572-9241
©2026 The Author(s). HemaSphere is published by John Wiley & Sons Ltd on behalf of European Hematology Association. This is an open access Abstract Book distributed under the Attribution-NonCommercial-NoDerivs (CC BY-NC-ND), which allows third parties to download the articles and share them with others as long as they credit the author and the Abstract Book, but they cannot change the content in any way or use them commercially.
**Abstract Book Citations:** Authors, Title, HemaSphere, 2026;10:(S1):pages. The abstract book DOIs can be found on the "Author Information" page.
**Disclaimer:** Articles published in the journal HemaSphere exclusively reflect the opinions of the authors. The authors are responsible for all content in their abstracts including accuracy of the facts, statements, citing resources, etc.

### Summary/Conclusion

This prospective study of 109 HSCT patients found that pre-transplant malnutrition (prevalence 59.6%) significantly increased the risk of early infection and mortality. Hypoalbuminemia and low phase angle were independent risk factors for both outcomes, highlighting that systematic pre-transplant nutritional assessment is essential for identifying high-risk patients and improving prognosis.

**Table 1 Logistic Regression Analysis of the Association between Pre-transplantation Nutritional Indicators and Early Infection**

| Variables | Univariate analyses | | Multivariate analysis | |
|---|---|---|---|---|
| | OR（95% *CI*） | p | OR（95% *CI*） | p |
| **PG-SGA（point）** | | | | |
| **<9** | 1 | | 1 | |
| **severe malnutrition，≥9** | --- | 0.789 | --- | 0.697 |
| **Albumin（g/L）** | | | | |
| **≥35** | 1 | | 1 | |
| **malnutrition,<35** | 3.733（1.501-9.288） | **0.005** | 3.191（1.303-7.816） | **0.011** |
| **Phase angle（for every 1 degree increase）** | 0.578（0.361-0.926） | **0.023** | 0.608（0.376-0.984） | **0.043** |
| **BMI, Body Mass Inde（kg/m²）** | | | | |
| **≥18.5** | 1 | | 1 | |
| **malnutrition,<18.5** | --- | 0.205 | | |
| **Fat Free Mass Index, FFMI（kg/m²）** | | | | |
| **male≥17 /female≥15** | 1 | | 1 | |
| **malnutrition, male<17 /female<15** | --- | 0.068 | -- | 0.062 |

**Note: Eight covariates were included in the analysis: age (less than 60 years old, greater than or equal to 60 years old), gender, history of chronic diseases, surgical history, type of transplantation, clinical diagnosis, ECOG score, and HCT-CI score; The PG-SGA refers to the Patient-Generated Subjective Global Assessment for Nutritional Status.**

**Table 2 COX regression analysis of the association between pre-transplantation nutritional indicators and early non-recurrence mortality**

| Variables | Univariate analyses | | Multivariate analysis | |
|---|---|---|---|---|
| | HR（95% *CI*） | p | HR（95% *CI*） | p |
| **PG-SGA（point）** | | | | |
| **<9** | 1 | | 1 | |
| **severe malnutrition，≥9** | --- | 0.082 | --- | 0.070 |
| **Albumin（g/L）** | | | | |
| **≥35** | 1 | | 1 | |
| **malnutrition,<35** | 3.087（1.154-8.256） | **0.025** | 3.537（1.232-10.154） | **0.019** |
| **Phase angle（for every 1 degree increase）** | 0.503（0.296-0.854） | **0.011** | 0.556（0.324-0.954） | **0.033** |
| **BMI, Body Mass Inde（kg/m²）** | | | | |
| **≥18.5** | 1 | | | |
| **malnutrition,<18.5** | --- | 0.056 | 2.912（1.057-8.021） | **0.039** |
| **Fat Free Mass Index, FFMI（kg/m²** | | | | |
| **male≥17 /female≥15** | 1 | | 1 | |
| **malnutrition, male<17 /female<15** | --- | 0.206 | -- | 0.062 |

**Note: Eight covariates were included in the analysis: age (less than 60 years old, greater than or equal to 60 years old), gender, history of chronic diseases, surgical history, type of transplantation, clinical diagnosis, ECOG score, and HCT-CI score; The PG-SGA refers to the Patient-Generated Subjective Global Assessment for Nutritional Status.**

**Copyright Information:** (Online) ISSN: 2572-9241
©2026 The Author(s). HemaSphere is published by John Wiley & Sons Ltd on behalf of European Hematology Association. This is an open access Abstract Book distributed under the Attribution-NonCommercial-NoDerivs (CC BY-NC-ND), which allows third parties to download the articles and share them with others as long as they credit the author and the Abstract Book, but they cannot change the content in any way or use them commercially.
**Abstract Book Citations:** Authors, Title, HemaSphere, 2026;10:(S1):pages. The abstract book DOIs can be found on the "Author Information" page.
**Disclaimer:** Articles published in the journal HemaSphere exclusively reflect the opinions of the authors. The authors are responsible for all content in their abstracts including accuracy of the facts, statements, citing resources, etc.

### (PF1153) PREDICTIVE VALUE OF EASIX SCORES AT DIFFERENT TIME POINTS FOR PTLD AND SURVIVAL IN PATIENTS WITH EBV REACTIVATION AFTER ALLOGENEIC HEMATOPOIETIC STEM CELL TRANSPLANTATION

**Topic:** 23. Stem cell transplantation - Clinical

Luxin Yang*[1, 2], Yi Luo[1, 2]

*[1]Zhejiang University, Institute of Hematology, Hangzhou, China;[2]Zhejiang Province Engineering Research Center for Stem Cell and Immunity Therapy, Hangzhou, China;*

### Background

EBV reactivation is a frequent complication after allo-HSCT, with widely varying reported rates of progression to post-transplant lymphoproliferative disorder (PTLD). Recent factors like the SARS-CoV-2 pandemic and letermovir prophylaxis may further increase EBV risks. Given PTLD's high mortality and the lack of early predictive biomarkers, new tools are urgently needed. The Endothelial Activation and Stress Index (EASIX), an endothelial damage marker linked to allo-HSCT outcomes and lymphoma-associated HLH survival, offers potential for early PTLD prediction and prognostication.

### Aims

Our aim was to validate whether EASIX scores at different time points after post-transplant EBV reactivation could predict the progression to PTLD and clinical overall survival (OS).

### Methods

A total of 126 patients who underwent allo-HSCT and developed EBV viremia between 2024 and 2025 in several transplantation centers of China were included in this study. EBV reactivation was identified when the viral load surpassed $1\times10^4$ copies/ml from whole blood samples. The diagnosis of PTLD was made in accordance with the guidelines established by the ECIL-6. A log transformation using base 2 (log2) was applied to EASIX scores to reduce skewness.

### Results

Among 126 allo-HSCT recipients who developed EBV viremia, 66 (52.4%) patients developed PTLD (28 probable and 38 proven). The median EASIX scores in PTLD patients were significantly higher than those in patients with EBV viremia alone at three key time points: the day of EBV reactivation (EASIX0) (median 2.6, IQR 1.3–4.2 vs. median 2.08, IQR 1.2–3.24), day 7 post-reactivation (EASIX7) (median 2.72, IQR 1.84–5.35 vs. median 2.16, IQR 1.41–3.92), and the day of peak EBV viral load (EASIXhighest) (median 4.92, IQR 2.24–51.92 vs. median 2.5, IQR 1.3–5.1). Both univariate and multivariate logistic regression analyses identified EASIX0 (per log2 increase: OR, 2.257 ; 95% CI, 1.512-3.371; P< 0.001), EASIX7 (per log2 increase: OR, 1.936; 95% CI, 1.378-2.72; P< 0.001), and EASIXhighest (per log2 increase: OR, 1.834; 95% CI, 1.384-2.43; P< 0.001) as independent risk factors for the development of PTLD. Donor type, time from transplantation to EBV reactivation, viral load, conditioning regimen, and patient age were not associated with the occurrence of PTLD. ROC curve analysis revealed that the AUCs for EASIX0, EASIX7, and EASIXhighest were 0.741, 0.747, and 0.78, respectively(Figure 1A). Multivariate Cox regression analysis identified higher EASIX0(per log2 increase: HR, 1.402 ; 95% CI, 1.146-1.714; P=0.001), EASIX7(per log2 increase: HR, 1.430 ; 95% CI, 1.191-1.716; P<0.001), and EASIXhighest(per log2 increase: HR, 1.315 ; 95% CI, 1.167-1.482; P<0.001) scores as independent risk factors for OS (Figure 1B-D).

**Copyright Information:** (Online) ISSN: 2572-9241
©2026 The Author(s). HemaSphere is published by John Wiley & Sons Ltd on behalf of European Hematology Association. This is an open access Abstract Book distributed under the Attribution-NonCommercial-NoDerivs (CC BY-NC-ND), which allows third parties to download the articles and share them with others as long as they credit the author and the Abstract Book, but they cannot change the content in any way or use them commercially.
**Abstract Book Citations:** Authors, Title, HemaSphere, 2026;10:(S1):pages. The abstract book DOIs can be found on the "Author Information" page.
**Disclaimer:** Articles published in the journal HemaSphere exclusively reflect the opinions of the authors. The authors are responsible for all content in their abstracts including accuracy of the facts, statements, citing resources, etc.

### Summary/Conclusion

In conclusion, EASIX scores at different time points following EBV viremia serve as effective predictors for PTLD. Crucially, EASIX0 and EASIX7 allow for the early assessment of disease severity, thereby enabling earlier pre-emptive interventions. Additionally, EASIX scores demonstrate a strong association with early mortality risk. Therefore, our findings support EASIX as a robust, universally accessible predictive marker for PTLD and a prognostic marker for outcome following EBV reactivation after allo-HSCT.

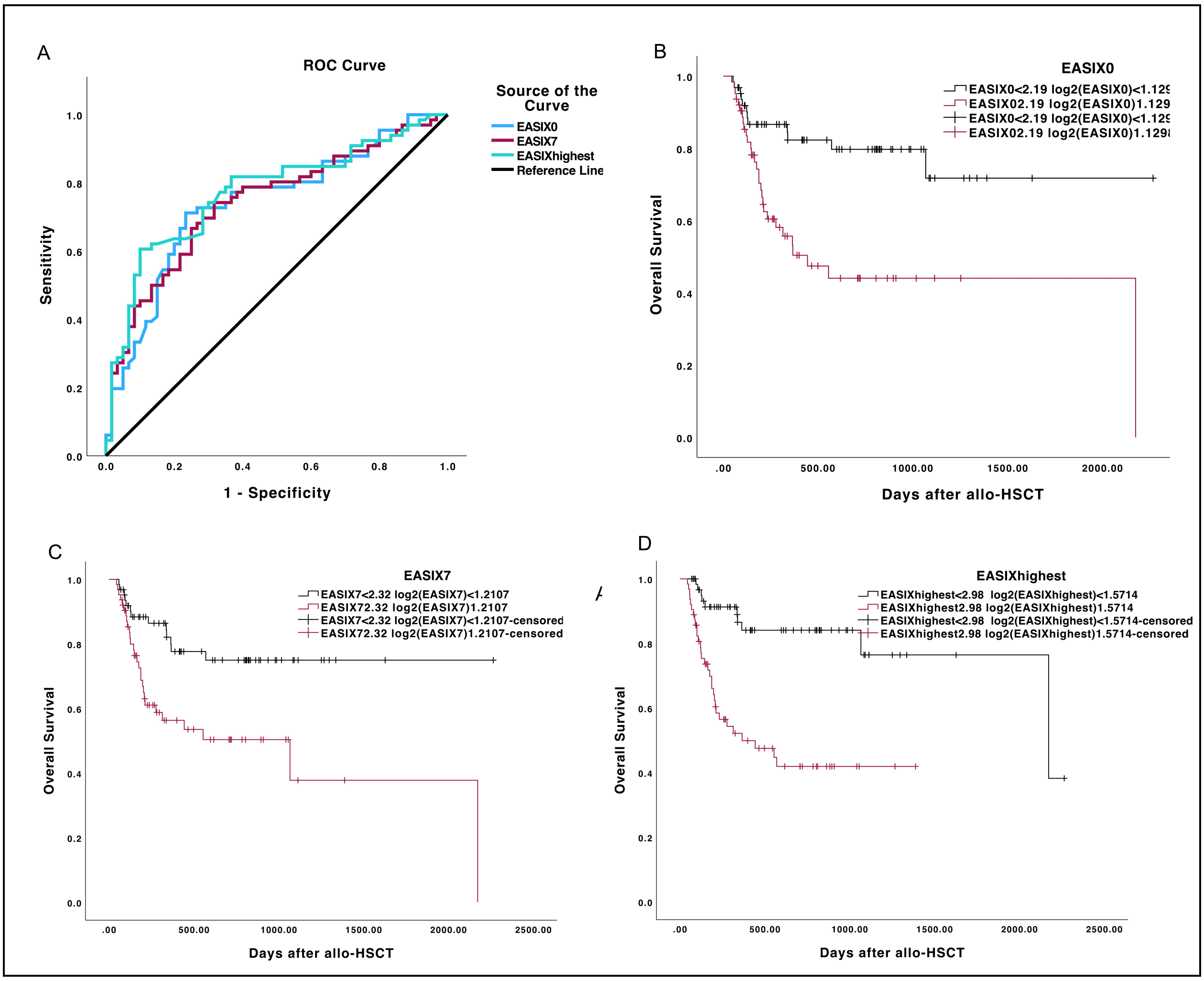

**Copyright Information:** (Online) ISSN: 2572-9241
©2026 The Author(s). HemaSphere is published by John Wiley & Sons Ltd on behalf of European Hematology Association. This is an open access Abstract Book distributed under the Attribution-NonCommercial-NoDerivs (CC BY-NC-ND), which allows third parties to download the articles and share them with others as long as they credit the author and the Abstract Book, but they cannot change the content in any way or use them commercially.
**Abstract Book Citations:** Authors, Title, HemaSphere, 2026;10:(S1):pages. The abstract book DOIs can be found on the "Author Information" page.
**Disclaimer:** Articles published in the journal HemaSphere exclusively reflect the opinions of the authors. The authors are responsible for all content in their abstracts including accuracy of the facts, statements, citing resources, etc.

### (PF1154) PIRCHE SCORES AND RECIPIENT HLA-B LEADER INFLUENCE RELAPSE AND CHRONIC GVHD AFTER 9/10 UNRELATED DONOR ALLO-HCT WITH CLASS I MISMATCH

**Topic:** 23. Stem cell transplantation - Clinical

Rodrigo Gil Manso*[1, 2], Francisco Javier Gil Etayo[1, 2, 3], Almudena Cabero[1, 2], Estefanía Perez-López[1, 2], Mónica Cabrero[1, 2], Alejandro Avendaño[1, 2], María del Carmen Gilabert Gutiérrez[1, 2], Ana García Bacelar[1, 2], Ana Africa Martin Lopez[1, 2], Mónica Baile-González[1, 2], Lourdes Vázquez-López[1, 2], Jenifer Alonso-Rodríguez[1, 2], Sara Marcos Asensio[1, 2], Pilar Terradillos Sánchez[1, 2], Irene Jiménez Hernán[1, 2], Ariadna Vicente Parra[1, 2], Ana Balanzategui Echevarría[1, 2], Nuria Calzada-Nieto[1, 2], Fermin Sanchez-Guijo[1, 2, 4, 5], Amalia Tejeda Velarde[1, 2, 3], Lucía López-Corral[1, 2, 5]

*[1]Hospital Universitario de Salamanca, Hematology, Salamanca, Spain;[2]Instituto de Investigación Biomédica de Salamanca (IBSAL), Salamanca, Spain; [3]Centro de Investigación del Cáncer (CIC, CSIC), Salamanca, Spain;[4]University of Salamanca, Salamanca, Spain; [5]Centro de Investigación Biomédica en Red del Cáncer (CIBERONC), Madrid, Spain;*

### Background

Allogeneic hematopoietic cell transplantation (allo-HCT) from 9/10 unrelated donors (UD) with HLA class I mismatch is associated with significant relapse and chronic graft-versus-host disease (cGVHD). Computational histocompatibility models may refine biological risk stratification beyond conventional HLA matching.

### Aims

To evaluate the impact of PIRCHE score (PS), HLA-B leader dimorphism and immunopeptidome-derived metrics on clinical outcomes after 9/10 UD allo-HCT.

### Methods

Retrospective single-center study including 84 adult patients undergoing 9/10 UD allo-HCT with class I mismatch. Median follow-up was 48.2 months. Endpoints were overall survival (OS), progression-free survival (PFS), graft-versus-host disease–free relapse-free survival (GRFS), relapse incidence (RI), non-relapse mortality (NRM), grade III–IV acute GVHD and chronic GVHD (cGVHD). Cox regression was used for OS, PFS and GRFS; Fine–Gray models for competing-risk endpoints.

**Copyright Information:** (Online) ISSN: 2572-9241
©2026 The Author(s). HemaSphere is published by John Wiley & Sons Ltd on behalf of European Hematology Association. This is an open access Abstract Book distributed under the Attribution-NonCommercial-NoDerivs (CC BY-NC-ND), which allows third parties to download the articles and share them with others as long as they credit the author and the Abstract Book, but they cannot change the content in any way or use them commercially.
**Abstract Book Citations:** Authors, Title, HemaSphere, 2026;10:(S1):pages. The abstract book DOIs can be found on the “Author Information” page.
**Disclaimer:** Articles published in the journal HemaSphere exclusively reflect the opinions of the authors. The authors are responsible for all content in their abstracts including accuracy of the facts, statements, citing resources, etc.

**Results**

Five-year OS was 59.5% (34 deaths). Very-high DRI independently predicted inferior OS (HR 5.72, p=0.006), PFS (HR 9.32, p<0.001) and GRFS (HR 4.83, p=0.005). HCT-CI ≥3 independently worsened OS (HR 2.72, p=0.013), PFS (HR 2.54, p=0.008) and NRM (sHR 9.45, p<0.001). Donor age independently increased the risk of mortality and NRM.

PIRCHE scores in the graft-versus-host (GvH) direction were associated with relapse in univariate analysis (PS-C GvH sHR 1.12 (p < 0.001), PS-I GvH sHR 1.06 (p < 0.001), PS-B GvH sHR 1.17 (p < 0.001), PS-T GvH sHR 1.02 (p=0.003)) as well as PIRCHE score in the host-versus-graf direction (HvG) (PS-II HvG sHR 1.02 (p = 0.027), PS-T HvG sHR 1.02 (p=0.045)). In multivariable competing-risk analysis, PS-T GvH remained independently associated with relapse (sHR 1.02 per unit, p=0.015) but not PS-T HvG (sHR 1.01 per unit, p=0.27). Interestingly, the dose of mononuclear cells infused was correlated with higher risk of relapse on multivariate anaylisis (sHR 1.20 per unit, p<0.001).

Higher PS-C GvH was significantly associated with increased risk of moderate/severe cGVHD in univariate analysis. Additionally, recipient HLA-B leader Met/Met genotype independently increased the risk of moderate/severe cGVHD even thougth the number of patients with this genotype was low (n=4)(sHR 7.09, p=0.010).

No significant associations were observed between immunopeptidome-derived metrics and any clinical endpoint. None of the evaluated computational algorithms independently influenced OS or GRFS after adjustment.

**Summary/Conclusion**

In 9/10 UD allo-HCT with class I mismatch, PIRCHE scores and recipient HLA-B leader genotype provide distinct biological signals. PS-T GvH independently predicts relapse, PS-C GvH associates with severe cGVHD, and recipient leader Met/Met genotype markedly increases severe cGVHD risk. Immunopeptidome metrics showed no prognostic value. These findings support selective integration of specific computational histocompatibility tools in donor selection for partially mismatched transplantation.

**Copyright Information:** (Online) ISSN: 2572-9241
©2026 The Author(s). HemaSphere is published by John Wiley & Sons Ltd on behalf of European Hematology Association. This is an open access Abstract Book distributed under the Attribution-NonCommercial-NoDerivs (CC BY-NC-ND), which allows third parties to download the articles and share them with others as long as they credit the author and the Abstract Book, but they cannot change the content in any way or use them commercially.
**Abstract Book Citations:** Authors, Title, HemaSphere, 2026;10:(S1):pages. The abstract book DOIs can be found on the "Author Information" page.
**Disclaimer:** Articles published in the journal HemaSphere exclusively reflect the opinions of the authors. The authors are responsible for all content in their abstracts including accuracy of the facts, statements, citing resources, etc.

### (PF1155) ADENOVIRUS INFECTION AFTER ALLO-HSCT: PERSISTENT VIRAL POSITIVITY INDICATES T-CELL DYSFUNCTION AND PREDICTS POOR OUTCOME

**Topic:** 23. Stem cell transplantation - Clinical

Zhihui Li*[1], Qinlong Zheng[2], Keyan Yang[2], Xianxuan Wang[1], Jing Li[1], Qian Fei[1], Jiahong Zhai[1], Zongyang Ming[1], Xiaopei Wen[1], Lei Wang[1], Tong Wu[1]

*[1]Department of Bone Marrow Transplantation Beijing Gobroad Boren Hospital, China, Beijing, China;[2]Department of Molecular Diagnostic Laboratory Beijing Gobroad Boren Hospital, China, Beijing, China;*

### Background

Adenovirus (ADV) infection following hematopoietic stem cell transplantation (HSCT) poses a significant clinical challenge, particularly in patients with hematologic malignancies. Its risk factors, clinical characteristics, and impact on prognosis require further elucidation.

### Aims

To investigate the risk factors, clinical features, pathogen characteristics, and prognostic impact of ADV infection in patients undergoing HSCT for hematologic malignancies.

### Methods

We enrolled 747 patients who underwent HSCT at Beijing GoBroad Boren Hospital between May 2020 and October 2025. The median follow-up time was 19.9 months (95% CI: 18.4-22.5 months). Among these, 72 patients developed AdV infection post-transplant (AdV+ group), matched with 675 contemporaneous patients without AdV infection (AdV- group). Categorical variables were compared using the Chi-square test or Fisher's exact test. Survival data were analyzed using Kaplan-Meier curves, with differences assessed by the log-rank test. Gray's test was used to evaluate differences in cumulative incidence of relapse.

### Results

The median time to ADV infection was 48.19 days (IQR: 26.62-91.59 days) post-transplant. Among ADV-infected patients, median age was 21.83 years (range: 0.35-75.59 years), including 48.6% children and 51.4% adults. The most common disease types were AML (38.9%), B-ALL (30.6%), and T-ALL (11.1%). We observed a significantly higher proportion of second transplants among patients with post-HSCT ADV infection (48.6% vs. 31.0%, p=0.004). Additionally, ADV+ patients had higher incidences of aGVHD (61.1% vs. 47.0%, p=0.031), bacterial infections (87.5% vs. 62.4%, p<0.001), and fungal infections (45.8% vs. 26.5%, p=0.001). The primary ADV pathogens were human mastadenovirus C (20.8%), adenovirus D (12.5%), and adenovirus A (6.9%). The main affected organs were the gastrointestinal tract (68.1%), blood (52.8%), and lungs (12.5%). Common viral co-infections included CMV (73.6%), EBV (55.6%), and BKV (34.7%).

ADV+ patients had significantly higher non-relapse mortality (NRM) compared to ADV- patients (2-year NRM 24.7% vs. 9.1%, p=0.0007), while the 2-year cumulative incidence of relapse was not significantly different (22.4% vs. 22.6%, p=0.78). Consequently, both disease-free survival (DFS) and overall survival (OS) were significantly worse in the ADV+ group (2-year DFS: 52.9% vs. 66.8%, p=0.036; 2-year OS: 56.1% vs. 72.5%, p=0.0079). Within the ADV+ cohort, clearance of ADV within 180 days post-infection was associated with significantly better DFS (p=0.0059) and OS (p=0.035). Patients with persistent ADV positivity had a significantly higher proportion of CD3+CD4+ T-cells (median: 18% vs. 9%, p=0.025) and a trend towards a higher CD4/CD8 ratio (p=0.066), suggesting potential accumulation of exhausted T-cells hindering viral clearance.

**Copyright Information:** (Online) ISSN: 2572-9241
©2026 The Author(s). HemaSphere is published by John Wiley & Sons Ltd on behalf of European Hematology Association. This is an open access Abstract Book distributed under the Attribution-NonCommercial-NoDerivs (CC BY-NC-ND), which allows third parties to download the articles and share them with others as long as they credit the author and the Abstract Book, but they cannot change the content in any way or use them commercially.
**Abstract Book Citations:** Authors, Title, HemaSphere, 2026;10:(S1):pages. The abstract book DOIs can be found on the “Author Information” page.
**Disclaimer:** Articles published in the journal HemaSphere exclusively reflect the opinions of the authors. The authors are responsible for all content in their abstracts including accuracy of the facts, statements, citing resources, etc.

**Summary/Conclusion**

AdV infection after allo-HSCT is associated with increased risks of aGVHD, concomitant infections, and higher NRM, resulting in significantly inferior survival. Persistent AdV positivity reflects impaired T-cell immunity, underscoring the need for early intervention and immune monitoring to improve outcomes in this high-risk population.

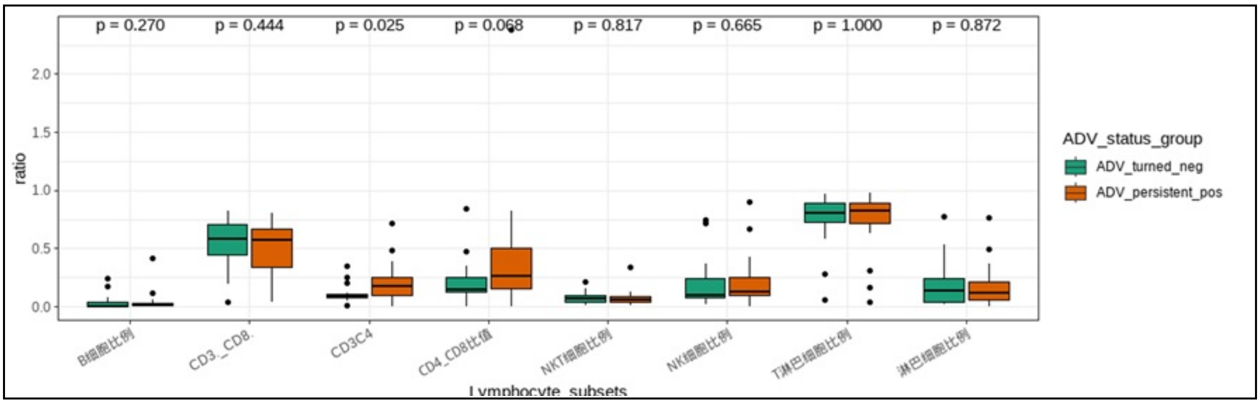

PatientswithpersistentlypositiveADVmayhaveahigherproportionofexhaustedTcellswhichcanaffecttheviralclearance

**Copyright Information:** (Online) ISSN: 2572-9241
©2026 The Author(s). HemaSphere is published by John Wiley & Sons Ltd on behalf of European Hematology Association. This is an open access Abstract Book distributed under the Attribution-NonCommercial-NoDerivs (CC BY-NC-ND), which allows third parties to download the articles and share them with others as long as they credit the author and the Abstract Book, but they cannot change the content in any way or use them commercially.
**Abstract Book Citations:** Authors, Title, HemaSphere, 2026;10:(S1):pages. The abstract book DOIs can be found on the "Author Information" page.
**Disclaimer:** Articles published in the journal HemaSphere exclusively reflect the opinions of the authors. The authors are responsible for all content in their abstracts including accuracy of the facts, statements, citing resources, etc.

### (PF1156) ALLOGENEIC HEMATOPOIETIC STEM CELL TRANSPLANTATION IN OLDER ADULTS: CLINICAL OUTCOMES AND INNOVATIVE USE OF PRE-EMPTIVE DONOR LYMPHOCYTE INFUSIONS

**Topic:** 23. Stem cell transplantation - Clinical

Davide Stella*[1], Alessandro Busca[1], Chiara Maria Dellacasa[1], Carolina Secreto[1], Camilla Gariazzo[1], Roberto Freilone[1], Luisa Giaccone[1], Victoria Potter[2]

*[1]Azienda Ospedaliero Universitaria Città della Salute e della Scienza di Torino, Torino, Italy;[2]King's College Hospital, London, United Kingdom;*

### Background

Allogeneic hematopoietic stem cell transplantation (HSCT) is increasingly performed in older adults with hematologic malignancies thanks to advances in donor selection, conditioning, and supportive care. However, data on outcomes in patients aged ≥60 years remain limited, especially for those ≥70 years.

### Aims

The primary aim of this study was to evaluate the feasibility and clinical outcomes of HSCT in patients aged ≥60 years, with a specific focus on those aged ≥70 years, in a contemporary single-centre cohort.
Secondary objectives were:

1. To assess overall survival (OS), non-relapse mortality (NRM), relapse incidence, graft-versus-host disease (GVHD), and secondary malignancies in this population.
2. To explore the impact of comorbidity burden, as assessed by the HCT-CI score, on transplant outcomes, particularly in patients aged ≥70 years.
3. To investigate the prognostic role of measurable residual disease (MRD) status at transplant.
4. To evaluate the effect of donor lymphocyte infusion (DLI), including pre-emptive administration for mixed chimerism, on survival and GVHD incidence.

By addressing these objectives, the study aimed to clarify whether chronological age alone should remain a limiting factor for HSCT candidacy in older adults and to identify modifiable factors that may optimize post-transplant outcomes in this expanding patient population.

### Methods

We conducted a retrospective single-centre analysis of all adults aged ≥60 years who underwent HSCT at King's College Hospital, London, between January 2022 and December 2024. Demographic, disease-related and transplant-related variables were collected, including HCT-CI score, MRD status, conditioning regimen, donor type and post-transplant interventions such as DLI. Outcomes included OS, NRM, relapse, GVHD and secondary malignancies.

**Copyright Information:** (Online) ISSN: 2572-9241
©2026 The Author(s). HemaSphere is published by John Wiley & Sons Ltd on behalf of European Hematology Association. This is an open access Abstract Book distributed under the Attribution-NonCommercial-NoDerivs (CC BY-NC-ND), which allows third parties to download the articles and share them with others as long as they credit the author and the Abstract Book, but they cannot change the content in any way or use them commercially.
**Abstract Book Citations:** Authors, Title, HemaSphere, 2026;10:(S1):pages. The abstract book DOIs can be found on the "Author Information" page.
**Disclaimer:** Articles published in the journal HemaSphere exclusively reflect the opinions of the authors. The authors are responsible for all content in their abstracts including accuracy of the facts, statements, citing resources, etc.

**Results**

Eighty-nine patients (90 HSCT procedures) were analysed (median age 66 years, range 60–75); 30 were aged 60–64, 39 aged 65–69 and 21 aged ≥70. AML (46%) and MDS (22%) were the most frequent indications; 92% received reduced-intensity conditioning and 83% had unrelated donors. Median follow-up was 19 months. One-year and three-year OS were 76% and 58%, respectively; NRM was 6.7% at day +100 and 18.2% at 24 months; relapse occurred in 26.7% of patients. No significant OS differences emerged between age groups (≈75% 1-year OS across all groups), although within the ≥70-year cohort a trend toward better OS was observed in patients with HCT-CI 0–2 versus ≥3 ($p = 0.07$). MRD negativity and receipt of DLI were both associated with improved OS (median 14 vs 5 months and 26 vs 7 months, respectively). Acute GVHD occurred in 39.8% and chronic GVHD in 14.8% of patients; secondary malignancies developed in 9%. Pre-emptive DLI was administered to 21% of patients, mainly for mixed chimerism; this approach, alongside its therapeutic use, was associated with a longer median OS compared with no DLI (26 vs 7 months) without a significant increase in GVHD incidence.

**Summary/Conclusion**

Allogeneic HSCT using mainly reduced-intensity conditioning is feasible in carefully selected patients aged ≥60 years and even in those ≥70 years, with acceptable early mortality and manageable GVHD. In this setting, comorbidity burden regains prognostic relevance beyond age 70. Importantly, proactive MRD monitoring and the use of pre-emptive DLI appear to improve disease control and survival without compromising safety, supporting their integration into post-transplant management of older HSCT candidates.

**Copyright Information:** (Online) ISSN: 2572-9241
©2026 The Author(s). HemaSphere is published by John Wiley & Sons Ltd on behalf of European Hematology Association. This is an open access Abstract Book distributed under the Attribution-NonCommercial-NoDerivs (CC BY-NC-ND), which allows third parties to download the articles and share them with others as long as they credit the author and the Abstract Book, but they cannot change the content in any way or use them commercially.
**Abstract Book Citations:** Authors, Title, HemaSphere, 2026;10:(S1):pages. The abstract book DOIs can be found on the “Author Information” page.
**Disclaimer:** Articles published in the journal HemaSphere exclusively reflect the opinions of the authors. The authors are responsible for all content in their abstracts including accuracy of the facts, statements, citing resources, etc.

### (PF1157) DEEP LEARNING–BASED PREDICTION OF CD34⁺ CELL MOBILIZATION IN HEALTHY DONORS: AN EXPLAINABLE ARTIFICIAL NEURAL NETWORK MODEL WITH SUPERIOR PREDICTIVE PERFORMANCE

**Topic:** 23. Stem cell transplantation - Clinical

Bedrettin Orhan*[1], Hilmi Erdem Gozden[1], Nihan Alkış[2]

*[1]Bursa Yüksek İhtisas Research and Training Hospital, Adult Hematology, Bursa, Türkiye;[2]Bursa VM Medical Park Hospital, Adult Hematology, Bursa, Türkiye;*

### Background

Peripheral blood stem cell (PBSC) mobilization with G-CSF is standard in allogeneic transplantation; however, $CD34^+$ yield shows marked inter-donor variability. Approximately 40% of donors require multiple apheresis sessions, and 2–5% fail to reach target doses, increasing donor burden and resource use. Conventional demographic-based prediction models demonstrate limited accuracy (AUC 0.67–0.74). The non-linear biological complexity of mobilization constrains traditional statistical approaches.

### Aims

This study aimed to develop an artificial neural network–based model to predict $CD34^+$ cell mobilization in healthy donors.

### Methods

This single-center retrospective observational study included 316 healthy allogeneic peripheral blood stem cell donors registered in the national donor registry (TÜRKÖK). All donors underwent mobilization with granulocyte colony-stimulating factor (G-CSF) alone, followed by standard apheresis. Eligible donors were aged 18–60 years with complete clinical and laboratory data; donors with comorbidities, alternative mobilization agents, incomplete procedures, or missing data were excluded. Donor variables included demographic characteristics, body mass index, smoking exposure, alcohol use, and COVID-19 infection and vaccination history. The primary outcome was total $CD34^+$ cell yield normalized to recipient body weight ($\times 10^6$/kg). A feed-forward artificial neural network was developed using the MATLAB Neural Network Toolbox with normalized inputs, ReLU activation, Adam (Adaptive Moment Estimation) optimization, five-fold cross-validation, and early stopping. Performance was compared with linear and logistic regression models. Model interpretability was assessed using Shapley Additive Explanations (SHAP).

### Results

The artificial neural network demonstrated strong predictive performance for $CD34^+$ cell mobilization. In regression analysis, the model achieved a root mean squared error of 3.21, a mean absolute error of 1.11, and an $R^2$ of 0.88, indicating that most of the variance in $CD34^+$ cell yield was explained. In classification analysis using a $\geq 5\times 10^6$/kg threshold, the model yielded an AUC of 0.81, with an accuracy of 0.78, sensitivity of 0.84, specificity of 0.76, and an F1-score of 0.89. Figure 1 shows observed versus AI-predicted $CD34^+$ cell yield in representative donors, along with the artificial neural network architecture and its clinical application interface. The high sensitivity highlights the model's effectiveness in identifying donors likely to achieve adequate mobilization. Overall, the ANN outperformed conventional logistic regression models reported in the literature (AUC 0.67–0.74).

**Copyright Information:** (Online) ISSN: 2572-9241
©2026 The Author(s). HemaSphere is published by John Wiley & Sons Ltd on behalf of European Hematology Association. This is an open access Abstract Book distributed under the Attribution-NonCommercial-NoDerivs (CC BY-NC-ND), which allows third parties to download the articles and share them with others as long as they credit the author and the Abstract Book, but they cannot change the content in any way or use them commercially.
**Abstract Book Citations:** Authors, Title, HemaSphere, 2026;10:(S1):pages. The abstract book DOIs can be found on the "Author Information" page.
**Disclaimer:** Articles published in the journal HemaSphere exclusively reflect the opinions of the authors. The authors are responsible for all content in their abstracts including accuracy of the facts, statements, citing resources, etc.

### Summary/Conclusion

Previous mobilization prediction models have primarily relied on linear statistical approaches with modest discriminatory power, while machine learning methods such as XGBoost have shown incremental improvements without sufficient transparency. To our knowledge, this study is the first to present a validated artificial neural network for CD34⁺ mobilization prediction with robust regression and classification performance combined with SHAP-based explainability. The single-center design and exclusive use of G-CSF–only mobilization represent limitations, necessitating external validation. Overall, this explainable framework offers a clinically meaningful, donor-centered decision-support tool with strong translational potential for stem cell transplantation.

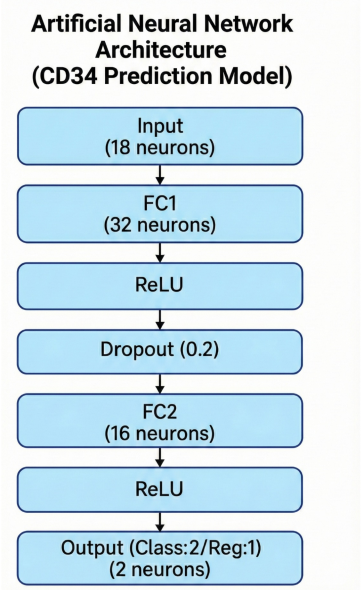

(A) Architecture of the artificial neural network used for CD34⁺ mobilization prediction, consisting of an 18-neuron input layer, two fully connected hidden layers with ReLU activation and dropout regularization, and a dual-output layer enabling regression and classification.

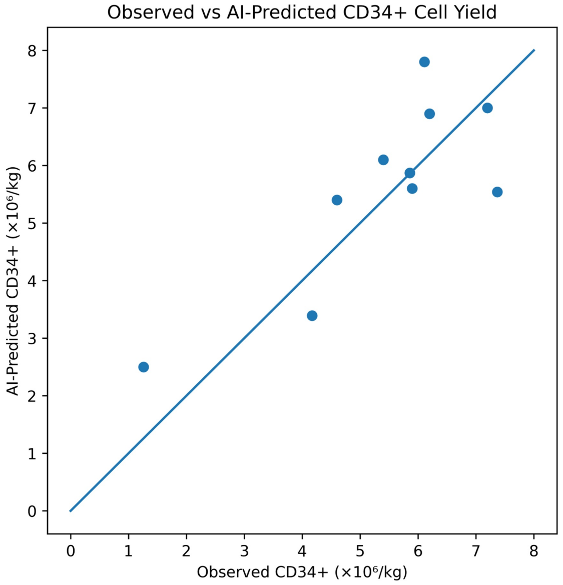

(B) Observed versus AI-predicted CD34⁺ cell yield in a representative subset of healthy donors, with the identity line (y = x) indicating ideal agreement.

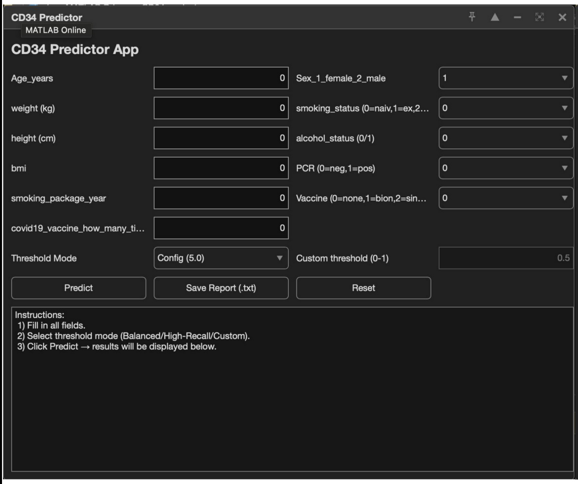

(C) Graphical user interface of the CD34 Predictor application allowing entry of donor demographic and clinical parameters.

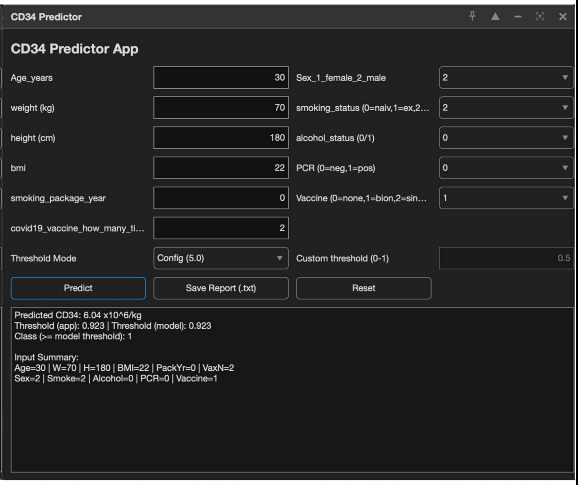

(D) Example output generated by the application, displaying predicted CD34⁺ cell yield and mobilization classification to support individualized donor assessment and apheresis planning.

**Copyright Information:** (Online) ISSN: 2572-9241
©2026 The Author(s). HemaSphere is published by John Wiley & Sons Ltd on behalf of European Hematology Association. This is an open access Abstract Book distributed under the Attribution-NonCommercial-NoDerivs (CC BY-NC-ND), which allows third parties to download the articles and share them with others as long as they credit the author and the Abstract Book, but they cannot change the content in any way or use them commercially.
**Abstract Book Citations:** Authors, Title, HemaSphere, 2026;10:(S1):pages. The abstract book DOIs can be found on the "Author Information" page.
**Disclaimer:** Articles published in the journal HemaSphere exclusively reflect the opinions of the authors. The authors are responsible for all content in their abstracts including accuracy of the facts, statements, citing resources, etc.

### (PF1158) MEASURABLE RESIDUAL DISEASE (MRD) EVALUATION AND OUTCOMES IN PATIENTS WITH ACUTE MYELOID LEUKEMIA (AML) UNDERGOING THEIR SECOND ALLOGENEIC STEM CELL TRANSPLANTATION

**Topic:** 23. Stem cell transplantation - Clinical

Jule Ussmann*[1, 2], Thuy My Le[1, 2], Donata Backhaus[1, 2], Lara Bischof[1, 2], Jacob Jendro[1, 2], Dominic Brauer[1, 2], Georg-Nikolaus Franke[1, 2], Vladan Vucinic[1, 2], Carmen Diana Herling[1, 2], Marco Herling[1, 2], Klaus Metzeler[1, 2], Sebastian Schwind[1, 2], Madlen Jentzsch[1, 2]

*[1]University of Leipzig Medical Center, Department for Hematology, Cell Therapy, Hemostaseology and Infectious Diseases, Leipzig, Germany; [2]Cancer Center Central Germany – CCCG, Leipzig, Germany;*

### Background

In selected patients (pts) with AML, a second (2$^{nd}$) allogeneic hematopoietic stem cell transplantation (HSCT) – either for graft rejection, AML relapse, or relapse of another myeloid neoplasm (MN) manifesting as AML – remains a curative treatment option. However, selecting pts who benefit from this intensive treatment  remains challenging, as prognostic factors that reliably predict outcomes in this context are scarce. MRD evaluation is an important prognostic tool, but little is known about its significance in the context of a 2$^{nd}$ HSCT.

### Aims

To evaluate outcomes in comparison to a 1$^{st}$ HSCT as well as the prognostic relevance of molecular MRD in AML pts receiving their 2$^{nd}$ HSCT.

### Methods

We retrospectively analyzed 925 consecutive HSCT performed between 1999 and 2024 at our center for an AML diagnosis, out of which 845 (91%) were given as 1$^{st}$, and 80 (9%) as 2$^{nd}$ HSCT. Reasons for 2$^{nd}$ HSCT were graft rejection (19%) and AML relapse or MN progression into AML (81%). According to local standards, the donor was changed for the 2$^{nd}$ HSCT in all but 2 pts without a suitable alternative donor. In pts with available samples, MRD prior to and after HSCT was evaluated by digital droplet PCR measuring mutations known at AML diagnosis. In pts without a mutation-based MRD result prior to HSCT, MRD measurements based on gene expression of *BAALC*, *MN1*, or *WT1* were used. 78% of HSCTs were performed in morphologic remission, of those 56% were MRD$^{neg}$ and 44% MRD$^{pos}$.

**Copyright Information:** (Online) ISSN: 2572-9241
©2026 The Author(s). HemaSphere is published by John Wiley & Sons Ltd on behalf of European Hematology Association. This is an open access Abstract Book distributed under the Attribution-NonCommercial-NoDerivs (CC BY-NC-ND), which allows third parties to download the articles and share them with others as long as they credit the author and the Abstract Book, but they cannot change the content in any way or use them commercially.
**Abstract Book Citations:** Authors, Title, HemaSphere, 2026;10:(S1):pages. The abstract book DOIs can be found on the "Author Information" page.
**Disclaimer:** Articles published in the journal HemaSphere exclusively reflect the opinions of the authors. The authors are responsible for all content in their abstracts including accuracy of the facts, statements, citing resources, etc.

**Results**

Compared to pts receiving their 1st HSCT, pts receiving their 2nd HSCT were more often male ($P$=.05), and more often had secondary AML ($P$=.05). They were younger (median 59 years *vs.* 55 years, $P$=.05), less often transplanted from a matched related ($P$<.001), and more often from a mismatched donor ($P$<.001). In contrast, neither the ELN2022 risk at diagnosis ($P$=.95) nor the MRD status at HSCT ($P$=.56) differed between pts receiving a 1st or 2nd HSCT. Compared to pts receiving their 1st HSCT, outcomes were not significantly worse for pts receiving their 2nd HSCT for graft rejection (cumulative incidence of relapse [CIR] $P$=.10, NRM $P$=.54, overall survival [OS] $P$=.20). However, pts who received their 2nd HSCT for relapse after 1st HSCT had similar CIR ($P$=.59), but higher non-relapse mortality (NRM, $P$<.001) and shorter OS ($P$<.001, Figure 1A) than pts after 1st HSCT.
MRD$^{pos}$ at HSCT associated with higher CIR and shorter OS in pts receiving their 1st HSCT ($P$<.001 and $P$<.001, respectively), while in pts receiving their 2nd HSCT, MRD$^{pos}$ showed only a trend for higher CIR ($P$=.15), but associated with shorter OS ($P$=.04, Figure 1B). Of note, irrespective of MRD status, CIR ($P$<.001) and OS ($P$<.001) were worse after 2ndHSCT than after 1st HSCT. Assessing molecular MRD during follow-up after 2nd HSCT allowed for a good relapse prediction within the next 28 days (AUC=.85) and 84 days (AUC=.74). However, this tended to be inferior – although not statistically significant - than relapse prediction after 1st HSCT (within 28 days, AUC=.96, $P$=.19, Figure 1C and within 84 days, AUC=.83, $P$=.12, Figure 1D).

**Summary/Conclusion**

Outcomes after 2nd HSCT for AML relapse were significantly worse compared to a 1st HSCT for AML – mainly due to a higher NRM. Still, detectable MRD at 2nd HSCT was a prognostic factor for OS, and MRD$^{pos}$ during follow-up after HSCT was a predictor of relapse. As NRM was especially high after a 2nd HSCT for relapsed disease, we should carefully select pts and optimize supportive treatments to improve outcomes.

**Copyright Information:** (Online) ISSN: 2572-9241
©2026 The Author(s). HemaSphere is published by John Wiley & Sons Ltd on behalf of European Hematology Association. This is an open access Abstract Book distributed under the Attribution-NonCommercial-NoDerivs (CC BY-NC-ND), which allows third parties to download the articles and share them with others as long as they credit the author and the Abstract Book, but they cannot change the content in any way or use them commercially.
**Abstract Book Citations:** Authors, Title, HemaSphere, 2026;10:(S1):pages. The abstract book DOIs can be found on the "Author Information" page.
**Disclaimer:** Articles published in the journal HemaSphere exclusively reflect the opinions of the authors. The authors are responsible for all content in their abstracts including accuracy of the facts, statements, citing resources, etc.

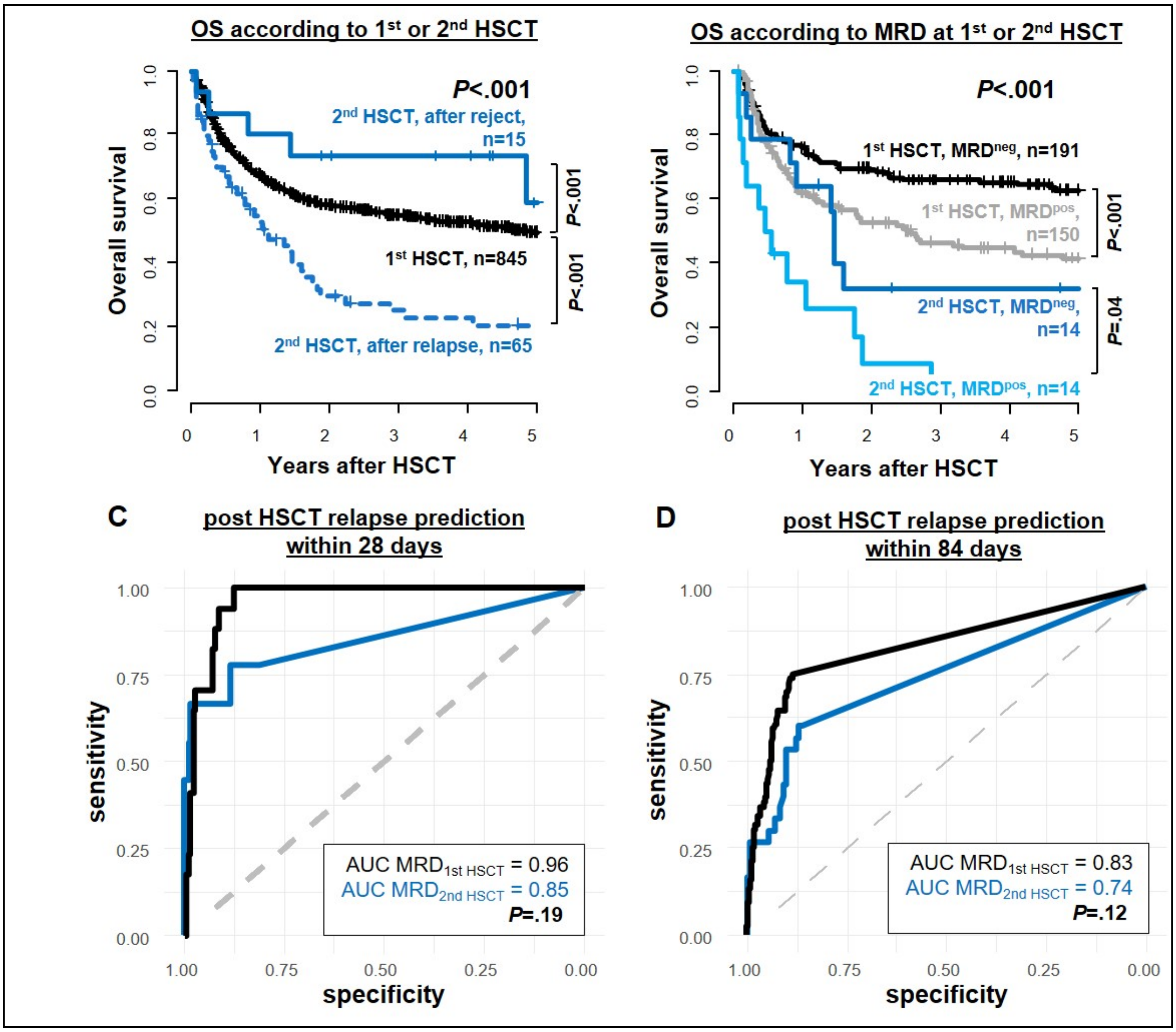

**Copyright Information:** (Online) ISSN: 2572-9241
©2026 The Author(s). HemaSphere is published by John Wiley & Sons Ltd on behalf of European Hematology Association. This is an open access Abstract Book distributed under the Attribution-NonCommercial-NoDerivs (CC BY-NC-ND), which allows third parties to download the articles and share them with others as long as they credit the author and the Abstract Book, but they cannot change the content in any way or use them commercially.
**Abstract Book Citations:** Authors, Title, HemaSphere, 2026;10:(S1):pages. The abstract book DOIs can be found on the "Author Information" page.
**Disclaimer:** Articles published in the journal HemaSphere exclusively reflect the opinions of the authors. The authors are responsible for all content in their abstracts including accuracy of the facts, statements, citing resources, etc.

### (PF1159) TWO-TUBE 12-COLOR FLOW CYTOMETRY–BASED PRE-TRANSPLANT IMMUNOPHENOTYPIC LSC-MRD STRATIFIES RELAPSE RISK AND SURVIVAL AFTER ALLO-HCT FOR AML

**Topic:** 23. Stem cell transplantation - Clinical

Tzu-Ting Chen*[1], Wen-Jyi Lo[2], Sing Ting Wang[1], Chia Yu Chen[1], Fu Ming Cheng[1], Chen-Yuan Lin[1], Chingchan Lin[1]

*[1]China Medical University Hospital, Division of Hematology and Oncology, Department of Internal Medicine, Taichung, Taiwan; [2]China Medical University, College of Medicine, Taichung, Taiwan;*

### Background

Allogeneic hematopoietic cell transplantation (allo-HCT) is the only curative option for many adults with acute myeloid leukemia (AML), yet post-transplant relapse remains the leading cause of treatment failure. Since 2022, we have implemented a two-tube 12-color flow cytometry panel to assess immunophenotypic leukemic stem cell–based MRD (iLSC-MRD), quantifying aberrant $CD34^+CD38^-$ LSC-enriched populations using a CD34/CD45/CD117/HLA-DR/CD38 backbone with additional LSC-associated and aberrant lineage markers.

### Aims

We aimed to determine, the prognostic value of pre-transplant iLSC-MRD for relapse, survival, and non-relapse complications in adult AML recipients of first allo-HCT.

### Methods

We retrospectively analyzed adults with AML who underwent allo-HCT at China Medical University Hospital between March 2022 and December 2025. Pre-transplant iLSC-MRD was assessed before conditioning using a standardized two-tube 12-color flow cytometry panel. Both tubes contained the myeloid backbone CD34, CD45, CD117, HLA-DR, and CD38; tube 1 additionally included CD15, CD45RA, CD123, CD33, CD13, CD44 and a combination aberrant-marker channel (CLL/T-cell–associated markers such as CD7, CD11b, CD22), while tube 2 included CD56, CD4, CD85k, CD19, CD64, CD71, and CD14. iLSC-MRD was defined by aberrant immunophenotypes within the $CD34^+CD38^-$ LSC-enriched compartment and classified as MRD-negative (MRD–) or MRD-positive (MRD+). Baseline variables included age, sex, cytogenetic risk, FLT3 status, donor type, conditioning intensity, and HCT-CI. Overall survival (OS) and relapse-free survival (RFS) were estimated using the Kaplan–Meier method and compared by log-rank test, while cumulative incidences of relapse, non-relapse mortality (NRM), GVHD, and viral events were evaluated with competing-risk methods.

### Results

Sixty-five adults were included; 43 were iLSC-MRD– and 22 were iLSC-MRD+ at transplant. Baseline age, donor type, conditioning intensity, and HCT-CI were broadly comparable between iLSC-MRD groups. Median OS was not reached in the iLSC-MRD– group and was 17.4 months in the iLSC-MRD+ group (log-rank p=0.018). Median RFS was likewise not reached in iLSC-MRD– patients and was 17.4 months in iLSC-MRD+ patients ( p=0.018). At 1 year, the cumulative incidence of relapse was approximately 0% in iLSC-MRD– patients versus 26% in iLSC-MRD+ patients ( p=0.001), with a shorter median time to relapse in the iLSC-MRD+ group (12.3 vs 20.6 months). In contrast, 1-year NRM was similar (cumulative incidence 15% vs 11% in iLSC-MRD– vs iLSC-MRD+; p=0.72). The incidences of grade II–IV aGVHD, cGVHD, EBV and CMV reactivation, and CMV disease did not differ meaningfully by iLSC-MRD status.

**Copyright Information:** (Online) ISSN: 2572-9241
©2026 The Author(s). HemaSphere is published by John Wiley & Sons Ltd on behalf of European Hematology Association. This is an open access Abstract Book distributed under the Attribution-NonCommercial-NoDerivs (CC BY-NC-ND), which allows third parties to download the articles and share them with others as long as they credit the author and the Abstract Book, but they cannot change the content in any way or use them commercially.
**Abstract Book Citations:** Authors, Title, HemaSphere, 2026;10:(S1):pages. The abstract book DOIs can be found on the "Author Information" page.
**Disclaimer:** Articles published in the journal HemaSphere exclusively reflect the opinions of the authors. The authors are responsible for all content in their abstracts including accuracy of the facts, statements, citing resources, etc.

**Summary/Conclusion**

In this single-center Asian cohort, pre-transplant immunophenotypic LSC-MRD positivity, determined by a two-tube 12-color flow cytometry assay with a CD34/CD45/CD117/HLA-DR/CD38 backbone and LSC-/aberrancy-oriented markers, identified AML patients at substantially higher 1-year relapse risk and inferior survival, without an increase in NRM, GVHD, or EBV/CMV complications. These findings support iLSC-based flow-cytometric MRD as a robust marker of leukemia-specific risk rather than global transplant vulnerability and provide a rationale for incorporating iLSC-MRD into pre-transplant risk stratification algorithms and MRD-adapted peri-transplant strategies—potentially including intensified or novel anti-leukemic approaches in iLSC-MRD+ patients—while maintaining acceptable toxicity.

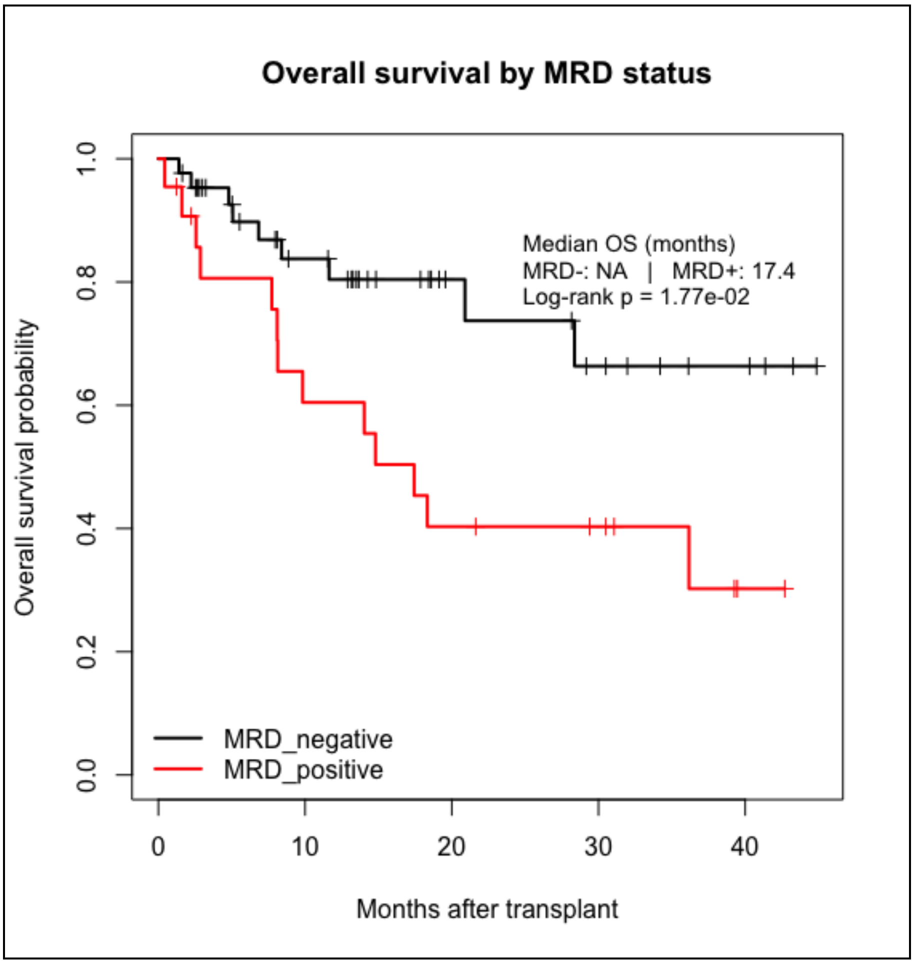

**Copyright Information:** (Online) ISSN: 2572-9241
©2026 The Author(s). HemaSphere is published by John Wiley & Sons Ltd on behalf of European Hematology Association. This is an open access Abstract Book distributed under the Attribution-NonCommercial-NoDerivs (CC BY-NC-ND), which allows third parties to download the articles and share them with others as long as they credit the author and the Abstract Book, but they cannot change the content in any way or use them commercially.
**Abstract Book Citations:** Authors, Title, HemaSphere, 2026;10:(S1):pages. The abstract book DOIs can be found on the "Author Information" page.
**Disclaimer:** Articles published in the journal HemaSphere exclusively reflect the opinions of the authors. The authors are responsible for all content in their abstracts including accuracy of the facts, statements, citing resources, etc.

### (PF1160) INTEGRATING DONOR CHIMERISM IN MACHINE LEARNING MODELS FOR DISEASE RELAPSE PREDICTION FOLLOWING ALLOGENEIC STEM CELL TRANSPLANTATION

**Topic:** 23. Stem cell transplantation - Clinical

Ray Mun Koo*[1, 2, 3], Farhad Goodarzy[4, 5], Eric Wong[1, 2, 6], Tony Papenfuss[4, 5], Rachel Koldej[1, 2], David Ritchie[1, 2, 3]

*[1]Royal Melbourne Hospital, Australian Cancer Research Foundation (ACRF) Translational Research Laboratory, Melbourne, Australia; [2]University of Melbourne, Faculty of Medicine, Dentistry and Health Sciences, Melbourne, Australia;[3]Peter MacCallum Cancer Centre - Royal Melbourne Hospital, Clinical Haematology, Melbourne, Australia; [4]Walter and Eliza Hall Institute of Medical Research, Melbourne, Australia; [5]University of Melbourne, Department of Medical Biology, Melbourne, Australia;[6]Austin Health, Clinical Haematology, Melbourne, Australia;*

### Background

Reliable and widely accessible biomarkers for early relapse prediction after allogeneic stem cell transplantation (alloSCT) are lacking, limiting risk-adapted monitoring and pre-emptive intervention. Donor chimerism is routinely performed but has insufficient predictive power for individual treatment decisions when used in isolation. We hypothesised that machine learning (ML) models integrating chimerism with transplant-related variables could enable clinically actionable relapse risk stratification.

### Aims

To develop and compare supervised ML models for relapse prediction in adults undergoing alloSCT for myeloid malignancies, with emphasis on early $CD3^+$ and $CD3^-$ donor chimerism.

### Methods

We studied 258 consecutive adults with acute myeloid leukaemia (AML), myelodysplasia-myeloproliferative neoplasm (MDS-MPN) or MDS undergoing first alloSCT (2016–2023). Donor, recipient and transplant variables were collated, including peripheral blood donor chimerism at day 30, 60 and 100 assessed by STR-PCR. Decision Tree (DT), Random Forest (RF), Support Vector Machine (SVM), Gradient Boosting Machine (GBM) and AdaBoost models were trained using 10-fold cross-validation with stratified sampling to preserve relapse class distribution. Feature importance was derived using the Gini index for tree-based models. Model performance was assessed by sensitivity, specificity, ROC-AUC and F1 score.

### Results

Cohort consisted predominantly of males (60%) with AML (70%) at a median age of 58 (range: 17-73). At a median follow-up of 817 days (range: 11-3078 days), 71 patients relapsed at a median of 224 days (2-year incidence 29%, 95% CI 23–35). Chimerism data were available in ≥84% of patients at all timepoints. Model-derived feature importance identified day 100 $CD3^+$ donor chimerism as the strongest predictor of relapse, followed by $CD34^+$ cell dose, day 100 $CD3^-$ donor chimerism, and donor and recipient age. For the entire cohort, DT achieved the highest sensitivity (60%), while SVM showed the highest specificity (97%). GBM and DT had the highest F1 scores (0.52 and 0.49) (**Figure 1**). When disease categories were analysed separately, DT had highest sensitivity for AML (54%) and MDS/MDS-MPN (53%), and specificity for all models were marginally higher in MDS/MDS-MPN compared to AML [median: 85% (range: 82-100) vs 82% (range: 72-100)]. RF demonstrated the best overall discrimination in the full cohort (ROC-AUC 0.64) and in MDS/MDS-MPN (0.76), while DT performed best in AML (ROC-AUC 0.62).

**Copyright Information:** (Online) ISSN: 2572-9241
©2026 The Author(s). HemaSphere is published by John Wiley & Sons Ltd on behalf of European Hematology Association. This is an open access Abstract Book distributed under the Attribution-NonCommercial-NoDerivs (CC BY-NC-ND), which allows third parties to download the articles and share them with others as long as they credit the author and the Abstract Book, but they cannot change the content in any way or use them commercially.
**Abstract Book Citations:** Authors, Title, HemaSphere, 2026;10:(S1):pages. The abstract book DOIs can be found on the "Author Information" page.
**Disclaimer:** Articles published in the journal HemaSphere exclusively reflect the opinions of the authors. The authors are responsible for all content in their abstracts including accuracy of the facts, statements, citing resources, etc.

### Summary/Conclusion

ML models incorporating standard-of-care variables and routinely available donor chimerism enable non-invasive post-alloSCT relapse risk stratification with high specificity, modest sensitivity and moderate discrimination. Rather than utilizing a single "best" ML model, incorporating multiple models into a "traffic light" risk stratification system leverages the unique strengths of different models, with the potential of providing clinicians with a pragmatic tool for relapse prediction. Further model optimization and prospective external validation is warranted.

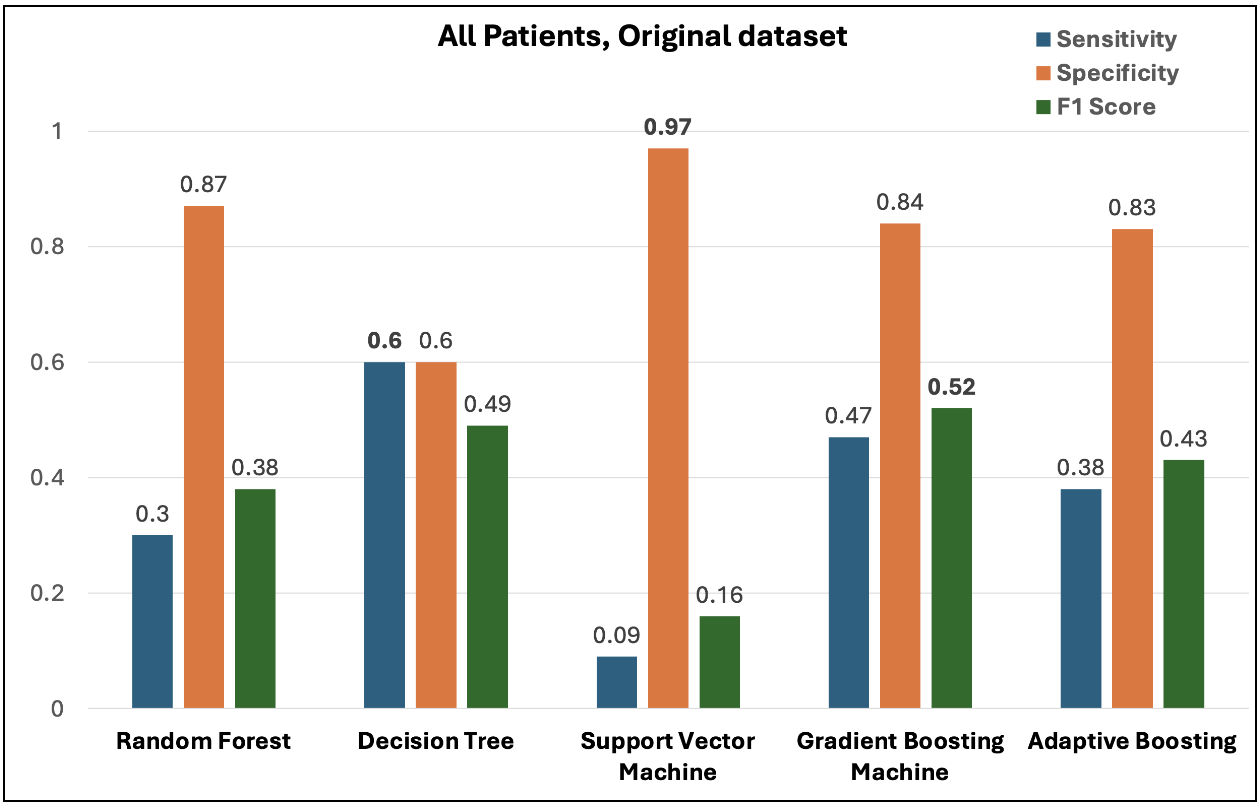

Figure 1. Model sensitivity, specificity and F1 score for the entire cohort.

**Copyright Information:** (Online) ISSN: 2572-9241
©2026 The Author(s). HemaSphere is published by John Wiley & Sons Ltd on behalf of European Hematology Association. This is an open access Abstract Book distributed under the Attribution-NonCommercial-NoDerivs (CC BY-NC-ND), which allows third parties to download the articles and share them with others as long as they credit the author and the Abstract Book, but they cannot change the content in any way or use them commercially.
**Abstract Book Citations:** Authors, Title, HemaSphere, 2026;10:(S1):pages. The abstract book DOIs can be found on the "Author Information" page.
**Disclaimer:** Articles published in the journal HemaSphere exclusively reflect the opinions of the authors. The authors are responsible for all content in their abstracts including accuracy of the facts, statements, citing resources, etc.

### (PF1161) HEMATOPOIETIC STEM CELL TRANSPLANTATION AND LATENT TUBERCULOSIS INFECTION

**Topic:** 23. Stem cell transplantation - Clinical

Alejandro Lada Colunga[1], Alberto Porro Camarero[1], Joud Zanabili Al Sibai[1], Ana Julia Gonzalez Huerta[1], Angel Ramírez Payer[1,1], Soledad Gonzalez Rodríguez[1], Pilar Palomo Moraleda[1], Jose Maria Garcia Gala[1], Carlos Fernandez Garcia[1], Adrian Sebastian Filipescu Stanciu[1], Rodrigo Garcia Alvarez[1], Lucia Morais Brais[1], Ana Pilar Gonzalez Rodríguez*[1]

[1]*HOSPITAL UNIVERSITARIO CENTRAL DE ASTURIAS, oviedo, Spain;*

### Background

Hematopoietic stem cell transplantation (HSCT) entails prolonged immunosuppression and increases the risk of latent tuberculosis infection (LTBI) reactivation. The optimal LTBI screening strategy in HSCT remains uncertain.

### Aims

To evaluate (i) concordance among LTBI screening tests (tuberculin skin test [TST] and interferon-gamma release assays [IGRAs]) and (ii) the tolerability and effectiveness of 9-month isoniazid preventive therapy (IPT).

### Methods

Single-center retrospective cohort of consecutive HSCT recipients (Hospital Universitario Central de Asturias, Spain). Screening differed by period: 2014–2015 (TST plus ELISA-based IGRA; ELISpot-based IGRA in a subset) and 2019–2020 (both IGRAs). LTBI was defined as any positive test after exclusion of active TB clinically and by chest imaging. LTBI cases received isoniazid for 9 months with adverse-event monitoring. Overall survival (OS) was analyzed by Kaplan–Meier.

### Results

We included 225 patients (median age 57 years; 62.2% male; 79.1% autologous and 20.9% allogeneic HSCT). LTBI prevalence was 17.8% (40/225), with 20/93 (21.5%) in 2014–2015 and 20/132 (15.2%) in 2019–2020. In 2014–2015, positivity rates were 9.0% for TST (8/89), 14.3% for ELISA-IGRA (13/91; 2 indeterminate), and 8.6% for ELISpot-IGRA (5/58; 4 indeterminate). TST and ELISA-IGRA were discordant (p=0.81), and combining assays captured cases missed by a single-test approach. **Pre-transplant lymphocyte counts correlated with TST induration size (p=0.04),** supporting greater dependence of TST on baseline cellular immunity. In 2019–2020, ELISA-IGRA was positive in 14.4% (19/132; 1 indeterminate) and ELISpot-IGRA in 13.3% (17/128; 10 indeterminate). All 40 LTBI patients received IPT; treatment was discontinued for toxicity in 1 patient. With a median follow-up of 59 months (through Nov 2024), no patient developed active TB. Four-year OS was ~60% and did not differ between LTBI and non-LTBI patients (p=0.8; HR 1.06).

### Summary/Conclusion

LTBI screening tests are frequently discordant in HSCT, and TST performance appears more influenced by baseline immune status than IGRA-based approaches. A complementary multi-assay strategy maximizes LTBI detection. Nine-month isoniazid IPT was feasible and well tolerated, and was associated with zero TB reactivation during long-term follow-up, without adverse impact on survival.

**Copyright Information:** (Online) ISSN: 2572-9241
©2026 The Author(s). HemaSphere is published by John Wiley & Sons Ltd on behalf of European Hematology Association. This is an open access Abstract Book distributed under the Attribution-NonCommercial-NoDerivs (CC BY-NC-ND), which allows third parties to download the articles and share them with others as long as they credit the author and the Abstract Book, but they cannot change the content in any way or use them commercially.
**Abstract Book Citations:** Authors, Title, HemaSphere, 2026;10:(S1):pages. The abstract book DOIs can be found on the "Author Information" page.
**Disclaimer:** Articles published in the journal HemaSphere exclusively reflect the opinions of the authors. The authors are responsible for all content in their abstracts including accuracy of the facts, statements, citing resources, etc.

### (PF1162) HIGH EFFICACY AND MANAGEABLE SAFETY OF MARIBAVIR AS FIRST LINE TREATMENT FOR CMV INFECTION IN ALLO-HSCT RECIPIENTS: A MULTICENTER, RETROSPECTIVE, CONTROLLED COHORT STUDY

**Topic:** 23. Stem cell transplantation - Clinical

Jiaxin Cao*[1], Lu Wang[2], Xueyao Li[1], Shunqing Wang[3], Liping Dou[4], Erlie Jiang[1], Aiming Pang[1]

*[1]Institute of Hematology & Blood Diseases Hospital, Chinese Academy of Medical Sciences & Peking Union Medical College, Tianjin, China; [2]The Fifth Medical Center of Chinese PLA General Hospital, Beijin, China;[3]Guangzhou First People's Hospital, guangzhou, China; [4]the First Medical Center of Chinese PLA General Hospital, Beijin, China;*

### Background

Cytomegalovirus (CMV) infection remains one of the most frequent and potentially life-threatening complications following allogeneic haematopoietic stem cell transplantation (allo-HSCT). Maribavir, an oral inhibitor targeting the CMV pUL97 kinase, offers a therapeutic alternative with a differentiated safety profile and activity against certain resistant viruses. Pivotal trials have established its role in refractory/resistant CMV disease. However, its real-world effectiveness across the spectrum of allo-HSCT patients, particularly as a first-line option, requires further investigation.

### Aims

To evaluate the efficacy and safety of maribavir as first-line therapy for cytomegalovirus (CMV) infection in patients following hematopoietic stem cell transplantation (HSCT).

### Methods

This  multicentre, retrospective, controlled cohort study included patients who received first-line maribavir (400 mg twice daily) for post-HSCT CMV infection at the Institute of Hematology & Blood Diseases Hospital, Chinese Academy of Medical Sciences, the Chinese PLA General Hospital and Guangzhou First People's Hospital (GFPH) between June 1, 2024, and July 31, 2025.To provide a comparative context, a control group was identified at IHCAMS, consisting of patients who received other first-line antiviral therapies (predominantly ganciclovir-based or foscarnet-based regimens) for CMV infection after allo-HSCT between Jan 1, 2023, and July 31, 2024. The primary endpoint was CMV clearance rate. Confirmed viremia clearance was defined as plasma CMV DNA levels below the lower limit of quantification in two consecutive post-baseline samples collected ≥5 days apart.

### Results

This study included 22 allo-HSCT recipients receiving first-line maribavir and 66 historical controls receiving non-maribavir antivirals. The maribavir group had higher baseline rates of neutropenia and renal impairment due to clinical selection. Viral clearance rates were similar (95.45% vs. 96.97%, P = 0.735). Median time to clearance was shorter with maribavir (7 vs. 10.5 days, P = 0.394). By intention-to-treat analysis, 14-day clearance showed a strong trend favoring maribavir (81.82% vs. 60.61%, P = 0.069). In the per-protocol analysis, which excluded patients who died within the 14-day observation period, this difference became statistically significant (85.71% vs. 61.54%, P = 0.040). Multivariate analysis confirmed first-line maribavir independently reduced risk of delayed clearance (OR 0.09, P = 0.010), while high peak viral load predicted delayed clearance. Six-month recurrence rates were comparable (15.31% vs. 11.3%, P = 0.590). Adjusted safety analyses showed higher crude adverse event rates in the maribavir group were attributable to baseline risk, not treatment effect.

**Copyright Information:** (Online) ISSN: 2572-9241
©2026 The Author(s). HemaSphere is published by John Wiley & Sons Ltd on behalf of European Hematology Association. This is an open access Abstract Book distributed under the Attribution-NonCommercial-NoDerivs (CC BY-NC-ND), which allows third parties to download the articles and share them with others as long as they credit the author and the Abstract Book, but they cannot change the content in any way or use them commercially.
**Abstract Book Citations:** Authors, Title, HemaSphere, 2026;10:(S1):pages. The abstract book DOIs can be found on the "Author Information" page.
**Disclaimer:** Articles published in the journal HemaSphere exclusively reflect the opinions of the authors. The authors are responsible for all content in their abstracts including accuracy of the facts, statements, citing resources, etc.

**Summary/Conclusion**

First-line maribavir achieves faster viral clearance than conventional antivirals in allo-HSCT recipients, with a safety profile driven by baseline patient risk rather than treatment-emergent toxicity. These findings support maribavir as an effective first-line option, particularly for patients with baseline comorbidities limiting conventional therapy.

**Copyright Information:** (Online) ISSN: 2572-9241
©2026 The Author(s). HemaSphere is published by John Wiley & Sons Ltd on behalf of European Hematology Association. This is an open access Abstract Book distributed under the Attribution-NonCommercial-NoDerivs (CC BY-NC-ND), which allows third parties to download the articles and share them with others as long as they credit the author and the Abstract Book, but they cannot change the content in any way or use them commercially.
**Abstract Book Citations:** Authors, Title, HemaSphere, 2026;10:(S1):pages. The abstract book DOIs can be found on the "Author Information" page.
**Disclaimer:** Articles published in the journal HemaSphere exclusively reflect the opinions of the authors. The authors are responsible for all content in their abstracts including accuracy of the facts, statements, citing resources, etc.

### (PF1163) SECONDARY MALIGNANCIES FOLLOWING ALLOGENEIC HEMATOPOIETIC STEM CELL TRANSPLANTATION IN FANCONI ANEMIA: A SINGLE-CENTER EXPERIENCE

**Topic:** 23. Stem cell transplantation - Clinical

Hanane Bouarab[1], Farih Mehdid[1], Baazizi Mounira[1], Nadia Rahmoune[1], Ait Ouali Dina[1], Sarah Zerkout[1], Fouzia Louar[1], Belkis Dekhili[1], Amira Fahem[1], Hanane Moussaoui[1], Sabrina Akhrouf[1], Farida Harieche[1], Rose Marie Hamladji[1], Redhouane Ahmed Nacer[1], Malek Benakli*[1]

[1]*Hematology and Bone Marrow Transplantation department, Pierre and Marie Curie Center, Algiers, Algeria;*

### Background

Fanconi anemia (FA) is the most common constitutional bone marrow failure syndrome, characterized by hematologic and extra-hematologic abnormalities and a high risk of secondary malignancies, including myelodysplastic syndrome, acute leukemia, and squamous cell carcinoma (SCC). While allogeneic hematopoietic stem cell transplantation (HSCT) remains the only curative treatment for hematologic manifestations, patients remain at high risk for secondary cancers post-transplant.

### Methods

From January 1998 to June 2024, 111 patients (pts) with FA underwent HSCT with sibling donors at our center. Secondary malignancies occurred in 6 pts (5.4%) post-transplant. The mean age was 8 years (3–13), with a male-to-female ratio of 2:1. Status at transplant included severe marrow aplasia (n=2) and moderate aplasia (n=4). The median interval from diagnosis to transplant was 24 months (4–46). Conditioning regimens consisted of chemotherapy-alone: Fludarabine, Cyclophosphamide, and reduced-dose ATG (n=4) or Busulfan and Cyclophosphamide (n=2). All pts received peripheral blood stem cells (PBSC) with a mean CD34+ cell dose of 8.4 x $10^6$/kg (4.54–16.7). GVHD prophylaxis included Methotrexate and Cyclosporine. As of June 30, 2025, the follow-up period ranged from 12 months to 27 years.

### Results

The mean duration of aplasia was 19 days (12–38). All pts required transfusions, with a mean of 3.5 packed red blood cell units/patient and 3 platelet units/patient. Acute GVHD was observed in 2 pts (33.3%), while chronic GVHD occurred in all 6 pts (100%), including 2 with extensive involvement. Secondary malignancies developed after a median post-transplant delay of 97 months (2–288). Four pts developed squamous cell carcinoma (esophagus: 2, oral cavity: 1, liver: 1), one patient presented with T/NK-cell lymphoma of the nasopharynx, and one patient developed acute myeloid leukemia. Despite treatment, 5 pts died within a mean of 2.5 months (1–6). One patient remains alive in complete remission with donor chimerism at 36 months of follow-up.

### Summary/Conclusion

Secondary malignancy is a major complication of FA and the leading cause of late mortality in transplanted pts. Myeloablative conditioning, chronic GVHD, prolonged immunosuppression, and UV exposure further increase this risk. These findings underscore the vital importance of long-term, specialized, and multidisciplinary surveillance for early detection of these life-threatening complications.

**Copyright Information:** (Online) ISSN: 2572-9241
©2026 The Author(s). HemaSphere is published by John Wiley & Sons Ltd on behalf of European Hematology Association. This is an open access Abstract Book distributed under the Attribution-NonCommercial-NoDerivs (CC BY-NC-ND), which allows third parties to download the articles and share them with others as long as they credit the author and the Abstract Book, but they cannot change the content in any way or use them commercially.
**Abstract Book Citations:** Authors, Title, HemaSphere, 2026;10:(S1):pages. The abstract book DOIs can be found on the "Author Information" page.
**Disclaimer:** Articles published in the journal HemaSphere exclusively reflect the opinions of the authors. The authors are responsible for all content in their abstracts including accuracy of the facts, statements, citing resources, etc.

### (PF1164) EVALUATION OF THE EAM CONDITIONING REGIMEN IN AUTOLOGOUS HEMATOPOIETIC STEM CELL TRANSPLANTATION FOR HODGKIN LYMPHOMA

**Topic:** 23. Stem cell transplantation - Clinical

Amal Bendimerad*[1], Soraya Benichou[1], Nawel Bounoua[1], Badra Enta Soltan[1], Nabil Yafour[1]

[1]*EHU 1er Novembre, Oran, Algeria;*

### Background

The combination of carmustine, etoposide, cytarabine, and melphalan (BEAM) is considered the standard conditioning regimen prior to autologous hematopoietic stem cell transplantation (ASCT) in patients with Hodgkin lymphoma (HL). However, the use of carmustine is associated with pulmonary toxicity, substantial cost, and periodic supply shortages. These limitations have prompted the development of carmustine-free conditioning strategies, most notably the EAM regimen, which substitutes carmustine and incorporates higher dose of cytarabine (total dose 8 g/m²).

### Aims

The objective of this study was to assess the efficacy and safety of the intensified EAM conditioning regimen in patients with HL undergoing ASCT at the Hematology Department in November 1st 1954 University Hospital, Oran, Algeria

### Methods

This single-center retrospective study was conducted over a 14-year period and included 120 patients with HL who received the EAM regimen as conditioning intensification prior ASCT.
CD34⁺ hematopoietic stem cells were mobilized using granulocyte colony-stimulating factor (G-CSF) alone (filgrastim or lenograstim) administered at 15 µg/kg/day for five consecutive days, or in combination with plerixafor in cases of mobilization failure. Collected HSCs were stored at +4°C.
Beginning in 2022, cryopreservation at −196°C was implemented on a case-by-case basis for grafts containing at least $3 \times 10^6$ CD34⁺ cells/kg. Cell viability was systematically assessed prior to reinfusion. The EAM conditioning regimen consisted of etoposide at a total dose of 800 mg/m², cytarabine at 8 g/m², and melphalan at 140 mg/m². Granulocyte colony-stimulating factor (G-CSF) was initiated on day +5 post conditioning and continued until hematologic recovery from aplasia. Date of assessment was 28/02/2026.

### Results

A total of 120 pts with HL were included between March 2011 and November 30th 2025. The median age was 28 years (range, 13–58 years), with a male-to-female ratio of 0.87 (56 males and 64 females).
The median time to absolute neutrophil count > 0.5 G/L was 14 days (range, 5–31 days), and to platelet recovery (> 20 G/L) was 13 days (range, 5–42 days). The median number of red blood cell concentrates transfused was 3 (range, 0–9), and the median number of platelet concentrates transfused was 3 (range, 0–9). At day 100 post-transplant, the TRM was 2,5% (3 pts). The relapse rate was 21,6% (26 pts). The overall survival at 5 years was 82,9% and the PFS was 78,8%.

### Summary/Conclusion

ASCT conditioned with EAM regimen appears to be a safe and effective therapeutic option for RRHL patients. The procedure was associated with low TRM, prompt hematologic recovery, favorable remission rates, and sustained long-term survival outcomes.

**Copyright Information:** (Online) ISSN: 2572-9241
©2026 The Author(s). HemaSphere is published by John Wiley & Sons Ltd on behalf of European Hematology Association. This is an open access Abstract Book distributed under the Attribution-NonCommercial-NoDerivs (CC BY-NC-ND), which allows third parties to download the articles and share them with others as long as they credit the author and the Abstract Book, but they cannot change the content in any way or use them commercially.
**Abstract Book Citations:** Authors, Title, HemaSphere, 2026;10:(S1):pages. The abstract book DOIs can be found on the "Author Information" page.
**Disclaimer:** Articles published in the journal HemaSphere exclusively reflect the opinions of the authors. The authors are responsible for all content in their abstracts including accuracy of the facts, statements, citing resources, etc.

### (PF1165) KANGFUXIN COMBINED WITH G-CSF ICE-CUBE CRYOTHERAPY EFFECTIVELY PREVENTS THIOTEPA-BASED CONDITIONING–INDUCED ORAL MUCOSITIS IN ALLOGENEIC HSCT: A PROSPECTIVE OPEN-LABEL COHORT STUDY

**Topic:** 23. Stem cell transplantation - Clinical

Li Wang*[1], Yimei Feng[1], Xiaoli Chen[1], Peiyan Kong[1], Aoxue Tan[1], Jia Liu[1], Xiaoqi Wang[1], Xi Zhang[1]

[1]*Xinqiao Hospital, Army Medical University, Chongqing, China;*

### Background

Thiotepa-containing conditioning regimens are widely used in allogeneic hematopoietic stem cell transplantation (allo-HSCT), particularly for hematologic malignancies with central nervous system involvement. However, oral mucositis (OM) remains a frequent and debilitating complication, negatively affecting treatment tolerance and quality of life. Currently available preventive strategies show limited efficacy.

### Aims

This prospective study (ChiCTR2300077686) aimed to evaluate the effectiveness and safety of Kangfuxin liquid combined with granulocyte colony-stimulating factor (G-CSF) ice-cube cryotherapy in preventing thiotepa-induced OM in allo-HSCT patients.

### Methods

A total of 89 patients undergoing allo-HSCT at Xinqiao Hospital were enrolled and assigned to either an experimental group (n=34) or a control group (n=55). The control group received conventional ice saltwater cryotherapy. The experimental group received cryotherapy using ice cubes prepared from 100 mL normal saline containing G-CSF (150 μg) and Kangfuxin liquid (100 mL). Cryotherapy was initiated 30 minutes before conditioning chemotherapy and continued until 30 minutes after completion. Primary endpoints included incidence and severity of OM (grade 3–4), pain intensity, duration of mucositis, and adverse events. Secondary endpoints included time to neutrophil and platelet engraftment.

### Results

The incidence of OM was significantly lower in the experimental group compared with the control group (67.6% vs 87.3%,$p$=0.03). Severe OM (grade 3–4) occurred in 14.7% of patients in the experimental group versus 58.2% in the control group ($p$<0.001). Mean oral pain scores were significantly reduced in the experimental group (2.18 ± 2.12 vs 4.51 ± 2.76, $p$<0.001).
The median duration of OM was markedly shorter in the experimental group (5.35 vs 11.91 days,$p$<0.001). No significant differences were observed between groups in time to neutrophil engraftment (15.74 ± 4.94 vs 15.88 ± 4.41 days, $p$=0.89) or platelet engraftment (14.36 ± 2.28 vs 14.59 ± 3.14 days, $p$=0.72). No cryotherapy-related adverse events were reported in either group.

### Summary/Conclusion

Kangfuxin combined with G-CSF ice-cube cryotherapy is a safe and effective strategy for preventing thiotepa-based conditioning–induced oral mucositis in allo-HSCT patients. This approach significantly reduces the incidence and severity of OM, alleviates pain, shortens mucositis duration, and improves patient quality of life, without impairing hematopoietic recovery. It represents a promising and easily applicable preventive intervention for clinical practice.

**Copyright Information:** (Online) ISSN: 2572-9241
©2026 The Author(s). HemaSphere is published by John Wiley & Sons Ltd on behalf of European Hematology Association. This is an open access Abstract Book distributed under the Attribution-NonCommercial-NoDerivs (CC BY-NC-ND), which allows third parties to download the articles and share them with others as long as they credit the author and the Abstract Book, but they cannot change the content in any way or use them commercially.
**Abstract Book Citations:** Authors, Title, HemaSphere, 2026;10:(S1):pages. The abstract book DOIs can be found on the "Author Information" page.
**Disclaimer:** Articles published in the journal HemaSphere exclusively reflect the opinions of the authors. The authors are responsible for all content in their abstracts including accuracy of the facts, statements, citing resources, etc.

### (PF1166) STUDY ON THE EFFECTS OF ENHANCED IMMUNE-NUTRIENT INTERVENTION IN PATIENTS UNDERGOING HEMATOPOIETIC STEM CELL TRANSPLANTATION

**Topic:** 23. Stem cell transplantation - Clinical

Xueqian Yan*[1], Zhuo Wan[1], Dan Fan[1], Yueru Ji[1], Qian Zhou[1], Jing Yan[1], Li Liu[1], Weiwei Qin[1]

[1] *Tangdu Hospital,Fourth Military Medical University, Hematology, Xi'an, China;*

### Background

Hematopoietic stem cell transplantation (HSCT) plays an important role in clinical practice. Malnutrition is common and severe complication, significantly affecting prognosis of HSCT patients.

### Aims

To explore the efficacy of enhanced immune nutrients (arginine, nucleotides, Omega-3 polyunsaturated fatty acids) in improving nutritional status, hematopoietic reconstitution and reducing transplant-related complications in HSCT patients, and to provide an optimized clinical nutritional support strategy for the peri-transplant period.

### Methods

A prospective single-arm study(ChiCTR2500106405) with 1:1 historical control was conducted. A total of 107 hematological patients undergoing HSCT (61 autologous, 46 allogeneic) from Jan-Dec 2025 were enrolled as the immunonutrition group;107 historical controls (2023–2024) with conventional nutritional intervention were matched by gender, age and transplantation type.After admission to the laminar ward, the immunonutrition group received weekly PG-SGA scoring, daily food intake monitoring,and intensive immune nutrient supplementation for energy gaps until discharge; the control group received routine nutritional support. Malnutrition indicators, hematopoietic reconstitution parameters, hospital stay and transplant-related complications were recorded.Subgroup analysis was performed on autologous transplant patients, and multivariate Logistic regression evaluated the independent effect of immunonutrition intervention on severe oral mucositis and severe malnutrition.

### Results

①Overall outcomes (Table 1):The immunonutrition group had significantly lower PG-SGA scores than the control group ( P<0.05), with notably reduced weight loss (P<0.05). Neutrophil engraftment time was significantly shorter in the immunonutrition group (P<0.05), and auto-HSCT patients in immunonutrition group had a significantly shorter hospital stay (P=0.004). The incidence of grade 3-4 oral mucositis was significantly lower in both auto-HSCT (P=0.019) and allo-HSCT (P=0.008) subgroups of the immunonutrition group. No significant differences were observed in platelet engraftment time, aGVHD incidence, or the severity of nausea, vomiting and diarrhea between the two groups(all P>0.05).②Subgroup analysis (Table 2):The multiple myeloma immunonutrition group had more comprehensive benefits, PG-SGA score (P=0.005), neutrophil implantation time (P=0.002), incidence of infection (P=0.005) were significantly better than those of the control group, and the degree of vomiting was significantly reduced (P=0.029).③ Multivariate analysis (Table 3): After adjusting for 9 confounding factors (age, gender, transplantation type, etc.), enhanced immunonutrition intervention was identified as an independent protective factor for grade 3–4 oral mucositis (OR=0.308, 95% CI: 0.157–0.606, P=0.001) and severe malnutrition (PG-SGA>9) (OR=0.333, 95% CI: 0.171–0.646, P=0.001) in HSCT patients.

**Copyright Information:** (Online) ISSN: 2572-9241
©2026 The Author(s). HemaSphere is published by John Wiley & Sons Ltd on behalf of European Hematology Association. This is an open access Abstract Book distributed under the Attribution-NonCommercial-NoDerivs (CC BY-NC-ND), which allows third parties to download the articles and share them with others as long as they credit the author and the Abstract Book, but they cannot change the content in any way or use them commercially.
**Abstract Book Citations:** Authors, Title, HemaSphere, 2026;10:(S1):pages. The abstract book DOIs can be found on the "Author Information" page.
**Disclaimer:** Articles published in the journal HemaSphere exclusively reflect the opinions of the authors. The authors are responsible for all content in their abstracts including accuracy of the facts, statements, citing resources, etc.

### Summary/Conclusion

Enhanced immunonutrient intervention significantly improves nutritional status, reduces weight loss, and promotes neutrophil hematopoietic reconstitution in HSCT patients. It lowers the risk of severe oral mucositis and shortens hospital stay in allogeneic transplant recipients. These beneficial effects are independent of clinical confounders and more prominent in multiple myeloma patients undergoing autologous transplantation. Further studies are warranted to validate these results. This regimen provides a clinical basis for precise nutritional support during the peri-transplant period of HSCT.

**Table 1 The effect of immune nutrient intervention in HSCT patients (n=214)**

| Outcome indicators | Autologous transplantation | | $t/\chi^2$ | P | Allogeneic transplantation | | $t/\chi^2$ | P |
|---|---|---|---|---|---|---|---|---|
| | Immune nutrition group (n=61) | Conventional nutrition group (n=61) | | | Immune nutrition group (n=46) | Conventional nutrition group (n=46) | | |
| PG-SGA(score) | 10.77±3.05 | 12.90±3.77 | -3.433 | 0.001 | 11.39±3.54 | 14.02±4.01 | -3.369 | 0.001 |
| Weight loss (kg) | -3.48±2.00 | -4.53±2.67 | -2.453 | 0.016 | -5.31±2.59 | -6.70±3.23 | -2.265 | 0.026 |
| Time of neutrophil engraftment (days) | 9.84±2.26 | 10.74±1.82 | -2.429 | 0.017 | 13.37±2.48 | 15.13±3.56 | 2.751 | 0.007 |
| Time of platelet engraftment (days) | 13.13±6.03 | 13.69±5.73 | -0.523 | 0.602 | 15.89±4.57 | 17.15±4.37 | 1.353 | 0.180 |
| Length of hospital stay (days) | 25.64±7.03 | 25.75±7.80 | 0.090 | 0.929 | 33.78±6.56 | 43.93±22.16 | 2.980 | 0.004 |
| aGVHD (cases) | --- | | | | 8 (17.39%) | 9 (19.57%) | 0.072 | 0.788 |
| Infection | 9 (14.75) | 18 (29.51%) | 3.853 | 0.050 | 15 (32.61%) | 17 (36.96%) | 0.192 | 0.662 |
| Oral mucositis (grade 3~4) | 6 (9.84%) | 16 (26.23%) | 5.545 | 0.019 | 9 (19.57%) | 21 (45.65%) | 7.123 | 0.008 |
| Nausea severity (score) | 1.89±0.76 | 1.75±0.91 | -0.868 | 0.387 | 1.89±0.57 | 1.93±0.71 | 0.324 | 0.747 |
| Vomiting severity (score) | 1.36±0.87 | 1.56±0.81 | -1.290 | 0.200 | 1.37±0.65 | 1.59±0.71 | 1.529 | 0.130 |
| Diarrhea severity (score) | 2.07±0.85 | 1.98±1.07 | -0.467 | 0.641 | 1.95±1.01 | 1.65±0.90 | 1.526 | 0.131 |

**Table 2 Subgroup analysis of the effect of immunenutrition intervention in patients with autologous HSCT(n=122)**

| Outcome indicators | Multiple myeloma | | $t/\chi^2$ | P | Lymphoma | | $t/\chi^2$ | P |
|---|---|---|---|---|---|---|---|---|
| | Immune nutrition group (n=31) | Conventional nutrition group (n=39) | | | Immune nutrition group (n=30) | Conventional nutrition group (n=22) | | |
| PG-SGA(score) | 11.00±2.72 | 13.26±3.71 | -2.938 | 0.005 | 10.36±3.59 | 12.53±3.86 | -2.062 | 0.044 |
| Weight loss (kg) | -3.30±1.79 | -4.39±2.77 | -1.989 | 0.051 | -3.66±2.21 | -4.76±2.50 | -1.672 | 0.101 |
| Time of neutrophil engraftment (days) | 9.44±1.47 | 10.74±1.95 | -3.201 | 0.002 | 10.55±3.14 | 10.73±1.70 | -0.277 | 0.783 |
| Time of platelet engraftment (days) | 12.15±5.09 | 12.29±4.83 | -0.114 | 0.910 | 14.86±7.21 | 15.13±6.30 | -0.143 | 0.886 |
| Length of hospital stay (days) | 22.58±5.32 | 23.74±6.38 | 0.814 | 0.418 | 28.80±7.26 | 29.32±7.03 | 0.258 | 0.798 |
| Infection | 2 (6.45%) | 15 (38.46%) | 7.962* | 0.005 | 7 (23.33%) | 3 (13.64%) | 0.271* | 0.603 |
| Oral mucositis (grade 3~4) | 3 (9.68%) | 7 (17.95%) | 0.965 | 0.326 | 3 (10.00%) | 9 (40.91%) | 6.831 | 0.009 |
| Nausea severity (score) | 1.84±0.86 | 1.59±0.88 | -1.187 | 0.239 | 1.93±0.64 | 2.05±0.90 | 0.526 | 0.601 |
| Vomiting severity (score) | 1.68±0.91 | 1.21±0.83 | -2.241 | 0.029 | 1.43±0.68 | 1.64±0.90 | 0.927 | 0.359 |
| Diarrhea severity (score) | 1.90±0.83 | 2.00±0.92 | -0.457 | 0.649 | 2.23±0.86 | 1.95±1.33 | -0.920 | 0.362 |

**Table 3 The effect of immune nutrients on severe oral mucositis and severe malnutrition in HSCT patients (n=214)**

| Dependent variable | Independent variable | B | SE | Wald $\chi^2$ | P | OR | 95%CI of OR |
|---|---|---|---|---|---|---|---|
| Oral mucositis (grade 3~4) | Conventional nutrition | | | | | 1 | |
| | Immunenutrition | -1.176 | 0.345 | 11.640 | 0.001 | 0.308 | 0.157~ 0.606 |
| PG-SGA＞9 | Conventional nutrition | | | | | 1 | |
| | Immunenutrition | -1.101 | 0.339 | 10.553 | 0.001 | 0.333 | 0.171 ~ 0.646 |

1.PG-SGA ≥9 scores indicate severe malnutrition;2.* represents the chi-square test with continuity correction.3.Adjusted for 9 variables including age, gender, drinking history, smoking history, chronic disease history, surgical history, clinical diagnosis, disease stage and transplantation type.

**Copyright Information:** (Online) ISSN: 2572-9241
©2026 The Author(s). HemaSphere is published by John Wiley & Sons Ltd on behalf of European Hematology Association. This is an open access Abstract Book distributed under the Attribution-NonCommercial-NoDerivs (CC BY-NC-ND), which allows third parties to download the articles and share them with others as long as they credit the author and the Abstract Book, but they cannot change the content in any way or use them commercially.
**Abstract Book Citations:** Authors, Title, HemaSphere, 2026;10:(S1):pages. The abstract book DOIs can be found on the "Author Information" page.
**Disclaimer:** Articles published in the journal HemaSphere exclusively reflect the opinions of the authors. The authors are responsible for all content in their abstracts including accuracy of the facts, statements, citing resources, etc.

### (PF1167) IMMUNE RECONSTITUTION KINETICS AND CLINICAL OUTCOMES AFTER ALLOGENEIC HSCT FOR CHRONIC MYELOMONOCYTIC LEUKEMIA: A MATCHED VERSUS HAPLOIDENTICAL DONOR COMPARISON

**Topic:** 23. Stem cell transplantation - Clinical

Mengtong Zang*[1, 2, 3, 4], Jianying Zhou[1, 2, 3, 4], Mengyu Xiao[1, 2, 3, 4], Lulu Wang[1, 2, 3, 4], Qiuyu Guo[1, 2, 3, 4], Haixia Fu[1, 2, 3, 4], Yuan-Yuan Zhang[1, 2, 3, 4], Xiao-Dong Mo[1, 2, 3, 4], Fengrong Wang[1, 2, 3, 4], Yuqian Sun[1, 2, 3, 4], Meng Lv[1, 2, 3, 4], Xiangyu Zhao[1, 2, 3, 4], Yu-Hong Chen[1, 2, 3, 4], Yu Wang[1, 2, 3, 4], Ying-Jun Chang[1, 2, 3, 4], Lan-Ping Xu[1, 2, 3, 4], Xiaojun Huang[1, 2, 3, 4], Xiaohui Zhang[1, 2, 3, 4]

*[1]Peking University People's Hospital, Peking University Institute of Hematology, Beijing, China, Beijing, China; [2]National Clinical Research Center for Hematologic Disease, Beijing, China, Beijing, China; [3]Beijing Key Laboratory of Cell and Gene Therapy for Hematologic Malignancies, Peking University, Beijing, China, Beijing, China; [4]Collaborative Innovation Center of Hematology,Peking University Institute of Hematology, Beijing, China, Beijing, China;*

### Background

Chronic myelomonocytic leukemia (CMML) is a poor-prognosis malignancy for which allogeneic hematopoietic stem cell transplantation (allo-HSCT) is the only potentially curative option. However, the impact of immune reconstitution (IR) on post-transplant outcomes in CMML remains poorly defined.

### Aims

This study aimed to characterize the dynamic kinetics of IR following HSCT in patients with CMML, and evaluated its impact on posttransplant clinical outcomes.

### Methods

In this longitudinal cohort study of 122 adult CMML patients who underwent allo-HSCT between 2009 and 2025, we quantitatively analyzed peripheral blood lymphocytes by flow cytometry, and simultaneously measured serum immunoglobulin levels on days 30, 60, 90, and 180 post-transplantation. IR dynamics were compared between patients receiving matched sibling donor(MSD) HSCT and haploidentical donor (HID) HSCT. Univariate Cox, logistic and competing risk regression were conducted to evaluate the association between IR on day +90 post-transplantation and clinical outcomes. All statistical tests were two-sided, with $P < 0.05$ considered statistically significant. Statistical analyses were conducted using R software (version 4.4.3).

**Copyright Information:** (Online) ISSN: 2572-9241
©2026 The Author(s). HemaSphere is published by John Wiley & Sons Ltd on behalf of European Hematology Association. This is an open access Abstract Book distributed under the Attribution-NonCommercial-NoDerivs (CC BY-NC-ND), which allows third parties to download the articles and share them with others as long as they credit the author and the Abstract Book, but they cannot change the content in any way or use them commercially.
**Abstract Book Citations:** Authors, Title, HemaSphere, 2026;10:(S1):pages. The abstract book DOIs can be found on the "Author Information" page.
**Disclaimer:** Articles published in the journal HemaSphere exclusively reflect the opinions of the authors. The authors are responsible for all content in their abstracts including accuracy of the facts, statements, citing resources, etc.

**Results**

Among the 122 CMML patients who underwent HSCT, 79 (64.75%) were male. A total of 43 patients received MSD-HSCT (including 34 matched related donor [MRD] and 9 matched unrelated donor [MUD] HSCT), while 79 patients underwent HID-HSCT. IR was compared between the two groups at days 30, 60, 90, and 180 post-transplantation. At day +30, MSD group exhibited significantly higher levels of CD3+, CD4+, CD3+CD4+CD28+, CD4+CD45RA+, CD4+CD45RO+, CD4+CD25+CD45RA+, CD4+CD25+CD45RO+, IgA, and IgM compared to HID group. These differences persisted for CD4+CD45RA+, CD4+CD25+CD45RA+, and IgM at days +60 and +90. By day +180, only CD4+CD45RA+ remained significantly elevated in the MSD group. Overall, most lymphocyte subsets exhibited an upward trajectory over time, whereas IgG and IgA did not.
Univariate logistic regression of day+90 IR showed no associations with 2-year overall survival (OS), relapse, progression-free survival (PFS), EBV reactivation, bacterial infection, or chronic graft-versus-host disease (cGVHD). Notably, at day +90, higher CD4+ and CD3+CD4+CD28+ counts were associated with reduced risks of CMV reactivation, fungal infection, and acute GVHD (aGVHD). CMV reactivation risk was also lower with elevated CD4+CD45RA+ and IgA. Fungal infection risk was inversely associated with CD3+ and CD4+CD45RO+ counts, while aGVHD protection was linked to higher CD4+CD45RO+ and all three immunoglobulin isotypes (IgG, IgA, IgM). CD4+CD45RA+ expansion was associated with improved PFS, consistently observed in subgroup analyses. Patients receiving HID-HSCT had a significantly higher risk of CMV infection. Furthermore, older patient age combined with failure to reconstitute CD4+CD45RA+ or higher IgG levels was associated with inferior OS. Among recipients of bone marrow plus peripheral blood grafts, those achieving CD4+CD45RA+ recovery demonstrated the most favorable PFS outcomes.

**Summary/Conclusion**

These findings demonstrate that IR impacts infection and GVHD post-transplant. While MSD recipients showed faster immune recovery and lower CMV reactivation, comparable outcomes across other endpoints support HID-HSCT as a safe and effective alternative for CMML. Prospective randomized trials are therefore warranted to validate these observations.

**Copyright Information:** (Online) ISSN: 2572-9241
©2026 The Author(s). HemaSphere is published by John Wiley & Sons Ltd on behalf of European Hematology Association. This is an open access Abstract Book distributed under the Attribution-NonCommercial-NoDerivs (CC BY-NC-ND), which allows third parties to download the articles and share them with others as long as they credit the author and the Abstract Book, but they cannot change the content in any way or use them commercially.
**Abstract Book Citations:** Authors, Title, HemaSphere, 2026;10:(S1):pages. The abstract book DOIs can be found on the “Author Information” page.
**Disclaimer:** Articles published in the journal HemaSphere exclusively reflect the opinions of the authors. The authors are responsible for all content in their abstracts including accuracy of the facts, statements, citing resources, etc.

### (PF1168) UMBILICAL CORD BLOOD MONONUCLEAR CELL INFUSION FOR SECONDARY POOR GRAFT FUNCTION AFTER ALLOGENEIC HEMATOPOIETIC STEM CELL TRANSPLANTATION: A PHASE I DOSE-ESCALATION STUDY

**Topic:** 23. Stem cell transplantation - Clinical

Yigeng Cao*[1], Rongli Zhang[1], Donglin Yang[1], Yi He[1], Aiming Pang[1], Erlie Jiang[1]

*[1]Institute of Hematology & Blood Diseases Hospital, National Clinical Research Center for Blood Diseases, Chinese Academy of Medical Sciences & Peking Union Medical College, Hematopoietic Stem Cell Transplantation Center, Tianjin, China;*

### Background

Secondary poor graft function (sPGF) is a life-threatening complication following allogeneic hematopoietic stem cell transplantation (allo-HSCT), characterized by persistent cytopenias despite complete donor chimerism. Currently, no standardized treatment is available for sPGF.

Umbilical cord blood mononuclear cells (UCB-MNCs) are heterogeneous, comprising hematopoietic stem cells, immune regulatory cells, and other functional subsets. These cells have been shown to secrete hematopoietic growth factors, promote tissue repair, and modulate the immune microenvironment, suggesting potential for restoring sPGF hematopoiesis.

### Aims

Conduct a prospective clinical study to evaluate the safety and optimal dosage of UCB-MNCs in the treatment of PGF.

### Methods

We conducted an open-label, phase 1 clinical trial (ClinicalTrials.gov, NCT06792682) using a standard 3+3 dose-escalation design to assess the safety and preliminary efficacy.

Eligible patients were ≥18 years of age, met the diagnostic criteria for sPGF and had an Eastern Cooperative Oncology Group (ECOG) performance status ≤2.

In the dose-escalation phase, patients were sequentially enrolled into three cohorts receiving UCB-MNCs at doses of $2.0\times10^6$, $3.5\times10^6$, and $5.0\times10^6$ cells/kg per infusion. All patients received four consecutive weekly intravenous infusions with standard supportive care. Dose-expansion phase will enroll an additional six patients at the recommended dose level, using the same administration schedule.

The primary endpoint was safety, graded according to the Common Terminology Criteria for Adverse Events (CTCAE) version 5.0. Secondary exploratory efficacy endpoints included time to neutrophil and platelet recovery.

Copyright Information: (Online) ISSN: 2572-9241
©2026 The Author(s). HemaSphere is published by John Wiley & Sons Ltd on behalf of European Hematology Association. This is an open access Abstract Book distributed under the Attribution-NonCommercial-NoDerivs (CC BY-NC-ND), which allows third parties to download the articles and share them with others as long as they credit the author and the Abstract Book, but they cannot change the content in any way or use them commercially.
**Abstract Book Citations:** Authors, Title, HemaSphere, 2026;10:(S1):pages. The abstract book DOIs can be found on the "Author Information" page.
**Disclaimer:** Articles published in the journal HemaSphere exclusively reflect the opinions of the authors. The authors are responsible for all content in their abstracts including accuracy of the facts, statements, citing resources, etc.

### Results

As of February 10, 2026, six patients (three each in the $2.0\times10^6$ cells/kg and $3.5\times10^6$ cells/kg cohorts) had completed the four-week UCB-MNCs infusion protocol and a minimum follow-up of 12 weeks.
During treatment and follow-up, two patients (33.3%) experienced adverse events, including one case of grade 2 cytomegalovirus (CMV), one case of grade 1 pulmonary infection, and one case of grade 2 chronic graft-versus-host disease (cGVHD). All adverse events were managed successfully with standard clinical interventions and resolved without long-term sequelae. None of the reported AEs were considered related to UCB-MNCs infusion.
No dose-limiting toxicities (DLTs) were observed in either cohort.No acute infusion-related adverse events occurred during or within 24 hours after UCB-MNCs administration. All patients remained alive at the time of last follow-up.
At the 6-month follow-up, the best post-treatment platelet count in the low-dose cohort was 78 (67–89) $\times10^9$/L (baseline: 19 [13-22] $\times10^9$/L) and the corresponding neutrophil count was 1.09 (0.59–2) $\times10^9$/L (baseline: 0.73 [0.73-1.03] $\times10^9$/L). No statistical data were available for the high-dose cohort as the follow-up time point was not reached.

### Summary/Conclusion

This phase 1 dose-escalation study indicate that repeated intravenous infusion of UCB-MNCs at doses of $2.0\times10^6$ and $3.5\times10^6$ cells/kg is well tolerated in sPGF patients. Ongoing dose-escalation and expansion phases will determine the recommended dose, further characterize long-term safety, and evaluate the hematopoietic efficacy of UCB-MNCs in this high-risk population.

**Copyright Information:** (Online) ISSN: 2572-9241
©2026 The Author(s). HemaSphere is published by John Wiley & Sons Ltd on behalf of European Hematology Association. This is an open access Abstract Book distributed under the Attribution-NonCommercial-NoDerivs (CC BY-NC-ND), which allows third parties to download the articles and share them with others as long as they credit the author and the Abstract Book, but they cannot change the content in any way or use them commercially.
**Abstract Book Citations:** Authors, Title, HemaSphere, 2026;10:(S1):pages. The abstract book DOIs can be found on the “Author Information” page.
**Disclaimer:** Articles published in the journal HemaSphere exclusively reflect the opinions of the authors. The authors are responsible for all content in their abstracts including accuracy of the facts, statements, citing resources, etc.

### (PF1169) COMPARISON OF TOTAL BODY IRRADIATION (TBI) AND NON-TBI CONDITIONING REGIMENS FOR ALLOGENEIC HEMATOPOIETIC STEM CELL TRANSPLANTATION IN PATIENTS WITH PRIMARY MYELOFIBROSIS

**Topic:** 23. Stem cell transplantation - Clinical

Yilin Liu[1], Jiali Wang[2], Aiming Pang[2], Yi He[2], Sizhou Feng[2], Mingzhe Han*[2], Erlie Jiang[2], Donglin Yang[2]

*[1]Institute of Hematology & Blood Diseases Hospital, Chinese Academy of Medical Science, Stem Cell Transplantation Center, Tianjin, China; [2]Institute of Hematology & Blood Diseases Hospital, Chinese Academy of Medical Science, Tianjin, China;*

### Background

Primary myelofibrosis (PMF) is associated with a dismal prognosis, and allogeneic hematopoietic stem cell transplantation (allo-HSCT) remains the sole potentially curative treatment. However, the optimal conditioning regimen remains controversial.

### Aims

This study aims to compare the differences in overall survival (OS), progression-free survival (PFS), and transplant-related mortality (NRM) between TBI-based and non-TBI regimens in patients with PMF, thereby providing evidence-based guidance for clinical practice.

### Methods

This retrospective study included 33 patients diagnosed with PMF who underwent allo-HSCT at our Stem Cell Transplantation Center between May 2012 and July 2025.

### Results

In this cohort, fourteen patients received a total body irradiation (TBI)-based regimen (TFMC, comprising TBI, fludarabine [FlU], melphalan [Mel] and cyclophosphamide [Cy]), whereas nineteen patients were administered a non-TBI regimen. Both groups shared an identical median follow-up duration of 23 months.
The TBI cohort exhibited a median age of 48.5 years (range, 25–60 years), compared to 42 years (range, 21–57 years) in the non-TBI cohort. Regarding donor characteristics, the median donor age was 25 years (range, 11–53 years) for the TBI group and 30 years (range, 11–58 years) for the non-TBI group. In terms of splenomegaly, 85.7% (12/14) of patients in the TBI cohort presented with splenomegaly prior to transplantation, with 28.6% (4/14) classified as having severe splenomegaly. In contrast, 79% (15/19) of the non-TBI cohort exhibited splenomegaly, with 21.1% (4/19) experiencing severe enlargement.
Hematopoietic Reconstitution: Neutrophil (ANC) engraftment within 28 days post-transplant reached 100% in both groups. However, the TBI group demonstrated a significantly faster neutrophil recovery ($p = 0.047$), with a median engraftment time of 12 days (range, 11–20 days) versus 16 days (range, 12–25 days) in the non-TBI group. Platelet (PLT) engraftment at day 28 was 76.9% in the TBI cohort and 73.7% in the non-TBI cohort, a difference that did not achieve statistical significance ($p = 0.56$).
Outcomes: Long-term survival analysis revealed a 2-year overall survival (OS) rate of 85.7% in the TBI cohort, compared to 68.4% in the non-TBI cohort ($p = 0.223$). Event-free survival (FFS) at 2 years was 85.7% for the TBI group and 62.7% for the non-TBI group ($p = 0.106$). Regarding early mortality risk, the TBI cohort exhibited a 100-day non-relapse mortality (NRM) of 7.1% and a 6-month NRM of 14.3%, whereas the non-TBI cohort displayed a 100-day NRM of 10.5% and a 6-month NRM of 26.3% ($p = 0.223$), indicating no statistically significant difference in early mortality between the two groups.

**Copyright Information:** (Online) ISSN: 2572-9241
©2026 The Author(s). HemaSphere is published by John Wiley & Sons Ltd on behalf of European Hematology Association. This is an open access Abstract Book distributed under the Attribution-NonCommercial-NoDerivs (CC BY-NC-ND), which allows third parties to download the articles and share them with others as long as they credit the author and the Abstract Book, but they cannot change the content in any way or use them commercially.
**Abstract Book Citations:** Authors, Title, HemaSphere, 2026;10:(S1):pages. The abstract book DOIs can be found on the "Author Information" page.
**Disclaimer:** Articles published in the journal HemaSphere exclusively reflect the opinions of the authors. The authors are responsible for all content in their abstracts including accuracy of the facts, statements, citing resources, etc.

### Summary/Conclusion

TBI cohort demonstrates a potential advantage in both survival and safety，although the differences in OS、FFS and NRM between the two groups did not reach statistical significance. We hypothesize that these findings may be limited by the relatively short follow-up duration and modest sample size of the current study. To confirm this potential clinical benefit, we propose conducting larger, prospective studies with extended follow-up periods.

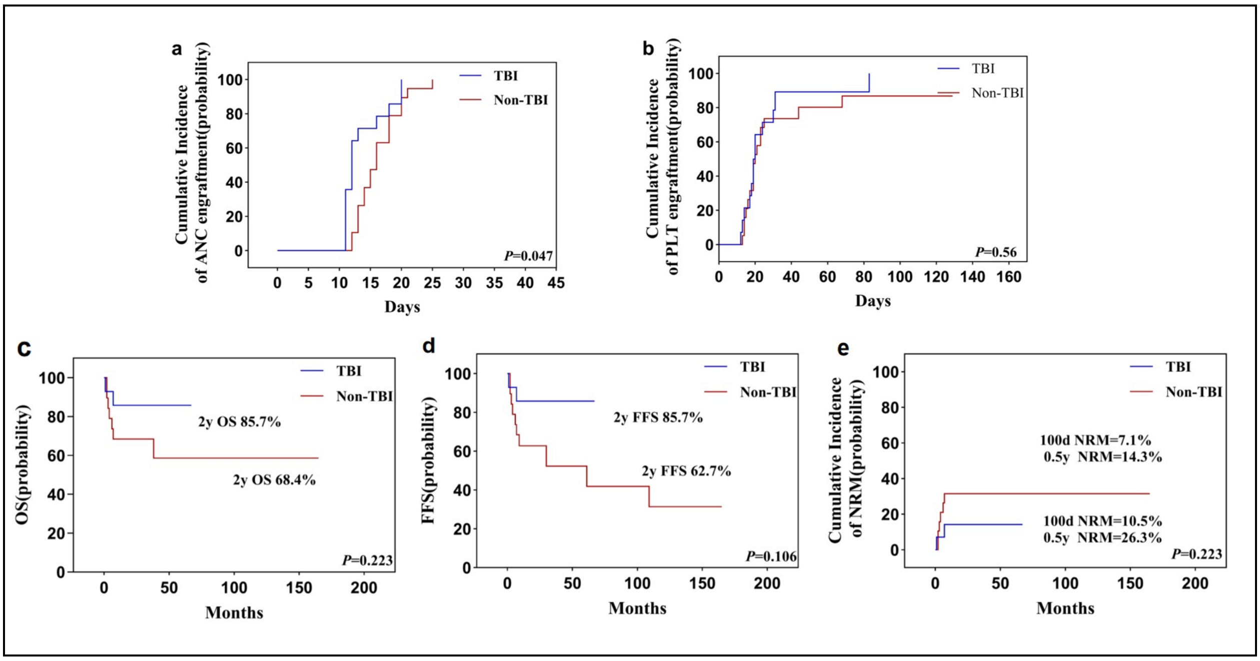

**Copyright Information:** (Online) ISSN: 2572-9241
©2026 The Author(s). HemaSphere is published by John Wiley & Sons Ltd on behalf of European Hematology Association. This is an open access Abstract Book distributed under the Attribution-NonCommercial-NoDerivs (CC BY-NC-ND), which allows third parties to download the articles and share them with others as long as they credit the author and the Abstract Book, but they cannot change the content in any way or use them commercially.
**Abstract Book Citations:** Authors, Title, HemaSphere, 2026;10:(S1):pages. The abstract book DOIs can be found on the "Author Information" page.
**Disclaimer:** Articles published in the journal HemaSphere exclusively reflect the opinions of the authors. The authors are responsible for all content in their abstracts including accuracy of the facts, statements, citing resources, etc.

### (PF1170) VENETOCLAX PLUS AZACITIDINE AS MAINTENANCE THERAPY TO REDUCE RELAPSE IN PEDIATRIC HIGH-RISK AML AFTER HAPLOIDENTICAL HEMATOPOIETIC STEM CELL TRANSPLANTATION: A DUAL-CENTER EXPERIENCE

**Topic:** 23. Stem cell transplantation - Clinical

Hongbo He*[1], Chenguang Jia[1], Bin Wang[1], Maoquan Qin[1], Yanhui Luo[1]

[1] *Beijing Children's Hospital, Hematology Center, Beijing, China;*

### Background

Allogeneic hematopoietic stem cell transplantation (HSCT) remains a cornerstone of curative therapy for pediatric high-risk acute myeloid leukemia (AML). However, post-transplant relapse persists as the primary cause of treatment failure. While Venetoclax combined with Azacitidine has shown promising activity in relapsed/refractory AML, its role as maintenance therapy following haploidentical HSCT in pediatric high-risk AML has not been systematically evaluated.

### Aims

This dual-center retrospective study aimed to assess the feasibility, safety, immune reconstitution profile, and anti-leukemic efficacy of Venetoclax plus Azacitidine (VEN+AZA) as post-transplant maintenance therapy in pediatric patients with high-risk AML undergoing haploidentical HSCT.

### Methods

A total of 72 pediatric patients with high-risk AML who received haploidentical HSCT were enrolled across two centers. Of these, 24 patients received VEN+AZA maintenance therapy post-transplantation (VEN+AZA group), while 48 patients did not (control group). Baseline demographics, disease characteristics, pre-transplant disease status, genetic alterations, and prior venetoclax treatment exposure were collected. Adverse events during maintenance therapy were graded per CTCAE v5.0. Post-transplant immune reconstitution was assessed longitudinally by quantifying lymphocyte subsets. Overall survival (OS), relapse-free survival (RFS), and cumulative incidence of relapse (CIR) were estimated using the Kaplan-Meier method, with comparisons between groups performed using the log-rank test.

### Results

Baseline Characteristics: The two cohorts were well-balanced regarding age, AML subtype, and genetic profiles. Notably, the VEN+AZA group had a significantly higher proportion of patients transplanted in second complete remission (CR2) (33.3% vs 6.3%, P=0.005), indicating a higher-risk baseline profile. Prior Venetoclax exposure was more frequent in the control group (66.7% vs 29.2%, P=0.005). Safety Profile: VEN+AZA maintenance was generally well-tolerated, with no treatment-related mortality (Grade 5=0). The most common grade 3/4 adverse events were neutropenia (79.2%) and leukopenia (54.2%). Non-hematologic toxicities were mild; grade 3/4 ALT elevation occurred in only 4.2% of patients. Severe infections were uncommon (4.2%), and no cases of sepsis or pancreatitis were documented. Immune Reconstitution: VEN+AZA maintenance did not compromise the recovery of total CD4+ T lymphocytes, B cells, or NK cells. However, a significant but transient delay in CD8+ T cells reconstitution was observed during the first year post-transplantation in the VEN+AZA group compared to controls ($P<0.05$ at multiple time points). By 24 months, CD8+ T cells counts had normalized to levels comparable with the control group, suggesting no long-term impairment of humoral immunity. Survival Outcomes: The VEN+AZA group achieved a 3-year OS of 95.8±4.1% and a 3-year RFS of 91.7±5.6%. Remarkably, VEN+AZA maintenance significantly reduced the cumulative incidence of relapse, with a 3-year CIR of 8.3% in the VEN+AZA group versus 24.8% in the control group, representing an absolute risk reduction of 16.5%.

**Copyright Information:** (Online) ISSN: 2572-9241
©2026 The Author(s). HemaSphere is published by John Wiley & Sons Ltd on behalf of European Hematology Association. This is an open access Abstract Book distributed under the Attribution-NonCommercial-NoDerivs (CC BY-NC-ND), which allows third parties to download the articles and share them with others as long as they credit the author and the Abstract Book, but they cannot change the content in any way or use them commercially.
**Abstract Book Citations:** Authors, Title, HemaSphere, 2026;10:(S1):pages. The abstract book DOIs can be found on the "Author Information" page.
**Disclaimer:** Articles published in the journal HemaSphere exclusively reflect the opinions of the authors. The authors are responsible for all content in their abstracts including accuracy of the facts, statements, citing resources, etc.

**Summary/Conclusion**

Venetoclax combined with Azacitidine represents a safe and feasible maintenance strategy following haploidentical HSCT in pediatric high-risk AML.

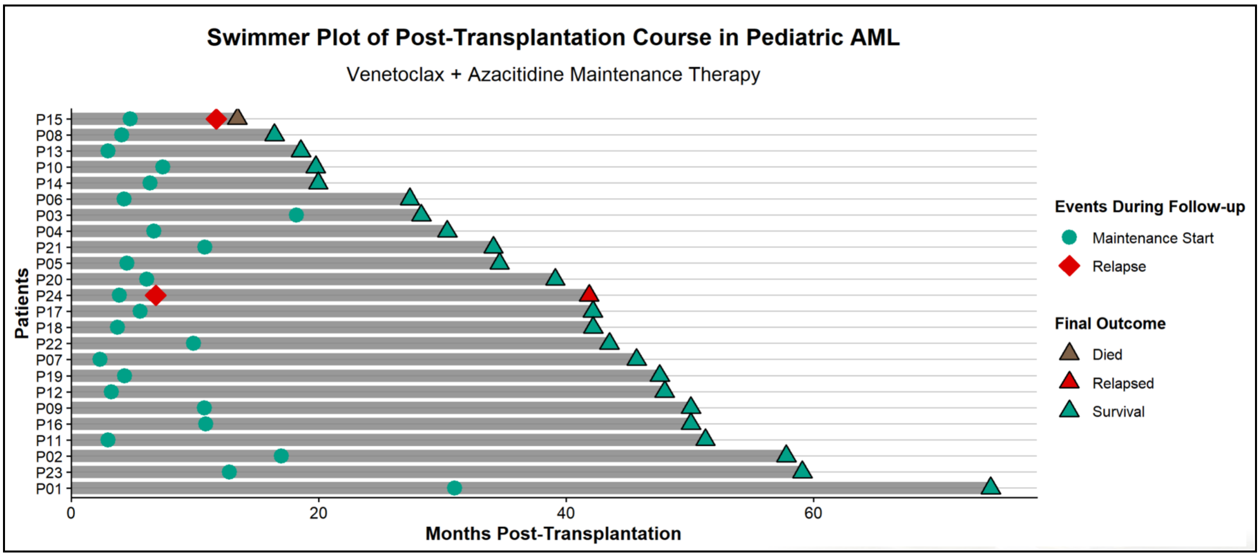

**Copyright Information:** (Online) ISSN: 2572-9241
©2026 The Author(s). HemaSphere is published by John Wiley & Sons Ltd on behalf of European Hematology Association. This is an open access Abstract Book distributed under the Attribution-NonCommercial-NoDerivs (CC BY-NC-ND), which allows third parties to download the articles and share them with others as long as they credit the author and the Abstract Book, but they cannot change the content in any way or use them commercially.
**Abstract Book Citations:** Authors, Title, HemaSphere, 2026;10:(S1):pages. The abstract book DOIs can be found on the "Author Information" page.
**Disclaimer:** Articles published in the journal HemaSphere exclusively reflect the opinions of the authors. The authors are responsible for all content in their abstracts including accuracy of the facts, statements, citing resources, etc.

### (PF1171) HIGH-SENSITIVITY NEXT GENERATION SEQUENCING (HS-NGS)-MEASURABLE RESIDUAL DISEASE (MRD) ASSAY AT DAY +100 POST- ALLOGENEIC HEMATOPOIETIC CELL TRANSPLANT (ALLO-HCT) PREDICTS RELAPSE RISK IN AML AND MDS

**Topic:** 23. Stem cell transplantation - Clinical

Madiha Iqbal*[1], Uyanik Mehmet[1], Michael Heckman[2], Judy Zhihui Fang[2], Talha Badar[1], Ernesto Ayala[1], Hemant Murthy[1], Mohamed Kharfan-Dabaja[1], Vivek Roy[1], Lauren Nguyen[3], Shuang Lu[3], Tracy Majewicz[3], Liuyan Jiang[3], Ke Li[3], James M Foran[1], Yao-Shan Fan[3]

*[1]Mayo Clinic, Division of Hematology-Oncology, Jacksonville, United States of America;[2]Mayo Clinic, Clinical Trials and Biostatistics, Jacksonville, United States of America; [3]Mayo Clinic, Lab Medicine and Pathology, Jacksonville, United States of America;*

### Background

Allo-HCT in remission is standard for AML/MDS at high relapse risk, yet post-HCT relapse is common. MRD is a strong prognostic biomarker; increasingly used to guide therapy and trial endpoints. Hs-NGS-based MRD assays can detect impending relapse post allo-HCT, but validated platforms are limited. The 2025 ELN–DAVID MRD recommendations endorse Hs-NGS-based MRD testing for *FLT3-ITD* pre-HCT, but a comprehensive post HCT assay is lacking

### Aims

Evaluate whether day +100 Hs NGS-MRD predicts relapse risk post allo-HCT in a cohort of 30 patients with AML and/or MDS.

### Methods

Patients in remission but with adverse ELN-2022 variants were included, plus 4 with *DDX41* or *PPM1D*. Pre-HCT MRD assessment used a diagnostic NGS assay (DNA SEQ. of 52 genes for variants and 35 for fusions; sensitivity 1%–5%). Day +100 MRD was asssessed using the Hs-NGS-MRD assay (DNA SEQ. of 34 genes for variants and RNA SEQ. of 43 for fusions; sensitivity 0.1%–0.05%). Baseline characteristics were compared using Wilcoxon/Fisher tests. OS and RFS from MRD assessment were estimated by Kaplan–Meier; associations between MRD status and outcomes were evaluated using Cox models with and without age adjustment.

**Copyright Information:** (Online) ISSN: 2572-9241
©2026 The Author(s). HemaSphere is published by John Wiley & Sons Ltd on behalf of European Hematology Association. This is an open access Abstract Book distributed under the Attribution-NonCommercial-NoDerivs (CC BY-NC-ND), which allows third parties to download the articles and share them with others as long as they credit the author and the Abstract Book, but they cannot change the content in any way or use them commercially.
**Abstract Book Citations:** Authors, Title, HemaSphere, 2026;10:(S1):pages. The abstract book DOIs can be found on the “Author Information” page.
**Disclaimer:** Articles published in the journal HemaSphere exclusively reflect the opinions of the authors. The authors are responsible for all content in their abstracts including accuracy of the facts, statements, citing resources, etc.

### Results

Among 30 patients, 14 (47%) were MRD-negative and 16 (53%) MRD-positive at day +100. Baseline charecteristics and pre-HCT mutation burden did not differ (P=0.91). *ASXL1* trended towards enrichment in MRD-negative patients (35.7% vs 6.3%; P=0.073) among 52 pre-HCT detected variants (median VAF 17.05). Among MRD-positive patients, 44% had one mutation, 31% two, and 25% ≥3 post-HCT. Thirty-seven post-HCT variants were identified (median VAF 0.16%); *DNMT3A* (56.3%) and *TP53* (37.5%) were most common and enriched in MRD-positive patients (P<0.001; P=0.019) (Table 1). The Hs-NGS-MRD assay identified 24 (65%) patients with VAF <2% (range 0.06–1.95), who would not have been detected by standard NGS. Only two patients had VAF >10. One-year cumulative incidence of relapse or death was 60.0% for MRD-positive vs 22.1% for MRD-negative. MRD-positivity was associated with increased relapse/death risk (unadjusted HR 3.39; 95% CI 0.91–12.55; P=0.068; age-adjusted HR 3.30; 95% CI 0.89–12.21; P=0.074).

| | Pre HCT | | | | Post HCT | |
|---|---|---|---|---|---|---|
| | MRD negative n (%) | MRD positive n (%) | p- value | | MRD positive n (%) | p-value |
| ***ASXL1*** | **5 (35.7)** | **1 (6.3)** | **0.073** | *ASXL1* | 1 (6.3) | 1 |
| *BCOR* | 1 (7.1) | 0 | 0.47 | *ABL1* | 1 (6.3) | 1 |
| *DDX41* | 2 (14.3) | 2 (12.5) | 1 | *DDX41* | 1 (6.3) | 1 |
| *DNMT3A* | 1 (7.1) | 2 (12.5) | 1 | ***DNMT3A*** | **9 (56.3)** | **<0.001** |
| *ETNK1* | 1 (7.1) | 0 | 0.47 | *EZH2* | 1 (6.3) | 1 |
| *FLT3* | 1 (7.1) | 0 | 0.47 | *IDH2* | 2 (12.5) | 0.49 |
| *IDH1* | 2 (14.3) | 0 | 0.21 | *JAK2* | 1 (6.3) | 1 |
| *IDH2* | 0 | 1 (6.3) | 1 | *KRAS* | 2 (12.5) | 0.49 |
| *JAK2* | 0 | 1 (6.3) | 1 | *PTPN11* | 1 (6.3) | 1 |
| *KRAS* | 0 | 1 (6.3) | 1 | *RUNX1* | 1 (6.3) | 1 |
| *NPM1* | 1 (7.1) | 1 (6.26) | 1 | *SF3B1* | 2 (12.5) | 0.49 |
| *NRAS* | 1 (7.1) | 0 | 0.47 | *SRSF2* | 3 (18.8) | 0.23 |
| *PPM1D* | 1 (7.1) | 2 (12.5) | 1 | ***TP53*** | **6 (37.5)** | **0.019** |
| *RUNX1* | 3 (21.4) | 1 (6.3) | 0.32 | *WT1* | 1 (6.3) | 1 |
| *SF3B1* | 2 (14.3) | 5 (31.3) | 0.40 | *WT2* | 1 (6.3) | 1 |
| *SRSF2* | 2 (14.3) | 2 (12.5) | 1 | | | |
| *TET2* | 1 (7.1) | 1 (6.3) | 1 | | | |
| *TP53* | 1 (7.1) | 4 (25) | 0.34 | | | |
| *U2AF1* | 2 (14.3) | 0 | 0.21 | | | |
| *WT1* | 0 | 1 (6.3) | 1 | | | |

**Table 1: Distribution of variants pre and post allo-HCT in the two groups.**

### Summary/Conclusion

Day +100 Hs-NGS MRD testing post allo-HCT identifies patients at increased risk of adverse outcomes, including most who would be missed by standard diagnostic NGS. Notably, 26/30 (87%) lacked typical qPCR-monitored lesions (*FLT3-ITD, NPM1, IDH1/2, JAK2*), underscoring the value of a broad Hs-NGS MRD assay. MRD-positive patients showed greater post-transplant mutational burden and clinically meaningful trends toward inferior OS and RFS. Findings support integrating early post-HCT Hs-NGS MRD into risk stratification, maintenance strategies, and longitudinal monitoring.

**Copyright Information:** (Online) ISSN: 2572-9241
©2026 The Author(s). HemaSphere is published by John Wiley & Sons Ltd on behalf of European Hematology Association. This is an open access Abstract Book distributed under the Attribution-NonCommercial-NoDerivs (CC BY-NC-ND), which allows third parties to download the articles and share them with others as long as they credit the author and the Abstract Book, but they cannot change the content in any way or use them commercially.
**Abstract Book Citations:** Authors, Title, HemaSphere, 2026;10:(S1):pages. The abstract book DOIs can be found on the "Author Information" page.
**Disclaimer:** Articles published in the journal HemaSphere exclusively reflect the opinions of the authors. The authors are responsible for all content in their abstracts including accuracy of the facts, statements, citing resources, etc.

**Figure 1**: Relapse-Free Survival by Measurable Residual Disease Status at Day +100

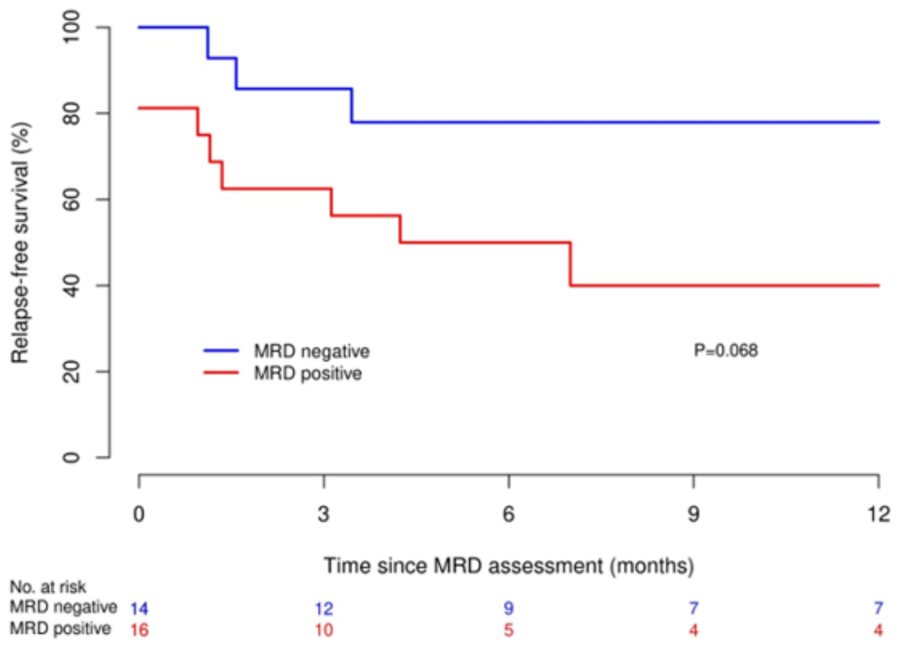

**Copyright Information:** (Online) ISSN: 2572-9241
©2026 The Author(s). HemaSphere is published by John Wiley & Sons Ltd on behalf of European Hematology Association. This is an open access Abstract Book distributed under the Attribution-NonCommercial-NoDerivs (CC BY-NC-ND), which allows third parties to download the articles and share them with others as long as they credit the author and the Abstract Book, but they cannot change the content in any way or use them commercially.
**Abstract Book Citations:** Authors, Title, HemaSphere, 2026;10:(S1):pages. The abstract book DOIs can be found on the "Author Information" page.
**Disclaimer:** Articles published in the journal HemaSphere exclusively reflect the opinions of the authors. The authors are responsible for all content in their abstracts including accuracy of the facts, statements, citing resources, etc.

### (PF1172) ASSOCIATION OF DONOR CHEMOKINE RECEPTOR GENE POLYMORPHISMS WITH THE RISK OF ACUTE GRAFT-VERSUS-HOST DISEASE

**Topic:** 23. Stem cell transplantation - Clinical

Heng Liu*[1], Tao Wu[1]

[1]*The 940th Hospital of Joint Logistics Support force of Chinese People's Liberation Army, hematology, Gansu, China;*

### Background

Allogeneic hematopoietic stem cell transplantation (allo-HSCT) serves as an effective therapeutic approach for curing various benign and malignant hematological disorders. However, post-transplant complications significantly impact treatment outcomes. Acute graft-versus-host disease (aGVHD), characterized by its high incidence and mortality rates, remains a major obstacle to the success of allo-HSCT. The recruitment of T lymphocytes into target organs constitutes a critical process in aGVHD pathogenesis, wherein multiple chemokine receptors expressed on lymphocytes—including C-C chemokine receptor type 5 (CCR5), C-C chemokine receptor type 6 (CCR6), and CXC chemokine receptor 3 (CXCR3)—play pivotal regulatory roles. Single nucleotide polymorphisms (SNPs), as common genetic variations in the genome, can influence gene expression levels and functional properties of encoded proteins at specific polymorphic sites. Therefore, this study investigated the polymorphisms in chemokine receptor genes (CCR5, CCR6, and CXCR3) of allo-HSCT donors and analyzed their association with the occurrence of aGVHD in recipients after allo-HSCT.

### Aims

To investigate the correlation between single nucleotide polymorphisms (SNPs) of chemokine receptor genes in allogeneic hematopoietic stem cell transplantation donors and the occurrence of acute graft-versus-host disease (aGVHD) in recipients.

### Methods

50 cases of allogeneic hematopoietic stem cell transplantation from February 2023 to December 2024 were selected to detect the polymorphism in the donor's chemokine receptor genes. Polymorphisms of donor chemokine receptor genes were analyzed, including CCR5 (rs2227010, rs2734648, rs1799987, rs1799988, rs1800023, rs1800024), CCR6 (rs1556413, rs3093024, rs1854853, rs3093023), and CXCR3 (rs2280964, rs34334103). Associations between specific genotypes and aGVHD development were evaluated.

### Results

Among the 50 recipients, 23 (46%) developed aGVHD. The CCR5-rs1800024 CC genotype in donors was associated with a lower incidence of aGVHD compared to the CT+TT genotypes (32.0% vs 60.0%, $P$ = 0.047) and reduced liver involvement (0.0% vs 20.0%, $P$ = 0.05). The heterozygous CT genotype correlated with a higher incidence of cutaneous aGVHD compared to homozygous genotypes (CC+TT) (60.9% vs 29.6%, $P$ = 0.027). CXCR3-rs34334103 CC genotype in donors showed a lower incidence of hepatic aGVHD compared to CT+TT genotypes (6.5% vs 50.0%, $P$ = 0.045). The CCR6-rs1854853 heterozygous AG genotype in donors was associated with a higher incidence of grade III–IV aGVHD compared to homozygous AA+GG genotypes (50.0% vs 0.0%, $P$ = 0.014). The CXCR3-rs2280964 CC genotype demonstrated superior treatment efficacy compared to CT+TT genotypes, with a higher overall response rate (100.0% vs 66.7%, $P$ = 0.047).

**Copyright Information:** (Online) ISSN: 2572-9241
©2026 The Author(s). HemaSphere is published by John Wiley & Sons Ltd on behalf of European Hematology Association. This is an open access Abstract Book distributed under the Attribution-NonCommercial-NoDerivs (CC BY-NC-ND), which allows third parties to download the articles and share them with others as long as they credit the author and the Abstract Book, but they cannot change the content in any way or use them commercially.
**Abstract Book Citations:** Authors, Title, HemaSphere, 2026;10:(S1):pages. The abstract book DOIs can be found on the "Author Information" page.
**Disclaimer:** Articles published in the journal HemaSphere exclusively reflect the opinions of the authors. The authors are responsible for all content in their abstracts including accuracy of the facts, statements, citing resources, etc.

**Summary/Conclusion**

The occurrence and clinical severity of aGVHD in recipients are significantly influenced by polymorphisms at four donor loci: CCR5-rs1800024, CXCR3-rs34334103, CCR6-rs1854853, and CXCR3-rs2280964. These findings highlight the potential role of chemokine receptor gene variants in modulating post-transplant immune responses and suggest avenues for personalized donor selection strategies.

**Copyright Information:** (Online) ISSN: 2572-9241
©2026 The Author(s). HemaSphere is published by John Wiley & Sons Ltd on behalf of European Hematology Association. This is an open access Abstract Book distributed under the Attribution-NonCommercial-NoDerivs (CC BY-NC-ND), which allows third parties to download the articles and share them with others as long as they credit the author and the Abstract Book, but they cannot change the content in any way or use them commercially.
**Abstract Book Citations:** Authors, Title, HemaSphere, 2026;10:(S1):pages. The abstract book DOIs can be found on the "Author Information" page.
**Disclaimer:** Articles published in the journal HemaSphere exclusively reflect the opinions of the authors. The authors are responsible for all content in their abstracts including accuracy of the facts, statements, citing resources, etc.

### (PF1173) EARLY IMMUNE RECONSTITUTION AFTER ALLOGENEIC HEMATOPOIETIC STEM CELL TRANSPLANTATION IN AML: ASSOCIATION WITH FLT3 STATUS AND POST-TRANSPLANT MIDOSTAURIN MAINTENANCE

**Topic:** 23. Stem cell transplantation - Clinical

Nevena Bešević*[1], Andrej Pešić[1], Nada Kraguljac-Kurtovic[1], Milena Todorovic Balint[1]

[1]*Clinic for hematology, University Clinical Center of Serbia, Stem cell unit, Belgrade, Serbia;*

### Background

Allogeneic hematopoietic stem cell transplantation (allo-HSCT) remains a central consolidative strategy in acute myeloid leukemia (AML). In patients with FLT3-mutated disease, post-transplant maintenance with midostaurin is increasingly implemented to mitigate relapse risk. While its anti-leukemic activity is established, its influence on post-transplant immune reconstitution remains unclear. Given the role of lymphocyte recovery in relapse control and immune regulation, this interaction is clinically relevant.

### Aims

The aim of the study was to evaluate whether post-transplant midostaurin maintenance therapy influences immune reconstitution in AML patients after allo-HSCT.

### Methods

This retrospective study included 39 AML patients who underwent allo-HSCT at our center between 2020 and 2025. Among them, seven patients had FLT3 ITD/TKD-positive AML. Thirty-six patients received myeloablative conditioning, while three patients were given reduced-intensity conditioning. All patients received anti-T-lymphocyte globulin for in vivo T-cell depletion (10mg/kg for sibling and 20mg/kg for unrelated donors on days -3, -2 and -1), along with cyclosporine A and methotrexate for graft-versus-host disease prophylaxis. Donor sources included matched sibling donors and unrelated donors (matched unrelated and mismatced unrelated). Peripheral blood was used as the graft source in all cases. Patients with FLT3-mutated AML received midostaurin maintenance therapy, initiated between days +30 and +60 post-transplant at a dose of 50mg twice daily. Immune reconstitution was assessed by peripheral blood immunophenotyping on days +28, +90 and +180 after transplantation. The analyzed lymphocyte subsets included CD4+ T lymphocytes, CD8+ T lymphocytes, B lymphocytes, natural killer (NK) cells, and NK-T lymphocytes. Differences in numerical variables were analyzed using the Mann-Whitney U test, while categorical variables were compared using Fisher's exact test.

### Results

A total of 39 patients with AML were included in the analysis. Seven patients were FLT3 ITD/TKD-positive and therefore received midostaurin maintenance therapy for at least six months after allo-HSCT. There were no significant differences between the groups regarding patient or donor age, patient gender, ELN 2022 risk stratification, conditioning regimen intensity, or donor distribution. Peripheral blood immune status assessed at day +28, before the initiation of midostaurin, showed no significant differences in lymphocyte subpopulation counts between the groups. When absolute lymphocyte counts were compared, patients receiving midostaurin showed a trend toward higher CD4+ T-lymphocyte counts at six months after transplantation, with a median count of 189/µL (IQR 133–219) compared to 123/µL (IQR 79–158) in patients not receiving the drug (P=0.06). No statistically significant differences were observed in CD4+ T-lymphocyte counts at day +90, or in other analyzed lymphocyte subpopulations at days +90 and +180.

**Copyright Information:** (Online) ISSN: 2572-9241
©2026 The Author(s). HemaSphere is published by John Wiley & Sons Ltd on behalf of European Hematology Association. This is an open access Abstract Book distributed under the Attribution-NonCommercial-NoDerivs (CC BY-NC-ND), which allows third parties to download the articles and share them with others as long as they credit the author and the Abstract Book, but they cannot change the content in any way or use them commercially.
**Abstract Book Citations:** Authors, Title, HemaSphere, 2026;10:(S1):pages. The abstract book DOIs can be found on the "Author Information" page.
**Disclaimer:** Articles published in the journal HemaSphere exclusively reflect the opinions of the authors. The authors are responsible for all content in their abstracts including accuracy of the facts, statements, citing resources, etc.

**Summary/Conclusion**

Post-transplant midostaurin maintenance therapy was not associated with significant differences in early lymphocyte subset recovery in AML patients undergoing allo-HSCT. A trend toward higher CD4+ T-lymphocyte counts at six months after transplantation was observed in patients receiving midostaurin, but without statistical significance. Further studies on larger cohorts are warranted to better elucidate the potential immunological impact of post-transplant FLT3-targeted maintenance therapy.

**Copyright Information:** (Online) ISSN: 2572-9241
©2026 The Author(s). HemaSphere is published by John Wiley & Sons Ltd on behalf of European Hematology Association. This is an open access Abstract Book distributed under the Attribution-NonCommercial-NoDerivs (CC BY-NC-ND), which allows third parties to download the articles and share them with others as long as they credit the author and the Abstract Book, but they cannot change the content in any way or use them commercially.
**Abstract Book Citations:** Authors, Title, HemaSphere, 2026;10:(S1):pages. The abstract book DOIs can be found on the "Author Information" page.
**Disclaimer:** Articles published in the journal HemaSphere exclusively reflect the opinions of the authors. The authors are responsible for all content in their abstracts including accuracy of the facts, statements, citing resources, etc.

### (PF1174) OUTCOMES OF HEMATOPOIETIC STEM CELL TRANSPLANTATION IN INBORN ERRORS OF IMMUNITY (IEI) IN A SINGLE CENTER FROM CORDOBA, ARGENTINA

**Topic:** 23. Stem cell transplantation - Clinical

Maria Laura Herrera Andino[1], Milagros Lopez Orozco[1], Maria Emilia Mas[1], Maria Victoria Suen[1], Ana Lisa Basquiera*[1], Adrian Kahn[1]

*[1]Hospital Privado Universitario de Córdoba, Córdoba, Córdoba, Argentina;*

### Background

Inborn errors of immunity (IEI) are genetic disorders clinically characterized by severe infections, immune dysregulation, and high early mortality. Allogeneic hematopoietic stem cell transplantation (HSCT) remains the only curative option for many patients with these disorders. Current outcomes data after HSCT from Argentina remain scarce.

### Aims

Describe transplant features and outcomes in a pediatric cohort of patients diagnosed with IEI undergoing HSCT at a single tertiary center from Cordoba, Argentina, between 2001 and 2025.

### Methods

This is a retrospective study including all consecutive pediatric patients (≤18 years) with IEI who underwent allogeneic HSCT at Hospital Privado Universitario de Córdoba between January 2001 and December 2025. Data were obtained from electronic medical records and included diagnosis, donor type, stem cell source, conditioning regimen, serotherapy, GVHD prophylaxis including post transplant cyclophosphamide (PTCy), engraftment, chimerism, and outcomes. Primary endpoints were overall survival (OS) and transplant-related mortality (TRM). Secondary endpoints included graft failure and acute and chronic graft-versus-host disease (GVHD). Survival was estimated using Kaplan–Meier survival curve.

### Results

Twenty-five patients (24 males, 1 female) were included. Median age at transplantation was 2 years old (range 0.3–16.5). The most frequent diagnoses were: Wiskott–Aldrich syndrome (24%), severe combined immunodeficiency (12%), hemophagocytic lymphohistiocytosis (12%), hyper-IgM syndrome (12%), chronic granulomatous disease (8%), and DOCK8 deficiency (8%). Median time from diagnosis to HSCT was 10.9 months. Most patients had low pre-transplant comorbidity scores (HCT-CI 0–1).All patients received myeloablative conditioning, mainly fludarabine–busulfan–based regimens. Donors were matched siblings in 12%, mismatched related in 8%, and unrelated in 68%. Stem cell sources were bone marrow (68%), peripheral blood (20%), and cord blood (12%). Serotherapy was administered in 48%, and PTCy was used in 16%.
Neutrophil engraftment was achieved in 84% of patients, with a median of 16 days; platelet recovery occurred at a median of 20 days. Secondary graft failure occurred in 8% and required retransplantation. By day 60, among the 15 patients with available follow-up, complete donor chimerism was observed in 40%, while mixed chimerism was detected in 60% of patients. Mucositis grade 2 or more occurred in 75% and febrile neutropenia in 68% of patients. Acute GVHD was observed in 56%, predominantly cutaneous, while chronic GVHD occurred in 8%. Cytomegalovirus reactivation was documented in 28% of cases. After a median follow-up of 47.2 months, 4-year overall survival was 76% (95%CI, 53-88). Six patients (24%) died from transplant-related causes within the first year. One-year OS was 76%, with similar 4-year survival, reflecting early mortality.

**Copyright Information:** (Online) ISSN: 2572-9241
©2026 The Author(s). HemaSphere is published by John Wiley & Sons Ltd on behalf of European Hematology Association. This is an open access Abstract Book distributed under the Attribution-NonCommercial-NoDerivs (CC BY-NC-ND), which allows third parties to download the articles and share them with others as long as they credit the author and the Abstract Book, but they cannot change the content in any way or use them commercially.
**Abstract Book Citations:** Authors, Title, HemaSphere, 2026;10:(S1):pages. The abstract book DOIs can be found on the "Author Information" page.
**Disclaimer:** Articles published in the journal HemaSphere exclusively reflect the opinions of the authors. The authors are responsible for all content in their abstracts including accuracy of the facts, statements, citing resources, etc.

**Summary/Conclusion**

Allogeneic HSCT achieved sustained engraftment in most patients with acceptable toxicity in our cohort. Transplant-related mortality remains the main limitation, with infections as the principal etiology. These findings support early referral for HSCT in IEI when indicated and highlight the need for continued optimization of supportive care and transplant strategies.

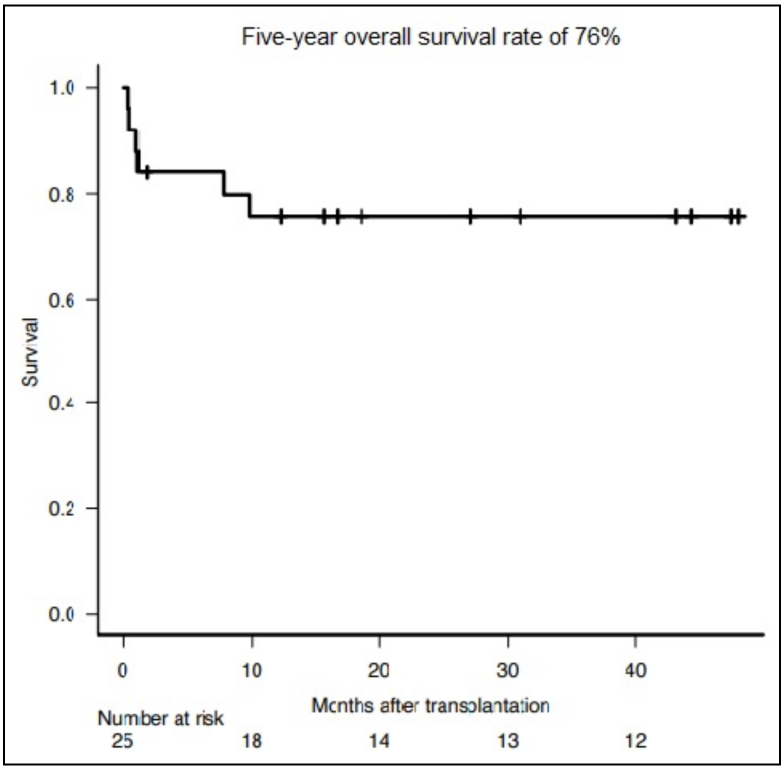

**Copyright Information:** (Online) ISSN: 2572-9241
©2026 The Author(s). HemaSphere is published by John Wiley & Sons Ltd on behalf of European Hematology Association. This is an open access Abstract Book distributed under the Attribution-NonCommercial-NoDerivs (CC BY-NC-ND), which allows third parties to download the articles and share them with others as long as they credit the author and the Abstract Book, but they cannot change the content in any way or use them commercially.
**Abstract Book Citations:** Authors, Title, HemaSphere, 2026;10:(S1):pages. The abstract book DOIs can be found on the "Author Information" page.
**Disclaimer:** Articles published in the journal HemaSphere exclusively reflect the opinions of the authors. The authors are responsible for all content in their abstracts including accuracy of the facts, statements, citing resources, etc.

### (PF1175) HIGH PRE-TRANSPLANT ANTI-A ANTIBODY TITER AND DELAYED LEUKOCYTE ENGRAFTMENT ARE INDEPENDENT RISK FACTORS FOR PURE RED CELL APLASIA AFTER HAPLOIDENTICAL HSCT IN AML PATIENTS

**Topic:** 23. Stem cell transplantation - Clinical

Dan Feng*[1], Zeheng Wu[1], Yigeng Cao[1], Rongli Zhang[1], Qiaoling MA[1], Yi He[1], Donglin Yang[1], Aiming Pang[1], Sizhou Feng[1], Mingzhe Han[1], Jialin Wei[1], Erlie Jiang[1]

*[1]State Key Laboratory of Experimental Hematology, National Clinical Research Center for Blood Diseases, Haihe Laboratory of Cell Ecosystem, Institute of Hematology & Blood Diseases Hospital, Chinese Academy of Medical Sciences &Peking Union Medical College, Tianjin, China, Tianjin, China;*

### Background

Haploidentical hematopoietic stem cell transplantation (HSCT) has markedly improved survival in patients with acute myeloid leukemia (AML). Nonetheless, post-transplant complications remain a major challenge. Delayed erythroid recovery and pure red cell aplasia (PRCA) are particularly common in ABO-incompatible donor-recipient pairs, and their mechanisms and optimal management remain poorly defined.

### Aims

To investigate the incidence, risk factors, and underlying mechanisms of delayed red blood cell recovery and pure red cell aplasia (PRCA) in AML patients undergoing haploidentical HSCT, with a particular focus on ABO incompatibility and pre-transplant anti-A/anti-B antibody titers.

### Methods

We analyzed 406 AML patients from the NICHE cohort who underwent haploidentical HSCT at the Chinese Academy of Medical Sciences Hematology Hospital between January 2027 and December 2023. Patients underwent serial complete blood counts including reticulocytes, and regular bone marrow morphology and chimerism assessments. Delayed erythroid recovery/PRCA was defined as reticulocyte count $<0.01\times10^{12}$/L and <1%, selective absence of erythroid lineage in the bone marrow, negative direct antiglobulin test, and full donor leukocyte chimerism, with normal leukocyte and platelet engraftment. Patients with infection, graft failure, or early relapse were excluded.

**Copyright Information:** (Online) ISSN: 2572-9241
©2026 The Author(s). HemaSphere is published by John Wiley & Sons Ltd on behalf of European Hematology Association. This is an open access Abstract Book distributed under the Attribution-NonCommercial-NoDerivs (CC BY-NC-ND), which allows third parties to download the articles and share them with others as long as they credit the author and the Abstract Book, but they cannot change the content in any way or use them commercially.
**Abstract Book Citations:** Authors, Title, HemaSphere, 2026;10:(S1):pages. The abstract book DOIs can be found on the "Author Information" page.
**Disclaimer:** Articles published in the journal HemaSphere exclusively reflect the opinions of the authors. The authors are responsible for all content in their abstracts including accuracy of the facts, statements, citing resources, etc.

**Results**

Of 406 patients, 234 were ABO-compatible and 172 ABO-incompatible (major mismatch 75, minor mismatch 73, bidirectional mismatch 24). The median age was 38 years (IQR 26.8–49.25). By day +60 post-transplant, 35 patients required red blood cell transfusions: 15/234 (6.4%) in ABO-compatible and 20/172 (11.6%) in ABO-incompatible patients (minor 12/73, 16.4%; major 8/75, 10.7%; bidirectional 0/24; p=0.021).
Among ABO-incompatible patients, delayed recovery occurred in 7 A→O and 2 B→O cases. Median pre-transplant anti-A titer was 128 [0–1024] in the delayed group versus 8 [0–64] in the well-engrafted group (p=0.068). Using a cutoff of 192, high anti-A titers were associated with a significantly higher risk of delayed erythroid recovery/PRCA (45% vs 15.1%, p=0.003). Median anti-B titer was 256 [40–384] versus 0 [0–32] in the well-engrafted group (p<0.001).
Delayed recovery was associated with later leukocyte engraftment (18.5 [16.8–21] vs 15.0 [14–18] days, p=0.001), while platelet engraftment and transplant interval were similar. Multivariable logistic regression identified high anti-A titer (OR=5.02, 95%CI 1.44–19.52, p=0.014) and delayed leukocyte engraftment (OR=1.21/day, 95%CI 1.06–1.40, p=0.007) as independent risk factors. Major mismatch was not significant (OR=1.31, 95%CI 0.38–4.86, p=0.675), and bidirectional mismatch could not be evaluated due to small numbers.

**Summary/Conclusion**

High pre-transplant anti-A antibody titers and delayed leukocyte engraftment independently increase the risk of delayed erythroid recovery and PRCA after haploidentical HSCT. These factors may serve as predictive markers to identify high-risk patients who could benefit from closer monitoring and early interventions to promote red cell recovery.

**Copyright Information:** (Online) ISSN: 2572-9241
©2026 The Author(s). HemaSphere is published by John Wiley & Sons Ltd on behalf of European Hematology Association. This is an open access Abstract Book distributed under the Attribution-NonCommercial-NoDerivs (CC BY-NC-ND), which allows third parties to download the articles and share them with others as long as they credit the author and the Abstract Book, but they cannot change the content in any way or use them commercially.
**Abstract Book Citations:** Authors, Title, HemaSphere, 2026;10:(S1):pages. The abstract book DOIs can be found on the “Author Information” page.
**Disclaimer:** Articles published in the journal HemaSphere exclusively reflect the opinions of the authors. The authors are responsible for all content in their abstracts including accuracy of the facts, statements, citing resources, etc.

### (PF1176) CONDITIONING DOSE ADJUSTMENT IN OBESE ADULT PATIENTS WITH SEVERE APLASTIC ANEMIA UNDERGOING HAPLOIDENTICAL ALLOGENEIC HEMATOPOIETIC STEM CELL TRANSPLANTATION: A PROPENSITY SCORE-MATCHED ANALYSIS

**Topic:** 23. Stem cell transplantation - Clinical

Wenni He*[1, 2, 3, 4], Tingting Han[1, 2, 3, 4], Yuan-Yuan Zhang[1, 2, 3, 4], Wei Han[1, 2, 3, 4], Chen-Hua Yan[1, 2, 3, 4], Xiao-Dong Mo[1, 2, 3, 4], Yu-Hong Chen[1, 2, 3, 4], Jing-Zhi Wang[1, 2, 3, 4], Fan Lin[1, 2, 3, 4], Fengrong Wang[1, 2, 3, 4], Zheng-Li Xu[1, 2, 3, 4], Feifei Tang[1, 2, 3, 4], Jinsong Jia[1, 2, 3, 4], Yu Wang[1, 2, 3, 4], Xiaohui Zhang[1, 2, 3, 4], Xiao-Jun Huang[1, 2, 3, 4], Lanping Xu[1, 2, 3, 4]

*[1]Peking University People's Hospital, Department of Hematology, Beijing, China;[2]Peking University Institute of Hematology, Beijing, China; [3]National Clinical Research Centre for Hematologic Disease, Beijing, China;[4]Beijing Key Laboratory of Hematopoietic Stem Cell Transplant, Beijing, China;*

### Background

Haploidentical hematopoietic stem cell transplantation (haplo-HSCT) has become an important curative treatment for patients with severe aplastic anemia (SAA), particularly in regions where matched sibling donors are unavailable. Obesity is associated with altered drug pharmacokinetics that may affect conditioning drug exposure, and dose adjustment is common in clinical practice. However, dosing strategies vary substantially across transplant centers, and no consensus exists regarding optimal conditioning dose adjustment in obese recipients. The 2014 EBMT and ASBMT guidelines reviewed available data but were unable to recommend a unified dosing approach because of heterogeneity across studies. As a result, data guiding conditioning dose adjustment in obese patients with SAA undergoing haplo-HSCT remain limited.

### Aims

This study aimed to evaluate the safety and clinical impact of conditioning dose adjustment in obese adult patients with SAA undergoing haplo-HSCT.

### Methods

We conducted a single-center retrospective study including 209 adult patients with SAA who underwent haplo-HSCT between January 2006 and December 2022. Obesity was defined as actual body weight ≥120% of ideal body weight. Obese patients received conditioning based on adjusted ideal body weight, whereas non-obese patients received actual body weight–based dosing. After propensity score matching, 104 patients (29 obese and 75 non-obese) were included.Transplant outcomes including engraftment, graft-versus-host disease (GVHD), viral reactivation, conditioning-related toxicity, overall survival (OS), and GVHD-free/relapse-free survival (GRFS) were evaluated.

**Copyright Information:** (Online) ISSN: 2572-9241
©2026 The Author(s). HemaSphere is published by John Wiley & Sons Ltd on behalf of European Hematology Association. This is an open access Abstract Book distributed under the Attribution-NonCommercial-NoDerivs (CC BY-NC-ND), which allows third parties to download the articles and share them with others as long as they credit the author and the Abstract Book, but they cannot change the content in any way or use them commercially.
**Abstract Book Citations:** Authors, Title, HemaSphere, 2026;10:(S1):pages. The abstract book DOIs can be found on the "Author Information" page.
**Disclaimer:** Articles published in the journal HemaSphere exclusively reflect the opinions of the authors. The authors are responsible for all content in their abstracts including accuracy of the facts, statements, citing resources, etc.

**Results**

Survival outcomes were comparable between obese and non-obese recipients. The 5-year overall survival (OS) rates were 85.4% and 77.3%, respectively (P = 0.29), and the 5-year GVHD-free/relapse-free survival (GRFS) rates were 72.1% and 69.2% (P = 0.77). In multivariable analysis, obesity was not associated with inferior survival, whereas Eastern Cooperative Oncology Group (ECOG) performance status ≥2 was an independent adverse prognostic factor for OS (P = 0.013). Hematopoietic engraftment was achieved in nearly all patients, with similar cumulative incidences of neutrophil engraftment by day +28 (97.0% vs. 93.0%, P = 0.14) and platelet engraftment by day +100 (86.0% vs. 84.0%, P > 0.9). The cumulative incidences of grade II–IV acute GVHD (31.0% vs. 16.0%, P = 0.076), grade III–IV acute GVHD (13.8% vs. 3.4%, P = 0.16), and chronic GVHD (44% vs. 30%, P = 0.32) were comparable between groups. The incidences of viral reactivation and conditioning-related cardiac toxicity were also similar between obese and non-obese recipients.

**Summary/Conclusion**

Conditioning dose adjustment using adjusted ideal body weight in obese patients with SAA undergoing haplo-HSCT was safe and did not compromise engraftment, GVHD incidence, toxicity, or survival outcomes. These findings support the safety and feasibility of dose-adjusted conditioning in obese patients with SAA undergoing haplo-HSCT.

**Copyright Information:** (Online) ISSN: 2572-9241
©2026 The Author(s). HemaSphere is published by John Wiley & Sons Ltd on behalf of European Hematology Association. This is an open access Abstract Book distributed under the Attribution-NonCommercial-NoDerivs (CC BY-NC-ND), which allows third parties to download the articles and share them with others as long as they credit the author and the Abstract Book, but they cannot change the content in any way or use them commercially.
**Abstract Book Citations:** Authors, Title, HemaSphere, 2026;10:(S1):pages. The abstract book DOIs can be found on the "Author Information" page.
**Disclaimer:** Articles published in the journal HemaSphere exclusively reflect the opinions of the authors. The authors are responsible for all content in their abstracts including accuracy of the facts, statements, citing resources, etc.

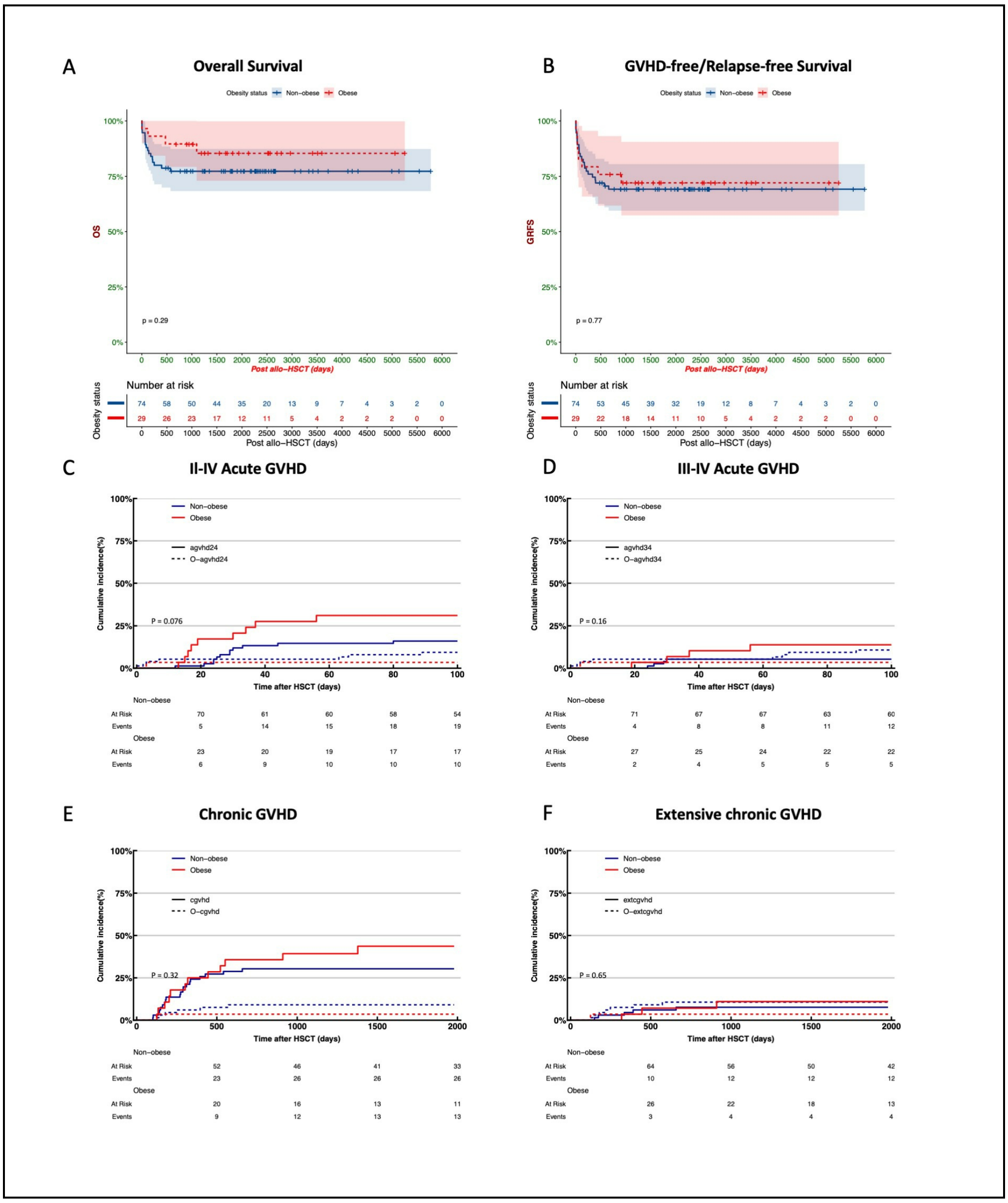

**Copyright Information:** (Online) ISSN: 2572-9241
©2026 The Author(s). HemaSphere is published by John Wiley & Sons Ltd on behalf of European Hematology Association. This is an open access Abstract Book distributed under the Attribution-NonCommercial-NoDerivs (CC BY-NC-ND), which allows third parties to download the articles and share them with others as long as they credit the author and the Abstract Book, but they cannot change the content in any way or use them commercially.
**Abstract Book Citations:** Authors, Title, HemaSphere, 2026;10:(S1):pages. The abstract book DOIs can be found on the "Author Information" page.
**Disclaimer:** Articles published in the journal HemaSphere exclusively reflect the opinions of the authors. The authors are responsible for all content in their abstracts including accuracy of the facts, statements, citing resources, etc.

### (PF1177) INVESTIGATION ON THE WILLINGNESS OF FAMILY MEMBERS OF HEMATOLOGICAL PATIENTS TO DONATE FOR HEMATOPOIETIC STEM CELL TRANSPLANTATION

**Topic:** 23. Stem cell transplantation - Clinical

Xue Wang*[1]

[1] *The Second Affiliated Hospital of the Army Medical University of the People's Liberation Army of China, ChongQing, China;*

### Background

Hematopoietic stem cell transplantation is a crucial treatment for many hematological malignancies and life-threatening diseases. Family members are often the primary potential donors, especially in the context of haploidentical transplantation. However, their donation willingness may be influenced by multiple factors such as cognition, psychology, and economy. Understanding these factors is essential for clinical nurses to provide targeted support and improve donor availability.

### Aims

To explore the willingness of family members of hematological patients to donate for hematopoietic stem cell transplantation and its influencing factors, providing an empirical basis for improving the success rate of related donor transplantation and optimizing clinical nursing education strategies.

### Methods

Methods: A convenience sampling method was used to select family members of inpatients from the Hematology Department of a tertiary hospital in Chongqing from May 2024 to May 2025 as the survey subjects. A cross-sectional survey was conducted using a self-designed "Questionnaire on Donation Willingness of Family Members for Hematopoietic Stem Cell Transplantation," covering general demographic characteristics, cognition level of hematopoietic stem cells, donation attitudes and concerns, and perceived social support. According to Kendall's sample size estimation principle and considering 20% invalid questionnaires, no less than 300 questionnaires were planned to be distributed. Multiple linear regression analysis was used to explore the independent influencing factors of donation willingness.

### Results

A total of 265 valid questionnaires were collected in this study. The survey showed that the donation willingness score of patients' family members was 38.5±6.8 points, with 56.8% being completely willing to donate. The main concerns of family members regarding donation were concentrated on "concerns about the safety of the donation process" (86.4%), "worry about affecting their own health" (78.2%), and "economic burden" (23.1%). Regression analysis results showed that the family members' cognition level of donation, their relationship with the patient (immediate family), and family monthly income were the main factors influencing their donation willingness ($P<0.05$).

### Summary/Conclusion

The willingness of family members of hematological patients to donate hematopoietic stem cells is influenced by multiple factors, with significant concerns existing particularly at the cognitive and psychological levels. Clinical nursing staff should pay attention to providing refined education for family members, offer targeted psychological and informational support, and establish a systematic pre-donation communication mechanism to effectively enhance the donation willingness of family members, thereby creating opportunities for more patients awaiting transplantation.

**Copyright Information:** (Online) ISSN: 2572-9241
©2026 The Author(s). HemaSphere is published by John Wiley & Sons Ltd on behalf of European Hematology Association. This is an open access Abstract Book distributed under the Attribution-NonCommercial-NoDerivs (CC BY-NC-ND), which allows third parties to download the articles and share them with others as long as they credit the author and the Abstract Book, but they cannot change the content in any way or use them commercially.
**Abstract Book Citations:** Authors, Title, HemaSphere, 2026;10:(S1):pages. The abstract book DOIs can be found on the "Author Information" page.
**Disclaimer:** Articles published in the journal HemaSphere exclusively reflect the opinions of the authors. The authors are responsible for all content in their abstracts including accuracy of the facts, statements, citing resources, etc.

### (PF1178) AGE-ADJUSTED MEL/FLU CONDITIONING WITH REDUCED-DOSE ATG IN MRD-POSITIVE AML:A PROSPECTIVE STUDY

**Topic:** 23. Stem cell transplantation - Clinical

Fengbo Jin*[1], Wanlu Tian[1], Wei Qian[1], Lixia Liu[1], Yingying Chen[1]

[1] *The First Affiliated Hospital of Anhui Medical University�Anhui Public Health Clinical Center, hefei,anhui, China;*

### Background

Minimal residual disease (MRD)-positive acute myeloid leukemia (AML) remains a high-risk population with poor prognosis, particularly in the setting of allogeneic hematopoietic stem cell transplantation (allo-HSCT). Optimizing conditioning regimens to reduce relapse while maintaining acceptable toxicity is critical. This prospective study evaluates the efficacy and safety of an age-adjusted Melphalan/Fludarabine (Mel/Flu) conditioning regimen combined with reduced-dose anti-thymocyte globulin (ATG) in patients with MRD-positive AML undergoing haploidentical transplantation.

### Aims

This prospective study evaluates the efficacy and safety of an age-adjusted Melphalan/Fludarabine (Mel/Flu) conditioning regimen combined with reduced-dose anti-thymocyte globulin (ATG) in patients with MRD-positive AML undergoing haploidentical transplantation.

### Methods

A total of 11 patients with MRD-positive AML were enrolled in this ongoing prospective study. All patients were classified as high-risk according to ELN risk stratification. The conditioning regimen consisted of busulfan 3.2 mg/kg/day on days -8 to -7, fludarabine 30 mg/m²/day on days -6 to -2, and melphalan at age-adjusted doses: 140 mg/m² for patients ≤60 years and 100 mg/m² for those >60 years on days -6 to -5. For haploidentical donors, ATG was administered at a reduced dose of 5 mg/kg total on days -3 to -2,and with low dose of post-cyclophosphamide (14.5mg/kg/d,+3d,+5d).

### Results

The median age of the cohort was 57 years (range 16–62), with 6 males and 5 females. All patients had AML with high-risk features. Pre-transplant MRD positivity was confirmed in all cases (Figure 1). The transplantation protocol characteristics, including HLA Match Level, Melphalan Dosage (mg/m²), ATG Dosage. Pre-transplant Gene MRD and Pre-transplant Flow MRD, are summarized in the table below (Table 1). Post-transplant complications included cytomegalovirus (CMV) viremia and acute graft-versus-host disease (aGVHD). Due to the small sample size, Kaplan-Meier survival analysis was not feasible. The current survival status is shown in the chart below (red: deceased, blue: alive) (Figure 2). Minimal residual disease (MRD) status in patients pre- and post-transplant, as measured by gene-based MRD detection and flow cytometry MRD detection. Positive and negative MRD results are represented by red and blue bars, respectively (Figure 3)�

### Summary/Conclusion

This preliminary analysis suggests that age-adjusted Mel/Flu conditioning with reduced-dose ATG is feasible in MRD-positive AML patients undergoing allo-HSCT. All 11 enrolled patients achieved complete remission after transplantation. The two deceased patients died due to severe infections. All patients remained in a state of continuous remission during the follow-up period.Further patient enrollment and longer follow-up are needed to fully assess long-term outcomes and transplant-related toxicities.

**Copyright Information:** (Online) ISSN: 2572-9241
©2026 The Author(s). HemaSphere is published by John Wiley & Sons Ltd on behalf of European Hematology Association. This is an open access Abstract Book distributed under the Attribution-NonCommercial-NoDerivs (CC BY-NC-ND), which allows third parties to download the articles and share them with others as long as they credit the author and the Abstract Book, but they cannot change the content in any way or use them commercially.
**Abstract Book Citations:** Authors, Title, HemaSphere, 2026;10:(S1):pages. The abstract book DOIs can be found on the "Author Information" page.
**Disclaimer:** Articles published in the journal HemaSphere exclusively reflect the opinions of the authors. The authors are responsible for all content in their abstracts including accuracy of the facts, statements, citing resources, etc.

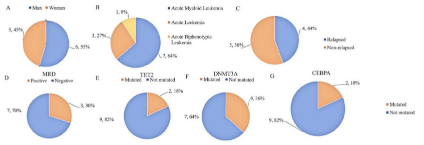

**Figure 1. Patient Baseline Characteristics**

(A) Gender distribution. (B) Distribution of disease types. (C) History of relapse. (D) MRD positivity rate pre-transplant. TET2(E)、DNMT3A(F)、CEBPA(G) mutation and distribution.

**Table 1 Transplantation Protocol Characteristics**

| Name/Sex | age | HLA Match Level | Melphalan Dosage (mg/m²) | ATG Dosage | Use of Letermovir | Pre-transplant Gene MRD | Pre-transplant Flow MRD(%) |
|---|---|---|---|---|---|---|---|
| 1/M | 57 | 6/12 | 140 | 5mg/kg | Yes | Positive | 2.83 |
| 2/F | 59 | 5/10 | 140 | 5mg/kg | Yes | Positive | 13.54 |
| 3/F | 16 | 12/12 | 100 | -- | Yes | Positive | 0.02 |
| 4/F | 53 | 10/12 | 100 | 5mg/kg | Yes | Positive | Negative |
| 5/M | 62 | 7/12 | 100 | 5mg/kg | Yes | Positive | Negative |
| 6/M | 54 | 6/12 | 140 | 5mg/kg | Yes | Positive | Negative |
| 7/M | 46 | 6/12 | 140 | 5mg/kg | Yes | Positive | 11.4 |
| 8/M | 32 | 6/12 | 140 | 5mg/kg | Yes | Positive | 10.5 |
| 9/M | 57 | 6/12 | 140 | 5mg/kg | Yes | Positive | 0.87 |
| 10/F | 57 | 6/12 | 140 | 5mg/kg | Yes | Positive | 2.07 |
| 11/F | 60 | 8/12 | 100 | 5mg/kg | Yes | Positive | 27.64 |

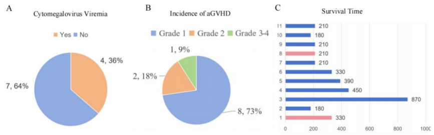

**Figure 2. Survival Status Chart**

(A) Cytomegalovirus ciremia after transplant. (B) Incidence of aGVHD. (C) Kaplan-Meier (KM) time of each patient、Survival Status Chart (Red: deceased, Blue: alive).

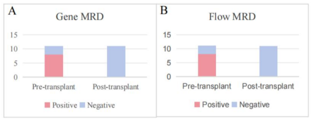

**Figure 3. Post-transplant MRD status**

Minimal residual disease (MRD) status in patients pre- and post-transplant, as measured by (A) gene-based MRD detection and (B) flow cytometry MRD detection. Positive and negative MRD results are represented by red and blue bars, respectively.

figure1,figure2,figure3,table 1

**Copyright Information:** (Online) ISSN: 2572-9241
©2026 The Author(s). HemaSphere is published by John Wiley & Sons Ltd on behalf of European Hematology Association. This is an open access Abstract Book distributed under the Attribution-NonCommercial-NoDerivs (CC BY-NC-ND), which allows third parties to download the articles and share them with others as long as they credit the author and the Abstract Book, but they cannot change the content in any way or use them commercially.
**Abstract Book Citations:** Authors, Title, HemaSphere, 2026;10:(S1):pages. The abstract book DOIs can be found on the "Author Information" page.
**Disclaimer:** Articles published in the journal HemaSphere exclusively reflect the opinions of the authors. The authors are responsible for all content in their abstracts including accuracy of the facts, statements, citing resources, etc.

### (PF1179) ASSOCIATION BETWEEN SERUM BIOMAKERS AND ACUTE GRAFT-VERSUS- HOST DISEASE.

**Topic:** 23. Stem cell transplantation - Clinical

Heng Liu*[1], Tao Wu[1]

*[1]The 940th Hospital of Joint Logistics Support force of Chinese People's Liberation Army, hematology, Gansu, China;*

### Background

Allogeneic hematopoietic stem cell transplantation (allo-HSCT) offers a curative option for patients with various malignant hematologic diseases, yet post-transplant complications exert a profound adverse impact on the ultimate treatment outcomes. Acute graft-versus-host disease (aGVHD) represents one of the most predominant complications and leading causes of mortality following allo-HSCT. Its early clinical manifestations lack specificity; thus, the early identification and prediction of aGVHD onset and its severity can facilitate more optimal clinical management of the disease, thereby reducing non-relapse mortality (NRM) after transplantation. In our center, we dynamically monitored the peripheral blood levels of cytokines including sST2, IL-6, IL-8, elafin, Reg-3α and sTNFR1 in patients undergoing allo-HSCT to investigate their correlations with the onset and severity of aGVHD, aiming to provide evidence for the identification of predictive biomarkers for aGVHD.

### Aims

This study aimed to explore the correlation of serum biomarkers-interleukin-6 (IL-6), interleukin-8 (IL-8), tumor necrosis factor receptor 1 (TNFR1), islet regeneration-derived factor 3α (REG-3α), soluble tumor suppressor protein 2 (sST2), and elafin with aGVHD following allogeneic hematopoietic stem cell transplantation (allo-HSCT).

### Methods

A retrospective analysis was conducted on the levels of IL-6, IL-8, sTNFR1, sST2, Reg3α, and elafin in 106 patients who underwent allo-HSCT, with measurements taken at 28, 56, and 90 days after transplantation. The cytokine levels were compared between patients who developed aGVHD and those who did not.

### Results

Among the participants, 49 patients (46.2%) developed aGVHD (aGVHD group), while 57 patients did not (control group). The sST2 levels in the aGVHD group were significantly higher than those in the control group at 28, 56, and 90 days post transplantation ($P < 0.05$). Additionally, the levels of sTNFR1 and Reg3α in the aGVHD group were elevated compared to those at 56 days post transplantation. No significant differences were observed in other biomarkers. Furthermore, the levels of sTNFR1, Reg3α, and elafin in patients with grade I aGVHD were significantly lower than those in patients with grade II-IV aGVHD.

### Summary/Conclusion

The serum biomarkers (IL-8, sST2, sTNFR1, Reg3α, elafin) are associated with the occurrence and severity of aGVHD. These indicators may be valuable for clinical diagnosis and intervention.

**Copyright Information:** (Online) ISSN: 2572-9241
©2026 The Author(s). HemaSphere is published by John Wiley & Sons Ltd on behalf of European Hematology Association. This is an open access Abstract Book distributed under the Attribution-NonCommercial-NoDerivs (CC BY-NC-ND), which allows third parties to download the articles and share them with others as long as they credit the author and the Abstract Book, but they cannot change the content in any way or use them commercially.
**Abstract Book Citations:** Authors, Title, HemaSphere, 2026;10:(S1):pages. The abstract book DOIs can be found on the "Author Information" page.
**Disclaimer:** Articles published in the journal HemaSphere exclusively reflect the opinions of the authors. The authors are responsible for all content in their abstracts including accuracy of the facts, statements, citing resources, etc.

### (PF1180) ACTIVATION OF VLA-4 SIGNAL REJUVENATES AGED HEMATOPOIETIC STEM CELLS BY CORRECTING MYELOID BIAS

**Topic:** 24. Hematopoiesis, stem cells and microenvironment

Jiayu Liu*[1], Qiaomei He[1], Fangfang Yu[1], Lianghua Shen[2], Xiaodan Ding[1], Yan Zhang[1], Xianmin Song[1]

[1]*Shanghai General Hospital, Shanghai, China;*[2]*Shanghai General Hospital, Shangha, China;*

### Background

Aging hematopoietic stem cells (HSCs) show reduced regenerative capacity and myeloid-biased differentiation, contributing to the impaired immunity and a higher risk of hematologic malignancy in older individuals. Our previous work demonstrated that deletion of *Itga4*, which encodes the α4 subunit of very late antigen-4 (VLA-4), drives myeloid skewing in HSCs. In contrast, activation of VLA-4 signal with the selective VLA-4 agonist 2 (A2) reduces HSC lineage skewing and promotes early lymphoid differentiation. These findings suggest that activation of VLA-4 signal may correct the myeloid bias and rejuvenate aged HSCs.

### Aims

We aimed to investigate whether activation of VLA-4 signal can reshape myeloid-biased differentiation of aged HSCs, thereby restoring hematopoietic function in aging.

### Methods

Based on our preliminary research, VLA-4 agonist (A2) was used as a chemical probe for activating VLA-4 signal. Young and aged mice received continuous A2 administration. We assessed hematopoietic and immunophenotypic changes in bone marrow (BM), the BM microenvironment, and spleen, including cytokine profiles in BM supernatant and cellular compositions of hematopoietic and stromal compartments. The regenerative capacities of aged HSCs were evaluated through competitive transplantation mouse models with purified HSCs or bone marrow cells. Additionally, 5-fluorouracil (5-FU) challenge was applied during continuous A2 administration to test whether VLA-4 activation rescues stress hematopoiesis and promotes hematopoietic recovery in aged mice.

### Results

In aging models, the hematopoiesis was rejuvenated with A2 treatment, characterized by reduced ineffective HSC expansion, attenuated myeloid skewing (decreased GMPs), and enhanced lymphoid potential (increased CLPs and naïve T cells). Additionally, the inflammatory cytokines in the aged bone marrow microenvironment were suppressed and the homeostasis of stromal cell subsets was restored with A2, thereby reshaping the aged BM niche. Competitive transplantation assays demonstrated that the self-renewal capacity of aged HSCs was enhanced and lymphoid output was promoted with A2. Under 5-FU-induced stress, VLA-4 activation recapitulated phenotypes observed under steady-state conditions, namely reducing senescence-induced overexpansion of HSCs and GMPs and decreasing inflammatory factors in bone marrow supernatant. Moreover, VLA-4 activation exerted cytoprotective effects, as evidenced by decreased DNA damage and reduced apoptosis.. Functionally, VLA-4 activation under 5-FU challenge extended survival of aged mice, facilitated recovery of long-term reconstitution capacity and enhanced lymphoid output of aged HSCs.

### Summary/Conclusion

Our data highlight an important role for VLA-4 signaling in regulating HSC aging and demonstrate that VLA-4 activation rejuvenates aged HSCs, at least in part, by correcting age-associated differentiation bias and remodeling the bone marrow microenvironment.

**Copyright Information:** (Online) ISSN: 2572-9241
©2026 The Author(s). HemaSphere is published by John Wiley & Sons Ltd on behalf of European Hematology Association. This is an open access Abstract Book distributed under the Attribution-NonCommercial-NoDerivs (CC BY-NC-ND), which allows third parties to download the articles and share them with others as long as they credit the author and the Abstract Book, but they cannot change the content in any way or use them commercially.
**Abstract Book Citations:** Authors, Title, HemaSphere, 2026;10:(S1):pages. The abstract book DOIs can be found on the "Author Information" page.
**Disclaimer:** Articles published in the journal HemaSphere exclusively reflect the opinions of the authors. The authors are responsible for all content in their abstracts including accuracy of the facts, statements, citing resources, etc.

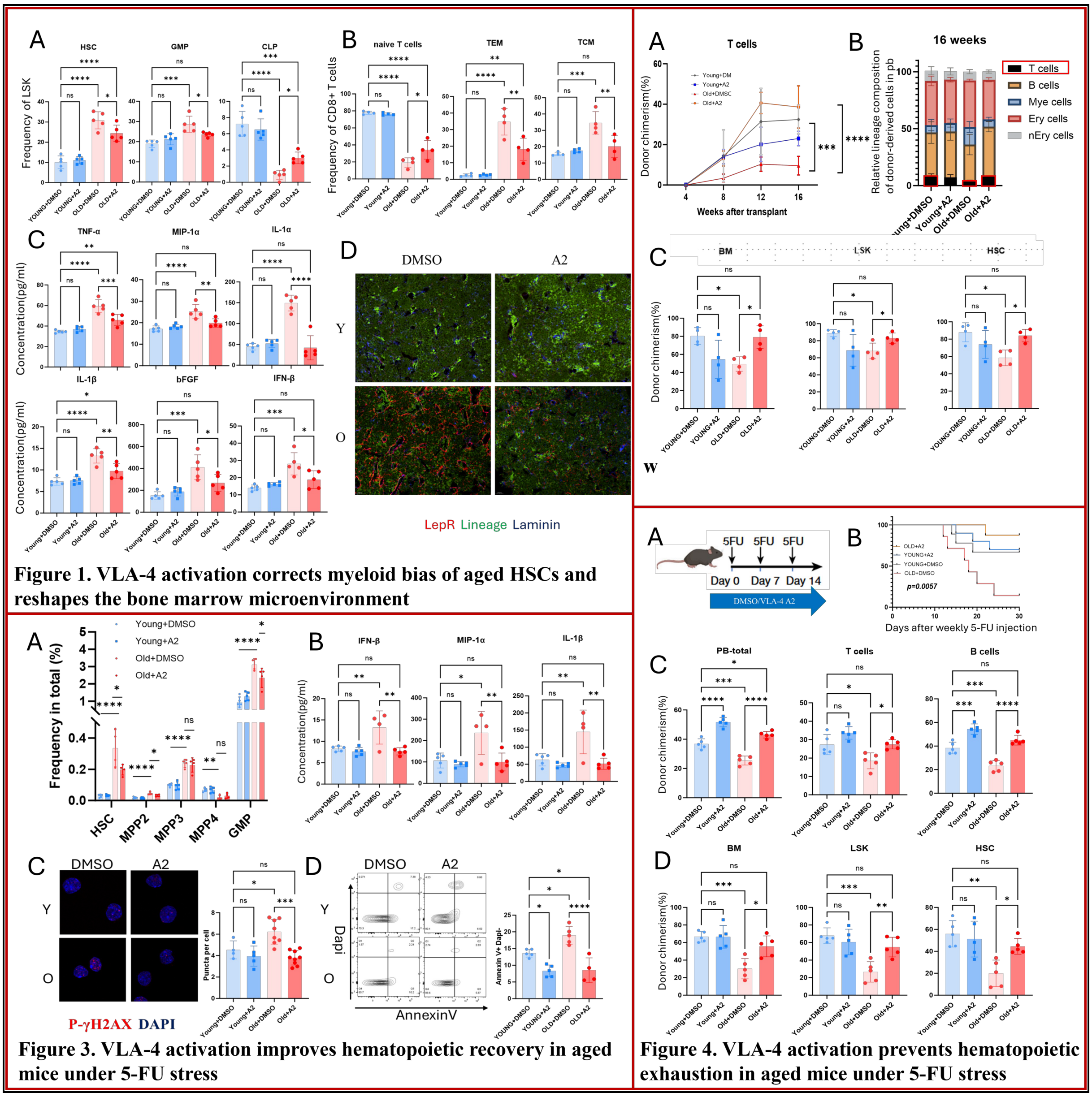

Figure 1. VLA-4 activation corrects myeloid bias of aged HSCs and reshapes the bone marrow microenvironment. Figure 2. VLA-4 activation improves self-renewal capacity and lymphoid output of aged HSCs. Figure 3. VLA-4 activation improves hematopoietic recovery in aged mice under 5-FU stress. Figure 4. VLA-4 activation prevents hematopoietic exhaustion in aged mice under 5-FU stress.

**Copyright Information:** (Online) ISSN: 2572-9241
©2026 The Author(s). HemaSphere is published by John Wiley & Sons Ltd on behalf of European Hematology Association. This is an open access Abstract Book distributed under the Attribution-NonCommercial-NoDerivs (CC BY-NC-ND), which allows third parties to download the articles and share them with others as long as they credit the author and the Abstract Book, but they cannot change the content in any way or use them commercially.
**Abstract Book Citations:** Authors, Title, HemaSphere, 2026;10:(S1):pages. The abstract book DOIs can be found on the "Author Information" page.
**Disclaimer:** Articles published in the journal HemaSphere exclusively reflect the opinions of the authors. The authors are responsible for all content in their abstracts including accuracy of the facts, statements, citing resources, etc.

### (PF1181) MUTUAL REGULATION BETWEEN ACUTE MYELOID LEUKAEMIA AND CHOLINERGIC SIGNALLING IN THE BONE MARROW MODULATES RESISTANCE TO INTENSIVE CHEMOTHERAPY

**Topic:** 24. Hematopoiesis, stem cells and microenvironment

Yexuan Zhu*[1], Claire Fielding[1], Simón Méndez-Ferrer[1, 2, 3]

*[1]University of Cambridge, Cambridge, United Kingdom; [2]NHS Blood and Transplant, Cambridge, United Kingdom; [3]University of Seville, Seville, Spain;*

### Background

Upon transformation, haematopoietic stem/progenitor cells (HSPCs) or granulocyte-monocyte-progenitors (GMPs) behave as leukaemia-initiating cells (LICs) with stem cell properties (LSCs) in acute myeloid leukaemia (AML). Quiescent LSCs can escape anti-proliferative chemotherapy and drive relapse. Previous studies showed that sympathetic cholinergic neurons stimulate bone marrow (BM) mesenchymal stromal cells (BMSCs) to release CXCL12, which promotes quiescence of normal and leukaemic HSCs (Nat Commun 2022;13:543). Preliminary evidence indicates that loss of cholinergic signals in the BM niche accelerates myeloid leukaemogenesis (HemaSphere 2022;6:S3;S250). Conversely, increased parasympathetic-derived acetylcholine (ACh) may directly increase AML LSC survival and maintenance (HemaSphere 2023;7:S3;P420).

### Aims

Dissect LSC-intrinsic and niche-dependent effects of cholinergic signals in AML.

### Methods

Inducible MLL-AF9 mice (Cancer Cell 2016;30:43) were used to model highly chemo-resistant AML. BMSCs were cultured as self-renewing mesenchymal spheres (mesenspheres). Superior cervical ganglion neurons were co-cultured with iMLL-AF9 blasts and/or mesenspheres. Direct and niche-mediated cholinergic regulation of AML were studied through leukaemic GMP (L-GMP) monocultures or co-cultures with mesenspheres treated with cholinergic modulators. LSC and LIC function were measured by flow cytometry, CFU assays and *in vivo* upon chemotherapy treatment.

### Results

AML blasts can reprogramme sympathetic neurons and induce the cholinergic marker GFRα2, more pronouncedly in the presence of BMSCs (a). Resembling the physiological cholinergic switch (Cell Stem Cell 2022;29:528), AML-dependent cholinergic induction was mediated by interleukin-6 (IL6) and inhibited by IL6 blockade (a). Similar results were observed using AML blast conditioned medium (b). Interestingly, divergent regulation was noted on AML cells and their niche: ACh triggered cell cycle entry of monocultured L-GMPs (c), while it preserved L-GMP quiescence in co-culture with BMSCs (d); both effects were likely transduced by the a7-nicotinic receptor as they were blocked by a-bungarotoxin (c-d).
To evaluate the contribution of cholinergic neural signals, we analysed GFRα2 knockout mice, which lack cholinergic innervation (Neuron 1999, 22:243). Endosteal BM L-HSPCs showed reduced quiescence in $GFR\alpha2^{-/-}$ mice after AraC (e); this was associated with delayed relapse after induction chemotherapy (f). Similarly, the peripheral a7-nicotinic receptor antagonist hexamethonium sensitised LSCs to chemotherapy, delayed AML relapse (g) and reduced leukaemic burden (h) and L-GMP frequency (i); consistently, combinatorial therapy showed decreased colony formation in replating colony assays and reduced long-term BM LICs (j-l). However, L-HSPCs (m) and L-GMPs (n) escaping combinatorial therapy showed cell cycle entry, formed larger colonies in replating CFU assays (o) and accelerated AML development in secondary recipients (p).

**Copyright Information:** (Online) ISSN: 2572-9241
©2026 The Author(s). HemaSphere is published by John Wiley & Sons Ltd on behalf of European Hematology Association. This is an open access Abstract Book distributed under the Attribution-NonCommercial-NoDerivs (CC BY-NC-ND), which allows third parties to download the articles and share them with others as long as they credit the author and the Abstract Book, but they cannot change the content in any way or use them commercially.
**Abstract Book Citations:** Authors, Title, HemaSphere, 2026;10:(S1):pages. The abstract book DOIs can be found on the "Author Information" page.
**Disclaimer:** Articles published in the journal HemaSphere exclusively reflect the opinions of the authors. The authors are responsible for all content in their abstracts including accuracy of the facts, statements, citing resources, etc.

### Summary/Conclusion

Our results reveal a double-edged sword effect of cholinergic regulation on AML LSCs. Direct ACh stimulation promotes AML proliferation whilst, in the BM niche, BMSC-mediated signalling protects quiescent LSCs from chemotherapy. Cholinergic blockade enhances chemosensitivity but also stimulates proliferation of residual LSCs escaping chemotherapy. We conclude that AML-induced cholinergic signals promote 1) leukaemogenesis acting directly on leukaemic cells and 2) chemotherapy resistance through modulation of BMSCs.

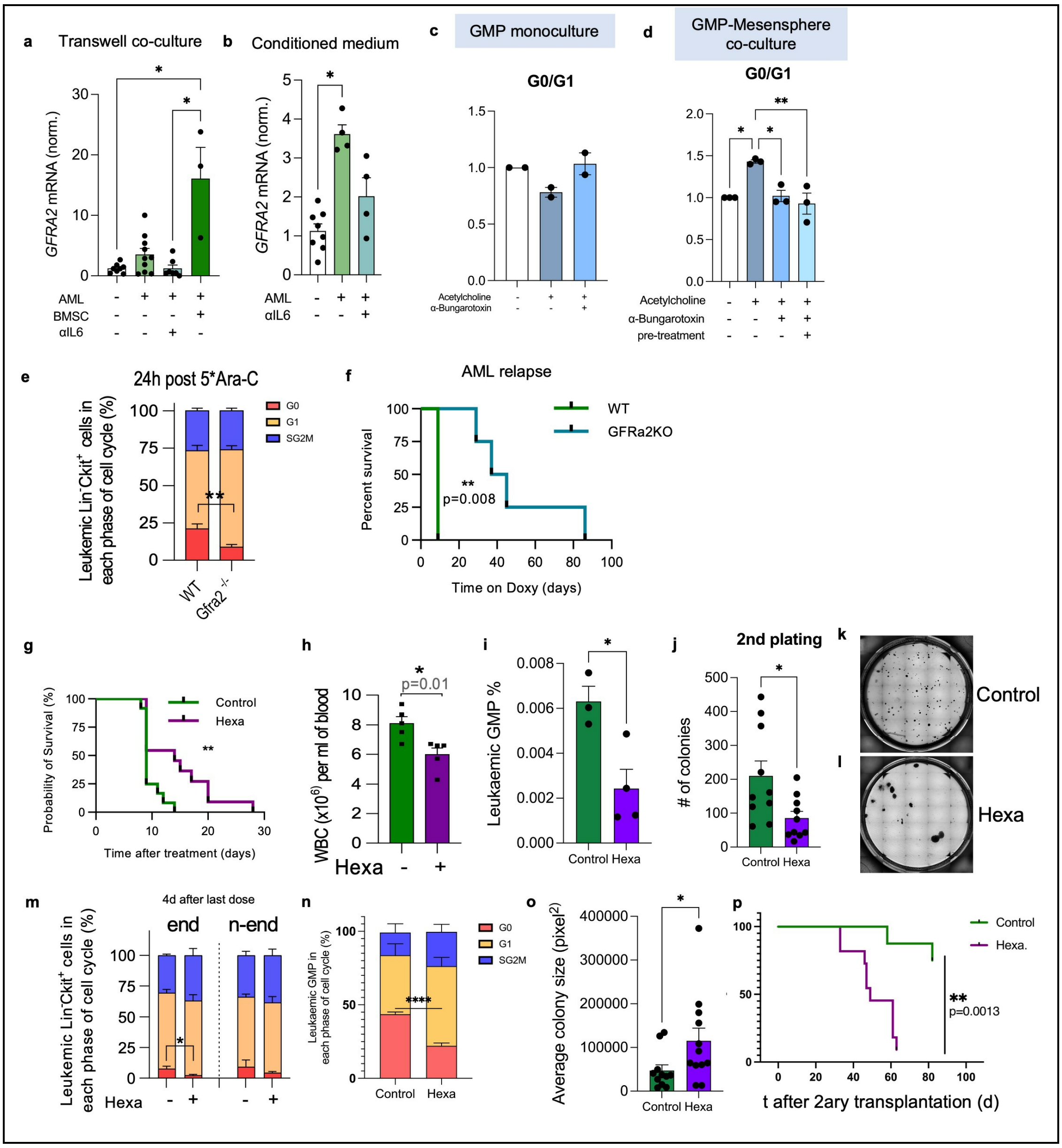

**Copyright Information:** (Online) ISSN: 2572-9241
©2026 The Author(s). HemaSphere is published by John Wiley & Sons Ltd on behalf of European Hematology Association. This is an open access Abstract Book distributed under the Attribution-NonCommercial-NoDerivs (CC BY-NC-ND), which allows third parties to download the articles and share them with others as long as they credit the author and the Abstract Book, but they cannot change the content in any way or use them commercially.
**Abstract Book Citations:** Authors, Title, HemaSphere, 2026;10:(S1):pages. The abstract book DOIs can be found on the "Author Information" page.
**Disclaimer:** Articles published in the journal HemaSphere exclusively reflect the opinions of the authors. The authors are responsible for all content in their abstracts including accuracy of the facts, statements, citing resources, etc.

### (PF1184) DRIVERS SHAPING PRE-NEOPLASTIC CLONAL EVOLUTION

**Topic:** 24. Hematopoiesis, stem cells and microenvironment

Kristina Kirschner*[1], Eric Latorre-Crespo[2], Neil Robertson[3], Gozde Kosebent[1], Mesbah Uddin[4], Eric Whitsel[5], Michael Honigberg[4], Alexander Bick[6], Alexander Reiner[7], Valeria Orru[8], Michele Marongiu[8], Franceso Cucca[8], Edoardo Fiorillo[8], Ian Deary[9], Sarah Harris[9], Simon Cox[9], Riccardo Marioni[9], Linus Schumacher[9], Tamir Chandra[1]

*[1]Mayo Clinic, Hematology, Rochester, United States of America;[2]Centre de Recerca Matemàtica, Barcelona, Spain;[3]Mayo Clinic, Rochester, United States of America; [4]Broad Institute, Boston, United States of America; [5]University of North Carolina, Chapel Hill, United States of America; [6]Vanderbilt University, Nashville, United States of America; [7]University of Washington, Seattle, United States of America; [8]National Research Council, Lanusei, United States of America; [9]University of Edinburgh, Edinburgh, United Kingdom;*

### Background

Clonal hematopoiesis (CH) is characterized by the expansion of blood cell clones carrying somatic mutations in otherwise healthy aged individuals and is associated with increased risk of hematologic malignancies, cardiovascular disease, and all-cause mortality. While CH mutations affect diverse genes commonly mutated in myeloid malignancies, the underlying mechanisms driving clonal expansion and their association with adverse outcomes remain incompletely understood. Traditional approaches focusing on single mutation properties and variant allele frequency (VAF) have shown limited predictive value for clinical risk stratification.

### Aims

To elucidate the fundamental mechanisms driving pre-neoplastic clonal evolution over the human lifespan and identify key factors that determine progression to adverse clinical outcomes.

### Methods

We analyzed longitudinal sequencing data from three independent aging cohorts comprising 713 individuals with CH (total observations = 2,341) to model clonal dynamics over time. We integrated mutational fitness measurements with co-mutational context analysis and estimated age at time of mutation acquisition (ATMA) using Bayesian computational modeling. Clonal competition dynamics were assessed by tracking VAF trajectories and examining fitness effects in different mutational backgrounds. Clinical outcome was measured as overall survival and correlated with our integrated evolutionary parameters

### Results

Our analysis reveals that clonal evolution is fundamentally shaped by mutational context rather than individual mutation properties alone. The same driver mutation demonstrated dramatically different fitness effects depending on co-occurring mutations, with a mean 10% difference in predicted clone size across different clonal contexts. This context-dependency explains the limited predictive utility of VAF measurements in isolation. We demonstrate that CH risk exists on a continuous spectrum determined by the complete mutational landscape within each individual. Critically, the timing of mutation acquisition emerged as a key determinant of clonal expansion potential. By integrating mutation fitness with ATMA, we achieved significant prediction of survival outcomes (p=0.04), while traditional parameters (VAF or fitness alone) showed no significant association. Our model explains the paradoxical behavior of specific mutations: DNMT3A mutations typically arise early in life but often remain relatively small clones without substantial late-life expansion, whereas splicing factor mutations emerge later but demonstrate more aggressive growth kinetics. These patterns reflect the interplay between the temporal window available for expansion and the selective pressures operating at different life stages

**Copyright Information:** (Online) ISSN: 2572-9241
©2026 The Author(s). HemaSphere is published by John Wiley & Sons Ltd on behalf of European Hematology Association. This is an open access Abstract Book distributed under the Attribution-NonCommercial-NoDerivs (CC BY-NC-ND), which allows third parties to download the articles and share them with others as long as they credit the author and the Abstract Book, but they cannot change the content in any way or use them commercially.
**Abstract Book Citations:** Authors, Title, HemaSphere, 2026;10:(S1):pages. The abstract book DOIs can be found on the "Author Information" page.
**Disclaimer:** Articles published in the journal HemaSphere exclusively reflect the opinions of the authors. The authors are responsible for all content in their abstracts including accuracy of the facts, statements, citing resources, etc.

**Summary/Conclusion**

Pre-neoplastic clonal evolution is governed by the dynamic interplay between mutational fitness, co-mutational context, and timing of acquisition. Our integrated framework substantially improves outcome prediction compared to conventional VAF-based approaches and provides mechanistic insight into CH heterogeneity. These findings have important implications for risk stratification and early intervention trial design.

**Copyright Information:** (Online) ISSN: 2572-9241
©2026 The Author(s). HemaSphere is published by John Wiley & Sons Ltd on behalf of European Hematology Association. This is an open access Abstract Book distributed under the Attribution-NonCommercial-NoDerivs (CC BY-NC-ND), which allows third parties to download the articles and share them with others as long as they credit the author and the Abstract Book, but they cannot change the content in any way or use them commercially.
**Abstract Book Citations:** Authors, Title, HemaSphere, 2026;10:(S1):pages. The abstract book DOIs can be found on the "Author Information" page.
**Disclaimer:** Articles published in the journal HemaSphere exclusively reflect the opinions of the authors. The authors are responsible for all content in their abstracts including accuracy of the facts, statements, citing resources, etc.

### (PF1185) GLUCOCORTICOID INDUCED LEUCINE ZIPPER REGULATES HEMATOPOIETIC STEM CELL QUIESCENCE VIA REPRESSION OF MTOR-MEDIATED ACTIVATION

**Topic:** 24. Hematopoiesis, stem cells and microenvironment

Oxana Bereshchenko*[1], Marta Febo[1], Zenobio Barros de Viana[1], Stefano Bruscoli[1]

[1]*University of Perugia, Perugia, Italy;*

**Background**

The life-long maintenance of hematopoietic stem cells (HSC) is linked to their quiescent state, while proliferation is associated with differentiation and a loss of stem cell potential. The balance between HSC quiescence and proliferation is tightly regulated by intrinsic and extrinsic cues in the bone marrow. This regulatory network may become altered by various stresses, leading to aberrant HSC cell cycling, loss of HSC function, and hematological malignancy. Glucocorticoids (GC) are steroid hormones that play a critical role in regulating vital body functions, including immune and stress responses. GC have been shown to regulate HSC development in zebrafish and homing of human HSC (1,2). Glucocorticoid-Induced Leucine Zipper (GILZ) is a gene rapidly induced by GC, which mediates many of GC' anti-proliferative and anti-inflammatory effects by similarly interfering with regulation of cell proliferation, survival and gene expression in many cell types (3-5). Its role in control of HSC function has not been yet tested.

**Aims**

The aim of this study is to evaluate the role of GILZ in HSC at steady-state and in response to GC using in vivo mouse models.

**Methods**

The role of GILZ in HSC was investigated using wild-type (WT), GILZ knock-out (KO), and GILZ-overexpressing mice (GILZ-TG). Competitive transplantation assays were performed using WT and GILZ-KO bone marrow cells. HSC frequency, number, proliferation status and signaling pathways activities were evaluated using flow cytometry; differential gene expression was analyzed by RNA Seq and qPCR methods; statistical analyses were performed using Prism software.

**Results**

We found that within HPSC compartment, GILZ is expressed at highest levels in most primitive HSC. We addressed GILZ's role in HSC function using GILZ knock-out (KO) and GILZ overexpressing transgenic (TG) mice. At steady state, GILZ-KO mice showed reduced proportion of HSC in G0 phase of the cell cycle and a reduced HSC number, associated with increased cell death by apoptosis. Cell intrinsic role of GILZ in controlling HSC proliferation was confirmed in competitive transplantation experiments. Comprehensive analysis of gene expression revealed that GILZ deficiency results in significantly reduced expression of genes implicated in HSC function/identity, an enrichment in the mTOR signaling signature and a significant up-regulation of Myc-target genes, suggesting that GILZ deficiency is associated with a loss of HSC quiescence and HSC activation. Consistently, GILZ deficient HSC showed enhanced proliferation as revealed by flow cytometry analysis of Ki67 expression and DNA content, associated with higher levels of mTOR signaling activity as evidenced by flow cytometry analysis of phosphorylated S6 protein. mTOR inhibition by rapamycin treatment in vivo was able to rescue the increased proliferation of GILZ KO HSC. Importantly, in the absence of GILZ, HSC did not respond to a growth-suppressing effect of dexamethasone (Dex) associated with downregulation of mTOR signaling in the HSC.

**Copyright Information:** (Online) ISSN: 2572-9241
©2026 The Author(s). HemaSphere is published by John Wiley & Sons Ltd on behalf of European Hematology Association. This is an open access Abstract Book distributed under the Attribution-NonCommercial-NoDerivs (CC BY-NC-ND), which allows third parties to download the articles and share them with others as long as they credit the author and the Abstract Book, but they cannot change the content in any way or use them commercially.
**Abstract Book Citations:** Authors, Title, HemaSphere, 2026;10:(S1):pages. The abstract book DOIs can be found on the "Author Information" page.
**Disclaimer:** Articles published in the journal HemaSphere exclusively reflect the opinions of the authors. The authors are responsible for all content in their abstracts including accuracy of the facts, statements, citing resources, etc.

**Summary/Conclusion**

Our data demonstrate that GC represent a novel mechanism of preserving HSC quiescence and function acting via its effector GILZ by suppression of mTOR-mediated regulation of the activation related genes. Thus, GILZ protein emerges as a novel factor preserving HSC quiescence.

**Copyright Information:** (Online) ISSN: 2572-9241
©2026 The Author(s). HemaSphere is published by John Wiley & Sons Ltd on behalf of European Hematology Association. This is an open access Abstract Book distributed under the Attribution-NonCommercial-NoDerivs (CC BY-NC-ND), which allows third parties to download the articles and share them with others as long as they credit the author and the Abstract Book, but they cannot change the content in any way or use them commercially.
**Abstract Book Citations:** Authors, Title, HemaSphere, 2026;10:(S1):pages. The abstract book DOIs can be found on the "Author Information" page.
**Disclaimer:** Articles published in the journal HemaSphere exclusively reflect the opinions of the authors. The authors are responsible for all content in their abstracts including accuracy of the facts, statements, citing resources, etc.

### (PF1186) CHRONIC INFLAMMATION DRIVES HSC AGING, CLONAL EXPANSION, AND ALTERED INFECTION RESPONSE IN MICE

**Topic:** 24. Hematopoiesis, stem cells and microenvironment

Srdjan Grusanovic*[1], Magdalena Havlova[1, 2], Matus Kolarik[3], Safaa Andarawi[1, 4], Irina Ribeiro Bas[1, 2], Sofia Bentivegna[5, 6], Kirsten Grønbæk[5, 6, 7], Julia Starkova[3], Meritxell Alberich-Jorda[1, 3]

*[1]Institute of Molecular Genetics of the Czech Academy of Sciences, Prague, Czechia;[2]Faculty of Science, Charles University, Prague, Czechia; [3]Childhood Leukaemia Investigation Prague, Department of Pediatric Haematology and Oncology, 2nd Faculty of Medicine, Charles University in Prague, University Hospital Motol and Homolka, Prague, Czechia; [4]Biomedical Center, Faculty of Medicine in Pilsen, Charles University, Pilsen, Czechia; [5]Rigshospitalet, Department of Hematology, Copenhagen, Denmark; [6]University of Copenhagen, Biotech Research and Innovation Centre, Copenhagen, Denmark; [7]Department of Clinical Medicine, Faculty of Health and Medical Sciences, University of Copenhagen, Copenhagen, Denmark;*

**Background**

Hematopoietic stem cells (HSCs) sustain lifelong blood production and, although largely quiescent at steady state, they must dynamically respond to inflammatory stress. Both acute and chronic inflammatory signals directly act on HSCs and alter their proliferation, differentiation, and self-renewal. Chronic inflammation, a hallmark of autoimmune and autoinflammatory diseases, exposes HSCs to persistent stress. Clinically, patients with sterile inflammatory disorders are more susceptible to infections, and age-associated low-grade inflammation is strongly linked to clonal hematopoiesis, an important risk factor for hematologic malignancies. While the immediate effects of inflammatory signaling on HSC activation have been extensively studied, far less is known about the long-term consequences of sustained sterile inflammation.

**Aims**

We aimed to determine whether sterile chronic inflammation accelerates HSC aging, promotes clonal hematopoiesis, and alters HSC responses to infection

**Methods**

Chronic multifocal osteomyelitis (CMO) mouse model, flow cytometry, murine chimeras, RNA sequencing, murine survival assays, *Candida albicans* infection and LPS administration *in vivo*, confocal imaging of H4K16ac, glucose uptake, seahorse metabolic profiling

**Copyright Information:** (Online) ISSN: 2572-9241
©2026 The Author(s). HemaSphere is published by John Wiley & Sons Ltd on behalf of European Hematology Association. This is an open access Abstract Book distributed under the Attribution-NonCommercial-NoDerivs (CC BY-NC-ND), which allows third parties to download the articles and share them with others as long as they credit the author and the Abstract Book, but they cannot change the content in any way or use them commercially.
**Abstract Book Citations:** Authors, Title, HemaSphere, 2026;10:(S1):pages. The abstract book DOIs can be found on the "Author Information" page.
**Disclaimer:** Articles published in the journal HemaSphere exclusively reflect the opinions of the authors. The authors are responsible for all content in their abstracts including accuracy of the facts, statements, citing resources, etc.

**Results**

CMO HSCs exhibited significantly reduced glucose uptake ability and a loss of H4K16ac polarity in comparison to WT, and the Seahorse metabolic profiling revealed increased total ATP production and elevated mitochondrial ATP synthesis in CMO c-Kit$^{+}$ cells, supporting a shift toward an aging-associated metabolic phenotype. The CMO inflammatory environment conferred a mild competitive advantage to Tet2$^{-/-}$ cells compared to WT environment, thereby promoting clonal expansion. This expansion was observed in both peripheral blood (PB) and bone marrow (BM) of recipient mice and was most pronounced in BM progenitors (MPP2, MPP3, MPP4, and ST-HSCs), while the LT-HSCs remained unaffected. RNA-seq analysis demonstrated that CMO-exposed Tet2$^{-/-}$ LSK cells upregulated IL-1 and IL-6 signaling pathways and showed enhanced differentiation and cell-cycle activity compared to WT-exposed Tet2$^{-/-}$ LSKs. Upon secondary transplantation, CMO-exposed Tet2$^{-/-}$ LSKs exhibited a transient engraftment advantage over WT-exposed Tet2$^{-/-}$ LSKs. However, this difference was no longer evident 16 weeks post-transplantation, further supporting that LT-HSC function was not permanently altered by CMO exposure. In response to infection, LPS-challenged CMO mice displayed a greater increase in PB granulocytes, reduced egress of BM granulocytes, and moderate LSK expansion compared to WT mice. Similarly, upon *Candida albicans* administration, CMO mice exhibited a similar granulocytic response as in the LPS challenge and showed improved overall survival relative to WT controls.

**Summary/Conclusion**

In conclusion, our data indicate that (i) CMO HSCs display signs of accelerated aging, (ii) CMO environment promotes expansion of Tet2-/- progenitors and imprints inflammatory and proliferative signatures without affecting the LT-HSCs, and (iii) CMO mice display enhanced resistance to infections, possibly due to the pre-existing granulocytic expansion. Altogether, our study highlights chronic sterile inflammation as a potential driver of hematopoietic aging, clonal expansion, and altered host defense with direct relevance to human autoinflammatory disease.

This project was partially supported by the Czech Science Foundation (GACR # 24-10938S) and EHA Junior Research Grant (# RG-202410-06934)

**Copyright Information:** (Online) ISSN: 2572-9241
©2026 The Author(s). HemaSphere is published by John Wiley & Sons Ltd on behalf of European Hematology Association. This is an open access Abstract Book distributed under the Attribution-NonCommercial-NoDerivs (CC BY-NC-ND), which allows third parties to download the articles and share them with others as long as they credit the author and the Abstract Book, but they cannot change the content in any way or use them commercially.
**Abstract Book Citations:** Authors, Title, HemaSphere, 2026;10:(S1):pages. The abstract book DOIs can be found on the “Author Information” page.
**Disclaimer:** Articles published in the journal HemaSphere exclusively reflect the opinions of the authors. The authors are responsible for all content in their abstracts including accuracy of the facts, statements, citing resources, etc.

### (PF1187) IMPACT OF THE BONE MARROW MICROENVIRONMENT IN GATA2 DEFICIENCY SYNDROME

**Topic:** 24. Hematopoiesis, stem cells and microenvironment

Pauline Enfedaque*[1], Antoine Nouhaud[1], Surya Cayre[2], Hedi Somai[1], Simon Boutinaud[3], Mathieu Bouttier[1], Guillaume Andrieu[4], Esmaa Sellam[1], Clémence Rouzier[1], Sylvie Hebrard[1], Stéphanie Dufrechou[5], Naïs Prade[5], Bastien Gerby[1], Christine Didier[1], Maria Carolina Florian[2], Marlène Pasquet[3], Eric Delabesse[1, 5], Cyril Broccardo[1], Laetitia Largeaud[1, 5]

*[1]Research Center in Cancer de Toulouse, Toulouse, France; [2]Idibell Institut d'Investigació Biomèdica de Bellvitge, Barcelone, Spain; [3]CHU Purpan, Toulouse, France; [4]Hôpital Necker-Enfants Malades, Paris, France; [5]IUCT Oncopole, Toulouse, France;*

### Background

GATA2 deficiency is a hereditary syndrome predisposing to hematologic malignancies, immune deficiency and vascular abnormalities, with nearly 80% of patients developing hematologic disorders before the age of 40.

### Aims

Although disease progression has largely been considered stem cell-intrinsic, the contribution of the bone marrow microenvironment to hematopoietic failure remains poorly defined.

### Methods

To address this, we investigate the structural, transcriptional and functional properties of the niche in a *Gata2*$^{R396Q/+}$ mouse model carrying a recurrent germline mutation, previously shown to disrupt hematopoietic stem and progenitor cells (HSPC) balance and function (Largeaud L *et al.*, 2025).

### Results

Spectral cytometry revealed a significant expansion of endothelial cell populations in mutant bone marrow. Three-dimensional bone imaging demonstrated vascular network disorganization, with increased arteriolar vessels. In parallel, LepR staining intensity was enhanced at the vascular interface, suggesting altered perivascular mesenchymal stromal cells (MSC) engagement and niche architecture remodeling.

To determine the molecular consequences of this remodeling, we performed single-cell RNAseq and bulk RNAseq on sorted HSPC, MSCs, endothelial cells and osteoblasts. Across all compartments, mutant cells exhibited downregulation of inflammataroy and immune response pathways, consistent with impaired responsiveness to environmental cues. Plus, we observed an enrichment of metabolic and proliferative programs, defining a transcriptional state of compensatory activation leading to exhaustion.

Functionally, mutant MSCs displayed impaired self-renewal and increased adipogenic and osteogenic differentiation potential. Bone imaging confirmed enhanced adipogenesis in the proximal metaphysis, consistent with premature niche aging.

Because hematopoietic stem cell transplantation (HSCT) is currently the only curative therapy for GATA2 patients, we next assessed whether the mutant niche could sustain normal hematopoiesis. WT HSPCs transplanted into Gata2$^{R396Q/+}$ recipient mice showed reduced frequency of early hematopoietic progenitors, indicating impaired maintenance of early hematopoietic compartments. Under chronic inflammatory stimulation, peripheral blood analysis revealed the emergence of a myeloid bias, consistent with stress-enforced differentiation.

**Copyright Information:** (Online) ISSN: 2572-9241
©2026 The Author(s). HemaSphere is published by John Wiley & Sons Ltd on behalf of European Hematology Association. This is an open access Abstract Book distributed under the Attribution-NonCommercial-NoDerivs (CC BY-NC-ND), which allows third parties to download the articles and share them with others as long as they credit the author and the Abstract Book, but they cannot change the content in any way or use them commercially.
**Abstract Book Citations:** Authors, Title, HemaSphere, 2026;10:(S1):pages. The abstract book DOIs can be found on the "Author Information" page.
**Disclaimer:** Articles published in the journal HemaSphere exclusively reflect the opinions of the authors. The authors are responsible for all content in their abstracts including accuracy of the facts, statements, citing resources, etc.

**Summary/Conclusion**

Together, these findings demonstrate that GATA2 deficiency is not only a hematopoietic cell-intrinsic disorder, but also a disease of maladaptive niche remodeling. The mutant microenvironment exhibits impaired inflammatory sensing, metabolic overactivation and premature differentiation driving exhaustion of both microenvironment cells and transplanted WT cells. These results provide a framework to explain post-transplantation vulnerabilities and highlight the importance of targeting microenvironment dysfunction to preserve long-term hematopoiesis in GATA2 patients.

**Copyright Information:** (Online) ISSN: 2572-9241
©2026 The Author(s). HemaSphere is published by John Wiley & Sons Ltd on behalf of European Hematology Association. This is an open access Abstract Book distributed under the Attribution-NonCommercial-NoDerivs (CC BY-NC-ND), which allows third parties to download the articles and share them with others as long as they credit the author and the Abstract Book, but they cannot change the content in any way or use them commercially.
**Abstract Book Citations:** Authors, Title, HemaSphere, 2026;10:(S1):pages. The abstract book DOIs can be found on the "Author Information" page.
**Disclaimer:** Articles published in the journal HemaSphere exclusively reflect the opinions of the authors. The authors are responsible for all content in their abstracts including accuracy of the facts, statements, citing resources, etc.

### (PF1188) BLOOD-BASED EPIGENETIC INSTABILITY LINKED TO HUMAN AGING AND DISEASE

**Topic:** 24. Hematopoiesis, stem cells and microenvironment

Salman Basrai[1, 2], Ido Nofech-Mozes[1, 2], Rajesh Detroja[3], Fernando Scolari[4], Mehran Bakhtiari[3], Andrea Arruda[3], Tracy Murphy[3], Scott Bratman[3, 5], Steven M Chan[3, 6], Mark Minden[3], Jae-Sook Ahn[7], Dennis Kim[3], Robert Kridel[3, 6], Phyllis Billia[8], Sagi Abelson*[1, 2]

*[1]Ontario Institute for Cancer Research, Toronto, Canada;[2]University of Toronto, Molecular Genetics, Toronto, Canada;[3]Princess Margaret Cancer Centre, Toronto, Canada; [4]Hospital de Clínicas de Porto Alegre, Porto Alegre, Brazil;[5]University of Toronto, Radiation Oncology, Toronto, Canada; [6]University of Toronto, Medical Biophysics, Toronto, Canada;[7]Chonnam National University, Hwasun, South Korea; [8]TGH Peter Munk Cardiac Centre, Toronto, Canada;*

### Background

Clonal hematopoiesis (CH) is a prevalent, age-associated state marked by expansion of genetically distinct hematopoietic clones and increased risk of hematologic malignancies, cardiovascular events, and mortality. Although CH has been predominantly studied through the lens of somatic driver mutations, emerging data demonstrate that CH without known genetic drivers is common and carries comparable health risks. These observations point to additional, non-genetic mechanisms contributing to declining hematopoietic diversity with age.

### Aims

To define and quantify epigenetic dysregulation in peripheral blood and determine its relationship to hematologic malignancy, aging, and cardiovascular outcomes - clinical phenotypes previously linked to CH.

### Methods

We analyzed blood DNA methylation profiles from 1,658 healthy 18-year-old individuals, a population with minimal clonal expansions. We identified 31,744 Epigenetically Stable Loci (ESLs) - CpG sites exhibiting remarkably consistent and predominantly low methylation levels across individuals. DNA methylation instability (DMI) was defined as deviation from the expected hypomethylation state at ESLs and was quantified in independent cohorts comprising healthy individuals (n=2,939), patients with diverse hematologic malignancies (n=3,159), and cardiovascular disease (n=2,788).

**Copyright Information:** (Online) ISSN: 2572-9241
©2026 The Author(s). HemaSphere is published by John Wiley & Sons Ltd on behalf of European Hematology Association. This is an open access Abstract Book distributed under the Attribution-NonCommercial-NoDerivs (CC BY-NC-ND), which allows third parties to download the articles and share them with others as long as they credit the author and the Abstract Book, but they cannot change the content in any way or use them commercially.
**Abstract Book Citations:** Authors, Title, HemaSphere, 2026;10:(S1):pages. The abstract book DOIs can be found on the “Author Information” page.
**Disclaimer:** Articles published in the journal HemaSphere exclusively reflect the opinions of the authors. The authors are responsible for all content in their abstracts including accuracy of the facts, statements, citing resources, etc.

**Results**

ESLs reflect stringent regulation: ESLs display consistent hypomethylation across blood cell types and are significantly enriched in CpG islands and promoter regions. ESLs identified in blood were also hypomethylated across diverse tissues and developmental stages, suggesting tight regulatory control established early in embryogenesis and maintained throughout life.
DMI marks clonal expansion: DMI was significantly elevated across multiple myeloid and lymphoid malignancies relative to non-cancer controls. In longitudinal leukemia samples, DMI tracked leukemic burden and mutations' allele frequencies during therapy and follow-up. Paired diagnosis–relapse analyses demonstrated persistence of ESL methylation patterns, enabling their use as markers for disease monitoring. Tracing patient-specific ESL methylation from relapse to diagnosis demonstrates that resistant epi-clones were already present before treatment initiation, indicating early establishment of DMI during clonal evolution.
DMI increases with aging and predicts cardiovascular risk: In cancer-free cohorts, DMI correlated positively with age, indicating that epigenetic destabilization is a general feature of aging hematopoiesis rather than a consequence of malignant transformation alone. Elevated DMI was associated with increased risk of coronary heart disease. Among patients with cardiogenic disease, high DMI levels stratified individuals into significantly higher mortality risk groups. The association between DMI and survival remained independent of CH mutations in *TET2* and *DNMT3A* and amplified their prognostic impact, indicating that DMI contributes to cardiovascular risk through mechanisms that are both distinct from and additive to canonical CH drivers.

**Summary/Conclusion**

We define progressive DNA methylation instability at promoter-enriched, developmentally constrained loci as a unifying epigenetic hallmark of aging, hematologic malignancy, and cardiovascular disease. Its quantification provides a scalable and biologically grounded biomarker platform for detecting hazardous epigenetic clonal expansion and guiding early detection and risk stratification.

**Copyright Information:** (Online) ISSN: 2572-9241
©2026 The Author(s). HemaSphere is published by John Wiley & Sons Ltd on behalf of European Hematology Association. This is an open access Abstract Book distributed under the Attribution-NonCommercial-NoDerivs (CC BY-NC-ND), which allows third parties to download the articles and share them with others as long as they credit the author and the Abstract Book, but they cannot change the content in any way or use them commercially.
**Abstract Book Citations:** Authors, Title, HemaSphere, 2026;10:(S1):pages. The abstract book DOIs can be found on the "Author Information" page.
**Disclaimer:** Articles published in the journal HemaSphere exclusively reflect the opinions of the authors. The authors are responsible for all content in their abstracts including accuracy of the facts, statements, citing resources, etc.

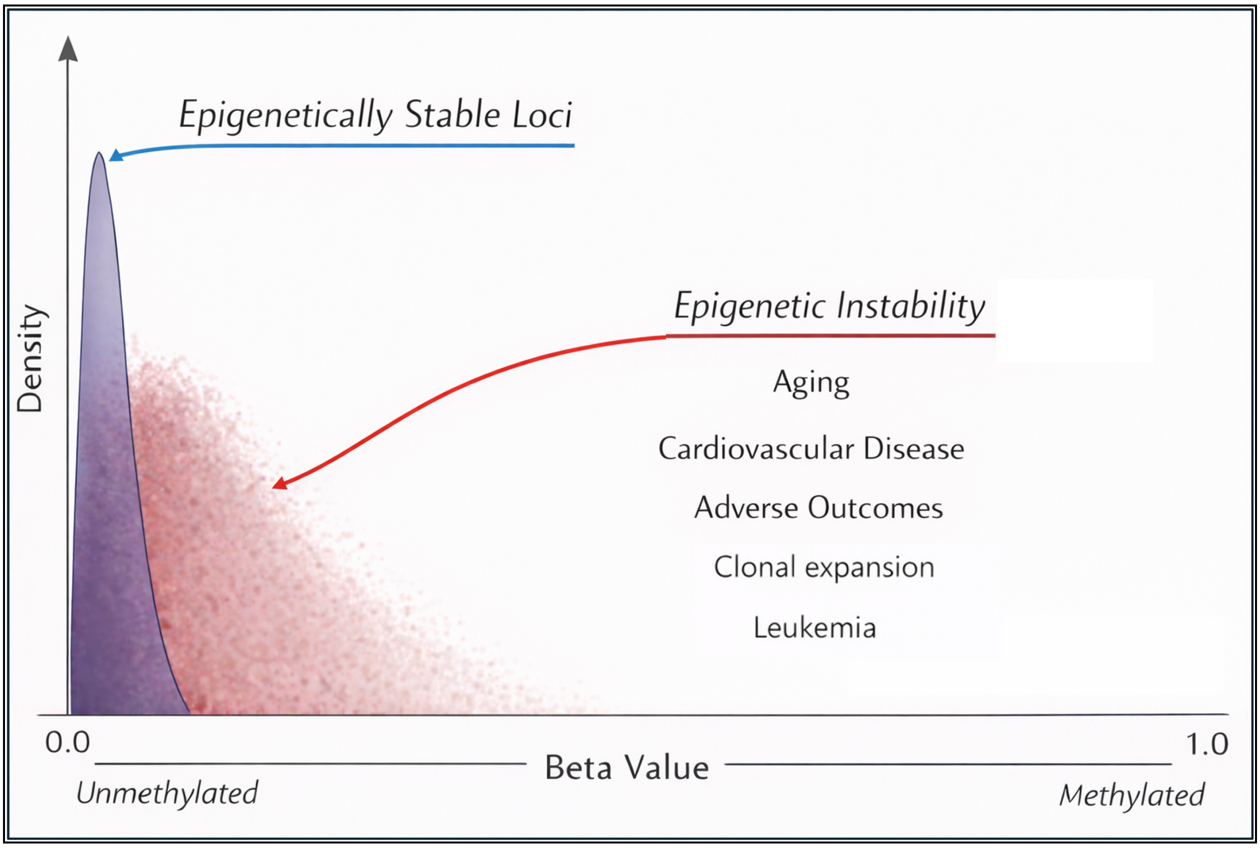

Copyright Information: (Online) ISSN: 2572-9241
©2026 The Author(s). HemaSphere is published by John Wiley & Sons Ltd on behalf of European Hematology Association. This is an open access Abstract Book distributed under the Attribution-NonCommercial-NoDerivs (CC BY-NC-ND), which allows third parties to download the articles and share them with others as long as they credit the author and the Abstract Book, but they cannot change the content in any way or use them commercially.
Abstract Book Citations: Authors, Title, HemaSphere, 2026;10:(S1):pages. The abstract book DOIs can be found on the "Author Information" page.
Disclaimer: Articles published in the journal HemaSphere exclusively reflect the opinions of the authors. The authors are responsible for all content in their abstracts including accuracy of the facts, statements, citing resources, etc.

### (PF1189) INVESTIGATING THE MAINTENANCE OF SELF-RENEWAL PROGRAMS BY AML LEUKEMIC STEM CELLS

**Topic:** 24. Hematopoiesis, stem cells and microenvironment

Júlia Aguadé-Gorgorió*[1], Chiara Perrod[1], Christian Romero[1], Marta Meroño[1], Paulo Mendoça[1], Feiyang MA[2], Yiqian Gu[2], Vincenzo Calvanese[1, 3]

*[1]Josep Carreras Leukemia Research Institute, Barcelona, Spain;[2]University of California Los Angeles, Los Angeles, United States of America; [3]University College London, London, United Kingdom;*

### Background

Survival rates of acute myeloid leukemia (AML) remain low despite meaningful improvements in therapeutic strategies. Chemotherapy resistance and high relapse rates are mainly caused by the persistence of leukemia stem cells (LSC), rare malignant cells that exploit the molecular pathways of healthy hematopoietic stem cells (HSC) to acquire self-renewal abilities. Therefore, understanding self-renewal mechanisms of AML LSC is essential for defeating this disease.

### Aims

We aim to dissect the regulatory programs co-opted by AML LSC to acquire stem cell properties, and their role in establishing self-renewal.

### Methods

We performed single cell 10x Multiome in primary healthy bone marrow and AML samples enriched for HSC/LSC populations (GPR56+CD34+) to characterize the transcriptional and gene regulatory networks in the context of healthy and malignant self-renewal. Because many transcription factors are transcribed from alternative promoters generating different isoforms, an aspect often overlooked in gene-focused single cell profiling, we performed long-read sequencing on human HSPC to define alternative promoters and isoforms and quantified them in 5'scRNAseq of the same primary samples, as well as in RNAseq data from normal hematopoietic stem and progenitor cells. To validate the findings from our limited dataset we also mined large AML gene expression databases as well as published scRNAseq datasets of primary AML.
To establish systems for functional profiling, we evaluated different in vitro culture methods (monoculture and coculture, different media and cytokine compositions from Pabst et al 2014, Fares et al 2014, Fleming et al 2025, Derevyanko et al 2025) and in vivo xenograft models (NSG-SGM3, NBSGW, NGS-W41 mice) for primary AML.

### Results

We identified 14 core transcription factors (TF, e.g. HLF, PRDM16, HOPX and NKX2-3) that are associated with normal HSC self-renewal, upregulated in AML LSC, and correlated with poor survival in AML. Most of these factors are enriched in AML with minimal differentiation and expressed at higher levels at relapse, suggesting their potential role as candidate regulators of LSC establishment and maintenance. 10x Multiome and 5'scRNAseq analysis revealed that AML LSC co-opt only part of the self-renewal TF and programs specific to HSC, with important patient-to-patient heterogeneity. Importantly, we uncovered differences in isoform expression of self-renewal TF between HSC and LSC, hinting at potential LSC-specific vulnerabilities. Isoform specific gain and loss of function studies (CRISPR, degron) in human HSC showed remarkable differences in self-renewal activity between isoforms.
We were able to expand patient-derived LSC in co-culture with MS5 cells and the media composition described by Derevyanko et al 2025, transduce them with lentiviral vectors, and engraft them into immunodeficient mice for generation of WT and modified patient derived xenografts. This will allow us to perform functional assays with clinically relevant samples in the future.

**Copyright Information:** (Online) ISSN: 2572-9241
©2026 The Author(s). HemaSphere is published by John Wiley & Sons Ltd on behalf of European Hematology Association. This is an open access Abstract Book distributed under the Attribution-NonCommercial-NoDerivs (CC BY-NC-ND), which allows third parties to download the articles and share them with others as long as they credit the author and the Abstract Book, but they cannot change the content in any way or use them commercially.
**Abstract Book Citations:** Authors, Title, HemaSphere, 2026;10:(S1):pages. The abstract book DOIs can be found on the "Author Information" page.
**Disclaimer:** Articles published in the journal HemaSphere exclusively reflect the opinions of the authors. The authors are responsible for all content in their abstracts including accuracy of the facts, statements, citing resources, etc.

**Summary/Conclusion**

While AML LSC and HSC share important self-renewal mechanisms, notable differences exist in the expression of stemness TF, particularly at the isoform level, and critical transcriptional programs. These preliminary findings highlight the importance of understanding the programs governing both healthy and malignant self-renewal and warrant further investigation into the specific roles of different TF isoforms, which could lead to novel targeting opportunities directed at AML LSC.

**Copyright Information:** (Online) ISSN: 2572-9241
©2026 The Author(s). HemaSphere is published by John Wiley & Sons Ltd on behalf of European Hematology Association. This is an open access Abstract Book distributed under the Attribution-NonCommercial-NoDerivs (CC BY-NC-ND), which allows third parties to download the articles and share them with others as long as they credit the author and the Abstract Book, but they cannot change the content in any way or use them commercially.
**Abstract Book Citations:** Authors, Title, HemaSphere, 2026;10:(S1):pages. The abstract book DOIs can be found on the "Author Information" page.
**Disclaimer:** Articles published in the journal HemaSphere exclusively reflect the opinions of the authors. The authors are responsible for all content in their abstracts including accuracy of the facts, statements, citing resources, etc.

### (PF1190) IMBALANCE OF THE YBX1–STING AXIS IN SENESCENT BONE MARROW MESENCHYMAL STROMAL CELLS DRIVES CHEMOSENSITIVITY IN ELDERLY ACUTE MYELOID LEUKEMIA

**Topic:** 24. Hematopoiesis, stem cells and microenvironment

Cong Wang*[1, 2], Jie Liu[2], Chengyan Chai[1], Zheng Wang[1, 2], Xi Zhang[1, 2]

*[1]Xinqiao Hospital of Army Medical University, Chongqing, China;[2]Jinfeng laboratory, Chongqing, China;*

### Background

Elderly acute myeloid leukemia (AML) is characterized by poor response to cytarabine-based chemotherapy and poor overall survival. While leukemic cell–intrinsic mutations contribute to treatment failure, increasing evidence suggests that the aged bone marrow microenvironment plays a critical regulatory role. Bone marrow mesenchymal stromal cells (BMSCs), as a central component of the hematopoietic niche, undergo senescence during aging and may reshape immune and inflammatory signaling.
Our preliminary data demonstrate that senescent BMSCs attenuate AML cell sensitivity to cytarabine and exhibit functional imbalance of the STING pathway, characterized by chronic low-level basal activation but impaired responsiveness to exogenous stimulation. Importantly, the immune regulatory mechanisms mediated by senescent BMSCs within the elderly bone marrow microenvironment remain largely unexplored.

### Aims

This study aims to elucidate the molecular mechanisms underlying functional imbalance of the STING pathway in senescent BMSCs, to define how the immune-low-reactive state of senescent BMSCs remodels the bone marrow microenvironment and reduces AML chemosensitivity, and to explore the therapeutic potential of targeting senescent BMSCs to enhance treatment response.

### Methods

Replicative senescence models of human BMSCs were established and co-cultured with AML cells to assess cytarabine sensitivity. Transcriptomic profiling was performed using RNA-seq to compare young and senescent BMSCs. Differential expression analysis and bioinformatic pathway enrichment were conducted to identify key regulators.
Mechanistic investigations were performed through molecular interventions including siRNA-mediated knockdown, pharmacologic inhibition, and plasmid-based overexpression. Protein–protein interactions were evaluated by co-immunoprecipitation and domain truncation assays.
Phenotypic and signaling analyses were conducted using RT-qPCR, Western blotting, flow cytometry, and senescence-associated β-galactosidase (SA-β-gal) staining.

**Copyright Information:** (Online) ISSN: 2572-9241
©2026 The Author(s). HemaSphere is published by John Wiley & Sons Ltd on behalf of European Hematology Association. This is an open access Abstract Book distributed under the Attribution-NonCommercial-NoDerivs (CC BY-NC-ND), which allows third parties to download the articles and share them with others as long as they credit the author and the Abstract Book, but they cannot change the content in any way or use them commercially.
**Abstract Book Citations:** Authors, Title, HemaSphere, 2026;10:(S1):pages. The abstract book DOIs can be found on the “Author Information” page.
**Disclaimer:** Articles published in the journal HemaSphere exclusively reflect the opinions of the authors. The authors are responsible for all content in their abstracts including accuracy of the facts, statements, citing resources, etc.

**Results**

Senescent BMSCs significantly decreased AML cell sensitivity to cytarabine in co-culture assays, indicating a microenvironment-mediated chemoprotective effect. Despite elevated basal cytosolic DNA levels, senescent BMSCs exhibited impaired activation of the STING pathway upon agonist challenge.
Transcriptomic profiling identified YBX1 as markedly downregulated in senescent BMSCs, with expression levels positively correlated with interferon-stimulated gene signatures. Restoration of YBX1 in senescent BMSCs rescued STING pathway activation and IFN-I production, whereas YBX1 in silencing young BMSCs attenuated signaling responsiveness, supporting a functional role for YBX1 in maintaining STING competence.
Mechanistically, co-immunoprecipitation assays confirmed inducible interaction between YBX1 and STING. Domain mapping further revealed that both the cold shock domain (CSD) and C-terminal domain (CTD) are required for efficient STING signal propagation, suggesting that YBX1 facilitates STING complex assembly and downstream signal amplification.

**Summary/Conclusion**

Our findings identify YBX1 as a critical regulator of STING signal amplification in BMSCs. Aging-associated downregulation of YBX1 leads to functional imbalance of the STING pathway, creating an immune-low-reactive niche that impairs chemotherapy responsiveness in elderly AML. Targeting the YBX1–STING axis may represent a novel microenvironment-directed strategy to enhance therapeutic responsiveness in elderly AML.

**Copyright Information:** (Online) ISSN: 2572-9241
©2026 The Author(s). HemaSphere is published by John Wiley & Sons Ltd on behalf of European Hematology Association. This is an open access Abstract Book distributed under the Attribution-NonCommercial-NoDerivs (CC BY-NC-ND), which allows third parties to download the articles and share them with others as long as they credit the author and the Abstract Book, but they cannot change the content in any way or use them commercially.
**Abstract Book Citations:** Authors, Title, HemaSphere, 2026;10:(S1):pages. The abstract book DOIs can be found on the “Author Information” page.
**Disclaimer:** Articles published in the journal HemaSphere exclusively reflect the opinions of the authors. The authors are responsible for all content in their abstracts including accuracy of the facts, statements, citing resources, etc.

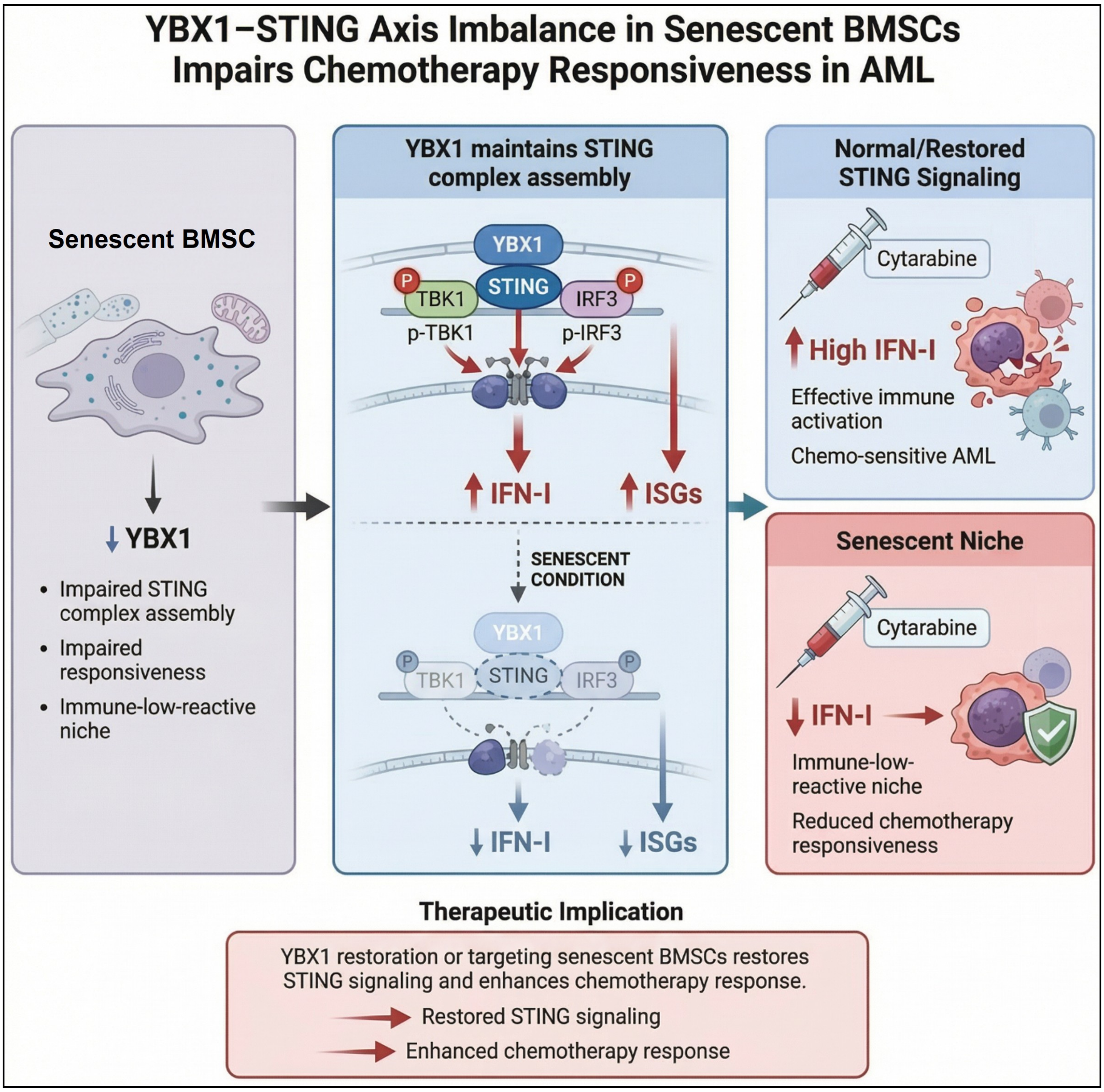

**Copyright Information:** (Online) ISSN: 2572-9241
©2026 The Author(s). HemaSphere is published by John Wiley & Sons Ltd on behalf of European Hematology Association. This is an open access Abstract Book distributed under the Attribution-NonCommercial-NoDerivs (CC BY-NC-ND), which allows third parties to download the articles and share them with others as long as they credit the author and the Abstract Book, but they cannot change the content in any way or use them commercially.
**Abstract Book Citations:** Authors, Title, HemaSphere, 2026;10:(S1):pages. The abstract book DOIs can be found on the "Author Information" page.
**Disclaimer:** Articles published in the journal HemaSphere exclusively reflect the opinions of the authors. The authors are responsible for all content in their abstracts including accuracy of the facts, statements, citing resources, etc.

### (PF1191) LIN28B PROMOTES B CELL DIFFERENTIATION LIMITING RECEPTOR EDITING EARLY IN LIFE

**Topic:** 24. Hematopoiesis, stem cells and microenvironment

Elena Boldrin*[1], Giorgia Montano[1], Niklas Segrén[1], Stefan Lang[1], Shamit Soneji[1], Joan Yuan[1]

[1]*Lund University, Lund, Sweden;*

### Background

B cell precursor acute lymphoblastic leukemia (BCP-ALL) is the most common malignancy of childhood and its originating genetic lesions can arise as early as in utero. However, the contribution of early life factors to malignant transformation is still underexplored. B cell development during perinatal life differs significantly from adulthood, enabling the maturation of a broader range of self and poly-reactive specificities. These differences are in part mediated by the developmentally restricted expression of the RNA-binding protein LIN28B.

### Aims

Our goal was to clarify the contribution of LIN28B to B cell differentiation early in life, and to explore its interactions with genetic lesions that predispose to leukemia.

### Methods

We analyzed in vivo and in vitro differentiation of BCPs from adult wildtype and LIN28B inducible mice, and neonatal wildtype or Lin28b knockout mice using single-cell RNA sequencing (scRNAseq) and flow cytometry. To model a leukemia-prone environment, we crossed these strains with PAX5 haploinsufficient mice (Pax5+/–).

### Results scRNAseq analyses revealed reduced representation of late small preB cells in LIN28B expressing and neonatal mice, hallmarked by immunoglobulin λ chain transcription. In adults, one major bottleneck in B cell development occurs during the small preB to immature B (ImmB) cell transition, when BCPs undergo sequential attempts at immunoglobulin light chain recombination to generate a functional yet innocuous B cell receptor (BCR). LIN28B expression reduced markers of receptor editing in both adult and neonate ImmB cells, limiting λ chain usage and distal Jκ segment recombination. Additionally, LIN28B reduced the ImmB cell ability to internalize the BCR and induce Rag in vitro in the context of both blunted tonic signaling and IgM crosslinking, indicating limited receptor editing. This was accompanied by accelerated ImmB cell maturation by LIN28B in vivo and in vitro, leading to increased B cell output from bone marrow to spleen. At the same time, LIN28B expression altered the quality of the emerging ImmB cells, which displayed increased markers of activation and self-reactivity. This is in line with enhanced AID activation in neonatal versus adult ImmB cells, previously reported in association with increased self-reactivity.
Remarkably, LIN28B expression in neonates and adults reduced the aberrant accumulation of CD25+ small preB cells imposed by PAX5 haploinsufficiency. Analysis of B cell differentiation by scRNAseq, and in vivo/in vitro assays confirmed that LIN28B expression alleviated a differentiation block in Pax5+/– BCPs, restoring ImmB cell maturation. Although further analyses are needed to clarify the mechanism, our data suggest that LIN28B does not rescue PAX5 expression or directly target pathways altered in Pax5+/– mice but rather restores differentiation by limiting receptor editing.

**Copyright Information:** (Online) ISSN: 2572-9241
©2026 The Author(s). HemaSphere is published by John Wiley & Sons Ltd on behalf of European Hematology Association. This is an open access Abstract Book distributed under the Attribution-NonCommercial-NoDerivs (CC BY-NC-ND), which allows third parties to download the articles and share them with others as long as they credit the author and the Abstract Book, but they cannot change the content in any way or use them commercially.
**Abstract Book Citations:** Authors, Title, HemaSphere, 2026;10:(S1):pages. The abstract book DOIs can be found on the "Author Information" page.
**Disclaimer:** Articles published in the journal HemaSphere exclusively reflect the opinions of the authors. The authors are responsible for all content in their abstracts including accuracy of the facts, statements, citing resources, etc.

**Summary/Conclusion**

We conclude that LIN28B short-circuits a major checkpoint in BCP differentiation in early life, dramatically reducing the overall time invested in light chain recombination and accelerating B cell output. These findings highlight distinct control of central tolerance mechanisms during ontogeny and shed new light on the forces that shape the unique neonatal B cell repertoire. Importantly, LIN28B dependent boost in differentiation persists in presence of genetically imposed differentiation blocks, suggesting potential implications for leukemia initiation in early life.

**Copyright Information:** (Online) ISSN: 2572-9241
©2026 The Author(s). HemaSphere is published by John Wiley & Sons Ltd on behalf of European Hematology Association. This is an open access Abstract Book distributed under the Attribution-NonCommercial-NoDerivs (CC BY-NC-ND), which allows third parties to download the articles and share them with others as long as they credit the author and the Abstract Book, but they cannot change the content in any way or use them commercially.
**Abstract Book Citations:** Authors, Title, HemaSphere, 2026;10:(S1):pages. The abstract book DOIs can be found on the "Author Information" page.
**Disclaimer:** Articles published in the journal HemaSphere exclusively reflect the opinions of the authors. The authors are responsible for all content in their abstracts including accuracy of the facts, statements, citing resources, etc.

### (PF1192) AN IPSC-LCH MODEL AS PLATFORM TO IDENTIFY NEW STRATEGIES IN TARGETING BRAFV600E HEMATOPOIETIC PROGENITORS

**Topic:** 24. Hematopoiesis, stem cells and microenvironment

Giulio Abagnale*[1], Raphaela Schwentner[1], Maki Sakuma[1], Philipp Ben Soussia-Weiss[1], Wouter van Midden[1], Caterina Sturtzel[1], Ulrike Pötschger[1], Magdalena Rados[1], Patrick Say[1], Sabine Taschner-Mandl[1], Ingrid Simonitsch-Klupp[2], Christoph Hafemeister[1], Florian Halbritter[1], Martin Distel[3], Sebastian Eder[1, 4], Caroline Hutter[1, 2, 4]

*[1]St. Anna Children´s Cancer Research Institute, Vienna, Austria;[2]Medical University of Vienna, Vienna, Austria;[3]The University of Utah, Salt Lake City, United States of America;[4]St. Anna Children´s Hospital, Vienna, Austria;*

### Background

Langerhans Cell Histiocytosis (LCH) is a rare pediatric inflammatory myeloid neoplasm characterized by a single oncogenic hit: an activating mutation of the MAPK pathway, with $BRAF^{V600E}$ being the most common one found in patients. The rarity of LCH and lack of proper models have limited its mechanistic understanding and advancement of new treatments. Although targeted therapies with MAPK pathway inhibitors (MAPKi) have improved the overall survival of high-risk LCH patients, evidence suggests these drugs alone cannot prevent relapses. Moreover, the persistence of malignant progenitors in the bone marrow is associated to higher incidence of neurodegeneration, a highly debilitating sequela of LCH that appears years after diagnosis, and thus their eradication has a great clinical relevance.

### Aims

We used a human iPSC-LCH model (Abagnale et al., Blood, 2025) as platform to inform treatment that can selectively eradicate $BRAF^{V600E}$ progenitors. Our main aim is to assess how $BRAF^{V600E}$ $CD34^{+}$ iPSC derived hematopoietic progenitors (iHPCs) respond to different MAPKi either alone or in combination with a second inhibitor or chemotherapeutic compound in order to improve treatment efficacy and reduce toxicity. Moreover, we wanted to understand if within the $CD34^{+}$ population there is heterogeneity in MAPK pathway activation in response to inhibitors.

### Methods iPSCs lines derived from a LCH patient and a healthy donor were edited via CRISPR/Cas9 to introduce the V600E mutation in one endogenous BRAF locus. We directed differentiation of these isogenic pairs of $BRAF^{V600E}$ and $BRAF^{WT}$ iPSCs lines toward the hematopoietic lineage and characterized their progeny by FACS and scRNA-seq. We tested responses of $CD34^{+}$ iHPCs to compound libraries and combinatorial drug treatments in a 384 well format via High Throughput Screens. Finally, we engineered a fluorescent ERK-Kinase Translocation Reporter (ERK-KTR) in $BRAF^{V600E}$ iPSCs, allowing us to follow MAPK pathway activity in single living cells during drug treatment.

**Copyright Information:** (Online) ISSN: 2572-9241
©2026 The Author(s). HemaSphere is published by John Wiley & Sons Ltd on behalf of European Hematology Association. This is an open access Abstract Book distributed under the Attribution-NonCommercial-NoDerivs (CC BY-NC-ND), which allows third parties to download the articles and share them with others as long as they credit the author and the Abstract Book, but they cannot change the content in any way or use them commercially.
**Abstract Book Citations:** Authors, Title, HemaSphere, 2026;10:(S1):pages. The abstract book DOIs can be found on the "Author Information" page.
**Disclaimer:** Articles published in the journal HemaSphere exclusively reflect the opinions of the authors. The authors are responsible for all content in their abstracts including accuracy of the facts, statements, citing resources, etc.

### Results

We have previously demonstrated that sensitivity of $BRAF^{V600E}$ iPSC-derived myeloid populations to MAPKi increases as cell differentiate, with immature progenitors being least affected. Our new effort focuses on selectively targeting $BRAF^{V600E}$ $CD34^+$ progenitors. Our results indicate that the combination of the RAF inhibitor (RAFi) Tovorafenib and MEK inhibitor (MEKi) Mirdametinib was the one with the strongest synergy and was most effective in targeting $BRAF^{V600E}$ $CD34^+$ iHPCs. Data also suggests that in presence of MEKi, dosage of Cladribine/Cytarabine can be reduced two fold without affecting efficacy. Finally, using ERK-KTR reporter we show that RAFi/MEKi combination induces a stronger reduction in MAPK pathway activity than RAFi alone. Notably we observed that MAPK pathway inhibition is heterogeneous in single $CD34^+$ mutated progenitors following treatment, with some cells achieving a broad decrease in pathway activity while others show a more moderate response.

### Summary/Conclusion

We employed an iPSC-LCH in vitro model to investigate the mechanistic limitations of current targeted therapies. We identified synergistic interaction between MAPKi in $CD34^+$ $BRAF^{V600E}$ cells and highlighted heterogeneity in MAPK inhibition on a single cell level, which can inform treatment options. In conclusion, our model provides a tool to investigate the efficacy of targeted therapies and facilitate the discovery of new treatments to eradicate malignant LCH progenitors.

**Copyright Information:** (Online) ISSN: 2572-9241
©2026 The Author(s). HemaSphere is published by John Wiley & Sons Ltd on behalf of European Hematology Association. This is an open access Abstract Book distributed under the Attribution-NonCommercial-NoDerivs (CC BY-NC-ND), which allows third parties to download the articles and share them with others as long as they credit the author and the Abstract Book, but they cannot change the content in any way or use them commercially.
**Abstract Book Citations:** Authors, Title, HemaSphere, 2026;10:(S1):pages. The abstract book DOIs can be found on the “Author Information” page.
**Disclaimer:** Articles published in the journal HemaSphere exclusively reflect the opinions of the authors. The authors are responsible for all content in their abstracts including accuracy of the facts, statements, citing resources, etc.

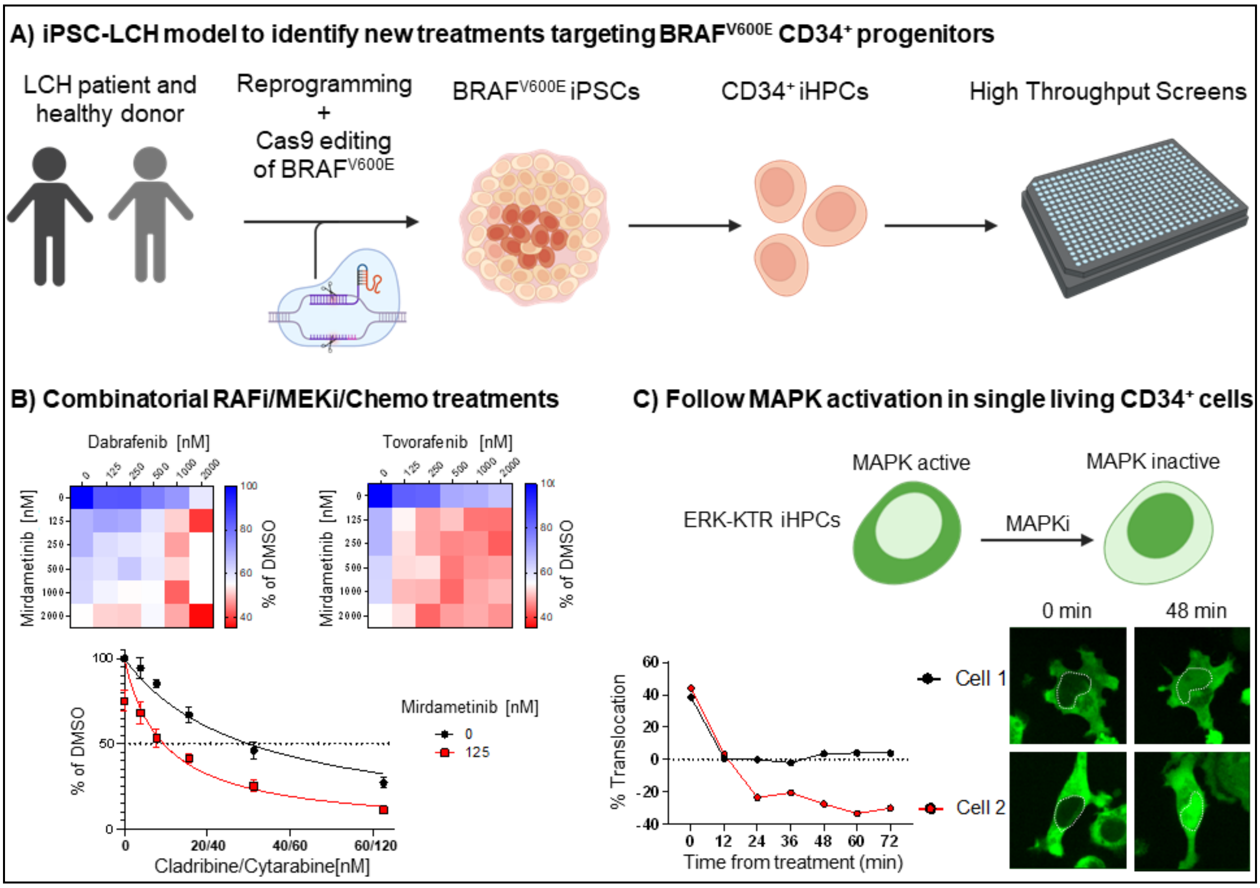

A) Outline of the project: healthy donor and patient derived iPSCs are edited with Cas9 to express the BRAFV600E mutation. Isogenic WT and V600E iPSCs lines are differentiated toward the hematopoietic lineage and CD34+ iPSC-derived progenitors are isolated and analyzed in High Throughput Screens. B) Top panel: 2-compound matrices showing that combination of Mirdametinib and Tovorafenib leads to stronger reduction in numbers of CD34+ mutated cells than a combination of Dabrafenib and Trametinib. Lower panel: dose of Cladribine/Cytarabine can be reduced two fold without affecting efficacy if combined with 125 nM of Mirdametinib. Values are normalized to DMSO (DMSO =100). C) An ERK-KTR fluorescent reporter is used to track activity of MAPK pathway in single living cells. When fluorescence is accumulated in the cytoplasm, MAPK pathway is active, viceversa it shuttles to the nucleus when the pathway is inactive. Treatment with a combination of Tovorafenib and Mirdametinib leads to signal shuttling to the nucleus, following MAPK pathway inhibition. Notably, some CD34+ mutated progenitors display a stronger inhibition (Cell 2, stronger nuclear fluorescence) than others, highlighting heterogeneity in their response to treatment.

**Copyright Information:** (Online) ISSN: 2572-9241
©2026 The Author(s). HemaSphere is published by John Wiley & Sons Ltd on behalf of European Hematology Association. This is an open access Abstract Book distributed under the Attribution-NonCommercial-NoDerivs (CC BY-NC-ND), which allows third parties to download the articles and share them with others as long as they credit the author and the Abstract Book, but they cannot change the content in any way or use them commercially.
**Abstract Book Citations:** Authors, Title, HemaSphere, 2026;10:(S1):pages. The abstract book DOIs can be found on the "Author Information" page.
**Disclaimer:** Articles published in the journal HemaSphere exclusively reflect the opinions of the authors. The authors are responsible for all content in their abstracts including accuracy of the facts, statements, citing resources, etc.

### (PF1193) MECHANOTRANSDUCTION DRIVEN BY PIEZO1 HYPERACTIVATION RESTRAINS PROPLATELET FORMATION VIA A CA2+-RHOA-ROCK AXIS

**Topic:** 24. Hematopoiesis, stem cells and microenvironment

Vittorio Abbonante*[1], Christian Di Buduo[1], Antonella Nostroso[2], Vanessa D'onofrio[2], Aurora Deacutis[3], Giovanni Vozzi[3, 3], Roberta Russo[2], Immacolata Andolfo[2, 2], Alessandra Balduini[1]

*[1]University of Pavia, Pavia, Italy; [2]Universityof Naples Federico II, Naples, Italy; [3]University of Pisa, Pisa, Italy;*

### Background

Megakaryocytes (Mk) integrate biochemical and biomechanical signals within the bone marrow niche to regulate platelet production. Intracellular $Ca^{2+}$ signaling is a key regulator of Mk differentiation and proplatelet formation and represents a central bridge between mechanical stimulation and downstream signal transduction. We previously demonstrated that the mechanosensitive cation channel PIEZO1 is expressed in Mk and upregulated in primary myelofibrosis, where its activation restrains Mk differentiation and platelet production (Am J Hematol 2024). Gain-of-function (GOF) PIEZO1 mutations cause Dehydrated Hereditary Stomatocytosis (DHS) and have recently been associated with bone marrow Mk hyperplasia with dysplastic features and platelet hyperreactivity; however, whether PIEZO1 hyperactivation directly impairs platelet biogenesis and through which signaling mechanism remains unknown.

### Aims

To define the signaling pathway through which PIEZO1 regulates platelet biogenesis and to validate its relevance in Mk derived from DHS patients harboring GOF PIEZO1 mutations.

### Methods

Using a multimodal approach, human mature Mk were subjected to PIEZO1 activation by YODA1 or cyclic mechanical stretch using a microengineered platform incorporating a biocompatible extracellular matrix–coated silk fibroin membrane to reproduce physiologic mechanical stimulation (50–600 mPa). Intracellular $Ca^{2+}$ dynamics were quantified by live-cell time-lapse imaging. Transcriptomic profiling, Western blot, capillary immunoassay, and active RhoA-GTP detection were performed. PIEZO1 dependency and functional rescue were validated by siRNA knockdown and pharmacologic inhibition.

### Results

PIEZO1 activation induced robust $Ca^{2+}$ influx, followed by reduced proplatelet formation and increased actomyosin contractility, as evidenced by enhanced stress fiber formation. RNA-seq revealed enrichment of $Ca^{2+}$-driven actomyosin contractility signatures, including ERK1/2 cascade, actin cytoskeleton organization, and small GTPase signaling (NES>1.8; FDR<0.02). Phospho-ERK and phospho-MLC2 levels increased ~2.5-fold ($p<0.01$) in a $Ca^{2+}$- and PIEZO1-dependent manner. PIEZO1 activation induced $Ca^{2+}$-dependent RhoA activation, and inhibition of downstream ROCK or myosin fully restored proplatelet formation.
The clinical cohort comprised 5 DHS patients with platelet counts within the low-normal range (median $174\times10^3$/µL, range 154–223). DHS-derived Mk exhibited ~3-fold increased $CD41^+$ output despite ~2-fold reduced CD42b expression, consistent with impaired maturation. Functionally, DHS Mk showed >50% reduction in proplatelet formation and >3-fold increase in cells displaying stress fibers. $Ca^{2+}$ dynamics were altered, with enhanced spontaneous oscillations, total $Ca^{2+}$ influx, and steeper $Ca^{2+}$ entry slopes following YODA1 stimulation. Active RhoA-GTP, phospho-ERK, and phospho-MLC2 were increased by ~2-fold compared to controls ($p<0.01$). ROCK inhibition rescued proplatelet formation ($p<0.05$).

**Copyright Information:** (Online) ISSN: 2572-9241
©2026 The Author(s). HemaSphere is published by John Wiley & Sons Ltd on behalf of European Hematology Association. This is an open access Abstract Book distributed under the Attribution-NonCommercial-NoDerivs (CC BY-NC-ND), which allows third parties to download the articles and share them with others as long as they credit the author and the Abstract Book, but they cannot change the content in any way or use them commercially.
**Abstract Book Citations:** Authors, Title, HemaSphere, 2026;10:(S1):pages. The abstract book DOIs can be found on the “Author Information” page.
**Disclaimer:** Articles published in the journal HemaSphere exclusively reflect the opinions of the authors. The authors are responsible for all content in their abstracts including accuracy of the facts, statements, citing resources, etc.

**Summary/Conclusion**

PIEZO1 functions as a mechanosensitive negative regulator of platelet biogenesis by activating a $Ca^{2+}$-dependent RhoA–ROCK–MLC2 axis that enhances actomyosin contractility and impairs proplatelet extension. DHS patient-derived Mk recapitulate this pathway ex vivo, demonstrating that PIEZO1 hyperactivation intrinsically limits platelet-forming capacity at the single-cell level. These findings provide a mechanistic basis for the apparent uncoupling between Mk expansion and circulating platelet numbers reported in DHS.

**Copyright Information:** (Online) ISSN: 2572-9241
©2026 The Author(s). HemaSphere is published by John Wiley & Sons Ltd on behalf of European Hematology Association. This is an open access Abstract Book distributed under the Attribution-NonCommercial-NoDerivs (CC BY-NC-ND), which allows third parties to download the articles and share them with others as long as they credit the author and the Abstract Book, but they cannot change the content in any way or use them commercially.
**Abstract Book Citations:** Authors, Title, HemaSphere, 2026;10:(S1):pages. The abstract book DOIs can be found on the "Author Information" page.
**Disclaimer:** Articles published in the journal HemaSphere exclusively reflect the opinions of the authors. The authors are responsible for all content in their abstracts including accuracy of the facts, statements, citing resources, etc.

### (PF1194) DISSECTING THE ROLE OF THE CXCL12–CXCR4–HMGB1 SIGNALING AXIS IN HSC-INDEPENDENT HEMATOPOIESIS

**Topic:** 24. Hematopoiesis, stem cells and microenvironment

Alessandro Muratore*[1], Giovanni Canu[2], Cristiana Barone[1], Veronica Bonalume[3], Giulia Quattrini[1], Sandesh Patel[1], Gloria Zambelli[1], Mahdieh Naghavi Alhosseini[1], Thea Milanesi[1], Francesca Caprioglio[4], Raffaella Meneveri[1], Silvia Brunelli[1], Christiana Ruhrberg[2], Marco Bianchi[4], Alessandro Fantin[3], Emanuele Azzoni[1]

*[1]University of Milano-Bicocca, School of Medicine and Surgery, Monza, Italy; [2]University College London, UCL Institute of Ophthalmology, London, United Kingdom; [3]University of Milan, Department of Bioscience,, Milano, Italy; [4]IRCCS Ospedale San Raffaele, Chromatin Dynamics Unit, Division of Genetics and Cell Biology, Milano, Italy;*

### Background

Embryogenesis relies on multiple signaling pathways that regulate blood vessel morphogenesis and the temporally associated emergence of hematopoietic progenitor cells. Early in development, erythro-myeloid progenitors (EMPs) emerge in the yolk sac and generate multilineage output, followed by fetal-restricted hematopoietic progenitors (including embryonic multipotent hematopoietic progenitors, eMPPs), which arise within the major arteries and contribute to blood formation also postnatally. Hematopoietic stem cells (HSCs) appear later and their contribution during fetal life is limited. Despite the essential role of the CXCL12-CXCR4 axis in adult hematopoiesis, its function during embryonic hematopoietic waves remains poorly defined. Moreover, High Mobility Group Box 1 (HMGB1) is a pro-inflammatory molecule that can form a heterocomplex with CXCL12 and enhance CXCR4 signaling.

### Aims

The impact of the CXCL12-CXCR4 axis during embryonic development is not fully understood. This study investigates its role in the generation, migration and expansion of hematopoietic progenitors.

### Methods

CXCL12 expression was mapped in CXCL12-eGFP reporter mice at embryonic days (E)8.5, E9.5 and E10.5 by confocal imaging. CXCL12-null embryos were used to assess ligand requirements, inducible endothelial-specific CXCR4 conditional knockout embryos (Cdh5CreER$^{T2}$;CXCR4$^{flox/flox}$;Rosa26$^{LSL\text{-}tdTomato}$) to evaluate receptor involvement, and HMGB1-null embryos to determine the impact of the additional CXCR4 ligand. All three knockout models were analyzed at E12.5 and E14.5 in the fetal liver (FL) and peripheral blood (PB) using flow cytometry, and the numbers of FL progenitors with clonogenic potential were scored using colony forming units (CFU) assay.

### Results

Confocal analysis revealed strong expression in the vitelline and umbilical arteries, which give rise to fetal-restricted progenitors. CXCL12-null embryos exhibited a reduction in the number of FL EMPs (Kit$^{+}$CD41$^{lo}$CD16/32$^{+}$) and delayed erythroid maturation in both FL and PB, consistent with a reduction in multilineage and erythroid CFUs. CXCR4 conditional knockout embryos did not impair immunophenotypic HSC frequency at E12.5, but multilineage CFUs were significantly reduced. HMGB1-null embryos displayed a reduction in the frequencies of FL EMPs and multipotent progenitors (MPPs, LSK$^{+}$CD48$^{+}$CD150$^{-}$) and a broad reduction in all clonogenic subsets without affecting HSC numbers. In these embryos, reductions in hematopoietic cell (Ter119$^{-}$CD45$^{+}$) and subsets frequencies at E12.5 were partially compensated by E14.5.

**Copyright Information:** (Online) ISSN: 2572-9241
©2026 The Author(s). HemaSphere is published by John Wiley & Sons Ltd on behalf of European Hematology Association. This is an open access Abstract Book distributed under the Attribution-NonCommercial-NoDerivs (CC BY-NC-ND), which allows third parties to download the articles and share them with others as long as they credit the author and the Abstract Book, but they cannot change the content in any way or use them commercially.
**Abstract Book Citations:** Authors, Title, HemaSphere, 2026;10:(S1):pages. The abstract book DOIs can be found on the "Author Information" page.
**Disclaimer:** Articles published in the journal HemaSphere exclusively reflect the opinions of the authors. The authors are responsible for all content in their abstracts including accuracy of the facts, statements, citing resources, etc.

**Summary/Conclusion**

Our data indicate that the CXCL12-CXCR4 axis is required for progenitor generation during the HSC-independent waves, particularly for EMPs, while HSC colonization of fetal liver appears largely CXCL12-CXCR4 independent, although later expansion may be affected. HMGB1 influences both EMPs and MPPs, and the broader phenotype observed in HMGB1-null embryos suggests that this ligand may act through mechanisms other than the CXCL12-CXCR4 axis. In HMGB1-deficient embryos, the partial recovery observed at E14.5 relative to E12.5 suggests that early HSC-independent MPPs require HMGB1, whereas later HSC-dependent MPPs may provide compensatory hematopoietic contribution. By continuing to explore this pathway, we will provide new insights with potential implications for congenital hematologic disorders, regenerative therapies and pediatric leukemias.

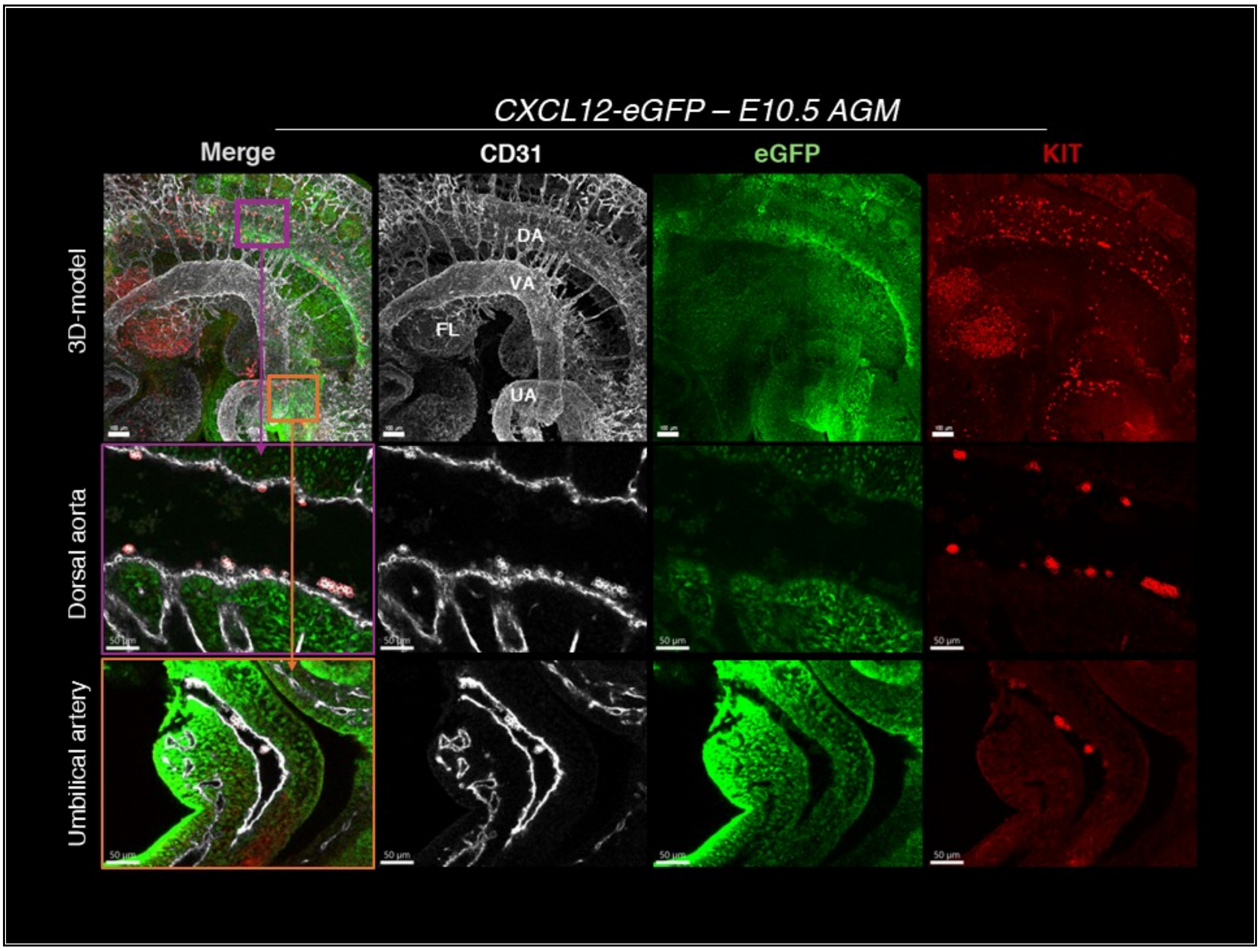

Representative confocal whole-mount immunofluorescence images of a CXCL12-eGFP embryo at E10.5. CD31⁺ vasculature is shown in white, Kit⁺ hematopoietic progenitors in red, and eGFP⁺ cells in green.

**Copyright Information:** (Online) ISSN: 2572-9241
©2026 The Author(s). HemaSphere is published by John Wiley & Sons Ltd on behalf of European Hematology Association. This is an open access Abstract Book distributed under the Attribution-NonCommercial-NoDerivs (CC BY-NC-ND), which allows third parties to download the articles and share them with others as long as they credit the author and the Abstract Book, but they cannot change the content in any way or use them commercially.
**Abstract Book Citations:** Authors, Title, HemaSphere, 2026;10:(S1):pages. The abstract book DOIs can be found on the "Author Information" page.
**Disclaimer:** Articles published in the journal HemaSphere exclusively reflect the opinions of the authors. The authors are responsible for all content in their abstracts including accuracy of the facts, statements, citing resources, etc.

### (PF1195) HIGHLY SELECTIVE JAK1 INHIBITION FOR HEMOPHAGOCYTIC LYMPHOHISTIOCYTOSIS IN MURINE MODELS WITH PRELIMINARY CLINICAL INVESTIGATION

**Topic:** 24. Hematopoiesis, stem cells and microenvironment

Yijun Wu[1], Xu Sun[1], Kai Kang[1], He Li[1], Yang Dai*[1], Ailin Zhao[1], Ting Niu[1]

[1] *West China Hospital, Sichuan University, China, China;*

### Background

Hemophagocytic lymphohistiocytosis (HLH) is a life-threatening hyperinflammatory syndrome characterized by an uncontrolled cytokine storm, in which JAK-STAT pathway is essential for inflammatory signaling. The JAK1/2 inhibitor ruxolitinib has shown some efficacy in HLH but is limited by non-selectivity, with remaining concerns regarding JAK2 blockade-associated hematological toxicities.

### Aims

This study aims to investigate the therapeutic potential of highly selective JAK1 inhibition in HLH and evaluate the preclinical efficacy and clinical translation value of the selective JAK1 inhibitor golidocitinib.

### Methods

RNA sequencing for peripheral blood samples was performed in a large cohort of HLH patients (n=73) with controls (n=70). Using murine models of primary (*Prf1*$^{-/-}$ mice infected with LCMV) and secondary (CpG-induced) HLH, the novel agent golidocitinib with high JAK1 selectivity was evaluated relative to unselective JAK inhibitors ruxolitinib (JAK1/2) and gecacitinib (pan-JAK). Single-cell RNA sequencing was performed for patient samples upon highly selective JAK1 inhibition.

### Results

Transcriptomic profiling revealed prominent JAK-STAT pathway activation in HLH patients, characterized by upregulated STAT1/STAT3 activity and inflammatory cytokines, validating the rationale for targeting JAK1. At doses established by *in vivo* escalation studies, highly selective JAK1 inhibitor golidocitinib was well-tolerated and demonstrated superior therapeutic effects in both primary and secondary HLH models, successfully ameliorating pancytopenia, splenomegaly, inflammation impairment, and hypercytokinemia, while ruxolitinib and gecacitinib performed suboptimally. In primary HLH, 100% survival was observed uniquely with golidocitinib treatment over the prolonged one-month period. Mechanistically, golidocitinib significantly attenuated pathogenic T cell activation in primary HLH and monocyte-related inflammation in secondary HLH, while concurrently lessening neutrophil hyperactivation across both settings. Consistent with its superior efficacy, golidocitinib exerted greatest inflammation-resolving transcriptional shifts in liver and spleen of primary mice, especially JAK-STAT-associated proinflammatory signaling. In three relapsed/refractory HLH patients associated with T cell malignancies (t-HLH), golidocitinib induced a rapid therapeutic response, suggesting preliminary clinical efficacy. Single-cell RNA sequencing of patient samples further pinpointed activated CST7$^{+}$/G0S2$^{+}$ neutrophils as potential targets, which exhibited reduced JAK-STAT activity following golidocitinib treatment, alongside its dual therapeutic mechanism of attenuating inflammation crosstalk and eliminating malignant T-cell clones in t-HLH.

### Summary/Conclusion

These findings highlight the therapeutic advantage of highly selective JAK1 inhibition and support golidocitinib as a promising candidate for HLH.

**Copyright Information:** (Online) ISSN: 2572-9241
©2026 The Author(s). HemaSphere is published by John Wiley & Sons Ltd on behalf of European Hematology Association. This is an open access Abstract Book distributed under the Attribution-NonCommercial-NoDerivs (CC BY-NC-ND), which allows third parties to download the articles and share them with others as long as they credit the author and the Abstract Book, but they cannot change the content in any way or use them commercially.
**Abstract Book Citations:** Authors, Title, HemaSphere, 2026;10:(S1):pages. The abstract book DOIs can be found on the “Author Information” page.
**Disclaimer:** Articles published in the journal HemaSphere exclusively reflect the opinions of the authors. The authors are responsible for all content in their abstracts including accuracy of the facts, statements, citing resources, etc.

### (PF1196) CHIP-ASSOCIATED MUTATIONS ARE LINKED TO POOR SURVIVAL IN SOLID MALIGNANCIES AND DRIVE PRO-TUMORIGENIC MACROPHAGE CROSSTALK

**Topic:** 24. Hematopoiesis, stem cells and microenvironment

Jan Vorwerk*[1], Lisa Harloff[1], Katrin Isaev[1], Benjamin Vieten[1], Lorenz Oelschläger[1], Theo Leitner[1], Leopold Schawe[1, 2], Helal Ahmed[1, 3], Subbaiah Chary Nimmagadda[1], Pradeep Kumar Patnana[1], Reza Akbarzadeh[4], Jens Humrich[4], Rüdiger Braun[5], Michael Dengler[6], Nikolaschristiancornelius Bubnoff[1], Cyrus Khandanpour[1, 7, 8]

*[1]Department of Hematology and Oncology, University Cancer Center Schleswig-Holstein (UCCSH), University Hospital Schleswig-Holstein (UKSH) – Campus Lübeck, Lübeck, Germany; [2]Lübeck Institute of Experimental Dermatology (LIED), University of Lübeck, Lübeck, Germany; [3]Faculty of Medicine and Health Sciences, Amran University, Amran, Yemen; [4]Department of Rheumatology and Clinical Immunology, University Hospital Schleswig-Holstein (UKSH) – Campus Lübeck, Lübeck, Germany; [5]Department of Surgery, University Hospital Schleswig-Holstein (UKSH) – Campus Lübeck, Lübeck, Germany; [6]Division of Oncology, Medical University of Graz, Graz, Austria; [7]Department of Hematology and Oncology, Klinikum Oldenburg, Oldenburg, Germany; [8]University Medicine Oldenburg, Carl von Ossietzky University of Oldenburg, Oldenburg, Germany;*

### Background

Clonal hematopoiesis of indeterminate potential (CHIP) refers to the age-associated acquisition and expansion of hematopoietic stem and progenitor cell clones carrying recurrent somatic mutations, in the absence of diagnostic criteria for hematologic malignancy. CHIP is linked to a systemic pro-inflammatory phenotype and has been consistently associated with an increased risk of cardio- and cerebrovascular events. In contrast, it remains insufficiently understood how CHIP mutations influence the biology and progression of solid malignancies.

### Aims

The aim of this translational study, therefore, was to compare survival outcomes of patients with solid tumors with and without CHIP and to investigate the underlying biological mechanisms using a murine model of clonal hematopoiesis.

### Methods

We first performed a retrospective analysis of whole-exome sequencing (WES) data from 585 patients with advanced solid malignancies discussed in our Molecular Tumor Board (MTB) and classified patients as CHIP-positive or CHIP-negative. CHIP status was correlated with clinical characteristics, genomic features, and survival. For mechanistic studies, murine lineage-negative ($Lin^{-}$) hematopoietic cells were transduced with the CHIP driver *Jak2-V617F* and transplanted into C57BL/6 recipient mice. Bone marrow (BM) cells were subsequently harvested, differentiated into macrophages using murine M-CSF, and co-cultured with the murine pancreatic adenocarcinoma cell line R254 or a lung adenocarcinoma cell line (KP) isolated from lesions developed in our *Kras*$^{LSL\text{-}G12D}$;*Tp53*$^{fl/fl}$ mouse model.

**Copyright Information:** (Online) ISSN: 2572-9241
©2026 The Author(s). HemaSphere is published by John Wiley & Sons Ltd on behalf of European Hematology Association. This is an open access Abstract Book distributed under the Attribution-NonCommercial-NoDerivs (CC BY-NC-ND), which allows third parties to download the articles and share them with others as long as they credit the author and the Abstract Book, but they cannot change the content in any way or use them commercially.
**Abstract Book Citations:** Authors, Title, HemaSphere, 2026;10:(S1):pages. The abstract book DOIs can be found on the "Author Information" page.
**Disclaimer:** Articles published in the journal HemaSphere exclusively reflect the opinions of the authors. The authors are responsible for all content in their abstracts including accuracy of the facts, statements, citing resources, etc.

**Results**

CHIP-associated mutations were detected in 15.7% of patients. Prevalence differed by tumor entity, being highest in hematologic malignancies (27.5%) and – among solid tumors – in sarcomas (22.7%), breast cancer (19.0%), colorectal cancer (17.2%), and pancreatic cancer (13.1%). Kaplan-Meier analysis showed significantly inferior overall survival (OS) for CHIP-positive vs CHIP-negative patients (median OS: 46.0 vs 93.0 months; log-rank p**=0.004). This association remained significant after adjustment for age, microsatellite instability (MSI), homologous recombination deficiency (HRD), number of prior therapy lines, and relevant clinical covariates. In mechanistic co-culture assays, R254 and KP tumor cells displayed a 11.5% (p*=0.038) and 14.9% (p*=0.020) increase in cell numbers, respectively, when cultured with *Jak2-V617F* macrophages compared with wild-type (WT) macrophages. Notably, treatment with JAK1/2i ruxolitinib or LSD1i bomedemstat selectively reduced *Jak2-V617F* macrophage abundance without affecting WT macrophages and consequently decreased R254 cell proliferation by 72.0% (ruxolitinib) and 50.0% (bomedemstat). Similar trends were observed in KP cells, albeit less pronounced.

**Summary/Conclusion**

CHIP-associated mutations are linked to inferior OS in patients with advanced malignancies and, in a murine model, promote tumor cell expansion via *Jak2-V617F*-driven macrophage-tumor interactions. These data suggest that CHIP can shape a pro-tumorigenic immune microenvironment and may represent a therapeutically actionable vulnerability, where targeting mutant immune cells complements tumor-directed therapies.

**Copyright Information:** (Online) ISSN: 2572-9241
©2026 The Author(s). HemaSphere is published by John Wiley & Sons Ltd on behalf of European Hematology Association. This is an open access Abstract Book distributed under the Attribution-NonCommercial-NoDerivs (CC BY-NC-ND), which allows third parties to download the articles and share them with others as long as they credit the author and the Abstract Book, but they cannot change the content in any way or use them commercially.
**Abstract Book Citations:** Authors, Title, HemaSphere, 2026;10:(S1):pages. The abstract book DOIs can be found on the "Author Information" page.
**Disclaimer:** Articles published in the journal HemaSphere exclusively reflect the opinions of the authors. The authors are responsible for all content in their abstracts including accuracy of the facts, statements, citing resources, etc.

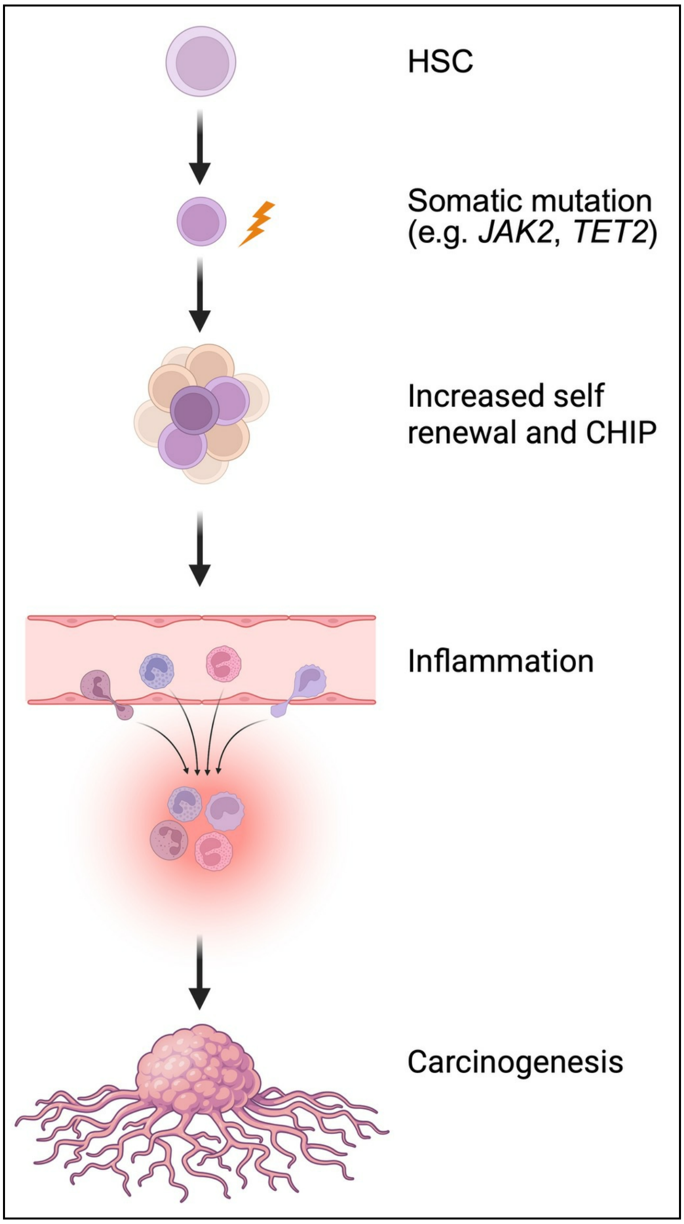

**Copyright Information:** (Online) ISSN: 2572-9241
©2026 The Author(s). HemaSphere is published by John Wiley & Sons Ltd on behalf of European Hematology Association. This is an open access Abstract Book distributed under the Attribution-NonCommercial-NoDerivs (CC BY-NC-ND), which allows third parties to download the articles and share them with others as long as they credit the author and the Abstract Book, but they cannot change the content in any way or use them commercially.
**Abstract Book Citations:** Authors, Title, HemaSphere, 2026;10:(S1):pages. The abstract book DOIs can be found on the “Author Information” page.
**Disclaimer:** Articles published in the journal HemaSphere exclusively reflect the opinions of the authors. The authors are responsible for all content in their abstracts including accuracy of the facts, statements, citing resources, etc.

### (PF1197) WHO SHAKES HANDS WITH THE ENEMY? UNDERSTANDING THE MECHANISMS OF STROMAL SUPPORT OF B PRECURSOR ACUTE LYMPHOBLASTIC LEUKAEMIA CELLS.

**Topic:** 24. Hematopoiesis, stem cells and microenvironment

Lucas Wing*[1], Jenny Chatzigerou[1, 2], Juma Ward[1, 2], Amy Simpson[1], Aditi Dey[1], Paul Genever[2], Adele Fielding[1, 2]

*[1]University of York, Biology, York, United Kingdom;[2]Hull York Medical School, York, United Kingdom;*

### Background

Bone marrow mesenchymal stromal cells (BM-MSCs) become cancer-associated fibroblasts (CAFs) after exposure to B-precursor acute lymphoblastic leukemia (B-ALL) cells or chemotherapy and create a protective niche. Our lab has shown that BM-MSCs protect B-ALL cells from chemotherapy agents through mitochondrial transfer via tunnelling nanotubes. The BM-MSC population is heterogeneous and the extent to which different subsets of BM-MSCs support ALL is unknown.

### Aims

To use hTERT immortalised BM-MSC clones isolated from the femoral head of a human donor, previously confirmed to represent existing BM-MSC subsets (James et al, Stem Cell Reports 2015), to investigate the ability of these subsets to protect ALL cells from chemotherapy and to determine the mechanisms underlying this.

### Methods

Flow cytometry was used to assess the apoptosis of the B-ALL cell line SEM with four distinct BM-MSC clones (Y101, Y102, Y201, Y202) in the presence of relevant B-ALL chemotherapy agents (daunorubicin, dexamethasone, vincristine, cytarabine). Flow cytometry was also used to quantify the amount of mitochondrial transfer from the BM-MSCs to ALL cell lines using the dye MitoTracker Deep Red. qPCR and cytokine quantification was used to assess CAF response previously characterised by Burt *et. al. (Blood Advances, 2024).* RNA sequencing was carried out on the BM-MSC clones to determine transcriptional differences between them.

### Results

Mapping RNAseq data from each of the four clones to a publicly available scRNAseq data from human bone marrow indicated that all of the clones were non-differentiated MSC with a fibro-MSC-like transcriptome. Despite this, there were notable morphological, functional and transcriptomic differences between the clones. Different BM-MSC clones protected the ALL cells from apoptosis in response to chemotherapy agents to differing degrees with 30.5% of ALL cells in late apoptosis in response to cytarabine challenge when cultured with Y201 cells vs 8.9% when cultured with Y202 cells (21.51% difference significant by two-Way Anova, $p<0.0001$). The difference could not be explained by the level of mitochondrial transfer, which was equal between all clones. Protection was ablated by transwell insert, implying contact dependence. All BM-MSC lines showed similar upregulation of CAF associated genes as quantified by a gene expression panel (including *MX2, OAS1, IL-6 & CXCL8)* and an increase in secretion of the CAF associated cytokines IL-6 and IL-8. Analysis of the bulk-RNA sequencing data on the BM-MSC lines revealed cell-cell adhesion pathways to be significantly overrepresented in the most chemoprotective BM-MSC lines (FDR p<1x10-11) with cadherin-mediated cell adhesion being prominent.

**Copyright Information:** (Online) ISSN: 2572-9241
©2026 The Author(s). HemaSphere is published by John Wiley & Sons Ltd on behalf of European Hematology Association. This is an open access Abstract Book distributed under the Attribution-NonCommercial-NoDerivs (CC BY-NC-ND), which allows third parties to download the articles and share them with others as long as they credit the author and the Abstract Book, but they cannot change the content in any way or use them commercially.
**Abstract Book Citations:** Authors, Title, HemaSphere, 2026;10:(S1):pages. The abstract book DOIs can be found on the "Author Information" page.
**Disclaimer:** Articles published in the journal HemaSphere exclusively reflect the opinions of the authors. The authors are responsible for all content in their abstracts including accuracy of the facts, statements, citing resources, etc.

**Summary/Conclusion**

Our data indicate that different BM-MSC populations differentially protect B-ALL cells, unrelated to their ability to transfer mitochondria which suggests additional mechanisms are in play. We are in the process of knocking out target molecules identified from our RNA sequencing data in our BM-MSCs to elucidate their role in chemoprotection.

**Copyright Information:** (Online) ISSN: 2572-9241
©2026 The Author(s). HemaSphere is published by John Wiley & Sons Ltd on behalf of European Hematology Association. This is an open access Abstract Book distributed under the Attribution-NonCommercial-NoDerivs (CC BY-NC-ND), which allows third parties to download the articles and share them with others as long as they credit the author and the Abstract Book, but they cannot change the content in any way or use them commercially.
**Abstract Book Citations:** Authors, Title, HemaSphere, 2026;10:(S1):pages. The abstract book DOIs can be found on the "Author Information" page.
**Disclaimer:** Articles published in the journal HemaSphere exclusively reflect the opinions of the authors. The authors are responsible for all content in their abstracts including accuracy of the facts, statements, citing resources, etc.

### (PF1198) INTERACTION BETWEEN BONE MARROW MESENCHYMAL STEM CELLS AND HEMATOPOIETIC STEM CELLS IN CLONAL HEMATOPOIESIS AND ACUTE MYELOID LEUKEMIA

**Topic:** 24. Hematopoiesis, stem cells and microenvironment

Mariia Bashaeva*[1, 2], Antonia Pailhes[1, 2], Yana Dragieva[1, 2], Bianka Ksienzyk[3], Maja Rothenberg-Thurley[3], Boris Holzapfel[4], Jörg Arnholdt[4], Alexander Paulus[4], Dominic Simon[4], Klaus Metzeler[5], Aikaterina Symeonidi[1, 6], Maria Solovey[7], Maria Colomé-Tatché[1, 6, 8], Frank Ziemann[2, 3, 9, 10]

*[1]Biomedical center, LMU Munich, Department of Physiological Chemistry, Munich, Germany;[2]ELLF - Experimentelle Leukämie- und Lymphomforschung, University Hospital LMU Munich, Munich, Germany; [3]Laboratory for Leukemia Diagnostics, Department of Internal Medicine III, LMU Munich, Munich, Germany; [4]University Hospital LMU Munich, Department of Orthopaedic Surgery, Munich, Germany; [5]Dept. of Hematology, Cell Therapy, Hemostaseology and Infectious Diseases, University Hospital Leipzig, Leipzig, Germany; [6]Helmholtz Center Munich, Munich, Germany; [7]Department of Internal Medicine III, University Hospital LMU Munich, Munich, Germany; [8]Hospital del Mar Research Institute (HMRIB), Barcelona, Spain;[9]German Cancer Consortium (DKTK), partner site Munich, Munich, Germany; [10]Bavarian Cancer Research Center (BKFZ), Muncih, Germany;*

### Background

Acute myeloid leukemia (AML) is a malignancy of hematopoietic stem cells (HSCs) with a poor prognosis despite many therapeutic options. Clonal hematopoiesis (CH) is a known myeloid disease precursor state that can precede AML. Understanding CH biology bears the potential to prevent AML occurrence.
Mesenchymal stem cells (MSCs) are part of the bone marrow niche supporting HSCs. In healthy bone marrow, MSCs are essential for maintaining normal hematopoiesis. In AML, MSCs are part of the tumor microenvironment and were shown to support leukemic blasts, e.g., by escaping chemotherapy. The role of MSCs in clonal hematopoiesis is not yet clear.

### Aims

We aimed to investigate the transcriptomic interaction between MSCs and HSCs by performing a series of autologous and cross-over co-culture experiments using bone marrow from healthy donors and donors with CH or AML. Using this approach, we want to understand the role of MSCs in the transition from CH to AML.

### Methods

We collected primary bone marrow cells from healthy individuals and individuals with clonal hematopoiesis from the ConNECT cohort, LMU Munich. Primary bone marrow samples of AML donors were provided by the Laboratory for Leukemia Diagnostics, LMU Munich. We co-cultured primary bone marrow MSCs and HSCs of healthy, CH and AML donors in different combinations: autologous co-cultures using cells coming from the same donor (healthy MSC + healthy HSC, CH MSC + CH HSC etc.), and cross-over co-cultures using cells from different donors (healthy MSC + CH HSC, healthy MSC + AML HSC etc.). We also included monoculture controls of each cell type and donor. After 7 days of culture, we performed bulk RNA sequencing on each cell type and condition. Data analysis was performed using standard analysis pipelines in R version 4.5.

**Copyright Information:** (Online) ISSN: 2572-9241
©2026 The Author(s). HemaSphere is published by John Wiley & Sons Ltd on behalf of European Hematology Association. This is an open access Abstract Book distributed under the Attribution-NonCommercial-NoDerivs (CC BY-NC-ND), which allows third parties to download the articles and share them with others as long as they credit the author and the Abstract Book, but they cannot change the content in any way or use them commercially.
**Abstract Book Citations:** Authors, Title, HemaSphere, 2026;10:(S1):pages. The abstract book DOIs can be found on the "Author Information" page.
**Disclaimer:** Articles published in the journal HemaSphere exclusively reflect the opinions of the authors. The authors are responsible for all content in their abstracts including accuracy of the facts, statements, citing resources, etc.

**Results**

We first compared monocultures to autologous co-cultures. Both HSC and MSC monocultures from all health statuses differed from their respective co-cultures (4744, 1041, 378, 83, 32 and 25 differentially expressed genes (DEGs), p.adj < 0.05 in healthy, CH and AML MSCs and healthy, CH and AML HSCs, respectively). As expected, common transcriptomic programs included cell-cell communication and extracellular matrix indicating interaction of HSC and MSC.

We then compared MSCs of different health statuses, and repeated this analysis with HSCs. With 64 and 8 DEGs, the MSCs from healthy and CH donors were transcriptomically different from AML cells. The same applied to the HSCs, with 115 and 44 DEGs for healthy and CH cells, respectively. The gene signatures featured regulation of developmental processes for MSCs and immune response for HSCs. The comparison of healthy and CD cells, however, did not reveal any statistically significant DEGs.

**Summary/Conclusion**

We successfully performed co-culture assays on primary human MSCs and HSCs from healthy, CH and AML individuals. The transcriptomic profile of hematopoietic and mesenchymal compartments showed differences between the health statuses, potentially linked to the AML development.
Of interest is the comparison of different cross-over co-cultures. This analysis will unravel the functionality of the mesenchymal niche, e.g., if CH MSCs support leukemic blasts in co-culture with AML HSCs, and if CH MSCs support normal hematopoiesis when co-cultured with healthy HSCs. Analysis of our cross-over co-culture experiments as well as exploration of cell-cell interaction will be presented at the meeting.

**Copyright Information:** (Online) ISSN: 2572-9241
©2026 The Author(s). HemaSphere is published by John Wiley & Sons Ltd on behalf of European Hematology Association. This is an open access Abstract Book distributed under the Attribution-NonCommercial-NoDerivs (CC BY-NC-ND), which allows third parties to download the articles and share them with others as long as they credit the author and the Abstract Book, but they cannot change the content in any way or use them commercially.
**Abstract Book Citations:** Authors, Title, HemaSphere, 2026;10:(S1):pages. The abstract book DOIs can be found on the “Author Information” page.
**Disclaimer:** Articles published in the journal HemaSphere exclusively reflect the opinions of the authors. The authors are responsible for all content in their abstracts including accuracy of the facts, statements, citing resources, etc.

### (PF1199) A MULTI-DIMENSIONAL STUDY OF BONE MARROW STROMA DRIVERS OF HEMATOPOIETIC RECOVERY AFTER CHEMOTHERAPY FOR ACUTE MYELOID LEUKEMIA

**Topic:** 24. Hematopoiesis, stem cells and microenvironment

Germaine Chia*[1, 2, 3], Gavin Tjin[4, 5], Nadia Fernandes[6], Nathalie Lambie[6], Floriane S. Tissot[3, 7], Cera Mai[3, 7], Sara González Antón[3, 7], Deena Iskander[1, 8], Nik Matthews[6], Louise E. Purton[4, 5], Andrew Innes[1, 2], Cristina Lo Celso[1, 3, 7]

*[1]Centre for Haematology, Department of Immunology and Inflammation, Faculty of Medicine, Imperial College London, London, United Kingdom; [2]Department of Haematology, Hammersmith Hospital, Imperial College Healthcare NHS Trust, London, United Kingdom; [3]The Francis Crick Institute, London, United Kingdom; [4]St. Vincent's Institute of Medical Research, Fitzroy, Victoria, Australia; [5]The Department of Medicine, The University of Melbourne, Parkville, Victoria, Australia;[6]NIHR Imperial BRC Genomics Facility, Imperial College London, London, United Kingdom; [7]Faculty of Natural Sciences, Department of Life Sciences, Imperial College London, London, United Kingdom; [8]Department of Paediatric Haematology, St Mary's Hospital, Imperial College Healthcare NHS Trust, London, United Kingdom;*

### Background

Acute myeloid leukemia (AML) is an aggressive, heterogeneous hematological malignancy, for which chemotherapy remains a cornerstone of treatment. Despite therapeutic advances and better supportive care, a significant proportion of patients relapse and die from their disease. Personalized medicine strategies supported by accurate risk stratification and targeted therapy development are thus urgently required. Complete remission (CR) with incomplete hematologic recovery (CRi) reflects poor bone marrow (BM) function after chemotherapy and has been shown to predict adverse outcomes in AML patients. Key drivers of poor hematopoietic recovery are not fully understood but elucidating this is critical for developing BMME-directed therapies aimed at improving treatment efficacy while reducing toxicity.

### Aims

Given the central role of the BM microenvironment (BMME) in supporting healthy hematopoiesis, we hypothesize that poor hematopoietic recovery after chemotherapy is linked to stroma loss/dysfunction. We aim to 1) Define BMME cellular characteristics associated with CRi; 2) Define molecular drivers of CRi after chemotherapy.

### Methods

Multiplex immunostaining of archival formalin fixed paraffin embedded bone marrow sections from Imperial College Hammersmith Hospital AML patients with CR (n=10)/CRi (n=9) was performed by cyclical heat-induced epitope retrieval, target detection with primary/secondary antibodies and Opal tyramide signal amplification. Images were acquired by spectral acquisition/linear unmixing with confocal/2-photon microscopy, and processed in Fiji/QuPath. The positive signal area was estimated by thresholding each channel and normalizing by cellular tissue area. Pixel and object classifiers trained in Ilastik using small regions from each image were used to quantify and characterize positive objects. For identification of molecular drivers of CRi, pilot spatial transcriptomics were run on NanoString GeoMx DSP and CosMx SMI platforms, using Whole Transcriptome panels. Differential gene expression was analyzed using manufacturer-recommended pipelines. Statistical analyses were performed in R.

**Copyright Information:** (Online) ISSN: 2572-9241
©2026 The Author(s). HemaSphere is published by John Wiley & Sons Ltd on behalf of European Hematology Association. This is an open access Abstract Book distributed under the Attribution-NonCommercial-NoDerivs (CC BY-NC-ND), which allows third parties to download the articles and share them with others as long as they credit the author and the Abstract Book, but they cannot change the content in any way or use them commercially.
**Abstract Book Citations:** Authors, Title, HemaSphere, 2026;10:(S1):pages. The abstract book DOIs can be found on the "Author Information" page.
**Disclaimer:** Articles published in the journal HemaSphere exclusively reflect the opinions of the authors. The authors are responsible for all content in their abstracts including accuracy of the facts, statements, citing resources, etc.

### Results

Using optimized panels of antibodies, we could visualize the human AML stromal and immune cellular BMME. We observed lower estimated VCAM1 staining in CRi compared to CR patients at diagnosis (6.86% ±5.04% vs 15.75%±9.27%, p=0.0196), with no significant differences in other targets. Quantification of VCAM1+ object characteristics confirmed these results. Between-group differences were abrogated in patients with myelodysplasia-related genetics when we stratified patients by their presence or absence. Early paired diagnostic/response sample analysis revealed heterogeneous responses to chemotherapy. Our pilot GeoMx spatial transcriptomics experiment detected possible subtle molecular differences in erythroid genes and CosMx data analysis is ongoing.

### Summary/Conclusion

We demonstrate a multi-dimensional approach to studying hematopoietic recovery after AML chemotherapy, linking clinical phenotype to biological processes. Synergistic cell-cell/molecular interactions may have stronger contributions to post-chemotherapy hematopoietic recovery, influenced by inter-individual and disease heterogeneity. Work is ongoing to explore hematopoiesis-related processes underpinning CRi including further immunohistology and analyzing AML patient cohorts receiving different induction protocols and from other centers.

**Copyright Information:** (Online) ISSN: 2572-9241
©2026 The Author(s). HemaSphere is published by John Wiley & Sons Ltd on behalf of European Hematology Association. This is an open access Abstract Book distributed under the Attribution-NonCommercial-NoDerivs (CC BY-NC-ND), which allows third parties to download the articles and share them with others as long as they credit the author and the Abstract Book, but they cannot change the content in any way or use them commercially.
**Abstract Book Citations:** Authors, Title, HemaSphere, 2026;10:(S1):pages. The abstract book DOIs can be found on the "Author Information" page.
**Disclaimer:** Articles published in the journal HemaSphere exclusively reflect the opinions of the authors. The authors are responsible for all content in their abstracts including accuracy of the facts, statements, citing resources, etc.

### (PF1200) PARALLEL MYELOID AND LYMPHOID CLONAL EVOLUTION IN JAK2 V617F–POSITIVE ESSENTIAL THROMBOCYTHEMIA AND CHRONIC LYMPHOCYTIC LEUKEMIA

**Topic:** 24. Hematopoiesis, stem cells and microenvironment

Hema Aremanda*[1], Amitoj Gill[2]

*[1]Sierra view Medical center, Internal Medicine, Porterville, United States of America;[2]Kaweah Health, Visalia, United States of America;*

### Background

Myeloproliferative neoplasms and lymphoid malignancies are traditionally regarded as biologically distinct hematologic disorders arising from separate differentiation pathways. Molecular and cytogenetic assessment has revealed clonal heterogeneity within hematopoietic neoplasia, including occasional coexistence of myeloid and lymphoid malignancies in the same patient. The biological relationships between concurrent clones remain incompletely defined. Integrated evaluation of hematologic trends with lineage-specific genetic findings may clarify clonal architecture and disease evolution.

### Aims

To describe the longitudinal molecular, cytogenetic, and hematologic evolution of coexisting myeloid and lymphoid neoplasms and highlight the value of lineage-specific monitoring.

### Methods

Longitudinal clinical, hematologic, molecular, and cytogenetic data were retrospectively reviewed over a 12-year period.Serial complete blood counts were correlated with peripheral blood morphology, flow cytometry, and fluorescence in situ hybridization. Molecular testing for myeloproliferative neoplasm–associated mutations, including JAK2 V617F, was performed. Clonal architecture and disease evolution were assessed through integrated analysis of hematologic and genetic data.

### Results

At initial evaluation, laboratory studies demonstrated persistent thrombocytosis with detection of a low-allele burden JAK2 V617F mutation, supporting a diagnosis of essential thrombocythemia. Longitudinal follow-up revealed stable hematologic control under cytoreductive therapy for several years.

Subsequently, progressive lymphocytosis emerged, accompanied by immunophenotypic findings consistent with chronic lymphocytic leukemia/small lymphocytic lymphoma. Flow cytometry identified a clonal B-cell population expressing CD19, CD20 (heterogeneous), CD5, and CD23 with light-chain restriction. Cytogenetic evaluation demonstrated monoallelic and biallelic 13q14 deletion, consistent with favorable-risk CLL.

Over time, divergent hematologic trajectories were observed, with expansion of the lymphoid clone occurring despite sustained myeloid disease control. Treatment modifications produced differential effects on hematologic parameters across lineages, supporting clonal independence with discordant lineage-specific genetics.

**Copyright Information:** (Online) ISSN: 2572-9241
©2026 The Author(s). HemaSphere is published by John Wiley & Sons Ltd on behalf of European Hematology Association. This is an open access Abstract Book distributed under the Attribution-NonCommercial-NoDerivs (CC BY-NC-ND), which allows third parties to download the articles and share them with others as long as they credit the author and the Abstract Book, but they cannot change the content in any way or use them commercially.
**Abstract Book Citations:** Authors, Title, HemaSphere, 2026;10:(S1):pages. The abstract book DOIs can be found on the "Author Information" page.
**Disclaimer:** Articles published in the journal HemaSphere exclusively reflect the opinions of the authors. The authors are responsible for all content in their abstracts including accuracy of the facts, statements, citing resources, etc.

**Summary/Conclusion**

This longitudinal case demonstrates the coexistence of JAK2 V617F–positive essential thrombocythemia and chronic lymphocytic leukemia with distinct clinical and genetic features evolving over time. Integrated hematologic, immunophenotypic, molecular, and cytogenetic assessment supports parallel myeloid and lymphoid clonal trajectories without evidence of lineage transformation. These findings highlight the importance of lineage-specific evaluation when new or sustained abnormalities emerge during follow-up and underscore the value of longitudinal profiling to interpret clonal architecture and guide disease-specific management.

**Figure 1.** Longitudinal hematologic trends and clonal evolution over 12 years. **Panel A** shows platelet count trends in JAK2 V617F–positive essential thrombocythemia with sustained cytoreduction on hydroxyurea, treatment interruption due to anemia, and re-initiation following rebound thrombocytosis. **Panel B** depicts absolute lymphocyte count progression in chronic lymphocytic leukemia with an initial watchful-waiting phase and subsequent treatment initiation. **Panel C** summarizes parallel myeloid and lymphoid disease trajectories without evidence of lineage transformation.

**Copyright Information:** (Online) ISSN: 2572-9241
©2026 The Author(s). HemaSphere is published by John Wiley & Sons Ltd on behalf of European Hematology Association. This is an open access Abstract Book distributed under the Attribution-NonCommercial-NoDerivs (CC BY-NC-ND), which allows third parties to download the articles and share them with others as long as they credit the author and the Abstract Book, but they cannot change the content in any way or use them commercially.
**Abstract Book Citations:** Authors, Title, HemaSphere, 2026;10:(S1):pages. The abstract book DOIs can be found on the “Author Information” page.
**Disclaimer:** Articles published in the journal HemaSphere exclusively reflect the opinions of the authors. The authors are responsible for all content in their abstracts including accuracy of the facts, statements, citing resources, etc.

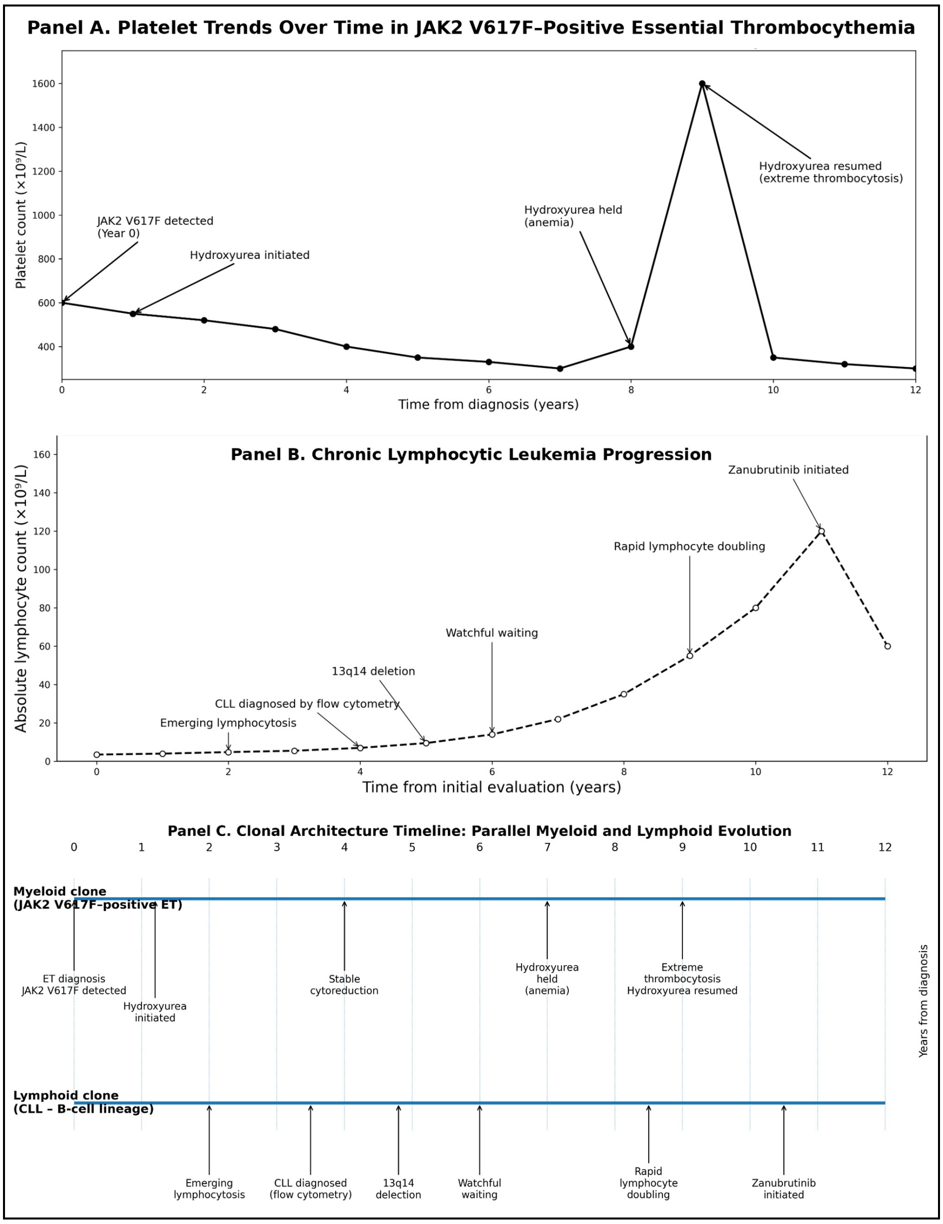

**Copyright Information:** (Online) ISSN: 2572-9241
©2026 The Author(s). HemaSphere is published by John Wiley & Sons Ltd on behalf of European Hematology Association. This is an open access Abstract Book distributed under the Attribution-NonCommercial-NoDerivs (CC BY-NC-ND), which allows third parties to download the articles and share them with others as long as they credit the author and the Abstract Book, but they cannot change the content in any way or use them commercially.
**Abstract Book Citations:** Authors, Title, HemaSphere, 2026;10:(S1):pages. The abstract book DOIs can be found on the "Author Information" page.
**Disclaimer:** Articles published in the journal HemaSphere exclusively reflect the opinions of the authors. The authors are responsible for all content in their abstracts including accuracy of the facts, statements, citing resources, etc.

### (PF1201) PREVALENCE OF CYTOPENIA AND CLONAL CYTOPENIA OF UNDETERMINED SIGNIFICANCE (CCUS) IN PATIENTS WITH CARDIAC ARRHYTHMIAS

**Topic:** 24. Hematopoiesis, stem cells and microenvironment

Jan Vorwerk*[1], Anna Traub[2], Charlotte Forner[1, 2], Lorenz Oelschläger[1], Sascha Hatahet[2], Fiona van Eetvelde[2], Naseer Mateen[2], Sorin Popescu[2, 3], Cara Seidel[1, 4], Tabea Kunz[1], Elias Rawish[3, 4], Dominik Rebafka[4], Jan-Per Wenzel[2], Charlotte Eitel[2, 3], Friederike Wortmann[1], Bruno Köhler[1, 5], Niklas Gebauer[1], Nikolaschristiancornelius Bubnoff[1], Roland Tilz[2, 3], Cyrus Khandanpour[1, 6, 7]

*[1]Department of Hematology and Oncology, University Cancer Center Schleswig-Holstein, University Hospital Schleswig-Holstein, Lübeck, Germany; [2]Department of Rhythmology, University Heart Center Lübeck, University Hospital Schleswig-Holstein (UKSH) – Campus Lübeck, Lübeck, Germany; [3]German Center for Cardiovascular Research (DZHK) – Partner Side Hamburg/Kiel/Lübeck, Lübeck, Germany; [4]Department of Cardiology, Angiology, and Intensive Care, University Heart Center Lübeck, University Hospital Schleswig-Holstein, Lübeck, Germany; [5]German Cancer Consortium (DKTK), German Cancer Research Center (DKFZ), Heidelberg, Germany; [6]Department of Hematology and Oncology, Klinikum Oldenburg, Oldenburg, Germany; [7]University Medicine Oldenburg, Carl von Ossietzky University of Oldenburg, Oldenburg, Germany;*

### Background

Cardiac arrhythmias increase steeply with age, yet biological links between aging and arrhythmogenesis remain incompletely defined. Clonal hematopoiesis may promote systemic inflammation and myocardial electrical remodeling. Data on clonal cytopenia of undetermined significance (CCUS) – defines as clonal hematopoiesis in the presence of persistent unexplained cytopenia – are sparse in patients with cardiac arrhythmias.

### Aims

To determine the prevalence of unexplained cytopenia and CCUS in patients with heart rhythm disorders, characterize the mutational spectrum, and explore clinical outcome including survival.

### Methods

Consecutive adult patients undergoing complete blood counts were screened at a tertiary rhythmology center. Unexplained cytopenia was defined on ≥2 independent measurements as hemoglobin <10.0 g/dL, leukocytes <2×$10^9$/L, neutrophils <1×$10^9$/L, or platelets <150×$10^9$/L after systematic exclusion of secondary causes. Patients with persistent unexplained cytopenia underwent targeted next-generation sequencing (NGS) of 159 myeloid genes. CCUS was defined as ≥1 pathogenic/likely pathogenic somatic variant in a clonal hematopoiesis-associated gene with variant allele frequency (VAF) ≥1%, excluding variants of uncertain significance and excluding baseline myeloid neoplasms. A supplementary external survival analysis was performed in TriNetX using 1:1 propensity-score matching to compare overall survival in arrhythmia subgroups with vs without cytopenia.

**Copyright Information:** (Online) ISSN: 2572-9241
©2026 The Author(s). HemaSphere is published by John Wiley & Sons Ltd on behalf of European Hematology Association. This is an open access Abstract Book distributed under the Attribution-NonCommercial-NoDerivs (CC BY-NC-ND), which allows third parties to download the articles and share them with others as long as they credit the author and the Abstract Book, but they cannot change the content in any way or use them commercially.
**Abstract Book Citations:** Authors, Title, HemaSphere, 2026;10:(S1):pages. The abstract book DOIs can be found on the "Author Information" page.
**Disclaimer:** Articles published in the journal HemaSphere exclusively reflect the opinions of the authors. The authors are responsible for all content in their abstracts including accuracy of the facts, statements, citing resources, etc.

### Results

Of 2,000 evaluable patients, any cytopenia was present in 42.6% and unexplained cytopenia in 8.5% (169/2,000) after excluding secondary etiologies. Thrombocytopenia predominated (83.4%). Among atrial fibrillation patients, unexplained cytopenia was associated with a higher proportion of persistent/permanent atrial fibrillation (72.2% vs 56.1%), corresponding to a 1.29-fold higher risk (95% CI 1.1–1.5). Among 51 sequenced patients, CCUS was identified in 49.0% (25/51) and increased with age (0% ≤50 years; 41.7% age 51–74; 54.0% ≥75). Extrapolated to the screened cohort, estimated CCUS prevalence was 4.2% (95% CI 3.00–5.33). Mutations were most frequent in *DNMT3A* (32.0%) and *TET2* (20.0%), followed by *TP53* (16.0%) and spliceosome genes (20.0%). 40.0% harbored ≥2 variants, and median VAF was 4.0%. In the external matched analysis, cytopenia was consistently associated with inferior survival across ventricular arrhythmias, bradyarrhythmias, atrial fibrillation, and heart failure (log-rank p<0.001 for all).

### Summary/Conclusion

Unexplained cytopenia is common among patients with heart rhythm disorders, and approximately half of sequenced patients with unexplained cytopenia meet criteria for CCUS, suggesting an under-recognized cardio-hematologic substrate. These findings support consideration of integrated evaluation pathways and prospective studies testing inflammation- or clone-informed risk stratification in arrhythmia populations.

**Copyright Information:** (Online) ISSN: 2572-9241
©2026 The Author(s). HemaSphere is published by John Wiley & Sons Ltd on behalf of European Hematology Association. This is an open access Abstract Book distributed under the Attribution-NonCommercial-NoDerivs (CC BY-NC-ND), which allows third parties to download the articles and share them with others as long as they credit the author and the Abstract Book, but they cannot change the content in any way or use them commercially.
**Abstract Book Citations:** Authors, Title, HemaSphere, 2026;10:(S1):pages. The abstract book DOIs can be found on the "Author Information" page.
**Disclaimer:** Articles published in the journal HemaSphere exclusively reflect the opinions of the authors. The authors are responsible for all content in their abstracts including accuracy of the facts, statements, citing resources, etc.

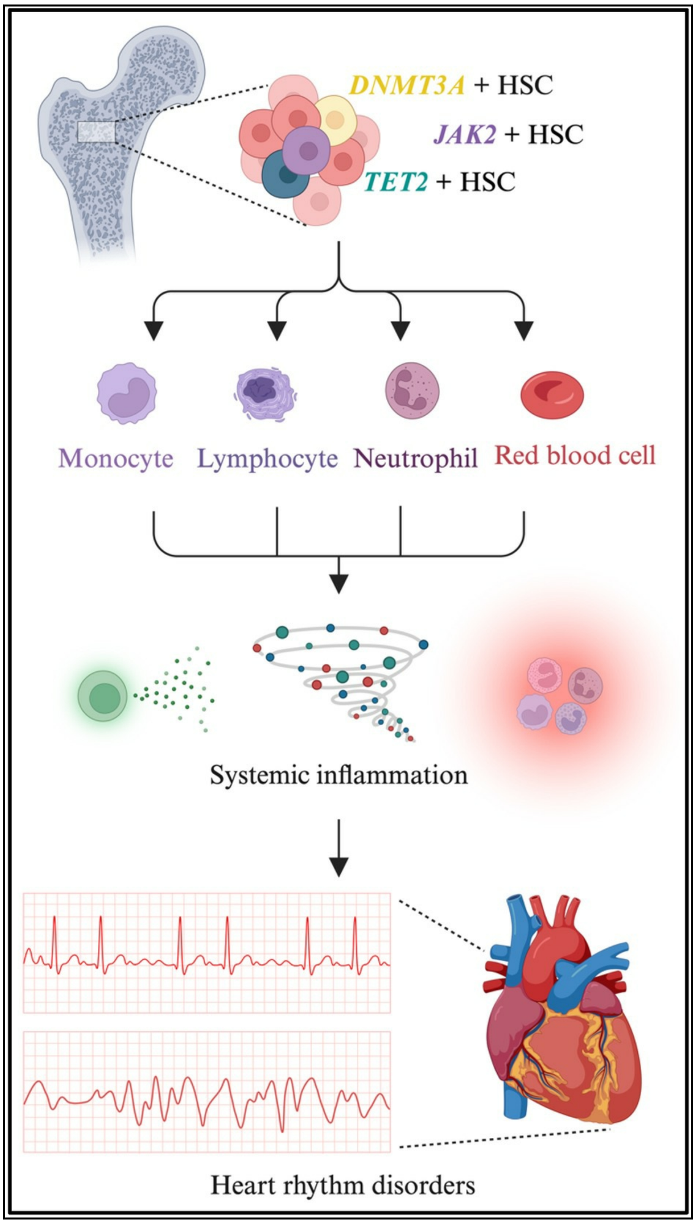

**Copyright Information:** (Online) ISSN: 2572-9241
©2026 The Author(s). HemaSphere is published by John Wiley & Sons Ltd on behalf of European Hematology Association. This is an open access Abstract Book distributed under the Attribution-NonCommercial-NoDerivs (CC BY-NC-ND), which allows third parties to download the articles and share them with others as long as they credit the author and the Abstract Book, but they cannot change the content in any way or use them commercially.
**Abstract Book Citations:** Authors, Title, HemaSphere, 2026;10:(S1):pages. The abstract book DOIs can be found on the "Author Information" page.
**Disclaimer:** Articles published in the journal HemaSphere exclusively reflect the opinions of the authors. The authors are responsible for all content in their abstracts including accuracy of the facts, statements, citing resources, etc.

### (PF1202) PROTEOMIC MAPPING OF NICHE COMMUNICATION IN INFANT KMT2A-REARRANGED LEUKEMIA

**Topic:** 24. Hematopoiesis, stem cells and microenvironment

Joanna Watral*[1], Jiaer Wang[1], Sudip Ghosh[1], Johannes Nilsson[1], Ariana Calderón[2], Mina Davoudi[1], Qinyu Zhang[2], Maria Jassinskaja[2], David Bryder[2], Charlotta Böiers[2], Jenny Hansson[1]

*[1]Lund University, Department of Experimental Medical Science, Lund, Sweden;[2]Lund University, Department of Laboratory Medicine, Lund, Sweden;*

**Background**

Infant leukemias driven by gene rearrangements of *KMT2A* (*MLL*) represent an aggressive form of childhood cancer, characterized by a high relapse rate and poor clinical outcome. While intrinsic molecular features of leukemia-initiating cells have been extensively studied, the role of the developmental microenvironment in shaping infant leukemia biology remains unclear. In particular, the communicating proteins in the fetal liver (FL) and neonatal bone marrow niches remain largely unexplored.

**Aims**

We aimed to define the proteomic alterations within the fetal leukemic niche during *KMT2A* fusion-driven transformation and to identify communication mechanisms between pre-leukemic cells and their developmental microenvironment.

**Methods**

Using inducible *KMT2A* fusion mouse models, we isolated hematopoietic stem cell and progenitor populations together with steady-state niche-associated compartments from FL. The hematopoietic cells were cultured in a PVA-based *ex vivo* system enabling controlled secretome profiling. Proteomic analysis of secretome and FL niche fractions was performed using DIA mass spectrometry (FAIMSPro–Exploris 480). Flow cytometry was used to characterize cellular remodeling and differentiation dynamics of hematopoietic and niche populations. Bioinformatic analyses were used for the identification of enriched signaling pathways and prediction of ligand–receptor interactions associated with leukemic initiation.

**Results**

We established a quantitative workflow to profile leukemia-associated extracellular proteomic changes within the fetal niche. *KMT2A* fusion induction resulted in marked cell- and time-dependent remodeling of extracellular protein composition, predominantly characterized by reduced secretion of specific protein clusters associated with neutrophil degranulation and lipid metabolism pathways, highlighting candidate regulators of early leukemic niche remodeling.
Proteomic characterization of steady-state FL niche compartments, including stromal, hepatoblast, mesenchymal, and endothelial populations, showed distinct cell type-specific proteomic signatures. Pathway enrichment analyses identified unique biological programs for each niche population, underscoring their functional heterogeneity and suggesting differential capacities to sense and respond to leukemia-derived signals. Ligand–receptor mapping identified candidate interaction pairs involved in leukemic initiation and progression in infancy.

**Copyright Information:** (Online) ISSN: 2572-9241
©2026 The Author(s). HemaSphere is published by John Wiley & Sons Ltd on behalf of European Hematology Association. This is an open access Abstract Book distributed under the Attribution-NonCommercial-NoDerivs (CC BY-NC-ND), which allows third parties to download the articles and share them with others as long as they credit the author and the Abstract Book, but they cannot change the content in any way or use them commercially.
**Abstract Book Citations:** Authors, Title, HemaSphere, 2026;10:(S1):pages. The abstract book DOIs can be found on the "Author Information" page.
**Disclaimer:** Articles published in the journal HemaSphere exclusively reflect the opinions of the authors. The authors are responsible for all content in their abstracts including accuracy of the facts, statements, citing resources, etc.

**Summary/Conclusion**

Our findings demonstrate that *KMT2A* fusion-driven transformation in infancy occurs within a uniquely structured developmental niche characterized by cell type-specific proteomic programs. These findings provide a proteomic framework for understanding developmental niche-leukemia interactions in infancy and uncover candidate microenvironmental vulnerabilities with therapeutic potential.

**Copyright Information:** (Online) ISSN: 2572-9241
©2026 The Author(s). HemaSphere is published by John Wiley & Sons Ltd on behalf of European Hematology Association. This is an open access Abstract Book distributed under the Attribution-NonCommercial-NoDerivs (CC BY-NC-ND), which allows third parties to download the articles and share them with others as long as they credit the author and the Abstract Book, but they cannot change the content in any way or use them commercially.
**Abstract Book Citations:** Authors, Title, HemaSphere, 2026;10:(S1):pages. The abstract book DOIs can be found on the "Author Information" page.
**Disclaimer:** Articles published in the journal HemaSphere exclusively reflect the opinions of the authors. The authors are responsible for all content in their abstracts including accuracy of the facts, statements, citing resources, etc.

### (PF1203) TRANSPLANTATION IN NEONATE MOUSE ENHANCES UMBILICAL CORD BLOOD CD34+ CELLS PERMISSIVENESS TO ETO2::GLIS2-DRIVEN LEUKEMOGENESIS

**Topic:** 24. Hematopoiesis, stem cells and microenvironment

Klaudia Galant[1], Vilma Barroca[1], Saiyirami Devanand[1], Didier Busso[1], Guillaume Piton[1], Nathalie Dechamps[1], Laurent Renou[1], Thomas Mercher[2], Stephanie Gachet*[1], Francoise Pflumio[1]

*[1]Inserm U1274, Commissariat à l'Energie Atomique et aux Energies Alternatives (CEA), Stabilité Génétique Cellules Souches et Radiations, Fontenay-aux-Roses, France; [2]Gustave Roussy Cancer Campus, Inserm U1170, Equipe Biologie des leucémies pédiatriques, Villejuif, France;*

### Background

Acute megakaryoblastic leukemia (AMKL) is a rare but aggressive acute myeloid leukemia (AML) subtype mostly found in children before the age of 3. Approximately 30% of pediatric de novo AMKL patients harbor the ETO2::GLIS2 (EG) fusion which is associated with poor prognosis and high relapse risk. Previous mouse and human models demonstrated that fetal cells are highly permissive to EG-driven transformation. Using human cord blood (CB) CD34+ cells, we further showed that specific human cytokines markedly enhance leukemogenesis, indicating that both the cell of origin and microenvironmental cues critically influence EG-driven leukemia emergence across developmental stages. However, pediatric AML is commonly modeled by transplanting oncogenic fusion-expressing human hematopoietic cells oncogenic into adult mice, potentially overlooking age-dependent niche effects of recipients. As fetal, newborn and adult hematopoietic cell microenvironments differ substantially in terms of anatomical localization, niche cell composition and capacity to support hematopoiesis, neonatal recipients may provide a more physiologically relevant context to model pediatric leukemia development.

### Aims

Taking advantage of our previous results showing that CB cells critically rely on microenvironmental cues for efficient EG-driven transformation, we aimed to determine whether neonatal recipients provide a more permissive microenvironmental settings than adult NSG mice for EG-driven leukemogenesis.

### Methods

EG-transduced CB hematopoietic and progenitor cells (HSPCs), including CD34+CD38-/CD38+ subsets, were transplanted into sublethally irradiated neonate or adult NSG mice using intrahepatic or intravenous routes, respectively. Cell dose escalations adjusted according to recipient size allow quantitative comparisons of leukemia-initiating cell frequencies.

### Results

We found that neonatal NSG mice supported EG-driven leukemia development more effectively than adult recipients, demonstrating that the intrinsic permissiveness of CB HSPCs to EG-driven transformation is strongly shaped by the developmental stage of the surrounding niche. Cell-dose escalation revealed that leukemia-initiating cell frequencies were approximately 10-fold higher in CD34+CD38-/+ subsets as compared to total CD34+ cells in all recipients, highlighting functional heterogeneity within CB cell populations in susceptibility to EG-driven leukemia. While neonatal microenvironments or transplanted autologous cells provided supportive signals facilitating leukemic development, recipient age did not significantly impact overall human cell engraftment dynamics or secondary transplant abilities.

**Copyright Information:** (Online) ISSN: 2572-9241
©2026 The Author(s). HemaSphere is published by John Wiley & Sons Ltd on behalf of European Hematology Association. This is an open access Abstract Book distributed under the Attribution-NonCommercial-NoDerivs (CC BY-NC-ND), which allows third parties to download the articles and share them with others as long as they credit the author and the Abstract Book, but they cannot change the content in any way or use them commercially.
**Abstract Book Citations:** Authors, Title, HemaSphere, 2026;10:(S1):pages. The abstract book DOIs can be found on the “Author Information” page.
**Disclaimer:** Articles published in the journal HemaSphere exclusively reflect the opinions of the authors. The authors are responsible for all content in their abstracts including accuracy of the facts, statements, citing resources, etc.

**Summary/Conclusion**

Collectively, our findings demonstrate that EG-driven leukemogenesis is strongly influenced by the developmental stage of the surrounding niche/microenvironment with neonatal NSG recipients being significantly more permissive than adults. Beyond EG-driven leukemogenesis, this age-relevant model enables to study pediatric leukemogenesis driven by oncogenic fusions with low cell inputs. More generally, this model can provide a valuable platform to dissect extrinsic clues, including cytokine-mediated autocrine and paracrine signaling, that facilitate pediatric leukemogenesis, and may reveal relevant cell and molecular targets for therapeutic intervention.

**Copyright Information:** (Online) ISSN: 2572-9241
©2026 The Author(s). HemaSphere is published by John Wiley & Sons Ltd on behalf of European Hematology Association. This is an open access Abstract Book distributed under the Attribution-NonCommercial-NoDerivs (CC BY-NC-ND), which allows third parties to download the articles and share them with others as long as they credit the author and the Abstract Book, but they cannot change the content in any way or use them commercially.
**Abstract Book Citations:** Authors, Title, HemaSphere, 2026;10:(S1):pages. The abstract book DOIs can be found on the "Author Information" page.
**Disclaimer:** Articles published in the journal HemaSphere exclusively reflect the opinions of the authors. The authors are responsible for all content in their abstracts including accuracy of the facts, statements, citing resources, etc.

### (PF1204) ROLE OF LINKER HISTONES AS EPIGENETIC REGULATORS OF HEMATOPOIESIS IN HEALTH AND DISEASE

**Topic:** 24. Hematopoiesis, stem cells and microenvironment

Daniel Soto Ortiz*[1], Raghad Shuwaikan[2], Marçal Vázquez-Valls[1], Fatimah Alsultan[1], Vincenzo Lagani[1], Felipe Prosper[3, 4, 5, 6], Wolfgang Fischle[1], Jesper Tegner[1], Mikel Hernaez[2, 4, 5, 7], Guillermo Serrano[1], Itziar Cenzano Armendariz[1], Nuria Planell[2], David Gomez Cabrero[1]

*[1]Biomedical Sciences Division (BioMed), King Abdullah University of Science and Technology (KAUST), Thuwal, Saudi Arabia; [2]Computational Biology Program, Cima Universidad de Navarra, Instituto de Investigación Sanitaria de Navarra (IdiSNA), Pamplona, Spain; [3]Hemato-Oncology Program, Cima Universidad de Navarra, Cancer Center Clínica Universidad de Navarra (CCUN), IdiSNA, Pamplona, Spain; [4]Centro de Investigación Biomedica en Red de Cancer (CIBERONC), Madrid, Spain; [5]Regenerative Medicine Program, Cima Universidad de Navarra, IdiSNA, Pamplona, Spain; [6]Hematology and Cell Therapy Department, Clinica Universidad de Navarra, CCUN, IdiSNA, Pamplona, Spain; [7]Data Science and Artificial Intelligence Institute (DATAI), Universidad de Navarra, Pamplona, Spain;*

### Background

Hematopoiesis relies on a tightly regulated balance between hematopoietic stem cell (HSC) self-renewal and lineage commitment, through coordinated transcriptional and epigenetic mechanisms. Among epigenetic regulators, linker histones (H1) shape chromatin organization and gene expression by controlling nucleosome spacing and DNA accessibility to transcription factors. Emerging evidence suggests a role for H1 in defining hematopoietic cell identity and guiding lineage specification. However, how H1 variant composition changes during hematopoietic differentiation and whether these patterns are altered in hematologic malignancies remain poorly understood.

### Aims

To characterize the distribution of H1 variants across the hematopoietic hierarchy and determine their association with lineage specification and differentiation. We further assessed whether H1-associated transcriptional patterns are disrupted in hematologic, using B-cell acute lymphoblastic leukemia (B-ALL) as a model.

### Methods

We analyzed four independent single-cell RNA sequencing datasets spanning early progenitors to mature cell populations to characterize H1 variants gene expression across hematopoietic differentiation. We quantified the contribution of H1-related expression signatures to identify stage and lineage using random forest. Associations between H1 variants and biological programs were assessed based on coordinated gene expression. To investigate H1-associated alterations in malignancy, we analyzed 15 B-ALL subtypes.

**Copyright Information:** (Online) ISSN: 2572-9241
©2026 The Author(s). HemaSphere is published by John Wiley & Sons Ltd on behalf of European Hematology Association. This is an open access Abstract Book distributed under the Attribution-NonCommercial-NoDerivs (CC BY-NC-ND), which allows third parties to download the articles and share them with others as long as they credit the author and the Abstract Book, but they cannot change the content in any way or use them commercially.
**Abstract Book Citations:** Authors, Title, HemaSphere, 2026;10:(S1):pages. The abstract book DOIs can be found on the "Author Information" page.
**Disclaimer:** Articles published in the journal HemaSphere exclusively reflect the opinions of the authors. The authors are responsible for all content in their abstracts including accuracy of the facts, statements, citing resources, etc.

**Results**

Notably, the most important feature identified as the strongest predictor of cell identity was the total amount of H1, that adds up the signal from all H1 variants. Additionally, distinct and reproducible H1 variant expression patterns were observed across the hematopoietic hierarchy, indicating coordinated regulation during differentiation. *H1-0* was enriched in HSCs compared to mature populations, suggesting a role in maintaining undifferentiated states. In contrast, differentiation toward the lymphoid lineage was associated with reduced *H1-0* expression and increased expression of replication-dependent H1 variants, suggesting a role of H1 genes for B-cell differentiation.
Variant-specific associations indicated functional specialization among H1 proteins. Cell cycle-dependent H1 variants were associated with chromosome organization and cell cycle pathways, whereas *H1-10* was linked to lymphocyte differentiation and correlated with B-cell-related transcription factors, including *TCF3*, *MEF2D*, and *KLF10*, highlighting the involvement of H1 in B-cell differentiation.

Analysis of 15 B-ALL subtypes revealed significant H1 dysregulation where several subtypes exhibited an inversion of the healthy H1 patterns, where malignant blasts showed aberrant *H1-5* downregulation and unexpected maintenance or upregulation of *H1-0*.

**Summary/Conclusion**

H1 variants show dynamic and lineage-specific expression during hematopoiesis, distinguishing stem and differentiated states and associating with lineage-defining transcriptional programs. The observed dysregulation of these patterns across B-ALL subtypes highlights the potential role of H1 variants in malignant transformation and warrants further investigation into their contribution to the leukemic differentiation block.

**Copyright Information:** (Online) ISSN: 2572-9241
©2026 The Author(s). HemaSphere is published by John Wiley & Sons Ltd on behalf of European Hematology Association. This is an open access Abstract Book distributed under the Attribution-NonCommercial-NoDerivs (CC BY-NC-ND), which allows third parties to download the articles and share them with others as long as they credit the author and the Abstract Book, but they cannot change the content in any way or use them commercially.
**Abstract Book Citations:** Authors, Title, HemaSphere, 2026;10:(S1):pages. The abstract book DOIs can be found on the "Author Information" page.
**Disclaimer:** Articles published in the journal HemaSphere exclusively reflect the opinions of the authors. The authors are responsible for all content in their abstracts including accuracy of the facts, statements, citing resources, etc.

### (PF1205) THERAPY INTENSITY SHAPES EPIGENETIC AGING TRAJECTORIES IN CAYA SURVIVORS OF HEMATOLOGIC MALIGNANCIES

**Topic:** 24. Hematopoiesis, stem cells and microenvironment

Sanem Özayral*[1], Moritz Kochanek[2], Jasper Feldkamp[2, 3], Daniel Teichmann[4], Celine Maslinski[1], Frederik Damm[1], Anja Borgmann-Staudt[5], Lars Bullinger[1], Bernhard Wörmann[1], David Capper[4, 6], Mareike Frick[1, 6]

*[1]Charité – Universitätsmedizin Berlin, Department of Hematology, Oncology, and Cancer Immunology, Campus Virchow Klinikum, Berlin, Germany; [2]Berlin Institute of Health at Charité – Universitätsmedizin Berlin, Berlin, Germany;[3]Charité - Universitätsmedizin Berlin, Department of Hematology, Oncology, and Cancer Immunology, Campus Charité Mitte, Berlin, Germany; [4]Charité – Universitätsmedizin Berlin, Department of Neuropathology, Berlin, Germany;[5]Charité – Universitätsmedizin Berlin, Department of Pediatric Oncology and Hematology, Berlin, Germany; [6]German Cancer Consortium (DKTK), Partner Site Berlin, German Cancer Research Center (DKFZ), Heidelberg, Germany;*

### Background

Cancer treatments in young individuals frequently lead to late and long term chronic health problems that are reminiscent of conditions known in elderly populations, supporting the idea of cancer- and therapy-induced accelerated biological aging. Measuring alterations in DNA methylation patterns is an established hallmark of aging and can be a powerful tool to detect and quantify accelerated aging processes. Several epigenetic clocks regressing CpG methylation to various endpoints have been developed to capture distinct aspects of aging.

### Aims

We aimed at capturing various epigenetic age measures in survivors of CAYA (children, adolescents and young adults) hematologic cancer to analyze different features of aging.

### Methods

DNA methylation levels were measured from whole blood using the Illumina Infinium MethylationEPIC arrays in 87 CAYA cancer survivors of hematologic cancer (ALL: n = 32, AML: n = 4, Hodgkin's lymphoma: n = 36, NHL: n = 15) excluding survivors who had received allogeneic stem cell transplantation (median age at sampling = 21.0 years, range = 17.9–42.9; median years after therapy = 9.5). Age and sex-matched healthy controls from GSE197676 served as controls. Methylation data were processed using the Bioconductor R packages *minfi* and *sesame*. Epigenetic ages were calculated for SystemsAge (including organ-specific epigenetic ages) and principal component-based clocks (PCHorvath1, PCHorvath2, PCGrimAge and PCPhenoAge). Epigenetic age acceleration (EAA) was defined as the residuals obtained from regressing epigenetic age on chronological age.

**Copyright Information:** (Online) ISSN: 2572-9241
©2026 The Author(s). HemaSphere is published by John Wiley & Sons Ltd on behalf of European Hematology Association. This is an open access Abstract Book distributed under the Attribution-NonCommercial-NoDerivs (CC BY-NC-ND), which allows third parties to download the articles and share them with others as long as they credit the author and the Abstract Book, but they cannot change the content in any way or use them commercially.
**Abstract Book Citations:** Authors, Title, HemaSphere, 2026;10:(S1):pages. The abstract book DOIs can be found on the "Author Information" page.
**Disclaimer:** Articles published in the journal HemaSphere exclusively reflect the opinions of the authors. The authors are responsible for all content in their abstracts including accuracy of the facts, statements, citing resources, etc.

### Results

Survivors had higher EAA in first generation clocks PCHorvath1 (mean 1.51 years, p = 0.006) and PCHorvath2 (mean 1.76 years, p = 0.004) compared to healthy controls, reflecting a cell intrinsic epigenetic aging in survivors. Second generation epigenetic clocks PCPhenoAge (mean 2.58 years, $p < 10^{-4}$) and PCGrimAge (mean 1.21 years, p = 0.001) trained to measure morbidity and mortality also detected higher EAA in CAYA survivors. Finally, the highest difference in EAA was observed for SystemsAge (mean 6.11 years, $p < 10^{-3}$), a meta-clock combining measures for aging processes in individual organs.

Stratification of cancer survivors by treatment intensity using a modified version of the Intensity of Treatment Rating Scale (Kazak et al., Pediatr Blood Cancer, 2012) demonstrated progressively increased epigenetic age acceleration with higher treatment intensity across all analyzed generations of epigenetic clocks. PCHorvath1 and PCHorvath2 demonstrated significantly and progressively higher EAA from low to high intensity treatment (p = 0.032 and p = 0.011, respectively for comparison of low to high treatment intensity). PCPhenoAge and PCGrimAge strengthened this finding by exhibiting similar patterns (p = 0.036 and p = 0.016, respectively). Finally, EAA of SystemsAge further supports the presence of treatment-associated epigenetic signatures indicative of multi-organ aging dependent on treatment intensity (p = 0.013). Additionally, the impact of different treatment modalities, including radiation and cytotoxic therapy, was analyzed.

### Summary/Conclusion

CAYA cancer survivors of hematologic malignancies demonstrate accelerated aging in epigenetic clocks of various generations, directly correlating with intensity of cancer treatment. These results underline that treatment intensity dependent molecular alterations associated with aging can be reliably detected many years after cancer therapy.

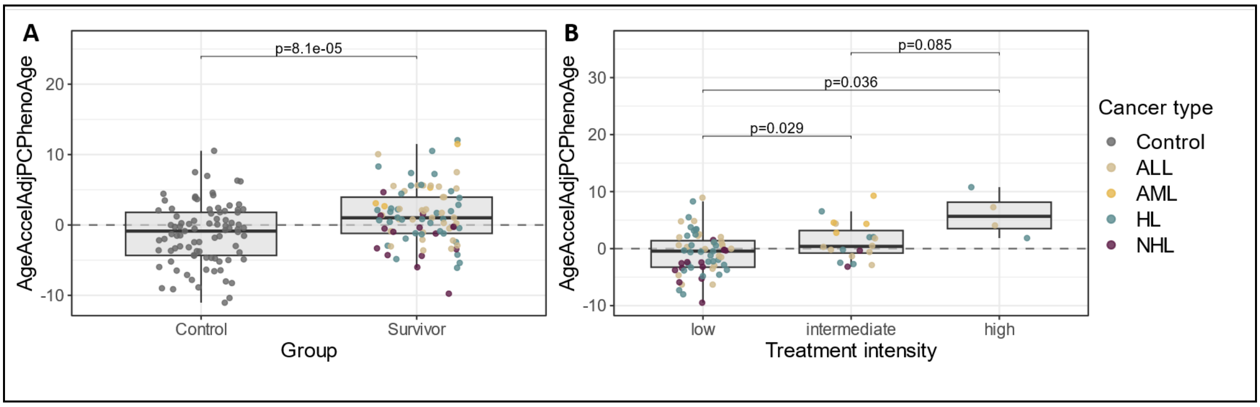

Figure 1: Adjusted epigenetic age acceleration (exemplified by PCPhenoAge). (A) Comparison of survivors and controls. (B) Comparison across treatment intensity groups (low, intermediate, high). Boxes represent the interquartile range (IQR) with the median indicated by the central line; whiskers extend to 1.5× IQR, and points represent individual samples. P values were calculated using Welch's two-sided t-test. ALL = acute lymphoblastic leukemia; AML = acute myeloid leukemia; HL = Hodgkin's lymphoma; NHL = Non-Hodgkin lymphoma. Similar results were observed using other epigenetic clocks.

**Copyright Information:** (Online) ISSN: 2572-9241
©2026 The Author(s). HemaSphere is published by John Wiley & Sons Ltd on behalf of European Hematology Association. This is an open access Abstract Book distributed under the Attribution-NonCommercial-NoDerivs (CC BY-NC-ND), which allows third parties to download the articles and share them with others as long as they credit the author and the Abstract Book, but they cannot change the content in any way or use them commercially.
**Abstract Book Citations:** Authors, Title, HemaSphere, 2026;10:(S1):pages. The abstract book DOIs can be found on the "Author Information" page.
**Disclaimer:** Articles published in the journal HemaSphere exclusively reflect the opinions of the authors. The authors are responsible for all content in their abstracts including accuracy of the facts, statements, citing resources, etc.

### (PF1206) SELF-ASSEMBLED IMMUNE CELL TUMOR ENGAGER (SITE) CONDUCTS THE IMMUNE ORCHESTRA: COORDINATION OF T CELLS, NK CELLS, AND MACROPHAGES FOR SYNERGISTIC ANTI-TUMOR RESPONSES IN MULTIPLE MYELOMA

**Topic:** 25. Gene therapy, cellular immunotherapy and vaccination - Biology & translational research

Shannuo Li*[1], Douglas Sborov[2], Jiyuan Yang[1], Jindřich Kopeček[1]

*[1]University of Utah, Department of Molecular Pharmaceutics, Salt Lake City, United States of America;[2]University of Utah, Huntsman Cancer Institute, Salt Lake City, United States of America;*

### Background

We developed the Self-Assembled Immune Cell Tumor Engager (SITE) platform, a novel modular immunotherapy designed for precision, adaptability, and controlled immune activation. SITE is a two-component, self-assembling system composed of a multiple myeloma (MM) cell-targeting Fab' fragment conjugated to a 25-base morpholino oligonucleotide (Fab'$_{MM}$-MORF1) targeting BCMA, SLAMF7, and CD38, and an immune cell-engaging Fab' fragment conjugated to a complementary morpholino (Fab'$_{CD3}$-MORF2 for T cells, Fab'$_{CD16a}$-MORF2 for NK cells, Fab'$_{CD89}$-MORF2 for macrophages (MΦ)). Hybridization of MORF1 and MORF2 brings immune effectors into proximity with MM cells. In this abstract, we further demonstrated that SITE enables coordinated engagement of adaptive and innate immune effectors, with the potential to produce synergistic anti-tumor activity through complementary cytotoxic mechanisms, immune amplification, and reduced tumor immune escape.

### Aims

To evaluate the therapeutic efficacy and mechanistic basis of SITE in MM, with emphasis on whether coordinated engagement of multiple immune effectors produces synergistic anti-tumor responses through complementary and cooperative immune functions.

### Methods

Fab'-MORF conjugates were synthesized by enzymatic digestion of antibodies and reduction to produce Fab'-thiols, and subsequent conjugation to MORF1 or MORF2. Conjugates and hybrid complexes were characterized by size exclusion chromatography. Primary immune cells were isolated from the whole blood of healthy donors. In vitro efficacy was evaluated in MM cell lines using flow cytometry-based cytotoxicity and mechanistic assays. Combination configurations were designed to test complementary recognition pathways (T + NK) and cooperative cytotoxic–phagocytic interactions (T + MΦ). In vivo studies were conducted in NRG mice intravenously inoculated with MM.1S cells for toxicity assessment or survival analysis.

### Results

MM-targeting (Fab'$_{MM}$-MORF1) and immune cells-engaging (Fab'$_{CD3/CD16a/CD89}$-MORF2) conjugates were successfully synthesized and demonstrated specific hybridization (Fig. 1A). In vitro, naïve T cells, NK cells and MΦ were robustly activated and mediated effective killing or phagocytosis of MM cells when engaged through SITE. Among tested patient samples, 14 out of 18 showed favorable responses to T or NK cell-based SITE. Multi-effector engagement produced enhanced anti-tumor activity compared with single-effector targeting. Combined T- and NK-cell recruitment resulted in superior cytotoxicity by complementary recognition, integrating antigen-specific killing and MHC-independent tumor elimination, thereby reducing immune escape (Fig.1B). T cells and MΦs co-engagement amplified tumor clearance through a cytotoxic–phagocytic feedback loop: MΦ-mediated tumor antigen processing enhanced T-cell activation, while T cell derived cytokines potentiated MΦ tumoricidal activity. In vivo, T-SITE therapy significantly improved survival in NRG mice.

**Copyright Information:** (Online) ISSN: 2572-9241
©2026 The Author(s). HemaSphere is published by John Wiley & Sons Ltd on behalf of European Hematology Association. This is an open access Abstract Book distributed under the Attribution-NonCommercial-NoDerivs (CC BY-NC-ND), which allows third parties to download the articles and share them with others as long as they credit the author and the Abstract Book, but they cannot change the content in any way or use them commercially.
**Abstract Book Citations:** Authors, Title, HemaSphere, 2026;10:(S1):pages. The abstract book DOIs can be found on the "Author Information" page.
**Disclaimer:** Articles published in the journal HemaSphere exclusively reflect the opinions of the authors. The authors are responsible for all content in their abstracts including accuracy of the facts, statements, citing resources, etc.

### Summary/Conclusion

The SITE platform enables coordinated recruitment of complementary immune effector populations, resulting in synergistic anti-MM activity. T+NK engagement broadens tumor recognition and limits immune escape, while T + MΦ co-engagement establishes immune amplification through reciprocal activation. By integrating multi-antigen targeting with adaptive and innate immune cooperation, SITE drives amplified and sustained tumor elimination and provides a strong foundation for next-generation personalized immunotherapy.

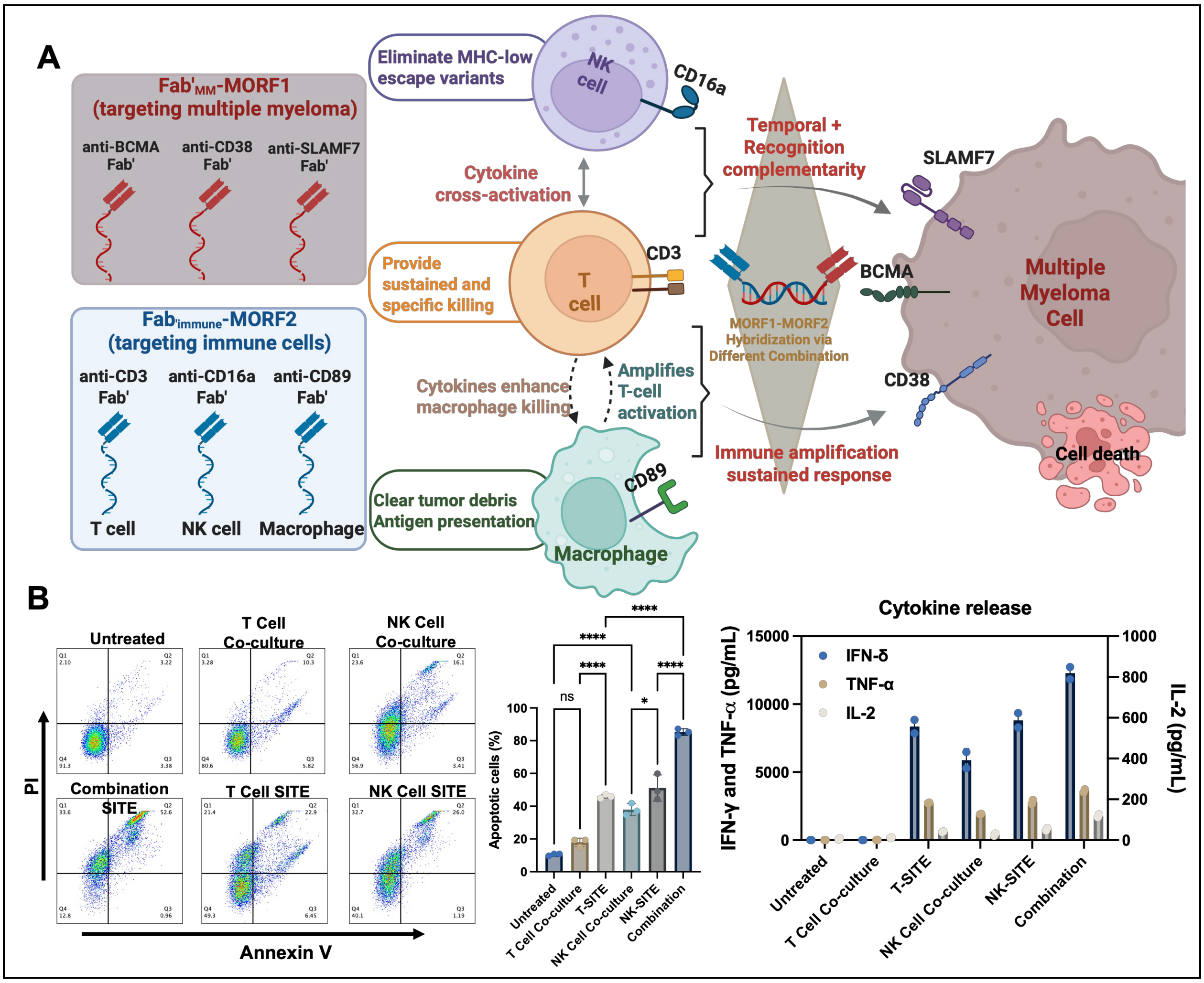

Figure 1. SITE enables coordinated multi-immune cell engagement for enhanced anti-tumor activity in multiple myeloma (MM). A) Schematic illustration of the Self-Assembled Immune Cell Tumor Engager (SITE) platform. Tumor-targeting Fab′-MORF1 modules recognize multiple myeloma antigens (BCMA, CD38, and SLAMF7), while immune cell-targeting Fab′-MORF2 modules recruit and activate T cells (CD3), NK cells (CD16a), and macrophages (CD89). Hybridization between MORF1 and MORF2 enables modular assembly at the tumor site, bringing immune effector cells into close proximity with MM cells. Coordinated engagement of T cells and NK cells by SITE promotes cytokine cross-activation and complementary recognition, integrating antigen-specific killing with MHC-independent tumor elimination. Coordinated activation of T cells and macrophages supports sustained cytotoxicity, clearance of tumor debris, and antigen presentation, leading to immune amplification and durable anti-tumor responses. B) Representative flow cytometry plots show enhanced immune cell-mediated tumor killing following combination SITE treatment of T cell and NK cell SITE compared with controls. Quantitative analyses of cytokine release demonstrate increased cytotoxicity and immune activation with multi-cell combination SITE engagement.

**Copyright Information:** (Online) ISSN: 2572-9241
©2026 The Author(s). HemaSphere is published by John Wiley & Sons Ltd on behalf of European Hematology Association. This is an open access Abstract Book distributed under the Attribution-NonCommercial-NoDerivs (CC BY-NC-ND), which allows third parties to download the articles and share them with others as long as they credit the author and the Abstract Book, but they cannot change the content in any way or use them commercially.
**Abstract Book Citations:** Authors, Title, HemaSphere, 2026;10:(S1):pages. The abstract book DOIs can be found on the “Author Information” page.
**Disclaimer:** Articles published in the journal HemaSphere exclusively reflect the opinions of the authors. The authors are responsible for all content in their abstracts including accuracy of the facts, statements, citing resources, etc.

### (PF1207) PRECLINICAL DEVELOPMENT OF AN ALLOGENEIC KIR3DL2-TARGETED CAR-T CELL THERAPY FOR MATURE T-CELL LYMPHOMAS

**Topic:** 25. Gene therapy, cellular immunotherapy and vaccination - Biology & translational research

Xiao Jing Ong*[1, 2], Thungoc Nguyen[1], Carrie van der Weyden[2, 3], Joshua Casan[2, 3], Vicky Mengfei Qin[1, 2], Arthur Xuan Wang[1, 2], Rebecca Castle[1], Emma O'rourke[1], Enola Roussel[1, 2], Karla Cowley[1, 2], Ada Koo[1, 2], Jennii Luu[1], Kaylene Simpson[1, 2], Paul Beavis[1, 2], H. Miles Prince[2, 3], Joe Jiang Zhu[1, 2], Criselle D'souza[1, 2], Paul Neeson[1, 2]

*[1]Peter MacCallum Cancer Centre, Melbourne, VIC, Australia;[2]The University of Melbourne, The Sir Peter MacCallum Department of Oncology, Faculty of Medicine, Dentistry and Health Science, Melbourne, VIC, Australia; [3]Peter MacCallum Cancer Centre and Royal Melbourne Hospital, Department of Haematology, Melbourne, VIC, Australia;*

### Background

Despite significant therapeutic advances in B-cell malignancies, patients with relapsed/refractory T-cell lymphoma (TCL) continue to face dismal outcomes, with response durations of less than 6 months and a 5-year survival below 30%. Although CAR-T cell therapy has revolutionized treatment for B-cell cancers, its translation to TCL has been challenging, in part due to the lack of tumor-restricted target antigens. Several CAR-T targets under investigation, including CD5 and CD7, are not only frequently downregulated in TCL cells but are also expressed by normal T cells, resulting in fratricide, T-cell aplasia, and limited therapeutic efficacy. Additional challenges include manufacturing barriers in heavily pre-treated patients and tumor escape driven by antigen heterogeneity.

### Aims

We sought to develop an allogeneic CAR-T cell therapy for TCL by targeting the KIR3DL2 receptor, a molecule highly expressed in TCL patient tumor cells but minimally expressed in healthy T (<1%) and NK cells (<15%). We further aimed to identify drug combinations that synergize with CAR-T cells to prevent tumor escape.

### Methods

To evaluate the efficacy of the CAR target, we generated second-generation KIR3DL2-directed CAR-T cells and assessed them in murine xenograft models that recapitulate cutaneous and leukemic TCL using HuT78 (cutaneous TCL) and DERL7 (peripheral TCL) lines. Allogeneic KIR3DL2 CAR-T cells were produced using CRISPR-Cas9 Homology-directed Repair (HDR) by double knock-in of the CAR into T-cell receptor alpha constant (TRAC), and HLA-E into the β2-microglobulin (β2M) gene locus. To address antigen heterogeneity, we performed a high-throughput drug screen of 667 compounds on cell lines with varying KIR3DL2 expression - HH (negative), SR786 (intermediate) and HuT78 (high).

**Copyright Information:** (Online) ISSN: 2572-9241
©2026 The Author(s). HemaSphere is published by John Wiley & Sons Ltd on behalf of European Hematology Association. This is an open access Abstract Book distributed under the Attribution-NonCommercial-NoDerivs (CC BY-NC-ND), which allows third parties to download the articles and share them with others as long as they credit the author and the Abstract Book, but they cannot change the content in any way or use them commercially.
**Abstract Book Citations:** Authors, Title, HemaSphere, 2026;10:(S1):pages. The abstract book DOIs can be found on the "Author Information" page.
**Disclaimer:** Articles published in the journal HemaSphere exclusively reflect the opinions of the authors. The authors are responsible for all content in their abstracts including accuracy of the facts, statements, citing resources, etc.

**Results**

KIR3DL2 CAR-T cells significantly controlled tumor growth in both subcutaneous and disseminated TCL xenograft models (**Figure**), with 80% of mice surviving over 60 days, compared to 15 days in the control group (n = 6, $p < 0.0001$, Mantel-Cox (log-rank) test). This efficacy is likely driven by CAR-T cell persistence observed in the spleen and blood of mice at day 60 post-infusion. Additionally, we showed that peripheral T cells from TCL patients were suboptimal starting material for CAR-T production, as malignant T cells can be transduced, and patient-derived CAR-T cells have reduced tumor-killing capacity. Given the limitations of autologous manufacturing, we generated allogeneic CAR-T cells with >50% double-knock-in and >80% double-knock-out efficiency and achieved a 60-fold expansion over 10 days. These allogeneic CAR-T cells exhibited robust anti-tumor potency in 16-hour coculture assays against *ex vivo* TCL patient tumor samples (n = 4) with a significant reduction in tumor cells at a low 1:1 E:T ratio compared to untransduced T cell control. To address antigen-low escape, we identified drugs that upregulated KIR3DL2, with Histone Deacetylase inhibitors as top hits. Preclinical evaluation of combination therapy against *ex vivo* TCL patient samples is currently underway.

**Summary/Conclusion**

In summary, we developed an off-the-shelf KIR3DL2-targeted CAR-T therapy with potent activity against TCL cell lines and patient tumor samples. Pharmacologic combination offers a rational strategy to mitigate KIR3DL2 expression heterogeneity and augment CAR-T activity. Collectively, our preclinical findings support allogeneic KIR3DL2-CAR-T cells as a promising therapeutic strategy for TCL.

**Copyright Information:** (Online) ISSN: 2572-9241
©2026 The Author(s). HemaSphere is published by John Wiley & Sons Ltd on behalf of European Hematology Association. This is an open access Abstract Book distributed under the Attribution-NonCommercial-NoDerivs (CC BY-NC-ND), which allows third parties to download the articles and share them with others as long as they credit the author and the Abstract Book, but they cannot change the content in any way or use them commercially.
**Abstract Book Citations:** Authors, Title, HemaSphere, 2026;10:(S1):pages. The abstract book DOIs can be found on the "Author Information" page.
**Disclaimer:** Articles published in the journal HemaSphere exclusively reflect the opinions of the authors. The authors are responsible for all content in their abstracts including accuracy of the facts, statements, citing resources, etc.

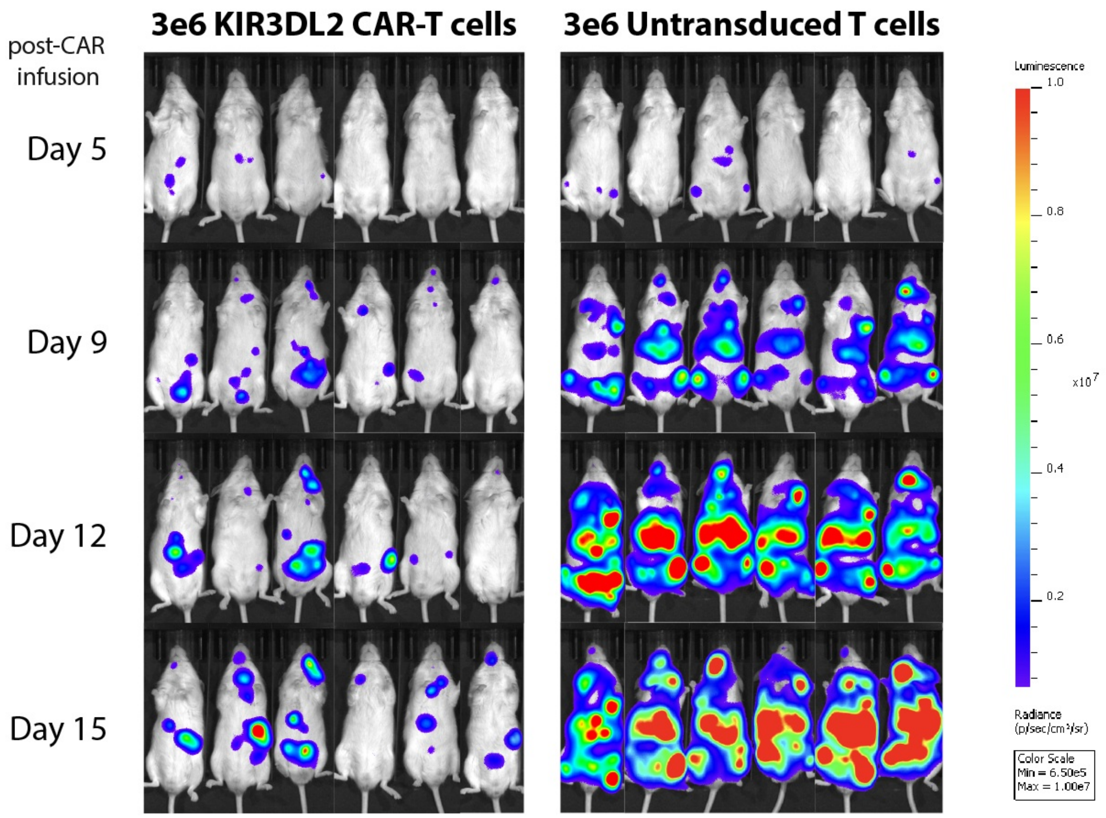

**Figure. Bioluminescence image of aggressive leukemic cutaneous T-cell lymphoma (CTCL) model in KIR3DL2-CAR-T treated or untransduced T cell treated groups**

**Copyright Information:** (Online) ISSN: 2572-9241
©2026 The Author(s). HemaSphere is published by John Wiley & Sons Ltd on behalf of European Hematology Association. This is an open access Abstract Book distributed under the Attribution-NonCommercial-NoDerivs (CC BY-NC-ND), which allows third parties to download the articles and share them with others as long as they credit the author and the Abstract Book, but they cannot change the content in any way or use them commercially.
**Abstract Book Citations:** Authors, Title, HemaSphere, 2026;10:(S1):pages. The abstract book DOIs can be found on the "Author Information" page.
**Disclaimer:** Articles published in the journal HemaSphere exclusively reflect the opinions of the authors. The authors are responsible for all content in their abstracts including accuracy of the facts, statements, citing resources, etc.

### (PF1208) A NEXT-GENERATION FCRL5/BCMA TRISPECIFIC ANTIBODY DEMONSTRATES SUPERIOR ACTIVITY OVER BISPECIFIC CONSTRUCTS AND SUPPORTS CLINICAL DEVELOPMENT IN B-CELL MALIGNANCIES

**Topic:** 25. Gene therapy, cellular immunotherapy and vaccination - Biology & translational research

Jessica Encinas Mayoral*[1], Jian Cui[1], Alice Mattinson[2], Edward King[2], Yulia Lampi[2], Simon Bornschein[2], Steve Treon[1], Zachary Hunter[1], Mehmet Samur[1], Mariateresa Fulciniti[1], Nikhil Munshi[1]

*[1]Dana Farber Cancer Institute, Boston, United States of America;[2]Coding Bio, Oxford, United Kingdom;*

### Background

BCMA-directed CAR T cell therapy has improved outcomes in multiple myeloma (MM), but their impact is limited by manufacturing challenges and relapses driven by antigen escape. In this context, trispecific T cell engagers (TriTE) represent an attractive alternative approach, enabling simultaneous targeting of multiple tumor antigens designed to broaden tumor coverage, reduce antigen escape, and potentially enhance depth of response. As an off-the-shelf therapy with simplified logistics and manageable toxicity profile, TriTEs represent a promising next-generation immunotherapeutic approach.
FcRL5 is a B-lineage-restricted surface antigen that supports malignant plasma cell differentiation. FcRL5 is universally expressed in MM cell lines and in primary samples from newly diagnosed and relapsed/refractory MM. It is also uniquely expressed in Waldenström macroglobulinemia (WM), where a protein-coding RNA isoform is absent in healthy B cells. Unlike GPRC5D, FcRL5 is not expressed in other healthy tissues, highlighting its potential as a selective therapeutic target. Based on these findings, we developed a TriTE targeting BCMA and FcRL5.

### Aims

The goal is to assess the preclinical efficacy of the next-generation BCMA/FcRL5 TriTE in models of MM and WM, and to establish the rationale that dual-antigen trispecific targeting can outperform BCMA-directed bispecific therapies.

### Methods

FcRL5 and BCMA expression in MM and WM cells was evaluated by flow cytometry, western blot, and RNA-seq. High-throughput TriTE generation and screening were performed. In vitro cytotoxicity and T-cell activation were evaluated using flow-based assays. In vivo efficacy was tested in humanized mouse models bearing subcutaneous NCI-H929 or intravenous U266B1 tumors, followed by PBMC infusion and Q7D×4 dosing, and benchmarked against commercial BCMA and FcRL5 bispecific antibodies.

### Results

We utilized a novel antibody discovery platform combining high-throughput functional screening with generative AI to select and optimize antibodies with the strongest immune response, independent of affinity. We generated BCMA/FcRL5 TriTEs that markedly enhanced tumor cell killing and T-cell response both against MM cell lines and primary patient MM cells compared with the other benchmark clinical BCMA or FcRL5 TCE. The activity extended to WM, where the TriTEs also mediated potent killing in WM cell lines and primary cells. TriTE demonstrated synergistic activity even when both antigens were expressed at low levels and remained effective in myeloma cells with BCMA knockout. In vivo, BCMA/FcRL5 TriTEs produced robust antitumor efficacy in humanized mice bearing subcutaneous NCI-H929 tumors, yielding tumor volume reduction in all treated animals. In the disseminated U266B1 MM model, dose-escalation studies showed that TriTE achieved superior antitumor activity at a 10-fold lower dose than Teclistamab and prolonged survival; median survival: 62 days for control, 82 days for Teclistamab while all TriTE-treated mice remained alive beyond 130 days, consistent with durable responses in this model.

**Copyright Information:** (Online) ISSN: 2572-9241
©2026 The Author(s). HemaSphere is published by John Wiley & Sons Ltd on behalf of European Hematology Association. This is an open access Abstract Book distributed under the Attribution-NonCommercial-NoDerivs (CC BY-NC-ND), which allows third parties to download the articles and share them with others as long as they credit the author and the Abstract Book, but they cannot change the content in any way or use them commercially.
**Abstract Book Citations:** Authors, Title, HemaSphere, 2026;10:(S1):pages. The abstract book DOIs can be found on the "Author Information" page.
**Disclaimer:** Articles published in the journal HemaSphere exclusively reflect the opinions of the authors. The authors are responsible for all content in their abstracts including accuracy of the facts, statements, citing resources, etc.

**Summary/Conclusion**

Leveraging a high-throughput discovery platform, we have generated BCMA/FcRL5 TriTE antibodies with superior antitumor activity and increased in vivo potency, outperforming BCMA-directed therapy even at lower doses in vivo. These results support dual-antigen targeting as a strategy to overcome the limitations of BCMA-only approaches and provide a strong rationale for clinical translation of our BCMA/FcRL5 TriTE in MM and WM.

**Copyright Information:** (Online) ISSN: 2572-9241
©2026 The Author(s). HemaSphere is published by John Wiley & Sons Ltd on behalf of European Hematology Association. This is an open access Abstract Book distributed under the Attribution-NonCommercial-NoDerivs (CC BY-NC-ND), which allows third parties to download the articles and share them with others as long as they credit the author and the Abstract Book, but they cannot change the content in any way or use them commercially.
**Abstract Book Citations:** Authors, Title, HemaSphere, 2026;10:(S1):pages. The abstract book DOIs can be found on the "Author Information" page.
**Disclaimer:** Articles published in the journal HemaSphere exclusively reflect the opinions of the authors. The authors are responsible for all content in their abstracts including accuracy of the facts, statements, citing resources, etc.

### (PF1209) CAR-T THERAPY RESPONSE AND RESISTANCE STUDIES BASED ON SUBCELLULAR AND MULTIPLEX SINGLE-CELL IMAGING

**Topic:** 25. Gene therapy, cellular immunotherapy and vaccination - Biology & translational research

Liting Chen*[1], Yuekun Fang[1], Yicheng Zhang[1]

[1]*Tongji Hospital, Tongji Medical College, Huazhong University of Science and Technology, Department of Hematology, Wuhan, China;*

### Background

CAR T-cell therapy has achieved remarkable success in treating hematological malignancies, yet patient outcomes remain highly variable (50-90% response rates). Efficacy is hindered by two intertwined factors: the immunosuppressive tumor microenvironment (TME) in DLBCL, whose role in CAR-T outcomes remains poorly understood, and the lack of long-term subcellular phenotyping of CAR-T cytotoxicity, which could identify efficacy-related biomarkers. A comprehensive approach integrating high-resolution dynamic imaging of CAR-T function and high-multiplex spatial TME analysis is needed.

### Aims

To dissect determinants of CAR-T response and resistance through: (1) establishing a high-throughput 3D live imaging platform for subcellular cytotoxicity dynamics, and (2) characterizing DLBCL microenvironment composition, state, and spatial architecture relative to clinical outcomes.

### Methods

We developed two complementary high-resolution imaging platforms. For subcellular imaging, we established a pipeline comprising: a microfluidic chip for large-scale co-culture of CAR-T and Nalm6 cells (monitoring >400 cell pairs); high-throughput Bessel oblique plane microscopy (HBOPM) enabling automated detection of immune cytotoxicity events and 3D subcellular imaging at 320 nm isotropic resolution over extended periods; and a spatial-spectrum phenotyping algorithm quantifying organelle phenotypes correlated with killing efficacy. For TME analysis, we designed a 31-marker imaging mass cytometry (IMC) panel for tumor sections from 13 relapsed/refractory DLBCL patients receiving CD19/22 CAR-T therapy. Twenty sections were sampled pre-infusion or post-relapse. Cells were segmented with CellProfiler, clustered with Rphenograph, visualized by t-SNE, and analyzed spatially with imcRtools.

### Results

HBOPM captured key subcellular events associated with cytolytic capacity, including immune synapse formation and sustained microtubule changes. Actin retrograde flow speed, actin depletion coefficient, microtubule polarization, and CAR-T/target contact area were identified as parameters strongly correlated with cytotoxic function, with dasatinib-treated controls validating platform effectiveness. IMC analysis identified 493,715 cells and 35 clusters, redefined into 10 metaclusters. CD4+ T-cell fraction positively associated with remission duration. Higher Ki67, CD57, TIM3 and lower CD69 on T cells—particularly CD8+/CD4+ Tem and Te subsets—were seen in poor outcomes. Immune-rich cellular neighborhoods correlated with longer remission, while fibroblasts and endothelial cells resided closer to tumor cells in poor responders.

**Copyright Information:** (Online) ISSN: 2572-9241
©2026 The Author(s). HemaSphere is published by John Wiley & Sons Ltd on behalf of European Hematology Association. This is an open access Abstract Book distributed under the Attribution-NonCommercial-NoDerivs (CC BY-NC-ND), which allows third parties to download the articles and share them with others as long as they credit the author and the Abstract Book, but they cannot change the content in any way or use them commercially.
**Abstract Book Citations:** Authors, Title, HemaSphere, 2026;10:(S1):pages. The abstract book DOIs can be found on the "Author Information" page.
**Disclaimer:** Articles published in the journal HemaSphere exclusively reflect the opinions of the authors. The authors are responsible for all content in their abstracts including accuracy of the facts, statements, citing resources, etc.

**Summary/Conclusion**

We established a multi-scale imaging strategy integrating subcellular live imaging with multiplex single-cell spatial analysis. HBOPM reveals cytotoxic function is governed by dynamic immune synapse events, while IMC demonstrates that pre-existing immunosuppressive TME landscape—characterized by specific cell compositions, activation/exhaustion states, and spatial architectures—significantly influences clinical outcomes. This combined approach establishes a powerful pipeline for quantifying T-cell function and predicting patient response, with broad applicability for advancing T-cell-based immunotherapies.

**Copyright Information:** (Online) ISSN: 2572-9241
©2026 The Author(s). HemaSphere is published by John Wiley & Sons Ltd on behalf of European Hematology Association. This is an open access Abstract Book distributed under the Attribution-NonCommercial-NoDerivs (CC BY-NC-ND), which allows third parties to download the articles and share them with others as long as they credit the author and the Abstract Book, but they cannot change the content in any way or use them commercially.
**Abstract Book Citations:** Authors, Title, HemaSphere, 2026;10:(S1):pages. The abstract book DOIs can be found on the “Author Information” page.
**Disclaimer:** Articles published in the journal HemaSphere exclusively reflect the opinions of the authors. The authors are responsible for all content in their abstracts including accuracy of the facts, statements, citing resources, etc.

### (PF1210) MICROENVIRONMENT-DIRECTED NANOBODY-BASED FAP CAR-T CELLS TARGET PATHOGENIC STROMA IN MYELOFIBROSIS

**Topic:** 25. Gene therapy, cellular immunotherapy and vaccination - Biology & translational research

Zhipeng Zhou*[1], Pei Xuying[1], Meng Lv[1], Zhifan Zhao[1], Zhuojun Liu[1], Yuxuan Du[1], Xiangyu Zhao[1], Xiaojun Huang[1]

[1]*Peking University People's Hospital, Beijing, China;*

### Background

Myelofibrosis (MF) is a life-threatening myeloproliferative neoplasm characterized by progressive bone marrow fibrosis, cytopenias, splenomegaly, and inferior survival. Although stem cell transplantation is potentially curative, its applicability is limited by patient age, comorbidities, and transplant-related toxicities. Standard JAK inhibitors effectively relieve symptoms but rarely reverse established fibrosis or eliminate the pathogenic stromal niche. Progressive marrow failure remains a major cause of death. Therefore, strategies that directly remodel the fibrotic microenvironment represent a critical unmet need. Fibroblast Activation Protein (FAP) is selectively expressed on activated fibroblasts while being minimally present in normal organs. We hypothesized that selective depletion of $FAP^+$ stromal cells using chimeric antigen receptor (CAR)-T cells could dismantle the fibrotic niche and provide a novel microenvironment-directed therapeutic strategy for MF.

### Aims

To evaluate the specificity, safety, and in vivo targeting efficacy of nanobody-based FAP CAR-T cells against MF-associated pathogenic stromal cells.

### Methods

We engineered human T cells to express a second-generation CAR comprising a high-affinity anti-FAP nanobody (VHH) linked to 4-1BB and CD3ζ domains. In vitro cytotoxicity was measured against FAP-expressing cells (K562-FAP, MRC-5, and primary MF patient-derived bone marrow stromal cells). Target specificity and safety were assessed against FAP-negative controls, including the wild-type K562 cell line and bone marrow mononuclear cells (BMMCs) from healthy donors. In vivo efficacy was evaluated in an NPG mouse model engrafted with FAP-expressing K562-Luciferase cells within the bilateral femur cavities to simulate the bone marrow niche, followed by intravenous infusion of FAP CAR-T cells.

### Results

Quantitative immunohistochemistry (Mean Optical Density) confirmed significant FAP upregulation in MF bone marrow stroma, whereas FAP expression was negligible in healthy donors. FAP CAR-T cells were generated with ~60% transduction efficiency and exhibited robust expansion. In vitro, FAP CAR-T cells demonstrated potent, dose-dependent cytotoxicity against K562-FAP and MRC-5 cells, as well as primary MF-derived BMSCs. At a 5:1 effector-to-target ratio, specific lysis exceeded 70% for $FAP^+$ targets. In contrast, minimal cytotoxicity was observed against wild-type K562 or healthy donor BMMCs, indicating high target specificity and a favorable safety profile. In the NPG bone marrow engraftment model, intravenously infused FAP CAR-T cells showed in vivo expansion between Days 14 and 28, resulting in significant reduction of BLI signal compared with PBS and mock T-cell controls. By Day 28, BLI intensity in the FAP CAR-T group was markedly decreased, indicating effective depletion of $FAP^+$ targets within the marrow cavity. Survival analysis further demonstrated superior outcomes in the FAP CAR-T–treated group compared with PBS or mock T-treated mice.

**Copyright Information:** (Online) ISSN: 2572-9241
©2026 The Author(s). HemaSphere is published by John Wiley & Sons Ltd on behalf of European Hematology Association. This is an open access Abstract Book distributed under the Attribution-NonCommercial-NoDerivs (CC BY-NC-ND), which allows third parties to download the articles and share them with others as long as they credit the author and the Abstract Book, but they cannot change the content in any way or use them commercially.
**Abstract Book Citations:** Authors, Title, HemaSphere, 2026;10:(S1):pages. The abstract book DOIs can be found on the “Author Information” page.
**Disclaimer:** Articles published in the journal HemaSphere exclusively reflect the opinions of the authors. The authors are responsible for all content in their abstracts including accuracy of the facts, statements, citing resources, etc.

**Summary/Conclusion**

Nanobody-based FAP CAR-T cells demonstrate potent, specific, and safe targeting of pathogenic stromal cells in MF. These findings provide compelling proof-of-concept that microenvironment-directed cellular immunotherapy can effectively deplete the fibrotic niche within the bone marrow. Targeting FAP⁺ stromal elements represents a novel therapeutic paradigm that extends CAR-T technology beyond malignant clone eradication toward direct remodeling of the tumor-supportive microenvironment in myelofibrosis.

**Copyright Information:** (Online) ISSN: 2572-9241
©2026 The Author(s). HemaSphere is published by John Wiley & Sons Ltd on behalf of European Hematology Association. This is an open access Abstract Book distributed under the Attribution-NonCommercial-NoDerivs (CC BY-NC-ND), which allows third parties to download the articles and share them with others as long as they credit the author and the Abstract Book, but they cannot change the content in any way or use them commercially.
**Abstract Book Citations:** Authors, Title, HemaSphere, 2026;10:(S1):pages. The abstract book DOIs can be found on the "Author Information" page.
**Disclaimer:** Articles published in the journal HemaSphere exclusively reflect the opinions of the authors. The authors are responsible for all content in their abstracts including accuracy of the facts, statements, citing resources, etc.

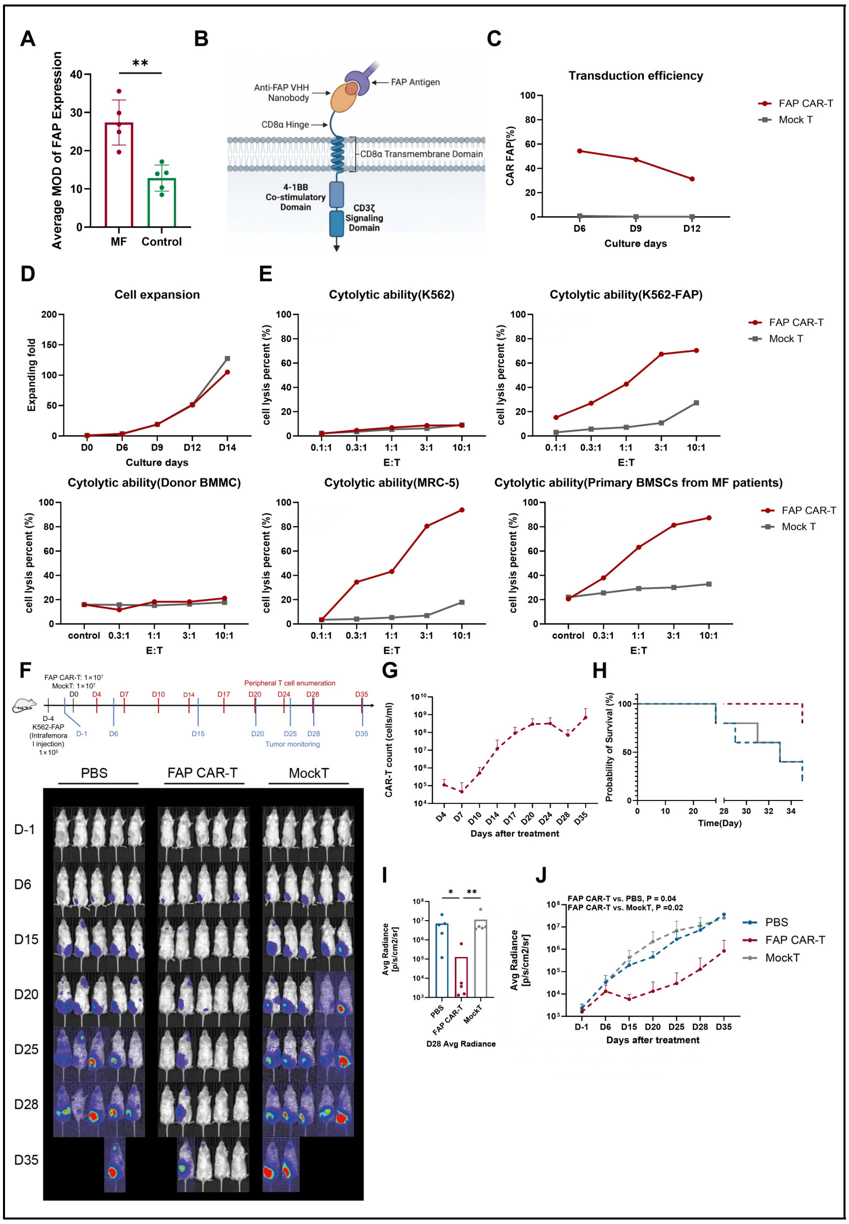

Preclinical evaluation of nanobody-based FAP CAR-T cells in myelofibrosis. (A) Quantitative IHC (Mean Optical Density) of FAP expression in bone marrow from MF patients and healthy controls. (B) Schematic of the anti-FAP VHH CAR structure. (C, D) Lentiviral transduction efficiency and ex vivo expansion kinetics. (E) In vitro specific cytotoxicity against FAP-negative (wild-type K562, healthy donor BMMCs) and FAP-positive targets (K562-FAP, MRC-5, primary MF-BMSCs) at indicated E:T ratios. (F) Experimental timeline (top) and representative bioluminescence imaging (BLI) of NPG mice intrafemorally engrafted with K562-FAP-Luc+ cells (bottom). (G) In vivo peripheral CAR-T cell enumeration over time. (H) Survival curves of treated mice. (I, J) Cross-sectional quantification of BLI at Day 28 (I) and longitudinal tumor burden monitoring (J).

**Copyright Information:** (Online) ISSN: 2572-9241
©2026 The Author(s). HemaSphere is published by John Wiley & Sons Ltd on behalf of European Hematology Association. This is an open access Abstract Book distributed under the Attribution-NonCommercial-NoDerivs (CC BY-NC-ND), which allows third parties to download the articles and share them with others as long as they credit the author and the Abstract Book, but they cannot change the content in any way or use them commercially.
**Abstract Book Citations:** Authors, Title, HemaSphere, 2026;10:(S1):pages. The abstract book DOIs can be found on the "Author Information" page.
**Disclaimer:** Articles published in the journal HemaSphere exclusively reflect the opinions of the authors. The authors are responsible for all content in their abstracts including accuracy of the facts, statements, citing resources, etc.

### (PF1211) GENERATION OF BONE MARROW–TARGETED FAS-SHIELDED CXCR4+ TREGS AS CELLULAR THERAPY FOR APLASTIC ANAEMIA.

**Topic:** 25. Gene therapy, cellular immunotherapy and vaccination - Biology & translational research

Karen Ileana Larios-Martinez*[1, 2], Rita Antunes Dos Reis[1], Syed Mian[2], Dominique Bonnet[2], Shahram Kordasti[1], Sophie Papa[1]

[1]*King's College London, London, United Kingdom;* [2]*The Francis Crick Institute, London, United Kingdom;*

### Background

Immune aplastic anaemia (iAA) is a rare, life-threatening haematological disorder with predominantly autoimmune features. In severe and very severe AA, standard treatment is immunosuppressive therapy (IST), with or without eltrombopag, or haematopoietic stem cell transplantation (HSCT), depending on patient factors and donor availability. However, these approaches can be associated with significant toxicity, and not all patients are eligible. iAA is associated with both quantitative and functional defects in regulatory T cells (Tregs), in part due to their susceptibility to Fas ligand (FasL)-mediated apoptosis. Building on these findings, an on-going phase I clinical trial is evaluating adoptive transfer of ex vivo expanded autologous Tregs as a potentially efficacious, low-toxicity therapy (TIARA trial, NCT05386264). To further improve persistence and bone marrow (BM) homing, we have engineered “shielded” Tregs that are resistant to Fas-mediated apoptosis and express the chemokine receptor CXCR4, which supports migration to and retention within the BM niche.

### Aims

To engineer shielded CXCR4 Tregs and evaluate their reduced Fas-L sensitivity and enhanced migration into the bone marrow.

### Methods

Modified Tregs were generated by lentiviral transduction of CD4+CD25+ Treg cells from blood of healthy donors. Cells were engineered with the CXCR4_miRshFAS construct to express two modifications: a miRNA-30-based shRNA against FAS (shFAS) and the chemokine receptor. Treg phenotype, Treg transduction, CXCR4 expression and CD95 knockdown were assessed via flow cytometry. Apoptosis resistance of Tregs was evaluated by CD95 (Fas) expression and FasL-induced apoptosis assay. Treg migration capacity was evaluated *in vitro* using a trans-well migration assay and *in vivo* using NSG c-kit mice. Briefly, 10X10^6 cells were injected intravenously into mice and tissues were processed after 16 hours to assess homing and after 7 days to assess persistence.

### Results

Tregs were successfully transduced with the CXCR4_miRshFAS construct and expanded *in vitro.* The shielded CXCR4 Tregs maintained a stable phenotype and remained able to suppress Teff proliferation in suppression assays. Moreover, shielded CXCR4 Tregs knocked-down the expression of Fas, which translated into resistance to Fas-L induced apoptosis (27.5%, **p=0.0078). These cells also showed higher surface expression of CXCR4, resulting in superior migration capacity in a trans-well migration assay (*p=0.0226). The enhanced BM migration was also observed in NSG-ckit mice after 16 hours of intravenous Treg injection (n=4, **p=0.008). Moreover, mice injected with shielded CXCR4 Tregs showed higher persistence in bone marrow when compared to conventional Tregs (n=4, *p=0.04).

**Copyright Information:** (Online) ISSN: 2572-9241
©2026 The Author(s). HemaSphere is published by John Wiley & Sons Ltd on behalf of European Hematology Association. This is an open access Abstract Book distributed under the Attribution-NonCommercial-NoDerivs (CC BY-NC-ND), which allows third parties to download the articles and share them with others as long as they credit the author and the Abstract Book, but they cannot change the content in any way or use them commercially.
**Abstract Book Citations:** Authors, Title, HemaSphere, 2026;10:(S1):pages. The abstract book DOIs can be found on the “Author Information” page.
**Disclaimer:** Articles published in the journal HemaSphere exclusively reflect the opinions of the authors. The authors are responsible for all content in their abstracts including accuracy of the facts, statements, citing resources, etc.

**Summary/Conclusion**

Shielded CXCR4 Tregs demonstrated higher resistance to Fas-L induced apoptosis without compromising their suppressive function and expansion. Additionally, the modified Tregs showed superior migration capacity in *vitro* and outperformed conventional expanded Tregs *in vivo*, with enhanced migration and persistence into the bone marrow. These findings support shielded CXCR4 Tregs as an innovative strategy to effectively direct engineered inflammatory-resistant Tregs to the site of inflammation. This approach should allow us to use a smaller number of Tregs that home to the site of inflammation, thereby substantially shortening ex vivo expansion and making future clinical trials both more feasible and potentially more effective.

**Copyright Information:** (Online) ISSN: 2572-9241
©2026 The Author(s). HemaSphere is published by John Wiley & Sons Ltd on behalf of European Hematology Association. This is an open access Abstract Book distributed under the Attribution-NonCommercial-NoDerivs (CC BY-NC-ND), which allows third parties to download the articles and share them with others as long as they credit the author and the Abstract Book, but they cannot change the content in any way or use them commercially.
**Abstract Book Citations:** Authors, Title, HemaSphere, 2026;10:(S1):pages. The abstract book DOIs can be found on the "Author Information" page.
**Disclaimer:** Articles published in the journal HemaSphere exclusively reflect the opinions of the authors. The authors are responsible for all content in their abstracts including accuracy of the facts, statements, citing resources, etc.

### (PF1212) ENGINEERING ENHANCED PRIME EDITORS WITH EXPANDED PAM SCOPE FOR PRECISE CORRECTION JAK2V617F MUTATION IN MYELOPROLIFERATIVE NEOPLASM

**Topic:** 25. Gene therapy, cellular immunotherapy and vaccination - Biology & translational research

Wei Lu*[1], Yue Liu[1], Xi Zhang[1]

[1]*Xinqiao Hospital of Army Medical University, Medical Center of Hematology, Chongqing, China;*

### Background

The JAK2V617F mutation is the most common driver in Philadelphia-negative myeloproliferative neoplasms (MPNs), making it an attractive therapeutic target. Prime editing enables precise genome modification without double-strand breaks, but its application at the JAK2V617F locus is constrained by limited protospacer adjacent motif (PAM) availability for pegRNA design. Recently developed prime editor variants with enhanced efficiency (PE7, PESB2) and expanded PAM recognition (PE-SPRY, PE-SpG) offer potential solutions to overcome these barriers.

### Aims

To engineer enhanced prime editors combining high efficiency with broad PAM compatibility, and to evaluate their capacity for precise correction of the JAK2V617F mutation.

### Methods

We generated dual-reporter stable cell lines (EGFP-mCherry for fluorescence-based detection; Fluc-Rluc for luciferase-based quantification) to benchmark prime editor efficiency. We fused the La and SB domains from PE7 and PESB2 with PE-SPRY and PE-SpG to create PE7-SPRY and PESB2-SpG variants. Editing efficiency was quantified by flow cytometry and dual-luciferase assays. A stable cell line integrating a fragment of the human *JAK2* gene containing the V617F mutation was established as a correction model. At this locus, we designed pegRNAs with systematically varied reverse transcription template (RTT) and primer binding site (PBS) lengths using PESB2. Additionally, we employed PE7-SPRY to screen ten pegRNAs targeting distinct PAM sequences including ACA, AGA, and CAG surrounding the JAK2V617F locus. Editing efficiencies were assessed by targeted next-generation sequencing (NGS).

### Results

In dual-reporter assays, PESB2 demonstrated significantly higher editing efficiency compared to PE7, consistent with published reports. Our newly engineered PE7-SPRY and PESB2-SpG variants substantially outperformed the parental PE-SPRY and PE-SpG editors, confirming successful domain fusion and enhanced activity. In the JAK2V617F sequence-integrated stable cell line, screening of two pegRNAs using PESB2 with varying RTT and PBS combinations yielded optimal editing efficiencies up to 2% by NGS analysis. To overcome PAM limitations restricting pegRNA design, we employed PE7-SPRY to test ten pegRNAs targeting distinct PAM sequences surrounding the mutation site, achieving detectable editing with the most efficient pegRNA at 0.1%. These results indicate that local chromatin context, pegRNA architecture, and PAM accessibility collectively influence editing outcomes at the JAK2V617F locus.

**Copyright Information:** (Online) ISSN: 2572-9241
©2026 The Author(s). HemaSphere is published by John Wiley & Sons Ltd on behalf of European Hematology Association. This is an open access Abstract Book distributed under the Attribution-NonCommercial-NoDerivs (CC BY-NC-ND), which allows third parties to download the articles and share them with others as long as they credit the author and the Abstract Book, but they cannot change the content in any way or use them commercially.
**Abstract Book Citations:** Authors, Title, HemaSphere, 2026;10:(S1):pages. The abstract book DOIs can be found on the "Author Information" page.
**Disclaimer:** Articles published in the journal HemaSphere exclusively reflect the opinions of the authors. The authors are responsible for all content in their abstracts including accuracy of the facts, statements, citing resources, etc.

### Summary/Conclusion

We successfully established robust quantitative platforms for evaluating and optimizing prime editing systems for JAK2V617F correction. Our engineered PE7-SPRY and PESB2-SpG variants demonstrate expanded PAM recognition with improved efficiency, representing valuable tools for therapeutic genome editing. While current editing efficiencies at the JAK2V617F locus remain limited (0.1-2%), this study provides critical insights into pegRNA design principles and identifies key parameters constraining editing efficiency. Ongoing investigations combining optimized pegRNAs with nickase-guided editing strategies aim to enhance editing rates. These findings establish a foundation for developing precision gene therapy approaches for JAK2V617F-driven myeloproliferative neoplasms and demonstrate the feasibility of targeting this clinically significant mutation using next-generation prime editing technologies.

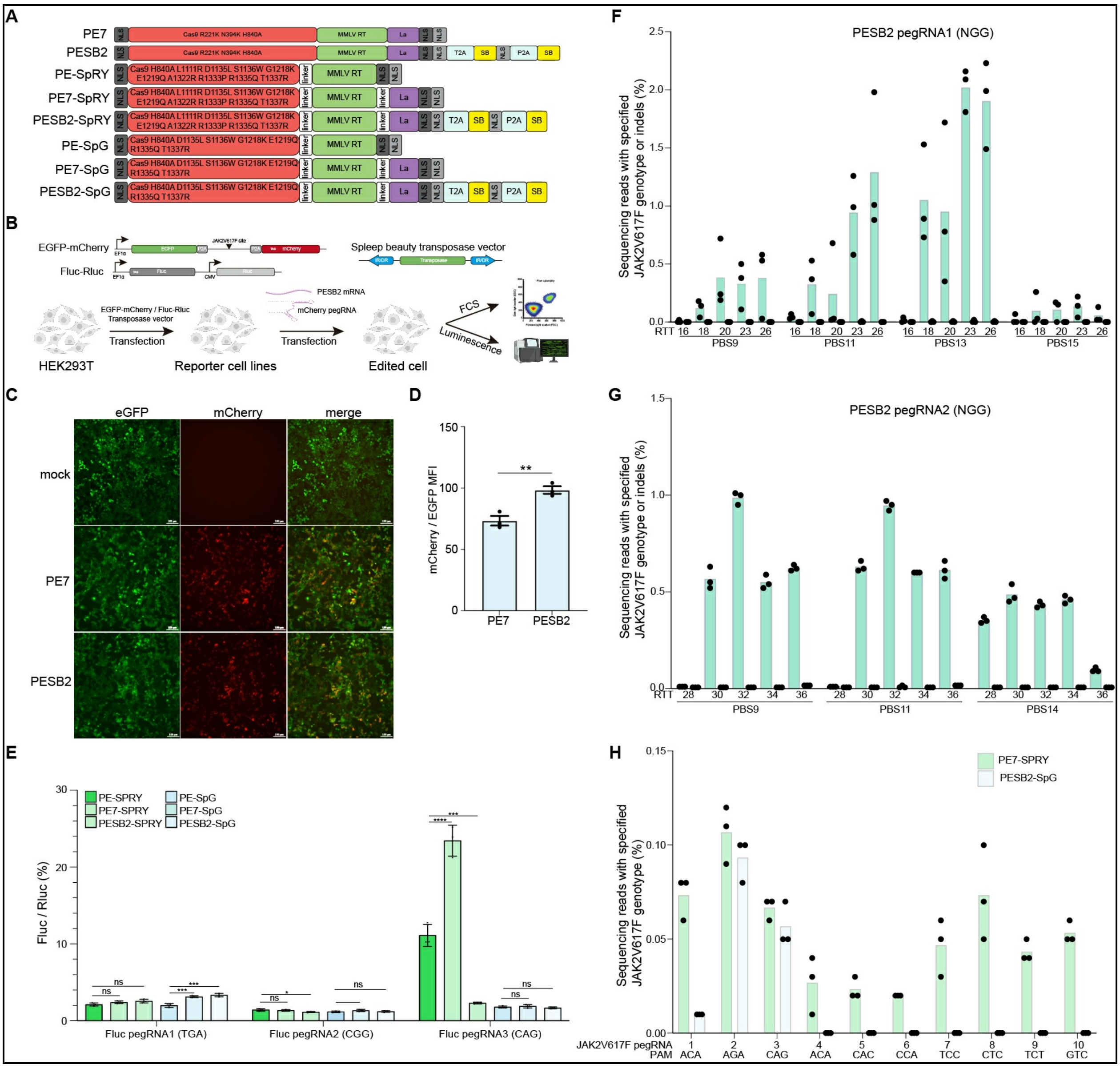

**Copyright Information:** (Online) ISSN: 2572-9241
©2026 The Author(s). HemaSphere is published by John Wiley & Sons Ltd on behalf of European Hematology Association. This is an open access Abstract Book distributed under the Attribution-NonCommercial-NoDerivs (CC BY-NC-ND), which allows third parties to download the articles and share them with others as long as they credit the author and the Abstract Book, but they cannot change the content in any way or use them commercially.
**Abstract Book Citations:** Authors, Title, HemaSphere, 2026;10:(S1):pages. The abstract book DOIs can be found on the "Author Information" page.
**Disclaimer:** Articles published in the journal HemaSphere exclusively reflect the opinions of the authors. The authors are responsible for all content in their abstracts including accuracy of the facts, statements, citing resources, etc.

### (PF1213) T-CELL RECEPTOR-ENGINEERED T CELLS EFFICIENTLY TARGET ACUTE MYELOID LEUKEMIA WITH DIFFERENT TYPES OF NUCLEOPHOSMIN 1 MUTATIONS.

**Topic:** 25. Gene therapy, cellular immunotherapy and vaccination - Biology & translational research

Eva Argiro*[1], Georgia Koutsoumpli[1], Michel Kester[1], Mirjam Heemskerk[1], Rosa de Groot[1], Constantijn Halkes[1], Marieke Griffioen[1]

[1]*Leiden University Medical Center, Department of Hematology, Leiden, The Netherlands;*

### Background

Acute myeloid leukemia (AML) is a life-threatening disease that requires further therapeutic options, especially for patients with relapsed disease. In 30% of AML patients, driver mutations in *NPM1* occur which are typically 4-base-pair frameshift mutations leading to an 11 amino acid alternative reading frame. The most prevalent *NPM1* mutations are type A (TCTG; 75-80%), B (CATG; 5-10%) and D (CCTG, 5-10%), which encode either the aberrant peptide sequence CLAVEEVSLRK or CMAVEEVSLRK. In previous research, we isolated a T cell clone from an HLA-A*02:01-positive healthy individual expressing a T cell receptor (TCR) that specifically binds to the NPM1 neoantigen CLAVEEVSL in HLA-A*02:01. T cells engineered with this TCR induced strong lysis of HLA-A*02:01-positive AML cells *in vitro* as well as *in vivo* in immunodeficient mice engrafted with the *NPM1*-mutated AML cell line OCI-AML3, holding promise for clinical translation.

### Aims

Here, we assessed the potential of NPM1-A2 TCR-T cells to bind and recognize different NPM1 neopeptides and their capacity to target primary AML with distinct *NPM1* mutations in HLA-A*02:01 as well as in other HLA-A*02 molecules.

### Methods

Based on *NPM1* mutation type and neopeptide frequencies in transcriptomics data and historical literature comprising 2703 patients, 14 NPM1 neopeptides were selected. These neopeptides were evaluated for HLA-A*02 binding affinity in fluorescence-based competitive binding assays and for recognition by NPM1-A2 TCR-T cells on peptide-loaded cells and primary AML samples by interferon gamma (IFN-γ) ELISA and in flow cytometry-based lysis assays.

### Results

Our studies demonstrated that 16% of *NPM1*-mutated AML patients have mutations encoding NPM1 neopeptides other than CLAVEEVSL. When exogenously loaded on T2 cells or Epstein-Barr virus (EBV)-transformed B lymphoblastoid cell lines, NPM1-A2 TCR-T cells were shown to recognize CLAVEEVSL and CMAVEEVSL in the context of HLA-A*02:01 and HLA-A*02:09. NPM1 neopeptide CQAVEEVSL, which is encoded by 9.2% of patients with non-ABD *NPM1* mutations, was exclusively recognized by NPM1-A2 TCR-T cells in HLA-A*02:06. Finally, our data showed that NPM1-A2 TCR-T cells efficiently recognized and similarly lysed all HLA-A*02:01-positive primary AML tested with type A, B, or D *NPM1* mutations *in vitro.*

### Summary/Conclusion

Our results supported the initiation of a first-in-human phase I/II clinical trial assessing safety and preliminary efficacy of NPM1-specific TCR-T cell therapy in HLA-A*02:01 positive patients with (molecularly) relapsed or refractory AML with *NPM1* mutation type A, B or D, which is currently open for patient recruitment (NCT06424340).

**Copyright Information:** (Online) ISSN: 2572-9241
©2026 The Author(s). HemaSphere is published by John Wiley & Sons Ltd on behalf of European Hematology Association. This is an open access Abstract Book distributed under the Attribution-NonCommercial-NoDerivs (CC BY-NC-ND), which allows third parties to download the articles and share them with others as long as they credit the author and the Abstract Book, but they cannot change the content in any way or use them commercially.
**Abstract Book Citations:** Authors, Title, HemaSphere, 2026;10:(S1):pages. The abstract book DOIs can be found on the "Author Information" page.
**Disclaimer:** Articles published in the journal HemaSphere exclusively reflect the opinions of the authors. The authors are responsible for all content in their abstracts including accuracy of the facts, statements, citing resources, etc.

### (PF1214) SL4Y: A PRIMARY-FAITHFUL, MULTIPLEX-EDITABLE PLATFORM FOR ALLOGENEIC CAR-T CELL ENGINEERING

**Topic:** 25. Gene therapy, cellular immunotherapy and vaccination - Biology & translational research

Katie Flight*[1], Laura Morton[1], Daisy Gresham[1], Yixi Zhang[1], Ciaran Acuna[1], Annabel Laidler[1], Elisabeth Nacheva[2], Paul Maclocia[1], Marc Mansour[1]

*[1]University College London, UCL Cancer Institute, London, United Kingdom; [2]University College London, Research Department of Pathology, London, United Kingdom;*

### Background

CAR-T cell therapies have revolutionised the treatment of B cell malignancies. However, autologous manufacture is costly, slow and yields variable products, driving intense interest in universal allogeneic approaches. Developing these requires extensive multiplex genetic engineering, yet the field still relies on finite, donor-variable primary T cells that are poorly suited to standardised iterative optimisation. Existing immortalised lines offer renewability but lack the biological fidelity of healthy donor T cells. There is a clear need for a renewable T cell platform that faithfully mirrors primary cells while supporting the engineering demands of next-generation CAR-T development.

### Aims

1. To develop a renewable human T cell platform that faithfully recapitulates primary donor-derived T cells
2. To assess suitability for the multi-layered genetic engineering required for allogeneic CAR-T development
3. To evaluate the translational potential of engineered immortalised T cells as a preclinical model for universal CAR-T therapies

### Methods

Primary human T cells were isolated from PBMCs and immortalised using Herpesvirus saimiri, a non-integrating simian herpesvirus, generating SL4Y cells. These were longitudinally characterised by immunophenotyping, cytogenetics, RNA and TCR sequencing, and qPCR for cellular identity, genomic stability, and transcriptional profiling. Genetic engineerability was tested via single and multiplex CRISPR/Cas9 knockout, single-cell cloning, and retro/lentiviral transduction, modelling the sequential edits required for allogeneic CAR-T manufacture (endogenous TCR ablation, MHC elimination, CAR introduction). Functional competence was assessed through TCR-driven stimulation assays and antigen-specific cytotoxicity co-cultures *in vitro*, and by adoptive transfer into mouse models *in vivo.*

**Copyright Information:** (Online) ISSN: 2572-9241
©2026 The Author(s). HemaSphere is published by John Wiley & Sons Ltd on behalf of European Hematology Association. This is an open access Abstract Book distributed under the Attribution-NonCommercial-NoDerivs (CC BY-NC-ND), which allows third parties to download the articles and share them with others as long as they credit the author and the Abstract Book, but they cannot change the content in any way or use them commercially.
**Abstract Book Citations:** Authors, Title, HemaSphere, 2026;10:(S1):pages. The abstract book DOIs can be found on the “Author Information” page.
**Disclaimer:** Articles published in the journal HemaSphere exclusively reflect the opinions of the authors. The authors are responsible for all content in their abstracts including accuracy of the facts, statements, citing resources, etc.

**Results**

SL4Y cells proliferated indefinitely, grew cytokine-independently, and recovered robustly from cryopreservation, establishing a renewable, expandable platform suitable for batch processing and QC. In contrast to immortalised cell lines, they maintained diploidy, diverse polyclonal TCR repertoires, donor-like $CD4^+$/$CD8^+$ subset architecture, and memory-associated phenotypes, without accumulating exhaustion markers- unlike primary T cells.
SL4Y cells tolerated the multiplex genetic engineering central to allogeneic CAR-T development: CRISPR/Cas9 efficiently ablated endogenous TCR, MHC class I, and II individually and in combination, while viral transduction enabled CAR and transgenic TCR expression. Engineered SL4Y cells retained intact signalling, mounted robust polyclonal responses, and demonstrated antigen-specific cytokine production and cytotoxicity via CAR engagement. Engineered cells expanded and engrafted *in vivo* (efficacy studies ongoing). Incorporation of an RQR8 suicide switch enabled rapid selective depletion, providing an integrated safety mechanism essential for any off-the-shelf cellular product.

**Summary/Conclusion**

SL4Y cells bridge a critical gap between finite primary donor cells and biologically compromised immortalised lines. By combining indefinite expansion, preserved polyclonality and effector function, tolerance of the multiplex editing demanded by allogeneic CAR-T manufacture, and integrated safety control, they provide a scalable and standardised platform for iterative T cell engineering and preclinical evaluation. These findings position SL4Y cells as a tractable foundation for accelerating the development of next-gen adoptive cell therapies.

**Copyright Information:** (Online) ISSN: 2572-9241
©2026 The Author(s). HemaSphere is published by John Wiley & Sons Ltd on behalf of European Hematology Association. This is an open access Abstract Book distributed under the Attribution-NonCommercial-NoDerivs (CC BY-NC-ND), which allows third parties to download the articles and share them with others as long as they credit the author and the Abstract Book, but they cannot change the content in any way or use them commercially.
**Abstract Book Citations:** Authors, Title, HemaSphere, 2026;10:(S1):pages. The abstract book DOIs can be found on the “Author Information” page.
**Disclaimer:** Articles published in the journal HemaSphere exclusively reflect the opinions of the authors. The authors are responsible for all content in their abstracts including accuracy of the facts, statements, citing resources, etc.

### (PF1215) SECOND PRIMARY MALIGNANCIES AFTER CAR-T-CELL THERAPY: A REAL-WORLD STUDY FROM THREE UNIVERSITY CENTERS

**Topic:** 25. Gene therapy, cellular immunotherapy and vaccination - Biology & translational research

Jessica Lechner[1], Inna Shaforostova*[1], Lina Kolloch[2], Markus Maulhardt[3], Evgenii Shumilov[2], Marie-Noëlle Kronig[1], Urban Novak[1], Ulrike Bacher[4], Gerald Wulf[3], Georg Lenz[2], Thomas Pabst[2]

*[1]Inselspital Bern University Hospital, Medical Oncology, Bern, Switzerland; [2]University Hospital Münster, Department of Medicine A, Hematology, Oncology and Pneumology, Muenster, Germany; [3]University Hospital Göttingen, Department of Hematology and Medical Oncology, Goettingen, Germany; [4]Inselspital Bern University Hospital, Department of Hematology, Bern, Switzerland;*

### Background

Chimeric antigen receptor cell (CAR-T-cell) therapy has significantly improved treatment outcomes in relapsed/refractory B-cell malignancies. However, the therapy may also be associated with significant complications. While data on efficacy and short-term immune-related toxicities are well established, information on long-term toxicities remains limited. Recent reports suggest an increased risk of second primary malignancies (SPMs) following CAR-T-cell therapy, however the data are still scarce.

### Aims

The aim of this retrospective multicenter study was to evaluate the risk of secondary malignancies after CAR-T-cell therapy and to assess patient (pt) outcomes.

### Methods

A total of 618 adult patients (pts) with hematologic malignancies treated with CAR-T-cell therapy at three centers in Switzerland and Germany between January 2019 and October 2025 were included.

### Results

The median follow-up was 37 months (range, 5-66).
34 pts (5.5%) developed SPMs. The median age was 70 years (range, 23-84), with 22 (65%) males and 12 (35%) females. The median onset time of SPM after CAR-T-cell infusion was 15 months (range, 2-61). The most common underlying diagnosis was diffuse large B-cell lymphoma (19 pts, 56%) followed by mantle cell lymphoma (6 pts,17%), multiple myeloma (5 pts,15 %), follicular lymphoma (2 pts, 6%) and B-ALL (2 pts, 6%). The median number of prior lines of therapy was 3 (range, 1-15).13 (38.2%) pts received radiation. 14 (41%) of pts underwent autologous or allogenic stem cell transplant. CAR T-cell products administered included axicabtagene ciloleucel in 13 (38%) pts, tisagenlecleucel in 8 pts (24%), brexucabtagene autoleucel in 6 pts (18%), idecabtagene vicleucel in 5 pts (15%), and lisocabtagen maraleucel in 2 pts (6%). No SPMs were observed in pts treated with ciltacabtagene autoleucel.
Focusing in more detail on the second primary malignancies, hematologic second primary malignancies (HSPMs) occurred in 10/34 (29% of pts), while 24/34 (71%) developed solid tumors. Among HSPMs, 8/10 of pts developed myelodysplastic syndrome (MDS), and 2/10 developed acute myeloid leukemia (AML). Among non-hematologic second primary malignancies the majority of pts had non-melanoma skin cancers (NMSCs) (9 pts, 26%). Other solid tumors included urological (3/34, 9%), breast (2/34, 6%), gastrointestinal (2/34, 6%), oropharyngeal (2/34, 6%), lung (1/34, 3%), ovarian (1/34, 3%), melanoma (1/34, 3%), and CUP (3/34, 9%). Six patients (6/34, 18%) developed multiple solid malignancies, all of which were NMSCs.
13 pts (38%) died by the last follow-up with SPMs as a cause of death in 6/13 (46%) pts. The 5-year progression-free and overall survival in whole cohort with SPMs were 36% (95% CI 18-73) and 38% (95% CI 18-79), respectively.

**Copyright Information:** (Online) ISSN: 2572-9241
©2026 The Author(s). HemaSphere is published by John Wiley & Sons Ltd on behalf of European Hematology Association. This is an open access Abstract Book distributed under the Attribution-NonCommercial-NoDerivs (CC BY-NC-ND), which allows third parties to download the articles and share them with others as long as they credit the author and the Abstract Book, but they cannot change the content in any way or use them commercially.
**Abstract Book Citations:** Authors, Title, HemaSphere, 2026;10:(S1):pages. The abstract book DOIs can be found on the “Author Information” page.
**Disclaimer:** Articles published in the journal HemaSphere exclusively reflect the opinions of the authors. The authors are responsible for all content in their abstracts including accuracy of the facts, statements, citing resources, etc.

**Summary/Conclusion**

In this large cohort of CAR-T cell therapy recipients, second primary malignancies were identified in 5.5% of patients at a median interval of 15 months (range, 2-61) from CAR-T-cell-infusion; mostly hematologic malignancies (mainly MDS, few AML) and non-melanoma skin cancers, with an incidence comparable to previous studies. Notably, no T-cell-lymphomas were diagnosed. Based on these and previous data, dermatological screening should be included in routine follow-up after CAR-T-cell therapy. Further studies are needed to identify risk factors for SPMs and to determine whether the risk is attributable solely to CAR-T-cell therapy or is influenced by prior treatments, particularly alkylating agents and radiation.

**Copyright Information:** (Online) ISSN: 2572-9241
©2026 The Author(s). HemaSphere is published by John Wiley & Sons Ltd on behalf of European Hematology Association. This is an open access Abstract Book distributed under the Attribution-NonCommercial-NoDerivs (CC BY-NC-ND), which allows third parties to download the articles and share them with others as long as they credit the author and the Abstract Book, but they cannot change the content in any way or use them commercially.
**Abstract Book Citations:** Authors, Title, HemaSphere, 2026;10:(S1):pages. The abstract book DOIs can be found on the "Author Information" page.
**Disclaimer:** Articles published in the journal HemaSphere exclusively reflect the opinions of the authors. The authors are responsible for all content in their abstracts including accuracy of the facts, statements, citing resources, etc.

### (PF1216) CD19-CD22 COREGULATION SHAPES ANTIGEN SELECTION IN MULTI-TARGET CAR T-CELL STRATEGIES FOR B-CELL MALIGNANCIES

**Topic:** 25. Gene therapy, cellular immunotherapy and vaccination - Biology & translational research

Aditya Ramdas Iyer*[1, 2, 3], Mehwish Nafiz[1, 2, 3], Shivani Singh[2], Gauspasha Deshmukh[2], Pragya Gupta[2], Sivaprakash Ramalingam[1]

*[1]Indian Institute of Technology Kanpur, Biological Sciences and Bioengineering, Kanpur, India;[2]CSIR Institute of Genomics and Integrative Biology, Delhi, India; [3]Academy of Scientific & Innovative Research, Ghaziabad, India;*

### Background

Anti-CD19 CAR T-cell therapy has transformed the treatment of B-cell malignancies, yet relapse due to CD19 downregulation or loss remains a major challenge. Dual- or multi-antigen CARs (e.g., targeting CD22 or CD20 alongside CD19) are under investigation, but their success varies and the biological basis for these differences is not fully defined.

### Aims

To generate an extensively validated model system to test CAR-T cells targeting B-cell malignancies, investigate potential co-regulation among key B-cell antigens, and determine how antigen interdependence between CD19, CD20, and CD22 influences CAR T cell efficacy.

### Methods

CRISPR Cas9-mediated single, double, and triple knockouts of CD19, CD20, and CD22 were generated in Raji cells to model antigen loss scenarios. Expression of CD19, CD20, and CD22 was analysed across multiple B-cell leukemia and lymphoma cell lines and primary B-cells. Functional CAR T cell cytotoxicity assays were performed using single- and dual-targeting CAR constructs in antigen-loss models and with primary B cells. Rescue experiments were conducted in antigen loss models. Transcriptomic and pathway analyses were conducted to identify signalling networks underlying antigen interdependence.

**Copyright Information:** (Online) ISSN: 2572-9241
©2026 The Author(s). HemaSphere is published by John Wiley & Sons Ltd on behalf of European Hematology Association. This is an open access Abstract Book distributed under the Attribution-NonCommercial-NoDerivs (CC BY-NC-ND), which allows third parties to download the articles and share them with others as long as they credit the author and the Abstract Book, but they cannot change the content in any way or use them commercially.
**Abstract Book Citations:** Authors, Title, HemaSphere, 2026;10:(S1):pages. The abstract book DOIs can be found on the "Author Information" page.
**Disclaimer:** Articles published in the journal HemaSphere exclusively reflect the opinions of the authors. The authors are responsible for all content in their abstracts including accuracy of the facts, statements, citing resources, etc.

### Results

During the generation of CRISPR Cas9-mediated knockout models, a strong co-regulation between CD19 and CD22 was first observed in Raji cells. CD19 knockout reduced CD22 surface expression by approximately 60 percent. To determine whether this observation was Raji-specific, CD19 knockout and knockdown experiments were performed in additional B-cell leukemia and lymphoma cell lines, as well as in primary human B cells. In both systems, loss or reduction of CD19 consistently resulted in a similar decrease in CD22 expression, confirming that the CD19–CD22 relationship was not a Raji-specific artifact. CD19 and CD22 expression were strongly correlated across cell lines (R = 0.88).
Functional cytotoxicity assays showed that anti-CD22 CAR T cells lysed only about 10 to 40 percent of CD19-deficient targets at 24 hours compared to approximately 95 percent lysis of wild-type cells at a 5:1 effector-to-target ratio, indicating that reduced CD22 levels impaired CAR-mediated killing. Similarly, bispecific CAR T cells targeting CD20 and CD19 achieved about 95 percent cytotoxicity compared with approximately 60 percent cytotoxicity for CAR T cells targeting CD22 and CD19 in the CD19 loss scenario. When B cell lines and primary B cells were co-cultured with CD19 CAR T cells and resistant populations were analysed, surviving cells showed coordinated reduction of both CD19 and CD22 expression, consistent with antigen modulation under CAR T cell pressure.
Rescue experiments further demonstrated that re-expression of CD19 in CD19-deficient Raji cells restored CD22 surface levels to approximately 100 percent of wild-type levels, confirming that CD22 downregulation occurred as a direct consequence of CD19 loss. Consistent with these observations, transcriptomic and proteomic analyses (RNA sequencing and mass spectrometry) also revealed concordant CD19 and CD22 co-regulation. Detailed analysis of the signalling pathways potentially mediating this relationship is currently underway.

### Summary/Conclusion

CD19–CD22 interdependence directly limits CD22-based CAR efficacy. Accounting for antigen co-regulation is essential for rational CAR design to overcome antigen escape and improve durability.

**Copyright Information:** (Online) ISSN: 2572-9241
©2026 The Author(s). HemaSphere is published by John Wiley & Sons Ltd on behalf of European Hematology Association. This is an open access Abstract Book distributed under the Attribution-NonCommercial-NoDerivs (CC BY-NC-ND), which allows third parties to download the articles and share them with others as long as they credit the author and the Abstract Book, but they cannot change the content in any way or use them commercially.
**Abstract Book Citations:** Authors, Title, HemaSphere, 2026;10:(S1):pages. The abstract book DOIs can be found on the "Author Information" page.
**Disclaimer:** Articles published in the journal HemaSphere exclusively reflect the opinions of the authors. The authors are responsible for all content in their abstracts including accuracy of the facts, statements, citing resources, etc.

### (PF1217) CD45 TRANS-ENGAGEMENT ENABLES STERIC IMMUNE SHIELDING AND DURABLE PERSISTENCE OF ALLOGENEIC NK CELLS

**Topic:** 25. Gene therapy, cellular immunotherapy and vaccination - Biology & translational research

Safa Bazaz*[1], Alamdar Hussain[1], Stefanie Renken[1], Charlotte Hållstrand[1], Stephan Meinke[1], Hazel Reilly[1], Sophia Borate[1], Nutsa Burduli[1], Didem Cakirsoy Akyoney[1], Nursen Cetrez[1], Carin Dahlberg[1], Arnika Wagner[1, 1], Quirin Hammer[1], Hans-Gustaf Ljunggren[1], Evren Alici[1]

*[1]Karolinska Institutet, Center for Hematology and Regenerative Medicine (HERM), Department of Medicine Huddinge, Karolinska Institutet, SE-141 83 Stockholm, Sweden, Stockholm, Sweden;*

### Background

Allogeneic NK and other retargeted cell therapies offer a scalable off-the-shelf immuno-oncology, yet their clinical durability remains constrained by rapid host-versus-graft immune rejection. Prevailing universal engineering strategies often require multi-locus HLA disruption combined with expression of inhibitory ligands such as HLA-E or CD47, increasing genetic complexity and raising concerns regarding manufacturing burden and impaired effector fitness. VY-UC is a simplified, trans-acting allo-evasion strategy based on steric immune shielding.

### Aims

In this study we evaluated VY-UC in stringent HLA-mismatched PBMC-challenge xenograft models. Vy-UC consists of a membrane-tethered anti-human CD45 VHH that engages CD45 on host leukocytes in trans, creating a local steric barrier designed to limit immune synapse formation and immune-mediated clearance without requiring HLA disruption.

### Methods

In a persistence setting, NSG-tg(hu-IL15) mice (N=12) received human PBMCs followed by unmodified NK or VY-UC-engineered NK cells, and were assigned to 4 experimental groups. Half of the mice were sacrificed on day 4 and the remaining half on day 21 for tissue harvest and analysis. To assess the therapeutic relevance under immune rejection pressure, we employed a leukemia immune-rejection model with 4 experimental groups (N=8, NSG-tg(hu-IL15) mice). Animals received Nalm6-Luc on day 0, PBMCs on day 3, and CAR-NK, VY-UC CAR-NK, CAR-T, or VY-UC CAR-T on day 6.

### Results

Early biodistribution was comparable between groups; however, VY-UC conferred a significant late persistence advantage. VY-UC NK cells remained readily detectable in spleen, blood, liver and bone marrow, whereas control NK cells were undetectable across tissues studied. In the leukemia immune-rejection model, at day 21 VY-UC increased CAR-NK persistence by 15-20-fold across tissues. Median persistence increased from 0 vs 33% in spleen, 0 to 29% in blood, 1-2% to 40% in liver, and bone marrow 5% to 40%, for CAR-NK vs VY-UC CAR-NK, respectively. VY-UC similarly enhanced CAR-T cell persistence.

### Summary/Conclusion

Together, these data establish VY-UC as a modular, single-step immune-stealth strategy that significantly enhances the durability of off-the-shelf NK cell therapies under stringent host immune rejection conditions. This approach is currently being advanced into a first-in-human clinical trial in patients with penta-refractory multiple myeloma.

**Copyright Information:** (Online) ISSN: 2572-9241
©2026 The Author(s). HemaSphere is published by John Wiley & Sons Ltd on behalf of European Hematology Association. This is an open access Abstract Book distributed under the Attribution-NonCommercial-NoDerivs (CC BY-NC-ND), which allows third parties to download the articles and share them with others as long as they credit the author and the Abstract Book, but they cannot change the content in any way or use them commercially.
**Abstract Book Citations:** Authors, Title, HemaSphere, 2026;10:(S1):pages. The abstract book DOIs can be found on the "Author Information" page.
**Disclaimer:** Articles published in the journal HemaSphere exclusively reflect the opinions of the authors. The authors are responsible for all content in their abstracts including accuracy of the facts, statements, citing resources, etc.

### (PF1218) PYRUVATE BOOSTS CAR-T CELL ANTITUMOR THERAPEUTIC EFFICACY VIA METABOLISM AND FATE REPROGRAMMING

**Topic:** 25. Gene therapy, cellular immunotherapy and vaccination - Biology & translational research

Longbin Zhang*[1], Yueyu Dai[1], Yue Liu[1], Xi Zhang[1]

[1]*Xinqiao Hospital of Army Medical University, Medical Center of Hematology, Chongqing, China;*

### Background

Recent studies have confirmed the remarkable efficacy of chimeric antigen receptor T (CAR-T) cell therapy in the treatment of hematological malignancies. However, a considerable proportion of patients ultimately experience tumor relapse and disease progression, primarily due to inadequate CAR-T cell persistence in vivo. To address this limitation, various strategies have been explored to enhance the long-term antitumor activity of CAR-T cells, among which modulating their metabolic characteristics has emerged as a highly promising research direction. The fate determination of T cells is highly dependent on their metabolic patterns. Although the role of glycolysis in T cell function has been extensively studied, whether TCA cycle-related metabolic substrates—core components of oxidative metabolism—can reshape CAR-T cell fate decisions through metabolic remodeling remains to be systematically elucidated.

### Aims

This study focused on investigating the impact of TCA cycle-related metabolic substrates on CAR-T cell memory differentiation and antitumor efficacy, as these substrates not only function as energy metabolites but may also possess immunomodulatory properties.

### Methods

Specifically, we selected pyruvate, a TCA cycle-related metabolite with dual functions in metabolism and immune regulation, as the research subject. Through screening of TCA cycle-related substrates, we found that pyruvate significantly upregulated CD62L expression in T cells while downregulating exhaustion-related genes and reducing reactive oxygen species (ROS) levels. Mechanistically, we explored the effects of pyruvate on T cell signaling and metabolic patterns. Subsequently, CAR-T cells pretreated with pyruvate were subjected to systematic analyses, including phenotypic characterization of T cell differentiation, metabolic profiling, and RNA sequencing, aiming to elucidate the metabolic remodeling and fate reprogramming induced by pyruvate. Furthermore, we conducted a comparative evaluation of the in vivo and in vitro antitumor efficacy between pyruvate-pretreated CAR-T cells and untreated controls.

### Results

Our results demonstrated that pyruvate pretreatment significantly increased the proportions of central memory (Tcm) and naïve (Tn) T cell subsets within the CAR-T cell population. Mechanistic studies revealed that pyruvate modulated the T cell receptor (TCR) and phosphatidylinositol 3-kinase (PI3K)-Akt signaling pathways, thereby influencing the mTOR signaling pathway. In vitro assays showed that pyruvate-pretreated CAR-T cells exhibited reduced exhaustion, enhanced persistence, and improved cytotoxic function. Targeted metabolomics analysis indicated that pyruvate suppressed glycolytic activity while promoting TCA cycle metabolism, inducing metabolic reprogramming that favored memory T cell differentiation. Moreover, in vivo experiments using a leukemia mouse model demonstrated that pyruvate-pretreated CAR-T cells achieved superior tumor control and significantly improved mouse survival rates compared to the control group.

**Copyright Information:** (Online) ISSN: 2572-9241
©2026 The Author(s). HemaSphere is published by John Wiley & Sons Ltd on behalf of European Hematology Association. This is an open access Abstract Book distributed under the Attribution-NonCommercial-NoDerivs (CC BY-NC-ND), which allows third parties to download the articles and share them with others as long as they credit the author and the Abstract Book, but they cannot change the content in any way or use them commercially.
**Abstract Book Citations:** Authors, Title, HemaSphere, 2026;10:(S1):pages. The abstract book DOIs can be found on the "Author Information" page.
**Disclaimer:** Articles published in the journal HemaSphere exclusively reflect the opinions of the authors. The authors are responsible for all content in their abstracts including accuracy of the facts, statements, citing resources, etc.

### Summary/Conclusion

In conclusion, this study provides systematic evidence that pyruvate pretreatment enhances the antitumor efficacy and in vivo persistence of CAR-T cells by remodeling metabolic patterns and promoting memory differentiation, offering a promising novel strategy for optimizing CAR-T cell therapy to achieve durable clinical responses.

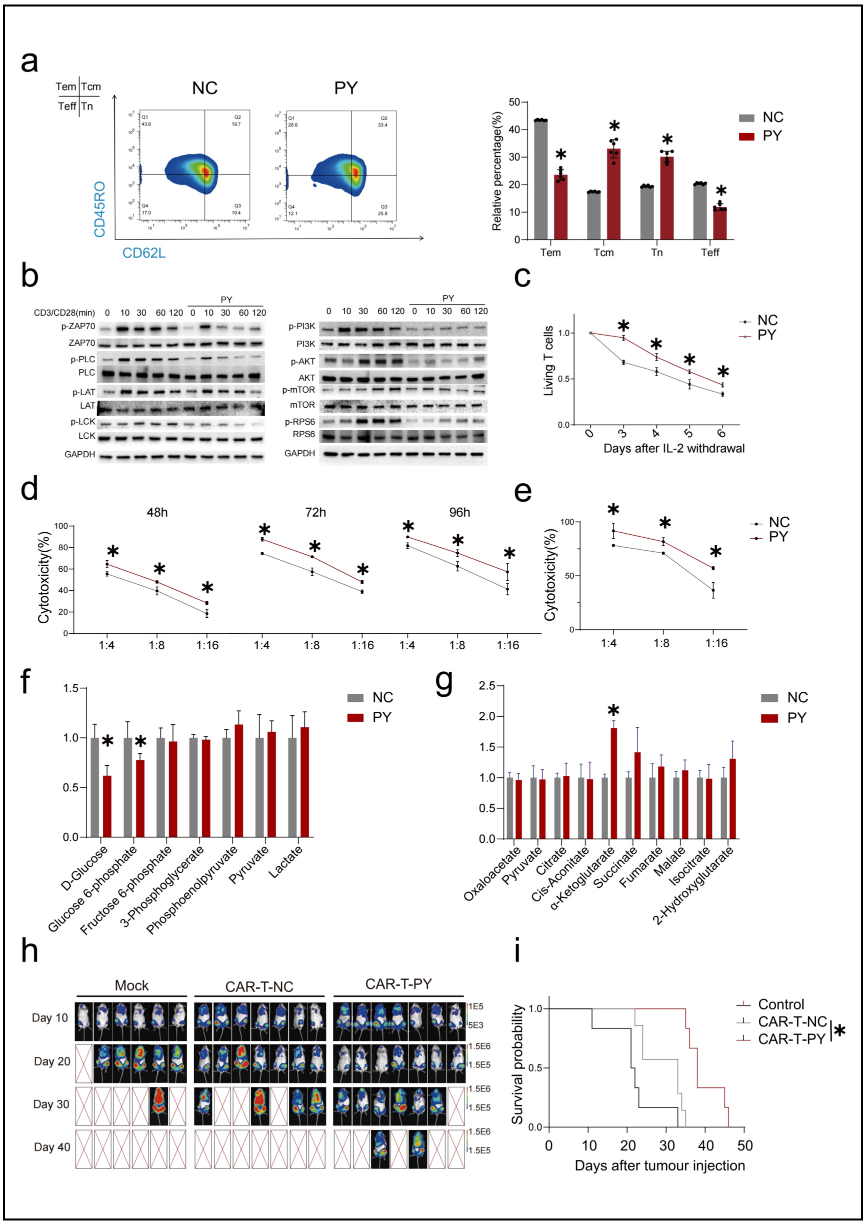

**Copyright Information:** (Online) ISSN: 2572-9241
©2026 The Author(s). HemaSphere is published by John Wiley & Sons Ltd on behalf of European Hematology Association. This is an open access Abstract Book distributed under the Attribution-NonCommercial-NoDerivs (CC BY-NC-ND), which allows third parties to download the articles and share them with others as long as they credit the author and the Abstract Book, but they cannot change the content in any way or use them commercially.
**Abstract Book Citations:** Authors, Title, HemaSphere, 2026;10:(S1):pages. The abstract book DOIs can be found on the "Author Information" page.
**Disclaimer:** Articles published in the journal HemaSphere exclusively reflect the opinions of the authors. The authors are responsible for all content in their abstracts including accuracy of the facts, statements, citing resources, etc.

### (PF1219) SHORT-TERM METABOLIC PRE-CONDITIONING WITH A FASTING-MIMICKING VERSUS KETOGENIC DIET DIFFERENTIALLY MODULATES MEMORY-LIKE T-CELL PHENOTYPES RELEVANT TO CAR T-CELL MANUFACTURING

**Topic:** 25. Gene therapy, cellular immunotherapy and vaccination - Biology & translational research

Benjamin Gyau*[1], Sylvia Crowder[2], Reginald Atkins[1], Meghan Menges[1], Emily Merritt[3], Luis Cuadrado Delgado[1], Nathan Parker[2], Tiara Boykins[4], Omar Castaneda Puglianini[5], Melissa Alsina[5], Ken Shain[6], Rachid Baz[6], Hien Liu[5], Brandon Blue[6], Taiga Nishihori[5], Ariel Grajales-Cruz[6], Doris Hansen[5], Jerald Noble[1], Tiffany Carson[2], Heather Jim[7], Frederick Locke[5], Ciara Freeman[5]

*[1]Moffitt Cancer Center and Research Institute, Clinical Lab Sciences, Tampa, United States of America; [2]Moffitt Cancer Center and Research Institute, Health Outcomes, Tampa, United States of America; [3]Moffitt Cancer Center and Research Institute, ICE-T Data Management, Tampa, United States of America; [4]Moffitt Cancer Center and Research Institute, Research Operations, Tampa, United States of America; [5]Moffitt Cancer Center and Research Institute, NTRO Blood and Marrow Transplant and Cellular Immunotherapy, Tampa, United States of America; [6]Moffitt Cancer Center and Research Institute, Malignant Hematology, Tampa, United States of America; [7]Moffitt Cancer Center and Research Institute, Population Science, Tampa, United States of America;*

### Background

Chimeric antigen receptor (CAR) T-cell therapy has dramatically improved outcomes in multiple myeloma (MM); however, disease-related T-cell exhaustion contributes to suboptimal leukapheresis starting material, a key and potentially modifiable constraint on CAR T-cell manufacturing and durability. Dietary interventions, including the fasting-mimicking diet (FMD) and ketogenic diet (KD), may rapidly remodel the immune system to physiologically optimize T cells prior to leukapheresis.

### Aims

To compare the effects of a ketogenic diet (KD) and a fasting-mimicking diet (FMD) on circulating T-cell phenotypes relevant to CAR T-cell biology.

### Methods

In this pilot study, healthy volunteers had PBMCs collected at baseline prior to dietary intervention, then sequentially completed 14 days on a ketogenic diet (KD), a washout period, and 5 days on a fasting-mimicking diet (FMD), with phenotypic analysis by expanded flow cytometry performed at each timepoint. In addition, multiple myeloma patients awaiting CAR T-cell therapy were enrolled to assess feasibility; consented patients selected their dietary intervention of choice (FMD or KD), and results from the first three enrolled patients (n=3) who chose FMD are reported. All meals and snacks compliant with each diet were fully provided by a research kitchen under dietitian supervision, and all subjects were closely monitored. Assays targeted biologically pre-defined T-cell memory and stemness compartments relevant to CAR T-cell manufacturing, including canonical memory subsets, TCF7 expression, and exhaustion markers (PD1, LAG3, TIGIT, CD39).

**Copyright Information:** (Online) ISSN: 2572-9241
©2026 The Author(s). HemaSphere is published by John Wiley & Sons Ltd on behalf of European Hematology Association. This is an open access Abstract Book distributed under the Attribution-NonCommercial-NoDerivs (CC BY-NC-ND), which allows third parties to download the articles and share them with others as long as they credit the author and the Abstract Book, but they cannot change the content in any way or use them commercially.
**Abstract Book Citations:** Authors, Title, HemaSphere, 2026;10:(S1):pages. The abstract book DOIs can be found on the "Author Information" page.
**Disclaimer:** Articles published in the journal HemaSphere exclusively reflect the opinions of the authors. The authors are responsible for all content in their abstracts including accuracy of the facts, statements, citing resources, etc.

### Results

In healthy volunteers, FMD was associated with an increased CD4:CD8 ratio, a change not observed with KD (Figure 1A). A directional increase in global $CD8^+$ T-cell stemness was observed with FMD but did not reach statistical significance (Figure 1B), with subset-specific analysis demonstrating a significant enrichment of $TCF7^+$ cells confined to the central memory (CM) compartment (Figure 1C), an effect not observed with KD. In addition, FMD, but not KD, reduced TIGIT expression on both $CD4^+$ and $CD8^+$ T cells (Figure 1D-E) and altered the overall exhaustion profile, increasing the proportion of T cells lacking exhaustion markers while reducing cells co-expressing multiple markers (LAG3, CD39, PD1, TIGIT, Figure 1F). In the MM cohort, early non-significant trends were directionally consistent with findings in healthy volunteers.

### Summary/Conclusion

Metabolic pre-conditioning with a fasting-mimicking diet (FMD) was associated with favorable pre-apheresis T-cell features, including reduced exhaustion marker expression, enrichment of memory-like subsets, and increased CD4:CD8 ratios, phenotypes previously linked to improved CAR T-cell fitness and durability. Directionally consistent, though non-significant, trends in a small initial cohort of patients with MM support the feasibility of dietary intervention prior to leukapheresis. Ongoing studies include expanded patient enrollment and evaluation of the impact of FMD-associated T-cell alterations on CAR T-cell manufacturing, phenotype, and function, identifying short-term dietary interventions as a feasible, non-pharmacologic strategy to enhance leukapheresis starting material.

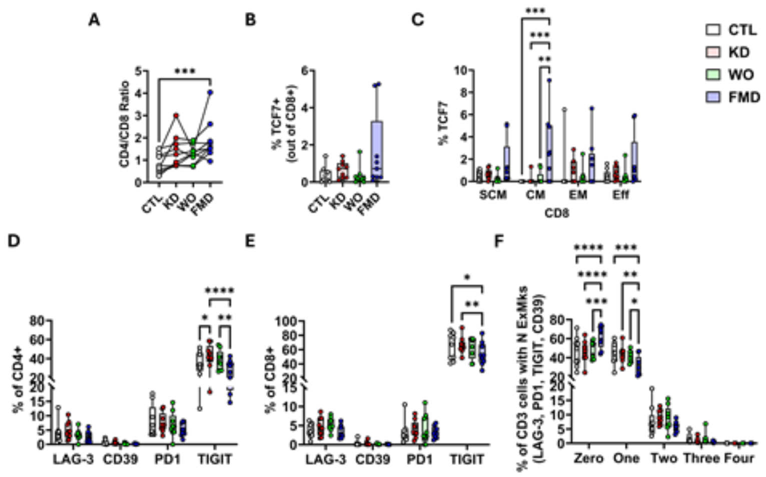

**Figure 1. Immunophenotypic remodeling of the T-cell compartment following metabolic pre-conditioning with a Fasting-Mimicking Diet (FMD). (A)** Changes in the CD4:CD8 T-cell ratio from baseline following dietary interventions, highlighting a significant increase post-FMD. **(B)** Evaluation of overall nuclear stemness, showing the percentage of CD45+CD3+CD8+TCF7+ T cells. **(C)** CD8+TCF7+ subset-specific quantification demonstrating a significant upregulation in the percentage of CD8+ Central Memory (CM; CCR7+CD45RO+) compartment following FMD. **(D-E)** FMD differentially suppresses the expression of the co-inhibitory receptor TIGIT on CD3+CD4+/CD8+ T-cells. **(F)** Shift in the overall T-cell exhaustion profile. The Box and whiskers plot illustrates the frequency of CD3+ T-cells expressing zero, one, or multiple co-inhibitory markers (LAG3, CD39, PD1, and TIGIT), revealing a significant FMD-mediated increase in non-exhausted T-cells and a concurrent reduction in cells co-expressing multiple exhaustion markers. The data were analyzed by a nonparametric one-way ANOVA Friedman test with Dunn's multiple comparison test (A-B) and an ordinary two-way ANOVA with Tukey's multiple comparison test (C-F). Data are presented as Mean ± SD. $p<0.05$ is considered significant. *$p<0.05$, **$p<0.01$, ***$p<0.001$, ****$p<0.0001$, statistics not shown = not significant.

**Copyright Information:** (Online) ISSN: 2572-9241
©2026 The Author(s). HemaSphere is published by John Wiley & Sons Ltd on behalf of European Hematology Association. This is an open access Abstract Book distributed under the Attribution-NonCommercial-NoDerivs (CC BY-NC-ND), which allows third parties to download the articles and share them with others as long as they credit the author and the Abstract Book, but they cannot change the content in any way or use them commercially.
**Abstract Book Citations:** Authors, Title, HemaSphere, 2026;10:(S1):pages. The abstract book DOIs can be found on the "Author Information" page.
**Disclaimer:** Articles published in the journal HemaSphere exclusively reflect the opinions of the authors. The authors are responsible for all content in their abstracts including accuracy of the facts, statements, citing resources, etc.

### (PF1220) A PROTEIN-BASED APPROACH TO GENERATE ALLOGENEIC CHIMERIC ANTIGEN RECEPTOR (CAR)-T CELLS

**Topic:** 25. Gene therapy, cellular immunotherapy and vaccination - Biology & translational research

Malika Hoekx*[1], Guoxiang Yang[2], Li-Chin Yao[2], Lydia Lee[1], Martin Pule[1], James Keck[2], Paul Maclocia[1]

[1]*University College London, Haematology, London, United Kingdom;* [2]*The Jackson Laboratory, Sacramento, United States of America;*

### Background

The use of allogeneic chimeric antigen receptor (CAR)-T cells from healthy donors could address many drawbacks of autologous CAR-T cell therapies including time delays, manufacturing failure, poor T cell quality, logistical issues, and high cost. Allogeneic CAR-T cells risk causing graft versus host disease (GvHD), mediated by T cell receptors (TCR) on donor CAR-T cells. They also risk rejection by the host immune system, largely mediated by recognition of foreign major histocompatibility complex (MHC)-I molecules on donor CAR-T cells, leading to limited persistence and treatment efficacy. Most previous approaches to allogeneic CAR-T cells use genome-editing to delete TCR and/or MHC-I, introducing complexity, added cost, and the potential for off-target genotoxicity.

### Aims

This study aims to express CAR and downregulate TCR and MHC-I in a single vector, eliminating the need for genome editing.

### Methods

A synthetic protein, VHH3-US11, was designed comprising an anti-CD3 single-domain VHH fused to the N-terminus of US11, a protein from human cytomegalovirus (HCMV) that binds MHC-I α-chains and directs them for ubiquitination and proteasomal degradation. VHH3-US11 was cloned into a tricistronic gamma-retroviral vector encoding the sort-suicide gene RQR8 and anti-CD19 CAR (CAR19). Surface expression of TCR and MHC-I was detected by flow cytometry. *In vitro* GvHD and allo-rejection were assessed by 10-day one-way mixed lymphocyte reactions (MLRs). Protection from alloreactive CD8 lysis was assessed by co-culture with HA-1 TCR-transduced effector cells. *In vitro* efficacy was assessed by 48hr co-culture assays with cytokine quantification by ELISA. *In vivo* efficacy was assessed using a Nalm6 NSG xenograft stress test model. *In vivo* GvHD was assessed in irradiated NSG-SGM3 mice receiving $5 \times 10^6$ purified CAR-T cells with exogenous IL-2.

### Results

Expression of VHH3-US11 led to robust downregulation of both TCR and MHC-I in primary T cells (Fig. 1A) and cell lines, maintained over 14 days and after co-culture with CD19+ targets (Fig. 1B). In MLRs, VHH3-US11 T cells showed significantly lower expansion and HLA-DR expression in response to allogeneic PBMCs compared to control T cells (Fig. 1C). Allogeneic PBMCs showed significantly lower expansion and HLA-DR expression in response to VHH3-US11 T cells (Fig. 1D). VHH3-US11 T cells were resistant to lysis by alloreactive HA-1 TCR-expressing CD8 T cells (Fig. 1E).
VHH3-US11 CAR19 cells demonstrated antigen-specific cytotoxicity (Fig. 1F) and IFN-γ secretion comparable to conventional CAR19 cells *in vitro*, with similar proliferation, differentiation, and exhaustion profiles. In the *in vivo* Nalm6 stress test, conventional CAR19 and VHH3-US11 CAR19 demonstrated comparable tumor regression and overall survival (Fig. 1G).
In the *in vivo* GvHD model, CAR19 recipients developed GvHD with median survival of 27 days, while CAR19-VHH3-US11 recipients showed significantly prolonged survival (median 38 days, $p<0.05$) with sustained CAR-T expansion but no GvHD until late outgrowth of residual TCR+ cells (Fig. 1H).

**Copyright Information:** (Online) ISSN: 2572-9241
©2026 The Author(s). HemaSphere is published by John Wiley & Sons Ltd on behalf of European Hematology Association. This is an open access Abstract Book distributed under the Attribution-NonCommercial-NoDerivs (CC BY-NC-ND), which allows third parties to download the articles and share them with others as long as they credit the author and the Abstract Book, but they cannot change the content in any way or use them commercially.
**Abstract Book Citations:** Authors, Title, HemaSphere, 2026;10:(S1):pages. The abstract book DOIs can be found on the "Author Information" page.
**Disclaimer:** Articles published in the journal HemaSphere exclusively reflect the opinions of the authors. The authors are responsible for all content in their abstracts including accuracy of the facts, statements, citing resources, etc.

### Summary/Conclusion

Anti-CD3 VHH fused to US11 allows for simultaneous downregulation of TCR and MHC-I from a single transgene without genome editing. MLRs and alloreactive killing assays demonstrate functional loss of TCR and MHC-I, preventing allo-recognition in both directions. CAR-T cells co-expressing VHH3-US11 are equally functional to conventional CAR-T cells *in vitro* and *in vivo*, and show significant protection from GvHD *in vivo*.

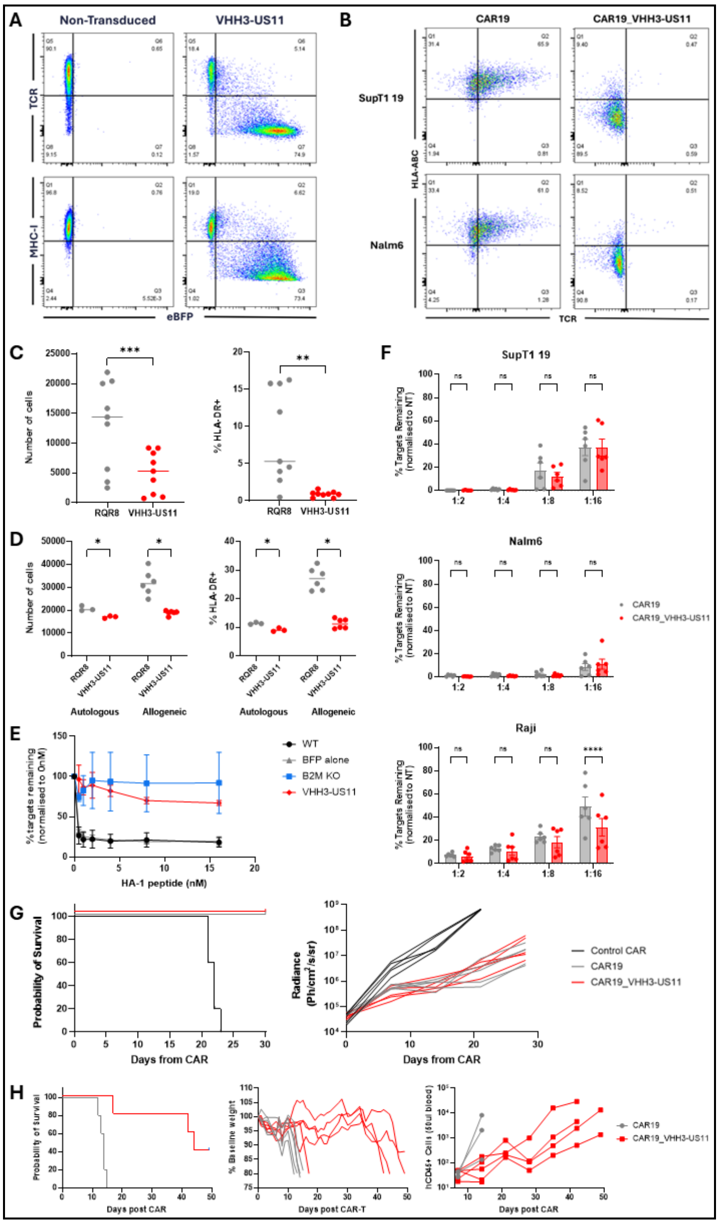

**Copyright Information:** (Online) ISSN: 2572-9241
©2026 The Author(s). HemaSphere is published by John Wiley & Sons Ltd on behalf of European Hematology Association. This is an open access Abstract Book distributed under the Attribution-NonCommercial-NoDerivs (CC BY-NC-ND), which allows third parties to download the articles and share them with others as long as they credit the author and the Abstract Book, but they cannot change the content in any way or use them commercially.
**Abstract Book Citations:** Authors, Title, HemaSphere, 2026;10:(S1):pages. The abstract book DOIs can be found on the "Author Information" page.
**Disclaimer:** Articles published in the journal HemaSphere exclusively reflect the opinions of the authors. The authors are responsible for all content in their abstracts including accuracy of the facts, statements, citing resources, etc.

### (PF1221) OVERCOMING LACTATE-MEDIATED T CELL SUPPRESSION VIA PTPN1 INHIBITION: A NOVEL STRATEGY TO ENHANCE CAR-T CELL EFFICACY IN MULTIPLE MYELOMA

**Topic:** 25. Gene therapy, cellular immunotherapy and vaccination - Biology & translational research

Siyin Chen[1], Xi Zhang[1], Aishun Jin*[2]

*[1]Xinqiao Hospital ARMY Medical University, Hematology department, Chongqing, China; [2]Chongqing medical university, Chongqing, China;*

### Background

Background: In multiple myeloma, tumor cell metabolism and hypoxia lead to lactate accumulation and an acidic microenvironment. Lactate suppresses T cell anti-tumor capabilities, including those of CAR-T cells. The limited clinical efficacy of CAR-T in multiple myeloma pressing for the need to uncover mechanisms that sustain T cell activity under lactate conditions.Identifying mechanisms that sustain T cell function under lactate conditions is therefore crucial for improving CAR-T cell efficacy in hematological cancers. This study identified genetic regulators that preserve T cell function under

### Aims

Aims: This study aims to identify key regulators that maintain T cell function under the immunosuppressive conditions of the multiple myeloma tumor microenvironment, specifically focusing on overcoming lactate-induced suppression. We will investigate the role of PTPN1 in T cell activation and effector function in the presence of lactate and evaluate its potential as a therapeutic target to enhance CAR-T cell efficacy.

### Methods

Methods: We performed a genome-wide CRISPR knockout (KO) screen in human Jurkat T cells to identify genes critical for maintaining CD25 expression under lactate stress. Cells were stimulated with anti-CD3 and anti-CD28 antibodies in the presence of 15 mM lactate, followed by sorting of $CD25^+$ and $CD25^-$ populations and next-generation sequencing (NGS). Candidate genes were validated through in vitro assays in primary T cells and, crucially, in ex vivo expanded T cells representative of those used in adoptive cell therapy.

### Results

Results: The CRISPR screen identified Protein Tyrosine Phosphatase Non-receptor type 1 (PTPN1) as a crucial regulator of T cell activation in the presence of lactate. Mechanistically, lactate suppressed $CD8^+$ T cell activation and cytotoxic function by reducing oxidative modification of PTPN1 at cysteine 215 (Cys215). This redox shift impaired TCR/IL-2/STAT5 signaling and effector cytokine production. Crucially, both genetic ablation of *PTPN1* and pharmacological inhibition using AC484 significantly rescued T cell signaling and restored the anti-tumor function of ex vivo expanded $CD8^+$ T cells, including their cytotoxic capacity, under lactate-rich conditions. These findings demonstrate that PTPN1 plays a critical role in mediating lactate-induced suppression of T cell effector function relevant to adoptive cell therapy.

**Copyright Information:** (Online) ISSN: 2572-9241
©2026 The Author(s). HemaSphere is published by John Wiley & Sons Ltd on behalf of European Hematology Association. This is an open access Abstract Book distributed under the Attribution-NonCommercial-NoDerivs (CC BY-NC-ND), which allows third parties to download the articles and share them with others as long as they credit the author and the Abstract Book, but they cannot change the content in any way or use them commercially.
**Abstract Book Citations:** Authors, Title, HemaSphere, 2026;10:(S1):pages. The abstract book DOIs can be found on the "Author Information" page.
**Disclaimer:** Articles published in the journal HemaSphere exclusively reflect the opinions of the authors. The authors are responsible for all content in their abstracts including accuracy of the facts, statements, citing resources, etc.

**Summary/Conclusion**

Conclusions: Lactate suppresses T cell function, including that of CAR-T cells, by shifting the redox state of PTPN1 towards a less oxidized conformation, thereby impairing T cell signaling. Targeting PTPN1 restores T cell activation and effector functions under lactate stress, highlighting PTPN1 as a potential therapeutic target to enhance CAR-T cell persistence and efficacy in the acidic TME of hematological malignancies. These findings offer a novel strategy to overcome metabolic barriers and improve the clinical success of CAR-T cell immunotherapy.

**Copyright Information:** (Online) ISSN: 2572-9241
©2026 The Author(s). HemaSphere is published by John Wiley & Sons Ltd on behalf of European Hematology Association. This is an open access Abstract Book distributed under the Attribution-NonCommercial-NoDerivs (CC BY-NC-ND), which allows third parties to download the articles and share them with others as long as they credit the author and the Abstract Book, but they cannot change the content in any way or use them commercially.
**Abstract Book Citations:** Authors, Title, HemaSphere, 2026;10:(S1):pages. The abstract book DOIs can be found on the "Author Information" page.
**Disclaimer:** Articles published in the journal HemaSphere exclusively reflect the opinions of the authors. The authors are responsible for all content in their abstracts including accuracy of the facts, statements, citing resources, etc.

### (PF1222) TARGETING EBV WITH TCR-REDIRECTED INKT CELLS: AN OFF-THE-SHELF THERAPY WITH ENHANCED EFFICACY AND SAFETY

**Topic:** 25. Gene therapy, cellular immunotherapy and vaccination - Biology & translational research

Lianghua Shen*[1], Changmeng Zhang[1], Dexian Quan[2], Yuhua Wan[2], Ziyu Chen[1], Jian Xu[1], Xiaodan Ding[1], Qiaomei He[1], Qiufang Guo[2], Liping Wan[1], Jian Hua[2], Pengran Wang[1], Xianmin Song[1], Yan Zhang[1]

*[1]Shanghai General Hospital, Shanghai Jiao Tong University School of Medicine, Department of Hematology, Shanghai, China;*
*[2]SPH Biotherapeutics (Shanghai) Co., Ltd., Shanghai, China;*

### Background

Epstein-Barr virus (EBV)-associated hematologic malignancies, including EBV-positive lymphomas such as Hodgkin lymphoma, diffuse large B-cell lymphoma, and post-transplant lymphoproliferative disorders, represent a significant clinical challenge with limited treatment options for relapsed/refractory patients. While allogeneic T cell-based immunotherapies targeting viral antigens have demonstrated clinical proof-of-concept, their widespread adoption is constrained by substantial safety risks.

### Aims

To develop an allogeneic “off-the-shelf” TCR-iNKT cell platform targeting EBV antigens and evaluate its anti-tumor efficacy and safety profile compared to conventional TCR-T therapy.

### Methods

We identified high-affinity TCRs targeting the EBV latent antigens EBNA3B (HLA-A*11:01-restricted). Using a lentiviral vector, these TCRs were expressed in invariant natural killer T (iNKT) cells derived from allogeneic hematopoietic stem cells (HSCs), creating an “off-the-shelf” TCR-iNKT platform. Their function was evaluated through *in vitro* activation, cytotoxicity, and transcriptomic profiling, and compared directly to conventional TCR-T cells. *In vivo* efficacy and safety were assessed using EBV antigen-positive xenograft models in NCG mice.

### Results

TCR-iNKT cells demonstrated superior antigen-specific activation, with higher expression of activation markers, and greater cytokines production compared to mock iNKT cells in *vitro*. RNA sequencing confirmed enhanced cytotoxic gene signatures in TCR-iNKT cells. In murine models, a single dose of TCR-iNKT cells mediated potent and sustained tumor control. Crucially, while equally efficacious, TCR-iNKT cell treatment resulted in significantly lower clinical GVHD scores, reduced pathological tissue damage, and better preservation of body weight than TCR-T therapy.

### Summary/Conclusion

We successfully developed a scalable process for generating allogeneic TCR-iNKT cells targeting EBV. This platform synergizes the precise targeting of TCRs with the inherent efficacy and favorable safety profile of iNKT cells, presenting a promising and readily available “off-the-shelf” immunotherapy for EBV-positive cancers.

**Copyright Information:** (Online) ISSN: 2572-9241
©2026 The Author(s). HemaSphere is published by John Wiley & Sons Ltd on behalf of European Hematology Association. This is an open access Abstract Book distributed under the Attribution-NonCommercial-NoDerivs (CC BY-NC-ND), which allows third parties to download the articles and share them with others as long as they credit the author and the Abstract Book, but they cannot change the content in any way or use them commercially.
**Abstract Book Citations:** Authors, Title, HemaSphere, 2026;10:(S1):pages. The abstract book DOIs can be found on the “Author Information” page.
**Disclaimer:** Articles published in the journal HemaSphere exclusively reflect the opinions of the authors. The authors are responsible for all content in their abstracts including accuracy of the facts, statements, citing resources, etc.

### (PF1223) DYNAMICS OF ANTI-SARS COV-2 T CELL IMMUNITY IN PATIENTS RECEIVING ANTI-CD19 CAR-T CELLS FOR B CELL MALIGNANCIES

**Topic:** 25. Gene therapy, cellular immunotherapy and vaccination - Biology & translational research

Ferhat Bayazit*[1], Melanie Gaudfrin[1], Adrien Lapoutge[1], Marc Muller[2], Marion Divoux[2], Arnaud Campidelli[3], Caroline Jacquet[3], Marie Drouilly[3], Pierre Feugier[3, 4], Natalia De Isla[1], David Moulin[1], Marie Therese Rubio[1, 2], Marcelo De Carvalho Bittencourt[1, 5], Simona Pagliuca[1, 2]

*[1]IMoPa - UMR7365- University of Lorraine, Vandoeuvre Les Nancy, France;[2]Genetic department, Nancy university hospital, vandoeuvre les nancy, France; [3]Department of hematology, Nancy university hospital, vandoeuvre les nancy, France;[4]inserm U1256 nutrition génétique et exposition aux risques environnementaux, université de lorraine, vandoeuvre les nancy, France; [5]laboratory of immunology, university hospital of nancy, vandoeuvre les nancy, France;*

### Background

SARS-CoV-2 has transitioned from a pandemic threat to an endemic pathogen, reshaping T cell immunity across populations. While vaccination and natural infections have attenuated COVID-19 severity, the extent to which CAR-T cell therapy recipients retain functional anti-SARS-CoV-2 T cell immunity remains poorly characterized.

### Aims

Here, our intention is to analyze the T cell receptor (TCR) β (TRB) repertoire in a post-pandemic CAR-T cell cohort whose immune reconstitution occurred during ubiquitous viral circulation and vaccination campaigns.

### Methods

We characterized the TCR repertoire of 123 longitudinal PBMC samples from 39 patients treated at our institution with anti CD19 CAR-T cells for relapsed/refractory B-cell malignancies from January 2020 to March 2023 (median 3 samples/patient; N=71 <3 months, N=20 3-6 months, N=32 >6 months) along with samples from 24 healthy controls. All patients received at least one dose of an mRNA-based anti–SARS-CoV-2 vaccine ≥3 months after CAR-T cell infusion, in accordance with institutional practice. Genomic libraries were generated with Invivoscribe LymphoTrack immunoassay and sequenced with a MiSeq or a NovaSeq 6000; clonotypes were called with MiXCR. SARS-CoV-2–reactive CDR3β sequences were identified by matching against a curated dataset of 146,576 COVID-associated sequences. Analyses were performed with R 4.5.1

**Copyright Information:** (Online) ISSN: 2572-9241
©2026 The Author(s). HemaSphere is published by John Wiley & Sons Ltd on behalf of European Hematology Association. This is an open access Abstract Book distributed under the Attribution-NonCommercial-NoDerivs (CC BY-NC-ND), which allows third parties to download the articles and share them with others as long as they credit the author and the Abstract Book, but they cannot change the content in any way or use them commercially.
**Abstract Book Citations:** Authors, Title, HemaSphere, 2026;10:(S1):pages. The abstract book DOIs can be found on the "Author Information" page.
**Disclaimer:** Articles published in the journal HemaSphere exclusively reflect the opinions of the authors. The authors are responsible for all content in their abstracts including accuracy of the facts, statements, citing resources, etc.

**Results**

Overall, TRB sequencing yielded 342,607 productive clonotypes, including 12,181 SARS-CoV-2–reactive clonotypes. Global repertoire architecture in CAR-T recipients was profoundly contracted compared with healthy controls, with 4.8-fold lower richness, 6-fold lower Inverse Simpson index (ISI), and a higher Gini index (GI), consistent with decreased diversity and increased clonal inequality (**A**). This global pauciclonal state did not correlate with time post-infusion (p=0.28) (**B**), supporting persistent impairment of overall diversity across time.
Although the prevalence of SARS-CoV-2–reactive sequences did not differ between CAR-T recipients and controls (5.8% vs 5.3% per sample; p=0.18), the COVID-specific compartment in CAR-T patients was less diverse (reduced richness and ISI) yet retained a similar dominance structure, with GI comparable to controls. Protein-specific analysis revealed that the proportion of Spike-reactive clonotypes increased over time in CAR-T recipients (31% at >3 months vs 20% at <3 months) (**C**), whereas clonotypes targeting nucleocapsid, membrane, and envelope decreased, consistent with relative enrichment of vaccine-driven responses and attenuation of non-Spike specificities over follow-up. Longitudinally, the diversity of the SARS-CoV-2–reactive repertoire remained stable after CAR-T infusion, with no association between ISI and time (r=0.097, p=0.288). However, between consecutive timepoints, clonal tracking showed that only 15.5% of SARS-CoV-2–reactive clonotypes persisted, corresponding to an 84.5% turnover rate, indicating continuous remodeling while maintaining a conserved overall architecture.

**Summary/Conclusion**

Here by means of immune repertoire sequencing, we showed that SARS-CoV-2 profoundly remodels the post–CAR-T TRB repertoire in the endemic era. While the proportion of SARS-CoV-2–reactive sequences is preserved, the COVID-specific repertoire is durably narrowed, with reduced diversity but maintained clonal hierarchy and high clonotype turnover over time, suggesting ongoing remodeling during immune reconstitution, consistent with preserved vaccine-shaped (Spike-biased) T cell responses.

**Copyright Information:** (Online) ISSN: 2572-9241
©2026 The Author(s). HemaSphere is published by John Wiley & Sons Ltd on behalf of European Hematology Association. This is an open access Abstract Book distributed under the Attribution-NonCommercial-NoDerivs (CC BY-NC-ND), which allows third parties to download the articles and share them with others as long as they credit the author and the Abstract Book, but they cannot change the content in any way or use them commercially.
**Abstract Book Citations:** Authors, Title, HemaSphere, 2026;10:(S1):pages. The abstract book DOIs can be found on the “Author Information” page.
**Disclaimer:** Articles published in the journal HemaSphere exclusively reflect the opinions of the authors. The authors are responsible for all content in their abstracts including accuracy of the facts, statements, citing resources, etc.

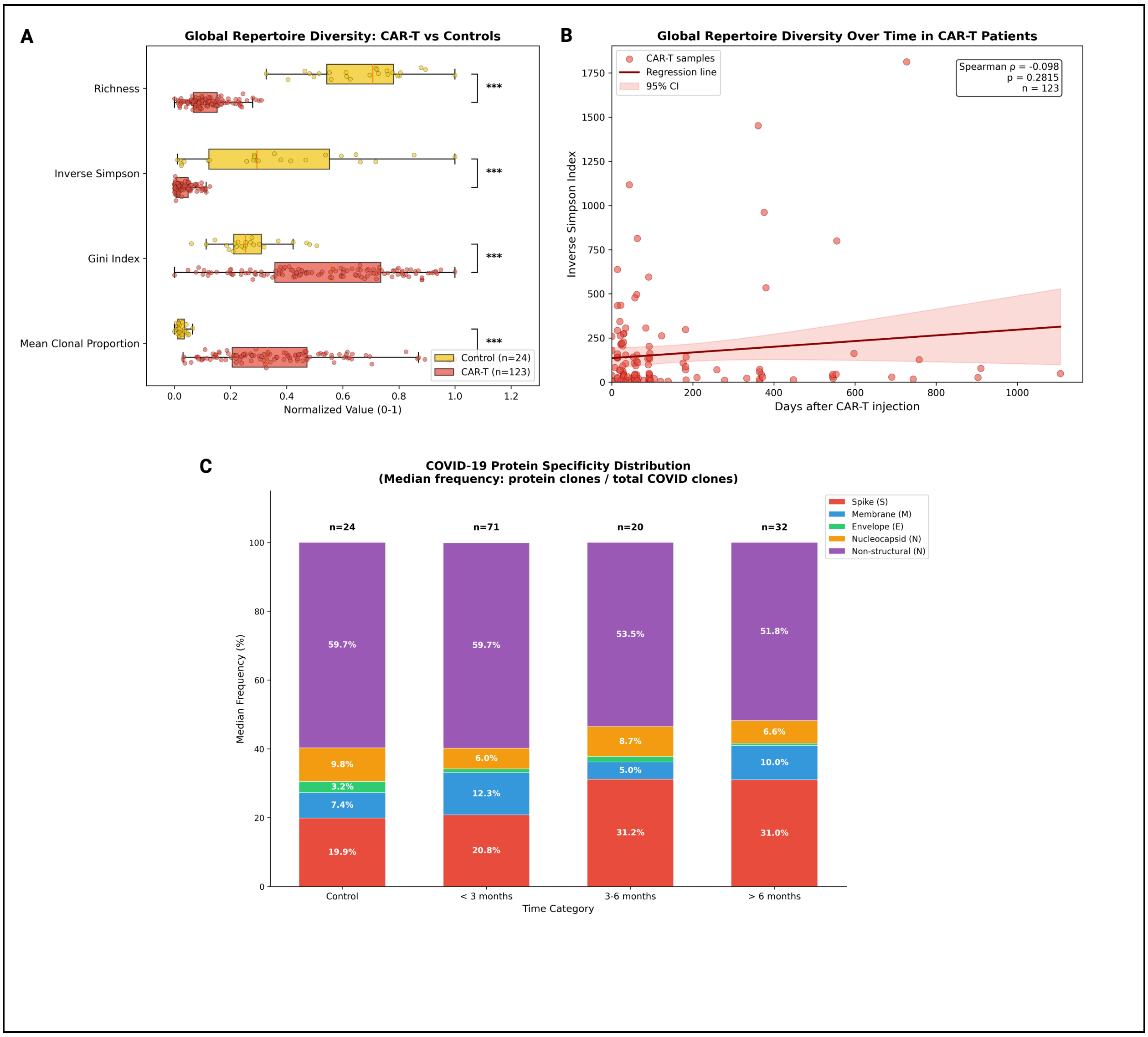

(A) Global TCR repertoire diversity in CAR-T recipients (red, n=123) versus controls (yellow, n=24). Boxplots (median, IQR; whiskers 1.5×IQR) show normalized (0–1) richness, inverse Simpson, Gini index, and mean clonal proportion; dots represent individual samples; group comparisons are significant (***). (B) Longitudinal change in global diversity after CAR-T infusion. Inverse Simpson index plotted versus days post-infusion for CAR-T samples (n=123); red line shows linear regression with 95% CI shading; Spearman correlation is reported. (C) Distribution of SARS-CoV-2–specific TCR clones by viral protein (S, M, E, N, non-structural) in controls and CAR-T samples stratified by time since infusion (<3 months, 3–6 months, >6 months). Stacked bars show median relative frequencies (percent of total COVID-19 clones); n indicates samples per group.

**Copyright Information:** (Online) ISSN: 2572-9241
©2026 The Author(s). HemaSphere is published by John Wiley & Sons Ltd on behalf of European Hematology Association. This is an open access Abstract Book distributed under the Attribution-NonCommercial-NoDerivs (CC BY-NC-ND), which allows third parties to download the articles and share them with others as long as they credit the author and the Abstract Book, but they cannot change the content in any way or use them commercially.
**Abstract Book Citations:** Authors, Title, HemaSphere, 2026;10:(S1):pages. The abstract book DOIs can be found on the "Author Information" page.
**Disclaimer:** Articles published in the journal HemaSphere exclusively reflect the opinions of the authors. The authors are responsible for all content in their abstracts including accuracy of the facts, statements, citing resources, etc.

### (PF1224) ARGINASE INHIBITION IN HUMAN GRANULOCYTES DRIVES HYPERACTIVATION AND ENHANCED EFFECTOR FUNCTION OF TUMOR ANTIGEN–SPECIFIC T CELLS

**Topic:** 25. Gene therapy, cellular immunotherapy and vaccination - Biology & translational research

David Charalambous*[1], Ann-Katrin Gebel[1], Stephanie Reichert[1], Matthias Bros[2], Matthias Theobald[1, 3, 4], Hakim Echchannaoui[1, 4], Markus Munder[1, 3, 5]

*[1]University Medical Center (UMC) Mainz, Department of Hematology and Medical Oncology, Mainz, Germany;[2]University Medical Center (UMC) Mainz, Department of Dermatology, Mainz, Germany; [3]Institute of Immunology, Research Center for Immunotherapy (FZI), Mainz, Germany; [4]German Cancer Consortium (DKTK), Partner Site Frankfurt/Mainz, Mainz, Germany; [5]Deaconess Foundation Hospital Speyer, Department of Hematology and Medical Oncology, Speyer, Germany;*

### Background

Arginine depletion mediated by myeloid cell-derived arginase 1 is a key immunosuppressive mechanism in the tumor microenvironment, limiting T cell activation and effector function. We recently identified a distinct hyperactivated state of human T cells upon pharmacologic inhibition of arginase released by human polymorphonuclear neutrophils (PMN).

### Aims

Our aim is to characterize the functional and metabolic effect of PMN-secretome (upon arginase inhibition) in tumor antigen-specific TCR-engineered T cells as a novel approach to improve T cell based immunotherapies.

### Methods

Human T cells were isolated from peripheral blood mononuclear cells (PBMCs) of healthy donors and retrovirally transduced with a tumor antigen-specific TCR and stimulated in a peptide-specific manner. T cells were cultured in PMN-derived supernatant, generated in the presence of the arginase inhibitor nor-NOHA (PMN+N) or in standard cell culture medium. Flow-cytometry based assays were used to characterize T cell phenotype changes and the associated mitochondrial mass and membrane potential parameters. Mitochondrial function and bioenergetic profiling were analyzed using a Seahorse XF Mito Stress Test. A comprehensive analysis of the cytokine secretion profile was performed by cytokine bead array. T cell cytotoxicity against target tumor cells was assessed using a luciferase-based killing assay and a colony formation assay. CD107a degranulation and intracellular granzyme B expression were also assessed (flow cytometry) to further characterize effector function.

### Results

Upon antigen-specific activation, exposure to PMN+N supernatant significantly increased the frequency of granzyme B–expressing T cells and enhanced degranulation compared to conventional culture conditions. Cytotoxic T cell luciferase-based killing assay and colony forming assay both demonstrated enhanced (25% relative increase and 33% increase) tumor-specific killing compared to conventional activation. On a metabolic level, mitochondrial membrane potential and mitochondrial mass (42% relative increase) were both enhanced, suggesting an improved mitochondrial fitness. Accordingly, Seahorse analysis revealed increased maximal respiration (3-fold) and spare respiratory capacity (6-fold) in PMN+N-conditioned T cells compared to controls. PMN+N-conditioned T cells exhibited markedly enhanced cytokine secretion, including IFNg, TNFa, and IL-4 by up to 4-fold.

**Copyright Information:** (Online) ISSN: 2572-9241
©2026 The Author(s). HemaSphere is published by John Wiley & Sons Ltd on behalf of European Hematology Association. This is an open access Abstract Book distributed under the Attribution-NonCommercial-NoDerivs (CC BY-NC-ND), which allows third parties to download the articles and share them with others as long as they credit the author and the Abstract Book, but they cannot change the content in any way or use them commercially.
**Abstract Book Citations:** Authors, Title, HemaSphere, 2026;10:(S1):pages. The abstract book DOIs can be found on the "Author Information" page.
**Disclaimer:** Articles published in the journal HemaSphere exclusively reflect the opinions of the authors. The authors are responsible for all content in their abstracts including accuracy of the facts, statements, citing resources, etc.

**Summary/Conclusion**

Our data demonstrate that inhibition of PMN-derived arginase unleashes potent PMN-mediated enhancement of T-cell function, resulting in augmented tumor antigen-specific effector responses. These findings provide a strong rationale for further preclinical evaluation of arginase inhibitors as potential novel antitumor therapeutic strategies.

**Copyright Information:** (Online) ISSN: 2572-9241
©2026 The Author(s). HemaSphere is published by John Wiley & Sons Ltd on behalf of European Hematology Association. This is an open access Abstract Book distributed under the Attribution-NonCommercial-NoDerivs (CC BY-NC-ND), which allows third parties to download the articles and share them with others as long as they credit the author and the Abstract Book, but they cannot change the content in any way or use them commercially.
**Abstract Book Citations:** Authors, Title, HemaSphere, 2026;10:(S1):pages. The abstract book DOIs can be found on the "Author Information" page.
**Disclaimer:** Articles published in the journal HemaSphere exclusively reflect the opinions of the authors. The authors are responsible for all content in their abstracts including accuracy of the facts, statements, citing resources, etc.

### (PF1225) BASELINE T-CELL REPERTOIRE SKEWING AND EFFECTOR CD8 T-CELL DYNAMICS IN PROLONGED CYTOPENIAS FOLLOWING ANTI-CD19 CAR T-CELL THERAPY

**Topic:** 25. Gene therapy, cellular immunotherapy and vaccination - Biology & translational research

Akhil Khera*[1, 2], Alicia Au[1], Michelle Tan[1], Amna Malik[1], Lino Carunchio[2], Nehal Joshi[1], Rocel Saputil[1], Camelia Vladescu[2], Edward Bataillard[2], Sasha Marks[2], Lucy Cook[1, 2], Nichola Cooper[1, 2]

[1]*Imperial College London, Department of Immunology and Inflammation, London, United Kingdom;*[2]*Imperial College Healthcare NHS Trust, Haematology, London, United Kingdom;*

### Background

Anti-CD19 CAR T-cell therapy has transformed outcomes in B-cell malignancies, but prolonged cytopenias occur in up to 50% of patients and pathophysiology remains poorly understood. In particular, the role of immune repertoire architecture and T-cell phenotypic states in prolonged cytopenias is unexplored.

### Aims

To investigate whether pre-treatment T-cell immune repertoire architecture and phenotype are associated with the development of prolonged cytopenias following CD19-directed CAR T-cell therapy.

### Methods

This single-centre prospective observational study utilised anonymised clinical data and peripheral blood mononuclear cells (PBMCs) from patients receiving autologous anti-CD19 CAR T-cell therapy. Prolonged cytopenia was defined as persistent grade ≥3 cytopenia lasting >30 days post-infusion (neutrophils <1.0 $\times 10^9$/L, platelets <50 $\times 10^9$/L, or haemoglobin <80 g/L). PBMCs were collected pre-lymphodepletion and CAR T-cell infusion for T-cell receptor beta (TRB) sequencing and flow cytometry. Longitudinal flow cytometry was performed at serial time points post-CAR T-cell infusion. Group comparisons were performed using non-parametric statistical tests.

### Results

Ten patients were included (median age 54 years, range 28-74; 7 male, 3 female), nine with relapsed/refractory Diffuse Large B-cell Lymphoma, and one with relapsed B-cell Acute Lymphoblastic Leukaemia. Five patients (50%) developed prolonged cytopenias. TRB Sequencing pre-lymphodepletion and pre-CAR T-cell demonstrated that the proportion of expanded T-cell clonotypes (occupying >1% of the T-cell repertoire) was significantly higher in patients who developed prolonged cytopenias (56% vs 22%, $p = 0.05$) compared to those without. T-cell repertoire diversity, measured by Shannon diversity index, was reduced in CAR T-cell recipients compared with healthy controls and was lowest in patients who developed prolonged cytopenias (median Shannon diversity index 4.5 vs 5.5, $p = 0.0005$). At baseline, patients who developed prolonged cytopenias showed numerically higher CD8:CD4 and CD8 TEMRA:CD4 regulatory T-cell (Tregs) ratios. This higher CD8 TEMRA:CD4 Tregs ratio persisted over 30 days post-CAR T-cell infusion, whereas patients without prolonged cytopenias maintained a lower ratio over time. The expanded CD8 TEMRA population displayed a highly differentiated CD57+CD28- phenotype, consistent with cytotoxic effector state.

**Copyright Information:** (Online) ISSN: 2572-9241
©2026 The Author(s). HemaSphere is published by John Wiley & Sons Ltd on behalf of European Hematology Association. This is an open access Abstract Book distributed under the Attribution-NonCommercial-NoDerivs (CC BY-NC-ND), which allows third parties to download the articles and share them with others as long as they credit the author and the Abstract Book, but they cannot change the content in any way or use them commercially.
**Abstract Book Citations:** Authors, Title, HemaSphere, 2026;10:(S1):pages. The abstract book DOIs can be found on the "Author Information" page.
**Disclaimer:** Articles published in the journal HemaSphere exclusively reflect the opinions of the authors. The authors are responsible for all content in their abstracts including accuracy of the facts, statements, citing resources, etc.

**Summary/Conclusion**

In this prospective study, prolonged cytopenias following anti-CD19 CAR T-cell therapy were associated with baseline immune repertoire skewing and altered T-cell dynamics. Patients who developed prolonged cytopenias exhibited greater clonal expansion and reduced T-cell repertoire diversity before CAR T-cell therapy. Skewing towards highly differentiated CD8+ TEMRA populations was a feature of patients with prolonged cytopenias, whereas those without prolonged cytopenias demonstrated more stable T-cell profiles. These findings suggest that pre-existing immune repertoire characteristics and effector T-cell dynamics may influence the likelihood of developing prolonged cytopenias after CAR T-cell therapy. Further investigation in larger cohorts is warranted to identify immune biomarkers that can define patients at risk of developing prolonged cytopenias.

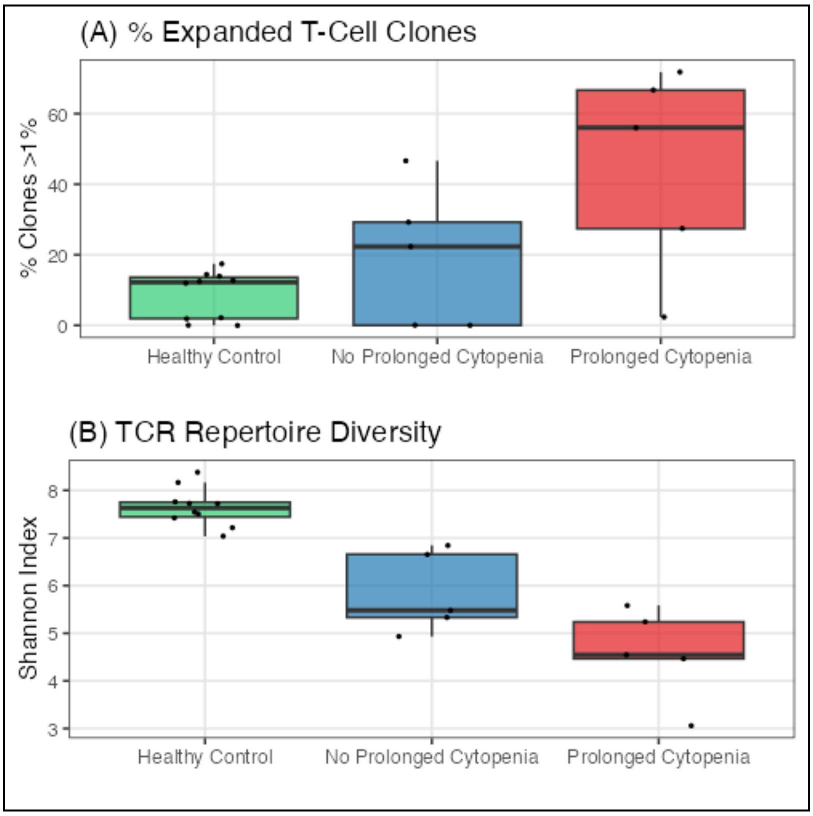

Expanded T-cell clonotype proportion (clones occupying >1% of the repertoire) and Shannon diversity index measured pre-lymphodepletion and pre-CAR T-cell infusion in healthy controls and CAR T-cell recipients grouped by prolonged cytopenia (>30 days) (expanded clonotypes p = 0.05; Shannon diversity index p = 0.0005).

**Copyright Information:** (Online) ISSN: 2572-9241
©2026 The Author(s). HemaSphere is published by John Wiley & Sons Ltd on behalf of European Hematology Association. This is an open access Abstract Book distributed under the Attribution-NonCommercial-NoDerivs (CC BY-NC-ND), which allows third parties to download the articles and share them with others as long as they credit the author and the Abstract Book, but they cannot change the content in any way or use them commercially.
**Abstract Book Citations:** Authors, Title, HemaSphere, 2026;10:(S1):pages. The abstract book DOIs can be found on the "Author Information" page.
**Disclaimer:** Articles published in the journal HemaSphere exclusively reflect the opinions of the authors. The authors are responsible for all content in their abstracts including accuracy of the facts, statements, citing resources, etc.

### (PF1226) IDENTIFICATION OF PREDICTIVE AND PROGNOSTIC BIOMARKERS IN PATIENTS UNDERGOING CAR-T CELL THERAPY FOR HAEMATOLOGICAL MALIGNANCIES

**Topic:** 25. Gene therapy, cellular immunotherapy and vaccination - Biology & translational research

Francesca Vaglio*[1, 2], Gianluca Storci[2], Daria Messelodi[2], Salvatore Nicola Bertuccio[2], Serena De Matteis[2], Marta Tassoni[1], Renée Concetta Duardo[2], Federica Panaccione[2], Cristiano Mauro[2], Giordana Scirè Calabrisotto[2], Giordana Orsino[2], Massimiliano Bonafé[1, 2], Francesca Bonifazi[2]

*[1]università di Bologna, Bologna, Italy; [2]IRCCS Azienda ospedaliero-universitaria di Bologna - Policlinico Sant'Orsola, Bologna, Italy;*

### Background

CAR-T cell expansion after infusion is associated with both clinical response and immune effector cell-associated neurotoxicity syndrome (ICANS). However, considering that expansion occurs concomitantly with toxicityit it has no predictive value. Moreover, robust CAR-T expansion does not invariably translate into clinical benefit, suggesting that the pre-infusion inflammatory status may influence cytotoxicity and therapeutic efficacy. The identification of early biomarkers predicting toxicity and response remains an unmet clinical need.

### Aims

To identify biomarkers predictive of ICANS and one-year clinical response in lymphoma patients treated with CAR-T cells through an integrated analysis of plasma extracellular vesicles (EVs), immune cell subsets and soluble mediators.

### Methods

84 patients with different non-Hodgkin lymphoma subtypes were enrolled. Plasma samples were collected at the pre-lymphodepletion (PLD) time point, prior to CAR-T infusion.

Plasma EVs were analyzed using two complementary flow cytometry approaches. On the BD FACSLyric platform, EVs identification was performed according to the Lanuti method using a lipophilic cationic probe and phalloidin exclusion strategy, followed by assessment of target-specific markers including enolase 2 (ENO2) and PD-L1. On the Beckman Coulter platform, EV size was defined using Megamix beads and ENO2 expression was evaluated within the EV population.

In a subset of 49 patients, T-cell and myeloid cell subpopulations were characterized in fresh peripheral PBMCs samples through multicolor flow cytometry at the PLD time point. In the same setting, soluble plasma and serum molecules were quantified using ELISA and Luminex assays.

Clinical response was evaluated one year post-infusion. Patients  who achieved a complete or partial response were classified as responders (R), whereas those with stable or progressive disease were classified as non-responders (NR). Statistical analyses were performed using Mann–Whitney test.

**Copyright Information:** (Online) ISSN: 2572-9241
©2026 The Author(s). HemaSphere is published by John Wiley & Sons Ltd on behalf of European Hematology Association. This is an open access Abstract Book distributed under the Attribution-NonCommercial-NoDerivs (CC BY-NC-ND), which allows third parties to download the articles and share them with others as long as they credit the author and the Abstract Book, but they cannot change the content in any way or use them commercially.
**Abstract Book Citations:** Authors, Title, HemaSphere, 2026;10:(S1):pages. The abstract book DOIs can be found on the “Author Information” page.
**Disclaimer:** Articles published in the journal HemaSphere exclusively reflect the opinions of the authors. The authors are responsible for all content in their abstracts including accuracy of the facts, statements, citing resources, etc.

**Results**

At PLD time point plasma levels of EVs positive for enolase 2 ($ENO2^+$), a neuronal stress-associated marker, were observed in patients who developed ICANS post-CAR-T infusion. The predictive value of $ENO2^+$ EVs was confirmed using both cytometric approaches, supporting that either technique can be reliably used depending on instrument availability. ICANS-developing patients also showed increased frequencies of senescent $CD8^+$ T cells and monocytic myeloid-derived suppressor cells (M-MDSCs), suggesting pre-existing immune dysregulation.
Regarding the prediction of treatment outcome, NR patients displayed significantly higher PLD levels of PD-$L1^+$ EVs and soluble PD-L1 compared to R patients. Increased proportions of senescent $CD8^+$ T cells and myeloid-derived suppressor cells (MDSCs), particularly polymorphonuclear myeloid-derived suppressor cells (PMN-MDSCs), were also observed in NR patients, compatible with a systemic immunosuppressive profile that may contribute to reduced CAR-T efficacy.

**Summary/Conclusion**

$ENO2^+$ EVs emerge as early predictive biomarker of ICANS, reflecting a pre-existing neuronal stress status in ICANS-developing patients. In parallel, a pre-infusion immunosuppressive milieu characterized by T-cell senescence, PMN-MDSCs expansion, and elevated PD-L1—both soluble and EV-associated—correlates with worse clinical response. Reducing systemic inflammation and preserving T-cell function could there represent useful strategies to improve CAR-T cell therapy response.

**Copyright Information:** (Online) ISSN: 2572-9241
©2026 The Author(s). HemaSphere is published by John Wiley & Sons Ltd on behalf of European Hematology Association. This is an open access Abstract Book distributed under the Attribution-NonCommercial-NoDerivs (CC BY-NC-ND), which allows third parties to download the articles and share them with others as long as they credit the author and the Abstract Book, but they cannot change the content in any way or use them commercially.
**Abstract Book Citations:** Authors, Title, HemaSphere, 2026;10:(S1):pages. The abstract book DOIs can be found on the “Author Information” page.
**Disclaimer:** Articles published in the journal HemaSphere exclusively reflect the opinions of the authors. The authors are responsible for all content in their abstracts including accuracy of the facts, statements, citing resources, etc.

### (PF1227) HIJACKING THE TUMOR MICROENVIRONMENT: CD209-DIRECTED CAR-T CELLS TO DEPLETE TUMOR-ASSOCIATED MACROPHAGES

**Topic:** 25. Gene therapy, cellular immunotherapy and vaccination - Biology & translational research

Marta Kłopotowska*[1], Julia Maroszek[2, 3], Anna Jurga[1, 4], Olga Malik[1, 4], Christopher Forcados[5, 6], Sebastien Wälchli[5], Przemyslaw Juszczynski[2], MacIej Szydłowski[2], Magdalena Winiarska[1], Małgorzata Bajor[1]

*[1]Department of Immunology, Mossakowski Medical Research Institute, Polish Academy of Sciences, Warsaw, Poland; [2]Department of Experimental Hematology, Institute of Hematology and Transfusion Medicine, Warsaw, Poland;[3]Centre of Postgraduate Medical Education, Doctoral School, Warsaw, Poland; [4]Immunooncology Students' Science Association, Medical University of Warsaw, Warsaw, Poland; [5]Translational Research Unit, Section of Cellular Therapy, Department of Oncology, Oslo University Hospital, Oslo, Norway; [6]Institute of Clinical Medicine, Faculty of Medicine, University of Oslo, Oslo, Norway;*

### Background

Tumor-associated macrophages (TAMs), a major immune population within the tumor microenvironment (TME), promote immune evasion and are associated with unfavorable prognosis and adverse clinical outcomes across diverse malignancies. In this study, we focused on classical Hodgkin lymphoma (cHL), a malignancy characterized by a highly immunosuppressive TME in which TAMs represent a key cellular component and are associated with reduced clinical benefit. Recent evidence indicates that paracrine interactions with Reed–Sternberg (RS) cells polarize macrophages toward a protumorigenic M2-like phenotype in a process accompanied by upregulation of different antigens. In polarized TAMs, we have identified CD209 (DC-SIGN) and explored it as a novel target for CAR-T cell-based immunotherapy. Accordingly, we designed and generated several CD209-directed CAR-T cell constructs and performed functional *in vitro* assays to assess their efficacy in eliminating macrophages.

### Aims

Development and evaluation of CD209-directed CAR-T cells for selective depletion of tumor-associated macrophages.

### Methods

Macrophages were obtained either from the human monocytic cell line THP1 or from primary human monocytes isolated from buffy coats of healthy blood donors, differentiated with PMA and M-CSF, respectively and polarized towards TAMs with IL-4, IL-10 or by coculture with L1236 and L428 - RS cells in a transwell system. CAR constructs were generated using three different scFvs with either CD28 or 41BB costimulatory domains. For CAR-T cell generation, PBMCs were stimulated with anti-CD3/CD28 antibodies and cultured with IL-2. Subsequently, CAR constructs were introduced by lentiviral transduction. CAR-T cell-mediated cytotoxicity against polarized macrophages or THP1 cells was assessed using flow cytometry and RTCA-based killing assays.

### Results

In *in vitro* RS-polarized TAMs, we identified multiple upregulated genes encoding surface proteins that may serve as potential CAR-T targets. From these candidates, we selected CD209, whose upregulation was validated in TAM-like macrophages *in vitro*. To identify the most effective CAR-T cell-based strategy, we tested six different CAR constructs incorporating three different scFvs against CD209 and two intracellular costimulatory domains (CD28 or 41BB). In contrast to 41BB variant, CAR-T cells containing the CD28 costimulatory domain demonstrated efficient TAMs killing, degranulation (CD107a expression), and cytokine production (IFNγ and TNFα), independently of the incorporated scFvs.

**Copyright Information:** (Online) ISSN: 2572-9241
©2026 The Author(s). HemaSphere is published by John Wiley & Sons Ltd on behalf of European Hematology Association. This is an open access Abstract Book distributed under the Attribution-NonCommercial-NoDerivs (CC BY-NC-ND), which allows third parties to download the articles and share them with others as long as they credit the author and the Abstract Book, but they cannot change the content in any way or use them commercially.
**Abstract Book Citations:** Authors, Title, HemaSphere, 2026;10:(S1):pages. The abstract book DOIs can be found on the “Author Information” page.
**Disclaimer:** Articles published in the journal HemaSphere exclusively reflect the opinions of the authors. The authors are responsible for all content in their abstracts including accuracy of the facts, statements, citing resources, etc.

**Summary/Conclusion**

We have developed CAR-T cells that effectively target CD209$^{+}$ TAM-like macrophages. We are currently investigating the applicability of this approach in classical Hodgkin lymphoma. In parallel, we are also testing this strategy across diverse tumor models characterized by an immunosuppressive TME. Importantly, CD209 (DC-SIGN) is enriched in the myeloid compartment in multiple cancers and correlates with unfavorable clinical outcomes. Therefore, we propose here that CD209-directed CAR-T cells may synergize with existing immunotherapies to improve clinical outcomes in patients with diverse tumor types.
Funding: This work was supported by the Polish National Science Centre within Grant Opus 2022/45/B/NZ6/02588.

**Copyright Information:** (Online) ISSN: 2572-9241
©2026 The Author(s). HemaSphere is published by John Wiley & Sons Ltd on behalf of European Hematology Association. This is an open access Abstract Book distributed under the Attribution-NonCommercial-NoDerivs (CC BY-NC-ND), which allows third parties to download the articles and share them with others as long as they credit the author and the Abstract Book, but they cannot change the content in any way or use them commercially.
**Abstract Book Citations:** Authors, Title, HemaSphere, 2026;10:(S1):pages. The abstract book DOIs can be found on the "Author Information" page.
**Disclaimer:** Articles published in the journal HemaSphere exclusively reflect the opinions of the authors. The authors are responsible for all content in their abstracts including accuracy of the facts, statements, citing resources, etc.

### (PF1228) RAPID POINT-OF-CARE MANUFACTURING OF LEUKAPHERESIS-FREE CD19 CAR-T CELLS

**Topic:** 25. Gene therapy, cellular immunotherapy and vaccination - Biology & translational research

Maria Giannaki*[1], Fani Stavridou[1], Ioulia Mavrikou[1], Kiriaki Paschoudi[1, 2], Ioanna Vallianou[1], Elina-Ioanna Latrovali[1, 2], Christina Beta[1, 2], Ioannis Kyriakou[1], Georgios Karavalakis[1], Ioanna Sakellari[1], Evangelia Yannaki[1], Anastasia Papadopoulou[1, 2]

*[1] "George Papanikolaou" Hospital, Gene and Cell Therapy Center, Hematology Department – Hematopoietic Cell Transplantation Unit, Thessaloniki, Greece; [2] Aristotle University of Thessaloniki, Department of Genetics, Development and Molecular Biology, School of Biology, Thessaloniki, Greece;*

### Background

CAR-T cell therapy has transformed the treatment of B-cell malignancies; yet, its widespread use is challenged by centralized, leukapheresis-dependent and prolonged production, high costs and access constraints. As CAR-T therapies expand toward earlier lines of treatment and additional indications, simplified, rapid and decentralized manufacturing that shortens time-to-treatment is needed.

### Aims

To develop and evaluate a rapid, leukapheresis-free, CAR-T cell manufacturing platform from small peripheral blood volumes using G-Rex bioreactors.

### Methods

Blood samples from healthy donors (n=3) and relapsed/refractory DLBCL patients (n=5) were processed. Two workflows using a third-generation CD19 γ-retroviral vector were evaluated: i) "Expansion-in-G-Rex", where anti-CD3/CD28–activated PBMCs were transduced in plates and expanded in G-Rex10M, and ii) "Transduction-in-G-Rex", in which activated PBMCs were transduced and expanded in G-Rex bioreactors. Three seeding densities (0.25, 0.5, 0.8x10$^6$ cells/cm²) and two vector volumes (0.4 and 0.75 ml/cm²) were tested. Expansion, phenotype, transduction efficiency, proliferative and cytotoxic capacity were assessed by flow cytometry, while antigen-specific responses were evaluated by ELISPOT.

**Copyright Information:** (Online) ISSN: 2572-9241
©2026 The Author(s). HemaSphere is published by John Wiley & Sons Ltd on behalf of European Hematology Association. This is an open access Abstract Book distributed under the Attribution-NonCommercial-NoDerivs (CC BY-NC-ND), which allows third parties to download the articles and share them with others as long as they credit the author and the Abstract Book, but they cannot change the content in any way or use them commercially.
**Abstract Book Citations:** Authors, Title, HemaSphere, 2026;10:(S1):pages. The abstract book DOIs can be found on the "Author Information" page.
**Disclaimer:** Articles published in the journal HemaSphere exclusively reflect the opinions of the authors. The authors are responsible for all content in their abstracts including accuracy of the facts, statements, citing resources, etc.

### Results

The "Expansion-in-G-Rex" protocol yielded high transduction efficiency (58–81%) and expansion (26–32 fold). However, it required 2–3 weeks to complete and involved multiple handlings. To overcome these limitations, we developed the "Transduction-in-G-Rex" protocol, in which transduction and expansion were performed in G-Rex. The optimal condition ($0.25x10^6$ cells/$cm^2$, 0.4 ml/$cm^2$ vector) enabled 75±26-fold CAR-T expansion within 7 days, demonstrating that transduction directly in G-Rex accelerates cell kinetics. GMP validation using PBMCs from one healthy donor and two DLBCL patients confirmed the method's robustness, resulting in 164±53-fold expansion within 8–9 days, producing $8.2±0.7×10^9$ cells with high CAR expression (67±3%), low vector copy number (1.4±0.2), and a polyclonal CD8+/CD4+ composition (48±14%/50±14%). Phenotypically, CAR-T cells were enriched in naïve (26±7%) and memory subsets (central memory: 32±5%, effector memory: 33±10%), expressed activation markers (CD69+: 59±14%, CD25+: 52±5%), and exhibited a favorable functional profile with increased CD27 and CD62L expression (68±3% and 58±10%, respectively) and low expression of exhaustion/senescence markers ($PD\text{-}1^+$: 11±2%, $CTLA\text{-}4^+$: 2.9±0.2%, $CD27^-$/$CD45RA^+$/$CD57^+$: 6±2%). Functionally, CAR-Ts showed robust proliferation upon CD3 restimulation compared to baseline (divided: 84% vs 0%, p<0.0001; proliferation index: 2.9 vs 0, p=0.0004) and strong, antigen-specific IFN-γ secretion and cytotoxicity against $CD19^+$ Raji cells compared to untransduced T cells [Spot forming cells (SFC)/25,000 cells: 795±51 vs 89±38, p=0.0004; % lysis Raji cells: 82±2% vs 31±6%, p<0.0001]. The cytotoxic activity was specific, as reactivity against $CD19^-$ K562 targets was minimal and similar to untransduced T cells (SFC/25,000 cells: 38±12 vs 21±16, p=0.98; % lysis: 43±7% vs 46±2%, p=0.7).

### Summary/Conclusion

We describe a rapid, simplified, GMP-compliant CAR-T cell manufacturing platform that supports decentralized, point-of-care production of CAR-T cells from small volumes of peripheral blood, without leukapheresis or upfront $CD3^+$ T-cell selection. This approach has the potential to shorten time to treatment, improve accessibility and facilitate broader implementation of CAR-T therapy across diverse clinical settings.

**Copyright Information:** (Online) ISSN: 2572-9241
©2026 The Author(s). HemaSphere is published by John Wiley & Sons Ltd on behalf of European Hematology Association. This is an open access Abstract Book distributed under the Attribution-NonCommercial-NoDerivs (CC BY-NC-ND), which allows third parties to download the articles and share them with others as long as they credit the author and the Abstract Book, but they cannot change the content in any way or use them commercially.
**Abstract Book Citations:** Authors, Title, HemaSphere, 2026;10:(S1):pages. The abstract book DOIs can be found on the "Author Information" page.
**Disclaimer:** Articles published in the journal HemaSphere exclusively reflect the opinions of the authors. The authors are responsible for all content in their abstracts including accuracy of the facts, statements, citing resources, etc.

### (PF1229) SCFV-BASED ANTI-CD72 IMMUNOTHERAPY AS A POTENTIAL TREATMENT FOR B-CELL MALIGNANCIES

**Topic:** 25. Gene therapy, cellular immunotherapy and vaccination - Biology & translational research

Klaudyna Fidyt*[1, 2, 3], Katsiaryna Marhelava[2, 4], Christopher Forcados[5], Marta Krawczyk[2, 4, 6], Marta Kłopotowska[4], Monika Pepek[2], Cristina Olivar-Fernández[7], Aleksandra Swiderska[8], Mercedes Guerrero-Murillo[1], Bartosz Perkowski[9], Łukasz Sędek[9], Daria Owczarek[10], Anna Szumera-Cieckiewicz[10], Norma Gutierrez Gutierrez[11, 12], Miguel Alcoceba Sánchez[11, 12], Pablo Menendez[1, 3, 12, 13, 14, 15], Sebastien Wälchli[5], Malgorzata Firczuk[2, 4], Magdalena Winiarska[2, 4]

*[1]Josep Carreras Leukaemia Research Institute, Barcelona, Spain;[2]Medical University of Warsaw, Department of Immunology, Warsaw, Poland; [3]Red Española de Terapias Avanzadas (TERAV), Instituto de Salud Carlos III (ISCIII), Barcelona, Spain; [4]Mossakowski Medical Research Institute, Polish Academy of Sciences, Department of Immunology, Warsaw, Poland;[5]Oslo University Hospital, Translational Research Unit, Section of Cellular Therapy, Department of Oncology, Oslo, Norway; [6]Mossakowski Medical Research Institute, Polish Academy of Sciences, Doctoral School of Translational Medicine, Warsaw, Poland; [7]Instituto Universitario de Oncología (IUOPA), Universidad de Oviedo, Departamento de Bioquímica y Biología Molecular, Oviedo, Spain; [8]Pure Biologics, Wroclaw, Poland; [9]Medical University of Silesia in Katowice, Department of Pediatric Hematology and Oncology, Zabrze, Poland; [10]Maria Skłodowska-Curie National Research Institute of Oncology, Warsaw, Poland; [11]Hospital Universitario de Salamanca (HUS/IBSAL) and Centro de Investigación del Cáncer-IBMCC (USAL-CSIC), Servicio de Hematología, Salamanca, Spain; [12]Centro de Investigación Biomédica en Red de Cáncer (CIBERONC), Madrid, Spain;[13]Institut de Recerca Hospital Sant Joan de Déu-Pediatric Cancer Center Barcelona (SJD-PCCB), Barcelona, Spain; [14]University of Barcelona, Department of Biomedicine, School of Medicine, Barcelona, Spain; [15]Institució Catalana de Recerca i Estudis Avançats (ICREA), Barcelona, Spain;*

### Background

*** Klaudyna Fidyt, Katsiaryna Marhelava, and Christopher Forcados contributed equally to this work**

Therapy of B-cell malignancies has undergone a significant transformation since the implementation of CD19-directed chimeric antigen receptor-modified T cells (CD19 CAR-T). Nevertheless, roughly half of the treated patients experience disease progression and relapse following CD19 CAR-T infusion. The resistance mechanisms are multifaceted and relate to CAR-T-cell dysfunction, a suppressive tumor microenvironment, or tumor intrinsic features, which may lead to target-antigen escape. One approach to treat CD19-negative relapses is to identify alternative tumor antigens and develop corresponding novel CARs. Herein, we propose scFv-based anti-CD72 immunotherapy as a potential treatment for relapsed B-cell malignancies following CD19-directed interventions.

### Aims

Development and validation of anti-CD72 immunotherapy for B-cell malignancies.

### Methods

Specific CD72 binders were selected from a human hyperimmune scFv library using the phage display technology. Identification of the most selective/active scFv-based CD72 CARs was performed utilizing Jurkat (J)-76-NFAT-GFP reporter assay. The killing potential of the generated CD72 CAR-T/BiTE was tested using flow cytometry-based method.

**Copyright Information:** (Online) ISSN: 2572-9241
©2026 The Author(s). HemaSphere is published by John Wiley & Sons Ltd on behalf of European Hematology Association. This is an open access Abstract Book distributed under the Attribution-NonCommercial-NoDerivs (CC BY-NC-ND), which allows third parties to download the articles and share them with others as long as they credit the author and the Abstract Book, but they cannot change the content in any way or use them commercially.
**Abstract Book Citations:** Authors, Title, HemaSphere, 2026;10:(S1):pages. The abstract book DOIs can be found on the "Author Information" page.
**Disclaimer:** Articles published in the journal HemaSphere exclusively reflect the opinions of the authors. The authors are responsible for all content in their abstracts including accuracy of the facts, statements, citing resources, etc.

**Results**

Using a transcriptomic approach, we identified 24 genes coding for membrane proteins and designed flow cytometry panels to evaluate their surface expression. In addition to CD19/CD22, CD72 revealed the most homogenous expression among the tested proteins in both B-cell acute lymphoblastic leukemia (B-ALL) and B-cell lymphoma cell lines. Importantly, the target expression was also confirmed in primary B-ALL and lymphoma samples at diagnosis and post-CD19 CAR-T treatment. In normal cells, CD72 was restricted to the immune compartment, as confirmed with the Tabula Sapiens single-cell transcriptomic atlas and by immunohistochemistry of healthy tissues. Using phage display with a hyperimmune scFv library, we selected eleven CD72 unique binders, designed eleven second-generation CAR constructs (CD8H/CD8TM/4-1BB-3z), and tested target-induced activation in the J-76-NFAT-GFP reporter system. We selected the top three active/specific constructs and observed only modest cytotoxicity towards target cell lines when CARs were expressed in primary T cells. Subsequently, by exchanging the CD8-hinge, CD8-transmembrane, and 4-1BB costimulatory domains for CD28 (CD72-28x3-3z), we significantly improved the killing capacity of CAR-T cells expressing one of the leading binders. Additionally, using a selected binder, we generated CD72 bispecific T-cell engager (BiTE), as another immunotherapy format. In vitro assays with primary T cells and CD72 BiTE showed potent killing of CD72-expressing target cells, including cell lines with confirmed resistance to CD19 CAR-T.

**Summary/Conclusion**

Our in vitro results demonstrate that CD72-directed scFv-based immunotherapy is a promising treatment of B-cell tumors, including those resistant to CD19 CAR-T therapy. Further in vivo studies are warranted to confirm the efficacy of CD72 immunotherapy in CAR-T and BiTE formats.

**Copyright Information:** (Online) ISSN: 2572-9241
©2026 The Author(s). HemaSphere is published by John Wiley & Sons Ltd on behalf of European Hematology Association. This is an open access Abstract Book distributed under the Attribution-NonCommercial-NoDerivs (CC BY-NC-ND), which allows third parties to download the articles and share them with others as long as they credit the author and the Abstract Book, but they cannot change the content in any way or use them commercially.
**Abstract Book Citations:** Authors, Title, HemaSphere, 2026;10:(S1):pages. The abstract book DOIs can be found on the “Author Information” page.
**Disclaimer:** Articles published in the journal HemaSphere exclusively reflect the opinions of the authors. The authors are responsible for all content in their abstracts including accuracy of the facts, statements, citing resources, etc.

### (PF1232) DURABLE CLINICAL BENEFITS WITH EXAGAMGLOGENE AUTOTEMCEL FOR OVER 6 YEARS IN PATIENTS AGED 12 TO 35 YEARS WITH SICKLE CELL DISEASE WITH RECURRENT VASO-OCCLUSIVE CRISES

**Topic:** 26. Gene therapy, cellular immunotherapy and vaccination - Clinical

Stephan Grupp*[1], Franco Locatelli[2], Akshay Sharma[3], Monica Bhatia[4], Markus Mapara[5], Lyndsay Molinari[6], Laurence Dedeken[7], Robert Liem[8], Kevin H.M. Kuo[9], Paul Telfer[10], Ami J. Shah[11], Marina Cavazzana[12], Selim Corbacioglu[13], Mariane de Montalembert[14], Stephan Lobitz[15], Damiano Rondelli[16], Martin Steinberg[17], Mark Walters[18], Kathleen Lee-Sarwar[19], Weiyu Zhou[19], Chris Lux[19], William Hobbs[19], Haydar Frangoul[20]

*[1]Children's Hospital of Philadelphia, Perelman School of Medicine at the University of Pennsylvania, Philadelphia, United States of America; [2]IRCCS, Ospedale Pediatrico Bambino Gesù Rome, Catholic University of the Sacred Heart, Rome, Italy;[3]St. Jude Children's Research Hospital, Department of Bone Marrow Transplantation and Cellular Therapy, Memphis, United States of America; [4]Columbia University Irving Medical Center, New York – Presbyterian-Morgan Stanley Children's Hospital, Department of Pediatrics, New York, United States of America; [5]Columbia University, Division of Hematology and Oncology, New York, United States of America; [6]Sarah Cannon Pediatric Transplant and Cellular Therapy Program at Methodist Children's Hospital, San Antonio, United States of America; [7]Hopital Universitaire des Enfants Reine Fabiola, Brussels, Belgium;[8]Ann & Robert H. Lurie Children's Hospital of Chicago, Chicago, United States of America; [9]University of Toronto, Division of Hematology, Toronto, Canada; [10]Royal London Hospital, Barts Health NHS Trust, London, United Kingdom; [11]Stanford University, Palo Alto, United States of America; [12]University of Paris, Necker-Enfants Malades Hospital, Assistance Publique-Hôpitaux de Paris (AP-HP), Paris, France; [13]University of Regensburg, Regensburg, Germany; [14]University of Paris-Cité, Necker-Enfants Malades Hospital, Assistance Publique-Hôpitaux de Paris (AP-HP), Paris, France; [15]Gemeinschaftsklinikum Mittelrhein, Koblenz, Germany; [16]University of Illinois at Chicago, Chicago, United States of America; [17]Boston University Chobanian & Avedisian School of Medicine, Boston, United States of America; [18]UCSF Benioff Children's Hospital, Oakland, United States of America; [19]Vertex Pharmaceuticals Incorporated, Boston, United States of America; [20]Sarah Cannon Research Institute at The Children's Hospital at TriStar Centennial, Nashville, United States of America;*

### Background

Exagamglogene autotemcel (exa-cel), a one-time, non-viral, *ex vivo* CRISPR/Cas9 gene-edited, autologous cell therapy is approved for treatment of sickle cell disease (SCD) patients aged ≥12 years with recurrent vaso-occlusive crises (VOCs). CLIMB SCD-121, a phase 3 study in participants with severe SCD, has completed and demonstrated the transformative benefits of exa-cel.

### Aims

Evaluate longer-term efficacy and safety of exa-cel in SCD participants aged 12 to 35 years.

### Methods

CLIMB SCD-121 was a 2-year, phase 3 study of exa-cel in participants aged 12 to 35 years with SCD and history of ≥2 VOCs/year for 2 years before screening. Following completion of CLIMB SCD-121, participants enrolled in long-term study CLIMB-131 for up to 15 years follow-up after exa-cel infusion. For CLIMB SCD-121, the primary endpoint was proportion of participants free of severe VOCs for ≥12 consecutive months (VF12); key secondary endpoint was proportion of participants free from inpatient hospitalization for severe VOCs for ≥12 consecutive months (HF12). Data are from July 20, 2025; updated data will be presented.

**Copyright Information:** (Online) ISSN: 2572-9241
©2026 The Author(s). HemaSphere is published by John Wiley & Sons Ltd on behalf of European Hematology Association. This is an open access Abstract Book distributed under the Attribution-NonCommercial-NoDerivs (CC BY-NC-ND), which allows third parties to download the articles and share them with others as long as they credit the author and the Abstract Book, but they cannot change the content in any way or use them commercially.
**Abstract Book Citations:** Authors, Title, HemaSphere, 2026;10:(S1):pages. The abstract book DOIs can be found on the "Author Information" page.
**Disclaimer:** Articles published in the journal HemaSphere exclusively reflect the opinions of the authors. The authors are responsible for all content in their abstracts including accuracy of the facts, statements, citing resources, etc.

**Results**

46 participants (12 adolescents, 34 adults [≥18 years]; mean age: 21.4 years [range: 12, 34]) with mean 4.2 VOCs/year at baseline received exa-cel and had a median follow-up of 3.7 years (range: 0.7, 6.1). The median number of mobilization/apheresis cycles was 2 (range: 1, 6); 92% of adolescents underwent ≤2 cycles as compared to 56% in adults. 45 participants completed CLIMB SCD-121 and enrolled in the ongoing CLIMB-131 trial. All 45 evaluable participants (100%) achieved both VF12 and HF12 with a median VOC-free duration of 3.2 years (range: 1.2, 5.9) and median hospitalization-free duration of 3.4 years (range: 1.6, 5.9) in CLIMB SCD-121 and CLIMB-131 combined. Durable increases in mean HbF to ~40% resulting in normal or near normal levels of total Hb were observed. Allelic editing was stable over time in bone marrow and peripheral blood. Clinically meaningful improvements in hemolysis markers (lactate dehydrogenase, haptoglobin, indirect bilirubin, reticulocyte count) were observed compared to baseline and were maintained in CLIMB-131 for ≥6 years at longest follow-up.
Exa-cel safety was consistent with myeloablative conditioning and autologous transplant. There was one death which occurred 0.7 years after exa-cel infusion, which was previously reported, from respiratory failure due to COVID-19 infection not related to exa-cel. There were no malignancies through follow-up in CLIMB-131.

**Summary/Conclusion**

Exa-cel demonstrated durable clinical benefits in adults and adolescents with severe SCD for up to 6.1 years of follow-up. 100% of participants achieved VOC-free in CLIMB SCD-121 and CLIMB-131 combined. All participants had durable increases in Hb and HbF levels and stable allelic editing. Clinically meaningful improvements were also observed in hemolysis markers. The safety profile is consistent with busulfan myeloablative conditioning and autologous transplant. These results continue to support exa-cel as a potential one-time functional cure for severe SCD.

**Copyright Information:** (Online) ISSN: 2572-9241
©2026 The Author(s). HemaSphere is published by John Wiley & Sons Ltd on behalf of European Hematology Association. This is an open access Abstract Book distributed under the Attribution-NonCommercial-NoDerivs (CC BY-NC-ND), which allows third parties to download the articles and share them with others as long as they credit the author and the Abstract Book, but they cannot change the content in any way or use them commercially.
**Abstract Book Citations:** Authors, Title, HemaSphere, 2026;10:(S1):pages. The abstract book DOIs can be found on the “Author Information” page.
**Disclaimer:** Articles published in the journal HemaSphere exclusively reflect the opinions of the authors. The authors are responsible for all content in their abstracts including accuracy of the facts, statements, citing resources, etc.

### (PF1233) AN EXPLORATORY STUDY OF YTS109 CELL THERAPY IN PATIENTS WITH AUTOIMMUNE HEMOLYTIC ANEMIA

**Topic:** 26. Gene therapy, cellular immunotherapy and vaccination - Clinical

Lele Zhang[1, 2], Ruonan Li[1, 2], Jing Zhang[1, 2], Weiwang Li[1, 2], Jingyu Zhao[1, 2], Xingxin Li[1, 2], Liyun Li[1, 2], Xiao Yu[1, 2], Zhexiang Kuang[1, 2], Yuechen Luo[1, 2], Liwei Fang[1, 2], Neng Nie[1, 2], Meili Ge[1, 2], Yang Li[3], Lei Lei[3], Shasha Zhang[3], Hongli Zheng[3], Xueqiang Zhao[3], Xin Lin[4], Jun Shi*[1, 2]

*[1]Institute of Hematology & Blood Diseases Hospital Chinese Academy of Medical Sciences & Peking Union Medical College, National Clinical Research Center for Blood Diseases, Tianjin, China; [2]Institute of Hematology & Blood Diseases Hospital, Chinese Academy of Medical Sciences & Peking Union Medical College, Red Blood Cell Diseases Center & Regenerative Medicine Clinic, Tianjin, China; [3]Bristar Immunotech Limited, beijing, China; [4]Tsinghua University School of Medicine, Department of Basic Medical Sciences, beijing, China;*

### Background

Autoimmune hemolytic anemia (AIHA) is a rare and heterogeneous autoimmune disorder characterized by the destruction of red blood cells mediated by autoantibodies. Patients with refractory AIHA (rAIHA) who fail multiple lines of therapy have limited treatment options and poor prognosis. Recent studies have demonstrated the potential of autologous CD19-directed CAR-T cell therapy in inducing sustained remission in rAIHA. However, the high cost and complex manufacturing process of autologous CAR-T products limit their widespread application. Allogeneic CAR-T therapies offer a promising alternative by enabling off-the-shelf availability. YTS109 is a novel allogeneic STAR-T product targeting CD19, which has previously demonstrated promising clinical efficacy and a favorable safety profile in patients with severe refractory systemic lupus erythematosus (SLE). Here, we report the first-in-human data of YTS109 in patients with AIHA.

### Aims

To evaluate the safety, tolerability, and preliminary efficacy of YTS109 in patients with AIHA who have failed at least three prior lines of therapy.

### Methods

This is an investigator-initiated, open-label, dose-escalation study (NCT06770504). Patients with rAIHA received a single infusion of YTS109 following lymphodepletion with fludarabine (25-30 mg/m²) and cyclophosphamide (1000 mg/m²). Three dose levels were explored: $0.5\times10^6$/kg (n=1), $1\times10^6$/kg (n=3), and $2\times10^6$/kg (n=3). Safety assessments included monitoring for cytokine release syndrome (CRS), immune effector cell-associated neurotoxicity syndrome (ICANS), and infections. Efficacy was evaluated by hemoglobin levels, hemolytic markers, and transfusion independence. Pharmacokinetics included peripheral blood STAR-T copy numbers and B-cell aplasia duration.

**Copyright Information:** (Online) ISSN: 2572-9241
©2026 The Author(s). HemaSphere is published by John Wiley & Sons Ltd on behalf of European Hematology Association. This is an open access Abstract Book distributed under the Attribution-NonCommercial-NoDerivs (CC BY-NC-ND), which allows third parties to download the articles and share them with others as long as they credit the author and the Abstract Book, but they cannot change the content in any way or use them commercially.
**Abstract Book Citations:** Authors, Title, HemaSphere, 2026;10:(S1):pages. The abstract book DOIs can be found on the “Author Information” page.
**Disclaimer:** Articles published in the journal HemaSphere exclusively reflect the opinions of the authors. The authors are responsible for all content in their abstracts including accuracy of the facts, statements, citing resources, etc.

### Results

As of January 30, 2026, seven patients with rAIHA (median age 45 years, range 24–70 years) had received YTS109 infusion. No dose-limiting toxicities (DLTs) were observed. The safety profile was favorable, with no grade ≥3 CRS or ICANS reported. The most common adverse events were grade 1 CRS (n=3). No severe infections or graft-versus-host disease (GvHD) occurred.
Preliminary efficacy data showed rapid and marked improvement in hemolytic parameters in 6 out of 7 patients. The overall response rate (ORR) was 6/7, with 5 patients achieving complete remission (CR/CRi) and 1 patient achieving partial remission (PR). At data cutoff, the longest responding patient has maintained sustained remission for over 9 months. STAR-T expansion was observed in all patients, with peak levels occurring between Day 4 and Day 7 post-infusion. Nearly all patients achieved B-cell depletion following infusion.

### Summary/Conclusion

This first-in-human study of allogeneic STAR-T therapy (YTS109) in refractory AIHA demonstrates a favorable safety profile and promising clinical activity. These early results support the potential of YTS109 as an off-the-shelf immunotherapeutic option for patients with rAIHA. Longer follow-up and expansion cohorts are warranted to confirm durability and optimize dosing.

**Copyright Information:** (Online) ISSN: 2572-9241
©2026 The Author(s). HemaSphere is published by John Wiley & Sons Ltd on behalf of European Hematology Association. This is an open access Abstract Book distributed under the Attribution-NonCommercial-NoDerivs (CC BY-NC-ND), which allows third parties to download the articles and share them with others as long as they credit the author and the Abstract Book, but they cannot change the content in any way or use them commercially.
**Abstract Book Citations:** Authors, Title, HemaSphere, 2026;10:(S1):pages. The abstract book DOIs can be found on the "Author Information" page.
**Disclaimer:** Articles published in the journal HemaSphere exclusively reflect the opinions of the authors. The authors are responsible for all content in their abstracts including accuracy of the facts, statements, citing resources, etc.

### (PF1234) POLYANTIGENIC DENDRITIC-CELL VACCINATION AGAINST RELAPSED OR REFRACTORY EBV-ASSOCIATED LYMPHOPROLIFERATIVE DISORDERS RESULTS FROM A PHASE I CLINICAL STUDY

**Topic:** 26. Gene therapy, cellular immunotherapy and vaccination - Clinical

Chunrui Li*[1], Di Wang[1], Atsuhiko Hasegawa[2], Huining Liu[3], Peiling Zhang[1], Yuhan Bao[1], Ning An[1], Mei Huang[1], Yi Xiao[1], Donghua Zhang[1], Miao Zheng[1], Wei Huang[1], Lijun Jiang[1]

*[1]Department of Hematology, Tongji Hospital, Tongji Medical College, Huazhong University of Science and Technology, Wuhan, China; [2]Tumor Cell Biology, Clinical Research Institute, National Hospital Organization Kyushu Cancer Center, Fukuoka, Japan; [3]KOUSAI Bio Co., Ltd.,, Shanghai, China;*

### Background

Epstein-Barr virus (EBV) is a highly prevalent human herpesvirus and was the first human tumor associated virus to be discovered. KSD-101 is an autologous dendritic cell (DC) vaccine loaded with EBV related antigen, designed to treat EBV-associated lymphoproliferative disorders (EBV-LPDs).

### Aims

The primary endpoint was safety and tolerability, assessed by the incidence, nature and severity of treatment-related adverse events (TRAEs) and dose-limiting toxicities (DLTs). Secondary endpoint was to explore clinical and immune responses.

### Methods

Eligible patients were aged 18-70 years with histologically or clinically confirmed relapsed/refractory EBV-LPDs. KSD-101 was administered subcutaneously on a schedule of approximately 2-week intervals for 3-5 doses. Dose escalation followed a standard 3 + 3 design.

### Results

Between January 16, 2023 and May 30, 2024, 19 patients (pts) were screened and 14 were enrolled and received at least one dose of KSD-101. Dose escalation evaluated $5.0 \times 10^6$ DCs per dose (n = 3) and $7.5 \times 10^6$ DCs per dose (n = 2). Based on cumulative safety, manufacturing feasibility and immunologic activity, $5.0 \times 10^6$ DCs per dose was selected as the biologically active recommended dose for expansion. In total, 12 pts received $5.0 \times 10^6$ DCs and two received $7.5 \times 10^6$ DCs per dose. KSD-101 was well tolerated at both dose levels, with no DLTs and no grade ≥3 TRAEs. The most frequent TRAEs were injection-site reactions (12/14, 85.7%) and transient fever (9/14, 64.3%), typically occurring within 24 hours of vaccination and resolving after a median of 10 and 3 days, respectively. Data cut-off was September 30, 2025, the median follow-up was 81.7 weeks (range, 7.7-130 weeks), and median overall survival (OS) and progression-free survival (PFS) had not been reached. Two hemophagocytic lymphohistiocytosis pts discontinued from the study due to rapid early-stage disease progression and subsequently received alternative therapeutic interventions. The objective response rate (ORR) was 91.7% (11/12) and the disease control rate (DCR) was 100.0% (12/12). In pts' blood, an early increase in $CD4^+$ T cells and $CD8^+$ T cells by week 4 and B cells by week 6 after vaccinations was observed. At week 6, the regulatory T cells reached a nadir. After infusion, EBV LMP2–specific $CD8^+$ T cells rose rapidly at week 6 and the peak proportion of EBV LMP2–specific $CD8^+$ T cells in each pt (average, 3.58%) was significantly increased ($p < 0.05$) compared to baseline (average, 0.69%).

**Copyright Information:** (Online) ISSN: 2572-9241
©2026 The Author(s). HemaSphere is published by John Wiley & Sons Ltd on behalf of European Hematology Association. This is an open access Abstract Book distributed under the Attribution-NonCommercial-NoDerivs (CC BY-NC-ND), which allows third parties to download the articles and share them with others as long as they credit the author and the Abstract Book, but they cannot change the content in any way or use them commercially.
**Abstract Book Citations:** Authors, Title, HemaSphere, 2026;10:(S1):pages. The abstract book DOIs can be found on the "Author Information" page.
**Disclaimer:** Articles published in the journal HemaSphere exclusively reflect the opinions of the authors. The authors are responsible for all content in their abstracts including accuracy of the facts, statements, citing resources, etc.

**Summary/Conclusion**

AS of now, KSD-101 has demonstrated good safety and efficacy and could activate immune system. Our study highlights its potential as a promising therapeutic agent for the treatment of EBV-LPDs. Larger prospective studies and a longer follow-up period is needed to be conducted. Clinical trial information: NCT05635591.

**Copyright Information:** (Online) ISSN: 2572-9241
©2026 The Author(s). HemaSphere is published by John Wiley & Sons Ltd on behalf of European Hematology Association. This is an open access Abstract Book distributed under the Attribution-NonCommercial-NoDerivs (CC BY-NC-ND), which allows third parties to download the articles and share them with others as long as they credit the author and the Abstract Book, but they cannot change the content in any way or use them commercially.
**Abstract Book Citations:** Authors, Title, HemaSphere, 2026;10:(S1):pages. The abstract book DOIs can be found on the "Author Information" page.
**Disclaimer:** Articles published in the journal HemaSphere exclusively reflect the opinions of the authors. The authors are responsible for all content in their abstracts including accuracy of the facts, statements, citing resources, etc.

### (PF1235) PHASE 1 STUDY OF SL0349, A BISPECIFIC NANOBODY BASED BCMA/GPRC5D CAR-T, IN RELAPSED OR REFRACTORY MULTIPLE MYELOMA

**Topic:** 26. Gene therapy, cellular immunotherapy and vaccination - Clinical

Su Li*[1], Shi'hui Lin[2], Junying Wang[3], Chengwei Jin[1], Wei Liu[1], Wei Sheng[2], Lin Wang[2], Wang Chun[1], Jian Hou[3], Jing Pan[4]

*[1]Liquan Hospital, GoBroad Medical Institute of Hematology (Shanghai Center), Department of Hematology, Shanghai, China; [2]Hebei Senlang Biotechnology Co., Ltd, Hebei, China; [3]Renji Hospital Affiliated With Shanghai Jiao Tong University School of Medicine, Shanghai, China; [4]State Key Laboratory of Experimental Hematology, Department of Hemato-oncology and Immunotherapy, Beijing Gobroad Hospital, Beijing, China;*

### Background

BCMA CAR-T have emerged as breakthrough treatments for patients with relapsed/refractory multiple myeloma (RRMM). However, the development of drug resistance remains inevitable, leaving patients with limited later-line options. SL0349 is a bispecific nanobody-based chimeric antigen receptor T cell (CAR-T) product targeting both BCMA and GPRC5D, engineered with VHH binders and an optimized structure. This dual-targeting strategy aims to overcome antigen escape due to mutation or downregulation of either target. This article reports preliminary data from a currently active phase 1 study (NCT07003568), evaluating the safety and efficacy of this therapeutic approach.

### Aims

This study aimed to evaluate the safety and preliminary efficacy of SL0349 in heavily pretreated patients with RRMM.

### Methods

This open label, single dose, exploratory phase I study was initiated on June 1, 2025, and was carried out at Shanghai Liquan Hospital (Shanghai, China). Eligible patients aged 18 to 75 years had a diagnosis of RRMM. Patients were administered SL0349 at dose of $1.0\times10^6$ cells/kg. The primary objective was to assess safety, defined by adverse events and laboratory abnormalities, as well as the efficacy evaluation was designated as the secondary objective. Analyses of safety and efficacy were done in all patients who received SL0349 according to the protocol.

**Copyright Information:** (Online) ISSN: 2572-9241
©2026 The Author(s). HemaSphere is published by John Wiley & Sons Ltd on behalf of European Hematology Association. This is an open access Abstract Book distributed under the Attribution-NonCommercial-NoDerivs (CC BY-NC-ND), which allows third parties to download the articles and share them with others as long as they credit the author and the Abstract Book, but they cannot change the content in any way or use them commercially.
**Abstract Book Citations:** Authors, Title, HemaSphere, 2026;10:(S1):pages. The abstract book DOIs can be found on the “Author Information” page.
**Disclaimer:** Articles published in the journal HemaSphere exclusively reflect the opinions of the authors. The authors are responsible for all content in their abstracts including accuracy of the facts, statements, citing resources, etc.

### Results

As of February 19, 2026, a total of 18 patients were infused with anti-BCMA/GPRC5D bispecific CAR T cells. The median age was 58.0 years old (Range 36-70). 8 were male and 10 were female patients, with 12 (66.7%) were Caucasian and 6 (33.3%) were Asian respectively. The median number of prior lines of therapy was 3 (range: 2-9). 13 patients (72.2%) were previously treated with stem-cell transplantation (SCT). 9 patients (50.0%) presented with extramedullary disease (EMD), of which 2 patients had central nerves system involvements. Among 12 patients with available cytogenetic assessment, 8 (66.7%) patients were diagnosed with high-risk cytogenetic profile.
The most common grade 3 or worse adverse events were hematologic toxicities in 94.44% (17/18) patients. Cytokine release syndrome occurred in 94.44% (17/18) patients, of which all cases were grade 1-2. Grade 1 immune effector cell-associated neurotoxicity syndrome occurred in 16.67% (3/18) patients. Off-target toxicities potentially related to GPRC5D expression in keratinized tissues were observed, including grade 1 nail changes (11.1%), grade 1 rash (11.1%). No cerebellar toxicities and grade 3 or above organ toxicities were observed.
The overall response rate was 100%, with CR (CR+sCR) rate of 83.3% (15/18). The measurable residual disease negativity rate was achieved in 100% (14/14) among available patients assessed by bone marrow flow cytometry. For patients with EMD, 66.67% (6/9) achieved sCR and 33.33% (3/9) achieved PR. Of the 13 patients previously treated with SCT, 76.92% (10/13) achieved sCR. The median time to response was 17 days (IQR 15-24) post-infusion.
The SL0349 CAR transgene concentrations (mean maximum concentration [$C_{max}$]=535071 copies/μg DNA) showed maximum peripheral expansion at a median of 13.5 (range: 10-21) days.

### Summary/Conclusion

This analysis of the phase 1 study establishes a positive risk–benefit profile for a single low-dose infusion of SL0349, yielding early, deep responses in heavily pretreated RRMM patients, particularly for patients with high-risk cytogenetic features and/or EMD lesions.

**Copyright Information:** (Online) ISSN: 2572-9241
©2026 The Author(s). HemaSphere is published by John Wiley & Sons Ltd on behalf of European Hematology Association. This is an open access Abstract Book distributed under the Attribution-NonCommercial-NoDerivs (CC BY-NC-ND), which allows third parties to download the articles and share them with others as long as they credit the author and the Abstract Book, but they cannot change the content in any way or use them commercially.
**Abstract Book Citations:** Authors, Title, HemaSphere, 2026;10:(S1):pages. The abstract book DOIs can be found on the “Author Information” page.
**Disclaimer:** Articles published in the journal HemaSphere exclusively reflect the opinions of the authors. The authors are responsible for all content in their abstracts including accuracy of the facts, statements, citing resources, etc.

### (PF1237) GENE-MODIFIED AUTOLOGOUS HEMATOPOIETIC STEM CELL THERAPY FOR TRANSFUSION-DEPENDENT Β-THALASSEMIA: A REAL-WORLD STUDY ON CLINICAL EFFICACY AND SAFETY IN 44 CHINESE PATIENTS

**Topic:** 26. Gene therapy, cellular immunotherapy and vaccination - Clinical

Peihong Wang*[1], Jinrong Zhao[1], Shunqing Wang[1]

[1] *Guangzhou First People's Hospital, Department of Hematology, Guangzhou, China;*

### Background

β-Thalassemia is a rare monogenic autosomal recessive hemoglobinopathy caused by mutations in the HBB gene. Its most severe phenotype, transfusion-dependent β-thalassemia (TDT), requires lifelong regular red blood cell (RBC) transfusions, which inevitably lead to severe iron overload and progressive multi-organ damage. In recent years, gene therapy has emerged as a highly promising innovative strategy: by collecting autologous $CD34^+$ hematopoietic stem and progenitor cells (HSPCs), transducing them ex vivo with a lentiviral vector encoding a functional β-globin gene, and reinfusing them into patients, durable cure can be achieved without immunological complications.

### Aims

To comprehensively evaluate the clinical efficacy, hematopoietic reconstitution kinetics, and long-term safety of KL003-based lentiviral gene therapy in a real-world cohort of Chinese patients with TDT.

### Methods

This single-center, retrospective real-world study included 44 TDT patients treated between March 1, 2025, and December 31, 2025. Genotypes were classified as $\beta^0/\beta^0$-like, $\beta^+/\beta^0$, or $\beta^+/\beta^+$. Mobilized peripheral blood $CD34^+$ HSPCs were transduced ex vivo with KL003, a self-inactivating (SIN) lentiviral vector encoding a functional β-globin gene, before reinfusion. Myeloablative conditioning regimens included busulfan (3.2 mg/kg/day × 4 days) or total marrow irradiation (TMI, 2 Gy twice daily on days −4 to −2) combined with fludarabine (Flu, 30 mg/m²/day on days −6 to −3). Primary endpoints were hematopoietic engraftment, overall survival (OS), treatment-related adverse events (TRAEs), and transfusion independence (TI), defined as maintaining Hb ≥90 g/L for ≥6 months without RBC transfusions. Secondary endpoints included time to TI, changes in Hb, vector copy number (VCN) kinetics, and clonal dominance risk.

### Results

Forty-four TDT patients (29 males, 15 females; median age 14 years, range 3–29) were analyzed. Genotype distribution: $\beta^0/\beta^0$-like (n = 10), $\beta^+/\beta^0$ (n = 27), and $\beta^+/\beta^+$ (n = 7). Pre-therapy median annualized transfusion burden was 315.6 mL/kg (range: 77.4–540.7 mL/kg). Twenty-nine patients received busulfan-based conditioning, while 15 received TMI + Flu. As of February 28, 2026, the median follow-up was 5.6 months (range: 2.2–11.4 months), with 100% OS (44/44). Median times to neutrophil (ANC ≥0.5 × $10^9$/L) and platelet (PLT ≥20 × $10^9$/L) engraftment were 15 days (8–22) and 19 days (10–85), respectively. Notably, 43 of 44 patients discontinued RBC transfusions. Among 15 patients with ≥6 months of follow-up, all achieved sustained TI, with a median transfusion-free duration of 7.2 months (range: 6.0–10.2 months). All patients experienced grade ≥3 hematologic toxicity (expected post-conditioning effect). No unexpected adverse events, replication-competent lentivirus (RCL), insertional oncogenesis, clonal dominance, or new malignancies were detected. Incomplete VCN data did not detract from the observed clinical improvement, supporting robust early efficacy.

**Copyright Information:** (Online) ISSN: 2572-9241
©2026 The Author(s). HemaSphere is published by John Wiley & Sons Ltd on behalf of European Hematology Association. This is an open access Abstract Book distributed under the Attribution-NonCommercial-NoDerivs (CC BY-NC-ND), which allows third parties to download the articles and share them with others as long as they credit the author and the Abstract Book, but they cannot change the content in any way or use them commercially.
**Abstract Book Citations:** Authors, Title, HemaSphere, 2026;10:(S1):pages. The abstract book DOIs can be found on the "Author Information" page.
**Disclaimer:** Articles published in the journal HemaSphere exclusively reflect the opinions of the authors. The authors are responsible for all content in their abstracts including accuracy of the facts, statements, citing resources, etc.

### Summary/Conclusion

This real-world study confirms the potential of KL003-based gene therapy in Chinese TDT patients. It provides rapid and stable hematopoietic reconstitution, freeing patients from transfusion dependence and demonstrating a reliable short- to medium-term safety profile. Longer follow-up is needed to verify durable molecular response and long-term safety.

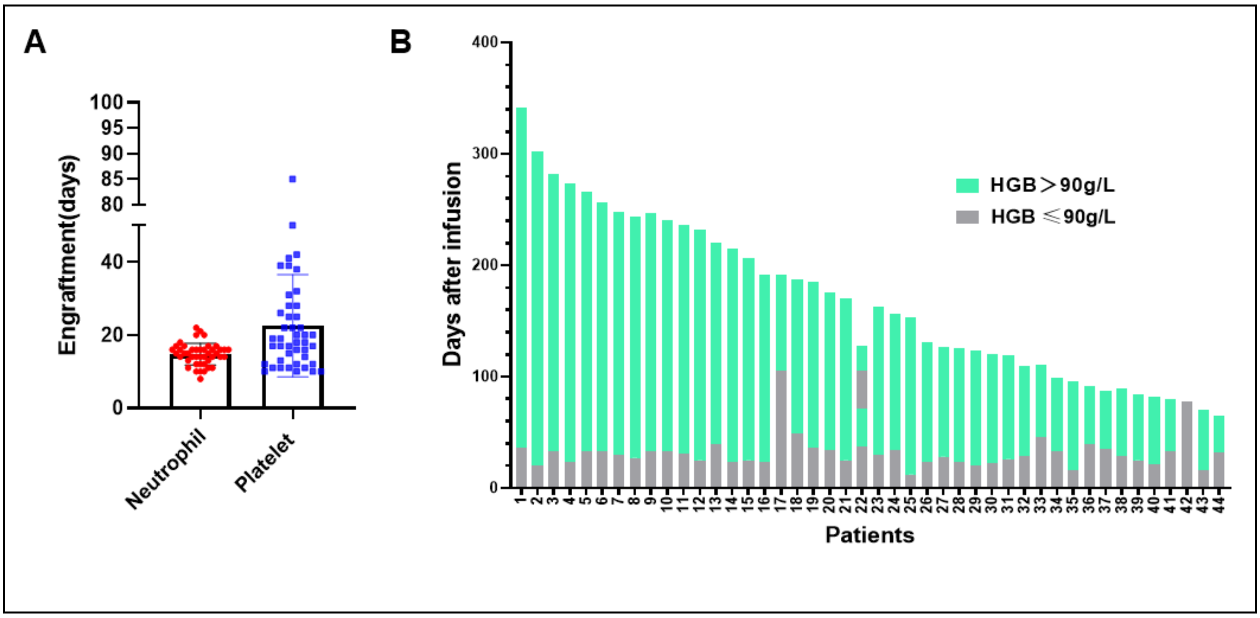

**Copyright Information:** (Online) ISSN: 2572-9241
©2026 The Author(s). HemaSphere is published by John Wiley & Sons Ltd on behalf of European Hematology Association. This is an open access Abstract Book distributed under the Attribution-NonCommercial-NoDerivs (CC BY-NC-ND), which allows third parties to download the articles and share them with others as long as they credit the author and the Abstract Book, but they cannot change the content in any way or use them commercially.
**Abstract Book Citations:** Authors, Title, HemaSphere, 2026;10:(S1):pages. The abstract book DOIs can be found on the “Author Information” page.
**Disclaimer:** Articles published in the journal HemaSphere exclusively reflect the opinions of the authors. The authors are responsible for all content in their abstracts including accuracy of the facts, statements, citing resources, etc.

### (PF1238) EFFICACY AND SAFETY OF ALLOGENEIC CD19-DIRECTED CAR-T CELL THERAPY DERIVED FROM UMBILICAL CORD BLOOD IN RELAPSED/REFRACTORY ACUTE LYMPHOBLASTIC LEUKEMIA

**Topic:** 26. Gene therapy, cellular immunotherapy and vaccination - Clinical

Xiao Wang*[1], Guangcui He[1], Xu-Pai Zhang[1], Sihan Lai[1], Shan Zhang[1], Yan Deng[1], Lei MA[1], Hai Yi[1]

[1] *The General Hospital of Western Theater Command, Chengdu, China;*

### Background

Chimeric antigen receptor (CAR) T-cell therapy has revolutionized the treatment landscape for patients with relapsed/refractory acute lymphoblastic leukemia (R/R ALL), offering unprecedented therapeutic promise. However, autologous CAR-T cells often exhibit functional impairment due to prior lines of therapy, characterized by limited expansion, terminal differentiation, exhaustion, and poor persistence. Furthermore,the individualized manufacturing process is time-consuming and costly, significantly restricting its clinical accessibility.

### Aims

In this study, we evaluated the efficacy and safety of allogeneic universal CD19 CAR-T cells derived from umbilical cord blood (UCB) in patients with R/R ALL.

### Methods

Cryopreserved UCB units from healthy donors were processed with a Pan-T isolation kit. CRISPR/Cas9 was used to disrupt TRBC, HLA-A and HLA-B, followed by lentiviral transduction encoding a CD19-directed CAR and cytomegalovirus IL-10 (cmvIL-10), generating allogeneic CD19 CAR-T cells (UCAR-T). Enrolled R/R ALL patients received lymphodepletion chemotherapy with fludarabine, cyclophosphamide, and etoposide prior to a single intravenous infusion of UCAR-T cells at a dose of 0.5~1 × $10^6$ cells/kg based on body weight. CAR-T cell expansion, bone marrow minimal residual disease (MRD), and adverse events were monitored longitudinally.

### Results

A total of 9 R/R ALL patients aged 14~61 years were enrolled. UCAR-T cell expansion was detected in the peripheral blood of 8 patients, among whom 7 achieved complete remission (CR) or CR with incomplete hematologic recovery (CRi) with concomitant bone marrow MRD negativity. Grade 1–2 cytokine release syndrome (CRS) occurred in 6 patients, and grade 1 immune effector cell-associated neurotoxicity syndrome (ICANS) was observed in 1 patient.

### Summary/Conclusion

UCB-derived UCAR-T cells exhibit robust in vivo expansion and potent antitumor activity. This preliminary clinical study suggests that UCAR-T therapy is associated with favorable efficacy and an acceptable safety profile in patients with R/R ALL, although further validation in large-scale clinical trials is warranted.

**Copyright Information:** (Online) ISSN: 2572-9241
©2026 The Author(s). HemaSphere is published by John Wiley & Sons Ltd on behalf of European Hematology Association. This is an open access Abstract Book distributed under the Attribution-NonCommercial-NoDerivs (CC BY-NC-ND), which allows third parties to download the articles and share them with others as long as they credit the author and the Abstract Book, but they cannot change the content in any way or use them commercially.
**Abstract Book Citations:** Authors, Title, HemaSphere, 2026;10:(S1):pages. The abstract book DOIs can be found on the "Author Information" page.
**Disclaimer:** Articles published in the journal HemaSphere exclusively reflect the opinions of the authors. The authors are responsible for all content in their abstracts including accuracy of the facts, statements, citing resources, etc.

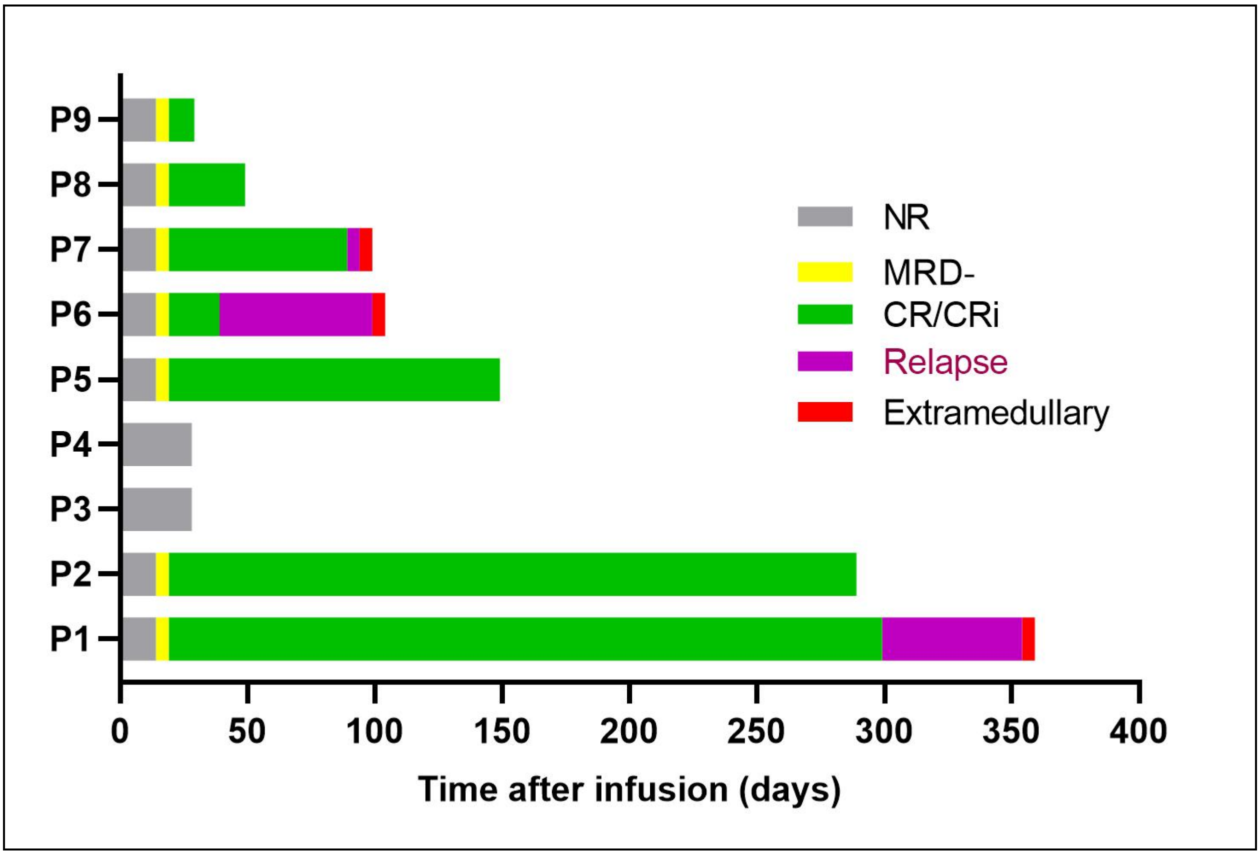

Swimmer plot of time-to-event outcomes for patients with R/R ALL treated with UCAR-T

**Copyright Information:** (Online) ISSN: 2572-9241
©2026 The Author(s). HemaSphere is published by John Wiley & Sons Ltd on behalf of European Hematology Association. This is an open access Abstract Book distributed under the Attribution-NonCommercial-NoDerivs (CC BY-NC-ND), which allows third parties to download the articles and share them with others as long as they credit the author and the Abstract Book, but they cannot change the content in any way or use them commercially.
**Abstract Book Citations:** Authors, Title, HemaSphere, 2026;10:(S1):pages. The abstract book DOIs can be found on the "Author Information" page.
**Disclaimer:** Articles published in the journal HemaSphere exclusively reflect the opinions of the authors. The authors are responsible for all content in their abstracts including accuracy of the facts, statements, citing resources, etc.

### (PF1239) RELAPSED OR REFRACTORY (R/R) EBV-RELATED POST-TRANSPLANT LYMPHOPROLIFERATIVE DISORDERS (PTLD): REAL-LIFE MULTICENTER ITALIAN EXPERIENCE WITH TABELECLEUCEL

**Topic:** 26. Gene therapy, cellular immunotherapy and vaccination - Clinical

Chiara Consoli*[1], Anna Maria Barbui[2], Monica Rondi[2], Chiara Nozzoli[3], Ilaria Cutini[3], Fabrizio Vianello[4], Daniele Avenoso[5], Cristina Tecchio[6], Giorgia Battipaglia[7], Cinzia Pellegrini[8], Enrico Maffini[8], Patrizia Chiusolo[9], Luisa Giaccone[1], Silvia Giorgi[1], Davide Camoirano[1], Michele Clerico[1], Francesca Patriarca[10], Gabriele Facchin[10], Benedetto Bruno[1], Federica Cavallo[1]

*[1]Division of Hematology and Stem Cell Transplant Unit, University of Torino, AOU Città della Salute e della Scienza - Turin, Italy, Department of Molecular Biotechnology and Health Sciences, University of Torino - Turin, Italy, Turin, Italy; [2]Azienda Socio Sanitaria Territoriale Papa Giovanni XXIII - Bergamo, Italy, Bergamo, Italy; [3]SODc Ematologia AAD Trapianto di midollo osseo - Azienda Ospedaliero-Universitaria Careggi - Firenze, Italy, Firenze, Italy; [4]IRCCS Istituto Oncologico Veneto - Oncologia 1 - Padova, Italy, Padova, Italy; [5]Unit of Blood Diseases and Bone Marrow Transplantation, Department of Clinical and Experimental Science, University of Brescia, ASST Spedali Civili di Brescia - Brescia, Italy, Brescia, Italy; [6]Dipartimento di Ingegneria per la Medicina di Innovazione, Sezione di Biomedicina Innovativa, Area Ematologia, Università di Verona, Italy, Verona, Italy; [7]Dipartimento di medicina clinica e chirurgia, Università degli studi di Napoli Federico II – Napoli, Italy, Napoli, Italy,[8]Istituto di Ematologia "Seràgnoli", IRCCS Azienda Ospedaliero-Universitaria di Bologna - Bologna, Italy, Bologna, Italy; [9]Università Cattolica del Sacro Cuore – Roma, Italy, Rome, Italy; [10]Division of Hematology and Stem Cell Transplantation, University Hospital ASUFC, Udine, Italy., Department of Medicine (DMED), University of Udine, Udine, Italy., Udine, Italy;*

### Background

Epstein Barr virus-associated post-transplant lymphoproliferative disease (EBV+PTLD) is a rare complication occurring after either allogeneic stem cell (HSCT) or solid organ transplantation (SOT), with a very poor outcome in relapsed/refractory (R/R) patients. Tabelecleucel is an allogeneic EBV-specific T-cell immunotherapy that targets EBV-positive cells based on human leukocyte antigen (HLA) restriction. The phase 3 ALLELE trial reported an overall response rate (ORR) of 51%, with complete remission (CR) in 28% and 12 months-overall survival (OS) of 84% among responders. Since February 2023, tabelecleucel became available in Italy for adult and paediatric R/R EBV+PTLD patients.

### Aims

This retrospective observational analysis describes the preliminary Italian real-life experience with use of tabelecleucel.

### Methods

We retrospectively analysed adult R/R PTLD patients candidated and referred to treatment with tebelecleucel within the compassionate use program and commercially available pathway.

**Copyright Information:** (Online) ISSN: 2572-9241
©2026 The Author(s). HemaSphere is published by John Wiley & Sons Ltd on behalf of European Hematology Association. This is an open access Abstract Book distributed under the Attribution-NonCommercial-NoDerivs (CC BY-NC-ND), which allows third parties to download the articles and share them with others as long as they credit the author and the Abstract Book, but they cannot change the content in any way or use them commercially.
**Abstract Book Citations:** Authors, Title, HemaSphere, 2026;10:(S1):pages. The abstract book DOIs can be found on the "Author Information" page.
**Disclaimer:** Articles published in the journal HemaSphere exclusively reflect the opinions of the authors. The authors are responsible for all content in their abstracts including accuracy of the facts, statements, citing resources, etc.

### Results

From January 2024 to February 2026, 23 patients were enrolled across 10 Italian centres. The median age at first tabelecleucel dose was 61 years (range 24–76); male and female patients were 12 (63.2%) and 11 (47.8%), respectively. Both allo-HSCT and SOT recipients were included: 10 (43.5%) and 13 (56.5%) patients, respectively. Histology was monomorphic in 90% of cases, with diffuse large B-cell lymphoma accounting for 60%. Extranodal involvement was observed in 18 patients (78.3%), including central nervous system localization in 2 cases. According to the PTLD-adapted prognostic index, 50% of patients were classified as high risk, and 16 patients (69.6%) presented with stage IV disease. The origin of PTLD was documented in 21 of 23 cases: 11 of recipient origin and 10 of donor origin. In 10 cases, this attribution was established based on high-resolution HLA typing. The median time from transplant to PTLD onset was 21 months (range 1-227) and 13 cases (56.5%) had early-onset disease (≤24 months). Median time from PTLD diagnosis to tabelecleucel was 3 months (range 0.9–131), while median time from PTLD relapse to tabelecleucel infusion was 33 days (range 7-78). Eighteen patients (78.3%) received rituximab monotherapy as first-line treatment, one patient benefited from immunoreduction alone as a first-line approach. The median number of tabelecleucel cycles was 1 (range 0–8): 3 patients did not receive tabelecleucel (1 early death, 2 unavailability of product). The ORR was 35%. Among SOT recipients, ORR was 54.5%, with CR in 5 out of 11 patients (45.5%); among HSCT recipients, ORR was 11.1% (only one out of 9 patient achieved CR). No significant treatment-related toxicities were reported. After a median follow-up of 6 months, 11 of 23 patients were alive (7 disease free and 4 with disease), while 10 patients (43.5%) died due to progressive disease.

### Summary/Conclusion

The preliminary Italian experience supports the efficacy and safety of tabelecleucel in patients with prior SOT, consistent with the results of the ALLELE trial, while outcome was worst in the cohort of HSCT recipients. Early referral and timely product allocation remain critical, as delays and product unavailability limited treatment delivery in a subset of patients. Longer follow-up and larger cohorts are needed to better define outcomes, particularly in HSCT recipients. Additional studies are warranted to define prognostic factors influencing response to tabelecleucel.

**Copyright Information:** (Online) ISSN: 2572-9241
©2026 The Author(s). HemaSphere is published by John Wiley & Sons Ltd on behalf of European Hematology Association. This is an open access Abstract Book distributed under the Attribution-NonCommercial-NoDerivs (CC BY-NC-ND), which allows third parties to download the articles and share them with others as long as they credit the author and the Abstract Book, but they cannot change the content in any way or use them commercially.
**Abstract Book Citations:** Authors, Title, HemaSphere, 2026;10:(S1):pages. The abstract book DOIs can be found on the “Author Information” page.
**Disclaimer:** Articles published in the journal HemaSphere exclusively reflect the opinions of the authors. The authors are responsible for all content in their abstracts including accuracy of the facts, statements, citing resources, etc.

### (PF1240) HYPER-FRACTIONATED RADIOTHERAPY AS A BRIDGING STRATEGY ATTENUATES T-CELL EXHAUSTION TO ENHANCE CD19 CAR-T EFFICACY IN RELAPSED/REFRACTORY DIFFUSE LARGE B-CELL LYMPHOMA

**Topic:** 26. Gene therapy, cellular immunotherapy and vaccination - Clinical

Jing Ruan*[1], Wei Zhang[1], Xiaorong Hou[1]

[1] *Peking Union Medical College Hospital, hematology, Beijing, China;*

### Background

Recent clinical studies have confirmed the safety and efficacy of radiotherapy as a bridging strategy prior to CAR-T therapy in R/R DLBCL. Conventional fractionation regimens remain the most commonly adopted, with a daily dose of 3–5 Gy per fraction. Hyper-fractionated radiotherapy, characterized by lower fractional doses and higher treatment frequency, allows effective target-dose delivery within a shorter timeframe while minimizing radiation-related toxicity. Beyond reducing tumor burden, this approach may also remodel the tumor microenvironment and potentiate the efficacy of immunotherapy.

### Aims

We aim to investigate the efficacy and safety of bridging hyper-fractionated radiotherapy in combination with CD19 CAR-T therapy for R/R DLBCL. The potential mechanisms would also be explored.

### Methods

This was a single-center, prospective clinical study (NCT05514327) enrolling patients with R/R DLBCL with measurable lesions. Hyper-fractionated radiotherapy was administered following T-cell apheresis. A total dose of 30 Gy was prescribed to all patients using a hyper-fractionated regimen of 1.5 Gy twice daily over approximately 10 treatment days (weekends excluded). CAR-T cell infusion started within 1-2 weeks after the completion of radiotherapy. The efficacy and adverse reactions at different time points after radiotherapy and CAR-T cell infusion were monitored and analyzed.

### Results

Between June 2022 and June 2025, 25 R/R DLBCL patients have been enrolled in our study. Baseline clinical characteristics are summarized in Table 1. Focal radiotherapy was delivered in 11 patients (44%), and comprehensive regional radiotherapy in 14 patients (56%). All of them achieved PR after the completion of hyper-fractionated radiotherapy. 11 (44%) patients had grade 1 CRS, 6 (24%) had grade 2 CRS and 4 (16%) had grade 3 CRS. 5 (20%) ICANS were recorded, 2 (8%) were grade 1, 2 (8%) were grade 3 and 1 (4%) was grade 4. 22 (88%) had grade 3-4 granulocytopenia and the median duration time was 5.5 (1-16) days. Till submission, the median follow-up time was 17 (1-44) months (Figure 1). At 1-month follow-up, 16/25 (64%) patients achieved CR, 4/25 (16%) had PR, 2/25 (8%) had SD and 3/25 (12%) had PD. The CR rate reached 19/25 (76%) at 6-month follow-up. The estimated 2-year PFS was 63.3% and the 2-year OS was 75%. Patients with TP53 mutations had lower 1-year PFS (0% vs 72.6%, p=0.005) and 1-year OS (0% vs 85.7%, p=0.004[illegible]Figure 2). Cox analysis showed that the ratio of higher exhausted T cells (CD3+PD1+TIM3+T and CD8+PD1+T) before CAR-T infusion were associated with inferior PFS and OS. Patients with CD8+PD1+T ratio[illegible]8.5% also had shorter 1-year PFS (0.125 vs 1, p<0.001) and 1-year OS (0.333 vs 1, p=0.006). Notably, hyper-fractionated radiotherapy significantly reduced both the absolute count ($135\times10^6$/L vs. $39\times10^6$/L, p=0.030) and proportion (15.8% vs. 10.0%, p=0.015) of $CD8^+PD\text{-}1^+$ T cells (Figure 3). No significant differences were observed in other immune cell populations, including Tregs, NK cells, and monocytes.

**Copyright Information:** (Online) ISSN: 2572-9241
©2026 The Author(s). HemaSphere is published by John Wiley & Sons Ltd on behalf of European Hematology Association. This is an open access Abstract Book distributed under the Attribution-NonCommercial-NoDerivs (CC BY-NC-ND), which allows third parties to download the articles and share them with others as long as they credit the author and the Abstract Book, but they cannot change the content in any way or use them commercially.
**Abstract Book Citations:** Authors, Title, HemaSphere, 2026;10:(S1):pages. The abstract book DOIs can be found on the "Author Information" page.
**Disclaimer:** Articles published in the journal HemaSphere exclusively reflect the opinions of the authors. The authors are responsible for all content in their abstracts including accuracy of the facts, statements, citing resources, etc.

### Summary/Conclusion

Hyper-fractionated radiotherapy as a bridging therapy can not only reduce tumor burden, but also reduce exhausted T cells (CD8+PD1+T). The lower exhausted T cells are significantly related to better PFS and OS. These findings suggest that hyper-fractionated radiotherapy acts synergistically with CAR-T therapy by modulating the host immune status, thereby facilitating durable long-term remission in patients with R/R DLBCL.

**Table 1: Clinical characteristics of the DLBCL patients**

| Basic characteristics | Value (N=25) |
|---|---|
| Age yr, median(range) | 55 (27-76) |
| Sex, n(%) | |
| male | 19 (76%) |
| female | 6 (24%) |
| Pathological and molecular characteristics, n(%) | |
| Germinal center type | 9 (36%) |
| Double expressor | 10 (40%) |
| TP53 mutation | 4 (16%) |
| Ann Arbor stage at diagnosis, n(%) | |
| I-II | 8 (32%) |
| III-IV | 17 (68%) |
| Bulky diseases, n(%) | 6 (24%) |
| R-IPI at diagnosis, n(%) | |
| 1-2 | 15 (60%) |
| 3-5 | 10 (40%) |
| Previous treatment | |
| Prior lines of therapy, n median(range) | 2 (1-5) |
| Primary refractory disease, n(%) | 13 (52%) |
| Prior autologous stem cell transplantation, n(%) | 2 (8%) |
| CAR-T cell product, n(%) | |
| relmacabtagene autoleucel | 10 (40%) |
| axicabtagene ciloleucel | 15 (60%) |

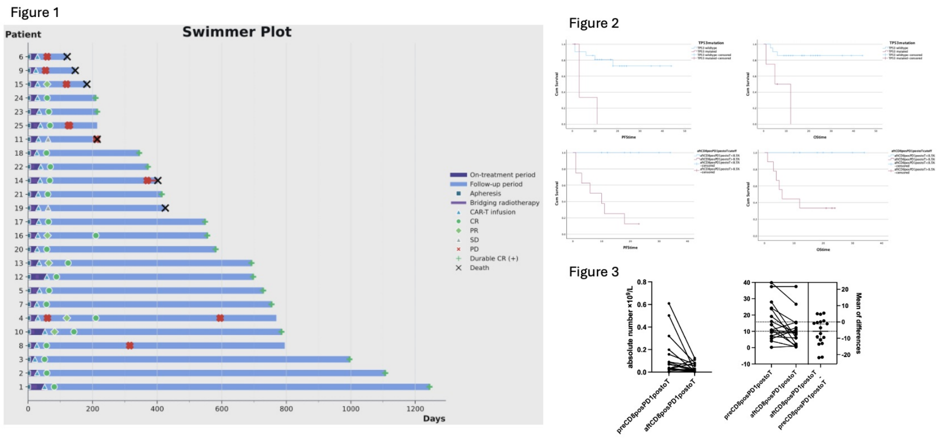

Table 1: the basic characteristics of the enrolled patients; Figure 1. Clinical outcome displayed by the swimmer plot; Figure 2. The Kaplan-Meier survival analysis indicated that TP53 mutation (A-B) and CD8+PD1+T [illegible]8.5% [illegible]C-D[illegible]were associated with inferior PFS and OS; Figure 3. The absolute count and proportion of $CD8^+PD\text{-}1^+$ T cells significantly decreased after hyper-fractionated radiotherapy.

**Copyright Information:** (Online) ISSN: 2572-9241
©2026 The Author(s). HemaSphere is published by John Wiley & Sons Ltd on behalf of European Hematology Association. This is an open access Abstract Book distributed under the Attribution-NonCommercial-NoDerivs (CC BY-NC-ND), which allows third parties to download the articles and share them with others as long as they credit the author and the Abstract Book, but they cannot change the content in any way or use them commercially.
**Abstract Book Citations:** Authors, Title, HemaSphere, 2026;10:(S1):pages. The abstract book DOIs can be found on the "Author Information" page.
**Disclaimer:** Articles published in the journal HemaSphere exclusively reflect the opinions of the authors. The authors are responsible for all content in their abstracts including accuracy of the facts, statements, citing resources, etc.

### (PF1241) SAFETY, CELLULAR KINETICS AND EARLY EFFICACY OF RAPCABTAGENE AUTOLEUCEL (YTB323), A RAPIDLY MANUFACTURED AUTOLOGOUS CD19 CAR-T THERAPY, IN SEVERE, REFRACTORY AUTOIMMUNE DISEASES

**Topic:** 26. Gene therapy, cellular immunotherapy and vaccination - Clinical

Pere Barba*[1], Dinesh Khanna[2], Mi Kwon[3], Sylvain Choquet[4], Edouard Forcade[5], Eva Wagner-Drouet[6], Ralph Wäsch[7], Thomas Pabst[8], Lionel Trueb[9], Luc Matthieu Fornecker[10], Xavier Andrade-Gonzalez[11], Avichai Shimoni[12], Ulf Schnetzke[13], Francesco Saraceni[14], Yunxin Chen[15, 16], Akiyo Yoshida[17], Yasuyuki Arai[18], John Moore[19], Matthew Matasar[20], Rohit Aggarwal[21], Vineeth Varanasi[22], Muhsen Alani[23, 24], Aimee Readie[23], Melissa Fernandes[25], Angeliki Giannelou[23], Yuejia Xu[25], Alexandra Kuznetsova[25], Fernando Kawakami[25], Bibha Das[25], Chi-Yung Sean Lee[26], Ewa Gatlik[25], Susanne Diehl[25], Anubhav Mathur[23], Ana-Laura Jordán Garrote[27], Steve Draikiwicz[23]

*[1]Vall d'Hebron University Hospital, Hematology, Barcelona, Spain;[2]University of Michigan, Ann Arbor, United States of America; [3]Hospital General Universitario Gregorio Marañón, Madrid, Spain;[4]Pitié-Salpêtrière Hospital, APHP Sorbonne Universite, Paris, France; [5]CHU Bordeaux, Pessac, France; [6]University Medical Center Mainz, Mainz, Germany; [7]University Medical Center, Freiburg, Freiburg, Germany; [8]University Hospital Inselspital Bern, Bern, Switzerland; [9]University Hospital of Lausanne (CHUV), Lausanne, Switzerland; [10]Hôpitaux Universitaires de Strasbourg, Strasbourg, France; [11]Avera Cancer Institute, Sioux Falls, United States of America; [12]Chaim Sheba Medical Center, Tel-Hashomer, Israel; [13]Klinik für Innere Medizin II, Abteilung für Hämatologie und Internistische Onkologie, Universitätsklinikum Jena, Jena, Germany; [14]Azienda Ospedaliero Universitaria delle Marche, Ancona, Italy; [15]Singapore General Hospital, Singapore, Singapore; [16]Duke-National University of Singapore, Singapore, Singapore; [17]Kanazawa University, Kanazawa, Japan; [18]Kyoto University, Kyoto, Japan; [19]St. Vincent's Hospital, Sydney, Australia; [20]Rutgers Cancer Institute, New Brunswick, United States of America;[21]University of Pittsburgh Medical Center, Pittsburgh, United States of America; [22]Novartis Healthcare Pvt Ltd, Hyderabad, India; [23]Novartis Pharmaceuticals, East Hanover, United States of America; [24]University of Washington, Department of Medicine, Division of Rheumatology, Seattle, United States of America; [25]Novartis Pharma AG, Basel, Switzerland; [26]Novartis Pharmaceuticals, Cambridge, United States of America; [27]Novartis Farmacéutica S.A., Barcelona, Spain;*

### Background

Chimeric antigen receptor (CAR)-T cell therapy has shown promise in inducing rapid and deep B cell depletion in patients (pts) with severe autoimmune diseases (sAIDs) refractory to available treatments, potentially enabling an 'immune reset' and inducing sustained remission. Rapcabtagene autoleucel (rap-cel) is a rapidly manufactured (next-generation T Charge™ platform), autologous CD19-targeted CAR-T cell therapy that is being investigated for the treatment of sAIDs and hematological malignancies.

### Aims

Safety, cellular kinetics and efficacy outcomes in pts with severe, refractory (sr) idiopathic inflammatory myopathies (IIM), diffuse cutaneous systemic sclerosis (dcSSc) or systemic lupus erythematosus (srSLE: SLE and lupus nephritis [LN]) after a single i.v. infusion of rap-cel.

### Methods

This analysis includes the single-arm safety cohorts of the Phase 2, controlled AUTOGRAPH studies in srIIM (NCT06665256) and srdcSSc (NCT06655896), and the Phase 1/2 single-arm study in srSLE (NCT05798117). Pts underwent immunosuppressant washout prior to leukapheresis. Pts received protocol-defined lymphodepleting chemotherapy (fludarabine and cyclophosphamide), followed by rap-cel infusion. All pts were hospitalized for ≥14 days post-infusion and monitored for adverse events (AEs). Safety, cellular kinetic and efficacy outcomes were assessed.

Copyright Information: (Online) ISSN: 2572-9241
©2026 The Author(s). HemaSphere is published by John Wiley & Sons Ltd on behalf of European Hematology Association. This is an open access Abstract Book distributed under the Attribution-NonCommercial-NoDerivs (CC BY-NC-ND), which allows third parties to download the articles and share them with others as long as they credit the author and the Abstract Book, but they cannot change the content in any way or use them commercially.
Abstract Book Citations: Authors, Title, HemaSphere, 2026;10:(S1):pages. The abstract book DOIs can be found on the "Author Information" page.
Disclaimer: Articles published in the journal HemaSphere exclusively reflect the opinions of the authors. The authors are responsible for all content in their abstracts including accuracy of the facts, statements, citing resources, etc.

**Results**

As of the data cut-off, a total of 33 pts: N=6 (IIM), N=6 (dcSSc), N=21 (srSLE: LN n=12, SLE n=9) were evaluable. Median (range) follow-up post-infusion was 156 (114–270), 170 (142–311), and 330 (126–637) days, respectively. At baseline (BL), 6/6 IIM pts (age range 43–61 years; 3/6 with interstitial lung disease [ILD]) had significant muscle weakness (median manual muscle testing 8 of 129 [range 98–133]); 6/6 dcSSc pts (age range 32–56 years; 3/6 history of ILD) had severe skin involvement (median modified Rodnan skin score [mRSS] of 36.5 [range 34–48]); and 21/21 srSLE pts (age range 22–55 years) had high disease activity (median SLE disease activity index 2000 [SLEDAI-2K] score of 12 [range 6–26]).

Rap-cel was generally well tolerated. Transient Grade (Gr) 3/4 cytopenia post-lymphodepletion (resolving ≤28 days post-infusion) was observed. Cytokine release syndrome occurred in most pts (23/33), all Gr 1 or 2, except 1 Gr 3 (IIM); all resolved without sequelae. Only 1 SLE pt presented an immune cell-associated neurotoxicity syndrome (Gr 2), which resolved within 24 hrs with corticosteroids. AEs, including infections, were manageable and aligned with the safety profile of CAR-T cell therapies (Table 1). A fatal case of pneumococcal meningitis was reported 11 months after treatment (srSLE pt unvaccinated before/after CAR-T therapy), occurring after data cut-off.

Median peak CAR-T cell expansion by flow cytometry was reached 10–12 (range 10–28) days after infusion. Pts demonstrated rapid and deep B cell depletion, followed by B cell recovery (~50–190 days post-infusion) with a predominantly naïve phenotype.

At data cut-off, 5/6 IIM pts achieved moderate–major improvement in Total Improvement Score. In dcSSc, % relative improvement from BL in mRSS and forced vital capacity % predicted in pts with SSc-ILD was
–46% (n=6) and +8% (n=3), respectively. Mean reduction from BL to Month 12 in SLEDAI-2K score was 12.6 (n=10) in srSLE pts.

**Summary/Conclusion**

Rap-cel demonstrates rapid CAR-T *in vivo* expansion, deep B cell depletion, promising initial signs of effective B cell reset and efficacy, and a favorable safety profile in pts with IIM, dcSSc or SLE/LN.

**Copyright Information:** (Online) ISSN: 2572-9241
©2026 The Author(s). HemaSphere is published by John Wiley & Sons Ltd on behalf of European Hematology Association. This is an open access Abstract Book distributed under the Attribution-NonCommercial-NoDerivs (CC BY-NC-ND), which allows third parties to download the articles and share them with others as long as they credit the author and the Abstract Book, but they cannot change the content in any way or use them commercially.
**Abstract Book Citations:** Authors, Title, HemaSphere, 2026;10:(S1):pages. The abstract book DOIs can be found on the "Author Information" page.
**Disclaimer:** Articles published in the journal HemaSphere exclusively reflect the opinions of the authors. The authors are responsible for all content in their abstracts including accuracy of the facts, statements, citing resources, etc.

**Table 1. Adverse events of special interest (AESI) after rap-cel infusion in the severe, refractory IIM, dcSSc and srSLE studies**

| | IIM (N=6) | | dcSSc (N=6) | | srSLE (N=21) | |
|---|---|---|---|---|---|---|
| | **All Grs, n (%)** | **Gr ≥3, n (%)** | **All Grs, n (%)** | **Gr ≥3, n (%)** | **All Grs, n (%)** | **Gr ≥3, n (%)** |
| **Number of patients with ≥1 AESI** | 6 (100.0) | 3 (50.0) | 6 (100.0) | 5 (83.3) | 21 (100.0) | 19 (90.5) |
| **Infections** | 4 (66.7) | 1 (16.7) | 5 (83.3) | 1 (16.7) | 15 (71.4) | 1 (4.8) |
| **Hematological disorders (including cytopenias)** | 3 (50.0) | 3 (50.0) | 4 (66.7) | 4 (66.7) | 19 (90.5) | 18 (85.7) |
| Neutropenia | 3 (50.0) | 3 (50.0) | 4 (66.7) | 3 (50.0) | N/A | 2 (9.5) |
| **Prolonged B cell depletion** | 0 | 0 | 0 | 0 | 2 (9.5) | 1 (4.8) |
| **Hypogammaglobulinemia** | 0 | 0 | 1 (16.7) | 0 | 6 (28.6) | 2 (9.5) |
| Received IVIG treatment | - | - | 0 | - | 4 | 1 |
| **CRS** | 5 (83.3) | 1 (16.7) | 6 (100.0) | 0 | 12 (57.1) | 0 |
| Received tocilizumab | 4 | 1 | 6 | - | 10 | - |
| Received vasopressors | 1 | 1 | 0 | - | 0 | - |
| Required ICU admission | 1 | 0 | 0 | - | 0 | - |
| Median time to onset (range), days[a] | 7 (4–8) | | 6 (5–14) | | 8 (6–15) | |
| Median duration (range), days[a] | 11 (9–14) | | 6 (1–14) | | 3.5 (1–10) | |
| **ICANS** | 0 | 0 | 0 | 0 | 1 (4.8)[b] | 0 |
| Received dexamethasone | - | - | - | - | 1 | - |
| Median time to onset (range), days | - | | - | | 6 | |
| Median duration (range), days | - | | - | | 8[c] | |

[a]Calculated using total number of events; [b]Gr 2; [c]Dexamethasone administered across multiple days (ICANS resolved within 24 hours with treatment).

AESI, adverse events of special interest; CRS, cytokine release syndrome; dcSSc, diffuse cutaneous systemic sclerosis; Gr, Grade; ICANS, immune cell-associated neurotoxicity syndrome; ICU, intensive care unit; IIM, idiopathic inflammatory myopathies; IVIG, intravenous immunoglobulin; N/A, not available; rap-cel, rapcabtagene autoleucel; srSLE, severe refractory systemic lupus erythematosus.

**Copyright Information:** (Online) ISSN: 2572-9241
©2026 The Author(s). HemaSphere is published by John Wiley & Sons Ltd on behalf of European Hematology Association. This is an open access Abstract Book distributed under the Attribution-NonCommercial-NoDerivs (CC BY-NC-ND), which allows third parties to download the articles and share them with others as long as they credit the author and the Abstract Book, but they cannot change the content in any way or use them commercially.
**Abstract Book Citations:** Authors, Title, HemaSphere, 2026;10:(S1):pages. The abstract book DOIs can be found on the "Author Information" page.
**Disclaimer:** Articles published in the journal HemaSphere exclusively reflect the opinions of the authors. The authors are responsible for all content in their abstracts including accuracy of the facts, statements, citing resources, etc.

### (PF1242) MINIMAL CARDIOVASCULAR COMPLICATIONS FOLLOWING CAR T-CELL THERAPY: INSIGHTS FROM REAL-WORLD DATA

**Topic:** 26. Gene therapy, cellular immunotherapy and vaccination - Clinical

Jakob Christoph Voran[1], Carolin Richard[1], Lars Fransecky[1], Hatim Seoudy[1], Manuel Hecht[1], Philipp Nakov[1], Guranda Chitadze[1], Oliver Mueller[1], Cornelia Baden[1], Tim Versteegen[1], Christiane Pott[1], Astrid Dempfle[1], Claudia Baldus[1], Friedrich Stölzel[1], Derk Frank[1], David Baden*[1]

*[1]University Hospital Schleswig-Holstein, Campus Kiel, Kiel, Germany;*

### Background

CAR T-cell therapy has transformed treatment of hematologic malignancies, targeting CD19 in ALL/lymphoma and BCMA in multiple myeloma. Despite high efficacy, complications such as CRS, ICANS, and cardiovascular events pose risks. Population-level data on cardiac outcomes remain limited.

### Aims

To evaluate the frequency and impact of cardiovascular complications after CAR T-cell therapy, we analyzed in-hospital outcomes in Germany (2019-2023), compared them with a U.S. cohort (2019-2024), and examined a patient cohort from our center representing a subset of the German population (2019-2023).

### Methods

German inpatients treated with CAR T cells were identified using OPS codes with data provided by the German federal statistical office DESTATIS. The U.S. cohort included adults with ALL, lymphoma, or multiple myeloma treated with CAR T-cells, identified via TriNetX, a platform offering anonymized EHR data from more than 275 million patients. Underlying diseases, comorbidities, CRS, ICANS, cardiovascular events, and outcomes were defined using ICD and OPS codes. For patients treated at our center, complete health records enabled a more detailed analysis.

### Results

In Germany, 2,545 CAR T-cell cases were identified, predominantly male (63%) with median age of 61 years. CAR-T-usage increased from 163 cases in 2019 to 1,052 in 2023. Major adverse cardiac events (MACE) defined as death, cardiac arrest, cardiogenic shock, and myocardial infarction occurred in 6%, declining from 9% to 4% over time. CRS occurred in 77%, ICANS in 22% with cardiovascular events being too infrequent to correlate with CRS or ICANS in this subgroup.
In the U.S., 1,335 adult CAR T-cell patients were analyzed. MACE within 30 days occurred in 2.7%, and within 3 years, 7% developed new atrial fibrillation/flutter or heart failure, 3% myocardial infarction. Patients with pre-existing cardiac disease had higher cardiovascular event rates and a trend toward lower 3-year survival (57% vs. 69%, p=0.063, Figure 1) while cardiovascular risk factors without manifest disease did not significantly increase event rates. CRS and ICANS occurred in 69% and 16%, respectively, with no significant association to cardiac risk.
At our center, 104 patients were treated with detailed records available. Only three developed symptomatic cancer therapy–related cardiac dysfunction, although 50% met criteria for cardiovascular toxicity, primarily due to elevated high-sensitivity troponin T or NT-proBNP levels.

**Copyright Information:** (Online) ISSN: 2572-9241
©2026 The Author(s). HemaSphere is published by John Wiley & Sons Ltd on behalf of European Hematology Association. This is an open access Abstract Book distributed under the Attribution-NonCommercial-NoDerivs (CC BY-NC-ND), which allows third parties to download the articles and share them with others as long as they credit the author and the Abstract Book, but they cannot change the content in any way or use them commercially.
**Abstract Book Citations:** Authors, Title, HemaSphere, 2026;10:(S1):pages. The abstract book DOIs can be found on the "Author Information" page.
**Disclaimer:** Articles published in the journal HemaSphere exclusively reflect the opinions of the authors. The authors are responsible for all content in their abstracts including accuracy of the facts, statements, citing resources, etc.

**Summary/Conclusion**

Across all cohorts, cardiovascular complications were rare, even as CAR T-cell use increased. Patients with pre-existing cardiac disease showed higher event rates, while those without risk factors had low complication rates. CRS and ICANS did not correlate with cardiovascular events, likely reflecting careful patient selection and management. Since elevated cardiac enzymes do not always reflect lasting injury or future risk, these results suggest possible overclassification in the ESC guideline and highlight the importance of focusing on clinical symptoms. Patients with pre-existing cardiac conditions may benefit from extended monitoring, while CAR T-cell therapy appears safe for adults, including elderly patients, with careful risk management.

**Figure 1: Overall survival of patients in the US CAR T-cell cohort after propensity score matching stratified by cardiac disease or risk factors prior to CAR T-cell therapy**

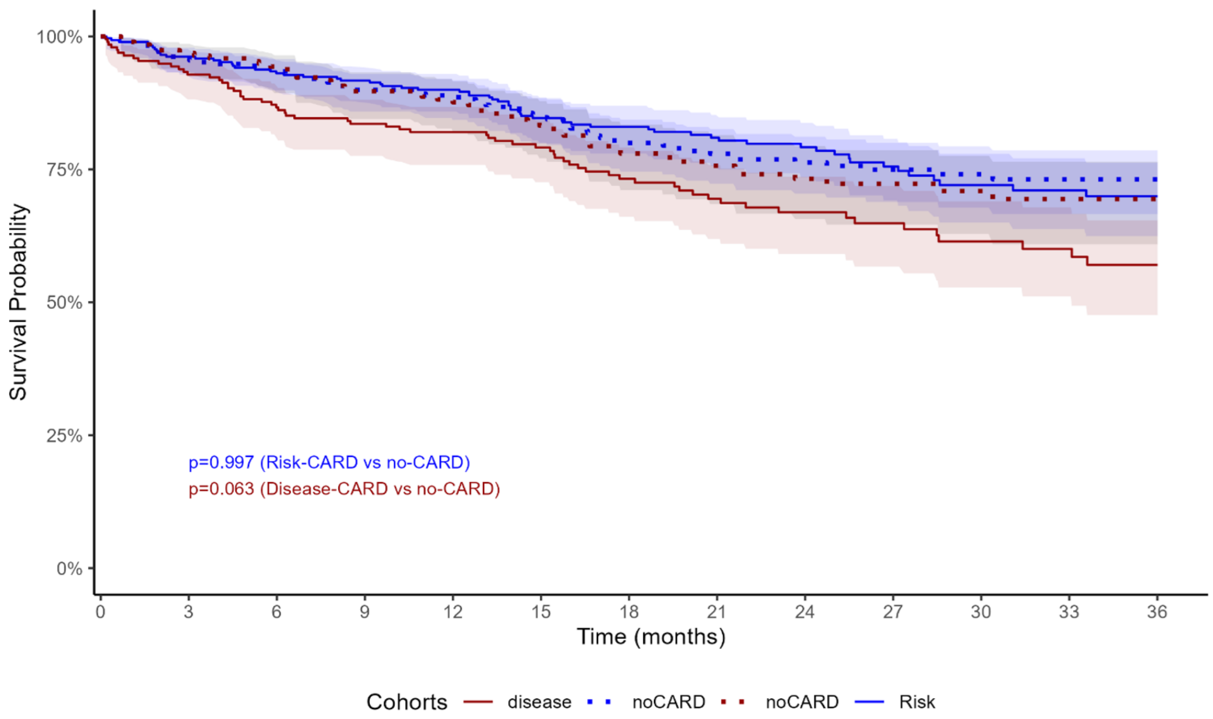

Figure 1: Kaplan-Meier survival analysis of US CAR T-cell patients, starting from therapy initiation. Groups included those with prior cardiac disease (Disease-CARD), those with cardiac risk factors (Risk-CARD), and those without either (No-CARD). After propensity score matching, groups were compared pairwise, with dotted lines representing the corresponding No-CARD cohort in the same color. Median OS was not reached, OS after 3 years was 70% vs 73% (N=290 per group, Risk-CARD vs No-CARD, p=0.997) and 57% vs 69% (N=195 per group, Disease-CARD vs No-CARD, p=0.063).

**Copyright Information:** (Online) ISSN: 2572-9241
©2026 The Author(s). HemaSphere is published by John Wiley & Sons Ltd on behalf of European Hematology Association. This is an open access Abstract Book distributed under the Attribution-NonCommercial-NoDerivs (CC BY-NC-ND), which allows third parties to download the articles and share them with others as long as they credit the author and the Abstract Book, but they cannot change the content in any way or use them commercially.
**Abstract Book Citations:** Authors, Title, HemaSphere, 2026;10:(S1):pages. The abstract book DOIs can be found on the "Author Information" page.
**Disclaimer:** Articles published in the journal HemaSphere exclusively reflect the opinions of the authors. The authors are responsible for all content in their abstracts including accuracy of the facts, statements, citing resources, etc.

### (PF1243) LENTIVIRAL HEMATOPOIETIC STEM AND PROGENITOR CELL GENE THERAPY (ETUVETIDIGENE AUTOTEMCEL) IN PATIENTS AFFECTED BY WISKOTT-ALDRICH SYNDROME: EARLY ACCESS EXPERIENCE

**Topic:** 26. Gene therapy, cellular immunotherapy and vaccination - Clinical

Sabina Cenciarelli*[1, 2, 3], Stefania Giannelli[2], Federico Fraschetta[2], Carmen Caputo[2], Davide Sala[2], Ketty Sinigaglia[2], Mariam Datukishvili[2], Elena Tomasetto[4], Francesca Ciotti[1], Maddalena Fraschini[1], Federica Salerio[2], Camilla Ferri[5], Federica Barzaghi[1, 2], Valeria Calbi[1, 2], Daniele Canarutto[1, 2, 6], Giulia Consiglieri[1, 2], Matteo Doglio[1, 2], Francesca Fumagalli[1, 2], Vera Gallo[1, 2], Maddalena Migliavacca[1, 2], Salvatore Recupero[1, 2, 3], Francesca Tucci[1, 2], Maria Ester Bernardo[1, 2, 6], Maria Pia Cicalese[1, 2, 6], Federica Miotto[4], Alessandro Aiuti[1, 2, 6], Francesca Ferrua[1, 2]

*[1]IRCCS San Raffaele Scientific Institute, Pediatric Immunohematology and Bone Marrow Transplant Unit, Milan, Italy; [2]IRCCS San Raffaele Scientific Institute, San Raffaele – Telethon Institute for Gene Therapy, Milan, Italy; [3]University of Rome Tor Vergata, PhD in Immunology, Molecular Medicine and Applied Biotechnology, Rome, Italy; [4]Fondazione Telethon ETS, Rome, Italy; [5]IRCCS San Raffaele Scientific Institute, Hospital Pharmacy, Milan, Italy; [6]Vita-Salute San Raffaele University, Milan, Italy;*

### Background

The efficacy and safety of a single infusion of Etuvetidigene autotemcel (etu-cel) for the treatment of Wiskott-Aldrich Syndrome (WAS) has been demonstrated by integrated analyses on the clinical development program cohort (including trials NTC01515462 and NTC03837483) (Ferrua et al, ASH 2025). Etu-cel showed a favorable benefit-risk profile, with sustained long-term significant clinical benefit, and became the first gene therapy (GT) to be approved by European Medicines Agency (EMA) and Food and Drug Administration (FDA) for the treatment of WAS, and the first to be licensed by a no-profit foundation. It consists of an autologous lentiviral hematopoietic stem and progenitor cell (HSPC) GT indicated for WAS patients aged 6 months and older who have a mutation in the WAS gene and for whom a hematopoietic stem cell transplant (HSCT) is appropriate, but no suitable matched-related HSPC donor is available.

### Aims

After the completion of the development program, etu-cel has been administered to 9 patients through an early access scheme in Italy. Here we describe their clinical outcome, with a focus on the 7 patients with a minimum follow-up (FU) of 6 months.

### Methods

All the patients presented severe thrombocytopenia (median platelet count pre-GT: $23.5 \times 10^9$/L, range: 9-31). Six patients had significant autoimmunity/autoinflammation signs prior to treatment (inflammatory bowel disease, post-Covid-19 Multisystem Inflammatory Syndrome in Children, vasculitis, severe refractory thrombocytopenia). To note, one patient had already experienced complete rejection of 3 unsuccessful and complicated allogeneic HSCTs followed by infusion of unmanipulated autologous back-up. All patients received rituximab and a reduced intensity conditioning based on fludarabine and busulfan before the infusion of the cryopreserved formulation of etu-cel, based on mobilized peripheral blood HSPC. Median age at treatment was 2 years (0.9-18.4). Drug product (DP)'s characteristics and dose were in line with the clinical development program.

**Copyright Information:** (Online) ISSN: 2572-9241
©2026 The Author(s). HemaSphere is published by John Wiley & Sons Ltd on behalf of European Hematology Association. This is an open access Abstract Book distributed under the Attribution-NonCommercial-NoDerivs (CC BY-NC-ND), which allows third parties to download the articles and share them with others as long as they credit the author and the Abstract Book, but they cannot change the content in any way or use them commercially.
**Abstract Book Citations:** Authors, Title, HemaSphere, 2026;10:(S1):pages. The abstract book DOIs can be found on the "Author Information" page.
**Disclaimer:** Articles published in the journal HemaSphere exclusively reflect the opinions of the authors. The authors are responsible for all content in their abstracts including accuracy of the facts, statements, citing resources, etc.

### Results

At the current FU (median: 6 months, range: 2-24), all patients are alive and in good clinical condition. None presented DP-related adverse events or reactions. All patients achieved engraftment of neutrophils (at a median of 26 days post-GT, range 23-48) and of gene-corrected cells. WASP expression increased in platelets (PLT) and lymphocytes.
Patients with a minimum FU of 6 months showed stable increase of PLT count after treatment. During FU, median PLT count resulted $105x10^9$/L at +3 months (n=6), $123x10^9$/L at +6 months (n=7), $123.5x10^9$/L at +1 year (n=4), $119x10^9$/L at +1.5 year (n=4) and $228x10^9$/L at +2 years (n=1). All patients are PLT-transfusion independent from the early post-GT phase and no moderate or severe bleeding events were recorded. Two patients received a thrombopoietin receptor agonist in the initial post-engraftment phase (PLT count censored accordingly). No severe infections were registered after 6 months post-GT. Antimicrobial prophylaxes were stopped in 3 patients and immunoglobulin replacement in 4 patients at +1 year FU, approximately. Eczema improved in all patients and disappeared starting from +1 year FU. No signs of new or previous autoimmunity were detected post-GT.

### Summary/Conclusion

After treatment, all patients experienced clinical benefit, with progressive improvement or resolution of WAS manifestations. In this early access scenario, etu-cel has confirmed the effectiveness and safety already shown in the clinical development program.

**Copyright Information:** (Online) ISSN: 2572-9241
©2026 The Author(s). HemaSphere is published by John Wiley & Sons Ltd on behalf of European Hematology Association. This is an open access Abstract Book distributed under the Attribution-NonCommercial-NoDerivs (CC BY-NC-ND), which allows third parties to download the articles and share them with others as long as they credit the author and the Abstract Book, but they cannot change the content in any way or use them commercially.
**Abstract Book Citations:** Authors, Title, HemaSphere, 2026;10:(S1):pages. The abstract book DOIs can be found on the "Author Information" page.
**Disclaimer:** Articles published in the journal HemaSphere exclusively reflect the opinions of the authors. The authors are responsible for all content in their abstracts including accuracy of the facts, statements, citing resources, etc.

### (PF1244) PREDICTIVE PERFORMANCE OF MODELS FOR ICAHT IN REAL-WORLD COHORTS OF RELAPSED/REFRACTORY B-CELL MALIGNANCY TREATED WITH CAR-T

**Topic:** 26. Gene therapy, cellular immunotherapy and vaccination - Clinical

Yuting Gong*[1], Yafang Guo[2], Zekai Mao[1], Li Zhu[1], Yicheng Zhang[1], Xiaoxi Zhou[1], Jia Wei[1]

[1]*Tongji Hospital, Tongji Medical College, Huazhong University of Science and Technology, Hematology, Wuhan, Hubei, China;* [2]*Shanxi Bethune Hospital, Shanxi Academy of Medical Sciences, Third Hospital of Shanxi Medical University, Tongji Shanxi Hospital, Taiyuan, China;*

### Background

Chimeric antigen receptor T-cell (CAR-T) therapy has significantly improved outcomes in relapsed or refractory hematologic malignancies but is frequently complicated by immune effector cell–associated hematotoxicity (ICAHT), a common and clinically significant toxicity characterized by severe cytopenias. Several predictive models, including CAR-HT, ALL-HT, eIPM (pre/post), and EASIX, have been proposed to identify patients at high risk. However, these models were developed in different disease contexts, and their comparative performance for predicting severe early and late ICAHT in real-world practice requires further validation in broader cohorts.

### Aims

This study aimed to compare the performance of five predictive models (CAR-HT, ALL-HT, eIPMpre, eIPMpost, and EASIX) in predicting severe early and late ICAHT in real-world patients with relapsed/refractory B-NHL and ALL/LBL treated with CAR-T.

### Methods

This real-world study included 557 patients with relapsed/refractory B-NHL and ALL/LBL who received CAR-T at Tongji Hospital, Tongji Medical College, Huazhong University of Science and Technology between June 2016 and May 2025. Patients who died or received additional systemic anti-tumor therapy before day 30 were excluded. ICAHT was graded according to 2023 EHA/EBMT criteria. CAR-HT, eIPMpre, eIPMpost, and EASIX were calculated in all patients, and ALL-HT in ALL/LBL cases. Predictive performance for grade 4 early and late ICAHT was assessed using ROC analysis and multivariable logistic regression, with AUC comparison by the DeLong test.

### Results

In the B cell lymphoma cohort, both CAR-HT and eIPMpost effectively predicted early grade 4 ICAHT, with CAR-HT emerging as the most robust model for early ICAHT, retaining independent predictive value in multivariable analysis (adjusted AUC = 0.814). In terms of late ICAHT, all four models demonstrated predictive ability, among which eIPMpost showed the best discriminatory performance (adjusted AUC = 0.854). Notably, the predictive effect of EASIX for early ICAHT was attenuated in the multivariable model, but it remained significant for late events. In the B-ALL/LBL cohort, predictive performance for early grade 4 ICAHT was uniformly strong across models, with ALL-HT demonstrating the best overall discrimination. For early ICAHT, the ALL-HT model exhibited the strongest predictive ability (adjusted AUC = 0.890, OR = 8.482). The CAR-HT, eIPMpre, eIPMpost, and EASIX models also demonstrated acceptable predictive value. For late ICAHT, no reliable predictions were made due to the limited sample size and statistical power.

**Copyright Information:** (Online) ISSN: 2572-9241
©2026 The Author(s). HemaSphere is published by John Wiley & Sons Ltd on behalf of European Hematology Association. This is an open access Abstract Book distributed under the Attribution-NonCommercial-NoDerivs (CC BY-NC-ND), which allows third parties to download the articles and share them with others as long as they credit the author and the Abstract Book, but they cannot change the content in any way or use them commercially.
**Abstract Book Citations:** Authors, Title, HemaSphere, 2026;10:(S1):pages. The abstract book DOIs can be found on the "Author Information" page.
**Disclaimer:** Articles published in the journal HemaSphere exclusively reflect the opinions of the authors. The authors are responsible for all content in their abstracts including accuracy of the facts, statements, citing resources, etc.

### Summary/Conclusion

In this real-world cohort, ICAHT risk prediction after CAR-T was disease-specific. CAR-HT and eIPMpost were most appropriate for early and late ICAHT in B-cell lymphoma, respectively, and ALL-HT for early ICAHT in B-ALL/LBL.

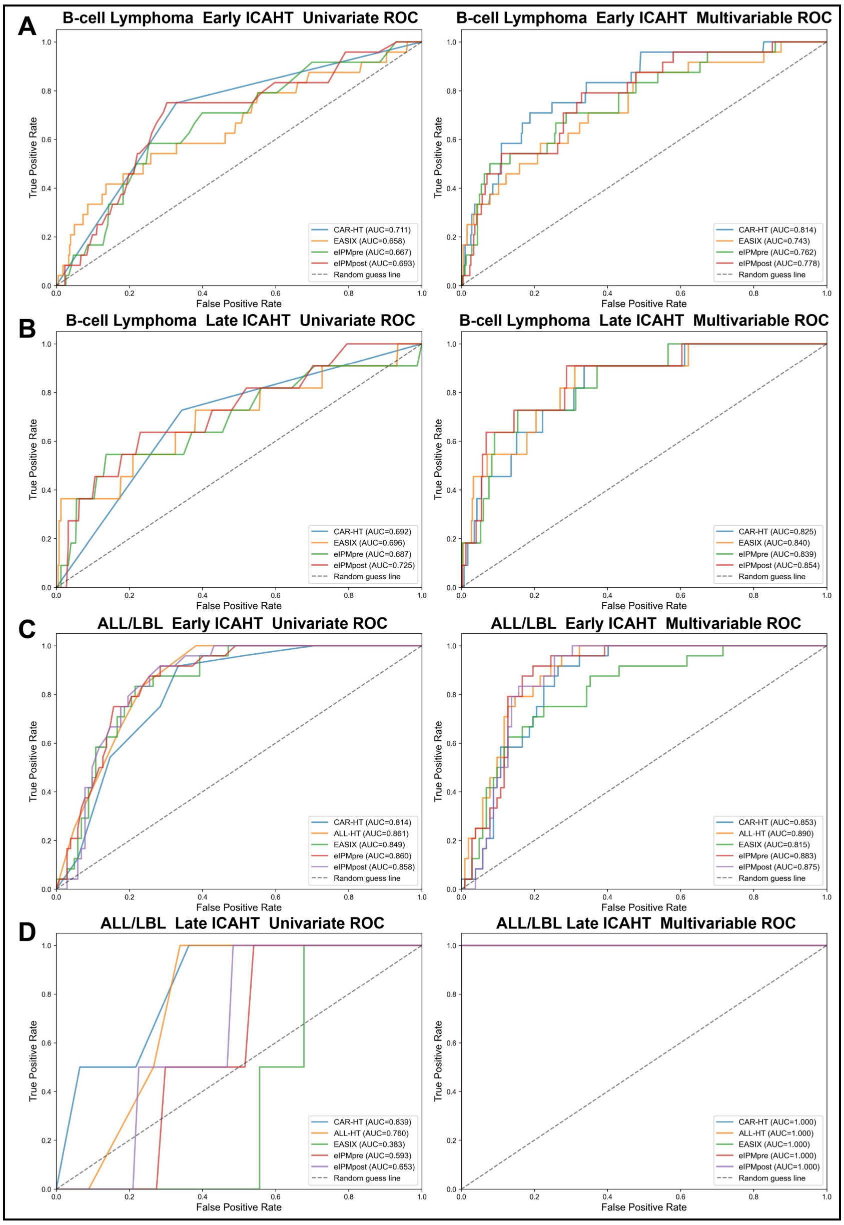

Figure 1. Predictive performance of models for early and late ICAHT in B-NHL and ALL/LBL patients treated with CAR-T. (A)ROC curves for early ICAHT within 30 days in B-cell lymphoma. (B)ROC curves for late ICAHT beyond 30 days in B-cell lymphoma. (C)ROC curves for early ICAHT within 30 days in ALL/LBL. (D)ROC curves for late ICAHT beyond 30 days in ALL/LBL.

**Copyright Information:** (Online) ISSN: 2572-9241
©2026 The Author(s). HemaSphere is published by John Wiley & Sons Ltd on behalf of European Hematology Association. This is an open access Abstract Book distributed under the Attribution-NonCommercial-NoDerivs (CC BY-NC-ND), which allows third parties to download the articles and share them with others as long as they credit the author and the Abstract Book, but they cannot change the content in any way or use them commercially.
**Abstract Book Citations:** Authors, Title, HemaSphere, 2026;10:(S1):pages. The abstract book DOIs can be found on the "Author Information" page.
**Disclaimer:** Articles published in the journal HemaSphere exclusively reflect the opinions of the authors. The authors are responsible for all content in their abstracts including accuracy of the facts, statements, citing resources, etc.

### (PF1245) L-IPM: A NOVEL MODEL FOR PREDICTING LATE NEUTROPENIA IMMUNE EFFECTOR CELL-ASSOCIATED HEMATOTOXICITY (N-ICAHT) AFTER CAR-T THERAPY WITH MULTICENTER VALIDATION

**Topic:** 26. Gene therapy, cellular immunotherapy and vaccination - Clinical

Ao-Ran Zhang*[1], Zihong Cai[2], Xin-Le Wang[1], Tong Su[1], Guohuizhi Ye[2], Bailin He[2], Xuying Pei[1], Xiangyu Zhao[1], Zhiling Yan[3], Hongsheng Zhou[2], Meng Lv[1]

*[1]Peking University People's Hosptial, Beijing, China; [2]Nanfang Hospital Affiliated to Southern Medical University, Guangzhou, China; [3]Affiliated Hospital of Xuzhou Medical University, Xuzhou, China;*

### Background

Late (≥day 30) neutropenia–associated immune effector cell–associated hematotoxicity (N-ICAHT) is a clinically significant complication after CAR-T therapy and is associated with infection and inferior outcomes. Existing models, including **CAR-HEMATOTOX(CAR-HT), ALL-HEMATOTOX(ALL-HT), and eIPM**, were primarily developed for **early toxicity** and show limited utility in predicting **late N-ICAHT**. Effective risk stratification tools for late events remain lacking.

### Aims

To develop and multicenter-validate a predictive model for late N-ICAHT using routinely available clinical variables.

### Methods

A consecutive cohort of 135 patients who received commercial CAR-T products (brexucabtagene autoleucel) or investigational CD19-, BCMA-, or CD7-targeted CAR-T therapies between 2022 and 2025 was included as the derivation cohort. An independent external validation cohort comprised 70 patients from two additional centers who received CD19- or CD7-targeted CAR-T therapy. Multivariable logistic regression was used to construct a **point-based risk score (Late Inflammatory Prognostic Model score, L-IPM$_{score}$)**, while LASSO regression was applied to develop a **machine learning–based model (L-IPM$_{index}$)** for predicting grade 3–4 late N-ICAHT. Candidate variables included age, sex, diagnosis, prior HSCT, CRS grade, ICANS grade, IEC-HS, early N-ICAHT grade, and log-transformed peak ferritin, IL-6, and D-dimer levels within 0–30 days post-infusion. Variables with *P*<0.10 in univariate analysis were entered into LASSO regression, with the optimal lambda selected by cross-validation (λ=0.033). Both models were externally validated in a multicenter cohort of 70 patients. Model discrimination was assessed using ROC analysis and compared using DeLong's test.

**Copyright Information:** (Online) ISSN: 2572-9241
©2026 The Author(s). HemaSphere is published by John Wiley & Sons Ltd on behalf of European Hematology Association. This is an open access Abstract Book distributed under the Attribution-NonCommercial-NoDerivs (CC BY-NC-ND), which allows third parties to download the articles and share them with others as long as they credit the author and the Abstract Book, but they cannot change the content in any way or use them commercially.
**Abstract Book Citations:** Authors, Title, HemaSphere, 2026;10:(S1):pages. The abstract book DOIs can be found on the "Author Information" page.
**Disclaimer:** Articles published in the journal HemaSphere exclusively reflect the opinions of the authors. The authors are responsible for all content in their abstracts including accuracy of the facts, statements, citing resources, etc.

| Characteristics | Derivation Cohort (n=135) | External Validation (n=70) | P Value |
|---|---|---|---|
| Age, median (IQR) | 41 (25-55) | 30 (21-44) | **<0.001*** |
| **Diagnosis** | | | **<0.001*** |
| **B-ALL/NHL, n(%)** | 73(54) | 63 (90) | |
| **T-ALL/NHL, n(%)** | 28 (21) | 7 (10) | |
| **MM, n(%)** | 34(25) | NA | |
| **Prior HSCT, Yes, n(%)** | 49(36) | 16 (23) | **0.050*** |
| **Early N-ICAHT, n(%)** | | | 0.29 |
| **1** | 41 (30) | 23 (33) | |
| **2** | 22 (16) | 14 (20) | |
| **3** | 47 (35) | 18 (26) | |
| **4** | 25 (19) | 15 (21) | |
| **CRS 0-1, n(%)** | 81 (60) | 30 (43) | **0.019*** |
| **CRS 2+, n(%)** | 54 (40) | 40 (57) | |
| **ICANS 0-1, n(%)** | 128 (95) | 67 (96) | 0.78 |
| **ICANS 2+, n(%)** | 7 (5) | 3 (4) | |

**Copyright Information:** (Online) ISSN: 2572-9241
©2026 The Author(s). HemaSphere is published by John Wiley & Sons Ltd on behalf of European Hematology Association. This is an open access Abstract Book distributed under the Attribution-NonCommercial-NoDerivs (CC BY-NC-ND), which allows third parties to download the articles and share them with others as long as they credit the author and the Abstract Book, but they cannot change the content in any way or use them commercially.
**Abstract Book Citations:** Authors, Title, HemaSphere, 2026;10:(S1):pages. The abstract book DOIs can be found on the "Author Information" page.
**Disclaimer:** Articles published in the journal HemaSphere exclusively reflect the opinions of the authors. The authors are responsible for all content in their abstracts including accuracy of the facts, statements, citing resources, etc.

**Results**

The L-IPM$_{score}$ incorporated four variables assessed within days 0–30 post-infusion: **peak D-dimer, IL-6, ferritin, and early N-ICAHT grade. One point was assigned for each variable exceeding predefined cutoffs (D-dimer ≥5,000 ng/mL; IL-6 ≥168 pg/mL; ferritin ≥2,000 ng/mL; early N-ICAHT grade 3–4).** Patients were stratified into **low- (0–1)** and **high-risk (≥2)** groups for late N-ICAHT.

The **LASSO-derived model (L-IPM$_{index}$) was defined as: 0.427 × (early N-ICAHT grade) + 0.155 × ln(peak IL-6) + 0.001 × ln(peak ferritin) – 2.330.**
In the derivation cohort, ROC analysis demonstrated incremental improvement across models. CAR-HEMATOTOX (AUC 0.673) and eIPMpre (AUC 0.689) showed modest discrimination. The **L-IPM$_{index}$ improved predictive accuracy (AUC 0.728), while the L-IPM$_{score}$ achieved the highest performance (AUC 0.887).** DeLong's test confirmed the superiority of L-IPM$_{score}$ over all comparator models (all $P$<0.001) **(Figure)**.
In the multicenter validation cohort, L-IPM$_{index}$ (AUC 0.637) and L-IPM$_{score}$ (AUC 0.604) demonstrated comparable performance to CAR-HT (0.558), ALL-HT (0.581), eIPMpre (0.605), and eIPMpost (0.646).

**Summary/Conclusion**

The L-IPM system represents a practical and user-friendly tool for predicting **late N-ICAHT after CAR-T therapy.** Further validation in larger, prospective cohorts is warranted.

**Copyright Information:** (Online) ISSN: 2572-9241
©2026 The Author(s). HemaSphere is published by John Wiley & Sons Ltd on behalf of European Hematology Association. This is an open access Abstract Book distributed under the Attribution-NonCommercial-NoDerivs (CC BY-NC-ND), which allows third parties to download the articles and share them with others as long as they credit the author and the Abstract Book, but they cannot change the content in any way or use them commercially.
**Abstract Book Citations:** Authors, Title, HemaSphere, 2026;10:(S1):pages. The abstract book DOIs can be found on the "Author Information" page.
**Disclaimer:** Articles published in the journal HemaSphere exclusively reflect the opinions of the authors. The authors are responsible for all content in their abstracts including accuracy of the facts, statements, citing resources, etc.

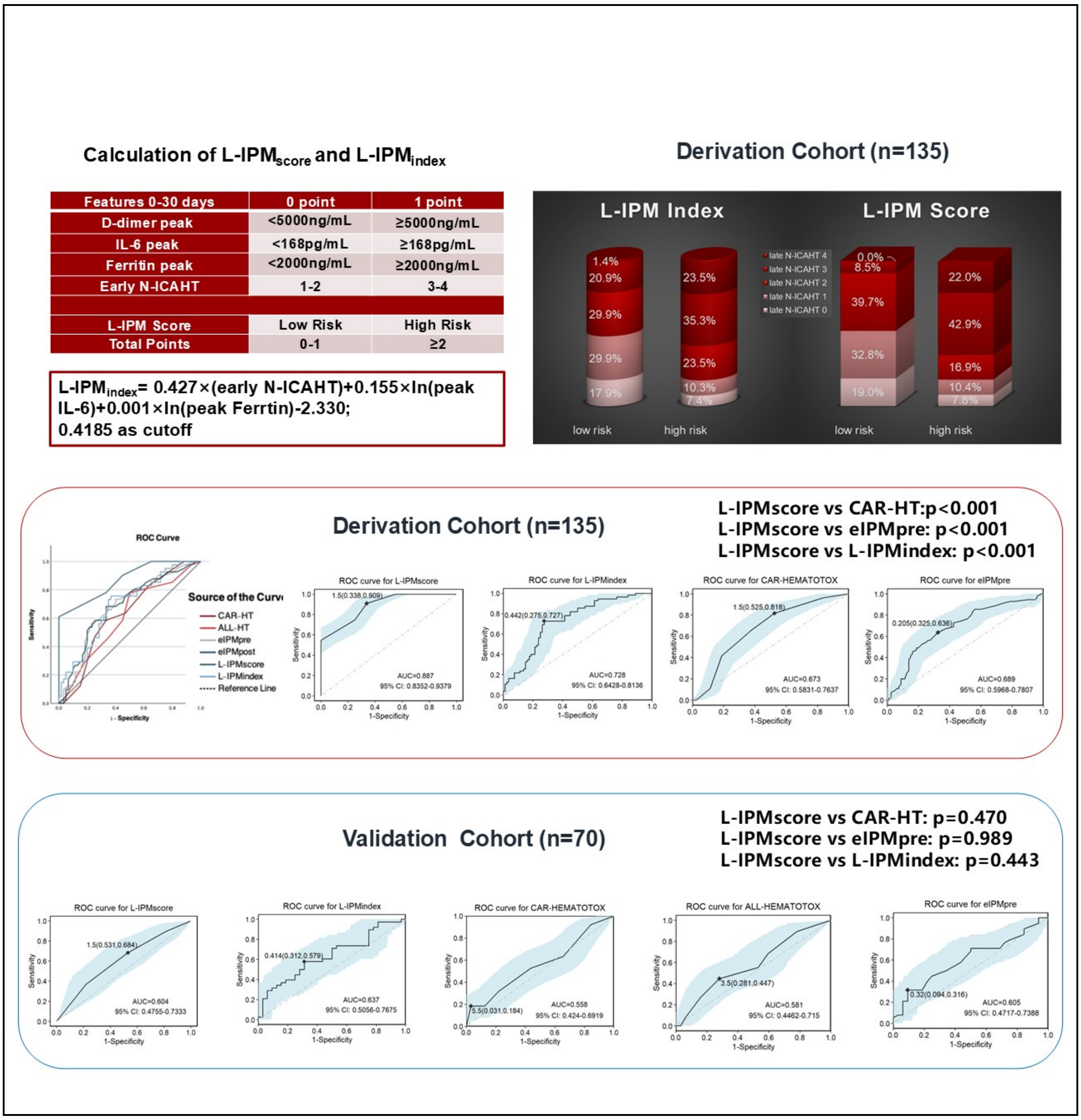

**Copyright Information:** (Online) ISSN: 2572-9241
©2026 The Author(s). HemaSphere is published by John Wiley & Sons Ltd on behalf of European Hematology Association. This is an open access Abstract Book distributed under the Attribution-NonCommercial-NoDerivs (CC BY-NC-ND), which allows third parties to download the articles and share them with others as long as they credit the author and the Abstract Book, but they cannot change the content in any way or use them commercially.
**Abstract Book Citations:** Authors, Title, HemaSphere, 2026;10:(S1):pages. The abstract book DOIs can be found on the "Author Information" page.
**Disclaimer:** Articles published in the journal HemaSphere exclusively reflect the opinions of the authors. The authors are responsible for all content in their abstracts including accuracy of the facts, statements, citing resources, etc.

### (PF1246) SECONDARY PERIPHERAL T-CELL LYMPHOMA IN A PATIENT OF DLBCL HARBORING CANCER SUSCEPTIBILITY GENE MUTATION FOLLOWING CD19/CD22 BISPECIFIC CAR-T CELL THERAPY

**Topic:** 26. Gene therapy, cellular immunotherapy and vaccination - Clinical

Xindi Liu[1], Qinlong Zheng[2], Liang Wang*[1]

[1]*Beijing Tongren Hospital, Capital Medical University, Beijing, China;*[2]*Beijing Gaobo Boren Hospital, Beijing, China;*

### Background

Chimeric antigen receptor (CAR)-T cell therapy has revolutionized treatment for relapsed/refractory B-cell malignancies, but rare secondary T-cell malignancies have emerged as a serious safety concern.

### Aims

We report a case of secondary peripheral T-cell lymphoma (PTCL) definitively proven to originate from infused CD19/CD22 bispecific CAR-T cells, with comprehensive molecular characterization of oncogenic mechanisms.

### Methods

A 66-year-old woman with diffuse large B-cell lymphoma (DLBCL) and history of breast and cervical cancers received autologous CD19/CD22 CAR-T cells in November 2024 following CNS relapse. Nine months post-infusion, she developed a lower lip mass diagnosed as PTCL, not otherwise specified (PTCL-NOS). Comparative genomic analysis of the CAR-T product and PTCL tissue was performed. Whole genome sequencing (WGS), T-cell receptor (TCR) clonality assessment, single-cell RNA sequencing (scRNA-seq), and targeted mutation analysis were conducted on pre-infusion CAR-T product, peripheral blood mononuclear cells (PBMCs), and PTCL tissue.

### Results

TCR sequencing identified identical clonal rearrangements (*TRBD*101/*TRBJ2701 and TRGV801/TRGJ101) in both CAR-T product and PTCL, confirming clonal origin. WGS revealed pathogenic somatic mutations in TET2 (p.R1261H, p.E1755*) with higher variant allele frequencies in PTCL versus CAR-T product, indicating pre-existing mutations with subsequent clonal expansion. The patient harbored a germline *BLM* p.L107Ffs*36 mutation, contributing to genomic instability. Additional oncogenic events included del(11)(q23.2q25) involving *KMT2A* and *ATM*, loss of chromosome X, and clonal CAR vector integrations within tumor suppressor genes (*NF1*, *CBX5*, *RNF213*). scRNA-seq and flow cytometry of the CAR-T product showed no malignant population at infusion, excluding pre-existing malignancy.

### Summary/Conclusion

This case provides unequivocal molecular evidence of CAR-T cell malignant transformation driven by three convergent factors: (1) pre-existing *TET2* driver mutations representing clonal hematopoiesis, (2) germline *BLM* deficiency exacerbating genomic instability, and (3) insertional mutagenesis from CAR vector integration. These findings underscore the critical importance of pre-infusion genomic screening of CAR-T products for CHIP-related mutations, cancer susceptibility gene testing in patients with prior malignancies, and lifelong surveillance for secondary malignancies in CAR-T recipients.

**Copyright Information:** (Online) ISSN: 2572-9241
©2026 The Author(s). HemaSphere is published by John Wiley & Sons Ltd on behalf of European Hematology Association. This is an open access Abstract Book distributed under the Attribution-NonCommercial-NoDerivs (CC BY-NC-ND), which allows third parties to download the articles and share them with others as long as they credit the author and the Abstract Book, but they cannot change the content in any way or use them commercially.
**Abstract Book Citations:** Authors, Title, HemaSphere, 2026;10:(S1):pages. The abstract book DOIs can be found on the "Author Information" page.
**Disclaimer:** Articles published in the journal HemaSphere exclusively reflect the opinions of the authors. The authors are responsible for all content in their abstracts including accuracy of the facts, statements, citing resources, etc.

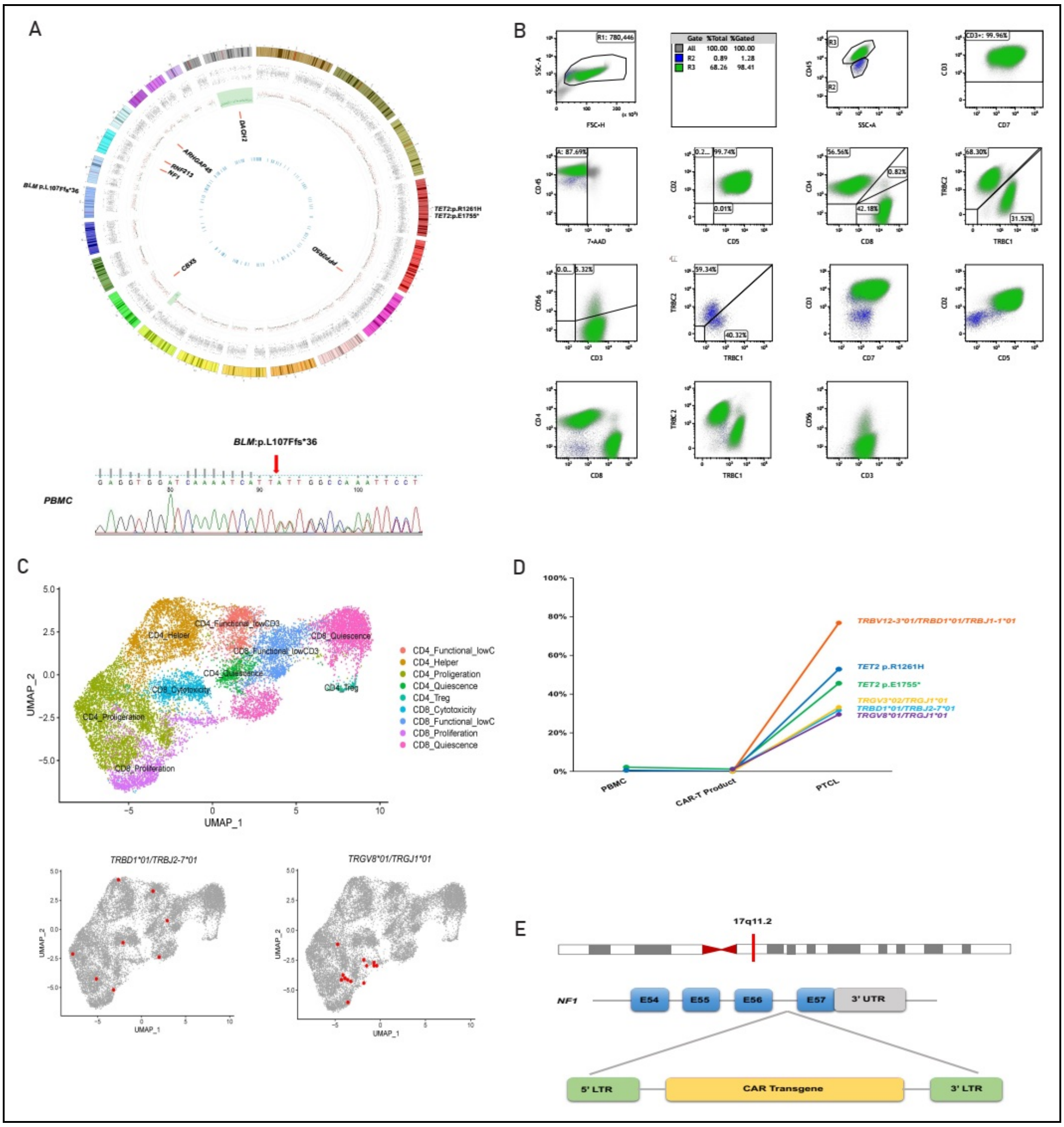

**Copyright Information:** (Online) ISSN: 2572-9241
©2026 The Author(s). HemaSphere is published by John Wiley & Sons Ltd on behalf of European Hematology Association. This is an open access Abstract Book distributed under the Attribution-NonCommercial-NoDerivs (CC BY-NC-ND), which allows third parties to download the articles and share them with others as long as they credit the author and the Abstract Book, but they cannot change the content in any way or use them commercially.
**Abstract Book Citations:** Authors, Title, HemaSphere, 2026;10:(S1):pages. The abstract book DOIs can be found on the "Author Information" page.
**Disclaimer:** Articles published in the journal HemaSphere exclusively reflect the opinions of the authors. The authors are responsible for all content in their abstracts including accuracy of the facts, statements, citing resources, etc.

### (PF1247) OUT-OF-SPECIFICATION ANTI-BCMA CAR-T IN HEAVILY PRETREATED MULTIPLE MYELOMA: OUTCOMES FROM A SINGLE TRANSPLANT CENTER

**Topic:** 26. Gene therapy, cellular immunotherapy and vaccination - Clinical

James Di Palma-Grisi*[1], Daniel Ambrosio[2], Erica Dececco[1], Jason Romancik[3], Noa Biran[3], David H. Vesole[3], Pooja Phull[3], Joseph Franz[3], Maureen McCarthy[3], David Siegel[3], Harsh Parmar[3]

*[1]Hackensack University Medical Center, Hackensack, United States of America;[2]Hackensack Meridian School of Medicine, Nutley, United States of America; [3]John Theurer Cancer Center, Hackensack, United States of America;*

### Background

Chimeric antigen receptor T-cell (CAR-T) therapy has rapidly transformed the therapeutic landscape for multiple myeloma (MM). Ciltacabtagene autoleucel (Cilta-cel) and idecabtagene vicleucel (Ide-cel) have become standard-of-care therapies for relapsed/refractory MM (RRMM). Manufacturing failure (MF) is estimated at 6-8% overall among patients receiving anti-BCMA CAR-T (Patel et al., 2024) and typically results in an out-of-specification (OOS) product that fails to meet viability, cell dose, sterility, potency, or transduction efficiency criteria (Dulobas et al., 2025).

### Aims

To examine the outcomes among patients with RRMM who received OOS anti-BCMA CAR-T compared with those who received in-specification (IS) anti-BCMA CAR-T.

### Methods

We performed a retrospective analysis of 250 patients who underwent apheresis for anti-BCMA CAR-T therapy at our institution, treated between June 2021 and January 2026. Baseline characteristics were recorded; response rates were measured according to International Myeloma Working Group criteria. Progression-free survival (PFS) and overall survival (OS) were analysed using the Kaplan-Meier method. Univariate and multivariate Cox proportional hazards models were used to identify predictors of PFS and OS.

### Results

Of the 250 patients who underwent apheresis, 28 CAR-T products (11.2%) were OOS, of which 20 were infused. The OOS rate was 16.2% (11/68) for ide-cel and 9.3% (17/182) for cilta-cel. Patients with OOS products were older (median 74.8 vs. 67.6 years, p = 0.037) and more heavily pretreated (median 6 vs. 5 prior lines of therapy, p = 0.046). There were no significant differences by sex (p = 0.1), cytogenetic risk (high-risk: 13.6% vs. standard-risk: 9.0%, p = 0.25), or EASIX score (median 1.0 vs. 1.1, p = 0.59). A higher proportion of OOS patients had ISS stage III disease at diagnosis (45.5% vs. 21.4%). There was no significant difference in the distribution of CRS grades (Pearson chi-square 7.70, p = 0.053) or ICANS grades (Pearson chi-square 1.76, p = 0.78) between the OOS and IS groups. The overall response rate (ORR) was 85% for OOS vs. 90.5% for IS (p = 0.45), and very good partial response or better was achieved by 70% versus 80.2% (p = 0.13). Median PFS was 13.1 months for OOS vs. 23.9 months for IS (p = 0.039). Median OS was 21.9 months for OOS vs. 44.9 months for IS (p = 0.17) (Figure 1). Univariate analysis of PFS is reported in Table 1. In multivariate analysis, OOS status was independently associated with inferior PFS (HR 2.05, p = 0.026), along with lymphodepletion (fludarabine/cyclophosphamide : bendamustine HR 0.42, p = 0.0001) and CAR-T product (cilta-cel : ide-cel HR 0.34, p < 0.0001).

**Copyright Information:** (Online) ISSN: 2572-9241
©2026 The Author(s). HemaSphere is published by John Wiley & Sons Ltd on behalf of European Hematology Association. This is an open access Abstract Book distributed under the Attribution-NonCommercial-NoDerivs (CC BY-NC-ND), which allows third parties to download the articles and share them with others as long as they credit the author and the Abstract Book, but they cannot change the content in any way or use them commercially.
**Abstract Book Citations:** Authors, Title, HemaSphere, 2026;10:(S1):pages. The abstract book DOIs can be found on the "Author Information" page.
**Disclaimer:** Articles published in the journal HemaSphere exclusively reflect the opinions of the authors. The authors are responsible for all content in their abstracts including accuracy of the facts, statements, citing resources, etc.

| Variable | Hazard Ratio | p-value |
|---|---|---|
| Age > 68 | 0.97 | 0.92 |
| Male sex | 0.79 | 0.26 |
| LD (FC) | 0.42 | 0.0002 |
| Cilta-cel | 0.32 | <0.0001 |
| ISS > 2 | 1.20 | 0.47 |
| EMD | 1.07 | 0.76 |
| Lines of therapy > 4 | 2.10 | 0.09 |
| OOS | 1.88 | 0.04 |

Table 1. Univariate analysis of PFS.

**Copyright Information:** (Online) ISSN: 2572-9241
©2026 The Author(s). HemaSphere is published by John Wiley & Sons Ltd on behalf of European Hematology Association. This is an open access Abstract Book distributed under the Attribution-NonCommercial-NoDerivs (CC BY-NC-ND), which allows third parties to download the articles and share them with others as long as they credit the author and the Abstract Book, but they cannot change the content in any way or use them commercially.
**Abstract Book Citations:** Authors, Title, HemaSphere, 2026;10:(S1):pages. The abstract book DOIs can be found on the "Author Information" page.
**Disclaimer:** Articles published in the journal HemaSphere exclusively reflect the opinions of the authors. The authors are responsible for all content in their abstracts including accuracy of the facts, statements, citing resources, etc.

### Summary/Conclusion

OOS anti-BCMA CAR-T products were associated with significantly shorter PFS compared with IS products, even after adjustment for confounders in multivariable analysis. OOS products still demonstrated meaningful clinical activity, with an ORR of 85% and a median PFS of 13.1 months. Patients who produced OOS products were older and more heavily pretreated, suggesting that host factors contributing to manufacturing failure may independently influence outcomes. These findings support the infusion of OOS CAR-T products in patients with RRMM who lack viable alternative treatments, as clinically meaningful responses can still be achieved.

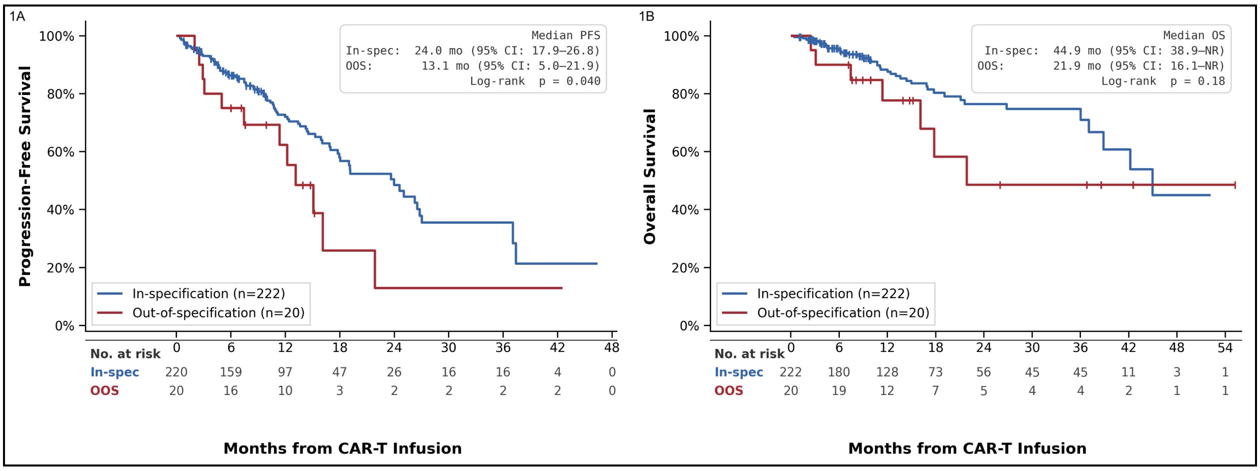

Figure 1. Kaplan-Meier curves for PFS (1A) and OS (1B) for OOS and IS groups.

**Copyright Information:** (Online) ISSN: 2572-9241
©2026 The Author(s). HemaSphere is published by John Wiley & Sons Ltd on behalf of European Hematology Association. This is an open access Abstract Book distributed under the Attribution-NonCommercial-NoDerivs (CC BY-NC-ND), which allows third parties to download the articles and share them with others as long as they credit the author and the Abstract Book, but they cannot change the content in any way or use them commercially.
**Abstract Book Citations:** Authors, Title, HemaSphere, 2026;10:(S1):pages. The abstract book DOIs can be found on the "Author Information" page.
**Disclaimer:** Articles published in the journal HemaSphere exclusively reflect the opinions of the authors. The authors are responsible for all content in their abstracts including accuracy of the facts, statements, citing resources, etc.

### (PF1248) A PROSPECTIVE, SINGLE-ARM CLINICAL STUDY OF CTDNA-GUIDED EARLY SECOND INFUSION OF AXI-CEL IN RELAPSED/REFRACTORY LARGE B-CELL LYMPHOMA

**Topic:** 26. Gene therapy, cellular immunotherapy and vaccination - Clinical

Sanfang Tu*[1], Kaixin Ou[1], Lei Wang[1], Zhao Liang[1], Yanjie He[1], Shaojie Wu[1], Jilong Yang[1], Chunyan Yue[1], Tianyou Yan[1], Yuhua Li[1]

[1]*Department of Hematology, Zhujiang Hospital of Southern Medical University, Guangzhou, China;*

### Background

CD19 chimeric antigen receptor T-cell (CAR-T) therapy has remarkably improved outcomes for relapsed or refractory large B-cell lymphoma (R/R LBCL). However, patients failing to achieve deep remission early after the first infusion face a persistently high risk of relapse. Historical data indicates that a second CAR-T infusion administered after relapse or progression generally yields poor salvage outcomes. Therefore, exploring preemptive consolidation interventions represents a critical unmet clinical need.

### Aims

To evaluate the efficacy, safety, and cellular kinetics of an early second CD19 CAR-T cell infusion as a consolidation therapy for R/R LBCL patients who show initial response but fail to achieve deep remission early after the first infusion.

### Methods

In this prospective, single-center, single-arm clinical trial, adult patients with R/R LBCL who achieved PR by PET-CT, or CR with ctDNA positivity within 60 days after the first Axicabtagene Ciloleucel(Axi-cel , $2 \times 10^6$ cells/kg) infusion were eligible for an early second axi-cel infusion. The second axi-cel infusion was administered at a dose of $2 \times 10^6$ cells/kg between 30 to 90 days after the first infusion. FC conditioning regimen was also administered prior to the second infusion. The study has been registered on clinicaltrial.gov (NCT06902012).

### Results

Until now, a total of 7 patients with a median age of 55 years (range, 38-64) were analyzed. All patients had high-risk disease features, including IPI scores of 3 to 4, double-expressor phenotype in 3 patients (42.9%), and central nervous system (CNS) involvement in 3 patients (42.9%). Among the 4 patients who were in CR but ctDNA-positive before the second infusion, 2 patients achieved ctDNA-negative CR at 1 month post-infusion and have maintained sustained remission for 396 and 257 days, respectively; the other 2 patients maintained CR but remained ctDNA-positive at 1 month with their 3-month evaluations pending. Among the 3 patients who were in PR before the second infusion, 1 patient with ctDNA-negative PR maintained this status at 1 month (3-month evaluation pending). Conversely, both patients with ctDNA-positive PR experienced early disease progression. The last follow-up date was February 15, 2026. With a median follow-up time of 93 days after the second CD19 CAR-T infusion, the median OS and median PFS were not reached.
The median time to peak expansion ($T_{max}$) was comparable between the first and second infusions (10 days vs. 12 days; P = 0.578). However, a trend toward a lower peak CAR-T cell count ($C_{max}$) was observed following the second infusion compared to the first (median 2.99 [range, 0.18-6.01] vs. 30.65 [range, 1.90-74.15] cells/μL; P = 0.078).
In safety, the early second CAR-T infusion was highly tolerable. Cytokine release syndrome (CRS) occurred in only 3 patients (42.8%), all of which were Grade 1. No cases of immune effector cell-associated neurotoxicity syndrome (ICANS) were observed. The most common hematologic adverse events were grade 4 neutropenia (6/7, 85.7%) and grade 3-4 thrombocytopenia (3/7, 42.9%). No treatment-related death or serious adverse events (SAE) occurred.

**Copyright Information:** (Online) ISSN: 2572-9241
©2026 The Author(s). HemaSphere is published by John Wiley & Sons Ltd on behalf of European Hematology Association. This is an open access Abstract Book distributed under the Attribution-NonCommercial-NoDerivs (CC BY-NC-ND), which allows third parties to download the articles and share them with others as long as they credit the author and the Abstract Book, but they cannot change the content in any way or use them commercially.
**Abstract Book Citations:** Authors, Title, HemaSphere, 2026;10:(S1):pages. The abstract book DOIs can be found on the "Author Information" page.
**Disclaimer:** Articles published in the journal HemaSphere exclusively reflect the opinions of the authors. The authors are responsible for all content in their abstracts including accuracy of the facts, statements, citing resources, etc.

### Summary/Conclusion

Early second infusion of CD19 CAR-T cells is feasible and demonstrates a highly favorable safety profile with no high-grade CRS or ICANS, while also demonstrating reliable in vivo CAR-T cell expansion. ctDNA-guided early intervention at minimal tumor burden is critical for successful second CAR-T infusions. Ongoing enrollment will validate these results.

| Patient | Age (years) | Gender | Diagnosis | IPI | ECOG | Prior Lines | COO Subtype | Disease Status | Pre-1st Infusion Status | CNS Involvement | Max Tumor Size (cm) | Involved Sites | Post-1st Infusion ctDNA | Post-1st Infusion PET-CT | Post-2nd Infusion ctDNA | Status at last follow-up |
|---|---|---|---|---|---|---|---|---|---|---|---|---|---|---|---|---|
| Pt 1 | 47 | Female | DLBCL | 3 | 4 | 3 | GCB | Relapsed | PR | Yes | 2.2 | Optic canal, parailiac vessels | Positive | CR | Negative | CR |
| Pt 2 | 57 | Male | DLBCL | 3 | 1 | 2 | GCB | Primary Refractory | PD | No | 2.6 | Pleura, neck, abdominopelvic cavity | Positive | CR | Negative | CR |
| Pt 3 | 38 | Male | DLBCL | 4 | 1 | 2 | GCB | Primary Refractory | PD | No | 4.4 | Abdomen, retroperitoneum, cardiophrenic angle | Positive | PR | unknown | death |
| Pt 4 | 62 | Male | PMBCL | 4 | 2 | 3 | non-GCB | Relapsed | PR | No | 3.8 | Mediastinum, femur, thyroid | Positive | CR | Positive | CR |
| Pt 5 | 64 | Male | DLBCL | 4 | 3 | 2 | non-GCB | Relapsed | PD | Yes | 2.0 | CNS | Positive | CR | Positive | CR |
| Pt 6 | 55 | Male | DLBCL | 4 | 4 | 2 | GCB | Relapsed | PD | Yes | 4.5 | CNS | Positive | PR | Positive | PD |
| Pt 7 | 46 | Male | DLBCL | 3 | 1 | 2 | non-GCB | Primary Refractory | PR | No | 3.5 | Paracaval, retroperitoneum, mesentery | Negative | PR | Negative | PR |

Patient Baseline Characteristics

Copyright Information: (Online) ISSN: 2572-9241
©2026 The Author(s). HemaSphere is published by John Wiley & Sons Ltd on behalf of European Hematology Association. This is an open access Abstract Book distributed under the Attribution-NonCommercial-NoDerivs (CC BY-NC-ND), which allows third parties to download the articles and share them with others as long as they credit the author and the Abstract Book, but they cannot change the content in any way or use them commercially.
Abstract Book Citations: Authors, Title, HemaSphere, 2026;10:(S1):pages. The abstract book DOIs can be found on the "Author Information" page.
Disclaimer: Articles published in the journal HemaSphere exclusively reflect the opinions of the authors. The authors are responsible for all content in their abstracts including accuracy of the facts, statements, citing resources, etc.

### (PF1249) PRE-TREATMENT WHITE MATTER CHANGES ON BRAIN MRI SUGGESTING SMALL VESSEL DISEASE PREDICT IMMUNE EFFECTOR CELL–ASSOCIATED NEUROTOXICITY AFTER CHIMERIC ANTIGEN RECEPTOR T-CELL THERAPY

**Topic:** 26. Gene therapy, cellular immunotherapy and vaccination - Clinical

Tomohiro Nagano*[1, 2], Hideaki Fujiwara[2], Aya Komura[2], Ryuichiro Hiyama[1, 2], Daisuke Ikeda[1, 2], Kenta Hayashino[1, 2], Toshiki Terao[1, 2], Chihiro Kamoi[2, 3], Takumi Kondo[2], Keisuke Seike[2], Shuntaro Ikegawa[2], Noboru Asada[2], Daisuke Ennishi[2, 4], Keiko Fujii[2, 5], Nobuharu Fujii[2, 3], Yoshinobu Maeda[2]

*[1]Okayama University Graduate School of Medicine, Dentistry, and Pharmaceutical Sciences, Hematology, Oncology and Respiratory Medicine, Okayama, Japan; [2]Okayama University Hospital, Hematology, Okayama, Japan; [3]Okayama University Hospital, Transfusion and Cell Therapy, Okayama, Japan; [4]Okayama University Hospital, Medical Oncology, Okayama, Japan; [5]Okayama University Hospital, Clinical Laboratory, Okayama, Japan;*

### Background

Immune effector cell–associated neurotoxicity syndrome (ICANS) is an important adverse effect following chimeric antigen receptor T-cell (CAR-T) therapy, and some patients experience prolonged neurologic symptoms. Therefore, prevention and early risk stratification are crucial; however, pre-treatment predictors of ICANS are limited. Because disruption of the blood–brain barrier with leakage of cytokines or inflammatory cells into the central nervous system (CNS) has been implicated in ICANS pathogenesis, we focused on small-vessel disease–related white matter changes as markers of vascular fragility and reduced resilience to neurotoxicity.

### Aims

We aimed to determine whether the pre-treatment Fazekas score (AJR Am J Roentgenol. 1987; 149:351), reflecting brain white matter changes, is associated with ICANS and prolonged neurologic symptoms after CAR-T therapy.

### Methods

We retrospectively analyzed 93 patients who underwent commercial CAR-T therapy for large B-cell lymphoma at Okayama University Hospital between December 2019 and June 2025 with brain magnetic resonance imaging (MRI). The Fazekas score (0–6, sum of periventricular and deep white matter ratings) was assessed on pre-infusion MRI, excluding lesions attributable to prior CNS events or lymphoma involvement, and categorized into three groups: low (0–2), intermediate (int; 3–4), and high (5–6). Prolonged neurologic symptoms were defined as lasting for ≥28 days.

**Copyright Information:** (Online) ISSN: 2572-9241
©2026 The Author(s). HemaSphere is published by John Wiley & Sons Ltd on behalf of European Hematology Association. This is an open access Abstract Book distributed under the Attribution-NonCommercial-NoDerivs (CC BY-NC-ND), which allows third parties to download the articles and share them with others as long as they credit the author and the Abstract Book, but they cannot change the content in any way or use them commercially.
**Abstract Book Citations:** Authors, Title, HemaSphere, 2026;10:(S1):pages. The abstract book DOIs can be found on the “Author Information” page.
**Disclaimer:** Articles published in the journal HemaSphere exclusively reflect the opinions of the authors. The authors are responsible for all content in their abstracts including accuracy of the facts, statements, citing resources, etc.

**Results**

Among 93 patients, including 15 ICANS events, 64, 20, and 9 were in the low, int, and high Fazekas categories, respectively. Higher Fazekas categories were associated with older age, a greater number of vascular risk factors (hypertension, diabetes, and dyslipidemia), and prior stroke, while other baseline characteristics, including CNS involvement, CAR-T products, prior therapies, and inflammatory markers, were comparable across groups. The cumulative incidences of ICANS in patients with low, int, and high scores were 11%, 20%, and 44%, respectively (p=0.03). The incidence of cytokine release syndrome (CRS), including grade ≥2 CRS, did not differ among groups (p=0.32 and p=0.87). In multivariable analysis adjusted for axi-cel use and $\log_2$ mEASIX (Blood Adv. 2021; 5:3397), the Fazekas score remained independently associated with ICANS (HR 1.51, 95% CI 1.09–2.10). In exploratory product-stratified analyses, the association between higher Fazekas categories and ICANS was particularly evident in axi-cel recipients (p=0.004), but not in tisa-cel and liso-cel (p=0.51), where ICANS events were relatively low. Prolonged neurologic symptoms were markedly enriched in the high Fazekas group, occurring in 1/64 patients (1.6%; low), 0/20 (0%; int), and 2/9 (22%; high) (p=0.046). Despite limited statistical power due to few ICANS events, a high score tended to be associated with poor recovery from ICANS. In contrast, progression-free and overall survival did not differ by Fazekas group (p=0.82 and p=0.70), suggesting a more specific association with neurotoxicity than with disease control.

**Summary/Conclusion**

Our data suggest that pre-existing brain white matter changes are associated with both the development of ICANS and the risk of prolonged neurologic symptoms. Incorporating brain MRI and Fazekas scoring before CAR-T therapy may help refine neurotoxicity risk stratification and could be particularly informative regarding CAR-T product selection and identification of patients who need closer ICANS surveillance.

**Copyright Information:** (Online) ISSN: 2572-9241
©2026 The Author(s). HemaSphere is published by John Wiley & Sons Ltd on behalf of European Hematology Association. This is an open access Abstract Book distributed under the Attribution-NonCommercial-NoDerivs (CC BY-NC-ND), which allows third parties to download the articles and share them with others as long as they credit the author and the Abstract Book, but they cannot change the content in any way or use them commercially.
**Abstract Book Citations:** Authors, Title, HemaSphere, 2026;10:(S1):pages. The abstract book DOIs can be found on the “Author Information” page.
**Disclaimer:** Articles published in the journal HemaSphere exclusively reflect the opinions of the authors. The authors are responsible for all content in their abstracts including accuracy of the facts, statements, citing resources, etc.

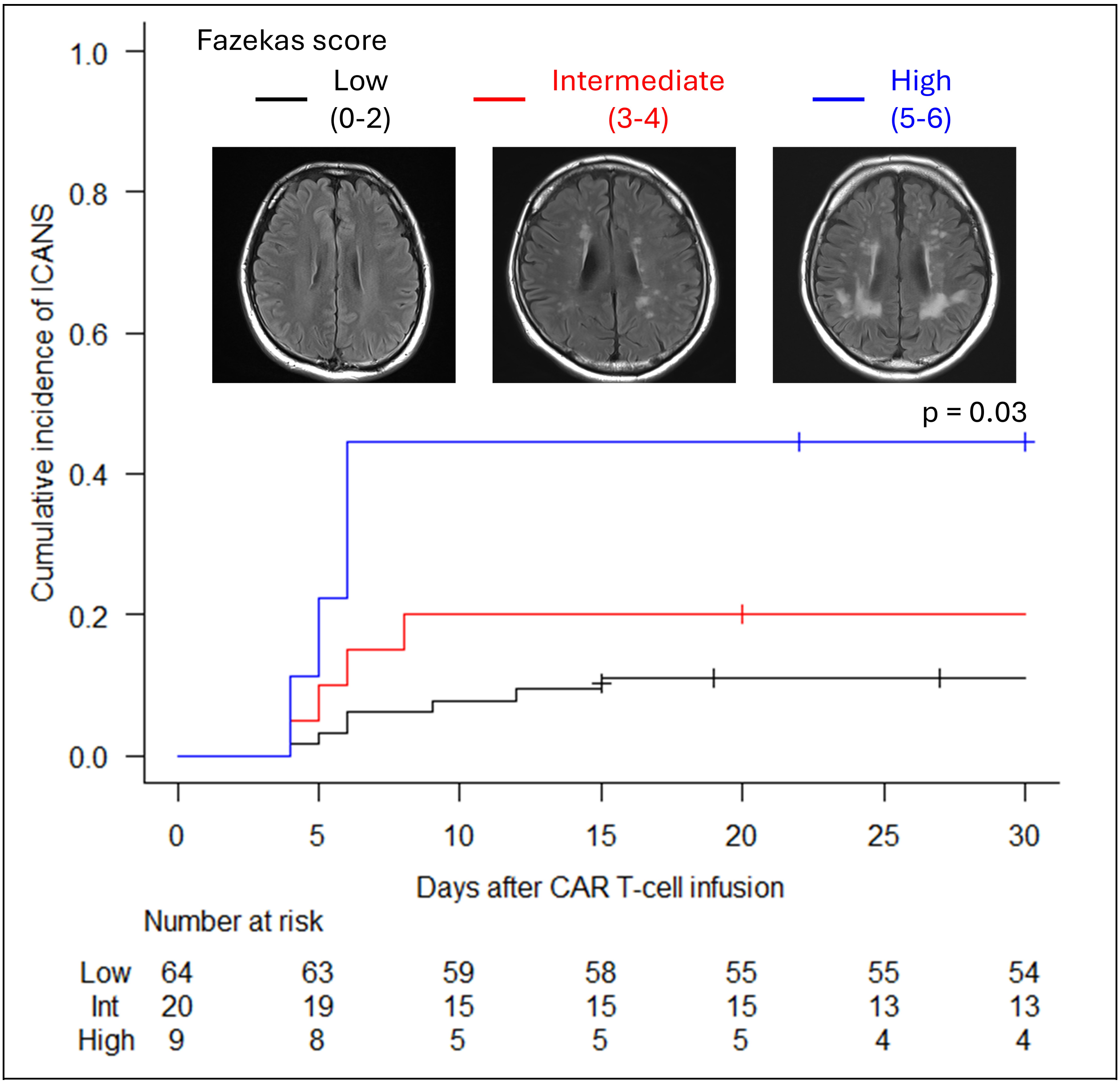

Copyright Information: (Online) ISSN: 2572-9241
©2026 The Author(s). HemaSphere is published by John Wiley & Sons Ltd on behalf of European Hematology Association. This is an open access Abstract Book distributed under the Attribution-NonCommercial-NoDerivs (CC BY-NC-ND), which allows third parties to download the articles and share them with others as long as they credit the author and the Abstract Book, but they cannot change the content in any way or use them commercially.
Abstract Book Citations: Authors, Title, HemaSphere, 2026;10:(S1):pages. The abstract book DOIs can be found on the "Author Information" page.
Disclaimer: Articles published in the journal HemaSphere exclusively reflect the opinions of the authors. The authors are responsible for all content in their abstracts including accuracy of the facts, statements, citing resources, etc.

### (PF1250) CURING TRANSFUSION-DEPENDENT HEMOGLOBIN H-CONSTANT SPRING VIA EX VIVO GENE EDITING OF AUTOLOGOUS HEMATOPOIETIC STEM CELLS

**Topic:** 26. Gene therapy, cellular immunotherapy and vaccination - Clinical

Li Wang[1], Hui Xu*[2], Junbin Liang[2], Yaoyun Li[1], Lin Cheng[2], Ying Luo[2], Liancheng Huang[2], Lihong Zeng[1], Rongrong Luo[1], Bin Xiao[1], Meiping Liu[1], Liping Huang[1], Zhendong Xiao[3], Xinhua Zhang[1], Junjiu Huang[4]

*[1]923rd Hospital of the People's Liberation Army, Nanning, China;[2]Reforgene Medicine, Guangzhou, China;[3]The Third Affiliated Hospital of Sun Yat-Sen University, Guangzhou, China;[4]Sun Yat-sen University, Guangzhou, China;*

### Background

Hemoglobin H Constant Spring (HbH-CS) (--/$\alpha^{CS}\alpha$) is the most common non-deletion α-thalassemia that can lead to severe health problems. The clinical severity of HbH-CS is widely variable and a considerable proportion of patients with HbH-CS need regular or irregular transfusion to support a normal growth, development and life. Currently, there is no cure for this type of disease worldwide. Because patients with HbH-CS has only a single base mutation in the *Hemoglobin Subunit Alpha* 2 (*HBA2*: c.427T>C) compared to that of asymptomatic carriers (--/αα), we speculate that repairing of the mutation by base editing may provide a potential cure for this type of disease.

### Aims

To repair the CS mutation of *HBA2* gene and increase the expression of normal α-globin chain in erythrocytes, we used *ex vivo* Cytosine Base Editor (CBE)–based gene editing to modify *HBA2* gene in hematopoietic stem and progenitor cells (HSPCs), producing RM004. NCT06107400 is an investigator initiated first-in-human study of edited patient cells (RM004) in transfusion-dependent HbH-CS. Here, we present the initial results from the first patient treated with RM004.

### Methods

Patients (12–35 y of age) with HbH-CS receiving packed red blood cell (pRBC) transfusions of ≥100 mL/kg/y or ≥10 units/y in the previous 2 y were eligible. Peripheral CD34+ HSPCs were collected by apheresis after mobilization with G-CSF and plerixafor. CD34+ cells were edited with CBEs using a guide RNA specific for the CS mutation. Prior to RM004 infusion, patient received myeloablative conditioning with busulfan form day-7 to day-3. Patient were monitored for stem cell engraftment/hematopoietic recovery, adverse events (AEs), Hb production, expression of HbA/HbH/Hb-CS, proportion of CS negative red blood cells and requirements for packed red blood cell (pRBC) transfusions.

**Copyright Information:** (Online) ISSN: 2572-9241
©2026 The Author(s). HemaSphere is published by John Wiley & Sons Ltd on behalf of European Hematology Association. This is an open access Abstract Book distributed under the Attribution-NonCommercial-NoDerivs (CC BY-NC-ND), which allows third parties to download the articles and share them with others as long as they credit the author and the Abstract Book, but they cannot change the content in any way or use them commercially.
**Abstract Book Citations:** Authors, Title, HemaSphere, 2026;10:(S1):pages. The abstract book DOIs can be found on the “Author Information” page.
**Disclaimer:** Articles published in the journal HemaSphere exclusively reflect the opinions of the authors. The authors are responsible for all content in their abstracts including accuracy of the facts, statements, citing resources, etc.

### Results

As of January 25, 2026, a 14-year-old female patient with HbH-CS was treated with RM004 and has been followed for over 21 months. Prior to enrollment, the patient required 29 units (178 mL/kg/year) of pRBC transfusions annually. After a single dose infusion of RM004 cells, the patient achieved neutrophils engraftment and platelets engraftment on Day 14 and Day 20, respectively. The last pRBC transfusion occurred on Day 13 after RM004 treatment, and the patient has remained transfusion-free for 21 months. Notably, total Hb increased to over 9 g/dL on Day 26 and remained between 9 and 11 g/dL during follow-up. Therefore, the patient has achieved the primary efficacy endpoint TI12.
Following RM004 infusion, the proportions of HbH and Hb-CS decreased from 3.8% and 1.1% to 0 and 0.2% (month 21), respectively. Concurrently, the proportion of $HbA_1$ increased from 93% to 97.9% (month 21). The proportion of CS-positive RBCs significantly decreased from 100% to less than 10%, indicating that over 90% of peripheral blood erythrocytes originated from successfully edited HSPCs. The safety profile was generally consistent with that of busulfan myeloablation and autologous hematopoietic stem cell transplantation. No serious adverse events were reported.

### Summary/Conclusion

These results present the first successful application of gene editing in treating a patient with transfusion-dependent α-thalassemia. The patient treated with RM004 demonstrated successful engraftment and a clinically meaningful increase in hemoglobin levels, leading to transfusion independence. This proof-of-concept study indicates that RM004 is a promising therapy for the treatment of HbH-CS.

**Copyright Information:** (Online) ISSN: 2572-9241
©2026 The Author(s). HemaSphere is published by John Wiley & Sons Ltd on behalf of European Hematology Association. This is an open access Abstract Book distributed under the Attribution-NonCommercial-NoDerivs (CC BY-NC-ND), which allows third parties to download the articles and share them with others as long as they credit the author and the Abstract Book, but they cannot change the content in any way or use them commercially.
**Abstract Book Citations:** Authors, Title, HemaSphere, 2026;10:(S1):pages. The abstract book DOIs can be found on the “Author Information” page.
**Disclaimer:** Articles published in the journal HemaSphere exclusively reflect the opinions of the authors. The authors are responsible for all content in their abstracts including accuracy of the facts, statements, citing resources, etc.

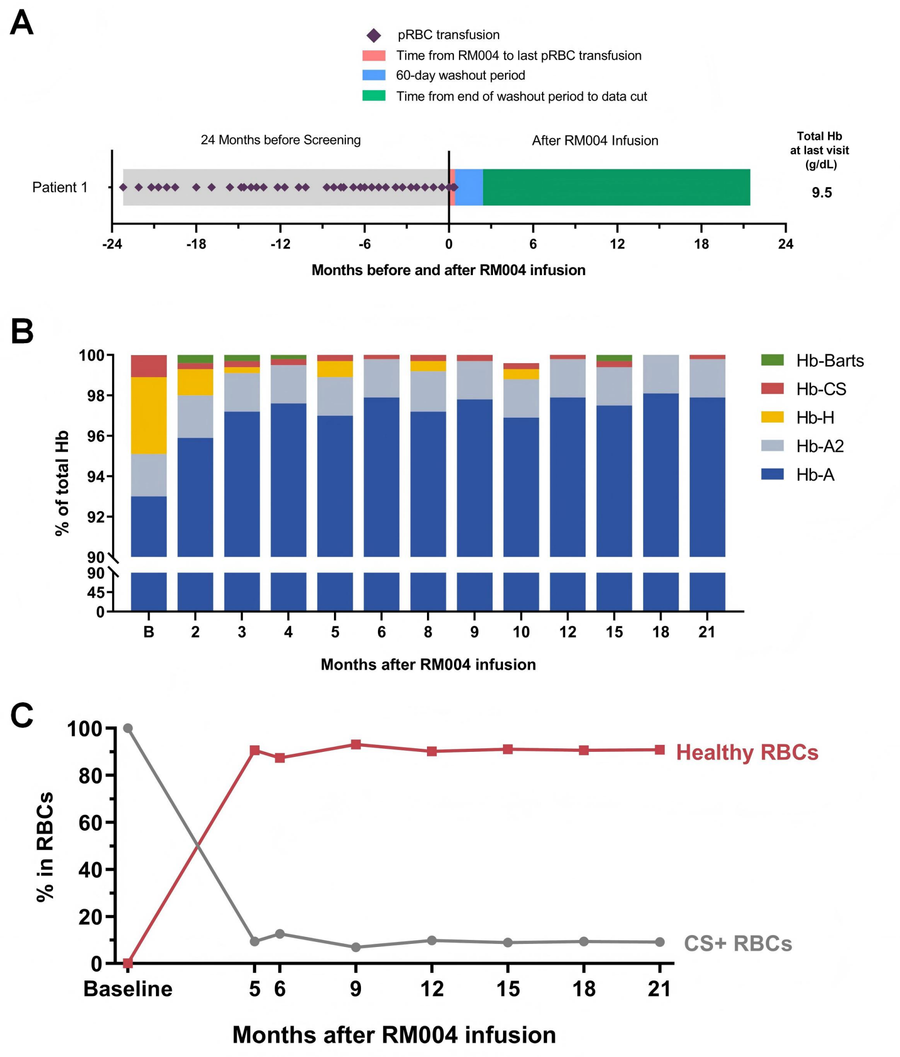

A. Prior transfusion frequency and time to transfusion-free status; B. Proportion of hemoglobin variants in peripheral blood; C. Ratio of CS+ erythrocytes and normal erythrocytes in the patient's peripheral blood pre- and post-treatment.

**Copyright Information:** (Online) ISSN: 2572-9241
©2026 The Author(s). HemaSphere is published by John Wiley & Sons Ltd on behalf of European Hematology Association. This is an open access Abstract Book distributed under the Attribution-NonCommercial-NoDerivs (CC BY-NC-ND), which allows third parties to download the articles and share them with others as long as they credit the author and the Abstract Book, but they cannot change the content in any way or use them commercially.
**Abstract Book Citations:** Authors, Title, HemaSphere, 2026;10:(S1):pages. The abstract book DOIs can be found on the "Author Information" page.
**Disclaimer:** Articles published in the journal HemaSphere exclusively reflect the opinions of the authors. The authors are responsible for all content in their abstracts including accuracy of the facts, statements, citing resources, etc.

### (PF1251) EFFICACY AND SAFETY OF AVATROMBOPAG IN THE MANAGEMENT OF PROLONGED CYTOPENIAS FOLLOWING CAR-T CELL THERAPY: A GETH-TC STUDY

**Topic:** 26. Gene therapy, cellular immunotherapy and vaccination - Clinical

José María Sánchez-Pina[1], Denis Zafra Torres[1], Daniel Gil-Alós*[1], Almudena Zaratiegui-Vergara[1], María Eva Mingot Castellano[2], Adolfo Jesus Saez Marin[1], Juan Luis Reguera Ortega[2], Maria Nieves Lopez Muñoz[1], Magdalena Corona[1], Maria Pilar Martinez Sanchez[1], Guillermo Ramos Moreno[1], Pascual Balsalobre[3], Joaquín Martinez-Lopez[1], Maria Calbacho Robles[1]

*[1]Hospital 12 de Octubre, Haematology, Madrid, Spain;[2]Hospital Universitario Virgen del Rocío, Haematology, Sevilla, Spain; [3]Grupo Español de Trasplante de Progenitores Hematopoyéticos y Terapia Celular, Madrid, Spain;*

### Background

CAR-T cell therapy has revolutionized the treatment of relapsed/refractory haematological malignancies. However, prolonged and severe cytopenias remain a significant complication, increasing the risk of life-threatening infections and haemorrhages while adding a substantial impact on healthcare resources. Management strategies lack of definitive consensus.

### Aims

To evaluate the clinical impact, safety profile, and haematological recovery outcomes of patients experiencing persistent cytopenias after CAR-T cell infusion treated with avatrombopag.

### Methods

A retrospective study was conducted on patients treated with CAR-T and avatrombopag for the management of post-infusion cytopenias. In accordance with institutional policy protocol, Avatrombopag was initiated from day 14 post infusion in patients with grade ≥ 3 thrombocytopenia and a decrease in platelet counts over two consecutive controls. Adverse events and cytopenias were graded according to the Common Terminology Criteria for Adverse Events (CTCAE) v6.0.

**Copyright Information:** (Online) ISSN: 2572-9241
©2026 The Author(s). HemaSphere is published by John Wiley & Sons Ltd on behalf of European Hematology Association. This is an open access Abstract Book distributed under the Attribution-NonCommercial-NoDerivs (CC BY-NC-ND), which allows third parties to download the articles and share them with others as long as they credit the author and the Abstract Book, but they cannot change the content in any way or use them commercially.
**Abstract Book Citations:** Authors, Title, HemaSphere, 2026;10:(S1):pages. The abstract book DOIs can be found on the "Author Information" page.
**Disclaimer:** Articles published in the journal HemaSphere exclusively reflect the opinions of the authors. The authors are responsible for all content in their abstracts including accuracy of the facts, statements, citing resources, etc.

**Results**

47 patients were treated with avatrombopag: 28 patients with DLBCL (Axi-cel), 14 patients with MM (12 Cilta-cel, 2 Ide-cel), 4 with FL (Axi-cel), and 1 with MCL (Brexu-cel). Starting dose was 20 mg; a dose of 60 mg was reached in 31, 40 mg in 11 patients, and 20 mg in 1 patient. Median platelet count at the start of avatrombopag treatment was 34,000 platelets/mm³ (6,000–50,000). In 14 patients (30%), platelet transfusion was avoided; among these patients, 84.61% achieved a recovery of ≥ 50,000 platelets in 12 days (6–114) from avatrombopag initiation. A total of 323 platelet units were administered for patients who required transfusion (median of 7.4 units, 4–39), median time from avatrombopag initiation to transfusion independence was 18 days (16–145). Currently, 18% have not yet reached a platelet count above 50,000/mm³. Thirteen patients (28.9%) presented with neutropenia (<500 neutrophils/mm³) at the start of avatrombopag treatment. Median time to recover >500 neutrophils/mm³ was 13 days (2–81) from avatrombopag initiation. All patients received support with G-CSF. Median haemoglobin level at the start of avatrombopag was 9.85 g/dL (7–13.2). 68% required transfusion. Median number of red blood cell units was 5.5 (1–15). Median time to achieve transfusion independence from avatrombopag initiation was 21 days (3–102). There were no significant differences between MM and NHL patients regarding transfusion requirements or the duration of cytopenias. Side effects included one case of grade 1 persisting nausea and another with grade 1 hepatitis, resolved with a 20 mg dose reduction. Bleeding events occurred in 5 patients: 4 were grade 1 and 1 grade 3, presenting as a traumatic subarachnoid haemorrhage secondary to a fall. Infections occurred in seven patients: 2 cases of cholecystitis, 1 *e. faecium* bacteraemia, 2 *e. coli* bacteraemia, 1 bacterial meningitis and 1 case of cellulitis. With a median follow-up of 3.71 months (0.66–13.53), 12 patients remain on treatment. Progressed disease has been observed in 15% of patients, and 92.5% remain alive. TRM=1, due to bacterial meningitis.

**Summary/Conclusion**

Avatrombopag appears to be an effective and safe therapeutic option for accelerating hematopoietic recovery and reducing transfusion requirements in patients with prolonged cytopenias post-CAR-T. Our findings suggest thar early intervention may be associated with improved clinical outcomes, although larger prospective studies are warranted to confirm these findings.

**Copyright Information:** (Online) ISSN: 2572-9241
©2026 The Author(s). HemaSphere is published by John Wiley & Sons Ltd on behalf of European Hematology Association. This is an open access Abstract Book distributed under the Attribution-NonCommercial-NoDerivs (CC BY-NC-ND), which allows third parties to download the articles and share them with others as long as they credit the author and the Abstract Book, but they cannot change the content in any way or use them commercially.
**Abstract Book Citations:** Authors, Title, HemaSphere, 2026;10:(S1):pages. The abstract book DOIs can be found on the "Author Information" page.
**Disclaimer:** Articles published in the journal HemaSphere exclusively reflect the opinions of the authors. The authors are responsible for all content in their abstracts including accuracy of the facts, statements, citing resources, etc.

### (PF1252) EFFICACY AND SAFETY OF CAR-T CELL THERAPY VERSUS BISPECIFIC ANTIBODIES IN MULTIPLE MYELOMA PATIENTS WITH EXTRAMEDULLARY DISEASE: A SYSTEMATIC REVIEW AND META-ANALYSIS

**Topic:** 26. Gene therapy, cellular immunotherapy and vaccination - Clinical

Jiajia Li*[1, 2], Shihui Ren[1], Mengjiao Li[1, 2], Yanling Li[1, 2], Ling Qiu[1], Dan Chen[1], Xiao Wang[1, 3], Mingqiang Ren[4], Hao Yao[1, 3, 5, 6], Fangyi Fan[1, 2]

*[1]Department of Hematology, The General Hospital of Western Theater Command, Chengdu, China;[2]Department of Clinical Medicine, North Sichuan Medical College, Nanchong, China; [3]College of Medicine, Southwest Jiaotong University, Chengdu, China; [4]Department of Hematology, Affiliated Hospital of Zunyi Medical Univercity, Zunyi, China;[5]Institute of Basic Medicine, North Sichuan Medical College, Nanchong, China; [6]Tissue Stress Injury and Functional Repair Key Laboratory of Sichuan Province, Chengdu, China;*

### Background

Extramedullary disease (EMD) in multiple myeloma (MM) represents an aggressive disease phenotype and is associated with poor prognosis under conventional therapies. Novel B-cell maturation antigen (BCMA)-targeted immunotherapies, including chimeric antigen receptor T-cell (CAR-T) therapy and bispecific antibodies (BsAbs), have demonstrated substantial efficacy in relapsed/refractory MM (RRMM). However, direct comparative evidence in the high-risk EMD subgroup remains limited.

### Aims

To systematically evaluate and compare the efficacy and safety of CAR-T therapy versus BsAbs in patients with RRMM presenting with EMD.

### Methods

A systematic search of PubMed, Embase, and Web of Science was conducted for studies published between January 1, 2020, and February 2026. Search terms combined controlled vocabulary and free-text keywords including “multiple myeloma,” “extramedullary,” “EMD,” “CAR-T,” and “bispecific antibody.” Clinical studies reporting outcomes of CAR-T therapies (e.g., idecabtagene vicleucel, ciltacabtagene autoleucel) or BsAbs (e.g., teclistamab, elranatamab) in MM patients with EMD were included, encompassing randomized trials, single-arm studies, and real-world data. In the absence of direct head-to-head comparisons, indirect comparisons based on pooled single-arm rates were performed. Primary endpoints included overall response rate (ORR), stringent complete response (sCR), progression-free survival (PFS), and overall survival (OS). Secondary endpoints comprised the incidence and severity of cytokine release syndrome (CRS) and immune effector cell-associated neurotoxicity syndrome (ICANS). Risk of bias was assessed using the Cochrane tool. Meta-analysis was performed using R software. Risk ratios (RRs) with 95% confidence intervals (CIs) were calculated for dichotomous outcomes, and hazard ratios (HRs) with 95% CIs for time-to-event outcomes. Heterogeneity was assessed using the $I^2$ statistic; a random-effects model was applied when $I^2 > 50\%$.

**Copyright Information:** (Online) ISSN: 2572-9241
©2026 The Author(s). HemaSphere is published by John Wiley & Sons Ltd on behalf of European Hematology Association. This is an open access Abstract Book distributed under the Attribution-NonCommercial-NoDerivs (CC BY-NC-ND), which allows third parties to download the articles and share them with others as long as they credit the author and the Abstract Book, but they cannot change the content in any way or use them commercially.
**Abstract Book Citations:** Authors, Title, HemaSphere, 2026;10:(S1):pages. The abstract book DOIs can be found on the “Author Information” page.
**Disclaimer:** Articles published in the journal HemaSphere exclusively reflect the opinions of the authors. The authors are responsible for all content in their abstracts including accuracy of the facts, statements, citing resources, etc.

### Results

A total of 873 RRMM patients with EMD from 18 studies were included (CAR-T: n = 521; BsAbs: n = 352). CAR-T therapy demonstrated significantly higher ORR (RR = 1.28, 95% CI 1.12–1.47, P < 0.001) and sCR rates (RR = 1.85, 95% CI 1.45–2.36, P < 0.001) compared with BsAbs. The pooled hazard ratio for disease progression or death favored CAR-T therapy (HR = 0.69, 95% CI 0.56–0.85, P < 0.001). No statistically significant difference in OS was observed (HR = 0.82, 95% CI 0.65–1.03, P = 0.09), although a numerical trend favored CAR-T. Regarding safety, BsAbs were associated with significantly lower incidence of any-grade CRS (RR = 0.62, 95% CI 0.51–0.76), grade ≥3 CRS (RR = 0.41, 95% CI 0.28–0.61), and any-grade ICANS (RR = 0.35, 95% CI 0.21–0.58) compared with CAR-T therapy.

### Summary/Conclusion

In RRMM patients with EMD, CAR-T therapy appears to achieve deeper responses and significantly prolong PFS compared with BsAbs, albeit at the expense of higher rates of severe CRS and ICANS. These findings highlight a trade-off between efficacy and toxicity, underscoring the need for individualized treatment selection in this high-risk population. Prospective head-to-head randomized trials are needed to confirm these results.

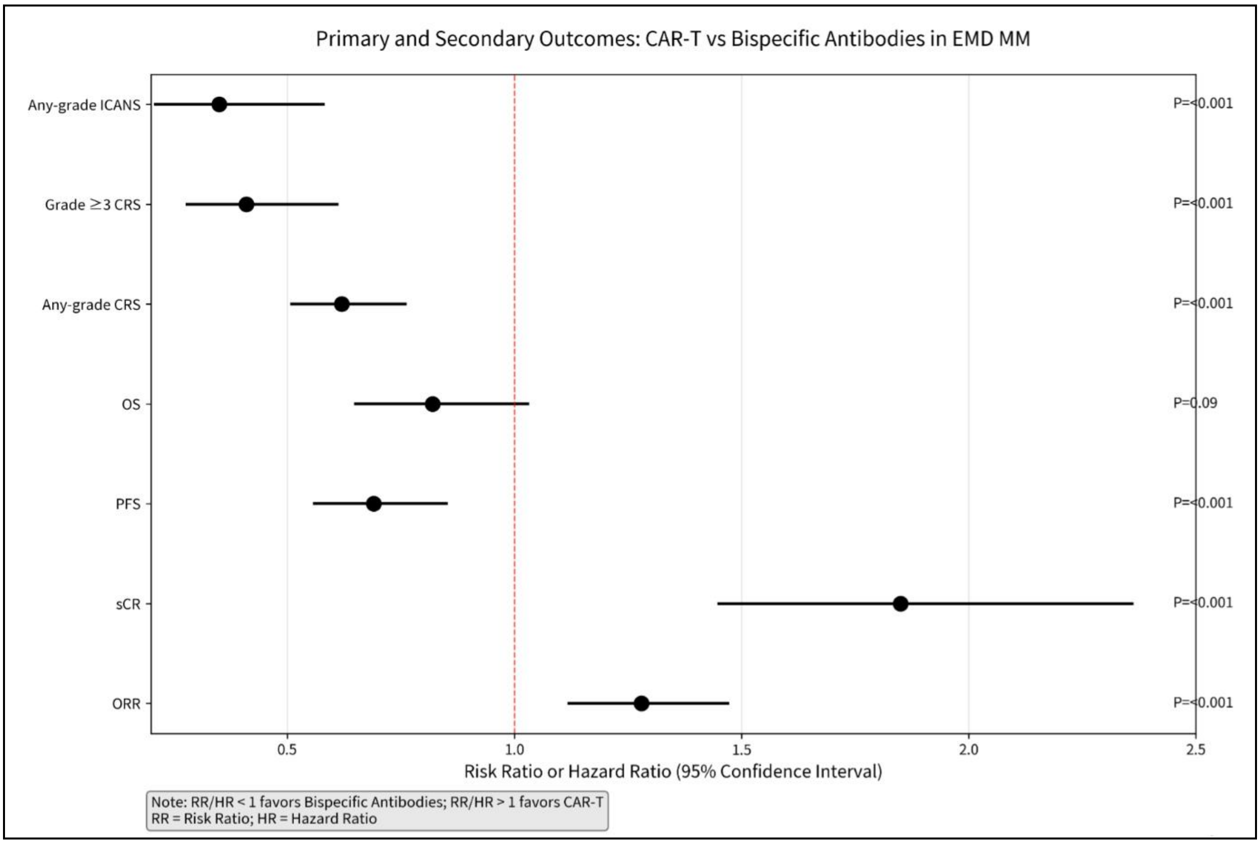

Figure. Pooled forest plots of efficacy and safety outcomes. Efficacy outcomes demonstrate significantly improved ORR, sCR, and PFS with CAR-T therapy compared with BsAbs, while no statistically significant difference in OS was observed, although a favorable trend toward CAR-T was noted. Safety analyses indicate significantly lower risks of CRS and ICANS with BsAbs. The red dashed line represents the line of no effect; black squares indicate pooled effect estimates, and horizontal lines denote 95% confidence intervals.

**Copyright Information:** (Online) ISSN: 2572-9241
©2026 The Author(s). HemaSphere is published by John Wiley & Sons Ltd on behalf of European Hematology Association. This is an open access Abstract Book distributed under the Attribution-NonCommercial-NoDerivs (CC BY-NC-ND), which allows third parties to download the articles and share them with others as long as they credit the author and the Abstract Book, but they cannot change the content in any way or use them commercially.
**Abstract Book Citations:** Authors, Title, HemaSphere, 2026;10:(S1):pages. The abstract book DOIs can be found on the "Author Information" page.
**Disclaimer:** Articles published in the journal HemaSphere exclusively reflect the opinions of the authors. The authors are responsible for all content in their abstracts including accuracy of the facts, statements, citing resources, etc.

### (PF1253) CAR-T FITNESS INDEX (CAR-FIT): INTEGRATING COMORBIDITY AND FRAILTY ASSESSMENT TO GUIDE RISK-ADAPTED DELIVERY IN PATIENTS WITH BORDERLINE PHYSIOLOGICAL RESERVE.

**Topic:** 26. Gene therapy, cellular immunotherapy and vaccination - Clinical

Kye Ling Wong*[1], Nicole-Ann Lim[1], Cheng Kiat Lawrence Ng[1], Francesca Lorraine Wei Inng Lim[1], Shin Yeu Ong[1], Tertius Tuy[1], Chieh Hwee Ang[1], Michelle Limei Poon[2], Esther Hian Li Chan[2], Victor Ling[2], William Chan[2], Edwin Thong[2], Chandramouli Nagarajan[1], William Hwang[1], Aloysius Ho[1], Yunxin Chen[1]

*[1]Singapore General Hospital, Haematology, Singapore, Singapore;[2]National University Hospital, Singapore, Singapore;*

### Background

Selecting patients for chimeric antigen receptor T-cell (CAR-T) therapy is essential to minimise preventable adverse outcomes and support appropriate use of healthcare resources, yet it remains largely subjective in current clinical settings. Existing tools to evaluate co-morbidities include the cumulative illness rating scale (CIRS), "Severe4" and Cellular Therapy Comorbidity Index (CTCI). However, individual scores may not reflect the complex interplay between disease burden and kinetics, frailty and pre-existing co-morbidities. We propose a stepwise framework that systematically integrates frailty and comorbidity assessments to enable objective stratification of patients along a fitness continuum. This approach aims to guide optimization of the "borderline fit" patients to access CAR-T therapy safely whilst minimizing undue risk in patients on the far end of the fitness spectrum.

### Aims

We aim to correlate comorbidity assessments with clinical outcomes and develop a framework that integrates comorbidity and frailty assessments to optimally balance safety and equity of CAR-T access in clinical practice.

### Methods

Data from 80 patients with relapsed refractory diffuse large B cell lymphoma treated with CAR-T therapy between 2020-2025 in two treating institutions in Singapore were retrospectively analysed. Outcomes included overall survival (OS), progression-free survival (PFS), and severe treatment-related complications, defined as Grade ≥3 cytokine release syndrome, immune-effector cell-associated neurotoxicity syndrome, or immune effector cell-associated haematotoxicity. Patients' fitness and comorbidities were assessed using ECOG, Karnofsky, CIRS, Severe4 and CTCI.

**Copyright Information:** (Online) ISSN: 2572-9241
©2026 The Author(s). HemaSphere is published by John Wiley & Sons Ltd on behalf of European Hematology Association. This is an open access Abstract Book distributed under the Attribution-NonCommercial-NoDerivs (CC BY-NC-ND), which allows third parties to download the articles and share them with others as long as they credit the author and the Abstract Book, but they cannot change the content in any way or use them commercially.
**Abstract Book Citations:** Authors, Title, HemaSphere, 2026;10:(S1):pages. The abstract book DOIs can be found on the "Author Information" page.
**Disclaimer:** Articles published in the journal HemaSphere exclusively reflect the opinions of the authors. The authors are responsible for all content in their abstracts including accuracy of the facts, statements, citing resources, etc.

### Results

The median age was 60 years. The majority of patients had ECOG 0–1 and Karnofsky scores of 90–100. (Table 1). Using novel prognostic indices, a subset of patients had higher comorbidity burden: 30% (n=24) had CIRS ≥7, 8.8% (n=7) had presence of Severe4, and 5% (n=4) had CTCI >3.
We propose a clinical algorithm to guide clinicians in using comorbidity assessments in daily practice. (Figure 1). With the suggested classification framework, 51.2% (N=41) of patients were fit, 28.8% (N=23) were borderline-fit and 20% (N=16) were unfit. There was a significant difference in median PFS among the fit, borderline and unfit groups (not reached at 60 months, 95% CI 33.7-NR; 12.2 months, 95% CI 6.03-NR; 8.7 months, 95% CI 6.27-NR), log-rank p = 0.027. A corresponding difference in OS at 36 months was also noted, fit (85.2%, 95% CI 70.6-100%), borderline-fit (66.7%, 95% CI 47.3-94.1%) and unfit (did not reach 36 months), p < 0.001.

**Table 1. Demographics, CAR-T cell product, adverse events.**

| Variable | Overall (N = 80) |
|---|---|
| Age, median (IQR) | 60 (51-70) |
| Gender, n (%) | |
| Male | 43 (53.8) |
| Female | 37 (46.2) |
| ECOG 0-1, n (%) | 71 (88.7) |
| Karnofsky, median (IQR) | 90 (80-100) |
| CAR-T product | |
| Tisagenlecleucel | 34 (42.5) |
| Axicabtagene ciloleucel | 36 (45.0) |
| Academic | 10 (12.5) |
| Adverse events | |
| CRS Grade ≥ 3, n (%) | 5 (6.2) |
| ICANS Grade ≥ 3, n (%) | 8 (10.0) |
| T-ICAHT Grade ≥ 3, n (%) | 20 (25.0) |
| N-ICAHT Grade ≥ 3, n (%) | 44 (55.0) |

### Summary/Conclusion

Combining CIRS, Severe4, and CTCI improves outcome stratification compared with any individual score alone. When integrated with frailty assessment, this approach can refine patient selection to allow safer access to potentially eligible candidates.

**Copyright Information:** (Online) ISSN: 2572-9241
©2026 The Author(s). HemaSphere is published by John Wiley & Sons Ltd on behalf of European Hematology Association. This is an open access Abstract Book distributed under the Attribution-NonCommercial-NoDerivs (CC BY-NC-ND), which allows third parties to download the articles and share them with others as long as they credit the author and the Abstract Book, but they cannot change the content in any way or use them commercially.
**Abstract Book Citations:** Authors, Title, HemaSphere, 2026;10:(S1):pages. The abstract book DOIs can be found on the "Author Information" page.
**Disclaimer:** Articles published in the journal HemaSphere exclusively reflect the opinions of the authors. The authors are responsible for all content in their abstracts including accuracy of the facts, statements, citing resources, etc.

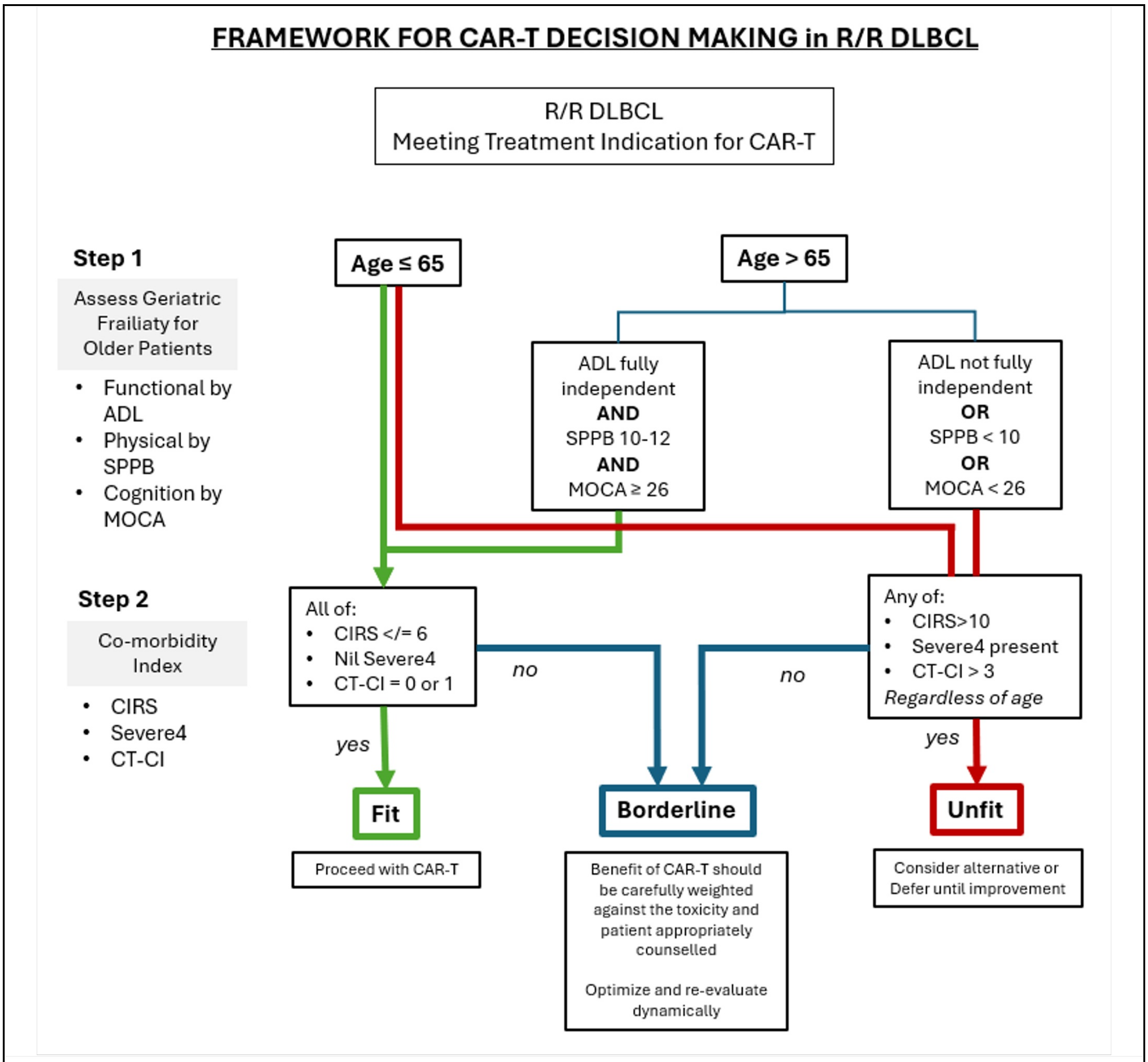

ADL - activities of daily living, SPPB - short physical performance battery, MOCA - Montreal cognitive assessment, CIRS - cumulative illness rating scale, CTCI - cellular therapy comorbidity index.

Figure 1. Framework for CAR-T decision making in RR DLBCL.

**Copyright Information:** (Online) ISSN: 2572-9241
©2026 The Author(s). HemaSphere is published by John Wiley & Sons Ltd on behalf of European Hematology Association. This is an open access Abstract Book distributed under the Attribution-NonCommercial-NoDerivs (CC BY-NC-ND), which allows third parties to download the articles and share them with others as long as they credit the author and the Abstract Book, but they cannot change the content in any way or use them commercially.
**Abstract Book Citations:** Authors, Title, HemaSphere, 2026;10:(S1):pages. The abstract book DOIs can be found on the "Author Information" page.
**Disclaimer:** Articles published in the journal HemaSphere exclusively reflect the opinions of the authors. The authors are responsible for all content in their abstracts including accuracy of the facts, statements, citing resources, etc.

### (PF1254) PREVALENCE AND PROGNOSTIC IMPACT OF FAMILIAL HEMATOLOGICAL MALIGNANCIES IN PATIENTS WITH B-CELL NEOPLASMS TREATED WITH CAR-T THERAPY

**Topic:** 26. Gene therapy, cellular immunotherapy and vaccination - Clinical

Ilaria Pansini*[1, 2], Roberto Maggi[1, 2], Eugenio Galli[1], Alessandro Corrente[2], Nicola Piccirillo[1, 2], Marcello Viscovo[1, 2], Stefan Hohaus[1, 2], Federica Sorà[1, 2], Patrizia Chiusolo[1, 2], Simona Sica[1, 2]

*[1]Fondazione Policlinico Universitario A. Gemelli IRCCS, Rome, Italy, Department of Laboratory and Hematological Sciences, Rome, Italy; [2]Università Cattolica del Sacro Cuore, Rome, Italy, Hematology Section, Department of Radiological and Hematological Sciences, Rome, Italy;*

### Background

A family history of haematological malignancy (FHHM) is a recognised risk factor for the development of B-cell neoplasms. Population-based studies report that individuals with a first-degree relative affected by a haematopoietic malignancy have a 1.7–1.8-fold increased risk of non-Hodgkin lymphoma (NHL), with stronger associations observed for specific subtypes such as chronic lymphocytic leukaemia. In large lymphoma cohorts, FHHM has been reported in approximately 20% of patients. However, its clinical impact in the setting of CAR-T therapy remains unclear.

### Aims

The aim of the study is to assess the prevalence of FHHM in patients with B-cell neoplasms treated with CAR-T cell therapy and to evaluate its impact on post–CAR-T outcomes.

### Methods

We performed a retrospective single-centre analysis of 152 adult patients with B-cell malignancies treated with CAR-T therapy between September 2019 and October 2025. Diagnoses included diffuse large B-cell lymphoma (DLBCL), mantle cell lymphoma (MCL), B-cell acute lymphoblastic leukaemia (B-ALL), and multiple myeloma (MM).
FHHM was defined as ≥1 affected first-degree relative or ≥2 affected second-degree relatives and was assessed at baseline from medical records. Categorical variables were compared using chi-square or Fisher's exact tests, as appropriate.
Progression-free survival (PFS) was defined as time from CAR-T infusion to progression or death. Survival was estimated using Kaplan–Meier methodology and compared according to familial status (Figure 1A).

**Copyright Information:** (Online) ISSN: 2572-9241
©2026 The Author(s). HemaSphere is published by John Wiley & Sons Ltd on behalf of European Hematology Association. This is an open access Abstract Book distributed under the Attribution-NonCommercial-NoDerivs (CC BY-NC-ND), which allows third parties to download the articles and share them with others as long as they credit the author and the Abstract Book, but they cannot change the content in any way or use them commercially.
**Abstract Book Citations:** Authors, Title, HemaSphere, 2026;10:(S1):pages. The abstract book DOIs can be found on the "Author Information" page.
**Disclaimer:** Articles published in the journal HemaSphere exclusively reflect the opinions of the authors. The authors are responsible for all content in their abstracts including accuracy of the facts, statements, citing resources, etc.

### Results

Of 152 patients, 32 (21%) met criteria for FHHM and 120 (79%) did not (Figure 1B).
In the FHHM group, diagnoses were DLBCL (80%), B-ALL (5%), MCL (7.5%) and MM (7.5%). Median age was 57 years (19–74), with 50% female patients. At infusion, 41.4% were in complete response or partial response (CR/PR) and 58.6% in stable disease or progressive disease (SD/PD). Median LDH was 202 U/L (124–727) and median prior lines were 2 (1–6).
In the non-FHHM group, diagnoses were DLBCL (74.5%), B-ALL (9.8%), MCL (12.0%) and MM (3.8%). Median age was 60 years (20–77), with 56.6% female patients. At infusion, 39.4% were in CR/PR and 60.6% in SD/PD. Median LDH was 209 U/L (101–3000) and median prior lines were 2 (1–7). Baseline characteristics did not significantly differ between groups (all p>0.05).
Post–CAR-T toxicities were comparable. In the FHHM group, CRS grade 0–1, 2 and 3–4 occurred in 47.5%, 45.5% and 7.5%, respectively; ICANS ≥1 occurred in 25%. In the non-FHHM group, CRS grade 0–1, 2 and 3–4 occurred in 43.6%, 49.6% and 6.8%, and ICANS ≥1 in 36.8%.
At 12 months, PFS was 60.0% without FHHM and 70.1% with FHHM; at 24 months, 52.3% and 64.7%, respectively. FHHM was not associated with inferior PFS (HR 0.64, 95% CI 0.31–1.30; p=0.217) (Figure 1A).

### Summary/Conclusion

In this CAR-T cohort, the prevalence of FHHM (21%) was comparable to that reported at diagnosis in lymphoma populations. Familial background was not associated with increased toxicity or inferior progression-free survival. Despite the established role of familial aggregation in disease risk, FHHM does not appear to adversely influence short-term outcomes after CAR-T therapy. Further studies are needed to clarify the biological basis of familial clustering in B-cell malignancies.

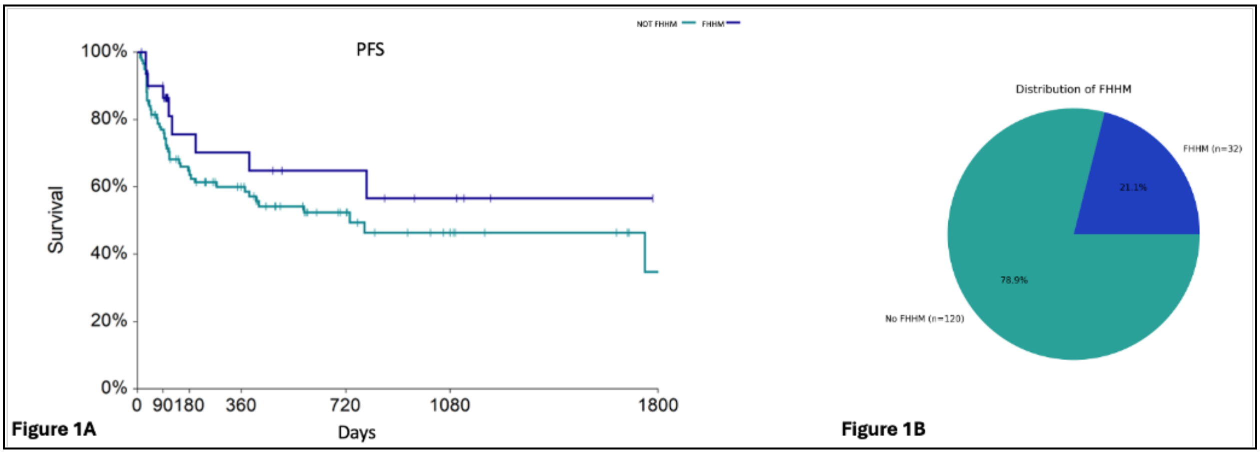

Figure 1. (A) Kaplan–Meier curves of progression-free survival (PFS) according to familial haematological malignancy (FHHM) status after CAR-T therapy. No significant difference was observed between patients with and without FHHM (HR 0.64, 95% CI 0.31–1.30; p=0.217). (B) Distribution of FHHM within the study cohort (32/152, 21%).

**Copyright Information:** (Online) ISSN: 2572-9241
©2026 The Author(s). HemaSphere is published by John Wiley & Sons Ltd on behalf of European Hematology Association. This is an open access Abstract Book distributed under the Attribution-NonCommercial-NoDerivs (CC BY-NC-ND), which allows third parties to download the articles and share them with others as long as they credit the author and the Abstract Book, but they cannot change the content in any way or use them commercially.
**Abstract Book Citations:** Authors, Title, HemaSphere, 2026;10:(S1):pages. The abstract book DOIs can be found on the "Author Information" page.
**Disclaimer:** Articles published in the journal HemaSphere exclusively reflect the opinions of the authors. The authors are responsible for all content in their abstracts including accuracy of the facts, statements, citing resources, etc.

### (PF1255) SILTUXIMAB PROPHYLAXIS FOR CYTOKINE RELEASE SYNDROME (CRS) AND IMMUNE EFFECTOR CELL–ASSOCIATED NEUROTOXICITY SYNDROME (ICANS): REAL-WORLD EVIDENCE (RWE)

**Topic:** 26. Gene therapy, cellular immunotherapy and vaccination - Clinical

Jana Mihályová*[1, 2], Michaela Hornakova[1], Juraj Ďuraš[1, 2], Michal Kascak[1, 2], Barbora Dluhosova[1, 2], Katerina Karasova[1], Katarina Hradská[1, 2], Martin Lachnit[1], Sandra Charvátová[1, 2], Petra Plonková[1], Tereza Popková[1, 2], Ludmila Muronova[1, 2], Hana Plonková[1], Ondrej Venglar[1, 2], Tomas Jelínek[1, 2], Milan Navrátil[1, 2], Zdenek Koristek[1, 2], Roman Hájek[1, 2]

*[1]University hospital Ostrava, Department of Hematooncology, Ostrava, Czechia;[2]University of Ostrava, Department of Hematooncology, Faculty of Medicine, Ostrava, Czechia;*

### Background

Management of CRS and ICANS is evolving from a more conservative approach toward earlier intervention and prophylactic strategies as experience with CAR T-cell therapy expands. Tocilizumab remains standard for CRS, corticosteroids for ICANS, and anakinra is increasingly incorporated for prophylaxis and treatment of ICANS (and CRS) (Park et al, 2023). Siltuximab, a monoclonal antibody targeting circulating IL-6, has demonstrated activity in refractory cases, and emerging data suggest that early or prophylactic use may reduce toxicity severity. However, evidence supporting prophylactic siltuximab remains limited (Bajwa et al, 2025; Narkhede et al, 2024; Denlinger et al, 2025). Here, we report real-world outcomes of patients receiving prophylactic siltuximab prior to anti-CD19 CAR T-cell therapy.

### Aims

To assess the frequency and severity of CRS and ICANS, overall response rate (ORR) and progression-free survival (PFS).

### Methods

We conducted a retrospective analysis of patients with B-cell non-Hodgkin lymphoma (B-NHL, n=23) and B-cell acute lymphoblastic leukemia (B-ALL, n=1) treated with anti-CD19 CAR T-cells (20 axi-cel, 2 brexu-cel, 1 tisa-cel, 1 zamto-cel) (October 2022 - November 2025). Patients treated with tocilizumab (8 mg/kg iv) at the onset of CRS grade ≥1 (control cohort) were compared with a consecutive cohort of patients who received prophylacitic siltuximab. A single dose of siltuximab (11 mg/kg iv) was administered on day –1 prior to CAR T-cell infusion. CRS/ICANS resolution was defined as the last day of toxicity-directed therapy. Group comparisons were performed using the Mann–Whitney U and Fisher's exact tests. PFS was estimated by Kaplan–Meier and compared by log-rank test (two-sided $p < 0.05$).

### Results

Twenty-four patients were included (siltuximab prophylaxis, n=11; control cohort n=13). Baseline characteristics were comparable, including age (median 62 vs 55 years, p=0.75), prior therapy (median 1 in both groups), ECOG, LDH, disease status, and CAR T-cell product distribution (10 axi-cel and 1 brexu-cel in both cohorts; 1 tisa-cel and 1 zamto-cel in the control group). CRS occurred less frequently in the siltuximab cohort compared with control cohort (55% vs 92%), approaching statistical significance (p=0.061). All CRS events were grade 1–2 in both groups. While overall ICANS incidence was similar (45% vs 54%, p=1.0), clinically relevant differences in severity were observed. No grade ≥2 ICANS occurred in the siltuximab cohort, whereas the control group included grade 2 (n=1, 8%) and grade 3 events (n=3, 23%). Median ICANS onset was day 6 vs 4 and median duration was 4 vs 8 days, respectively. ORR (≥PR) were comparable (73% vs 76%, p=1.0). Median PFS was 13.8 months (1–15 months) in the siltuximab group and not reached in control group (1–28.7 months; p=0.76). Importantly, siltuximab prophylaxis was not associated with an increased risk of infection (36% vs 62%, p=0.41).

**Copyright Information:** (Online) ISSN: 2572-9241
©2026 The Author(s). HemaSphere is published by John Wiley & Sons Ltd on behalf of European Hematology Association. This is an open access Abstract Book distributed under the Attribution-NonCommercial-NoDerivs (CC BY-NC-ND), which allows third parties to download the articles and share them with others as long as they credit the author and the Abstract Book, but they cannot change the content in any way or use them commercially.
**Abstract Book Citations:** Authors, Title, HemaSphere, 2026;10:(S1):pages. The abstract book DOIs can be found on the "Author Information" page.
**Disclaimer:** Articles published in the journal HemaSphere exclusively reflect the opinions of the authors. The authors are responsible for all content in their abstracts including accuracy of the facts, statements, citing resources, etc.

**Summary/Conclusion**

To our knowledge, this is the first real-world analysis evaluating prophylactic siltuximab administered prior to anti-CD19 CAR T-cell therapy. Siltuximab prophylaxis was associated with a lower incidence of CRS (55%) without grade ≥3 events. Although overall ICANS incidence was comparable to control cohort, no grade ≥2 ICANS occurred. These findings are consistent with results from the phase I siltuximab study
(Denlinger et al, 2025). Our data warrants further exploration of siltuximab as a prophylactic agent for CRS and ICANS in CAR T-cell therapy.

**Copyright Information:** (Online) ISSN: 2572-9241
©2026 The Author(s). HemaSphere is published by John Wiley & Sons Ltd on behalf of European Hematology Association. This is an open access Abstract Book distributed under the Attribution-NonCommercial-NoDerivs (CC BY-NC-ND), which allows third parties to download the articles and share them with others as long as they credit the author and the Abstract Book, but they cannot change the content in any way or use them commercially.
**Abstract Book Citations:** Authors, Title, HemaSphere, 2026;10:(S1):pages. The abstract book DOIs can be found on the "Author Information" page.
**Disclaimer:** Articles published in the journal HemaSphere exclusively reflect the opinions of the authors. The authors are responsible for all content in their abstracts including accuracy of the facts, statements, citing resources, etc.

### (PF1256) DIVERGENT PATHWAYS: COMPARATIVE LOGISTICAL AND PERCEPTUAL BARRIERS TO GENE THERAPY ADOPTION IN HEMOPHILIA VERSUS HEMOGLOBINOPATHIES IN THE UNITED STATES

**Topic:** 26. Gene therapy, cellular immunotherapy and vaccination - Clinical

Sarah Hendry[1], Sam Young*[1]

[1]*Spherix Global Insights, Exton, United States of America;*

### Background

Gene therapy represents a paradigm shift in hematology, offering a potentially curative, one-time treatment for inherited blood disorders. Yet despite simultaneous regulatory progress across hemophilia, sickle cell disease (SCD), and beta-thalassemia, clinical uptake remains uneven. While hemoglobinopathy programs report increasing referral momentum, adoption in hemophilia lags behind. Understanding these divergences is essential for optimizing access and implementation across indications.

### Aims

The aim of this study is to compare physician-reported logistical, perceptual, and systemic barriers influencing gene therapy adoption across hemophilia, sickle cell disease, and thalassemia, using cross-indication data from Spherix Global Insights (2024–2025) and expert perspectives from field hematologists in the United States.

### Methods

Mixed-method analysis integrating quantitative data from U.S.-based hematologists treating ≥5 patients with each condition (n=197), including referral likelihood, perceived candidate volume, and operational barriers, qualitative insights from 17 in-depth physician interviews and expert commentary, thematic synthesis of perceptual and infrastructural determinants affecting gene therapy decision-making.

### Results

Across conditions, 83% of hematologists agreed that the efficacy of gene therapy outweighs long-term risks. However, logistical and perceptual divides emerged. In hemoglobinopathies (SCD, thalassemia), adoption constrained primarily by systemic logistics—prior authorization, coordination of services, and payer readiness. 82% of surveyed hematologists cited institutional capacity as limiting uptake. Patient motivation, caregiver engagement, and established transplant infrastructures facilitate readiness despite administrative bottlenecks. In hemophilia, barriers skew toward psychological and clinical inertia. Nearly half (45%) of physicians reported diminished urgency due to effective non-gene-modifying therapies. Logistical complexity (immunosuppression, monitoring) and institutional hesitancy persist, with 43% citing staff burden as a deterrent to referrals. Cost was a dominant constraint across all indications, perceived as more prohibitive in hemophilia due to single-patient contracting and center certification demands.

### Summary/Conclusion

While hemoglobinopathy gene therapy adoption is slowed by logistical hurdles, hemophilia faces deeper perceptual and operational resistance—stemming from therapeutic alternatives, infrastructure immaturity, and physician caution. Realizing equitable access requires disease-specific strategies: for hemophilia, focus needs to center on institutional enablement with re-education. In th United States, te forthcoming CMS Cell and Gene Therapy Access Model may alleviate economic barriers but will not alone bridge the perceptual divide between these patient communities.

**Copyright Information:** (Online) ISSN: 2572-9241
©2026 The Author(s). HemaSphere is published by John Wiley & Sons Ltd on behalf of European Hematology Association. This is an open access Abstract Book distributed under the Attribution-NonCommercial-NoDerivs (CC BY-NC-ND), which allows third parties to download the articles and share them with others as long as they credit the author and the Abstract Book, but they cannot change the content in any way or use them commercially.
**Abstract Book Citations:** Authors, Title, HemaSphere, 2026;10:(S1):pages. The abstract book DOIs can be found on the "Author Information" page.
**Disclaimer:** Articles published in the journal HemaSphere exclusively reflect the opinions of the authors. The authors are responsible for all content in their abstracts including accuracy of the facts, statements, citing resources, etc.

### (PF1257) GOING FOR SECONDS: CLINICAL OUTCOMES AND RESPONSE PREDICTORS FOLLOWING SECOND CD19 CAR T-CELL INFUSION

**Topic:** 26. Gene therapy, cellular immunotherapy and vaccination - Clinical

Kayleen Shi*[1, 2], Samar Shamas[1, 2], Tobias Tix[1, 2], Linus Kruk[1, 2], Linus Roessiger[3], Julia Frimmel[4], Florentien Kolbig[1], Adrian Gottschlich[1, 5], Simon Völkl[3], Fabian Müller[3], Christiane Pott[4], Malte von Bonin[6], Michael von Bergwelt[1], Veit Bücklein[1, 5, 7, 8], Kai Rejeski[1, 2, 7, 9], Marion Subklewe[1, 5, 7, 8]

*[1]Department of Medicine III, LMU University Hospital, LMU Munich, Munich, Germany; [2]Laboratory for Precision Immunotherapy, Department of Medicine III, University Hospital, LMU Munich, Munich, Germany; [3]Department of Internal Medicine 5-Hematology and Oncology, University Hospital, FAU Erlangen-Nuremberg, Erlangen-Nuremberg, Germany; [4]Division of Stem Cell Transplantation and Cellular Immunotherapies, Department of Internal Medicine II, University Hospital Schleswig Holstein, University Kiel, Kiel, Germany; [5]Laboratory for Translational Cancer Immunology, LMU Gene Center, Munich, Germany; [6]Department of Hematology/Oncology, University Hospital Dresden, Dresden, Germany; [7]German Cancer Consortium (DKTK) and German Cancer Research Center (DKFZ), Heidelberg, Germany; [8]Laboratory for Leukemia Diagnostics (LFL), LMU Munich, Munich, Germany; [9]The Bavarian Cancer Research Center (BZKF), Munich, Germany;*

### Background

CD19-directed CAR T-cell therapy has transformed outcomes in relapsed or refractory B-cell malignancies. Nevertheless, a substantial proportion of patients relapse after an initial response to CAR T cell therapy (CAR1) and prognosis after progression remains poor. In selected patients with late relapse, reinfusion of a cryopreserved second bag of the identical CAR-T product (CAR2) has been employed as a salvage strategy. This approach allows interrogation of disease- and host-intrinsic determinants of restored sensitivity to the same CART product. However, clinical experience remains limited, and the biological factors underlying differential outcomes after CAR2 are poorly understood.

### Aims

We sought to identify determinants of restored therapeutic sensitivity to CAR2 by contrasting durable responders with patients experiencing repeat relapse.

### Methods

We performed a multicenter retrospective observational cohort study of adults with B-cell malignancies relapsing after CAR1 who received CAR2 infusion. 12 patients were included (DLBCL n=7, FL n=1, B-ALL n=4) treated with axi-cel (n=6), tisa-cel (n=2), liso-cel (n=1) or MB-CART19.1 (n=3). 10 of 12 patients received a back-up bag (2nd bag) of the leukapheresis product collected prior to CAR1. The remaining two patients underwent another round of leukapheresis after CAR1. Variables captured for both infusions included toxicity profiles, best response according to Lugano criteria as well as PFS and OS.

**Copyright Information:** (Online) ISSN: 2572-9241
©2026 The Author(s). HemaSphere is published by John Wiley & Sons Ltd on behalf of European Hematology Association. This is an open access Abstract Book distributed under the Attribution-NonCommercial-NoDerivs (CC BY-NC-ND), which allows third parties to download the articles and share them with others as long as they credit the author and the Abstract Book, but they cannot change the content in any way or use them commercially.
**Abstract Book Citations:** Authors, Title, HemaSphere, 2026;10:(S1):pages. The abstract book DOIs can be found on the "Author Information" page.
**Disclaimer:** Articles published in the journal HemaSphere exclusively reflect the opinions of the authors. The authors are responsible for all content in their abstracts including accuracy of the facts, statements, citing resources, etc.

### Results

Median time from CAR1 to relapse was 10.3 months (range 0.1–35.2) and the median interval from relapse to CAR2 was 1.8 months (range 0.5–12.9). The best overall response after CAR2 was 91% (CR 58%, PR 33%). At a median follow-up of 11.9 months (range 0.9–58.7) after CAR2, median PFS was 9.2 months (range 0.9–58.7). Seven deaths occurred, five of which were related to relapse/progression. The patient with the longest duration of response to CAR1 had a NRM event 27 days after CAR2.
In exploratory analyses, extended PFS after CAR2 was associated with better response to CAR1 (median PFS after CAR2 in patients with following responses to CAR1: CR 382 days vs PR 201 days vs PD 27 days). Additionally, patients with CAR2 progression at data cut-off had a shorter duration of response after CAR1 than those without CAR2 progression (149 vs 667 days). Of interest, maximum CRS and ICANS grades decreased from CAR1 to CAR2 (for CRS 50% and for ICANS 40% of patients with lower grade after CAR2). When comparing differences in baseline features between CAR2 responders vs. non-responders, we noted elevated LDH levels in non-responders at the time of CAR2 reinfusion.

### Summary/Conclusion

In this real-world cohort, CAR2 appears to be a feasible salvage strategy in selected patients. Late CAR1 relapse and better response to CAR1 emerge as predictors of response to CAR2. In addition, differences in LDH levels at CAR2 reinfusion are observed between responders vs non-responders while toxicity severity diminished between CAR1 and CAR2 within the same patient. Further analyses investigating biological mechanisms underlying these associations are ongoing and will be presented.

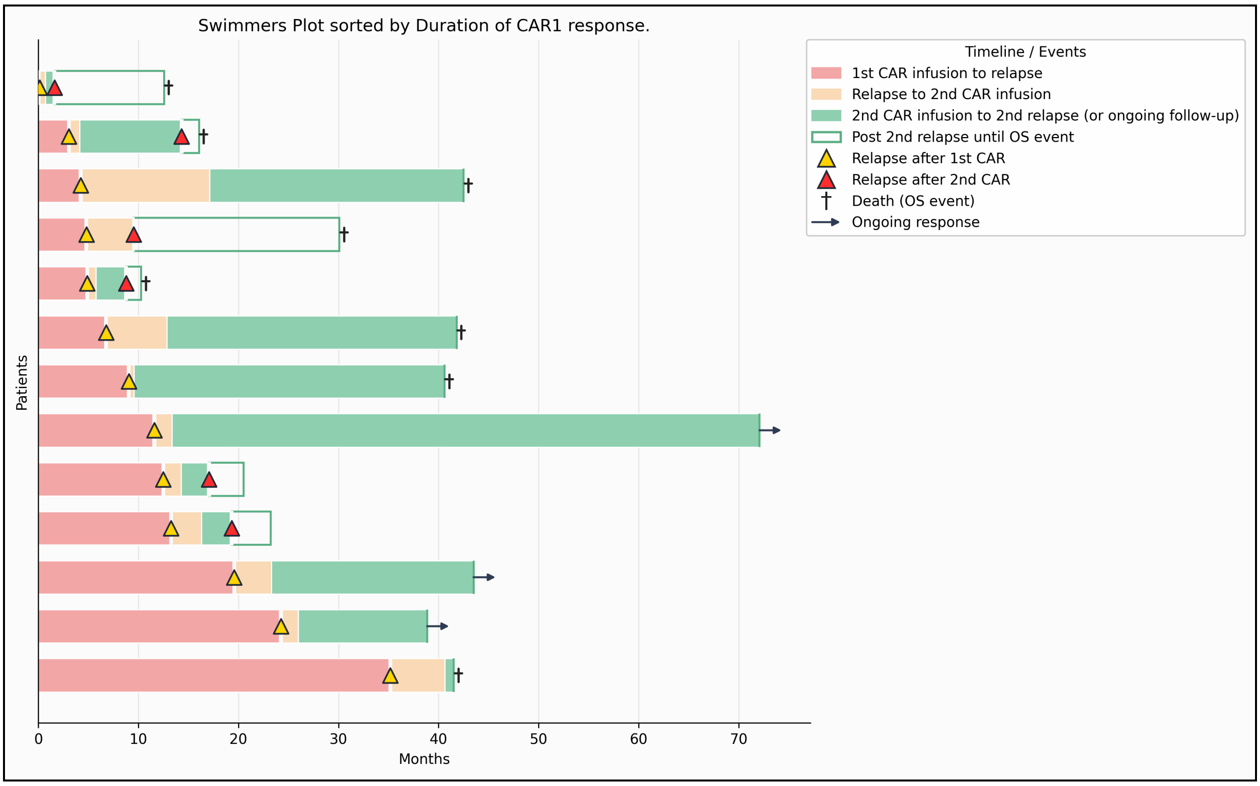

Copyright Information: (Online) ISSN: 2572-9241
©2026 The Author(s). HemaSphere is published by John Wiley & Sons Ltd on behalf of European Hematology Association. This is an open access Abstract Book distributed under the Attribution-NonCommercial-NoDerivs (CC BY-NC-ND), which allows third parties to download the articles and share them with others as long as they credit the author and the Abstract Book, but they cannot change the content in any way or use them commercially.
Abstract Book Citations: Authors, Title, HemaSphere, 2026;10:(S1):pages. The abstract book DOIs can be found on the "Author Information" page.
Disclaimer: Articles published in the journal HemaSphere exclusively reflect the opinions of the authors. The authors are responsible for all content in their abstracts including accuracy of the facts, statements, citing resources, etc.

### (PF1258) CURRENT CLINICAL TRIALS FOR NEW THERAPIES IN SICKLE CELL DISEASE DO NOT ADEQUATELY REPRESENT THE PATIENT POPULATION

**Topic:** 27. Sickle cell disease

Najibah Galadanci[1], Jill Hancock[1], Allison Plaxco[2], Gerhard Hellemann[2], Charles Abrams[3], Kathleen Torres[4], Julie Kanter*[1]

*[1]University of Alabama at Birmingham, Lifespan Comprehensive Sickle Cell Center, Birmingham, United States of America; [2]University of Alabama at Birmingham, Department of Biostatistics, School of Public Health, Birmingham, United States of America; [3]Perelman School of Medicine, University of Pennsylvania, Department of Medicine, Philadelphia, United States of America; [4]ASH Research Collaborative, Washington DC, United States of America;*

**Background**

Sickle cell disease (SCD) is a progressive disorder characterized by acute pain crisis (APC), organ damage, and early mortality. Despite the (few) disease-modifying therapies available, many individuals experience inadequate symptom control or treatment intolerance. Treatment effectiveness varies by genotypes, age, and social contexts, highlighting SCD heterogeneity. The gold standard for clinical trials (CT) (randomized-placebo control with APC as an endpoint) may be suboptimal and has contributed to unsuccessful trials. Further, two recently approved SCD therapies were withdrawn from Europe after real-world or later-stage data raised concerns about safety and efficacy.

**Aims**

To assess the generalizability of commonly used inclusion and exclusion criteria in CT for SCD relative to real-world patients.

**Methods**

Using validated hash-based tokenization to generate unique hash-ID, we linked and de-duplicated subjects across three registries to identify the population of patients potentially eligible for an outpatient CT for SCD (Galadanci, Blood Advances 2026). Databases used: ALSTATE (the Alabama SCD data collection surveillance registry funded by the Centers of Disease Control, GRNDaD (registry of the National Alliance of SCD Centers) and the ASH RC Data Hub. We identified subjects' SCD phenotype, organ complications, current treatment and number of APC (emergency department visits or hospitalizations) in 2024. We also reviewed clinicaltrials.gov to identify active and/or recruiting industry trials and key eligibility criteria. (Table 1)

**Copyright Information:** (Online) ISSN: 2572-9241
©2026 The Author(s). HemaSphere is published by John Wiley & Sons Ltd on behalf of European Hematology Association. This is an open access Abstract Book distributed under the Attribution-NonCommercial-NoDerivs (CC BY-NC-ND), which allows third parties to download the articles and share them with others as long as they credit the author and the Abstract Book, but they cannot change the content in any way or use them commercially.
**Abstract Book Citations:** Authors, Title, HemaSphere, 2026;10:(S1):pages. The abstract book DOIs can be found on the "Author Information" page.
**Disclaimer:** Articles published in the journal HemaSphere exclusively reflect the opinions of the authors. The authors are responsible for all content in their abstracts including accuracy of the facts, statements, citing resources, etc.

### Results

There were 6606 unique subjects after de-duplication. GRNDaD is the most highly-phenotyped and smallest data set (n=456). These subjects were linked via hash-ID to other registries. We selected only adults 18-65 years (n=385) and only those currently alive in Alabama (n=363). Using genotype as a proxy for hemoglobin (Hb), only subjects with HbSS/HbSB0 were included (n=251). Exclusion included chronic red cell transfusions (65), prior stem cell transplant (17) and ESRD (6) leaving 160 individuals. Applying common trial APC criteria (2-12 APC/year) only 45 subjects (9.9% of the original GRNDaD cohort) were potentially eligible for most SCD trials (figure). When excluding persons on hydroxyurea, only 8 persons remained eligible.

| NCT Number | Age range (years) | Key inclusion criteria (APC/YR) | Key exclusion criteria | Hb criteria (g/dl) |
|---|---|---|---|---|
| **NCT07431398** | 18–65 | ≥4 | no HU | ≥ 5.5 and ≤ 12 |
| **NCT06546670** | 12-55 | 2-10 | no HU | n/a |
| **NCT06924970** | 16-55 | 2-10 | >10 APC/yr | ≥5.5 and ≤10.5 |
| **NCT05031780** | 16-55 | 2-10 | >10 APC/yr | ≥5.5 and ≤10.5 |
| **NCT04624659** | 12-65 | 2–15 | >15 VOC/yr | ≥5.0 and ≤10.0 |
| **NCT06612268** | >/=12 | 1-15 | >15 VOC and no crizanlizumab | ≥5.0 and ≤10.0 |
| **NCT06975865** | 10-65 | 2-10 SCA only | no anticoagulation | n/a |
| **NCT06439082** | >/=12 | 4-12 | No clinical stroke | n/a |
| **NCT05431088** | 12-65 | 2-10 | n/a | n/a |
| **NCT06481306** | >/=18 | ≥ 4 | >8 APC | ≥ 5.5 and ≤ 12 |

### Summary/Conclusion

Current CT eligibility criteria exclude the majority of real-world adults with SCD. At this SCD center, ~90% of the population are excluded with these criteria (and would not be included in a drug label). These findings suggest 1). New trial designs with broader inclusion criteria and better endpoints are urgently needed 2). New medications would not be available for most people with SCD who need them and 3). Widespread (off label) use of these therapies in the post-marketing period can lead to detrimental results. Continued development of safe, effective, and accessible therapies is essential to reduce morbidity, and improve survival and quality of life for individuals living with SCD.

**Copyright Information:** (Online) ISSN: 2572-9241
©2026 The Author(s). HemaSphere is published by John Wiley & Sons Ltd on behalf of European Hematology Association. This is an open access Abstract Book distributed under the Attribution-NonCommercial-NoDerivs (CC BY-NC-ND), which allows third parties to download the articles and share them with others as long as they credit the author and the Abstract Book, but they cannot change the content in any way or use them commercially.
**Abstract Book Citations:** Authors, Title, HemaSphere, 2026;10:(S1):pages. The abstract book DOIs can be found on the "Author Information" page.
**Disclaimer:** Articles published in the journal HemaSphere exclusively reflect the opinions of the authors. The authors are responsible for all content in their abstracts including accuracy of the facts, statements, citing resources, etc.

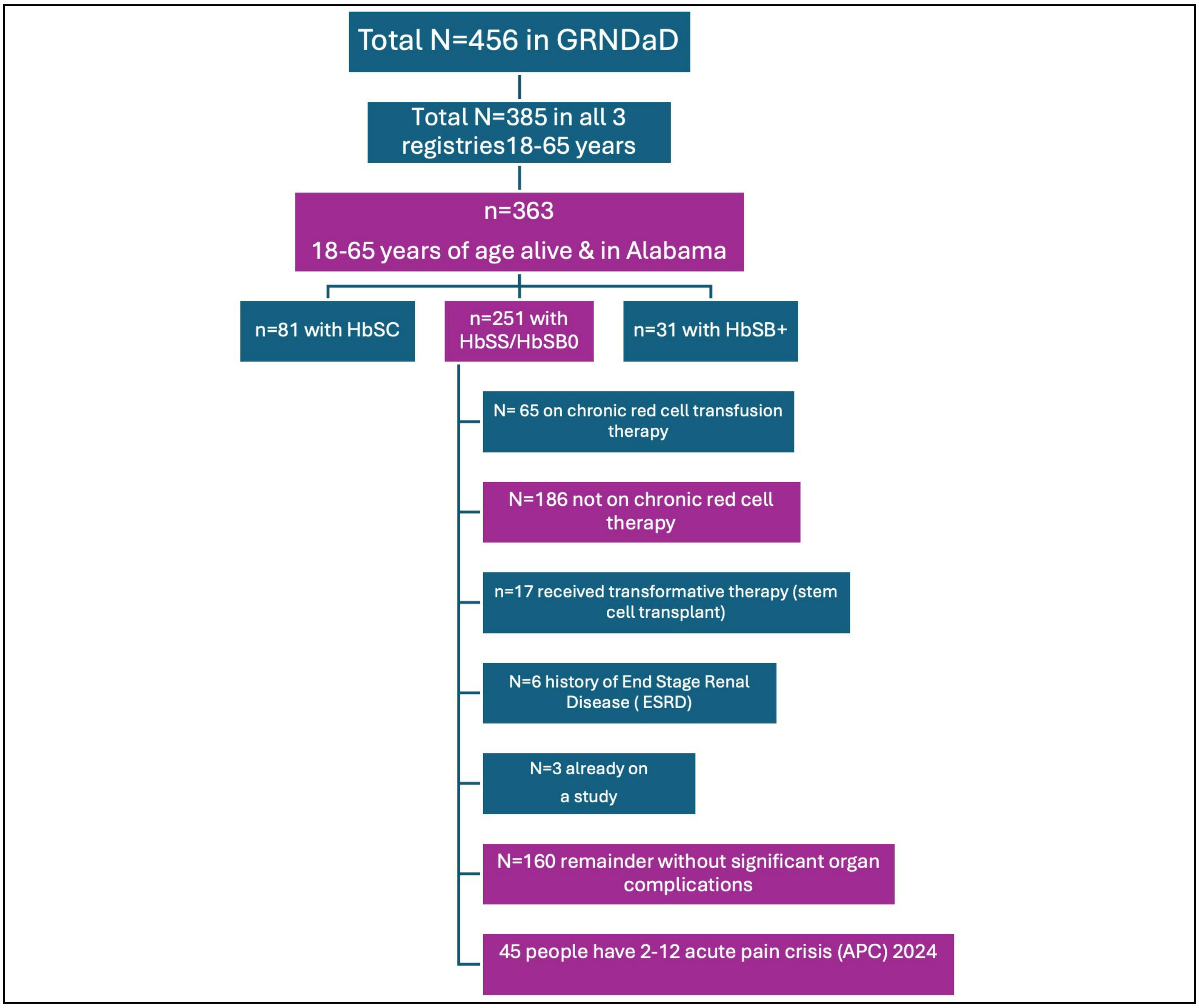

Adults With SCD Identified Using Hash-ID in Three Registries Potentially Eligible for Clinical Trials Based on Common Inclusion/Exclusion Criteria

**Copyright Information:** (Online) ISSN: 2572-9241
©2026 The Author(s). HemaSphere is published by John Wiley & Sons Ltd on behalf of European Hematology Association. This is an open access Abstract Book distributed under the Attribution-NonCommercial-NoDerivs (CC BY-NC-ND), which allows third parties to download the articles and share them with others as long as they credit the author and the Abstract Book, but they cannot change the content in any way or use them commercially.
**Abstract Book Citations:** Authors, Title, HemaSphere, 2026;10:(S1):pages. The abstract book DOIs can be found on the "Author Information" page.
**Disclaimer:** Articles published in the journal HemaSphere exclusively reflect the opinions of the authors. The authors are responsible for all content in their abstracts including accuracy of the facts, statements, citing resources, etc.

### (PF1259) TARGETING DCN1 OVERCOMES DELETERIOUS EFFECTS OF BROAD NEDDYLATION INHIBITION AND ENABLES FETAL HEMOGLOBIN INDUCTION IN SICKLE CELL DISEASE

**Topic:** 27. Sickle cell disease

Sriram Krishnamoorthy*[1], Samuel Miller[1], Anthony Monti[1], Enhua Zhou[1], Chi Hua Sarah Lin[1], Orna Lynch[1], Sayani Mukherjee[1], Cassie Hei[1], Robert Nicewonger[1], Colin Diner[1], Alejandra MacIas[1], Kristen Marino[1], Na Li[1], Ravi Ujjinamatada[1], Akshay Salegaonkar[1], Sunil Kuppasani[1], Maximilian Grazado[1], Corey Hayford[1], Frank Lovering[1], Qin Tang[1], Emily Ye[1], Wei Zhang[1], Timothy Fulton[1], Jennifer O'brien[1], Xiaoji Sun[1], Atli Thorarensen[1], Mauricio Cortes[1], Cameron Trenor Iii[1]

[1]*Cellarity, Somerville, United States of America;*

### Background

Sickle cell disease (SCD) is a genetic disorder caused by a point mutation in the beta-globin gene characterized by hemolysis, vaso-occlusive crisis (VOC), and pain. Human genetic studies and therapeutic approaches have demonstrated that increasing fetal hemoglobin (HbF) reduces VOCs and ameliorates SCD progression. The standard of care, hydroxyurea, induces HbF, but has side effects such as myelosuppression and genotoxicity with high variability in patient responses. Recent approval of an autologous cell-based gene therapy, targeting the BCL11A enhancer, has demonstrated the curative potential of HbF in SCD. However, this therapy remains limited due to inaccessibility and high cost and risk of toxicity. Unmet need in SCD remains high, with a strong interest in oral HbF inducers potentially overcoming access challenges.

### Results

We dissected human erythropoiesis using single cell transcriptomics and derived cellular trajectories with discovery of neddylation as a regulator of HbF. Pan-neddylation inhibition using MLN4924 or knockout of ubiquitin conjugating enzyme E2M (UBE2M) or Cullin 3 (CUL3) induced HbF accompanied with cytotoxicity. Using single cell transcriptomics, we identified that pan-neddylation inhibition, though efficacious in inducing HBG, also caused erythropoiesis to skew toward an aberrant cell state. We therefore investigated CUL3 regulation to preserve robust HbF induction avoiding lineage skewing.
We explored a neddylation protein-protein interaction sub-network pruned of nodes with significant impact on cell viability using the cancer Dependency Map CRISPR viability dataset. This revealed Defective in Cullin Neddylation 1 (DCN1), a scaffold protein bridging UBC12 and CUL3 facilitating neddylation. Genetic knockout and pharmacological inhibition of DCN1 in CD34 cells, induced both HBG and HbF. Pharmacologic inhibition of DCN1 led to a 2.5-fold increase in HBG expression (EC50 = 17.5nM)25% HbF by HPLC, and a 3-fold increase in HBG/HBB ($p<0.001$). Mechanistic deconvolution revealed that DCN1 inhibition selectively induces HBG via globin switching through partial suppression of CUL3 neddylation, without impairment of erythropoiesis. DCN1 inhibition resulted in differential regulation, higher chromatin accessibility and transcriptional activity at the HBG loci, via known activators such as NFYA. These observations provide the first evidence linking DCN1 inhibition and globin locus regulation.
Using structure guided chemistry, we generated a first-in-class DCN1 inhibitor, CLY-124. In NBSGW mice engrafted with human CD34 cells, CLY-124 administration led to a 4 -fold induction in HBG and up to 30% HBG over total globin gene expression ($p<0.0001$), 2-fold greater than hydroxyurea and approaching levels reported for BCL11A enhancer knockout. In addition, DCN1 inhibition combined with hydroxyurea induced >25% HbF *in vitro* and a robust synergistic induction *in vivo*.

**Copyright Information:** (Online) ISSN: 2572-9241
©2026 The Author(s). HemaSphere is published by John Wiley & Sons Ltd on behalf of European Hematology Association. This is an open access Abstract Book distributed under the Attribution-NonCommercial-NoDerivs (CC BY-NC-ND), which allows third parties to download the articles and share them with others as long as they credit the author and the Abstract Book, but they cannot change the content in any way or use them commercially.
**Abstract Book Citations:** Authors, Title, HemaSphere, 2026;10:(S1):pages. The abstract book DOIs can be found on the “Author Information” page.
**Disclaimer:** Articles published in the journal HemaSphere exclusively reflect the opinions of the authors. The authors are responsible for all content in their abstracts including accuracy of the facts, statements, citing resources, etc.

**Summary/Conclusion**

In summary, we identified DCN1 as a novel target for the treatment of SCD, and discovered a first-in-class inhibitor, CLY-124, that induces HbF without cytotoxicity as monotherapy and shows synergy with hydroxyurea. At pharmacologically active doses in preclinical studies, CLY-124 exhibited a favorable safety profile with no observed toxicities or cytopenias. CLY-124 is being evaluated in a first-in-human study assessing safety, pharmacokinetics, and HbF induction in healthy volunteers and participants with SCD.

**Copyright Information:** (Online) ISSN: 2572-9241
©2026 The Author(s). HemaSphere is published by John Wiley & Sons Ltd on behalf of European Hematology Association. This is an open access Abstract Book distributed under the Attribution-NonCommercial-NoDerivs (CC BY-NC-ND), which allows third parties to download the articles and share them with others as long as they credit the author and the Abstract Book, but they cannot change the content in any way or use them commercially.
**Abstract Book Citations:** Authors, Title, HemaSphere, 2026;10:(S1):pages. The abstract book DOIs can be found on the "Author Information" page.
**Disclaimer:** Articles published in the journal HemaSphere exclusively reflect the opinions of the authors. The authors are responsible for all content in their abstracts including accuracy of the facts, statements, citing resources, etc.

### (PF1260) AFTER TWO DECADES OF HYDROXYUREA: LEUKEMIC RISK IN SICKLE CELL DISEASE ASSESSED BY INTEGRATED MARROW MORPHOLOGY, CYTOGENETICS AND GENOMICS

**Topic:** 27. Sickle cell disease

Aurelio Maggio*[1], Rosario Di Maggio[2], Disma Renda[2], Vincenzo Luca Lentini[3], Alessandra Santoro[4], Angela Vitrano[2], Francesca Garofano[5], Alessandro Inzerillo[6], Mariasanta Napolitano[7]

*[1]Foundation Franco and Piera Cutino, Palermo, Italy; [2]AOR Villa Sofia V. Cervello, Palermo, Italy; [3]Ospedali Riuniti Villa Sofia-Cervello, Palermo, Italy; [4]AOR Villa Sofia Cervello, Palermo, Italy; [5]Università degli Studi di Palermo, Palermo, Italy; [6]AOR Villa Sofia V, Palermo, Italy; [7]Direttore UOC Ematologia e Malattie rare, PO "Cervello, Palermo, Italy;*

### Background

Hydroxyurea (HU) is the main disease-modifying therapy in sickle cell disease (SCD), yet concerns about leukemogenicity after very prolonged exposure persist. Data beyond 20 years and analyses integrating marrow morphology, cytogenetics and high-resolution genomics are scarce. The relative contribution of chronic inflammatory stress, clonal hematopoiesis (CHIP), inherited susceptibility, and treatment exposure in ultra-long-term treated SCD patients remains undefined.

### Aims

To characterize clonal architecture and myeloid neoplasm evolution in SCD patients treated with HU for ≥20 years compared with a shorter-duration cohort (<20 years).

### Methods

Single-center pilot study of 15 SCD patients aged >50 years receiving HU (n=4 <20 years; n=11 ≥20 years). Bone marrow (BM) morphology, conventional karyotype, array-CGH and targeted NGS (including Indel/SNP calling) were performed; analyses were descriptive. Retrospective study authorization was obtained by IRB.

### Results

In the ≥20-year cohort, median BM cellularity was 64% and megakaryocytic dysplasia/atypia was observed in 8/11 (73%). Two myeloid transformations occurred, both in the ≥20-year cohort: one myelodysplastic neoplasm with excess blasts (treated with decitabine) and one MDS/AML with fibrosis carrying a likely pathogenic TP53 variant, a recognized high-risk CHIP-associated driver. Targeted NGS showed heterogeneous clonal profiles, including JAK2 V617F (VAF 15%), TP53 and BRCA2 variants, alongside additional variants in genes not classically linked to CHIP (e.g., PRSS1, HFE, CDCA8, NCOA3). One clinically stable patient carried JAK2 and BRCA2 variants without morphologic progression. Notably, the patient with excess blasts had a sister with an identical NGS profile despite <20 years of HU exposure and no hematologic transformation, suggesting familial clonal susceptibility. Across the cohort, no trend toward higher variant burden or increased copy-number/structural genomic complexity was observed with ≥20 years of HU.

**Copyright Information:** (Online) ISSN: 2572-9241
©2026 The Author(s). HemaSphere is published by John Wiley & Sons Ltd on behalf of European Hematology Association. This is an open access Abstract Book distributed under the Attribution-NonCommercial-NoDerivs (CC BY-NC-ND), which allows third parties to download the articles and share them with others as long as they credit the author and the Abstract Book, but they cannot change the content in any way or use them commercially.
**Abstract Book Citations:** Authors, Title, HemaSphere, 2026;10:(S1):pages. The abstract book DOIs can be found on the "Author Information" page.
**Disclaimer:** Articles published in the journal HemaSphere exclusively reflect the opinions of the authors. The authors are responsible for all content in their abstracts including accuracy of the facts, statements, citing resources, etc.

### Summary/Conclusion

Very long-term HU exposure in SCD was associated with isolated transformation events but not with a consistent signature of cumulative genomic instability. The coexistence of CHIP-associated drivers and familial mutational concordance supports a model in which intrinsic clonal susceptibility within a chronically inflamed and stress-activated hematopoietic milieu characteristic of SCD biology may be more relevant than HU duration per se. HU may act as a selective pressure rather than a direct mutagen. Prospective longitudinal surveillance integrating molecular and inflammation-related biomarkers is warranted to refine risk stratification beyond time-based treatment metrics.

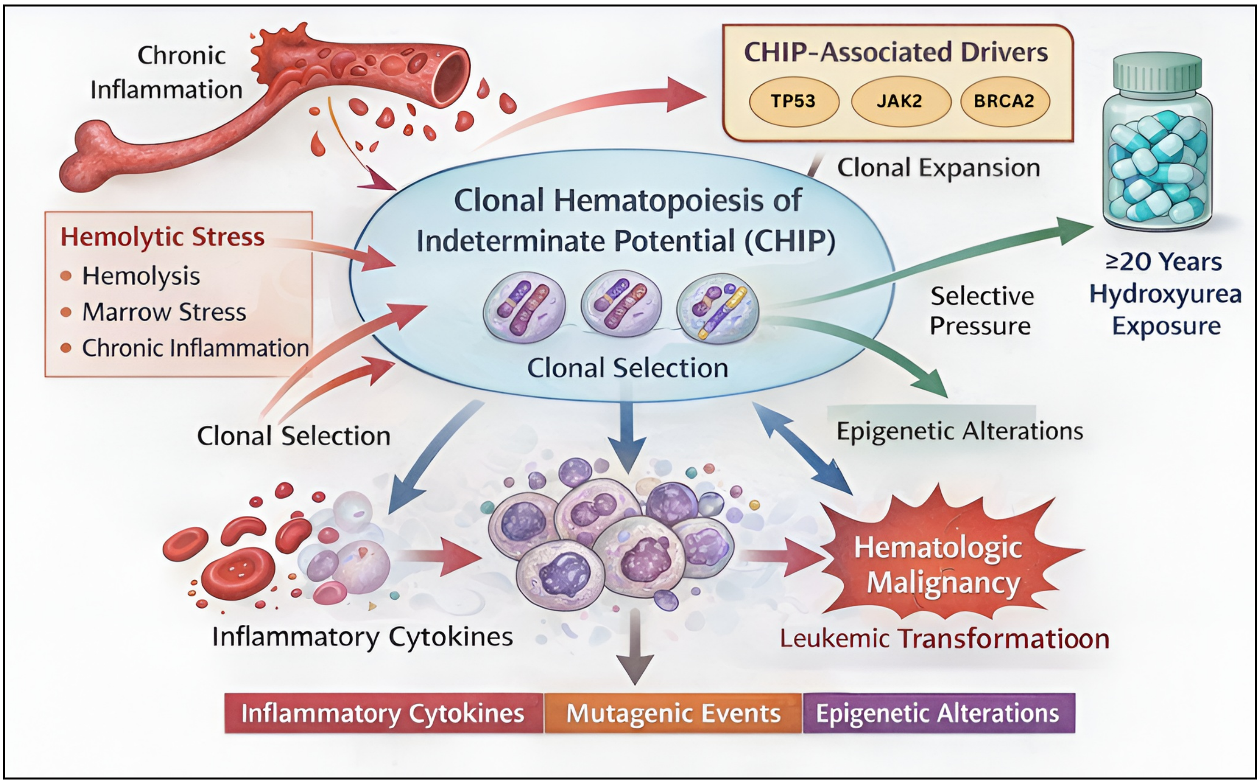

Integrated conceptual model of leukemic risk after ≥20 years of hydroxyurea exposure in SCD. Chronic inflammation, hemolytic/marrow stress and CHIP-associated drivers may promote clonal evolution; hydroxyurea may act as selective pressure rather than a direct mutagenic trigger.

**Copyright Information:** (Online) ISSN: 2572-9241
©2026 The Author(s). HemaSphere is published by John Wiley & Sons Ltd on behalf of European Hematology Association. This is an open access Abstract Book distributed under the Attribution-NonCommercial-NoDerivs (CC BY-NC-ND), which allows third parties to download the articles and share them with others as long as they credit the author and the Abstract Book, but they cannot change the content in any way or use them commercially.
**Abstract Book Citations:** Authors, Title, HemaSphere, 2026;10:(S1):pages. The abstract book DOIs can be found on the "Author Information" page.
**Disclaimer:** Articles published in the journal HemaSphere exclusively reflect the opinions of the authors. The authors are responsible for all content in their abstracts including accuracy of the facts, statements, citing resources, etc.

### (PF1261) EMERGENCY IMPLEMENTATION OF A SICLOPEDIE-BASED TELEMEDICINE HOTLINE FOR CHILDREN AND PREGNANT WOMEN WITH SICKLE CELL DISEASE AND THALASSEMIA AFTER CYCLONE CHIDO IN MAYOTTE

**Topic:** 27. Sickle cell disease

Elena Properzi*[1, 2, 3], Ibtissem Butez[3], Cindy Mescam[1], Jean-Christophe Nahon[1], Vincent Malcor Deydier De Pierrefeu[1], Mariama Nouroudine[3], Cécile Daumesnil[4], Audrey Douanier[3], Estelle Mechain[2], Abdourahim Chamouine[3], Pablo Bartolucci[1]

*[1]CHU Mondor, Unité maladie génétiques globule rouge, Créteil, France;[2]Centre Hospitalier Intercommunal de Créteil, Pediatrics, Créteil, France; [3]Centre Hospitalier de Mayotte, Pediatrics, Mamoudzou, Mayotte;[4]CHU Rouen, Rouen, France;*

### Background

Cyclone Chido struck Mayotte on December 14, 2024, causing major destruction, particularly in informal settlements inhabited by low-income and migrant populations. Transportation systems were disrupted and the local hospital partially damaged. French government deployed the Élément de Sécurité Civile Rapide d'Intervention Médicale, a field hospital dedicated to acute disaster-related conditions. While emergency response structures focused primarily on trauma and acute medical needs, continuity of care for chronic diseases was not initially organized. Sickle cell disease (SCD), due to its severity and unpredictability, represented a major concern, especially for children and pregnant women. All major triggers of vaso-occlusive complications were present: dehydration, climatic stress, increased infectious risk due to water shortages, and psychological stress.

### Aims

To describe the emergency deployment of a structured hotline and telemedicine program, coordinated by RED France using the Siclopedie electronic medical record, to ensure systematic follow-up of children and pregnant women with SCD and thalassemia in Mayotte.

### Methods

All patients were previously registered in Siclopedie. From January 3 to 23, trained nurses and research staff contacted families by phone (2-hour time difference with mainland France; 1 hour with Réunion Island).
Patients were triaged into three risk-based categories:

- Red: acute warning signs (infection, vaso-occlusive crisis (VOC), respiratory symptoms, altered general condition), prompting immediate physician contact and same-day teleconsultation.
- Orange: moderate symptoms (fatigue, reduced intake, jaundice/pallor, splenomegaly, dark urine, gastrointestinal symptoms) or predefined high-risk profiles (infants <1 year, pregnant women, children aged 1–5 years on hydroxyurea or chronic transfusion programs, previously transfused patients, or limited disease education).
- Green: no concerning criteria.

Following teleconsultation, patients were classified as stable, requiring organized outpatient care (hospital or mobile clinic), or urgently referred with direct physician-to-physician communication.
Food and water insecurity were assessed, and NGOs were alerted when necessary. A mobile medical unit enabled on-site consultations in remote villages.

**Copyright Information:** (Online) ISSN: 2572-9241
©2026 The Author(s). HemaSphere is published by John Wiley & Sons Ltd on behalf of European Hematology Association. This is an open access Abstract Book distributed under the Attribution-NonCommercial-NoDerivs (CC BY-NC-ND), which allows third parties to download the articles and share them with others as long as they credit the author and the Abstract Book, but they cannot change the content in any way or use them commercially.
**Abstract Book Citations:** Authors, Title, HemaSphere, 2026;10:(S1):pages. The abstract book DOIs can be found on the "Author Information" page.
**Disclaimer:** Articles published in the journal HemaSphere exclusively reflect the opinions of the authors. The authors are responsible for all content in their abstracts including accuracy of the facts, statements, citing resources, etc.

### Results

Between January 3 and January 23, 235 patients were contacted. Multilingual teleconsultations were provided when needed.

We triaged 10 red, 27 orange, and 111 green cases, leading to 60 teleconsultations (including follow-up).
Mean delay from triage to teleconsultation was 1.3 h for red cases, 39h for orange cases.
A 12 years-old with VOC and gastroenteritis, a toddler with gastroenteritis and a 12 years-old with infected dog bites were hospitalized. Mean delay from triage to hospitalization has been 3.2 h
From January 6 to 10, the mobile unit conducted 60 consultations across 4 health zones.

### Summary/Conclusion

This three-step model—remote triage, teleconsultation, and coordinated local intervention—enabled rapid and structured care delivery despite disaster-related disruption. By reducing geographic and social barriers, it supported vulnerable chronic disease cohorts while alleviating pressure on damaged local healthcare facilities.
Siclopedie-based telemedicine hotlines may represent a scalable strategy for chronic disease management in disaster settings. The program remains active to support understaffed local teams.

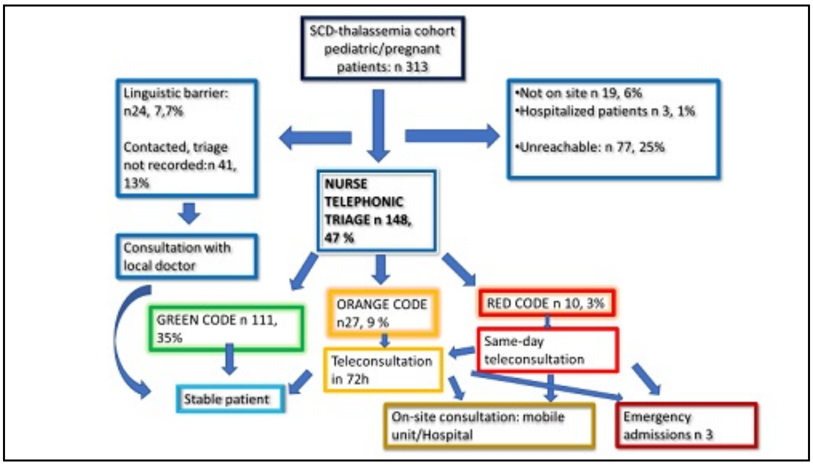

**Copyright Information:** (Online) ISSN: 2572-9241
©2026 The Author(s). HemaSphere is published by John Wiley & Sons Ltd on behalf of European Hematology Association. This is an open access Abstract Book distributed under the Attribution-NonCommercial-NoDerivs (CC BY-NC-ND), which allows third parties to download the articles and share them with others as long as they credit the author and the Abstract Book, but they cannot change the content in any way or use them commercially.
**Abstract Book Citations:** Authors, Title, HemaSphere, 2026;10:(S1):pages. The abstract book DOIs can be found on the "Author Information" page.
**Disclaimer:** Articles published in the journal HemaSphere exclusively reflect the opinions of the authors. The authors are responsible for all content in their abstracts including accuracy of the facts, statements, citing resources, etc.

### (PF1262) THROMBOTIC AND BLEEDING OUTCOMES DURING PREGNANCY AND THE POSTPARTUM PERIOD AMONG PEOPLE WITH SICKLE CELL DISEASE: A MULTI-CENTER RETROSPECTIVE ANALYSIS

**Topic:** 27. Sickle cell disease

Ryan Hunt[1], Krista Latta[1], Layla Van Doren[2], Alexandra Galls[3], Bria Carrithers[2], Valentina Restrepo[2], Maureen Achebe[4], Sharl Azar[3], Nancy Berliner[4], Jean Connors[5], Elizabeth Klings[6], Arielle L. Langer[4], Thomas McElrath[4], Patrick McGann[7], Deborah Siegal[8], Molly Stout[1], Lauren Merz*[1]

*[1]University of Michigan, Ann Arbor, United States of America; [2]Yale University, New Haven, United States of America; [3]Massachusetts General Hospital, Boston, United States of America; [4]Brigham and Women's Hospital, Boston, United States of America; [5]Dana-Farber Cancer Institute, Boston, United States of America; [6]Boston University, Boston, United States of America; [7]Brown University, Providence, United States of America; [8]University of Ottawa, Ottawa, Canada;*

### Background

During pregnancy and the postpartum period, people with sickle cell disease (SCD) are at high risk for both venous thromboembolism (VTE) and postpartum hemorrhage (PPH). However, the reported rates are broad (VTE: 2%-17%; PPH: 5-24%) and do not delineate rates by SCD genotype, thromboprophylaxis or aspirin use, or other key variables. This uncertainty confounds management decisions around thromboprophylaxis in this medically complex population.

### Aims

To describe the incidence and risk factors associated with VTE and PPH in a multi-center pregnant and postpartum SCD cohort.

### Methods

We conducted an IRB-approved multi-institutional retrospective cohort study of SCD pregnancies from 2015 to 2025 across three academic centers: University of Michigan (UM), Yale University, and Mass General Brigham (MGB). A universal case report form was used to capture baseline demographic and clinical characteristics, obstetric and SCD variables during pregnancy through 90 days postpartum, and potential risk factors for thrombotic or bleeding complications. The primary endpoints were the incidence of VTE and PPH (stratified by severity of estimated blood loss [EBL] ≥ 500 and ≥ 1,000 mL). Univariate and multivariable logistic regression models were used to identify independent predictors of VTE and PPH.

**Copyright Information:** (Online) ISSN: 2572-9241
©2026 The Author(s). HemaSphere is published by John Wiley & Sons Ltd on behalf of European Hematology Association. This is an open access Abstract Book distributed under the Attribution-NonCommercial-NoDerivs (CC BY-NC-ND), which allows third parties to download the articles and share them with others as long as they credit the author and the Abstract Book, but they cannot change the content in any way or use them commercially.
**Abstract Book Citations:** Authors, Title, HemaSphere, 2026;10:(S1):pages. The abstract book DOIs can be found on the “Author Information” page.
**Disclaimer:** Articles published in the journal HemaSphere exclusively reflect the opinions of the authors. The authors are responsible for all content in their abstracts including accuracy of the facts, statements, citing resources, etc.

**Results**

Demographic information, pregnancy management, and primary outcomes of 165 pregnancies among 117 people are shown in **Table 1.** The overall VTE incidence was 7.9% (13/165). Among individuals with HbSS and HbSβ⁰-thalassemia, 8.6% (8/93) of pregnancies were complicated by VTE compared to 6.9% (5/72) of pregnancies among individuals with other genotypes. VTE occurred both antepartum (6/13) and postpartum (7/13). 38.5% (5/13) of patients were prescribed anticoagulation at the time of the event, but adherence to therapy was not assessed. There was a significant difference in VTE incidence by site (0 events at Yale to 15.6% at MGB, p=0.002) as well as significant variation in planned transfusion support and timing of thromboprophylaxis across sites (p<0.001).

Postpartum hemorrhage ≥ 500 mL occurred in 55.4% (77/139) of pregnancies with recorded EBL while PPH ≥1000 mL occurred in 14.4% (20/139) of pregnancies with no significant difference across sites (p=0.09). In multivariable logistic regression, Caesarean section was the only significant independent predictor of PPH (aOR 8.25; 95% CI 3.7 - 18.3; p < .0001). After adjusting for delivery mode and disease severity, neither anticoagulant (aOR 1.11, p = 0.78) nor aspirin prescription (aOR 1.20, p = 0.64) was significantly associated with increased PPH risk.

**Summary/Conclusion**

Incidence of VTE and PPH are significantly higher in pregnant and postpartum patients with SCD compared to the general population (7.9% vs 0.2%; 14.4% vs 5%). In the United States there is heterogeneity in aspirin use, timing and use of thromboprophylaxis, and red blood cell transfusion strategies during pregnancy, reflecting the lack of guidelines for this population. VTE incidence varies widely across institutions, possibly due to differences in SCD genotype, severity, and prevention strategies. Reassuringly, aspirin and anticoagulant use were not independently associated with risk of PPH. Our data emphasizes the high risk of both VTE and PPH in pregnant and postpartum patients with SCD, and the need to prospectively evaluate the safety and efficacy of interventions to reduce the incidence, morbidity, and mortality of both PPH and VTE in this complex population.

**Copyright Information:** (Online) ISSN: 2572-9241
©2026 The Author(s). HemaSphere is published by John Wiley & Sons Ltd on behalf of European Hematology Association. This is an open access Abstract Book distributed under the Attribution-NonCommercial-NoDerivs (CC BY-NC-ND), which allows third parties to download the articles and share them with others as long as they credit the author and the Abstract Book, but they cannot change the content in any way or use them commercially.
**Abstract Book Citations:** Authors, Title, HemaSphere, 2026;10:(S1):pages. The abstract book DOIs can be found on the "Author Information" page.
**Disclaimer:** Articles published in the journal HemaSphere exclusively reflect the opinions of the authors. The authors are responsible for all content in their abstracts including accuracy of the facts, statements, citing resources, etc.

**Table 1: Baseline Genotypes, Demographics, Management, and Outcomes by Site.**

| Characteristic | MGB | UM | Yale | p-value |
|---|---|---|---|---|
| *Persons (Pregnancy count)* | 43 (64) | 29 (39) | 45 (62) | -- |
| <u>*Genotype*</u> | | | | |
| *HbSS or HbSβº* | 30 (69.8%) | 22 (79.3%) | 19 (42.2%) | -- |
| *HbSC, HbSβ⁺, other* | 13 (30.2%) | 6 (20.7%) | 26 (57.8%) | 0.002 |
| <u>*Demographics*</u> | | | | |
| *Age at delivery (median)* | 32 | 28 | 29 | 0.015 |
| *Body Mass Index (mean)* | 25.6 | 25.8 | 28 | 0.030 |
| <u>*Clinical History*</u> | | | | |
| *Prior VTE (%)* | 9 (14.1%) | 5 (12.8%) | 4 (6.9%) | 0.425 |
| *History of spontaneous pregnancy loss* | 20 (46.5%) | 14 (48.2%) | 9 (20.0%) | 0.012 |
| <u>*Management*</u> | | | | |
| *Use of planned RBC transfusion* | 9 (14.1%) | 14 (35.9%) | 5 (8.1%) | < 0.001 |
| *RBC transfusion given peri-partum* | 32 (50.0%) | 20 (51.3%) | 18 (29.0%) | 0.026 |
| *Aspirin Use* | 44 (68.8%) | 20 (51.3%) | 27 (43.6%) | 0.020 |
| *Use of anticoagulation, any time* | 24 (37.5%) | 19 (48.7%) | 23 (37.1%) | 0.375 |
| *anticoagulation antepartum +postpartum* | 17 (26.5%) | 11 (28.2%) | 2 (3.2%) | < 0.001 |
| *anticoagulation postpartum only* | 7 (10.9%) | 7 (17.9%) | 15 (24.2%) | < 0.001 |
| <u>*Clinical Outcomes*</u> | | | | |
| *VTE occurrence* | 10 (15.6%) | 3 (7.7%) | 0 (0.0%) | 0.002 |
| *Postpartum blood loss ≥ 500 mL** | 33 (55.9%) | 17 (55%) | 27 (55.1%) | 0.99 |
| *Postpartum blood loss ≥ 1000 mL** | 10 (16.9%) | 7 (22.6%) | 3 (6.1%) | 0.094 |

*calculated among births with recorded/ available EBL

**Copyright Information:** (Online) ISSN: 2572-9241
©2026 The Author(s). HemaSphere is published by John Wiley & Sons Ltd on behalf of European Hematology Association. This is an open access Abstract Book distributed under the Attribution-NonCommercial-NoDerivs (CC BY-NC-ND), which allows third parties to download the articles and share them with others as long as they credit the author and the Abstract Book, but they cannot change the content in any way or use them commercially.
**Abstract Book Citations:** Authors, Title, HemaSphere, 2026;10:(S1):pages. The abstract book DOIs can be found on the "Author Information" page.
**Disclaimer:** Articles published in the journal HemaSphere exclusively reflect the opinions of the authors. The authors are responsible for all content in their abstracts including accuracy of the facts, statements, citing resources, etc.

### (PF1263) CHRONIC KIDNEY DISEASE IN ADULTS WITH SICKLE CELL DISEASE: INSIGHTS FROM A LARGE UK MULTI-CENTRE COHORT

**Topic:** 27. Sickle cell disease

Naomi Cornish*[1], Sara Stuart-Smith[2, 3], Sheila Azouji-Benjamin[4], Claire Sharpe[3, 5], Kate Gardner[2, 4]

*[1]University of Bristol, Bristol, United Kingdom; [2]King's College London, London, United Kingdom; [3]King's College Hospital NHS Foundation Trust, London, United Kingdom; [4]Guy's and St Thomas' NHS Foundation Trust, London, United Kingdom; [5]University of Nottingham School of Medicine, Nottingham, United Kingdom;*

### Background

Chronic kidney disease (CKD) is a common complication of sickle cell disease (SCD). Treatment with angiotensin-converting enzyme inhibitors (ACEi) or angiotensin receptor blockers (ARB) is recommended for patients with significant proteinuria to reduce risks of progression to end-stage renal failure.

### Aims

To evaluate prevalence and risk factors for CKD and management of proteinuria in patients with SCD.

### Methods

We analysed data from The Sickle Natural History Study, a multi-centre prospective cohort of adults with SCD in the United Kingdom recruited between 2021-2024. All participants provided informed consent. CKD was defined using UK Kidney Association criteria (Table 1), based on urinary albumin:creatinine ratio (ACR) and estimated glomerular filtration rate (eGFR), calculated using the CKD-EPI (2021) equation. Cross-sectional analyses were performed using steady-state clinical and laboratory measurements from routine hospital visits along with the documented medical history. Associations between 25 variables and CKD were assessed using multivariable logistic regression adjusted for age, sex, *HBB* genotype and sickle cell disease-modifying therapy (defined as hydroxycarbamide and/or regular transfusions); p-values were adjusted for multiple testing using false discovery rate (FDR) correction.

### Results

From a cohort of 697 recruited patients, we excluded 8 patients who were on haemodialysis or who had received a renal transplant, 22 patients with a history of malignancy and 8 patients who were missing essential clinical data. Of the remaining 659 patients (median age 37 years), 266 (40%) had CKD of any stage; 18 (3%) had moderate-severe CKD (eGFR<45ml/min). Risk factors independently associated with higher odds of CKD in multivariable regression analyses (FDR-P<0.05) included HbSS/HbS$ß^0$ genotype, increasing age, hypertension and higher serum LDH; higher haemoglobin levels were associated with lower odds of CKD (Figure 1). Assessment of CKD management revealed that, of 120 patients with either urinary ACR>30mg/mmol or urinary protein:creatinine ratio>50mg/mmol documented on at least two occasions; only 36 (30%) were prescribed an ACEi or ARB and 73 (61%) were receiving hydroxycarbamide and/or transfusions.

**Copyright Information:** (Online) ISSN: 2572-9241
©2026 The Author(s). HemaSphere is published by John Wiley & Sons Ltd on behalf of European Hematology Association. This is an open access Abstract Book distributed under the Attribution-NonCommercial-NoDerivs (CC BY-NC-ND), which allows third parties to download the articles and share them with others as long as they credit the author and the Abstract Book, but they cannot change the content in any way or use them commercially.
**Abstract Book Citations:** Authors, Title, HemaSphere, 2026;10:(S1):pages. The abstract book DOIs can be found on the "Author Information" page.
**Disclaimer:** Articles published in the journal HemaSphere exclusively reflect the opinions of the authors. The authors are responsible for all content in their abstracts including accuracy of the facts, statements, citing resources, etc.

### Summary/Conclusion

In this large cohort of patients with sickle cell disease, CKD was highly prevalent and associated with well-known risk factors. However, only a minority of patients received ACEi/ARB therapy as management of proteinuria. Identifying and addressing barriers to implementation of guidelines for CKD treatment may improve outcomes in this group of patients.

**Table 1.** Cohort distribution across CKD risk categories, stratified by eGFR and urinary ACR (UKKA classification). Participants with eGFR < 60 or urine ACR > 3mg/mmol were defined as having CKD

| | Urine ACR category | | | | |
|---|---|---|---|---|---|
| eGFR category (GFR ml/min/1.73m²) | A1 <3 mg/mmol | A2 3-30mg/mmol | A3 >30mg/mmol | Unknown | Total |
| G1: Normal or high (eGFR>90) | 225 | 142 | 51 | 105 | 523 |
| G2: Mildly decreased (eGFR 60-89) | 51 | 23 | 16 | 12 | 102 |
| G3a: Mild-moderately decreased (eGFR 45-59) | 3 | 5 | 7 | 1 | 16 |
| G3b: Moderately-severely decreased (eGFR 30-44) | 0 | 2 | 6 | 0 | 8 |
| G4: Severely decreased (eGFR 15-29) | 0 | 1 | 2 | 0 | 3 |
| G5: Kidney failure (eGFR<15) | 0 | 0 | 7 | 0 | 7 |
| Total | 279 | 173 | 89 | 118 | 659 |

**Copyright Information:** (Online) ISSN: 2572-9241
©2026 The Author(s). HemaSphere is published by John Wiley & Sons Ltd on behalf of European Hematology Association. This is an open access Abstract Book distributed under the Attribution-NonCommercial-NoDerivs (CC BY-NC-ND), which allows third parties to download the articles and share them with others as long as they credit the author and the Abstract Book, but they cannot change the content in any way or use them commercially.
**Abstract Book Citations:** Authors, Title, HemaSphere, 2026;10:(S1):pages. The abstract book DOIs can be found on the "Author Information" page.
**Disclaimer:** Articles published in the journal HemaSphere exclusively reflect the opinions of the authors. The authors are responsible for all content in their abstracts including accuracy of the facts, statements, citing resources, etc.

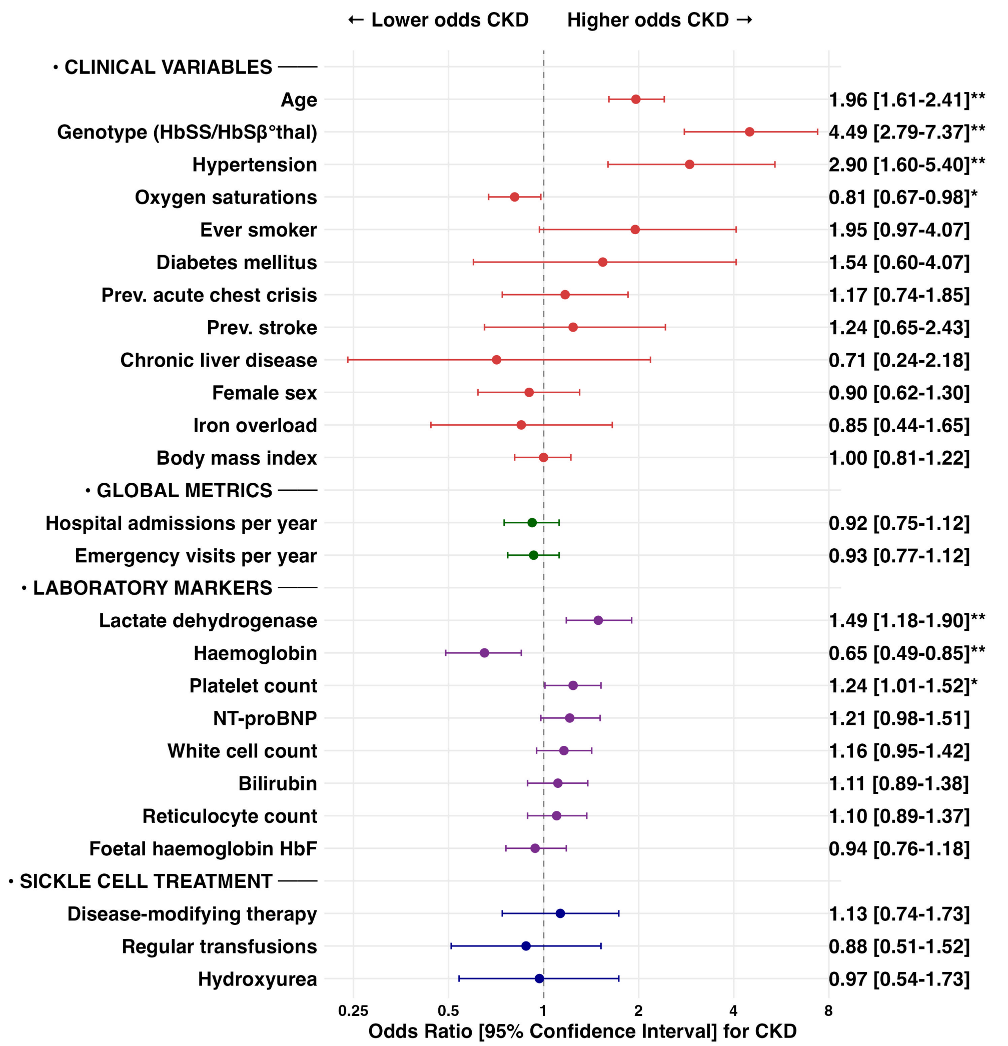

**Figure 1:** Forest plot showing associations with chronic kidney disease (CKD). Laboratory markers were log-transformed if right-skewed and all continuous variables were scaled to one standard deviation (SD). Odds ratios (ORs) and 95% confidence intervals (CIs) for CKD are shown per category for binary variables and per SD increase for continuous variables, derived from multivariable logistic regression. *P<0.05; **FDR P<0.05.

**Copyright Information:** (Online) ISSN: 2572-9241
©2026 The Author(s). HemaSphere is published by John Wiley & Sons Ltd on behalf of European Hematology Association. This is an open access Abstract Book distributed under the Attribution-NonCommercial-NoDerivs (CC BY-NC-ND), which allows third parties to download the articles and share them with others as long as they credit the author and the Abstract Book, but they cannot change the content in any way or use them commercially.
**Abstract Book Citations:** Authors, Title, HemaSphere, 2026;10:(S1):pages. The abstract book DOIs can be found on the "Author Information" page.
**Disclaimer:** Articles published in the journal HemaSphere exclusively reflect the opinions of the authors. The authors are responsible for all content in their abstracts including accuracy of the facts, statements, citing resources, etc.

### (PF1264) METABOLIC DYSREGULATION OF ENERGY, ARGININE METABOLISM AND INFLAMMATION ASSOCIATED WITH POINT OF SICKLING IN PATIENTS WITH SICKLE CELL ANEMIA

**Topic:** 27. Sickle cell disease

Amira Idrizovic*[1], Sigrid van der Veen[2], Marissa Traets[3], Betzabel Cajiao Garcia[3], Cesare Rollo[4], Emmanuel Adu[5], Emmanuelle Bourdelier[6], Elisabetta Mezzalira[7], Mirco D'agnolo[7], Mickael Marin[6], Tiziana Sanavia[4], Anna Collado Gimbert[8], Sara Isabel Reidel[1], David Beneitez Pastor[9], Bart Biemond[10], Erfan Nur[10], Karin Fijnvandraat[10], Saskia Schols[11], Anita Rijneveld[12], Marjon Cnossen[12], Aida Kidane[13], Nicolas Hebert[14], Maria Paola Boaro[7], Slimane Allali[15], Caroline L. V. Kim[16], Anna Ruiz-Llobet[17], Piero Fariselli[4], Raffaella Colombatti[7], Pablo Bartolucci[5], Mariane de Montalembert[15], Richard van Wijk[3], Judith Jans[3], Nanda Verhoeven- Duif[3], Minke Rab[3], Maria del Mar Mañú Pereira[1], Eduard J. van Beers[2]

*[1]Vall d´Hebron Research Institute, Rare anemia disorders research laboratory, Barcelona, Spain;[2]University Medical Center Utrecht, Divisie Interne Geneeskunde en Dermatologie, Van Creveldkliniek, Utrecht, The Netherlands; [3]University Medical Center Utrecht, Utrecht, The Netherlands;[4]University of Torino, Torino, Italy; [5]Université Paris-Est Créteil, Paris, France;[6]INSERM, BIGR, Paris, France;[7]University of Padova, Padova, Italy;[8]Vall d'Hebron Hospital / Vall d'Hebron Institut de Recerca, Barcelona, Spain;[9]Hospital Universitari Vall d'Hebron, Barcelona, Spain;[10]Amsterdam UMC, Amsterdam, The Netherlands;[11]Radboud University Medical Center, Nijmegen, The Netherlands;[12]Erasmus Medical Center Cancer Institute, Rotterdam, The Netherlands;[13]Sophia Children's Hospital Erasmus MC, Rotterdam, The Netherlands;[14]Etablissement Français du Sang, Creteil, France;[15]Hôpital Necker, Paris, France;[16]Université Paris Cité, Paris, France;[17]Hospital Sant Joan De Déu, Barcelona, Spain;*

### Background

Sickle cell anemia (SCA) exhibits large interindividual variability in clinical presentation. Oxygen-gradient ektacytometry (oxygenscan) measures the point of sickling (PoS), which reflects patient-specific sickling and is associated with the severity of the clinical phenotype, while untargeted metabolomics potentially reveals biochemical pathways linked to disease severity.

### Aims

Identify metabolites and metabolic pathways associated with PoS.

### Methods

Steady-state SCA patients (HbSS) (>3 years) were recruited via ERN-EuroBloodNet centers in France, Italy, Spain, and the Netherlands. Patients with chronic or occasional transfusion or missing HbF data were excluded. Hematological analyses, oxygenscan and dried blood spot untargeted metabolomics (direct-infusion MS) were performed on the same blood sample. Metabolites (isomers) were Z-score normalized to healthy controls. Because HbF correlated with PoS, PoS was adjusted for HbF by linear regression; HbF-independent residuals were used for quartile stratification (Q2-Q3 merged). Multivariate PLS-DA (1309 metabolites), ANOVA (A-FDR≤0.05; MetaboAnalyst 6.0), and linear regression (lr) analyses (1876 metabolites) adjusted for HbF, age and country were performed (FDR≤0.05; R4.3.1).

### Results

After HbF correction, 368 SCA patients (88% on hydroxyurea) were stratified by PoS into three groups: high (median=56, being the least favourable for patient), intermediate (median=36.7) and low (median=23.8, being the most favourable). PLS-DA analyses partially separated groups (Fig.1A); 118 metabolites differed significantly across groups (ANOVA) and 36 remained independently associated with PoS in regression analyses (Fig.1C).

Patients with high PoS showed an altered energy metabolism, including increased glycolytic intermediates (glyceraldehyde-3-phosphate [A-FDR <0.001], 2,3-DPG [lr +0.05, (A)-FDR=0.003]), pentose phosphate pathway (D-ribulose-5-phosphate [A-FDR <0.001]), TCA(oxalosuccinic acid [lr +0.03 FDR=0.04]) and multiple carnitine-related metabolites (all FDR<0.05). Glutathione metabolism (cystein glutathione disulfide [A-FDR =0.001] and cysteinglycine disulfide [A-FDR=0.01]) and arginine metabolism (N-alpha-Acetyl-L-citrulline [A-FDR=<0.001; lr +0.10, FDR=0.01], spermidine [lr +0.11, FDR =0.03] and N1,N8-Diacetylspermidine [lr +0.02, FDR= 0.03] associated with high PoS. Accompanied by higher platelet counts (high 335x109/L vs. low PoS 295x109/L; p =0.05) and signs of increased platelet activation (prostaglandin metabolite; lr +0.03, FDR=0.03). High PoS correlated with upstream accumulation in the tryptophan metabolism (N-acetyl tryptophan [lr +0.04, (A-)FDR=0.04]), while Low PoS associated with downstream accumulation of quinaldic acid and indole lactic acid (both A-FDR<0.01). Low PoS also associated with regulation of inflammation, including bile acid-related metabolites (FDR<0.05)(Fig.1B).

### Summary/Conclusion

In this large European SCA cohort, high PoS was associated with a disturbed energy metabolism and changes in arginine, glutathione and tryptophan metabolism. Those reflect metabolic responses to chronic inflammation and oxidative stress. Low PoS was associated with metabolites linked to regulation of inflammation, where tryptophan and bile acid metabolites suggest a role for microbiome-associated immune and metabolic pathways. These results link well-described metabolic pathways associated with disease severity as shown by PoS. These insights might lead to identifying metabolic pathways as potential therapeutic targets in SCA.

**Copyright Information:** (Online) ISSN: 2572-9241
©2026 The Author(s). HemaSphere is published by John Wiley & Sons Ltd on behalf of European Hematology Association. This is an open access Abstract Book distributed under the Attribution-NonCommercial-NoDerivs (CC BY-NC-ND), which allows third parties to download the articles and share them with others as long as they credit the author and the Abstract Book, but they cannot change the content in any way or use them commercially.
**Abstract Book Citations:** Authors, Title, HemaSphere, 2026;10:(S1):pages. The abstract book DOIs can be found on the "Author Information" page.
**Disclaimer:** Articles published in the journal HemaSphere exclusively reflect the opinions of the authors. The authors are responsible for all content in their abstracts including accuracy of the facts, statements, citing resources, etc.

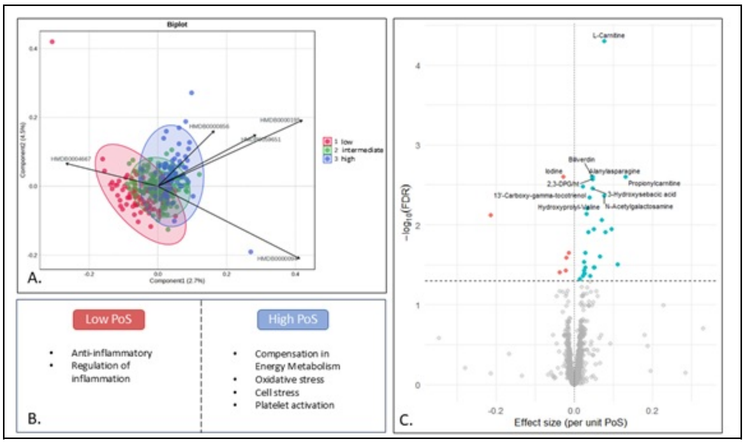

PLS-DA, linear regression and generic results. A. PLS-DA plot of SCA patients grouped by the adjusted PoS. Group 1: Low adjusted PoS, group 2: Intermediate adjusted PoS, group 3: high adjusted PoS. B. Overview of processes and pathways affected by metabolites in patients with a low or high PoS. C. Results of the linear regression analysis (adjusted for age, HbF, country); gray are metabolites with no significant association, orange is a negative association and blue is a positive association. The top 10 metabolites (by FDR) are listed by name in the plot.

**Copyright Information:** (Online) ISSN: 2572-9241
©2026 The Author(s). HemaSphere is published by John Wiley & Sons Ltd on behalf of European Hematology Association. This is an open access Abstract Book distributed under the Attribution-NonCommercial-NoDerivs (CC BY-NC-ND), which allows third parties to download the articles and share them with others as long as they credit the author and the Abstract Book, but they cannot change the content in any way or use them commercially.
**Abstract Book Citations:** Authors, Title, HemaSphere, 2026;10:(S1):pages. The abstract book DOIs can be found on the “Author Information” page.
**Disclaimer:** Articles published in the journal HemaSphere exclusively reflect the opinions of the authors. The authors are responsible for all content in their abstracts including accuracy of the facts, statements, citing resources, etc.

### (PF1265) DOUBLE-BLIND, RANDOMIZED, PLACEBO-CONTROLLED STUDY OF CROVALIMAB AS ADJUNCT TREATMENT FOR THE PREVENTION OF VASO-OCCLUSIVE EPISODES IN SICKLE CELL DISEASE: RESULTS FROM THE PHASE 2A CROSSWALK-C TRIAL

**Topic:** 27. Sickle cell disease

Pablo Bartolucci*[1], Laine Alvarenga[2], Ali Bülent Antmen[3], Rodolfo Delfini[4], Beng Fuh[5], Lucia De Franceschi[6], Jeffrey Glassberg[7], Birol Guvenc[8], Adlette Inati[9], Elena Cela[10], Doreen Karimi[11], Clarisse Lobo[12], Alexandre Lorenzetti[13], Johnny Mahlangu[14], Ana Cristina Silva Pinto[15], Selma Ünal[16], Amparo Yovanna Castro Sánchez[17], Simon Buatois[17], Asad Charania[17], Diana Gradinaru[17], Thomas Perretti[17], Pontus Lundberg[17], Sasha Sreckovic[18], Fredrick Chite Asirwa[19]

*[1]Hôpital Universitaire Necker Enfants-Malades, GH Paris Centre, Biotherapy Department and Centre d'Investigation Clinique-Biothérapie, Paris, France; [2]Hospital São Rafael/IDOR, Salvador, Brazil; [3]Adana Acibadem Hospital, Department of Pediatric Hematology, Adana, Türkiye; [4]Hospital Samaritano, Sao Paulo, Brazil; [5]East Carolina University, Division of Pediatric Hematology/Oncology, Greenville, NC, United States of America; [6]University of Verona and Azienda Ospedaliera Universitaria Verona, Department of Medicine, Verona, Italy; [7]Icahn School of Medicine at Mount Sinai, Department of Emergency Medicine, New York, United States of America; [8]Cukurova University, Department of Hematology, Medical Faculty, Adana, Türkiye; [9]University of Balamand, NINI Hospital, Faculty of Medicine and Medical Sciences, Balamand, Lebanon;[10]Hospital General Universitario Gregorio Marañón, Universidad Complutense de Madrid, Madrid, Spain; [11]Gertrude's Children's Hospital, Department of Hematology/Oncology, Nairobi, Kenya; [12]Instituto Estadual de Hematologia do Rio de Janeiro HEMORIO, Rio de Janeiro, Brazil; [13]Hospital de Base de São José do Rio Preto, Serviço de Hematologia e Hemoterapia, São Paulo, Brazil; [14]University of the Witwatersrand, Department of Molecular Medicine and Haematology, Johannesburg, South Africa; [15]Regional Blood Center, Ribeirão Preto School of Medicine, University of São Paulo, Department of Medical Imaging, Hematology, and Oncology, São Paulo, Brazil; [16]Mersin University, Department of Pediatric Hematology, Faculty of Medicine, Mersin, Türkiye; [17]F. Hoffmann-La Roche Ltd, Basel, Switzerland; [18]Genentech, Inc., South San Francisco, United States of America; [19]International Cancer Institute, Eldoret, Kenya;*

### Background

Sickle cell disease (SCD) is characterized by chronic hemolysis, inflammation, and microvascular occlusions, which present as acute painful vaso-occlusive episodes (VOEs). VOEs are the most common cause of healthcare utilization among patients (pts) with SCD. The pathogenesis of vaso-occlusion is complex and multifactorial; yet, available nonclinical data suggest a multimodal role for complement dysregulation in SCD pathophysiology and end organ damage.

Crovalimab (crova) is a novel, humanized anti-C5 monoclonal antibody that allows for small-volume subcutaneous (SC) self-injection. Crova demonstrated rapid and sustained complement inhibition in paroxysmal nocturnal hemoglobinuria (PNH) clinical trials and is approved for the treatment of PNH.

### Aims

To evaluate crova vs placebo (pbo) for VOE prevention in SCD from CROSSWALK-c (NCT05075824).

**Copyright Information:** (Online) ISSN: 2572-9241
©2026 The Author(s). HemaSphere is published by John Wiley & Sons Ltd on behalf of European Hematology Association. This is an open access Abstract Book distributed under the Attribution-NonCommercial-NoDerivs (CC BY-NC-ND), which allows third parties to download the articles and share them with others as long as they credit the author and the Abstract Book, but they cannot change the content in any way or use them commercially.
**Abstract Book Citations:** Authors, Title, HemaSphere, 2026;10:(S1):pages. The abstract book DOIs can be found on the "Author Information" page.
**Disclaimer:** Articles published in the journal HemaSphere exclusively reflect the opinions of the authors. The authors are responsible for all content in their abstracts including accuracy of the facts, statements, citing resources, etc.

### Methods

Pts aged 12–55 years with SCD (HbSS or HbSβ0) and 2–10 documented VOEs in the prior 12 months were randomized 1:1 to crova or pbo. Pts on a stable dose of concurrent SCD-directed therapy were eligible. The crova arm received a weight-based loading dosing regimen then SC maintenance (Q4W). A 48-week treatment period was followed by a 46-week safety follow-up. The primary objective was to evaluate the efficacy of crova vs pbo based on the annualized rate (AR) of medical facility (MF) VOEs in the ITT population. Secondary efficacy endpoints included the annualized number of days hospitalized for an MF VOE, and the ARs of uncomplicated MF VOEs and acute chest syndrome. All efficacy endpoints were assessed in the ITT and an exploratory efficacy population. Pharmacokinetics (PK), pharmacodynamics (PD), biomarkers, and safety were assessed.

### Results

At the primary analysis (clinical cutoff: June 12, 2025), 94 pts were randomized (46 to crova; 48 to pbo). The study did not meet its primary endpoint. The AR of MF VOEs in the ITT was 2.2 (90% CI: 1.49, 3.22) with crova and 2.8 (90% CI: 1.82, 4.18) with pbo, a 21% AR reduction with crova vs pbo (AR ratio, 0.79; 90% CI: 0.514, 1.224; $P$=0.3799). Key secondary endpoints consistently showed no clear crova benefit (**Table**).
Among crova-treated pts, 89.1% (41/46) remained above the crova serum concentration target threshold for complete terminal complement inhibition (~100 µg/mL) throughout the treatment period. 100% (45/45) achieved full C5 target binding (target <0.0001 g/L) and 96% (43/45) achieved full terminal complement inhibition (CH50 target <30 U/mL) at Day 1, and all patients had full C5 inhibition and clinically relevant terminal complement inhibition throughout study treatment. No impact of anti-drug antibodies on PK/PD was observed. No changes in hemoglobin or markers of hemolysis were observed.
Adverse events (AEs) occurred in 95.7% (Grade 3–5: 78.3%) of crova-treated and 95.8% (Grade 3–5: 85.4%) of pbo-treated pts; there was 1 fatal AE per arm (crova: pulmonary hypertension; pbo: hypovolemic shock [occurred in follow-up]), neither treatment related. Severe infections occurred in 47.8% of crova-treated and 41.7% of pbo-treated pts. There were no meningococcal infections.

### Summary/Conclusion

The primary efficacy endpoint was not met and crova showed no clear benefit over pbo. Crova was well tolerated and achieved sustained C5 target engagement and complete complement inhibition, suggesting complement blockade is insufficient to prevent VOEs. The risk–benefit profile does not support crova as an adjunct treatment for VOE prevention in SCD.

**Copyright Information:** (Online) ISSN: 2572-9241
©2026 The Author(s). HemaSphere is published by John Wiley & Sons Ltd on behalf of European Hematology Association. This is an open access Abstract Book distributed under the Attribution-NonCommercial-NoDerivs (CC BY-NC-ND), which allows third parties to download the articles and share them with others as long as they credit the author and the Abstract Book, but they cannot change the content in any way or use them commercially.
**Abstract Book Citations:** Authors, Title, HemaSphere, 2026;10:(S1):pages. The abstract book DOIs can be found on the "Author Information" page.
**Disclaimer:** Articles published in the journal HemaSphere exclusively reflect the opinions of the authors. The authors are responsible for all content in their abstracts including accuracy of the facts, statements, citing resources, etc.

**Table. Efficacy outcomes**

| | ITT (N=94) | | EEP (N=84) | |
|---|---|---|---|---|
| | Pbo (n=48) | Crova (n=46) | Pbo (n=45) | Crova (n=39) |
| **AR of MF VOEs** | | | | |
| Rate (90% CI) | 2.8 (1.82, 4.18) | 2.2 (1.49, 3.22) | 2.0 (1.36, 2.90) | 1.8 (1.25, 2.64) |
| AR ratio (90% CI) | 0.79 (0.514, 1.224)* | | 0.91 (0.609, 1.373) | |
| **Annualized number of days hospitalized for a MF VOE** | | | | |
| Mean (SD) | 9.87 (11.67) | 13.69 (21.92) | 9.87 (11.90) | 13.67 (22.22) |
| Median (range) | 5.42 (0–44) | 6.96 (0–106.2) | 5.42 (0–44) | 5.40 (0–106.2) |
| **AR of uncomplicated MF VOEs** | | | | |
| Rate (90% CI) | 2.6 (1.67, 3.95) | 2.0 (1.31, 2.95) | 1.9 (1.29, 2.84) | 1.7 (1.16, 2.54) |
| AR ratio (90% CI) | 0.77 (0.490, 1.202) | | 0.90 (0.586, 1.374) | |
| **AR of acute chest syndrome** | | | | |
| Rate (90% CI) | 0.2 (0.05, 0.42) | 0.2 (0.07, 0.43) | 0.1 (0.03, 0.30) | 0.1 (0.03, 0.29) |
| AR ratio (90% CI) | 1.16 (0.364, 3.694) | | 0.99 (0.306, 3.169) | |

* Primary endpoint was not met (P=0.3799). P value was calculated using a Wald test conducted at 2-sided Type I error level of 0.1. No statistical testing was performed for secondary endpoints. AR, annualized rate; CI, confidence interval; crova, crovalimab; EEP, exploratory efficacy population (patients who met inclusion/exclusion criteria relevant to the primary endpoint and received ≥80% expected crova exposure); ITT, intention-to-treat; MF, medical facility; pbo, placebo; SD, standard deviation; VOE, vaso-occlusive episode.

**Copyright Information:** (Online) ISSN: 2572-9241
©2026 The Author(s). HemaSphere is published by John Wiley & Sons Ltd on behalf of European Hematology Association. This is an open access Abstract Book distributed under the Attribution-NonCommercial-NoDerivs (CC BY-NC-ND), which allows third parties to download the articles and share them with others as long as they credit the author and the Abstract Book, but they cannot change the content in any way or use them commercially.
**Abstract Book Citations:** Authors, Title, HemaSphere, 2026;10:(S1):pages. The abstract book DOIs can be found on the "Author Information" page.
**Disclaimer:** Articles published in the journal HemaSphere exclusively reflect the opinions of the authors. The authors are responsible for all content in their abstracts including accuracy of the facts, statements, citing resources, etc.

### (PF1266) MEMAGEM PHASE IIA/IIB CLINICAL TRIAL: RESPONSES OF RED BLOOD CELLS OF ADULT AND ADOLESCENT PATIENTS WITH SICKLE CELL DISEASE TO MEMANTINE TREATMENT

**Topic:** 27. Sickle cell disease

Anna Bogdanova*[1], Leonid Livshits[2], Fabio Valeri[3], Sivan Raz[4], Ariel Koren[5], Carina Levin[5], Max Gassmann[1]

*[1]University of Zurich, Institute of Veterinary Physiology, Vetsuisse Faculty, Zurich, Switzerland;[2]MIGAL Galilee Research Institute, Kiryat Shmona, Israel; [3]University of Zurich, Zurich, Switzerland; [4]The Max Stern Yezreel Valley College, Jezreel Valley, Israel; [5]Emek Medical Center, Pediatric Hematology Unit, Afula, Israel;*

### Background

In a recent phase IIa/IIb MeMAGEM clinical trial, the safety and tolerability of the N-methyl-D-aspartate (NMDA) receptor antagonist memantine were confirmed in adolescents and young adults with sickle cell disease (SCD) (1). Memantine is a drug used to treat dementia and was recently suggested for off-label use in patients with SCD (2). The MeMAGEN trial protocol was approved by the local Helsinki Committee (EMC-0071-17), and informed consent to participate was obtained for all participants or their parents. The dose-escalation single-center study involved 17 children and young adults with a history of SCD. The drug was safe and well-tolerated over the daily doses ranging from 5 to 20 mg in adults and up to 15 mg in adolescents when used in combination with hydroxyurea (HU) therapy. Fewer hospital admissions were observed during the combined treatment period compared to the two years of HU monotherapy. Cognitive function improvements were reported (3).

### Aims

The aim of this study was to understand the cellular and molecular mechanisms of responses of RBCs of patients to memantine therapy.

### Methods

Red blood cell (RBC) indices and standard biochemistry parameters were obtained monthly during the study, along with exploratory parameters reflecting $K^+$ transport, hydration, density, and membrane stability of RBCs.

**Copyright Information:** (Online) ISSN: 2572-9241
©2026 The Author(s). HemaSphere is published by John Wiley & Sons Ltd on behalf of European Hematology Association. This is an open access Abstract Book distributed under the Attribution-NonCommercial-NoDerivs (CC BY-NC-ND), which allows third parties to download the articles and share them with others as long as they credit the author and the Abstract Book, but they cannot change the content in any way or use them commercially.
**Abstract Book Citations:** Authors, Title, HemaSphere, 2026;10:(S1):pages. The abstract book DOIs can be found on the “Author Information” page.
**Disclaimer:** Articles published in the journal HemaSphere exclusively reflect the opinions of the authors. The authors are responsible for all content in their abstracts including accuracy of the facts, statements, citing resources, etc.

**Results**

Based on these tests, the patients were stratified into two groups: those with baseline high $K^+$ leak from RBCs and those with lower $K^+$ leak, mediated mainly by a hyperactive $Ca^{2+}$-sensitive $K^+$ (Gardos) channel. These two groups differed in mean corpuscular hemoglobin concentration (mean±SD: 32.9±1.8 vs 31.2±1.5 g/dL) and HbF levels (medians: 13.5% vs 23.5%), both of which were lower in SCD patients with leaky RBCs. Further differences were observed in serum iron and ferritin levels that showed a strong positive association ($p<0.005$) with $K^+$ leak in patients with leaky RBCs. Memantine treatment targets excessive $Ca^{2+}$ uptake, caused by the abnormally high abundance and/or activity of several $Ca^{2+}$-permeable ion channels, including NMDA receptors. Inhibition of NMDA receptors with memantine caused a dose-dependent reduction in $K^+$ leak in patients presenting initially with high $K^+$ loss from RBCs. Prevention of $K^+$ leak led to increased MCV and MCHC, and to higher intracellular $K^+$, which was particularly important for SCD patients with lower HbF despite HU treatment. Ferritin and serum iron levels decreased as the $K^+$ leak dropped in response to memantine treatment. Higher intracellular $K^+$ prevented terminal dehydration and, hence, irreversible HbS aggregation in SCD patients, observed as a reduction in terminally dehydrated RBCs in our study. In patients with low $K^+$ leak, hemoglobin was proportional to the intracellular $K^+$ levels.

**Summary/Conclusion**

Taken together, these observations strongly suggest that combining HU and memantine treatment is beneficial for patients suffering from SCD.

**Copyright Information:** (Online) ISSN: 2572-9241
©2026 The Author(s). HemaSphere is published by John Wiley & Sons Ltd on behalf of European Hematology Association. This is an open access Abstract Book distributed under the Attribution-NonCommercial-NoDerivs (CC BY-NC-ND), which allows third parties to download the articles and share them with others as long as they credit the author and the Abstract Book, but they cannot change the content in any way or use them commercially.
**Abstract Book Citations:** Authors, Title, HemaSphere, 2026;10:(S1):pages. The abstract book DOIs can be found on the "Author Information" page.
**Disclaimer:** Articles published in the journal HemaSphere exclusively reflect the opinions of the authors. The authors are responsible for all content in their abstracts including accuracy of the facts, statements, citing resources, etc.

### (PF1267) EFFECT OF HEMATOPOIETIC CELL TRANSPLANTATION ON INFLAMMATORY AND ANGIOGENIC PLASMA PROTEINS IN SICKLE CELL DISEASE

**Topic:** 27. Sickle cell disease

Maud Zwolsman*[1, 2], Lydian De Ligt[1, 2], Sanjay Thakoerdin[1], Elisabeth Dovern[1], Bart Biemond[1], Erfan Nur[1, 2]

[1]*Amsterdam UMC, Department of Hematology, Amsterdam, The Netherlands;*[2]*Sanquin Reseach and Landsteiner Laboratory, Amsterdam, The Netherlands;*

### Background

Sickle cell disease (SCD) is characterized by hemolysis and vaso-occlusion, resulting in chronic inflammation and ischemia-driven abnormal angiogenesis. Multiple organ complications have been linked to increased levels of pro-inflammatory and pro-angiogenic cytokines, including IL-18 (cardiomyopathy), CXCL12 (pulmonary hypertension), and increased ANGPT-2/ANGPT-1 ratio (retinopathy). While allogeneic hematopoietic cell transplantation (HCT) provides a cure for patients with SCD, its effect on the underlying pro-inflammatory and pro-angiogenic pathways remains unclear.

### Aims

We aimed to assess the effect of HCT on proteins involved in pro-inflammatory response, anti-inflammatory/immunoregulatory response, angiogenesis and hypoxic response. Furthermore, we examined associations between the values of these proteins and pre-transplantation disease characteristics and treatment. We also assessed associations with post-transplantation complications and donor-related factors.

### Methods

Plasma samples of adult patients with SCD were collected at baseline and after 12 months post-transplantation. A selection of 24 plasma proteins were quantified using highly sensitive and specific proximity extension assay technique (Olink, Thermo Fisher Scientific). Baseline protein values from our cohort were compared to reference values of 15 healthy controls provided by Olink using the one-sample Wilcoxon test. Either a paired t-test or Wilcoxon signed-rank test was used as appropriate based on normality to assess protein changes after HCT. The Mann-Whitney U test was used to test for associations between protein levels and binary clinical parameters at baseline (e.g. genotype, RBC transfusion, hydroxyurea use) or follow-up (e.g. GvHD, sirolimus use, donor carrier status). Benjamini-Hochberg's false discovery rate correction was applied across all proteins simultaneously.

### Results

A total of 34 adults (50% female, median age 27 years [range 18 – 49]) undergoing either nonmyeloablative matched sibling donor transplantation (n=23), or reduced-intensity haploidentical donor transplantation (n=11) were included in this study. At baseline, we found elevated proteins involved in pro-inflammatory response (including IL-1β, IL-8, and IL-18), anti-inflammatory response (IL-10 and IL-1RN), angiogenic dysregulation (ANGPT-2 and VEGF-A) and hypoxic response (EPO and EGLN1), as compared to reference values. At >12 months post-transplantation, significant decreases in levels of proteins involved in inflammation (IL-18, PTX3, TACI), angiogenesis (ANGPT-2, CD200R1, CXCL12) and hypoxic responses (EPO) were observed (Figure 1). These findings were accompanied by a normalization of hemolysis (hemoglobin, reticulocytes, bilirubin, LDH). While no graft failure occurred in our cohort, patients that experienced declining donor chimerism warranting intervention to avert graft failure exhibited significantly higher IL-18 levels at baseline compared to those with stable chimerism (median 4194 vs 1855 pg/mL, $p<0.05$).

**Copyright Information:** (Online) ISSN: 2572-9241
©2026 The Author(s). HemaSphere is published by John Wiley & Sons Ltd on behalf of European Hematology Association. This is an open access Abstract Book distributed under the Attribution-NonCommercial-NoDerivs (CC BY-NC-ND), which allows third parties to download the articles and share them with others as long as they credit the author and the Abstract Book, but they cannot change the content in any way or use them commercially.
**Abstract Book Citations:** Authors, Title, HemaSphere, 2026;10:(S1):pages. The abstract book DOIs can be found on the "Author Information" page.
**Disclaimer:** Articles published in the journal HemaSphere exclusively reflect the opinions of the authors. The authors are responsible for all content in their abstracts including accuracy of the facts, statements, citing resources, etc.

### Summary/Conclusion

Our study shows that patients with SCD have a profound pro-inflammatory and pro-angiogenic profile, with significant reduction of key inflammatory and angiogenic proteins following allogeneic HCT. Resolution of hemolysis and ischemia-reperfusion injury are likely the main drivers of amelioration of chronic inflammation and angiogenesis post-transplantation, likely contributing to clinical benefits and vascular stabilization.

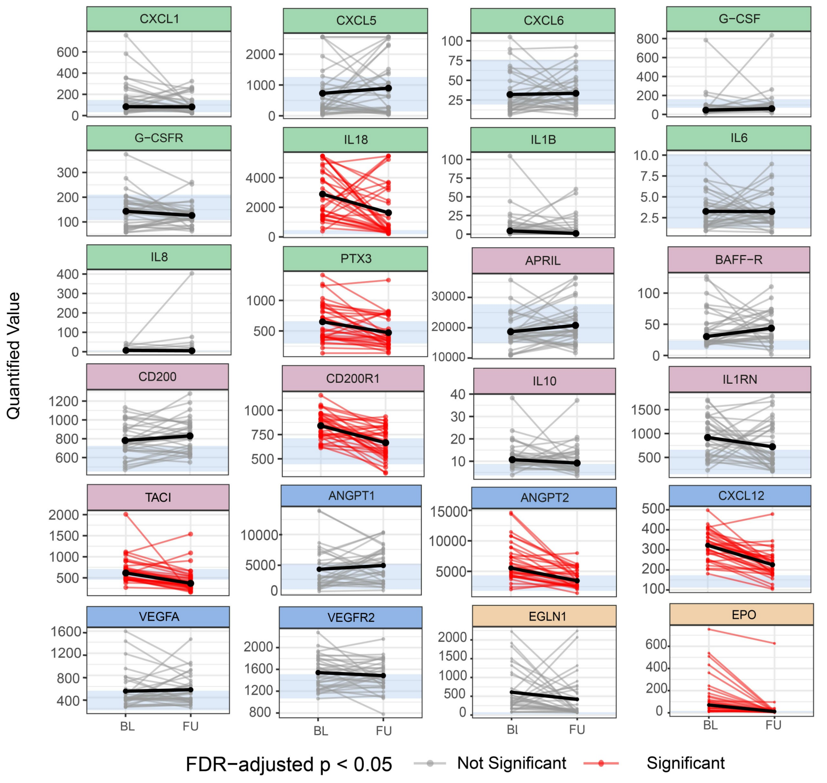

**Figure 1. Longitudinal individual plasma protein dynamics at baseline and after allogeneic hematopoietic cell transplantation (n=34).** Green-labeled plots represent markers of inflammation, pink-labeled plots represent anti-inflammatory markers, blue-labeled plots represent markers of angiogenesis, and orange-labeled plots represent markers of hypoxic regulation. Black lines show mean (normally distributed delta) or median (non−normally distributed delta) as appropriate. Red trajectories indicate significant protein changes after false discovery rate (FDR) correction (p<0.05). Light blue shaded lines represent reference ranges (10th-90th percentile) from healthy controls (n=15). Y-axis shows quantified values in pg/mL, except for EPO which is expressed in mU/mL.

*Abbreviations: Angiopoietin-1 (ANGPT-1); Angiopoietin-2 (ANGPT-2); OX-2 membrane glycoprotein (CD200, MOX1) Cell surface glycoprotein CD200 receptor 1 (CD200R1); Egl nine homolog 1 (EGLN1); Erythropoietin (EPO); Vascular endothelial growth factor receptor 2 (KDR, VEGFR2); Vascular endothelial growth factor (VEGF-A); Stromal cell-derived factor 1 (SDF1, CXCL12); Interleukin-8(IL-8, CXCL8); Interleukin-10 (IL-10); Interleukin-18 (IL-18); Interleukin-1 beta (IL-1β); Interleukin-1 receptor antagonist protein (IL-1RN); Interleukin-6 (IL-6); Granulocyte colony-stimulating factor (G-CSF); Granulocyte colony-stimulating factor receptor (G-CSFR); Growth-regulated alpha protein (CXCL1); C-X-C motif chemokine 5 (CXCL5); C-X-C motif chemokine (CXCL6); Pentraxin-related protein (PTX3); Tumor necrosis factor receptor superfamily member 13B (TNFRSF13B, TACI); Tumor necrosis factor receptor superfamily member 13C (TNFRSF13C, BAFF-R); Tumor necrosis factor ligand superfamily member 13 (TNFSF13, APRIL).*

**Copyright Information:** (Online) ISSN: 2572-9241
©2026 The Author(s). HemaSphere is published by John Wiley & Sons Ltd on behalf of European Hematology Association. This is an open access Abstract Book distributed under the Attribution-NonCommercial-NoDerivs (CC BY-NC-ND), which allows third parties to download the articles and share them with others as long as they credit the author and the Abstract Book, but they cannot change the content in any way or use them commercially.
**Abstract Book Citations:** Authors, Title, HemaSphere, 2026;10:(S1):pages. The abstract book DOIs can be found on the "Author Information" page.
**Disclaimer:** Articles published in the journal HemaSphere exclusively reflect the opinions of the authors. The authors are responsible for all content in their abstracts including accuracy of the facts, statements, citing resources, etc.

### (PF1268) INFLAMMATORY AP-1/MYC AXIS DRIVES PREMATURE AGING AND SENESCENCE OF HAEMATOPOIETIC STEM CELLS IN SICKLE CELL DISEASE

**Topic:** 27. Sickle cell disease

Julien Grenier*[1], Barbara Peyrard[1], Auria Godard[1], Chloe Carrie[1], Marie-Laurence Deligny[1], Sophie Beley[1], Benoit Meunier[1, 2], Thierry Cheyrol[3], Rachel Rignault[4], Julien Cheyrol[3], Imane Agouti[1, 2], Pascale Poullin[2], Thiago MacIel[4], Wassim El Nemer[1], Maria de Grandis[1]

*[1]Aix Marseille Univ, CNRS, EFS, ADES, Labex GR-Ex, Marseille, France; [2]Service d'Hémaphérèse et d'Autotransfusion, Hôpital de la Conception, APHM, Marseille, France; [3]Aix-Marseill univ, CEFOS, Marseille, France; [4]Laboratory of Molecular Mechanisms of Hematological Disorders and Therapeutic Implications, Institut IMAGINE, INSERM UMR1163 Université Paris Cité, Paris, France;*

### Background

Sickle cell disease (SCD) is a common hereditary hemoglobinopathy characterized by chronic haemolytic anaemia, inflammation, and progressive multi-organ damage. Although traditionally viewed as a peripheral red blood cell disorder, growing evidence indicates substantial bone marrow dysfunction, including ineffective erythropoiesis and niche alterations. Chronic inflammation and metabolic stress profoundly influence haematopoietic stem cell (HSC) behaviour. However, the intrinsic impact of SCD on the HSC compartment remains poorly understood.

### Aims

We aimed to delineate the molecular and functional landscape of hematopoietic stem and progenitor cell dysfunction in SCD and to identify the underlying mechanisms driving these alterations in order to uncover potential therapeutic targets.

### Methods

We conducted an integrated longitudinal analysis of haematopoiesis in the Townes SCD mouse model, combining in vivo and in vitro approaches, including flow cytometry, single-cell RNA sequencing, self-renewal assays, and transplantation experiments. Mice were also treated to assess the reversibility of stem cell alterations. To ensure translational relevance, parallel studies were performed on circulating CD34$^{+}$ cells from SCD patients, including in vitro treatment assays and bulk RNA sequencing.

### Results

SCD mice exhibited a marked loss of long-term HSCs, with expansion of MPP3 and MEP progenitors, reflecting erythro-myeloid skewing. Functionally, HSCs showed enhanced cycling, reduced self-renewal, and exhaustion in ex vivo culture and colony assays, with severely impaired repopulation in transplantation experiments. Decreased BM CXCL12, splenomegaly, extramedullary haematopoiesis, and increased circulating HSPCs indicate niche disruption and pathological mobilization. Single-cell RNA sequencing of LSK cells revealed transcriptional signatures of accelerated aging, including AP-1–driven inflammatory programs. SCD HSCs also displayed hallmarks of premature senescence, such as iron overload, altered mitochondrial membrane potential (TMRM), and elevated β-galactosidase activity. Antioxidant treatment (NAC) partially restored LT-HSC frequency and attenuated inflammatory signatures, implicating oxidative stress as a key driver of dysfunction. In parallel, circulating CD34$^{+}$ cells from SCD patients showed increased erythroid/myeloid repopulation, consistent with HSC mobilization. Transcriptomics revealed AP-1–driven inflammation, DNA repair activation, and dysregulation of HSC maintenance factors, including MYC upregulation at the protein level. Pharmacologic MYC inhibition reduced proliferation while preserving viable CD34$^{+}$ cells and enhanced clonogenic potential in patient-derived samples.

**Copyright Information:** (Online) ISSN: 2572-9241
©2026 The Author(s). HemaSphere is published by John Wiley & Sons Ltd on behalf of European Hematology Association. This is an open access Abstract Book distributed under the Attribution-NonCommercial-NoDerivs (CC BY-NC-ND), which allows third parties to download the articles and share them with others as long as they credit the author and the Abstract Book, but they cannot change the content in any way or use them commercially.
**Abstract Book Citations:** Authors, Title, HemaSphere, 2026;10:(S1):pages. The abstract book DOIs can be found on the "Author Information" page.
**Disclaimer:** Articles published in the journal HemaSphere exclusively reflect the opinions of the authors. The authors are responsible for all content in their abstracts including accuracy of the facts, statements, citing resources, etc.

**Summary/Conclusion**

Our findings reveal that SCD drives early and broad alterations in the hematopoietic stem cell compartment, with premature HSC senescence emerging as a key functional consequence of chronic inflammation. Mechanistically, chronic inflammatory signals lead to ROS accumulation and iron overload, activating AP-1 pathways and upregulating MYC. This cascade disrupts HSC quiescence, promotes repeated cell cycling, and ultimately results in exhaustion and accelerated stem cell aging. Targeting these inflammatory and metabolic pathways may preserve HSC fitness and improve outcomes of stem cell–based and gene therapies in SCD.

**Copyright Information:** (Online) ISSN: 2572-9241
©2026 The Author(s). HemaSphere is published by John Wiley & Sons Ltd on behalf of European Hematology Association. This is an open access Abstract Book distributed under the Attribution-NonCommercial-NoDerivs (CC BY-NC-ND), which allows third parties to download the articles and share them with others as long as they credit the author and the Abstract Book, but they cannot change the content in any way or use them commercially.
**Abstract Book Citations:** Authors, Title, HemaSphere, 2026;10:(S1):pages. The abstract book DOIs can be found on the "Author Information" page.
**Disclaimer:** Articles published in the journal HemaSphere exclusively reflect the opinions of the authors. The authors are responsible for all content in their abstracts including accuracy of the facts, statements, citing resources, etc.

### (PF1269) IMPAIRED FUNCTION OF PYRUVATE KINASE IS ASSOCIATED WITH HEMOLYSIS-RELATED INFLAMMATION, INEFFECTIVE ERYTHROPOIESIS, AND ENDOTHELIAL DYSFUNCTION IN ADULTS WITH SICKLE CELL ANEMIA

**Topic:** 27. Sickle cell disease

Marissa Traets[1], Jennifer Bos[1], Sigrid van der Veen[2], Brigitte van Oirschot[1], Saskia Schols[3], Marjon Cnossen[4], Erfan Nur[5], Bart Biemond[5], Eduard J. van Beers[2], Anita Rijneveld[6], Richard van Wijk[1], Minke Rab*[1, 6]

*[1]University Medical Center Utrecht, Central Diagnostic Laboratory-Research, Utrecht, The Netherlands;[2]University Medical Center Utrecht, Center for Benign Hematology, Thrombosis and Hemostasis - Van Creveldkliniek, Utrecht, The Netherlands; [3]Radboud University Medical Center, Department of Hematology, Nijmegen, The Netherlands;[4]Erasmus University Medical Center Rotterdam, Department of Pediatric Hematology, Erasmus MC- Sophia Children's Hospital, Rotterdam, The Netherlands; [5]Amsterdam University Medical Centers, Hematology, Amsterdam, The Netherlands; [6]Erasmus University Medical Center Rotterdam, Hematology, Rotterdam, The Netherlands;*

### Background

Sickle cell anemia (SCA) is characterized by red blood cell (RBC) sickling due to hemoglobin S polymerization, leading to vaso-occlusive episodes and chronic hemolytic anemia. Intravascular hemolysis contributes to oxidative stress, endothelial dysfunction, and inflammation. Besides hydroxyurea (HU) and transfusion, new therapeutic approaches include pyruvate kinase (PK) activators, which may increase ATP, improve membrane health, and reduce sickling.

### Aims

Is impaired PK function is associated with disease modifiers (fetal hemoglobin, α-thalassemia) and laboratory markers of hemolysis, ineffective erythropoiesis, inflammation, and endothelial dysfunction in adults with SCA.

### Methods

PK activity was measured and related to hexokinase (HK) activity, another RBC age-dependent enzyme, in adults with HbSS or HbSβ⁰-thalassemia at steady state and without transfusion 2 months prior to enrollment. PK thermostability was expressed as % residual PK activity after exposure to 53°C (1 hour). Plasma levels of soluble transferrin receptor (sTFR), hemopexin, heme oxygenase-1 (HO-1), CD40 Ligand (CD40L), myeloperoxidase (MPO), and VEGF were measured with Meso Scale Discovery ELISA. Plasma proinflammatory cytokines were measured with OLINK technology. RBC adhesion to laminin (Biolamina) was investigated on IBIDI µ-slides I 0.4. Correlations were tested using the Spearman's correlation and group comparisons with Mann-Whitney U test ($p<0.05$, GraphPad Prism v10).

**Copyright Information:** (Online) ISSN: 2572-9241
©2026 The Author(s). HemaSphere is published by John Wiley & Sons Ltd on behalf of European Hematology Association. This is an open access Abstract Book distributed under the Attribution-NonCommercial-NoDerivs (CC BY-NC-ND), which allows third parties to download the articles and share them with others as long as they credit the author and the Abstract Book, but they cannot change the content in any way or use them commercially.
**Abstract Book Citations:** Authors, Title, HemaSphere, 2026;10:(S1):pages. The abstract book DOIs can be found on the “Author Information” page.
**Disclaimer:** Articles published in the journal HemaSphere exclusively reflect the opinions of the authors. The authors are responsible for all content in their abstracts including accuracy of the facts, statements, citing resources, etc.

### Results

Seventy-seven subjects were included (median age 31 years; 57% female; 60% on HU). Neither HU use or concomitant α-thalassemia was associated with PK thermostability or PK/HK ratio. Individuals with HbF >15% showed higher PK thermostability (76.5% vs 71.5%, p<0.05) and PK/HK ratio (6.5 vs 5.5, p<0.05).
Next, we explored if PK characteristics were associated with markers of hemolysis and inflammation. PK thermostability correlated with erythroblast count (r= -0.325, p<0.01), and sTFR (r=-0.381, p<0.001), ferritin (r=-0.329, p=0.004), and absolute reticulocyte count (r=-0.429, p=0.0001). PK/HK ratio associated with bilirubin levels (r=-0.392, p=0.001), hemopexin (r=0.254, p=0.026), and hemoglobin (r=0.364, p=0.001, Figure 1A-C). These findings indicate that impaired PK activity and thermostability are associated with increased hemolysis and enhanced erythropoiesis. Furthermore, PK thermostability correlated with WBC (r=-0.316, p<0.011), neutrophils (r=-0.433, p<0.001), monocytes (r=-0.235, p<0.05) and CD40L (r=-0.337, p=0.003). The association with CD40L, a platelet-associated pro-inflammatory molecule linked to endothelial cell activation, indicates increased endothelial dysfunction in individuals with reduced PK thermostability. This was supported by an association of PK thermostability with P-selectin (r=-0.242, p<0.05) and RBC adhesion (r=-0.338, p<0.05, Figure 1D-E). In adults without HU therapy an association with IL-18, that has been linked to cardiomyopathy, and PK/HK ratio (r=-0.433, p<0.05) was found. In addition, we found a significant correlation with PK-thermostability and interferon- γ (r=-0.428, p<0.05) and sVCAM (r=-0.420, p<0.05, Figure 1G-I).

### Summary/Conclusion

Impaired PK function, reflected by reduced PK thermostability and lower PK/HK ratio, is associated with several markers of increased hemolysis, ineffective erythropoiesis, inflammation, and endothelial dysfunction in adults with SCD. Future research is needed to determine if these findings translate to changes in these markers after in vivo activation of PK.

**Copyright Information:** (Online) ISSN: 2572-9241
©2026 The Author(s). HemaSphere is published by John Wiley & Sons Ltd on behalf of European Hematology Association. This is an open access Abstract Book distributed under the Attribution-NonCommercial-NoDerivs (CC BY-NC-ND), which allows third parties to download the articles and share them with others as long as they credit the author and the Abstract Book, but they cannot change the content in any way or use them commercially.
**Abstract Book Citations:** Authors, Title, HemaSphere, 2026;10:(S1):pages. The abstract book DOIs can be found on the “Author Information” page.
**Disclaimer:** Articles published in the journal HemaSphere exclusively reflect the opinions of the authors. The authors are responsible for all content in their abstracts including accuracy of the facts, statements, citing resources, etc.

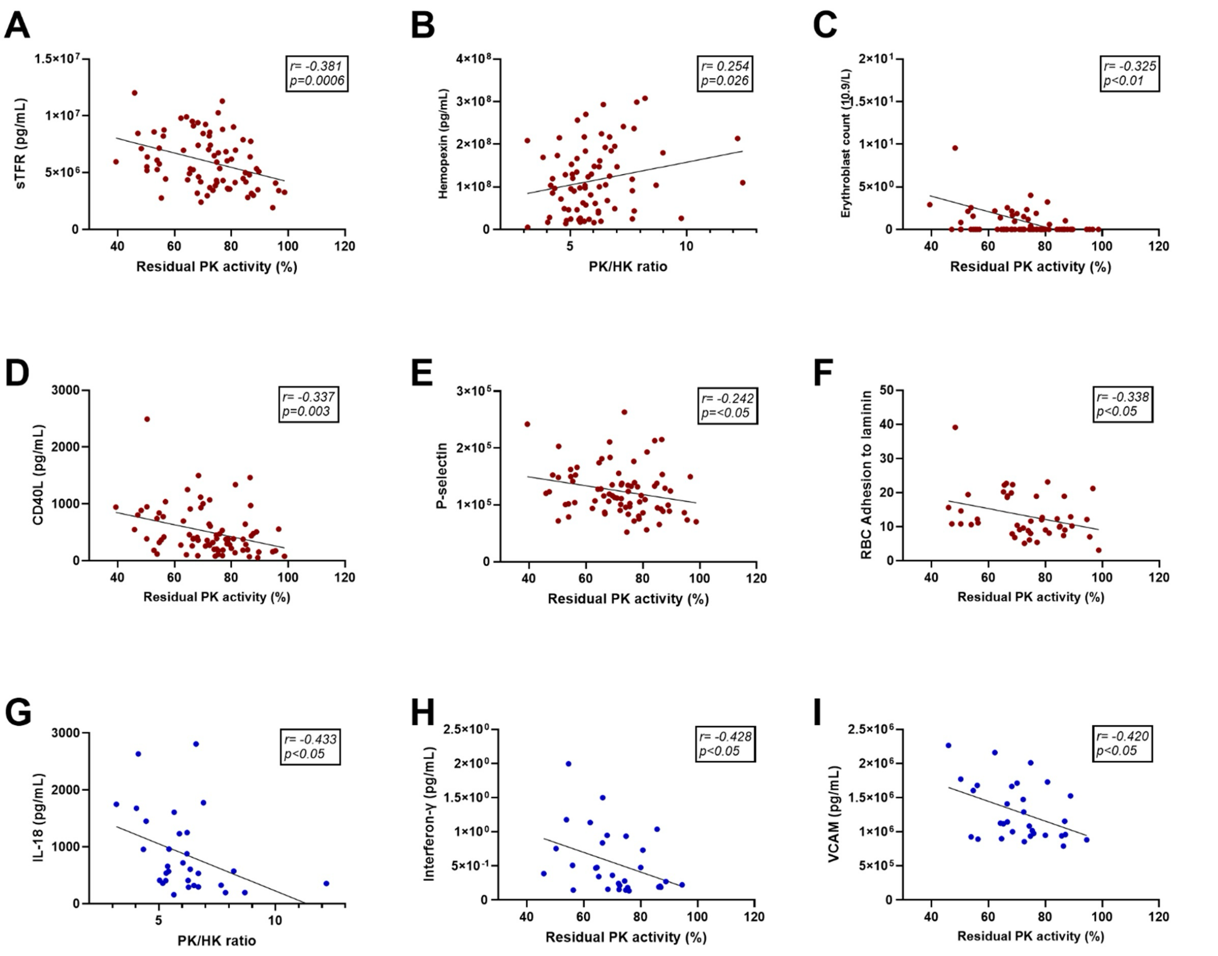

**Figure 1. Associations between relative pyruvate kinase (PK) deficiency, reflected by a reduced PK/ hexokinase (HK) activity ratio or PK thermostability with markers of hemolysis, ineffective erythropoiesis, red blood cell (RBC) adhesion and inflammation.** Panel A-F display associations found in a cohort of 77 sickle cell anemia patients. Panel G-I display significant associations in a subgroup of 31 SCS patients without hydroxyurea therapy.

**Copyright Information:** (Online) ISSN: 2572-9241
©2026 The Author(s). HemaSphere is published by John Wiley & Sons Ltd on behalf of European Hematology Association. This is an open access Abstract Book distributed under the Attribution-NonCommercial-NoDerivs (CC BY-NC-ND), which allows third parties to download the articles and share them with others as long as they credit the author and the Abstract Book, but they cannot change the content in any way or use them commercially.
**Abstract Book Citations:** Authors, Title, HemaSphere, 2026;10:(S1):pages. The abstract book DOIs can be found on the "Author Information" page.
**Disclaimer:** Articles published in the journal HemaSphere exclusively reflect the opinions of the authors. The authors are responsible for all content in their abstracts including accuracy of the facts, statements, citing resources, etc.

### (PF1270) PRESERVATION OF ADAPTIVE IMMUNITY AFTER NONMYELOABLATIVE HEMATOPOIETIC CELL TRANSPLANTATION IN ADULTS WITH SICKLE CELL DISEASE (PROTECT STUDY)

**Topic:** 27. Sickle cell disease

Lisa Dovern*[1], Maud Zwolsman[1], Abraham Goorhuis[1], Bart Biemond[1], Katja C. Wolthers[1], Erfan Nur[1, 2]

*[1]Amsterdam UMC, Hematology, Amsterdam, The Netherlands; [2]Sanquin Research, Amsterdam, The Netherlands;*

### Background

Recipients of allogeneic hematopoietic cell transplantations (HCT) generally lose vaccination-derived immunity, necessitating revaccination. Patients with sickle cell disease (SCD) undergoing matched sibling donor (MSD) transplantation with nonmyeloablative alemtuzumab/total body irradiation (TBI) conditioning develop mixed lymphoid chimerism, potentially preserving their own immunity. We hypothesized that mixed chimerism after non-myeloablative MSD transplantation results in preservation of immunity, rendering revaccination potentially unnecessary.

### Methods

Preservation of previously developed immunity against measles, rubella, and *Streptococcus pneumoniae* was prospectively evaluated in SCD patients undergoing non-myeloablative transplantation (mixed chimerism; n=24) as compared to patients undergoing haploidentical HCT with a more intensive conditioning regimen (full donor chimerism; n=14). To confirm that the preservation of pre-transplant immunity was recipient-derived, we evaluated immunity against HBV vaccination in a subgroup of patients undergoing MSD (n=6) or haploidentical (n=6) transplantation and in non-transplant control SCD patients (n=6). None of the donors had been infected by or vaccinated against HBV. All patients received HBV vaccinations in an accelerated schedule before transplantation. Additionally, immune reconstitution was assessed by measuring lymphocyte subsets (CD3+, CD4+, CD8+, CD19+, and natural killer cells), and total IgG levels at baseline and multiple timepoints after transplantation.

### Results

After 24 months, immunity preservation was higher in MSD recipients than in haploidentical recipients for measles (86% vs 70%, p=0.36, Figure 1A) and rubella (95% vs 67%, p=0.053, Figure 1B). Pneumococcal immunity remained intact in 95% of MSD recipients compared to only 17% of haploidentical recipients (p=0.005, Figure 1C). Of the patients vaccinated with HBV prior to HCT, three patients (50%) in the MSD cohort and two patients (33%) in the haploidentical cohort lost protective anti-HBs titers, with results in the MSD cohort likely confounded by rituximab treatment shortly after transplantation (Figure 1D). In the non-transplant control cohort, one patient remained seronegative and another patient with initial response lost protection against HBV. Immune reconstitution after HCT followed similar trajectories in both groups, with CD3+, CD4+, CD8+, and CD19+ counts falling below reference values after 3 months and recovering to normal levels between 12 and 24 months. However, throughout follow-up, MSD recipients maintained significantly higher total IgG levels, most pronounced at +3 months (16.1 vs 8.1 g/L, p<0.001).

**Copyright Information:** (Online) ISSN: 2572-9241
©2026 The Author(s). HemaSphere is published by John Wiley & Sons Ltd on behalf of European Hematology Association. This is an open access Abstract Book distributed under the Attribution-NonCommercial-NoDerivs (CC BY-NC-ND), which allows third parties to download the articles and share them with others as long as they credit the author and the Abstract Book, but they cannot change the content in any way or use them commercially.
**Abstract Book Citations:** Authors, Title, HemaSphere, 2026;10:(S1):pages. The abstract book DOIs can be found on the “Author Information” page.
**Disclaimer:** Articles published in the journal HemaSphere exclusively reflect the opinions of the authors. The authors are responsible for all content in their abstracts including accuracy of the facts, statements, citing resources, etc.

**Summary/Conclusion**

In conclusion, we demonstrate that in SCD patients, nonmyeloablative MSD transplantation leads to more durable preservation of pre-transplant immunity compared to haploidentical transplantation with RIC. Immune reconstitution was comparable between both groups. The preservation of anti-HBV response between the transplant groups and the non-transplant controls was also comparable, indicating that the preservation of pre-transplant immunity is recipient-derived rather than donor-derived, as all donors were not exposed to HBV (vaccination). While revaccination programs might be delayed to at least 2 years post-transplantation, a prospective study using titer-guided revaccinations might establish whether revaccination after nonmyeloablative Mwas comparablentation in SCD patients can be entirely abandoned.

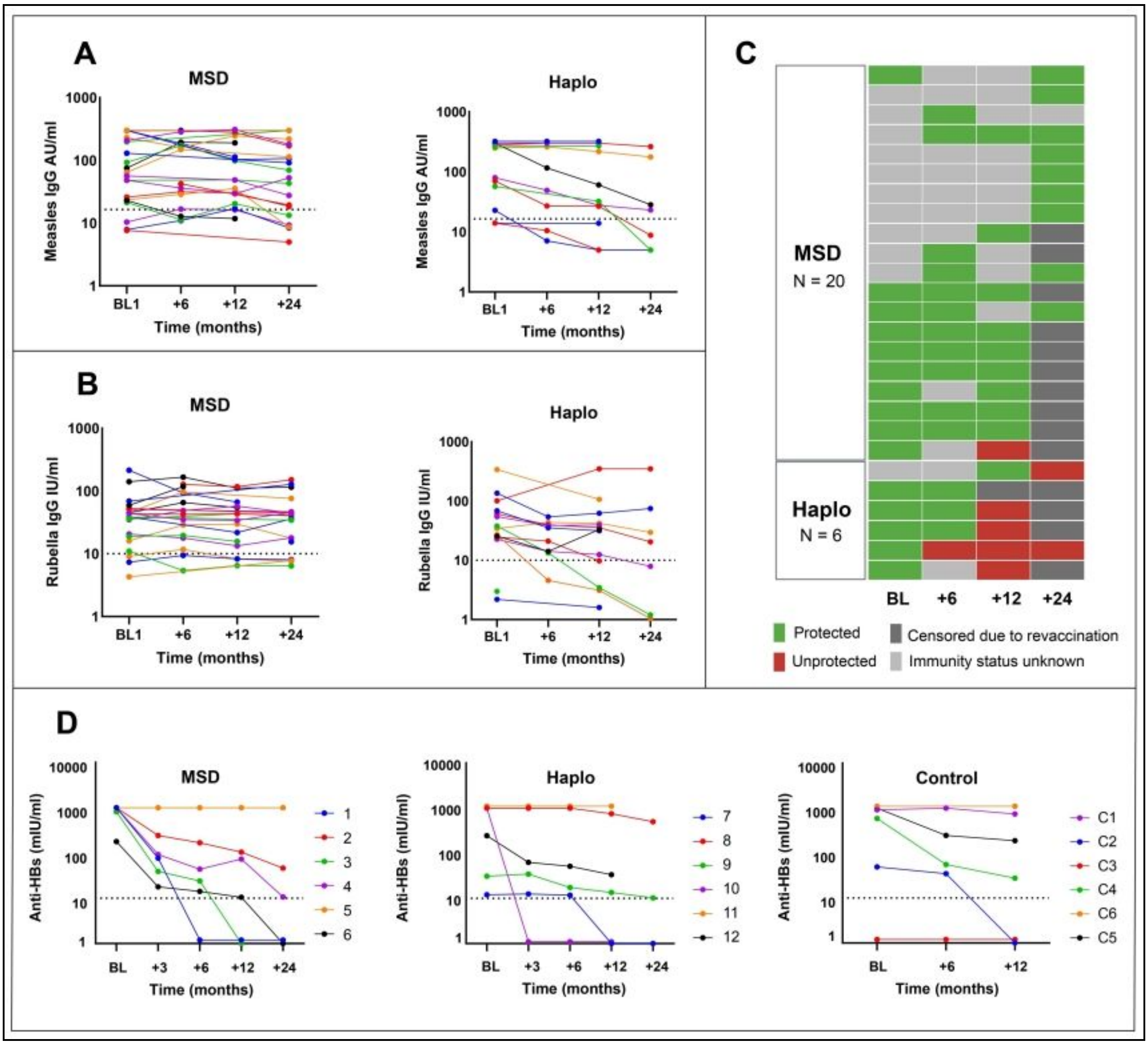

**Copyright Information:** (Online) ISSN: 2572-9241
©2026 The Author(s). HemaSphere is published by John Wiley & Sons Ltd on behalf of European Hematology Association. This is an open access Abstract Book distributed under the Attribution-NonCommercial-NoDerivs (CC BY-NC-ND), which allows third parties to download the articles and share them with others as long as they credit the author and the Abstract Book, but they cannot change the content in any way or use them commercially.
**Abstract Book Citations:** Authors, Title, HemaSphere, 2026;10:(S1):pages. The abstract book DOIs can be found on the "Author Information" page.
**Disclaimer:** Articles published in the journal HemaSphere exclusively reflect the opinions of the authors. The authors are responsible for all content in their abstracts including accuracy of the facts, statements, citing resources, etc.

### (PF1271) STEROID PRE-BUSULFAN REDUCED INTENSITY CONDITIONING MATCH RELATED HEMATOPOIETIC STEM CELL TRANSPLANTATION OUTCOME OF PATIENTS WITH SICKLE CELL DISEASE

**Topic:** 27. Sickle cell disease

Abdulrahman Nasiri*[1], Sara Samarkandi[2], Reem Alasbali[2], Ahmed Alahmdi[2], Huda Alfattah[2], Tariq Alzahrani[3], Hadeel Samarkandi[2], Ahmed Abdrabou[2], Mostafa Mohammed Saleh[2], Riad El Fakih[2], Amr Suleiman Hanbali[2], Hazzaa Alzahrani[2], Aljurf Mahmoud[2], Fahed Almhareb[2], Ali Alahmari[2]

*[1]Imam Mohammad Ibn Saud Islamic University, College of Medicine, Riyadh, Saudi Arabia;[2]King Faisal Specialist Hospital & Research Centre, Department of Hematology, Riyadh, Saudi Arabia; [3]Saudi Food and Drug Authority, Riyadh, Saudi Arabia;*

### Background

Sickle cell disease (SCD) is a complex genetic disorder causing significant morbidity and reduced life expectancy. Hematopoietic stem cell transplantation (HSCT) remains the only widely available curative option for SCD. However, its efficacy and safety in adult patients are still under-explored, with most studies focusing on pediatric populations. We present one of the largest single-center experiences of HLA-identical sibling HSCT for SCD, with a particular focus on adult recipients.

### Methods

We retrospectively analyzed 115 consecutive SCD patients who underwent HLA-identical sibling HSCT between 2013 and 2023 at King Faisal Specialist Hospital & Research Centre, Riyadh, Saudi Arabia The majority (76.5%, 88 patients) were adults (≥18 years), with a median age of 23 years (range 14-43). Patients were considered for HSCT if they met specific criteria, including hemoglobin S level <30%, platelet count >30 x 10^9/L, and adequate organ function.
Conditioning regimen was IVBU14/FLU/ATG for all patients (100%). GVHD prophylaxis consisted of tacrolimus/MTX (42.6%, 49 patients) or CSA/MTX (57.4%, 66 patients). Bone marrow was the sole stem cell source (100%, 115 patients).

### Results

With a median follow-up of 12.5 months (range 0.7-60), we observed a remarkably high overall survival rate of 96.5% (111 out of 115 patients alive at last follow-up). Engraftment was robust across the cohort. The median time to neutrophil recovery (>500 × 10^9/L) was 16 days (range 9-30), and platelet recovery (>50 × 10^9/L) occurred at a median of 20 days (range 13-30).
The incidence of acute graft-versus-host disease (aGVHD) was 19.1% (22 patients), while chronic GVHD (cGVHD) occurred in 10.4% (12 patients) of the cohort. These rates are within acceptable ranges for allogeneic HSCT.
Regimen-related toxicities were observed, with mucositis being the most common, affecting 74.8% of patients (86 individuals).
Serious complications were relatively infrequent. Hemorrhagic cystitis and veno-occlusive disease were rare, each affecting only 0.9% of the cohort (1 patient each). Renal toxicity, as evidenced by elevated creatinine levels, was observed in 12.2% of patients (14 individuals).
Infection rates were notable, with CMV reactivation occurring in 63.5% of patients (73 individuals). Fungal infections were less common, affecting 3.5% of the cohort (4 patients).

**Copyright Information:** (Online) ISSN: 2572-9241
©2026 The Author(s). HemaSphere is published by John Wiley & Sons Ltd on behalf of European Hematology Association. This is an open access Abstract Book distributed under the Attribution-NonCommercial-NoDerivs (CC BY-NC-ND), which allows third parties to download the articles and share them with others as long as they credit the author and the Abstract Book, but they cannot change the content in any way or use them commercially.
**Abstract Book Citations:** Authors, Title, HemaSphere, 2026;10:(S1):pages. The abstract book DOIs can be found on the "Author Information" page.
**Disclaimer:** Articles published in the journal HemaSphere exclusively reflect the opinions of the authors. The authors are responsible for all content in their abstracts including accuracy of the facts, statements, citing resources, etc.

**Summary/Conclusion**

This cohort study demonstrates remarkably high survival rates and acceptable toxicity profiles for HLA-identical sibling HSCT in SCD, particularly in adult patients. The overall survival rate of 96.5% at a median follow-up of 12.5 months surpasses many previously reported survival rates in SCD transplantation.
The use of bone marrow as the sole stem cell source aligns with current best practices in SCD transplantation and may have contributed to the relatively low incidence of chronic GVHD observed in our cohort.
While overall complication rates were low, the high incidence of CMV reactivation (63.5%) underscores the need for rigorous viral monitoring and pre-emptive therapy protocols in the post-transplant period. Future studies should focus on optimizing CMV management strategies in this population.
These findings may significantly impact treatment paradigms for adult SCD patients, offering hope for a curative option to a broader patient population.

**Copyright Information:** (Online) ISSN: 2572-9241
©2026 The Author(s). HemaSphere is published by John Wiley & Sons Ltd on behalf of European Hematology Association. This is an open access Abstract Book distributed under the Attribution-NonCommercial-NoDerivs (CC BY-NC-ND), which allows third parties to download the articles and share them with others as long as they credit the author and the Abstract Book, but they cannot change the content in any way or use them commercially.
**Abstract Book Citations:** Authors, Title, HemaSphere, 2026;10:(S1):pages. The abstract book DOIs can be found on the "Author Information" page.
**Disclaimer:** Articles published in the journal HemaSphere exclusively reflect the opinions of the authors. The authors are responsible for all content in their abstracts including accuracy of the facts, statements, citing resources, etc.

### (PF1272) CHRONIC PAIN, FUNCTIONAL IMPAIRMENT, AND GENERALIZED MECHANICAL HYPERSENSITIVITY IN ADULTS WITH SICKLE CELL DISEASE

**Topic:** 27. Sickle cell disease

Fábio Galvão[1, 2], Ana Vieira Pansani[1, 2], Bruno Benites[1, 2], Manoella Galafassi[1], Mariana Rosa[1], Bianca Possidio Campelo[1], Amanda Lopes[1], João Vitor Facco[1, 2], Glenda Feldberg*[1, 2], Sara Olalla Saad[1, 2]

[1]*University of Campinas (UNICAMP), Hematology and Transfusion Medicine Center (Hemocentro UNICAMP), Campinas, Brazil;*
[2]*Blood National Institute of Science and Technology (INCT Sangue), Campinas, Brazil;*

### Background

Pain is the most characteristic clinical manifestation of sickle cell disease (SCD) and is associated with increased morbidity and mortality. Although vaso-occlusive crises are the hallmark of the disease and account for most acute hospitalizations, chronic pain is increasingly recognized, particularly in adults. More than half of adults with SCD report pain on most days, suggesting a persistent pattern overlapping with acute crises. Quantitative sensory testing indicates alterations in nociceptive processing in this population. Given the high burden of chronic pain and possible mechanical hypersensitivity, objective assessment of pain sensitivity may improve clinical characterization of pain in SCD.

### Aims

To characterize chronic pain, functional impairment, and generalized mechanical hypersensitivity in adults with SCD and, secondarily, to compare pressure pain threshold (PPT) with healthy individuals.

### Methods

This cross-sectional study included adults with SCD followed at a specialized outpatient clinic. Pain, stiffness, and functional status were assessed using the Western Ontario and McMaster Universities Osteoarthritis Index (WOMAC). Pressure pain threshold (PPT) was measured by digital algometry at six bilateral anatomical sites (shoulders, hips, and lumbar region).
Within-group analyses were performed using the Mann–Whitney U test for continuous variables and the chi-square test for categorical variables. Differences in PPT between SCD and healthy controls were evaluated using a linear mixed-effects model adjusted for age and sex, accounting for repeated measures. Site-specific analyses were conducted using linear regression with Sidak correction. Statistical significance was set at $p < 0.05$.

### Results

Ninety-nine adults with SCD were evaluated (median age 45 years [18–73]; 69.7% female). Chronic pain was present in 69.7% of participants. Median WOMAC scores were 5 (0–17) for pain, 2 (0–8) for stiffness, and 12 (0–60) for physical function. Individuals without chronic pain had significantly worse WOMAC scores for pain (6.0 [0–17.0] vs 1.5 [0–14.0]; p=0.003), stiffness (3.0 [0–8.0] vs 0.5 [0–8.0]; p=0.004), and physical function (17.0 [0–60.0] vs 4.5 [0–58.0]; p=0.002). Chronic pain prevalence did not differ according to genotype severity (p=0.665).
Seventy healthy controls were included in the comparative analysis. In adjusted analyses, individuals with SCD showed significantly lower global PPT compared with controls ($\beta = -26.3$ N; 95% CI −31.8 to −20.8; p<0.001), with consistent reductions across all sites.

**Copyright Information:** (Online) ISSN: 2572-9241
©2026 The Author(s). HemaSphere is published by John Wiley & Sons Ltd on behalf of European Hematology Association. This is an open access Abstract Book distributed under the Attribution-NonCommercial-NoDerivs (CC BY-NC-ND), which allows third parties to download the articles and share them with others as long as they credit the author and the Abstract Book, but they cannot change the content in any way or use them commercially.
**Abstract Book Citations:** Authors, Title, HemaSphere, 2026;10:(S1):pages. The abstract book DOIs can be found on the “Author Information” page.
**Disclaimer:** Articles published in the journal HemaSphere exclusively reflect the opinions of the authors. The authors are responsible for all content in their abstracts including accuracy of the facts, statements, citing resources, etc.

**Summary/Conclusion**

Adults with SCD exhibit a high frequency of chronic pain and functional impairment, accompanied by a generalized reduction in pressure pain threshold. Persistence of this finding after adjustment for age and sex, and its independence from genotype severity, suggest that mechanical hypersensitivity represents a consistent clinical feature of pain in SCD. These findings reinforce the importance of evaluating chronic pain beyond acute vaso-occlusive crises. Incorporating objective pain sensitivity measures may improve clinical characterization of pain in SCD and support individualized management strategies. Further studies are warranted to clarify the clinical relevance of altered pain sensitivity in SCD.

**Copyright Information:** (Online) ISSN: 2572-9241
©2026 The Author(s). HemaSphere is published by John Wiley & Sons Ltd on behalf of European Hematology Association. This is an open access Abstract Book distributed under the Attribution-NonCommercial-NoDerivs (CC BY-NC-ND), which allows third parties to download the articles and share them with others as long as they credit the author and the Abstract Book, but they cannot change the content in any way or use them commercially.
**Abstract Book Citations:** Authors, Title, HemaSphere, 2026;10:(S1):pages. The abstract book DOIs can be found on the "Author Information" page.
**Disclaimer:** Articles published in the journal HemaSphere exclusively reflect the opinions of the authors. The authors are responsible for all content in their abstracts including accuracy of the facts, statements, citing resources, etc.

### (PF1273) IDENTIFYING THE ROLE OF SOCIOECONOMIC AND ENVIRONMENTAL DETERMINANTS OF HEALTH IN SICKLE CELL DISEASE FROM PATIENTS' LIVED EXPERIENCES IN THE UK

**Topic:** 27. Sickle cell disease

Chandni N. Hindocha[1], Frédéric B. Piel*[1]

[1] *Imperial College London, School of Public Health, London, United Kingdom;*

### Background

Sickle cell disease (SCD) is characterised by a remarkable phenotypic heterogeneity which can only be partly explained by genetic factors. There is relatively limited conclusive evidence of the role of wider determinants of health in SCD, including socioeconomic deprivation, environmental exposure, and inequitable healthcare access. Identifying patient-reported experience of these determinants is key to guide future research in this area.

### Aims

To evaluate the impact of socioeconomic and environmental determinants of health in individuals living with SCD, and to identify priority domains for future research.

### Methods

A cross-sectional patient-reported outcomes online survey was co-developed with patient representatives. Participants rated the influence of 19 socioeconomic (income stability, employment, housing quality, access to financial support, and mental health services) and 14 environmental (temperature variability, cold exposure, air quality, humidity, and housing-related environmental conditions) determinants using Likert scales. These were complemented by open text answers and online workshops to provide more details. Quantitative summaries were then conducted to identify lived-experience priorities. Qualitative free-text responses were analysed using thematic synthesis to contextualise quantitative findings and capture lived experience relevant to supportive care pathways.

### Results

A total 108 participants responded to our survey. They were predominantly of Black, Black British, Caribbean, or African heritage (81%) but included a few from Asian or White backgrounds. There were slightly more female participants (54%) than males. The survey reflected the complexity of influences of socioeconomic and environmental factors in SCD, as all the factors listed (**Figure 1**) were considered to have a strong or very strong influence by more than 50% of the participants. Access to prescription medications, timely pain relief, housing quality and financial and professional support were the main socioeconomic linked to complications in SCD, while the general level of education was considered to be the least relevant of the factors considered. Temperature and exposure to cold were by far considered as the most relevant environmental determinants, while noise pollution and humidity were considered to have less influence. Further analysis of the qualitative data is ongoing.

### Summary/Conclusion

These findings, based on lived experience, provide more rigorous evidence of the influence of a wide range of socioeconomic and environmental determinants on complications in SCD. They are providing valuable evidence to inform future research priorities on these topics in the UK and beyond. As some of these factors are modifiable, studies could identify practical actions that people with SCD could take to improve the management of their condition.

**Copyright Information:** (Online) ISSN: 2572-9241
©2026 The Author(s). HemaSphere is published by John Wiley & Sons Ltd on behalf of European Hematology Association. This is an open access Abstract Book distributed under the Attribution-NonCommercial-NoDerivs (CC BY-NC-ND), which allows third parties to download the articles and share them with others as long as they credit the author and the Abstract Book, but they cannot change the content in any way or use them commercially.
**Abstract Book Citations:** Authors, Title, HemaSphere, 2026;10:(S1):pages. The abstract book DOIs can be found on the "Author Information" page.
**Disclaimer:** Articles published in the journal HemaSphere exclusively reflect the opinions of the authors. The authors are responsible for all content in their abstracts including accuracy of the facts, statements, citing resources, etc.

**Figure 1.** Influence of socioeconomic (top) and environmental (bottom) determinants of health based on the lived experience of 108 patients surveyed in the UK

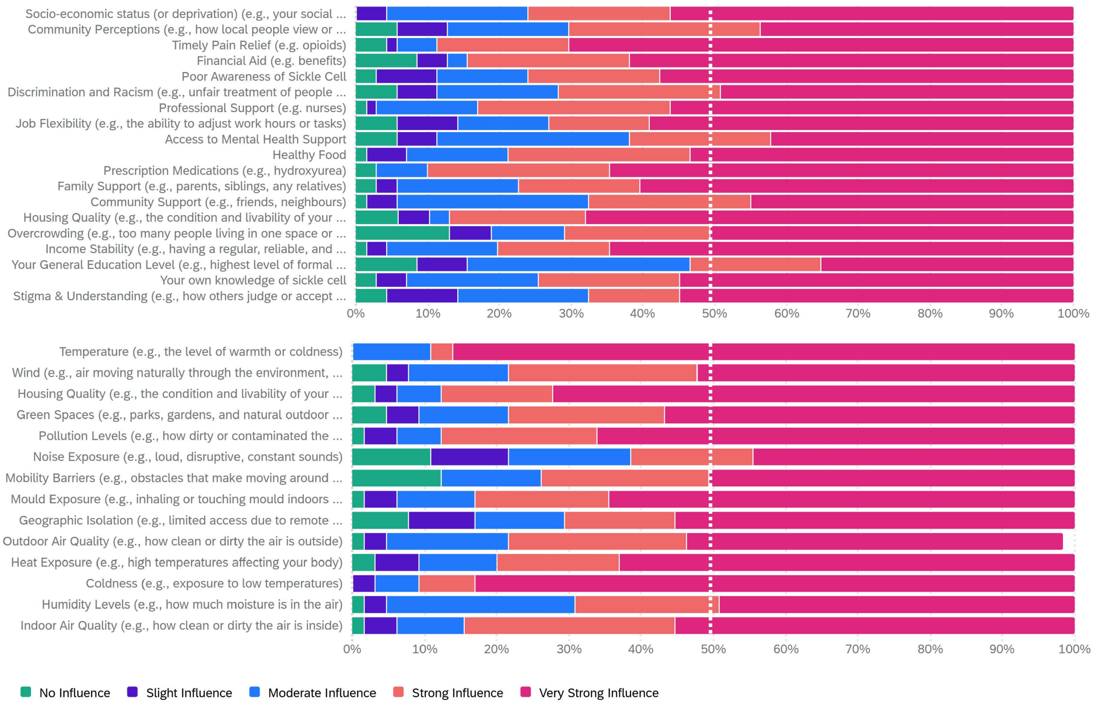

Figure 1. Influence of socioeconomic (top) and environmental (bottom) determinants of health based on the lived experience of 108 patients surveyed in the UK

**Copyright Information:** (Online) ISSN: 2572-9241
©2026 The Author(s). HemaSphere is published by John Wiley & Sons Ltd on behalf of European Hematology Association. This is an open access Abstract Book distributed under the Attribution-NonCommercial-NoDerivs (CC BY-NC-ND), which allows third parties to download the articles and share them with others as long as they credit the author and the Abstract Book, but they cannot change the content in any way or use them commercially.
**Abstract Book Citations:** Authors, Title, HemaSphere, 2026;10:(S1):pages. The abstract book DOIs can be found on the "Author Information" page.
**Disclaimer:** Articles published in the journal HemaSphere exclusively reflect the opinions of the authors. The authors are responsible for all content in their abstracts including accuracy of the facts, statements, citing resources, etc.

### (PF1274) INFLAMMATION DRIVES AGE-PROGRAMMED REMODELING OF HEMATOPOIETIC STEM CELLS IN SICKLE CELL DISEASE

**Topic:** 27. Sickle cell disease

Alice Corsia[1, 2, 3], Sara El Hoss[1, 3], Steicy Sobrino[4], Rachel Rignault[1, 3], Adeline Denis[4], Margot Tragin[5], Laure Joseph[1, 2, 3], Sandra Manceau[1, 2, 3], Jean-Benoît Arlet[1, 3, 6], Pablo Bartolucci[7], Jacques Pariat[8], Benedicte Neven[9], Annarita Miccio[4], Emmanuelle Six[4], Olivier Hermine[1, 3, 10], Marina Cavazzana[11, 12], Thiago MacIel*[1, 3]

*[1]Laboratory of Molecular Mechanisms of Hematological Disorders and Therapeutic Implications, Institut Imagine, INSERM UMR 1163, Université Paris Cité, Paris, France; [2]Hôpital Necker- Enfants malades, Assistance-Publique-Hôpitaux de Paris, Paris, France; [3]Initiatives IdEx Globule Rouge d'Excellence (InIdex GR-Ex), Université Paris Cité, Paris, France; [4]Laboratory of Chromatin and Gene Regulation During Development, Institut Imagine, INSERM UMR 1163, Université Paris Cité, Paris, France; [5]Bioinformatics Facilities, SFR Necker, Institut Imagine, INSERM UMR 11, Paris, France; [6]French Sickle Cell Referral Center, Internal Medicine Department, Georges Pompidou European Hospital, and Université de Paris-Cité, Paris, France; [7]Univ Paris Est Créteil, Hôpitaux Universitaires Henri Mondor, APHP, Sickle cell and Red cell disorders referral center - UMGGR, Créteil, France; [8]Department of Orthopedic Surgery, AP-HP, Henri Mondor Hospital, Créteil, France; [9]Pediatric Immunology, Hematology and Rheumatology Department, Hôpital Necker-Enfants Malades, Assistance Publique-Hôpitaux de Paris, Paris, France; [10]Department of Hematology, Assistance Publique - Hôpitaux de Paris Necker Hospital, Paris, France; [11]Departement of Biotherapy Necker Children's Hospital, Assistance Publique-Hôpitaux de Paris (AP-HP) Groupe Hospitalier Universitaire Centre, Université Paris Cité, Paris, France; [12]Biotherapy Clinical Investigation Center, AP-HP, INSERM, Institut Imagine, Paris, France;*

### Background

Sickle cell disease (SCD) is characterized by recurrent vaso-occlusive crises and chronic intravascular hemolysis, leading to sustained systemic inflammation. Hemolysis-associated damage signals activate NF-kB and inflamasome pathways, contributing to a pro-inflammatory bone marrow microenvironment. While inflammatory stress is known to perturb hematopoietic stem cell (HSC) function, whether SCD induces progressive, age-dependent inflammatory programming of the stem cell compartment remains unknown.

### Aims

We sought to define age-specific inflammatory signatures and functional alterations of HSCs in SCD patients and to determine whether pharmacologic inhibition of NF-
kB-driven inflammatory cascades restores stem cell integrity across disease stages.

### Methods

Hematopoietic stem and progenitor cells (HSPCs) were isolated from bone marrow samples of pediatric (n=5), adolescent (n=3), and adult (n=4) SCD patients and age-matched healthy donors. Human samples were obtained after informed consent in accordance with the Declaration of Helsinki and with approval from the local institutional review board. Single-cell RNA sequencing combined with functional assays was performed to characterize inflammatory states and stem cell fitness. To assess conservation of inflammatory signatures across species and disease progression,  age-stratified HSPCs from the Townes SCD mouse model (4-40 weeks) were analyzed by bulk RNA-seq and flow cytometry. In vivo pharmacologic inhibition of NF-kB-dependent inflammatory signaling was performed to evaluate reversibility of stem cell alterations.

**Copyright Information:** (Online) ISSN: 2572-9241
©2026 The Author(s). HemaSphere is published by John Wiley & Sons Ltd on behalf of European Hematology Association. This is an open access Abstract Book distributed under the Attribution-NonCommercial-NoDerivs (CC BY-NC-ND), which allows third parties to download the articles and share them with others as long as they credit the author and the Abstract Book, but they cannot change the content in any way or use them commercially.
**Abstract Book Citations:** Authors, Title, HemaSphere, 2026;10:(S1):pages. The abstract book DOIs can be found on the "Author Information" page.
**Disclaimer:** Articles published in the journal HemaSphere exclusively reflect the opinions of the authors. The authors are responsible for all content in their abstracts including accuracy of the facts, statements, citing resources, etc.

**Results**

Single cell transcriptomic profiling identified interferon-α/γ responses and TNF-α/NF-kB signaling as dominant inflammatory hallmarks of SCD HSCs. Pathway analysis uncovered a previously unrecognized biphasic TNF/NF-kB transcriptional program: pediatric HSCs displayed robust activation of NF-kB target genes, whereas adolescent and adult HSCs exhibited relative pathway repression, consistent with age-dependent inflammatory reprogramming of the stem cell compartment. These transcriptional signatures were associated with activation of inflammasome-related pathways and cytokines, consistent with pro-inflammatory bone marrow microenvironment. Age-dependent inflammatory programs were conserved in the Townes SCD mouse model (SS vs AA), where bulk RNA-seq analyses across young (4-8 weeks), adult (16 weeks), and aged mice (40 weeks) demonstratedsimilar dynamics, supporting translational relevance. Importantly, in vivo targeted inhibition of pro-inflammatory cytokine signaling restored HSC composition and normalized inflammatory transcriptional profiles, demonstrating a causal and reversible link between chronic inflammatory activation in stem cell dysfunction.

**Summary/Conclusion**

We identify chronic inflammation as a central, age-programmed driver of HSC remodeling in SCD. Our data uncover a dynamic and reversible inflammatory rewiring of the stem cell compartment in SCD, characterized by distinct pediatric and adult states, and demonstrate that targeting pro-inflammatory signaling can restore stem cell composition and attenuate inflammatory transcriptional programs. These findings have direct implications for gene therapy in SCD, providing a strong rationale for integrating anti-inflammatory strategies to enhance stem cell fitness and improve therapeutic outcomes across disease stages.

**Copyright Information:** (Online) ISSN: 2572-9241
©2026 The Author(s). HemaSphere is published by John Wiley & Sons Ltd on behalf of European Hematology Association. This is an open access Abstract Book distributed under the Attribution-NonCommercial-NoDerivs (CC BY-NC-ND), which allows third parties to download the articles and share them with others as long as they credit the author and the Abstract Book, but they cannot change the content in any way or use them commercially.
**Abstract Book Citations:** Authors, Title, HemaSphere, 2026;10:(S1):pages. The abstract book DOIs can be found on the "Author Information" page.
**Disclaimer:** Articles published in the journal HemaSphere exclusively reflect the opinions of the authors. The authors are responsible for all content in their abstracts including accuracy of the facts, statements, citing resources, etc.

### (PF1275) RELATIVE CONTRIBUTION OF VASO-OCCLUSIVE CRISES AND NON-CRISIS SYMPTOMS TO DAILY DISEASE BURDEN IN ADULTS WITH SICKLE CELL DISEASE: REGIONAL ANALYSIS FROM THE GLOBAL LISTEN SURVEY

**Topic:** 27. Sickle cell disease

Johnny Mahlangu*[1], Shashidhar Rao[2], Rohan Kolla[2], Emma Tan[2], Biree Andemariam[3]

*[1]Department of Molecular Medicine and Haematology, Faculty of Health Science, University of the Witwatersrand, Johannesburg, South Africa, Johannesburg, South Africa; [2]Novo Nordisk Health Care AG, Zurich, Switzerland; [3]University of Connecticut Health, Farmington, Connecticut, United States of America;*

### Background

Clinical management and trial endpoints in sickle cell disease (SCD) have historically prioritised vaso-occlusive crises (VOCs). However, accumulating evidence suggests that chronic and non-crisis symptoms substantially contribute to overall disease burden. Robust cross-regional data quantifying the relative contribution of these symptoms to daily functioning remain limited.

### Aims

To quantify and compare the patient-reported impact of VOCs and non-crisis symptoms on daily life among adults with SCD across six regions.

### Methods

LISTEN was a multinational, cross-sectional, quantitative survey conducted between Oct-2022 and Aug-2023 in 17 countries. Adults with SCD (≥18 years) who self-declared a formal diagnosis from a healthcare professional were recruited via patient advocacy networks and clinical sites. All respondents provided informed consent. The survey instrument incorporated structured symptom frequency and impact scales. VOCs were categorised by frequency (every day to several times a week/once to several times a month/less often than once a month/rarely or never/not sure), and the impact of symptoms on daily life was assessed using five-categories (not at all/a little/moderately/very much/a lot). Descriptive statistics were generated by region: Sub-Saharan Africa (SSA), Europe (EUR), Latin America (LATAM), Middle East and North Africa (MENA), North America (NA), and South Asia (SA). Between-region comparisons were exploratory, and descriptive given the cross-sectional design.

**Copyright Information:** (Online) ISSN: 2572-9241
©2026 The Author(s). HemaSphere is published by John Wiley & Sons Ltd on behalf of European Hematology Association. This is an open access Abstract Book distributed under the Attribution-NonCommercial-NoDerivs (CC BY-NC-ND), which allows third parties to download the articles and share them with others as long as they credit the author and the Abstract Book, but they cannot change the content in any way or use them commercially.
**Abstract Book Citations:** Authors, Title, HemaSphere, 2026;10:(S1):pages. The abstract book DOIs can be found on the "Author Information" page.
**Disclaimer:** Articles published in the journal HemaSphere exclusively reflect the opinions of the authors. The authors are responsible for all content in their abstracts including accuracy of the facts, statements, citing resources, etc.

### Results

A total of 1,145 adults (58% female; median age: 30 years; IQR: 24–38) completed the survey (SSA n=307; EUR n=242; LATAM n=122; MENA n=142; SA n=78; NA n=254). Frequent acute VOCs were reported by 52–68% of participants across regions and 59–83% of participants rated this pain as “moderately/very much & a lot” affecting their daily life. Beyond acute VOCs, fatigue was noted as the most consistently impactful non-crisis symptom, with 70–84% of respondents across all regions, while anaemia-related symptoms (e.g. dyspnoea/palpitations/dizziness) and chronic pain affected 33–77% and 58–74%, respectively, with notable regional variation Table 1. All regions, except SA, reported greater percentage impact of fatigue than acute VOCs.

**Table 1. Most Impactful Symptoms Reported by Adults with Sickle Cell Disease and Their Regional Differences**

| | Sub-Saharan Africa (n=307) | Europe (n=242) | Latin America (n=122) | Middle East and North Africa (n=142) | South Asia (n=78) | North America (n=254) |
|---|---|---|---|---|---|---|
| **Symptoms (%)*** | | | | | | |
| Fatigue | 76 | 70 | 84 | 80 | 76 | 73 |
| Acute VOCs | 69 | 59 | 74 | 72 | 83 | 65 |
| Symptoms related to anaemia | 65 | 62 | 68 | 77 | 33 | 46 |
| Chronic pain | 58 | 59 | 74 | 65 | 59 | 64 |
| *Percentage of respondents who reported impact of their symptoms in the category “moderately/very much/a lot” | | | | | | |

### Summary/Conclusion

In this large, geographically diverse adult SCD cohort, daily disease burden extends beyond episodic VOCs and is consistently driven by fatigue, anaemia-related symptoms and chronic pain. These non-crisis symptoms substantially shape patients’ daily disease experience. These findings support broader incorporation of validated patient-reported symptom measures in clinical practice and trial design. While cross-sectional and self-reported, the consistency of the findings across regions underscores the need to move beyond crisis-centric outcome frameworks to more comprehensively address total disease burden in SCD.

**Copyright Information:** (Online) ISSN: 2572-9241
©2026 The Author(s). HemaSphere is published by John Wiley & Sons Ltd on behalf of European Hematology Association. This is an open access Abstract Book distributed under the Attribution-NonCommercial-NoDerivs (CC BY-NC-ND), which allows third parties to download the articles and share them with others as long as they credit the author and the Abstract Book, but they cannot change the content in any way or use them commercially.
**Abstract Book Citations:** Authors, Title, HemaSphere, 2026;10:(S1):pages. The abstract book DOIs can be found on the “Author Information” page.
**Disclaimer:** Articles published in the journal HemaSphere exclusively reflect the opinions of the authors. The authors are responsible for all content in their abstracts including accuracy of the facts, statements, citing resources, etc.

### (PF1276) IMPACT OF SGLT2 INHIBITOR THERAPY (DAPAGLIFLOZIN), ON RENAL OUTCOMES IN PATIENTS WITH SICKLE CELL DISEASE : A SINGLE-CENTER PILOT STUDY.

**Topic:** 27. Sickle cell disease

Fatima Bensiradj*[1], Yosr Zaouali[1], Vincent Malcor Deydier De Pierrefeu[1], Jamila Alhamrouni[2], Habibi Anoosha[1], Anais Bourguignon D'herbigny[1], Gonzalo De Luna[1], Geoffroy Volle[3], Pablo Bartolucci[1], Vincent Audard[4]

*[1]Sickle cell referral center, Créteil, France;[2]GHSIF site Melun-Senart, Melun, France;[3]Créteil intercommunal Hospital, Créteil, France; [4]Henri Mondor Hospital, Nephrology and Renal Transplant Department, Créteil, France;*

### Background

The optimal therapeutic management for sickle cell disease(SCD)patients with renal impairment has yet to be determined.It currently relies on angiotensin-converting enzyme inhibitors(ACEi) or angiotensin II receptor blockers(ARBs).Hydroxyurea is also potentially contributing to proteinuria reduction.Given the well-established renoprotective effects of SGLT2 inhibitors in diabetic and non-diabetic chronic kidney disease(CKD),it was relevant to propose this therapy to sickle cell patients with persistently progressive kidney disease.

### Aims

This pilot study aims to assess the safety and the effect of Dapagliflozin on renal outcomes in SCD patients.

### Methods

We conducted a prospective,observational study at sickle cell referral center(Henri Mondor Hospital).Adult patients (≥18 years)with SCD receiving a stable dose(≥3 months)of an ACEi or ARBs,with an eGFR of 35–75mL/min/1.73m² and a urinary albumin-to-creatinine ratio(ACR) of 20–500mg/mmol,were eligible.For patients receiving hydroxyurea,the dosage was required to be stable for at least 3months.All participants received Dapagliflozin 10mg once daily.Renal function and serum electrolytes were assessed weekly during the first month and subsequently at months1,3,6,and12.We compared the outcomes of eGFR and ACR with these parameters one and two years before inclusion.

### Results

Among the 40 screened patients,16 fulfilled the inclusion criteria.Fortheen patients(88%)were homozygous(SS).The median age was 55y[37-71].Renal function baseline at M0 showed a mean eGFR of 48ml/min/1.73 m²(SD +/-13).An early eGFR reduction was observed shortly after treatment initiation,with a mean decline of 2ml/min/1.73m²(n = 10).This initial drop exceeded 20%of baseline eGFR in five patients,prompting adjustment of diuretic therapy.Five patients prematurely stopped treatment:one for a vaso-occlusive-crisis,three for personal or follow-up difficulties,and one was lost to follow-up***(Flowchart)***.Before starting Dapaglifozin,the annual decline in eGFR was between–3.2 to –4.5 mL/min/1.73m².Following treatment initiation,the rate of decline slowed to–1.6mL/min/1.73m²(n=10)over 12months.The ACR increased from 68 to 100 mg/mmol prior to baseline(M0).After treatment initiation,ACR stabilized with a modest reduction(–7.6 to –10.7 mg/mmol), remaining within a range of 74 to 89mg/mmol(n=10).The main adverse events observed included nocturia,genital infection,abdominal pain.No urinary tract infection was reported.One patient was hospitalized for an acute kidney injury,3 months after treatment initiation and permanently discontinued Dapagliflozin.Two patients required temporary discontinuation due to congestive renal failure or functional acute kidney injury related to dehydration.

**Copyright Information:** (Online) ISSN: 2572-9241
©2026 The Author(s). HemaSphere is published by John Wiley & Sons Ltd on behalf of European Hematology Association. This is an open access Abstract Book distributed under the Attribution-NonCommercial-NoDerivs (CC BY-NC-ND), which allows third parties to download the articles and share them with others as long as they credit the author and the Abstract Book, but they cannot change the content in any way or use them commercially.
**Abstract Book Citations:** Authors, Title, HemaSphere, 2026;10:(S1):pages. The abstract book DOIs can be found on the “Author Information” page.
**Disclaimer:** Articles published in the journal HemaSphere exclusively reflect the opinions of the authors. The authors are responsible for all content in their abstracts including accuracy of the facts, statements, citing resources, etc.

**Summary/Conclusion**

Given the limited sample size of the study,no definitive conclusions can be drawn.Nevertheless,the observed trends suggest a potential attenuation of CKD progression following Dapagliflozin initiation. Treatment was associated with stabilization of µalbuminuria,supporting a possible glomerular protective effect,as well as a numerical slowing of eGFR decline,corresponding to an approximate 60% reduction in the annual rate of deterioration during follow-up period.On the other hand,caution is advised in patients with reduced renal function,especially with cardiac dysfunction or diuretic therapy.These preliminary findings provide a signal that SGLT2 inhibition may offer renoprotective benefits in SCD associated nephropathy and justify further investigation in larger,controlled studies.

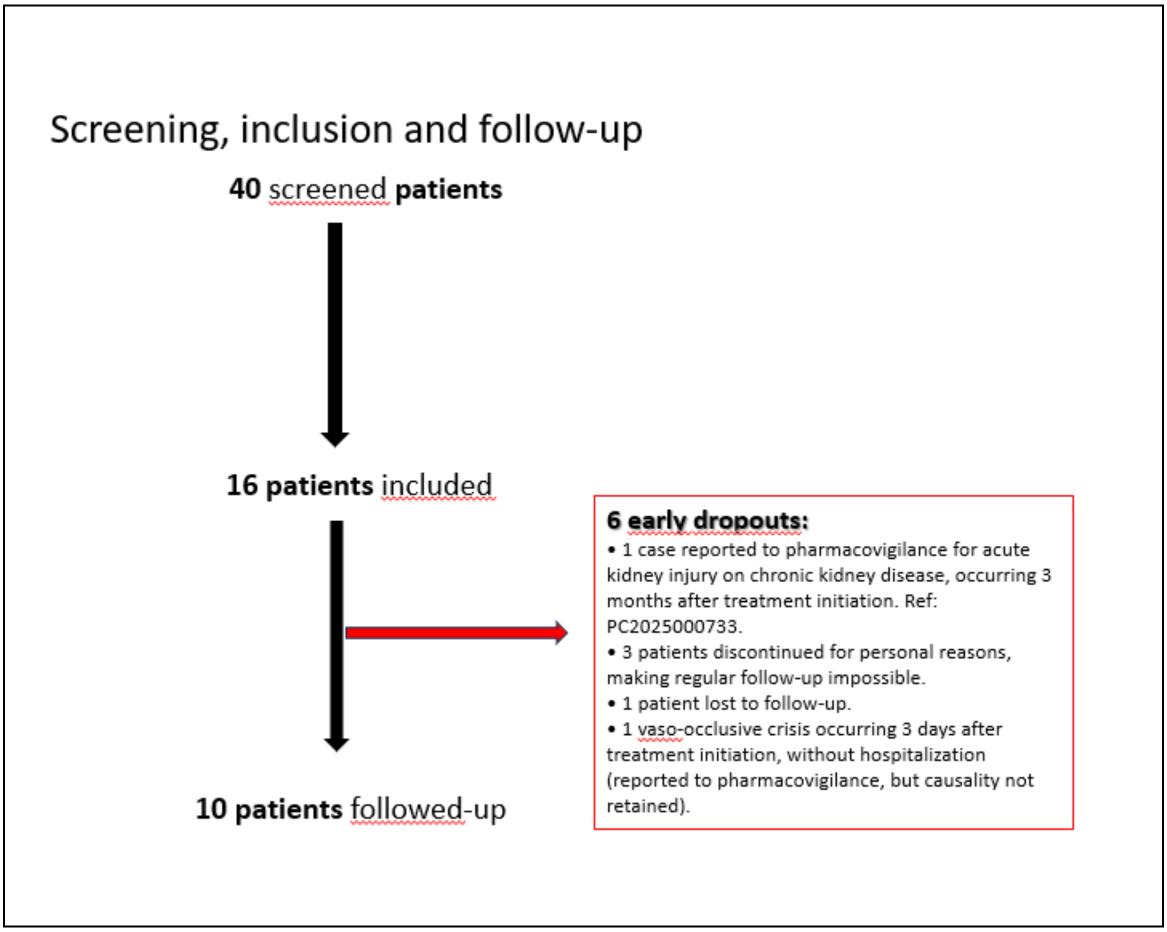

Flowchart

**Copyright Information:** (Online) ISSN: 2572-9241
©2026 The Author(s). HemaSphere is published by John Wiley & Sons Ltd on behalf of European Hematology Association. This is an open access Abstract Book distributed under the Attribution-NonCommercial-NoDerivs (CC BY-NC-ND), which allows third parties to download the articles and share them with others as long as they credit the author and the Abstract Book, but they cannot change the content in any way or use them commercially.
**Abstract Book Citations:** Authors, Title, HemaSphere, 2026;10:(S1):pages. The abstract book DOIs can be found on the "Author Information" page.
**Disclaimer:** Articles published in the journal HemaSphere exclusively reflect the opinions of the authors. The authors are responsible for all content in their abstracts including accuracy of the facts, statements, citing resources, etc.

### (PF1277) SYSTEMATIC INTENSIFICATION OF RED CELL EXCHANGE WITH HYDROXYUREA DECREASED DIRECT TRANSFUSION-RELATED COSTS IN ADULTS WITH SICKLE CELL DISEASE

**Topic:** 27. Sickle cell disease

Maria Alexandra Nica*[1, 2], Alexie Pellan-Sayegh[1], Jules Mercier-Ross[2], Nicolas Mercure-Corriveau[3], Yves Pastore[2, 4], Anne-Sophie Lemay[3], Benjamin Rioux-Massé[3], Stéphanie Forté[1, 5]

*[1]Université de Montréal, Faculty of Medicine, Montreal, Canada;[2]Centre hospitalier de l'Université de Montréal, Department of Medicine, Montreal, Canada; [3]Centre hospitalier de l'Université de Montréal, Division of Hematology-Transfusion Medicine, Montreal, Canada; [4]Centre hospitalier universitaire Sainte-Justine, Department of Pediatrics, Montreal, Canada;[5]Centre hospitalier de l'Université de Montréal, Division of Medical Oncology and Hematology, Montreal, Canada;*

### Background

Chronic red cell exchange (RCE), which replaces abnormal red blood cells (RBCs) with healthy donor cells, is an effective strategy to prevent complications in adults with sickle cell disease (SCD), particularly those with neurological involvement. However, RCE is invasive, resource-intensive, and associated with substantial healthcare costs. Since 2020, patients undergoing chronic RCE at our institution have been systematically offered hydroxyurea (HU) intensification with the aim to extend RCE intervals and reduce transfusion burden while maintaining clinical and biological efficacy. While clinical outcomes of this strategy have been previously described, its economic impact has not been evaluated.

### Aims

To assess the impact of systematic HU intensification on transfusion burden and to estimate the associated direct transfusion-related cost differences from a hospital-based healthcare perspective.

### Methods

This retrospective single-center cohort study included adults with SCD undergoing chronic RCE who initiated HU between June 2020 and June 2025. A pre–post design was used, with each participant serving as their own control. Two 12-month periods before and after HU initiation were compared, excluding the first 3 months post-HU for stabilization. Transfusion burden was assessed by cumulative annual RBC units. Comparisons were performed using paired t-tests and linear mixed-effects models. Direct transfusion-related costs were estimated using institutional transfusion medicine data for fiscal year 2025–2026. The acquisition cost of one standard RBC unit was 528.12 CAD, with an additional 265.40 CAD for extensive phenotyping routinely required in SCD, for a total product cost of 793.52 CAD per unit. To better reflect hospital expenditures, an additional 80% of the standard acquisition cost (422.50 CAD) was added to account for laboratory and nursing time and associated processes. Annual cost differences were calculated based on mean unit reduction per patient.

**Copyright Information:** (Online) ISSN: 2572-9241
©2026 The Author(s). HemaSphere is published by John Wiley & Sons Ltd on behalf of European Hematology Association. This is an open access Abstract Book distributed under the Attribution-NonCommercial-NoDerivs (CC BY-NC-ND), which allows third parties to download the articles and share them with others as long as they credit the author and the Abstract Book, but they cannot change the content in any way or use them commercially.
**Abstract Book Citations:** Authors, Title, HemaSphere, 2026;10:(S1):pages. The abstract book DOIs can be found on the "Author Information" page.
**Disclaimer:** Articles published in the journal HemaSphere exclusively reflect the opinions of the authors. The authors are responsible for all content in their abstracts including accuracy of the facts, statements, citing resources, etc.

### Results

Forty-two patients met the inclusion criteria; 15 were excluded because of non-adherence to HU, and 27 were included in the final cohort (70% male, 82% HbSS/HbSβ⁰ genotype, mean age 44.5 years). As previously shown, this therapeutic strategy was deemed safe and no statistically significant difference was observed in the intervals between RCE before and after HU initiation and in the number of RBC units exchanged per session. However, the cumulative number of RBC units over 12 months decreased significantly, from a mean of 81.7 units per patient in the year prior to HU to 76.2 units afterward (-5.5 units, $p = 0.007$). Based on a unit cost of 1,216.02 CAD, this reduction corresponded to an estimated direct cost difference of 6,688 CAD per patient per year. For the 27 patients included, this represents an estimated annual reduction of approximately 180,000 CAD in direct transfusion-related costs, assuming stable transfusion requirements over time.

### Summary/Conclusion

In adults with SCD undergoing chronic RCE, systematic HU intensification was associated with a modest but significant reduction in annual transfusion burden, translating into meaningful direct transfusion-related cost differences from a hospital perspective. These findings suggest that HU intensification may contribute to optimization of healthcare resource utilization in highly transfusion-dependent SCD populations. Further studies should evaluate long-term economic impact, including procedural and indirect costs, as well as patient-centered economic outcomes.

**Copyright Information:** (Online) ISSN: 2572-9241
©2026 The Author(s). HemaSphere is published by John Wiley & Sons Ltd on behalf of European Hematology Association. This is an open access Abstract Book distributed under the Attribution-NonCommercial-NoDerivs (CC BY-NC-ND), which allows third parties to download the articles and share them with others as long as they credit the author and the Abstract Book, but they cannot change the content in any way or use them commercially.
**Abstract Book Citations:** Authors, Title, HemaSphere, 2026;10:(S1):pages. The abstract book DOIs can be found on the "Author Information" page.
**Disclaimer:** Articles published in the journal HemaSphere exclusively reflect the opinions of the authors. The authors are responsible for all content in their abstracts including accuracy of the facts, statements, citing resources, etc.

### (PF1278) NON-MALIGNANT CLONAL HEMATOPOIETIC EXPANSION DRIVEN BY 11P15 IMPRINTING MAY EXPLAIN LATE-ONSET SICKLE CELL DISEASE

**Topic:** 27. Sickle cell disease

Lucie Donaty*[1], Muriel Giansily Blaizot[1], Bertchansky Ivan[2], Jérôme Moreaux[1], Patricia Aguilar Martinez[1, 2]

[1]*Montpellier University Hospital (CHU), Department of biological hematology, Montpellier, France;*[2]*Montpellier University Hospital (CHU), Reference Centre for rare red blood cell disorders, Montpellier, France;*

### Background

Sickle cell disease (SCD) is an autosomal recessive disorder typically diagnosed in early childhood. Exceptional cases of late-onset have been reported in individuals carrying sickle cell trait (AS), due to mosaic segmental uniparental disomy (UPD) of chromosome 11. This chromosome contains the *HBB* gene and a region with imprinted genes involved in growth regulation. Although clonal hematopoietic expansion is classically linked to age-related clonal hematopoiesis or hematologic malignancies, this case illustrates how non-malignant somatic events can similarly confer selective advantages and drive the progressive clonal outgrowth of S-homozygous erythroid precursors.

### Aims

We report an unusual case of SCD diagnosed during adolescence in a patient with progressive clinical worsening. Genetic analyses revealed a mosaic loss of heterozygosity (LOH) on chromosome 11. We compared this case with previously documented UDP-associated hemoglobinopathies.

### Methods

A 16-year-old girl from Congo, with no medical history during childhood, presented with vaso-occlusive crises, hepatosplenomegaly, microcytic anemia and thrombocytosis. Hemoglobin (Hb) studies and molecular analysis of the beta and alpha globin genes were performed. Chromosomal microarray analysis was used to investigate copy-neutral LOH.

### Results

Hemoglobin studies revealed 73% HbS and 21% HbA without transfusion, suggesting an initial diagnosis of HbS/beta-plus thalassemia. Molecular testing instead demonstrated near-homozygosity for HbS with a residual wild-type allele. Homozygous α-thalassemia (a-$^{3.7}$/a-$^{3.7}$) was also identified. SNP-array analysis detected an approximately 52 Mb segmental copy-neutral LOH of the short arm of chromosome 11 (11p15.4-p11.12), consistent with mosaic paternal uniparental isodisomy. This somatic event results in a double paternal imprinting of the *IGF2/H19* region, leading to overexpression of the paternal growth enhancer *IGF2* and the under expression of the maternal growth suppressor *H19*, contributing to selective advantage of the ßS erythroid clones. This may have led to the progressive replacement of AS cells by SS cells, explaining the delayed onset of SCD symptoms.
Clinically, the patient experienced increasingly frequent vaso-occlusive crises, transfusion requirements with moderate iron overload, and early bilateral cataracts before 30 years of age.
A literature review confirms that mosaic 11p15 UPD associated with hemoglobinopathy remains exceptional. Only a small number of cases have been reported globally, including only two late-onset SCD cases and several cases (~5) of late-onset β-thalassemia major driven by similar mechanisms of clonal UPD-mediated conversion from a carrier to a disease phenotype.

**Copyright Information:** (Online) ISSN: 2572-9241
©2026 The Author(s). HemaSphere is published by John Wiley & Sons Ltd on behalf of European Hematology Association. This is an open access Abstract Book distributed under the Attribution-NonCommercial-NoDerivs (CC BY-NC-ND), which allows third parties to download the articles and share them with others as long as they credit the author and the Abstract Book, but they cannot change the content in any way or use them commercially.
**Abstract Book Citations:** Authors, Title, HemaSphere, 2026;10:(S1):pages. The abstract book DOIs can be found on the "Author Information" page.
**Disclaimer:** Articles published in the journal HemaSphere exclusively reflect the opinions of the authors. The authors are responsible for all content in their abstracts including accuracy of the facts, statements, citing resources, etc.

**Summary/Conclusion**

This case illustrates a rare, non-malignant clonal hematopoietic expansion caused by somatic UPD 11p15 leading to "functional" *HBB* S-homozygosity and delayed-onset SCD. It highlights an under-recognized mechanism of clonal evolution driven by imprinting dysregulation *(IGF2/H19)* outside classical oncogenic pathways. Clinicians should consider somatic mosaicism when evaluating atypical or late presentations of hemoglobinopathies or unexplained hematologic imbalance.

**Copyright Information:** (Online) ISSN: 2572-9241
©2026 The Author(s). HemaSphere is published by John Wiley & Sons Ltd on behalf of European Hematology Association. This is an open access Abstract Book distributed under the Attribution-NonCommercial-NoDerivs (CC BY-NC-ND), which allows third parties to download the articles and share them with others as long as they credit the author and the Abstract Book, but they cannot change the content in any way or use them commercially.
**Abstract Book Citations:** Authors, Title, HemaSphere, 2026;10:(S1):pages. The abstract book DOIs can be found on the "Author Information" page.
**Disclaimer:** Articles published in the journal HemaSphere exclusively reflect the opinions of the authors. The authors are responsible for all content in their abstracts including accuracy of the facts, statements, citing resources, etc.

### (PF1279) ANALYSIS OF PSYCHOSOCIAL FACTORS ASSOCIATED WITH INCREASEDEMERGENCY DEPARTMENT VISITS FOR OPIOID USE IN PATIENTS WITHSICKLE CELL DISEASE

**Topic:** 27. Sickle cell disease

Luiz Gabriel de Oliveira Correia[1], Dahra Teles Soares Cruz*[2], Mauricéa Maria de Santana[3], Alessandra Ferraz de Sá[4], Ludmilla Medeiros Costa Vasconcelos[5], camila Oliveira Aguiar Correia[6]

*[1]Hemope, Recife, PE, Brazil, Recife, Brazil; [2]Universidade de Pernambuco, Recife, Brazil; [3]FIOCRUZ, Recife, Brazil; [4]Hemope, Recife, PE, Brazil, recife, Brazil; [5]Secretaria de Saúde do Recife, Recife, Brazil; [6]Hospital Barão de Lucena, Recife, Brazil;*

### Background

Sickle cell disease is characterized by episodes of acute pain that can lead to emergency room visits and opioid use. The literature shows that a minority of patients are responsible for a significant portion of these visits. The psychosocial factors associated with this increased use are not yet fully understood, especially in the local context.

### Aims

To characterize and comparatively analyze psychosocial factors associated with emergency room visits for opioid use in adults with sickle cell disease followed up at a referral unit.

### Methods

Cross-sectional study conducted at the Hematology and Hemotherapy Foundation of Pernambuco, in Brazil, between July 2025 and January 2026. Ninety-five adult patients participated, divided according to the number of emergency room visits in the previous 12 months (≤2 vs. ≥3) and subdivided into groups of extremes of use (≤1 vs. ≥5). The data collection included two questionnaires: the first on sociodemographic profile, use of the health network, self-promotion strategies for health, and subjective aspects related to opioid use; and the second, the Self Report Questionnaire (SRQ-20), a
validated scale for identifying and quantifying mental distress, using a cutoff point of ≥ 7 points for mental distress. Comparisons were performed using Pearson's Chi-square or Fisher's Exact test (5%).

### Results

In the total sample, high socioeconomic vulnerability was observed, with the majority unemployed (79.7%), with family income up to 1 minimum wage (64.4%), and receiving government assistance (74.6%). Significant fragility in the support network was also observed, with the majority being single (86.3%) and a high percentage having gone unaccompanied on their last visit to the emergency room for pain (42.1%). Furthermore, mental distress was highly prevalent and similar across groups (>70%), with a higher proportion of loss of appetite ($p = 0.013$) and inability to find satisfaction
in daily activities ($p = 0.049$) among participants in the subgroup with ≥5 visits to the emergency room in the last 12 months. This proved relevant when weaknesses in self-promotion of health were identified, such as a high degree of sedentary lifestyle (77.9%) and a considerable percentage of absence of collective spiritual activities (37%) despite a high rate of individual spiritual activities (78.9%). Among those with the highest frequency of visits to the emergency room, despite good adherence to the hematology outpatient clinic (69.6%), there was a lower proportion of individuals > 45 years old ($p = 0.049$), a higher expectation of receiving opioids ($p = 0.027$), and a greater fear of not being able to cope with pain without these medications ($p = 0.027$). Regarding the interview location, a higher proportion of interviewees were found in inpatient beds in the group with the highest frequency of visits to the emergency room (76.1%), and a higher proportion of interviewees were found in the outpatient clinic in the group with the lowest use (75.5%) ($p < 0.001$) – a finding that also showed statistical significance when comparing extreme subgroups ($p < 0.001$).

**Copyright Information:** (Online) ISSN: 2572-9241
©2026 The Author(s). HemaSphere is published by John Wiley & Sons Ltd on behalf of European Hematology Association. This is an open access Abstract Book distributed under the Attribution-NonCommercial-NoDerivs (CC BY-NC-ND), which allows third parties to download the articles and share them with others as long as they credit the author and the Abstract Book, but they cannot change the content in any way or use them commercially.
**Abstract Book Citations:** Authors, Title, HemaSphere, 2026;10:(S1):pages. The abstract book DOIs can be found on the "Author Information" page.
**Disclaimer:** Articles published in the journal HemaSphere exclusively reflect the opinions of the authors. The authors are responsible for all content in their abstracts including accuracy of the facts, statements, citing resources, etc.

**Summary/Conclusion**

High socioeconomic vulnerability and prevalence of mental suffering were identified. The differences were concentrated in aspects related to opioid use and the healthcare network's usage profile, reinforcing the need for strategies targeted at the usage profile and integrated into mental health care.

**Copyright Information:** (Online) ISSN: 2572-9241
©2026 The Author(s). HemaSphere is published by John Wiley & Sons Ltd on behalf of European Hematology Association. This is an open access Abstract Book distributed under the Attribution-NonCommercial-NoDerivs (CC BY-NC-ND), which allows third parties to download the articles and share them with others as long as they credit the author and the Abstract Book, but they cannot change the content in any way or use them commercially.
**Abstract Book Citations:** Authors, Title, HemaSphere, 2026;10:(S1):pages. The abstract book DOIs can be found on the "Author Information" page.
**Disclaimer:** Articles published in the journal HemaSphere exclusively reflect the opinions of the authors. The authors are responsible for all content in their abstracts including accuracy of the facts, statements, citing resources, etc.

### (PF1280) DATA-DRIVEN CLUSTERING AND EXOME ANALYSIS REVEAL POLYGENIC BURDEN IN NON-TRANSFUSION-DEPENDENT Β-THALASSEMIA

**Topic:** 28. Thalassemias

Filippo Russo*[1, 2], Anthony Iscaro[1, 2], Simona Leoni[3, 4], Barbara Eleni Rosato[1, 2], Valentina Brancaleoni*[4], Roberta Marra[1, 2], Antonella Nostroso[1, 2], Natalia Scaramellini[3, 4], Immacolata Andolfo[1, 2], Irene Motta[3, 4], Roberta Russo[1, 2]

*[1]Department of Molecular Medicine and Medical Biotechnology, University of Naples Federico II, Naples, Italy;[2]CEINGE - Advanced Biotechnology Franco Salvatore, Naples, Italy; [3]Dipartimento di Scienze Cliniche e di Comunità, Dipartimento di Eccellenza 2023-2027, University of Milan, Milan, Italy; [4]SC Medicina ad Indirizzo Metabolico, Fondazione IRCCS Ca' Granda Ospedale Maggiore Policlinico, Milan, Italy;*

### Background

β-thalassemia (BT) is a genetic disorder characterized by marked clinical heterogeneity (Taher et al., 2021). While primary and secondary modifiers are well established, the broader genetic basis of this phenotypic variability remains unknown. We recently demonstrated that additional variants in hereditary red blood cell disorder (HRBCD)-related genes exacerbate the clinical phenotype in BT carriers (Pinto et al. 2023). However, the cumulative impact of novel modifiers is yet to be elucidated.

### Aims

This study aimed to stratify a cohort of BT patients into distinct phenotypic subgroups using an unsupervised, data-driven clustering approach. To identify genetic modifiers determining clinical severity, functionally weighted whole-exome sequencing (WES) data were further investigated in subgroups composed primarily of patients with non-transfusion-dependent thalassemia (NTDT) identified in the previous analysis.

### Methods

We analysed 114 adult BT patients, genotypically characterized through a composite severity score (Thal_SCORE). Phenotypic stratification was performed using principal component analysis (PCA) and hierarchical clustering (Ward's method) on twelve haemato-biochemical parameters. To identify genetic modifiers, we performed a SKAT-O analysis on WES data from a subset of patients following linkage disequilibrium pruning. Variant weights were defined as the sigmoid-transformed score derived from the American College of Medical Genetics and Genomics (ACMG) classification criteria. The model was adjusted by including age, binary sex, and genetic ancestry. Firth's logistic regression assessed variant-level associations (minor allele counts ≥2) within candidate genes.

### Results

Unsupervised clustering identified 5 distinct phenotypic groups (PERMANOVA, $p$=0.001). Interestingly, this data-driven partition spontaneously segregated patients based on transfusion requirement, with Clusters 3 and 5 composed almost entirely of NTDT individuals. Notably, we observed significantly more severe anaemic phenotype in Cluster 5 (n=15; median Hb=8.4 g/dL) compared to Cluster 3 (n=17; median Hb=10.2 g/dL). In these latter two subgroups, SKAT-O analysis identified 561 candidate genes ( $p$≤0.01). Firth regression isolated 44 significant variants closely associated with clinical phenotypes: 23 variants associated with Cluster 5 (β>0) had a higher pathogenic burden (mean ACMG score: -5.3), compared to the 21 Cluster 3-related variants (β<0, mean ACMG score: -10.3) (Mann-Whitney, $p$=0.014). This suggested that clinical worsening may be driven by a true enrichment of functionally deleterious variants. After applying a filter to select pathogenicity-prone variants (ACMG score>0), four key variants emerged ($p$≤0.05, FDR=0.08): missense variants in *PC*, *DAGLA*, and *SLC29A1* were significantly enriched in Cluster 5, while a variant in *GPALPP1* was associated with Cluster 3.

**Copyright Information:** (Online) ISSN: 2572-9241
©2026 The Author(s). HemaSphere is published by John Wiley & Sons Ltd on behalf of European Hematology Association. This is an open access Abstract Book distributed under the Attribution-NonCommercial-NoDerivs (CC BY-NC-ND), which allows third parties to download the articles and share them with others as long as they credit the author and the Abstract Book, but they cannot change the content in any way or use them commercially.
**Abstract Book Citations:** Authors, Title, HemaSphere, 2026;10:(S1):pages. The abstract book DOIs can be found on the "Author Information" page.
**Disclaimer:** Articles published in the journal HemaSphere exclusively reflect the opinions of the authors. The authors are responsible for all content in their abstracts including accuracy of the facts, statements, citing resources, etc.

**Summary/Conclusion**

The significantly increased burden of functionally deleterious variants in the cohort of patients with a more severe and predominantly NTDT phenotype suggested an additive polygenic mechanism, potentially involving combined alteration of cellular metabolism and membrane stability, which could exacerbate severity. Pending further functional validation, these findings provide a new framework for understanding the phenotypic heterogeneity of thalassemia and enabling patients' risk stratification and management.

**Copyright Information:** (Online) ISSN: 2572-9241
©2026 The Author(s). HemaSphere is published by John Wiley & Sons Ltd on behalf of European Hematology Association. This is an open access Abstract Book distributed under the Attribution-NonCommercial-NoDerivs (CC BY-NC-ND), which allows third parties to download the articles and share them with others as long as they credit the author and the Abstract Book, but they cannot change the content in any way or use them commercially.
**Abstract Book Citations:** Authors, Title, HemaSphere, 2026;10:(S1):pages. The abstract book DOIs can be found on the "Author Information" page.
**Disclaimer:** Articles published in the journal HemaSphere exclusively reflect the opinions of the authors. The authors are responsible for all content in their abstracts including accuracy of the facts, statements, citing resources, etc.

### (PF1281) EFFICACY OF MITAPIVAT IN PATIENTS WITH NON–TRANSFUSION-DEPENDENT ALPHA OR BETA-THALASSEMIA WITH BASELINE HEMOGLOBIN ≥9.5 G/DL: SUBGROUP ANALYSIS FROM THE PHASE 3 ENERGIZE TRIAL

**Topic:** 28. Thalassemias

Khaled M. Musallam*[1, 2, 3], Lauren Czapla[4], Jeremie H. Estepp[4], Gavi Price[4], Shihao Shen[4], Ali T. Taher[5], Kevin H. M. Kuo[6]

*[1]Center for Research on Rare Blood Disorders and Thalassemia & Sickle Cell Center, Burjeel Cancer Institute, Abu Dhabi, United Arab Emirates; [2]Department of Public Health & Epidemiology, Khalifa University, Abu Dhabi, United Arab Emirates;[3]Division of Hematology and Oncology, Department of Pediatrics, Weill Cornell Medicine, New York, United States of America; [4]Agios Pharmaceuticals, Inc., Cambridge, United States of America; [5]Division of Hematology and Oncology, Department of Internal Medicine, American University of Beirut Medical Center, Beirut, Lebanon; [6]Division of Hematology, Department of Medicine, University of Toronto, Toronto, Canada;*

### Background

Patients (pts) with non–transfusion-dependent (NTD) α- or β-thalassemia have a wide range of anemia severity leading to complications and diminished quality of life. Each 1.0 g/dL hemoglobin (Hb) increase is associated with reduced morbidity risk, and Thalassaemia International Federation guidelines recommend long-term therapy to raise Hb by ≥1.0 g/dL. Mitapivat, a first-in-class oral allosteric activator of pyruvate kinase, including the red blood cell (RBC)-specific PKR isoform and the PKM2 isoform, is approved to be used in adults with α- or β-thalassemia, regardless of transfusion needs, in the US and Saudi Arabia. In the randomized, double-blind, placebo-controlled, phase 3 ENERGIZE trial in pts with NTD α- or β-thalassemia (NCT04770753; Eudra CT: 2021-000211-23), 42.3% of mitapivat-treated pts achieved the primary endpoint of Hb response vs 1.6% of placebo-treated pts during the 24-week (wk) double-blind period, with improved Functional Assessment of Chronic Illness Therapy (FACIT)-Fatigue subscale score over Wks 12-24 vs placebo (least-squares mean [LSM] difference [95% CI], 3.40 [1.2, 5.6]).

### Aims

To assess efficacy of mitapivat in pts with NTD α- or β-thalassemia with baseline (BL) Hb ≥9.5 g/dL in a post hoc subgroup analysis of ENERGIZE.

### Methods

Protocol inclusion criteria included pts aged ≥18 years with NTD α- or β-thalassemia and BL Hb ≤10 g/dL. Pts were randomized 2:1 to mitapivat 100 mg twice daily or placebo for 24 wks. NTD thalassemia was defined as ≤5 RBC units in the 24 wks before randomization, no RBC transfusion ≤8 wks before informed consent, and no RBC transfusion during screening. For this subgroup analysis in pts with BL Hb ≥9.5 g/dL, the endpoints were Hb response (≥1.0 g/dL increase in average Hb over Wks 12-24 vs BL), change from BL in mean Hb, and change from BL in mean FACIT-Fatigue subscale score over Wks 12-24. BL Hb was defined as the mean of all assessments ≤42 days before randomization or treatment initiation. Hb response was analyzed using Mantel–Haenszel (no stratification); changes in Hb and FACIT-Fatigue were compared using ANCOVA (treatment as factor; BL as covariate).

**Copyright Information:** (Online) ISSN: 2572-9241
©2026 The Author(s). HemaSphere is published by John Wiley & Sons Ltd on behalf of European Hematology Association. This is an open access Abstract Book distributed under the Attribution-NonCommercial-NoDerivs (CC BY-NC-ND), which allows third parties to download the articles and share them with others as long as they credit the author and the Abstract Book, but they cannot change the content in any way or use them commercially.
**Abstract Book Citations:** Authors, Title, HemaSphere, 2026;10:(S1):pages. The abstract book DOIs can be found on the “Author Information” page.
**Disclaimer:** Articles published in the journal HemaSphere exclusively reflect the opinions of the authors. The authors are responsible for all content in their abstracts including accuracy of the facts, statements, citing resources, etc.

### Results

Of 194 randomized pts (mitapivat, n=130; placebo, n=64); 29 (mitapivat, n=18; placebo, n=11; 58.6% female; 72.4% White) had BL Hb ≥9.5 g/dL. Mean (SD) age and BL Hb were 43.3 (12.0) years and 9.8 (0.3) g/dL, respectively.
In this analysis, a greater proportion of pts treated with mitapivat vs placebo achieved Hb response (38.9% [7/18] vs 0% [0/11]; 95% CI, 16.4%, 61.4%; **Table**). In the 7 Hb responders with mitapivat, mean (SD) change from BL in Hb over Wks 12-24 was 1.5 (2.0) g/dL. LSM change from BL (95% CI) in Hb over Wks 12-24 was greater in the mitapivat vs placebo group (0.9 [0.6, 1.2] vs –0.3 [–0.6, 0.1] g/dL), as was LSM change from BL (95% CI) in FACIT-Fatigue score (5.1 [2.4, 7.7] vs 0.8 [–2.5, 4.1]; higher scores indicate improvement in fatigue).

### Summary/Conclusion

In pts with BL Hb ≥9.5 g/dL in ENERGIZE, almost 40% of mitapivat-treated pts were Hb responders vs 0 placebo-treated pts. Mitapivat-treated pts also had greater improvement in fatigue vs placebo-treated pts. These findings align with results from the overall ENERGIZE population, suggesting mitapivat also provides therapeutic benefit for pts with higher BL Hb, as demonstrated by clinically meaningful improvements in both anemia and fatigue.

| | Mitapivat (n=18) | Placebo (n=11) |
|---|---|---|
| **Hb response[a], n (%)** | 7 (38.9) | 0 (0) |
| Response rate difference, % | 38.9 | |
| 95% CI | 16.4, 61.4 | |
| **Change from BL in Hb concentration (Wks 12-24), g/dL[b]** | | |
| LSM (SE) | 0.9 (0.1) | –0.3 (0.2) |
| 95% CI | 0.6, 1.2 | –0.6, 0.1 |
| **Change from BL in FACIT-Fatigue score (Wks 12-24)[b,c]** | | |
| LSM (SE) | 5.1 (1.3) | 0.8 (1.6) |
| 95% CI | 2.4, 7.7 | –2.5, 4.1 |

Table. Treatment outcomes among patients with BL Hb ≥9.5 g/dL. Footnote a: Analyzed using a Mantel–Haenszel method without adjusting for randomization stratification factors. Footnote b: Based on an ANCOVA model with average change from BL over Wks 12–24 as the dependent variable, treatment group as the independent variable, and BL as a covariate. Footnote c: In ENERGIZE, the meaningful within-person change in FACIT-Fatigue was estimated to be a ≥4.5-point improvement from BL in average score over Wks 12-24. ANCOVA, analysis of covariance; BL, baseline; FACIT, Functional Assessment of Chronic Illness Therapy; Hb, hemoglobin; LSM, least-squares mean; SE, standard error; Wk, week.

**Copyright Information:** (Online) ISSN: 2572-9241
©2026 The Author(s). HemaSphere is published by John Wiley & Sons Ltd on behalf of European Hematology Association. This is an open access Abstract Book distributed under the Attribution-NonCommercial-NoDerivs (CC BY-NC-ND), which allows third parties to download the articles and share them with others as long as they credit the author and the Abstract Book, but they cannot change the content in any way or use them commercially.
**Abstract Book Citations:** Authors, Title, HemaSphere, 2026;10:(S1):pages. The abstract book DOIs can be found on the “Author Information” page.
**Disclaimer:** Articles published in the journal HemaSphere exclusively reflect the opinions of the authors. The authors are responsible for all content in their abstracts including accuracy of the facts, statements, citing resources, etc.

### (PF1282) LIVER STIFFNESS AND ITS DETERMINANTS IN TRANSFUSION-DEPENDENT THALASSEMIA: A CROSS-SECTIONAL STUDY USING TRANSIENT ELASTOGRAPHY

**Topic:** 28. Thalassemias

Antonella Meloni*[1], Laura Pistoia[1], Rosamaria Rosso[2], Michela Chiodi[3], Luciana Rigoli[4], Vincenza Rossi[5], Roberto Mattei[6], Alberto Cossu[7], Priscilla Fina[8], Giuseppe Peritore[9], Vincenzo Positano[1], Andrea Barison[1]

*[1]Fondazione G. Monasterio CNR-Regione Toscana, Pisa, Italy; [2]Azienda Ospedaliero-Universitaria Policlinico "Vittorio Emanuele", Catania, Italy; [3]Ospedale "San Donato", Arezzo, Italy; [4]Policlinico "G. Martino", Messina, Italy; [5]Ospedale di Cosenza, Cosenza, Italy; [6]A.U.L.S.S. 5 Polesani, Adria (RO), Italy; [7]Azienda Ospedaliero-Universitaria "S. Anna", Cona (FE), Italy; [8]Ospedale "Sandro Pertini", Roma, Italy; [9]"ARNAS" Civico, Di Cristina Benfratelli, Palermo, Italy;*

### Background

Accurate assessment of liver fibrosis is crucial in transfusion-dependent β-thalassemia (TDT). Transient elastography (TE) is a validated, noninvasive method for estimating liver stiffness, but its relationship with liver iron concentration (LIC) in TDT remains controversial, with conflicting results potentially influenced by hepatitis C virus (HCV) status.

### Aims

The aim of this multicenter cross-sectional study was to evaluate liver stiffness measurements (LSMs) and to identify their determinants, including demographic and clinical factors, liver enzymes, hepatic iron load, and endocrine complications in a well-managed cohort of patients with TDT.

### Methods

A total of 163 TDT patients (42.06±9.07 years; 46% females) enrolled in the Extension–Myocardial Iron Overload Network were included. All participants underwent magnetic resonance imaging (MRI) for iron quantification within three months of TE. Clinical and biochemical data were collected, and endocrine complications were defined according to established clinical criteria.

### Results

The mean LSM was 6.78±3.49 kPa (range 2.80–25.80 kPa), and advanced fibrosis (LSM≥8 kPa) was identified in 23.3% of patients. LSMs were not associated with sex, splenectomy status, age, age at initiation of transfusions, or age at initiation of chelation therapy. Patients with chronic HCV infection had higher LSMs than never-infected and past-infected patients (p=0.003 and p=0.018, respectively), while no difference was observed between the latter two groups (p=0.233), which were therefore combined as non-chronic HCV. LSMs correlated positively with body mass index (R=0.238; p=0.003), mean serum ferritin (R=0.175; p=0.030), alanine aminotransferase (ALT) (R=0.381; p<0.0001), aspartate aminotransferase (AST) (R=0.371; p<0.0001), and gamma-glutamyl transferase (GGT) (R=0.285; p=0.001), but not with pre-transfusion hemoglobin or AST/ALT ratio. No association was observed between LSMs and MRI-derived liver iron concentration (LIC), either in the overall cohort or after stratification by fibrosis stage or HCV status. Hepatic iron overload was not associated with increased LSMs. Among endocrine complications, hypogonadism (58.3%) was associated with higher LSMs (p=0.031). Although LSMs were similar in patients with and without diabetes, a positive correlation with fasting glucose was observed in non-diabetic patients (R=0.306; p=0.001). LSMs were higher in patients with at least one endocrinopathy compared with those without (7.22±3.89 vs. 5.91±2.30 kPa; p=0.015).
In univariate analysis, chronic HCV infection, ferritin, liver enzymes, and endocrine complications predicted LSMs. In multivariable analysis, AST, GGT, and chronic HCV infection remained independently associated with liver stiffness (p<0.0001).

**Copyright Information:** (Online) ISSN: 2572-9241
©2026 The Author(s). HemaSphere is published by John Wiley & Sons Ltd on behalf of European Hematology Association. This is an open access Abstract Book distributed under the Attribution-NonCommercial-NoDerivs (CC BY-NC-ND), which allows third parties to download the articles and share them with others as long as they credit the author and the Abstract Book, but they cannot change the content in any way or use them commercially.
**Abstract Book Citations:** Authors, Title, HemaSphere, 2026;10:(S1):pages. The abstract book DOIs can be found on the "Author Information" page.
**Disclaimer:** Articles published in the journal HemaSphere exclusively reflect the opinions of the authors. The authors are responsible for all content in their abstracts including accuracy of the facts, statements, citing resources, etc.

**Summary/Conclusion**

In a contemporary, well-managed TDT cohort, chronic HCV infection and biochemical markers of hepatocellular injury (AST and GGT) were independently associated with increased liver stiffness, whereas hepatic iron burden was not. These results underscore the multifactorial pathogenesis of liver fibrosis in TDT and support integrated surveillance strategies combining elastography with comprehensive clinical evaluation to improve risk stratification and optimize patient management.

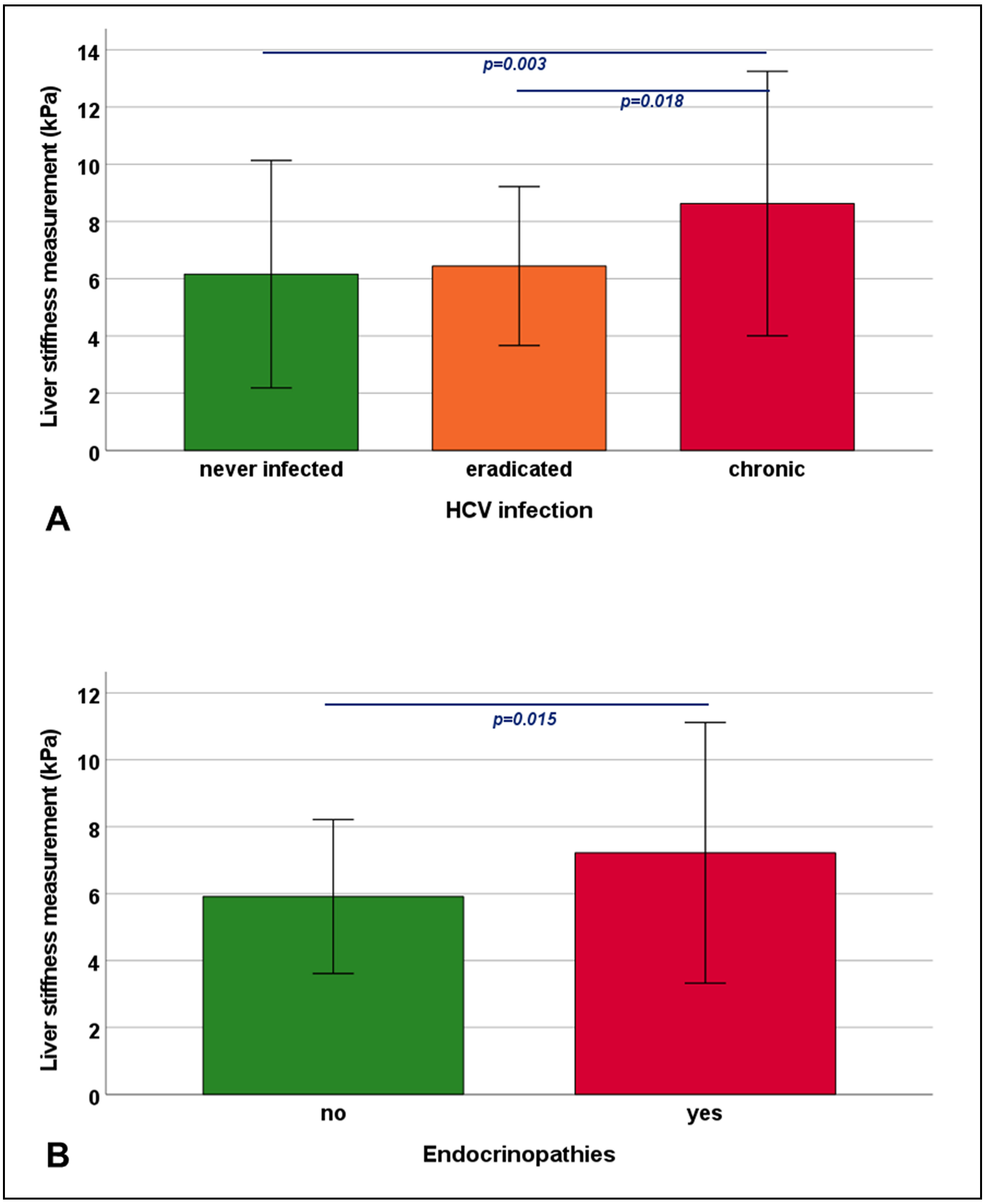

A) Mean liver stiffness measurements in the three groups of patients identified based on the HCV infection status: never contracted, eradicated, and chronic. B) Mean liver stiffness measurements in patients without and with endocrinopathies (diabetes, hypothyroidism, hypoparathyroidism, and hypogonadism). In both graphs, the bars in the boxes represent the standard deviation and the horizontal blue lines indicate a significant difference between two groups.

**Copyright Information:** (Online) ISSN: 2572-9241
©2026 The Author(s). HemaSphere is published by John Wiley & Sons Ltd on behalf of European Hematology Association. This is an open access Abstract Book distributed under the Attribution-NonCommercial-NoDerivs (CC BY-NC-ND), which allows third parties to download the articles and share them with others as long as they credit the author and the Abstract Book, but they cannot change the content in any way or use them commercially.
**Abstract Book Citations:** Authors, Title, HemaSphere, 2026;10:(S1):pages. The abstract book DOIs can be found on the "Author Information" page.
**Disclaimer:** Articles published in the journal HemaSphere exclusively reflect the opinions of the authors. The authors are responsible for all content in their abstracts including accuracy of the facts, statements, citing resources, etc.

### (PF1283) EARLY CARDIAC REMODELING WITH SUBTLE LV STRAIN IMPAIRMENT IN CHILDREN WITH TDT: A CASE–CONTROL SPECKLE-TRACKING STUDY

**Topic:** 28. Thalassemias

Daniella Hodroj[1], Rim Salameh[1], Rana Zareef[1], Hani Tamim[2], Maha Makki[2], Fadi Bitar[1], Miguel R. Abboud*[1], Ziad Bulbul[1]

*[1]American University of Beirut Medical center, Pediatrics and Adolescent Medicine, Beirut, Lebanon;[2]American University of Beirut Medical center, Clinical research institute, Beirut, Lebanon;*

### Background

Transfusion dependent beta thalassemia (TDT) is a chronic hemoglobinopathy in which sustained anemia and treatment-related exposures, particularly transfusions and iron loading, may drive early cardiac remodeling and subclinical myocardial dysfunction. Conventional echocardiography can remain reassuring despite evolving impairment; speckle-tracking echocardiography (STE) may detect earlier changes in myocardial deformation.

### Aims

To detect and characterize subclinical ventricular dysfunction in children with TDT using STE by comparing left and right ventricle (LV/RV) deformation and conventional echocardiographic indices with healthy controls; to identify clinical, laboratory, and echocardiographic correlates of LV/RV strain; and to evaluate longitudinal changes in strain in children with serial echocardiograms.

### Methods

We conducted a retrospective single-center study including children with TDT who underwent transthoracic echocardiography between January 2015 and December 2023 and healthy controls with normal echocardiograms. Demographic and clinical data (age, sex, body surface area, therapies including transfusion and chelation, and available hemoglobin and ferritin) were extracted from medical records. Conventional echocardiographic parameters, Doppler diastolic indices, maximal tricuspid regurgitation velocity (TRv), and STE-derived LV global longitudinal strain (GLS) and RV free-wall strain (FWS) were analyzed. Correlation analyses and multivariable linear regression were performed; longitudinal changes in a subset with serial studies were assessed.

### Results

Ninety-six children were included (39 TDT; 57 age- and sex-matched controls). Mean age was 9.8 ± 5 years and 54.2% were female; mean follow-up was 3.67 ± 1.98 years. Age and sex did not differ between groups. Median ferritin level was 1617 [1149–2274], with a median hemoglobin level of 9.9 [8.2–11.4]. Compared with controls, TDT patients had a larger left atrial volume index (24.6 vs 16.0 mL/m², p<0.001) and larger indexed LV end-diastolic diameter (42.8 vs 36.3 mm/m², p=0.002), with lower thickness (IVSd 5.3 ± 1.7 vs 6.2 ± 1.2 mm, p=0.007; LVPWd 4.9 ± 1.2 vs 6.6 ± 1.6 mm, p<0.001). Diastolic indices suggested higher filling burden (MV E p<0.001; E/A p=0.006; IVRT p<0.001), with higher TRv (2.2 vs 2.0 m/s, p<0.001). LVEF was comparable, but LV GLS was modestly less negative in TDT (–21.9% vs –22.4%, p=0.038). RV size indices, FAC, and RV strain were similar between groups. Hemoglobin and ferritin levels, use of iron chelation or folic acid, and transfusion frequency were not associated with LV or RV strain. In a longitudinal subset (n=24), LV strain worsened slightly over time (–21.5% to –21.0%; p=0.001), while RV strain remained stable.

**Copyright Information:** (Online) ISSN: 2572-9241
©2026 The Author(s). HemaSphere is published by John Wiley & Sons Ltd on behalf of European Hematology Association. This is an open access Abstract Book distributed under the Attribution-NonCommercial-NoDerivs (CC BY-NC-ND), which allows third parties to download the articles and share them with others as long as they credit the author and the Abstract Book, but they cannot change the content in any way or use them commercially.
**Abstract Book Citations:** Authors, Title, HemaSphere, 2026;10:(S1):pages. The abstract book DOIs can be found on the “Author Information” page.
**Disclaimer:** Articles published in the journal HemaSphere exclusively reflect the opinions of the authors. The authors are responsible for all content in their abstracts including accuracy of the facts, statements, citing resources, etc.

**Summary/Conclusion**

TDT group demonstrates echocardiographic evidence of remodeling and increased filling burden with a small reduction in LV deformation, while RV deformation is preserved. Overall, these findings support ongoing cardiac follow-up but do not necessarily imply the need for very frequent surveillance in all patients. Larger prospective studies are needed to define risk-stratification markers and identify which children require closer monitoring.

**Copyright Information:** (Online) ISSN: 2572-9241
©2026 The Author(s). HemaSphere is published by John Wiley & Sons Ltd on behalf of European Hematology Association. This is an open access Abstract Book distributed under the Attribution-NonCommercial-NoDerivs (CC BY-NC-ND), which allows third parties to download the articles and share them with others as long as they credit the author and the Abstract Book, but they cannot change the content in any way or use them commercially.
**Abstract Book Citations:** Authors, Title, HemaSphere, 2026;10:(S1):pages. The abstract book DOIs can be found on the "Author Information" page.
**Disclaimer:** Articles published in the journal HemaSphere exclusively reflect the opinions of the authors. The authors are responsible for all content in their abstracts including accuracy of the facts, statements, citing resources, etc.

### (PF1284) MATCHED SIBLING VS ALTERNATIVE DONOR HSCT FOR Β-THALASSEMIA MAJOR: A SYSTEMATIC REVIEW AND META-ANALYSIS

**Topic:** 28. Thalassemias

Shankar Biswas*[1], Yashasvi Srivastava[1]

[1]*Ivano-Frankivsk National Medical University, Internal Medicine, Ivano-Frankivsk, Ukraine;*

### Background

Allogeneic hematopoietic stem cell transplantation (HSCT) remains the only established curative therapy for transfusion-dependent β-thalassemia major. While matched sibling donors (MSD) are preferred, only 25-30% of patients have an available MSD, necessitating alternative donors including matched unrelated (MUD), mismatched unrelated (MMUD), and mismatched related donors (MMRD). Individual studies comparing donor types report variable outcomes, and no prior meta-analysis has quantified the comparative efficacy and safety across these donor categories.

### Aims

To systematically compare transplant outcomes between MSD and alternative donor HSCT in β-thalassemia major, with thalassemia-free survival (TFS) and overall survival (OS) as primary endpoints, and acute/chronic graft-versus-host disease (GVHD) and graft failure as secondary endpoints.

### Methods

We searched PubMed, Embase, CENTRAL, and Web of Science through January 2025 following PRISMA guidelines (PROSPERO: pending). Comparative cohort studies reporting outcomes by donor type in β-thalassemia major patients were eligible. Risk of bias was assessed using Newcastle-Ottawa Scale. Pairwise meta-analyses used random-effects models (REML, Knapp-Hartung adjustment) with risk difference (RD) as the effect measure. Sensitivity analyses excluded studies with potential cohort overlap. Heterogeneity was quantified by $I^2$ and Cochran's Q.

### Results

Seven studies (3,253 patients; 6 countries; 2008-2024) were included. Six scored ≥7/9 on NOS (Good quality). For MSD vs MUD (5 studies, 1,424 patients): TFS was significantly higher with MSD (pooled RD: +8.4%; 95%CI: 1.4-15.3; p=0.028; $I^2$=0%), while OS showed a non-significant trend favoring MSD (RD: +3.8%; 95%CI: -2.9-10.5; p=0.134; $I^2$=0%). MUD recipients had significantly higher acute GVHD grade II-IV (RD: -15.3%; 95%CI: -24.4 to -6.3; p=0.030; $I^2$=0%). Chronic GVHD trended higher with MUD (RD: -14.9%; 95%CI: -30.6-0.8; p=0.053). Single-arm pooled proportions demonstrated a gradient of decreasing TFS across donor categories: MSD 89.8%, MUD 82.6%, MMUD 78.5%, MMRD 67.4%. Sensitivity analysis excluding potentially overlapping cohorts yielded consistent results (TFS RD: +7.2%; p=0.098). Heterogeneity was zero across all comparisons.

### Summary/Conclusion

This first meta-analysis comparing donor types in β-thalassemia HSCT confirms MSD superiority for TFS with an 8.4 percentage-point advantage over MUD, driven primarily by higher GVHD rates with alternative donors. The consistent donor-type gradient (MSD>MUD>MMUD>MMRD) provides quantitative evidence to guide donor selection algorithms. Despite improving MUD outcomes, these findings support prioritizing MSD when available and highlight the need for enhanced GVHD prophylaxis strategies in alternative donor transplants.

**Copyright Information:** (Online) ISSN: 2572-9241
©2026 The Author(s). HemaSphere is published by John Wiley & Sons Ltd on behalf of European Hematology Association. This is an open access Abstract Book distributed under the Attribution-NonCommercial-NoDerivs (CC BY-NC-ND), which allows third parties to download the articles and share them with others as long as they credit the author and the Abstract Book, but they cannot change the content in any way or use them commercially.
**Abstract Book Citations:** Authors, Title, HemaSphere, 2026;10:(S1):pages. The abstract book DOIs can be found on the “Author Information” page.
**Disclaimer:** Articles published in the journal HemaSphere exclusively reflect the opinions of the authors. The authors are responsible for all content in their abstracts including accuracy of the facts, statements, citing resources, etc.

### (PF1285) LONGITUDINAL EVALUATION OF ANEMIA SEVERITY AND MORBIDITY IN PATIENTS WITH NON-TRANSFUSION DEPENDENT Α-THALASSEMIA (HBH THALASSEMIA)

**Topic:** 28. Thalassemias

Konstantinos Bistas*[1, 2], Polyxeni Delaporta[1, 2], Elena Chatzikalil[1, 2], Vasiliki Maravgaki[1, 2], Vasiliki Chouliara[1, 2], Efthalia-Faidra Agiomavriti-Stefanopoulou[1, 2], Vangelis Mitros[1, 2], Aikaterini Evangelatou[1, 2], Sofirela Berdalli[1, 2], Stavroula Mylona[3], Dimitra Kyriakopoulou[1, 2], Antonis Kattamis[1, 2]

*[1]Division of Pediatric Hematology-Oncology, First Department of Pediatrics, National and Kapodistrian University of Athens, Athens, Greece; [2]"Aghia Sophia" Children's Hospital, ERN-EuroBloodNet Center, Athens, Greece;[3]Hematology Department, "Aghia Sophia" Children's Hospital, Athens, Greece, Athens, Greece;*

### Background

Hemoglobinopathy H (HbH) is an α-thalassemia phenotype characterized by loss of function of >2 α-globin genes, chronic hemolysis and anemia. α-Thalassemia variants are either gene deletion(s) (del) involving one or both α-globin genes, or non-deletions (ndel) which are usually more severe than one α-globin gene deletions.

### Aims

Investigation of longitudinal anemia and complication burden evolution in HbH patients (pts) with various genotypes.

### Methods

A retrospective study included all HbH pts, with available molecular analysis, evaluated between 1975-2025 and followed for ≥6 months. Longitudinal clinical and laboratory parameters, including growth indices, hemoglobin (Hb) and ferritin, as well as time to first severe anemia episode (SA) (Hb<8g/dl), splenomegaly (SM) and transfusion history were recorded. Growth (height, weight, BMI) and anemia were evaluated by calculating the z-scores from the age and gender-matched reference population. Analysis was done on R-Studio and a two-sided p-value α<0.05 was considered significant.

### Results

Out of 148 HbH pts registered in the unit, sufficient data were available for 95 pts (male:37,female:58). Genotype distribution was: 54.7% del/del, 25.3% del/ndel and 20% ndel/ndel [most prevalent:-$\alpha^{3.7}$/--$^{MED}$(33.7%)]. Median follow up was 9.7 years [range (R): 0.53, 42.84]. Age at diagnosis was significantly lower in ndel/ndel compared to del/del(0.90 vs 2.65 years, $p_a$=0.001). Across z-score analysis, standardized growth metrics were aligned with population norms (median at last visit:Height-z:0.67(R: -1.18,2.50), Weight-z: 0.41(R:-1.27,2.11), BMI-z:0.29(R:-5.99,2.35) and did not differ between genotypes and genders. Hb at diagnosis was significantly lower in ndel/ndel [mean(SD):8.47g/dl(1.08)] compared to both del/del [9.04g/dl(1.01), p=0.027] and del/ndel [9.39g/dl(1.07), p=0.005]. Longitudinal Hb analysis indicated that: ndel/ndel have significantly lower Hb across all ages compared to del/del(meanΔ=-0.70g/dl, p<0.001); no difference was found between del/ndel and del/del; Hb-z demonstrated a nonlinear trajectory with significant genotype-related variation, with anemia severity worsening at early childhood(Hbz:-5.6 to-7.8SD), improving by adolescence (Hbz:-4.2 to-6.1SD) and being more affected in ndel/ndel compared to other genotypes (Figure). Relatively to del/del, ndel/ndel exhibit earlier onset of SA (HR=5.8,p=0.01), first transfusion (HR=3.7,p=0.001), ferritin levels> 300ng/dL (HR=3.3,p=0.020) and SM (HR=4.6,p<0.001). Del/ndel had a higher risk compared to del/del for transfusion requirements (HR=3.1,p=0.008) while for SM and SA they share the same risk with del/del (p=0.592, p=0.281) but deteriorate by puberty (p=0.041, p=0.004 respectively). Gender did not affect outcomes.

**Copyright Information:** (Online) ISSN: 2572-9241
©2026 The Author(s). HemaSphere is published by John Wiley & Sons Ltd on behalf of European Hematology Association. This is an open access Abstract Book distributed under the Attribution-NonCommercial-NoDerivs (CC BY-NC-ND), which allows third parties to download the articles and share them with others as long as they credit the author and the Abstract Book, but they cannot change the content in any way or use them commercially.
**Abstract Book Citations:** Authors, Title, HemaSphere, 2026;10:(S1):pages. The abstract book DOIs can be found on the "Author Information" page.
**Disclaimer:** Articles published in the journal HemaSphere exclusively reflect the opinions of the authors. The authors are responsible for all content in their abstracts including accuracy of the facts, statements, citing resources, etc.

### Summary/Conclusion

Our data showed that ndel/ndel is associated with more severe phenotype in regard to anemia and complications. Del/del tends to follow a stable trajectory while del/ndel oscillates from milder to more severe phenotypes. All HbH pts present chronic anemia with longitudinal stable Hb trajectories, but with significant intergroup heterogeneity. Anemia severity relative to healthy population worsens in early childhood and stabilizes by adolescence, emphasizing the need for longitudinal close monitoring for all genotype groups, particularly during childhood.

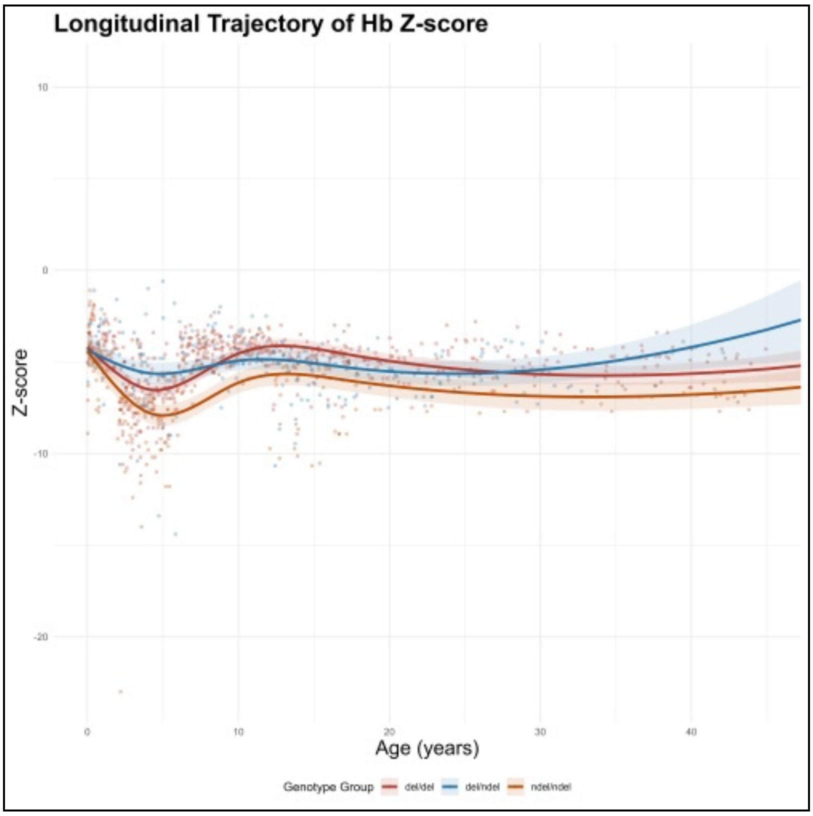

Figure: Points represent observed Hb Z-scores. Lines show model-based predictions. Shaded areas indicate 95% confidence intervals. Hb Z-score demonstrated a nonlinear trajectory with significant genotype-related variation, with anemia severity worsening at childhood improving by adolescence and being more affected in ndel/ndel compared to other genotypes.

**Copyright Information:** (Online) ISSN: 2572-9241
©2026 The Author(s). HemaSphere is published by John Wiley & Sons Ltd on behalf of European Hematology Association. This is an open access Abstract Book distributed under the Attribution-NonCommercial-NoDerivs (CC BY-NC-ND), which allows third parties to download the articles and share them with others as long as they credit the author and the Abstract Book, but they cannot change the content in any way or use them commercially.
**Abstract Book Citations:** Authors, Title, HemaSphere, 2026;10:(S1):pages. The abstract book DOIs can be found on the “Author Information” page.
**Disclaimer:** Articles published in the journal HemaSphere exclusively reflect the opinions of the authors. The authors are responsible for all content in their abstracts including accuracy of the facts, statements, citing resources, etc.

### (PF1286) EFFECT OF SGLT2 INHIBITORS ON PRE-TRANSFUSION HEMOGLOBIN AND TRANSFUSION REQUIREMENTS IN TRANSFUSION-DEPENDENT Β-THALASSEMIA: A LONGITUDINAL CASE SERIES STUDY

**Topic:** 28. Thalassemias

Theodora Maria Venou*[1], Konstantina Voura[1], Efthymia Vlachaki[1]

[1]*Adult Thalassemia Unit, 2nd Department of Internal Medicine, Hippokration Geneneral Hospital, Thessaloniki, Greece;*

### Background

Sodium–glucose cotransporter-2 (SGLT2) inhibitors increase hemoglobin levels in several clinical settings, likely through improved renal oxygen sensing, stimulation of erythropoietin production, plasma volume contraction due to glycosuria, and modulation of iron metabolism. Their effects in transfusion-dependent β-thalassemia remain unknown.

### Aims

Aim of the present study was to evaluate longitudinal changes in pre-transfusion hemoglobin and transfusion burden in patients with transfusion-dependent β-thalassemia after initiation of SGLT2 inhibitor therapy.

### Methods

We performed a single-center longitudinal observational study including six adults with transfusion-dependent β-thalassemia (median age: 47.5 years, IQR: 44.3–60.5) receiving regular transfusions at Adult Thalassemia Unit of Hippokration General Hospital in Thessaloniki, Greece who initiated SGLT2 inhibitor therapy (dapagliflozin) for approved clinical indications (diabetes mellitus, heart failure, or both). Baseline values represented the mean of measurements obtained during the 6 months prior to treatment, while follow-up values reflected period means at 0–3, 3–6, and 6–12 months. Outcomes included pre-transfusion hemoglobin (Hb), transfusion burden (packed RBC volume, mL/month), ferritin, LDH, and indirect bilirubin. Continuous variables are presented as median (IQR). Longitudinal comparisons used Friedman tests and paired comparisons used Wilcoxon signed-rank test. Statistical analysis was performed using SPSS 29.0.1.

### Results

Median pre-transfusion Hb increased from 10.6 g/dL (10.38–10.6) at baseline to 10.80 g/dL (10.53–11.07) at 3–6 months and 10.90 g/dL (10.75–11.05) at 6–12 months. Global longitudinal testing showed a borderline time effect (Friedman $\chi^2$=7.74, p=0.052), while paired comparison demonstrated a significant increase from baseline to 12 months (Wilcoxon, p=0.042), with improvement observed in 5 out of 6 patients. Median transfusion burden decreased from 815 mL/month (687–857) at baseline to 714 mL/month (638–809) at 3–6 months and 702 mL/month (587–830) at 6–12 months, without statistical significance (Friedman $\chi^2$=2.08, p=0.555). Median ferritin declined from 649 ng/mL (520–999) to 400 ng/mL (261–859) at 12 months (p=0.167), in the absence of changes in chelation therapy. LDH remained stable (p=0.293) while indirect bilirubin fluctuated over time (p=0.236). Median albuminuria decreased from 261 to 132 mg/24h, while urinary glucose excretion increased from 144 to 4547 mg/24h, consistent with the expected pharmacodynamic effects of SGLT2 inhibition.

**Copyright Information:** (Online) ISSN: 2572-9241
©2026 The Author(s). HemaSphere is published by John Wiley & Sons Ltd on behalf of European Hematology Association. This is an open access Abstract Book distributed under the Attribution-NonCommercial-NoDerivs (CC BY-NC-ND), which allows third parties to download the articles and share them with others as long as they credit the author and the Abstract Book, but they cannot change the content in any way or use them commercially.
**Abstract Book Citations:** Authors, Title, HemaSphere, 2026;10:(S1):pages. The abstract book DOIs can be found on the "Author Information" page.
**Disclaimer:** Articles published in the journal HemaSphere exclusively reflect the opinions of the authors. The authors are responsible for all content in their abstracts including accuracy of the facts, statements, citing resources, etc.

**Summary/Conclusion**

SGLT2 inhibitor therapy was associated with a consistent signal toward improved pre-transfusion hemoglobin, reaching statistical significance at 12 months, and a non-significant reduction in transfusion requirements. This effect may reflect combined mechanisms, including osmotic diuresis–related plasma volume contraction secondary to glycosuria, as well as potential stimulation of erythropoiesis and modulation of iron metabolism. Hemolysis biomarkers remained discordant and do not support a definitive effect on hemolysis. Larger prospective studies are warranted to confirm these findings, integrating comprehensive erythropoietic and iron-regulatory biomarkers, including erythropoietin, hepcidin and HIF- pathway mediators.

**Copyright Information:** (Online) ISSN: 2572-9241
©2026 The Author(s). HemaSphere is published by John Wiley & Sons Ltd on behalf of European Hematology Association. This is an open access Abstract Book distributed under the Attribution-NonCommercial-NoDerivs (CC BY-NC-ND), which allows third parties to download the articles and share them with others as long as they credit the author and the Abstract Book, but they cannot change the content in any way or use them commercially.
**Abstract Book Citations:** Authors, Title, HemaSphere, 2026;10:(S1):pages. The abstract book DOIs can be found on the “Author Information” page.
**Disclaimer:** Articles published in the journal HemaSphere exclusively reflect the opinions of the authors. The authors are responsible for all content in their abstracts including accuracy of the facts, statements, citing resources, etc.

### (PF1287) EFFICACY OF THALIDOMIDE IN TRANSFUSION DEPENDENT THALASSEMIA: RESULTS FROM A RANDOMIZED DOUBLE BLIND PLACEBO CONTROLLED TRIAL

**Topic:** 28. Thalassemias

Rekhasri V*[1], Lalit Meena[2], Upinder Kaur[3], Priyanka Aggarwal[1], Vineeta Gupta[1]

*[1]Institute of Medical Sciences, Dept of Pediatrics, Varanasi, India;[2]Institute of Medical Sciences, Department of General Medicine, Varanasi, India; [3]Institute of Medical Sciences, Department of Pharmacology, Varanasi, India;*

### Background

Transfusion-dependent thalassemia (TDT) carries substantial transfusion burden and long-term morbidity despite optimal supportive care. Thalidomide has shown potential to improve hemoglobin levels and reduce transfusion needs, but high quality randomized controlled evidence remains limited.

### Aims

To evaluate the efficacy and safety of thalidomide in reducing transfusion requirements in patients with TDT.

### Methods

This randomized, double-blind, placebo-controlled trial was conducted at the Thalassemia Unit, IMS-BHU, Varanasi, from August 2024 to February 2026.
Eligible patients aged 12–25 years with transfusion-dependent homozygous β-thalassemia or HbE-β thalassemia were randomized (1:1) to receive thalidomide (2–3 mg/kg/day) or placebo for 6 months in addition to standard care.
The primary outcome was change in transfusion requirement assessed by transfusion interval and mean hemoglobin level. Secondary outcomes included hematological, biochemical, and nutritional parameters, response rates, and adverse events. Treatment response was categorized according to hemoglobin levels maintained after intervention.

**Copyright Information:** (Online) ISSN: 2572-9241
©2026 The Author(s). HemaSphere is published by John Wiley & Sons Ltd on behalf of European Hematology Association. This is an open access Abstract Book distributed under the Attribution-NonCommercial-NoDerivs (CC BY-NC-ND), which allows third parties to download the articles and share them with others as long as they credit the author and the Abstract Book, but they cannot change the content in any way or use them commercially.
**Abstract Book Citations:** Authors, Title, HemaSphere, 2026;10:(S1):pages. The abstract book DOIs can be found on the “Author Information” page.
**Disclaimer:** Articles published in the journal HemaSphere exclusively reflect the opinions of the authors. The authors are responsible for all content in their abstracts including accuracy of the facts, statements, citing resources, etc.

### Results

Fifty-six patients were randomized (28 per group). Baseline demographic, clinical, hematological, biochemical, and nutritional parameters were comparable.
The primary outcome of transfusion requirement improved significantly in the thalidomide group, with increased mean hemoglobin (7.42 ± 0.80 to 8.07 ± 1.00 g/dL; $p = 0.004$) and prolonged transfusion interval ($p = 0.004$). Significant improvements were observed in MCHC, serum LDH, and serum uric acid, while no significant changes occurred in the placebo group. Based on the response criteria, there were 6(21.4%) excellent and 14(50%) good and 3 (10.8%) partial and 5(17.8%) non responders. Major adverse events leading to drug withdrawal occurred in three patients receiving thalidomide.
Results are summarized in Table 1.

**Table 1. Effect of thalidomide on transfusion requirement and laboratory parameters (Group A)**

| Parameters | Baseline | 6 months | p value |
|---|---|---|---|
| Interval between two transfusions (in days) Median (IQR) | 15 (15-15) | 15 (15-21.25) | 0.004 |
| Rate of fall of Hb (g/wk) | 0.33± 0.16 | 0.26 ± 0.14 | 0.13 |
| Mean Hb (g/dl) | 7.42 ± 0.8 | 8.07 ± 1.00 | 0.004 |
| Mean MCV(fL) | 78.07 ± 0.722 | 77.8 ± 0.903 | 0.21 |
| Mean MCHC (g/dl) | 33.66 ± 0.287 | 34.55 ± 0.769 | <0.001 |
| S.LDH (U/L) | 292.8 ± 35.81 | 274.8 ± 35.6 | 0.03 |
| S.Uric acid (mg/dl) | 4.67 ± 0.572 | 4.14 ± 0.409 | <0.001 |
| S.Ferritin (ng/ml) Median (IQR) | 2204 (1534.3–2479) | 2375 (1628.4 –2711.0) | 0.14† |

† Wilcoxon signed-rank test.

### Summary/Conclusion

Thalidomide therapy in TDT resulted in statistically significant yet clinically modest improvement in hemoglobin levels and transfusion requirements without achieving transfusion independence. With careful patient selection and monitoring, thalidomide may serve as an adjunct therapy aimed at reducing transfusion burden rather than disease modification or cure.

**Copyright Information:** (Online) ISSN: 2572-9241
©2026 The Author(s). HemaSphere is published by John Wiley & Sons Ltd on behalf of European Hematology Association. This is an open access Abstract Book distributed under the Attribution-NonCommercial-NoDerivs (CC BY-NC-ND), which allows third parties to download the articles and share them with others as long as they credit the author and the Abstract Book, but they cannot change the content in any way or use them commercially.
**Abstract Book Citations:** Authors, Title, HemaSphere, 2026;10:(S1):pages. The abstract book DOIs can be found on the "Author Information" page.
**Disclaimer:** Articles published in the journal HemaSphere exclusively reflect the opinions of the authors. The authors are responsible for all content in their abstracts including accuracy of the facts, statements, citing resources, etc.

### (PF1288) HEPATIC FAT AND IRON QUANTIFICATION BY MRI IN NON-TRANSFUSION-DEPENDENT THALASSEMIA: RESULTS FROM A MULTICENTER COHORT

**Topic:** 28. Thalassemias

Antonella Meloni*[1], Vincenzo Positano[1], Laura Pistoia[1], Domenico Visceglie[2], Roberto Lisi[3], Alessandra Salvo[4], Maria Pafundi[5], Gennaro Restaino[6], Luigi Barbuto[7], Stefania Renne[8], Antonino Vallone[9], Andrea Barison[1]

*[1]Fondazione G. Monasterio CNR-Regione Toscana, Pisa, Italy; [2]Ospedale "Di Venere", Bari, Italy; [3]Presidio Ospedaliero Garibaldi-Centro - ARNAS Garibaldi, Catania, Italy; [4]Presidio Ospedaliero "Umberto I", Siracusa, Italy; [5]Azienda Ospedaliera Regionale San Carlo di Potenza, Potenza, Italy; [6]Responsible Research Hospital, Campobasso, Italy; [7]Azienda Ospedaliera di Rilievo Nazionale Antonio Cardarelli, Napoli, Italy; [8]Presidio Ospedaliero "Giovanni Paolo II", Lamezia Terme (CZ), Italy; [9]Azienda Ospedaliera "Garibaldi" Presidio Ospedaliero Nesima, Catania, Italy;*

### Background

In non-transfusion-dependent thalassemia (NTDT), ineffective erythropoiesis and subsequent hepcidin suppression drive increased intestinal iron absorption, leading to preferential iron deposition within the hepatic parenchyma. This chronic siderosis promotes a pro-inflammatory and oxidative environment conducive to progressive liver injury. However, as therapeutic advancements extend life expectancy, the clinical spectrum of NTDT is increasingly complicated by metabolic comorbidities, including dysglycemia and hepatic steatosis. Reflecting global trends in metabolic-associated steatotic liver disease (MASLD), these disturbances impose an additional pathological burden on the thalassemic liver, necessitating a clearer understanding of their prevalence and clinical correlates in this population.

### Aims

This study used quantitative magnetic resonance imaging (MRI) to assess hepatic fat fraction (FF) and its correlates in a well-characterized cohort of NTDT patients.

### Methods

We evaluated 70 NTDT patients (50% female; mean age 43.79±12.91 years) from the Extension-Myocardial Iron Overload (E-MIOT) Network who were either transfusion-naïve or sporadically transfused.
MRI was used to simultaneously quantify hepatic FF and liver iron concentration (LIC). Results were compared with healthy volunteers.

### Results

Hepatic FF did not differ significantly between NTDT patients and healthy subjects (3.87±8.11 vs. 0.92±1.11, p=0.888), whereas LIC was significantly higher in the NTDT group (3.29±1.72 vs. 1.11±0.12 mg/g dw, $p<0.0001$).
Hepatic FF showed no association with sex, age, splenectomy status, serum ferritin, ALT/AST ratio, or lipid profile. However, a weak but significant correlation was observed between body mass index (BMI) and hepatic FF (R=0.216, p=0.047). Hepatic FF correlated positively with LIC (R=0.296, p=0.013), and patients with significant iron overload (LIC>3 mg/g dw) exhibited significantly higher FF values than those with normal LIC (5.98 ±10.36 vs.1.63±3.68 %, p=0.032).
Dysglycemia was present in 7.4% of patients. No significant differences were found between patients with normal and altered glucose metabolism regarding LIC or hepatic FF.

**Copyright Information:** (Online) ISSN: 2572-9241
©2026 The Author(s). HemaSphere is published by John Wiley & Sons Ltd on behalf of European Hematology Association. This is an open access Abstract Book distributed under the Attribution-NonCommercial-NoDerivs (CC BY-NC-ND), which allows third parties to download the articles and share them with others as long as they credit the author and the Abstract Book, but they cannot change the content in any way or use them commercially.
**Abstract Book Citations:** Authors, Title, HemaSphere, 2026;10:(S1):pages. The abstract book DOIs can be found on the "Author Information" page.
**Disclaimer:** Articles published in the journal HemaSphere exclusively reflect the opinions of the authors. The authors are responsible for all content in their abstracts including accuracy of the facts, statements, citing resources, etc.

### Summary/Conclusion

In NTDT patients, hepatic fat accumulation is significantly linked to the severity of iron overload and BMI. The correlation with LIC suggests that hepatocyte-specific iron loading may promote lipid accumulation via oxidative stress and mitochondrial dysfunction. These results emphasize that, in the absence of regular transfusions, managing iron overload remains the primary intervention to mitigate both siderosis and secondary metabolic liver changes.

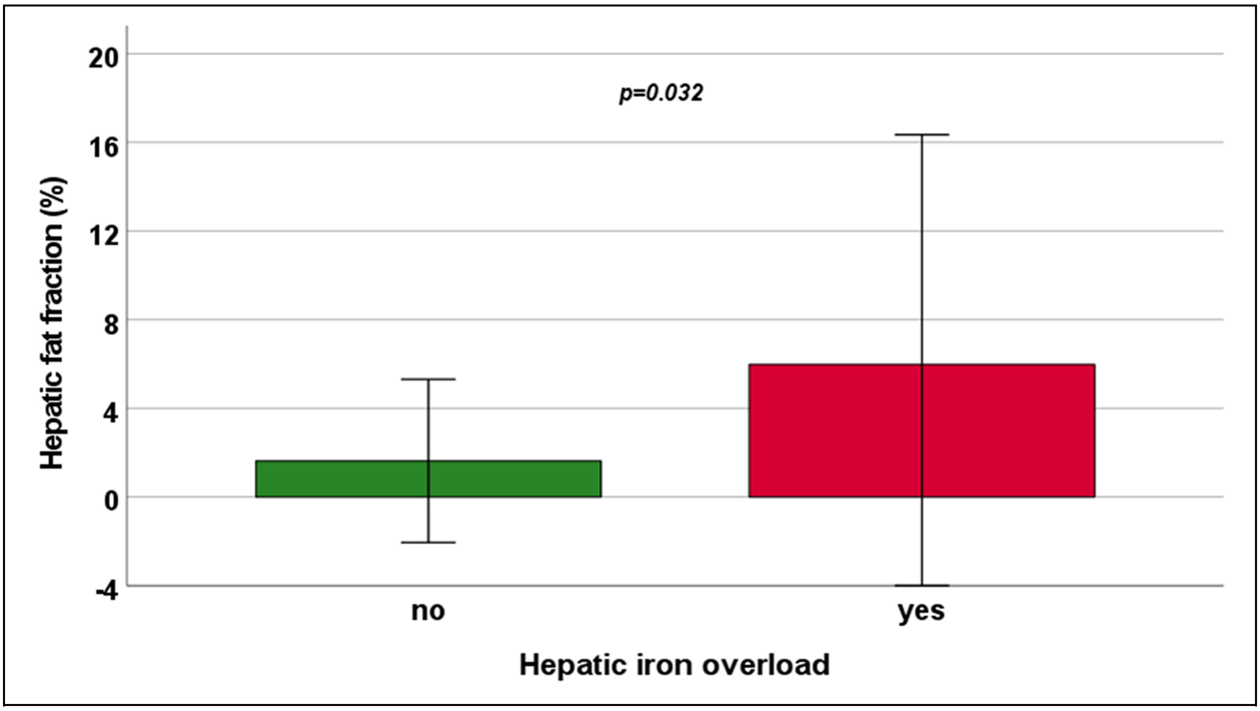

Comparison of hepatic fat fraction between non-transfusion-dependent thalassemia patients without and with hepatic iron overload. Bar graphs show mean ± standard deviation.

**Copyright Information:** (Online) ISSN: 2572-9241
©2026 The Author(s). HemaSphere is published by John Wiley & Sons Ltd on behalf of European Hematology Association. This is an open access Abstract Book distributed under the Attribution-NonCommercial-NoDerivs (CC BY-NC-ND), which allows third parties to download the articles and share them with others as long as they credit the author and the Abstract Book, but they cannot change the content in any way or use them commercially.
**Abstract Book Citations:** Authors, Title, HemaSphere, 2026;10:(S1):pages. The abstract book DOIs can be found on the "Author Information" page.
**Disclaimer:** Articles published in the journal HemaSphere exclusively reflect the opinions of the authors. The authors are responsible for all content in their abstracts including accuracy of the facts, statements, citing resources, etc.

### (PF1289) REPLICATING HEMATOPATHOLOGIST REASONING: A HYBRID AI PIPELINE FOR COST-EFFECTIVE HEMOGLOBINOPATHY TRIAGE USING CBC AND HPLC

**Topic:** 28. Thalassemias

Rahul Bhargav*[1], Shrinidhi Nathany[1], Moin Makda[2], Swarsat Kaushik Nath[1], Kamlesh Prajapati[1], Hrithik Bagga[1], Vikas Dua[1], Siddharth Sagar[1], Haristuti Verma[1]

*[1]Fortis Memorial Research Institute, Gurugram, India; [2]Netaji Subhas University of Technology, New Delhi, India;*

### Background

India harbors >42 million β-thalassemia carriers, with HbE up to 50% in Northeastern tribes and HbS up to 35% in Central-Western tribal populations. Over 160,000 Ayushman Bharat centres generate CBC daily, yet <1,200 perform HPLC/CE. Iron deficiency in >50% of screening-age women confounds CBC-based detection, and α-thalassemia, which is undetectable by routine HPLC, remains unscreened. We developed a two-stage diagnostic pipeline, coupling a rule-based decision tree with a QLoRA fine-tuned Llama 3.1-8B augmented by RAG.

### Aims

To validate a two-stage diagnostic pipeline against HPLC-confirmed diagnoses in 30,193 reports from a single-centre Indian reports, evaluating whether a RAG-augmented fine-tuned LLM replicating stepwise hematopathologist reasoning achieves expert-level carrier detection, including flagging suspected α-thalassemia undetectable by HPLC.

### Methods

In a retrospective cohort study conducted at a tertiary hematology reference laboratory in New Delhi, 30,193 unique HPLC reports (Bio-Rad VARIANT II) were split into training (n=21,135), validation (n=3,019), and held-out test (n=6,039) sets using stratified group k-fold cross-validation. CBC data were available for 100% of abnormal and 24.5% of normal cases. The ground truth for the 12 diagnostic categories was defined by a three-tier consensus: ICSH thresholds, dual hematopathologist review, and final adjudication (κ=0.924). Stage 1: Deterministic decision tree formulated upon ICSH/BCSH guidelines, with borderline thresholds iteratively refined on a selectively sampled, class-balanced subset (n=15,000) to prevent natural prevalence bias and accurately capture IDA+trait dual pathology. Stage 2: Flagged cases were processed by a QLoRA fine-tuned (r=64, NF4) Llama 3.1-8B trained on 5,247 expert-annotated case-interpretation pairs from the training set only, replicating stepwise hematopathologist reasoning, integrating CBC, computing discriminant indices, generating ranked 12-class differentials. RAG corpus: 1,212 expert-annotated edge cases, also exclusively from training data, in six tiers: borderline HbA2 (412), iron-deficient trait (287), rare variants (198), co-elution artefacts (134), compound heterozygotes (89), population-specific priors (92). When evidence was insufficient, cases were flagged for automated review. For suspected α-thalassemia (microcytic + normal HPLC + replete iron), the LLM flagged for molecular confirmation. For ultra-rare classes, we employed leave-one-out cross-validation (LOOCV) on the training data, ensuring the left-out case was excluded from the RAG corpus, alongside a temporal hold-out set evaluated on the final two month

### Results

Stage 1 (test, n=6,039): sensitivity 99.85% (2,014/2,017), NPV 99.92%. LOOCV on ultra-rare training classes: HPFH 80.8%, sickle-β-thal 83.9%, HbE/β-thal 87.2%. QLoRA+RAG concordance: 93.4% vs 82.1% zero-shot. RAG regional priors improved top-1 accuracy 4–7% over non-RAG baseline. The clinical hallucination rate was strictly constrained to 4.1%. Full pipeline test set: macro-AUROC 0.9847, weighted F1 0.9534.

**Copyright Information:** (Online) ISSN: 2572-9241
©2026 The Author(s). HemaSphere is published by John Wiley & Sons Ltd on behalf of European Hematology Association. This is an open access Abstract Book distributed under the Attribution-NonCommercial-NoDerivs (CC BY-NC-ND), which allows third parties to download the articles and share them with others as long as they credit the author and the Abstract Book, but they cannot change the content in any way or use them commercially.
**Abstract Book Citations:** Authors, Title, HemaSphere, 2026;10:(S1):pages. The abstract book DOIs can be found on the “Author Information” page.
**Disclaimer:** Articles published in the journal HemaSphere exclusively reflect the opinions of the authors. The authors are responsible for all content in their abstracts including accuracy of the facts, statements, citing resources, etc.

**Summary/Conclusion**

This study demonstrates that decision-tree triage plus RAG-augmented QLoRA Llama 3.1-8B , with tiered expert-annotated retrieval from training data, and CBC-integrated reasoning , achieves expert-level concordance for carrier detection from routine CBC across 30,193 Indian cases.

**Copyright Information:** (Online) ISSN: 2572-9241
©2026 The Author(s). HemaSphere is published by John Wiley & Sons Ltd on behalf of European Hematology Association. This is an open access Abstract Book distributed under the Attribution-NonCommercial-NoDerivs (CC BY-NC-ND), which allows third parties to download the articles and share them with others as long as they credit the author and the Abstract Book, but they cannot change the content in any way or use them commercially.
**Abstract Book Citations:** Authors, Title, HemaSphere, 2026;10:(S1):pages. The abstract book DOIs can be found on the "Author Information" page.
**Disclaimer:** Articles published in the journal HemaSphere exclusively reflect the opinions of the authors. The authors are responsible for all content in their abstracts including accuracy of the facts, statements, citing resources, etc.

### (PF1290) MULTIOMIC PROFILING UNCOVERS HEME METABOLISM DYSFUNCTION IN CONGENITAL DYSERYTHROPOIETIC ANEMIAS

**Topic:** 29. Enzymopathies, membranopathies and other red blood cell disorders

Antonella Nostroso*[1, 2], Barbara Eleni Rosato[1, 2], Roberta Marra[1, 2], Valeria Pennella[1, 2], Federica Maria Esposito[1, 2], Vanessa D'onofrio[1, 2], Alessia Perrotta[1, 2], Vincenza Cerbone[2], Julie Reisz[3], Achille Iolascon[1, 2], Angelo D'alessandro[3], Immacolata Andolfo[1, 2], Roberta Russo[1, 2]

*[1]University of Naples Federico II, Napoli, Italy;[2]Ceinge Biotecnologie Avanzate Franco Salvatore, Napoli, Italy; [3]University of Colorado Anschutz Medical Campus, Department of Biochemistry and Molecular Genetics, Aurora, CO, United States of America;*

### Background

Congenital dyserythropoietic anemias (CDAs) are inherited disorders characterized by ineffective erythropoiesis and iron overload, primarily due to impaired terminal differentiation of erythroid precursors. CDA type I (CDA I) results from biallelic defects in *CDAN1* or *CDIN1*, while CDA type II (CDA II) is caused by pathogenic variants in *SEC23B*. Despite distinct genetic causes, both conditions converge on impaired erythroid maturation. However, the molecular mechanisms underlying this defect remain poorly understood.

### Aims

This study sheds light on deregulated processes driving ineffective erythropoiesis across CDA subtypes through the establishment and characterization of cellular models of CDA I and CDA II.

### Methods

HUDEP-2 (Human Umbilical cord blood–Derived Erythroid Progenitor-2) cells were transduced with lentiviral small hairpin RNAs (shRNAs) stably silencing *CDIN1* or *SEC23B* (shCDIN1 and shSEC23B). Erythroid differentiation was induced by erythropoietin for 10 days, and cells were harvested at days 0, 2, 4, 7, and 10. Erythroid maturation was assessed using flow cytometry, defining early ($CD36^+/GPA^-$), intermediate ($CD36^+/GPA^+$), and late ($CD36^-/GPA^+$) erythroblasts. RNA sequencing, proteomics, and metabolomics were employed for comprehensive molecular profiling. Computational deconvolution was conducted using CIBERSORTx software, which decomposes bulk RNA-seq data into distinct cellular components.

**Copyright Information:** (Online) ISSN: 2572-9241
©2026 The Author(s). HemaSphere is published by John Wiley & Sons Ltd on behalf of European Hematology Association. This is an open access Abstract Book distributed under the Attribution-NonCommercial-NoDerivs (CC BY-NC-ND), which allows third parties to download the articles and share them with others as long as they credit the author and the Abstract Book, but they cannot change the content in any way or use them commercially.
**Abstract Book Citations:** Authors, Title, HemaSphere, 2026;10:(S1):pages. The abstract book DOIs can be found on the “Author Information” page.
**Disclaimer:** Articles published in the journal HemaSphere exclusively reflect the opinions of the authors. The authors are responsible for all content in their abstracts including accuracy of the facts, statements, citing resources, etc.

**Results**

HUDEP-2 shCDIN1 and shSEC23B were induced to undergo erythroid differentiation. Flow cytometry analysis showed a pronounced accumulation of early and intermediate erythroblasts and a reduction in late erythroblasts, particularly on day 7 and day 10, confirming a maturation defect. To gain further insight into pathomechanisms leading to impaired differentiation, a multiomic approach was employed. Deconvolution analysis of RNA-seq data confirmed a significant increase in early progenitors and a reduction in orthochromatic erythroblasts at day 7, indicating a failure to progress to terminal stages of differentiation. Trend analysis of gene expression revealed that the main changes occur during the transition from day 4 to day 7. At this point, integration of transcriptomic and proteomic analyses highlighted a strong deregulation of heme biosynthesis (GO:0006783) in both shCDIN1 and shSEC23B cells. Specifically, we observed a strong deregulation of key proteins involved in this process, including upregulation of CPOX and FECH, key enzymes of heme biosynthesis, as well as upregulation of SLC25A37, the mitochondrial iron transporter. Indeed, metabolomic profiling of differentiating cells revealed significant alterations in mitochondrial metabolism and redox balance, with increased levels of tricarboxylic acid cycle intermediates and pentose phosphate pathway metabolites, indicating a compensatory metabolic shift due to disrupted heme biosynthesis and impaired iron metabolism.

**Summary/Conclusion**

These findings underscore the critical role of heme metabolism and mitochondrial stress in the pathophysiology of CDA I and II. Despite distinct genetic defects in CDIN1 and SEC23B, both models converge on a common pathway of impaired erythroid maturation, characterized by delayed heme biosynthesis, mitochondrial dysfunction, and redox imbalance. These insights highlight the potential for targeting heme biosynthesis and mitochondrial function in future therapeutic strategies, opening new avenues for the treatment of CDA I and II.

**Copyright Information:** (Online) ISSN: 2572-9241
©2026 The Author(s). HemaSphere is published by John Wiley & Sons Ltd on behalf of European Hematology Association. This is an open access Abstract Book distributed under the Attribution-NonCommercial-NoDerivs (CC BY-NC-ND), which allows third parties to download the articles and share them with others as long as they credit the author and the Abstract Book, but they cannot change the content in any way or use them commercially.
**Abstract Book Citations:** Authors, Title, HemaSphere, 2026;10:(S1):pages. The abstract book DOIs can be found on the “Author Information” page.
**Disclaimer:** Articles published in the journal HemaSphere exclusively reflect the opinions of the authors. The authors are responsible for all content in their abstracts including accuracy of the facts, statements, citing resources, etc.

### (PF1291) CLINICAL CHARACTERISTICS, TREATMENT LANDSCAPE, AND LONG-TERM OUTCOMES OF 597 PATIENTS WITH AUTOIMMUNE HEMOLYTIC ANEMIA: A STUDY BASED ON THE LARGEST ASIAN RETROSPECTIVE COHORT

**Topic:** 29. Enzymopathies, membranopathies and other red blood cell disorders

Junan Zhang*[1], Jiewen Qiu[1], Meili Ge[1], Yizhou Zheng[1], Fengkui Zhang[1], Jun Shi[1], Xin Zhao[1]

*[1]National Clinical Research Center for Blood Diseases, Institute of Hematology & Blood Diseases Hospital, Chinese Academy of Medical Sciences & Peking Union Medical College. State Key Laboratory of Experimental Hematology, Haihe Laboratory of Cell Ecosystem, Red Blood Cell Diseases Center and Regenerative Medicine Clinic, Tianjin, China;*

### Background

Autoimmune hemolytic anemia (AIHA) is the most prevalent form of acquired hemolytic anemia, representing a heterogeneous group of anemias.

### Aims

As the largest retrospective study of AIHA in Asia, this study aimed to characterize clinical features, treatment patterns, response rates, and long-term outcomes across major subtypes, thereby providing benchmarks for risk stratification and evaluation of novel therapies.

### Methods

This retrospective cohort study analyzed 597 AIHA patients hospitalized atour institution from January 2014 to December 2023, including 449 warm AIHA (wAIHA), 117 mixed AIHA (mAIHA), and 31 direct antiglobulin test (DAT)-negative AIHA. With a median follow-up of 57 months, we evaluated baseline characteristics, treatment responses, and long-term outcomes including overall survival (OS), event-free survival (EFS, composite endpoint of relapse or death), and complications. Survival was analyzed by Kaplan-Meier and Cox regression models.

### Results

Analysis of 597 AIHA patients revealed distinct features across subtypes. Compared to wAIHA, mAIHA exhibited earlier onset, poorer erythropoiesis, cold agglutinin-like features, and a higher proportion of secondary forms (39.3% vs 22.5%). In contrast, the DAT-negative cohort was predominantly primary (83.9%) and demonstrated a favorable prognosis, with a mortality rate of 9.7%. Among wAIHA and mAIHA patients (median onset age 48 years; 70% female), the 1-, 3-, and 5-year OS rates were 96%, 92%, and 89%, respectively. Multivariable Cox regression identified five independent risk factors: age ≥60 years (HR=3.04), glucocorticoids (GCs) resistance (HR=2.45), opportunistic infections (HR=2.13), hemoglobin (Hb) <6 g/dL (HR=1.98), and bone marrow responsiveness index <121 (HR=1.73). Serological analysis revealed that IgM-mediated and IgM+IgA-mediated subtypes were associated with significantly worse OS. The median EFS was 17 months, with relapses primarily triggered by treatment tapering (>70%) or infections (>20%). Thrombotic events occurred in approximately 10% of cases, with a clustering during active hemolysis and within the first month following diagnosis. First-line GCs demonstrated high efficacy across all subtypes, with overall response rates all exceeding 90%. In wAIHA/mAIHA patients, second-line therapies (64.5%) comprised rituximab (54.5%, with comparable efficacy between standard-dose and low-dose), cyclosporine (17.5%), androgens (13.2%), cyclophosphamide (4.7%), mycophenolate mofetil (3.0%), splenectomy (2.5%) and others. Notably, 6 patients with multi-refractory AIHA achieved complete response and durable treatment-free remission following autologous CD19 CAR T-cell therapy.

**Copyright Information:** (Online) ISSN: 2572-9241
©2026 The Author(s). HemaSphere is published by John Wiley & Sons Ltd on behalf of European Hematology Association. This is an open access Abstract Book distributed under the Attribution-NonCommercial-NoDerivs (CC BY-NC-ND), which allows third parties to download the articles and share them with others as long as they credit the author and the Abstract Book, but they cannot change the content in any way or use them commercially.
**Abstract Book Citations:** Authors, Title, HemaSphere, 2026;10:(S1):pages. The abstract book DOIs can be found on the “Author Information” page.
**Disclaimer:** Articles published in the journal HemaSphere exclusively reflect the opinions of the authors. The authors are responsible for all content in their abstracts including accuracy of the facts, statements, citing resources, etc.

**Summary/Conclusion**

This largest Asian cohort study confirms that mAIHA presents with earlier onset, more secondary forms, cold agglutinin-like features, and poorer bone marrow compensation than wAIHA. Independent risk factors of OS (age ≥60, GCs resistance, opportunistic infections, Hb <6 g/dL, impaired erythropoiesis) were validated. IgM-mediated wAIHA (with/without IgA) had worse OS, while cold agglutinin titers showed limited prognostic value. After rigorous exclusion, DAT-negative AIHA follows a relatively favorable course. A nomogram model was developed to facilitate risk stratification. Documentation of treatment patterns and responses illustrates the landscape of AIHA in Asian populations. Multiple emerging therapies, particularly CAR-T, are progressively reshaping the therapeutic paradigm of AIHA.

**Copyright Information:** (Online) ISSN: 2572-9241
©2026 The Author(s). HemaSphere is published by John Wiley & Sons Ltd on behalf of European Hematology Association. This is an open access Abstract Book distributed under the Attribution-NonCommercial-NoDerivs (CC BY-NC-ND), which allows third parties to download the articles and share them with others as long as they credit the author and the Abstract Book, but they cannot change the content in any way or use them commercially.
**Abstract Book Citations:** Authors, Title, HemaSphere, 2026;10:(S1):pages. The abstract book DOIs can be found on the “Author Information” page.
**Disclaimer:** Articles published in the journal HemaSphere exclusively reflect the opinions of the authors. The authors are responsible for all content in their abstracts including accuracy of the facts, statements, citing resources, etc.

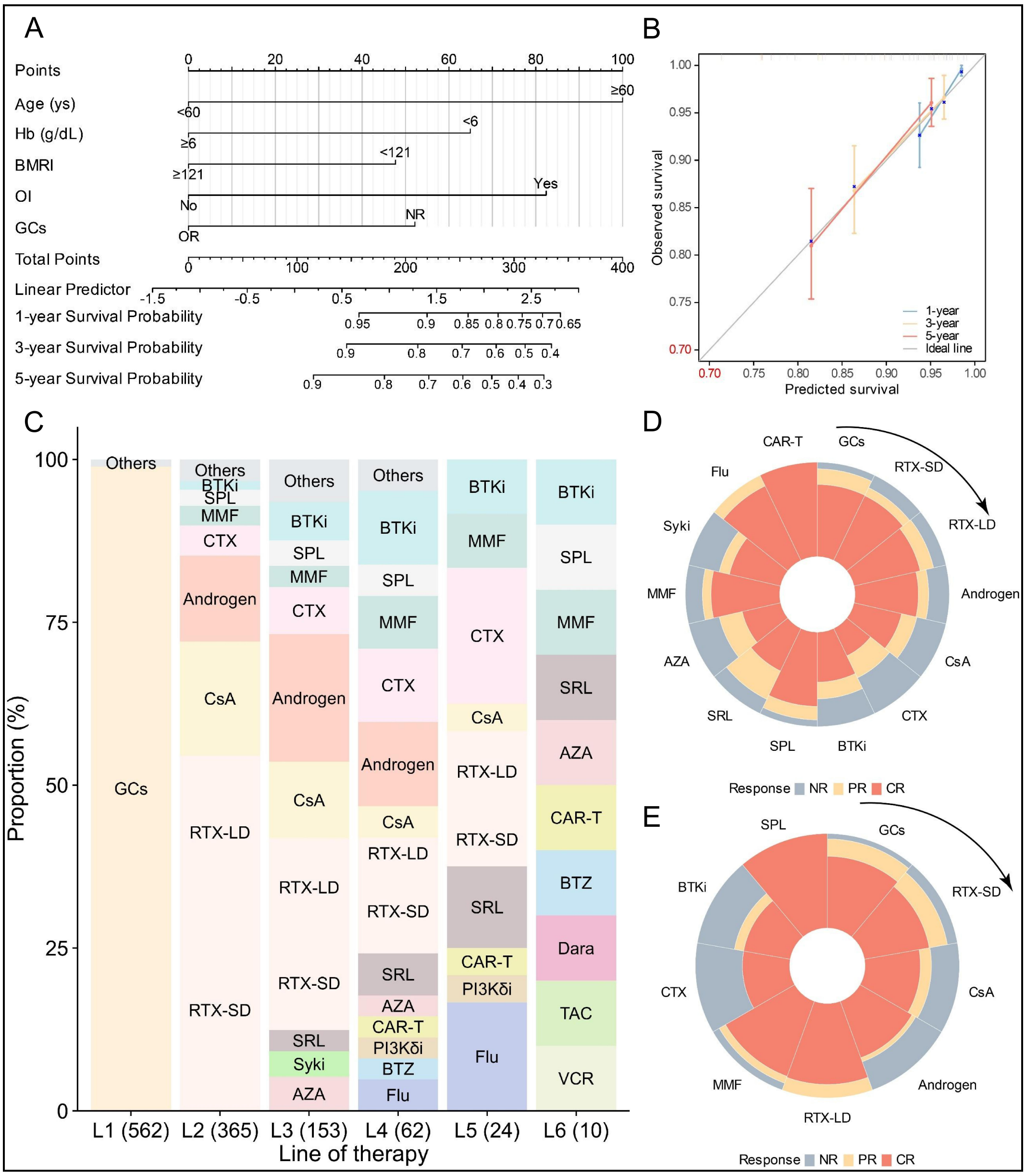

Figure. Prognostic nomogram for overall survival, therapeutic landscape, and treatment responses in patients with wAIHA/mAIHA. (A) Prognostic nomogram integrating five independent predictors derived from the multivariable model. The total points calculated from these variables correspond to the predicted probability of 1-, 3-, and 5-year overall survival. (B) Calibration plots displaying the consistency between nomogram-predicted (x-axis) and observed (y-axis) survival rates. The dashed diagonal line (45°) represents the ideal prediction model, indicating satisfactory agreement within the study cohort. (C) Distribution of therapeutic regimens across sequential lines of therapy (L1–L6), illustrating the heterogeneity and evolution of treatment patterns. (D, E) Evaluation of therapeutic efficacy in (D) primary and (E) secondary AIHA. Regimens are arranged clockwise starting from GCs (indicated by the arrow) and are ranked by frequency of use. Stacked bars represent the proportion of complete response (CR), partial response (PR), and no response (NR) for each regimen. Abbreviations: OS, overall survival; Hb, hemoglobin at onset; BMRI, bone marrow responsiveness index; OI, opportunistic infections; GCs, glucocorticoids; OR, overall response; NR, no response (or non-response); PR, partial response; CR, complete response; RTX-SD, rituximab standard dose; RTX-LD, rituximab low dose; CsA, cyclosporine A; CTX, cyclophosphamide; MMF, mycophenolate mofetil; AZA, azathioprine; SPL, splenectomy; BTKi, Bruton tyrosine kinase inhibitor; Syki, spleen tyrosine kinase inhibitor; SRL, sirolimus; BTZ, bortezomib; Dara, daratumumab; PI3Kδi, phosphatidylinositol 3-kinase delta inhibitor; CAR-T, chimeric antigen receptor T-cell therapy; Flu, fludarabine; TAC, tacrolimus; VCR, vincristine; Others, splenic artery embolization, B-cell activating factor receptor (BAFF-R) inhibitors, bendamustine, thalidomide, etc.

**Copyright Information:** (Online) ISSN: 2572-9241
©2026 The Author(s). HemaSphere is published by John Wiley & Sons Ltd on behalf of European Hematology Association. This is an open access Abstract Book distributed under the Attribution-NonCommercial-NoDerivs (CC BY-NC-ND), which allows third parties to download the articles and share them with others as long as they credit the author and the Abstract Book, but they cannot change the content in any way or use them commercially.
**Abstract Book Citations:** Authors, Title, HemaSphere, 2026;10:(S1):pages. The abstract book DOIs can be found on the “Author Information” page.
**Disclaimer:** Articles published in the journal HemaSphere exclusively reflect the opinions of the authors. The authors are responsible for all content in their abstracts including accuracy of the facts, statements, citing resources, etc.

### (PF1292) FERTILITY AND PREGNANCY OUTCOMES IN WOMEN WITH CONGENITAL HEMOLYTIC ANEMIAS: A SINGLE CENTRE RETROSPECTIVE STUDY

**Topic:** 29. Enzymopathies, membranopathies and other red blood cell disorders

Elisa Sambruna*[1], Marta Bortolotti[2], Giacinto Pedone[2, 3], Paola Bianchi[2], Elisa Fermo[2], Anna Paola Marcello[2], Anna Zaninoni[2], Cristina Vercellati[2], Francesca Filippi[4], Edgardo Somigliana[4, 5], Wilma Barcellini[2], Francesco Passamonti[1, 2], Bruno Fattizzo[1, 2]

*[1]Department of Oncology and Onco-hematology, University of Milan., Milan, Italy;[2]Hematology Unit, Fondazione IRCCS Ca' Granda Ospedale Maggiore Policlinico., Milan, Italy; [3]Department of Pathophysiology and Transplantation, University of Milan., Milan, Italy; [4]Infertility Unit, Fondazione IRCCS Ca' Granda Ospedale Maggiore Policlinico., Milan, Italy; [5]Department of Clinical Sciences and Community Health, University of Milan., Milan, Italy;*

### Background

Congenital hemolytic anemias (CHAs) are a group of rare inherited disorders characterized by a variable degree of anemia, chronic hemolysis, splenomegaly, gallstones, and iron overload. Severe anemia and iron overload may impact normal growth and disrupt hormonal secretion, resulting in endocrine dysfunctions, such as hypogonadism. Furthermore, severe anemia, biliary colic, thrombosis, and splenomegaly may complicate pregnancy. Unlike other more common congenital anemias (i.e., thalassemia and sickle cell disease), few data about fertility and pregnancy outcomes are available for these disorders.

### Aims

To evaluate fertility and pregnancy outcomes in a cohort of women with CHAs followed at our center.

### Methods

A questionnaire to investigate fertility and pregnancy outcomes in CHAs was created for administration via in-person or telephone interviews to women with CHAs, followed at a tertiary hematology center in Milan, Italy. Descriptive statistical methods were adopted to analyze data.

### Results

In the last 15 years, we collected a cohort of 253 women with a diagnosis of CHAs (160 hereditary spherocytosis, HS; 18 hereditary elliptocytosis, HE; 18 hereditary stomatocytosis, HSt; 14 congenital dyserythropoietic anemias, CDA; 19 pyruvate kinase deficiency, PKD; 24 multiple hemolytic defects). In this abstract, we present preliminary data from 43 women (31 HS, 1 HSt, 2 HE, 4 CDA, 5 PKD), for a total of 65 pregnancies. The mean age at the time of the interview was 55 ± 19 years. Eleven patients were splenectomized, and no one presented with iron overload.
Menstrual cycle was preserved in 95% of patients (median duration was 28 days, min-max 20-45). 8 women never tried to conceive. Among the others, 29 (83%) got spontaneously pregnant, 3 (9%) conceived through medically assisted reproduction (2 ovarian stimulation, 1 IVF), whilst 3 women (9%) were unable to conceive. 3 out of the 6 patients with fertility disorders were CDA women, with an infertility rate in this population of 75%.
Maternal complications were mainly related to anemia, and transfusions were necessary in 18% of pregnancies, reaching 83% in PKD; no thrombotic events occurred, and thrombotic prophylaxis was performed in 12% of pregnancies and only in 3/11 splenectomized women. Obstetrics outcomes were overall favorable, with most pregnancies reaching term; fetal complications were uncommon (4%), and the miscarriage rate was only slightly higher than that of the general population (17%).

**Copyright Information:** (Online) ISSN: 2572-9241
©2026 The Author(s). HemaSphere is published by John Wiley & Sons Ltd on behalf of European Hematology Association. This is an open access Abstract Book distributed under the Attribution-NonCommercial-NoDerivs (CC BY-NC-ND), which allows third parties to download the articles and share them with others as long as they credit the author and the Abstract Book, but they cannot change the content in any way or use them commercially.
**Abstract Book Citations:** Authors, Title, HemaSphere, 2026;10:(S1):pages. The abstract book DOIs can be found on the "Author Information" page.
**Disclaimer:** Articles published in the journal HemaSphere exclusively reflect the opinions of the authors. The authors are responsible for all content in their abstracts including accuracy of the facts, statements, citing resources, etc.

| | All | HS, HSt, HE | CDAs | PKD |
|---|---|---|---|---|
| n° of women | 43 | 34 | 4 | 5 |
| n° of pregnancy | 65 | 53 | 6 | 6 |
| Infertility, n (%) | 6 (14) | 3 (9) | 3 (75) | 0 |
| Outcome of pregnancies, n (%)<br>- Childbirth<br>- Miscarriage<br>- Voluntary termination of pregnancy | 51 (78)<br>11 (17)<br>3 (5) | 42 (79)<br>10 (19)<br>1 (2) | 4 (66)<br>1 (17)<br>1 (17) | 5 (83)<br>0<br>1 (17) |
| Maternal complications, n (%)<br>- Worsening of anemia with transfusion need<br>- Biliary colic<br>- Renal colic<br>- Preeclampsia<br>- Hypothyroidism<br>- Gestational diabetes | 12 (18)<br>1<br>1<br>1<br>2<br>1 | 7 (13)<br>0<br>1<br>1<br>1<br>1 | 0 | 5 (83)<br>1<br>0<br>0<br>1<br>0 |
| Fetal complications, n (%)<br>- Fetal ascites<br>- Pyloric stenosis | 2 (4)<br>1<br>1 | 2<br>1<br>1 | 0 | 0 |

### Summary/Conclusion

Preliminary results of this study suggest that fertility is generally preserved in hereditary membrane disorders and PKD, whilst it seems markedly affected in CDA patients. Maternal and fetal outcomes were overall favorable except for the increased transfusion need, particularly in PKD women. Broadening the study, we hope to give more insights about fertility and pregnancy outcomes in women with these rare disorders.

**Copyright Information:** (Online) ISSN: 2572-9241
©2026 The Author(s). HemaSphere is published by John Wiley & Sons Ltd on behalf of European Hematology Association. This is an open access Abstract Book distributed under the Attribution-NonCommercial-NoDerivs (CC BY-NC-ND), which allows third parties to download the articles and share them with others as long as they credit the author and the Abstract Book, but they cannot change the content in any way or use them commercially.
**Abstract Book Citations:** Authors, Title, HemaSphere, 2026;10:(S1):pages. The abstract book DOIs can be found on the "Author Information" page.
**Disclaimer:** Articles published in the journal HemaSphere exclusively reflect the opinions of the authors. The authors are responsible for all content in their abstracts including accuracy of the facts, statements, citing resources, etc.

### (PF1293) RAPID, DOSE-DEPENDENT REDUCTION OF PLASMA PROTOPORPHYRIN IX WITH PORT-77 IN ADULTS WITH ERYTHROPOIETIC PROTOPORPHYRIA: RESULTS FROM THE PHASE 2A GATEWAY STUDY

**Topic:** 29. Enzymopathies, membranopathies and other red blood cell disorders

Melissa Vadnais*[1], Amy Chinn[1], Bhavik Shah[2], Alex Vo[1], Pete Schmidt[1]

*[1]Portal Therapeutics, Palo Alto, United States of America;[2]Bridge Bio, Palo Alto, United States of America;*

### Background

Erythropoietic protoporphyria (EPP) is a rare genetic disorder of heme biosynthesis caused by an autosomal recessive mutation in FECH, the enzyme in the heme biosynthesis pathway that inserts $Fe^{2+}$ into protoporphyrin IX (PPIX) to form heme. This reduction in FECH activity results in accumulation of PPIX in marrow reticulocytes and circulating erythrocytes. PPIX is actively transported from erythrocytes into plasma by the ABCG2 transporter which leads to its deposition in liver and skin. The accumulated PPIX in skin, when activated by sunlight, generates singlet oxygen-mediated free radicals which lead to cutaneous damage and excruciating pain. In the liver, excess PPIX can cause cholestasis and progressive hepatobiliary injury. PORT-77 is an investigational, orally administered inhibitor of the ABCG2 transporter designed to block erythrocyte efflux of PPIX into plasma, thereby reducing deposition in the liver and skin. Therapies that directly reduce plasma PPIX may modify disease biology.

### Aims

To evaluate the safety, pharmacokinetics (PK), and pharmacodynamic (PD) effects of PORT-77 on plasma PPIX in adults with EPP.

### Methods

The Phase 2a GATEWAY study was conducted to evaluate the safety, tolerability, PK, and PD effects on plasma PPIX of PORT-77 in adults with EPP. GATEWAY was a blinded, randomized, placebo-controlled crossover study in which 19 adults with EPP received placebo in Period 1 followed by PORT-77 oral suspension at 180 mg once daily (QD) or 300 mg twice daily (BID) in Period 2. Plasma PPIX levels were quantified using LC-MS/MS. Safety was evaluated through adverse event (AE) monitoring, laboratory assessments, physical examinations, and discontinuations. PK parameters were assessed using noncompartmental methods, and dose-dependent reductions in plasma PPIX were evaluated as a pharmacodynamic endpoint.

**Copyright Information:** (Online) ISSN: 2572-9241
©2026 The Author(s). HemaSphere is published by John Wiley & Sons Ltd on behalf of European Hematology Association. This is an open access Abstract Book distributed under the Attribution-NonCommercial-NoDerivs (CC BY-NC-ND), which allows third parties to download the articles and share them with others as long as they credit the author and the Abstract Book, but they cannot change the content in any way or use them commercially.
**Abstract Book Citations:** Authors, Title, HemaSphere, 2026;10:(S1):pages. The abstract book DOIs can be found on the "Author Information" page.
**Disclaimer:** Articles published in the journal HemaSphere exclusively reflect the opinions of the authors. The authors are responsible for all content in their abstracts including accuracy of the facts, statements, citing resources, etc.

### Results

PORT-77 produced rapid, dose-dependent, and highly significant reductions in plasma PPIX, with mean decreases of ~60% (180 mg oral suspension QD), ~80% (300 mg oral suspension BID) (Figure 1). Reductions were observed within hours of treatment initiation and were consistent across a broad range of baseline PPIX levels (8–121 µg/dL). Pharmacokinetics were dose-proportional, and steady state was achieved within 4 days of dosing. PORT-77 was well tolerated, with no serious AEs and no treatment discontinuations. AEs occurred more frequently during the placebo period than with active treatment (Table 1). Participants were enrolled from three countries (AUS, UK, US) and ranged in age from 20 to 58 years.

| | **Placebo (N=20)** | **PORT-77 (N=19)** |
|---|---|---|
| Participants with ≥1 TEAE | 9 (45%) | 6 (32%) |
| Drug-related TEAEs | 7 (35%) | 4 (21%) |
| Serious TEAEs | 0 | 0 |
| Serious drug-related TEAEs | 0 | 0 |
| TEAEs leading to discontinuation | 0 | 0 |
| Drug-related discontinuations | 0 | 0 |
| Fatal TEAEs | 0 | 0 |

Table 1. Safety in the GATEWAY Study (Safety Population)

### Summary/Conclusion

PORT-77 produced rapid, substantial, and dose-dependent reductions in plasma PPIX, with consistent effects across a wide range of baseline levels and steady state achieved within days. Combined with a favorable safety profile, with no tolerability signals identified, these findings support its continued development as a potential disease-modifying therapy for EPP and XLP in a global Phase 2b/3 program.

**Copyright Information:** (Online) ISSN: 2572-9241
©2026 The Author(s). HemaSphere is published by John Wiley & Sons Ltd on behalf of European Hematology Association. This is an open access Abstract Book distributed under the Attribution-NonCommercial-NoDerivs (CC BY-NC-ND), which allows third parties to download the articles and share them with others as long as they credit the author and the Abstract Book, but they cannot change the content in any way or use them commercially.
**Abstract Book Citations:** Authors, Title, HemaSphere, 2026;10:(S1):pages. The abstract book DOIs can be found on the "Author Information" page.
**Disclaimer:** Articles published in the journal HemaSphere exclusively reflect the opinions of the authors. The authors are responsible for all content in their abstracts including accuracy of the facts, statements, citing resources, etc.

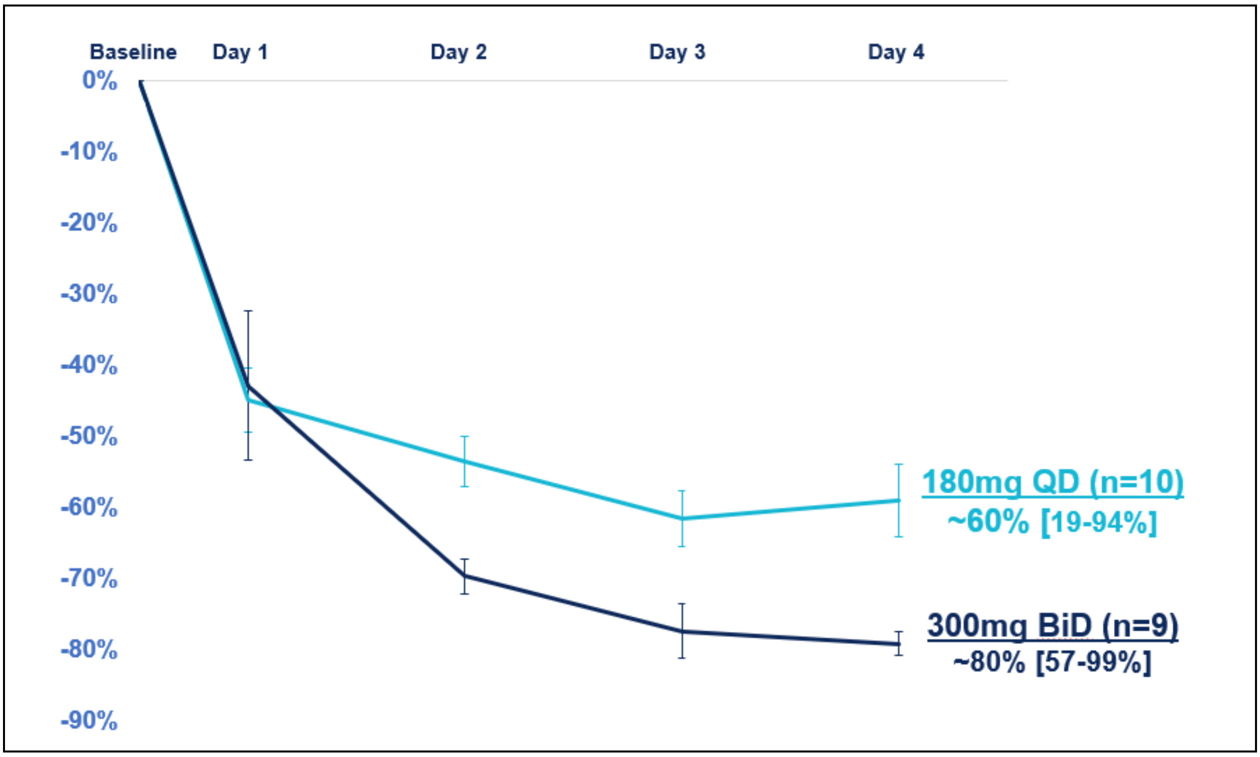

Figure 1. Mean Percent Reduction in Plasma PPIX Rapid, dose-dependent reductions in plasma PPIX, with greater lowering at 300 mg BID than 180 mg QD, consistent with dose-proportional PK and on-target suppression of plasma PPIX.

**Copyright Information:** (Online) ISSN: 2572-9241
©2026 The Author(s). HemaSphere is published by John Wiley & Sons Ltd on behalf of European Hematology Association. This is an open access Abstract Book distributed under the Attribution-NonCommercial-NoDerivs (CC BY-NC-ND), which allows third parties to download the articles and share them with others as long as they credit the author and the Abstract Book, but they cannot change the content in any way or use them commercially.
**Abstract Book Citations:** Authors, Title, HemaSphere, 2026;10:(S1):pages. The abstract book DOIs can be found on the “Author Information” page.
**Disclaimer:** Articles published in the journal HemaSphere exclusively reflect the opinions of the authors. The authors are responsible for all content in their abstracts including accuracy of the facts, statements, citing resources, etc.

### (PF1294) IMMUNOGENETIC AND MOLECULAR CHARACTERIZATION OF COLD AGGLUTININ DISEASE: POTENTIAL INSIGHTS BEYOND THE DETECTABLE B-CELL CLONE

**Topic:** 29. Enzymopathies, membranopathies and other red blood cell disorders

Matteo Bellia*[1], Riccardo Moia[1], Giacinto Pedone[2, 3], Anna Zaninoni[2], Marta Bortolotti[2], Mohammad Almasri[1], Jana Nabki[1], Luca Cividini[1], Bashar Al Deeban[1], Chiara Cosentino[1], Nawar Maher[1], Marco Gallazzi[1], Fatemeh Bokaei[1], Silvia Rasi[1], Francesca Maiellaro[1], Eleonora Secomandi[1], Wilma Barcellini[2], Gianluca Gaidano[1], Bruno Fattizzo[2, 4], Andrea Patriarca[1]

*[1]Division of Hematology, Department of Translational Medicine, Università del Piemonte Orientale, Novara, Italy;[2]Fondazione IRCCS Ca' Granda Ospedale Maggiore Policlinico, Milan, Italy; [3]Department of Pathophysiology and Transplantation, Università degli Studi di Milano, Milan, Italy; [4]Department of Oncology and Haemato-Oncology, University of Milan, Milan, Italy;*

### Background

Although cold agglutinin disease (CAD) is defined as a clonal disorder, such B-cell clones may be difficult to characterize by immunophenotypic features and the molecular lesions are largely unknown. In addition, the frequency of coexisting age-related clonal hematopoiesis in CAD remains unknown.

### Aims

By combining gene mutations (both lymphoid and myeloid genes) and immunoglobulin (IG) gene rearrangement analysis (both heavy and light chains) in CAD patients' samples, we aimed to further dissect the disease molecular pathogenesis.

### Methods

A multicenter real-life cohort of CAD patients provided with clinical and laboratory data has been tested. gDNA from bone marrow (BM) and peripheral blood (PB) was analysed by CAPP-Seq, targeting 146 lymphoid and myeloid genes. Heavy and light IG gene rearrangements were evaluated using an amplicon-based next-generation sequencing approach.

### Results

A total of 17 (9F and 8M) newly diagnosed CAD patients composed the study cohort. All samples were analysed on gDNA from BM aspirates and 8 (47%) patients were also provided with coupled PB samples. The cohort median age was 73 years (IQR 67 - 82), median bone marrow recovery index (BMRI) was 119 (IQR 96 – 160), median haemoglobin was 9.9 g/dL (IQR 8.0 – 10.6), and median LDH was 558 U/L (IQR 262 – 617). In all cases a C3-direct antiglobulin test resulted positive. A B-cell lymphoproliferative clone was identified in 8 (47%) patients by flow cytometry on BM aspirate and/or immunohistochemistry on BM biopsy. By evaluating BM samples, all patients harbored a clonal IG gene rearrangement. IGHV 4-34 rearrangement was the most frequent among IGHV genes, occurring in 12 (76%) patients. Regarding IG light chains, all patients presented K rearrangement, with IGKV 3-20 being the most frequently observed in 11/17 (65%) patients followed by IGVK 4-1 in 2/17 (12%) patients. CAPP-Seq revealed at least one somatic nonsynonymous mutation in 11 (65%) patients. The most frequently mutated *bona fide* lymphoid genes were *KMT2D* in 4 (23%) patients, *KLHL6* in 3 (18%), *CARD11* and *TP53* in 2 (12%) (Fig1A). Notably, in patients with no evidence of B-cell lymphoproliferative disorder by flow cytometry or BM biopsy, IGHV rearrangements and/or lymphoid gene mutations were found in every case, reinforcing the concept that CAD originates from a lymphoid clone, either from an overt B-cell malignancy or from lymphoid clonal haematopoiesis. Albeit limited by the absence of sorted cells, myeloid clonal haematopoiesis was also co-existing in these patients, as documented by detectable *bona fide* myeloid gene mutations of *TET2*, *DNMT3A*, and *ASXL1* mutations in 6 (35.3%) patients. Among the 8 cases provided with both PB and BM samples, all gene mutations detected in PB were identified in BM, and 23 mutations out of 29 (79.3%) mutations detected on BM were also confirmed in PB (Fig1B).

**Copyright Information:** (Online) ISSN: 2572-9241
©2026 The Author(s). HemaSphere is published by John Wiley & Sons Ltd on behalf of European Hematology Association. This is an open access Abstract Book distributed under the Attribution-NonCommercial-NoDerivs (CC BY-NC-ND), which allows third parties to download the articles and share them with others as long as they credit the author and the Abstract Book, but they cannot change the content in any way or use them commercially.
**Abstract Book Citations:** Authors, Title, HemaSphere, 2026;10:(S1):pages. The abstract book DOIs can be found on the "Author Information" page.
**Disclaimer:** Articles published in the journal HemaSphere exclusively reflect the opinions of the authors. The authors are responsible for all content in their abstracts including accuracy of the facts, statements, citing resources, etc.

### Summary/Conclusion

This study demonstrates that IG clonality and lymphoid somatic mutations are detectable in CAD irrespective of the presence of an overt bone marrow B-cell clone, reinforcing the underlying clonal nature of the disorder. Also, PB analysis reflects, in most cases, the molecular profile identified in BM. These findings support the use of integrated molecular profiling (IG clonality and gene mutations) to better characterize CAD pathogenesis and to refine diagnostic and therapeutic strategies guided by specific pathway vulnerabilities.

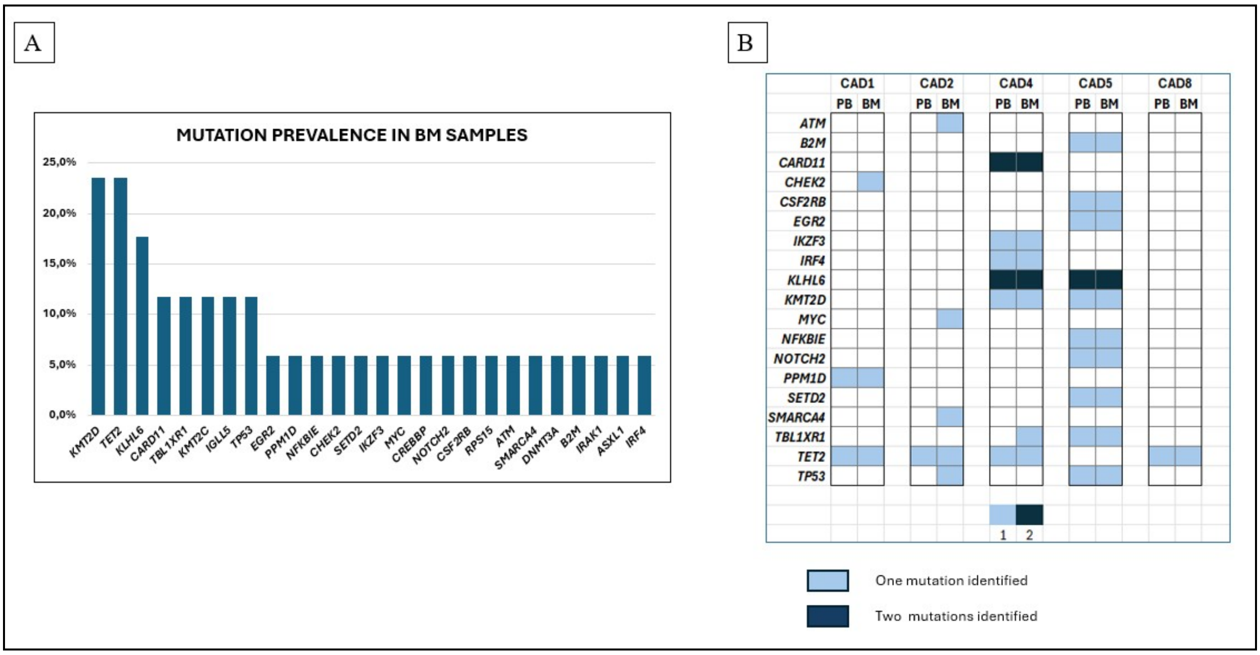

Panel A: prevalence of somatic nonsynonimous mutations identified in BM samples from study cohort patients. Panel B: correspondance of mutations identified in patients provided with both PB and BM samples

Copyright Information: (Online) ISSN: 2572-9241
©2026 The Author(s). HemaSphere is published by John Wiley & Sons Ltd on behalf of European Hematology Association. This is an open access Abstract Book distributed under the Attribution-NonCommercial-NoDerivs (CC BY-NC-ND), which allows third parties to download the articles and share them with others as long as they credit the author and the Abstract Book, but they cannot change the content in any way or use them commercially.
Abstract Book Citations: Authors, Title, HemaSphere, 2026;10:(S1):pages. The abstract book DOIs can be found on the "Author Information" page.
Disclaimer: Articles published in the journal HemaSphere exclusively reflect the opinions of the authors. The authors are responsible for all content in their abstracts including accuracy of the facts, statements, citing resources, etc.

### (PF1295) DARATUMUMAB FOR REFRACTORY IMMUNE-MEDIATED ANEMIAS: A REAL-WORLD MULTICENTRE EXPERIENCE

**Topic:** 29. Enzymopathies, membranopathies and other red blood cell disorders

Alessandro Cellini*[1], Sabrina Giammarco[2], Maria Assunta Limongiello[2], Simona Sica[2], Bruno Fattizzo[3], Giacinto Pedone[3], Andrea Aroldi[4], Marilena Fedele[4], Laura Pavan[1], Francesco Angotzi[1], Livio Trentin[1], Andrea Visentin[1]

*[1]Università di Padova, Department of Medicine, Padova, Italy; [2]Università Cattolica del Sacro Cuore-Roma, Department of Hematology, Rome, Italy; [3]Università Statale di Milano, Department of Oncology and Hemato-Oncology, Milano, Italy; [4]IRCCS San Gerardo dei Tintori, Hematology Division, Monza, Italy;*

### Background

Immune-mediated anemias (IA) refractory to standard care represent an unmet clinical need, as their management is hindered both by the lack of effective therapeutic options and the cumulative toxicity from prolonged corticosteroid use and other rescue medications. Daratumumab (Dara), an anti-CD38 antibody, has been proven to have activity in immune-related diseases by targeting both long-lived plasma cells and CD38+ T cells, and reducing autoantibody production.

### Aims

To evaluate the efficacy and safety of Dara in a cohort of patients treated for relapsed/refractory IA.

### Methods

Patients treated with Dara for relapsed/refractory IA within a compassionate use program between Nov 2022 and May 2025 were enrolled. Patients were required to have a diagnosis of primary or secondary IA, defined as warm or cold autoimmune haemolytic anemia (wAIHA or cAIHA) or pure red cell aplasia (PRCA). The primary endpoint of the study was overall response rate (ORR) at 24 weeks, with responses defined as an increase of haemoglobin (Hb) of at least 20 g/L in AIHA and appropriate reticulocyte count and reduction in transfusion dependence for PRCA.

**Copyright Information:** (Online) ISSN: 2572-9241
©2026 The Author(s). HemaSphere is published by John Wiley & Sons Ltd on behalf of European Hematology Association. This is an open access Abstract Book distributed under the Attribution-NonCommercial-NoDerivs (CC BY-NC-ND), which allows third parties to download the articles and share them with others as long as they credit the author and the Abstract Book, but they cannot change the content in any way or use them commercially.
**Abstract Book Citations:** Authors, Title, HemaSphere, 2026;10:(S1):pages. The abstract book DOIs can be found on the “Author Information” page.
**Disclaimer:** Articles published in the journal HemaSphere exclusively reflect the opinions of the authors. The authors are responsible for all content in their abstracts including accuracy of the facts, statements, citing resources, etc.

### Results

A total of 16 patients were enrolled, of which 8 (50%) had AIHA (3 with cAIHA, 4 with wAIHA, 1 with Evan's syndrome), of which 3 had a secondary form of AIHA and 8 (50%) had PRCA (Table). Among patients with AIHA, median age at Dara start was 59 yrs (49-70), and the median number of previous therapies was 4 (2-7), with all patients having received at least one course of Rituximab. Three (37.5%) subjects had a partial response (PR) to the last line of therapy, whereas the remainder were no responders. Median Hb at Dara start was 77.5 g/L (40-125), and ORR at 24 weeks was 62.5%, with 3 (37.5%) patients experiencing a complete response (CR), 3 PR and 2 non-responders (1 Evan's syndrome, 1 multirefractory wAIHA secondary to Lymphoplasmacytic Lymphomas).
All but one patient with PRCA experienced such cytopenia as complication of allogeneic stem cell transplantation. In this group, median age at Dara start was 61.5 yrs (45-69), with no individual being a responder to the previous line of therapy. Median Hb at Dara start was 72 g/L (66-88), and ORR at 24 weeks was 87.5%, with all responders reaching a CR.
After a median follow-up of 16 months (3-37) in the whole cohort, all but 2 patients completed the planned cycles of Daratumumab. Among patients with AIHA, 2 patients required a new line of treatment after a median of 19 months, the 1-yr time to next treatment (TTNT) was 75% (50.3-100) and a median TTNT of 19 months. Within the group affected by PRCA no patient required a further line of treatment.
After 196 Dara infusions, no infusion reactions were documented, 3 infectious events were recorded (1 grade 5 septic shock, 1 G2 pneumonia and 1 G3 pneumonia).

### Summary/Conclusion

Daratumumab was associated with a favorable safety profile in heavily pretreated IA, exhibiting a particularly high response rate in refractory PRCA, and a favorable safety profile in this small compassionate-use cohort. Prospective studies are warranted to define optimal patient selection and treatment schedule.

| | AIHA | PRCA |
|---|---|---|
| Age - Median (range) | 59 (49-70) | 61.5 (45-69) |
| Male - No. (%) | 4 (50) | 3 (100) |
| No. previous lines - Median (range) | 4 (2-7) | 7 (6-10) |
| Secondary cytopenia - No. (%) | 3 (37.5) | 0 (0) |
| Hb (g/L) - median (range) | 77.5 (40-125) | 72 (65-88) |
| 24-week ORR - No. (%) | 5 (62.5) | 7 (87.5) |
| 24-week CR - No. (%) | 3 (37.5) | 7 (87.5) |

Copyright Information: (Online) ISSN: 2572-9241
©2026 The Author(s). HemaSphere is published by John Wiley & Sons Ltd on behalf of European Hematology Association. This is an open access Abstract Book distributed under the Attribution-NonCommercial-NoDerivs (CC BY-NC-ND), which allows third parties to download the articles and share them with others as long as they credit the author and the Abstract Book, but they cannot change the content in any way or use them commercially.
Abstract Book Citations: Authors, Title, HemaSphere, 2026;10:(S1):pages. The abstract book DOIs can be found on the "Author Information" page.
Disclaimer: Articles published in the journal HemaSphere exclusively reflect the opinions of the authors. The authors are responsible for all content in their abstracts including accuracy of the facts, statements, citing resources, etc.

### (PF1296) PATIENT-DERIVED CELL-BASED MODEL OF CONGENITAL DYSERYTHROPOIETIC ANEMIA TYPE II FOR STUDYING AND RESCUING DISEASE PHENOTYPES

**Topic:** 29. Enzymopathies, membranopathies and other red blood cell disorders

Ravina Taak*[1], Dinesh Babu[2], Manu Jamwal[1], Dhavapriya Palani[2], Prashant Sharma[1], Prateek Bhatia[1], Deepak Bansal[1], Rv Shaji[2], Reena Das[1]

*[1]Postgraduate Institute Of Medical Education And Research Chandigarh, Hematology, Chandigarh, India;[2]Christian Medical College, Vellore, India;*

### Background

Congenital dyserythropoietic anemias (CDAs) are rare inherited disorders characterized by ineffective erythropoiesis and abnormal erythroblast morphology. CDA type II (CDA II), the most common subtype, is caused by biallelic mutations in *SEC23B*, a critical component of the COPII vesicular trafficking complex required for endoplasmic reticulum–Golgi transport. Clinically, CDA II presents with variable severity, ranging from mild anemia to transfusion dependence, and is associated with reticulocytopenia, jaundice, hepatosplenomegaly, and iron overload. Bone marrow examination typically reveals erythroid hyperplasia with dyserythropoiesis, including 10–30% binucleated erythroblasts. Although the genetic basis of CDA II is established, the cellular mechanisms driving ineffective erythropoiesis remain incompletely defined, and existing in vivo models do not fully recaptulate the human erythroid phenotype.

### Aims

To develop a stable patient-derived immortalized erythroid progenitor cell (iEPC) model of CDA II for functional characterization of *SEC23B* variants and assessment of phenotypic rescue.

### Methods

Peripheral blood samples were collected after informed consent from genetically confirmed CDA II patients. Targeted next-generation sequencing identified compound heterozygous *SEC23B* variants. Patient 1 carried NM_006363.4:c.1043A>G (rs2060144762) and NM_006363.4:c.1898delC (novel) and had a severe, transfusion-dependent phenotype. Patient 2 harbored NM_006363.4:c.1589G>A (rs368545054) and NM_006363.4:c.1905+3G>T (novel) with a milder clinical course. Peripheral blood mononuclear cells isolated were transduced with a lentiviral vector expressing HPV E6/E7 and cultured in erythroid expansion medium to generate stable iEPC lines. Erythroid differentiation and ineffective erythropoiesis were evaluated using flow cytometry, Giemsa staining, transmission electron microscopy, and Western blot analysis.

**Copyright Information:** (Online) ISSN: 2572-9241
©2026 The Author(s). HemaSphere is published by John Wiley & Sons Ltd on behalf of European Hematology Association. This is an open access Abstract Book distributed under the Attribution-NonCommercial-NoDerivs (CC BY-NC-ND), which allows third parties to download the articles and share them with others as long as they credit the author and the Abstract Book, but they cannot change the content in any way or use them commercially.
**Abstract Book Citations:** Authors, Title, HemaSphere, 2026;10:(S1):pages. The abstract book DOIs can be found on the “Author Information” page.
**Disclaimer:** Articles published in the journal HemaSphere exclusively reflect the opinions of the authors. The authors are responsible for all content in their abstracts including accuracy of the facts, statements, citing resources, etc.

**Results**

The established CDA II–derived iEPCs expressed the erythroid markers CD71 and CD235a and predominantly corresponded to the proerythroblast and basophilic erythroblast stages.
Upon cytokine-induced differentiation, CDA II iEPCs exhibited altered expression kinetics of CD71, CD105, and CD235a, along with significantly reduced enucleation compared with wild-type (WT) controls, consistent with ineffective erythropoiesis. Increased cell death and a higher rate of apoptosis were observed in CDA II iEPCs relative to WT iEPCs. Giemsa staining demonstrated frequent binucleation at the orthochromatic erythroblast stage. Transmission electron microscopy revealed discontinuous double membranes in mature erythroblasts. Notably, differentiation arrest was evident at the terminal stage of erythroid maturation.
Western blot analysis confirmed reduced SEC23B protein expression in CDA II iEPCs. Importantly, lentiviral-mediated re-expression of wild-type SEC23B partially rescued the disease phenotype, as indicated by reduced binucleation, improved cellular proliferation, and enhanced terminal erythroid differentiation.

**Summary/Conclusion**

We established a patient-derived CDA II iEPC model from a small volume of peripheral blood that recapitulates key morphological, molecular, and functional features of the disease. Genetic complementation with wild-type *SEC23B* ameliorated the observed defects, supporting the pathogenicity of the identified variants and demonstrating the utility of this platform for mechanistic studies and therapeutic evaluation in rare red cell disorders.

**Copyright Information:** (Online) ISSN: 2572-9241
©2026 The Author(s). HemaSphere is published by John Wiley & Sons Ltd on behalf of European Hematology Association. This is an open access Abstract Book distributed under the Attribution-NonCommercial-NoDerivs (CC BY-NC-ND), which allows third parties to download the articles and share them with others as long as they credit the author and the Abstract Book, but they cannot change the content in any way or use them commercially.
**Abstract Book Citations:** Authors, Title, HemaSphere, 2026;10:(S1):pages. The abstract book DOIs can be found on the “Author Information” page.
**Disclaimer:** Articles published in the journal HemaSphere exclusively reflect the opinions of the authors. The authors are responsible for all content in their abstracts including accuracy of the facts, statements, citing resources, etc.

### (PF1297) PHARMACODYNAMIC EFFECT OF NIPOCALIMAB IN WARM AUTOIMMUNE HEMOLYTIC ANEMIA (WAIHA) AND CORRELATION WITH CLINICAL IMPROVEMENT

**Topic:** 29. Enzymopathies, membranopathies and other red blood cell disorders

Bruno Fattizzo[1, 2], Irina Murakhovskaya[3], Yasutaka Ueda*[4], Josephine Vos[5, 6], Eugene Myshkin[7], Vani Lakshminarayanan[8], Kristen Brown[8], Leona Ling[7], Emile van den Akker[9], Kerly Fu[9], Cathye Shu[8], Kristen Sweet[8], Carolyn A. Cuff[7], Sheng Gao[8], Marcia Teixeira Dos Santos[8]

*[1]Hematology Unit, Fondazione IRCCS Ca' Granda Ospedale Maggiore Policlinico, Milan, Italy; [2]Department of Oncology and Hemato-Oncology, University of Milan, Milan, Italy; [3]Albert Einstein College of Medicine/Montefiore Medical Center, Bronx, NY, United States of America; [4]The University of Osaka, Suita, Osaka, Japan;[5]Department of Hematology and Amsterdam Institute for Infection and Immunity, Amsterdam UMC, University of Amsterdam, Amsterdam, The Netherlands; [6]Department of Immunohematology Diagnostics, Sanquin Diagnostics Services, Amsterdam, The Netherlands; [7]Johnson & Johnson, Cambridge, MA, United States of America; [8]Johnson & Johnson, Spring House, PA, United States of America;[9]Sanquin Research and Landsteiner Laboratory, Amsterdam UMC, University of Amsterdam, Amsterdam, The Netherlands;*

### Background

Warm autoimmune hemolytic anemia (wAIHA) is a rare, potentially life-threatening disease characterized by autoantibody (typically IgG)-mediated premature destruction of red blood cells (RBCs). Nipocalimab is a fully human monoclonal antibody that binds FcRn with high specificity and affinity to block the IgG-recycling pathway, thus lowering total IgG (including pathogenic anti-RBC IgG autoantibodies) without impacting other key humoral and cellular immune functions.

### Aims

This analysis characterized the pharmacodynamic (PD) effect of nipocalimab on total IgG and anti-RBC IgG and the correlation with the change in hemoglobin (Hgb) and hemolytic markers during the 24-week double blind (DB) period of the phase 2/3 ENERGY trial (ClinicalTrials.gov Identifier: NCT04119050).

### Methods

Adults with wAIHA (Hgb <10 g/dL, laboratory evidence of hemolysis, and positive direct antiglobulin test) who received ≥1 prior therapy and consented to participate were randomized 1:1:1 to intravenous nipocalimab 30 mg/kg q4w, 15 mg/kg q2w, or placebo. Serum total IgG and anti-RBC IgG levels were measured in the DB period, and percent change from baseline was determined at all timepoints with available samples. Total IgG was measured with an immunonephelometric assay at a central lab. A semiquantitative multiplex flow cytometry based-assay was used to measure binding of anti-RBC IgG at 37$^{o}$C to 12 reference RBC populations (representing warm autoantibodies pathogenic in wAIHA). The median percent change values for all positive reference RBC types were used to represent the overall percent change in anti-RBC IgG. Spearman correlations of change in total and anti-RBC IgG levels with changes in Hgb and hemolytic markers (ie, reticulocyte counts, indirect bilirubin) were determined.

**Copyright Information:** (Online) ISSN: 2572-9241
©2026 The Author(s). HemaSphere is published by John Wiley & Sons Ltd on behalf of European Hematology Association. This is an open access Abstract Book distributed under the Attribution-NonCommercial-NoDerivs (CC BY-NC-ND), which allows third parties to download the articles and share them with others as long as they credit the author and the Abstract Book, but they cannot change the content in any way or use them commercially.
**Abstract Book Citations:** Authors, Title, HemaSphere, 2026;10:(S1):pages. The abstract book DOIs can be found on the "Author Information" page.
**Disclaimer:** Articles published in the journal HemaSphere exclusively reflect the opinions of the authors. The authors are responsible for all content in their abstracts including accuracy of the facts, statements, citing resources, etc.

### Results

Of the 118 patients in the ENERGY trial, 114 were included in the total IgG analysis (38, 37, and 39 in nipocalimab 30 mg/kg q4w, 15 mg/kg q2w, and placebo groups, respectively) and 89 were included in the anti-RBC IgG analysis (30, 31, and 28 in nipocalimab 30 mg/kg q4w, 15 mg/kg q2w, and placebo groups, respectively) based on patient consent and sample availability. Baseline clinical characteristics for individuals in the PD analysis set were similar between treatment groups and to those of the overall study population.

Rapid and persistent reduction in total IgG was observed from Week 1 (W1) through W24 in the nipocalimab groups. Similarly, rapid and persistent reduction in anti-RBC IgG was observed in nipocalimab treatment groups, with consistent trends across the 12 reference RBC types. The median overall reduction in anti-RBC IgG was 41% and 35% at W1 and 23% and 16% at W24 for the 30 mg/kg q4w and 15 mg/kg q2w groups, respectively.

Correlations were observed between total IgG reduction and Hgb improvement (R=–0.22; *P*<0.0001) as well as anti-RBC IgG reduction and Hgb improvement (R=–0.33, *P*<0.0001). In addition, anti-RBC IgG reduction was correlated with improvement in hemolytic markers.

### Summary/Conclusion

Nipocalimab reduced both total IgG as well as pathogenic anti-RBC IgG autoantibodies in wAIHA, supporting its mechanism of action as an FcRn blocker. A correlation was observed between anti-RBC IgG reduction and improvement in disease biomarkers, most notably increase in Hgb, consistent with nipocalimab targeting autoantibody-mediated disease mechanisms in wAIHA.

**Copyright Information:** (Online) ISSN: 2572-9241
©2026 The Author(s). HemaSphere is published by John Wiley & Sons Ltd on behalf of European Hematology Association. This is an open access Abstract Book distributed under the Attribution-NonCommercial-NoDerivs (CC BY-NC-ND), which allows third parties to download the articles and share them with others as long as they credit the author and the Abstract Book, but they cannot change the content in any way or use them commercially.
**Abstract Book Citations:** Authors, Title, HemaSphere, 2026;10:(S1):pages. The abstract book DOIs can be found on the “Author Information” page.
**Disclaimer:** Articles published in the journal HemaSphere exclusively reflect the opinions of the authors. The authors are responsible for all content in their abstracts including accuracy of the facts, statements, citing resources, etc.

### (PF1298) CLINICAL VARIABILITY OF ATP11C-RELATED HEMOLYTIC ANEMIA: EXPANDING THE PHENOTYPIC AND DIAGNOSTIC SPECTRUM

**Topic:** 29. Enzymopathies, membranopathies and other red blood cell disorders

Vanessa D'onofrio*[1, 2], Federica Maria Esposito[1, 2], Roberta Marra[2], Barbara Eleni Rosato[1, 2], Antonella Nostroso[1, 2], Alessia Perrotta[1, 2], Eli Matsell[3], Mariangela Manno[2], Anthony Iscaro[1, 2], Manuela Dionisi[2], Stefania Martone[4], Francesco Arcioni[5], Antonio Barbato[6], Lorenzo Chiariotti[1, 2], Rosa Della Monica[2], Brigitte van Oirschot[7], Richard van Wijk[7], Robert Molday[3], Achille Iolascon[1, 2], Roberta Russo[1, 2], Immacolata Andolfo[1, 2]

*[1]University of Naples Federico II, Department of Molecular Medicine and Medical Biotechnology, Naples, Italy;[2]CEINGE Advanced Biotechnology Franco Salvatore, Naples, Italy; [3]University of British Columbia, Department of Biochemistry and Molecular Biology, Vancouver, Canada; [4]Federico II University Hospital, DAI of Laboratory and Transfusion Medicine, Naples, Italy; [5]Santa Maria della Misericordia Hospital, Pediatric Oncology-Hematology Unit, Perugia, Italy;[6]University of Naples Federico II, Department of Clinical Medicine and Surgery, Naples, Italy; [7]Central Diagnostic Laboratory - Research, University Medical Center Utrecht, Utrecht, The Netherlands;*

### Background

*ATP11C*, located on the X-chromosome, encodes the major phosphatidylserine flippase in human red blood cells (RBC), maintaining membrane phospholipid asymmetry crucial for cell survival. *ATP11C* variants have recently been linked to a novel form of X-linked congenital hemolytic anemia.

### Aims

The aim of our study is to expand the clinical spectrum of ATP11C-related hemolytic anemia, including adult-onset disease and hepatic iron overload, and to evaluate functional assays and X-chromosome inactivation (XCI) for variant interpretation.

### Methods

Targeted NGS; osmotic gradient ektacytometry; peripheral blood RNA qRT-PCR; RBC membrane immunoblotting; HUMARA assay; ex vivo flippase assay on purified RBC; recombinant mutant ATP11C expression and ATPase activity in HEK293 cells.

**Copyright Information:** (Online) ISSN: 2572-9241
©2026 The Author(s). HemaSphere is published by John Wiley & Sons Ltd on behalf of European Hematology Association. This is an open access Abstract Book distributed under the Attribution-NonCommercial-NoDerivs (CC BY-NC-ND), which allows third parties to download the articles and share them with others as long as they credit the author and the Abstract Book, but they cannot change the content in any way or use them commercially.
**Abstract Book Citations:** Authors, Title, HemaSphere, 2026;10:(S1):pages. The abstract book DOIs can be found on the “Author Information” page.
**Disclaimer:** Articles published in the journal HemaSphere exclusively reflect the opinions of the authors. The authors are responsible for all content in their abstracts including accuracy of the facts, statements, citing resources, etc.

**Results**

Three novel *ATP11C* variants were functionally validated in patients referred for suspected hereditary RBC and/or iron disorders. The first patient (P1) is a male neonate with mild hemolytic anemia with no relevant family history. Genetic analysis revealed the presence of the *ATP11C* variant c.1399C>T (p.R467C), which localizes within a region critical for its catalytic function. Functional studies conducted in HEK293 cells demonstrated that the R467C variant leads to significantly reduced ATP11C protein expression and a concomitant decrease in its catalytic activity. These findings, supported by structural analysis, are consistent with a LoF mechanism and may explain the early neonatal onset of the phenotype observed in this patient.
The second patient (P2) is a 53-year-old male with suspected hereditary hemochromatosis due to hepatic iron overload. The identification of a splice-site variant in *ATP11C*, c.2226-1G>C, combined with clinical re-evaluation, revealing markers of hemolysis, led to a revised diagnosis of compensated hemolysis with iron overload. To our knowledge, this is the first ATP11C case presenting in adulthood and the first associated with hepatic iron overload. In silico tools predicted disruption of the canonical splice site. In line with this, functional studies demonstrated reduced ATP11C protein expression in RBC plasma membrane and a reduced flippase activity of purified RBC, supporting a LoF mechanism.
The third patient (P3) is a 68-year-old female with mild anemia and hemolysis. Genetic testing identified the heterozygous *ATP11C* variant c.1826A>T (p.D609V). Notably, she is also a carrier of a beta-thalassemia trait, which may contribute to anemia but does not explain the hemolytic features observed. HUMARA analysis showed skewed XCI with preferential expression of the mutant *ATP11C* allele. The identified variant localizes within the N domain of the ATP11C protein, which is critical for its function. In line with this, functional studies in HEK293 cells revealed reduced ATP11C protein expression, although catalytic activity appeared to be preserved. Concordantly, ex vivo flippase activity measured on purified RBCs did not differ from controls, supporting preserved enzymatic function despite reduced protein levels.

**Summary/Conclusion**

Our data show that ATP11C-related disease can manifest even in late adulthood and may include previously unrecognized phenotype such as hepatic iron overload. These findings broaden the clinical spectrum of ATP11C-associated anemia with variable degree of hemolysis (from compensated to severe) and provide a strong rationale for the routine inclusion of *ATP11C* in diagnostic NGS panels for hereditary RBC disorders and unexplained iron metabolism abnormalities.

**Copyright Information:** (Online) ISSN: 2572-9241
©2026 The Author(s). HemaSphere is published by John Wiley & Sons Ltd on behalf of European Hematology Association. This is an open access Abstract Book distributed under the Attribution-NonCommercial-NoDerivs (CC BY-NC-ND), which allows third parties to download the articles and share them with others as long as they credit the author and the Abstract Book, but they cannot change the content in any way or use them commercially.
**Abstract Book Citations:** Authors, Title, HemaSphere, 2026;10:(S1):pages. The abstract book DOIs can be found on the "Author Information" page.
**Disclaimer:** Articles published in the journal HemaSphere exclusively reflect the opinions of the authors. The authors are responsible for all content in their abstracts including accuracy of the facts, statements, citing resources, etc.

### (PF1299) UROLITHIN A UNCOUPLES IRON TOXICITY FROM HEPATIC FIBROSIS BY DISRUPTING THE NETOSIS–HMGB1/TLR4 AXIS

**Topic:** 30. Iron metabolism, deficiency and overload

Hangjie Fu*[1], Wenxia Li[2]

*[1]School of Public Health, Zhejiang Chinese Medical University, Hangzhou, China;[2]Institute of Large-Scale Scientific Facility, Beihang University, hangzhou, China;*

### Background

Iron overload drives hepatic fibrogenesis through catalytic oxidative stress. However, the specific immunopathological relays that convert this initial chemical insult into the sustained, organized sterile inflammation required for fibrosis remain elusive. Current chelation therapies primarily target the source of toxicity but often fail to arrest established inflammatory cascades. Urolithin A (UA), a gut-derived postbiotic metabolite of dietary ellagitannins, possesses unique mitochondrial quality-control and anti-inflammatory properties, yet its efficacy against iron overload-induced liver injury and the underlying mechanisms remain to be defined.

### Aims

This study aimed to delineate the hierarchical immune-stromal relays linking iron toxicity to sterile inflammation and to evaluate whether UA can therapeutically uncouple this pathogenic cascade to prevent hepatic fibrosis.

### Methods

We employed an iron dextran–induced murine liver fibrosis model coupled with unbiased transcriptomic profiling to identify dominant pathogenic signatures. To dissect intercellular communication, we established a hierarchical in vitro co-culture system comprising neutrophils, macrophages, and hepatic stellate cells (HSCs). This was complemented by epistasis-guided gain- and loss-of-function assays targeting the NETosis–HMGB1/TLR4 axis using specific inhibitors, neutralizing antibodies, and recombinant proteins.

### Results

Phenotypically, UA administration significantly ameliorated hepatic iron accumulation, oxidative injury, and fibrotic remodeling, while concurrently restoring mitochondrial ultrastructure and systemic iron homeostasis. Unbiased transcriptomic analysis identified neutrophil extracellular trap (NET) formation as a critical upstream trigger of sterile inflammation suppressed by UA. Mechanistically, we demonstrated that iron-dependent lipid peroxidation acts as the primary trigger for NETosis. These NETs promote the extracellular release of the alarmin HMGB1, which subsequently engages Toll-like receptor 4 (TLR4) signaling on macrophages. This interaction licenses macrophage polarization toward a pro-inflammatory phenotype (elevated TNF-α and IL-6), which in turn drives HSC activation and collagen production. Crucially, UA uncoupled iron toxicity from immune amplification by dampening the upstream redox–NETosis trigger, thereby effectively blocking the downstream HMGB1–TLR4–mediated macrophage polarization and fibrogenic crosstalk. Analysis of human clinical datasets further confirmed that signatures of the NETosis–HMGB1/TLR4 axis correlate with fibrosis severity.

**Copyright Information:** (Online) ISSN: 2572-9241
©2026 The Author(s). HemaSphere is published by John Wiley & Sons Ltd on behalf of European Hematology Association. This is an open access Abstract Book distributed under the Attribution-NonCommercial-NoDerivs (CC BY-NC-ND), which allows third parties to download the articles and share them with others as long as they credit the author and the Abstract Book, but they cannot change the content in any way or use them commercially.
**Abstract Book Citations:** Authors, Title, HemaSphere, 2026;10:(S1):pages. The abstract book DOIs can be found on the "Author Information" page.
**Disclaimer:** Articles published in the journal HemaSphere exclusively reflect the opinions of the authors. The authors are responsible for all content in their abstracts including accuracy of the facts, statements, citing resources, etc.

### Summary/Conclusion

This study identifies the redox–NETosis–HMGB1/TLR4 axis as a critical driver converting iron toxicity into fibrotic tissue remodeling. By targeting mitochondrial dysfunction and inhibiting this upstream NETosis relay, Urolithin A emerges as a promising dual-action postbiotic therapeutic for managing iron overload-associated chronic liver diseases.

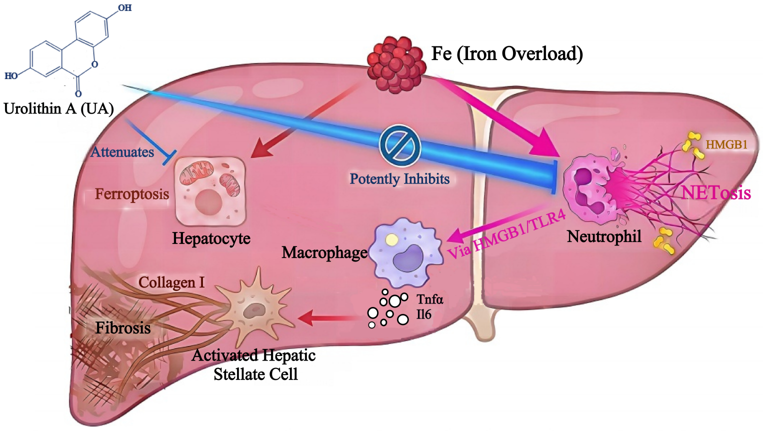

Pathologically, excess iron accumulation (Iron Overload) targets two cell populations: it drives hepatocyte ferroptosis and triggers neutrophils to undergo NETosis. The formation of neutrophil extracellular traps (NETs) releases the alarmin HMGB1, which subsequently binds to TLR4 on macrophages. This signaling axis polarizes macrophages to secrete pro-inflammatory cytokines (TNF-α and IL-6), thereby “licensing” activated hepatic stellate cells (HSCs) to produce Collagen I and drive fibrogenesis. As depicted, UA exerts a hepatoprotective effect by attenuating ferroptosis and, critically, by potently inhibiting the upstream iron-induced NETosis. By disrupting this neutrophil–macrophage–HSC relay, UA effectively blocks the cascade from chemical iron toxicity to sustained fibrosis.

**Copyright Information:** (Online) ISSN: 2572-9241
©2026 The Author(s). HemaSphere is published by John Wiley & Sons Ltd on behalf of European Hematology Association. This is an open access Abstract Book distributed under the Attribution-NonCommercial-NoDerivs (CC BY-NC-ND), which allows third parties to download the articles and share them with others as long as they credit the author and the Abstract Book, but they cannot change the content in any way or use them commercially.
**Abstract Book Citations:** Authors, Title, HemaSphere, 2026;10:(S1):pages. The abstract book DOIs can be found on the “Author Information” page.
**Disclaimer:** Articles published in the journal HemaSphere exclusively reflect the opinions of the authors. The authors are responsible for all content in their abstracts including accuracy of the facts, statements, citing resources, etc.

### (PF1300) NCOA4 ORCHESTRATES HEPATIC METABOLIC REPROGRAMMING

**Topic:** 30. Iron metabolism, deficiency and overload

Valeria Furiosi[1], Mariateresa Pettinato[1, 2], Rossana Carleo[1, 2], Giorgia Federico[3], Francesca Carlomagno[3], Antonella Nai[1, 2], Alessia Pagani[1, 2], Laura Silvestri*[1, 2]

*[1]Ospedale San Raffaele, Milano, Italy; [2]Vita-Salute San Raffaele University, Milano, Italy; [3]University of Naples Federico II, Napoli, Italy;*

### Background

Nuclear receptor coactivator 4 (NCOA4) is a selective autophagy receptor that mediates ferritinophagy, the lysosomal degradation of ferritin complexes. This process releases labile iron from ferritin nanocages, replenishing the cytosolic iron pool required for cellular functions. In mice, NCOA4-dependent ferritinophagy is essential for erythropoiesis, and Ncoa4 knockout (KO) mice exhibit impaired iron-dependent erythropoiesis. While these roles are well established in erythroid tissues, the impact of NCOA4 on non-hematopoietic organs, particularly the liver, a central organ for metabolic regulation, remains poorly understood.

### Aims

This study aimed to investigate how dysregulated ferritinophagy affects hepatic bioenergetics and susceptibility to metabolic stressors, such as high-fat diets.

### Methods

Adult male wild-type and Ncoa4 KO mice were maintained on either a chow diet or a Western diet (WD) for 20 weeks. Hematological and biochemical analyses, and hepatic metabolic characterization were conducted at the end of the dietary treatment.

### Results

In basal conditions, Ncoa4 KO mice accumulated ferritin in the liver and exhibited functional iron deficiency, evidenced by upregulation of transferrin receptor 1 (TfR1) and reduced hepcidin and Bmp6 mRNA levels. Due to ferritin accumulation, total liver iron increased. This iron-restricted state induced redox imbalance, characterized by glutathione depletion (reduced GSH, increased GSSG), a decreased GSH/GSSG ratio, and elevated $NAD^+$/NADH and $NADP^+$/NADPH ratios, indicative of oxidative stress. Fatty acid metabolism was reprogrammed, with reduced β-oxidation, impaired lipolysis, and increased de novo lipogenesis, resulting in elevated hepatic triglycerides and cholesterol. Mitochondria exhibited bioenergetic deficits, including reduced TCA cycle and OXPHOS activity, coupled with increased mitochondrial ROS. These effects were partially iron-independent, since mice experiencing a liver iron deficiency due to hepcidin upregulation did not show the same metabolic reprogramming. Feeding Ncoa4 KO mice a Western diet further exacerbated these phenotypes.

### Summary/Conclusion

NCOA4 is critical for hepatic metabolic homeostasis, maintaining iron bioavailability to support redox balance, lipid catabolism, and mitochondrial function. Although functional iron deficiency contributes to the observed phenotype, some effects persist independently, suggesting that NCOA4 may scaffold additional pathways.

**Copyright Information:** (Online) ISSN: 2572-9241
©2026 The Author(s). HemaSphere is published by John Wiley & Sons Ltd on behalf of European Hematology Association. This is an open access Abstract Book distributed under the Attribution-NonCommercial-NoDerivs (CC BY-NC-ND), which allows third parties to download the articles and share them with others as long as they credit the author and the Abstract Book, but they cannot change the content in any way or use them commercially.
**Abstract Book Citations:** Authors, Title, HemaSphere, 2026;10:(S1):pages. The abstract book DOIs can be found on the “Author Information” page.
**Disclaimer:** Articles published in the journal HemaSphere exclusively reflect the opinions of the authors. The authors are responsible for all content in their abstracts including accuracy of the facts, statements, citing resources, etc.

### (PF1301) DARPP-32 (PPP1R1B) LIMITS TRANSFERRIN-DEPENDENT IRON UPTAKE: A NEW NODE IN IRON HOMEOSTASIS

**Topic:** 30. Iron metabolism, deficiency and overload

Pedro Fuentes*[1], Sandra M. Fernández[1], Laura Calvo López[1], Gonzalo Hernandez Viedma[1], Clara Suarez-Quintana[1, 2], Marta Ramila Carreño[1], Mayka Sanchez[1]

*[1]Universitat Internacional de Catalunya (UIC), Iron Metabolism: Regulation and Diseases, Department of Biomedical Sciences, Sant Cugat del Vallès, Spain; [2]Universitat Internacional de Catalunya (UIC), Department of Basic Sciences, Computational RNA Biology Group, Sant Cugat del Vallés, Spain;*

**Background**

Cellular iron availability is controlled by uptake through the transferrin–transferrin receptor 1 (TF–TFR1) pathway and by post-transcriptional iron sensing via the IRP/IRE network. While many components are well defined, additional layers that tune TF–TFR1 trafficking and intracellular iron supply remain unclear. PPP1R1B (DARPP-32), a phosphoregulator best known in neuronal signaling and cancer biology, has emerged as a candidate node connecting phosphorylation control with iron-linked phenotypes.

**Aims**

To determine whether DARPP-32 (PPP1R1B) impacts intracellular iron availability by modulating TF–TFR1-dependent iron acquisition, and to identify features consistent with an endocytic contribution.

**Methods**

Iron availability was modulated using ferric ammonium citrate (FAC) and deferoxamine (DFO), incorporating FAC pre-loading prior to chelation. Experiments were performed in HeLa, SH-SY5Y and HuTu-80 cells. DARPP-32 and tDARPP-32 were transiently expressed, and Thr34 variants (T34A non-phosphorylatable and T34D phosphomimetic) were included. Total cellular iron was quantified by ferrozine colorimetry (protein-normalized) and by inductively coupled plasma–optical emission spectroscopy (ICP-OES). Iron export was assessed using a doxycycline-inducible HEK-293T-TREx ferroportin-GFP system. TF–TFR1 trafficking was assessed by flow cytometry using Alexa-conjugated transferrin, quantifying internalized versus surface-bound fractions using acid stripping and temperature blockade; imatinib served as an uptake-inhibition control. Transferrin recycling was evaluated by chase kinetics, and early endosomal architecture was examined by EEA1 immunofluorescence.

**Results**

DARPP-32 (PPP1R1B) exhibited iron-responsive behavior in human cellular models, with protein levels increasing under iron supplementation and decreasing upon iron chelation, consistent with integration into iron-adaptive pathways. Across complementary functional readouts, expression of DARPP-32 isoforms was associated with a reduction in total intracellular iron content and with a context-dependent decrease in transferrin uptake, supporting the notion that DARPP-32 can act as a negative modulator of transferrin-mediated iron acquisition. Kinetic analyses were consistent with the uptake phenotype not being explained by accelerated transferrin recycling to the plasma membrane, pointing instead to alterations in internalization and/or early trafficking steps. In line with this, DARPP-32 expression was accompanied by remodeling of the early endosomal compartment, reflected by a reduction in the size of EEA1-positive early endosomal structures.

**Copyright Information:** (Online) ISSN: 2572-9241
©2026 The Author(s). HemaSphere is published by John Wiley & Sons Ltd on behalf of European Hematology Association. This is an open access Abstract Book distributed under the Attribution-NonCommercial-NoDerivs (CC BY-NC-ND), which allows third parties to download the articles and share them with others as long as they credit the author and the Abstract Book, but they cannot change the content in any way or use them commercially.
**Abstract Book Citations:** Authors, Title, HemaSphere, 2026;10:(S1):pages. The abstract book DOIs can be found on the “Author Information” page.
**Disclaimer:** Articles published in the journal HemaSphere exclusively reflect the opinions of the authors. The authors are responsible for all content in their abstracts including accuracy of the facts, statements, citing resources, etc.

**Summary/Conclusion**

DARPP-32 (PPP1R1B) can constrain TF–TFR1-dependent iron entry and reduce intracellular iron availability in a context-dependent manner, accompanied by changes in early endosome architecture. These findings position PPP1R1B at the interface of phosphoregulation, endocytic trafficking and iron homeostasis, motivating loss-of-function and pathway-level studies to define its contribution to iron-dependent phenotypes in hematologic disease.

**Copyright Information:** (Online) ISSN: 2572-9241
©2026 The Author(s). HemaSphere is published by John Wiley & Sons Ltd on behalf of European Hematology Association. This is an open access Abstract Book distributed under the Attribution-NonCommercial-NoDerivs (CC BY-NC-ND), which allows third parties to download the articles and share them with others as long as they credit the author and the Abstract Book, but they cannot change the content in any way or use them commercially.
**Abstract Book Citations:** Authors, Title, HemaSphere, 2026;10:(S1):pages. The abstract book DOIs can be found on the "Author Information" page.
**Disclaimer:** Articles published in the journal HemaSphere exclusively reflect the opinions of the authors. The authors are responsible for all content in their abstracts including accuracy of the facts, statements, citing resources, etc.

### (PF1302) FERRITIN DYNAMICS AND BURDEN OF IRON DEFICIENCY IN PREGNANCY: RESULTS OF A PAIRED, REAL-WORLD, SINGLE CENTER COHORT

**Topic:** 30. Iron metabolism, deficiency and overload

Richard C. Godby*[1], Myra Wick[1], Mingchun Liu[1], Rachael F. Grace[2], Julie Lamppa[1], Sara Gasner[1], Vanessa Torbenson[1], Katherine Talbott[1], Ronald Go[1], Meera Sridharan[1], Danette Bruns[1], Jen Burt[1], Jessica Gonzalez[1], Jamie Petsch[1], Matthew Warner[1]

*[1]Mayo Clinic, Rochester, United States of America; [2]Dana-Farber/Boston Children's Cancer and Blood Disorders Center, Boston, United States of America;*

### Background

Iron deficiency (ID) is a global epidemic with discrepant definitions and should be corrected in pregnancy to avoid adverse maternal-fetal outcomes. Current guidelines recommend only screening for ID during pregnancy in the presence of anemia despite the high prevalence of ID in reproductive-age females and availability of ferritin as a high-value biomarker. While it is known that iron requirements increase during pregnancy, the magnitude of ferritin change and burden of iron deficiency during pregnancy have not been fully defined.

### Aims

To address this gap in understanding, we aim to define the magnitude of ferritin change and describe the burden of iron deficiency in pregnancy.

### Methods

In March 2024 at the American Society of Hematology Quality Improvement Training Institute, our multidisciplinary team developed a quality improvement (QI) initiative to standardize an approach to screening for ID in pregnancy. After local dissemination and education of the standardized care pathway via patient/provider handouts and divisional presentations, obstetric providers paired ferritin testing with two standard hemoglobin assessments in early (8-12 weeks) and late (24-28 weeks) gestation to minimize lab draws and optimize logistics. The process was facilitated by modifying prenatal order sets in the electronic medical record. ID was defined as a ferritin <50 mcg/L given that hepcidin levels normalize, gastrointestinal absorption of iron decreases, and clinical fatigue improves at this threshold.

### Results

Obstetric data were captured post-intervention from March 2024 – December 2025 (n=3373 pregnancies). Of those pregnancies, 25% experienced anemia (hemoglobin <11 g/dL) despite 92% having a documented prenatal vitamin containing iron and 32% having additional oral iron supplementation.

After omitting patients with duplicative testing within the same gestational period, 33% (1129/3373) had a ferritin checked in early gestation and 56% (1905/3373) had a ferritin checked in late gestation. Further stratification of only those with paired ferritin testing in both early and late gestation resulted in a total of 708 pregnancies for evaluation.

Median ferritin decreased from 63mcg/L in early gestation to 21mcg/L in late gestation, representing a 67% decrease in ferritin during pregnancy (**Figure**) [p<0.001, Wilcoxon Signed-Rank test]. In this paired cohort, 86% (611/708) of pregnancies had ID by late gestation.

**Copyright Information:** (Online) ISSN: 2572-9241
©2026 The Author(s). HemaSphere is published by John Wiley & Sons Ltd on behalf of European Hematology Association. This is an open access Abstract Book distributed under the Attribution-NonCommercial-NoDerivs (CC BY-NC-ND), which allows third parties to download the articles and share them with others as long as they credit the author and the Abstract Book, but they cannot change the content in any way or use them commercially.
**Abstract Book Citations:** Authors, Title, HemaSphere, 2026;10:(S1):pages. The abstract book DOIs can be found on the "Author Information" page.
**Disclaimer:** Articles published in the journal HemaSphere exclusively reflect the opinions of the authors. The authors are responsible for all content in their abstracts including accuracy of the facts, statements, citing resources, etc.

**Summary/Conclusion**

ID in pregnancy is underrecognized and undertreated. There is a significant decrease in ferritin from early to late gestation, with over 80% of pregnancies being ID as they approach delivery despite almost universal iron supplementation with a prenatal vitamin and/or additional oral iron therapies. Given the potential implications for reevaluation of appropriate reference ranges and equitable prenatal care globally, additional studies are warranted to better characterize ID in pregnancy. Simultaneously, discussions are needed around current guidelines and recommendations for both screening and treating ID in pregnancy.

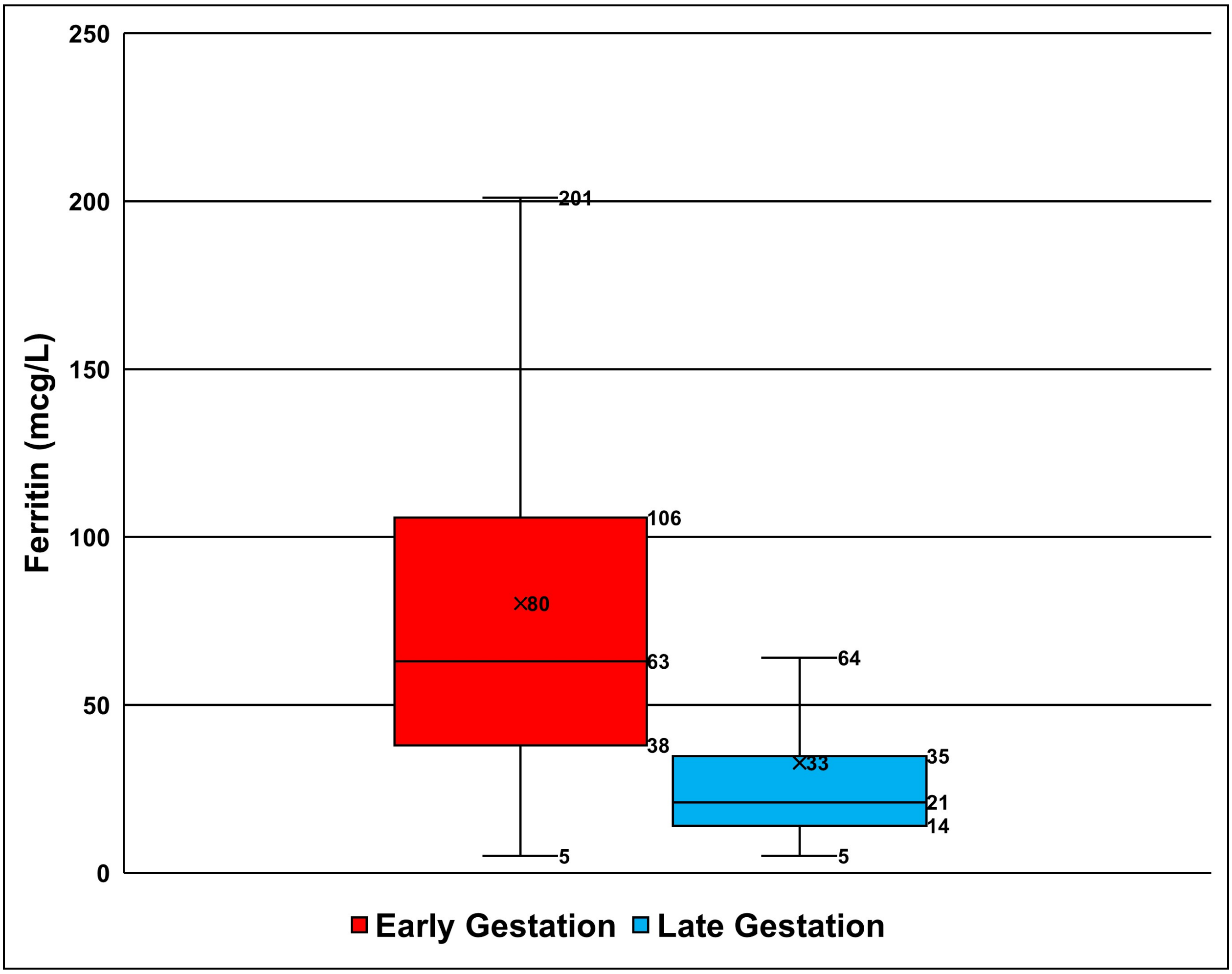

**Copyright Information:** (Online) ISSN: 2572-9241
©2026 The Author(s). HemaSphere is published by John Wiley & Sons Ltd on behalf of European Hematology Association. This is an open access Abstract Book distributed under the Attribution-NonCommercial-NoDerivs (CC BY-NC-ND), which allows third parties to download the articles and share them with others as long as they credit the author and the Abstract Book, but they cannot change the content in any way or use them commercially.
**Abstract Book Citations:** Authors, Title, HemaSphere, 2026;10:(S1):pages. The abstract book DOIs can be found on the "Author Information" page.
**Disclaimer:** Articles published in the journal HemaSphere exclusively reflect the opinions of the authors. The authors are responsible for all content in their abstracts including accuracy of the facts, statements, citing resources, etc.

### (PF1303) ERYTHROPOIETIN IS REDUCED IN HEREDITARY IRON OVERLOAD AND INVERSELY ASSOCIATED WITH IRON INDICES

**Topic:** 30. Iron metabolism, deficiency and overload

Elena Chatzikalil*[1, 2], Polyxeni Delaporta[1, 2], Konstantinos Bistas[1, 2], Stavros Glentis[1, 2], Aimilia Mantzou[1, 2], Ilona Binenbaum[1, 2], Vasiliki Chouliara[1, 2], Vangelis Mitros[1, 2], Vasiliki Maravgaki[1, 2], Efthalia-Faidra Agiomavriti-Stefanopoulou[1, 2], Sofirela Berdalli[1, 2], Dimitra Kyriakopoulou[1, 2], Ioannis Papassotiriou[1, 2], Antonis Kattamis[1, 2]

*[1]Thalassemia Unit, First Department of Pediatrics, National and Kapodistrian University of Athens, Athens, Greece;[2]"Aghia Sophia" Children's Hospital, ERN-EuroBloodNet Center, Athens, Greece;*

### Background

Erythropoietin (EPO), the stimulator of red blood cell (RBC) production, and iron, essential for hemoglobin (Hb) formation, are main components of erythropoiesis. The stimulatory effects of excess EPO levels on erythropoiesis have been well evaluated, however, the effects of excess iron on EPO regulation remain insufficiently characterized, with conflicting evidence reported.

### Aims

To evaluate EPO levels in patients (pts) with primary iron overload and to investigate potential correlations with other biochemical markers of iron overload and erythropoiesis.

### Methods

20 individuals (19 families) (15 males, 5 females) aged 23.6±18.4 years, with primary iron overload (Hereditary Hemochromatosis (HH) type 1: 5 pts, HH type 2A: 6 pts, Ferroportin disease: 3 pts, HH heterozygotes: 7 pts (*HFE*:4, *STAB1*:1, *TFR2*:1)) were included. Complete hematology and biochemistry assays, to exclude chronic inflammation were performed. Iron metabolism was assessed by serum ferritin (SF), iron and transferrin saturation (TSAT). Soluble transferrin receptor (sTfR) was measured by enzyme-linked immunosorbent assay (ELISA) and compared to aged-matched controls. Genetic analysis for iron overload was performed by Next Generation Sequencing (NGS) targeted panel (Illumina MiSeq) followed by Sanger sequencing for confirming NGS identified mutations. EPO concentration was measured by chem-iluminescent technique (Immulite 2000, Siemens). Statistical analysis was performed using RStudio v.4.4.1. Values of $p \leq 0.05$ were considered statistically significant.

### Results

EPO (mIU/mL) 13.9±19.8 and sTfR (mg/L) 1.3±0.9 were significantly decreased ($p<0.01$ and $p<0.05$ respectively) compared with healthy controls, while mean SF (ng/dL) 1528,5±1022,6, iron (g/dL) 169.5±84.1 and TSAT (%) 57.3±26.8, were significantly ($p<0.05$) elevated compared with the reference values. Mean values for Hb (g/dL) and RBC were 12.2±1.9 4.6±0.6 x$10^3$ respectively, with 5 pts being anemic. A strong significant negative correlation between EPO and serum iron ($p<0.001$) and TSAT ($p<0.001$), and a strong significant positive correlation between EPO and sTfR ($p<0.05$) were observed. sTfR was significantly negatively correlated with serum iron ($p<0.001$) and TSAT ($p<0.05$). HH (type 1 and 2A) homozygotes had significantly higher SF ($p=0.05$) and TSAT ($p<0.01$) levels and significantly lower EPO levels ($p<0.001$), compared with other pts groups.

**Copyright Information:** (Online) ISSN: 2572-9241
©2026 The Author(s). HemaSphere is published by John Wiley & Sons Ltd on behalf of European Hematology Association. This is an open access Abstract Book distributed under the Attribution-NonCommercial-NoDerivs (CC BY-NC-ND), which allows third parties to download the articles and share them with others as long as they credit the author and the Abstract Book, but they cannot change the content in any way or use them commercially.
**Abstract Book Citations:** Authors, Title, HemaSphere, 2026;10:(S1):pages. The abstract book DOIs can be found on the "Author Information" page.
**Disclaimer:** Articles published in the journal HemaSphere exclusively reflect the opinions of the authors. The authors are responsible for all content in their abstracts including accuracy of the facts, statements, citing resources, etc.

**Summary/Conclusion**

Our study demonstrates that primary iron overload is associated with low circulating EPO levels, indicating a relative suppression of erythropoietic drive, possibly based on current decreased hematopoietic needs. We further suggest EPO has a negative effect on the probands' erythropoietic activity, based on its strong positive correlation with sTfR. Remarkably, pts with HH type 1 and 2A who have significant higher iron load compared to the other groups, present a pronounced EPO reduction. The exact clinical significance of iron overload-related genetic alterations in decreasing EPO remains uncertain, however, the strong negative effect of *HFE* and *HJV* alterations on EPO levels might provide the basis for further research into the potential independent influence of the *HFE/HJV*-related pathways on EPO regulation.

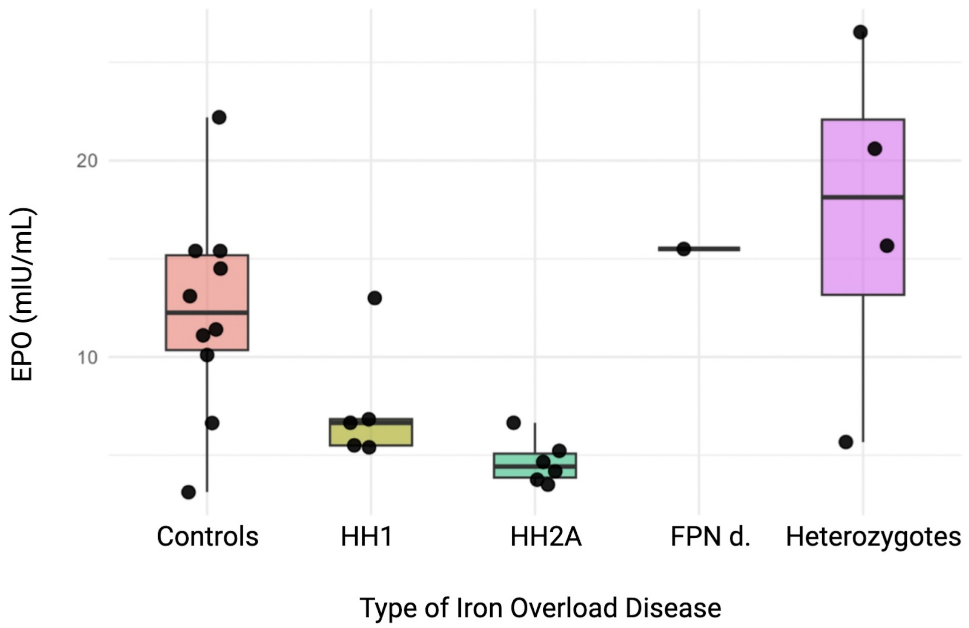

**Figure 1.** Comparison of EPO (mIU/mL) levels among patients with different types of primary iron overload disease and healthy controls. EPO levels are decreased in homozygous patients with hereditary diseases of iron overload (HH type 1 and 2A). [EPO: erythropoietin, FPN d.: ferroportin disease, HH1: hereditary hemochromatosis type 1, HH2A: hereditary hemochromatosis type 2A]

**Copyright Information:** (Online) ISSN: 2572-9241
©2026 The Author(s). HemaSphere is published by John Wiley & Sons Ltd on behalf of European Hematology Association. This is an open access Abstract Book distributed under the Attribution-NonCommercial-NoDerivs (CC BY-NC-ND), which allows third parties to download the articles and share them with others as long as they credit the author and the Abstract Book, but they cannot change the content in any way or use them commercially.
**Abstract Book Citations:** Authors, Title, HemaSphere, 2026;10:(S1):pages. The abstract book DOIs can be found on the "Author Information" page.
**Disclaimer:** Articles published in the journal HemaSphere exclusively reflect the opinions of the authors. The authors are responsible for all content in their abstracts including accuracy of the facts, statements, citing resources, etc.

### (PF1304) CLINICAL CHARACTERISTICS AND NOVEL PREDICTORS OF IRON DEPOSITION IN CHINESE PATIENTS WITH UNEXPLAINED HYPERFERRITINEMIA: A SINGLE-CENTER RETROSPECTIVE STUDY

**Topic:** 30. Iron metabolism, deficiency and overload

Junyu Zhang*[1], Sichun Xiang[1], Cangjian Zhang[1], Jinjian Lan[1]

[1]*Lishui Central Hospital, Department of Hematology, Lishui, China;*

### Background

Serum ferritin, a key marker of body iron stores, is commonly elevated in conditions such as liver disease, malignancy, and metabolic syndrome. However, a subset of patients exhibits persistent hyperferritinemia without an identifiable underlying cause, classified as "unexplained hyperferritinemia." These individuals may be at risk for occult iron overload. Currently, there is a paucity of systematic data on the clinical characteristics and actual prevalence of iron overload in this population within China.

### Aims

This study aimed to characterize the clinical profile of patients with unexplained hyperferritinemia and to identify clinical and laboratory features associated with the progression from hyperferritinemia to iron deposition disease.

### Methods

This retrospective analysis included 14,667 individuals who underwent health screening between June 1, 2024, and May 31, 2025, after excluding secondary causes of elevated ferritin. From those with ferritin >600 ng/mL, 56 were randomly selected for in-depth evaluation. Data collected included lifestyle factors, laboratory parameters, hepatic and cardiac magnetic resonance imaging.

### Results

Among the 14,667 participants (8,097 males; median age 49 years), the median serum ferritin level was 201.5 ng/mL (range 2–1675.6). 3,505 had ferritin levels of 300–600 ng/mL, 713 had 600–1000 ng/mL, and 178 had ≥1000 ng/mL. Male sex, urea, total cholesterol, high-density lipoprotein, fasting glucose, gamma-glutamyl transferase and aspartate aminotransferase ($P < 0.001$), creatinine ($P = 0.002$), triglycerides ($P = 0.005$), low-density lipoprotein ($P = 0.002$) as independent risk factors for ferritin ≥1000 ng/mL.
In the randomly selected subcohort with ferritin >600 ng/mL (n = 56; 53 males; median age 58 years, the median ferritin level was 1178.6 ng/mL. MRI revealed organ iron deposition in 22 individuals (39.3%): cardiac deposition in 16, hepatic deposition in 15, and splenic deposition in 2. Whole-exome sequencing (n = 9) identified variants in iron metabolism-related genes (HFE, APOB, STAB1, ABCG8) in 6 cases. Regression analysis identified LDL/AFP ($P = 0.001$), TG/AFP ($P = 0.008$), FIB/AFP ($P = 0.043$), TC/AFP ($P = 0.047$), TC/CA199 ($P = 0.048$), AFP/Ca ($P = 0.004$), AFP/K ($P = 0.032$), HDL/Mg ($P = 0.016$), Na ($P = 0.003$), and P ($P = 0.018$) as independent risk factors for cardiac iron deposition. ROC analysis for LDL/AFP yielded an optimal cutoff of 1.139 for predicting cardiac iron deposition (sensitivity 66.7%, specificity 85.0%). Independent risk factors for hepatic iron deposition included age/ALP ($P = 0.005$), TG/P ($P = 0.005$), TG/Mg ($P = 0.035$), TC/CEA ($P = 0.037$), ALP/HA ($P = 0.044$), BUN/CEA ($P = 0.037$), and HA ($P = 0.049$). The optimal cutoff for age/ALP in predicting hepatic iron deposition was 0.867 (sensitivity 90.9%, specificity 61.4%).

**Copyright Information:** (Online) ISSN: 2572-9241
©2026 The Author(s). HemaSphere is published by John Wiley & Sons Ltd on behalf of European Hematology Association. This is an open access Abstract Book distributed under the Attribution-NonCommercial-NoDerivs (CC BY-NC-ND), which allows third parties to download the articles and share them with others as long as they credit the author and the Abstract Book, but they cannot change the content in any way or use them commercially.
**Abstract Book Citations:** Authors, Title, HemaSphere, 2026;10:(S1):pages. The abstract book DOIs can be found on the "Author Information" page.
**Disclaimer:** Articles published in the journal HemaSphere exclusively reflect the opinions of the authors. The authors are responsible for all content in their abstracts including accuracy of the facts, statements, citing resources, etc.

Summary/Conclusion

Unexplained hyperferritinemia is highly prevalent in the healthy population undergoing routine health examinations. Marked hyperferritinemia (≥1000 ng/mL) is predominantly associated with male sex and underlying metabolic dysregulation. Among individuals with serum ferritin levels exceeding 600 ng/mL, the prevalence of organ iron deposition is substantial (39.3%). An LDL/AFP ratio >1.139 and an age/ALP ratio >0.86 serve as useful predictors for cardiac and hepatic iron deposition, respectively. These readily accessible biomarkers may facilitate early identification of individuals at risk for iron overload and could guide targeted clinical surveillance and intervention strategies.

**Copyright Information:** (Online) ISSN: 2572-9241
©2026 The Author(s). HemaSphere is published by John Wiley & Sons Ltd on behalf of European Hematology Association. This is an open access Abstract Book distributed under the Attribution-NonCommercial-NoDerivs (CC BY-NC-ND), which allows third parties to download the articles and share them with others as long as they credit the author and the Abstract Book, but they cannot change the content in any way or use them commercially.
**Abstract Book Citations:** Authors, Title, HemaSphere, 2026;10:(S1):pages. The abstract book DOIs can be found on the “Author Information” page.
**Disclaimer:** Articles published in the journal HemaSphere exclusively reflect the opinions of the authors. The authors are responsible for all content in their abstracts including accuracy of the facts, statements, citing resources, etc.

### (PF1305) THE FERROCLEAR PHASE 2, MULTICENTER, RANDOMIZED, DOUBLE-BLIND STUDY: ADDRESSING PATIENT NEEDS IN HEREDITARY HEMOCHROMATOSIS THROUGH A NOVEL TARGETED APPROACH

**Topic:** 30. Iron metabolism, deficiency and overload

Domenico Girelli*[1], Deya Cherpokova,[2], Sonya Abraham,[3], Gregory Kato[3], Jens-Alexander Fuchs,[4], Heng Zou,[3], Vania Manolova,[5], Nataliya Doliba,[3], Charlotte Pollet,[6], Kris Kowdley,[7], Uta Merle,[8], John Olynyk,[9], Jeremy Shearman[10]

*[1]Department of Medicine, Section of Internal Medicine and EuroBloodNet Referral Center, University of Verona and Azienda Ospedaliera Universitaria Integrata of Verona, Verona, Italy; [2]CSL Innovation GmbH, Marburg, Germany; [3]CSL Behring, King of Prussia, Pennsylvania, United States of America; [4]CSL Behring, Glattbrugg, Switzerland; [5]CSL Behring, Schlieren, Switzerland; [6]CSL Vifor, Glattbrugg, Switzerland; [7]Liver Institute Northwest, Elson S. Floyd College of Medicine, Washington State University, Seattle, Washington, United States of America; [8]Department of Internal Medicine IV, Gastroenterology & Hepatology, Medical University of Heidelberg, Heidelberg, Germany; [9]Curtin Medical Research Institute, Curtin University, Bentley, and Fiona Stanley Hospital, Murdoch, Western Australia, Australia; [10]Department of Gastroenterology, South Warwickshire University NHS Foundation Trust and Warwick Medical School, Warwick, United Kingdom;*

### Background

Hereditary hemochromatosis (HH) is a genetic disorder caused by mutations in the homeostatic iron regulator (*HFE*) gene that result in reduced levels of circulating hepcidin. Hepcidin is the central regulator of systemic iron metabolism, and its deficiency in HH results in excessive dietary iron absorption and progressive iron accumulation in several organs. This can lead to significant complications over time, including liver disease (cirrhosis and hepatocellular carcinoma), arthritis, diabetes mellitus, and cardiac failure. Therapeutic phlebotomy (venesection) is the current accepted treatment to reduce the consequences of iron overload. However, adherence to guideline-recommended phlebotomy frequency can be suboptimal due to patient intolerance, insufficient therapeutic response, or interference with daily activities. Iron chelation therapy and erythrocytapheresis are recommended when phlebotomy is not suitable but have significant side effects. Currently, no targeted oral therapies are available for HH. Due to the pivotal role of the hepcidin-ferroportin (FPN) axis in iron homeostasis, FPN represents a potential therapeutic target in HH. Vamifeport is a small-molecule, oral FPN inhibitor that acts as a hepcidin mimetic and has been shown to reduce dietary iron absorption and restore balanced iron distribution in organs.

### Aims

To provide proof-of-concept that vamifeport can reduce liver iron overload in patients with *HFE*-HH.

### Methods

This phase 2, multicenter, randomized, placebo-controlled, double-blind study (ClinicalTrials.gov: NCT07332091; FERROCLEAR) is being conducted in adult patients (≥18 years) with a confirmed diagnosis of *HFE*-HH and documented evidence of iron overload. Eligible patients are randomized to receive either vamifeport (low or high dose) or a matching placebo for 12 months. The primary endpoint is the reduction in liver iron concentration as measured by magnetic resonance imaging (MRI) using FerriScan® at 12 months of treatment. FerriScan® is a standardized, United States Food and Drug Administration (FDA)-approved, European (CE)-marked method of using MRI scanners to measure liver iron concentration. Secondary endpoints include safety, pharmacokinetics, changes in iron parameters, and patient-reported outcomes.

**Copyright Information:** (Online) ISSN: 2572-9241
©2026 The Author(s). HemaSphere is published by John Wiley & Sons Ltd on behalf of European Hematology Association. This is an open access Abstract Book distributed under the Attribution-NonCommercial-NoDerivs (CC BY-NC-ND), which allows third parties to download the articles and share them with others as long as they credit the author and the Abstract Book, but they cannot change the content in any way or use them commercially.
**Abstract Book Citations:** Authors, Title, HemaSphere, 2026;10:(S1):pages. The abstract book DOIs can be found on the "Author Information" page.
**Disclaimer:** Articles published in the journal HemaSphere exclusively reflect the opinions of the authors. The authors are responsible for all content in their abstracts including accuracy of the facts, statements, citing resources, etc.

### Results

The study is currently active and plans to recruit in 18 countries (details available in Figure 1). The recruitment status and site locations are available at ClinicalTrials.gov (NCT07332091).

### Summary/Conclusion

Vamifeport is a potential oral alternative to existing therapies that could provide a more targeted approach to iron regulation in patients with *HFE*-HH. The FERROCLEAR study will provide data on whether modulating the FPN axis with a targeted pharmacologic approach can effectively reduce liver iron concentration and improve patient outcomes.

**Figure 1.** The global recruitment sites for the FERROCLEAR phase 2 study in patients with *HFE*-HH

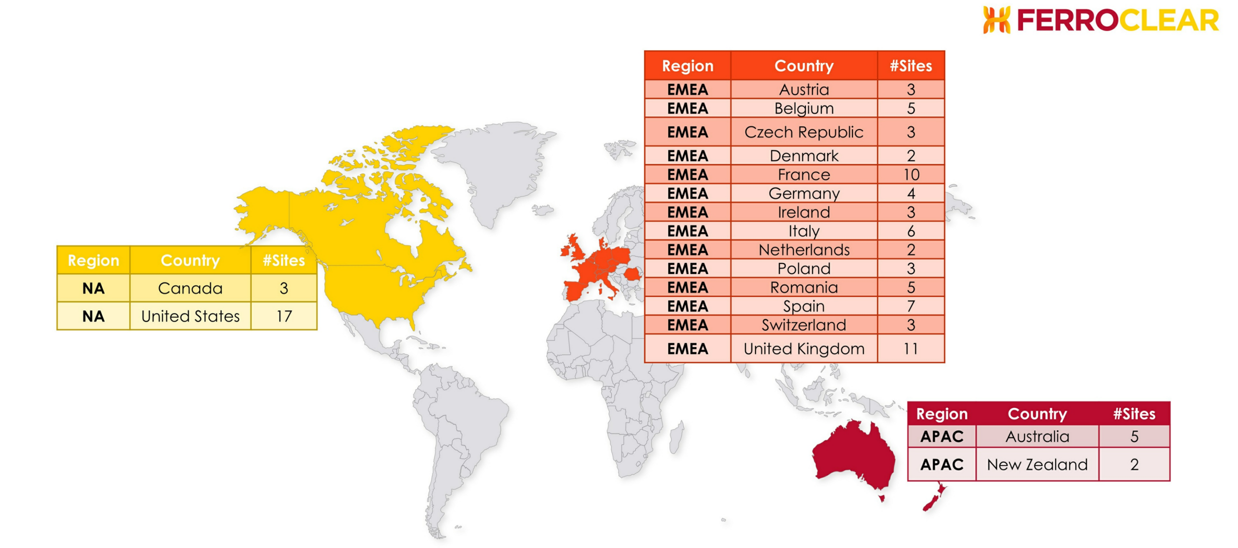

| Region | Country | #Sites |
|---|---|---|
| NA | Canada | 3 |
| NA | United States | 17 |

| Region | Country | #Sites |
|---|---|---|
| EMEA | Austria | 3 |
| EMEA | Belgium | 5 |
| EMEA | Czech Republic | 3 |
| EMEA | Denmark | 2 |
| EMEA | France | 10 |
| EMEA | Germany | 4 |
| EMEA | Ireland | 3 |
| EMEA | Italy | 6 |
| EMEA | Netherlands | 2 |
| EMEA | Poland | 3 |
| EMEA | Romania | 5 |
| EMEA | Spain | 7 |
| EMEA | Switzerland | 3 |
| EMEA | United Kingdom | 11 |

| Region | Country | #Sites |
|---|---|---|
| APAC | Australia | 5 |
| APAC | New Zealand | 2 |

The study details are available at ClinicalTrials.gov (NCT07332091).

**Copyright Information:** (Online) ISSN: 2572-9241
©2026 The Author(s). HemaSphere is published by John Wiley & Sons Ltd on behalf of European Hematology Association. This is an open access Abstract Book distributed under the Attribution-NonCommercial-NoDerivs (CC BY-NC-ND), which allows third parties to download the articles and share them with others as long as they credit the author and the Abstract Book, but they cannot change the content in any way or use them commercially.
**Abstract Book Citations:** Authors, Title, HemaSphere, 2026;10:(S1):pages. The abstract book DOIs can be found on the "Author Information" page.
**Disclaimer:** Articles published in the journal HemaSphere exclusively reflect the opinions of the authors. The authors are responsible for all content in their abstracts including accuracy of the facts, statements, citing resources, etc.

### (PF1306) ORDINAL LOGISTIC REGRESSION MODEL PREDICTING HEPATIC IRON OVERLOAD BASED ON FERRITIN AND TSAT IN PATIENTS WITH HYPERFERRITINEMIA AND HFE MUTATIONS WITHOUT HEREDITARY HEMOCHROMATOSIS

**Topic:** 30. Iron metabolism, deficiency and overload

Iván Andreu Vilarroig*[1], Nuria Claros Barrachina[1], María Mas Esteve[1], Katiusca Buelvas de la Ossa[1], Laura Belenguer de la Asunción[1], Víctor Blasco Ibáñez[1], Dolores Beltrán Archilés[1], Anna Ferrer Rodrigo[1], David Arias Ibáñez[1], Alexandra Alinyo Dies[1], Clara Espinosa Arnandis[1], Mónica Lisseth Torres MacIas[1], Ana Facal Malvar[1], Luís Serrano Picazo[1], Patricia Lizama Pérez[1], Carolina Cañigral Ortiz[1], Manuel Fernández-Delgado Momparler[1], Eva Mas Esteve[1], Raimundo Garcia Boyero[1]

*[1]General University Hospital of Castellon, Castelló de la Plana, Spain;*

### Background

Hepatic magnetic resonance imaging (liver MRI) is the most widely used technique to assess hepatic iron overload in patients with hyperferritinemia and plays a key role in treatment decision-making (1). In the absence of other predictive factors, its use may currently be overindicated.

### Aims

To predict the degree of hepatic iron overload assessed by liver MRI based on predictive factors in patients with hyperferritinemia with a genetic component without hereditary hemochromatosis.

### Methods

Retrospective study of patients with hyperferritinemia and non–C282Y homozygous HFE mutations seen in the Hematology outpatient clinics of Hospital General de Castellón in 2023, who underwent liver MRI and laboratory testing up to 3 months before or after imaging, without acute illness or prior phlebotomy or erythroapheresis. Data were extracted from electronic medical records (OrionClinic®). The degree of hepatic iron overload was categorized using the Osatek-SEDIA method (2). Its association with ferritin (ng/mL), transferrin saturation (TSAT, %), hypertransaminasemia, steatosis, chronic liver disease, age, sex, and active alcohol use was analyzed (3). The prediction of iron hepatic overload was made using an ordinal logistic regression model in RStudio®.
The Brant–Wald test was performed to assess the proportional odds assumption. Predictive performance was evaluated using global accuracy metrics and comparison with the No Information Rate (NIR). Heatmaps were generated to visualize the estimated probability of belonging to each hepatic iron overload category according to ferritin and TSAT levels.

**Copyright Information:** (Online) ISSN: 2572-9241
©2026 The Author(s). HemaSphere is published by John Wiley & Sons Ltd on behalf of European Hematology Association. This is an open access Abstract Book distributed under the Attribution-NonCommercial-NoDerivs (CC BY-NC-ND), which allows third parties to download the articles and share them with others as long as they credit the author and the Abstract Book, but they cannot change the content in any way or use them commercially.
**Abstract Book Citations:** Authors, Title, HemaSphere, 2026;10:(S1):pages. The abstract book DOIs can be found on the “Author Information” page.
**Disclaimer:** Articles published in the journal HemaSphere exclusively reflect the opinions of the authors. The authors are responsible for all content in their abstracts including accuracy of the facts, statements, citing resources, etc.

### Results

A total of 137 patients were included. Median age was 65 years (22–87). Of these, 85.4% were men and 14.6% were women. Two patients had died at the time of analysis.
There was a statistically significant correlation between the degree of iron overload and ferritin (rho = 0.524; $p < 0.000001$), TSAT (rho = 0.33; $p = 0.00006$), and hypertransaminasemia ($p = 0.04$). Other variables, including age, sex, HFE genotype, alcohol consumption, hepatic steatosis, and chronic liver disease, did not show significant associations.
The final ordinal logistic regression model included ferritin and TSAT as predictors, as hypertransaminasemia was ultimately excluded due to its low impact (AIC = 250.4 vs. 250.8).
**The model estimated that for each 1 ng/mL increase in ferritin, the probability of being in a higher iron overload category increased by 0.43%, and for each 1% increase in TSAT, the probability increased by 4.4%.** Probability tables were created to reflect the likelihood of belonging to each category (image 1).
The proportional odds assumption was confirmed, and internal validation showed an overall accuracy of 63%, significantly higher than the NIR ($p = 0.002$).

### Summary/Conclusion

- Serum ferritin and TSAT were significantly associated with the degree of hepatic iron overload on MRI in patients with genetically driven hyperferritinemia other than C282Y homozygosity.
- An ordinal logistic regression model accurately estimated the probability of each hepatic iron overload category, enabling the creation of probability tables.
- These tools may help clinicians optimize the selection of patients for hepatic MRI, improving resource use.
- Model interpretation is limited by the retrospective design, and prospective validation is still required.

**Copyright Information:** (Online) ISSN: 2572-9241
©2026 The Author(s). HemaSphere is published by John Wiley & Sons Ltd on behalf of European Hematology Association. This is an open access Abstract Book distributed under the Attribution-NonCommercial-NoDerivs (CC BY-NC-ND), which allows third parties to download the articles and share them with others as long as they credit the author and the Abstract Book, but they cannot change the content in any way or use them commercially.
**Abstract Book Citations:** Authors, Title, HemaSphere, 2026;10:(S1):pages. The abstract book DOIs can be found on the "Author Information" page.
**Disclaimer:** Articles published in the journal HemaSphere exclusively reflect the opinions of the authors. The authors are responsible for all content in their abstracts including accuracy of the facts, statements, citing resources, etc.

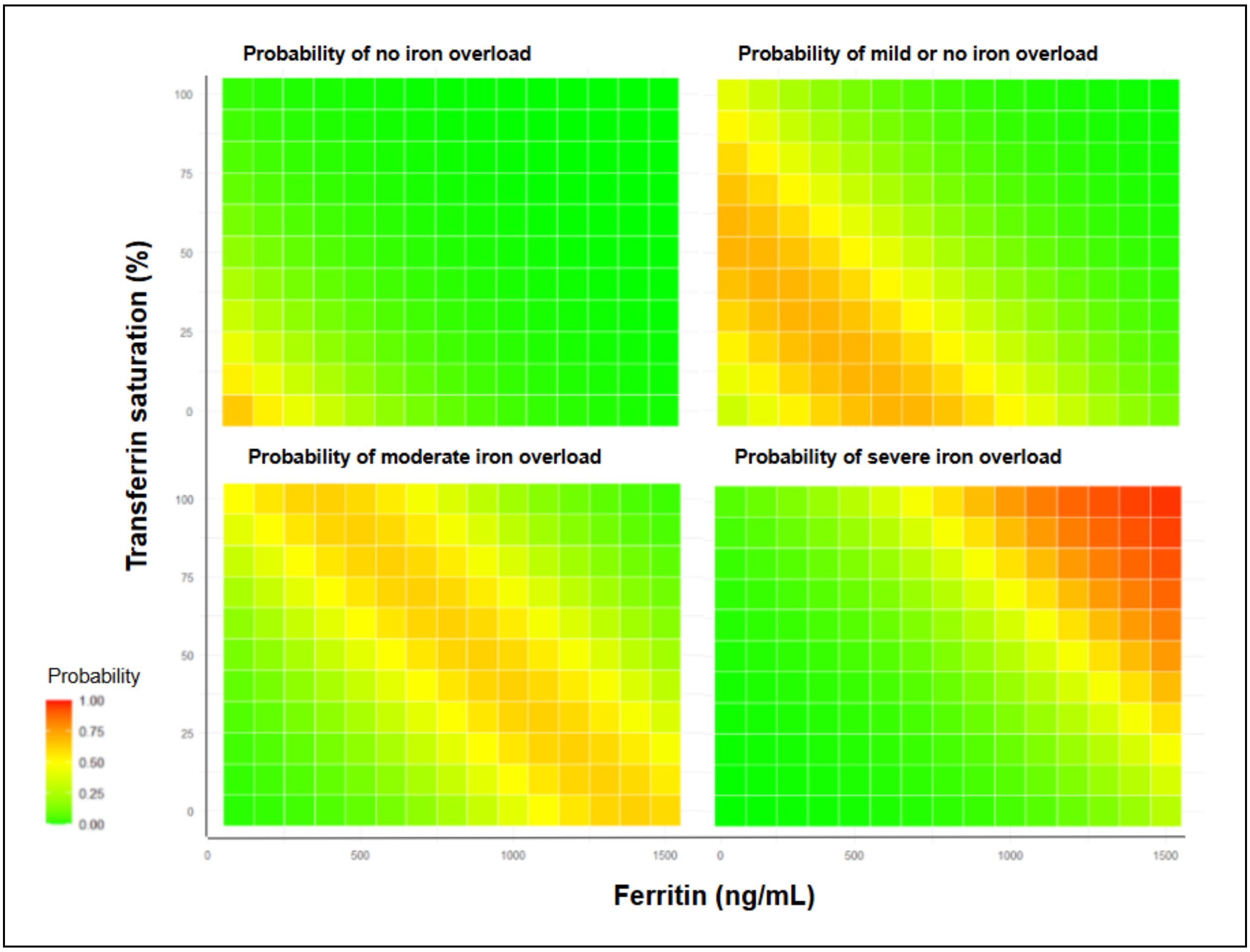

**Copyright Information:** (Online) ISSN: 2572-9241
©2026 The Author(s). HemaSphere is published by John Wiley & Sons Ltd on behalf of European Hematology Association. This is an open access Abstract Book distributed under the Attribution-NonCommercial-NoDerivs (CC BY-NC-ND), which allows third parties to download the articles and share them with others as long as they credit the author and the Abstract Book, but they cannot change the content in any way or use them commercially.
**Abstract Book Citations:** Authors, Title, HemaSphere, 2026;10:(S1):pages. The abstract book DOIs can be found on the “Author Information” page.
**Disclaimer:** Articles published in the journal HemaSphere exclusively reflect the opinions of the authors. The authors are responsible for all content in their abstracts including accuracy of the facts, statements, citing resources, etc.

### (PF1307) MANGANESE DEFICIENCY INDEPENDENTLY CONTRIBUTES TO CHRONIC ANEMIA IN ADULTS

**Topic:** 30. Iron metabolism, deficiency and overload

Karina Basmajian*[1], Aren Dermarderosian[2], Andreas Lau[3], Kevin Bera[3], Daniel Park[4], Alec Chan-Golston[5], Camron Farjami[6], Arya Mostofi[7], Elham Akhtari[8], Sarmen Sarkissian[9], Mojtaba Akhtari[3]

*[1]Arrowhead Regional Medical Center, Colton, United States of America;[2]University of California, Los Angeles, Los Angeles, United States of America; [3]Loma Linda University Medical Center, Loma Linda, United States of America;[4]Harbor-UCLA Medical Center, Torrance, United States of America; [5]University of California, Merced, Merced, United States of America;[6]Palos Verdes Peninsula High School, Palos Verdes, United States of America; [7]7University of California, San Diego, San Diego, United States of America; [8]School of Persian Medicine, Iran University of Medical Sciences, Tehran, Iran;[9]Ventura County Medical Center, Ventura, United States of America;*

**Background**

Chronic anemia affects nearly 10% of U.S. adults and is commonly attributed to iron, folate, or vitamin B12 deficiency. When common deficiencies are ruled out, consideration of less frequently assessed trace elements may be warranted given their role in erythropoiesis. Manganese (Mn) is one such element, contributing to erythropoiesis through antioxidant defense via manganese superoxide dismutase (MnSOD) and regulation of hypoxia-inducible factor (HIF) pathway, which influences erythropoietin transcription. Despite this, the impact of Mn deficiency in chronic anemia remains unclear.

**Aims**

To evaluate whether Mn deficiency is associated with anemia and whether it occurs independently of other micronutrient deficiencies and hematologic diseases.

**Methods**

We conducted a retrospective single-center analysis of 202 adult patients (median age 67 years; range 20-96) with measured serum Mn levels from an outpatient hematology clinic between October 2019 to December 2024. Mn deficiency was defined as ≤ 0.6 µg/L. Data collected include demographics, hematologic diagnoses, chronic kidney disease, albumin, and hemoglobin (Hgb). Additional deficiencies were defined as iron (ferritin < 30 ng/mL, transferrin saturation < 20%), copper (copper level < 73 mcg/dL), zinc (zinc < 60 µg/dL), selenium (selenium < 10 µg/dL), and chromium (chromium < 1.4 µg/dL). Statistical analyses included Fisher's exact test, chi-square test, and logistic regression to evaluate independent associations.

**Results**

Mn deficiency was present in 76.7% (n=155) of patients. Within the Mn-deficient group, 78.7% (n=122) had anemia (Hgb < 12 g/dL in women, Hgb < 13 g/dL in men). Patients ≥ 69 years had 2.49 times higher odds of Mn deficiency (95% CI: 1.24-5.02, *p*=0.010). Low hemoglobin (*p* <0.001) and diagnosis of multiple myeloma (*p*=0.028) were independently associated with Mn deficiency. Iron deficiency prevalence did not differ significantly between Mn-replete and Mn-deficient groups (43.12% vs 43.01%, *p*=0.238). No significant associations were found between Mn deficiency and other trace element deficiencies, including copper (*p*=0.843), zinc (*p*=0.624), and selenium (*p*=0.323). Sensitivity analysis excluding anemic patients yielded similar non-significant results for iron deficiency (OR=1.55, 95% CI: 0.43-5.60, *p*=0.506).

**Copyright Information:** (Online) ISSN: 2572-9241
©2026 The Author(s). HemaSphere is published by John Wiley & Sons Ltd on behalf of European Hematology Association. This is an open access Abstract Book distributed under the Attribution-NonCommercial-NoDerivs (CC BY-NC-ND), which allows third parties to download the articles and share them with others as long as they credit the author and the Abstract Book, but they cannot change the content in any way or use them commercially.
**Abstract Book Citations:** Authors, Title, HemaSphere, 2026;10:(S1):pages. The abstract book DOIs can be found on the "Author Information" page.
**Disclaimer:** Articles published in the journal HemaSphere exclusively reflect the opinions of the authors. The authors are responsible for all content in their abstracts including accuracy of the facts, statements, citing resources, etc.

**Summary/Conclusion**

Mn deficiency is prevalent in adults and may represent an underrecognized factor in chronic anemia, particularly when common causes have been ruled out. We found no significant associations between Mn deficiency and deficiencies in other trace elements, suggesting Mn deficiency may contribute to anemia through independent mechanisms. The significant association with multiple myeloma is notable and warrants further investigation. Older age predicted Mn deficiency, even after adjusting for confounders, possibly reflecting age-related changes in intake, absorption, or physiological requirements. These findings highlight the importance of evaluating Mn levels in patients with chronic anemia, especially among older adults or those with known risk factors for trace element deficiencies. Future prospective and mechanistic studies are warranted to better understand the role of Mn deficiency in anemia.

**Copyright Information:** (Online) ISSN: 2572-9241
©2026 The Author(s). HemaSphere is published by John Wiley & Sons Ltd on behalf of European Hematology Association. This is an open access Abstract Book distributed under the Attribution-NonCommercial-NoDerivs (CC BY-NC-ND), which allows third parties to download the articles and share them with others as long as they credit the author and the Abstract Book, but they cannot change the content in any way or use them commercially.
**Abstract Book Citations:** Authors, Title, HemaSphere, 2026;10:(S1):pages. The abstract book DOIs can be found on the "Author Information" page.
**Disclaimer:** Articles published in the journal HemaSphere exclusively reflect the opinions of the authors. The authors are responsible for all content in their abstracts including accuracy of the facts, statements, citing resources, etc.

### (PF1308) IDENTIFYING MF PATIENTS AT HIGH INFECTION RISK DURING RUXOLITINIB: THE HIDDEN ROLE OF TRANSFUSIONS, IRON OVERLOAD, AND INFLAMMATORY STATUS

**Topic:** 31. Infections in hematology (incl. supportive care/therapy)

Francesca Palandri*[1], Chiara Sartor[1], Filippo Branzanti[2], Massimo Breccia[3], Massimiliano Bonifacio[4], Elena Elli[5], Elisabetta Abruzzese[6], Mario Tiribelli[7], Erika Morsia[8], Mirko Farina[9], Giulia Benevolo[10], Eloise Beggiato[11], Novella Pugliese[12], Alessia Tieghi[13], Emilia Scalzulli[3], Gianni Binotto[14], Francesco Cavazzini[15], Alessandra Iurlo[16], Monica Crugnola[17], Andrea Duminuco[18], Alessandra Dedola[1, 2], Flavia Salvatore[1, 2], Daniele Cavallina[1, 2], Nicola Severi[1, 2], Daniele Cattaneo[16], Bruno Martino[19], Daniela Cilloni[20], Monica Bocchia[21], Fabrizio Pane[12], Florian Heidel[22], Giuseppe Palumbo[23], Giovanni Caocci[24]

*[1]IRCCS Azienda Ospedaliero-Universitaria di Bologna, Bologna, Italy;[2]Università di Bologna, Dipartimento di Scienze Mediche e Chirurgiche, Bologna, Italy; [3]Az. Policlinico Umberto I-Sapienza University, Hematology, Department of Translational and Precision Medicine, Roma, Italy; [4]University of Verona, Department of Engineering for Innovation Medicine, Section of Innovation Biomedicine, Hematology Area, Verona, Italy; [5]Fondazione IRCCS San Gerardo dei Tintori, Divisione di ematologia e unità trapianto di midollo, Monza, Italy; [6]Ospedale S. Eugenio, Division of Hematology, Roma, Italy;[7]University of Udine, Division of Hematology and BMT, Department of Medical Area, Udine, Italy; [8]Università Politecnica delle Marche, Hematology Unit, Department of Clinical and Molecular Sciences, DISCLIMO, Ancona, Italy; [9]University of Brescia, ASST Spedali Civili of Brescia, Unit of Blood Diseases and Stem Cells Transplantation, Department of Clinical and Experimental Sciences, Brescia, Italy; [10]Città della Salute e della Scienza Hospital, University Hematology Division, Torino, Italy; [11]University of Torino, Unit of Hematology, Department of Oncology, Torino, Italy; [12]Federico II University Medical School, Department of Clinical Medicine and Surgery, Napoli, Italy; [13]Azienda USL - IRCCS di Reggio Emilia, Reggio Emilia, Department of Hematology, Reggio Emilia, Italy; [14]University of Padova, Unit of Hematology and Clinical Immunology, Padova, Italy; [15]University of Ferrara, Division of Hematology, Ferrara, Italy; [16]Foundation IRCCS Ca' Granda Ospedale Maggiore Policlinico, Hematology Division, Milano, Italy; [17]Azienda Ospedaliero-Universitaria di Parma, Haematology and BMT Centre, Parma, Italy;[18]AOU Policlinico "G. Rodolico" – San Marco, UO Ematologia, Catania, Italy; [19]Azienda Ospedaliera 'Bianchi Melacrino Morelli', Division of Hematology, Reggio Calabria, Italy; [20]University of Turin, Department of Clinical and Biological Sciences, Torino, Italy;[21]Azienda Ospedaliera Universitaria Senese, University of Siena, Hematology Unit, Siena, Italy; [22]Hannover Medical School (MHH), Hematology, Hemostasis, Oncology and Stem Cell Transplantation, Hannover, Germany; [23]University of Catania, Department of Scienze Mediche, Chirurgiche e Tecnologie Avanzate "G.F. Ingrassia", Catania, Italy; [24]Ospedale Businco, Università degli studi di Cagliari, Ematologia, Cagliari, Italy;*

### Background

Infections are a lead cause of morbidity and mortality in myelofibrosis (MF) patients (pts). Red blood cell (RBC) transfusions, iron overload (IO), and systemic inflammation—reflected by an elevated neutrophil-to-lymphocyte ratio (NLR)—are associated with increased infection risk.  Their impact has not been evaluated in a cohort homogeneously treated with ruxolitinib (RUX).

### Aims

To assess predictors of infections including transfusion burden, IO, and NLR in RUX-treated MF pts.

**Copyright Information:** (Online) ISSN: 2572-9241
©2026 The Author(s). HemaSphere is published by John Wiley & Sons Ltd on behalf of European Hematology Association. This is an open access Abstract Book distributed under the Attribution-NonCommercial-NoDerivs (CC BY-NC-ND), which allows third parties to download the articles and share them with others as long as they credit the author and the Abstract Book, but they cannot change the content in any way or use them commercially.
**Abstract Book Citations:** Authors, Title, HemaSphere, 2026;10:(S1):pages. The abstract book DOIs can be found on the "Author Information" page.
**Disclaimer:** Articles published in the journal HemaSphere exclusively reflect the opinions of the authors. The authors are responsible for all content in their abstracts including accuracy of the facts, statements, citing resources, etc.

### Methods

This sub-analysis of the RUX-MF real-world study included 968 MF pts with available data on infections, vaccine/antimicrobial prophylaxis before and during RUX. Transfusion dependency (TD) was defined as ≥3 RBC units (RBCU) in the 12 wks before RUX; transfusion requirement (TR) as ≥1 RBCU in the prior year not meeting TD criteria. Normal NLR was defined as ≤3.53 (*Forget et al*, 2017) and IO as ferritin>1000 ng/mL and/or RBCU>20. Competing risk regression was used to assess predictors of infection, including IO, DIPSS Int2/High (vs Int1), PMF (vs SMF), age ≥65 yrs, platelets (Plt)<100×10⁹/L, NLR>3.53, spleen≥10cm, pre-RUX infections. IO was used instead of TR/TD for collinearity and broader clinical applicability.

### Results

Of 968 pts, 51.7% had PMF and 50.0% DIPSS int-2/high. At baseline, 9.8% had Plt <100×10⁹/L and 39.6% Hb<10g/dL. Most (75.3%) pts had high NLR (>3.53). TR and TD occurred in 62 (6.4%) and 169 (17.5%) pts; mean pre-RUX RBCU was 10±8 and mean ferritin was 775.0±895.5ng/mL. Overall, 111 pts (11.5%) had IO.
Pre-RUX infections were documented in 163 pts, mainly respiratory (39.9%), followed by genitourinary (GU, 13.5%), HBV (9.2%), gastrointestinal (GI; 8.6%), skin/soft tissue (6.7%) and VZV (6.1%). Latent TB was identified in 21.5% of pts.
Among 459 evaluable pts, vaccination rates were: recombinant VZV 25.1%, HBV 10.9%, meningococcal 13.3%, pneumococcal 19.6%, and type b *Haemophilus influenzae* 11.3%. Antimicrobial prophylaxis (acyclovir/isoniazid/lamivudine) was used in 127 (13.1%) pts.
After a median 2.5 yrs on RUX, 281 pts (29.0%) experienced ≥1 infection for a total of 370 events (incidence rate, IR: 11.4 %p-y), mostly grade 1–2 (62.4%); in 28 pts infection was the ultimate cause of death.
Respiratory infections were the most common (45.4%), followed by GI (13.5%) and GU (12.7%). VZV reactivations occurred in 12.4% of pts, sepsis in 5.7%, skin/soft tissue infections in 4.3% and opportunistic/atypical in 3.8%.
At 5 yrs, the cumulative incidence functions of first infection by Fine-Gray model with death as competing risk was 43.5% in TD, 36.8% in TR and 29.4% in non-TR pts (sub distribution hazard ratio, SHR 1.3, 95%CI 1.2–1.5, p<.001) (**Figure**).
In univariate competing risk regression, IO (SHR 1.9, p=.003), DIPSS Int-2/High (1.4, p=.002), NLR>3.53 (1.4, p=.04) and pre-RUX infection (2.37, p<.001) were linked to higher infection risk. In multivariate analysis, all variables remained significant. Notably, in the 229 pts achieving an IWG-MRT spleen response at 6 mos, IR of infections was lower vs. non-responders (8.5 vs 11.6%p-y, p=.02).

### Summary/Conclusion

Infection risk on RUX was driven by prior infectious history and by an increased inflammatory and transfusion burden, whereas early spleen response was protective. Elevated NLR and IO independently increased infection risk, emerging as simple surrogate markers of infectious vulnerability. These factors may help guide therapeutic choices, including anemia-directed approaches, and targeted prophylaxis.

**Copyright Information:** (Online) ISSN: 2572-9241
©2026 The Author(s). HemaSphere is published by John Wiley & Sons Ltd on behalf of European Hematology Association. This is an open access Abstract Book distributed under the Attribution-NonCommercial-NoDerivs (CC BY-NC-ND), which allows third parties to download the articles and share them with others as long as they credit the author and the Abstract Book, but they cannot change the content in any way or use them commercially.
**Abstract Book Citations:** Authors, Title, HemaSphere, 2026;10:(S1):pages. The abstract book DOIs can be found on the "Author Information" page.
**Disclaimer:** Articles published in the journal HemaSphere exclusively reflect the opinions of the authors. The authors are responsible for all content in their abstracts including accuracy of the facts, statements, citing resources, etc.

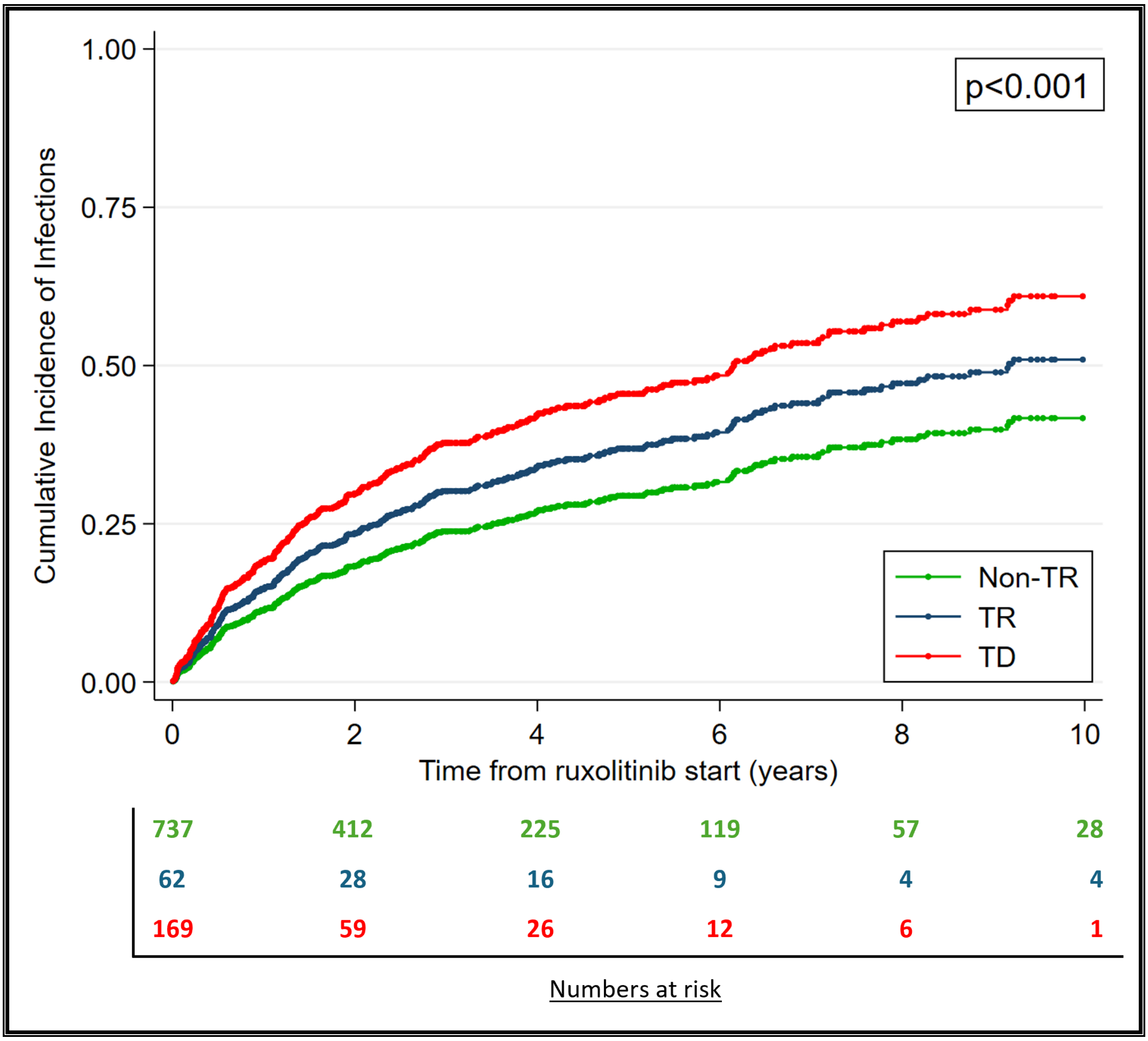

Cumulative Incidence Function of Infections estimated by Fine–Gray model (death as competing risk) by transfusion burden stratification.

**Copyright Information:** (Online) ISSN: 2572-9241
©2026 The Author(s). HemaSphere is published by John Wiley & Sons Ltd on behalf of European Hematology Association. This is an open access Abstract Book distributed under the Attribution-NonCommercial-NoDerivs (CC BY-NC-ND), which allows third parties to download the articles and share them with others as long as they credit the author and the Abstract Book, but they cannot change the content in any way or use them commercially.
**Abstract Book Citations:** Authors, Title, HemaSphere, 2026;10:(S1):pages. The abstract book DOIs can be found on the "Author Information" page.
**Disclaimer:** Articles published in the journal HemaSphere exclusively reflect the opinions of the authors. The authors are responsible for all content in their abstracts including accuracy of the facts, statements, citing resources, etc.

### (PF1309) DISENTANGLING INFECTION FROM IMMUNE TOXICITY: PLASMA MICROBIAL CELL-FREE DNA SEQUENCING DURING CAR T-CELL–ASSOCIATED CRS

**Topic:** 31. Infections in hematology (incl. supportive care/therapy)

Sigrun Einarsdottir*[1], Silvia Escribano-Serrat[1], Marina Gomez-Llobell[1], Jonathan Peled[1], Sapir Israeli[1], Teng Fei[1], Melvili Cintro Cotto[1], Mini Kamboj[1], Elena Mead[1], M. Lia Palomba[1], Jae Park[1], Gunjan Shah[1], Kai Rejeski[1], Miguel-Angel Perales[1], Roni Shouval[1], Zainab Shahid[1]

[1]*Memorial Sloan Kettering Cancer Center, New York, United States of America;*

### Background

Fever is a defining feature of cytokine release syndrome (CRS) after CAR T-cell therapy, creating a persistent challenge in distinguishing immune-mediated toxicity from superimposed or distinct infection. Standard microbiologic testing often provides limited pathogen detection, leading to extended empiric antimicrobial exposure, associated with negative impact on the microbiome and potential toxicity. Plasma microbial cell-free DNA (mcfDNA) sequencing represents a promising adjunctive tool, but its clinical utility and interpretability during CRS remain unclear.

### Methods

We retrospectively analyzed 38 patients with relapsed/refractory B-cell lymphoma treated with CD19 CAR T-cell therapy (2019–2024) who developed grade ≥2 CRS at a single center. Archived plasma samples obtained between day −6 and +28 were serially tested using the Karius Test, a sequencing assay that identifies circulating mcfDNA from bacteria, fungi, and DNA viruses. Findings were compared with clinician-adjudicated infections (per Shahid 2024), blood cultures, and plasma viral PCR performed during routine clinical care within a ±7-day window. Results were blinded to clinicians and adjudicators. Each mcfDNA detection was classified by clinical context at sampling (CRS/fever within ±3 days of fever ≥38°C; clinical infection syndrome; routine surveillance) and by potential clinical actionability (could change therapy; prompt testing or imaging; uncertain clinical relevance).

### Results

Median age was 66 years (IQR, 52–73); 55% were male. CRS was grade 2 in 33 (87%). Products included axicabtagene ciloleucel (n=25), lisocabtagene maraleucel (n=7), and tisagenlecleucel (n=6). Among 236 blood culture sets (601 bottles) obtained from 37/38 patients, only 3 bacteremias were identified (in 3/37 patients [8%]). Clinician-adjudicated infections occurred in 23/38 patients (61%), comprising 31 episodes (10 bacterial, 10 viral, and 11 clinical syndromes without an identified pathogen).
Across 131 mcfDNA tests (median, 3 per patient; IQR, 2–4), 36 (27%) were positive. Of these, 36 (27%) were positive yielding 61 detections of 31 unique organisms. mcfDNA identified concordant pathogens in 2 of 3 evaluable bacteremias and 2 of 3 DNA viremias. In this low-prevalence setting, negative mcfDNA results were rarely followed by bacteremia (92/93 evaluable instances). During CRS/fever, 30% of mcfDNA samples were positive (**Figure 1A**) and 67% of detections were retrospectively classified as potentially actionable (*vs* 57% during routine surveillance). Detections were predominantly gram-negative bacteria (*P. aeruginosa*, *E. coli*, *S. maltophilia*, *Klebsiella* spp.). DNA viral reactivations (CMV, BK virus, HHV-6B), likely to result in confirmatory testing, were detected across clinical contexts, consistent with subclinical DNAemia during immunosuppression. Across all contexts, a substantial proportion of detections (>30%) were rare environmental and commensal organisms with uncertain clinical significance (**Figure 1**).

**Copyright Information:** (Online) ISSN: 2572-9241
©2026 The Author(s). HemaSphere is published by John Wiley & Sons Ltd on behalf of European Hematology Association. This is an open access Abstract Book distributed under the Attribution-NonCommercial-NoDerivs (CC BY-NC-ND), which allows third parties to download the articles and share them with others as long as they credit the author and the Abstract Book, but they cannot change the content in any way or use them commercially.
**Abstract Book Citations:** Authors, Title, HemaSphere, 2026;10:(S1):pages. The abstract book DOIs can be found on the "Author Information" page.
**Disclaimer:** Articles published in the journal HemaSphere exclusively reflect the opinions of the authors. The authors are responsible for all content in their abstracts including accuracy of the facts, statements, citing resources, etc.

### Summary/Conclusion

This is the first study evaluating mcfDNA sequencing during CAR T-cell–associated CRS. During febrile episodes, mcfDNA testing identified potentially actionable pathogens in over half of positive results, including gram-negative bacteria and DNA viral reactivations not otherwise monitored. Given the recognized limitations of conventional diagnostics in antibiotic-exposed patients, our data support prospective evaluation of mcfDNA as an adjunctive tool in infection diagnostics in CRS.

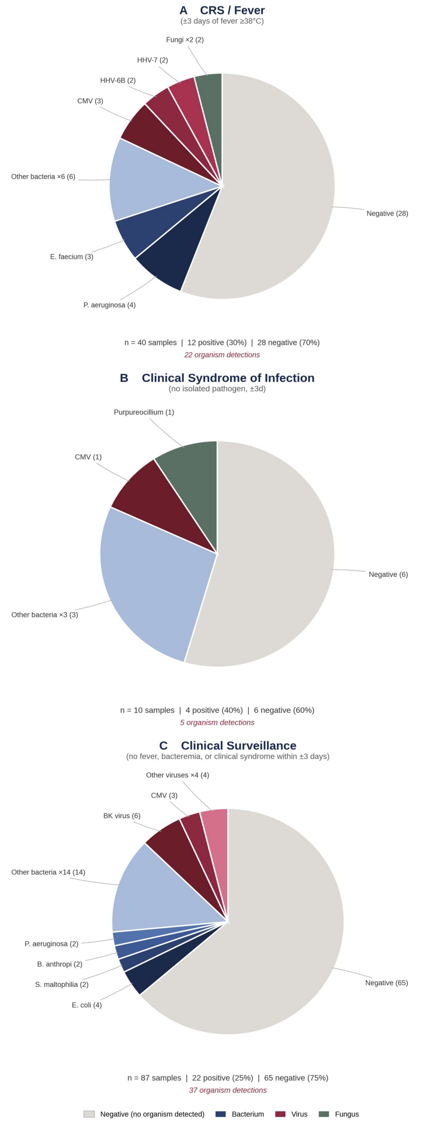

Figure 1. Karius mcfDNA detections by clinical context at time of sampling. Pie charts showing the distribution of plasma mcfDNA detections stratified by clinical context. Samples were collected between day −6 and +28 relative to CAR-T infusion and classified by clinical context within a ±3-day window. (A) CRS/Fever: samples obtained within ±3 days of documented fever ≥38°C. (B) Clinical syndrome of infection: samples within ±3 days of a clinically diagnosed infection without an isolated pathogen. (C) Clinical surveillance: samples with no fever, bacteremia, or clinical syndrome within ±3 days. Individual organisms are labeled where count ≥2; remaining organisms are grouped as "Other." Colors indicate organism type: blue = bacterium, red = virus, green = fungus, gray = negative (no organism detected). Categories are non-mutually exclusive. Exophiala dermatitidis excluded per manufacturer quality review.

**Copyright Information:** (Online) ISSN: 2572-9241
©2026 The Author(s). HemaSphere is published by John Wiley & Sons Ltd on behalf of European Hematology Association. This is an open access Abstract Book distributed under the Attribution-NonCommercial-NoDerivs (CC BY-NC-ND), which allows third parties to download the articles and share them with others as long as they credit the author and the Abstract Book, but they cannot change the content in any way or use them commercially.
**Abstract Book Citations:** Authors, Title, HemaSphere, 2026;10:(S1):pages. The abstract book DOIs can be found on the "Author Information" page.
**Disclaimer:** Articles published in the journal HemaSphere exclusively reflect the opinions of the authors. The authors are responsible for all content in their abstracts including accuracy of the facts, statements, citing resources, etc.

### (PF1310) STEMNESS EROSION AND METABOLIC OVERLOAD OF TCF7⁺ GAMMA-DELTA T CELLS DRIVE OPPORTUNISTIC VIRAL INFECTIONS FOLLOWING CD5- AND CD7-TARGETED CAR-T THERAPY

**Topic:** 31. Infections in hematology (incl. supportive care/therapy)

Ya Luo[1], Ming-Kun Wang*[1], Zhuojun Liu[1], Yi-Han Yang[1], Ao-Ran Zhang[1], Tong Su[1], Xiao-Han Su[1], Su-Xuan Liu[1], Xuying Pei[1], Xiangyu Zhao[1], Pin-Pin Sui[1], Meng Lv[1]

*[1]Peking University People's Hosptial, Beijing, China;*

**Background**

CAR-T therapies targeting CD7 or CD5 have transformed relapsed/refractory T-ALL/LBL and PTCL treatment (*Blood 2022, ASCO 2024*). However, unlike B-cell-directed therapies, CD5- or CD7-CAR-T induce profound cellular immunodeficiency and pan-T-cell aplasia. The resulting virus reactivation mirrors allo-HSCT, representing a major driver of non-relapse morbidity. The specific immune determinants of this vulnerability remain undefined.

**Aims**

To identify shared immune signatures governing viral susceptibility after CD5/CD7 CAR-T therapy, aiming to uncover predictive biomarkers for clinical viral control.

**Methods**

We conducted longitudinal single-cell RNA-seq of 50 PBMC samples collected 1–3 months post CD7 or CD5 CAR-T in 3 trials (NCT06136364; NCT05170568; NCT06874946). Data integration was performed using Harmony, followed by differential abundance, trajectory, and gene set enrichment analyses. Findings were validated by flow cytometry.

**Copyright Information:** (Online) ISSN: 2572-9241
©2026 The Author(s). HemaSphere is published by John Wiley & Sons Ltd on behalf of European Hematology Association. This is an open access Abstract Book distributed under the Attribution-NonCommercial-NoDerivs (CC BY-NC-ND), which allows third parties to download the articles and share them with others as long as they credit the author and the Abstract Book, but they cannot change the content in any way or use them commercially.
**Abstract Book Citations:** Authors, Title, HemaSphere, 2026;10:(S1):pages. The abstract book DOIs can be found on the "Author Information" page.
**Disclaimer:** Articles published in the journal HemaSphere exclusively reflect the opinions of the authors. The authors are responsible for all content in their abstracts including accuracy of the facts, statements, citing resources, etc.

**Results**

Nineteen patients (CD7 n=11; CD5 n=8) were included (median age 33, range 20–50; 74% male). Most had T-ALL/LBL (n=16), and 3 had PTCL–mycosis fungoides. Bone marrow involvement was present in 79%, and EMD in 74%. Viral infection occurred in 63% (n=12), including CMV, EBV, ADV, HPIV, HSV, and parvovirus B19. No bridging transplantation occurred within 3 months post–CAR-T.

Recovery of conventional $CD4^+$ and $CD8^+$ αβ T cells was comparable between patients with and without viral infection. In contrast, the target-negative ($CD5^-/CD7^-$) γδ T-cell compartment demonstrated significantly attenuated longitudinal expansion in patients who developed viral reactivation. Trajectory inference analysis localized this defect to a failure in maintaining a stem-like $TCF7^+$ progenitor reservoir. The frequency of $TCF7^+$ γδ T cells was significantly reduced in infected patients (P=0.038, Figure A–B), indicating a selective impairment of this progenitor population rather than global T-cell deficiency.

At the transcriptional level, virus-susceptible patients exhibited a >4-fold downregulation of core stemness-associated genes **TCF7** (P=0.0085) and **SELL** (P=0.0047), accompanied by upregulation of the terminal effector molecule **GZMB** (P=0.048). This coordinated shift suggests erosion of progenitor identity and skewing toward premature terminal differentiation, thereby limiting the self-renewal capacity of the γδ T-cell compartment.GSEA further revealed pronounced metabolic reprogramming in γδ T cells from infected patients. Pathways related to **Oxidative Phosphorylation** (NES=3.19, P<0.001) and **Apoptosis** (NES=1.87, P=0.015) were significantly enriched, consistent with metabolic hyperactivation and increased apoptotic signaling. These findings support a model in which excessive metabolic demand contributes to accelerated attrition of the $TCF7^+$ progenitor pool.

In an independent pilot cohort, spectral flow cytometry confirmed an approximately 3.5-fold reduction in $TCF7^+$ γδ T-cell frequency in patients who developed viral infection (P=0.0079), recapitulating the single-cell observations and supporting the clinical reproducibility of this immune signature (Figure C).

**Summary/Conclusion**

Opportunistic viral susceptibility after CD5- and CD7-targeted CAR-T therapy is associated with selective collapse of the $TCF7^+$ γδ T-cell progenitor compartment and metabolic overload, decoupling antiviral control from absolute T-cell counts. Monitoring the $TCF7^+$ γδ reservoir provides a biologically grounded approach for risk stratification and may inform individualized antiviral prevention strategies.

**Copyright Information:** (Online) ISSN: 2572-9241
©2026 The Author(s). HemaSphere is published by John Wiley & Sons Ltd on behalf of European Hematology Association. This is an open access Abstract Book distributed under the Attribution-NonCommercial-NoDerivs (CC BY-NC-ND), which allows third parties to download the articles and share them with others as long as they credit the author and the Abstract Book, but they cannot change the content in any way or use them commercially.
**Abstract Book Citations:** Authors, Title, HemaSphere, 2026;10:(S1):pages. The abstract book DOIs can be found on the "Author Information" page.
**Disclaimer:** Articles published in the journal HemaSphere exclusively reflect the opinions of the authors. The authors are responsible for all content in their abstracts including accuracy of the facts, statements, citing resources, etc.

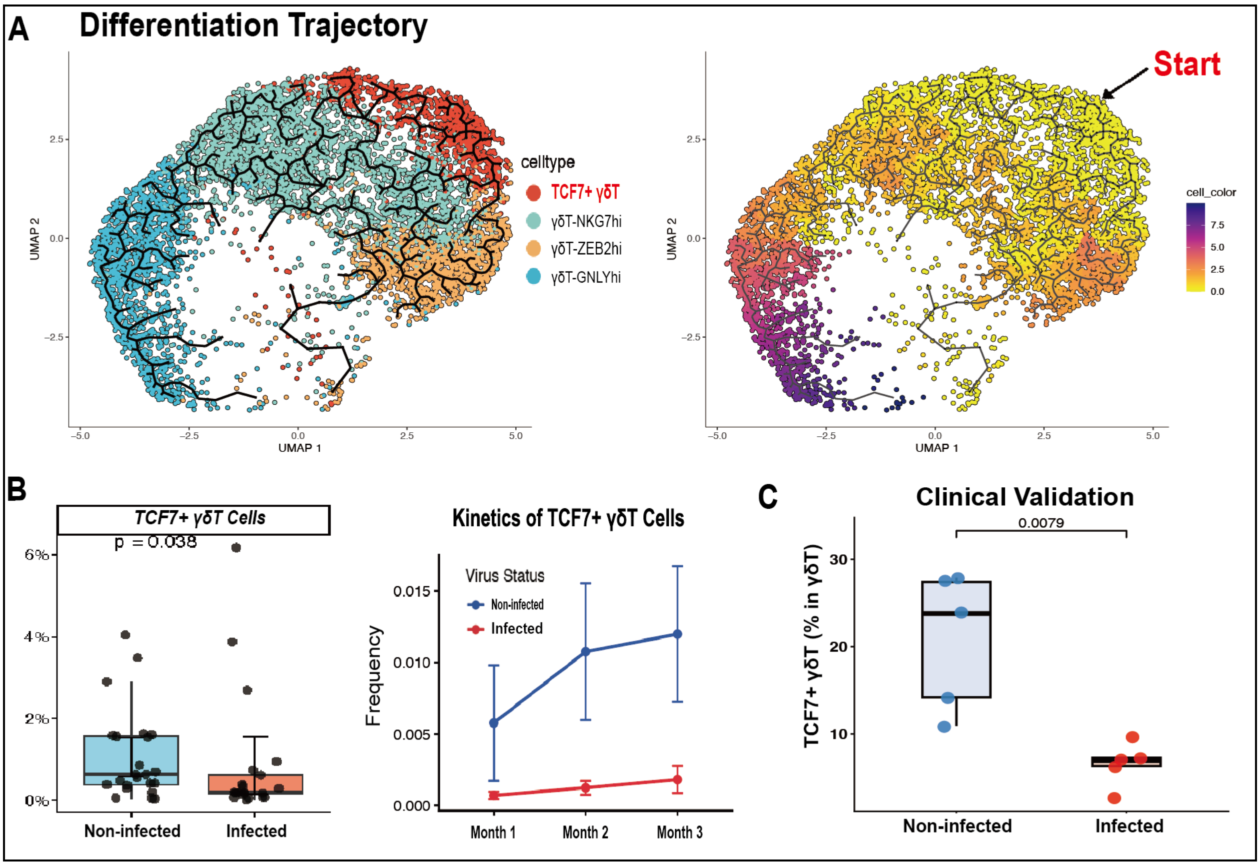

Copyright Information: (Online) ISSN: 2572-9241
©2026 The Author(s). HemaSphere is published by John Wiley & Sons Ltd on behalf of European Hematology Association. This is an open access Abstract Book distributed under the Attribution-NonCommercial-NoDerivs (CC BY-NC-ND), which allows third parties to download the articles and share them with others as long as they credit the author and the Abstract Book, but they cannot change the content in any way or use them commercially.
Abstract Book Citations: Authors, Title, HemaSphere, 2026;10:(S1):pages. The abstract book DOIs can be found on the "Author Information" page.
Disclaimer: Articles published in the journal HemaSphere exclusively reflect the opinions of the authors. The authors are responsible for all content in their abstracts including accuracy of the facts, statements, citing resources, etc.

### (PF1311) MODERN AML THERAPY SAVES LIVES, BUT INFECTION STILL DRIVES MORTALITY: EPIAMLINF EXPERIENCE FROM SWG IN AML AND INFECTIONS

**Topic:** 31. Infections in hematology (incl. supportive care/therapy)

Jon Salmanton-García*[1], Francesco Marchesi[2], Jannik Stemler[3], Alessandra Romano[4], Dario Leotta[4], Roberta de Marchi[5], Davide Facchinelli[6], Adele Santoni[7], Lubos Drgona[8], Milan Navrátil[9], Uluhan Sili[10], Nikola Pantic[11], Luana Fianchi[12], Oliver A. Cornely[1], Livio Pagano[12]

*[1]Institute of Translational Research, University of Cologne, Cologne, Germany;[2]2. IRCCS Regina Elena National Cancer Institute,, Rome,, Italy; [3]1. Institute of Translational Research, University of Cologne,, Cologne,, Germany;[4]3. Azienda Ospedaliero-Universitaria Policlinico 'G. Rodolico-S.Marco',, Catania,, Italy; [5]4. Instituto Oncologico Veneto IRCCS, sede di Castelfranco Veneto, ,, Castelfranco Veneto, Italy; [6]5. Ospedale San Bortolo, ,, Vicenza, Italy; [7]6. Azienda ospedaliero-universitaria Senese, ,, Siena, Italy; [8]7. Department of Oncohematology, Faculty of Medicine, Comenius University and National Cancer Institute, ,, Bratislava, Slovakia; [9]8. Department of Hematology, Faculty Hospital Ostrava, ;, Ostrava, Czechia; [10]9. Department of Infectious Diseases and Clinical Microbiology, School of Medicine, Marmara University, ,, Istanbul, Türkiye; [11]10. Clinic of Hematology, University Clinical Center, , ;, Belgrade, Serbia; [12]11. Fondazione Policlinico Universitario Agostino Gemelli - IRCCS,, Rome,, Italy;*

### Background

Despite major advances in acute myeloid leukaemia (AML) therapy, infections remain a critical determinant of outcome. However, contemporary multinational real-world data integrating modern treatment regimens and microbiological epidemiology are scarce.

### Aims

To define the current infectious landscape and its impact on survival in AML patients treated in the modern therapeutic era.

### Methods

This multicentre observational study included patients with AML diagnosed between March 1, 2025 and February 28, 2026, with a follow-up period of 45 days. Relapsed cases were eligible only if the initial AML diagnosis occurred within this timeframe. For patients undergoing allogeneic hematopoietic stem cell transplantation (allo-HSCT), only infections occurring after AML relapse were considered eligible. Data collected included clinical characteristics, treatment phase and regimen, comorbidities, antimicrobial prophylaxis, infectious presentation, microbiological findings, anti-infective therapy, and clinical outcomes.

**Copyright Information:** (Online) ISSN: 2572-9241
©2026 The Author(s). HemaSphere is published by John Wiley & Sons Ltd on behalf of European Hematology Association. This is an open access Abstract Book distributed under the Attribution-NonCommercial-NoDerivs (CC BY-NC-ND), which allows third parties to download the articles and share them with others as long as they credit the author and the Abstract Book, but they cannot change the content in any way or use them commercially.
**Abstract Book Citations:** Authors, Title, HemaSphere, 2026;10:(S1):pages. The abstract book DOIs can be found on the "Author Information" page.
**Disclaimer:** Articles published in the journal HemaSphere exclusively reflect the opinions of the authors. The authors are responsible for all content in their abstracts including accuracy of the facts, statements, citing resources, etc.

### Results

A total of 574 patients with AML from 14 countries were included. AML subtypes were distributed as follows: AML with defining genetic abnormalities (48%), AML with myelodysplasia-related changes (AML-MR, 23%), AML defined by differentiation (21%), and therapy-related AML (8%). Most patients underwent induction therapy (61%) or consolidation (29%). Treatment regimens included azacitidine–venetoclax (19%), CPX-351 (13%), high/intermediate-dose cytarabine (11%), and intensive combination chemotherapy (e.g., “3+7”) (23% overall).

Comorbidities were common, particularly cardiac (42%) and metabolic conditions (31%), although 25% of patients had no comorbidities. Prophylactic antimicrobial use was frequent, with antifungals administered in 79%, antivirals in 40%, and antibiotics in 22% of patients, respectively.

Microbiologically documented infections occurred in 36% of patients, clinically documented infections in 14%, and fever of unknown origin in 21%; 29% of patients had no documented infection. Overall, 67% received antimicrobial therapy, with 50% of infections involving the lungs.

Among microbiologically documented infections, bacterial pathogens predominated, with 25% of cases showing coinfections. The most common bacterial isolates were *Escherichia* spp. (15%), *Enterococcus* spp. (10%), *Klebsiella* spp. (8%), *Staphylococcus* spp. (7%), and *Pseudomonas* spp. (5%). Viral infections, including SARS-CoV-2 and influenza A, were identified in 1.5–2.0% of patients, while fungal infections, primarily *Aspergillus* spp. and *Candida* spp., were less frequent.

Infections resolved in in 47% of cases. Overall survival was 83%, with 26% of deaths attributed solely to infection and 25% to a combination of infection and AML. Complete remission of AML was achieved in 58% of patients.

### Summary/Conclusion

In this contemporary AML cohort treated with both intensive chemotherapy and less intensive regimens, infections remain highly prevalent. Bacterial pathogens predominate, and coinfections are common. Although overall survival reflects therapeutic advances, infection continues to account for nearly one in two deaths. These findings underscore the need for improved infectious risk stratification and integrated haematology–infectious disease management in modern AML care.

**Copyright Information:** (Online) ISSN: 2572-9241
©2026 The Author(s). HemaSphere is published by John Wiley & Sons Ltd on behalf of European Hematology Association. This is an open access Abstract Book distributed under the Attribution-NonCommercial-NoDerivs (CC BY-NC-ND), which allows third parties to download the articles and share them with others as long as they credit the author and the Abstract Book, but they cannot change the content in any way or use them commercially.
**Abstract Book Citations:** Authors, Title, HemaSphere, 2026;10:(S1):pages. The abstract book DOIs can be found on the “Author Information” page.
**Disclaimer:** Articles published in the journal HemaSphere exclusively reflect the opinions of the authors. The authors are responsible for all content in their abstracts including accuracy of the facts, statements, citing resources, etc.

### (PF1312) RATE OF INFECTIOUS COMPLICATIONS IN AZACITIDINE-VENETOCLAX TREATED AML PATIENTS WITH OR WITHOUT FLUOROQUINOLONE AND POSACONAZOLE PROPHYLAXIS: INTERIM ANALYSIS OF REAL LIFE PROVENZA STUDY

**Topic:** 31. Infections in hematology (incl. supportive care/therapy)

Adele Santoni*[1], Chiara Carrara[1], Gabriele Giuliano[1], Michela Rondoni[2], Angela Consagra[3], Elisabetta Todisco[4], Monica Crugnola[5], Benedetta Cambo'[5], Daniela Cilloni[6], Rachele Giubbolini[7], Bianca Mecacci[8], Roberta Giachetti[9], Alessandra Caremani[10], Federica Pilo[11], Roberto La Tagliata[12], Vincenzo Sammartano[13], Mario Annunziata[14], Maria Ilaria DEL Principe[15], Nicoletta D'angelo[1], Corrado Zuanelli Brambilla[1], Mario Tumbarello[1], Monica Bocchia[1]

*[1]Azienda Ospedaliero Universitaria Senese, University of Siena, Siena, Italy;[2]Azienda Unità Sanitaria Locale della Romagna, Ravenna, Italy; [3]Ausl Toscana Centro Ospedale San Giovanni di Dio, University of Florence, Firenze, Italy;[4]ASST Valle Olona, Busto Arsizio, Italy; [5]Azienda Ospedaliero Universitaria di Parma, Parma, Italy;[6]AO Ordine Mauriziano di Torino, University of Turin, Torino, Italy; [7]Azienda USL Toscana Nord Ovest, Livorno, Italy;[8]Azienda USL Toscana Nord Ovest, Versilia Camaiore, Italy; [9]USL Toscana Centro, Prato, Pescia, Empoli, Italy; [10]San Donato Hospital, Arezzo, Italy; [11]"A. Businco" Oncology Hospital, Cagliari, Italy; [12]Santa Rosa Hospital, Viterbo, Italy; [13]A.O.U. Federico II, Napoli, Italy; [14]AORN Cardarelli, Napoli, Italy; [15]S. Eugenio Hospital, Tor Vergata University, Roma, Italy;*

### Background

Azacitidine with Venetoclax (AZA/VEN) is the standard of care in newly diagnosed (ND) elderly/unfit Acute Myeloid Leukemia (AML) patients (pts). Despite a relative safety profile, it often causes prolonged cytopenia, exposing pts to bacterial and fungal infection and increasing infectious related mortality (IRM). Thus, infections management is crucial and fluoroquinolone (FQ) and antifungal (AF) prophylaxis (PPX) are still recommended by several guidelines, although there is no consensus about their use in this setting. Moreover, considering multidrug resistant bacteria and fungi increase, especially in Italy, PPX role in neutropenic AML pts should be reassessed and several Italian Hematology Unit started to omit it, as local policy.

### Aims

PROVENZA study is a retrospective observational multicentric real-life study aiming to evaluate bacterial and fungal infection rate and IRM in ND AML adult pts undergoing AZA/VEN therapy, with or without FQ and AF PPX.

### Methods

Up to date, 237 AML pts treated with induction therapy with AZA/VEN in 15 Italian hematological centers were enrolled.

**Copyright Information:** (Online) ISSN: 2572-9241
©2026 The Author(s). HemaSphere is published by John Wiley & Sons Ltd on behalf of European Hematology Association. This is an open access Abstract Book distributed under the Attribution-NonCommercial-NoDerivs (CC BY-NC-ND), which allows third parties to download the articles and share them with others as long as they credit the author and the Abstract Book, but they cannot change the content in any way or use them commercially.
**Abstract Book Citations:** Authors, Title, HemaSphere, 2026;10:(S1):pages. The abstract book DOIs can be found on the "Author Information" page.
**Disclaimer:** Articles published in the journal HemaSphere exclusively reflect the opinions of the authors. The authors are responsible for all content in their abstracts including accuracy of the facts, statements, citing resources, etc.

**Results**

Figure 1 reports pts characteristics: median age was 76 years, M/F was 144/93. Inpatients were 47%, while outpatients were 53%. Regarding FQ PPX, 60/237(25%) pts received it, while in 177/237(75%) it was omitted. Median length of neutropenia was similar in FQ and not FQ group (19 and 20 days, respectively, p=0.089), although GCSF was used in 26.7% FQ PPX pts and in 54.2% not FQ PPX (p<0.001). Of note, of the 47% pts receiving induction therapy as inpatients, 52% belonged to not FQ group (93/177) and 30% to FQ one (18/60) (p=0.002) yet with similar hospitalization length (26 vs 27 days, p=0.885). Interestingly, 73.3% of FQ pts did not show any infectious event at all, compared to 54.2% not FQ pts, (p<0.001). Indeed, bloodstream infections were documented in 1/60(7.7%) FQ pt and 17/177(21.3%) not FQ pts (p=0,251), while organ infections were detected in 5/60(8.3%) FQ pts and in 20/177(11.3%) not FQ ones (p=0.519), without differences. However, no septic shock in FQ PPX pts was observed while we documented a significantly higher rate (7.3%) in the group without FQ (p=0.031). Finally considering AF PPX, 149/237(62.9%) pts received posaconazole, while in 88/237(37.1%) it was omitted. Despite that, in the whole cohort we did not record any fungal infection, regardless AF PPX use. Overall 60-day mortality was 8.9%(21/237). At univariable analysis, variables associated with 60-day mortality were bacterial organ infections (7/21death(33.3%)vs18/216alive(8.3%);p<0.001) and septic shock (8/21(38.31%)vs5/216(2.3%);p<0.001). Cox regression analysis, adjusted for age and GCSF use, confirmed these results, without showing any correlation between mortality and both FQ (p=0.856) nor AF PPX (p=0.951). To identify risk factors for infections, we performed a multivariate analysis. A longer hospitalization was significantly associated with an increased infection risk (p=0.038), instead FQ PPX (p=0.084) and G-CSF use (p=0.062) seemed to be protective factors, without achieving statistical significance, likely due to the small sample size.

**Summary/Conclusion**

Interim analysis of PROVENZA study doesn't show that FQ and AF PPX omission impacts on mortality in elderly/unfit AML pts treated with AZA/VEN, even if FQ could reduce bacterial infections. However, ongoing accrual and additional data are mandatory to confirm these results and ultimately clarify PPX role in this setting.

**Copyright Information:** (Online) ISSN: 2572-9241
©2026 The Author(s). HemaSphere is published by John Wiley & Sons Ltd on behalf of European Hematology Association. This is an open access Abstract Book distributed under the Attribution-NonCommercial-NoDerivs (CC BY-NC-ND), which allows third parties to download the articles and share them with others as long as they credit the author and the Abstract Book, but they cannot change the content in any way or use them commercially.
**Abstract Book Citations:** Authors, Title, HemaSphere, 2026;10:(S1):pages. The abstract book DOIs can be found on the "Author Information" page.
**Disclaimer:** Articles published in the journal HemaSphere exclusively reflect the opinions of the authors. The authors are responsible for all content in their abstracts including accuracy of the facts, statements, citing resources, etc.

**Figure 1**

| | TOTAL 237 | FQ 60 | non FQ 177 | p-value |
|---|---|---|---|---|
| Median age (years) (range) | 76 (72-79) | 76.5 (73-82) | 74 (71-78) | 0.075 |
| Male (%)<br>Female (%) | 144 (61)<br>93 (39) | 37 (61)<br>23 (39) | 107 (61)<br>70 (39) | 0.0868 |
| PS 0-1 (%)<br>PS 2-3 (%) | 190 (80.2)<br>47 (19.8) | 52 (86.7)<br>8 (13.3) | 138 (78.0)<br>39 (22.0) | 0.144<br>0.144 |
| De Novo AML (%)<br>Secondary AML (%) | 147 (62%)<br>90 (38%) | 41 (68.3)<br>19 (31.7) | 106 (59.9)<br>71 (40.1) | 0.244 |
| ELN risk<br>Low (%)<br>Intermediate (%)<br>High (%) | <br>33/211 (16)<br>79/211 (37)<br>99/211 (47) | <br>4/49 (8)<br>15/49 (31)<br>30/49 (61) | <br>29/162 (18)<br>64/162 (39)<br>69/162 (43) | <br>0.1<br>0.26<br>**0.022** |
| Inpatient treatment (%) | 111 (46.8) | 18 (30.0) | 93 (52.5) | **0.002** |
| Median hospitalization (days) (range) | 26 (15-39) | 27 (16-34) | 26 (15-40) | 0.885 |
| Median Neutropenia (days) (range) | 20 (8-28) | 19 (9-29) | 20 (11-28) | 0.089 |
| GCSF (%) | 112 (47.3) | 16 (26.7) | 96 (54.2) | **<0.001** |
| Mucosal toxicity (%) | 14 (5.9) | 1 (1.7) | 13 (7.3) | 0.107 |
| FN readmission (%) | 25 (10.5) | 7 (11.7) | 18 (10.2) | 0.744 |
| NO events (%) | 140 (59.1) | 44 (73.3) | 96 (54.2) | **<0.001** |
| FUO (%) | 54 (57.4) | 10 (71.4) | 44 (55.0) | 0.521 |
| BSI (%) | 18 (19.4) | 1 (7.7) | 17 (21.3) | 0.251 |
| Organ infections (%) | 25 (10.5) | 5 (8.3) | 20 (11.3) | 0.519 |
| Septic shock (%) | 13 (5.5) | 0 (0) | 13 (7.3) | **0.031** |
| 60 days response (%) | 139 (64.2) | 39 (68.4) | 99 (62.7) | 0.437 |
| 60 days mortality (%)<br>IRM (%) | 21 (8.9)<br>15 (68.2) | 3 (5.0)<br>1 (33.3) | 18 (10.2)<br>14 (73.7) | 0.223<br>0.227 |

FQ: fluoroquinolone; AF: antifungal; PS: performance status; AML: acute myeloid leukemia; ELN: European Leukemia Net; GCSF: granulocyte colony stimulating factor; FN: febrile neutropenia; FUO: fever of unknown origin; BSI: bloodstream infections; IRM: infectious related mortality

Figure 1

Copyright Information: (Online) ISSN: 2572-9241
©2026 The Author(s). HemaSphere is published by John Wiley & Sons Ltd on behalf of European Hematology Association. This is an open access Abstract Book distributed under the Attribution-NonCommercial-NoDerivs (CC BY-NC-ND), which allows third parties to download the articles and share them with others as long as they credit the author and the Abstract Book, but they cannot change the content in any way or use them commercially.
Abstract Book Citations: Authors, Title, HemaSphere, 2026;10:(S1):pages. The abstract book DOIs can be found on the “Author Information” page.
Disclaimer: Articles published in the journal HemaSphere exclusively reflect the opinions of the authors. The authors are responsible for all content in their abstracts including accuracy of the facts, statements, citing resources, etc.

### (PF1313) NEBULIZED AMPHOTERICIN B VERSUS POSACONAZOLE FOR PROPHYLAXIS OF INVASIVE FUNGAL DISEASE DURING POST-CHEMOTHERAPY NEUTROPENIA IN ACUTE LEUKEMIA: A PROPENSITY SCORE–MATCHED NONINFERIORITY COHORT STUDY

**Topic:** 31. Infections in hematology (incl. supportive care/therapy)

Jingyu Wang*[1], Zhenzhen Wang[2], Lihua Wu[2], Chunhua Liu[2], Xiyan Wang[2], Hong Liu[2], Runxia Gu[2], Shuning Wei[2], Ying Wang[2]

*[1]Haihe Laboratory of Cell Ecosystem, Tianjin Medical University, Tianjin, China;[2]Institute of Hematology & Blood Disease Hospital, China Academy of Medical Science & Peking Union Medical College, Tianjin, China;*

### Background

Acute leukemia patients frequently develop post-chemotherapy neutropenia and are at high risk for invasive fungal disease (IFD). Pulmonary IFD is common and associated with poor outcomes. Triazole prophylaxis may be limited by drug–drug interactions, exposure variability requiring monitoring, and cost. Nebulized amphotericin B (AmB) provides site-directed pulmonary prophylaxis with relatively few systemic interactions.

### Aims

To compare the real-world effectiveness and safety of nebulized AmB versus posaconazole (POS) for preventing IFD during the post-chemotherapy neutropenic period in acute leukemia, and to assess the non-inferiority of nebulized AmB for the primary endpoint.

### Methods

We conducted a single-center retrospective cohort study including acute leukemia patients who received antifungal prophylaxis during chemotherapy-associated neutropenia between January 2022 and April 2024.Patients on voriconazole were excluded from primary comparisons. Outcomes were classified by international criteria. Primary endpoint: proven/probable IFD; secondary: overall IFD (proven/probable/possible). Propensity score matching (PSM) was primary analysis; effect measure was risk difference (RD=AmB–POS) with prespecified non-inferiority margin Δ=6%. Sensitivity analyses used weighting and regression adjustment (age, BMI, performance status). The study was IRB-approved with waiver of informed consent.

**Copyright Information:** (Online) ISSN: 2572-9241
©2026 The Author(s). HemaSphere is published by John Wiley & Sons Ltd on behalf of European Hematology Association. This is an open access Abstract Book distributed under the Attribution-NonCommercial-NoDerivs (CC BY-NC-ND), which allows third parties to download the articles and share them with others as long as they credit the author and the Abstract Book, but they cannot change the content in any way or use them commercially.
**Abstract Book Citations:** Authors, Title, HemaSphere, 2026;10:(S1):pages. The abstract book DOIs can be found on the "Author Information" page.
**Disclaimer:** Articles published in the journal HemaSphere exclusively reflect the opinions of the authors. The authors are responsible for all content in their abstracts including accuracy of the facts, statements, citing resources, etc.

### Results

After propensity score matching, 93 patients in the nebulized amphotericin B group were matched to 70 patients in the posaconazole group, with substantial improvement in covariate balance (standardized mean difference reduced from 1.183 to 0.024). Proven or probable invasive fungal infection occurred in 3.23% (3/93) in the nebulized amphotericin B group and 5.71% (4/70) in the posaconazole group (RD −2.49%; 95% CI −9.47% to 3.79%); the upper confidence bound (3.79%) was below the prespecified 6% margin, meeting the criterion for non-inferiority. Sensitivity analysis yielded an RD of 1.00% (95% CI −5.18% to 5.84%), consistent with non-inferiority. For the secondary endpoint, overall invasive fungal infection occurred in 9.68% versus 17.14%, respectively (RD −7.47%; 95% CI −18.81% to 3.22%). Weighted analyses did not demonstrate a clear statistical difference between groups (weighted RD 1.61%; 95% CI −7.38% to 9.86%; weighted regression RD 2.26%; 95% CI −7.58% to 10.45%). No prophylaxis-related serious adverse events were observed.

**Table 1** Non-inferiority Analysis of the Primary Outcome

| **Analysis** | **AmB group(%)** | **POS group (%)** | **RD(%)** | **95% CI** |
|---|---|---|---|---|
| Primary analysis (unweighted) | 3.23% | 5.71% | −2.49% | −9.47% ~ 3.79% |
| Sensitivity analysis（Weighted LPM） | 3.46% | 2.46% | 1.00% | −5.18% ~ 5.84% |

RD was defined as the event rate in the AmB group minus that in the POS group. Non-inferiority was concluded when the upper bound of the 95% CI for the RD was <6 percentage points (Δ=6%).

### Summary/Conclusion

In this real-world cohort of acute leukemia patients receiving antifungal prophylaxis during the post-chemotherapy neutropenic period, nebulized AmB was non-inferior to POS for preventing proven or probable IFD and was not associated with prophylaxis-related serious adverse events. When triazole prophylaxis is constrained by drug–drug interactions, intolerance, limitations of oral administration, or cost, nebulized amphotericin B may be a feasible alternative or adjunct prophylactic strategy. Prospective multicenter studies are warranted to confirm these findings and to refine patient selection and dosing regimens.

**Copyright Information:** (Online) ISSN: 2572-9241
©2026 The Author(s). HemaSphere is published by John Wiley & Sons Ltd on behalf of European Hematology Association. This is an open access Abstract Book distributed under the Attribution-NonCommercial-NoDerivs (CC BY-NC-ND), which allows third parties to download the articles and share them with others as long as they credit the author and the Abstract Book, but they cannot change the content in any way or use them commercially.
**Abstract Book Citations:** Authors, Title, HemaSphere, 2026;10:(S1):pages. The abstract book DOIs can be found on the "Author Information" page.
**Disclaimer:** Articles published in the journal HemaSphere exclusively reflect the opinions of the authors. The authors are responsible for all content in their abstracts including accuracy of the facts, statements, citing resources, etc.

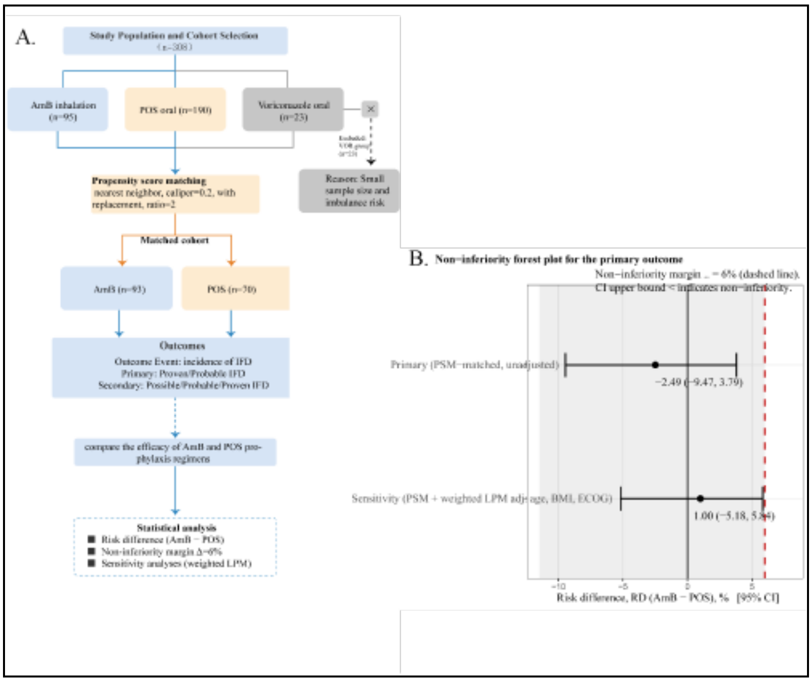

(A) Flow chart of study population, cohort selection, and propensity score matching. (B) Non-inferiority forest plot for the primary endpoint. Points indicate the risk difference (RD; AmB minus POS) and horizontal bars denote 95% confidence intervals (CIs). The solid vertical line indicates the null effect (RD=0), and the red dashed line marks the prespecified non-inferiority margin (Δ=6%). Non-inferiority of nebulized amphotericin B versus posaconazole was concluded when the upper bound of the 95% CI was below Δ.

**Copyright Information:** (Online) ISSN: 2572-9241
©2026 The Author(s). HemaSphere is published by John Wiley & Sons Ltd on behalf of European Hematology Association. This is an open access Abstract Book distributed under the Attribution-NonCommercial-NoDerivs (CC BY-NC-ND), which allows third parties to download the articles and share them with others as long as they credit the author and the Abstract Book, but they cannot change the content in any way or use them commercially.
**Abstract Book Citations:** Authors, Title, HemaSphere, 2026;10:(S1):pages. The abstract book DOIs can be found on the "Author Information" page.
**Disclaimer:** Articles published in the journal HemaSphere exclusively reflect the opinions of the authors. The authors are responsible for all content in their abstracts including accuracy of the facts, statements, citing resources, etc.

### (PF1314) DOES MASK TYPE MATTER? A THREE-COHORT COMPARISON OF NO MASKING, FFP2, AND SURGICAL MASKS IN ALLOGENEIC HEMATOPOIETIC STEM CELL TRANSPLANTATION

**Topic:** 31. Infections in hematology (incl. supportive care/therapy)

Tim Richardson*[1], Philipp Gödel[1], Udo Holtick[1], Elisa Carpinteiro[1], Christian Funke[1], Christoph Scheid[1]

[1] *University hospital of cologne, Cologne, Germany;*

### Background

Viral respiratory infections (RVIs) are a major cause of morbidity in patients undergoing allogeneic hematopoietic stem cell transplantation (allo-HSCT). Our previously published data demonstrated that consistent FFP2 masking reduced RVI incidence from 20.7% to 2.0% on a dedicated allo-HSCT ward (Richardson et al., Sci Rep 2024). Whether surgical masks — more comfortable, cheaper, and logistically simpler — offer meaningful protection remains an open and clinically pressing question.

### Aims

To compare RVI incidence and clinical outcomes across three consecutive cohorts of 150 allo-HSCT patients each: no masking (01/2018–09/2020), FFP2 masking (10/2020–10/2022), and surgical masking (11/2023–12/2025). All three periods were matched for winter months to control for seasonal variation in respiratory pathogen circulation.

### Methods

Single-centre retrospective analysis of 450 consecutive allo-HSCT patients. Primary endpoint: in-hospital RVI incidence. Secondary endpoints: length of hospital stay, bronchoalveolar lavage (BAL) rate, and hospital survival. Chi-square tests were used for categorical comparisons; Mann-Whitney U for continuous variables. Ethics approval was obtained.

### Results

RVI incidence differed significantly across all three cohorts ($\chi^2=30.8$, $p<0.001$): 20.7% (no mask), 7.3% (surgical mask), and 2.0% (FFP2 mask). Compared to no masking, surgical masks significantly reduced RVIs ($p=0.002$). The difference between surgical and FFP2 masks did not reach statistical significance ($p=0.055$), yet the absolute gap of 5.3 percentage points represents a clinically relevant difference that should not be dismissed on statistical grounds alone. Median hospital stay was 26 days (no mask), 23.5 days (FFP2), and 23 days (surgical mask). BAL procedures were performed in 14 (9.3%), 5 (3.3%), and 9 (6.0%) patients respectively (overall $p=0.098$), reflecting a trend toward reduced diagnostic burden with any masking. ICU transfer and other clinical endpoints did not differ significantly across cohorts. Predominant pathogens in the surgical mask era were SARS-CoV-2 (55%), Rhinovirus (27%), and Parainfluenza (18%). No significant differences in hospital survival were observed.

### Summary/Conclusion

Any mask beats no mask — but mask type matters. Surgical masks offer meaningful protection against RVIs in allo-HSCT patients, yet FFP2 masks appear superior in absolute terms. Given the logistical and tolerability advantages of surgical masks, they may serve as a pragmatic compromise in centres where consistent FFP2 adherence is difficult to maintain. These data provide a rare head-to-head evidence base for mask-type decisions in one of the most immunocompromised patient populations in medicine and should inform future infection control guidelines.

**Copyright Information:** (Online) ISSN: 2572-9241
©2026 The Author(s). HemaSphere is published by John Wiley & Sons Ltd on behalf of European Hematology Association. This is an open access Abstract Book distributed under the Attribution-NonCommercial-NoDerivs (CC BY-NC-ND), which allows third parties to download the articles and share them with others as long as they credit the author and the Abstract Book, but they cannot change the content in any way or use them commercially.
**Abstract Book Citations:** Authors, Title, HemaSphere, 2026;10:(S1):pages. The abstract book DOIs can be found on the "Author Information" page.
**Disclaimer:** Articles published in the journal HemaSphere exclusively reflect the opinions of the authors. The authors are responsible for all content in their abstracts including accuracy of the facts, statements, citing resources, etc.

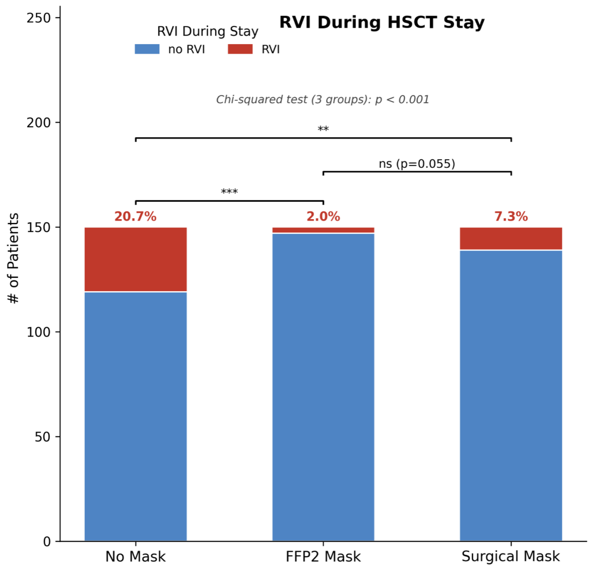

**Copyright Information:** (Online) ISSN: 2572-9241
©2026 The Author(s). HemaSphere is published by John Wiley & Sons Ltd on behalf of European Hematology Association. This is an open access Abstract Book distributed under the Attribution-NonCommercial-NoDerivs (CC BY-NC-ND), which allows third parties to download the articles and share them with others as long as they credit the author and the Abstract Book, but they cannot change the content in any way or use them commercially.
**Abstract Book Citations:** Authors, Title, HemaSphere, 2026;10:(S1):pages. The abstract book DOIs can be found on the "Author Information" page.
**Disclaimer:** Articles published in the journal HemaSphere exclusively reflect the opinions of the authors. The authors are responsible for all content in their abstracts including accuracy of the facts, statements, citing resources, etc.

### (PF1315) MARIBAVIR FOR PRE-EMPTIVE TREATMENT OF CYTOMEGALOVIRUS REACTIVATION IN CHIMERIC ANTIGEN RECEPTOR T-CELL THERAPY RECIPIENTS

**Topic:** 31. Infections in hematology (incl. supportive care/therapy)

Cristian Gutierrez-Padilla[1], Jose María Sánchez-Pina[1], Adolfo Jesus Saez Marin[1], María Sánchez Tabernero[1], Guillermo Ramos Moreno[1], Magdalena Corona[1], M Nieves Lopez[1], Pilar Martínez Sánchez[1], Daniel Gil Alós[1], Ana Cuervo-Fonseca[1], Almudena Zaratiegui-Vergara*[1], Silvia Chacon[1], Maria Puy Goyache[1], Susana Cortijo[1], Maria Calbacho[1], Joaquín Martinez-Lopez[1]

[1]*Hospital Universitario 12 de Octubre, Hematology, Madrid, Spain;*

### Background

Clinically significant cytomegalovirus (CMV) reactivation in patients undergoing CAR-T therapy represents a therapeutic challenge due to the combined toxicities of both the cellular product and conventional antivirals. Evidence regarding the use of maribavir in this population is currently lacking, with clinical experience derived almost exclusively from hematopoietic stem cell and solid organ transplantation settings.

### Aims

To evaluate the effectiveness and safety of maribavir for pre-emptive treatment of CMV reactivation in CAR-T recipients.

### Methods

We conducted a single-center retrospective study of CMV-seropositive CAR-T recipients who developed CMV reactivation and received pre-emptive maribavir between September 2020 and September 2025. CMV viral load was monitored twice weekly during the first six weeks post-infusion. Maribavir was initiated for DNAemia ≥1,500 copies/mL (plasma quantitative polymerase chain reaction) with a viral doubling time ≤ 2 days. Viral clearance was defined as at least one undetectable viral load.

### Results

| *Table 1.* **BASELINE CHARACTERISTICS (N=25)** | | |
|---|---|---|
| | **n (%)** | **Median (IQR)** |
| **Sex** | | |
| Female | 15 (60) | |
| **Age (y)** | | 68 (64-73) |
| **Underlying disease** | | |

**Copyright Information:** (Online) ISSN: 2572-9241
©2026 The Author(s). HemaSphere is published by John Wiley & Sons Ltd on behalf of European Hematology Association. This is an open access Abstract Book distributed under the Attribution-NonCommercial-NoDerivs (CC BY-NC-ND), which allows third parties to download the articles and share them with others as long as they credit the author and the Abstract Book, but they cannot change the content in any way or use them commercially.
**Abstract Book Citations:** Authors, Title, HemaSphere, 2026;10:(S1):pages. The abstract book DOIs can be found on the "Author Information" page.
**Disclaimer:** Articles published in the journal HemaSphere exclusively reflect the opinions of the authors. The authors are responsible for all content in their abstracts including accuracy of the facts, statements, citing resources, etc.

| | | |
|---|---|---|
| NHL-B | 19 (76) | |
| MM | 6 (24) | |
| **Prior lines** | | 2 (1-2) |
| **CAR-T product** | | |
| Ide-cel | 1 (4) | |
| Ari0002h | 2 (8) | |
| Axi-cel | 20 (80) | |
| Cilta-cel | 2 (8) | |
| **Bridging therapy** | 23 (92) | |
| **CRS ≥ 2** | 11 (44) | |
| **ICANS ≥ 2** | 8 (32) | |
| **Tocilizumab** | 18 (72) | |
| **Tocilizumab dose (mg)** | | 1200 (600–1600) |
| **Corticosteroids** | 23 (92) | |
| **Corticosteroid dose (dexamethasone-equivalent, mg)** | | 180 (70-360) |
| **Other CRS/ICANS therapy** | 14 (58) | |
| **Siltuximab dose (mg)** | | 1540 (1200–1550) |
| **Anakinra dose (mg)** | | 2450 (1775–3125) |
| **ICU admission** | 8 (32) | |

**Copyright Information:** (Online) ISSN: 2572-9241
©2026 The Author(s). HemaSphere is published by John Wiley & Sons Ltd on behalf of European Hematology Association. This is an open access Abstract Book distributed under the Attribution-NonCommercial-NoDerivs (CC BY-NC-ND), which allows third parties to download the articles and share them with others as long as they credit the author and the Abstract Book, but they cannot change the content in any way or use them commercially.
**Abstract Book Citations:** Authors, Title, HemaSphere, 2026;10:(S1):pages. The abstract book DOIs can be found on the "Author Information" page.
**Disclaimer:** Articles published in the journal HemaSphere exclusively reflect the opinions of the authors. The authors are responsible for all content in their abstracts including accuracy of the facts, statements, citing resources, etc.

Twenty-five patients were included: 19 with B-cell non-Hodgkin lymphoma (NHL-B) and 6 with multiple myeloma (MM). The most frequently infused CAR-T product was axicabtagene ciloleucel (80%). Grade ≥2 CRS and ICANS occurred in 44% and 32% of patients, respectively, with frequent use of corticosteroids (92%), tocilizumab (72%) and other immunomodulators (58%). Baseline characteristics are summarized in Table 1.
Following CAR-T infusion, CMV reactivation occurred at a median of 16 days (IQR, 5–23), with a median viral load of 1,499 copies/mL (IQR, 1,045–2,477). Maribavir was used as first-line antiviral therapy in 84% of patients. *Figure 1* shows the distribution of CMV viral load at the time of maribavir initiation (median 2,477 copies/mL; IQR, 1,613–3,573). Maribavir was primarily used for grade 3–4 cytopenias (80%), followed by toxicity (12%) and antiviral refractoriness (8%).
Viral clearance was achieved in 100% of patients after a median of 8 days of treatment (IQR, 6–15). Median treatment duration was 14 days (IQR, 11–18). CMV reactivation after initial clearance occurred in 12% of patients, with no primary treatment failures. No maribavir-related toxicities, CMV-related hospitalizations, CMV disease, or CMV-related deaths were observed.
The estimated 12-month PFS was 55.1% (95% CI, 36.9–82.5%) *(Figure 2),* and 12-month OS was 67% (95% CI, 47–95.4%) *(Figure 3).* Median follow-up was 220 days (IQR, 97–335). A total of 7 patients (28%) died during follow-up, mainly due to progression of the underlying disease (16%), with additional deaths attributed to non-CMV infections (4%) and other causes (8%).

**Summary/Conclusion**

In this small retrospective cohort, maribavir provided effective viral control with minimal toxicity, supporting its use as a safe pre-emptive option for CMV reactivation in heavily immunocompromised CAR-T recipients. Larger prospective studies with comparative cohorts are needed to confirm these results.

**Copyright Information:** (Online) ISSN: 2572-9241
©2026 The Author(s). HemaSphere is published by John Wiley & Sons Ltd on behalf of European Hematology Association. This is an open access Abstract Book distributed under the Attribution-NonCommercial-NoDerivs (CC BY-NC-ND), which allows third parties to download the articles and share them with others as long as they credit the author and the Abstract Book, but they cannot change the content in any way or use them commercially.
**Abstract Book Citations:** Authors, Title, HemaSphere, 2026;10:(S1):pages. The abstract book DOIs can be found on the “Author Information” page.
**Disclaimer:** Articles published in the journal HemaSphere exclusively reflect the opinions of the authors. The authors are responsible for all content in their abstracts including accuracy of the facts, statements, citing resources, etc.

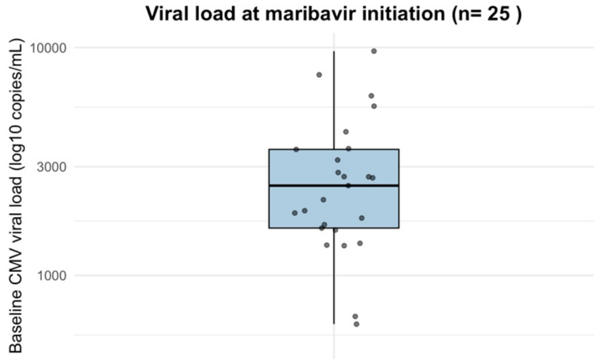

*Figure 1*

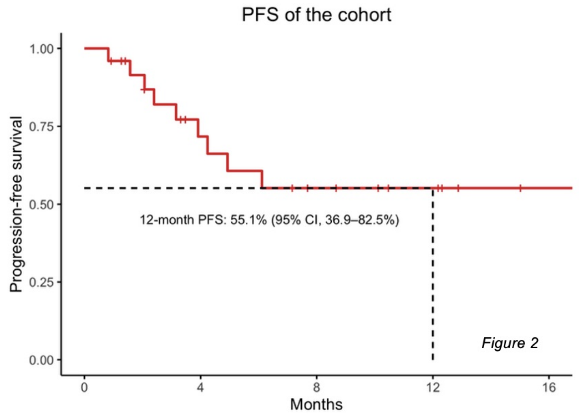

*Figure 2*

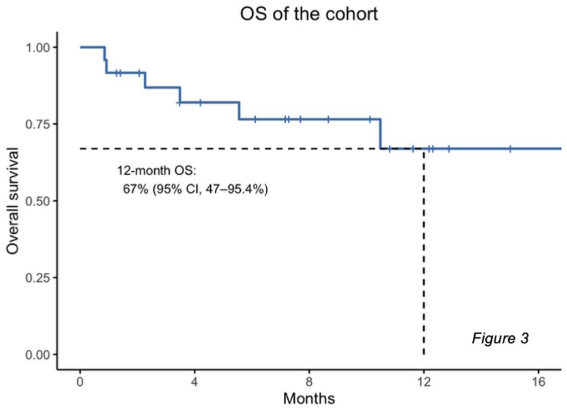

*Figure 3*

**Copyright Information:** (Online) ISSN: 2572-9241
©2026 The Author(s). HemaSphere is published by John Wiley & Sons Ltd on behalf of European Hematology Association. This is an open access Abstract Book distributed under the Attribution-NonCommercial-NoDerivs (CC BY-NC-ND), which allows third parties to download the articles and share them with others as long as they credit the author and the Abstract Book, but they cannot change the content in any way or use them commercially.
**Abstract Book Citations:** Authors, Title, HemaSphere, 2026;10:(S1):pages. The abstract book DOIs can be found on the "Author Information" page.
**Disclaimer:** Articles published in the journal HemaSphere exclusively reflect the opinions of the authors. The authors are responsible for all content in their abstracts including accuracy of the facts, statements, citing resources, etc.

### (PF1316) EFFICACY OF AN ANTIMICROBIAL STEWARDSHIP INTERVENTION FOR EARLY DISCONTINUATION OF ANTIBIOTIC THERAPY IN PATIENTS WITH FEBRILE NEUTROPENIA RECEIVING CELLULAR THERAPIES

**Topic:** 31. Infections in hematology (incl. supportive care/therapy)

Camilla Gariazzo*[1], Roberto Passera[2], Carolina Secreto[1], Chiara Maria Dellacasa[1], Sara Muccio[1], Francesca Bassi[1], Davide Stella[1], Scaldaferri Matilde[3], Silvia Corcione[4], Francesco Giuseppe Derosa[4], Alessandro Busca[1]

*[1]AOU Città della Salute e della Scienza, Stem Cell Transplant Unit, Torino, Italy;[2]University of Torino, Department of Medical Sciences, Torino, Italy; [3]AOU Città della Salute e della Scienza, S.C. Clinical Pharmacology, Torino, Italy;[4]AOU Città della Salute e della Scienza, Unit of Infectious Diseases, Torino, Italy;*

### Background

Febrile neutropenia (FN) is a frequent complication during cellular therapies but no consensus has been reached concerning optimal extent of empiric antibiotic treatment (EAT). Recently, scant studies demonstrated safety and efficacy of antibiotic cessation regardless of neutrophil recovery in hematologic patients.

### Aims

In October 2023, the Stem Cell Transplant Unit in Turin implemented a protocol for antimicrobial stewardship in neutropenic patients, in order to evaluate how the standardization of early EAT discontinuation would affect the total duration of treatment and impact patients' outcome.

### Methods

Patients receiving hematopoietic stem cell transplant (HSCT) or CAR-T therapy were enrolled in an intervention study for antibiotic discontinuation upon the first episode of FN, provided that they had no evidence of clinically (CDI) or microbiologically documented infection (MDI) and that they have been afebrile for more than 72 hours while on EAT. Data were compared to an historical group of patients who received cell therapy before the protocol development.
Primary endpoints were the difference of antibiotic days during the first episode of FN and the total number of days on treatment during hospitalization.
Infection-related mortality, fever relapse, rates of CDI, MDI and ICU admission and carbapenem use were identified as secondary endpoints.

**Copyright Information:** (Online) ISSN: 2572-9241
©2026 The Author(s). HemaSphere is published by John Wiley & Sons Ltd on behalf of European Hematology Association. This is an open access Abstract Book distributed under the Attribution-NonCommercial-NoDerivs (CC BY-NC-ND), which allows third parties to download the articles and share them with others as long as they credit the author and the Abstract Book, but they cannot change the content in any way or use them commercially.
**Abstract Book Citations:** Authors, Title, HemaSphere, 2026;10:(S1):pages. The abstract book DOIs can be found on the "Author Information" page.
**Disclaimer:** Articles published in the journal HemaSphere exclusively reflect the opinions of the authors. The authors are responsible for all content in their abstracts including accuracy of the facts, statements, citing resources, etc.

**Results**

Overall, 59 patients were eligible for early antibiotic discontinuation: 40 received HSCT and 19 received CAR-T; of the 59 patients eligible in the control group, 42 received HSCT and 17 CAR-T. Patients in the intervention and control group were analyzed separately according to the type of cell therapy (HSCT vs CAR-T).
Median absolute neutrophil count (ANC) at fever onset and at EAT discontinuation, as the median duration of neutropenia were superimposable between the study and control group, although HSCT patients experienced significantly longer median duration of neutropenia and lower ANC compared to CAR-T's.
Interestingly, median duration of EAT at first episode of FN was significantly lower in the intervention compared to the control group in both populations (6 days vs 8 days, p 0.001 for HSCT and 6 days vs 9 days, $p<0.001$ for CAR-T), as well as the total duration of antibiotic treatment during hospitalization (13 days vs 15 days, p 0.005 for HSCT and 6 days vs 9 days, $p<0.001$ for CAR-T).
Fever recurrence was more frequent in the intervention group receiving HSCT (62% vs 37%, p 0.051), while it was not different in the CAR-T group (0% vs 6%, p 0.472), although this did not translate into higher rates of CDI or MDI.
Additionally, there was a trend towards reduced use of carbapenems in the intervention group in patients receiving CAR-T therapy (41% vs 5%, p 0.016), while this was not confirmed for HSCT (48% vs 40%, p 0.513).
Median follow up was 12 vs 42 months in the study and control group, and KM estimates of OS were 84.7% and 66.1%, respectively.
Infection-related mortality occurred only in two patients in the intervention group, and no increase in ICU admissions was observed.

**Summary/Conclusion**

Our study demonstrated how antimicrobial stewardship was able to mitigate antibiotic duration both upon the first episode of FN and during the whole hospital stay in patients receiving cellular therapies. This effect did not translate into higher rates of MDI or CDI and showed a shift towards carbapenem sparing, providing proof of sustained efficacy of EAT and preserved safety in the hematological setting.

**Copyright Information:** (Online) ISSN: 2572-9241
©2026 The Author(s). HemaSphere is published by John Wiley & Sons Ltd on behalf of European Hematology Association. This is an open access Abstract Book distributed under the Attribution-NonCommercial-NoDerivs (CC BY-NC-ND), which allows third parties to download the articles and share them with others as long as they credit the author and the Abstract Book, but they cannot change the content in any way or use them commercially.
**Abstract Book Citations:** Authors, Title, HemaSphere, 2026;10:(S1):pages. The abstract book DOIs can be found on the “Author Information” page.
**Disclaimer:** Articles published in the journal HemaSphere exclusively reflect the opinions of the authors. The authors are responsible for all content in their abstracts including accuracy of the facts, statements, citing resources, etc.

### (PF1317) HBV-DNA–GUIDED MONITORING FOR HEPATITIS B VIRUS REACTIVATION ENABLES SAFE MANAGEMENT WITHOUT ROUTINE ANTIVIRAL PROPHYLAXIS AFTER CAR T-CELL THERAPY IN R/R LBCL

**Topic:** 31. Infections in hematology (incl. supportive care/therapy)

Risa Nishiyama*[1], Shinichi Makita[1], Wataru Takeda[2], Shotaro Watanabe[1], Yuta Ito[1], Noriko Iwaki[1], Suguru Fukuhara[1], Wataru Munakata[1], Koji Izutsu[1]

*[1]National Cancer Center Hospital, Department of Hematology, Tokyo, Japan;[2]National Cancer Center Hospital, Department of Hematopoietic Stem Cell Transplantation, Tokyo, Japan;*

### Background

Data regarding hepatitis B virus (HBV) reactivation after chimeric antigen receptor (CAR) T-cell therapy are limited, and optimal strategies for monitoring and prophylaxis remain unclear. In the US and Europe, prophylactic antiviral therapy has been recommended for patients (pts) with B-cell lymphoma who are positive for hepatitis B core antibody (HBcAb). In contrast, Japanese guidelines recommend HBV-DNA–guided monitoring for patients receiving anti-CD20 antibody-containing chemotherapy; however, specific recommendations for CAR-T therapy remain unestablished.

### Aims

This study aimed to clarify the incidence and clinical features of HBV reactivation after CAR T-cell therapy and to assess the feasibility of HBV-DNA–guided monitoring without routine antiviral prophylaxis.

### Methods

We retrospectively analyzed pts with large B-cell lymphoma who received anti-CD19 CAR T-cell therapy at National Cancer Center Hospital, Tokyo, Japan between December 2020 and January 2026. HBV serological status was confirmed in all pts prior to CAR T-cell infusion. According to the Japanese hepatitis B treatment guidelines, pts positive for either HBcAb or hepatitis B surface antibody (HBsAb) underwent serial HBV-DNA monitoring, except for those with isolated HBsAb positivity associated with prior HBV vaccination. We excluded pts with 1) isolated HBsAb positivity due to vaccination, 2) hepatitis B surface antigen (HBsAg) positivity requiring nucleotide analog therapy, 3) a history of HBV reactivation requiring nucleotide analog therapy prior to CAR T-cell therapy, or 4) early disease progression preventing HBV-DNA testing after CAR T-cell therapy.

### Results

A total of 163 pts underwent CAR T-cell therapy. At baseline, 131 pts were negative for all HBV serological markers. Among the 32 pts with positivity for at least one HBV serological marker, seven were excluded from analysis based on the criteria above (isolated HBsAb positivity with vaccination, n=1; HBsAg positivity, n=2; prior nucleotide analog therapy for reactivation, n=3; early disease progression, n=1). The remaining 25 pts underwent serial HBV-DNA monitoring; five pts were positive for HBsAb alone, four for HBcAb alone, and sixteen for both HBsAb and HBcAb. HBV-DNA reactivation occurred in two of 25 pts after CAR T-cell infusion at 1.2 and 12 months, respectively. Both pts were successfully treated with nucleotide analog therapy, resulting in decreased HBV-DNA levels promptly. No cases of hepatitis related to HBV reactivation were observed. Twenty-three pts maintained persistently negative HBV-DNA throughout follow-up. The median follow-up period for HBV-DNA monitoring was 16.9 months (range, 1.3–62.4), including 17 pts followed for more than one year.

**Copyright Information:** (Online) ISSN: 2572-9241
©2026 The Author(s). HemaSphere is published by John Wiley & Sons Ltd on behalf of European Hematology Association. This is an open access Abstract Book distributed under the Attribution-NonCommercial-NoDerivs (CC BY-NC-ND), which allows third parties to download the articles and share them with others as long as they credit the author and the Abstract Book, but they cannot change the content in any way or use them commercially.
**Abstract Book Citations:** Authors, Title, HemaSphere, 2026;10:(S1):pages. The abstract book DOIs can be found on the "Author Information" page.
**Disclaimer:** Articles published in the journal HemaSphere exclusively reflect the opinions of the authors. The authors are responsible for all content in their abstracts including accuracy of the facts, statements, citing resources, etc.

**Summary/Conclusion**

Although HBV reactivation can occur following CAR T-cell therapy, the incidence was low (2/25, 8%) in our cohort. Reactivation was successfully controlled through HBV-DNA–guided monitoring and timely nucleotide analog therapy, and no cases of hepatitis. Among pts with resolved HBV infection managed with HBV-DNA monitoring alone, 23 pts maintained persistently negative HBV-DNA levels and did not require prophylactic nucleotide analog. These findings highlight the clinical benefit of avoiding unnecessary long-term nucleotide analog exposure and suggest that an HBV-DNA–based monitoring strategy without routine prophylaxis represents a feasible management option after CAR T-cell therapy, particularly in regions where HBV-DNA testing is readily accessible.

Figure 1. Patient flow of HBV serological screening and outcomes of HBV-DNA–guided monitoring after CAR T-cell therapy

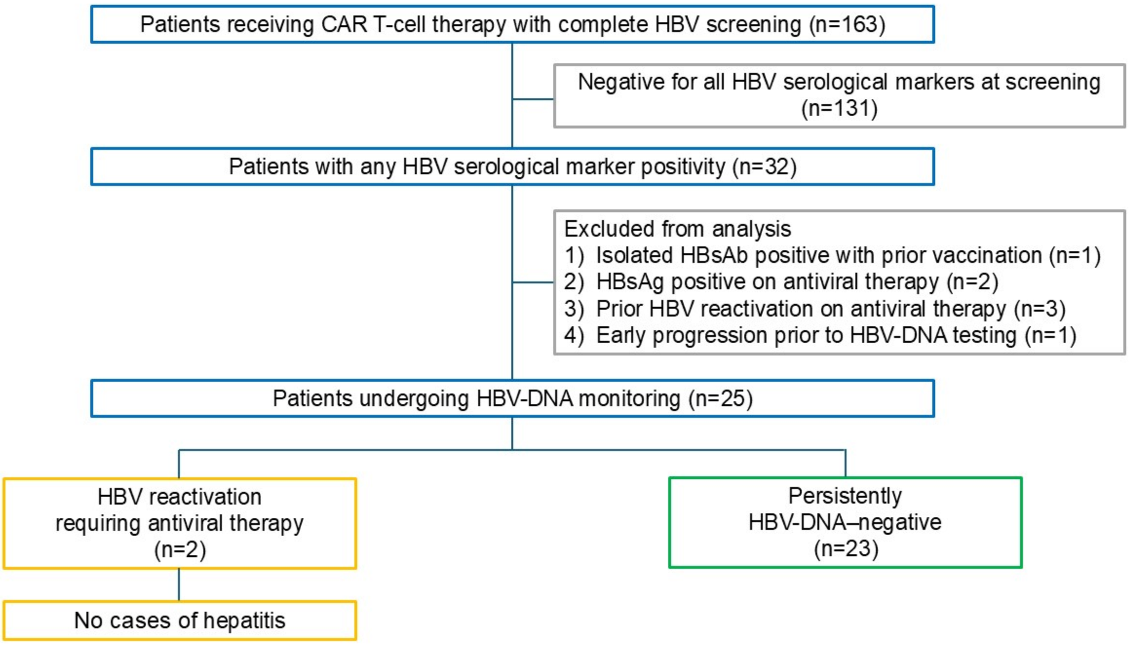

**Copyright Information:** (Online) ISSN: 2572-9241
©2026 The Author(s). HemaSphere is published by John Wiley & Sons Ltd on behalf of European Hematology Association. This is an open access Abstract Book distributed under the Attribution-NonCommercial-NoDerivs (CC BY-NC-ND), which allows third parties to download the articles and share them with others as long as they credit the author and the Abstract Book, but they cannot change the content in any way or use them commercially.
**Abstract Book Citations:** Authors, Title, HemaSphere, 2026;10:(S1):pages. The abstract book DOIs can be found on the "Author Information" page.
**Disclaimer:** Articles published in the journal HemaSphere exclusively reflect the opinions of the authors. The authors are responsible for all content in their abstracts including accuracy of the facts, statements, citing resources, etc.

### (PF1318) CLINICAL CHARACTERISTICS AND OUTCOMES OF SAPROCHAETE CLAVATA INFECTIONS IN PATIENTS WITH HEMATOLOGIC MALIGNANCIES: A SINGLE-CENTER SERIES

**Topic:** 31. Infections in hematology (incl. supportive care/therapy)

Ibrahim Talha Sisman*[1], Derya Demirtas[1], Fatma Arikan[1], Asu Fergün Aydın[1], Isik Atagunduz[1], Tayfur Toptas[1]

[1]*Department of Hematology, Marmara University School of Medicine, Istanbul, Türkiye;*

### Background

*Saprochaete clavata* is a rare opportunistic yeast increasingly recognized in patients with hematologic malignancies, particularly during profound or prolonged neutropenia and intensive supportive care. Limited clinical experience and isolation from both sterile and non-sterile sites create diagnostic uncertainty and management challenges, often leading to poor outcomes.

### Aims

This study describes host factors, specimen categories , antifungal management and early outcomes among patients with hematologic malignancies with culture positive *Saprochaete clavata* at a single center.

### Methods

Consecutive patients with hematologic malignancies and culture-positive *S. clavata* (Jan 2020–July 2025) were retrospectively reviewed. Variables included malignancy, transplant status, supportive care, and neutropenia duration. Episodes were categorized as sterile-site positive (blood/catheter), non-sterile only (urine/respiratory), or multiple sites. Antifungal therapy and 14-/30-day all-cause mortality were reported as n/N (%) or median (range).

### Results

Twelve patients were reviewed (6/12 male, 50%; median age 47 years [33–79]). Underlying malignancies were AML (4/12, 33%), ALL (3/12, 25%), CLL (2/12, 17%), HL (2/12, 17%), and T-ALL (1/12, 8%); 5/12 (42%) occurred after allogeneic HSCT. Supportive-care exposures were common: central venous catheter 11/12 (92%), mucositis 4/12 (33%), TPN 4/12 (33%), and mechanical ventilation 4/12 (33%). Median neutropenia duration before the first positive culture was 10 days (0–135) for ANC <500/µL  and 2 days (0–116) for ANC <100/µL ; post-culture medians were 6 days (0–19) and 3 days (0–19), respectively. Episodes were sterile-site positive in 6/12 (50%) and non-sterile only in 6/12 (50%), with ≥2 specimen sources in 4/12 (33%). Bloodstream infection without additional focus and suspected dissemination were each recorded in 3/12 (25%); galactomannan was positive in 5/12 (42%). Targeted antifungal therapy was given in 8/12 (67%) and withheld in 4/12 (33%) when isolates were initially interpreted as colonization. Initial therapy was amphotericin B in 5/12 (42%) and voriconazole in 3/12 (25%); overall exposure was 8/12 (67%) and 4/12 (33%), respectively. All-cause mortality was 5/12 (42%) at 14 days and 8/12 (67%) at 30 days; median culture-to-death was 19 days (1–192) among deaths (n=11).

**Copyright Information:** (Online) ISSN: 2572-9241
©2026 The Author(s). HemaSphere is published by John Wiley & Sons Ltd on behalf of European Hematology Association. This is an open access Abstract Book distributed under the Attribution-NonCommercial-NoDerivs (CC BY-NC-ND), which allows third parties to download the articles and share them with others as long as they credit the author and the Abstract Book, but they cannot change the content in any way or use them commercially.
**Abstract Book Citations:** Authors, Title, HemaSphere, 2026;10:(S1):pages. The abstract book DOIs can be found on the "Author Information" page.
**Disclaimer:** Articles published in the journal HemaSphere exclusively reflect the opinions of the authors. The authors are responsible for all content in their abstracts including accuracy of the facts, statements, citing resources, etc.

### Summary/Conclusion

In this series, *Saprochaete clavata* presented as a highly lethal opportunistic pathogen in patients with hematologic malignancies, characterized by substantial 14-day and 30-day all-cause mortality rates of 42% and 67%, respectively.  The high prevalence of central venous catheter use and prolonged neutropenia underscores the vulnerability of this cohort. Notably, the galactomannan positivity highlights a significant diagnostic pitfall, as cross-reactivity may lead to the misidentification of invasive aspergillosis. Furthermore, the fact that one-third of patients received no targeted therapy due to isolates being interpreted as colonization, despite nearly universal mortality,demonstrates an urgent need for standardized diagnostic criteria to differentiate colonization from invasive disease. Given the high mortality and the rapid disease course, early clinical suspicion and prompt initiation of appropriate antifungal therapy are critical once *S. clavata* is identified in high-risk neutropenic and post-transplant patients.

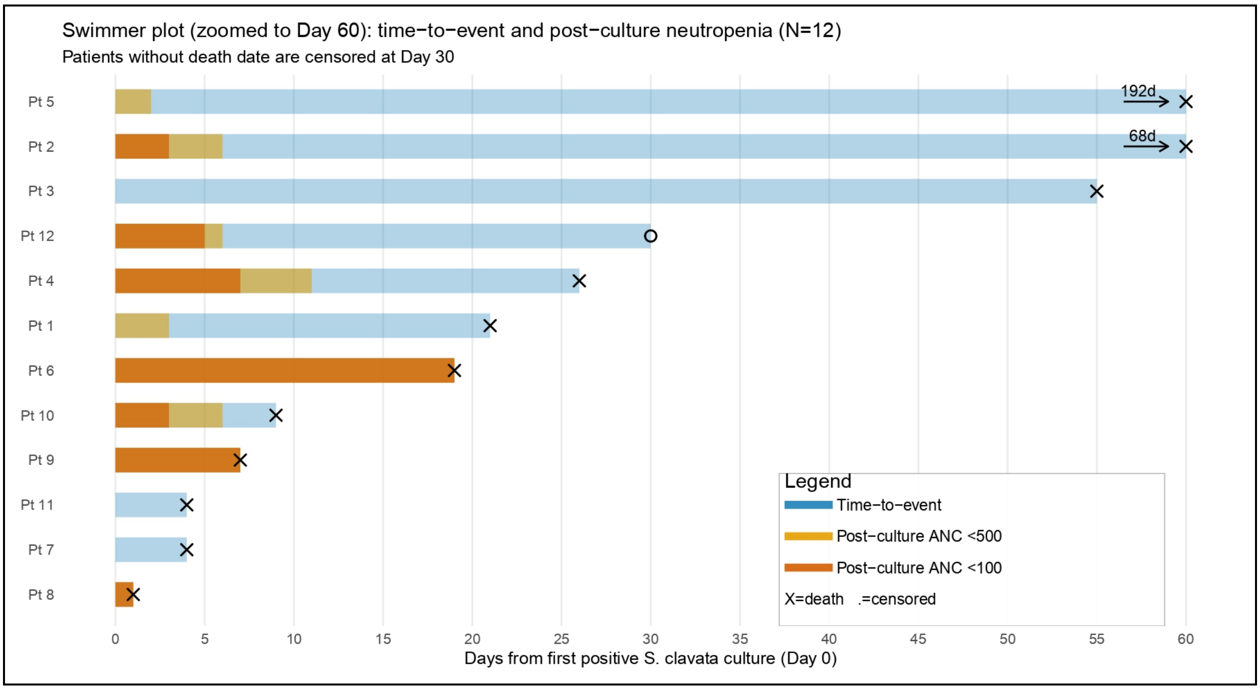

Figure 1. Swimmer plot showing time from first Saprochaete clavata culture (Day 0) to death (X) or censoring (○).

**Copyright Information:** (Online) ISSN: 2572-9241
©2026 The Author(s). HemaSphere is published by John Wiley & Sons Ltd on behalf of European Hematology Association. This is an open access Abstract Book distributed under the Attribution-NonCommercial-NoDerivs (CC BY-NC-ND), which allows third parties to download the articles and share them with others as long as they credit the author and the Abstract Book, but they cannot change the content in any way or use them commercially.
**Abstract Book Citations:** Authors, Title, HemaSphere, 2026;10:(S1):pages. The abstract book DOIs can be found on the "Author Information" page.
**Disclaimer:** Articles published in the journal HemaSphere exclusively reflect the opinions of the authors. The authors are responsible for all content in their abstracts including accuracy of the facts, statements, citing resources, etc.

### (PF1319) COMMON RESPIRATORY VIRUS DETECTION BY TARGETED NEXT-GENERATION SEQUENCING IN PEDIATRIC HEMATOLOGICAL PATIENTS: A STRATIFIED ANALYSIS BY HSCT STATUS AND UNDERLYING DISEASE

**Topic:** 31. Infections in hematology (incl. supportive care/therapy)

Changwen Xue*[1], Chunhui Xu[1], Yang Wan[1], Wenyu Yang[1], Xiaofan Zhu[1]

[1] *Institute of Hematology & Blood Diseases Hospital, Chinese Academy of Medical Sciences, Tianjin, China;*

### Background

Pediatric patients with hematological diseases are at high risk for respiratory viral infections. Targeted next-generation sequencing (tNGS) enables comprehensive virus detection, but the epidemiology of common respiratory viruses across different clinical contexts remains to be elucidated.

### Aims

To compare the detection rates, profiles, and co-infection patterns of common respiratory viruses (influenza virus, SARS-CoV-2, human coronavirus, parainfluenza virus, respiratory syncytial virus, rhinovirus, human metapneumovirus, bocavirus, and adenovirus) between samples from hematopoietic stem cell transplantation (HSCT) and non-HSCT pediatric hematological patients with suspected respiratory infections, with further stratification by underlying disease type.

### Methods

We retrospectively analyzed 970 tNGS samples from 579 pediatric hematological patients with suspected respiratory infections at the Institute of Hematology & Blood Diseases Hospital, Chinese Academy of Medical Sciences, Tianjin, China. Samples were stratified by HSCT status at the time of sampling: HSCT group (136 patients, 230 samples) and non-HSCT group (497 patients, 740 samples); 54 patients contributed samples to both groups. Samples were further stratified by underlying disease type (benign vs. malignant). Detection rates of nine common respiratory viruses and viral co-infection patterns were compared between groups. Samples with no common respiratory virus detected were classified as negative for the purpose of this analysis.

### Results

The overall detection rate of common respiratory viruses was comparable between HSCT and non-HSCT samples (38.7% vs. 34.6%, P=0.284). However, malignant disease samples had significantly higher virus detection than benign disease samples (37.4% vs. 25.3%, P=0.004). The highest detection rate was observed in HSCT-malignant samples (41.7%), followed by non-HSCT-malignant (36.3%), HSCT-benign (29.9%), and non-HSCT-benign samples (21.8%). Viral co-infections (≥2 viruses) were significantly more frequent in HSCT than non-HSCT samples (21.3% vs. 10.9%, P=0.011). Pathogen profiles differed significantly: adenovirus (4.3% vs. 1.1%, P=0.001) and bocavirus (2.6% vs. 0.5%, P=0.009) were more frequent in HSCT samples. Within the HSCT group, rhinovirus was more common in malignant than benign samples (16.6% vs. 4.5%, P=0.013), while human coronavirus showed a trend toward being more frequent in benign samples (9.0% vs. 3.7%, P=0.091). Within the non-HSCT group, parainfluenza virus and SARS-CoV-2 showed trends toward higher detection in malignant samples (P=0.084 and P=0.059, respectively).

**Copyright Information:** (Online) ISSN: 2572-9241
©2026 The Author(s). HemaSphere is published by John Wiley & Sons Ltd on behalf of European Hematology Association. This is an open access Abstract Book distributed under the Attribution-NonCommercial-NoDerivs (CC BY-NC-ND), which allows third parties to download the articles and share them with others as long as they credit the author and the Abstract Book, but they cannot change the content in any way or use them commercially.
**Abstract Book Citations:** Authors, Title, HemaSphere, 2026;10:(S1):pages. The abstract book DOIs can be found on the "Author Information" page.
**Disclaimer:** Articles published in the journal HemaSphere exclusively reflect the opinions of the authors. The authors are responsible for all content in their abstracts including accuracy of the facts, statements, citing resources, etc.

### Summary/Conclusion

Targeted next-generation sequencing reveals distinct patterns of common respiratory virus detection in pediatric hematological patients stratified by HSCT status and underlying disease type. Malignant disease and HSCT status are the risk factors for respiratory virus infections. The higher rates of viral co-infections and adenovirus/bocavirus detection in HSCT samples, and the predominance of rhinovirus in HSCT-malignant patients, suggest virus-specific vulnerabilities that may inform early surveillance and prophylactic treatment strategies in this vulnerable population.

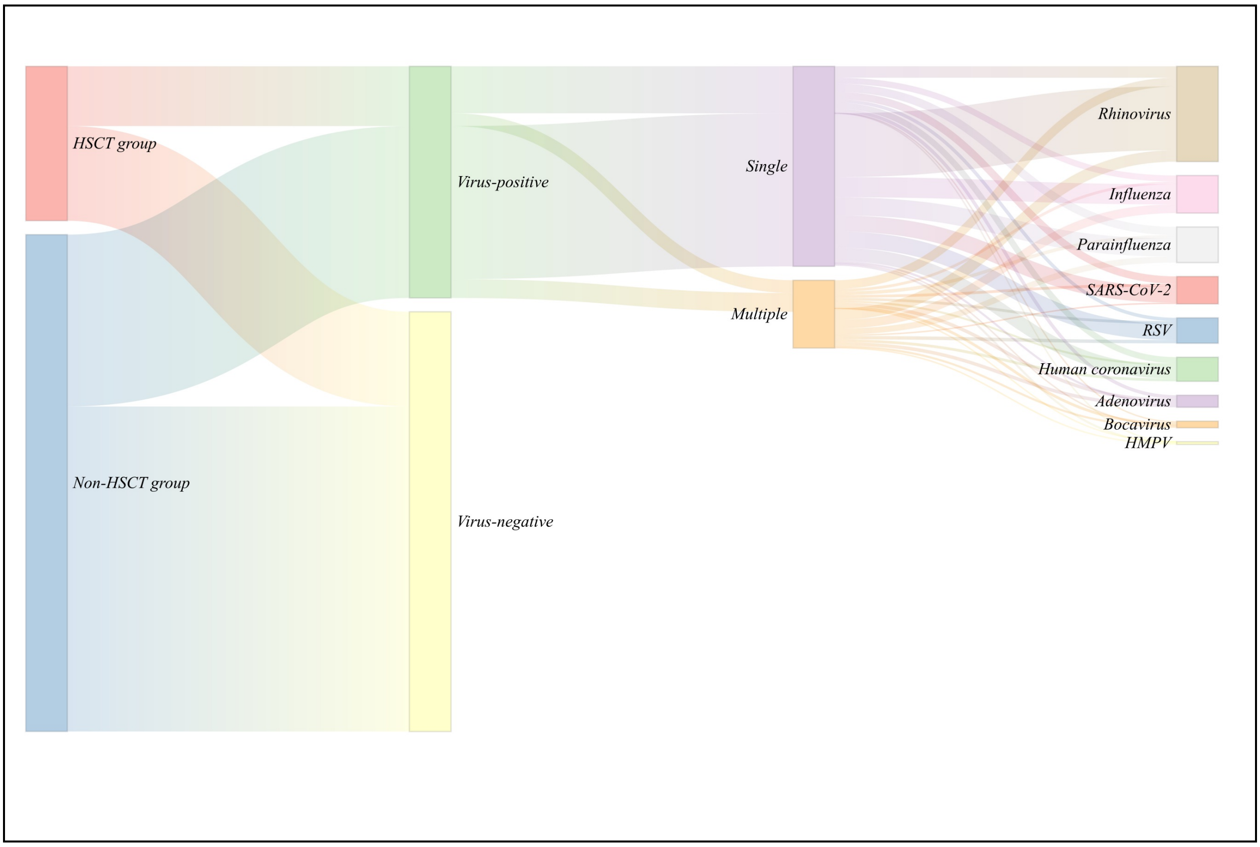

Figure 1. Common Respiratory Virus Detection by Targeted Next-Generation Sequencing in HSCT versus Non-HSCT Pediatric Hematological Patients. Sankey diagram illustrating the flow from 970 tNGS samples to virus detection outcomes, stratified by hematopoietic stem cell transplantation (HSCT) status at the time of sampling. Node widths are proportional to sample numbers. For "multiple" node, each sample may be counted multiple times.

**Copyright Information:** (Online) ISSN: 2572-9241
©2026 The Author(s). HemaSphere is published by John Wiley & Sons Ltd on behalf of European Hematology Association. This is an open access Abstract Book distributed under the Attribution-NonCommercial-NoDerivs (CC BY-NC-ND), which allows third parties to download the articles and share them with others as long as they credit the author and the Abstract Book, but they cannot change the content in any way or use them commercially.
**Abstract Book Citations:** Authors, Title, HemaSphere, 2026;10:(S1):pages. The abstract book DOIs can be found on the "Author Information" page.
**Disclaimer:** Articles published in the journal HemaSphere exclusively reflect the opinions of the authors. The authors are responsible for all content in their abstracts including accuracy of the facts, statements, citing resources, etc.

### (PF1320) INTRAVENOUS IMMUNOGLOBULIN REPLACEMENT THERAPY IS ASSOCIATED WITH REDUCTION IN BOTH SEVERE AND ALL GRADE INFECTIONS IN PATIENTS WITH B-CELL MALIGNANCIES

**Topic:** 31. Infections in hematology (incl. supportive care/therapy)

Elly Stylianou[1], Johanna Sikl[1], Charlotte Schostok[1], Elisa Schulze[1], Vadim Lesan[2], Lorenz Thurner[1], Igor Age Kos[1], Onur Cetin[1], Sarah Altmeyer[1], Jörg Thomas Bittenbring[1], Philipp Staber[1, 3], Konstantinos Christofyllakis*[1, 4]

*[1]Saarland University Medical Center, Homburg, Germany; [2]Hopitaux Schuman, Luxembourg, Luxembourg; [3]Medical University Vienna, Vienna, Austria; [4]Frankfurt Oder Academic Hospital, Frankfurt Oder, Germany;*

### Background

Patients with hematologic malignancies are at high risk for hypogammaglobinemia which can result in high infection rates and reduced survival, either through infection-related morbidity and therefore reduced tolerance to anti-cancer therapy, or mortality. Immunoglobulin replacement therapy (IgRT) can be offered to patients who exhibit either proven specific antibody failure or a serum IgG level of <4 g/L and suffer from severe or recurrent infections. IgRT can also be given off-label preemptively in patients at high risk for severe infections such as those receiving CAR-T or bispecific antibodies.

### Aims

To determine the degree of benefit of IgRT for patients with lymphoproliferative diseases of B cell origin.

### Methods

We performed a retrospective single centre analysis in a universtity hospital in the post SARS-CoV-2 pandemic era. We included all patients diagnosed with lymphoproliferative diseases of B cell origin between 2023 and 2024. Patients without at least one follow up data point after diagnosis and those with primary immunodeficiency were excluded. The primary endpoints were rate of infections as well as severe infections (grade 3-5 according to CTCAE 5.0). The 3- and 6-month intervals prior to and after the first immunoglobulin dose were compared with regard to infection rates. A paired t-test was performed for values that followed the normal distribution, otherwise a Wilcoxon signed-rank test was used.

**Copyright Information:** (Online) ISSN: 2572-9241
©2026 The Author(s). HemaSphere is published by John Wiley & Sons Ltd on behalf of European Hematology Association. This is an open access Abstract Book distributed under the Attribution-NonCommercial-NoDerivs (CC BY-NC-ND), which allows third parties to download the articles and share them with others as long as they credit the author and the Abstract Book, but they cannot change the content in any way or use them commercially.
**Abstract Book Citations:** Authors, Title, HemaSphere, 2026;10:(S1):pages. The abstract book DOIs can be found on the “Author Information” page.
**Disclaimer:** Articles published in the journal HemaSphere exclusively reflect the opinions of the authors. The authors are responsible for all content in their abstracts including accuracy of the facts, statements, citing resources, etc.

**Results**

The database search identified a total of 870 eligible patients. Of those, 78 (9.0%) received IgRT and were included in the analysis (chronic lymphatic leukaemia 23%, follicular lymphoma 9%, mantle cell lymphoma 9%, M. Waldenström 9%, marginal zone lymphoma 6%, large B cell lymphoma 13%, transformed indolent B-cell lymphomas 8%, B-cell acute lymphoblastic leukaemia 10%, other 13%). Median age was 63.5 years, and 41% of patients were female. Treatment regimens included: 86% anti-CD20 antibody, 7% anti-CD19 antibody, 17% anti-CD20/CD3 bispecific antibody, 34% had received CD19 directed CAR-T cells, 13% allogeneic and 14% autologous hematopoietic stem cell transplant. Median IgG levels prior to start of IgRT were 369.5 mg/dl, and 32 (53%) of patients had IgG levels lower than 4 g/L. A total of 193 infections were recorded (median per patient 2.0, range 0-9). Of those, 100 events were grade 3-5, median per patient 1.0, (range 0-6). In patients receiving IgRT, infection rate dropped from 0.78 (±0.92) in the 3 months prior to 0.40 (±0.65) in the 3-month interval after the first immunoglobulin dose (p = .005). A similar decrease was shown for the 6 months interval (1.04 (±1.20) infections prior vs 0.56 (±0.80) after, p = .004). Accordingly, grade 3-5 infection rate also decreased after IgRT (3-m: 0.49 (±0.80) grade 3-5 infections prior vs 0.17 (±0.41) after, p = .003, 6-m: 0.62 (±0.96) infections prior vs 0.23 (±0.53) after, p = .003). In the sensitivity analysis excluding patients receiving CAR-T, bispecific antibodies or stem cell transplantation (n = 37), infection rates significantly decreased after IgRT at 3 months (p = .003) and 6 months (p = .002). Grade 3-5 infections also decreased significantly at 3 months (p = .008), while the reduction at 6 months showed a statistical trend but did not reach significance (p = .052).

**Summary/Conclusion**

Immunoglobulin replacement therapy is associated with a significant decrease of infection rate in patients with B-cell malignancies.

**Copyright Information:** (Online) ISSN: 2572-9241
©2026 The Author(s). HemaSphere is published by John Wiley & Sons Ltd on behalf of European Hematology Association. This is an open access Abstract Book distributed under the Attribution-NonCommercial-NoDerivs (CC BY-NC-ND), which allows third parties to download the articles and share them with others as long as they credit the author and the Abstract Book, but they cannot change the content in any way or use them commercially.
**Abstract Book Citations:** Authors, Title, HemaSphere, 2026;10:(S1):pages. The abstract book DOIs can be found on the "Author Information" page.
**Disclaimer:** Articles published in the journal HemaSphere exclusively reflect the opinions of the authors. The authors are responsible for all content in their abstracts including accuracy of the facts, statements, citing resources, etc.

### (PF1321) E-IMPACT: EUROPEAN MULTICENTER PRACTICE ASSESSMENT OF CELL THERAPY AND BISPECIFICS – CHARACTERIZING INFECTION MANAGEMENT AND PROPHYLAXIS IN THE REAL-WORLD SETTING.

**Topic:** 31. Infections in hematology (incl. supportive care/therapy)

Maria Stefania Infante*[1], Jung-Ah Kim[2], Andrea Gutierrez Villanueva[3], Alejandro Diaz Diaz[4], Alejandro Avendaño Pita[5], Andrea Murtas[6], Angela Loizidou[7], Antonjia Verhaz[8], Wing Yan Au[9], Avinash Aujayeb[10], Nargiza Babakhanova[11], Bahar Uncu Ulu[12], Ioanna Sakellari[13], Boudhayan Bhattacharjee[14], Qing Cao[15], Charikleia Kelaidi[16], Chidiebere Ndubueze[17], Chizaram Onyeaghala[18], Alberto Garcia-Salido[19], Claudio Cerchione[20], Dafna Yahav[21], Adoracion Gonzalez[22], Delia Dima[23], Christian Poulsen[24], Soumyadip Chatterji[25], Nikola Pantic[26], Maria Eduarda Ruiz Pena[27], Elena Rossi[28], Eleni Gavrilaki[29], Fabiana Dellai[30], Fabio Forghieri[31], Francesca Farina[32], Filippo Luciani[33], Güldane Cengiz Seval[12], Gulhadiye Acvu[34], Abubakar Gwarzo[35], Habip Gedik[36], Abubakar Mursal[37], Ildefonso Espigado[38], Alexey Maximov[39], José-Angel Hernández-Rivas[1], Josia Fauser[40], Juan Pablo Cabrera Guerrero[41], Arda Isik[42], Koray Yalçın[43], Lorenzo Chiusaroli[44], Lubos Drgona[45], Lucrecia Yáñez[46], Mathias Brehon[47], Anastasia Athanasiadou[29], Manuela Spadea[48], Margherita Malchiodi[49], Mario Virgilio Papa[44], Martin Čerňan[50], Paloma Merino[51], Nikolaos Syrmos[29], Milan Navrátil[52], Carolina Miranda[53], Raeseok Lee[54], Marcio Nucci[55], Andrea Mohorea-Neata[56], Marta Sitges Arriaga[57], Nazish Arshad[58], Nuno Principe[59], Raluca Papagheorghe[60], Rogelio de Jesus Treviño-Rangel[61], Rosanne Sprute[62], Rui Bergantim[63], Sanjay Bhattacharya[64], Sapura Ibragimova[65], Sonia Pi Ruano[66], Jiří Šrámek[67], Stephen Ogbere[68], Syed Abdullah[69], Terenzio Cosio[70], Toine Mercier[71], Toni Valkovic[72], Ulrich Jaeger[73], Vanesa Castro[74], Barbora Weinbergerova[75], Zaira Palacios Baena[38], Mi Zhou[76], Oliver A. Cornely[77], Livio Pagano[78], Jon Salmanton-García[79]

*[1]University Hospital Infanta Leonor, Madrid, Spain; [2]Soonchunhyang University Hospital, Seoul, South Korea; [3]Hospital Puerta de Hierro, Madrid, Spain; [4]Hospital Pablo Tobon Uribe, Medellin, Colombia; [5]Hospital Universitario de Salamanca, Salamanca, Spain; [6]azienda ospedaliero-universitario di Sassari, Sassari, Italy; [7]Institut jules bordet, Brussels, Belgium; [8]Clinic for Infectious diseases, Banja Luka, Bosnia and Herzegovina; [9]Hong Kong Sanatorium, Hong Kong, Hong Kong; [10]northumbria Helathcare NHS Foundation Trust, Newcastle, United Kingdom; [11]Tashkent Hospital, Tashkent, Uzbekistan; [12]Anakara Oncology Hospital, Ankara, Türkiye; [13]general hospital george papanikolaou, Thessaloniki, Greece; [14]School of tropical medicine, Kolkata, India; [15]Shangai Children medical hospital, shangai, China; [16]aghia sophia children hospital, athens, Greece; [17]University of Nigeria Teaching hospital, Enugu, Nigeria; [18]University of Port Harcourt Teaching hospital, Port harcourt, Nigeria; [19]Hospital Infantil Universitario Niño Jesus, Madrid, Spain; [20]IRST IRCCS hematology unit, meldola, Italy; [21]sheba medical center, ramat gan, Israel; [22]instituto valenciano de oncologia, valencia, Spain; [23]Ion Chiricuta Oncology Institute, cluj-napoca, Romania; [24]Zealand University Hospital, Rosklinde, Denmark; [25]Tata Medical Center, Kolkata, India; [26]University clinical center of Serbia, Belgrade, Serbia; [27]Pedro Hispano Hospital, Matosinhos, Portugal; [28]Hospital Gemelli, Rome, Italy; [29]aristotle university hospital, thessaloniki, Greece; [30]ospedale santa maria della misericordia, udine, Italy; [31]AOU Modena, Modena, Italy; [32]IRCSS San Raffaele, Milano, Italy; [33]azienda sanitaria provinciale di cosenza, cosenza, Italy; [34]ege university hospital, izmir, Türkiye; [35]federal university dutse, Jigawa state, Nigeria; [36]University of health science, instanbul, Türkiye; [37]university of maiduguri teaching hospital, maiduguri, Nigeria; [38]HU Virgen Macarena, Sevilla, Spain; [39]AF Tsyb scientific center, obninsk, Russia; [40]Innsbruck Hospital, Innsbruck, Austria; [41]Hospital regional de alta especialidad, mexico city, Mexico; [42]Instanbul Medeniyet University, instanbul, Türkiye; [43]Bahcesehir University, instanbul, Türkiye; [44]university of padua, padua, Italy; [45]Department of Oncohematology, Comenius University and National Cancer Institute, bratislava, Slovenia; [46]University Hospital Marques de Valdecilla, santander, Spain; [47]Medipole Hopital Prive, villeurbanne, France; [48]University of Turin, turin, Italy; [49]Università degli Studi di Siena, siena, Italy; [50]Palacký University Olomouc and University Hospital Olomouc, olomouc, Czechia; [51]hospital clinico san carlos, madrid, Spain; [52]University Hospital Ostrava, ostrava, Czechia; [53]hospital universitario rey juan carlos, mostoles, Spain; [54]seoul st. mary's hospital, seul, South Korea; [55]Universidade Federal do Rio de Janeiro, rio de janeiro, Brazil; [56]fundeni clinical institute, bucarest, Romania; [57]ICO GIRONA, girona, Spain; [58]dow university of health science, karachi, Pakistan; [59]unidade local de saude sao joao, porto, Portugal; [60]coltea clinical hospital, bucarest, Romania; [61]universidad autonoma de nuevo leon, monterrey, Mexico; [62]university hospital cologne, cologne, Germany; [63]Hospital Lusiadas Porto, porto, Portugal; [64]tata medical center, kolkata, India; [65]center of pediatric oncology, tashkent, Uzbekistan; [66]hospital arnau de vilanova-Lliria,*

Copyright Information: (Online) ISSN: 2572-9241
©2026 The Author(s). HemaSphere is published by John Wiley & Sons Ltd on behalf of European Hematology Association. This is an open access Abstract Book distributed under the Attribution-NonCommercial-NoDerivs (CC BY-NC-ND), which allows third parties to download the articles and share them with others as long as they credit the author and the Abstract Book, but they cannot change the content in any way or use them commercially.
Abstract Book Citations: Authors, Title, HemaSphere, 2026;10:(S1):pages. The abstract book DOIs can be found on the "Author Information" page.
Disclaimer: Articles published in the journal HemaSphere exclusively reflect the opinions of the authors. The authors are responsible for all content in their abstracts including accuracy of the facts, statements, citing resources, etc.

valencia, Spain; [67]University Hospital Plzeň - Lochotín, pilsen, Czechia; [68]federal medical center, yenagoa, Nigeria; [69]dr ziauddin university hospital, karachi, Pakistan; [70]universita cattolica del sacro cuore, roma, Italy; [71]AZ Sint-Maarten / KU Leuven, mechelen, Belgium; [72]general hopspital pula, pula, Croatia; [73]medical university of vienna, wien, Austria; [74]hospital de salnes, vilagarcia, Spain; [75]masaryk university hospital brno, brno, Czechia; [76]children hospital of soochow university, suzhou, China; [77]Department I for Internal Medicine, University Hospital of Cologne, cologne, Germany; [78]Fondazione Policlinico A. Gemelli – IRCCS – Universita' Cattolica Del Sacro Cuor, roma, Italy; [79]Institute of Translational Research, University of Cologne, cologne, Germany;

### Background

**Background:** Bispecific antibodies (BsAbs) and CAR-T cell therapies have significantly improved outcomes in relapsed/refractory hematologic malignancies but are associated with profound and prolonged immunosuppression. This includes B-cell aplasia, T-cell depletion, and hypogammaglobulinemia, resulting in a high risk of opportunistic infections. Current clinical management varies significantly due to a lack of harmonized international guidelines.

### Aims

To evaluate current European and international clinical practices for infection prophylaxis, monitoring, and management in patients treated with BsAbs and CAR-T therapies.

### Methods

The E-IMPACT study is a multicenter survey-based assessment. An anonymous questionnaire was distributed to 74 hematology units globally, primarily from Spain (16.2%), Italy (12.1%) and Turkey (8.1%). The survey evaluated protocols for bacterial, viral, fungal, and *Pneumocystis jirovecii* (PJP) prophylaxis, alongside cytopenia and hypogammaglobulinemia management

### Results

The survey revealed significant heterogeneity in management strategies:

- **Viral and PJP Prophylaxis:** Prophylaxis for PJP is nearly universal for both BsAbs (95.6%) and CAR-T (96.5%). Antiviral prophylaxis (acyclovir/valacyclovir) is highly implemented, though more frequent in CAR-T (91.4%) than BsAbs (75.0%).
- **Antibacterial and Antifungal:** Antibacterial prophylaxis is used in 75.9% of CAR-T vs. 66.2% of BsAbs patients; cotrimoxazole is the most common agent for BsAbs (45.6%). Antifungal prophylaxis is more frequent in CAR-T (86.2%) compared to BsAbs (63.2%), primarily targeted at patients with severe/prolonged neutropenia.
- **Prophylaxis duration:** Antibacterial prophylaxis is mantained until neutropenia resolves in 29.9% of BsAbs vs 31% of CAR-T patients. Duration of PJP and viral prophylaxis remains inconsistent across institutions.
- **CMV Management:** Monitoring for CMV-DNA by PCR is not performed in 27.6% of BsAbs centers and 12.1% of CAR-T centers. Weekly monitoring is more common in CAR-T (22.4%) than BsAbs (13.8%). A pre-emptive strategy is the most frequent approach (45.6% for BsAbs, 39.7% for CAR-T).
- **Immunodeficiencies and Cytopenias:** IgG monitoring is standard (93% for BsAbs; 96% for CAR-T), with most centers checking monthly. However, replacement therapy is largely restrictive: 59.7% (BsAbs) and 47.4% (CAR-T) initiate IgG replacement only when levels fall below a specific threshold (typically <400 mg/dL). G-CSF is used routinely by 37.3% of centers for BsAbs and 35.1% for CAR-T.

- **Infection Frequency:** Bacterial infections were perceived as the most frequent complication across both therapy types.

**Copyright Information:** (Online) ISSN: 2572-9241
©2026 The Author(s). HemaSphere is published by John Wiley & Sons Ltd on behalf of European Hematology Association. This is an open access Abstract Book distributed under the Attribution-NonCommercial-NoDerivs (CC BY-NC-ND), which allows third parties to download the articles and share them with others as long as they credit the author and the Abstract Book, but they cannot change the content in any way or use them commercially.
**Abstract Book Citations:** Authors, Title, HemaSphere, 2026;10:(S1):pages. The abstract book DOIs can be found on the "Author Information" page.
**Disclaimer:** Articles published in the journal HemaSphere exclusively reflect the opinions of the authors. The authors are responsible for all content in their abstracts including accuracy of the facts, statements, citing resources, etc.

**Summary/Conclusion**

E-IMPACT demonstrates that while PJP and viral prophylaxis are well-established, there is substantial variability in CMV surveillance, antifungal use, and immunoglobulin replacement criteria.
The disparities in management across centers highlight the urgent need for standardized European clinical guidelines to optimize the safety and therapeutic efficacy of these innovative therapies.

**Copyright Information:** (Online) ISSN: 2572-9241
©2026 The Author(s). HemaSphere is published by John Wiley & Sons Ltd on behalf of European Hematology Association. This is an open access Abstract Book distributed under the Attribution-NonCommercial-NoDerivs (CC BY-NC-ND), which allows third parties to download the articles and share them with others as long as they credit the author and the Abstract Book, but they cannot change the content in any way or use them commercially.
**Abstract Book Citations:** Authors, Title, HemaSphere, 2026;10:(S1):pages. The abstract book DOIs can be found on the "Author Information" page.
**Disclaimer:** Articles published in the journal HemaSphere exclusively reflect the opinions of the authors. The authors are responsible for all content in their abstracts including accuracy of the facts, statements, citing resources, etc.

### (PF1322) HOW TO IMPLEMENT VACCINATION COVERAGE IN MULTIPLE MYELOMA PATIENTS: A SINGLE-CENTER EXPERIENCE

**Topic:** 31. Infections in hematology (incl. supportive care/therapy)

Mohammadreza Khosropoor[1], Antonella Ippolito[1], Gabriele Giorgianni[2], Bruno Garibaldi[1], Claudia Bellofiore[1], Concetta Conticello[1], Francesco Di Raimondo[1, 3], Rosalia Ragusa[1], Alessandra Romano*[1, 3]

*[1]AOU Policlinico Rodolico San Marco, UOC Ematologia, Catania, Italy;[2]AOU Policlinico Rodolico San Marco, UO Radiologia, Catania, Italy; [3]Università degli Studi di Catania, Dipartimento di Chirurgia e Specialità Medico Chirurgiche, Catania, Italy;*

### Background

Infections are a major clinical problem in the management of multiple myeloma (MM) due to both disease- and treatment-related factors. Measures to prevent infection, such as vaccinations, are therefore of paramount importance in the clinical care of MM patients.

### Aims

Since data regarding the burden of Respiratory Syncytial virus (RSV) infection is of particular interest given the recent approval and full reimbursable of two immunogenic prefusion F-vaccines against RSV in Sicily, Italy, we designed a survey to verify the coverage and efficacy of VRS vaccination.

### Methods

First, we retrospectively analyzed the vaccination coverage in 716 MM/MGUS patients registered in the Catania–Messina–Enna Integrated Cancer Registry between 2000 and 2023. Then, we prospectively collected data about vaccination rate in 297 consecutive MM patients referring to our center between since October 2024. Seasonal vaccination against flu (FV) and S. pneumoniae (PV) was recommended to all patients, independently from age, disease status or treatment in co-administration associated to VRS, H. Zoster and COVID-19. All vaccinations were given by general practitioner according to Italian law for prevention activities and but from October 2024 our Center offered vaccination in the hematological center.

### Results

In the retrospective study, 419 subjects (58.5%) had received at least one recommended vaccination. However, vaccination coverage was suboptimal: influenza 30.9%, SARS-CoV-2 39%, pneumococcus 10.3%, herpes zoster 6%, meningococcus 1.4%; anti-VRS vaccination was not yet available. All-cause mortality was significantly higher in the unvaccinated than in the vaccinated subjects (53.5% vs. 14.1%; p<0.0001). Kaplan–Meier curves showed a significant benefit associated with vaccination in both MGUS and MM patients (log-rank p<0.0001). In patients with MGUS, 5-year survival was 82.5% in the vaccinated compared to 43.2% in the unvaccinated; in patients with MM, it was approximately 58.1% vs. 24.5%. In the multivariate Cox regression model, vaccination was associated with a marked reduction in the risk of death (HR 0.21; 95% CI 0.15–0.29; p<0.001).
From October 2024, vaccination coverage was still suboptimal but improved: influenza 74.7%, pneumococcus 43.1%, herpes zoster 27.2%, VRS 5.1%. Adherence was higher (85.2 vs 10.2%) for patients who have chosen to get vaccinated in hospital, where a dedicated schedule of co-administration was offered in 2-months interval.

**Copyright Information:** (Online) ISSN: 2572-9241
©2026 The Author(s). HemaSphere is published by John Wiley & Sons Ltd on behalf of European Hematology Association. This is an open access Abstract Book distributed under the Attribution-NonCommercial-NoDerivs (CC BY-NC-ND), which allows third parties to download the articles and share them with others as long as they credit the author and the Abstract Book, but they cannot change the content in any way or use them commercially.
**Abstract Book Citations:** Authors, Title, HemaSphere, 2026;10:(S1):pages. The abstract book DOIs can be found on the “Author Information” page.
**Disclaimer:** Articles published in the journal HemaSphere exclusively reflect the opinions of the authors. The authors are responsible for all content in their abstracts including accuracy of the facts, statements, citing resources, etc.

**Summary/Conclusion**

Vaccination against respiratory pathogens is associated to reduced risk of death in MGUS and MM patients. However, vaccination coverage among MM patients remains insufficient, underscoring the need for targeted educational campaigns and stronger public health strategies to reduce infection-related morbidity and mortality. Offering vaccination in the hematological center can overcome logistics issues and vaccination hesitation.

**Copyright Information:** (Online) ISSN: 2572-9241
©2026 The Author(s). HemaSphere is published by John Wiley & Sons Ltd on behalf of European Hematology Association. This is an open access Abstract Book distributed under the Attribution-NonCommercial-NoDerivs (CC BY-NC-ND), which allows third parties to download the articles and share them with others as long as they credit the author and the Abstract Book, but they cannot change the content in any way or use them commercially.
**Abstract Book Citations:** Authors, Title, HemaSphere, 2026;10:(S1):pages. The abstract book DOIs can be found on the "Author Information" page.
**Disclaimer:** Articles published in the journal HemaSphere exclusively reflect the opinions of the authors. The authors are responsible for all content in their abstracts including accuracy of the facts, statements, citing resources, etc.

### (PF1323) CLINICAL FEATURES, OUTCOMES, AND SEROLOGIC FINDINGS OF TOXOPLASMOSIS AFTER ALLOGENEIC HEMATOPOIETIC STEM CELL TRANSPLANTATION: A JAPANESE SINGLE-CENTER STUDY

**Topic:** 31. Infections in hematology (incl. supportive care/therapy)

Takahide Ara*[1], Tomoyuki Endo[1, 2], Masahiro Onozawa[1], Keito Suto[1], Yuta Hasegawa[1], Toshihiro Matsukawa[1], Souichi Shiratori[1], Hideki Goto[1], Masao Nakagawa[1], Kaoru Kahata[1], Daigo Hashimoto[1], Takanori Teshima[1]

*[1]Hokkaido University Hospital, Department of Hematology, Sapporo, Japan;[2]Hokkaido University Hospital, Department of Infection Control and Prevention, Sapporo, Japan;*

### Background

Toxoplasmosis is a rare but life-threatening complication after allogeneic hematopoietic stem cell transplantation (allo-SCT), and early-onset disease can be a medical emergency. Trimethoprim/sulfamethoxazole (TMP-SMX) is recommended for primary prophylaxis; however, prolonged cytopenia after transplantation often precludes timely re-initiation. Because Toxoplasma seroprevalence varies geographically, region-specific risk assessment and management strategies are needed.

### Aims

To evaluate Toxoplasma IgG seroprevalence, the incidence and characteristics of post-transplant toxoplasmosis, and prophylaxis practices in allo-SCT recipients, and to inform an effective prevention strategy.

### Methods

We retrospectively analyzed 619 allo-SCT procedures performed between January 2012 and August 2024. Possible, probable, and definite toxoplasmosis were defined according to ECIL-9 criteria. In patients with repeated Toxoplasma IgG testing during treatment for hematologic diseases, IgG levels were evaluated longitudinally using the ratio to the upper limit of normal.

### Results

During the study period, 619 allo-SCT procedures were performed in 571 patients (median age 50 years, range 17–70). Stem cell sources were bone marrow (n=90), peripheral blood (n=356), and cord blood (n=173).
Eight patients developed toxoplasmosis (definite n=1, probable n=5, possible n=2), with a 2-year cumulative incidence of 1.4% (Figure A). Median onset was day 50.5 after allo-SCT (range 28–389). Clinical involvement included central nervous system (n=7), lung (n=2), lymph node (n=1), and heart (n=1). Two patients had acute GVHD at onset, and six were receiving systemic corticosteroids. None of the 8 patients were receiving TMP-SMX prophylaxis at toxoplasmosis onset. Peripheral blood Toxoplasma qPCR was performed in 4 patients as part of a clinical research protocol, and in 3 of them, qPCR became positive about one week before clinical diagnosis. Prognosis after toxoplasmosis was poor, with a 1-year overall survival of only 18.8%, and toxoplasmosis was the direct cause of death in 5 of 8 cases (Figure B).
Among 480 transplants (447 patients) with available peri-transplant serology, 66 (13.8%) were Toxoplasma IgG-positive. Of the 8 patients who developed toxoplasmosis, 7 had pre-transplant IgG assessed: one remained consistently seronegative on repeated testing, and one showed seroreversion from initially positive to negative before transplantation. In patients with serial IgG measurements and an initially positive result (n=16), IgG levels decreased over time during treatment (p=0.044), and 5 of 16 patients became seronegative during follow-up (Figure C). In IgG-positive recipients, pre-transplant TMP-SMX exposure tended to be associated with a lower risk of post-transplant toxoplasmosis (2/39 with exposure vs 5/27 without), although this did not reach statistical significance (p=0.113).

**Copyright Information:** (Online) ISSN: 2572-9241
©2026 The Author(s). HemaSphere is published by John Wiley & Sons Ltd on behalf of European Hematology Association. This is an open access Abstract Book distributed under the Attribution-NonCommercial-NoDerivs (CC BY-NC-ND), which allows third parties to download the articles and share them with others as long as they credit the author and the Abstract Book, but they cannot change the content in any way or use them commercially.
**Abstract Book Citations:** Authors, Title, HemaSphere, 2026;10:(S1):pages. The abstract book DOIs can be found on the "Author Information" page.
**Disclaimer:** Articles published in the journal HemaSphere exclusively reflect the opinions of the authors. The authors are responsible for all content in their abstracts including accuracy of the facts, statements, citing resources, etc.

### Summary/Conclusion

Post-allo-SCT toxoplasmosis frequently occurred early, when restarting TMP-SMX prophylaxis is challenging, and was associated with dismal outcomes. Pre-transplant Toxoplasma IgG screening is useful for risk stratification; however, IgG levels may wane during treatment for hematologic diseases, and seroreversion may occur, potentially leading to under-recognition of at-risk recipients. Screening at diagnosis of the underlying disease may mitigate this risk. Risk-adapted pre-transplant TMP-SMX prophylaxis may reduce post-transplant toxoplasmosis, although larger studies are needed.

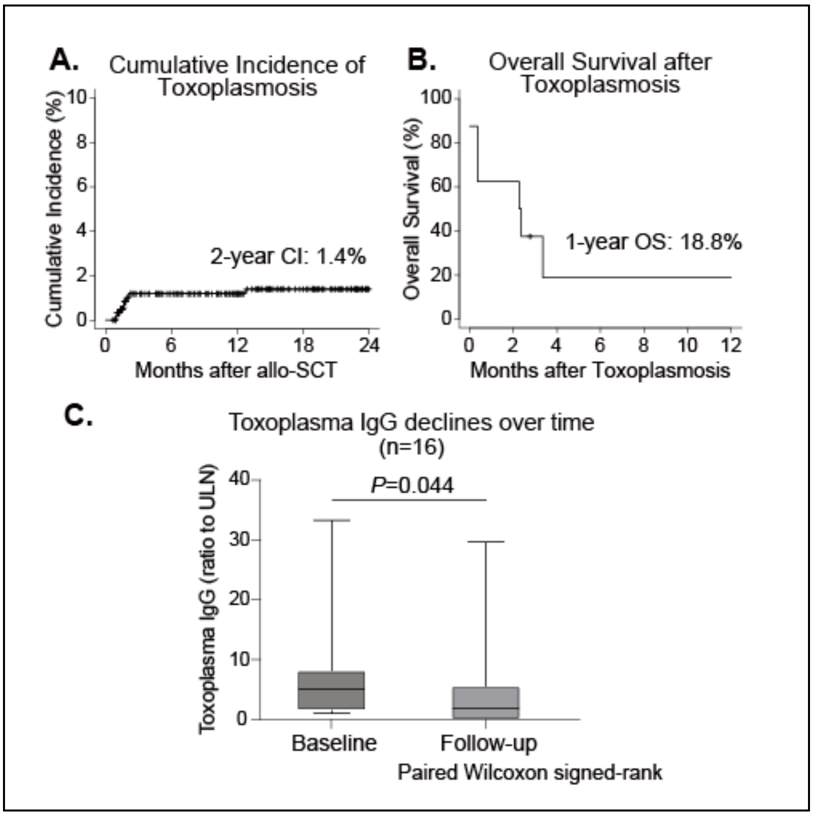

**Copyright Information:** (Online) ISSN: 2572-9241
©2026 The Author(s). HemaSphere is published by John Wiley & Sons Ltd on behalf of European Hematology Association. This is an open access Abstract Book distributed under the Attribution-NonCommercial-NoDerivs (CC BY-NC-ND), which allows third parties to download the articles and share them with others as long as they credit the author and the Abstract Book, but they cannot change the content in any way or use them commercially.
**Abstract Book Citations:** Authors, Title, HemaSphere, 2026;10:(S1):pages. The abstract book DOIs can be found on the "Author Information" page.
**Disclaimer:** Articles published in the journal HemaSphere exclusively reflect the opinions of the authors. The authors are responsible for all content in their abstracts including accuracy of the facts, statements, citing resources, etc.

### (PF1324) THE HEALTHCARE BURDEN OF SECONDARY CARE INFECTIONS IN MM AND CLL: A REAL-WORLD ANALYSIS IN SWEDEN

**Topic:** 31. Infections in hematology (incl. supportive care/therapy)

Ingrid Glimelius*[1, 2], Love Tätting[3], Jonatan Freilich[4, 5], Marta Natalia Stelmaszuk[4], Qian Yang[6], Anna Deleskog[7], Sigurdur Yngvi Kristinsson[8]

*[1]Uppsala University, Department of Immunology, Genetics and Pathology, Uppsala, Sweden; [2]Uppsala University Hospital, Blood, Oncology and Tumor Department, Akademiska sjukhuset, Uppsala, Sweden; [3]Linköping University, Department of Haematology in Linköping, and Department of Biomedical and Clinical Sciences, Linköping, Sweden; [4]Parexel Sweden AB, Access Consulting – RWE, Stockholm, Sweden; [5]Umeå University, Department of Public Health and Clinical Medicine, Umeå, Sweden; [6]Parexel Sweden AB, RWE Consulting – Epidemiology and COA, Stockholm, Sweden; [7]Takeda Pharma AB, Stockholm, Sweden; [8]University of Iceland, Department of Hematology, Faculty of Medicine, Reykjavik, Iceland;*

### Background

Patients with multiple myeloma (MM) and chronic lymphocytic leukaemia (CLL) are immunocompromised owing to underlying disease processes and use of immunosuppressive therapies. Patients are highly susceptible to infections, resulting in hospitalisation and high morbidity.

### Aims

To quantify the healthcare burden of infections in MM and CLL by evaluating hospital visits and lengths of inpatient stays.

### Methods

Patients diagnosed with MM or CLL (January 2010–December 2021) were identified from the Swedish Cancer Registry, National Patient Register, Prescribed Drug Register and Cause of Death Register. Patients were followed from the date of first diagnosis until the date of death, end of data capture or emigration. Infection events were identified using International Classification of Diseases 10th Revision codes. Hospital resource use was determined by the number of outpatient and inpatient visits and length of inpatient stays per person-year. Patients were grouped by whether or not they had a secondary care infection (ie, those occurring in hospital or during visits to specialised outpatient clinics) within the first year of index date. *P*-values were calculated using a *t*-test. Those with <1 year of follow-up were excluded.

**Copyright Information:** (Online) ISSN: 2572-9241
©2026 The Author(s). HemaSphere is published by John Wiley & Sons Ltd on behalf of European Hematology Association. This is an open access Abstract Book distributed under the Attribution-NonCommercial-NoDerivs (CC BY-NC-ND), which allows third parties to download the articles and share them with others as long as they credit the author and the Abstract Book, but they cannot change the content in any way or use them commercially.
**Abstract Book Citations:** Authors, Title, HemaSphere, 2026;10:(S1):pages. The abstract book DOIs can be found on the “Author Information” page.
**Disclaimer:** Articles published in the journal HemaSphere exclusively reflect the opinions of the authors. The authors are responsible for all content in their abstracts including accuracy of the facts, statements, citing resources, etc.

### Results

In total, 8532 (MM) and 7234 (CLL) patients were included in the analysis. Most were male (MM: 57%; CLL: 62%) with a median age of 71 years (interquartile range: 64–78). Patients were followed up for a median (range) 3.3 (0–13) and 4.8 (0–13) years, respectively. Charlson Comorbidity Index was ≥5 in 37% and 25% of MM and CLL patients, respectively. History of secondary care infection ≤10 years prior to MM or CLL diagnosis was confirmed in 36% and 30% of patients, respectively. Over the follow-up period, 49% (MM) and 30% (CLL) of patients had ≥2 secondary care infections. At least one anti-infection dispensation was recorded for 63% (MM) and 68% (CLL) of patients, with 76% and 39%, respectively, receiving anti-infective prophylaxis at follow-up. Of those hospitalised with secondary care infections (MM: 71%/CLL: 60%), length of stay was >3 days in 71% and 66% of patients, respectively. Patients with a secondary care infection during the first year of diagnosis experienced significantly more inpatient/outpatient visits than those who did not (*P*<0.0001; Table); inpatient stays were also significantly longer.

**Table. Hospital resource use in patients with MM or CLL and secondary care infections**

| | **MM** | | | **CLL** | | |
|---|---|---|---|---|---|---|
| | **No infection in first year since diagnosis (n=4463)** | **Infection in first year after diagnosis (n=2621)** | ***P*-value** | **No infection in first year since diagnosis (n=5773)** | **Infection in first year after diagnosis (n=966)** | ***P*-value** |
| Inpatient visits per PY (mean, SD visits)[a] | 1.5 (2.8) | 2.4 (5.8) | <0.0001 | 0.9 (1.8) | 2.1 (3.9) | <0.0001 |
| Length of inpatient stay per PY, days (mean, SD)[a] | 10.6 (23.0) | 16.5 (33.3) | <0.0001 | 5.3 (13.7) | 14.0 (31.8) | <0.0001 |
| Outpatient visits per PY (mean, SD visits)[a] | 9.8 (10.1) | 12.5 (16.0) | <0.0001 | 5.4 (6.0) | 8.2 (9.4) | <0.0001 |

[a]Only visits attended by a doctor are recorded in the National Patient Register.
CLL, chronic lymphocytic leukaemia; MM, multiple myeloma; PY, person-year; SD, standard deviation.

### Summary/Conclusion

Infections in patients with MM or CLL impose a substantial strain on healthcare systems. We provide strong evidence supporting infection control strategies to improve standards of care and outcomes in these patients.

This study was funded by Takeda Pharma AB, Sweden. Medical writing support was provided by Parexel and was funded by Takeda.

**Copyright Information:** (Online) ISSN: 2572-9241
©2026 The Author(s). HemaSphere is published by John Wiley & Sons Ltd on behalf of European Hematology Association. This is an open access Abstract Book distributed under the Attribution-NonCommercial-NoDerivs (CC BY-NC-ND), which allows third parties to download the articles and share them with others as long as they credit the author and the Abstract Book, but they cannot change the content in any way or use them commercially.
**Abstract Book Citations:** Authors, Title, HemaSphere, 2026;10:(S1):pages. The abstract book DOIs can be found on the "Author Information" page.
**Disclaimer:** Articles published in the journal HemaSphere exclusively reflect the opinions of the authors. The authors are responsible for all content in their abstracts including accuracy of the facts, statements, citing resources, etc.

### (PF1325) SPECTRUM OF EBV-ASSOCIATED EVENTS AFTER ALLOGENEIC HSCT: INCIDENCE, MANAGEMENT STRATEGY AND OUTCOMES IN A SINGLE-CENTER COHORT

**Topic:** 31. Infections in hematology (incl. supportive care/therapy)

Oleksandr Lysytsia[1], Olha Karpenko*[1], Oleksandr Istomin[1], Nataliia Bindiuzhenko[1], Hanna Brudna[1], Andrii Budzyn[1]

[1]*National Children's Specialized Hospital "Ohmatdyt", Kyiv, Ukraine;*

**Background**

After allogeneic HSCT, EBV DNA-emia is frequently detected during routine monitoring, but only a subset of patients develop EBV disease, most often classified as probable EBV disease. Management is guided by viral load kinetics, immunosuppression (IST) reduction, and rituximab in preemptive or therapeutic settings. Real-world data on structured single-center strategies remain limited.

**Aims**

To assess the incidence, management, and survival of EBV-associated events requiring intervention after allogeneic HSCT in a single-center cohort.

**Methods**

We conducted a retrospective single-center study of consecutive adult and pediatric patients undergoing allogeneic HSCT over five years. EBV DNA-emia and EBV disease were defined per ECIL-6 criteria; in the absence of biopsy, cases were classified as probable EBV disease. EBV monitoring was performed by quantitative PCR at predefined intervals, with viral load expressed in copies/mL. Preemptive therapy was initiated for EBV DNA-emia >1,000 copies/mL on two consecutive tests; patients meeting criteria for probable EBV disease received immediate treatment. Management included immunosuppression reduction and rituximab. Overall survival (primary endpoint) and mortality were assessed from HSCT using Kaplan–Meier and log-rank testing. Categorical variables were analyzed with $\chi^2$ or Fisher's exact test.

**Results**

Among 239 consecutive allo-HSCT recipients, 17 (7.1%) developed EBV-related events requiring intervention. Nine patients (3.8% of the cohort; 52.9% of EBV cases) had probable PTLD. Eleven events occurred in pediatric patients (64.7%) and six in adults (35.3%). Median follow-up was 9.4 months; median time to first EBV DNA-emia was 66 days post-HSCT (IQR 52–79). Most events followed matched unrelated donor HSCT (88%). ATG was used in 82% and PTCy in 41%, with overlap in selected cases. Management included IST reduction in all cases and rituximab in 88.2% (median 1 dose, IQR 1–2). Median peak EBV load was 2884 copies/mL (IQR 1980–4076). Two deaths occurred in the EBV group (11.8%), none EBV-related (TA-TMA/organ toxicity).. One-year OS was 91.7% vs 83.2% in patients without EBV events (log-rank p=0.84); longer-term survival was limited by follow-up.

**Summary/Conclusion**

In our center's analysis, IST reduction combined with risk-adapted rituximab resulted in effective control of EBV-related events without EBV-related mortality. EBV DNA-guided treatment allowed for timely intervention while limiting the risks of tapering and without compromising overall survival. The optimal timing and intensity of rituximab require prospective validation.

**Copyright Information:** (Online) ISSN: 2572-9241
©2026 The Author(s). HemaSphere is published by John Wiley & Sons Ltd on behalf of European Hematology Association. This is an open access Abstract Book distributed under the Attribution-NonCommercial-NoDerivs (CC BY-NC-ND), which allows third parties to download the articles and share them with others as long as they credit the author and the Abstract Book, but they cannot change the content in any way or use them commercially.
**Abstract Book Citations:** Authors, Title, HemaSphere, 2026;10:(S1):pages. The abstract book DOIs can be found on the "Author Information" page.
**Disclaimer:** Articles published in the journal HemaSphere exclusively reflect the opinions of the authors. The authors are responsible for all content in their abstracts including accuracy of the facts, statements, citing resources, etc.

Table 1. Patient and transplant characteristics (2021-2025)

| Characteristic | Overall Cohort (239) |
|---|---|
| Male, n (%) | 140 (58,6) |
| Female, n (%) | 99 (41,4) |
| Pediatric patients, n (%) | 132 (55,2) |
| Adult patients, n (%) | 107 (44,8) |
| Matched unrelated donor, n (%) | 114 (47,6) |
| Other types of donors, n (%) | 125 (52,4) |
| Median follow-up, months | 9,4 |

Table 2. EBV-Associated Events Subgroup

| Characteristic | Value |
|---|---|
| Incidence, n (%) | 17 (7,1) |
| Probable EBV disease, n (%) | 9 (52.9% of EBV events; 3.8% of cohort) |
| Pediatric patients, n (%) | 11 (64,7) |
| MUD, n (%) | 15 (88) |
| ATG exposure, n (%) | 14 (82) |
| PTCy exposure, n (%) | 7 (41,2), n (%) |
| Median time to EBV DNA-emia, days | 66 (IQR 52–79) |
| Rituximab use, n (%) | 15 (88,2) |
| Median rituximab doses | 1 (IQR 1–2) |
| One-year OS, % (EBV / overall) | 91,7 / 83,2% |

**Copyright Information:** (Online) ISSN: 2572-9241
©2026 The Author(s). HemaSphere is published by John Wiley & Sons Ltd on behalf of European Hematology Association. This is an open access Abstract Book distributed under the Attribution-NonCommercial-NoDerivs (CC BY-NC-ND), which allows third parties to download the articles and share them with others as long as they credit the author and the Abstract Book, but they cannot change the content in any way or use them commercially.
**Abstract Book Citations:** Authors, Title, HemaSphere, 2026;10:(S1):pages. The abstract book DOIs can be found on the "Author Information" page.
**Disclaimer:** Articles published in the journal HemaSphere exclusively reflect the opinions of the authors. The authors are responsible for all content in their abstracts including accuracy of the facts, statements, citing resources, etc.

### (PF1326) PATTERNS OF INFECTION AND THEIR IMPACT ON THE OUTCOMES OF NEXT-GENERATION B-CELL DEPLETING IMMUNOTHERAPIES IN NON-HODGKIN'S LYMPHOMAS: FINDINGS FROM A MULTICENTER RETROSPECTIVE REAL-LIFE COHORT.

**Topic:** 31. Infections in hematology (incl. supportive care/therapy)

Lorenzo Nigri*[1, 2], Simone Ragaini[1, 2], Mattia Novo[3], Lorenzo Comba[4, 5], Luca Legato[3], Matteo Bisio[3], Laura Bertolotti[4], Michele Clerico[1, 2], Filippo Fasano[3], Elia Boccellato[4], Federica Cavallo[1, 2], Barbara Botto[3], Claudia Castellino[4], Luisa Giaccone[1, 2], Carola Boccomini[3], Sara Bringhen[4], Benedetto Bruno[1, 2], Roberto Freilone[3], Massimo Massaia[1, 4], Simone Ferrero[1, 2], Alessia Castellino[1, 4]

*[1]University of Torino, Department of Molecular Biotechnology and Health Sciences, Torino, Italy;[2]AOU Città della salute e della scienza, Division of Hematology and Stem Cell Transplant Unit, University of Torino, Torino, Italy; [3]AOU Città della salute e della scienza, Division of Hematology, Torino, Italy; [4]A.O. Santa Croce e Carle, Division of Hematology, Cuneo, Italy;[5]A.O. Santa Croce e Carle, Division of Immunohematology and Blood Transfusion, Cuneo, Italy;*

### Background

Novel immunotherapies have changed the outcome of relapsed/refractory (R/R) non-Hodgkin's lymphomas (NHL) patients (pts), but with increased susceptibility to infections.

### Aims

This observational retrospective multicenter study evaluated the incidence, type, timing, and outcome of NHL pts treated with novel immunotherapies.

### Methods

NHL patients from three Italian centers who started treatment between December 1, 2019, and October 31, 2025, were included.

**Copyright Information:** (Online) ISSN: 2572-9241
©2026 The Author(s). HemaSphere is published by John Wiley & Sons Ltd on behalf of European Hematology Association. This is an open access Abstract Book distributed under the Attribution-NonCommercial-NoDerivs (CC BY-NC-ND), which allows third parties to download the articles and share them with others as long as they credit the author and the Abstract Book, but they cannot change the content in any way or use them commercially.
**Abstract Book Citations:** Authors, Title, HemaSphere, 2026;10:(S1):pages. The abstract book DOIs can be found on the "Author Information" page.
**Disclaimer:** Articles published in the journal HemaSphere exclusively reflect the opinions of the authors. The authors are responsible for all content in their abstracts including accuracy of the facts, statements, citing resources, etc.

**Results**

158 NHL pts received 195 lines of immunotherapies, including 108 with anti-CD19 CAR-T cells [58 (54%) Axi-cel, 33 (31%) Tisa-cel, 16 (14%) Brexu-cel, 1 (1%) Liso-cel], 40 with antiCD20/antiCD3 bispecific MoAbs [13 (33%) glofitamab, 18 (45%) epcoritamab, 9 (22%) mosunetuzumab], 26 with ADC [11 (42%) anti-CD19 ADC: loncastuximab, 15 (58%) anti-CD79b ADC polatuzumab with rituximab-bendamustine], 21 with naked MoAbs (tafasitamab-lenalidomide). Of the 158 patients, 24 received two, five received three, and one received four consecutive immunotherapy lines. Median age was 67 years (IQ, 58–74). The main clinical characteristics were: advanced stage in 84%, elevated LDH in 62%, bulky disease in 33%, extranodal involvement in 75%, IPI 3-5 in 69%, and ECOG PS $\geq$ 2 in 20%. The median number of previous treatments was 2 (range 1–7), with 42 pts receiving ASCT, and 3 allo-SCT. Total infective events were 162: 93 (57%) bacterial, 48 (30%) viral, 4 (2.5%) mixed viral and bacterial, 15 (9%) FUO, and 2 fungal infections, with higher viral events in ADC-treated pts (p 0.017). Infection grade was 1-2 in 70 (43%), 3-4 in 35 (22%), and 5 in 6 (4%) cases. Hospitalization was needed in 46% of patients, while treatment modification in 21%. Median time from immunotherapy start to first infection was 22 days, with 76% and 59% of infections in first 100 and 30 days. Median onset time was shorter for CAR-T and bispecific MoAbs (15 and 17 days) than for naked MoAbs or ADC (30 and 70 days, p 0.069). Among 195 immunotherapies, 47% had no infections, 28% had one and 25% had 2 or more (range 1–6), with higher infection rates in bispecific MoAbs group (p 0.035). Very early infections (< 30 days) were 93 (59%, mostly bacterial), early infections (31-100 days) were 26 (17%), and late infections (> 100 days) were 38 (24%, with equal bacterial or viral causes). GCS-F was given in 63% and IGIV in 43% of cases, with higher use in CAR-T cells or bispecific MoAB patients (p < 0.001). Two patients received levofloxacin, while all but one received anti-PJ (99%) and antiviral (97%) prophylaxis. Univariate analysis showed bispecific MoAbs (HR 3.38, 95% CI 1.22-10, p 0.023) and IVIG administration (HR 2.32, 95% CI 1.30-1.48, p 0.005) correlated with higher infection risk, while progressive disease showed lower risk. In multivariate analysis, only IGIV administration (HR 2.14, 95% CI 1.12- 4.16, p 0.023) remained significant, indicating patients who experienced more infections probably received as consequence more IVIG support. Of 74 deaths, 56 were from lymphoma progression and 6 from infections.

**Summary/Conclusion**

Infections are common in novel immunotherapy for NHL; however, most cases occurred early (< 100 days) and were mild-to-moderate and manageable. In R/R NHL pts, the main cause of death remains lymphoma progression.

**Copyright Information:** (Online) ISSN: 2572-9241
©2026 The Author(s). HemaSphere is published by John Wiley & Sons Ltd on behalf of European Hematology Association. This is an open access Abstract Book distributed under the Attribution-NonCommercial-NoDerivs (CC BY-NC-ND), which allows third parties to download the articles and share them with others as long as they credit the author and the Abstract Book, but they cannot change the content in any way or use them commercially.
**Abstract Book Citations:** Authors, Title, HemaSphere, 2026;10:(S1):pages. The abstract book DOIs can be found on the “Author Information” page.
**Disclaimer:** Articles published in the journal HemaSphere exclusively reflect the opinions of the authors. The authors are responsible for all content in their abstracts including accuracy of the facts, statements, citing resources, etc.

### (PF1327) ORAL CONTEZOLID FOR GRAM-POSITIVE BLOODSTREAM INFECTIONS IN HEMATOLOGIC PATIENTS: A PROPENSITY SCORE STUDY

**Topic:** 31. Infections in hematology (incl. supportive care/therapy)

Nuobing Yang*[1], Yumeng Zhao[2], Sizhou Feng[2], Wenjing Guo[2]

*[1]State Key Laboratory of Experimental Hematology, National Clinical Research Center for Blood Diseases, Haihe Laboratory of Cell Ecosystem, Institute of Hematology & Blood Diseases Hospital, Chinese Academy of Medical Sciences & Peking Union Medical College, Tianjin, China; [2]State Key Laboratory of Experimental Hematology, National Clinical Research Center for Blood Diseases, Haihe Laboratory of Cell Ecosystem, Institute of Hematology & Blood Diseases Hospital, Chinese Academy of Medical Sciences & Peking Union Medical College, Tianjin, China, Tianjin, China;*

### Background

Management of Gram-positive bloodstream infections (BSIs) in patients with hematologic diseases is challenging due to nephrotoxicity, hematologic toxicity, and the need for intravenous administration of existing therapies. Contezolid, a novel oral oxazolidinone with improved hematologic safety, has not been adequately evaluated in this vulnerable population.

### Aims

To evaluate the effectiveness and safety of oral contezolid for Gram-positive bloodstream infections (BSIs) in patients with hematologic diseases.

### Methods

We conducted a retrospective cohort study at a specialized hematology hospital in China. Hematologic patients with Gram-positive BSIs receiving oral contezolid as primary targeted therapy were compared with those receiving other antibiotics. Propensity score (PS) matching and inverse probability of treatment weighting (IPTW) were applied to balance baseline characteristics.

### Results

A total of 334 patients were included (44 contezolid; 290 controls). Patients receiving contezolid were older and more likely to have recent allogeneic hematopoietic stem cell transplantation, complicated BSIs, and concurrent pneumonia. After PS matching (41 contezolid vs. 106 control), 30-day death or relapse (17.1% vs. 8.5%, p=0.229), 90-day relapse (2.4% vs. 4.7%, p=0.872) and treatment modification rates (14.6% vs. 17.0%, p=0.923) were comparable. IPTW analysis also showed no significant association of contezolid with 30-day death or relapse (aHR=1.65, p=0.400). Septic shock, complicated BSIs, and concurrent pneumonia were independent predictors of 30-day poor outcomes. No contezolid-related adverse events leading to treatment modification were observed.

### Summary/Conclusion

Oral contezolid demonstrated effectiveness and safety comparable to standard therapies after adjustment for baseline differences and may represent a viable alternative for hematologic patients with Gram-positive BSIs.

**Copyright Information:** (Online) ISSN: 2572-9241
©2026 The Author(s). HemaSphere is published by John Wiley & Sons Ltd on behalf of European Hematology Association. This is an open access Abstract Book distributed under the Attribution-NonCommercial-NoDerivs (CC BY-NC-ND), which allows third parties to download the articles and share them with others as long as they credit the author and the Abstract Book, but they cannot change the content in any way or use them commercially.
**Abstract Book Citations:** Authors, Title, HemaSphere, 2026;10:(S1):pages. The abstract book DOIs can be found on the "Author Information" page.
**Disclaimer:** Articles published in the journal HemaSphere exclusively reflect the opinions of the authors. The authors are responsible for all content in their abstracts including accuracy of the facts, statements, citing resources, etc.

### (PF1328) IMMUNOGLOBULIN PROPHYLAXIS REDUCES SEVERE INFECTIONS IN MULTIPLE MYELOMA: A SYSTEMATIC REVIEW AND META-ANALYSIS

**Topic:** 31. Infections in hematology (incl. supportive care/therapy)

Federico Spataro*[1], Antonio Solimando[2], Vanessa Desantis[1], Alessandro Andriano[1], Giulia de Martino[1], Angelo Vacca[2], Roberto Ria[2]

*[1]University of Bari Aldo Moro, Department of Precision and Regenerative Medicine and Ionian Area - DiMePRe-J, Section of Pharmacology, Bari, Italy; [2]University of Bari Aldo Moro, Department of Precision and Regenerative Medicine and Ionian Area - DiMePRe-J, Guido Baccelli Unit of Internal Medicine, School of Medicine, Bari, Italy;*

### Background

Multiple myeloma (MM) is characterized by profound immune dysfunction, which predisposes patients to severe and recurrent infections. Immunoglobulin replacement therapy (IgRT) may reduce infection risk; however, available evidence is heterogeneous and largely limited to intravenous administration. Its clinical impact, particularly in patients receiving modern immunotherapies such as bispecific T-cell engagers (BiTEs), remains insufficiently defined.

### Aims

To quantify the effect of prophylactic IgRT on the risk of severe infections in patients with MM.

### Methods

We conducted a systematic review and meta-analysis - according to PRISMA guidelines and registered in PROSPERO (CRD420261277656) - of studies comparing MM patients receiving IgRT as preemptive or primary prophylaxis with those receiving secondary prophylaxis or no IgRT. MEDLINE and LILACS were searched through December 30, 2025. Both intravenous (IVIg) and subcutaneous (SCIg) administration routes were included. Comparative studies reporting hazard ratios (HRs) for severe (grade 3-4) infections, or providing sufficient data for HR estimation, were eligible. Pooled HRs were calculated using random-effects models.

### Results

Eight studies comprising 358 IgRT-treated patients and 430 controls were included, with a weighted mean follow-up of 10.6 months Most studies evaluated IVIg; one study exclusively assessed SCIg, and one included both IVIg and SCIg.Overall, IgRT prophylaxis was associated with a marked reduction in the incidence of severe infections, with a pooled HR of 0.25 (95% CI 0.13-0.49; p<0.0001), corresponding to an approximately 75% relative risk reduction (Figure A). Among patients treated with BiTEs, the pooled HR of 0.32 (95% CI 0.18-0.56; p<0.0001), as showed in Figure B. Within this subgroup, the strongest effect was observed in studies evaluating BCMA-directed BiTEs, with a pooled HR of 0.19 (95% CI 0.07-0.51). Findings were consistent across study designs and sensitivity analyses, with low heterogeneity observed after leave-one-out analysis.

**Copyright Information:** (Online) ISSN: 2572-9241
©2026 The Author(s). HemaSphere is published by John Wiley & Sons Ltd on behalf of European Hematology Association. This is an open access Abstract Book distributed under the Attribution-NonCommercial-NoDerivs (CC BY-NC-ND), which allows third parties to download the articles and share them with others as long as they credit the author and the Abstract Book, but they cannot change the content in any way or use them commercially.
**Abstract Book Citations:** Authors, Title, HemaSphere, 2026;10:(S1):pages. The abstract book DOIs can be found on the "Author Information" page.
**Disclaimer:** Articles published in the journal HemaSphere exclusively reflect the opinions of the authors. The authors are responsible for all content in their abstracts including accuracy of the facts, statements, citing resources, etc.

**Summary/Conclusion**

IgRT prophylaxis is associated with a substantial reduction in severe infections in MM, including high-risk patients receiving BiTEs therapy. The observed benefit is clinically meaningful and primarily supported by evidence from IVIg studies, although limited data suggest comparable effectiveness with SCIg. These findings support systematic consideration of IgRT prophylaxis in high-risk MM populations, while prospective studies are warranted to define optimal timing, patient selection, and administration strategies.

This study was funded by and by European Union—Next Generation EU—PNRR M6C2— “Investimento 2.1 Potenziamento e rafforzamento della ricerca biomedica nel SSN” (project n. PNRR-POC-2022-12375862 FUSION-TARGET) to AGS. Moreover, protected time and access to the bibliographic and scientific resources were supported by contributions from “Fondo per il Programma Nazionale di Ricerca e Progetti di Rilevante Interesse Nazionale - PRIN” (code 2022ZKKWLW to AGS), and from the “Società Italiana di Medicina Interna—SIMI” 2023 Research Award (CAMEL to AGS). Finally, this study was also funded by the Italian network of excellence for advanced diagnosis -INNOVA-,“Ministero della Salute” (code PNC-E3-2022-23683266 PNC-HLS-DA, to VD and AGS), and PNRR-MCNT1-2023-12377893 DEMMO-“Deciphering multiple myeloma using multiomic approaches for immunotherapy modeling“ to VD and AGS.

**Copyright Information:** (Online) ISSN: 2572-9241
©2026 The Author(s). HemaSphere is published by John Wiley & Sons Ltd on behalf of European Hematology Association. This is an open access Abstract Book distributed under the Attribution-NonCommercial-NoDerivs (CC BY-NC-ND), which allows third parties to download the articles and share them with others as long as they credit the author and the Abstract Book, but they cannot change the content in any way or use them commercially.
**Abstract Book Citations:** Authors, Title, HemaSphere, 2026;10:(S1):pages. The abstract book DOIs can be found on the “Author Information” page.
**Disclaimer:** Articles published in the journal HemaSphere exclusively reflect the opinions of the authors. The authors are responsible for all content in their abstracts including accuracy of the facts, statements, citing resources, etc.

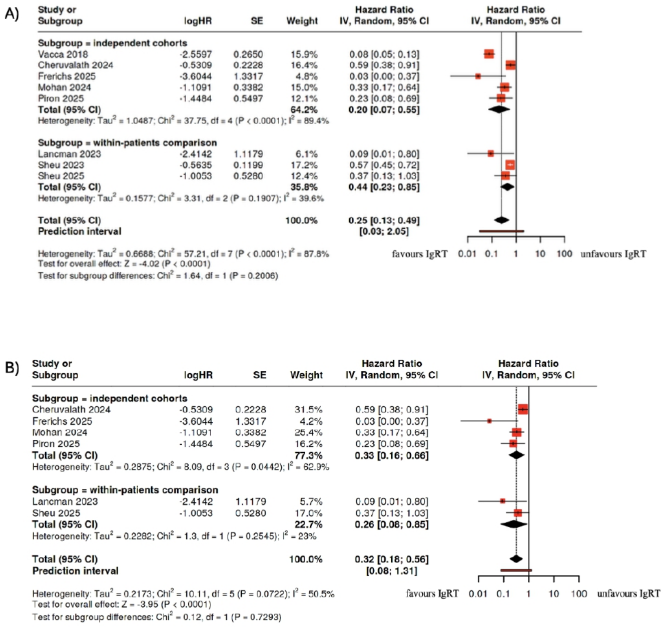

A) Forest plot of the overall meta-analysis. B) Forest plot of the BiTEs subgroup meta-analysis.

**Copyright Information:** (Online) ISSN: 2572-9241
©2026 The Author(s). HemaSphere is published by John Wiley & Sons Ltd on behalf of European Hematology Association. This is an open access Abstract Book distributed under the Attribution-NonCommercial-NoDerivs (CC BY-NC-ND), which allows third parties to download the articles and share them with others as long as they credit the author and the Abstract Book, but they cannot change the content in any way or use them commercially.
**Abstract Book Citations:** Authors, Title, HemaSphere, 2026;10:(S1):pages. The abstract book DOIs can be found on the "Author Information" page.
**Disclaimer:** Articles published in the journal HemaSphere exclusively reflect the opinions of the authors. The authors are responsible for all content in their abstracts including accuracy of the facts, statements, citing resources, etc.

### (PF1329) LIBERAL VERSUS RESTRICTIVE BLOOD TRANSFUSION STRATEGIES IN NEUROCRITICAL CARE: A SYSTEMATIC REVIEW AND META-ANALYSIS OF RANDOMIZED CONTROLLED TRIALS

**Topic:** 32. Transfusion medicine

Aymar Akilimali*[1]

[1]*Medical Research Circle, Research, Goma, Congo (the Democratic Republic of the);*

### Background

Neurocritical care patients, including those with traumatic brain injury, subarachnoid hemorrhage, and intracerebral hemorrhage, often develop anemia, compromising brain oxygen delivery and increasing morbidity and mortality. Blood transfusion strategies, either liberal or restrictive, are commonly used to manage anemia in these patients, but the optimal approach remains unclear due to mixed results in existing studies.

### Methods

A systematic search of PubMed, Cochrane Library, ScienceDirect, and Google Scholar from inception to December 2024 for randomized controlled trials (RCTs) evaluating restrictive versus liberal transfusion strategies in adult neurocritical care patients. Outcomes included mortality, Glasgow Outcome Scale (GOS), red blood cell (RBC) units transfused, sepsis, intensive care unit (ICU)/hospital length of stay, and secondary complications. The study is registered with PROSPERO (CRD42025635426).

### Results

The analysis included seven RCTs with 1941 patients. The restrictive strategy significantly reduced the number of RBC units transfused per patient (MD: 2.36; 95% CI: 1.08-3.64; $p = 0.0003$) and was associated with a lower incidence of sepsis (RR: 0.73; 95% CI: 0.56-0.96; $p = 0.02$). There were no significant differences between restrictive and liberal strategies for ICU (RR 0.74; 95% CI 0.28-1.91; $p = 0.53$), in-hospital (RR 0.77; 95% CI 0.35-1.68), 30-day (RR 0.91; 95% CI 0.70-1.18), 6-month (RR 0.98; 95% CI 0.67-1.44), or long-term mortality (RR 1.00; 95% CI 0.80-1.24). GOS scores at 6 months showed no significant difference (RR 0.94; 95% CI 0.83-1.07). ICU and hospital length of stay were also comparable between strategies. Secondary outcomes, including stroke, brain hypoxia, intracranial hypertension, and other non-neurological complications, showed no significant differences between the two strategies.

### Summary/Conclusion

Restrictive transfusion strategies are as effective as liberal strategies in terms of mortality and neurological complications, with additional benefits such as fewer RBC transfusions and lower sepsis rates. These findings support restrictive strategies as a safer approach to managing anemia in neurocritical care, though further research on long-term outcomes is needed.

**Copyright Information:** (Online) ISSN: 2572-9241
©2026 The Author(s). HemaSphere is published by John Wiley & Sons Ltd on behalf of European Hematology Association. This is an open access Abstract Book distributed under the Attribution-NonCommercial-NoDerivs (CC BY-NC-ND), which allows third parties to download the articles and share them with others as long as they credit the author and the Abstract Book, but they cannot change the content in any way or use them commercially.
**Abstract Book Citations:** Authors, Title, HemaSphere, 2026;10:(S1):pages. The abstract book DOIs can be found on the “Author Information” page.
**Disclaimer:** Articles published in the journal HemaSphere exclusively reflect the opinions of the authors. The authors are responsible for all content in their abstracts including accuracy of the facts, statements, citing resources, etc.

### (PF1330) GENOMIC CHARACTERIZATION OF BLOOD CELL ANTIGEN DIVERSITY IN SOUTH-EAST ASIAN POPULATION WITH EUROPEAN AND AFRICAN COMPARATORS

**Topic:** 32. Transfusion medicine

Natasha Anthony*[1], Michael Ng[2], Connie Chen[1], Eugene Fan[1, 3, 4, 5]

*[1]Department of Haematology, Tan Tock Seng Hospital, Singapore, Singapore, Singapore;[2]Health Sciences Authority, Singapore, Singapore, Singapore; [3]Department of Laboratory Medicine, Khoo Teck Puat Hospital, Singapore, Singapore, Singapore;[4]Lee Kong Chian School of Medicine, Nanyang Technological University, Singapore, Singapore, Singapore; [5]Yong Loo Lin School of Medicine, National University of Singapore, Singapore, Singapore, Singapore;*

### Background

Genetic diversity influences alloimmunization risk, transfusion reactions, and transfusion practice. However, comprehensive population-based data on transfusion-relevant variants in Southeast Asian populations remain limited.

### Methods

We analyzed allele frequencies of transfusion-relevant variants in the SG10K_Health cohort (9,770 healthy Singaporeans of Chinese, Malay and Indian ancestry) [Figure 1 not included]. Variants included selected platelet (HPA), neutrophil (HNA), and red cell antigen polymorphisms defined by well-characterized single-nucleotide substitutions and reliably captured in the SG10K data. Frequencies stratified by genetic ancestry were extracted from the CHORUS variant browser and compared with non-Finnish European and African data from gnomAD. Allele frequencies reflect coding variant distribution and are not equivalent to serologically determined antigen prevalence, though they correlate with expected phenotype frequency under Hardy–Weinberg assumptions.

### Results

Marked inter-population differences were observed [**Table 1**], particularly for platelet and neutrophil antigens. The near absence of HPA-1b in Chinese individuals (and low frequency in Malays) explains the relative rarity of anti-HPA-1 neonatal autoimmune thrombocytopenia in Singapore. In contrast, HPA-5b is present at low but detectable frequency in local populations, permitting clinically relevant HPA-5 mismatch. HPA-6b was detected at low frequency (2–3%) in Chinese and Indian individuals. HPA-30b was observed at low frequency overall, with relative enrichment in individuals of Indian ancestry (3%). In contrast to other HPA systems where one allele predominates, HPA-15 demonstrates near-balanced allele frequencies globally, likely reflecting an older, functionally tolerated polymorphism.
HNA-3 and HNA-4 are antigen-defining missense variants with relatively simple genotype–phenotype correlation to enable population-level comparison. The relatively higher HNA-3b allele frequency observed in Chinese individuals may have greater clinical relevance than HNA-4b in this population in transfusion associated lung injury.
For most blood group systems included, analyzed single nucleotide polymorphisms (SNPs) were antigen-defining missense variants. The ACKR1 promoter Duffy-null variant is highly prevalent in African populations but rare in most non-African ethnicities. The Kell K allele was uncommon in Asian populations compared with Europeans, whereas Kidd frequencies were broadly similar. Systems not defined by a single SNP (e.g. M/N) were excluded.

**Copyright Information:** (Online) ISSN: 2572-9241
©2026 The Author(s). HemaSphere is published by John Wiley & Sons Ltd on behalf of European Hematology Association. This is an open access Abstract Book distributed under the Attribution-NonCommercial-NoDerivs (CC BY-NC-ND), which allows third parties to download the articles and share them with others as long as they credit the author and the Abstract Book, but they cannot change the content in any way or use them commercially.
**Abstract Book Citations:** Authors, Title, HemaSphere, 2026;10:(S1):pages. The abstract book DOIs can be found on the “Author Information” page.
**Disclaimer:** Articles published in the journal HemaSphere exclusively reflect the opinions of the authors. The authors are responsible for all content in their abstracts including accuracy of the facts, statements, citing resources, etc.

### Summary/Conclusion

Substantial ethnic differences in transfusion-relevant variants, particularly platelet and neutrophil antigens, underscore the need for population-specific genomic reference data to inform transfusion risk assessment in multi-ethnic healthcare settings.

| Antigen | Gene | Chr | Nucleotide | Protein Change | dbSNP | Indian | Malay | Chinese | European | African |
|---|---|---|---|---|---|---|---|---|---|---|
| HPA **1b** | ITGB3 | 17 | T>C | L33P | rs5918 | 0.1134 | 0.0216 | 0.00 | 0.1529 | 0.1064 |
| HPA **2b** | GP1BA | 17 | C>T | T145M | rs6065 | 0.0542 | 0.0503 | 0.0354 | 0.0765 | 0.2266 |
| HPA **3b** | ITGA2B | 17 | T>G | I843S | rs5911 | 0.3379 | 0.5000 | 0.4479 | 0.3767 | 0.3856 |
| HPA **4b** | ITGB3 | 17 | G>A | R143Q | rs5917 | 0.0000 | 0.0014 | 0.0010 | 0.0001 | 0.0005 |
| HPA **5b** | ITGA2 | 5 | G>A | E505K | rs1801106 | 0.0889 | 0.0596 | 0.0188 | 0.0878 | 0.1902 |
| HPA **6b** | ITGB3 | 17 | G>A | R489Q | rs13306487 | 0.0015 | 0.0286 | 0.0233 | 0.0001 | 0.0000 |
| HPA **15b** | CD109 | 8 | C>A | S682Y | rs10455097 | 0.4021 | 0.5479 | 0.4289 | 0.4880 | 0.3459 |
| HPA **30b** | ITGA2B | 17 | G>C | Q806H | rs377753373 | 0.0254 | 0.0014 | 0.0000 | 0.0000 | 0.0000 |
| HNA **3b** | SLC44A2 | 19 | G>A | R154Q | rs2288904 | 0.2161 | 0.2527 | 0.3114 | 0.2201 | 0.0640 |
| HNA **4b** | ITGAM | 16 | G>A | R77H | rs1143679 | 0.0454 | 0.0418 | 0.0045 | 0.1053 | 0.1066 |
| Kell (**K**) | KEL | 7 | C>T | M193T | rs8176058 | 0.0080 | 0.0000 | 0.0005 | 0.0400 | 0.0100 |
| Duffy (**Fy$^b$**) | ACKR1 | 1 | G>A | G42D | rs12075 | 0.3360 | 0.1316 | 0.0424 | 0.5800 | # |
| Duffy-null (**Fy[a−b−]**) | ACKR1 | 1 | T>C | No protein change | rs2814778 | 0.0019 | 0.0013 | 0.0005 | 0.0022 | 0.8380 |
| Kidd (**Jk$^b$**) | SLC14A1 | 18 | G>A | D280N | rs1058396 | 0.4040 | 0.4532 | 0.5490 | 0.4816 | 0.2675 |
| Dombrock (**Do$^b$**) | ART4 | 12 | A>G | N265D | rs11276 | 0.4034 | 0.1173 | 0.0938 | 0.3936 | 0.2754 |
| Colton (**Co$^b$**) | AQP1 | 7 | C>T | A45V | rs28362692 | 0.0082 | 0.0013 | 0.0005 | 0.0439 | 0.0090 |
| Cartwright (**Yt$^b$**) | ACHE | 7 | C>A | H322N | rs1799805 | 0.0518 | 0.0051 | 0.0862 | 0.0399 | 0.0060 |
| Scianna (**Sc$^b$**) | ERMAP | 1 | G>A | R77H | rs56025238 | 0.0012 | 0.0014 | 0.0000 | 0.0033 | 0.0003 |
| Knops (**Kn$^b$**) | CR1 | 1 | G>A | V1561M | rs17047660 | 0.0000 | 0.0013 | 0.0000 | 0.0008 | 0.2369 |
| Xg (**Xg$^a$ negative**)^ | XG | X | C>G | No protein change | rs311103 | 0.0244 | 0.0100 | 0.0299 | 0.3198 | 0.4304 |
| S/s (**S**) | GYPB | 4 | C>T | T48M | rs7683365 | 0.3159 | 0.0796 | 0.0404 | 0.2952 | 0.1898 |

**Table 1.** Allele Frequencies of Transfusion-Relevant Genetic Variants by Ancestry in the SG10K_Health Cohort with European and African Comparison

*# High Fyb allele frequency in Africans does not translate phenotypic Fyb+ expression, as Duffy-null regulatory variant suppresses red cell antigen expression*

*^ (XG) is a regulatory variant influencing Xg$^a$ expression; there is no antithetical Xg$^b$ antigen. As XG is X-linked, allele frequency does not directly correspond to phenotype prevalence*

Table 1

**Copyright Information:** (Online) ISSN: 2572-9241
©2026 The Author(s). HemaSphere is published by John Wiley & Sons Ltd on behalf of European Hematology Association. This is an open access Abstract Book distributed under the Attribution-NonCommercial-NoDerivs (CC BY-NC-ND), which allows third parties to download the articles and share them with others as long as they credit the author and the Abstract Book, but they cannot change the content in any way or use them commercially.
**Abstract Book Citations:** Authors, Title, HemaSphere, 2026;10:(S1):pages. The abstract book DOIs can be found on the "Author Information" page.
**Disclaimer:** Articles published in the journal HemaSphere exclusively reflect the opinions of the authors. The authors are responsible for all content in their abstracts including accuracy of the facts, statements, citing resources, etc.

### (PF1331) FEASIBILITY AND SAFETY OF HOME-BASED BLOOD COUNT MEASUREMENT AND TRANSFUSIONS IN SYSTEMATICALLY TRAINED PATIENTS WITH ACUTE MYELOID LEUKEMIA

**Topic:** 32. Transfusion medicine

Iben Nielsen*[1], Caecilie Ingrisch[1], Christian Landbo[1], Lennart Friis-Hansen[2], Judith Rindum[3], Kristina Noerskov[4, 5], Randa Zo El-Ghina[6], Morten Hansen[6], Mary Jarden[1, 7], Lars Kjeldsen[1]

*[1]Copenhagen University Hospital Rigshospitalet, Department of Haematology, Copenhagen, Denmark;[2]Odense University Hospital, Department of Clinical Biochemistry, Odense, Denmark; [3]The Capital Region of Denmark, Center for Health, Hillerød, Denmark; [4]Zealand University Hospital, Haematology, Roskilde, Denmark; [5]University of Southern Denmark, Department of Regional Health Research, Odense, Denmark; [6]Copenhagen University Hospital Rigshospitalet, Department of Clinical Immunology, Copenhagen, Denmark; [7]University of Copenhagen, Faculty of Health and Medical Sciences, Copenhagen, Denmark;*

### Background

Acute myeloid leukemia (AML) requires intensive chemotherapy that causes profound cytopenia and frequent transfusions. In Denmark, AML care has increasingly shifted from inpatient to outpatient management, supported by patient education, central venous catheter (CVC) self-care, and home administration of intravenous chemotherapy. Although this approach reduces hospitalization, patients still attend the day-hospital several times weekly for complete blood count monitoring (CBC) and transfusions. Despite low transfusion reaction rates, research on home-transfusions has rarely examined patient self-administration. To advance outpatient care, it is important to evaluate the feasibility, safety, and experiences of patients and caregivers.

### Aims

To investigate the feasibility and safety, including complication rates, of systematically trained patients ≥ 18 years diagnosed with AML receiving intensive induction and consolidation chemotherapy, performing CBC self-monitoring and self-administration of blood and platelet transfusions at home.

### Methods

This study included an in-hospital training phase followed by home implementation. In Phase 1 during induction treatment, patients received structured education and certification in CBC self-measurement, transfusion administration, and vital-sign monitoring. Training included CVC blood sampling, use of the Hemoscreen™, transfusion procedures, and post-medication. Patients performed supervised transfusions until competency was achieved. In Phase 2, during reinduction and consolidation, certified patients independently conducted CBCs, transfusions, and vital-sign monitoring at home, with 24-hour clinical support available. CBC results were transferred to the electronic health record to guide same-day delivery of blood products for self-transfusion.

### Results

Of 34 eligible patients, 21 were recruited (62%). Five withdrew due to prolonged hospitalization, inability to complete training, or personal reasons (retention 76%). All remaining participants (n=15) completed home CBCs, and all but one performed home transfusions. Participants engaged for a mean of 46.7 days (range 19–150), covering cytopenic periods after one or two reinduction/consolidation cycles. They completed a mean of 8.6 (range 3–23) home CBCs and 6.2 (range 0–19) home transfusions, thus avoiding in average 8 visits to the hospital. Non-adherence was mainly due to Hemoscreen™ technical issues. No adverse events or transfusion-related complications were observed. As secondary outcomes, quality of life increased during the treatment trajectory, symptom burden decreased, patient empowerment was maintained, and patient satisfaction was high.

**Copyright Information:** (Online) ISSN: 2572-9241
©2026 The Author(s). HemaSphere is published by John Wiley & Sons Ltd on behalf of European Hematology Association. This is an open access Abstract Book distributed under the Attribution-NonCommercial-NoDerivs (CC BY-NC-ND), which allows third parties to download the articles and share them with others as long as they credit the author and the Abstract Book, but they cannot change the content in any way or use them commercially.
**Abstract Book Citations:** Authors, Title, HemaSphere, 2026;10:(S1):pages. The abstract book DOIs can be found on the “Author Information” page.
**Disclaimer:** Articles published in the journal HemaSphere exclusively reflect the opinions of the authors. The authors are responsible for all content in their abstracts including accuracy of the facts, statements, citing resources, etc.

**Summary/Conclusion**

Home-based CBC monitoring and self-administration of transfusions were feasible, safe and well accepted, among trained AML patients. High adherence and no adverse events support integrating this model into outpatient AML care. These findings may extend to other transfusion-dependent cancer populations by reducing outpatient visits, inpatient stays, and unnecessary healthcare contact.

**Copyright Information:** (Online) ISSN: 2572-9241
©2026 The Author(s). HemaSphere is published by John Wiley & Sons Ltd on behalf of European Hematology Association. This is an open access Abstract Book distributed under the Attribution-NonCommercial-NoDerivs (CC BY-NC-ND), which allows third parties to download the articles and share them with others as long as they credit the author and the Abstract Book, but they cannot change the content in any way or use them commercially.
**Abstract Book Citations:** Authors, Title, HemaSphere, 2026;10:(S1):pages. The abstract book DOIs can be found on the "Author Information" page.
**Disclaimer:** Articles published in the journal HemaSphere exclusively reflect the opinions of the authors. The authors are responsible for all content in their abstracts including accuracy of the facts, statements, citing resources, etc.

### (PF1332) DONOR-INTRINSIC MEMBRANE PROTEOME ALTERATIONS PRECEDE HIGH END-STORAGE HEMOLYSIS IN STORED RED BLOOD CELLS

**Topic:** 32. Transfusion medicine

Katerina Nikolaidou*[1], Effrosyni Zontou[1], Nikoleta Baka[1], Monika Dzieciatkowska[2], Molly Boettiger[2], Julie Reisz[2], Ioannis Kereres[1], Sotirios Fortis[3], Konstantinos Stamoulis[4], Anastasios Kriebardis[3], Angelo D'alessandro[2], Alkmini Anastasiadi[1], Vasileios Tzounakas[1]

*[1]University of Patras, School of Medicine, Department of Biochemistry, Patras, Greece; [2]University of Colorado Anschutz Medical Campus, Department of Biochemistry and Molecular Genetics, Aurora CO, United States of America; [3]University of West Attica, School of Health and Caring Sciences, Department of Biomedical Sciences, Laboratory of Reliability and Quality Control in Laboratory Hematology, Egaleo, Greece; [4]Hellenic National Blood Transfusion Centre, Acharnes, Greece;*

### Background

Red blood cell (RBC) transfusion is the most common life-sustaining intervention for numerous pathologies. Increasing evidence suggests that donor-to-donor variability significantly influences the quality of packed RBC units, with in-bag hemolysis being closely associated with donor-specific characteristics.

### Aims

The aim of the study was the retrospective identification of biomarkers associated with distinct patterns of end-of-storage hemolysis.

### Methods

Nineteen leukoreduced RBC units (CPD/SAGM) from healthy donors were assessed at the beginning (day 2) and the end (day 42) of the storage period for RBC integrity, redox homeostasis and cellular aging, along with comprehensive metabolomic and proteomic profiling through UHPLC/MS. Cluster analysis was applied to classify RBC units based on end-of-storage hemolysis. Two clusters arose (silhouette coefficient 0.9): a high hemolysis (H-group; n=10; 120.68±11.77 mg Hb/dL), and a low hemolysis one (L-group; n=9; 51.9 ±15.23 mg Hb/dL). Significance was accepted at $p<0.05$.

### Results

At the onset of storage, both groups were resilient to induced and spontaneous hemolysis, yet the H-group displayed subtle molecular signatures distinguishing it from the L-group. Membrane proteomics revealed increased hemoglobin (Hb) binding (1.6-fold), alongside reduced translocation of small GTPases (e.g., Rac1 1.4-fold), and decreased levels of actin, stomatin, and Piezo-1 ($p<0.05$). In terms of metabolism, pyruvate kinase and lactate dehydrogenase were elevated in the H-group membrane, while aldolase and GAPDH were reduced. This pattern was accompanied by perturbations in glycolysis, with trends for elevated intermediates (e.g., 2,3-DPG 2.8-fold), without corresponding increases in terminal products. Enhanced purine catabolism was also evident (e.g., increased allantoate), alongside slightly elevated phosphatidylserine exposure, consistent with lower levels of flippase ATP11c in the membrane. The H-group exhibited a trend toward increased reactive oxygen species (ROS) production following oxidative challenge yet maintained a trend for higher glutathione levels and more membrane-associated antioxidant enzymes, including peroxiredoxin and catalase. At the end of storage, the pattern of increased Hb membrane binding and reduced relocation of small GTPases persisted in high-hemolyzers, alongside a similar decrease in the membrane-associated T-complex. Overall, the early subtle signatures evolved into overt pathological phenotypes, since extracellular $K^+$ levels were slightly elevated and redox homeostasis was further disrupted, as evidenced by increased ROS generation (6387±2318 vs 4358±497 MFI/mg, $p<0.05$) and hemolysis (19.9±6.7 vs 13.5±5.7 mg Hb/dL, $p<0.05$) following oxidative challenge.

**Copyright Information:** (Online) ISSN: 2572-9241
©2026 The Author(s). HemaSphere is published by John Wiley & Sons Ltd on behalf of European Hematology Association. This is an open access Abstract Book distributed under the Attribution-NonCommercial-NoDerivs (CC BY-NC-ND), which allows third parties to download the articles and share them with others as long as they credit the author and the Abstract Book, but they cannot change the content in any way or use them commercially.
**Abstract Book Citations:** Authors, Title, HemaSphere, 2026;10:(S1):pages. The abstract book DOIs can be found on the “Author Information” page.
**Disclaimer:** Articles published in the journal HemaSphere exclusively reflect the opinions of the authors. The authors are responsible for all content in their abstracts including accuracy of the facts, statements, citing resources, etc.

**Summary/Conclusion**

High end-of-storage hemolysis is preceded by subtle molecular signatures detectable weeks before overt hemolytic phenotypes emerge. High-hemolyzers exhibit persistent membrane alterations, like increased Hb binding and reduced GTPase translocation, alongside transient metabolic and redox perturbations. The latter pattern likely reflects an early but ultimately insufficient compensatory response to oxidative stress, resulting in redox imbalance over time. The persistent membrane Hb binding and impaired GTPase dynamics represent promising intrinsic markers distinguishing poor-storing units from storage onset.
This research was supported by Grant #84111 from the Research Committee of the University of Patras via "C. CARATHEODORI" program.

**Copyright Information:** (Online) ISSN: 2572-9241
©2026 The Author(s). HemaSphere is published by John Wiley & Sons Ltd on behalf of European Hematology Association. This is an open access Abstract Book distributed under the Attribution-NonCommercial-NoDerivs (CC BY-NC-ND), which allows third parties to download the articles and share them with others as long as they credit the author and the Abstract Book, but they cannot change the content in any way or use them commercially.
**Abstract Book Citations:** Authors, Title, HemaSphere, 2026;10:(S1):pages. The abstract book DOIs can be found on the "Author Information" page.
**Disclaimer:** Articles published in the journal HemaSphere exclusively reflect the opinions of the authors. The authors are responsible for all content in their abstracts including accuracy of the facts, statements, citing resources, etc.

### (PF1333) OPTIMISING BLOOD PRODUCT STEWARDSHIP: AN AUDIT OF INACCURATE IRRADIATED BLOOD COMPONENT FLAGGING AND IMPLEMENTATION OF TIME-LIMITED REFERRALS

**Topic:** 32. Transfusion medicine

Elena Bax*[1], Elaina Pasangha[1, 2], Dennis Famili[1], Anita Arasaretnam[1]

[1]*University Hospitals Sussex NHS Foundation Trust, Brighton and Hove, United Kingdom;*[2]*King's College Hospital NHS Foundation Trust, LONDON, United Kingdom;*

### Background

Irradiated blood components are essential for preventing transfusion-associated graft-versus-host disease (TA-GVHD). They are indicated lifelong post treatment with purine analogues and Hodgkin’s lymphoma and for a fixed duration post bone marrow transplant (BMT). However, they carry higher costs, shorter shelf-lives, and longer preparation times compared to standard components. This may lead to unacceptable delay in delivering blood components or rescheduling of surgical procedures. Effective stewardship requires restricting their use to active clinical indications.

### Aims

This audit assessed the accuracy of the "irradiated blood required" flag at Royal Sussex County Hospital to quantify patients no longer requiring these products. We aimed to identify diagnoses associated with inaccurate flagging and implement a referral pathway requiring prescribers to specify indications and durations, thereby reducing inappropriate irradiated blood product administration.

### Methods

We conducted a retrospective audit of all patients (n=724) flagged for irradiated products at the Royal Sussex County Hospital. We reviewed electronic health records to establish diagnosis and status. Valid ongoing requirements followed British Society of Haematology guidelines including, post-allograft (post 6 months arbitrarily, lymphocyte count $<1.0\times10^9$/L, active GVHD or on immunosuppression), BMT donors 7 days prior to harvest, post-autograft ( from initiation of conditioning until 3 months post-transplant/ 6 months if total body irradiation is used), Hodgkin’s lymphoma, purine analogue treatment, and aplastic anaemia with immunosuppression treatment. Patients outside these parameters were classified as "not needing ongoing irradiated blood".

### Results

Of 724 records, 308 (42.5%) were deceased. Among 416 living patients, 279 (67.1%) had a valid ongoing requirement, while 129 (31.0%) did not. Data was missing for 8 patients (1.9%).
Among appropriately flagged patients (n=279), common indications were Hodgkin’s lymphoma (31.1%), non-Hodgkin’s lymphoma (29.7%), and CLL/SLL (13.2%)- the latter two including those who treated with purine analogues or within the suggested timeframe post BMT.
In the inaccurately flagged cohort (n=129), the majority had expired time-limited indications including AML/ALL post-allograft (20.9%) and Multiple Myeloma post-autograft (15.4%). NHL post-autograft and patients who had previously required neonatal/intrauterine transfusions accounted for 3.9% and 5.4% respectively.

**Copyright Information:** (Online) ISSN: 2572-9241
©2026 The Author(s). HemaSphere is published by John Wiley & Sons Ltd on behalf of European Hematology Association. This is an open access Abstract Book distributed under the Attribution-NonCommercial-NoDerivs (CC BY-NC-ND), which allows third parties to download the articles and share them with others as long as they credit the author and the Abstract Book, but they cannot change the content in any way or use them commercially.
**Abstract Book Citations:** Authors, Title, HemaSphere, 2026;10:(S1):pages. The abstract book DOIs can be found on the “Author Information” page.
**Disclaimer:** Articles published in the journal HemaSphere exclusively reflect the opinions of the authors. The authors are responsible for all content in their abstracts including accuracy of the facts, statements, citing resources, etc.

**Summary/Conclusion**

The irradiated blood flag was inaccurate in 31%, driven by a failure to update records after temporary indications resolved. Causes included prescriber and historical "blanket" referrals with the blood bank.
To address this, we updated the referral system done at the time of initiation of chemotherapy to include a mandatory time-bound component. Flags are now removed automatically after the indicated duration unless clinically renewed with communication sent through. Pre-transfusion checklists were updated to include a checkbox for irradiated blood products to trigger review of indication prior to each blood transfusion. Changes to the induction booklet and education sessions with both Resident Doctors and Haematology team members are underway. We plan to re-audit after implementation to assess accuracy of flags and impact of change.

**Copyright Information:** (Online) ISSN: 2572-9241
©2026 The Author(s). HemaSphere is published by John Wiley & Sons Ltd on behalf of European Hematology Association. This is an open access Abstract Book distributed under the Attribution-NonCommercial-NoDerivs (CC BY-NC-ND), which allows third parties to download the articles and share them with others as long as they credit the author and the Abstract Book, but they cannot change the content in any way or use them commercially.
**Abstract Book Citations:** Authors, Title, HemaSphere, 2026;10:(S1):pages. The abstract book DOIs can be found on the "Author Information" page.
**Disclaimer:** Articles published in the journal HemaSphere exclusively reflect the opinions of the authors. The authors are responsible for all content in their abstracts including accuracy of the facts, statements, citing resources, etc.

### (PF1334) PLATELET CONCENTRATES CONSERVE THEIR CAPABILITY TO RESTORE HAEMOSTASIS DESPITE UNDERGOING PHENOTYPIC CHANGES DURING COLD STORAGE

**Topic:** 32. Transfusion medicine

Nina Wolska[1], Irene Marini[1], Brigitte Weidenthaler[1], Günalp Uzun*[1], Karoline Kleck[1], Tamam Bakchoul[1]

[1] *Institute for Clinical and Experimental Transfusion Medicine, Tuebingen, Germany;*

### Background

Platelet concentrates (PCs) are used to treat or prevent bleeding in patients with impaired platelet function or after injury. These products are currently stored at room temperature (RT) only for 4-5 days due to high risk of bacterial infection. Therefore, cold storage has been investigated as alternative storage temperature to extend the storage time and reduce the risk of bacterial contamination.

### Aims

To analyse the cellular and molecular changes in platelet concentrates stored at RT and 4°C and evaluate their*in vitro* functionality.

### Methods

Apheresis PCs (n=10) were collected from healthy donors and separated into 2 equivalent GMP conforming bags. One PC bag was stored at RT and the other at 4°C, both under agitation. Flow cytometry analysis was performed to determine platelet activation and metabolic status: CD62P, CD63 expression, phosphatidylserine (PS) exposure, mitochondrial membrane depolarization were detected, with and without agonists´ stimulation. Next, aggregometry and thromboelastography (TEG) assays were performed. A novel flow chamber *ex vivo* system, mimicking the physiological blood flow, was used to assess clot forming ability in a spick-in PCs system. All tests were performed after 1, 6 and 10 days of storage.

### Results

All PCs were valid over the entire storage period at both temperatures according to the quality parameters: platelet count, pH, product weight, and sterility at endpoint. Cold storage maintained slightly higher platelet counts. PCs stored at 4°C displayed enhanced PS exposure, decreased mitochondrial membrane potential and spontaneous degranulation compared to RT-PCs, but did not show lower viability. Nevertheless, the responsiveness of 4°C-PCs, upon TRAP incubation, was not affected. A general time-dependent decrease of platelet responsiveness, at both conditions, was observed during the entire storage period. Moreover, improved aggregation was detected in PCs stored at 4°C upon stimulation with arachidonic acid, ADP, and TRAP-6. While comparable aggregation ability was observed after collagen or ristocetin incubation. TEG showed similar results for both storage temperatures. Finally, in the *ex vivo* thrombus formation assay, upon stimulation, all products at both storage conditions showed normal thrombus formation, without indication of thrombotic phenotype.

### Summary/Conclusion

Our findings indicate that cold storage, despite inducing changes at the molecular level, does not affect the haemostatic functionality of PCs. Therefore, cold storage of PCs might be a promising strategy to extend the storage time, reduce the risk of bacterial infection post transfusion, without either impairing the cell functionality or enhancing the thrombotic risk.

**Copyright Information:** (Online) ISSN: 2572-9241
©2026 The Author(s). HemaSphere is published by John Wiley & Sons Ltd on behalf of European Hematology Association. This is an open access Abstract Book distributed under the Attribution-NonCommercial-NoDerivs (CC BY-NC-ND), which allows third parties to download the articles and share them with others as long as they credit the author and the Abstract Book, but they cannot change the content in any way or use them commercially.
**Abstract Book Citations:** Authors, Title, HemaSphere, 2026;10:(S1):pages. The abstract book DOIs can be found on the “Author Information” page.
**Disclaimer:** Articles published in the journal HemaSphere exclusively reflect the opinions of the authors. The authors are responsible for all content in their abstracts including accuracy of the facts, statements, citing resources, etc.

### (PF1335) FERRITIN AND HEMOGLOBIN LEVELS AS CRITERIA FOR ADMISSION TO BLOOD DONATION THAT PREVENT THE DEVELOPMENT OF IRON DEFICIENCY IN REGULAR BLOOD DONORS

**Topic:** 32. Transfusion medicine

Tetiana Kalynychenko*[1], Elena Malygon[2], Belousov Andrey[2], Maryna Yagovdik[1], Parubets Lidiia[1]

*[1]State Institution "National Research Center for Radiation Medicine, Hematology and Oncology of the National Academy of Medical Sciences of Ukraine", Kyiv, Ukraine; [2]Kharkiv National Medical University, Kharkiv, Ukraine;*

### Background

The significant loss of iron from hemoglobin that occurs during each donation poses a threat to the development of a deficiency of this trace element in regular blood donors. There are different approaches for preventing these conditions used by blood supply organizations in European countries. At the same time, the lack of unified scientifically based recommendations, as well as the presence of historically established discrepancies in this field complicate prevention. We studied the effectiveness of one such strategy operating in the Ukrainian blood service and is based on a combination of tests, including regular hemoglobin (Hb) level screening before each blood donation and ferritin content monitoring in serum or plasma after every 4th consecutive donation.

### Aims

The aim of the study was to analyze the effectiveness of Hb and ferritin as criteria for donation eligibility among regular blood donors in connection with the risk of developing iron deficiency conditions for this category of individuals.

### Methods

The studied group consisted of regular whole blood donors (n=100), 57% of whom were women (mean age of donors: 42±12 years; men — 41±13 years; women — 43±11 years). One third of the women were over 55 years of age (mean age 59±3 years). Donors' ferritin and Hb levels were analyzed to determine eligibility for blood donation (threshold values for indicators: ferritin ≥ 15 μg/L; Hb ≥ 125 g/L for women and ≥ 135 g/L for men). Donors were divided into subgroups by donation history, gender, and age.

### Results

It was found that 18% of donors had ferritin levels below 15 μg/L (10.6±1.7 μg/L). 83% of these donors were young and middle-aged women (20 to 55 years old). Men with low serum ferritin in the donor history had more than 7 donations in the last 2 years, unlike women, in whom low ferritin levels were detected immediately after the first donations. The average Hb level in male and female donors with ferritin levels below 15 μg/L was 147±5.9 g/L and 131.7±7.9 g/L, respectively (p=0.009). 14% of the study group had ferritin values ranging from 15 to 30 μg/L. The latter had a history of 5 to 9 donations. Overall, the ferritin level was significantly higher in male donors (2.2 times) than in female donors (med): 80.1 (30.7-131.4) μg/l (max 280 μg/l) versus 36.5 (16.7-74.2) μg/l (max 160 μg/l) (p<0.0001).

**Copyright Information:** (Online) ISSN: 2572-9241
©2026 The Author(s). HemaSphere is published by John Wiley & Sons Ltd on behalf of European Hematology Association. This is an open access Abstract Book distributed under the Attribution-NonCommercial-NoDerivs (CC BY-NC-ND), which allows third parties to download the articles and share them with others as long as they credit the author and the Abstract Book, but they cannot change the content in any way or use them commercially.
**Abstract Book Citations:** Authors, Title, HemaSphere, 2026;10:(S1):pages. The abstract book DOIs can be found on the "Author Information" page.
**Disclaimer:** Articles published in the journal HemaSphere exclusively reflect the opinions of the authors. The authors are responsible for all content in their abstracts including accuracy of the facts, statements, citing resources, etc.

**Summary/Conclusion**

The frequency of ferritin monitoring after every fourth donation before the fifth (which has been currently set by regulatory authorities in many countries), as well as the regulatory Hb cutoff level, do not fully allow for the detection of the development of latent iron deficiency in regular blood donors. Determination of the donor's baseline ferritin level and personalized adjustment of donation interval recommendations should be considered as possible actions to reduce risks. In particular, systematic ferritin testing of individuals with previously detected moderately low ferritin levels (≤30 μg/L) is considered necessary, as such regular donors who donate blood every few months are at increased risk of developing iron deficiency. This is especially true for young and middle-aged female donors. Further in-depth research on the development of an optimal model adapted to the needs of Ukraine during the war, as well as in the post-war development of the blood supply industry, is essential from the perspective of analyzing both the demographic and physiological characteristics of different groups of regular blood donors.

**Copyright Information:** (Online) ISSN: 2572-9241
©2026 The Author(s). HemaSphere is published by John Wiley & Sons Ltd on behalf of European Hematology Association. This is an open access Abstract Book distributed under the Attribution-NonCommercial-NoDerivs (CC BY-NC-ND), which allows third parties to download the articles and share them with others as long as they credit the author and the Abstract Book, but they cannot change the content in any way or use them commercially.
**Abstract Book Citations:** Authors, Title, HemaSphere, 2026;10:(S1):pages. The abstract book DOIs can be found on the “Author Information” page.
**Disclaimer:** Articles published in the journal HemaSphere exclusively reflect the opinions of the authors. The authors are responsible for all content in their abstracts including accuracy of the facts, statements, citing resources, etc.

### (PF1336) TRANSFUSION BURDEN IS THE DOMINANT MODIFIABLE DETERMINANT OF CLINICALLY SIGNIFICANT ALLOIMMUNIZATION IN HEMOLYTIC ANEMIAS: A 30-YEAR INSTITUTIONAL REGISTRY FROM A BRAZILIAN REFERRAL CENTER

**Topic:** 32. Transfusion medicine

Daniely Fagundes*[1], Luiz Amorim[1], Thayanne Bezerra[1], Victoria Pires[2], Victoria Florentino[2], Mariana Leonor[2], Municki Correa[1], Vanessa Souza[1]

*[1]HEMORIO, Hematology, Rio de Janeiro, Brazil; [2]Universo, Medicine, Rio de Janeiro, Brazil;*

### Background

Hemolytic anemias comprise heterogeneous immune and non-immune disorders frequently requiring red blood cell (RBC) transfusion for severe anemia. Alloimmunization remains a major transfusion-related complication, increasing immunohematologic complexity and delaying compatible blood provision. Integrated real-world data combining sociodemographic characteristics, etiologic subtypes, and independent predictors of clinically significant alloimmunization remain limited, particularly in genetically diverse populations.

### Aims

To characterize sociodemographic profile, hemolytic anemia subtypes, transfusion exposure, alloantibody patterns, and independent determinants of clinically significant alloimmunization using a 30-year institutional registry from a Brazilian tertiary referral center.

### Methods

This retrospective analytical registry study included 306 unique patients recorded between 1995 and 2025. Sociodemographic variables (age, sex), etiologic subtype, and transfusion data were extracted. Subtypes included warm autoimmune hemolytic anemia (AIHA), cold-mediated forms, secondary immune hemolysis, and non-immune etiologies. All patients underwent direct antiglobulin testing; chronic cases received extended immunohematologic investigation. Clinically significant alloantibodies were defined as antibodies directed against Rh, Kell, Kidd, Duffy, and MNS systems. Associations were evaluated using chi-square or Fisher's exact tests and Pearson correlation. Multivariable logistic regression restricted to transfused patients incorporated Firth bias-reduced adjustment.

### Results

Median age was in the sixth decade, with female predominance. Warm AIHA represented the most frequent subtype, followed by cold-mediated and secondary immune forms. Seventy-eight patients (25.5%) required RBC transfusion. Compared with the overall population, transfused individuals were older and more frequently presented with active immune-mediated disease. Clinically significant alloimmunization occurred in 52 patients overall and in 25 transfused individuals. A clear dose–response relationship was observed between cumulative transfusion exposure and alloimmunization risk ($p<0.05$). In multivariable Firth regression, log(1 + cumulative RBC units) emerged as the sole independent predictor, whereas age, sex, disease subtype, and calendar period were not independently associated. Count modeling confirmed transfusion exposure as the primary determinant of alloantibody multiplicity. Although transfusion rates decreased over time, the strength of association between transfusion burden and alloimmunization remained stable across decades.

**Copyright Information:** (Online) ISSN: 2572-9241
©2026 The Author(s). HemaSphere is published by John Wiley & Sons Ltd on behalf of European Hematology Association. This is an open access Abstract Book distributed under the Attribution-NonCommercial-NoDerivs (CC BY-NC-ND), which allows third parties to download the articles and share them with others as long as they credit the author and the Abstract Book, but they cannot change the content in any way or use them commercially.
**Abstract Book Citations:** Authors, Title, HemaSphere, 2026;10:(S1):pages. The abstract book DOIs can be found on the "Author Information" page.
**Disclaimer:** Articles published in the journal HemaSphere exclusively reflect the opinions of the authors. The authors are responsible for all content in their abstracts including accuracy of the facts, statements, citing resources, etc.

**Summary/Conclusion**

Across three decades of institutional registry data from a genetically diverse population, cumulative transfusion exposure—not demographic factors or disease subtype—was the dominant and modifiable determinant of clinically significant alloimmunization in hemolytic anemias. These findings support early risk-adapted extended antigen matching and proactive immunohematologic surveillance in patients anticipated to require repeated transfusions.

**Copyright Information:** (Online) ISSN: 2572-9241
©2026 The Author(s). HemaSphere is published by John Wiley & Sons Ltd on behalf of European Hematology Association. This is an open access Abstract Book distributed under the Attribution-NonCommercial-NoDerivs (CC BY-NC-ND), which allows third parties to download the articles and share them with others as long as they credit the author and the Abstract Book, but they cannot change the content in any way or use them commercially.
**Abstract Book Citations:** Authors, Title, HemaSphere, 2026;10:(S1):pages. The abstract book DOIs can be found on the "Author Information" page.
**Disclaimer:** Articles published in the journal HemaSphere exclusively reflect the opinions of the authors. The authors are responsible for all content in their abstracts including accuracy of the facts, statements, citing resources, etc.

### (PF1338) SECONDARY FINDINGS FROM WHOLE GENOME SEQUENCING: A CAUSE FOR CONCERN?

**Topic:** 33. Platelet disorders

Nehal Joshi*[1], Alice Hart[2], Spencer MacKie[2], Hannah Hendry[3], Nichola Cooper[1]

[1]*Imperial College London, Immunology & Inflammation, London, United Kingdom;* [2]*IMPERIAL COLLEGE HEALTHCARE NHS TRUST, Haematology, London, United Kingdom;* [3]*North Thames Genomic Laboratory Hub, London, United Kingdom;*

### Background

The reporting of secondary findings (SF) identified during whole genome sequencing (WGS) remains controversial. International guidance varies. The American College of Medical Genetics and Genomics recommends active interrogation and reporting of pathogenic variants in a defined list of actionable genes, with patient opt-out at consent. In contrast, European guidance advocates a more cautious approach, particularly in children and for late-onset conditions. Within the NHS Genomic Medicine Service (GMS), SF were evaluated in the 100,000 Genomes Project but are not routinely sought or reported in current clinical WGS pathways. However, phenotype-driven variant prioritisation tools (e.g. Exomiser) used in interpretation pipelines may highlight pathogenic variants unrelated to the primary indication for testing. The practical impact of this in haematology is unclear.

### Aims

To evaluate the frequency and reporting of secondary findings identified through clinical WGS in patients with immune thrombocytopenia (ITP), and to explore how these findings are managed within UK practice.

### Methods

We performed WGS in 130 patients (100 adults; 30 children) with a clinical diagnosis of ITP to investigate potential inborn errors of immunity or hereditary thrombocytopenia. Variants were analysed using standard NHS GMS pipelines including phenotype-driven prioritisation.

### Results

Pathogenic secondary findings unrelated to the thrombocytopenia phenotype were identified in 1% of adults (MSH6) and 10% of children (TP53, PALB2, RYR1). Most involved cancer predisposition genes; RYR1 confers susceptibility to malignant hyperthermia. Specific variants are detailed in the accompanying figure.
In addition, variants explaining the presenting phenotype but also conferring malignancy risk were identified in 4% of adults (NFKB1, NFKB2, ANKRD26) and 3% of children (ETV6). These findings blur the distinction between primary and secondary results, as they carry broader health implications beyond thrombocytopenia.
Although the NHS GMS does not routinely report SF, in our centre secondary findings were reviewed by a Clinical Genetics multidisciplinary team (MDT), with case-by-case decisions regarding disclosure. Reporting was therefore dependent on variant characteristics and MDT consensus rather than a predefined national framework.

**Copyright Information:** (Online) ISSN: 2572-9241
©2026 The Author(s). HemaSphere is published by John Wiley & Sons Ltd on behalf of European Hematology Association. This is an open access Abstract Book distributed under the Attribution-NonCommercial-NoDerivs (CC BY-NC-ND), which allows third parties to download the articles and share them with others as long as they credit the author and the Abstract Book, but they cannot change the content in any way or use them commercially.
**Abstract Book Citations:** Authors, Title, HemaSphere, 2026;10:(S1):pages. The abstract book DOIs can be found on the "Author Information" page.
**Disclaimer:** Articles published in the journal HemaSphere exclusively reflect the opinions of the authors. The authors are responsible for all content in their abstracts including accuracy of the facts, statements, citing resources, etc.

**Summary/Conclusion**

Clinically significant secondary findings occur in a meaningful proportion of patients undergoing WGS for haematological indications, particularly in paediatric cohorts. The absence of clear national guidance to SF reporting creates variability in practice and potential uncertainty for clinicians and patients.
These findings emphasise the need for:

1. Ethical, robust and consistent consent processes addressing the possibility of incidental results;
2. consistent policy regarding disclosure of actionable variants and clear national guidance for clinicians requesting WGS;
3. structured MDT involvement including clinical geneticists and genetic counsellors;
4. appropriate resourcing to support post-test counselling and long-term management.

As genomic testing becomes embedded in data-driven haematology, the ethical and practical implications of secondary findings cannot be overlooked. Further research is required to evaluate clinician and public perspectives and to inform policy development internationally. The impact of reporting secondary findings must not be underestimated, it has significant and far-reaching implications for patients and their families, as well the entire health service.

| Secondary findings | | Variants explaining thrombocytopenia but carrying risk of malignancy |
|---|---|---|
| PALB2 c.1027C>T p.Gln343Ter | | ETV6 c.135del p.(Asp46Thrfs*21) |
| TP53 c.375G>A p.(Thr125=) | | NFKB1 c.850C>T p.(Arg284*) |
| MSH6 c.3863_3865dup p.(Lys1288_Phe1289Ter) | | NFKB2 c.722G>A p.(Arg241Gln) |
| RYR1 c.1615T>C p.(Phe539Leu) | | ANKRD26 c.-128G>C |
| | | ANKRD26 – c.-116C>G |

Variants identified as 'secondary findings' or 'explaining phenotype and conferring malignancy risk'.

Copyright Information: (Online) ISSN: 2572-9241
©2026 The Author(s). HemaSphere is published by John Wiley & Sons Ltd on behalf of European Hematology Association. This is an open access Abstract Book distributed under the Attribution-NonCommercial-NoDerivs (CC BY-NC-ND), which allows third parties to download the articles and share them with others as long as they credit the author and the Abstract Book, but they cannot change the content in any way or use them commercially.
**Abstract Book Citations:** Authors, Title, HemaSphere, 2026;10:(S1):pages. The abstract book DOIs can be found on the "Author Information" page.
**Disclaimer:** Articles published in the journal HemaSphere exclusively reflect the opinions of the authors. The authors are responsible for all content in their abstracts including accuracy of the facts, statements, citing resources, etc.

### (PF1339) REDUCED EXPRESSION OF SRSF1 PROMOTES LCK EXON 8 SKIPPING TO ATTENUATE TCR SIGNALING AND TREG INDUCTION IN IMMUNE THROMBOCYTOPENIA

**Topic:** 33. Platelet disorders

Yan Liu*[1], Xiang Hu[1], Shaoqiu Leng[1], Shuwen Wang[1], Jun Peng[1]

[1]*Qilu Hospital Of Shandong University, Department Of Hematology, Jinan, China;*

### Background

Immune thrombocytopenia (ITP) is an autoimmune disorder characterized by antibody-mediated platelet destruction and defective regulatory T-cell (Treg) function, yet its molecular basis remains poorly understood.

### Aims

This study aims to elucidate how dysregulated RNA splicing contributes to Treg deficiency in ITP.

### Methods

We analyzed CD4⁺ naïve T cells from ITP patients and healthy controls using RNA-sequencing to identify splicing alterations. An LCK minigene was constructed and combined with RNA pull-down mass spectrometry and RNA immunoprecipitation assays to identify splicing regulators. Functional validation included overexpression and knockdown experiments, antisense oligonucleotide (ASO) targeting, and adoptive transfer of genetically modified CD4⁺ naïve T cells into CD61-knockout mice in an active ITP model. Protein interaction studies were performed using engineered Jurkat cells.

### Results

CD4⁺ naïve T cells from ITP patients showed diminished TCR signaling and impaired Treg induction. Transcriptome analysis revealed widespread splicing dysregulation, most notably exon 8 skipping in *LCK*. Splicing factor SRSF1 was identified as a direct upstream regulator of *LCK* exon 8 skipping and was found to be downregulated in ITP patient cells. Restoring SRSF1 expression rescued Treg induction in vitro, while ASO-mediated targeting of SRSF1 enhanced exon skipping and suppressed TCR signaling. In vivo, adoptive transfer of CD4⁺ naïve T cells expressing exon 8–skipped murine *Lck* exacerbated ITP features, including reduced Treg proportions and platelet counts. Mechanistically, the exon 8–skipped LCK isoform exhibited markedly reduced binding to ZAP70 and CD3ζ.

### Summary/Conclusion

CD4⁺ naïve T cells from ITP patients showed diminished TCR signaling and impaired Treg induction. Transcriptome analysis revealed widespread splicing dysregulation, most notably exon 8 skipping in *LCK*. Splicing factor SRSF1 was identified as a direct upstream regulator of *LCK* exon 8 skipping and was found to be downregulated in ITP patient cells. Restoring SRSF1 expression rescued Treg induction in vitro, while ASO-mediated targeting of SRSF1 enhanced exon skipping and suppressed TCR signaling. In vivo, adoptive transfer of CD4⁺ naïve T cells expressing exon 8–skipped murine *Lck* exacerbated ITP features, including reduced Treg proportions and platelet counts. Mechanistically, the exon 8–skipped LCK isoform exhibited markedly reduced binding to ZAP70 and CD3ζ.

**Copyright Information:** (Online) ISSN: 2572-9241
©2026 The Author(s). HemaSphere is published by John Wiley & Sons Ltd on behalf of European Hematology Association. This is an open access Abstract Book distributed under the Attribution-NonCommercial-NoDerivs (CC BY-NC-ND), which allows third parties to download the articles and share them with others as long as they credit the author and the Abstract Book, but they cannot change the content in any way or use them commercially.
**Abstract Book Citations:** Authors, Title, HemaSphere, 2026;10:(S1):pages. The abstract book DOIs can be found on the "Author Information" page.
**Disclaimer:** Articles published in the journal HemaSphere exclusively reflect the opinions of the authors. The authors are responsible for all content in their abstracts including accuracy of the facts, statements, citing resources, etc.

### (PF1340) EFFECT OF IANALUMAB PLUS ELTROMBOPAG ON PATIENT-REPORTED OUTCOMES IN PRIMARY IMMUNE THROMBOCYTOPENIA: RESULTS FROM THE VAYHIT2 PHASE 3 TRIAL

**Topic:** 33. Platelet disorders

Nichola Cooper*[1], Adam Cuker[2], Tuphan Dolai[3], Waleed Ghanima[4, 5], David Gómez Almaguer[6], Arpad Illes[7], Marc Michel[8], Luminita Ocroteala[9], Sue Pavord[10], Veronica Privitera[11], Fahri Sahin[12], Thomas Stauch[13], Marek Trněný[14], Raymond Wong[15], Tie-Nan Zhu[16], Martina Crysandt[17], Rahim Lalji[18], Marine Weill[19], Patrick Urban[19], Justyna Fronczek[19], Matthew Foster[20], Hanny Al-Samkari[21]

*[1]Imperial College London, Centre for Haematology, Department of Immunology and Inflammation, London, United Kingdom; [2]Perelman School of Medicine, University of Pennsylvania, Department of Medicine and Department of Pathology and Laboratory Medicine, Philadelphia, PA, United States of America; [3]Nil Ratan Sircar Medical College and Hospital, Department of Haematology, Kolkata, India; [4]Østfold Hospital Trust, Kalnes, Norway;[5]University of Oslo, Oslo, Norway; [6]Hospital Universitario "Dr José Eleuterio González", Universidad Autónoma de Nuevo León, Department of Hematology, Monterrey, Mexico; [7]University of Debrecen, Department of Hematology, Faculty of Medicine, Debrecen, Hungary; [8]Henri Mondor University Hospital, Assistance Publique-Hôpitaux de Paris, Paris-Est Créteil University, Department of Internal Medicine, Créteil, France; [9]Municipal Clinical Hospital Filantropia Craiova, Craiova, Romania; [10]Oxford University Hospitals NHS Trust, Oxford University, Department of Haematology, Oxford, United Kingdom; [11]Hospital Italiano de Buenos Aires, Buenos Aires, Argentina;[12]Ege University, Ege Adult Haemophilia and Thrombosis Center, İzmir, Türkiye; [13]University Hospital Jena, Department of Internal Medicine II, Hematology and Oncology, Jena, Germany; [14]Charles University, General Hospital, First Faculty of Medicine, Prague, Czechia; [15]Prince of Wales Hospital, Sir Y.K. Pao Centre for Cancer and Department of Medicine & Therapeutics, Hong Kong, Hong Kong; [16]Peking Union Medical College Hospital, Beijing, China; [17]RWTH Aachen University & Center for Integrated Oncology Aachen Bonn Cologne Düsseldorf (CIO ABCD), Department of Hematology, Oncology, Hemostaseology and Stem Cell Transplantation, Medical Faculty, Aachen, Germany; [18]Novartis Pharmaceuticals UK Ltd., London, United Kingdom; [19]Novartis Pharma AG, Basel, Switzerland; [20]Novartis Pharmaceuticals Corporation, East Hanover, NJ, United States of America;[21]Massachusetts General Hospital, Harvard Medical School, Division of Hematology Oncology, Boston, MA, United States of America;*

### Background

In the 2018 immune thrombocytopenia (ITP) World Impact Survey, between one-third and one-half of patients reported a meaningful impact on their health-related quality of life (HRQoL), including fatigue, reduced ability to exercise, and high symptom burden.[1] The phase 3 VAYHIT2 trial (NCT05653219) demonstrated prolonged time to treatment failure and improved stable response rates at 6 months with ianalumab plus eltrombopag vs placebo plus eltrombopag in patients with ITP who had insufficient response to or relapsed after first-line corticosteroids. In addition, improvement in fatigue was observed using the Patient-Reported Outcomes Measurement Information System Short Form v1.0 Fatigue 13a.[2]

### Aims

To further characterize the impact of ianalumab plus eltrombopag on patient-reported outcomes (PROs) using a disease-specific instrument validated to measure the burden of ITP symptoms and its treatment.

**Copyright Information:** (Online) ISSN: 2572-9241
©2026 The Author(s). HemaSphere is published by John Wiley & Sons Ltd on behalf of European Hematology Association. This is an open access Abstract Book distributed under the Attribution-NonCommercial-NoDerivs (CC BY-NC-ND), which allows third parties to download the articles and share them with others as long as they credit the author and the Abstract Book, but they cannot change the content in any way or use them commercially.
**Abstract Book Citations:** Authors, Title, HemaSphere, 2026;10:(S1):pages. The abstract book DOIs can be found on the "Author Information" page.
**Disclaimer:** Articles published in the journal HemaSphere exclusively reflect the opinions of the authors. The authors are responsible for all content in their abstracts including accuracy of the facts, statements, citing resources, etc.

### Methods

Patients (N=152) were randomized (1:1:1) to receive ianalumab 9 or 3 mg/kg or placebo, plus eltrombopag. Ianalumab or placebo was administered as four once-monthly intravenous infusions, alongside daily eltrombopag for 16 weeks. This was followed by an 8-week eltrombopag tapering period until discontinuation in eligible patients by week 25. PROs were collected at on-treatment visits and during post-treatment follow-up until end of study and assessed using the Immune Thrombocytopenic Purpura Patient Assessment Questionnaire (ITP-PAQ), a 44-item, 10-scale instrument for HRQoL in adults with ITP. Each scale was scored from 0 to 100, with higher scores indicating better HRQoL. Scores were analyzed using repeated linear mixed models, with least-square (LS) mean changes from baseline calculated for each treatment group. This analysis focused on the ITP-PAQ scales of Symptoms, Fatigue, Bother, and Activity, as these were secondary endpoints of interest.

### Results

At data cutoff (June 20, 2025), the median duration of follow-up was 12.9, 13.6, and 11.6 months with ianalumab 9 mg/kg plus eltrombopag (hereafter referred to as the ianalumab 9 mg/kg group), ianalumab 3 mg/kg plus eltrombopag (ianalumab 3 mg/kg group), and placebo plus eltrombopag (placebo group), respectively. Across ITP-PAQ scales of Symptoms, Fatigue, Bother, and Activity, scores at most time points were highest in the ianalumab 9 mg/kg group (**Figure**); however, improvements were observed in all treatment groups, beginning early in the treatment period and extending through eltrombopag tapering and discontinuation (week 25), with LS-mean (95% confidence interval) changes from baseline for ITP-PAQ Symptoms being 21.6 (17.6-25.7) and 18.4 (14.2-22.5) with ianalumab 9 and 3 mg/kg, respectively, and 16.7 (12.8-20.6) with placebo; for Fatigue, these figures were 21.2 (15.3-27.2), 12.9 (6.6-19.2), and 13.6 (7.7-19.6). Corresponding improvements from baseline at week 25 for Bother were 26.4 (20.9-31.8), 25.4 (19.7-31.2), and 19.0 (13.5-24.4); and 25.0 (18.0-32.1), 22.8 (15.3-30.3), and 20.5 (13.4-27.5) for Activity.

### Summary/Conclusion

Improvements in the ITP-specific PROs across the Symptoms, Fatigue, Bother, and Activity scales were observed in all groups, with greater and more consistent improvements observed in the ianalumab 9 mg/kg group. There was no indication of increased treatment-related bother or reduced HRQoL due to the addition of ianalumab.

### References:

1. Cooper N, et al. *Am J Hematol.* 2021;96:188–198.
2. Cuker A, et al. *N Engl J Med.* 2025. doi:10.1056/NEJMoa2515168.

**Copyright Information:** (Online) ISSN: 2572-9241
©2026 The Author(s). HemaSphere is published by John Wiley & Sons Ltd on behalf of European Hematology Association. This is an open access Abstract Book distributed under the Attribution-NonCommercial-NoDerivs (CC BY-NC-ND), which allows third parties to download the articles and share them with others as long as they credit the author and the Abstract Book, but they cannot change the content in any way or use them commercially.
**Abstract Book Citations:** Authors, Title, HemaSphere, 2026;10:(S1):pages. The abstract book DOIs can be found on the "Author Information" page.
**Disclaimer:** Articles published in the journal HemaSphere exclusively reflect the opinions of the authors. The authors are responsible for all content in their abstracts including accuracy of the facts, statements, citing resources, etc.

Figure: ITP-PAQ Scale Changes from Baseline for Physical Health (Symptoms, Fatigue, Bother, and Activity) Domains

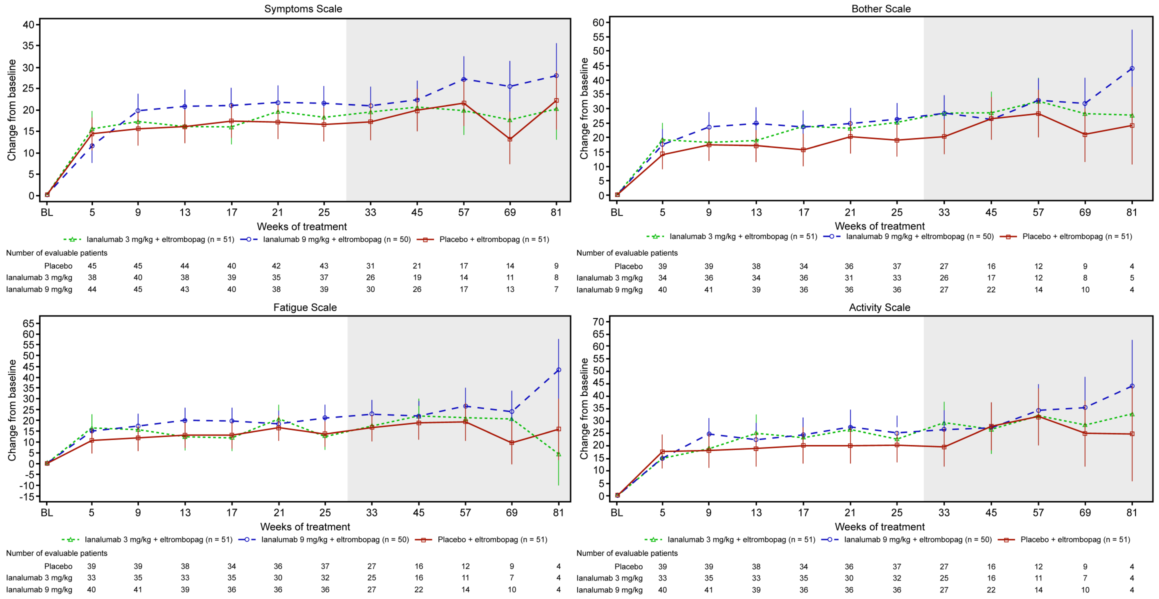

The symbols at each time point for each treatment group indicate least-square means. The vertical lines indicate 95% confidence intervals. Changes over time were estimated using a mixed-effect repeated measurement analysis of changes from baseline. The gray-shaded areas indicate the time period during which scheduled assessments were missing for more than 40% of patients, as ongoing patients have not yet reached these time points.

**Copyright Information:** (Online) ISSN: 2572-9241
©2026 The Author(s). HemaSphere is published by John Wiley & Sons Ltd on behalf of European Hematology Association. This is an open access Abstract Book distributed under the Attribution-NonCommercial-NoDerivs (CC BY-NC-ND), which allows third parties to download the articles and share them with others as long as they credit the author and the Abstract Book, but they cannot change the content in any way or use them commercially.
**Abstract Book Citations:** Authors, Title, HemaSphere, 2026;10:(S1):pages. The abstract book DOIs can be found on the "Author Information" page.
**Disclaimer:** Articles published in the journal HemaSphere exclusively reflect the opinions of the authors. The authors are responsible for all content in their abstracts including accuracy of the facts, statements, citing resources, etc.

### (PF1341) SOFT MECHANICS AND MICROGEOMETRY OF 3D SILK NICHES IMPROVE HUMAN THROMBOPOIESIS IN VITRO

**Topic:** 33. Platelet disorders

Christian Di Buduo*[1], Elisa Gabinaud[1], Matteo Migliavacca[1], Claudia Del Fante[2, 2], Alessandra Balduini[1]

*[1]University of Pavia, Pavia, Italy; [2]IRCCS Policlinico San Matteo Foundation, Pavia, Italy;*

### Background

Efficient ex vivo production of human platelets remains limited by conventional culture systems' inability to support megakaryocyte maturation and coordinated proplatelet formation. Flat 2D environments fail to reproduce the confinement, mechanical compliance, and spatial organization, resulting in incomplete maturation and heterogeneous, functionally immature platelets. Silk-based biomaterials, owing to their tunable mechanics, porosity, and long-term stability, are fundamental building blocks to engineer bone marrow–like niches that enhance megakaryocyte differentiation and platelet biogenesis.

### Aims

To engineer and validate silk-based bone marrow niches in which the intrinsic mechanical and structural properties of the biomaterial actively promote terminal megakaryocyte maturation, proplatelet formation, and efficient generation of human platelets ex vivo.

### Methods

Porous 3D scaffolds were fabricated from Bombyx mori silk fibroin and tuned to bone marrow-like mechanics and microarchitecture. HSPCs, from circulating adult and mobilized peripheral blood, were differentiated toward megakaryocytes under parallel flat 2D and 3D conditions. Megakaryocyte survival, maturation, proplatelet formation, and platelet production under controlled flow were assessed using quantitative imaging, immunophenotyping, and functional assays.

### Results

The engineered silk niche provided a reproducible soft microenvironment that redirected physiological thrombopoiesis. Scaffold stiffness and elasticity were tuned to match native bone marrow compliance, while pore size and porosity recapitulated the architecture of marrow tissue and supported efficient nutrient and oxygen exchange. Differentiation was initiated from early progenitors, allowing progressive adaptation to the 3D environment before lineage commitment and terminal maturation. Immunophenotyping revealed a uniform cellular distribution throughout the scaffold and the generation of enlarged, polyploid $CD61^+CD42b^+$ megakaryocytes, consistent with advanced maturation. The 3D niche significantly improved megakaryocyte survival during prolonged culture and increased the density of thrombopoietic regions. Proplatelet formation increased up to fivefold compared with 2D cultures, with over 80% of megakaryocytes forming highly branched networks, whereas flat cultures produced sparse, truncated structures. Transcriptomic profiling identified 3D-specific signatures linked to cytoskeletal remodeling and terminal maturation programs. Platelets generated in the 3D silk model displayed discoid morphology, size distribution, granule content, mitochondrial abundance, and preserved sialylation comparable to peripheral blood platelets. They retained robust agonist-induced activation and adhesion-dependent cytoskeletal remodeling.

**Copyright Information:** (Online) ISSN: 2572-9241
©2026 The Author(s). HemaSphere is published by John Wiley & Sons Ltd on behalf of European Hematology Association. This is an open access Abstract Book distributed under the Attribution-NonCommercial-NoDerivs (CC BY-NC-ND), which allows third parties to download the articles and share them with others as long as they credit the author and the Abstract Book, but they cannot change the content in any way or use them commercially.
**Abstract Book Citations:** Authors, Title, HemaSphere, 2026;10:(S1):pages. The abstract book DOIs can be found on the "Author Information" page.
**Disclaimer:** Articles published in the journal HemaSphere exclusively reflect the opinions of the authors. The authors are responsible for all content in their abstracts including accuracy of the facts, statements, citing resources, etc.

**Summary/Conclusion**

A physiologically defined silk bone marrow niche enables efficient megakaryocyte maturation and generation of functionally competent human platelets. By isolating the contribution of physical microenvironmental and biochemical cues, the silk system provides a unique scalable foundation for translational platelet manufacturing, disease modeling, and personalized transfusion strategies.

**Copyright Information:** (Online) ISSN: 2572-9241
©2026 The Author(s). HemaSphere is published by John Wiley & Sons Ltd on behalf of European Hematology Association. This is an open access Abstract Book distributed under the Attribution-NonCommercial-NoDerivs (CC BY-NC-ND), which allows third parties to download the articles and share them with others as long as they credit the author and the Abstract Book, but they cannot change the content in any way or use them commercially.
**Abstract Book Citations:** Authors, Title, HemaSphere, 2026;10:(S1):pages. The abstract book DOIs can be found on the "Author Information" page.
**Disclaimer:** Articles published in the journal HemaSphere exclusively reflect the opinions of the authors. The authors are responsible for all content in their abstracts including accuracy of the facts, statements, citing resources, etc.

### (PF1342) EARLY PREDICTION OF THROMBOTIC THROMBOCYTOPENIC PURPURA FROM ROUTINE LABORATORY DATA USING MACHINE LEARNING

**Topic:** 33. Platelet disorders

Ella Derkzen*[1], Jenyvette Hsia[1], Benjamin Hedley[2], Amy Tai[3], Michael Samuel[2], Rui Su[2], Cyrus Hsia[2, 4], Benjamin Chin-Yee[2, 4]

*[1]Western University, Faculty of Engineering, London, Canada;[2]London Health Sciences Centre, Department of Pathology and Laboratory Medicine, London, Canada; [3]University of Waterloo, Department of Computer Science, Waterloo, Canada;[4]London Health Sciences Centre, Division of Hematology, Department of Medicine, London, Canada;*

### Background

Thrombotic thrombocytopenic purpura (TTP) is a rare, life-threatening thrombotic microangiopathy with mortality approaching 90% without urgent plasma exchange, reduced to approximately 10% with early treatment. Diagnosis requires ADAMTS13 activity level <10% in the appropriate clinical context; however, prolonged turnaround times may delay therapy. Early recognition is further complicated by nonspecific clinical and laboratory features shared with other microangiopathic hemolytic anemias (MAHAs), including hemolytic uremic syndrome (HUS), disseminated intravascular coagulation, and atypical HUS.

### Aims

To develop a laboratory-based prediction tool using routinely available data to improve patient outcomes through earlier identification, diagnosis and treatment.

### Methods

We developed and evaluated machine learning models to predict TTP, defined by ADAMTS13 activity <10%. All patients who underwent ADAMTS13 testing at Canadian tertiary care centre from 2012-2024 and who had ≥70% of predefined laboratory parameters available within seven days of testing were included. Candidate predictors included demographics, comorbidities, and routinely available laboratory variables. Data were randomly split into 75% training and 25% testing sets. A scikit-learn pipeline was used for median imputation of missing values and feature scaling. Hyperparameters were optimized via 100-iteration RandomizedSearchCV, class imbalance was addressed using scale_pos_weight, and probabilities were calibrated with isotonic regression. XGBoost served as the primary classifier and was compared with Random Forest and LightGBM using identical preprocessing.  Cross-validation will be performed by splitting the training data into 10 groups, 9 will be used for training and 1 will be used to validate its performance on unseen data, this is done 10 times by switching what data is in each group for every iteration.

### Results

The final dataset comprised 441 tests, including 91 (21%) classified as TTP (ADAMTS13 <10%) and 350 (79%) classified as non-TTP MAHAs. Twenty-seven variables were used for model development, including age, sex, comorbidities (active cancer, prior solid organ or stem cell transplant), complete blood count indices, coagulation and hemolysis markers, and renal function. On the test set, the XGBoost model achieved better discrimination (ROC AUC 0.9323) compared with Random Forest (0.9234) and LightGBM (0.8090). Test accuracy, sensitivity, specificity, precision, and F1 score were 0.9009, 0.8261, 0.9205, 0.7308, and 0.7755, respectively, for the XGBoost model. Feature importance analysis identified active cancer and creatinine as the strongest predictors, followed by INR, fibrinogen, LDH, and platelet count (Figure 1).

**Copyright Information:** (Online) ISSN: 2572-9241
©2026 The Author(s). HemaSphere is published by John Wiley & Sons Ltd on behalf of European Hematology Association. This is an open access Abstract Book distributed under the Attribution-NonCommercial-NoDerivs (CC BY-NC-ND), which allows third parties to download the articles and share them with others as long as they credit the author and the Abstract Book, but they cannot change the content in any way or use them commercially.
**Abstract Book Citations:** Authors, Title, HemaSphere, 2026;10:(S1):pages. The abstract book DOIs can be found on the "Author Information" page.
**Disclaimer:** Articles published in the journal HemaSphere exclusively reflect the opinions of the authors. The authors are responsible for all content in their abstracts including accuracy of the facts, statements, citing resources, etc.

**Summary/Conclusion**

A machine learning model trained on routinely available laboratory and clinical variables accurately predicts TTP with low ADAMTS13, using features known to correlate with disease biology, and may support earlier diagnosis and treatment. Such a tool could be integrated into laboratory information systems to enable automated risk flagging rather than relying on bedside assessment. Multicentre and prospective evaluation is planned to assess generalizability and clinical impact.

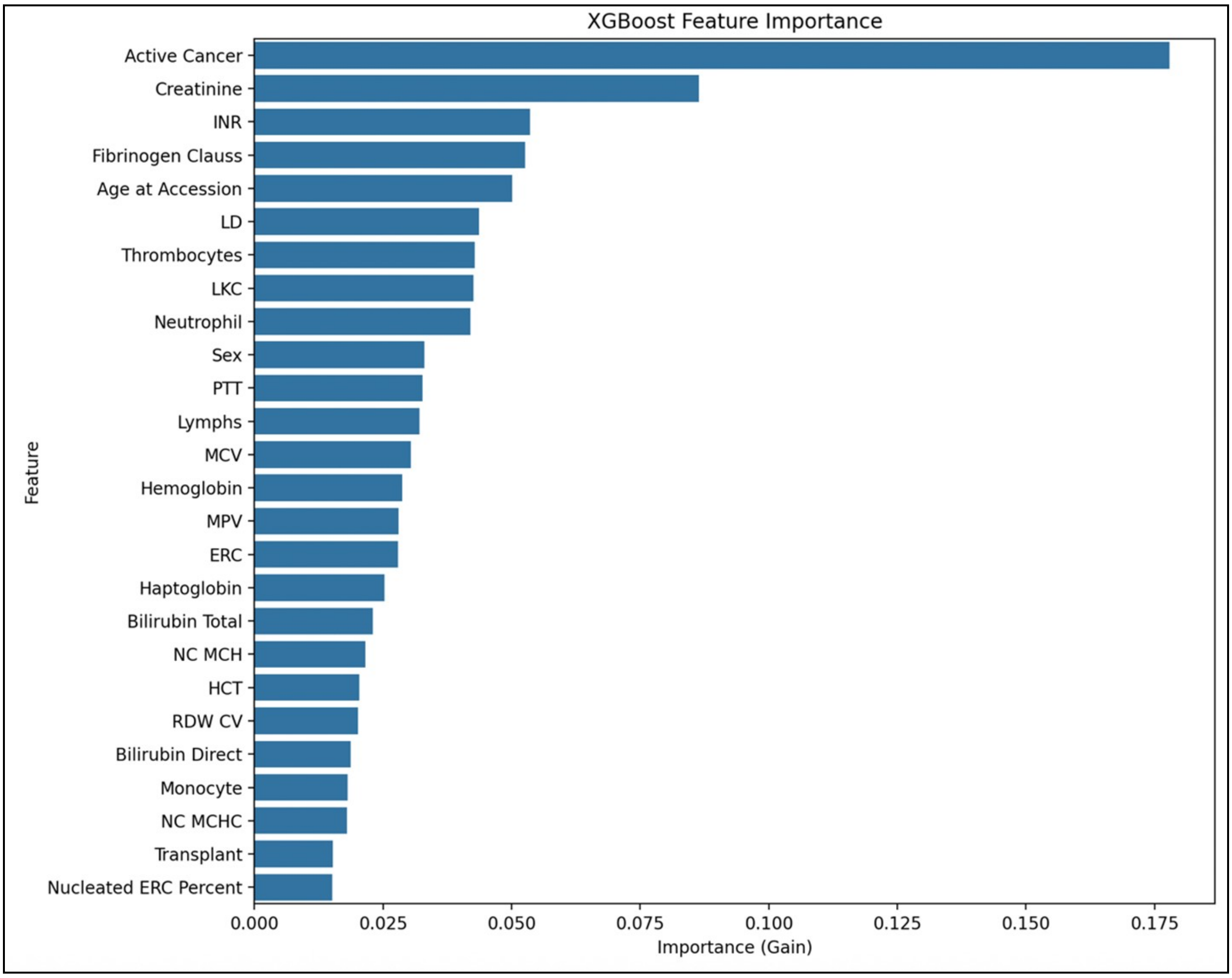

Figure 1 Feature importance

**Copyright Information:** (Online) ISSN: 2572-9241
©2026 The Author(s). HemaSphere is published by John Wiley & Sons Ltd on behalf of European Hematology Association. This is an open access Abstract Book distributed under the Attribution-NonCommercial-NoDerivs (CC BY-NC-ND), which allows third parties to download the articles and share them with others as long as they credit the author and the Abstract Book, but they cannot change the content in any way or use them commercially.
**Abstract Book Citations:** Authors, Title, HemaSphere, 2026;10:(S1):pages. The abstract book DOIs can be found on the "Author Information" page.
**Disclaimer:** Articles published in the journal HemaSphere exclusively reflect the opinions of the authors. The authors are responsible for all content in their abstracts including accuracy of the facts, statements, citing resources, etc.

### (PF1343) CHARACTERISTICS AND MANAGEMENT OF HIV-ASSOCIATED IMMUNE THROMBOCYTOPENIA: UPDATE IN THE COMBINED ANTIRETROVIRAL THERAPY ERA

**Topic:** 33. Platelet disorders

Clément Viguier[1, 2], Matthieu Godinot[3], Etienne Crickx[4, 5], Matthieu Mahevas[4, 5], Karamba Sylla[6], Valérie Pourcher[7], Victor Hemar[8], Adrien Lemaignen[9], Gwenael Le Moal[10], Helene Bazus[11], Camille Mettler[5, 12], Mathilde Devaux[13], Lou MacAux[14], Marc Olivier Vareil[15], Marie Dermit[1], Valérie Garrait[16], Fanshen Lionetto[17], Pierre Yves Jeandel[18], Cedric Joseph[19], Marc Michel[4, 5], Guillaume Martin-Blondel[1, 2], Bertrand Godeau[4, 5], Pierre Delobel[1, 2], Guillaume Moulis*[2, 5, 20]

*[1]Toulouse University Hospital, Tropical and infectious diseases, Toulouse, France; [2]Toulouse University Hospital, NEO-I3D research group, Toulouse, France; [3]Hospices civils de Lyon, Tropical and infectious diseases, Lyon, France; [4]Henri Mondor University Hospital, Internal Medicine and Clinical Immunology, Créteil, France; [5]Henri Mondor University Hospital, National Reference Centre for Adult Immune Cytopenias (CeReCAI), Créteil, France; [6]French Guiana University Hospital, Tropical and infectious diseases, Cayenne, France; [7]Pitié-Salpêtrière University Hospital, Tropical and infectious diseases, Paris, France; [8]Bordeaux University Hospital, Internal Medicine and Infectious Diseases Department, Bordeaux, France; [9]Centre Hospitalier Universitaire de Tours, Infectious Disease and Internal Medicine Unit, Tours, France; [10]Poitiers University Hospital, Tropical and infectious diseases, Poitiers, France; [11]Lens Hospital, Tropical and infectious diseases, Lens, France; [12]Cochin University Hospital, Internal medicine, Paris, France; [13]Poissy-Saint-Germain-en-Laye Hospital, Internal medicine, Saint-Germain-en-Laye, France; [14]Avicenne Hospital, Tropical and infectious diseases, Bobigny, France; [15]Côte Basque Hospital, Tropical and infectious diseases, Bayonne, France; [16]Centre Hospitalier Intercommunal de Créteil, Internal medicine, Créteil, France; [17]Toulon Hospital, Tropical and infectious diseases, Toulon, France; [18]Nice University Hospital, Internal medicine, Nice, France; [19]University Hospital of Amiens-Picardie, Tropical and infectious diseases, Amiens, France; [20]Toulouse University Hospital, Internal medicine, Toulouse, France;*

### Background

Despite major advances in HIV care, HIV-associated immune thrombocytopenia (HIV-ITP) remains a poorly described condition, with substantial gaps in its management. Available data are scarce, derived from small cohorts (n<45), lacking strict diagnostic criteria and predating 2013. Therefore, they do not reflect recent major changes in HIV infection (widespread use of combination antiretroviral therapy – cART) and ITP management.

### Aims

This study aimed to describe HIV-ITP characteristics, assess the real-world effectiveness and safety of its treatments, and evaluate the impact of sustained viral suppression on ITP progression.

### Methods

We conducted a national, multicentre, retrospective cohort study including patients diagnosed with HIV-ITP between 2013-2023, with HIV infection preceding or coinciding with ITP, platelet count $<30\times10^9/L$ and documented response to corticosteroids and/or intravenous immunoglobulin confirming the immunological mechanism of thrombocytopenia. Patients were identified in the French nationwide CARMEN-France ITP registry and through a national call for cases to French autoimmune cytopenia (CeReCAI) and infectious disease society networks.

**Copyright Information:** (Online) ISSN: 2572-9241
©2026 The Author(s). HemaSphere is published by John Wiley & Sons Ltd on behalf of European Hematology Association. This is an open access Abstract Book distributed under the Attribution-NonCommercial-NoDerivs (CC BY-NC-ND), which allows third parties to download the articles and share them with others as long as they credit the author and the Abstract Book, but they cannot change the content in any way or use them commercially.
**Abstract Book Citations:** Authors, Title, HemaSphere, 2026;10:(S1):pages. The abstract book DOIs can be found on the "Author Information" page.
**Disclaimer:** Articles published in the journal HemaSphere exclusively reflect the opinions of the authors. The authors are responsible for all content in their abstracts including accuracy of the facts, statements, citing resources, etc.

**Results**

We included 72 patients with a median follow-up of 4.2 years. HIV-ITP occurred predominantly in individuals with poor immunovirological status (52.8% with advanced HIV disease). Severe bleeding was rare (<1.5%), even in cases of profound thrombocytopenia. Short corticosteroid courses demonstrated a favourable safety profile, including in severely immunocompromised individuals. Thrombopoietin receptor agonists (TPO-RAs) were highly effective (response rate: 29/33) but two thrombotic events occurred. Other maintenance therapies (rituximab, fostamatinib) appeared effective (2/3; 2/2), with no increased rates of infection or thrombosis. Assessment of the association between ITP progression and HIV viral load identified two patterns: some patients developed ITP (14/72) or required maintenance ITP therapy despite sustained undetectable viral load (11/60), whereas durable virologic control reduced ITP treatment needs in 37/42 patients.

**Summary/Conclusion**

HIV-ITP is a heterogeneous condition. When indicated, short-course corticosteroids appear safe and effective as first-line therapy, even in patients with advanced HIV disease. Effective virologic suppression with cART is recommended, since it can induce ITP remission in some patients. However, many still require maintenance treatment for ITP, which should be individualised based on infectious and thrombotic risk factors. TPO-RAs were highly effective.

**Copyright Information:** (Online) ISSN: 2572-9241
©2026 The Author(s). HemaSphere is published by John Wiley & Sons Ltd on behalf of European Hematology Association. This is an open access Abstract Book distributed under the Attribution-NonCommercial-NoDerivs (CC BY-NC-ND), which allows third parties to download the articles and share them with others as long as they credit the author and the Abstract Book, but they cannot change the content in any way or use them commercially.
**Abstract Book Citations:** Authors, Title, HemaSphere, 2026;10:(S1):pages. The abstract book DOIs can be found on the "Author Information" page.
**Disclaimer:** Articles published in the journal HemaSphere exclusively reflect the opinions of the authors. The authors are responsible for all content in their abstracts including accuracy of the facts, statements, citing resources, etc.

### (PF1344) IDENTIFICATION OF POTENTIAL PREDICTIVE FACTORS OF NON-RESPONSE TO RITUXIMAB DURING IMMUNE THROMBOCYTOPENIA

**Topic:** 33. Platelet disorders

Roman Praliaud*[1], Baptiste Lamarthee[1], Alexis Varin[1], Noémie Herrmann[1], Marion Ciudad[1], Claudie Cladiere[1], Romain Boidot[2], Maxime Samson[1], Bernard Bonnotte[1], Sylvain Audia[1]

*[1]University of Burgundy Europe, Dijon University Hospital, French Reference Centre for Autoimmune Cytopenia, INSERM UMR 1098, Internal Medicine and Clinical Immunology, Dijon, France; [2]Georges François Leclerc Centre, INSERM UMR 1231, Dijon, France;*

**Background**

Immune thrombocytopenia (ITP) is a rare autoimmune disorder with a risk of severe bleeding. First-line therapies rapidly increase platelet counts but do not modify disease course, with about 70% of adults progressing to chronic ITP and requiring additional treatments. Second-line options mainly rely on thrombopoietin receptor agonists and B cell targeting therapies. Rituximab (RTX) yields response rates of 60% at one year and 30% at five years, but no reliable clinical or biological predictors of response have been identified yet.

**Aims**

To identify biological predictive factors of response to rituximab, we performed a translational multi-omics approach to characterize the immune profile of ITP patients before treatment.

**Methods**

This study was conducted using a biobank of cryopreserved peripheral blood mononuclear cells (PBMC) samples from adult patients with ITP, prospectively enrolled in between 2010 and 2020. All PBMC used were collected prior to RTX administration. To achieve a high robustness, long-term response at 5 years was considered, based on platelet count $>30\times10^9$/L with at least a two-fold increase from baseline and without additional ITP therapy. Others were classified as non-responders (NR). Based on these criteria, 20 responders (R) and 18 NR were identified.
Single-cell RNA sequencing (scRNA-seq) was first performed on PBMC from 4 R and 4 NR (matched for age, sex, ITP duration and prior therapies). This unbiased approach revealed certain immunopathogenic mechanisms of interest.
To validate these findings and identify biomarkers suitable for routine clinical use, a comprehensive immunophenotyping of circulating immune cells by flow cytometry and serum cytokine measurements using Luminex and ELISA assays were performed across the entire cohort. Additionally, a transcriptomic signature was identified by bulk RNA-seq, and was subsequently confirmed by quantitative PCR (qPCR).

**Copyright Information:** (Online) ISSN: 2572-9241
©2026 The Author(s). HemaSphere is published by John Wiley & Sons Ltd on behalf of European Hematology Association. This is an open access Abstract Book distributed under the Attribution-NonCommercial-NoDerivs (CC BY-NC-ND), which allows third parties to download the articles and share them with others as long as they credit the author and the Abstract Book, but they cannot change the content in any way or use them commercially.
**Abstract Book Citations:** Authors, Title, HemaSphere, 2026;10:(S1):pages. The abstract book DOIs can be found on the “Author Information” page.
**Disclaimer:** Articles published in the journal HemaSphere exclusively reflect the opinions of the authors. The authors are responsible for all content in their abstracts including accuracy of the facts, statements, citing resources, etc.

### Results scRNA-seq analysis identified cellular subpopulations associated with non-response to RTX, most particularly CD8 T cell subsets, showing more advanced activation and differentiation, with enrichment of activation and cytokine-related pathways. These results were confirmed by flow cytometry immunophenotyping.
Cell-to-cell communication analysis further revealed 28 ligand-receptor pairs overexpressed in NR, that were quantified in serum by Luminex and ELISA.
Pseudobulk RNA-seq identified a transcriptomic signature associated with NR to RTX, refined and validated using bulk RNA-seq. The expression of the 13 selected genes was subsequently measured by qPCR.
Finally, through this multi-omics approach, three blood-derived candidate biomarkers of non-response to RTX were retained: a lymphocyte subpopulation (2.6% [2-3.8] vs. 1.4% [0.9-1.9], $p$=0.0002, area under the curve [AUC]=0.84 [95%CI 0.7-0.9]), a serum cytokine (242.7 pg/ml [120.7-378.2] vs. 159.8 pg/ml [118.5-207.9], $p$=0.02, AUC=0.74 [95%CI 0.6-0.9]), and the normalized expression of a mRNA transcript ($75 \times 10^{-5}$ [44-140] vs. $37 \times 10^{-5}$ [25-65], $p$=0.006, AUC=0.76 [95%CI 0.6-0.9]).

### Summary/Conclusion

The lack of biomarkers to guide therapeutic decision-making in ITP is a recognized unmet need. Our multi-omics approach has led to the identification of three candidate biomarkers. They are easily measurable in clinical practice and could potentially be used in combination. Multivariate statistical analyses are ongoing to further validate these biomarkers prior to confirmation in an external cohort.

**Copyright Information:** (Online) ISSN: 2572-9241
©2026 The Author(s). HemaSphere is published by John Wiley & Sons Ltd on behalf of European Hematology Association. This is an open access Abstract Book distributed under the Attribution-NonCommercial-NoDerivs (CC BY-NC-ND), which allows third parties to download the articles and share them with others as long as they credit the author and the Abstract Book, but they cannot change the content in any way or use them commercially.
**Abstract Book Citations:** Authors, Title, HemaSphere, 2026;10:(S1):pages. The abstract book DOIs can be found on the “Author Information” page.
**Disclaimer:** Articles published in the journal HemaSphere exclusively reflect the opinions of the authors. The authors are responsible for all content in their abstracts including accuracy of the facts, statements, citing resources, etc.

### (PF1345) EFFECTIVENESS AND SAFETY OF FIRST-LINE TREATMENT WITH DEXAMETHASONE VERSUS PREDNISONE IN ELDERLY PATIENTS WITH IMMUNE THROMBOCYTOPENIA: AN EMULATED TRIAL IN THE CARMEN-FRANCE REGISTRY

**Topic:** 33. Platelet disorders

Aude Legal[1], Matthieu Mahevas[2], Thibault Comont[1], Jean Francois Viallard[3], Sylvain Audia[4], Stephane Cheze[5], Miguel Carreiro[6], Mikael Ebbo[7], Natacha Brun[8], Joséphine Thomazeau[9], Delphine Gobert[10], Clément Gourguechon[11], Jehane Faldlallah[12], Sarah Khatibi[13], Yann Leveneur[14], Serge Madaule[15], Quitterie Reynaud[16], Etienne Riviere[17], Ailsa Robbins[18], Mathilde Roumier[19], Marc Ruivard[20], Albanie Toledano[21], Yoann Zadro[1], Marianne Lepetit[1], Laurie Chabbert[1], Bernard Bonnotte[4], Marc Michel[2], Bertrand Godeau[2], Margaux Lafaurie[1], Guillaume Moulis*[1]

*[1]Toulouse University hospital, Toulouse, France; [2]Henri Mondor University hospital, Créteil, France; [3]Bordeaux University hospital, Bordeaux, France; [4]Dijon University hospital, Dijon, France; [5]Caen University hospital, Caen, France; [6]Montauban hospital, Montauban, France; [7]Marseille University hospital, Marseille, France; [8]Rodez hospital, Rodez, France; [9]Foix hospital, Foix, France; [10]Saint Antoine University hospital, Paris, France; [11]Amiens University hospital, Amiens, France; [12]Saint Louis University hospital, Paris, France; [13]Castres hospital, Castres, France; [14]Tarbes hospital, Tarbes, France; [15]Albi hospital, Albi, France; [16]Lyon Universisty hospital, Lyon, France; [17]CHU de Bordeaux, Pessac, France; [18]Reims University hospital, Reims, France; [19]Foch hospital, Suresnes, France; [20]Clermont-Ferrand University hospital, Clermont-Ferrand, France; [21]Ducuing hospital, Toulouse, France;*

### Background

The first-line treatment for primary ITP currently consists of dexamethasone (DEX) or prednisone (PDN), with or without intravenous immunoglobulin (IVIg) depending on the bleeding manifestations. There are no comparative data on the efficacy and safety of these treatments in elderly patients, even though the median age at diagnosis of ITP is 60 years. Furthermore, no study has evaluated the efficacy of DEX at a dose of 20 mg/day for 4 days in ITP.

### Aims

The objective of this study was to compare the effectiveness and safety of DEX and PDN as initial treatment of primary ITP in elderly patients.

### Methods

We conducted an emulated trial in the CARMEN-France registry. The study population consisted in patients aged ≥65 years with newly diagnosed primary ITP between 2013 and 2023, platelet count <30 x $10^9$/L, bone smear consistent with peripheral thrombocytopenia and no life-threatening bleeding. Patients were treated with DEX 40 or 20 mg/day for 4 days ± a new course between Day 10 and Day 21, and matched 1:1 to patients treated with PDN 1 mg/kg/day for a maximum of 6 weeks (matched for age, antiplatelet or anticoagulant therapy, concomitant exposure to IVIg, bleeding and diabetes). The efficacy endpoints were response (platelet count ≥30 x $10^9$/L without new treatment) on Day 5 ± 2 days, Day 28 ± 1 week, Month 6 ± 1 month and Month 12 ± 1 month. Adverse drug reactions related to corticosteroid therapy were described.

**Copyright Information:** (Online) ISSN: 2572-9241
©2026 The Author(s). HemaSphere is published by John Wiley & Sons Ltd on behalf of European Hematology Association. This is an open access Abstract Book distributed under the Attribution-NonCommercial-NoDerivs (CC BY-NC-ND), which allows third parties to download the articles and share them with others as long as they credit the author and the Abstract Book, but they cannot change the content in any way or use them commercially.
**Abstract Book Citations:** Authors, Title, HemaSphere, 2026;10:(S1):pages. The abstract book DOIs can be found on the "Author Information" page.
**Disclaimer:** Articles published in the journal HemaSphere exclusively reflect the opinions of the authors. The authors are responsible for all content in their abstracts including accuracy of the facts, statements, citing resources, etc.

**Results**

Seventy patients were included (35 in each arm). The frequency of responders was similar between the DEX and PDN groups on Day 5 (70.6% and 77.1%, respectively). On Day 28, there were fewer responders in the DEX arm (34.3% versus 57.1%). However, in this real world registry, 10 patients who responded to a first cycle of DEX relapsed between Day 10 and Day 21 and received maintenance therapy or PDN instead of a second cycle of DEX, and were therefore classified as non-responders at Day 28 in this analysis. There were fewer responders on DEX at M6 (22.9% versus 40.0%) and M12 (20.0% versus 37.1%). The frequency of adverse events was similar between the two groups. The proportions of diabetes and bacterial infections were higher in the DEX arm. Subject to limited numbers, the 20 mg/day DEX dose (n=9) seems as effective as the 40 mg/day dose (n=26) with only one adverse event reported at 20 mg/day versus 9 at 40 mg/day.

**Summary/Conclusion**

In elderly patients with newly diagnosed ITP, DEX does not appear to be more effective or safe than PDN. Further large-scale studies are needed to clarify the role of DEX, particularly at a dose of 20 mg/day, which may be better tolerated and equally effective as 40 mg/day in this population.

**Copyright Information:** (Online) ISSN: 2572-9241
©2026 The Author(s). HemaSphere is published by John Wiley & Sons Ltd on behalf of European Hematology Association. This is an open access Abstract Book distributed under the Attribution-NonCommercial-NoDerivs (CC BY-NC-ND), which allows third parties to download the articles and share them with others as long as they credit the author and the Abstract Book, but they cannot change the content in any way or use them commercially.
**Abstract Book Citations:** Authors, Title, HemaSphere, 2026;10:(S1):pages. The abstract book DOIs can be found on the "Author Information" page.
**Disclaimer:** Articles published in the journal HemaSphere exclusively reflect the opinions of the authors. The authors are responsible for all content in their abstracts including accuracy of the facts, statements, citing resources, etc.

### (PF1346) REDUCED ETS-2 EXPRESSION IN T HELPER CELLS IS ASSOCIATED WITH TH1/TH17 SKEWING AND REGULATORY T CELL DYSFUNCTION IN PRIMARY IMMUNE THROMBOCYTOPENIA

**Topic:** 33. Platelet disorders

Ioanna Aggeletopoulou[1], Athanasia Mouzaki*[1]

[1]*University of Patras, Medical School,, Internal Medicine, Patras, Greece;*

### Background

In primary immune thrombocytopenia (pITP), autoreactive effector B and T cells initiate and sustain platelet destruction in pro-inflammatory milieu characterized by T helper (Th) 1 and Th17 effector cell polarization. Concurrently, regulatory Th cells (Tregs) are numerically and functionally impaired, leading to loss of immune tolerance. We previously demonstrated that in healthy individuals the IL-2 gene is repressed in naive Th cells by the transcription factor Ets-2 through direct binding to the IL-2 promoter. Ets-2 expression is downregulated upon naive Th cell activation and in memory Th cells, suggesting that Ets-2 critically regulates early events in Th cell differentiation.

### Aims

To investigate the role of Ets-2 in pITP pathogenesis by analyzing its expression and associated cytokine gene profiles in naive and memory effector Th cells (Teffs) and Tregs from patients and healthy controls.

### Methods

Peripheral blood samples were obtained from 9 patients with pITP and 10 age- and sex-matched healthy controls. Peripheral blood mononuclear cells (PBMCs) were isolated and immunophenotyped. Naive ($CD4^+CD45RA^+CD25^-$) and memory ($CD4^+CD45RO^+CD25^-$) Teffs, as well as Tregs ($CD4^+CD25^+$), were sorted by flow cytometry. Bioinformatic analysis was performed to identify putative Ets-2 binding sites within promoter regions of Th cytokine genes other than IL-2. Cytokine mRNA expression was quantified by qPCR. Immunofluorescence analysis was used to evaluate nuclear localization of Ets-2.

### Results

Phenotypic analysis showed increased frequencies of naive Teffs and decreased frequencies of memory Teffs and Tregs in pITP patients compared to controls. Bioinformatic analysis revealed multiple Ets-2 binding sites in promoter regions of various Th cell cytokines including IFN-γ, IL-4, IL-5, IL-10, and IL-17. Naive Teffs from pITP patients exhibited significantly reduced Ets-2 mRNA and protein expression compared to controls. These cells constitutively expressed IL-2 and IFN-γ, whereas control naive Teffs did not. Memory Teffs from pITP patients constitutively expressed IL-17, in contrast to controls. Notably, pITP Tregs constitutively expressed IL-2 and IFN-γ and showed reduced IL-10 mRNA levels compared with control Tregs. Immunofluorescence analysis demonstrated markedly reduced nuclear localization of Ets-2 in Teffs from pITP patients (Figure 1).

### Summary/Conclusion

Our results strongly suggest that low expression and synthesis of Ets-2 in naive Teffs of pITP patients leads to impaired downstream events in Th cell plasticity. This manifests as high constitutive gene expression of Th1/Th17 cytokines in naive and memory Teffs and atypical cytokine gene expression in Tregs.

**Copyright Information:** (Online) ISSN: 2572-9241
©2026 The Author(s). HemaSphere is published by John Wiley & Sons Ltd on behalf of European Hematology Association. This is an open access Abstract Book distributed under the Attribution-NonCommercial-NoDerivs (CC BY-NC-ND), which allows third parties to download the articles and share them with others as long as they credit the author and the Abstract Book, but they cannot change the content in any way or use them commercially.
**Abstract Book Citations:** Authors, Title, HemaSphere, 2026;10:(S1):pages. The abstract book DOIs can be found on the "Author Information" page.
**Disclaimer:** Articles published in the journal HemaSphere exclusively reflect the opinions of the authors. The authors are responsible for all content in their abstracts including accuracy of the facts, statements, citing resources, etc.

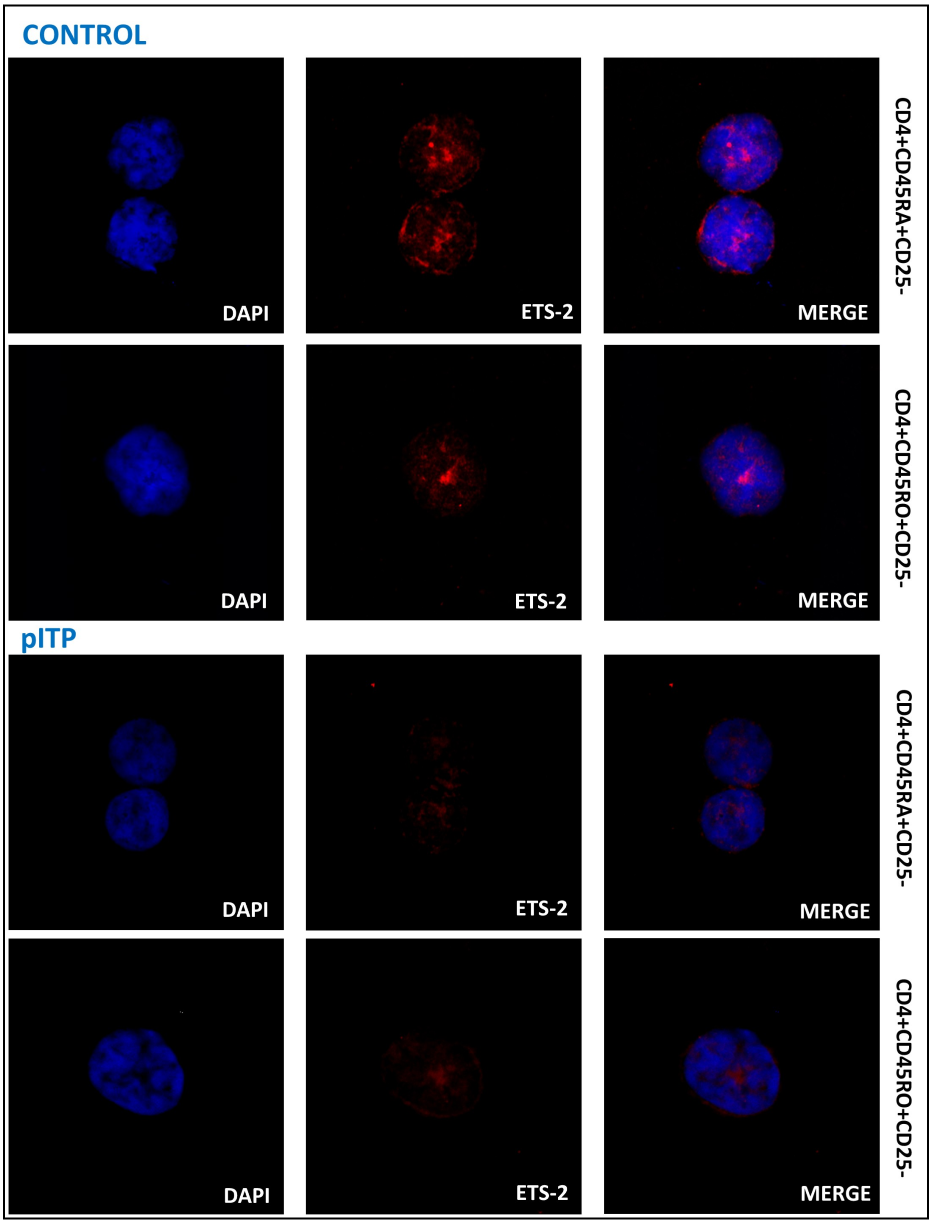

Figure 1. Representative immunofluorescence analysis of Ets-2 expression in human naive Th cells (CD4+CD45RA+CD25-) and memory Th cells (CD4+CD45RO+CD25-) from a healthy control and a patient with pITP. Ets-2 was detected using a mouse monoclonal anti-Ets-2 antibody (IgG1 isotype), followed by an Alexa Fluor® 561-conjugated goat anti-mouse IgG secondary antibody (red). Nuclei were counterstained with DAPI (blue).

**Copyright Information:** (Online) ISSN: 2572-9241
©2026 The Author(s). HemaSphere is published by John Wiley & Sons Ltd on behalf of European Hematology Association. This is an open access Abstract Book distributed under the Attribution-NonCommercial-NoDerivs (CC BY-NC-ND), which allows third parties to download the articles and share them with others as long as they credit the author and the Abstract Book, but they cannot change the content in any way or use them commercially.
**Abstract Book Citations:** Authors, Title, HemaSphere, 2026;10:(S1):pages. The abstract book DOIs can be found on the "Author Information" page.
**Disclaimer:** Articles published in the journal HemaSphere exclusively reflect the opinions of the authors. The authors are responsible for all content in their abstracts including accuracy of the facts, statements, citing resources, etc.

### (PF1347) CLINICAL OUTCOMES OF ROMIPLOSTIM IN CHEMOTHERAPY-INDUCED THROMBOCYTOPENIA: A SINGLE INSTITUTION EXPERIENCE

**Topic:** 33. Platelet disorders

Kelly Meza-Capcha*[1], Mridula Nadamuni[2], Hugo Lara-Martinez[3], Michael Kroll[3], Effrosyni Apostolidou[3]

*[1]Baylor College of Medicine, UT MD Anderson Cancer Center, Houston, United States of America;[2]UT MD Anderson Cancer Center, Houston, United States of America; [3]UT MD Anderson Cancer Center, Section of Hematology, Houston, United States of America;*

### Background

Chemotherapy-induced thrombocytopenia (CIT) leads to dose reductions or treatment delays, compromising delivery of cytotoxic chemotherapy and targeted therapies. Delays or dose reductions in treatments are known to affect cancer-related outcomes, including progression free survival and overall survival (OS). Romiplostim, a thrombopoietin receptor agonist, has emerged as a supportive strategy for CIT. However, real-world data regarding efficacy of romiplostim, predictors of response and survival outcomes remain limited.

### Aims

To evaluate the efficacy of romiplostim in achieving platelet recovery in patients with CIT. Secondary objectives included its impact on chemotherapy continuation, time to platelet recovery, predictors of response, and OS.

### Methods

We conducted an IRB approved, retrospective, single-center study of adult patients who received romiplostim for CIT at MD Anderson Cancer Center between January 1, 2018 and January 1, 2025. A total of 110 patients were included. Patients treated with romiplostim for myeloid malignancies or in the post-stem cell transplantation setting were excluded. Clinical characteristics were compared using chi-square and t-tests, as appropriate. Multivariable analyses evaluated predictors of response, and survival analyses evaluated OS.

**Copyright Information:** (Online) ISSN: 2572-9241
©2026 The Author(s). HemaSphere is published by John Wiley & Sons Ltd on behalf of European Hematology Association. This is an open access Abstract Book distributed under the Attribution-NonCommercial-NoDerivs (CC BY-NC-ND), which allows third parties to download the articles and share them with others as long as they credit the author and the Abstract Book, but they cannot change the content in any way or use them commercially.
**Abstract Book Citations:** Authors, Title, HemaSphere, 2026;10:(S1):pages. The abstract book DOIs can be found on the “Author Information” page.
**Disclaimer:** Articles published in the journal HemaSphere exclusively reflect the opinions of the authors. The authors are responsible for all content in their abstracts including accuracy of the facts, statements, citing resources, etc.

### Results

Among 110 patients (median age 56 years [18–83]; 55% women), 97 (88%) had solid tumors and 13 (12%) had non-myeloid hematologic malignancies (multiple myeloma or lymphoma). The most common solid tumors were sarcoma, lung, gynecologic, and breast; 78 patients (71%) had metastatic disease. Patients received a median of 2 prior line of therapy (0–9). Over a median follow-up of 6.2 months (IQR 3.3–14), patients received a median of 8 weekly romiplostim doses (1–206) at doses of 0.5-10 mcg/kg (median 3 mcg/kg) administered subcutaneously. Median PLT counts increased from 53×10⁹/L (IQR 35–75) to 79×10⁹/L (IQR 45–126) during treatment ($p<0.001$). Romiplostim enabled continuation of roughly 2 additional chemotherapy cycles (0–74), corresponding to approximately 4.5 weeks (1–5) of extended treatment. Overall, 60 patients (55%) achieved PLT count ≥75×10⁹/L typically within 14 days (IQR 7–26), predominantly among solid tumors (58/97 solid tumors vs 2/13 hematologic). PLT increases were significant in solid tumors (median 87 vs 57×10⁹/L, $p<0.001$) but not hematologic malignancies (35 vs 23×10⁹/L, $p=0.06$). Baseline thrombocytopenia severity influenced response: patients with grade 1–2 thrombocytopenia (n=58) achieved higher PLT counts than those with grade 3–4 (n=53) (104 vs 52×10⁹/L, $p<0.001$). 38.4% achieved PLT counts ≥100×10⁹/L within 18 days (IQR 7–49). In multivariable analysis, baseline grade 3–4 thrombocytopenia and bone marrow involvement were associated with reduced likelihood of response, though these associations did not reach statistical significance ($p\geq0.07$). Median OS from romiplostim initiation was 10 months (95% CI 7–12), with 6- and 12-month OS rates of 63% and 41%, respectively. 19% had a history of venous or arterial thromboembolism within 6 months; no recurrent events occurred during romiplostim therapy.

### Summary/Conclusion

Romiplostim is a safe and effective therapy for CIT, enabling continuation of cancer treatment, particularly in patients with solid tumors. Severe baseline thrombocytopenia and bone marrow involvement were associated with lower response rates. These results support targeted use of romiplostim and emphasize the need for prospective studies to refine predictors of treatment response.

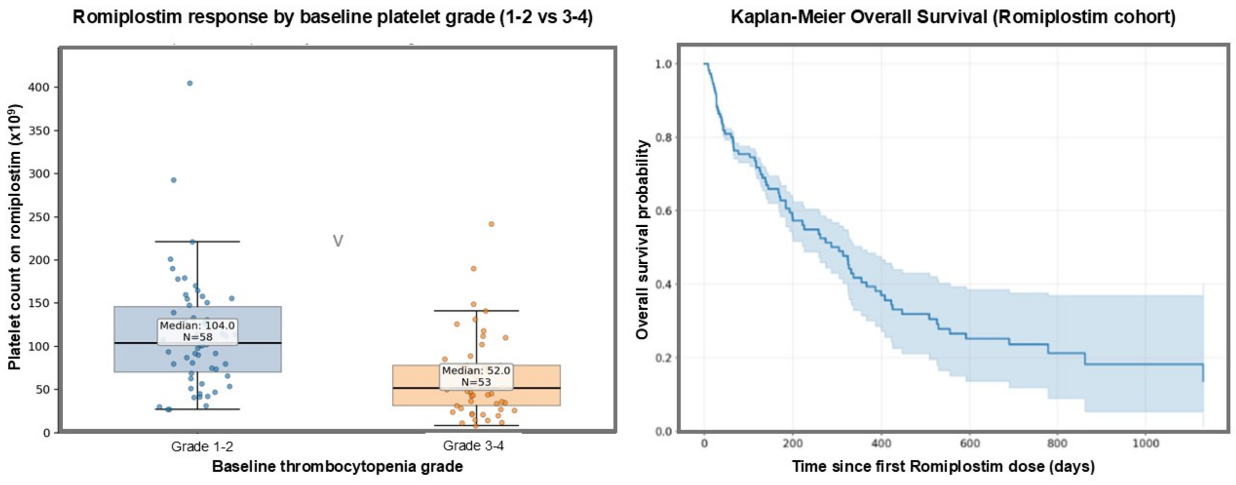

**Copyright Information:** (Online) ISSN: 2572-9241
©2026 The Author(s). HemaSphere is published by John Wiley & Sons Ltd on behalf of European Hematology Association. This is an open access Abstract Book distributed under the Attribution-NonCommercial-NoDerivs (CC BY-NC-ND), which allows third parties to download the articles and share them with others as long as they credit the author and the Abstract Book, but they cannot change the content in any way or use them commercially.
**Abstract Book Citations:** Authors, Title, HemaSphere, 2026;10:(S1):pages. The abstract book DOIs can be found on the "Author Information" page.
**Disclaimer:** Articles published in the journal HemaSphere exclusively reflect the opinions of the authors. The authors are responsible for all content in their abstracts including accuracy of the facts, statements, citing resources, etc.

### (PF1348) FATIGUE IN ADULTS WITH IMMUNE THROMBOCYTOPENIA (ITP): A CROSS-SECTIONAL OBSERVATIONAL STUDY

**Topic:** 33. Platelet disorders

David J. Kuter*[1], Sandhya Panch[2], Caroline Piatek[3], Maria Belen Rodriguez[4], Ahmed Daak[4], Julie Kissell[4], Anna Compagnoni[5], Howard Liebmann[6], Nichola Cooper[7]

*[1]Hematology Division, Massachusetts General Hospital, Harvard Medical School, Boston, United States of America;[2]Department of Medicine, University of Washington, Seattle, United States of America; [3]Division of Hematology, Department of Medicine, Keck School of Medicine of USC, Los Angeles, United States of America; [4]Sanofi, Cambridge, United States of America; [5]Cytel, Cambridge, MA, USA; on behalf of Sanofi R&D, Cambridge, United States of America; [6]University of Southern California, Los Angeles, United States of America; [7]Hammersmith Hospital, London, United Kingdom;*

### Background

ITP is a rare disease characterized by complex immune dysregulation, autoantibody-mediated destruction and impaired platelet production, resulting in greater risk of bleeding. Although low platelet counts and bleeding events have traditionally guided disease management, patients consistently report a broader symptom burden. Fatigue is among the most common debilitating symptoms of ITP, significantly contributing to overall disease burden and reduced HRQOL. In clinical research settings, assessments such as the FACIT-Fatigue scale and ITP Patient Assessment Questionnaire (ITP-PAQ) are often used to characterize the severity and impact of fatigue in ITP and guide approaches to improve HRQOL. In particular, ITP-PAQ Item 10 has been used to measure the effect of treatment on fatigue in an ITP trial.

### Aims

Assess fatigue in adults with ITP and evaluate correlations with bleeding and clinical/laboratory values.

### Methods

Adults aged ≥18 y with chronic (>1 y) primary ITP who had an initial response to first-line therapy and were either receiving ITP treatment (regardless of platelet counts) or in remission/observation without treatment for ≤1 y were eligible. At enrollment, clinical outcome assessments measured fatigue, bleeding severity using the IBLS bleeding scale (scale: 0 [no bleeding] to 2 [marked bleeding]), and clinical factors. Fatigue was assessed by using FACIT-Fatigue, a 13-item tool that measures fatigue and its impact on daily life (score: 0-52; lower score = greater fatigue) and ITP-PAQ, a disease-specific tool to measure HRQOL (score: 0-100; higher score = better HRQOL). Correlations were assessed by Pearson or Spearman rank correlation.

**Copyright Information:** (Online) ISSN: 2572-9241
©2026 The Author(s). HemaSphere is published by John Wiley & Sons Ltd on behalf of European Hematology Association. This is an open access Abstract Book distributed under the Attribution-NonCommercial-NoDerivs (CC BY-NC-ND), which allows third parties to download the articles and share them with others as long as they credit the author and the Abstract Book, but they cannot change the content in any way or use them commercially.
**Abstract Book Citations:** Authors, Title, HemaSphere, 2026;10:(S1):pages. The abstract book DOIs can be found on the “Author Information” page.
**Disclaimer:** Articles published in the journal HemaSphere exclusively reflect the opinions of the authors. The authors are responsible for all content in their abstracts including accuracy of the facts, statements, citing resources, etc.

**Results**

As of 23Jan2026 data cutoff, 104 patients were screened; 97 were eligible for the study and completed FACIT-Fatigue and ITP-PAQ assessments (65% USA, 35% UK). Median age at enrollment was 54 years (Q1-Q3, 41-66); 65% were female. Median age at ITP diagnosis was 43 years (Q1-Q3, 25-55), with a median duration of ITP of 10 years (Q1-Q3, 4-18); fatigue was the symptom prompting ITP diagnosis for 8/88 patients. Eighty-two patients reported receiving active ITP treatment, including TPO-RAs (n=67), CS (n=18), other immunosuppressants (n=12), fostamatinib (n=6), rituximab (n=2), and/or other (n=3). Median (Q1-Q3) was $143\times10^9$/L ($85-222\times10^9$/L) for platelet count, 139 (130-150) g/L for hemoglobin, 151 (84-274) pmol/L for ferritin, 15 (11-18) µmol/L for iron, and 63 (58-71) µmol/L for total iron binding capacity (TIBC). Mean (SD) IBLS score was 0.04 (0.10).
The mean (SD) FACIT-Fatigue score was 37 (12); 26 (27%) patients had a score of ≤30 and 50% scored ≤40 (Table 1). ITP-PAQ scores by domain are also shown in Table 1. Strong correlations were observed between FACIT-Fatigue and ITP-PAQ Item 10 (which evaluates physical fatigue due to ITP or its treatment; $\rho=0.7150$; $P<0.05$) and ITP-PAQ Emotional Health-Psychological Health Domain ($\rho=0.6298$; $P<0.05$). No correlation was observed between FACIT-Fatigue and iron, TIBC, or ferritin levels.

**Summary/Conclusion**

Although most patients received active ITP treatment and had adequate platelet counts, minimal bleeding, and other labs within normal limits at the time of index visit, a proportion of patients still reported notable levels of fatigue. These findings underscore an unmet need for therapies that more effectively address the multifaceted burden of ITP. Further research is needed to better understand factors contributing to fatigue in ITP.

**Table 1. FACIT-Fatigue and ITP-PAQ Scores**

| Scale | Score Mean (SD) |
|---|---|
| **FACIT-Fatigue*** | 37 (12) |
| **ITP-PAQ Domains** | |
| Symptom | 78 (16) |
| Physical Health-Bother | 79 (25) |
| Physical Health-Activity | 76 (29) |
| Fatigue/Sleep (includes Item 10)† | 71 (25) |
| Emotional Health-Psychological Health | 76 (26) |
| Fear | 84 (18) |
| Social Activity | 82 (21) |
| Work | 83 (24) |
| Fertility | 72 (28) |
| Menstrual Symptoms | 68 (34) |
| Overall QOL | 65 (26) |

N=97 except for ITP-PAQ Emotional Health-Psychological Health (n=96), Work (n=41), Fertility (n=23), and Menstrual Symptoms (n=23).
*A score of 43 has been reported as a threshold for distinguishing anemic cancer patients from the general population (Cella et al. *Cancer.* 2002). Mean (SD) of FACIT-Fatigue scores reported in other conditions: 36 (12) for cancer (n=297), 38 (10) for stroke survivors (n=51), and 34 (13) among HIV positive patients (n=51) (Butt et al. *J Psychosom Res.* 2013).
†Fatigue/sleep domain includes Item 10, which evaluates physical fatigue and subsequent details.
ITP, immune thrombocytopenia; QOL, quality of life.

**Copyright Information:** (Online) ISSN: 2572-9241
©2026 The Author(s). HemaSphere is published by John Wiley & Sons Ltd on behalf of European Hematology Association. This is an open access Abstract Book distributed under the Attribution-NonCommercial-NoDerivs (CC BY-NC-ND), which allows third parties to download the articles and share them with others as long as they credit the author and the Abstract Book, but they cannot change the content in any way or use them commercially.
**Abstract Book Citations:** Authors, Title, HemaSphere, 2026;10:(S1):pages. The abstract book DOIs can be found on the “Author Information” page.
**Disclaimer:** Articles published in the journal HemaSphere exclusively reflect the opinions of the authors. The authors are responsible for all content in their abstracts including accuracy of the facts, statements, citing resources, etc.

### (PF1349) PROSPECTIVE EVALUATION OF A MODIFIED AVATROMBOPAG INITIATION DOSE TO REDUCE PLATELET OVERSHOOTING IN IMMUNE THROMBOCYTOPENIA

**Topic:** 33. Platelet disorders

Noor Al-Musawi[1], Alaa Alwan[2], Camilla Jørgensen[3], Mazin Shubber[1], Alaadin Naji[1], Bassam Matti[1], Lamya Garabet[3], Heidi Pettersen[4], Eirik Tjønnfjord[4], Alaadin Sahham[5], Mohammad Saleem[6], Tareq Saleh[1], Waleed Ghanima*[4]

*[1]Hematology and Transplant Center, Baghdad Medical City, Baghdad, Iraq; [2]The National Center Of Hematology/ Mustansiriya University, Baghdad, Iraq; [3]Østfold Hospital, Grålum, Norway; [4]Østfold Hospital, Hematooncology, Grålum, Norway; [5]Baghdad Medical College, University of Baghdad, Baghdad, Iraq; [6]Hematology and Bone Marrow Transplant Center, Medical city Teaching Hospital, Baghdad, Iraq;*

### Background

Avatrombopag is an oral thrombopoietin receptor agonist (TPO-RA) commonly used in immune thrombocytopenia (ITP) to increase platelet counts. The recommended starting dose is 20 mg daily. However, overshooting of the platelets to counts exceeding 400 × $10^9$/L has been reported to occur in 20–40% following treatment initiation. Such increase in platelet count platelet necessitates dose reduction or temporary interruption and may predispose to thrombosis.

### Aims

We aimed to prospectively evaluate the time to response, incidence of platelet overshooting, and initial and durable response rates following a reduced starting regimen of avatrombopag.

### Methods

This was a single-arm, single-center, prospective study (NCT07133659) including TPO-RA–naïve adults with primary ITP, platelet count <30 × $10^9$/L, and indication for a second-line therapy. Patients received avatrombopag 20 mg every other day during the first 2 weeks. Thereafter, dosing was titrated to maintain platelet counts between 50–150 × $10^9$/L over a period of 20 weeks.
Platelet overshooting was defined as platelet count >400 × $10^9$/L within the first 4 weeks after treatment initiation. Response at day 8 was defined as the proportion of patients achieving platelet counts ≥50 × $10^9$/L at day 8; response at 6 weeks was defined as the proportion of patients achieving platelet counts ≥50 × $10^9$/L in 2 consecutive platelet counts between weeks 6 and 8; durable response was defined as 4 consecutive platelet counts ≥50 × $10^9$/L between weeks 12 and 20.
The study was approved by the Institutional Review Board at the Hematology and Transplant Center, Medical City Teaching Hospital, Baghdad, Iraq.

**Copyright Information:** (Online) ISSN: 2572-9241
©2026 The Author(s). HemaSphere is published by John Wiley & Sons Ltd on behalf of European Hematology Association. This is an open access Abstract Book distributed under the Attribution-NonCommercial-NoDerivs (CC BY-NC-ND), which allows third parties to download the articles and share them with others as long as they credit the author and the Abstract Book, but they cannot change the content in any way or use them commercially.
**Abstract Book Citations:** Authors, Title, HemaSphere, 2026;10:(S1):pages. The abstract book DOIs can be found on the "Author Information" page.
**Disclaimer:** Articles published in the journal HemaSphere exclusively reflect the opinions of the authors. The authors are responsible for all content in their abstracts including accuracy of the facts, statements, citing resources, etc.

### Results

Thirty patients were enrolled between Jul 2025 and Jan 2026. Two patients were excluded for meeting predefined exclusion criteria. Twenty-eight patients were included in the analysis. Median (Q1–Q3) age was 43.0 (20.5-62.5) years, and 22 (78.6%) were female. All patients had previously received steroids; 9 and 2 patients had received 2 and ≥3 prior individual treatments, respectively.
Median time to first platelet count ≥50 × 10⁹/L was 7 (Q1–Q3; 7-7) days. Response by day 8 was achieved in 22 (78.6%) patients. Platelet overshooting occurred in 3/28 patients (10.7%). During the study period, the starting dose was increased in 13 patients, remained unchanged in 2, and decreased in 13 (Figure 1A). Median (Q1-Q3) number of dose modifications was 3.0 (2.0-5.5). Median platelet count during 20 weeks of the study period is shown in Figure 1B. Twenty-five patients were evaluable for response at week 6, as one patient died, one withdrew consent at week 3 and in one blood tests were not performed. Of these, 20 (80.0%) patients achieved response at week 6. Durable response was achieved in 13 of 18 (72.2%) evaluable patients who had completed 20 weeks.
Adverse events (AE) were reported in 9 patients. Most common AE were arthralgia and fever. One 83-year-old patient died in hospital; bleeding was excluded in this patient. One patient developed a deep vein thrombosis 8 weeks after avatrombopag initiation; platelet count at the event was 140 × 10⁹/L.

### Summary/Conclusion

This pilot study indicates the reduced initial avatrombopag dose was associated with lower incidence of platelet overshooting compared with historical data, while maintaining high initial response rates. The regimen was generally well tolerated. A randomized controlled trial is warranted to confirm these findings.

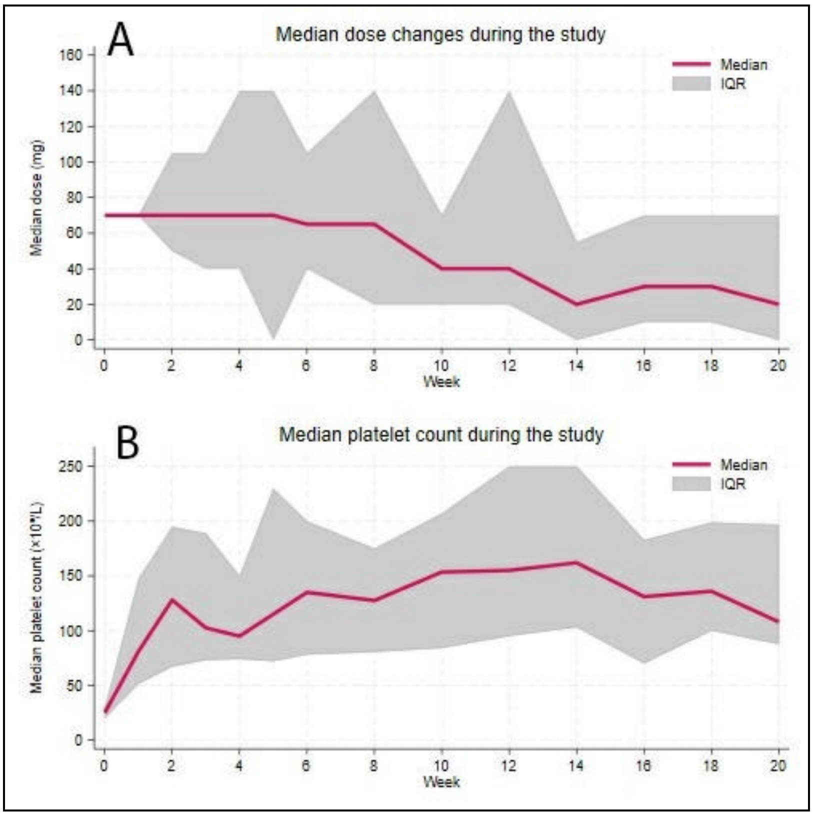

Median Avatrombopag dose (A) and platelet count (B) during the study

**Copyright Information:** (Online) ISSN: 2572-9241
©2026 The Author(s). HemaSphere is published by John Wiley & Sons Ltd on behalf of European Hematology Association. This is an open access Abstract Book distributed under the Attribution-NonCommercial-NoDerivs (CC BY-NC-ND), which allows third parties to download the articles and share them with others as long as they credit the author and the Abstract Book, but they cannot change the content in any way or use them commercially.
**Abstract Book Citations:** Authors, Title, HemaSphere, 2026;10:(S1):pages. The abstract book DOIs can be found on the "Author Information" page.
**Disclaimer:** Articles published in the journal HemaSphere exclusively reflect the opinions of the authors. The authors are responsible for all content in their abstracts including accuracy of the facts, statements, citing resources, etc.

### (PF1351) PLATELET FUNCTION DEFECTS IN DEPRESSION: LIGHT TRANSMISSION AGGREGOMETRY PATTERNS IN PATIENTS TREATED WITH SSRIS AND SNRIS

**Topic:** 33. Platelet disorders

Nikolaos Kotsiou*[1], Stamatia Theodoridou[2], Paschalis Evangelidis[1], Genovefa Mantzou[2], Athanasios Vyzantiadis[2], Charalampia Avgerinou[2], Ioanna Tsairidou[1], Panagiotis Kalmoukos[1], Iraklis Leonidis[1], Zacharo Ntova[2], Ioannis Eftychidis[1], Sofia Chissan[1], Eleni Gavriilaki[1], Sofia Vakalopoulou[1]

*[1]Aristotle University of Thessaloniki, 2nd Propedeutic Department of Internal Medicine, Hippokration General Hospital, Thessaloniki, Greece; [2]Hippokration General Hospital, Hemoglobinopathy Prevention Unit, Thessaloniki, Greece;*

### Background

Selective Serotonin Reuptake Inhibitors (SSRIs) and Serotonin-Norepinephrine Reuptake Inhibitors (SNRIs) are primary depression treatments. Mechanistically, they block platelet serotonin transporters (SERT), depleting dense-granule serotonin crucial for secondary hemostasis. While bleeding risks are established,specific in vitro aggregation patterns and their link to clinical bleeding and hematological markers remain underexplored.

### Aims

The primary aim of this study was to characterize platelet function profiles using Light Transmission Aggregometry (LTA) and examine the bleeding phenotype of patients receiving long-term antidepressant therapy, while evaluating hematological parameters as biomarkers for occult bleeding.

### Methods

We conducted a retrospective analysis of 17 patients evaluated between October 2022 and January 2026, who were receiving stable monotherapy or combination therapy with SSRIs (e.g., Sertraline, Fluoxetine) or SNRIs (e.g., Venlafaxine). Patients taking nonsteroidal anti-inflammatory drugs (NSAIDs) or antiplatelet agents were excluded. Platelet counts were assessed in both Whole Blood (Coulter) and Platelet-Rich Plasma (PRP) to ensure validity. LTA was performed using a standard panel of agonists: ADP, Epinephrine, Collagen, Arachidonic Acid and Ristocetin. A secretion defect was defined as maximal aggregation <60% compared to the daily control. Clinical bleeding was quantified using the ISTH-BAT score.

**Copyright Information:** (Online) ISSN: 2572-9241
©2026 The Author(s). HemaSphere is published by John Wiley & Sons Ltd on behalf of European Hematology Association. This is an open access Abstract Book distributed under the Attribution-NonCommercial-NoDerivs (CC BY-NC-ND), which allows third parties to download the articles and share them with others as long as they credit the author and the Abstract Book, but they cannot change the content in any way or use them commercially.
**Abstract Book Citations:** Authors, Title, HemaSphere, 2026;10:(S1):pages. The abstract book DOIs can be found on the "Author Information" page.
**Disclaimer:** Articles published in the journal HemaSphere exclusively reflect the opinions of the authors. The authors are responsible for all content in their abstracts including accuracy of the facts, statements, citing resources, etc.

### Results

Baseline characteristics and hematological parameters of the 17 evaluated patients are summarized in Table 1. Median ISTH-BAT score was 3.0 (IQR: 3.0–8.0, range: 2–10), indicating a highly variable, predominantly mild-to-moderate clinical bleeding tendency. Overall, LTA analysis revealed widespread inhibition of secondary hemostasis; notably, only 3 patients exhibited entirely normal platelet functionality,while the remaining 14 (82.4%) demonstrated at least one distinct secretion or aggregation defect. Epinephrine-induced aggregation was the most frequently affected pathway, showing a clinically significant defect (<60% of control) in 7 out of 11 evaluated patients (63.6%). Arachidonic acid response was also markedly impaired in 6 out of 17 evaluated patients (35.3%), mimicking an aspirin-like effect despite confirmed absence of NSAID intake.This suggests that SSRIs/SNRIs may exert inhibitory effects extending beyond dense-granule serotonin depletion, potentially affecting downstream signal transduction pathways. Collagen and ADP responses were impaired in 5 out of 16 (31.2%) and 4 out of 17 (23.5%) evaluated cases, respectively. Regarding hematological parameters, while anemia (Hb<11.5 g/dL) was present in only 17.6% of cases (n=3),an elevated RDW-CV (>14.5%) was observed in 41.2% of the cohort (n=7). This pronounced discordance, high RDW with preserved median Hemoglobin (13.2 g/dL), suggests that RDW may serve as a highly sensitive,early biomarker for occult gastrointestinal blood loss in this patient population, before a manifest drop in hemoglobin becomes apparent.

### Summary/Conclusion

Long-term treatment with SSRIs and SNRIs causes profound platelet dysfunction, notably impairing Epinephrine and Arachidonic Acid aggregation pathways. Furthermore, elevated RDW in non-anemic patients indicates a risk of subclinical chronic blood loss. These findings highlight the clinical utility of LTA in identifying at-risk patients, urging caution when planning invasive procedures or prescribing concurrent antithrombotics.

**Copyright Information:** (Online) ISSN: 2572-9241
©2026 The Author(s). HemaSphere is published by John Wiley & Sons Ltd on behalf of European Hematology Association. This is an open access Abstract Book distributed under the Attribution-NonCommercial-NoDerivs (CC BY-NC-ND), which allows third parties to download the articles and share them with others as long as they credit the author and the Abstract Book, but they cannot change the content in any way or use them commercially.
**Abstract Book Citations:** Authors, Title, HemaSphere, 2026;10:(S1):pages. The abstract book DOIs can be found on the “Author Information” page.
**Disclaimer:** Articles published in the journal HemaSphere exclusively reflect the opinions of the authors. The authors are responsible for all content in their abstracts including accuracy of the facts, statements, citing resources, etc.

Table 1: Baseline Characteristics and Hematological Parameters (N=17)

| Parameter | Value |
|---|---|
| **Demographics** | |
| Age (years), Mean (± SD) | 51.0 ± (12.9) |
| **Sex, n (%)** | |
| Female, n (%) | 16 (94.1%) |
| Male, n (%) | 1 (5.9%) |
| **Medication Class, n (%)** | |
| SSRIs | 10 (58.8%) |
| SNRIs | 3 (17.6%) |
| Other psychotropics | 4 (23.6%) |
| **Clinical and Laboratory Data** | |
| ISTH-BAT Score, Median (IQR) | 3.0 (3.0 - 8.0) |
| Hemoglobin (g/dL), Median (IQR) | 13.2 (12.3 - 13.5) |
| RDW-CV (%), Median (IQR) | 14.0 (13.1 - 14.7) |
| PDW (%), Median (IQR) | 17.1 (16.4 - 17.7) |
| MPV (fL), Mean (± SD) | 8.7 (± 0.8) |
| Platelet Count Whole Blood (10^3/µL), Mean (± SD) | 254.9 (± 72.9) |
| Platelet Count PRP (10^3/µL), Mean ± SD | 193.5 (± 82.7) |

*SD: Standard Deviation, SSRIs: Selective Serotonin Reuptake Inhibitors, SNRIs: Serotonin-Norepinephrine Reuptake Inhibitors, RDW-CV: Red Cell Distribution Width Coefficient of Variation, IQR: Interquartile Range, PDW: Platelet Distribution Width, MPV: Mean Platelet Volume*

**Copyright Information:** (Online) ISSN: 2572-9241
©2026 The Author(s). HemaSphere is published by John Wiley & Sons Ltd on behalf of European Hematology Association. This is an open access Abstract Book distributed under the Attribution-NonCommercial-NoDerivs (CC BY-NC-ND), which allows third parties to download the articles and share them with others as long as they credit the author and the Abstract Book, but they cannot change the content in any way or use them commercially.
**Abstract Book Citations:** Authors, Title, HemaSphere, 2026;10:(S1):pages. The abstract book DOIs can be found on the "Author Information" page.
**Disclaimer:** Articles published in the journal HemaSphere exclusively reflect the opinions of the authors. The authors are responsible for all content in their abstracts including accuracy of the facts, statements, citing resources, etc.

### (PF1352) EFFICACY AND SAFETY OF LUSUTROMBOPAG IN PERSISTENT OR CHRONIC PRIMARY IMMUNE THROMBOCYTOPENIA: A PROSPECTIVE, OPEN-LABEL, SINGLE-ARM PHASE II STUDY

**Topic:** 33. Platelet disorders

Rongfeng Fu[1, 2], Xiaofan Liu*[1, 2], Xinmiao Qu[1, 2], Xin Wang[1, 2], Yueshen MA[1, 2], Feng Xue[1, 2], Yunfei Chen[1, 2], Wei Liu[1, 2], Mankai Ju[1, 2], Ting Sun[1, 2], Xinyue Dai[1, 2], Huan Dong[1, 2], Wenjing Gu[1, 2], Yanmei Xu[1, 2], Anqi Zhang[1, 2], Huan Wang[3], Junren Chen[1, 2], Renchi Yang[1, 2], Lei Zhang[1, 2]

*[1]State Key Laboratory of Experimental Hematology, National Clinical Research Center for Blood Diseases, Haihe Laboratory of Cell Ecosystem, Tianjin Key Laboratory of Gene Therapy for Blood Diseases, CAMS Key Laboratory of Gene Therapy for Blood Diseases, Institute of Hematology & Blood Diseases Hospital, Chinese Academy of Medical Sciences & Peking Union Medical College, Tianjin, China; [2]Tianjin Institutes of Health Science, Tianjin, China;[3]Eddingpharm, Shanghai, China;*

### Background

Lusutrombopag is a novel oral thrombopoietin receptor agonist (TPO-RA) with a favorable safety profile, approved for thrombocytopenia in chronic liver disease. However, clinical evidence of lusutrombopag in primary immune thrombocytopenia (ITP) remains limited.

### Aims

To evaluate the efficacy, safety, and dose-titration feasibility of lusutrombopag in patients with persistent or chronic primary ITP.

### Methods

This prospective, single-center, single-arm, open-label trial (NCT06287567) enrolled Chinese adult patients with persistent or chronic primary ITP who had received at least one prior line of therapy and had a baseline platelet (PLT) count $<30\times10^9$/L (or $<50\times10^9$/L if on stable steroid therapy). Eligible participants received an initial dose of 3 mg lusutrombopag once daily for up to 4 weeks during the fixed-dose phase, followed by an 8-week dose-titration phase where doses were adjusted based on platelet counts (ranging from 3 mg weekly to 6 mg daily) for target PLT of 50-150×$10^9$/L, and a 4-week safety follow-up period. The primary endpoint was the proportion of patients achieving a PLT response, defined as a PLT count $\geq50\times10^9$/L at Week 4 or $\geq250\times10^9$/L at least once within 4 weeks. Secondary endpoints included the proportions of patients with PLT counts $\geq50\times10^9$/L at Week 6 and Week 12, time to first achieving a PLT count $\geq50\times10^9$/L, sustained responses (defined as PLT counts maintaining $\geq50\times10^9$/L for ≥75% of measurements), reduction in the use of concomitant ITP medications, and adverse events (AEs).

**Copyright Information:** (Online) ISSN: 2572-9241
©2026 The Author(s). HemaSphere is published by John Wiley & Sons Ltd on behalf of European Hematology Association. This is an open access Abstract Book distributed under the Attribution-NonCommercial-NoDerivs (CC BY-NC-ND), which allows third parties to download the articles and share them with others as long as they credit the author and the Abstract Book, but they cannot change the content in any way or use them commercially.
**Abstract Book Citations:** Authors, Title, HemaSphere, 2026;10:(S1):pages. The abstract book DOIs can be found on the "Author Information" page.
**Disclaimer:** Articles published in the journal HemaSphere exclusively reflect the opinions of the authors. The authors are responsible for all content in their abstracts including accuracy of the facts, statements, citing resources, etc.

**Results**

From April 2024 to November 2024, a total of 17 patients were enrolled, with a mean age of 47.4 years and 70.6% (12/17) being female. The median duration from ITP diagnosis to study entry was 17.2 months (range, 3.7-357.7), and none of the patients had prior splenectomy. The median cumulative dose was 159.0 mg (range, 63.0-402.0), and the median daily dose was 2.2 mg (range, 0.8-4.7). The primary endpoint was achieved in 15 patients (88.2%; 95% confidence interval [CI], 63.6-98.5) (Figure 1A). A rapid response was observed, with a median time to first achieving a PLT count ≥50×$10^9$/L of 8.0 days (range, 7-15). The proportions of patients with PLT ≥50×$10^9$/L were 75% (12/16) at Week 6 and 87.5% (14/16) at Week 12. The median PLT among all patients increased from 25.0×$10^9$/L at baseline to 80.0×$10^9$/L at Week 4, 89.0×$10^9$/L at Week 6, and 92.0×$10^9$/L at Week 12 (Figure 1B). The overall sustained response rate was 58.8% (10/17), and among patients who received an average daily dose <3 mg, the sustained response was observed in 80% (8/10). The median cumulative duration with a PLT ≥50×$10^9$/L was 10 weeks across the 12-week treatment period. Among patients receiving concomitant ITP medications, 62.5% (5/8) achieved a reduction in these medications during the study. Treatment-emergent AEs occurred in 14 patients (82.4%), with only one (5.9%) treatment-related AE recorded (grade 2 headache). No hepatotoxicity or treatment-related thromboembolic events were observed, and no patient experienced World Health Organization (WHO) grade 2 or higher bleeding events during the study.

**Summary/Conclusion**

In Chinese adult patients with persistent or chronic primary ITP, lusutrombopag provides rapid and sustained platelet response with a favorable safety profile. The study suggests that lusutrombopag, with a dose-titration strategy, is a promising therapeutic option for primary ITP, warranting further validation in large-scale randomized controlled trials.

**Copyright Information:** (Online) ISSN: 2572-9241
©2026 The Author(s). HemaSphere is published by John Wiley & Sons Ltd on behalf of European Hematology Association. This is an open access Abstract Book distributed under the Attribution-NonCommercial-NoDerivs (CC BY-NC-ND), which allows third parties to download the articles and share them with others as long as they credit the author and the Abstract Book, but they cannot change the content in any way or use them commercially.
**Abstract Book Citations:** Authors, Title, HemaSphere, 2026;10:(S1):pages. The abstract book DOIs can be found on the “Author Information” page.
**Disclaimer:** Articles published in the journal HemaSphere exclusively reflect the opinions of the authors. The authors are responsible for all content in their abstracts including accuracy of the facts, statements, citing resources, etc.

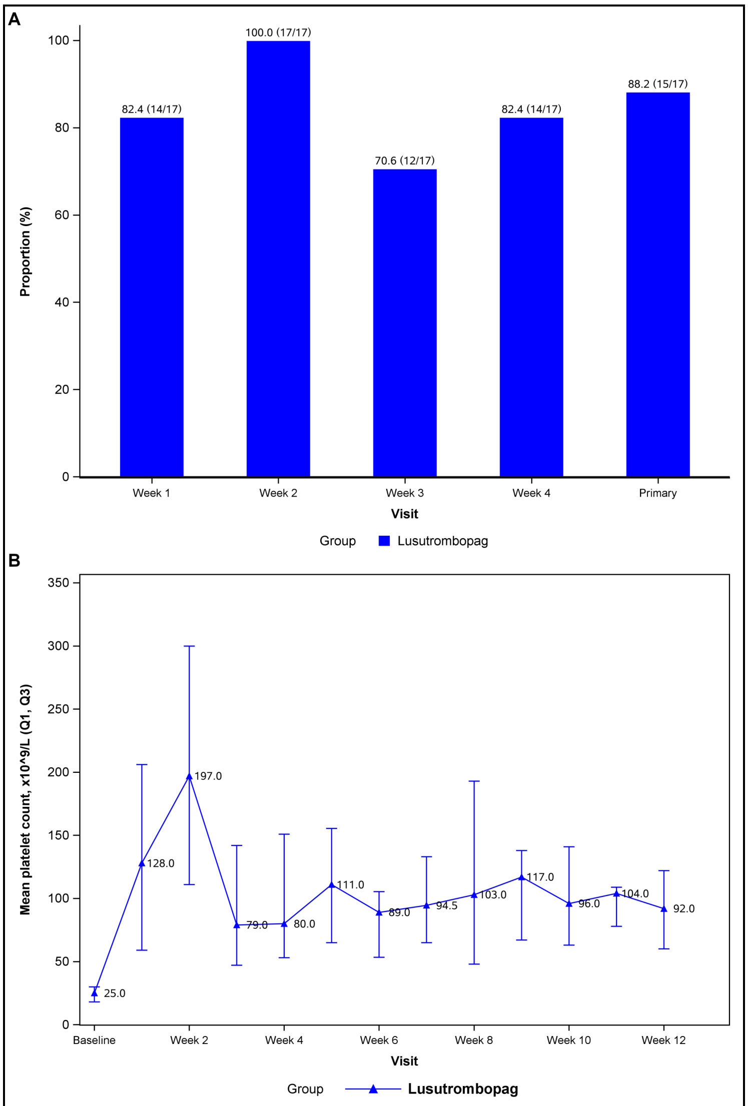

**Figure 1. Clinical efficacy outcomes.** Proportions of patients achieving platelet counts ≥50×$10^9$/L within 4 weeks (A) and median platelet levels within 12 weeks (B). *Primary (primary endpoint): proportions of patients achieving either a platelet count ≥50×$10^9$/L at Week 4 or a platelet count ≥250×$10^9$/L at least once within 4 weeks.

**Copyright Information:** (Online) ISSN: 2572-9241
©2026 The Author(s). HemaSphere is published by John Wiley & Sons Ltd on behalf of European Hematology Association. This is an open access Abstract Book distributed under the Attribution-NonCommercial-NoDerivs (CC BY-NC-ND), which allows third parties to download the articles and share them with others as long as they credit the author and the Abstract Book, but they cannot change the content in any way or use them commercially.
**Abstract Book Citations:** Authors, Title, HemaSphere, 2026;10:(S1):pages. The abstract book DOIs can be found on the "Author Information" page.
**Disclaimer:** Articles published in the journal HemaSphere exclusively reflect the opinions of the authors. The authors are responsible for all content in their abstracts including accuracy of the facts, statements, citing resources, etc.

### (PF1353) COMBINATION OF ANTI-CD38 AND ANTI-CD20 MONOCLONAL ANTIBODIES IN THE TREATMENT OF REFRACTORY/RELAPSED IMMUNE THROMBOCYTOPENIA: A PROSPECTIVE CLINICAL STUDY

**Topic:** 33. Platelet disorders

Chen Li*[1], Yimei Feng[1], Jia Liu[1], Aoxue Tan[1], Xiaoqi Wang[1], Xi Zhang[1]

[1]*Xinqiao Hospital, Army Medical University, Chongqing, China;*

### Background

Immune thrombocytopenia (ITP) is an acquired autoimmune disorder characterized by B cell–mediated production of autoantibodies against platelets, leading to increased platelet destruction or impaired platelet production. In addition, long-lived plasma cells (CD38+), which derive from B cells, serve as continuous sources of autoantibodies and may contribute to disease persistence and refractoriness. Although anti-CD20 monoclonal antibody therapy is effective as a second-line treatment for ITP, recent studies suggest that high-dose anti-CD38 monotherapy may be an option for patients with relapsed or refractory (R/R) ITP. However, high-dose monotherapy with anti-CD20 or anti-CD38 antibodies is associated with increased infection risk and limited durability

### Aims

We initiated a prospective, single-arm, single-center clinical trial aiming at evaluating the safety and efficacy of a low-dose combination of anti-CD20 and anti-CD38 monoclonal antibodies in patients with R/R ITP.

### Methods

From May 2025 to January 2026, 10 female patients (median age was 44.5 years ranging from 17 to 73 years) with refractory/relapsed ITP were enrolled. All Patients were treated with a combination of anti-CD20 monoclonal antibody 200mg and anti-CD38 monoclonal antibody 100mg weekly for four weeks. The outcome of treatment was assessed as platelet count response: Complete response (CR), PLT count ≥ $100\times10^{9}$/L and the absence of bleeding; Response (R), PLT count ≥ $30\times10^{9}$/L with an increase of at least 2-fold of baseline and the absence of bleeding; No response (NR), platelet count < $30\times10^{9}$/L, or less than 2-fold of baseline, or the presence of bleeding; Relapse (RE), after achieved the effective treatment, the platelet count reduces to less than $30\times10^{9}$/L, or less than 2-fold of the baseline, or bleeding symptoms occur.

### Results

After 4 weeks of therapy, the proportion of CD20+ lymphocytes decreased from 23.97% to 0.3% (P < 0.05), and CD38+ lymphocytes from 6.20% to 0% (P < 0.05). The median baseline platelet count was $12\times10^{9}$/L. At week 4, seven patients (70%) achieved complete response (CR), two (20%) achieved response (R), and one had no response (NR), resulting in an overall response rate (ORR) of 90%. Treatment was well tolerated, with no severe adverse events observed. Among responders, the median platelet count increased to $120\times10^{9}$/L (P < 0.05). The median time to response was 1 week (range, 1–4), and the median duration of response (DoR) was 3.5 months (range, 1–9). The median bleeding score decreased from 1 at baseline to 0 after treatment (P < 0.05). During follow-up, relapse occurred in two patients, while seven maintained sustained CR. All patients had received multiple prior therapies, including corticosteroids, immunoglobulins, TPO-RAs, sirolimus, ATRA, cyclosporine, traditional Chinese medicine, and splenectomy. Following anti-CD20 plus anti-CD38 therapy, the mean number of maintenance treatments per patient decreased from 3 to 1.5 (P < 0.05). The two patients with prior splenectomy showed NR or relapse.

**Copyright Information:** (Online) ISSN: 2572-9241
©2026 The Author(s). HemaSphere is published by John Wiley & Sons Ltd on behalf of European Hematology Association. This is an open access Abstract Book distributed under the Attribution-NonCommercial-NoDerivs (CC BY-NC-ND), which allows third parties to download the articles and share them with others as long as they credit the author and the Abstract Book, but they cannot change the content in any way or use them commercially.
**Abstract Book Citations:** Authors, Title, HemaSphere, 2026;10:(S1):pages. The abstract book DOIs can be found on the “Author Information” page.
**Disclaimer:** Articles published in the journal HemaSphere exclusively reflect the opinions of the authors. The authors are responsible for all content in their abstracts including accuracy of the facts, statements, citing resources, etc.

**Summary/Conclusion**

Combination of anti-CD20 and anti-CD38 monoclonal antibodies may offer a safe and effective approach to increase platelet count and minimize maintenance therapy in R/R ITP. However, these preliminary findings require the confirmation through larger amount of data with longer periods for observation.

**Copyright Information:** (Online) ISSN: 2572-9241
©2026 The Author(s). HemaSphere is published by John Wiley & Sons Ltd on behalf of European Hematology Association. This is an open access Abstract Book distributed under the Attribution-NonCommercial-NoDerivs (CC BY-NC-ND), which allows third parties to download the articles and share them with others as long as they credit the author and the Abstract Book, but they cannot change the content in any way or use them commercially.
**Abstract Book Citations:** Authors, Title, HemaSphere, 2026;10:(S1):pages. The abstract book DOIs can be found on the "Author Information" page.
**Disclaimer:** Articles published in the journal HemaSphere exclusively reflect the opinions of the authors. The authors are responsible for all content in their abstracts including accuracy of the facts, statements, citing resources, etc.

### (PF1354) PLATELET COUNT DYNAMICS AFTER COVID19 INFECTION VS. COVID19 VACCINATION IN PATIENTS WITH ITP: DATA FROM THE ITP NATURAL HISTORY STUDY REGISTRY

**Topic:** 33. Platelet disorders

Taylor Bowles*[1, 2], David Ciemnecki[2], Caroline Kruse[2]

*[1]OU Hudson College of Public Health, Biostatistics & Epidemiology, Oklahoma City, United States of America;[2]Platelet Disorder Support Association, Cleveland, United States of America;*

### Background

Immune thrombocytopenia (ITP) is an immune-mediated disorder in which patients commonly experience platelet fluctuations after infection or vaccination. Previous analysis of the PDSA ITP Registry have reported platelet changes following COVID-19 infection or vaccination. This study expands these findings by examining platelet increases versus decreases, recovery times, and age-related trends.

### Aims

To compare patient-reported platelet count changes and recovery times following COVID-19 infection versus COVID-19 vaccination among patients with ITP.

### Methods

Self-reported survey data from 871 participants who completed the COVID-19 & ITP survey between February 2021 and February 2026 were analyzed. Descriptive statistics were generated for key variables, including age, sex, and splenectomy status. Platelet count changes after COVID-19 infection and vaccination were categorized as increased or decreased for recovery time comparisons and as change or no change for age-wise comparisons. Platelet count recovery time was dichotomized as <4 weeks and ≥4 weeks. Cross-tabulations compared recovery time by platelet response direction for both infection and vaccination.

### Results

Participants were mostly female (75%, 629/841), diagnosed with ITP as their primary rare disease (99%, 861/868), and most reported that their ITP was not in remission (57%, 497/869). Forty percent (344/870) reported a confirmed COVID-19 infection, while 86% (745/869) received at least one COVID-19 vaccine dose. Splenectomy was reported by 20% (174/867) of respondents. Following COVID-19 infection, 28% (89/314) reported a platelet decrease, 12% (38/314) reported an increase, and 56% (177/314) reported no change. Among those reporting a change, 75% of participants with a platelet increase recovered within 4 weeks versus 64% of those with a decrease. Following COVID-19 vaccination, 25% (166/664) reported a platelet decrease, 11% (72/664) reported an increase, and 62% (410/664) reported no change. Among participants reporting a change after vaccination, 77% of those with a platelet increase recovered within 4 weeks versus 66% of those with a decrease. In the subset with complete covid impact and age data (N=310), participants under the age of 40 were more likely to report a platelet change (an increase or decrease) than those over 40 years of age (50%,57/114 vs. 34%, 67/196). Conversely, no change was reported by 50% (57/114) of participants <40 and 66% (129/196) of participants ≥40 (Pearson $\chi^2$=7.51, df=1, p=0.006; N=310). There was no significant difference in recovery time when comparing infection-related versus vaccine-related platelet changes. Across both exposures, recovery was consistently faster following platelet increases than platelet decreases, though not significant.

**Copyright Information:** (Online) ISSN: 2572-9241
©2026 The Author(s). HemaSphere is published by John Wiley & Sons Ltd on behalf of European Hematology Association. This is an open access Abstract Book distributed under the Attribution-NonCommercial-NoDerivs (CC BY-NC-ND), which allows third parties to download the articles and share them with others as long as they credit the author and the Abstract Book, but they cannot change the content in any way or use them commercially.
**Abstract Book Citations:** Authors, Title, HemaSphere, 2026;10:(S1):pages. The abstract book DOIs can be found on the “Author Information” page.
**Disclaimer:** Articles published in the journal HemaSphere exclusively reflect the opinions of the authors. The authors are responsible for all content in their abstracts including accuracy of the facts, statements, citing resources, etc.

**Summary/Conclusion**

Among registry participants, platelet count decreases were reported more frequently than increases following both COVID-19 infection and vaccination, with most changes resolving within 4 weeks. Recovery within 4 weeks was more frequently reported after platelet increases than after decreases for both exposures. Participants younger than 40 years were significantly more likely to report a platelet change following COVID-19 exposure than those over 40 years. Recovery time did not differ significantly between infection-related and vaccine-related exposures. These findings suggest clinicians should consider age-informed monitoring and personalized follow-up for patients following either exposure.

**Copyright Information:** (Online) ISSN: 2572-9241
©2026 The Author(s). HemaSphere is published by John Wiley & Sons Ltd on behalf of European Hematology Association. This is an open access Abstract Book distributed under the Attribution-NonCommercial-NoDerivs (CC BY-NC-ND), which allows third parties to download the articles and share them with others as long as they credit the author and the Abstract Book, but they cannot change the content in any way or use them commercially.
**Abstract Book Citations:** Authors, Title, HemaSphere, 2026;10:(S1):pages. The abstract book DOIs can be found on the "Author Information" page.
**Disclaimer:** Articles published in the journal HemaSphere exclusively reflect the opinions of the authors. The authors are responsible for all content in their abstracts including accuracy of the facts, statements, citing resources, etc.

### (PF1356) ADAPT-BLEED: A PRACTICAL MACHINE LEARNING–BASED BLEEDING RISK SCORE WITH A BEDSIDE DIGITAL APPLICATION

**Topic:** 34. Bleeding disorders (congenital and acquired)

Tamar Tadmor*[1, 2], Ron Likvornik[3], Bracha Shapira[3], Noam Benyamini[4], Anatoly Nemets[5], Galia Stemer[6], Orit Sofer[7], Maya Koren-Michowitz[8], Lior Rokach[3]

*[1]Bnai Zion medical center, Hematology, Haifa, Israel; [2]The Ruth and Bruce Rappaport Faculty of Medicine, Technion,, Haifa, Israel; [3]Ben-Gurion University of the Negev, Department of Software and Information Systems Engineering, Beer Sheva, Israel; [4]Tzafon Medical Center (Poriya), Teberia, Israel; [5]Barzilai Medical Center,, Ashkelon, Israel; [6]Galil Medical Center,, Naharia, Israel; [7]Hillel Yaffe Medical Center,, Hadera, Israel;[8]Shamir Medical Center, Beer Yaacov, Israel;*

### Background

The use of anticoagulation (AC) has been much expanded, and indication for chronic AC use includes a broad range of patients mainly with a diagnosis of: arrhythmia mainly atrial fibrillation, or patients who developed venous thromboembolism (VTE) and in some instance in patients who developed arterial thrombosis.
Taking inconsideration possible bleeding - complications in patients using chronic oral-AC, several scores have been generated to predict bleeding risk: HAS-BLED Score, HEMORR2HAGES score, or Orbit score
However, a comprehensive and dynamic model integrating different indications for the use of AC, drug interactions, and lab values is lacking,

### Aims

We aimed to develop and validate a machine learning–based model to improve prediction of major bleeding among anticoagulated patients and to translate it into a practical clinical tool.

### Methods

We analyzed electronic health records from 153,540 adults receiving anticoagulation therapy across six Israeli hospitals between 2010 and 2023, using the Kineret national clinical data infrastructure. Major bleeding within 12 months served as the primary outcome.
An XGBoost model was trained to capture complex, context-dependent interactions between clinical variables.
Model performance was compared with established bleeding risk scores.
To enhance clinical usability, we derived a simplified point-based score using the SHAPoint framework and implemented it as a user-friendly digital application.

### Results

Among 153,540 patients in the final analytical cohort, 4,000 (2.61%) experienced major bleeding within 12 months. The XGBoost model achieved an AUC of 0.732 (95% CI 0.715–0.750), significantly outperforming HAS-BLED (0.638), ORBIT (0.633), ATRIA (0.653), and the DOAC score (0.656). Key predictors included prior stroke, hypertension, chronic anemia, chronic kidney disease, and recent clinical events, with clinically meaningful interaction effects. The derived point-based ADAPT-BLEED score achieved an AUC of 0.682, retaining 93.1% of the full model’s discriminative capacity. Risk stratification demonstrated a 9.7-fold gradient between the lowest- and highest-risk groups (0.68% vs. 6.59% annual bleeding rate). The accompanying digital application enables rapid bedside calculation and individualized risk estimation for routine clinical use.

**Copyright Information:** (Online) ISSN: 2572-9241
©2026 The Author(s). HemaSphere is published by John Wiley & Sons Ltd on behalf of European Hematology Association. This is an open access Abstract Book distributed under the Attribution-NonCommercial-NoDerivs (CC BY-NC-ND), which allows third parties to download the articles and share them with others as long as they credit the author and the Abstract Book, but they cannot change the content in any way or use them commercially.
**Abstract Book Citations:** Authors, Title, HemaSphere, 2026;10:(S1):pages. The abstract book DOIs can be found on the “Author Information” page.
**Disclaimer:** Articles published in the journal HemaSphere exclusively reflect the opinions of the authors. The authors are responsible for all content in their abstracts including accuracy of the facts, statements, citing resources, etc.

**Summary/Conclusion**

ADAPT-BLEED improves prediction of major bleeding in anticoagulated patients compared with existing clinical scores. By integrating machine learning accuracy with interpretability and a deployable clinical application, this approach provides an actionable and scalable tool for personalized bleeding risk assessment.

**Copyright Information:** (Online) ISSN: 2572-9241
©2026 The Author(s). HemaSphere is published by John Wiley & Sons Ltd on behalf of European Hematology Association. This is an open access Abstract Book distributed under the Attribution-NonCommercial-NoDerivs (CC BY-NC-ND), which allows third parties to download the articles and share them with others as long as they credit the author and the Abstract Book, but they cannot change the content in any way or use them commercially.
**Abstract Book Citations:** Authors, Title, HemaSphere, 2026;10:(S1):pages. The abstract book DOIs can be found on the "Author Information" page.
**Disclaimer:** Articles published in the journal HemaSphere exclusively reflect the opinions of the authors. The authors are responsible for all content in their abstracts including accuracy of the facts, statements, citing resources, etc.

### (PF1357) DISTINCT CLINICAL PROFILES AND BLEEDING SEVERITY IN CONGENITAL AND ACQUIRED FACTOR XIII DEFICIENCY: A SINGLE-CENTER EXPERIENCE

**Topic:** 34. Bleeding disorders (congenital and acquired)

Alfadil Haroon*[1], Tusneem Alhassan[1], Hazzaa Alzahrani[1]

[1]*Cancer Center of Excellence, King Faisal Specialist Hospital and Research Center, Adult Hematology, stem cell transplant and cellular therapy section, Riyad, Saudi Arabia;*

**Background**

Factor XIII deficiency (FXIIID) is a rare coagulopathy arising from congenital quantitative or acquired qualitative defects in the FXIII A-subunit. Acquired FXIIID, though uncommon, carries significant hemorrhagic risk and frequently complicates malignant or autoimmune conditions.Comparative clinical data between both subtypes remain scarce

**Aims**

To compare the clinical characteristics, bleeding patterns, laboratory profiles and outcomes of congenital versus acquired factor XIII deficiency, and to identify factor XIII level predicting bleeding severity

**Methods**

We retrospectively analyzed 25 patients with FXIIID, including 13(52.0%) congenital and 12(48.0%) acquired cases. receiver operating characteristic (ROC) analysis was performed to identify optimal FXIII thresholds predicting severe bleeding

**Copyright Information:** (Online) ISSN: 2572-9241
©2026 The Author(s). HemaSphere is published by John Wiley & Sons Ltd on behalf of European Hematology Association. This is an open access Abstract Book distributed under the Attribution-NonCommercial-NoDerivs (CC BY-NC-ND), which allows third parties to download the articles and share them with others as long as they credit the author and the Abstract Book, but they cannot change the content in any way or use them commercially.
**Abstract Book Citations:** Authors, Title, HemaSphere, 2026;10:(S1):pages. The abstract book DOIs can be found on the "Author Information" page.
**Disclaimer:** Articles published in the journal HemaSphere exclusively reflect the opinions of the authors. The authors are responsible for all content in their abstracts including accuracy of the facts, statements, citing resources, etc.

**Results**

Of 25 patients with FXIIID 13 (52%) were females and 12 (48%) males. The overall median age at report was 35 years (IQR 28–60). Congenital FXIIID group  diagnosed in early childhood and significantly younger at the time of report (median 31 years, IQR 26–35) compared to those with acquired disease (median 60 years, IQR 35–65)
Acquired FXIIID was associated with malignancy in 91.6% of cases, predominantly myeloid malignancies (66.6%), with cytogenetic and molecular abnormalities including TP53, RUNX1, SRSF2, and KMT2A-MLL rearrangement. In contrast, congenital cases were more commonly associated with inherited or chronic conditions such as iron deficiency anemia and hypothyroidism
The median FXIII activity at diagnosis was 0.10 IU/mL (IQR 0.10–0.30). Congenital patients had lower activity levels (median 0.10, range 0.06–0.15 IU/mL) compared to acquired cases (median 0.30, range 0.10–0.50 IU/mL). Long-term prophylactic FXIII replacement was administered to 92.3% of congenital patients but only 16.7% of acquired cases. ROC analysis demonstrated moderate predictive ability of FXIII activity level for severe bleeding (AUC = 0.702), with an optimal cutoff of 0.13 IU/mL (sensitivity 58.8%, specificity 75.0%, Youden's J = 0.338)

Severe bleeding was documented in 68.0% of all patients, with a significantly higher prevalence in acquired deficiency (91.7%) than congenital (46.2%). Gastrointestinal bleeding was more frequent in acquired cases (41.6% vs. 15.3%), muscle or joint bleeding (33.3% vs. 15.0%). CNS bleeding rates were comparable between groups (16.6% vs. 15.0%), while mucosal bleeding was observed exclusively in congenital patients (15.0%)
Combined FXIII concentrate and antifibrinolytic therapy was used to treat bleeding episodes in both groups (76.9% vs. 75.0%), while cryoprecipitate plus tranexamic acid was used in 25.0% of acquired cases.Noncomplicated vaginal delivery was achieved in 3(23.1%) congenital patients and recurrent abortion was reported in 2(23.1%)congenital  and 1 acquired patient (8.3%).Hemostatic coverage for surgical procedures consisted primarily of FXIII plus tranexamic acid

**Summary/Conclusion**

Congenital and acquired FXIIID exhibit distinct clinical profiles. Congenital disease presents earlier with uniformly low FXIII activity and responds well to long-term prophylaxis. Acquired deficiency occurs at an advanced age is predominantly associated with hematologic malignancy and carries a substantially higher risk of severe bleeding despite relatively preserved factor levels. ROC analysis demonstrated only moderate predictive accuracy for bleeding severity, highlighting the need to integrate clinical context and etiology into risk stratification and management decisions

**Copyright Information:** (Online) ISSN: 2572-9241
©2026 The Author(s). HemaSphere is published by John Wiley & Sons Ltd on behalf of European Hematology Association. This is an open access Abstract Book distributed under the Attribution-NonCommercial-NoDerivs (CC BY-NC-ND), which allows third parties to download the articles and share them with others as long as they credit the author and the Abstract Book, but they cannot change the content in any way or use them commercially.
**Abstract Book Citations:** Authors, Title, HemaSphere, 2026;10:(S1):pages. The abstract book DOIs can be found on the “Author Information” page.
**Disclaimer:** Articles published in the journal HemaSphere exclusively reflect the opinions of the authors. The authors are responsible for all content in their abstracts including accuracy of the facts, statements, citing resources, etc.

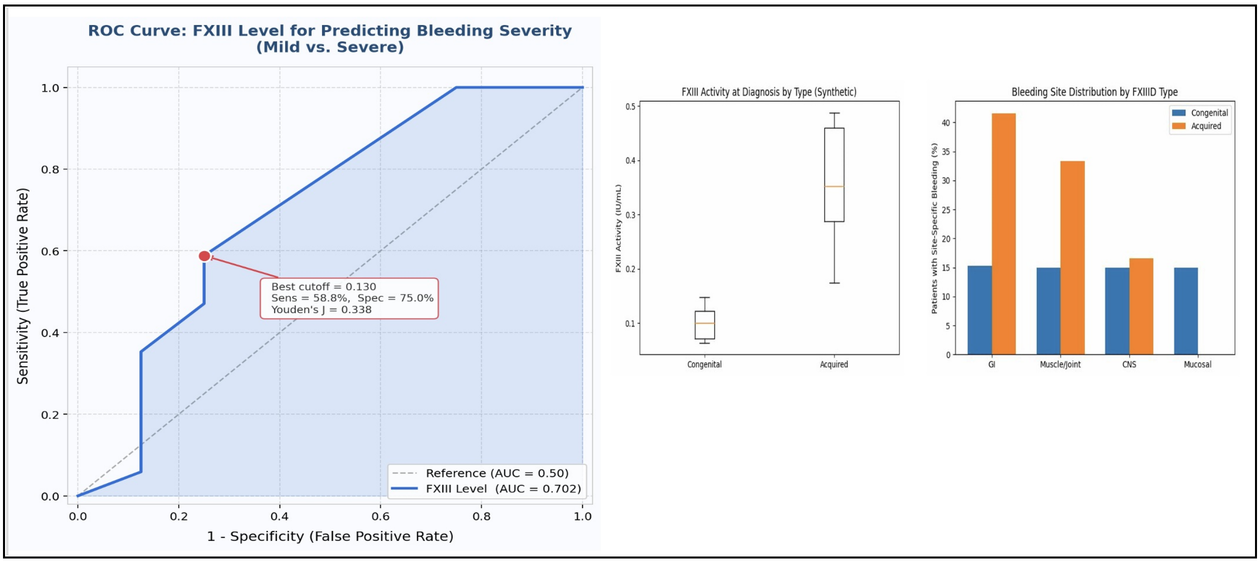

**Copyright Information:** (Online) ISSN: 2572-9241
©2026 The Author(s). HemaSphere is published by John Wiley & Sons Ltd on behalf of European Hematology Association. This is an open access Abstract Book distributed under the Attribution-NonCommercial-NoDerivs (CC BY-NC-ND), which allows third parties to download the articles and share them with others as long as they credit the author and the Abstract Book, but they cannot change the content in any way or use them commercially.
**Abstract Book Citations:** Authors, Title, HemaSphere, 2026;10:(S1):pages. The abstract book DOIs can be found on the "Author Information" page.
**Disclaimer:** Articles published in the journal HemaSphere exclusively reflect the opinions of the authors. The authors are responsible for all content in their abstracts including accuracy of the facts, statements, citing resources, etc.

### (PF1358) DIAGNOSTIC VALUE OF THROMBIN GENERATION USING DIFFERENT TRIGGER REAGENTS IN PATIENTS WITH BLEEDING DISORDER OF UNKNOWN CAUSE

**Topic:** 34. Bleeding disorders (congenital and acquired)

Emma Urlings*[1, 2], Amaury Monard[1, 2], Inge Merry[1], Floor Derikx[1], René van Oerle[3], Erik Beckers[1, 3], Yvonne Henskens[2, 4], Henri Spronk[2, 3], Floor Heubel-Moenen[1, 2]

*[1]Maastricht University Medical Center+, Internal Medicine, Maastricht, The Netherlands; [2]Maastricht University, Cardiovascular Research Institute Maastricht, Maastricht, The Netherlands; [3]Maastricht University, Biochemistry, Maastricht, The Netherlands; [4]Maastricht University Medical Center+, Central Diagnostic Laboratory, Maastricht, The Netherlands;*

### Background

The majority of patients with an increased bleeding tendency remain undiagnosed after extensive laboratory testing and are classified as having a bleeding disorder of unknown cause (BDUC). Global hemostasis assays, such as the plasma thrombin generation assay (TGA), may provide enhanced sensitivity and provide a rationale for the diminished coagulation profile.

### Aims

To assess whether TGA can identify a bleeding tendency in BDUC patients and to determine the most sensitive TGA trigger reagent for this patient population.

### Methods

BDUC patients (n=83) from the *Predictors of Bleeding Evaluation in Adult Hematologic Patients* (ProBe-AHP) study were analyzed. Patients had clinically relevant bleeding symptoms (elevated ISTH-BAT scores or based on clinical gestalt) and normal standard hemostasis test results. TGA was performed using the Calibrated Automated Thrombography (CAT) method (Stago BNL) with three different trigger reagents: PPP Reagent INT (intrinsic activation), PPP Reagent LOW (low tissue factor (TF) activation), and PPP Reagent LOW 1:5 PL (low TF and diluted phospholipids). Reference values were obtained from 107 healthy volunteers.

### Results

TGA was performed in a total of 83 BDUC patients and the lag time (LT), velocity index (VI), peak height (PH), and endogenous thrombin potential (ETP) were analyzed. Compared with healthy volunteers, BDUC patients had a longer LT in all three trigger reagents, and in PPP Reagent LOW 1:5 PL, ETP, PH, and VI were also decreased. Overall, 21/83 (25%) BDUC patients had one or more abnormal TGA parameters suggestive of a bleeding tendency. All three reagents demonstrated comparable sensitivity in detecting a bleeding phenotype. PPP Reagent INT and PPP Reagent LOW each identified 10 patients with abnormalities suggestive of a bleeding tendency, while PPP Reagent LOW 1:5 PL identified 8 patients. Multiple coagulation factors demonstrated moderate to strong correlations with TGA parameters: fibrinogen correlated with LT and PH across reagents, FII showed the strongest associations with PH and VI (PPP Reagent INT), and FVIII, FIX, and FXI correlated moderately with ETP and PH.

### Summary/Conclusion

While TGA identified hemostatic abnormalities in one-quarter of BDUC patients, trigger reagent modifications provided no added diagnostic advantage in this patient population.

**Copyright Information:** (Online) ISSN: 2572-9241
©2026 The Author(s). HemaSphere is published by John Wiley & Sons Ltd on behalf of European Hematology Association. This is an open access Abstract Book distributed under the Attribution-NonCommercial-NoDerivs (CC BY-NC-ND), which allows third parties to download the articles and share them with others as long as they credit the author and the Abstract Book, but they cannot change the content in any way or use them commercially.
**Abstract Book Citations:** Authors, Title, HemaSphere, 2026;10:(S1):pages. The abstract book DOIs can be found on the "Author Information" page.
**Disclaimer:** Articles published in the journal HemaSphere exclusively reflect the opinions of the authors. The authors are responsible for all content in their abstracts including accuracy of the facts, statements, citing resources, etc.

### (PF1359) MATCHING-ADJUSTED INDIRECT COMPARISON OF RIX-FP VERSUS MARSTACIMAB FOR THE PROPHYLAXIS OF SEVERE HEMOPHILIA B

**Topic:** 34. Bleeding disorders (congenital and acquired)

Benoit Guillet[1, 2], Songkai Yan*[3], Douglass Drelich[3], Becky Hooper[4], Kevin Hou[4], Sarah Organ[4], Hongseok Kim[3], Amrita Debnath[4], Harry Odia[4], Korinna Karampampa[5], Maria Elisa Mancuso[6]

*[1]University Hospital, Rennes, CRH, CRC-MHC (Centre de Référence de l'Hémophilie, Centre de Ressource et de Compétence Des Maladies Hémorragiques Constitutionnelles), Rennes, France; [2]Univ Rennes, CHU Rennes, Inserm, EHESP, Irset (Institut de Recherche en santé, Environnement et Travail), Rennes, France; [3]CSL Behring, King of Prussia, United States of America; [4]Eversana, Victoria, BC, Canada; [5]CSL Behring, Stockholm, Sweden; [6]IRCCS Humanitas Research Hospital and Humanitas University, Milan, Italy;*

**Background**

Prophylactic factor IX replacement therapy has been a standard of care for individuals with hemophilia (hem) B. Recombinant factor IX albumin fusion protein (rIX-FP) is a common prophylaxis treatment approved in many countries, including the US, in the EU, Asia, and Middle East for children and adults with hem B, with PROLONG-9FP being one of the pivotal trials. Marstacimab has recently been approved in the US, EU, and Japan for prophylaxis in adolescents and adults with hem A and hem B without inhibitors based on the pivotal trial BASIS. No trial has directly compared rIX-FP and marstacimab in the treatment of hem B.

**Aims**

The aim of this study is to compare the efficacy of rIX-FP versus marstacimab in the treatment of adults with severe hem B who received prior routine prophylaxis, using rigorous matching-adjusted indirect comparison (MAIC) methods.

**Methods**

Unanchored MAICs leveraged individual patient data (IPD) from PROLONG-9FP and publicly available summary-level data (SLD) from BASIS for subjects without inhibitors who received routine prophylaxis. Due to the lack of hem B subgroup data available in the primary publication of BASIS, publicly available SLD was obtained from the German Gemeinsamer Bundesausschuss (G-BA) submission dossier and French Haute Autorité de santé (HAS) decision document. Patients from PROLONG-9FP were first matched to BASIS by excluding patients from the PROLONG-9FP IPD who would not have been eligible to enroll in BASIS. Patients in PROLONG-9FP without severe hem B were also excluded to align with the BASIS hem B population. Next, patients in PROLONG-9FP who received routine prophylaxis were weighted to align with BASIS on age, body mass index, and geographic region. Three efficacy outcomes were assessed based on data availability for the hem B population of BASIS: mean annualized bleeding rate (ABR), mean annualized spontaneous bleeding rate (AsBR), and mean annualized joint bleeding rate (AjBR). A secondary analysis was conducted including only patients from PROLONG-9FP who received weekly prophylaxis for 26 weeks followed by dosing regimen adjustment to every 14-day prophylaxis. Results of comparative efficacy are reported as rate ratios (RRs) with 95% confidence intervals (CIs). Annualized rates are reported separately for each therapy.

**Copyright Information:** (Online) ISSN: 2572-9241
©2026 The Author(s). HemaSphere is published by John Wiley & Sons Ltd on behalf of European Hematology Association. This is an open access Abstract Book distributed under the Attribution-NonCommercial-NoDerivs (CC BY-NC-ND), which allows third parties to download the articles and share them with others as long as they credit the author and the Abstract Book, but they cannot change the content in any way or use them commercially.
**Abstract Book Citations:** Authors, Title, HemaSphere, 2026;10:(S1):pages. The abstract book DOIs can be found on the "Author Information" page.
**Disclaimer:** Articles published in the journal HemaSphere exclusively reflect the opinions of the authors. The authors are responsible for all content in their abstracts including accuracy of the facts, statements, citing resources, etc.

### Results

After adjustment for key factors, rIX-FP was associated with a statistically significant lower mean ABR, AsBR, and AjBR versus marstacimab (Table 1). Results were consistent in the secondary analysis.

Table 1. MAIC Results of rIX-FP versus Marstacimab

| Outcome | Estimate | Marstacimab | rIX-FP | |
|---|---|---|---|---|
| | | | Naïve | MAIC |
| Mean ABR | RR (95% CI) | - | **0.34 (0.16,0.74)** | **0.33 (0.14,0.77)** |
| | Annualized Rate | 4.60* | 1.56 | 1.54 |
| Mean ABR | RR (95% CI) | - | **0.33 (0.15,0.72)** | **0.33 (0.14,0.75)** |
| | Annualized Rate | 4.71** | 1.56 | 1.54 |
| Mean AsBR | RR (95% CI) | - | **0.25 (0.12,0.57)** | **0.25 (0.10,0.59)** |
| | Annualized Rate | 2.88* | 0.71 | 0.71 |
| Mean AjBR | RR (95% CI) | - | **0.29 (0.13,0.64)** | **0.31 (0.13,0.74)** |
| | Annualized Rate | 3.87* | 1.12 | 1.18 |

Note: Confidence intervals excluding 1 indicate a statistically significant result and are bolded. Rate ratios less than 1 indicate a favourable result for rIX-FP.
* Outcome as reported by the G-BA submission dossier.
** Outcome as reported by the HAS decision document.

**Copyright Information:** (Online) ISSN: 2572-9241
©2026 The Author(s). HemaSphere is published by John Wiley & Sons Ltd on behalf of European Hematology Association. This is an open access Abstract Book distributed under the Attribution-NonCommercial-NoDerivs (CC BY-NC-ND), which allows third parties to download the articles and share them with others as long as they credit the author and the Abstract Book, but they cannot change the content in any way or use them commercially.
**Abstract Book Citations:** Authors, Title, HemaSphere, 2026;10:(S1):pages. The abstract book DOIs can be found on the "Author Information" page.
**Disclaimer:** Articles published in the journal HemaSphere exclusively reflect the opinions of the authors. The authors are responsible for all content in their abstracts including accuracy of the facts, statements, citing resources, etc.

**Summary/Conclusion**

The MAICs suggest favourable efficacy for routine prophylaxis with rIX-FP versus marstacimab across all outcomes assessed. Results were consistent in the secondary analysis. This suggests rIX-FP may be associated with reduced bleeding compared to marstacimab in patients with severe hem B.

**Copyright Information:** (Online) ISSN: 2572-9241
©2026 The Author(s). HemaSphere is published by John Wiley & Sons Ltd on behalf of European Hematology Association. This is an open access Abstract Book distributed under the Attribution-NonCommercial-NoDerivs (CC BY-NC-ND), which allows third parties to download the articles and share them with others as long as they credit the author and the Abstract Book, but they cannot change the content in any way or use them commercially.
**Abstract Book Citations:** Authors, Title, HemaSphere, 2026;10:(S1):pages. The abstract book DOIs can be found on the "Author Information" page.
**Disclaimer:** Articles published in the journal HemaSphere exclusively reflect the opinions of the authors. The authors are responsible for all content in their abstracts including accuracy of the facts, statements, citing resources, etc.

### (PF1360) WHEN PLATELETS TURN AGAINST HEMOSTASIS: PREDICTORS OF ACQUIRED VON WILLEBRAND SYNDROME IN MPN AND ISTH-BAT SCORE-BASED BLEEDING RISK

**Topic:** 34. Bleeding disorders (congenital and acquired)

Antonio Pagana[1], Miriana Limoli[1], Veronica Vecchio[1], Stephanie Grasso[1, 2], Salvatore La Penta[1], Annalisa Santisi[1], Arianna Sbriglione[1], Andrea Duminuco[1], Francesco Di Raimondo[1], Giuseppe Alberto Maria Palumbo[1], Gaetano Giuffrida[1, 2], Uros Markovic*[1, 2]

*[1]Hematology and Bone Marrow Transplatation Unit, A.O.U. Policlinico "G. Rodolico - S.Marco", Catania, Italy; [2]Rare Hematological Diseases Unit, AOU Policlinico "G.Rodolico-S.Marco", Catania, Italy;*

### Background

Acquired von Willebrand syndrome (aVWS) is an adult-onset bleeding disorder characterized by predominantly muco-cutaneous hemorrhagic manifestations of variable severity. aVWS is frequent in Philadelphia chromosome–negative myeloproliferative neoplasms (MPNs), such as essential thrombocythemia (ET), polycythemia vera (PV), and myelofibrosis (MF),particularly in case of extreme thrombocytosis, with platelets (PLT) exceeding 1.300–1.500 x $10^9$/L.

### Aims

Evaluate the prevalence of aVWS in MPN patients and identify clinical, hematologic, and molecular factors associated with its occurrence and bleeding manifestations.

### Methods

A cohort of 202 patients with MPNs (47% male; median age: 66 years; range: 26–89 years) underwent screening, regardless of bleeding history. Assessments included von Willebrand factor antigen (vWF:Ag), vWF ristocetin cofactor (vWF:RCo), vWF:RCo/Ag ratio, and Factor VIII levels. A diagnosis of aVWS was established in case of reduced vWF:RCo (<50 IU/dL) combined with ratio <0.7. Complete blood counts were obtained concurrently, while baseline MPN characteristics were retrieved from medical records. aVWS positive patients (aVWS+) were also evaluated using the ISTH-BAT score to assess bleeding manifestations.

**Copyright Information:** (Online) ISSN: 2572-9241
©2026 The Author(s). HemaSphere is published by John Wiley & Sons Ltd on behalf of European Hematology Association. This is an open access Abstract Book distributed under the Attribution-NonCommercial-NoDerivs (CC BY-NC-ND), which allows third parties to download the articles and share them with others as long as they credit the author and the Abstract Book, but they cannot change the content in any way or use them commercially.
**Abstract Book Citations:** Authors, Title, HemaSphere, 2026;10:(S1):pages. The abstract book DOIs can be found on the "Author Information" page.
**Disclaimer:** Articles published in the journal HemaSphere exclusively reflect the opinions of the authors. The authors are responsible for all content in their abstracts including accuracy of the facts, statements, citing resources, etc.

**Results**

Sixty-one patients were aVWS+ (30%), with median age 63 years, male prevalence (54%), median RCo level of 32 IU/dL and ratio 0.36. Ten patients (11%) also had values of vWF:Ag and/or FVIII <50 IU/dL, associated with lower median RCo (25 IU/dL). Half of them had MF, 25% were TE, while others had PV or not otherwise specified MPN, and in more than 60% splenomegaly was present at time of evaluation. Around 70% were positive for *JAK2 V617F*, while 20% had *CALR* mutation. 25 patients (40%) received hydroxyurea,ten (16%) ruxolitinib, while three patients received either momelotinib or anagrelide. In 23 patients (38%), no cytoreductive drugs were administered, and around 20% of aVWS+ cases did not receive antiaggregants due to prior bleeding. Conversely, 141 aVWS-negative patients (aVWS-) had a median age of 68 years, 55% females, a median RCo of 95.5 IU/dL, and a 0.53 ratio.

Higher PLTs (median 562 x $10^9$/L vs 377 x $10^9$/L, p=0.0001) (Fig. 1) and younger age (median 63 vs 68 years, p=0.01) were more present in aVWS+ patients, using Mann-Whitney test. Platelet count confirmed its impact in multivariate logistic regression (p=0.0002). Diagnosis of ET or MF was associated with increased risk of aVWS compared with PV ($\chi$2 test p=0.03, RR 1.66, 95%CI 1.02-2.69). Splenomegaly was more frequent in aVWS+ patients ($\chi$2 test p=0.01, RR = 1.18, 95%CI 1.13-2.86), and the same was for *CALR* positive patients (p=0.04 and RR 1.75, 95%CI 1.07-2.87). Diagnosis of MF/TE confirmed positive trend for aVWS using multivariate logistic regression (p=0.078), probably due to higher platelet count.
Following linear regression analysis of ISTH-BAT score based on RCo levels in aVWS+ patients only, the most frequent bleeding manifestations were following tooth extractions (p=0.04), and muscle hematomas (p=0.06). Spearman's rank correlation analysis confirmed a strong statistical correlation between RCo and muscle hematoma (p=0.01).

**Summary/Conclusion**

The study highlights the contributing roles of moderate thrombocytosis, CALR mutation, splenomegaly, and younger age in aVWS diagnosis of MPN patients. Furthermore, a warranty is needed in aVWS+ patients, specifically in case of surgery, given the higher frequency of bleeding following dental procedures.

**Copyright Information:** (Online) ISSN: 2572-9241
©2026 The Author(s). HemaSphere is published by John Wiley & Sons Ltd on behalf of European Hematology Association. This is an open access Abstract Book distributed under the Attribution-NonCommercial-NoDerivs (CC BY-NC-ND), which allows third parties to download the articles and share them with others as long as they credit the author and the Abstract Book, but they cannot change the content in any way or use them commercially.
**Abstract Book Citations:** Authors, Title, HemaSphere, 2026;10:(S1):pages. The abstract book DOIs can be found on the "Author Information" page.
**Disclaimer:** Articles published in the journal HemaSphere exclusively reflect the opinions of the authors. The authors are responsible for all content in their abstracts including accuracy of the facts, statements, citing resources, etc.

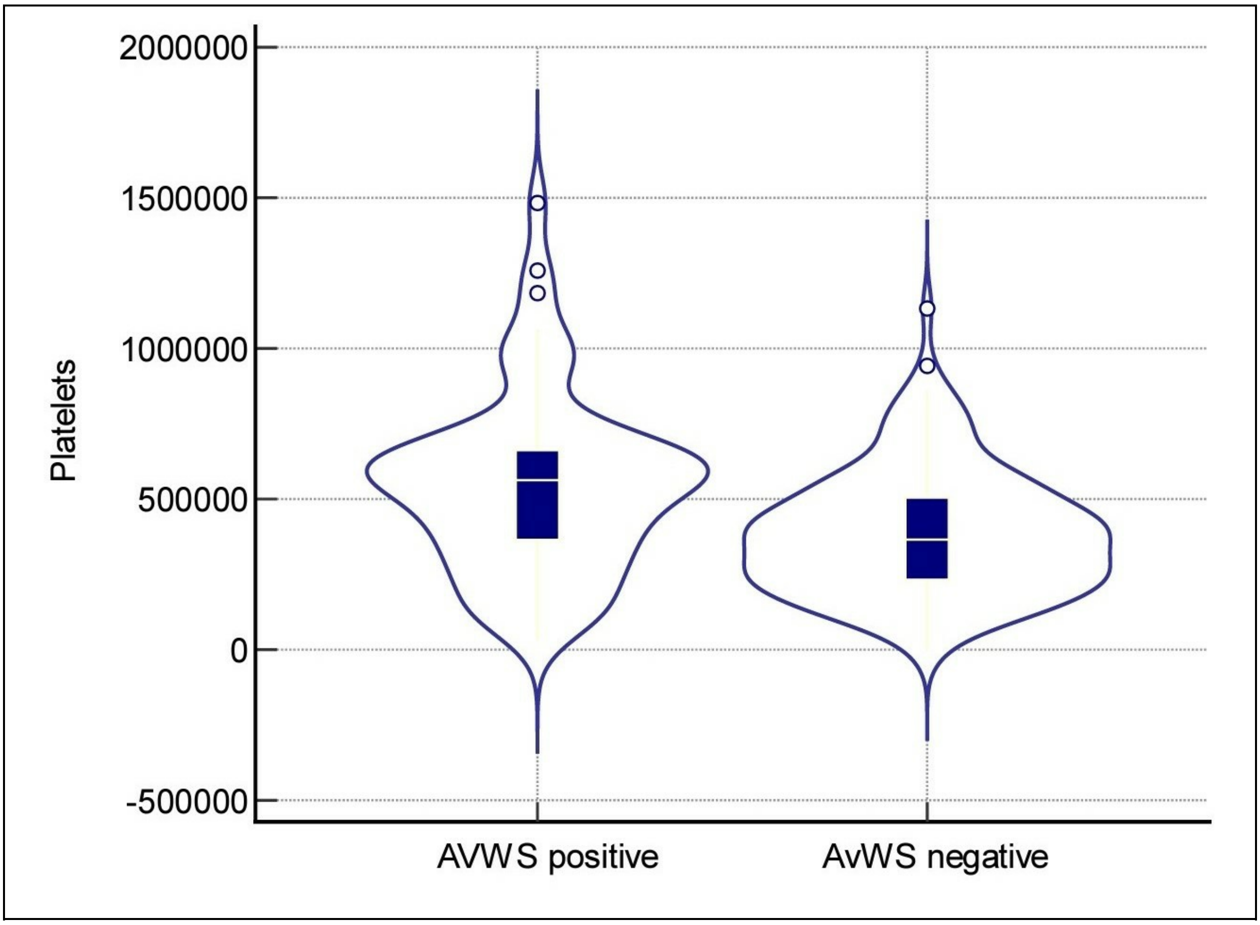

Figure 1. Violin plots showing Platelet Count Distribution in Patients positive and negative for Acquired von Willebrand Syndrome (AVWS); Figure caption - The width of each violin represents the density of observations at different platelet count values. The embedded boxplots indicate the median and interquartile range (IQR), while individual points represent observed values

**Copyright Information:** (Online) ISSN: 2572-9241
©2026 The Author(s). HemaSphere is published by John Wiley & Sons Ltd on behalf of European Hematology Association. This is an open access Abstract Book distributed under the Attribution-NonCommercial-NoDerivs (CC BY-NC-ND), which allows third parties to download the articles and share them with others as long as they credit the author and the Abstract Book, but they cannot change the content in any way or use them commercially.
**Abstract Book Citations:** Authors, Title, HemaSphere, 2026;10:(S1):pages. The abstract book DOIs can be found on the "Author Information" page.
**Disclaimer:** Articles published in the journal HemaSphere exclusively reflect the opinions of the authors. The authors are responsible for all content in their abstracts including accuracy of the facts, statements, citing resources, etc.

### (PF1361) REAL-WORLD EVIDENCE: LEUKEMIA BURDEN DRIVES HEMORRHAGIC RISK IN IBRUTINIB-TREATED CLL PATIENTS

**Topic:** 34. Bleeding disorders (congenital and acquired)

Jelena Ivanović*[1], Sofija Kozarac[1], Vojin Vukovic[1, 2], Tamara Bibic[1], Darko Aleksic[1], Darko Antic[1, 2]

[1]*Clinic for Hematology, University Clinical Center of Serbia, Belgrade, Serbia;*[2]*Faculty od Medicine, University of Belgrade, Belgrade, Serbia;*

### Background

Bleeding is a common adverse event during Bruton tyrosine kinase inhibitor (BTKi) therapy in chronic lymphocytic leukemia (CLL), frequently attributed to drug-induced platelet dysfunction, concomitant antithrombotic therapy (ATT) or cardiovascular comorbidities (CVC). However, real-world determinants of hemorrhagic risk in this vulnerable population remain insufficiently defined.

### Aims

To identify determinants of bleeding during ibrutinib therapy in real-world CLL patients and to define independent predictorsacross bleeding severities.

### Methods

We retrospectively analyzed 111 consecutive CLL patients treated with ibrutinib at the University Clinical Center of Serbia. Bleeding events were classified according to International Society on Thrombosis and Haemostasis (ISTH) criteria and further stratified into non-significant and severe (clinically significant minor and major) events. Univariable analyses used chi-square/Fisher's exact and t-test/Mann–Whitney U tests. Multivariable logistic regression identified independent predictors of overall bleeding.

### Results

Bleeding occurred in 48.6% (54/111) of patients during ibrutinib therapy. Of these, 34 patients (30.6% of the total cohort) experienced non-significant bleeding, while 7 patients (6.3% of the total cohort) experienced severe bleeding. Univariable analyses revealed no significant association between bleeding incidence and age (p=0.862), number of prior therapies (p=0.490), pre-existing CVC (e.g., atrial fibrillation p=0.364, hypertension p=0.364, heart failure p=1.000), or concomitant ATT (p=0.482 for anticoagulants, p=0.773 for antiplatelets). Only 5.2% of patients with bleeding were on anticoagulants, and 12.3% on antiplatelets at the time of bleeding. Instead, markers of leukemia burden were significantly associated with an increased risk of bleeding: advanced Rai stage (p=0.008), presence of TP53 mutation (p=0.033), lower baseline hemoglobin (p=0.020; mean Hgb 115.76±19.93 g/L vs 125.26±22.44 g/L for non-bleeding patients), and lower baseline platelet count (p=0.038; median Plt: 120.5 vs 152x$10^9$/L for non-bleeding patients). Multivariable logistic regression confirmed advanced Rai stage as the sole independent predictor of overall bleeding incidence (OR 1.76 per stage increase; 95% CI 1.11–2.80; p=0.017). Bleeding led to permanent ibrutinib discontinuation in 4.5% of patients. Hemorrhagic events significantly contributed to healthcare utilization (HCU) within the first 6 months of therapy, with a mean of 0.30 Medical Events of Interest, 0.06 emergency department visit, and 0.05 inpatient admissions per patient, with an average length of stay of 0.44 days. Further univariable and multivariable analyses failed to identify independent predictors specifically for clinically significant or major bleeding events.

**Copyright Information:** (Online) ISSN: 2572-9241
©2026 The Author(s). HemaSphere is published by John Wiley & Sons Ltd on behalf of European Hematology Association. This is an open access Abstract Book distributed under the Attribution-NonCommercial-NoDerivs (CC BY-NC-ND), which allows third parties to download the articles and share them with others as long as they credit the author and the Abstract Book, but they cannot change the content in any way or use them commercially.
**Abstract Book Citations:** Authors, Title, HemaSphere, 2026;10:(S1):pages. The abstract book DOIs can be found on the "Author Information" page.
**Disclaimer:** Articles published in the journal HemaSphere exclusively reflect the opinions of the authors. The authors are responsible for all content in their abstracts including accuracy of the facts, statements, citing resources, etc.

### Summary/Conclusion

In this real-world cohort, bleeding during ibrutinib therapy was frequent, but not associated with antithrombotic exposure or CV comorbidity. Instead, bleeding risk correlated with leukemia burden, with advanced Rai stage independently predicting its incidence. While major bleeding occurred in 6.3% of patients and contributed to HCU, reliable predictors of severe hemorrhages remain elusive. These findings challenge conventional bleeding risk assumptions in CLL, suggesting that hemorrhage during ibrutinib therapy may primarily reflect disease-related vulnerability rather than purely drug-induced platelet dysfunction, highlighting the unmet need for better tools to predict severe bleeding.

**Copyright Information:** (Online) ISSN: 2572-9241
©2026 The Author(s). HemaSphere is published by John Wiley & Sons Ltd on behalf of European Hematology Association. This is an open access Abstract Book distributed under the Attribution-NonCommercial-NoDerivs (CC BY-NC-ND), which allows third parties to download the articles and share them with others as long as they credit the author and the Abstract Book, but they cannot change the content in any way or use them commercially.
**Abstract Book Citations:** Authors, Title, HemaSphere, 2026;10:(S1):pages. The abstract book DOIs can be found on the "Author Information" page.
**Disclaimer:** Articles published in the journal HemaSphere exclusively reflect the opinions of the authors. The authors are responsible for all content in their abstracts including accuracy of the facts, statements, citing resources, etc.

### (PF1362) HEMORRHAGIC RISK EVALUATION AND MANAGEMENT OF PEDIATRIC PATIENTS AFFECTED BY MILD FACTOR VII DEFICIENCY UNDERGOING SURGERY

**Topic:** 34. Bleeding disorders (congenital and acquired)

Beatrice Borsellino*[1], Tiziano Martini[1], Irene Ricca[1], Roberto Albiani[1], Berardino Pollio[1]

[1]*Hemophilia Centre, Immune-Hematology and Transfusion Medicine, University Hospital "Città della Salute e della Scienza", Turin, Italy, Turin, Italy;*

### Background

Identifying perioperative bleeding risk in a pediatric population undergoing surgery is quite complex, as clinical history is often not particularly informative and laboratory tests can be influenced by multiple pre-analytical variables.

### Aims

The aim of the study was to evaluate the perioperative bleeding risk in a cohort of children with a borderline Factor VII deficiency (ranging from 20% to 40%) and to assess the potential need for preoperative prophylaxis.

### Methods

In this retrospective observational study, we evaluated consecutive pediatric patients undergoing surgery between January 2017 and February 2020 at the “Regina Margherita Hospital", Turin, Italy. A multivariate statistical analysis was conducted to assess whether there were variables capable of influencing the clinician's choice about the preference of a preoperative prophylaxis strategy used only in case of abnormal perioperative bleeding versus a wait-and-see approach with Factor VII available in the operating room. Written informed consent was obtained from the parents/guardians of all patients. The analysis was conducted starting from a cohort of 64 children undergoing surgery (adenoid and tonsil surgery 59.3%; urological surgery for 21.9%; general surgery 4.7%; neurosurgery 4.7%; heart surgery 1.6%; high airway surgery 3.2%; toracic surgery 1.6%; ortopedic surgery 3.2%), divided into two groups based on preoperative treatment: 41 children were not subjected to prophylaxis, with rVIIa factor available in the operating room to be used in case of abnormal bleeding, while 23 children underwent preventive treatment with rVIIa factor before surgery. All patients underwent the ISTH-BAT score evaluation (ISTH-BAT 0: 85.9%; 1: 7.8%; 2: 4.7%; 3: 1.6%) (Table 1).

### Results

The group of patients who did not receive a prophylaxis, showed a higher tendency of bleeding (3/41: 7.3%), whereas in the groups of patients who received anti-haemorrhagic prophylaxis with rVIIa factor there were no bleeding episodes. Even if the bleeding is present only in the not-treated group, the difference between the two groups was not statistically significant (p= 0.29). Multivariate analysis revealed that preoperative dosages of Factor VII and hemoglobin (Hb) are two independent variables: patients subjected to preoperative treatment had a more significant Factor VII deficiency (30.9% ± 6.4) compared to patients not subjected to a prophylactic treatment (40.5% ± 6.5); this difference between the groups resulted to be statistically significant (p<0.0001). Moreover, patients subjected to prophylaxis had a lower Hb value than untreated patients, with a value of 12.1 +/- 1.3 gr/dl, which also resulted to be statistically significant (p<0.03).

**Copyright Information:** (Online) ISSN: 2572-9241
©2026 The Author(s). HemaSphere is published by John Wiley & Sons Ltd on behalf of European Hematology Association. This is an open access Abstract Book distributed under the Attribution-NonCommercial-NoDerivs (CC BY-NC-ND), which allows third parties to download the articles and share them with others as long as they credit the author and the Abstract Book, but they cannot change the content in any way or use them commercially.
**Abstract Book Citations:** Authors, Title, HemaSphere, 2026;10:(S1):pages. The abstract book DOIs can be found on the “Author Information” page.
**Disclaimer:** Articles published in the journal HemaSphere exclusively reflect the opinions of the authors. The authors are responsible for all content in their abstracts including accuracy of the facts, statements, citing resources, etc.

### Summary/Conclusion

Our study showed a high prevalence of perioperative bleeding (3/64: 4.7%), confirming the insidiousness of Factor VII deficiency. Both the approaches of keeping Factor VII available in the operating room in case of bleeding and the preventive treatment with rVIIa factor before surgery are effective and safe in children with mild Factor VII deficiency between 20% and 40%. Even if both the approaches can be considered valid, it is noteworthy that bleeding was observed only in the group of untreated patients. Our results, although not statistically significant due to the limited number of patients, seem to indicate that a prophylactic approach in patients with Factor VII value < 31% is preferable to a watchful waiting approach.

**Table 1. Characteristics of the study population**

| | Pts not undergoing FVII prophylaxis (n=41) | Pts undergoing FVII prophylaxis (n=23) |
|---|---|---|
| Age range (years) | 6.0 ± 3.5 | 5.7 ± 3.1 |
| Gender (male/female) | 26/15 | 16/7 |
| Type of surgery, no (%): | | |
| • adenoid and tonsil surgery | 23 (56.1) | 15 (65.2) |
| • urological surgery | 11 (26.8) | 3 (13) |
| • general surgery | 1 (2.4) | 2 (8.7) |
| • neurosurgery | 2 (4.9) | 1 (4.3) |
| • heart surgery | 1 (2.4) | 0 (0) |
| • high airway surgery | 1 (2.4) | 1 (4.3) |
| • toracic surgery | 0 (0) | 1 (4.3) |
| • ortopedic surgery | 2 (4.9) | 0 (0) |
| ISTH-BAT, no (%): | | |
| • 0 | 37 (90.2) | 18 (78.3) |
| • 1 | 3 (7.3) | 2 (8.7) |
| • 2 | 1 (2.4) | 2 (8.7) |
| • 3 | 0 (0) | 1 (4.3) |
| Perioperative bleeding, no (%) | 3 (7.3) | 0 (0) |

**Copyright Information:** (Online) ISSN: 2572-9241
©2026 The Author(s). HemaSphere is published by John Wiley & Sons Ltd on behalf of European Hematology Association. This is an open access Abstract Book distributed under the Attribution-NonCommercial-NoDerivs (CC BY-NC-ND), which allows third parties to download the articles and share them with others as long as they credit the author and the Abstract Book, but they cannot change the content in any way or use them commercially.
**Abstract Book Citations:** Authors, Title, HemaSphere, 2026;10:(S1):pages. The abstract book DOIs can be found on the "Author Information" page.
**Disclaimer:** Articles published in the journal HemaSphere exclusively reflect the opinions of the authors. The authors are responsible for all content in their abstracts including accuracy of the facts, statements, citing resources, etc.

### (PF1363) CONGENITAL FACTOR VII DEFICIENCY: LESSONS FROM CLINICAL, LABORATORY, AND GENETIC DATA

**Topic:** 34. Bleeding disorders (congenital and acquired)

Carina Brito*[1], Miguel de Oliveira[1], Maria Pereira Coutinho[1], Fernanda Leite[1], Eugénia Cruz[1], Sara Morais[1]

[1] *Unidade Local de Saúde de Santo António, Centro de Coagulopatias Congénitas, Unidade de Trombose e Hemostase, Serviço de Imuno-hemoterapia, Porto, Portugal;*

### Background

Congenital factor VII (FVII) deficiency is a rare and clinically heterogeneous coagulopathy, with a weak correlation between plasma levels and bleeding risk. Levels above 5% are generally considered to be hemostatic and adequate to prevent major bleeding. Comprehensive clinical and laboratory characterization is essential to understand this variability and guide management.

### Aims

To describe a cohort of patients with FVII deficiency followed at our center, assessing the relationship between laboratory parameters and clinical phenotype.

### Methods

Retrospective analysis of patients with FVII deficiency using electronic medical records. Data are reported as median (range).

### Results

Forty patients were included [24 men, 16 women], median age 29 years (4–80), median follow-up 5 years (0–45). Median FVII level was 24% (<1 - 50%) [4 patients ≤1%, 1 patient >1 - ≤10%, 11 patients >10 - ≤20%, 24 patients >20%], with median age at diagnosis of 16 years (3-79). Bleeding symptoms occurred in 32%, most commonly epistaxis (46%) and abnormal uterine bleeding (AUB) (46%), the latter affecting 78% of symptomatic women. Events were more frequent in patients with FVII ≤1% (43%) and >10 - ≤20% (38%) but were mostly minor and managed with oral antifibrinolytics; only 4 patients had abnormal ISTH-BAT scores. One severe event - traumatic intracranial hemorrhage - occurred in a patient with FVII ≤1%. Two patients (one with epistaxis, one with AUB and dental extraction) required red blood cell transfusion, both with FVII ≤1%. A total of 37 major and 18 minor surgeries were performed. Recombinant FVIIa was used in 4 major surgeries (3 patients ≤1%, 1 patient >10 - ≤20%) and 2 minor surgeries (both ≤1%); all others were managed with antifibrinolytics in patients with FVII >1%. No perioperative bleeding complications were reported. FVIIa was also administered in 3 patients with FVII ≤1% (hemarthrosis, intracranial hemorrhage, and postpartum AUB). Genetic testing was performed in 17 patients, identifying pathogenic variants in 15, predominantly exon 9 mutations (12 patients). All symptomatic patients with identified mutations carried exon 9 variants.

### Summary/Conclusion

This cohort highlights the marked heterogeneity of FVII deficiency and the weak overall correlation between plasma levels and bleeding phenotype. Although very low FVII levels increase bleeding risk - mostly minor - the variability underscores the need for individualized clinical assessment. The absence of perioperative complications, even in patients undergoing multiple surgeries, demonstrates the effectiveness of tailored prophylactic strategies. These findings emphasize the importance of personalized risk stratification, optimized perioperative management, and timely access to specific hemostatic therapies to ensure safe, effective care.

**Copyright Information:** (Online) ISSN: 2572-9241
©2026 The Author(s). HemaSphere is published by John Wiley & Sons Ltd on behalf of European Hematology Association. This is an open access Abstract Book distributed under the Attribution-NonCommercial-NoDerivs (CC BY-NC-ND), which allows third parties to download the articles and share them with others as long as they credit the author and the Abstract Book, but they cannot change the content in any way or use them commercially.
**Abstract Book Citations:** Authors, Title, HemaSphere, 2026;10:(S1):pages. The abstract book DOIs can be found on the "Author Information" page.
**Disclaimer:** Articles published in the journal HemaSphere exclusively reflect the opinions of the authors. The authors are responsible for all content in their abstracts including accuracy of the facts, statements, citing resources, etc.

### (PF1364) PROPHYLACTIC ANTICOAGULATION IN PREGNANT WOMEN WITH HEREDITARY THROMBOPHILIA AND OBSTETRIC COMPLICATIONS: SYSTEMATIC REVIEW OF THE LITERATURE

**Topic:** 34. Bleeding disorders (congenital and acquired)

Peterson Gontijo*[1, 2], Glaciano Ribeiro[2], Marcia T Delamain[1], Sabrina Brandi[1], Sophia Gonca;ves[1]

*[1]Ciencias Medicas de Minas Gerais, Hematology, Belo Horizonte, Brazil;[2]Grupo Pulsa, Hematology, Belo Horizonte, Brazil;*

### Background

Hereditary thrombophilia is associated with obstetric complications, but the benefit of prophylactic anticoagulation during pregnancy remains controversial. International guidelines present divergent recommendations, especially for pregnant women with hereditary thrombophilia and a history of obstetric complications, in the absence of previous venous thromboembolism. The scarcity of studies targeting this population contributes to uncertainties in clinical practice.

### Aims

To evaluate whether prophylactic anticoagulation during pregnancy reduces maternal or fetal outcomes in pregnant women with hereditary thrombophilia and a history of obstetric complications, compared to non-use

### Methods

Systematic review according to the PRISMA protocol, with searches in the PubMed/MEDLINE, Cochrane Library, LILACS, and SciELO databases, using MeSH, DeCS descriptors, and free terms. Randomized clinical trials, cohort studies, and case-control studies published in the last 10 years in English, Portuguese, or Spanish were included. Pregnant women with hereditary thrombophilia, without previous venous thromboembolism, and with pregnancy loss or other obstetric complications were included. Isolated acquired thrombophilia, previous venous thromboembolism, systematic reviews, case reports, and studies that did not evaluate maternal or fetal outcomes were excluded. Data selection and extraction were performed by two independent reviewers.

### Results

Five studies were included, four cohort studies and one randomized clinical trial. The cohort studies suggested an association between low molecular weight heparin and improved fetal outcomes, mainly a reduction in pregnancy loss. However, they had methodological limitations, such as small sample size and lack of randomization. The most recent randomized clinical trial did not demonstrate a benefit in reducing maternal or fetal outcomes, including the live birth rate.

### Summary/Conclusion

Although observational studies suggest a possible fetal benefit of prophylactic anticoagulation in pregnant women with hereditary thrombophilia and a history of obstetric complications, this effect has not been confirmed by randomized clinical trials. Therefore, there is no consistent scientific support for the systematic indication of prophylactic anticoagulation in this population, reinforcing the need for an individualized clinical approach and risk profile assessment, in addition to new well-designed clinical trials.

**Copyright Information:** (Online) ISSN: 2572-9241
©2026 The Author(s). HemaSphere is published by John Wiley & Sons Ltd on behalf of European Hematology Association. This is an open access Abstract Book distributed under the Attribution-NonCommercial-NoDerivs (CC BY-NC-ND), which allows third parties to download the articles and share them with others as long as they credit the author and the Abstract Book, but they cannot change the content in any way or use them commercially.
**Abstract Book Citations:** Authors, Title, HemaSphere, 2026;10:(S1):pages. The abstract book DOIs can be found on the "Author Information" page.
**Disclaimer:** Articles published in the journal HemaSphere exclusively reflect the opinions of the authors. The authors are responsible for all content in their abstracts including accuracy of the facts, statements, citing resources, etc.

### (PF1365) ELICITING EXPERT RECOMMENDATIONS ON THE DOSING OF FACTOR REPLACEMENT THERAPY WITH MARSTACIMAB FOR BREAKTHROUGH BLEEDING & PERIOPERATIVE SITUATIONS IN HAEMOPHILIA: INTERIM RESULTS OF A DELPHI STUDY

**Topic:** 34. Bleeding disorders (congenital and acquired)

Danielle Riley[1], Charlotte Oswald[1], Luis Val-Maranes[1], Lucy Nuttall[1], Priya Patel[2], Leila Lackey[2], Jennifer Schumacher[2], Hae Kyung Kim*[3]

*[1]Adelphi Values PROVE, Bollington, United Kingdom; [2]Pfizer, New York, United States of America; [3]Pfizer, Madrid, Spain;*

### Background

Marstacimab, an anti-Tissue Factor Pathway Inhibitor (TFPI) monoclonal antibody, has been approved for routine prophylaxis to prevent or reduce the frequency of bleeding episodes in adult and adolescent patients aged ≥12 years with haemophilia A and B without inhibitors. The use of anti-TFPI products has been associated with the development of thromboembolic events in patients exposed to additional haemostatic agents. While clinical trials of marstacimab have shown reductions in bleeding episodes, breakthrough bleeding management (BTB) during the pivotal study was approached pragmatically, with investigators instructed to use the lowest effective dose of a product to manage bleeding. However, this approach yielded variability in terms of clinical practice and product availability. Therefore, standardised guidance using empirical evidence from these trials and real-world experience (RW)was warranted.

### Aims

Building on data from pivotal studies, as well as panellists' clinical experience with marstacimab, this study seeks to elicit expert recommendations on the dosing, frequency, and duration of haemostatic agents when used with marstacimab for the management of BTB and surgical situations in patients with haemophilia.

### Methods

Haematologists were recruited globally based on pre-defined screening criteria, including clinical experience with marstacimab or subject-matter expertise in haemophilia, to participate in a double-blinded modified Delphi comprising two survey rounds and an online consensus meeting (**Fig 1**). The Delphi methodology allows iterative consensus generation, with consensus defined as ≥80% agreement. The first-round was used to elicit recommended ranges for dosing, frequency and duration for BTB management (mild, moderate and severe) and in surgical situations (minor vs major). Responses were analysed descriptively and are presented as median (interquartile range, IQR). Subsequent Delphi rounds will seek to further refine recommended dosing and duration ranges, explore additional scenarios (i.e., extended half-life use, other haemostatic agents), and elicit consensus recommendations.

**Copyright Information:** (Online) ISSN: 2572-9241
©2026 The Author(s). HemaSphere is published by John Wiley & Sons Ltd on behalf of European Hematology Association. This is an open access Abstract Book distributed under the Attribution-NonCommercial-NoDerivs (CC BY-NC-ND), which allows third parties to download the articles and share them with others as long as they credit the author and the Abstract Book, but they cannot change the content in any way or use them commercially.
**Abstract Book Citations:** Authors, Title, HemaSphere, 2026;10:(S1):pages. The abstract book DOIs can be found on the "Author Information" page.
**Disclaimer:** Articles published in the journal HemaSphere exclusively reflect the opinions of the authors. The authors are responsible for all content in their abstracts including accuracy of the facts, statements, citing resources, etc.

### Results

Interim responses from the first-round survey on clinical considerations and recommended dosing, frequency, and duration of standard half-life factor replacement therapies (FRT) for managing BTB events and in surgical situations in non-inhibitor patients with haemophilia are presented in Table 1.

**Table 1. Preliminary recommended dosing and duration ranges among non-inhibitor patients; first-round Delphi panel survey responses (N=16)**

| On-demand FRT (SHL) | Median minimum and maximum dose, IU/kg | IQR for minimum and maximum dose combined, IU/kg | Median duration of treatment range, hours |
|---|---|---|---|
| **BTB** | | | |
| **Mild** | | | |
| FVIII | 15–30 | 15–30 | 24–48 |
| FIX | 25–40 | 25–40 | 24–48 |
| **Moderate** | | | |
| FVIII | 20–40 | 20–40 | 24–72 |
| FIX | 30–50 | 30–50 | 24–72 |
| **Severe** | | | |
| FVIII | 35–50 | 30–50 | 60–120 |
| FIX | 50–75 | 40–80 | 48–120 |
| **Surgical situations** | | | |
| **Minor** | | | |
| FVIII | 20–30 | 20–30 | 24–48 |
| FIX | 30–50 | 22.5–50 | 24–36 |
| **Major** | | | |
| FVIII | 35–50 | 28.75–50 | 48–96 |
| FIX | 35–60 | 30–80 | 48–108 |

FRT: Factor replacement therapy; IQR: Interquartile Range. SHL: standard half-life. FVIII: Factor VIII; FIX: Factor IX.

### Summary/Conclusion

Preliminary findings provide early insights into dosing recommendations for SHL FRT with marstacimab in the management of BTB and perioperative situations. The dosing ranges recommended in the first-round survey align with management practices observed in marstacimab clinical studies.

**Copyright Information:** (Online) ISSN: 2572-9241
©2026 The Author(s). HemaSphere is published by John Wiley & Sons Ltd on behalf of European Hematology Association. This is an open access Abstract Book distributed under the Attribution-NonCommercial-NoDerivs (CC BY-NC-ND), which allows third parties to download the articles and share them with others as long as they credit the author and the Abstract Book, but they cannot change the content in any way or use them commercially.
**Abstract Book Citations:** Authors, Title, HemaSphere, 2026;10:(S1):pages. The abstract book DOIs can be found on the "Author Information" page.
**Disclaimer:** Articles published in the journal HemaSphere exclusively reflect the opinions of the authors. The authors are responsible for all content in their abstracts including accuracy of the facts, statements, citing resources, etc.

**Fig 1. Modified Delphi Study Design involving two survey rounds followed by an online consensus meeting to iteratively explore and elicit consensus recommendations**

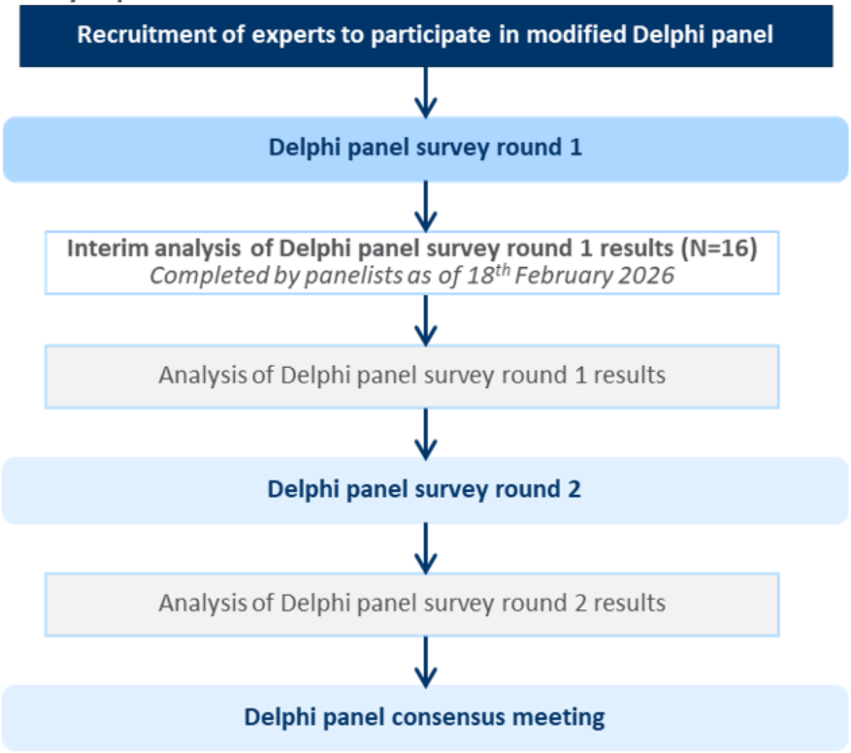

**Copyright Information:** (Online) ISSN: 2572-9241
©2026 The Author(s). HemaSphere is published by John Wiley & Sons Ltd on behalf of European Hematology Association. This is an open access Abstract Book distributed under the Attribution-NonCommercial-NoDerivs (CC BY-NC-ND), which allows third parties to download the articles and share them with others as long as they credit the author and the Abstract Book, but they cannot change the content in any way or use them commercially.
**Abstract Book Citations:** Authors, Title, HemaSphere, 2026;10:(S1):pages. The abstract book DOIs can be found on the "Author Information" page.
**Disclaimer:** Articles published in the journal HemaSphere exclusively reflect the opinions of the authors. The authors are responsible for all content in their abstracts including accuracy of the facts, statements, citing resources, etc.

### (PF1366) FUNCTIONAL OUTCOMES IN PEDIATRIC HEMOPHILIA A: EVALUATING THE PEDHAL SCORE AND ITS DETERMINANTS IN A DEVELOPING HEALTHCARE SETTING

**Topic:** 34. Bleeding disorders (congenital and acquired)

Mohamed Elshinawy*[1], Noor Elshinawy[2], Niveen Alansary[3]

*[1]Faculty of Medicine, University of Alexandria, Pediatric Hematology, Alexandria, Egypt;[2]Faculty of Medicine, University of Alexandria, Alexandria, Egypt; [3]Medical Research Institute, University of Alexandria, Clinical Hematology, Alexandria, Egypt;*

### Background

Historically, pediatric patients with hemophilia A in developing nations have faced significant barriers to optimal care. However, as replacement therapies become increasingly accessible, there is a visible shift toward improved functional outcomes.

### Aims

This study aims to evaluate the Pediatric Hemophilia Activities List (PedHAL) score in children with hemophilia A and identify the primary factors influencing this score.

### Methods

An 18-month cross-sectional study (July 2024 – December 2025) was conducted, involving all patients aged 4–18 years attending our pediatric hematology clinic. Functional assessment was performed using the 22-item $PedHAL_{short}$. Despite being concise, this version retains all seven functional domains of the original 53-item questionnaire. The study adhered to ethical standards, with informed consent obtained from all participants or guardians.

### Results

Of the 102 patients recruited, 91 provided complete questionnaire data. The cohort consisted of 65 severe and 26 moderate hemophilia A cases. Patients exhibited significantly lower $PedHAL_{short}$ scores compared to healthy controls (69.1±8.4 vs. 97.4±2.2), $p < 0.0001$. The domains (leisure activities & sports, Sitting/kneeling/standing and leg functions) had the lowest average scores (44.3, 51.1 and 54.6 respectively). Multiple regression analysis identified adherence to prophylaxis in severe cases as the strongest predictor of superior functional scores (B = 0.62, p = 0.005). Additionally, negative inhibitor status, absence of target joints, and higher socioeconomic standing were significantly associated with better outcomes (p = 0.012, 0.016 and 0.041 respectively). Conversely, age of bleeding onset, family history, and the use of recombinant products showed no significant correlation with PedHAL score.

### Summary/Conclusion

Children with hemophilia A in our population demonstrate significantly impaired functional scores. Factors ensuring consistent hemostasis—specifically prophylaxis adherence and socioeconomic stability—are critical for better outcomes. To optimize care, it is essential to establish a National Hemophilia Registry and foster collaborations with international healthcare bodies.

**Copyright Information:** (Online) ISSN: 2572-9241
©2026 The Author(s). HemaSphere is published by John Wiley & Sons Ltd on behalf of European Hematology Association. This is an open access Abstract Book distributed under the Attribution-NonCommercial-NoDerivs (CC BY-NC-ND), which allows third parties to download the articles and share them with others as long as they credit the author and the Abstract Book, but they cannot change the content in any way or use them commercially.
**Abstract Book Citations:** Authors, Title, HemaSphere, 2026;10:(S1):pages. The abstract book DOIs can be found on the "Author Information" page.
**Disclaimer:** Articles published in the journal HemaSphere exclusively reflect the opinions of the authors. The authors are responsible for all content in their abstracts including accuracy of the facts, statements, citing resources, etc.

### (PF1367) COMPARATIVE EFFECTIVENESS AND SAFETY OF DIRECT ORAL ANTICOAGULANTS VERSUS WARFARIN IN PATIENTS WITH ATRIAL FIBRILLATION AND HEMATOLOGIC MALIGNANCY: A REAL-WORLD COHORT STUDY

**Topic:** 35. Thrombosis and vascular biology

Cher Ying Foo*[1], Pallavi Matai[2], Samuel Asirvatham[3], Peter Noseworthy[3], Ojasav Sehrawat[3]

*[1]Rochester General Hospital, Rochester, United States of America;[2]UPMC Harrisburg, Internal Medicine, Harrisburg, United States of America; [3]Mayo Clinic, Rochester, United States of America;*

### Background

Patients with hematologic malignancies and atrial fibrillation (AF) face competing risks of thromboembolism and bleeding, complicating anticoagulation decisions. While direct oral anticoagulants (DOACs) show favorable outcomes in mixed cancer populations, hematologic malignancies remain underrepresented. This is the first study comparing DOAC versus warfarin outcomes specifically in this population.

### Aims

To compare the effectiveness and safety of DOACs versus warfarin in patients with AF and hematologic malignancies using real-world data.

### Methods

We conducted a retrospective cohort study using the TriNetX Global Collaborative Network. Adults with AF or atrial flutter, a hematologic malignancy (leukemias, lymphomas, multiple myeloma, myelodysplastic syndromes, or myeloproliferative neoplasms), and newly initiated oral anticoagulation were included. Patients with a history of mechanical heart valve, moderate-to-severe mitral stenosis, prior venous thromboembolism, or any prior occurrence of a study outcome were excluded. Cohort A received a DOAC (apixaban, rivaroxaban, dabigatran, or edoxaban); Cohort B received warfarin. Propensity score matching (1:1, nearest neighbor) adjusted for demographics, cardiovascular comorbidities, malignancy subtype, cytopenias, chemotherapy, immunotherapy, transfusions, antiplatelet agents, and estimated glomerular filtration rate. Follow-up was 5 years from index. The primary outcome was a composite of ischemic stroke, intracranial hemorrhage, or arterial embolism. Secondary outcomes included individual components of the primary composite and major gastrointestinal bleeding.

### Results

Among 52,373 eligible patients, 10,567 initiated warfarin and 41,806 initiated DOACs. After 1:1 matching, 10,254 patients remained per cohort with good covariate balance (standardized mean differences <0.10 for most variables). Warfarin was associated with significantly higher risk of the primary composite outcome compared with DOACs (4.7% vs 2.0%; HR 2.08, 95% CI 1.76–2.45; p<0.001). Ischemic stroke rates were 2.4% vs 0.7% (HR 3.13, 95% CI 2.39–4.09; p<0.001), arterial embolism 0.6% vs 0.2% (HR 2.84, 95% CI 1.74–4.65; p<0.001), and intracranial hemorrhage 2.0% vs 1.3% (HR 1.41, 95% CI 1.13–1.75; p=0.002), all favoring DOACs. Major GI bleeding was comparable between groups (14.0% vs 12.5%; HR 1.01, 95% CI 0.94–1.10; p=0.75). On subgroup analysis, warfarin was associated with higher composite outcome risk across key subgroups including females (HR 2.34, 95% CI 1.79–3.07), males (HR 2.70, 95% CI 1.90–3.82), age <75 years (HR 1.80, 95% CI 1.23–2.62), age ≥75 years (HR 1.72, 95% CI 1.45–2.05), BMI ≥30 (HR 2.04, 95% CI 1.60–2.60), thrombocytopenia (HR 1.49, 95% CI 1.05–2.11), and CKD stage ≥3 (HR 1.90, 95% CI 1.53–2.37), all p<0.05.

**Copyright Information:** (Online) ISSN: 2572-9241
©2026 The Author(s). HemaSphere is published by John Wiley & Sons Ltd on behalf of European Hematology Association. This is an open access Abstract Book distributed under the Attribution-NonCommercial-NoDerivs (CC BY-NC-ND), which allows third parties to download the articles and share them with others as long as they credit the author and the Abstract Book, but they cannot change the content in any way or use them commercially.
**Abstract Book Citations:** Authors, Title, HemaSphere, 2026;10:(S1):pages. The abstract book DOIs can be found on the “Author Information” page.
**Disclaimer:** Articles published in the journal HemaSphere exclusively reflect the opinions of the authors. The authors are responsible for all content in their abstracts including accuracy of the facts, statements, citing resources, etc.

**Summary/Conclusion**

In the largest dedicated cohort to date of patients with AF and hematologic malignancies, warfarin was associated with substantially higher thromboembolic and intracranial hemorrhage risk compared with DOACs, without a meaningful difference in major bleeding, consistently across key clinical subgroups. These real-world findings support DOACs as the preferred anticoagulation strategy in this high-risk population.

**Copyright Information:** (Online) ISSN: 2572-9241
©2026 The Author(s). HemaSphere is published by John Wiley & Sons Ltd on behalf of European Hematology Association. This is an open access Abstract Book distributed under the Attribution-NonCommercial-NoDerivs (CC BY-NC-ND), which allows third parties to download the articles and share them with others as long as they credit the author and the Abstract Book, but they cannot change the content in any way or use them commercially.
**Abstract Book Citations:** Authors, Title, HemaSphere, 2026;10:(S1):pages. The abstract book DOIs can be found on the "Author Information" page.
**Disclaimer:** Articles published in the journal HemaSphere exclusively reflect the opinions of the authors. The authors are responsible for all content in their abstracts including accuracy of the facts, statements, citing resources, etc.

### (PF1368) EFFECTIVENESS AND SAFETY OF PRIMARY THROMBOPROPHYLAXIS IN ANTIPHOSPHOLIPID ANTIBODY CARRIERS ACCORDING TO THE SEROLOGICAL 2023 ACR/EULAR DOMAINS

**Topic:** 35. Thrombosis and vascular biology

Rodrigo Cantera Estefanía*[1], Rafael Gálvez[2], Irene Gorostidi Álvarez[3], Héctor Cruz Barquín[3], Raquel García Ruiz[3], Marina Herrero López[3], Jose Flores García[3], Maria Oviedo Madrid[3], María Abando Casuso[3], Ariadna García Ascacíbar[4], Ligia Gabrie Rodríguez[2], Lucrecia Yáñez[3], Marcos López Hoyos[5], Juan José Domínguez-García[3], María Luisa González Ponte[3], Belén González-Mesones Galán[3], Gala Aglaia Méndez Navarro[3], Juan Irure[5], Jose Luis Hernández Hernández[6], Victor Manuel Martínez Taboada[2]

*[1]Hospital Universitario de Navarra, Hematology and Hemotherapy, Pamplona, Spain;[2]Hospital Universitario Marqués de Valdecilla, Rheumatology, Santander, Spain; [3]Hospital Universitario Marqués de Valdecilla, Hematology and Hemotherapy, Santander, Spain; [4]Hospital Universitario Araba, Hematology and Hemotherapy, Vitoria, Spain; [5]Hospital Universitario Marqués de Valdecilla, Immunology, Santander, Spain; [6]Hospital Universitario Marqués de Valdecilla, Internal Medicine, Santander, Spain;*

### Background

The 2023 ACR/EULAR APS criteria reduced IgM weighting and introduced a category for isolated lupus anticoagulant, assigning one point per laboratory domain and creating uncertainty regarding primary thromboprophylaxis in aPL carriers with <3 points, while also defining a new thrombotic risk score.

### Aims

Evaluate effectiveness and safety of primary thromboprophylaxis and assess of the new ACR/EULAR thrombotic risk score.

### Methods

All asymptomatic carriers with confirmed lupus anticoagulant (LA), anticardiolipin (aCL), or anti-β2-glycoprotein I (anti-β2GPI) antibodies between January 2018 and March 2024, except for isolated LA, in accordance with the 2023 ACR/EULAR criteria. Primary outcomes were thrombotic-free survival (TFS), grade ≥3 bleeding-free survival (BFS), and bleeding events per ISTH criteria. Logistic and Cox models were used, and the Altman–Andersen method estimated 5-year NNT and NNH when statistically significant.

**Copyright Information:** (Online) ISSN: 2572-9241
©2026 The Author(s). HemaSphere is published by John Wiley & Sons Ltd on behalf of European Hematology Association. This is an open access Abstract Book distributed under the Attribution-NonCommercial-NoDerivs (CC BY-NC-ND), which allows third parties to download the articles and share them with others as long as they credit the author and the Abstract Book, but they cannot change the content in any way or use them commercially.
**Abstract Book Citations:** Authors, Title, HemaSphere, 2026;10:(S1):pages. The abstract book DOIs can be found on the "Author Information" page.
**Disclaimer:** Articles published in the journal HemaSphere exclusively reflect the opinions of the authors. The authors are responsible for all content in their abstracts including accuracy of the facts, statements, citing resources, etc.

**Results**

A total of 985 patients were analysed (main characteristics in table 1) with a median follow-up of 2.84 [IQR 1,12-4,74] years. Incidence of venous (VTE) and arterial (ATE) thromboses were 4.6% and 4.1%, respectively. Among all patients, acetylsalicylic acid (ASA) improved TFS (HR 0.51; *p*=0.001) revealing a 5-years NNT of 24.9 patients, without affecting BFS (HR 0.96;*p*=0.81). P2Y12 inhibitors were associated with higher ATE (OR 13.17; *p*<0.001) and minor bleeding (OR 4.875; *p*=0.007) with a 5-year NNH of 6.6 patients.
Among 458 carriers with <3 laboratory points, ASA remained protective (HR 0.53;*p*=0.047, NNT 23.2 patients) without increasing minor or major bleeding (image 1). P2Y12 inhibitors showed no benefit in TFS and were linked to decreased BFS (HR 4.211; p=0.008, NNH 5.6 patients).
The 2023 ACR/EULAR high-risk VTE profile was associated with VTE (OR 2.416; p=0.005) and worse TFS (HR 2.378; p<0.001). In contrast, high risk ATE profile showed non-significant trend toward association with ATE (OR 1.926, p=0.075, HR 1.436; p=0.094). Hypertension <180/110 mmHg was associated with ATE (OR 2.18; p=0.045), while hospitalization with complete immobilization was associated with VTE (OR 3.355; p=0.042). Regarding combined TFS, the following factors were associated with worse outcomes: hypertension <180/110 mmHg (HR 1.659; p=0.047), active cancer (HR 3.038; p<0.001), and hospitalization with partial immobilization (HR 2.566; p=0.021).

Table1. Main characteristics of included patients.

| | | Total aPL carriers N=985 | | <3 points carriers N=458 | |
|---|---|---|---|---|---|
| | | N | % | N | % |
| Median age [IQR], years | | 53.0 [41–65] | | 53 [40.5-65.5] | |
| Female sex | | 686 | 69.6 | 327 | 71.4 |
| **D7** | Isolated LA | 232 | 23.6 | 187 | 40.8 |
| | Persistent LA | 354 | 35.9 | 0 | 0.0 |
| **D8** | lgM high/moderate titers (aCL or aβ2gpI) | 410 | 41.6 | 307 | 67.0 |
| | lgG moderate titers (aCL and/or aβ2gpI) | 57 | 5.8 | 0 | 0.0 |
| | lgG high titers (aCL or aβ2gpI) | 100 | 10.2 | 0 | 0.0 |
| | lgG high titers (aCL and aβ2gpI) | 72 | 7.3 | 0 | 0.0 |
| **Treatment** | Anticoagulant therapy | 5 | 0.7 | 2 | 0.4 |
| | VKA | 4 | 0.4 | 2 | 0.4 |
| | DOACs | 0 | 0.0 | 0 | 0.0 |
| | LMWH | 1 | 0.1 | 0 | 0.0 |
| | Antiagregated | 612 | 62.1 | 247 | 53.9 |
| | ASA | 601 | 61.0 | 241 | 52.6 |
| | P2Y12 inhibitors | 17 | 1.7 | 8 | 1.7 |

D: domain. DM: diabetes mellitus. DOACs: direct oral anticoagulants. LMWH: low molecular weight heparins. VKA: vitamin K antagonists.

**Summary/Conclusion**

Low-dose aspirin may lower thrombotic risk without increasing bleeding in asymptomatic aPL carriers, even in those with <3 laboratory points. P2Y12 inhibitors demonstrated reduced efficacy and an increased risk of bleeding, warranting confirmation in future studies. High risk VTE profiles are associated with first episode of VTE.

**Copyright Information:** (Online) ISSN: 2572-9241
©2026 The Author(s). HemaSphere is published by John Wiley & Sons Ltd on behalf of European Hematology Association. This is an open access Abstract Book distributed under the Attribution-NonCommercial-NoDerivs (CC BY-NC-ND), which allows third parties to download the articles and share them with others as long as they credit the author and the Abstract Book, but they cannot change the content in any way or use them commercially.
**Abstract Book Citations:** Authors, Title, HemaSphere, 2026;10:(S1):pages. The abstract book DOIs can be found on the "Author Information" page.
**Disclaimer:** Articles published in the journal HemaSphere exclusively reflect the opinions of the authors. The authors are responsible for all content in their abstracts including accuracy of the facts, statements, citing resources, etc.

**Image 1.** Forest plots of regression models among <3 points carriers.

**A)** Logistic regression for VTE. **B)** Logistic regression for ATE. **C)** Cox regression for TFS. **D)** Logistic regression for minor bleeding. **E)** Logistic regression for major bleeding. **F)** Cox regression for BFS.

*OR: odds ratio. HR: hazard ratio. CI: confidence interval.*

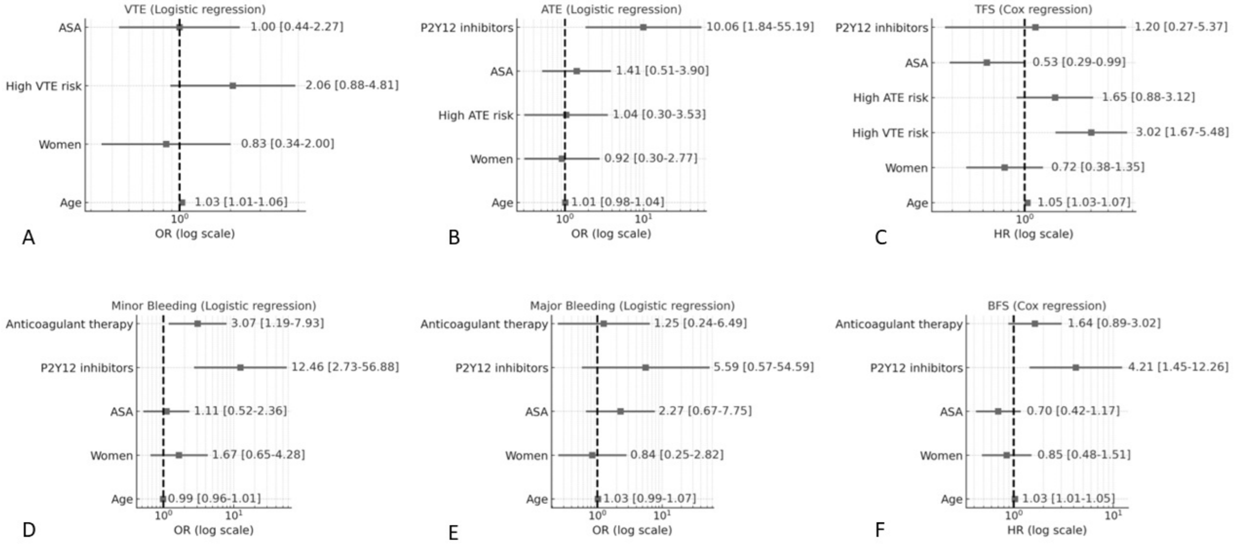

**Copyright Information:** (Online) ISSN: 2572-9241
©2026 The Author(s). HemaSphere is published by John Wiley & Sons Ltd on behalf of European Hematology Association. This is an open access Abstract Book distributed under the Attribution-NonCommercial-NoDerivs (CC BY-NC-ND), which allows third parties to download the articles and share them with others as long as they credit the author and the Abstract Book, but they cannot change the content in any way or use them commercially.
**Abstract Book Citations:** Authors, Title, HemaSphere, 2026;10:(S1):pages. The abstract book DOIs can be found on the "Author Information" page.
**Disclaimer:** Articles published in the journal HemaSphere exclusively reflect the opinions of the authors. The authors are responsible for all content in their abstracts including accuracy of the facts, statements, citing resources, etc.

### (PF1369) REAL-WORLD THROMBOTIC OUTCOMES AND MANAGEMENT STRATEGIES IN SEVERE SECONDARY ERYTHROCYTOSIS: A RETROSPECTIVE COHORT STUDY

**Topic:** 35. Thrombosis and vascular biology

Shamsher Sandhu*[1], Abdulla Ahmed[1], Elia Arbana[1], Benjamin Chin-Yee[2, 3], Cyrus Hsia[2, 3]

[1]*Western University, Schulich School of Medicine and Dentistry, London, Canada;*[2]*London Health Sciences Centre, Division of Hematology, Department of Medicine, London, Canada;* [3]*London Health Sciences Centre, Department of Pathology and Laboratory Medicine, London, Canada;*

### Background

Secondary erythrocytosis (SE) is a common condition encountered in hematology clinics, with a subset of patients presenting with severe SE, hemoglobin (Hb) ≥ 200 g/L. Uncertainty remains regarding risk factors for severe SE and whether, compared to mild or moderate SE, severe SE is associated with higher rates of arterial and venous thrombosis. Moreover, there are no clear management guidelines for severe SE, owing to lack of evidence.

### Aims

To conduct a retrospective cohort study to assess risk factors for severe SE, its association with thrombotic outcomes, and management strategies in comparison to patients with mild or moderate SE.

### Methods

We conducted a retrospective cohort study at an academic tertiary care centre in Ontario, Canada. All consecutive patients with erythrocytosis (Hb ≥160 g/L females; Hb ≥165 g/L males) who underwent next-generation sequencing which confirmed absence of pathogenic *JAK2* mutations and who were not diagnosed with a myeloproliferative neoplasm were included. Eligible patients were divided into two groups: severe SE (Hb ≥ 200 g/L), and mild/moderate SE (Hb ≥165 g/L males;≥160 g/L females and <200 g/L throughout follow-up). The first documented elevated hemoglobin meeting inclusion criteria was designated time 0. The primary outcome was the occurrence of arterial or venous thrombosis, independently confirmed by two physician reviewers. Secondary analyses compared baseline demographic and clinical risk factors for SE, and management strategies between cohorts. Thrombosis-free survival was assessed using survival analysis, and thrombosis rates were compared between groups. Risk factor analysis was performed using chi-square testing to identify variables associated with severe SE.

### Results

A total of 581 patients with SE were included, comprising 66 patients (92.4% male) with severe SE and 515 patients (72.2% male) with mild/moderate SE. Compared to patients with mild/moderate SE, patients with severe SE had significantly higher rates of smoking (67% vs 51%; p=0.01), chronic obstructive pulmonary disease (COPD, 24% vs. 14%; p=0.025), and androgen use (17% vs 9%; p=0.034). Over median follow-up of 7.12 and 6.18 years, thrombotic events occurred in 11 patients and 66 patients in the severe and mild/moderate SE group, respectively, with corresponding incidence rates of 1.94 and 1.23 events per 100 patient-years. Kaplan-Meier estimates of thrombosis-free survival were similar between groups at 1, 3, and 5 years following diagnosis. Cox proportional hazards analysis showed no significant association between severe SE and thrombosis-free survival (HR 1.26, 95% CI 0.66-2.40; p=0.477), with no violation of proportional hazards assumption (p=0.30). Compared to mild/moderate SE, patients with severe SE were more likely to receive phlebotomy (41% vs. 9%; p<0.001) and antiplatelet therapy (50% vs. 35%; p=0.02).

**Copyright Information:** (Online) ISSN: 2572-9241
©2026 The Author(s). HemaSphere is published by John Wiley & Sons Ltd on behalf of European Hematology Association. This is an open access Abstract Book distributed under the Attribution-NonCommercial-NoDerivs (CC BY-NC-ND), which allows third parties to download the articles and share them with others as long as they credit the author and the Abstract Book, but they cannot change the content in any way or use them commercially.
**Abstract Book Citations:** Authors, Title, HemaSphere, 2026;10:(S1):pages. The abstract book DOIs can be found on the "Author Information" page.
**Disclaimer:** Articles published in the journal HemaSphere exclusively reflect the opinions of the authors. The authors are responsible for all content in their abstracts including accuracy of the facts, statements, citing resources, etc.

### Summary/Conclusion

In this single-centre retrospective cohort study, severe SE was not associated with higher rates of thrombosis or reduced thrombosis-free survival compared to mild/moderate SE. Smoking, COPD, and androgen use were associated with development of severe SE. Patients with severe SE were more likely to receive phlebotomy and antiplatelet therapy; whether these interventions attenuated thrombotic risk in this group remains uncertain. These findings underscore the need for prospective studies to clarify thrombotic risk and establish evidence-based management strategies for patients with severe SE.

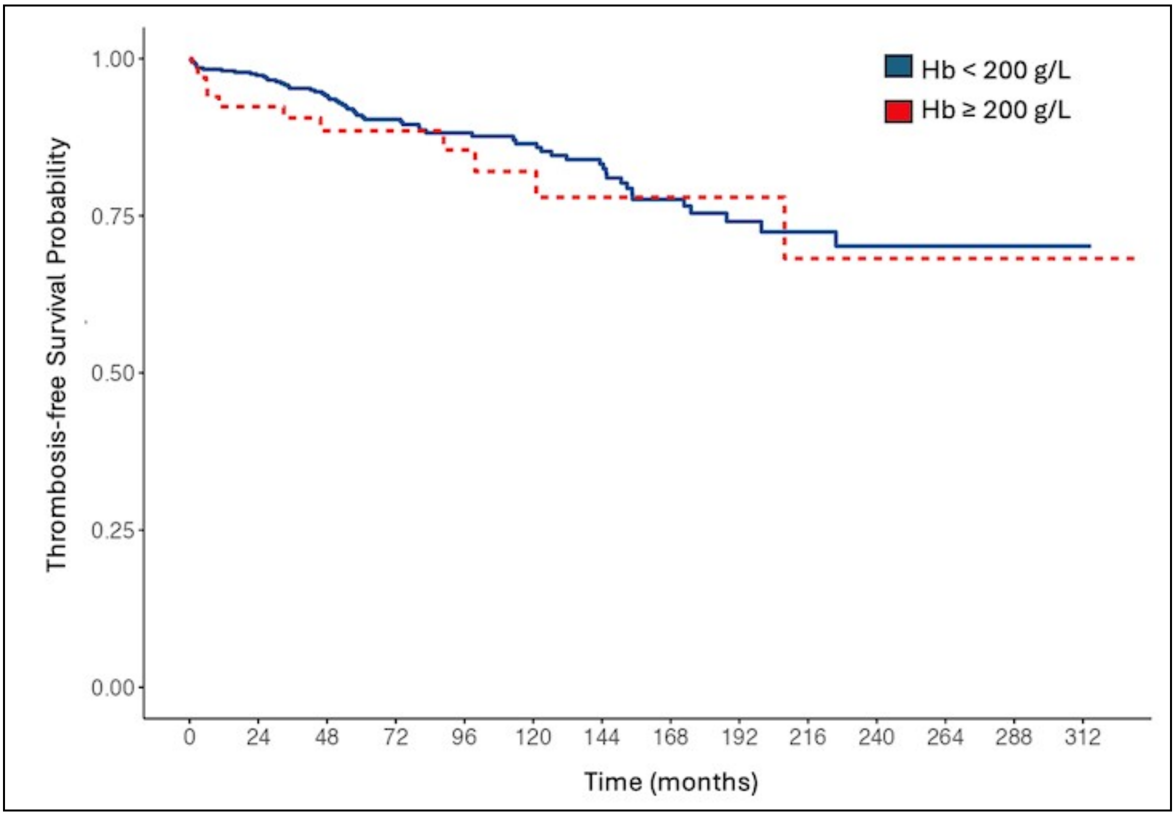

Figure 1. Kaplan-Meier thrombosis-free survival curve

Copyright Information: (Online) ISSN: 2572-9241
©2026 The Author(s). HemaSphere is published by John Wiley & Sons Ltd on behalf of European Hematology Association. This is an open access Abstract Book distributed under the Attribution-NonCommercial-NoDerivs (CC BY-NC-ND), which allows third parties to download the articles and share them with others as long as they credit the author and the Abstract Book, but they cannot change the content in any way or use them commercially.
Abstract Book Citations: Authors, Title, HemaSphere, 2026;10:(S1):pages. The abstract book DOIs can be found on the "Author Information" page.
Disclaimer: Articles published in the journal HemaSphere exclusively reflect the opinions of the authors. The authors are responsible for all content in their abstracts including accuracy of the facts, statements, citing resources, etc.

### (PF1370) TARGETING TPO/MPL SIGNALING TO MITIGATE JAK2V617F-DRIVEN CARDIAC MICROVASCULAR DISEASE

**Topic:** 35. Thrombosis and vascular biology

Xiaoxi Yang[1], Kyla Masarik[1], Xiaochuan Sun[1], Fangfang Zhang[1], Kirsten Zheng[2], Haoyi Zheng[3], Huichun Zhan*[4, 5]

*[1]Stony Brook School of Medicine, Stony Brook, United States of America;[2]Harborfields High School, Greenlawn, United States of America; [3]Saint Francis Hospital, Roslyn, United States of America;[4]Stony Brook Medicine, Stony Brook, United States of America; [5]Northport VA Medical Center, Northport, United States of America;*

### Background

Individuals with JAK2V617F-mutant myeloproliferative neoplasms (MPNs) or clonal hematopoiesis of indeterminate potential (CHIP) have a markedly increased risk of cardiovascular disease (CVD), yet the mechanisms linking mutant hematopoiesis to vascular and cardiac dysfunction remain unclear. The thrombopoietin receptor MPL is essential for hematopoiesis and is also expressed in endothelial cells (ECs), but its role in JAK2V617F-associated cardiovascular complications is unknown.

### Aims

We investigated whether JAK2V617F-mutant hematopoiesis promotes cardiac microvascular injury and maladaptive remodeling under cardiometabolic stress, and whether pharmacologic MPL inhibition mitigates this risk.

### Methods

Chimeric mice with JAK2V617F-mutant hematopoiesis and wild-type ECs were generated by bone marrow transplantation. After full donor engraftment, mice were assigned to: (1) chow diet, (2) high-fat/high-cholesterol diet (HFD), or (3) HFD plus anti-MPL neutralizing antibody AMM2 (0.1mg/kg i.v. every 10 days for ~40 days). Chimeric mice with wild-type hematopoietic cells on either chow or HFD served as controls.

### Results

Recipients of mutant marrow developed an MPN phenotype with elevated blood counts. Mice harboring JAK2V617F-mutant hematopoietic cells developed a distinct cardiac microvascular phenotype, both at baseline and after HFD challenge, characterized by increased left ventricular (LV) mass with preserved ejection fraction (LVEF). Under HFD stress, LVEF declined modestly, accompanied by endocardial endothelial dysfunction. Histology showed coronary arteriole stenosis, perivascular fibrosis, endocardial disruption, and reduced microvascular density — hallmarks of cardiac microvascular dysfunction — in the absence of epicardial coronary artery disease. This phenotype resembles ischemia with non-obstructive coronary arteries (INOCA) and heart failure with preserved ejection fraction (HFpEF), conditions in which coronary microvascular dysfunction is central. Consistently, observational studies link CHIP to coronary microvascular dysfunction and show a stronger with HFpEF than with HFrEF.

Immunohistochemistry demonstrated that MPL is predominantly expressed in a subset of endocardial ECs, a population critical for coronary vasculature formation and cardiac repair. Single-cell RNA-seq identified these cells as primary targets of mutant hematopoiesis under cardiometabolic stress, with activation of inflammatory, stress-response, and EndMT programs and evidence of endocardial injury. TPO/MPL signaling was upregulated in endocardial ECs of JAK2V617F-mutant mice. Treatment with AMM2 suppressed inflammatory and stress pathways in endocardial ECs, reduced endocardial injury, increased coronary microvascular density, and improved cardiac function despite persistent systemic inflammation. Notably, the AMM2 used did not affect peripheral blood counts in wild-type mice.

**Copyright Information:** (Online) ISSN: 2572-9241
©2026 The Author(s). HemaSphere is published by John Wiley & Sons Ltd on behalf of European Hematology Association. This is an open access Abstract Book distributed under the Attribution-NonCommercial-NoDerivs (CC BY-NC-ND), which allows third parties to download the articles and share them with others as long as they credit the author and the Abstract Book, but they cannot change the content in any way or use them commercially.
**Abstract Book Citations:** Authors, Title, HemaSphere, 2026;10:(S1):pages. The abstract book DOIs can be found on the "Author Information" page.
**Disclaimer:** Articles published in the journal HemaSphere exclusively reflect the opinions of the authors. The authors are responsible for all content in their abstracts including accuracy of the facts, statements, citing resources, etc.

**Summary/Conclusion**

JAK2V617F-mutant hematopoiesis drives cardiac microvascular injury and maladaptive remodeling, predisposing to INOCA- and HFpEF-like phenotypes, particularly under metabolic stress. Selective vulnerability of endocardial ECs and activation of TPO/MPL signaling are central to this process. Pharmacologic MPL inhibition enhances endocardial EC–mediated vascular repair and improves cardiac function, identifying TPO/MPL signaling as a potential therapeutic target to reduce cardiovascular risk in JAK2V617F-positive CHIP or MPNs.

**Copyright Information:** (Online) ISSN: 2572-9241
©2026 The Author(s). HemaSphere is published by John Wiley & Sons Ltd on behalf of European Hematology Association. This is an open access Abstract Book distributed under the Attribution-NonCommercial-NoDerivs (CC BY-NC-ND), which allows third parties to download the articles and share them with others as long as they credit the author and the Abstract Book, but they cannot change the content in any way or use them commercially.
**Abstract Book Citations:** Authors, Title, HemaSphere, 2026;10:(S1):pages. The abstract book DOIs can be found on the "Author Information" page.
**Disclaimer:** Articles published in the journal HemaSphere exclusively reflect the opinions of the authors. The authors are responsible for all content in their abstracts including accuracy of the facts, statements, citing resources, etc.

### (PF1371) EFFECTIVENESS AND SAFETY OF DIRECT ORAL ANTICOAGULANTS IN HIGH-RISK CANCER PATIENTS WITH ATRIAL FIBRILLATION AND/OR VENOUS THROMBOEMBOLISM: REAL-WORLD EVIDENCE

**Topic:** 35. Thrombosis and vascular biology

Agata Ogłoza-Puchowska*[1], Ewa Lewicka[2], Andrzej Mital[1], Marta Ziembińska[1], Jan MacIej Zaucha[1]

*[1]Medical University of Gdańsk, University Clinical Centre of Gdańsk, Department of Hematology, Transplantology and Cellular Therapies, Gdańsk, Poland; [2]Medical University of Gdańsk, University Clinical Centre of Gdańsk, Department of Cardiology and Electrotherapy, Gdańsk, Poland;*

### Background

Current guidelines recommend direct oral anticoagulants (DOACs) for stroke prevention in atrial fibrillation (AF) and for the treatment and secondary prevention of venous thromboembolism (VTE). In patients with cancer, however, the net clinical benefit of DOAC therapy remains a concern because of an elevated bleeding risk. Real-world data evaluating the effectiveness and safety of DOACs in high-risk oncologic populations are therefore needed.

### Aims

To evaluate the real-world effectiveness and safety of direct oral anticoagulants (DOACs) in a high-risk cohort of cancer patients with atrial fibrillation and/or venous thromboembolism during a predefined 12-month follow-up.

### Methods

This single-center, prospective observational study included consecutive patients with active or prior cancer who were treated with DOACs for AF and/or VTE for at least 12 months. Patients received apixaban 5 mg twice daily, rivaroxaban 20 mg once daily, dabigatran 150 mg twice daily, or reduced DOAC doses at the discretion of the treating physician. Patients were followed for 12 months, with scheduled visits every 3 months. The primary effectiveness outcome was objectively confirmed stroke or systemic embolism, or recurrent VTE. The primary safety outcome was clinically relevant bleeding, defined as a composite of major and clinically relevant non-major bleeding.

### Results

A total of 200 cancer patients were included: 141 (70.5%) treated for AF, 53 (26.5%) for VTE, and 6 (3.0%) for both conditions. At baseline, 40 patients (20%) had a HAS-BLED score ≥3 (median score, 2). Nearly all patients (98.1%) had a $CHA_2DS_2$-VA score ≥3, with 33% having a score ≥4 and 5.5% ≥6 (median score, 3). A cancer-associated thrombosis (CAT) risk score >8% was observed in 19 patients. Active cancer was present in 74% of the cohort, reflecting a high oncologic risk profile. Median follow-up was 11.99 months (IQR 9.94–13.01). No strokes or systemic embolic events occurred. Recurrent VTE was observed in 3 patients (2.23%) receiving full-dose DOACs and in 1 patient (1.51%) receiving reduced doses. Clinically relevant bleeding occurred in 18 patients (13.43%) in the full-dose group and in 8 patients (12.12%) in the reduced-dose group.

### Summary/Conclusion

In this real-world cohort of high-risk cancer patients with AF and/or VTE, DOAC therapy was associated with very low rates of thromboembolic events and an acceptable bleeding risk. Comparable safety and effectiveness outcomes were observed with reduced-dose regimens. These findings support individualized DOAC dosing strategies in oncologic patients, tailored to balance thrombotic risk, bleeding risk, and overall clinical complexity.

**Copyright Information:** (Online) ISSN: 2572-9241
©2026 The Author(s). HemaSphere is published by John Wiley & Sons Ltd on behalf of European Hematology Association. This is an open access Abstract Book distributed under the Attribution-NonCommercial-NoDerivs (CC BY-NC-ND), which allows third parties to download the articles and share them with others as long as they credit the author and the Abstract Book, but they cannot change the content in any way or use them commercially.
**Abstract Book Citations:** Authors, Title, HemaSphere, 2026;10:(S1):pages. The abstract book DOIs can be found on the "Author Information" page.
**Disclaimer:** Articles published in the journal HemaSphere exclusively reflect the opinions of the authors. The authors are responsible for all content in their abstracts including accuracy of the facts, statements, citing resources, etc.

### (PF1372) DIAGNOSTIC PERFORMANCE OF DEEP LEARNING-BASED ARTIFICIAL INTELLIGENCE VERSUS RADIOLOGISTS FOR THE DETECTION OF PULMONARY EMBOLISM ON COMPUTED TOMOGRAPHY: A SYSTEMATIC REVIEW AND META-ANALYSIS

**Topic:** 35. Thrombosis and vascular biology

Rahul Falodia*[1]

[1] *All India Institute of Medical Sciences, Jodhpur, India;*

### Background

Pulmonary embolism (PE) remains a leading cause of preventable hospital mortality, yet diagnostic accuracy is often compromised by high case volumes, radiologist fatigue, and "inattentional blindness" on non-dedicated scans. While Deep Learning (DL) algorithms show promise, their comparative performance against standard clinical reporting in real-world settings remains debated.

### Aims

We performed a systematic review and meta-analysis to quantify the diagnostic accuracy of DL models versus radiologists, with a specific focus on incidental PE detection.

### Methods

We searched PubMed, EMBASE, and Cochrane Library for studies published between January 2022 and February 2026 that directly compared DL model performance with human interpretation for PE detection on CT. The primary outcome was diagnostic sensitivity. Data were pooled using a random-effects model (GLMM). Subgroup analyses were performed for incidental PE on cardiac/oncologic CT and ultra-low-dose protocols.

### Results

Thirteen studies comprising 19,866 patients met inclusion criteria.

- Sensitivity: The DL group demonstrated a pooled random-effects sensitivity of 92.4% [95% CI: 86.0-96.0] , compared to 83.1% [95% CI: 71.1-90.8] for radiologists. Subgroup analysis showed a significant difference under the common-effect model (p < 0.0001).
- Specificity: Both groups achieved exceptionally high specificity. The DL group pooled specificity was 99.7% [95% CI: 99.1-99.9] , while radiologists achieved 99.8% [95% CI: 99.3-100.0].
- Accuracy: Overall diagnostic accuracy was comparable, with the DL group at 98.9% [95% CI: 97.9-99.4] and radiologists at 98.5% [95% CI: 97.1-99.2], indicating that the significant gain in AI sensitivity did not come at the cost of excessive false positives.. Significant heterogeneity was observed across all metrics (I^2 > 90%). Crucially, in the subgroup of incidental PE on cardiac and cancer staging CTs (Brin et al., 2025; Wiklund et al., 2023), human sensitivity dropped to 21.3-45.5% due to inattentional blindness, whereas AI maintained a sensitivity of 90.7-100%, correctly identifying over 60 emboli that were missed in the original clinical reports. In terms of workflow efficiency, AI integration was found to significantly reduce the time-to-diagnosis for positive PE cases, with one included study (Ambrogio et al., 2025) reporting a reduction in notification time from a potential 4.5 days (routine reporting backlog) to under 24 hours.

**Copyright Information:** (Online) ISSN: 2572-9241
©2026 The Author(s). HemaSphere is published by John Wiley & Sons Ltd on behalf of European Hematology Association. This is an open access Abstract Book distributed under the Attribution-NonCommercial-NoDerivs (CC BY-NC-ND), which allows third parties to download the articles and share them with others as long as they credit the author and the Abstract Book, but they cannot change the content in any way or use them commercially.
**Abstract Book Citations:** Authors, Title, HemaSphere, 2026;10:(S1):pages. The abstract book DOIs can be found on the “Author Information” page.
**Disclaimer:** Articles published in the journal HemaSphere exclusively reflect the opinions of the authors. The authors are responsible for all content in their abstracts including accuracy of the facts, statements, citing resources, etc.

### Summary/Conclusion

Deep learning algorithms demonstrate superior sensitivity compared to radiologists for the detection of PE on CT, while maintaining non-inferior specificity and overall accuracy. These findings suggest that AI can serve as a highly effective screening and triage tool to assist radiologists, potentially reducing the rate of missed diagnoses in acute clinical conditions

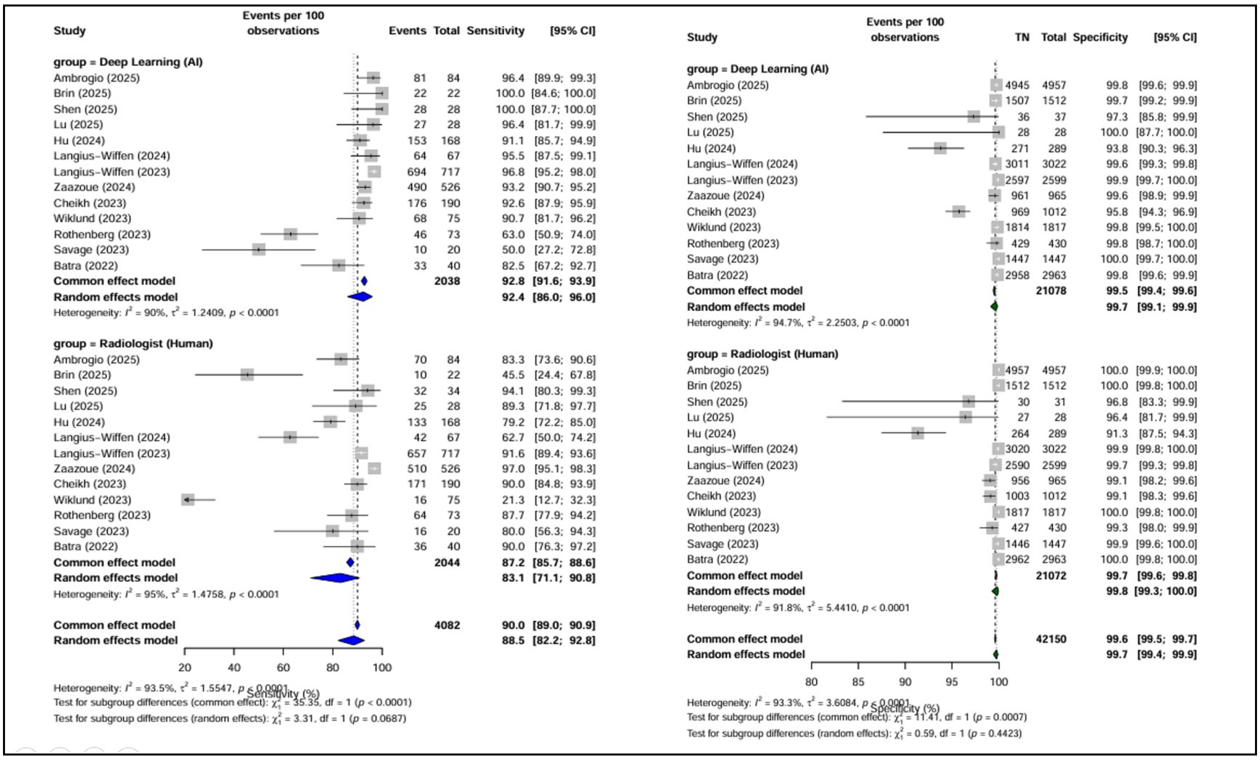

**Copyright Information:** (Online) ISSN: 2572-9241
©2026 The Author(s). HemaSphere is published by John Wiley & Sons Ltd on behalf of European Hematology Association. This is an open access Abstract Book distributed under the Attribution-NonCommercial-NoDerivs (CC BY-NC-ND), which allows third parties to download the articles and share them with others as long as they credit the author and the Abstract Book, but they cannot change the content in any way or use them commercially.
**Abstract Book Citations:** Authors, Title, HemaSphere, 2026;10:(S1):pages. The abstract book DOIs can be found on the "Author Information" page.
**Disclaimer:** Articles published in the journal HemaSphere exclusively reflect the opinions of the authors. The authors are responsible for all content in their abstracts including accuracy of the facts, statements, citing resources, etc.

### (PF1373) PREVENT THE PREVENTABLE: A MULTIFACETED QUALITY IMPROVEMENT INITIATIVE TO REDUCE HOSPITAL-ACQUIRED THROMBOSIS

**Topic:** 35. Thrombosis and vascular biology

Abdulrahman Al Raizah*[1, 1], Ayman Hejazi[1], Aimn W Abdujawad[1], Rawan Al Mohamady[1], Ayman Almozaini[1], Sumayyah Altamimi[1], Khalid Aldosari[1], Ayel Yahya[1], Mohammed Bakkar[1], Ayman Ibrahim[1], Abdullah Alsaleh[1], Husam Alsadi[1], Bader Alahmari[1], Abdul Rauf Ghori[1], Hind Salama[1], Mohsen Alzahrani[1], Ahmed Alaskar[1]

*[1]Oncology department, Ministry of National Guard Health Affairs, King Abdullah International Medical Research Center, King Saud bin Abdulaziz University of Health Sciences, Saudi society of bone marrow transplant, Riyadh, Saudi Arabia;*

### Background

Hospital-acquired pulmonary embolism (PE) remains a preventable cause of morbidity and mortality in surgical patients. In early 2024, internal surveillance at King Abdulaziz Medical City identified hospital-acquired PE rates exceeding benchmarks from comparable tertiary-care centers.

### Aims

This quality improvement project was initiated with the aim of reducing the rate of hospital-acquired PE.

### Methods

It was conducted at King Abdulaziz Medical City, a tertiary care center. All patients admitted under surgical departments for more than 48 hours were included,no exclusions. PDSA cycles guided
implementation. The interventions began in October 2024 and included:

1. Education of Healthcare Providers Targeted education sessions were delivered to surgical teams focusing on proper VTE prophylaxis indications.

2. Weekly audit-and-feedback system: patient-level data are automatically extracted, reviewed, and email feedback is sent to clinical teams to correct deviations from VTE prophylaxis guidelines.

3. EHR Pop-Up Alert with Embedded VTE Prophylaxis Algorithm A real-time decision-support alert was integrated into the EHR to guide correct agent and dosing based on weight, renal function, and VTE risk.

The pre-intervention period spanned January to September 2024, and the post-intervention period covered October 2024 to September 2025. The process measure was the proportion of patients receiving appropriate VTE prophylaxis, and the outcome measure was the rate of hospital-acquired pulmonary embolism per 1,000 admissions. Statistical process control (SPC) charts were constructed to assess process stability and identify special cause variation.

**Copyright Information:** (Online) ISSN: 2572-9241
©2026 The Author(s). HemaSphere is published by John Wiley & Sons Ltd on behalf of European Hematology Association. This is an open access Abstract Book distributed under the Attribution-NonCommercial-NoDerivs (CC BY-NC-ND), which allows third parties to download the articles and share them with others as long as they credit the author and the Abstract Book, but they cannot change the content in any way or use them commercially.
**Abstract Book Citations:** Authors, Title, HemaSphere, 2026;10:(S1):pages. The abstract book DOIs can be found on the “Author Information” page.
**Disclaimer:** Articles published in the journal HemaSphere exclusively reflect the opinions of the authors. The authors are responsible for all content in their abstracts including accuracy of the facts, statements, citing resources, etc.

### Results

A total of 12,370 surgical admissions were included: 5,091 during the pre-intervention period and 7,279 during the post-intervention period.

**Process measure – appropriate VTE prophylaxis:**

The proportion of patients receiving appropriate VTE prophylaxis increased from 40.8% in the pre-intervention period to 58.9% post-intervention, representing an absolute improvement of 18.1 percentage points (relative increase 44%; $p < 0.0001$, Chi-square test). Statistical process control charts demonstrated a clear shift in the centerline beginning shortly after implementation, with sustained improvement over time. Multiple Nelson rules (Rules 1, 2, 3, 4, and 8) were triggered, consistent with special-cause variation rather than random fluctuation.(figurre 1&2).

**Outcome measure – hospital-acquired pulmonary embolism:**

The rate of hospital-acquired PE decreased from 13.6 to 9.3 per 1,000 admissions, corresponding to an absolute reduction of 4.3 events per 1,000 admissions and a rate ratio of 0.68 (95% CI 0.49–0.96; $p = 0.03$, Poisson regression).( Figure3)

### Summary/Conclusion

A multifaceted quality improvement intervention—combining education, EHR decision support, and audit-and-feedback—improved the appropriateness of VTE prophylaxis, which translated into a reduction in hospital-acquired PE rates. Future efforts will focus on sustaining process reliability and evaluating scalability across additional centers.

**Copyright Information:** (Online) ISSN: 2572-9241
©2026 The Author(s). HemaSphere is published by John Wiley & Sons Ltd on behalf of European Hematology Association. This is an open access Abstract Book distributed under the Attribution-NonCommercial-NoDerivs (CC BY-NC-ND), which allows third parties to download the articles and share them with others as long as they credit the author and the Abstract Book, but they cannot change the content in any way or use them commercially.
**Abstract Book Citations:** Authors, Title, HemaSphere, 2026;10:(S1):pages. The abstract book DOIs can be found on the "Author Information" page.
**Disclaimer:** Articles published in the journal HemaSphere exclusively reflect the opinions of the authors. The authors are responsible for all content in their abstracts including accuracy of the facts, statements, citing resources, etc.

Figure 1

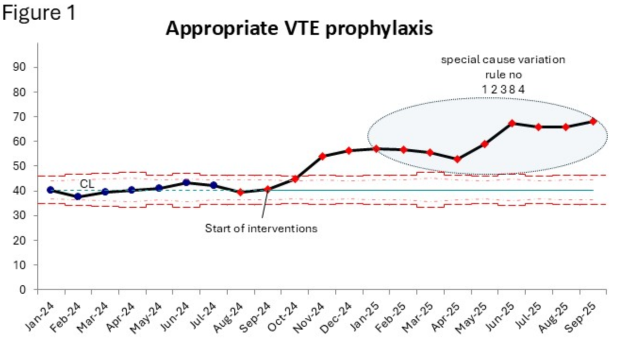

Figure 2

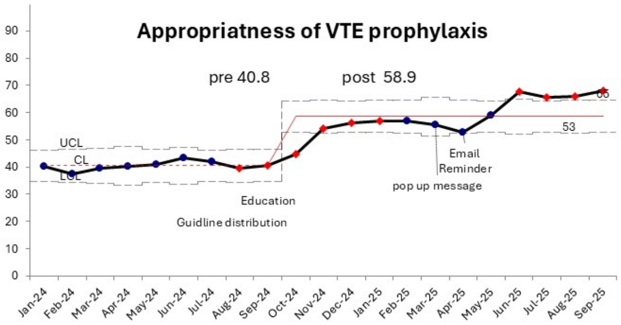

Figure 3

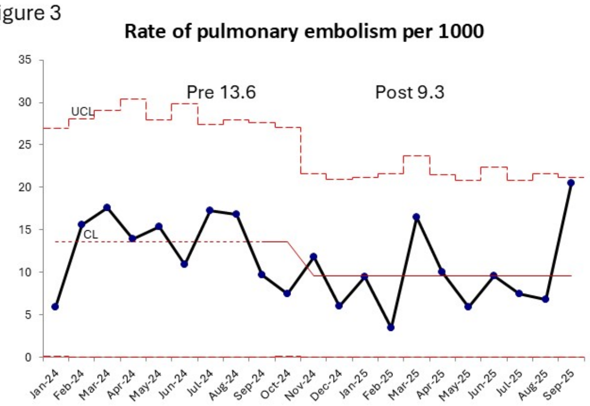

**Copyright Information:** (Online) ISSN: 2572-9241
©2026 The Author(s). HemaSphere is published by John Wiley & Sons Ltd on behalf of European Hematology Association. This is an open access Abstract Book distributed under the Attribution-NonCommercial-NoDerivs (CC BY-NC-ND), which allows third parties to download the articles and share them with others as long as they credit the author and the Abstract Book, but they cannot change the content in any way or use them commercially.
**Abstract Book Citations:** Authors, Title, HemaSphere, 2026;10:(S1):pages. The abstract book DOIs can be found on the "Author Information" page.
**Disclaimer:** Articles published in the journal HemaSphere exclusively reflect the opinions of the authors. The authors are responsible for all content in their abstracts including accuracy of the facts, statements, citing resources, etc.

### (PF1374) UTILIZATION AND EFFICACY OF THERAPEUTIC PLASMA EXCHANGE IN THROMBOTIC MICROANGIOPATHIES: A REAL-WORLD ANALYSIS FROM A NATIONWIDE REGISTRY

**Topic:** 35. Thrombosis and vascular biology

Paschalis Evangelidis*[1], Eleni Gavriilaki[1], Nikolaos Kotsiou[1], Panagiotis Dolgyras[2], Apostolia Papalexandri[2], Katerina Kosmidou[2], Chrysavgi Lalagianni[2], Zoi Bousiou[2], Iliana Stamatiou[3], Vasiliki Sakka[3], Emmanouil Panagiotopoulos[3], Vassiliki Labropoulou[4], Angelos Sarantopoulos[4], Valera Krisela[4], Vasileios Bolanos[5], Anthi Bouchla[6], Anastasia Banti[7], Panagiotis Zikos[8], Ioannis Papakonstantinou[8], Ifigeneia Tzannou[9], Loukia Ligdi[9], Maria Liga[10, 10], Christina Kaitantzoglou[11], Epameinondas Kantidakis[12], Christina Kalpadaki[12], Maria Krithinaki[12], Maria Ximeri[12], Charalampos Pontikoglou[12], Marios Papasotiriou[13], Georgia Kanellopoulou[14], Ioannis Saridakis[15], Anastasia Sioni[16], Stathis Tsiakas[17], Aglaia Chalkia[18], Theodoros Ntrinias[19], Maria Angelopoulou[20], Vasiliki Pappa[6], Konstantinos Liapis[3], Ioannis Kotsianidis[3], Evdokia Mandala[21], Efrosyni Nomikou[5], Sofia Vakalopoulou[1], Ioannis Baltadakis[9], Ioanna Sakellari[2], Elisavet Grouzi[22]

*[1]Aristotle University of Thessaloniki, Second Propedeutic Department of Internal Medicine, Thessaloniki, Greece; [2]G. Papanicolaou Hospital, Hematology Department - BMT Unit, Thessaloniki, Greece; [3]Democritus University of Thrace, Department of Hematology, Alexandroupolis, Greece; [4]University of Patras, Department of Internal Medicine, Division of Hematology, Patras, Greece; [5]Hippokration General Hospital of Athens, Blood Bank and Haemophilia Unit, Athens, Greece; [6]National and Kapodistrian University of Athens, Medical School, University General Hospital 'Attikon', Second Department of Internal Medicine and Research Institute, Hematology Unit, Athens, Greece; [7]Papageorgiou General Hospital, Hematology Department, Thessaloniki, Greece; [8]Agios Andreas General Hospital of Patras, Hematology Department, Patras, Greece; [9]Evangelismos General Hospital, Hematology and Lymphomas Department and Bone Marrow Transplantation Unit, Athens, Greece; [10]University of Patras, Hematology Department, Bone Marrow Transplantation Unit and Institute of Cell Therapy, Patras, Greece; [11]Evangelismos Hospital, Nephrology Department, Athens, Greece; [12]University of Crete School of Medicine & University Hospital of Heraklion, Department of Hematology, Heraklion, Greece; [13]University Hospital of Patras, University of Patras, Department of Nephrology and Kidney Transplantation, Patras, Greece; [14]Laiko General Hospital, National and Kapodistrian University of Athens, Second Regional Blood Donation Center, Athens, Greece; [15]University General Hospital of Heraklion, Rheumatology–Immunology Department, Heraklion, Greece; [16]Agios Savvas Cancer Hospital, Hematology Department, Athens, Greece; [17]Nephrology and Kidney Transplant Department, Nephrology and Kidney Transplant Department, Athens, Greece; [18]Hippokration General Hospital of Athens, Nephrology Department, Athens, Greece; [19]University General Hospital of Patras, Nephrology and Transplant Center, Patras, Greece; [20]Laiko General Hospital, Hematology Clinic and Bone Marrow Transplant Unit, Athens, Greece; [21]Hippokration Hospital, Aristotle University of Thessaloniki, Thessaloniki, 4th Department of Internal Medicine, Thessaloniki, Greece; [22]Agios Savvas Cancer Hospital, Blood Transfusion Department, Athens, Greece;*

### Background

Thrombotic microangiopathies (TMAs), including thrombotic thrombocytopenic purpura (TTP), hemolytic uremic syndrome (HUS), secondary TMAs (sTMAs), in subjects with autoimmune disorders, and transplant-associated TMA (TA-TMA), constitute rare and life-threatening syndromes. Beyond their high mortality and morbidity, TMA survivors face several long-term complications. The Hellenic Hematology Association, recognizing the rarity of these disorders and the burden that these patients experience, has organized a nationwide registry, aiming to collect data from adult patients diagnosed with the aforementioned syndromes, with ongoing follow-up. Moreover, therapeutic plasma exchange (TPE) is considered a standard of care for the management of patients with TTP. However, factors associated with TPE outcomes in real-world TTP patients remain poorly understood, along with its efficacy in patients with other TMAs.

**Copyright Information:** (Online) ISSN: 2572-9241
©2026 The Author(s). HemaSphere is published by John Wiley & Sons Ltd on behalf of European Hematology Association. This is an open access Abstract Book distributed under the Attribution-NonCommercial-NoDerivs (CC BY-NC-ND), which allows third parties to download the articles and share them with others as long as they credit the author and the Abstract Book, but they cannot change the content in any way or use them commercially.
**Abstract Book Citations:** Authors, Title, HemaSphere, 2026;10:(S1):pages. The abstract book DOIs can be found on the "Author Information" page.
**Disclaimer:** Articles published in the journal HemaSphere exclusively reflect the opinions of the authors. The authors are responsible for all content in their abstracts including accuracy of the facts, statements, citing resources, etc.

### Aims

Therefore, our objective is to investigate the real-world utilization and efficacy of TPE in TMA patients and investigate factors associated with response to TPE.

### Methods

Patients with a diagnosis of TMA and ongoing follow-up were enrolled in the registry. Clinical/laboratory data were collected at the time of TMA diagnosis and during ongoing follow-up visits. Response to TPE was defined as platelets (PLTs)$\geq$150×10⁹/L, lactate dehydrogenase <1.5×upper limit of normal, and no new/progressive ischemic organ damage. TTP relapse was considered as recurrent PLTs<150×10⁹/L (excluding other causes) and/or organ ischemia and ADAMTS13 <20%.

### Results

From 2024, 156 patients were enrolled in our registry from 19 different centers. In the current analysis, we included 147 patients with TMAs: 114 (77.5%) with TTP, 22 (15%) with TA-TMA, 5 (3.4%) with sTMA, and 6 (4.1%) with HUS. The characteristics of patients of different groups are presented in Table 1a. Concerning TTP patients, during the treatment of the first acute episode, TPE was applied to 105 (92.1%), with a median number of 8 (interquartile range [IQR] 5-13) sessions. Among patients treated with TPE, 101 (96.2%) were also treated with corticosteroids, 27 (25.7%) with caplacizumab, and 58 (55.2%) with rituximab. Moreover, 81/105 (77.1%) responded to TPE during the first TTP episode. TPE-responders were found to have a better Karnofsky performance status (p=0.017) and lower creatinine levels (p=0.015) (Table 1b). In 97/105 (92.4%) patients, remission of the TTP episode was achieved. With a median follow-up of 10 (6-62.3) months, TTP relapse was observed in 35/114 (30.7%) patients, with a median of 1 (1-2) episodes. In total, 68 relapse episodes were reported: TPE was used in 53 (77.9%). Among these 53 episodes, caplacizumab was also used in 13 (24.5%), and rituximab in 44 (83%). No-response to TPE was observed only in 3/53 (5.7%) relapse episodes. Additionally, all sTMA patients received TPE (median number of TPE sessions 8 [3-21]) and responded. Other treatment approaches included: rituximab in 2 (40%) and corticosteroids in 4 (80%). Remission of TMA was achieved in all of them. In the HUS cohort, 3 subjects were treated with TPE, with 2 of them responding. Furthermore, among them, 2 received C5 complement inhibitors, with achievement of remission in both. We did not analyse the characteristics of TA-TMA subjects.

### Summary/Conclusion

Our nationwide registry confirms high real-world TPE efficacy in TTP and identifies outcome-associated factors, highlighting also the critical role of systematic data collection in peripheral settings.

**Copyright Information:** (Online) ISSN: 2572-9241
©2026 The Author(s). HemaSphere is published by John Wiley & Sons Ltd on behalf of European Hematology Association. This is an open access Abstract Book distributed under the Attribution-NonCommercial-NoDerivs (CC BY-NC-ND), which allows third parties to download the articles and share them with others as long as they credit the author and the Abstract Book, but they cannot change the content in any way or use them commercially.
**Abstract Book Citations:** Authors, Title, HemaSphere, 2026;10:(S1):pages. The abstract book DOIs can be found on the "Author Information" page.
**Disclaimer:** Articles published in the journal HemaSphere exclusively reflect the opinions of the authors. The authors are responsible for all content in their abstracts including accuracy of the facts, statements, citing resources, etc.

| Table 1a. Baseline Clinical and Laboratory Characteristics of the Study Population. | | | | | | |
|---|---|---|---|---|---|---|
| | Total cohort (n=147) | TTP (n=114) | TA-TMA (n=22) | sTMA (n=5) | HUS (n=6) | P-value |
| Median age at diagnosis (IQR) | 46 (34-58) | 47 (36-58) | 46 (32-58) | 39 (25-54) | 28 (18-37) | 0.008 |
| Sex, n (%)<br>Male<br>Female | <br>67 (45.6)<br>80 (54.4) | <br>49 (43)<br>65 (57) | <br>12 (54.5)<br>10 (45.5) | <br>1 (20)<br>4 (80) | <br>5 (83.3)<br>1 (16.7) | 0.14 |
| Median BMI (IQR) | 27 (24.6-30.4) | 28.1 (25.6-31.1) | 25.1 (21.8-27.8) | 22.7 (19.7-29.3) | 21.7 (20.3-23.9) | <0.001 |
| KPS category, n (%)<br>100-80<br>70-50<br>40-0 | <br>116 (78.9)<br>14 (9.5)<br>17 (11.6) | <br>90 (78.9)<br>10 (8.8)<br>14 (12.3) | <br>15 (68.2)<br>4 (18.2)<br>3 (13.6) | <br>5 (100)<br>0<br>0 | <br>6 (100)<br>0<br>0 | 0.71 |
| Mean Hb (g/dL) (SD) | 9.3 (2.3) | 9.4 (2.2) | 8.6 (1.4) | 7.8 (2.6) | 13.4 (1.9) | <0.001 |
| Median MCV (fL) (IQR) | 87.4 (83.4-91) | 87.2 (83.4-90.1) | 90 (85-94.6) | 87.5 (79-97.1) | 83.7 (82.7-84.9) | 0.11 |
| Median PLTs (x10$^9$/L) (IQR) | 17 (10-28.8) | 18 (10-28.5) | 16 (11-22.5) | 6.5 (1-93) | 70 (24.5-284) | 0.039 |
| Median Schistocytes (%) (IQR) | 5 (3-8) | 6 (3-9) | 3.3 (1.2-5.6) | 2 (0-16) | 0 (0-0) | <0.001 |
| Median creatinine (mg/dL) (IQR) | 1 (0.8-1.3) | 1 (0.9-1.2) | 0.9 (0.6-1.5) | 0.9 (0.9-1) | 1.4 (1-3.5) | 0.29 |
| CNS involvement, n (%) | 32 (21.8) | 30 (26.3) | 2 (9.1) | 0 | 0 | 0.1 |
| Hypertension, n (%) | 25 (17) | 22 (19.3) | 2 (9.1) | 0 | 0 | 0.6 |

| Table 1b. Comparison of clinical, laboratory, and treatment characteristics between responders and non-responders to TPE during the first TTP episode. | | | |
|---|---|---|---|
| | Responders to TPE (n=81) | Non-responders (n=24) | P-value |
| Median age at diagnosis (IQR) | 46 (35-58) | 52 (38-60) | 0.20 |
| Sex, n (%)<br>Male<br>Female | <br>33 (40.7)<br>48 (59.3) | <br>11 (45.8)<br>13 (54.2) | 0.81 |
| Median BMI (IQR) | 27.5 (25.6-31.1) | 28.5 (25-30.7) | 0.71 |
| KPS cat, n (%)<br>100-80<br>70-50<br>40-0 | <br>66 (81.5)<br>4 (4.9)<br>11 (13.6) | <br>16 (66.7)<br>6 (25)<br>2 (8.3) | 0.017 |
| Mean Hb (g/dL) (SD) | 9.1 (2.2) | 9.3 (2.2) | 0.69 |
| Median MCV (fL) (IQR) | 87.1 (82.3-90.5) | 86.7 (83-89.6) | 0.67 |
| Median PLTs (x10$^9$/L) (IQR) | 18 (8.5-30) | 15 (10.3-23.5) | 0.81 |
| Median Schistocytes (%) (IQR25-75) | 6 (3-10) | 5 (4.1-8) | 0.68 |
| Median creatinine (mg/dL) (IQR) | 0.9 (0.8-1.2) | 1.1 (1-1.6) | 0.015 |
| CNS involvement, n (%) | 21 (25.9) | 7 (29.2) | 0.78 |
| Hypertension, n (%) | 13 (16) | 5 (20.8) | 0.55 |
| Caplacizumab, n (%) | 24 (29.6) | 3 (12.5) | 0.09 |
| Median TPE sessions (IQR) | 8 (5-12) | 9 (6-20) | 0.062 |

**Abbreviations:** BMI: body mass index, CNS: central nervous system, Hb: hemoglobin, HUS: hemolytic uremic syndrome, IQR: interquartile range, KPS: Karnofsky performance status, MCV: mean corpuscular volume, NS: not stated, PLTs: platelets, SD: standard deviation, sTMA: secondary thrombotic microangiopathy, TA-TMA: transplant-associated thrombotic microangiopathy, TMA: thrombotic microangiopathy, TPE: therapeutic plasma exchange, TTP: thrombotic thrombocytopenic purpura

**Copyright Information:** (Online) ISSN: 2572-9241
©2026 The Author(s). HemaSphere is published by John Wiley & Sons Ltd on behalf of European Hematology Association. This is an open access Abstract Book distributed under the Attribution-NonCommercial-NoDerivs (CC BY-NC-ND), which allows third parties to download the articles and share them with others as long as they credit the author and the Abstract Book, but they cannot change the content in any way or use them commercially.
**Abstract Book Citations:** Authors, Title, HemaSphere, 2026;10:(S1):pages. The abstract book DOIs can be found on the "Author Information" page.
**Disclaimer:** Articles published in the journal HemaSphere exclusively reflect the opinions of the authors. The authors are responsible for all content in their abstracts including accuracy of the facts, statements, citing resources, etc.

### (PF1375) CEREBRAL VENOUS SINUS THROMBOSIS IN MYELOPROLIFERATIVE NEOPLASMS: A PREDOMINANCE IN YOUNGER JAK2-MUTATED FEMALES WITH POLYCYTHAEMIA VERA AND A POTENTIAL RISK OF LATE RECURRENT THROMBOSIS

**Topic:** 35. Thrombosis and vascular biology

Tarik Rosun*[1], Natalie Myatt[1], Katherine Makris[1], Craig Lennie[1], Claire Woodley[1], Natalia Curto-Garcia[1], Claire Harrison[1], Andrew Doyle[2], Priya Sriskandarajah[1]

*[1]Guy's and St Thomas' NHS Foundation Trust, Department of Haematology, London, United Kingdom;[2]Guy's and St Thomas' NHS Foundation Trust, Department of Haemostasis and Thrombosis, London, United Kingdom;*

### Background

Cerebral venous sinus thrombosis (CVST) is a rare but potentially serious complication of myeloproliferative neoplasms (MPNs). Limited data exist on long term thrombosis risk, recurrence patterns, and optimal anticoagulation strategies in this population, particularly beyond the acute phase.

### Aims

To describe the clinical characteristics, management, and long-term outcomes of patients with MPN associated CVST, including the incidence and timing of thrombotic and haemorrhagic events, in a single UK tertiary centre.

### Methods

We conducted a retrospective study of all MPN patients with an associated CVST, managed at Guy's and St Thomas' hospital between 2013 and 2025. Clinical data were collected including patient demographic, MPN subtype, mutation status, cytoreductive treatment and complications. Data on CVST diagnosis, anticoagulation therapy, and long-term outcomes including recurrent thrombosis, were analysed.

**Copyright Information:** (Online) ISSN: 2572-9241
©2026 The Author(s). HemaSphere is published by John Wiley & Sons Ltd on behalf of European Hematology Association. This is an open access Abstract Book distributed under the Attribution-NonCommercial-NoDerivs (CC BY-NC-ND), which allows third parties to download the articles and share them with others as long as they credit the author and the Abstract Book, but they cannot change the content in any way or use them commercially.
**Abstract Book Citations:** Authors, Title, HemaSphere, 2026;10:(S1):pages. The abstract book DOIs can be found on the "Author Information" page.
**Disclaimer:** Articles published in the journal HemaSphere exclusively reflect the opinions of the authors. The authors are responsible for all content in their abstracts including accuracy of the facts, statements, citing resources, etc.

### Results

Thirty patients were included (24/30 females, 80% and 6/30 males, 20%) with a median age of 45 years (range 15-71). MPN subtypes were polycythaemia vera (PV) in 20/30 (67%) and essential thrombocythaemia (ET) in 10/30 patients (33%). Driver mutations included *JAK2*V617F in 27/30 (90%), *CALR* in 1/30 (3%) and 2/30 (7%) triple negative. Additional venous thromboembolism risk factors were present in 12/30 (40%) including pregnancy (2/30; 7%), prior VTE (5/30; 17%) and thrombophilia (lupus anticoagulant 5/30; 17%)
CVST preceded MPN diagnosis in 3/30 (10%), was concurrent in 13/30 (43%), and occurred after established MPN in 14/30 (47%) (median 7 years post-diagnosis); 5/14 (36%) were receiving cytoreductive therapy at the time of CVST (Hydroxycarbamide (3/5); Pegylated Interferon (2/5)). Headache was the most common presenting symptom in 20/30 (67%), and a sagittal sinus involvement occurred in 20/30 (67%). Six patients (20%) had a concomitant thrombotic event at diagnosis (4 splanchnic vein, 1 vertebral artery, 1 cerebrovascular).

Initial anticoagulation included low molecular weight heparin (10/30, 33%), warfarin (11/30, 37%), and direct oral anticoagulants (DOACs; 5/30, 17%). Concomitant antiplatelet therapy (APT) was used in 13/30 (43%). Long term anticoagulation was continued in 25/30 (83%), predominantly with DOACs (19/25, 76%). A temporal shift toward DOAC use was observed (median year of CVST diagnosis: 2012 for warfarin vs 2020 for DOACs). Over a median follow-up of 8 years (range 2-43), 5/30 (17%) patients developed recurrent thrombosis (2 arterial, 3 venous), with a median time to recurrence of 7 years (range 1-17). Cerebral haemorrhagic complications occurred in 2/30 patients (7%). There were no deaths or persistent neurological sequelae.

### Summary/Conclusion

In this single-centre cohort with prolonged follow-up, MPN-associated CVST predominantly affected younger female patients with *JAK2* mutated disease, most commonly polycythaemia vera. Despite anticoagulation and cytoreductive therapy, a risk of late recurrent thrombosis was observed, highlighting the importance of prolonged clinical vigilance and further studies to optimise long term management strategies in this population.

**Copyright Information:** (Online) ISSN: 2572-9241
©2026 The Author(s). HemaSphere is published by John Wiley & Sons Ltd on behalf of European Hematology Association. This is an open access Abstract Book distributed under the Attribution-NonCommercial-NoDerivs (CC BY-NC-ND), which allows third parties to download the articles and share them with others as long as they credit the author and the Abstract Book, but they cannot change the content in any way or use them commercially.
**Abstract Book Citations:** Authors, Title, HemaSphere, 2026;10:(S1):pages. The abstract book DOIs can be found on the "Author Information" page.
**Disclaimer:** Articles published in the journal HemaSphere exclusively reflect the opinions of the authors. The authors are responsible for all content in their abstracts including accuracy of the facts, statements, citing resources, etc.

### (PF1376) ADAMTS13 INHIBITOR TESTING: EVALUATION OF A CHEMILUMINESCENCE BASED MIXING ASSAY FOR MONITORING IMMUNE THROMBOTIC THROMBOCYTOPENIC PURPURA. STUDY OF THE SPANISH APHERESIS GROUP (GEA)

**Topic:** 35. Thrombosis and vascular biology

Cristina Pascual-Izquierdo*[1], Jorge M. Nieto[2], Inés Gómez-Seguí[3], Teresa Fidalgo[4], Alicia Ortiz Lopez[1], Patricia Martinho[4], Silvia Escribano-Serrat[5], Julia Martinez-Sanchez[5], Ana Belén Moreno-Castaño[5], Antonio Moscardo[3], Patricia Gonzalez-Marugan[1], Marta M. Carracedo[2], Maribel Díaz-Ricart[5]

*[1]Hospital General Universitario Gregorio Marañon.Instituto de Investigación Gregorio Marañón, Hematology department, Madrid, Spain; [2]Hospital Universiario Clinico San Carlos, Madrid, Spain; [3]Hospital Universitario La Fe, Hematology department, Valencia, Spain; [4]Centro Hospitalar Universitário de Coimbra, Coimbra, Spain; [5]Hospital Cinic-IDIBAPS, Hematology department, Barcelona, Spain;*

### Background

Thrombotic thrombocytopenic purpura (TTP) is a haematological emergency caused by severe ADAMTS13 deficiency, which may be congenital (cTTP) or inmune due to anti-ADAMTS13 antibodies (iTTP). Distinguishing between these forms is essential, as management strategies differ significantly. Detection of ADAMTS13 inhibitors is commonly performed using a plasma mixing assay based on a modified Bethesda method, with ELISA and FRETS-VWF73 considered reference techniques.
A rapid chemiluminescence-based assay (CLIA) for ADAMTS13 activity has recently been standardized. However, CLIA-based methods have been reported to yield lower activity values than reference techniques. In this multicentre international study, we investigated whether this analytical bias affects the performance of CLIA-based mixing assays, potentially increasing false-positive inhibitor results.

### Aims

To evaluate the performance of a CLIA-based mixing assay compared with reference methods in patients with iTTP.

### Methods

A total of 73 inhibitor measurements from 21 patients diagnosed with iTTP were analysed across five centres in Spain and Portugal. All patients were evaluated at diagnosis, and an additional 52 measurements corresponded to early follow-up samples collected on days +7, +14, and +21.
Mixing assays were performed by combining patient plasma 1:1 with normal pooled plasma. ADAMTS13 activity in the mixture (test) was compared with that of normal plasma mixed 1:1 with buffer (expected value). Inhibitor titres were expressed in Bethesda units (BU), with values >0.5 BU considered positive. Reference techniques included ELISA (TECHNOZYM ADAMTS13 Activity, Technoclone) and in-house FRETS-VWF73 assays. Quantitative agreement was assessed using Spearman's rank correlation coefficient (ρ), while qualitative agreement was evaluated using Cohen's kappa (κ), sensitivity, specificity, and positive and negative predictive values.

### Results

CLIA-based mixing assays showed a very strong quantitative correlation with reference methods both at diagnosis and during follow-up (ρ = 0.93 and 0.92, respectively). However, qualitative agreement was moderate (κ = 0.662). At diagnosis, CLIA yielded false-positive results in 9.5% of cases (2/21), with no false negatives observed. In contrast, false-negative results were identified exclusively during follow-up, occurring in 9.6% of samples (5/52). (Figure1)

**Copyright Information:** (Online) ISSN: 2572-9241
©2026 The Author(s). HemaSphere is published by John Wiley & Sons Ltd on behalf of European Hematology Association. This is an open access Abstract Book distributed under the Attribution-NonCommercial-NoDerivs (CC BY-NC-ND), which allows third parties to download the articles and share them with others as long as they credit the author and the Abstract Book, but they cannot change the content in any way or use them commercially.
**Abstract Book Citations:** Authors, Title, HemaSphere, 2026;10:(S1):pages. The abstract book DOIs can be found on the "Author Information" page.
**Disclaimer:** Articles published in the journal HemaSphere exclusively reflect the opinions of the authors. The authors are responsible for all content in their abstracts including accuracy of the facts, statements, citing resources, etc.

### Summary/Conclusion

CLIA-based mixing assays demonstrate excellent quantitative correlation with reference techniques; however, their performance decrease when clinical cut-offs are applied. False-positive CLIA results at diagnosis may have relevant clinical implications, nevertheless, the discrepancy observed at diagnosis should be validated in a control cohort including healthy subjects and/or patients with cTTP. At present, positive inhibitor results obtained by CLIA should be confirmed using reference methods.

**Figure 1: Performance of the MIX 50/50 CLIA mixing assay compared with reference methods**

| | **MIX 50/50 CLIA (N = 73)** | |
|---|---|---|
| | Positive | Negative |
| **MIX50/50 ELISA/FRET** (Positive) | 51 (69.9%) | 5 (6.8%) |
| **MIX50/50 ELISA/FRET** (Negative) | 4 (5.5%) | 13 (17.8%) |

Agreement between methods: Cohen's kappa = 0.662

| | **MIX 50/50 CLIA (N = 21) at Day 0** | |
|---|---|---|
| | Positive | Negative |
| **MIX50/50 ELISA/FRET** (Positive) | 19 (90.5%) | 0 (0%) |
| **MIX50/50 ELISA/FRET** (Negative) | 2 (9.5%) | 0 (0%) |

Agreement between methods: Cohen's kappa = *

| | **MIX 50/50 CLIA (N = 52) at follow-up** | |
|---|---|---|
| | Positive | Negative |
| **MIX50/50 ELISA/FRET** (Positive) | 32 (61.5%) | 5 (9.6%) |
| **MIX50/50 ELISA/FRET** (Negative) | 2 (3.8%) | 13 (25%) |

Agreement between methods: Cohen's kappa = 0.69

*Qualitative agreement between MIX 50/50 CLIA and MIX 50/50 ELISA/FRET mixing assays for ADAMTS13 inhibitor detection in the overall cohort (N = 73), at diagnosis (Day 0, N = 21), and during follow-up (N = 52). Results are expressed as number of samples and percentage. Agreement between methods was assessed using Cohen's kappa coefficient (κ). *At diagnosis, κ could not be calculated due to the absence of negative results by CLIA.*

**Copyright Information:** (Online) ISSN: 2572-9241
©2026 The Author(s). HemaSphere is published by John Wiley & Sons Ltd on behalf of European Hematology Association. This is an open access Abstract Book distributed under the Attribution-NonCommercial-NoDerivs (CC BY-NC-ND), which allows third parties to download the articles and share them with others as long as they credit the author and the Abstract Book, but they cannot change the content in any way or use them commercially.
**Abstract Book Citations:** Authors, Title, HemaSphere, 2026;10:(S1):pages. The abstract book DOIs can be found on the "Author Information" page.
**Disclaimer:** Articles published in the journal HemaSphere exclusively reflect the opinions of the authors. The authors are responsible for all content in their abstracts including accuracy of the facts, statements, citing resources, etc.

### (PF1377) DEMOGRAPHIC CHARACTERISTICS OF OVARIAN VEIN THROMBOSIS INCLUDING RISK FACTORS AND OUTCOME IN OMANI PATIENTS: A RETROSPECTIVE STUDY

**Topic:** 35. Thrombosis and vascular biology

Dina Al Hinai*[1], Huda Al Nazwani[2], Khalid Nasser Alhashmi[2]

*[1]Oman Medical Speciality Board, Muscat, Oman; [2]Armed Forces Hospital, Muscat, Oman;*

### Background

Ovarian vein thrombosis (OVT) is a rare but serious type of venous thromboembolism (VTE) that occurs in the female gonad veins which can cause fatal complications. Compared to lower limb deep vein thrombosis (DVT), OVT is around 60 times less common but recently in the last few decades. There are many contributing clinical risk factors and the clinical presentation varies from asymptomatic to non-specific abdominal pain. Using anticoagulation for treatment of OVT has been suggested, but no clear guidelines had been published. A better understanding of patients with OVT in all aspects is need to give us an insight for optimal management and prevention of major complications that can be avoided by initiation of the proper management at time of diagnosis.

### Aims

This study aims to describe the demographic data and clinical characteristics of Omani patients diagnosed with (OVT), identify the risk factors and symptoms at presentation, and determine the diagnostic methods used for OVT. Secondary objectives include assessing the use of anticoagulants, particularly direct oral anticoagulants (DOACs), for OVT and evaluating the patient's outcome.

### Methods

A retrospective descriptive study was conducted at Royal Hospital and Armed Forced Hospital (January 2010 to December 2023). A convenient non-random sampling method was used. The inclusion criteria were Omani females of all ages with diagnoses confirmed by radiological imaging. Categorical variables were presented as frequencies and percentages, continuous variables were presented as means and data were analyzed using SPSS version 29.0.

### Results

We included 80 female patients with a median age of 42 years (IQR: 19–89). Among them, 60% were incidentally diagnosed while 40% presented with symptoms. The majority had right-sided OVT (57.5%). Presenting symptoms included abdominal pain (28.8%) and other gastrointestinal symptoms (8.8%). The primary risk factor was malignancy within six months of diagnosis (45%) and recent surgery (17.5%). Other factors included idiopathic (21.3%), pregnancy related (6.3%), using oral contraceptives (2.5%), and miscarriages (3.8%). Only one patient had a pelvic infection. The diagnosis was primarily via CT scan (92.5%). Eight patients had elevated D-dimer (0.5 mg/L). Five patients were tested for inherited thrombophilia, six for antiphospholipid, and twelve for lupus anticoagulant test. Results were negative, except for tow patients with positive lupus anticoagulant. Treatment varied: 38.8% did not receive treatment while others were treated with warfarin, low molecular weight heparin, and unfractionated heparin. Only 10% of patients were treated with DOACs. Anticoagulation duration ranged from less than three months to more than six months, three patients experienced non-significant bleeding. Follow-up was inconsistent; 23.8% of patients had no follow-up and the rest had follow-up with different intervals (less than six months to one year). CT scans were the most commonly used imaging modality during follow-up (48.8%). Only six patients developed pulmonary embolism and one patient had thrombosis progression, the rest had no complications.

**Copyright Information:** (Online) ISSN: 2572-9241
©2026 The Author(s). HemaSphere is published by John Wiley & Sons Ltd on behalf of European Hematology Association. This is an open access Abstract Book distributed under the Attribution-NonCommercial-NoDerivs (CC BY-NC-ND), which allows third parties to download the articles and share them with others as long as they credit the author and the Abstract Book, but they cannot change the content in any way or use them commercially.
**Abstract Book Citations:** Authors, Title, HemaSphere, 2026;10:(S1):pages. The abstract book DOIs can be found on the "Author Information" page.
**Disclaimer:** Articles published in the journal HemaSphere exclusively reflect the opinions of the authors. The authors are responsible for all content in their abstracts including accuracy of the facts, statements, citing resources, etc.

**Summary/Conclusion**

OVT is a rare clinical entity with nonspecific symptoms and is often diagnosed incidentally. Using anticoagulation for the treatment of OVT has been suggested, but no clear guidelines exist. Further prospective, multicenter studies with long-term follow-up are needed to define treatment indications and the role of thrombophilia testing.

**Copyright Information:** (Online) ISSN: 2572-9241
©2026 The Author(s). HemaSphere is published by John Wiley & Sons Ltd on behalf of European Hematology Association. This is an open access Abstract Book distributed under the Attribution-NonCommercial-NoDerivs (CC BY-NC-ND), which allows third parties to download the articles and share them with others as long as they credit the author and the Abstract Book, but they cannot change the content in any way or use them commercially.
**Abstract Book Citations:** Authors, Title, HemaSphere, 2026;10:(S1):pages. The abstract book DOIs can be found on the "Author Information" page.
**Disclaimer:** Articles published in the journal HemaSphere exclusively reflect the opinions of the authors. The authors are responsible for all content in their abstracts including accuracy of the facts, statements, citing resources, etc.

### (PF1378) NOVEL VARIANTS (P.H401Q AND P.K402E) IN Β-SHEET A CAUSE ANTITHROMBIN DEFICIENCY VIA IMPAIRED ALLOSTERIC ACTIVATION

**Topic:** 35. Thrombosis and vascular biology

Ke Zhang*[1], Changming Chen[1], Xi Wu[1], Wenman Wu[1], Xuefeng Wang[1], Jing Dai[1, 2]

[1]*Department of Laboratory Medicine, Ruijin Hospital, Shanghai Jiao Tong University School of Medicine, Shanghai, China;*
[2]*Shanghai Institute of Hematology, Ruijin Hospital, Shanghai Jiao Tong University School of Medicine, Shanghai, China;*

### Background

Antithrombin (AT) is a key serine protease inhibitor that maintains hemostatic balance. Its inhibitory activity is heparin-dependent: heparin binding to AT induces conformational changes that accelerate the inhibition thrombin (FIIa) and factor Xa (FXa). The β-sheet A domain is a crucial structural element that undergoes conformational rearrangement to accommodate reactive center loop (RCL) insertion during the inhibitory process. Variants in this domain may disrupt the allosteric activation pathway and impair inhibitory function.

### Aims

This study aimed to elucidate the molecular pathological mechanisms underlying the effects of two rare missense variants inβ-sheet A domain, p.H401Q and p.K402E (located in exon 6), on antithrombin structure, heparin affinity, and inhibitory function.

### Methods

Variants were confirmed in probands by Sanger sequencing. Thrombotic potential was assessed via thrombin generation test (TGT) in patient plasma. Recombinant wild-type (WT), H401Q, and K402E proteins (based on the S137A β-AT enriched background) were expressed. Protein expression, oligomerization, and modification were analyzed by Coomassie staining and Western blot under reducing/non-reducing conditions without/with N-deglycosylation. Inhibition of FIIa and FXa by WT and variant proteins was quantified by determining second-order rate constants ($k_2$) without/with unfractionated heparin or pentasaccharide (H5), and inhibitory efficiency was evaluated by stoichiometry of Inhibition (SI). AT-protease complex formation was analyzed by non-reducing Coomassie staining.

### Results

Sanger sequencing confirmed two probands carrying c.1203T>A (p.H401Q) with cerebral venous sinus thrombosis and c.1204A>G (p.K402E) with a history of assisted reproductive failure, with plasma AT activities of 73% (anti-Xa/IIa) and 60%/61% (anti-Xa/IIa), respectively, consistent with type II PE deficiency **(Figure A)**. TGT in patient plasma showed shortened lag time, increased peak thrombin, and elevated endogenous thrombin potential (ETP) ± heparin, indicating impaired anticoagulation **(Figure B)**. Recombinant mutant proteins formed oligomers under non-reducing conditions; K402E exhibited aberrant high-molecular-weight bands under reducing/non-reducing conditions persisting after N-deglycosylation, suggesting structural alteration **(Figure C-D)**. Functional assays revealed markedly reduced inhibition of FIIa/FXa ($k_2$) for both mutants, especially without heparin, with impaired heparin enhancement (H401Q: 41.41%/13.29% FIIa/FXa without heparin vs. 30.77%/32.66% with heparin; K402E: 26.18%/2.53% without heparin vs. 15.22%/11.69% with heparin) **(Figure E-G)**. SI was abnormally elevated (WT ≈1.1; H401Q: 2.66–4.10; K402E: 9.51–24.56), indicating profound inefficiency and substrate-like behavior **(Figure H-M)**. Complex formation analysis showed that WT formed stable covalent complexes with FIIa/FXa ± heparin, whereas mutants predominantly exhibited cleaved AT bands, with K402E showing virtually no complex formation, confirming defective target enzyme engagement **(Figure N-O)**.

**Copyright Information:** (Online) ISSN: 2572-9241
©2026 The Author(s). HemaSphere is published by John Wiley & Sons Ltd on behalf of European Hematology Association. This is an open access Abstract Book distributed under the Attribution-NonCommercial-NoDerivs (CC BY-NC-ND), which allows third parties to download the articles and share them with others as long as they credit the author and the Abstract Book, but they cannot change the content in any way or use them commercially.
**Abstract Book Citations:** Authors, Title, HemaSphere, 2026;10:(S1):pages. The abstract book DOIs can be found on the “Author Information” page.
**Disclaimer:** Articles published in the journal HemaSphere exclusively reflect the opinions of the authors. The authors are responsible for all content in their abstracts including accuracy of the facts, statements, citing resources, etc.

**Summary/Conclusion**

p.H401Q and p.K402E variants destabilize the β-sheet A domain, leading to abnormal polymerization, defective heparin binding/activation, and severely impaired inhibition of FIIa and FXa. This molecular dysfunction underlies thrombotic events and potentially coagulation-related reproductive failure, primarily by disrupting the normal inhibitory mechanism and target enzyme binding.

**Copyright Information:** (Online) ISSN: 2572-9241
©2026 The Author(s). HemaSphere is published by John Wiley & Sons Ltd on behalf of European Hematology Association. This is an open access Abstract Book distributed under the Attribution-NonCommercial-NoDerivs (CC BY-NC-ND), which allows third parties to download the articles and share them with others as long as they credit the author and the Abstract Book, but they cannot change the content in any way or use them commercially.
**Abstract Book Citations:** Authors, Title, HemaSphere, 2026;10:(S1):pages. The abstract book DOIs can be found on the “Author Information” page.
**Disclaimer:** Articles published in the journal HemaSphere exclusively reflect the opinions of the authors. The authors are responsible for all content in their abstracts including accuracy of the facts, statements, citing resources, etc.

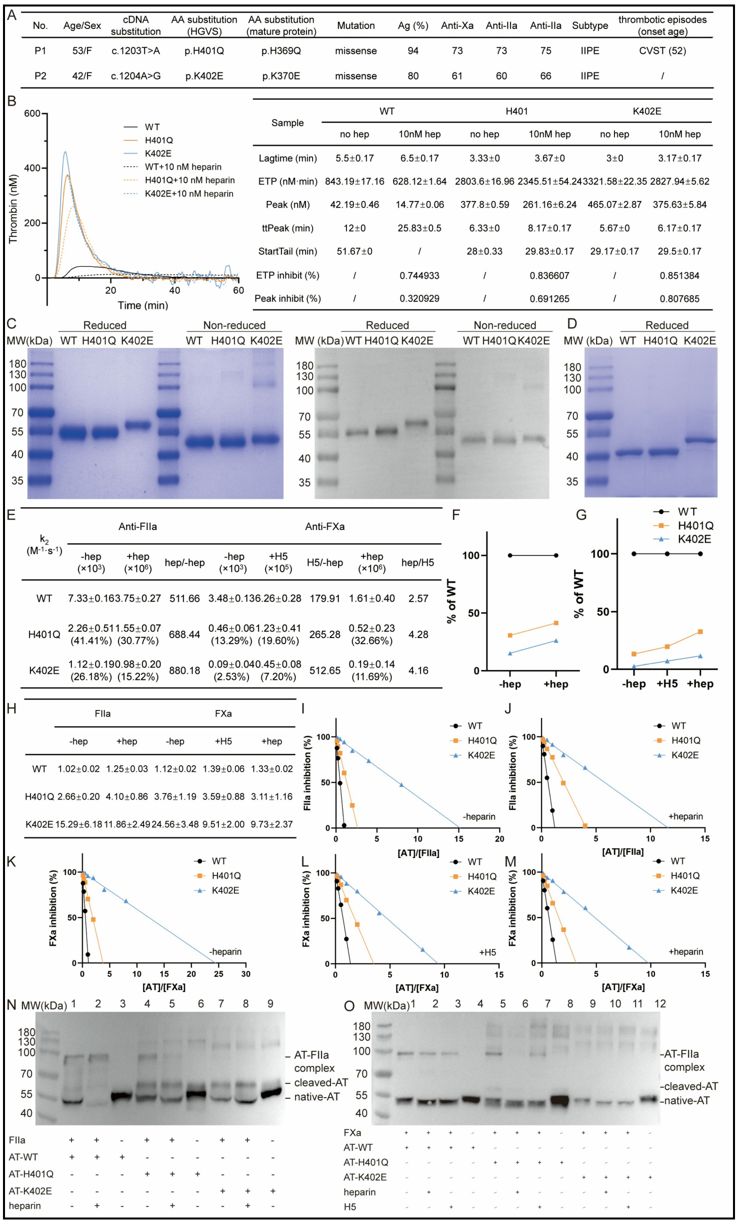

(A) Schematic of two probands. (B) TGT in normal human and patients plasma ± 10 nM heparin. (C) Western blot and Coomassie staining of recombinant WT and variant proteins under reducing and non-reducing conditions. (D) Western blot of WT and variant proteins after PNGase F treatment under reducing conditions. (E) k2 for inhibition of FIIa and FXa by WT and variant proteins ± heparin (hep) or H5. Data are mean ± SD. (F-G) Residual k2 values of variants expressed as percentage of WT activity for FIIa and FXa under indicated conditions. (H-M) SI for WT and variant proteins against FIIa ± hep or against FXa ± hep/H5. (N-O) Non-reducing SDS-PAGE analysis of covalent complex formation between AT (WT or variants) and FIIa ± hep or FXa ± hep/H5.

Copyright Information: (Online) ISSN: 2572-9241
©2026 The Author(s). HemaSphere is published by John Wiley & Sons Ltd on behalf of European Hematology Association. This is an open access Abstract Book distributed under the Attribution-NonCommercial-NoDerivs (CC BY-NC-ND), which allows third parties to download the articles and share them with others as long as they credit the author and the Abstract Book, but they cannot change the content in any way or use them commercially.
Abstract Book Citations: Authors, Title, HemaSphere, 2026;10:(S1):pages. The abstract book DOIs can be found on the “Author Information” page.
Disclaimer: Articles published in the journal HemaSphere exclusively reflect the opinions of the authors. The authors are responsible for all content in their abstracts including accuracy of the facts, statements, citing resources, etc.

### (PF1379) THE EVALUATION OF ANTI-FACTOR XA LEVELS IN HOSPITALISED PATIENTS RECEIVING LOW MOLECULAR WEIGHT HEPARIN (LMWH): A PROSPECTIVE STUDY

**Topic:** 35. Thrombosis and vascular biology

Nancy Okinda*[1], Marcus Lance[1], Laura Kirui[1], Tandzile Simelane[1]

[1]*AGA KHAN UNIVERSITY, PATHOLOGY, Nairobi, Kenya;*

### Background

Venous thromboembolism (VTE) is a major cause of morbidity in hospitalized patients. While low molecular weight heparin (LMWH) is the standard for prophylaxis, standard dosing may not achieve adequate anticoagulation in all patient populations, especially those with altered pharmacokinetics. Individuals of African descent have a significantly higher incidence of VTE, yet there is a paucity of data regarding LMWH efficacy in this setting.

### Aims

The study aimed to evaluate the proportion of hospitalized patients in Nairobi, Kenya, who achieved prophylactic anti-Factor Xa levels while receiving standard-dose low molecular weight heparin (LMWH). Additionally, it sought to identify specific patient characteristics—such as BMI, creatinine clearance, and vasopressor use—associated with these anti-Factor Xa levels

### Methods

This single-center, prospective cross-sectional study at the Aga Khan University Hospital, Nairobi, evaluated anti-Factor Xa levels in 92 adult inpatients receiving standard-dose LMWH prophylaxis (enoxaparin 40mg). Blood samples were collected four hours after the third dose and analyzed using a chromogenic assay. Statistical analysis focused on the association between patient characteristics—such as BMI, creatinine clearance (CrCl), and vasopressor use—and anti-Factor Xa levels.

### Results

Of the 90 patients with analyzable results, a vast majority (87.8%) had subprophylactic anti-Factor Xa levels (<0.2 IU/mL). Only 7.8% achieved the prophylactic target (0.2–0.5 IU/mL), and 4.4% were in the supraprophylactic range. Multinomial logistic regression revealed that higher creatinine clearance was significantly associated with subprophylactic levels (OR=1.03, p=0.033), with a 2.9% increase in the odds of being subprophylactic for every 1 mL/min increase in CrCl. BMI and vasopressor use were not significantly associated with anti-Factor Xa levels.

### Summary/Conclusion

Most patients receiving standard-dose LMWH prophylaxis in this African inpatient population did not reach target prophylactic anti-Factor Xa levels, particularly those with normal or augmented renal function. These findings highlight the potential inadequacy of standard dosing and support the need for individualized dosing strategies and further outcome-focused research to establish evidence-based monitoring protocols in this population.

**Copyright Information:** (Online) ISSN: 2572-9241
©2026 The Author(s). HemaSphere is published by John Wiley & Sons Ltd on behalf of European Hematology Association. This is an open access Abstract Book distributed under the Attribution-NonCommercial-NoDerivs (CC BY-NC-ND), which allows third parties to download the articles and share them with others as long as they credit the author and the Abstract Book, but they cannot change the content in any way or use them commercially.
**Abstract Book Citations:** Authors, Title, HemaSphere, 2026;10:(S1):pages. The abstract book DOIs can be found on the “Author Information” page.
**Disclaimer:** Articles published in the journal HemaSphere exclusively reflect the opinions of the authors. The authors are responsible for all content in their abstracts including accuracy of the facts, statements, citing resources, etc.

### (PF1380) HOW CERTAIN CAN THE DASH AND HERDOO2 SCORES IDENTIFY LOW-RISK PATIENTS?

**Topic:** 35. Thrombosis and vascular biology

B Bhoelan[1], Jaime Borjas Howard[2], Tessa Elling[1], Karina Meijer[1], Jasper van Miert*[1]

[1]*University Medical Center Groningen, Hematology, Groningen, The Netherlands;* [2]*UK Health Security Agency, London, United Kingdom;*

### Background

Guidelines prescribe indefinite anticoagulation for patients with unprovoked venous thromboembolism (VTE). Identifying patients with low recurrence risk could save them from the bleeding risk associated with treatment.

### Aims

Quantify the certainty that risk tools identify low-risk groups.

### Methods

We searched PubMed and Embase on February 3rd, 2023, for studies validating the DASH and HERDOO2 risk scores to predict recurrence after a first unprovoked VTE. Using a Bayesian meta-analysis, we assessed whether low-risk groups are truly low-risk according to ISTH SSC criteria (Kearon c.s., 2010): the 1-year recurrence risk should be <5%, and <15% for 5 years. In addition, the upper limit of the 95% confidence interval should be below 8% and 24%, respectively. We translate this as a 97.5% certainty the recurrence risk is below 8% and 24%, respectively. We used a non-informative prior for recurrence risk and a prior based on Turner c.s., 2012, for between-study heterogeneity.

### Results

We identified 8 studies evaluating DASH and included 3 that provided 1 or 5-year follow-up and cumulative recurrence for each score. For HERDOO2, we found 5 studies and included 2 with suitable follow-up.
Those with DASH score ≤0 fulfil the low-risk criteria: at 5 years, it was 100% certain recurrence risk was <15%. Whether patients with score 1 are low-risk can be debated: it provides a 37% certainty of recurrence <15% (although 100% below 24%). The estimated cumulative recurrence in this group was 15.7% (95% highest density credible interval 11.3–20.1%). The recurrence risk with DASH score 2 was marginally higher: 16.8% (95% CrI  11.2–23.1%), providing certainties of 25% <15% and 98% <24% cumulative recurrence. Higher scores indicated higher recurrence risks (see figure). No externally validated data were available for 1-year follow-up.
In HERDOO2, those with score ≤1 had 97% certainty of a 1-year recurrence risk <5% and 99% ≤8%. No externally validated data were available for 5-year follow-up.

### Summary/Conclusion

A DASH score ≤0 identifies low recurrence risk at 5 years with sufficient certainty; for scores 1 and 2 this is questionable and depends on the certainty required by patients and physicians. A HERDOO2 score of ≤1 identifies low-risk patients at 1 year. Future studies should provide recurrence data for each score group and at timepoints 1 and 5 years, to substantiate their certainty and clinical usefulness.

**Copyright Information:** (Online) ISSN: 2572-9241
©2026 The Author(s). HemaSphere is published by John Wiley & Sons Ltd on behalf of European Hematology Association. This is an open access Abstract Book distributed under the Attribution-NonCommercial-NoDerivs (CC BY-NC-ND), which allows third parties to download the articles and share them with others as long as they credit the author and the Abstract Book, but they cannot change the content in any way or use them commercially.
**Abstract Book Citations:** Authors, Title, HemaSphere, 2026;10:(S1):pages. The abstract book DOIs can be found on the “Author Information” page.
**Disclaimer:** Articles published in the journal HemaSphere exclusively reflect the opinions of the authors. The authors are responsible for all content in their abstracts including accuracy of the facts, statements, citing resources, etc.

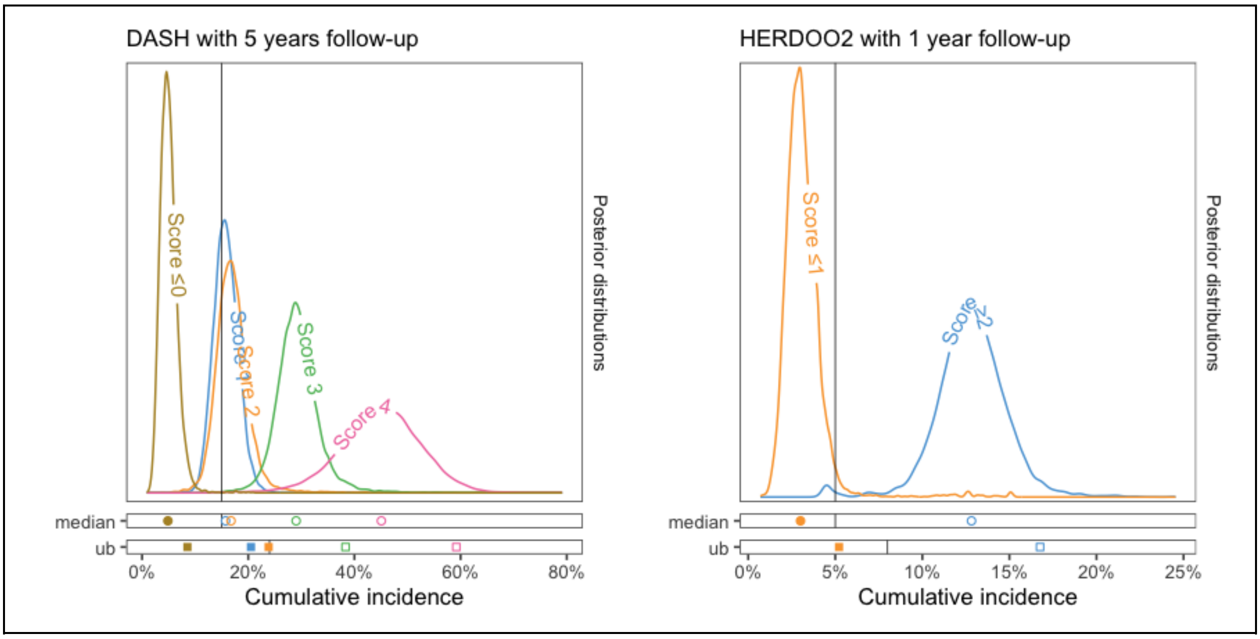

Posterior distributions of the cumulative recurrence of venous thromboembolism (VTE) after a first unprovoked VTE, as assessed by different risk scores

**Copyright Information:** (Online) ISSN: 2572-9241
©2026 The Author(s). HemaSphere is published by John Wiley & Sons Ltd on behalf of European Hematology Association. This is an open access Abstract Book distributed under the Attribution-NonCommercial-NoDerivs (CC BY-NC-ND), which allows third parties to download the articles and share them with others as long as they credit the author and the Abstract Book, but they cannot change the content in any way or use them commercially.
**Abstract Book Citations:** Authors, Title, HemaSphere, 2026;10:(S1):pages. The abstract book DOIs can be found on the "Author Information" page.
**Disclaimer:** Articles published in the journal HemaSphere exclusively reflect the opinions of the authors. The authors are responsible for all content in their abstracts including accuracy of the facts, statements, citing resources, etc.

### (PF1381) EVALUATION OF EFFECTIVENESS AND SAFETY OF DIRECT ORAL ANTICOAGULANTS IN SPLANCHNIC VENOUS THROMBOSIS

**Topic:** 35. Thrombosis and vascular biology

Rodrigo Cantera Estefanía*[1], Julia Coll[1], Leire Armesto Aguado[1], María Lisón Madrona[1], Jorge Guillermo Abarzuza Armendáriz[1], Ibone Ajuria Fondado[1], Daniel Vinicio Reyes Cevallos[1], Ainhoa Sánchez Pastor[2], Iñigo García Erice[1], María Victoria Aznar Moreno[1], Sara Labari Jiménez[1], María José Paloma Mora[1], Ana Margarita Redondo Izal[1]

*[1]Hospital Universitario de Navarra, Hematology and Hemotherapy, Pamplona, Spain;[2]Hospital Universitario de Navarra, Internal Medicine, Pamplona, Spain;*

### Background

Direct oral anticoagulants (DOACs) are increasingly used in Splanchnic vein thrombosis (SVT), yet comparative data against vitamin K antagonists (VKA) and low-molecular-weight heparin (LMWH) remains limited.

### Aims

To evaluate the effectiveness, safety, and survival outcomes associated with DOACs compared with VKA and LMWH.

### Methods

Retrospective cohort study of patients with SVT treated between 2020 and 2024.

### Results

A total of 356 patients were analysed, 112 women (31.5%), with median age of 62.53 years (IQR 54.31–73.29) and median follow-up of 2.72 years (IQR 1.09–5.01) (**table 1**). No differences were observed in recanalization (DOACs 35.5% vs VKA 52.1% vs 42.4%, p=0.077), nor overall bleeding rates (30.6% DOACs, 21.4% VKA, 23.7% LMWH; p=0.379). In multivariable analyses adjusted for potential confounders, no significant differences were identified in recanalization or bleeding (**table 2**).
In the multivariable overall survival (OS) analysis, compared with LMWH, DOACs (HR 0.528; p=0.013) and VKA (HR 0.420; p=0.001) were associated with improved OS.
In the multivariable Cox regression model, no significant differences were observed in thrombotic-free survival (DOACs HR 0.853; p=0.401; VKA HR 0.740; p=0.073), nor in an independent model assessing direct comparison between DOACs and VKA (HR 0.868; p=0.512).
Regarding bleeding-free survival (BFS), DOACs (HR 0.597; p=0.018) and VKA (HR 0.506; p=0.001) were associated with improved BFS compared with LMWH. In a separate model for direct comparison between DOACs and VKA, no differences were identified (VKA HR 0.847; p=0.523).
Within the DOAC subgroup, multivariable logistic regression showed no significant differences among individual DOAC agents in recanalization or bleeding.

*Table 1. Characteristics of patients.*

**Copyright Information:** (Online) ISSN: 2572-9241
©2026 The Author(s). HemaSphere is published by John Wiley & Sons Ltd on behalf of European Hematology Association. This is an open access Abstract Book distributed under the Attribution-NonCommercial-NoDerivs (CC BY-NC-ND), which allows third parties to download the articles and share them with others as long as they credit the author and the Abstract Book, but they cannot change the content in any way or use them commercially.
**Abstract Book Citations:** Authors, Title, HemaSphere, 2026;10:(S1):pages. The abstract book DOIs can be found on the “Author Information” page.
**Disclaimer:** Articles published in the journal HemaSphere exclusively reflect the opinions of the authors. The authors are responsible for all content in their abstracts including accuracy of the facts, statements, citing resources, etc.

| | | DOACs (N=62) | VKA (N=117) | LMWH (N=177) | p |
|---|---|---|---|---|---|
| | Age (median, [IQR]) | 66.39 [50.85-74.71] | 61.53 [51.60-70.87] | 62.30 [55.67-73.75] | 0.095 |
| | Women | 23 (37.1%) | 30 (25.6%) | 59 (33.3%) | 0.219 |
| Clinic | Asymptomatic | 19 (30.6%) | 28 (23.9%) | 47 (26.6%) | 0.624 |
| | Chronic | 17 (27.4%) | 37 (31.6%) | 68 (38.4%) | 0.222 |
| Localization | Mesenteric | 17 (27.4%) | 57 (48.7%) | 66 (37.3%) | 0.016 |
| | Portal | 51 (82.3%) | 78 (66.7%) | 122 (68.9%) | 0.076 |
| | Splenic | 20 (32.3%) | 38 (32.5%) | 55 (31.1%) | 0.964 |
| | Budd-Chiari | 1 (1.6%) | 2 (1.7%) | 9 (5.1%) | 0.204 |
| Aetiology | Cancer | 32 (51.6%) | 11 (9.4%) | 92 (52.0%) | 0.000 |
| | Cirrhosis | 11 (17.7%) | 18 (15.4%) | 36 (20.3%) | 0.557 |
| | Abdominal surgery | 3 (4.8%) | 7 (6.0%) | 8 (4.5%) | 0.851 |
| | Pancreatitis | 3 (4.8%) | 29 (24.8%) | 31 (17.5%) | 0.004 |
| | Abdominal infection | 11 (17.7%) | 28 (23.9%) | 29 (16.4%) | 0.261 |
| | Inflammatory intestinal disease | 1 (1.6%) | 2 (1.7%) | 1 (0.6%) | 0.609 |
| | Myeloproliferative neoplasms | 5 (8.1%) | 4 (3.4%) | 3 (1.7%) | 0.057 |
| | Thrombophilia | 4 (6.5%) | 18 (15.4%) | 8 (4.5%) | 0.004 |
| | Hormonal treatment | 0 (0.0%) | 2 (1.7%) | 1 (0.6%) | 0.419 |
| | Pregnancy-related | 0 (0.0%) | 1 (0.9%) | 0 (0.0%) | 0.359 |
| | Behçet's Disease | 0 (0.0%) | 1 (0.9%) | 0 (0.0%) | 0.359 |
| | Idiopathic | 3 (4.8%) | 13 (11.1%) | 3 (1.7%) | 0.002 |

*Table 2. Logistic regression.*

| Outcome | Variable | p | OR | 95% CI for OR | |
|---|---|---|---|---|---|
| | | | | Inferior | Superior |
| No Recanalization | Women | 0.273 | 1.310 | 0.808 | 2.122 |
| | Age | 1.000 | 1.000 | 0.986 | 1.014 |
| | LMWH [ref] | 0.605 | - | - | - |
| | DOACs | 0.354 | 1.347 | 0.718 | 2.528 |
| | VKA | 0.946 | 0.982 | 0.576 | 1.673 |
| | Portal vein | 0.051 | 0.588 | 0.345 | 1.001 |
| | Cancer | 0.014 | 1.967 | 1.148 | 3.369 |
| | Pancreatitis | 0.005 | 0.383 | 0.197 | 0.746 |
| | Chronic | 0.052 | 1.604 | 0.995 | 2.585 |
| Bleeding (at least minor) | Women | 0.872 | 1.046 | 0.601 | 1.821 |
| | Age | 0.000 | 1.040 | 1.019 | 1.060 |
| | LMWH [ref] | 0.519 | - | - | - |
| | DOACs | 0.252 | 1.491 | 0.752 | 2.955 |
| | VKA | 0.707 | 1.121 | 0.617 | 2.037 |
| | Portal vein | 0.071 | 1.756 | 0.954 | 3.233 |
| | Intraabdominal infection | 0.012 | 0.354 | 0.158 | 0.794 |
| | Chronic | 0.022 | 1.888 | 1.094 | 3.256 |

### Summary/Conclusion

DOACs appear to be effective and safe in patients with SVT. Prospective studies are warranted to confirm these findings.

**Copyright Information:** (Online) ISSN: 2572-9241
©2026 The Author(s). HemaSphere is published by John Wiley & Sons Ltd on behalf of European Hematology Association. This is an open access Abstract Book distributed under the Attribution-NonCommercial-NoDerivs (CC BY-NC-ND), which allows third parties to download the articles and share them with others as long as they credit the author and the Abstract Book, but they cannot change the content in any way or use them commercially.
**Abstract Book Citations:** Authors, Title, HemaSphere, 2026;10:(S1):pages. The abstract book DOIs can be found on the "Author Information" page.
**Disclaimer:** Articles published in the journal HemaSphere exclusively reflect the opinions of the authors. The authors are responsible for all content in their abstracts including accuracy of the facts, statements, citing resources, etc.

**Figure 1**. Regression curves of Overall Survival, Thrombotic free survival and Bleeding free survival. DOACs: direct oral anticoagulants. LMWH: low molecular weight heparins. VKA: vitamin K antagonists.

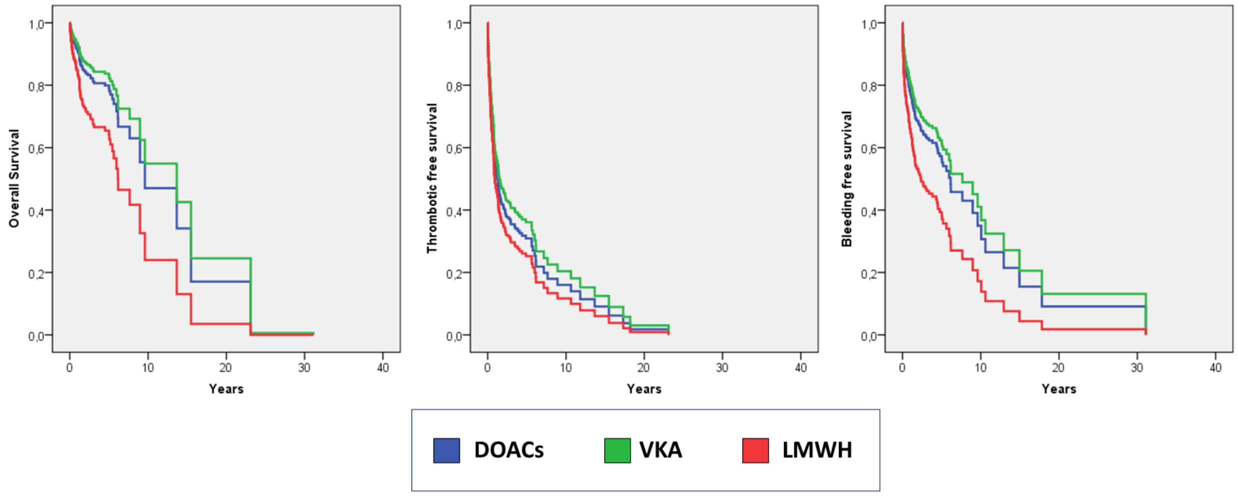

Copyright Information: (Online) ISSN: 2572-9241
©2026 The Author(s). HemaSphere is published by John Wiley & Sons Ltd on behalf of European Hematology Association. This is an open access Abstract Book distributed under the Attribution-NonCommercial-NoDerivs (CC BY-NC-ND), which allows third parties to download the articles and share them with others as long as they credit the author and the Abstract Book, but they cannot change the content in any way or use them commercially.
Abstract Book Citations: Authors, Title, HemaSphere, 2026;10:(S1):pages. The abstract book DOIs can be found on the "Author Information" page.
Disclaimer: Articles published in the journal HemaSphere exclusively reflect the opinions of the authors. The authors are responsible for all content in their abstracts including accuracy of the facts, statements, citing resources, etc.

### (PF1382) MANAGEMENT OF VENOUS THROMBOEMBOLISM (VTE) IN SICKLE CELL DISEASE: A COMPARATIVE STUDY ON THE USE OF DIRECT ORAL ANTICOAGULANTS (DOACS) AND WARFARIN

**Topic:** 35. Thrombosis and vascular biology

Turki Alshuaibi*[1], Mosaad Almegren[2], Bader Alrawahi[3]

*[1]King Fahad General Hospital, Medicine -Division of Hematology, Jeddah, Saudi Arabia;[2]Imam Mohammed Ibn Saud University, riyadh, Saudi Arabia; [3]Sultan Qaboos University Hospital, Muscat, Oman;*

### Background

SCD is often complicated by a vaso-occlusive crisis (VOC), a state in which sickle-shaped red blood cells obstruct blood flow, leading to end-organ damage . Owing to the turbidity of blood flow, VOCs are associated with a hypercoagulable state, which predisposes patients to the development of venous thromboembolism (VTE) . Furthermore, increased levels of circulating tissue factors, increased platelet activation and thrombin generation, and decreased levels of natural anticoagulants in SCD create a high risk of thrombosis . These factors are further exacerbated by the use of central venous catheters in patients who require frequent blood transfusions. An estimated 11% of SCD patients experience a VTE event before they reach 40 years of age, with a five- year recurrence rate reaching up to 36% . VTE is a significant cause of morbidity and mortality in patients with SCD . Data on the use of direct oral anticoagulants (DOACs) in patients with sickle cell disease (SCD) and Venous Thromboembolism (VTE) are limited . We aimed to compare DOACs and warfarin in SCD patients with Venous Thromboembolism (VTE).

### Aims

This study aimed to compare the clinical outcomes of direct oral anticoagulants (DOACs) with warfarin in the management of patients with SCD and first (VTE event).

### Methods

This multicenter retrospective study was conducted at three tertiary care hospitals in Saudi Arabia, Oman and Kuwait. This study included adult patients ≥ 18 years of age with SCD who developed their first episode of VTE event. Recurrent thrombosis and bleeding were compared between patients treated with DOACs and warfarin. Data were analyzed using IBM Statistical Package for the Social Sciences (SPSS) version 21.

### Results

This study included 99 patients with SCD and venous thrombosis, of whom 67 (67.7%) were on DOACs, and 32 (32.3%) received warfarin therapy. The DOACs used were rivaroxaban in 44 patients,
apixaban in 21 and dabigatran in 2 patients. Mean age of all study participants was 32.8 ± 9.9 years and majority (60.6%) were women. Sixty-three patients (63.6 %) were homozygous for hemoglobin S, the mutated form of hemoglobin responsible for SCD.Baseline risk of bleeding was similar for the two groups of patients receiving DOACs or warfarin.
Median follow-up time was 44 (1-130) months. Pulmonary embolism was the most common type of thrombosis observed in 64 patients (64.6 %). Three patients developed recurrent VTE within six months of the first episode, whereas six patients developed recurrent thrombosis after one year. No significant difference was noted among patients on either type of anticoagulation in terms of major bleeding episodes (OR=1.1; 95%CI 1.1-1.8; p: 1.00), recurrence of thrombosis (OR=0.68; 95%CI 0.03-11.2; p: 0.68) or mortality (OR=0.46; 95%CI 0.06-3.4; p: 0.59). Clinically relevant non-major bleeding was significantly lower in patients on DOACs than those on warfarin (OR=0.06; 95%CI 0.01-0.52; p: 0.01)

**Copyright Information:** (Online) ISSN: 2572-9241
©2026 The Author(s). HemaSphere is published by John Wiley & Sons Ltd on behalf of European Hematology Association. This is an open access Abstract Book distributed under the Attribution-NonCommercial-NoDerivs (CC BY-NC-ND), which allows third parties to download the articles and share them with others as long as they credit the author and the Abstract Book, but they cannot change the content in any way or use them commercially.
**Abstract Book Citations:** Authors, Title, HemaSphere, 2026;10:(S1):pages. The abstract book DOIs can be found on the “Author Information” page.
**Disclaimer:** Articles published in the journal HemaSphere exclusively reflect the opinions of the authors. The authors are responsible for all content in their abstracts including accuracy of the facts, statements, citing resources, etc.

**Summary/Conclusion**

This study indicates that DOACs are associated with similar clinical outcomes, comparable recurrence rates, and a relatively lower risk of bleeding than warfarin in the management of patients with SCD
with venous thrombosis. However, randomized controlled trials comparing the safety and efficacy of DOACs and warfarin should be conducted for further confirmation.

**Copyright Information:** (Online) ISSN: 2572-9241
©2026 The Author(s). HemaSphere is published by John Wiley & Sons Ltd on behalf of European Hematology Association. This is an open access Abstract Book distributed under the Attribution-NonCommercial-NoDerivs (CC BY-NC-ND), which allows third parties to download the articles and share them with others as long as they credit the author and the Abstract Book, but they cannot change the content in any way or use them commercially.
**Abstract Book Citations:** Authors, Title, HemaSphere, 2026;10:(S1):pages. The abstract book DOIs can be found on the "Author Information" page.
**Disclaimer:** Articles published in the journal HemaSphere exclusively reflect the opinions of the authors. The authors are responsible for all content in their abstracts including accuracy of the facts, statements, citing resources, etc.

### (PF1383) LINKAGE OF RS6903608 TO HLA CLASS II RISK HAPLOTYPES IN IMMUNE-MEDIATED THROMBOTIC THROMBOCYTOPENIC PURPURA: INSIGHTS FROM A TURKISH COHORT

**Topic:** 35. Thrombosis and vascular biology

Semra Cemre Atalar*[1], Hayriye Şentürk Çiftçi[2], Dilan Karaca[1], Demet Kıvanç İzgi[2], Cevat İlteriş Kıkılı[3], Fatma Oğuz[2], Muhlis Cem Ar[4], Sevgi Kalayoglu Besisik[5]

*[1]Istanbul University Faculty of Medicine, Department of Internal Medicine, Istanbul, Türkiye;[2]Istanbul University Faculty of Medicine, Department of Medical Biochemistry, Istanbul, Türkiye; [3]Koc University Hospital, Department of Medical Oncology, Istanbul, Türkiye; [4]Istanbul University-Cerrahpasa, Cerrahpasa Faculty Of Medicine, Department of Hematology, Istanbul, Türkiye; [5]Istanbul University, Faculty of Medicine, Department of Hematology, Istanbul, Türkiye;*

### Background

Severe ADAMTS13 deficiency in immune-mediated thrombotic thrombocytopenic purpura (iTTP) results from an autoimmune response against ADAMTS13. The HLA-region variant rs6903608 has been reported as the strongest genetic susceptibility locus in European cohorts and associated with relapse risk. However, the absence of association in Japanese patients suggests population-specific genetic architecture. Whether this susceptibility signal is transferable across ethnically distinct populations remains unclear, with potential implications for genetic risk stratification in iTTP.

### Aims

To evaluate whether the rs6903608 susceptibility locus previously reported in European cohorts is reproducible in a Turkish iTTP population and to investigate its potential association with disease course and relapse.

### Methods

71 adult patients diagnosed with iTTP (ADAMTS13 activity <10%) from two tertiary centers and 100 healthy controls were genotyped for rs6903608. Clinical data, including relapse history, laboratory parameters, organ involvement, and treatment characteristics, were collected retrospectively. Genotype distributions were compared between patients and controls, and associations with relapse were analyzed.

**Copyright Information:** (Online) ISSN: 2572-9241
©2026 The Author(s). HemaSphere is published by John Wiley & Sons Ltd on behalf of European Hematology Association. This is an open access Abstract Book distributed under the Attribution-NonCommercial-NoDerivs (CC BY-NC-ND), which allows third parties to download the articles and share them with others as long as they credit the author and the Abstract Book, but they cannot change the content in any way or use them commercially.
**Abstract Book Citations:** Authors, Title, HemaSphere, 2026;10:(S1):pages. The abstract book DOIs can be found on the "Author Information" page.
**Disclaimer:** Articles published in the journal HemaSphere exclusively reflect the opinions of the authors. The authors are responsible for all content in their abstracts including accuracy of the facts, statements, citing resources, etc.

### Results

Genotype distribution did not differ between iTTP patients and healthy controls (homozygous variant detected in 10/71 patients and 12/100 controls), arguing against a primary susceptibility effect.
Relapse occurred in 6 of 10 homozygous-variant patients, compared with 25 of 61 non-homozygous patients. Although statistical significance was not reached, the distribution suggested a possible association with disease course rather than disease onset.

Table A – Susceptibility

| Genotype (rs6903608) | iTTP Patients (n=71) | Controls (n=100) |
|---|---|---|
| Homozygous variant | 10 | 12 |
| Others | 61 | 88 |

Table B – Disease course (relapse within patients)

| Genotype | Relapse | No relapse |
|---|---|---|
| Homozygous variant | 6 | 4 |
| Others | 25 | 36 |

*Preliminary analysis showed no statistically significant association; final p value is undergoing formal verification.*

### Summary/Conclusion

In this Turkish cohort, rs6903608 was not associated with disease susceptibility, supporting population-dependent genetic effects. The higher relapse frequency among homozygous variant patients suggests a potential role as a modifier of disease activity rather than a primary risk factor. These findings indicate that HLA-linked variants may influence chronic immune persistence rather than the initial development of disease. Genetic risk models derived from European cohorts may therefore not be universally applicable and could affect follow-up and preemptive treatment strategies in iTTP. Larger multi-ethnic studies are required for validation.

**Copyright Information:** (Online) ISSN: 2572-9241
©2026 The Author(s). HemaSphere is published by John Wiley & Sons Ltd on behalf of European Hematology Association. This is an open access Abstract Book distributed under the Attribution-NonCommercial-NoDerivs (CC BY-NC-ND), which allows third parties to download the articles and share them with others as long as they credit the author and the Abstract Book, but they cannot change the content in any way or use them commercially.
**Abstract Book Citations:** Authors, Title, HemaSphere, 2026;10:(S1):pages. The abstract book DOIs can be found on the “Author Information” page.
**Disclaimer:** Articles published in the journal HemaSphere exclusively reflect the opinions of the authors. The authors are responsible for all content in their abstracts including accuracy of the facts, statements, citing resources, etc.

### (PF1384) COMPOSITE ENDOTHELIAL STRESS AND INFLAMMATORY INDICES AS PREDICTORS OF STROKE SEVERITY AND FUNCTIONAL OUTCOME

**Topic:** 35. Thrombosis and vascular biology

Michail Makris*[1], Eleftheria Ztriva[1], Michail Diamantidis[2], Vasiliki Gougoula[1], Vasileios Patriarcheas[1], Michail Giannakakis[1], Marina Psoma[1], Stavroula Bountola[1], Vasileios Perifanis[1], Eleni Gavriilaki[3], George Ntaios[1], Christos Savopoulos[1], Georgia Kaiafa[1]

*[1]Aristotle University of Thessaloniki, First Propaedeutic Department of Internal Medicine, THESSALONIKI, Greece;[2]Thalassemia and Sickle Cell Disease Unit, Department of Hematology, Larissa, Greece; [3]Aristotle University of Thessaloniki, 2nd Propedeutic Department of Internal Medicine, Aristotle University of Thessaloniki, Thessaloniki, Greece;*

### Background

Endothelial dysfunction and thromboinflammation play key roles in the pathophysiology of stroke. Composite laboratory indices integrating routine biomarkers may provide clinically meaningful prognostic information beyond traditional risk factors.

### Aims

To evaluate the association of composite endothelial and inflammatory indices with stroke severity, functional outcome, and mortality.

### Methods

We retrospectively studied 100 patients aged ≥ 18 years admitted with acute ischemic stroke (AIS) or transient ischemic attack (TIA) between January 2020 and July 2024. Composite indices were calculated from routine laboratory parameters, including NLR (neutrophil-to-lymphocyte ratio), PLR (platelet-to-lymphocyte ratio), SII (platelets × neutrophils/lymphocytes), FAR (fibrinogen/albumin), FPR (fibrinogen/platelets), and HALP (hemoglobin × albumin × lymphocytes/platelets), along with the endothelial stress indices EASIX (LDH × creatinine/platelets) and mEASIX (LDH × creatinine × C-reactive protein/platelets). Stroke severity was assessed by the NIHSS, and disability assessed at 12 months by the modified Rankin scale (mRS). Outcomes also included in-hospital and 12-month mortality.

### Results

Median age was 82 years; 56% were female. HALP was associated with lower NIHSS at admission (B=−1.807, p=0.047). At discharge, higher NLR (B=2.467, p=0.008), PLR (B=2.548, p=0.013), and SII (B=2.243, p=0.011) were independently associated with higher NIHSS, whereas HALP remained inversely associated (B=−2.594, p=0.005). At 6 months, PLR independently predicted unfavorable functional outcome (mRS 3–6, OR=3.058, p=0.019), whereas HALP demonstrated a protective association (OR=0.283, p=0.007). mEASIX independently predicted both in-hospital mortality (OR=1.749, p=0.010) and 6-month mortality (OR=1.721, p=0.003). Higher NLR (OR=2.903, p=0.020) and SII (OR=3.904, p=0.006) were also independently associated with increased in-hospital mortality, whereas HALP was associated with lower risk of death (OR=0.362, p=0.035). At 6 months, NLR (OR=2.424, p=0.025), PLR (OR=2.317, p=0.038), and SII (OR=2.467, p=0.015) independently predicted mortality, while HALP remained inversely associated with mortality risk (OR=0.363, p=0.009).

**Copyright Information:** (Online) ISSN: 2572-9241
©2026 The Author(s). HemaSphere is published by John Wiley & Sons Ltd on behalf of European Hematology Association. This is an open access Abstract Book distributed under the Attribution-NonCommercial-NoDerivs (CC BY-NC-ND), which allows third parties to download the articles and share them with others as long as they credit the author and the Abstract Book, but they cannot change the content in any way or use them commercially.
**Abstract Book Citations:** Authors, Title, HemaSphere, 2026;10:(S1):pages. The abstract book DOIs can be found on the “Author Information” page.
**Disclaimer:** Articles published in the journal HemaSphere exclusively reflect the opinions of the authors. The authors are responsible for all content in their abstracts including accuracy of the facts, statements, citing resources, etc.

**Summary/Conclusion**

Composite inflammatory and endothelial indices reflect different processes involved in stroke. Inflammatory markers (NLR, PLR, SII) were predominantly associated with neurological severity and functional disability, whereas the endothelial stress marker mEASIX independently predicted mortality. HALP showed a consistent inverse association across outcomes. Overall, these findings underline the role of thromboinflammation and endothelial dysfunction in stroke outcomes and the potential utility of composite indices for risk assessment.

**Copyright Information:** (Online) ISSN: 2572-9241
©2026 The Author(s). HemaSphere is published by John Wiley & Sons Ltd on behalf of European Hematology Association. This is an open access Abstract Book distributed under the Attribution-NonCommercial-NoDerivs (CC BY-NC-ND), which allows third parties to download the articles and share them with others as long as they credit the author and the Abstract Book, but they cannot change the content in any way or use them commercially.
**Abstract Book Citations:** Authors, Title, HemaSphere, 2026;10:(S1):pages. The abstract book DOIs can be found on the "Author Information" page.
**Disclaimer:** Articles published in the journal HemaSphere exclusively reflect the opinions of the authors. The authors are responsible for all content in their abstracts including accuracy of the facts, statements, citing resources, etc.

### (PF1385) DEVELOPMENT OF AN EORTC INSTRUMENT TO ASSESS THE HEALTH-RELATED QUALITY OF LIFE OF PATIENTS WITH HAEMATOLOGICAL MALIGNANCIES WHO RECEIVED CHIMERIC ANTIGEN RECEPTOR T-CELL THERAPY OR T-CELL ENGAGERS

**Topic:** 36. Quality of life, ethics, supportive and palliative care

Kyra Michielsen*[1], Charalampia Kyriakou[2], Anne Spanjaart[3], Susanne Singer[4], Isabella Werry[5], Oliver Bayer[5], Marija Stanić Damić[6], Duska Petranovic[6], Maria Ramos Lopes Gomes Da Silva[7], Catriona Parker[8], Zoe McQuilten[8], Sophie Paget-Bailly[9], Natacha Bolaños[10], Eilidh Duncan[11], Jhulia Salviano[11], Marie José Kersten[3], Simone Oerlemans[1]

*[1]Netherlands Comprehensive Cancer Organisation (IKNL), Department of Research and Development, Utrecht, The Netherlands; [2]University College Hospital, Department of Haematology, London, United Kingdom; [3]Amsterdam UMC, Location University of Amsterdam, Department of Haematology, Cancer Center Amsterdam, Amsterdam, The Netherlands; [4]Comprehensive Cancer Center Mecklenburg-Vorpommern, Department of Quality of Life, Rostock, Germany; [5]University Medical Centre Mainz, Institute of Medical Biostatistics, Epidemiology and Informatics (IMBEI), Division of Epidemiology and Health Services Research, Mainz, Germany; [6]University Clinical Hospital Centre Rijeka, Department of Haematology, Rijeka, Croatia; [7]Portuguese Institute of Oncology, Department of Haematology, Lisbon, Portugal; [8]Transfusion Research Unit, School of Public Health and Preventive Medicine, Monash University, Melbourne, Australia; [9]University hospital of Besançon, Methodology and Quality of Life Unit in Oncology, Besançon, France; [10]Lymphoma Coalition, Madrid, Spain; [11]Myeloma Patients Europe, Brussels, Belgium;*

### Background

Chimeric Antigen Receptor (CAR) T-cell therapy and bispecific T-cell engagers (BiTEs) are emerging therapies for patients with lymphoma, multiple myeloma (MM) and leukaemia. These therapies may impact patients' quality of life (QoL) both through their effects on the disease itself and through their associated adverse effects. To date, no validated multilingual treatment-specific questionnaires are available to assess QoL in patients receiving these treatments.

### Aims

This study describes the Phase I development by the European Organisation for Research and Treatment of Cancer (EORTC) Quality of Life Group (QLG) of a QoL instrument for patients receiving CAR T-cell therapy or BiTEs as a supplement to the EORTC QLQ-C30 and disease specific modules for MM or aggressive lymphoma (i.e. EORTC QLQ-MY20 and NHL29).

### Methods

This is phase I of the four-phase EORTC QLG questionnaire development process and was performed according to the EORTC QLG guidelines in Australia, Croatia, France, Germany, Portugal, the Netherlands and the United Kingdom. In Phase I, an exhaustive list of relevant QoL issues was generated through a structured literature review and insights from meetings with clinicians and patient organisations. In addition, patients and health care professionals (HCP) interviews are being conducted to identify missing issues. Patients and HCPs subsequently will score all issues on relevance and importance to patients having undergone CAR T-cell therapy or BiTEs. Issues with the highest relevance and importance scores will be selected and converted in items in Phase II of the EORTC questionnaire development. Data will be stratified for treatment (type and product), haematologic malignancy group and time since treatment.

**Copyright Information:** (Online) ISSN: 2572-9241
©2026 The Author(s). HemaSphere is published by John Wiley & Sons Ltd on behalf of European Hematology Association. This is an open access Abstract Book distributed under the Attribution-NonCommercial-NoDerivs (CC BY-NC-ND), which allows third parties to download the articles and share them with others as long as they credit the author and the Abstract Book, but they cannot change the content in any way or use them commercially.
**Abstract Book Citations:** Authors, Title, HemaSphere, 2026;10:(S1):pages. The abstract book DOIs can be found on the "Author Information" page.
**Disclaimer:** Articles published in the journal HemaSphere exclusively reflect the opinions of the authors. The authors are responsible for all content in their abstracts including accuracy of the facts, statements, citing resources, etc.

**Results**

Over 90 issues were identified through literature review and meetings with collaborators. Of these issues about half address psychosocial well-being (e.g. Behavioural change, Cognitive function, Emotional distress). The other issues relate to physical well-being (e.g. Mobility, muscles, joints & bones, Neurological & nervous system symptoms) (see Figure 1). The issue list was presented to 45 patients and 38 HCPs during interviews spread over the countries listed above. Patients' and HCPs ratings of the relevance and importance of these issues will be used to inform which items should be included in the Phase II item list and provisional questionnaire.

**Summary/Conclusion**

This project seeks to address the current gap in multilingual PROMs that adequately capture the experiences of patients undergoing CAR T-cell therapy and BiTEs. Continued use of generic PROMs may fail to reflect the full range of patient-reported experiences. The identification of new issues in this Phase I study will highlight the necessity of further developing a treatment-specific instrument. In Phase II, these issues will be systematically refined and operationalized in candidate questionnaire items.

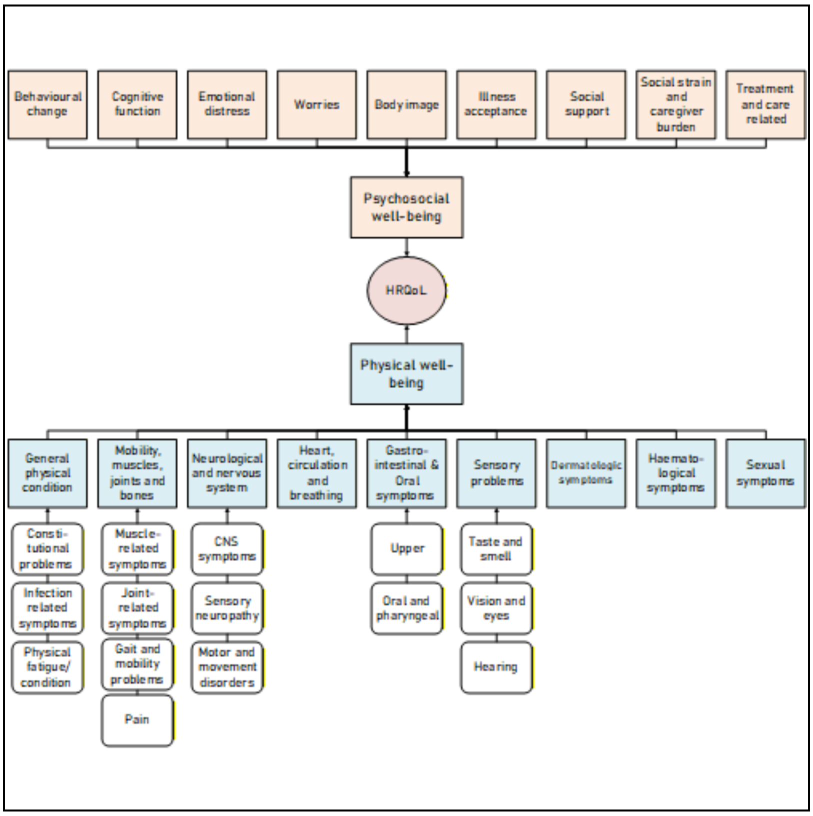

Figure 1: Conceptual Framework of the Phase I issue List for the EORTC QLG Module Development for CAR T-Cell Therapy and Bispecific T-Cell Engagers

Copyright Information: (Online) ISSN: 2572-9241
©2026 The Author(s). HemaSphere is published by John Wiley & Sons Ltd on behalf of European Hematology Association. This is an open access Abstract Book distributed under the Attribution-NonCommercial-NoDerivs (CC BY-NC-ND), which allows third parties to download the articles and share them with others as long as they credit the author and the Abstract Book, but they cannot change the content in any way or use them commercially.
Abstract Book Citations: Authors, Title, HemaSphere, 2026;10:(S1):pages. The abstract book DOIs can be found on the "Author Information" page.
Disclaimer: Articles published in the journal HemaSphere exclusively reflect the opinions of the authors. The authors are responsible for all content in their abstracts including accuracy of the facts, statements, citing resources, etc.

### (PF1386) PATIENT-REPORTED OUTCOMES OF PIRTOBRUTINIB VS. BENDAR IN UNTREATED CLL/SLL: FINDINGS FROM BRUIN-CLL-313 PHASE 3 STUDY

**Topic:** 36. Quality of life, ethics, supportive and palliative care

Tomasz Wrobel*[1], Michal Kwiatek[2], Ederson Roberto de Mattos[3], Ki-Seong Eom[4], Alexander Egle[5], Anna Panovská[6], Zhanet Grudeva-Popova[7], Hsuan-Jen Shih[8], Luis Felipe Casado Montero[9], Paolo Sportoletti[10], Vu Minh Hua[11], Shinsuke Iida[12], Naleen Raj Bhandari[13], Rong Qi[13], Weiji Su[13], Amy S. Ruppert[13], Alejandro Levy[13], Katherine Bao[13], Sarang Abhyankar[13], Rodrigo Ito[13], Wojciech Jurczak[14]

*[1]Wroclaw Medical University, Wroclaw, Poland; [2]AIDPORT Clinical Trials Hospital, Skorzewo, Poland; [3]Hematology Department, Hospital Amaral Carvalho - Jau, São Paulo, Brazil; [4]Catholic Hematology Hospital, Seoul St. Mary's Hospital, The Catholic University of Korea, Seoul, South Korea; [5]Third Medical Department for Hematology, Oncology, Hemostaseology, Infectious Disease and Rheumatology, Paracelsus University Hospital, Salzburg, Austria; [6]Department of Internal Medicine, Hematology and Oncology, Masaryk University, Brno, Czechia; [7]Department of Clinical Oncology, Medical University of Plovdiv, Plovdiv, Bulgaria; [8]Division of Hematology, Department of Internal Medicine, Linkou Chang Gung Memorial Hospital, Taoyuan 33305, Taiwan, Taiwan; [9]HM CIOCC MADRID (Centro Integral Oncológico Clara Campal), Instituto de Investigación Sanitaria HM Hospitales, Madrid, Spain; [10]Department of Medicine and Surgery Institute of Hematology Centro di Ricerca Emato- Oncologica (CREO) University of Perugia, Perugia, Italy; [11]Liverpool Hospital, New South Wales, Australia; [12]Department of Hematology and Oncology, Nagoya City University Graduate School of Medical Sciences, Nagoya, Japan; [13]Eli Lilly and Company, Indianapolis, IN, United States of America; [14]National Research Institute of Oncology, Krakow, Poland;*

### Background

BRUIN-CLL-313 (NCT05023980) is an open label, global, randomized phase 3 study comparing pirtobrutinib with bendamustine plus rituximab (BendaR) in untreated CLL/SLL. BRUIN-CLL-313 showed superior PFS with pirtobrutinib vs BendaR (HR: 0.199, 95%CI: 0.107, 0.367; p<0.0001). Secondary and exploratory endpoints assessing time-to-worsening (TTW) of patient-reported outcomes (PROs) are reported.

### Methods

The design of BRUIN-CLL-313 has been reported elsewhere (Jurczak et al., 2025). Participants completed PRO assessments at baseline (cycle 1 day 1), then Q4W (CLL/SLL-related symptoms, physical functioning [PF], fatigue) or Q8W (overall quality of life [QoL]) through cycle 8, followed by Q16W for the first 2 yrs and every Q24W thereafter until disease progression or up to 4 yrs. CLL/SLL-related symptoms and fatigue were assessed using 13 and 6 items, respectively, from the EORTC Item Library. PF and overall QoL were measured using PF and global health status/QoL subscales of the EORTC Quality of Life Questionnaire Core-30. Each PRO is scored from 0-100; higher scores represent worse CLL/SLL-related symptoms and fatigue but better PF and QoL. TTW of CLL/SLL-related symptoms and PF (secondary) and TTW of fatigue and overall QoL (exploratory) were defined as time from randomization until first instance of PRO worsening, which was required to be sustained for ≥1 subsequent assessment, or death. TTW was estimated by Kaplan-Meier method; corresponding hazard ratios (HRs), and 95% confidence intervals (95%CI) were estimated by Cox proportional hazard models, stratified by randomization strata (Rai stage and IGHV mutation status). 2-sided p-values ≤0.05 were considered statistically significant but not adjusted for multiplicity. Data cutoff was 11 July 2025.

**Copyright Information:** (Online) ISSN: 2572-9241
©2026 The Author(s). HemaSphere is published by John Wiley & Sons Ltd on behalf of European Hematology Association. This is an open access Abstract Book distributed under the Attribution-NonCommercial-NoDerivs (CC BY-NC-ND), which allows third parties to download the articles and share them with others as long as they credit the author and the Abstract Book, but they cannot change the content in any way or use them commercially.
**Abstract Book Citations:** Authors, Title, HemaSphere, 2026;10:(S1):pages. The abstract book DOIs can be found on the "Author Information" page.
**Disclaimer:** Articles published in the journal HemaSphere exclusively reflect the opinions of the authors. The authors are responsible for all content in their abstracts including accuracy of the facts, statements, citing resources, etc.

### Results

A total of 282 participants were randomized to pirtobrutinib (n=141) or BendaR (n=141). Baseline characteristics were comparable, including median age (65 yrs vs 66 yrs) and ECOG performance status 1 or 2 (52% vs 61%). Baseline PRO completion rate was 80% for pirtobrutinib vs 72% for BendaR. Median PRO follow-up in TTW analysis was 25 months (pirtobrutinib) and 15 months (BendaR) and should be considered in interpretation of TTW results. Median TTW was not reached for any PRO endpoints. Pirtobrutinib was associated with prolongation of TTW of CLL/SLL-related symptoms, with HR (95%CI) of 0.50 (0.30, 0.86), p=0.01 (**Fig.**) and the worsening-free survival rate (95%CI) at 18mo was 81.7% (73.1%, 87.8%) for pirtobrutinib vs 68.1% (57.5%, 76.5%) for BendaR. For TTW of PF, the HR was 0.58 (0.30, 1.10), p=0.09 and 18mo worsening-free survival rate was 87.3% (79.5%, 92.3%) for pirtobrutinib vs 77.5% (67.5%, 84.7%) for BendaR. For TTW of fatigue, HR was 0.56 (0.32, 1.00), p=0.04 and 18mo worsening-free survival rate was 81.8% (73.3%, 87.9%) for pirtobrutinib vs 72.2% (61.8%, 80.2%) for BendaR. For TTW of overall QoL, HR was 0.56 (0.28, 1.12), p=0.10 and 18mo worsening-free survival rate was 88.0% (80.2%, 92.9%) for pirtobrutinib vs 80.7% (70.8%, 87.6%) for BendaR.

### Summary/Conclusion

In participants with untreated CLL/SLL, pirtobrutinib monotherapy confers sustained benefit in controlling disease-related symptoms and fatigue, as evidenced by a significant reduction in worsening vs BendaR. While not statistically significant, TTW of other PROs trended similarly. These findings reinforce the clinical efficacy of pirtobrutinib, offering patient-centered evidence that complements previously reported clinical outcomes.

Jurczak W et al. JCO. 2025

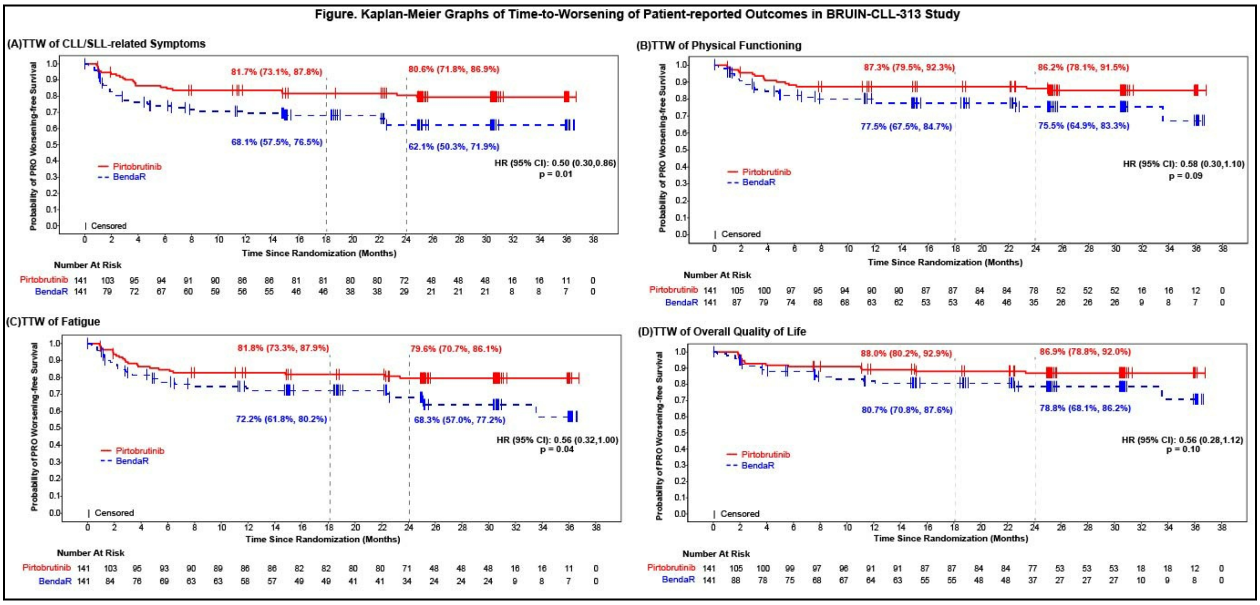

Figure. Kaplan-Meier Graphs of Time-to-Worsening of Patient-reported Outcomes in BRUIN-CLL-313 Study

**Copyright Information:** (Online) ISSN: 2572-9241
©2026 The Author(s). HemaSphere is published by John Wiley & Sons Ltd on behalf of European Hematology Association. This is an open access Abstract Book distributed under the Attribution-NonCommercial-NoDerivs (CC BY-NC-ND), which allows third parties to download the articles and share them with others as long as they credit the author and the Abstract Book, but they cannot change the content in any way or use them commercially.
**Abstract Book Citations:** Authors, Title, HemaSphere, 2026;10:(S1):pages. The abstract book DOIs can be found on the "Author Information" page.
**Disclaimer:** Articles published in the journal HemaSphere exclusively reflect the opinions of the authors. The authors are responsible for all content in their abstracts including accuracy of the facts, statements, citing resources, etc.

### (PF1387) SELF-REPORTED ADVERSE EVENTS USING PRO-CTCAE IN PATIENTS WITH LYMPHOMA AND MULTIPLE MYELOMA IN A REAL-WORLD SETTING

**Topic:** 36. Quality of life, ethics, supportive and palliative care

Zoë Kamperman*[1], Lonneke van de Poll-Franse[2, 3], Djamila Issa[4], Eduardus F.M. Posthuma[1, 5, 6], Afke Ekels[1], Corina van den Hurk[7, 8], Marie José Kersten[9, 10, 11], Simone Oerlemans[1]

*[1]the Netherlands Comprehensive Cancer Organisation (IKNL), Research and Development, Utrecht, The Netherlands; [2]Tilburg University, Department of Medical and Clinical Psychology, Tilburg, The Netherlands; [3]the Netherlands Cancer Institute (NKI), Department of Psychosocial Research and Epidemiology, Amsterdam, The Netherlands; [4]Jeroen Bosch Ziekenhuis, Department of Hematology, Den Bosch, The Netherlands; [5]Reinier de Graaf Gasthuis, Department of Internal Medincine, Delft, The Netherlands; [6]Leiden University Medical Center, Department of Hematology, Leiden, The Netherlands; [7]Catherina Hospital, Eindhoven, The Netherlands; [8]Santeon, Utrecht, The Netherlands; [9]Amsterdam University Medical Center, location University of Amsterdam, Department of Hematology, Amsterdam, The Netherlands; [10]Cancer Center Amsterdam, Amsterdam, The Netherlands; [11]LYMMCARE (Lymphoma and Myeloma Center Amsterdam), Amsterdam, The Netherlands;*

### Background

Treatment-related adverse events (AEs), such as polyneuropathy, fatigue and dyspnea, are common in patients with lymphoma and multiple myeloma (MM) and can negatively affect their health-related quality of life and treatment adherence. Traditional AE reporting relies on clinicians, yet many symptomatic AEs are best known by patients themselves. The Patient-Reported Outcomes version of the Common Terminology Criteria for Adverse Events (PRO-CTCAE) was developed to systematically capture these symptomatic AEs. However, real-world PRO-CTCAE-reported AEs have not been studied in patients with lymphoma and MM, despite the high prevalence of and need for early detection of AEs.

### Aims

To evaluate patient-reported AEs in patients with lymphoma and MM treated in routine clinical practice from the start of first line therapy until one year after.

### Methods

Newly diagnosed patients in 2020-2024 with indolent or aggressive lymphoma and MM, aged ≥18 years were included in the Dutch PROFILES (Patient Reported Outcomes Following Initial treatment and Long-term Evaluation of Survivorship) real-world cohort. From January 2023 onwards, patients receiving active anti-cancer treatment could voluntarily monitor their AEs on a weekly basis through a selected list of 20 PRO-CTCAE items. Data were linked directly to clinical data from the Netherlands Cancer Registry.

Standardized 5-point PRO-CTCAE scale scores were classified as no AE (grade 0) to severe AEs requiring medical intervention (grade 3). Proportions of any AE (≥ grade 1) and severe (grade 2-3) AEs were calculated over time.

**Copyright Information:** (Online) ISSN: 2572-9241
©2026 The Author(s). HemaSphere is published by John Wiley & Sons Ltd on behalf of European Hematology Association. This is an open access Abstract Book distributed under the Attribution-NonCommercial-NoDerivs (CC BY-NC-ND), which allows third parties to download the articles and share them with others as long as they credit the author and the Abstract Book, but they cannot change the content in any way or use them commercially.
**Abstract Book Citations:** Authors, Title, HemaSphere, 2026;10:(S1):pages. The abstract book DOIs can be found on the "Author Information" page.
**Disclaimer:** Articles published in the journal HemaSphere exclusively reflect the opinions of the authors. The authors are responsible for all content in their abstracts including accuracy of the facts, statements, citing resources, etc.

### Results

A total of 2,712 PRO-CTCAE questionnaires of 117 patients were included in this study (median age 62 yr (IQR 52-68), 68% male). Patients were diagnosed with indolent NHL (27%), aggressive NHL (50%) or MM (22%). 75% of patients had a partner and 49% were highly educated; 33% had multiple comorbidities. Patients completed a median of 21 questionnaires (IQR 13-34).
All patients (100%) reported at least one AE of any grade during the first year after start of initial treatment, with fatigue (97%), muscle weakness (89%), dyspnea (86%), and polyneuropathy (85%) being the most common (Table 1). Over time, proportions of grade 2/3 AEs generally remained below 20%, except for fatigue, which ranged between 15–50%, and pain, which reached 30% during the first 5 weeks (Figure 1).
Polyneuropathy showed an emergence pattern during the first 2-3 months, whereas pain, sadness and fatigue declined during the first 20 weeks. Dyspnea and muscle weakness remained relatively stable throughout follow-up. In depth analyses on associated socio-demographic, clinical and treatment characteristics, as well as time-to-event and area-under-the-curve analyses will be presented at EHA 2026.

**Table 1 Proportion (%) of patients having ever reported AEs from start of first treatment until 1yr after**

| AE | Grade 1-3 | Grade 2-3 |
|---|---|---|
| Polyneuropathy | 85% | 36% |
| Dyspnea | 86% | 50% |
| Muscle weakness | 89% | 44% |
| Pain | 82% | 59% |
| Sadness | 80% | 42% |
| Fatigue | 97% | 88% |

### Summary/Conclusion

Findings indicate a high overall prevalence of self-reported AEs during the first year after treatment initiation, with fatigue being the most prevalent and persistent AE. While several AEs stabilize or decline over time, a substantial proportion of patients continue to experience persistent AEs even a year after initiation of first line treatment. This emphasizes the fact that consequences of cancer (treatment) may be long-lasting for many patients, which should be considered when informing patients and planning survivorship care.

**Copyright Information:** (Online) ISSN: 2572-9241
©2026 The Author(s). HemaSphere is published by John Wiley & Sons Ltd on behalf of European Hematology Association. This is an open access Abstract Book distributed under the Attribution-NonCommercial-NoDerivs (CC BY-NC-ND), which allows third parties to download the articles and share them with others as long as they credit the author and the Abstract Book, but they cannot change the content in any way or use them commercially.
**Abstract Book Citations:** Authors, Title, HemaSphere, 2026;10:(S1):pages. The abstract book DOIs can be found on the "Author Information" page.
**Disclaimer:** Articles published in the journal HemaSphere exclusively reflect the opinions of the authors. The authors are responsible for all content in their abstracts including accuracy of the facts, statements, citing resources, etc.

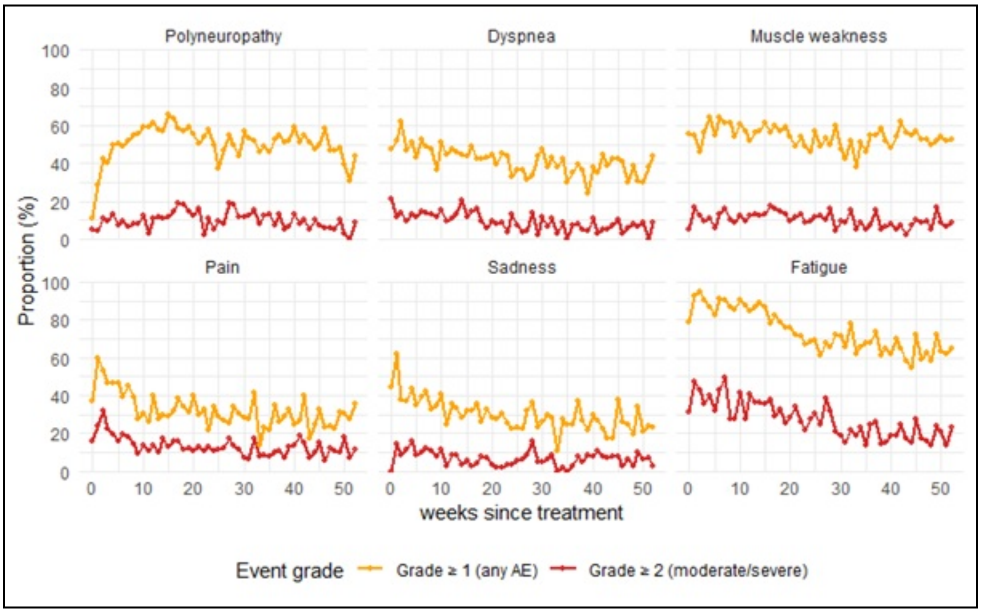

Figure 1 Results from patient reported AEs using PRO-CTCAE reported in the SYMPRO-app by patients diagnosed with lymphoma or multiple myeloma receiving active anti-cancer treatment; proportion (%) event of any grade (1,2,3) and proportion of grade 2-3 (severe) from start of treatment until one year after.

**Copyright Information:** (Online) ISSN: 2572-9241
©2026 The Author(s). HemaSphere is published by John Wiley & Sons Ltd on behalf of European Hematology Association. This is an open access Abstract Book distributed under the Attribution-NonCommercial-NoDerivs (CC BY-NC-ND), which allows third parties to download the articles and share them with others as long as they credit the author and the Abstract Book, but they cannot change the content in any way or use them commercially.
**Abstract Book Citations:** Authors, Title, HemaSphere, 2026;10:(S1):pages. The abstract book DOIs can be found on the "Author Information" page.
**Disclaimer:** Articles published in the journal HemaSphere exclusively reflect the opinions of the authors. The authors are responsible for all content in their abstracts including accuracy of the facts, statements, citing resources, etc.

### (PF1388) ASSISTING PATIENTS DURING CAR-T THERAPY: A DYNAMIC SCORE-BASED ANALYSIS OF NURSING BURDEN, CLINICAL COMPLEXITY, AND FUNCTIONAL DECLINE

**Topic:** 36. Quality of life, ethics, supportive and palliative care

Eugenio Galli*[1], Elisabetta Schifitto[1], Ilaria Pansini[1, 2], Alessandro Corrente[2], Alessio Capitani[1], Teresa Laviano[1], Cinzia Sechi[1], Marco Cioce[1], Federica Sora[1, 2], Patrizia Chiusolo[1, 2], Simona Sica[1, 2]

*[1]Fondazione Policlinico Universitario A. Gemelli IRCCS, Rome, Italy;[2]Università Cattolica del Sacro Cuore, Rome, Italy;*

### Background

CAR-T therapy for relapsed/refractory B-cell malignancies offers high efficacy but is associated with severe toxicities (CRS, ICANS, cytopenias, infections) requiring intensive nursing care. The first two weeks post-infusion are critical, with functional decline, hemodynamic instability, and increased complication risk. Quantitative data on evolving nursing needs remain limited.

### Aims

To evaluate the applicability and dynamic evolution of validated nursing assessment scales in CAR-T patients and identify peak periods of clinical vulnerability and nursing workload to guide structured monitoring and resource allocation.

### Methods

Sixty adult patients receiving CAR-T therapy at Fondazione Policlinico Universitario A. Gemelli IRCCS (Rome) between June 2024–September 2025 were prospectively assessed at admission, day −5/−4, day 0, day +7, day +14, and discharge. Six validated scales were used: **NRS-2002** (nutritional risk), **Conley** (fall risk), **Braden** (pressure injury risk), **Barthel Index** (independence in daily activities), **IDA** (nursing workload), and **MEWS** (early clinical deterioration). CRS and ICANS were graded per EBMT/EHA guidelines. Descriptive statistics and ANOVA analyzed temporal variations.

### Results

At admission, nursing burden was low: Conley 0.7 (7% at risk of falls), Braden 21.9 (low pressure injury risk), Barthel 97.3 (92% fully independent), IDA 26.9; 32% had NRS-2002 ≥3, being at risk of malnutrition. **After CAR-T infusion, all scores worsened** (Figure 1A-E), reflecting increasing nursing needs. **Fall risk (Conley)** rose to 1.7 at day 7 (52% at risk) and 1.9 at day 14 (62% at risk; 20 patients discharged before day 14), partially recovering to 1.45 at discharge (37%). **Pressure injury risk** increased progressively: Braden decreased from 20.9 at day 0 to 19.1 at day 7 and 18.6 at day 14, indicating higher vulnerability, then partially improved to 21 at discharge. **Independence (Barthel)** declined from 98.5 at day 0 to 76.2 at day 7 and 74.0 at day 14, with 13–16% of patients totally dependent, recovering to 93.2 at discharge. **Nursing workload (IDA)** peaked at day 8 (19.4) from 26.9 at admission, reflecting intensified care interventions, monitoring, and support, then partially improved at discharge. **MEWS** scores showed **early deterioration risk** peaking at day 5 (mean 1.1, 13% at risk), in line with CRS/ICANS kinetics. Overall, the period between day 0 and day 14 represents the highest nursing burden; although partial recovery occurs by discharge, functional impairment and dependency persist in a subset of patients, underscoring the need for continued monitoring and multidisciplinary support.

**Copyright Information:** (Online) ISSN: 2572-9241
©2026 The Author(s). HemaSphere is published by John Wiley & Sons Ltd on behalf of European Hematology Association. This is an open access Abstract Book distributed under the Attribution-NonCommercial-NoDerivs (CC BY-NC-ND), which allows third parties to download the articles and share them with others as long as they credit the author and the Abstract Book, but they cannot change the content in any way or use them commercially.
**Abstract Book Citations:** Authors, Title, HemaSphere, 2026;10:(S1):pages. The abstract book DOIs can be found on the "Author Information" page.
**Disclaimer:** Articles published in the journal HemaSphere exclusively reflect the opinions of the authors. The authors are responsible for all content in their abstracts including accuracy of the facts, statements, citing resources, etc.

**Summary/Conclusion**

CAR-T therapy induces a dynamic increase in nursing burden, peaking between days 7–14, coinciding with inflammatory toxicities. Functional decline, dependency, and care intensity remain elevated at discharge. Score-based monitoring allows timely interventions, resource optimization (time-allocations), and coordinated interdisciplinary care, supporting patient safety during the acute CAR-T treatment phase.

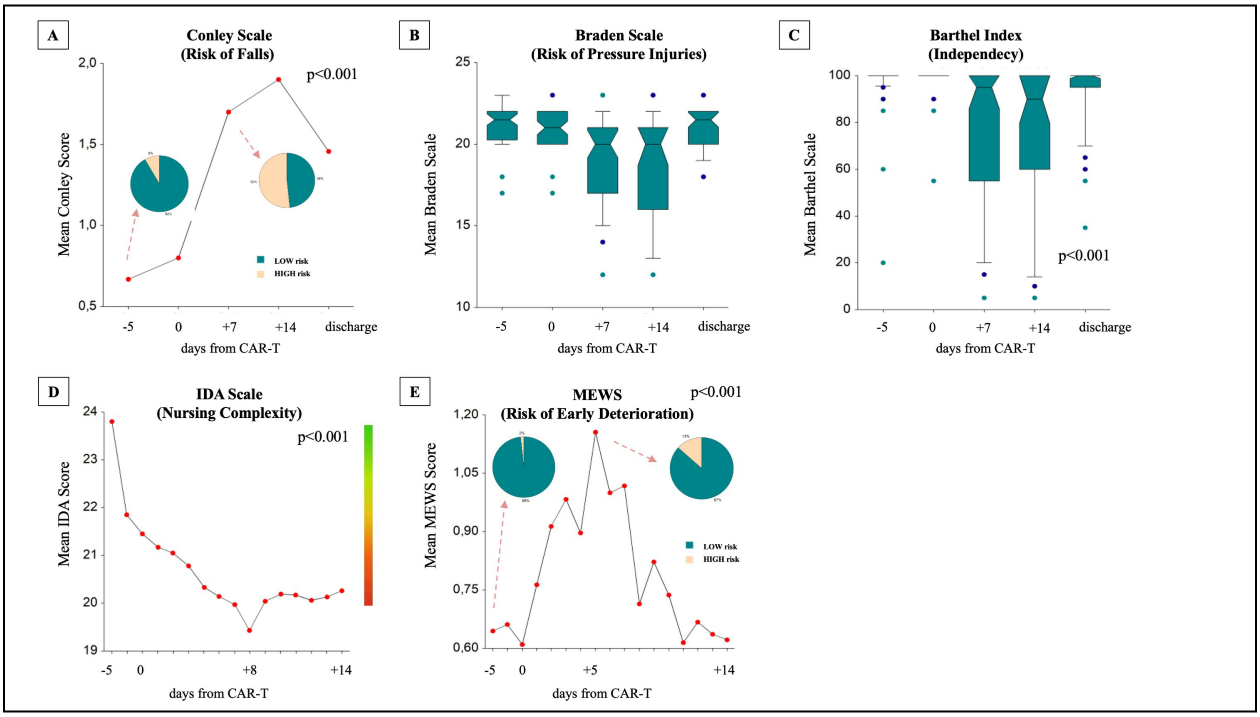

**Copyright Information:** (Online) ISSN: 2572-9241
©2026 The Author(s). HemaSphere is published by John Wiley & Sons Ltd on behalf of European Hematology Association. This is an open access Abstract Book distributed under the Attribution-NonCommercial-NoDerivs (CC BY-NC-ND), which allows third parties to download the articles and share them with others as long as they credit the author and the Abstract Book, but they cannot change the content in any way or use them commercially.
**Abstract Book Citations:** Authors, Title, HemaSphere, 2026;10:(S1):pages. The abstract book DOIs can be found on the "Author Information" page.
**Disclaimer:** Articles published in the journal HemaSphere exclusively reflect the opinions of the authors. The authors are responsible for all content in their abstracts including accuracy of the facts, statements, citing resources, etc.

### (PF1389) AMBULATORY DELIVERY OF BISPECIFIC ANTIBODIES IN RELAPSED/REFRACTORY MULTIPLE MYELOMA (RRMM) VIA A VIRTUAL WARD PATHWAY: REDUCED INPATIENT BED-DAYS AND PRESERVED SAFETY

**Topic:** 36. Quality of life, ethics, supportive and palliative care

Victor Ling*[1], Veronica Lewis[1], Megan Graham[1], Sarah Foody[1], Ioana Tudor[1], Claire Chapman[1], Anna Mayhew[1], Iain Goodhart[1], Helen Bentley[1], Elena Ganendra[1], Michael Chapman[1], Charles Crawley[1], Faisal Basheer[1], Andrew King[1], Marquita Camilleri[1]

[1]*Cambridge University Hospitals NHS Foundation Trust, Cambridge, United Kingdom;*

### Background

Bispecific antibodies (BsAbs) offer effective therapy for RRMM. The increased risk of cytokine release syndrome (CRS) and immune effector cell-associated neurotoxicity syndrome (ICANS) during step-up dosing initially needed inpatient monitoring, causing bed pressures, access delays, and patient burden. Our first-in-the-UK ambulatory care pathway using a Virtual Ward (VW), supported by early oral dexamethasone for grade 1 CRS, provides a safe outpatient strategy that optimises healthcare resources and reduces direct costs.

### Aims

To evaluate the impact of VW implementation (from 04/2025) on inpatient length of stay (LOS), bed-day utilisation, pathway efficiency, direct costs during BsAb priming, safety and CRS management in a real-world cohort.

### Methods

Retrospective UK single-centre analysis carried out 06/2022-01/2026, comparing pre-VW (n=21) vs post-VW (n=20) of 41 BsAb priming episodes in 39 patients. Endpoints included pathway utilisation (fully inpatient vs fully ambulatory/step-down/step-up), median LOS, actual vs expected inpatient bed-days, CRS/ICANS incidence and management, and barriers to ambulatory eligibility.

**Copyright Information:** (Online) ISSN: 2572-9241
©2026 The Author(s). HemaSphere is published by John Wiley & Sons Ltd on behalf of European Hematology Association. This is an open access Abstract Book distributed under the Attribution-NonCommercial-NoDerivs (CC BY-NC-ND), which allows third parties to download the articles and share them with others as long as they credit the author and the Abstract Book, but they cannot change the content in any way or use them commercially.
**Abstract Book Citations:** Authors, Title, HemaSphere, 2026;10:(S1):pages. The abstract book DOIs can be found on the “Author Information” page.
**Disclaimer:** Articles published in the journal HemaSphere exclusively reflect the opinions of the authors. The authors are responsible for all content in their abstracts including accuracy of the facts, statements, citing resources, etc.

**Results**

Median age was 64 years (range 48–82); 67% patients were male and 85% had prior autograft. Treatment comprised elranatamab (73%), teclistamab (22%), and talquetamab (5%). ISS was available at diagnosis in 41% of patients (I 12%, II 22%, III 7%). Cytogenetic risk was standard in 10% and adverse with ≥1 high-risk feature in 27% (data unavailable in 63%). Disease responses were: 46% CR, 24% VGPR, 12% progressive disease/deceased; 5% remain in early follow-up.

Before 04/2025 all 21 patients underwent inpatient step-up dosing (median LOS 8 days, IQR 6-10). After VW implementation, 20% (4/20) were fully ambulatory, 10% (2/20) transitioned from inpatient care to VW, and 15% (3/20) stepped up from VW to inpatient care; 65% (13/20) remained fully inpatient. Common eligibility barriers to VW ambulatory care included renal/organ dysfunction (n=3), living alone (n=2), distance/transport constraints (n=3), and clinical trial participation (n=1). Four patients eligible for VW chose full inpatient management.

Median post-VW median LOS was reduced to 6 days (IQR 0–9). Using the pre-pathway median inpatient LOS of 8 days, expected utalisation for 20 patients was 160 bed days. Observed utalisation was 110 bed days, corresponding to 50 inpatient bed days avoided and 31% reduction in inpatient bed-days. Based on institutional estimates (£350 per inpatient night vs £100 per VW night), this corresponds to an approximate direct cost saving of £12,500 across the post-VW cohort.

CRS occurred in 63% (26/41): 18% grade 1, 15% grade 2, 4% grade 3. No ICANS was observed. Post-VW, 89% of grade 1 CRS events were managed with early oral dexamethasone only (vs 33% pre-VW); 33% of CRS events managed entirely without admission. Tocilizumab use was low (17%).

**Summary/Conclusion**

45% patients received ambulatory or step-down care following use of VW, reducing median inpatient LOS to save 50 bed-days and corresponding to an estimated £12,500 direct cost saving. Severe CRS was low, with 33% low-grade events managed with early oral dexamethasone alone without admission. As the first structured UK implementation of a VW pathway for BsAb priming, this practical and reproducible model can support increasing BsAb demand and constrained inpatient capacity, while highlighting the importance of addressing common eligibility barriers for broader adoption.

**Copyright Information:** (Online) ISSN: 2572-9241
©2026 The Author(s). HemaSphere is published by John Wiley & Sons Ltd on behalf of European Hematology Association. This is an open access Abstract Book distributed under the Attribution-NonCommercial-NoDerivs (CC BY-NC-ND), which allows third parties to download the articles and share them with others as long as they credit the author and the Abstract Book, but they cannot change the content in any way or use them commercially.
**Abstract Book Citations:** Authors, Title, HemaSphere, 2026;10:(S1):pages. The abstract book DOIs can be found on the “Author Information” page.
**Disclaimer:** Articles published in the journal HemaSphere exclusively reflect the opinions of the authors. The authors are responsible for all content in their abstracts including accuracy of the facts, statements, citing resources, etc.

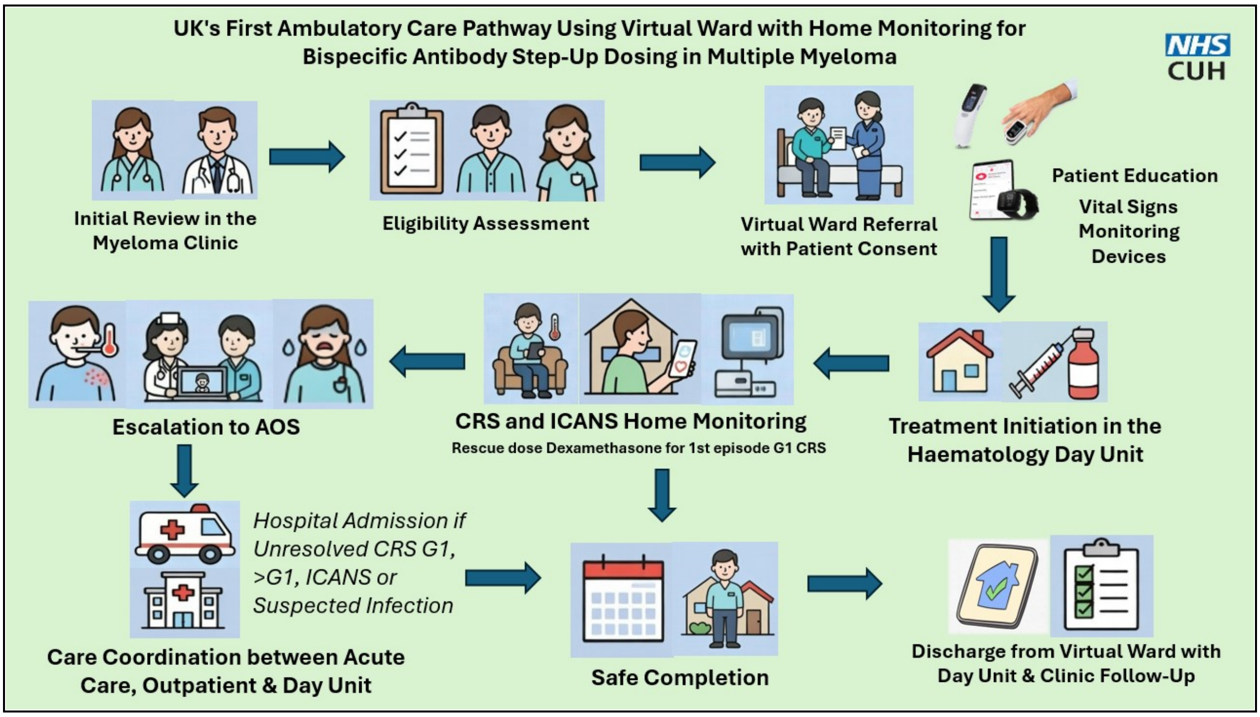

UK's First Ambulatory Care Pathway Using Virtual Ward with Home Monitoring for Bispecific Antibody Step-Up Dosing in Multiple Myeloma

**Copyright Information:** (Online) ISSN: 2572-9241
©2026 The Author(s). HemaSphere is published by John Wiley & Sons Ltd on behalf of European Hematology Association. This is an open access Abstract Book distributed under the Attribution-NonCommercial-NoDerivs (CC BY-NC-ND), which allows third parties to download the articles and share them with others as long as they credit the author and the Abstract Book, but they cannot change the content in any way or use them commercially.
**Abstract Book Citations:** Authors, Title, HemaSphere, 2026;10:(S1):pages. The abstract book DOIs can be found on the “Author Information” page.
**Disclaimer:** Articles published in the journal HemaSphere exclusively reflect the opinions of the authors. The authors are responsible for all content in their abstracts including accuracy of the facts, statements, citing resources, etc.

### (PF1390) QUALITY-ADJUSTED TIME WITHOUT SYMPTOMS OR TOXICITY (Q-TWIST) OF QUIZARTINIB (QUIZ) VS PLACEBO (PBO) IN PATIENTS WITH NEWLY DIAGNOSED FLT3-ITD+ ACUTE MYELOID LEUKEMIA (AML) IN THE QUANTUM-FIRST TRIAL

**Topic:** 36. Quality of life, ethics, supportive and palliative care

Jorge Cortes*[1], Mikkael A. Sekeres[2], Jes B. Hansen[3], Kristen Tecson[4], Sudhir Unni[4], Yvonne Duong[4], Akash Nahar[4], Kristy Burns[4], Esther N. Oliva[5]

*[1]Georgia Cancer Center at Augusta University, Augusta, GA, United States of America;[2]Sylvester Comprehensive Cancer Center, University of Miami Health System, Miami, FL, United States of America; [3]Daiichi Sankyo Europe GmbH, Munich, Germany; [4]Daiichi Sankyo Inc., Basking Ridge, United States of America;[5]Hematology Department, London North West University Healthcare NHS Trust, London, United Kingdom;*

### Background

In QuANTUM-First, Quiz, a selective FLT3 inhibitor, significantly prolonged overall survival (OS) vs PBO when combined with standard chemotherapy and as maintenance monotherapy in patients (pts) with newly diagnosed *FLT3*-ITD+ AML (median OS 31.9 vs 15.1 mo; hazard ratio 0.78; *P*=0.032). Rates of Grade ≥3 treatment-emergent adverse events (TEAEs) were comparable between arms (Quiz 92%, PBO 90%), as were pt-reported outcomes (PROs) and healthcare resource utilization (HRU); overall, the rate of serious adverse events (SAEs) was higher for Quiz (54% vs 46%) but lower during maintenance (34% vs 37%). The Q-TWiST method provides a framework to evaluate the quality of time without disease or need for additional therapy, with a relative gain in Q-TWiST of ≥10% defined as clinically important.

### Aims

To evaluate the quality-adjusted survival benefit of Quiz vs PBO in QuANTUM-First.

### Methods

Pts received standard induction and consolidation chemotherapy plus Quiz or PBO, with optional allo-hematopoietic cell transplant during consolidation. Pts achieving remission could receive single-agent maintenance for ≤3 y. Q-TWiST analyses were conducted on the intent-to-treat population. The primary analysis partitioned time from randomization until death or last follow-up in these health states: time with toxicity (TOX; defined by Grade ≥3 TEAE), time without symptoms or toxicity before relapse (TWiST), and time from relapse (REL; defined by refractory disease or loss of composite complete remission [CRc]). Mean health state durations were derived from Kaplan–Meier estimates using a 55.8-mo restricted mean survival time. Health state durations were adjusted for health-related quality of life by multiplying by the corresponding mean health utility weights from EQ-5D-5L scores from the trial; Q-TWiST is the sum of the quality-adjusted times. Two prespecified sensitivity analyses redefined either TOX using SAE, or REL as refractory disease or loss of CR.

**Copyright Information:** (Online) ISSN: 2572-9241
©2026 The Author(s). HemaSphere is published by John Wiley & Sons Ltd on behalf of European Hematology Association. This is an open access Abstract Book distributed under the Attribution-NonCommercial-NoDerivs (CC BY-NC-ND), which allows third parties to download the articles and share them with others as long as they credit the author and the Abstract Book, but they cannot change the content in any way or use them commercially.
**Abstract Book Citations:** Authors, Title, HemaSphere, 2026;10:(S1):pages. The abstract book DOIs can be found on the “Author Information” page.
**Disclaimer:** Articles published in the journal HemaSphere exclusively reflect the opinions of the authors. The authors are responsible for all content in their abstracts including accuracy of the facts, statements, citing resources, etc.

**Results**

In the primary analysis, the mean Q-TWiST for Quiz (n=268) was numerically longer than PBO (n=271) (25.1 vs 22.2 mo; difference: 2.9 mo; *P*=0.058), primarily driven by more time in TOX (7.4 vs 3.6 mo; *P*<0.01), reflecting a clinically important 10.5% relative Q-TWiST gain (**Fig A**). The SAE-based sensitivity analysis showed a significant Q-TWiST gain for Quiz vs PBO (25.2 vs 22.1 mo; difference 3.1 mo; *P*<0.05; 11.0% relative gain), driven by more time in TWiST (20.3 vs 15.0 mo; *P*<0.01), as the TOX difference was no longer significant (1.2 vs 2.1 mo; *P*=0.28) (**Fig B**). When REL was redefined by loss of CR, Quiz achieved a significantly longer mean Q-TWiST vs PBO (25.3 vs 22.2 mo; difference 3.1 mo; *P*<0.05; 10.9% relative gain), despite pts spending longer in TOX (5.0 vs 2.5 mo; *P*=0.01). Threshold analyses supported the robustness of the sensitivity analyses, with the benefit for Quiz primarily associated with the utility for TOX. A limitation is potential bias from the dependence of TOX duration on treatment time, as total exposure was longer for Quiz (208.2 pt-y [PY]) vs PBO (174.4 PY).

**Summary/Conclusion**

Despite nominal differences in toxicity between Quiz and PBO, the OS benefit with Quiz is accompanied by a quality-adjusted survival advantage, with relative gains in Q-TWiST exceeding the clinically important 10% threshold in the two sensitivity analyses. These findings, along with comparable PRO and HRU across treatment arms, underscore the clinical value of Quiz in newly diagnosed *FLT3*-ITD+ AML.

**Figure. Q-TWiST Health State Durations**

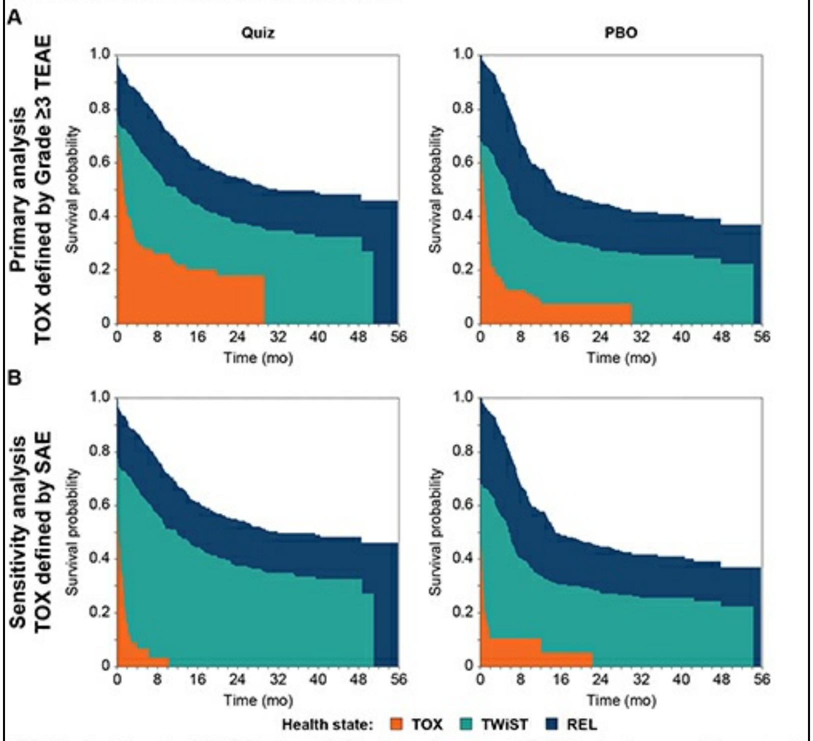

PBO, placebo; Quiz, quizartinib; REL, relapse; SAE, serious adverse event; TEAE, treatment-emergent adverse event; TOX, toxicity; TWiST, time without symptoms or toxicity before relapse.

**Copyright Information:** (Online) ISSN: 2572-9241
©2026 The Author(s). HemaSphere is published by John Wiley & Sons Ltd on behalf of European Hematology Association. This is an open access Abstract Book distributed under the Attribution-NonCommercial-NoDerivs (CC BY-NC-ND), which allows third parties to download the articles and share them with others as long as they credit the author and the Abstract Book, but they cannot change the content in any way or use them commercially.
**Abstract Book Citations:** Authors, Title, HemaSphere, 2026;10:(S1):pages. The abstract book DOIs can be found on the “Author Information” page.
**Disclaimer:** Articles published in the journal HemaSphere exclusively reflect the opinions of the authors. The authors are responsible for all content in their abstracts including accuracy of the facts, statements, citing resources, etc.

### (PF1391) ADAPTIVE, STEPPED-CARE FOR CANCER-RELATED PTSD IN HCT SURVIVORS: RESULTS FROM A SEQUENTIAL MULTIPLE ASSIGNMENT RANDOMIZED TRIAL (SMART)

**Topic:** 36. Quality of life, ethics, supportive and palliative care

Sophia Smith*[1], Eric Kuhn[2], Tamara Somers[3], Eric Laber[3], Cole Manschot[4], Anthony Sung[5], Sarah Kelleher[3], Catherine Majestic[1], Karen Syrjala[6], Deborah Maryer[7], Sergio Giralt[8], Andrea Pires[1], Rebecca Gebert[9], Jason Owen[2], Jean Yi[6], Allison Applebaum[10]

*[1]Duke University, School of Nursing, Durham, United States of America;[2]National Center for PTSD, Palo Alto, CA, United States of America; [3]Duke University, Durham, NC, United States of America;[4]Merck, Rahway, NJ, United States of America;[5]University of Kansas, Kansas City, KS, United States of America; [6]Fred Hutchinson Cancer Center, Seattle, WA, United States of America; [7]University of North Carolina, Chapel Hill, NC, United States of America;[8]Memorial Sloan Kettering, New York, NY, United States of America; [9]Boston University, Boston, MA, United States of America; [10]Mount Sinai School of Medicine, Brookdale Department of Geriatrics and Palliative Medicine, New York, NY, United States of America;*

### Background

Cancer-related posttraumatic stress disorder (PTSD) is common among hematopoietic cell transplantation (HCT) survivors, yet access to trauma-focused care remains limited. Scalable digital tools and adaptive treatment models may improve symptoms and personalize care delivery.

### Aims

To evaluate an adaptive stepped-care model for cancer-related PTSD using a sequential multiple assignment randomized trial (SMART) and determine which treatment pathways yield the greatest symptom improvement.

### Methods

HCT survivors 1–5 years post-transplant (N=477) screening positive for probable or subthreshold PTSD (PCL-5) were randomized to the Cancer Distress Coach (CaDC) app or Usual Care. At Week 4, non-responders (<5-point PCL-5 reduction) were re-randomized to CaDC+Coaching or therapist-delivered CBT-PTSD. PTSD symptoms (primary outcome), depression, and anxiety were assessed at baseline, Week 4, Month 3 (primary endpoint), and Month 6. Analyses compared adaptive regimens using global and pairwise tests. This trial is registered at ClinicalTrials.gov (NCT04058795).

### Results

All participants showed PTSD symptom reductions by Month 3, with significant differences across adaptive regimens (global p=.017). The most effective pathway (CaDC → CBT-PTSD) achieved the greatest PTSD reduction (mean −17.2 points; d=0.96), significantly outperforming Usual Care → CaDC+Coaching (p<.04). Improvements were sustained through Month 6 (global p=.002). Depression (d=0.45) and anxiety (d=0.47) also improved significantly, with CaDC → CBT-PTSD yielding the strongest effects (global p<.001). Early differences between CaDC and Usual Care at Week 4 were not statistically significant; however, pathways beginning with CaDC demonstrated more robust and sustained improvement over time.

**Copyright Information:** (Online) ISSN: 2572-9241
©2026 The Author(s). HemaSphere is published by John Wiley & Sons Ltd on behalf of European Hematology Association. This is an open access Abstract Book distributed under the Attribution-NonCommercial-NoDerivs (CC BY-NC-ND), which allows third parties to download the articles and share them with others as long as they credit the author and the Abstract Book, but they cannot change the content in any way or use them commercially.
**Abstract Book Citations:** Authors, Title, HemaSphere, 2026;10:(S1):pages. The abstract book DOIs can be found on the "Author Information" page.
**Disclaimer:** Articles published in the journal HemaSphere exclusively reflect the opinions of the authors. The authors are responsible for all content in their abstracts including accuracy of the facts, statements, citing resources, etc.

**Summary/Conclusion**

A stepped-care model beginning with a digital intervention (CaDC) and escalating to CBT-PTSD effectively reduced PTSD, depression, and anxiety among HCT survivors. Findings highlight the value of adaptive digital-first strategies for scalable and personalized trauma-focused care in cancer survivorship. Research reported in this publication was supported by the National Cancer Institute of the National Institutes of Health under Award Number R01-CA244172.

**Copyright Information:** (Online) ISSN: 2572-9241
©2026 The Author(s). HemaSphere is published by John Wiley & Sons Ltd on behalf of European Hematology Association. This is an open access Abstract Book distributed under the Attribution-NonCommercial-NoDerivs (CC BY-NC-ND), which allows third parties to download the articles and share them with others as long as they credit the author and the Abstract Book, but they cannot change the content in any way or use them commercially.
**Abstract Book Citations:** Authors, Title, HemaSphere, 2026;10:(S1):pages. The abstract book DOIs can be found on the "Author Information" page.
**Disclaimer:** Articles published in the journal HemaSphere exclusively reflect the opinions of the authors. The authors are responsible for all content in their abstracts including accuracy of the facts, statements, citing resources, etc.

### (PF1392) REAL-WORLD OUTCOMES OF SICKLE CELL DISEASE PATIENTS TRANSITIONING FROM PEDIATRIC TO ADULT CARE: IMPACT ON ACUTE CARE UTILIZATION

**Topic:** 36. Quality of life, ethics, supportive and palliative care

Heidi Cho*[1], Olga Tesarova[2], Orlando Esparza[2], Tinu Lufadeju[1], Michael Mapp[1], Nancy Lunney[1], Vladimir Otasevic[1, 1]

*[1]Parexel, Global Medical Services, Raleigh, United States of America;[2]Parexel, Medical Services, Raleigh, United States of America;*

### Background

The transition from pediatric to adult care is a vulnerable period for individuals with sickle cell disease (SCD). Pediatric programs typically provide coordinated, multidisciplinary care with proactive complication screening and strong psychosocial support. In contrast, adult care systems may be less integrated, with greater variability in access to hematology specialists, care coordination, and preventive services. Prior studies show that young adults with SCD experience increased morbidity, higher acute care utilization, and elevated mortality during this transition, reflecting gaps in continuity of care and transition readiness. Despite these concerns, real-world data (RWD) quantifying changes in emergency department (ED) visits and hospitalizations during the transition remain limited.

### Aims

This study evaluates real-world outcomes among SCD patients transitioning from pediatric to adult care, with a primary focus on whether acute care utilization, including ED visits and hospitalizations, increases after transfer. Given the loss of care continuity and reduced access to specialized adult hematology services during this period, the analysis aims to quantify how these systemic changes translate into measurable differences in acute care use.

### Methods

A retrospective, multicenter RWD analysis was conducted using the TriNetX Global Network on February 16, 2026, drawing from electronic medical records of 247,636 patients across 110 healthcare organizations. SCD was defined using ICD-10 code D57. Two cohorts were identified: patients ≤17 years old (Cohort 1) and patients ≥18 years old (Cohort 2). The mean age at index event (ED visit or hospitalization) was 2.74±3.78 years in Cohort 1 and 34.2±17.4 years in Cohort 2; 52.38% of Cohort 1 and 28.96% of Cohort 2 were male. Outcomes were assessed from index event through 10 years. Acute care utilization was compared between cohorts, and Cox proportional hazards models were used to estimate risk.

### Results

Adults with SCD demonstrated a measurable increase in acute care utilization following transition to adult care. Compared with pediatric patients, adults experienced higher rates of ED visits and unplanned hospitalizations. Adults exhibited a 9.94% higher risk of inpatient hospitalization, corresponding to an adjusted relative risk (RR) of 1.19 (95% CI: 1.477–1.53; p<0.0001). Early analyses suggest that patients without structured transition planning, lacking documented readiness assessments, or experiencing delays in establishing adult hematology care show the greatest increases in acute care use. These findings align with prior literature linking inadequate transition preparation to increased acute care utilization and preventable complications.

**Copyright Information:** (Online) ISSN: 2572-9241
©2026 The Author(s). HemaSphere is published by John Wiley & Sons Ltd on behalf of European Hematology Association. This is an open access Abstract Book distributed under the Attribution-NonCommercial-NoDerivs (CC BY-NC-ND), which allows third parties to download the articles and share them with others as long as they credit the author and the Abstract Book, but they cannot change the content in any way or use them commercially.
**Abstract Book Citations:** Authors, Title, HemaSphere, 2026;10:(S1):pages. The abstract book DOIs can be found on the “Author Information” page.
**Disclaimer:** Articles published in the journal HemaSphere exclusively reflect the opinions of the authors. The authors are responsible for all content in their abstracts including accuracy of the facts, statements, citing resources, etc.

**Summary/Conclusion**

RWD indicates that the transition from pediatric to adult care is associated with increased acute care utilization among individuals with SCD, likely reflecting gaps in transition readiness, care coordination, and access to specialized adult services. Planned subgroup analyses will examine the impact of documented transition planning, continuity of care, and time to first adult hematology visit. These findings underscore the need for standardized transition pathways, earlier preparation during adolescence, and improved integration between pediatric and adult hematology programs. Strengthening transition processes may reduce preventable acute care use and improve long-term outcomes for young adults with SCD.

**Copyright Information:** (Online) ISSN: 2572-9241
©2026 The Author(s). HemaSphere is published by John Wiley & Sons Ltd on behalf of European Hematology Association. This is an open access Abstract Book distributed under the Attribution-NonCommercial-NoDerivs (CC BY-NC-ND), which allows third parties to download the articles and share them with others as long as they credit the author and the Abstract Book, but they cannot change the content in any way or use them commercially.
**Abstract Book Citations:** Authors, Title, HemaSphere, 2026;10:(S1):pages. The abstract book DOIs can be found on the "Author Information" page.
**Disclaimer:** Articles published in the journal HemaSphere exclusively reflect the opinions of the authors. The authors are responsible for all content in their abstracts including accuracy of the facts, statements, citing resources, etc.

### (PF1393) FAVORABLE HEALTHCARE RESOURCE UTILIZATION (HCRU) WITH TAGRAXOFUSP (TAG) VS VENETOCLAX (VEN) IN PATIENTS WITH BLASTIC PLASMACYTOID DENDRITIC CELL NEOPLASM (BPDCN): RESULTS OF A REAL-WORLD (RW) ANALYSIS

**Topic:** 36. Quality of life, ethics, supportive and palliative care

Anthony Stein*[1], Guru Subramanian Guru Murthy[2], Pankit Vachhani[3], Sunil Iyer[4], Deborah Goldschmidt[5], Yipeng Gao[6], John Katsetos[7], Corey Pelletier[7], Tibor Kovacsovics[8]

*[1]City of Hope, Duarte, United States of America;[2]Medical College of Wisconsin, Milwaukee, United States of America; [3]University of Alabama at Birmingham, Birmingham, United States of America;[4]Columbia University, New York, United States of America; [5]Analysis Group, New York, United States of America; [6]Analysis Group, Boston, United States of America; [7]Menarini Group, New York, United States of America; [8]City of Hope, Goodyear, United States of America;*

### Background

TAG, a first-in-class CD123-targeted therapy, is the only drug approved to treat BPDCN, an aggressive orphan hematologic malignancy characterized by cells expressing CD123 and other markers. The first-line BPDCN treatment goal is to rapidly induce a complete response, without prolonged myelosuppression, to enable hematopoietic cell transplantation (HCT) for eligible patients. In clinical and RW settings, TAG showed high rates of rapid, durable responses, allowing effective bridge to HCT following a TAG-induced remission with associated long-term survival (Pemmaraju, JCO 2022; Herling, Ann Hematol 2025). Although VEN is not approved for BPDCN, limited RW use has been reported (Gangat, Am J Hematol 2022; Pemmaraju, Blood Neoplasia 2025). Given this practice, we examined RW outcomes and reported a clinically meaningful longer median overall survival (OS) with TAG- vs VEN-based regimens (35.3 vs 10.5 mo; Stein, ASH 2025) in a RW claims analysis.

### Aims

Examine differences in HCRU metrics of pts with BPDCN treated with TAG- vs VEN-based regimens in a RW claims analysis.

### Methods

The US Komodo Research Database was used to retrospectively analyze claims data from pts with ≥1 BPDCN diagnosis between Jan 2016 and Dec 2023 and subsequent initiation of treatment with a TAG- or VEN-based regimen. Index date was defined as first inpatient (IP) dosing of TAG or start of VEN-based therapy. Pts on TAG were matched 1:1 with pts on VEN by line of therapy (LOT). This analysis describes pt characteristics during the 6-month (mo) preindex period and follow-up HCRU metrics from index to earliest end of index LOT, end of data availability, or death, up to 6 mo. HCRU metrics and primary diagnoses of emergency room (ER) visits were summarized separately for mo 1 and mos 2-6 postindex and reported per pt per mo (PPPM).

**Copyright Information:** (Online) ISSN: 2572-9241
©2026 The Author(s). HemaSphere is published by John Wiley & Sons Ltd on behalf of European Hematology Association. This is an open access Abstract Book distributed under the Attribution-NonCommercial-NoDerivs (CC BY-NC-ND), which allows third parties to download the articles and share them with others as long as they credit the author and the Abstract Book, but they cannot change the content in any way or use them commercially.
**Abstract Book Citations:** Authors, Title, HemaSphere, 2026;10:(S1):pages. The abstract book DOIs can be found on the "Author Information" page.
**Disclaimer:** Articles published in the journal HemaSphere exclusively reflect the opinions of the authors. The authors are responsible for all content in their abstracts including accuracy of the facts, statements, citing resources, etc.

**Results**

The TAG cohort (mean age, 61 y; male, 72%; mean Charlson Comorbidity Index [CCI] score, 0.8) and VEN cohort (mean age, 67 y; male, 83%; mean CCI score, 1.1) each comprised 47 pts. The most common CCI comorbidities in the TAG and VEN cohorts, respectively, were diabetes (21% vs 30%), chronic pulmonary disease (21% vs 15%), and peripheral vascular disease (13% vs 26%). At 1 mo postindex, TAG and VEN pts had the same median total IP duration (10.8 days), but TAG pts had fewer transfusions than VEN pts (19% vs 34%; Fig 1). At 2-6 mo postindex, similar proportions of TAG and VEN pts had ≥1 IP admission (36% vs 29%) or ER visit (24% vs 27%); the most common primary diagnosis of ER visit was hypertension (33%) for TAG and neutropenia (38%) for VEN. During mos 2-6 postindex, median total IP duration was 3 days for TAG and 5.8 days for VEN; the median number of outpatient (OP) visits was 8.8 for TAG and 6.6 for VEN. The TAG cohort had less transfusion burden than VEN during mos 2-6 (3% vs 32%).

**Summary/Conclusion**

During months 2-6, TAG showed reduced median total IP duration (3 vs 5.8 days) and substantially reduced transfusion rates (3% vs 32%) compared with VEN, demonstrating rapid hematopoietic recovery and absence of cumulative myelotoxicity. During this same period, TAG and VEN had similar IP and ER admission rates. Together with TAG's outcomes from a well-controlled prospective trial, European RW setting, and the previously reported prolonged RW OS with TAG- vs VEN-based regimens, this HCRU analysis affirms TAG as the first-line standard of care for all eligible patients with BPDCN and the backbone of future combination regimens.

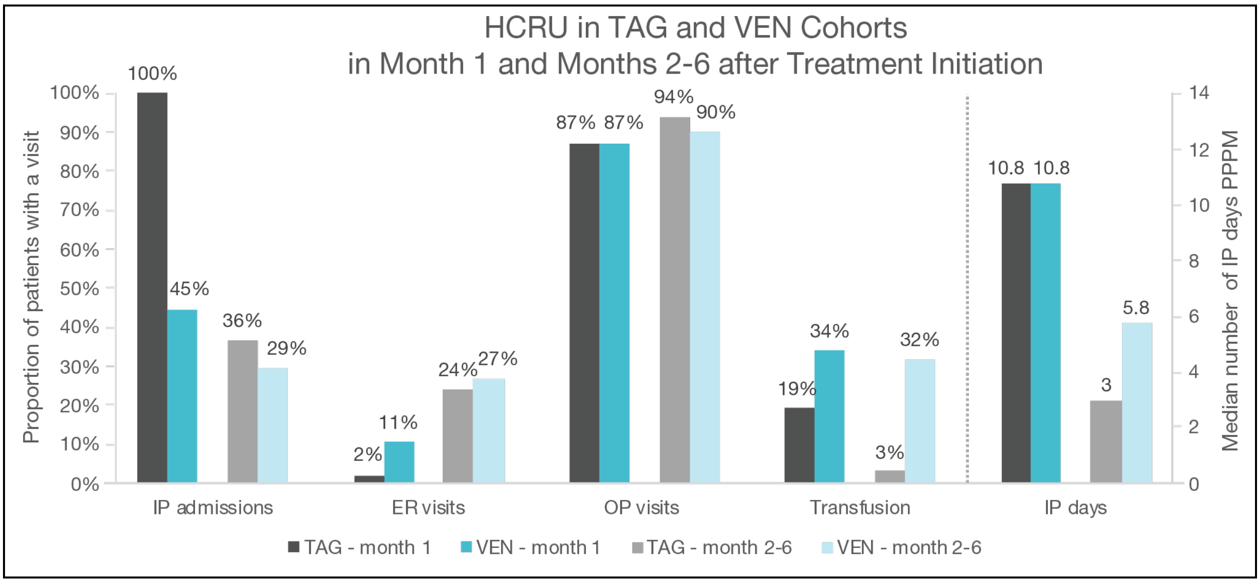

Copyright Information: (Online) ISSN: 2572-9241
©2026 The Author(s). HemaSphere is published by John Wiley & Sons Ltd on behalf of European Hematology Association. This is an open access Abstract Book distributed under the Attribution-NonCommercial-NoDerivs (CC BY-NC-ND), which allows third parties to download the articles and share them with others as long as they credit the author and the Abstract Book, but they cannot change the content in any way or use them commercially.
Abstract Book Citations: Authors, Title, HemaSphere, 2026;10:(S1):pages. The abstract book DOIs can be found on the "Author Information" page.
Disclaimer: Articles published in the journal HemaSphere exclusively reflect the opinions of the authors. The authors are responsible for all content in their abstracts including accuracy of the facts, statements, citing resources, etc.

### (PF1394) SLEEP DISTURBANCES AFTER HEMATOLOGICAL MALIGNANCIES DIAGNOSIS: PREVALENCEAND SEVERITY IN A PATIENT SURVEY

**Topic:** 36. Quality of life, ethics, supportive and palliative care

Esther N. Oliva*[1], Matteo Fattizzo[2, 3], Roberta Siclari[3], Maria Teresa Voso[4], Carmelo Gurnari[4], Chiara Frairia[5], Pasquale Niscola[6], Giuseppe Ianni[2], Vaitsa Katsomitrou[1], Ravi Bimolah[1], Cindy Carasig[1], Marife Colis[1], Giorgia Francesconi[1], Rhodora Relucio[1], Muna Rimal[1], Samantha Nier[7]

*[1]London North West Healthcare University NHS Trust, Hematology Department, London, United Kingdom;[2]Associazione QOL-ONE, Reggio Calabria, Italy; [3]Associazione QOL-ONE, Italia, Reggio Calabria, Italy; [4]Universita' di Roma Tor Vergata, Department of Biomedicine and Prevention, Roma, Italy; [5]University Hospital Città della Salute e della Scienza,Torino, Italy, Hematology, Torino, Italy; [6]S. Eugenio Hospital, ASL Roma 2, Rome, Italy, Hematology Unit, Roma, Italy;[7]Acute Leukemia Advocates Network, Bern, Switzerland;*

### Background

Hematological malignancies and their treatments may disrupt sleep patterns through pro-inflammatory cytokine release and psychological distress. Conversely, chronic sleep deprivation impairs immune surveillance and circadian regulation of hematopoiesis, potentially compromising clinical outcomes and exacerbating cancer-related fatigue in patients with hematological malignancies (HMs).

### Aims

To evaluate prevalence, severity and clinical correlates of sleep disturbances after diagnosis of HMs and to quantify unmet supportive care needs.

### Methods

An anonymous multilingual online survey is being conducted over 8 weeks among adults with HMs recruited through clinics in Italy and the UK and patient advocacy websites. The questionnaire assessed disease characteristics, diagnosis communication, pre- and post-diagnosis sleep patterns, treatment-related sleep impact and support needs. Pre/post differences were analyzed using McNemar-Bowker tests. The Pittsburgh Sleep Quality Index (PSQI) was optionally completed and component scores and Global score (>5) were analyzed using Chi-square tests.

**Copyright Information:** (Online) ISSN: 2572-9241
©2026 The Author(s). HemaSphere is published by John Wiley & Sons Ltd on behalf of European Hematology Association. This is an open access Abstract Book distributed under the Attribution-NonCommercial-NoDerivs (CC BY-NC-ND), which allows third parties to download the articles and share them with others as long as they credit the author and the Abstract Book, but they cannot change the content in any way or use them commercially.
**Abstract Book Citations:** Authors, Title, HemaSphere, 2026;10:(S1):pages. The abstract book DOIs can be found on the "Author Information" page.
**Disclaimer:** Articles published in the journal HemaSphere exclusively reflect the opinions of the authors. The authors are responsible for all content in their abstracts including accuracy of the facts, statements, citing resources, etc.

**Results**

At the present analysis, 201 patients (52% female), mean age 56 years (25–88), participated. Diagnoses included chronic myeloid leukemia (65%), acute leukemias (10%), lymphoma (5%), chronic lymphocytic leukemia (5%), chronic myeloproliferative neoplasms (4%), myeloma (3%), VEXAS (2%) and myelodysplastic neoplasms (1%). Although 76% felt emotionally supported at diagnosis, 54% reported negative impact. Sustained sleep deterioration occurred in 74% within the first week post-diagnosis (31% significant). Pre-diagnosis, 66% rated sleep as good/very good and post-diagnosis, 60% reported significant worsening of sleep duration ($p<0.0001$) : difficulty initiating and maintaining sleep was reported by 80% and 84%, respectively, with 29% experiencing early awakening most nights. Furthermore, overall sleep-related quality of life was poor-very poor in 33%. Fatigue (47%), night sweats (38%), anxiety (37%) and pain (37%) were the main sleep-disrupting symptoms. Targeted therapies (27%) and chemotherapy (21%) were most frequently associated with sleep impact. Seventy-one percent reported relapse-related worry affecting sleep. Sleep aid medication use increased significantly after diagnosis ($p<0.0001$; 15 to 54 cases). Importantly, 81% reported little/ no clinical attention to sleep and 65% expressed a need for structured support.
Among 117 (58%) patients completing PSQI, 78% confirmed poor global sleep quality, with frequent impairment in duration (62%), disturbances (62%), latency (51%) and efficiency (56%). Patient-reported complaints were significantly associated with corresponding PSQI domains ($p<0.05$).

**Summary/Conclusion**

Sleep disturbance emerges early after HM diagnosis and persists across the disease trajectory. Nearly four out of five patients exhibited objectively poor global sleep quality. Despite its high prevalence and clear clinical impact, sleep remains systematically under-recognized in routine hematology care. These findings identify sleep as a modifiable and actionable determinant of patient-reported outcomes (PROs). Integrating routine screening and structured behavioral and psychosocial sleep interventions into standard hematological pathways represents a high-impact strategy to improve PROs and strengthen comprehensive supportive care.

**Copyright Information:** (Online) ISSN: 2572-9241
©2026 The Author(s). HemaSphere is published by John Wiley & Sons Ltd on behalf of European Hematology Association. This is an open access Abstract Book distributed under the Attribution-NonCommercial-NoDerivs (CC BY-NC-ND), which allows third parties to download the articles and share them with others as long as they credit the author and the Abstract Book, but they cannot change the content in any way or use them commercially.
**Abstract Book Citations:** Authors, Title, HemaSphere, 2026;10:(S1):pages. The abstract book DOIs can be found on the “Author Information” page.
**Disclaimer:** Articles published in the journal HemaSphere exclusively reflect the opinions of the authors. The authors are responsible for all content in their abstracts including accuracy of the facts, statements, citing resources, etc.

### (PF1395) THE AGGRESSIVENESS OF END-OF-LIFE CARE IN JAEMATOLOGY-ONCOLOGY PATIENTS AND ITS ASSOCIATION WITH QUALITY OF DYING AND DEATH

**Topic:** 36. Quality of life, ethics, supportive and palliative care

Michal Braun[1, 2], Adir Shaulov[1], May Bergman[2], Guy Lichtenfeld[2], Ilana Nili Hellmann[3], Tamar Tadmor[4], Nira Bonneh[5], Shelly Rado[6], Ariel Aviv*[5]

*[1]Hadassah Medical Center, Jeruslaem, Israel;[2]The Academic college of Tel Aviv Yaffo, Yaffo, Israel;[3]Gray Faculty of Medical and Health sciences, Tel Aviv University, Tel Aviv, Israel;[4]Bnai-Zion Medical Center, Haifa, Israel;[5]Ha'Emek medical center, Afula, Israel;[6]Hillel Yaffe Medical Center, Hadera, Israel;*

### Background

Assessing the quality of end of life (EOL) care in cancer patients is complex. Earle's criteria were first published in 2008 and they are composed of six different items, each indicating an overly aggressive EOL care. These criteria were developed for retrieval from solid tumor patients' large datasets and have since become the standard for measuring quality of EOL care in cancer. Earle's criteria can be calculated dichotomously (i.e any positive item defines death as aggressive) or as sum of positive items per patient from 0 to 6 (Hui modification). Trials comparing hematology and oncology patients using these criteria have repeatedly shown a worse score in hematology than in oncology, as an indicator of more aggressive EOL care in hematology.

The QODD tool is a questionnaire developed to evaluate the subjective components of dying. This validated tool uses interviews with bereaved family members as proxy respondents and is composed of 31 questions focusing on 6 conceptual domains regarding the deceased last month of life. The score is presented on a 0-100 scale (100 being the best possible experience) and can be presented both as each domain separately and as the sum of all domains. This questionnaire is not specific to cancer, and to the best of our knowledge has never been tested in hematology.

### Aims

Our study had 2 objectives: first, to describe the aggressiveness of EOL care in hemato-oncology patients in Israel according to Earle’s criteria, and second to examine the correlation between these criteria and the subjective experience using the QODD questionnaire.

### Methods

We reviewed the clinical records of 644 deceased hemato-oncologic patients in 4 Israeli hospitals between 2019 and 2021 and extracted all items regarding care at the last month of life.
We then interviewed 45 family members of deceased patients several months after death using the QODD questionnaire.
Quantitative and statistical analyses were applied to Earle score, along with various analyses of QODD scores, enabling cross-validation between objective data and family-reported experiences.

**Copyright Information:** (Online) ISSN: 2572-9241
©2026 The Author(s). HemaSphere is published by John Wiley & Sons Ltd on behalf of European Hematology Association. This is an open access Abstract Book distributed under the Attribution-NonCommercial-NoDerivs (CC BY-NC-ND), which allows third parties to download the articles and share them with others as long as they credit the author and the Abstract Book, but they cannot change the content in any way or use them commercially.
**Abstract Book Citations:** Authors, Title, HemaSphere, 2026;10:(S1):pages. The abstract book DOIs can be found on the “Author Information” page.
**Disclaimer:** Articles published in the journal HemaSphere exclusively reflect the opinions of the authors. The authors are responsible for all content in their abstracts including accuracy of the facts, statements, citing resources, etc.

### Results

The mean age of death in our cohort was 74 (range 19-103), 58.5% were males. The main diseases in our cohort were multiple myeloma (21.3%), acute leukemia (18.1%) and aggressive NHL (17.1%).
Using the dichotomous approach, 87.3% of patients were categorized as having received aggressive EOL care. However, in the graded analysis, most were in the low-to-moderate range (median of 2 out of 6). The most common contributor was death in hospital, which occurred in 72% of patients.
Younger patients and men tended to receive more aggressive EOL care.
The overall average QODD score was 62.5 (moderate experience). Subdomain scores were relatively high for "moment of death" (average score of 75) and "time with others" (average score 72.7). The lowest score was for "symptom control" subdomain – 45.7 **(Figure 1)**
There was no significant correlation between total QODD score and the level of treatment aggressiveness according to Earle criteria, though some subdomain associations were found.

### Summary/Conclusion

This is the first study ever trying to correlate Earle criteria with subjective end of life experience. In contrary to the initial hypothesis, death aggressiveness was generally low to moderate among hemato-oncology patients in Israel. Earle's criteria may not adequately reflect the quality of dying in this population.
The absence of correlation with QODD underscores the need for hematology-specific indices.

**Copyright Information:** (Online) ISSN: 2572-9241
©2026 The Author(s). HemaSphere is published by John Wiley & Sons Ltd on behalf of European Hematology Association. This is an open access Abstract Book distributed under the Attribution-NonCommercial-NoDerivs (CC BY-NC-ND), which allows third parties to download the articles and share them with others as long as they credit the author and the Abstract Book, but they cannot change the content in any way or use them commercially.
**Abstract Book Citations:** Authors, Title, HemaSphere, 2026;10:(S1):pages. The abstract book DOIs can be found on the "Author Information" page.
**Disclaimer:** Articles published in the journal HemaSphere exclusively reflect the opinions of the authors. The authors are responsible for all content in their abstracts including accuracy of the facts, statements, citing resources, etc.

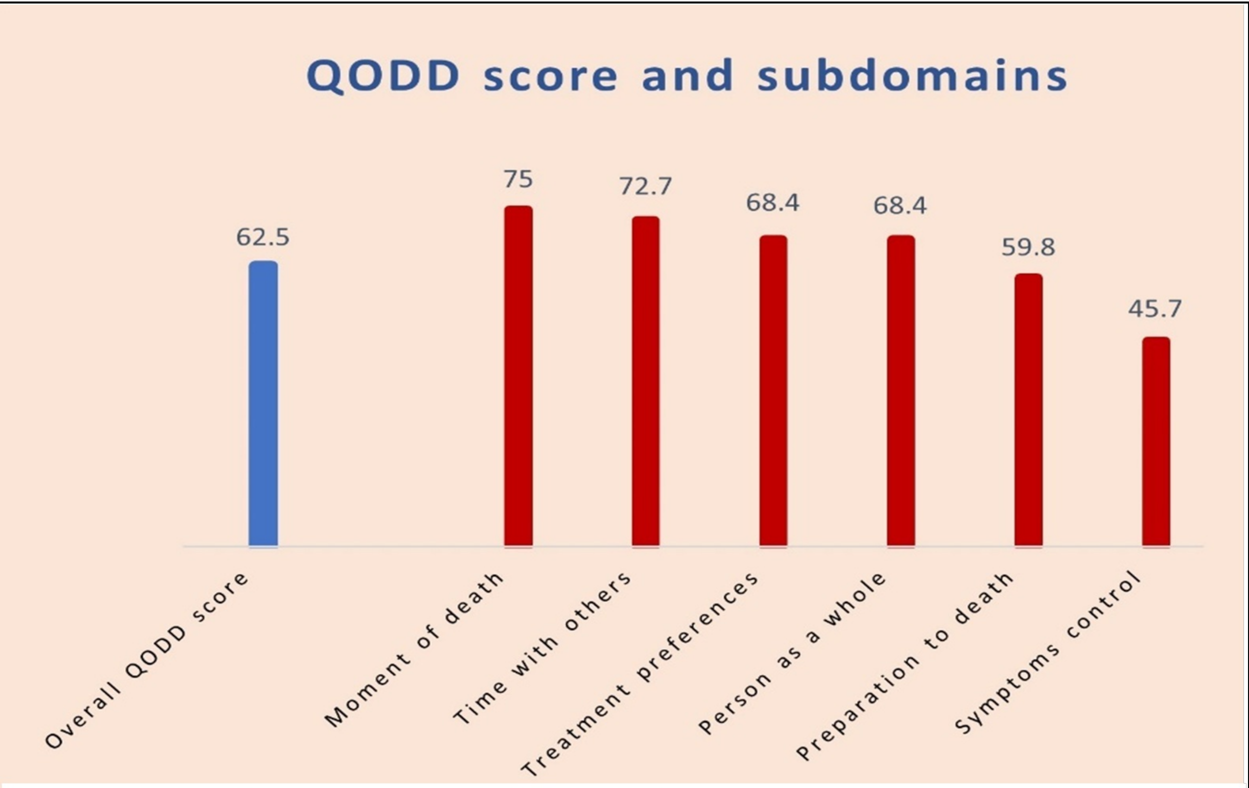

***Figure 1*** *: End of life experience according to QODD as assessed by caregivers of hematology patients.*

**Copyright Information:** (Online) ISSN: 2572-9241
©2026 The Author(s). HemaSphere is published by John Wiley & Sons Ltd on behalf of European Hematology Association. This is an open access Abstract Book distributed under the Attribution-NonCommercial-NoDerivs (CC BY-NC-ND), which allows third parties to download the articles and share them with others as long as they credit the author and the Abstract Book, but they cannot change the content in any way or use them commercially.
**Abstract Book Citations:** Authors, Title, HemaSphere, 2026;10:(S1):pages. The abstract book DOIs can be found on the "Author Information" page.
**Disclaimer:** Articles published in the journal HemaSphere exclusively reflect the opinions of the authors. The authors are responsible for all content in their abstracts including accuracy of the facts, statements, citing resources, etc.

### (PF1396) EMOTIONAL DISTRESS DOMINATES QUALITY OF LIFE BURDEN IN CML: PSYCHOMETRIC VALIDATION OF PATIENT REPORTED OUTCOMES IN A GLOBAL LEUKEMIA EXPERIENCE SURVEY

**Topic:** 36. Quality of life, ethics, supportive and palliative care

Samantha Nier*[1], Denis Costello[2], Matteo Fattizzo[3], Giuseppe Ianni[3], Esther N. Oliva[4]

*[1]Acute Leukemia Advocates Network, Bern, Switzerland; [2]CML Advocates Network, Bern, Switzerland; [3]Associazione QOL-ONE, Reggio Calabria, Italy; [4]Hematology Dept, London North West University Healthcare NHS Trust, London, United Kingdom;*

### Background

Chronic Myeloid Leukemia (CML) is a form of leukemia requiring lifelong therapy, predominantly with tyrosine kinase inhibitors (TKIs). Although survival has markedly improved, many patients experience persistent physical symptoms, treatment-related adverse events and psychological distress. Ongoing concerns about disease progression, long-term toxicity, employment, relationships, body image and financial burden may substantially impair quality of life (QoL). Therefore, systematic evaluation of patient-reported outcomes (PROs) using validated, disease-specific instruments is essential in contemporary CML management.

### Aims

To evaluate the psychometric performance of the Hematological Malignancy PRO (HM-PRO) instrument in a CML cohort from the a global leukemia experience survey

### Methods

This global anonymous online survey was conducted from August 19, 2023 to January 5, 2024 in 13 languages. Patients were recruited through the Acute Leukemia Advocates Network (ALAN), CML Advocates Network (CMLAN) and CLL Advocates Network (CLLAN). Non-sensitive demographics, CML characteristics and HM-PRO responses were collected. Reliability and internal consistency were evaluated across HM-PRO domains: Physical Behavior (PB, 7 items), Emotional Behavior (EB, 11 items), Social Well-Being (SW, 3 items), Eating and Drinking (ED, 2 items), Part A (sum of domains) and Symptom Scale (SS, 18 items). Score banding was defined as: 0–7.5 no effect; >7.5–25.5 small; >25.5–41.5 moderate; >41.5–74.5 very large; >74.5 extremely large.

**Copyright Information:** (Online) ISSN: 2572-9241
©2026 The Author(s). HemaSphere is published by John Wiley & Sons Ltd on behalf of European Hematology Association. This is an open access Abstract Book distributed under the Attribution-NonCommercial-NoDerivs (CC BY-NC-ND), which allows third parties to download the articles and share them with others as long as they credit the author and the Abstract Book, but they cannot change the content in any way or use them commercially.
**Abstract Book Citations:** Authors, Title, HemaSphere, 2026;10:(S1):pages. The abstract book DOIs can be found on the "Author Information" page.
**Disclaimer:** Articles published in the journal HemaSphere exclusively reflect the opinions of the authors. The authors are responsible for all content in their abstracts including accuracy of the facts, statements, citing resources, etc.

**Results**

Overall, 660 CML patients (57% females; median age=55 years; age range=20–84; median disease duration=5 years; disease duration range=0–27; employment status: 53% employed, 25% retired, 13% unemployed, 2% students and 7% reported other status; 79% lived with a partner or family member) participated in the survey. Among 549 treated patients, most were receiving TKIs.

Median HM-PRO scores indicated a moderate overall impact, primarily driven by EB (Table). Standardized Cronbach's alpha coefficients demonstrated strong internal consistency across domains (Table).

Small effects were reported for PB (63%), most frequently difficulty with physical activity/sport, SW (59%) driven by problems with sexual life in 46% and ED (60%) due to changes in eating and drinking habits. In contrast, EB showed moderate to extremely large effects in 75% of respondents. Prominent concerns included worry about future health (85%), sleep disturbances (73%), difficulty concentrating (71%), fear of being a burden (70%), anxiety (68%), treatment-related worry (67%), fear of dying (60%), distress (57%) and concerns about appearance (52%).

Although SS overall indicated no to small effect in 98% of cases, fatigue and related physical symptoms were consistently reported by patients. Fatigue was reported majority of respondents, 46% experiencing mild fatigue, 29% severe fatigue, 75% reduced energy. Other highly prevalent symptoms included body pain (59%), back pain (53%), skin problems (53%), hair loss (46%), headaches (44%), stomach ache (43%), night sweats (37%), diarrhea (37%) and nausea (35%).

**Summary/Conclusion**

The HM-PRO demonstrated strong internal reliability in CML patients, supporting its validity as a disease-specific PRO instrument. Emotional distress emerged as the most compromised domain and the primary contributor to overall impact (Part A), underscoring the importance of structured psychosocial and QoL assessment within multidisciplinary CML care.

**Table**

| Variable | Median (range) | Cronbach's Alpha |
|---|---|---|
| PB | 14.29 | 0.832 |
| EB | 40.91 | 0.881 |
| SW | 16.67 | 0.742 |
| ED | 25.00 | 0.731 |
| Part A | 26.14 | 0.757 |
| SS | 2.78 | 0.807 |

Table

**Copyright Information:** (Online) ISSN: 2572-9241
©2026 The Author(s). HemaSphere is published by John Wiley & Sons Ltd on behalf of European Hematology Association. This is an open access Abstract Book distributed under the Attribution-NonCommercial-NoDerivs (CC BY-NC-ND), which allows third parties to download the articles and share them with others as long as they credit the author and the Abstract Book, but they cannot change the content in any way or use them commercially.
**Abstract Book Citations:** Authors, Title, HemaSphere, 2026;10:(S1):pages. The abstract book DOIs can be found on the "Author Information" page.
**Disclaimer:** Articles published in the journal HemaSphere exclusively reflect the opinions of the authors. The authors are responsible for all content in their abstracts including accuracy of the facts, statements, citing resources, etc.

### (PF1397) IMPACT OF TRIAL BLINDING ON DIFFERENCES IN PATIENT-REPORTED OUTCOMES IN RANDOMIZED CLINICAL TRIALS OF HEMATOLOGIC MALIGNANCIES

**Topic:** 36. Quality of life, ethics, supportive and palliative care

Fabio Efficace*[1], David Cella[2], Massimo Di Maio[3], Neil Aaronson[4], Francesco Sparano[1], Rajshekhar Chakraborty[5], Darshi Shah[6], Veer Shah[7], Lorenzo Santilli[1], Chiara Pallisco[3], Giorgia Novero[3], Valentina Ponti[3], Paola Sabbadini[3], Anna Amela Valsecchi[3], Gary Collins[8, 9], Amelie Anota[10]

*[1]Italian Group for Adult Hematologic Diseases (GIMEMA), Data Center and Health Outcomes Research Unit, Rome, Italy, Rome, Italy; [2]Department of Medical Social Sciences, Northwestern University Feinberg School of Medicine, Chicago, United States of America; [3]Department of Oncology, University of Torino, AOU Città della Salute e della Scienza, Torino, Italy;[4]Division of Psychosocial Research and Epidemiology, Netherlands Cancer Institute, Amsterdam, The Netherlands; [5]Multiple Myeloma and Amyloidosis Program, Columbia University Irving Medical Center, New York, United States of America; [6]Renaissance School of Medicine, Stony Brook University, Stony Brook, United States of America; [7]Icahn School of Medicine at Mount Sinai, New York, United States of America; [8]Department of Applied Health Sciences. School of Health Sciences, College of Medicine and Health, University of Birmingham, Edgbaston, United Kingdom; [9]NIHR Birmingham Biomedical Research Centre, Birmingham, University Hospitals Birmingham NHS Foundation Trust and University of Birmingham, Birmingham, United Kingdom; [10]Biostatistics Unit, Direction of Clinical Research and Innovation, and French National Platform Quality of Life and Cancer, Centre Léon Bérard, Lyon, France;*

### Background

Patient-reported outcomes (PROs) play a critical role in informing benefit–risk assessments in randomized controlled trials (RCTs) of hematologic malignancies. However, concerns remain that lack of blinding in this setting may influence PRO results.

### Aims

To evaluate whether open-label design is associated with a higher likelihood of PRO results favoring the experimental arm compared with blinded design in RCTs of hematologic malignancies.

### Methods

We conducted a systematic literature review to identify RCTs with PRO endpoints in hematologic malignancies published between 2015 and 2025. This study is part of the IMPROvE project (PROSPERO: CRD420251061655).
Extracted data included trial characteristics, direction of PRO results, and completeness of PRO reporting using the CONSORT-PRO checklist. Trials were classified as favoring the experimental or control arm if the majority of statistically significant differences in any PRO scale, at any time point, indicated better outcomes in the respective arm. If an equal number of PRO differences favored both arms, or if no statistically significant differences were observed, trials were classified as equivalent. A CONSORT-PRO score was computed based on published scoring criteria, with higher scores indicating greater completeness of PRO reporting.
Univariable and multivariable logistic regression models were used to estimate the association between study design (i.e., open-label vs blinded) and the likelihood of identifying statistically significant PRO differences favoring the experimental arm. Multivariable analysis was adjusted for the following covariates: study setting, industry sponsorship, sample size, year of publication, disease type, treatment line (newly diagnosed/first-line vs other), type of experimental treatment (immunotherapy vs other), mode of therapy administration (intravenous vs other), CONSORT-PRO score, PRO endpoint, and publication of a secondary paper on PRO results.

**Copyright Information:** (Online) ISSN: 2572-9241
©2026 The Author(s). HemaSphere is published by John Wiley & Sons Ltd on behalf of European Hematology Association. This is an open access Abstract Book distributed under the Attribution-NonCommercial-NoDerivs (CC BY-NC-ND), which allows third parties to download the articles and share them with others as long as they credit the author and the Abstract Book, but they cannot change the content in any way or use them commercially.
**Abstract Book Citations:** Authors, Title, HemaSphere, 2026;10:(S1):pages. The abstract book DOIs can be found on the "Author Information" page.
**Disclaimer:** Articles published in the journal HemaSphere exclusively reflect the opinions of the authors. The authors are responsible for all content in their abstracts including accuracy of the facts, statements, citing resources, etc.

**Results**

We identified 143 RCTs conducted in multiple myeloma (n = 55), lymphomas and chronic lymphocytic leukemia (n = 42), leukemias and myelodysplastic neoplasms (n = 27), and myeloproliferative neoplasms (n = 19). Most trials (71%) were open-label. Completeness of PRO reporting varied substantially across studies.
In the overall sample, PRO results favored the experimental arm in 51% of open-label trials and 34% of blinded trials. However, among RCTs that pre-specified primary PRO scales in the statistical plan (n=64, 45%), analyses limited to those primary scales yielded proportions of 54% and 44%, respectively.
In univariable logistic regression, blinded (vs open-label) trials were not statistically significantly associated with the likelihood of PRO results favoring the experimental arm (OR 0.50, 95% CI 0.23–1.05; p=0.07) and this finding remained non-significant in multivariable analysis.
The only factor independently associated with a higher likelihood that statistically significant PRO differences favored the experimental treatment was higher completeness of PRO reporting (OR 1.17, 95% CI 1.07–1.28; p<0.001).

**Summary/Conclusion**

Although numerical differences were observed, open-label design was not statistically significantly associated with a higher likelihood of PRO results favoring the experimental arm. Our findings also underscore the importance of high-quality reporting to support appropriate interpretation of PRO data in RCTs of hematological malignancies.

**Copyright Information:** (Online) ISSN: 2572-9241
©2026 The Author(s). HemaSphere is published by John Wiley & Sons Ltd on behalf of European Hematology Association. This is an open access Abstract Book distributed under the Attribution-NonCommercial-NoDerivs (CC BY-NC-ND), which allows third parties to download the articles and share them with others as long as they credit the author and the Abstract Book, but they cannot change the content in any way or use them commercially.
**Abstract Book Citations:** Authors, Title, HemaSphere, 2026;10:(S1):pages. The abstract book DOIs can be found on the "Author Information" page.
**Disclaimer:** Articles published in the journal HemaSphere exclusively reflect the opinions of the authors. The authors are responsible for all content in their abstracts including accuracy of the facts, statements, citing resources, etc.

### (PF1398) A LONGITUDINAL ANALYSIS OF PATIENTS WITH CLL/SLL WITH IMPAIRED HEALTH-RELATED QUALITY OF LIFE SCORES AT BASELINE WHO WERE TREATED WITH ZANUBRUTINIB VERSUS IBRUTINIB: A POST HOC ANALYSIS OF ALPINE

**Topic:** 36. Quality of life, ethics, supportive and palliative care

Loic Ysebaert*[1], Timothy Victor[2, 3], Frederick B. Barnes[2], Rasika Korde[2], Emmanuelle Ferrant[4], Jennifer R. Brown[5], Tommi Salmi[6], G. Rhys Williams[7], Gisoo Barnes[7], Mazyar Shadman[8]

*[1]IUCT-Oncopole, Toulouse, France; [2]BeOne Medicines, Ltd., San Carlos, United States of America; [3]University of Pennsylvania, Philadelphia, United States of America; [4]CHU de Lyon-Sud, Lyon, France; [5]Dana-Farber Cancer Institute, Boston, United States of America; [6]BeOne Medicines Ltd., Basel, Switzerland; [7]BeOne Medicines Ltd., San Carlos, United States of America; [8]Fred Hutchinson Cancer Center, Seattle, United States of America;*

### Background

In relapsed/refractory chronic lymphocytic leukemia (CLL) or small lymphocytic lymphoma (SLL), baseline health-related quality of life (HRQoL) impairment may reflect greater disease burden and heightened vulnerability to treatment-related symptoms. Prior analyses demonstrated a significant progression-free survival (PFS) advantage for zanubrutinib versus ibrutinib regardless of baseline HRQoL impairment, as assessed using the visual analog scale of the EuroQol 5 dimension questionnaire (EQ VAS) (Ysebaert et al. *Blood* 2025).

### Aims

To evaluate longitudinal patient-reported HRQoL with zanubrutinib versus ibrutinib and assess whether effects differed by baseline HRQoL impairment in patients with CLL/SLL in the Phase 3 ALPINE trial (NCT03734016).

### Methods

Patients provided informed consent and completed two validated patient-reported outcome (PRO) instruments to assess HRQoL: the EQ VAS and the European Organisation for Research and Treatment of Cancer Quality of Life Questionnaire Core 30 (QLQ-C30) fatigue scale. Baseline HRQoL impairment was defined separately using two prespecified clinician-informed thresholds: EQ VAS, <70 (impaired) and ≥70 (not impaired); QLQ-C30 fatigue, >39 (impaired) and ≤39 (not impaired). Lower scores in EQ VAS and higher scores in QLQ-C30 fatigue indicated greater impairment. PROs were analyzed longitudinally using a mixed model for repeated measures (MMRM) with visit (time) treated nominally, incorporating all available post-baseline PRO assessments. Prespecified contrasts evaluated overall treatment effects, treatment-by-impairment differences, and month-specific treatment effects by impairment status at Months 6 and 12 (treatment × baseline impairment status × month). *P*-values for prespecified contracts were Tukey–Kramer adjusted.

**Copyright Information:** (Online) ISSN: 2572-9241
©2026 The Author(s). HemaSphere is published by John Wiley & Sons Ltd on behalf of European Hematology Association. This is an open access Abstract Book distributed under the Attribution-NonCommercial-NoDerivs (CC BY-NC-ND), which allows third parties to download the articles and share them with others as long as they credit the author and the Abstract Book, but they cannot change the content in any way or use them commercially.
**Abstract Book Citations:** Authors, Title, HemaSphere, 2026;10:(S1):pages. The abstract book DOIs can be found on the “Author Information” page.
**Disclaimer:** Articles published in the journal HemaSphere exclusively reflect the opinions of the authors. The authors are responsible for all content in their abstracts including accuracy of the facts, statements, citing resources, etc.

### Results

Overall, 573 patients were included in the EQ VAS analysis (not impaired n=416; impaired n=157) and 575 in the QLQ-C30 fatigue analysis (not impaired n=365; impaired n=210). In the MMRM, zanubrutinib was associated with significantly better EQ VAS scores versus ibrutinib (−3.52 [95% confidence interval {CI}: −5.58 to −1.46]; *P*=0.0008), regardless of baseline impairment status. Among baseline-impaired patients, the between-group difference in EQ VAS score favored zanubrutinib versus ibrutinib (−6.08 [95% CI: −10.40 to −1.76]; *P*=0.0018). In month-specific contrasts within the impaired subgroup, EQ VAS scores significantly favored zanubrutinib versus ibrutinib at month 6 (−6.73 [95% CI: −12.49 to −0.96]; *P*=0.0098), with a directionally consistent, clinically relevant between-arm difference observed at month 12 (−5.43 [95% CI: −11.21 to 0.35]; *P*=0.0833) (Fig 1). For QLQ-C30 fatigue, no overall or month-specific between-arm differences were observed.

### Summary/Conclusion

Treatment with zanubrutinib resulted in better overall health status (EQ VAS) compared with ibrutinib among patients with baseline HRQoL impairment. Taken together with prior evidence that zanubrutinib provided consistent PFS benefit regardless of baseline HRQoL impairment status, these empirical results identify baseline HRQoL impairment as a critical factor at the time of treatment initiation and may help inform early symptom monitoring and supportive care planning in patients with CLL/SLL.

**Fig 1. Treatment × Baseline Impairment Status × Month**

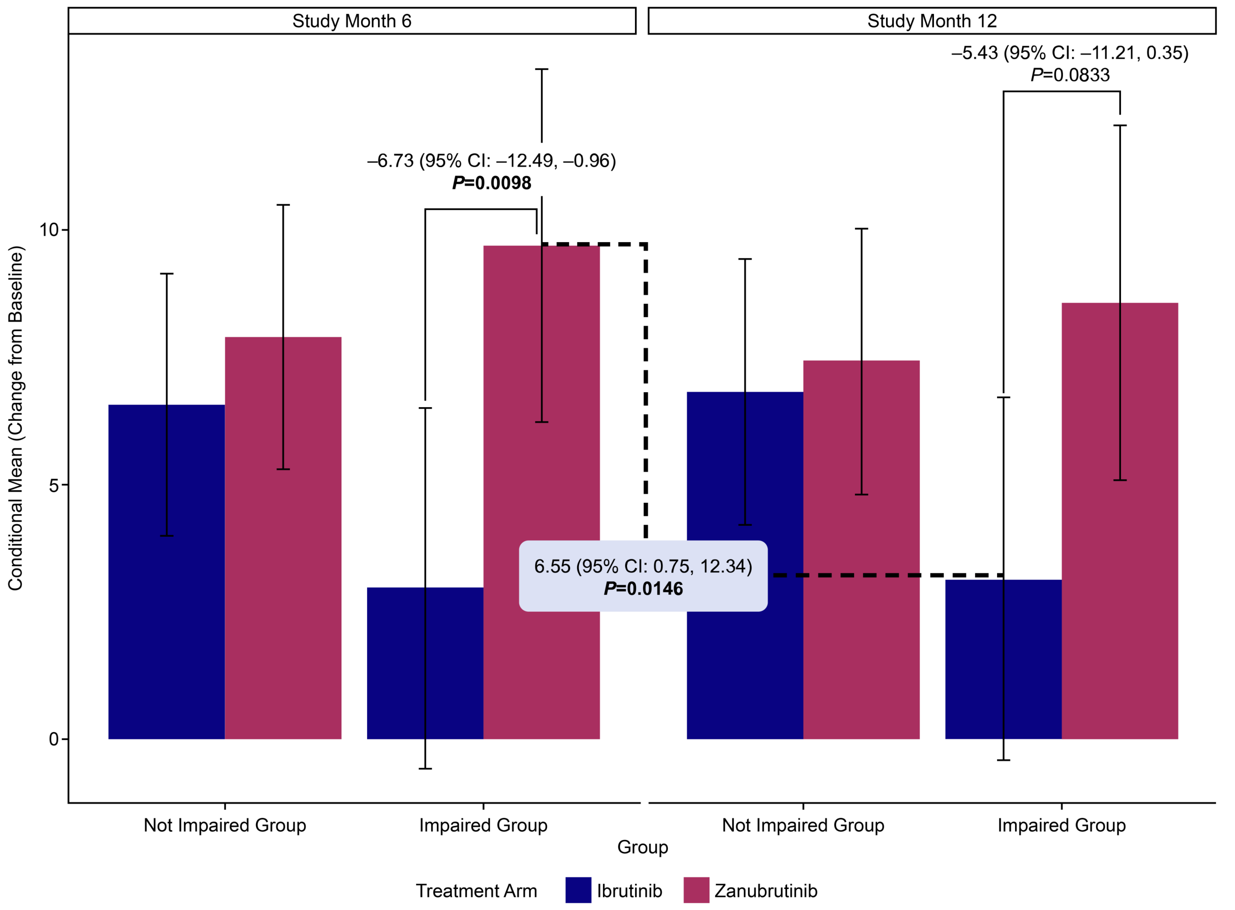

**Copyright Information:** (Online) ISSN: 2572-9241
©2026 The Author(s). HemaSphere is published by John Wiley & Sons Ltd on behalf of European Hematology Association. This is an open access Abstract Book distributed under the Attribution-NonCommercial-NoDerivs (CC BY-NC-ND), which allows third parties to download the articles and share them with others as long as they credit the author and the Abstract Book, but they cannot change the content in any way or use them commercially.
**Abstract Book Citations:** Authors, Title, HemaSphere, 2026;10:(S1):pages. The abstract book DOIs can be found on the "Author Information" page.
**Disclaimer:** Articles published in the journal HemaSphere exclusively reflect the opinions of the authors. The authors are responsible for all content in their abstracts including accuracy of the facts, statements, citing resources, etc.

### (PF1399) FAMILY CARER BURDEN AND THEIR PROXY CONCORDANCE OF PATIENT-REPORTED OUTCOMES: A GLOBAL LEUKEMIA EXPERIENCE SURVEY

**Topic:** 36. Quality of life, ethics, supportive and palliative care

Samantha Nier*[1], Matteo Fattizzo[2], Giuseppe Ianni[2], Tatyana Ionova[3], Sam Salek[4], Esther N. Oliva[5]

*[1]Acute Leukemia Advocates Network, Bern, Switzerland; [2]Associazione QOL-ONE, Reggio Calabria, Italy; [3]St.Petersburg State University Hospital, St.Petersburg, Russia; [4]University of Hertfordshire, Hatfield, United Kingdom; [5]Hematology Dept, London North West University Healthcare NHS Trust, London, United Kingdom;*

### Background

Patients with leukemia require sustained medical, practical and emotional support. Family carers often assume major responsibilities in daily care, healthcare coordination and psychosocial assistance. This multidimensional burden may affect carers' wellbeing and influence how they perceive and report patient-reported outcomes (PROs). Evaluating carer burden and concordance with PROs is therefore critical to ensure comprehensive leukemia care.

### Aims

To evaluate carer burden and concordance between proxy assessment and patients' own assessment of PROs using the Hematological Malignancy specific PRO measure (HM-PRO).

### Methods

A global anonymous online survey was conducted between August 19, 2023 and January 5, 2024 in 13 languages. Patients with acute and chronic leukemia and their carers were recruited through the Acute Leukemia Advocates Network (ALAN), CML Advocates Network (CMLAN) and CLL Advocates Network (CLLAN). Non-sensitive demographic, clinical, treatment and HM-PRO data were collected.
HM-PRO Part A assesses quality of life (QoL) domains: Physical Behaviour (PB, 7 items), Emotional Behaviour (EB, 11 items), Social Wellbeing (SW, 3 items), Eating and Drinking (ED, 2 items), and total Part A score. Part B evaluates signs and symptoms (SS, 18 items). In addition, a dedicated section evaluated carer burden and included proxy HM-PRO completion. Agreement was assessed using Cohen's kappa (κ) and paired differences using the Wilcoxon signed-rank test.

**Copyright Information:** (Online) ISSN: 2572-9241
©2026 The Author(s). HemaSphere is published by John Wiley & Sons Ltd on behalf of European Hematology Association. This is an open access Abstract Book distributed under the Attribution-NonCommercial-NoDerivs (CC BY-NC-ND), which allows third parties to download the articles and share them with others as long as they credit the author and the Abstract Book, but they cannot change the content in any way or use them commercially.
**Abstract Book Citations:** Authors, Title, HemaSphere, 2026;10:(S1):pages. The abstract book DOIs can be found on the "Author Information" page.
**Disclaimer:** Articles published in the journal HemaSphere exclusively reflect the opinions of the authors. The authors are responsible for all content in their abstracts including accuracy of the facts, statements, citing resources, etc.

**Results**

205 carers (51% females; median age=56; age range=15-84; employment status: 48% employed, 27% retired, 5% homemaker, 2% student, 18% unemployed; 84% lived with relative patient and 7% were full-time family carers) participated in the survey. Diagnoses of patients (57.3% females; median age = 53; age range=20–64) being cared were as follows: acute lymphoblastic leukemia (20%), acute myeloid leukemia (26%), chronic lymphocytic leukemia (26%), chronic myeloid leukemia (26%) and others (<3%).
Care activities included emotional support (84%), household tasks (73%), accompanying appointments (68%), shopping (51%), healthcare interaction (42%), prescription collection (40%), personal care (34%), financial management (32%), medication administration (27%) and childcare (20%). 27% provided >36 hours/week of care.
Overall concordance showed moderate agreement for PB (κ=0.47), SW (κ=0.45), and SS (κ=0.53) and fair agreement for EB (κ=0.21), ED (κ=0.35) and Part A (κ=0.37). Carers generally underestimated patient-reported impact, except for PB and SW (Figure).
In acute leukemias, moderate agreement was observed for PB (κ=0.44), with weak to moderate agreement for SW, Part A, and SS (κ≈0.36–0.39). Significant differences were found for SS (p=0.012) and EB (κ=0.17; p=0.04).
In chronic leukemias, substantial agreement was observed for PB (κ=0.63), moderate for SW (κ=0.49–0.51), and fair for ED, EB and Part A (κ≈0.26–0.37), with significant paired differences.

**Summary/Conclusion**

Carers of leukemia patients experience considerable practical and emotional burden, with 27% providing intensive care (>36 hours/week). While physical and symptom domains showed moderate concordance, emotional and overall QoL domains demonstrated only fair agreement, particularly in acute leukemia. Family carers tended to underestimate psychosocial impact. Integrating both patient and carers assessments may improve holistic management and identify unmet supportive care needs. Proxy reporting should therefore not replace direct PRO, especially for emotional wellbeing.

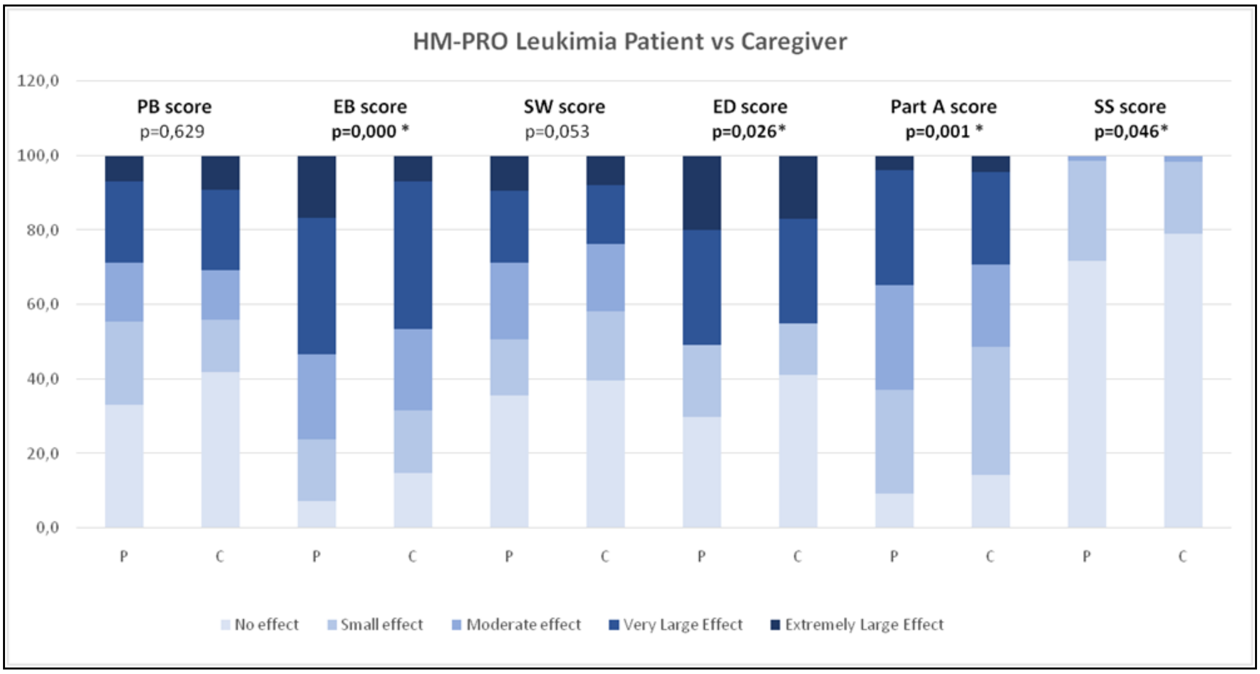

Figure

**Copyright Information:** (Online) ISSN: 2572-9241
©2026 The Author(s). HemaSphere is published by John Wiley & Sons Ltd on behalf of European Hematology Association. This is an open access Abstract Book distributed under the Attribution-NonCommercial-NoDerivs (CC BY-NC-ND), which allows third parties to download the articles and share them with others as long as they credit the author and the Abstract Book, but they cannot change the content in any way or use them commercially.
**Abstract Book Citations:** Authors, Title, HemaSphere, 2026;10:(S1):pages. The abstract book DOIs can be found on the "Author Information" page.
**Disclaimer:** Articles published in the journal HemaSphere exclusively reflect the opinions of the authors. The authors are responsible for all content in their abstracts including accuracy of the facts, statements, citing resources, etc.

### (PF1400) THE IDIOPATHIC MULTICENTRIC CASTLEMAN DISEASE SYMPTOM BURDEN SCALE (ISBUS): PRELIMINARY FINDINGS IN THE GENERATION OF A NOVEL INSTRUMENT

**Topic:** 36. Quality of life, ethics, supportive and palliative care

Philip Powell*[1], Anju Keetharuth[1], Se Frances[1], Jill Carlton[1], Antonio Adolfo Guerra Soares Brandão[2], Karthik Ramasamy[3], Francis Shupo[4], Kelley Dacus[4], Yildiz Kelahmetoglu[4], Kelly Makarounas-Kirchmann[5, 6], Annmarie Bosco[7], Satyen Gohil[8], H. Miles Prince[9], Luke Chen[10], Sudipto Mukherjee[11]

*[1]The University of Sheffield, ScHARR, Sheffield, United Kingdom;[2]Hospital das Clinicas da Faculdade de Medicina da Universidade de Sao Paulo, Sao Paulo, Brazil; [3]Oxford University Hospitals NHS Foundation Trust, Oxford, United Kingdom; [4]Recordati Rare Diseases, Hammersmith, United Kingdom; [5]Monash University, Public Health, Melbourne, Australia;[6]KMC Health Care, Melbourne, Australia; [7]Prince of Wales Hospital, Sydney, Australia;[8]University College London Hospitals, London, United Kingdom; [9]Epworth Healthcare and University of Melbourne, Melbourne, Australia; [10]Dalhousie University, Halifax, Canada; [11]Cleveland Clinic, Cleveland, United States of America;*

### Background idiopathic multicentric Castleman Disease (iMCD) is a rare, life-threatening, non-neoplastic lymphoproliferative disorder characterized by generalized lymphadenopathy and a chronic hyperinflammatory state. Patients with iMCD have a high symptom burden, impacting their quality of life (QoL). No existing patient-reported outcome measure (PROM) comprehensively captures iMCD symptom burden for use in clinical monitoring or trials.

### Aims

The iMCD symptom burden study (ISBUS) is a multi-institution, international initiative to develop a novel PROM to quantify symptom burden and support longitudinal symptom monitoring and evaluation of treatment response.

### Methods

A four-stage PROM development process is being undertaken in accordance with established best-practice guidance, as outlined by the US Food and Drug Administration (FDA), including: (1) item generation; (2) cognitive debriefing interviews; (3) cross-sectional psychometric survey; and (4) preliminary assessment of meaningful change. Initial item generation was based on existing evidence, and patient and clinician input. In stage 2, the comprehensibility, relevance, and comprehensiveness of the draft PROM, comprising 42 items, was assessed through cognitive interviews with 10 patients with iMCD. Transcripts were analysed using framework analysis to assess content validity and inform item refinement. In Stage 3, a revised version of the PROM is being administered online to a target sample of 50+ patients with iMCD, alongside established QoL and generic symptom measures. Due to the rarity of the condition, a multi-country approach to recruitment has been adopted, involving patients from the UK, US, Canada, Australia, and New Zealand. Final item selection will be guided by psychometric performance, content validity, and multidisciplinary expert consensus, including people with lived experience. Stage 4 involves a repeat survey after two months and qualitative interviews with patients to determine preliminary meaningful change thresholds.

**Copyright Information:** (Online) ISSN: 2572-9241
©2026 The Author(s). HemaSphere is published by John Wiley & Sons Ltd on behalf of European Hematology Association. This is an open access Abstract Book distributed under the Attribution-NonCommercial-NoDerivs (CC BY-NC-ND), which allows third parties to download the articles and share them with others as long as they credit the author and the Abstract Book, but they cannot change the content in any way or use them commercially.
**Abstract Book Citations:** Authors, Title, HemaSphere, 2026;10:(S1):pages. The abstract book DOIs can be found on the "Author Information" page.
**Disclaimer:** Articles published in the journal HemaSphere exclusively reflect the opinions of the authors. The authors are responsible for all content in their abstracts including accuracy of the facts, statements, citing resources, etc.

**Results**

Following cognitive debriefing, 30 of 42 items were retained with good comprehensibility, relevance, and comprehensiveness. Minor revisions were made to improve clarity (e.g., replacing “weight loss” with “unintentional weight loss”). Items with limited relevance (e.g., “cough”) were removed. From a preliminary analysis of 44 participants in the psychometric survey (US=22, UK=8, Australia=5, Canada=9), symptom prevalence and perceived impact differed across the cohort. The mean number of symptoms reported was 16.5/30 (SD=8.5). The most endorsed symptoms included “tiredness (i.e., either physical and/or mental tiredness)”, “low energy”, “difficulty concentrating”, “physical weakness”, and “headaches”. Whereas symptoms such as “unintentional weight loss” and “fever” were experienced least. Symptoms including “tiredness”, “low energy” and “exhaustion” had the biggest perceived impact on patients’ lives. Further recruitment is ongoing. These emerging data will be used to inform final item selection for ISBUS alongside involvement of patient and clinical advisors.

**Summary/Conclusion**

A novel PROM for quantifying iMCD symptom burden (ISBUS) will be produced, featuring rigorous methods and lived experience throughout. Preliminary evidence supports content validity. Ongoing psychometric analyses will evaluate symptom prevalence, item redundancy, and structural validity to inform item selection and the reliability and construct validity of the final measure.

**Copyright Information:** (Online) ISSN: 2572-9241
©2026 The Author(s). HemaSphere is published by John Wiley & Sons Ltd on behalf of European Hematology Association. This is an open access Abstract Book distributed under the Attribution-NonCommercial-NoDerivs (CC BY-NC-ND), which allows third parties to download the articles and share them with others as long as they credit the author and the Abstract Book, but they cannot change the content in any way or use them commercially.
**Abstract Book Citations:** Authors, Title, HemaSphere, 2026;10:(S1):pages. The abstract book DOIs can be found on the “Author Information” page.
**Disclaimer:** Articles published in the journal HemaSphere exclusively reflect the opinions of the authors. The authors are responsible for all content in their abstracts including accuracy of the facts, statements, citing resources, etc.

### (PF1401) MENTAL HEALTH OF ADULT PATIENTS WITH MASTOCYTOSIS: A DANISH NATIONWIDE MATCHED COHORT STUDY

**Topic:** 36. Quality of life, ethics, supportive and palliative care

Maren Jørgensen*[1, 2], Andreas Kiesbye Øvlisen[1], Jonas Jensen[1], Tarec El-Galaly[3], Hanne Vestergaard[4, 5], Sigurd Broesby-Olsen[5, 6], Marianne Severinsen[1, 2]

[1]*Aalborg University Hostpial, Department of Hematology, Gistrup, Denmark;*[2]*Aalborg University, Department of Clinical Medicine, Gistrup, Denmark;* [3]*Aarhus University Hospital, Department of Hematology, Aarhus, Denmark;*[4]*Odense University Hospital, Department of Hematology, Odense, Denmark;* [5]*Odense University Hospital, Mastocytosis Centre, Odense, Denmark;* [6]*Odense University Hospital, Department of Dermatology and Allergy, Odense, Denmark;*

### Background

Mastocytosis is a clonal mast cell disorder characterized by chronic mediator-related symptoms and heterogeneous systemic involvement. Neuropsychiatric symptoms are frequently reported, but population-based evidence on incident depression and anxiety is limited. Psychotropic drug (PD) prescriptions may serve as a proxy for clinically treated affective and anxiety disorders.

### Aims

To investigate the risk of incident pharmacologically treated depression or anxiety among patients with mastocytosis compared with matched comparators from the general Danish population.

### Methods

In this nationwide matched cohort study, adult patients (≥18 years old) diagnosed with mastocytosis between January 1, 2005, and December 31, 2023, in Denmark were identified and matched 1:5 on age, sex, Charlson Comorbidity Index, and educational level. Individuals with any PD prescription or psychiatric hospital contact in the 10 years prior to diagnosis were excluded. The primary outcome was first PD prescription (antidepressants, anxiolytics, or antipsychotic) after diagnosis. Cumulative risks were estimated using competing risk methods. Cox proportional hazards models were used to estimate five-year hazard ratios (HRs) with 95% confidence intervals (CIs). Secondary outcomes included psychiatric hospital contact, private tax-funded psychologist/psychiatrist contact, and intentional self-harm or suicidal events.

### Results

We included 874 patients with mastocytosis and 4370 matched comparators. Patients had a significantly higher risk of incident PD prescription over a period of 10 years after diagnosis ($p<0.001$). The five-year cumulative risk of PD initiation was 13.1% in patients and 8.4% in comparators. The HR for incident PD use, with comparators as reference, was 1.72 (95% CI 1.38-2.15). Results were consistent in sensitivity analyses of risk of second PD and landmark analyses at one-, two-, and five-years post index. Risk factors for PD initiation among patients with mastocytosis were higher age, comorbidity, and low educational level. Risk of anxiolytic prescriptions was higher among patients with advanced mastocytosis. A sensitivity analysis of timing of first PD in the period 5 years before to 5 years after diagnosis showed a slightly higher risk of incident anxiolytic prescription among patients in the years preceding diagnosis, whereas no difference was found in the risk of antidepressant prescriptions prior to diagnosis. No difference was found in risk of psychiatric hospital contact ($p=0.28$), private psychologist/psychiatrist contact ($p=0.73$) or intentional self-harm or suicidal events ($p=0.32$).

**Copyright Information:** (Online) ISSN: 2572-9241
©2026 The Author(s). HemaSphere is published by John Wiley & Sons Ltd on behalf of European Hematology Association. This is an open access Abstract Book distributed under the Attribution-NonCommercial-NoDerivs (CC BY-NC-ND), which allows third parties to download the articles and share them with others as long as they credit the author and the Abstract Book, but they cannot change the content in any way or use them commercially.
**Abstract Book Citations:** Authors, Title, HemaSphere, 2026;10:(S1):pages. The abstract book DOIs can be found on the "Author Information" page.
**Disclaimer:** Articles published in the journal HemaSphere exclusively reflect the opinions of the authors. The authors are responsible for all content in their abstracts including accuracy of the facts, statements, citing resources, etc.

### Summary/Conclusion

Mastocytosis is associated with an increased risk of incident pharmacologically treated depression or anxiety. These findings underscore the need for mental health assessment in patients with mastocytosis.

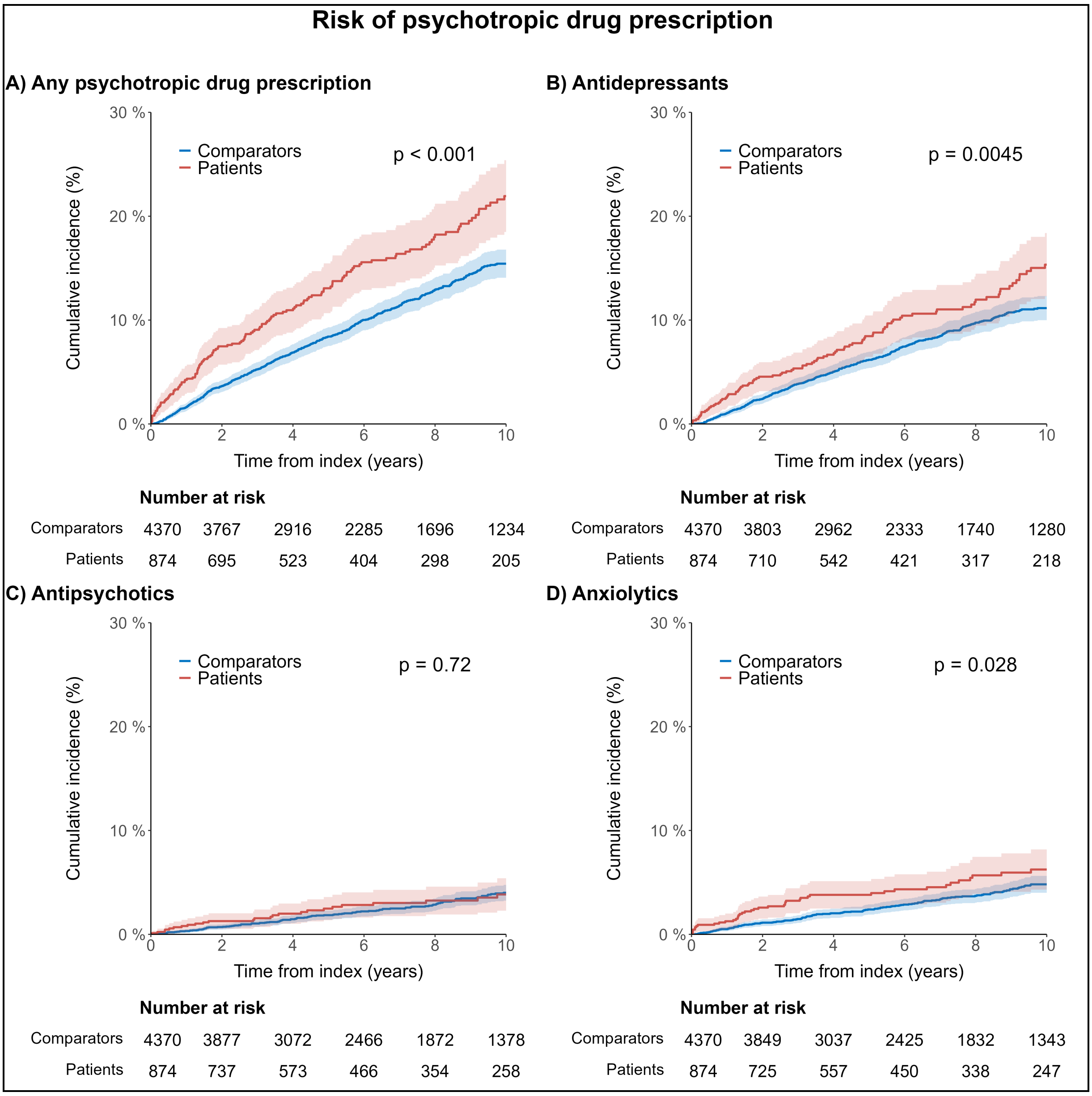

Risk of first redeemed psychotropic drug (PD, antidepressant, anxiolytic, or antipsychotic) prescriptions between patients with mastocytosis and matched comparators. Patients and comparators were excluded if they received any PD, had contact with psychiatric hospital departments, or with private psychologist/psychiatrist 10 years prior to index. Greys test was used to investigate differences in cumulated incidences. The cumulative incidences of any PD, antidepressant, and anxiolytic prescription were significantly higher among patients than comparators.

**Copyright Information:** (Online) ISSN: 2572-9241
©2026 The Author(s). HemaSphere is published by John Wiley & Sons Ltd on behalf of European Hematology Association. This is an open access Abstract Book distributed under the Attribution-NonCommercial-NoDerivs (CC BY-NC-ND), which allows third parties to download the articles and share them with others as long as they credit the author and the Abstract Book, but they cannot change the content in any way or use them commercially.
**Abstract Book Citations:** Authors, Title, HemaSphere, 2026;10:(S1):pages. The abstract book DOIs can be found on the “Author Information” page.
**Disclaimer:** Articles published in the journal HemaSphere exclusively reflect the opinions of the authors. The authors are responsible for all content in their abstracts including accuracy of the facts, statements, citing resources, etc.

### (PF1402) OUTCOME FOLLOWING CARDIAC ARREST AND/OR INTENSIVE CARE IN PATIENTS WITH HEMATOLOGICAL MALIGNANCIES: A SWEDISH NATIONWIDE REGISTRY-BASED STUDY

**Topic:** 36. Quality of life, ethics, supportive and palliative care

Malin Petersson*[1, 2], Johan Malmgren[3, 4], Linda Block[3, 4], Lena Von Bahr[1, 5]

*[1]University of Gothenburg, Sahlgrenska Academy, Institute of Medicine, Gothenburg, Sweden;[2]Växjö Hospital, Department of Internal Medicine, Växjö, Sweden; [3]Sahlgrenska University Hospital, Department of Anaesthesia and Intensive Care, Gothenburg, Sweden; [4]University of Gothenburg, Sahlgrenska Academy, Institute of Clinical Sciences, Department of Anaesthesiology and Intensive Care, Gothenburg, Sweden; [5]Sahlgrenska University Hospital, Department of Hematology, Gothenburg, Sweden;*

### Background

In patients with hematological malignancies, prognosis can be difficult to ascertain. Although SAPS3, a mortality risk stratification score often used in intensive care, includes hematological malignancy as one of several criteria, it does not differentiate between types of hematological malignancies. Individualised prognostication in the setting of cardiac arrest and/or intensive care may provide information on which patients might benefit from such interventions. Such data could potentially inform shared decision-making with patients on goals of care.

### Aims

1) To describe outcomes of cardiac arrest or intensive care across different hematological malignancies and to investigate potential predictive factors.
2) To assess the validity of SAPS3 across different hematological malignancies.

### Methods

Patients aged 18 years or older registered in any of the patient registries for ALL, AML, CLL, CML, MDS, myeloma and lymphoma who experienced a cardiac arrest (registered in the Swedish Cardiopulmonary Resuscitation Registry 2008-2023) or an intensive care admission (registered in the Swedish Intensive Care Registry 2021 to 2023), were identified. Data from the registries were linked to the patient registry to obtain comorbidity data. Basic characteristics are presented as means and standard deviations for continuous variables and as numbers and percentages for categorical variables. Correlation between predicted and observed mortality was assessed using Pearson correlation coefficients.

### Results

In total, 1021 in-hospital cardiac arrests were reported between 2008 and 2023. At arrest, the average age was 72.3 (SD 11.3) years, and 68% were males (n=697). The overall 30-day mortality was 74% (n=757), comparable across diagnoses (range 67% in MDS to 83% in CML). For intensive care, 2416 admissions for 1895 unique patients were observed between 2021 and 2023. The average age was 70 (SD 13) years at admission, and the 30-day mortality 37% (n=891, range 22% in CML to 45% in AML). Across subgroups, the predicted 30-day mortality based on SAPS3 was higher than the observed mortality, with a weak to moderate correlation (**Figure 1**). Septic shock was the cause for admission in 21% of patients (n=510). In patients with septic schock the 30-day mortality was somewhat higher, overall 43% (range 17% in ALL to to 53% in MDS). The predicted 30-day mortality based on SAPS3 was higher than the observed mortality also in patients with septic shock across subgroups.

**Copyright Information:** (Online) ISSN: 2572-9241
©2026 The Author(s). HemaSphere is published by John Wiley & Sons Ltd on behalf of European Hematology Association. This is an open access Abstract Book distributed under the Attribution-NonCommercial-NoDerivs (CC BY-NC-ND), which allows third parties to download the articles and share them with others as long as they credit the author and the Abstract Book, but they cannot change the content in any way or use them commercially.
**Abstract Book Citations:** Authors, Title, HemaSphere, 2026;10:(S1):pages. The abstract book DOIs can be found on the "Author Information" page.
**Disclaimer:** Articles published in the journal HemaSphere exclusively reflect the opinions of the authors. The authors are responsible for all content in their abstracts including accuracy of the facts, statements, citing resources, etc.

### Summary/Conclusion

We describe preliminary results from a large nationwide study on outcomes after cardiac arrest and/or intensive care across different hematological malignancies. While the overall mortality after cardiac arrest is high across subgroups, the mortality after intensive care admissions varies. Thus, indicating that the type of hematological malignancy may be important to consider when deciding on intensive care admission. Further, SAPS3 predicted mortality was higher than observed mortality, potentially indicating an overestimation of the actual mortality in patients with hematological malignancies across subgroups. Analyses of treatment- and diagnosis-related characteristics and their impact on survival are planned.

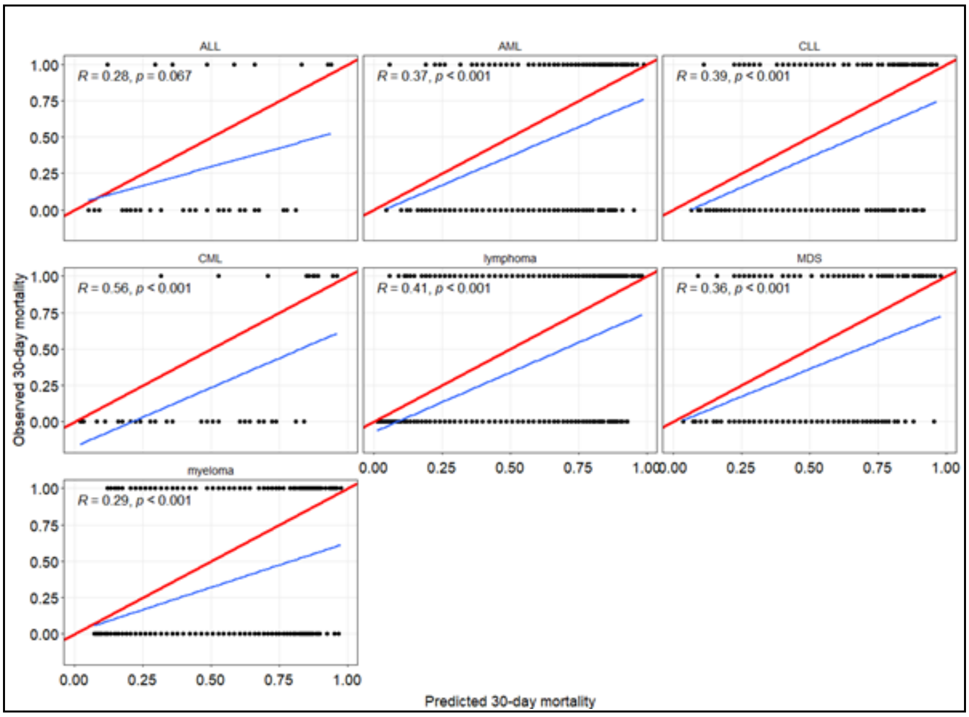

Predicted 30-day mortality based on SAPS3 compared with observed 30-day mortality across hematological malignancies. The red line corresponds to R=1, and the blue line displays the observed correlation. Pearson correlation coefficients and p-values are noted in the graph.

**Copyright Information:** (Online) ISSN: 2572-9241
©2026 The Author(s). HemaSphere is published by John Wiley & Sons Ltd on behalf of European Hematology Association. This is an open access Abstract Book distributed under the Attribution-NonCommercial-NoDerivs (CC BY-NC-ND), which allows third parties to download the articles and share them with others as long as they credit the author and the Abstract Book, but they cannot change the content in any way or use them commercially.
**Abstract Book Citations:** Authors, Title, HemaSphere, 2026;10:(S1):pages. The abstract book DOIs can be found on the "Author Information" page.
**Disclaimer:** Articles published in the journal HemaSphere exclusively reflect the opinions of the authors. The authors are responsible for all content in their abstracts including accuracy of the facts, statements, citing resources, etc.

### (PF1403) OPERATIONAL INTEGRATION OF T-CELL–ENGAGING BISPECIFIC ANTIBODIES IN COMMUNITY HEMATOLOGY: A LONGITUDINAL MIXED-METHODS ANALYSIS

**Topic:** 36. Quality of life, ethics, supportive and palliative care

Carmine Deluca*[1], Ben Johnson[1], Michael Beyer[1], Marina Wanes[1], Hans C. Lee[2], Amy Goodrich[3], Jeff Yorio[4]

*[1]PeerView Institute for Medical Education, New York, United States of America;[2]Sarah Cannon Research Institute, Nashville, United States of America; [3]Johns Hopkins University, Kimmel Cancer Center, Baltimore, United States of America;[4]Texas Oncology, Austin, United States of America;*

### Background

The recent regulatory approvals of T-cell–engaging bispecific antibodies in diffuse large B-cell lymphoma (DLBCL), follicular lymphoma (FL), and multiple myeloma (MM) have transformed treatment algorithms and introduced novel operational demands in community hematology settings. Despite emerging clinical guidance, practical integration strategies—particularly regarding step-up dosing logistics, toxicity monitoring, and outpatient delivery—remain incompletely standardized in community practice (Crombie J et al. *Blood.* 2024;143:1565-157; Garfall A et al. Front Oncol. 2025;15:1630146).

### Aims

We evaluated longitudinal, multi-platform interventions designed to assess and improve community clinician preparedness for implementing bispecific therapy across B-cell malignancies.

### Methods

Interventions included 10 Project ECHO® sessions, 15 meetings within US Oncology Network practices, and 3 live programs at medical congresses, with corresponding enduring activities. Qualitative analysis of expert-facilitated peer-to-peer dialogues was conducted to identify adoption behaviors, operational strategies, and barriers to bispecific implementation. Quantitative learner assessments contextualized qualitative findings. Overall, 3,059 learners participated, including hematologist-oncologists, nurse practitioners, physician associates, and oncology nurses. Pre- and post-intervention assessments included case-based and practice-pattern questions designed to evaluate knowledge and skills in clinical decision-making and operational integration of bispecific antibodies.

### Results

Baseline knowledge and skills related to operational integration of bispecific antibodies were limited (37%; n=1,524), particularly in domains related to step-up dosing decision-making and outpatient monitoring. Following structured interprofessional interventions, the proportion of learners demonstrating evidence-aligned clinical decision-making and operational preparedness increased to 71% (absolute increase +34%; n=1,215). Qualitative analyses (n=396) revealed progressive shifts from foundational familiarity with bispecific mechanisms toward operational workflow optimization and outpatient transition planning. Many clinics reported active implementation of outpatient delivery pathways and dosing de-escalation strategies to mitigate toxicity. Clinicians expressed willingness to adopt co-management models with academic centers but identified persistent barriers, including step-up dosing complexity, limited outpatient monitoring infrastructure, and constrained supportive care resources.

**Copyright Information:** (Online) ISSN: 2572-9241
©2026 The Author(s). HemaSphere is published by John Wiley & Sons Ltd on behalf of European Hematology Association. This is an open access Abstract Book distributed under the Attribution-NonCommercial-NoDerivs (CC BY-NC-ND), which allows third parties to download the articles and share them with others as long as they credit the author and the Abstract Book, but they cannot change the content in any way or use them commercially.
**Abstract Book Citations:** Authors, Title, HemaSphere, 2026;10:(S1):pages. The abstract book DOIs can be found on the “Author Information” page.
**Disclaimer:** Articles published in the journal HemaSphere exclusively reflect the opinions of the authors. The authors are responsible for all content in their abstracts including accuracy of the facts, statements, citing resources, etc.

**Summary/Conclusion**

Community clinicians are increasingly incorporating bispecific antibodies into routine care for DLBCL, FL, and MM; however, significant logistical and skill-based barriers remain. As bispecific antibodies become foundational across B-cell malignancies, development of scalable outpatient workflows, step-up dosing protocols, and inter-institutional co-management pathways will be essential to ensure safe and equitable implementation in community practice.

**Copyright Information:** (Online) ISSN: 2572-9241
©2026 The Author(s). HemaSphere is published by John Wiley & Sons Ltd on behalf of European Hematology Association. This is an open access Abstract Book distributed under the Attribution-NonCommercial-NoDerivs (CC BY-NC-ND), which allows third parties to download the articles and share them with others as long as they credit the author and the Abstract Book, but they cannot change the content in any way or use them commercially.
**Abstract Book Citations:** Authors, Title, HemaSphere, 2026;10:(S1):pages. The abstract book DOIs can be found on the "Author Information" page.
**Disclaimer:** Articles published in the journal HemaSphere exclusively reflect the opinions of the authors. The authors are responsible for all content in their abstracts including accuracy of the facts, statements, citing resources, etc.

### (PF1404) REAL-WORLD EXPERIENCES OF FAMILY CARERS SUPPORTING PEOPLE WITH MYELOMA

**Topic:** 36. Quality of life, ethics, supportive and palliative care

Eilidh Duncan*[1], Silene Ten Seldam[1], Diane Loening-Martens[1], Xavier Leleu[2], Charlotte Pawlyn[3], Séverine Wollenschneider[1], Katie Joyner[1], Kate Morgan[1]

*[1]Myeloma Patients Europe, Brussels, Belgium; [2]Hôpital La Mileterie, Poitiers, France; [3]The Institute of Cancer Research, London, United Kingdom;*

### Background

The course of myeloma is often complex, typically involving several lines of treatment and alternating phases of remission and relapse. Informal or family carers play a critical role in supporting myeloma patients throughout their disease and treatments, but their needs and challenges are often under-represented in research and in the provision of support.

### Aims

This research aimed to generate evidence on the experiences of family carers supporting people with myeloma.

### Methods

A pan-European online survey was distributed via a network of myeloma patient organisations in eight languages. Eligible participants were over 18, and supporting someone diagnosed with myeloma who had received (or was currently receiving) at least one treatment. Data collected included demographics, Quality of Life (QoL), support provided by family carers, and the impact of myeloma treatments.

Participants rated their own health and their own quality of life on a scale of 1(very poor) to 7(excellent). The impact of treatment on the family carer was rated across seven domains (frequency, effort, precautions, emotional, daily activity, family life and financial) on a scale of 0 (no impact) to 10 (considerable impact). The total impact for each treatment type was summed across all burden domains with a maximum possible score of 70.

### Results

The survey achieved 169 responses from 14 European countries. Family carers ranged in age from 21-79 years old (mean 52 years) and the majority were female (79%).

The majority of participants supported their spouse or partner (56%) but there were also participants supporting a parent (31%), sibling (6%), daughter/son (4%), friend (2%) and grandparent (1%). Most (68%) were living in the same household but for those who weren't, 48% were living 20 or more minutes away from the person they were supporting.

The most common types of support provided by participants were i) providing emotional support, ii) getting information about myeloma and treatments, iii) accompanying to appointments, iv) doing what they can to maintain quality of life, and v) helping with management of symptoms and side-effects.

The greatest proportion of participants rated their own health and their own quality of life around the mid-point on the scale; 58% rated their health and 56% rated their QoL at 4 or 5. Most participants (76%) reported having adjusted their own work commitments to allow them to support their relative or friend.

Family carers reported the impact that different treatment administration types had on them. Infusion treatments were rated as having the greatest impact overall (median overall burden score =42) and oral treatments the least (median=25). Across all treatment types, the impact was felt most in terms of emotional well-being, and on daily and family life (median scores 5-7; 4-7 and 4-7 respectively).

**Copyright Information:** (Online) ISSN: 2572-9241
©2026 The Author(s). HemaSphere is published by John Wiley & Sons Ltd on behalf of European Hematology Association. This is an open access Abstract Book distributed under the Attribution-NonCommercial-NoDerivs (CC BY-NC-ND), which allows third parties to download the articles and share them with others as long as they credit the author and the Abstract Book, but they cannot change the content in any way or use them commercially.
**Abstract Book Citations:** Authors, Title, HemaSphere, 2026;10:(S1):pages. The abstract book DOIs can be found on the "Author Information" page.
**Disclaimer:** Articles published in the journal HemaSphere exclusively reflect the opinions of the authors. The authors are responsible for all content in their abstracts including accuracy of the facts, statements, citing resources, etc.

**Summary/Conclusion**

Family carers play an extensive multi-dimensional role in myeloma care including providing emotional, practical and logistical support. Many carers rated their own health and quality of life to be only mid-level suggesting they are experiencing strain. A large proportion had adjusted their work commitments indicating disruption to daily life. Myeloma treatment was consistently reported to affect the emotional wellbeing, daily activity and family life of family carers. These findings point to the need for improved recognition, resources and systemic support tailored to the needs of people supporting someone with myeloma.

**Copyright Information:** (Online) ISSN: 2572-9241
©2026 The Author(s). HemaSphere is published by John Wiley & Sons Ltd on behalf of European Hematology Association. This is an open access Abstract Book distributed under the Attribution-NonCommercial-NoDerivs (CC BY-NC-ND), which allows third parties to download the articles and share them with others as long as they credit the author and the Abstract Book, but they cannot change the content in any way or use them commercially.
**Abstract Book Citations:** Authors, Title, HemaSphere, 2026;10:(S1):pages. The abstract book DOIs can be found on the "Author Information" page.
**Disclaimer:** Articles published in the journal HemaSphere exclusively reflect the opinions of the authors. The authors are responsible for all content in their abstracts including accuracy of the facts, statements, citing resources, etc.

### (PF1405) SYMPTOM BURDEN AND LONGITUDINAL QUALITY OF LIFE DURING INTENSIVE INDUCTION THERAPY FOR ACUTE MYELOID LEUKAEMIA

**Topic:** 36. Quality of life, ethics, supportive and palliative care

Kevin D. Hofer*[1], Nadine Muggler[1], Sebastian Stolz[1], Wiebke Rösler[1], Max Jan Rieger[1], Markus G. Manz[1], Jeremy Deuel[1]

[1]*University Hospital Zurich, Medical Oncology and Haematoloy, Zurich, Switzerland;*

### Background

Intensive induction chemotherapy for acute myeloid leukaemia (AML) is associated with considerable toxicities, which may impair quality of life (QoL). Several studies have described the long-term impact of treatment; however, little is known on the course of immediate side effects, their impact on QoL during induction chemotherapy, and how they relate to leukaemia response.

### Aims

We conducted a prospective, longitudinal, observational study to assess patient-reported outcomes once weekly during induction therapy through a validated leukaemia-specific (FACT-Leu) and dedicated mucositis questionnaire (OMDQ).

### Methods

Data analysis included descriptive statistics for demographic characteristics and Fisher's exact test or Wilcoxon's rank-sum test to evaluate differences in patient-reported outcomes across cohorts, as appropriate.

### Results

We enrolled 33 patients with acute leukaemia (30 AML, 2 acute undifferentiated leukaemia, 1 mixed-phenotype acute leukaemia), receiving intensive induction chemotherapy with daunorubicin and cytarabine. Mean age was 57 years (range 20-71) and 18 patients were female (55%). Twenty-nine percent were classified as adverse risk based on ELN2022 guidelines and 36% received add-on treatment with the FLT3 inhibitor midostaurin. Overall QoL, represented by mean FACT-Leu total scores, was strongly impaired at baseline (96.9/176, with higher scores indicating higher QoL) with substantial variability among patients (coefficient of variation 13.5 - 30.9% across timepoints). Total scores increased numerically during treatment, largely driven by improvements in emotional well-being (Figure A and B) and leukaemia-related concerns (leukaemia subscale). Physical and social subscales remained stable during treatment, while functional impairment was most pronounced at week 2 (mean score 11.2/28). The most prevalent symptoms across all timepoints were the inability to perform usual activities (mean: 0.76/4) and perceived lack of support from friends (0.87/4) and family (0.96/4). Scores for mouth and throat soreness were significantly higher during treatment in patients with complete remission at day 28 ($P = 0.006$ at week 3, Figure C). Responders also experienced earlier peaks and more prolonged diarrhoea, evidenced by higher rates of moderate-to-severe mucositis and/or diarrhoea (>3 on a numerical rating scale from 0-10) at week 3 ($P = 0.004$).

### Summary/Conclusion

This longitudinal QoL analysis during intensive induction chemotherapy demonstrates a high burden of emotional and physical symptoms that substantially impair QoL and highlight unmet supportive care and psychosocial needs in this population. Notably, overall QoL improves early during treatment, likely reflecting control of emotional distress and leukaemia-specific symptoms, despite the negative impact of treatment-related toxicities on well-being. Furthermore, our findings suggest a potential association between gastrointestinal side effects and therapeutic response, which merits further investigation.

**Copyright Information:** (Online) ISSN: 2572-9241
©2026 The Author(s). HemaSphere is published by John Wiley & Sons Ltd on behalf of European Hematology Association. This is an open access Abstract Book distributed under the Attribution-NonCommercial-NoDerivs (CC BY-NC-ND), which allows third parties to download the articles and share them with others as long as they credit the author and the Abstract Book, but they cannot change the content in any way or use them commercially.
**Abstract Book Citations:** Authors, Title, HemaSphere, 2026;10:(S1):pages. The abstract book DOIs can be found on the "Author Information" page.
**Disclaimer:** Articles published in the journal HemaSphere exclusively reflect the opinions of the authors. The authors are responsible for all content in their abstracts including accuracy of the facts, statements, citing resources, etc.

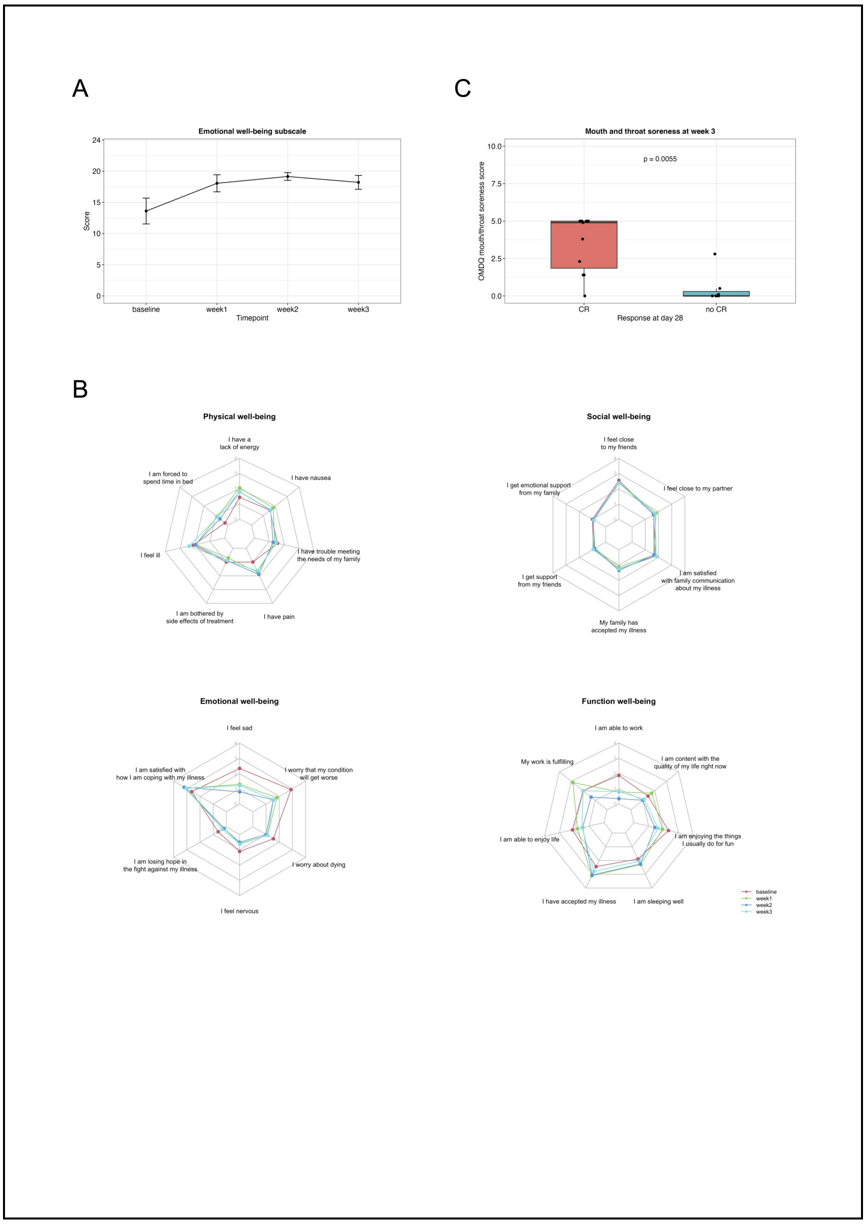

A, Emotional well-being subscale (score range 0-24) of the FACT-Leu questionnaire by timepoint of induction therapy. Higher scores reflect higher QoL. Error bars indicate standard errors from the mean. B, Radar plots on specific subscales of the FACT-Leu questionnaire stratified by timepoint. Mean scores indicate to which extent a statement applies for the previous 7 days (0 = Not at all; 1 = A little bit; 2 = Somewhat; 3 = Quite a bit; 4 = Very much). Scores of negative statements are reversed for subscale scoring. C, Mouth and throat soreness as assessed through the OMDQ at week 3, stratified by bone marrow response at day 28 (n=18). Symptoms are evaluated by a numerical rating scale from 0-10 with higher scores indicating higher symptom burden. CR, complete response including patients with complete response and incomplete or partial hematologic recovery.

**Copyright Information:** (Online) ISSN: 2572-9241
©2026 The Author(s). HemaSphere is published by John Wiley & Sons Ltd on behalf of European Hematology Association. This is an open access Abstract Book distributed under the Attribution-NonCommercial-NoDerivs (CC BY-NC-ND), which allows third parties to download the articles and share them with others as long as they credit the author and the Abstract Book, but they cannot change the content in any way or use them commercially.
**Abstract Book Citations:** Authors, Title, HemaSphere, 2026;10:(S1):pages. The abstract book DOIs can be found on the "Author Information" page.
**Disclaimer:** Articles published in the journal HemaSphere exclusively reflect the opinions of the authors. The authors are responsible for all content in their abstracts including accuracy of the facts, statements, citing resources, etc.

### (PF1406) SYMPTOM BURDEN OF PATIENTS WITH MULTIPLE MYELOMA DURING THE LAST WEEK OF LIFE COMPARED TO OTHER HAEMATOLOGICAL MALIGNANCIES- A POPULATION-BASED REGISTRY STUDY

**Topic:** 36. Quality of life, ethics, supportive and palliative care

Emma Freij*[1, 2], Christel Hedman[3, 4, 5], Ellis Slotman[5, 6], Staffan Lundström[4, 7], Lena Von Bahr[8, 9], Linda Björkhem-Bergman[4, 10]

*[1]NVS, Huddinge, Sweden; [2]Karolinska Universitetssjukhuset Hud, ME Hematology, Huddinge, Sweden; [3]Karolinska Institutet-Solna, Solna, Sweden; [4]Stockholm Sjukhem, Palliative Medicine and R&D unit, Stockholms Stad, Sweden; [5]Lund University, Institute for Palliative Care, Lund, Sweden; [6]Netherlands Comprehensive Cancer Organisation,, Department of Research and Development, Utrecht, The Netherlands; [7]Karolinska Institutet, Department of Oncology-Pathology, Stockholm, Sweden; [8]University of Gothenburg, Sahlgrenska Academy, Gothenburg, Sweden;[9]Sahlgrenska University Hospital, Section of hematology and coagulation, Gothenburg, Sweden; [10]Karolinska Institutet, Department of Neurobiology, Care Sciences and Society (NVS), Stockholm, Sweden;*

### Background

Haematological malignancies (HM) consist of a heterogenous group of diseases with a large symptom burden at the end of life. Pain is especially prominent for patients with Multiple Myeloma (MM), however there is limited research of the symptom burden at the end of life for patients with MM compared to other HM.

### Aims

The aim of this study was to map symptoms during the last week of life in patients with MM compared to other HM divided into two groups: aggressive and indolent HM. Secondary aims included comparing place of death as well as if end-of-life conversations had occurred.

### Methods

Data from the Swedish Register of Palliative Care on HM patients who died between 2011-2023 was analysed. The patients were divided into three groups: MM (n=4310), aggressive HM (n=8974) and indolent HM (n=6085). Symptoms and symptom relief in the last week of life were analysed and compared between the three groups, as well as end-of-life conversations and place of death. Uni- and multivariable analysis adjusting for sex, age and place of death were performed.

### Results

Patients with MM had a significantly higher rate of pain during the last week of life compared to both patients with aggressive and indolent HM, 77% compared to 73% and 71% (p<0.001 for both); adjusted OR 1.29 (95%CI 1.19-1.41) and 1.35 (95%CI 1.23-1.48). The pain for MM patients was less likely be fully relieved compared to the other HM (p<0.001 for both). The patients with aggressive HM were more likely to die in specialised palliative care (SPC) compared to the other groups (p<0.001) and more likely to have end-of-life conversations (p<0.001).

***Table 1** Demographic characteristics of the patients with MM aggressive and indolent HM as well as symptoms in the last week of life, if end-of-life conversation occurred and place of death.* *** p<0.001, **p<0.01, *p<0.05 comparing MM with aggressive or indolent HM , ¤ p<0.001 comparing aggressive and Indolent HM. Abbreviations: SPC= inpatient specialised palliative care

| | Multiple myeloma | Aggressive | Indolent |
|---|---|---|---|

**Copyright Information:** (Online) ISSN: 2572-9241
©2026 The Author(s). HemaSphere is published by John Wiley & Sons Ltd on behalf of European Hematology Association. This is an open access Abstract Book distributed under the Attribution-NonCommercial-NoDerivs (CC BY-NC-ND), which allows third parties to download the articles and share them with others as long as they credit the author and the Abstract Book, but they cannot change the content in any way or use them commercially.
**Abstract Book Citations:** Authors, Title, HemaSphere, 2026;10:(S1):pages. The abstract book DOIs can be found on the "Author Information" page.
**Disclaimer:** Articles published in the journal HemaSphere exclusively reflect the opinions of the authors. The authors are responsible for all content in their abstracts including accuracy of the facts, statements, citing resources, etc.

| | n=4310 | n=8974 | n=6085 |
|---|---|---|---|
| **Sex** | | | |
| Male n, (%) | 2357 (54.6%) | 4703 (52.4%***¤) | 3548 (58.3%***¤) |
| Female n, (%) | 1953 (45.3%) | 4271 (47.5%) | 2537 (41.6%) |
| Age, median, IQR | 78 (71-84) | 78 (71-84) | 82***¤ (75-88) |
| 0-17 years | 0 (0%) | 28 (0.3%) | 7 (0.1%) |
| 18-64 years | 500 (11.6%) | 1190 (13.3%) | 363 (6.0%) |
| 65-74 years | 1117 (25.9%) | 2137 (23.8%) | 1097 (18.0%) |
| 75-84 years | 1686 (39.1%) | 3469 (38.7%) | 2320 (38.1%) |
| >85 years | 1007 (23.4%) | 2150 (24.0%) | 2298 (37.8%) |
| Pain n,(%) | 3334 (77.4%) | 6539***(72.9%) | 4295***(70.6%) |
| Nausea n,(%) | 568 (13.2%) | 1435***(16.0%) | 836¤(13.7%) |
| Breathlessness n,(%) | 994 (23.1%) | 2392***(26.7%) | 1667***(27.4%) |
| Anxiety n,(%) | 2325 (53.9%) | 4911 (54.7%) | 3180*¤(52.3%) |
| Confusion / Delirium n,(%) | 1310 (30.4%) | 2268***(25.3%) | 1520***(25.0%) |
| Death rattle n,(%) | 2370 (55.0%) | 3839***(42.8%) | 2777***¤(45.6%) |

**Copyright Information:** (Online) ISSN: 2572-9241
©2026 The Author(s). HemaSphere is published by John Wiley & Sons Ltd on behalf of European Hematology Association. This is an open access Abstract Book distributed under the Attribution-NonCommercial-NoDerivs (CC BY-NC-ND), which allows third parties to download the articles and share them with others as long as they credit the author and the Abstract Book, but they cannot change the content in any way or use them commercially.
**Abstract Book Citations:** Authors, Title, HemaSphere, 2026;10:(S1):pages. The abstract book DOIs can be found on the "Author Information" page.
**Disclaimer:** Articles published in the journal HemaSphere exclusively reflect the opinions of the authors. The authors are responsible for all content in their abstracts including accuracy of the facts, statements, citing resources, etc.

| | | | |
|---|---|---|---|
| **End of life discussions n, (%)** | | | |
| "yes" | 2816 (65.3%) | 6232***(69.4%) | 3649***¤(60.0%) |
| Not known | 476 (11.0%) | 851 (9.5%) | 663 (10.9%) |
| **Place of death n, (%)** | | | |
| Hospital n,(%) | 1745 (40.5%) | 3582 (39.9%) | 2440 (40.1%) |
| SPC n,(%) | 886 (20.6%) | 2205***(24.6%) | 1034***¤(17.0%) |
| Home n,(%) | 791 (18.4%) | 1732 (19.3%) | 917***¤(15.1%) |
| Other n,(%) | 888 (20.6%) | 1455***(16.2%) | 1694***¤(27.8%) |

### Summary/Conclusion

Pain is more prevalent for patients with MM compared to aggressive and indolent HM and less likely to be fully relieved. Patients with aggressive HM are more likely to die in SPC and have end-of-life conversations compared to patients with indolent HM or MM. Future research should investigate how to best manage the complex pain needs of MM and how to achieve timely palliative care and end-of-life conversations for all HM.

**Copyright Information:** (Online) ISSN: 2572-9241
©2026 The Author(s). HemaSphere is published by John Wiley & Sons Ltd on behalf of European Hematology Association. This is an open access Abstract Book distributed under the Attribution-NonCommercial-NoDerivs (CC BY-NC-ND), which allows third parties to download the articles and share them with others as long as they credit the author and the Abstract Book, but they cannot change the content in any way or use them commercially.
**Abstract Book Citations:** Authors, Title, HemaSphere, 2026;10:(S1):pages. The abstract book DOIs can be found on the "Author Information" page.
**Disclaimer:** Articles published in the journal HemaSphere exclusively reflect the opinions of the authors. The authors are responsible for all content in their abstracts including accuracy of the facts, statements, citing resources, etc.

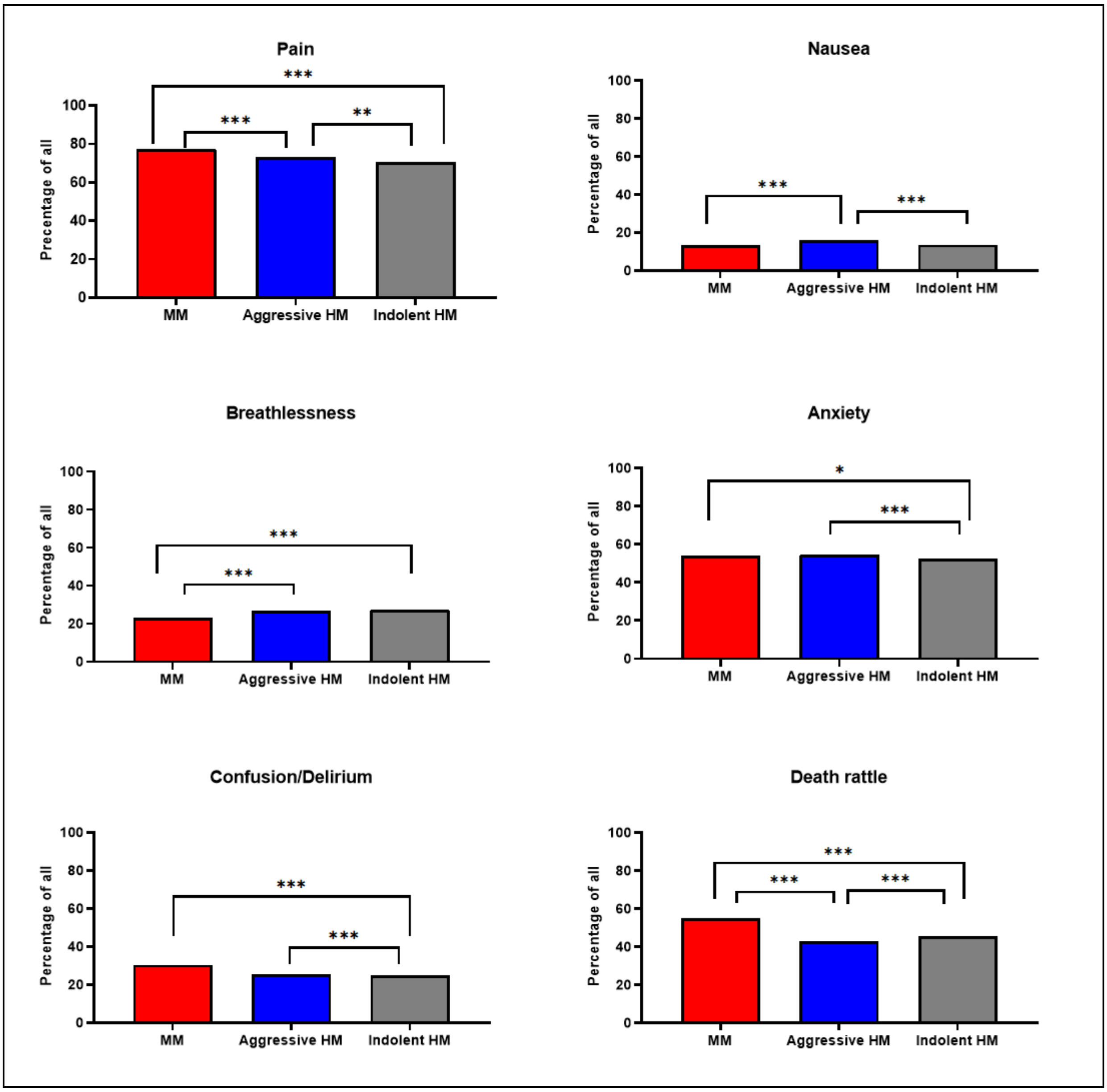

Prevalence of symptoms in patients with Multiple Myeloma, aggressive and indolent haematological malignancies the last week in life. ***p<0.001, **p<0.01, *p<0.05

**Copyright Information:** (Online) ISSN: 2572-9241
©2026 The Author(s). HemaSphere is published by John Wiley & Sons Ltd on behalf of European Hematology Association. This is an open access Abstract Book distributed under the Attribution-NonCommercial-NoDerivs (CC BY-NC-ND), which allows third parties to download the articles and share them with others as long as they credit the author and the Abstract Book, but they cannot change the content in any way or use them commercially.
**Abstract Book Citations:** Authors, Title, HemaSphere, 2026;10:(S1):pages. The abstract book DOIs can be found on the "Author Information" page.
**Disclaimer:** Articles published in the journal HemaSphere exclusively reflect the opinions of the authors. The authors are responsible for all content in their abstracts including accuracy of the facts, statements, citing resources, etc.

### (PF1407) REAL - WORLD EVIDENCE ON THE IMPACT OF RELATIVE DOSE INTENSITY (RDI) ON SURVIVAL IN PATIENTS WITH HEMATOLOGICAL MALIGNANCIES IN A MIDDLE – INCOME COUNTRY.

**Topic:** 36. Quality of life, ethics, supportive and palliative care

Carlos Gomez - Calcetero*[1], Juanita Granados - Diaz[1], Adriana Aya -Porto[2], Paola Omaña - Orduz[1], Jorge Daza - Buitrago[1], Virginia Abello - Polo[1]

*[1]Luis Carlos Sarmiento Angulo Cancer Treatment and Research Centre (CTIC), Research Group GIGA, CTIC/Universidad El Bosque., Bogotá, Colombia; [2]Luis Carlos Sarmiento Angulo Cancer Treatment and Research Centre (CTIC), Research and Education Institute, Bogotá, Colombia;*

### Background

Patients with hematological neoplasms in Low- and Middle-income countries often experience treatment delays due to disparities in access to care, diagnostic gaps, economic constraints, geographic barriers, social factors and treatment – related toxicities. Delays in dose administration lead to a reduction in relative dose intensity (RDI). The RDI has been shown to be a surrogate for overall survival (OS) in patients with solid tumors; however, evidence regarding the impact of RDI on survival in hematologic malignancies remains limited.

### Aims

To evaluate the association between RDI and OS in patients with hematologic malignancies treated at a referral center in a middle-income country in Latin America.

### Methods

We conducted a retrospective analytical observational cohort study including patients ≥18 years with an incident diagnosis of hematologic malignancies Hodgkin lymphoma (HL), non-Hodgkin lymphoma (NHL), multiple myeloma (MM), and acute leukemias (AL) treated at Fundación CTIC (Bogotá, Colombia) between January 2023 and December 2024. All patients were enrolled in the prospective institutional observational registry EVA (Ev idence–Verification–Analysis) and had provided informed consent. RDI was calculated as (total dose received/total planned dose) ×100.
A tracer first-line agent with predefined RDI targets was selected per disease based on available evidence: bortezomib (MM), doxorubicin (HL/NHL), cytarabine (fit AML), 5-azacitidine (unfit AML), L-asparaginase (AYA B-ALL), and cyclophosphamide (>39 years B-ALL). Target RDI thresholds were ≥95% (MM), ≥85% (lymphomas), and ≥75% (AL).

Associations between RDI status and clinical variables were assessed using Pearson's $\chi^2$ test. Crude risk ratios (cRR) were calculated for failure to achieve target RDI (exposure) and mortality (outcome). Stratified analyses were performed by disease risk/stage. Absolute risk reduction (ARR) and number needed to treat (NNT) were estimated. Survival was analyzed using Kaplan–Meier curves and Cox proportional hazards models (HR). Log-rank tests compared survival curves. Significance was set at $p<0.05$. Analyses were performed in Python 3.11.

**Copyright Information:** (Online) ISSN: 2572-9241
©2026 The Author(s). HemaSphere is published by John Wiley & Sons Ltd on behalf of European Hematology Association. This is an open access Abstract Book distributed under the Attribution-NonCommercial-NoDerivs (CC BY-NC-ND), which allows third parties to download the articles and share them with others as long as they credit the author and the Abstract Book, but they cannot change the content in any way or use them commercially.
**Abstract Book Citations:** Authors, Title, HemaSphere, 2026;10:(S1):pages. The abstract book DOIs can be found on the "Author Information" page.
**Disclaimer:** Articles published in the journal HemaSphere exclusively reflect the opinions of the authors. The authors are responsible for all content in their abstracts including accuracy of the facts, statements, citing resources, etc.

### Results

Among 194 patients, 134 (69%) achieved target RDI. Baseline characteristics were similar between groups (table 1). Failure to achieve RDI was associated with higher mortality (cRR=3.08; 95% CI 2.24–4.25; p<0.05). Stratified analysis showed stronger associations in advanced/high-risk disease (RR=3.45; 95% CI, 2.38–5.0; p<0.05) ) compared with early/low-intermediate risk (RR=2.5; 95% CI, 1.45–4.35; p<0.05), suggesting potential interaction; however, this was not confirmed due to lack of statistical heterogeneity. Achieving RDI was associated with lower mortality (HR=0.27; 95% CI 0.17–0.43; p<0.001). Absolute mortality reduction was 21% in early-stage (NNT=5) and 29% in advanced-stage disease (NNT=3). Median OS for the cohort was 7 months (log-rank p<0.001), figure1.

### Summary/Conclusion

Achieving the target of RDI in a cohort of patients with hematological malignancies was associated with lower risk of death. The findings suggest that disease risk/stage acts as an effect modifier in the relationship between RDI attainment and mortality, with a more pronounced effect observed in the high – risk subgroup.

Copyright Information: (Online) ISSN: 2572-9241
©2026 The Author(s). HemaSphere is published by John Wiley & Sons Ltd on behalf of European Hematology Association. This is an open access Abstract Book distributed under the Attribution-NonCommercial-NoDerivs (CC BY-NC-ND), which allows third parties to download the articles and share them with others as long as they credit the author and the Abstract Book, but they cannot change the content in any way or use them commercially.
Abstract Book Citations: Authors, Title, HemaSphere, 2026;10:(S1):pages. The abstract book DOIs can be found on the “Author Information” page.
Disclaimer: Articles published in the journal HemaSphere exclusively reflect the opinions of the authors. The authors are responsible for all content in their abstracts including accuracy of the facts, statements, citing resources, etc.

**Table 1. Sociodemographic and Clinical characteristics of the cohort.**

| Categories | Total (n=194) | Treatment completed according to RDI (n=134) | Treatment incomplete according to RDI (n=60) | p value* |
|---|---|---|---|---|
| **Social Demographics characteristics** | | | | |
| **Age, years, n (%)** | | | | |
| *>60* | 95(49) | 60(40,8) | 35(58,3) | |
| *27-59* | 88(45,3) | 68(50,7) | 20(33,3) | 0,0668 |
| *18-26* | 11(5,7) | 6(4,5) | 5(8,3) | |
| **Sex, n(%)** | | | | |
| *Male* | 108(55,7) | 77(57,5) | 31(51,7) | 0,552 |
| *Female* | 86(44,3) | 57(42,5) | 29(48,3) | |
| **Diagnosis characteristics** | | | | |
| **Hematological diagnosis, n(%)** | | | | |
| *Lymphoma ‡* | 105(54,1) | 73(54,5) | 32(53,3) | |
| *Multiple myeloma* | 52(26,8) | 36(26,9) | 16(26,7) | 0,9754 |
| *Acute leukemias †* | 37(19,1) | 25(18,7) | 12(20) | |
| **Risk, n(%) §** | | | | |
| *High* | 37(19,1) | 25(18,7) | 12(20) | |
| *Intermediate* | 10(5,2) | 1(0,7) | 9(15) | 0,4526 |
| *Low* | 4(2,1) | 4(3) | - | |
| **Staging, n(%)** | | | | |
| *Advanced §§* | 120(61,9) | 75(56) | 45(75) | |
| *Early §§§* | 23(11,9) | 16(11,9) | 7(11,7) | 0,2406 |
| *Unknown* | 7(3,6) | 2(1,5) | 5(8,3) | |
| **Mortality, n(%)** | | | | |
| *No* | 113(58,2) | 100(74,6) | 13(21,7) | **0,000** |
| *Yes* | 81(41,8) | 34(25,4) | 47(78,3) | |

* Pearson's $\chi^2$ test. † Acute myeloid leukemia and acute lymphoblastic leukemia. ‡ Hodgkin and non-Hodgkin lymphomas. § for acute leukemias based on molecular risk. §§ for MM R-ISS II and III, for HL and NHL Ann Arbor III and IV. §§§ for MM R-ISS I, for HL and NHL Ann Arbor I and II.

**Figure 1.**

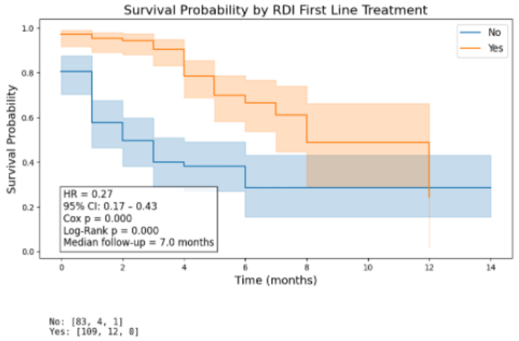

**Copyright Information:** (Online) ISSN: 2572-9241
©2026 The Author(s). HemaSphere is published by John Wiley & Sons Ltd on behalf of European Hematology Association. This is an open access Abstract Book distributed under the Attribution-NonCommercial-NoDerivs (CC BY-NC-ND), which allows third parties to download the articles and share them with others as long as they credit the author and the Abstract Book, but they cannot change the content in any way or use them commercially.
**Abstract Book Citations:** Authors, Title, HemaSphere, 2026;10:(S1):pages. The abstract book DOIs can be found on the "Author Information" page.
**Disclaimer:** Articles published in the journal HemaSphere exclusively reflect the opinions of the authors. The authors are responsible for all content in their abstracts including accuracy of the facts, statements, citing resources, etc.

### (PF1408) ACCESS AND OUTCOMES FOR 2ND LINE AXICABTAGENE CILOLEUCEL IN PATIENTS WITH LARGE B-CELL LYMPHOMA ACCORDING TO SOCIOECONOMIC DEPRIVATION, DISTANCE AND ETHNICITY IN THE UK

**Topic:** 37. Health economics

Emil Kumar*[1], Amy Kirkwood[2], Sotiria Dimou-Besikli[1], Carolyn Maharaj[3], Jenny Le[4], Lisanne Schoutens[5], Martin Loader[6], Muhammad Farhan[7], Robin Sanderson[1], Wendy Osborne[8], Sridhar Chaganti[9], Euan Haynes[8], Ben Uttenthal[6], Katerina Panopoulou[5], Nikesh Dhiraj[10], Michael Northend[3], Jane Norman[7], Andrea Kuhnl[1], Bernard Maybury[5]

*[1]King's College Hospital, Department of Haematology, London, United Kingdom;[2]CRUK & UCL Cancer Clinical Trials Centre, UCL Cancer Institute, UCL, London, United Kingdom; [3]University College London Hospitals, London, Department of Haematology, London, United Kingdom; [4]Bristol Haematology and Oncology Centre, Bristol, United Kingdom; [5]Oxford University Hospitals, Department of Haematology, Oxford, United Kingdom; [6]Cambridge University Hospitals, Department of Haematology, Cambridge, United Kingdom; [7]Manchester Royal Infirmary, Manchester, United Kingdom;[8]The Newcastle upon Tyne Hospitals NHS Foundation Trust and Newcastle University, Newcastle upon Tyne, UK, Department of Haematology, Newcastle, United Kingdom; [9]Queen Elizabeth Hospital, Department of Haematology, Birmingham, United Kingdom; [10]University Hospitals Bristol and Weston, Department of Haematology, Bristol, United Kingdom;*

### Background

We previously showed that for large B cell lymphoma (LBCL) patients treated with 3$^{rd}$ line (3L) CD19 CAR-T at three UK National Health Service (NHS) CAR-T centres in 2019-2022, 25% of patients travelled >100km; and that the most deprived patients experienced poorer survival, driven by higher rates of pre-infusion drop-out and COVID-19 death (Dragoi et al, BJHaem, 2025).

### Aims

We aimed to evaluate the impact of distance to CAR-T centre, socio-economic deprivation, and ethnicity on LBCL outcomes following 2L CAR-T in the UK.

### Methods

We analysed these factors in a cohort of 204 patients approved for 2L axicabtagene ciloleucel at eight CAR-T centres in England between May 2023 and November 2024, with median follow-up of 16.4 months (IQR 12.0-20.1). Postcode-level data on deprivation were derived using the English “Index of Multiple Deprivation” (IMD) 2019, with patients grouped by high (IMD 1-3), intermediate (IMD 4-7) and low (IMD 8-10) deprivation.

**Copyright Information:** (Online) ISSN: 2572-9241
©2026 The Author(s). HemaSphere is published by John Wiley & Sons Ltd on behalf of European Hematology Association. This is an open access Abstract Book distributed under the Attribution-NonCommercial-NoDerivs (CC BY-NC-ND), which allows third parties to download the articles and share them with others as long as they credit the author and the Abstract Book, but they cannot change the content in any way or use them commercially.
**Abstract Book Citations:** Authors, Title, HemaSphere, 2026;10:(S1):pages. The abstract book DOIs can be found on the “Author Information” page.
**Disclaimer:** Articles published in the journal HemaSphere exclusively reflect the opinions of the authors. The authors are responsible for all content in their abstracts including accuracy of the facts, statements, citing resources, etc.

### Results

The distribution by deprivation was: 58 Low (28.9%), 102 Medium (50.7%), and 41 High (20.4%). The distribution by ethnicity was: 166 White (89.2%), 15 Asian (8.1%), 5 Black (2.7%) (18 Unknown). There was a trend to greater proportion of ethnic minority patients from more deprived areas (Low 7.3%, Medium 10.6%, High 17.5%, p=0.14). Patients from the most deprived areas travelled less for CAR-T (median distance by IMD: Low 42.6km, Medium 43.1km, High 29.6km, p=0.026), likely reflecting the inner-city location of most centres. 8% of patients travelled >100km.
There were no significant differences in baseline characteristics (including age, stage, LDH, bulk, IPI) according to deprivation. 94.7% of patients received bridging therapy, with no significant differences by deprivation. Importantly, there were no differences in vein-to-vein time, or drop-out rate by deprivation (92.0% infused for whole cohort).
In the low deprivation group, there was a trend to superior response to bridging (overall response rate by IMD: Low 68.0%, Medium 43.1%, High 44.1%, p=0.099), and more grade 3+ CRS events (% by IMD: Low 9.4%, Medium 3.2%, High 0%, p=0.024); but no significant difference in grade 3+ ICANS, tocilizumab/steroid use or ICU admission. There were no significant differences in complete metabolic response rate, PFS/OS, or non-relapse mortality by deprivation. There were no significant differences in toxicity / efficacy outcomes in ethnic minority patients, or for patients travelling >100km. There were no reported COVID-19 deaths in this cohort, highlighting the evolution in COVID-19 risk.

### Summary/Conclusion

Our data suggest similar outcomes for patients receiving 2L axi-cel irrespective of socio-economic deprivation, ethnicity or distance to centre in a diverse UK cohort, contrasting with findings at 3L. Moving CAR-T to earlier treatment line, more pro-active bridging strategies, and the decline in the COVID-19 pandemic, may have helped mitigate competing risks felt by more deprived patients; although deprivation, ethnicity and distance may impact on patient experience and outcomes in ways not captured here. Furthermore, the effect of these factors on rate of referral for CAR-T was not examined and warrants separate analysis. We saw fewer patients travelling >100km - attributable to an increase in the number of UK CAR-T centres - but for those still facing this barrier, outcomes were equivalent, underlining that they should still be offered CAR-T and the necessary support to travel.

**Copyright Information:** (Online) ISSN: 2572-9241
©2026 The Author(s). HemaSphere is published by John Wiley & Sons Ltd on behalf of European Hematology Association. This is an open access Abstract Book distributed under the Attribution-NonCommercial-NoDerivs (CC BY-NC-ND), which allows third parties to download the articles and share them with others as long as they credit the author and the Abstract Book, but they cannot change the content in any way or use them commercially.
**Abstract Book Citations:** Authors, Title, HemaSphere, 2026;10:(S1):pages. The abstract book DOIs can be found on the "Author Information" page.
**Disclaimer:** Articles published in the journal HemaSphere exclusively reflect the opinions of the authors. The authors are responsible for all content in their abstracts including accuracy of the facts, statements, citing resources, etc.

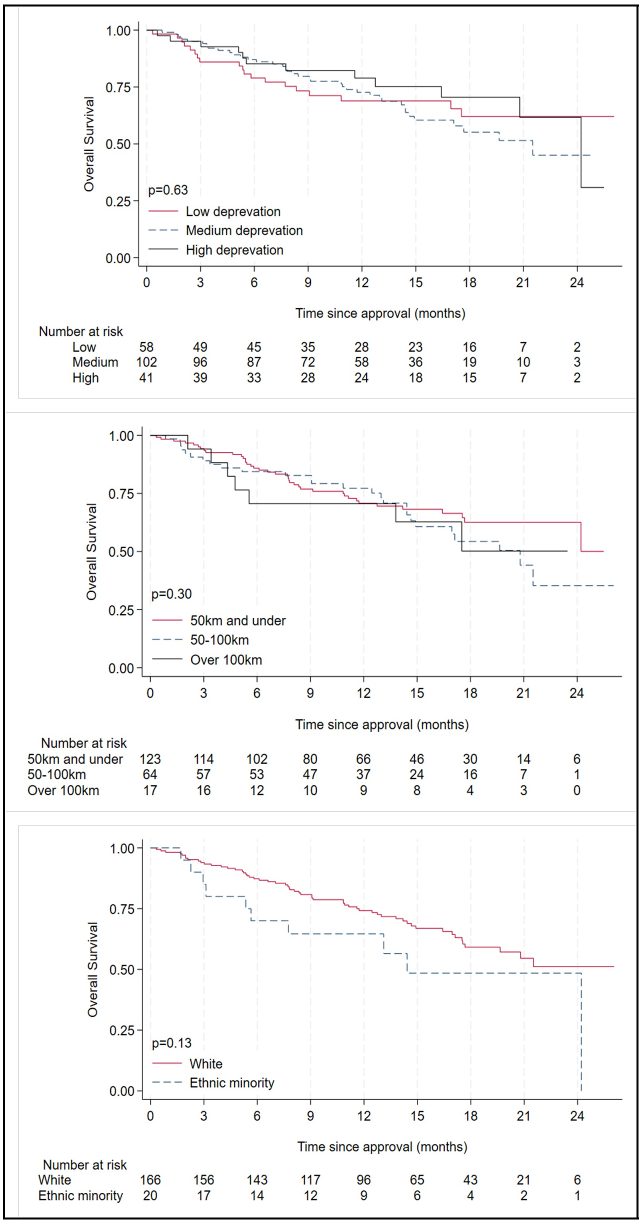

**Copyright Information:** (Online) ISSN: 2572-9241
©2026 The Author(s). HemaSphere is published by John Wiley & Sons Ltd on behalf of European Hematology Association. This is an open access Abstract Book distributed under the Attribution-NonCommercial-NoDerivs (CC BY-NC-ND), which allows third parties to download the articles and share them with others as long as they credit the author and the Abstract Book, but they cannot change the content in any way or use them commercially.
**Abstract Book Citations:** Authors, Title, HemaSphere, 2026;10:(S1):pages. The abstract book DOIs can be found on the "Author Information" page.
**Disclaimer:** Articles published in the journal HemaSphere exclusively reflect the opinions of the authors. The authors are responsible for all content in their abstracts including accuracy of the facts, statements, citing resources, etc.

### (PF1409) TIME BURDEN, TIME SPENT AT HOME, AND PERSISTENCE WITH AZACITIDINE OR DECITABINE PLUS VENETOCLAX IN NEWLY DIAGNOSED ACUTE MYELOID LEUKEMIA IN THE UNITED STATES

**Topic:** 37. Health economics

Amer M. Zeidan*[1], Ruizhi Zhao[2], Arthur Voegel[3], Dina Christensen[2], Maryia Zhdanava[4], Anabelle Tardif-Samson[4], Claire Vanden Eynde[4], Harold Keer[2], Dominic Pilon[4], Fang Sun[2]

*[1]Yale School of Medicine, New Haven, CT, United States of America;[2]Taiho Oncology, Inc., Princeton, NJ, United States of America; [3]Analysis Group, Inc., New York, United States of America;[4]Analysis Group, Montreal, Canada;*

### Background

For patients with newly diagnosed acute myeloid leukemia (AML) ineligible for intensive chemotherapy in the United States (US), the standard of care is a parenteral hypomethylating agent (HMA) in combination with oral venetoclax (VEN). This includes azacitidine + VEN (AZA+VEN) and decitabine + VEN (DEC+VEN), which require 7 and 5 days of in-clinic administration per 28-day treatment cycle, respectively. Repeated clinic visits impose a substantial time burden on patients and caregivers, potentially affecting their quality of life and treatment persistence. Real-world evidence characterizing time burden, time spent at home, and treatment persistence by AZA+VEN and DEC+VEN regimens remains sparse.

### Aims

To evaluate time burden, time spent at home, and treatment persistence following initiation of AZA+VEN or DEC+VEN among patients with newly diagnosed AML.

### Methods

This retrospective cohort study used the Humana Healthcare Research Database (01/2019–12/2024), a US claims database of Medicare Advantage beneficiaries, to identify adults with newly diagnosed AML who initiated AZA+VEN or DEC+VEN (index date = first HMA claim). A 12-month period of continuous insurance eligibility before the index date was used to assess baseline characteristics. Outcomes were assessed from the index date until the end of continuous insurance eligibility, data availability, or death. Time burden was defined as the number of distinct days with healthcare encounters, including inpatient (IP) days, emergency room (ER) visits, and outpatient (OP) visits, with OP visits subcategorized as HMA administration, transfusion support, other AML-related care, or other OP services. Time spent at home was defined as the proportion of days not spent in an IP or ER setting (any cause) and without cancer-related OP visits. Treatment persistence was assessed overall and for label-conforming HMA cycles operationally defined to reflect real-world treatment patterns as ≥3 days of DEC or ≥4 days of AZA, with ≥16 days of VEN per cycle. Treatment persistence was assessed using Kaplan–Meier methods as time to discontinuation or death (TTDD).

### Results

A total of 519 patients initiated AZA+VEN and 218 initiated DEC+VEN (mean age: 76.7 and 75.6 years; female: 45.9% and 41.7%; White: 84.6% and 79.8%; other active cancer: 22.2% and 27.5%). During mean follow-up of 10.6 months (AZA+VEN) and 11.4 months (DEC+VEN), patients spent a mean of 12.0 and 11.8 days per month, respectively, on healthcare visits, including 3.4 and 2.9 days spent on HMA administration. The mean proportion of time spent at home was 65.0% for AZA+VEN and 64.0% for DEC+VEN. Median TTDD was 4.7 months with AZA+VEN and 5.8 months with DEC+VEN (**Figure 1**). When restricted to label-conforming cycles, median TTDD was 2.6 and 3.0 months, respectively. At 6 months, 43.6% (AZA+VEN) and 48.7% (DEC+VEN) remained on treatment, and 21.2% and 23.6% remained on label-conforming cycles.

**Copyright Information:** (Online) ISSN: 2572-9241
©2026 The Author(s). HemaSphere is published by John Wiley & Sons Ltd on behalf of European Hematology Association. This is an open access Abstract Book distributed under the Attribution-NonCommercial-NoDerivs (CC BY-NC-ND), which allows third parties to download the articles and share them with others as long as they credit the author and the Abstract Book, but they cannot change the content in any way or use them commercially.
**Abstract Book Citations:** Authors, Title, HemaSphere, 2026;10:(S1):pages. The abstract book DOIs can be found on the “Author Information” page.
**Disclaimer:** Articles published in the journal HemaSphere exclusively reflect the opinions of the authors. The authors are responsible for all content in their abstracts including accuracy of the facts, statements, citing resources, etc.

**Summary/Conclusion**

Despite differing HMA administration schedules, AZA+VEN and DEC+VEN were associated with comparable time burden, time spent at home, and treatment persistence patterns, including shorter label-conforming persistence potentially reflecting real-world dose modifications. These findings underscore the need for alternative effective treatment options that minimize clinical visits and optimize care delivery across both HMA + VEN regimens for patients with newly diagnosed AML ineligible for intensive chemotherapy.

Figure 1. Treatment Persistence

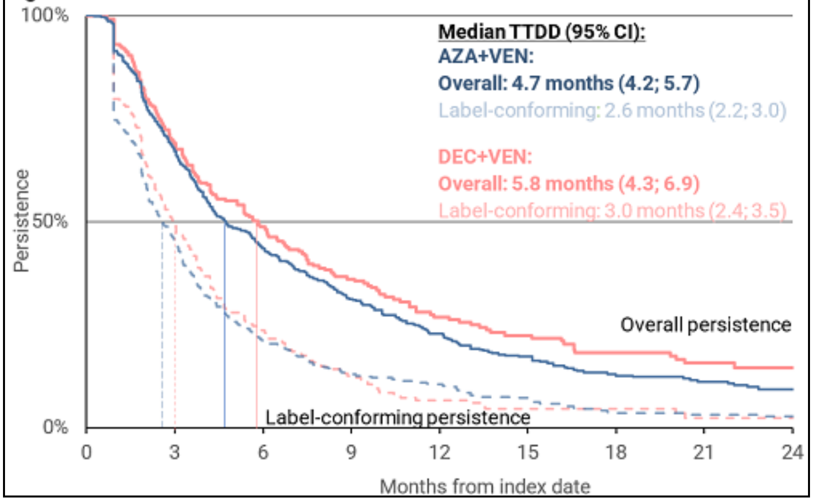

AZA: azacitidine; CI: confidence interval; DEC: decitabine; HMA: hypomethylating agent; VEN: venetoclax; TTDD: time to discontinuation or death.

**Copyright Information:** (Online) ISSN: 2572-9241
©2026 The Author(s). HemaSphere is published by John Wiley & Sons Ltd on behalf of European Hematology Association. This is an open access Abstract Book distributed under the Attribution-NonCommercial-NoDerivs (CC BY-NC-ND), which allows third parties to download the articles and share them with others as long as they credit the author and the Abstract Book, but they cannot change the content in any way or use them commercially.
**Abstract Book Citations:** Authors, Title, HemaSphere, 2026;10:(S1):pages. The abstract book DOIs can be found on the "Author Information" page.
**Disclaimer:** Articles published in the journal HemaSphere exclusively reflect the opinions of the authors. The authors are responsible for all content in their abstracts including accuracy of the facts, statements, citing resources, etc.

### (PF1410) EPCORITAMAB HOME-BASED CARE PATHWAYS IN RELAPSED/REFRACTORY DLBCL VERSUS CONVENTIONAL EPCORITAMAB AND GLOFITAMAB HOSPITAL-BASED CARE PATWHAY : A COST-MINIMIZATION AND BUDGET IMPACT ANALYSIS

**Topic:** 37. Health economics

Côme Bommier*[1], Benoit Tessoulin[2], Julie Abraham[3], Sophie Bernard[4], Stephanie Guidez[5], Lucie Oberic[6], Charaf Souihi[7], Ségolène Pettre[8], Kavita Sail[9], Jeremie Zerbit[10]

*[1]Hemato-Oncology Department (DEPHI), Hôpital St Louis, Université Paris Cité, Paris, France;[2]Hematology Department, CHU Nantes & CRCI2NA,, Nantes, France; [3]Department of Hematology, Dupuytren Hospital, Limoges University Hospital,, Limoges, France; [4]Department of hematology, Centre Hospitalier de la Côte Basque,, Bayonne, France;[5]Department of Hematology, Poitiers University Hospital,, Poitiers, France; [6]Department of hematology, Univ Toulouse, CHU Toulouse, IUCT-Oncopole,, toulouse, France; [7]HEOR, AbbVie,, Boulogne-Billancourt, France; [8]Direction médicale, AbbVie,, Boulogne-Billancourt, France; [9]HEOR, AbbVie Inc,, Chicago, France; [10]Hospitalisation à Domicile, AP-HP, Paris, Paris, France;*

### Background

In France, epcoritamab and glofitamab in monotherapy have received a favorable reimbursement opinion from the French National Authority for Health (HAS) and are currently available through an early access program for the treatment of adults with relapsed or refractory (R/R) diffuse large B-cell lymphoma (DLBCL) after at least two prior systemic therapies, who are ineligible for all available treatments or who have failed CAR-T cell therapies. These CD3xCD20 bispecific antibodies primarily differ by their mode and duration of administration: epcoritamab is administered subcutaneously until disease progression and/or unacceptable toxicity while glofitamab is administered intravenously for a fixed duration of 12 cycles. Both treatments are delivered in a Health Care Center (HCC); however, only epcoritamab is eligible for a Hospitalization at Home (HaH) administration pathway, due to its shorter administration time. In a context of capacity and budget constraints and an expected increasing use of bispecific antibodies, HCCs need to develop alternate care pathways. Moreover, allowing patients to remain in their home environment throughout their care pathway is likely to improve quality of life.

### Aims

To assess the economic impact of epcoritamab HaH care pathway versus epcoritamab and glofitamab usual HCC care pathways for the treatment of patients with R/R DLBCL.

### Methods

Four care pathways were compared: (1) epcoritamab administered exclusively in HCC (reference), (2) epcoritamab with HaH starting from cycle 2, (3) epcoritamab with HaH from cycle 1, and (4) glofitamab administered exclusively in HCC. Pathway definitions were informed in 2025 by a French clinician advisory panel (n=7) to ensure alignment with routine clinical practice in France. A cost-minimization model was developed from the perspective of the National Health Insurance, including administration, follow-up, and transportation costs over a two-year horizon. A budget impact model was developed using a multi-cohort incident approach over five years. Treatment duration was extrapolated using a Weibull model from time-to-discontinuation (TTD) data from EPCORE NHL-1, a phase I/II trial of epcoritamab monotherapy in R/R large B-cell lymphoma. To focus on care pathway costs, efficacy was assumed equivalent across pathways as between epcoritamab and glofitamab.

**Copyright Information:** (Online) ISSN: 2572-9241
©2026 The Author(s). HemaSphere is published by John Wiley & Sons Ltd on behalf of European Hematology Association. This is an open access Abstract Book distributed under the Attribution-NonCommercial-NoDerivs (CC BY-NC-ND), which allows third parties to download the articles and share them with others as long as they credit the author and the Abstract Book, but they cannot change the content in any way or use them commercially.
**Abstract Book Citations:** Authors, Title, HemaSphere, 2026;10:(S1):pages. The abstract book DOIs can be found on the "Author Information" page.
**Disclaimer:** Articles published in the journal HemaSphere exclusively reflect the opinions of the authors. The authors are responsible for all content in their abstracts including accuracy of the facts, statements, citing resources, etc.

### Results

For a patient treated with epcoritamab, the total cost of a HCC-based pathway was estimated at 12,922€, compared with 10,145€ for HaH from cycle 2 (–21.5%) and 7,858€ for HaH from cycle 1 (–39.1%). The savings for national health insurance ranged from 2,777€ to 5,064€ per patient over two years. For an HCC managing 50 patients, shifting progressively to HaH from cycle 1 could yield cumulative savings of approximately 673,000€ over five years. The total cost of a HCC-based glofitamab pathway was estimated at 7,268€, similar to the cost of epcoritamab in HaH from cycle 1 (7,858€), despite more frequent administrations with epcoritamab (Figure 1).

### Summary/Conclusion

HaH for the administration of epcoritamab in patients with R/R DLBCL reduces costs by 21–39% compared with HCC-based care, leading to total pathway costs in the same range as in-hospital glofitamab (7,858€ vs 7,268€), despite glofitamab involving fewer administrations. These findings support the organizational and economic relevance of HaH, whenever feasible, for patients with R/R DLBCL receiving epcoritamab, highlighting potential gains in patient quality of life and healthcare system efficiency

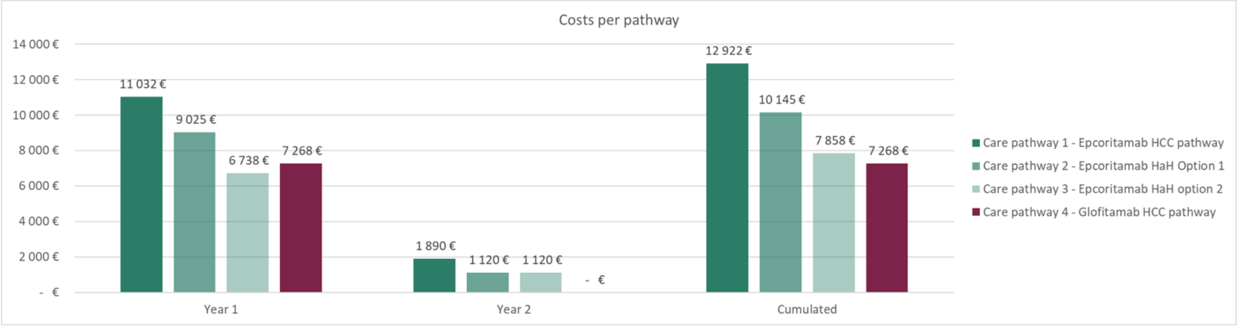

*Figure 1: Yearly costs per pathway*

**Copyright Information:** (Online) ISSN: 2572-9241
©2026 The Author(s). HemaSphere is published by John Wiley & Sons Ltd on behalf of European Hematology Association. This is an open access Abstract Book distributed under the Attribution-NonCommercial-NoDerivs (CC BY-NC-ND), which allows third parties to download the articles and share them with others as long as they credit the author and the Abstract Book, but they cannot change the content in any way or use them commercially.
**Abstract Book Citations:** Authors, Title, HemaSphere, 2026;10:(S1):pages. The abstract book DOIs can be found on the "Author Information" page.
**Disclaimer:** Articles published in the journal HemaSphere exclusively reflect the opinions of the authors. The authors are responsible for all content in their abstracts including accuracy of the facts, statements, citing resources, etc.

### (PF1411) RACIAL DISPARITIES IN ACCESS TO AUTOLOGOUS STEM CELL TRANSPLANTATION FOR MULTIPLE MYELOMA: A POPULATION-BASED REGISTRY ANALYSIS

**Topic:** 37. Health economics

Mridula Talari*[1], Ajay Iyer[1], Chelsie MacKanos[2], Cherian Verghese[1]

[1]*University of Missouri, Hematology-Medical Oncology, Columbia, United States of America;*[2]*University of Missouri, Columbia, United States of America;*

### Background

Health disparities in multiple myeloma (MM) remain a global challenge, with race, socioeconomic status, and geography affecting outcomes worldwide. African American (AA) patients have higher MM incidence compared to Caucasian Americans (CA), yet remain underrepresented in autologous stem cell transplantation (ASCT) utilization. In the United States, ASCT utilization is higher among CA patients than among AA patients, and this disparity persists even after referral to transplant centers. Importantly, prior studies have shown that when AA patients receive ASCT, they demonstrate superior outcomes, suggesting access barriers rather than biology-driven mortality disparities. We examined racial disparities in MM incidence, ASCT utilization and mortality outcomes in Missouri using population-based registry data.

### Aims

To examine racial disparities in Multiple Myeloma incidence, ASCT utilization, and mortality outcomes between African American (AA) and Caucasian American (CA) patients, using population-based registry data.

### Methods

A retrospective population-based analysis was conducted using data from the Missouri Cancer Registry and BMT InfoNet for MM patients treated between January 2020 and December 2025. We analyzed MM incidence and mortality rates (age-adjusted per 100,000 population) by race (AA, CA, Other). ASCT procedures performed at designated transplant centers in Missouri were analyzed by race during the 5-year study period. Odds ratio (OR) with 95% confidence intervals (CI) were calculated using chi-square analysis to compare ASCT utilization between AA and CA.

**Copyright Information:** (Online) ISSN: 2572-9241
©2026 The Author(s). HemaSphere is published by John Wiley & Sons Ltd on behalf of European Hematology Association. This is an open access Abstract Book distributed under the Attribution-NonCommercial-NoDerivs (CC BY-NC-ND), which allows third parties to download the articles and share them with others as long as they credit the author and the Abstract Book, but they cannot change the content in any way or use them commercially.
**Abstract Book Citations:** Authors, Title, HemaSphere, 2026;10:(S1):pages. The abstract book DOIs can be found on the "Author Information" page.
**Disclaimer:** Articles published in the journal HemaSphere exclusively reflect the opinions of the authors. The authors are responsible for all content in their abstracts including accuracy of the facts, statements, citing resources, etc.

### Results

A total of 5164 MM cases were identified statewide between January 2020 and December 2025. The mean age at diagnosis was 67 years for the entire cohort. AA patients had a higher incidence (12-20 per 100,000) than CA patients (5-8 per 100,000). Mortality was higher among AA patients (5-7 per 100,000) compared to CA patients (2-4 per 100,000). There were 1359 ASCT procedures statewide (AA: 217 vs CA: 1130 vs Other: 12). ASCT utilization was 27.4% among CA patients compared to 21.6% in AA patients in Missouri (OR 0.73, 95% CI 0.62-0.85, p<0.001). AA patients were 27% less likely to receive ASCT compared to CA patients, consistent with national patterns. Prior studies have shown that when AA patients receive ASCT, they achieve outcomes equivalent to or superior to that of CA patients, suggesting that access disparities rather than biology drive outcomes.

**Table 1. Racial Disparities in Multiple Myeloma (MM) Incidence, Mortality, and ASCT Utilization in Missouri (2020-2025)**

| **Parameter** | **CA (n=4,119)** | **AA (n=1,004)** | **P-value** |
|---|---|---|---|
| Incidence (per 100,000) | 5-8 | 12-20 | 0.001 |
| Mortality (per 100,000) | 2-4 | 5-7 | 0.001 |
| ASCT Utilization, % | 27.4 | 21.6 | 0.001 |
| Odds Ratio for ASCT (95% CI) | 1.0 (Reference) | 0.73 (0.62-0.85) | 0.001 |

**Abbreviations:** CA = Caucasian American; AA = African American; ASCT = Autologous stem cell transplantation

### Summary/Conclusion

Significant racial disparities in MM incidence, ASCT utilization, and mortality outcomes were observed in the population database from the Missouri Cancer Registry in the United States. Given lower ASCT utilization among AA patients despite documented equivalent or superior outcomes when transplanted, targeted interventions are needed to improve equitable access and mortality outcomes. These findings have national and global implications, as similar socioeconomic and ethnic disparities in transplant access and disease outcomes exist worldwide.

**Copyright Information:** (Online) ISSN: 2572-9241
©2026 The Author(s). HemaSphere is published by John Wiley & Sons Ltd on behalf of European Hematology Association. This is an open access Abstract Book distributed under the Attribution-NonCommercial-NoDerivs (CC BY-NC-ND), which allows third parties to download the articles and share them with others as long as they credit the author and the Abstract Book, but they cannot change the content in any way or use them commercially.
**Abstract Book Citations:** Authors, Title, HemaSphere, 2026;10:(S1):pages. The abstract book DOIs can be found on the "Author Information" page.
**Disclaimer:** Articles published in the journal HemaSphere exclusively reflect the opinions of the authors. The authors are responsible for all content in their abstracts including accuracy of the facts, statements, citing resources, etc.

### (PF1412) SOCIOECONOMIC AND EMPLOYMENT IMPACT OF ONCOHEMATOLOGICAL DISEASES IN SPAIN: 2026 PATIENT-REPORTED OUTCOMES FROM AELCLÉS

**Topic:** 37. Health economics

Soraya Garcia Egido[1], María Anciones Polo[2], Isaac de Íscar López[2], Ascensión Hernández Encinas[1, 2], María del Carmen López Avilés[1], Delia Blanco Lorenzo[1], Elena Palma[1], Ana Belén Ramos García[1], Celia Marín Rancel*[1]

*[1]AELCLÉS, Agrupación Española de entidades de Lucha Contra la Leucemia y Enfermedades de la Sangre, Salamanca, Spain;*
*[2]University of Salamanca, Salamanca, Spain;*

### Background

Advances in treatment have improved survival in oncohematological diseases; however, longer survival has shifted part of the burden toward employment, economic, and social consequences. Patients often experience prolonged work disruption, income reduction, and additional out-of-pocket expenses. The economic impact varies by diagnosis and is greater in conditions requiring long-term care, hospitalization, or associated with persistent treatment-related sequelae.

### Aims

To generate patient-reported evidence on the employment, economic, and social impact of oncohematological diseases in Spain, including consequences for informal caregivers.

### Methods

A multiregional online questionnaire targeted adults diagnosed with hematological diseases and their caregivers, collecting 204 complete questionnaires between 2024 and 2025. The measurement instrument assessed employment before and after diagnosis, work disability applications, perceived labor impact, monthly out-of-pocket costs, and caregiver employment effects. Data analysis comprised four phases: data cleaning and organization; variable recoding and categorization of open responses; descriptive and inferential analyses; identification of patterns using multivariate techniques (External Logistic Biplot, HJ Biplot, CHAID); and translation of findings into actionable evidence.

**Copyright Information:** (Online) ISSN: 2572-9241
©2026 The Author(s). HemaSphere is published by John Wiley & Sons Ltd on behalf of European Hematology Association. This is an open access Abstract Book distributed under the Attribution-NonCommercial-NoDerivs (CC BY-NC-ND), which allows third parties to download the articles and share them with others as long as they credit the author and the Abstract Book, but they cannot change the content in any way or use them commercially.
**Abstract Book Citations:** Authors, Title, HemaSphere, 2026;10:(S1):pages. The abstract book DOIs can be found on the “Author Information” page.
**Disclaimer:** Articles published in the journal HemaSphere exclusively reflect the opinions of the authors. The authors are responsible for all content in their abstracts including accuracy of the facts, statements, citing resources, etc.

### Results

**Economic impact:** Most participants reported financial deterioration, affecting 82.6% of chronic leukemias, 78.9% of acute lymphoblastic leukemia, 75.0% of Hodgkin lymphoma, and 70% of non-Hodgkin lymphoma. In acute myeloid leukemia, 34.8% reported monthly out-of-pocket expenses exceeding €300, and 26.3% of acute lymphoblastic leukemia did as well. In chronic leukemias and Hodgkin lymphoma, expenses most frequently ranged from €101 to €200. Acute conditions were therefore associated with a higher direct financial burden.

**Employment impact:** Over 65% reported a negative effect on employment, particularly in Hodgkin lymphoma (78.1%), chronic leukemias (73.9%), non-Hodgkin lymphoma (73.3%), and acute myeloid leukemia (69.6%). Disability applications were higher in multiple myeloma (45.5%), Hodgkin lymphoma (43.8%), non-Hodgkin lymphoma (43.3%), and acute myeloid leukemia (37.0%), but lower in non-malignant disorders (14.3%). Current work disability was substantial: acute lymphoblastic leukemia (42.1%), non-Hodgkin lymphoma (40%), multiple myeloma (36.4%), acute myeloid leukemia (34.8%), and Hodgkin lymphoma (31.3%). Multiple myeloma also showed 39.4% retired or early-retired and 12.1% with difficulties returning to work.

**Social and caregiver impact:** Work discontinuation reflects disease-, treatment-, care-, and organization-related barriers. A significant proportion of caregivers abandoned or modified employment for full-time care, including reduced hours, leave, or temporary disability, particularly in acute myeloid leukemia (43.5%), acute lymphoblastic leukemia (36.8%), and multiple myeloma (30.3%).

### Summary/Conclusion

Oncohematological diseases generate a sustained socioeconomic burden on patients and caregivers. Findings support integrated policies on employment protection, structured reintegration, caregiver support, and systematic assessment of socioeconomic impact within hematology care. Further research stratified by population sectors and age groups is needed to clarify differential impacts, long-term employment trajectories, and unmet support needs.

**Copyright Information:** (Online) ISSN: 2572-9241
©2026 The Author(s). HemaSphere is published by John Wiley & Sons Ltd on behalf of European Hematology Association. This is an open access Abstract Book distributed under the Attribution-NonCommercial-NoDerivs (CC BY-NC-ND), which allows third parties to download the articles and share them with others as long as they credit the author and the Abstract Book, but they cannot change the content in any way or use them commercially.
**Abstract Book Citations:** Authors, Title, HemaSphere, 2026;10:(S1):pages. The abstract book DOIs can be found on the “Author Information” page.
**Disclaimer:** Articles published in the journal HemaSphere exclusively reflect the opinions of the authors. The authors are responsible for all content in their abstracts including accuracy of the facts, statements, citing resources, etc.

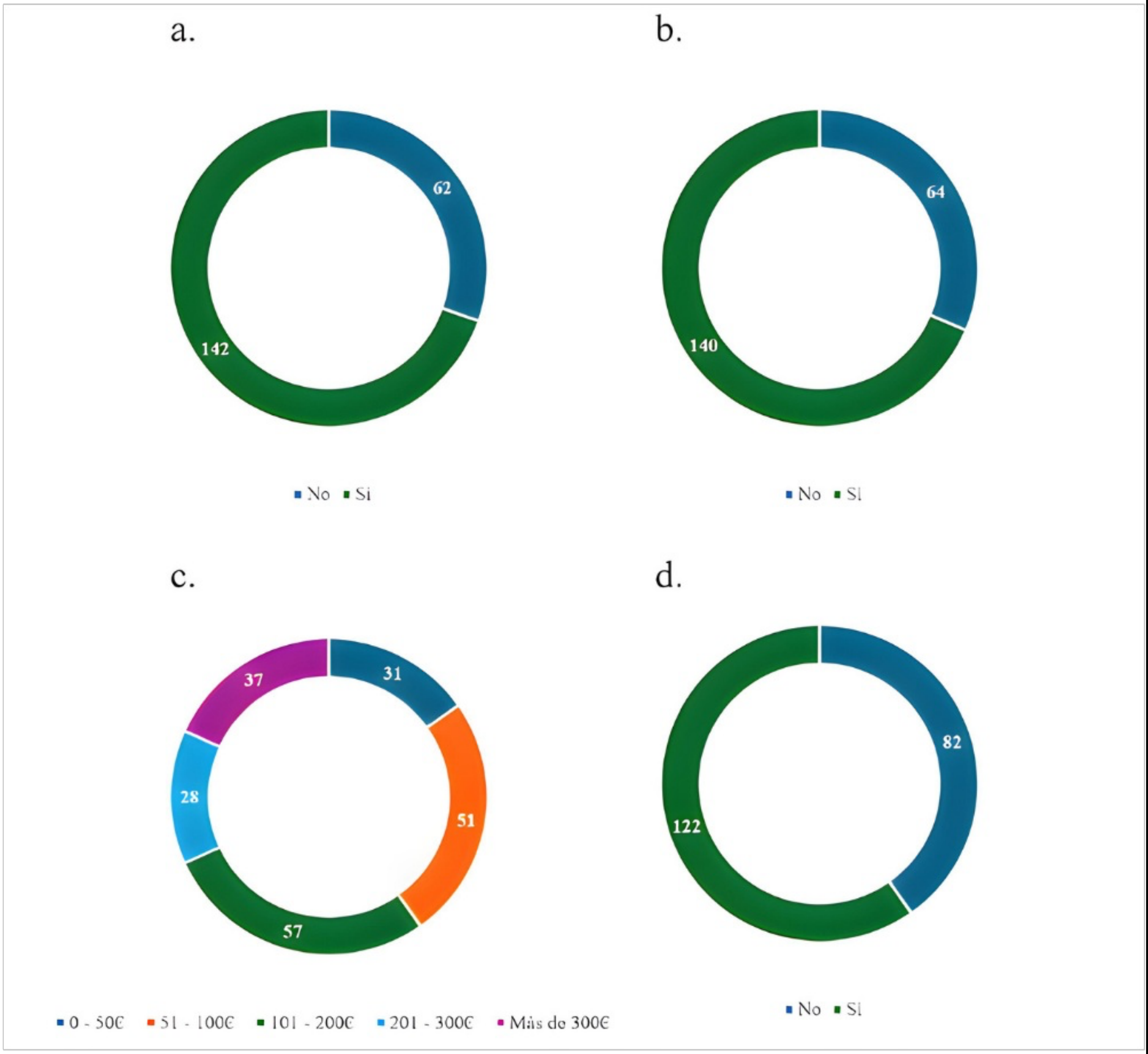

***Figure 1. Absolute distribution of the impact of diagnosis on work, economic, and social aspects: (a) employment status, (b) economic situation, (c) additional monthly expenses, and (d) disability application.***

**Copyright Information:** (Online) ISSN: 2572-9241
©2026 The Author(s). HemaSphere is published by John Wiley & Sons Ltd on behalf of European Hematology Association. This is an open access Abstract Book distributed under the Attribution-NonCommercial-NoDerivs (CC BY-NC-ND), which allows third parties to download the articles and share them with others as long as they credit the author and the Abstract Book, but they cannot change the content in any way or use them commercially.
**Abstract Book Citations:** Authors, Title, HemaSphere, 2026;10:(S1):pages. The abstract book DOIs can be found on the "Author Information" page.
**Disclaimer:** Articles published in the journal HemaSphere exclusively reflect the opinions of the authors. The authors are responsible for all content in their abstracts including accuracy of the facts, statements, citing resources, etc.

### (PF1413) ECONOMIC IMPACT OF ISATUXIMAB AS FIRST-LINE TREATMENT FOR NEWLY DIAGNOSED MULTIPLE MYELOMA: A BUDGET IMPACT ANALYSIS

**Topic:** 37. Health economics

Isabell Kaufhold*[1], Katja Hesse[1], Florian Kron[1, 2, 3]

*[1]VITIS Healthcare Group, Health Economics and Outcomes Research, Cologne, Germany;[2]University Hospital Cologne, Department I of Internal Medicine, Center for Integrated Oncology Cologne, Faculty of Medicine, Cologne, Germany; [3]FOM University of Applied Sciences, Essen, Germany;*

### Background

Multiple myeloma is a heterogeneous hematologic malignancy with approximately 7,300 incident cases annually in Germany. Since the introduction of autologous stem cell transplantation, the treatment landscape has evolved rapidly, with multiple novel regimens improving long-term survival and response rates. Isatuximab, a novel monoclonal anti-CD38 antibody, in combination with bortezomib, lenalidomide, and dexamethasone, demonstrated a significant improvement in minimal residual disease and achievement of complete response and is approved for first-line treatment for transplant-eligible patients as induction therapy before autologous stem cell transplantation and transplant-ineligible patients. However, novel regimens may be associated with high drug acquisition costs, potentially increasing financial pressure on the German statutory health insurance.

### Aims

This study aims to assess the budgetary impact of introducing IsaVRd for first-line treatment of multiple myeloma from the perspective of the German statutory health insurance.

### Methods

A budget-impact model with annual cycles, a time horizon of five years and no discounting was developed following German and international health economic modelling guidelines. The treatment mix was determined in accordance with the current multiple myeloma guidelines of the European Hematology Association (EHA) and the European Myeloma Network (EMN). For transplant-eligible patients, isatuximab or daratumumab combined with bortezomib, lenalidomide and dexamethasone (IsaVRd or DaraVRd) were considered. For transplant-ineligible patients, IsaVRd, DaraVRd or daratumumab combined with lenalidomide and dexamethasone (DaraRd) were included. Transplant-eligible patients are assumed to proceed to autologous stem cell transplantation after induction, thus limiting treatment duration to induction cycles, whereas median treatment duration for transplant-ineligible patients was derived from pivotal approval trials. Population estimates were obtained from German cancer registry data and prospectively modelled. Market shares were assumed for each treatment regimen and prospectively varied in three scenarios (low, moderate and high uptake of IsaVRd). Costs included drug acquisition, healthcare resource use and adverse events management based on German drug price databases and outpatient and inpatient reimbursement tariffs. Deterministic sensitivity analyses were conducted to test robustness of results.

### Results

Over a five-year time horizon, the introduction of IsaVRd resulted in cumulative savings ranging from approximately € 218 million (low uptake scenario) to € 389 million (high uptake scenario), primarily driven by substitution of alternative first-line regimens and reduced drug acquisition costs of IsaVRd.

**Copyright Information:** (Online) ISSN: 2572-9241
©2026 The Author(s). HemaSphere is published by John Wiley & Sons Ltd on behalf of European Hematology Association. This is an open access Abstract Book distributed under the Attribution-NonCommercial-NoDerivs (CC BY-NC-ND), which allows third parties to download the articles and share them with others as long as they credit the author and the Abstract Book, but they cannot change the content in any way or use them commercially.
**Abstract Book Citations:** Authors, Title, HemaSphere, 2026;10:(S1):pages. The abstract book DOIs can be found on the "Author Information" page.
**Disclaimer:** Articles published in the journal HemaSphere exclusively reflect the opinions of the authors. The authors are responsible for all content in their abstracts including accuracy of the facts, statements, citing resources, etc.

**Summary/Conclusion**

From the perspective of the German statutory health insurance, the introduction of isatuximab-based first-line treatment for newly diagnosed multiple myeloma may generate substantial five-year budget savings for the German statutory health insurance, highlighting the affordability of adopting this regimen in routine care.

**Copyright Information:** (Online) ISSN: 2572-9241
©2026 The Author(s). HemaSphere is published by John Wiley & Sons Ltd on behalf of European Hematology Association. This is an open access Abstract Book distributed under the Attribution-NonCommercial-NoDerivs (CC BY-NC-ND), which allows third parties to download the articles and share them with others as long as they credit the author and the Abstract Book, but they cannot change the content in any way or use them commercially.
**Abstract Book Citations:** Authors, Title, HemaSphere, 2026;10:(S1):pages. The abstract book DOIs can be found on the "Author Information" page.
**Disclaimer:** Articles published in the journal HemaSphere exclusively reflect the opinions of the authors. The authors are responsible for all content in their abstracts including accuracy of the facts, statements, citing resources, etc.

### (PF1414) COST-EFFECTIVENESS OF AXICABTAGENE CILOLEUCEL IN TAIWANESE PATIENTS WITH RELAPSED/REFRACTORY DIFFUSE LARGE B-CELL LYMPHOMA AND PRIMARY MEDIASTINAL B-CELL LYMPHOMA AFTER TWO LINES OF SYSTEMIC THERAPY

**Topic:** 37. Health economics

Kuan-Der Lee*[1, 2, 3], Yu-Chiao Wang[4], Wen-Wen Yang[5], Tza Kai Diong[6], Bor-Sheng Ko[7, 8, 9, 10], Raoh-Fang Pwu[11]

*[1]Cell Therapy and Regenerative Medicine Center and Department of Oncology, Taichung Veterans General Hospital, Taichung, Taiwan; [2]Taipei Medical University, International Ph.D. Program for Cell Therapy and Regeneration Medicine, College of Medicine, School of Medicine, Taipei, Taiwan; [3]National Chung Hsing University, Department of Post-Baccalaureate Medicine, College of Medicine, Taichung, Taiwan; [4]Yuanpei University of Medical Technology, Department of Healthcare Management, Hsinchu, Taiwan; [5]Fu Jen Catholic University, Data Science Center, New Taipei, Taiwan; [6]Gilead Sciences Singapore, Singapore, Singapore; [7]National Taiwan University Cancer Center, Department of Hematological Oncology, Taipei, Taiwan; [8]National Taiwan University, Tai-Chen Cell Therapy Center, Taipei, Taiwan; [9]National Taiwan University, Department of Internal Medicine, College of Medicine, Taipei, Taiwan; [10]National Taiwan University Hospital, Department of Internal Medicine, Taipei, Taiwan; [11]Taipei Medical University, School of Health Care Administration, Taipei, Taiwan;*

### Background

Diffuse large B-cell lymphoma (DLBCL) is the most common aggressive B-cell lymphoma in Taiwan and continues to contribute substantially to the national lymphoma disease burden. Although a proportion of patients can achieve long-term remission with frontline therapy, a clinically important subgroup progresses through multiple lines of treatment. In the third-line and beyond (3L+) setting, effective therapeutic options remain limited, resulting in poor long-term survival. The management of 3L+ DLBCL therefore represents a significant clinical and policy challenge, particularly in the context of emerging one-time cellular therapies and the need to prioritize healthcare resources.

In Taiwan, chimeric antigen receptor T-cell (CAR T-cell) therapies have been introduced into clinical practice, yet access differs by product. Currently, the National Health Insurance (NHI) reimburses tisagenlecleucel (tisa-cel) for 3L+ treatment, while axicabtagene ciloleucel (axi-cel) remains unreimbursed, underscoring the need for robust local economic evidence to inform reimbursement decisions.

### Aims

This study aims to assess the cost-effectiveness of axi-cel compared to tisa-cel as a 3L+ treatment for DLBCL and primary mediastinal B-cell lymphoma (PMBCL) in Taiwan from the perspective of National Health Insurance.

### Methods

To account for potential long-term survival and cure effects associated with CAR T-cell therapy, a partitioned survival mixture cure model (PS-MCM) adapted with Taiwan-specific parameters was used to evaluate the clinical and economic outcomes of axi-cel and tisa-cel. The model included three health states: pre-progression survival, post-progression survival, and death. Clinical efficacy inputs were derived from trial data adjusted using a matching-adjusted indirect comparison (MAIC) to enable comparative analysis in the absence of head-to-head trials. Costs and resource use were informed by the Taiwan NHI database, local clinical expert opinion, and published literature. Background mortality was based on the 2023 Taiwan life tables. A lifetime horizon (40 years), monthly cycles, and a 3% annual discount rate were applied. Measured outcomes included total costs, quality-adjusted life years (QALYs), and incremental cost-effectiveness ratios (ICERs).

**Copyright Information:** (Online) ISSN: 2572-9241
©2026 The Author(s). HemaSphere is published by John Wiley & Sons Ltd on behalf of European Hematology Association. This is an open access Abstract Book distributed under the Attribution-NonCommercial-NoDerivs (CC BY-NC-ND), which allows third parties to download the articles and share them with others as long as they credit the author and the Abstract Book, but they cannot change the content in any way or use them commercially.
**Abstract Book Citations:** Authors, Title, HemaSphere, 2026;10:(S1):pages. The abstract book DOIs can be found on the “Author Information” page.
**Disclaimer:** Articles published in the journal HemaSphere exclusively reflect the opinions of the authors. The authors are responsible for all content in their abstracts including accuracy of the facts, statements, citing resources, etc.

### Results

In the base-case analysis, axi-cel demonstrated superior clinical outcomes at lower total discounted costs compared with tisa-cel, indicating that axi-cel is a dominant option. Undiscounted life-years were higher with axi-cel than with tisa-cel (11.63 vs. 7.86 years), corresponding to an incremental gain of 3.77 years, while quality-adjusted life-years (QALYs) were also increased (6.84 vs. 4.33 QALYs; +2.51 QALYs). Total discounted costs were marginally lower for axi-cel (NTD 9.24M [USD 307,991]) compared with tisa-cel (NTD 9.26M [USD 308,557]), based on an exchange rate of 1 USD to 30 NTD. This slight cost reduction was primarily driven by lower expenditures related to apheresis and hospitalization for axi-cel, assuming identical drug acquisition costs for both CAR T-cell therapies. Sensitivity analyses confirmed that axi-cel remained cost-effective across key model assumptions.

### Summary/Conclusion

This analysis shows that axi-cel delivers greater health benefits at lower overall costs than tisa-cel in patients with 3L+ R/R DLBCL and PMBCL, representing a dominant treatment option. These findings further support axi-cel's value for reimbursement consideration in Taiwan.

**Table: Base-case cost-effectiveness results**

| Results | Axi-cel | Tisa-cel | Difference |
|---|---|---|---|
| **Undiscounted** | | | |
| Lifetime costs | NTD 9,453,702 [USD 315,123] | NTD 9,423,813 [USD 314,127] | NTD 29,889 [USD 996] |
| QALYs | 9.30 | 5.86 | 3.45 |
| ICER (Axi-cel vs. Tisa-cel) | **NTD 8,672 [USD 289] per QALY gained** | | |
| **Discounted** | | | |
| Lifetime costs | NTD 9,239,742 [USD 307,991] | NTD 9,256,721 [USD 308,557] | NTD -16,978 [USD -566] |
| QALYs | 6.84 | 4.33 | 2.51 |
| ICER (Axi-cel vs. Tisa-cel) | | | **Dominant** |

Abbreviations: Axi-cel, axicabtagene ciloleucel; ICER, incremental cost-effectiveness ratio; NTD, New Taiwan dollar; QALY, quality-adjusted life-year; SoC, standard of care; USD, United States dollar.
Note: Costs were converted from New Taiwan dollars (NTD) to US dollars (USD) at an exchange rate of 1 USD = 30 NTD (approximate annual average based on data from the Central Bank of the Republic of China [Taiwan]) and rounded to the nearest whole number.

Base-case cost-effectiveness results

Copyright Information: (Online) ISSN: 2572-9241
©2026 The Author(s). HemaSphere is published by John Wiley & Sons Ltd on behalf of European Hematology Association. This is an open access Abstract Book distributed under the Attribution-NonCommercial-NoDerivs (CC BY-NC-ND), which allows third parties to download the articles and share them with others as long as they credit the author and the Abstract Book, but they cannot change the content in any way or use them commercially.
Abstract Book Citations: Authors, Title, HemaSphere, 2026;10:(S1):pages. The abstract book DOIs can be found on the "Author Information" page.
Disclaimer: Articles published in the journal HemaSphere exclusively reflect the opinions of the authors. The authors are responsible for all content in their abstracts including accuracy of the facts, statements, citing resources, etc.

### (PF1415) CAR T CELL THERAPY IN CLINICAL ROUTINE: QUANTIFICATION OF REAL-WORLD HOSPITAL RESOURCE USE ACROSS CAR T CARE PATHWAY IN GERMANY

**Topic:** 37. Health economics

Katja Hesse*[1], Isabell Kaufhold[1], Markus Finzsch[2], Florian Kron[1, 3, 4]

*[1]VITIS Healthcare Group, Health Economics & Outcomes Research, Cologne, Germany;[2]Gilead Sciences GmbH, Market Access, Martinsried, Germany; [3]University Hospital Cologne, Department I of Internal Medicine, Center for Integrated Oncology Cologne, Faculty of Medicine, Cologne, Germany; [4]FOM University of Applied Sciences, Essen, Germany;*

### Background

Chimeric antigen receptor T cell (CAR T) therapies have substantially expanded treatment options for hematologic malignancies. CAR T provision represents a resource-intensive care pathway, complex coordination requirements, individualized product handling, and the involvement of various professional groups within the hospital. With increasing CAR T adoption and anticipated indication expansions, European hospitals face challenges in organizing care and ensuring adequate capacity. Economic evaluations of CAR T predominantly focus on drug acquisition, while evidence on process-based hospital resource use remains limited.

### Aims

This study aims to systematically quantify hospital resource use associated with CAR T therapy and to elucidate the principal process-level and stakeholder-specific determinants of resource consumption. Given evidence-based care organization and capacity planning in the context of anticipated indication expansions, the analysis also aims to inform discussions of hospital reimbursement beyond drug acquisition costs.

### Methods

A process-based analysis of hospital resource use was conducted at a German university hospital, performing more than 50 treatments annually across different approved CAR T products and indications. CAR T specific activities were identified along predefined steps of the CAR T treatment pathway derived from literature, refined through standard operating procedures, complemented by expert input, and classified as case-based or overarching activities. Personnel time was assessed through structured evaluations and interviews with representatives of CAR T coordination, medical staff, laboratory, pharmacy, and medical controlling.

**Copyright Information:** (Online) ISSN: 2572-9241
©2026 The Author(s). HemaSphere is published by John Wiley & Sons Ltd on behalf of European Hematology Association. This is an open access Abstract Book distributed under the Attribution-NonCommercial-NoDerivs (CC BY-NC-ND), which allows third parties to download the articles and share them with others as long as they credit the author and the Abstract Book, but they cannot change the content in any way or use them commercially.
**Abstract Book Citations:** Authors, Title, HemaSphere, 2026;10:(S1):pages. The abstract book DOIs can be found on the “Author Information” page.
**Disclaimer:** Articles published in the journal HemaSphere exclusively reflect the opinions of the authors. The authors are responsible for all content in their abstracts including accuracy of the facts, statements, citing resources, etc.

### Results

In total, 82 case-based activities were identified. Cumulative personnel time amounted to 1,647 minutes (27.5 hours) per CAR T case. Table 1 presents the major contributors to case-based personnel time across process steps and professions.

| Category | Share of Total Personnel Time |
|---|---|
| **Process steps** | |
| Shipping to clinic | 27% |
| Administration of CAR T cells | 13% |
| Clarification of cost coverage | 12% |
| **Professions** | |
| Medical staff | 33% |
| CAR T coordinator | 25% |
| Laboratory staff | 20% |

*Table 1 TOP 3 Contributors to Case-Based Personnel Time*

Resource use was unevenly distributed along the treatment pathway. Organizational and documentation-related activities associated with shipping to clinic emerged as a major driver, including coordination of product delivery and receipt with the laboratory and pharmacy and related logistical processes. Certification and staff training were identified as resource-intensive overarching activities not directly attributable to individual CAR T cases.

### Summary/Conclusion

This study provides a first detailed characterization of hospital resource use associated with CAR T therapy beyond drug acquisition costs, based on analysis of a single-center high-volume German university hospital. While resource use may vary across centers, indications and local treatment pathways the analysis suggests that case-based resource consumption is driven by organizational, coordination, and documentation-related activities. By identifying key process- and stakeholder-specific resource drivers, the findings support process optimization and capacity management as CAR T services expand to higher treatment volumes and additional indications. Moreover, process-based quantification of real-world resource use enhances transparency regarding hospital services required for CAR T delivery and provides evidence to inform hospital reimbursement discussions.

**Copyright Information:** (Online) ISSN: 2572-9241
©2026 The Author(s). HemaSphere is published by John Wiley & Sons Ltd on behalf of European Hematology Association. This is an open access Abstract Book distributed under the Attribution-NonCommercial-NoDerivs (CC BY-NC-ND), which allows third parties to download the articles and share them with others as long as they credit the author and the Abstract Book, but they cannot change the content in any way or use them commercially.
**Abstract Book Citations:** Authors, Title, HemaSphere, 2026;10:(S1):pages. The abstract book DOIs can be found on the "Author Information" page.
**Disclaimer:** Articles published in the journal HemaSphere exclusively reflect the opinions of the authors. The authors are responsible for all content in their abstracts including accuracy of the facts, statements, citing resources, etc.

### (PF1416) COMPREHENSIVE CHARACTERIZATION OF HEALTHCARE RESOURCE UTILIZATION AFTER OUTPATIENT ADMINISTRATION OF LISOCABTAGENE MARALEUCEL IN THE REAL-WORLD SETTING (THE HELIOS STUDY)

**Topic:** 37. Health economics

Jennifer Huang*[1, 2], Jenna Voutsinas[1], Ryan Basom[1], Ismat Jahan[1], Kelly Crebs[1], Samuel Albert[1], Cindy Sidwa[1], Alex Susser[1], Andrew Portuguese[1, 2], Emily Liang[1, 2], Alexandre Hirayama[1, 2], Zachary Yaffe[1, 2], Christy Khouderchah[1], Lorenzo Iovino[1, 2], Houston E Warren[1, 2], Fei Fei Liu[3], Christine Fu[3], Jinender Kumar[3], Mazyar Shadman[1, 2], Vicky Wu[1], Jordan Gauthier[1, 2]

*[1]Fred Hutchinson Cancer Center, Seattle, United States of America;[2]University of Washington, Seattle, United States of America;* *[3]Bristol Myers Squibb, Lawrence Township, United States of America;*

### Background

Lisocabtagene maraleucel (liso-cel) is FDA- and EMA-approved for multiple B-cell malignancies. While its favorable safety profile potentially enables patient management in the outpatient setting, the safety and healthcare resource utilization (HCRU) associated with outpatient treatment with liso-cel in the real-world setting has not been well characterized across the spectrum of B-cell malignancies.

### Aims

To characterize HCRU after SOC liso-cel treatment in the outpatient setting and identify factors predictive of HCRU.

### Methods

We retrospectively analyzed data from 177 consecutive patients (pts) with B-cell malignancies with planned infusion of FDA-approved liso-cel or non-conforming product at Fred Hutchinson Cancer Center (FHCC) from April 2021 to September 2025. We assessed hospitalization frequency, and transfusion and granulocyte colony-stimulating factor (G-CSF) support from day 0 through day +30 after liso-cel infusion and applied univariate regression models to identify factors predictive of HCRU. Per FHCC standard practice, any grade CRS prompted inpatient admission.

**Copyright Information:** (Online) ISSN: 2572-9241
©2026 The Author(s). HemaSphere is published by John Wiley & Sons Ltd on behalf of European Hematology Association. This is an open access Abstract Book distributed under the Attribution-NonCommercial-NoDerivs (CC BY-NC-ND), which allows third parties to download the articles and share them with others as long as they credit the author and the Abstract Book, but they cannot change the content in any way or use them commercially.
**Abstract Book Citations:** Authors, Title, HemaSphere, 2026;10:(S1):pages. The abstract book DOIs can be found on the "Author Information" page.
**Disclaimer:** Articles published in the journal HemaSphere exclusively reflect the opinions of the authors. The authors are responsible for all content in their abstracts including accuracy of the facts, statements, citing resources, etc.

**Results**

Of 177 pts who underwent leukapheresis, 99% completed lymphodepletion (n=175), all of whom received liso-cel. Seventy-three percent of pts (n=127) underwent outpatient liso-cel administration while 27% received liso-cel inpatient (planned, 9.7% [n=17]; unplanned, 17.7% [n=31]). Characteristics of the outpatient liso-cel administration cohort are detailed in Fig 1A. In this 127-pt cohort, we observed that 38% (n=48) of pts remained in the outpatient setting from day 0 to day +30. Of pts admitted between days 0 to +14, the median time to hospitalization was 4.0 days (IQR, 2-8; one admission, 52%; two admissions, 9%) with a median length of stay (LOS) of 6 days (Interquartile range [IQR]=4-8). There were 6 intensive care unit (ICU) admissions (median ICU LOS, 2 days; IQR, 2-4). During the day +15 to +30 period, 6% of pts were admitted (n=8; single admission in all pts; median LOS, 3 days; IQR, 2-4), including one ICU admission.
We next analyzed key supportive care measures contributing to HCRU in the outpatient liso-cel infusion cohort, stratifying by the early vs. late treatment period after liso-cel administration. In the day 0 to +14 period, 33% of pts (n=42) required packed red blood cell (pRBC) transfusions (median, 1 unit [U]; IQR, 1-2U); 5% (n=6) received platelet transfusions (median, 4.5U; IQR, 3-6U), and 76% (n=96) of patients were given G-CSF. We observed decreased HCRU during the day +15 to +30 period; 16% (n=20) of pts required pRBC transfusions (median 1.5U; IQR, 1-2.5U); 13% of pts (n=16) received platelet transfusions; and 26% (n=33) of pts were given G-CSF (Fig 2B).
Unadjusted univariate analyses of factors predictive of inpatient admission after outpatient liso-cel administration showed an association between lower pre-leukapheresis hemoglobin in those who were hospitalized (OR, 0.81; 95% CI 0.65-0.98; p=0.036). We could not confirm associations between age, disease type, pre-leukapheresis LDH, CRP, ferritin, absolute neutrophil count, absolute lymphocyte count, and platelet count.

**Summary/Conclusion**

Liso-cel was administered in the outpatient setting in 73% of liso-cel recipients at FHCC; among these, 38% did not require subsequent hospitalization. Admissions were typically limited to a single and short hospitalization within the first two weeks. HCRU was minimal after day +15. Results indicate that liso-cel can be administered safely in an outpatient setting, with limited HCRU.

**Copyright Information:** (Online) ISSN: 2572-9241
©2026 The Author(s). HemaSphere is published by John Wiley & Sons Ltd on behalf of European Hematology Association. This is an open access Abstract Book distributed under the Attribution-NonCommercial-NoDerivs (CC BY-NC-ND), which allows third parties to download the articles and share them with others as long as they credit the author and the Abstract Book, but they cannot change the content in any way or use them commercially.
**Abstract Book Citations:** Authors, Title, HemaSphere, 2026;10:(S1):pages. The abstract book DOIs can be found on the "Author Information" page.
**Disclaimer:** Articles published in the journal HemaSphere exclusively reflect the opinions of the authors. The authors are responsible for all content in their abstracts including accuracy of the facts, statements, citing resources, etc.

**A**

| Characteristic | Outpatient N = 127 |
|---|---|
| **Age at time of CAR T-cell infusion**[1] | 69 (37, 83) |
| **Gender** | |
| Male | 79 (62%) |
| Female | 48 (38%) |
| **Disease type** | |
| Large B Cell Lymphoma | 103 (81%) |
| Mantle Cell Lymphoma | 9 (7.1%) |
| Follicular Lymphoma | 12 (9.4%) |
| Chronic Lymphocytic Leukemia | 2 (1.6%) |
| Plasmablastic lymphoma | 1 (0.8%) |
| **Race** | |
| White | 103 (81%) |
| Asian | 13 (10%) |
| Unknown | 4 (3.1%) |
| Black or African American | 2 (1.6%) |
| Multiple | 2 (1.6%) |
| American Indian or Alaska Native | 2 (1.6%) |
| Native Hawaiian or Other Pacific Islander | 1 (0.8%) |
| **Ethnicity** | |
| Not Hispanic or Latino | 117 (92%) |
| Hispanic or Latino | 8 (6.3%) |
| Unknown | 2 (1.6%) |
| **ECOG performance score-** | |
| 0 | 69 (58%) |
| 1 | 42 (35%) |
| 2 | 5 (4.2%) |
| 3 | 3 (2.5%) |
| 4 | 0 (0%) |
| Missing | 8 |

[1]Median (Min, Max); n (%)

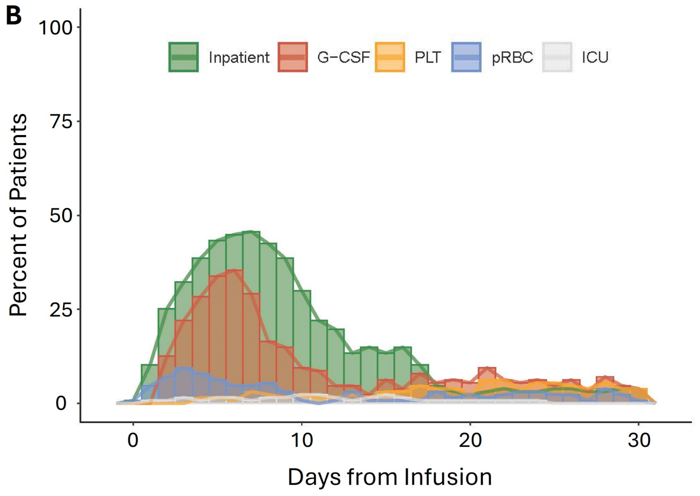

**A)** Patient characteristics of those who received their liso-cel infusion inpatient versus outpatient. **B)** Healthcare utilization by day for patients who received outpatient administration of liso-cel. Utilization of inpatient hospitalization, ICU stay, transfusion support, and G-CSF support.

G-CSF: granulocyte colony stimulating factor. ICU: intensive care unit. Plt: platelets. pRBCs: packed red blood cells.

A) Patient characteristics of those who received their liso-cel infusion inpatient versus outpatient. B) Healthcare utilization by day for patients who received outpatient administration of liso-cel. Utilization of inpatient hospitalization, ICU stay, transfusion support, and G-CSF support. G-CSF: granulocyte colony stimulating factor. ICU: intensive care unit. Plt: platelets. pRBCs: packed red blood cells.

**Copyright Information:** (Online) ISSN: 2572-9241
©2026 The Author(s). HemaSphere is published by John Wiley & Sons Ltd on behalf of European Hematology Association. This is an open access Abstract Book distributed under the Attribution-NonCommercial-NoDerivs (CC BY-NC-ND), which allows third parties to download the articles and share them with others as long as they credit the author and the Abstract Book, but they cannot change the content in any way or use them commercially.
**Abstract Book Citations:** Authors, Title, HemaSphere, 2026;10:(S1):pages. The abstract book DOIs can be found on the "Author Information" page.
**Disclaimer:** Articles published in the journal HemaSphere exclusively reflect the opinions of the authors. The authors are responsible for all content in their abstracts including accuracy of the facts, statements, citing resources, etc.

### (PF1417) ECONOMIC BURDEN OF CHRONIC GRAFT-VERSUS-HOST DISEASE AFTER ALLOGENEIC STEM CELL TRANSPLANT IN SPAIN

**Topic:** 37. Health economics

Jordi Aparici Espot[1], Julia Fernandez Morata*[2], Marcos Valdés-Álvarez[3], Carlos Crespo[4, 5], Mónica Cerezales[3]

*[1]Sanofi Aventis SA, Barcelona, Spain;[2]Sanofi Aventis SA, Madrid, Spain;[3]Axentiva Solutions S.L, Oviedo, Spain;[4]Axentiva Solutions S.L, Barcelona, Spain; [5]University of Barcelona, Department G.M. Statistics, Barcelona, Spain;*

### Background

Allogeneic Hematopoietic Stem Cell Transplantation (allo-HSCT) involves transplanting hematopoietic stem cells from a donor to restore the patient's hematopoietic function. However, allo-HSCT carries significant risks and complications such as Graft-versus-Host Disease (GVHD). GVHD is a multisystem disease caused by the recipient's cells recognizing the donor tissues as foreign, triggering an inflammatory and fibrotic immune response. GVHD is classified as acute and chronic (cGVHD) based on its pathophysiology and clinical presentation. Its diagnosis, management, and treatment are complex, which might significantly increase the costs associated with transplantation.

### Aims

The objective was to estimate the economic burden of allo-HSCT, including the management of cGVHD in 2024 from the National Healthcare System (NHS) perspective in Spain.

### Methods

An economic burden model from an incidence approach was built based on the patient journey designed from a pragmatic literature review, from allo-HSCT procedure to cGVHD's third line (3L) of treatment. Clinical inputs (e.g. incidence of complications, distribution of treatments for cGVHD) were sourced from the literature. A bottom-up approach was followed, including costs for diagnosis-related groups for in-hospital care and micro-costing based on tariffs and drug costs after discharge. Number of patients subjected to allo-HSCT in Spain in 2024 was obtained from the transplant registry *(ONT)*. The overall burden of HSCT procedure and cGVHD, as well as the average cost per patient, in general and specifically for those achieving each treatment line, were estimated. Costs were inflated to 2025 euros.

### Results

The patient journey starts with the conditioning phase before allo-HSCT and extends up to two years, including follow-up after discharge, incidence of complications post allo-HSCT (including cGVHD -diagnosis, follow-up, and treatment) (Figure 1).
According to ONT in 2024, 1,598 patients were subjected to allo-HSCT, of which 591 (37%) were estimated to have developed cGVHD (431 moderate-severe cGVHD). For this cohort, total costs were €57,777,597; more than 50% attributed to conditioning and transplant (€30,853,366), while follow-up after discharge accounted for >25% of the costs (€14,592,425). cGVHD's diagnosis and treatment represented €5,809,116, being 7.8% of the total costs assigned to treatment (€4,554,052).
The total mean cost per patient in this cohort was €97,719, representing cGVHD's diagnosis €2,123, and its treatment €7,646. Cost for patients achieving third line is €112,695.

**Copyright Information:** (Online) ISSN: 2572-9241
©2026 The Author(s). HemaSphere is published by John Wiley & Sons Ltd on behalf of European Hematology Association. This is an open access Abstract Book distributed under the Attribution-NonCommercial-NoDerivs (CC BY-NC-ND), which allows third parties to download the articles and share them with others as long as they credit the author and the Abstract Book, but they cannot change the content in any way or use them commercially.
**Abstract Book Citations:** Authors, Title, HemaSphere, 2026;10:(S1):pages. The abstract book DOIs can be found on the "Author Information" page.
**Disclaimer:** Articles published in the journal HemaSphere exclusively reflect the opinions of the authors. The authors are responsible for all content in their abstracts including accuracy of the facts, statements, citing resources, etc.

### Summary/Conclusion

This study showed that the largest driver of health care costs is associated with the allo-HSCT procedure itself, with management of cGvHD complications accounting for less than 8%.

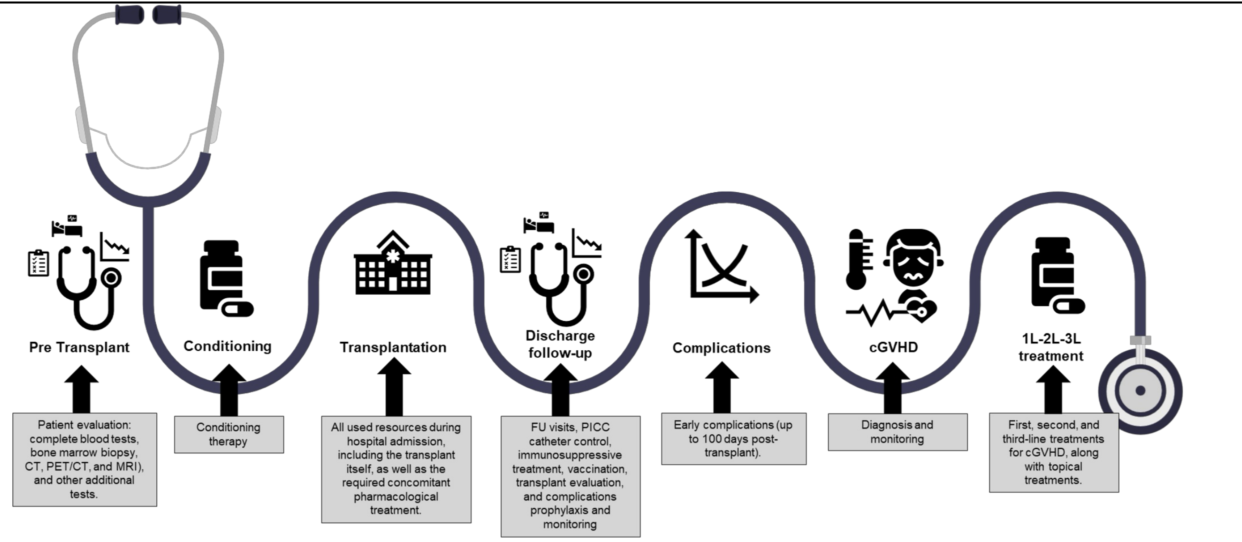

Figure 1: Patient Journey of a patient from Allogeneic Hematopoietic Stem Cell transplant to cGVHD and subsequent lines of treatment; including all phases of the patient journey and the specific resources used in each phase. Acronyms: cGVHD, chronic graft versus host disease; CT, computed tomography; FU, follow-up; MRI, magnetic resonance imaging; PET/CT, positron emission tomography and computed tomography; PICC, peripherally inserted central catheter.

**Copyright Information:** (Online) ISSN: 2572-9241
©2026 The Author(s). HemaSphere is published by John Wiley & Sons Ltd on behalf of European Hematology Association. This is an open access Abstract Book distributed under the Attribution-NonCommercial-NoDerivs (CC BY-NC-ND), which allows third parties to download the articles and share them with others as long as they credit the author and the Abstract Book, but they cannot change the content in any way or use them commercially.
**Abstract Book Citations:** Authors, Title, HemaSphere, 2026;10:(S1):pages. The abstract book DOIs can be found on the "Author Information" page.
**Disclaimer:** Articles published in the journal HemaSphere exclusively reflect the opinions of the authors. The authors are responsible for all content in their abstracts including accuracy of the facts, statements, citing resources, etc.

### (PF1418) THE COST OF SAFETY: A REAL-WORLD COMPARATIVE ANALYSIS OF EARLY HEALTH CARE UTILIZATION IN CLL PATIENTS TREATED WITH FIRST- VS. SECOND-GENERATION BTK INHIBITORS

**Topic:** 37. Health economics

Jelena Ivanović*[1], Sofija Kozarac[1], Vojin Vukovic[1, 2], Tamara Bibic[1], Darko Aleksic[1], Darko Antic[1, 2]

*[1]Clinic for Hematology, University Clinical Center of Serbia, Belgrade, Serbia;[2]Faculty od Medicine, University of Belgrade, Belgrade, Serbia;*

### Background

Second-generation Bruton tyrosine kinase inhibitors (BTKi), such as acalabrutinib, were developed to mitigate the off-target toxicity profile of ibrutinib, particularly cardiovascular events (CVe). While clinical trials show improved safety, it remains uncertain whether this translates into reduced healthcare burden in real-world practice, where safer agents are expected to decrease medical contacts.

### Aims to quantify early healthcare utilization (HCU) in chronic lymphocytic leukemia (CLL) patients and determine whether the choice of BTKi generation significantly impacts the immediate resource burden during the critical first 6 months of therapy in a real-world academic center.

### Methods

This retrospective single-center study analyzed 125 consecutive CLL patients treated with BTKi at the University Clinical Center of Serbia (111 ibrutinib, 14 acalabrutinib). HCU was assessed over a standardized 6-month observation period, chosen to focus on early event patterns and mitigate potential biases from varying follow-up durations and heterogeneous cohort sizes. Key HCU metrics included Medical Events of Interest (MEOI: hemorrhagic, CV, infectious), emergency department (ED) visits, hospital admissions (HA), and length of hospital stay (LoS). Statistical comparisons used Mann–Whitney U and Chi-square/Fisher’s exact tests.

### Results

During the first 6 months, mean MEOI burden was 1.57 events per patient. Patients averaged 0.22 ED visits and 0.30 HA. Mean ED visits were 0.23 (ibrutinib) vs 0.14 (acalabrutinib), and HA 0.29 vs 0.36, respectively. Despite the introduction of a second-generation agent and substantial imbalance in cohort sizes (111/14), no statistically significant difference was observed in median MEOI (1.0 vs 1.0; p=0.637), ED visits (p=0.815), or HA (p=0.503). CVe were numerically more frequent with ibrutinib but without statistical significance (atrial fibrillation, AF: 4/111 vs 0/14, p=0.62; hypertension, HT: 7/111 vs 0/14, p=0.43; heart failure, HF: 2/111 vs 0/14, p=1.00). Hemorrhagic events were the only safety outcome demonstrating a statistically significant early difference, occurring only in the ibrutinib cohort (37/111 vs 0/14; p=0.006), but no reduction in ED visits or HA was observed, reflecting predominantly clinically non-significant bleeding. Across the entire follow-up during therapy, acalabrutinib showed consistently lower numerical rates of AF (0% vs 12.6%; p=0.364), HT (0% vs 12.6%; p=0.364), HF (0% vs 6.3%; p=1.000), and any bleeding (21.4% vs 48.6%; p=0.085), yet none reached statistical significance. Notably, infectious complications proved to be a persistent driver of HCU regardless of the agent. The acalabrutinib cohort exhibited a trend toward prolonged hospitalization driven by severe infections (mean LoS: 12.79days vs 2.92days for ibrutinib), including outliers with extended admissions up to 131days.

**Copyright Information:** (Online) ISSN: 2572-9241
©2026 The Author(s). HemaSphere is published by John Wiley & Sons Ltd on behalf of European Hematology Association. This is an open access Abstract Book distributed under the Attribution-NonCommercial-NoDerivs (CC BY-NC-ND), which allows third parties to download the articles and share them with others as long as they credit the author and the Abstract Book, but they cannot change the content in any way or use them commercially.
**Abstract Book Citations:** Authors, Title, HemaSphere, 2026;10:(S1):pages. The abstract book DOIs can be found on the “Author Information” page.
**Disclaimer:** Articles published in the journal HemaSphere exclusively reflect the opinions of the authors. The authors are responsible for all content in their abstracts including accuracy of the facts, statements, citing resources, etc.

### Summary/Conclusion

In this real-world analysis, transition to a second-generation BTKi didn't significantly reduce early HCU. While acalabrutinib demonstrated a favorable safety profile, the total burden on the healthcare system—driven largely by infectious complications and patient complexity—remained comparable to ibrutinib. These findings challenge the assumption that improved safety automatically translate into immediate resource savings. For health economics modeling, the "cost of safety" must account for persistent infectious risks and patient characteristics that dictate resource use as much as the drug's selectivity.

**Copyright Information:** (Online) ISSN: 2572-9241
©2026 The Author(s). HemaSphere is published by John Wiley & Sons Ltd on behalf of European Hematology Association. This is an open access Abstract Book distributed under the Attribution-NonCommercial-NoDerivs (CC BY-NC-ND), which allows third parties to download the articles and share them with others as long as they credit the author and the Abstract Book, but they cannot change the content in any way or use them commercially.
**Abstract Book Citations:** Authors, Title, HemaSphere, 2026;10:(S1):pages. The abstract book DOIs can be found on the "Author Information" page.
**Disclaimer:** Articles published in the journal HemaSphere exclusively reflect the opinions of the authors. The authors are responsible for all content in their abstracts including accuracy of the facts, statements, citing resources, etc.

### (PF1419) CALM: BUILDING A COMPREHENSIVE ATLAS OF LEUKEMIA AND ITS MICROENVIRONMENT TO ENABLE MECHANISTIC AND THERAPEUTIC DISCOVERIES FOR PRECISION MEDICINE

**Topic:** 38. Novel technologies, techniques and digital analytical tools in hematology

Bofei Wang[1], Yu-An Kuo[1], Wei Xiao[1], Enes Dasdemir[2], Elisabeth Rebboah[1], Hussein Abbas*[1]

*[1]UNIVERSITY OF TEXAS MD ANDERSON CANCER CENTER, Houston, United States of America;[2]UNIVERSITY OF HOUSTON, Houston, United States of America;*

### Background

Despite recent therapeutic advances in targeted inhibitors and immunotherapies, significant heterogeneity in leukemia biology and treatment response persists. Relapses are driven by the heterogeneity in cell populations in the bone marrow microenvironment: including their dynamic cellular states, diverse genetic profiles, evolving transcriptional programs, and complex interplay. Current molecular diagnostics capture only a fraction of this complexity. Additionally, leukemic cells originate from immune or hematopoietic precursors, making their interaction with the immune system inherently complex and their distinction from normal compartments obscured by shared lineage markers and functional mimicry. Recent advances in single-cell RNA, immune profiling, and spatial transcriptomics have yielded valuable mechanistic insights. However, unlike in solid cancers, current research fails to establish an integrated atlas with comprehensive pictures of genotype, cellular states across diverse treatment modalities, biological subgroups, hematopoietic hierarchies, and longitudinal disease trajectories to enable comparative analyses of therapeutic response, dynamic lineage development and plasticity.

### Aims

In this study, we aim to build a publicly accessible Comprehensive Atlas of Leukemia and its Microenvironment (CALM), serving as a shared resource for rapid query of gene expression and correlation, systematic mapping of cellular compartments, inference of cellular interactions, and longitudinal tracking of transcriptional and clonal dynamics.

### Methods

A total of 467,095 high-quality cells from 73 acute myeloid leukemia (AML) patient samples and 9 healthy donors (HD) linked to extensive clinical, cytogenetic, molecular, and treatment-response annotations were integrated into a shiny-based portal for analytics and visualization.

### Results

We leveraged CALM to perform comparative analysis of surface proteins across AML cells with distinct phenotypes and with healthy compartments. This analysis identified proteins uniquely expressed on AML cells with stem cell phenotype, which may serve as potential targets for immune therapy. Further focused analysis of the immune compartment revealed diverse T cell distribution across disease status, where patients with persistent disease displayed higher diversity and a more differentiated phenotype. These patients also exhibited greater clonal expansion, particularly in the CD8 effector population. We further applied machine learning models on conventional T cells from HD and newly diagnosed AML patients and identified a unique signature pertinent to AML and patient outcomes. This signature was validated in two independent cohorts, where patients with higher score showed worse overall survival. Ongoing work includes single-cell multimodal profiling of 78 chronic lymphocytic leukemia samples receiving triplet therapies and 22 acute lymphocytic leukemia samples, which will be integrated into CALM together with other disease samples to cover a full spectrum of classic and rare leukemias.

**Copyright Information:** (Online) ISSN: 2572-9241
©2026 The Author(s). HemaSphere is published by John Wiley & Sons Ltd on behalf of European Hematology Association. This is an open access Abstract Book distributed under the Attribution-NonCommercial-NoDerivs (CC BY-NC-ND), which allows third parties to download the articles and share them with others as long as they credit the author and the Abstract Book, but they cannot change the content in any way or use them commercially.
**Abstract Book Citations:** Authors, Title, HemaSphere, 2026;10:(S1):pages. The abstract book DOIs can be found on the "Author Information" page.
**Disclaimer:** Articles published in the journal HemaSphere exclusively reflect the opinions of the authors. The authors are responsible for all content in their abstracts including accuracy of the facts, statements, citing resources, etc.

### Summary/Conclusion

Collectively, our well-annotated atlas provides in-depth analysis and visualization that will be publicly available via web portal and support real-time data updates to support hypothesis generation, therapy optimization and biomarker discovery in leukemia.

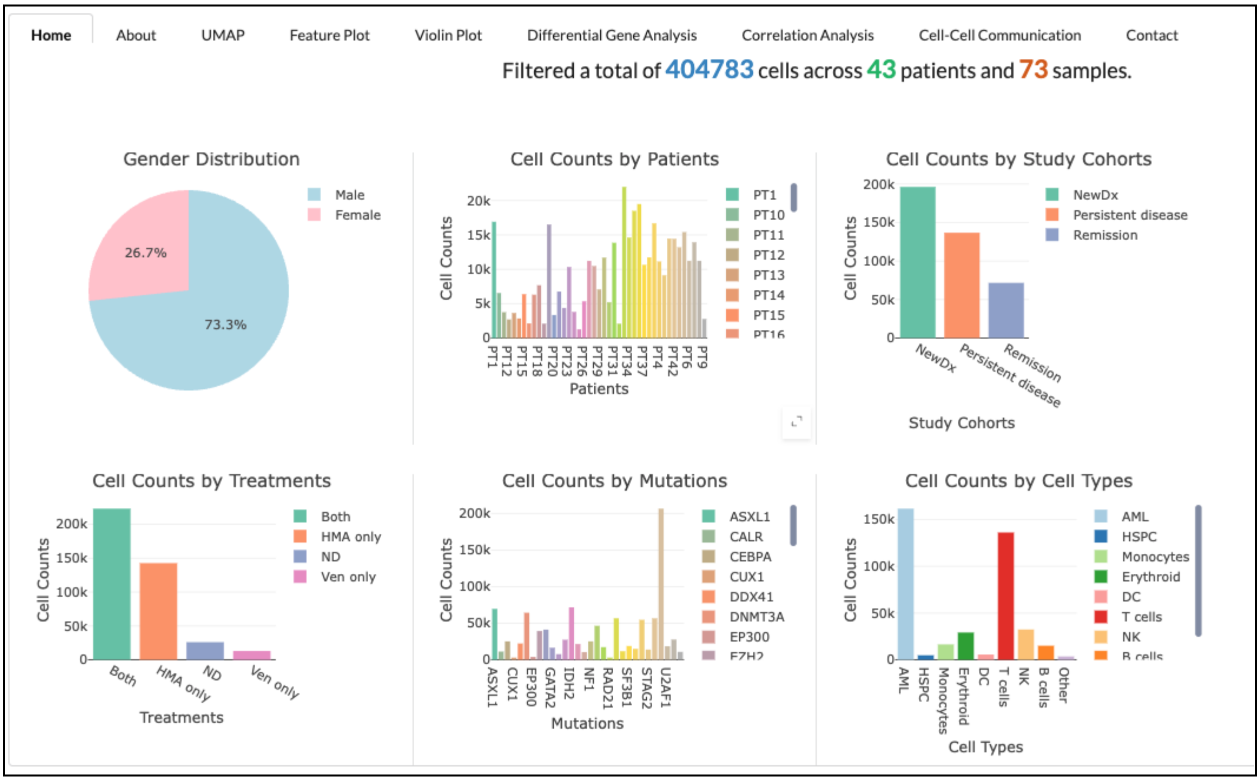

Example of the web portal application

**Copyright Information:** (Online) ISSN: 2572-9241
©2026 The Author(s). HemaSphere is published by John Wiley & Sons Ltd on behalf of European Hematology Association. This is an open access Abstract Book distributed under the Attribution-NonCommercial-NoDerivs (CC BY-NC-ND), which allows third parties to download the articles and share them with others as long as they credit the author and the Abstract Book, but they cannot change the content in any way or use them commercially.
**Abstract Book Citations:** Authors, Title, HemaSphere, 2026;10:(S1):pages. The abstract book DOIs can be found on the "Author Information" page.
**Disclaimer:** Articles published in the journal HemaSphere exclusively reflect the opinions of the authors. The authors are responsible for all content in their abstracts including accuracy of the facts, statements, citing resources, etc.

### (PF1420) UNSUPERVISED CLUSTERING FOR MRD FLOW CYTOMETRY IN AML: MULTICENTRIC VALIDATION AND PROGNOSTIC IMPACT AT SUB-ELN THRESHOLDS.

**Topic:** 38. Novel technologies, techniques and digital analytical tools in hematology

Aguirre Mimoun*[1], Bouchra Badaoui[2], Veronique Saada[3], Nicolas Lechevalier[1], Igor Sedorov[1], Vincent Olivier[4], Audrey Bidet[1], Emilie Klein[1], Thibaut Leguay[1], Edouard Forcade[1], Arnaud Pigneux[1], Stéphane Ducassou[1], Pierre, Yves Dumas[1], Jean-Philippe Vial[1]

*[1]Bordeaux university hospital, Bordeaux, France; [2]CHU Henri Mondor, Créteil, France; [3]Gustave Roussy, Villejuif, France; [4]CHRU Brest, Brest, France;*

### Background

Measurable residual disease (MRD) by flow cytometry (FCM) is increasingly guiding therapeutic decisions in acute myeloid leukemia (AML). ELN 2025 guidelines define a positivity threshold at 0.1% and a vigilance zone from 0.01%. However, manual gating limits reliable detection at low levels due to inter-operator variability. Unsupervised clustering may improve reproducibility and sensitivity, but its analytical reliability versus manual gating and prognostic value at sub-ELN thresholds have not been established.

### Aims

To validate the analytical reliability of unsupervised clustering (FlowSOM) versus manual gating in a multicenter blinded setting, and to evaluate the prognostic impact of lowering the MRD-FCM detection threshold.

### Methods

Two complementary retrospective cohorts were analyzed:
**Cohort A (analytical validation, 2023-2025):** 52 AML patients, 13-color ELN 2025 panel, 120 bone marrow samples analyzed blindly by 3 centers (1 FlowSOM, 2 manual gating). Concordance assessed by Fleiss kappa; diagnostic performance evaluated against molecular MRD when available.
**Cohort B (prognostic validation, 2019-2023)**: 208 adults with AML treated with intensive chemotherapy, 9-color ELN panel, FlowSOM analysis. MRD evaluated after 1 (n=144) or 2 (n=77) intensive chemotherapy. Allogeneic transplantation (47.2%) modeled as time-dependent variable. Median follow-up: 36.3 months.
For both cohort informed consent was obtained; data recorded in DATAML-Bdx database approved by French regulatory authorities (CCTIRS, CNIL).

**Copyright Information:** (Online) ISSN: 2572-9241
©2026 The Author(s). HemaSphere is published by John Wiley & Sons Ltd on behalf of European Hematology Association. This is an open access Abstract Book distributed under the Attribution-NonCommercial-NoDerivs (CC BY-NC-ND), which allows third parties to download the articles and share them with others as long as they credit the author and the Abstract Book, but they cannot change the content in any way or use them commercially.
**Abstract Book Citations:** Authors, Title, HemaSphere, 2026;10:(S1):pages. The abstract book DOIs can be found on the "Author Information" page.
**Disclaimer:** Articles published in the journal HemaSphere exclusively reflect the opinions of the authors. The authors are responsible for all content in their abstracts including accuracy of the facts, statements, citing resources, etc.

### Results

**Cohort A:** At 0.1%, FlowSOM-manual gating concordance (79-81%, kappa 0.33-0.46) was equivalent to inter-manual concordance (79%, kappa 0.25). Using molecular MRD as reference (49 samples, 22 patients), FlowSOM showed high PPV (87%), comparable to manual gating (80-92%). Among 26 molecular MRD-positive samples, 10 (38.5%) were undetected by any FCM method at 0.1%, involving significantly lower molecular levels (median 0.52% vs 8.0%, *p*=0.003). Lowering the FCM threshold to 0.01% reduced false negatives by 40%.
**Cohort B:** The optimal threshold identified by log-likelihood maximization was **0.03%**. At this threshold, MRD positivity after first induction was associated with decreased relapse-free survival (HR=2.08, 95%CI 1.18-3.65, *p*=0.007) and increased cumulative incidence of relapse (sHR=2.23, *p*=0.008), while the standard 0.1% threshold was less discriminant (HR=1.71, 95%CI 1.03-2.84, *p*=0.032). In multivariate analysis adjusting for age, sex, *NPM1*, *FLT3-ITD* and *CBF* fusions, MRD at 0.03% remained significant (*p*=0.044). Concordant results were observed after second induction (HR=2.47, *p*=0.009). PPV versus molecular MRD (n=63) was ~95% regardless of threshold. NPV improved from 18% at 0.1% to 26% at 0.03%, and to 42% at 0.01%, aligned with the ELN 2025 vigilance zone.

**Positive predictive value (PPV)/Negative predictive Value (NPV) by threshold - Cohort B vs molecular MRD**

| Threshold | PPV vs molecular MRD | NPV vs molecular MRD |
|---|---|---|
| 0.1% | 95% | 18% |
| 0.03% | 95% | 26% |
| 0.01% | 96% | 42% |

### Summary/Conclusion

Unsupervised clustering is analytically equivalent to manual gating in a decentralized multicenter setting.
It enables reliable lowering of the MRD-FCM detection threshold by one log below the ELN standard with improved relapse prediction and enhanced NPV. The high PPV supports MRD-guided treatment intensification, although validation across broader molecular subgroups is warranted. These findings support integration of unsupervised clustering in multicenter MRD strategies for AML.

**Copyright Information:** (Online) ISSN: 2572-9241
©2026 The Author(s). HemaSphere is published by John Wiley & Sons Ltd on behalf of European Hematology Association. This is an open access Abstract Book distributed under the Attribution-NonCommercial-NoDerivs (CC BY-NC-ND), which allows third parties to download the articles and share them with others as long as they credit the author and the Abstract Book, but they cannot change the content in any way or use them commercially.
**Abstract Book Citations:** Authors, Title, HemaSphere, 2026;10:(S1):pages. The abstract book DOIs can be found on the “Author Information” page.
**Disclaimer:** Articles published in the journal HemaSphere exclusively reflect the opinions of the authors. The authors are responsible for all content in their abstracts including accuracy of the facts, statements, citing resources, etc.

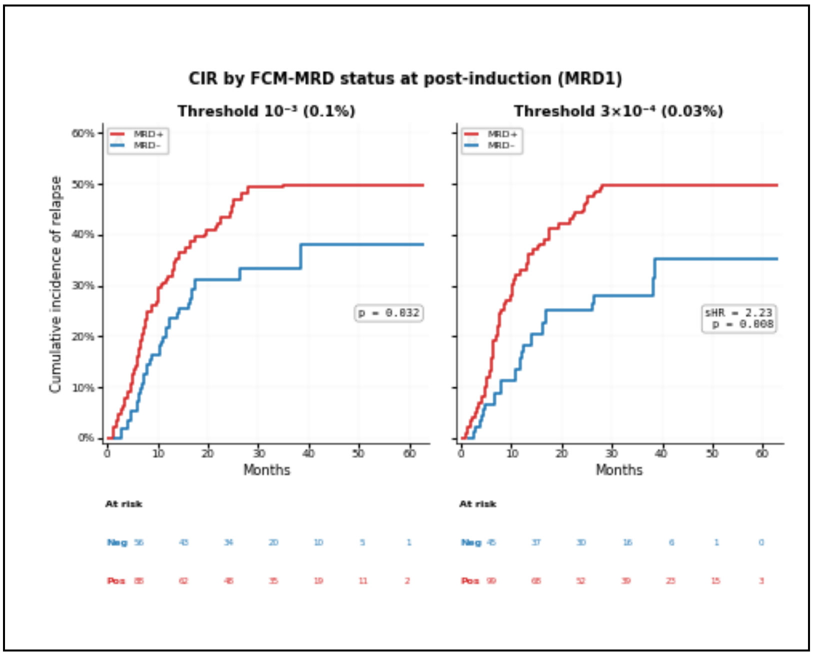

**Copyright Information:** (Online) ISSN: 2572-9241
©2026 The Author(s). HemaSphere is published by John Wiley & Sons Ltd on behalf of European Hematology Association. This is an open access Abstract Book distributed under the Attribution-NonCommercial-NoDerivs (CC BY-NC-ND), which allows third parties to download the articles and share them with others as long as they credit the author and the Abstract Book, but they cannot change the content in any way or use them commercially.
**Abstract Book Citations:** Authors, Title, HemaSphere, 2026;10:(S1):pages. The abstract book DOIs can be found on the "Author Information" page.
**Disclaimer:** Articles published in the journal HemaSphere exclusively reflect the opinions of the authors. The authors are responsible for all content in their abstracts including accuracy of the facts, statements, citing resources, etc.

### (PF1421) DISTINGUISHING MYELOPROLIFERATIVE NEOPLASMS AND PREDICTING JAK2 AND CALR MUTATION STATUS FROM BONE MARROW WHOLE SLIDE IMAGES USING SELF-SUPERVISED VISION TRANSFORMERS AND MULTI-INSTANCE LEARNING

**Topic:** 38. Novel technologies, techniques and digital analytical tools in hematology

Jan-Niklas Eckardt*[1, 2, 3, 4], Chethan Babu V. Reddy[4], Holger Hauspurg[5], Sebastian Riechert[4], Tim Schmittmann[4], Ishan Srivastava[1], Susann Winter[1], Markus Badstübner[4], Martin Bornhäuser[1, 6, 7], Markus Tiemann[5], Jan Moritz Middeke[1, 2, 3]

*[1]Department of Internal Medicine I, University Hospital Carl Gustav Carus, Technical University Dresden, Dresden, Germany; [2]Else Kröner Fresenius Center for Digital Health, Technical University Dresden, Dresden, Germany;[3]Mildred Scheel Cancer Center, University Hospital Dresden, Technical University Dresden, Dresden, Germany; [4]Cancilico GmbH, Dresden, Germany; [5]Institute for Hematopathology Hamburg, Hamburg, Germany;[6]German Cancer Consortium (DKTK), Partner Site Dresden, and German Cancer Research Center (DKFZ), Heidelberg, Germany; [7]National Center for Tumor Diseases Dresden (NCT/UCC), a partnership between DKFZ, Faculty of Medicine and University Hospital Dresden, TUD Dresden University of Technology, and Helmholtz-Zentrum Dresden-Rossendorf (HZDR), Dresden, Germany;*

### Background

Philadelphia-(Ph-) negative Myeloproliferative Neoplasms (MPN) encompass a set of hematopoietic stem cell disorders that share genetic driver alterations in *JAK2*, *CALR*, and/or *MPL*, impacting signaling pathways critical for hematopoiesis. The evaluation of bone marrow morphology is crucial in the diagnostic workup and requires experienced hematopathologists. Artificial intelligence (AI)-based computer vision may support image-based diagnostics and predict digital biomarkers directly from images.

### Aims

To develop and test an AI model distinguishing polycythemia vera (PV), essential thrombocythemia (ET), and primary myelofibrosis (PMF) in bone marrow smears (BMS) and predicting mutation status of *JAK2* and *CALR*.

### Methods

Whole slide images (WSI) from 473 BMS were digitized using a Sysmex P1000 scanner at 20x and 40x magnification. Tile extraction was performed using SlideFlow with a tile size of 224 × 224 pixels. Blur-based filtering and Otsu thresholding was used to exclude regions with poor scanning focus and/or background areas. For feature extraction, we used Virchow, a self-supervised transformer-based vision encoder foundation model trained specifically on large-scale histopathology data which leverages knowledge distillation and contrastive learning principles to learn semantically rich, domain-specific image features without requiring manual annotations. The resulting 2048-dimensional feature vectors were subsequently used for slide-level classification using an attention-based multi-instance-learning (MIL) model. Binary cross-entropy with logits loss was used as the objective function, learning rate was governed by the Adam optimizer, and hyperparameters were tuned with the Optuna framework.

**Copyright Information:** (Online) ISSN: 2572-9241
©2026 The Author(s). HemaSphere is published by John Wiley & Sons Ltd on behalf of European Hematology Association. This is an open access Abstract Book distributed under the Attribution-NonCommercial-NoDerivs (CC BY-NC-ND), which allows third parties to download the articles and share them with others as long as they credit the author and the Abstract Book, but they cannot change the content in any way or use them commercially.
**Abstract Book Citations:** Authors, Title, HemaSphere, 2026;10:(S1):pages. The abstract book DOIs can be found on the “Author Information” page.
**Disclaimer:** Articles published in the journal HemaSphere exclusively reflect the opinions of the authors. The authors are responsible for all content in their abstracts including accuracy of the facts, statements, citing resources, etc.

### Results

Training and internal validation were conducted with a 2:1 split. For Ph-negative MPN subtyping, classification performance was superior at 40x magnification compared to 20x, yielding a mean macro-averaged receiver operating characteristic area under the curve (ROC-AUC) of 0.84 (standard deviation [std.] ± 0), a mean macro-averaged F1 score of 0.68 (std ± 0.01), and a mean accuracy of 0.747 (std ± 0.02). PV was most reliably detected with a ROC-AUC of 0.92 (std ± 0.01), while ET and PMF yielded ROC-AUCs of 0.81 (std ± 0.01) and 0.78 (std ± 0.01), respectively. Image-based mutation prediction was also more accurate at 40x magnification. Given subtype specific differences in frequencies of *JAK2*, *CALR*, and *MPL* mutations showing either highly dysbalanced data (*JAK2* in PV) or very few training samples (*MPL* in all subtypes, *CALR* in PV), not all possible combinations could be tested. For *JAK2* prediction in ET, we obtained a macro-averaged ROC-AUC of 0.72 (std. ± 0.03), a mean macro-averaged F1 score of 0.62 (std ± 0.03), and a mean accuracy of 0.66 (std ± 0.03). For *CALR* in ET, macro-averaged ROC-AUC was 0.74 (std. ± 0.03), mean macro-averaged F1 score was 0.66 (std ± 0.08), and a mean accuracy of 0.71 (std ± 0.08). For *JAK2* in PMF, macro-averaged ROC-AUC was 0.68 (std. ± 0.02), mean macro-averaged F1 score was 0.65 (std ± 0.02), and a mean accuracy of 0.64 (std ± 0.03). Lastly *CALR* prediction in PMF showed a macro-averaged ROC-AUC of 0.68 (std. ± 0.03), a mean macro-averaged F1 score of 0.63 (std ± 0.01), and a mean accuracy of 0.66 (std ± 0.05).

### Summary/Conclusion

AI-based computer vision can reliably distinguish between PV, ET, and MF and predict*JAK2* and *CALR* mutation status in MF and ET based on bone marrow smear image data.

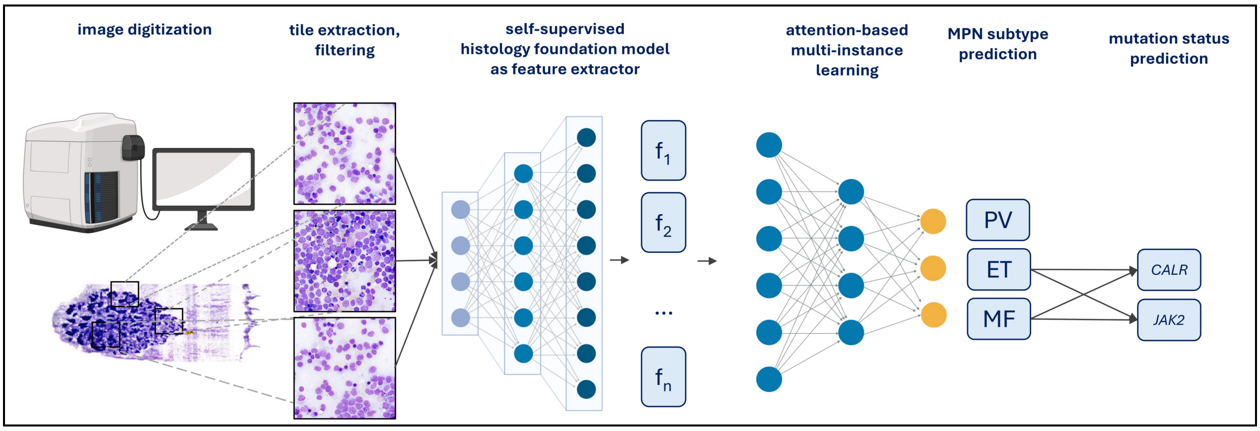

**Copyright Information:** (Online) ISSN: 2572-9241
©2026 The Author(s). HemaSphere is published by John Wiley & Sons Ltd on behalf of European Hematology Association. This is an open access Abstract Book distributed under the Attribution-NonCommercial-NoDerivs (CC BY-NC-ND), which allows third parties to download the articles and share them with others as long as they credit the author and the Abstract Book, but they cannot change the content in any way or use them commercially.
**Abstract Book Citations:** Authors, Title, HemaSphere, 2026;10:(S1):pages. The abstract book DOIs can be found on the "Author Information" page.
**Disclaimer:** Articles published in the journal HemaSphere exclusively reflect the opinions of the authors. The authors are responsible for all content in their abstracts including accuracy of the facts, statements, citing resources, etc.

### (PF1422) COMET: MUTATION-AGNOSTIC DETECTION OF CLONAL HEMATOPOIESIS FROM DNA METHYLATION

**Topic:** 38. Novel technologies, techniques and digital analytical tools in hematology

Samuel Crofts[1, 2], Caleb Grenko[1], Yossawat Suwanlikit[1, 3], Emma Yang[1], Neil Robertson[1], Riccardo Marioni[2], Simon Cox[4], Kristina Kirschner[1], Tamir Chandra*[1]

*[1]Mayo Clinic, Robert and Arlene Kogod Center on Aging, Rochester, United States of America;[2]University of Edinburgh, Institute for Genetics and Cancer (IGC), Edinburgh, United Kingdom; [3]Mahidol University, Department of Pharmacology, Bangkok, Thailand; [4]University of Edinburgh, Lothian Birth Cohorts, Department of Psychology, Edinburgh, United Kingdom;*

### Background

Clonal hematopoiesis of indeterminate potential (CHIP) is associated with cardiovascular disease, hematologic malignancy, and mortality. Current detection relies on targeted sequencing with manual curation, limiting scalability and missing clonal expansions driven by mutations outside standard gene panels. Meanwhile, CHIP's relationship with neurodegeneration remains controversial, with prior cross-sectional studies reporting conflicting findings.

### Aims

We developed COMET (Clonal Observation from METhylation), an algorithm that quantifies total clonal burden from DNA methylation arrays in a mutation-agnostic manner. We validated COMET against sequencing-based detection and applied it to determine whether CHIP burden associates with progressive brain structural changes.

### Methods

COMET exploits CpG sites that faithfully transmit allelic methylation status across cell divisions. As clones expand, methylation proportions shift predictably toward discrete values (0%, 50%, 100%), creating a detectable epigenetic signature independent of driver mutation identity. We validated COMET against targeted sequencing in Generation Scotland (n=15,900) and applied it to the Lothian Birth Cohort 1936, which provides blood DNA methylation and structural brain MRI at ages 70, 73, 76, and 79 years.

### Results

COMET predictions correlated strongly with sequencing-based variant allele fractions ($R^2$=0.80) and accurately tracked longitudinal clonal dynamics (mean error 1.7%). The algorithm detected CHIP regardless of driver mutation type, including DNMT3A, TET2, ASXL1, and mosaic chromosomal alterations. Applied to Generation Scotland, COMET replicated known associations with age, smoking, and COPD, and GWAS identified TCL1A and NRIP1 as genetic determinants of clonal burden. In the Lothian Birth Cohort, higher COMET-predicted clonal burden associated with reduced grey matter volume cross-sectionally, with no significant white matter association. Longitudinally, baseline clonal burden predicted accelerated cortical atrophy over 9 years, particularly in frontal and temporal regions, with increasing COMET scores tracking progressive grey matter loss.

### Summary/Conclusion

COMET enables scalable, mutation-agnostic detection of clonal hematopoiesis from standard methylation arrays, validated against sequencing and replicating known epidemiological and genetic associations. Application to a dual-longitudinal cohort demonstrates that CHIP burden associates with accelerated grey matter atrophy, suggesting clonal hematopoiesis contributes to brain aging with potential implications for risk stratification.

**Copyright Information:** (Online) ISSN: 2572-9241
©2026 The Author(s). HemaSphere is published by John Wiley & Sons Ltd on behalf of European Hematology Association. This is an open access Abstract Book distributed under the Attribution-NonCommercial-NoDerivs (CC BY-NC-ND), which allows third parties to download the articles and share them with others as long as they credit the author and the Abstract Book, but they cannot change the content in any way or use them commercially.
**Abstract Book Citations:** Authors, Title, HemaSphere, 2026;10:(S1):pages. The abstract book DOIs can be found on the "Author Information" page.
**Disclaimer:** Articles published in the journal HemaSphere exclusively reflect the opinions of the authors. The authors are responsible for all content in their abstracts including accuracy of the facts, statements, citing resources, etc.

### (PF1423) PROTEOME-WIDE MENDELIAN RANDOMIZATION IDENTIFIES HOST PROTEIN PATHWAYS SHAPING GVHD SEVERITY AND RELAPSE AFTER ALLOGENEIC HSCT

**Topic:** 38. Novel technologies, techniques and digital analytical tools in hematology

Xinghao Yu*[1], Yiyin Chen[1], Depei Wu[1], Yang Xu[1]

[1] *The First Affiliated Hospital of Soochow University, Suzhou, China;*

### Background

Acute and chronic graft-versus-host disease (GVHD) remain major complications after allogeneic hematopoietic stem cell transplantation (allo-HSCT), yet biomarker signals are often confounded by inflammation, treatment, and reverse causation. Genetic instruments for circulating proteins provide an opportunity to prioritize host pathways that may shape GVHD susceptibility and the GVHD–relapse balance.

### Aims

To systematically evaluate potential causal effects of circulating proteins on GVHD-related outcomes and relapse, and to identify reproducible, translation-oriented protein modules across independent proteomic sources.

### Methods

We performed two-sample Mendelian randomization (MR) using population-based proteomic GWAS (pQTL) as exposures from three independent sources (UK Biobank, deCODE, and Finland). Cis-pQTL instruments were prioritized; cis+trans instruments were used for sensitivity. Outcomes included GWAS summary statistics for aGVHD (grade 2/2b/3), cGVHD, gut GVHD, and relapse in allo-HSCT recipients, with analyses conducted in overall and donor-type strata (ALL/FS/URD where available). MR estimates were derived using Wald ratio (single-SNP) or inverse-variance weighted (IVW) methods (multi-SNP), followed by false discovery rate (FDR) control. Robustness was assessed by heterogeneity/pleiotropy diagnostics and directionality checks; colocalization was used to assess locus-level credibility for prioritized proteins when feasible.

### Results

Across endpoints, we identified 37 proteins significantly associated with GVHD and/or relapse that replicated across at least two proteomic sources at FDR $q<0.05$ with consistent direction. Several established GVHD biomarkers showed concordant genetic evidence, including IL1RL1 (ST2) and PI3 (Elafin), supporting their relevance beyond reactive inflammation. Notably, multiple proteins mapped to biologically coherent modules: (i) epithelial/barrier and tissue-protective signals (e.g., TIMP2) were associated with lower risk of severe aGVHD (including grade 3); (ii) endothelial activation/adhesion pathways (e.g., SELP/SELE) were linked to GVHD-related outcomes; and (iii) innate immune sensing markers highlighted a GVHD–relapse trade-off. Specifically, genetically predicted higher soluble CD14 was associated with reduced aGVHD severity but increased relapse risk, suggesting a host "trajectory" axis relevant to balancing inflammatory tissue injury versus graft-versus-leukemia activity. For several proteins, MR significance was not consistently accompanied by strong colocalization, indicating that some signals should be interpreted as locus-/pathway-level prioritization rather than definitive causal effector proteins.

**Copyright Information:** (Online) ISSN: 2572-9241
©2026 The Author(s). HemaSphere is published by John Wiley & Sons Ltd on behalf of European Hematology Association. This is an open access Abstract Book distributed under the Attribution-NonCommercial-NoDerivs (CC BY-NC-ND), which allows third parties to download the articles and share them with others as long as they credit the author and the Abstract Book, but they cannot change the content in any way or use them commercially.
**Abstract Book Citations:** Authors, Title, HemaSphere, 2026;10:(S1):pages. The abstract book DOIs can be found on the "Author Information" page.
**Disclaimer:** Articles published in the journal HemaSphere exclusively reflect the opinions of the authors. The authors are responsible for all content in their abstracts including accuracy of the facts, statements, citing resources, etc.

### Summary/Conclusion

Proteome-wide MR across three independent pQTL sources prioritizes reproducible host protein pathways influencing GVHD phenotypes and relapse after allo-HSCT. Beyond reinforcing established biomarkers (ST2/Elafin), the analysis highlights tissue-protective and endothelial modules as potentially “GVHD-and-relapse-friendly” targets, and identifies a CD14-centered trade-off axis that may inform individualized prophylaxis and post-transplant surveillance. These findings motivate targeted measurement and functional validation to translate genetically anchored protein modules into actionable risk stratification and intervention strategies.

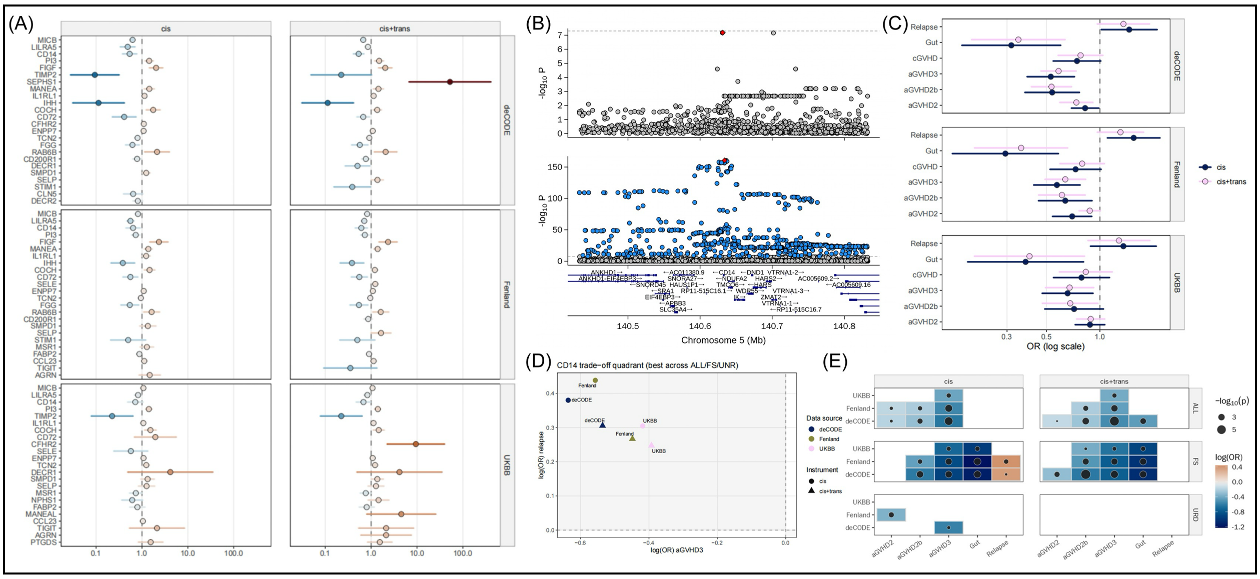

**Copyright Information:** (Online) ISSN: 2572-9241
©2026 The Author(s). HemaSphere is published by John Wiley & Sons Ltd on behalf of European Hematology Association. This is an open access Abstract Book distributed under the Attribution-NonCommercial-NoDerivs (CC BY-NC-ND), which allows third parties to download the articles and share them with others as long as they credit the author and the Abstract Book, but they cannot change the content in any way or use them commercially.
**Abstract Book Citations:** Authors, Title, HemaSphere, 2026;10:(S1):pages. The abstract book DOIs can be found on the “Author Information” page.
**Disclaimer:** Articles published in the journal HemaSphere exclusively reflect the opinions of the authors. The authors are responsible for all content in their abstracts including accuracy of the facts, statements, citing resources, etc.

### (PF1424) SCALING CYTOMORPHOLOGICAL ANALYSIS: AN UNSUPERVISED APPROACH TO PHENOTYPING SINGLE CELLS FROM BONE MARROW SMEARS

**Topic:** 38. Novel technologies, techniques and digital analytical tools in hematology

Henri Sundquist*[1, 2, 3], Stas Tatun[1, 2, 3], Oscar Brück[1, 2, 3]

*[1]Helsinki University Hospital, Hematoscope Lab, Comprehensive Cancer Center, Helsinki, Finland;[2]University of Helsinki, Department of Oncology, Helsinki, Finland; [3]University of Helsinki and Helsinki University Hospital, Department of Clinical Chemistry, HUS Diagnostic Center, Helsinki, Finland;*

### Background

Cytomorphological assessment of bone marrow (BM) aspirate smears remains the primary examination for diagnosing acute myeloid leukemia (AML). However, inter-observer variability and time constraints restrict cytomorphological assessment to primary diagnostic indices such as the proportion of myeloblasts and the presence of dysplasia. To unlock novel biomarkers from these accessible samples, we need scalable new tools that move beyond traditional cytomorphology into high-detail quantitative analysis.

### Aims

- To develop an unsupervised approach for discovering semantic cytomorphological features at the single-cell level.
- Correlate cytomorphological features with the traditional French-American-British (FAB) classification and common genomic and cytogenetic alterations.

### Methods

We digitized 5919 May-Grünwald-Giemsa-stained BM smears at 100x magnification from patients diagnosed with AML at the Helsinki University Hospital (749 pre-treatment and 5170 follow-up samples from 661 patients). We analyzed the samples with Cellbytes, a medical software suite for analyzing BM samples based on a vision transformer (ViT) backbone, known for its performance in image analysis, followed by a variety of classification models. We extracted over 2.3 million immature myeloid cells classified as myeloblasts, promyelocytes, or promonocytes by Cellbytes.
To obtain single-cell-level embeddings specific to immature myeloid cells in AML, we trained a smaller ViT (DeiT-Ti architecture) using DINOv2. Next, to discover the interpretable cytomorphological features, we trained a sparse autoencoder on the 2.3 million cells embedded by our DeiT-Ti model and extracted sparse features for each cell.

### Results

The sparse autoencoder identified semantic features corresponding to cytomorphological features such as the nuclear-cytoplasmic ratio, vacuoles, granularity, and nucleus shape (Figure 1). We then studied the associations between the features and clinical data from pre-treatment samples that were available for 605 patients. We calculated the proportion of cells activating each semantic feature sample-wise and found 8 cytomorphological clusters by applying the HDBSCAN algorithm to UMAP-reduced representations. We found a cluster containing most samples with an M3 FAB classification, which was also enriched for the *PML-RARA* fusion gene ($p=0.002$, Bonferroni corrected, Pearson's chi-squared test), Another cluster was enriched for patients with translocations involving chromosome 11 ($p<0.001$).

**Copyright Information:** (Online) ISSN: 2572-9241
©2026 The Author(s). HemaSphere is published by John Wiley & Sons Ltd on behalf of European Hematology Association. This is an open access Abstract Book distributed under the Attribution-NonCommercial-NoDerivs (CC BY-NC-ND), which allows third parties to download the articles and share them with others as long as they credit the author and the Abstract Book, but they cannot change the content in any way or use them commercially.
**Abstract Book Citations:** Authors, Title, HemaSphere, 2026;10:(S1):pages. The abstract book DOIs can be found on the "Author Information" page.
**Disclaimer:** Articles published in the journal HemaSphere exclusively reflect the opinions of the authors. The authors are responsible for all content in their abstracts including accuracy of the facts, statements, citing resources, etc.

### Summary/Conclusion

We developed an unsupervised pipeline for BM cytomorphology analysis capable of finding semantic features from single cells. By clustering the samples with activation rates of the semantic features, we could partly recapitulate the FAB classification. Moreover, we found that the clusters associated with clinically relevant variables such as the *PML-RARA* fusion gene or translocations involving chromosome 11.

Ultimately, this approach is a proof-of-concept demonstrating how computational cytomorphological analysis can be used to produce quantifiable data and could in the future be used to identify explainable treatment biomarkers and track temporal disease dynamics alongside molecular genetics.

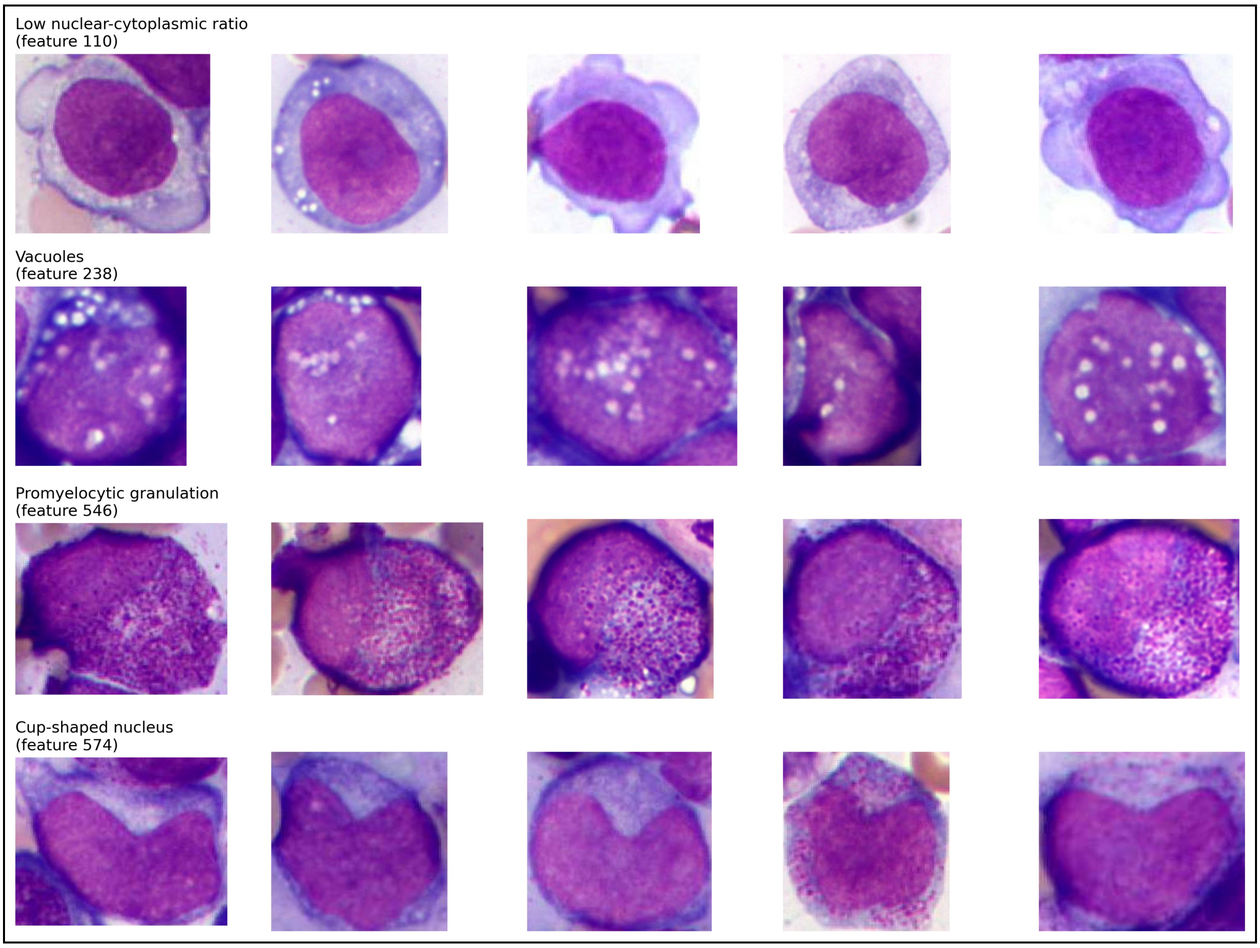

Figure 1. Top 5 cells with highest activation scores for a selection of semantic features

**Copyright Information:** (Online) ISSN: 2572-9241
©2026 The Author(s). HemaSphere is published by John Wiley & Sons Ltd on behalf of European Hematology Association. This is an open access Abstract Book distributed under the Attribution-NonCommercial-NoDerivs (CC BY-NC-ND), which allows third parties to download the articles and share them with others as long as they credit the author and the Abstract Book, but they cannot change the content in any way or use them commercially.
**Abstract Book Citations:** Authors, Title, HemaSphere, 2026;10:(S1):pages. The abstract book DOIs can be found on the "Author Information" page.
**Disclaimer:** Articles published in the journal HemaSphere exclusively reflect the opinions of the authors. The authors are responsible for all content in their abstracts including accuracy of the facts, statements, citing resources, etc.

### (PF1425) THE ELECTRONIC FRAILTY INDEX IDENTIFIES HIGH-RISK OLDER ADULTS RECEIVING CHIMERIC ANTIGEN RECEPTOR T-CELL THERAPY

**Topic:** 38. Novel technologies, techniques and digital analytical tools in hematology

Justine Enns*[1], Mark Korst[1], Isabel Neckermann[1], Olivia Cheung[1], Bharati Kochar[2], Ariela Orkaby[3], Nora Horick[2], Matthew Frigault[1], Areej El-Jawahri[1], Richard A. Newcomb[1], P. Connor Johnson[1]

*[1]Mass General Brigham Cancer Institute, Boston, United States of America; [2]Massachusetts General Hospital, Boston, United States of America; [3]Brigham & Women's Hospital, Boston, United States of America;*

### Background

Chimeric antigen receptor T-cell therapy (CAR-T) has transformed outcomes in hematologic malignancies and can be effective in older adults. Frailty, a syndrome of vulnerability common in older adults, is associated with poor outcomes but is difficult to measure in clinical practice. Moreover, the relationship between frailty and CAR-T outcomes is poorly understood.

### Aims

We aimed to assess whether an automated frailty index (MGB-eFI) is associated with survival and toxicity in older adults receiving CAR-T cell therapy.

### Methods

We conducted a retrospective analysis of 260 patients age ≥65 years receiving commercial CAR-T at Massachusetts General Brigham (MGB) for multiple myeloma and B cell non-Hodgkin lymphoma. We abstracted patient characteristics and clinical outcomes from the electronic health record (EHR). Using EHR data from the Research Patient Data Registry, we calculated the MGB-eFI, a validated electronic frailty score based upon a deficit accumulation approach to frailty. The MGB-eFI assesses 31 age-related health deficits and yields a score ranging 0–1 (<0.1 is robust, 0.1–0.2 is pre-frail, 0.2–0.3 is frail, and >0.3 is very frail). We then evaluated associations of the eFI (categorical variable of robust/prefrail vs frail vs very frail) with overall survival (OS), event-free (death, progression, or start of new treatment) survival (EFS), cytokine release syndrome (CRS), immune cell-associated neurotoxicity syndrome (ICANS), and infection within 30 days of infusion using univariate and multivariable Cox and logistic regression models controlling for confounders given event sample size. For grade 3+ CRS, only Fisher's exact test could be run given the number of events (n=7).

### Results

Among 260 patients (median age 73 years [range 65–91], 45% female), the most common CAR-T products used were liso-cel (35%), tisa-cel (18%), ide-cel (18%), cilta-cel (12%), and axi-cel (10%). With the eFI, 10% of patients were robust, 25% prefrail, 28% frail, and 37% very frail. In univariate analyses compared to robust/prefrail patients, being frail was not associated with worse OS or EFS but was associated with higher rates of ICANS, grade 3+ ICANS, and infection. Being very frail was associated with worse OS and EFS and higher rates of ICANS, grade 3+ ICANS, and infection. Frailty was not associated with risk of CRS, but was associated with grade 3+ CRS (robust/prefrail=0%, frail=1%, very frail=6%, p=0.02). In multivariable analyses, being frail was not associated with OS (hazard ratio [HR] 1.20, p=0.481) or EFS (HR=1.40, p=0.127), but being very frail was associated with numerically worse OS (HR=1.55, p=0.072) and significantly worse EFS (HR=2.01, p=0.001). In multivariable analyses, both frail (odds ratio [OR]=3.29, p=0.047) and very frail patients (OR=4.83, p=0.006) had higher odds of grade 3+ ICANS and infections (frail: OR=2.37, p=0.026; very frail: OR=2.18, p=0.035).

**Copyright Information:** (Online) ISSN: 2572-9241
©2026 The Author(s). HemaSphere is published by John Wiley & Sons Ltd on behalf of European Hematology Association. This is an open access Abstract Book distributed under the Attribution-NonCommercial-NoDerivs (CC BY-NC-ND), which allows third parties to download the articles and share them with others as long as they credit the author and the Abstract Book, but they cannot change the content in any way or use them commercially.
**Abstract Book Citations:** Authors, Title, HemaSphere, 2026;10:(S1):pages. The abstract book DOIs can be found on the "Author Information" page.
**Disclaimer:** Articles published in the journal HemaSphere exclusively reflect the opinions of the authors. The authors are responsible for all content in their abstracts including accuracy of the facts, statements, citing resources, etc.

### Summary/Conclusion

The automated eFI identifies frail and very frail patients, both of whom have higher risk for high-grade ICANS and infections. Very frail patients represent an especially high-risk group with worse EFS and higher odds of CAR-T toxicities, despite controlling for other clinical and patient factors. The eFI holds promise as a tool that if integrated into the EHR can help clinicians with risk stratification and CAR-T decision-making for older adults with hematologic malignancies.

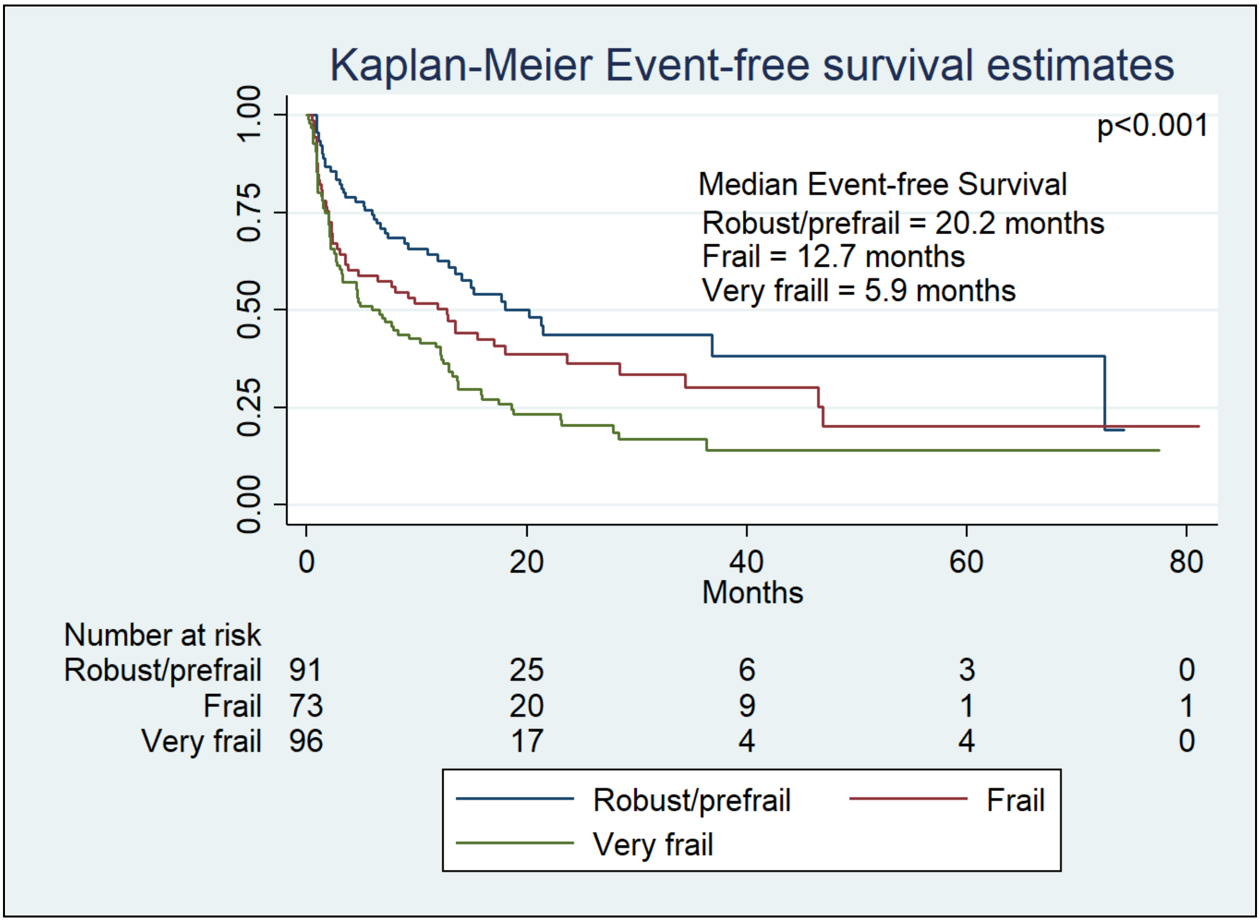

Event-free survival by frailty category in older adults receiving CAR-T cell therapy

Copyright Information: (Online) ISSN: 2572-9241
©2026 The Author(s). HemaSphere is published by John Wiley & Sons Ltd on behalf of European Hematology Association. This is an open access Abstract Book distributed under the Attribution-NonCommercial-NoDerivs (CC BY-NC-ND), which allows third parties to download the articles and share them with others as long as they credit the author and the Abstract Book, but they cannot change the content in any way or use them commercially.
Abstract Book Citations: Authors, Title, HemaSphere, 2026;10:(S1):pages. The abstract book DOIs can be found on the "Author Information" page.
Disclaimer: Articles published in the journal HemaSphere exclusively reflect the opinions of the authors. The authors are responsible for all content in their abstracts including accuracy of the facts, statements, citing resources, etc.

### (PF1426) DYNAMIC REMODELING OF HSC LINEAGE BIAS DURING INFECTION

**Topic:** 38. Novel technologies, techniques and digital analytical tools in hematology

Katerina Vecerkova*[1, 2], Irina Ribeiro Bas[3, 4], Michal Kolar[1, 2], Meritxell Alberich-Jorda[3]

*[1]Institute of Molecular Genetics of the Czech Academy of Sciences, Laboratory of Genomics and Bioinformatics, Prague, Czechia; [2]University of Chemistry and Technology, Prague, Department of Informatics and Chemistry, Prague, Czechia;[3]Institute of Molecular Genetics of the Czech Academy of Sciences, Laboratory of Haematooncology, Prague, Czechia; [4]Faculty of Science, Charles University, Prague, Czechia;*

### Background

Systemic infection induces stress on the hematopoietic system, requiring rapid adaptation of hematopoietic stem cells (HSCs) to sustain immune cell production. During infection, HSCs undergo transcriptional and functional reprogramming that biases lineage output and supports emergency myelopoiesis. However, most single-cell studies of infection-associated hematopoiesis focus on non-infection conditions or limited early timepoints, providing limited insight into the dynamic HSC state changes over the course of infection.

### Aims

To assess the dynamics of emergency granulopoiesis following systemic infection and to characterize how HSC transcriptional states and differentiation biases evolve over time using longitudinal single-cell profiling.

### Methods

Wild-type C57BL/6 mice were intravenously infected with *Candida albicans* or PBS control. Hematopoietic stem and progenitor cells (HSPCs) and mature populations were analyzed by flow cytometry at 4 hours, 3 days, 1 week, 1 month, and 2 months post-infection, alongside assessment of fungal burden. Long-term HSCs were isolated by FACS and profiled by single-cell RNA sequencing at matching timepoints. Differentiation bias, lineage priming, fate tendencies, cell-cell communication, and latent transcriptional programs were inferred using integrative computational analyses.

### Results

Systemic *C. albicans* infection induced rapid and dynamic remodeling of hematopoiesis over a two-month period. Emergency myelopoiesis was detectable as early as 4 hours post-infection, followed a bell-shaped trajectory, and peaked at one week before returning to baseline by two months. At peak response, HSCs, LSKs, and myeloid-biased MPP3s were expanded, while lymphoid-biased MPP4s were reduced. These HSPC changes were mirrored by coordinated increases in neutrophils and monocytes in bone marrow and blood and closely tracked fungal burden, which peaked at one week and was fully resolved by two months. Longitudinal single-cell profiling identified ten transcriptionally distinct HSC clusters with defined lineage-associated biases. Transcriptional and proportional changes were detectable as early as 4 hours post-infection, driven by the transient expansion of an inflammatory-biased HSC cluster that returned toward baseline after two months. Trajectory inference revealed dynamic lineage priming with altered differentiation probabilities across timepoints. Analysis of cell-cell communication highlighted time-dependent remodeling of cytokine and immune signaling pathways, while latent gene programs captured coordinated immune, stress, and differentiation-associated responses. Notably, cluster-specific transcriptional changes persisted at late timepoints, suggesting long-term alteration of HSC state.

**Copyright Information:** (Online) ISSN: 2572-9241
©2026 The Author(s). HemaSphere is published by John Wiley & Sons Ltd on behalf of European Hematology Association. This is an open access Abstract Book distributed under the Attribution-NonCommercial-NoDerivs (CC BY-NC-ND), which allows third parties to download the articles and share them with others as long as they credit the author and the Abstract Book, but they cannot change the content in any way or use them commercially.
**Abstract Book Citations:** Authors, Title, HemaSphere, 2026;10:(S1):pages. The abstract book DOIs can be found on the "Author Information" page.
**Disclaimer:** Articles published in the journal HemaSphere exclusively reflect the opinions of the authors. The authors are responsible for all content in their abstracts including accuracy of the facts, statements, citing resources, etc.

**Summary/Conclusion**

Systemic *C. albicans* infection induces highly dynamic reprogramming of HSCs, affecting both intrinsic differentiation bias and extrinsic signaling interactions. Longitudinal single-cell analysis reveals temporal coordination of lineage priming, signaling pathways, and gene programs that are not apparent from steady-state or early timepoint analyses and establishes a framework for studying long-term infection-associated alterations in stem cell state, including transcriptional programs suggestive of persistent inflammatory memory.

**Copyright Information:** (Online) ISSN: 2572-9241
©2026 The Author(s). HemaSphere is published by John Wiley & Sons Ltd on behalf of European Hematology Association. This is an open access Abstract Book distributed under the Attribution-NonCommercial-NoDerivs (CC BY-NC-ND), which allows third parties to download the articles and share them with others as long as they credit the author and the Abstract Book, but they cannot change the content in any way or use them commercially.
**Abstract Book Citations:** Authors, Title, HemaSphere, 2026;10:(S1):pages. The abstract book DOIs can be found on the "Author Information" page.
**Disclaimer:** Articles published in the journal HemaSphere exclusively reflect the opinions of the authors. The authors are responsible for all content in their abstracts including accuracy of the facts, statements, citing resources, etc.

### (PF1427) PREDICTION OF SF3B1 MUTATION FROM DIGITIZED BONE MARROW ASPIRATION SMEARS OF MYELODYSPLASTIC SYNDROMES USING SUPERVISED MACHINE LEARNING

**Topic:** 38. Novel technologies, techniques and digital analytical tools in hematology

Theerin Lanamtieng*[1], Oscar Brück[1], Mikko Purhonen[1], Stas Tatun[1]

[1]*University of Helsinki, Hematoscope Lab, Comprehensive Cancer Center, Department of Oncology, Department of Clinical Chemistry, HUS Diagnostic Center, Helsinki University Hospital, Helsinki, Finland;*

### Background

*SF3B1* mutations define a biologically and clinically distinct subset of myelodysplastic syndromes (MDS).To better understand the impact of SF3B1 on bone marrow cell composition and morphology beyond classical ring sideroblasts, we investigated whether *SF3B1* mutation status can be predicted directly from digitized bone marrow aspiration smears from MDS patients using image-derived single-cell features and supervised machine learning.

### Methods

A total of 568 MDS bone marrow aspiration smear samples from 103 *SF3B1*-mutated (18%) and 465 wild-type cases (82%) were included from Helsinki University Hospital, Finland. Smears were digitized at 100x magnification and analyzed using a foundation model to extract high-dimensional features at the single-cell level. For each sample, a median of 844 cells per sample were analyzed. The dataset was split into independent training (n = 397) and test (n = 171) sets with preserved class balance. An XGBoost classifier was trained on the training set and evaluated on the test set. Cell-level mutation probabilities were generated and aggregated using the mean probability of the top 20 most confident cells to derive sample-level predictions. Model performance was assessed using sensitivity, specificity, precision, accuracy, F1 score, area under the receiver operating characteristic curve (ROC-AUC), and area under the precision–recall curve (PR-AUC). Model explainability was explored using SHAP values to identify the most predictive morphological features.

### Results

In the independent test set, the model achieved a ROC-AUC of 0.84 and PR-AUC of 0.63. Performance metrics included sensitivity 0.86, specificity 0.76, precision 0.41, accuracy 0.78, and F1 score 0.86. SHAP analysis identified a limited set of highly informative image-derived features, reflecting distinct cellular morphology patterns associated with *SF3B1* mutation in adult MDS. The top 5 SHAP-ranked features were range of nuclear/cytoplastic (N/C) ratio of erythroblast, proportion of basophil, proportion of vacuolated eosinophil, range of neutrophil perimeter and proportion of vacuolated proerythroblast. The top 5 positive correlation features with SF3B1 were proportion of basophil, median of N/C ratio of erythroblast, proportion of erythroblast, proportion of erythropoietic cell, range of N/C ratio of lymphocyte.

### Summary/Conclusion

This study demonstrates that *SF3B1* mutation status can be predicted from digitized bone marrow aspiration smears from MDS patients using single-cell features and gradient boosting. The combination of predictive performance and interpretable morphology-based explanations highlights the potential of machine learning-assisted cytomorphology as a complementary tool for molecular stratification in MDS.

**Copyright Information:** (Online) ISSN: 2572-9241
©2026 The Author(s). HemaSphere is published by John Wiley & Sons Ltd on behalf of European Hematology Association. This is an open access Abstract Book distributed under the Attribution-NonCommercial-NoDerivs (CC BY-NC-ND), which allows third parties to download the articles and share them with others as long as they credit the author and the Abstract Book, but they cannot change the content in any way or use them commercially.
**Abstract Book Citations:** Authors, Title, HemaSphere, 2026;10:(S1):pages. The abstract book DOIs can be found on the "Author Information" page.
**Disclaimer:** Articles published in the journal HemaSphere exclusively reflect the opinions of the authors. The authors are responsible for all content in their abstracts including accuracy of the facts, statements, citing resources, etc.

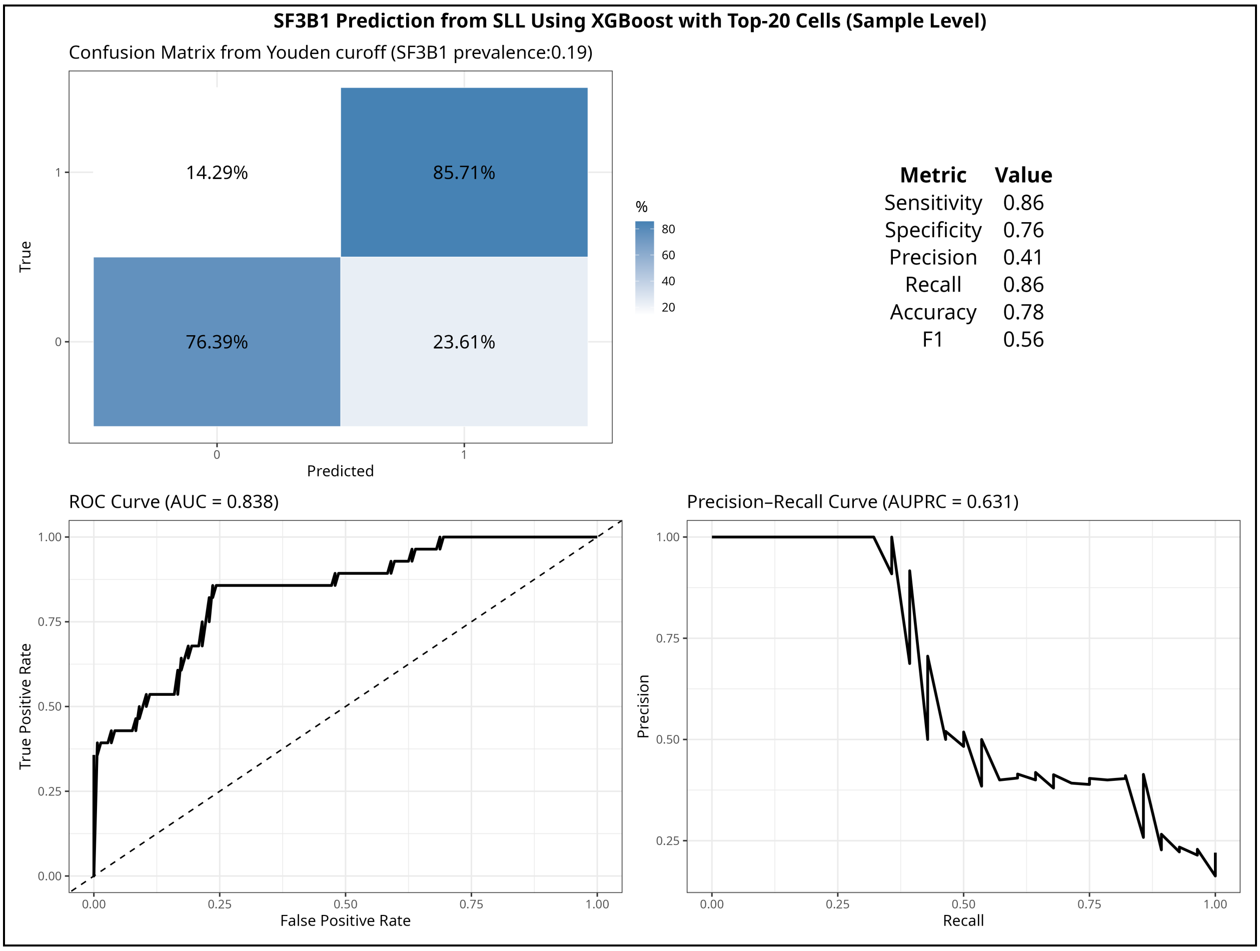

**Copyright Information:** (Online) ISSN: 2572-9241
©2026 The Author(s). HemaSphere is published by John Wiley & Sons Ltd on behalf of European Hematology Association. This is an open access Abstract Book distributed under the Attribution-NonCommercial-NoDerivs (CC BY-NC-ND), which allows third parties to download the articles and share them with others as long as they credit the author and the Abstract Book, but they cannot change the content in any way or use them commercially.
**Abstract Book Citations:** Authors, Title, HemaSphere, 2026;10:(S1):pages. The abstract book DOIs can be found on the "Author Information" page.
**Disclaimer:** Articles published in the journal HemaSphere exclusively reflect the opinions of the authors. The authors are responsible for all content in their abstracts including accuracy of the facts, statements, citing resources, etc.

### (PF1428) EVALUATION OF RAPID EPIGENOMICS PROFILING OF ACUTE LEUKEMIA BY NANOPORE SEQUENCING

**Topic:** 38. Novel technologies, techniques and digital analytical tools in hematology

Ka Ngai Lau*[1], Tsz Fung Wong[1], Sze Fai Yip[1]

[1]*Tuen Mun Hospital, Clinical Pathology, Hong Kong, Hong Kong;*

### Background

Risk stratification and clinical management for acute myeloid leukemia (AML) relies heavily on its genomic profiling. Conventional cytogenetic study is currently the cornerstone in AML genomic profiling but has limitations. Whole genome sequencing (WGS) has been shown to provide greater diagnostic yield and more efficient risk stratification. Oxford Nanopore Sequencing has the advantage of identifying structural variations (SVs) while providing copy number variations (CNVs), single nucleotide variants (SNVs), indels and methylation detection simultaneously. Recently, a methylation-based classification of acute leukemia by sparse sequencing data (MARLIN) was developed by the Dana-Farber Cancer Institute which provides rapid acute leukemia classification within 2 hours of nanopore sequencing.

### Aims

To evaluate the clinical utility of Nanopore Sequencing with adaptive sampling and methylation-based classification in providing rapid epigenomic profiling for AML patients.

### Methods

DNA was extracted from bone marrow samples from 11 patients with AML and a patient with mixed phenotype acute leukemia (MPAL), B/myeloid. Nanopore sequencing with adaptive sampling targeting 800 regions was performed with Promethion P2 Solo device using FLO-PRO114M flow cells. SVs, CNVs and myeloid panel SNVs and indels were detected by SVIM, EPI2ME and DeepVariant tools respectively. The results were integrated and compared with the cytogenetic and myeloid NGS results and targeted RNA-sequencing in selected cases at diagnosis. Modified basecalling with detection of 5mC and 5hmC bases at CpG sites was performed retrospectively by Dorado. Methylation calls at the 357,340 CpG sites from the MARLIN reference cohort were extracted using modkit. The MARLIN classifier with a prediction threshold of 80% was utilized to predict their respective methylation classes.

### Results

The fastest time from DNA extraction to reporting of genomic profile took 24 hours. Mean coverage at the target regions was 23.3X. All 5 cases with clinically significant chromosomal translocations, such as *CBFB*::*MYH11*, *RUNX1*::*RUNX1T1*, *NUP98*::*NSD1*, *MECOM* rearrangement and *KMT2A*-partial tandem duplication (PTD) were detected. All CNVs events (>5Mbs) detected were concordant. A clonal evolution event del(13)(q12q14) was missed. For myeloid panel SNVs and indels detection, sensitivity and specificity were 91.7% (22/24) and 100% respectively. The two variants not detected were *CEBPA* b-ZIP domain in-frame deletion variant and subclonal *PTPN11* missense variant. For *FLT3*-ITD, 2/4 variants were detected. Additional clinically significant findings were detected by nanopore sequencing in 3 cases, namely *NUP98*::*NSD1* fusion, Xp11.4 microdeletion and *KMT2A*-PTD. MARLIN successfully predicted methylation classes in 10/12 cases (Figure 1b). The predicted methylation classes in 6 cases were highly concordant to the genetic driver alterations. In case AML2, the predicted *CEBPA* methylation class complemented the missed *CEBPA* b-ZIP variant by nanopore sequencing. The *HOX* Group 4 was genetically heterogeneous and required further genomic profiling to identify any *HOX*-activating driver mutations such as *NUP98*::*NSD1.* MARLIN failed to predict methylation class in two cases, namely AML with atypical *MECOM* rearrangement and MAPL, B/myeloid with complex karyotype and *KMT2A* amplification.

**Copyright Information:** (Online) ISSN: 2572-9241
©2026 The Author(s). HemaSphere is published by John Wiley & Sons Ltd on behalf of European Hematology Association. This is an open access Abstract Book distributed under the Attribution-NonCommercial-NoDerivs (CC BY-NC-ND), which allows third parties to download the articles and share them with others as long as they credit the author and the Abstract Book, but they cannot change the content in any way or use them commercially.
**Abstract Book Citations:** Authors, Title, HemaSphere, 2026;10:(S1):pages. The abstract book DOIs can be found on the "Author Information" page.
**Disclaimer:** Articles published in the journal HemaSphere exclusively reflect the opinions of the authors. The authors are responsible for all content in their abstracts including accuracy of the facts, statements, citing resources, etc.

### Summary/Conclusion

Nanopore sequencing with adaptive sampling complemented with MARLIN methylation class prediction can rapidly provide comprehensive epigenomic profiling for AML patients.

**Figure 1**

**a) Workflow of rapid genomic profiling of acute leukaemia by Nanopore Sequencing** **b)**

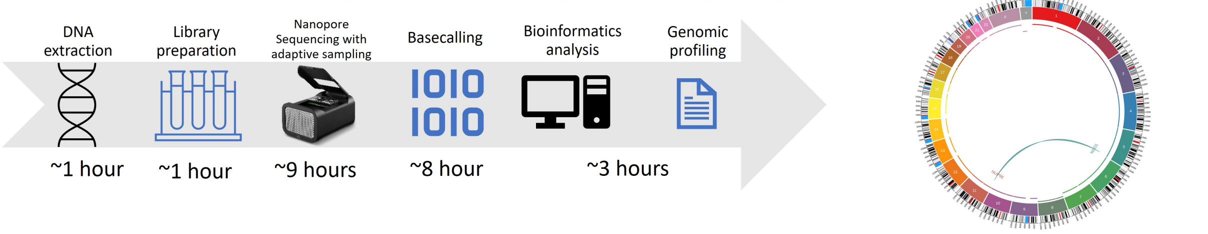

Genomic profile of case AML4 with *NUP98*::NSD1 and trisomy 8

**c) Methylation class predicted by MARLIN**

| Case | Lineage | Genetic driver alterations and myeloid gene mutations | MARLIN lineage | Methylation family | Methylation class | CpG coverage | Prediction score |
|---|---|---|---|---|---|---|---|
| AML1 | AML | *CBFB* ::*MYH11* | AML | *CBFB* ::*MYH11* | *CBFB* ::*MYH11* | 90221 | 99.8% |
| AML2 | AML | *CEBPA* bZIP inframe deletion* | AML | *CEBPA* | *CEBPA* | 54438 | 96.1% |
| AML3 | AML | *IDH2* | AML | *HOX NPM1* | *HOX* Grp 3 | 48067 | 99.4% |
| AML4 | AML | *NUP98* ::*NSD1,* trisomy 8 | AML | *HOX NPM1* | *HOX* Grp 4 | 105285 | 99.7% |
| AML5 | AML | *RUNX1* ::*RUNX1T1* | AML | *RUNX1* ::*RUNX1T1* | *RUNX1* ::*RUNX1T1* | 51619 | 99.9% |
| AML6 | AML | Atypical *MECOM* rearrangement: t(3;3)(p22-24;q26) | AML | *HOX NPM1* | *HOX* Grp 4 | 45593 | 78.0% |
| AML7 | AML | Trisomy 21, *RUNX1* , *BCORL1* , *TET2* , *SRSF2* | AML | Myelodysplasia | *TP53* Aneuploidy | 90421 | 91.7% |
| AML8 | AML | *NPM1* , *DNMT3A, PTPN11* * , *SUZ12* | AML | *HOX NPM1* | *HOX* Grp 1 | 58148 | 93.2% |
| AML9 | AML | Monosomy 7, trisomy 11, *KMT2A* -PTD, *DNMT3A* , *IDH2* , *NRAS* , *PTPN11* | AML | *HOX NPM1* | *HOX* Grp 4 | 55058 | 97.8% |
| AML10 | AML | *NUP98* ::*NSD1* | AML | *HOX NPM1* | *HOX* Grp 4 | 51431 | 94.7% |
| AML11 | AML | *IDH1* , *KMT2A* -PTD | AML | *HOX NPM1* | *HOX* Grp 3 | 52909 | 99.2% |
| MPAL12 | MPAL, B/myeloid | Complex karytype, *KMT2A* gene amplification, *TP53* | B-ALL | *KMT2A* -R B-ALL | *KMT2A* -R B-ALL | 113913 | 72.3% |

*Variants that were missed by nanopore sequencing.
Methylation classes in cyan color were highly concordant with the genetic drivers, i.e. *CBFB*::*MYH11*, *RUNX1*::*RUNX1T1*, *CEBBPA*, *NPM1*, *IDH1* and *IDH2*. *HOX* Grp 4 and *TP53* aneuploidy were genetically heterogeneous and require genomic profiling to identify the driver alterations. MARLIN failed to predict the methylation class in two cases, namely AML with atypical *MECOM* rearrangement and MPAL, B/myeloid with complex karyotype and *KMT2A* gene amplification.

**Copyright Information:** (Online) ISSN: 2572-9241
©2026 The Author(s). HemaSphere is published by John Wiley & Sons Ltd on behalf of European Hematology Association. This is an open access Abstract Book distributed under the Attribution-NonCommercial-NoDerivs (CC BY-NC-ND), which allows third parties to download the articles and share them with others as long as they credit the author and the Abstract Book, but they cannot change the content in any way or use them commercially.
**Abstract Book Citations:** Authors, Title, HemaSphere, 2026;10:(S1):pages. The abstract book DOIs can be found on the "Author Information" page.
**Disclaimer:** Articles published in the journal HemaSphere exclusively reflect the opinions of the authors. The authors are responsible for all content in their abstracts including accuracy of the facts, statements, citing resources, etc.

### (PF1430) DATA DRIVEN CLINICAL-GENOMIC UNSUPERVISED CLUSTERING IDENTIFIES PROGNOSTIC AML SUBTYPES

**Topic:** 38. Novel technologies, techniques and digital analytical tools in hematology

Hadil Zureigat*[1, 1], Tian Shulan[2], Oudai Alkabbani[1], Rong He[1], Dragan Jevremovic[1], Patricia Greipp[1], Antoine Saliba[1], Aasiya Matin[1], Mehrdad Hefazi[1], Abhishek Mangaonkar[1], Naseema Gangat[1], William Hogan[1], Hassan Alkhateeb[1], Cecilia Arana Yi[3], James M Foran[4], Mark Litzow[1], Mithun Shah[1], Kebede Begna[1], Mrinal Patnaik[1], Aref Al-Kali[1], Tariq Kewan[1]

*[1]Mayo Clinic, Rochester, United States of America; [2]Mayo Clinic, Quantitative Health Sciences, Rochester, United States of America; [3]Mayo Clinic, Scottsdale, United States of America; [4]Mayo Clinic, Jacksonville, United States of America;*

### Background

Somatic mutations (MTs) drive myeloid leukemogenesis in acute myeloid leukemia (AML), but current risk models over-emphasize select MT regardless of clone size or associated co-mutations (CoM).

### Aims

Apply machine learning clustering to comprehensive mutational profiles, incorporating variant allele frequency (VAF), to identify novel molecular/clinical AML subgroups.

### Methods

Unsupervised hierarchical clustering of molecular profiles was performed using Euclidean distance and Ward's minimum variance linkage method. Optimal cluster number was determined by the highest average silhouette score. Feature importance was ranked by the mean decrease in accuracy. Cox regression model was used to assess overall survival (OS) differences between clusters.

**Copyright Information:** (Online) ISSN: 2572-9241
©2026 The Author(s). HemaSphere is published by John Wiley & Sons Ltd on behalf of European Hematology Association. This is an open access Abstract Book distributed under the Attribution-NonCommercial-NoDerivs (CC BY-NC-ND), which allows third parties to download the articles and share them with others as long as they credit the author and the Abstract Book, but they cannot change the content in any way or use them commercially.
**Abstract Book Citations:** Authors, Title, HemaSphere, 2026;10:(S1):pages. The abstract book DOIs can be found on the "Author Information" page.
**Disclaimer:** Articles published in the journal HemaSphere exclusively reflect the opinions of the authors. The authors are responsible for all content in their abstracts including accuracy of the facts, statements, citing resources, etc.

**Results**

512 AML patients were identified with median age of 68.5 (IQR: 60-75) years, 60% were male, 67% had abnormal karyotypes, and 92% had somatic MTs, with 65% (n=331) having multiple MTs. Most common MTs were *TP53* (31%), *TET2* (19%) A*SXL1* (18%), *NPM1* (17%), *DNMT3A* (16%), *RUNX1* (16%), *SRSF2* (15%), and *IDH2* (14%). Intensive chemotherapy (IC) was given in 42%, lower intensity regimens (LIR; hypomethylating agent +/- venetoclax) in 47%, and 146 (29%) received hematopoietic stem cell transplantation (HSCT).

Six molecular clusters were identified (C1-C6); C4 had 43% (n= 219) and C6 only had 4% (n=20) of cases. MT VAF carried the most weight in cluster assignment. *TP53*, followed by *NPM1*, were the most important. C1 was most notable for *ASXL1* (81%) and *SRSF2* (66%) MTs and had higher *ASXL1* median VAF (mVAF) than other clusters (p=0.009). Most (85%) C2 cases had normal karyotypes and *NPM1* MT (92%), mVAF of which was higher (p=0.008). C3 was notable for *TET2* MT (97%) and *SRSF2* MT (*46%).* All (99%) C5 cases had complex karyotypes and *TP53* MT, mVAF of which was higher (p=0.0001). Most C6 (90%) had *DNMT3A* and (75%) *IDH2* MT, while 30% had *RUNX1* MT. Lastly, C4 harbored low frequency MT, including *CSF3R*, *NRAS*, *DDX41*, *KIT*, *U2AF1*, *SF3B1*, and *WT1* (Figure-1A). Importantly, some patients with the same MTs were assigned to different clusters based on associated CoM. E.g., most *IDH1* MT assigned to C4 (64%), while 24% were in C2 (*NPM1* CoM). *IDH2* MTs were distributed to C1 (*ASXL1* CoM), C2 (*NPM1* CoM), and C6 (*DNMT3A* CoM).

At diagnosis, C3 and C1 were older, C1 had higher white blood cell count (20 x109/L) and C5 had lower platelets (34 x109/L) ($p < 0.001$). All C5 cases, and most of C1 (84%), C3 (72%) and C6 (65%) were adverse risk by ELN 2022 risk.C2 and C4 included patients with intermediate (50% and 28%) or favorable (41% and 23%) risk. The median follow-up time was 51 (95%CI: 45-57) months (mo). Clusters had different OS whether treated with IC ($p <0.001$) or LIR ($p = 0.001$) (Figure-1B). Adjusted for age, type of treatment, and HSCT, C2 had better OS vs. C1 (hazard ratio [HR] 0.46, 95%CI 0.30,0.71), while C5 had worse OS vs. C1 (HR 1.88 95%CI 1.31, 2.69). Those with favorable or adverse ELN risk had different OS across clusters, while intermediate-risk patients assigned to different clusters had similar OS. In C2 and C4, those treated with IC had longer median OS compared to LIR, $p = 0.003$ and $p<0.001$, respectively.

**Summary/Conclusion**

Unsupervised clustering identified six clinically distinct AML subgroups with OS differences independent of ELN 2022 risk classification. Cluster assignment was driven not only by dominant mutations but also by VAF and coM patterns, highlighting molecular heterogeneity beyond single-gene models.

**Copyright Information:** (Online) ISSN: 2572-9241
©2026 The Author(s). HemaSphere is published by John Wiley & Sons Ltd on behalf of European Hematology Association. This is an open access Abstract Book distributed under the Attribution-NonCommercial-NoDerivs (CC BY-NC-ND), which allows third parties to download the articles and share them with others as long as they credit the author and the Abstract Book, but they cannot change the content in any way or use them commercially.
**Abstract Book Citations:** Authors, Title, HemaSphere, 2026;10:(S1):pages. The abstract book DOIs can be found on the "Author Information" page.
**Disclaimer:** Articles published in the journal HemaSphere exclusively reflect the opinions of the authors. The authors are responsible for all content in their abstracts including accuracy of the facts, statements, citing resources, etc.

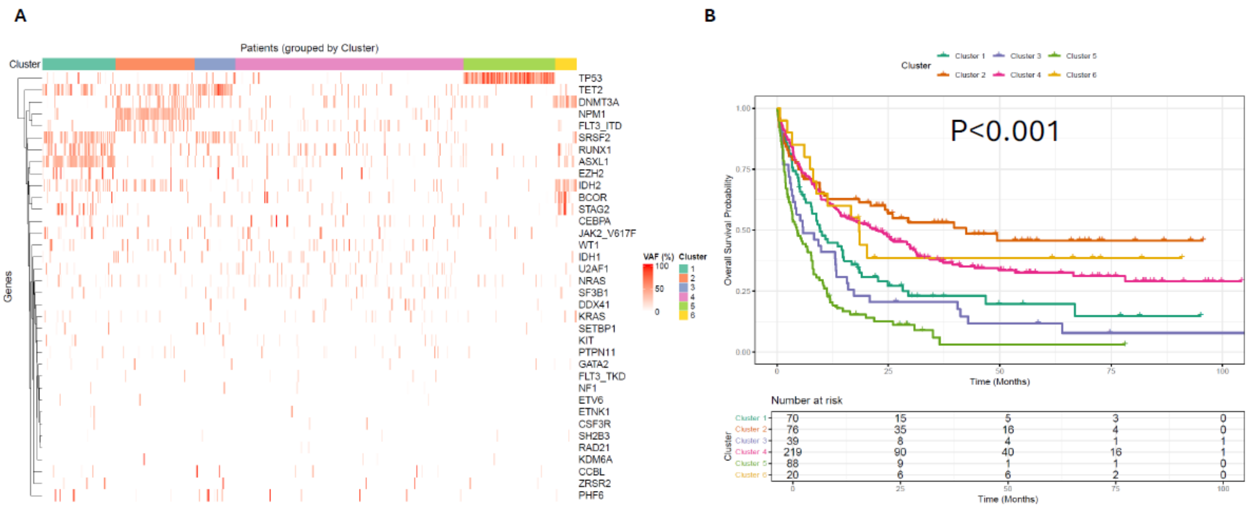

**Figure 1. A:** Molecular features based on mutations variant allele frequency (VAF) of AML patients using hierarchical clustering. **B.** Kaplan–Meier analysis of overall survival (OS) among patients with acute myeloid leukemia based on the identified clusters (C1-C6).

**Copyright Information:** (Online) ISSN: 2572-9241
©2026 The Author(s). HemaSphere is published by John Wiley & Sons Ltd on behalf of European Hematology Association. This is an open access Abstract Book distributed under the Attribution-NonCommercial-NoDerivs (CC BY-NC-ND), which allows third parties to download the articles and share them with others as long as they credit the author and the Abstract Book, but they cannot change the content in any way or use them commercially.
**Abstract Book Citations:** Authors, Title, HemaSphere, 2026;10:(S1):pages. The abstract book DOIs can be found on the “Author Information” page.
**Disclaimer:** Articles published in the journal HemaSphere exclusively reflect the opinions of the authors. The authors are responsible for all content in their abstracts including accuracy of the facts, statements, citing resources, etc.

### (PF1431) SAME AI, DIFFERENT DOCTORS: EXPERTISE DETERMINES RESPONSE TO TREATMENT RECOMMENDATIONS IN LYMPHOMA

**Topic:** 38. Novel technologies, techniques and digital analytical tools in hematology

Francesco Malaspina*[1], Alessandro Lucchesi[1], Pietro Rossi[1], Jessica Rosa[1], Eliana Valentina Liardo[1], Margherita Parolini[1], Marianna Norata[1], Michela Ceccolini[1], Sonia Ronconi[1], Selene Guerzoni[1], Giorgia Micucci[1], Benedetta Giannini[1], Sofia Cortesi[1], Monica Poggiaspalla[1], Gerardo Musuraca[1]

*[1]IRCCS Istituto Romagnolo per lo Studio dei Tumori (IRST) "Dino Amadori", Hematology, Meldola, Italy;*

### Background

Relapsed/refractory DLBCL management involves rapidly evolving therapies (CAR-T, bispecific antibodies, ADCs) often lacking comparative evidence. LLMs are explored as decision-support tools, yet their clinical acceptability and influence on physicians with varying expertise remain poorly characterized. While LLMs achieve high scores on medical benchmarks, their readiness for clinical deployment remains uncertain.

### Aims

To evaluate clinical acceptability of LLM-generated R/R DLBCL treatment recommendations, assess their influence on hematologist decision-making, and characterize patterns of unacceptable recommendations.

### Methods

We developed "Ariadne", a RAG-augmented LLM with curated R/R DLBCL treatment database and guidelines. Fifty validated cases were evaluated by paired Lymphoma Subspecialists (LS) and General Hematologists (GH) (100 evaluations). Experts provided treatment recommendations, reviewed LLM outputs, and rated acceptability. Rejection reasons were collected. Analysis used GEE with cluster-robust standard errors.

### Results

The LLM achieved 83% acceptability (95% CI: 74.5-89.1%). Concordance analysis revealed asymmetry: LS rejected 8 recommendations (16%) that GH accepted versus 1 (2%) reverse (McNemar p=0.039). Behavioral influence differed markedly: GH modified recommendations significantly more than LS (32% vs 6%, p=0.002), a 5-fold difference.

Rejection causes were divided in two groups based on their potential for harm, with sub-optimal or too toxic treatment choices considered as high risk and regulatory or other minor concerns rated as low risk. In total, 17 rejection evaluations occurred across 13 unique cases (4 rejected by both experts, 9 by one expert only). Among these 13 rejected cases, 10 (77%) involved harm potential (suboptimal treatment or toxicity concerns). Notably, among 8 cases where LS rejected but GH accepted, 5 (63%) also involved harm potential. GH changed treatment choiche to align to the LLM in 4 asymmetric cases, with 2 (50%) deemed harmful.

A specific error emerged in 2 cases: when narratives contained both time-from-diagnosis and time-from-treatment-end metrics (one >12mo, one <12mo), the LLM selected the clinically irrelevant interval and thus proposed a sub-optimal treatment (ASCT instead of CAR-T). Stress-testing revealed inconsistencies, with specific stylistic choices that favoured misinterpretation.

**Copyright Information:** (Online) ISSN: 2572-9241
©2026 The Author(s). HemaSphere is published by John Wiley & Sons Ltd on behalf of European Hematology Association. This is an open access Abstract Book distributed under the Attribution-NonCommercial-NoDerivs (CC BY-NC-ND), which allows third parties to download the articles and share them with others as long as they credit the author and the Abstract Book, but they cannot change the content in any way or use them commercially.
**Abstract Book Citations:** Authors, Title, HemaSphere, 2026;10:(S1):pages. The abstract book DOIs can be found on the "Author Information" page.
**Disclaimer:** Articles published in the journal HemaSphere exclusively reflect the opinions of the authors. The authors are responsible for all content in their abstracts including accuracy of the facts, statements, citing resources, etc.

**Summary/Conclusion**

RAG-enhanced LLMs achieved 83% acceptability in R/R DLBCL recommendations, comparable to their performance on health benchmarks. However, important limitations challenge clinical deployment readiness.

The 5-fold differential in influence (GH modifying 32% vs LS 6%) has critical implications. The asymmetric concordance pattern suggests subspecialist expertise provides important safeguards. Importantly, 77% of rejected cases involved clinically meaningful deficiencies rather than regulatory or minor preferences. When the LLM fails, it predominantly fails in potentially harmful ways. The temporal information processing error, though rare, represents a qualitatively different failure than knowledge gaps and may not be addressable through knowledge base expansion.

Despite high acceptability comparable to benchmarks, the combination of clinically meaningful deficiencies in most rejected cases (77%), differential influence creating vulnerability in generalists, and error patterns requiring architectural solutions indicates current technology requires substantial safeguards before deployment in high-stakes decisions.

**Copyright Information:** (Online) ISSN: 2572-9241
©2026 The Author(s). HemaSphere is published by John Wiley & Sons Ltd on behalf of European Hematology Association. This is an open access Abstract Book distributed under the Attribution-NonCommercial-NoDerivs (CC BY-NC-ND), which allows third parties to download the articles and share them with others as long as they credit the author and the Abstract Book, but they cannot change the content in any way or use them commercially.
**Abstract Book Citations:** Authors, Title, HemaSphere, 2026;10:(S1):pages. The abstract book DOIs can be found on the "Author Information" page.
**Disclaimer:** Articles published in the journal HemaSphere exclusively reflect the opinions of the authors. The authors are responsible for all content in their abstracts including accuracy of the facts, statements, citing resources, etc.

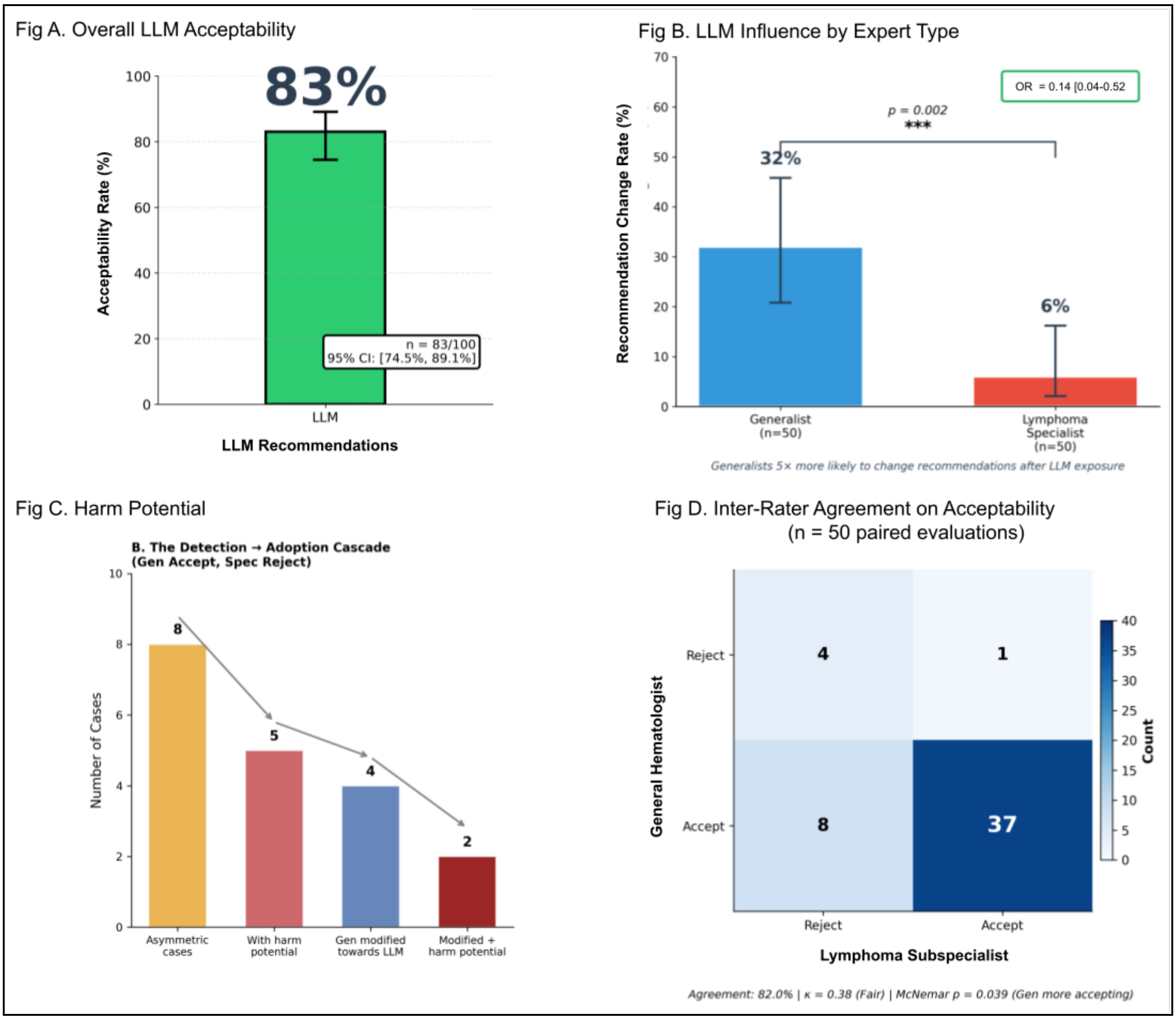

A) Overall acceptability of LLM proposed treatments by human experts. B) Differential inlfuence of LLM recommendations on human experts stratified by experience in lymphoma treatment. C) Harm potential of LLM recommendations accepted by general hematologist but rejected by lymphoma subspecialists. D) Inter-Rater agreement matrix of acceptability between expert types

**Copyright Information:** (Online) ISSN: 2572-9241
©2026 The Author(s). HemaSphere is published by John Wiley & Sons Ltd on behalf of European Hematology Association. This is an open access Abstract Book distributed under the Attribution-NonCommercial-NoDerivs (CC BY-NC-ND), which allows third parties to download the articles and share them with others as long as they credit the author and the Abstract Book, but they cannot change the content in any way or use them commercially.
**Abstract Book Citations:** Authors, Title, HemaSphere, 2026;10:(S1):pages. The abstract book DOIs can be found on the "Author Information" page.
**Disclaimer:** Articles published in the journal HemaSphere exclusively reflect the opinions of the authors. The authors are responsible for all content in their abstracts including accuracy of the facts, statements, citing resources, etc.

### (PF1432) AN INTEGRATED COMPUTATIONAL AND SINGLE-CELL SCREENING WORKFLOW FOR RAPID GENERATION OF NOVEL CAR CONSTRUCTS

**Topic:** 38. Novel technologies, techniques and digital analytical tools in hematology

Alexandra Mayr*[1], Monika Hexel[1], Alexandra Tuch[1], Katharina von Werthern[1], Daniel Gievers[1], Sylvia Martin[1], Dirk Jäger[1], Patrick Schmidt[1]

*[1]NCT Heidelberg, Heidelberg, Germany;*

### Background

The establishment of CAR T-cell therapy in haematological diseases has transformed clinical practice. Effective CAR T-cell therapy requires the generation of potent and selective binders to specific antigens. Identification of these binders is achieved by screening of large libraries of CAR-constructs.

### Aims

Our workflow demonstrates the application of specific software tools and the use of artificial intelligence to generate novel CAR constructs for a given antigen within short time.

### Methods

As a model target structure for the in-silico design of new CARs, we used a previously in-vitro confirmed target of CAR T-cell therapy in cardiovascular disease. A living CAR T-cell library was screened against this specific target. Using single cell optofluidics, the library enabled us to identify CAR T-cells which specifically bind to the target. Analysis of these specific single CAR T-cells gave precise sequences of highly represented CARs on the cell surface. Instead of testing all read CAR sequences in vitro, we used software such as IMGT, VIDJIL and CircosPlot to modulate the CAR structure. We gained detailed insights into the respective CAR structure such as frequency of pairing of specific heavy and light chains this way. To increase reliability of data analysis, we aligned the given data of the different tools and included those which were compatible. In a further step, in-silico generated CAR sequences were run by the AI tool “Alpha Fold 3”. This software allowed us to make statements about the protein structure and to predict binding probabilities of these CAR constructs to the target.

### Results

In-silico generated CARs that showed the highest binding probability were cloned into plasmids for validation. The CAR expression of newly generated CAR T-cells was analyzed and compared between various CARs.

### Summary/Conclusion

Our approach of using computational and experimental tools enables fast and efficient screening for novel CAR constructs in short time. Detailed insight into the DNA sequences of the CARs allows a glimpse on the huge impact minor changes can have on binding efficiency. This may lead to more precise and hence more specific and more often successful CAR T-cell therapy beyond its original hematological application in fields such as cardiovascular or autoimmune diseases. This emphasizes the broad application and usage our technology offers for future CAR T-cell therapy, not only for hematological diseases but also other indications. We conclude that usage of software and AI tools will further enhance the application of CAR T-cell therapy for the benefit of patients.

**Copyright Information:** (Online) ISSN: 2572-9241
©2026 The Author(s). HemaSphere is published by John Wiley & Sons Ltd on behalf of European Hematology Association. This is an open access Abstract Book distributed under the Attribution-NonCommercial-NoDerivs (CC BY-NC-ND), which allows third parties to download the articles and share them with others as long as they credit the author and the Abstract Book, but they cannot change the content in any way or use them commercially.
**Abstract Book Citations:** Authors, Title, HemaSphere, 2026;10:(S1):pages. The abstract book DOIs can be found on the “Author Information” page.
**Disclaimer:** Articles published in the journal HemaSphere exclusively reflect the opinions of the authors. The authors are responsible for all content in their abstracts including accuracy of the facts, statements, citing resources, etc.

### (PF1433) HEMATRACK ALL: UMI-CORRECTED NANOPORE SEQUENCING FOR HIGHLY SENSITIVE MEASURABLE RESIDUAL DISEASE (MRD) MONITORING IN ALL

**Topic:** 38. Novel technologies, techniques and digital analytical tools in hematology

Claudia Pahlke*[1], Vineeth Surendranath[1], Madlen Pahlke[1], Thomas Lingl[1], Dingquan Yu[1], Tom Kasper[1], Pritha Dasgupta[2], Shekhar Krishnan[3], Doreen Tolksdorf[1], Gerhard Schoefl[1], Anja Klussmeier[1], Cornelia Eckert[4], Vaskar Saha[5], Vinzenz Lange[1], Ulf-Peter Guenther[1]

*[1]DKMS Life Science Lab, Dresden, Germany; [2]Tata Translational Cancer Research Centre, Kolkata, India; [3]DKMS Life Science Lab India, Kolkata, India; [4]Charité - Universitätsmedizin Berlin, Department of Pediatric Oncology/Hematology, Berlin, Germany; [5]The University of Manchester, Division of Cancer Sciences, Manchester, United Kingdom;*

### Background

In acute lymphoblastic leukemia (ALL), measurable residual disease (MRD) is a key parameter for risk-adapted stratification and is regarded as the most robust prognostic indicator of treatment response and overall survival. However, conventional MRD detection methods such as flow cytometry and real-time quantitative PCR (RQ-PCR) are constrained by limitations in standardization, sensitivity, and scalability, although RQ-PCR remains the clinical gold standard. Next-generation sequencing (NGS) of immunoglobulin (IG) and T-cell receptor (TR) rearrangements can address several of these limitations. While there have been efforts to establish NGS-based MRD assessment on Illumina by the EuroMRD consortium and others, the comparatively high raw read error rate of Oxford Nanopore Technologies (ONT) has so far prevented its use for highly sensitive MRD detection.

### Aims

We aimed to develop and validate a nanopore-based assay that employs UMI-mediated error correction to enable highly sensitive, quantitative, and cost-efficient MRD monitoring for ALL based on IG/TR rearrangements, using Illumina-based NGS as the NGS reference and RQ-PCR as clinical gold standard.

### Methods

HemaTrack ALL combines UMI-tagging of immunoglobulin (IGH, IGK) and T-cell receptor genes (TRD, TRG, TRB) in a multiplexed format with an automated analysis pipeline (QuaSIR) optimized for ONT data. UMI families are used to build high-fidelity consensus sequences, correcting platform-specific sequencing errors and enabling direct molecule-level quantification of leukemic clonotypes. Analytical performance of the assay was evaluated in dilution series of ALL cell lines in matched Illumina–ONT datasets generated from the same UMI-tagged libraries. Subsequently, clinical performance was evaluated in a clinical cohort of biobanked ALL samples with prior RQ-PCR-based MRD assessment.

### Results

In dilution studies, HemaTrack ALL achieved highly linear and precise quantification of ALL cell line clonotypes down to the lowest tested dilution of 1 in 10,000 cells ($10^{-4}$). UMI-based consensus building efficiently rescued ONT-specific base-calling errors, resulting in complete sequence-level concordance with matched Illumina libraries across all leukemic clonotypes. In a first clinical cohort, NGS-based MRD quantification showed concordance with RQ-PCR in 17 of 19 MRD measurements. The two discrepant cases involved markers that were either potentially non-specific according to HemaTrack ALL but positive by RQ-PCR, or negative by HemaTrack ALL while being detectable only below the RQ-PCR limit of quantification. Across patients, the markedly increased number of informative rearrangements per case is expected to improve robustness of longitudinal MRD tracking.

**Copyright Information:** (Online) ISSN: 2572-9241
©2026 The Author(s). HemaSphere is published by John Wiley & Sons Ltd on behalf of European Hematology Association. This is an open access Abstract Book distributed under the Attribution-NonCommercial-NoDerivs (CC BY-NC-ND), which allows third parties to download the articles and share them with others as long as they credit the author and the Abstract Book, but they cannot change the content in any way or use them commercially.
**Abstract Book Citations:** Authors, Title, HemaSphere, 2026;10:(S1):pages. The abstract book DOIs can be found on the "Author Information" page.
**Disclaimer:** Articles published in the journal HemaSphere exclusively reflect the opinions of the authors. The authors are responsible for all content in their abstracts including accuracy of the facts, statements, citing resources, etc.

**Summary/Conclusion**

HemaTrack ALL demonstrates that UMI-corrected nanopore sequencing can serve as a robust end-to-end solution for accurate MRD monitoring in ALL. Using Illumina-based NGS as an established reference platform and RQ-PCR as clinical gold standard, the assay achieves high concordance across technologies while exploiting the specific advantages of ONT sequencing, such as higher flexibility, shorter turnaround times, and reduced costs. A prospective multicenter study has recently been initiated to further explore the clinical utility of this nanopore-based MRD assay.

**Copyright Information:** (Online) ISSN: 2572-9241
©2026 The Author(s). HemaSphere is published by John Wiley & Sons Ltd on behalf of European Hematology Association. This is an open access Abstract Book distributed under the Attribution-NonCommercial-NoDerivs (CC BY-NC-ND), which allows third parties to download the articles and share them with others as long as they credit the author and the Abstract Book, but they cannot change the content in any way or use them commercially.
**Abstract Book Citations:** Authors, Title, HemaSphere, 2026;10:(S1):pages. The abstract book DOIs can be found on the "Author Information" page.
**Disclaimer:** Articles published in the journal HemaSphere exclusively reflect the opinions of the authors. The authors are responsible for all content in their abstracts including accuracy of the facts, statements, citing resources, etc.

### (PF1434) FLUX2TARGET: TOOLKIT FOR EXPLORATION OF METABOLIC ACTIVITY PATTERNS AT BULK AND SINGLE CELL LEVEL IN LEUKEMIA

**Topic:** 38. Novel technologies, techniques and digital analytical tools in hematology

Buddika Jayasingha*[1], Konstantin Ivanov[1], Arghavan Alizadeh Fekejvar[1], Valtteri Puttonen[1], Viivi Väisänen[1], Dominique Sternadt[2], Sui Huang[1], Jan Jacob Schuringa[2], Merja Heinäniemi[1]

*[1]University of Eastern Finland, Institute of Biomedicine, Kuopio, Finland;[2]University Medical center Groningen, University of Groningen, Groningen, The Netherlands;*

### Background

Leukemia is life threating malignancy, shaped not only by genetic alterations but also by metabolic reprogramming that affects growth and therapy response. High-resolution single-cell analyses of gene expression in combination with metabolic flux estimation can provide systems-level view of functional rewiring, enabling identification of novel therapeutic vulnerabilities. This approach holds promise to resolve intra-patient variability and target cell state evolution and adaptation during treatment.

### Aims

We set out to characterize metabolic vulnerabilities in leukemic cell states based on flux profiles inferred from gene expression data. Genome-scale models include thousands of reactions while genomics studies involve comparisons across different cell states and donors, posing a challenge. We developed a computational framework that facilitates such analysis through interactive visualization and knowledge graph-guided drug target prediction.

### Methods

We present a Python package and a web interface to enable interactive analysis of cellular metabolism based on different flux estimation tools, knowledge graphs and literature retrieval with AI-agents. To benchmark the analysis, we used MetaFlux for bulk transcriptome data from an IDH2$^{R140Q}$ mutant vs WT AML cell line models (TF1) with available cellular lipidome and medium metabolome profiles. The conversion of α-KG and NADPH into 2-HG and $NADP^{+}$ was included to the model. To demonstrate application in large datasets, we analysed with MetaFlux and scFEA two datasets: public drug screen profiles from blood cells and CITE-seq data that we generated from primary patient samples representing different leukemia types (n=42), including AML (n=13) bone marrow (BM) profiles. Healthy reference data (n=8) was included to analyze disease-specific vulnerabilities.

**Copyright Information:** (Online) ISSN: 2572-9241
©2026 The Author(s). HemaSphere is published by John Wiley & Sons Ltd on behalf of European Hematology Association. This is an open access Abstract Book distributed under the Attribution-NonCommercial-NoDerivs (CC BY-NC-ND), which allows third parties to download the articles and share them with others as long as they credit the author and the Abstract Book, but they cannot change the content in any way or use them commercially.
**Abstract Book Citations:** Authors, Title, HemaSphere, 2026;10:(S1):pages. The abstract book DOIs can be found on the “Author Information” page.
**Disclaimer:** Articles published in the journal HemaSphere exclusively reflect the opinions of the authors. The authors are responsible for all content in their abstracts including accuracy of the facts, statements, citing resources, etc.

**Results**

In IDH2$^{R140Q}$ mutant AML cells, reactions involved in fatty acid oxidation (FAO) displayed elevated flux, consistent with lipidomic and metabolomic data indicating alterations in long-chain acylcarnitines (CAR) and transcriptome data indicating higher expression levels of the fatty acid importer CD36 and enzymes related to FAO in *IDH2*$^{R140Q}$ cells. In addition, pentose phosphate and glutathione pathways exhibited higher flux, likely to compensate for NADPH depletion and maintain redox balance. This activity profile suggests reduced glycolysis dependence, consistent with experimental results.

Matching of the primary AML samples against healthy BM reference data indicated that majority of cases (11 of 13) harbored leukemic cells clusters corresponding to multiple phenotypes, most prevalently stem/progenitor- and monocytes-like cells. We sought to identify the metabolic differences relevant for the primitive to differentiated state transitions and found 480 significant flux changes (adj. pval <0.05). Knowledge graphs and large language models enabled linking of these metabolic alterations to potential therapeutic agents, exemplified by pentose phosphate pathway reactions catalyzed by G6PD were connected to methotrexate, while creatine biosynthesis reactions catalyzed by GATM and GAMT enzymes were associated with novel drugs tested in solid cancers. Integration of healthy bone marrow data further allows prioritizing targets with AML-specific expression.

**Summary/Conclusion**

We present a versatile toolkit for analysis of metabolic adaptations which can be used to discover new drug targets in leukemias and other malignancies.

**Copyright Information:** (Online) ISSN: 2572-9241
©2026 The Author(s). HemaSphere is published by John Wiley & Sons Ltd on behalf of European Hematology Association. This is an open access Abstract Book distributed under the Attribution-NonCommercial-NoDerivs (CC BY-NC-ND), which allows third parties to download the articles and share them with others as long as they credit the author and the Abstract Book, but they cannot change the content in any way or use them commercially.
**Abstract Book Citations:** Authors, Title, HemaSphere, 2026;10:(S1):pages. The abstract book DOIs can be found on the “Author Information” page.
**Disclaimer:** Articles published in the journal HemaSphere exclusively reflect the opinions of the authors. The authors are responsible for all content in their abstracts including accuracy of the facts, statements, citing resources, etc.

### (PS1435) MENINGEAL STROMAL NICHE REWIRES THYMIDYLATE SYNTHESIS AND THYMIDINE SALVAGE IN METHOTREXATE-RESISTANT CENTRAL NERVOUS SYSTEM ACUTE LYMPHOBLASTIC LEUKEMIA

**Topic:** 01. Acute lymphoblastic leukemia - Biology & translational research

Shirley Lam*[1], Engy Shokry[2], Yi-Hsia Liu[1], Tom Gilbey[2], Katerina Himonas[3], Vignir Helgason[1], Saverio Tardito[4], David Sumpton[2], Chris Halsey[1]

*[1]School of Cancer Sciences, University of Glasgow, United Kingdom;[2]Cancer Research UK Scotland Institute, Glasgow, United Kingdom; [3]The Cyprus Institute of Neurology & Genetics, Nicosia, Cyprus;[4]Medical University of Vienna, Vienna, Austria;*

### Background

Chemo-resistant acute lymphoblastic leukemia (ALL) cells can persist within the leptomeningeal niche through attachment to the meningeal stromal cells. In this compartment, blasts are bathed in nutrient-poor cerebrospinal fluid (CSF) and exposed to CNS-directed methotrexate (MTX). While MTX can induce cancer cell-intrinsic metabolic changes, how meningeal stroma and CSF environment jointly alter leukemic metabolism and drive resistance to treatment remains poorly defined.

### Aims

To investigate whether meningeal stromal contact enables ALL cells cultured under physiological CSF-like conditions to reprogram MTX-perturbed metabolic pathways and thereby tolerate MTX-induced cytotoxicity.

### Methods

We established co-cultures of ALL cell lines with primary meningeal stromal cells in custom CSF-like media. Drug responses to MTX and cytarabine were quantified using dose-response cell viability assays. MTX-resistant CD19+ ALL cells were isolated from stromal cells by flow cytometric sorting. We performed LC-MS/MS metabolomics, Western blotting, shRNA-mediated knockdown of TYMS or TK1, EdU labelling with flow-cytometric analysis, thymidine rescue experiments, and nucleoside transporter inhibition.

### Results

Meningeal stromal-attached ALL cells (N = 3 cell lines) were significantly protected from MTX, with $IC_{50}$ values ≥ 3.3-fold higher than matched mono-cultured cells. This protective effect was not observed with cytarabine, suggesting an MTX-selective tolerance mechanism. Under low-dose MTX (0.04μM) versus DMSO, the EdU+ S-phase fraction among viable cells increased similarly in adherent and mono-cultured conditions (2.3 vs 2.6-fold; $p < 0.01$). However, only adherent ALL cells showed elevated protein levels of MTR-perturbed folate/thymidylate synthesis, dihydrofolate reductase (DHFR) (13.0-fold; $p < 0.001$) and thymidylate synthase (TYMS) (2.4-fold; $p < 0.01$), in response to low-dose MTX. In parallel, adherent cells exhibited 60.3-fold higher dTMP:dUMP ratio than non-adherent cells ($p < 0.05$), indicating active thymidylate synthesis mediated by stromal cells under MTX exposure. Disrupting *de novo* thymidylate synthesis via TYMS knockdown in ALL cells reduced stromal-mediated MTX tolerance (2.2-fold decrease in $IC_{50}$). This is phenocopied by exogenous thymidine, which fully rescued ALL cell viability across 0.04μM – 10μM MTX. Stromal contact also produced a trend toward increased thymidine kinase 1 (TK1) protein in MTX-resistant ALL cells (3.9-fold; $p = 0.170$; n = 3), suggesting activation of thymidine salvage. Consistent with this, TK1 knockdown in adherent cells restored MTX sensitivity (1.5-fold decrease in $IC_{50}$) compared to scrambled shRNA control. Finally, combining MTX with a nucleoside transporter inhibitor NBMPR (0.5μM) markedly reduced survival of MTX-resistant ALL cells (N = 3 cell lines), decreasing mean residual viability by 84.6%.

**Copyright Information:** (Online) ISSN: 2572-9241
©2026 The Author(s). HemaSphere is published by John Wiley & Sons Ltd on behalf of European Hematology Association. This is an open access Abstract Book distributed under the Attribution-NonCommercial-NoDerivs (CC BY-NC-ND), which allows third parties to download the articles and share them with others as long as they credit the author and the Abstract Book, but they cannot change the content in any way or use them commercially.
**Abstract Book Citations:** Authors, Title, HemaSphere, 2026;10:(S1):pages. The abstract book DOIs can be found on the "Author Information" page.
**Disclaimer:** Articles published in the journal HemaSphere exclusively reflect the opinions of the authors. The authors are responsible for all content in their abstracts including accuracy of the facts, statements, citing resources, etc.

**Summary/Conclusion**

Under CSF-like conditions, meningeal stromal contact primes ALL cells to withstand MTX by promoting *de novo* thymidylate synthesis and thymidine salvage. Uptake of scarce environmental nucleosides in CSF may become conditionally critical for ALL cells under MTX-imposed nucleotide stress. Ongoing work is focused on identifying the environmental source of thymidine and assessing whether CNS-penetrant inhibitors of nucleoside uptake can potentiate MTX efficacy in preclinical CNS-ALL models.

**Copyright Information:** (Online) ISSN: 2572-9241
©2026 The Author(s). HemaSphere is published by John Wiley & Sons Ltd on behalf of European Hematology Association. This is an open access Abstract Book distributed under the Attribution-NonCommercial-NoDerivs (CC BY-NC-ND), which allows third parties to download the articles and share them with others as long as they credit the author and the Abstract Book, but they cannot change the content in any way or use them commercially.
**Abstract Book Citations:** Authors, Title, HemaSphere, 2026;10:(S1):pages. The abstract book DOIs can be found on the "Author Information" page.
**Disclaimer:** Articles published in the journal HemaSphere exclusively reflect the opinions of the authors. The authors are responsible for all content in their abstracts including accuracy of the facts, statements, citing resources, etc.

### (PS1436) A DIETARY PAN-AMINO ACID DEPRIVATION SCREEN IN VIVO REVEALS A CRITICAL ROLE FOR HISTIDINE IN T-ALL

**Topic:** 01. Acute lymphoblastic leukemia - Biology & translational research

Komal Mandleywala*[1], Simona Ulrich[2], Victoria Da Silva Diz[1], Christopher Thai[1], Raphael Morscher[2], Daniel Herranz[1]

*[1]Rutgers Cancer Institute, Pharmacology, New Brunswick, United States of America;[2]University of Zurich, Pediatric Cancer Metabolism, Zurich, Switzerland;*

### Background

T-cell acute lymphoblastic leukemia (T-ALL) is an aggressive hematological malignancy accounting for 10–15% of pediatric and 25% of adult ALL cases. Despite improved outcomes with intensified chemotherapy, relapse and refractory disease remain major challenges, underscoring the need for novel therapeutic strategies. Moreover, the longstanding clinical success of targeted metabolic therapies highlights the importance of further exploring metabolic vulnerabilities in leukemia. Additionally, dietary modulation of systemic metabolism has shown promising antitumor effects in preclinical models and is under clinical evaluation; however, most studies have examined single interventions and not in primary orthotopic settings. We therefore hypothesized that altering dietary nutrient composition to modulate T-ALL metabolism could elicit therapeutic effects. Using an orthotopic, immunocompetent, high-throughput T-ALL mouse model, we performed an unbiased *in vivo* screen removing each of the 20 amino acids from the diet. Unexpectedly, histidine restriction (HR) produced the strongest antileukemic effect.

### Aims

The overall aim of this study was to identify novel metabolic vulnerabilities in T-ALL through a comprehensive, unbiased pan–amino acid dropout screen in an orthotopic, immunocompetent NOTCH1-driven T-ALL mouse model. Upon identifying histidine as a critical requirement for leukemia progression, we sought to elucidate the molecular mechanisms underlying the antileukemic effects of histidine restriction using integrated multi-omic approaches, including transcriptomics, proteomics, and ribosome profiling.

### Methods

We conducted a comprehensive pan–amino acid deprivation screen in an orthotopic, immunocompetent, high-throughput mouse model of primary NOTCH1-driven T-ALL. Leukemic mice were fed diets lacking each of the 20 proteinogenic amino acids. Survival, leukemia burden, and tolerability were assessed. HR was further evaluated using graded diets and validated in immunodeficient mice engrafted with human T-ALL patient-derived xenografts (PDXs) harboring distinct driver mutations. Mechanistic studies included transcriptomics, proteomics, and ribosome profiling. Dietary cholesterol rescue experiments were performed to functionally interrogate downstream effects.

### Results

Among all amino acid deprivations, histidine restriction uniquely and robustly extended survival of leukemic mice in a dose-dependent manner while remaining comparatively well tolerated. Antileukemic effects were confirmed in human T-ALL PDX models, indicating tumor cell–intrinsic vulnerability. Mechanistically, HR induced ribosome stalling and activation of the integrated stress response kinase GCN2. Integrated multiomic profiling revealed consistent suppression of cholesterol biosynthesis at transcriptional, translational, and proteomic levels. Critically, dietary cholesterol supplementation partially rescued the antileukemic effects of HR *in vivo*, mechanistically confirming that cholesterol pathway suppression mediates its therapeutic effects.

**Copyright Information:** (Online) ISSN: 2572-9241
©2026 The Author(s). HemaSphere is published by John Wiley & Sons Ltd on behalf of European Hematology Association. This is an open access Abstract Book distributed under the Attribution-NonCommercial-NoDerivs (CC BY-NC-ND), which allows third parties to download the articles and share them with others as long as they credit the author and the Abstract Book, but they cannot change the content in any way or use them commercially.
**Abstract Book Citations:** Authors, Title, HemaSphere, 2026;10:(S1):pages. The abstract book DOIs can be found on the "Author Information" page.
**Disclaimer:** Articles published in the journal HemaSphere exclusively reflect the opinions of the authors. The authors are responsible for all content in their abstracts including accuracy of the facts, statements, citing resources, etc.

**Summary/Conclusion**

Our unbiased *in vivo* amino acid deprivation screen identifies histidine as a previously unrecognized metabolic vulnerability in T-ALL. Dietary histidine restriction exerts dose-dependent antileukemic effects by coupling ribosome stalling and GCN2 activation to suppression of cholesterol biosynthesis. These findings establish a novel regulatory axis linking amino acid availability, translational control, and lipid metabolism in leukemia.

**Copyright Information:** (Online) ISSN: 2572-9241
©2026 The Author(s). HemaSphere is published by John Wiley & Sons Ltd on behalf of European Hematology Association. This is an open access Abstract Book distributed under the Attribution-NonCommercial-NoDerivs (CC BY-NC-ND), which allows third parties to download the articles and share them with others as long as they credit the author and the Abstract Book, but they cannot change the content in any way or use them commercially.
**Abstract Book Citations:** Authors, Title, HemaSphere, 2026;10:(S1):pages. The abstract book DOIs can be found on the "Author Information" page.
**Disclaimer:** Articles published in the journal HemaSphere exclusively reflect the opinions of the authors. The authors are responsible for all content in their abstracts including accuracy of the facts, statements, citing resources, etc.

### (PS1437) PH+B-ALL IS DEFINED BY BCR::ABL1-INDUCED ENHANCER REPROGRAMMING AND HYPERSENSITIVITY TO ENHANCER-TARGETING DRUGS

**Topic:** 01. Acute lymphoblastic leukemia - Biology & translational research

Han Leng Ng[1], Trudy Glaser[1], Jintao Zhu[1], Valeriya Malysheva[2], Mikhail Spivakov[2], Nicholas T Crump[1], Andrew Innes[1], Richard Burt[1], Niklas Feldhahn*[1]

*[1]Imperial College London, Department for Immunology & Inflammation, Centre for Haematology, LONDON, United Kingdom;*
*[2]Imperial College London, MRC London Institute of Medical Sciences, LONDON, United Kingdom;*

### Background

B-lineage acute-lymphoblastic leukemia (B-ALL) subtypes are defined and driven by distinct initiating genetic lesions and disease-defining transcriptional programs. Both are often interconnected, as most initiating genetic lesions in B-ALL deregulate transcription factor (TF)-encoding genes. As TFs largely mediate their function through enhancers, cancer initiation and progression are frequently attributed to aberrant enhancer function. Given the importance of enhancer deregulation in cancer, enhancer-targeting drugs have been explored over the last two decades as a therapeutic strategy for cancer treatment, with drug candidates being currently in trials for several cancers. However, for cancers not driven by TF-related lesions, it is less clear how their transcriptional programs are established, if it involves enhancer-deregulation, and if they are consecutively sensitive to enhancer-targeting.

### Aims

To unveil how leukaemias not driven by TF-related lesions establish their transcriptional programs, and if it involves enhancer-deregulation and associated sensitivity to enhancer-targeting drugs.

### Methods

As an example of a leukemia not driven by a TF-related lesion, we studied Ph+B-ALL, the most common B-ALL in adults with historically poor prognosis driven by the BCR::ABL1 tyrosine kinase. We performed a multi-omics approach to study the transcriptome, enhancer activities, and 3D enhancers interactions using ChIP-Seq, RNA-Seq, and Hi-C-based methods to link enhancers to promoters they regulate. For functional experiments, we interfered with enhancer function and BCR::ABL1-regulated TFs using enhancer-targeting drugs, targeted proteasomal degradation and RNA interference. Lastly, we tested the sensitivity of Ph+B-ALL cells to enhancer inhibition using single and combined treatment of enhancer-targeting drugs with or without clinically relevant dosages of BCR::ABL1 kinase inhibitors (TKIs).

### Results

We report that malignant transformation and transcriptional reprogramming by BCR::ABL1 are defined by enhancer reprogramming and that enhancer signatures differentiate Ph+B-ALL from other leukemias. We show that Ph+B-ALL key genes are enhancer regulated and that activation of enhancers that physically interact with BCR::ABL1-regulated genes is dependent on BCR::ABL1 kinase activity. Consequently, BCR::ABL1-induced genes are especially sensitive to enhancer-targeting drugs. We show that BCR::ABL1 utilizes STAT5, ETV5 and MYC to promote enhancer activation, though most BCR::ABL1-induced enhancers were regulated independent of them. Most importantly, we show that Ph+B-ALL cells are hypersensitive to enhancer-targeting drugs and that their combined use with TKIs used for Ph+B-ALL therapy improves their efficacy, especially in cells from *IKZF1*$^{\mathrm{PLUS}}$ patients that most frequently relapse from current treatment.

**Copyright Information:** (Online) ISSN: 2572-9241
©2026 The Author(s). HemaSphere is published by John Wiley & Sons Ltd on behalf of European Hematology Association. This is an open access Abstract Book distributed under the Attribution-NonCommercial-NoDerivs (CC BY-NC-ND), which allows third parties to download the articles and share them with others as long as they credit the author and the Abstract Book, but they cannot change the content in any way or use them commercially.
**Abstract Book Citations:** Authors, Title, HemaSphere, 2026;10:(S1):pages. The abstract book DOIs can be found on the "Author Information" page.
**Disclaimer:** Articles published in the journal HemaSphere exclusively reflect the opinions of the authors. The authors are responsible for all content in their abstracts including accuracy of the facts, statements, citing resources, etc.

### Summary/Conclusion

We provide new insights into how BCR::ABL1 induces transcriptional reprogramming to promote Ph+B-ALL. We show that enhancer regulation is part of malignant transformation by BCR::ABL1 and a defining feature of Ph+B-ALL. Given that BCR::ABL1 itself executes enhancer activation, BCR::ABL1-regulated genes are highly sensitive to enhancer inhibition and Ph+B-ALL cells hypersensitive to enhancer-targeting drugs. Importantly, as combination of enhancer-targeting drugs with TKIs improves their efficacy, our work provides a basis for their potential, future addition to current therapy.

**Copyright Information:** (Online) ISSN: 2572-9241
©2026 The Author(s). HemaSphere is published by John Wiley & Sons Ltd on behalf of European Hematology Association. This is an open access Abstract Book distributed under the Attribution-NonCommercial-NoDerivs (CC BY-NC-ND), which allows third parties to download the articles and share them with others as long as they credit the author and the Abstract Book, but they cannot change the content in any way or use them commercially.
**Abstract Book Citations:** Authors, Title, HemaSphere, 2026;10:(S1):pages. The abstract book DOIs can be found on the “Author Information” page.
**Disclaimer:** Articles published in the journal HemaSphere exclusively reflect the opinions of the authors. The authors are responsible for all content in their abstracts including accuracy of the facts, statements, citing resources, etc.

### (PS1438) LIPIDOMIC REPROGRAMMING LINKS VEGF-DEPENDENT CNS ADAPTATION TO FERROPTOSIS SENSITIVITY IN ACUTE LYMPHOBLASTIC LEUKEMIA

**Topic:** 01. Acute lymphoblastic leukemia - Biology & translational research

Vera Muench*[1], Rolf Köhler[2], Alireza Abaei[3], Volker Rasche[3], Shuo Liu[1], Alexandra Niedermayer[1, 4], Stefanie Enzenmueller[1], Aigerim Zkrina[1], Gudrun Strauss[1, 4], Daniel Tews[1, 4], Pamela Fischer-Posovszky[1, 4], Johann Kraus[5], Hans Kestler[4, 5], Klaus-Michael Debatin[1, 4], Lüder Hinrich Meyer[1]

*[1]Ulm University Medical Center, Department of Pediatrics and Adolescent Medicine, Ulm, Germany; [2]University of Heidelberg, Institute of Human Genetics, Heidelberg, Germany; [3]Ulm University, ULMTeC Core Facility Small Animal Imaging, Ulm, Germany; [4]German Center for Child and Adolescent Health (DZKJ), partner site Ulm, Ulm, Germany; [5]Ulm University, Institute of Medical Systems Biology, Ulm, Germany;*

### Background

Central nervous system (CNS) involvement in pediatric acute lymphoblastic leukemia (ALL) remains a major clinical challenge. Subclinical CNS disease occurs in all patients and requires intensive therapy to prevent relapse, yet current treatments cause significant neurotoxicity, highlighting the need for targeted therapies. We previously found increased vascular endothelial growth factor A (VEGF) expression in CNS ALL and reduction of CNS leukemia *in vivo* by VEGF blockade. However, the role of VEGF signaling in CNS entry via VEGF receptor 2 (VEGFR2)-mediated transendothelial migration (TEM) and in sustaining ALL survival within the CNS remains unclear.

### Aims

In this study, we aimed to elucidate the contribution of VEGF to TEM, to ALL survival within the CNS, and to other mechanisms.

### Methods

We employed a patient-derived xenograft (PDX) mouse model and defined the time point of manifest CNS involvement ($CNS^{onset}$) in three PDX samples. Anti-VEGF treatment with bevacizumab was initiated either prior to $CNS^{onset}$ to assess effects on CNS entry or upon manifest CNS ALL to evaluate niche-dependent maintenance in these models. TEM was further examined using knockdown, antibody-mediated blockade, and pharmacologic inhibition of VEGFR2 *in vitro*. In addition, broader VEGF signaling using the broad spectrum tyrosine kinase inhibitor Sorafenib was tested *in vivo*, followed by *in vitro* analyses of TEM and ALL survival. Ferroptosis induction, a lipid peroxidation-driven form of cell death previously linked to Sorafenib and CNS ALL, was assessed using BODIPY™ 581/591 C11. To define compartment-specific lipid signatures associated with lipid peroxidation vulnerability, lipidomic profiling of CNS- and BM-derived ALL cells was performed.

**Copyright Information:** (Online) ISSN: 2572-9241
©2026 The Author(s). HemaSphere is published by John Wiley & Sons Ltd on behalf of European Hematology Association. This is an open access Abstract Book distributed under the Attribution-NonCommercial-NoDerivs (CC BY-NC-ND), which allows third parties to download the articles and share them with others as long as they credit the author and the Abstract Book, but they cannot change the content in any way or use them commercially.
**Abstract Book Citations:** Authors, Title, HemaSphere, 2026;10:(S1):pages. The abstract book DOIs can be found on the “Author Information” page.
**Disclaimer:** Articles published in the journal HemaSphere exclusively reflect the opinions of the authors. The authors are responsible for all content in their abstracts including accuracy of the facts, statements, citing resources, etc.

**Results**

Pre-CNS$^{onset}$ bevacizumab treatment showed only modest reduction in CNS ALL, and VEGFR2 blockade failed to alter ALL cell TEM *in vitro*, indicating that VEGF signaling only has a minor contribution to CNS entry. However, bevacizumab administered upon manifest CNS ALL consistently reduced CNS ALL, highlighting its importance for ALL maintenance in the CNS.

Post-CNS$^{onset}$ treatment with Sorafenib also significantly decreased CNS ALL in all PDX models. While Sorafenib reduced TEM *in vitro*, consistent with inhibition of VEGFR2 downstream signalling, it also directly mediated cell death by lipid peroxidation in ALL cell lines, which was rescued by ferrostatin-1, indicating induction of ferroptosis as mechanism of cell death induction.
To further explore a metabolic basis of this vulnerability, we compared lipid profiles of CNS- and BM-derived ALL cells and identified a prototype CNS ALL profile characterized by enrichment in phospholipids, along with a significantly higher peroxidizability index in CNS-derived ALL cells. These findings of an increased susceptibility to lipid peroxidation may provide a mechanistic rationale for therapeutic approaches involving targeted induction of ferroptosis in CNS ALL.

**Summary/Conclusion**

In summary, our data show that VEGF signaling is critical for CNS ALL maintenance and that Sorafenib exerts pronounced CNS activity through two complementary mechanisms, reducing TEM and inducing ferroptosis. CNS-specific lipid remodeling increases peroxidation susceptibility, priming ALL cells for ferroptosis and revealing a compartment-specific vulnerability. These findings highlight the translational potential of therapies targeting VEGF signaling and inducing ferroptosis for CNS ALL, providing a rationale for future CNS-directed ALL strategies.

**Copyright Information:** (Online) ISSN: 2572-9241
©2026 The Author(s). HemaSphere is published by John Wiley & Sons Ltd on behalf of European Hematology Association. This is an open access Abstract Book distributed under the Attribution-NonCommercial-NoDerivs (CC BY-NC-ND), which allows third parties to download the articles and share them with others as long as they credit the author and the Abstract Book, but they cannot change the content in any way or use them commercially.
**Abstract Book Citations:** Authors, Title, HemaSphere, 2026;10:(S1):pages. The abstract book DOIs can be found on the "Author Information" page.
**Disclaimer:** Articles published in the journal HemaSphere exclusively reflect the opinions of the authors. The authors are responsible for all content in their abstracts including accuracy of the facts, statements, citing resources, etc.

### (PS1439) IKZF1 LOSS ESTABLISHES A SELF-RENEWING THYMOCYTE POOL DRIVING T-ALL

**Topic:** 01. Acute lymphoblastic leukemia - Biology & translational research

Maaike Van Trimpont*[1, 2], Sara T'sas[1, 2], Els Van Ammel[1, 2], Pieter Van Vlierberghe[2, 3], Cedric Tremblay[4], Tim Pieters[1, 2], Steven Goossens[1, 2]

*[1]Ghent University, Unit for Translational Research in Oncology (UTRiO), Department of Diagnostic Sciences (Faculty of Medicine and Health Sciences), Ghent, Belgium; [2]Cancer Research Institute Ghent (CRIG), Ghent, Belgium; [3]Ghent University, Department of Biomolecular Medicine, Ghent, Belgium; [4]University of Manitoba, Winnipeg, Canada;*

### Background

T-cell acute lymphoblastic leukemia (T-ALL) is an aggressive hematologic malignancy driven by (epi)genetic alterations that disrupt normal T-cell development. Despite intensive chemotherapy and hematopoietic stem cell transplantation, relapse occurs in approximately 15% of pediatric and 40% of adult patients. Relapsed T-ALL patients have a long-term survival of <10%, highlighting a high clinical need for alternative salvage strategies. However, the molecular mechanisms that drive relapse and therapy resistance in T-ALL are poorly understood. IKZF1, also known as Ikaros, is a zinc finger transcription factor essential for lymphoid development and thymocyte differentiation. Its alterations, observed in 5-15% of early T-cell precursor ALL (ETP-ALL) cases, correlate with poor survival and increased relapse risk.

### Aims

IKZF1 is a tumor suppressor whose loss is associated with poor outcomes and higher relapse rates in T-ALL. We aim to evaluate the role of IKZF1 in T-ALL initiation, thymocyte self-renewal, and response to therapy, with the goal of identifying potential therapeutic vulnerabilities in high-risk and relapsed patients.

### Methods

We generated a conditional Ikzf1 knockout mouse model to examine the effects of Ikzf1 loss, alone and in combination with Lmo2 overexpression. Mice were monitored for spontaneous leukemia, and Kaplan-Meier analyses assessed disease latency and penetrance. Immunophenotyping of Ikzf1-deficient tumors and pre-leukemic thymocytes was performed using multiparameter flow cytometry. Functional self-renewal was evaluated by transplanting CD45.2 Ikzf1-deficient thymocytes into sublethally irradiated CD45.1 recipients. Additionally, *in vitro* T-cell co-cultures were performed using pre-leukemic Ikzf1-deficient lineage-negative (Lin-), Sca-1-positive (Sca-1+), and c-Kit-positive (c-Kit+) (LSK) cells on mouse OP9-DL1 stromal cells to evaluate T-cell differentiation arrest and developmental progression.

### Results

Loss of IKZF1 drives thymocyte self-renewal and promotes aggressive immature T-ALL with full penetrance and short latency, independent of Lmo2. Ikzf1-null leukemias expressed high levels of Lyl1, consistent with an immature T-ALL phenotype. Functionally, Ikzf1-deficient thymocytes efficiently repopulated the thymus upon transplantation, whereas wild-type cells failed to engraft. Notably, unlike pre-leukemic CD2-Lmo2 thymi enriched for CD4–CD8– double-negative cells, Ikzf1-null thymi maintained normal double-negative proportions but exhibited increased mature CD3+ populations. *In vitro* co-culture of pre-leukemic Ikzf1-deficient LSK cells on OP9-DL1 stroma revealed impaired T-cell differentiation, with an abnormal accumulation of CD3+ CD4+ T cells.

**Copyright Information:** (Online) ISSN: 2572-9241
©2026 The Author(s). HemaSphere is published by John Wiley & Sons Ltd on behalf of European Hematology Association. This is an open access Abstract Book distributed under the Attribution-NonCommercial-NoDerivs (CC BY-NC-ND), which allows third parties to download the articles and share them with others as long as they credit the author and the Abstract Book, but they cannot change the content in any way or use them commercially.
**Abstract Book Citations:** Authors, Title, HemaSphere, 2026;10:(S1):pages. The abstract book DOIs can be found on the “Author Information” page.
**Disclaimer:** Articles published in the journal HemaSphere exclusively reflect the opinions of the authors. The authors are responsible for all content in their abstracts including accuracy of the facts, statements, citing resources, etc.

**Summary/Conclusion**

Our findings demonstrate that Ikzf1-null thymocytes can repopulate and self-renew the thymus. To our knowledge, IKZF1 represents the first tumor suppressor linked to thymocyte self-renewal. These self-renewing thymocytes display relative chemotolerance and may serve as a reservoir for relapse. Understanding the molecular pathways downstream of IKZF1 loss could reveal novel therapeutic vulnerabilities, and targeting aberrant self-renewal programs may provide innovative strategies for high-risk T-ALL patients with IKZF1 alterations.

**Copyright Information:** (Online) ISSN: 2572-9241
©2026 The Author(s). HemaSphere is published by John Wiley & Sons Ltd on behalf of European Hematology Association. This is an open access Abstract Book distributed under the Attribution-NonCommercial-NoDerivs (CC BY-NC-ND), which allows third parties to download the articles and share them with others as long as they credit the author and the Abstract Book, but they cannot change the content in any way or use them commercially.
**Abstract Book Citations:** Authors, Title, HemaSphere, 2026;10:(S1):pages. The abstract book DOIs can be found on the “Author Information” page.
**Disclaimer:** Articles published in the journal HemaSphere exclusively reflect the opinions of the authors. The authors are responsible for all content in their abstracts including accuracy of the facts, statements, citing resources, etc.

### (PS1440) MULTICENTER VALIDATION OF AN OPEN ACADEMIC PROTOCOL FOR NGS-BASED DETECTION OF MEASURABLE RESIDUAL DISEASE (MRD) IN ACUTE LYMPHOBLASTIC LEUKEMIA: A JOINT EUROCLONALITY-NGS AND EUROMRD STUDY

**Topic:** 01. Acute lymphoblastic leukemia - Biology & translational research

Eva Fronkova*[1], Michael Svaton[1], Daniel Dulla[1], Andrea Rennerova[1], Leona Rezkova Reznickova[1], Michaela Kotrova[2], Ema Joudalova[1], Nikos Darzentas[2], Constantin Proske[2], Heiko Trautmann[2], Onno J Verhagen[3], Silvia Armuzzi[4, 5], Carolina Terragna[4], Roberta Cavagna[6], Cornelia Eckert[7], Jona Van der Straeten[8], Grazia Fazio[9], Masashi Sanada[10], Helen Vålerhaugen[11], Rathana Kim[12], Domenico Salemi[13], Patrick Villarese[14], Aurore Touzart[14], Aurélie Caye-Eude[15], Vincent van der Velden[16], Allen Eng Juh Yeoh[17, 18, 19], Yi Lu[20], Tomas Reigl[21, 22], Anton Langerak[16], Monika Brüggemann[2], Jan Trka[1]

*[1]CLIP, Department of Paediatric Haematology and Oncology, 2nd Faculty of Medicine, Charles University and University Hospital Motol and Homolka, Prague, Czechia; [2]Unit for Hematological Diagnostics, Department of Internal Medicine II, University Hospital Schleswig-Holstein, Kiel, Germany; [3]Department of Immunocytology, Sanquin Diagnostic Services, Amsterdam, The Netherlands; [4]IRCCS Azienda Ospedaliero-Universitaria di Bologna Istituto di Ematologia "Seràgnoli", Bologna, Italy;[5]DIMEC, University of Bologna, Bologna, Italy; [6]Hematology and Bone Marrow Transplant Unit, ASST Papa Giovanni XXIII, Bergamo, Italy; [7]Department of Pediatric Oncology/Hematology, Charité-Universitätsmedizin Berlin, Berlin, Germany;[8]Molecular Hematology Laboratory, Vrije Universiteit Brussel, Universitair Ziekenhuis Brussel, Brussels, Belgium; [9]Tettamanti Center, Fondazione IRCCS, San Gerardo dei Tintori, Monza, Italy; [10]Department of Advanced Diagnosis, Clinical Research Center, NHO Nagoya Medical Center, Nagoya, Japan; [11]Department of Pathology, The Norwegian Radium Hospital, Oslo University Hospital, Oslo, Norway; [12]Laboratory of Hematology, Hôpital Universitaire Saint Louis, AP-HP and Université Paris Cité, Paris, France;[13]UOSD Laboratorio Oncoematologia, Divisione Ematologia con UTMO, A.O. Ospedali Riuniti Villa Sofia-Cervello, Palermo, Italy; [14]Laboratory of Onco-Hematology, Hôpital Universitaire Necker Enfants Malades, AP-HP and Université Paris Centre, Paris, France; [15]Developmental Genetics Department, Molecular Genetics Laboratory, Hôpital Universitaire Robert Debré, AP-HP and Université Paris Cité, Paris, France; [16]Department of Immunology, Laboratory of Medical Immunology, Erasmus MC, University Medical Centre Rotterdam, Rotterdam, The Netherlands; [17]Department of Pediatrics, Yong Loo Lin School of Medicine, National University of Singapore, Singapore, Singapore; [18]VIVA-University Children's Cancer Center, Khoo Teck Puat-National University Children's Medical Institute, National University Hospital, National University Health System, Singapore, Singapore; [19]National University Cancer Institute, National University Hospital, National University Health System, Singapore, Singapore; [20]VIVA-NUS Centre for Translational Research in Acute Leukaemia, Department of Pediatrics, Yong Loo Lin School of Medicine, National University of Singapore, Singapore, Singapore; [21]Institute of Medical Genetics and Genomics, Faculty of Medicine, Masaryk University, Brno, Czechia; [22]Central European Institute of Technology, Masaryk University, Brno, Czechia;*

### Background

MRD detection is a key driver of treatment decisions in acute lymphoblastic leukemia (ALL). Clonal rearrangements of immunoglobulin (IG) and T-cell receptor (TR) genes at the DNA level have long served as universal MRD targets, detected first by quantitative PCR and more recently by next-generation sequencing (NGS). However, non-commercial IG/TR NGS-MRD detection has not yet been fully standardized or multicentrically validated for clinical use. Within the EuroClonality-NGS working group, we developed and validated a standard operating procedure for MRD detection in ALL, building on a previously published NGS-based approach for MRD target identification.

**Copyright Information:** (Online) ISSN: 2572-9241
©2026 The Author(s). HemaSphere is published by John Wiley & Sons Ltd on behalf of European Hematology Association. This is an open access Abstract Book distributed under the Attribution-NonCommercial-NoDerivs (CC BY-NC-ND), which allows third parties to download the articles and share them with others as long as they credit the author and the Abstract Book, but they cannot change the content in any way or use them commercially.
**Abstract Book Citations:** Authors, Title, HemaSphere, 2026;10:(S1):pages. The abstract book DOIs can be found on the “Author Information” page.
**Disclaimer:** Articles published in the journal HemaSphere exclusively reflect the opinions of the authors. The authors are responsible for all content in their abstracts including accuracy of the facts, statements, citing resources, etc.

### Aims

Our aim was to multicentrically validate the newly developed NGS-based MRD method through a series of inter-laboratory comparisons within the EuroMRD consortium. Specifically: (1) to standardize the selection of IG/TR targets, including cross-lineage rearrangements, while avoiding public clones; (2) establish rules for contamination prevention and reduce the rate of false positivity across participating laboratories; (3) test assay sensitivity (multicentrically to $10^{-5}$; centrally to $10^{-6}$); and (4) achieve accuracy in quantification, including in the setting of B-lineage aplasia after B-cell depleting immunotherapy.

### Methods

From 2022 to 2025, we conducted seven inter-laboratory comparisons involving sample distribution or *in silico* data evaluations, with regular participation of 16 laboratories. The wet-lab part included MRD quantification in the distributed samples with predefined dilutions; a polyclonal control (buffy coat) was always included. Primers for library preparation and spiked-in amplification controls (cIT-QC) were centrally provided. The *in silico* part included assessment of the suitability of the given targets for MRD detection using our newly developed IG/TR sequence database for public clone identification, and analysis of the provided NGS data with ARResT/Interrogate.

### Results

The proportion of laboratories reporting false-positive results decreased from 25% to 0% in 2025 after key risk factors had been identified and addressed. One important measure was the assignment of two different index combinations (both i5 and i7) when analyzing multiple MRD samples together. Another critical factor was the identification of public clones, which should be avoided due to their contribution to false positive results. In the multi-center setting, a $10^{-5}$ dilution was tested across four *IGH* targets in two patient samples distributed to 16 laboratories. Positivity in at least one biallelic target was detected by 15/16 laboratories for the first sample and by all laboratories for the second. We centrally tested a $10^{-6}$ dilution using samples from B-ALL patients with various *IGH* rearrangements and found positive results in 27 of 30 targets. Applicability of cross-lineage TR rearrangements in the same samples was complicated by the high prevalence of public clones (40% for *TRG*, 20% for *TRB* and 38% for *TRD*). For unique TR rearrangements, a sensitivity of $10^{-6}$ was achieved in 70% of samples. Testing of 17 samples from ALL patients after B-cell-depleting therapy showed that cIT-QC normalization is necessary, as unnormalized NGS-MRD significantly overestimated MRD levels compared with qPCR.

### Summary/Conclusion

Our new academic protocol for NGS-MRD detection achieves robust results thanks to inter-laboratory standardization, especially for the reliable detection of MRD negativity with high sensitivity.
*M.B. and J.T. contributed equally*

**Copyright Information:** (Online) ISSN: 2572-9241
©2026 The Author(s). HemaSphere is published by John Wiley & Sons Ltd on behalf of European Hematology Association. This is an open access Abstract Book distributed under the Attribution-NonCommercial-NoDerivs (CC BY-NC-ND), which allows third parties to download the articles and share them with others as long as they credit the author and the Abstract Book, but they cannot change the content in any way or use them commercially.
**Abstract Book Citations:** Authors, Title, HemaSphere, 2026;10:(S1):pages. The abstract book DOIs can be found on the "Author Information" page.
**Disclaimer:** Articles published in the journal HemaSphere exclusively reflect the opinions of the authors. The authors are responsible for all content in their abstracts including accuracy of the facts, statements, citing resources, etc.

### (PS1441) IN VIVO ISOGENIC MODELLING UNCOVERS TP53-ASSOCIATED RELAPSE TRAJECTORIES IN T-CELL ACUTE LYMPHOBLASTIC LEUKEMIA

**Topic:** 01. Acute lymphoblastic leukemia - Biology & translational research

Stephanie Gachet*[1], Samuel Quentin[2], Lucie Hernandez[1], Loïc Maillard[1], Marie Passet[2], Rathana Kim[2], Hugo Bergugnat[1], Melha Benlebna[1], Rémy Jelin[3], Marine Aglave[3], Maxime Boy[1], Véronique Parietti[1], Pierre Fenaux[4], Andre Baruchel[5], Hervé Dombret[6], Nicolas Boissel[6], François Sigaux[2], Hugues Dethé[2], Emmanuelle Clappier[2], Jean Soulier[2]

*[1]Inserm UMR 1342, Institut de Recherche Saint-Louis (IRSL), Université Paris Cité, Paris, France;[2]Inserm UMR 1342, Institut de Recherche Saint-Louis (IRSL), Université Paris Cité, Hôpital Saint-Louis, Laboratoire Hématologie, APHP, Paris, France; [3]Gustave Roussy, Plateforme de bioinformatique, Villejuif, France; [4]Hôpital Saint-Louis, Service Hématologie Senior, APHP, Paris, France; [5]Hôpital Robert Debré, APHP, Université Paris Cité, Paris, France;[6]Hôpital Saint-Louis, APHP, Paris, France;*

### Background

T-cell Acute Lymphoblastic Leukemia (T-ALL) is an aggressive hematological malignancy in which relapse remains frequent and associated with poor prognosis. While exhaustive genetic and immunophenotypic landscape studies have enabled to identify a large variety of T-ALL subtypes, these only partially correlate with the onset of relapse and the mechanisms driving relapse remain largely elusive. Therefore, comprehensive understanding of the functional mechanisms underlying relapse is a major unmet need.

While rarely detected at diagnosis (2-3%), *TP53* alterations (*TP53*$^{alt}$, mutations or deletions) are more frequently detected at relapse (10-25%). However, the functional cellular mechanisms by which *TP53*$^{alt}$ or other cause of relapse, including possibly non-genetic cause that contribute to treatment resistance remain largely elusive, in part due to the lack of faithful experimental models. Importantly, *TP53*$^{alt}$ cells are genetically trackable, allowing to monitor the mutant cells at various stages of T-ALL progression.

### Aims

In this work, we leveraged isogenic, patient-derived xenograft (PDX) pairs generated from diagnosis and *TP53*$^{alt}$ relapse T-ALL cases to functionally address the underlying cellular mechanisms driving relapse.

### Methods

We generated isogenic PDX models from paired diagnosis and relapse samples of four T-ALL patients harboring *TP53*$^{alt}$ at relapse. We performed deep genomics, bulk and single-cell transcriptomics, on diagnosis-relapse primary and PDXs pairs, combined with functional analyses such as serial transplants, leukemia-initiating activity (LIC) evaluation, gene silencing, and mitochondrial respiration measurements.

### Results

Isogenic PDXs faithfully recapitulated the genomic, transcriptional and functional features of the primary leukemias at both diagnosis and relapse. Beyond inter-patient heterogeneity, functional analyses revealed a unique cell-intrinsic relapse phenotype that includes greater LIC activity which can be experimentally conferred to diagnosis cells by *TP53* silencing. Transcriptomic profiling linked this phenotype to deregulated cell metabolism, cell signalling, MYC pathways and stemness programs.
Integration of single-cell profiling, deep genomics and *TP53*-mutant backtracking at diagnosis, remission and relapse uncovered ultra-rare pre-relapse cells at diagnosis expressing a relapse-like transcriptional profile that sequentially evolved upon therapy towards biallelic *TP53* inactivation and overt relapse.

**Copyright Information:** (Online) ISSN: 2572-9241
©2026 The Author(s). HemaSphere is published by John Wiley & Sons Ltd on behalf of European Hematology Association. This is an open access Abstract Book distributed under the Attribution-NonCommercial-NoDerivs (CC BY-NC-ND), which allows third parties to download the articles and share them with others as long as they credit the author and the Abstract Book, but they cannot change the content in any way or use them commercially.
**Abstract Book Citations:** Authors, Title, HemaSphere, 2026;10:(S1):pages. The abstract book DOIs can be found on the “Author Information” page.
**Disclaimer:** Articles published in the journal HemaSphere exclusively reflect the opinions of the authors. The authors are responsible for all content in their abstracts including accuracy of the facts, statements, citing resources, etc.

### Summary/Conclusion

Collectively, our findings support a model in which T-ALL relapses emerge from a selected pre-existing transcriptional state characterized by deregulated metabolism that favors subsequent *TP53* inactivation. Beyond T-ALL and *TP53*$^{alt}$, this work may be of broad interest for monitoring prospects and early therapeutic intervention for relapse in acute leukemia.

**Copyright Information:** (Online) ISSN: 2572-9241
©2026 The Author(s). HemaSphere is published by John Wiley & Sons Ltd on behalf of European Hematology Association. This is an open access Abstract Book distributed under the Attribution-NonCommercial-NoDerivs (CC BY-NC-ND), which allows third parties to download the articles and share them with others as long as they credit the author and the Abstract Book, but they cannot change the content in any way or use them commercially.
**Abstract Book Citations:** Authors, Title, HemaSphere, 2026;10:(S1):pages. The abstract book DOIs can be found on the "Author Information" page.
**Disclaimer:** Articles published in the journal HemaSphere exclusively reflect the opinions of the authors. The authors are responsible for all content in their abstracts including accuracy of the facts, statements, citing resources, etc.

### (PS1442) DOWN SYNDROME FETAL LIVER HAEMATOPOIETIC STEM AND PROGENITOR CELLS FAIL TO PROVIDE A PERMISSIVE CELL CONTEXT FOR KMT2A::AFF1 INFANT ALL INITIATION

**Topic:** 01. Acute lymphoblastic leukemia - Biology & translational research

Lucy Hamer*[1], Thomas Jackson[2], Natalina Elliott[2], Emily Neil[1, 2], Zhenhua Li[3], Jun J Yang[3], Ananya Mahadevan[2], Joe Cross[2], Irene Roberts[1, 2], Anindita Roy[1, 2]

[1]*University of Oxford, MRC Molecular Haematology Unit, Weatherall Institute of Molecular Medicine, Oxford, United Kingdom;* [2]*University of Oxford, Department of Paediatrics, Oxford, United Kingdom;* [3]*St. Jude Children's Research Hospital, Department of Pharmacy and Pharmaceutical Sciences, Memphis, United States of America;*

### Background

Infant acute lymphoblastic leukaemia (iALL) is a high-risk leukaemia that arises *in utero* through a single-hit, most commonly *KMT2A::AFF1* translocation. While there is a 27-fold increased risk of childhood ALL in Down Syndrome (DS), there are no reports of iALL. We hypothesise that DS fetal haematopoietic stem and progenitor cells (HSPCs) do not provide the necessary fetal context for *KMT2A::AFF1* iALL transformation.

### Aims

Assess transformation capacity of DS primary human fetal liver (FL) HSPCs upon *KMT2A::AFF1* translocation induction.

### Methods

*KMT2A::AFF1* translocation was induced in DS-FL CD34+ cells through CRISPR-Cas9 editing at *KMT2A* and *AFF1* breakpoints. Age-matched disomic (normal) FL (N-FL) was edited in parallel, with mock-edited cells serving as a Cas9 control. Digital droplet PCR (ddPCR) was performed 12h post-electroporation to measure the frequency of translocation. Edited $^{CRISPR}$*KMT2A::AFF1* cells were co-cultured on MS-5 stroma and/or injected into sub-lethally irradiated NSG mice to model leukaemic transformation. Transformation capacity of specific FAC-sorted N and DS-FL HSPC populations (HSC, MPP and LMPP) was also assessed *in vitro*. Transformed populations were characterised by immunophenotyping and qPCR to detect the *KMT2A::AFF1* transcript. Bone marrow (BM) harvested from leukemic $^{CRISPR}$*KMT2A::AFF1* xenografts at cull was analysed by RNA sequencing and compared to primary patient *KMT2A::AFF1* B-ALL data.

**Copyright Information:** (Online) ISSN: 2572-9241
©2026 The Author(s). HemaSphere is published by John Wiley & Sons Ltd on behalf of European Hematology Association. This is an open access Abstract Book distributed under the Attribution-NonCommercial-NoDerivs (CC BY-NC-ND), which allows third parties to download the articles and share them with others as long as they credit the author and the Abstract Book, but they cannot change the content in any way or use them commercially.
**Abstract Book Citations:** Authors, Title, HemaSphere, 2026;10:(S1):pages. The abstract book DOIs can be found on the "Author Information" page.
**Disclaimer:** Articles published in the journal HemaSphere exclusively reflect the opinions of the authors. The authors are responsible for all content in their abstracts including accuracy of the facts, statements, citing resources, etc.

**Results**

The *KMT2A::AFF1* translocation could be induced in DS-FL samples (n=9), with a similar editing efficiency as in N-FL (n=9) (1.4% vs 1.3% respectively, p=0.9076). However, the transformation efficiency of $^{CRISPR}$*KMT2A::AFF1* DS-FL was significantly reduced *in vitro* (p<0.001) with 28.2% of wells (30/104 wells, n= 5 biological samples) transforming compared to 91.1% of wells in N-FL (68/82 wells, n=6). Transformed wells from both N and DS-FL displayed substantial B-lineage expansion of *KMT2A::AFF1*+ populations. For both N and DS-FL, the LMPP population was most amenable to $^{CRISPR}$*KMT2A::AFF1* transformation *in vitro*. N-FL $^{CRISPR}$*KMT2A::AFF1* xenografts efficiently recapitulate key features of ProB iALL (7/8 mice, median latency 23 weeks), with dominance of CD19+ CD10- blasts in the BM, splenomegaly and CNS infiltration. DS-FL $^{CRISPR}$*KMT2A::AFF1* xenografts displayed much lower incidence of leukaemia (2/9 mice) and median latency was significantly prolonged (38 weeks, p<0.001). Mice with DS-FL $^{CRISPR}$*KMT2A::AFF1* ALL presented with less aggressive disease: no splenomegaly or CNS involvement. Other DS-FL $^{CRISPR}$*KMT2A::AFF1* primary mice developed preleukaemia (4/9), where blasts were present in the BM but did not cause symptomatic disease or serially engraft. Transcriptionally, whilst N-FL $^{CRISPR}$*KMT2A::AFF1* xenografts closely resemble high-risk iALL patient samples, DS-FL $^{CRISPR}$*KMT2A::AFF1* ALL blasts share characteristics of childhood *KMT2A::AFF1* ALL (chALL). There are differences in the expression of some key oncogenes, with *HOXA* genes upregulated in DS $^{CRISPR}$*KMT2A::AFF1* ALL.

**Summary/Conclusion**

*KMT2A::AFF1* ALL transformation occurred with reduced efficiency in DS-FL, both *in vitro* and *in vivo*. DS-FL $^{CRISPR}$*KMT2A::AFF1* ALL presented with less aggressive features *in vivo* and had a more chALL-like transcriptional profile. Our functional and molecular data indicate that DS FL HSPC do not provide optimal fetal context to permit aggressive iALL initiation.

**Copyright Information:** (Online) ISSN: 2572-9241
©2026 The Author(s). HemaSphere is published by John Wiley & Sons Ltd on behalf of European Hematology Association. This is an open access Abstract Book distributed under the Attribution-NonCommercial-NoDerivs (CC BY-NC-ND), which allows third parties to download the articles and share them with others as long as they credit the author and the Abstract Book, but they cannot change the content in any way or use them commercially.
**Abstract Book Citations:** Authors, Title, HemaSphere, 2026;10:(S1):pages. The abstract book DOIs can be found on the “Author Information” page.
**Disclaimer:** Articles published in the journal HemaSphere exclusively reflect the opinions of the authors. The authors are responsible for all content in their abstracts including accuracy of the facts, statements, citing resources, etc.

### (PS1443) MENIN INHIBITION SENSITIZES KMT2A-REARRANGED B-ALL TO FERROPTOSIS THROUGH SLC7A11 DOWNREGULATION

**Topic:** 01. Acute lymphoblastic leukemia - Biology & translational research

Martina Maria Capelletti*[1], Martina Failla[1], Lorenzo Manfreda[1], Giorgia Scotellaro[1], Grazia Fazio[1, 2], Stefano Rebellato[2], Domenico Gaspari[2], Jolanda Sarno[1], Chiara Buracchi[3], Luigia Valsecchi[2], Michela Bardini[2], Giovanni Cazzaniga[1, 2], Angela Maria Savino[1]

*[1]University of Milan-Bicocca, School of Medicine and Surgery,, Monza, Italy; [2]Tettamanti Center, Fondazione IRCCS San Gerardo dei Tintori, Monza, Italy; [3]Pediatrics, Fondazione IRCCS San Gerardo dei Tintori, Monza, Italy;*

### Background

Among the genomically defined high-risk subtypes of B-ALL are those with *KMT2A*-rearrangement (r) which are associated with inferior outcomes with chemotherapy-based approaches. In this context, menin inhibitors have emerged as a promising targeted therapeutic strategy for KMT2A-rearranged leukemias.

### Aims

In this study, we investigated whether menin inhibition primes KMT2A-rearranged B-ALL to metabolic stress and identified dysregulation of glutathione redox homeostasis as a key consequence of menin inhibition, thereby sensitizing leukemic cells to ferroptosis.

### Methods

Bulk RNAseq was performed on SEM cell line using the Universal Plus Total RNAseq library kit. Libraries were analyzed on the NextSeq 2000 Illumina platform (2x paired-end). Drugs used included RSL3, Revumenib and MI-463 (Cayman Chemicals). In vitro, KMT2Ar cell lines SEM and KOPN8 were used. Cell viability after treatments was assessed by CelltiterGlo (Promega). ROS levels were measured with Bodipy C11 probes (ThermoFisher). KMT2A-r PDXs were generated by transplanting KMT2Ar primary cells into NSG mice, with ex vivo drug treatments on fresh patient samples. α-rabbit SLC7A11, α-rabbit total NRF2, α-mouse KEAP1 for Western Blot (WB) and immunofluorescent (IF) analysis were purchased from ProteinTech.

### Results

To characterize transcriptional programs induced by menin inhibition, bulk RNA sequencing was performed on SEM cells treated with Revumenib (100 nM), MI-463 (1.5 μM), or vehicle. Differential expression analysis identified 1914 genes significantly modulated by Revumenib and 1290 by MI-463, with 1061 commonly deregulated. Gene set enrichment analysis (GSEA) showed concordant downregulation of mTORC1 signaling, hypoxia, glycolysis, and serine/glycine metabolism pathways, including key metabolic genes (*SLC7A11, PSAT1, PHGDH*). Conversely, gene sets related to glutathione metabolism and oxidative stress response were enriched. Based on transcriptomic results and literature curation, 21 ferroptosis-related genes were further analyzed, revealing marked modulation of ferroptotic regulatory networks in menin inhibitor–treated cells compared with controls. Among the most significantly downregulated transcripts, SLC7A11 suppression was consistently observed across KMT2A-rearranged cell line models and confirmed at the protein level by immunofluorescence and Western blot. Functionally, menin inhibition resulted in reduced GSH/GSSG ratio and increased NADP+/NADPH ratio, indicating impaired redox homeostasis. Consistently, analysis of antioxidant regulators showed NRF2 downregulation and KEAP1 upregulation. Combination with the ferroptosis inducer RSL3 demonstrated synergistic cytotoxicity *in vitro* and *ex vivo* in PDX models of KMT2Ar B-ALL.

**Copyright Information:** (Online) ISSN: 2572-9241
©2026 The Author(s). HemaSphere is published by John Wiley & Sons Ltd on behalf of European Hematology Association. This is an open access Abstract Book distributed under the Attribution-NonCommercial-NoDerivs (CC BY-NC-ND), which allows third parties to download the articles and share them with others as long as they credit the author and the Abstract Book, but they cannot change the content in any way or use them commercially.
**Abstract Book Citations:** Authors, Title, HemaSphere, 2026;10:(S1):pages. The abstract book DOIs can be found on the "Author Information" page.
**Disclaimer:** Articles published in the journal HemaSphere exclusively reflect the opinions of the authors. The authors are responsible for all content in their abstracts including accuracy of the facts, statements, citing resources, etc.

### Summary/Conclusion

Mechanistically, menin inhibition distrupts the transcriptional program sustaining redox homeostasis. Pharmacological blockade of menin leads to downregulation of NRF2 and its downstream target SLC7A11, leading to increased lipid peroxidation and reduced GSH levels. These alterations translated into increased ferroptosis susceptibility. Therefore, combining menin inhibitors with ferroptotic inducers resulted in synergistic antileukemic activity in KMT2A-rearranged B-ALL, both *in vitro* and *ex vivo*. Collectively, these findings demonstrate that menin inhibitors induce both direct antileukemic effects and metabolic reprogramming thereby creating new opportunities for rational combination therapies.

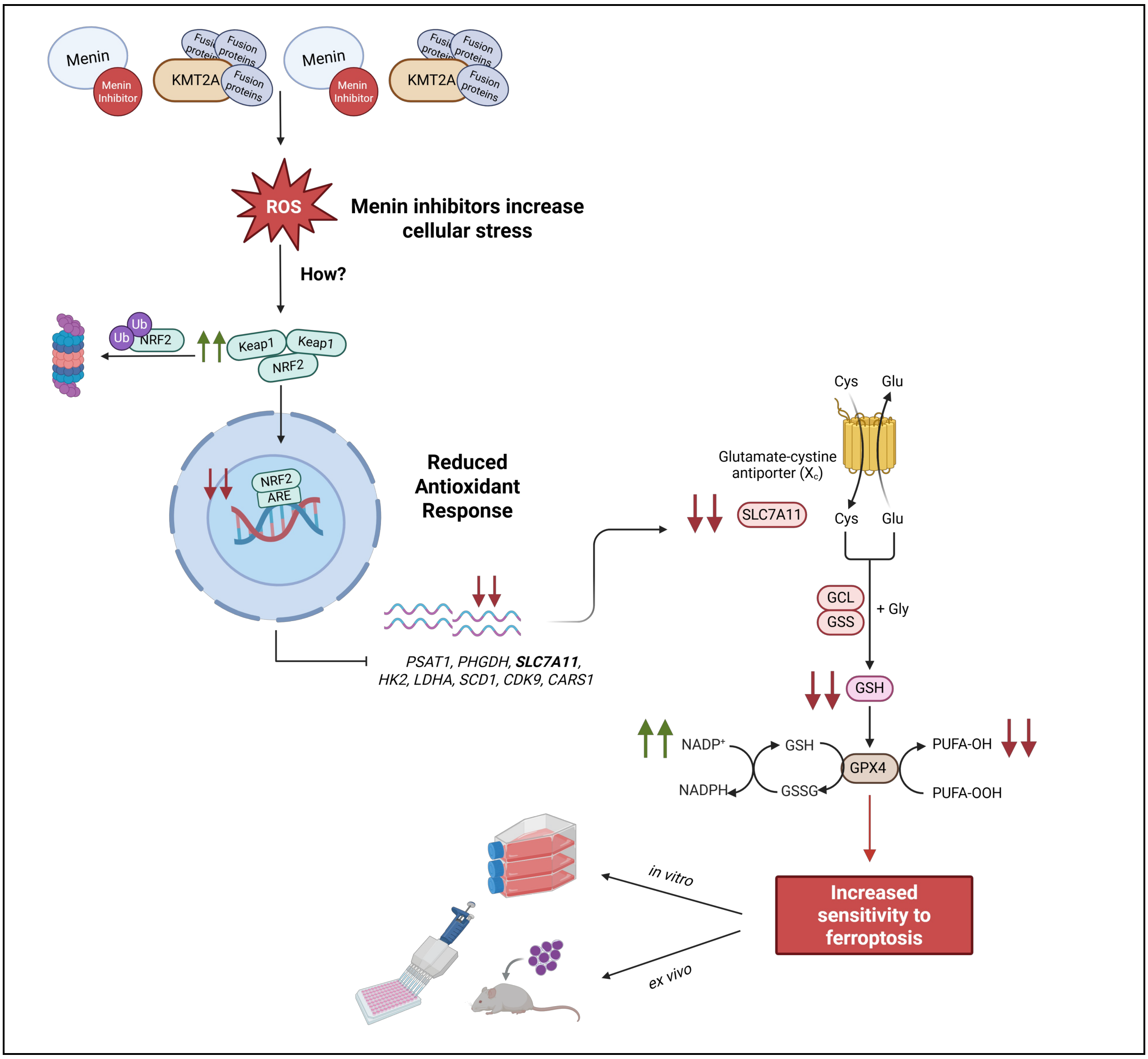

Graphical Abstract

**Copyright Information:** (Online) ISSN: 2572-9241
©2026 The Author(s). HemaSphere is published by John Wiley & Sons Ltd on behalf of European Hematology Association. This is an open access Abstract Book distributed under the Attribution-NonCommercial-NoDerivs (CC BY-NC-ND), which allows third parties to download the articles and share them with others as long as they credit the author and the Abstract Book, but they cannot change the content in any way or use them commercially.
**Abstract Book Citations:** Authors, Title, HemaSphere, 2026;10:(S1):pages. The abstract book DOIs can be found on the "Author Information" page.
**Disclaimer:** Articles published in the journal HemaSphere exclusively reflect the opinions of the authors. The authors are responsible for all content in their abstracts including accuracy of the facts, statements, citing resources, etc.

### (PS1444) SMARCA4 MUTATIONS PROMOTE CHEMOTHERAPY RESISTANCE IN T-ACUTE LYMPHOBLASTIC LEUKEMIA THROUGH ALTERED CHROMATIN REMODELING

**Topic:** 01. Acute lymphoblastic leukemia - Biology & translational research

Violeta García-Hernández*[1, 2, 3], María Maqueda[2, 3], Alba Vidal[2, 3], Giulia Faccincani[2, 4], Jessica González[2, 3], Arnau Iglesias[2, 3], Sarah Bray[1], Anna Bigas[2, 3, 4]

*[1]University of Cambridge, Cambridge, United Kingdom;[2]Hospital del Mar Research Institute, Barcelona, Spain;[3]CIBERONC, Barcelona, Spain;[4]Josep Carreras Leukemia Research Institute, Barcelona, Spain;*

### Background

Relapse and therapy resistance remain the major barriers to curing T-cell acute lymphoblastic leukemia (T-ALL). Despite advances in precision medicine, treatment still relies largely on intensive chemotherapy, and actionable resistance mechanisms remain poorly defined. Our genomic analyses of paired diagnosis–relapse T-ALL cohorts revealed a marked enrichment of alterations in chromatin regulators at relapse (Sentis et al, 2020). Among these, heterozygous loss-of-function mutations in the SWI/SNF ATPase SMARCA4 occur in ~10% of relapsed cases and are largely absent at diagnosis. Reconstruction of clonal size further concluded that relapse clones often pre-exist at diagnosis at very low frequencies, suggesting tolerance to chemotherapy.

### Aims

We hypothesized that SMARCA4 relapse-associated mutations contribute to therapy tolerance in a subset of T-ALL. We aimed to define their functional impact on treatment response and leukemic cell state, as a model to study chromatin-mediated chemotherapy resistance.

### Methods

We generated CRISPR/Cas9-edited isogenic T-ALL models in multiple genetic backgrounds, introducing heterozygous SMARCA4 mutations (RPMI8402, CCRF-CEM) or reverting an endogenous mutation to wild type, WT (Jurkat). We assessed proliferation, apoptosis, and response to conventional T-ALL treatments in vitro and in xenograft in vivo models. To investigate mechanisms of resistence, we performed bulk and single-cell transcriptomic and epigenomic profiling at baseline and during early chemotherapy exposure. As a complementary model of SMARCA4 inactivation, we also profiled SMARCA4-knockdown RPMI8402 cells.

**Copyright Information:** (Online) ISSN: 2572-9241
©2026 The Author(s). HemaSphere is published by John Wiley & Sons Ltd on behalf of European Hematology Association. This is an open access Abstract Book distributed under the Attribution-NonCommercial-NoDerivs (CC BY-NC-ND), which allows third parties to download the articles and share them with others as long as they credit the author and the Abstract Book, but they cannot change the content in any way or use them commercially.
**Abstract Book Citations:** Authors, Title, HemaSphere, 2026;10:(S1):pages. The abstract book DOIs can be found on the "Author Information" page.
**Disclaimer:** Articles published in the journal HemaSphere exclusively reflect the opinions of the authors. The authors are responsible for all content in their abstracts including accuracy of the facts, statements, citing resources, etc.

**Results**

Heterozygous SMARCA4 mutations conferred a reproducible survival advantage under multiple chemotherapy agents, including induction-like regimens (vincristine, dexamethasone, L-asparaginase), methotrexate or doxorubicin. Mutant RPMI8402 and Jurkat cells displayed competitive outgrowth under chemotherapy both in vivo and in vitro, without a baseline proliferative advantage.
By contrast, SMARCA4 knockdown reduced baseline proliferation and MYC/NOTCH signaling. This is in line with reported anti-leukemic effects of SMARCA4 inhibition in T-ALL, highlighting a context-dependent distinction between heterozygous relapse mutations and near-complete loss of SMARCA4.
Mechanistically, WT cells underwent rapid chemotherapy-induced chromatin remodeling, activating stress-response transcriptional programs while repressing cellular fitness pathways. In contrast, SMARCA4-mutant cells showed attenuated chromatin accessibility changes and impaired activation of stress-response regulons, including JUN/FOS and SP-associated networks. Mutant cells partially maintained accessibility and expression of survival and metabolic fitness programs, indicating preservation of a drug-tolerant chromatin state.
Preliminary single-cell multiome analyses further confirm differential regulation of fitness- and stress-related pathways. Ongoing profiling will define how SMARCA4 mutations shape epigenetic and transcriptional heterogeneity associated with the drug-tolerant state.

**Summary/Conclusion**

Relapse-associated SMARCA4 mutations modulate chemotherapy-induced chromatin and transcriptional responses, promoting tolerance to cytotoxic stress in T-ALL cells.
These findings support a context-dependent role for SMARCA4 in T-ALL and provide a framework to further dissect chromatin-mediated mechanisms of relapse, with potential implications for epigenetically disrupted cancer.

**Copyright Information:** (Online) ISSN: 2572-9241
©2026 The Author(s). HemaSphere is published by John Wiley & Sons Ltd on behalf of European Hematology Association. This is an open access Abstract Book distributed under the Attribution-NonCommercial-NoDerivs (CC BY-NC-ND), which allows third parties to download the articles and share them with others as long as they credit the author and the Abstract Book, but they cannot change the content in any way or use them commercially.
**Abstract Book Citations:** Authors, Title, HemaSphere, 2026;10:(S1):pages. The abstract book DOIs can be found on the "Author Information" page.
**Disclaimer:** Articles published in the journal HemaSphere exclusively reflect the opinions of the authors. The authors are responsible for all content in their abstracts including accuracy of the facts, statements, citing resources, etc.

### (PS1445) LONGITUDINAL IMMUNE REMODELING IN ADULT PH+ ACUTE LYMPHOBLASTIC LEUKEMIA PATIENTS TREATED WITH FRONTLINE PONATINIB AND BLINATUMOMAB

**Topic:** 01. Acute lymphoblastic leukemia - Biology & translational research

Nadia Peragine*[1], Michela Ansuinelli[1], Giulia Radice[1], Mariangela Di Trani[1], Maria Stefania De Propris[1], Giada Almici[1], Francesco Di Raimondo[2], Maria Paola Martelli[3], Daniele Mattei[4], Erika Borlenghi[5], Daniela Pietrasanta[6], Daniele Vallisa[7], Sabina Chiaretti[1], Anna Rita Guarini[1], Robin Foà[1]

*[1]Sapienza University, Division of Hematology, Department of Translational and Precision Medicine, Rome, Italy;[2]A.O.U. Policlinico "G.Rodolico-San Marco", Division of Hematology and Bone Marrow Transplant, Catania, Italy; [3]Università degli Studi di Perugia - Santa Maria Misericordia Hospital, Department of Medicine and Surgery, Perugia, Italy; [4]ASOS Croce and Carlo, Division of Hematology, Cuneo, Italy; [5]ASST Spedali Civili di Brescia, Hematology, Brescia, Italy; [6]Azienda Ospedaliera SS Arrigo e Biagio e Cesare Arrigo, Division of Hematology, Alessandria, Italy; [7]Ospedale Guglielmo da Saliceto, Hematology Unit, Department of Oncology and Hematology, Piacenza, Italy;*

### Background

Frontline chemotherapy-free strategies combining tyrosine kinase inhibitors (TKIs) and blinatumomab have substantially improved the remission rates and survival of adult $Ph^+$ acute lymphoblastic leukemia ($Ph^+$ ALL) patients. Treatment-induced modulation of immune cell subsets remains poorly evaluated and may be biologically linked to treatment response.

### Aims

To investigate the long-term immunomodulatory effects of the frontline ponatinib and blinatumomab (pona+blina) association in adult $Ph^+$ ALL, providing a comprehensive longitudinal characterization of the lymphoid immune landscape in both the peripheral blood (PB) and bone marrow (BM) compartments.

### Methods

We analyzed 126 adult $Ph^+$ ALL patients enrolled in the experimental arm of the GIMEMA ALL2820 phase III trial (NCT04722848), where frontline pona+blina was used. PB and BM mononuclear cells (MCs) were collected at the end of ponatinib induction (T0) and after 2 (T2), 4 (T4) and 5 (T5) blinatumomab cycles. Immunophenotyping was performed via multiparametric flow cytometry. Functional activity was assessed by measuring intracellular cytokine production (IFNγ, TNFα), cytolytic protein expression (perforin, granzyme B) and MC cytotoxicity against the K562 cell line.

**Copyright Information:** (Online) ISSN: 2572-9241
©2026 The Author(s). HemaSphere is published by John Wiley & Sons Ltd on behalf of European Hematology Association. This is an open access Abstract Book distributed under the Attribution-NonCommercial-NoDerivs (CC BY-NC-ND), which allows third parties to download the articles and share them with others as long as they credit the author and the Abstract Book, but they cannot change the content in any way or use them commercially.
**Abstract Book Citations:** Authors, Title, HemaSphere, 2026;10:(S1):pages. The abstract book DOIs can be found on the "Author Information" page.
**Disclaimer:** Articles published in the journal HemaSphere exclusively reflect the opinions of the authors. The authors are responsible for all content in their abstracts including accuracy of the facts, statements, citing resources, etc.

### Results

Our data show that the pona+blina combination induces a significant and sustained expansion of the PB T-cell compartment (both CD8 and CD4 conventional T-cells) at each post-blinatumomab timepoint compared to T0. Specifically, median T cells rose from 980 cells/mL at T0 to 1260 cells/mL at T2 (p<0.0001), 1270 cells/mL at T4 (p<0.0001) and 1280 cells/mL at T5 (p=0.005). In contrast, NK and T-NK innate cells showed a limited expansion. Longitudinal flow cytometry revealed a progressive shift towards T and NK effector phenotypes, as documented by an increase in T effector memory ($T_{EM}$ T0 *vs* T4 p=0.006; T0 *vs* T5 p=0.007) and T effector memory re-expressing CD45RA ($T_{EMRA}$ T0 *vs* T5 p=0.002) populations (Fig. 1A,B), as well as in mature $CD56^{dim}KIR^{+}NKG2A^{-}CD57^{+}$ NK cells (T0 *vs* T2 p=0.044; T0 *vs* T4 p=0.002; T0 *vs* T5 p=0.033) (Fig. 1C). This maturation was accompanied by a progressive rise in IFNγ and TNFα production by $CD4^{+}$ T (IFNγ T0 *vs* T4, p=0.01; T0 *vs* T5, p=0.013; TNFα T0 *vs* T4, p=0.039; T0 *vs* T5, p=0.016) and NK cells (IFNγ T0 *vs* T2, p=0.015; T0 *vs* T4, p=0.002; T0 *vs* T5, p=0.029; TNFα T0 *vs* T2, p=0.044; T0 *vs* T4, p=0.014; T0 *vs* T5, p=0.020). BM analysis showed a concordant NK cell maturation but a delayed T and NK cytokine production. A significantly increased production of NK cell perforin (PB T0 *vs* T4 p=0.029, T0 *vs* T5 p=0.016; BM T0 *vs* T4 p=0.032, T0 *vs* T5 p=0.021) and granzyme B (PB T0 *vs* T5 p=0.047) was recorded, leading to improved NK mediated K562 cell killing in both the PB and BM (T0 *vs* T4, 25:1 E:T ratio p<0.001 and p=0.008, respectively). Finally, patients in complete molecular response (CMR) exhibited significantly lower $TIM3^{+}$ and $TIM3^{+}PD1^{+}$ T cells compared to non-CMR patients at T0 (p=0.031 and p=0.019, respectively) and superior IFNγ/TNFα production by $CD8^{+}$ T and NK cells at T2 (IFNγ p=0.033 and p=0.022; TNFα p=0.029 and p=0.034, respectively).

### Summary/Conclusion

This study shows that in adult $Ph^{+}$ ALL frontline pona+blina treatment can induce a measurable *in vivo* immune remodeling, characterized by an enrichment of terminally differentiated T and NK cells with enhanced functional competence, providing additional mechanistic insights into the clinical success of this combination.

NP and MA contributed equally to this work.

**Copyright Information:** (Online) ISSN: 2572-9241
©2026 The Author(s). HemaSphere is published by John Wiley & Sons Ltd on behalf of European Hematology Association. This is an open access Abstract Book distributed under the Attribution-NonCommercial-NoDerivs (CC BY-NC-ND), which allows third parties to download the articles and share them with others as long as they credit the author and the Abstract Book, but they cannot change the content in any way or use them commercially.
**Abstract Book Citations:** Authors, Title, HemaSphere, 2026;10:(S1):pages. The abstract book DOIs can be found on the "Author Information" page.
**Disclaimer:** Articles published in the journal HemaSphere exclusively reflect the opinions of the authors. The authors are responsible for all content in their abstracts including accuracy of the facts, statements, citing resources, etc.

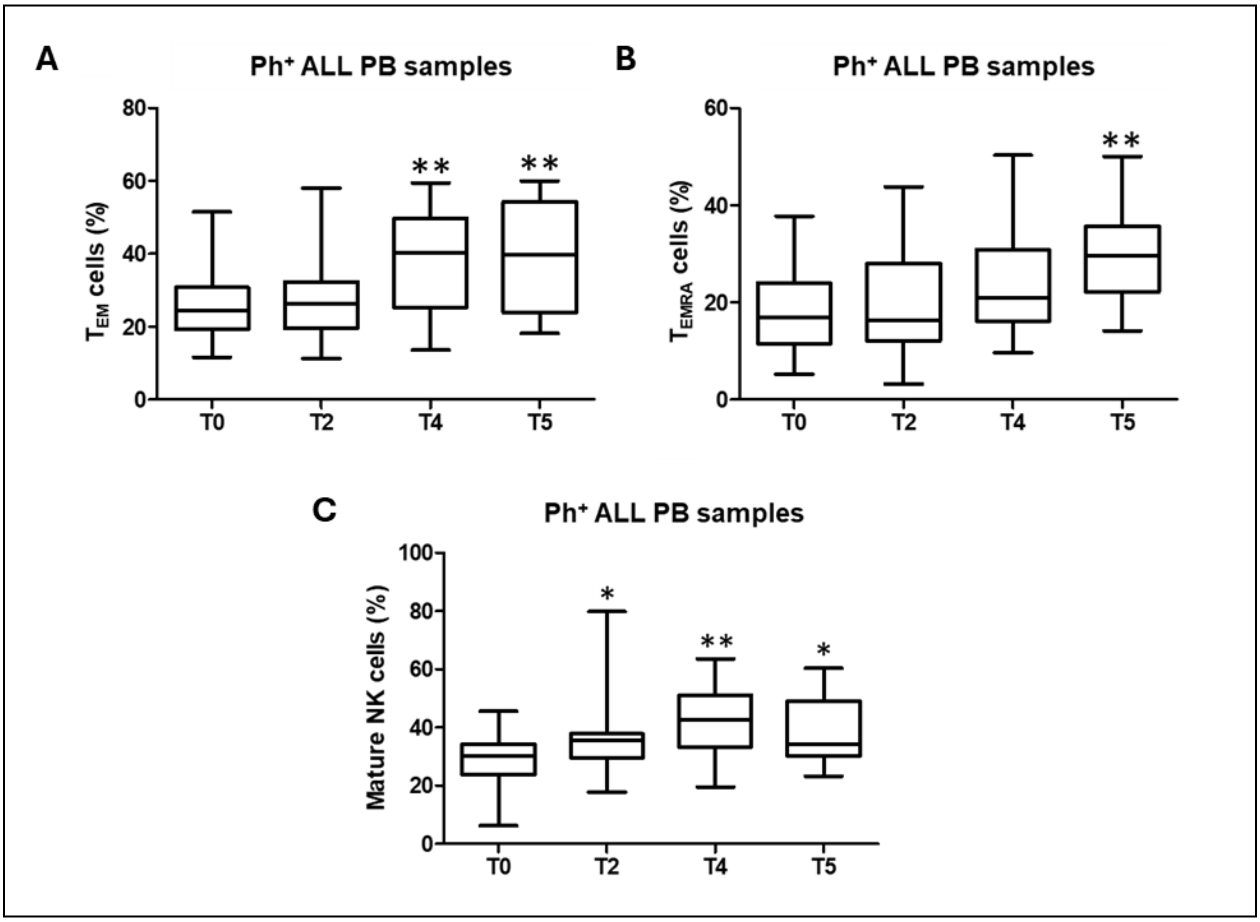

Figure 1. Effects of pona+blina treatment on PB effector memory and TEMRA T cells (A, B) and mature NK cell (C) frequencies.

**Copyright Information:** (Online) ISSN: 2572-9241
©2026 The Author(s). HemaSphere is published by John Wiley & Sons Ltd on behalf of European Hematology Association. This is an open access Abstract Book distributed under the Attribution-NonCommercial-NoDerivs (CC BY-NC-ND), which allows third parties to download the articles and share them with others as long as they credit the author and the Abstract Book, but they cannot change the content in any way or use them commercially.
**Abstract Book Citations:** Authors, Title, HemaSphere, 2026;10:(S1):pages. The abstract book DOIs can be found on the "Author Information" page.
**Disclaimer:** Articles published in the journal HemaSphere exclusively reflect the opinions of the authors. The authors are responsible for all content in their abstracts including accuracy of the facts, statements, citing resources, etc.

### (PS1446) A TARGETABLE, TWO-LEVEL POSITIVE FEEDBACK LOOP REGULATES MYC ONCOGENE EXPRESSION IN SIGNALING-INDUCED LEUKEMIA

**Topic:** 01. Acute lymphoblastic leukemia - Biology & translational research

Trudy Glaser[1], Jintao Zhu[1], Yousif Almemar[1], Han Leng Ng[1], Niklas Feldhahn*[1]

[1] *Imperial College London, Department for Immunology & Inflammation, Centre for Haematology, LONDON, United Kingdom;*

### Background

While controlled *MYC* expression is essential for immune cell activation, differentiation and quiescence, uncontrolled *MYC* can promote cancer. To ensure control, healthy cells induce *MYC* through transient stimuli thereby allowing temporary/limited expression. However, cancers can hijack this mechanism of control by adapting genetic lesions that continuously activate pathways that induce *MYC*. Signaling-induced leukemias, such as Philadelphia chromosome-positive B-lineage leukemia (Ph+B-ALL), Ph-like B-ALL and chronic myeloid leukemia (CML), are such examples. They are driven by ABL1 class or CRLF2 alterations that constitutively activate PI3K/AKT, JAK/STAT, RAS/MEK/ERK and SRC kinase signaling, which all can induce MYC. However, while oncogenic signaling and MYC expression in these leukemias has been investigated, the relative contribution of each pathway to *MYC* induction remains unclear. Likewise, the molecular basis for its maintenance has been little explored.

### Aims

We aimed to define the respective contribution individual signaling pathways to *MYC* expression in CML, Ph+ and Ph-like B-ALL cells and explore transcriptional dependencies of deregulated MYC expression.

### Methods

We performed drug sensitivity assays combined with monitoring of MYC expression by Western blot and qRT-PCR, and *MYC* locus analysis using H3K27ac- and MYC-ChIP-Seq combined with MYC degrader/PROTAC treatment for functional investigation.

### Results

We show that in signaling-induced leukemia *MYC* expression is amplified by a transcriptional positive feedback loop. MYC protein binds to its own super-enhancer (*MYC*-SE/BENC) known to amplify the transcriptional output of the *MYC* gene, and its loss through targeted protein degradation reduces both, *MYC*-SE activation and *MYC* mRNA expression. This mechanism is not leukemia specific as prostate cancer cells treated with the MYC inhibitor MYCi975 show similar effects. However, signaling-induced leukemias constantly feed this loop by MYC-inducing oncogenic signaling. Notably, different leukemia types use different signaling pathways to induce *MYC* and pathway inhibition sensitivities (i.e., their effect on proliferation/survival) directly correlate with their abilities to induce MYC. Importantly, in all three leukemia types including CRLF2-driven Ph-like B-ALL, MYC expression can be abolished by Ponatinib treatment, suggesting a potential new avenue of treatment for this poor prognosis leukemia.

### Summary/Conclusion

We postulate a yet undescribed, two-level mechanism of *MYC* induction in signaling-induced leukemia and describe a possible way for its therapeutic inhibition.

**Copyright Information:** (Online) ISSN: 2572-9241
©2026 The Author(s). HemaSphere is published by John Wiley & Sons Ltd on behalf of European Hematology Association. This is an open access Abstract Book distributed under the Attribution-NonCommercial-NoDerivs (CC BY-NC-ND), which allows third parties to download the articles and share them with others as long as they credit the author and the Abstract Book, but they cannot change the content in any way or use them commercially.
**Abstract Book Citations:** Authors, Title, HemaSphere, 2026;10:(S1):pages. The abstract book DOIs can be found on the “Author Information” page.
**Disclaimer:** Articles published in the journal HemaSphere exclusively reflect the opinions of the authors. The authors are responsible for all content in their abstracts including accuracy of the facts, statements, citing resources, etc.

### (PS1447) A NOVEL INTRONIC ENHANCER REGULATES BCR::ABL1 EXPRESSION IN PHILADELPHIA CHROMOSOME–POSITIVE B-ALL AND CML

**Topic:** 01. Acute lymphoblastic leukemia - Biology & translational research

Jintao Zhu*[1], Mahnoor Usman[1], Nicholas T Crump[1], Niklas Feldhahn[1]

[1] *Imperial College London, Dept. of Immunology and Inflammation; Centre for Haematology, London, United Kingdom;*

### Background

*BCR::ABL1* is the driver of Philadelphia chromosome-positive B-ALL (Ph+B-ALL), the most common form of B-ALL in adults and historically associated with a poor prognosis. Ph+B-ALL arises from the t(9;22) translocation, resulting in a constitutively active *ABL1* kinase. Although tyrosine kinase inhibitors combined with immunotherapies have largely improved clinical outcomes, resistance remains a major therapeutic challenge, with up to 20-65% of patients failing to archieve durable remission or ultimately relapsing. As *BCR::ABL1* can be detected in healthy individuals, leukaemogenesis likely requires its transcriptional upregulation to oncogenic levels. Consistent with this, in two mouse models, *BCR::ABL1* expression driven solely by the native *BCR* promoter was insufficient to induce leukaemia. In contrast, mouse models that developed disease showed a substantial increase in *BCR::ABL1* expression, suggesting that transcriptional upregulation of the fusion gene is required for disease initiation. However, the transcriptional control elements that regulate *BCR::ABL1* expression remain poorly understood, and thus, elucidating them could uncover new therapeutic targets for *BCR::ABL1*-driven leukaemias.

### Aims

This study investigates a putative intronic enhancer, termed *iBCR*, in the regulation of *BCR::ABL1* expression and leukemic cell behaviour, and identifies the transcription factors (TFs) that execute its function.

### Methods

H3K27ac ChIP-seq was performed in Ph+ and Ph-negative B-ALL cell lines to assess*iBCR* and *BCR* promoter activity. *iBCR* function was examined in KCL-22 cells using CRISPR interference (CRISPRi) and CRISPR/Cas9-mediated silencing or deletion. *BCR::ABL1* expression was measured by qRT-PCR, and cell proliferation was assessed using cell competition and Cell Titer-Glo assays. Candidate TFs (MYC) were degraded using a targeted protein degradation, with effects on *BCR::ABL1* expression and *iBCR* activity analyzed by qRT-PCR and H3K27ac ChIP-seq.

### Results

We identified a candidate intronic enhancer within the *BCR* gene located in the *BCR::ABL1*$^{p190}$ translocation region. Integration of published translocation breakpoints with ChIP-seq data shows that over 75% of breakpoints occur downstream of this region and therefore do not delete it, and only a few occasions lead to its complete loss. This indicates a selective pressure in *BCR::ABL1*-driven leukemia cells to retain this element (named *iBCR*). *iBCR*-like regions are similarly present in *BCR::ABL1*-driven chronic myeloid leukaemia (CML) cells, though less conserved compared to Ph+B-ALL cells. H3K27ac ChIP-seq analysis further showed increased *iBCR* and *BCR* promoter activity in Ph+ compared to Ph–negative B-ALL, with *BCR* promoter and *iBCR* activity strongly correlating ($R^2$ = 0.71). CRISPRi-mediated *iBCR* silencing decreased *BCR::ABL1* expression to a similar extent as *BCR* promoter targeting (p = 0.0036) and decreased cell proliferation in cell competition assays. Consistently, cell clones with CRISPR-induced *iBCR* deletion showed reduced *BCR::ABL1* expression ($p = 1 \times 10^{-6}$) and decreased cell proliferation. We further demonstrate that the TF MYC binds to the *iBCR* and that its degrader-induced loss reduces *BCR::ABL1* expression and diminishes *iBCR* activity by H3K27ac ChIP-seq.

**Copyright Information:** (Online) ISSN: 2572-9241
©2026 The Author(s). HemaSphere is published by John Wiley & Sons Ltd on behalf of European Hematology Association. This is an open access Abstract Book distributed under the Attribution-NonCommercial-NoDerivs (CC BY-NC-ND), which allows third parties to download the articles and share them with others as long as they credit the author and the Abstract Book, but they cannot change the content in any way or use them commercially.
**Abstract Book Citations:** Authors, Title, HemaSphere, 2026;10:(S1):pages. The abstract book DOIs can be found on the "Author Information" page.
**Disclaimer:** Articles published in the journal HemaSphere exclusively reflect the opinions of the authors. The authors are responsible for all content in their abstracts including accuracy of the facts, statements, citing resources, etc.

**Summary/Conclusion**

Our findings establish *iBCR* as a key regulatory element that sustains *BCR::ABL1* expression and promotes proliferation in Ph+ B-ALL and CML cells. The dependence of *iBCR* on MYC further highlights a potential therapeutic target in *BCR::ABL1*-driven leukaemias.

**Copyright Information:** (Online) ISSN: 2572-9241
©2026 The Author(s). HemaSphere is published by John Wiley & Sons Ltd on behalf of European Hematology Association. This is an open access Abstract Book distributed under the Attribution-NonCommercial-NoDerivs (CC BY-NC-ND), which allows third parties to download the articles and share them with others as long as they credit the author and the Abstract Book, but they cannot change the content in any way or use them commercially.
**Abstract Book Citations:** Authors, Title, HemaSphere, 2026;10:(S1):pages. The abstract book DOIs can be found on the "Author Information" page.
**Disclaimer:** Articles published in the journal HemaSphere exclusively reflect the opinions of the authors. The authors are responsible for all content in their abstracts including accuracy of the facts, statements, citing resources, etc.

### (PS1448) SERTRALINE MODULATES CHOLESTEROL METABOLISM AND SYNERGIZES WITH PROTEASOME AND HDAC INHIBITORS TO TARGET SERINE/GLYCINE METABOLIC ADDICTION IN T-CELL MALIGNANCIES

**Topic:** 01. Acute lymphoblastic leukemia - Biology & translational research

Anaís Sánchez-Castillo*[1, 2], Paulien Verstraete[1, 2], João Fontela[1, 2], Elien Heylen[1, 2], Lies Matthys[1, 2], Sofie Meykens[1, 2], Daan Dierickx[2, 3], Linde Van Aerschot[1, 2], Michiel Vandenbosch[4], Gabriella Dow[5], Ilaria Elia[5], Marlies Vanden Bempt[2, 3], Jan Cools[2, 6], Kim Kampen[1, 2, 7], Kim de Keersmaecker[1, 2]

*[1]KU Leuven, Laboratory for Disease Mechanisms in Cancer, Department of Oncology, Leuven, Belgium;[2]Leuven Cancer Institute (LKI), Leuven, Belgium; [3]KU Leuven, Laboratory for Experimental Hematology, Department of Oncology, Leuven, Belgium; [4]Maastricht University, The Maastricht MultiModal Molecular Imaging (M4I) institute, Division of Imaging Mass Spectrometry (IMS), Maastricht, The Netherlands; [5]KU Leuven, Lab of Metabolic Regulation of Cell function, Department of Cellular and Molecular Medicine, Leuven, Belgium; [6]VIB Center for Cancer Biology, Laboratory for the Molecular biology of Leukemia, Leuven, Belgium; [7]Maastricht University Medical Center, Department of Radiation Oncology (MAASTRO), GROW School for Oncology and Reproduction, Maastricht, The Netherlands;*

### Background

T-ALL and PTCL are aggressive T-cell cancers with major unmet clinical needs. Despite improved survival in T-ALL, relapse and treatment toxicity remain significant challenges. PTCL has poor outcomes (~30% 5-year survival) and frequent relapses, highlighting the need for safer and more effective therapies.
Over 70% of T-ALL and 50% of PTCL tumors overexpress serine/glycine synthesis pathway (SSP) enzymes, creating a metabolic addiction that is mostly absent in healthy cells. The antidepressant sertraline is a clinically used drug inhibiting SSP enzymes SHMT1/2, reducing proliferation in SSP-dependent cancers. However, as monotherapy, sertraline is insufficient to fully block disease progression *in vivo*.

### Aims

To identify synergistic combination therapies that target SSP-addicted T-cell malignancies, and to delineate the mode of action of these combination therapies.

### Methods

Evaluation of drug effects using T-ALL cell lines, *ex vivo* cultured cells from PDXs and MYCN PTCL mouse model and *in vivo* PDX and MYCN PTCL models. Mechanistic studies entailing mass spectrometry-based $^{13}C_6$-glucose tracing, lipidomics, proteomics, and RNA-seq, followed by validation assays.

**Copyright Information:** (Online) ISSN: 2572-9241
©2026 The Author(s). HemaSphere is published by John Wiley & Sons Ltd on behalf of European Hematology Association. This is an open access Abstract Book distributed under the Attribution-NonCommercial-NoDerivs (CC BY-NC-ND), which allows third parties to download the articles and share them with others as long as they credit the author and the Abstract Book, but they cannot change the content in any way or use them commercially.
**Abstract Book Citations:** Authors, Title, HemaSphere, 2026;10:(S1):pages. The abstract book DOIs can be found on the "Author Information" page.
**Disclaimer:** Articles published in the journal HemaSphere exclusively reflect the opinions of the authors. The authors are responsible for all content in their abstracts including accuracy of the facts, statements, citing resources, etc.

### Results

In a screen with FDA-approved drugs, we identified strong synergy between sertraline and either proteasome inhibitors (PIs; bortezomib, carfilzomib) or histone deacetylase inhibitors (HDACis; romidepsin, vorinostat). Sertraline combined with HDACis or PIs synergistically suppressed proliferation and induced apoptosis in SSP-dependent T-ALL and PTCL cells, with minimal effects on healthy PBMCs  and SSP-independent cells.
In addition to inhibiting the SSP, proteomics and RNA-seq analyses revealed sertraline-driven metabolic rewiring towards cholesterol biosynthesis. Sertraline-treated SSP-dependent cell lines showed upregulation of cholesterol biosynthesis enzymes (HMGCS1, SQLE, FASN), resulting in elevated intracellular cholesterol levels. Interestingly, carfilzomib upregulated cholesterol efflux proteins and reduced total lipid levels, further limiting nutrient availability in T-ALL cells treated with sertraline/carfilzomib combination therapy. Moreover, sertraline/carfilzomib impaired mitochondrial respiration, and increased ROS and DNA damage, leading to apoptosis. In a highly aggressive MYCN-overexpressing PTCL mouse model, the addition of carfilzomib significantly enhanced the therapeutic efficacy of sertraline. Notably, this drug combination also elevated the presence of natural killer T-cells (NKT), neutrophils and eosinophils in this immunocompetent model.
When combining sertraline with HDACi romidepsin, RNA-seq showed synergistic upregulation of the cholesterol biosynthesis pathway, as well as downregulation of MYC targets. Furthermore, the addition of sertraline further increased several histone acetylation marks (H3K27ac, H3K56ac, H4ac) compared to romidepsin monotherapy. As acetyl-CoA fuels both cholesterol synthesis and histone acetylation, these findings support a combined metabolic/epigenetic mode of drug synergy. Sertraline combined with romidepsin significantly reduced disease burden in a MYCN-driven PTCL model and in a T-ALL PDX model.

### Summary/Conclusion

We identified sertraline combined with PIs or HDACis as synergistic drug combinations targeting SSP-addicted T-cell malignancies and unraveled the metabolic/epigenetic axis underlying drug synergy. All these agents are clinically used, which should facilitate clinical translation of our findings.

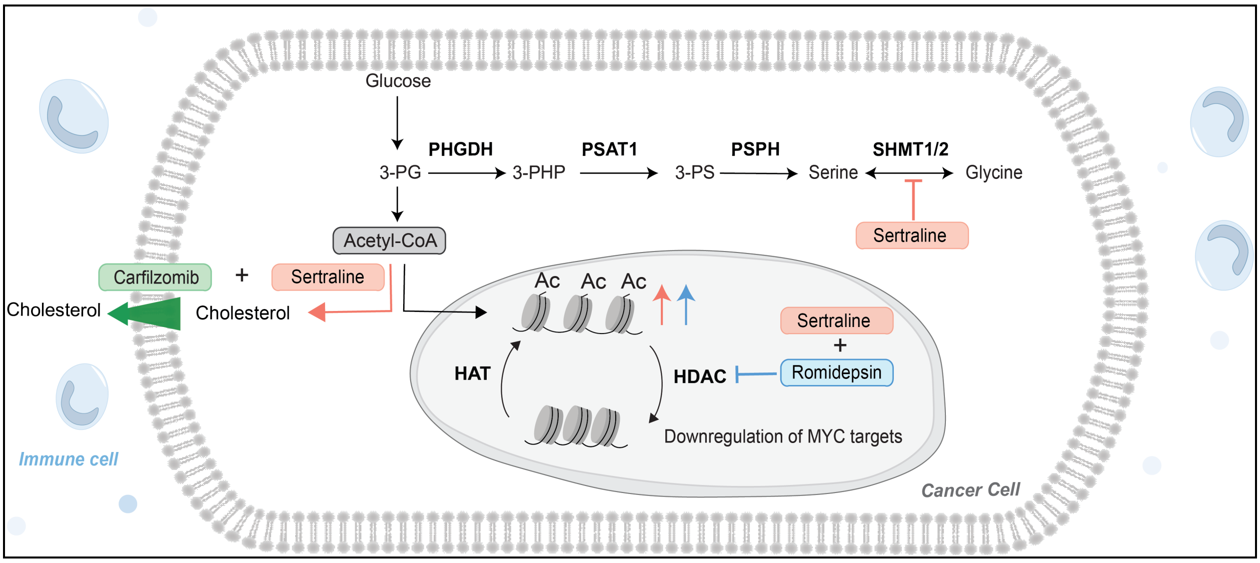

Proposed sertraline-based combination therapies with either PIs (carfilzomib) or HDACis (romidepsin) to target SSP-addicted T-cell malignancies, and the metabolic/epigenetic mechanisms underlying drug synergy.

**Copyright Information:** (Online) ISSN: 2572-9241
©2026 The Author(s). HemaSphere is published by John Wiley & Sons Ltd on behalf of European Hematology Association. This is an open access Abstract Book distributed under the Attribution-NonCommercial-NoDerivs (CC BY-NC-ND), which allows third parties to download the articles and share them with others as long as they credit the author and the Abstract Book, but they cannot change the content in any way or use them commercially.
**Abstract Book Citations:** Authors, Title, HemaSphere, 2026;10:(S1):pages. The abstract book DOIs can be found on the "Author Information" page.
**Disclaimer:** Articles published in the journal HemaSphere exclusively reflect the opinions of the authors. The authors are responsible for all content in their abstracts including accuracy of the facts, statements, citing resources, etc.

### (PS1449) REVEALING THERAPEUTIC VULNERABILITY IN HIGHLY CHEMORESISTANT TCF3::HLF B-ALL: GENOME-WIDE CRISPR/CAS9 SCREENING IDENTIFIES CRITICAL MOLECULAR DETERMINANTS OF RESPONSE TO THE NEW PRODRUG ACHM-025

**Topic:** 01. Acute lymphoblastic leukemia - Biology & translational research

Zahra Sheybani*[1], Faezeh Khalili[1], Bartimée Galvan[2], Aneta Drakul[2], Jean-Pierre Bourquin[2], Antoine De Weck[1], Yizhou Huang[1], Richard Lock[1], Charles De Bock[1], Hansen Kosasih[1]

*[1]Children's Cancer Institute at Minderoo Children's Comprehensive Cancer Centre, School of Clinical Medicine, UNSW Sydney, Sydney, Australia; [2]Kinderspital Zürich, Zürich, Switzerland;*

### Background

ACHM-025 is a new prodrug that is activated by the enzyme AKR1C3 to generate a potent DNA cross-linking agent. Previously, we have demonstrated the profound and curative *in vivo* efficacy of ACHM-025 in patient-derived xenograft (PDX) models of T-cell acute lymphoblastic leukaemia (T-ALL). Consistent with the mechanism of action, its therapeutic efficacy was directly correlated with AKR1C3 expression. To broaden the utility of ACHM-025 across other leukaemia subtypes, we analysed publicly available sequencing data and identified *TCF3::HLF*-positive B-cell ALL with equivalent levels of high *AKR1C3* expression observed in T-ALL. This is of clinical significance, as *TCF3::HLF*-positive B-ALL is a rare high-risk and chemoresistant subtype.

### Aims

1. Determine the sensitivity of *TCF3::HLF*-positive B-ALL to ACHM-025
2. Use genome-wide CRISPR/Cas9 screening to identify candidate genes conferring sensitivity or resistance to ACHM-025

### Methods

AKR1C3 protein levels in *TCF3::HLF* cell lines and PDX cells were assessed by Western blot. Sensitivity to ACHM-025 was measured by DAPI exclusion using flow cytometry. A genome-wide CRISPR/Cas9 knockout screen was carried out on the *TCF3::HLF*-positive cell line, UoC-B1, treated with ACHM-025 at IC30 for 22 days. SgRNA sequences were PCR-amplified from genomic DNA and deep sequenced. Selected candidate genes were individually knocked out, and single-gene knockout cell populations were longitudinally tracked over 14 days under ACHM-025 treatment, followed by combination synergy testing for the prioritised hit.

**Copyright Information:** (Online) ISSN: 2572-9241
©2026 The Author(s). HemaSphere is published by John Wiley & Sons Ltd on behalf of European Hematology Association. This is an open access Abstract Book distributed under the Attribution-NonCommercial-NoDerivs (CC BY-NC-ND), which allows third parties to download the articles and share them with others as long as they credit the author and the Abstract Book, but they cannot change the content in any way or use them commercially.
**Abstract Book Citations:** Authors, Title, HemaSphere, 2026;10:(S1):pages. The abstract book DOIs can be found on the "Author Information" page.
**Disclaimer:** Articles published in the journal HemaSphere exclusively reflect the opinions of the authors. The authors are responsible for all content in their abstracts including accuracy of the facts, statements, citing resources, etc.

**Results**

Western blots showed high AKR1C3 in *TCF3::HLF*-positive cell lines UoC-B1 and HAL-01 and PDX samples. Cell lines and PDX samples showed sensitivity to ACHM-025 (UoC-B1 = 130 nM, HAL-01 = 200 nM, PDX ALL77 = 35 nM, PDX ALL7 = 50 nM) comparable to that observed in sensitive T-ALL. The genome-wide CRISPR/Cas9 screening followed by pathway enrichment and gene set enrichment analyses identified chromatin organisation and heterochromatin assembly components as mediators of ACHM-025 sensitivity. Subsequent single-gene knockout tracking experiments validated four candidate genes, ARID2, CNOT4, SUV39H1, and TBL1XR1. Conversely, genes involved in interstrand crosslink (ICL) repair and cohesin complex function were significantly depleted. Two significant dropout hits that were subsequently validated included the deubiquitinase USP1, a key regulator of the Fanconi anaemia/ICL repair pathway, and the cohesin-associated factor PDS5B. Furthermore, pharmacological inhibition of USP1 phenocopied the genetic loss, where treatment with the selective inhibitor KSQ-4279 demonstrated synergistic activity in combination with ACHM-025 in UoC-B1 cells.

**Summary/Conclusion**

This study establishes *TCF3::HLF* B-ALL as a biologically defined target of ACHM-025 and delineates pathways governing therapeutic sensitivity and resistance. The genome-wide screening identified the Fanconi anaemia/ICL axis, highlighted by USP1, as a key vulnerability to ACHM-025–induced DNA damage. These findings implicate DNA damage repair capacity and chromosomal architecture maintenance as critical determinants of therapeutic escape under ACHM-025 crosslinking stress. The observed synergy between ACHM-025 and USP1 inhibition supports a rational combination strategy to enhance anti-leukaemic efficacy in this fatal subtype. ACHM-025 is currently being evaluated *in vivo* using a single mouse trial (SMT) format across 15 independent *TCF3::HLF* PDX samples.

**Copyright Information:** (Online) ISSN: 2572-9241
©2026 The Author(s). HemaSphere is published by John Wiley & Sons Ltd on behalf of European Hematology Association. This is an open access Abstract Book distributed under the Attribution-NonCommercial-NoDerivs (CC BY-NC-ND), which allows third parties to download the articles and share them with others as long as they credit the author and the Abstract Book, but they cannot change the content in any way or use them commercially.
**Abstract Book Citations:** Authors, Title, HemaSphere, 2026;10:(S1):pages. The abstract book DOIs can be found on the “Author Information” page.
**Disclaimer:** Articles published in the journal HemaSphere exclusively reflect the opinions of the authors. The authors are responsible for all content in their abstracts including accuracy of the facts, statements, citing resources, etc.

### (PS1450) TARGETING HMOX1 EXPLOITS A FERROPTOSIS VULNERABILITY IN HIGH-RISK ETP-ALL

**Topic:** 01. Acute lymphoblastic leukemia - Biology & translational research

Martina Portaluri*[1], Valeria Tosello[1], Costanzo Padovano[2], Vincenzo Giambra[2], Gianluigi Ubaldo Mazzoccoli[2], Giovanni Roti[3], Stefano Indraccolo[1, 4], Erich Piovan[4, 5]

*[1]Istituto Oncologico Veneto - IRCCS, UOSD Oncologia di base sperimentale e traslazionale, Padova, Italy;[2]Casa Sollievo della Sofferenza - IRCCS, San Giovanni Rotondo, Italy; [3]Università di Parma, Parma, Italy;[4]Università di Padova, Dipartimento di Scienze Chirurgiche, Oncologiche e Gastroenterologiche, Sezione di Oncologia, Padova, Italy; [5]Istituto Oncologico veneto - IRCCS, UOC Immunologia e Diagnostica Molecolare Oncologica, Padova, Italy;*

### Background

Early T-cell precursor acute lymphoblastic leukemia (ETP-ALL) is a high-risk T-ALL subtype characterized by therapy resistance and inferior outcomes. While its genomic landscape has been partially defined, subtype-specific metabolic dependencies remain poorly understood. HMOX1, a key effector of NRF2-mediated redox adaptation, regulates heme degradation and iron homeostasis and may critically influence ferroptosis susceptibility. Its functional role in ETP-ALL has not been investigated.

### Aims

To define redox and iron metabolic dependencies in ETP-ALL and evaluate HMOX1 as a subtype-specific therapeutic vulnerability.

### Methods

Public metabolomic datasets from primary T-ALL (n=22; 9 ETP, 13 non-ETP) were re-analyzed and integrated with metabolomic profiling of patient-derived xenografts (n=11). Bulk RNA-seq datasets (ETP n=110; near-ETP n=168; non-ETP n=863) were interrogated using METAFlux to derive metabolic reaction activity scores. Functional metabolic profiling was performed in ETP (n=3) and non-ETP (n=9) cell lines, assessing mitochondrial ROS, lipid peroxidation, labile iron pool, glutathione levels, and mitochondrial function. HMOX1 dependency was evaluated by shRNA silencing. Ferroptosis sensitivity was assessed using RSL3 and Erastin. Single-cell metabolic heterogeneity was interrogated by SCENITH.

### Results

ETP-ALL exhibited a distinct metabolic program characterized by enrichment of glutathione/cysteine metabolism and lipid biosynthesis pathways, alongside reduced nucleotide biosynthesis compared to non-ETP cases. Transcriptomic flux modeling confirmed increased activity of redox buffering reactions and iron-handling pathways.
Functionally, ETP cells displayed elevated basal lipid peroxidation and labile iron levels, consistent with a ferroptosis-primed phenotype. Genetic silencing of HMOX1 significantly increased mitochondrial ROS and lipid peroxidation, inducing compensatory MYC and G2/M checkpoint programs.
Pharmacologically, ETP-ALL cells demonstrated enhanced sensitivity to ferroptosis inducers (RSL3, Erastin) compared to non-ETP models, with further potentiation upon HMOX1 depletion. Single-cell metabolic profiling identified a CD117$^{+}$ subpopulation with high fatty acid oxidation capacity, suggesting metabolically plastic reservoirs of ferroptosis susceptibility.

**Copyright Information:** (Online) ISSN: 2572-9241
©2026 The Author(s). HemaSphere is published by John Wiley & Sons Ltd on behalf of European Hematology Association. This is an open access Abstract Book distributed under the Attribution-NonCommercial-NoDerivs (CC BY-NC-ND), which allows third parties to download the articles and share them with others as long as they credit the author and the Abstract Book, but they cannot change the content in any way or use them commercially.
**Abstract Book Citations:** Authors, Title, HemaSphere, 2026;10:(S1):pages. The abstract book DOIs can be found on the “Author Information” page.
**Disclaimer:** Articles published in the journal HemaSphere exclusively reflect the opinions of the authors. The authors are responsible for all content in their abstracts including accuracy of the facts, statements, citing resources, etc.

**Summary/Conclusion**

ETP-ALL is characterized by a redox-adapted yet ferroptosis-vulnerable metabolic state. HMOX1 acts as a metabolic gatekeeper limiting iron-driven lipid peroxidation and sustaining glutathione-dependent detoxification. Its inhibition exposes a dependency on the GPX4–GSH axis and enhances ferroptosis sensitivity. These findings nominate HMOX1 as a rational combinatorial target with ferroptosis induction strategies in high-risk ETP-ALL.

**Copyright Information:** (Online) ISSN: 2572-9241
©2026 The Author(s). HemaSphere is published by John Wiley & Sons Ltd on behalf of European Hematology Association. This is an open access Abstract Book distributed under the Attribution-NonCommercial-NoDerivs (CC BY-NC-ND), which allows third parties to download the articles and share them with others as long as they credit the author and the Abstract Book, but they cannot change the content in any way or use them commercially.
**Abstract Book Citations:** Authors, Title, HemaSphere, 2026;10:(S1):pages. The abstract book DOIs can be found on the "Author Information" page.
**Disclaimer:** Articles published in the journal HemaSphere exclusively reflect the opinions of the authors. The authors are responsible for all content in their abstracts including accuracy of the facts, statements, citing resources, etc.

### (PS1452) EPITP - AN EPIGENETIC BIOMARKER OF EARLY T-CELL PRECURSOR ACUTE LYMPHOBLASTIC LEUKEMIA

**Topic:** 01. Acute lymphoblastic leukemia - Biology & translational research

Fernanda Schäfer Hackenhaar*[1], Melanie M. Hagleitner[2], Nina Refhagen[1], Valerie De Haas[2], Mattias Landfors[1], Sanna Siitonen[3], Vesa Juvonen[4], Frank Van Leeuwen[2], Pia Osterman[1], Kjeld Schmiegelow[5], Trond Flægstad[6], Olafur Jonsson[7], Jukka Kanerva[3], Jonas Abrahamsson[8], Mats Heyman[9], Ulrika Norén-Nyström[1], Hanne Marquart[10], Magnus Hultdin[1], Sofie Degerman[1]

*[1]Umeå Universitet, Umeå, Sweden; [2]Prinses Máxima Centrum Voor Kinderoncologie, Utrecht, The Netherlands; [3]University of Helsinki, Helsinki, Finland; [4]University of Turku-Main building, Turku, Finland; [5]University of Copenhagen, København K, Denmark; [6]University Hospital Of North Norway, Tromsø, Norway; [7]Landsspítali í Fossvogi, Reykjavík, Iceland; [8]Gothenburg University, Gothenburg, Sweden; [9]Astrid Lindgrens Barnsjukhus, Solna, Sweden; [10]Copenhagen University Hospital, København Ø, Denmark;*

### Background

Improved diagnostic biomarkers for biological subtypes of T-cell acute lymphoblastic leukemia (T-ALL) are needed for further development of personalized treatment strategies.

### Aims

The objective of this study was to discover biological subtypes of T-ALL by integrating multiple omics datasets, with a particular focus on DNA methylation profiling, which previous classification methods have not addressed.

### Methods

An unsupervised machine learning multi-omics factor analysis (MOFA) was applied to transcriptomic and DNA methylation data from 108 pediatric Nordic NOPHO-ALL2008 T-ALL diagnostic samples. Key MOFA factors were correlated with existing phenotypes and clinical markers. The identified epigenetic biomarker of early T-cell precursor ALL ($E_{pi}$TP-ALL) was validated in an independent cohort of 156 Dutch DCOG-ALL10/11 pediatric diagnostic T-ALL samples.

### Results

MOFA-Factor1 matched the well-established CpG island methylator phenotype (CIMP), while MOFA-Factor2 was associated with cell-of-origin markers and the early T-cell precursor (ETP)-ALL immunophenotype. Focusing on the DNA methylation signatures associated with MOFA-Factor2, an $E_{pi}$TP-ALL classifier was defined. An $E_{pi}$TP-ALL subtype was found in about 30% of NOPHO samples, confirmed by the DCOG validation cohort results. The $E_{pi}$TP subtype displayed methylation and expression signatures similar to $CD34^{+}$ stem cells and immature T-cells, and shared methylation features with mixed-phenotype acute leukemia and acute myeloid leukemia. The $E_{pi}$TP-ALLs demonstrated expression of genes associated with immature T-cells and myeloid lineages, while non-$E_{pi}$TPs exhibited expression of mature T-cell genes, including several that may serve as potential drug targets in both subtypes. $E_{pi}$TP-ALL cases included both immunophenotype-defined ETPs and additional samples, suggesting a broadened classification of ETP-ALL. The $E_{pi}$TP and non-$E_{pi}$TP profiles were also observed in adult T-ALLs, with a higher frequency of samples classified as $E_{pi}$TP-ALL.

**Copyright Information:** (Online) ISSN: 2572-9241
©2026 The Author(s). HemaSphere is published by John Wiley & Sons Ltd on behalf of European Hematology Association. This is an open access Abstract Book distributed under the Attribution-NonCommercial-NoDerivs (CC BY-NC-ND), which allows third parties to download the articles and share them with others as long as they credit the author and the Abstract Book, but they cannot change the content in any way or use them commercially.
**Abstract Book Citations:** Authors, Title, HemaSphere, 2026;10:(S1):pages. The abstract book DOIs can be found on the "Author Information" page.
**Disclaimer:** Articles published in the journal HemaSphere exclusively reflect the opinions of the authors. The authors are responsible for all content in their abstracts including accuracy of the facts, statements, citing resources, etc.

**Summary/Conclusion**

Methylation-based $E_{pi}$TP-ALL subtyping at the time of diagnosis enables the identification of an expanded subtype of immature ETP-ALL cases, and may provide valuable guidance for selecting targeted therapies.

**Copyright Information:** (Online) ISSN: 2572-9241
©2026 The Author(s). HemaSphere is published by John Wiley & Sons Ltd on behalf of European Hematology Association. This is an open access Abstract Book distributed under the Attribution-NonCommercial-NoDerivs (CC BY-NC-ND), which allows third parties to download the articles and share them with others as long as they credit the author and the Abstract Book, but they cannot change the content in any way or use them commercially.
**Abstract Book Citations:** Authors, Title, HemaSphere, 2026;10:(S1):pages. The abstract book DOIs can be found on the “Author Information” page.
**Disclaimer:** Articles published in the journal HemaSphere exclusively reflect the opinions of the authors. The authors are responsible for all content in their abstracts including accuracy of the facts, statements, citing resources, etc.

### (PS1453) BCG MOBILIZES SYSTEMIC IMMUNITY TO RESTRAIN B-CELL ACUTE LYMPHOBLASTIC LEUKEMIA

**Topic:** 01. Acute lymphoblastic leukemia - Biology & translational research

Mina Nf Morcos*[1], Franziska Auer[1], Irfan R Akhtar[1], Annika Semeth[1], Tiina Tuononen[2], Aleksandra Pandyra[3], Arndt Borkhardt[3], Merja Heinäniemi[2], Julia Hauer[1, 4, 5]

*[1]Department of Pediatrics, School of Medicine, Technical University of Munich; 80804, Munich, Germany;[2]Institute of Biomedicine, School of Medicine, University of Eastern Finland, Yliopistonranta 1, FI-70211, Kuopio, Finland; [3]Department of Pediatric Oncology, Hematology and Clinical Immunology, Heinrich-Heine University Duesseldorf, Medical Faculty., Duesseldorf, Germany; [4]German Cancer Consortium (DKTK), 81675, Munich, Germany;[5]German Center for Child and Adolescent Health (DZKJ), partner site Munich, Munich, Germany;*

### Background

B-cell acute lymphoblastic leukemia (B-ALL) is the most common pediatric cancer. It peaks at 2-6 years, coinciding with daycare/school entry. An interplay between genetic and environmental factors (e.g. delayed infection) drives B-ALL from preleukemic clones. Strategies enhancing immune surveillance can control infections and, potentially, eradicate preleukemic clones, ultimately preventing B-ALL. Trained immunity (TI) describes innate immune cells' immunological memory induced by a training agent (as BCG or ß-glucan), that confers nonspecific protection against secondary infections and solid tumors. Epidemiological data suggest a protective role of BCG against B-ALL but lack experimental support.

### Aims

We set out to evaluate whether BCG-induced TI can protect against B-ALL.

### Methods

B-ALL cells from Pax5$^{+/-}$ WBMC transplantation (Tx) were cultured *in vitro* and GFP-transduced. Tx-experiments of these cells in mice that were either pre-trained or subsequently treated with BCG or PBS were evaluated using survival studies, flow cytometric immune phenotyping, cytokine profiling, and single-cell RNA sequencing (scRNA-seq).

**Copyright Information:** (Online) ISSN: 2572-9241
©2026 The Author(s). HemaSphere is published by John Wiley & Sons Ltd on behalf of European Hematology Association. This is an open access Abstract Book distributed under the Attribution-NonCommercial-NoDerivs (CC BY-NC-ND), which allows third parties to download the articles and share them with others as long as they credit the author and the Abstract Book, but they cannot change the content in any way or use them commercially.
**Abstract Book Citations:** Authors, Title, HemaSphere, 2026;10:(S1):pages. The abstract book DOIs can be found on the "Author Information" page.
**Disclaimer:** Articles published in the journal HemaSphere exclusively reflect the opinions of the authors. The authors are responsible for all content in their abstracts including accuracy of the facts, statements, citing resources, etc.

**Results**

A single BCG vaccination 7d or 28d prior to B-ALL Tx resulted in significantly lower leukemic burden in peripheral blood (PB), spleen, and bone marrow (BM) at 5-6 weeks post-Tx, compared to disseminated leukemia in PBS controls (28d, n= 4 vs 4, BM: 8.95%±17.9 vs 91.2%±5.3, $p<0.001$). This led to prolonged survival for 28d BCG-trained mice (Median survival (MS) : 71.5d vs 39d). This effect was dose-dependent, as 1/10 the standard BCG training dose (equivalent to infant vaccination) remained protective, but with a lower magnitude (MS: 48d vs 40d).
Leukemia reduction in 7d BCG-trained mice was accompanied by induced myelopoiesis from early hematopoietic progenitors to mature myeloid cells in the BM and PB, a TI hallmark. Also, $CD4^{+}$ and $CD8^{+}$ T-cells were significantly increased, together with elevated IL-1a and IFN-γ levels in trained animals' BM. Preservation of BCG-antileukemic effects in IFN-$g^{-/-}$ mice or following IFN-g neutralization suggests IFN-g as a marker of immune activation rather than a driver of protection. scRNA-seq of sort-purified B-ALL cells from BCG mice revealed distinct clustering, partial loss of B-cell identity, and increased plasticity compared to PBS controls.
Moreover, we investigated the crosstalk between innate and adaptive immunity in 28d BCG-trained B-ALL-transplanted mice (n= 5 vs 5). Two weeks after-Tx, BM macrophages exhibited a pro-inflammatory phenotype, characterized by increased CD64 and MHCII and reduced CD206 and F4/80 levels. Interestingly, those cells showed significantly lower PD-L1 levels, along with a significant increase in $CX3CR1^{+}$ $CD8^{+}$ T-cells, highlighting reduced inhibitory signaling and enhanced effector differentiation.
To assess BCG's therapeutic potential in early relapses, we transplanted mice with B-ALL cells and treated them 3d after engraftment with BCG or PBS. PB analysis 2 weeks later revealed comparable low $GFP^{+}$ B-ALL (BCG 5.1%±2.6 vs PBS 8.3%±4.3, ns). Those cells increased 6x in PBS mice PB at 4 weeks, compared to only 1.6x in BCG-treated mice. Monocytes, granulocytes, and T-cells were significantly increased in the PB of BCG-treated mice, along with prolonged survival (MS: 48d vs 37d).

**Summary/Conclusion**

In summary, we provide experimental proof-of-concept for BCG's systemic immune stimulation and protection against B-ALL, from preventive and therapeutic perspectives, enabling future therapeutic intervention.

**Copyright Information:** (Online) ISSN: 2572-9241
©2026 The Author(s). HemaSphere is published by John Wiley & Sons Ltd on behalf of European Hematology Association. This is an open access Abstract Book distributed under the Attribution-NonCommercial-NoDerivs (CC BY-NC-ND), which allows third parties to download the articles and share them with others as long as they credit the author and the Abstract Book, but they cannot change the content in any way or use them commercially.
**Abstract Book Citations:** Authors, Title, HemaSphere, 2026;10:(S1):pages. The abstract book DOIs can be found on the "Author Information" page.
**Disclaimer:** Articles published in the journal HemaSphere exclusively reflect the opinions of the authors. The authors are responsible for all content in their abstracts including accuracy of the facts, statements, citing resources, etc.

HemaSphere

### (PS1454) COOPERATIVITY OF KMT2A-MLLT1 WITH KMT2C LOSS ENABLES DUAL MYELOID-LYMPHOID LEUKEMIC POTENTIAL AND INDUCES CYTOSKELETAL REPROGRAMMING IN A ZEBRAFISH MODEL OF INFANT LEUKEMIA

**Topic:** 01. Acute lymphoblastic leukemia - Biology & translational research

Sarada Ketharnathan*[1], Keon Collett[2], Anna Pawelko[1], Serkan Dogan[1], Sujata Pokharel[1], Sergey Prykhozhij[1], Benno Orr[3], Jason Berman[1, 4]

*[1]CHEO Research Institute, Ottawa, Canada; [2]University of Calgary, Cumming School of Medicine, Calgary, Canada; [3]University of Toronto, Department of Molecular Genetics, Toronto, Canada; [4]University of Ottawa, Departments of Pediatrics, Ottawa, Canada;*

### Background

Infant leukemia (IL) presents in children with aggressive features such as high white blood cell counts, hepatosplenomegaly and central nervous system involvement. Chromosomal rearrangements involving the *KMT2A* gene, termed '*KMT2A-r*' are seen in 80% of IL patients. Of the common binding partners, MLLT1 is associated with the worst outcomes, with a 5-year event-free survival of 25% (vs. 34% for AFF1 and 68% for MLLT3). However, *KMT2A-r* alone is not sufficient to induce rapid-onset leukemia. Heterozygous germline mutations in the *KMT2C* gene were found to be enriched in *KMT2A-r* IL patients. The zebrafish is an ideal model for studying IL on account of its highly conserved hematopoietic system with easy access to embryonic progenitor cells.

### Aims

To evaluate the molecular mechanisms underlying the cooperativity between the *KMT2A-MLLT1* fusion and *kmt2ca* loss in driving rapid-onset IL.

### Methods

Whole mount-in situ hybridization (WISH) was used to characterize developmental hematopoiesis using the following lineage markers: erythroid *hbbe3*, myeloid *spi1b (pu.1)* and pan-leukocyte *lcp1.* To study adult hematopoiesis, we performed flow cytometry on isolated whole kidney marrows (WKMs, analogous to the human bone marrow). Epigenetic and transcriptomic analysis was performed using Bulk Assay for Transposase-Accessible Chromatin using Sequencing and RNA sequencing, respectively.

**Copyright Information:** (Online) ISSN: 2572-9241
©2026 The Author(s). HemaSphere is published by John Wiley & Sons Ltd on behalf of European Hematology Association. This is an open access Abstract Book distributed under the Attribution-NonCommercial-NoDerivs (CC BY-NC-ND), which allows third parties to download the articles and share them with others as long as they credit the author and the Abstract Book, but they cannot change the content in any way or use them commercially.
**Abstract Book Citations:** Authors, Title, HemaSphere, 2026;10:(S1):pages. The abstract book DOIs can be found on the "Author Information" page.
**Disclaimer:** Articles published in the journal HemaSphere exclusively reflect the opinions of the authors. The authors are responsible for all content in their abstracts including accuracy of the facts, statements, citing resources, etc.

**Results**

We expressed the human *KMT2A-MLLT1* fusion in the background of wildtype or *kmt2ca* loss-of-function zebrafish mutants generated using CRISPR-Cas9 editing. Characterization of primitive hematopoiesis showed reduced *hbbe3*$^{+}$ erythrocytes and *spi1b*$^{+}$ myeloid precursors in both *kmt2ca*$^{-/-}$ and *KMT2A-MLLT1* embryos at 22 hours post-fertilization. This reduction was further exacerbated in *kmt2ca-/-;KMT2A-MLLT1* compound mutants. By 4 days post-fertilization, we observed increased *lcp1*$^{+}$ lymphoblasts in the developing thymus and *lcp1*$^{+}$ granulocytes in the caudal hematopoietic tissue of *kmt2ca*$^{-/-}$ and *KMT2A-MLLT1* larvae, suggesting leukocyte lineage bias. Flow cytometry of kidney marrows at 12 months revealed a lymphoid bias in *KMT2A:MLLT1* fish with no changes in the hematopoietic stem cell (HSC) compartment. In contrast, ~50% of *kmt2ca*$^{-/-}$*;KMT2A-MLLT1* compound mutants exhibited lymphoid expansion, with 1 of 3 fish showing concurrent HSC expansion, suggesting lymphoid leukemia. The remaining fish showed expanded myeloid and HSC compartments, suggesting myeloid leukemia. These findings are consistent with the known association of *KMT2A-MLLT1* with both lymphoid and myeloid leukemias in IL patients. Epigenetic analysis at 24 hpf revealed alterations in genes involved in leukocyte migration and infiltration, RAS kinase signaling and organization of the cytoskeleton. Concurrently, Rho GTPAse signalling and leukocyte migratory pathways were transcriptionally dysregulated.

**Summary/Conclusion**

In summary, our data suggests a role for cytoskeletal signaling in accelerating disease progression in*KMT2A-r* IL for the first time, potentially by modulating leukocyte behavior. The conserved ability of *KMT2A-r* to drive both lymphoid and myeloid disease in the zebrafish makes it an ideal model to study IL biology with the aim of identifying novel therapeutic candidates to improve treatment outcomes for this incredibly vulnerable patient population.

**Copyright Information:** (Online) ISSN: 2572-9241
©2026 The Author(s). HemaSphere is published by John Wiley & Sons Ltd on behalf of European Hematology Association. This is an open access Abstract Book distributed under the Attribution-NonCommercial-NoDerivs (CC BY-NC-ND), which allows third parties to download the articles and share them with others as long as they credit the author and the Abstract Book, but they cannot change the content in any way or use them commercially.
**Abstract Book Citations:** Authors, Title, HemaSphere, 2026;10:(S1):pages. The abstract book DOIs can be found on the "Author Information" page.
**Disclaimer:** Articles published in the journal HemaSphere exclusively reflect the opinions of the authors. The authors are responsible for all content in their abstracts including accuracy of the facts, statements, citing resources, etc.

### (PS1455) METABOLIC REPROGRAMMING TOWARD OXIDATIVE PHOSPHORYLATION DRIVES RESISTANCE IN B-ALL

**Topic:** 01. Acute lymphoblastic leukemia - Biology & translational research

Maximilian Mönnig*[1], Magdalena Pajonk[1], Sergi Beneyto-Calabuig[2, 3], Fabian Schulte[1], Aykut Demir[1], Valérie Marot-Lassauzaie[4, 5], Laleh Haghverdi[4, 5], Carsten Müller-Tidow[1], Lars Velten[2, 3], Simon Raffel[1]

*[1]Department of Hematology, Oncology and Rheumatology - University Hospital Heidelberg, Heidelberg, Germany; [2]Centre for Genomic Regulation (CRG), The Barcelona Institute of Science and Technology, Barcelona, Spain; [3]Universitat Pompeu Fabra, Barcelona, Spain; [4]Berlin Institute Of Health, Charité – Universitätsmedizin Berlin, Berlin, Germany; [5]Max Delbrück Center - Berlin Institute For Medical Systems Biology, Berlin, Germany;*

### Background

Despite major therapeutic advances, a substantial proportion of adult patients with B-cell acute lymphoblastic leukemia (B-ALL) relapse or develop refractory disease. Proposed resistance mechanisms include clonal evolution and metabolic adaptation, yet their interaction at the single-cell level during disease progression remains unclear. Integrated analysis of clonal hierarchy, transcriptional programs, and metabolic states across longitudinal stages may elucidate resistance dynamics.

### Aims

We aimed to dissect clonal evolution and metabolic rewiring during B-ALL progression using a novel single-cell multiomic approach that simultaneously integrates clonal architecture, transcriptome, and metabolic state at single-cell resolution, with validation in independent bulk transcriptomic datasets and functional modeling in B-ALL cell lines.

### Methods

Cryopreserved bone marrow aspirates collected at nine disease timepoints from a BCR-ABL–positive patient were processed using the MutaSeq protocol, enabling amplification of driver mutations and transcriptomic profiling. Index-sorting allowed the integration of single-cell transcriptomes with six metabolic fluorescence parameters, including ROS, intracellular pH, autophagy, lipid droplets, mitochondrial mass, and mitochondrial potential. Clonal hierarchies were reconstructed using CloneTracer, and lowly expressed driver mutations were assigned to established clones. Public bulk RNA-seq datasets from adult and pediatric B-ALL cohorts were analyzed to validate relapse-associated programs, and transcriptional activity was inferred using the decoupler ULM model. Inducible TXNIP knockdown models were generated in two B-ALL cell lines via lentiviral shRNA transduction.

### Results

Single-cell multiomic profiling of a single BCR-ABL–positive B-ALL patient across nine longitudinal disease timepoints enabled reconstruction of the clonal hierarchy based on the BCR-ABL translocation and mitochondrial mutations. A T315I-mutant clone emerged at minimal residual disease (MRD) before allogeneic transplantation. At relapse, two dominant clones were identified, with both showing increased mitochondrial mass. Transcriptomic analysis revealed upregulated MYC activity and oxidative phosphorylation (OXPHOS) in both clones. A subpopulation was carrying the transcriptional changes already at MRD. Two independent bulk RNA-seq datasets of matched B-ALL samples confirmed enrichment of OXPHOS and MYC-associated programs at relapse, and higher OXPHOS scores correlated with inferior survival in relapse samples. TXNIP showed an inverse correlation with MYC activity in single-cell and external cohorts, and low TXNIP expression was associated with adverse prognosis. Functional perturbation in B-ALL cell lines supported a role for MYC and TXNIP in regulating mitochondrial metabolic reprogramming.

**Copyright Information:** (Online) ISSN: 2572-9241
©2026 The Author(s). HemaSphere is published by John Wiley & Sons Ltd on behalf of European Hematology Association. This is an open access Abstract Book distributed under the Attribution-NonCommercial-NoDerivs (CC BY-NC-ND), which allows third parties to download the articles and share them with others as long as they credit the author and the Abstract Book, but they cannot change the content in any way or use them commercially.
**Abstract Book Citations:** Authors, Title, HemaSphere, 2026;10:(S1):pages. The abstract book DOIs can be found on the "Author Information" page.
**Disclaimer:** Articles published in the journal HemaSphere exclusively reflect the opinions of the authors. The authors are responsible for all content in their abstracts including accuracy of the facts, statements, citing resources, etc.

**Summary/Conclusion**

Longitudinal single-cell multiomics revealed clonal evolution and metabolic rewiring in BCR-ABL–positive B-ALL, identifying relapse-associated clones with distinct metabolic features and elevated MYC activity. Bulk datasets confirmed OXPHOS upregulation at relapse and its association with inferior survival. Functional modulation of MYC and TXNIP recapitulated key metabolic phenotypes in vitro, highlighting metabolic reprogramming as a potential therapeutic vulnerability in relapsed B-ALL.

**Copyright Information:** (Online) ISSN: 2572-9241
©2026 The Author(s). HemaSphere is published by John Wiley & Sons Ltd on behalf of European Hematology Association. This is an open access Abstract Book distributed under the Attribution-NonCommercial-NoDerivs (CC BY-NC-ND), which allows third parties to download the articles and share them with others as long as they credit the author and the Abstract Book, but they cannot change the content in any way or use them commercially.
**Abstract Book Citations:** Authors, Title, HemaSphere, 2026;10:(S1):pages. The abstract book DOIs can be found on the "Author Information" page.
**Disclaimer:** Articles published in the journal HemaSphere exclusively reflect the opinions of the authors. The authors are responsible for all content in their abstracts including accuracy of the facts, statements, citing resources, etc.

### (PS1456) NEXT-GENERATION FLT3-DIRECTED CHIMERIC ANTIGEN RECEPTOR T CELL (FLT3CART) IMMUNOTHERAPY FOR KMT2A-REARRANGED ACUTE LYMPHOBLASTIC LEUKEMIA (KMT2A-R ALL)

**Topic:** 01. Acute lymphoblastic leukemia - Biology & translational research

Shubhmita Bhatnagar*[1], Michael Yarnell[2, 3], Camilo Arenas Merizalde[1], Sarah Haines[1], Terry Fry[2, 3], Sarah Tasian[1, 4, 5]

*[1]Children's Hospital Of Philadelphia, Division of Oncology and Center for Childhood Research, Philadelphia, United States of America; [2]University of Colorado Anschutz Medical Campus, Department of Pediatrics, Aurora, United States of America; [3]Children's Hospital Colorado, Center for Cancer and Blood Disorders, Aurora, United States of America;[4]University of Pennsylvania School of Medicine, Department of Pediatrics and Abramson Cancer Center, Philadelphia, United States of America; [5]Princess Máxima Center for Pediatric Oncology, Utrecht, The Netherlands;*

### Background

Relapsed B-ALL remains the leading cause of cancer-related mortality in children and adolescents/young adults (AYAs). Many patients with high-risk B-ALL, particularly those with *KMT2A* rearrangements, face poor outcomes and limited access to effective salvage therapies. Although CD19-directed chimeric antigen receptor T cells (CD19CART) can induce deep remissions, approximately 50% of patients relapse again due to poor T cell persistence, antigen loss (including lineage switch [LS]), immune escape, and other mechanisms. We previously demonstrated robust preclinical activity of FLT3CART comprised of an NC7 binder scFv and 4-1BB/CD3ζ costimulatory domains (NC7-CART) against *KMT2A*-R ALL and lineage switch (LS) acute myeloid leukemia (AML) (Niswander *Haematologica* 2023). However, our preclinical studies showed that NC7-FLT3CART induces weaker IFN-γ and Th1 responses than those of CD19CART, suggesting that durable remissions will likely require enhanced strategies.

### Aims

We aim to optimize next-generation FLT3CARTs with alternative binders and costimulatory domains to enhance efficacy and overcome potential immunotherapeutic resistance in translational *KMT2A*-R ALL and AML models.

### Methods

Utilizing a human B cell-derived phage library and further optimizations, we generated fully human scFv or single domain IgG variable heavy chain (VH) binders targeting a distinct FLT3 extracellular domain. Each binder was then incorporated into second-generation CAR constructs containing CD3ζ and CD28 or 4-1BB endodomains. Lead candidate next-generation FLT3CARTs were selected based in vitro activity against ALL and AML cell lines. Patient-derived xenograft (PDX) mouse models of infant, pediatric, and young adult *KMT2A-R* ALL and AML models were treated intravenously with saline, mock T cells, NC-7 FLT3CART, or one of four next-generation FLT3CARTs. Human CART and leukemia cells were quantified weekly via flow cytometry (FC) analysis of murine peripheral blood and in end-study bone marrow and/or spleens.

### Results

Initial screening identified greatest functionality of 2G5 and H5 VH FLT3 binders. Affinity-enhanced versions of 2G5 and H5 (2G5-F7 and H5-A10) were generated through CDR3 amino acid substitutions, resulting in a range of FLT3-binding affinities. These lead candidate fully human FLT3CARTs demonstrated superior Type 1 cytokine production and comparable or superior *in vivo* anti-leukemia activity versus our original NC7-FLT3CART in a MOLM14 AML cell line xenograft model. Finally, superior anti-leukemia activity of next-generation FLT3CARTs with greater *in vivo* expansion and cytokine production was observed in *KMT2A*-R ALL (n=2) and *FLT3*-mutant AML (n=1) PDX models. The dsNC7-FLT3CART particularly induced IFN-γ production and most potently inhibited *in vivo* leukemia proliferation (representative data in **Figure 1**).

**Copyright Information:** (Online) ISSN: 2572-9241
©2026 The Author(s). HemaSphere is published by John Wiley & Sons Ltd on behalf of European Hematology Association. This is an open access Abstract Book distributed under the Attribution-NonCommercial-NoDerivs (CC BY-NC-ND), which allows third parties to download the articles and share them with others as long as they credit the author and the Abstract Book, but they cannot change the content in any way or use them commercially.
**Abstract Book Citations:** Authors, Title, HemaSphere, 2026;10:(S1):pages. The abstract book DOIs can be found on the "Author Information" page.
**Disclaimer:** Articles published in the journal HemaSphere exclusively reflect the opinions of the authors. The authors are responsible for all content in their abstracts including accuracy of the facts, statements, citing resources, etc.

### Summary/Conclusion

Novel cysteine-substituted FLT3CARTs demonstrated enhanced cytokine production, *in vivo* expansion, and superior anti-leukemia activity compared to the original NC7-FLT3CART in *KMT2A*-rearranged ALL and AML models. Additional studies in *KMT2A*-R ALL and LS AML PDX models are underway to validate and extend these findings in preparation for a phase 1 clinical trial in development.

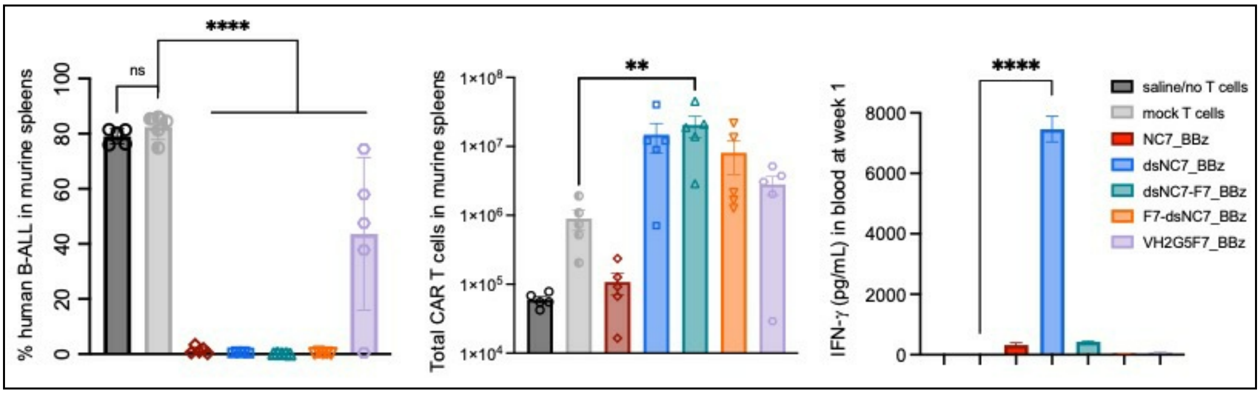

Figure 1. Next-generation FLT3CARTs inhibit in vivo KMT2A-R ALL proliferation with greater in vivo expansion and cytokine production than original NC7-FLT3CART. Human CD19+/CD45+ ALL cells (left) and CD3+ T cells (center) were quantified in saline, mock, or CART-treated ALL PDX model mice in end-study spleens. Greatest IFN-γ production was induced by the dsNC7-FLT3CART product (blue). Statistical analysis was performed via one-way ANOVA with Dunnett's post-test for multiple comparisons using the mock T cell cohort (light grey) as comparator. **p<0.01, ****p<0.0001

**Copyright Information:** (Online) ISSN: 2572-9241
©2026 The Author(s). HemaSphere is published by John Wiley & Sons Ltd on behalf of European Hematology Association. This is an open access Abstract Book distributed under the Attribution-NonCommercial-NoDerivs (CC BY-NC-ND), which allows third parties to download the articles and share them with others as long as they credit the author and the Abstract Book, but they cannot change the content in any way or use them commercially.
**Abstract Book Citations:** Authors, Title, HemaSphere, 2026;10:(S1):pages. The abstract book DOIs can be found on the "Author Information" page.
**Disclaimer:** Articles published in the journal HemaSphere exclusively reflect the opinions of the authors. The authors are responsible for all content in their abstracts including accuracy of the facts, statements, citing resources, etc.

### (PS1457) VENETOCLAX-BASED COMBINATIONS TARGET HIGH-RISK ADULT ACUTE LYMPHOBLASTIC LEUKEMIA: SYNERGISTIC ACTIVITY WITH PONATINIB AND BLINATUMOMAB ACROSS MOLECULAR SUBGROUPS

**Topic:** 01. Acute lymphoblastic leukemia - Biology & translational research

Federico Lo Schiavo*[1, 2], Chiara Salvesi[1], Lorenzo Ledda[3], Andrea Ghelli Luserna di Rosà[4], Eugenio Fonzi[5], Asia Spedaluzzi[1], Maria Benedetta Giannini[6], Cristina Papayannidis[7], Michela Rondoni[8], Giovanni Marconi[8, 9], Anna Maria Mianulli[10], Sara Galimberti[11], Prassede Salutari[12], Fabio Giglio[13], Crescenza Pasciolla[14], Monica Fumagalli[15], Annalisa Imovilli[16], Endri Mauro[17], Giovanni Martinelli[7], Giorgia Simonetti[1], Anna Ferrari[1]

*[1]IRCCS Istituto Romagnolo per lo Studio dei Tumori IRST "Dino Amadori, Biosciences Laboratory, Meldola, Italy; [2]Università degli Studi del Sannio, Dipartimento di Scienze e Tecnologie, Benevento, Italy; [3]Azienda Ospedaliera Santa Maria di Terni, Terni, Italy; [4]Fondazione Pisana per Scienza ONLUS, Pisa, Italy;[5]IRCCS Istituto Romagnolo per lo Studio dei Tumori IRST "Dino Amadori", Unit of Biostatistics and Clinical Trials, Meldola, Italy; [6]IRCCS Istituto Romagnolo per lo Studio dei Tumori (IRST) "Dino Amadori", SSD Ematologia e Trapianti CSE, Meldola, Italy; [7]IRCCS Azienda Ospedaliero-Universitaria di Bologna, Istituto di Ematologia "Seràgnoli", Bologna, Italy; [8]Ospedale Santa Maria delle Croci, U.O. Ematologia, Ravenna, Italy;[9]Alma Mater Studiorum Università di Bologna, Bologna, Italy; [10]Ospedale Infermi, U.O. Ematologia, Rimini, Italy; [11]AOUP Santa Chiara, Pisa, Italy; [12]Ospedale Civile Santo Spirito, UOC Ematologia Clinica, Pescara, Italy; [13]IRCCS San Raffaele Scientific Institute, Milano, Italy; [14]Istituto Oncologico IRCCS "Giovanni Paolo II", Bari, Italy; [15]IRCCS Ospedale San Gerardo, Ematologia, Monza, Italy; [16]AUSL - IRCCS di Reggio Emilia, S.C. Ematologia, Reggio Emilia, Italy; [17]Ospedale Ca' Foncello, Treviso, Italy;*

### Background

Despite new therapy advancements, in adult acute lymphoblastic leukemia (ALL), long-term survival remains limited (30–60%), particularly in relapsed/refractory (R/R) disease. Venetoclax (Ven) has shown efficacy in acute myeloid leukemia (AML) combinations and preclinical/early-clinical activity in B/T-ALL. Ponatinib (Pona) and blinatumomab (Blina), widely used in B-ALL (Ph+, Ph−, Ph-like), are rational partners for Ven in ALL.

### Aims

To assess Ven activity across ALL models, define molecular predictors of response, and evaluate its combination with Pona and Blina in ALL.

### Methods

A comprehensive preclinical panel included 19 acute leukemia (AL) cell lines (4 AML, 2 T-ALL, 12 B-ALL) and 37 primary pt cells [33 B-ALL (24 Diagnosis;9 Rel), 4 T-ALL, 1 mixed AL]. Cell viability and proliferation were assessed using the RealTime-Glo. Co-culture experiments with healthy donor CD3⁺ T cells were performed to assess Blina efficacy (24h). RNA-seq was performed in 31 adult B-ALL patients and 19 donors; digitalMLPA-ALL copy number alteration CNA analysis was conducted in 25 DNA.

**Copyright Information:** (Online) ISSN: 2572-9241
©2026 The Author(s). HemaSphere is published by John Wiley & Sons Ltd on behalf of European Hematology Association. This is an open access Abstract Book distributed under the Attribution-NonCommercial-NoDerivs (CC BY-NC-ND), which allows third parties to download the articles and share them with others as long as they credit the author and the Abstract Book, but they cannot change the content in any way or use them commercially.
**Abstract Book Citations:** Authors, Title, HemaSphere, 2026;10:(S1):pages. The abstract book DOIs can be found on the "Author Information" page.
**Disclaimer:** Articles published in the journal HemaSphere exclusively reflect the opinions of the authors. The authors are responsible for all content in their abstracts including accuracy of the facts, statements, citing resources, etc.

### Results

Ven exhibited heterogeneous activity across AL cell lines, with 72.2% classified as sensitive (detailed in Fig. 1A). Both T-ALL cell lines were resistant to ven, as were OCI-AML3 (*NPM1*-mut), MUTZ-5 (adult Ph-like), and KOPN8 (*KMT2A*r B-ALL) cell lines. Importantly, marked sensitivity to ven was observed across multiple B-ALL subgroups (e.g KASUMI-10, JIH-5, and REH were the most sensitive; Fig 1A), including high-risk subtypes. Importantly, the Ven–Pona combination showed synergistic effects in SUP-B15 cells and additive effects in other B-ALL subgroups, including MHH-CALL4 and NALM-19. *BCL2* expression was evaluated in 31/33 treated primary B-ALL samples and compared with healthy donors, identifying distinct high- (n 7; 6 Ph- and 1 Ph+) and low-expressing subsets (n 12; 6 Ph+ and 6 Ph–). No significant correlation was observed between high *BCL2* expression and treatment response. dMLPA-ALL reveal and enrichment of 20q13.12/13 deletions in responder group, while the non-resp one is enriched in large chr17 CNAs. Based on the median response, interestingly 5/9 relapse were classified as sensitive (3 Ph–, including one *BCL2*r case, and 2 Ph+, both harboring *ABL1* T315I) and 4 (Ph-like, MPAL *KMT2A*r, Ph+, *CEBPE*r) were non-resp and exhibited marked resistance, with high $IC_{50}$ (16 to 99 µM). Importantly, the Ven–Pona combo (n=4) showed evidence of synergistic/additive interactions in 3/4 pts samples (2 Ph-,1 Ph+; Fig. 1B), with the higher syn score in a relapsed Ph-like case. The resistant case was a *DUX4*r case, which relapsed 7 years after achieving CR. Notably, in preliminary co-culture experiments using NALM-6 and healthy CD3+ T cells, the addition of Ven enhanced NALM-6 sensitivity to T CD3+ cytotoxicity. Remarkably, the ven-blina combo further potentiated T-cell killing.

### Summary/Conclusion

Ven showed strong activity across most AL cell line molecular subgroups. In our cohort of treated 33 B-ALL pts, a distinct highly resistant subset emerged (warranting further characterization), with non-responder observed at both diagnosis and relapse. Ven was effective in most relapsed pts, including high-risk subgroups such as *BCL2*r and *ABL1* T315I cases, representing a valuable therapeutic option. Preliminary data on Ven combined with Pona/Blina show promising activity and may support a future therapeutic strategy. Supported by PNRR-POC-2022-12375862 FUSION-TARGET

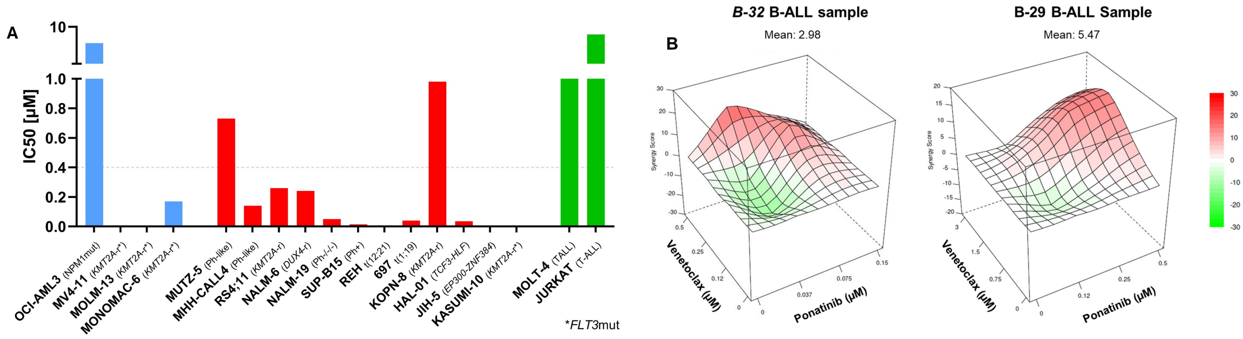

**Fig.1. (A)** *In vitro* 72h response to venetoclax in 19 acute leukemia cell lines (4 AML, 2 T-ALL, and 12 B-ALL). Cell lines with an $IC_{50}$ lower than 0.4 µM (grey dashed line) were classified as sensitive. Among AML cell lines (blue), MV4-11 and MOLM-13 were the most sensitive, both harboring *KMT2A*-r and *FLT3*-mut, followed by MONOMAC-6 (acute monocytic leukemia, *KMT2A*-r and *FLT3*-mut). OCI-AML3, carrying *NPM1* and *DNMT3A* mutations, was the most resistant AML cell line, with an $IC_{50}$ of 6.5 µM. B-ALL cell lines (red) showed a more heterogeneous response: KASUMI-10, REH, and JIH-5 were the most sensitive ($IC_{50}$ range 0.001-0.0001 µM), whereas MUTZ-5 and KOPN-8 were particularly resistant, with $IC_{50}$ values of 0.73 and 0.98 µM, respectively. The two T-ALL cell lines (green), MOLT-4 and JURKAT, were both highly resistant to venetoclax, with $IC_{50}$ values of 1.06 and 8.37 µM, respectively. * *FLT3*-mutated cell lines. (B) Selection of ZIP synergy score calculated using SynergyFinder+ for two primary B-ALL patient samples. In primary Ph- B-ALL sample B32 (left; *BCL2L1–NRIP1* fusion, hyperdiploid karyotype, and presence of myeloid markers) and in primary Ph+ B-ALL sample B29 (right), the combination at 72h showed an overall additive effect with synergistic interactions at specific dose combinations.

**Copyright Information:** (Online) ISSN: 2572-9241
©2026 The Author(s). HemaSphere is published by John Wiley & Sons Ltd on behalf of European Hematology Association. This is an open access Abstract Book distributed under the Attribution-NonCommercial-NoDerivs (CC BY-NC-ND), which allows third parties to download the articles and share them with others as long as they credit the author and the Abstract Book, but they cannot change the content in any way or use them commercially.
**Abstract Book Citations:** Authors, Title, HemaSphere, 2026;10:(S1):pages. The abstract book DOIs can be found on the “Author Information” page.
**Disclaimer:** Articles published in the journal HemaSphere exclusively reflect the opinions of the authors. The authors are responsible for all content in their abstracts including accuracy of the facts, statements, citing resources, etc.

### (PS1458) FLT3 AND BCL2 AS THERAPEUTIC TARGETS IN PAX5-ALTERED PEDIATRIC B-CELL PRECURSOR LYMPHOBLASTIC LEUKEMIA

**Topic:** 01. Acute lymphoblastic leukemia - Biology & translational research

Alessia Curto*[1, 2], Rebecca Rapisarda[1, 2], Nicolò Peccatori[1, 2], Domenico Gaspari[3], Luigia Valsecchi[2], Simona Procopio[1, 2], Stefano Rebellato[2], Silvia Rasetti[1, 2], Chiara Palmi[2], Michela Bardini[2], Andrea Biondi[1, 2], Giovanni Cazzaniga[1, 2], Grazia Fazio[1, 2]

*[1]University of Milano-Bicocca, School of Medicine and Surgery, Monza, Italy;[2]Tettamanti Center, Fondazione IRCCS San Gerardo dei Tintori, Monza, Italy; [3]IRCCS San Gerardo dei Tintori, Monza, Italy;*

### Background

B-Cell Precursor Acute Lymphoblastic Leukemia (BCP-ALL) is the most common pediatric cancer, with survival rates exceeding 85%. However, the prognosis remains poor for patients who are refractory to 1$^{st}$ line treatment or who experience relapse after multi-chemotherapy but also after CD19-directed immunotherapy. PAX5, a transcriptional factor that plays a crucial role in B-cell development, is frequently altered in BCP-ALL (*PAX5*-alt). *PAX5*-alt ALL is characterized by a specific gene expression profile and is commonly associated with various *PAX5* alterations, including rearrangements (*PAX5*r). These rearrangements disrupt the physiological PAX5 transcriptional program, leading to the repression of genes typically activated by wild-type PAX5 and the upregulation of genes normally repressed by PAX5. The fms-like tyrosine kinase 3 (FLT3) gene has been previously identified among PAX5 repressed genes, suggesting its potential role in sustaining leukemia cell survival in *PAX5*r ALL. In addition, B-ALL frequently exhibits dysregulation of the anti-apoptotic B-cell lymphoma 2 (BCL-2) family proteins, shifting the apoptotic balance toward cell survival and contributing leukemogenesis.

### Aims

This study aimed to evaluate, in preclinical models of *PAX5*r BCP-ALL, the therapeutic efficacy of FLT3 and BCL-2 inhibition using gilteritinib and venetoclax, respectively.

### Methods

Gilteritinib and venetoclax were first tested *in-vitro* in NALL-1 (*PAX5::ETV6*), SEM (*KMT2A::AFF1*), 697 (*TCF3::PBX1*), and RCH-ACV (*TCF3::PBX1*) cell lines, and *ex-vivo* in five *PAX5*r PDX samples (*PAX5::JAK2, PAX5::SOX5, PAX5::DACH2, PAX5::AUTS2*). Apoptosis was assessed by Annexin V/7-AAD FACS staining. For *in-vivo* studies, female NSG mice were transplanted with *PAX5r* (*PAX5::SOX5*) leukemic cells and treated with gilteritinib (10 or 20 mg/kg) once ≥10% hCD10$^+$/CD19$^+$ blasts were detected. Tumor burden, defined as white blood cells count x % hCD10$^+$/CD19$^+$ blasts, was evaluated in the spleen (SP), bone marrow (BM), and central nervous system (CNS). FACS analysis was performed to assess FLT3 expression and activation.

### Results

FLT3 protein expression was higher in NALL-1 compared with 697 and RCH-ACV cell lines, which were used as negative controls (MFI 399 vs 86.3 vs 72.8). Consistently, gilteritinib was more effective in reducing cell viability in NALL-1 than in 697 cells (IC50 1014 nM vs 7170 nM, p<0.02). *Ex-vivo* 72-hour treatment with gilteritinib in *PAX5r* PDX blasts at nanomolar concentrations induced potent cytotoxicity (IC50 317-724.5 nM, p<0.01). Similarly, venetoclax reduces cell viability in NALL-1 cells (IC50 6.76 nM, p<0.0001) and in *PAX5r* PDX blasts (IC50 1.87-29.87 nM, p<0.001). *In-vivo*, treatment with gilteritinib at 20 mg/kg significantly reduced leukemic hCD10$^+$/CD19$^+$ blasts in bone marrow (p<0.0001), spleen (p<0.0001) and central nervous system (p<0.01). A trend toward reduced phosphorylated FLT3 (pFLT3) was also observed in BM hCD10$^+$ blasts (p=n.s.).

**Copyright Information:** (Online) ISSN: 2572-9241
©2026 The Author(s). HemaSphere is published by John Wiley & Sons Ltd on behalf of European Hematology Association. This is an open access Abstract Book distributed under the Attribution-NonCommercial-NoDerivs (CC BY-NC-ND), which allows third parties to download the articles and share them with others as long as they credit the author and the Abstract Book, but they cannot change the content in any way or use them commercially.
**Abstract Book Citations:** Authors, Title, HemaSphere, 2026;10:(S1):pages. The abstract book DOIs can be found on the "Author Information" page.
**Disclaimer:** Articles published in the journal HemaSphere exclusively reflect the opinions of the authors. The authors are responsible for all content in their abstracts including accuracy of the facts, statements, citing resources, etc.

**Summary/Conclusion**

FLT3 represents a novel potential therapeutic target in pediatric *PAX5*r BCP-ALL. Gilteritinib and venetoclax induced apoptosis in monotherapy in *ex-vivo PAX5*r models. *In-vivo*, gilteritinib demonstrated antileukemic activity, likely mediated by a reduction in FLT3 activation. In the future, it will be interesting to evaluate the combination of these drugs for the treatment of *PAX5*r BCP-ALL and to investigate the underlying molecular mechanism using innovative techniques such as CyTOF and RNA-sequencing.

**Copyright Information:** (Online) ISSN: 2572-9241
©2026 The Author(s). HemaSphere is published by John Wiley & Sons Ltd on behalf of European Hematology Association. This is an open access Abstract Book distributed under the Attribution-NonCommercial-NoDerivs (CC BY-NC-ND), which allows third parties to download the articles and share them with others as long as they credit the author and the Abstract Book, but they cannot change the content in any way or use them commercially.
**Abstract Book Citations:** Authors, Title, HemaSphere, 2026;10:(S1):pages. The abstract book DOIs can be found on the “Author Information” page.
**Disclaimer:** Articles published in the journal HemaSphere exclusively reflect the opinions of the authors. The authors are responsible for all content in their abstracts including accuracy of the facts, statements, citing resources, etc.

### (PS1459) TARGETABLE, RELAPSE SPECIFIC MUTATIONS ARE SIGNIFICANTLY ENRICHED IN ACUTE LYMPHOBLASTIC LEUKEMIA DURING INDUCTION THERAPY AND CAN ATTAIN CLONAL DOMINANCE

**Topic:** 01. Acute lymphoblastic leukemia - Biology & translational research

Rabab Allawati*[1], Julie Irving[1], James Allan[1], Dino Masic[1]

[1]*Newcastle University, Newcastle upon tyne, United Kingdom;*

### Background

Childhood acute lymphoblastic leukaemia (ALL) is the most common paediatric cancer. While cure rates are good, relapsed ALL remains a common cause of death in children with cancer.
Genomic analyses of paired presentation and relapse samples have revealed extensive clonal diversity and shown that in most cases, leukaemic cells at relapse are related to a minor clone present at diagnosis. These low-level mutated clones persist during treatment and there is anecdotal evidence for targetable mutated genes such as *JAK* and *RAS* being detectable in the MRD.

### Aims

This study aimed to track relapse-associated mutations from diagnosis through MRD to relapse to investigate the dynamics of clonal evolution and opportunities for therapeutic targeting of MRD to avert relapse.

### Methods

Triplet samples (diagnosis, MRD and relapse) were obtained from the Australian Sydney Children's Hospital Biobank (n=20). Whole exome sequencing (WES) of diagnosis–relapse pairs was used to identify relapse-associated mutations, which were subsequently tracked in matched diagnosis, MRD, and relapse samples using droplet digital PCR (ddPCR). Cancer cell fraction (CCF) was estimated by integrating variant allele frequency with blast percentage.

### Results

WES identified 19 relapse-associated mutations in 15 patients which were then tracked from diagnosis through MRD and relapse using ddPCR. Most mutations (17/19) were detectable at very low level at diagnosis (CCF, mean and SD, 1.85 ± 3.8%), prior to therapy (Figure 1). However, these subclones showed significant expansion during the first few weeks of therapy, with the mean CCF increasing from 1.85 ± 3.8% to 30.09 ± 45.10% within the MRD sample. Several mutations in a variety of genes reached full clonal dominance within the MRD; they included *KRAS, TET2, TSHZ2, NF1,* and *NT5C3A*. In contrast, 2 gene mutations; *JAK2* and *ZFHX3* were undetectable at diagnosis and during therapy but emerged at relapse, suggesting *de novo* acquisition. In Patient 2, low-level *TET2* and *KRAS* mutations present at diagnosis became clonally dominant by day 18 of treatment, consistent with expansion of a single therapy-resistant clone. Similarly, in patient 3, three relapse-associated mutations (*FLT3, NOTCH1,* and *DCC*) displayed comparable CCFs at day 14, supporting their presence within the same dominant clone.

### Summary/Conclusion

We show for the first time that mutations 'appearing' at relapse are invariably present at diagnosis and are significantly increased during the first few weeks of therapy, with some achieving clonal dominance even at this early stage of therapy. Many mutations are therapeutically tractable suggesting that genetic analyses of the MRD in high-risk patients may identity targeted agents that can be used to avert relapse.

**Copyright Information:** (Online) ISSN: 2572-9241
©2026 The Author(s). HemaSphere is published by John Wiley & Sons Ltd on behalf of European Hematology Association. This is an open access Abstract Book distributed under the Attribution-NonCommercial-NoDerivs (CC BY-NC-ND), which allows third parties to download the articles and share them with others as long as they credit the author and the Abstract Book, but they cannot change the content in any way or use them commercially.
**Abstract Book Citations:** Authors, Title, HemaSphere, 2026;10:(S1):pages. The abstract book DOIs can be found on the "Author Information" page.
**Disclaimer:** Articles published in the journal HemaSphere exclusively reflect the opinions of the authors. The authors are responsible for all content in their abstracts including accuracy of the facts, statements, citing resources, etc.

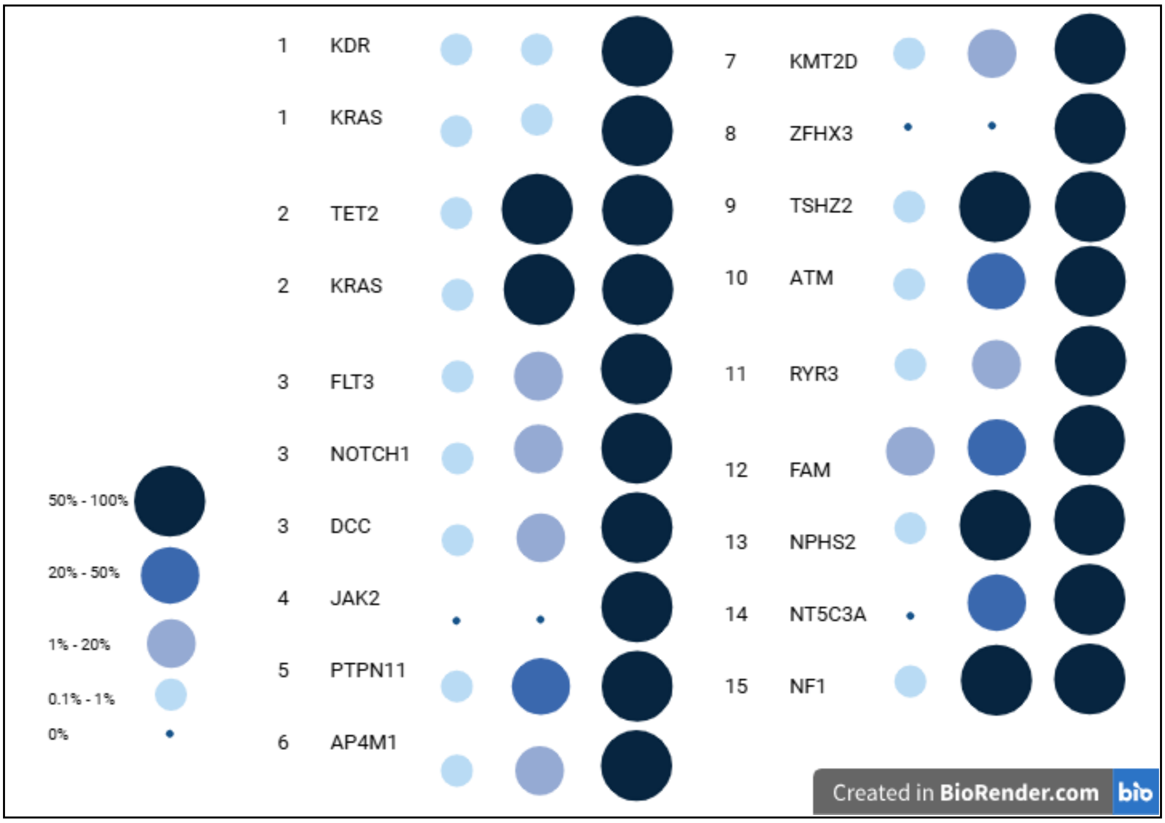

CCF values at the tree timelines of matched Dx (diagnosis), MRD and relapse samples of different gene mutations detected by ddPCR. Diameter and colour of circles are proportional to the CCF% of each mutation in individual sample as shown in the box on the left.

**Copyright Information:** (Online) ISSN: 2572-9241
©2026 The Author(s). HemaSphere is published by John Wiley & Sons Ltd on behalf of European Hematology Association. This is an open access Abstract Book distributed under the Attribution-NonCommercial-NoDerivs (CC BY-NC-ND), which allows third parties to download the articles and share them with others as long as they credit the author and the Abstract Book, but they cannot change the content in any way or use them commercially.
**Abstract Book Citations:** Authors, Title, HemaSphere, 2026;10:(S1):pages. The abstract book DOIs can be found on the "Author Information" page.
**Disclaimer:** Articles published in the journal HemaSphere exclusively reflect the opinions of the authors. The authors are responsible for all content in their abstracts including accuracy of the facts, statements, citing resources, etc.

### (PS1460) DIVERGENCE BETWEEN LEUKEMOGENIC BASAL T-CELL RECEPTOR SIGNALING AND ANTIGEN-INDUCED ACTIVATION IN MOUSE MODELS OF T-LYMPHOBLASTIC LEUKEMIA/LYMPHOMA

**Topic:** 01. Acute lymphoblastic leukemia - Biology & translational research

Marina Baessa*[1, 2], Telmo Catarino[1], Ivette Pacheco-Leyva[1], Telma Costa[1], Daniel Lopes[1], Juliana Cunha[1], Nuno R. Dos Santos[1]

*[1]i3S - Instituto de Investigação e Inovação em Saúde da Universidade do Porto, Porto, Portugal;[2]ICBAS - Instituto de Ciências Biomédicas Abel Salazar da Universidade do Porto, Porto, Portugal;*

### Background

T-lymphoblastic leukemia/lymphoma (T-ALL/LBL) is a malignant disorder characterized by development arrest of immature T cells, followed by rapid proliferation and dissemination of these aberrant T lymphocytes. The T cell receptor (TCR) plays an essential role in T cell development, allowing the interaction with MHC-bound antigens, which defines T cell fate during positive and negative selection.

### Aims

Since many T-ALL/LBL cases exhibit TCR on their leukemic cells, we aimed to understand the TCR role in leukemia development, and the impact of leukemic cell antigenic stimulation.

### Methods

To do so, we explored disease development and TCR targeting in transgenic TCR mouse models, namely the Marilyn and OT-I mice. The Marilyn mice express a transgenic TCR specific for the HY male antigen bound to MHC class II, leading to positive selection of CD4+ thymocytes in females and negative selection in males. The OT-I mice bear a transgenic TCR which is responsive to the SIINFEKL chicken ovalbumin peptide (OVA). OTI-expressing T cells are positively selected in both male and females.

### Results

Marilyn *Rag2-/-* females but not males developed T cell leukemia that, when transplanted, led to higher tumor burden in females *Rag2-/-* recipient mice. Moreover, we found delayed disease progression in female recipient mice treated with the male antigen cognate peptide (DBY), which led to leukemic cell death as assessed by cleaved Caspase-3 expression in the spleen. In addition, antigenic targeting of this transgenic TCR translated into prolonged survival of recipient mice. Around 50% of TCR OT-I *Rag2-/-* mice developed a T-ALL/LBL disease, characterized mainly by thymic lymphoma, increase of white cells in the bloodstream and leukemic cell dissemination to lymphoid and non-lymphoid organs. We also found that mice carrying two copies of the OT-I transgene had increased T-ALL/LBL incidence than mice with a single copy. This malignant transformation appears to be triggered by basal TCR signaling, since the ablation of its interaction with MHC class I (achieved by β2-microglobulin gene knockout) impaired spontaneous disease development in this mouse model. To confirm their malignant nature, OT-I *Rag2-/-* leukemic cells were injected into *Rag2-/-* mice. These mice rapidly developed fatal leukemia with the same immunophenotypic features. Both leukemic and non-leukemic OT-I *Rag2-/-* T cells were stimulated *in vitro* with OVA to assess their response to activation of the TCR pathway. Surprisingly, when treated with OVA , most OT-I *Rag2-/-* leukemic cells did not upregulate the CD69 activation marker and those that did had low levels compared to healthy OT-I *Rag2-/-* thymocytes. To further investigate the possible loss of TCR function, these cells were also stimulated with an antibody targeting surface CD3, part of the TCR/CD3 complex, and PMA/Ionomycin, which activates the TCR signaling pathway through PKCθ activation and increased levels of cationic calcium, respectively. Once again, leukemic OT-I cells were less responsive to these stimuli compared to healthy OT-I thymocytes.

**Copyright Information:** (Online) ISSN: 2572-9241
©2026 The Author(s). HemaSphere is published by John Wiley & Sons Ltd on behalf of European Hematology Association. This is an open access Abstract Book distributed under the Attribution-NonCommercial-NoDerivs (CC BY-NC-ND), which allows third parties to download the articles and share them with others as long as they credit the author and the Abstract Book, but they cannot change the content in any way or use them commercially.
**Abstract Book Citations:** Authors, Title, HemaSphere, 2026;10:(S1):pages. The abstract book DOIs can be found on the "Author Information" page.
**Disclaimer:** Articles published in the journal HemaSphere exclusively reflect the opinions of the authors. The authors are responsible for all content in their abstracts including accuracy of the facts, statements, citing resources, etc.

### Summary/Conclusion

These findings show that transgenic TCR OT-I leads to T cell leukemia through basal TCR signaling by presentation of self-peptides to the receptor. However, upon malignant transformation, the transgenic TCR becomes less responsive to antigenic stimulation. Overall, our data demonstrate that transgenic TCR expression leads to T-ALL/LBL development and that strong stimulation of TCR suppresses this disease.

**Copyright Information:** (Online) ISSN: 2572-9241
©2026 The Author(s). HemaSphere is published by John Wiley & Sons Ltd on behalf of European Hematology Association. This is an open access Abstract Book distributed under the Attribution-NonCommercial-NoDerivs (CC BY-NC-ND), which allows third parties to download the articles and share them with others as long as they credit the author and the Abstract Book, but they cannot change the content in any way or use them commercially.
**Abstract Book Citations:** Authors, Title, HemaSphere, 2026;10:(S1):pages. The abstract book DOIs can be found on the "Author Information" page.
**Disclaimer:** Articles published in the journal HemaSphere exclusively reflect the opinions of the authors. The authors are responsible for all content in their abstracts including accuracy of the facts, statements, citing resources, etc.

### (PS1461) SINGLE-CELL ANALYSIS REVEALS DISTINCT CLONAL ORIGINS OF CML-LIKE AND TYPICAL BCR::ABL1-POSITIVE ALL

**Topic:** 01. Acute lymphoblastic leukemia - Biology & translational research

Lenka Hovorkova*[1], Lucie Winkowska[1], Barbora Dvorakova[1], Eliska Potuckova[1], Jan Stuchly[1], Martina Vaskova[1], Jan Starý[1], Gunnar Cario[2], Giovanni Cazzaniga[3, 4], Katerina MacHova Polakova[5, 6], Jan Trka[1], Marketa Zaliova[1], Jan Zuna[1]

*[1]CLIP (Childhood Leukaemia Investigation Prague), Second Faculty of Medicine, Charles University, Department of Paediatric Haematology and Oncology, University Hospital Motol and Homolka, Prague, Czechia; [2]Department of Pediatrics I, Pediatric Hematology and Oncology, ALL-BFM Study Group, University Medical Center Schleswig-Holstein, Kiel, Germany; [3]School of Medicine and Surgery, University of Milano-Bicocca, Milan, Italy; [4]Tettamanti Center, Fondazione IRCCS San Gerardo dei Tintori, Monza, Italy; [5]Institute of Hematology and Blood Transfusion, Prague, Czechia;[6]Institute of Pathological Physiology, First Faculty of Medicine, Charles University, Prague, Czechia;*

### Background

BCR::ABL1-positive acute lymphoblastic leukaemia (ALL) remains one of the most prognostically unfavourable childhood ALL subtypes, despite the introduction of tyrosine kinase inhibitors. In our previous work, we identified two subtypes within this entity: chronic myeloid leukaemia (CML)-like and Typical Ph+ ALL. These subtypes were defined based on MRD assessment using clonal IG/TR rearrangements and BCR::ABL1 quantification at the transcript or genomic level. CML-like cases are characterised by higher levels of BCR::ABL1 relative to IG/TR, reflecting the presence of the primary fusion outside the main ALL clone, typically in myeloid or non-ALL lymphoid cells. More recently, efforts to distinguish these subtypes at diagnosis have led to a transcriptome-based classification, identifying “multilineage” and “lymphoid” subgroups.

### Aims

The aim of this study was to establish a genomics-based approach that would provide a more precise and comprehensive understanding of the distinct biological features underlying the CML-like and Typical Ph+ ALL subtypes.

### Methods

Eight BCR::ABL1-positive patients were analysed using a modified single-cell TARGET-seq protocol (1 CML in lymphoid blast crisis, 3 Typical Ph+ ALL, 4 CML-like ALL). This approach enabled parallel genomic detection of patient-specific BCR::ABL1 intronic fusions, clonal IG/TR rearrangements, and additional leukaemia-associated markers (e.g. IKZF1 intragenic deletions). Multiple cell populations were analysed at single-cell resolution, allowing detailed mapping of clonal architecture and lineage involvement. The method enabled assessment of intrapatient heterogeneity and comparison across subtypes.

### Results

In patients with Typical Ph+ ALL, the BCR::ABL1 fusion was consistently detected in the same cells as clonal IG/TR rearrangements. Importantly, in the Typical Ph+ ALL cases showing partly myeloid phenotype (by flow cytometry – co-expression of CD13 and/or CD33 on leukaemic blasts – or by whole transcriptome sequencing – clustering in a multilineage cluster) the BCR::ABL1 fusion was detected also in myeloid subpopulations, but always together with IG/TR clonal rearrangements and other analysed markers. In contrast, patients with the CML-like phenotype showed a distinct pattern; a proportion of cells outside the main ALL clone carried the BCR::ABL1 fusion as the sole detectable leukaemic marker, without concurrent IG/TR rearrangements or other leukaemia-specific targets. This observation confirms the transformation occurring at an earlier stage of haematopoietic differentiation in CML-like cases as well as a plasticity of fully leukaemic BCR::ABL1 ALL cells towards myeloid lineage.

**Copyright Information:** (Online) ISSN: 2572-9241
©2026 The Author(s). HemaSphere is published by John Wiley & Sons Ltd on behalf of European Hematology Association. This is an open access Abstract Book distributed under the Attribution-NonCommercial-NoDerivs (CC BY-NC-ND), which allows third parties to download the articles and share them with others as long as they credit the author and the Abstract Book, but they cannot change the content in any way or use them commercially.
**Abstract Book Citations:** Authors, Title, HemaSphere, 2026;10:(S1):pages. The abstract book DOIs can be found on the “Author Information” page.
**Disclaimer:** Articles published in the journal HemaSphere exclusively reflect the opinions of the authors. The authors are responsible for all content in their abstracts including accuracy of the facts, statements, citing resources, etc.

**Summary/Conclusion**

Integrated with transcriptomic profiling, our single-cell genomic analyses provide an additional layer of biological resolution in BCR::ABL1-positive ALL, further underscore the marked heterogeneity across the CML-like/multilineage/Typical Ph+ ALL/lymphoid subgroups and provides explanation of differences between transcriptomic-based classification and MRD or single-cell -based definitions. Supported by Czech Health Research Council NW25-03-00276.

**Copyright Information:** (Online) ISSN: 2572-9241
©2026 The Author(s). HemaSphere is published by John Wiley & Sons Ltd on behalf of European Hematology Association. This is an open access Abstract Book distributed under the Attribution-NonCommercial-NoDerivs (CC BY-NC-ND), which allows third parties to download the articles and share them with others as long as they credit the author and the Abstract Book, but they cannot change the content in any way or use them commercially.
**Abstract Book Citations:** Authors, Title, HemaSphere, 2026;10:(S1):pages. The abstract book DOIs can be found on the "Author Information" page.
**Disclaimer:** Articles published in the journal HemaSphere exclusively reflect the opinions of the authors. The authors are responsible for all content in their abstracts including accuracy of the facts, statements, citing resources, etc.

### (PS1462) MYC/BCL2-REARRANGED B-CELL NEOPLASMS WITH PRECURSOR FEATURES: INTEGRATED MOLECULAR PROFILING FOR IMPROVED DIAGNOSTIC CLASSIFICATION

**Topic:** 01. Acute lymphoblastic leukemia - Biology & translational research

Cecilia Bozzetti*[1, 2], Johanna-Maria Horns[2], Sonja Bendig[2, 3], Tassja Rugenstein[2], Sandra Bufe[2], Thomas Beder[2, 3], Michaela Kotrova[2], José Ignacio Martín-Subero[4], Carlos López Pleguezuelos[4], Anna Stengel[5], Saskia Kohlscheen[2], Stefan Schwartz[6], Boris Böll[7], Edgar Jost[8], Max Topp[9], Sven Neubert[9], Julia Alten[10], Christiane Pott[1, 2], Anne Wilke[11], Nicola Gökbuget[11], Wencke Walter[5], Lorenz Bastian[1, 2, 3], Nikos Darzentas[2], Claudia Baldus[1, 2, 3], Monika Brüggemann[1, 2, 3]

*[1]University Cancer Center Schleswig-Holstein (UCCSH), University Hospital Schleswig-Holstein, Kiel, Germany;[2]University Hospital Schleswig-Holstein, Internal Medicine II, Kiel, Germany; [3]Clinical Research Unit "CATCH ALL" (KFO 5010) funded by the Deutsche Forschungsgemeinschaft, Kiel, Germany; [4]Fundació de Recerca Clínic Barcelona-Institut d'Investigacions Biomèdiques August Pi i Sunyer (FRCB-IDIBAPS), Barcelona, Spain; [5]MLL Munich Leukemia Laboratory, Munich, Germany; [6]Charité - Universitätsmedizin Berlin, Campus Benjamin Franklin, Berlin, Germany;[7]Center for Integrated Oncology Aachen Bonn Cologne Dusseldorf, University of Cologne, Cologne, Germany; [8]University Hospital Aachen & Center for Integrated Oncology Aachen Bonn Cologne Düsseldorf, Aachen, Germany; [9]Universitätsklinikums Würzburg, Würzburg, Germany; [10]Berlin-Frankfurt-Münster ALL Study Group Germany (BFM-G), University Medical Center Schleswig-Holstein, Kiel, Germany; [11]University Hospital Frankfurt, Frankfurt, Germany;*

### Background

B-cell precursor acute lymphoblastic leukaemia (B-ALL) with *MYC/BCL2* rearrangements is a rare molecular subgroup introduced in the WHO 2022 classification and is associated with poor prognosis. These cases harbour *MYC* translocations and frequently *BCL2* rearrangements, leading to overlap with aggressive mature B-cell lymphomas. Distinguishing *de novo* precursor B-ALL with *MYC/BCL2* rearrangements from leukaemic presentations of mature B-cell neoplasms remains challenging and is clinically crucial due to therapeutic implications.

### Aims

To characterise molecular, immunogenomic, and epigenetic features of *MYC/BCL2*-rearranged (*MYC/BCL2*-r) B-cell neoplasms with precursor features to better distinguish *de novo MYC/BCL2-r* B-ALL from transformed/leukaemic mature B-cell malignancies.

### Methods

We analysed 25 patients with *MYC/BCL2*-r B-cell malignancies (24 adults, 1 infant, 10 male, 15 female, median age 61y [0.9-87]) referred for routine diagnostics with suspicion of B-ALL. DNA-based capture sequencing with EC-NDC-panel (Univ8® Genomics) was performed on diagnostic samples (18 bone marrow, 6 blood, 1 other) to identify clonal IG/TR rearrangements, somatic variants (SNVs) in 82 lymphoid-associated genes, and structural variants (SV) involving selected regions. RNA sequencing was performed for 24 samples. Sequencing data was analysed using the ARResT/Interrogate and the IntegrateALL pipeline. EPIC-Arrays were used for genome-wide DNA methylation profiling (n=25). Flow cytometry data was available for 19, histology/FISH testing for 15 and MRD/clinical follow-up data for 14 patients treated according to the GMALL protocols.

**Copyright Information:** (Online) ISSN: 2572-9241
©2026 The Author(s). HemaSphere is published by John Wiley & Sons Ltd on behalf of European Hematology Association. This is an open access Abstract Book distributed under the Attribution-NonCommercial-NoDerivs (CC BY-NC-ND), which allows third parties to download the articles and share them with others as long as they credit the author and the Abstract Book, but they cannot change the content in any way or use them commercially.
**Abstract Book Citations:** Authors, Title, HemaSphere, 2026;10:(S1):pages. The abstract book DOIs can be found on the "Author Information" page.
**Disclaimer:** Articles published in the journal HemaSphere exclusively reflect the opinions of the authors. The authors are responsible for all content in their abstracts including accuracy of the facts, statements, citing resources, etc.

### Results

Clonal IG/TR rearrangements were detected in 25/25 (median 4 [1-8]), predominantly IGKVJ (68%) and IGHVJ (64%). Cross-lineage rearrangements were observed in 40% of cases.

All cases harboured SNVs, most frequently affecting *KMT2D* (44%), *MYC* (32%), *KRAS* (28%) and *TP53* (24%). Mutations characteristic of mature B-cell neoplasms, i.e. *CCND3* and *CREBBP*, were present in 28%. SV involving *MYC* and *IG* were identified in 72% of cases. *BCL2* rearrangements occurred in 48% and *BCL6* in 12%. Double-hit constellations were observed in 20% (*MYC/BCL2*), 8% (*MYC/BCL6)* and 4% (*BCL2/BCL6)*, respectively. *MYC* breakpoints predominantly mapped to exon 1, with *IGH* partner breakpoints mainly located in the constant region (28%) and class switch region (24%). *IGL* and *IGK* partners were detected in 12% and 4%, respectively. Genomic features of a mature cell of origin (i.e. somatic hypermutation, breakpoint in the IGHswc region) were found in 64%.

Gene expression profiling revealed a distinct molecular signature clearly separating these cases from other B-ALL molecular subgroups. DNA methylation analysis revealed divergent epigenetic profiles, separating patients into two groups: one group (n=8) clustering with a B-ALL reference cohort (n=86), and a second group (n=17) with Burkitt leukaemia samples (n=26). Immunophenotyping was most frequently consistent with common/pre-B ALL (17/19 - 89%). Clinical follow-up (median 281d [26–2331]) confirmed a high relapse rate (8/14 - 57%).

### Summary/Conclusion

*MYC/BCL2*-r B-cell neoplasms with precursor features show heterogeneous molecular profiles spanning from immature to mature origins. Integrated genomic and epigenomic profiling improves classification and is essential for distinguishing de novo *MYC/BCL2*-r B-ALL from leukaemic presentations of mature B-cell neoplasms, guiding accurate risk stratification and treatment.

**Copyright Information:** (Online) ISSN: 2572-9241
©2026 The Author(s). HemaSphere is published by John Wiley & Sons Ltd on behalf of European Hematology Association. This is an open access Abstract Book distributed under the Attribution-NonCommercial-NoDerivs (CC BY-NC-ND), which allows third parties to download the articles and share them with others as long as they credit the author and the Abstract Book, but they cannot change the content in any way or use them commercially.
**Abstract Book Citations:** Authors, Title, HemaSphere, 2026;10:(S1):pages. The abstract book DOIs can be found on the “Author Information” page.
**Disclaimer:** Articles published in the journal HemaSphere exclusively reflect the opinions of the authors. The authors are responsible for all content in their abstracts including accuracy of the facts, statements, citing resources, etc.

### (PS1463) T-MAPS: NOVEL SURFACE MARKERS FOR MEASURABLE RESIDUAL DISEASE MONITORING IN PEDIATRIC T-CELL ACUTE LYMPHOBLASTIC LEUKEMIA

**Topic:** 01. Acute lymphoblastic leukemia - Biology & translational research

Adela Vavrova*[1, 2], Daniela Kužílková[1, 2, 3], Michaela Reiterova[1, 2, 3], Marketa Zaliova[1, 2, 3], Jan Zuna[1, 2, 3], Jan Trka[1, 2, 3], Tomas Kalina[1, 2, 3]

*[1]CLIP (Childhood Leukaemia Investigation Prague), Prague, Czechia;[2]Department of Paediatric Haematology and Oncology, Second Faculty of Medicine, Charles University, Prague, Czechia; [3]Motol and Homolka University Hospital, Prague, Czechia;*

### Background

Pediatric T-cell acute lymphoblastic leukemia (T-ALL) is an aggressive malignancy. Despite improved prognosis over recent decades, relapse still occurs in approximately 20 % of patients and is associated with poor outcome. The level of measurable residual disease (MRD) on day 15, assessed by flow cytometry, serves as a key prognostic marker. However, accurate MRD evaluation is often complicated by phenotypic overlap between malignant and non-malignant T cells, particularly in T-ALL cases with surface CD3 (sCD3) expression. Another diagnostically challenging subtype is early T-cell precursor (ETP) ALL, which exhibits an immature T-cell phenotype and aberrant expression of myeloid/stem cell markers.

### Aims

This study aims to identify new surface markers of T-ALL cells that could serve for flow cytometry-based MRD monitoring.

### Methods

Full-spectrum flow cytometry was used to evaluate the expression of surface markers (n=307) in pediatric T-ALL cases (n=42). Of those, n=13 were of ETP-ALL immunophenotype and another n=15 showed sCD3 expression. These expression profiles were compared to healthy donor peripheral blood (HDPB, n=11) T cells, as well as to developmental stages of T cells in non-malignant thymus (n=3) and bone marrow (BM, n=3) samples. For unsupervised analysis principal component analysis (PCA) was employed. RNA-seq data available for a subset of our T-ALL cohort (n=34) were used for marker confirmation. For statistical comparison, Wilcoxon rank-sum tests were performed, $P<0.05$ was considered statistically significant.

### Results

First, aberrantly expressed markers of T-ALL cells were identified based on the following criteria: (I) at least a two-fold difference in median expression between T lymphoblasts and both HDPB T cells and non-malignant BM T cells, with expression of >1,000 molecules per cell in at least one of the populations; and (II) non-overlapping expression patterns between T lymphoblasts and both HDPB T cells and non-malignant BM T cells. In sum, four markers (CD38, CD71, CD82, CD98) were over-expressed and three markers (CD6, CD52, CD229) exhibited decreased expression in at least 75 % of the investigated samples. The expression level of CD38, CD52, and CD98 did not correspond to any recognized physiological stage of T-cell development, suggesting an immunophenotypic pattern preferentially associated with pediatric T-ALL.
Next, we focused on T-ALL subtypes posing challenges for MRD assessment. We identified four over-expressed markers (CD97, CD166, CD200, CD312) and two under-expressed markers (CD53, CD352) observed solely in more than 75 % of ETP-ALL samples. Except for CD53, all these markers were ranked among the seven most significant parameters discriminating ETP ALL samples from HDPB samples in PCA analysis. Except for CD200, all markers showed a significant difference in RNA expression in ETP ALL samples compared to other T-ALL samples. Subsequently, we identified CD44 as under-expressed in more than 75 % of sCD3+ T-ALL samples.

**Copyright Information:** (Online) ISSN: 2572-9241
©2026 The Author(s). HemaSphere is published by John Wiley & Sons Ltd on behalf of European Hematology Association. This is an open access Abstract Book distributed under the Attribution-NonCommercial-NoDerivs (CC BY-NC-ND), which allows third parties to download the articles and share them with others as long as they credit the author and the Abstract Book, but they cannot change the content in any way or use them commercially.
**Abstract Book Citations:** Authors, Title, HemaSphere, 2026;10:(S1):pages. The abstract book DOIs can be found on the "Author Information" page.
**Disclaimer:** Articles published in the journal HemaSphere exclusively reflect the opinions of the authors. The authors are responsible for all content in their abstracts including accuracy of the facts, statements, citing resources, etc.

**Summary/Conclusion**

In conclusion, we identified seven CD markers potentially suitable for MRD monitoring in at least 75 % of pediatric T-ALL samples. Moreover, we deeply characterized ETP-ALL and sCD3+ T-ALL subgroups. Selected markers are currently being tested prospectively to assess their treatment stability and potential utility. This work was supported by GAUK 318225 and Ministry of Health of the Czech Republic, grant nr NU23J-03-00026.

**Copyright Information:** (Online) ISSN: 2572-9241
©2026 The Author(s). HemaSphere is published by John Wiley & Sons Ltd on behalf of European Hematology Association. This is an open access Abstract Book distributed under the Attribution-NonCommercial-NoDerivs (CC BY-NC-ND), which allows third parties to download the articles and share them with others as long as they credit the author and the Abstract Book, but they cannot change the content in any way or use them commercially.
**Abstract Book Citations:** Authors, Title, HemaSphere, 2026;10:(S1):pages. The abstract book DOIs can be found on the "Author Information" page.
**Disclaimer:** Articles published in the journal HemaSphere exclusively reflect the opinions of the authors. The authors are responsible for all content in their abstracts including accuracy of the facts, statements, citing resources, etc.

### (PS1464) EFFICACY OF BIOMIMETIC NANODRUG CX4945 IN B-ALL PATIENT-DERIVED XENOGRAFT MOUSE MODEL

**Topic:** 01. Acute lymphoblastic leukemia - Biology & translational research

Yan Wang[1], Qi Han[1], Huimin Xiang[1], Mingze Lu[2], Fei Wang[2], Haoan Wu[2], Chunhua Song[3], Yu Zhang[2], Zheng Ge*[1]

*[1]Zhongda Hospital Southeast University, Institute of Hematology Southeast University, Nanjing, China; [2]State Key Laboratory of Digital Medical Engineering, Jiangsu Key Laboratory for Biomaterials and Devices, School of Biological Science and Medical Engineering & Basic Medicine Research and Innovation Center of Ministry of Education, Southeast University, Nanjing, China; [3]Division of Hematology, The Ohio State University Wexner Medical Center, the James Cancer Hospital, Columbus, United States of America;*

### Background

Acute lymphoblastic leukemia (B-ALL) is a high-risk malignancy characterized by the abnormal proliferation of B-lymphocytes. It is often associated with dysfunction of the IKAROS transcription factor encoded by *IKZF1* gene. The CK2 kinase inhibitor CX4945 has shown potential in the treatment of acute leukemia by reducing the phosphorylation of IKAROS and restoring its function. To overcome the limitations of CX4945, which exhibits poor bioavailability and rapid metabolic clearance, we developed a novel biomimetic nanotherapeutic formulation. Designated as CX4945@PLGA-PEG@CM, this nanosystem was surface-modified with bone marrow stromal cell membrane to optimize its pharmacokinetic properties.

### Aims

This study aimed to evaluate the in vivo therapeutic efficacy of the CX4945@PLGA-PEG@CM nanodrug in the B-ALL patient-derived xenograft (PDX) mouse model, with the underlying mechanism involving targeted inhibition of the CXCR4/CXCL12 axis to disrupt leukemic cell homing.

### Methods

Two patient samples from high-risk B-ALL patients with CXCR4 expression were respectively injected into NCG mice intravenously at a quantity of $4\times10^6$ / $2\times10^7$ cells per mouse. Treatment groups were assigned after peripheral blood monitoring in the PDX mouse models exceeded 5%. The mice were treated for 27 days with the drug (CX4945 equivalent concentration of 20 mg/kg, every 3 day, i.v.) (scheme see Fig. 1A, 2A). When control mice met early culling criteria, including severe locomotor impairment or hindlimb paralysis, half of the mice in each group were euthanized. Single cells from spleen and bone marrow tissues were collected and analyzed by flow cytometry to assess leukemia burden. The remaining half of the mice were used for Kaplan-Meier survival curves constructed for statistical assessment.

### Results

PDX1 and PDX2 results showed that treatment with CX4945@PLGA-PEG@CM (CX4945 equivalent concentration of 20 mg/kg) significantly prolonged mouse survival compared to either CX4945 or CX4945@PLGA-PEG control (Fig1.B and Fig2.B), significantly reduced the spleen size and weight (Fig1. C and D) in the order of CX4945@PLGA-PEG@CM<CX4945@PLGA-PEG<free CX4945<control group. Additionally, the % $hCD45^+hCD19^+$ leukemia cells are significantly reduced in bone marrow and spleen with the order of CX4945@PLGA-PEG@CM < CX4945@PLGA-PEG < free CX4945 drug < control group. These data indicate that the CX4945@PLGA-PEG@CM bionanomimetic nanomedicine significantly enhanced CX4945 efficacy in B-ALL.

**Copyright Information:** (Online) ISSN: 2572-9241
©2026 The Author(s). HemaSphere is published by John Wiley & Sons Ltd on behalf of European Hematology Association. This is an open access Abstract Book distributed under the Attribution-NonCommercial-NoDerivs (CC BY-NC-ND), which allows third parties to download the articles and share them with others as long as they credit the author and the Abstract Book, but they cannot change the content in any way or use them commercially.
**Abstract Book Citations:** Authors, Title, HemaSphere, 2026;10:(S1):pages. The abstract book DOIs can be found on the "Author Information" page.
**Disclaimer:** Articles published in the journal HemaSphere exclusively reflect the opinions of the authors. The authors are responsible for all content in their abstracts including accuracy of the facts, statements, citing resources, etc.

### Summary/Conclusion

Our results demonstrated the therapeutic efficacy of CX4945@PLGA-PEG@CM in high-risk B-ALL. Our data also revealed that CXCL12 on the biomimetic membrane of CX4945@PLGA-PEG@CM specifically targets the CXCR4-expressed B-ALL cells and enhances the drug efficacy, and also blocks the tumor cell homing.

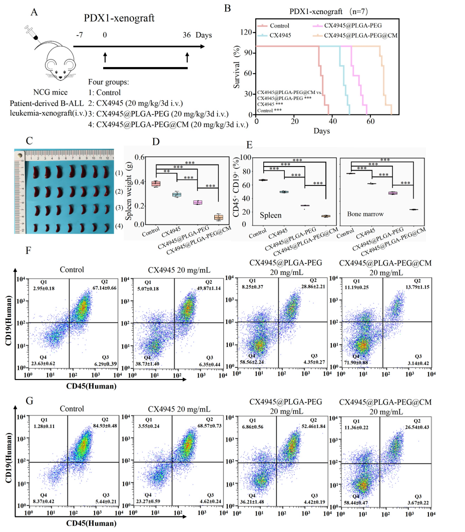

Fig.1 (A)Schematic Diagram of Patient-derived B-ALL leukemia-xenograft (PDX1). (B) Kaplan-Meier survival curves for the xenograft mouse model (PDX1). (C-D)Spleen images (C) and body weights (D) of four groups of mice treated with different drug formulations: (1) Control; (2) CX4945; (3) CX4945@PLGA-PEG; (4) CX4945@PLGA-PEG@CM. (E-G) Percentage of human CD45$^{+}$/CD19$^{+}$cells in mouse spleen and bone marrow following treatment with different types of drugs.

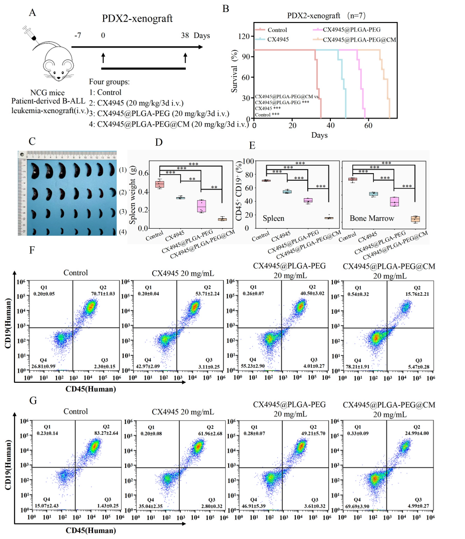

Fig.2 (A)Schematic Diagram of Patient-derived B-ALL leukemia-xenograft (PDX2). (B) Kaplan-Meier survival curves for the xenograft mouse model (PDX2). (C-D)Spleen images (C) and body weights (D) of four groups of mice treated with different drug formulations: (1) Control; (2) CX4945; (3) CX4945@PLGA-PEG; (4) CX4945@PLGA-PEG@CM. (E-G) Percentage of human CD45$^{+}$/CD19$^{+}$cells in mouse spleen and bone marrow following treatment with different types of drugs.

**Copyright Information:** (Online) ISSN: 2572-9241
©2026 The Author(s). HemaSphere is published by John Wiley & Sons Ltd on behalf of European Hematology Association. This is an open access Abstract Book distributed under the Attribution-NonCommercial-NoDerivs (CC BY-NC-ND), which allows third parties to download the articles and share them with others as long as they credit the author and the Abstract Book, but they cannot change the content in any way or use them commercially.
**Abstract Book Citations:** Authors, Title, HemaSphere, 2026;10:(S1):pages. The abstract book DOIs can be found on the "Author Information" page.
**Disclaimer:** Articles published in the journal HemaSphere exclusively reflect the opinions of the authors. The authors are responsible for all content in their abstracts including accuracy of the facts, statements, citing resources, etc.

### (PS1465) SHORT PEPTIDE INHIBITORS AGAINST DUX4-R LEUKEMIA

**Topic:** 01. Acute lymphoblastic leukemia - Biology & translational research

Mara Salomè*[1, 2], Maria Pannese[2], Daniele Campolungo[2], Beatrice Biferali[2], Valeria Runfola[2], Paola Ghezzi[2], Michela Carlet[3, 4], Chiara Zucchelli[2], Andrea Berardi[2], Giovanna Musco[2], Irmela Jeremias[3, 5], Davide Gabellini[2]

*[1]Università Vita-Salute San Raffaele, Milan, Italy; [2]IRCCS Ospedale San Saffaele, Division of Genetics and Cell Biology, Milan, Italy; [3]Helmholtz Zentrum München-German Research Center for Environmental Health, Research Unit Apoptosis in Hematopoietic Stem Cells, Munich, Germany; [4]Medical University Innsbruck, Institute of Bioinformatics, Biocenter, Innsbruck, Austria; [5]Dr. von Hauner Children's Hospital, Ludwig Maximilians University (LMU), Department of Pediatric Hematology/Oncology, Munich, Germany;*

### Background

Translocations of the embryonic transcription factor DUX4, giving rise to the rearranged oncogenic driver DUX4-r, are responsible for up to 10% of acute lymphoblastic leukemia (ALL) cases. Due to the cryptic nature of the translocation and the biological features of the disease, DUX4-r ALL is an under-diagnosed and an under-studied subtype of ALL. Since no targeted treatment is available, non-responders/relapsed patients face a dismal prognosis. Hence, novel therapeutic strategies are urgently needed.
We and other showed that the ability to bind DNA and activate gene expression is required for DUX4-r leukemogenic potential. Therefore, identifying inhibitors of DUX4-r transcriptional activity and test their activity in ALL could help the development of effective therapeutic strategies in a personalized medicine perspective. Through proteomics, we identified matrin 3 (MATR3) for its ability to bind directly to DUX4-r DNA-binding domain (dbd) and inhibit its transcriptional potential.

### Aims

The aim of this study is to test the inhibitory potential of MATR3-based DUX4-r targeting strategies in relevant cellular and animal models of DUX4-r ALL.

### Methods

MATR3 or MATR3 fragments were delivered by means of lentiviral vectors in human leukemia cell lines to test the effects on proliferation, target gene expression and tumor spheroid formation driven by DUX4-r. Moreover, a MATR3 fragment was overexpressed in PDX derived from DUX4-r+ B-ALL patients to evaluate the effects on leukemia cell expansion and leukemia latency in vivo. To assess the impact of MATR3 in an established disease model, an inducible MATR3 construct was induced only after leukemia development. Through structure/function studies, we identified MATR3-based Short Peptide Inhibitors (SPI) sufficient to bind DUX4-r and designed drug-like PROteolisis TArgeting Chimera (PROTAC).

### Results

We found that MATR3 and MATR3 fragments decrease DUX4-r dependent transactivation and target gene expression. Delivery of a MATR3 fragment selectively blunts DUX4-r leukemia cell adhesion and proliferation. Importantly, negligent effects were observed by MATR3 delivery to DUX4-r negative cells. In vivo, the MATR3 fragment significantly reduces leukemia burden delaying the expansion of DUX4-r PDX cells even upon expression in an already established leukemia model. Interestingly, NMR studies suggest that MATR3 competes with DNA for DUX4-r binding, acting as a direct inhibitor of transcription. Finally, we successfully generated PROTACs inducing DUX4-r protein ubiquitin proteasome-dependent degradation in ALL cells.

**Copyright Information:** (Online) ISSN: 2572-9241
©2026 The Author(s). HemaSphere is published by John Wiley & Sons Ltd on behalf of European Hematology Association. This is an open access Abstract Book distributed under the Attribution-NonCommercial-NoDerivs (CC BY-NC-ND), which allows third parties to download the articles and share them with others as long as they credit the author and the Abstract Book, but they cannot change the content in any way or use them commercially.
**Abstract Book Citations:** Authors, Title, HemaSphere, 2026;10:(S1):pages. The abstract book DOIs can be found on the "Author Information" page.
**Disclaimer:** Articles published in the journal HemaSphere exclusively reflect the opinions of the authors. The authors are responsible for all content in their abstracts including accuracy of the facts, statements, citing resources, etc.

**Summary/Conclusion**

Collectively, our results demonstrate that MATR3 is a natural inhibitor of DUX4-r that binds directly to its DNA-binding domain and prevents activation of DUX4-r targets, blocking DUX4-r oncogenic activity. Our data also support the possibility to generate drug-like mimicking peptides and DUX4-r bifunctional inhibitors to be tested in pre-clinical settings.

**Copyright Information:** (Online) ISSN: 2572-9241
©2026 The Author(s). HemaSphere is published by John Wiley & Sons Ltd on behalf of European Hematology Association. This is an open access Abstract Book distributed under the Attribution-NonCommercial-NoDerivs (CC BY-NC-ND), which allows third parties to download the articles and share them with others as long as they credit the author and the Abstract Book, but they cannot change the content in any way or use them commercially.
**Abstract Book Citations:** Authors, Title, HemaSphere, 2026;10:(S1):pages. The abstract book DOIs can be found on the "Author Information" page.
**Disclaimer:** Articles published in the journal HemaSphere exclusively reflect the opinions of the authors. The authors are responsible for all content in their abstracts including accuracy of the facts, statements, citing resources, etc.

### (PS1466) IGH::ID4-REARRANGEMENTS ARE THE MOST FREQUENT DRIVER ABERRATIONS OF ADULT PAX5ALT B-ALL AND DEFINE A DISTINCT MOLECULAR ENTITY

**Topic:** 01. Acute lymphoblastic leukemia - Biology & translational research

Sonja Bendig*[1, 2], Thomas Beder[1], Johanna-Maria Horns[1], Malwine J. Barz[1, 2], Nadine Wolgast[1, 2], Alina Marie Hartmann[1, 2], Fabio Steffen[3], Loredana Cantoni[3], Britta Kehden[1], Franziska Westendorf[4], Björn Steffen[5], Edgar Jost[6], Simon Raffel[7], Guranda Chitadze[1, 2], Beat Bornhauser[3], Jean-Pierre Bourquin[3], Nicola Gökbuget[5], Claudia Baldus[1, 2], Lorenz Bastian[1, 2], Monika Brüggemann[1, 2]

*[1]University Hospital Schleswig-Holstein, Medical Department II, Hematology and Oncology, Kiel, Germany;[2]Clinical Research Unit 'CATCH ALL' (KFO 5010) funded by the Deutsche Forschungsgemeinschaft, Kiel, Germany; [3]University Children's Hospital, Department of Oncology and Children's Research Center, Zurich, Switzerland; [4]University Medical Center Hamburg-Eppendorf, Department of Oncology, Hematology and Bone Marrow Transplantation with Section Pneumology, University Cancer Center Hamburg, Hamburg, Germany; [5]Goethe University, Department of Medicine II, Hematology and Oncology, Frankfurt, Germany; [6]Medical Faculty, RWTH Aachen University, Department of Hematology, Oncology, Hemostaseology and Stem Cell Transplantation, Aachen, Germany; [7]Heidelberg University Hospital, Department of Internal Medicine V, Heidelberg, Germany;*

### Background

The 2022 International Consensus Classifications defines PAX5alt B acute lymphoblastic leukemia (B-ALL) as provisional diagnostic entity by a variety of *PAX5* alterations and gene expression profiling. However, reproducible consensus definitions are missing. The *IGH::ID4* rearrangement has been described as a rare recurrent driver in B-ALL by cytogenetic analyses, but its role in current RNA-seq-based B-ALL subtype definitions has not been established.

### Aims

To comprehensively characterize *IGH::ID4* rearranged B-ALL and to determine its relationship to PAX5alt cases.

### Methods

1,887 adult B-ALL patients were analyzed with the EuroClonality NGS DNA Capture Assay (n=1,261), RNA-seq (n=774), or both (n=148). *IGH::ID4* and PAX5alt patients were additionally analyzed with ex vivo drug response profiling (n=6) and Infinium MethylationEPIC Arrays (n=11) compared to a published B-ALL reference (n=84), MLPA (n= 19), SNP Array (n=6), immunophenotyping (n=8) and scRNA Seq (n=4).

**Copyright Information:** (Online) ISSN: 2572-9241
©2026 The Author(s). HemaSphere is published by John Wiley & Sons Ltd on behalf of European Hematology Association. This is an open access Abstract Book distributed under the Attribution-NonCommercial-NoDerivs (CC BY-NC-ND), which allows third parties to download the articles and share them with others as long as they credit the author and the Abstract Book, but they cannot change the content in any way or use them commercially.
**Abstract Book Citations:** Authors, Title, HemaSphere, 2026;10:(S1):pages. The abstract book DOIs can be found on the "Author Information" page.
**Disclaimer:** Articles published in the journal HemaSphere exclusively reflect the opinions of the authors. The authors are responsible for all content in their abstracts including accuracy of the facts, statements, citing resources, etc.

### Results

From a total of 1,887 adult B-ALL patients, we identified 13 with an *IGH::ID4* gene rearrangement and absence of subtype defining drivers or other recurrent SNVs (9 male, 4 female, median age 30 years (range 18-64)). Breakpoints were observed 0.7 – 200kb (median: 29kb) downstream of *ID4* and the rearrangement was missed by RNA-Seq fusion calling in 8/11 cases. In all MLPA profiled cases (n=11), *CDKN2A* and *PAX5* deletions were detected. All *IGH::ID4* patients with RNA-Seq (n=11) were predicted as PAX5alt with high-confidence (n=6) or as candidates (n=5) (ALLCatchR). Of the remaining RNA-seq cases, 39/799 had high-confidence PAX5alt predictions and/or *PAX5*alt driver events, most frequently *ETV6::PAX5* (n=3), other *PAX5* rearrangements (n=4), *PAX5* SNVs (n=12) or combinations (n=1). Analysis of DNA-methylation, gene expression and ex vivo drug response profiling put *IGH::ID4* cases in a continuum with remaining PAX5alt patients. Both groups were comparable in age and sex distribution and demonstrated excellent early treatment response, with the majority attaining MRD negativity after Induction I (*IGH::ID4*: 9/12; PAX5alt: 19/30) and Consolidation I (10/10; 24/27). However, *IGH::ID4* patients displayed subtle but consistent differences in the methylation profile (1,390 and 112,171 differentially methylated CpGs compared to PAX5alt and remaining B-ALL cases, FDR<0.01) and unsupervised gene expression analysis grouped *IGH::ID4* cases together within the PAX5alt cluster. A machine learning classifier trained on seven *IGH::ID4* patients successfully identified the remaining four cases without false positives. Applied to published cohorts, it identified 25 additional cases. In the largest cohort (Gu et al., 2019), 11/12 cases were predicted as PAX5alt without defining drivers and 1/12 as B-other. Recurrent features included 9p aberrations (7/9) and t(6;14)/*IGH::ID4* (5/12), consistent with our profile of *IGH::ID4*-rearranged B-ALL. Machine learning classification thus enables the identification of this patient group even in the absence of genomic rearrangement data.

### Summary/Conclusion

*IGH::ID4* rearrangements are the most frequent driver aberration of PAX5alt B-ALL in adults. While therearrangement is frequently missed in RNA-seq fusion calling, *IGH::ID4* cases can be identified by a distinct gene expression signature. Clinical and functional characterization positions *IGH::ID4*-rearranged B-ALL in the continuum of PAX5-altered B-ALL while supporting its recognition as a distinct entity within PAX5alt B-ALL.

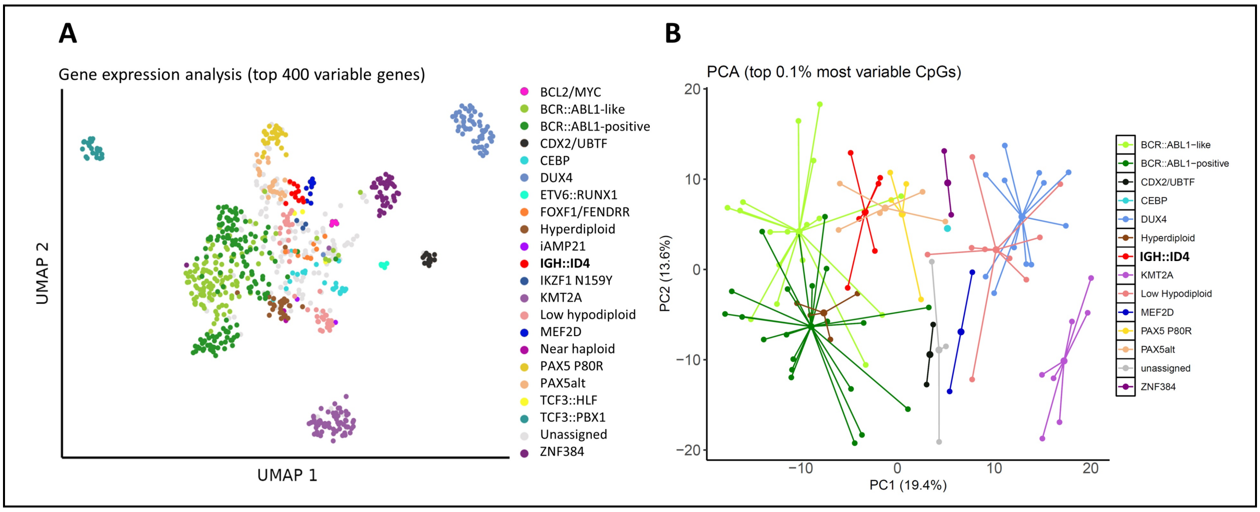

Figure 1A). Unsupervised gene expression analysis of adult B-ALL patients (n=774). IGH::ID4 patients (n=11, red) have a distinct gene expression signature and group together within the PAX5alt cluster. Other PAX5alt patients are shown in peach (n=39). 1B) Unsupervised DNA Methylation Analysis of adult B-ALL patients (n=95). IGH::ID4 patients (n= 6, red) are put in continuum with remaining PAX5alt patients (n=5, peach), with a largely overlapping profile but consistent subtle differences.

**Copyright Information:** (Online) ISSN: 2572-9241
©2026 The Author(s). HemaSphere is published by John Wiley & Sons Ltd on behalf of European Hematology Association. This is an open access Abstract Book distributed under the Attribution-NonCommercial-NoDerivs (CC BY-NC-ND), which allows third parties to download the articles and share them with others as long as they credit the author and the Abstract Book, but they cannot change the content in any way or use them commercially.
**Abstract Book Citations:** Authors, Title, HemaSphere, 2026;10:(S1):pages. The abstract book DOIs can be found on the “Author Information” page.
**Disclaimer:** Articles published in the journal HemaSphere exclusively reflect the opinions of the authors. The authors are responsible for all content in their abstracts including accuracy of the facts, statements, citing resources, etc.

### (PS1467) INFLUENCE OF AGE AND CYTOGENETIC RISK GROUP ON THE PROGNOSTIC EFFECT OF KEY COPY NUMBER ALTERATIONS IN B-CELL ACUTE LYMPHOBLASTIC LEUKAEMIA, INSIGHTS FROM THE HARMONY PLATFORM

**Topic:** 01. Acute lymphoblastic leukemia - Biology & translational research

Anthony Moorman*[1], Sulaiman Lawal[1], Jude Gibson[1], Sophia Polychronopoulou[2], Martin Zimmermann[3], Jordi Ribera[4], Hester de Groot-Kruseman[5], Michael Doubek[6], Ana García-Gambín[7], Amir Enshaei[1]

*[1]Newcastle University, Translational and Clinical Research Institute, Newcastle Upon Tyne, United Kingdom; [2]AGHIA SOPHIA Children's Hospital, Haematology, Athens, Greece; [3]Hannover Medical School, Pediatric Hematology/Oncology, Hannover, United Kingdom; [4]Josep Carreras Leukaemia Research Institute, PETHEMA, Barcelona, Spain;[5]Princess Máxima Center, Utrecht, The Netherlands; [6]University Hospital and Masaryk University, Brno, Czechia; [7]HARMONY Alliance Foundation, Salamanca, Spain;*

**Background**

Copy number alterations (CNA) are recognised prognostic markers in B-cell acute lymphoblastic leukaemia (B-ALL) and increasingly used to make treatment decisions.

**Aims**

To further explore the prognostic relevance of CNA profiles specifically in relation with age and cytogenetic risk group (CRG)

**Methods**

Data was extracted from the HARMONY Platform for 3,062 patients with B-ALL where the copy number status of the following 8 commonly affected gene/regions was known: *EBF1*, *IKZF1*, *CDKN2A/B*, *PAX5*, *ETV6*, *BTG1*, *RB1* and PAR1. Patients were classified into 6 groups: (1) No-Deletion (NoDel): No deletions involving these 8 genes/regions (n=1166); (2) Isolated Minor Deletion (IsoMinDel): Isolated deletions of *BTG1*, *ETV6*, or *PAX5* (n=356); (3) ETV6-Plus (ETV6+) (n=135): *ETV6* deletion plus one additional deletion involving *BTG1*, *CDKN2A/B*, or *PAX5*; (4) IKZF1plus (IKZF1+): *IKZF1* deletion together with at least one additional deletion involving *CDKN2A/B*, *PAX5*, or PAR1 (n=270); (5) IKZF1-Other (IKZF1del): *IKZF1* deletions not included in IKZF1+ (n=287); (6) All other deletion combinations (OtherDel) (n=848). Among the IKZF1+ cases, *ERG* deletion status was normal (n=55) or unknown (n=215). CRG was defined as good (*ETV6::RUNX1*,high hyperdiploid) (gCRG), high (*BCR*::ABL1, *KMT2A*, iAMP21, haploid, low hypodiploid, *TCF3::HLF*) (hCRG) and intermediate (all other abnormalities) (iCRG).

**Copyright Information:** (Online) ISSN: 2572-9241
©2026 The Author(s). HemaSphere is published by John Wiley & Sons Ltd on behalf of European Hematology Association. This is an open access Abstract Book distributed under the Attribution-NonCommercial-NoDerivs (CC BY-NC-ND), which allows third parties to download the articles and share them with others as long as they credit the author and the Abstract Book, but they cannot change the content in any way or use them commercially.
**Abstract Book Citations:** Authors, Title, HemaSphere, 2026;10:(S1):pages. The abstract book DOIs can be found on the "Author Information" page.
**Disclaimer:** Articles published in the journal HemaSphere exclusively reflect the opinions of the authors. The authors are responsible for all content in their abstracts including accuracy of the facts, statements, citing resources, etc.

**Results**

CNA groups were strongly correlated with age and CRG. The combined frequency of NoDel/IsoMinDel/ETV6+ was 62% in younger children (1-9y) versus 33% in adults (20-83y). In contrast the frequency of IKZF1+ and IKZF1del increased with age: 5% to 20% and 6% to 18% respectively. Among gCRG patients, 72% had a NoDel/IsoMinDel/ETV6+ profile while only 1% had IKZF1+ and 4% IKZF1del. Most patients with IKZF1+ (73%) were in iCRG emphasising its utility as a risk stratifier. One third of patients in hCRG had NoDel/IsoMinDel/ETV6+. The proportion of patients achieving MRD <0.01% by the end of induction varied significantly by CNA group: ETV6+ 67%, IsoMinDel 60%, NoDel 44%, OtherDel 45%, IKZF1+ 27% and IKZF1del 23%. The difference in MRD by CNA group is likely due to the proportion of cases in gCRG in each group: 78%, 66%, 38%, 29%, 5% and 16% respectively. As the patients in this cohort were treated over several decades and with different protocols, we focussed on the relative outcome by CNA group within age and CRG. There was no evidence for a differential outcome for NoDel, IsoMinDel and ETV6+ groups by age. Patients with IKZF1+ had the worst outcome in all age groups. Among children (1-14y), there was no evidence for a difference in outcome between the IKZF1del and OtherDel groups. There was little evidence for a differential prognostic effect by CNA group among adolescents (15-19y) and adults (20-83y); although the number of patients in the NoDel, IsoMinDel, or ETV6+ groups was limited. There was no evidence that CNA group affected outcome among *ETV6::RUNX1* cases whereas patients with high hyperdiploidy and IKZF1+/IKZF1del/OtherDel had an inferior outcome. Among patients in iCRG, IKZF1+ was the strong indicator of adverse outcome, whereas among patients in hCRG, both IKZF1+ and IKZF1del were equally adverse.

**Summary/Conclusion**

The frequency of these CNA groups correlates with age and CRG. The prognostic effect of CNA groups is strongest in younger children and those in iCRG and less pronounced among adolescents and adults.

**Copyright Information:** (Online) ISSN: 2572-9241
©2026 The Author(s). HemaSphere is published by John Wiley & Sons Ltd on behalf of European Hematology Association. This is an open access Abstract Book distributed under the Attribution-NonCommercial-NoDerivs (CC BY-NC-ND), which allows third parties to download the articles and share them with others as long as they credit the author and the Abstract Book, but they cannot change the content in any way or use them commercially.
**Abstract Book Citations:** Authors, Title, HemaSphere, 2026;10:(S1):pages. The abstract book DOIs can be found on the “Author Information” page.
**Disclaimer:** Articles published in the journal HemaSphere exclusively reflect the opinions of the authors. The authors are responsible for all content in their abstracts including accuracy of the facts, statements, citing resources, etc.

### (PS1468) SINGLE-CELL MULTIOMICS CAPTURES THE DYNAMICS OF LINEAGE TRANSDIFFERENTIATION IN PAX5 P80R PEDIATRIC ACUTE LYMPHOBLASTIC LEUKEMIA

**Topic:** 01. Acute lymphoblastic leukemia - Biology & translational research

Alyssa Alvaro*[1], Vojen Sadilek[1], Barbora Cabalková[1], Aneta Skotnicova[1], Michaela Reiterova[1], Martina Vášková[1], Marketa Zaliova[1], Ondrej Hrusak[1], Jan Trka[1], Tomas Kalina[1], Eva Fronkova[1], Ester Mejstrikova[1], Jan Stuchly[1]

*[1]Second Faculty of Medicine and University Hospital Motol and Homolka, CLIP - Department of Paediatric Haematology and Oncology, Prague, Czechia;*

### Background

Certain subtypes of pediatric B-cell precursor acute lymphoblastic leukemia (BCP ALL) are prone to undergo a myeloid lineage switch, most often displaying a monocytic phenotype. This switch usually happens during the early phase of treatment but can also be present at diagnosis (Dx). This unusual but well-documented phenomenon can cause diagnostic problems, particularly in the assessment of response to treatment. The cause of the lineage switch and its dynamics are not yet fully understood. Single-cell multiomics offers unprecedented insight into the dynamics of the putative transdifferentiation between lineages. In this study we focus on the PAX5 P80R subtype of BCP ALL. This subtype frequently manifests an abrupt change in phenotype in the early phase of treatment, which makes the investigation of the dynamics of the process challenging. At the same time, however, lineage instability is often already evident at Dx. We therefore focused on the in-depth analysis of the bone marrow (BM) samples at Dx.

### Aims

The aim of the presented study was a detailed understanding of the mechanism of lineage instability in PAX5 P80R BCP ALL and, in particular, to describe the process of transdifferentiation by means of computational biology tools.

### Methods

We used the 10x Genomics Single Cell 5' v2 platform to simultaneously evaluate gene expression, cell surface protein expression, and BCR rearrangements at the single-cell level from BM samples of six pediatric patients with PAX5 P80R BCP ALL at Dx. We used state-of-the-art computational tools to analyze the topological and geometric structure of the multimodal data, probabilistic cell-state dynamics, and RNA splicing rates in order to capture and describe the switching process.

### Results

All six patients manifested a phenotypic switch towards a monocytoid phenotype in the course of treatment. Given the rapid dynamics of lineage switching in PAX5 P80R BCP ALL, we expected heterogeneity across patients, reflecting differences in underlying genetic background and potential infection-related triggers.
In two cases the switch was already apparent at the protein level (gradual loss of CD19 and gain of CD33). At the RNA level a clear myeloid priming in phenotypically B-blast populations and evidence of a transdifferentiation process were discovered in four Dx samples. These populations were immunophenotypically consistent with B-blasts, yet exhibited a shared myeloid-primed transcriptomic signature (e.g., CSF2RA, CEBPD, CD86, CD68, CYBB, S100A4, TYROBP, SRGN, CLEC12A). We were able to define gene expression programs and model the transdifferentiation dynamics by gathering evidence across all measured modalities. In two patients the results remain inconclusive.

**Copyright Information:** (Online) ISSN: 2572-9241
©2026 The Author(s). HemaSphere is published by John Wiley & Sons Ltd on behalf of European Hematology Association. This is an open access Abstract Book distributed under the Attribution-NonCommercial-NoDerivs (CC BY-NC-ND), which allows third parties to download the articles and share them with others as long as they credit the author and the Abstract Book, but they cannot change the content in any way or use them commercially.
**Abstract Book Citations:** Authors, Title, HemaSphere, 2026;10:(S1):pages. The abstract book DOIs can be found on the "Author Information" page.
**Disclaimer:** Articles published in the journal HemaSphere exclusively reflect the opinions of the authors. The authors are responsible for all content in their abstracts including accuracy of the facts, statements, citing resources, etc.

**Summary/Conclusion**

While the exact dynamics and/or presence of the switching phenomenon at Dx differs between patients, in most cases we identified intermediate cellular populations exhibiting common features and gradual lineage transdifferentiation before the apparent immunophenotypic shift. This RNA-protein discordance supports our transdifferentiation model, further reinforced by computational interpretations, which positioned these intermediate populations before the appearance of monocytic cells in all samples.

Supported by NU23-05-00353, NW24-07-00264, UNCE/24/MED/003, LX22NPO510, NU23-07-00170 and Marie Sklodowska-Curie Actions Grant Agreement nr. 101167512.

**Copyright Information:** (Online) ISSN: 2572-9241
©2026 The Author(s). HemaSphere is published by John Wiley & Sons Ltd on behalf of European Hematology Association. This is an open access Abstract Book distributed under the Attribution-NonCommercial-NoDerivs (CC BY-NC-ND), which allows third parties to download the articles and share them with others as long as they credit the author and the Abstract Book, but they cannot change the content in any way or use them commercially.
**Abstract Book Citations:** Authors, Title, HemaSphere, 2026;10:(S1):pages. The abstract book DOIs can be found on the "Author Information" page.
**Disclaimer:** Articles published in the journal HemaSphere exclusively reflect the opinions of the authors. The authors are responsible for all content in their abstracts including accuracy of the facts, statements, citing resources, etc.

### (PS1469) ETV6::JAK2 FUSION PROMOTES CENTRAL NERVOUS SYSTEM INVASION IN MURINE B-CELL ACUTE LYMPHOBLASTIC LEUKEMIA

**Topic:** 01. Acute lymphoblastic leukemia - Biology & translational research

Telma Costa*[1, 2], Telmo Catarino[1], Ivette Pacheco-Leyva[1], Nuno R. Dos Santos[1]

*[1]i3S - Instituto de Investigação e Inovação em Saúde da Universidade do Porto, Porto, Portugal;[2]ICBAS - Instituto de Ciências Biomédicas Abel Salazar da Universidade do Porto, Porto, Portugal;*

### Background

Acute lymphoblastic leukemia (ALL) is the most frequent pediatric malignancy and remains an important cause of mortality and morbidity in all ages. A subgroup of B-cell precursor ALL (BCP-ALL) patients with bad prognosis carries specific genetic alterations, including *JAK2* fusions and *CDKN2A* deletions. Disease relapse in these patients is often associated with central nervous system (CNS) invasion.

### Aims

Here, we hypothesized that *JAK2* fusions are a risk factor for development of aggressive CNS-infiltrating BCP-ALL.

### Methods

To study the impact of constitutively active JAK2 kinase signaling in CNS involvement by B-ALL, we generated mice expressing an *ETV6::JAK2* fusion in the lymphoid lineage, in a leukemia-prone genetic background (*Rag2* and *Cdkn2a* deficiency).

### Results

Indeed, in comparison to $Rag2^{-/-}$;$Cdkn2a^{-/-}$ littermates, ETV6::JAK2;$Rag2^{-/-}$;$Cdkn2a^{-/-}$ mice exhibited earlier onset of B-ALL and presented more frequent CNS invasion (e.g., paraparesis/paraplegia of hind limbs). Immunohistochemical analyses of diseased ETV6::JAK2;$Rag2^{-/-}$;$Cdkn2a^{-/-}$ and $Rag2^{-/-}$;$Cdkn2a^{-/-}$ mice revealed leptomeningeal invasion by $B220^{+}PAX5^{+}$ cells expressing phosphorylated STAT5, a JAK kinase substrate. *In vitro* treatment of primary leukemic cells with AZD1480 (JAK2 kinase inhibitor) significantly impaired the survival of ETV6::JAK2;$Rag2^{-/-}$;$Cdkn2a^{-/-}$ cells but not $Rag2^{-/-}$;$Cdkn2a^{-/-}$ cells. Moreover, treatment of $Rag2^{-/-}$;$Il2rg^{-/-}$ mice transplanted with ETV6::JAK2-driven B-ALL with FDA-approved JAK1/JAK2 inhibitor Ruxolitinib (50 mg/kg/daily) resulted in significant reduction of circulating B-ALL cells and concomitant increased number of apoptotic cells in the spleen, in comparison to vehicle group. In parallel, to grasp the dynamics of CNS invasion by B-ALL, we infused CD45.2-expressing ETV6::JAK2;$Rag2^{-/-}$;$Cdkn2a^{-/-}$ and $Rag2^{-/-}$;$Cdkn2a^{-/-}$ cells in $Rag2^{-/-}$;$CD45.1^{+}$ recipients and detected leukemic cells in the cerebrospinal fluid (CSF) at different timepoints, via flow cytometry analysis. Mice injected with ETV6::JAK2-driven B-ALL presented earlier and significantly higher invasion of CSF by $CD45.2^{+}B220^{+}$ cells compared to those transplanted with $Rag2^{-/-}$;$Cdkn2a^{-/-}$ leukemia, but similar tumor burden in the bone marrow.

Moreover, transcriptomic analysis of leukemic cells from ETV6::JAK2;$Rag2^{-/-}$;$Cdkn2a^{-/-}$ mice revealed upregulation of genes (*Selplg* and *Ccr5)* and signaling pathways (unfolded protein response and cholesterol synthesis) related to CNS invasion in B-ALL, compared to $Rag2^{-/-}$;$Cdkn2a^{-/-}$ leukemia.

**Copyright Information:** (Online) ISSN: 2572-9241
©2026 The Author(s). HemaSphere is published by John Wiley & Sons Ltd on behalf of European Hematology Association. This is an open access Abstract Book distributed under the Attribution-NonCommercial-NoDerivs (CC BY-NC-ND), which allows third parties to download the articles and share them with others as long as they credit the author and the Abstract Book, but they cannot change the content in any way or use them commercially.
**Abstract Book Citations:** Authors, Title, HemaSphere, 2026;10:(S1):pages. The abstract book DOIs can be found on the "Author Information" page.
**Disclaimer:** Articles published in the journal HemaSphere exclusively reflect the opinions of the authors. The authors are responsible for all content in their abstracts including accuracy of the facts, statements, citing resources, etc.

### Summary/Conclusion

In conclusion, the *ETV6::JAK2* fusion promotes leukemia survival, accelerates disease development, and confers increased neurotropism to leukemic cells.

**Copyright Information:** (Online) ISSN: 2572-9241
©2026 The Author(s). HemaSphere is published by John Wiley & Sons Ltd on behalf of European Hematology Association. This is an open access Abstract Book distributed under the Attribution-NonCommercial-NoDerivs (CC BY-NC-ND), which allows third parties to download the articles and share them with others as long as they credit the author and the Abstract Book, but they cannot change the content in any way or use them commercially.
**Abstract Book Citations:** Authors, Title, HemaSphere, 2026;10:(S1):pages. The abstract book DOIs can be found on the "Author Information" page.
**Disclaimer:** Articles published in the journal HemaSphere exclusively reflect the opinions of the authors. The authors are responsible for all content in their abstracts including accuracy of the facts, statements, citing resources, etc.

### (PS1470) PREVALENCE AND IMPACT OF INCIDENTALLY DETECTED GERMLINE VARIANTS IN PEDIATRIC ACUTE LYMPHOBLASTIC LEUKEMIA

**Topic:** 01. Acute lymphoblastic leukemia - Biology & translational research

Borbála Péterffy*[1, 2], Emese Zsigrai[3], Bálint Egyed[1, 4], Szilvia Krizsán[1, 2, 4], Gábor Bedics[1, 2], Dániel Erdélyi[4], Judit Müller[4], Lajos Hegyi[1, 2], Anna Bekő[1, 2], Lili Anna Kenéz[1, 2], Krisztina Csanádi[5], György Péter[5], Gábor Ottóffy[6], Katalin Csernus[6], Ágnes Vojcek[6], Lilla Tiszlavicz[7], Lilla Tiszlavicz[7], Iván Patricza[7], Ágnes Kelemen[8], Péter Hauser[9], Krisztián Kállay[9], Gabriella Kertész[9], Anna Rakonczay[9], Zsuzsanna Gaál[3], Miklós Petrás[3], István Szegedi[3], Gábor Barna[10], Ágnes Márk[10], Botond Timár[10], Irén Haltrich[4], Zsuzsanna Hevessy[11], Anikó Ujfalusi[11], Béla Kajtár[12], Gergely Krivan[9], András Matolcsy[10], Csongor Kiss[3], Gábor Kovács[4], Donát Alpár[1, 2], Csaba Bödör[1, 2]

*[1]Semmelweis University, HCEMM-SE Molecular Oncohematology Research Group, Department of Pathology and Experimental Cancer Research, Budapest, Hungary; [2]Semmelweis University, MTA-SE „Lendület" Molecular Oncohematology Research Group, Department of Pathology and Experimental Cancer Research, Budapest, Hungary; [3]University of Debrecen, Division of Pediatric Hematology-Oncology, Institute of Pediatrics, Debrecen, Hungary; [4]Semmelweis University, Pediatric Center, Budapest, Hungary; [5]Heim Pál National Pediatric Institute, Hemato-Oncology Unit, Budapest, Hungary; [6]University of Pécs, Department of Pediatrics, Oncohaematology Division, Pécs, Hungary; [7]University of Szeged, Department of Pediatrics and Pediatric Health Care Center, Szeged, Hungary; [8]Borsod-Abaúj-Zemplén County Central Hospital and University Teaching Hospital, Velkey László Child's Health Center, Miskolc, Hungary; [9]Central Hospital of Southern Pest, National Institute of Hematology and Infectious Diseases, Pediatric Hematology and Stem Cell Transplantation Department, Budapest, Hungary; [10]Semmelweis University, Department of Pathology and Experimental Cancer Research, Budapest, Hungary; [11]University of Debrecen, Department of Laboratory Medicine, Debrecen, Hungary; [12]University of Pécs, Department of Pathology, Pécs, Hungary;*

### Background

Among childhood malignancies, inherited cancer predisposition is considered relatively uncommon in pediatric acute lymphoblastic leukemia (ALL). Despite extensive characterization of the somatic genetic landscape of ALL, evidence regarding germline alterations detected during routine diagnostic testing remain limited.

### Aims

We sought to investigate the prevalence, spectrum and clinical significance of germline alterations incidentally discovered during standard diagnostic tumor genetic testing in a real-world cohort of consecutively diagnosed children with ALL.

### Methods

Diagnostic bone marrow samples from 192 children diagnosed with B-ALL (n=150), B-LBL (n=3), T-ALL (n=30), or T-LBL (n=9) were investigated. Mutations were identified using a custom QIASeq Targeted DNA Panel covering 102 disease-relevant genes. Variants with minor allele frequency >1% in The Genome Aggregation Database (gnomAD 2.1) were excluded. Putative germline alterations with a variant allele frequency of 45-55% were further examined in remission samples, and germline origin was confirmed or excluded by Sanger sequencing. All alterations included in further analysis were classified as pathogenic (P), likely pathogenic (LP) or variant of unknown significance (VUS) by at least two of the three databases used (Franklin, VarSome, ClinVar).

**Copyright Information:** (Online) ISSN: 2572-9241
©2026 The Author(s). HemaSphere is published by John Wiley & Sons Ltd on behalf of European Hematology Association. This is an open access Abstract Book distributed under the Attribution-NonCommercial-NoDerivs (CC BY-NC-ND), which allows third parties to download the articles and share them with others as long as they credit the author and the Abstract Book, but they cannot change the content in any way or use them commercially.
**Abstract Book Citations:** Authors, Title, HemaSphere, 2026;10:(S1):pages. The abstract book DOIs can be found on the "Author Information" page.
**Disclaimer:** Articles published in the journal HemaSphere exclusively reflect the opinions of the authors. The authors are responsible for all content in their abstracts including accuracy of the facts, statements, citing resources, etc.

**Results**

Twenty-six patients (13.5%) harbored a total of 28 germline alterations, 10.7% of which were classified as pathogenic or likely pathogenic, while the remainder as VUS. A pathogenic *TP53* c.916C>T mutation was discovered in a patient with hypodiploid ALL and a pathogenic *USH2A* c.3623G>A variant was detected in one B-other case. A likely pathogenic frameshift *CDKN2A* variant was identified in a patient with *KMT2A*-rearranged B-ALL. A single VUS was detected in *ETV6*, a gene linked to germline predisposition to ALL. Germline variants were most frequently observed in *SH2B3* (n=3). Notably, two of the three *SH2B3*-altered cases carried the identical c.622G>C alteration. Among the 20 patients in the total cohort who experienced relapse, six carried germline alterations, including the *TP53* and *USH2A* pathogenic variants, as well as VUS in *SH2B3, DNMT3A, MYC* and *NR3C1.* One patient with an *SH2B3* c.1085A>C germline alteration and *ETV6::RUNX1* fusion experienced two relapses, with the second one being restricted to the central nervous system. In this case, the presence of the germline variant influenced donor selection for hematopoietic stem cell transplantation, since one related candidate also carried the variant.

**Summary/Conclusion**

Germline alterations discovered during standard tumor diagnostic workup of children with ALL can have a substantial impact on clinical decision-making. Inherited variants hold particular significance in the context of treatment intensity or hematopoietic stem cell transplantation, as donor selection may be directly affected. Furthermore, the presence of these constitutive alterations should be taken into account during follow-up. Confirmation of the germline origin and careful interpretation of potential clinical consequences are therefore essential, and genetic counseling should be considered in clinically relevant scenarios.

**Copyright Information:** (Online) ISSN: 2572-9241
©2026 The Author(s). HemaSphere is published by John Wiley & Sons Ltd on behalf of European Hematology Association. This is an open access Abstract Book distributed under the Attribution-NonCommercial-NoDerivs (CC BY-NC-ND), which allows third parties to download the articles and share them with others as long as they credit the author and the Abstract Book, but they cannot change the content in any way or use them commercially.
**Abstract Book Citations:** Authors, Title, HemaSphere, 2026;10:(S1):pages. The abstract book DOIs can be found on the "Author Information" page.
**Disclaimer:** Articles published in the journal HemaSphere exclusively reflect the opinions of the authors. The authors are responsible for all content in their abstracts including accuracy of the facts, statements, citing resources, etc.

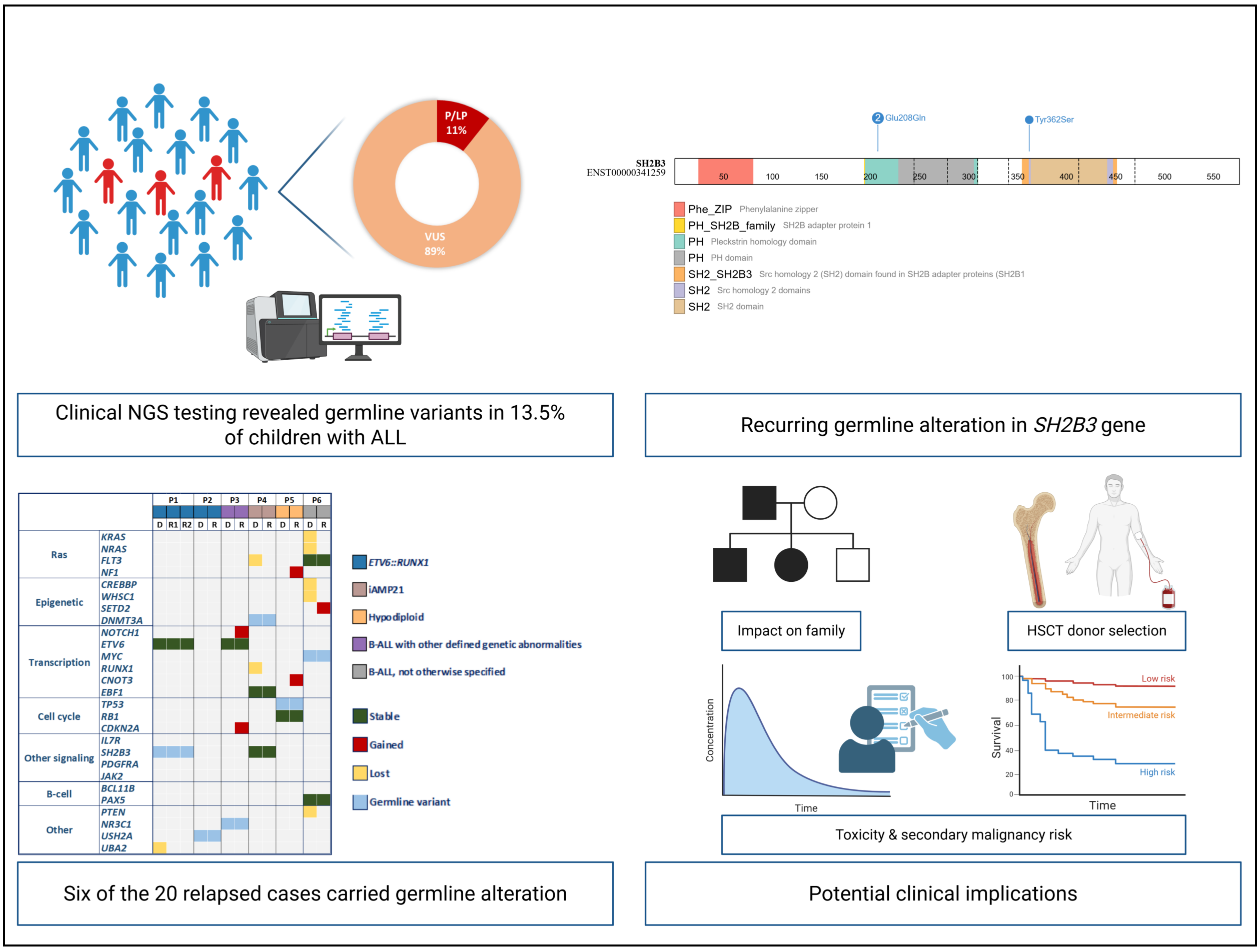

**Copyright Information:** (Online) ISSN: 2572-9241
©2026 The Author(s). HemaSphere is published by John Wiley & Sons Ltd on behalf of European Hematology Association. This is an open access Abstract Book distributed under the Attribution-NonCommercial-NoDerivs (CC BY-NC-ND), which allows third parties to download the articles and share them with others as long as they credit the author and the Abstract Book, but they cannot change the content in any way or use them commercially.
**Abstract Book Citations:** Authors, Title, HemaSphere, 2026;10:(S1):pages. The abstract book DOIs can be found on the "Author Information" page.
**Disclaimer:** Articles published in the journal HemaSphere exclusively reflect the opinions of the authors. The authors are responsible for all content in their abstracts including accuracy of the facts, statements, citing resources, etc.

### (PS1471) DEVELOPMENT OF ANTIGEN-SPECIFIC CAR-T CELLS TARGETING LEUKEMIA-INITIATING CELLS IN HUMAN T-CELL ACUTE LYMPHOBLASTIC LEUKEMIA

**Topic:** 01. Acute lymphoblastic leukemia - Biology & translational research

Serena di Iasio*[1], Elisabetta De Santis[1], Chiara Di Nunzio[1], Valentina Canistro[1], Patrizio Panelli[2], Mattia Miroballo[1], Gaja Bruno[1], Mattia Colucci[1], Costanzo Padovano[1], Giuseppe Miscio[3], Angelo Michele Carella[4], Andrew Weng[5], Vincenzo Giambra[1]

*[1]Institute for Stem Cell Biology, Regenerative Medicine and Innovative Therapies (ISBReMIT), Fondazione IRCCS Casa Sollievo della Sofferenza, San Giovanni Rotondo (FG), Italy; [2]Josep Carreras Leukaemia Research Institute Barcelona, Spain, Barcelona, Spain; [3]Clinical Laboratory Analysis and Transfusional Medicine, Fondazione IRCCS Casa Sollievo della Sofferenza, San Giovanni Rotondo (FG), Italy; [4]IRCCS Casa Sollievo della Sofferenza, Department of Hematology and Stem Cell Transplant Unit, San Giovanni Rotondo (FG), Italy; [5]Terry Fox Laboratory, BC Cancer Research Institute, Vancouver, BC V5Z 1L3, Canada, Vancouver, Canada;*

### Background

T-cell acute lymphoblastic leukemia (T-ALL) is an aggressive hematologic malignancy driven by uncontrolled expansion of immature T cells and sustained by the persistence of leukemia-initiating cells (LICs). Recent advances in adoptive immunotherapy have facilitated the development of chimeric antigen receptor (CAR)-engineered killer cells designed to target antigen-expressing tumor populations. However, effective CAR-based strategies that selectively eradicate LIC-enriched subsets in T-ALL remain largely underdeveloped.

### Aims

Building on recent progress and our previous findings, we hypothesize that specific regulatory pathways drive the progression and maintenance of LICs in human T-ALL, and that distinct LIC-enriched subsets display distinct immunophenotypic profiles, making them promising targets for optimized CAR T-cell therapies. Accordingly, we propose the following specific aims: (1) Characterize Wnt-active cell populations in human T-ALL; (2) Generate and optimize CAR-engineered killer cells targeting antigen-expressing leukemic cells; and (3) Define the genotypic, transcriptional, and proteomic alterations in leukemia cells following exposure to CAR T cells.

### Methods

Leukemia cells from three different patient-derived xenografts (PDXs) were transduced with a fluorescent Wnt/β-catenin reporter lentiviral construct (7TGC) and FACS-sorted prior to transplantation into immunodeficient (NSG) mice to assess LIC activity. Tumor heterogeneity was investigated performing single-cell RNA sequencing on 7TGC-transduced PDXs. To develop a targeted immunotherapeutic approach, second-generation CD7-directed CAR constructs were generated, a CRISPR/Cas9-mediated CD7 knockout strategy was optimized to eliminate CD7 expression on the CAR-T cells to overcome the fratricide issue with CD7, and *in vitro* cytotoxicity of CAR-killer cells was evaluated against a CD7+ leukemia cell line.

**Copyright Information:** (Online) ISSN: 2572-9241
©2026 The Author(s). HemaSphere is published by John Wiley & Sons Ltd on behalf of European Hematology Association. This is an open access Abstract Book distributed under the Attribution-NonCommercial-NoDerivs (CC BY-NC-ND), which allows third parties to download the articles and share them with others as long as they credit the author and the Abstract Book, but they cannot change the content in any way or use them commercially.
**Abstract Book Citations:** Authors, Title, HemaSphere, 2026;10:(S1):pages. The abstract book DOIs can be found on the “Author Information” page.
**Disclaimer:** Articles published in the journal HemaSphere exclusively reflect the opinions of the authors. The authors are responsible for all content in their abstracts including accuracy of the facts, statements, citing resources, etc.

**Results**

Flow cytometry of leukemic cells from moribund mice showed that less than 4% had active Wnt signaling. However, Wnt-active ($GFP^+$) cells demonstrated a higher LIC frequency than Wnt-inactive ($GFP^−$) cells in limiting dilution transplantation assays. Single-cell transcriptomic profiling identified two distinct $GFP^+$ clusters characterized by differential expression of CD7, CADM1, and TSPAN13, a finding validated in PDXs and primary patient samples. Following CD7 knockout strategy, αCD7 CAR-T cells exhibit potent cytotoxic activity against CD7+ leukemia cells compared to control. By integrating single-cell transcriptomic profiling with proteomic assays and high-dimensional immunophenotyping, we aim to generate a comprehensive molecular and cellular map of leukemia cells for the development of more effective and personalized CAR-based treatments for T-ALL.

**Summary/Conclusion**

These results provide a comprehensive framework for LIC-specific CAR-T development in T-ALL, supporting strategies to enhance selectivity, reduce toxicity, and counteract tumor heterogeneity.

**Copyright Information:** (Online) ISSN: 2572-9241
©2026 The Author(s). HemaSphere is published by John Wiley & Sons Ltd on behalf of European Hematology Association. This is an open access Abstract Book distributed under the Attribution-NonCommercial-NoDerivs (CC BY-NC-ND), which allows third parties to download the articles and share them with others as long as they credit the author and the Abstract Book, but they cannot change the content in any way or use them commercially.
**Abstract Book Citations:** Authors, Title, HemaSphere, 2026;10:(S1):pages. The abstract book DOIs can be found on the “Author Information” page.
**Disclaimer:** Articles published in the journal HemaSphere exclusively reflect the opinions of the authors. The authors are responsible for all content in their abstracts including accuracy of the facts, statements, citing resources, etc.

### (PS1472) PROTEOMIC EXPLORATION OF LINEAGE-SPECIFIC HDAC3 VULNERABILITIES IN KMT2A-REARRANGED ACUTE LEUKEMIA

**Topic:** 01. Acute lymphoblastic leukemia - Biology & translational research

Jiaer Wang*[1], Johannes Nilsson[1], Jens Christiansen[1], Joanna Watral[1], Ariana Calderón[1], Mina Davoudi[1], Sudip Ghosh[1, 1], Tamara C.A.I. Verbeek[2], Ronald Stam[2], Charlotta Böiers[1], Marcus Järås[1], Jenny Hansson[1]

*[1]Lund Stem Cell Center, Lund University, Lund, Sweden;[2]Princess Maxima Center,, Utrecht, The Netherlands;*

### Background

Acute leukemia is a heterogeneous disease, with clinical outcomes and molecular features shaped by its developmental origin and lineage commitment. Emerging evidence indicates that this heterogeneity is underpinned by stage-specific proteomic programs that confer distinct therapeutic vulnerabilities. Histone deacetylase 3 (HDAC3) plays a critical role in epigenetic regulation and has emerged as a therapeutic target in acute leukemia. Our recent study identified elevated HDAC3 expression specifically in fetal pre-leukemic progenitor cells compared to their adult counterparts.

### Aims

We aimed to i) determine the role of HDAC3 in governing cell fate and therapeutic vulnerability in leukemic progression, ii) compare lineage-specific HDAC3 dependency in acute lymphoblastic leukemia (ALL) versus acute myeloid leukemia (AML) malignancies, and iii) decipher proteomic networks underlying age- and lineage-specific responses to design HDAC3 inhibitor-based synergistic therapies.

### Methods

We utilized fetal and adult hematopoietic stem and progenitor cells (Lin$^-$Sca-1$^+$c-Kit$^+$ (LSK)), isolated from *KMT2A*-rearranged transgenic mice, with doxycycline induction *in vitro* to distinguish pre-leukemic from healthy states, alongside human *KMT2A*-rearranged ALL and AML cell lines, to model age- and lineage-specific contexts. HDAC3 inhibition was achieved using specific pharmacological inhibitors with efficacy validated by a fluorescence-based activity assay. Quantitative proteomic profiling was performed to identify context-specific alterations. HDAC3 inhibition effects on cell fate (mouse LSKs) and drug sensitivity (IC50, apoptosis, viability) were assessed by flow cytometry and WST-1assay to define age- and lineage-dependent responses.

### Results

HDAC3 inhibition revealed age-specific vulnerability, with fetal mouse healthy and pre-leukemic cells showing higher apoptosis than adult bone marrow populations following treatment. Moreover, preliminary results suggested divergent lineage priming upon HDAC3 inhibition, with fetal healthy and pre-leukemic cells tending toward CD19+ B-cell differentiation and the adult counterparts toward the CD11b+ myeloid lineage. In human cell lines, ALL cells demonstrated greater sensitivity to HDAC3 inhibition than AML cells, evidenced by reduced viability at high-dose short-term treatment and lower IC50 values in dose-response assays. Consistently, basal HDAC3 enzymatic activity was lower in ALL lines and was further reduced upon inhibitor treatment, correlating with their heightened susceptibility. These differential sensitivities across age and lineage contexts suggest distinct proteomic composition driving HDAC3 dependency.

### Summary/Conclusion

Our study investigated age- and lineage-specific vulnerabilities to HDAC3 inhibition in *KMT2A*-rearranged acute leukemia. Fetal and adult healthy and pre-leukemic cells exhibited distinct lineage priming upon HDAC3 inhibition, with fetal cells showing a B-lymphoid bias. ALL cells demonstrated greater dependency on HDAC3 than AML cells, correlating with lower baseline enzymatic activity. Initial proteomic profiling of inhibitor-treated lines supports this notion and is guiding the identification of rational combination strategies with HDAC3 inhibition.

**Copyright Information:** (Online) ISSN: 2572-9241
©2026 The Author(s). HemaSphere is published by John Wiley & Sons Ltd on behalf of European Hematology Association. This is an open access Abstract Book distributed under the Attribution-NonCommercial-NoDerivs (CC BY-NC-ND), which allows third parties to download the articles and share them with others as long as they credit the author and the Abstract Book, but they cannot change the content in any way or use them commercially.
**Abstract Book Citations:** Authors, Title, HemaSphere, 2026;10:(S1):pages. The abstract book DOIs can be found on the “Author Information” page.
**Disclaimer:** Articles published in the journal HemaSphere exclusively reflect the opinions of the authors. The authors are responsible for all content in their abstracts including accuracy of the facts, statements, citing resources, etc.

### (PS1473) SAFETY AND PRELIMINARY EFFICACY OF OLVEREMBATINIB (HQP1351) COMBINED WITH LISAFTOCLAX (APG-2575) IN PEDIATRIC PATIENTS WITH RELAPSED/REFRACTORY (R/R PH+ ALL): RESULTS OF A PHASE 1B STUDY

**Topic:** 02. Acute lymphoblastic leukemia - Clinical

Jingliao Zhang*[1], Junxia Wang[1], Tianfeng Liu[1], Xiaoyan Zhang[1], Ranran Zhang[1], Ruan Min[1], Lixian Chang[1], Xiaoming Liu[1], Ren Yuanyuan[1], Chen Xia[1], Huanshan Guo[2], Hengbang Wang[3], Zi Chen[3], Yingchi Zhang[1], Yumei Chen[1], Li Zhang[1], Ye Guo[1], Fang Liu[1], Xiaojuan Chen[1], Yao Zou[1], Wenyu Yang[1], Yifan Zhai[2, 3, 4], Xiaofan Zhu[1]

*[1]State Key Laboratory of Experimental Hematology, National Clinical Research Center for Blood Diseases, Haihe Laboratory of Cell Ecosystem, Institute of Hematology and Blood Diseases Hospital, Chinese Academy of Medical Sciences and Peking Union Medical College, Tianjin, China; [2]Guangzhou Healthquest Pharma Co., Ltd., Guangzhou, China; [3]Ascentage Pharma (Suzhou) Co., Ltd., Suzhou, China; [4]Ascentage Pharma Group Inc., Rockville, MD, United States of America;*

### Background

Pediatric Ph$^+$ ALL is a high-risk subtype with limited treatments after TKI failure. Olverembatinib is a third-generation TKI active against *BCR::ABL1* variants including T315I. Lisaftoclax is a new selective BCL-2 inhibitor with encouraging activities in hematologic malignancies.

### Aims

This study evaluated the safety, preliminary efficacy, and pharmacokinetics of olverembatinib plus lisaftoclax in pediatric patients with R/R Ph$^+$ ALL.

### Methods

This open-label, dose-escalation and expansion study (NCT05495035) enrolled patients aged <18 with R/R Ph$^+$ ALL resistant or intolerant to ≥1 prior TKI using a 3+3 dose-escalation design. Patients received olverembatinib monotherapy (40 mg adult-equivalent dose [AED] QOD, days 1-14), followed by combination therapy with lisaftoclax (200, 400, or 600 mg AED daily, with 3-day dose ramp-up) and dexamethasone (6 mg/m²/day, days 15-42).

**Copyright Information:** (Online) ISSN: 2572-9241
©2026 The Author(s). HemaSphere is published by John Wiley & Sons Ltd on behalf of European Hematology Association. This is an open access Abstract Book distributed under the Attribution-NonCommercial-NoDerivs (CC BY-NC-ND), which allows third parties to download the articles and share them with others as long as they credit the author and the Abstract Book, but they cannot change the content in any way or use them commercially.
**Abstract Book Citations:** Authors, Title, HemaSphere, 2026;10:(S1):pages. The abstract book DOIs can be found on the “Author Information” page.
**Disclaimer:** Articles published in the journal HemaSphere exclusively reflect the opinions of the authors. The authors are responsible for all content in their abstracts including accuracy of the facts, statements, citing resources, etc.

### Results

From Sep 2022 through Feb 2026, 18 patients were screened, and 17 treated (200 mg, n=4; 400 mg, n=4; 600 mg, n=9). Six (40%) of 15 patients harbored baseline *ABL1* kinase domain (KD) mutations, including L248N (n=1), T315I (n=4), and F317L (n=1). Median age was 13.0 (range 9-15) years. One patient discontinued on C1D1 because of a seizure, which was not treatment related. Sixteen eligible patients (14/16, R/R; 6/16, intolerant) received lisaftoclax at AEDs of 200 (n=3), 400 (n=4), or 600 mg (n=9). Twelve of 17 patients (70.6%) experienced grade ≥3 hematologic TEAEs, including neutropenia or anemia (41.2%, n=7 each), thrombocytopenia (17.6%, n=3), and elevated ALT or AST (11.8%, n=2). Two (11.8%) discontinued because of AEs (one seizure on C1D1, one lisaftoclax-related hepatotoxicity). TRAEs occurred in 16 (94.1%) patients, with grade ≥3 TRAEs in 11 (64.7%). Frequent grade ≥3 TRAEs were neutrophil count decreased (41.2%) and anemia or platelet count decreased (17.6%, n=3 each). ALT, AST, or bilirubin elevations occurred in 41.2% (n=7, any grade), with one serious adverse event (5.9%) reported. No treatment-related deaths occurred. Among nine evaluable patients, the ORR (CR+CR with incomplete count recovery [CRi]) was 55.6% (5/9; 3 CR, 2 CRi) after olverembatinib monotherapy (EOM). By C2D15 and end of combination therapy (C2D27), the ORR increased to 88.9% (8/9; all CR). Among patients evaluable for response depth, CCyR (by FISH) increased from 40.0% (4/10) at EOM to 88.9% (8/9) at C2D15. MRD negativity (by cytometry) increased from 25.0% (3/12) at EOM to 66.7% (8/12) at C2D15 and C2D28. MMR or better (via quantitative PCR) was achieved by 93.3% (14/15) of patients by C2D28, with complete molecular response observed in 21.4% (3/14) at C2D15 and 20.0% (3/15) at C2D28. Importantly, responses occurred across dose levels and *ABL1* genetic variants. Pharmacokinetic data demonstrated predictable and comparable exposure to adult profiles for both agents, with no significant drug accumulation or drug-drug interactions. Olverembatinib had a half-life of ~13-16 hours and lisaftoclax ~4-6 hours. Both agents were detectable in CSF, indicating CNS penetration.

### Summary/Conclusion

Olverembatinib plus lisaftoclax induces rapid, deep, and high-quality remissions in pediatric R/R $Ph^+$ ALL with manageable toxicity and favorable pharmacokinetics. The chemotherapy-free regimen demonstrated antileukemic activity in *ABL1* KD mutants and evidence of CNS penetration, positioning this oral dual-targeted regimen as a potentially viable strategy for this high-risk population.

**Copyright Information:** (Online) ISSN: 2572-9241
©2026 The Author(s). HemaSphere is published by John Wiley & Sons Ltd on behalf of European Hematology Association. This is an open access Abstract Book distributed under the Attribution-NonCommercial-NoDerivs (CC BY-NC-ND), which allows third parties to download the articles and share them with others as long as they credit the author and the Abstract Book, but they cannot change the content in any way or use them commercially.
**Abstract Book Citations:** Authors, Title, HemaSphere, 2026;10:(S1):pages. The abstract book DOIs can be found on the "Author Information" page.
**Disclaimer:** Articles published in the journal HemaSphere exclusively reflect the opinions of the authors. The authors are responsible for all content in their abstracts including accuracy of the facts, statements, citing resources, etc.

### (PS1474) DIFFERENCES IN GENOMIC AND TRANSCRIPTOMIC FEATURES BETWEEN ADULTS WITH NEWLY DIAGNOSED BCR::ABL1-POSITIVE ACUTE LYMPHOBLASTIC LEUKEMIA WITH MULTILINEAGE AND LYMPHOID-ONLY INVOLVEMENT

**Topic:** 02. Acute lymphoblastic leukemia - Clinical

Zhenzhen Jiang*[1], Mei Bao[1], Xiaoshuai Zhang[1], Chengcheng Yan[2], Ye Li[1], Ya-Zhen Qin[1], Ya-Zhe Wang[1], Qian Jiang[1]

*[1]Peking University People's Hospital, Beijing, China;[2]Tianjin Kingmed Diagnostics Laboratory, Tianjin, China;*

### Background

The differences in outcomes between *BCR::ABL1*-positive acute lymphoblastic leukemia (ALL) with multilineage and lymphoid-only involvement remain controversial. There are limited data on these two disease types.

### Aims

To explore the genomic and transcriptomic differences between adults with newly diagnosed *BCR::ABL1*-positive ALL with multilineage and lymphoid-only involvement and identify molecular features associated with outcomes.

### Methods

A retrospective study on adults with *BCR::ABL1*-positive ALL who had genomic and/or transcriptomic data was designed. Cox regression analysis was used to identify molecular and clinical covariates associated with outcomes. Event-free survival (EFS) was defined as the interval from the starting TKI-therapy to failure to achieve complete remission (CR) after a 4-week induction, BCR-ABL >1% at 3 months, loss of response, or death.

### Results

Data of 191 patients (62 multilineage and 129 lymphoid-only) were included. 94 (49%) patients were male. Median age was 47 (IQR, 34-59) years. Median follow-up was 20 (IQR, 9-34) months. 182 (95%) patients achieved CR; 148 (77%), complete molecular remission. 91 (48%) underwent transplant. 3-year probabilities of EFS and OS were 61% (95% CI, 53-70%) and 82% (74-90%), respectively. There were no significant differences in EFS and OS by disease type (multilineage or lymphoid-only involvement).
*RUNX1* (31% *vs.* 2%, $p < 0.001$) and *BCORL1* (18% *vs.* 2%, $p < 0.001$) mutations were more common in patients with multilineage involvement; while *IKZF1*$^{plus}$ (10% *vs.* 30%, $p = 0.002$), in those with lymphoid-only involvement. Patients with multilineage (*vs.* lymphoid-only) involvement had higher WBC and platelet counts, and lower blasts at baseline. In multivariate analysis *WT1* mutation (HR = 2.8 [1.0-8.0], $p = 0.046$) and trisomy 8 (HR = 2.5 [1.0-6.2], $p = 0.048$) were significantly-associated with inferior EFS independent of higher WBC count, initial imatinib use (*vs.* 3G-TKI), and non-transplant.
In the RNAseq data from 94 patients (33 multilineage and 61 lymphoid-only), GSVA showed that multilineage (*vs.* lymphoid-only) involvement exhibited activation of pathways related to leukemia stem cell properties, cell chemotaxis, metabolism. Immune infiltration analyses revealed that multilineage involvement had higher proportion of monocytes, eosinophils, resting mast cells, and Tregs; while lymphoid-only involvement, B cell naive, T cell CD4 memory resting, T cell CD8, and T cells follicular helper. Clustering analysis identified two distinct clusters including Cluster 1 (more pronounced FGFR ligand binding) and Cluster 2 (more pronounced leukemia stem cells characteristics, cell chemotaxis, metabolism, and suppressive immune responses). In multivariate analysis Cluster 2 (HR = 2.7 [1.3-5.4], $p = 0.006$), trisomy 8 (HR = 3.9 [1.2-12.8], $p = 0.027$), and higher WBC count (HR = 1.0 [1.0-1.0], $p = 0.029$) were significantly-associated with inferior EFS. Neither genomic nor transcriptomic data, disease type had no impact on outcomes in multivariate analysis.

**Copyright Information:** (Online) ISSN: 2572-9241
©2026 The Author(s). HemaSphere is published by John Wiley & Sons Ltd on behalf of European Hematology Association. This is an open access Abstract Book distributed under the Attribution-NonCommercial-NoDerivs (CC BY-NC-ND), which allows third parties to download the articles and share them with others as long as they credit the author and the Abstract Book, but they cannot change the content in any way or use them commercially.
**Abstract Book Citations:** Authors, Title, HemaSphere, 2026;10:(S1):pages. The abstract book DOIs can be found on the "Author Information" page.
**Disclaimer:** Articles published in the journal HemaSphere exclusively reflect the opinions of the authors. The authors are responsible for all content in their abstracts including accuracy of the facts, statements, citing resources, etc.

**Summary/Conclusion**

There were significant differences in genomic and transcriptomic signatures as well as clinical characteristics between patients with multilineage and lymphoid-only involvement *BCR::ABL1*-positive ALL, while outcomes were comparable. Certain molecular features were identified to predict high risk population which may help to improve the management and stratification of *BCR::ABL1*-positive ALL.

**Copyright Information:** (Online) ISSN: 2572-9241
©2026 The Author(s). HemaSphere is published by John Wiley & Sons Ltd on behalf of European Hematology Association. This is an open access Abstract Book distributed under the Attribution-NonCommercial-NoDerivs (CC BY-NC-ND), which allows third parties to download the articles and share them with others as long as they credit the author and the Abstract Book, but they cannot change the content in any way or use them commercially.
**Abstract Book Citations:** Authors, Title, HemaSphere, 2026;10:(S1):pages. The abstract book DOIs can be found on the "Author Information" page.
**Disclaimer:** Articles published in the journal HemaSphere exclusively reflect the opinions of the authors. The authors are responsible for all content in their abstracts including accuracy of the facts, statements, citing resources, etc.

### (PS1475) THE APPLICATION AND STUDY OF USING NGS FOR DETECTING IG/TCR GENE REARRANGEMENTS IN BONE MARROW FOR MRD MONITORING IN PEDIATRIC ACUTE LYMPHOBLASTIC LEUKEMIA

**Topic:** 02. Acute lymphoblastic leukemia - Clinical

Yang Li*[1], Peifang Xiao[2]

*[1]Children's Hospital of Soochow University, Department of Hematology, Suzhou, Jiangsu, China;[2]Children's Hospital of Soochow University, Department of Pediatric Hematology and Oncology, Suzhou, China;*

### Background

Pediatric acute lymphoblastic leukemia (ALL) is the most common childhood cancer, and relapse remains a major challenge. Minimal residual disease (MRD) is a critical prognostic factor, but conventional detection methods have limited sensitivity. Next-generation sequencing (NGS) of immunoglobulin (Ig) and T-cell receptor (TCR) gene rearrangements offers a highly sensitive approach for MRD monitoring. Yet, its clinical utility in pediatric ALL requires further validation.

### Aims

This study aims to evaluate the clinical application value of NGS-based detection of Ig/TCR gene rearrangements for MRD monitoring in pediatric ALL, and to compare its performance with conventional multiparameter flow cytometry (MFC) and real-time quantitative PCR (RQ-PCR) methods.

### Methods

This retrospective study enrolled 237 pediatric patients newly diagnosed with B/T-ALL between March 2023 and October 2025. Bone marrow samples were collected at diagnosis, day 19 (D19), and day 46 (D46) of induction therapy. Genomic DNA was subjected to ultra-deep NGS targeting Ig/TCR gene rearrangements, achieving a sensitivity of $10^{-6}$. MRD was also assessed by MFC (sensitivity $10^{-4}$) and RQ-PCR for fusion genes (sensitivity $10^{-4}$-$10^{-5}$). Concordance among methods was evaluated using Cohen's kappa coefficient. Prognostic value was assessed by Kaplan-Meier survival analysis, Cox regression, and ROC curve analysis.

**Copyright Information:** (Online) ISSN: 2572-9241
©2026 The Author(s). HemaSphere is published by John Wiley & Sons Ltd on behalf of European Hematology Association. This is an open access Abstract Book distributed under the Attribution-NonCommercial-NoDerivs (CC BY-NC-ND), which allows third parties to download the articles and share them with others as long as they credit the author and the Abstract Book, but they cannot change the content in any way or use them commercially.
**Abstract Book Citations:** Authors, Title, HemaSphere, 2026;10:(S1):pages. The abstract book DOIs can be found on the "Author Information" page.
**Disclaimer:** Articles published in the journal HemaSphere exclusively reflect the opinions of the authors. The authors are responsible for all content in their abstracts including accuracy of the facts, statements, citing resources, etc.

### Results

Among 237 patients (56.5% male; median age 5.83 years; B-ALL 91.6%, T-ALL 8.4%), NGS identified at least one clonal Ig/TCR rearrangement in 218 patients (92.0%), with a median of 2 clones per patient. NGS-MRD positivity rates were 78.9% at D19 and 23.6% at D46. In comparison, MFC-MRD positivity rates were 57.4% at D19 and 5.5% at D46, while RQ-PCR-MRD (fusion gene-positive subgroup, n=147) showed positivity rates of 60.5% at D19 and 14.3% at D46. Concordance between NGS and MFC was low at D19 (κ=0.271) and D46 (κ=0.316). NGS identified additional MRD-positive cases in 65 patients at D19 and 43 at D46 that were missed by MFC.

Survival analysis revealed that D46 NGS-MRD status was a powerful prognostic indicator. Patients with D46 NGS-MRD positivity had significantly inferior 2-year EFS (79.6% vs. 96.5%, P<0.0001) and OS (93.4% vs. 100.0%, P=0.0012). Univariate Cox regression demonstrated D46 NGS-MRD positivity was strongly associated with relapse risk (HR=8.46), with a higher hazard ratio compared to D46 MFC-MRD (HR=5.30) and D46 RQ-PCR-MRD (HR=5.46). Multivariable analysis confirmed D46 NGS-MRD as an independent predictor of relapse (HR=11.74). ROC analysis for predicting relapse yielded an AUC of 0.742 for D46 NGS-MRD, outperforming D19 NGS-MRD (0.546) and MFC-MRD at both time points (0.594, 0.571).

Dynamic analysis identified three patient groups: early clearance/stable negative (D19-/D46-, relapse 4.0%), delayed clearance (D19+/D46-, relapse 2.3%), and persistent positivity (D19+/D46+, relapse 19.6%). The persistent positivity group had significantly higher relapse rates (P=9.00×10^-5). Clonal evolution analysis showed IGH remained predominant, but frequencies of IGL, TRB, and TRG increased from D19 to D46, suggesting selective pressure during therapy.

### Summary/Conclusion

NGS-based MRD monitoring demonstrates superior sensitivity in pediatric ALL compared to conventional methods. MRD status at D46 serves as a powerful independent predictor of relapse, with superior prognostic discrimination. Multi-target tracking capability helps mitigate false-negative results due to clonal evolution.

**Copyright Information:** (Online) ISSN: 2572-9241
©2026 The Author(s). HemaSphere is published by John Wiley & Sons Ltd on behalf of European Hematology Association. This is an open access Abstract Book distributed under the Attribution-NonCommercial-NoDerivs (CC BY-NC-ND), which allows third parties to download the articles and share them with others as long as they credit the author and the Abstract Book, but they cannot change the content in any way or use them commercially.
**Abstract Book Citations:** Authors, Title, HemaSphere, 2026;10:(S1):pages. The abstract book DOIs can be found on the "Author Information" page.
**Disclaimer:** Articles published in the journal HemaSphere exclusively reflect the opinions of the authors. The authors are responsible for all content in their abstracts including accuracy of the facts, statements, citing resources, etc.

### (PS1476) COMBINATION OF VENETOCLAX AND CHEMOTHERAPY IN NEWLY DIAGNOSED ADULT T-CELL ACUTE LYMPHOBLASTIC LEUKEMIA: INTERIM ANALYSIS OF A MULTICENTER PROSPECTIVE STUDY

**Topic:** 02. Acute lymphoblastic leukemia - Clinical

Lijun Peng*[1], Weiyang Liu[1], Jiayi Ren[1], Ruirui Shi[1], Yuanfang Liu[1], Jie Hao[2], Bing-Bing Zhao[3], Weiping Zhang[1], Jin Wang[1], Jian-qing Mi[1]

*[1]Shanghai Institute of Hematology, State Key Laboratory of Medical Genomics, National Research Center for Translational Medicine at Shanghai, Ruijin Hospital, Shanghai jiao Tong University School of Medicine, 200025 Shanghai, China; Shanghai Key Laboratory of Gene Editing and Cell-based Immunotherapy for Hematological Diseases, Ruijin Hospital, Shanghai jiao Tong University School of Medicine, 200025., Shanghai, China; [2]Department of Hematology, Shanghai Jing'an District Beizhan Hospital, Shanghai, China; [3]Shanghai 411 Hospital, China RongTong Medical Healthcare Group Co.Ltd, Shanghai, China;*

### Background

Adult T-cell acute lymphoblastic leukemia (T-ALL) remains a biologically heterogeneous disease with suboptimal outcomes under conventional intensive chemotherapy. Early achievement of measurable residual disease (MRD) negativity after induction therapy is a key prognostic factor for long-term survival. Venetoclax, a selective BCL-2 inhibitor, has demonstrated promising antileukemic activity in lymphoid malignancies. However, prospective data on venetoclax-based regimens in newly diagnosed adult T-ALL are still limited.

### Aims

To define the efficacy (MRD negativity rate) and safety profile of venetoclax in combination with chemotherapy in newly diagnosed adult T-ALL.

### Methods

This is an ongoing, multicenter, phase II prospective clinical trial evaluating the efficacy and safety of venetoclax in combination with a pediatric-inspired chemotherapy regimen in adults with newly diagnosed T-ALL. Induction therapy consisted of vincristine, idarubicin, prednisone/prednisolone, cyclophosphamide and pegaspargase. Venetoclax was administered orally with a ramp-up schedule of 100 mg on day 4, 200 mg on day 5, and 400 mg from day 6 to day 14 during induction. Patients with poor early response, defined as day 14 bone marrow blasts >25% (M3 marrow), continued venetoclax at 400 mg daily until day 21. During the consolidation phase, venetoclax (400 mg on days 1–7) was combined with alternating hyper-CVAD courses A and B. High-risk T-ALL patients with suitable donors were planned to proceed to allogeneic hematopoietic stem cell transplantation (allo-HSCT) after achieving MRD negativity.

Primary endpoint was MRD negativity after induction therapy. Secondary endpoints included complete remission (CR) rate, overall survival (OS), event-free survival (EFS) and allo-HSCT rate.

Multiparameter flow cytometry was employed for MRD monitoring, negative MRD was defined as less than 0.01% (MRD<$10^{-4}$). Safety assessments included the evaluation of adverse events, which were graded according to CTCAE (version 5.0).

**Copyright Information:** (Online) ISSN: 2572-9241
©2026 The Author(s). HemaSphere is published by John Wiley & Sons Ltd on behalf of European Hematology Association. This is an open access Abstract Book distributed under the Attribution-NonCommercial-NoDerivs (CC BY-NC-ND), which allows third parties to download the articles and share them with others as long as they credit the author and the Abstract Book, but they cannot change the content in any way or use them commercially.
**Abstract Book Citations:** Authors, Title, HemaSphere, 2026;10:(S1):pages. The abstract book DOIs can be found on the “Author Information” page.
**Disclaimer:** Articles published in the journal HemaSphere exclusively reflect the opinions of the authors. The authors are responsible for all content in their abstracts including accuracy of the facts, statements, citing resources, etc.

### Results

Between August 2024 and December 2025, 27 patients were enrolled, and 23 were evaluable. The median age was 34 years (range, 17–62), 65.2% were male, and 47.8% had early T-cell precursor acute lymphoblastic leukemia (ETP-ALL). Four patients received extended venetoclax for 21 days during induction.

After one induction cycle, the overall response rate (CR and CR with incomplete peripheral blood count recovery) was 87.0% (20/23), with comparable responses in ETP-ALL (90.9%, 10/11) and non-ETP ALL (83.3%, 10/12) (p=1.0). Among patients achieving CR, the MRD-negative rate was 75.0% (15/20), including 70.0% (7/10) in ETP-ALL and 80.0% (8/10) in non-ETP ALL (p=1.0). Four relapses occurred (all prior to transplantation). Twelve (52.2%) patients proceeded to allogeneic stem cell transplantation. At the data cutoff in January 2026, all patients were alive except for one patient who died during the consolidation phase due to concomitant autoimmune encephalitis. Survival data analyses will be presented later.

The most frequent all-grade adverse events were leukocytopenia and thrombocytopenia (100%). Median times to platelet and neutrophil recovery were 22 days and 21 days, respectively. Only one patient (1/23, 4.3%) developed tumor lysis syndrome, which resolved after symptomatic treatment. No early death was observed.

### Summary/Conclusion

Venetoclax-based combination regimens could further enhance the efficacy of intensive chemotherapy, particularly in patients with ETP-ALL.

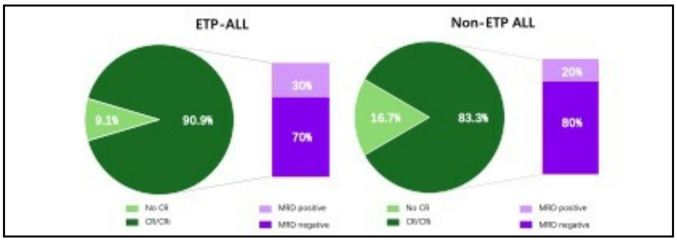

Figure 1. Treatment response in ETP-ALL (n=11) and non-ETP ALL (n=12). CR, complete remission; CRi, complete remission with incomplete peripheral recovery; MRD, minimal residual disease.

**Copyright Information:** (Online) ISSN: 2572-9241
©2026 The Author(s). HemaSphere is published by John Wiley & Sons Ltd on behalf of European Hematology Association. This is an open access Abstract Book distributed under the Attribution-NonCommercial-NoDerivs (CC BY-NC-ND), which allows third parties to download the articles and share them with others as long as they credit the author and the Abstract Book, but they cannot change the content in any way or use them commercially.
**Abstract Book Citations:** Authors, Title, HemaSphere, 2026;10:(S1):pages. The abstract book DOIs can be found on the "Author Information" page.
**Disclaimer:** Articles published in the journal HemaSphere exclusively reflect the opinions of the authors. The authors are responsible for all content in their abstracts including accuracy of the facts, statements, citing resources, etc.

### (PS1477) LATE RELAPSE AND PROGNOSTIC HETEROGENEITY IN ETV6-ALTERED PEDIATRIC B-ALL: A RETROSPECTIVE COHORT STUDY

**Topic:** 02. Acute lymphoblastic leukemia - Clinical

Xiaoyu Chen*[1], Shaoyan Hu[1]

[1]*Children's Hospital of Soochow University, Department of Hematology and Oncology, Suzhou, China;*

### Background

*ETV6::RUNX1* is a common pediatric B-ALL subtype with favorable prognosis, routinely classified as low-risk. However, the clinical implications of *ETV6* mutations in B-ALL remain poorly characterized and require further investigation.

### Aims

In this study, we aimed to systematically investigate the clinical and prognostic significance of*ETV6*alterations in pediatric B-ALL. We sought to delineate clinical characteristics of patients with *ETV6::RUNX1*fusion and isolated *ETV6*mutations, compare EFS and OS across these subgroups with *ETV6*-wild-type and other genetic subtypes, and analyze relapse patterns, including timing and post-relapse outcomes, between *ETV6*-altered and wild-type patients. A particular focus was to determine whether the prognostic effect of *ETV6* mutations depends on the presence of *ETV6::RUNX1*fusion.

### Methods

We retrospectively analyzed 653 pediatric patients with newly diagnosed B-ALL from the CCLG2008, CCCG2015, and CCCG2020 cohorts who underwent whole-transcriptome sequencing. Patients were categorized into *ETV6::RUNX1* fusion, *ETV6* mutation, and *ETV6*-wild-type groups. Clinical characteristics, outcomes, and relapse patterns were compared.

### Results

Among all 653 patients with a median follow-up of 3.31 (IQR: 1.92–4.61) years, the 5-year OS and EFS for the entire cohort were 96.4% ± 1.8% and 85.3% ± 3.4%. *ETV6::RUNX1* fusion was identified in 150 patients, isolated *ETV6* mutations in 20 patients, and 7 harbored concurrent *ETV6::RUNX1* fusion and *ETV6* mutation. Compared with *ETV6*-wild-type patients, those with *ETV6::RUNX1* had significantly lower proportions of age at diagnosis <10 years ($p$<0.001), WBC <50×10⁹/L ($p$<0.001), and Day19 MRD≥1% ($p$=0.038), and a significantly higher proportion classified as low risk ($p$<0.001). No specific clinical characteristics were observed in *ETV6* mutated patients. *ETV6::RUNX1* was associated with favorable outcomes (5-year OS: 98.5%±1.0%; 5-year EFS: 93.4%±2.5%), with significantly better EFS than *ETV6*-wild-type patients ($p$=0.003) but comparable OS between the two groups. Patients with isolated *ETV6* mutations demonstrated significantly inferior outcomes (5-year OS: 85.7%±9.4%; 5-year EFS: 73.0%±10.4%). Their prognosis was worse than that of patients with *ETV6::RUNX1* fusion or the hyperdiploid subtype (EFS: $p$=0.043 and $p$=0.032, respectively; OS: $p$=0.012 and $p$=0.003, respectively), but remained comparable to that of *ETV6*-wild-type patients. Relapse occurred later in patients with *ETV6* alterations (median 2.57 vs. 1.51 years, $p$=0.016) than *ETV6*-wild-type patients, but post-relapse survival did not differ. First, when patients with concurrent *ETV6::RUNX1* fusion and *ETV6* mutation were combined with those harboring *ETV6::RUNX1* fusion alone, this combined cohort showed significantly better OS and EFS than patients with isolated *ETV6* mutations ($p$=0.012, $p$=0.043, respectively). In contrast, no significant differences in OS or EFS were observed between patients with *ETV6::RUNX1* fusion alone and the combined cohort comprising patients with isolated *ETV6* mutations together with those harboring both alterations.

**Copyright Information:** (Online) ISSN: 2572-9241
©2026 The Author(s). HemaSphere is published by John Wiley & Sons Ltd on behalf of European Hematology Association. This is an open access Abstract Book distributed under the Attribution-NonCommercial-NoDerivs (CC BY-NC-ND), which allows third parties to download the articles and share them with others as long as they credit the author and the Abstract Book, but they cannot change the content in any way or use them commercially.
**Abstract Book Citations:** Authors, Title, HemaSphere, 2026;10:(S1):pages. The abstract book DOIs can be found on the "Author Information" page.
**Disclaimer:** Articles published in the journal HemaSphere exclusively reflect the opinions of the authors. The authors are responsible for all content in their abstracts including accuracy of the facts, statements, citing resources, etc.

**Summary/Conclusion**

Although patients with *ETV6::RUNX1* had a favorable prognosis and those with isolated *ETV6* mutations did not, both groups exhibited a distinct tendency toward late relapse, captured due to our cohort's substantial follow-up. Notably, the adverse prognostic effect of *ETV6* mutation was reversed when it co-occurred with *ETV6::RUNX1* fusion, resulting in outcomes comparable to fusion alone.

**Copyright Information:** (Online) ISSN: 2572-9241
©2026 The Author(s). HemaSphere is published by John Wiley & Sons Ltd on behalf of European Hematology Association. This is an open access Abstract Book distributed under the Attribution-NonCommercial-NoDerivs (CC BY-NC-ND), which allows third parties to download the articles and share them with others as long as they credit the author and the Abstract Book, but they cannot change the content in any way or use them commercially.
**Abstract Book Citations:** Authors, Title, HemaSphere, 2026;10:(S1):pages. The abstract book DOIs can be found on the "Author Information" page.
**Disclaimer:** Articles published in the journal HemaSphere exclusively reflect the opinions of the authors. The authors are responsible for all content in their abstracts including accuracy of the facts, statements, citing resources, etc.

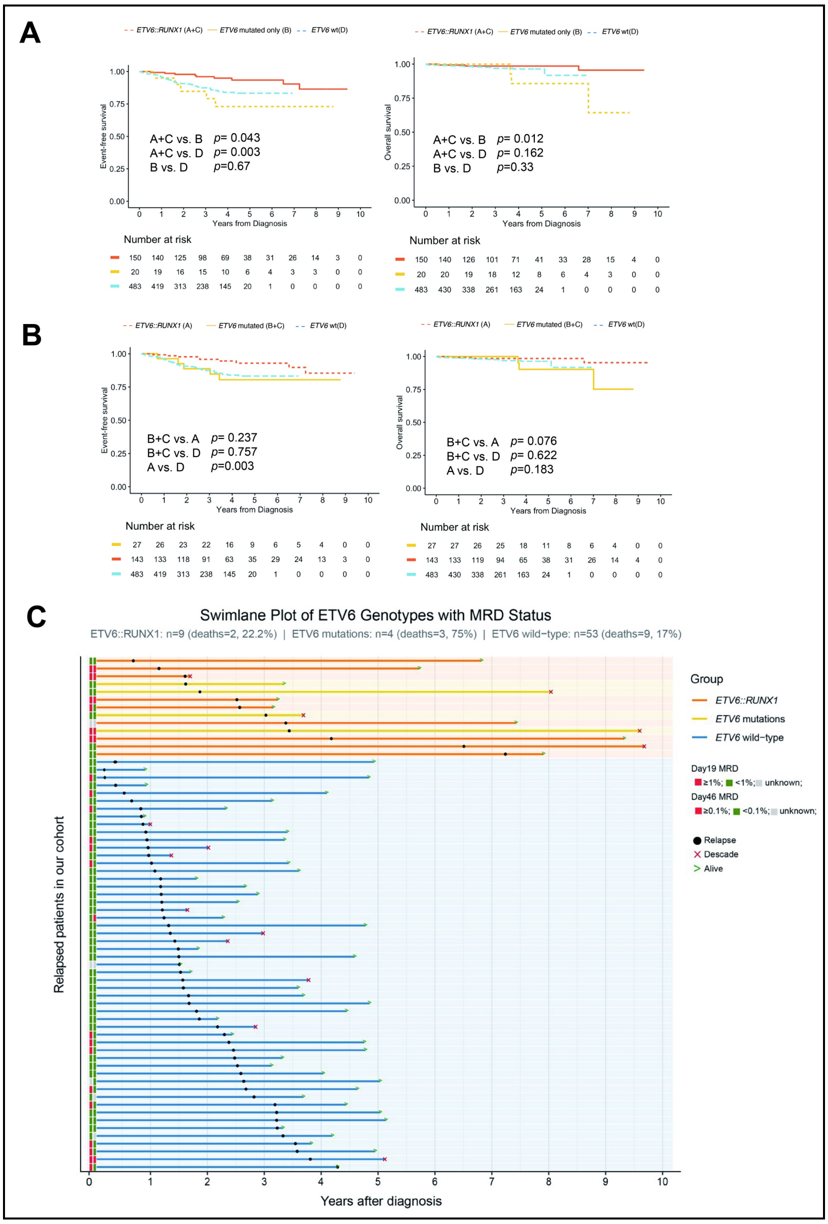

(A) Event-free survival (EFS) and overall survival (OS) among patients with ETV6::RUNX1 fusion with or without concomitant ETV6 mutation (A + C, n = 150), isolated ETV6 mutation without ETV6::RUNX1 fusion (B, n = 20), and ETV6-wild-type (D, n = 483). (B) EFS and OS among patients with ETV6::RUNX1 fusion alone (A, n = 143), ETV6 mutation with or without ETV6::RUNX1 fusion (B + C, n = 27), and ETV6-wild-type (D, n = 483). (C) Swimmer plot of relapsed patients in our study showing MRD status, time to relapse, and post-relapse outcomes. Each horizontal bar represents one patient, with the x-axis indicating follow-up time (years). Patients are color-coded as follows: orange, ETV6::RUNX1 fusion (with or without mutation); gold, isolated ETV6 mutation; blue, ETV6-wild-type. Black dots denote time of relapse. Green arrows at the end of a bar indicate the patient was alive at last follow-up; red crosses indicate death. Minimal residual disease (MRD) status at day 19 and day 46 is also shown for each patient.

**Copyright Information:** (Online) ISSN: 2572-9241
©2026 The Author(s). HemaSphere is published by John Wiley & Sons Ltd on behalf of European Hematology Association. This is an open access Abstract Book distributed under the Attribution-NonCommercial-NoDerivs (CC BY-NC-ND), which allows third parties to download the articles and share them with others as long as they credit the author and the Abstract Book, but they cannot change the content in any way or use them commercially.
**Abstract Book Citations:** Authors, Title, HemaSphere, 2026;10:(S1):pages. The abstract book DOIs can be found on the "Author Information" page.
**Disclaimer:** Articles published in the journal HemaSphere exclusively reflect the opinions of the authors. The authors are responsible for all content in their abstracts including accuracy of the facts, statements, citing resources, etc.

### (PS1478) A PHASE 2 STUDY OF VENETOCLAX PLUS MINI-HYPER-CVD IN OLDER ADULTS WITH DE NOVO PHILADELPHIA NEGATIVE ALL

**Topic:** 02. Acute lymphoblastic leukemia - Clinical

Marlise Luskin*[1], Julia Keating[1], Malgorzata McMasters[2], Julie Asch[3], Hanno Hock[4], Shai Shimony[1], Caner Saygin[5], Evan Chen[1], Yael Flamand[1], Ilene Galinsky[1], Howard Weiner[5], Jeremy Ryan[1], Richard Stone[1], Virginia Volpe[1], Anthony Letai[1], Eric Winer[1], Bradley D. Hunter[3], Marina Konopleva[6], Jacqueline Garcia[1], Nitin Jain[7], Wendy Stock[5], Daniel J. Deangelo[1]

*[1]Dana-Farber Cancer Institute, Boston, United States of America; [2]Beth Israel Deaconess Medical Center, Boston, United States of America; [3]Intermountain Health, Salt Lake City, United States of America; [4]Massachusetts General Hospital, Boston, United States of America; [5]University of Chicago, Chicago, United States of America; [6]Albert Einstein College of Medicine, New York City, United States of America; [7]The University of Texas MD Anderson Cancer Center, Houston, United States of America;*

### Background

Older adults with Philadelphia chromosome-negative (Ph-) acute lymphoblastic leukemia (ALL) have poor outcomes due to treatment resistance and toxicity. Lymphoblasts are BCL-2 dependent and vulnerable to BCL-2 inhibition with venetoclax (VEN). Our phase 1b study of VEN plus mini-hyper-CVD chemotherapy (CTx) demonstrated safety in relapsed/refractory and newly diagnosed (older adult) ALL.

### Aims

Evaluate the efficacy of VEN plus mini-hyper-CVD CTx in older patients (pts) with newly diagnosed Ph- ALL, including rate of complete response (CR) and rate of measurable residual disease (MRD)-negativity by flow cytometry (FC) by cycle 2 (C2) of CTx. Secondary aims evaluate durability of response and overall survival (OS)

### Methods

This multi-center Phase II study (NCT03319901) enrolled pts ≥55 yrs (≥50 if BMI ≥35 kg/m$^2$) with newly diagnosed B- or T-ALL. Informed consent was obtained from all patients. Treatment: 3-day VEN ramp-up (100/200/400 mg/day) followed by up to 8 courses of mini-hyper-CVD CTx (alternating 28-day “A” and “B” cycles, with intra-thecal CTx; rituximab if CD20 ≥1%) with VEN for 21 days/cycle. An amendment introduced 4 blinatumomab (blin) cycles in lieu of C5-8 of CTx for B-ALL, limited CTx for pts ≥70 yrs, and decreased VEN to 14 days beginning C2. CTx (+/- blin) is followed by VEN plus POMP for 2 yrs. Pts proceeded to hematopoietic transplant (HCT) per physician discretion. MRD was evaluated by FC (sensitivity 0.01%). Survival analyses were not censored for transplant.

**Copyright Information:** (Online) ISSN: 2572-9241
©2026 The Author(s). HemaSphere is published by John Wiley & Sons Ltd on behalf of European Hematology Association. This is an open access Abstract Book distributed under the Attribution-NonCommercial-NoDerivs (CC BY-NC-ND), which allows third parties to download the articles and share them with others as long as they credit the author and the Abstract Book, but they cannot change the content in any way or use them commercially.
**Abstract Book Citations:** Authors, Title, HemaSphere, 2026;10:(S1):pages. The abstract book DOIs can be found on the “Author Information” page.
**Disclaimer:** Articles published in the journal HemaSphere exclusively reflect the opinions of the authors. The authors are responsible for all content in their abstracts including accuracy of the facts, statements, citing resources, etc.

### Results

This fully accrued study enrolled 50 pts (09/2021-09/2025), with median age of 66.3 yrs (range, 52.5-81.3, 15 [30%] ≥70 yrs), 28 (56%) female, 6 (12%) Hispanic, and 3 (6%) Black. ALL subtype included 42 (84%) B-ALL (48% CD20+, n=24) and 8 (16%) T-ALL (4 early T-cell precursor [ETP]). In B-ALL, genetic groups included 11 (26%) hypodiploid (all *TP53* mut), 6 (14%) *KMT2A*-r, and 11 (26%) Ph-like. Seven (20%) B-ALL pts had multiple myeloma in CR (5 prior auto HCT, all prior lenalidomide).
Median CTx cycles received was 3 (range 1-8, most [n=41, 82%] 2-4). Median time from start of C1 to start of C2 was 34 days (range 27-47), and from C2 to C3 was 32 days (range 26-55). Blin was received by 26 (61.9%) B-ALL pts. HCT in CR1 was pursued in 15 (30%) pts.
By end of C2, CR/CRp was achieved in 44 (88%, 43 CR, 1 CRp) pts, with 5 (10%) refractory (3 Ph-like, 1*KMT2A*-r), and 1 (2%) early death. Among pts achieving CR/CRp, FC MRD negativity was achieved by C2 in 37/44 (84%) (most, 34/44 [77%] by C1): 11/11 (100%) hypodiploid, 3/6 (50%) *KMT2A*-r, 6/11 (54.5%) Ph-like, 4/4 (100%) ETP, 2/4 (50%) non-ETP T-ALL. Among pts achieving CR/CRp with available NGS-assessed MRD (n= 35), best MRD response by C2 was $<10^{-6}$ (n=18/35, 51%), $<10^{-5}$ (n=25/35, 71%), and $<10^{-4}$ (n=28, 80%). Death in CR occurred in 5 pts, 4 at least possibly related to study treatment (3 sepsis – C2, C4, maintenance; 1 C2 sudden death). Grade 3+ infections (inclusive of febrile neutropenia) occurred in 14/121 (11.6%) of post C1 cycles.
With a median follow-up of 21.8 months (95% CI 13.0, 26.2), the estimated 1-yr and 2-yr OS were 82% (95% CI 71, 94) and 75% (95% CI 61, 89), respectively. The 1-yr and 2-yr DFS were 69% (95% CI 54, 83) and 57% (95% CI 39, 74), respectively. For pts with hypodiploid ALL (n=11), both 1- and 2-year OS were 91% (95% CI 74, 100) and both 1- and 2-year DFS were 67% (95% CI 36, 99).

### Summary/Conclusion

VEN plus mini-hyper-CVD was an effective treatment for older adults with newly diagnosed Ph-neg ALL, including hypodiploid B-ALL.

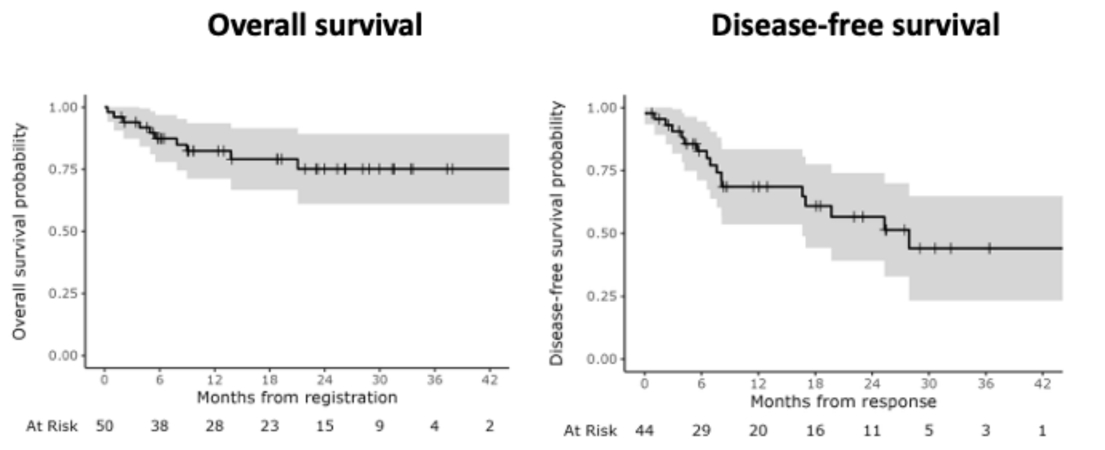

**FIGURE:** Overall and disease-free survival of patients receiving venetoclax plus mini-hyper-CVD

**Copyright Information:** (Online) ISSN: 2572-9241
©2026 The Author(s). HemaSphere is published by John Wiley & Sons Ltd on behalf of European Hematology Association. This is an open access Abstract Book distributed under the Attribution-NonCommercial-NoDerivs (CC BY-NC-ND), which allows third parties to download the articles and share them with others as long as they credit the author and the Abstract Book, but they cannot change the content in any way or use them commercially.
**Abstract Book Citations:** Authors, Title, HemaSphere, 2026;10:(S1):pages. The abstract book DOIs can be found on the "Author Information" page.
**Disclaimer:** Articles published in the journal HemaSphere exclusively reflect the opinions of the authors. The authors are responsible for all content in their abstracts including accuracy of the facts, statements, citing resources, etc.

### (PS1479) UPDATED RESULTS OF POLARIS-1 (PART 1), A GLOBAL REGISTRATIONAL PHASE 3 STUDY: OLVEREMBATINIB COMBINED WITH LOW-INTENSITY CHEMOTHERAPY IN NEWLY DIAGNOSED PH+ ALL

**Topic:** 02. Acute lymphoblastic leukemia - Clinical

Suning Chen*[1], Yu Wu[2], Jianyu Weng[3], Cheng Zhang[4], Hongsheng Zhou[5], Vadim Doronin[6], Mei Hong[7], Ying Wang[8], Xudong Wei[9], Yurii Osipov[10], Julia Alexeeva[10], Bo Huang[11], Zi Chen[11], Wei Chu[11], Lichuang Men[11], Lixin Jiang[12], Dengkun Xiong[11], Weili Zhao[13], Yifan Zhai[11, 12, 14]

*[1]The First Affiliated Hospital of Soochow University, Suzhou, China;[2]West China Hospital Sichuan University, Chengdu, China; [3]Guangdong Provincial People's Hospital, Guangzhou, China;[4]Xinqiao Hospital Army Medical University, Chongqing, China; [5]NanFang Hospital, Guangzhou, China;[6]Moscow SBHI City Clinical Hospital Named After S.P. Botkin of the Moscow Healthcare Department, Moscow, Russia; [7]Union Hospital, Tongji Medical College, Huazhong University of Science and Technology, Department of Hematology, Wuhan, China; [8]Institute of Hematology & Blood Diseases Hospital, Chinese Academy of Medical Sciences, Tianjin, China; [9]Henan Cancer Hospital, Zhengzhou, China; [10]National Medical Research Center named after V.A. Almazov, Saint Petersburg, Russia; [11]Ascentage Pharma (Suzhou) Co., Ltd., Suzhou, China; [12]Ascentage Pharma Group Inc., Rockville, MD, United States of America; [13]Shanghai Institute of Hematology, State Key Laboratory of Medical Genomics, National Research Center for Translational Medicine at Shanghai, Ruijin Hospital Affiliated to Shanghai Jiao Tong University School of Medicine, Shanghai, China; [14]Guangzhou Healthquest Pharma Co., Ltd., Guangzhou, China;*

### Background

Philadelphia chromosome–positive acute lymphoblastic leukemia ($Ph^+$ ALL) is the most common genetic subtype of adult ALL with high recurrence risk and poor outcomes. Olverembatinib is a third-generation tyrosine kinase inhibitor with activity against wild-type and mutant *BCR::ABL1*.

### Aims

POLARIS-1 is a global, multicenter, registrational phase 3 study evaluating olverembatinib in combination with reduced-intensity chemotherapy in newly diagnosed (ND) $Ph^+$ ALL.

### Methods

Part 1 of the POLARIS-1 trial (ClinicalTrials.gov ID: NCT06051409) enrolled patients with ND $Ph^+$ ALL, *BCR::ABL1* variants, ECOG PS ≤ 2, and adequate organ function. Olverembatinib 30 or 40mg was administered orally QOD in repeated 28-day cycles. Induction comprised 3 cycles of vincristine (1.4 mg/m$^2$ on days 1, 8, 15, and 22), dexamethasone (10 mg on days 1-14, 5 mg days 15-21, 2.5 mg days 22-28), ± daunorubicin (25 mg/m$^2$ days 1-3). Consolidation alternated 3 cycles of HD-MTX (1.0 g/m$^2$) with 3 cycles of cytarabine (1,000 mg/m$^2$ every 12 hours, 3 days). Maintenance continued for 12 cycles. Primary endpoint was MRD negativity (*BCR::ABL/ABL1* ≤ 0.01% by qPCR) by end of induction. Correlative analyses used baseline genetic profiles.

**Copyright Information:** (Online) ISSN: 2572-9241
©2026 The Author(s). HemaSphere is published by John Wiley & Sons Ltd on behalf of European Hematology Association. This is an open access Abstract Book distributed under the Attribution-NonCommercial-NoDerivs (CC BY-NC-ND), which allows third parties to download the articles and share them with others as long as they credit the author and the Abstract Book, but they cannot change the content in any way or use them commercially.
**Abstract Book Citations:** Authors, Title, HemaSphere, 2026;10:(S1):pages. The abstract book DOIs can be found on the "Author Information" page.
**Disclaimer:** Articles published in the journal HemaSphere exclusively reflect the opinions of the authors. The authors are responsible for all content in their abstracts including accuracy of the facts, statements, citing resources, etc.

### Results

A total of 55 patients were enrolled as of January 10, 2026 (median age 43 years, 63.6% female). *BCR::ABL1* subtypes were p190 (65.5%), p210 (29.1%), p230 (1.8%), and p190+p210 (3.6%); median treatment duration was 11.6 months. Among 54 efficacy-evaluable patients, 94.4% achieved CR/CRi, 64.8% MRD negativity, and 63.0% MRD-negative CR by the end of induction (EOI). MRD-negativity rates at the end of cycles 3, 6, and 9 were 65.9%, 90.6%, and 93.1%. All patients experienced ≥1 TEAE. Common TEAEs (≥20%) included neutropenia (76.4%), anemia (74.5%), hypokalemia (72.4%), leukopenia (70.9%), and thrombocytopenia (67.3%). Grade ≥ 3 TEAEs (≥20%) included neutropenia (69.1%), thrombocytopenia (61.8%), leukopenia (60.0%), anemia (60.0%), pneumonia (34.5%), lymphocyte count decreased (23.6%), and hypokalemia (21.8%). Two deaths from TEAEs (pneumonia; pulmonary edema with septic shock) were unrelated/unlikely related to olverembatinib. One patient had grade 2 coronary artery disease possibly related to olverembatinib. Treatment discontinuation was due to HSCT (9.1%), progression (7.3%), and/or toxicity (10.9%). No significant differences in efficacy or safety were observed between olverembatinib 30 and 40 mg. The PK profile of olverembatinib plus chemotherapy was similar to that of monotherapy. Among 50 patients with baseline genetic testing, 10 had *IKZF1*$^{plus}$ genotype; 9 achieved MRD negativity by EOI. All 7 patients with *IKZF1* and *BTG1* deletions achieved MRD negativity during induction.

### Summary/Conclusion

Olverembatinib in combination with low-intensity chemotherapy achieved 63.0% MRD-negative CR in ND Ph$^{+}$ ALL by EOI, including patients with inferior prognostic genotypes, with a favorable safety profile.

**Copyright Information:** (Online) ISSN: 2572-9241
©2026 The Author(s). HemaSphere is published by John Wiley & Sons Ltd on behalf of European Hematology Association. This is an open access Abstract Book distributed under the Attribution-NonCommercial-NoDerivs (CC BY-NC-ND), which allows third parties to download the articles and share them with others as long as they credit the author and the Abstract Book, but they cannot change the content in any way or use them commercially.
**Abstract Book Citations:** Authors, Title, HemaSphere, 2026;10:(S1):pages. The abstract book DOIs can be found on the "Author Information" page.
**Disclaimer:** Articles published in the journal HemaSphere exclusively reflect the opinions of the authors. The authors are responsible for all content in their abstracts including accuracy of the facts, statements, citing resources, etc.

HemaSphere eha

### (PS1480) EARLY B-CELL RECOVERY AFTER BREXUCABTAGENE AUTOLEUCEL DOES NOT PREDICT INFERIOR EVENT-FREE SURVIVAL IN ADULT B-ALL

**Topic:** 02. Acute lymphoblastic leukemia - Clinical

Kriti Gera*[1], Jasmine Nadayil[1], Ayda Soltanian[1], Rawan Faramand[2], Bijal D. Shah[2]

*[1]Moffitt Cancer Center/University of South Florida, Tampa, United States of America;[2]H. Lee Moffitt Cancer Center and Research Institute, Tampa, United States of America;*

### Background

B-cell aplasia (BCA) is commonly used as a surrogate marker of CAR T-cell persistence in CD19-directed therapy, and early B-cell recovery has historically been associated with increased relapse risk. However, the prognostic impact of early B-cell recovery following brexucabtagene autoleucel (brexu-cel) in adult B-cell acute lymphoblastic leukemia (B-ALL) remains unclear.

### Methods

We performed a retrospective single-center analysis of adults with B-ALL who received brexu-cel (January 201-April 2025). Event-free survival (EFS) was defined as the time from infusion to relapse or death, censored at last follow-up. MRD was assessed by clonoSEQ at D30 and D90 bone marrow assessment. Loss of BCA was defined as first detection of any CD19+ event by flow cytometry (limit of detection 0.025 k/µL). Duration of BCA was the time from infusion to loss of BCA and estimated by Kaplan–Meier (KM) methods. Early B-cell recovery was defined as recovery by D90.

**Copyright Information:** (Online) ISSN: 2572-9241
©2026 The Author(s). HemaSphere is published by John Wiley & Sons Ltd on behalf of European Hematology Association. This is an open access Abstract Book distributed under the Attribution-NonCommercial-NoDerivs (CC BY-NC-ND), which allows third parties to download the articles and share them with others as long as they credit the author and the Abstract Book, but they cannot change the content in any way or use them commercially.
**Abstract Book Citations:** Authors, Title, HemaSphere, 2026;10:(S1):pages. The abstract book DOIs can be found on the "Author Information" page.
**Disclaimer:** Articles published in the journal HemaSphere exclusively reflect the opinions of the authors. The authors are responsible for all content in their abstracts including accuracy of the facts, statements, citing resources, etc.

### Results

Forty-nine patients were included (Table 1). Among 48 evaluable for pre-infusion BCA, 17 (35%) had no BCA. At a median follow-up of 468 days (range, 7–1321), 32 of 49 patients (65%) experienced B-cell recovery. Median time to recovery was 183 days, with no difference by prior BCA status. Only one patient without prior BCA recovered by Day 30 and remained relapse-free, undergoing alloHSCT 6.6 months post-infusion. Seven additional patients recovered by Day 90 (4 with prior BCA; 3 without). Two underwent consolidative alloHSCT approximately 4 months post-CAR T. Only one patient with early recovery relapsed (245 days post-infusion). All (n=8) had low burden disease (<5% marrow blasts) and were MRD negative at D90.

Median EFS for the overall cohort was 442 days, with 6- and 12-month EFS of 70.9% and 55.0%, respectively. Early B-cell recovery was associated with superior EFS compared with no early recovery (median not reached vs 354 days;12-month 83.3% vs 49.5%; log-rank p=0.039). When stratified by prior BCA, early recovery was not associated with significantly improved EFS among patients with prior BCA (N=31;12-month 50% vs 43%; p=0.217), whereas among patients without prior BCA early recovery was associated with significantly superior EFS (N=17;12-month EFS 100% vs 35%, p=0.048).

At the D90 landmark (n=39), early B-cell recovery demonstrated a borderline association with improved EFS (log-rank p=0.052). However, among MRD-negative patients at D90 (n=27), early recovery was not associated with improved EFS (p=0.132). No significant differences were observed at D180 or D360 landmarks (p=0.182 and p=0.229).

| Characteristic | N (%) |
|---|---|
| Median Age at apheresis (range) | 45.0 (7, 77) |
| Male | 29 (59.2%) |
| White | 41 (83.7%) |
| KPS ≥90 | 30 (61.2%) |
| Marrow blasts at apheresis | |
| · <5% | 33 (67.3%) |
| · 6–50% | 7 (14.3%) |
| · >50% | 6 (12.2%) |
| Prior Blinatumomab | 27 (55.1%) |
| Prior Inotuzumab | 18 (36.7%) |
| Prior AlloHSCT | 11 (22.4%) |
| Bridging therapy | 41 (83.7%) |

Table 1: Baseline characteristics

### Summary/Conclusion

In this single-center brexu-cel cohort, early B-cell recovery was not associated with inferior EFS. After adjustment by landmark and MRD status, early recovery did not independently predict relapse risk, suggesting that BCA kinetics alone may be insufficient as a biomarker of CAR T persistence or disease control in adult B-ALL treated with brexu-cel. Larger multicenter studies are warranted to validate these observations.

**Copyright Information:** (Online) ISSN: 2572-9241
©2026 The Author(s). HemaSphere is published by John Wiley & Sons Ltd on behalf of European Hematology Association. This is an open access Abstract Book distributed under the Attribution-NonCommercial-NoDerivs (CC BY-NC-ND), which allows third parties to download the articles and share them with others as long as they credit the author and the Abstract Book, but they cannot change the content in any way or use them commercially.
**Abstract Book Citations:** Authors, Title, HemaSphere, 2026;10:(S1):pages. The abstract book DOIs can be found on the "Author Information" page.
**Disclaimer:** Articles published in the journal HemaSphere exclusively reflect the opinions of the authors. The authors are responsible for all content in their abstracts including accuracy of the facts, statements, citing resources, etc.

### (PS1481) SUBCUTANEOUS BLINATUMOMAB FOR PATIENTS WITH RELAPSED/REFRACTORY PHILADELPHIA CHROMOSOME–POSITIVE B-CELL ACUTE LYMPHOBLASTIC LEUKEMIA: RESULTS FROM A PHASE 1/2 STUDY

**Topic:** 02. Acute lymphoblastic leukemia - Clinical

Elias Jabbour*[1], Federico Lussana[2], Alessandro Rambaldi[2, 3], Cristina Papayannidis[4], Maria Pilar Martinez Sanchez[5], Jose Juan Rifon Roca[6], Mar Tomo[7], Stefan Schwartz[8], Vladan Vucinic[9], Boris Böll[10], Anita Rijneveld[11], Yuliya Katlinskaya[12], Jessica Choudhry[12], Erik Rasmussen[12], Erin Guest[12], Hagop Kantarjian[1]

*[1]The University of Texas MD Anderson Cancer Center, Houston, United States of America;[2]Department of Oncology-Hematology, University of Milan and Azienda Socio Sanitaria Territoriale Papa Giovanni XXIII, Milan and Bergamo, Italy; [3]Foundation of Clinical Research-FROM Ospedale Papa Giovanni XXIII, Bergamo, Italy;[4]IRCCS, Azienda Ospedaliero-Universitaria di Bologna, Istituto di Ematologia "Seràgnoli", Bologna, Italy; [5]Hospital Universitario 12 de Octubre, Hematology Department, Madrid, Spain; [6]Clínica Universidad de Navarra, Hematology and Hemotherapy Department, Pamplona, Spain; [7]Hospital Clínico Universitario de Valencia, Instituto de Investigación Sanitaria INCLIVA, Hematology Department, Valencia, Spain; [8]Department of Hematology, Oncology and Tumor Immunology, Campus Benjamin Franklin, Charité–Universitätsmedizin Berlin, corporate member of Freie Universität and Humboldt-Universität zu Berlin, Berlin, Germany; [9]Universitätsklinikum Leipzig, Department of Hematology, Cellular Therapy, Hemostaseology and Infectious Diseases, Leipzig, Germany; [10]University Hospital of Cologne, First Department of Internal Medicine I, Cologne, Germany; [11]Erasmus University Medical Center Cancer Institute, Department of Hematology, Rotterdam, The Netherlands; [12]Amgen Inc., Thousand Oaks, United States of America;*

### Background

Blinatumomab, an anti-CD19 bispecific T-cell engager (BiTE®), is approved for the treatment of patients with relapsed or refractory (R/R) B-cell acute lymphoblastic leukemia (B-ALL) and is administered as a 28-day continuous intravenous (cIV) infusion. In patients with R/R Philadelphia chromosome–positive (Ph+) B-ALL, blinatumomab is often combined with a tyrosine kinase inhibitor (TKI) to target the chromosomal rearrangement. Considering the high efficacy and acceptable safety profile of subcutaneous (SC) blinatumomab in patients with R/R B-ALL (Jabbour et al. 2025), we report outcomes of an ad hoc analysis, specifically from the Ph+ patient subset in the dose escalation, dose expansion, and pharmacokinetic evaluation cohorts of this phase 1/2 study (NCT04521231) evaluating two select dosing regimens.

### Aims

To evaluate the efficacy of SC blinatumomab in adults with R/R Ph+ B-ALL.

### Methods

In this multicenter phase 1/2 study, adults with R/R Ph+ B-ALL received SC blinatumomab injection (0.3–1.2 mL) for 2–5 cycles; each treatment cycle consisted of 4 weeks of treatment followed by 1 week treatment-free interval. In each cycle, patients received either 250 µg once-daily (QD) for week 1 followed by 500 µg thrice-weekly (TIW) (250/500), or 500 µg QD for week 1 followed by 1000 µg TIW (500/1000). For dose expansion, the primary endpoint was complete remission (CR) or CR with partial hematologic recovery (CRh). Secondary endpoints were CR/CRh/CR with incomplete hematologic recovery (CRi) and measurable residual disease (MRD) response rate. Data on subsequent hematopoietic stem cell transplant (HSCT) following SC blinatumomab were also collected.

**Copyright Information:** (Online) ISSN: 2572-9241
©2026 The Author(s). HemaSphere is published by John Wiley & Sons Ltd on behalf of European Hematology Association. This is an open access Abstract Book distributed under the Attribution-NonCommercial-NoDerivs (CC BY-NC-ND), which allows third parties to download the articles and share them with others as long as they credit the author and the Abstract Book, but they cannot change the content in any way or use them commercially.
**Abstract Book Citations:** Authors, Title, HemaSphere, 2026;10:(S1):pages. The abstract book DOIs can be found on the "Author Information" page.
**Disclaimer:** Articles published in the journal HemaSphere exclusively reflect the opinions of the authors. The authors are responsible for all content in their abstracts including accuracy of the facts, statements, citing resources, etc.

### Results

At data cut-off on November 13, 2025, 16 adults with R/R Ph+ B-ALL were included (250/500: n=8; 500/1000: n=8). Median age was 49 years (range, 19–72), 11 patients (69%) were male, 15 (94%) non-Hispanic, and 13 (81%) White. Median bone marrow blasts were 60% (range, 5–95). Median prior lines of therapy were 3.5 (range, 1–7); 8 (50%) had prior HSCT, 6 (38%) had prior chimeric antigen receptor T-cell (CAR-T) therapy, and 6 (38%) received prior cIV blinatumomab. All patients received prior TKI therapy (median 3; range, 1–5). Eight patients (50%) received a TKI concurrently with SC blinatumomab (250/500: ponatinib, n=4; dasatinib, n=1; imatinib, n=1; 500/1000: ponatinib, n=2) with no new safety signals observed. Nine patients (7 concurrent and 2 post-treatment initiations) continued TKI after SC blinatumomab for a median of 9.5 months at data cutoff.
Patients completed a median of 2 cycles (range, 0–5) of SC blinatumomab. Overall, CR/CRh was achieved within 2 cycles in 13/16 patients (81%; 80% confidence interval [CI], 63%–93%) and CR/CRh/CRi in 15/16 patients (94%; 80% CI, 78%–99%). MRD negativity ($<10^{-4}$; polymerase chain reaction or flow cytometry) was achieved in 10/13 responders. A total of five patients (31%) underwent subsequent HSCT (250/500: 2/8 [29%]; 500/1000: 3/8 [38%]), of whom one in 500/1000 also received CAR-T therapy. Two patients in 250/500 received CAR-T therapy without HSCT following SC blinatumomab. Nine patients were alive at last contact; deaths were due to disease progression (n=4), sepsis, multi-organ failure, and embolic infarcts (n=1, each).

### Summary/Conclusion

SC blinatumomab resulted in high remission and MRD-negative response rates in heavily pre-treated patients with R/R Ph+ B-ALL receiving concomitant TKIs. These data support further evaluation of SC blinatumomab across Ph+ B-ALL settings.

**Figure: Treatment duration and clinical outcomes following SC blinatumomab in patients with R/R Ph+ B-ALL.**

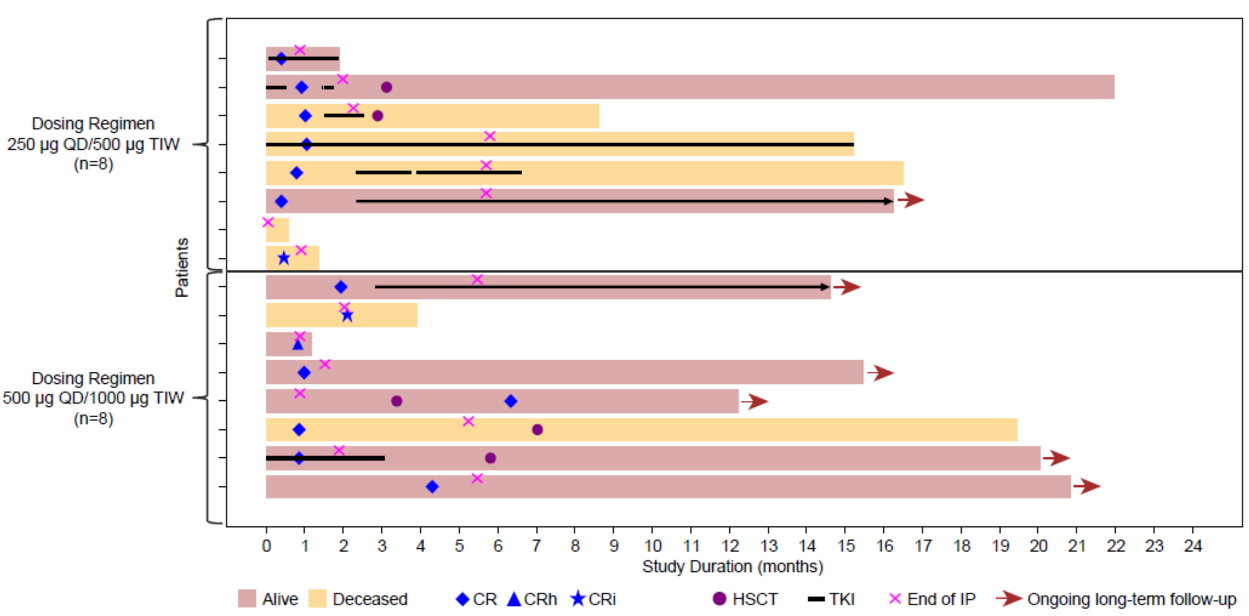

B-ALL, B-cell acute lymphoblastic leukemia; CR, complete remission; CRh, complete remission with partial hematologic recovery; CRi, complete remission with incomplete hematologic recovery; HSCT, hematopoietic stem cell transplantation; IP, investigational product; Ph+, Philadelphia chromosome–positive; R/R, relapsed or refractory; SC, subcutaneous; TIW, thrice weekly; TKI, tyrosine kinase inhibitor; QD, once daily

**Copyright Information:** (Online) ISSN: 2572-9241
©2026 The Author(s). HemaSphere is published by John Wiley & Sons Ltd on behalf of European Hematology Association. This is an open access Abstract Book distributed under the Attribution-NonCommercial-NoDerivs (CC BY-NC-ND), which allows third parties to download the articles and share them with others as long as they credit the author and the Abstract Book, but they cannot change the content in any way or use them commercially.
**Abstract Book Citations:** Authors, Title, HemaSphere, 2026;10:(S1):pages. The abstract book DOIs can be found on the "Author Information" page.
**Disclaimer:** Articles published in the journal HemaSphere exclusively reflect the opinions of the authors. The authors are responsible for all content in their abstracts including accuracy of the facts, statements, citing resources, etc.

### (PS1482) REAL-WORLD EFFICACY AND SAFETY OF INOTUZUMAB OZOGAMICIN IN B-CELL ACUTE LYMPHOBLASTIC LEUKEMIA: A MULTICENTER OK-1 COHORT STUDY

**Topic:** 02. Acute lymphoblastic leukemia - Clinical

Jiaxin Zheng*[1], Fen Zhou[2], Feifei Tang[3], Wenhao Zhong[1], Bailin He[1], Zhixiang Wang[1], Jieyu Ye[1], Heng Mei[4], Hongsheng Zhou[1]

*[1]Department of Hematology, Nanfang Hospital, Southern Medical University, Guangzhou, China;[2]Department of Pediatrics, Wuhan Union Hospital, Tongji Medical College, Huazhong University of Science and Technology, Wuhan, China; [3]Peking University People's Hospital, Peking University Institute of Hematology, National Clinical Research Center for Hematologic Disease, Beijing Key Laboratory of Hematopoietic Stem Cell Transplantation, Peking University, Beijing, China; [4]Union Hospital, Tongji Medical College, Huazhong University of Science and Technology, Wuhan, China;*

### Background

Relapsed/refractory B-cell acute lymphoblastic leukemia (R/R B-ALL) is associated with poor prognosis. While inotuzumab ozogamicin (InO) has demonstrated efficacy in clinical trials, real-world data across distinct disease states remain limited.

### Aims

This study aimed to evaluate the real-world efficacy and safety of InO in patients with B-ALL and to compare outcomes across different clinical subgroups based on disease status at treatment initiation.

### Methods

This multicenter retrospective cohort study included 69 patients with B-ALL who received at least one dose of InO between June 2022 and September 2025. Patients received either fixed-dose InO (1 mg on days 1, 8, and 15; n=64) or weight-based dosing (1.8 mg/m²/cycle; n=5). Based on baseline disease status, patients were categorized into four subgroups (categories were not mutually exclusive): isolated bone marrow relapse without extramedullary disease (EMD,Group A, n=27), isolated MRD positivity without morphologic relapse or EMD (Group B, n=18), presence of EMD with or without marrow involvement (Group C, n=24), and InO administered as bridging therapy to chimeric antigen receptor T-cell therapy (CAR-T) or hematopoietic stem cell transplantation (HSCT) (Group D, n=24). Complete remission (CR) was defined as bone marrow blasts <5% without EMD, and MRD negativity was defined as <0.01% by multiparameter flow cytometry, with the additional requirement of BCR::ABL1 transcript level <0.01% for patients with Ph-positive (Ph+) disease. Assessed outcomes included CR rate, MRD negativity rate, EMD response rate, progression-free survival (PFS), overall survival (OS), and treatment-related toxicities; survival was analyzed by Kaplan-Meier method with log-rank tests.

**Copyright Information:** (Online) ISSN: 2572-9241
©2026 The Author(s). HemaSphere is published by John Wiley & Sons Ltd on behalf of European Hematology Association. This is an open access Abstract Book distributed under the Attribution-NonCommercial-NoDerivs (CC BY-NC-ND), which allows third parties to download the articles and share them with others as long as they credit the author and the Abstract Book, but they cannot change the content in any way or use them commercially.
**Abstract Book Citations:** Authors, Title, HemaSphere, 2026;10:(S1):pages. The abstract book DOIs can be found on the "Author Information" page.
**Disclaimer:** Articles published in the journal HemaSphere exclusively reflect the opinions of the authors. The authors are responsible for all content in their abstracts including accuracy of the facts, statements, citing resources, etc.

**Results**

Median age was 31 years (range, 13-75), 52% were male, R/R disease accounted for 88% of patients (n=61) (Fig.A).Among 67 evaluable patients, the overall CR rate was 80.6% (54/67). Patients with isolated MRD positivity (Group B) achieved an MRD negativity rate of 66.7% (12/18). Those with isolated bone marrow relapse (Group A) had a CR rate of 69.2% (18/26). Among patients with EMD (Group C), the marrow CR rate was 78.3% (18/23) and the EMD response rate was 42.1% (8/19)(Fig.B). All 24 patients receiving InO as bridging therapy (Group D) successfully proceeded to planned HSCT (n=15) or CAR-T (n=9). After a median follow-up of 15 months, median OS was not reached; 1-year and 2-year OS rates were 80.8% and 77.4%, respectively (Fig.C).Median progression-free survival (PFS) was 7 months for Group A, 4 months for Group C, and not reached for Groups B and D. (P<0.001) (Fig.D).Patients receiving bridging therapy (Group D) demonstrated significantly superior 2-year OS compared to those who did not undergo bridging (95.5% vs. 63.9%; HR 0.18; P=0.008). R/R versus non-R/R 2-year OS was 73.9% vs. 100% (P=0.139). Sinusoidal obstruction syndrome (SOS) occurred in 2 patients (2.9%); no treatment-related deaths occurred.

**Summary/Conclusion**

In this real-world cohort, InO demonstrated high overall remission rates with a favorable safety profile. Patients with isolated MRD positivity derived the greatest benefit. While EMD response was modest, survival outcomes were comparable to non-EMD patients. InO effectively served as bridging therapy, enabling successful transition to HSCT or CAR-T in all intended patients with significantly improved long-term survival.

**Copyright Information:** (Online) ISSN: 2572-9241
©2026 The Author(s). HemaSphere is published by John Wiley & Sons Ltd on behalf of European Hematology Association. This is an open access Abstract Book distributed under the Attribution-NonCommercial-NoDerivs (CC BY-NC-ND), which allows third parties to download the articles and share them with others as long as they credit the author and the Abstract Book, but they cannot change the content in any way or use them commercially.
**Abstract Book Citations:** Authors, Title, HemaSphere, 2026;10:(S1):pages. The abstract book DOIs can be found on the “Author Information” page.
**Disclaimer:** Articles published in the journal HemaSphere exclusively reflect the opinions of the authors. The authors are responsible for all content in their abstracts including accuracy of the facts, statements, citing resources, etc.

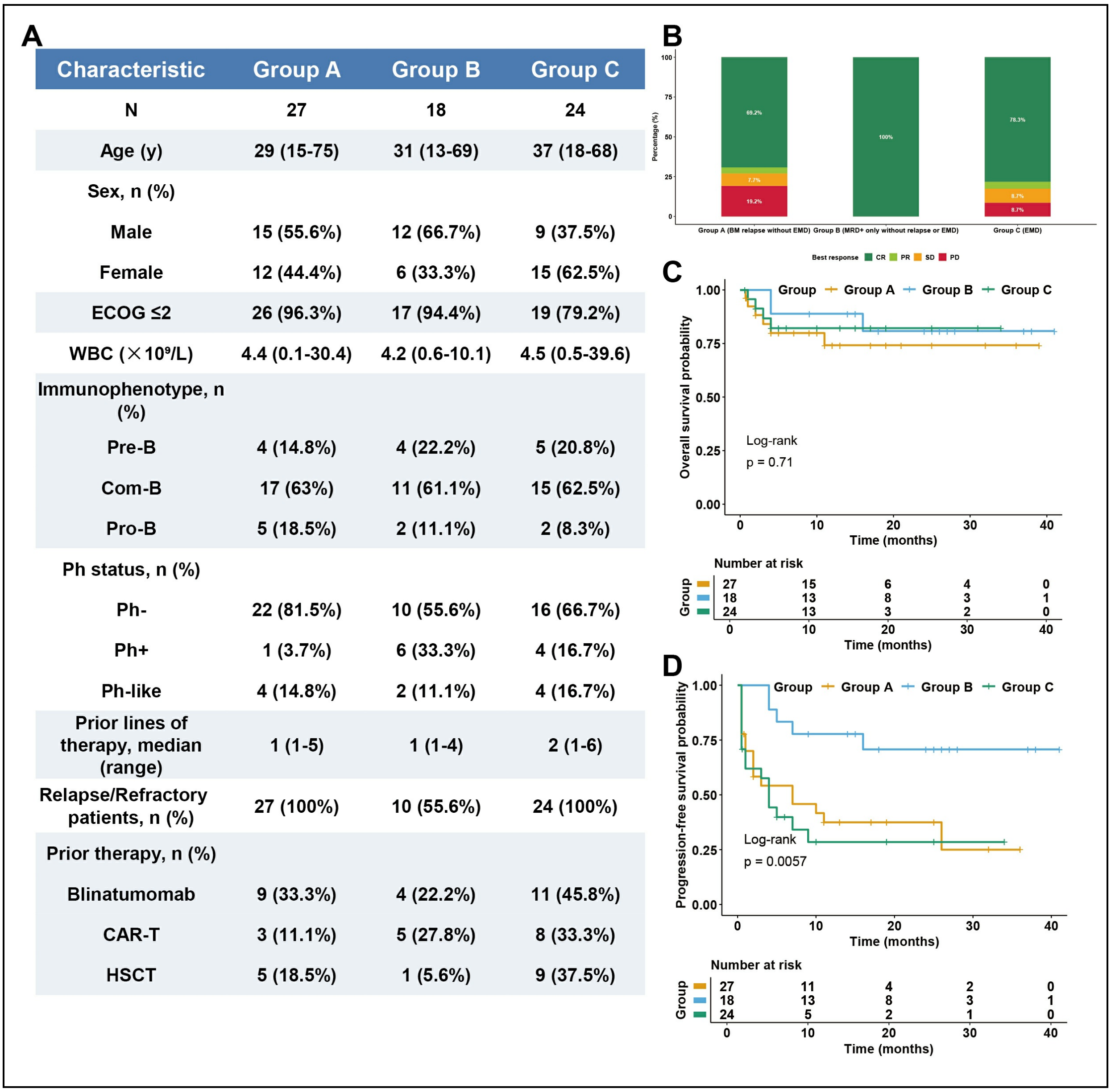

A

| Characteristic | Group A | Group B | Group C |
|---|---|---|---|
| N | 27 | 18 | 24 |
| Age (y) | 29 (15-75) | 31 (13-69) | 37 (18-68) |
| Sex, n (%) | | | |
| Male | 15 (55.6%) | 12 (66.7%) | 9 (37.5%) |
| Female | 12 (44.4%) | 6 (33.3%) | 15 (62.5%) |
| ECOG ≤2 | 26 (96.3%) | 17 (94.4%) | 19 (79.2%) |
| WBC (×10⁹/L) | 4.4 (0.1-30.4) | 4.2 (0.6-10.1) | 4.5 (0.5-39.6) |
| Immunophenotype, n (%) | | | |
| Pre-B | 4 (14.8%) | 4 (22.2%) | 5 (20.8%) |
| Com-B | 17 (63%) | 11 (61.1%) | 15 (62.5%) |
| Pro-B | 5 (18.5%) | 2 (11.1%) | 2 (8.3%) |
| Ph status, n (%) | | | |
| Ph- | 22 (81.5%) | 10 (55.6%) | 16 (66.7%) |
| Ph+ | 1 (3.7%) | 6 (33.3%) | 4 (16.7%) |
| Ph-like | 4 (14.8%) | 2 (11.1%) | 4 (16.7%) |
| Prior lines of therapy, median (range) | 1 (1-5) | 1 (1-4) | 2 (1-6) |
| Relapse/Refractory patients, n (%) | 27 (100%) | 10 (55.6%) | 24 (100%) |
| Prior therapy, n (%) | | | |
| Blinatumomab | 9 (33.3%) | 4 (22.2%) | 11 (45.8%) |
| CAR-T | 3 (11.1%) | 5 (27.8%) | 8 (33.3%) |
| HSCT | 5 (18.5%) | 1 (5.6%) | 9 (37.5%) |

Patient characteristics and efficacy of Inotuzumab Ozogamicin in different clinical subgroups.Patient characteristics.(A) Best response to Inotuzumab Ozogamicin by subgroup.(B) Overall Survival (C) and Progression-Free Survival (D) by subgroup after Inotuzumab Ozogamicin treatment.

Copyright Information: (Online) ISSN: 2572-9241
©2026 The Author(s). HemaSphere is published by John Wiley & Sons Ltd on behalf of European Hematology Association. This is an open access Abstract Book distributed under the Attribution-NonCommercial-NoDerivs (CC BY-NC-ND), which allows third parties to download the articles and share them with others as long as they credit the author and the Abstract Book, but they cannot change the content in any way or use them commercially.
Abstract Book Citations: Authors, Title, HemaSphere, 2026;10:(S1):pages. The abstract book DOIs can be found on the "Author Information" page.
Disclaimer: Articles published in the journal HemaSphere exclusively reflect the opinions of the authors. The authors are responsible for all content in their abstracts including accuracy of the facts, statements, citing resources, etc.

### (PS1483) PHASE I CLINICAL TRIAL OF CD19 CAR -T CELLS SECRETING PD-1-TARGETING IL-21 IN RELAPSED/REFRACTORY B-CELL ACUTE LYMPHOBLASTIC LEUKEMIA

**Topic:** 02. Acute lymphoblastic leukemia - Clinical

Lei Lv[1], Meng Li[1], Jingjing Yang[1], Qingyang Liu*[1], Yanan Wen[1], Yu Jing[1], Liping Dou[1]

[1] *The Fifth Medical Center of Chinese PLA General Hospital, Beijing, China;*

### Background

Patients with relapsed or refractory B-cell acute lymphoblastic leukemia (R/R B-ALL) have an extremely poor prognosis. Although CD19-targeted CAR-T cell therapy can induce high initial response rates, T cell exhaustion often leads to relapse, with the PD-1/PD-L1 axis being a key mechanism.

### Aims

This study aimed to evaluate the safety and efficacy of a novel bispecific armored CAR-T cell therapy (PZ04) for R/R B-ALL. These engineered cells constitutively secrete a PD-1Ab21 fusion entity, designed to locally block the immune checkpoint and enhance T-cell function.

### Methods

This single-center, Phase I study (ChiCTR2300072572) enrolled 17 patients with R/R B-ALL. Patients received PZ04 cell infusion following lymphodepleting chemotherapy with an FC regimen (fludarabine/cytarabine). Primary endpoint was safety and the second endpoint was complete remission (CR) rate.

### Results

The results demonstrated a CR rate of 81.3% (13/16), with all responders achieving minimal residual disease (MRD)-negative status. The 1-year overall survival (OS) rate was 53.0%. Among six patients with baseline central nervous system involvement, four (83.3%) achieved complete remission. Regarding safety, the incidence of grade ≥3 cytokine release syndrome (CRS) and immune effector cell-associated neurotoxicity syndrome (ICANS) was 18.8% each (3/16 for each). No severe adverse events related to systemic immune checkpoint inhibitors were observed. Further analysis indicated a trend toward superior long-term survival in patients (n=6) who underwent consolidative allogeneic hematopoietic stem cell transplantation (allo-HSCT) following CAR-T therapy (1-year event-free survival 53.3% vs. 21.8%, P=0.075).

### Summary/Conclusion

Conclusion: The bispecific CD19-targeted CAR-T cells secreting the PD-1Ab21 fusion entity (PZ04) demonstrate a high response rate and a manageable safety profile in patients with R/R B-ALL. Its innovative design provides a novel strategy to counteract T-cell exhaustion and shows potential as an effective bridge to transplantation. Larger studies are warranted to validate its long-term efficacy.

**Copyright Information:** (Online) ISSN: 2572-9241
©2026 The Author(s). HemaSphere is published by John Wiley & Sons Ltd on behalf of European Hematology Association. This is an open access Abstract Book distributed under the Attribution-NonCommercial-NoDerivs (CC BY-NC-ND), which allows third parties to download the articles and share them with others as long as they credit the author and the Abstract Book, but they cannot change the content in any way or use them commercially.
**Abstract Book Citations:** Authors, Title, HemaSphere, 2026;10:(S1):pages. The abstract book DOIs can be found on the “Author Information” page.
**Disclaimer:** Articles published in the journal HemaSphere exclusively reflect the opinions of the authors. The authors are responsible for all content in their abstracts including accuracy of the facts, statements, citing resources, etc.

### (PS1484) THE EFFECT OF OBECABTAGENE AUTOLEUCEL ON ADULT PATIENTS WITH RELAPSED/REFRACTORY B-CELL ACUTE LYMPHOBLASTIC LEUKEMIA AND EXTRAMEDULLARY DISEASE

**Topic:** 02. Acute lymphoblastic leukemia - Clinical

Jae H. Park*[1], Karamjeet S. Sandhu[2], Claire Roddie[3], Bijal D. Shah[4], Eleni Tholouli[5], Paul Shaughnessy[6], Pere Barba[7], Manuel Guerreiro[8], Deborah Yallop[9], Aaron Logan[10], Mehrdad Abedi[11], Michael Bishop[12], Daniel J. Deangelo[13], Pierre Lao-Sirieix[14], Ping Wang[15], Wolfram Brugger[16], Elias Jabbour[17]

*[1]Memorial Sloan Kettering Cancer Center, New York, United States of America;[2]City of Hope National Medical Center, Duarte, United States of America; [3]University College London Cancer Institute, London, United Kingdom; [4]Moffitt Cancer Center, Tampa, United States of America; [5]Manchester Royal Infirmary, Manchester, United Kingdom;[6]Sarah Cannon Transplant and Cellular Therapy Program, Methodist Hospital, San Antonio, United States of America; [7]Hospital Universitari Vall d'Hebron-Universitat Autónoma de Barcelona, Barcelona, Spain; [8]Hospital Universitari i Politècnic La Fe, Valencia, Spain;[9]King's College Hospital NHS Foundation Trust, London, United Kingdom; [10]Hematology, Blood and Marrow Transplantation, and Cellular Therapy Program, University of California San Francisco, San Francisco, United States of America; [11]University of California Davis, Davis, United States of America; [12]The David and Etta Jonas Center for Cellular Therapy, University of Chicago, Chicago, United States of America; [13]Department of Medical Oncology, Dana-Farber Cancer Institute, Boston, United States of America;[14]Autolus Therapeutics, London, United Kingdom; [15]Autolus Therapeutics, Rockville, United States of America; [16]Autolus Therapeutics, Weil am Rhein, Germany; [17]University of Texas MD Anderson Cancer Center, Houston, United States of America;*

### Background

Patients with relapsed/refractory B-cell acute lymphoblastic leukemia (R/R B-ALL) and extramedullary disease (EMD) have limited treatment options and are a population with an unmet need. Obecabtagene autoleucel (obe-cel) is an autologous chimeric antigen receptor (CAR) T-cell therapy with a fast off-rate CAT19 binding domain and a 4-1BB-ζ co-stimulatory domain designed to improve persistence and reduce severe immunotoxicity.

### Aims

To report a post-hoc analysis of the Phase Ib/II FELIX study (NCT04404660), evaluating efficacy and safety of obe-cel in patients with R/R B-ALL, by EMD status at lymphodepletion.

### Methods

Enrolled adult patients with R/R B-ALL provided written informed consent and, following lymphodepletion, received obe-cel using a tumor burden–guided dosing strategy to minimize toxicity. Overall remission rate (ORR; complete remission [CR]/CR with incomplete hematologic recovery [CRi]), event-free survival (EFS), overall survival (OS), and safety are reported for patients with or without EMD.

**Copyright Information:** (Online) ISSN: 2572-9241
©2026 The Author(s). HemaSphere is published by John Wiley & Sons Ltd on behalf of European Hematology Association. This is an open access Abstract Book distributed under the Attribution-NonCommercial-NoDerivs (CC BY-NC-ND), which allows third parties to download the articles and share them with others as long as they credit the author and the Abstract Book, but they cannot change the content in any way or use them commercially.
**Abstract Book Citations:** Authors, Title, HemaSphere, 2026;10:(S1):pages. The abstract book DOIs can be found on the "Author Information" page.
**Disclaimer:** Articles published in the journal HemaSphere exclusively reflect the opinions of the authors. The authors are responsible for all content in their abstracts including accuracy of the facts, statements, citing resources, etc.

### Results

Of 127 obe-cel infused patients, 27 (21%) had EMD at lymphodepletion and 100 (79%) did not. At screening, the median age (range) was 36.0 years (20–73) in patients with EMD, and 50.5 years (20–81) in patients without EMD. Of the patients with EMD, 13 (48%) were male and 11 (41%) were Hispanic/Latino; of those without EMD, 53 (53%) were male and 27 (27%) were Hispanic/Latino. The median number of prior lines of therapy (range) was 3.0 (1–6) and 2.0 (1–6) for patients with and without EMD at lymphodepletion, respectively; prior stem cell transplant (SCT) was received by 12 (44%) and 44 (44%) patients, respectively. At lymphodepletion, the median bone marrow (BM) blast percentage (range) was 54% (0–100) in patients with EMD and 39% (0–100) in patients without EMD; Philadelphia chromosome-positive disease was observed in 6 (22%) and 30 (30%) patients, respectively. At 32.8 months' median follow-up (range 19.9–52.8), the ORR (95% confidence interval [CI]) was 59% (39–78) in patients with EMD and 83% (74–90) in patients without EMD. Median duration of remission (DoR; 95% CI) among responders was 42.5 months (3.4–not evaluable [NE]) and NE, in those with (n=16) and without (n=83) EMD at lymphodepletion, respectively (**Figure**). Overall, median EFS (95% CI) was 4.5 months (0.0–NE) and 14.3 months (9.0–NE) in patients with and without EMD at lymphodepletion, respectively; however, median EFS appeared to be comparable in responders with (44.6 months [6.0–NE]) and without EMD (NE). Overall, median OS (95% CI) was 15.3 months (7.85–NE) and 21.0 months (13.2–NE) in patients with and without EMD at lymphodepletion, respectively. The incidence of Grade ≥3 cytokine release syndrome in patients with and those without EMD was 3.7% and 2.0%; the incidence of Grade ≥3 immune effector cell-associated neurotoxicity syndrome was 19% and 4.0%, respectively. Cerebrospinal fluid pharmacokinetic analyses are underway; data will be presented.

### Summary/Conclusion

Obe-cel treatment demonstrated favorable efficacy and safety outcomes in patients with and without EMD in the FELIX trial. Patients with EMD were less likely to achieve remission and had numerically shorter median EFS versus those without EMD; however, DoR among responders was comparable between the two groups of patients. Overall, these findings support a positive benefit–risk profile for obe-cel, irrespective of EMD status at lymphodepletion.

**Copyright Information:** (Online) ISSN: 2572-9241
©2026 The Author(s). HemaSphere is published by John Wiley & Sons Ltd on behalf of European Hematology Association. This is an open access Abstract Book distributed under the Attribution-NonCommercial-NoDerivs (CC BY-NC-ND), which allows third parties to download the articles and share them with others as long as they credit the author and the Abstract Book, but they cannot change the content in any way or use them commercially.
**Abstract Book Citations:** Authors, Title, HemaSphere, 2026;10:(S1):pages. The abstract book DOIs can be found on the "Author Information" page.
**Disclaimer:** Articles published in the journal HemaSphere exclusively reflect the opinions of the authors. The authors are responsible for all content in their abstracts including accuracy of the facts, statements, citing resources, etc.

**Duration of remission in patients with R/R B-ALL, by EMD status at lymphodepletion**

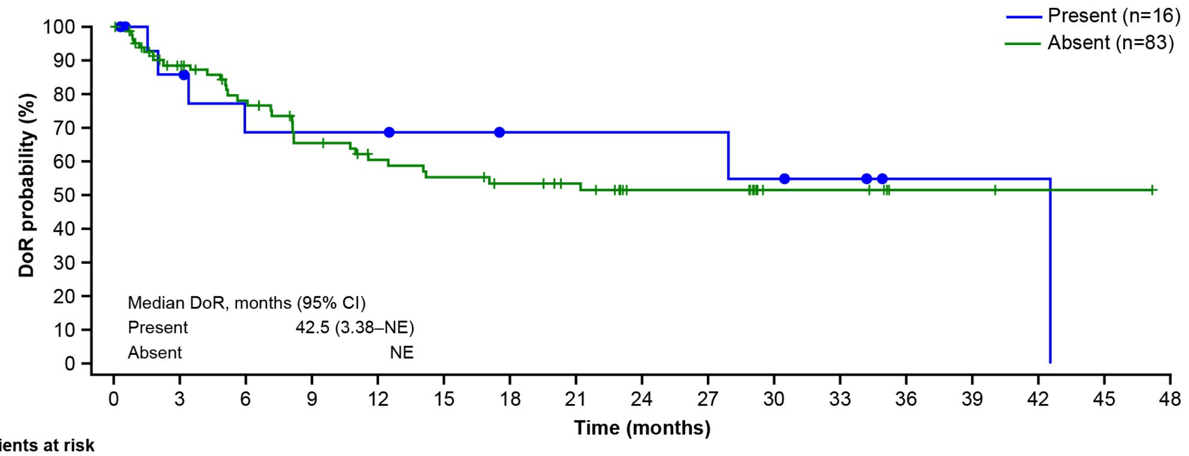

**Patients at risk**

| | | | | | | | | | | | | | | | | | |
|---|---|---|---|---|---|---|---|---|---|---|---|---|---|---|---|---|---|
| Present (n=16) | 16 | 12 | 8 | 8 | 8 | 7 | 5 | 5 | 5 | 5 | 4 | 3 | 1 | 1 | 1 | 0 | 0 |
| Absent (n=83) | 83 | 64 | 51 | 41 | 35 | 32 | 29 | 26 | 16 | 16 | 7 | 7 | 2 | 2 | 1 | 1 | 0 |

Censoring for new non-protocol anticancer therapies

CI, confidence interval; DoR, duration of remission; EMD, extramedullary disease; NE, not estimable; R/R B-ALL, relapsed/refractory B-cell acute lymphoblastic leukemia.

©2026 American Society of Clinical Oncology, Inc. Reused with permission. This abstract was accepted and previously presented at the 2026 ASCO Annual Meeting. All rights reserved.

**Copyright Information:** (Online) ISSN: 2572-9241
©2026 The Author(s). HemaSphere is published by John Wiley & Sons Ltd on behalf of European Hematology Association. This is an open access Abstract Book distributed under the Attribution-NonCommercial-NoDerivs (CC BY-NC-ND), which allows third parties to download the articles and share them with others as long as they credit the author and the Abstract Book, but they cannot change the content in any way or use them commercially.
**Abstract Book Citations:** Authors, Title, HemaSphere, 2026;10:(S1):pages. The abstract book DOIs can be found on the "Author Information" page.
**Disclaimer:** Articles published in the journal HemaSphere exclusively reflect the opinions of the authors. The authors are responsible for all content in their abstracts including accuracy of the facts, statements, citing resources, etc.

### (PS1485) COMBINATION THERAPY WITH BLINATUMOMAB AND ASCIMINIB FOR PHILADELPHIA CHROMOSOME-POSITIVE ACUTE LYMPHOBLASTIC LEUKEMIA: RESULTS FROM A PHASE I/II STUDY

**Topic:** 02. Acute lymphoblastic leukemia - Clinical

Nicholas Short*[1], Manuel Maroun[1], Courtney Dinardo[1], Miriam Garcia[1], David McCall[1], Branko Cuglievan[1], Omer Karrar[1], Maria Hachem[1], Wei Qiao[1], Xuelin Huang[1], Maro Ohanian[1], Alessandra Ferrajoli[1], Koichi Takahashi[1], Mahesh Swaminathan[1], Kara Stevens[1], Elias Jabbour[1], Hagop Kantarjian[1]

[1]*University of Texas MD Anderson Cancer Center, Leukemia, Houston, United States of America;*

### Background

Despite improvements in frontline therapy for Philadelphia chromosome-positive acute lymphoblastic leukemia (Ph+ ALL), the outcomes of relapsed Ph+ ALL remain poor. We hypothesized that the combination of blinatumomab + asciminib, a potent allosteric inhibitor of BCR::ABL1, may lead to deep and durable remissions in patients (pts) with relapsed/refractory (R/R) Ph+ ALL after failure of standard therapies.

### Aims

This is a phase I/II study evaluating the combination of blinatumomab + asciminib in pts ≥12 years of age with R/R Ph+ ALL or CML-LBP.

### Methods

In the phase I portion, pts receive up to 5 cycles of asciminib at a dose of 80mg, 120mg or 200mg orally daily in combination with continuous infusion blinatumomab in a standard 4-week on/2-week off schedule (42-day cycles). CNS prophylaxis with 6-8 doses of IT chemotherapy is recommended. For pts not proceeding with allo-HSCT or CAR T-cell therapy, asciminib is continued indefinitely. The primary endpoint of the phase I study is the minimum safe and biologically effective dose of asciminib in combination with blinatumomab.

**Copyright Information:** (Online) ISSN: 2572-9241
©2026 The Author(s). HemaSphere is published by John Wiley & Sons Ltd on behalf of European Hematology Association. This is an open access Abstract Book distributed under the Attribution-NonCommercial-NoDerivs (CC BY-NC-ND), which allows third parties to download the articles and share them with others as long as they credit the author and the Abstract Book, but they cannot change the content in any way or use them commercially.
**Abstract Book Citations:** Authors, Title, HemaSphere, 2026;10:(S1):pages. The abstract book DOIs can be found on the "Author Information" page.
**Disclaimer:** Articles published in the journal HemaSphere exclusively reflect the opinions of the authors. The authors are responsible for all content in their abstracts including accuracy of the facts, statements, citing resources, etc.

### Results

As of February 2026, 10 pts have been treated in the phase I portion of the study (9 with Ph+ ALL and 1 with CML-LBP). The median age is 47.5 years (range, 15-65). Five pts had received ≥2 prior TKIs, 5 pts had received prior blinatumomab + ponatinib, and 1 pt had undergone prior allo-HSCT.
Three pts received asciminib 80mg, 3 received 120mg and 4 received 200mg. No DLTs nor any grade 3 or higher related adverse events were observed. Four pts experienced CRS (all grade 1) and 2 pts experienced neurotoxicity (grade 1 tremor in both). There was one on-study death (due to CNS relapse).

All 10 pts (100%) achieved CR, all after the first cycle. Eight pts had a trackable sequence for NGS IG/TR MRD (sensitivity: $10^{-6}$): 5/8 (63%) achieved NGS MRD negativity after cycle 1 and 7/8 (88%) as best response; the other pt had NGS MRD below the level of detection as best response. The 2 pts without a trackable IG/TR achieved PCR negativity for *BCR::ABL1* after cycle 1.
The median follow-up is 10.7 months (range, 0.6-17.8 months). The median number of cycles received is 3.5 (range, 1-5). Among the 10 treated pts, 2 have relapsed (1 of whom was nonadherent to therapy) and 8 are in ongoing continuous remission. One relapse was bone marrow-only and 1 was CNS-only (5.1 and 6.5 months from start of therapy, respectively). The asciminib doses in the relapsed pts were 80mg and 120mg, respectively. Among the 8 pts in continuous remission at last follow-up, 1 received allo-HSCT (pt with CML-LBP), 4 received CD19 CAR T-cell consolidation (1 brexu-cel and 3 obe-cel), and the 3 have not received consolidation therapy (2 still receiving combination therapy and 1 receiving asciminib maintenance).
As of last follow-up, 1 pt has died (CNS relapse). The estimated 1-year RFS and OS rates are 75% and 88%, respectively.

### Summary/Conclusion

In this ongoing phase I/II study, the combination of blinatumomab + asciminib is safe and highly effective in pts with R/R Ph+ ALL, including those with prior exposure to blinatumomab + ponatinib. No DLTs have been observed at doses of asciminib up to 200mg daily. Responses have been deep, with NGS MRD negativity in 88% of evaluable pts. The study continues to accrue R/R pts. Given the encouraging activity observed in the R/R population, the study has been expanded to also enroll frontline pts ≥18 years of age.

**Copyright Information:** (Online) ISSN: 2572-9241
©2026 The Author(s). HemaSphere is published by John Wiley & Sons Ltd on behalf of European Hematology Association. This is an open access Abstract Book distributed under the Attribution-NonCommercial-NoDerivs (CC BY-NC-ND), which allows third parties to download the articles and share them with others as long as they credit the author and the Abstract Book, but they cannot change the content in any way or use them commercially.
**Abstract Book Citations:** Authors, Title, HemaSphere, 2026;10:(S1):pages. The abstract book DOIs can be found on the "Author Information" page.
**Disclaimer:** Articles published in the journal HemaSphere exclusively reflect the opinions of the authors. The authors are responsible for all content in their abstracts including accuracy of the facts, statements, citing resources, etc.

### (PS1486) CD19 CAR-T CELLS FEATURING A CD8Α HINGE GLYCINE DELETION EXHIBIT FAVORABLE SAFETY AND EFFICACY IN R/R ALL

**Topic:** 02. Acute lymphoblastic leukemia - Clinical

Weiwei Tian*[1, 2, 3], Linyu Li[1], Shaoli Zhang[2], Jia Wei[2, 4]

*[1]Third Hospital of Shanxi Medical University, Shanxi Bethune Hospital, Shanxi Academy of Medical Sciences, Tongji Shanxi Hospital, Taiyuan, China; [2]Shanxi Bethune Hospital, Shanxi Academy of MedicalSciences, Third Hospital of Shanxi Medical University, TongiShanxi Hospital, Taiyuan, China; [3]The Key Laboratory of Molecular Diagnosis and Treatment of Hematological Diseases of Shanxi Province, Taiyuan, China; [4]Tongji Hospital, Tongji Medical College, Huazhong University of Science and Technology, Wuhan, China;*

### Background

To evaluate the safety and efficacy of CD19 chimeric antigen receptor T (CAR-T) cells featuring a glycine–glycine deletion (GG-deletion) in the CD8α hinge region in patients with relapsed or refractory B-cell acute lymphoblastic leukemia (R/R B-ALL).

### Aims

This study aims to provide evidence to support further clinical development of this modified CAR-T platform.

### Methods

Patients with R/R ALL who received CD19(GG) CAR-T cell infusion were enrolled. CAR-T cells were administered at dose levels of $1\times10^6$/kg and $2\times10^6$/kg. The primary endpoint was safety, including the incidence and severity of cytokine release syndrome (CRS) and immune effector cell–associated neurotoxicity syndrome (ICANS). Secondary endpoints included objective response rate , complete remission rate, minimal residual disease negativity rate, event-free survival, and in vivo expansion kinetics of CAR-T cells.

### Results

Between November 2022 and November 2025, a total of 16 patients were enrolled. The median age was 30 years (range, 14–60), and the median number of prior treatment lines was 3 (range, 2–7). CRS occurred in 62.5% of patients, with grade 3 CRS observed in only 6.3%; no grade 4–5 adverse events were reported. ICANS occurred in 12.5% of patients, all of which were grade 1. At 1 month after infusion, the objective response rate was 100%, and the rate of complete remission or complete remission with incomplete hematologic recovery was 93.8%. The minimal residual disease-negative rate was 93.3%. With a median follow-up of 14.2 months (95% CI, 12.104–16.269), the 1-year event-free survival rate was 59.2%, and the 1-year overall survival rate was 87.5%. The peak expansion of CAR-T cells reached 36,596.1 copies/μg DNA (range, 4,580–259,437).

### Summary/Conclusion

CD19(GG) CAR-T cell therapy demonstrated a favorable safety profile with a low incidence of severe toxicities and induced deep remissions in patients with R/R ALL, representing a promising therapeutic option for this population.

**Copyright Information:** (Online) ISSN: 2572-9241
©2026 The Author(s). HemaSphere is published by John Wiley & Sons Ltd on behalf of European Hematology Association. This is an open access Abstract Book distributed under the Attribution-NonCommercial-NoDerivs (CC BY-NC-ND), which allows third parties to download the articles and share them with others as long as they credit the author and the Abstract Book, but they cannot change the content in any way or use them commercially.
**Abstract Book Citations:** Authors, Title, HemaSphere, 2026;10:(S1):pages. The abstract book DOIs can be found on the “Author Information” page.
**Disclaimer:** Articles published in the journal HemaSphere exclusively reflect the opinions of the authors. The authors are responsible for all content in their abstracts including accuracy of the facts, statements, citing resources, etc.

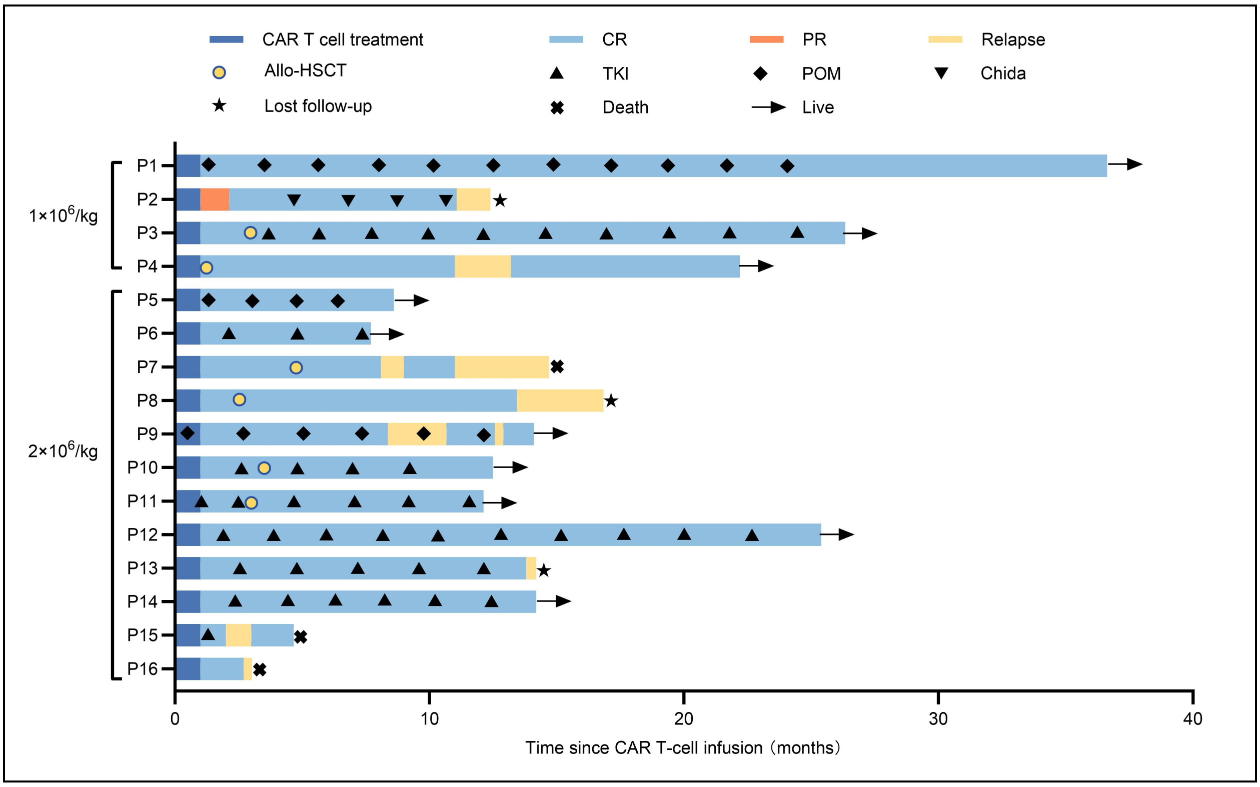

Figure. Swimmer plot of follow-up and clinical outcomes after CD19(GG) CAR-T cell therapy Each horizontal bar represents an individual patient treated with CD19(GG) CAR-T cells, plotted from the day of infusion to the last follow-up or occurrence of an event. Patients are ordered by infused CAR-T cell dose from lowest to highest, and bar length indicates follow-up duration. Colors and symbols denote disease status and key clinical events: color changes specifically reflect transitions in disease status; circles indicate allogeneic hematopoietic stem cell transplantation (allo-HSCT); triangles and squares denote maintenance therapy with tyrosine kinase inhibitors (TKIs), pomalidomide, or chidamide; crosses indicate death. Patients alive and event-free at last follow-up are shown as censored. This figure is descriptive and not intended for statistical inference of dose–outcome relationships.

**Copyright Information:** (Online) ISSN: 2572-9241
©2026 The Author(s). HemaSphere is published by John Wiley & Sons Ltd on behalf of European Hematology Association. This is an open access Abstract Book distributed under the Attribution-NonCommercial-NoDerivs (CC BY-NC-ND), which allows third parties to download the articles and share them with others as long as they credit the author and the Abstract Book, but they cannot change the content in any way or use them commercially.
**Abstract Book Citations:** Authors, Title, HemaSphere, 2026;10:(S1):pages. The abstract book DOIs can be found on the "Author Information" page.
**Disclaimer:** Articles published in the journal HemaSphere exclusively reflect the opinions of the authors. The authors are responsible for all content in their abstracts including accuracy of the facts, statements, citing resources, etc.

### (PS1487) EVALUATION OF PATIENT SPECIFIC STRUCTURAL VARIANTS AS MEASURABLE RESIDUAL DISEASE MARKERS IN PEDIATRIC ACUTE LYMPHOBLASTIC LEUKAEMIA

**Topic:** 02. Acute lymphoblastic leukemia - Clinical

Karin Sollander*[1], Millaray Marincevic[2], Anett Ketscher[3], Leonie Saft[4], Rose-Marie Amini[2], Gisela Barbany[1], Lucia Cavelier[1]

*[1]Karolinska Institutet, Department of Molecular Medicine and Surgery, Stockholm, Sweden; [2]Uppsala University, Department of Immunology, Genetics and Pathology, Uppsala, Sweden; [3]Karolinska University Hospital, Clinical Pathology and Cancer Diagnostic, Solna, Sweden; [4]Karolinska Institutet, Department of Oncology-Pathology, Solna, Sweden;*

### Background

Measurable residual disease (MRD) is an important prognostic factor during treatment of acute lymphoblastic leukaemia (ALL). Currently, flow cytometry and IG/TR RQ-PCR are the gold-standard MRD methods. In a small proportion of patients, suitable markers are absent at diagnosis or lack sufficient sensitivity for MRD analysis. In rare situations, such as mixed-lineage disease, these markers may also disappear or change during treatment.
Structural genomic rearrangements, known as structural variants, result in the disease-driving events and represent stable MRD markers.

### Aims

To evaluate ddPCR targeting patient-specific structural variants as an alternative MRD method in whom gold-standard assays are suboptimal.

### Methods

Structural variants were identified in diagnostic whole-genome sequencing (WGS) data from eleven pediatric patients with ALL treated according to the ALLTogether protocol. The cohort represented diverse ALL subtypes (4 B-ALL, 4 T-ALL, 1 ETP ALL and 2 MPAL, T/myeloid) with at least three follow-up time points (day 15, 29 and 78). For each patient in the cohort, one structural variant was chosen as an MRD marker. Selection was prioritized as follows: subgroup-defining aberrations according to ICC 2022; alterations defining provisional entities; lesions included in the UK-ALL-CNA risk profile; other structural variants present in all leukemic blasts.
Patient-specific assays were applied to follow-up samples and compared with flow cytometry and IG/TR RQ-PCR. Within the cohort, two patients showed discrepancies between the standard MRD methods, with only one method detecting the leukemic clone in follow-up samples (one B-ALL and one MPAL).

### Results

Structural variants were identified, and ddPCR assays were successfully established for all eleven patients. MRD measurements using patient-specific ddPCR showed high concordance with flow cytometry and IG/TR RQ-PCR. When classified as MRD-positive or MRD-negative, concordance between flow cytometry and ddPCR was 80% (91 samples, total number of samples from 11 patients with 3-17 samples), and between RQ-PCR and ddPCR 82% (66 samples, 10 patients with 1-16 samples), comparable to the concordance between flow cytometry and RQ-PCR (85%, 87 samples from 10 patients with 1-26 samples).
Among the quantifiable MRD-positive samples, the Pearson correlation coefficient was $R^2 = 0.89$ (flow cytometry vs ddPCR) and $R^2 = 0.92$ (RQ-PCR vs ddPCR) (Figure 1). In the discrepant patients, ddPCR confirmed all available MRD-positive measurements.

**Copyright Information:** (Online) ISSN: 2572-9241
©2026 The Author(s). HemaSphere is published by John Wiley & Sons Ltd on behalf of European Hematology Association. This is an open access Abstract Book distributed under the Attribution-NonCommercial-NoDerivs (CC BY-NC-ND), which allows third parties to download the articles and share them with others as long as they credit the author and the Abstract Book, but they cannot change the content in any way or use them commercially.
**Abstract Book Citations:** Authors, Title, HemaSphere, 2026;10:(S1):pages. The abstract book DOIs can be found on the “Author Information” page.
**Disclaimer:** Articles published in the journal HemaSphere exclusively reflect the opinions of the authors. The authors are responsible for all content in their abstracts including accuracy of the facts, statements, citing resources, etc.

### Summary/Conclusion ddPCR targeting patient-specific structural variants provides MRD measurements highly concordant with current gold-standard methods in a small cohort of B-ALL, T-ALL, ETP-ALL and MPAL. Assays were successfully designed for all eleven patients, including two in whom one of the standard methods was inadequate. When classifying follow-up samples as MRD-positive or -negative, concordance between ddPCR, flow cytometry, and RQ-PCR was 80–85%. Quantitative comparisons of MRD-positive samples showed strong correlations, with $R^2 = 0.89$ for flow cytometry vs ddPCR and $R^2 = 0.92$ for RQ-PCR vs ddPCR.
These results highlight the potential of structural variant–based ddPCR as a reliable MRD strategy, particularly for patients in whom conventional methods are difficult to apply or interpret. Because the monitored variants represent the genetic drivers of ALL, this approach may offer both biologically and clinically meaningful disease monitoring.

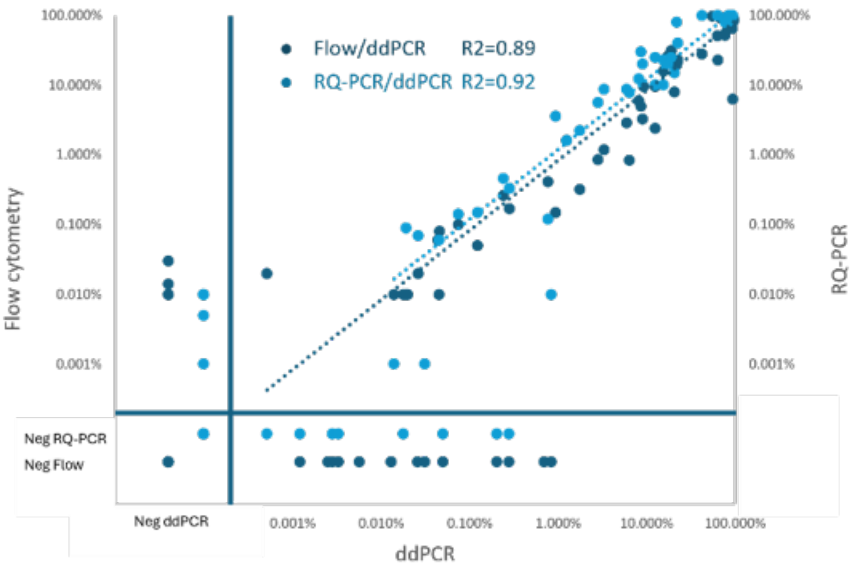

| | Flow + | Flow - | RQ-PCR + | RQ-PCR - |
|---|---|---|---|---|
| ddPCR + | 57 | 14 | 41 | 8 |
| ddPCR - | 4 | 16 | 4 | 13 |

Figure 1:. Correlation of MRD measurements in follow-up samples between ddPCR and the gold-standard methods, flow cytometry and IG/TR RQ-PCR. Pearson correlation coefficients were calculated for paired samples quantified by both methods. The table indicates the number of patients within each quadrant of the graph.

Copyright Information: (Online) ISSN: 2572-9241
©2026 The Author(s). HemaSphere is published by John Wiley & Sons Ltd on behalf of European Hematology Association. This is an open access Abstract Book distributed under the Attribution-NonCommercial-NoDerivs (CC BY-NC-ND), which allows third parties to download the articles and share them with others as long as they credit the author and the Abstract Book, but they cannot change the content in any way or use them commercially.
Abstract Book Citations: Authors, Title, HemaSphere, 2026;10:(S1):pages. The abstract book DOIs can be found on the “Author Information” page.
Disclaimer: Articles published in the journal HemaSphere exclusively reflect the opinions of the authors. The authors are responsible for all content in their abstracts including accuracy of the facts, statements, citing resources, etc.

### (PS1488) DEEPER REMISSION ACHIEVEMENT AND LONGER LEUKEMIA-FREE SURVIVAL WITH INATI-CEL AS CONSOLIDATION THERAPY IN ALLOGENEIC TRANSPLANT-INELIGIBLE ADOLESCENT AND ADULT PATIENTS WITH B-ALL

**Topic:** 02. Acute lymphoblastic leukemia - Clinical

Sanfang Tu*[1], Minhong Huang[1], Xuan Zhou[1], Chunyan Yue[1], Lei Wang[1], Shaojie Wu[1], Zhao Liang[1], Rui Huang[1], Yuxian Huang[1], Yanjie He[1], Chaoyang Song[1], Yuhua Li[1]

[1]*Department of Hematology, Zhujiang Hospital of Southern Medical University, Guangzhou, China;*

### Background

Allogeneic hematopoietic stem cell transplantation (allo-HSCT) remains the standard consolidation strategy for patients with B-cell acute lymphoblastic leukemia (B-ALL) in remission. However, a proportion of patients are ineligible due to donor unavailability, comorbidities, or individual risk considerations. CD19-directed chimeric antigen receptor T-cell (CAR-T) therapy has demonstrated high remission rates, although durable leukemia-free survival remains suboptimal, particularly in patients with higher disease burden. Inaticabtagene autoleucel (Inati-cel) is a CD19 CAR-T product incorporating a distinct single-chain variable fragment construct.

### Aims

To evaluate the efficacy and safety of Inati-cel as consolidation therapy in adolescent and adult B-ALL patients in remission and ineligible for allo-HSCT.

### Methods

This single-center prospective interventional study enrolled patients aged 14–80 years with B-ALL in CR/CRi (bone marrow blasts <5%, no extramedullary disease) deemed unsuitable for allo-HSCT. The study was registered at ClinicalTrials.gov (NCT07072494). After leukapheresis, patients received lymphodepletion with fludarabine (30 mg/m²/day for 3 days) and cyclophosphamide (500 mg/m²/day for 2 days), followed by infusion of $0.6\times10^8$ viable Inati-cel cells. Bone marrow morphology and measurable residual disease (MRD) were evaluated at 1, 3, and 12 months post-infusion. CAR-T expansion kinetics were monitored by flow cytometry. Primary endpoints were 1-year overall survival (OS) and leukemia-free survival (LFS) rate. Secondary endpoints included 1-year cumulative incidence of relapse (CIR), MRD conversion, and safety.

**Copyright Information:** (Online) ISSN: 2572-9241
©2026 The Author(s). HemaSphere is published by John Wiley & Sons Ltd on behalf of European Hematology Association. This is an open access Abstract Book distributed under the Attribution-NonCommercial-NoDerivs (CC BY-NC-ND), which allows third parties to download the articles and share them with others as long as they credit the author and the Abstract Book, but they cannot change the content in any way or use them commercially.
**Abstract Book Citations:** Authors, Title, HemaSphere, 2026;10:(S1):pages. The abstract book DOIs can be found on the “Author Information” page.
**Disclaimer:** Articles published in the journal HemaSphere exclusively reflect the opinions of the authors. The authors are responsible for all content in their abstracts including accuracy of the facts, statements, citing resources, etc.

### Results

Eighteen patients were enrolled, including 10 males and 8 females, with a median age of 33.5 years (range, 15–67). Twelve patients received CAR-T cell infusion during CR1, five during CR2, and one during CR3. Before infusion, 14 patients (77.8%) were MRD-negative and 4 were MRD-positive. Median follow-up was 212 days (last follow-up January 2026)The estimated 1-year cumulative OS and LFS rates were 91.7% and 77.4%, respectively, and the 1-year cumulative CIR was 14.9%. Median OS and LFS were not reached.Two patients experienced recurrence at 82 days and 101 days after the CAR-T infusion, respectively. Two patients died from recurrence and septic shock, respectively.

A median time to peak of CAR-T cell expansion (Tmax) of 14 days (range: 10–60 days) as detected by flow cytometry, with a median absolute CAR-T cell count of 56.14 cells/μL (range: 0.98–1011.74 cells/μL). The median duration of CAR-T cell persistence was 133 days (range: 10–554 days), and all patients retained detectable CAR-T cells through the end of the follow-up period.

All patients developed cytokine release syndrome (CRS), including grade 1 (n=12), grade 2 (n=4), and grade 3 (n=2). Two patients developed immune effector cell-associated neurotoxicity syndrome (ICANS). One patient experienced grade 3 ICANS which resolved completely following treatment with dexamethasone. The other patient had grade 1 ICANS . Cytopenias occurred in all patients and recovered with supportive care. Five patients (27.8%) developed mild-to-moderate infections, primarily pulmonary or urinary, with one died from septic shock at day 142 after CAR-T infusion .

### Summary/Conclusion

Inati-cel demonstrated encouraging efficacy and manageable toxicity as consolidation therapy in adolescent and adult B-ALL patients in remission who were ineligible for allo-HSCT.

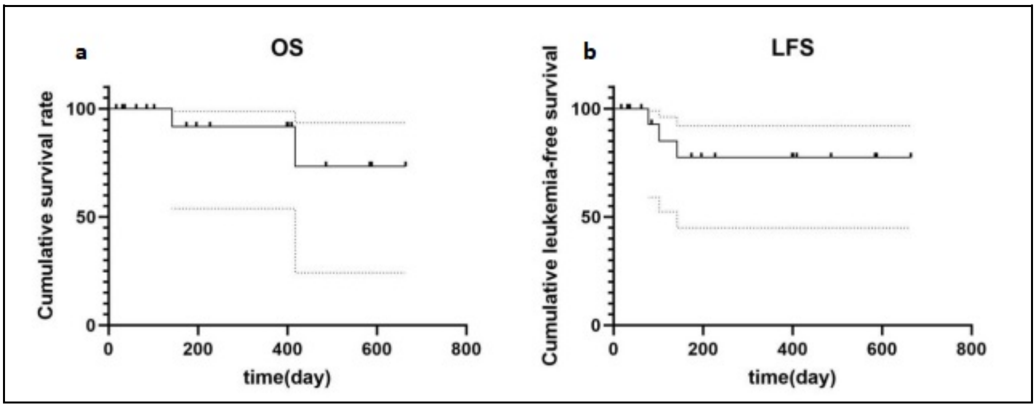

Fig.a Kaplan Meier plot for OS Fig.b Kaplan Meier plot for LFS

**Copyright Information:** (Online) ISSN: 2572-9241
©2026 The Author(s). HemaSphere is published by John Wiley & Sons Ltd on behalf of European Hematology Association. This is an open access Abstract Book distributed under the Attribution-NonCommercial-NoDerivs (CC BY-NC-ND), which allows third parties to download the articles and share them with others as long as they credit the author and the Abstract Book, but they cannot change the content in any way or use them commercially.
**Abstract Book Citations:** Authors, Title, HemaSphere, 2026;10:(S1):pages. The abstract book DOIs can be found on the "Author Information" page.
**Disclaimer:** Articles published in the journal HemaSphere exclusively reflect the opinions of the authors. The authors are responsible for all content in their abstracts including accuracy of the facts, statements, citing resources, etc.

### (PS1489) PHASE II STUDY OF DOSE-ADJUSTED EPOCH ± RITUXIMAB (R) + TAFASITAMAB (TAFA) IN NEWLY-DIAGNOSED (ND) ADULTS WITH PHILADELPHIA CHROMOSOME NEGATIVE (PH-) B-CELL ACUTE LYMPHOBLASTIC LEUKEMIA (B-ALL)

**Topic:** 02. Acute lymphoblastic leukemia - Clinical

Noam Kopmar[1], Jonathan Fromm[2], Noah Pinke[1], Ted Gooley[3], Christen Martino[1], Ashtyn Sherrow[1], Cristina Ghiuzeli[1], Jason Cooper[1], Erik Kimble[1], Johnnie Orozco[1], Mary-Elizabeth Percival[1], Roland Walter[1], Anna B. Halpern[1], Paul Hendrie[1], Raya Mawad[1], Vivian G. Oehler[1], Bart Scott[1], Jacob Appelbaum[1], Joshua Veatch[1], Ryan Cassaday*[1]

*[1]University of Washington and Fred Hutch Cancer Center, Seattle, United States of America;[2]University of Washington, Department of Laboratory Medicine & Pathology, Seattle, United States of America; [3]Fred Hutch Cancer Center, Public Health Sciences Division, Biostatistics Program, Seattle, United States of America;*

### Background

For adults with Ph- B-ALL, frontline regimens now often include the CD3-CD19 bispecific blinatumomab (blin) upon achieving measurable residual disease negativity (MRD-; <$10^{-4}$). However, only about 50% of patients (pts) reach this depth of response (Litzow, et al, *NEJM*, 2024), blin is not available globally, and its administration is challenging. Dose-adjusted etoposide, prednisone, vincristine, cyclophosphamide, and doxorubicin (DA-EPOCH) ± R is safe and active in these pts (Cassaday, et al, *Leuk Lymphoma*, 2023) and easier to deliver. Tafa is a CD19 monoclonal antibody active in B-cell lymphomas.

### Aims

We hypothesized that the addition of tafa to DA-EPOCH ± R in Ph- B-ALL will yield higher rates of MRD- without increasing toxicity while remaining feasible.

### Methods

This is a phase II investigator-initiated study (NCT05453500) in adults with ND CD19+ Ph- B-ALL not eligible for a pediatric regimen. All pts gave informed consent. DA-EPOCH ± R was given as cited above; tafa 12 mg/kg was given IV on day (d) 1, 8, and 15 of each cycle (C). After blin consolidation was FDA approved, pts could receive this off-study. The primary endpoint was MRD- by multiparameter flow cytometry (MFC) per EuroFlow (~<$10^{-4}$) after C1 (~d 20); secondary endpoints included MRD- by MFC after C4 (~d 80); non-hematologic adverse events (AEs, CTCAE v5); and relapse-free (RFS) and overall survival (OS). Exploratory endpoints included high-throughput sequencing (HTS; <$10^{-6}$) by clonoSEQ in marrow and cerebrospinal fluid (CSF). If ≥13 of 30 evaluable pts (≥43%) are MRD- after C1, the primary endpoint is met (vs 28% with DA-EPOCH ± R alone; 80% power and one-sided $\alpha = 0.05$).

### Results

From 3/2023 to 1/2026, 31 pts enrolled: 2 pts did not finish C1 due to grade (G) 4 AST elevation (related) and G5 *C. perfringens* sepsis (unrelated); 29 were evaluable for response and are the focus here. Baseline features are in the Table. Complete response (CR) rate was 86% (25/29); MRD- by MFC after C1 was 48% (14/29) and 82% (23/28) by C4 in pts with sufficient follow-up (f/u). In pts MRD- by MFC, 53% (8/15) were MRD- by HTS. Other G3+ AEs seen in >2 pts included febrile neutropenia (10); infection (9); hypotension (8); low fibrinogen (6); hypoxia (4); atrial fibrillation (3); and syncope (3). Allotransplant (HCT) was performed in first CR in 4 pts. Initial CSF MFC was positive in 3 pts; HTS was positive in these plus 4 more (7 total). With a median f/u among survivors of 8 mo (1-35), there were 3 relapses: 1 post-HCT and 1 after only 2 treatment cycles; all were CD19+, and 0 involved the

**Copyright Information:** (Online) ISSN: 2572-9241
©2026 The Author(s). HemaSphere is published by John Wiley & Sons Ltd on behalf of European Hematology Association. This is an open access Abstract Book distributed under the Attribution-NonCommercial-NoDerivs (CC BY-NC-ND), which allows third parties to download the articles and share them with others as long as they credit the author and the Abstract Book, but they cannot change the content in any way or use them commercially.
**Abstract Book Citations:** Authors, Title, HemaSphere, 2026;10:(S1):pages. The abstract book DOIs can be found on the "Author Information" page.
**Disclaimer:** Articles published in the journal HemaSphere exclusively reflect the opinions of the authors. The authors are responsible for all content in their abstracts including accuracy of the facts, statements, citing resources, etc.

central nervous system (CNS). Only 4 pts received blin consolidation after they were MRD- by MFC. 6 pts died: 4 non-relapse causes (3 post-HCT), 2 refractory ALL, and 0 from study treatment. 1-year estimates of RFS and OS were 77% and 78%, respectively.

| Baseline Features (N = 29) | n/Median (%/Range) |
|---|---|
| Age (years), median | 70 (44-84) |
| ≥70 years old | 15 (52%) |
| Sex, Male | 21 (72%) |
| Race | |
| White | 24 (79%) |
| Asian | 2 (7%) |
| Ethnicity, Hispanic/Latino | 5 (17%) |
| ECOG Status | |
| 0-1 | 23 (79%) |
| 2-3 | 6 (21%) |
| Prior Chemo/Radiation for Cancer | 13 (45%) |
| WBC (x$10^3$/μL), median | 2.9 (0.5-65.9) |
| Platelets (x$10^3$/μL), median | 55 (11-180) |
| NCCN Genetic Risk, Poor | 19 (66%) |

### Summary/Conclusion

DA-EPOCH ± R + tafa yields higher rates of MRD- than historical controls. This study has met its primary endpoint, with enrollment expected to end before the congress. F/U is immature, but early survival rates are comparable to more complex strategies; updates will be provided. HTS on CSF can identify CNS disease more frequently.

**Copyright Information:** (Online) ISSN: 2572-9241
©2026 The Author(s). HemaSphere is published by John Wiley & Sons Ltd on behalf of European Hematology Association. This is an open access Abstract Book distributed under the Attribution-NonCommercial-NoDerivs (CC BY-NC-ND), which allows third parties to download the articles and share them with others as long as they credit the author and the Abstract Book, but they cannot change the content in any way or use them commercially.
**Abstract Book Citations:** Authors, Title, HemaSphere, 2026;10:(S1):pages. The abstract book DOIs can be found on the "Author Information" page.
**Disclaimer:** Articles published in the journal HemaSphere exclusively reflect the opinions of the authors. The authors are responsible for all content in their abstracts including accuracy of the facts, statements, citing resources, etc.

### (PS1490) LONG-TERM SURVIVAL AND IMMUNOGLOBULIN PATTERNS FOLLOWING BLINATUMOMAB IN INFANTS WITH KMT2A-REARRANGED ALL

**Topic:** 02. Acute lymphoblastic leukemia - Clinical

Miguel Vieira Martins*[1], Paola de Lorenzo[2, 3], Rishi Kotecha[4, 5], Andishe Attarbaschi[6], Gabriele Escherich[7, 8], Karsten Nysom[9], Jan Starý[10, 11], Alina Ferster[12], Benoit Brethon[13], Franco Locatelli[14], Martin Schrappe[15], Peggy Scholte-van Houtem[1], Maria Grazia Valsecchi[2, 16], Robrecht Pieters[1], Inge Van Der Sluis[1]

*[1]Princess Maxima Center for Pediatric Oncology, Research - Pieters Group, Utrecht, The Netherlands;[2]Fondazione IRCCS San Gerardo dei Tintori,, Biostatistics and Clinical Epidemiology, Monza, Italy; [3]Tettamanti Center, Fondazione IRCCS San Gerardo dei Tintori, Monza, Italy; [4]Australian and New Zealand Children's Hematology and Oncology Group, Perth, Australia; [5]Children's Hospital and University of Western Australia,, Perth, Australia; [6]St. Anna Children's Hospital, Pediatric Hematology and Oncology, Vienna, Austria; [7]German Cooperative Study Group for Childhood Acute Lymphoblastic Leukemia, Hamburg, Germany; [8]University Medical Center Hamburg-Eppendorf, Clinic of Pediatric Hematology and Oncology, Hamburg, Germany; [9]Rigshospitalet Copenhagen University Hospital,, Department of Pediatrics and Adolescent Medicine, Copenhagen, Denmark; [10]Czech Working Group for Pediatric Hematology and CLIP (Childhood Leukemia Investigation Prague), Prague, Czechia; [11]Second Faculty of Medicine, Charles University and University Hospital Motol, Department of Pediatric Hematology and Oncology, Prague, Czechia; [12]Hôpital Universitaire des Enfants Reine Fabiola, Bruxelles, Belgium; [13]University Robert Debre Hospital, Assistance Publique-Hôpitaux de Paris, Paris, France; [14]IRCCS Ospedale Pediatrico Bambino Gesù, Catholic University of the Sacred Heart, Department of Pediatric Hematology-Oncology and Cell and Gene Therapy, Rome, Italy; [15]ALL-Berlin-Frankfurt-Münster (BFM) Group, University Medical Center Schleswig-Holstein, Campus Kiel, Kiel, Germany; [16]University of Milano-Bicocca, School of Medicine and Surgery, Milano, Italy;*

### Background

Infant *KMT2A*-rearranged acute lymphoblastic leukemia (*KMT2A*-r ALL) remains a dire disease with poor prognosis despite treatment intensification in the latest protocols. Blinatumomab, a bispecific T-cell engager that targets CD19 redirecting $CD3^{+}$ T-cells to trigger a cytotoxic response, has shown promise in improving outcomes for infants with *KMT2A*-r ALL when added to standard of care chemotherapy. Blinatumomab is expected to impair immunoglobulin G (lgG) production, which can increase susceptibility to infection. Little is, however, known about the impact of IgG depletion and recovery post-blinatumomab in infants.

### Aims

Our study intended to show the long-term survival of infants with *KMT2A*-r ALL following the addition of blinatumomab to standard chemotherapy, as well as describe patterns of immunoglobulin depletion and recovery.

### Methods

Our study included 30 infants who received Interfant-06 protocol chemotherapy, supplemented by one course of blinatumomab (28-day continuous infusion, 15μg/$m^2$/day) added post-induction. IgG serum levels (g/L) were monitored systematically from the start of the blinatumomab cycle through to the start of Protocol IB. Subsequent measurements were performed at investigators' discretion. IVIg supplementation and infections were also reported.

**Copyright Information:** (Online) ISSN: 2572-9241
©2026 The Author(s). HemaSphere is published by John Wiley & Sons Ltd on behalf of European Hematology Association. This is an open access Abstract Book distributed under the Attribution-NonCommercial-NoDerivs (CC BY-NC-ND), which allows third parties to download the articles and share them with others as long as they credit the author and the Abstract Book, but they cannot change the content in any way or use them commercially.
**Abstract Book Citations:** Authors, Title, HemaSphere, 2026;10:(S1):pages. The abstract book DOIs can be found on the "Author Information" page.
**Disclaimer:** Articles published in the journal HemaSphere exclusively reflect the opinions of the authors. The authors are responsible for all content in their abstracts including accuracy of the facts, statements, citing resources, etc.

### Results

Adding blinatumomab to upfront standard therapy resulted in a sustained beneficial outcome for infants with *KMT2A*-r ALL with a 4-year DFS and OS of 83.3% and 93.3%, compared to 44.0% and 60.2% in Interfant-06-selected controls, respectively (Fig. 1A-B). A total of 169 IgG values were documented during and after treatment. IgG levels decreased steadily over the course of blinatumomab, reaching a median nadir of 2.05 g/L ( Z-score -1.29) at the beginning of the next treatment phase - Protocol IB (Fig 1C). A gradual increase in IgG was then subsequently noted until maintenance, where all available values normalized (>4 g/L). Nearly two-thirds of patients (n=19/30, 63%) were supplemented with IVIg during the study, predominantly during the blinatumomab cycle (n=12, 40%). Infections remained minimal during blinatumomab accounting for 8% (n=8/95) of all infections reported during protocol, with no post-induction infectious deaths, as opposed to 4% in Interfant-06.

### Summary/Conclusion

Adding blinatumomab to upfront standard therapy resulted in marked improvements to survival outcomes for infants with *KMT2A*-r ALL. While we demonstrated that this approach consistently led to a decrease in immunoglobulin levels we identified a shorter nadir and earlier recovery of IgG in infants compared to adults, where limited evidence points to a late recovery with a nadir around 57 weeks post-blinatumomab. Findings of this study can guide pre-emptive strategies for IVIg replacement to prevent significant treatment-related hypogammaglobulinemia. Since immunoglobulin levels vary widely in infants, a decision to supplement should consider infant age-specific reference ranges. Moreover, delayed immunoglobulin recovery appears to persist beyond the end of blinatumomab, which suggests a combined cumulative effect with subsequent chemotherapy. Despite higher infection rates post-blinatumomab, there were no infectious deaths resulting from the combined treatment regimen.
Ongoing trials involving blinatumomab should validate outcomes in infants with KMT2A-r ALL, monitoring immunoglobulin recovery throughout treatment to guide infection-prevention strategies.

1A)

1B)

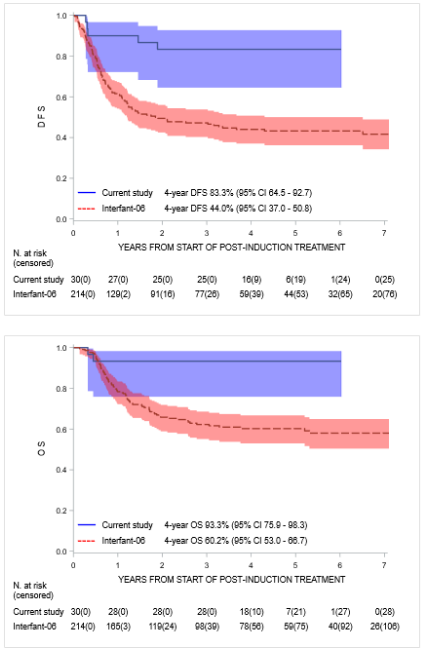

1C)

| Phase | Patients with IgG levels (n) | Available IgG levels, n (%) | IgG levels (g/L) | IgG levels z-score |
|---|---|---|---|---|
| Blin Day 1 | 30 | 30 (17.8) | 2.82 (0.90-11.10) | - 0.72 (-1.61; 0.48) |
| Blin Day 15 | 26 | 26 (15.4) | 3.35 (0.70-5.80) | -0.67 (-1.72; 0.15) |
| Blin Day 29 | 27 | 27 (16.0) | 2.20 (0.60-7.92) | -0.97 (-1.96; 0.80) |
| IB | 18 | 18 (10.7) | 2.05 (0.61-9.02) | -1.29 (-1.99; 0.98) |
| MM | 10 | 15 (8.9) | 2.85 (0.70-11.10) | -0.94 (-1.94; 0.35) |
| OD | 7 | 14 (8.3) | 3.62 (0.40-6.50) | -0.91 (-1.85; -0.10) |
| MT | 11 | 27 (16.0) | 4.29 (1.20-9.50) | -0.72 (-1.62; 0.25) |
| AEoT | 7 | 12 (7.1) | 7.99 (4.30-10.45) | 0.10 (-0.60; 0.28) |

***Figure 1. Survival outcomes and variability of IgG levels during and after treatment.*** **A) Disease-free survival,** current study vs. Interfant-06; **B) Overall survival,** current study vs. Interfant-06; **C) Descriptive statistics of measured IgG levels during treatment.** Data on IgG levels and respective z-scores are presented as median (range). Z-scores were calculated according to reference values.. Blin: Blinatumomab; IB: Protocol IB; MM: MARMA; OD: OCTADAD; MT: maintenance; AEoT: After End of Treatment.

**Copyright Information:** (Online) ISSN: 2572-9241
©2026 The Author(s). HemaSphere is published by John Wiley & Sons Ltd on behalf of European Hematology Association. This is an open access Abstract Book distributed under the Attribution-NonCommercial-NoDerivs (CC BY-NC-ND), which allows third parties to download the articles and share them with others as long as they credit the author and the Abstract Book, but they cannot change the content in any way or use them commercially.
**Abstract Book Citations:** Authors, Title, HemaSphere, 2026;10:(S1):pages. The abstract book DOIs can be found on the "Author Information" page.
**Disclaimer:** Articles published in the journal HemaSphere exclusively reflect the opinions of the authors. The authors are responsible for all content in their abstracts including accuracy of the facts, statements, citing resources, etc.

### (PS1491) CLINICAL EFFICACY ANALYSIS OF CAR-T THERAPY IN PATIENTS WITH RELAPSED/REFRACTORY B-ALL WITH CENTRAL NERVOUS SYSTEM LEUKEMIA

**Topic:** 02. Acute lymphoblastic leukemia - Clinical

Yajie Shi[1], Biyi Li[1], Yuehui Lin[1], Shuang You Liu[1], Biping Deng[2], Chunrong Tong[1], Tong Wu[3], Zhao Defeng*[1]

[1]*Department of Hematology, Beijing Boren Hospital, Beijing, China;*[2]*Cell Culture Laboratory, Beijing Boren Hospital, BEIJING, China;* [3]*Department of Hematopoietic Stem Cell Transplantation, Beijing Boren Hospital, BEIJING, China;*

### Background

With the rapid development of immunotherapy, chimeric antigen receptor T-cell(CAR-T) therapy has demonstrated significant efficacy in relapsed/refractory(R/R) B-cell acute lymphoblastic leukemia (B-ALL). However, central nervous system leukemia (CNSL) remains a major challenge in B-ALL treatment.  CAR-T therapy  precise efficacy and safety for CNSL are not yet fully elucidated.Therefore, a thorough analysis of the efficacy of CAR-T therapy in B-ALL  with CNSL holds significant clinical  value.

### Aims

To investigate the clinical efficacy of CAR-T therapy in patients with R/R B-ALL with CNSL

### Methods

This study included 62 patients with R/R B-ALL with CNSL admitted to Beijing Boren Hospital between January 2018 and December 2024. The median age was 21 years (range 2-63), with 38 males (61.3%) and 24 females (38.7%).20 patients had isolated CNS relapse,42 patients had relapse both  bone marrow and CNS.All patients received bridging therapy  prior to CAR-T infusion to minish burthen of tumor,and 36 achieved CSF complete remission.CAR-T targets were selected based on flow cytometry antigen expression: CD19 CAR-T (n=57), CD22 CAR-T (n=4), and CD19+CD22 CAR-T (n=1). The median infused CAR-T cell dose was $8.95\times10^5$/kg. CAR-T cells were detected by flow cytometry in the CSF within one month post-infusion,with a detection rate of 65.1%. Follow-up ended on December 31, 2025, with a median follow-up of 32.5 months. Disease-free survival (DFS) and overall survival (OS) were the primary endpoints.

**Copyright Information:** (Online) ISSN: 2572-9241
©2026 The Author(s). HemaSphere is published by John Wiley & Sons Ltd on behalf of European Hematology Association. This is an open access Abstract Book distributed under the Attribution-NonCommercial-NoDerivs (CC BY-NC-ND), which allows third parties to download the articles and share them with others as long as they credit the author and the Abstract Book, but they cannot change the content in any way or use them commercially.
**Abstract Book Citations:** Authors, Title, HemaSphere, 2026;10:(S1):pages. The abstract book DOIs can be found on the "Author Information" page.
**Disclaimer:** Articles published in the journal HemaSphere exclusively reflect the opinions of the authors. The authors are responsible for all content in their abstracts including accuracy of the facts, statements, citing resources, etc.

**Results**

20 patients with isolated CNS relapse achieved CSF complete remission (response rate 100%). Among 42 patients with both bone marrow and CNS relapse, 39 achieved complete remission (response rate 92.9%). At the last follow-up, 45 patients were alive, with 41 in continuous remission (66.12%). The 1-, 2-, and 3-year OS rates were 82.3%, 79.%, and 71.8%;the 1-year DFS rate was 78.7%, the 2-and 3-year DFS rates were both 71.65%. Median OS and DFS were not reached.

Among 62 B-ALL, following the initial CAR-T , 28 opted for sequential CAR-T with a different target, 20 underwent bridging allo-HSCT, and 14 received maintenance therapy with targeted agents combined with chemotherapy. The Sequential CAR-T group had 1-, 2-, and 3-year OS rates of 96.4%, and 1-year DFS of 96.4%, with 2-and 3-year DFS rates of 92.6%; median OS and DFS were not reached. The allo-HSCT group had 1-year OS of 80.0%, 2-and 3-year OS of 75.0%, 1-year DFS of 73.6%, and 2-year and 3-year DFS of 67.9%;median OS and DFS were not reached. The targeted agents combined with chemotherapy group had 1-year OS of 87.1%, 2-year OS of 50.0%, 3-year OS of 20.05%, and median OS of 15.1 months,1-year DFS was 42.9%, 2-and 3-year DFS were 34.3%, and median DFS was 5.6 months. The Sequential CAR-T group showed significantly higher OS and DFS rates compared to both the allo-HSCT and targeted agents combined with chemotherapy group ($P<0.05$ for both comparisons).
The incidence of cytokine release syndrome (CRS) was 77.42%: Grade 1 n=44, Grade 2 n=2, Grade 3 n=1, and Grade 4 n=1. The incidence of immune effector cell-associated neurotoxicity syndrome (ICANS) was 19.35% (Grade 1: n=11,Grade 2: n=1).

**Summary/Conclusion**

1.CAR-T therapy can achieve high response rates and prolong survival in patients with R/R B-ALL with CNSL.
2.For R/R B-ALL patients who achieve remission after CAR-T therapy, sequential CAR-T therapy demonstrates superior efficacy compared to bridging allo-HSCT or targeted agents combined with chemotherapy.

**Copyright Information:** (Online) ISSN: 2572-9241
©2026 The Author(s). HemaSphere is published by John Wiley & Sons Ltd on behalf of European Hematology Association. This is an open access Abstract Book distributed under the Attribution-NonCommercial-NoDerivs (CC BY-NC-ND), which allows third parties to download the articles and share them with others as long as they credit the author and the Abstract Book, but they cannot change the content in any way or use them commercially.
**Abstract Book Citations:** Authors, Title, HemaSphere, 2026;10:(S1):pages. The abstract book DOIs can be found on the “Author Information” page.
**Disclaimer:** Articles published in the journal HemaSphere exclusively reflect the opinions of the authors. The authors are responsible for all content in their abstracts including accuracy of the facts, statements, citing resources, etc.

### (PS1492) DONOR AGE ≥50 YEARS COMBINED WITH DONOR-CI ≥3 IDENTIFIES A HIGH-RISK COHORT FOR ADVERSE OUTCOMES AFTER ALLOGENEIC HEMATOPOIETIC STEM CELL TRANSPLANTATION IN B-CELL ACUTE LYMPHOBLASTIC LEUKEMIA

**Topic:** 02. Acute lymphoblastic leukemia - Clinical

Ning Wu[1], Jun Kong[1], Meng Lv[1], Xiao-Dong Mo[1], Yuqian Sun[1], Yifei Cheng[1], Lanping Xu[1], Xiaohui Zhang[1], Xiao-Jun Huang[1], Yu Wang*[1]

*[1]Peking University People's Hospital, Peking University Institute of Hematology, National Clinical Research Center for Hematologic Disease, Beijing Key Laboratory of Cell and Gene Therapy for Hematologic Malignancies, Peking University, Beijing, China., Beijing, China;*

### Background

Allogeneic hematopoietic stem cell transplantation (allo-HSCT) is curative for B-cell acute lymphoblastic leukemia (B-ALL), yet outcomes are limited by GVHD and relapse. Donor age is a well-established prognostic factor, but the prognostic value of donor comorbidities (Donor-CI) alone is unclear. We hypothesized that Donor-CI synergizes with advanced donor age to stratify transplant risk, driven by the combined effects of immunosenescence and comorbidity-associated chronic inflammation.

### Aims

The primary aim of this study was to validate the ability of a composite metric integrating Donor-CI and donor age to identify a high-risk donor cohort for adverse post-transplant outcomes in patients with B-ALL undergoing allo-HSCT.

### Methods

Single-center retrospective study of 458 consecutive B-ALL patients who underwent allo-HSCT with haploidentical/matched sibling donors (2019–2021) was conducted. Donor comorbidities were scored via the standardized HCT-CI system, with data from pre-donation health records and structured questionnaires (96.7% component capture). Donors were stratified by Donor-CI (0-2 vs. ≥3) and age (<50 vs. ≥50 years, ROC-derived cutoff) into four groups: Group1 (CI≥3+age≥50, n=20), Group2 (CI≥3+age<50, n=56), Group3 (CI0-2+age≥50, n=93), Group4 (CI0-2+age<50, n=289). Primary endpoint was overall survival (OS); secondary endpoints included GVHD, relapse, and leukemia-free survival (LFS). Statistical analyses used Kaplan-Meier, Fine-Gray, Cox regression models, and bootstrap resampling (1,000 iterations) for Group1.

**Copyright Information:** (Online) ISSN: 2572-9241
©2026 The Author(s). HemaSphere is published by John Wiley & Sons Ltd on behalf of European Hematology Association. This is an open access Abstract Book distributed under the Attribution-NonCommercial-NoDerivs (CC BY-NC-ND), which allows third parties to download the articles and share them with others as long as they credit the author and the Abstract Book, but they cannot change the content in any way or use them commercially.
**Abstract Book Citations:** Authors, Title, HemaSphere, 2026;10:(S1):pages. The abstract book DOIs can be found on the "Author Information" page.
**Disclaimer:** Articles published in the journal HemaSphere exclusively reflect the opinions of the authors. The authors are responsible for all content in their abstracts including accuracy of the facts, statements, citing resources, etc.

### Results

- Donor age alone (≥50 years, n=113) was associated with higher grade II-IV aGVHD (36.3% vs.30.1%, *P*=0.046), cGVHD (47.8% vs.39.4%, *P*=0.028), inferior LFS (77.0% vs.83.2%, *P*=0.039) and OS (81.4% vs.86.7%, *P*=0.041) vs. donors <50 years.
- Donor-CI alone (0, 1-2, ≥3 points) showed no statistically significant differences in post-transplant outcomes (all*P*>0.05).
- The composite Donor-CI/age metric identified Group1 as a high-risk cohort: vs. combined Groups2-4 (n=438), Group1 had higher grade II-IV aGVHD (40.0% vs.31.6%, *P*=0.030), grade III-IV aGVHD (15.0% vs.8.6%, *P*=0.045), relapse (25.0% vs.14.2%, *P*=0.033), inferior LFS (70.0% vs.81.7%, *P*=0.027) and OS (75.0% vs.85.0%, *P*=0.034).
- Multivariate analysis (adjusting for recipient age, donor source, high-risk cytogenetics) confirmed Group1 independently predicted worse OS (HR=2.85, 95%CI=1.52-5.35, *P*=0.035), LFS (HR=2.42, 95%CI=1.34-4.37, *P*=0.041) and higher relapse risk (HR=3.16, 95%CI=1.45-6.89, *P*=0.022) vs. Group4 (reference); Groups2/3 had no significant risk (all *P*>0.05). Relapse was the predominant death cause in Group1 (80.0%, 4/5 deaths).

### Summary/Conclusion

Donor-CI ≥3 combined with donor age ≥50 years identifies a high-risk cohort for adverse outcomes after allo-HSCT in B-ALL, with independently increased risks of severe GVHD, relapse, and mortality. This composite metric provides a practical, simple tool for donor risk assessment and informed donor selection in related donor allo-HSCT. Limitations include the retrospective single-center design and small Group1 sample size, necessitating validation in large multi-center cohorts.

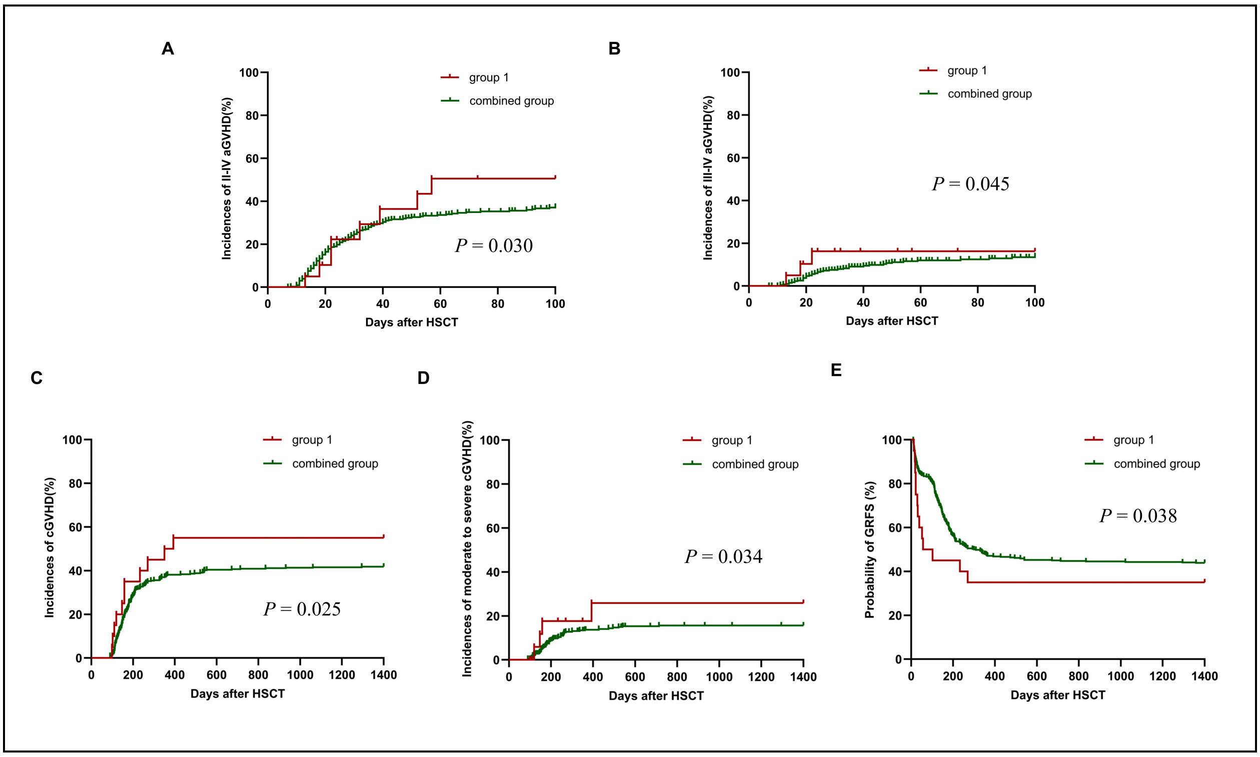

**Copyright Information:** (Online) ISSN: 2572-9241
©2026 The Author(s). HemaSphere is published by John Wiley & Sons Ltd on behalf of European Hematology Association. This is an open access Abstract Book distributed under the Attribution-NonCommercial-NoDerivs (CC BY-NC-ND), which allows third parties to download the articles and share them with others as long as they credit the author and the Abstract Book, but they cannot change the content in any way or use them commercially.
**Abstract Book Citations:** Authors, Title, HemaSphere, 2026;10:(S1):pages. The abstract book DOIs can be found on the "Author Information" page.
**Disclaimer:** Articles published in the journal HemaSphere exclusively reflect the opinions of the authors. The authors are responsible for all content in their abstracts including accuracy of the facts, statements, citing resources, etc.

### (PS1493) THE TREATMENT OF B-LYMPHOBLASTIC LYMPHOMA IN THE GERMAN MULTICENTER STUDY GROUP FOR ADULT ALL (GMALL): A RETROSPECTIVE ANALYSIS

**Topic:** 02. Acute lymphoblastic leukemia - Clinical

Markus Liesenfeld*[1], Julius Enssle[1], Veit Bücklein[2], Simon Raffel[3], Christoph Faul[4], Folker Schneller[5], Andreas Viardot[6], Jan Schleicher[7], Hendrik Poeck[8], Jürgen Krauter[9], Hubert Serve[1], Thomas Oellerich[1], Nicola Gökbuget[1], Björn Steffen[1]

*[1]Goethe University Frankfurt, Department of Medicine, Hematology and Oncology, University Hospital, Frankfurt am Main, Germany; [2]LMU, Department of Medicine III, University Hospital of Munich, München, Germany; [3]University Hospital Heidelberg, Department of Medicine, Hematology, Oncology and Rheumatology, Heidelberg, Germany; [4]University Hospital Tübingen, Department of Hematology, Oncology, Clinical Immunology, and Rheumatology, Tübingen, Germany; [5]TU München, Klinikum rechts der Isar, Medical Department III, München, Germany; [6]University Hospital of Ulm, Department of Internal Medicine III, Ulm, Germany; [7]Klinikum Stuttgart, Klinik für Hämatologie, Onkologie, Palliativmedizin und Stammzelltransplantation, Stuttgart, Germany; [8]University Hospital Regensburg, Department of Internal Medicine III, Hematology & Internal Oncology, Regensburg, Germany; [9]Klinikum Braunschweig, Hematology and Oncology, Braunschweig, Germany;*

### Background

B-lymphoblastic lymphoma (B-LBL) is extremely rare and biologically closely related to acute B-lymphoblastic leukemia (BCP-ALL). Cases often present with distinct clinical characteristics and may follow a heterogenous clinical course. Current treatment approaches follow ALL-based strategies. Here, we report on a large cohort of adult B-LBL pts prospectively treated with intensive pediatric-based protocols.

### Aims

This study analyses overall survival (OS) in patients (pts) with B-LBL, treated within different protocols of the GMALL study group.

### Methods

Pts with B-LBL prospectively registered between 2002 and 2022 were included. Pts were stratified into four groups according to age (≤55 years: younger, >55 years: older) and treatment strategy which was historically the B-ALL/NHL protocol (Hoelzer et al, Blood 2014) vs. currently ALL-based protocols (e.g. Goekbuget et al, Blood 2021). Pts characteristics were analysed to describe outcomes and to identify potential risk factors.

**Copyright Information:** (Online) ISSN: 2572-9241
©2026 The Author(s). HemaSphere is published by John Wiley & Sons Ltd on behalf of European Hematology Association. This is an open access Abstract Book distributed under the Attribution-NonCommercial-NoDerivs (CC BY-NC-ND), which allows third parties to download the articles and share them with others as long as they credit the author and the Abstract Book, but they cannot change the content in any way or use them commercially.
**Abstract Book Citations:** Authors, Title, HemaSphere, 2026;10:(S1):pages. The abstract book DOIs can be found on the "Author Information" page.
**Disclaimer:** Articles published in the journal HemaSphere exclusively reflect the opinions of the authors. The authors are responsible for all content in their abstracts including accuracy of the facts, statements, citing resources, etc.

**Results**

A total of 85 pts with a median age of 48 (range 16-80) years (yrs) were included from 51 centers. 74% of the pts had extranodal involvement and 45% had bone involvement. Treatment according to ALL protocols was performed in 24 younger pts (group A) and 11 older pts (group B). 14 of the younger pts were treated in the GMALL08 study. The B-ALL/NHL protocol was administered in 33 younger pts (group C) and 17 older pts (group D) respectively.
In the total cohort (n=85) complete response (CR) rate after induction was 74% with the median OS (mOS) not reached at a median follow up (FU) of 43.4 months (mo) (interquartile range [IQR] 10.81 - 49.51). The OS at 1 and 3 yrs was 85% (95% confidence interval [CI]: 77-93) and 64% (95% CI: 54-77), respectively. After CR 26 pts (31%) relapsed. The median time to relapse was 7.9 mo (IQR: 4.91 - 19.57).
In A-D the CR rate was 79%, 88%, 64%, 47% respectively. The mOS was not reached for A and D. The mOS for B and C was 11.7 and 80.3 mo. (Fig 1). Pts treated in the GMALL08 study had an overall response rate (ORR) of 92% (CR 71%, partial Remission [PR] 21%).
Higher age at initial diagnosis was associated with inferior OS (Hazard ratio [HR] 1.03, [95% CI: 1.01–1.06, p = 0.02). Elevated LDH (>1xULN) and a disseminated disease with extranodal and nodal manifestations at diagnosis trended toward inferior OS (n=69; HR 2.02, CI 95%: 0.88 - 4.64, p=0,1 and n=82; HR 2.15, CI 95%: 0.97 - 4.75, p=0.06, respectively).

**Summary/Conclusion**

This is the largest cohort of adult B-LBL pts prospectively registered in a standardized treatment setting. Response rates were variable due to a large proportion of PRs. This underlines the need for refined response assessment as in the GMALL08 trial. The protocols show comparable outcomes, but the use of B-NHL protocols is no longer recommended in the GMALL group. Overall, B-LBL had a favourable outcome with intensive chemotherapy-based protocols. The outcomes with current protocols including immunotherapy remain to be evaluated.

**Copyright Information:** (Online) ISSN: 2572-9241
©2026 The Author(s). HemaSphere is published by John Wiley & Sons Ltd on behalf of European Hematology Association. This is an open access Abstract Book distributed under the Attribution-NonCommercial-NoDerivs (CC BY-NC-ND), which allows third parties to download the articles and share them with others as long as they credit the author and the Abstract Book, but they cannot change the content in any way or use them commercially.
**Abstract Book Citations:** Authors, Title, HemaSphere, 2026;10:(S1):pages. The abstract book DOIs can be found on the "Author Information" page.
**Disclaimer:** Articles published in the journal HemaSphere exclusively reflect the opinions of the authors. The authors are responsible for all content in their abstracts including accuracy of the facts, statements, citing resources, etc.

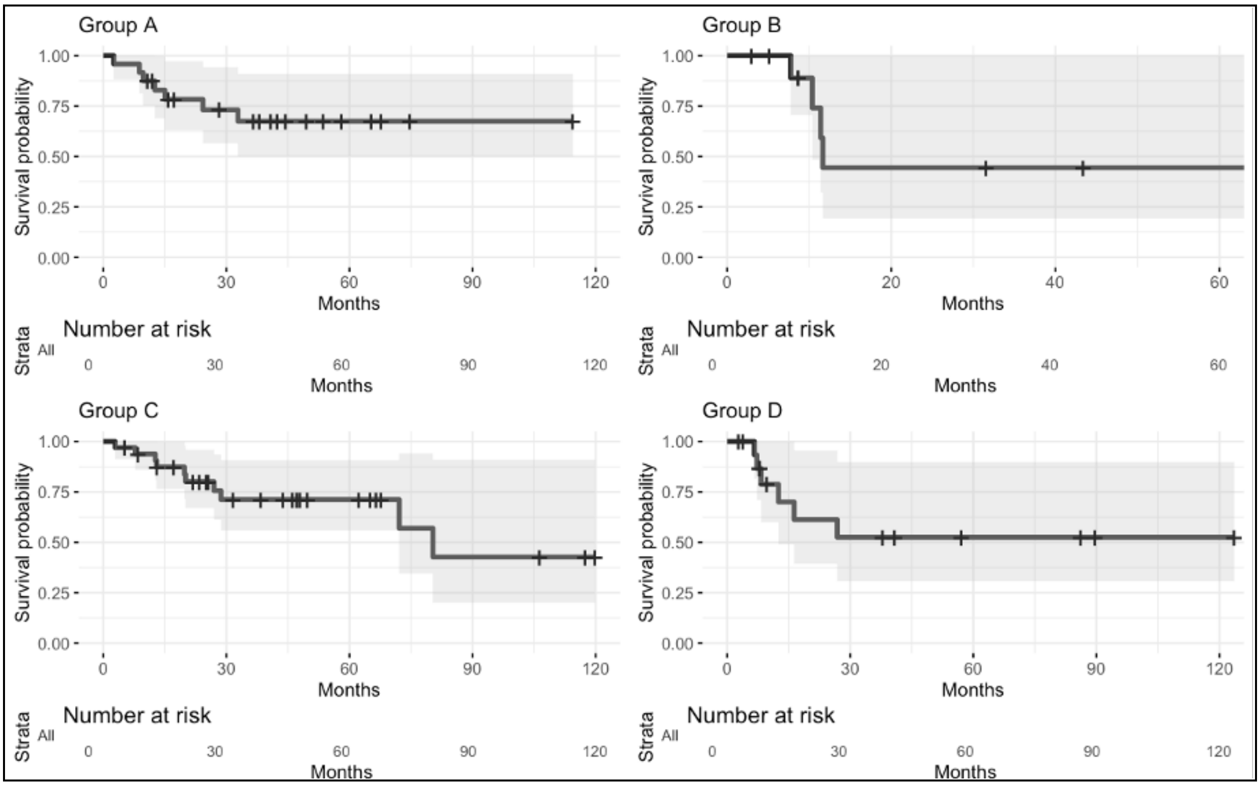

Fig. 1: Kaplan-Meier estimates of OS, A: ALL-protocol ≤55 FU 42,4 mo (IQR 14.4 - 50.53), B: ALL-protocol >55 FU 31,6 mo (IQR 8.18 - 21.62), C: B-ALL/NHL protocol ≤55 FU 47,1 mo (IQR 17.12 - 62.29) D: >55 FU 40,8 mo (IQR 7.82 - 40.83), +censored

**Copyright Information:** (Online) ISSN: 2572-9241
©2026 The Author(s). HemaSphere is published by John Wiley & Sons Ltd on behalf of European Hematology Association. This is an open access Abstract Book distributed under the Attribution-NonCommercial-NoDerivs (CC BY-NC-ND), which allows third parties to download the articles and share them with others as long as they credit the author and the Abstract Book, but they cannot change the content in any way or use them commercially.
**Abstract Book Citations:** Authors, Title, HemaSphere, 2026;10:(S1):pages. The abstract book DOIs can be found on the "Author Information" page.
**Disclaimer:** Articles published in the journal HemaSphere exclusively reflect the opinions of the authors. The authors are responsible for all content in their abstracts including accuracy of the facts, statements, citing resources, etc.

### (PS1494) A REAL-WORLD STUDY OF INOTUZUMAB OZOGAMICIN COMBINED WITH VENETOCLAX AS FRONTLINE THERAPY FOR NEWLY-DIAGNOSED PH-NEGATIVE B-CELL ACUTE LYMPHOBLASTIC LEUKEMIA PATIENTS WITH SEVERE COMORBIDITY

**Topic:** 02. Acute lymphoblastic leukemia - Clinical

Yuchen Yan*[1], Cheng Wang[1], Weiyang Liu[1], Li-Jun Peng[1], Jiayi Ren[1], Yuanfang Liu[1], Jie Hao[2], Duohui Jing[1], Weiping Zhang[1], Jian-qing Mi[1], Jin Wang[1]

*[1]Shanghai Institute of Hematology, State Key Laboratory of Medical Genomics, National Research Centre for Translational Medicine at Shanghai, Ruijin Hospital Affiliated to Shanghai Jiao Tong University School of Medicine, Shanghai, China;*
*[2]Shanghai North Station Hospital, Shanghai, China;*

### Background

Chemotherapy is globally applicable for induction therapy of newly-diagnosed Ph-Negative B-cell acute lymphoblastic leukemia(B-ALL). However, some patients couldn't tolerate chemotherapy due to severe comorbidity. Inotuzumab ozogamicin(INO) is an antibody-drug conjugate for CD22-positive B-ALL. Previous study proved combination of INO and venetoclax (VEN) as chemo-free therapy had high efficacy in salvage therapy of relapse/refractory B-ALL. However, the efficacy and safety of the combination of INO and VEN as frontline therapy has not been reported.

### Aims

Herein, we present the clinical outcomes of the combination of INO and VEN for newly-diagnosed adult B-ALL patients with severe comorbidity in real-world study. (ClinicalTrials.gov number: NCT07238907)

### Methods

In this study, newly-diagnosed B-ALL patients treated by INO+VEN as chemo-free induction therapy were analyzed. 1 cycle of induction cycle consists of INO 1mg at day1, day8, day15; VEN 100mg at day1, 200mg at day2, 400mg from day3 to day21 orally; Methylprednisolone 1mg/kg for 2 weeks followed by a 2-weeks taper. CD22 positivity is defined as CD22 expression ≥20% on bone marrow lymphoblasts by multiparameter flow cytometry (MFC). Efficacy evaluation was assessed after 1 cycle of INO+VEN therapy. Minimal residual disease (MRD) is routinely monitored by MFC, and MRD negativity is defined as no detectable leukemic cells with a sensibility of $10^{-4}$. Adverse events (AEs) were assessed according to CTCAE v5.0.

**Copyright Information:** (Online) ISSN: 2572-9241
©2026 The Author(s). HemaSphere is published by John Wiley & Sons Ltd on behalf of European Hematology Association. This is an open access Abstract Book distributed under the Attribution-NonCommercial-NoDerivs (CC BY-NC-ND), which allows third parties to download the articles and share them with others as long as they credit the author and the Abstract Book, but they cannot change the content in any way or use them commercially.
**Abstract Book Citations:** Authors, Title, HemaSphere, 2026;10:(S1):pages. The abstract book DOIs can be found on the "Author Information" page.
**Disclaimer:** Articles published in the journal HemaSphere exclusively reflect the opinions of the authors. The authors are responsible for all content in their abstracts including accuracy of the facts, statements, citing resources, etc.

**Results**

From March 2025 to December 2025, a total of 8 newly-diagnosed Ph-negative B-ALL patients received INO+VEN as chemo-free induction therapy. The median age was 41 years old (range, 15-74), and 6 patients were female. All patients were CD22 positive, 6 (75%) patients were classified as genetic high-risk. Eligible patients received allogeneic hematopoietic stem cell transplantation (allo-HSCT) after consolidation therapy. After 1 cycle, overall response rate (ORR) was 100%. complete remission (CR) was achieved in 5 patients (62.5%), and complete remission with incomplete hematologic recovery (CRi) was achieved in 3 patients (37.5%). The MRD was negative in 5 patients (62.5%). After 2 cycles, MRD negativity was promoted to 87.5%. The baseline characteristics and responses of patients are shown in the table 1.
Up to February 2026, the median follow-up time was 6 months. The 6-months Overall Survival (OS) and Relapse-Free Survival (RFS) were both 100%. 1 patient (12.5%) was bridged to allo-HSCT after consolidation therapy, 7 patients received consolidation therapy. The safety profile of INO+VEN was acceptable, with no observed veno-occlusive disease (VOD) or tumor lysis syndrome (TLS). Grade 3–4 AEs were predominantly hematological, including grade 4 neutropenia (n=4) and grade 3–4 thrombocytopenia (n=3). Non-hematological AEs were mostly grade 1–2 and reversible, such as elevated transaminase (n=3) and bilirubin (n=2). No treatment-related mortality occurred.

**Summary/Conclusion**

Here, we first reported the combination of INO and VEN as chemo-free induction therapy for newly-diagnosed Ph- B-ALL patients with severe comorbidity. Despite most patients were genetic high-risk, this chemotherapy-free regimen achieved a high CR rate and demonstrated a favorable safety. These findings will be further validated through a large-scale prospective study.

**Copyright Information:** (Online) ISSN: 2572-9241
©2026 The Author(s). HemaSphere is published by John Wiley & Sons Ltd on behalf of European Hematology Association. This is an open access Abstract Book distributed under the Attribution-NonCommercial-NoDerivs (CC BY-NC-ND), which allows third parties to download the articles and share them with others as long as they credit the author and the Abstract Book, but they cannot change the content in any way or use them commercially.
**Abstract Book Citations:** Authors, Title, HemaSphere, 2026;10:(S1):pages. The abstract book DOIs can be found on the "Author Information" page.
**Disclaimer:** Articles published in the journal HemaSphere exclusively reflect the opinions of the authors. The authors are responsible for all content in their abstracts including accuracy of the facts, statements, citing resources, etc.

| Number | Age | Gender | WBC count at diagnosis (×$10^9$/L) | Comorbidity | Molecular abberation | Response after 1 cycle | MRD after 1 cycle |
|---|---|---|---|---|---|---|---|
| 1 | 46 | female | 4.6 | Bilateral suppurative otitis media | MTAP::CDKN2B-AS1, PAX5 p.G183S (VAF=78.2%) | CRi | Negative |
| 2 | 53 | female | 1.86 | Active Crohn's disease | None | CRi | Negative |
| 3 | 74 | female | 2.97 | Active tuberculosis | IDH1 p.R132G (VAF=2%) | CRi | Negative |
| 4 | 63 | female | 6.06 | pulmonary embolism, Deep vein thrombosis of the lower extremities | KMT2A::AFF1 | CR | Negative |
| 5 | 15 | male | 8.27 | Paraneoplastic syndrome, cachexia | TP53 p.R280G (VAF=11.3%) | CR | 0.03% |
| 6 | 51 | female | 1.11 | Intracranial arterial malformation, Intracranial atherosclerosis | CRLF2::IGH, JAK1 p.Y940F (VAF=33.9%), JAK2 p.R683K (VAF=2.9%) | CR | Negative |
| 7 | 60 | male | 17.5 | gastric malignancies, cachexia | PAX5::JAK2 | CR | 1.10% |
| 8 | 27 | female | 24.29 | Abortion after 24 weeks' gestation | KMT2A::AFF1 | CR | 0.08% |

Table 1. Baseline characteristics and responses of all patients

Copyright Information: (Online) ISSN: 2572-9241
©2026 The Author(s). HemaSphere is published by John Wiley & Sons Ltd on behalf of European Hematology Association. This is an open access Abstract Book distributed under the Attribution-NonCommercial-NoDerivs (CC BY-NC-ND), which allows third parties to download the articles and share them with others as long as they credit the author and the Abstract Book, but they cannot change the content in any way or use them commercially.
Abstract Book Citations: Authors, Title, HemaSphere, 2026;10:(S1):pages. The abstract book DOIs can be found on the "Author Information" page.
Disclaimer: Articles published in the journal HemaSphere exclusively reflect the opinions of the authors. The authors are responsible for all content in their abstracts including accuracy of the facts, statements, citing resources, etc.

### (PS1495) FATIGUE AND HEALTH-RELATED QUALITY OF LIFE IN PATIENTS WITH NEWLY DIAGNOSED PH+ ALL TREATED WITH FRONTLINE PONATINIB PLUS BLINATUMOMAB. RESULTS OF THE PHASE III GIMEMA ALL2820 TRIAL

**Topic:** 02. Acute lymphoblastic leukemia - Clinical

Fabio Efficace*[1], Laura Cannella[1], Daniela Krepper[1], Cristina Skert[2], Erika Borlenghi[3], Francesco Di Raimondo[4], Ludovica Fucci[5], Federico Lussana[6], Caterina Alati[7], Patrizia Chiusolo[8], Daniele Mattei[9], Valentina Mancini[10], Barbara Scappini[11], Maria Paola Martelli[12], Bianca Serio[13], Ernesta Audisio[14], Mario Luppi[15], Antonella Cucca[16], Prassede Salutari[17], Daniele Vallisa[18], Alessandro Rambaldi[6], Paola Fazi[1], Robin Foà[5], Sabina Chiaretti[5]

*[1]Italian Group for Adult Hematologic Diseases (GIMEMA), Data Center and Health Outcomes Research Unit, Rome, Italy, Rome, Italy; [2]Hematology Unit, Azienda Ulss3 Serenissima, Ospedale dell'Angelo, Mestre Venezia, Italy; [3]Department of Hematology, ASST Spedali Civili of Brescia, Brescia, Italy; [4]Division of Hematology, Azienda Policlinico-S. Marco, University of Catania, Catania, Italy; [5]Hematology, Department of Translational and Precision Medicine, Sapienza University, Rome, Italy; [6]Department of Oncology and Hematology, University of Milan and ASST Papa Giovanni XXIII, Bergamo, Italy; [7]Hematology and Stem Cell Transplantation and Cellular Therapies Unit (CTMO), Department of Hemato-Oncology and Radiotherapy, Bianchi-Melacrino-Morelli Hospital, Reggio Calabria, Italy; [8]Department of Radiological and Hematological Sciences, A. Gemelli University Polyclinic Foundation IRCCS, Catholic University of the Sacred Heart, Rome, Italy; [9]Complex Structure of Hematology, ASO S. Croce e Carle, Cuneo, Italy; [10]Department of Hematology and Oncology, ASST Grande Ospedale Metropolitano Niguarda, Milan, Italy; [11]Hematology Department, Azienda Ospedaliero-Universitaria Careggi, Florence, Italy; [12]Hematology and Clinical Immunology Division, Department of Medicine and Surgery, University of Perugia, Perugia, Italy; [13]Hematology and Bone Marrow Transplantation Unit, AOU San Giovanni di Dio Ruggi d'Aragona, Department of Medicine and Surgery, University of Salerno, Salerno, Italy; [14]SC Hematology, AOU Città della Salute e della Scienza, Turin, Italy; [15]Section of Hematology, Department of Medical and Surgical Sciences, University of Modena and Reggio Emilia, AOU Modena, Modena, Italy; [16]Hematology and Bone Marrow Transplant Unit, POU San Francesco Hospital, Nuoro, Italy; [17]Department of Hematology, Ospedale Civile Spirito Santo, Pescara, Italy; [18]Hematology and Bone Marrow Transplant Unit, Department of Oncology and Hematology, Guglielmo da Saliceto Hospital, Piacenza, Italy;*

### Background

The phase III GIMEMA ALL2820 trial, designed for newly-diagnosed Philadelphia–positive acute lymphoblastic leukemia (Ph+ ALL) adult patients with no upper age limit, is showing a significantly superior clinical efficacy of a targeted chemo-free approach based on ponatinib plus blinatumomab compared to imatinib plus chemotherapy (Chiaretti S, et al. ASH 2025, abs 439). Evidence regarding the impact of a chemo-free strategy on patient-reported outcomes (PROs) remains very limited.

### Aims

Fatigue and health-related quality of life (HRQoL) were prespecified secondary endpoints of the phase III GIMEMA ALL2820 trial. We report the comparative findings between treatment arms.

**Copyright Information:** (Online) ISSN: 2572-9241
©2026 The Author(s). HemaSphere is published by John Wiley & Sons Ltd on behalf of European Hematology Association. This is an open access Abstract Book distributed under the Attribution-NonCommercial-NoDerivs (CC BY-NC-ND), which allows third parties to download the articles and share them with others as long as they credit the author and the Abstract Book, but they cannot change the content in any way or use them commercially.
**Abstract Book Citations:** Authors, Title, HemaSphere, 2026;10:(S1):pages. The abstract book DOIs can be found on the "Author Information" page.
**Disclaimer:** Articles published in the journal HemaSphere exclusively reflect the opinions of the authors. The authors are responsible for all content in their abstracts including accuracy of the facts, statements, citing resources, etc.

### Methods

Patients were randomized in a 2:1 fashion to ponatinib (45/30 mg according to age) plus ≥2 cycles of blinatumomab or to imatinib (800/600 mg according to age) plus systemic chemotherapy. The pre-specified primary PRO endpoint was fatigue, measured using the validated FACIT-Fatigue questionnaire. This 13-item measure assesses self-reported tiredness, weakness, and difficulty in participating in usual activities due to fatigue. Based on established thresholds, a difference in the FACIT-Fatigue scores of at least 3 points was considered to be clinically meaningful. In addition, HRQoL was assessed with the EORTC QLQ-C30 questionnaire. PRO assessments were performed at pre-specified timepoints. Mean trajectories from baseline to day +70 (end of induction) and day +133 (after 2 cycles of blinatumomab/therapy) were evaluated in this report. For the purpose of this analysis, between-arm differences in EORTC QLQ-C30 scales at day +133 are also presented. Longitudinal trajectories and between-arm differences were estimated using linear mixed-effects models. All patients provided written informed consent.

### Results

A total of 236 patients were randomized in a 2:1 ratio to the chemo-free experimental arm (n = 158) and to the chemotherapy control arm (n = 78). The median age was 56 years (IQR 45.3–66.7); 125 patients (53.0%) were male. Overall questionnaire compliance was 95.3% at baseline, 83.9% at day +70, and 78.7% at day +133. From baseline to day +133, mean change of the FACIT-Fatigue score was 0.6 points in the chemotherapy arm and 4.4 points in the chemo-free arm, yielding a between-arm difference in change of 3.8 points, exceeding the pre-defined threshold for clinical meaningfulness and reaching statistical significance ($p = 0.018$). At day +133 patients treated with ponatinib–blinatumomab reported significantly lower fatigue (higher FACIT-F scores) compared with imatinib plus chemotherapy (mean difference 4.6 points; $p = 0.005$). Statistically and clinically significant differences favoring the ponatinib–blinatumomab approach were also observed at day +133 for the following EORTC QLQ-C30 scales: global health status/QoL ($\Delta$ =7.8 points; $p = 0.044$), role functioning ($\Delta$ = 11.4 points; $p = 0.022$), fatigue ($\Delta$ = 9.4 points; $p = 0.022$), nausea/vomiting ($\Delta$ = 8.4 points; $p = 0.001$), and diarrhea ($\Delta$ = 6.3 points; $p = 0.020$).

### Summary/Conclusion

The frontline ponatinib plus blinatumomab strategy is associated with significantly improved patient-reported fatigue and HRQoL compared to imatinib plus chemotherapy in newly diagnosed adult Ph+ ALL patients. These findings further support the adoption of this chemotherapy-free strategy as a new standard of care in this setting.

**Copyright Information:** (Online) ISSN: 2572-9241
©2026 The Author(s). HemaSphere is published by John Wiley & Sons Ltd on behalf of European Hematology Association. This is an open access Abstract Book distributed under the Attribution-NonCommercial-NoDerivs (CC BY-NC-ND), which allows third parties to download the articles and share them with others as long as they credit the author and the Abstract Book, but they cannot change the content in any way or use them commercially.
**Abstract Book Citations:** Authors, Title, HemaSphere, 2026;10:(S1):pages. The abstract book DOIs can be found on the "Author Information" page.
**Disclaimer:** Articles published in the journal HemaSphere exclusively reflect the opinions of the authors. The authors are responsible for all content in their abstracts including accuracy of the facts, statements, citing resources, etc.

### (PS1496) EFFICACY AND SAFETY OF FLUMATINIB COMBINED WITH CHEMOTHERAPY IN NEWLY DIAGNOSED ADULT PHILADELPHIA CHROMOSOME-POSITIVE ACUTE LYMPHOBLASTIC LEUKEMIA

**Topic:** 02. Acute lymphoblastic leukemia - Clinical

Weimin Wang*[1], Shuyu Han[1], Yan Yang[1], Yanan Wang[1], Jieke Cui[1], Yuxia Dai[1], Silin Gan[1], Hui Sun[1]

[1] *the first affiliated hospital of Zhengzhou University, Zhengzhou, China;*

### Background

Philadelphia chromosome-positive acute lymphoblastic leukemia (Ph+ ALL) is a highly aggressive hematologic malignancy and the most common subtype of adult ALL. The integration of tyrosine kinase inhibitors (TKIs) has significantly improved clinical outcomes, establishing TKI-based combination chemotherapy as the standard first-line therapy. Flumatinib, a domestically developed second-generation TKI, has demonstrated promising clinical efficacy; however, its comprehensive safety and efficacy profile in newly diagnosed adult Ph+ ALL requires further elucidation.

### Aims

This study investigates the efficacy and safety of Flumatinib Combined with Chemotherapy in Newly Diagnosed Adult Ph+ ALL.

### Methods

We retrospectively collected clinical data from 73 adult patients newly diagnosed with Ph+ ALL between April 2020 and June 2025 at The First Affiliated Hospital of Zhengzhou University, utilizing inpatient and outpatient medical records. Baseline characteristics included age, gender, leukocyte count, bone marrow blast percentage, fusion transcript type, cytogenetics, mutational status, and transplant history. Treatment responses were evaluated, encompassing the 3-month complete molecular response (CMR) rate, minimal residual disease (MRD) negativity, overall survival (OS), relapse-free survival (RFS), relapse incidence, adverse events, and central nervous system leukemia (CNSL) status, to identify prognostic determinants.

### Results

Among 73 newly diagnosed adult Ph+ ALL patients (median age 45 years [range 15-68]; 48% male), the median initial WBC was $26.47\times10^9$/L (range $0.35$-$365.92\times10^9$/L), with hyperleukocytosis ($\geq30\times10^9$/L) in 42.5%. The average percentage of bone marrow blasts was 89.8%,(range 24.8% to 99.5%). BCR-ABL1 isoforms: p190 in 68.5%, p210 in 28.7%, and rare E1A3 in 2.8%.
Cytogenetics: normal karyotype 12.1%, isolated Ph 48.5%, and complex karyotype 17.8%. Among 59 patients with next-generation sequencing, 30.1% harbored IKZF1 mutations, including 4 cases (5.5%) of IKZF1plus genotype (IKZF1 deletion plus CDKN2A/B/PAX5/PAR1 loss without ERG deletion). Allo-HSCT was performed in 32.8%.
Flumatinib (600 mg/d) combined with multiagent chemotherapy achieved CR rates of 100%, 98.6%, and 93.8% at 28 days, 3 months, and 6 months, respectively. MRD negativity by flow cytometry was 86.3%, 95.7%, and 92.1% at these timepoints. Major molecular response (MMR) rates were 67.1%, 84.3%, and 90.5%, while CMR rates reached 45.2%, 67.1%, and 79.4%.
The median follow-up duration was 32 months, The OS rates at 1 year, 2 years, and 3 years were 91.7%, 85.1%, and 81.2%, RFS rates were 80.5%, 67.1%, and 59.6%. IKZF1 mutation, advanced age, ABL1 kinase mutation, and CNSL are adverse prognostic factors for survival.
Hematologic adverse events were notable, with 91.8% of patients developing grade 3-4 neutropenia and 83.6% experiencing thrombocytopenia, with 100% grade 1-2 myelosuppression. Non-hematologic toxicities included gastrointestinal reactions (31.5% grade 1-2, 68.5% grade 3-4) and transaminase elevation (58.9% grade 1-2, 4% grade 3-4), all manageable with supportive care.

**Copyright Information:** (Online) ISSN: 2572-9241
©2026 The Author(s). HemaSphere is published by John Wiley & Sons Ltd on behalf of European Hematology Association. This is an open access Abstract Book distributed under the Attribution-NonCommercial-NoDerivs (CC BY-NC-ND), which allows third parties to download the articles and share them with others as long as they credit the author and the Abstract Book, but they cannot change the content in any way or use them commercially.
**Abstract Book Citations:** Authors, Title, HemaSphere, 2026;10:(S1):pages. The abstract book DOIs can be found on the "Author Information" page.
**Disclaimer:** Articles published in the journal HemaSphere exclusively reflect the opinions of the authors. The authors are responsible for all content in their abstracts including accuracy of the facts, statements, citing resources, etc.

**Summary/Conclusion**

Flumatinib-based combination chemotherapy represents an effective and safe therapeutic option for Ph+ ALL, further multicenter prospective validation for long-term efficacy optimization.

**Copyright Information:** (Online) ISSN: 2572-9241
©2026 The Author(s). HemaSphere is published by John Wiley & Sons Ltd on behalf of European Hematology Association. This is an open access Abstract Book distributed under the Attribution-NonCommercial-NoDerivs (CC BY-NC-ND), which allows third parties to download the articles and share them with others as long as they credit the author and the Abstract Book, but they cannot change the content in any way or use them commercially.
**Abstract Book Citations:** Authors, Title, HemaSphere, 2026;10:(S1):pages. The abstract book DOIs can be found on the "Author Information" page.
**Disclaimer:** Articles published in the journal HemaSphere exclusively reflect the opinions of the authors. The authors are responsible for all content in their abstracts including accuracy of the facts, statements, citing resources, etc.

### (PS1497) THE BLINATUMOMAB ERA IN PHILADELPHIA CHROMOSOME POSITIVE B-ACUTE LYMPHOBLASTIC LEUKEMIA (PH+ B-ALL): A SINGLE-CENTER ANALYSIS OF TREATMENT EVOLUTION AND SURVIVAL

**Topic:** 02. Acute lymphoblastic leukemia - Clinical

Valerie Hui Shan Tan*[1], Jing Yuan Tan[1], Zhentang Lao[1], Esmeralda Teo[1], Chieh Hwee Ang[1], Jeffrey Quek[1], Francesca Lorraine Wei Inng Lim[1], Than Hein[1], Tertius Tuy[1], Lawrence Cheng Kiat Ng[1], Gee Fung How[1], William Hwang[1], Aloysius Ho[1], Yeh Ching Linn[1], Gee Chuan Wong[1], Abdul Halim Nurul Aidah[1]

[1]*Singapore General Hospital, Haematology, Singapore, Singapore;*

### Background

Ph+ B-ALL has historically been associated with dismal outcomes (<20% 5-year survival). The advent of BCR-ABL tyrosine kinase inhibitors (TKIs), such as imatinib, dasatinib, ponatinib, and introduction of blinatumomab have greatly improved survival.

### Aims

We analyzed our center's 15-year experience to assess the impact of these advances on patient outcomes.

### Methods

Our center saw a total of 107 adult Ph+ B-ALL patients from 2010 to 2025. For this analysis, we included patients who attained CR with induction chemotherapy and remained alive at 3 months after diagnosis. Overall survival (OS) and progression-free survival (PFS) were analyzed by Kaplan–Meier methods and log-rank tests. Outcomes were evaluated at a 3-year landmark to account for differences in follow-up duration between historical (Imatinib) and modern (Dasatinib/Blinatumomab) treatment eras (median follow-up: 68 months). Complete molecular response (CMR) was defined as BCR-ABL ≤ 0.01%.

### Results

89 patients were included for analysis. Median age was 48.2 (Range 18.1–78.6). 42 (47.2%) were male. 55 patients (62%) underwent allogeneic transplant (alloHSCT) at CR1. 25 (36.20%) patients received hyperCVAD + imatinib, 45 (63.80%) patients received hyperCVAD + dasatinib, 23 (25.8%) patients had blinatumomab incorporated into frontline treatment (median 2 cycles). Patients with blinatumomab incorporated into frontline treatment had significantly higher 3-year OS (91.3% vs 59.4%, p=0.0468) and 3-year PFS (85.6% vs 40.0%, p=0.0042) (Figures 1A and 1B). In the transplanted subgroup (n=55), patients who received blinatumomab pre-transplant had significantly higher 3-year PFS (90.0% vs 39.9%, p=0.0485) and a trend toward improved OS (90.0% vs 65.7%, p=0.177) though this was non significant. Among blinatumomab-treated patients, 3-year OS did not differ between those who underwent alloHSCT (n=10) and those who did not (n=13) (90.0% vs 92.3%, p=0.605) (Figures 1C and 1D). Among patients receiving hyperCVAD + TKI, 90-day CMR was higher with dasatinib than imatinib (77.3% vs 56.0%, p=0.082). Overall, 61 patients (69%) achieved 90-day CMR; within this group, 3-year OS did not differ between transplanted and non-transplanted patients (66.0% vs 62.9%, p=0.593). Rates of bacteremia and fungemia did not differ by blinatumomab use or TKI. The only notable difference was higher pre-transplant CMV in blinatumomab-treated patients (30.0% vs 4.4%, p=0.037).
CNS relapse occurred in 6 patients (6.7%), 2 received hyperCVAD + imatinib, 3 received hyperCVAD + dasatinib and 1 patient received EWALL + dasatinib. Of note, 2 out of 6 patients had CNS involvement at diagnosis. None of the 6 patients had received blinatumomab in frontline. Despite improved CNS penetration of dasatinib, sanctuary-site relapse remains a clinically relevant pattern of treatment failure, underscoring the continued importance of CNS-directed prophylaxis and disease control strategies.

**Copyright Information:** (Online) ISSN: 2572-9241
©2026 The Author(s). HemaSphere is published by John Wiley & Sons Ltd on behalf of European Hematology Association. This is an open access Abstract Book distributed under the Attribution-NonCommercial-NoDerivs (CC BY-NC-ND), which allows third parties to download the articles and share them with others as long as they credit the author and the Abstract Book, but they cannot change the content in any way or use them commercially.
**Abstract Book Citations:** Authors, Title, HemaSphere, 2026;10:(S1):pages. The abstract book DOIs can be found on the "Author Information" page.
**Disclaimer:** Articles published in the journal HemaSphere exclusively reflect the opinions of the authors. The authors are responsible for all content in their abstracts including accuracy of the facts, statements, citing resources, etc.

### Summary/Conclusion

Overall, survival outcomes for Ph+ B-ALL has significantly improved with the frontline incorporation of blinatumomab. Our study is one of the few real world Asian studies evaluating survival outcomes and supports continued evolution toward frontline TKI–immunotherapy strategies in Ph+ ALL.

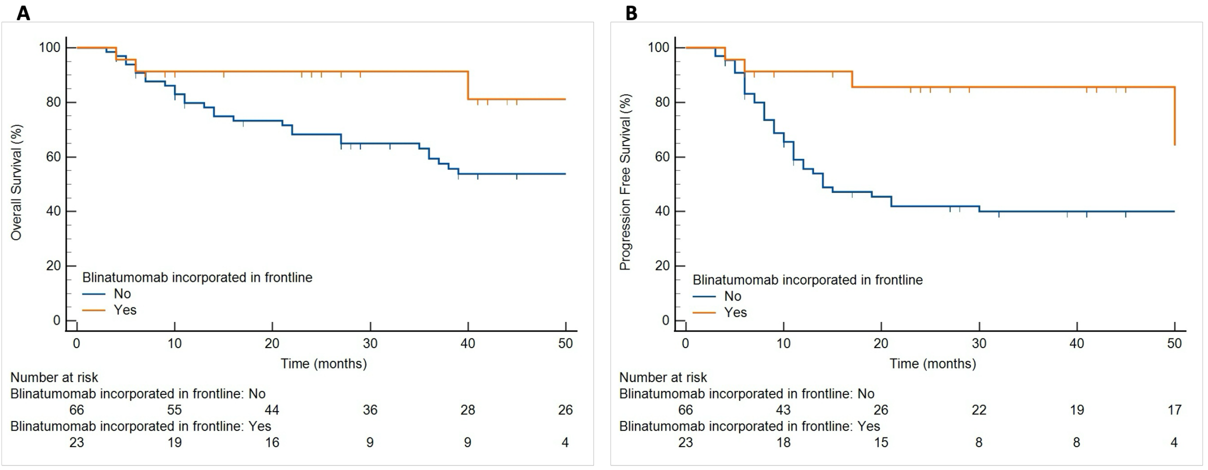

**Figures 1A and 1B:** Survival outcomes for patients receiving front-line blinatumomab vs those who did not

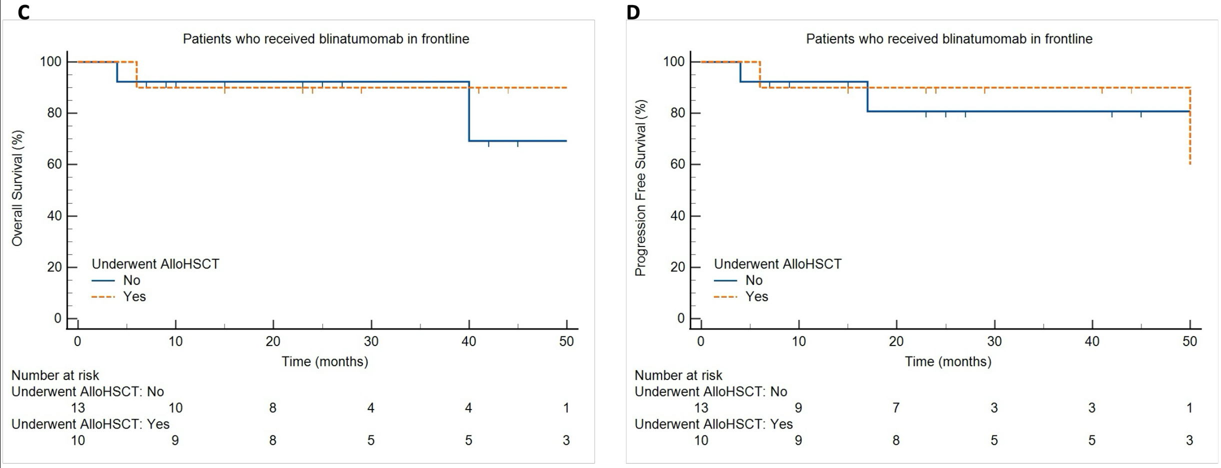

**Figures 1C and 1D**: Survival outcomes of patients who received front-line blinatumomab and underwent AlloHSCT vs those who did not.

**Copyright Information:** (Online) ISSN: 2572-9241
©2026 The Author(s). HemaSphere is published by John Wiley & Sons Ltd on behalf of European Hematology Association. This is an open access Abstract Book distributed under the Attribution-NonCommercial-NoDerivs (CC BY-NC-ND), which allows third parties to download the articles and share them with others as long as they credit the author and the Abstract Book, but they cannot change the content in any way or use them commercially.
**Abstract Book Citations:** Authors, Title, HemaSphere, 2026;10:(S1):pages. The abstract book DOIs can be found on the "Author Information" page.
**Disclaimer:** Articles published in the journal HemaSphere exclusively reflect the opinions of the authors. The authors are responsible for all content in their abstracts including accuracy of the facts, statements, citing resources, etc.

### (PS1498) ADJUSTED COMPARISON OF PEDIATRIC AND ADULT TREATMENT REGIMENS IN ACUTE LYMPHOBLASTIC LEUKEMIA (ALL): RESULTS OF A MAIC ANALYSIS IN A LATIN AMERICAN COHORT

**Topic:** 02. Acute lymphoblastic leukemia - Clinical

Claudia Valeria Silva Tirado*[1], Janeth Elena Araujo Cárdenas[2], Oscar Bustos Salgado[1], Alvaro Cabrera[1], Christian Ramos[1], Gilberto Israel Barranco Lampon[1]

*[1]HOSPITAL GENERAL DE MEXICO, CIUDAD DE MEXICO, CDMX, Mexico;[2]INSTITUTO NACIONAL DE CANCEROLOGIA, CIUDAD DE MEXICO, CDMX, Mexico;*

### Background

In adolescent and young adult (AYA) patients with acute lymphoblastic leukemia (ALL), pediatric-inspired regimens have demonstrated superior efficacy compared with treatment protocols originally designed for adults. In particular, the CALGB 10403 protocol has shown higher complete remission rates and improved overall survival compared with regimens such as Hyper-CVAD. However, in Latin America, its implementation faces significant challenges, including limited access to key therapeutic agents, constraints in specialized treatment units, and the lack of standardized molecular monitoring.

### Aims

Compare clinical outcomes (mortality, relapse, and complete remission) among adults with acute lymphoblastic leukemia (ALL) treated with pediatric-inspired regimens (CALGB 10403) versus adult regimens (Hyper-CVAD) across four public hospitals in Mexico, adjusting for prognostic variables through a Matching-Adjusted Indirect Comparison (MAIC) analysis.

### Methods

This retrospective, observational, and descriptive study included adults diagnosed with acute lymphoblastic leukemia (ALL) who were treated between 2018 and 2023 at Hospital General de México, Instituto Nacional de Cancerología (INCan), Hospital Regional de Alta Especialidad de Ixtapaluca (HRAEI), and Hospital Juárez de México. Patients who received treatment with either CALGB 10403 or Hyper-CVAD were included. To ensure comparability between groups, a Matching-Adjusted Indirect Comparison (MAIC) analysis was performed, adjusting for age, clinical risk category, immunophenotype, presence of BCR-ABL1, baseline leukocytosis, central nervous system (CNS) involvement, and measurable residual disease (MRD) status. Adjusted odds ratios (ORs) were calculated for mortality, relapse, and complete remission.

### Results

A total of 511 adult patients with acute lymphoblastic leukemia (ALL) were analyzed. Of these, 71.6% (n = 366) were treated with adult regimens and 28.4% (n = 145) with a pediatric-inspired regimen. Sex distribution was balanced (49.1% female, 50.9% male). The B-cell immunophenotype was the most frequent (95.4%), while the T-cell variant was observed in 4.4% of cases. BCR-ABL1 positivity was identified in 9.3% of patients. Patients treated with CALGB 10403 showed lower mortality compared with those receiving Hyper-CVAD (OR 0.24; 95% CI: 0.16–0.37; $p < 0.001$). No significant differences were observed in relapse rates (OR 1.20; 95% CI: 0.80–1.81; $p = 0.379$). Age under 35 years was associated with lower mortality (OR 0.49) but with a higher risk of relapse (OR 1.81). Positive measurable residual disease (MRD) was associated with increased mortality (OR 3.85) and higher relapse risk (OR 4.63); lack of MRD assessment was also associated with increased mortality (OR 2.39). After MAIC adjustment, lower mortality with CALGB 10403 persisted (adjusted OR 0.38; 95% CI: 0.22–0.66; $p < 0.001$), with no significant differences in relapse (OR 1.14; 95% CI: 0.74–1.76) or complete remission rates (60.0% vs. 57.1%).

**Copyright Information:** (Online) ISSN: 2572-9241
©2026 The Author(s). HemaSphere is published by John Wiley & Sons Ltd on behalf of European Hematology Association. This is an open access Abstract Book distributed under the Attribution-NonCommercial-NoDerivs (CC BY-NC-ND), which allows third parties to download the articles and share them with others as long as they credit the author and the Abstract Book, but they cannot change the content in any way or use them commercially.
**Abstract Book Citations:** Authors, Title, HemaSphere, 2026;10:(S1):pages. The abstract book DOIs can be found on the “Author Information” page.
**Disclaimer:** Articles published in the journal HemaSphere exclusively reflect the opinions of the authors. The authors are responsible for all content in their abstracts including accuracy of the facts, statements, citing resources, etc.

**Summary/Conclusion**

The pediatric-inspired regimen CALGB 10403 was associated with a significant reduction in mortality among young adults with acute lymphoblastic leukemia (ALL), without an increase in relapse risk, even after adjustment for clinical risk factors. These findings support its adoption in the AYA population in resource-limited settings, provided that adequate access to essential medications and measurable residual disease (MRD) monitoring is ensured.

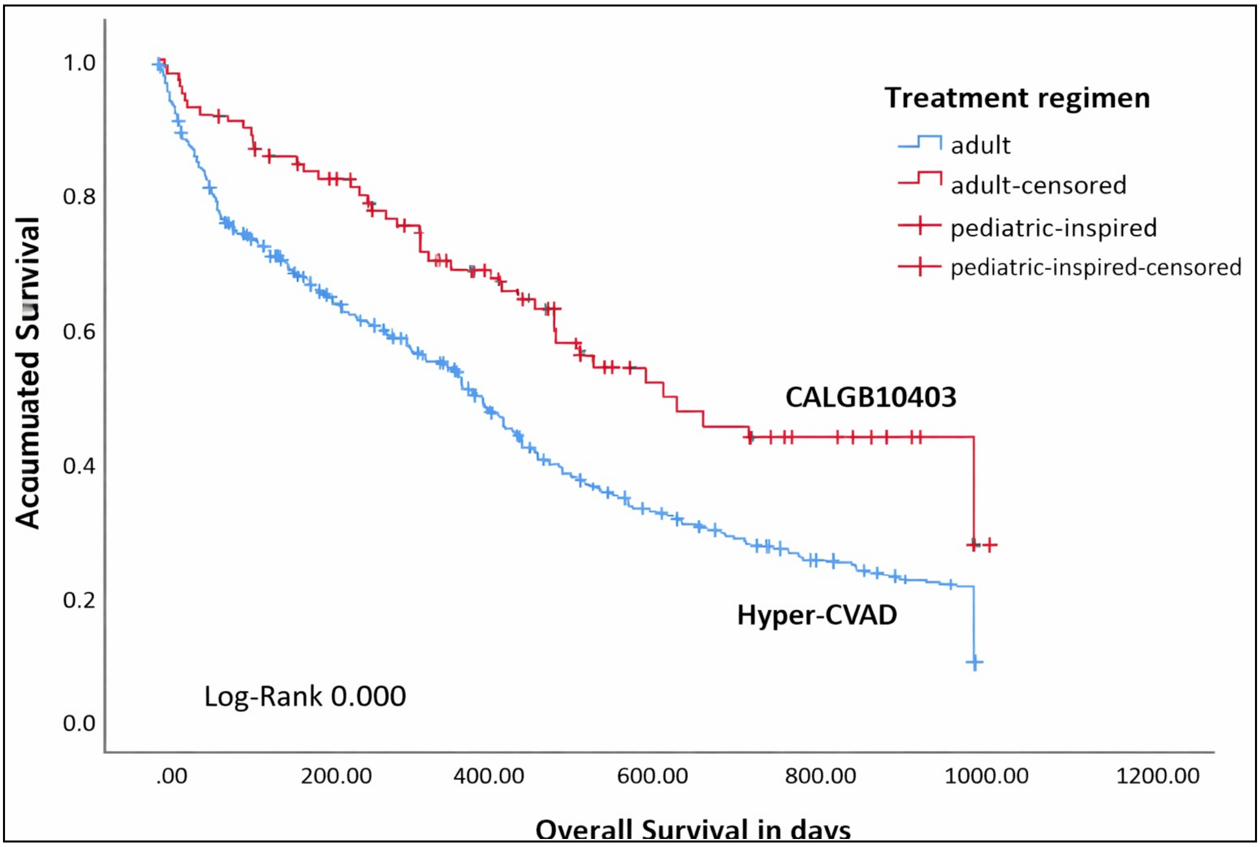

**Copyright Information:** (Online) ISSN: 2572-9241
©2026 The Author(s). HemaSphere is published by John Wiley & Sons Ltd on behalf of European Hematology Association. This is an open access Abstract Book distributed under the Attribution-NonCommercial-NoDerivs (CC BY-NC-ND), which allows third parties to download the articles and share them with others as long as they credit the author and the Abstract Book, but they cannot change the content in any way or use them commercially.
**Abstract Book Citations:** Authors, Title, HemaSphere, 2026;10:(S1):pages. The abstract book DOIs can be found on the "Author Information" page.
**Disclaimer:** Articles published in the journal HemaSphere exclusively reflect the opinions of the authors. The authors are responsible for all content in their abstracts including accuracy of the facts, statements, citing resources, etc.

### (PS1499) DONOR-DERIVED CAR-T THERAPY FOR RELAPSE OF B-ALL AFTER SECOND ALLOGENEIC HEMATOPOIETIC STEM CELL TRANSPLANTATION

**Topic:** 02. Acute lymphoblastic leukemia - Clinical

Biyi Li[1], Yuehui Lin[1], Weiya Li[2], Shuang You Liu[1], Biping Deng[1], Chunrong Tong[1], Tong Wu[1], Fengyan Jin[2], Zhao Defeng*[1]

*[1]Department of Hematology, Beijing Boren Hospital, Beijing, China;[2]Department of Hematology, the First Hospital of Jilin University, Hematology, Changchun, China;*

### Background

Patients with B-cell acute lymphoblastic leukemia (B-ALL) who relapse after a second allogeneic hematopoietic stem cell transplantation (allo-HSCT) have an extremely poor prognosis, and evidence regarding donor-derived CAR-T therapy in this setting remains limited.

### Aims

To investigate the efficacy and safety of donor-derived CAR-T cell therapyfor relapse of B-ALL after second allogeneic hematopoietic stem cell transplantation

### Methods

We analyzed 10 patients with relapsed B-ALL after second allo-HSCT who received donor-derived CD19- or CD22-directed CAR-T therapy. Target selection was based on antigen expression at relapse. Bridging therapy and individualized lymphodepletion were permitted. Efficacy and safety were evaluated following CAR-T infusion.

### Results

The median age was 33 years (range, 5-58); 6 patients were male. One patient (10%) harbored TP53 mutation, 2 (20%) had BCR::ABL1, and 4 (40%) had extramedullary disease (EMD). At first HSCT, 7 patients received haploidentical grafts and 3 received matched unrelated donor (MUD) transplants; at second HSCT, 9 underwent haploidentical and one MUD transplantation. Median time from first and second HSCT to relapse was 8.4 months (range, 2.3-29.9) and 5.3 months (range, 0-17.7), respectively. Patients had received a median of four prior treatment lines (range, 2-8), and seven had prior CAR-T exposure.
All patients (100%) achieved bone marrow complete remission (CR), including 8 (80%) with MRD negativity. Among 4 patients with EMD, 2 achieved CR, one was not evaluable, and one with CD19/CD22-negative myeloid sarcoma progressed. Median time to best response was 14 days (range, 14-60). With a median follow-up of 24.3 months, median disease-free survival (DFS) was 2.9 months (95%CI, 1.4-not reached) and median overall survival (OS) was 10.9 months (95%CI, 5.1-not reached). Toxicities were manageable. Cytokine release syndrome occurred in 10 patients (100%), predominantly grade 1-2, and no treatment-related mortality was observed. One patient experienced exacerbation of pre-existing grade 2 skin acute GVHD after infusion.

### Summary/Conclusion

Donor-derived CAR-T therapy is a feasible and effective salvage option for patients with B-ALL relapsing after a second allo-HSCT, with manageable toxicity.

**Copyright Information:** (Online) ISSN: 2572-9241
©2026 The Author(s). HemaSphere is published by John Wiley & Sons Ltd on behalf of European Hematology Association. This is an open access Abstract Book distributed under the Attribution-NonCommercial-NoDerivs (CC BY-NC-ND), which allows third parties to download the articles and share them with others as long as they credit the author and the Abstract Book, but they cannot change the content in any way or use them commercially.
**Abstract Book Citations:** Authors, Title, HemaSphere, 2026;10:(S1):pages. The abstract book DOIs can be found on the "Author Information" page.
**Disclaimer:** Articles published in the journal HemaSphere exclusively reflect the opinions of the authors. The authors are responsible for all content in their abstracts including accuracy of the facts, statements, citing resources, etc.

### (PS1500) PROGNOSIS AND TREATMENT-RELATED TOXICITIES IN DOWN SYNDROME ACUTE LYMPHOBLASTIC LEUKEMIA IN JCCG ALL-B12

**Topic:** 02. Acute lymphoblastic leukemia - Clinical

Shunsuke Yamamoto*[1], Motohiro Kato[2], Yuka Iijima-Yamashita[3], Masashi Sanada[3], Toshihiko Imamura[4], Akiko Kada[3, 5], Akiko M. Saito[3], Katsuyoshi Koh[6], Yasuhiro Okamoto[7]

*[1]Graduate School of Medical Sciences, Kyushu University, Department of Pediatrics, Fukuoka, Japan;[2]The University of Tokyo, Department of Pediatrics, Tokyo, Japan; [3]NHO Nagoya Medical Center, Clinical Research Center, Nagoya, Japan;[4]Kyoto Prefectural University of Medicine, Department of Pediatrics, Kyoto, Japan; [5]Fujita Health University, Center For Translational Research, Toyoake, Japan; [6]Saitama Children's Medical Center, Department of Hematology/Oncology, Saitama, Japan; [7]Kagoshima University Hospital, Department of Pediatrics, Kagoshima, Japan;*

### Background

Children with Down syndrome (DS) have an elevated risk of acute lymphoblastic leukemia (ALL), and survival remains inferior to that of non-DS-ALL.

### Aims

To assess prognosis, relapse patterns, and treatment-related toxicities of DS-ALL within the nationwide, risk-adapted JCCG ALL-B12 trial.

### Methods

The cohort included 1,804 patients enrolled between November 2012 and November 2017, including 44 DS-ALL. Risk was assigned as standard (SR), intermediate (IR), or high (HR) using protocol-defined clinical, genetic, and minimal residual disease (MRD) criteria. Treatment followed ALL-B12, and non-DS SR received reduced-intensity induction and 2 g/m² high-dose methotrexate (HD-MTX) in consolidation; IR received early intensification with augmented asparaginase and 5 g/m² HD-MTX; HR entered a randomized comparison between block-type consolidation and an HD-MTX regimen at 5 g/m² per cycle. DS-ALL received protocol-specified MTX modifications (0.5 g/m² for the first consolidation course, then 1.0 g/m²). MRD was measured at the end of induction (TP1) and the end of consolidation (TP2), with $10^{-4}$ as the threshold. *CRLF2* rearrangement (CRLF2r) was determined by targeted DNA capture sequencing and MLPA of pseudoautosomal region 1. We performed analyses excluding cases with canonical favorable or unfavorable genetic subtypes. Primary endpoints were 5-year event-free survival (EFS) and overall survival (OS), estimated using the Kaplan–Meier method and the log-rank test. Grade ≥3 toxicities were graded by CTCAE v4.0 and summarized by treatment phase after allocation. The study was approved by institutional review boards and conducted in accordance with the Declaration of Helsinki.

**Copyright Information:** (Online) ISSN: 2572-9241
©2026 The Author(s). HemaSphere is published by John Wiley & Sons Ltd on behalf of European Hematology Association. This is an open access Abstract Book distributed under the Attribution-NonCommercial-NoDerivs (CC BY-NC-ND), which allows third parties to download the articles and share them with others as long as they credit the author and the Abstract Book, but they cannot change the content in any way or use them commercially.
**Abstract Book Citations:** Authors, Title, HemaSphere, 2026;10:(S1):pages. The abstract book DOIs can be found on the "Author Information" page.
**Disclaimer:** Articles published in the journal HemaSphere exclusively reflect the opinions of the authors. The authors are responsible for all content in their abstracts including accuracy of the facts, statements, citing resources, etc.

**Results**

Among 44 DS-ALL patients, 20, 18, and 3 were classified as SR, IR, and HR, respectively: three lacked risk assignment due to severe induction events, including two induction deaths. Five-year EFS and OS in DS-ALL were 70.5% and 81.8%, versus 85.6% and 94.6% in non-DS-ALL ($P<0.001$ for both), with the difference apparent in SR. DS-ALL exhibited fewer favorable genetics and a relapse pattern dominated by isolated bone marrow events; no CNS relapses occurred. At TP1, MRD did not separate outcomes in DS-ALL. At TP2, all three DS-ALL patients with MRD $\geq 10^{-4}$ relapsed and died within three years; even with TP2 MRD $<10^{-4}$, DS-ALL outcomes remained inferior to non-DS with comparable MRD. Data on CRLF2r were available in all DS-ALL (15/44 overall; 14/36 in the marker-agnostic subset) and showed a non-significant trend toward lower EFS/OS. Grade ≥3 toxicities were more frequent in DS-ALL: hyperglycemia in HD-MTX–containing intensified therapy and reinduction, mucositis in induction, HD-MTX–containing intensified therapy and reinduction, and pulmonary infections in induction and early intensification. No treatment-related mortality occurred after induction in DS-ALL.

**Summary/Conclusion**

Under a national regimen, DS-ALL showed inferior survival, distinctive relapse distribution, and higher toxicities. TP2 MRD $\geq 10^{-4}$ identified a high-risk subgroup, while CRLF2r showed a non-significant adverse trend. Supportive care was effective, but relapse risk persisted. DS status may carry adverse risk beyond conventional factors, highlighting limits of multi-agent chemotherapy and the need to add immune therapies such as blinatumomab to reduce relapse and complications.

**Copyright Information:** (Online) ISSN: 2572-9241
©2026 The Author(s). HemaSphere is published by John Wiley & Sons Ltd on behalf of European Hematology Association. This is an open access Abstract Book distributed under the Attribution-NonCommercial-NoDerivs (CC BY-NC-ND), which allows third parties to download the articles and share them with others as long as they credit the author and the Abstract Book, but they cannot change the content in any way or use them commercially.
**Abstract Book Citations:** Authors, Title, HemaSphere, 2026;10:(S1):pages. The abstract book DOIs can be found on the “Author Information” page.
**Disclaimer:** Articles published in the journal HemaSphere exclusively reflect the opinions of the authors. The authors are responsible for all content in their abstracts including accuracy of the facts, statements, citing resources, etc.

### (PS1501) MYELOABLATIVE CONDITIONING REGIMENS CONTAINING MELPHALAN OR THIOTEPA IMPROVE SURVIVAL OUTCOMES OF ALLO-HSCT IN CHILDREN WITH RELAPSED ACUTE LYMPHOBLASTIC LEUKEMIA

**Topic:** 02. Acute lymphoblastic leukemia - Clinical

Huili Zhu[1], Zhijie Wei*[1]

[1]*Hebei Yanda Lu Daopei Hospital, Langfang, China;*

### Background

Acute lymphoblastic leukemia (ALL) is the most prevalent hematological malignancy in the pediatric population..For children who experience early first relapse or second or greater relapse, outcomes remain poor despite the availability of HSCT The combination of thiotepa or melphalan on the basis of the myeloablative conditioning regimen has a stronger anti-leukemia effect and penetration, effectively preventing recurrence.

### Aims

Evaluate the effectiveness and safety of the intensified myeloablative conditioning regimen.

### Methods

A retrospective cohort study was conducted on 258 pediatric patients with relapsed ALL who underwent allo-HSCT at our institution between July 1, 2019, and September 30, 2024. Eligibility criteria included a pre-transplant disease status of ≥ complete remission 2 (CR2). All patients received MAC regimens: 195 patients were administered total body irradiation (TBI)-based MAC, while 63 received busulfan-based MAC. To augment anti-leukemic efficacy and reduce relapse risk, 50 patients were assigned to a intensified myeloablative conditioning (iMAC) regimen (MAC combined with melphalan or thiotepa). Among these 50 patients, 32 received melphalan (110 mg/m²) and 18 received thiotepa (5.0–7.5 mg/kg). 7 patients exhibiting positive minimal residual disease (MRD) (median MRD: 0.04%). The remaining 208 patients received conventional myeloablative conditioning (cMAC). Their median age was 8.62 years;  20 patients having positive pre-transplant MRD (median MRD: 0.02%). The median follow-up duration was 50.60 months (range: 0.43–78.00 months).

### Results

Among 208 patients who received the conventional myeloablative regimen cMAC The 5-year overall survival (OS) rate was 73.50%, the 5-year recurrence rate was 13.60%, and the 5-year non-relapse mortality (NRM) rate was 17.75%. The incidence of grade III-IV acute graft-versus-host disease (aGVHD) after transplantation was 17.30%. The incidence of cytomegalovirus (CMV) infection within 100 days was 47.10%, and the incidence of Epstein-Barr virus (EBV) infection was 13.50%. During the follow-up period, 26 patients relapsed and 55 patients died, among which 20 died of the primary disease and infection was another major cause of death. Among 50 patients who received the intensified myeloablative conditioning iMAC,  The 5-year OS rate was 86.40% (equal to disease-free survival), which was significantly higher than that of tthe cMAC group(p=0.01). The 5-year recurrence rate was 6.30%, The 5-year NRM rate was 7.87%, The incidence of grade III-IV aGVHD after transplantation was 10.00%, which was lower than that of the cMAC group. The incidence of CMV infection within 100 days was 38.00%, and the incidence of EBV infection was 8.00%, both of which were lower than those of the cMAC group,although there was no statistical difference. During the follow-up period, 3 patients relapsed, all of whom had bone marrow and extramedullary relapse. During the follow-up period, 5 patients died, among which 2 died of the primary disease, 2 died of infection, and 1 died of graft-versus-host disease.

**Copyright Information:** (Online) ISSN: 2572-9241
©2026 The Author(s). HemaSphere is published by John Wiley & Sons Ltd on behalf of European Hematology Association. This is an open access Abstract Book distributed under the Attribution-NonCommercial-NoDerivs (CC BY-NC-ND), which allows third parties to download the articles and share them with others as long as they credit the author and the Abstract Book, but they cannot change the content in any way or use them commercially.
**Abstract Book Citations:** Authors, Title, HemaSphere, 2026;10:(S1):pages. The abstract book DOIs can be found on the “Author Information” page.
**Disclaimer:** Articles published in the journal HemaSphere exclusively reflect the opinions of the authors. The authors are responsible for all content in their abstracts including accuracy of the facts, statements, citing resources, etc.

**Summary/Conclusion**

For pediatric and adolescent patients with ALL in pre-transplant status ≥ CR2, the intensified myeloablative conditioning regimen significantly reduces post-transplant relapse risk without increasing the incidence of grade III–IV aGVHD, NRM, or viral infections. This regimen represents a promising therapeutic strategy for improving survival outcomes in this high-risk population.

**Copyright Information:** (Online) ISSN: 2572-9241
©2026 The Author(s). HemaSphere is published by John Wiley & Sons Ltd on behalf of European Hematology Association. This is an open access Abstract Book distributed under the Attribution-NonCommercial-NoDerivs (CC BY-NC-ND), which allows third parties to download the articles and share them with others as long as they credit the author and the Abstract Book, but they cannot change the content in any way or use them commercially.
**Abstract Book Citations:** Authors, Title, HemaSphere, 2026;10:(S1):pages. The abstract book DOIs can be found on the "Author Information" page.
**Disclaimer:** Articles published in the journal HemaSphere exclusively reflect the opinions of the authors. The authors are responsible for all content in their abstracts including accuracy of the facts, statements, citing resources, etc.

### (PS1502) TREATMENT PATTERNS AND OUTCOMES WITH OLUTASIDENIB AFTER VENETOCLAX IN IDH1-MUTATED AML USING REAL-WORLD DATA FROM CHART REVIEW

**Topic:** 02. Acute lymphoblastic leukemia - Clinical

Pinkal Desai*[1], Ashwin Kishtagari[2], Namrata Chandhok[3], Timothy Pardee[4], Amber Thomassen[5], Aaron Sheppard[5], Catherine Lai[6]

*[1]Weill Cornell Medicine, Department of Medicine, Hematology/Oncology, New York, United States of America;[2]Vanderbilt-Ingram Cancer Center, Nashville, United States of America; [3]University of Miami, Sylvester Comprehensive Cancer Center, Miami, United States of America; [4]Wake Forest University School of Medicine, Winston-Salem, United States of America;[5]Rigel Pharmaceuticals, Inc., South San Francisco, United States of America; [6]University of Pennsylvania, Division of Hematology & Oncology, Abramson Cancer Center, Philadelphia, United States of America;*

### Background

Olutasidenib, a selective oral inhibitor of mutant IDH1 (m*IDH1*), is a key treatment option for patients with m*IDH1* mutated acute myeloid leukemia (AML). Targeted agents are commonly used for patients following failure of chemotherapy or front-line venetoclax-based regimens. Olutasidenib is approved for relapsed/refractory (R/R) m*IDH1* AML. In the pivotal Phase 2 trial (NCT02719574), olutasidenib achieved CR/CRh in 35% of patients, with a median duration of CR/CRh of 25.3 months and acceptable tolerability; however, real-world data on olutasidenib utilization and outcomes post-venetoclax remain limited.

### Aims

The objectives of this study were to describe the clinical characteristics, treatment patterns, safety, and real-world effectiveness of adults with R/R *mIDH1* AML receiving olutasidenib following a venetoclax-based therapy in routine practice.

### Methods

This retrospective cohort study used data from a physician-based chart review. Eligible patients were ≥18 years old and had R/R AML with an *IDH1* mutation, prior treatment with venetoclax, initiated olutasidenib between December 2022 – March 2025 and were assessed for a response. Patients were excluded if they received prior IDH1 targeted therapy or >1 additional therapy between receipt of venetoclax and olutasidenib. Patient disease characteristics, prior treatment history, and effectiveness and safety outcomes were summarized using descriptive statistics. Duration of response was estimated using Kaplan-Meier (KM) methods.

**Copyright Information:** (Online) ISSN: 2572-9241
©2026 The Author(s). HemaSphere is published by John Wiley & Sons Ltd on behalf of European Hematology Association. This is an open access Abstract Book distributed under the Attribution-NonCommercial-NoDerivs (CC BY-NC-ND), which allows third parties to download the articles and share them with others as long as they credit the author and the Abstract Book, but they cannot change the content in any way or use them commercially.
**Abstract Book Citations:** Authors, Title, HemaSphere, 2026;10:(S1):pages. The abstract book DOIs can be found on the “Author Information” page.
**Disclaimer:** Articles published in the journal HemaSphere exclusively reflect the opinions of the authors. The authors are responsible for all content in their abstracts including accuracy of the facts, statements, citing resources, etc.

### Results

Fifty-one charts were collected from 18 physicians. The median patient age at diagnosis was 61 years and 78% were male. The median follow-up time from diagnosis was 23.6 months. ECOG PS was 0-1 in 54.9% and 2-3 in 45.1% of patients at the time of olutasidenib initiation and 15.7% had AML with antecedent MDS. Most patients (60.8%) harbored at least one co-mutation, the most common being *DNMT3A* (43.1%), *FLT3* (35.3%) and *NPM1* (35.3%).
Most patients (90.2%) received venetoclax-based therapy in the first line; ORR was 81.3% and CR/CRi rate was 47.9%. The median duration of prior venetoclax treatment was 4.2 months (IQR, 1.6-16.2 months). Olutasidenib was mostly given as monotherapy (96.1%) and in the second line (72.5%), with 27.5% given as third-line treatment. Median olutasidenib treatment duration was 12.6 months (IQR, 7.2-20.1 months). The ORR for olutasidenib was 90.2%, with 60.8% CR/CRh and 57% CR, with a median CR/CRh DOR of 30.3 months. Of the 36 (70.6%) patients who were RBC and/or platelet transfusion dependent at baseline, 20 (55.6%) achieved transfusions independence while on olutasidenib. Of the 12 patients discontinuing olutasidenib, the primary reason was disease progression (41.7%). The most common adverse events were fatigue (11.8%), infection, neutropenia, and nausea (each 7.8%).

### Summary/Conclusion

Olutasidenib demonstrated robust real-world effectiveness in adults with R/R m*IDH1* AML previously treated with venetoclax, a subgroup that historically has very poor outcomes, with a CR/CRh rate of 60.8% and a median response duration of 30.3 months. Substantial reductions in transfusion dependence further support olutasidenib as a viable post-venetoclax therapeutic option. These findings suggest that earlier sequencing of olutasidenib in the treatment paradigm may optimize patient outcomes.

© 2026 American Society of Clinical Oncology, Inc. Reused with permission. This abstract was accepted and previously presented at the 2026 ASCO Annual Meeting. All rights reserved.

**Copyright Information:** (Online) ISSN: 2572-9241
©2026 The Author(s). HemaSphere is published by John Wiley & Sons Ltd on behalf of European Hematology Association. This is an open access Abstract Book distributed under the Attribution-NonCommercial-NoDerivs (CC BY-NC-ND), which allows third parties to download the articles and share them with others as long as they credit the author and the Abstract Book, but they cannot change the content in any way or use them commercially.
**Abstract Book Citations:** Authors, Title, HemaSphere, 2026;10:(S1):pages. The abstract book DOIs can be found on the “Author Information” page.
**Disclaimer:** Articles published in the journal HemaSphere exclusively reflect the opinions of the authors. The authors are responsible for all content in their abstracts including accuracy of the facts, statements, citing resources, etc.

### (PS1503) IMPACT OF MRD AND BRIDGING THERAPHY BEFORE ALLO-SCT IN B-ALL: 20-YEAR REAL-WORLD DATA

**Topic:** 02. Acute lymphoblastic leukemia - Clinical

Jorge Nuñez Zuno*[1], Jose Velasco Alvarez[1], Hugo Nieto-Gascon[1], M Jesus Figueroa-Nombela[1], Jesus Lozano-Sanchez[1], Estefanía Perez-López[1], Almudena Cabero[1], Rodrigo Gil-Manso[1], Alejandro Avendaño[1], Ana Africa Martin Lopez[1], Ana García Bacelar[1], Alberto Hernández-Sánchez[1], Magdalena Sierra[1], Lucía López-Corral[1], Fermin Sanchez-Guijo[1], Jesus Hernandez-Rivas[1], Mónica Cabrero[1]

*[1]Salamanca University Hospital-IBSAL, Hematology, Salamanca, Spain;*

### Background

Allogeneic-stem cell transplant (alloSCT) is a well-established curative treatment for acute lymphoblastic leukemia (ALL), although relapse remains the main cause of treatment failure. In this setting, minimal residual disease (MRD) is useful for identifying high-risk patients, and the availability of new drugs with minimal toxicity offers a feasible bridging therapy (BrT) for those with positive pre-alloSCT MRD. However, exact impact of pre-alloSCT MRD and optimal strategy for bridging therapy in the era of monoclonal antibodies (MoAbs) remains controversial.

### Aims

To evaluate the impact of pre-alloSCT MRD and BrT in the outcomes of ALL patients undergoing alloSCT in our center over a 20-year period.

### Methods

We retrospectively included consecutive patients aged ≥14 years with B-ALL who underwent alloSCT between 2005 and 2025. Among 139 patients in our historical ALL cohort who received alloSCT, 98 met the inclusion criteria. We analyzed baseline characteristics, overall survival (OS), progression-free survival (PFS), cumulative incidence of relapse (CIR), and bridging therapy use.

### Results

Characteristics of patients are detailed in **Table 1**. Twenty-four patients received BrT with Blinatumomab in 75% (n=18), chemotherapy in 17% (n=4) or Inotuzumab in 8% (n=2); Median blast percentage before BrT was 0.17% (0.001 – 10%). MRD-negativity rate after BrT was 79% (n=19/24), 75% (3/4) for chemotherapy, 78% (14/18) for Blinatumomab and 100% for Inotuzumab (2/2).

**Copyright Information:** (Online) ISSN: 2572-9241
©2026 The Author(s). HemaSphere is published by John Wiley & Sons Ltd on behalf of European Hematology Association. This is an open access Abstract Book distributed under the Attribution-NonCommercial-NoDerivs (CC BY-NC-ND), which allows third parties to download the articles and share them with others as long as they credit the author and the Abstract Book, but they cannot change the content in any way or use them commercially.
**Abstract Book Citations:** Authors, Title, HemaSphere, 2026;10:(S1):pages. The abstract book DOIs can be found on the "Author Information" page.
**Disclaimer:** Articles published in the journal HemaSphere exclusively reflect the opinions of the authors. The authors are responsible for all content in their abstracts including accuracy of the facts, statements, citing resources, etc.

Table 1. Characteristics of patients

| | No bridging therapy (n=74) | Bridging therapy (n=24) | *p*-value |
|---|---|---|---|
| **Age, median (range)** | 42 (15-70) | 34 (16-72) | *0.251* [1] |
| **Age ≥ 55y, n (%)** | 18 (24) | 5 (21) | *0.726* [2] |
| **Male sex, n (%)** | 39 (53) | 15 (63) | *0.402* [2] |
| **Ph-positive, n (%)** | 28 (38) | 4 (17) | *0.079* [3] |
| **AlloSCT in 1st CR, n (%)** | **54 (73)** | **11 (46)** | ***0.041*** [2] |
| **Pre-alloSCT MRD-negative, n (%)** | 63 (85) | 19 (79) | *0.329* [3] |
| **Matched related donor, n (%)** | **47 (63)** | **9 (38)** | ***0.033*** [2] |
| **Myeloablative conditioning, n (%)** | 51 (69) | 19 (79) | *0.378* [2] |
| **Peripheral blood source, n (%)** | 69 (93) | 22 (92) | *1.000* [2] |

[1] Mann-Whitney U test, [2]Pearson Chi-square test, [3]Fishers exact test

After a median follow-up of 59 months (3-245), 3 and 5-year PFS/OS were 51/65% and 50/64%, respectively. CIR was 18.9% and 27.3% at 12 and 24 months, In the case of MRD positive, CIR was 40.7% at 12 months vs 16.6% for those with MRD negative (NS).

Pre-alloSCT MRD stratifies survival in three prognostic groups (figure 1): 5y OS was 72.4, 53.2 and 40% for those MRD negative at CR, MRD negative after BrT, and MRD positive respectively (p=0.023). For those groups of patients, 5y PFS was 55.4, 42 and 35% (p=0.09).

In multivariate analysis, MRD positivity and conditioning regimen revealed significant impact on OS: MRD positivity pre-alloSCT HR 2.9 (1.3-6.8); p=0.01; MAC HR 0.44 (0.21-0.91); p=0.028.

### Summary/Conclusion

-MRD negative before alloSCT is associated with superior OS, while conditioning regimen also has a significant impact on outcomes.
-Bridging therapy may improve outcomes in MRD-positive patients; however, MRD conversion does not fully overcome the adverse biology associated with persistent disease after induction.
-Patients with persistent MRD-positivity represent a very-high risk population, for whom novel therapeutic approaches are needed.

**Copyright Information:** (Online) ISSN: 2572-9241
©2026 The Author(s). HemaSphere is published by John Wiley & Sons Ltd on behalf of European Hematology Association. This is an open access Abstract Book distributed under the Attribution-NonCommercial-NoDerivs (CC BY-NC-ND), which allows third parties to download the articles and share them with others as long as they credit the author and the Abstract Book, but they cannot change the content in any way or use them commercially.
**Abstract Book Citations:** Authors, Title, HemaSphere, 2026;10:(S1):pages. The abstract book DOIs can be found on the "Author Information" page.
**Disclaimer:** Articles published in the journal HemaSphere exclusively reflect the opinions of the authors. The authors are responsible for all content in their abstracts including accuracy of the facts, statements, citing resources, etc.

### (PS1504) CLINICAL OUTCOMES OF RELAPSED/REFRACTORY B-CELL ACUTE LYMPHOBLASTIC LEUKEMIA IN PATIENTS AFTER ALLOGENEIC HEMATOPOIETIC STEM CELL TRANSPLANTATION FOLLOWING BLINATUMOMAB TREATMENT: CORBLIN-T

**Topic:** 02. Acute lymphoblastic leukemia - Clinical

Akihito Shinohara*[1], Hirotoshi Sakaguchi[2], Atsumi Yanagisawa[3], Satoshi Matsushima[4], Satoshi Nishiwaki[5], Takayoshi Tachibana[6], Yasuyuki Arai[7], Itaru Kato[8], Motohiro Kato[9], Takuro Nishikawa[10], Noriko Doki[11], Mamiko Sakata-Yanagimoto[12], Utako Oba[13], Tomoaki Ueda[14], Yuta Katayama[15], Junya Kanda[16], Takahiro Fukuda[17], Takanori Teshima[18], Fumiko Ueki[4], Eiji Kawahara[4], Yoshiko Atsuta[3]

*[1]Tokyo Women's Medical University, Department of Hematology, Tokyo, Japan;[2]National Center for Child Health and Development, Children's Cancer Center, Tokyo, Japan; [3]Japanese Data Center for Hematopoietic Cell Transplantation, Nagakute, Japan; [4]Amgen K.K., Medical Affairs, Tokyo, Japan;[5]Nagoya University Hospital, Department of Advanced Medicine, Nagoya, Japan; [6]Yokohama City University Hospital, Department of Hematology, Rheumatology and Infectious Diseases, Yokohama, Japan; [7]Kyoto University Hospital, Department of Cytotherapy, Kyoto, Japan;[8]Kyoto University, Department of Pediatrics, Kyoto, Japan; [9]The University of Tokyo Hospital, Department of Pediatrics, Tokyo, Japan; [10]Kagoshima University, Creation of Community Pediatric Medicine, Graduate School of Medical and Dental Sciences, Kagoshima, Japan; [11]Tokyo Metropolitan Cancer and Infectious Diseases Center, Komagome Hospital, Hematology Division, Tokyo, Japan; [12]University of Tsukuba Hospital, Department of Hematology, Tsukuba, Japan; [13]Kyushu University, Department of Pediatrics, Graduate School of Medical Sciences, Fukuoka, Japan; [14]The University of Osaka, Department of Hematology and Oncology, Graduate School of Medicine, Suita, Japan; [15]Hiroshima Red Cross Hospital and Atomic-bomb Survivors Hospital, Department of Hematology, Hiroshima, Japan; [16]Kyoto University, Department of Hematology, Graduate School of Medicine, Kyoto, Japan;[17]National Cancer Center Hospital, Department of Hematopoietic Stem Cell Transplantation, Tokyo, Japan; [18]Hokkaido University, Department of Hematology, Faculty of Medicine, Sapporo, Japan;*

### Background

The treatment of relapsed or refractory acute lymphoblastic leukemia (R/R ALL) remains a major clinical challenge. Blinatumomab (BLN), a CD3/CD19-directed bispecific T-cell engager molecule, has demonstrated high remission rates and deep molecular responses in R/R B-cell ALL (R/R B-ALL). BLN has broadened eligibility for allogeneic hematopoietic cell transplantation (HSCT); however, post-transplant outcomes and safety following BLN remain unclear.

### Aims

We aimed to evaluate the clinical outcomes of HSCT after BLN treatment in a large nationwide cohort in Japan using data from the Transplant Registry Unified Management Program (TRUMP).

### Methods

This retrospective study used TRUMP registry data with additional data collected for this study. We analyzed patients with B-ALL who underwent HSCT between 2019 and 2020 after BLN. Patients were included if the analyzed HSCT was the first transplantation performed after BLN, even if it was the second HSCT in the patient's treatment history. Patients were included regardless of genetic abnormalities, whereas those with lymphoblastic lymphoma were excluded. This collaborative study involving the Japanese Society for Transplantation and Cellular Therapy, the Japanese Data Center for Hematopoietic Cell Transplantation, and Amgen Inc. was approved by the Institutional Review Committee of Tokyo Women's Medical University.

**Copyright Information:** (Online) ISSN: 2572-9241
©2026 The Author(s). HemaSphere is published by John Wiley & Sons Ltd on behalf of European Hematology Association. This is an open access Abstract Book distributed under the Attribution-NonCommercial-NoDerivs (CC BY-NC-ND), which allows third parties to download the articles and share them with others as long as they credit the author and the Abstract Book, but they cannot change the content in any way or use them commercially.
**Abstract Book Citations:** Authors, Title, HemaSphere, 2026;10:(S1):pages. The abstract book DOIs can be found on the "Author Information" page.
**Disclaimer:** Articles published in the journal HemaSphere exclusively reflect the opinions of the authors. The authors are responsible for all content in their abstracts including accuracy of the facts, statements, citing resources, etc.

**Results**

We analyzed 181 patients from 76 institutions. Among survivors, the median follow-up after HSCT was 1663 days. The median age at HSCT was 29 years; 54 patients (29.8%) were <16 years, 107 (59.1%) were male, and 31 (17.1%) harbored the BCR/ABL1 fusion gene. Overall, 147 cases (81.2%) were not in first complete remission at HSCT, and 134 patients (74.0%) underwent HSCT as their first transplantation. Stem cell sources were related bone marrow (BM), related peripheral blood (PB), unrelated BM, unrelated PB, and cord blood (CB) in 15 (8.3%), 42 (23.2%), 43 (23.8%), 11 (6.1%), and 70 (38.7%) patients, respectively. Excluding CB donors and one case with missing data, HLA-matched, HLA-mismatched, and haploidentical donors accounted for 52 (46.8%), 31 (27.9%), and 27 (24.3%) cases, respectively. The median time to neutrophil engraftment was 17 days, with a cumulative incidence (CI) by day 30 of 90.6% (95% CI, 85.3–94.1). At 3 years, the leukemia-free survival and overall survival rates (OS) were 41.8% (95% CI, 34.5–48.9) and 57.3% (95% CI, 49.6–64.2), respectively, whereas the 3-year CI of relapse and non-relapse mortality (NRM) were 38.6% (95% CI, 31.5–45.7) and 19.5% (95% CI, 14.1–25.7). The CI of grade 2–4 acute GVHD by day 100 was 40.5% (95% CI, 33.3–47.6), and the one-year CI of chronic and extensive chronic GVHD was 12.9% (95% CI, 8.5–18.2) and 7.8% (95% CI, 4.5–12.4), respectively. Post-transplant relapse occurred in 70 patients (38.7%). Regarding HSCT complications, the CI of sinusoidal obstruction syndrome (SOS) by one year was 11.7% (95% CI, 7.5–16.8); 57 of 181 patients (31.5%) had prior exposure to inotuzumab ozogamicin (InO).

**Summary/Conclusion**

In this analysis, HSCT following BLN for R/R B-ALL demonstrated acceptable OS. Although this population was heavily pretreated, NRM was lower than expected based on previous reports, suggesting that HSCT after BLN is a feasible and potentially safe approach. The incidence of SOS was notable; however, it should be considered that more than 30% of the study population had prior exposure to InO, which may have contributed to this finding.

**Copyright Information:** (Online) ISSN: 2572-9241
©2026 The Author(s). HemaSphere is published by John Wiley & Sons Ltd on behalf of European Hematology Association. This is an open access Abstract Book distributed under the Attribution-NonCommercial-NoDerivs (CC BY-NC-ND), which allows third parties to download the articles and share them with others as long as they credit the author and the Abstract Book, but they cannot change the content in any way or use them commercially.
**Abstract Book Citations:** Authors, Title, HemaSphere, 2026;10:(S1):pages. The abstract book DOIs can be found on the “Author Information” page.
**Disclaimer:** Articles published in the journal HemaSphere exclusively reflect the opinions of the authors. The authors are responsible for all content in their abstracts including accuracy of the facts, statements, citing resources, etc.

### (PS1505) EFFICACY AND TOXICITY OF INOTUZUMAB OZOGAMICIN BRIDGING TO CELLULAR THERAPIES IN A REAL-WORLD SETTING

**Topic:** 02. Acute lymphoblastic leukemia - Clinical

Mohit Vijayakumar*[1], Rodothea Amerikanou[1, 2], Marc Mansour[1, 2], Adele Fielding[3], Jenny O'nions[1], Rachael Hough[1], Richard Burt[1, 4]

*[1]University College London Hospitals, Haematology, London, United Kingdom; [2]University College London, Haematology, London, United Kingdom; [3]University of York, York Biomedical Research Institute, London, United Kingdom; [4]Imperial College London, Haematology, London, United Kingdom;*

### Background

Inotuzumab Ozogamicin (InO), is an anti-CD22-drug conjugate with activity in B-Acute Lymphoblastic Leukaemia (B-ALL). Real-world outcomes, especially in patients with extramedullary disease (EMD) and those bridged to cellular therapies, remain incompletely described.

### Aims

We aimed to evaluate real-world efficacy, EMD responses, bridging to cellular therapies, and toxicity in adults treated with InO at a tertiary United Kingdom centre.

### Methods

We conducted a retrospective analysis of adult patients treated with InO between 1st December 2018 and 31st July 2025. Event free survival (EFS) was defined as the time from the first InO dose (C1D1) to refractory disease, relapse or death. Overall survival (OS) was from C1D1 to death.

**Copyright Information:** (Online) ISSN: 2572-9241
©2026 The Author(s). HemaSphere is published by John Wiley & Sons Ltd on behalf of European Hematology Association. This is an open access Abstract Book distributed under the Attribution-NonCommercial-NoDerivs (CC BY-NC-ND), which allows third parties to download the articles and share them with others as long as they credit the author and the Abstract Book, but they cannot change the content in any way or use them commercially.
**Abstract Book Citations:** Authors, Title, HemaSphere, 2026;10:(S1):pages. The abstract book DOIs can be found on the "Author Information" page.
**Disclaimer:** Articles published in the journal HemaSphere exclusively reflect the opinions of the authors. The authors are responsible for all content in their abstracts including accuracy of the facts, statements, citing resources, etc.

### Results

Twenty-six patients received InO. Median age was 51 years (range 19–79), 17 (65%) were male and 19 (73.1%) were White. Six (23%) had Philadelphia positive (Ph+) disease, and 8 (31%) had EMD prior Ino, 5 with concomitant bone marrow (BM) disease. The commonest EMD site was bone (n=4, 50%).
InO was prescribed as a median salvage line of 1.5 (range 1–4) and 18 (69%) had 2 or more cycles administered. Prior salvage or consolidation therapy included Blinatumomab in 14 (54%), chimeric antigen receptor T cells (CART) in 4 (15%) and allogeneic stem cell transplant (alloSCT) in 6 (23%).
Complete response (CR) was achieved in 19 (73%), with 14/19 patients (74%) measurable residual disease negative by quantitative polymerase chain reaction or flow cytometry. Among the 8 patients with EMD, 5 (63%) achieved complete metabolic response, 1 had stable disease, 1 had clinical resolution and 1 was not reassessed with imaging. Five patients (19%) were refractory.
InO facilitated bridging to cellular therapy in 12 (46%) patients (9 alloSCT, 3 CART), while 9 (35%) were treated with a palliative intent. Five (29% of intention to treat with cellular therapy) patients did not proceed to planned cellular therapy (n=2 alloSCT, n=3 CART). Failure to proceed to CART was due to progressive disease (n=2) and cerebrovascular accident (n=1), while failure to proceed to alloSCT was due to sinusoidal obstructive syndrome (SOS) and BM necrosis accompanied by haemophagocytic lymphohistiocytosis.
At a median follow-up of 10.4 months, median EFS was 14.4 months, and median OS was 23.8 months. The 2-year EFS was 32.9% and the 2-year OS was 45.8%.
Severe hepatotoxicity occurred in 5 (19%) patients, including 2 cases of post-alloSCT SOS and 3 SOS presentations outside the transplant setting. Hepatic steatosis (n=4) and siderosis (n=3) were noted among these patients. Further toxicity included 3 vascular events (deep vein thrombosis, cerebrovascular accident, myocardial infarction) and prolonged cytopenias resulting in treatment delay (n=4).
To date 12 (46%) have died with progression/relapse of B-ALL as the most common cause (n=10, 38%). Of the 10 with progressive disease, 4 (40%) were refractory to InO.

### Summary/Conclusion

InO demonstrated good activity in EMD and facilitated bridging to cellular therapies.Hepatotoxicity with SOS was observed in 19% (n=5) of patients, comparable to published literature. However, surprisingly 3/5 of the cases occurred without an allo-SCT. Notably, 4/5 patients had underlying hepatic abnormalities. We propose that hepatic steatosis and siderosis may predict increased SOS risk and should be screened for prior to InO. We also noted non-SOS vascular events in 3 patients, potentially suggesting endothelial activation due to InO may not be isolated to the liver.

**Copyright Information:** (Online) ISSN: 2572-9241
©2026 The Author(s). HemaSphere is published by John Wiley & Sons Ltd on behalf of European Hematology Association. This is an open access Abstract Book distributed under the Attribution-NonCommercial-NoDerivs (CC BY-NC-ND), which allows third parties to download the articles and share them with others as long as they credit the author and the Abstract Book, but they cannot change the content in any way or use them commercially.
**Abstract Book Citations:** Authors, Title, HemaSphere, 2026;10:(S1):pages. The abstract book DOIs can be found on the “Author Information” page.
**Disclaimer:** Articles published in the journal HemaSphere exclusively reflect the opinions of the authors. The authors are responsible for all content in their abstracts including accuracy of the facts, statements, citing resources, etc.

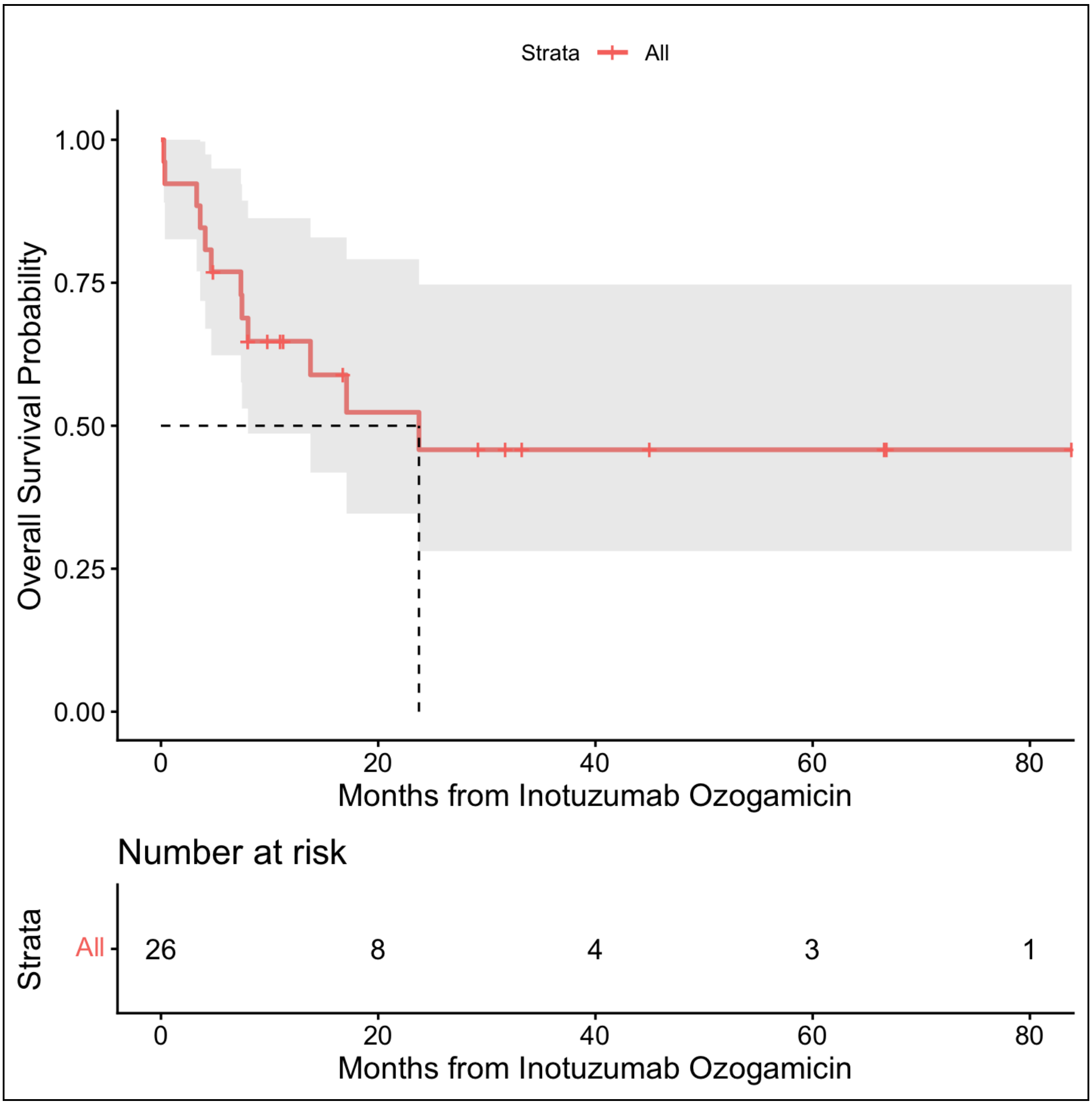

Overall Survival Curve of Inotuzumab Ozogamicin in Relapsed/Refractory B-ALL

**Copyright Information:** (Online) ISSN: 2572-9241
©2026 The Author(s). HemaSphere is published by John Wiley & Sons Ltd on behalf of European Hematology Association. This is an open access Abstract Book distributed under the Attribution-NonCommercial-NoDerivs (CC BY-NC-ND), which allows third parties to download the articles and share them with others as long as they credit the author and the Abstract Book, but they cannot change the content in any way or use them commercially.
**Abstract Book Citations:** Authors, Title, HemaSphere, 2026;10:(S1):pages. The abstract book DOIs can be found on the “Author Information” page.
**Disclaimer:** Articles published in the journal HemaSphere exclusively reflect the opinions of the authors. The authors are responsible for all content in their abstracts including accuracy of the facts, statements, citing resources, etc.

### (PS1506) EVALUATION OF BONE METABOLISM PARAMETERS AND BONE MINERAL DENSITY IN CHILDREN WITH ACUTE LYMPHOBLASTIC LEUKEMIA: A RETROSPECTIVE SINGLE-CENTER STUDY

**Topic:** 02. Acute lymphoblastic leukemia - Clinical

Ugur Mete*[1], Yeter Düzenli Kar[1], Numan Katmer[1], Melike Sezgİn Evİm[1], Adalet Meral Güneş[1]

[1]*Bursa Uludag University, Pediatric Hematology, Bursa, Türkiye;*

### Background

Acute lymphoblastic leukemia (ALL) is the most common childhood malignancy. While survival rates have improved substantially, ALL treatment ”including corticosteroids, methotrexate, and radiotherapy” exerts significant negative effects on bone metabolism. Osteopenia and osteoporosis are well-recognized complications during and after therapy. However, the differential impact of disease subtypes (B-ALL vs. T-ALL) and risk stratification on skeletal health remains insufficiently explored. This study aimed to evaluate bone metabolism parameters and bone mineral density (BMD) changes across ALL subtypes and risk groups throughout treatment.

### Aims

This retrospective study aimed and included children diagnosed with ALL between January 2018 and December 2023 at the Pediatric Hematology Clinic of Bursa Uludağ University.

### Methods

Demographic data, disease subtype, risk classification (standard, intermediate, high, transplant), and radiotherapy status were recorded. Serum vitamin D, calcium, phosphorus, parathyroid hormone (PTH), and alkaline phosphatase (ALP) levels were measured at diagnosis, pre and post-maintenance. BMD was assessed using dual-energy X-ray absorptiometry (DEXA) Z-scores. Longitudinal changes were analyzed using linear mixed-effects models adjusting for sex, age, radiotherapy, and baseline biochemical values. Both four-level and two-level (low-risk vs. high-risk) analyses were performed.

### Results

A total of 125 patients were included (57.6% male; mean age 6.19 ± 4.34 years); 102 had B-ALL and 23 had T-ALL. T-ALL patients were older, predominantly male, more frequently in high-risk group (60.9% vs. 28.4%), and had markedly elevated PTH at diagnosis (280.4 ± 276.9 vs. 27.7 ± 27.2 pg/mL). Pre-maintenance DEXA Z-scores were lower in T-ALL (−1.36 vs. −0.83) with higher osteoporosis rates (29.4% vs. 21.9%). DEXA Z-scores showed a stepwise decline across risk groups (medians: standard −0.73, intermediate −0.62, high −1.41, transplant −2.08). Post-maintenance normal BMD ranged from 78.9% (standard) to 30.0% (transplant). Phosphorus decreased significantly over time ($p<0.001$). PTH differed among risk groups ($p=0.001$), with radiotherapy as an independent predictor ($p<0.001$). ALP showed a significant group-by-time interaction in the two-level analysis ($p=0.006$), increasing in low-risk ($p=0.018$) but not in high-risk. Post-maintenance DEXA Z-scores were significantly higher in low-risk versus high-risk cases ($p=0.031$).

**Copyright Information:** (Online) ISSN: 2572-9241
©2026 The Author(s). HemaSphere is published by John Wiley & Sons Ltd on behalf of European Hematology Association. This is an open access Abstract Book distributed under the Attribution-NonCommercial-NoDerivs (CC BY-NC-ND), which allows third parties to download the articles and share them with others as long as they credit the author and the Abstract Book, but they cannot change the content in any way or use them commercially.
**Abstract Book Citations:** Authors, Title, HemaSphere, 2026;10:(S1):pages. The abstract book DOIs can be found on the “Author Information” page.
**Disclaimer:** Articles published in the journal HemaSphere exclusively reflect the opinions of the authors. The authors are responsible for all content in their abstracts including accuracy of the facts, statements, citing resources, etc.

**Summary/Conclusion**

These findings demonstrate that bone metabolism change across ALL subtypes and risk groups. Elevated PTH in T-ALL may reflect greater disease burden or paraneoplastic effects. The stepwise DEXA Z-score decline highlights the cumulative skeletal impact of intensive treatment. The universal phosphorus decline likely reflects prolonged corticosteroid and methotrexate exposure. Notably, ALP increased significantly only in the low-risk group, suggesting active bone recovery, while its absence in the high-risk group may indicate suppressed osteoblastic activity. Radiotherapy consistently emerged as an independent predictor of bone parameter alterations. Children with ALL demonstrate significant bone metabolism alterations throughout treatment, with greater skeletal compromise in higher-risk groups and T-ALL patients. Systematic bone health monitoring and early intervention are essential, particularly in high-risk patients and those receiving radiotherapy, to mitigate long-term skeletal morbidity in childhood ALL survivors.

**Copyright Information:** (Online) ISSN: 2572-9241
©2026 The Author(s). HemaSphere is published by John Wiley & Sons Ltd on behalf of European Hematology Association. This is an open access Abstract Book distributed under the Attribution-NonCommercial-NoDerivs (CC BY-NC-ND), which allows third parties to download the articles and share them with others as long as they credit the author and the Abstract Book, but they cannot change the content in any way or use them commercially.
**Abstract Book Citations:** Authors, Title, HemaSphere, 2026;10:(S1):pages. The abstract book DOIs can be found on the "Author Information" page.
**Disclaimer:** Articles published in the journal HemaSphere exclusively reflect the opinions of the authors. The authors are responsible for all content in their abstracts including accuracy of the facts, statements, citing resources, etc.

### (PS1507) OUTCOMES OF ADOLESCENTS AND YOUNG ADULTS ACUTE LYMPHOBLASTIC LEUKEMIA TREATED WITH THE PEDIATRIC EORTC 58951 PROTOCOL

**Topic:** 02. Acute lymphoblastic leukemia - Clinical

Imen Frikha*[1], Ines Turki[1], Khouloud Mezghanni[1], Rahma Mallek[1], Nour Louati[2], Ikram Dammak[3], Aicha Ben Kahla[1], Faten Kallel[1], Maha Charfi[1], Yossra Fakhfakh[1], Imen Ben Amor[1], Fatma Turki[4], Asma Koubaa[1], Nour Siala[1], Hanen Charfi[1], Henda Elleuch[3], Ikram Ben Amor[2], Nour Ben Abdeljalil[5], Moez Medhaffar[1], Moez Elloumi[1]

*[1]Hedi Chaker Hospital, Hematology, Sfax, Tunisia; [2]Regional Center of Transfusion, Sfax, Tunisia; [3]Laboratory of Hematology, Hedi Chaker Hos^pital, Sfax, Tunisia; [4]Hedi Chaker Hospital, Laboratory of Cytogenetic, Sfax, Tunisia; [5]National Bone Marrow Transplant Center, Tunis, Tunisia;*

### Background

Adolescents and young adults (AYA) acute lymphoblastic leukemia (ALL) have shown inferior outcomes compared with children. Pediatric-inspired regimens have been associated with improved survival in this population, including the EORTC 58951 protocol.

### Aims

This study aimed to evaluate treatment outcomes, survival and toxicity in AYA patients treated according to this pediatric protocol.

### Methods

We conducted a retrospective, single-center study including AYA patients aged 16-30 years, diagnosed with ALL and treated according to the EORTC 58951 protocol between 2000 and 2023. ALL were assigned to 4 risk-groups. Patients aged >20 years were classified at least to the average risk 2 (AR2) group. Baseline clinical, immunophenotypic, and cytogenetic characteristics were collected. We analysed corticosensitivity (CS), complete remission (CR), mesurable residual desease (MRD) positivity as $\geq 10^{-2}$ for MRD1 and $\geq 10^{-3}$ for MRD2 (assessed by flow cytometry), relapse rate, mortality rate, overall survival (OS), and event free survival (EFS).

### Results

A total of 103 AYA were included, with a median age of 20 years (16-30). Sex ratio was 1,5. There are B-ALL in 53% and T-ALL in 47% of cases . Central nervous system involvement was documented in 3 patients (3%). Bcr-Abl rearrangement was identified in 4 (3,9%), and MLL-AF4 in 2 patients (1,9%). Cytogenetic abnormalities include : hyperdiploidy>50 (n=3), t(9,22) (n=3), 11q23 (n=6), and complexe karyotype (n=11). Corticosensitivity was observed in 75 patients (73%). CR was achieved in 94 pts (91%). MRD negativity was achieved in 71% at MRD1 and 74% at MRD2. Finally, ALL were stratified into AR1 (15%), AR2 (54%), and HR (31%) groups. Fifteen patients underwent allogenic stem cell transplantation (ASCT) of the 32 indicated. Mortality rate was 13,5%. Within a median follow-up of 166 months, relapse rate was 32% (36% in AR1, 30% in AR2 and 33% in HR group, p=0,88). The ASCT did not reduce relapse rate (39 vs 34%, p=0,87). Five years OS and EFS were 49.5% and 47,6%, respectively.

### Summary/Conclusion

In our cohort, AYA ALL treated with pediatric-inspired protocols achieved better outcomes than those reported with adult regimens, including lower relapse rates (30% vs 35-50%), and comparable survival. However, these results remain inferior to those achieved in childern, due to higher mortality.

**Copyright Information:** (Online) ISSN: 2572-9241
©2026 The Author(s). HemaSphere is published by John Wiley & Sons Ltd on behalf of European Hematology Association. This is an open access Abstract Book distributed under the Attribution-NonCommercial-NoDerivs (CC BY-NC-ND), which allows third parties to download the articles and share them with others as long as they credit the author and the Abstract Book, but they cannot change the content in any way or use them commercially.
**Abstract Book Citations:** Authors, Title, HemaSphere, 2026;10:(S1):pages. The abstract book DOIs can be found on the "Author Information" page.
**Disclaimer:** Articles published in the journal HemaSphere exclusively reflect the opinions of the authors. The authors are responsible for all content in their abstracts including accuracy of the facts, statements, citing resources, etc.

### (PS1508) LABORATORY'S ROLE IN PEDIATRIC ACUTE LYMPHOBLASTIC LEUKEMIA (ALL) IN A REAL WORLD SETTING. EXPERIENCE OF THE ALLIC 2010 PROTOCOL. GATLA GROUP

**Topic:** 02. Acute lymphoblastic leukemia - Clinical

Tania Encina*[1], María Florencia Sanchez[1], María Paula Iommi[1], Cecilia Lang[1], Marisa Sandoval[2], Horacio Daniel Caferri[3], Mónica Hernández[3], Brenda Kaltenbach[4], Maria Alejandra Cedola[5], María Luz Cosentini[6], Gimena Gil[3], Sergio Gómez[7], María Cecilia Murray[8], Emilse Caballero[8], Silvia Ines Do Santos[8], Florencia Gutierrez[9], Claudia Mabel Ruiz[7], Noelia Franco[6], María Constanza Drozdowski[5], Hector Torres[10], María Noelia Cabero[10], Evangelina Agriello[1]

*[1]Laboratorio de Especialidades Bioquímicas, Bahía Blanca, Argentina;[2]Universidad Nacional del Sur, Bahía Blanca, Argentina;[3]Hospital Interzonal General de Agudos (HIGA) Dr. José Penna, Bahía Blanca, Argentina;[4]Hospital Italiano Regional del Sur, Bahía Blanca, Argentina; [5]Clínica pediátrica San Lucas, Neuquén, Argentina; [6]HOSPITAL MATERNO INFANTIL DR HECTOR QUINTANA, Jujuy, Argentina; [7]Clínica del Niño de la Plata, La Plata, Argentina;[8]Hospital Interzonal Especializado Materno Infantil Tetamanti, Mar del Plata, Argentina; [9]Hospital Regional Comodoro Rivadavia, Comodoro Rivadavia, Argentina; [10]Hospital Materno Infantil de Salta, Salta, Argentina;*

### Background

Genetic alterations at diagnosis and MRD at specific stages of treatment are prognostic factors that define the outcome of pediatric patients with ALL. The immunophenotype and the determination of genetic/molecular alterations at diagnosis have been agreed upon and determined in the ALLIC 2010 protocol according to the BFM Study Group. During treatment, quantitative detection of MRD on days 15 and 33 post-induction and on day 78 post-consolidation are instances of review in the assignment of risk groups in pediatric patients with ALL.

### Aims

To evaluate the EFS and OS of our case series over the years of implementation of the ALLIC 2010 pediatric protocol, with centralized studies from different institutions in our country.

### Methods

A total of 449 pediatric patients (263 males and 186 females) from nine centers in the country, studied using bone marrow and/or peripheral blood samples. The participating centers were coded, and the results are presented anonymized. Diagnostic studies were performed: multiparametric flow cytometry (MPFC) according to BFM Study Group criteria, expanded with panels proposed by Euroflow, DNA content analysis (ploidy), and cytogenetic analysis. The following were evaluated using FISH/RT-PCR: BCR::ABL1, KMT2A::AFF1 and ETV6::RUNX1, TCF3::PBX1. MRD was determined by FCM on days 15 and 33 post-induction, including cellularity staining (Syto 16), and on day 78. The EFS and OS of patients from different centers were performed using IBM SSPS Statistics software.

**Copyright Information:** (Online) ISSN: 2572-9241
©2026 The Author(s). HemaSphere is published by John Wiley & Sons Ltd on behalf of European Hematology Association. This is an open access Abstract Book distributed under the Attribution-NonCommercial-NoDerivs (CC BY-NC-ND), which allows third parties to download the articles and share them with others as long as they credit the author and the Abstract Book, but they cannot change the content in any way or use them commercially.
**Abstract Book Citations:** Authors, Title, HemaSphere, 2026;10:(S1):pages. The abstract book DOIs can be found on the “Author Information” page.
**Disclaimer:** Articles published in the journal HemaSphere exclusively reflect the opinions of the authors. The authors are responsible for all content in their abstracts including accuracy of the facts, statements, citing resources, etc.

### Results

449 ALL cases were included, 404 B-lymphoid precursor and 45 T-precursor. Among B-ALL cases, 55/449 (12.2%) were determined to be ETV6::RUNX1, 21/449 (4.70%) BCR::ABL1, 9/449 (2.0%) KMT2A::AFF1, 12/449 (2.7%) TCF3::PBX1, among the most common molecular alterations. The risk distribution according to diagnostic and follow-up studies was: 81/449 (18.0%) standard risk, 255/449 (56.8%) intermediate risk, and 113/449 (25.2%) high risk.
The event-free survival (EFS) and overall survival (OS) obtained in patients for each risk group are detailed in the following table:

| | *SR* | *IR* | *HR* | *GLOBAL (%)* |
|---|---|---|---|---|
| *EFS (%)* | 80.2 | 82.7 | 69.0 | 78.8 |
| *OS (%)* | 81.5 | 76.5 | 48.7 | 70.4 |

The OS according to risk group in each center is presented in the following table:

| | *RE % (n)* | *RI % (n)* | *RA % (n)* | *SG % (n)* |
|---|---|---|---|---|
| *Center 1* | 100.0 (7) | 80.3 (30) | 40.0 (5) | 81.0 (43) |
| *Center 2* | 80.0 (5) | 70.4 (27) | 40.0 (10) | 64.3 (42) |
| *Center 3* | 83.3 (18) | 86.4 (44) | 60.0 (25) | 78.2 (97) |
| *Center 4* | -(0) | 85.7 (7) | 33.3 (3) | 70.0 (10)* |
| *Center 5* | 100.0 (2) | 85.7 (7) | 100.0 (1) | 90.0 (10)* |
| *Center 6* | 76.2 (21) | 76.5 (51) | 53.8 (26) | 70.4 (98) |
| *Center 7* | 78.6 (14) | 63.0 (46) | 45.8 (24) | 60.7 (84) |
| *Center 8* | 84.6 (13) | 75.0 (36) | 36.8 (19) | 66.2 (68) |
| *Center 9* | 0 (1) | 85.7 (7) | -(0) | 75.0 (8)* |

### Summary/Conclusion

The detection of genetic alterations at diagnosis, as well as the correct quantification of MRD at specific moments during treatment, allow for the objective designation of the risk group at diagnosis and its subsequent recategorization in order to adapt (decrease/intensify) the therapeutic regimen. EFS and OS were comparable to those reported for Latin American groups treated under the 2010 protocol. A different distribution of patient numbers across institutions was observed; therefore, sample size impacts the estimation of OS and does not reflect differences in quality of care. Consequently, these results should be considered exploratory. Each center had the overall and individual results available for evaluation and discussion. The overall trend is consistent with the expected behavior of the disease and the risk group, which validates the collaborative work.

**Copyright Information:** (Online) ISSN: 2572-9241
©2026 The Author(s). HemaSphere is published by John Wiley & Sons Ltd on behalf of European Hematology Association. This is an open access Abstract Book distributed under the Attribution-NonCommercial-NoDerivs (CC BY-NC-ND), which allows third parties to download the articles and share them with others as long as they credit the author and the Abstract Book, but they cannot change the content in any way or use them commercially.
**Abstract Book Citations:** Authors, Title, HemaSphere, 2026;10:(S1):pages. The abstract book DOIs can be found on the "Author Information" page.
**Disclaimer:** Articles published in the journal HemaSphere exclusively reflect the opinions of the authors. The authors are responsible for all content in their abstracts including accuracy of the facts, statements, citing resources, etc.

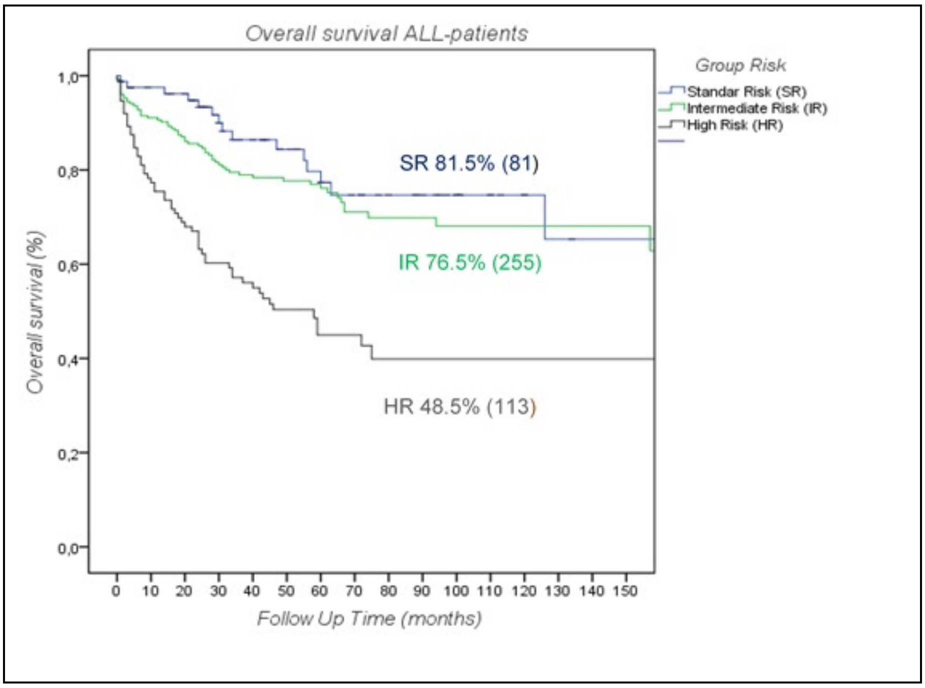

Overall survival (OS) obtained in ALL-patients for each risk group

**Copyright Information:** (Online) ISSN: 2572-9241
©2026 The Author(s). HemaSphere is published by John Wiley & Sons Ltd on behalf of European Hematology Association. This is an open access Abstract Book distributed under the Attribution-NonCommercial-NoDerivs (CC BY-NC-ND), which allows third parties to download the articles and share them with others as long as they credit the author and the Abstract Book, but they cannot change the content in any way or use them commercially.
**Abstract Book Citations:** Authors, Title, HemaSphere, 2026;10:(S1):pages. The abstract book DOIs can be found on the "Author Information" page.
**Disclaimer:** Articles published in the journal HemaSphere exclusively reflect the opinions of the authors. The authors are responsible for all content in their abstracts including accuracy of the facts, statements, citing resources, etc.

### (PS1509) TARGETING RIBOSOMAL RNA 2´-O-METHYLATION SELECTIVELY IMPAIRS LEUKAEMIA STEM CELLS

**Topic:** 03. Acute myeloid leukemia - Biology & translational research

Jia Yan*[1, 2], Christian Rohde[1], Aline Sähr[1], Marina Scheller[1], Viral Shah[1], Friederike Schreiter[3], Johannes Backs[3], Carsten Müller-Tidow[1, 2], Fengbiao Zhou[1, 2]

*[1]University Hospital Heidelberg, Heidelberg, Germany; [2]EMBL Heidelberg, Molecular Medicine Partnership Unit (MMPU), Heidelberg, Germany; [3]Institute of Experimental Cardiology Heidelberg University, Medical Faculty Heidelberg, Heidelberg, Germany;*

### Background

Acute myeloid leukemia (AML) is initiated and sustained by leukemia stem cells (LSCs), which are responsible for disease propagation, relapse, and therapeutic resistance. Therefore, effective elimination of LSCs represents a critical therapeutic strategy for AML. Dysregulation of RNA modifications plays an important role in leukemogenesis. We recently demonstrated that 2´-O-methylation (2´-O-Me) of ribosomal RNA (rRNA) is increased at multiple sites in human LSCs (Zhou et al., Cancer Discovery 2023). Notably, 2´-O-Me at G1447 (Gm1447) in the 18S ribosomal small subunit is dynamically regulated in primary AML and is closely associated with LSC phenotypes. Gm1447 is directed by snoRNA SNORD127. Gm1447/SNORD127 may represent a novel and potentially targetable vulnerability in AML. We aim to investigate the role of Gm1447 in leukemia and healthy hematopoiesis.

### Aims

To investigate the role of the SNORD127/Gm1447 in LSC self-renewal and normal hematopoiesis, and to evaluate its potential as a therapeutic target for acute myeloid leukemia.

### Methods

Snord127 knockout (Snord127$^{-/-}$) mice were generated to investigate the function of Snord127/Gm1447. The rRNA 2´-O-Me pattern was determined by RiboMethSeq in hematopoietic cells and multiple other organs. MLL-AF9-driven leukemia was induced to assess leukemia propagation by serial transplantation. The LSC compartment was analyzed by flow cytometry and functionally quantified by *in vivo* limiting dilution transplantation. The translation activity in LSCs was quantified by an O-propargyl-puromycin (OP-Puro) incorporation assay. The impact of Gm1447/Snord127 on normal hematopoiesis was evaluated by competitive transplantation and multilineage reconstitution assays. In parallel, SNORD127-targeting antisense oligonucleotides (ASOs) were designed, and their anti-leukemic efficacy was tested in multiple human AML cell lines.

**Copyright Information:** (Online) ISSN: 2572-9241
©2026 The Author(s). HemaSphere is published by John Wiley & Sons Ltd on behalf of European Hematology Association. This is an open access Abstract Book distributed under the Attribution-NonCommercial-NoDerivs (CC BY-NC-ND), which allows third parties to download the articles and share them with others as long as they credit the author and the Abstract Book, but they cannot change the content in any way or use them commercially.
**Abstract Book Citations:** Authors, Title, HemaSphere, 2026;10:(S1):pages. The abstract book DOIs can be found on the “Author Information” page.
**Disclaimer:** Articles published in the journal HemaSphere exclusively reflect the opinions of the authors. The authors are responsible for all content in their abstracts including accuracy of the facts, statements, citing resources, etc.

### Results

Mice with heterozygous or homozygous deletion of Snord127 were born at expected Mendelian ratios. Snord127$^{-/-}$ and Snord127$^{+/-}$ mice were healthy without overt abnormalities. The 2′-O-Me at Gm1447, the target site of Snord127, was specifically and completely abolished in Snord127$^{-/-}$ cells. Snord127$^{-/-}$ mice displayed normal blood cell counts. Hematopoietic stem cell function was preserved as demonstrated by long-term competitive transplantation. In the MLL-AF9-induced leukemia model, loss of Snord127 delayed AML progression in secondary transplantation (median survival: Snord127$^{+/+}$ 22 days, Snord127$^{+/-}$ 37 days, Snord127$^{-/-}$ 71 days; $P < 0.0001$). Consistent with impaired leukemia-propagating capacity, the GMP-like LSC-enriched compartment in leukemic bone marrow was reduced in Snord127-deficient leukemia ($P < 0.005$). Limiting dilution transplantation showed a marked reduction in functional LSC frequency (Snord127$^{+/+}$ 1 in 2,701; Snord127$^{+/-}$ 1 in 31,486, P = 0.00888; Snord127$^{-/-}$ 1 in 61,590, P = 0.00052). Mechanistically, OP-Puro incorporation revealed a much higher protein synthesis rate in LSCs than in non-LSC compartments, and Snord127 loss significantly reduced protein synthesis in LSCs. Importantly, SNORD127-targeting ASOs reduced clonogenic growth in multiple human AML cell lines, including Kasumi-1, HL-60, and THP-1.

### Summary/Conclusion

SNORD127-guided 18S rRNA Gm1447 is essential for leukemia stem cell maintenance but dispensable for normal hematopoiesis. These findings suggest that therapeutic targeting of leukemic stem cells by inhibiting SNORD127 via antisense oligonucleotides is a promising targeting approach.

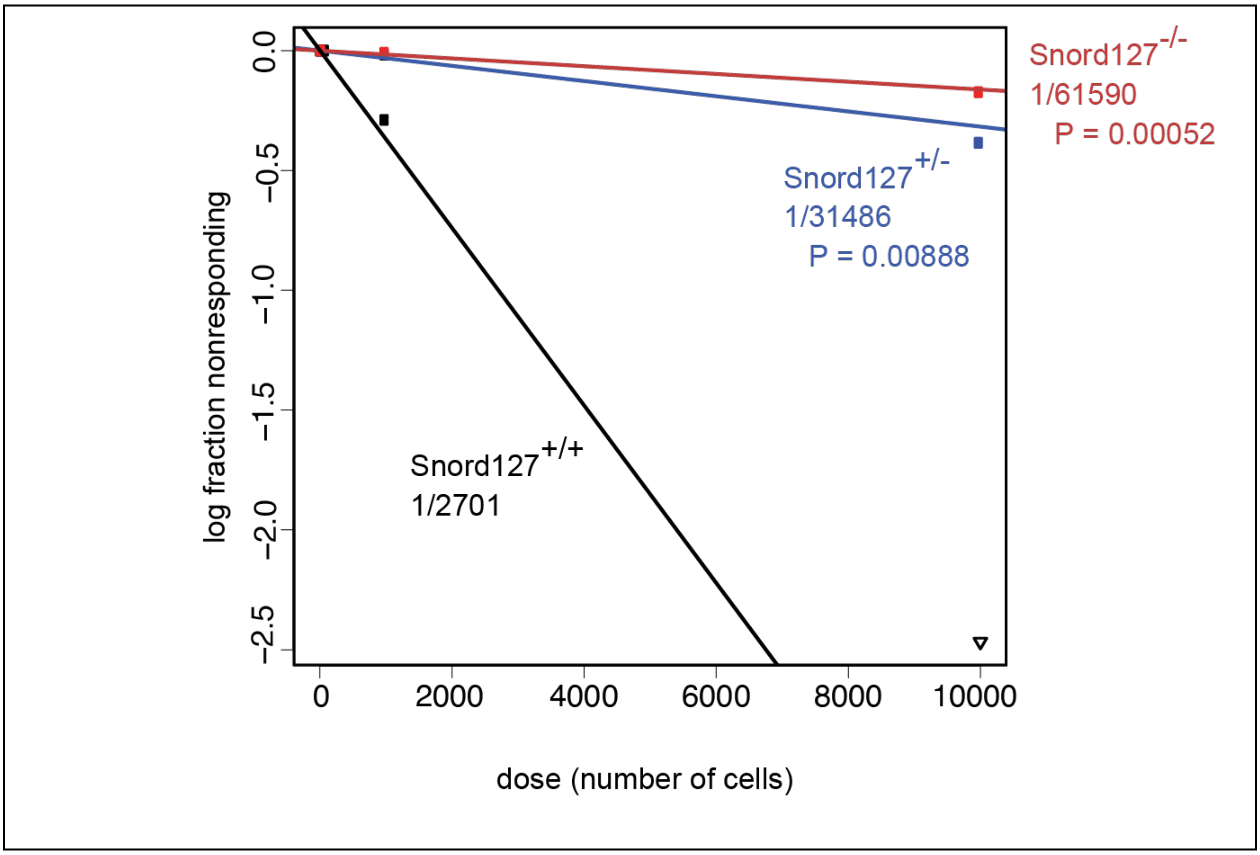

**Copyright Information:** (Online) ISSN: 2572-9241
©2026 The Author(s). HemaSphere is published by John Wiley & Sons Ltd on behalf of European Hematology Association. This is an open access Abstract Book distributed under the Attribution-NonCommercial-NoDerivs (CC BY-NC-ND), which allows third parties to download the articles and share them with others as long as they credit the author and the Abstract Book, but they cannot change the content in any way or use them commercially.
**Abstract Book Citations:** Authors, Title, HemaSphere, 2026;10:(S1):pages. The abstract book DOIs can be found on the “Author Information” page.
**Disclaimer:** Articles published in the journal HemaSphere exclusively reflect the opinions of the authors. The authors are responsible for all content in their abstracts including accuracy of the facts, statements, citing resources, etc.

### (PS1510) CSN5I-3 TARGETING OF JAB1 DISRUPTS FLT3-ITD SIGNALING AND IMPAIRS LEUKEMIC PROGRESSION IN ACUTE MYELOID LEUKEMIA

**Topic:** 03. Acute myeloid leukemia - Biology & translational research

Nan Zhang[1], Teng Wang[1], Qian Wang[2], Jian-Chuan Deng*[1]

*[1]The Second Affiliated Hospital of Chongqing Medical University, Department of hematology, Chongqing, China;[2]Sichuan Provincial People's Hospital, Department of hematology, Chengdu, China;*

### Background

FLT3-ITD mutations define a high-risk subset of acute myeloid leukemia (AML) characterized by persistent oncogenic signaling and frequent relapse despite the availability of FLT3 inhibitors. Strategies that directly target FLT3 kinase activity often fail to fully abrogate leukemic dependency. Targeting upstream regulatory mechanisms that sustain FLT3-ITD expression may represent a complementary therapeutic approach.

### Aims

To determine whether pharmacologic inhibition of Jab1 (CSN5), an upstream regulator of oncogenic signaling, can disrupt FLT3-ITD–driven leukemogenesis.

### Methods

FLT3-ITD AML cell lines and primary samples were subjected to Jab1 silencing and CSN5i-3 treatment. RNA-seq, MeRIP-seq, and ChIP-seq were performed to define transcriptional and epitranscriptomic regulatory programs. RNA stability assays assessed FLT3 transcript dynamics. Mx1-Cre–mediated conditional Jab1 deletion models were used to evaluate hematopoietic selectivity. Xenograft models assessed leukemic burden and survival following genetic or pharmacologic targeting.

### Results

Jab1 was significantly overexpressed in FLT3-ITD AML and associated with inferior clinical outcomes. Integrated RNA-seq and ChIP-seq analyses demonstrated that Jab1 directly occupied regulatory regions of IGF2BP3 and promoted its transcription, whereas no evidence supported direct transcriptional activation of FLT3. Consistently, Jab1 depletion reduced IGF2BP3 expression without affecting FLT3 primary transcript levels. MeRIP-seq revealed that loss of Jab1 led to a global reduction in m6A enrichment, including diminished m6A modification on FLT3 transcripts. RNA immunoprecipitation confirmed that IGF2BP3 bound FLT3 mRNA in an m6A-dependent manner, and Jab1 depletion significantly shortened FLT3 mRNA half-life, indicating impaired transcript stabilization rather than reduced transcription. Consequently, FLT3 protein abundance and downstream PI3K/AKT and MAPK signaling were markedly attenuated. Functionally, Jab1 silencing selectively impaired proliferation and induced apoptosis in FLT3-ITD AML cells. In vivo, Mx1-Cre–mediated Jab1 deletion significantly suppressed leukemic progression and prolonged survival without compromising normal hematopoiesis. Pharmacologic inhibition with CSN5i-3 recapitulated the genetic phenotype, reducing IGF2BP3 expression, destabilizing FLT3 transcripts, suppressing leukemic burden, and extending survival in FLT3-ITD xenograft models.

**Copyright Information:** (Online) ISSN: 2572-9241
©2026 The Author(s). HemaSphere is published by John Wiley & Sons Ltd on behalf of European Hematology Association. This is an open access Abstract Book distributed under the Attribution-NonCommercial-NoDerivs (CC BY-NC-ND), which allows third parties to download the articles and share them with others as long as they credit the author and the Abstract Book, but they cannot change the content in any way or use them commercially.
**Abstract Book Citations:** Authors, Title, HemaSphere, 2026;10:(S1):pages. The abstract book DOIs can be found on the "Author Information" page.
**Disclaimer:** Articles published in the journal HemaSphere exclusively reflect the opinions of the authors. The authors are responsible for all content in their abstracts including accuracy of the facts, statements, citing resources, etc.

**Summary/Conclusion**

Our findings identify Jab1 as a critical upstream regulator sustaining FLT3-ITD leukemic dependency through an IGF2BP3-mediated m6A stabilization mechanism rather than direct transcriptional control. Both genetic deletion and pharmacologic inhibition with CSN5i-3 disrupt this epitranscriptomic axis, attenuate oncogenic signaling, and confer significant survival benefit in vivo without impairing normal hematopoiesis. These data position Jab1 as a therapeutically actionable vulnerability in FLT3-ITD AML and support further development of CSN5-directed strategies as a complementary approach to current FLT3-targeted therapies.

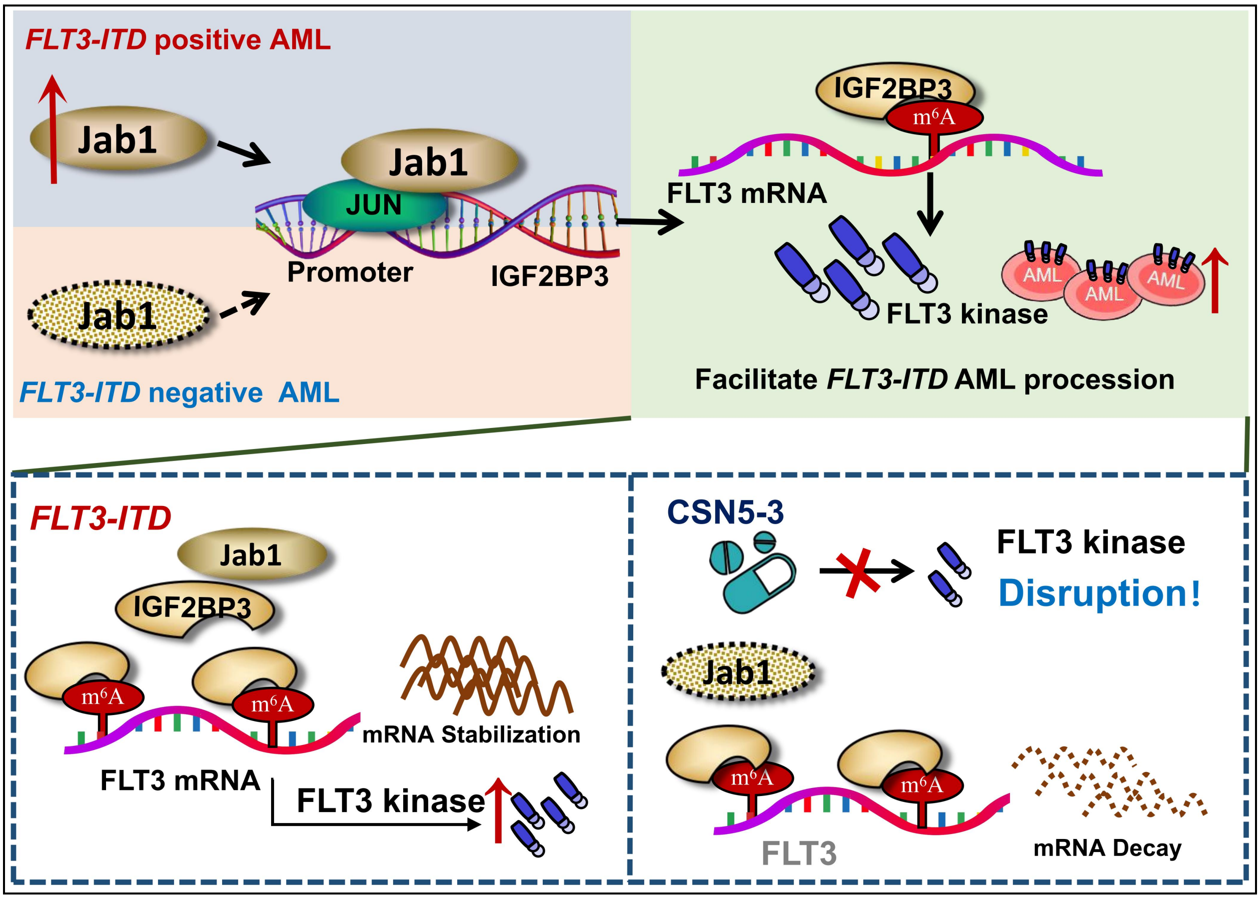

FLT3-ITD leukemogenesis is sustained by Jab1 mediated upregulation of IGF2BP3 reinforcing m6A dependent stabilization of FLT3 transcripts and amplifying oncogenic signaling, whereas pharmacologic inhibition with CSN5i-3 disrupts this axis suppresses leukemic progression and prolongs survival in vivo

**Copyright Information:** (Online) ISSN: 2572-9241
©2026 The Author(s). HemaSphere is published by John Wiley & Sons Ltd on behalf of European Hematology Association. This is an open access Abstract Book distributed under the Attribution-NonCommercial-NoDerivs (CC BY-NC-ND), which allows third parties to download the articles and share them with others as long as they credit the author and the Abstract Book, but they cannot change the content in any way or use them commercially.
**Abstract Book Citations:** Authors, Title, HemaSphere, 2026;10:(S1):pages. The abstract book DOIs can be found on the "Author Information" page.
**Disclaimer:** Articles published in the journal HemaSphere exclusively reflect the opinions of the authors. The authors are responsible for all content in their abstracts including accuracy of the facts, statements, citing resources, etc.

### (PS1511) HPCAL1-PPIL2 INTERACTION DRIVES GLYCOLYSIS AND REPRESENTS A THERAPEUTIC TARGET IN AML

**Topic:** 03. Acute myeloid leukemia - Biology & translational research

Linna Cheng*[1], Huiyang Zhang[1], Mengni Li[1], Qing Zhang[2], Hongchen Di[1], Rick Thorne[2], Zunmin Zhu[1]

[1] *Institution of Hematology，Henan Engineering Research Center for CAR-T Cell Therapy and Translational Applications，Henan Key Laboratory of Stem Cell Clinical Application and Key Technology，Henan Provincial People's Hospital of Zhengzhou University，School of Clinical Medicine, Henan University, Zhengzhou, China;* [2] *Translational Research Institute, Henan Provincial People's Hospital, Academy of Medical Science, Zhengzhou University, Zhengzhou, China;*

### Background

Acute myeloid leukemia (AML) continues to carry a poor prognosis, with long-term survival rates remaining discouragingly low despite recent therapeutic advances. This clinical reality underscores the pressing need to identify novel drivers of leukemogenesis that may serve as both prognostic biomarkers and therapeutic targets. In this study, we investigate Hippocalcin Like 1 (HPCAL1), a neuronal calcium sensor protein with no previously characterized function in hematopoiesis or myeloid malignancy, focusing on its potential role in AML pathogenesis and metabolic reprogramming.

### Aims

We aimed to define the clinical significance of HPCAL1 in AML, characterize its biological function in leukemic cell growth, and dissect the underlying molecular mechanism, with particular emphasis on its involvement in cancer metabolism.

### Methods

We examined HPCAL1 expression using publicly available transcriptomic datasets (GEO, TCGA) and primary patient samples, and assessed its prognostic relevance. Functional studies were performed using genetic knockdown and overexpression in human AML cell lines, with assays for proliferation, apoptosis, and colony formation.  In vivo leukemogenic potential was examined using both NSG xenograft and C57 AML murine models, while the role of HPCAL1 in normal hematopoiesis was investigated in systemic Hpcal1-knockout mice  Mechanistic investigations integrated RNA-sequencing, Seahorse metabolic flux analysis, and co-immunoprecipitation coupled with mass spectrometry to identify downstream pathways and interacting partners.

### Results

HPCAL1 was significantly upregulated in AML relative to normal hematopoietic tissues, and high expression independently predicted inferior overall survival. Functionally, HPCAL1 knockdown suppressed AML cell proliferation and induced apoptosis, whereas its overexpression conferred a growth advantage. In xenograft models, HPCAL1 overexpression accelerated disease progression, increased leukemic burden in the bone marrow, and shortened recipient survival. Notably, Hpcal1 knockout mice exhibited normal peripheral blood counts and intact hematopoietic stem/progenitor compartments, indicating that HPCAL1 is dispensable for normal hematopoiesis. Mechanistically, HPCAL1 overexpression enhanced glycolytic flux in AML cells, and proteomic analysis identified Peptidylprolyl Isomerase Like 2 (PPIL2) as a direct binding partner. PPIL2 knockdown abolished HPCAL1-driven glycolysis and proliferation, positioning the HPCAL1-PPIL2 axis as a critical regulator of Warburg metabolism in AML.

**Copyright Information:** (Online) ISSN: 2572-9241
©2026 The Author(s). HemaSphere is published by John Wiley & Sons Ltd on behalf of European Hematology Association. This is an open access Abstract Book distributed under the Attribution-NonCommercial-NoDerivs (CC BY-NC-ND), which allows third parties to download the articles and share them with others as long as they credit the author and the Abstract Book, but they cannot change the content in any way or use them commercially.
**Abstract Book Citations:** Authors, Title, HemaSphere, 2026;10:(S1):pages. The abstract book DOIs can be found on the "Author Information" page.
**Disclaimer:** Articles published in the journal HemaSphere exclusively reflect the opinions of the authors. The authors are responsible for all content in their abstracts including accuracy of the facts, statements, citing resources, etc.

**Summary/Conclusion**

We identify HPCAL1 as a previously unrecognized oncoprotein and independent prognostic factor in AML. HPCAL1 promotes leukemogenesis through physical interaction with PPIL2, leading to enhanced glycolytic reprogramming. The selective dependency of AML cells on this axis, contrasted with its dispensability in normal hematopoiesis, defines a potential therapeutic window. Targeting the HPCAL1-PPIL2 interaction may therefore represent a novel strategy for AML treatment, and ongoing efforts are focused on developing small-molecule inhibitors against this protein-protein interface.

**Copyright Information:** (Online) ISSN: 2572-9241
©2026 The Author(s). HemaSphere is published by John Wiley & Sons Ltd on behalf of European Hematology Association. This is an open access Abstract Book distributed under the Attribution-NonCommercial-NoDerivs (CC BY-NC-ND), which allows third parties to download the articles and share them with others as long as they credit the author and the Abstract Book, but they cannot change the content in any way or use them commercially.
**Abstract Book Citations:** Authors, Title, HemaSphere, 2026;10:(S1):pages. The abstract book DOIs can be found on the "Author Information" page.
**Disclaimer:** Articles published in the journal HemaSphere exclusively reflect the opinions of the authors. The authors are responsible for all content in their abstracts including accuracy of the facts, statements, citing resources, etc.

### (PS1512) ULTRA-HIGH-SENSITIVITY NEXT-GENERATION SEQUENCING–BASED MRD PREDICTS OUTCOME IN INTENSIVELY TREATED OLDER PATIENTS WITH ACUTE MYELOID LEUKEMIA: RESULTS FROM THE ALFA-1200 COHORT

**Topic:** 03. Acute myeloid leukemia - Biology & translational research

Augustin Boudry*[1], Laurene Fenwarth[1], Raphael Itzykson[2], Thorsten Braun[3], Lionel Ades[2], Elise Fournier[1], Céline Berthon[4], Delphine Lebon[5], Stephane de Botton[6], Régis Peffault de Latour[7], Cécile Pautas[3], Hervé Dombret[8], Emmanuel Raffoux[2], Claude Gardin[3], Karine Cellilebras[8], Claude Preudhomme[1], Nicolas Duployez[1]

*[1]Laboratory of Hematology, Biology and Pathology Center, CHU Lille, Lille, France;[2]Service Hématologie Adultes, Assistance Publique-Hôpitaux de Paris (APHP), Hôpital Saint-Louis, F-75010, Paris, France; [3]Department of Hematology, Hôpital Avicenne, AP-HP, Bobigny, France; [4]Department of Hematology, Claude Huriez Hospital, CHU Lille, Lille, France;[5]Department of Hematology and Cell Therapy, CHU Amiens-Picardie, Amiens, France; [6]Département d'Hématologie, Institut Gustave Roussy, Faculté Paris-Saclay, Villejuif, France; [7]Department of Stem Cell Transplantation, Hôpital Saint-Louis, AP-HP, Paris, France; [8]ALFA Coordination, Paris, France;*

### Background

Measurable residual disease (MRD) is a major biomarker of remission depth and relapse risk in acute myeloid leukemia (AML). Ultra-high-sensitivity next-generation sequencing (UHS-NGS) enables sensitive, multi-target detection of residual leukemic clones, but its prognostic value has mainly been established in younger intensively treated patients. Dedicated data in older AML patients, characterized by distinct molecular landscapes and frequent secondary AML associated mutations, remain limited.

### Aims

Evaluation of the prognostic value of UHS-NGS MRD in intensively treated AML patients aged ≥60 years enrolled in the ALFA-1200 trial.

### Methods

Among 506 patients enrolled in ALFA-1200, 369 achieved CR/CRp. Post-IDAC2 bone marrow samples were available for 113 patients; 93 had available diagnostic NGS and at least one trackable molecular marker. MRD was assessed after the second intermediate-dose cytarabine consolidation using an error-corrected UHS-NGS approach at a sensitivity threshold of $10^{-4}$, after exclusion of *DNMT3A*, *TET2*, *ASXL1*, and *IDH1/2* variants (*BJH 2025 Boudry et al.*). Myelodysplasia-related gene (MRG) mutations were defined according to ELN 2022 (*BCOR*, *EZH2*, *STAG2*, *RUNX1*, *SF3B1*, *U2AF1*, *SRSF2*, *ZRSR2*). Cumulative incidence of relapse (CIR) was modeled using Fine-Gray regression with death as a competing event, while relapse-free survival (RFS) and overall survival (OS) were analyzed using extended Cox models including allogeneic transplantation in first remission as a time-dependent covariate.

**Copyright Information:** (Online) ISSN: 2572-9241
©2026 The Author(s). HemaSphere is published by John Wiley & Sons Ltd on behalf of European Hematology Association. This is an open access Abstract Book distributed under the Attribution-NonCommercial-NoDerivs (CC BY-NC-ND), which allows third parties to download the articles and share them with others as long as they credit the author and the Abstract Book, but they cannot change the content in any way or use them commercially.
**Abstract Book Citations:** Authors, Title, HemaSphere, 2026;10:(S1):pages. The abstract book DOIs can be found on the "Author Information" page.
**Disclaimer:** Articles published in the journal HemaSphere exclusively reflect the opinions of the authors. The authors are responsible for all content in their abstracts including accuracy of the facts, statements, citing resources, etc.

**Results**

MRD was detectable in 49/93 patients (52.7%). The proportion of MRD-positive patients was 53%, 36%, and 70% in ELN 2022 favorable, intermediate, and adverse-risk groups, respectively (p=0.03). MRD-positive patients had higher 2-year CIR (73% vs 38%, p=0.003), inferior RFS (23% vs 55%, p=0.008), and lower OS (54% vs 79%, p=0.041).
Among patients with only MRG mutations detectable in remission, associated mutations detectable in remission, those with >1 persistent mutation had markedly inferior outcomes compared with those harboring a single mutation (2-year CIR 100% vs 50%, p<0.001; RFS 0% vs 50%, p<0.001; OS 43% vs 84%, p=0.038).
Based on these findings, an integrated MRD classification was defined as persistence of ≥1 non–MRG mutation or ≥2 MRG mutations. Using this model, MRD-positive patients had inferior 2-year CIR (81% vs 41%), RFS (13% vs 54%), and OS (42% vs 81%) (all p<0.001). In multivariable analyses, integrated MRD positivity remained associated with relapse (sHR 3.49), RFS (HR 3.26), and OS (HR 2.75) (all p<0.001).

**Summary/Conclusion**

Error-corrected UHS-NGS MRD retains strong prognostic value in intensively treated older AML patients. Beyond binary MRD assessment, quantitative interpretation of MRG mutations and integrated molecular models may improve risk stratification. These findings support the development of adapted MRD frameworks for elderly AML patients, but warrant confirmation in larger, independent cohorts.

**Copyright Information:** (Online) ISSN: 2572-9241
©2026 The Author(s). HemaSphere is published by John Wiley & Sons Ltd on behalf of European Hematology Association. This is an open access Abstract Book distributed under the Attribution-NonCommercial-NoDerivs (CC BY-NC-ND), which allows third parties to download the articles and share them with others as long as they credit the author and the Abstract Book, but they cannot change the content in any way or use them commercially.
**Abstract Book Citations:** Authors, Title, HemaSphere, 2026;10:(S1):pages. The abstract book DOIs can be found on the “Author Information” page.
**Disclaimer:** Articles published in the journal HemaSphere exclusively reflect the opinions of the authors. The authors are responsible for all content in their abstracts including accuracy of the facts, statements, citing resources, etc.

### (PS1513) ERADICATION OF LEUKEMIC STEM CELLS IN ACUTE MYELOID LEUKEMIA BY INDUCTION OF EXCESSIVE PRO-INFLAMMATORY SIGNALING RESULTS IN DEEP AND DURABLE REMISSION IN AML PDX MODELS

**Topic:** 03. Acute myeloid leukemia - Biology & translational research

Sayantanee Dutta[1], Ulrike Höckendorf[2], Arnold Kloos[3], Stefanie Prein[1], Marah Runtsch[1, 4], Carina Zötsch[4], Sebastian Vosberg[5], Yongjie Wang[6], Sophie Kienreich[1], Bettina Flasch[1], Grazia Malovan[1], Vanessa Jäger[1], Stefanie Stanzer[1], Timo Odinius[7], Celina Wagner[2], Lars Buschhorn[2], Veronika Dill[2], Bianca Perfler[1], Torsten Haferlach[8], Konstanze Döhner[9], Katharina Götze[2], Jürgen Ruland[2], Florian Bassermann[2], Adam Wahida[10], Mathias Heikenwälder[11], Caterina Branca[12], Johannes Schmoellerl[13], Johannes Zuber[13], Ann-Cathrin Burk[14], Robert Zeiser[14], Heinz Sill[1], Ashok Jayavelu[15], Armin Zebisch[1], Michael Heuser[16], Michael Dengler[1], Philipp Jost*[1]

*[1]Medical University of Graz, Graz, Austria; [2]Technical University of Munich, München, Germany; [3]Medizinische Hochschule Hannover, Hannover, Germany; [4]Cycuria Therapeutics, Graz, Austria; [5]LMU Munich, Munich, Germany; [6]University of Heidelberg, Heidelberg, Germany; [7]University Hospital Zürich, Zürich, Switzerland; [8]Munich Leukemia Laboratory, Munich, Germany; [9]Uniklinik Ulm, Ulm, Germany; [10]Massachusetts' General Hospital (MGH), Boston, United States of America; [11]German Cancer Research Center (DKFZ), Heidelberg, Germany; [12]University of Florida, GAINESVILLE, United States of America; [13]Research Institute of Molecular Pathology, Vienna, Austria; [14]Freiburg University Medical Center, Freiburg, Germany; [15]DKFZ, Hopp Children's Cancer Center (KiTZ), University of Heidelberg, Heidelberg, Germany; [16]Martin-Luther-Universität, Halle, Germany;*

### Background

Leukemic stem cells (LSCs) arise from hematopoietic progenitors through acquisition of oncogenic mutations and represent a major driver of relapse in acute myeloid leukemia (AML) due to their persistence during chemotherapy. Their close physiological resemblance to normal hematopoietic stem and progenitor cells (HSPCs) renders selective therapeutic targeting challenging. Increasing evidence indicates that inflammatory signaling, both intrinsic to LSCs and within their niche, critically regulates clonal selection, maintenance, and survival.

### Aims

To define the impact of tumor necrosis factor (TNF)–driven inflammatory signaling on LSCs and normal HSPCs.

### Methods

We utilized gene-targeted mouse models and syngeneic bone marrow transplantation of murine leukemia, complemented by AML patient-derived xenograft (PDX) models and ex vivo primary AML samples together with healthy HSPCs to study inflammatory signaling in AML. In addition, we utilized CRISPR-mediated gene editing in AML cell lines. Molecular analyses included transcriptomic analysis and analysis of published single-cell RNA sequencing datasets from AML patients and healthy donors.

**Copyright Information:** (Online) ISSN: 2572-9241
©2026 The Author(s). HemaSphere is published by John Wiley & Sons Ltd on behalf of European Hematology Association. This is an open access Abstract Book distributed under the Attribution-NonCommercial-NoDerivs (CC BY-NC-ND), which allows third parties to download the articles and share them with others as long as they credit the author and the Abstract Book, but they cannot change the content in any way or use them commercially.
**Abstract Book Citations:** Authors, Title, HemaSphere, 2026;10:(S1):pages. The abstract book DOIs can be found on the “Author Information” page.
**Disclaimer:** Articles published in the journal HemaSphere exclusively reflect the opinions of the authors. The authors are responsible for all content in their abstracts including accuracy of the facts, statements, citing resources, etc.

**Results**

TNF receptor agonists, including TNF and lymphotoxin-α (LTα3), induced cell death across 14 AML cell lines, with variable responses and marked sensitivity in a distinct subset. Genetic deletion of murine *Lta* resulted in aggressive, serially transplantable AML with an expanded pool of primitive blasts in a syngeneic transplantation model. *Ex vivo* treatment with recombinant human LTα3 (rhLTα3) induced cell death and/or differentiation in $Lin^{-}CD99^{+}$ LSCs and $CD34^{+}$ AML progenitors across multiple genetic subgroups (n = 30), whereas healthy HSPCs (n = 68) were largely unaffected and instead exhibited a beneficial proliferative response. *In vivo*, administration of LTα3 resulted in sustained disease remission in both AML PDX models and syngeneic bone marrow transplantation models of murine leukemia. Mechanistically, LTα3 engages TNFR1 and TNFR2 and induces depletion of the adaptor protein TRAF2, destabilizing the pro-survival TNFR1 signaling complex. As a consequence, canonical pro-survival signaling is attenuated, promoting cell death and differentiation programs. Genetically engineered AML cells lacking TRAF2 showed increased sensitivity to rhLTα3, which was restored upon re-expression of TRAF2. Early transcriptional responses to LTα3 were marked by inflammatory pathway activation and concomitant suppression of MYC and oxidative phosphorylation programs in sensitive AML cell lines and primary patient samples. Compared with TNF, LTα3 induced qualitatively similar but quantitatively attenuated transcriptional changes in AML and healthy HSPCs, suggesting a broader therapeutic window for LTα3.

**Summary/Conclusion**

Our findings uncover a selective vulnerability of LSCs in AML driven by excessive cytokine-dependent inflammatory signaling while preserving healthy hematopoiesis, thereby offering a strategy to decouple AML therapy from hematopoietic toxicity.

**Copyright Information:** (Online) ISSN: 2572-9241
©2026 The Author(s). HemaSphere is published by John Wiley & Sons Ltd on behalf of European Hematology Association. This is an open access Abstract Book distributed under the Attribution-NonCommercial-NoDerivs (CC BY-NC-ND), which allows third parties to download the articles and share them with others as long as they credit the author and the Abstract Book, but they cannot change the content in any way or use them commercially.
**Abstract Book Citations:** Authors, Title, HemaSphere, 2026;10:(S1):pages. The abstract book DOIs can be found on the "Author Information" page.
**Disclaimer:** Articles published in the journal HemaSphere exclusively reflect the opinions of the authors. The authors are responsible for all content in their abstracts including accuracy of the facts, statements, citing resources, etc.

### (PS1514) MULTI-OMICS CLASSIFICATION OF ACUTE MYELOID LEUKEMIA GUIDES DRUG COMBINATIONS TO OVERCOME VENETOCLAX RESISTANCE

**Topic:** 03. Acute myeloid leukemia - Biology & translational research

Runyu Yang*[1], Hui Feng[1], Fan Niu[1], Peng-Cheng He[1]

[1] *The First Affiliated Hospital of Xi'an Jiaotong University, Department of Hematology, Xi'an, China;*

### Background

Acute myeloid leukemia (AML) is an aggressive hematological malignancy. Conventional risk stratification in AML fails to predict patient responses to targeted therapies like Venetoclax, hindering precision medicine and the development of strategies to overcome drug resistance.

### Aims

Integrating multi-omics data to identify Venetoclax-resistant subgroups in AML using artificial intelligence and screen for feasible combination therapies.

### Methods

We established an integrated multi-omics framework incorporating mRNA/lncRNA expression, DNA methylation, copy number alterations, and somatic mutation data. Using nine complementary clustering algorithms, we identified molecular subtypes in a discovery cohort, validated in independent external cohorts. The multi-omics classification subsequently guided the screening of high risk subtypes specific sensitizers to Venetoclax, with candidate efficacy validated through in *vitro* and in *vivo* experiments.

### Results

We identified three molecularly distinct AML subtypes with unique clinical manifestations, a classification subsequently validated in independent external cohorts. Cluster 2 demonstrated the most favorable prognosis, while Cluster 3, characterized by high TP53 mutation frequency and significant immune infiltration, exhibited the poorest outcomes and pronounced resistance to Venetoclax. Multi-omics-guided drug screening revealed that Cluster 3 displays particular sensitivity to both Elesclomol and the clinically available proteasome inhibitor Bortezomib. Through comprehensive in *vitro* and in *vivo* validation, we demonstrated that both agents significantly enhance the therapeutic efficacy of Venetoclax. Of particular translational relevance, the Venetoclax-Bortezomib combination leverages an agent with an established safety profile, presenting a readily tractable strategy for near-future clinical translation.

### Summary/Conclusion

Our study establishes a novel multi-omics classification system that provides a robust foundation for investigating biological heterogeneity, elucidating resistance mechanisms, and developing effective combination strategies to achieve personalized therapy in AML.

**Copyright Information:** (Online) ISSN: 2572-9241
©2026 The Author(s). HemaSphere is published by John Wiley & Sons Ltd on behalf of European Hematology Association. This is an open access Abstract Book distributed under the Attribution-NonCommercial-NoDerivs (CC BY-NC-ND), which allows third parties to download the articles and share them with others as long as they credit the author and the Abstract Book, but they cannot change the content in any way or use them commercially.
**Abstract Book Citations:** Authors, Title, HemaSphere, 2026;10:(S1):pages. The abstract book DOIs can be found on the "Author Information" page.
**Disclaimer:** Articles published in the journal HemaSphere exclusively reflect the opinions of the authors. The authors are responsible for all content in their abstracts including accuracy of the facts, statements, citing resources, etc.

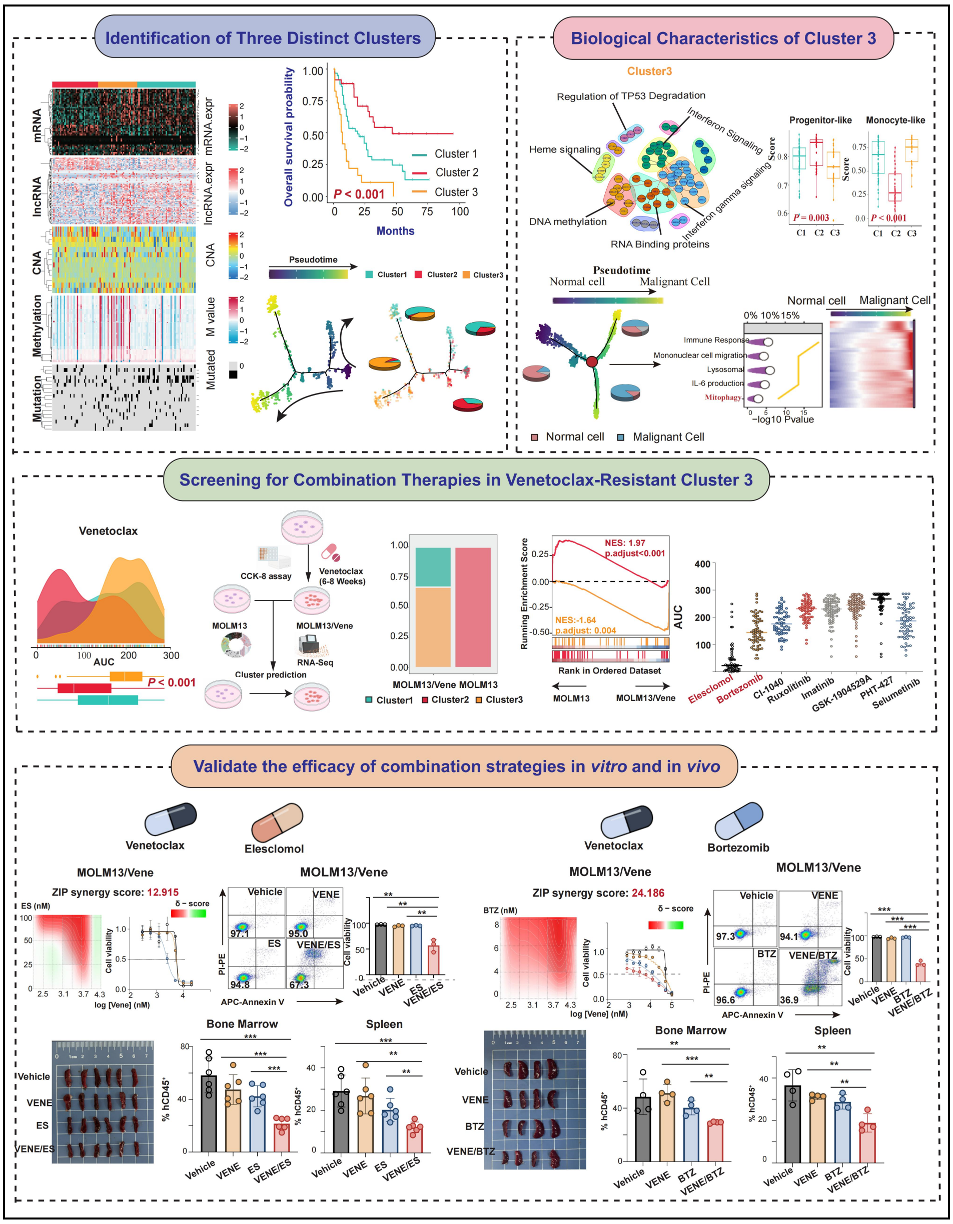

**Copyright Information:** (Online) ISSN: 2572-9241
©2026 The Author(s). HemaSphere is published by John Wiley & Sons Ltd on behalf of European Hematology Association. This is an open access Abstract Book distributed under the Attribution-NonCommercial-NoDerivs (CC BY-NC-ND), which allows third parties to download the articles and share them with others as long as they credit the author and the Abstract Book, but they cannot change the content in any way or use them commercially.
**Abstract Book Citations:** Authors, Title, HemaSphere, 2026;10:(S1):pages. The abstract book DOIs can be found on the "Author Information" page.
**Disclaimer:** Articles published in the journal HemaSphere exclusively reflect the opinions of the authors. The authors are responsible for all content in their abstracts including accuracy of the facts, statements, citing resources, etc.

### (PS1515) CD96 AS A THERAPEUTIC TARGET FOR CAR T-CELL THERAPY IN ACUTE MYELOID LEUKEMIA

**Topic:** 03. Acute myeloid leukemia - Biology & translational research

Xiaotong Gao*[1, 2, 3], Hailing Liu[1], Guokun Li[4], Yali Zhou[4], Yan Dong[2, 5], Xiao Chen[1], Wen Fan[2], Erbing Li[4], Sanmei Wang[1], Qinyao Luan[4], Jing Zhang[4], Hui Jin[1], Hui Liao[3], Yizhou Luo[3], Li Liu[2], Weiwei Qin[2], Jiangtao Ren[4], Jianyong Li[1, 6, 7, 8], Lei Fan[1, 6, 7]

*[1]Jiangsu Province Hospital, the First Affiliated Hospital with Nanjing Medical University, Department of Hematology, Nanjing, China; [2]Tangdu Hospital, Air Force Medical University, Department of Hematology, Xi'an, China;[3]Air Force Hospital of Eastern Theater, Department of Oncology and Hematology, Nanjing, China; [4]Nanjing Bioheng Biotech Co., Ltd, Nanjing, China; [5]Shanghai Institute of Hematology, State Key Laboratory of Medical Genomics, National Research Center for Translational Medicine at Shanghai, Ruijin Hospital Affiliated to Shanghai Jiao Tong University School of Medicine, Shanghai, China; [6]Key Laboratory of Hematology of Nanjing Medical University, Nanjing, China; [7]Jiangsu Key Lab of Cancer Biomarkers, Prevention and Treatment, Collaborative Innovation Center for Personalized Cancer Medicine, Nanjing Medical University, Nanjing, China; [8]National Clinical Research Center for Hematologic Diseases, the First Affiliated Hospital of Soochow University, Suzhou, China;*

### Background

Therapies leveraging chimeric antigen receptor (CAR) T cells for acute myeloid leukemia (AML) are limited by the scarcity of leukemia stem cell (LSC)-specific antigens.

### Aims

This study aimed to identify and validate CD96 as a novel immunotherapy target for AML, and to develop and optimize CD96-directed CAR T cells. A further objective was to design a combinatorial strategy to address antigen-low AML while preserving normal hematopoiesis.

### Methods

CD96 expression was interrogated in patient-derived AML cells and normal hematopoietic stem/progenitor cells (HSPCs). We engineered CD96-specific CAR T cells with different structural domains and evaluated their function against AML models in vitro and in vivo. Efficacy against CD96-low AML was augmented by co-expressing a CD33-targeted chimeric costimulatory receptor (CCR), and potential hematologic toxicity was assessed.

### Results

We found that CD96 is expressed in 48.3% of AML patients, with higher expression on LSCs than blasts, and is absent on normal HSPCs. We developed a panel of CD96-CAR T cells using single-chain variable fragments derived from various monoclonal antibodies, incorporating distinct transmembrane and costimulatory domains. Treatment with CD96-CAR T cells confers specific anti-leukemic activity correlated with CD96 expression. Notably, CAR T cells featuring a CD28 transmembrane and costimulatory domain (CD96-28z) exhibit enhanced proliferation and cytotoxic capabilities in vitro. In vivo, CD96-28z potently eliminated AML cells and prolonged survival in mice bearing CD96-high, but not CD96-low, AML. To address CD96-low AML, we combined CD96-28z with a CD33-CCR, thereby increasing cytotoxic efficacy. Importantly, CD96-CAR T cells did not inhibit colony formation by HSPCs during manufacturing.

**Copyright Information:** (Online) ISSN: 2572-9241
©2026 The Author(s). HemaSphere is published by John Wiley & Sons Ltd on behalf of European Hematology Association. This is an open access Abstract Book distributed under the Attribution-NonCommercial-NoDerivs (CC BY-NC-ND), which allows third parties to download the articles and share them with others as long as they credit the author and the Abstract Book, but they cannot change the content in any way or use them commercially.
**Abstract Book Citations:** Authors, Title, HemaSphere, 2026;10:(S1):pages. The abstract book DOIs can be found on the "Author Information" page.
**Disclaimer:** Articles published in the journal HemaSphere exclusively reflect the opinions of the authors. The authors are responsible for all content in their abstracts including accuracy of the facts, statements, citing resources, etc.

**Summary/Conclusion**

These findings indicate that CD96 is a promising target for AML immunotherapy, and the combination of CD96-CAR and CD33-CCR may represent a potent strategy for patients with CD96-positive AML while preserving normal hematopoiesis.

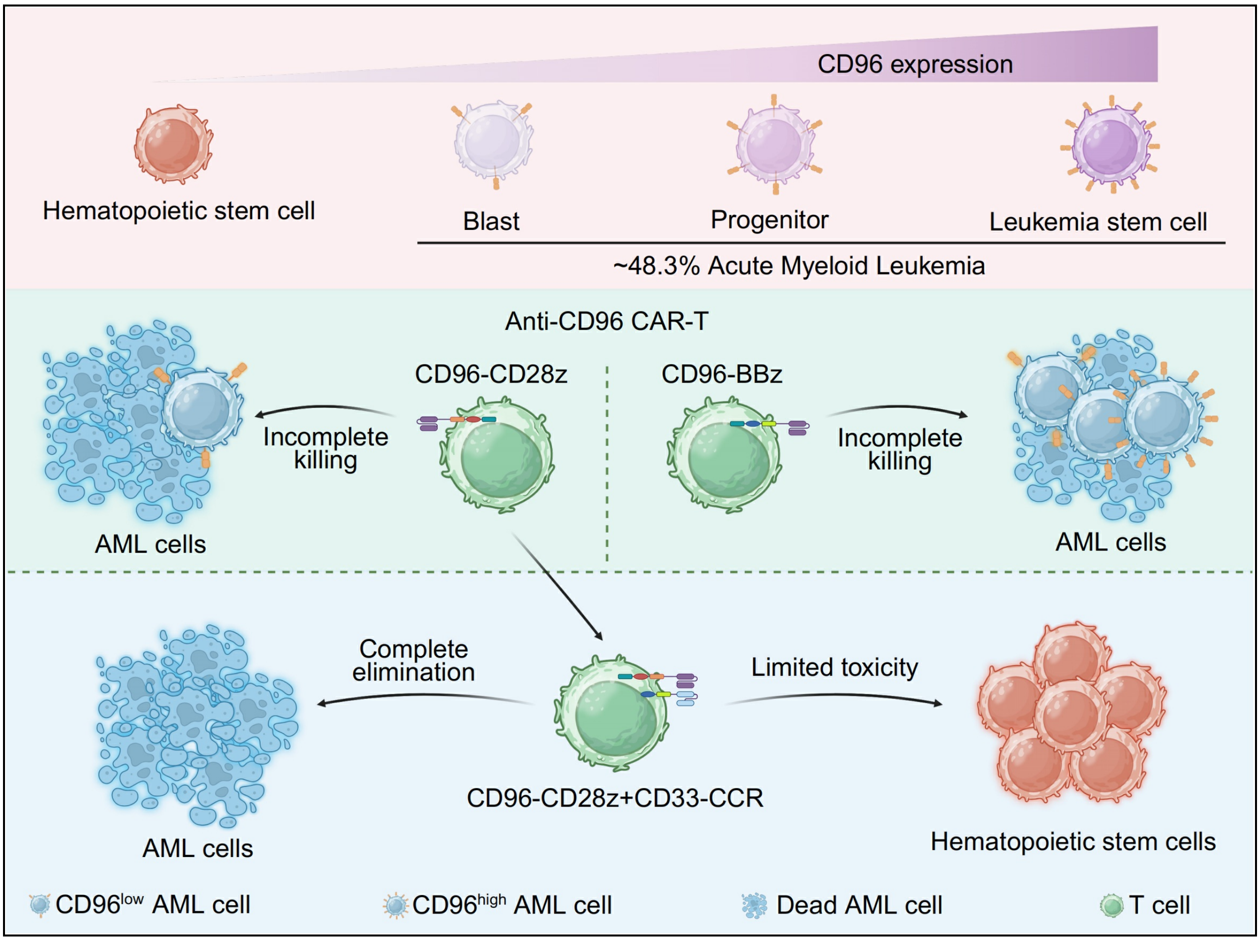

**Copyright Information:** (Online) ISSN: 2572-9241
©2026 The Author(s). HemaSphere is published by John Wiley & Sons Ltd on behalf of European Hematology Association. This is an open access Abstract Book distributed under the Attribution-NonCommercial-NoDerivs (CC BY-NC-ND), which allows third parties to download the articles and share them with others as long as they credit the author and the Abstract Book, but they cannot change the content in any way or use them commercially.
**Abstract Book Citations:** Authors, Title, HemaSphere, 2026;10:(S1):pages. The abstract book DOIs can be found on the "Author Information" page.
**Disclaimer:** Articles published in the journal HemaSphere exclusively reflect the opinions of the authors. The authors are responsible for all content in their abstracts including accuracy of the facts, statements, citing resources, etc.

### (PS1516) FLT3-ITD REPROGRAMS LEUCINE ANABOLISM TO DRIVE ACUTE MYELOID LEUKEMIA PROGRESSION

**Topic:** 03. Acute myeloid leukemia - Biology & translational research

Jun Long*[1], Xinjie Chen[2], Guilin Xu[1], Yan Shen[3], Xiu Luo[1], Wenjun Zhang[1], Ping Li[1], Jiong Hu[2], Aibin Liang[1]

*[1]Tongji hospital affiliated to Tongji University, Shanghai, China;[2]Shanghai Institute of Hematology, State Key Laboratory of Medical Genomics, National Research Center for Translational Medicine at Shanghai, Ruijin Hospital, Shanghai Jiao Tong University School of Medicine, Shanghai, China; [3]Ruijin hospital, Shanghai, China;*

### Background

FLT3 internal tandem duplication (FLT3-ITD) mutations are among the most frequent genetic alterations in acute myeloid leukemia (AML), driving aggressive disease progression and poor prognosis. Although FLT3 inhibitors show initial efficacy, resistance often develops, highlighting the need for novel therapeutic strategies. Metabolic reprogramming is a hallmark of cancer, and FLT3-ITD has been shown to alter various metabolic pathways in AML, including serine, glutamine, and fatty acid metabolism. However, the intrinsic metabolic differences between FLT3-ITD and FLT3-WT AML cells remain poorly understood.

### Aims

(1) To investigate how FLT3-ITD reprograms metabolism in AML, focusing on branched-chain amino acid (BCAA) metabolism. (2) To identify metabolic vulnerabilities specific to FLT3-ITD AML using CRISPR/Cas9 screens. (3) To evaluate the therapeutic potential of targeting leucine anabolism, either through dietary restriction or pharmacological inhibition of the leucine transporter SLC7A5. (4) To explore combinatorial strategies involving leucine restriction and FLT3 or BCL2 inhibition to overcome resistance.

### Methods

Mouse Models: Generated MLL-rearranged, FLT3-ITD-driven AML models and performed transcriptomic (RNA-seq) and metabolomic analyses. CRISPR Screens: Conducted in vitro and in vivo CRISPR/Cas9 metabolic screens to identify FLT3-ITD-specific vulnerabilities. Functional Validation: Used genetic ablation (SLC7A5 knockout) and pharmacological inhibition (BCH) to assess the role of leucine transport in FLT3-ITD AML. Dietary Intervention: Tested graded dietary leucine restriction in AML mouse models. Mechanistic Studies: Integrated ribosome profiling (Ribo-seq) and RNA-seq to explore leucine's role in mitochondrial electron transport chain (ETC) complex biosynthesis. Combination Therapy: Evaluated synergies between leucine restriction and FLT3 (quizartinib) or BCL2 (venetoclax) inhibitors.

### Results

FLT3-ITD upregulated BCAA metabolism, particularly leucine uptake via SLC7A5, leading to increased intracellular leucine levels. *In vitro* and *in vivo* metabolic CRISPR screens identified SLC7A5 as a top vulnerability in FLT3-ITD AML, with its loss selectively impairing leukemia progression. FLT3-ITD AML cells exhibited heightened sensitivity to leucine restriction, which reduced mitochondrial ETC complex translation and compromised energy metabolism. Dietary leucine restriction delayed AML progression and synergized with FLT3 or BCL2 inhibition to enhance survival, even in resistant disease.

**Copyright Information:** (Online) ISSN: 2572-9241
©2026 The Author(s). HemaSphere is published by John Wiley & Sons Ltd on behalf of European Hematology Association. This is an open access Abstract Book distributed under the Attribution-NonCommercial-NoDerivs (CC BY-NC-ND), which allows third parties to download the articles and share them with others as long as they credit the author and the Abstract Book, but they cannot change the content in any way or use them commercially.
**Abstract Book Citations:** Authors, Title, HemaSphere, 2026;10:(S1):pages. The abstract book DOIs can be found on the “Author Information” page.
**Disclaimer:** Articles published in the journal HemaSphere exclusively reflect the opinions of the authors. The authors are responsible for all content in their abstracts including accuracy of the facts, statements, citing resources, etc.

**Summary/Conclusion**

This study reveals that FLT3-ITD reprograms leucine anabolism to sustain AML progression by fueling mitochondrial ETC biosynthesis. SLC7A5-mediated leucine uptake emerges as a critical metabolic dependency, and its inhibition—either genetically or through dietary restriction—selectively targets FLT3-ITD AML while sparing normal hematopoiesis. Combining leucine restriction with FLT3 or BCL2 inhibitors offers a promising strategy to overcome therapeutic resistance. These findings highlight the potential of targeting leucine metabolism as a novel approach for FLT3-ITD AML treatment.

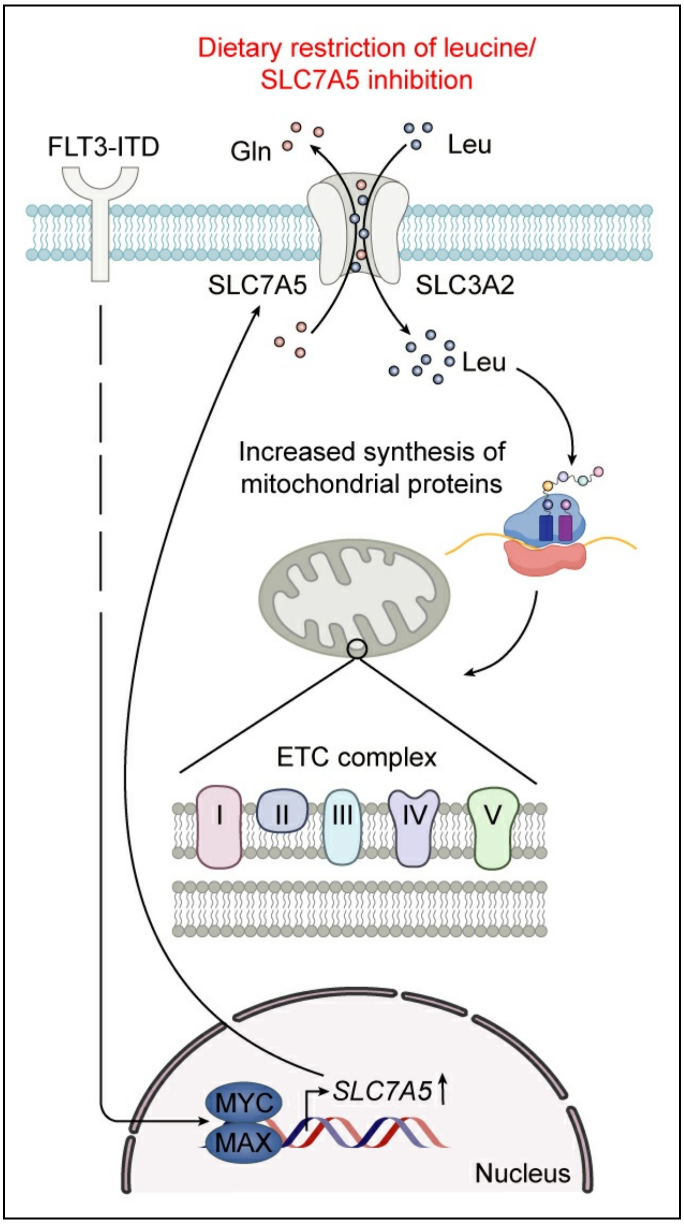

**Copyright Information:** (Online) ISSN: 2572-9241
©2026 The Author(s). HemaSphere is published by John Wiley & Sons Ltd on behalf of European Hematology Association. This is an open access Abstract Book distributed under the Attribution-NonCommercial-NoDerivs (CC BY-NC-ND), which allows third parties to download the articles and share them with others as long as they credit the author and the Abstract Book, but they cannot change the content in any way or use them commercially.
**Abstract Book Citations:** Authors, Title, HemaSphere, 2026;10:(S1):pages. The abstract book DOIs can be found on the "Author Information" page.
**Disclaimer:** Articles published in the journal HemaSphere exclusively reflect the opinions of the authors. The authors are responsible for all content in their abstracts including accuracy of the facts, statements, citing resources, etc.

### (PS1517) KMT2E/SWI-SNF-INDUCED CHROMATIN REMODELING ENHANCES TRANSFORMATION BUT ALSO ENABLES ATRA-INDUCED DIFFERENTIATION IN ACUTE MYELOID LEUKEMIA

**Topic:** 03. Acute myeloid leukemia - Biology & translational research

Isabel Weinhäuser*[1, 2, 3], Diego A Pereira-Martins[1, 2, 3], Luise Albuquerque-Simões[1, 2, 4], Luciana Yamamoto Almeida[2], Vincent van den Boom[1], Fatemeh Mojallali[1], Dominique Sternadt[1], Prodromos Chatzikyriakou[3], Lucio Henrique Sousa Pinheiro[2, 4], Lynn Quek[3], Gerwin Huls[1], Eduardo Rego[2, 4], Jan Jacob Schuringa[1]

*[1]University Medical Center Groningen, Experimental Hematology, Groningen, The Netherlands; [2]University of Sao Paulo and Hemocentro Ribeirao Preto, Department of Internal Medicine and the Center for Cell Based Therapy, Ribeirao Preto, Brazil; [3]King's College London, Myeloid Leukaemia Genomics and Biology Group, School of Cancer and Pharmaceutical Sciences, London, United Kingdom; [4]University of São Paulo Medical School, Laboratory of Medical Investigation in Pathogenesis and Targeted Therapy in Onco-Immuno-Hematology (LIM-31), Department of Internal Medicine, Hematology Division, Sao Paulo, Brazil;*

### Background

*KMT2E* was initially proposed as a histone lysine methyltransferase 2 (KMT2) family member based on domain homology, but unlike KMT2A–D it lacks methyltransferase activity and instead binds H3K4me3 via its PHD domain. Its location on chromosome 7q, frequently lost in myeloid malignancies, suggested a tumor-suppressive role, yet our previous work showed that KMT2E overexpression (OE) accelerates proliferation and increases tumor burden in AML, pointing to a more complex oncogenic function.

### Aims

Here, we investigated the molecular mechanisms by which KMT2E promotes leukemic progression and explored therapeutic vulnerabilities.

### Methods

We performed scRNA-seq on 29 AML samples (105,842 cells) to map *KMT2E* expression across cell types and compared transcriptional programs in *KMT2E* high vs low across the LSPC subtypes. We validated these programs in KMT2E-OE cells by RNA-seq. To interrogate chromatin regulation, we integrated ATACseq with H3K4me3/H3K27ac ChIP-seq in several AML models upon KMT2E-OE. Endogenous KMT2E-interacting partners were identified by pull-down and LC-MS/MS. *In vivo*, NSG mice were xenografted subcutaneously with U937 control vs KMT2E-OE cells and treated with ATRA.

**Copyright Information:** (Online) ISSN: 2572-9241
©2026 The Author(s). HemaSphere is published by John Wiley & Sons Ltd on behalf of European Hematology Association. This is an open access Abstract Book distributed under the Attribution-NonCommercial-NoDerivs (CC BY-NC-ND), which allows third parties to download the articles and share them with others as long as they credit the author and the Abstract Book, but they cannot change the content in any way or use them commercially.
**Abstract Book Citations:** Authors, Title, HemaSphere, 2026;10:(S1):pages. The abstract book DOIs can be found on the "Author Information" page.
**Disclaimer:** Articles published in the journal HemaSphere exclusively reflect the opinions of the authors. The authors are responsible for all content in their abstracts including accuracy of the facts, statements, citing resources, etc.

### Results

*KMT2E* was broadly expressed across AML cell types. Pseudobulk analysis of *KMT2E*$^{high}$ LSPCs revealed 509 genes commonly upregulated across HSC, LMPP and early GMP-like cells, including multiple SWI/SNF (BAF) complex members (SMARCA2, ARID1A, EP300) and oxidative phosphorylation (OXPHOS)-related genes, indicating increased reliance on mitochondrial metabolism. OCI-AML3 KMT2E-OE recapitulated this BAF/OXPHOS signature, and chromatin profiling showed that KMT2E-OE induces global chromatin opening at loci linked to cell-cycle progression and OXPHOS. Functionally, KMT2E-OE models displayed increased mitochondrial respiration, confirming OXPHOS dependency. KMT2E interactome revealed physical interactions with canonical BAF components (ARID1A, SMARCA4, SMARCA2) and DNA damage repair proteins (PARP1), consistent with a scaffold role recruiting chromatin-remodeling machinery. Integration of SMARCA4 and H3K27ac ChIP-seq with ATAC-seq data showed enhanced accessibility at BAF-regulated enhancers in KMT2E-OE cells. KMT2E ChIP-seq further confirmed direct binding at the *ARID1A* locus, co-localizing with H3K4me3 and H3K27ac marks, supporting BAF-mediated KMT2E chromatin remodeling. Given the requirement of the ARID1A–BAF axis for myeloid maturation, we tested whether KMT2E-driven recruitment of this complex re-sensitizes AML to differentiation therapy. In xenografts, KMT2E-OE U937 cells formed larger tumors than controls, but this advantage was abrogated by ATRA treatment. ATAC-seq revealed increased chromatin accessibility at retinoic acid receptor (RAR/RXR) target genes in KMT2E-OE cells, indicating an epigenetically primed state for ATRA-induced granulocytic differentiation. Consistently, ChIP-seq on ATRA treated cells showed enhanced RARA binding at downstream targets such as *CEBPA* in KMT2E-OE models.

### Summary/Conclusion

KMT2E acts as a critical regulator of chromatin accessibility by recruiting the BAF complex, driving OXPHOS-dependent leukemic progression while maintaining open chromatin at myeloid differentiation loci. This dual role creates a therapeutic vulnerability whereby KMT2E$^{high}$ AML cells become re-sensitized to differentiation-inducing agents such as ATRA. Thus, KMT2E functions as an epigenetic primer that reinforces leukemic fitness yet exposes an opportunity for targeted differentiation therapy.

**Copyright Information:** (Online) ISSN: 2572-9241
©2026 The Author(s). HemaSphere is published by John Wiley & Sons Ltd on behalf of European Hematology Association. This is an open access Abstract Book distributed under the Attribution-NonCommercial-NoDerivs (CC BY-NC-ND), which allows third parties to download the articles and share them with others as long as they credit the author and the Abstract Book, but they cannot change the content in any way or use them commercially.
**Abstract Book Citations:** Authors, Title, HemaSphere, 2026;10:(S1):pages. The abstract book DOIs can be found on the “Author Information” page.
**Disclaimer:** Articles published in the journal HemaSphere exclusively reflect the opinions of the authors. The authors are responsible for all content in their abstracts including accuracy of the facts, statements, citing resources, etc.

### (PS1518) OVERCOMING THE UNDRUGGABLE: AN AI-DRIVEN DESIGN PEPTIDE INHIBITOR TARGETING THE TBK1-OPTN INTERACTION SUPPRESSES AML AND REVERSES VENETOCLAX RESISTANCE

**Topic:** 03. Acute myeloid leukemia - Biology & translational research

Runyu Yang[1], Hui Feng[1], Peng-Cheng He[1], Fan Niu*[1]

[1] *The First Affiliated Hospital of Xi'an Jiaotong University, Department of Hematology, Xi'an, China;*

### Background

Our previous studies have established that the TBK1/OPTN interaction enhances mitophagy, thereby promoting AML cell proliferation and resistance to Venetoclax, highlighting the TBK1/OPTN axis as a potential therapeutic target in AML. However, crystallographic analysis reveals that the TBK1-OPTN interaction is primarily mediated by hydrophobic contacts between α-helices, forming a classic protein-protein interaction (PPI) interface. Such targets are often considered challenging to drug due to the absence of well defined small-molecule binding pockets, posing a significant barrier to clinical translation.

### Aims

Peptide inhibitors, characterized by their extensive interaction interfaces and high specificity, offer unique advantages in targeting PPIs such as the TBK1/OPTN complex. Leveraging precise structural information of the TBK1/OPTN interface, we employed artificial intelligence to design and optimize an inhibitory peptide aimed at overcoming the undruggability of this target.

### Methods

Based on the crystal structure of the TBK1/OPTN complex (PDB: 5EOF), we identified key residues at the binding interface through molecular dynamics (MD) simulations, free energy calculations (MM/GBSA), and hotspot analysis. The core OPTN-binding region was subsequently truncated from TBK1. After sequence alignment and solubility optimization, the deep learning model ProteinMPNN was utilized to generate high-affinity inhibitory peptides, designated as TOIP. The peptide was delivered using our established selenium nanoparticle delivery system. The in *vitro* efficacy of TOIP against AML cell lines and primary patient samples was assessed using CCK-8 assays and flow cytometry. In *vivo* anti-leukemic activity was evaluated in MOLM13 systemic xenograft models and patient-derived xenograft (PDX) models. The impact of TOIP on mitophagy and mitochondrial function was investigated using Seahorse analysis and mito-Keima assays. Finally, the ability of TOIP to sensitize Venetoclax was validated in both in *vitro* and in *vivo* Venetoclax-resistant models.

### Results

We successfully designed an inhibitory peptide targeting the TBK1/OPTN interface and achieved efficient intracellular delivery using a selenium nanoparticle system. TOIP effectively disrupted the TBK1-OPTN interaction, concomitantly reducing mitophagy and oxidative phosphorylation levels while increasing mitochondrial reactive oxygen species (Mito-Sox) accumulation in AML cells. TOIP demonstrated significant cytotoxicity against a panel of AML cell lines representing diverse FAB subtypes. In *vivo*, TOIP treatment reduced leukemic burden and prolonged survival in both MOLM13 CDX and PDX models. Importantly, TOIP exhibited a favorable safety profile, showing no off-target toxicity against $CD34^+$ hematopoietic stem cells from cord blood in *vitro* and no significant hepatorenal or organ toxicity at therapeutic doses in *vivo.* Furthermore, TOIP effectively resensitized Venetoclax-resistant AML cells to Venetoclax treatment in *vitro*, an effect that was corroborated in an in *vivo* Venetoclax-resistant model.

**Copyright Information:** (Online) ISSN: 2572-9241
©2026 The Author(s). HemaSphere is published by John Wiley & Sons Ltd on behalf of European Hematology Association. This is an open access Abstract Book distributed under the Attribution-NonCommercial-NoDerivs (CC BY-NC-ND), which allows third parties to download the articles and share them with others as long as they credit the author and the Abstract Book, but they cannot change the content in any way or use them commercially.
**Abstract Book Citations:** Authors, Title, HemaSphere, 2026;10:(S1):pages. The abstract book DOIs can be found on the "Author Information" page.
**Disclaimer:** Articles published in the journal HemaSphere exclusively reflect the opinions of the authors. The authors are responsible for all content in their abstracts including accuracy of the facts, statements, citing resources, etc.

### Summary/Conclusion

TOIP disrupts the TBK1-OPTN interaction, suppresses mitophagy, and exerts anti-leukemic effects while enhancing sensitivity to Venetoclax, with a favorable safety profile at therapeutic doses. TOIP represents a promising translational strategy derived from our previous mechanistic insights into the role of OPTN in AML.

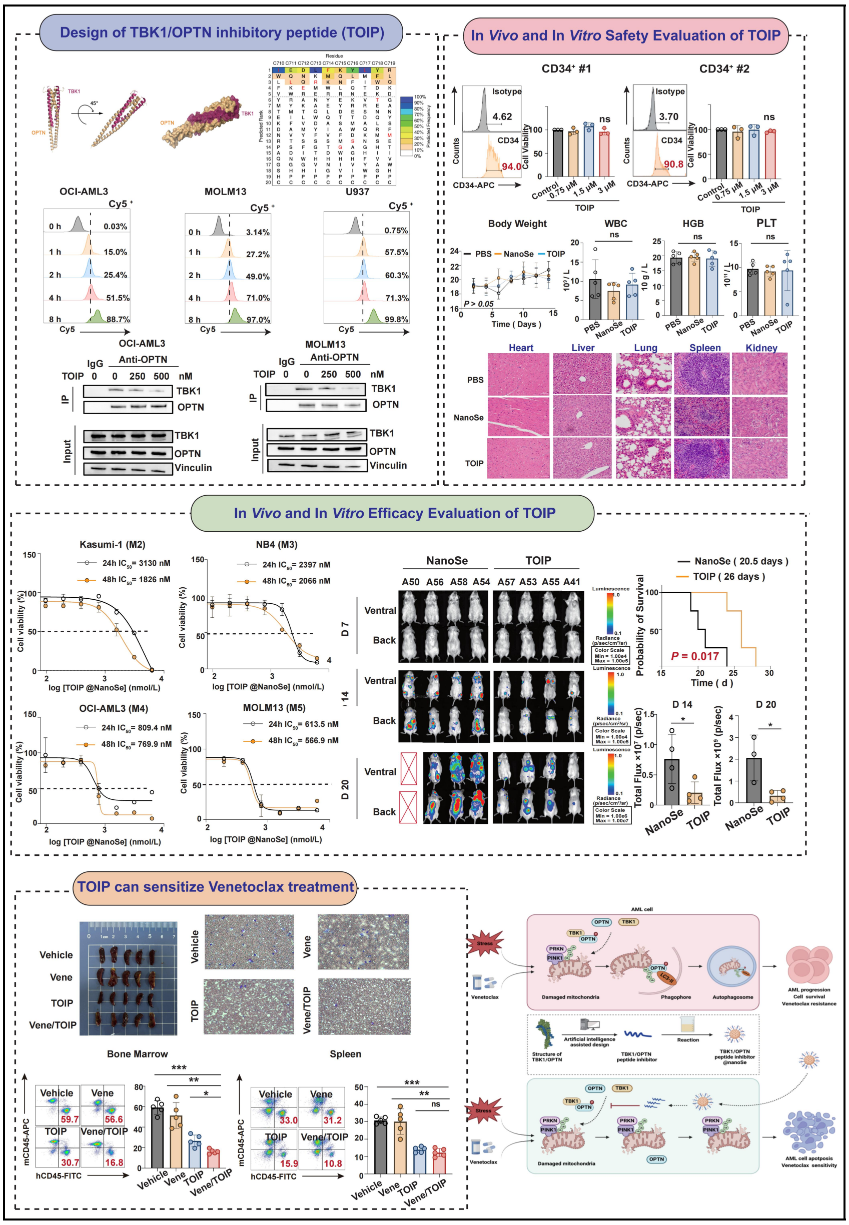

**Copyright Information:** (Online) ISSN: 2572-9241
©2026 The Author(s). HemaSphere is published by John Wiley & Sons Ltd on behalf of European Hematology Association. This is an open access Abstract Book distributed under the Attribution-NonCommercial-NoDerivs (CC BY-NC-ND), which allows third parties to download the articles and share them with others as long as they credit the author and the Abstract Book, but they cannot change the content in any way or use them commercially.
**Abstract Book Citations:** Authors, Title, HemaSphere, 2026;10:(S1):pages. The abstract book DOIs can be found on the "Author Information" page.
**Disclaimer:** Articles published in the journal HemaSphere exclusively reflect the opinions of the authors. The authors are responsible for all content in their abstracts including accuracy of the facts, statements, citing resources, etc.

### (PS1519) DECODING THE IMMUNE GEOGRAPHY OF ACUTE MYELOID LEUKEMIA IN THE LUNG

**Topic:** 03. Acute myeloid leukemia - Biology & translational research

Varvara Paraskevopoulou*[1], Ziyan Lin[1], Marta Casado-Pelaez[2], Daniela Grases[2], Wafa Al-Santli[1], Juan Carlos Balandrán[1], Fang Zhou[1], Ali Rashidfarrokhi[1], Michael Cross[1], Stephen Yeung[1], Konstantinos Ntatsoulis[1], Tejas Patel[1], Xufeng Chen[1], Deedra Nicolet[3], Marc Escobosa[2], Eduard Porta[2], Jennifer Trowbridge[4], Kamal Khanna[1], Thales Papagiannakopoulos[1], Andre Moreira[1], Rashmi Kanagal-Shamanna[5], Sanam Loghavi[5], Aris Tsirigos[1], Ann-Kathrin Eisfeld[3], Manel Esteller[6], Ioannis Aifantis[1]

*[1]NYU Grossman School of Medicine, New York, United States of America;[2]Josep Carreras Leukaemia Research Institute, Badalona, Spain; [3]Clara D. Bloomfield Center for Leukemia Outcomes Research, The Ohio State University Comprehensive Cancer Center, Columbus, United States of America; [4]The Jackson Laboratory, Bar Harbor, United States of America; [5]The University of Texas MD Anderson Cancer Center, Houston, United States of America; [6]Sant Pau Research Institute, Cancer Epigenetics Group, Barcelona, Spain;*

### Background

Acute myeloid leukemia (AML) is the most common acute leukemia in adults, with poor survival (Hayat et al. 2007). Research has focused on AML-bone marrow interactions (Baryawno et al. 2019; van Galen et al. 2019; Beneyto-Calabuig et al. 2023; Lasry et al. 2023; Yeaton et al. 2022); however, AML also exits the marrow and infiltrates solid tissues, and we know little about how leukemia interacts with extramedullary microenvironments. In the acute setting, respiratory failure occurs frequently due to leukemia infiltration of the lungs (Azoulay et al. 2003) and is a leading cause of early deaths, with autopsy studies reporting involvement in ~60% of patients (Kovalski et al. 1990; Hildebrand et al. 1990; Bodey et al. 1966). The underlying mechanisms remain unexplored, and therapeutic interventions are only empiric.

### Aims

We aimed to comprehensively characterize the AML lung tumor microenvironment (TME) and identify mechanisms driving pulmonary infiltration and respiratory compromise.

### Methods

We combined single-cell RNA sequencing with high-resolution spatial transcriptomics (Xenium 5k plex, 10x Genomics) and *in vivo* functional studies to define the cellular composition, spatial organization, and cell–cell interactions within the AML lung TME. *In silico* proximity mapping and ligand–receptor interaction analyses identified candidate regulators of cell–cell communication, which were functionally tested in murine models to investigate their role in lung infiltration.Clinical observations from AML patients with respiratory compromise were also evaluated to assess translational relevance.

**Copyright Information:** (Online) ISSN: 2572-9241
©2026 The Author(s). HemaSphere is published by John Wiley & Sons Ltd on behalf of European Hematology Association. This is an open access Abstract Book distributed under the Attribution-NonCommercial-NoDerivs (CC BY-NC-ND), which allows third parties to download the articles and share them with others as long as they credit the author and the Abstract Book, but they cannot change the content in any way or use them commercially.
**Abstract Book Citations:** Authors, Title, HemaSphere, 2026;10:(S1):pages. The abstract book DOIs can be found on the "Author Information" page.
**Disclaimer:** Articles published in the journal HemaSphere exclusively reflect the opinions of the authors. The authors are responsible for all content in their abstracts including accuracy of the facts, statements, citing resources, etc.

### Results

AML infiltration profoundly remodeled the lung microenvironment, characterized by expansion of fibroblasts and myofibroblasts, loss of capillary aerocytes, a marked increase in non-classical monocytes, and depletion of lymphoid lineages. These alterations were associated with impaired barrier integrity, compromised gas exchange, and activation of inflammatory pathways. Spatial analyses revealed preferential localization of AML cells at vascular–alveolar interfaces enriched for alveolar Type 1 epithelial cells, endothelial cells, and non-classical monocytes, forming permissive inflammatory niches. Targeting inflammation with glucocorticoids significantly reduced inflammatory responses and leukemic lung infiltration *in vivo*. Importantly, AML patients with suspected leukemic lung involvement experienced rapid improvement in oxygenation and pulmonary function following short courses of steroid administration. Mechanistically, we identified the S-type lectin Galectin-9 (Lgals9) (Rabinovich and Toscano 2009) as an inflammation-induced mediator of cell–cell interactions within the infiltrated lungs. The IL-33/IL-33 receptor axis also contributed to leukemic–niche crosstalk. Both *LGALS9* and *IL1RL1* were highly expressed in AML patients. Targeting either the Lgals9 or IL-33 axis significantly decreased lung infiltration by modulating both the immune landscape and AML cells themselves.

### Summary/Conclusion

We provide the first single-cell and spatial atlas of AML in the lung, representing the first leukemia map outside of the bone marrow for any blood cancer. AML-driven inflammation and stromal remodeling impair lung integrity and function, while Galectin-9 and IL-33 signaling emerge as critical regulators of leukemic lung infiltration, identifying key inflammatory immune mediators that can be targeted to prevent pulmonary complications and early mortality in AML.

**Copyright Information:** (Online) ISSN: 2572-9241
©2026 The Author(s). HemaSphere is published by John Wiley & Sons Ltd on behalf of European Hematology Association. This is an open access Abstract Book distributed under the Attribution-NonCommercial-NoDerivs (CC BY-NC-ND), which allows third parties to download the articles and share them with others as long as they credit the author and the Abstract Book, but they cannot change the content in any way or use them commercially.
**Abstract Book Citations:** Authors, Title, HemaSphere, 2026;10:(S1):pages. The abstract book DOIs can be found on the "Author Information" page.
**Disclaimer:** Articles published in the journal HemaSphere exclusively reflect the opinions of the authors. The authors are responsible for all content in their abstracts including accuracy of the facts, statements, citing resources, etc.

### (PS1520) THE HOXA9/METTL3/BMI1 AXIS PROMOTES ANTHRACYCLINE RESISTANCE IN ACUTE MYELOID LEUKEMIA THROUGH MRNA M6A MODIFICATION OF BMI1

**Topic:** 03. Acute myeloid leukemia - Biology & translational research

Hao Wang*[1, 2], Liping Dou[2]

[1]*Medical School of Chinese PLA, Beijing, China, Beijing, China;*[2]*State Key Laboratory of Experimental Hematology, Senior Department of Hematology, The Fifth Medical Center of PLA General Hospital, Beijing, China, Beijing, China;*

### Background

Despite the recent approval of several novel agents for acute myeloid leukemia (AML), relapse due to anthracycline resistance remains a major therapeutic challenge. Epigenetic alterations have been implicated in anthracycline-resistant AML, yet the precise underlying mechanisms are not fully understood.

### Aims

This study aims to delineate the role of the HOXA9/METTL3/BMI1 axis in driving anthracycline resistance in acute myeloid leukemia (AML). We seek to elucidate its mechanism—from HOXA9-mediated epigenetic activation of METTL3 to METTL3-driven, m$^6$A-dependent BMI1 stabilization—and to leverage this axis as a therapeutic target to overcome chemoresistance.

### Methods

To elucidate chromatin-level mechanisms of resistance, we performed comparative ATAC-seq and RNA-seq profiling in paired anthracycline-sensitive and -resistant AML cells. Transcriptional regulators of the m$^6$A methyltransferase METTL3 were predicted via motif enrichment analysis of accessible chromatin regions and integrated with expression data. HOXA9 was prioritized as a top candidate based on its strong correlation with METTL3 expression and its association with clinical drug resistance and poor prognosis. The functional role of the HOXA9/METTL3 axis was validated through lentiviral-mediated knockdown and overexpression, and by using the selective METTL3 inhibitor STM2457. To identify downstream effectors, we conducted MeRIP-seq, which identified BMI1 as a key m6A target. METTL3-dependent regulation of BMI1 was further confirmed by MeRIP-qPCR and RNA stability assays. The therapeutic potential of targeting this axis was evaluated by assessing cell viability and apoptosis following pharmacological or genetic inhibition of METTL3 and BMI1.

### Results

Anthracycline-resistant AML cells exhibited globally reduced chromatin accessibility, yet showed significantly increased accessibility at the METTL3 promoter region. This localized chromatin opening correlated with elevated METTL3 expression at both mRNA and protein levels in resistant cells and patient samples. Motif analysis and functional experiments identified HOXA9 as a transcriptional activator of METTL3 via direct binding to the “TAACGTG” motif within its proximal promoter. Clinically, HOXA9 was upregulated in drug-resistant and relapsed/refractory AML and correlated with poor prognosis. Functionally, HOXA9 depletion suppressed METTL3 expression and impaired leukemic proliferation. METTL3 overexpression conferred anthracycline resistance by increasing m$^6$A levels. Mechanistically, MeRIP-seq identified BMI1 as a direct m6A-modified target of METTL3. METTL3-mediated m6A modification stabilized BMI1 transcripts and increased its expression. More importantly, targeting BMI1 reversed the survival advantage conferred by the HOXA9/METTL3/BMI1 axis and restored anthracycline sensitivity both in vitro and in vivo.

**Copyright Information:** (Online) ISSN: 2572-9241
©2026 The Author(s). HemaSphere is published by John Wiley & Sons Ltd on behalf of European Hematology Association. This is an open access Abstract Book distributed under the Attribution-NonCommercial-NoDerivs (CC BY-NC-ND), which allows third parties to download the articles and share them with others as long as they credit the author and the Abstract Book, but they cannot change the content in any way or use them commercially.
**Abstract Book Citations:** Authors, Title, HemaSphere, 2026;10:(S1):pages. The abstract book DOIs can be found on the “Author Information” page.
**Disclaimer:** Articles published in the journal HemaSphere exclusively reflect the opinions of the authors. The authors are responsible for all content in their abstracts including accuracy of the facts, statements, citing resources, etc.

### Summary/Conclusion

Our findings define a critical HOXA9/METTL3/BMI1 axis that drives anthracycline resistance in AML. This axis operates through HOXA9-mediated chromatin remodeling to transactivate METTL3, which in turn stabilizes *BMI1* mRNA via $m^6A$ modification to promote cell survival. Targeted disruption of this pathway, through inhibition of METTL3 or BMI1, represents a promising therapeutic strategy for overcoming chemoresistance in AML.

**Copyright Information:** (Online) ISSN: 2572-9241
©2026 The Author(s). HemaSphere is published by John Wiley & Sons Ltd on behalf of European Hematology Association. This is an open access Abstract Book distributed under the Attribution-NonCommercial-NoDerivs (CC BY-NC-ND), which allows third parties to download the articles and share them with others as long as they credit the author and the Abstract Book, but they cannot change the content in any way or use them commercially.
**Abstract Book Citations:** Authors, Title, HemaSphere, 2026;10:(S1):pages. The abstract book DOIs can be found on the “Author Information” page.
**Disclaimer:** Articles published in the journal HemaSphere exclusively reflect the opinions of the authors. The authors are responsible for all content in their abstracts including accuracy of the facts, statements, citing resources, etc.

### (PS1521) LOSS OF FBXO11 DRIVES A STEM CELL PROGRAM IN ACUTE MYELOID LEUKEMIA VIA DYSREGULATION OF MITOCHONDRIAL LONP1

**Topic:** 03. Acute myeloid leukemia - Biology & translational research

Franziska Mey*[1, 2], Hayle Kincross[1, 2], Ya-Chi Angela Mo[2], Xuan Wang[2], Linda Chang[2], Gerben Duns[2], Jihong Jiang[2], Grace Cheng[2], Ramon Klein Geltink[3], Aly Karsan[1, 2, 4]

*[1]University of British Columbia, Vancouver, Canada; [2]British Columbia Cancer Research Centre, Vancouver, Canada; [3]B C Women's Hospital & Health Centre, Vancouver, Canada; [4]Vancouver General Hospital, Vancouver, Canada;*

### Background

Acute Myeloid Leukemia (AML) is an aggressive hematologic malignancy with poor clinical outcomes. AML is genetically and biologically heterogeneous, characterized by impaired differentiation and expansion of leukemic stem and progenitor cells. Although recurrent mutations in transcription factors, epigenetic regulators, and signalling pathways have been well described, less is known about the contribution of the ubiquitin pathway to AML ontogeny. Through integrative proteogenomic analyses, we identified recurrent mutations and downregulation in ubiquitin pathway genes, with the F-box protein FBXO11 being the most significantly downregulated component of the SKP1-CUL1-F-box (SCF) E3 ubiquitin ligase complex in AML. SCF E3 ubiquitin ligase complexes confer substrate specificity through F-box proteins, suggesting loss of *FBXO11* may dysregulate the activity of $SCF^{FBXO11}$ target proteins in AML. These observations suggest a potentially important role for *FBXO11* in regulating stem cell programs and leukemic transformation.

### Aims

We sought to define the consequences of dysregulated ubiquitination following *FBXO11* loss in hematopoietic stem and progenitor cells (HSPC). We assessed how loss of *FBXO11* contributes to leukemogenesis, including cooperation with established oncogenic drivers.

### Methods

We analyzed sequencing data from large AML cohorts (1727 unique cases) to identify ubiquitin pathway aberrations and expression changes. *FBXO11* expression was experimentally suppressed in primary human $CD34^{+}$ HSPCs or leukemic cell lines using lentiviral shRNA or CRISPR-Cas9 KO. Effects on cell cycle, differentiation, transcriptional programs, and mitochondrial function were assessed by flow cytometry, colony-forming assays, RNA sequencing, and Seahorse XF Analyzer. Protein–protein interaction assays were performed by mass spectrometry or confocal microscopy to identify and validate FBXO11 substrates. Finally, human xenograft models combining AML1-ETO and $KRAS^{G12D}$ mutations with or without *FBXO11* depletion were employed to evaluate leukemogenic potential in vivo.

**Copyright Information:** (Online) ISSN: 2572-9241
©2026 The Author(s). HemaSphere is published by John Wiley & Sons Ltd on behalf of European Hematology Association. This is an open access Abstract Book distributed under the Attribution-NonCommercial-NoDerivs (CC BY-NC-ND), which allows third parties to download the articles and share them with others as long as they credit the author and the Abstract Book, but they cannot change the content in any way or use them commercially.
**Abstract Book Citations:** Authors, Title, HemaSphere, 2026;10:(S1):pages. The abstract book DOIs can be found on the "Author Information" page.
**Disclaimer:** Articles published in the journal HemaSphere exclusively reflect the opinions of the authors. The authors are responsible for all content in their abstracts including accuracy of the facts, statements, citing resources, etc.

**Results**

Approximately 11% of adult AML cases harboured mutations in ubiquitin pathway genes. FBXO11 interacted with LONP1 and catalyzed its K63-linked ubiquitination, a modification required for efficient mitochondrial localization. *FBXO11* depletion impaired LONP1-mediated assembly of electron transport chain Complex IV, resulting in reduced mitochondrial respiration. Functionally, *FBXO11* loss drove a myeloid-biased, quiescent stem cell phenotype in vitro, with enrichment for HSPC transcriptional programs and increased myeloid over megakaryocytic or erythroid output.

In vivo, *FBXO11*depletion cooperated with AML1-ETO and $KRAS^{G12D}$ to induce a myeloid malignancy, demonstrating that *FBXO11* loss enhances leukemic initiation and self-renewal capacity.

**Summary/Conclusion**

Our findings establish *FBXO11* as a critical regulator of mitochondrial metabolism and stem cell fate in human hematopoiesis. Loss of *FBXO11* impairs LONP1-dependent mitochondrial function, enforcing a metabolically reprogrammed, quiescent stem cell state that cooperates with oncogenic drivers to initiate leukemia. This work reveals a leukemogenic mechanism linking ubiquitin-mediated regulation of mitochondrial metabolism with stem cell programs, offering potential avenues for targeted metabolic or ubiquitin pathway interventions in AML.

**Copyright Information:** (Online) ISSN: 2572-9241
©2026 The Author(s). HemaSphere is published by John Wiley & Sons Ltd on behalf of European Hematology Association. This is an open access Abstract Book distributed under the Attribution-NonCommercial-NoDerivs (CC BY-NC-ND), which allows third parties to download the articles and share them with others as long as they credit the author and the Abstract Book, but they cannot change the content in any way or use them commercially.
**Abstract Book Citations:** Authors, Title, HemaSphere, 2026;10:(S1):pages. The abstract book DOIs can be found on the "Author Information" page.
**Disclaimer:** Articles published in the journal HemaSphere exclusively reflect the opinions of the authors. The authors are responsible for all content in their abstracts including accuracy of the facts, statements, citing resources, etc.

### (PS1522) TREATING ACUTE MYELOID LEUKEMIA BY COMBINING CAR T CELL THERAPY AND LILRB2 TARGETING

**Topic:** 03. Acute myeloid leukemia - Biology & translational research

Silvan Brunn*[1], Morgane Chambovey[1], Marianna Ponzo[1], Laura Raas[1], Chiara F Magnani[1]

[1]*Universitätsspital Zürich, Department of Medical Oncology and Hematology, Zürich, Switzerland;*

### Background

CAR T-cell therapies have shown limited efficacy in Acute myeloid leukemia (AML), despite their established clinical benefit in other hematologic malignancies. This is due to the similarity between tumor cells and hematopoietic stem and progenitor cells, posing a safety risk of myeloablation, as well as the strong immunosuppressive tumor microenvironment in AML. In this context, the leukocyte immunoglobulin-like receptor subfamily B member 2 (LILRB2) has emerged as an immunosuppressive receptor in AML and other cancers. To date, the impact of LILRB2 on anti-AML CAR T-cell function remains unknown, and its potential as target for CAR T cells is not fully established.

### Aims

In this project, we combine targeting of the LILRB2 inhibitory receptor with already established anti-AML CAR T-cell approaches to increase their therapeutic efficacy against AML. Furthermore, we have designed and manufactured a CAR T cell directly targeting LILRB2 and exhibiting promising anti-AML function in vitro and in vivo.

### Results

We found that LILRB2 is overexpressed in approximately 45% of patient samples' blasts, highly expressed on tumor-associated myeloid cells, and present in only a minor fraction of healthy HSPCs. Using LILRB2 knockout (KO) and overexpressing cell-lines, we showed that CD33 CAR T-cell killing, expansion and cytokine production is impaired in response to LILRB2-overexpressing leukemia, associated with increased memory differentiation and expression of exhaustion molecules by CAR T cells. Mechanistically, LILRB2 expression by tumor cells leads to downregulation of gene sets associated with immune response and T cell activation in effector and target cells. This could result in decreased functionality, ultimately leading to exhaustion. LILRB2 inhibition by blocking antibodies partially restored reduced CD33 CAR T-cell functionality. Faster tumor outgrowth was observed in the bone marrow of CD33 CAR T-cell treated NSG mice bearing a LILRB2 overexpressing tumor compared to those bearing LILRB2 KO tumors. This finding was associated with increased effector-cell differentiation and exhaustion, suggesting an impact of high LILRB2 expression on disease control *in vivo*.

Having shown that AML suppresses CAR T cells through LILRB2, we investigated CAR T cells targeting LILRB2 as a strategy to target AML blasts while alleviating T-cell inhibition. We showed cytotoxic activity and increased cytokine production against two AML cell-lines (THP1 and MOLM14) modified to express LILRB2 in vitro and strongly reduced tumor growth in mice engrafted with LILRB2+ THP1 cells in vivo. In a second in vivo experiment using LILRB2+ MOLM14 cells, treatment with anti-LILRB2 CAR T cells achieved complete tumor eradication, comparable to the killing activity of already established anti-CD33 CAR T cells.

### Summary/Conclusion

Altogether, our study demonstrates that LILRB2 expression impairs CAR T-cell functional response in AML. Targeting LILRB2, either through pharmacological inhibition or direct targeting by anti-LILRB2 CAR T cells, therefore represents a promising therapeutic option to enhance CAR T-cell therapies in AML.

**Copyright Information:** (Online) ISSN: 2572-9241
©2026 The Author(s). HemaSphere is published by John Wiley & Sons Ltd on behalf of European Hematology Association. This is an open access Abstract Book distributed under the Attribution-NonCommercial-NoDerivs (CC BY-NC-ND), which allows third parties to download the articles and share them with others as long as they credit the author and the Abstract Book, but they cannot change the content in any way or use them commercially.
**Abstract Book Citations:** Authors, Title, HemaSphere, 2026;10:(S1):pages. The abstract book DOIs can be found on the "Author Information" page.
**Disclaimer:** Articles published in the journal HemaSphere exclusively reflect the opinions of the authors. The authors are responsible for all content in their abstracts including accuracy of the facts, statements, citing resources, etc.

### (PS1523) SYNERGISTIC EFFECT OF COMBINED CK2 AND XPO1 INHIBITION IN PRECLINICAL MODELS OF ACUTE MYELOID LEUKEMIA: MECHANISM AND THERAPEUTIC POTENTIAL

**Topic:** 03. Acute myeloid leukemia - Biology & translational research

Huideng Long[1], Qi Han[1], Chunhua Song[2], Zheng Ge*[1]

*[1]Zhongda Hospital Southeast University, Institute of Hematology Southeast University, Department of Hematology, Nanjing, China; [2]Division of Hematology, The Ohio State University Wexner Medical Center, The James Cancer Hospital, Columbus, United States of America;*

### Background

Therapeutic resistance in acute myeloid leukemia (AML) remains a major clinical challenge, necessitating novel strategies targeting non-redundant pathways. Casein kinase 2 (CK2) and exportin 1 (XPO1) are promising targets; CK2 is a pro-survival kinase, while XPO1 mediates nuclear export of tumor suppressors. Single-agent inhibitors CX-4945 (CK2i) and selinexor (KPT-330, XPO1i) have shown limited efficacy. Our previous in vitro experiments demonstrated that CX-4945 and KPT-330 synergistically inhibit proliferation and induce apoptosis in AML. The mechanistic basis for this synergy and its efficacy in vivo are not fully elucidated.

### Aims

This study aims to evaluate the anti-tumor efficacy in vivo of the CK2 inhibitor CX-4945 and the XPO1 inhibitor KPT-330, alone and in combination, and to investigate the underlying molecular mechanisms in preclinical models of AML.

### Methods

In vivo efficacy was assessed using cell-derived xenograft (CDX) and patient-derived xenograft (PDX) mouse models, analyzing tumor burden and splenic weight. In vitro, flow cytometry and protein/mRNA expression (western blot, qRT-PCR) were evaluated in AML cell lines (U937, MV4-11). Immunohistochemistry (IHC) was performed on AML tissue samples.

**Copyright Information:** (Online) ISSN: 2572-9241
©2026 The Author(s). HemaSphere is published by John Wiley & Sons Ltd on behalf of European Hematology Association. This is an open access Abstract Book distributed under the Attribution-NonCommercial-NoDerivs (CC BY-NC-ND), which allows third parties to download the articles and share them with others as long as they credit the author and the Abstract Book, but they cannot change the content in any way or use them commercially.
**Abstract Book Citations:** Authors, Title, HemaSphere, 2026;10:(S1):pages. The abstract book DOIs can be found on the “Author Information” page.
**Disclaimer:** Articles published in the journal HemaSphere exclusively reflect the opinions of the authors. The authors are responsible for all content in their abstracts including accuracy of the facts, statements, citing resources, etc.

**Results**

We observed the therapeutic efficacy of the combination of CX-4945 (CK2i) and selinexor (KPT-330, XPO1i) in the CDX and PDX models. Results showed that the spleen size and weight of the mice in the combined treatment group were significantly lower than those in the control and monotherapy groups (Fig. 1A-B), and the % $hCD13^{+}hCD45^{+}$ leukemia cells in the spleen and bone marrow were much lower in the combination group in CDX and PDX mouse models (Fig. 1C-D). Similarly, in the PDX model, the combination significantly reduced the human leukemia cell burden in the spleen and bone marrow (Fig. 1E-F). IHC staining further validated the significant suppression of leukemic infiltration in vivo (Fig. 1G-H). CX-4945 treatment significantly reduced XPO1 mRNA expression in a dose-dependent manner (Fig. 1I). Chromatin immunoprecipitation (ChIP) analysis confirmed that IKAROS directly binds to the XPO1 promoter region (Fig. 1J). The dual luciferase reporter assay demonstrated that IKAROS overexpression significantly inhibited XPO1 promoter activity (Fig. 1K). ChIP-qPCR analysis further confirmed that IKAROS binding to the XPO1 promoter was significantly enhanced after CX-4945 treatment (Fig. 1L). Importantly, knockdown of IKAROS (shIKZF1) significantly reversed the inhibitory effect of CX-4945 on XPO1 mRNA expression (Fig. 1M), indicating IKAROS-dependency. Overexpression and knockdown of IKAROS further confirmed IKAROS' direct regulation of XPO1 (Fig. 1N-O). Moreover, knockdown of XPO1 significantly reduced AKT phosphorylation (p-AKT) while upregulating the tumor suppressor FOXO3 (Fig. 1P). The combination therapy also significantly inhibited AKT phosphorylation and upregulated FOXO3 expression (Fig. 1Q). Compared with normal controls, FOXO3 mRNA expression was significantly downregulated in AML samples (Fig. 1R). AML patients with low FOXO3 expression had significantly shorter overall survival (Fig. 1S). A molecular mechanism model is proposed (Fig. 1T)

**Summary/Conclusion**

Our data demonstrate that combined inhibition of CK2 and XPO1 exerts a synergistic anti-tumor effect in preclinical models of AML, providing a strong rationale for the further clinical development of this combination therapy.

**Copyright Information:** (Online) ISSN: 2572-9241
©2026 The Author(s). HemaSphere is published by John Wiley & Sons Ltd on behalf of European Hematology Association. This is an open access Abstract Book distributed under the Attribution-NonCommercial-NoDerivs (CC BY-NC-ND), which allows third parties to download the articles and share them with others as long as they credit the author and the Abstract Book, but they cannot change the content in any way or use them commercially.
**Abstract Book Citations:** Authors, Title, HemaSphere, 2026;10:(S1):pages. The abstract book DOIs can be found on the "Author Information" page.
**Disclaimer:** Articles published in the journal HemaSphere exclusively reflect the opinions of the authors. The authors are responsible for all content in their abstracts including accuracy of the facts, statements, citing resources, etc.

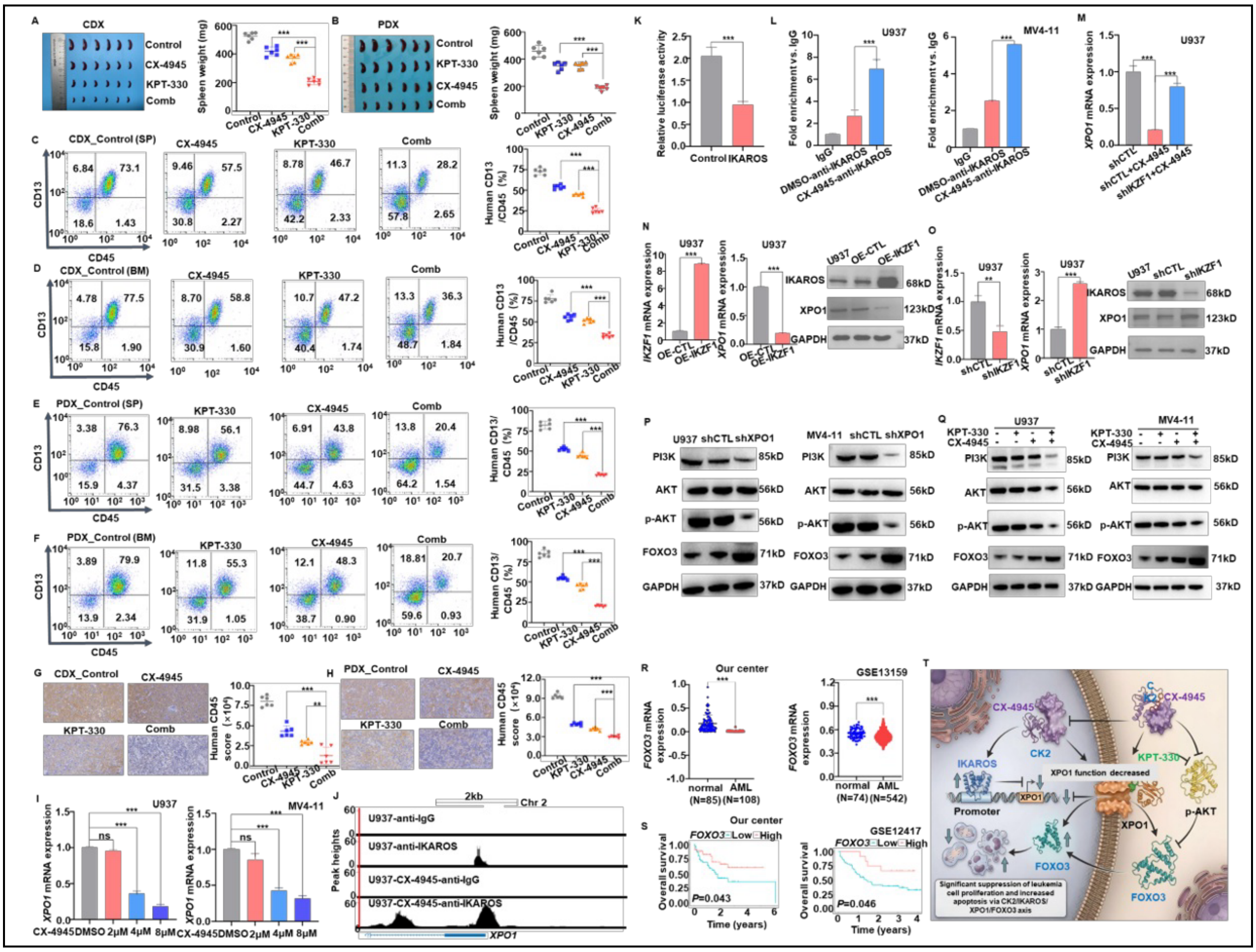

Figure 1. Synergistic anti-tumor effects of combined targeting of CK2 and XPO1 by CX-4945 and KPT-330 in acute myeloid leukemia preclinical models.(A) In the CDX model, spleen weight was significantly reduced in the combination treatment group compared to the control, CX-4945 alone, and KPT-330 alone groups. (B) In the PDX model, spleen weight was significantly reduced in the combination treatment group compared to the control, KPT-330 alone, and CX-4945 alone groups. (C, D) Flow cytometric analysis of the CDX model showed that the combination treatment group significantly reduced the percentage of human CD13+/CD45+ leukemia cells in the spleen (SP) and bone marrow (BM) compared to the control, CX-4945 alone, and KPT-330 alone groups. (E, F) Similarly, flow cytometric analysis of the PDX model demonstrated that the combination treatment group significantly reduced the percentage of human CD13+/CD45+ leukemia cells in the SP and BM. (G, H) Representative IHC images and quantification of human CD45 expression in tumor tissues from (G) CDX and (H) PDX mouse models. Mice were treated with vehicle, CX-4945, KPT-330, or the combination. The human CD45 score was calculated based on staining intensity and percentage of positive cells. (I) CX-4945 treatment dose-dependently decreased XPO1 mRNA expression levels in U937 and MV4-11 cells. (J) ChIP-seq map of the binding of IKAROS to the XPO1 gene. After U937 cells were treated with CX-4945, IKAROS-specific chromatin immunopre-cipitation and sequencing were performed. The peak diagram shows the binding signal intensity of IKAROS near the XPO1 gene locus, and the CX-4945 treatment group shows a stronger IKAROS binding peak. (K) The effect of IKAROS overexpression on the activity of the XPO1 promoter. (L) ChIP-qPCR analysis showed that IKAROS antibody (anti-IKAROS) significantly enriched the XPO1 promoter region compared to the control antibody (IgG) in both U937 and MV4-11 cells. Furthermore, CX-4945 treatment further enhanced IKAROS binding to this region. (M) Knockdown of IKAROS (shIKZF1) significantly reversed the inhibitory effect of CX-4945 on XPO1 mRNA expression in U937 cells. (N) In U937 cells, overexpression of IKAROS (OE-IKZF1) significantly upregulated IKZF1 mRNA levels and significantly downregulated XPO1 mRNA and protein expression. (O) Conversely, knockdown of IKAROS (shIKZF1) significantly downregulated IKZF1 mRNA levels and significantly upregulated XPO1 mRNA and protein expression. (P) Knockdown of XPO1 (shXPO1) significantly increased the phosphorylation level of AKT (p-AKT) and upregulated the protein expression of FOXO3 in AML cells. (Q) Combination treatment with KPT-330 and CX-4945 significantly increased the p-AKT and upregulated the protein expression of FOXO3 in AML cells. (R) FOXO3 mRNA expression levels were significantly lower in AML patients compared to normal controls in our center cohort and the GEO dataset GSE13159. (S) Kaplan-Meier survival analysis showed that AML patients with low FOXO3 expression had significantly shorter overall survival in our center cohort and the GEO dataset GSE12417. (T) Anti-tumor mechanism model of the combination in AML.

**Copyright Information:** (Online) ISSN: 2572-9241
©2026 The Author(s). HemaSphere is published by John Wiley & Sons Ltd on behalf of European Hematology Association. This is an open access Abstract Book distributed under the Attribution-NonCommercial-NoDerivs (CC BY-NC-ND), which allows third parties to download the articles and share them with others as long as they credit the author and the Abstract Book, but they cannot change the content in any way or use them commercially.
**Abstract Book Citations:** Authors, Title, HemaSphere, 2026;10:(S1):pages. The abstract book DOIs can be found on the “Author Information” page.
**Disclaimer:** Articles published in the journal HemaSphere exclusively reflect the opinions of the authors. The authors are responsible for all content in their abstracts including accuracy of the facts, statements, citing resources, etc.

### (PS1524) GB3226, A FIRST-IN-CLASS DUAL ENL-YEATS/FLT3 INHIBITOR, SUPPRESSES SEC-DRIVEN TRANSCRIPTION AND ONCOGENIC SIGNALING IN PRECLINICAL AML MODELS

**Topic:** 03. Acute myeloid leukemia - Biology & translational research

Louis Renzetti*[1], Stacia Kargman[1], Joseph Vacca[2], Mayumi Sugita[3], Sebastien Degorce[4], Tammy Ladduwahetty[4], Bradley Sherborne[4], Simon Taylor[4], Gillian Watt[4], Bhupinder Singh[1], Susan Tantawi[1], Monica Guzman[3], Brian Jacoby[1]

*[1]Galecto Inc., Copenhagen, Denmark; [2]Bridge Medicines, New York, United States of America; [3]Weill Cornell Medicine, New York, United States of America; [4]Pharmaron, Hertfordshire, United Kingdom;*

### Background

Relapsed/refractory (R/R) acute myeloid leukaemia (AML) remains associated with poor outcomes, particularly in molecular subtypes characterised by persistent SEC-dependent transcriptional programs and activating FLT3 mutations (~30% of adult AML). The YEATS domain of ENL (MLLT1) sustains oncogenic transcription of HOXA9, MEIS1 and MYC, while FLT3 mutations drive leukaemic proliferation and relapse. We hypothesised that concurrent inhibition of ENL-YEATS-mediated transcription and FLT3 signalling may provide a superior therapeutic strategy capable of constraining both leukaemic stemness and proliferative signalling.

### Aims

Here, we describe the preclinical pharmacology and activity of GB3226, a first-in-class, orally bioavailable, small molecule, dual inhibitor targeting the ENL-YEATS domain and FLT3.

### Methods

GB3226 was developed through structure-based drug design as an optimized analog of a selective ENL-YEATS tool compound (TDI-11055). Biochemical binding assays assessed ENL-YEATS and FLT3 inhibition. Kinase selectivity was evaluated across 370 kinases. Anti-proliferative activity was assessed in AML cell lines and primary AML samples. Transcriptomic analyses evaluated suppression of SEC target genes. In vivo efficacy was evaluated in subcutaneous and disseminated MV4;11 xenograft models. Pharmacokinetics and safety were assessed in multi-species nonclinical studies.

### Results

GB3226 potently inhibited ENL-YEATS (IC50=185 nM) and FLT3 WT (Kd=3nM) and clinically relevant mutant variants, including FLT3-ITD and D835 substitutions (Kd=3–27 nM), with high selectivity across epigenetic and kinase panels. GB3226 was inactive in a bromodomain panel. In a kinase panel of 370 kinases, GB3226 was active (IC50=40-60 nM) vs c-KIT, CLK1, CLK4, MELK and TRKC and was inactive vs the remaining kinases. In AML cell lines (MV4;11, MOLM13), GB3226 potently inhibited the proliferation (IC50=18-32 nM) and demonstrated additive to synergistic activity with cytarabine, venetoclax, gilteritinib, and revumenib. In primary AML patient samples, GB3226 exhibited anti-proliferative activity across multiple genotypes, including FLT3+, ASXL1+, NPM1c+ and TET2+. In vivo, oral administration of GB3226 (25, 50 and 100 mg/kg, BID or QD) induced rapid tumour regression in subcutaneous models and significantly reduced leukaemia burden and prolonged survival in disseminated models. The efficacy was statistically superior to gilteritinib and revumenib. Anti-leukaemic activity correlated with suppression of SEC target genes (HOXA9, MEIS1, MYC) in tumour tissue. GB3226 demonstrated favourable pharmacokinetics supporting once-daily administration, no hERG inhibition (IC50>30 µM), no QTc prolongation in canine studies, and an acceptable safety profile in 28-day repeat-dose GLP toxicology studies.

**Copyright Information:** (Online) ISSN: 2572-9241
©2026 The Author(s). HemaSphere is published by John Wiley & Sons Ltd on behalf of European Hematology Association. This is an open access Abstract Book distributed under the Attribution-NonCommercial-NoDerivs (CC BY-NC-ND), which allows third parties to download the articles and share them with others as long as they credit the author and the Abstract Book, but they cannot change the content in any way or use them commercially.
**Abstract Book Citations:** Authors, Title, HemaSphere, 2026;10:(S1):pages. The abstract book DOIs can be found on the "Author Information" page.
**Disclaimer:** Articles published in the journal HemaSphere exclusively reflect the opinions of the authors. The authors are responsible for all content in their abstracts including accuracy of the facts, statements, citing resources, etc.

**Summary/Conclusion**

GB3226 is a first-in-class dual inhibitor of ENL-YEATS and FLT3 that simultaneously suppresses SEC-driven oncogenic transcription and FLT3-mediated signalling. Preclinical studies demonstrate that GB3226 exhibits potent anti-leukaemic activity and has the potential for combination with multiple therapeutic agents across diverse AML genotypes. These findings support clinical evaluation of GB3226 as a differentiated therapeutic strategy in AML.

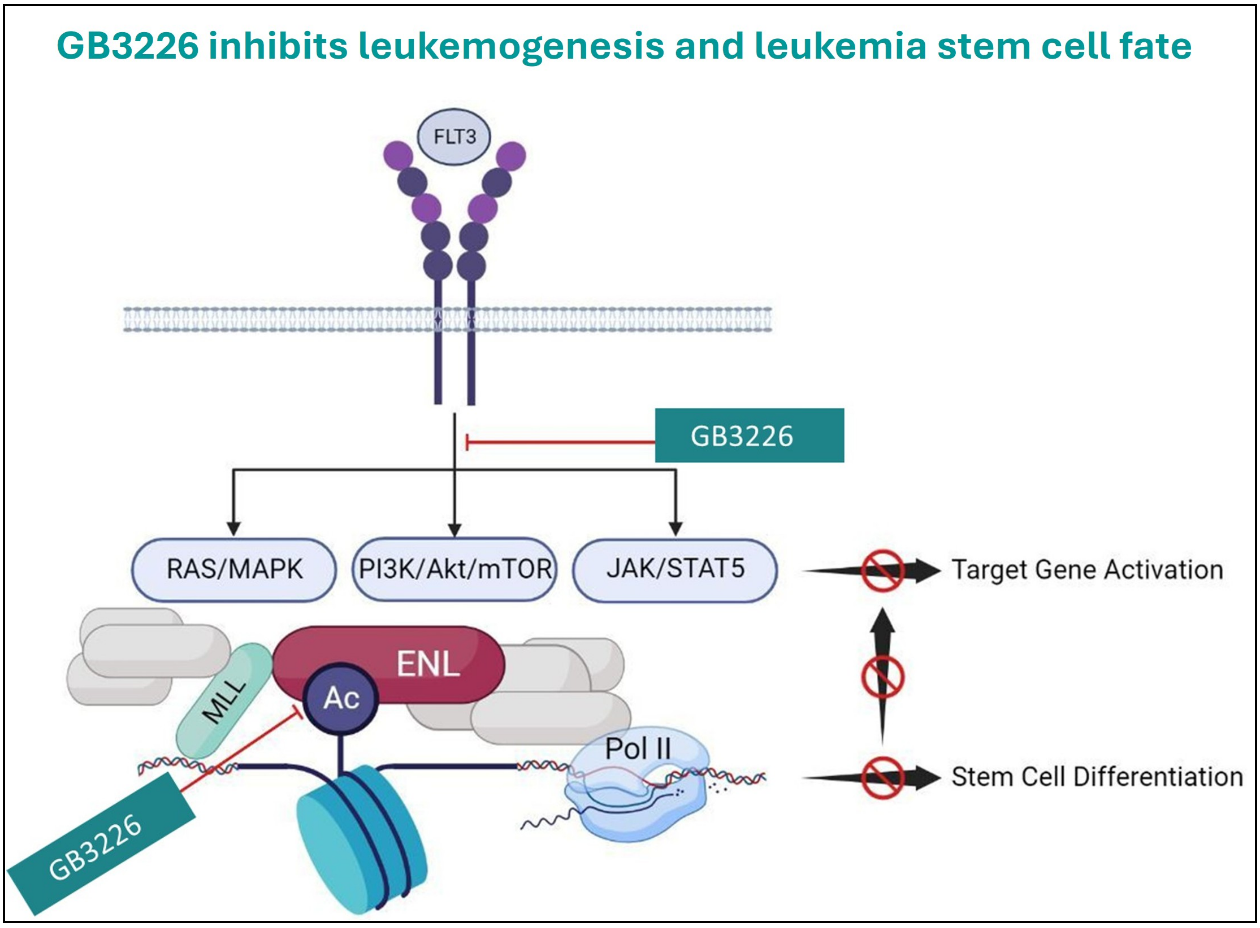

GB3226 is a potent, dual inhibitor of ENL-YEATS and FLT3

**Copyright Information:** (Online) ISSN: 2572-9241
©2026 The Author(s). HemaSphere is published by John Wiley & Sons Ltd on behalf of European Hematology Association. This is an open access Abstract Book distributed under the Attribution-NonCommercial-NoDerivs (CC BY-NC-ND), which allows third parties to download the articles and share them with others as long as they credit the author and the Abstract Book, but they cannot change the content in any way or use them commercially.
**Abstract Book Citations:** Authors, Title, HemaSphere, 2026;10:(S1):pages. The abstract book DOIs can be found on the "Author Information" page.
**Disclaimer:** Articles published in the journal HemaSphere exclusively reflect the opinions of the authors. The authors are responsible for all content in their abstracts including accuracy of the facts, statements, citing resources, etc.

### (PS1525) INTEGRATED ANALYSIS OF DRUG RESPONSE ACROSS TRANSCRIPTOMIC CELL STATES IDENTIFIES VENETOCLAX-BASED COMBINATION STRATEGIES IN AML

**Topic:** 03. Acute myeloid leukemia - Biology & translational research

Jie Zhu*[1]

[1]*FIMM – Institute for Molecular Medicine Finland, the University of Helsinki, Helsinki, Finland;*

### Background

Acute myeloid leukemia (AML) is a heterogeneous malignancy with poor survival despite advances in genomic profiling and risk stratification. Venetoclax, a selective BCL2 inhibitor, has improved outcomes in elderly and unfit patients, yet resistance and relapse remain common. Emerging evidence suggests that AML differentiation state influences venetoclax sensitivity: primitive stem/progenitor-like cells are highly BCL2-dependent and sensitive, whereas differentiated monocytic populations display relative resistance through alternative anti-apoptotic programs and activation of MAPK/ERK, JAK–STAT, and NF-κB signaling. We integrated ex vivo drug sensitivity and transcriptomic data from two independent AML cohorts (592 primary samples) to systematically map differentiation-associated drug vulnerabilities. Venetoclax was most effective in primitive subtypes, while MEK inhibitors and the HDAC inhibitor panobinostat preferentially targeted differentiated states and showed broad synergy with venetoclax. Mechanistic studies identified FOXC1 as a regulator of HDAC inhibitor response. These findings support differentiation-guided, biomarker-driven venetoclax combination strategies in AML.

### Aims

To integrate multi-cohort drug sensitivity and transcriptomic data to map differentiation-associated therapeutic dependencies in AML, functionally validate synergistic venetoclax combinations, and uncover molecular regulators of combination response.

### Methods

We integrated ex vivo drug sensitivity and transcriptomic data from two AML cohorts (BeatAML and FIMM; 592 samples, 104 shared compounds). Drug response (DSS) was correlated with differentiation states inferred by ssGSEA. scRNA-seq of venetoclax-treated patients (>80,000 cells) defined six malignant states. Venetoclax combinations were tested in AML cell lines and primary samples with synergy quantified by HSA. RNA-seq and CRISPR knockout identified FOXC1 as a regulator of panobinostat response.

### Results

Integration of ex vivo drug sensitivity data from BeatAML and FIMM cohorts (592 samples; 104 shared compounds) revealed differentiation-state–specific vulnerabilities. Venetoclax was most effective in primitive FAB M0–M1 subtypes, whereas differentiated M4–M5 cases were relatively resistant but more sensitive to MEK inhibitors and panobinostat. scRNA-seq (>80,000 cells) showed post-treatment shifts toward differentiated states, with refractory disease enriched for promonocyte/monocyte-like cells. Venetoclax response correlated positively with primitive signatures and negatively with differentiated signatures, while MEK inhibitors and panobinostat showed the opposite pattern. Venetoclax + panobinostat and venetoclax + trametinib demonstrated broad synergy in AML cell lines and primary samples (>56% of patients). Resistant models showed MAPK/JAK–STAT and NF-κB activation; combinations suppressed these pathways. FOXC1 was identified as a regulator of panobinostat response, and its activity correlated with HDAC inhibitor sensitivity across independent cohorts.

**Copyright Information:** (Online) ISSN: 2572-9241
©2026 The Author(s). HemaSphere is published by John Wiley & Sons Ltd on behalf of European Hematology Association. This is an open access Abstract Book distributed under the Attribution-NonCommercial-NoDerivs (CC BY-NC-ND), which allows third parties to download the articles and share them with others as long as they credit the author and the Abstract Book, but they cannot change the content in any way or use them commercially.
**Abstract Book Citations:** Authors, Title, HemaSphere, 2026;10:(S1):pages. The abstract book DOIs can be found on the “Author Information” page.
**Disclaimer:** Articles published in the journal HemaSphere exclusively reflect the opinions of the authors. The authors are responsible for all content in their abstracts including accuracy of the facts, statements, citing resources, etc.

**Summary/Conclusion**

AML differentiation state determines venetoclax response. Primitive cells are highly sensitive, whereas differentiated monocytic populations exhibit resistance driven by MAPK, JAK–STAT, and NF-κB signaling. Venetoclax combined with panobinostat or trametinib showed broad synergy in cell lines and primary samples. FOXC1 was identified as a regulator of HDAC inhibitor response and a potential biomarker for stratified venetoclax-based combination therapy.

**Copyright Information:** (Online) ISSN: 2572-9241
©2026 The Author(s). HemaSphere is published by John Wiley & Sons Ltd on behalf of European Hematology Association. This is an open access Abstract Book distributed under the Attribution-NonCommercial-NoDerivs (CC BY-NC-ND), which allows third parties to download the articles and share them with others as long as they credit the author and the Abstract Book, but they cannot change the content in any way or use them commercially.
**Abstract Book Citations:** Authors, Title, HemaSphere, 2026;10:(S1):pages. The abstract book DOIs can be found on the "Author Information" page.
**Disclaimer:** Articles published in the journal HemaSphere exclusively reflect the opinions of the authors. The authors are responsible for all content in their abstracts including accuracy of the facts, statements, citing resources, etc.

### (PS1526) ER–MITOCHONDRIAL $CA^{2+}$ DYSREGULATION UNDERLIES THE ANTI-LEUKEMIC EFFECTS OF TAMOXIFEN IN MYELOID NEOPLASMS

**Topic:** 03. Acute myeloid leukemia - Biology & translational research

Luise Albuquerque-Simões*[1, 2, 3, 4], Abdelrahman A.F. Elsayed[5], Diego A Pereira-Martins[1, 6], Vincent van den Boom[1], Jesse van der Velde[1, 5], Dominique Sternadt[1], Manuela Albuquerque de Melo[1, 3, 7], Artur Bernardo Ribeiro Bellomi[3, 7], Antonio Bruno Alves-Silva[3, 7], Livia Bassani Lins De Miranda[1], Isabel Weinhauser[1, 6], Prodromos Chatzikyriakou[6], Chunlong Zhao[5], Petra E. van der Wouden[5], Peter Fodran[5], Eduardo M. Rego[2, 3, 4], Emanuele Ammatuna[1], Lynn Quek[6], Gerwin Huls[1], Fabiola Traina[3, 7], Frank J. Dekker[5], Jan Jacob Schuringa[1]

*[1]University Medical Centre Groningen, Department of Hematology, Groningen, The Netherlands;[2]Faculdade De Medicina Da Universidade De São Paulo, Department of Hematology, LIM31, São Paulo, Brazil; [3]Hemocentro de Ribeirão Preto, Center for Cell-based Therapy, Ribeirão Preto, Brazil; [4]Instituto do Câncer do Estado de São Paulo, Department of Hematology, São Paulo, Brazil; [5]University Medical Center Groningen, Department of Chemical and Pharmaceutical Biology, Groningen, The Netherlands; [6]King's College London, Myeloid Leukaemia Genomics and Biology Group, School of Cancer and Pharmaceutical Sciences, London, United Kingdom; [7]Ribeirao Preto Medical School, University of Sao Paulo, Department of Medical Imaging, Hematology, and Clinical Oncology, Ribeirão Preto, Brazil;*

### Background

Therapy resistance in acute myeloid leukemia (AML) is linked to increased reliance on oxidative phosphorylation (OXPHOS). The selective estrogen receptor modulator tamoxifen (Tam) has recently been shown to reduce mutant allele burden in myeloproliferative neoplasms (MPNs) via mitochondrial inhibition and activation of the integrated stress response, but its mechanistic effects in AML remain poorly defined.

### Aims

We aimed to determine if and how Tam mediates anti-leukemic effects in AML.

### Methods

Primary AML blasts (n=30; $CD34^+$/$CD117^+$) and cell lines (n=12) were treated *ex vivo* with Tam, and viability and metabolic readouts were assessed by flow cytometry. We generated a biotinylated Tam probe to define Tam localization and the LC-MS/MS-based Tam interactome in AML.

**Copyright Information:** (Online) ISSN: 2572-9241
©2026 The Author(s). HemaSphere is published by John Wiley & Sons Ltd on behalf of European Hematology Association. This is an open access Abstract Book distributed under the Attribution-NonCommercial-NoDerivs (CC BY-NC-ND), which allows third parties to download the articles and share them with others as long as they credit the author and the Abstract Book, but they cannot change the content in any way or use them commercially.
**Abstract Book Citations:** Authors, Title, HemaSphere, 2026;10:(S1):pages. The abstract book DOIs can be found on the "Author Information" page.
**Disclaimer:** Articles published in the journal HemaSphere exclusively reflect the opinions of the authors. The authors are responsible for all content in their abstracts including accuracy of the facts, statements, citing resources, etc.

### Results

In primary AML, we identified a subset of cases (33%) with marked sensitivity to Tam. To delineate associated programs, we performed single-cell RNA sequencing on 7 AML samples (3 non-responders, 4 responders; 100,213 cells). Extensive transcriptional differences in HSC/LMPP populations were identified, with 671 genes being upregulated in responders, including *ATF3*, *CD34*, and *VDAC1/2.* Gene set enrichment analyses revealed enrichment of stem-like and OXPHOS-related programs. Cluster proportion analysis confirmed increased HSC/MPP fractions in responders and relative depletion of erythroid cells in non-responders. Over-representation analysis of the 671 shared genes highlighted mitochondrial pathways, ER-protein–containing complexes and unfolded protein response, suggesting a metabolically primed state. Interactome profiling revealed that Tam-binding proteins were enriched at ER–mitochondria contact site proteins, including VDAC1-3 and ATP2A2, implicating an impact on $Ca^{2+}$ homeostasis. Localization studies showed that Tam accumulates at the interface between ER and mitochondria. Tam triggered robust ER-to-mitochondria $Ca^{2+}$ efflux in AML cells, causing mitochondrial stress, loss of mitochondrial membrane potential (MPP), ROS accumulation and mitochondrial permeability transition pore disruption, which was not observed in breast cancer cells, indicating tissue-specific Tam mechanisms. Tam sensitivity strongly correlated with the magnitude of $Ca^{2+}$ release across AML cell lines (Tam AUC vs $Ca^{2+}$ release, r=-0.8) and primary samples (r=-0.6), establishing $Ca^{2+}$ dysregulation as a central driver of its anti-leukemic effect. Using DepMap CRISPR dependency scores, we identified *JAK2* as a key dependency in Tam-responsive models. Given that *JAK2* mutations are frequent in AML post-MPN and treated with the JAK inhibitor ruxolitinib (Ruxo), we tested if Tam–Ruxo combinations would result in superior effects compared to the monotherapies. In HEL (*JAK2*$^{V617F}$) cells, Ruxo+Tam combination treatment was synergistic, and Ruxo treatment amplified the Tam-induced $Ca^{2+}$ efflux, MMP loss and cell death. *In vivo*, lethally irradiated $CD45.1^{+}$ mice transplanted with *Jak2*$^{V617F}$ $CD45.2^{+}$ BM cells were significantly more sensitive to combination treatment, with an associated decreased MMP, indicating mitochondrial targeting in mutant cells.

### Summary/Conclusion

Tam disrupts $Ca^{2+}$-regulated mitochondrial metabolism in AML independently of classical estrogen receptor signaling. Measuring $Ca^{2+}$ flux magnitude emerges as a functional biomarker of Tam sensitivity and provides a rationale for combination treatment strategies, such as JAK inhibition, to exploit $Ca^{2+}$- and mitochondria-dependent vulnerabilities in AML.

**Copyright Information:** (Online) ISSN: 2572-9241
©2026 The Author(s). HemaSphere is published by John Wiley & Sons Ltd on behalf of European Hematology Association. This is an open access Abstract Book distributed under the Attribution-NonCommercial-NoDerivs (CC BY-NC-ND), which allows third parties to download the articles and share them with others as long as they credit the author and the Abstract Book, but they cannot change the content in any way or use them commercially.
**Abstract Book Citations:** Authors, Title, HemaSphere, 2026;10:(S1):pages. The abstract book DOIs can be found on the "Author Information" page.
**Disclaimer:** Articles published in the journal HemaSphere exclusively reflect the opinions of the authors. The authors are responsible for all content in their abstracts including accuracy of the facts, statements, citing resources, etc.

### (PS1527) DISTINCT MEN1 MUTATIONAL SIGNATURES ARE ASSOCIATED WITH ACQUIRED RESISTANCE DURING MENIN-KMT2A INHIBITION IN A PHASE 1 ENZOMENIB TRIAL

**Topic:** 03. Acute myeloid leukemia - Biology & translational research

Jevon Cutler*[1], Curtis Allred[2], Ernest Duah[2], Steven L Warner[2], Jason Foulks[2], Nitika Fnu[2], Matthew Hitron[2], Jatin Shah[2], Emma Searle[3], Naval Daver[4], Joshua Zeidner[5]

*[1]Oregon Health and Science University, Portland, United States of America;[2]Sumitomo Pharma America, Inc., Marlborough, United States of America; [3]The Christie Hospital & University of Manchester, Manchester, United Kingdom;[4]The University of Texas MD Anderson Cancer Center, Houston, United States of America; [5]University of North Carolina, Lineberger Comprehensive Cancer Center, Chapel Hill, United States of America;*

### Background

Menin inhibitors (MIs) represent a paradigm shift in treatment for patients (pts) with acute leukemias (ALs) with NPM1 mutations (*NPM1*m), KMT2A rearrangements (*KMT2A*r), and potentially other genomic alterations. Previous clinical trials in relapsed/refractory (R/R) pts treated with MIs reported ~1/3 of responding pts ultimately relapse with acquired *MEN1* mutations (*MEN1*m) (Perner, 2023). Our prior preclinical work identified candidate *MEN1*m using 5 different MIs and predicted that all will likely produce *MEN1*m clonal outgrowth (Bourgeois, 2024). Additionally, certain MIs appear to retain activity against select *MEN1*m, and thus by rational sequential administration of MIs, it may be possible to deepen or prolong responses. In Phase 1 clinical studies, enzomenib monotherapy exhibited promising activity in both R/R *KMT2Ar* and *NPM1m* AML (Daver, 2025). Previous preclinical work indicated that of the 5 MIs, enzomenib was among the least affected by known *MEN1*m, but predicted a new E368 mutation, leading to resistance specific to enzomenib.

### Aims

To characterize the spectrum and frequency of acquired *MEN1*m at relapse, and to evaluate whether mutational patterns are consistent with preclinical predictions, we performed serial molecular profiling of R/R AL pts at baseline and at relapse who were receiving enzomenib.

### Methods

Samples were obtained from the Phase 1 portion of the DSP-5336-101 trial (NCT04988555). RNA was isolated from subject bone marrow (BM) aspirates and sequenced for *MEN1*m using a custom ddPCR assay (LoD 0.01%) covering 13 previously reported mutations in 5 amino acid hotspots; M327, G331, T349, S160, and E368. MI-naïve pts (n=13) who responded and then relapsed on enzomenib were profiled at baseline and at the aspirate time point closest to relapse.

### Results

Of the 13 MI-naïve pts who subsequently relapsed, 6 (46%) acquired detectable *MEN1*m. Mutations previously shown to drive drug resistance to revumenib (Perner, 2023) were undetected, and M327 was detected in only 2 pts (15%), both at low variant allele frequency (VAF < 0.3%). Of the 6 pts who relapsed with *MEN1*m, mutations to E368 were detected in 5 (38%), including 1 pt with a concomitant M327I at 0.3%, suggesting E368 as the likely predominant acquired mutation associated with enzomenib resistance. Notably, all pts with detectable E368K mutations experienced dose interruptions (4/5) and/or received a potentially sub-efficacious dose (2/5), which suggests interrupted or insufficient exposure may promote the emergence of resistant *MEN1*m clones. Further preclinical and clinical analyses and studies to corroborate these findings are ongoing.

**Copyright Information:** (Online) ISSN: 2572-9241
©2026 The Author(s). HemaSphere is published by John Wiley & Sons Ltd on behalf of European Hematology Association. This is an open access Abstract Book distributed under the Attribution-NonCommercial-NoDerivs (CC BY-NC-ND), which allows third parties to download the articles and share them with others as long as they credit the author and the Abstract Book, but they cannot change the content in any way or use them commercially.
**Abstract Book Citations:** Authors, Title, HemaSphere, 2026;10:(S1):pages. The abstract book DOIs can be found on the "Author Information" page.
**Disclaimer:** Articles published in the journal HemaSphere exclusively reflect the opinions of the authors. The authors are responsible for all content in their abstracts including accuracy of the facts, statements, citing resources, etc.

**Summary/Conclusion**

We report the first clinical evidence of acquired *MEN1*m following enzomenib monotherapy identified in 6/13 R/R AL pts. E368K was the predominant resistance mutation, while mutations in M327, T349 and G331 were infrequent, supporting preclinical predictions. Importantly, the association between dose interruptions or sub-efficacious exposure and E368K emergence supports preclinical data suggesting that consistent, adequate dosing may reduce *MEN1*m frequency. These findings are important and novel given that E368 mutations appear to only impact enzomenib in contrast to other mutations (i.e. M327) which are detrimental to all currently available MIs. Collectively, these findings support further investigation of rational sequential MI therapy to improve clinical outcomes.

**Copyright Information:** (Online) ISSN: 2572-9241
©2026 The Author(s). HemaSphere is published by John Wiley & Sons Ltd on behalf of European Hematology Association. This is an open access Abstract Book distributed under the Attribution-NonCommercial-NoDerivs (CC BY-NC-ND), which allows third parties to download the articles and share them with others as long as they credit the author and the Abstract Book, but they cannot change the content in any way or use them commercially.
**Abstract Book Citations:** Authors, Title, HemaSphere, 2026;10:(S1):pages. The abstract book DOIs can be found on the “Author Information” page.
**Disclaimer:** Articles published in the journal HemaSphere exclusively reflect the opinions of the authors. The authors are responsible for all content in their abstracts including accuracy of the facts, statements, citing resources, etc.

### (PS1528) MATURE OSTEOBLAST-DERIVED CITRATE INDUCES A METABOLIC COLLAPSE IN ACUTE MYELOID LEUKEMIA.

**Topic:** 03. Acute myeloid leukemia - Biology & translational research

Lotfi Laaouimir*[1], Hernan Tirado[1], Nick van Gastel[1, 2]

*[1]UCLouvain, DeDuve Institute, brussels, Belgium; [2]WEL research institute, WELBIO, Wavre, Belgium;*

### Background

Acute myeloid leukemia (AML) is a malignancy of hematopoietic stem and progenitor cells. Chemoresistance in AML is believed to be attributable to many interacting factors, including metabolic abnormalities and the crosstalk between AML and the cells of the bone marrow microenvironment. Among the different reported bone marrow stromal cell-induced metabolic changes in AML, glycolytic and mitochondrial metabolism and their relation to chemoresistance has received growing interest in the field. However, which stromal cell subset is responsible for these metabolic changes and chemoprotection in AML remains unclear.

### Results

Co-culturing AML cells with variety of cell lines representing different stromal cell subsets revealed that osteoprogenitors are the most chemoprotective ($p<0.001$). Intriguingly, mature osteoblasts are not chemoprotective and induce apoptosis in AML cells ($p<0.0001$). Furthermore, RNA sequencing analysis revealed that both glycolytic genes and oxidative phosphorylation genes are significantly reduced in AML cells after coculture with mature osteoblasts ($p<0.01$).
Evaluating the secreted metabolome of mature osteoblasts using GC-MS revealed that they release citrate ($p<0.05$), a metabolite well-known as a glycolytic inhibitor. When exposing AML cells to citrate, we observed a significant decrease in the glycolytic capacity ($p<0.01$) and induction of apoptosis ($p<0.0001$). Knockdown of the citrate transporter Slc13a5 in AML cells prevented the citrate-induced cell death. We unexpectedly found that citrate also decreases mitochondrial activity in AML cells ($p<0.01$). Moreover, we found that citrate-treated AML cells upregulate the activity of cytosolic aconitase ($p<0.01$) by activating a mitochondrial protein called MitoNEET. Interestingly, blocking MitoNEET reverses the citrate-induced ($p<0.001$) and the mature osteoblast-induced cell death in AML cells ($p<0.01$). In addition, inducing AML in *Slc13a5*$^{fl/fl}$*Ocn*$^{Cre+}$ mice, which have more bone marrow citrate, or treating mice daily with citrate, decreased the leukemic burden ($p<0.001$).

### Summary/Conclusion

These findings indicate that activating MitoNEET could have therapeutic potential in AML, while increasing the levels of mature osteoblasts at the expense of osteoprogenitors could render chemotherapy more effective in AML.

**Copyright Information:** (Online) ISSN: 2572-9241
©2026 The Author(s). HemaSphere is published by John Wiley & Sons Ltd on behalf of European Hematology Association. This is an open access Abstract Book distributed under the Attribution-NonCommercial-NoDerivs (CC BY-NC-ND), which allows third parties to download the articles and share them with others as long as they credit the author and the Abstract Book, but they cannot change the content in any way or use them commercially.
**Abstract Book Citations:** Authors, Title, HemaSphere, 2026;10:(S1):pages. The abstract book DOIs can be found on the "Author Information" page.
**Disclaimer:** Articles published in the journal HemaSphere exclusively reflect the opinions of the authors. The authors are responsible for all content in their abstracts including accuracy of the facts, statements, citing resources, etc.

### (PS1529) TARGETING UGCG SENSITIZES AML CELLS TO VENETOCLAX THROUGH RAB32-MEDIATED ENDOPLASMIC RETICULUM-MITOCHONDRIA COMMUNICATION

**Topic:** 03. Acute myeloid leukemia - Biology & translational research

Xiaofan Sun[1], Yue Li[2], Danqi Pan[2], Caiping Wu[2], Weihao Xiao[2], Juan Du[2], Hui Zeng*[1]

[1]*Guangdong Provincial People's Hospital (Guangdong Academy of Medical Sciences), Southern Medical University, Guangzhou, China;* [2]*The First Affiliated Hospital of Jinan University, Guangzhou, China;*

### Background

Acute myeloid leukemia is a malignant and aggressive hematologic malignancy. The first-generation BCL-2 inhibitor venetoclax has become a first-line treatment for AML patients, significantly improving prognosis. However, drug resistance and relapse remain common, and research into its molecular mechanisms has been a persistent focus and challenge in the field of leukemia. Screening of AML cell lines and a large number of clinical samples revealed that the expression of UGCG, a key enzyme in sphingolipid metabolism, is significantly upregulated in AML patients resistant to venetoclax. Database analysis further showed that among venetoclax-resistant patients, those with higher UGCG expression have poorer survival, indicating that alterations in sphingolipid metabolism can affect therapeutic efficacy and lead to drug resistance. This underscores the necessity of further investigating combination therapeutic strategies involving venetoclax.

### Aims

Exploring the biological role and underlying mechanisms of targeting UGCG to enhance AML sensitivity to Venetoclax, thereby providing new insights for the treatment of AML.

### Methods

Utilizing AML cell lines, primary patient samples, and mouse leukemia models, we evaluated the anti-leukemic effects of UGCG inhibitors in combination with Venetoclax, either as single agents or in combination. The combination treatment was found to induce ceramide accumulation, which subsequently triggered endoplasmic reticulum stress (ERS), leading to increased CHOP expression and apoptosis in AML cells. Biological techniques such as laser confocal microscopy and transmission electron microscopy were employed to clarify the impact of the drug combination on endoplasmic reticulum-mitochondria interactions.

### Results

The combination of the UGCG inhibitor Eliglustat and Venetoclax reduced cell viability and induced apoptosis in AML cells. In vivo studies further demonstrated that the combination alleviated tumor burden and prolonged survival in xenograft mouse models. We found that the combination therapy induced endoplasmic reticulum ceramide accumulation, leading to mitochondrial dysfunction and activation of the ER stress pathway GRP78/PERK/CHOP, thereby promoting apoptosis in AML cells. Mechanistically, this effect was achieved through the activation of RAB32, which enhanced endoplasmic reticulum-mitochondria communication and triggered DRP1-dependent mitochondrial fission.

**Copyright Information:** (Online) ISSN: 2572-9241
©2026 The Author(s). HemaSphere is published by John Wiley & Sons Ltd on behalf of European Hematology Association. This is an open access Abstract Book distributed under the Attribution-NonCommercial-NoDerivs (CC BY-NC-ND), which allows third parties to download the articles and share them with others as long as they credit the author and the Abstract Book, but they cannot change the content in any way or use them commercially.
**Abstract Book Citations:** Authors, Title, HemaSphere, 2026;10:(S1):pages. The abstract book DOIs can be found on the "Author Information" page.
**Disclaimer:** Articles published in the journal HemaSphere exclusively reflect the opinions of the authors. The authors are responsible for all content in their abstracts including accuracy of the facts, statements, citing resources, etc.

**Summary/Conclusion**

UGCG is an enzyme that glycosylates ceramide and blunts its pro-apoptotic activity in cancer cells. Targeting UGCG sensitizes solid cancer cells to chemotherapy. However, whether targeting UGCG can sensitize AML cells to Venetoclax remains unclear. Here, we found that the inhibition of UGCG genetically or with its inhibitor Eliglustat efficiently suppressed growth and promoted apoptosis in AML cells. Moreover, Eliglustat in combination with Venetoclax increased apoptosis, reduced AML cell viability, and inhibited AML effectively both for primary AML cells and xenograft models. Mechanistically, the combination induced ceramide accumulation which activated the ER stress-GRP78/PERK/CHOP axis. Interestingly, combinatory treatment activated RAB32, which led to mitochondrial fission through ER-mitochondria communication and DRP1 activation. These findings demonstrate that targeting UGCG in combination with Venetoclax is an alternative combinatory strategy to treat AML, and provide insights for ceramide-mediated cell death in anti-cancer therapies.

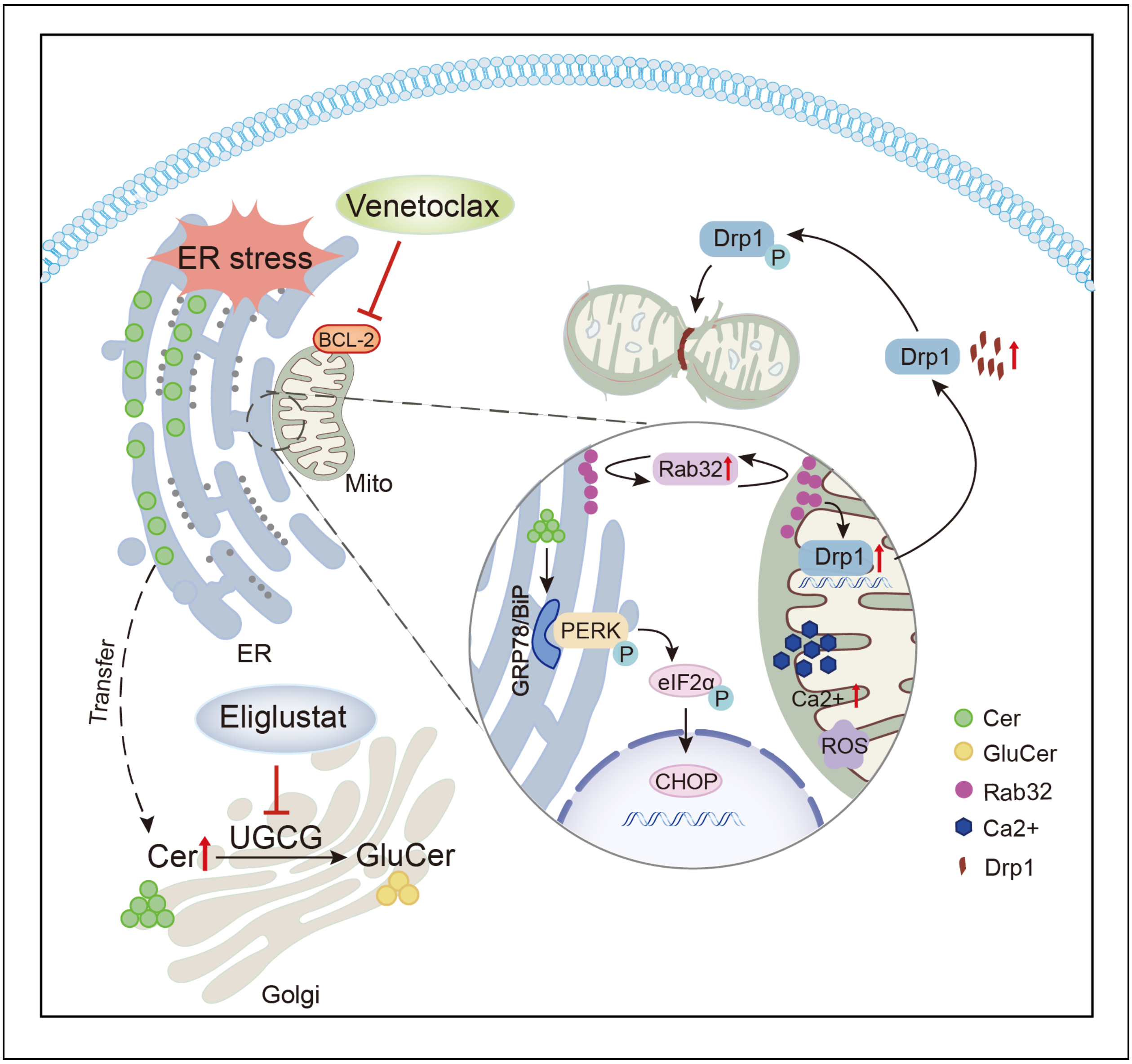

**Copyright Information:** (Online) ISSN: 2572-9241
©2026 The Author(s). HemaSphere is published by John Wiley & Sons Ltd on behalf of European Hematology Association. This is an open access Abstract Book distributed under the Attribution-NonCommercial-NoDerivs (CC BY-NC-ND), which allows third parties to download the articles and share them with others as long as they credit the author and the Abstract Book, but they cannot change the content in any way or use them commercially.
**Abstract Book Citations:** Authors, Title, HemaSphere, 2026;10:(S1):pages. The abstract book DOIs can be found on the "Author Information" page.
**Disclaimer:** Articles published in the journal HemaSphere exclusively reflect the opinions of the authors. The authors are responsible for all content in their abstracts including accuracy of the facts, statements, citing resources, etc.

### (PS1530) USP7 IS A THERAPEUTIC TARGET IN TP53-MUTATED ACUTE MYELOID LEUKEMIA

**Topic:** 03. Acute myeloid leukemia - Biology & translational research

Maximilian R. Zuleeg*[1, 2], Kirsten Peter[2], Arunima Murgai[2], Valeriia Sapozhnikova[3], Patricia Lemnitzer[2], Carlotta Pioppini[1], Milen Sterev[1], Nina Heisterkamp[2], Tatjana Krautloher[4], Jan Barinka[2, 5, 6], Simon Haas[2, 5, 6, 7, 8, 9, 10], Lars Bullinger[2, 7, 8], Ulrich Keller[2, 3, 7, 8, 9], Christian Steinebach[11], Philipp Mertins[3], Stephan Bohl[2], Jan Krönke[1, 2, 7]

*[1]Internal Medicine C, Hematology, Oncology, Stem Cell Transplantation and Palliative Care, University Medicine Greifswald, Greifswald, Germany; [2]Department of Hematology, Oncology and Cancer Immunology, Charité - Universitätsmedizin Berlin, corporate member of Freie Universität Berlin and Humboldt-Universität zu Berlin, Berlin, Germany; [3]Max Delbrück Center for Molecular Medicine, Berlin, Germany; [4]AstraZeneca, Hamburg, Germany; [5]Berlin Institute for Medical Systems Biology, Max Delbrück Center for Molecular Medicine in the Helmholtz Association, Berlin, Germany; [6]Berlin Institute of Health, Berlin, Germany; [7]German Cancer Consortium (DKTK) partner site Berlin and German Cancer Research Center (DKFZ), Heidelberg, Germany; [8]National Center for Tumor diseases (NCT) Berlin, a partnership between DKFZ and Charité-University Hospital, Berlin, Germany; [9]Cluster of Excellence ImmunoPreCept, Charité - Universitätsmedizin Berlin, Berlin, Germany;[10]Precision Healthcare University Research Institute, Queen Mary University of London, London, United Kingdom; [11]Institute of Pharmacy, University of Greifswald, Greifswald, Germany;*

### Background

Mutations in *TP53* confer resistance to chemotherapy and the BCL2 inhibitor venetoclax, creating an urgent need for alternative therapeutic strategies in AML. Inhibitors of Ubiquitin-specific Peptidase 7 (USP7), the deubiquitinating enzyme of MDM2, demonstrated activity in cancer, which was linked to the induction of p53. We found that USP7 inhibition remains active in *TP53* mutant AML, indicating additional substrates and downstream signaling.

### Aims

Characterization of p53-independent mechanisms of USP7 in AML and evaluation of USP7 inhibition as a therapeutic strategy.

### Methods

We studied the role of USP7 in AML by CRISPR/Cas9-mediated genetic inactivation and pharmacologic inhibition in AML cell lines and primary cells to evaluate its impact on leukemic cell growth and sensitivity to anti-leukemic drugs *in vitro* and *in vivo*. USP7-dependent changes in protein and transcript levels were assessed by mass spectrometry and RNA sequencing respectively, and validated by Western blot.

**Copyright Information:** (Online) ISSN: 2572-9241
©2026 The Author(s). HemaSphere is published by John Wiley & Sons Ltd on behalf of European Hematology Association. This is an open access Abstract Book distributed under the Attribution-NonCommercial-NoDerivs (CC BY-NC-ND), which allows third parties to download the articles and share them with others as long as they credit the author and the Abstract Book, but they cannot change the content in any way or use them commercially.
**Abstract Book Citations:** Authors, Title, HemaSphere, 2026;10:(S1):pages. The abstract book DOIs can be found on the “Author Information” page.
**Disclaimer:** Articles published in the journal HemaSphere exclusively reflect the opinions of the authors. The authors are responsible for all content in their abstracts including accuracy of the facts, statements, citing resources, etc.

**Results**

A focused CRISPR/Cas9 screen of 99 deubiquitinating enzymes identified USP7 as a critical vulnerability in AML, irrespective of *TP53* status. Both, small-molecule inhibition and proteolysis targeting chimera (PROTAC)-mediated degradation of USP7, induced AML cell death. USP7 inhibition showed strong synergy with anti-leukemic drugs, including chemotherapy and the BCL2 inhibitor venetoclax, in AML cell lines and primary patient cells, including those with *TP53* mutations. The combination of venetoclax and USP7 inhibition significantly reduced tumor burden and prolonged survival, compared with single agent treatment in an AML PDX mouse model *in vivo*.
Applying global proteomic analyses in *TP53* null HL60 cells, we found the proteins NCF1 and NCF2, components of the reactive oxygen species (ROS)-producing NADPH oxidase complex 2 (NOX2), to be reduced after USP7 inhibition. Co-immunoprecipitation and ubiquitination analyses revealed that NCF1 is a substrate of USP7. Consistently, NCF1 degradation mediated by USP7 inhibition, led to lower ROS levels. Direct inhibition of NOX2, like USP7 inhibition, resulted in a transcriptional downregulation of the anti-apoptotic protein BCL2A1. In primary AML single cell data, expression of NOX2 members highly correlated with *BCL2A1* expression. Furthermore, BCL2A1 overexpression conferred resistance to venetoclax in AML cell lines. Prognostically, high NOX2 expression levels were associated with impaired overall survival in the TCGA-AML dataset.

**Summary/Conclusion**

USP7 is a p53-independent vulnerability in AML that modulates a NOX2–BCL2A1 signaling pathway. USP7 inhibition synergizes with venetoclax, including in *TP53*-mutant AML, providing a strong rationale for clinical development of USP7-targeting strategies and biomarker-driven combination therapies.

**Copyright Information:** (Online) ISSN: 2572-9241
©2026 The Author(s). HemaSphere is published by John Wiley & Sons Ltd on behalf of European Hematology Association. This is an open access Abstract Book distributed under the Attribution-NonCommercial-NoDerivs (CC BY-NC-ND), which allows third parties to download the articles and share them with others as long as they credit the author and the Abstract Book, but they cannot change the content in any way or use them commercially.
**Abstract Book Citations:** Authors, Title, HemaSphere, 2026;10:(S1):pages. The abstract book DOIs can be found on the “Author Information” page.
**Disclaimer:** Articles published in the journal HemaSphere exclusively reflect the opinions of the authors. The authors are responsible for all content in their abstracts including accuracy of the facts, statements, citing resources, etc.

### (PS1531) SIPA1 DEFICIENCY IN HOST IMMUNE NICHE UNLOCKS ITS ANTI-AML ACTIVITY

**Topic:** 03. Acute myeloid leukemia - Biology & translational research

Runqing Zhang*[1], Lakshmi Sandhow[1], Huan Cai[1], Hong Qian[1]

[1]*Karolinska Institutet, Center for Hematology and Regenerative Medicine (HERM), Department of Medicine Huddinge, Huddinge, Sweden;*

### Background

Signal-induced proliferation-associated gene 1 (Sipa1), a RAP1 GTPase-activating protein in hematopoietic and stromal cells, negatively regulates G-protein signaling pathways involved in cell adhesion and proliferation. We have demonstrated that *Sipa1* loss induces alterations in bone marrow (BM) mesenchymal stem and progenitor cells (MSPCs) driving myeloproliferative neoplasm development (Xiao et al., Blood Adv. 2018;2(5):534-548) and that *Sipa1* reduction in BM MSPCs occurs in mice with acute myeloid leukemia (AML) (Xiao et al., Blood Adv. 2018;2(12):1480-1494). However, the impact and mechanisms of *Sipa1* in BM niches on AML progression remain undefined. Our recent findings showed delayed or blocked AML development after transplanting AML cells into *Sipa1*$^{-/-}$ recipients (ASH Abstract, 2025), indicating Sipa1-deficient hosts provide an unfit niche for AML cells, though mechanisms remain unclear.

### Aims

To elucidate the impact and mechanisms of *Sipa1* deficiency on AML progression.

### Methods

We investigated *Sipa1* expression impact in host BM mesenchymal and immune cell niches by transplanting MLL-AF9 AML cells into *Sipa1*$^{-/-}$ mice, followed by: 1) determining leukemogenicity through serial transplantation; 2) profiling immune cell function using co-culture assays, transplantation, and flow cytometry; 3) characterizing the mesenchymal niche by RNA sequencing; and 4) determining clinical correlations between *SIPA1* expression in BM mononuclear cells and treatment outcomes in AML patients.

**Copyright Information:** (Online) ISSN: 2572-9241
©2026 The Author(s). HemaSphere is published by John Wiley & Sons Ltd on behalf of European Hematology Association. This is an open access Abstract Book distributed under the Attribution-NonCommercial-NoDerivs (CC BY-NC-ND), which allows third parties to download the articles and share them with others as long as they credit the author and the Abstract Book, but they cannot change the content in any way or use them commercially.
**Abstract Book Citations:** Authors, Title, HemaSphere, 2026;10:(S1):pages. The abstract book DOIs can be found on the “Author Information” page.
**Disclaimer:** Articles published in the journal HemaSphere exclusively reflect the opinions of the authors. The authors are responsible for all content in their abstracts including accuracy of the facts, statements, citing resources, etc.

**Results**

In primary recipients, 30% of *Sipa1*$^{-/-}$ mice remained disease-free, whereas all *Sipa1*$^{+/+}$ mice developed AML. Notably, residual AML cells persisted in BM of disease-free *Sipa1*$^{-/-}$ primary recipients. Upon serial transplantation, none of the *Sipa1*$^{-/-}$ secondary recipients developed AML while all *Sipa1*$^{+/+}$ recipients did, suggesting a suppressive role of *Sipa1*$^{-/-}$ niches.
In vivo depletion of NK or CD4⁺ T cells by antibody neutralization abolished the AML-suppressive effect in*Sipa1*$^{-/-}$ recipients, indicating *Sipa1* deficiency enhances NK- and CD4⁺ T cell-mediated anti-AML responses. Mechanistically, spleen derived *Sipa1*$^{-/-}$ NK cells exhibited enhanced maturation when cocultured with AML cells regardless of CD4⁺ T cell presence and displayed increased cytotoxic activity in vitro compared to *Sipa1*$^{+/+}$ NK cells.
RNA sequencing revealed that at steady-state, *Sipa1*$^{-/-}$ stromal cells displayed upregulated inflammatory signatures compared with *Sipa1*$^{+/+}$ stromal cells. Upon AML challenge, stromal cells from disease-free *Sipa1*$^{-/-}$ recipients further upregulated immune-regulatory and metabolic gene signatures relative to steady-state *Sipa1*$^{-/-}$ stromal cells, potentially contributing to AML-suppressive niche formation.
Consistent with mouse findings, *SIPA1* expression in BM mononuclear cells from AML patients was an independent prognostic risk factor across most FAB subclasses and mutational subtypes. Upon stratification into cytolytic-high and cytolytic-low cohorts (Dufva et al., Cancer Cell. 2020;38(3):380-399), SIPA1 was a significant risk factor exclusively in the cytolytic-high cohort, highlighting its critical role in immune-mediated anti-AML activity (TCGA-LAML).

**Summary/Conclusion**

Our study demonstrates that *Sipa1* loss in the host microenvironment potentiates anti-AML responses primarily via NK and CD4⁺ T cells. Together with clinical correlation between low *SIPA1* expression and improved survival in AML patients, these results warrant further investigation into therapeutic potential of *Sipa1*-deficient immune cells in AML.

**Copyright Information:** (Online) ISSN: 2572-9241
©2026 The Author(s). HemaSphere is published by John Wiley & Sons Ltd on behalf of European Hematology Association. This is an open access Abstract Book distributed under the Attribution-NonCommercial-NoDerivs (CC BY-NC-ND), which allows third parties to download the articles and share them with others as long as they credit the author and the Abstract Book, but they cannot change the content in any way or use them commercially.
**Abstract Book Citations:** Authors, Title, HemaSphere, 2026;10:(S1):pages. The abstract book DOIs can be found on the “Author Information” page.
**Disclaimer:** Articles published in the journal HemaSphere exclusively reflect the opinions of the authors. The authors are responsible for all content in their abstracts including accuracy of the facts, statements, citing resources, etc.

### (PS1532) MYELOID MICROENVIRONMENT REMODELING AND IFITM-RIG-I HYPERACTIVATION DRIVE AML-ASSOCIATED HEMOPHAGOCYTIC LYMPHOHISTIOCYTOSIS, RATHER THAN CYTOTOXIC DEFICIENCY

**Topic:** 03. Acute myeloid leukemia - Biology & translational research

Guangshuai Teng*[1], Ke Shang[1], Yuhui Zhang[1], Anna Li[1], Chenxiao Du[1], Yan Wang[1], Yanqi Li[1], Huiqin Zhang[1], Yifan Duan[1], Xuelian Cheng[2], Yuan Zhou[2], Yajing Chu[2], Weiping Yuan[1, 2], Jie Bai[1]

*[1]The Second Hospital of Tianjin Medical University, Department of Hematology, Tianjin, China;[2]Institute of Hematology & Blood Diseases Hospital, Chinese Academy of Medical Sciences & Peking Union Medical College, State Key Laboratory of Experimental Hematology, National Clinical Research Center for Blood Diseases, Tianjin, China;*

### Background

Acute myeloid leukemia (AML) and other myeloid neoplasms (MNs)-associated hemophagocytic lymphohistiocytosis (sHLH) is a life-threatening hyperinflammatory condition with dismal prognosis, yet its pathogenesis remains incompletely understood. Current diagnostic and therapeutic paradigms assume cytotoxic function deficiency, but clinical outcomes remain unsatisfactory.

### Aims

To characterize the clinical features, genetic landscape, and cellular alterations in sHLH, and to identify key cellular subsets and molecular mechanisms within the bone marrow microenvironment that drive this syndrome.

### Methods

We enrolled 16 patients with sHLH (including AML, MDS, and MPN) and 48 matched MN patients without HLH (non-HLH). We integrated survival analysis, targeted gene sequencing, plasma cytokine profiling, and single-cell RNA sequencing (scRNA-seq) of bone marrow mononuclear cells (3 sHLH, 3 non-HLH). Mechanistic validation was performed using flow cytometry and Western blotting.

### Results sHLH patients exhibited significantly shorter median overall survival (3 vs. 15 months, $p<0.001$), and standard HLH-directed therapies showed limited efficacy. The sHLH cohort had a higher frequency of TP53 mutations and significantly elevated plasma CXCL10 levels ($p<0.05$). scRNA-seq revealed enhanced crosstalk between leukemic stem cells and immune cells in the bone marrow of sHLH patients. Pseudotime trajectory analysis identified a pathological branch in monocyte-to-macrophage differentiation in sHLH samples, resulting in macrophages with increased phagocytic activity and marked upregulation of cytokine-related genes.
Natural killer (NK) cells in sHLH patients did not exhibit impaired cytotoxicity; instead, they showed hyperactivation of the RIG-I pathway, accompanied by significant upregulation of IFITM1/3 and cytokine hypersecretion. IFITM-RIG-I axis activation correlated positively with proinflammatory cytokine levels. Cell-cell communication analysis confirmed marked upregulation of the CCL3-CCR1 ligand-receptor pair between NK cells and monocytes/macrophages. Western blotting and flow cytometry validation confirmed significantly higher expression of IFITM1/3 and RIG-I in NK cells from sHLH patients compared to non-HLH controls; high-throughput sequencing showed significantly higher CCR1 expression in the sHLH group.

**Copyright Information:** (Online) ISSN: 2572-9241
©2026 The Author(s). HemaSphere is published by John Wiley & Sons Ltd on behalf of European Hematology Association. This is an open access Abstract Book distributed under the Attribution-NonCommercial-NoDerivs (CC BY-NC-ND), which allows third parties to download the articles and share them with others as long as they credit the author and the Abstract Book, but they cannot change the content in any way or use them commercially.
**Abstract Book Citations:** Authors, Title, HemaSphere, 2026;10:(S1):pages. The abstract book DOIs can be found on the “Author Information” page.
**Disclaimer:** Articles published in the journal HemaSphere exclusively reflect the opinions of the authors. The authors are responsible for all content in their abstracts including accuracy of the facts, statements, citing resources, etc.

### Summary/Conclusion

This study demonstrates that sHLH is driven not by cytotoxic deficiency, but by an inflammatory process mediated by IFITM-RIG-I axis activation and myeloid microenvironment remodeling via CCL3-CCR1 signaling. These findings challenge the conventional paradigm and provide a mechanistic rationale for targeted therapies directed at this self-amplifying inflammatory loop.

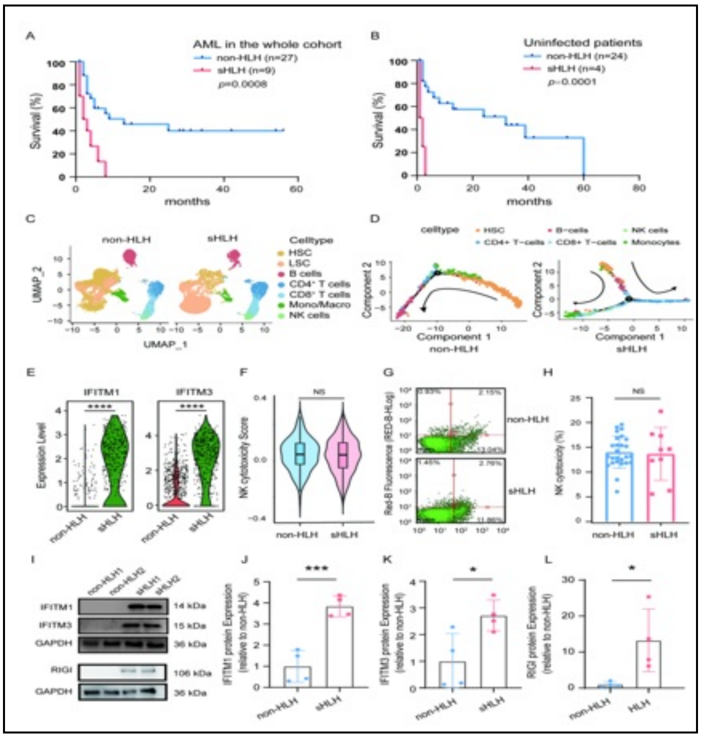

Figure. Survival and mechanistic analyses of non-HLH and sHLH patients with MN. (A-B) Kaplan-Meier curves comparing overall survival between non-HLH and sHLH groups in AML patients. (C) UMAP visualization of various cell populations of 6 cases. (D) Pseudotime trajectory of monocyte-to-macrophage differentiation (Monocle2). (E) Violin plot showing IFITM1/3 levels in monocyte/macrophage. (F) Violin plot showing NK cell cytotoxicity-related gene expression. (G) Representative flow cytometry of NK cell cytotoxicity. (H) Quantitative analysis of NK cell cytotoxicity (non-HLH, n=26; sHLH, n=9). (I) Western blot of IFITM1, IFITM3, and RIG-I proteins in NK cells. (J-L) Quantification of IFITM1, IFITM3, and RIG-I protein expression. Data are presented as mean±SD, n=4/group. Mann-Whitney U test. * p<0.05, ** p<0.01, *** p<0.001.

**Copyright Information:** (Online) ISSN: 2572-9241
©2026 The Author(s). HemaSphere is published by John Wiley & Sons Ltd on behalf of European Hematology Association. This is an open access Abstract Book distributed under the Attribution-NonCommercial-NoDerivs (CC BY-NC-ND), which allows third parties to download the articles and share them with others as long as they credit the author and the Abstract Book, but they cannot change the content in any way or use them commercially.
**Abstract Book Citations:** Authors, Title, HemaSphere, 2026;10:(S1):pages. The abstract book DOIs can be found on the “Author Information” page.
**Disclaimer:** Articles published in the journal HemaSphere exclusively reflect the opinions of the authors. The authors are responsible for all content in their abstracts including accuracy of the facts, statements, citing resources, etc.

### (PS1533) DNMT3AR878H-MUTATED QUIESCENT LEUKAEMIA STEM CELLS ARE TARGETABLE BY COMBINED VENETOCLAX AND AZACITIDINE THERAPY OR CELL-CYCLE PRIMING IN NPM1C-FLT3ITD AML

**Topic:** 03. Acute myeloid leukemia - Biology & translational research

Paniz Tavakoli Shirazi*[1, 2], Jasmin Straube[1, 2], Victoria Ling[1, 2], Emily Cooper[1], Siu Hang Natalie Chan[1], Rohit Haldar[1], Megan Bywater[1, 2], Steven Lane[1, 2, 3]

*[1]QIMR Berghofer Medical Research Institute, Cancer Research Program, Brisbane, Australia;[2]The University of Queensland, School of Biomedical Sciences, Brisbane, Australia; [3]Royal Brisbane and Women's Hospital, Cancer Care Services, Brisbane, Australia;*

### Background

Chemoresistance in acute myeloid leukaemia (AML) is driven by genetic alterations in leukaemia stem cells (LSCs). In *NPM1*$^{c}$-*FLT3*$^{ITD}$ AML, co-mutation with *DNMT3A*$^{R882H}$ defines a high-risk cohort with increased relapse and inferior survival (5-year survival 38% vs 55%). Using murine *Npm1*$^{c}$-*Flt3*$^{ITD}$ AML with or without *Dnmt3a*$^{R878H}$ (homologous to human R882H), we have shown that *Dnmt3a*$^{R878H}$ LSCs resist chemotherapy through epigenetically enforced quiescence. We hypothesise that these chemoresistant, quiescent LSCs retain sensitivity to cell-cycle independent therapies, and that driving cell cycle entry could restore chemosensitivity.

### Aims

- Determine whether *Dnmt3a*$^{R878H}$-mutated LSCs retain sensitivity to cell-cycle independent therapy.
- Determine whether driving cell-cycle entry restores chemosensitivity of *Dnmt3a*$^{R878H}$-mutated LSCs.

### Methods

To assess sensitivity to cell-cycle independent therapy, *Dnmt3a*$^{R878H}$-*Npm1*$^{c}$-*Flt3*$^{ITD}$ (DNF) and *Npm1*$^{c}$-*Flt3*$^{ITD}$(NF) mice received venetoclax + azacitidine (Ven+Aza; 100 mg/kg + 2 mg/kg), a standard-of-care regimen for patients unfit for intensive chemotherapy, or vehicle control. To induce cycling in stem cells, DNF mice received pegylated interferon-α (pegIFNα; 600 ng) or saline 48 hours prior to doxorubicin (Dox; 1.5 mg/kg) + cytarabine (Arac; 50 mg/kg). Disease burden and stem/progenitor compartments were quantified by flow cytometry. Quiescent LSCs were defined as Ki-67$^{-}$. LSC transcriptional programs were analysed by RNA sequencing and gene set enrichment analysis.

**Copyright Information:** (Online) ISSN: 2572-9241
©2026 The Author(s). HemaSphere is published by John Wiley & Sons Ltd on behalf of European Hematology Association. This is an open access Abstract Book distributed under the Attribution-NonCommercial-NoDerivs (CC BY-NC-ND), which allows third parties to download the articles and share them with others as long as they credit the author and the Abstract Book, but they cannot change the content in any way or use them commercially.
**Abstract Book Citations:** Authors, Title, HemaSphere, 2026;10:(S1):pages. The abstract book DOIs can be found on the "Author Information" page.
**Disclaimer:** Articles published in the journal HemaSphere exclusively reflect the opinions of the authors. The authors are responsible for all content in their abstracts including accuracy of the facts, statements, citing resources, etc.

**Results**

Ven+Aza treatment significantly reduced AML burden in peripheral blood, spleen, and bone marrow in DNF and NF mice. This was accompanied by a significant reduction in myeloid blasts (blasts ($x10^6$); vehicle vs Ven+Aza; DNF=8 vs 0.7; NF=7 vs 0.4, p<0.0001). In contrast to the chemoresistance previously demonstrated, DNF LSCs exhibit similar sensitivity to Ven+Aza as NF LSCs (residual LSCs ($x10^5$); vehicle vs Ven+Aza, DNF=12 vs 1.3; NF=10 vs 0.4, p<0.0001), representing comparable reductions in LSC frequency (%DNF vs NF LSC reduction; 88 vs 95). Ven+Aza treatment induced gene expression changes consistent with myeloid maturation (NES=1.8, p=1.3E-05), reduced stemness (NES=-1.7, p=2.6E-05) and quiescence (NES=-1.5, p=1.4E-02) in DNF LSCs; however, residual *Dnmt3a*$^{R878H}$-treated LSCs retained a quiescent transcriptional profile compared to NF, indicating persistence of a therapy-tolerant reservoir of disease.

To overcome the chemoresistance in DNF AML, pegIFNα priming was used prior to Dox+Arac treatment to induce cell cycling. PegIFNa plus Dox+Arac significantly enhanced depletion of quiescent DNF LSCs (%quiescent LSCs, pegIFNα+Dox+Arac=9.5 vs Dox+Arac=16.4, p=0.002). This was accompanied by marked reductions in AML burden, with significant decrease of myeloid blasts in the circulation and bone marrow. PegIFNα treatment also achieved a substantial contraction of the LSC compartment in DNF mice (residual LSCs ($x10^5$); pegIFNα+Dox+Arac=4 vs Dox+Arac=7, p=0.01).

**Summary/Conclusion**

*Dnmt3a*$^{R878H}$-mutant LSCs are resistant to cell-cycle dependent chemotherapy but remain sensitive to cell-cycle independent therapies such as Ven+Aza, supporting its use as an upfront therapeutic strategy in *DNMT3A*$^{R882H}$-*FLT3*$^{ITD}$-*NPM1*$^{c}$ AML. Alternatively, driving cell-cycle entry represents a strategy to overcome chemotherapy resistance, demonstrating LSC quiescence as a targetable vulnerability that may prevent relapse long-term and improve outcomes in *DNMT3A*-mutant AML.

**Copyright Information:** (Online) ISSN: 2572-9241
©2026 The Author(s). HemaSphere is published by John Wiley & Sons Ltd on behalf of European Hematology Association. This is an open access Abstract Book distributed under the Attribution-NonCommercial-NoDerivs (CC BY-NC-ND), which allows third parties to download the articles and share them with others as long as they credit the author and the Abstract Book, but they cannot change the content in any way or use them commercially.
**Abstract Book Citations:** Authors, Title, HemaSphere, 2026;10:(S1):pages. The abstract book DOIs can be found on the "Author Information" page.
**Disclaimer:** Articles published in the journal HemaSphere exclusively reflect the opinions of the authors. The authors are responsible for all content in their abstracts including accuracy of the facts, statements, citing resources, etc.

### (PS1534) AML-INDUCED MACROPHAGE-TREG CROSSTALK CREATES A SELF-REINFORCING IMMUNOSUPPRESSIVE LOOP VIA CD44 AND TIM-3

**Topic:** 03. Acute myeloid leukemia - Biology & translational research

Alicia Perzolli*[1, 2], Merel van der Meulen[1, 2], C. Michel Zwaan[1, 2], Olaf Heidenreich[1, 3]

*[1]Prinses Máxima Centrum Voor Kinderoncologie, Utrecht, The Netherlands;[2]Erasmus MC Locatie Sophia, Pediatric Oncology, Rotterdam, The Netherlands; [3]University Medical Center Utrecht, Utrecht, The Netherlands;*

### Background

Acute myeloid leukemia (AML) patients have seen limited benefit from novel immunotherapies, largely due to an immunosuppressive bone marrow microenvironment. However, the specific cellular interactions driving this evasion remain elusive. Using single-cell spatial transcriptomics (Xenium platform), we identified significantly increased co-localization of AML blasts, macrophages, and regulatory T cells (Tregs) in both pediatric and adult AML patients compared to non-leukemic controls, suggesting a structured immunosuppressive niche.

### Aims

To characterize the immunosuppressive crosstalk between AML cells, macrophages, and T cells, and to identify actionable molecular targets to disrupt these interactions.

### Methods

We established a multi-cellular co-culture system using naïve $CD4^{+}$ T cells and macrophages derived from human CD14+ monocytes that had been isolated from healthy PBMCs. Macrophages and/or naïve $CD4^{+}$ T cells were cultured with or without AML patient-derived xenografts (PDX) containing *KMT2A*-rearranged (*KMT2A*r) or *RUNX1::RUNX1T1* fusion genes. Treg induction ($CD25^{high}$ $FOXP3^{high}$ $CD127^{low}$), immune checkpoints, and macrophage polarization markers were analyzed via flow cytometry. Functional suppression was evaluated through T cell proliferation, killing assays, and cytokine profiling (TNF, IFNg, IL2). CD44 and TIM-3-mediated interactions were validated using blocking antibodies, and Treg recruitment was assessed via transwell migration assays.

### Results

Direct co-culture, but not transwell separation, between naïve CD4+ T cells and macrophages induced robust differentiation of induced Tregs (CD4+CD25+FOXP3+), reaching ~40% at day 2 and ~80% at day 4 (1:1 T cell:macrophage ratio). Inclusion of *KMT2Ar* PDX cells, but not *RUNX1::RUNX1T1*, significantly enhanced iTreg induction (+25% at day 2). These iTregs exhibited marked upregulation of CTLA4, PD-1, and LAG3. Notably, macrophages pre-incubated with PDX cells did not increase iTreg frequency but significantly enhanced the expression intensity of CD25 and FOXP3.

Functionally, these iTregs suppressed effector $CD3^{+}$ T cell proliferation and reduced pro-inflammatory cytokine production (TNF, IFNγ, IL2). Reciprocally, iTregs shifted macrophages toward a mixed pro-/anti-inflammatory phenotype. Importantly, both macrophages preincubated with AML cells and macrophage/AML-induced Treg cells impaired cytotoxic T cell activity against AML cells, demonstrating the functional relevance of the respective phenotypic changes. We identified strong upregulation of TIM-3 and CD44 on iTregs following co-culture. Dual blockade of CD44 and TIM-3 significantly impaired iTreg induction. Finally, migration assays confirmed that AML-associated macrophages actively recruit Tregs, completing a recruitment-differentiation circuit.

**Copyright Information:** (Online) ISSN: 2572-9241
©2026 The Author(s). HemaSphere is published by John Wiley & Sons Ltd on behalf of European Hematology Association. This is an open access Abstract Book distributed under the Attribution-NonCommercial-NoDerivs (CC BY-NC-ND), which allows third parties to download the articles and share them with others as long as they credit the author and the Abstract Book, but they cannot change the content in any way or use them commercially.
**Abstract Book Citations:** Authors, Title, HemaSphere, 2026;10:(S1):pages. The abstract book DOIs can be found on the "Author Information" page.
**Disclaimer:** Articles published in the journal HemaSphere exclusively reflect the opinions of the authors. The authors are responsible for all content in their abstracts including accuracy of the facts, statements, citing resources, etc.

**Summary/Conclusion**

Our findings identify macrophages as central orchestrators of Treg recruitment and differentiation in the AML niche. We demonstrate a "feedback loop" where AML cells prime macrophages to drive T cell exhaustion and Treg expansion, which in turn modifies the macrophage phenotype to sustain suppression. Disrupting this axis via CD44 or TIM-3 inhibition represents a promising therapeutic strategy to dismantle the AML immune-protective niche and enhance the efficacy of immunotherapies.

**Copyright Information:** (Online) ISSN: 2572-9241
©2026 The Author(s). HemaSphere is published by John Wiley & Sons Ltd on behalf of European Hematology Association. This is an open access Abstract Book distributed under the Attribution-NonCommercial-NoDerivs (CC BY-NC-ND), which allows third parties to download the articles and share them with others as long as they credit the author and the Abstract Book, but they cannot change the content in any way or use them commercially.
**Abstract Book Citations:** Authors, Title, HemaSphere, 2026;10:(S1):pages. The abstract book DOIs can be found on the "Author Information" page.
**Disclaimer:** Articles published in the journal HemaSphere exclusively reflect the opinions of the authors. The authors are responsible for all content in their abstracts including accuracy of the facts, statements, citing resources, etc.

### (PS1535) A DUAL STRATEGY FOR AML: CAR T CELLS DERIVED FROM A NOVEL, HIGH-AFFINITY ANTI-CLL-1 MONOCLONAL ANTIBODY AND SENSITIZATION BY A MENIN INHIBITOR

**Topic:** 03. Acute myeloid leukemia - Biology & translational research

Shuhei Kida*[1, 2], Makiko Suga[2], Yuta Yamaguchi[2], Shunya Ikeda[2], Yosuke Kogue[3], Kumi Shibata[2], Kazuhito Tsutsumi[2], Hiraku Murakami[2], Emiko Mizuta[2], Yo Mizutani[2], Naoki Hosen[2]

[1]*Osaka General Medical Center, Hematology and Oncology, Osaka, Japan;* [2]*The University of Osaka Graduate School of Medicine, Suita, Japan;* [3]*Osaka Research Center for Drug Discovery, Otsuka Pharmaceutical Company, Ltd., Minoh, Japan;*

### Background

CAR T cells targeting C-type lectin-like molecule-1 (CLL-1) have shown anti-leukemia efficacy in patients with acute myeloid leukemia (AML). However, CLL-1$^{low/-}$ AML cells are present in a subset of patients, suggesting that highly sensitive CAR T cells are required to eradicate all AML clones; alternatively, approaches to enhance CLL-1 expression should be explored to extend the application of CLL-1 CAR T cells to more AML patients.

### Aims

To identify a novel, high-affinity anti-CLL-1 monoclonal antibody (mAb) that enables sensitive CAR T-cell recognition, and to test whether pharmacologic upregulation of CLL-1 can sensitize CLL-1$^{low/-}$ AML cells to CLL-1 CAR T-cell cytotoxicity.

### Methods

We generated 13 new mAbs against human CLL-1 by immunizing BALB/c mice with Ba/F3 cells expressing human CLL-1 and screening hybridoma supernatants by flow cytometry using U937 cells and CLL-1-deficient U937 cells. Variable regions were cloned to construct second-generation CARs (CD28–CD3ζ), co-expressing truncated EGFR (tEGFR) as a transduction marker. CAR T cells from healthy donors were produced by retroviral transduction and compared by matching CAR$^{+}$ cell numbers based on tEGFR staining. Function was assessed by cytokine release and cytotoxicity. To model antigen heterogeneity, we used CRISPR-generated CLL-1-knockout U937 cells and shRNA-generated U937 clones with graded CLL-1 expression. In vivo efficacy was evaluated in a disseminated AML xenograft model. To explore CLL-1 upregulation, AML cell lines harboring MLL fusions (MOLM13, THP-1) or NPM1 mutation (OCI-AML3) were treated with revumenib (menin inhibitor), azacitidine, or decitabine.

**Copyright Information:** (Online) ISSN: 2572-9241
©2026 The Author(s). HemaSphere is published by John Wiley & Sons Ltd on behalf of European Hematology Association. This is an open access Abstract Book distributed under the Attribution-NonCommercial-NoDerivs (CC BY-NC-ND), which allows third parties to download the articles and share them with others as long as they credit the author and the Abstract Book, but they cannot change the content in any way or use them commercially.
**Abstract Book Citations:** Authors, Title, HemaSphere, 2026;10:(S1):pages. The abstract book DOIs can be found on the "Author Information" page.
**Disclaimer:** Articles published in the journal HemaSphere exclusively reflect the opinions of the authors. The authors are responsible for all content in their abstracts including accuracy of the facts, statements, citing resources, etc.

**Results**

Among 13 candidates, CAR T cells derived from mAb 2-23 (hereafter referred to as 2-23 CAR T cells) demonstrated the greatest potential for IL-2 production upon co-culture with CLL-1^+ AML cell lines, with no reactivity to CLL-1-deficient targets. Therefore, we selected 2-23 as the most promising mAb for further characterization. 2-23 showed higher affinity for CLL-1 than M26, a CLL-1 mAb used in many previous studies (Fig. 1A). The 2-23 CAR T cells produced higher amounts of IFN-g and IL-2 when co-cultured with any of the U937 leukemia cell clones. Notably, IL-2 production was detectable when the 2-23 CAR T cells were co-cultured with some of the CLL-1-low clones, although it was not detectable when CAR T cells derived from mAb M26 (M26 CAR T cells) were co-cultured with these clones (Fig. 1B). These results suggest that 2-23CAR T cells are more sensitive than M26 CAR T cells, whereas both 2-23 CAR T cells and M26 CAR T cells exhibited cytotoxicity against the U937 cell clones with low CLL-1 expression. In vivo, 2-23 CAR T cells eradicated leukemia cells, and survival was significantly prolonged compared with control T cells (Fig. 1C). In addition, revumenib markedly increased CLL-1 expression in AML cells with MLL fusions or NPM1 mutation, whereas azacitidine and decitabine showed little or no induction (Fig. 1D). Therefore, revumenib pretreatment increased the susceptibility of MOLM13 cells to 2-23 CAR T cells, leading to increased cytokine release and enhanced cytotoxicity in vitro (Fig. 1E).

**Summary/Conclusion**

CAR T cells derived from the new, high-affinity anti-CLL-1 mAb 2-23 demonstrated significant anti-AML efficacy against CLL-1-positive AML cells. Furthermore, we propose combining a menin inhibitor with CLL-1 CAR T cell therapy to potentially treat some patients with AML who lack or have low CLL-1 expression.

**Copyright Information:** (Online) ISSN: 2572-9241
©2026 The Author(s). HemaSphere is published by John Wiley & Sons Ltd on behalf of European Hematology Association. This is an open access Abstract Book distributed under the Attribution-NonCommercial-NoDerivs (CC BY-NC-ND), which allows third parties to download the articles and share them with others as long as they credit the author and the Abstract Book, but they cannot change the content in any way or use them commercially.
**Abstract Book Citations:** Authors, Title, HemaSphere, 2026;10:(S1):pages. The abstract book DOIs can be found on the "Author Information" page.
**Disclaimer:** Articles published in the journal HemaSphere exclusively reflect the opinions of the authors. The authors are responsible for all content in their abstracts including accuracy of the facts, statements, citing resources, etc.

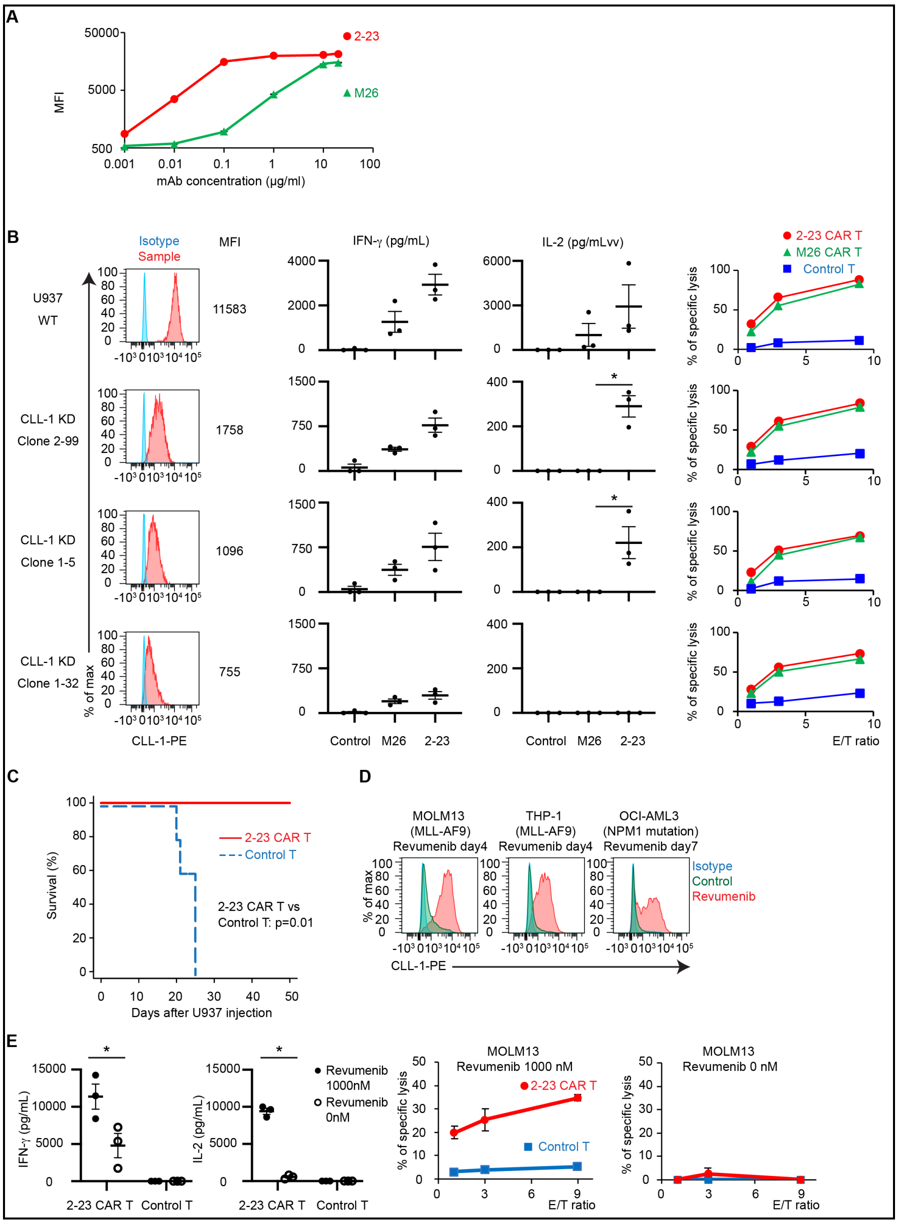

Figure 1. High-affinity 2-23 CLL-1 CAR T cells and revumenib-mediated CLL-1 upregulation support a strategy to enhance CLL-1 CAR T cell efficacy in AML.

**Copyright Information:** (Online) ISSN: 2572-9241
©2026 The Author(s). HemaSphere is published by John Wiley & Sons Ltd on behalf of European Hematology Association. This is an open access Abstract Book distributed under the Attribution-NonCommercial-NoDerivs (CC BY-NC-ND), which allows third parties to download the articles and share them with others as long as they credit the author and the Abstract Book, but they cannot change the content in any way or use them commercially.
**Abstract Book Citations:** Authors, Title, HemaSphere, 2026;10:(S1):pages. The abstract book DOIs can be found on the "Author Information" page.
**Disclaimer:** Articles published in the journal HemaSphere exclusively reflect the opinions of the authors. The authors are responsible for all content in their abstracts including accuracy of the facts, statements, citing resources, etc.

### (PS1536) TARGETING MCL-1 WITH CARDIO-SAFE DEGRADER CT-03P EXERTS ACTIVITY IN AML AND OVERCOMES VENETOCLAX RESISTANCE

**Topic:** 03. Acute myeloid leukemia - Biology & translational research

Mak Po Yee[1], Pawel Dobrzanski[2], Abhishek Maiti[3], Tomasz Tomczyk[2], Sylvain Cottens[2], Michal Walczak[2], Michael Andreeff[1], Bing Carter*[1]

*[1]The University of Texas MD Anderson Cancer Center, Section of Molecular Hematology and Therapy, Department of Leukemia, Houston, United States of America; [2]Captor Therapeutics, Wroclaw, Poland; [3]The University of Texas MD Anderson Cancer Center, Department of Leukemia, Houston, United States of America;*

### Background

BCL-2 inhibitor venetoclax/hypomethylating agent regimen are highly effective in inducing remissions in AML. However, majority of patients relapse due to the activation of oncogenic signaling pathways that stabilize MCL-1, a key resistance factor to venetoclax, or through TP53 mutation-mediated downregulation of pro-apoptotic proteins. We reported that MCL-1 not only directly protects leukemia cells from apoptosis but also exerts other functions that support leukemia growth and survival including regulating metabolic activities (Carter et al., Haematologica 2022). Several MCL-1 inhibitors have been developed and entered clinical trials in AML. However, increased troponin levels, suggesting potential cardiotoxicity in patients treated with MCL-1 inhibitors curbed their clinical development. Unlike MCL-1 inhibitors that increase stability and prevent degradation of MCL-1, which triggers cardiotoxicity, CT-03p, a cereblon-based MCL-1 bifunctional degrader (Captor Therapeutics) does not affect non-human primate cardiac function in vivo at doses exceeding Degradation Dose 50 by over 100 times. Cardio-safety has been established by troponin I and D analyses and in the histopathological examination.

### Aims

Investigate the therapeutic potential of CT-03p in AML cells, stem/progenitor cells, and AML cells resistance to venetoclax.

### Methods

Viable cells and cell death were measured by flow cytometry after treated cells were stained with annexin V and 7-aminoactinomycin D in the presence of count beads. Protein levels were determined by Western blot and oxidative phosphorylation and fatty acid oxidation by Seahorse metabolic assays.

**Copyright Information:** (Online) ISSN: 2572-9241
©2026 The Author(s). HemaSphere is published by John Wiley & Sons Ltd on behalf of European Hematology Association. This is an open access Abstract Book distributed under the Attribution-NonCommercial-NoDerivs (CC BY-NC-ND), which allows third parties to download the articles and share them with others as long as they credit the author and the Abstract Book, but they cannot change the content in any way or use them commercially.
**Abstract Book Citations:** Authors, Title, HemaSphere, 2026;10:(S1):pages. The abstract book DOIs can be found on the "Author Information" page.
**Disclaimer:** Articles published in the journal HemaSphere exclusively reflect the opinions of the authors. The authors are responsible for all content in their abstracts including accuracy of the facts, statements, citing resources, etc.

### Results

MCL-1 degrader CT-03p effectively degraded MCL-1 and induced cell death in MV4-11 cells in vitro and in vivo in a MV4-11 xenograft mouse model. Additionally, CT-03p is highly active in MV4-11 cells with acquired resistance to venetoclax.
We observed that like venetoclax, CT-03p tends to be less active in TP53-mutant compared to TP53 wild-type (WT) cells. To assess the impact of TP53 status on CT-03p activity, we treated TP53 isogenic Molm13 cells with CT-03p and found that cells with TP53 defect are less sensitive to MCL-1 degrader compared to WT controls. Utilizing the hitherto sole targetable TP53-Y220C mutation model, we demonstrated that converting Y220C-p53 to p53-WT conformation by a p53-Y220C reactivator enhanced CT-03p activity in TP53-Y220C Molm13 cells, supporting p53 induces apoptosis and TP53 mutations impair efficacy of agents targeting BCL-2 proteins. Combination of CT-03p with venetoclax synergistically induced cell
death in CT-03p or venetoclax insensitive AML cell lines and AML blasts and/or stem/progenitor cells from patients with AML, even with TP53 mutations..
Metabolic analysis showed that CT-03p reduced oxidative phosphorylation and fatty acid oxidation in AML cells, which both contribute to venetoclax and chemotherapy resistance.

### Summary/Conclusion

CT-03p degrades MCL-1, exerts antileukemia activities by inducing cell death and targeting metabolic activities, and overcomes venetoclax resistance in AML cells. TP53 mutations impair the efficacy of the MCL-1 degrader. Combination with venetoclax enhances CT-03p activities in venetoclax resistant and TP53-mutant AML cells and stem/progenitor cells. The importance of MCL-1 in therapy resistance and the lack of cardiotoxicity of CT-03p warrants future development of this degrader.

**Copyright Information:** (Online) ISSN: 2572-9241
©2026 The Author(s). HemaSphere is published by John Wiley & Sons Ltd on behalf of European Hematology Association. This is an open access Abstract Book distributed under the Attribution-NonCommercial-NoDerivs (CC BY-NC-ND), which allows third parties to download the articles and share them with others as long as they credit the author and the Abstract Book, but they cannot change the content in any way or use them commercially.
**Abstract Book Citations:** Authors, Title, HemaSphere, 2026;10:(S1):pages. The abstract book DOIs can be found on the "Author Information" page.
**Disclaimer:** Articles published in the journal HemaSphere exclusively reflect the opinions of the authors. The authors are responsible for all content in their abstracts including accuracy of the facts, statements, citing resources, etc.

### (PS1537) PROGNOSTIC VALIDATION OF POST CONSOLIDATION MEASURABLE RESIDUAL DISEASE AND CD34+CD38- ACUTE MYELOID LEUKEMIA STEM CELLS: TOOLS FOR CLINICAL INTERVENTION

**Topic:** 03. Acute myeloid leukemia - Biology & translational research

Kasper Croese*[1, 2], Tom Reuvekamp[1, 2, 3], Lukas Haaksma[1, 2], Angèle Kelder[1, 2], Daphne den Hartog[1, 2], Sven de Regt[1, 2], Dimitri Breems[4], Thomas Fischer[5], Bjørn T. Gjertsen[6], Laimonas Griškevičius[7], Gunnar Juliusson[8], Johan Maertens[9], Markus G. Manz[10, 11], Thomas Pabst[11, 12], Jakob Passweg[11, 13], Kimmo Porkka[14], Patrycja Gradowska[15, 16], Petrus Valk[15], Bob Löwenberg[15], Gert Ossenkoppele[1, 2], Arjan Van de Loosdrecht[1, 2], Costa Bachas[1, 2], Mette Hazenberg[2, 3, 17], David de Leeuw[1, 2], Jacqueline Cloos[1, 2]

*[1]Amsterdam UMC, location Vrije Universiteit Amsterdam, Department of Hematology, Amsterdam, The Netherlands;[2]Cancer Center Amsterdam, Amsterdam, The Netherlands; [3]Amsterdam UMC, location Universiteit van Amsterdam, Department of Hematology, Amsterdam, The Netherlands; [4]Ziekenhuis aan de Stroom, Department of Hematology, Antwerpen, Belgium;[5]Otto von Guericke University Hospital Magdeburg, Magdeburg, Germany; [6]Haukeland University Hospital, Bergen, Norway; [7]Vilnius University Hospital Santaros Klinikos and Vilnius University, Vilnius, Lithuania; [8]Skanes University Hospital, Lund, Sweden; [9]University Hospital Gasthuisberg, Leuven, Belgium; [10]University Hospital Zurich, Zurich, Switzerland; [11]Swiss Group for Clinical Cancer Research, Bern, Switzerland; [12]University Hospital Inselspital, Department of Medical Oncology, Bern, Switzerland; [13]University Hospital Basel, Basel, Switzerland; [14]Helsinki University Hospital Cancer Center, Helsinki, Finland; [15]Erasmus Medical Center Cancer Institute, Rotterdam, The Netherlands; [16]HOVON Foundation, Rotterdam, The Netherlands; [17]Sanquin Research, Amsterdam, The Netherlands;*

### Background

Multiparameter flow cytometry (MFC) based measurable residual disease (MRD) assessment in patients with acute myeloid leukemia (AML) after two cycles of intensive chemotherapy is highly prognostic for survival and routinely used in clinical practice. However, the value of MRD in the post consolidation setting remains less established. The addition of CD34+CD38- leukemia stem cell (LSC) assessment has been shown to further refine prognostication at diagnosis and after induction, but has not been studied after chemotherapy consolidation and only to a limited extent in the post-transplant setting.

### Aims

To investigate the prognostic impact of post consolidation MFC-MRD and LSC frequency.

### Methods

Intensively treated AML patients from the HOVON-42A, -81, -92, -102, -103, and -132 trials were screened for inclusion, in addition to transplanted patients from the Amsterdam UMC. MRD and LSC assessments were performed according to trial protocols and as previously described (Cloos JOVE 2018). MRD and LSC cutoffs were calculated from the white blood cell compartment, and primary endpoints were overall survival (OS) and cumulative incidence of relapse (CIR).

**Copyright Information:** (Online) ISSN: 2572-9241
©2026 The Author(s). HemaSphere is published by John Wiley & Sons Ltd on behalf of European Hematology Association. This is an open access Abstract Book distributed under the Attribution-NonCommercial-NoDerivs (CC BY-NC-ND), which allows third parties to download the articles and share them with others as long as they credit the author and the Abstract Book, but they cannot change the content in any way or use them commercially.
**Abstract Book Citations:** Authors, Title, HemaSphere, 2026;10:(S1):pages. The abstract book DOIs can be found on the “Author Information” page.
**Disclaimer:** Articles published in the journal HemaSphere exclusively reflect the opinions of the authors. The authors are responsible for all content in their abstracts including accuracy of the facts, statements, citing resources, etc.

### Results

Post consolidation MRD measurements were available for 687 patients within 4 months after completion of consolidation therapy, and LSC data were available for 357 patients.
**Following chemotherapy consolidation**, MRD positivity (cut-off: 0.1%) was associated with inferior OS (3-year OS, 60% MRDpos vs 78% MRDneg; hazard ratio [HR]: 1.81; 95% CI, 1.20–2.75; *P*<0.01; Fig 1A) and increased CIR (subdistribution HR [sHR]: 1.93; 95%CI, 1.30–2.86; *P*=0.001). In this cohort, LSC positivity (cut-off: 0.0001%) was likewise adverse, with worse survival (3-year OS, 64% LSCpos vs 83% LSCneg; HR: 2.00; 95% CI, 1.23–3.24; *P*<0.05; Fig 1B) and increased relapse rate (sHR: 2.17; 95% CI, 1.32–3.57; *P*<0.01). **After hematopoietic cell transplantation** (HCT), MRD positivity (cut-off: 0.1%) was associated with poor survival (3-year OS, 31% MRDpos vs 67% MRDneg; HR: 2.72; 95% CI, 1.78–4.15; *P*<0.0001; Fig 1C) and had a sHR of 1.59 (95% CI, 0.79–3.20; *P*=0.20) for relapse incidence. LSC positive (cut-off: 0.0003%) patients in this cohort also showed inferior OS (3-year OS, 28% LSCpos vs 72% LSCneg; HR: 4.0; 95% CI, 2.3–7.2; *P*<0.0001; Fig 1D) and increased CIR (sHR: 3.48; 95% 1.56–7.80; *P*<0.01). When stratifying for combined MRD and LSC status, a positive result of either or both markers resulted in an adverse prognosis (MRDpos/LSCneg, MRDneg/LSCpos and MRDpos/LSCpos). Multivariable analysis was performed separately for MRD and LSC and per consolidation cohort. After adjustment for age, sex, ELN-risk, time-dependent salvage HCT (after initial chemotherapy consolidation), conditioning regimen and donor type (for HCT), both MRD and LSC emerged as strong independent predictors of survival across consolidation cohorts.

### Summary/Conclusion

In intensively treated AML patients, post consolidation MRD and LSC are strong predictors of survival and relapse across consolidation strategies. Combined analysis of MRD and LSC refines risk stratification by identifying distinct single or double positive subgroups at high risk of poor outcome. Together, these findings corroborate post consolidation MRD and LSC as key clinical biomarkers that may serve as signals to consider pre-emptive therapeutic intervention, such as guiding to transplant, tapering immunosuppression, donor lymphocyte infusion, or introducing hypomethylating agents or targeted therapy.

**Copyright Information:** (Online) ISSN: 2572-9241
©2026 The Author(s). HemaSphere is published by John Wiley & Sons Ltd on behalf of European Hematology Association. This is an open access Abstract Book distributed under the Attribution-NonCommercial-NoDerivs (CC BY-NC-ND), which allows third parties to download the articles and share them with others as long as they credit the author and the Abstract Book, but they cannot change the content in any way or use them commercially.
**Abstract Book Citations:** Authors, Title, HemaSphere, 2026;10:(S1):pages. The abstract book DOIs can be found on the “Author Information” page.
**Disclaimer:** Articles published in the journal HemaSphere exclusively reflect the opinions of the authors. The authors are responsible for all content in their abstracts including accuracy of the facts, statements, citing resources, etc.

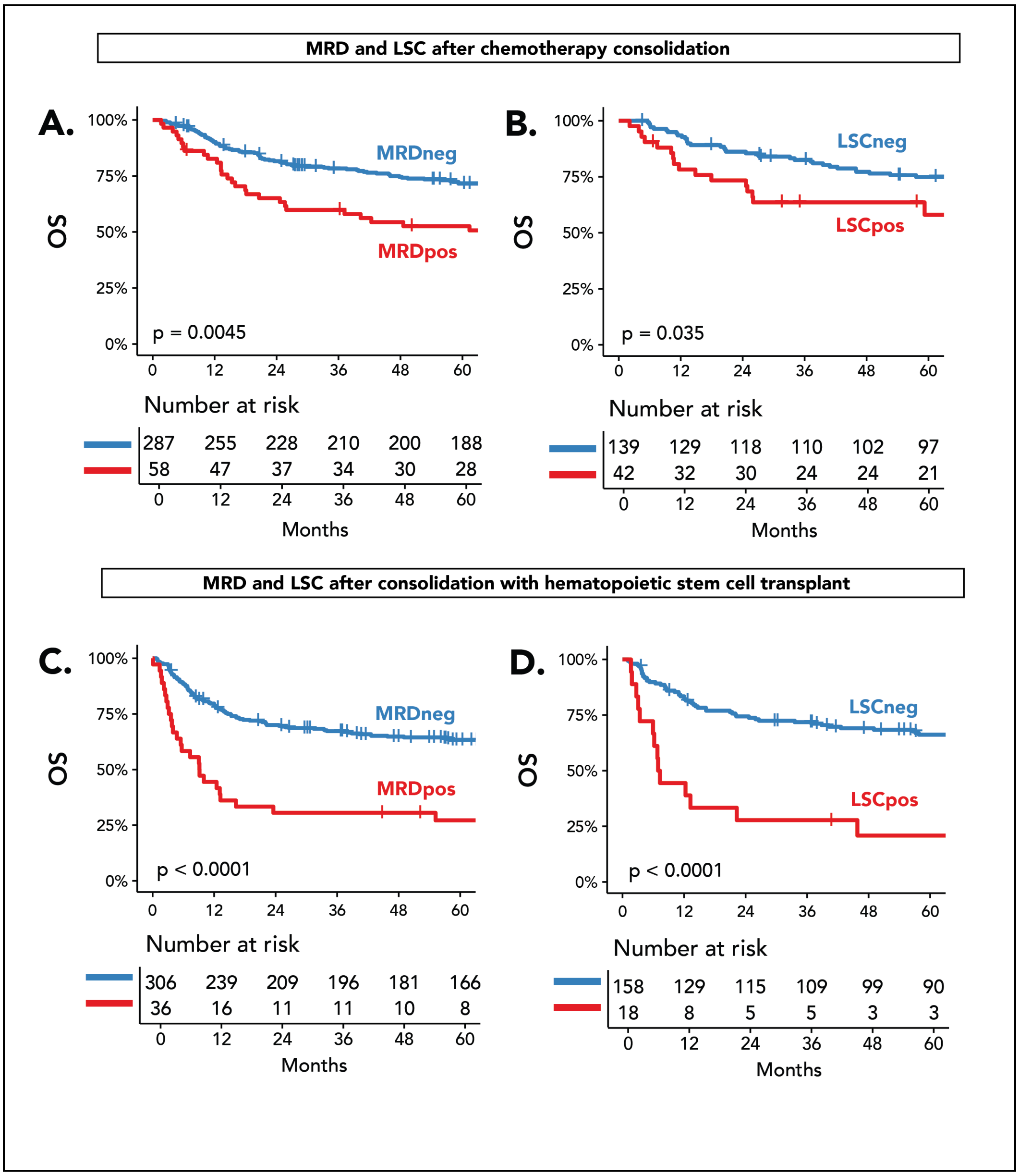

Figure 1. Survival outcomes by post consolidation MRD and LSC status. (A) Overall survival by post consolidation MRD (n = 345) and (B) LSC status (n = 181) in patients treated with chemotherapy consolidation; (C) overall survival by post consolidation MRD (n = 342) and (D) LSC (n = 176) in patients who received HCT (start time analysis: time of MRD or LSC measurement).

**Copyright Information:** (Online) ISSN: 2572-9241
©2026 The Author(s). HemaSphere is published by John Wiley & Sons Ltd on behalf of European Hematology Association. This is an open access Abstract Book distributed under the Attribution-NonCommercial-NoDerivs (CC BY-NC-ND), which allows third parties to download the articles and share them with others as long as they credit the author and the Abstract Book, but they cannot change the content in any way or use them commercially.
**Abstract Book Citations:** Authors, Title, HemaSphere, 2026;10:(S1):pages. The abstract book DOIs can be found on the "Author Information" page.
**Disclaimer:** Articles published in the journal HemaSphere exclusively reflect the opinions of the authors. The authors are responsible for all content in their abstracts including accuracy of the facts, statements, citing resources, etc.

### (PS1538) TRANSCRIPTIONAL REWIRING TO A MONOCYTIC STATE REVEALS THERAPEUTIC VULNERABILITIES IN MENIN INHIBITOR RESISTANCE AML

**Topic:** 03. Acute myeloid leukemia - Biology & translational research

Lanpeng Chen*[1], Bart Van Lith[1], Siyu Liu[2], Pearl Lee[1], Alwin Schuller[3], Lisa Drew[3]

*[1]Acerta Pharma, AstraZeneca, Haematology R&D Small Molecule Discovery, Oss, The Netherlands; [2]AstraZeneca, Oncology Data Sciences, Waltham, United States of America; [3]AstraZeneca, Haematology R&D Small Molecule Discovery, Waltham, United States of America;*

### Background

In clinical trials, Menin inhibitors (MENi) produce robust pharmacodynamic effects in relapsed/refractory acute myeloid leukemia (AML), notably downregulating the *HOXA/MEIS1* axis and inducing myeloid differentiation. However, clinical resistance to MENi has been observed by acquired *MEN1* mutations as well as other mechanisms. Together, these findings suggest that alternative, Menin-independent oncogenic pathways can emerge to bypass Menin-dependency.

### Aims

To characterize the molecular drivers of acquired MENi resistance and identify targetable therapeutic vulnerabilities in leukemic cells that have evaded Menin suppression.

### Methods

We generated three MOLM-13 acute myeloid leukemia (*KMT2A*-rearranged AML) cell line models with acquired resistance to clinical-stage MENi (Revumenib, Bleximenib, and Ziftomenib) via stepwise dose escalation. Transcriptomic profiling was performed by RNASeq to identify a conserved MENi-resistance signature using weighted gene co-expression network analysis (WGCNA) (Langfelder & Horvath, 2008) and GeneAgent, an LLM-based gene-set analysis tool (Wang et al., 2025). These findings were integrated with large-scale patient multi-omic datasets (BeatAML) to assess how resistance-associated genes correlate with specific genetic backgrounds and transcriptional AML phenotypes.

**Copyright Information:** (Online) ISSN: 2572-9241
©2026 The Author(s). HemaSphere is published by John Wiley & Sons Ltd on behalf of European Hematology Association. This is an open access Abstract Book distributed under the Attribution-NonCommercial-NoDerivs (CC BY-NC-ND), which allows third parties to download the articles and share them with others as long as they credit the author and the Abstract Book, but they cannot change the content in any way or use them commercially.
**Abstract Book Citations:** Authors, Title, HemaSphere, 2026;10:(S1):pages. The abstract book DOIs can be found on the “Author Information” page.
**Disclaimer:** Articles published in the journal HemaSphere exclusively reflect the opinions of the authors. The authors are responsible for all content in their abstracts including accuracy of the facts, statements, citing resources, etc.

**Results**

MENi-resistant (MENi-r) MOLM-13 cell lines mirrored on-target clinical effects, maintaining suppressed *HOX* gene expression and increased myeloid differentiation. Notably, no mutations were found in the *MEN1* gene, while the resistant cells exhibited a shift in cell viability to MENi, consistent with reduced drug sensitivity.
Transcriptomic analysis of overlapping changes revealed a complex resistance program. MENi-r cells underwent transcriptional rewiring characterized by myeloid differentiation, activation of MAPK and stress regulation pathways, and remodeling of cell-cycle control, while maintaining suppression of *HOXA/MEIS1* axis. Altogether, resistance appears to arise via adaptive lineage plasticity, shifting cells toward an inflammatory monocytic phenotype. This reprogrammed state likely bypasses the Menin blockade by activating compensatory PI3K/AKT and MAPK signaling.
Using WGCNA and GeneAgent, a MENi resistance gene signature was developed from genes robustly upregulated in the resistant lines. When integrated with BeatAML patient data, this MENi-r gene signature was significantly enriched in a subset of *NPM1c* and *KMT2A*-rearranged AML patients who exhibited a "differentiation-shifted" phenotype, defined by elevated myeloid gene signature expression (van Galen et al., 2019) . This subgroup of patients exhibited a high frequency of *RAS* mutations, MAPK pathway activation and dependency, and resistance to venetoclax. Of note, several immune receptors (e.g. CSF1R) were prominent within the MENi resistance signature and highly expressed in both the MENi-r models and the patient subgroup, highlighting potential targetable vulnerabilities in combination with MENi.

**Summary/Conclusion**

MENi resistance in AML is context-dependent and aligns with the AML hierarchical state; it can be defined as a differentiation-shifted, RAS/MAPK-driven state supported by inflammatory and metabolic adaptation. The overlap identifies rational combination strategies, potentially the use of MEK inhibitors, to overcome resistance. Future studies will focus on validating transcriptomic RAS activity and cell-state signatures as actionable biomarkers and evaluate these combinations in vitro and in vivo.

**Copyright Information:** (Online) ISSN: 2572-9241
©2026 The Author(s). HemaSphere is published by John Wiley & Sons Ltd on behalf of European Hematology Association. This is an open access Abstract Book distributed under the Attribution-NonCommercial-NoDerivs (CC BY-NC-ND), which allows third parties to download the articles and share them with others as long as they credit the author and the Abstract Book, but they cannot change the content in any way or use them commercially.
**Abstract Book Citations:** Authors, Title, HemaSphere, 2026;10:(S1):pages. The abstract book DOIs can be found on the “Author Information” page.
**Disclaimer:** Articles published in the journal HemaSphere exclusively reflect the opinions of the authors. The authors are responsible for all content in their abstracts including accuracy of the facts, statements, citing resources, etc.

### (PS1539) A PREVIOUSLY UNRECOGNIZED ROLE OF HUMAN SKIN MESENCHYMAL STEM CELLS IN CHEMOTHERAPY RESPONSE OF ACUTE MYELOID LEUKEMIA STEM CELLS

**Topic:** 03. Acute myeloid leukemia - Biology & translational research

Elory Leonard*[1], Lakshmi Sandhow[1], Huan Cai[1], Bei Lu[1], Hong Qian[1]

[1]*Karolinska Institutet, Center for Hematology and Regenerative Medicine, Department of Medicine, Huddinge, Stockholm, Sweden;*

**Background**

Acute myeloid leukemia (AML) is an aggressive blood cancer with a poor prognosis. The AML cells often infiltrate into extramedullary tissues or outside of bone marrow (BM), such as skin and brain. The leukemic cell infiltration in skin, called leukemia cutis, signifies a poorer prognosis in patients. Nevertheless, the skin-resident AML cells have been overlooked in clinic and research; consequently, little is known how the AML cells are maintained and protected in the skin.

**Aims**

We have previously in an AML mouse model demonstrated that skin mesenchymal stem cells (MSCs) can maintain and protect AML stem cells from chemotherapy (Sandhow et al., J. Exp. Med, 2023). However, it remains unexplored whether and how human skin MSCs also contribute to chemoprotection of AML cells.

**Methods**

In this study, we have evaluated the protective function and underlying mechanisms of human skin MSCs for AML cells using *in vitro* co-culture of human AML cell line THP-1 and primary AML patient cells with skin MSCs previously isolated from healthy donors, followed by flow cytometry, RNA-sequencing, metabolic and colony assays.

**Copyright Information:** (Online) ISSN: 2572-9241
©2026 The Author(s). HemaSphere is published by John Wiley & Sons Ltd on behalf of European Hematology Association. This is an open access Abstract Book distributed under the Attribution-NonCommercial-NoDerivs (CC BY-NC-ND), which allows third parties to download the articles and share them with others as long as they credit the author and the Abstract Book, but they cannot change the content in any way or use them commercially.
**Abstract Book Citations:** Authors, Title, HemaSphere, 2026;10:(S1):pages. The abstract book DOIs can be found on the “Author Information” page.
**Disclaimer:** Articles published in the journal HemaSphere exclusively reflect the opinions of the authors. The authors are responsible for all content in their abstracts including accuracy of the facts, statements, citing resources, etc.

**Results**

Interestingly, human skin MSCs displayed a superior protective effect towards THP-1 compared to their BM MSCs counterparts during cytarabine (Ara-C) treatment, reflected in the increased residual CD36+ chemo-resistant AML cells (p=0.0065). This finding was further validated by colony assays for assessing AML stem cells. Specifically, there was significantly more residual THP-1-derived cobblestone area-forming cells (CAFCs) (p=0.0298) and colony-forming unit cell (p=0.038) in the co-cultures of THP-1 with skin MSCs than that with BM MSCs. Most importantly, further work using primary patient AML cells suggested that human skin MSCs could also protect primary patient cells from Ara-C treatment in the co-cultures, displayed by more more AML-derived CAFCs and phenotypically defined AML stem and progenitor cells (CD33+CD38-) after Ara-C treatment, compared to BM MSCs counterparts.

Mechanistically, RNA-sequencing revealed human skin MSCs highly expressed genes associated with fatty acid metabolism in human skin MSCs. In line with this, single cell metabolic assay indicated increased capacity of fatty acid oxidation but less glucose dependence in the AML cells co-cultured with human skin MSCs relative to that with BM MSCs during Ara-C treatment (p=0.0197), providing a functional mechanism of the enhanced chemoprotective function of skin MSCs for AML cells.

In addition, human skin MSCs could maintain more AML cells in quiescence (G0 fraction) than BM MSCs *in vitro* (p=0.003). *In vivo*, skin-resident AML cells are more in quiescence than that in BM in *MLL-AF9* AML mouse model. Strikingly, we detected a higher fraction of quiescent KIT+ AML cells, enriched with AML stem cells, in the skin than that in BM (p=0.0132 and p=0.0487, respectively). This quiescent state of skin resident AML cells might represent another mechanism contributing to the better protection of skin mesenchymal niche toward AML cells against Ara-C treatment.

**Summary/Conclusion**

Taken together, our findings indicate human skin MSCs can protect AML by providing better metabolic support to AML stem cells and maintaining them in a quiescent state. The molecular characteristics of residual skin-resident AML cells and their impact on AML treatment and relapse in patients are currently under investigation.

**Copyright Information:** (Online) ISSN: 2572-9241
©2026 The Author(s). HemaSphere is published by John Wiley & Sons Ltd on behalf of European Hematology Association. This is an open access Abstract Book distributed under the Attribution-NonCommercial-NoDerivs (CC BY-NC-ND), which allows third parties to download the articles and share them with others as long as they credit the author and the Abstract Book, but they cannot change the content in any way or use them commercially.
**Abstract Book Citations:** Authors, Title, HemaSphere, 2026;10:(S1):pages. The abstract book DOIs can be found on the “Author Information” page.
**Disclaimer:** Articles published in the journal HemaSphere exclusively reflect the opinions of the authors. The authors are responsible for all content in their abstracts including accuracy of the facts, statements, citing resources, etc.

### (PS1540) AGE-DRIVEN REMODELLING OF THE TRANSCRIPTOMIC CELL-CELL INTERACTION LANDSCAPE IN THE BONE MARROW NICHE OF TP53-AML PATIENTS

**Topic:** 03. Acute myeloid leukemia - Biology & translational research

Rebecca Andersson*[1, 2], Miltiadis Tsesmelis[3], Alba Ferrer-Perez[1, 2], Sara Montserrat-Vazquez[1, 2], Eva Mejia-Ramirez[1, 2], Antonella Luciana Sturla[4, 5], Clara Maluquer[4, 5], Helena Pomares[4], Jasson Andres Villarreal Hernandez[4], Montserrat Arnan Sangerman[4, 5], Medhanie Mulaw[3], Maria Carolina Florian[1, 2, 6]

*[1]Institut d'Investigació Biomèdica de Bellvitge (IDIBELL), Stem Cell Aging Group, Regenerative Medicine Program, L'Hospitalet de Llobregat, Spain; [2]Program for advancing the Clinical Translation of Regenerative Medicine of Catalonia (P-CMR[C]), L'Hospitalet de Llobregat, Spain; [3]Universität Ulm, Institute of Molecular Medicine, Ulm, Germany; [4]Institut Català d'Oncologia, Hematology Department, L'Hospitalet de Llobregat, Spain; [5]Institut d'Investigació Biomèdica de Bellvitge (IDIBELL), Hematopoietic and Lymphoid tumors group, Molecular Mechanisms and Experimental Therapy in Oncology Program, L'Hospitalet de Llobregat, Spain; [6]ICREA, Barcelona, Spain;*

### Background

Acute Myeloid Leukaemia (AML) incidence increases drastically after 60 years of age, and the prognosis of elderly patients is very poor, with less than 20 % of patients 65 years or older surviving for more than 1 year. Advanced age increases the incidence of genetic lesions associated with dismal outcomes, of which *TP53* mutations are a major entity. *TP53*-mutated AML (*TP53*-AML) induces distinct biological changes, not just in the leukemic stem cells (LSCs) but also in the bone marrow niche (BMN) that supports hematopoietic stem/progenitor cells (HSPCs). The BMN is also altered upon ageing. These age-related changes are unexplored in the context of *TP53*-AML, but their identification can provide deeper understanding of the mechanisms of disease development and uncover novel druggable targets for *TP53*-AML treatment in the elderly.

### Aims

The aim of this study is to identify changes in the cell-cell interaction landscape between the BMN and LSCs in young vs. aged*TP53*-AML patients.

### Methods

HSPCs/LSCs (CD34+) and BMN cells (CD45/CD235a/CD71-depleted) from diagnostic BM aspirates of 4 young (≥18 - ≤61 yo, avg. 58.5 yo) and 4 elderly (≥64 yo, avg. 75 yo) *TP53*-AML patients were profiled using SHARE-seq, to analyse the transcriptome and chromatin accessibility at single-cell resolution. Sequencing data was processed in an in-house SHARE-seq pipeline with public datasets as annotation references. Further manual annotation combined with a Random Forest prediction (RFP) model was used to identify LSCs within the HSPCs, which was verified in a *TP53*-AML patient-derived xenograft (PDX) mouse model. Cell-cell interaction analysis of the sequencing data was performed using CellChatDB v. 1.6.1.

**Copyright Information:** (Online) ISSN: 2572-9241
©2026 The Author(s). HemaSphere is published by John Wiley & Sons Ltd on behalf of European Hematology Association. This is an open access Abstract Book distributed under the Attribution-NonCommercial-NoDerivs (CC BY-NC-ND), which allows third parties to download the articles and share them with others as long as they credit the author and the Abstract Book, but they cannot change the content in any way or use them commercially.
**Abstract Book Citations:** Authors, Title, HemaSphere, 2026;10:(S1):pages. The abstract book DOIs can be found on the "Author Information" page.
**Disclaimer:** Articles published in the journal HemaSphere exclusively reflect the opinions of the authors. The authors are responsible for all content in their abstracts including accuracy of the facts, statements, citing resources, etc.

**Results**

37922 HSPC/LSCs and 11260 BMN cells with a median of ~500 genes/cell were used in the CellChat analysis. Dimensionality reduction using UMAP revealed that HSPC- and BMN-derived cells cluster separately from each other, and 21 cell types in the shared UMAP were annotated. Cell identities from all major contributors of bone marrow tissue could be identified. The mixed manual and RFP model for LSC annotation identified a cluster of probable LSCs stemming from Multipotent progenitors (MPP), characterised amongst others by a high expression of *FLT3* and *CD133.* Verification of the selected LSC-markers in the PDX mouse model is ongoing. Analysis of the differential interactions between MPPs and LSCs vs. BMN cells from aged patients revealed that the interactions between the sinusoidal endothelial cells (SECs) and vascular smooth muscle cells (VSMCs) were increased both in number and strength to LSCs, while the Non-LSC MPPs had more numerous interactions with the Mesenchymal stem cell (MSC) subpopulations. This was also true in the young context, which highlights the reliance on the vascular niche for LSCs in *TP53*-AML. When making a direct comparison between the LSCs from the young vs. the aged cohort, a different pattern emerged. Here we observed a gain in the number of incoming and outgoing interactions with the MSCs with increasing age, especially towards osteolineage MSCs (Osteo-MSCs).

**Summary/Conclusion**

There is a remodelling of the transcriptional networks involved in cell-cell interactions in the BM of *TP53*-AML patients with advanced age. While LSCs from both young and elderly *TP53*-AML patients interact with the vascular niche, the interactions between LSCs and Osteo-MSCs increase with age, suggesting an age-related change in the BMN that may offer a new way to treat older *TP53*-AML patients.

**Copyright Information:** (Online) ISSN: 2572-9241
©2026 The Author(s). HemaSphere is published by John Wiley & Sons Ltd on behalf of European Hematology Association. This is an open access Abstract Book distributed under the Attribution-NonCommercial-NoDerivs (CC BY-NC-ND), which allows third parties to download the articles and share them with others as long as they credit the author and the Abstract Book, but they cannot change the content in any way or use them commercially.
**Abstract Book Citations:** Authors, Title, HemaSphere, 2026;10:(S1):pages. The abstract book DOIs can be found on the "Author Information" page.
**Disclaimer:** Articles published in the journal HemaSphere exclusively reflect the opinions of the authors. The authors are responsible for all content in their abstracts including accuracy of the facts, statements, citing resources, etc.

### (PS1541) MODELLING, DECIPHERING AND THERAPEUTIC TARGETING OF THE CD47–SIRPΑ AXIS IN THE PHAGOCYTIC NICHE OF ACUTE MYELOID LEUKEMIA

**Topic:** 03. Acute myeloid leukemia - Biology & translational research

Chloé Terrier*[1, 2], Sarah Barelier[2, 3], Célia Torrielli[1, 2], Magali Colazet[1, 2], Xavier Morelli[2, 4, 5], Yves Collette[2, 3, 4], Sylvain Garciaz[2, 4], Patrick Chames[1, 2], Thomas Miller[2, 4]

*[1]Aix Marseille University, Cancer Research Center of Marseille, Marseille, France;[2]Cancer Research Center of Marseille, Marseille, France; [3]Inserm, U1068, Marseille, France; [4]Institut Paoli Calmettes, Cancer Research Center of Marseille, Marseille, France; [5]CNRS, UMR7258, Marseille, France;*

### Background

Acute myeloid leukemia (AML) remains an aggressive malignancy with poor prognosis, and a critical need for effective immunotherapies. The CD47/SIRPα “don’t eat me” axis, is a validated innate immune checkpoint, however clinical success with CD47-blocking agents like magrolimab has been limited. This failure highlights still poorly understood mechanisms and underscores the need to further elucidate the biological factors modulating the efficacy of CD47/SIRPα targeting in AML, as well as the urgent necessity to develop new strategies that go beyond simple competitive blockade of this axis. We recently identified a dynamic conformational equilibrium in SIRPα between a "closed" state (CD47-binding) and an "open" state (incompatible with CD47). This discovery enables a "conformational trapping" strategy to functionally restore phagocytosis.

### Aims

We aimed to develop and characterize the first allosteric modulators of the CD47/SIRPα checkpoint by:

1. **Generating SIRPα-specific single-domain antibodies** (SdAbs) that stabilize the "open" conformation.
2. **Performing biochemical, biophysical, and structural characterization** to define their allosteric mechanism.
3. **Evaluating their functional capacity to restore macrophage-mediated phagocytosis** in AML models.

Collectively, these objectives establish a comprehensive mechanistic and functional framework to understand and optimize allosteric targeting of SIRPα in AML, spanning from molecular engineering and biophysical validation to preclinical assessment of therapeutic potential.

### Methods

SdAbs were generated via phage display technology using recombinant human SIRPαV1/V2 and CHO-transfected cells. Specificity was confirmed by ELISA and flow cytometry on THP-1 and CHO cells. Interaction kinetics and the ability to interfere with CD47-Fc binding were assessed. The molecular mechanism was resolved using X-ray crystallography. A robust 3-hour *in vitro* phagocytosis assay was developed using GFP-expressing THP-1 cells or primary human macrophages co-cultured with Azacitidine-treated, fluorescently labeled leukemic cell lines. Internalization was quantified by flow cytometry (GFP+/CellTracker+) and microscopy.

**Copyright Information:** (Online) ISSN: 2572-9241
©2026 The Author(s). HemaSphere is published by John Wiley & Sons Ltd on behalf of European Hematology Association. This is an open access Abstract Book distributed under the Attribution-NonCommercial-NoDerivs (CC BY-NC-ND), which allows third parties to download the articles and share them with others as long as they credit the author and the Abstract Book, but they cannot change the content in any way or use them commercially.
**Abstract Book Citations:** Authors, Title, HemaSphere, 2026;10:(S1):pages. The abstract book DOIs can be found on the “Author Information” page.
**Disclaimer:** Articles published in the journal HemaSphere exclusively reflect the opinions of the authors. The authors are responsible for all content in their abstracts including accuracy of the facts, statements, citing resources, etc.

**Results**

Five unique SdAb clones were identified with nanomolar affinity for SIRPαV2. X-ray crystallography confirmed that these binders act as allosteric modulators, targeting an epitope distinct from the CD47-binding interface. Notably, we identified two clones with opposite functional effects: **1E4** stabilizes the "closed" state (enhancing CD47 binding), while **D11** induces conformational trapping in the "open" state (inhibiting CD47 binding). In functional assays, D11 markedly enhanced macrophage-mediated phagocytosis of leukemic cells. Critically, the combination of Azacitidine and D11 showed efficacy comparable to, or exceeding, the Azacitidine-Magrolimab association. 1E4, conversely, significantly reduced phagocytosis.

**Summary/Conclusion**

These data validate a first-in-class mechanism for **modulating the CD47/SIRPα axis via allosteric conformational changes**. By shifting the SIRPα equilibrium to an "open" state, D11 provides a potent alternative to competitive blockade. Likewise, the reduction of phagocytosis in the presence of 1E4 establishes the strong dependence on the CD47-SIRPα checkpoint in our model. This study establishes a mechanistic framework to optimize allosteric targeting of myeloid checkpoints in AML. Ultimately, we aim to elucidate the molecular and cellular determinants governing the efficacy of this innovative SIRPα-targeting strategy.

**Copyright Information:** (Online) ISSN: 2572-9241
©2026 The Author(s). HemaSphere is published by John Wiley & Sons Ltd on behalf of European Hematology Association. This is an open access Abstract Book distributed under the Attribution-NonCommercial-NoDerivs (CC BY-NC-ND), which allows third parties to download the articles and share them with others as long as they credit the author and the Abstract Book, but they cannot change the content in any way or use them commercially.
**Abstract Book Citations:** Authors, Title, HemaSphere, 2026;10:(S1):pages. The abstract book DOIs can be found on the "Author Information" page.
**Disclaimer:** Articles published in the journal HemaSphere exclusively reflect the opinions of the authors. The authors are responsible for all content in their abstracts including accuracy of the facts, statements, citing resources, etc.

HemaSphere

### (PS1543) PRMT2 IS INVOLVED IN CYTOSKELETAL REMODELING AND JNK SIGNALING THROUGH VAV1 AND WASP INTERACTIONS IN ACUTE MYELOID LEUKEMIA

**Topic:** 03. Acute myeloid leukemia - Biology & translational research

Denis Masnikov*[1], Manon Paccagnini[1], Kassandra Ammouri[1], Aziza Aznague[1], Marc Maynadie[1], Cedric Rossi[1], Mary Callanan[1], Bastie Jean Noël[1], Romain Aucagne[1], Laurent Delva[1]

*[1]Université Bourgogne Europe, UFR des Science de Santé Inserm UMR1231, Dijon, France;*

### Background

Acute myeloid leukemia (AML) is maintained by a subpopulation of leukemic stem and progenitor cells (LSPCs), well known to drive therapy resistance, relapse, and immune evasion. These cells rely on dynamic cytoskeletal remodeling and adaptive stress signaling to maintain adhesion and survival within the inflammatory bone marrow niche. Protein arginine methyltransferases (PRMTs) catalyze arginine methylation, a key post-translational modification that modulates protein–protein interactions, RNA binding, and signal transduction. Among them, PRMT2 is structurally unique due to its N-terminal SH3 domain, suggesting a scaffolding role in cytoplasmic signaling complexes. While other PRMTs have established roles in transcription and splicing, the function of PRMT2 in hematologic malignancies remains poorly defined.

### Aims

This study aimed to define the functional role of PRMT2 in AML and to determine how its SH3-domain-dependent interactions and methyltransferase activity regulate cytoskeletal remodeling, inflammatory stress signaling, and leukemic stem/progenitor cell maintenance, with particular emphasis on their contribution to leukemia progression in vivo.

### Methods

We combined transcriptomic profiling (single-cell and bulk RNA-seq), interactome analysis (co-IP/MS), CRISPR/Cas9-mediated knockout in AML cells, biochemical interaction studies, and in vivo models, i.e. MLL-AF9-driven AML system in immunocompetent mice and xenograft model with immunodeficient mice, to dissect PRMT2-dependent signaling networks in AML.

### Results

PRMT2 is significantly enriched in LSPC-like populations and correlates with poor patient survival across various AML cohorts. Proteomic and biochemical analyses reveal that PRMT2 assembles a cytoplasmic complex with VAV1, WASP, and WIPF1, contributing to a core actin-regulatory module. Through SH3-domain-mediated interactions, PRMT2 promotes actin polymerization and JNK pathway activation, both crucial for maintaining cortical F-actin structure, cell adhesion, and inflammatory stress signaling. Loss of PRMT2 disrupts actin architecture, represses adhesion-associated gene programs, and significantly reduces stromal interaction in AML cells. Functionally, PRMT2 deletion delays leukemia development in vivo.

### Summary/Conclusion

PRMT2 orchestrates a methylation-dependent cytoskeletal and stress signaling program in AML by scaffolding and regulating the VAV1–WASP–WIPF1 axis. Its essential role in sustaining LSPC functions and its direct impact on disease progression in both mouse models and human datasets position PRMT2 as a mechanistically validated therapeutic target in AML.

**Copyright Information:** (Online) ISSN: 2572-9241
©2026 The Author(s). HemaSphere is published by John Wiley & Sons Ltd on behalf of European Hematology Association. This is an open access Abstract Book distributed under the Attribution-NonCommercial-NoDerivs (CC BY-NC-ND), which allows third parties to download the articles and share them with others as long as they credit the author and the Abstract Book, but they cannot change the content in any way or use them commercially.
**Abstract Book Citations:** Authors, Title, HemaSphere, 2026;10:(S1):pages. The abstract book DOIs can be found on the "Author Information" page.
**Disclaimer:** Articles published in the journal HemaSphere exclusively reflect the opinions of the authors. The authors are responsible for all content in their abstracts including accuracy of the facts, statements, citing resources, etc.

### (PS1544) OVERCOMING GENETIC OR ADAPTIVE RESISTANCE TO MENIN INHIBITORS IN MUTANT NPM1 AML WITH NOVEL ‘DRUGGABLE’ TARGETS IDENTIFIED VIA NPM1A-TURBOID FUSION AND MASS SPECTROMETRY

**Topic:** 03. Acute myeloid leukemia - Biology & translational research

Christopher Mill*[1], John Davis[1], Christine Birdwell[2], Hanxi Hou[1], Sanam Loghavi[1], Tapan Kadia[1], Naval Daver[1], Courtney Dinardo[1], Branko Cuglievan[1], Claes Wahlestedt[3], Xiaoping Su[1], David McCall[1], Ghayas Issa[1], Warren Fiskus[1]

*[1]UT MD Anderson Cancer Center, Houston, TX, United States of America;[2]UT Southwestern Medical Center, Dallas, TX, United States of America; [3]University of Miami Miller School of Medicine, Miami, FL, United States of America;*

### Background

In mutant (mt) NPM1 AML cells, treatment with Menin inhibitors (MI) disrupts the binding of Menin to MLL1, leading to reduced levels/activity of HOXA9 and MEIS1 and other MLL1 target genes. While treatment with Menin inhibitors (MI) is able induce complete remissions, most patients either fail to respond to MI or relapse after achieving remission. MI-resistance in AML is either due to mutations in the MI-binding domain of MEN1, or more commonly, adaptive, dysregulated epigenome and transcriptome regulated by MLL1.

### Aims

To identify novel ‘druggable’ targets in mtNPM1 AML that are effective against Menin inhibitor sensitive and resistant mtNPM1 AML.

### Methods

We knocked in TurboID by CRISPR/Cas9 into the C-terminus of mtNPM1 in OCI-AML3 cells. Mass spectrometry analysis of biotinylated proteins identified previously unknown, putative, mtNPM1 interactors (**Figure 1**). We utilized a CRISPR modified cell line in which mtNPM1 was knocked-in to assess differential sensitivity of therapeutics against the novel identified targets. To explore transcriptomic and proteomic alterations caused by these drugs, RNA-Seq and mass spectrometry were performed. Furthermore, the lethal activity of these agents was determined by burden and survival of NSG mice engrafted with mtNPM1 AML.

**Copyright Information:** (Online) ISSN: 2572-9241
©2026 The Author(s). HemaSphere is published by John Wiley & Sons Ltd on behalf of European Hematology Association. This is an open access Abstract Book distributed under the Attribution-NonCommercial-NoDerivs (CC BY-NC-ND), which allows third parties to download the articles and share them with others as long as they credit the author and the Abstract Book, but they cannot change the content in any way or use them commercially.
**Abstract Book Citations:** Authors, Title, HemaSphere, 2026;10:(S1):pages. The abstract book DOIs can be found on the “Author Information” page.
**Disclaimer:** Articles published in the journal HemaSphere exclusively reflect the opinions of the authors. The authors are responsible for all content in their abstracts including accuracy of the facts, statements, citing resources, etc.

### Results

Besides known interactors such as XPO1 and KMT2A, we discovered SMARCA2, eIF4A, AURKA, CDK9, PLK1, IRAK4, PARP, and SF3B1 as mtNPM1 interactor proteins. Next, utilizing our OCI-AML2 NPM1A KI, we determined the differential sensitivity of targeting novel mtNPM1 interactors. Rocaglamide A (RocA), an eIF4A inhibitor, and talazoparib (TLZ), a PARP inhibitor, induced greater loss of viability in OCI-AML2-mtNPM1 cells relative to parental cells. Moreover, co-treatment with MI and RocA or talazoparib induced synergistic lethality in MI-sensitive mtNPM1 AML models. When combined with a dual BET/HAT inhibitor (NEO2734/EP31670 - EP), both RocA and TLZ induced synergistic lethality in sensitive OCI-AML2-NPM1A KI and OCI-AML3, as well as MI-resistant (OCI-AML3-Menin-M327I or -MI-tolerant/resistant) cells.
RocA treatment induced log2 fold-reduction in mRNA expression of cytokines and stem cell markers, FLT3, IRF8, and CD244 while inducing cell cycle arrest and death genes, CDKN1A, GADD45A, and BAK1. Furthermore, RNA-Seq and mass spectrometry analysis showed reduced DNA repair and cell cycle gene sets. Treatment with RocA also downregulated myeloid transcription factors such as c-Myc, c-Myb, and PU.1, while inducing p21 protein expressions. EP treatment exhibited similar effects on protein expression of myeloid transcription factors. Moreover, EP reduced gene sets of transcription and translation while it increased apoptosis.
Ex vivo treatment with EP and RocA or TAZ induced synergistic loss of viability in patient-derived (N = 4) mtNPM1 AML cells. In NSG mice engrafted with luciferized mtNPM1 + FLT3-ITD AML PDX cells, compared to vehicle control, monotherapy with EP (7.5 mg/kg, QD), TAZ (0.25 mg/kg, QD) or RocA (0.5 mg/kg, 3x/wk) significantly reduced AML burden. Furthermore, compared to monotherapy, co-treatment of EP and RocA or TAZ significantly improved overall survival of the NSG mice.

### Summary/Conclusion

Collectively, these findings highlight the preclinical efficacy of eIF4A and PARP inhibitors and strongly support the rationale to further evaluate the efficacy of combinations involving these agents against mtNPM1 Menin inhibitor sensitive and resistant AML.

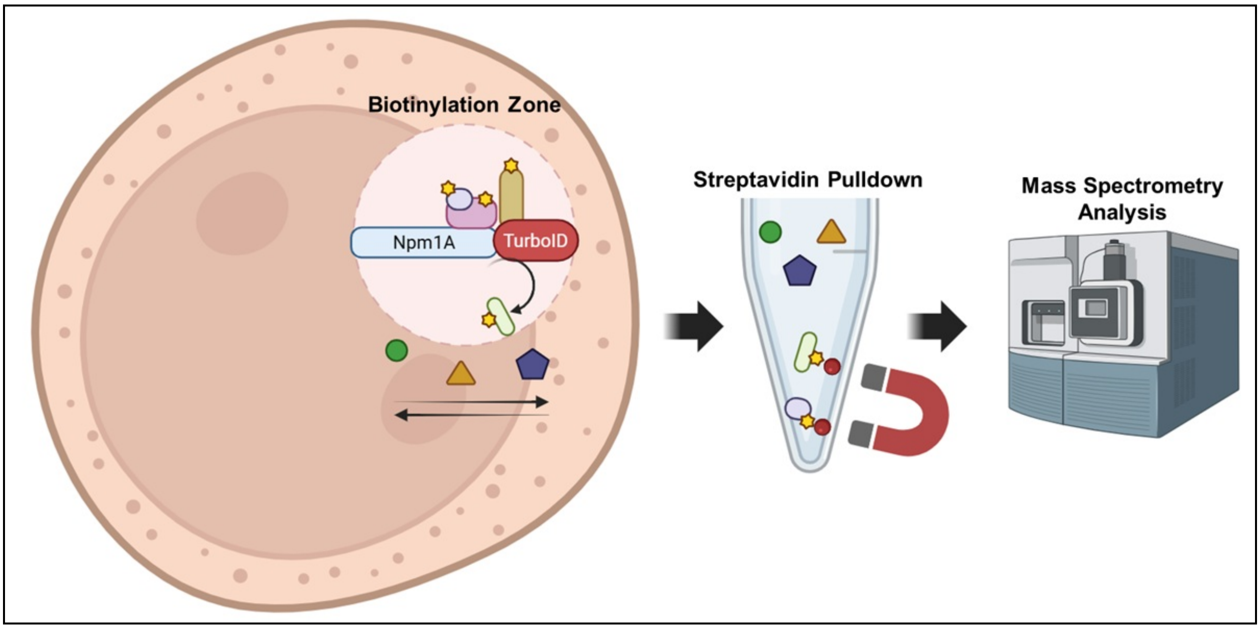

Model for Identifying Putative mtNpm1 Protein Interactors by CRISPR Knock-In (KI) of TurboID into the Npm1A Gene in OCI-AML3 Cells.

**Copyright Information:** (Online) ISSN: 2572-9241
©2026 The Author(s). HemaSphere is published by John Wiley & Sons Ltd on behalf of European Hematology Association. This is an open access Abstract Book distributed under the Attribution-NonCommercial-NoDerivs (CC BY-NC-ND), which allows third parties to download the articles and share them with others as long as they credit the author and the Abstract Book, but they cannot change the content in any way or use them commercially.
**Abstract Book Citations:** Authors, Title, HemaSphere, 2026;10:(S1):pages. The abstract book DOIs can be found on the "Author Information" page.
**Disclaimer:** Articles published in the journal HemaSphere exclusively reflect the opinions of the authors. The authors are responsible for all content in their abstracts including accuracy of the facts, statements, citing resources, etc.

### (PS1545) SH2B3 EXPRESSION PREDICTS SELINEXOR SENSITIVITY IN ACUTE MYELOID LEUKEMIA

**Topic:** 03. Acute myeloid leukemia - Biology & translational research

Shiyuan Zhang[1], Longlong Liu[2], Bin Xu[1], Xiwen Tong[1, 3], Yi Zhu[1], Andie Fu[1], Shuai Su[1], Yuling Wan[1], Zhuoxin Wen[1], Xuntao Lai[2], Zhenqian Huang[2], Jia Wei[1], Donghua Zhang*[1]

*[1]Tongji Hospital, Tongji Medical College, Huazhong University of Science and Technology, Hematology, Wuhan, China;[2]The First Affiliated Hospital of Guangzhou Medical University, Hematology, Guangzhou, China; [3]Sir Run Run Shaw Hospital, Zhejiang University School of Medicine, Hematology and Cell Therapy, Hangzhou, China;*

### Background

Although significant advances have been made in the treatment of acute myeloid leukemia (AML), patients (pts) continue to experience poor outcomes and high rates of relapse. Preclinical studies have demonstrated that selinexor induces cell cycle arrest and apoptosis in AML cell lines. Selinexor has shown efficacy and acceptable tolerability in AML. However, the responses among pts vary greatly, suggesting the need for biomarkers of selinexor sensitivity.
LNK, encoded by *SH2B3*, is a member of the SH2B adaptor family and highly expressed in hematopoietic stem cells, and negatively regulates thrombopoietin and erythropoietin signaling via JAK2 binding.

### Aims

Investigate whether *SH2B3* expression levels predict selinexor sensitivity in AML, with the aim of improving patient stratification and therapeutic outcomes.

### Methods

First, the sensitivity of eight human AML cell lines (MV4-11,OCI-AML3,MOLM-13,K562,THP-1,U937,HL-60,SKM-1) to selinexor was assessed. Cell viability following selinexor treatment was measured using CCK-8 assays, and apoptosis was quantified by annexin V/propidium iodide staining with flow cytometry. Baseline transcriptomic data of AML cell lines were retrieved from the Cell Model Passports database. Differential gene expression analysis between selinexor sensitive and resistant AML cells was performed in R using the limma package (thresholds: adjusted $P < 0.05$, $|\log_2 \text{fold-change}| > 1$). SH2B3 knockdown and overexpression were achieved via lentiviral transduction using pLKO.1 and pCDH vectors, respectively; transduction efficiency was verified by qRT-PCR and Western blot. A mouse subcutaneous AML tumor model was established for in vivo experiments. The mRNA expression level of SH2B3 was evaluated in bone marrow samples obtained from pts with newly diagnosed (ND) AML.

### Results

AML cell lines segregated into high-sensitivity (MV4-11,OCI-AML3,MOLM-13,K562) and low sensitivity (THP-1,U937,HL-60,SKM-1) groups based on half-maximal inhibitory concentration ($IC_{50}$) values and apoptosis response to selinexor. Comparison of transcriptome sequencing data identified *SH2B3* as the most significantly differentially expressed gene between these groups. Both in vitro and in vivo experiments confirmed that functional modulation of *SH2B3* altered selinexor response: *SH2B3*-knockdown AML models attenuated drug efficacy, whereas *SH2B3*-overexpression AML models enhanced proliferation inhibition and apoptotic induction of selinexor. Loss of *SH2B3* expression led to hyperactivation of the *JAK2-STAT5* pathway. Notably, treatment with the *JAK2* inhibitor (ruxolitinib) ameliorated the reduced sensitivity to selinexor observed in *SH2B3*-knockdown models. Furthermore, analysis of bone marrow samples from 20 pts with ND AML revealed that those with low baseline *SH2B3* expression also exhibited a poor response to selinexor therapy.

**Copyright Information:** (Online) ISSN: 2572-9241
©2026 The Author(s). HemaSphere is published by John Wiley & Sons Ltd on behalf of European Hematology Association. This is an open access Abstract Book distributed under the Attribution-NonCommercial-NoDerivs (CC BY-NC-ND), which allows third parties to download the articles and share them with others as long as they credit the author and the Abstract Book, but they cannot change the content in any way or use them commercially.
**Abstract Book Citations:** Authors, Title, HemaSphere, 2026;10:(S1):pages. The abstract book DOIs can be found on the “Author Information” page.
**Disclaimer:** Articles published in the journal HemaSphere exclusively reflect the opinions of the authors. The authors are responsible for all content in their abstracts including accuracy of the facts, statements, citing resources, etc.

**Summary/Conclusion**

Our data demonstrate that *SH2B3* expression correlates with selinexor responsiveness in AML. Modulation of*SH2B3* alters the cell growth and apoptosis induced by selinexor, suggesting its potential utility as a predictive biomarker. Importantly, these findings were validated not only through in vitro and in vivo experiments but also in clinical patient samples. The effect of *SH2B3* on selinexor sensitivity in AML is likely mediated via the *JAK2-STAT5* pathway, as resistance to selinexor resulting from *SH2B3* loss could be ameliorated by the addition of the *JAK2* inhibitor ruxolitinib. These insights provide a rationale for optimizing clinical treatment strategies.

**Copyright Information:** (Online) ISSN: 2572-9241
©2026 The Author(s). HemaSphere is published by John Wiley & Sons Ltd on behalf of European Hematology Association. This is an open access Abstract Book distributed under the Attribution-NonCommercial-NoDerivs (CC BY-NC-ND), which allows third parties to download the articles and share them with others as long as they credit the author and the Abstract Book, but they cannot change the content in any way or use them commercially.
**Abstract Book Citations:** Authors, Title, HemaSphere, 2026;10:(S1):pages. The abstract book DOIs can be found on the "Author Information" page.
**Disclaimer:** Articles published in the journal HemaSphere exclusively reflect the opinions of the authors. The authors are responsible for all content in their abstracts including accuracy of the facts, statements, citing resources, etc.

### (PS1546) EPIGENETIC REGULATION OF CD52 INFLUENCES THE EXPRESSION OF AKT AND MODULATES THE CELLULAR RESPONSE TO VENETOCLAX AND UPROSERTIB IN ACUTE MYELOID LEUKEMIA

**Topic:** 03. Acute myeloid leukemia - Biology & translational research

Carlos Quero-Dotor*[1], Eloy Santos-Pujol[1], Oriol de Barrios[1], Olga Collazo[1], Maribel Parra[1], Manel Esteller[2]

*[1]Josep Carreras Leukaemia Research Institute, Badalona, Barcelona, Spain;[2]Sant Pau Research Institute, Barcelona, Spain;*

### Background

Acute myeloid leukemia (AML) is a hematological malignancy characterized by frequent relapse and poor overall survival (OS). Venetoclax-based regimens targeting BCL-2 are a standard of care for AML patients; however, resistance mechanisms remain unknown, leading to the search for novel strategies in clinical trials. Similarly, other clinical approaches aim to inhibit dysregulated proliferative pathways, including MAPK and Akt. Interestingly, CD52 has emerged as a prognostic biomarker in AML, but its high expression in normal immune cells impairs its direct targeting. Preliminary data indicate that *CD52* is regulated by DNA methylation in AML (**Figure 1**) and its methylation status could represent a feasible marker to predict response to targeted agents. This study explores whether *CD52* epigenetic regulation could stratify patients and guide combination therapies with Venetoclax (Ven) and the Akt inhibitor Uprosertib (Upr).

### Aims

To evaluate *CD52* methylation status as a prognostic marker in AML and assess the efficacy of a therapeutic strategy combining Venetoclax and Uprosertib based on *CD52* epigenetic profile.

### Methods

DNA methylation, RNA-seq and survival data from AML patients were obtained from the public TCGA-LAML cohort. *CD52* was silenced in two hypomethylated AML cell lines (MUTZ-8 and SET-2) by short hairpin RNAs (shRNA). MUTZ-8 empty vector (EV) or CD52-depleted cells were injected into the bone marrow (BM) of NSG mice. Leukemic dissemination was monitored weekly in peripheral blood (PB), with terminal BM evaluation. RNA-seq was performed on the MUTZ-8 model (EV vs shRNA) to identify differentially expressed signaling pathways. Total and phosphorylated Akt were assessed by Western blot in 13 AML cell lines and primary leukocytes. Drug sensitivity to Upr and Ven, alone or combined, was examined in both shRNA models by MTT proliferation and Annexin V apoptosis assays. Synergy was quantified with Bliss analysis using proliferation data.

### Results

AML patients with lower *CD52* methylation levels exhibited poor OS rates than those with higher methylation levels ($p<0.05$). In xenograft models, mice injected with CD52-expressing cells presented higher PB dissemination ($p<0.01$), BM engraftment ($p<0.05$) and reduced OS ($p<0.001$), mirroring patients. RNA-seq analyses in patients and the MUTZ-8 model revealed that increased *CD52* levels correlated with a strong upregulation of the Akt signaling pathway (adj. $p<0.01$) and *Akt* expression ($p<0.01$). Besides, patients with high *CD52* and *Akt* expression exhibited a reduced OS ($p<0.05$). Across 13 AML cell lines, total Akt and pAkt levels inversely correlated with *CD52* methylation, while remaining low in normal leukocytes. Treatment with Upr increased apoptosis and reduced proliferation in CD52-positive cells across silencing models ($p<0.05$), while Ven alone showed no differential effect. The combination of Upr and Ven exerted a synergistic effect when *CD52* was expressed, notably decreasing proliferation ($p<0.05$) and increasing apoptosis ($p<0.001$), as confirmed by Bliss analysis.

**Copyright Information:** (Online) ISSN: 2572-9241
©2026 The Author(s). HemaSphere is published by John Wiley & Sons Ltd on behalf of European Hematology Association. This is an open access Abstract Book distributed under the Attribution-NonCommercial-NoDerivs (CC BY-NC-ND), which allows third parties to download the articles and share them with others as long as they credit the author and the Abstract Book, but they cannot change the content in any way or use them commercially.
**Abstract Book Citations:** Authors, Title, HemaSphere, 2026;10:(S1):pages. The abstract book DOIs can be found on the "Author Information" page.
**Disclaimer:** Articles published in the journal HemaSphere exclusively reflect the opinions of the authors. The authors are responsible for all content in their abstracts including accuracy of the facts, statements, citing resources, etc.

### Summary/Conclusion

*CD52* expression inversely correlates with promoter methylation in AML patients and predicts poor prognosis in patients and cell-derived xenograft mice models. Its epigenetic regulation is linked to the expression of pivotal targets, notably Akt. Dual Akt/BCL-2 targeting shows synergistic anti-leukemic effects in CD52-expressing cells, supporting this combination as a promising alternative for AML patients resistant to conventional Venetoclax-based therapies.

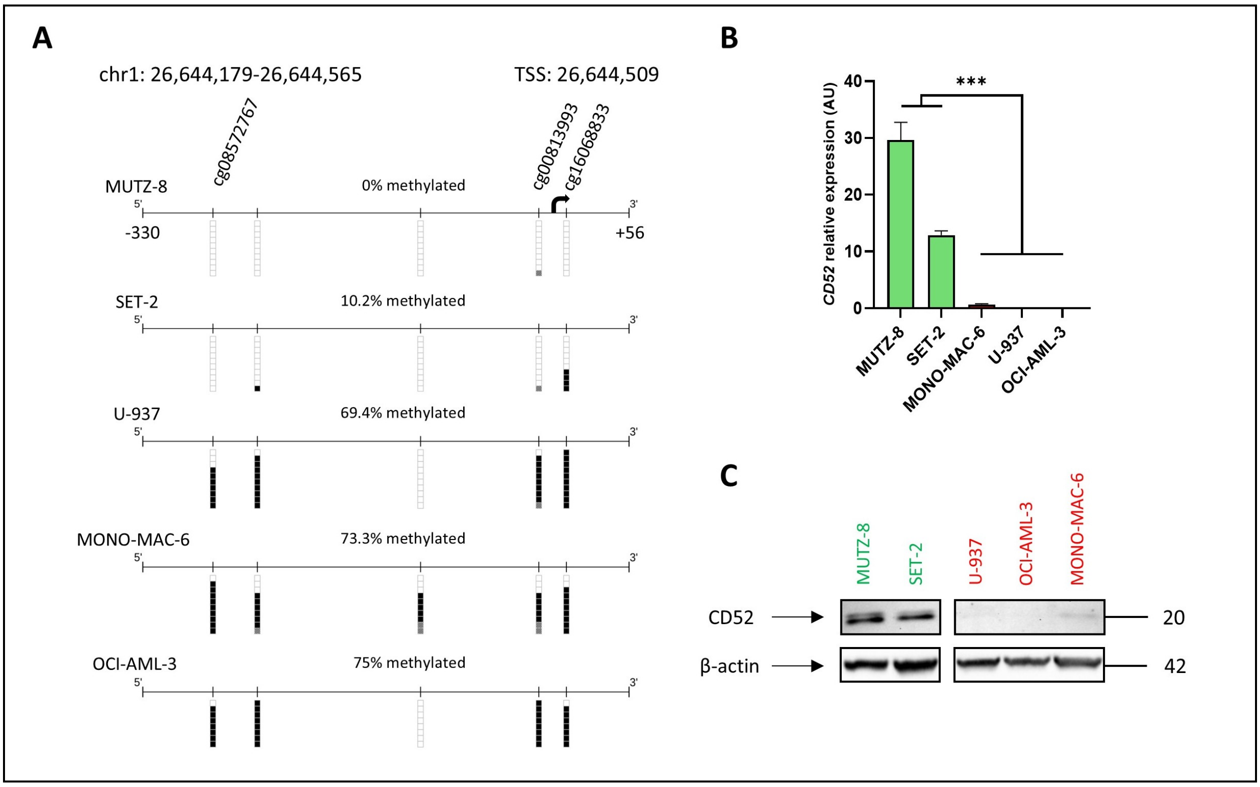

Figure 1. Epigenetic regulation of CD52 promoter in AML cell lines. A. Bisulfite sequencing of CD52 promoter in five AML cell lines: hypomethylated (MUTZ-8 and SET-2) and hypermethylated (U-937, MONO-MAC-6 and OCI-AML-3). CpGs sites are considered as hypomethylated when β-value < 0.66. B. mRNA levels of CD52 in AML cell lines. Green: hypomethylated; red: hypermethylated. C. Protein levels of CD52 in AML cell lines.

**Copyright Information:** (Online) ISSN: 2572-9241
©2026 The Author(s). HemaSphere is published by John Wiley & Sons Ltd on behalf of European Hematology Association. This is an open access Abstract Book distributed under the Attribution-NonCommercial-NoDerivs (CC BY-NC-ND), which allows third parties to download the articles and share them with others as long as they credit the author and the Abstract Book, but they cannot change the content in any way or use them commercially.
**Abstract Book Citations:** Authors, Title, HemaSphere, 2026;10:(S1):pages. The abstract book DOIs can be found on the “Author Information” page.
**Disclaimer:** Articles published in the journal HemaSphere exclusively reflect the opinions of the authors. The authors are responsible for all content in their abstracts including accuracy of the facts, statements, citing resources, etc.

### (PS1547) A NOVEL DECITABINE ANALOGUE WITH ENHANCED ANTI-LEUKEMIA ACTIVITY AND REDUCED TOXICITY IN VIVO

**Topic:** 03. Acute myeloid leukemia - Biology & translational research

Annika Fröhlich[1, 2], Binje Vick[1, 2, 3], Maike Däther[1, 4], Sogol Fatourechi[1, 4], Matthias Heiss[1, 4], Elsa Peev[1, 4], Franziska Traube[1, 4, 5], Thomas Carell[1, 4], Irmela Jeremias*[1, 2, 3, 6]

*[1]Cluster for Nucleic Acid Therapeutics Munich (CNATM), Munich, Germany;[2]Research Unit Apoptosis in Hematopoietic Stem Cells, Helmholtz Munich, Munich, Germany; [3]German Cancer Consortium (DKTK), partner site Munich, a partnership between DKFZ and University Hospital LMU Munich, Munich, Germany, Munich, Germany; [4]Institute of Chemical Epigenetics, Department of Chemistry, Ludwig-Maximilians-Universität München, Munich, Germany; [5]Institute of Biochemistry and Technical Biochemistry, Universität Stuttgart, Stuttgart, Germany; [6]Department of Pediatrics, Dr. von Hauner Children's Hospital, University Hospital, Ludwig-Maximilians-Universität München, Munich, Germany;*

**Background**

Hypomethylating agents like the DNMT inhibitor Decitabine have emerged as important pillar of therapy for patients with acute myeloid leukemia (AML); however, their clinical application is characterized by limited efficacy, adverse effects such as hematotoxicity and acquired resistance. Our academic collaboration partners developed the novel Decitabine analogue Carbacitabine.

**Aims**

We aimed to determine the efficiency and toxicity of Carbacitabine in patient-derived xenograft (PDX) models ofAML *in vivo*, in comparison to Decitabine.

**Methods**

We used our recently established serially transplantable, robust and genetically engineered PDX (*PDX) models of AML, expressing luciferase as a transgene, to compare the anti-tumor efficacy of Azacitidine, Decitabine and Carbacitabine both *in vitro* (n=6) and *in vivo* (n=5). In parallel to the *in vivo* experiments, *PDX AML cells were cultured *ex vivo* and treated with 0.3 to 3µM of Carbacitabine or Decitabine and apoptosis was measured by flow cytometry. Upon transplantation of Luciferase-expressing *PDX cells into immunodeficient NSG mice, mice were treated for four consecutive weeks with maximal tolerated doses (MTD) and tumor burden was monitored over time using *in vivo* bioluminescence imaging. Furthermore, hematotoxycity was compared by blood analysis of both drugs in immune-competent mice to define MTD.

**Copyright Information:** (Online) ISSN: 2572-9241
©2026 The Author(s). HemaSphere is published by John Wiley & Sons Ltd on behalf of European Hematology Association. This is an open access Abstract Book distributed under the Attribution-NonCommercial-NoDerivs (CC BY-NC-ND), which allows third parties to download the articles and share them with others as long as they credit the author and the Abstract Book, but they cannot change the content in any way or use them commercially.
**Abstract Book Citations:** Authors, Title, HemaSphere, 2026;10:(S1):pages. The abstract book DOIs can be found on the "Author Information" page.
**Disclaimer:** Articles published in the journal HemaSphere exclusively reflect the opinions of the authors. The authors are responsible for all content in their abstracts including accuracy of the facts, statements, citing resources, etc.

**Results**

*In vitro,* treatment with Carbacitabine led to a significantly higher induction of apoptosis across six independent *PDX AML models compared to Decitabine at the same dosage. Complementary proteomic analyses confirmed a clinically relevant upregulation of pro-apoptotic proteins following Carbacitabine exposure.
Toxicity studies in six adult C57BL/6J mice demonstrated that Carbacitabine was well tolerated at doses up to 100-fold higher than Decitabine, suggesting a substantially improved safety profile, including markedly reduced hematotoxicity.
*In vivo*, Carbacitabine exhibited superior anti-leukemic activity compared to Decitabine and Azacitidine at MTD in all tested *PDX models; tumor burden was reduced dramatically, reaching levels below minimal residual disease (MRD). After stop of therapy, tumor regression continued and persisted over several weeks, both in Decitabine and Carbacitabine treated mice. Imaging kinetics further revealed that tumor cell proliferation was clearly reduced at re-growth, suggesting a long-lasting epigenetic modification of the cells. Remarkably, in the *PDX AML-491 model, three out of six mice treated with Carbacitabine achieved low tumor levels as monitored with bioluminescence imaging and were considered as cured. Importantly, even *PDX models resistant towards Decitabine or Azacitidine responded to Carbacitabine treatment, indicating that the improved therapeutic window might open new treatment options even in resistant disease.

**Summary/Conclusion**

Our results demonstrate that Carbacitabine combines improved anti-leukemia activity with significantly reduced off-target toxicity *in vivo,* highlighting how structural optimization of DNMT-inhibiting antimetabolites can achieve a more favorable therapeutic balance that may benefit AML patients and improve future treatment strategies.

**Copyright Information:** (Online) ISSN: 2572-9241
©2026 The Author(s). HemaSphere is published by John Wiley & Sons Ltd on behalf of European Hematology Association. This is an open access Abstract Book distributed under the Attribution-NonCommercial-NoDerivs (CC BY-NC-ND), which allows third parties to download the articles and share them with others as long as they credit the author and the Abstract Book, but they cannot change the content in any way or use them commercially.
**Abstract Book Citations:** Authors, Title, HemaSphere, 2026;10:(S1):pages. The abstract book DOIs can be found on the “Author Information” page.
**Disclaimer:** Articles published in the journal HemaSphere exclusively reflect the opinions of the authors. The authors are responsible for all content in their abstracts including accuracy of the facts, statements, citing resources, etc.

### (PS1548) ACUTE MYELOID LEUKEMIA–ASSOCIATED INFLAMMATION PROMOTES BLOOD–BRAIN BARRIER DISRUPTION AND NEUROINFLAMMATION

**Topic:** 03. Acute myeloid leukemia - Biology & translational research

Oxana Bereshchenko*[1], Marta Febo[1], Paolo Cogliati[1], Daniele Sorcini[1], Paolo Sportoletti[1], Daniele Santoro[1], Artemii Frolkov[1], Vjola Tusha[1], Cataldo Arcuri[1], Stefano Bruscoli[1]

[1]*University of Perugia, Perugia, Italy;*

### Background

Acute myeloid leukemia (AML) is an aggressive hematologic malignancy frequently driven, in cytogenetically normal AML, by cooperating mutations in the NPM1 and FLT3 genes. AML is associated with systemic inflammation characterized by elevated pro-inflammatory cytokines and innate immune activation. Although inflammation has been linked to a wide range of neurological and psychiatric disorders, including major depressive disorder, it remains unclear whether AML-associated inflammatory signals may lead to alterations in the central nervous system (CNS). Given emerging evidence that clonal hematopoiesis can modulate neuroinflammation, we investigated whether AML induces CNS inflammation.

### Aims

To determine whether systemic inflammation in AML extends beyond the hematopoietic system and affects the CNS. Specifically, we assessed whether AML progression is associated with CNS inflammatory changes, blood–brain barrier (BBB) dysfunction, and glial activation, and explored the clinical relevance of these findings in AML patients.

### Methods

We used a murine NPMc$^+$/Flt3-ITD AML model. CNS leukocyte infiltration was analyzed by flow cytometry and immunofluorescence. Brain inflammatory mediators were quantified by qPCR. BBB integrity was assessed by Western blot analysis of occludin and claudin expression and by Evans Blue permeability assays. Mmp2 and Mmp9 expression levels were measured by qPCR. Glial activation was evaluated in cortex and hippocampus using GFAP and Iba1 immunostaining and flow cytometry. Human HMC3 microglia were co-cultured with OCI-AML3 or MOLM-13 AML cells in direct and transwell systems to assess cytokine production, activation markers, and morphology. MMP9 mRNA expression was analyzed in PBMCs from AML patients and healthy donors. Plasma neuronal injury markers (NSE, NF-H) were measured by multiplex assay.

### Results

Leukemic mice showed increased brain infiltration by CD4$^+$ and CD8$^+$ T cells, CD11b$^+$CD45hi myeloid cells, and increased microglial numbers. Reduced occludin levels and elevated Evans Blue permeability demonstrated BBB disruption. Mmp9 was upregulated in leukemic mice and MMP9 mRNA was elevated in AML patients, suggesting a role in tight junction degradation and enhanced leukocyte trafficking into the CNS. Brains of leukemic mice displayed a pro-inflammatory profile consistent with altered immune regulation. These molecular changes were accompanied by microglial and astroglial activation. In vitro, AML-conditioned media induced IL-1β and IL-6 production in human microglia and increased CD11b, CD14, and CD80 expression, with morphological changes consistent with activation, indicating that AML-derived soluble factors promote microglial inflammation. Plasma levels of NSE and NF-H were elevated in AML patients, supporting possible CNS involvement and neuronal stress.

**Copyright Information:** (Online) ISSN: 2572-9241
©2026 The Author(s). HemaSphere is published by John Wiley & Sons Ltd on behalf of European Hematology Association. This is an open access Abstract Book distributed under the Attribution-NonCommercial-NoDerivs (CC BY-NC-ND), which allows third parties to download the articles and share them with others as long as they credit the author and the Abstract Book, but they cannot change the content in any way or use them commercially.
**Abstract Book Citations:** Authors, Title, HemaSphere, 2026;10:(S1):pages. The abstract book DOIs can be found on the “Author Information” page.
**Disclaimer:** Articles published in the journal HemaSphere exclusively reflect the opinions of the authors. The authors are responsible for all content in their abstracts including accuracy of the facts, statements, citing resources, etc.

**Summary/Conclusion**

Collectively, these findings demonstrate that AML-associated systemic inflammation is accompanied by BBB disruption, leukocyte infiltration, glial activation, and molecular signatures of neuroinflammation, with supportive evidence from human samples. AML-associated inflammation may represent a previously underrecognized driver of neuroinflammation and neuronal injury, identifying CNS-directed inflammatory pathways as potential therapeutic targets to improve neurological outcomes and potentially alleviate psychological distress in AML.

**Copyright Information:** (Online) ISSN: 2572-9241
©2026 The Author(s). HemaSphere is published by John Wiley & Sons Ltd on behalf of European Hematology Association. This is an open access Abstract Book distributed under the Attribution-NonCommercial-NoDerivs (CC BY-NC-ND), which allows third parties to download the articles and share them with others as long as they credit the author and the Abstract Book, but they cannot change the content in any way or use them commercially.
**Abstract Book Citations:** Authors, Title, HemaSphere, 2026;10:(S1):pages. The abstract book DOIs can be found on the "Author Information" page.
**Disclaimer:** Articles published in the journal HemaSphere exclusively reflect the opinions of the authors. The authors are responsible for all content in their abstracts including accuracy of the facts, statements, citing resources, etc.

### (PS1549) INTEGRATIVE FUNCTIONAL SCREENING AND SINGLE-CELL LIPIDOMICS REVEAL METABOLIC ADAPTATION IN PU.1-DEFICIENT AML

**Topic:** 03. Acute myeloid leukemia - Biology & translational research

Füsun Boydere*[1], Lena Teschke[2], Jens Soltwisch[2], Frank Rosenbauer[1]

*[1]Institute of Molecular Tumor Biology, Münster, Germany;[2]Institute of Hygiene, Münster, Germany;*

### Background

Acute myeloid leukemia (AML) is sustained by transcriptional rewiring that alters lineage identity and survival control. In our previous work, PU.1 downregulation remodeled chromatin binding of its partner transcription factor RUNX1, activating an autophagy-predominated stem cell gene program that functionally supports leukemic survival. While this transcriptional and functional shift from cytokine dependency to autophagy reliance has been defined, its integration with stage-specific metabolic adaptation during leukemogenesis remains unclear.

### Aims

We aimed to define transcription factor dependencies and associated metabolic adapatations across wild-type, preleukemic, and leukemic states downstream of PU.1 loss and upregulation of autophagy.

### Methods

Building on the established PU.1-RUNX1-autophagy axis, we performed a pooled shRNA screen targeting all transcription factors expressed in murine neutrophilic myeloid cells. The goal was to identify regulators of metabolic programs that influence autophagy-dependent survival. Screening was conducted across wild-type, preleukemic, and leukemic contexts and analyzed using MAGeCK-based ranking and differential log-fold change comparisons.
Single-cell lipidomics using MALDI-based mass spectrometry imaging was applied to characterize lipid composition, abundance, and cellular heterogeneity across disease stages.

### Results

Consistent with prior findings, leukemic PU.1-low states displayed dependency patterns aligned with RUNX1-driven autophagy upregulation. In addition, the loss-of-function screen identified that PU.1-low cells exhibit reliance on entire transcription factor networks linked to metabolic and stress-response pathways that modulate survival in this context. In particular, we could identify essential dependencies on transcriptional regulators associated with lipid metabolic programs, suggesting that autophagy in PU.1-low cells is embedded within a broader metabolic regulatory network.
Based on these findings, we performed single-cell lipidomic analyses. This revealed phospholipid remodeling in preleukemic cells, indicating early reshaping of membrane lipid composition during PU.1-driven transformation. Given the central role of membrane dynamics in autophagy, these data support a model in which transcriptional reprogramming toward autophagy is accompanied by metabolic adaptation during leukemogenesis.

**Copyright Information:** (Online) ISSN: 2572-9241
©2026 The Author(s). HemaSphere is published by John Wiley & Sons Ltd on behalf of European Hematology Association. This is an open access Abstract Book distributed under the Attribution-NonCommercial-NoDerivs (CC BY-NC-ND), which allows third parties to download the articles and share them with others as long as they credit the author and the Abstract Book, but they cannot change the content in any way or use them commercially.
**Abstract Book Citations:** Authors, Title, HemaSphere, 2026;10:(S1):pages. The abstract book DOIs can be found on the "Author Information" page.
**Disclaimer:** Articles published in the journal HemaSphere exclusively reflect the opinions of the authors. The authors are responsible for all content in their abstracts including accuracy of the facts, statements, citing resources, etc.

**Summary/Conclusion**

Our data suggest that PU.1 downregulation induces RUNX1-driven autophagy that is sustained by transcriptional regulators of metabolic pathways. Early membrane lipid remodeling in preleukemic cells indicates that metabolic adaptation accompanies this survival rewiring during leukemogenesis. Together, these findings connect transcription factor redistribution to metabolic regulation and highlight stage-specific vulnerabilities in AML. Given that PU.1 activity is frequently diminished in human AML, disruption of PU.1-controlled transcriptional networks may expose metabolic dependencies that create therapeutic vulnerabilities in leukemic cells.

**Copyright Information:** (Online) ISSN: 2572-9241
©2026 The Author(s). HemaSphere is published by John Wiley & Sons Ltd on behalf of European Hematology Association. This is an open access Abstract Book distributed under the Attribution-NonCommercial-NoDerivs (CC BY-NC-ND), which allows third parties to download the articles and share them with others as long as they credit the author and the Abstract Book, but they cannot change the content in any way or use them commercially.
**Abstract Book Citations:** Authors, Title, HemaSphere, 2026;10:(S1):pages. The abstract book DOIs can be found on the "Author Information" page.
**Disclaimer:** Articles published in the journal HemaSphere exclusively reflect the opinions of the authors. The authors are responsible for all content in their abstracts including accuracy of the facts, statements, citing resources, etc.

### (PS1550) OFF-THE-SHELF ENGINEERED INVARIANT NATURAL KILLER T CELLS (INKT CELLS) AS A NOVEL APPROACH FOR ACUTE MYELOID LEUKEMIA TREATMENT

**Topic:** 03. Acute myeloid leukemia - Biology & translational research

Chaiyaporn Boonchalermvichian*[1, 2, 3], Hao Yan[2], Jeanette Baker[2], Quenton Bubb[4], Agnieszka Czechowicz[5], Robert Negrin[2]

*[1]Indiana University, Hematology/Oncology Division, Department of Medicine, Indianapolis, United States of America;[2]Stanford University, Division of Blood and Marrow Transplantation & Cellular Therapy, Stanford, United States of America; [3]Indiana University School of Medicine, Indiana University Melvin and Bren Simon Comprehensive Cancer Center, Indianapolis, United States of America; [4]Stanford University, Stem Cell Biology and Regenerative Medicine Graduate Program, Medical Scientist Training Program, Stanford, United States of America; [5]Stanford University, Division of Hematology, Oncology, Stem Cell Transplantation and Regenerative Medicine, Stanford, United States of America;*

### Background

Acute myeloid leukemia (AML) remains a challenging hematologic malignancy with limited effective therapies. We investigated a chimeric antigen receptor (CAR)-based strategy employing engineered invariant natural killer T (iNKT) cells that specifically target CD117, a well-established AML surface marker. iNKT cells, defined by their invariant T-cell receptor, exhibit potent cytotoxicity against cancer cells and uniquely mitigate graft-versus-host disease (GVHD), positioning them as promising “off-the-shelf” candidates for adoptive cell therapy across hematologic and solid tumors.

### Aims

To establish the therapeutic potential of CD117-CAR-iNKT cells in the management of AML and to explore their effectiveness as a novel adoptive cell therapy option.

### Methods

Peripheral blood mononuclear cells were isolated via gradient centrifugation from buffy coats using Ficoll Pague Plus Media, followed by enrichment using anti-iNKT microbeads. iNKT cells were stimulated with irradiated allogeneic PBMCs, α-Galcer, and key cytokines (IL2, IL15, IL21) on Days 0 and 10 to promote their expansion. After 14 days, we achieved a 67-80-fold increase in iNKT cell numbers with purity of 90-98%. These cells were then transduced with an MSGV1 gamma-retroviral vector modified to include a CAR sequence consisting of a single-chain variable fragment (scFv) that specifically binds to CD117. The retroviral transduction process, enhanced by fibronectin, resulted in transfection efficiencies ranging from 72% to 91.2%. We assessed the *in vitro* cytotoxicity of either CD117-CAR-iNKT cells or CD117-CAR-T cells by co-culturing effector cells with luciferase-transduced CD117 leukemia cell lines (Kasumi-1) for 24 hours. In addition, the multiplex cytokine quantification assay was done to determine the levels of secreted cytokines. The assay will be assessed by co-culturing effector cells with luciferase-transduced CD117 leukemia cell lines for 24 hours. The levels of cytokines in supernatants will be measured using Th1/Th2/Th9/Th17 Cytokine 18-Plex Human ProcartaPlex™ Panel (Invitrogen). *In vivo* efficacy was evaluated using NSG mice, which were irradiated (250 cGy/mouse) on Day 0 and subsequently engrafted with luciferase-transduced CD117 leukemia cell line on Day 4. Three days post-engraftment, mice were treated with either 2.0 x10e7 CD117-CAR-iNKT cells or untransduced iNKT cells, supplemented by intraperitoneal IL2 (1,000 units/animal) daily for three days and then every other day for two weeks. We monitored disease progression and tumor burden through bioluminescent imaging and clinical evaluations. Animals exhibiting severe illness were euthanized, and H&E-stained tissue samples were analyzed to determine the cause of death.

**Copyright Information:** (Online) ISSN: 2572-9241
©2026 The Author(s). HemaSphere is published by John Wiley & Sons Ltd on behalf of European Hematology Association. This is an open access Abstract Book distributed under the Attribution-NonCommercial-NoDerivs (CC BY-NC-ND), which allows third parties to download the articles and share them with others as long as they credit the author and the Abstract Book, but they cannot change the content in any way or use them commercially.
**Abstract Book Citations:** Authors, Title, HemaSphere, 2026;10:(S1):pages. The abstract book DOIs can be found on the “Author Information” page.
**Disclaimer:** Articles published in the journal HemaSphere exclusively reflect the opinions of the authors. The authors are responsible for all content in their abstracts including accuracy of the facts, statements, citing resources, etc.

**Results**

CD117-CAR-iNKT cells demonstrated dose-dependent cytotoxicity comparable to CD117-CAR-T cells and significantly superior to untransduced iNKT cells. Co-culture assays revealed robust key cytokine secretion as compared to untransduced iNKT cells ($p$ values: GM-CSF, IFN-γ, IL-2, $p < 0.0001$; TNF-α, IL-4; $p = 0.005$ and 0.0002, respectively). *In vivo*, treatment with 2.0 × 10^7 CD117-CAR-iNKT cells significantly improved survival ($p = 0.0099$) without evidence of GVHD at any dose.

**Summary/Conclusion**

CD117-CAR-iNKT cells exhibit potent, specific cytotoxicity against AML, robust key cytokine secretion, and significant survival benefit *in vivo*, while maintaining a favorable safety profile with no GVHD. These findings highlight CD117-CAR-iNKT cells as a promising off-the-shelf cellular therapy for AML.

**Copyright Information:** (Online) ISSN: 2572-9241
©2026 The Author(s). HemaSphere is published by John Wiley & Sons Ltd on behalf of European Hematology Association. This is an open access Abstract Book distributed under the Attribution-NonCommercial-NoDerivs (CC BY-NC-ND), which allows third parties to download the articles and share them with others as long as they credit the author and the Abstract Book, but they cannot change the content in any way or use them commercially.
**Abstract Book Citations:** Authors, Title, HemaSphere, 2026;10:(S1):pages. The abstract book DOIs can be found on the "Author Information" page.
**Disclaimer:** Articles published in the journal HemaSphere exclusively reflect the opinions of the authors. The authors are responsible for all content in their abstracts including accuracy of the facts, statements, citing resources, etc.

### (PS1551) INHIBITORS OF IRE1A ENHANCE VENETOCLAX ACTIVITY IN DE NOVO AND SECONDARY ACUTE MYELOID LEUKEMIA

**Topic:** 03. Acute myeloid leukemia - Biology & translational research

Samuele Cortellazzi[1], Rossana Vairo[1], Francesca Zampino[1], Livia Micaela Pasini[2], Giulia Pozzi[1], Judith Sokei[3], Mary Basse[3], Nancy Arthur[3], Raffaella Zamponi[1], Marco Vitale[4], Giovanni Roti[1], Francesca Ferraro[3], Cecilia Carubbi[1], Elena Masselli[1], Stephen M. Sykes[3], Orsola di Martino*[1]

*[1]University of Parma, Parma, Italy;[2]Parma University Hospital (AOUPR), Hematology and BMT Unit, Parma, Italy;[3]Washington University of Saint Louis, Saint Louis, United States of America;[4]Vita-Salute IRCSS San Raffaele, Milano, Italy;*

### Background

Venetoclax (VEN) has emerged as a promising option for AML patients who are ineligible for or have relapsed after standard-of-care therapies. Although ~75% initially respond, most eventually relapse due to acquired resistance, underscoring the urgent need to identify mechanisms of resistance and strategies to overcome them.

### Aims

The central goal of this study is to identify chemical strategies for improving VEN efficacy in*de novo* AML (pAML) and secondary AML (sAML).

### Methods

We developed a patient-derived AML (PD-AML) *ex vivo* platform to identify compounds that enhance VEN efficacy. Briefly, we collect fresh bone marrow samples/peripheral blood from AML patients at both diagnosis and relapse. Samples are then treated with 1.25 µM VEN alone or in combination focused 45-compound library enriched for pathways implicated in VEN resistance, such as metabolic reprogramming, inflammation, and stress adaptation. Pre- and post- drug treatment analyses include AML cell viability/abundance, immunophenotype, and genetic subtype. Compound interaction with VEN is calculated using SynergyFinder.

**Copyright Information:** (Online) ISSN: 2572-9241
©2026 The Author(s). HemaSphere is published by John Wiley & Sons Ltd on behalf of European Hematology Association. This is an open access Abstract Book distributed under the Attribution-NonCommercial-NoDerivs (CC BY-NC-ND), which allows third parties to download the articles and share them with others as long as they credit the author and the Abstract Book, but they cannot change the content in any way or use them commercially.
**Abstract Book Citations:** Authors, Title, HemaSphere, 2026;10:(S1):pages. The abstract book DOIs can be found on the "Author Information" page.
**Disclaimer:** Articles published in the journal HemaSphere exclusively reflect the opinions of the authors. The authors are responsible for all content in their abstracts including accuracy of the facts, statements, citing resources, etc.

### Results

To date, we have analyzed 29 PD-AML samples and observed VEN response patterns that are consistent with clinical outcomes: 38% of PD-AML are strongly responsive to VEN alone (>65% killing), 34% show intermediate (INT) sensitivity (~65-30% killing), and 28% are weakly (WEAK) responsive to VEN (<30% killing). Importantly, intermediate and refractory samples are enriched for *KRAS*, *NRAS*, *FLT3-ITD*, or *TP53* mutations, aligning with patient-derived data. From our library, two of the top-scoring compounds are KIRA6 and 4µ8C, both of which inhibit the enzyme IRE1α, a key component of the unfolded protein response. VEN combined with KIRA6 or 4µ8C killed PD-AML cells significantly more than VEN alone, with the greatest increase in cytotoxicity observed in INT and WEAK VEN responders. In patients, VEN is used in combination with 5-azacytidine (AZA). Although both AZA and KIRA6 enhance VEN cytotoxicity, VEN+KIRA6 is significantly more effective than VEN + AZA in eliminating WEAK PD-AML cells.

We also tested VEN+IRE1α inhibitors in sAML (n=4), which also showed heterogeneous VEN sensitivity. One MPN-derived sAML (sAML_1: $TP53^{WT}$; $IDH2^{R140Q}$/$JAK2^{V617F}$/$SRSF2^{P95H}$) showed near-complete loss of viable cells with low-dose VEN, whereas therapy-related sAML with complex karyotype and TP53 deletion (sAML_2: del(7q); TP53 loss ~60% nuclei) remained >70% viable after VEN alone. However, sAML_2 was highly responsive to the combination of VEN and KIRA6 (Figure A). Two additional MDS/MPN-derived sAMLs showed intermediate VEN sensitivity that improved with IRE1α inhibitors. At the protein level, we observed that VEN reduced IRE1α protein in VEN-sensitive sAML_1, while IRE1α persisted after VEN exposure in VEN-resistant sAML_2. Using, two AML cell lines that represent VEN-sensitivity (MOLM-13) and VEN-resistance (HNT-34), we found that VEN alone depleted IRE1a in MOLM-13 cells, not HNT-34. Notably, VEN+KIRA6 reduced IRE1α and MCL-1, a known VEN-resistance mechanism, and enhanced apoptosis induction in HNT-34 (Figure B).

### Summary/Conclusion sAML remains an area of major unmet need, with frequent resistance to VEN-based therapy. Our data indicate that IRE1α inhibition enhances VEN activity and can overcome VEN resistance, including high-risk TP53-altered sAML. Persistence of IRE1α after VEN exposure may serve as a functional biomarker of resistance in sAML. Cohort expansion is ongoing, focusing on TP53-altered sAML

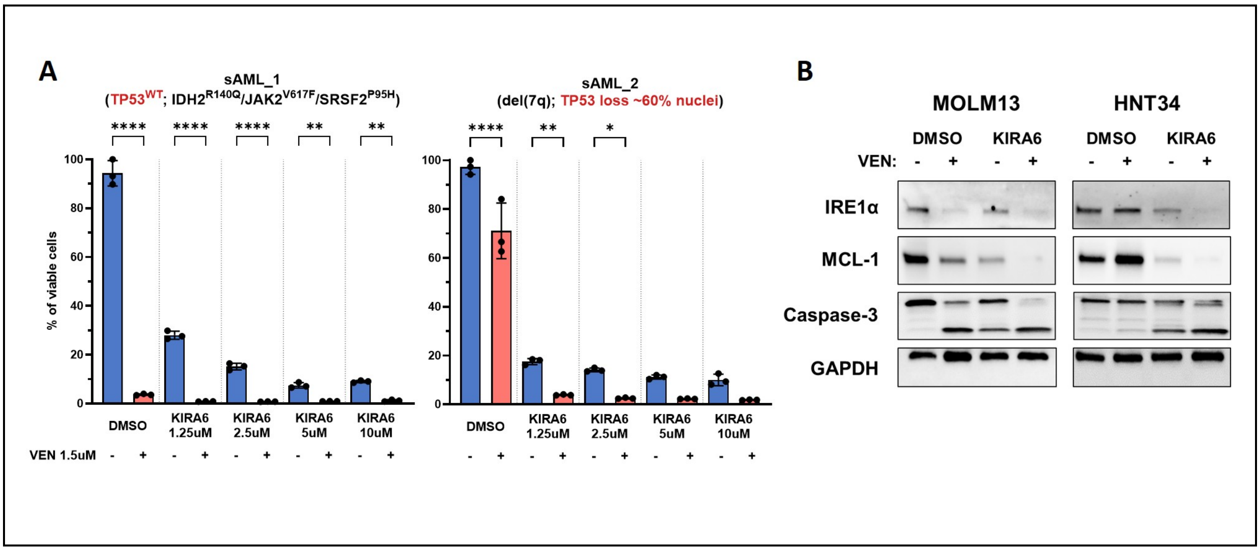

Figure. (A) Fresh sAML samples were treated ex vivo with VEN and KIRA6, alone or in combination, as indicated, with DMSO used as the negative control. Viability was assessed at 96 h using propidium iodide (PI) to discriminate live and dead cells. (B) Protein expression levels in MOLM-13 and HNT-34 cell lines were evaluated after 12 h of treatment with VEN and KIRA6, alone or in combination, as indicated.

**Copyright Information:** (Online) ISSN: 2572-9241
©2026 The Author(s). HemaSphere is published by John Wiley & Sons Ltd on behalf of European Hematology Association. This is an open access Abstract Book distributed under the Attribution-NonCommercial-NoDerivs (CC BY-NC-ND), which allows third parties to download the articles and share them with others as long as they credit the author and the Abstract Book, but they cannot change the content in any way or use them commercially.
**Abstract Book Citations:** Authors, Title, HemaSphere, 2026;10:(S1):pages. The abstract book DOIs can be found on the "Author Information" page.
**Disclaimer:** Articles published in the journal HemaSphere exclusively reflect the opinions of the authors. The authors are responsible for all content in their abstracts including accuracy of the facts, statements, citing resources, etc.

### (PS1552) SPATIAL CHARACTERIZATION OF THE IMMUNE MICROENVIRONMENT IN BONE MARROW AND EXTRAMEDULLARY ACUTE MYELOID LEUKEMIA

**Topic:** 03. Acute myeloid leukemia - Biology & translational research

Hossein Emami*[1, 2], Sophie Steinhäuser[1, 2], Karin Huber[1, 2, 3], Miriam Denker[1, 2], Jan Hahn[4, 5], Manuela Moritz[4], Ayham Moustafa[4, 5, 6], Julia Frimmel[1, 2], Florian Tran[7, 8], Annette I Garbe[1, 2], Friedrich Stölzel[1, 2, 3]

*[1]University Hospital Schleswig-Holstein, Department of Internal Medicine II (Hematology/Oncology), Kiel, Germany;[2]University Cancer Center Schleswig-Holstein (UCCSH), University Hospital Schleswig-Holstein, Kiel, Germany; [3]Clinical Research Unit "CATCH ALL" (KFO 5010/1), Kiel, Germany; [4]University Medical Center Hamburg-Eppendorf, Section of Mass Spectrometric Proteomics, Hamburg, Germany; [5]University Medical Center Hamburg-Eppendorf, Mildred Scheel Cancer Career Center HaTriCS4, Hamburg, Germany; [6]University Medical Center Hamburg-Eppendorf, Department of Radiotherapy and Radiooncology, Hamburg, Germany; [7]University Hospital Schleswig-Holstein and Institute of Clinical Molecular Biology, Christian-Albrechts-University, Kiel, Germany; [8]University Hospital Schleswig-Holstein, Hematopathology Section and Lymph Node Registry, Department of Pathology,, Kiel, Germany;*

### Background

Acute myeloid leukemia (AML) is a highly heterogeneous malignancy characterized by complex molecular alterations and somatic driver mutations. Beyond genetic drivers that drive clonal evolution, the bone marrow (BM) microenvironment is a critical regulator of leukemic progression, immune evasion, and therapy resistance. Far less is known about microenvironmental mechanisms in extramedullary (EM) AML, an aggressive manifestation marked by leukemic infiltration of peripheral tissues and frequently associated with adverse outcomes. Addressing this gap requires spatially resolved analyses that preserve tissue architecture and enable direct comparison of leukemic microenvironment interactions in BM versus EM sites.

### Aims

The aim of our study is a comparative analysis of paired BM and EM samples to identify EM-specific signatures that can be translated into unique vulnerabilities and identify novel therapeutic options. Particularly, we aim to map microenvironment-specific proteomic signatures in BM and EM AML and identify niche-dependent gene programs, interaction networks, as well as molecular features specific to EM AML (to elucidate how the leukemic microenvironment shapes disease biology).

### Methods

An innovative and cost-effective image-guided laser ablation system, 3D-MiTi-LAb, was used to define microenvironment-specific proteomic signatures and map BM AML proteomes at near single-cell resolution. We compared the proteome of BM AML blasts proximal and distal to vasculature across three spatial regions (n=3) of each category within one FFPE BM tissue sample, followed by validation using a co-culture model. To identify EM AML-specific niche components with potential therapeutic relevance, we performed spatially resolved transcriptome analysis from an independent patient sample using the Visium HD.

**Copyright Information:** (Online) ISSN: 2572-9241
©2026 The Author(s). HemaSphere is published by John Wiley & Sons Ltd on behalf of European Hematology Association. This is an open access Abstract Book distributed under the Attribution-NonCommercial-NoDerivs (CC BY-NC-ND), which allows third parties to download the articles and share them with others as long as they credit the author and the Abstract Book, but they cannot change the content in any way or use them commercially.
**Abstract Book Citations:** Authors, Title, HemaSphere, 2026;10:(S1):pages. The abstract book DOIs can be found on the "Author Information" page.
**Disclaimer:** Articles published in the journal HemaSphere exclusively reflect the opinions of the authors. The authors are responsible for all content in their abstracts including accuracy of the facts, statements, citing resources, etc.

### Results

Using 3D-MiTi-LAb, AML blasts proximal (vWF+) and distal (vWF-) to vasculature were selectively laser-microdissected from a patient-derived BM section and profiled by mass spectrometry. We identified 159 differentially expressed proteins in vascular-proximal versus -distal AML blasts, and protein set enrichment analysis (PSEA) revealed enriched pro-leukemic signatures, with the MYC Targets V1 showing the dominant enrichment. For *in vitro* validation, differential gene expression of the representative MYC target V1 genes was assessed in a monoblastic AML cell line, THP-1, cultured alone or in co-culture with the HUVEC endothelial cell line. RT-qPCR analysis identified deregulation of MYC target genes *DDX18*, *HSPD1*, *HSPE1*, *RPL6*, and *NPM1*, confirming the directional expression observed in the spatial proteomics data. Spatially resolved transcriptome analysis of EM AML revealed distinct myeloid and stromal niche clusters and cellular composition, indicating structured spatial organization of leukemic and microenvironmental cells.

### Summary/Conclusion

These results provide an initial indication of endothelial-driven activation of MYC-associated programs in AML BM and highlight the relevance of microenvironmental cues in shaping pro-leukemic signaling in AML. Spatial transcriptomics of EM AML revealed distinct myeloid and stromal niches, suggesting an organized microenvironmental architecture. Together, these spatial proteomic and transcriptomic analyses delineate niche-associated programs across anatomical sites and provide the foundation to systematically define EM AML-specific signatures that may translate into site-adapted therapeutic strategies targeting niche-specific interactions.

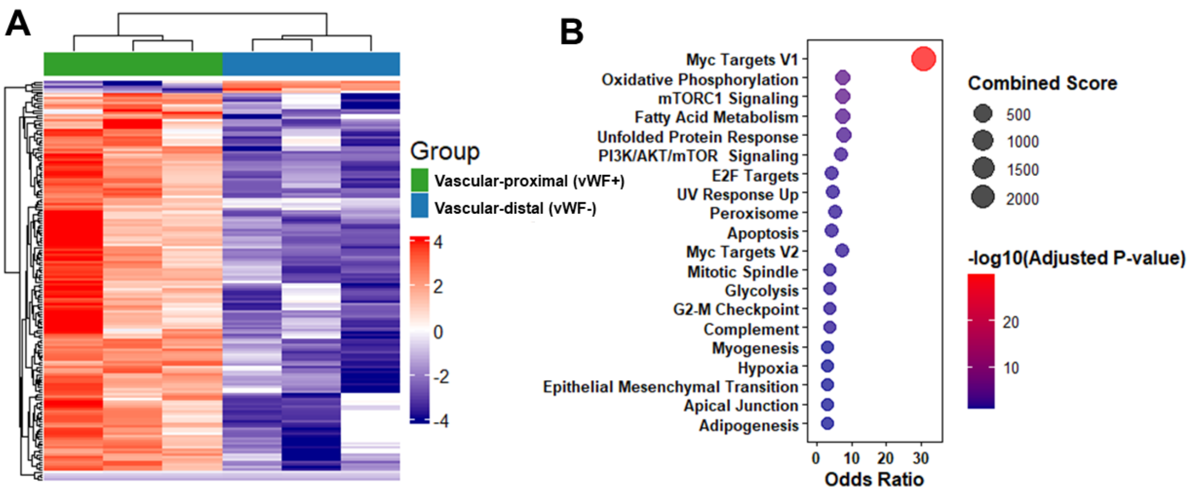

**Figure 1**: **Altered protein expression among vascular-proximal AML BM blasts. A)** Heatmap visualization of the 159 differentially changed proteins in vascular-proximal (vWF+) vs. vascular-distal (vWF-) AML BM blasts (log2FC > 1/< -1, p-val < 0.05). **B)** Pathway enrichment analysis of highly abundant proteins in vWF+ blasts revealed that MYC target V1 is the most enriched pathway.

**Copyright Information:** (Online) ISSN: 2572-9241
©2026 The Author(s). HemaSphere is published by John Wiley & Sons Ltd on behalf of European Hematology Association. This is an open access Abstract Book distributed under the Attribution-NonCommercial-NoDerivs (CC BY-NC-ND), which allows third parties to download the articles and share them with others as long as they credit the author and the Abstract Book, but they cannot change the content in any way or use them commercially.
**Abstract Book Citations:** Authors, Title, HemaSphere, 2026;10:(S1):pages. The abstract book DOIs can be found on the "Author Information" page.
**Disclaimer:** Articles published in the journal HemaSphere exclusively reflect the opinions of the authors. The authors are responsible for all content in their abstracts including accuracy of the facts, statements, citing resources, etc.

### (PS1553) CASP1-EGR4 AXIS REGULATES MACROPHAGE REPOLARIZATION: A MECHANISTIC LINK TO IMMUNOSUPPRESSION IN AML

**Topic:** 03. Acute myeloid leukemia - Biology & translational research

Yi Qian[1], Lijuan Li[1], Liansheng Zhang*[1]

[1] *The Second Hospital and Clinical Medical School, Lanzhou University, Department of Hematology, Lanzhou, China;*

### Background

Caspase-1 (CASP1), a key inflammasome component, is implicated in cancer progression, yet its role in shaping the acute myeloid leukemia (AML) immune microenvironment remains incompletely defined. Tumor-associated macrophages (TAMs) exhibiting an M2-like phenotype critically promote AML progression, but the mechanisms by which AML cells orchestrate this polarization are poorly understood.

### Aims

This study aimed to: (1) investigate the clinical significance of CASP1 in AML, including its diagnostic value and association with immune landscape; (2) elucidate the mechanistic role of AML cell-intrinsic CASP1 in regulating macrophage polarization and immunosuppression.

### Methods

We integrated clinical cohort analysis (57 AML patients, 16 non-cancer controls) with in vitro and in vivo mechanistic studies. CASP1 expression was measured by RT-qPCR in bone marrow samples. Immune profiling of peripheral blood was performed by flow cytometry to quantify major lymphocyte subsets, including T cells ($CD3^+$), B cells ($CD19^+$), NK cells ($CD56^+$), and $CD4^+$ and $CD8^+$ T-cell subpopulations, as well as regulatory T cells (Tregs, defined as $CD4^+CD25^+CD127^{low}$), multiplex immunoassay (12 cytokines), and macrophage polarization markers (CD163, CD206, CD86). Stable CASP1 knockdown (KD) AML cells (THP-1, MOLM-13) were generated via lentiviral shRNA. Conditioned medium experiments assessed paracrine effects on THP-1-derived macrophage polarization using RT-qPCR (M1/M2 gene panels), Western blot (CD86, CD206), flow cytometry ($CD163^+$, $CD86^+$), and immunofluorescence (CD206). Transcriptomic sequencing identified downstream mediators, validated by rescue experiments with EGR4 siRNA. In vivo effects were evaluated in NOD/SCID xenograft models (MOLM-13 CASP1 KD, ) with immunohistochemical analysis (Ki67, EGR4, CD206, CD86, IL-10, p-STAT3).

### Results

CASP1 was significantly upregulated in AML  and correlated with elevated tumor burden (blast%, WBC, LDH), FLT3-ITD mutations , and an immunosuppressive phenotype characterized by elevated M2-associated markers (CD163, CD206), reduced peripheral $CD8^+$ T cells, increased $CD4^+/CD8^+$ ratio, and elevated pro-inflammatory cytokines (IL-2, IL-4, IL-8, TNF-α). Mechanistically, CASP1 KD in AML cells shifted their secretome, significantly suppressing M2 polarization markers ($CD163^+$ cells, CD206 protein) and increasing M1-associated CD86 in macrophages . Transcriptomic analysis identified EGR4 as a key downstream target negatively regulated by CASP1, with consistent upregulation at mRNA and protein levels upon CASP1 KD. Rescue experiments demonstrated that EGR4 is required for these effects, with concurrent modulation of the IL-10/p-STAT3 pathway. In vivo, CASP1 KD suppressed tumor growth, upregulated EGR4, reduced M2 markers (CD206) and IL-10/p-STAT3 signaling, and increased M1-associated CD86 in the tumor microenvironment.

**Copyright Information:** (Online) ISSN: 2572-9241
©2026 The Author(s). HemaSphere is published by John Wiley & Sons Ltd on behalf of European Hematology Association. This is an open access Abstract Book distributed under the Attribution-NonCommercial-NoDerivs (CC BY-NC-ND), which allows third parties to download the articles and share them with others as long as they credit the author and the Abstract Book, but they cannot change the content in any way or use them commercially.
**Abstract Book Citations:** Authors, Title, HemaSphere, 2026;10:(S1):pages. The abstract book DOIs can be found on the "Author Information" page.
**Disclaimer:** Articles published in the journal HemaSphere exclusively reflect the opinions of the authors. The authors are responsible for all content in their abstracts including accuracy of the facts, statements, citing resources, etc.

**Summary/Conclusion**

This study identifies CASP1 as a diagnostic biomarker associated with high-risk AML and reveals a novel CASP1-EGR4 axis that promotes M2 macrophage polarization through modulation of the IL-10/p-STAT3 pathway, contributing to an immunosuppressive microenvironment. These findings establish the CASP1-EGR4 axis as a potential therapeutic target for TAM reprogramming in AML.

**Copyright Information:** (Online) ISSN: 2572-9241
©2026 The Author(s). HemaSphere is published by John Wiley & Sons Ltd on behalf of European Hematology Association. This is an open access Abstract Book distributed under the Attribution-NonCommercial-NoDerivs (CC BY-NC-ND), which allows third parties to download the articles and share them with others as long as they credit the author and the Abstract Book, but they cannot change the content in any way or use them commercially.
**Abstract Book Citations:** Authors, Title, HemaSphere, 2026;10:(S1):pages. The abstract book DOIs can be found on the "Author Information" page.
**Disclaimer:** Articles published in the journal HemaSphere exclusively reflect the opinions of the authors. The authors are responsible for all content in their abstracts including accuracy of the facts, statements, citing resources, etc.

### (PS1554) NOVEL FRAGMENTATION ASSAY ENABLES COMPREHENSIVE FLT3-INTERNAL TANDEM DUPLICATION DETECTION IN AML: AT DIAGNOSIS AND IN FOLLOW-UP

**Topic:** 03. Acute myeloid leukemia - Biology & translational research

Lisanne Beugelink*[1], Ayşenur Göker[1], Jeanine Gastreich[1], Melissa Rijken[1], Jolinda Konijnenburg[1], Petrus Valk[1]

[1]*Erasmus MC Cancer Institute, University Medical Center Rotterdam, Hematology, Rotterdam, The Netherlands;*

### Background

*FLT3* internal tandem duplications (*FLT3*-ITD) are among the most common mutations in acute myeloid leukemia (AML) and are associated with inferior prognosis. Standard treatment of *FLT3*-ITD AML patients now includes the addition of *FLT3* inhibitors, which makes adequate detection of all patient-specific *FLT3*-ITDs at diagnosis essential. Because *FLT3*-ITD measurable residual disease (MRD) in complete remission (CR) predicts higher relapse risk and inferior survival, international guidelines advise routine *FLT3*-ITD MRD testing. Fragment-length analysis is the gold standard for *FLT3*-ITD detection at diagnosis, but its 1–5% limit of detection (LOD) makes it unsuitable for MRD assessment. Amplicon-based next generation sequencing (NGS) enables much higher sensitivity (down to $10^{-6}$), yet its performance depends on the ITD characteristics (size and position). As a result, a subset of *FLT3*-ITDs cannot be reliably detected with current amplicon-based NGS MRD assays, emphasizing the need for an alternative approach.

### Aims

We aimed to determine if random fragmentation of *FLT3* amplicons enables reliable detection of *FLT3*-ITDs, missed by amplicon-based NGS assays, and whether this approach is suitable for *FLT3*-ITD MRD detection.

### Methods

Genomic DNA from bone marrow or peripheral blood of *FLT3*-ITD AML patients was isolated and quantified. Fragment-length analysis of *FLT3* exons 14/15 served as the reference method, alongside the ultrasensitive *FLT3*-ITD MRD amplicon-based modified GetITD NGS assay. The fragmentation assay was carried out on 50 ng DNA (diagnosis) or 500 ng (MRD). The *FLT3* exon 14/15 region was amplified using newly designed primers. After purification, bead-linked transposomes were applied to randomly fragment the amplicons and ligate adapter sequences. A second PCR step added index adapters, and the resulting libraries were sequenced (250 cycles) on a NovaSeq platform (Illumina).

### Results

Fragment-length analysis identified 78 *FLT3*-ITDs across 37 diagnostic *FLT3*-ITD AML samples (range 15-282 bp, median 84 bp). 18 *FLT3*-ITDs were not detected by the amplicon-based NGS assay (range 111–284 bp, median 192 bp). All *FLT3*-ITDs, including those missed by amplicon-based NGS, were detected with the fragmentation assay. The VAFs showed a strong positive correlation between the fragmentation assay and fragment-length analysis (Spearman $\rho = 0.954$, $p < 0.001$). To evaluate the LOD, serial dilutions up to $10^{-6}$ were generated from 3 diagnostic patient samples harboring 5 distinct *FLT3*-ITDs (48-198bp). The fragmentation assay reliably detected *FLT3*-ITDs down to a VAF of $1.7*10^{-5}$ (range $1.2*10^{-4}$ to $1.7*10^{-5}$). Next, we evaluated the assay in a set of 14 patient CR cycle 2 samples. 5 of these samples carried MRD as shown by the fragmentation assay, with the lowest detectable VAF of $1.04*10^{-4}$. The assay's performance was consistent with results of the amplicon-based NGS approach for 2 *FLT3*-ITDs (123 and 171bp), whereas the 3 longer *FLT3*-ITDs were not detected with the amplicon-based NGS approach (177-204bp, VAF range $4.66*10^{-2}$ to $1.04*10^{-4}$). The other 9 samples were MRD negative with both assays.

**Copyright Information:** (Online) ISSN: 2572-9241
©2026 The Author(s). HemaSphere is published by John Wiley & Sons Ltd on behalf of European Hematology Association. This is an open access Abstract Book distributed under the Attribution-NonCommercial-NoDerivs (CC BY-NC-ND), which allows third parties to download the articles and share them with others as long as they credit the author and the Abstract Book, but they cannot change the content in any way or use them commercially.
**Abstract Book Citations:** Authors, Title, HemaSphere, 2026;10:(S1):pages. The abstract book DOIs can be found on the "Author Information" page.
**Disclaimer:** Articles published in the journal HemaSphere exclusively reflect the opinions of the authors. The authors are responsible for all content in their abstracts including accuracy of the facts, statements, citing resources, etc.

**Summary/Conclusion**

The fragmentation-based *FLT3*-ITD assay showed robust performance, detecting all ITDs identified by fragment-length analysis at diagnosis as well as ITDs in CR, including relatively large *FLT3*-ITDs missed by amplicon-based NGS. Its high concordance in VAF quantification and ability to capture long ITDs support its suitability as a complementary method for comprehensive *FLT3*-ITD detection.

**Copyright Information:** (Online) ISSN: 2572-9241
©2026 The Author(s). HemaSphere is published by John Wiley & Sons Ltd on behalf of European Hematology Association. This is an open access Abstract Book distributed under the Attribution-NonCommercial-NoDerivs (CC BY-NC-ND), which allows third parties to download the articles and share them with others as long as they credit the author and the Abstract Book, but they cannot change the content in any way or use them commercially.
**Abstract Book Citations:** Authors, Title, HemaSphere, 2026;10:(S1):pages. The abstract book DOIs can be found on the “Author Information” page.
**Disclaimer:** Articles published in the journal HemaSphere exclusively reflect the opinions of the authors. The authors are responsible for all content in their abstracts including accuracy of the facts, statements, citing resources, etc.

### (PS1555) DEVELOPMENT AND VALIDATION OF AN RWD-BASED MACHINE LEARNING MODEL PREDICTING FEBRILE NEUTROPENIA WITH DAY-LEVEL ACCURACY IN PATIENTS WITH NEWLY DIAGNOSED AML UNDERGOING INTENSIVE CHEMOTHERAPY

**Topic:** 03. Acute myeloid leukemia - Biology & translational research

Anna Mervaala-Muroke*[1, 2, 3], Laura Toivanen[1, 2, 3], Pia-Sofia Ettala[4], Riikka Räty[5], Mikko Keränen[5], Oscar Brück[1, 2, 3]

*[1]Hematoscope Lab, Comprehensive Cancer Center, Helsinki University Hospital, Helsinki, Finland;[2]Department of Oncology, University of Helsinki, Helsinki, Finland; [3]Department of Clinical Chemistry, HUS Diagnostic Center, Helsinki University Hospital, Helsinki, Finland; [4]Department of Clinical Hematology and Stem Cell Transplantations, Turku University Hospital, Turku, Finland; [5]Department of Hematology, Comprehensive Cancer Center, Helsinki University Hospital, Helsinki, Finland;*

### Background

Neutropenic infections represent a major source of morbidity and mortality in patients with acute myeloid leukemia (AML) undergoing intensive chemotherapy. However, clinicians lack a reliable tool to anticipate the onset of neutropenic infection, thereby enabling the timely implementation of preventive measures.

### Aims

The objective of this study was to develop and externally validate a real-world data-based machine learning model that generates daily predictions of febrile neutropenia (FN) using a 48-hour prediction window.

### Methods

We conducted a retrospective observational study, collecting data from two Finnish university hospital districts. The study population comprised all adult patients with newly diagnosed AML receiving intensive chemotherapy, excluding those with acute promyelocytic leukemia. We developed a machine learning model employing the Extreme Gradient Boosting algorithm using over 1.2 million clinical data points, including demographic characteristics, molecular and cytogenetic alterations, body temperature measurements, routine laboratory tests, and their temporal dynamics. We evaluated the importance and impact of each variable using Shapley additive explanations (SHAP). We assessed model performance using F1 score (the harmonic mean of positive predictive value and sensitivity), the area under the receiver operating characteristic curve (AUROC), and the calibration curve. We first optimized a full-variable model and subsequently simplified and retrained it into a clinically useful model (**Figure 1**).

**Copyright Information:** (Online) ISSN: 2572-9241
©2026 The Author(s). HemaSphere is published by John Wiley & Sons Ltd on behalf of European Hematology Association. This is an open access Abstract Book distributed under the Attribution-NonCommercial-NoDerivs (CC BY-NC-ND), which allows third parties to download the articles and share them with others as long as they credit the author and the Abstract Book, but they cannot change the content in any way or use them commercially.
**Abstract Book Citations:** Authors, Title, HemaSphere, 2026;10:(S1):pages. The abstract book DOIs can be found on the "Author Information" page.
**Disclaimer:** Articles published in the journal HemaSphere exclusively reflect the opinions of the authors. The authors are responsible for all content in their abstracts including accuracy of the facts, statements, citing resources, etc.

### Results

We included 271 patients in the development cohort and 136 patients in the external validation cohort, observing 451 and 161 episodes of febrile neutropenia, respectively. Neutropenia (ANC ≤0.5 × $10^9$/L) began on average on day 8 during induction and on day 11 during consolidation.

Performance comparable to the full 272-variable model was maintained with only the 6 top SHAP-ranked variables: treatment phase, time since treatment initiation, body temperature, white blood cell count, absolute neutrophil count, and 3-day C-reactive protein trend. The simplified model achieved an AUROC of 0.92 and a correlation coefficient of 0.90 in the development cohort's test set (n=55). Model performance was superior during induction compared with that observed during consolidation (F1 score: 0.79 vs. 0.60), likely due to closer daily monitoring and a higher FN incidence. We observed an increased risk of FN during the induction phase, on days 6–18, at WBC values <1.4 × $10^9$/L, during severe neutropenia (ANC <0.2 × $10^9$/L), and with rising CRP levels. Strikingly, FN risk increased when body temperature rose >36.6°C, indicating that not only subfebrile levels but even normal-range values may be clinically informative.

External validation in the independent cohort (n=136) demonstrated an AUROC of 0.78 and a correlation coefficient of 0.62, suggesting promising utility despite substantially higher antibiotic exposure before FN onset (90.2%) than in the development cohort (55.6%, p<0.001).

### Summary/Conclusion

We developed a real-time FN screening algorithm with 6 easily available variables, enabling accurate and realistic risk assessment in patients with AML undergoing intensive chemotherapy. Although prospective multicenter validation is still needed, implementing the model in clinical practice could enable more timely antimicrobial use and potentially mitigate infectious complications.

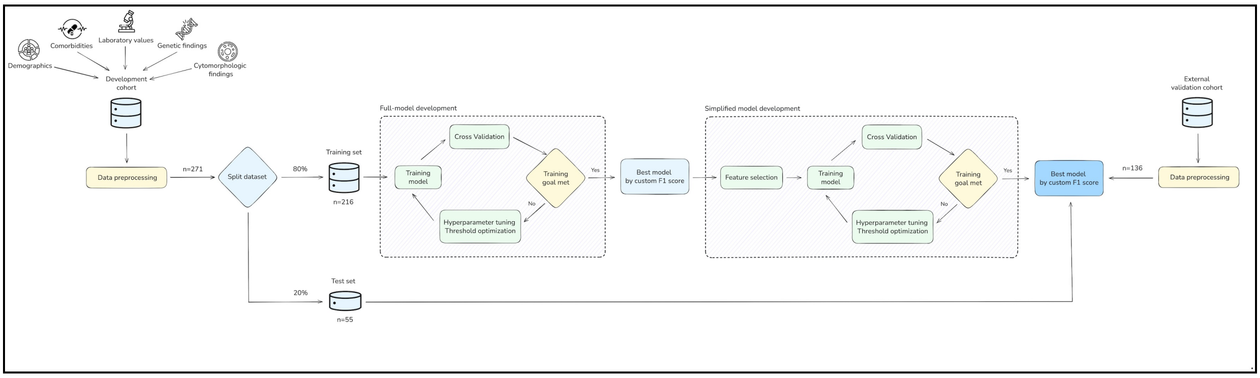

Figure 1. Study design.

**Copyright Information:** (Online) ISSN: 2572-9241
©2026 The Author(s). HemaSphere is published by John Wiley & Sons Ltd on behalf of European Hematology Association. This is an open access Abstract Book distributed under the Attribution-NonCommercial-NoDerivs (CC BY-NC-ND), which allows third parties to download the articles and share them with others as long as they credit the author and the Abstract Book, but they cannot change the content in any way or use them commercially.
**Abstract Book Citations:** Authors, Title, HemaSphere, 2026;10:(S1):pages. The abstract book DOIs can be found on the "Author Information" page.
**Disclaimer:** Articles published in the journal HemaSphere exclusively reflect the opinions of the authors. The authors are responsible for all content in their abstracts including accuracy of the facts, statements, citing resources, etc.

### (PS1556) DUAL BET/HAT INHIBITOR–BASED COMBINATIONS EXERT SYNERGISTIC LETHALITY AGAINST MYELOPROLIFERATIVE NEOPLASMS (MPN) TRANSFORMED TO AML

**Topic:** 03. Acute myeloid leukemia - Biology & translational research

Warren Fiskus*[1], Christopher Mill[1], Lucia Masarova[1], Naveen Pemmaraju[1], Prithviraj Bose[1], Hanxi Hou[1], Taghi Manshouri[1], Sanam Loghavi[1], Claes Wahlestedt[2], Courtney Dinardo[1]

*[1]UT MD Anderson Cancer Center, Leukemia, Houston, United States of America;[2]University of Miami Miller School of Medicine, Miami, United States of America;*

### Background

In MPN stem/progenitor cells, the co-occurrence of driver mutations in JAK2, CALR or MPL and co-mutations in chromatin/transcriptional regulators creates a dysregulated transcriptome and proteome. This dysregulation leads to increased growth, cell survival, transformation to secondary AML (sAML), therapy refractoriness and poor outcomes. We have shown that targeting BET proteins with BET inhibitors (i) disrupted RNAP2-mediated transcription and depleted c-Myc, PIM1, CDK6 and BCL2 but increased p21, p27 and HEXIM1 expression in post-MPN sAML cells. BETi treatment resulted in growth inhibition and apoptosis of sAML cells. Additionally, treatment with BETi (e.g., birabresib) reduced leukemia burden and induced clinical remissions. CRISPR-Cas9 dropout screens have shown that BRD4 and histone acetyltransferase EP300 are dependencies in post-MPN sAML cells, supporting the rationale to inhibit both proteins with a single targeted agent.

### Aims

To determine the effects of dual BET/HAT inhibitor monotherapy and combination with JAK inhibitor or CDK7 inhibitor in post-MPN sAML cells that are sensitive or persister/resistant to JAK or BET inhibitor.

### Methods

We determined the effects of treatment with the dual BET/HAT inhibitor EP31670 (EP) on the transcriptome, proteome, cell cycle and viability of post-MPN sAML cells. We also determined the effects of treatment with EP and JAK or CDK7 inhibitor in post-MPN sAML cells that are sensitive or resistant to JAK or BET inhibitor.

**Copyright Information:** (Online) ISSN: 2572-9241
©2026 The Author(s). HemaSphere is published by John Wiley & Sons Ltd on behalf of European Hematology Association. This is an open access Abstract Book distributed under the Attribution-NonCommercial-NoDerivs (CC BY-NC-ND), which allows third parties to download the articles and share them with others as long as they credit the author and the Abstract Book, but they cannot change the content in any way or use them commercially.
**Abstract Book Citations:** Authors, Title, HemaSphere, 2026;10:(S1):pages. The abstract book DOIs can be found on the "Author Information" page.
**Disclaimer:** Articles published in the journal HemaSphere exclusively reflect the opinions of the authors. The authors are responsible for all content in their abstracts including accuracy of the facts, statements, citing resources, etc.

**Results**

In present studies, we elucidated the gene-expression alterations and lethal activity of dual BET/HAT inhibitor EP31670 alone and in combination with other targeted agents to achieve superior preclinical efficacy in post-MPN sAML cells that are sensitive or resistant to JAKi or BETi. We determined, by RNA-Seq, that EP treatment negatively enriched multiple gene sets including MYC_TARGETS and repressed MYC, MYB, CDK6 and PIM1 but induced DCXR, HEXIM1 and p21 mRNA levels in sAML cells. Treatment with EP also reduced protein levels of c-Myb, c-Myc, CDK6, and PIM1, with increased expression of HEXIM1, p21, and p27. Mass spectrometry analysis following EP also showed negative enrichment of HALLMARK_MYC_TARGETS, IL6_JAK_STAT3_SIGNALING and IL2_STAT5_SIGNALING in SET2 cells. Notably, co-treatment with EP and ruxolitinib induced synergistic lethality in cultured and patient-derived (PD) post-MPN sAML. Pre-treatment with EP significantly increased the sensitivity of JAKi-resistant sAML cells to JAKi-induced lethality and BETi-resistant cells to BETi-induced lethality. These results suggested that EP overcomes resistance to JAKi and BETi in MPN-sAML cells. We also determined that co-treatment with EP and CDK7i SY-5609 (SY) was synergistically lethal in sAML cell lines including those resistant to JAKi or BETi, as well as PD MPN-sAML cells. We next determined in vivo efficacy of EP monotherapy or its co-treatment with SY in NSG mice engrafted with SET2. EP significantly reduced sAML burden and improved the survival of the mice. Co-treatment with EP and SY resulted in significantly greater reduction in leukemia burden and further improved the overall survival of the mice than treatment with each agent alone ($p < 0.05$).

**Summary/Conclusion**

These findings demonstrate promising preclinical activity of EP against MPN-sAML cells and strongly support the rationale to further evaluate the efficacy of EP-based combinations against advanced MPN with excess blasts or MPN-sAML.

**Copyright Information:** (Online) ISSN: 2572-9241
©2026 The Author(s). HemaSphere is published by John Wiley & Sons Ltd on behalf of European Hematology Association. This is an open access Abstract Book distributed under the Attribution-NonCommercial-NoDerivs (CC BY-NC-ND), which allows third parties to download the articles and share them with others as long as they credit the author and the Abstract Book, but they cannot change the content in any way or use them commercially.
**Abstract Book Citations:** Authors, Title, HemaSphere, 2026;10:(S1):pages. The abstract book DOIs can be found on the “Author Information” page.
**Disclaimer:** Articles published in the journal HemaSphere exclusively reflect the opinions of the authors. The authors are responsible for all content in their abstracts including accuracy of the facts, statements, citing resources, etc.

### (PS1557) DISSECTING THE ROLE AND MODULATION OF TNF RECEPTOR SIGNALING IN ACUTE MYELOID LEUKEMIA

**Topic:** 03. Acute myeloid leukemia - Biology & translational research

Sayantanee Dutta*[1], Ulrike Höckendorf[2], Stefanie Prein[1], Sophie Kienreich[1], Stefanie Stanzer[1], Michele Spanu[1], Vanessa Jäger[1], Bianca Perfler[1], Armin Zebisch[1], Ashok Jayavely[3], Katarina Vizar-Cisarova[1], Michael Dengler[1], Philipp Jost[1]

*[1]Medical University of Graz, Division of Oncology, Graz, Austria;[2]Technical University of Munich, Munich, Germany;[3]German Cancer Research Center (DKFZ), Heidelberg, Germany, Heidelberg, Germany;*

### Background

Tumor necrosis Factor receptor (TNFR)–mediated signaling constitutes a key regulatory axis in inflammation and immune homeostasis and is critically involved in both steady-state and stress-induced (emergency) hematopoiesis. Inflammation has emerged as a key pathogenic driver of myeloid malignancies, shaping clonal architecture, modulating immune responses, influencing therapeutic vulnerability, and ultimately impacting patient outcomes. We recently demonstrated that induction of TNFR-driven inflammatory signaling using lymphotoxin-α (LTα) selectively eliminates leukemic stem cells (LSCs) in AML while sparing healthy hematopoietic stem and progenitor cells (HSPCs) causing durable remission *in vivo*. LTα is a pro-inflammatory cytokine from TNF-superfamily which signals via its cognate receptors TNF receptor 1 (TNFR1) and HVEM (Herpesvirus entry mediator).

### Aims

Here, we extended our investigation to define the role of TNFR signaling in healthy and leukemic cells, focusing on the differential contributions of individual TNF receptors and their downstream pathways. An additional aim was to define the differences between LTα- and TNF-mediated signaling.

### Methods

Using CRISPR-engineered AML cell lines with targeted alterations in TNFR family members, we defined receptor-specific functions. TNFR signaling was modulated with receptor-neutralizing antibodies, and transcriptional consequences were analyzed by bulk RNA sequencing from AML cell lines and primary healthy and leukemic cells.

### Results

Treatment with TNFR agonists (TNF and LTα3) triggered cell death across AML cell lines, with heterogeneous responses and a clearly sensitive subgroup. Blocking TNFR1 with a neutralizing antibody in LTα sensitive cell lines and primary AML samples induced marked cellular stress, suggesting their functional dependency on pro-inflammatory TNFR1 signaling. TRAF2 (TNF receptor associated factor 2), a central adaptor downstream of multiple TNFR superfamily members—including TNFR1, TNFR2, CD40, and TNFRSF9—was depleted following LTα3 exposure. Genetic deletion of TNFR1, and TNFR2, restored LTα3-mediated TRAF2 depletion in some AML cell lines, whereas deletion of HVEM had no significant effect. At the transcriptional level, LTα3 not only activated TNFR1-dependent pathways but also induced TNFR2- and CD40-associated signaling and upregulated **TNFRSF9** expression in AML cell lines and primary samples. We propose that following LTα3 treatment, concurrent activation of multiple TRAF2-interacting receptors increases competition for TRAF2, creating heightened demand and functional depletion. Reduced TRAF2 availability destabilizes the TNFR1 signaling complex, shifting signaling from pro-survival pathways toward cell death or differentiation. Proteomic analysis showed that, compared with AML cells, healthy HSPCs express lower levels of TNFR2 and higher levels of TRAF2 protein. This expression profile likely renders them less susceptible to LTα3-induced TRAF2 depletion and the associated shift from pro-survival signaling toward cell death. Compared with TNF, LTα3 induced qualitatively similar but quantitatively attenuated transcriptional changes in AML and healthy HSPCs.

**Copyright Information:** (Online) ISSN: 2572-9241
©2026 The Author(s). HemaSphere is published by John Wiley & Sons Ltd on behalf of European Hematology Association. This is an open access Abstract Book distributed under the Attribution-NonCommercial-NoDerivs (CC BY-NC-ND), which allows third parties to download the articles and share them with others as long as they credit the author and the Abstract Book, but they cannot change the content in any way or use them commercially.
**Abstract Book Citations:** Authors, Title, HemaSphere, 2026;10:(S1):pages. The abstract book DOIs can be found on the “Author Information” page.
**Disclaimer:** Articles published in the journal HemaSphere exclusively reflect the opinions of the authors. The authors are responsible for all content in their abstracts including accuracy of the facts, statements, citing resources, etc.

**Summary/Conclusion**

Our data demonstrate that AML cells, but not healthy HSPCs, depend on tightly balanced pro-survival TNFR1 signaling. Both inhibition and hyperactivation of this pathway disrupt this balance, promoting cell death or differentiation in AML cells. These findings identify TNFR1 signaling as a selective therapeutic vulnerability in AML.

**Copyright Information:** (Online) ISSN: 2572-9241
©2026 The Author(s). HemaSphere is published by John Wiley & Sons Ltd on behalf of European Hematology Association. This is an open access Abstract Book distributed under the Attribution-NonCommercial-NoDerivs (CC BY-NC-ND), which allows third parties to download the articles and share them with others as long as they credit the author and the Abstract Book, but they cannot change the content in any way or use them commercially.
**Abstract Book Citations:** Authors, Title, HemaSphere, 2026;10:(S1):pages. The abstract book DOIs can be found on the "Author Information" page.
**Disclaimer:** Articles published in the journal HemaSphere exclusively reflect the opinions of the authors. The authors are responsible for all content in their abstracts including accuracy of the facts, statements, citing resources, etc.

### (PS1558) HARNESSING THE BONE MARROW STROMA IN AML: FAP-TARGETED COSTIMULATORY AGONISTS POTENTIATE T-CELL BISPECIFIC ANTIBODY ACTIVITY AND PRESERVE T-CELL FITNESS

**Topic:** 03. Acute myeloid leukemia - Biology & translational research

Anne-Sophie Neumann*[1, 2], Gerulf Hänel[1, 2], Irene Caporali[1, 2], Lis Winter[1, 2], Amelie Muth[1, 2], Tobias Straub[3], Eva Briem[4], Daniel Richter[4], Martina Rudelius[5], Vesna Kramar[6], Maria Amann[6], Veit Bücklein[1, 2, 7, 8], Christian Klein[6, 9], Marion Subklewe[1, 2, 7, 8]

*[1]Department of Medicine III, University Hospital, LMU Munich, Munich, Germany;[2]Laboratory for Translational Cancer Immunology, LMU Gene Center, Munich, Germany; [3]Bioinformatics Unit, Biomedical Center, LMU Munich, Martinsried, Germany; [4]Faculty of Biology, Anthropology & Human Genomics, LMU Munich, Munich, Germany;[5]Institute of Pathology, LMU Munich, Munich, Germany; [6]Roche Innovation Center Zurich, Roche Pharma Research and Early Development (pRED), Schlieren, Switzerland; [7]German Cancer Consortium (DKTK), partner site Munich, a partnership between DKFZ and LMU University Hospital, Munich, Germany; [8]Bavarian Cancer Research Center (BZKF), Munich, Germany;[9]Department of Biochemistry, Faculty of Chemistry and Pharmacy, LMU Munich, Munich, Germany;*

### Background

T-cell-engaging bispecific antibodies (TCBs) have demonstrated remarkable clinical efficacy in lymphoid malignancies, yet their therapeutic potential in acute myeloid leukemia (AML) remains limited. TCBs activate T cells through CD3 crosslinking alone, bypassing costimulatory pathways required for full effector functions. As a result, their efficacy may be particularly constrained in the immunosuppressive bone marrow niche in AML, where endogenous costimulatory signals are often limited.

### Aims

We hypothesized that combining TCBs with bone marrow-targeted costimulatory agonists represents a rational strategy to overcome this deficit. Accordingly, the AML-targeting Wilms tumor 1 (WT1)-TCB was evaluated in combination with costimulatory agonistic bispecific antibodies targeting 4-1BB or OX40, directed to the bone marrow niche by targeting fibroblast activation protein (FAP) expressed on bone marrow mesenchymal stromal cells.

### Methods

The costimulatory ligand expression on primary AML blasts and monocyte-derived dendritic cells (moDCs) was compared by MPFC. Efficacy of WT1-TCB + FAP-4-1BBL or FAP-OX40 was evaluated in short-term coculture assays (4 days) with human AML cell lines or murine Ba/F3 cell lines expressing hu4-1BBL or huCD86, healthy donor T cells, and an NIH-3T3 fibroblast cell line expressing FAP. To address T-cell exhaustion in the context of continuous target antigen exposure, we additionally performed long-term coculture assays over 28 days. Lastly, we investigated the impact of costimulation in an *ex vivo* setting with primary AML blasts. T-cell activation, phenotype, cytotoxicity, and metabolic fitness were assessed by MPFC, Seahorse assays, and bulk RNA sequencing.

**Copyright Information:** (Online) ISSN: 2572-9241
©2026 The Author(s). HemaSphere is published by John Wiley & Sons Ltd on behalf of European Hematology Association. This is an open access Abstract Book distributed under the Attribution-NonCommercial-NoDerivs (CC BY-NC-ND), which allows third parties to download the articles and share them with others as long as they credit the author and the Abstract Book, but they cannot change the content in any way or use them commercially.
**Abstract Book Citations:** Authors, Title, HemaSphere, 2026;10:(S1):pages. The abstract book DOIs can be found on the “Author Information” page.
**Disclaimer:** Articles published in the journal HemaSphere exclusively reflect the opinions of the authors. The authors are responsible for all content in their abstracts including accuracy of the facts, statements, citing resources, etc.

**Results**

Primary AML blasts showed highly heterogeneous expression of the costimulatory ligands CD86, 4-1BBL, and OX40L. Notably, levels of all three costimulatory ligands were considerably lower on primary AML blasts than on moDCs, suggesting that additional costimulation may enhance TCB efficacy. Indeed, the addition of FAP-4-1BBL and FAP-OX40 significantly enhanced WT1-TCB-mediated cytotoxicity against diverse AML cell lines. Our results could be further validated using primary AML samples in both allogeneic and autologous settings. Mechanistic dissection using the murine Ba/F3 system demonstrated that high CD86 expression on target cells did not diminish the costimulatory benefit. However, 4-1BBL expression at high levels abrogated the effect of exogenous costimulation, suggesting a saturation threshold. Importantly, long-term cultures mimicking continuous TCB application in the clinic demonstrated that both combinatorial approaches preserved T-cell fitness, including proliferation, cytokine secretion, cytotoxicity, and metabolic activity. Here, FAP-4-1BBL showed superior and more durable effects compared to FAP-OX40. Transcriptomic profiling revealed that FAP-4-1BBL costimulation sustains proliferative, metabolic, and cytotoxic effector gene programs while promoting an effector T-cell phenotype.

**Summary/Conclusion**

In conclusion, combining a WT1-targeting TCB with a FAP-targeted 4-1BB agonist significantly enhanced T-cell-mediated anti-leukemic activity and preserved T-cell fitness during continuous TCB stimulation. By directing costimulation to the bone marrow niche via FAP, this strategy addresses both the insufficient costimulatory ligand expression on AML blasts and the immunosuppressive bone marrow niche, representing a promising approach to overcome the barriers limiting TCB efficacy in AML.

**Copyright Information:** (Online) ISSN: 2572-9241
©2026 The Author(s). HemaSphere is published by John Wiley & Sons Ltd on behalf of European Hematology Association. This is an open access Abstract Book distributed under the Attribution-NonCommercial-NoDerivs (CC BY-NC-ND), which allows third parties to download the articles and share them with others as long as they credit the author and the Abstract Book, but they cannot change the content in any way or use them commercially.
**Abstract Book Citations:** Authors, Title, HemaSphere, 2026;10:(S1):pages. The abstract book DOIs can be found on the "Author Information" page.
**Disclaimer:** Articles published in the journal HemaSphere exclusively reflect the opinions of the authors. The authors are responsible for all content in their abstracts including accuracy of the facts, statements, citing resources, etc.

### (PS1559) DRUG REPURPOSING SCREENING IDENTIFIES NSC319726 AS A SELECTIVE ANTI-LEUKEMIC AGENT THAT TRIGGERS AN INTEGRATED STRESS RESPONSE-MEDIATED DEATH PROGRAM IN AML

**Topic:** 03. Acute myeloid leukemia - Biology & translational research

Peiyao Jiang*[1], Xianmin Song[1]

[1]*Shanghai General Hospital, Shanghai, China;*

### Background

Acute myeloid leukemia (AML) remains a therapeutic challenge due to relapse and limited curative options. Drug repurposing can rapidly identify compounds with unexpected anti-leukemic mechanisms.

### Aims

We combined an FDA-approved anti-tumor drug set with a cuproptosis-focused small-molecule collection to uncover hits that exploit metabolic and stress-response vulnerabilities in AML.

### Methods

A 406-compound library was screened at 1 µM for 48 h in five AML cell lines (THP-1, MOLM-13, MV4-11, HL-60, SKNO-1). Cell viability was assessed by CCK-8 and CellTiter-Glo assays. Selectivity was evaluated in healthy donor PBMCs (n=3). Mechanistic studies included transcriptomic profiling, qPCR, SunSET assays, JC-10 mitochondrial staining, and western blotting. Activity was validated in primary AML samples (n=2) and an NCG mouse xenograft model (n=6 per group) treated with NSC319726 daily via intraperitoneal injection. Tumor growth was analyzed using two-way ANOVA and endpoint comparisons.

### Results

NSC319726 emerged as a potent hit, exhibiting nanomolar IC50 values across multiple AML cell lines (range: 1.92–8.42 nM). In contrast, NSC319726 exhibited low toxicity toward PBMCs, yielding a selectivity index of up to 1640. RNA-seq revealed robust activation of the integrated stress response (ISR), with marked upregulation of DDIT3, PPP1R15A, and APOL6. Consistently, NSC319726 induced eIF2α phosphorylation and elevated ATF4/CHOP protein levels. ISR over-activation was accompanied by global translation suppression and pronounced mitochondrial depolarization. Notably, the pan-caspase inhibitor z-VAD-fmk failed to rescue viability, indicating a caspase-independent death mechanism. In vivo, NSC319726 demonstrated potent dose-dependent efficacy; by day 14, tumor growth inhibition (TGI) reached 60.5% (1 mg/kg) and 89.3% (5 mg/kg) (both $P < 0.0001$), with no observable systemic toxicity.

### Summary/Conclusion

Through targeted drug repurposing, we identified NSC319726 as a highly selective anti-AML agent. Its efficacy is mediated by ISR over-activation, translation collapse, and mitochondrial dysfunction. These findings provide a strong rationale for the further translational evaluation of NSC319726 in AML.

**Copyright Information:** (Online) ISSN: 2572-9241
©2026 The Author(s). HemaSphere is published by John Wiley & Sons Ltd on behalf of European Hematology Association. This is an open access Abstract Book distributed under the Attribution-NonCommercial-NoDerivs (CC BY-NC-ND), which allows third parties to download the articles and share them with others as long as they credit the author and the Abstract Book, but they cannot change the content in any way or use them commercially.
**Abstract Book Citations:** Authors, Title, HemaSphere, 2026;10:(S1):pages. The abstract book DOIs can be found on the “Author Information” page.
**Disclaimer:** Articles published in the journal HemaSphere exclusively reflect the opinions of the authors. The authors are responsible for all content in their abstracts including accuracy of the facts, statements, citing resources, etc.

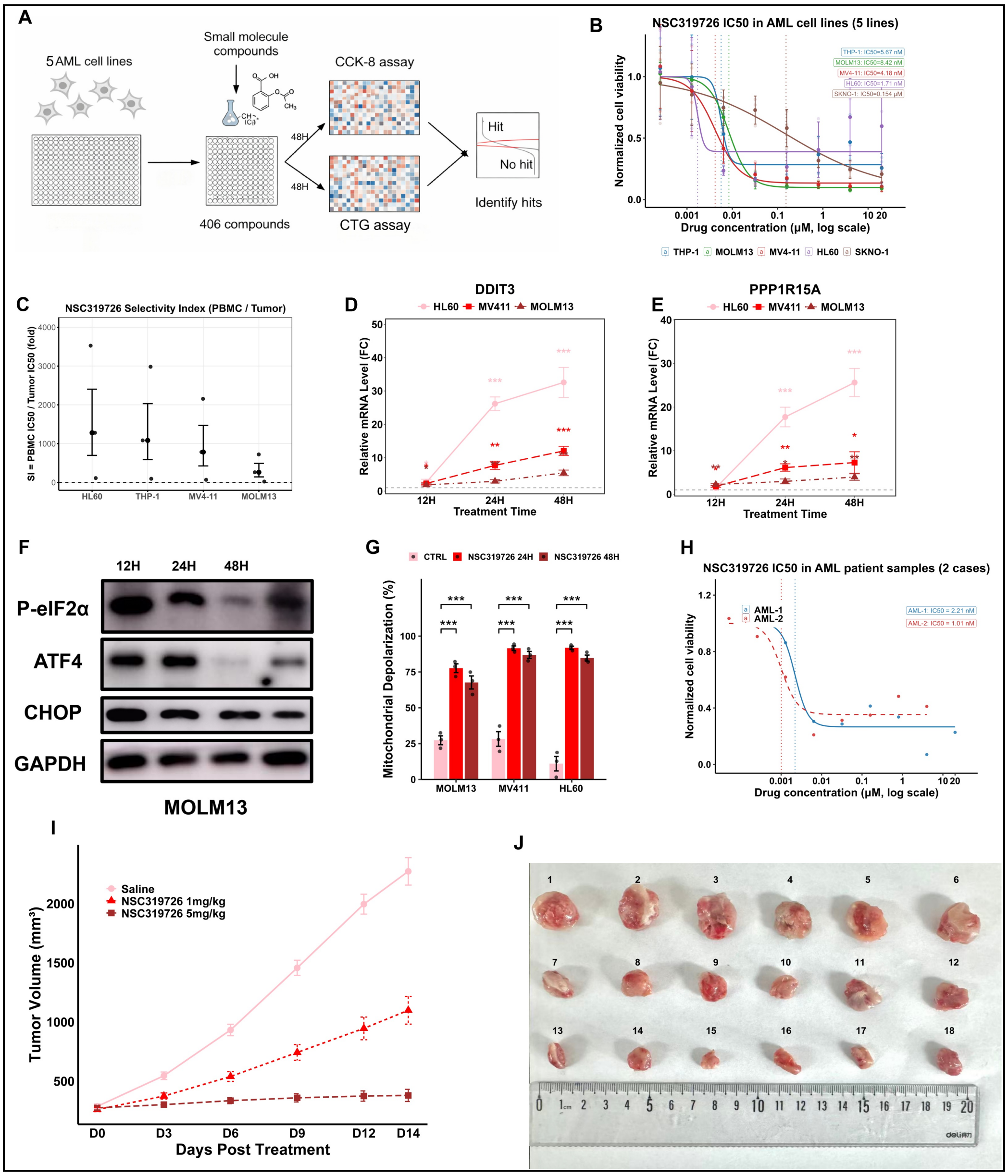

Figure. Identification and characterization of NSC319726 as a selective anti-AML agent. (A) Schematic of the screening workflow: 5 acute myeloid leukemia (AML) cell lines were screened with 406 small molecules (1 μM, 48 h) and read out by Cell Counting Kit-8 (CCK-8) and CellTiter-Glo (CTG) assays to identify hits. (B) Dose–response curves of NSC319726 in five AML cell lines. (C) Selectivity index (SI) calculated as peripheral blood mononuclear cell (PBMC) IC50 / tumor IC50. (D–E) qPCR time-course of indicated integrated stress response (ISR) genes following NSC319726 treatment. (F) Immunoblot showing ISR activation (p-eIF2α, ATF4, CHOP) in MOLM13 at indicated time points. (G) NSC319726 induces mitochondrial depolarization assessed by JC-10 staining. (H) Dose–response curves in primary AML patient samples (n=2). (I–J) In vivo efficacy in an MOLM13 xenograft model treated with NSC319726 (1 or 5 mg/kg) versus saline: tumor growth curves (I) and representative excised tumors at endpoint (J). Data are shown as mean ± SD/SEM as indicated; statistical significance is shown in panels where applicable (*P<0.05, **P<0.01, ***P<0.001).

**Copyright Information:** (Online) ISSN: 2572-9241
©2026 The Author(s). HemaSphere is published by John Wiley & Sons Ltd on behalf of European Hematology Association. This is an open access Abstract Book distributed under the Attribution-NonCommercial-NoDerivs (CC BY-NC-ND), which allows third parties to download the articles and share them with others as long as they credit the author and the Abstract Book, but they cannot change the content in any way or use them commercially.
**Abstract Book Citations:** Authors, Title, HemaSphere, 2026;10:(S1):pages. The abstract book DOIs can be found on the "Author Information" page.
**Disclaimer:** Articles published in the journal HemaSphere exclusively reflect the opinions of the authors. The authors are responsible for all content in their abstracts including accuracy of the facts, statements, citing resources, etc.

### (PS1560) MESENCHYMAL STROMAL CELLS PROTECT ACUTE MYELOID LEUKEMIA CELLS FROM VENETOCLAX-INDUCED APOPTOSIS THROUGH A CD90-MEDIATED ADHESION PATHWAY

**Topic:** 03. Acute myeloid leukemia - Biology & translational research

Marilena Ciciarello*[1, 2, 3], Karyna Volkava[4], Giulia Corradi[5], Manuela Piazzi[6], Vittoria Cenni[6], Samantha Bruno[7], Antonio Curti[8]

*[1]National Research Council (CNR), "Luigi Luca Cavalli-Sforza", Institute of Molecular Genetics, Bologna, Italy;[2]University of Bologna, Medical and Surgical Sciences (DIMEC), Bologna, Italy; [3]IRCCS Istituto Ortopedico Rizzoli, Bologna, Italy; [4]University of Bologna, Bo, Italy; [5]University of Chieti-Pescara, Medicine and Aging Science, Pescara, Italy;[6]National Research Council (CNR), Bologna, Italy; [7]University of Bologna, Bologna, Italy; [8]IRCCS Azienda Ospedaliero-Universitaria di Bologna, Istituto di Ematologia "Seràgnoli", Bologna, Italy;*

### Background

Despite the clinical impact of venetoclax (VEN) combined with azacitidine (AZA), resistance and relapse remain the rule rather than the exception in acute myeloid leukemia (AML). Increasing evidence indicates that the bone marrow microenvironment, particularly mesenchymal stromal cells (MSCs), actively protects leukemic cells from apoptosis. However, the molecular determinants of MSC-driven VEN resistance are incompletely understood.

### Aims

To dissect the cellular and molecular mechanisms by which MSCs confer resistance to VEN in AML.

### Methods

We established and optimized an MSC/AML co-culture model using the VEN-sensitive MV4-11 cell line, enabling discrimination between VEN-sensitive AML cells and MSC-protected AML cells. Co-cultured cells were subjected to apoptosis assays, flow cytometry, and electron microscopy, then sorted for transcriptomic profiling and protein analysis to characterize MSC/AML cell interactions and resistance-associated programs.

### Results

MSC co-culture generated two functionally distinct AML cell subpopulations: tightly adherent cells in direct contact with MSCs and floating cells in suspension. Strikingly, only adherent AML cells were protected from AZA/VEN-induced apoptosis, underscoring the requirement for physical niche engagement. Electron microscopy revealed intimate MSC/AML contacts accompanied by reciprocal morphological remodeling.
Transcriptomic profiling identified 663 differentially expressed genes ($|FC| \geq 2$; $p \leq 0.05$) between adherent and floating AML cells. Principal component analysis demonstrated that floating cells clustered with AML cells cultured alone, whereas adherent cells displayed a distinct transcriptional program. Enrichment analysis highlighted pathways related to adhesion, migration, and proliferation.
Among the most significantly upregulated genes in adherent cells was CD90/Thy-1, an adhesion molecule previously associated with adverse-risk AML and poor survival. Flow cytometry confirmed increased CD90 expression in adherent versus floating AML cells. Functional blockade of CD90 with an inactivating antibody significantly abrogated MSC-mediated protection from VEN. Consistently, protein analyses showed activation of CD90-associated pro-survival pathways in adherent AML cells, which were suppressed upon CD90 inhibition.

**Copyright Information:** (Online) ISSN: 2572-9241
©2026 The Author(s). HemaSphere is published by John Wiley & Sons Ltd on behalf of European Hematology Association. This is an open access Abstract Book distributed under the Attribution-NonCommercial-NoDerivs (CC BY-NC-ND), which allows third parties to download the articles and share them with others as long as they credit the author and the Abstract Book, but they cannot change the content in any way or use them commercially.
**Abstract Book Citations:** Authors, Title, HemaSphere, 2026;10:(S1):pages. The abstract book DOIs can be found on the "Author Information" page.
**Disclaimer:** Articles published in the journal HemaSphere exclusively reflect the opinions of the authors. The authors are responsible for all content in their abstracts including accuracy of the facts, statements, citing resources, etc.

### Summary/Conclusion

Our data identify CD90-dependent cell–cell adhesion as a critical mediator of MSC-driven VEN resistance in AML. These findings reveal a contact-dependent survival program operating within the leukemic niche and nominate CD90 and its downstream signaling network as actionable targets to overcome microenvironment-induced therapy resistance.

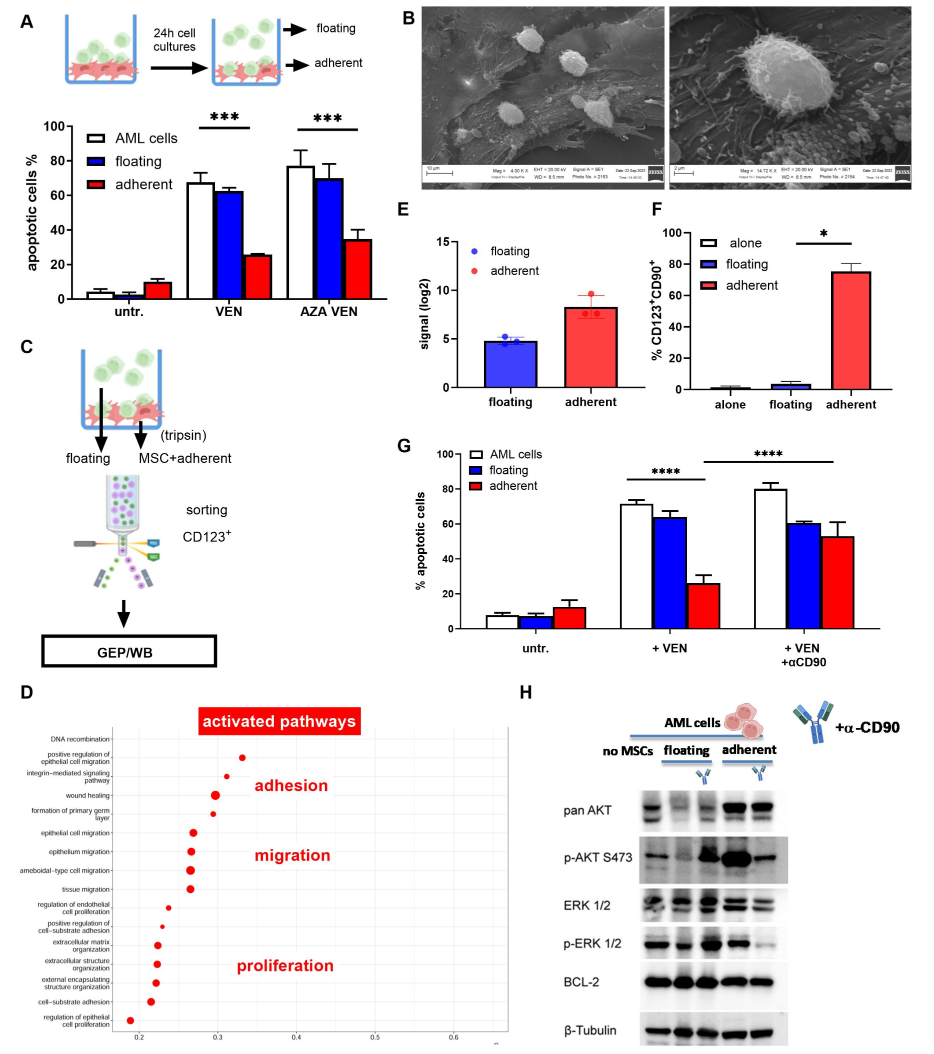

AML cells were seeded in contact with MSCs. We distinguished a population suspended in the medium (floating) and a cell population that remained adherent to the MSCs (adherent). VEN-induced apoptosis was significantly reduced in adherent cells compared with floating cells and in cultured AML cells alone (A). Electron microscopy revealed AML cells tightly bound to MSCs (B). Adherent AML cells were separated from floating cells using a Cell sorter (C). Pathway enrichment analysis revealed that DEGs were mainly involved in cell migration, adhesion, and proliferation (D). Among the DEGs upregulated in AML adherent cells, we identified CD90/Thy-1, an adhesion molecule (E). Flow cytometry analysis confirmed that adherent vs. floating AML cells showed significantly higher CD90 expression (F). The addition of an anti-CD90 inactivating antibody significantly decreased the MSC-protective effect on adherent cells (G). CD90-downstream pro-survival pathways were activated in adherent-sorted cells and suppressed by CD90 antibody addition (H).

**Copyright Information:** (Online) ISSN: 2572-9241
©2026 The Author(s). HemaSphere is published by John Wiley & Sons Ltd on behalf of European Hematology Association. This is an open access Abstract Book distributed under the Attribution-NonCommercial-NoDerivs (CC BY-NC-ND), which allows third parties to download the articles and share them with others as long as they credit the author and the Abstract Book, but they cannot change the content in any way or use them commercially.
**Abstract Book Citations:** Authors, Title, HemaSphere, 2026;10:(S1):pages. The abstract book DOIs can be found on the "Author Information" page.
**Disclaimer:** Articles published in the journal HemaSphere exclusively reflect the opinions of the authors. The authors are responsible for all content in their abstracts including accuracy of the facts, statements, citing resources, etc.

### (PS1561) CSF3R MEDIATES VENETOCLAX RESISTANCE IN ACUTE MYELOID LEUKEMIA VIA ACTIVATION OF THE JAK-STAT PATHWAY

**Topic:** 03. Acute myeloid leukemia - Biology & translational research

Yan Qin[1], Yue-Hui Liu[2], Jian-Hong Shi[3], Dongfeng Zeng[3], Zi-Yuan Nie*[2]

*[1]Affiliated Hospital of Hebei University, Baoding, China;[2]Department of Hematology, The Second Hospital of Hebei Medical University, Shijiazhuang, China; [3]Department of Haematology, Daping Hospital, Army Medical University, Chongqing, China;*

### Background

Venetoclax (VEN), a BCL-2 inhibitor, has significantly improved outcomes in patients with acute myeloid leukemia (AML). However, acquired resistance occurs at a high rate, limiting its long-term efficacy. Colony-stimulating factor 3 receptor (CSF3R) is a key regulator of myeloid differentiation and proliferation. Although its mutations have been well characterized in chronic neutrophilic leukemia, its role in AML, particularly in drug resistance, remains poorly understood.

### Aims

To investigate the role and underlying mechanism of CSF3R in VEN-resistant AML.

### Methods

Bone marrow mononuclear cells (BMMNCs) from VEN-sensitive and VEN-resistant AML patients were collected for single-cell RNA sequencing (scRNA-seq) using the 10x Genomics platform. CSF3R expression was validated by qRT-PCR and Western blot in patient samples and VEN-resistant cell lines (U937R, HL60R, MV4-11R). CSF3R-specific siRNA was transfected into VEN-resistant cells. Cell viability was assessed by CCK-8 assay, and apoptosis was detected by Annexin V/PI double staining flow cytometry. Expression of BCL-2 family proteins and key molecules of the JAK-STAT pathway were examined by Western blot. STAT5 nuclear translocation was observed by immunofluorescence.

### Results

Single-cell RNA sequencing revealed that CSF3R was significantly upregulated in leukemic cells from VEN-resistant AML patients compared to VEN-sensitive counterparts. qRT-PCR and Western blot validation confirmed that both mRNA and protein levels of CSF3R were significantly elevated in BMMNCs from VEN-resistant patients. Furthermore, CSF3R expression was increased in VEN-resistant cell lines (U937R, HL60R, and MV4-11R) established by concentration gradient method, compared to their parental cells. Functional assays demonstrated that phosphorylation levels of JAK2 and STAT5 were significantly higher in VEN-resistant cells than in parental cells, and immunofluorescence showed increased STAT5 nuclear translocation. Knockdown of CSF3R significantly inhibited JAK2 and STAT5 phosphorylation, reduced STAT5 nuclear translocation, and downregulated the expression of its downstream target genes MCL-1 and BCL-xL, thereby promoting apoptosis and inhibiting proliferation.

### Summary/Conclusion

This study is the first to elucidate the role of CSF3R in VEN-resistant AML. Overexpressed CSF3R mediates VEN resistance by activating the JAK-STAT pathway and upregulating MCL-1/BCL-xL/BCL-2. Targeting the CSF3R-JAK-STAT axis may represent a novel therapeutic strategy to overcome VEN resistance in AML.

**Copyright Information:** (Online) ISSN: 2572-9241
©2026 The Author(s). HemaSphere is published by John Wiley & Sons Ltd on behalf of European Hematology Association. This is an open access Abstract Book distributed under the Attribution-NonCommercial-NoDerivs (CC BY-NC-ND), which allows third parties to download the articles and share them with others as long as they credit the author and the Abstract Book, but they cannot change the content in any way or use them commercially.
**Abstract Book Citations:** Authors, Title, HemaSphere, 2026;10:(S1):pages. The abstract book DOIs can be found on the "Author Information" page.
**Disclaimer:** Articles published in the journal HemaSphere exclusively reflect the opinions of the authors. The authors are responsible for all content in their abstracts including accuracy of the facts, statements, citing resources, etc.

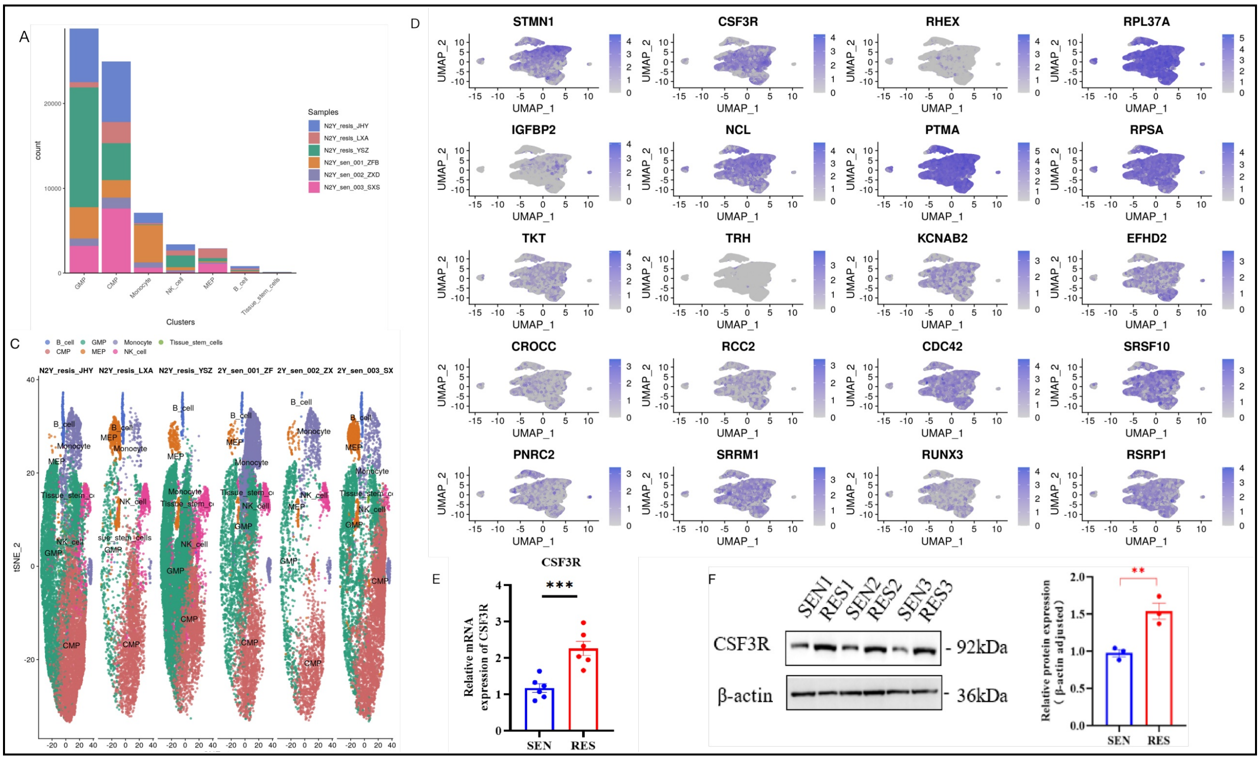

**Copyright Information:** (Online) ISSN: 2572-9241
©2026 The Author(s). HemaSphere is published by John Wiley & Sons Ltd on behalf of European Hematology Association. This is an open access Abstract Book distributed under the Attribution-NonCommercial-NoDerivs (CC BY-NC-ND), which allows third parties to download the articles and share them with others as long as they credit the author and the Abstract Book, but they cannot change the content in any way or use them commercially.
**Abstract Book Citations:** Authors, Title, HemaSphere, 2026;10:(S1):pages. The abstract book DOIs can be found on the "Author Information" page.
**Disclaimer:** Articles published in the journal HemaSphere exclusively reflect the opinions of the authors. The authors are responsible for all content in their abstracts including accuracy of the facts, statements, citing resources, etc.

### (PS1562) ULTRA-DEEP NGS MRD ASSESSMENT AROUND HSCT FOR RELAPSE PREDICTION IN AML.

**Topic:** 03. Acute myeloid leukemia - Biology & translational research

Ismael De La Iglesia San Sebastián[1], Raquel Fernández-González[1], Carlos Jimenez-Chillon[1], Miguel López-Esteban[1], Rebeca Bailen[1], Sara Fernández de Córdoba Oñate[1], Ignacio Alberto Gómez Centurión[1], Ana Maria Pérez Corral[1], Gabriela Rodríguez Macías[1], Mi Kwon[1], Ramón García-Sanz[1], Ismael Buño[1], Carolina Martinez Laperche*[1]

[1]*Hospital General Universitario Gregorio Marañón, Instituto de Investigación Sanitaria Gregorio Marañón, Hematology, Madrid, Spain;*

### Background

Relapse remains the leading cause of treatment failure after allogeneic hematopoietic stem cell transplantation (HSCT) in acute myeloid leukemia (AML). Measurable residual disease (MRD) assessment before and after HSCT is critical for relapse prediction, however, conventional techniques may be limited by both suboptimal sensitivity and the restricted number of markers assessed. Ultra-deep next-generation sequencing (UD-NGS) may improve MRD detection.

### Aims

To evaluate the impact of UD-NGS-based MRD assessment before and after HSCT on relapse prediction in AML patients and compare its performance with conventional techniques.

### Methods

We conducted a retrospective study of a consecutive cohort of AML patients who underwent HSCT at our institution between 2021-2024. A total of 63 AML patients were included. Diagnostic molecular markers were identifiable in 48/63 patients (76%). In 10/56 (18%) had no stored material available. Therefore, **38 patients** were included finally. Across 3 timepoints (pre-HSCT, day +30, and day +90 after HSCT), 114 samples were considered; adequate stored material was available for analysis in 108/114. Conventional MRD was performed using multiparametric flow cytometry (MFC) and molecular assays (quantitative PCR for *NPM1* (n=17) and *WT1* (n=15), ; six patients had no available marker for molecular monitoring.
UD-NGS was performed using SureSeq Myeloid MRD Plus NGS Panel (OGT, UK), covering hotspot exons in 13 AML-relevant genes, with a limit of detection of 0.05%. Analysis was performed with the manufacturer's proprietary software, tracking only variants identified at diagnosis. Statistical analysis was performed in RStudio.

**Copyright Information:** (Online) ISSN: 2572-9241
©2026 The Author(s). HemaSphere is published by John Wiley & Sons Ltd on behalf of European Hematology Association. This is an open access Abstract Book distributed under the Attribution-NonCommercial-NoDerivs (CC BY-NC-ND), which allows third parties to download the articles and share them with others as long as they credit the author and the Abstract Book, but they cannot change the content in any way or use them commercially.
**Abstract Book Citations:** Authors, Title, HemaSphere, 2026;10:(S1):pages. The abstract book DOIs can be found on the "Author Information" page.
**Disclaimer:** Articles published in the journal HemaSphere exclusively reflect the opinions of the authors. The authors are responsible for all content in their abstracts including accuracy of the facts, statements, citing resources, etc.

### Results

Median age was 54 years, 50% were female, and patients had received a median of two prior therapy lines. Donor type was haploidentical in 42% and HLA-identical in 58%; all patients received post-transplant cyclophosphamide. Median PFS was 23.2 months (3-57) and OS 25 months (3-61)
Median sequencing depth was 12,000 reads/base, within a range of 4,000 – 42,000. The most frequently selected molecular markers for longitudinal monitoring were *NPM1* (n=17), *FLT3*-ITD (n=17), *RUNX1* (n=9), *TP53* (n=6), *IDH2* (n=5), *FLT3*-TKD (n=5), *IDH1* (n=4), *SF3B1* (n=3), *JAK2* (n=2), and *CKIT* (n=1). 64% (24/38) of our cohort had two or more markers for the follow-up.
In total, 25 samples were UD-NGS-positive (12/PRE, 4/+30 and 9/+90, , of which 12 were negative by conventional techniques. Variants most frequently associated with UD-NGS+/conventional– discordance included *FLT3*-ITD, *FLT3*-TKD, *IDH2*, RUNX1, and *JAK2*. Conversely, **13** samples were positive by conventional techniques but negative by UD-NGS; mainly driven by *NPM1*.
Across all assessed time points (pre-HSCT, day +30, and day +90), MRD positivity by conventional methods (MFC and/or *NPM1*/ *WT1* qPCR) was associated with inferior PFS (HR 3.4, 95% CI 1.06–10.9; p=0.029). MRD positivity by UD-NGS showed a even stronger association with poor PFS (HR 12.7, 95% CI 2.8–57; p<0.001), suggesting improved prognostic stratification compared with conventional techniques (Figure 1A). The figure 1B summarizes longitudinal follow-up across all patients by relapse status.

### Summary/Conclusion

UD-NGS identified additional MRD-positive cases missed by conventional methods and provided substantially stronger risk discrimination for post-HSCT progression across pre- and early post-transplant time points. These findings support incorporating UD-NGS into longitudinal MRD monitoring to improve prognostic assessment after HSCT in AML.

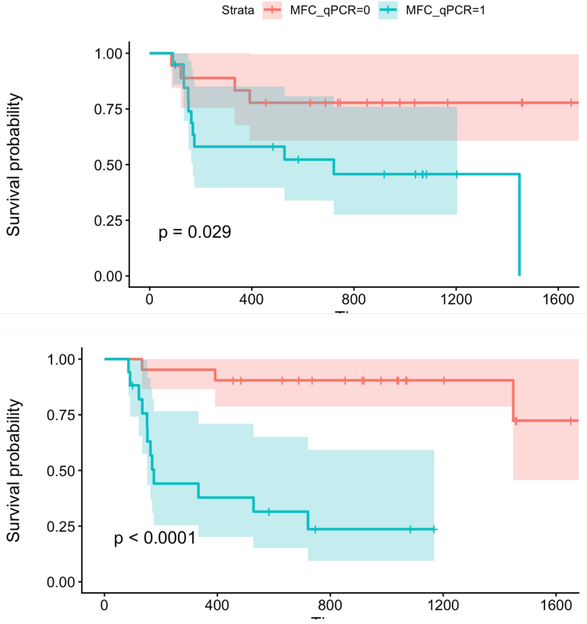

**Figure 1.** (A) Progression-free survival (PFS) stratified by conventional MRD status (MFC and/or NPM1/WT1 qPCR positive vs negative). (B) PFS stratified by UD-NGS MRD status (positive vs negative).

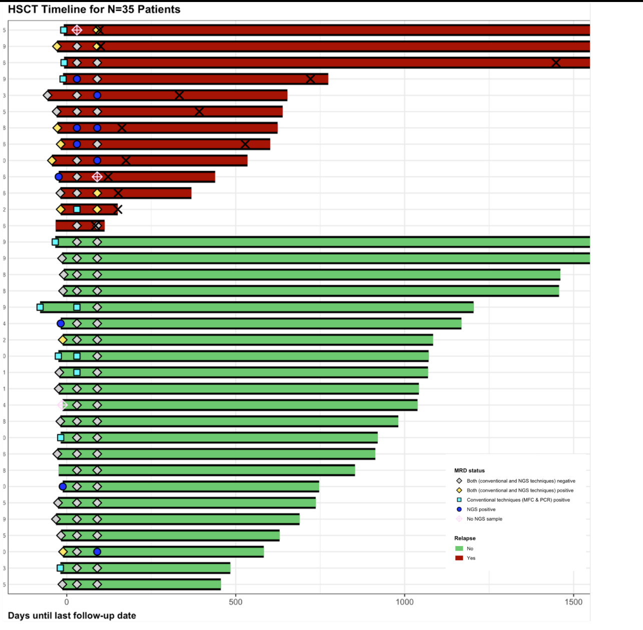

**Figure 2.** Longitudinal follow-up of MRD across all patients, displayed over time and grouped by relapse status.

**Copyright Information:** (Online) ISSN: 2572-9241
©2026 The Author(s). HemaSphere is published by John Wiley & Sons Ltd on behalf of European Hematology Association. This is an open access Abstract Book distributed under the Attribution-NonCommercial-NoDerivs (CC BY-NC-ND), which allows third parties to download the articles and share them with others as long as they credit the author and the Abstract Book, but they cannot change the content in any way or use them commercially.
**Abstract Book Citations:** Authors, Title, HemaSphere, 2026;10:(S1):pages. The abstract book DOIs can be found on the "Author Information" page.
**Disclaimer:** Articles published in the journal HemaSphere exclusively reflect the opinions of the authors. The authors are responsible for all content in their abstracts including accuracy of the facts, statements, citing resources, etc.

### (PS1563) TARGETING PFKFB4 TO DISRUPT ANTIOXIDANT HOMEOSTASIS AND OXPHOS DEPENDENCY IN ACUTE MYELOID LEUKEMIA

**Topic:** 03. Acute myeloid leukemia - Biology & translational research

Aysegul Erdem*[1, 2], Chie Yanase[2], Francesco Caligiore[3], Youzhong Liu[1], Claudia Morganti[2], Lotfi Laaouimir[1], Guido Bommer[3], Keisuke Ito[2], Nick van Gastel[1]

*[1]de Duve Institute, UCLouvain, Cellular Metabolism and Microenvironment Laboratory, Brussels, Belgium; [2]Ruth L. and David S. Gottesman Institute for Stem Cell and Regenerative Medicine Research, Albert Einstein College of Medicine, Bronx, United States of America; [3]de Duve Institute, UCLouvain, Biochemistry and Metabolic Research Group, Brussels, Belgium;*

### Background

Acute myeloid leukemia (AML) cells display an abnormal reliance on mitochondrial oxidative phosphorylation (OXPHOS), which is supported by a robust antioxidant system that promotes survival and contributes to chemoresistance. Although complete inhibition of OXPHOS can effectively eliminate AML cells, this strategy presents clinical challenges due to toxicity and limited therapeutic windows. Therefore, alternative approaches that indirectly disrupt mitochondrial metabolism and redox homeostasis are needed. The pentose phosphate pathway (PPP) is closely linked to mitochondrial function, as it generates NADPH required for antioxidant defense and ribose-5-phosphate necessary for nucleotide synthesis—two processes essential for leukemic cell proliferation and persistence.

### Aims

Here, we aimed to identify PFKFB4 (6-phosphofructo-2-kinase/fructose-2,6-biphosphatase 4), an enzyme that redirects the glycolytic flux towards the PPP, as a governer of this redox-OXPHOS axis in AML and to determine whether targeting this enzyme could expose a therapeutic vulnerability.

### Methods

We investigated the role of PFKFB4 using both pharmacological inhibition and genetic knockdown approaches in AML cell lines and primary patient samples (n = 19). Cellular viability, apoptosis, and mitochondrial function were assessed following PFKFB4 targeting. To characterize metabolic consequences, we performed metabolomic profiling, metabolic flux analysis, and RNA sequencing. Changes in antioxidant capacity and mitochondrial parameters were evaluated alongside PPP activity. To further probe the role of NADPH in AML survival, intracellular NADPH was depleted using *E.coli* soluble transhydrogenase (EcSTH). Finally, therapeutic efficacy was evaluated *in vivo* using an MLL-AF9–driven AML mouse model treated with the PFKFB4 inhibitor 5MPN, alone or in combination with induction chemotherapy

### Results

Inhibition or knockdown of PFKFB4 significantly reduced viability and induced apoptosis in AML cell lines and primary patient samples. Integrated metabolomic, fluxomic, and transcriptomic analyses revealed that targeting PFKFB4 disrupted cellular redox balance, resulting in NADPH depletion, impaired OXPHOS activity, and reduced mitochondrial volume. Genetic silencing of PFKFB4 phenocopied the pharmacological inhibition, producing comparable defects in mitochondrial respiration and PPP metabolism. Importantly, further depletion of intracellular NADPH through EcSTH expression markedly enhanced apoptosis induced by PFKFB4 inhibition, highlighting the central role of redox homeostasis in AML cell survival. In vivo, treatment with the PFKFB4 inhibitor 5MPN significantly prolonged survival in the MLL-AF9 AML mouse model. Therapeutic efficacy was further improved when combined with induction chemotherapy, suggesting effective targeting of chemoresistant leukemic populations.

**Copyright Information:** (Online) ISSN: 2572-9241
©2026 The Author(s). HemaSphere is published by John Wiley & Sons Ltd on behalf of European Hematology Association. This is an open access Abstract Book distributed under the Attribution-NonCommercial-NoDerivs (CC BY-NC-ND), which allows third parties to download the articles and share them with others as long as they credit the author and the Abstract Book, but they cannot change the content in any way or use them commercially.
**Abstract Book Citations:** Authors, Title, HemaSphere, 2026;10:(S1):pages. The abstract book DOIs can be found on the "Author Information" page.
**Disclaimer:** Articles published in the journal HemaSphere exclusively reflect the opinions of the authors. The authors are responsible for all content in their abstracts including accuracy of the facts, statements, citing resources, etc.

**Summary/Conclusion**

Together, these findings identify PFKFB4 as a key regulator linking glycolytic flux, the PPP, and mitochondrial redox control in AML. By sustaining NADPH production and supporting OXPHOS-dependent metabolism, PFKFB4 enables leukemic cells to maintain antioxidant defenses and survive therapeutic stress. Targeting PFKFB4 disrupts this metabolic axis, leading to mitochondrial dysfunction, oxidative imbalance, and leukemic cell death. Our results highlight PFKFB4 as a promising metabolic vulnerability and provide a rationale for therapeutic strategies aimed at disrupting the OXPHOS–redox network to overcome chemoresistance in AML.

**Copyright Information:** (Online) ISSN: 2572-9241
©2026 The Author(s). HemaSphere is published by John Wiley & Sons Ltd on behalf of European Hematology Association. This is an open access Abstract Book distributed under the Attribution-NonCommercial-NoDerivs (CC BY-NC-ND), which allows third parties to download the articles and share them with others as long as they credit the author and the Abstract Book, but they cannot change the content in any way or use them commercially.
**Abstract Book Citations:** Authors, Title, HemaSphere, 2026;10:(S1):pages. The abstract book DOIs can be found on the "Author Information" page.
**Disclaimer:** Articles published in the journal HemaSphere exclusively reflect the opinions of the authors. The authors are responsible for all content in their abstracts including accuracy of the facts, statements, citing resources, etc.

### (PS1564) UNRAVELLING THE MECHANISM OF CHEMORESISTANCE IN AML: THE ROLE OF ATF4-PPP1R15A AXIS

**Topic:** 03. Acute myeloid leukemia - Biology & translational research

Christina Sotiropoulou[1], Christos Kontos[1], Anthi Bouchla[2], Theodora Chatzilygeroudi[2], Papageorgiou Sotirios[2], Pinelopi Vryttia[2], Christina Apostolopoulou[2], Ioulia Markaki[2], Georgia Karamanou[2], Theodoros Karantanos[3], Vasiliki Pappa*[2]

*[1]National and Kapodistrian University of Athens, Department of Biochemistry and Molecular Biology, Faculty of Biology, ATHENS, Greece; [2]Attikon University General Hospital, Hematology Unit, Second Department of Internal Medicine and Research Institute, ATHENS, Greece; [3]Johns Hopkins University, Hematologic Malignancies Sidney Kimmel Comprehensive Cancer Center, Baltimore, MD, United States of America;*

### Background

Acute myeloid leukemia (AML) is often characterized by relapses driven in part by adaptive resistance of leukemic cells to chemotherapy-induced stress. The DNA Damage Response (DDR) promotes the survival of leukemic cells and contributes to chemoresistance. *PPP1R15A* (*GADD34*), a DDR gene, is a stress-induced phosphatase regulatory subunit and direct transcriptional target of ATF4. Our group has shown that *PPP1R15A* is upregulated in AML blasts and associated with chemotherapy resistance.

### Aims

This study aims to investigate the mechanistic contribution of *PPP1R15A* to chemotherapy-induced stress in AML and to evaluate whether pharmacologic inhibition of its upstream regulator, ATF4, impairs this adaptive pathway and enhances chemosensitivity.

### Methods

TF-1 AML cells were genetically modified to generate *PPP1R15A* knockout clones using lentiviral CRISPR/Cas9 (pLKO.1-puro). After puromycin selection, clonal cells were qPCR validated for target gene expression and treated with idarubicin (10 nM) or cytarabine (100 nM) for 24 h. RNA was extracted, rRNA-depleted, and sequenced (DNBSEQ-G400, PE100). The data were aligned to GRCh38 with STAR, and differential expression analysis was then performed with DESeq2, followed by GO/KEGG enrichment. ATF4 inhibition studies were performed in MOLM-13 and KASUMI-1 cells. Dose–response assays were conducted to determine 24-hour $IC_{50}$ values. Cells were subsequently treated with 0.5×, 1×, and 2× $IC_{50}$ concentrations of the ATF4 inhibitor for 6 and 12 hours. Whole-cell lysates were then subjected to Western blot analysis to determine ATF4 protein levels, p-eIF2α and total eIF2α. Transcriptional output was assessed by RT-qPCR for ATF4 target genes, including *PPP1R15A*, *DDIT3*, *ASNS*, and *ATF3*. Specifically, we measured the relative expression levels using the 2^−ΔΔCt method.

**Copyright Information:** (Online) ISSN: 2572-9241
©2026 The Author(s). HemaSphere is published by John Wiley & Sons Ltd on behalf of European Hematology Association. This is an open access Abstract Book distributed under the Attribution-NonCommercial-NoDerivs (CC BY-NC-ND), which allows third parties to download the articles and share them with others as long as they credit the author and the Abstract Book, but they cannot change the content in any way or use them commercially.
**Abstract Book Citations:** Authors, Title, HemaSphere, 2026;10:(S1):pages. The abstract book DOIs can be found on the "Author Information" page.
**Disclaimer:** Articles published in the journal HemaSphere exclusively reflect the opinions of the authors. The authors are responsible for all content in their abstracts including accuracy of the facts, statements, citing resources, etc.

### Results

The results of RNA sequencing of PPP1R15A-knockout TF-1 cells treated with idarubicin or cytarabine revealed remodeling of DDR and stress-related pathways. Compared to treated control cells, *PPP1R15A*-knockout cells exhibited significant downregulation of core DDR regulators, including *TP53*, *RAD51*, *PARP1*, *MSH2*, and *XRCC1*. Cell cycle checkpoint mediators such as *CDK1* and *CDC25C* were also reduced. Cytarabine-treated *PPP1R15A*-knockout cells showed suppression of mitochondrial oxidative phosphorylation genes (*MT-ND4*, *MT-CO2*, *MT-CO3*) and increased expression of stress regulators *DDIT4* and *ATF3*. Idarubicin treatment induced stress-associated transcripts such as *FTL* and *EEF1A1*, while reducing replication-associated genes, including *MCM2* and *PCNA*, respectively. Pathway enrichment analysis confirmed significant downregulation of p53 signaling, homologous recombination, mismatch repair, and oxidative phosphorylation pathways, alongside activation of unfolded protein response and ER stress–mediated apoptosis. ATF4 targeting significantly reduced AML cell viability, with $IC_{50}$ values ranging from 45–55 nM in MOLM-13 and KASUMI-1 cells. Pharmacologic inhibition led to a dose-dependent decrease in ATF4 protein levels while p-eIF2α and total eIF2α remained unchanged. RT-qPCR analysis revealed suppression of canonical ATF4 transcriptional targets, including *PPP1R15A*, *DDIT3* (*CHOP*), *ASNS*, and *ATF3*, confirming functional disruption of the ATF4-dependent stress-adaptive program.

### Summary/Conclusion

Our findings unravel that the ATF4–*PPP1R15A* axis sustains DDR, metabolic stability, and stress adaptation in AML. Its disruption impairs chemoresistance and sensitizes leukemic cells to genotoxic therapy.

**Copyright Information:** (Online) ISSN: 2572-9241
©2026 The Author(s). HemaSphere is published by John Wiley & Sons Ltd on behalf of European Hematology Association. This is an open access Abstract Book distributed under the Attribution-NonCommercial-NoDerivs (CC BY-NC-ND), which allows third parties to download the articles and share them with others as long as they credit the author and the Abstract Book, but they cannot change the content in any way or use them commercially.
**Abstract Book Citations:** Authors, Title, HemaSphere, 2026;10:(S1):pages. The abstract book DOIs can be found on the "Author Information" page.
**Disclaimer:** Articles published in the journal HemaSphere exclusively reflect the opinions of the authors. The authors are responsible for all content in their abstracts including accuracy of the facts, statements, citing resources, etc.

### (PS1565) TARGETING INFLAMMATION VIA P38MAPK INHIBITION TO IMPROVE AZACITIDINE EFFICACY IN AGED AML

**Topic:** 03. Acute myeloid leukemia - Biology & translational research

Ana Vitlic*[1], Wenjing Yang[1], Zimeng Jia[1], Shiva Nickaria[1], Sara Menegatti[1], Liam Tremble[2], Paula Maguire[2], Simona Valletta[1]

*[1]University of Manchester, Division of Cancer Sciences, Manchester, United Kingdom;[2]Poolbeg Pharma, London, United Kingdom;*

### Background

Acute myeloid leukaemia (AML) is a disease of elderly, with a median age at diagnosis > 65 years. Aging is accompanied by systemic low-grade inflammation and profound changes within the bone marrow microenvironment (BMM), alongside comorbidities that limit tolerance to standard treatments, such as azacitidine (AZA). Targeting age-related inflammatory pathways may enhance therapeutic efficacy. Appropriate aged preclinical AML models with preserved BMM that reflect age-associated changes are key for evaluating novel treatment combinations.

### Aims

To investigate the contribution of aging to immune-BMM interactions in AML and assess the therapeutic efficacy of AZA combined with the novel anti-inflammatory p38MAPK inhibitor POLB 001 (Poolbeg Pharma) in an aged AML mouse model.

### Methods

A non-conditioned MLL-AF9 AML model preserving the bone marrow niche was used. Old (18-month-old) and young (8-10 weeks old) mice were injected with MLL-AF9 and analysed 21 days post-injection for engraftment, immune populations, and niche composition including central BM (CBM) and bone lining (BL) by flow cytometry.
Separately, in aged AML mice, AZA and/or POLB 001 were administered for 5 days starting 15 days post-engraftment, with assessment on Day 20. Engraftment was analysed and immune components within BMM were phenotyped. Functional co-culture assays using splenocytes from MLL-AF9 mice were used to evaluate $CD8^{+}$ T-cell effector function and memory.

### Results

Comparable engraftment levels were observed in young and old mice. Young AML mice exhibited higher frequencies of B cells, Ly-$6C^{+}$ effector $CD8^{+}$ T cells and NKT cells in the BMM, whereas aged mice showed increased Tregs. CBM of young AML mice showed higher proportion of LeptinR+ mesenchymal stem cells (MSC), whilst aged leukaemic mice showed an increased vascular endothelial:MSC ratio in the BL niche, consistent with poorer prognosis features.
In aged AML mice, AZA induced a robust therapeutic response. POLB 001 was well tolerated both alone and in combination. The combination significantly reduced engraftment compared with AZA alone, suggesting synergistic effects. High dimensional immune profiling revealed marked immune remodelling in AZA-treated groups characterised by reduced inflammatory monocytes and neutrophils and increased B cells. Combination therapy further increased effector $CD8^{+}$ T cells, NK and NKT populations compared with AZA alone. Functionally, CD8+ T cells isolated from spleens of aged leukaemic mice retained the capacity to produce IFN-g following in vitro rechallenge with MLL-AF9.

**Copyright Information:** (Online) ISSN: 2572-9241
©2026 The Author(s). HemaSphere is published by John Wiley & Sons Ltd on behalf of European Hematology Association. This is an open access Abstract Book distributed under the Attribution-NonCommercial-NoDerivs (CC BY-NC-ND), which allows third parties to download the articles and share them with others as long as they credit the author and the Abstract Book, but they cannot change the content in any way or use them commercially.
**Abstract Book Citations:** Authors, Title, HemaSphere, 2026;10:(S1):pages. The abstract book DOIs can be found on the "Author Information" page.
**Disclaimer:** Articles published in the journal HemaSphere exclusively reflect the opinions of the authors. The authors are responsible for all content in their abstracts including accuracy of the facts, statements, citing resources, etc.

### Summary/Conclusion

This non-conditioned aged AML model recapitulates key age-associated alterations in the BMM and immune landscape, providing a clinically relevant platform for therapeutic testing. Addition of the p38MAPK inhibitor POLB 001 to AZA enhances immunomodulatory effects and reduces leukaemic burden in aged AML, supporting its potential as a combinatorial strategy to improve outcomes in elderly patients.

**Copyright Information:** (Online) ISSN: 2572-9241
©2026 The Author(s). HemaSphere is published by John Wiley & Sons Ltd on behalf of European Hematology Association. This is an open access Abstract Book distributed under the Attribution-NonCommercial-NoDerivs (CC BY-NC-ND), which allows third parties to download the articles and share them with others as long as they credit the author and the Abstract Book, but they cannot change the content in any way or use them commercially.
**Abstract Book Citations:** Authors, Title, HemaSphere, 2026;10:(S1):pages. The abstract book DOIs can be found on the "Author Information" page.
**Disclaimer:** Articles published in the journal HemaSphere exclusively reflect the opinions of the authors. The authors are responsible for all content in their abstracts including accuracy of the facts, statements, citing resources, etc.

### (PS1566) THERAPEUTIC EFFICACY AND SAFETY OF KIT-TARGETED APTAMER–MONOMETHYL AURISTATIN E CONJUGATE IN ACUTE MYELOID LEUKEMIA

**Topic:** 03. Acute myeloid leukemia - Biology & translational research

Siyu Lin[1], Tingting Liu*[1]

[1]*Renji Hospital, School of Medicine, Shanghai JiaoTong University, Department of Hematology, Shanghai, China;*

### Background

Aptamer-drug conjugates (ApDCs) offer advantages including low immunogenicity, high affinity, and rapid in vivo absorption, making them promising agents for cancer therapy.

### Aims

This study aimed to synthesize a KIT-targeting aptamer-drug conjugate linked to monomethyl auristatin E (MMAE) and to evaluate its therapeutic efficacy and safety in KIT-positive acute myeloid leukemia (AML) in vitro and in vivo.

### Methods

A KIT-targeted aptamer was identified by SELEX and then covalently linked to MMAE to generate KIT-MMAE. Its therapeutic effect and safety were assessed in KIT-positive AML cell lines, subcutaneous xenograft models, and patient-derived xenograft (PDX) models.

### Results

KIT-MMAE specifically recognizes and binds to the KIT-positive AML cell line Kasumi-1, but shows no significant binding to the KIT-negative HL-60 cells. Treatment with specific endocytosis pathway inhibitors confirmed that KIT-MMAE primarily enters target cells via the caveolae-mediated endocytosis pathway. KIT-MMAE significantly inhibited the proliferation of KIT-positive Kasumi-1 cells (IC50 decreased by 50% compared to the control group) and promoted their apoptosis. Cy5-labeled KIT-MMAE accumulated specifically in tumors of xenograft mice ($p < 0.05$ vs. control), whereas no specific accumulation was observed with control Cy5-Conseq-MMAE in the fluorescence images obtained with an in vivo imaging system. Compared with controls, KIT-MMAE-treated mice exhibited slower tumor growth and prolonged survival ($p < 0.05$). No significant histopathological abnormalities were observed in the heart, liver, spleen, lungs, or kidneys of treated mice.

### Summary/Conclusion

KIT-MMAE demonstrates high specificity, potent cytotoxicity, and an excellent safety profile against KIT-positive AML cells in vitro and in vivo, offering a promising precision therapy for KIT-positive AML.

**Copyright Information:** (Online) ISSN: 2572-9241
©2026 The Author(s). HemaSphere is published by John Wiley & Sons Ltd on behalf of European Hematology Association. This is an open access Abstract Book distributed under the Attribution-NonCommercial-NoDerivs (CC BY-NC-ND), which allows third parties to download the articles and share them with others as long as they credit the author and the Abstract Book, but they cannot change the content in any way or use them commercially.
**Abstract Book Citations:** Authors, Title, HemaSphere, 2026;10:(S1):pages. The abstract book DOIs can be found on the "Author Information" page.
**Disclaimer:** Articles published in the journal HemaSphere exclusively reflect the opinions of the authors. The authors are responsible for all content in their abstracts including accuracy of the facts, statements, citing resources, etc.

### (PS1567) PROTEOMICS REVEALS HSP90-ENRICHED FERROEVS ASSOCIATED WITH SERIAL TRANSMISSION OF FERROPTOSIS IN AML CELLS

**Topic:** 03. Acute myeloid leukemia - Biology & translational research

Hiroki Doi*[1], Sumie Fujii[2, 3], Masaya Hirayama[4], Kazuya Shiogama[4], Ryota Miyachi[3], Hideaki Matsuura[1, 3], Yasuo Miura[1, 2, 3]

*[1]Department of Cellular and Molecular Biology, Fujita Health University, Toyoake, Japan;[2]Department of Transfusion Medicine and Cell Therapy, Fujita Health University, Toyoake, Japan; [3]Department of Blood Transfusion, Fujita Health University, Toyoake, Japan; [4]Department of Pathology and Cytopathology, Fujita Health University, Toyoake, Japan;*

### Background

Ferroptosis is a regulated cell death mode distinct from apoptosis. Although ferroptosis contributes to tumor suppression, how ferroptotic cells communicate with neighboring cells and propagate death signals remains unclear. Extracellular vesicles (EVs) mediate intercellular communication by transferring bioactive cargo between cells. Here we defined EVs released from ferroptotic cells as FerroEVs and investigated their transmission potential and cargo determinants by quantitative proteomics.

### Aims

To characterize FerroEVs and elucidate the mechanisms of intercellular ferroptotic signaling using quantitative proteomic profiling.

### Methods

Human leukemia U937 cells were treated with erastin (1–50 µM, 24 h) with or without ferrostatin-1 (1–20 µM). Cell viability, intracellular ROS, mitochondrial membrane potential, and lipid peroxidation were quantified using standard assays. EVs were isolated from culture supernatants by differential ultracentrifugation and characterized by nanoparticle tracking, CD63-capture ELISA, and transmission electron microscopy (TEM). For recipient-cell assays, EVs doses were normalized to EVs protein levels quantified by ELISA. Proteomic differences between FerroEVs and control EVs were analyzed by label-free liquid chromatography–tandem mass spectrometry (LC–MS/MS).

### Results

Erastin induced ferroptosis in U937 cells in a dose-dependent manner ($IC_{50}$ = 19.6 µM). TEM revealed shrunken, electron-dense mitochondria that were reversed by ferrostatin-1. FerroEVs were $CD63^+$ exosome-like particles with a mean diameter of approximately 200 nm. When administered to naïve cells, FerroEVs significantly reduced viability (78.0 ± 3.3% of control; $p < 0.01$), increased intracellular ROS, and enhanced lipid peroxidation without elevating Annexin V positivity, indicating non-apoptotic mode of cell death. Notably, ferroptosis did not end in the first recipient population: Recipient cells that were exposed to the initial FerroEVs and underwent ferroptosis subsequently released a second generation of EVs (secondary FerroEVs). These secondary FerroEVs exhibited comparable ferroptosis-inducing capacity and further reduced the viability of new target cells. These findings suggest a serial transmission of ferroptotic stress via EV-associated factors. Label-free LC–MS/MS proteomics identified 3,794 proteins, with HSP90 showing the most prominent enrichment in FerroEVs (3.63-fold, $p = 0.036$). Given that HSP90 stabilizes FACL4 and promotes GPX4 degradation through chaperone-mediated autophagy, its marked enrichment within FerroEVs suggests a pivotal role in ferroptotic signaling and mediating its intercellular transmission.

**Copyright Information:** (Online) ISSN: 2572-9241
©2026 The Author(s). HemaSphere is published by John Wiley & Sons Ltd on behalf of European Hematology Association. This is an open access Abstract Book distributed under the Attribution-NonCommercial-NoDerivs (CC BY-NC-ND), which allows third parties to download the articles and share them with others as long as they credit the author and the Abstract Book, but they cannot change the content in any way or use them commercially.
**Abstract Book Citations:** Authors, Title, HemaSphere, 2026;10:(S1):pages. The abstract book DOIs can be found on the "Author Information" page.
**Disclaimer:** Articles published in the journal HemaSphere exclusively reflect the opinions of the authors. The authors are responsible for all content in their abstracts including accuracy of the facts, statements, citing resources, etc.

### Summary/Conclusion

Ferroptosis-induced leukemia cells release CD63⁺ FerroEVs that transmit ferroptotic stress to neighboring cells and trigger secondary FerroEVs release, supporting serial transmission of ferroptosis. Proteomics identified HSP90 enrichment in FerroEV cargo, nominating an HSP90–FerroEVs axis as a candidate mediator of intercellular ferroptosis and a potential therapeutic target.

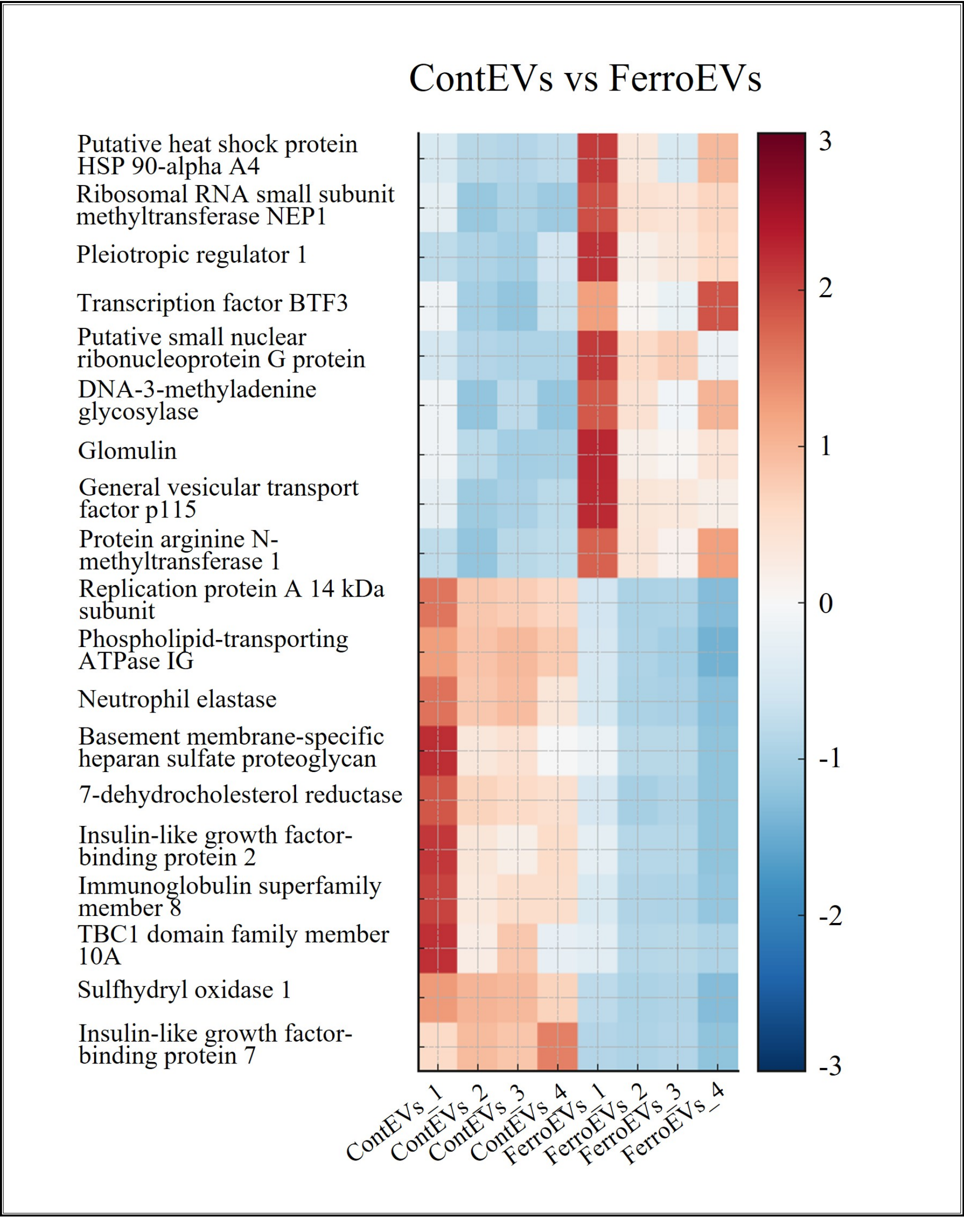

**Copyright Information:** (Online) ISSN: 2572-9241
©2026 The Author(s). HemaSphere is published by John Wiley & Sons Ltd on behalf of European Hematology Association. This is an open access Abstract Book distributed under the Attribution-NonCommercial-NoDerivs (CC BY-NC-ND), which allows third parties to download the articles and share them with others as long as they credit the author and the Abstract Book, but they cannot change the content in any way or use them commercially.
**Abstract Book Citations:** Authors, Title, HemaSphere, 2026;10:(S1):pages. The abstract book DOIs can be found on the "Author Information" page.
**Disclaimer:** Articles published in the journal HemaSphere exclusively reflect the opinions of the authors. The authors are responsible for all content in their abstracts including accuracy of the facts, statements, citing resources, etc.

### (PS1568) UNLOCKING VULNERABILITIES: BCL2 INHIBITORS TARGET BCOR-MUTATED AML

**Topic:** 03. Acute myeloid leukemia - Biology & translational research

Fredéric Courtier[1], Maxence Bourgoin[2], Remy Castellano[3], Benoit Goutorbe[1], Norbert Vey[1], Yves Collette[3], Arnaud Jacquel[2], Sandrine Roulland[4], Sylvain Garciaz*[1]

*[1]Institut Paoli-Calmettes, Marseille, France; [2]C3M, Nice, France; [3]CRCM, Marseille, France; [4]CIML, Marseille, France;*

**Background**

Acute myeloid leukaemia (AML) results from genetic mutations, characterised by abnormal cell growth that disrupts normal blood cell production. Treatment traditionally involves chemotherapy and stem cell transplants, but newer therapies venetoclax have recently been introduced. Venetoclax is a drug that inhibits BCL-2, thereby increasing the activation of BAX and BAK, which leads to apoptosis. However, some patients develop resistance to venetoclax due to mutations such as TP53, RAS or BAX. In contrast, mutations in NPM1 and IDH2 are consistently linked to favourable prognosis according to recent classification. Despite this, there remains an urgent need to refine and expand the identification of predictive biomarkers for venetoclax responsiveness, enabling more tailored and effective treatment strategies for AML patients.

**Aims**

to investigate which genetic alterations impact resistance or sensitivity to venetoclax, and to pinpoint biomarkers that predict venetoclax responsiveness in AML patients

**Methods**

We utilised a whole genome CRISPR loss of function screen employing the Brunello library on MV4;11 AML cell line and confirmed our results through additional independent validation techniques.

**Results**

Consistent with findings from previous CRISPR screen studies, our results demonstrate that deletion of TP53 and BAX leads to resistance against venetoclax. Notably, among the most prominent genes that enhance sensitivity, we observed that deletion of BCOR is linked to an increased response to venetoclax (**Figure**). Secondary mutations are generally associated with intermediate outcomes following venetoclax treatment, but their individual effects on response remain unclear. BCOR loss-of-function mutations are found in around 5% of AML cases. BCOR, located on Xp11.4, acts as a BCL6 corepressor and is part of the PRC1.1 complex, which represses gene expression by ubiquitinylating H2AK119. To confirm this observation, we created MV4-11 cells with BCOR knockout (KO) and cells with a non-targeted gene (NT), then conducted a competitive assay using either venetoclax treatment or the vehicle control (DMSO). Knocking out BCOR made the MV4-11 cell line 2-fold more susceptible to venetoclax. Subsequently, we utilised the HEMATO-BIO-IPC 2013-01 cohort for validation purposes. This cohort comprises approximately 650 individuals diagnosed with AML, ALL, and MDS, for whom clinical data and samples were collected at diagnosis, during complete remission, and at relapse. Out of these, 392 patients had AML, with next-generation sequencing (NGS) and BCOR status available for 307 cases. Additional analyses accessible for this cohort include RNA sequencing, drug sensitivity resistance profiling (DRSP), and BH3 profiling. DSRP found that BCORmut samples were 4-fold more sensitive to BCL2 inhibition and BH3 profiling confirms accurate BCL2 dependency revealed by mitochondrial depolarization to low dose BAD peptide treatment. In contrast, no BCORmut samples had MCL1 dependency. RNA seq identified that BCOR mutated have an higher expression of BCL-2/BCL2-xL and lower or same expression of MCL-1/BCL2-W/BCL2A1 than the non BCOR mutated counterpart. Transcriptionnal response to venetoclax in BCOR KO MV4:11 cells was associated with a differencial proapoptotic signature.

**Copyright Information:** (Online) ISSN: 2572-9241
©2026 The Author(s). HemaSphere is published by John Wiley & Sons Ltd on behalf of European Hematology Association. This is an open access Abstract Book distributed under the Attribution-NonCommercial-NoDerivs (CC BY-NC-ND), which allows third parties to download the articles and share them with others as long as they credit the author and the Abstract Book, but they cannot change the content in any way or use them commercially.
**Abstract Book Citations:** Authors, Title, HemaSphere, 2026;10:(S1):pages. The abstract book DOIs can be found on the "Author Information" page.
**Disclaimer:** Articles published in the journal HemaSphere exclusively reflect the opinions of the authors. The authors are responsible for all content in their abstracts including accuracy of the facts, statements, citing resources, etc.

**Summary/Conclusion**

AML cases with BCOR mutations exhibit a distinctive proapoptotic vulnerability, which can be leveraged to guide the choice of targeted therapies.

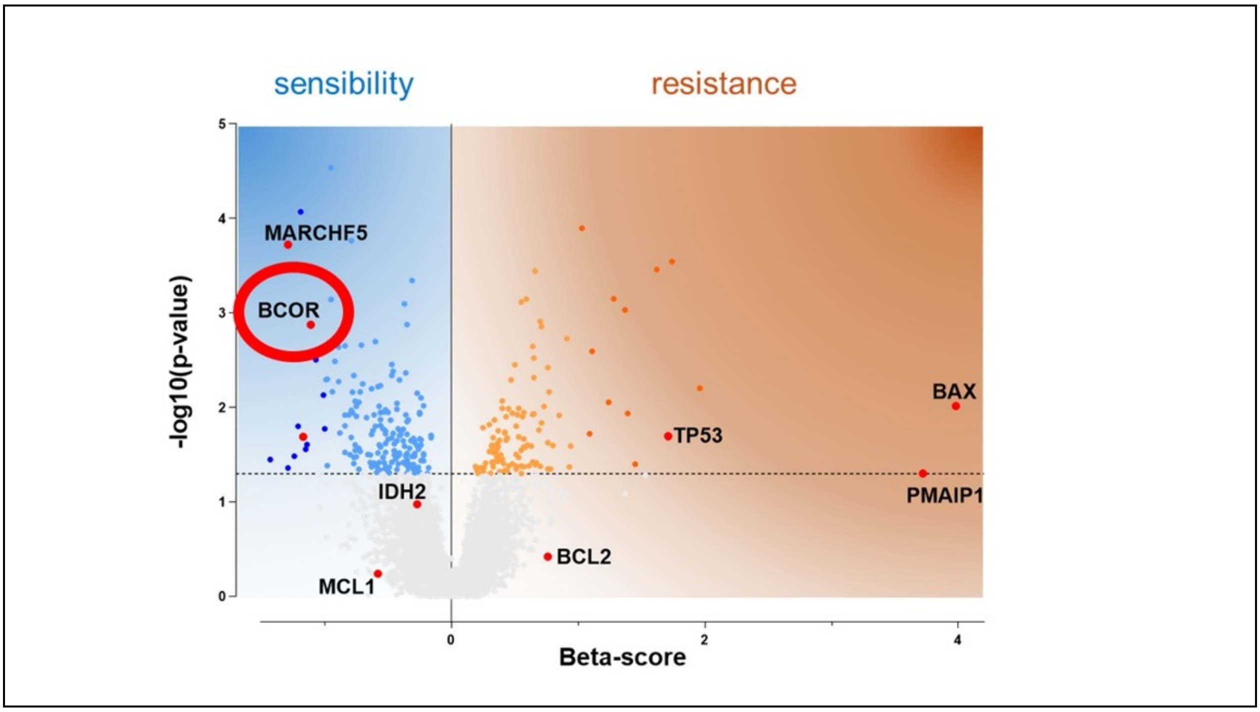

**Copyright Information:** (Online) ISSN: 2572-9241
©2026 The Author(s). HemaSphere is published by John Wiley & Sons Ltd on behalf of European Hematology Association. This is an open access Abstract Book distributed under the Attribution-NonCommercial-NoDerivs (CC BY-NC-ND), which allows third parties to download the articles and share them with others as long as they credit the author and the Abstract Book, but they cannot change the content in any way or use them commercially.
**Abstract Book Citations:** Authors, Title, HemaSphere, 2026;10:(S1):pages. The abstract book DOIs can be found on the "Author Information" page.
**Disclaimer:** Articles published in the journal HemaSphere exclusively reflect the opinions of the authors. The authors are responsible for all content in their abstracts including accuracy of the facts, statements, citing resources, etc.

### (PS1569) EFFECTS AND MECHANISTIC STUDY OF ARTESUNATE IN ACUTE MYELOID LEUKEMIA HARBORING SRSF2 P95H MUTATIONS

**Topic:** 03. Acute myeloid leukemia - Biology & translational research

Jiaxin Cheng*[1], Dengju Li[1]

[1]*Huazhong University of Science and Technology, Tongji Hospital, Wuhan, China;*

### Background

Acute myeloid leukemia (AML) is the most common acute leukemia in adults, representing a group of hematologic malignancies characterized by poor prognosis and high heterogeneity. The overall 5-year survival rate for adult patients is less than 30%. In recent years, genetic characteristics have been recognized as one of the most critical factors influencing the prognosis of AML patients. Among these, SRSF2 mutations, as the most common spliceosome mutation, indicate an unfavorable prognosis in AML patients. Therefore, exploring novel drugs in this field holds promise for providing more treatment options for AML patients.

### Aims

Artesunate, an antimalarial agent, has shown emerging anti-cancer potential. Here, we investigated its efficacy and mechanism against SRSF2-mutant AML.

### Methods

We utilized the CRISPR/Cas9 system to construct SRSF2-mutant cell lines. Following in vitro treatment of AML cell lines with artesunate, we assessed proliferation efficiency using the CCK-8 assay and measured apoptosis rates and cell cycle distribution via flow cytometry. To evaluate gene expression changes, RNA sequencing and qPCR were performed before and after drug treatment, and fluorescent probes were employed to detect cellular reactive oxygen species (ROS) levels.

**Copyright Information:** (Online) ISSN: 2572-9241
©2026 The Author(s). HemaSphere is published by John Wiley & Sons Ltd on behalf of European Hematology Association. This is an open access Abstract Book distributed under the Attribution-NonCommercial-NoDerivs (CC BY-NC-ND), which allows third parties to download the articles and share them with others as long as they credit the author and the Abstract Book, but they cannot change the content in any way or use them commercially.
**Abstract Book Citations:** Authors, Title, HemaSphere, 2026;10:(S1):pages. The abstract book DOIs can be found on the "Author Information" page.
**Disclaimer:** Articles published in the journal HemaSphere exclusively reflect the opinions of the authors. The authors are responsible for all content in their abstracts including accuracy of the facts, statements, citing resources, etc.

### Results

Our study demonstrates that artesunate exhibits antitumor effects against SRSF2-mutant AML cell lines. Engineered SRSF2 P95H mutant cell lines (SKM-1, Molm13, OCI-AML3) exhibited enhanced sensitivity to artesunate compared to wild-type controls. Artesunate inhibited proliferation, induced G0/G1 arrest, and promoted apoptosis in AML cells, particularly under serum-free conditions.The IC50 values for artesunate in SRSF2-mutant SKM-1 cells were 5.212 μM, which were significantly lower than in wild-type cells (8.248 μM), and further decreased to 2.792 μM under serum-free conditions.

| **IC50/μM** | | **Artesunate** | **Artesunate(serum-free)** |
|---|---|---|---|
| SKM-1 | WT | 8.248 | 5.549 |
| | SRSF2mut | 5.212 | 2.792 |
| molm13 | NC | 3.833 | 1.582 |
| | SRSF2mut | 1.455 | 0.786 |
| OCI-AML3 | NC | 5.214 | 3.456 |
| | SRSF2mut | 1.794 | 1.083 |

Mechanistically, artesunate downregulated mitochondrial apoptosis-related proteins and increased ROS levels, particularly in SRSF2-mutant cell lines and under serum-free conditions.It also modulated the expression of apoptosis-related proteins (reduced Bcl-2, elevated BAX and caspases), suggesting activation of the mitochondrial pathway of apoptosis.
Molecular docking revealed stronger binding of artesunate to mutant SRSF2 P95H, and artesunate treatment reduced SRSF2 expression in AML cells, possibly contributing to its selective efficacy. Clinically, low SRSF2 expression correlated with better prognosis in AML cohorts, and artesunate synergized with DA chemotherapy to enhance apoptosis in primary AML cells ex vivo. These findings support the repurposing potential of artesunate for SRSF2-mutant AML.

### Summary/Conclusion

Our study demonstrates the efficacy of artesunate against SRSF2-mutant AML by provoking oxidative mitochondrial apoptosis and interacting with the SRSF2 mutant protein, offering a compelling rationale for its therapeutic repurposing in this high-risk AML subset.

**Copyright Information:** (Online) ISSN: 2572-9241
©2026 The Author(s). HemaSphere is published by John Wiley & Sons Ltd on behalf of European Hematology Association. This is an open access Abstract Book distributed under the Attribution-NonCommercial-NoDerivs (CC BY-NC-ND), which allows third parties to download the articles and share them with others as long as they credit the author and the Abstract Book, but they cannot change the content in any way or use them commercially.
**Abstract Book Citations:** Authors, Title, HemaSphere, 2026;10:(S1):pages. The abstract book DOIs can be found on the "Author Information" page.
**Disclaimer:** Articles published in the journal HemaSphere exclusively reflect the opinions of the authors. The authors are responsible for all content in their abstracts including accuracy of the facts, statements, citing resources, etc.

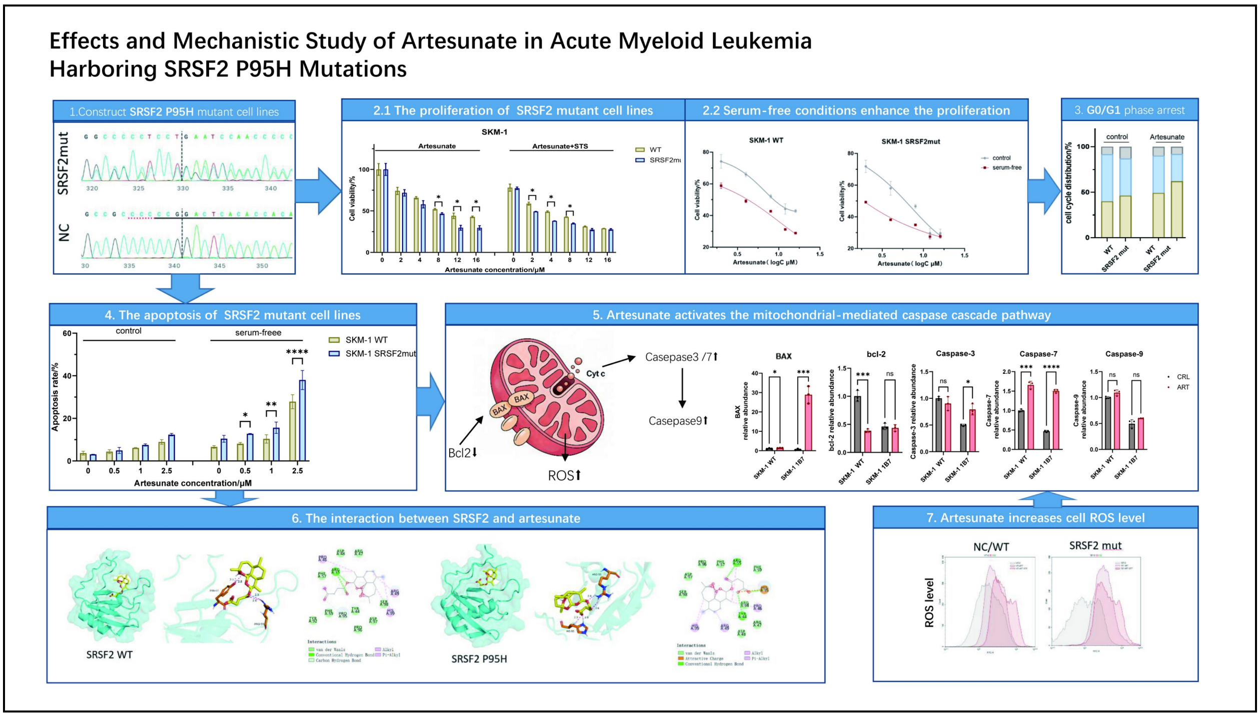

Due to space limitations, only the experimental results for the SKM-1 cell line are presented in this figure. Fig. 1 shows the DNA sequencing validation results following the construction of the SRSF2 P95H cell line, confirming the occurrence of the point mutation. Fig. 2.0 illustrates the growth inhibition effects of different concentrations of artesunate on the SKM-1 cell line under both conventional and serum-free conditions, with a comparison between wild-type and SRSF2-mutant cell lines. Furthermore, Fig. 2.1 builds upon this by performing linear fitting to compare growth inhibition under conventional and serum-free conditions. Fig. 3 elucidates the mechanism by which artesunate induces growth inhibition, attributing it to cell cycle arrest at the G0/G1 phase, with the proportion of arrested cells in the SRSF2-mutant cell line being approximately 10% higher than in the wild-type. Fig. 4 depicts the apoptosis levels in the SKM-1 cell line following treatment with varying concentrations of artesunate under conventional and serum-free conditions, with a higher apoptosis rate observed in the SRSF2-mutant cell line. Fig. 5 presents the expression levels of BAX, Bcl-2, and caspase-3/7/9, confirming that artesunate exerts its effects through the activation of the mitochondrial apoptosis pathway. Fig. 6 demonstrates the interaction between artesunate and both the wild-type SRSF2 and the P95H mutant. Following the mutation of proline to histidine in the SRSF2 protein, histidine forms hydrogen bonds with artesunate, enhancing binding affinity and suggesting a mechanism underlying the sensitivity of the SRSF2-mutant cell line to artesunate. Fig. 7 shows elevated intracellular reactive oxygen species (ROS) levels after artesunate treatment, with a further increase under serum-free conditions, validating mitochondrial damage caused by artesunate.

**Copyright Information:** (Online) ISSN: 2572-9241
©2026 The Author(s). HemaSphere is published by John Wiley & Sons Ltd on behalf of European Hematology Association. This is an open access Abstract Book distributed under the Attribution-NonCommercial-NoDerivs (CC BY-NC-ND), which allows third parties to download the articles and share them with others as long as they credit the author and the Abstract Book, but they cannot change the content in any way or use them commercially.
**Abstract Book Citations:** Authors, Title, HemaSphere, 2026;10:(S1):pages. The abstract book DOIs can be found on the "Author Information" page.
**Disclaimer:** Articles published in the journal HemaSphere exclusively reflect the opinions of the authors. The authors are responsible for all content in their abstracts including accuracy of the facts, statements, citing resources, etc.

### (PS1570) FOXC1 MEDIATES DRUG RESISTANCE AND MHC-II SUPPRESSION: A NOVEL DUAL MECHANISM IN RELAPSED/REFRACTORY AML

**Topic:** 03. Acute myeloid leukemia - Biology & translational research

Xiaoxuan Lu*[1], Xiaosu Zhao[1, 2]

[1]*Peking University People's Hospital, Peking University Institute of Hematology, National Clinical Research Center for Hematologic Disease, Beijing Key Laboratory of Cell and Gene Therapy for Hematologic Malignan-cies, Peking University, Beijing, China;* [2]*Collaborative Innovation Center of Hematology, Peking University, Beijing, China;*

### Background

Acute myeloid leukemia (AML) is a hematologic malignancy characterized by clonal expansion of abnormally differentiated myeloid blasts. Relapsed/refractory (R/R) AML remains a major cause of treatment failure and mortality. Overexpression of FOXC1 has been closely associated with the development and progression of multiple solid tumors. Previous mouse models have demonstrated that FOXC1 cooperates with HOXA9 to promote leukemogenesis. However, the role of FOXC1 in R/R AML remains unclear.

### Aims

To characterize the expression pattern of FOXC1 in AML and investigate potential mechanisms by which FOXC1 may contribute to R/R AML.

### Methods

We performed single-cell transcriptomic profiling of bone marrow samples from 8 R/R AML patients stratified by post-transplant outcome (refractory/relapsed/CR), and prioritized FOXC1 for further study based on its higher expression in the refractory group than in the relapsed group. FOXC1 knockdown was established in U937 and THP-1 cell lines. We then assessed responses to chemotherapeutic agents using cytotoxicity assays and evaluated changes in CIITA and MHC class II (MHC-II) related gene expression by qPCR and flow cytometry.

### Results

Single-cell RNA-seq (scRNA-seq) analysis showed that FOXC1 expression was enriched in AML blasts, with extremely low or no expression in other hematopoietic cell clusters (Fig. 1a). AML blasts were re-clustered into seven subclusters, and FOXC1 was detected across all subclusters, with the highest expression in the HSPC-like blast subcluster. As leukemia stem cell (LSC)-like populations are associated with chemoresistance and relapse in AML, this pattern supports a potential link between FOXC1 overexpression and chemotherapy resistance (Fig. 1b). In vitro, FOXC1 knockdown increased the sensitivity of AML cells to daunorubicin after 48 h of treatment, as indicated by a lower IC50 (Fig. 1d). FOXC1 knockdown also increased venetoclax sensitivity and apoptosis in AML cells. Together, these results support a role for FOXC1 in drug resistance in AML. In addition, we performed differential gene expression analysis and found significant downregulation of multiple MHC-II genes in FOXC1$^{+}$ HSPC-like AML blasts, and functional enrichment analyses consistently highlighted MHC-II antigen presentation pathways (Fig. 1c). In agreement with these findings, in vitro experiments further confirmed that FOXC1 knockdown significantly upregulated the expression of MHC-II molecules as well as CIITA, the master regulator of MHC-II expression, compared with control cells (Fig. 1e). In paired bone marrow samples from AML patients at our institution, FOXC1 expression varied with disease status and tended to show the highest levels in relapsed/refractory disease, intermediate levels at diagnosis, and the lowest levels in remission.

**Copyright Information:** (Online) ISSN: 2572-9241
©2026 The Author(s). HemaSphere is published by John Wiley & Sons Ltd on behalf of European Hematology Association. This is an open access Abstract Book distributed under the Attribution-NonCommercial-NoDerivs (CC BY-NC-ND), which allows third parties to download the articles and share them with others as long as they credit the author and the Abstract Book, but they cannot change the content in any way or use them commercially.
**Abstract Book Citations:** Authors, Title, HemaSphere, 2026;10:(S1):pages. The abstract book DOIs can be found on the "Author Information" page.
**Disclaimer:** Articles published in the journal HemaSphere exclusively reflect the opinions of the authors. The authors are responsible for all content in their abstracts including accuracy of the facts, statements, citing resources, etc.

**Summary/Conclusion**

FOXC1 overexpression has been linked to relapse/refractory disease in multiple solid tumors, but its role in R/R AML remains unclear. Here, scRNA-seq analysis showed that FOXC1 is preferentially expressed in HSPC-like AML blasts, a LSC-like population associated with chemoresistance. In addition, FOXC1 may contribute to immune evasion by suppressing CIITA/MHC-II-related genes. Together, these findings suggest that FOXC1 may promote R/R AML through a dual mechanism involving chemoresistance and immune evasion, identify FOXC1 as a potential therapeutic target, and warrant further mechanistic and functional validation.

**Copyright Information:** (Online) ISSN: 2572-9241
©2026 The Author(s). HemaSphere is published by John Wiley & Sons Ltd on behalf of European Hematology Association. This is an open access Abstract Book distributed under the Attribution-NonCommercial-NoDerivs (CC BY-NC-ND), which allows third parties to download the articles and share them with others as long as they credit the author and the Abstract Book, but they cannot change the content in any way or use them commercially.
**Abstract Book Citations:** Authors, Title, HemaSphere, 2026;10:(S1):pages. The abstract book DOIs can be found on the "Author Information" page.
**Disclaimer:** Articles published in the journal HemaSphere exclusively reflect the opinions of the authors. The authors are responsible for all content in their abstracts including accuracy of the facts, statements, citing resources, etc.

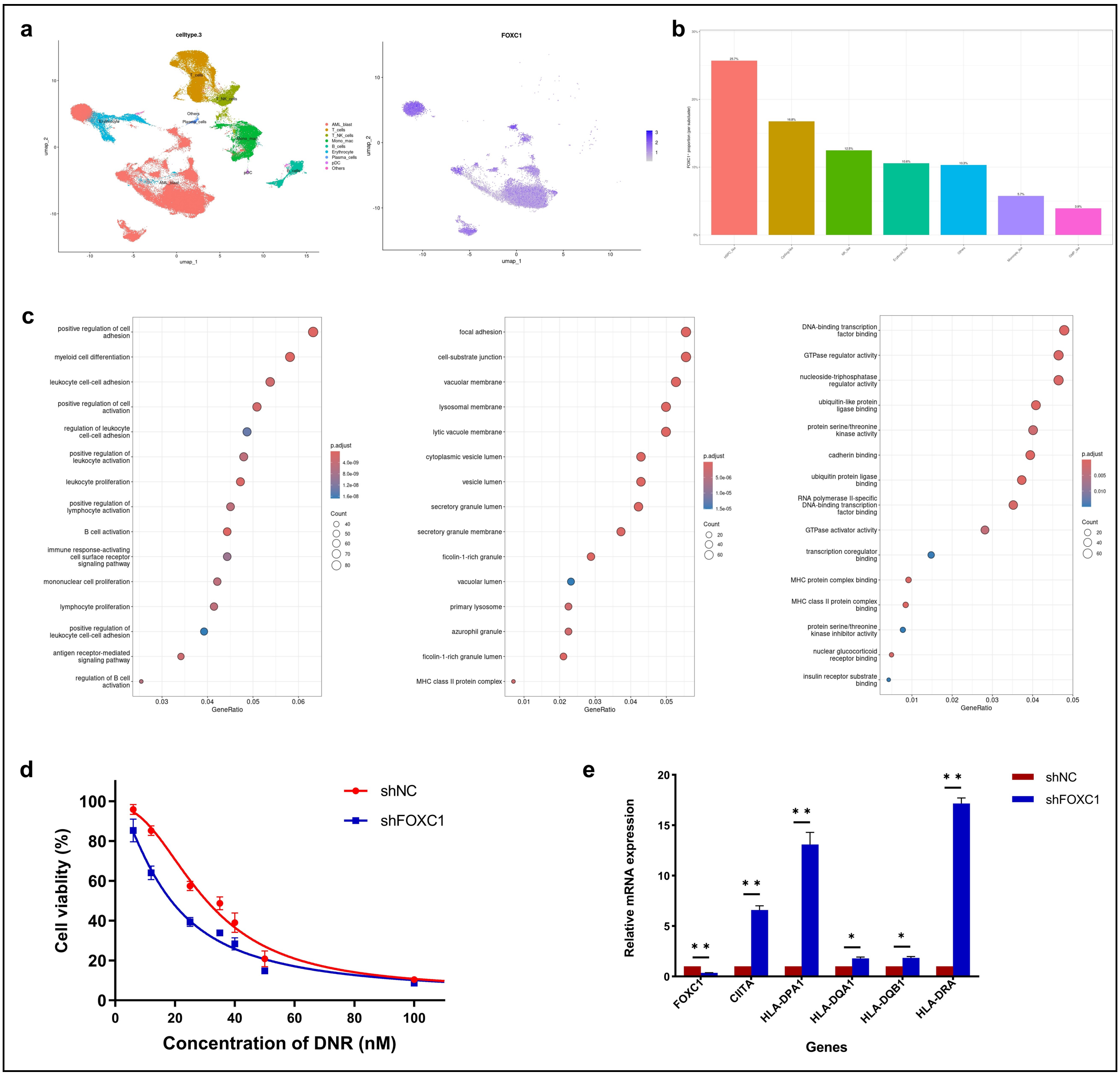

Figure 1. Expression pattern of FOXC1 in AML and its association with drug response and MHC-II suppression (a) UMAP projection of single-cell transcriptomes from bone marrow samples of 8 AML patients, annotated by cell type (left) and colored by FOXC1 expression level (right). (b) Histogram showing FOXC1 expression in HSPC-like AML cells from bone marrow samples of the 8 AML patients. (c) Differential gene expression analysis between FOXC1⁺ and FOXC1⁻ HSPC-like AML blasts followed by GO enrichment analysis revealed enrichment of multiple MHC-II–related pathways. (d) CCK8 dose-response curves after 48h of daunorubicin (DNR) treatment indicated increased sensitivity to DNR in shFOXC1 U937 cells (IC50: shNC, 29.88±0.6757nM; shFOXC1, 17.35±2.731nM; $P < 0.01$). (e) qPCR analysis of CIITA and MHC-II–related genes in shFOXC1 vs control (shNC) U937 cells.

**Copyright Information:** (Online) ISSN: 2572-9241
©2026 The Author(s). HemaSphere is published by John Wiley & Sons Ltd on behalf of European Hematology Association. This is an open access Abstract Book distributed under the Attribution-NonCommercial-NoDerivs (CC BY-NC-ND), which allows third parties to download the articles and share them with others as long as they credit the author and the Abstract Book, but they cannot change the content in any way or use them commercially.
**Abstract Book Citations:** Authors, Title, HemaSphere, 2026;10:(S1):pages. The abstract book DOIs can be found on the "Author Information" page.
**Disclaimer:** Articles published in the journal HemaSphere exclusively reflect the opinions of the authors. The authors are responsible for all content in their abstracts including accuracy of the facts, statements, citing resources, etc.

### (PS1571) SINGLE-CELL LANDSCAPE OF CEBPANTERM-MUTATED ZEBRAFISH REVEALS PROGENITOR DIFFERENTIATION BLOCK AND GRANULOCYTE DYSREGULATION

**Topic:** 03. Acute myeloid leukemia - Biology & translational research

Zhangjie Chen*[1, 2], Alexandra Lubin[1], Tanvi Tambaku[1], Katie-Jo Thorpe[1], Catherine Zhu[1], Elspeth Payne[1]

*[1]University College London Cancer Institute, London, United Kingdom; [2]Institute of Hematology & Blood Diseases Hospital, Chinese Academy of Medical Sciences & Peking Union Medical College, Tianjin, China;*

### Background

*CEBPA* is a transcription factor mutated in 10-15% of acute myeloid leukaemia (AML). Notably, 10% of these cases are inherited, typically involving a truncating germline mutation at the N-terminus (*CEBPA*$^{Nterm}$) as the founder event, followed by the second hit mutation in the BZIP domain of the other CEBPA allele (*CEBPA*$^{Cterm}$) driving progression to AML. However, the early cellular and molecular abnormalities initiated by the *CEBPA*$^{Nterm}$ mutation remain poorly defined.

### Aims

Utilizing single-cell RNA sequencing (scRNA-seq) in a *CEBPA*$^{Nterm}$-mutated zebrafish model, we aimed to map the abnormal cellular transitions and biological pathways that establish a pre-leukemic state.

### Methods

We performed scRNA-seq (Parse Biosciences Evercode WT) on heterozygous *CEBPA*$^{Nterm}$ and wild type (WT) zebrafish at 4 days, 2 weeks, 6 months and 16 months. Single-cell suspensions were prepared from whole larvae (4d, 2w) and adult kidney marrow (6m, 16m). After data integration, granulocytes and progenitor cells were subsetted for high-resolution annotation and further analysis.

### Results

Twenty distinct clusters were identified and annotated, including 4 HSPC and 3 granulocyte clusters (Figure 1A). While transcriptomatic profile of WT granulocytes remained stable between 6 and 16 months, *CEBPA*$^{Nterm}$ granulocytes exhibited progressive dysregulation. At both stages, *CEBPA*$^{Nterm}$ granulocytes showed significantly down-regulation of ribosomal and protein translation genes comparing to WT. Notably, compared to 6-month baseline, 16-month *CEBPA*$^{Nterm}$ granulocytes demonstrated further ribosomal and translation function suppression, along with increased pro-inflammatory and cell death pathways, including NOD-like receptor, MAPK signaling and necroptosis (Figure 1B).

Within the progenitor compartment, we identified HSPC1 as a cluster of interest, characterized by high expression of granulocyte lineage markers including *csf3r*, *spi1b*, and *cebpa*. At 6 months, *CEBPA*$^{Nterm}$ cells within this cluster exhibited a hyper-proliferative but dysfunctional state. Specifically, they showed an enrichment in DNA replication and mitotic cell cycle pathways, with a significant down-regulation of pathways associated with mature granulocyte function compared to WT. We further conducted a longitudinal DEG analysis between 2-week and 6-month stages. While WT progenitors showed only 23 down-regulated genes, the *CEBPA*$^{Nterm}$ group exhibited a much broader suppression of 111 genes. An enrichment analysis of the 91 genes exclusively down-regulated in *CEBPA*$^{Nterm}$ group focused almost entirely on normal granulocyte functional pathways.

Ligand-receptor analysis identified NECTIN3-LIGIT axis as a stable and consistent interaction between HSPC1 and granulocytes in WT. In contrast, this signaling was uniquely lost in *CEBPA*$^{Nterm}$ group at all stages except a transient presence at 2 weeks.

**Copyright Information:** (Online) ISSN: 2572-9241
©2026 The Author(s). HemaSphere is published by John Wiley & Sons Ltd on behalf of European Hematology Association. This is an open access Abstract Book distributed under the Attribution-NonCommercial-NoDerivs (CC BY-NC-ND), which allows third parties to download the articles and share them with others as long as they credit the author and the Abstract Book, but they cannot change the content in any way or use them commercially.
**Abstract Book Citations:** Authors, Title, HemaSphere, 2026;10:(S1):pages. The abstract book DOIs can be found on the “Author Information” page.
**Disclaimer:** Articles published in the journal HemaSphere exclusively reflect the opinions of the authors. The authors are responsible for all content in their abstracts including accuracy of the facts, statements, citing resources, etc.

### Summary/Conclusion

Our findings indicate that *CEBPA*$^{Nterm}$ mutation induces progressive granulocyte abnormalities in zebrafish, characterized by a progenitor differentiation block, the loss of NECTIN-TIGIT interaction and a shift toward pro-inflammatory and cell death pathways in mature granulocytes.

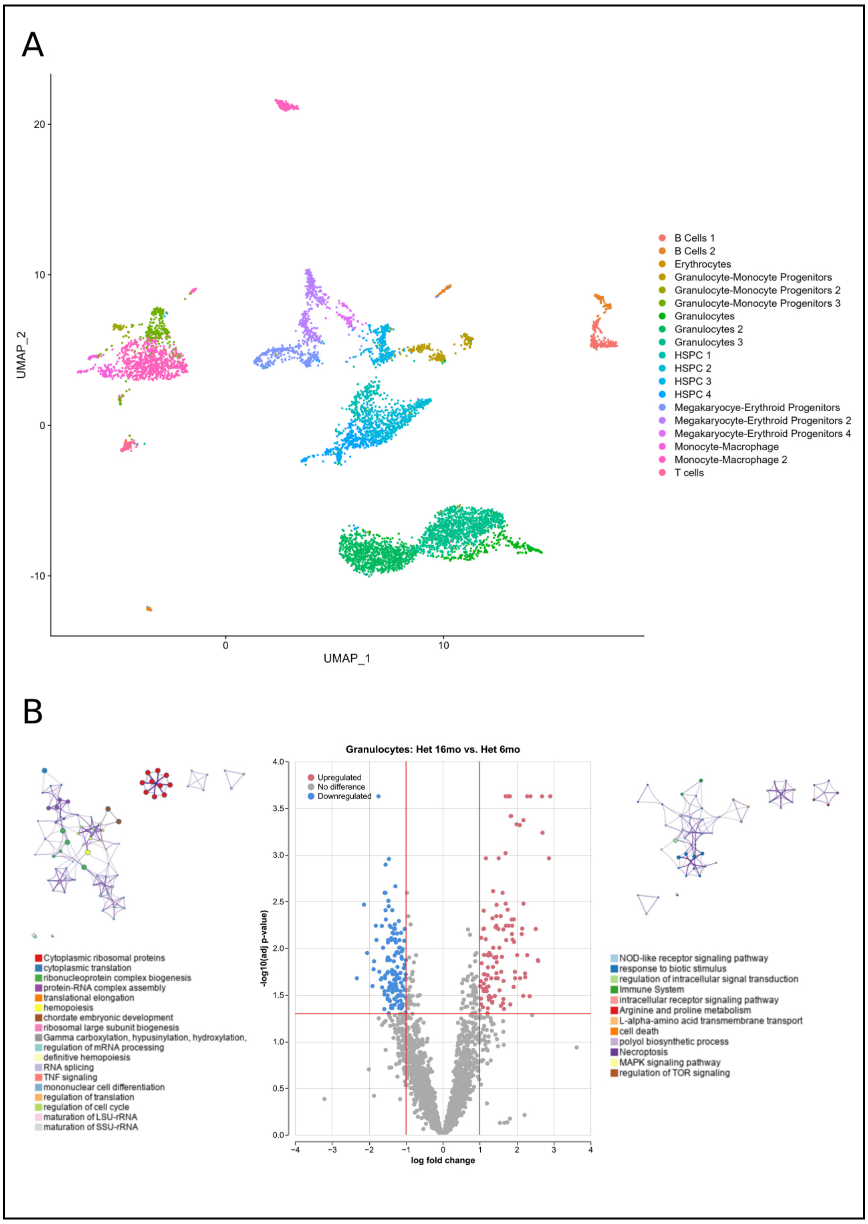

Figure 1. (A) UMAP of all clusters across 4d, 2w, 6mo, and 16mo cohorts. (B) DEG and enrichment analysis comparing 16-month vs. 6-month granulocytes in CEBPA Nterm group.

**Copyright Information:** (Online) ISSN: 2572-9241
©2026 The Author(s). HemaSphere is published by John Wiley & Sons Ltd on behalf of European Hematology Association. This is an open access Abstract Book distributed under the Attribution-NonCommercial-NoDerivs (CC BY-NC-ND), which allows third parties to download the articles and share them with others as long as they credit the author and the Abstract Book, but they cannot change the content in any way or use them commercially.
**Abstract Book Citations:** Authors, Title, HemaSphere, 2026;10:(S1):pages. The abstract book DOIs can be found on the "Author Information" page.
**Disclaimer:** Articles published in the journal HemaSphere exclusively reflect the opinions of the authors. The authors are responsible for all content in their abstracts including accuracy of the facts, statements, citing resources, etc.

### (PS1572) C-TERMINAL CEBPA MUTATIONS IMPOSE A DOSE-DEPENDENT MYELOID DIFFERENTIATION BLOCKADE IN ISOGENIC IPSC-DERIVED AML MODELS

**Topic:** 03. Acute myeloid leukemia - Biology & translational research

Antonio Benedetto Ventura*[1], Lucia Deligio[1], Tiziana Loconte[1], Antonio Negri[1], Licia Sanfelice[1], Eleonora Montenegro[1], Luigi Viggiano[2], P Garcia[3], Lorena Arranz[4], Daria Di Molfetta[2], Amer Ahmed[2], Sabino Ciavarella[1], Giuseppe Fiermonte[2], Giacomo Volpe[1, 5]

*[1]IRCCS Istituto Tumori "Giovanni Paolo II", Hematology and Cell Therapy Unit, Bari, Italy; [2]University of Bari "Aldo Moro", Department of Bioscience, Biotechnology and Environment, Bari, Italy; [3]University of Birmingham, Institute of Cancer and Genomic Sciences, College of Medical and Dental Sciences, Birmingham, United Kingdom; [4]University of Oslo, Norwegian Center for Molecular Medicine (NCMM), Oslo, Norway; [5]University of Bari "Aldo Moro", Department of Pharmacy – Pharmaceutical Sciences, Bari, Italy;*

### Background

The *CEBPA* (CCAAT/enhancer-binding protein alpha) gene encodes a leucine zipper transcription factor that regulates cell cycle arrest and drives commitment of hematopoietic progenitors (HSPCs) toward the granulocytic lineage. Its loss or functional alteration contributes to the differentiation block that characterizes acute myeloid leukemia (AML). C-terminal mutations affecting the bZIP domain impair dimerization and DNA binding. Monoallelic and biallelic mutations are associated with distinct clinical outcomes and may influence therapy response, underscoring the need to clarify genotype-specific mechanisms. Current AML cell lines and murine models do not permit controlled evaluation of CEBPA mutations on an identical genetic background, limiting the dissection of mutation-specific effects. Combining CRISPR/Cas9-mediated genome editing with induced pluripotent stem cell (iPSC) modeling provides an isogenic platform to dissect the molecular and functional consequences of distinct genotypes.

### Aims

This study aimed at generating isogenic iPSC lines harboring C-terminal *CEBPA* mutations, and to evaluate the impact of monoallelic and biallelic genotypes on HSPCs maturation and subsequent myeloid differentiation, defining genotype-specific functional consequences.

### Methods

Guide RNAs and CRISPR/Cas9 were used to introduce indels in the C-terminal region of *CEBPA* in hiPSCs BYS0112 (ACS-1026™, ATCC) and fibroblast-derived hiPSCs (cat. IPS17-00012, Stem Cell Technology Center, Radboudumc, the Netherlands). The obtained wild type (WT), monoallelic, and biallelic clones were differentiated into CD34+ HSPCs ( STEMdiff™ Hematopoietic Kit) and subsequently induced toward monocytes and granulocytes differentiation by Sigma-Aldrich Stemline® II Hematopoietic Stem Cell Expansion Medium supplemented with SCF, IL-3, Flt-3l, GM-CSF and G-CSF, then was assessed by flow cytometry and cytomorphology by optical microscopy. HSPCs were profiled by single-cell RNA sequencing (scRNA-seq) to characterize genotype-associated transcriptional changes and seeded in semisolid medium for colony-forming assays.

**Copyright Information:** (Online) ISSN: 2572-9241
©2026 The Author(s). HemaSphere is published by John Wiley & Sons Ltd on behalf of European Hematology Association. This is an open access Abstract Book distributed under the Attribution-NonCommercial-NoDerivs (CC BY-NC-ND), which allows third parties to download the articles and share them with others as long as they credit the author and the Abstract Book, but they cannot change the content in any way or use them commercially.
**Abstract Book Citations:** Authors, Title, HemaSphere, 2026;10:(S1):pages. The abstract book DOIs can be found on the "Author Information" page.
**Disclaimer:** Articles published in the journal HemaSphere exclusively reflect the opinions of the authors. The authors are responsible for all content in their abstracts including accuracy of the facts, statements, citing resources, etc.

**Results**

iPSCs efficiently generated CD34+ HSPCs across all genotypes, indicating preserved early hematopoietic attitude. During myeloid maturation, WT clones produced mature granulocytes and monocytes, whereas monoallelic mutants showed a marked reduction in mature myeloid output and biallelic mutants exhibited a substantial differentiation block. Cytospin-based morphological analysis confirmed the accumulation of immature progenitors in mutant conditions, accompanied by a reduction of terminally differentiated myeloid cells. scRNA-seq of CD34+ cells revealed that WT lines expressed a transcriptional fingerprint of both myeloid and lymphoid progenitors, whereas monoallelic and biallelic mutants retained lymphoid potential while displaying a progressive, dose-dependent depletion of granulocytic features. Colony-forming assays corroborated these findings displaying that - as compared with WT - mutant clones generated higher and larger colonies, mainly composed of CFU-GEMM and CFU-GM progenitors, with a reduced frequency of CFU-G and CFU-M colonies.

**Summary/Conclusion**

These findings underscore the central role of *CEBPA* in granulopoiesis and provide mechanistic insights into potential genotype-specific differentiation defects. Importantly, this study focuses exclusively on C-terminal mutations and establishes a iPSC-based platform for the functional characterization of this subset of CEBPA alterations in AML.

**Copyright Information:** (Online) ISSN: 2572-9241
©2026 The Author(s). HemaSphere is published by John Wiley & Sons Ltd on behalf of European Hematology Association. This is an open access Abstract Book distributed under the Attribution-NonCommercial-NoDerivs (CC BY-NC-ND), which allows third parties to download the articles and share them with others as long as they credit the author and the Abstract Book, but they cannot change the content in any way or use them commercially.
**Abstract Book Citations:** Authors, Title, HemaSphere, 2026;10:(S1):pages. The abstract book DOIs can be found on the "Author Information" page.
**Disclaimer:** Articles published in the journal HemaSphere exclusively reflect the opinions of the authors. The authors are responsible for all content in their abstracts including accuracy of the facts, statements, citing resources, etc.

### (PS1573) COMPUTATIONAL IDENTIFICATION OF A MONOCYTIC-METABOLIC VENETOCLAX-RESISTANT AML SUBTYPE WITH CDK INHIBITOR VULNERABILITY

**Topic:** 03. Acute myeloid leukemia - Biology & translational research

Vakul Mohanty[1], Natalia Baran*[2], Patryk Firmanty[3], Shubhankar Dash[3], Monika Chomczyk[3], Akansha Jalota[4], Merve Dede[1], Yuefan Huang[1], Cassandra Ramage[1], Laurie Cooper[1], Shan He[1], Sarah Pelletier[1], May Daher[1], Anna Stachurska-Skrodzka[5], Anna Skwarska[4], Jeffrey Tyner[6], Gordon Mills[7], Courtney Dinardo[1], Ing Soo Tiong[8], Andrew Wei[8], Marina Konopleva[4], Ken Chen[1]

*[1]MD Anderson Cancer Center, Houston, United States of America;[2]InselSpital University of Bern, Hematology and hematological central laboratory, Bern, Switzerland; [3]Institute of Hematology and Transfusion Medicine, Warsaw, Poland;[4]Albert Einstein College of Medicine, Department of Oncology, Bronx, United States of America; [5]Centre of Postgraduate Medical Education, Department of Cell Biology and Immunology, Warsaw, Poland; [6]Knight Cancer Institute, Oregon Health & Science University, Department of Cell, Developmental & Cancer Biology, Portland, United States of America; [7]Oregon Health & Science University, Division of Oncological Sciences, Portland, United States of America; [8]Peter MacCallum Cancer Centre and Royal Melbourne Hospital and University of Melbourne, Department of Haematology, Melbourne, Australia;*

### Background

Venetoclax (VEN), a BCL2 inhibitor with hypomethylating agents, represents standard induction therapy for older acute myeloid leukemia (AML) patients. However, primary and acquired resistance limits long-term cure rates to <25%. Recent studies have linked monocytic differentiation to VEN resistance through metabolic reprogramming and MCL1 dependence.

### Aims

We comprehensively characterize a distinct VEN-resistant AML subtype defined by monocytic differentiation and metabolic activation, and identify CDK inhibition as a feasible therapeutic vulnerability.

### Methods

We performed integrative analysis of transcriptomic and *ex vivo* drug response data from 23 leukemia cell lines to derive a VEN resistance signature using non-negative matrix factorization (NMF). The signature was validated in BeatAML, TCGA AML, and 6 single-cell RNA-seq datasets. We characterized the monocytic-metabolic (MM) resistance cluster through gene set enrichment analysis, mutational profiling, and drug sensitivity screening. Mechanistic validation was performed using dose-response matrices, Bliss synergy analysis, flow cytometry, immunoblotting assays in cell lines and primary patient samples, and colony-forming unit assay in cord blood-derived CD34+ cells.

**Copyright Information:** (Online) ISSN: 2572-9241
©2026 The Author(s). HemaSphere is published by John Wiley & Sons Ltd on behalf of European Hematology Association. This is an open access Abstract Book distributed under the Attribution-NonCommercial-NoDerivs (CC BY-NC-ND), which allows third parties to download the articles and share them with others as long as they credit the author and the Abstract Book, but they cannot change the content in any way or use them commercially.
**Abstract Book Citations:** Authors, Title, HemaSphere, 2026;10:(S1):pages. The abstract book DOIs can be found on the "Author Information" page.
**Disclaimer:** Articles published in the journal HemaSphere exclusively reflect the opinions of the authors. The authors are responsible for all content in their abstracts including accuracy of the facts, statements, citing resources, etc.

**Results**

We identified MM-AML as a distinct VEN-resistant cluster in 23% of all VEN-resistant cases, enriched for DNMT3A, IDH1/2, and monosomy 7. MM-AML exhibited monocytic differentiation with transcriptional activation of energy metabolism pathways and MYC-driven proliferation (both FDR<0.001). Dose-response matrices demonstrated significant synergy between VEN and CDK7/9 inhibitor SNS-032 in MM-AML like cells. IC50 values were strongly reduced upon combination treatment. Mechanistically, SNS-032, reduced expression of MCL1, CDK2, CDK7 and CDK9 proteins at 24h compared to vehicle, further significantly increased cleaved PARP and induced apoptosis (Annexin V+/DAPI+) when combined with VEN (p<0.001). Of note, CCLE CRISPR dependency analysis revealed MM-AML-like cell lines had significantly higher CDK7 and CDK9 dependency scores. *Ex vivo* treatment of heavly pretreated relapsed/refractory AML primary samples demonstrated promising efficacy upon SNS032-based treatment. Normal cord blood CD34+ cells showed strong sensitivity to higher doses of SNS032, suggesting a potential therapeutic window at doses efficacious against AML while sparing healthy progenitors. MM-AML classification was reproducible across BeatAML cohort 2, TCGA, and single-cell datasets. The latter revealed MM-AML- cells coexist with other resistance states in 38/46 patients (83%), with bulk classification matching dominant single-cell population in 80% of cases. Patients with high intra-tumor heterogeneity (>3 resistance states) had lower VEN response rates (p=0.03) and shorter PFS (p=0.02).

**Summary/Conclusion**

Taking together, we defined monocytic-metabolic VEN-resistant AML subtype characterized by DNMT3A/IDH mutations, metabolic reprogramming, and MYC-driven proliferation. CDK inhibition represents a feasible therapeutic vulnerability that transcriptionally suppresses MCL1 and synergizes with VEN *in vitro* and *ex vivo*. VEN+CDK inhibitor combinations demonstrate promising activity in relapsed/refractory MM-AML patient samples with acceptable selectivity for normal hematopoietic cells. These findings establish a precision medicine framework for overcoming VEN resistance in monocytic AML and warrant further clinical investigation of VEN+CDK inhibitor combinations in biomarker-selected patients.

**Copyright Information:** (Online) ISSN: 2572-9241
©2026 The Author(s). HemaSphere is published by John Wiley & Sons Ltd on behalf of European Hematology Association. This is an open access Abstract Book distributed under the Attribution-NonCommercial-NoDerivs (CC BY-NC-ND), which allows third parties to download the articles and share them with others as long as they credit the author and the Abstract Book, but they cannot change the content in any way or use them commercially.
**Abstract Book Citations:** Authors, Title, HemaSphere, 2026;10:(S1):pages. The abstract book DOIs can be found on the “Author Information” page.
**Disclaimer:** Articles published in the journal HemaSphere exclusively reflect the opinions of the authors. The authors are responsible for all content in their abstracts including accuracy of the facts, statements, citing resources, etc.

### (PS1574) CHEMOTHERAPY MODULATES THE EPIGENETIC LANDSCAPE AND THE IMMUNE MICROENVIRONMENT IN MLL-AF9-DRIVEN ACUTE MYELOID LEUKAEMIA

**Topic:** 03. Acute myeloid leukemia - Biology & translational research

Alessia Zangari*[1], Antonella Santoro[1, 2], Nicolò Gualandi[1], Edoardo Carsana[3], Tatiana Volpari[1], Simona Fusco[1], Kety Giannetti[1], Francesca Clemente[4], Achille Anselmo[4], Cristina Lo Celso[5, 6, 7], Raffaella Di Micco[1, 2]

*[1]San Raffaele Telethon Institute for Gene Therapy (SR-TIGET), IRCCS San Raffaele Scientific Institute, Unit of Senescence in Stem Cell Aging, Differentiation and Cancer, Milan, Italy; [2]University School for Advanced Studies IUSS, Pavia, Italy; [3]Human Technopole, Technology development IU, National Facility for Genomics, Milan, Italy; [4]IRCCS San Raffaele Scientific Institute, Flow Cytometry Resource, Advanced Cytometry Technical Application Laboratory, Milan, Italy; [5]Imperial College London, Department of Life Sciences, London, United Kingdom; [6]The Francis Crick Institute, London, United Kingdom; [7]Imperial College London, Centre for Haematology, Department of Immunology and Inflammation, London, United Kingdom;*

### Background

Acute Myeloid Leukaemia (AML) is an aggressive hematologic malignancy characterized by the expansion of immature myeloid cells in the bone marrow (BM), leading to hematopoietic failure. AML remains associated with early treatment failure and high relapse rates following intensive chemotherapy. AML is considered a “cold” tumour, with a poorly responsive immune microenvironment (IME), low neoantigen presentation, and limited efficacy of immune checkpoint inhibitors. Therapy-induced senescence (TIS) is a DNA Damage Response mediated growth arrest. This phenomenon shows promise in reshaping the immune landscape within solid tumours, offering hope for cancer cell eradication. Recent findings from our laboratory identified the Polycomb Repressive Complex 2 (PRC2) as a possible inhibitor of TIS establishment in AML patient blasts following ex-vivo chemotherapy. We therefore hypothesise that PRC2 inhibition enhances TIS in AML, promotes immune cell recruitment and activation, and ultimately improves chemotherapy response while reducing relapse risk.

### Aims

1. Explore responses to chemotherapy in a murine model of AML at single cell resolution.
2. Explore the epigenetic rewiring of leukemic cells upon chemotherapy.
3. Investigate remodelling of the IME.

### Methods

MLL-AF9-transformed murine AML cells were transplanted into immunocompetent mice without myeloablation, followed by a standard chemotherapy regimen (3+5 days of Daunorubicin and Cytarabine). BM was analysed two days post-treatment using high-throughput spectral flow cytometry of >30 markers. Single-cell RNA sequencing (scRNA-seq) was used to examine transcriptional changes after chemotherapy. Histone modifications and their target genes were assessed via Western blot and Cut&Tag analysis. EZH2 inhibition was performed through gene-editing knockout (KO) model and via pharmacological inhibition.

**Copyright Information:** (Online) ISSN: 2572-9241
©2026 The Author(s). HemaSphere is published by John Wiley & Sons Ltd on behalf of European Hematology Association. This is an open access Abstract Book distributed under the Attribution-NonCommercial-NoDerivs (CC BY-NC-ND), which allows third parties to download the articles and share them with others as long as they credit the author and the Abstract Book, but they cannot change the content in any way or use them commercially.
**Abstract Book Citations:** Authors, Title, HemaSphere, 2026;10:(S1):pages. The abstract book DOIs can be found on the “Author Information” page.
**Disclaimer:** Articles published in the journal HemaSphere exclusively reflect the opinions of the authors. The authors are responsible for all content in their abstracts including accuracy of the facts, statements, citing resources, etc.

### Results

Chemotherapy reduced overall leukemic burden, while none of the tumour clusters identified through scRNA-seq upregulated senescence signatures. AML clusters generally downregulated inflammatory and interferon (IFN) signatures post-treatment, while the non-leukemic compartment showed no significant activation of these genes. Quantitative Flow cytometry analysis revealed an immunosuppressive tumour microenvironment— increased PD-L1$^+$ monocytes, pDCs, exhausted NK cells, and reduced activated (IgM$^+$) B cells compared to healthy mice—that is maintained or even enhanced after chemotherapy. Genes linked to H3K27me3 histone modification, primarily mediated by the PRC2 catalytic subunit EZH2, were transcriptionally downregulated following treatment, with ongoing CUT&Tag analysis aimed at defining their identity. These changes corresponded to increased H3K27me3 protein levels in MLL-AF9 blasts. Parallel studies using EZH2 KO MLL-AF9 blasts and pharmacological PRC2 inhibition will determine whether PRC2 contributes to the reduced immune response and suppression of TIS signature after treatment.

### Summary/Conclusion

Our data suggest that PRC2 limits TIS and immune activation in AML, maintaining an immunosuppressive microenvironment. The increase in H3K27me3 and the downregulation of inflammatory pathways following chemotherapy support our hypothesis that PRC2 inhibition could enhance TIS, modulate immune responses, and improve treatment outcomes.

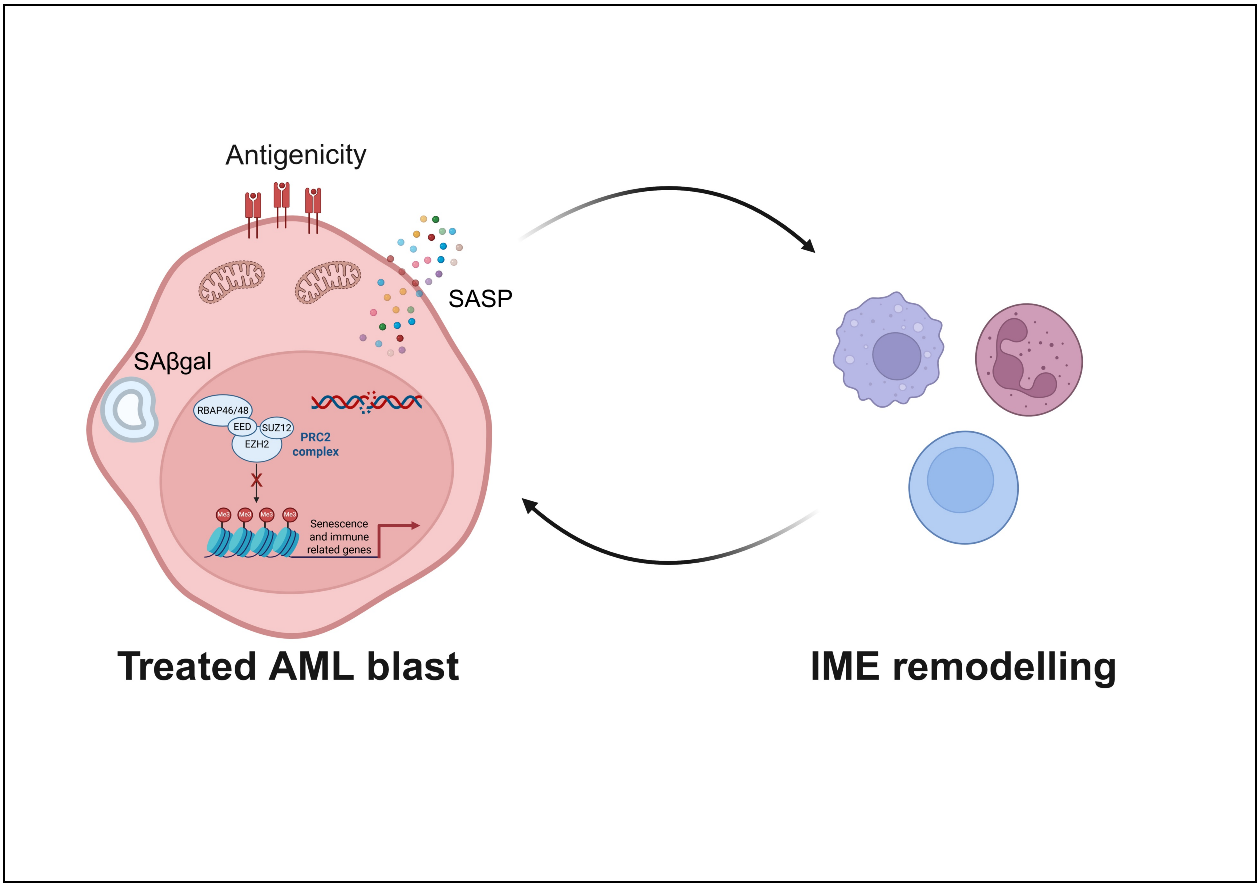

Graphical abstract

Copyright Information: (Online) ISSN: 2572-9241
©2026 The Author(s). HemaSphere is published by John Wiley & Sons Ltd on behalf of European Hematology Association. This is an open access Abstract Book distributed under the Attribution-NonCommercial-NoDerivs (CC BY-NC-ND), which allows third parties to download the articles and share them with others as long as they credit the author and the Abstract Book, but they cannot change the content in any way or use them commercially.
Abstract Book Citations: Authors, Title, HemaSphere, 2026;10:(S1):pages. The abstract book DOIs can be found on the "Author Information" page.
Disclaimer: Articles published in the journal HemaSphere exclusively reflect the opinions of the authors. The authors are responsible for all content in their abstracts including accuracy of the facts, statements, citing resources, etc.

### (PS1575) SINGLE-HIT AND MULTI-HIT DNMT3A, TET2 AND ASXL1 MUTATIONS SHAPE DISTINCT PATHWAYS TO AML

**Topic:** 03. Acute myeloid leukemia - Biology & translational research

Wencke Walter*[1], Manja Meggendorfer[1], Torsten Haferlach[1], Anna Stengel[1]

[1]*MLL Munich Leukemia Laboratory, Munich, Germany;*

### Background

Clonal hematopoiesis, an age-related process, emerges from somatic mutations in key hematopoietic genes (*DNMT3A*, *TET2*, *ASXL1*; DTA) with a variant allele fraction ≥2%, influenced by age-related inflammatory signalling. While single mutations (mut) in DTA-genes are common, the impact of multiple hits in these genes on malignant hematopoiesis and the progression to AML is less well known.

### Aims

We aimed to assess the frequency and impact of single and multi-hit DTA gene mutations in a large AML patient cohort.

### Methods

We performed genetic analysis – chromosome banding analysis, FISH, and targeted gene panel analysis - of 3,100 AML patients (pts) with *DNMT3A*, *TET2* and/or *ASXL1* muts.

**Copyright Information:** (Online) ISSN: 2572-9241
©2026 The Author(s). HemaSphere is published by John Wiley & Sons Ltd on behalf of European Hematology Association. This is an open access Abstract Book distributed under the Attribution-NonCommercial-NoDerivs (CC BY-NC-ND), which allows third parties to download the articles and share them with others as long as they credit the author and the Abstract Book, but they cannot change the content in any way or use them commercially.
**Abstract Book Citations:** Authors, Title, HemaSphere, 2026;10:(S1):pages. The abstract book DOIs can be found on the “Author Information” page.
**Disclaimer:** Articles published in the journal HemaSphere exclusively reflect the opinions of the authors. The authors are responsible for all content in their abstracts including accuracy of the facts, statements, citing resources, etc.

### Results

We identified 1,409 AML pts with *DNMT3A*mut, 951 pts with *TET2*mut and 740 pts with *ASXL1*mut. Multiple alterations (mh) were frequent for *TET2*, while *DNMT3A* and *ASXL1* were dominated by single hit (sh): *TET2* 46.7% sh vs. 53.3% mh; *DNMT3A* 86.9% sh vs. 13.1% mh; *ASXL1* 96.4% sh vs. 3.6% mh. Multi-hits were mainly mut+mut, although mut+loh contributed a significant number of cases to *DNMT3A*mh and *TET2*mh (Fig.).

35% of the AML *DNMT3A*sh cases belonged to the AML-*NPM1* subgroup and 20.4% to AML-MR. *TET2*sh and *ASXL1*sh showed lower frequencies of AML-*NPM1* (23% and 3% vs. 35%) and higher frequencies of AML-MR (37% and 43.3% vs. 20%). AMLs with other defining genetic alterations were rare. Interestingly, AML-*NPM1* with *DNMT3A*sh had a younger median age compared to *TET2*sh (63y vs. 70y). In contrast to *DNMT3A*mh, *TET2*mh contained a higher frequency of AML-*NPM1* (32% vs. 18.8%), with AML-*NPM1 DNMT3A*mh being again younger than AML-*NPM1 TET2*mh (64y vs. 74y).

535/1224 of *DNMT3A*sh AML carried the R882 variant and 51% of these cases were co-mutated with *NPM1*. In line with this, *DNMT3A*sh R882 showed a higher frequency of *FLT3*-ITD compared to nonR882 (28.4% vs 17.7%). Moreover, pts with *DNMT3A*sh R882 showed a higher proportion of AML-*NPM1* compared to *DNMT3A*mut+mut R882 (40.4% vs. 17.3%). The majority of *DNMT3A*mut+loh cases were AML-MR (50%) lacking the R882 hotspot mut. Interestingly, *TP53* mutations co-occurred more frequently with *DNMT3A*sh non-R882 compared to *DNMT3A*sh R882 cases (16.2% vs. 3.7%), with the majority of these cases associated with myelodysplastic changes and a complex karyotype (13/18, 72%).

AML *DNMT3A*mh pts generally exhibited a distinct co-mutation profile, showing increased frequencies of *IDH2* (34% vs. 17.3%), *BCOR* (21.6% vs. 9.8%), and *IDH1* (16.7% vs. 11%) mutations, alongside the decrease in *NPM1* mutations. Excluding *NPM1*, the co-mutation profiles of *TET2*sh and *TET2*mh were similar, with high frequencies of *DNMT3A* (32%, 22%), *RUNX1* (27%, 17.5%), *SRSF2* (26%, 35%), and *ASXL1* (26%, 28.6%) mutations — a profile more akin to AML-MR than to de novo AML. Among DTA genes, *ASXL1*sh mutations co-occurred more frequently with *TET2* (24.3%) than with *DNMT3A* (11.2%). However, the most frequent co-mutations were *RUNX1* (41.6%) and *SRSF2* (39.8%), which can synergistically affect the cell's epigenetic and splicing machinery, leading to a more aggressive disease phenotype.

### Summary/Conclusion

DTA gene mutation patterns, especially *DNMT3A* R882 vs. non-R882 and single vs. multi-hit status, define distinct molecular and clinical pathways in AML. *DNMT3A* R882 mutations link to early-onset, *NPM1*/*FLT3*-ITD positive AML, while non-R882 and multi-hit profiles suggest a secondary AML/MDS phenotype, underlining the significant role of DTA mutations in hematologic malignancy risk and evolution.

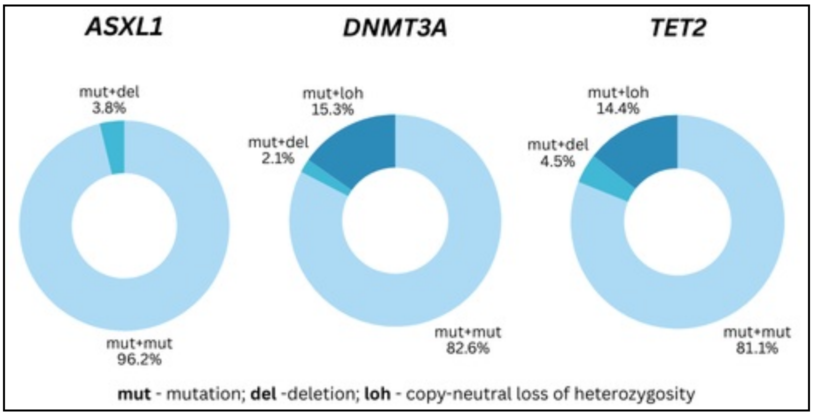

**Copyright Information:** (Online) ISSN: 2572-9241
©2026 The Author(s). HemaSphere is published by John Wiley & Sons Ltd on behalf of European Hematology Association. This is an open access Abstract Book distributed under the Attribution-NonCommercial-NoDerivs (CC BY-NC-ND), which allows third parties to download the articles and share them with others as long as they credit the author and the Abstract Book, but they cannot change the content in any way or use them commercially.
**Abstract Book Citations:** Authors, Title, HemaSphere, 2026;10:(S1):pages. The abstract book DOIs can be found on the “Author Information” page.
**Disclaimer:** Articles published in the journal HemaSphere exclusively reflect the opinions of the authors. The authors are responsible for all content in their abstracts including accuracy of the facts, statements, citing resources, etc.

### (PS1576) ACUTE MYELOID LEUKEMIA WITH NORMAL KARYOTYPE AND MYELODYSPLASIA-RELATED MUTATIONS: PROGNOSTIC IMPACT OF A PATHWAY-BASED STRATIFICATION

**Topic:** 03. Acute myeloid leukemia - Biology & translational research

Guadalupe Oñate*[1, 2], Albert Cortés[3], Montserrat Arnan Sangerman[4], Mar Tomo[5, 6], Antonia Sampol[7], Susana Vives Polo[8], Juan José Bargay Lleonart[9], Ferran Vall-Llovera Calmet[10], Antonio Garcia Guiñon[11], Rosa Coll Jorda[12], Sara Garcia Ávila[13], Marta Cervera Calvo[14], Sergio Mojal[1], Guillermo Ramil[1], Anna Giné-Vilalta[1], Laura Ranera[1], Helena Castellet[1], Josep Nomdedeu[1], Ana Garrido Diaz[1], Jorge Sierra Gil[1], Jordi Esteve Reyner[15], Marta Pratcorona[1]

*[1]Hospital de la Santa Creu i Sant Pau, Hematology, Barcelona, Spain;[2]Institut de recerca Sant Pau (IIB Sant Pau), Barcelona, Spain; [3]Hospital Clinic, Barcelona, Spain; [4]Institut Català d'Oncologia Hospital Duran i Reynals, L'Hospitalet de Llobregat, Spain; [5]Hospital Clínico Universitario, Valencia, Spain; [6]Instituto de Investigación INCLIVA, Valencia, Spain; [7]Hospital Son Espases, Palma de Mallorca, Spain; [8]Institut Català d'Oncologia Hospital Germans Trias i Pujol, Badalona, Spain;[9]Hospital Son Llatzer, Palma de Mallorca, Spain; [10]Hospital Mutua Terrassa, Terrassa, Spain; [11]Hospital Universitari Arnau de Vilanova, Lleida, Spain; [12]Institut Català d'Oncologia Hospital Trueta, Girona, Spain; [13]Hospital del Mar, Barcelona, Spain; [14]Institut Català d'Oncologia Hospital Joan XXIII, Tarragona, Spain; [15]Institute of Cancer & Blood Disorders (ICAMS), IDIBAPS, Hospital Clinic, Barcelona, Spain;*

### Background

The latest WHO and ICC acute myeloid leukemia (AML) classifications describe a myelodysplasia-related gene mutations category (AML-MRG), based on an 8-gene signature described by Lindsey and refined in a 10-gene category by Papaemmanuil. These genes can be grouped into 4 cellular pathways: splicing (*SF3B1, SRSF2, U2AF1, ZRSR2*), cohesin complex (*STAG2*), chromatin modifiers (*ASXL1, BCOR, EZH2, MLL*-PTD), or transcription factors (*RUNX1*). AML-MRG patients often harbour additional mutations at diagnosis across multiple pathways, resulting in a complex mutational landscape. Of note, these patients are considered adverse in ELN-22 stratification, but their outcome is often heterogeneous.

### Aims

To characterize the pathway-based mutational landscape of AML-MRG without additional recurrent genetic lesions and identify clinically distinct subsets.

### Methods

All adult AML patients diagnosed within the Spanish CETLAM cooperative group between 2017 and 2024 were centrally characterized at diagnosis with a 42-gene custom NGS panel. Inclusion criteria were: normal karyotype, absence of recurrent genetic abnormalities and ≥1 of the 10 dysplasia-related genes described by Papaemmanuil. All mutations detected by NGS were grouped according to their main cellular pathway **(Figure A)**. The relationship between variables was established using $\chi 2$ or Kruskal-Wallis tests. Survival analysis were performed with Kaplan-Meier and log-rank tests.

**Copyright Information:** (Online) ISSN: 2572-9241
©2026 The Author(s). HemaSphere is published by John Wiley & Sons Ltd on behalf of European Hematology Association. This is an open access Abstract Book distributed under the Attribution-NonCommercial-NoDerivs (CC BY-NC-ND), which allows third parties to download the articles and share them with others as long as they credit the author and the Abstract Book, but they cannot change the content in any way or use them commercially.
**Abstract Book Citations:** Authors, Title, HemaSphere, 2026;10:(S1):pages. The abstract book DOIs can be found on the "Author Information" page.
**Disclaimer:** Articles published in the journal HemaSphere exclusively reflect the opinions of the authors. The authors are responsible for all content in their abstracts including accuracy of the facts, statements, citing resources, etc.

### Results

A total of 95 out of 711 AML patients were selected (13%). Median age at diagnosis was 64 years (24-89), and 63% of patients were male (n=60). Most patients (71%, n=67) received intensive therapy. Median number of mutations detected by NGS was 4 (1-8). The most frequent mutated genes were *RUNX1* (48%), *ASXL1* (38%), and *DNMT3A* (32%). AML-MRG patients presented with a median of 3 cellular pathways altered at the time of diagnosis **(Figure B)**, involving methylation genes (23%), chromatin modifiers (22%), followed by transcription factors (17%), splicing (13%), kinases (10%), cohesin (6%), tumor suppressors (6%), and RAS (3%). There were no significant differences in the number of mutation nor pathways according to age or sex, but we observed a significant association between the number of mutations and the number of pathways **(Figure C)**. With a median follow-up of 25 months (95%CI 16-34) the 2-yr OS of the cohort was 51±6% with an EFS of 40±7%. There were no differences in OS regarding intensive or non-intensive therapy (2yr OS 50% vs 56% respectively; p=0.8). In the whole cohort, we observed similar outcomes according to the number of mutations at diagnosis, but significant differences when pathways were considered: patients with ≤2 pathways had a 2yr OS of 67±12% vs 46±8% in patients with >2 pathways (p=0.045; **Figure D**) that was sustained in the multivariate analysis (HR for OS in ≤2 pathways 0.38 (95%CI 0.14-0.98; p=0.046).

### Summary/Conclusion

This study examined a well-defined cohort of AML-MRG patients and integrated all diagnostic data from a standard myeloid NGS panel to identify biologically distinct subsets and evaluate their prognostic significance. AML-MRG patients exhibited mutations across multiple pathways, and those with less complex clonal profiles (fewer than two altered pathways) showed superior survival compared with patients harboring alterations in multiple pathways. Although these findings require validation in larger prospective cohorts, they may support improved risk stratification within a subgroup currently classified as adverse.

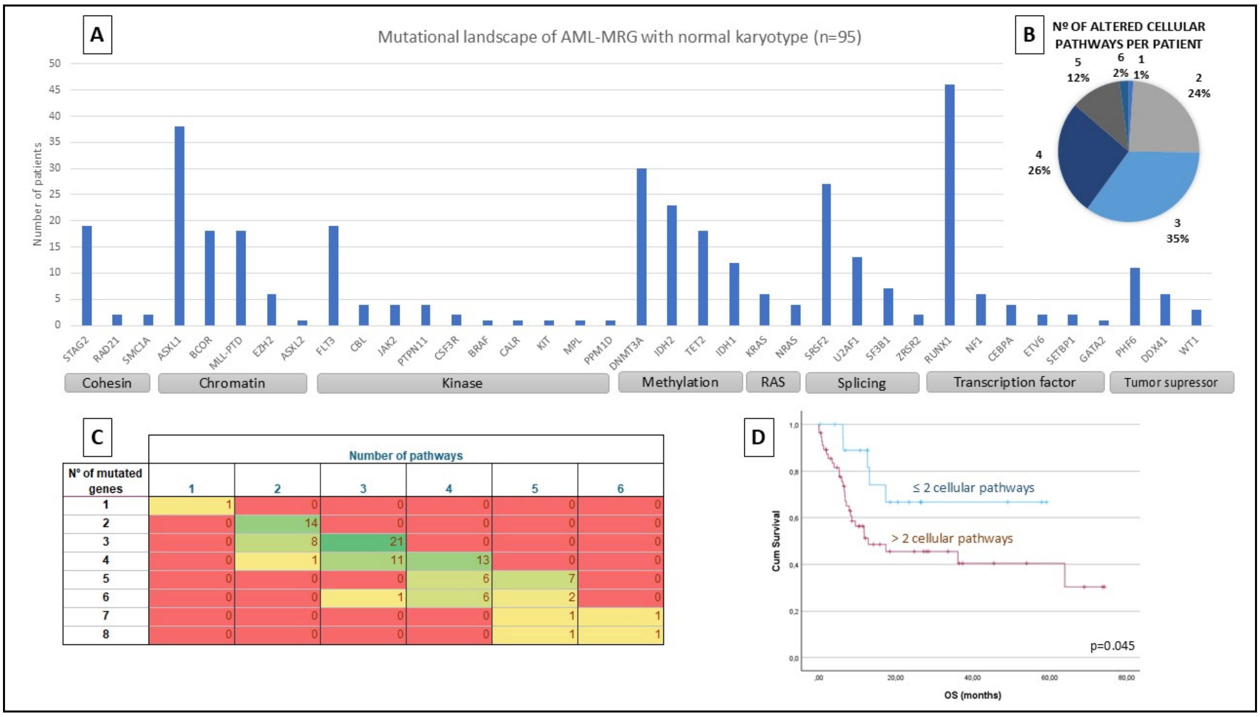

| Nº of mutated genes | Number of pathways 1 | 2 | 3 | 4 | 5 | 6 |
|---|---|---|---|---|---|---|
| 1 | 1 | 0 | 0 | 0 | 0 | 0 |
| 2 | 0 | 14 | 0 | 0 | 0 | 0 |
| 3 | 0 | 8 | 21 | 0 | 0 | 0 |
| 4 | 0 | 1 | 11 | 13 | 0 | 0 |
| 5 | 0 | 0 | 0 | 6 | 7 | 0 |
| 6 | 0 | 0 | 1 | 6 | 2 | 0 |
| 7 | 0 | 0 | 0 | 0 | 1 | 1 |
| 8 | 0 | 0 | 0 | 0 | 1 | 1 |

**Copyright Information:** (Online) ISSN: 2572-9241
©2026 The Author(s). HemaSphere is published by John Wiley & Sons Ltd on behalf of European Hematology Association. This is an open access Abstract Book distributed under the Attribution-NonCommercial-NoDerivs (CC BY-NC-ND), which allows third parties to download the articles and share them with others as long as they credit the author and the Abstract Book, but they cannot change the content in any way or use them commercially.
**Abstract Book Citations:** Authors, Title, HemaSphere, 2026;10:(S1):pages. The abstract book DOIs can be found on the "Author Information" page.
**Disclaimer:** Articles published in the journal HemaSphere exclusively reflect the opinions of the authors. The authors are responsible for all content in their abstracts including accuracy of the facts, statements, citing resources, etc.

### (PS1577) PATHOGENIC ROLE AND THERAPEUTIC IMPLICATIONS OF CYTOPLASMIC P53 AGGREGATES IN TP53-MUTATED ACUTE MYELOID LEUKEMIA AND MYELODYSPLASTIC SYNDROMES

**Topic:** 03. Acute myeloid leukemia - Biology & translational research

Tadashi Okamura*[1], Hiroki Hosoi[1], Midori Ikezaki[2], Daisuke Motooka[3], Ibu Matsuzaki[4], Yuichi Tochino[1], Motomi Osato[5], Shin-Ichi Murata[4], Yoshito Ihara[2], Jun Nakata[6], Kazuchika Nishitsuji[2], Takashi Sonoki[1]

*[1]Wakayama Medical University, Department of Hematology/Oncology, Wakayama, Japan;[2]Wakayama Medical University, Department of Biochemistry, School of Medicine, Wakayama, Japan; [3]Osaka University, Department of Infection Metagenomics, Osaka, Japan; [4]Wakayama Medical University, Department of Human Pathology, Wakayama, Japan;[5]Kumamoto University, International Research Center for Medical Sciences, Kumamoto, Japan; [6]Osaka University, Department of Clinical Laboratory and Biomedical Sciences, Osaka, Japan;*

### Background

*TP53* mutations are associated with poor outcomes in acute myeloid leukemia and myelodysplastic syndromes (AML/MDS); however, treating *TP53*-mutated AML/MDS remains a challenge. We previously showed that cytoplasmic p53 aggregates are associated with a poor prognosis in solid tumors. Here, we analyzed the correlation between cytoplasmic p53 aggregates and prognosis in AML/MDS. We also investigated the anti-tumor effect of p53 aggregation inhibition in AML cell lines.

### Aims

To elucidate the clinicopathological significance of cytoplasmic p53 aggregation in AML/MDS.

### Methods

We retrospectively analyzed 16 *TP53*-mutated and 30 *TP53*-wild-type cases of AML/MDS. The *TP53* mutation was determined by targeted sequencing. Cytoplasmic p53 aggregates in patients' bone marrow samples were evaluated by immunofluorescence staining, followed by confocal microscopy (Figure A). Induction chemotherapy response, overall survival (OS), and progression-free survival (PFS) were compared according to the presence or absence of cytoplasmic p53 aggregates. For the *in vitro* assay, cytoplasmic p53 aggregates were examined in AML cell lines (HL-60 [p53-negative], NB4 [p53 wild-type], KY821 [R175H mutated p53], and MOLM-16 [V173M/C238S-mutated p53]). ReACp53, a peptide inhibitor of p53 aggregation, was used to inhibit p53 aggregation and assess changes in p53 protein levels, subcellular localization, and p21 expression. Cell proliferation after treatment with ReACp53, alone or in combination with cytarabine (Ara-C), was assessed by an MTT assay. RNA sequencing was performed in KY821 cells following ReACp53 treatment.

**Copyright Information:** (Online) ISSN: 2572-9241
©2026 The Author(s). HemaSphere is published by John Wiley & Sons Ltd on behalf of European Hematology Association. This is an open access Abstract Book distributed under the Attribution-NonCommercial-NoDerivs (CC BY-NC-ND), which allows third parties to download the articles and share them with others as long as they credit the author and the Abstract Book, but they cannot change the content in any way or use them commercially.
**Abstract Book Citations:** Authors, Title, HemaSphere, 2026;10:(S1):pages. The abstract book DOIs can be found on the "Author Information" page.
**Disclaimer:** Articles published in the journal HemaSphere exclusively reflect the opinions of the authors. The authors are responsible for all content in their abstracts including accuracy of the facts, statements, citing resources, etc.

### Results

Among 46 cases, cytoplasmic p53 aggregates were observed in 14 cases (Figure A). Cytoplasmic p53 aggregates positive cases had a significantly lower complete response rate and a significantly poorer prognosis than negative cases (response rate: 79% vs 50%, $p$=0.047; 1-year OS: 11% vs 67%, $p$=0.00137; and 1-year PFS: 10% vs 73%, $p$<0.001) (Figure B). The presence of cytoplasmic p53 aggregates was significantly associated with *TP53* mutations in AML/MDS patients (specificity 96.7%; sensitivity 81.3%; $p$ < 0.001). Cytoplasmic p53 aggregates were observed in KY821 and MOLM-16 cells, whereas no nuclear aggregates were detected (Figure C). Treatment with ReACp53 reduced total p53 protein levels, cytoplasmic p53 aggregate formation, and promoted p53 nuclear translocation. ReACp53 also increased *p21* expression, indicating partial recovery of p53 function. Accordingly, ReACp53 induced dose-dependent growth inhibition in both cell lines and enhanced cytotoxicity when combined with Ara-C, suggesting that p53 aggregation is involved in chemoresistance. RNA-seq showed that inhibition of p53 aggregation is associated with gene expression changes consistent with enhanced myeloid differentiation and inflammatory response signatures, together with downregulation of cell cycle–related pathways, supporting partial recovery of p53 function (Figure D).

### Summary/Conclusion

In summary, we found that *TP53* mutation is highly correlated with cytoplasmic p53 aggregation in AML/MDS. The presence of cytoplasmic p53 aggregates was associated with poor prognosis and a poor response to chemotherapy in AML/MDS. An *in vitro* assay suggested that ReACp53 treatment allocates p53 to nucleus, resulting in partial recovery of p53 function. Our findings suggest that cytoplasmic p53 aggregates in *TP53*-mutated AML/MDS might be a therapeutic target that can be addressed by inhibiting aggregation.

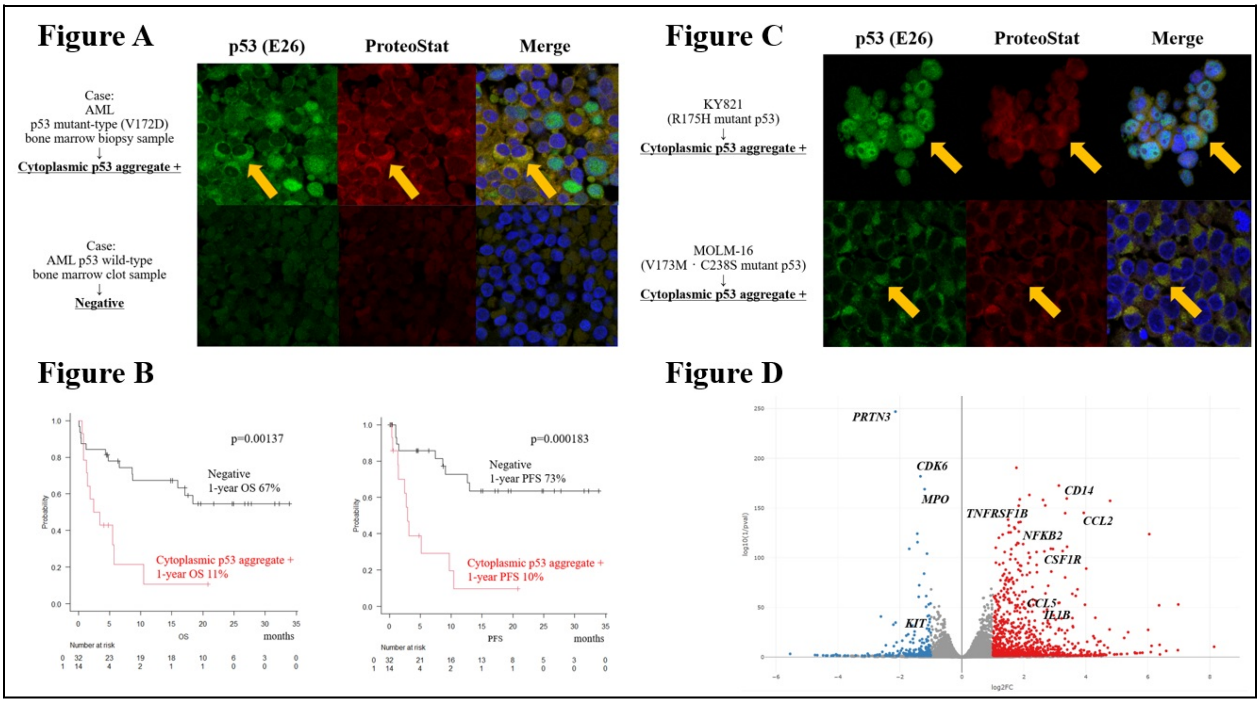

**Copyright Information:** (Online) ISSN: 2572-9241
©2026 The Author(s). HemaSphere is published by John Wiley & Sons Ltd on behalf of European Hematology Association. This is an open access Abstract Book distributed under the Attribution-NonCommercial-NoDerivs (CC BY-NC-ND), which allows third parties to download the articles and share them with others as long as they credit the author and the Abstract Book, but they cannot change the content in any way or use them commercially.
**Abstract Book Citations:** Authors, Title, HemaSphere, 2026;10:(S1):pages. The abstract book DOIs can be found on the "Author Information" page.
**Disclaimer:** Articles published in the journal HemaSphere exclusively reflect the opinions of the authors. The authors are responsible for all content in their abstracts including accuracy of the facts, statements, citing resources, etc.

### (PS1578) SHIKONIN EXERTS ANTI-LEUKEMIC ACTIVITY AGAINST FLT3-ITD-MUTATED ACUTE MYELOID LEUKEMIA BY PROMOTING CBL-MEDIATED DEGRADATION OF THE FLT3 RECEPTOR

**Topic:** 03. Acute myeloid leukemia - Biology & translational research

Yanfang Chen[1, 2], Long Su[1, 2], Jintong Chen[1, 2], Tianxue Qin[1, 2], Sujun Gao*[1, 2]

*[1]The First Hospital of Jilin University, Department of Hematology, Changchun, China;[2]The First Hospital of Jilin University, Key Laboratory of Hematology Precision Medicine of Jilin Province, Changchun, China;*

### Background

Mutations in the FMS-like tyrosine kinase 3 (FLT3) receptor gene are one of the most prevalent genetic abnormalities in AML, and internal tandem duplication (*FLT3*-ITD) mutations are recognized as an adverse prognostic marker for AML. Recently, FLT3 receptor inhibitors have achieved substantial progress in the treatment of AML. Neverthelss, drug resistance remains a major bottleneck limiting their clinical application. Thus, there is an urgent clinical need to develop new therapeutic agents for *FLT3*-ITD-mutated AML. In previous studies of our group, we found that shikonin (SHK) suppresses FLT3 kinase activity and blocks its downstream signaling pathways, thereby displaying selective cytotoxicity against *FLT3*-ITD-mutated AML cell lines.

### Aims

This study aims to investigate the anti-leukemic effects and its underlying molecular mechanisms of SHK on *FLT3*-ITD-mutated AML.

### Methods

AML cell lines with wild-type (OCI and Kasumi-1) and mutated *FLT3* gene (MV4-11 and MOLM-13) were used, and primary bone marrow samples from patients were collected. Western blot (WB) and quantitative polymerase chain reaction (q-PCR) were employed to quantitatively analyze protein and mRNA expression, respectively. MV4-11 cell lines stably transfected with CBL-knockdown virus were constructed to verify the role of CBL, while MV4-11 cell lines stably transfected with viruses targeting K612, K624, K633, and combined sites were established to validate the ubiquitination sites. The effects of SHK on cell proliferation and apoptosis were explored by CCK-8 assay and flow cytometry. Colony formation assay was performed to evaluate the safety of SHK.

### Results

SHK showed a dose-dependent manner of cytotoxicity against both leukemia cell lilnes and primary AML samples especially in those with FLT3-ITD mutations, which is comparable with commercial FLT3 inhitors (Midostaurin). SHK exhibited a stronger effect in down-regulating FLT3 receptor and phosphorylated FLT3 (p-FLT3) levels in mutant leukemia cell lines compared with wild-type cell lines. Its anti-leukemic activity was not mediated by downregulating FLT3 mRNA expression, but rather by promoting the degradation of FLT3 receptor protein. The E3 ubiquitin ligase CBL was identified as the key molecule involved in this process, with K612 and K633 identified as the major sites mediating FLT3 ubiquitination and degradation. Further studies demonstrated that SHK had no significant effect on CBL expression at either the transcriptional or translational level; instead, it enhanced the binding affinity between CBL and the FLT3 receptor. This enhanced interaction thereby promoted FLT3 protein degradation, ultimately exerting effects of inhibiting cell proliferation and inducing cell apoptosis. Moreover, SHK had no obvious impact on the proliferation of normal mouse bone marrow cells, indicating a certain degree of safety.

**Copyright Information:** (Online) ISSN: 2572-9241
©2026 The Author(s). HemaSphere is published by John Wiley & Sons Ltd on behalf of European Hematology Association. This is an open access Abstract Book distributed under the Attribution-NonCommercial-NoDerivs (CC BY-NC-ND), which allows third parties to download the articles and share them with others as long as they credit the author and the Abstract Book, but they cannot change the content in any way or use them commercially.
**Abstract Book Citations:** Authors, Title, HemaSphere, 2026;10:(S1):pages. The abstract book DOIs can be found on the "Author Information" page.
**Disclaimer:** Articles published in the journal HemaSphere exclusively reflect the opinions of the authors. The authors are responsible for all content in their abstracts including accuracy of the facts, statements, citing resources, etc.

**Summary/Conclusion**

SHK exerts its anti-leukemic effects against *FLT3*-ITD-mutated AML possibly by enhancing the binding affinity between the E3 ubiquitin ligase CBL and the FLT3 receptor. Investigation of this mechanism provides new possibilities for the clinical treatment of AML.

**Copyright Information:** (Online) ISSN: 2572-9241
©2026 The Author(s). HemaSphere is published by John Wiley & Sons Ltd on behalf of European Hematology Association. This is an open access Abstract Book distributed under the Attribution-NonCommercial-NoDerivs (CC BY-NC-ND), which allows third parties to download the articles and share them with others as long as they credit the author and the Abstract Book, but they cannot change the content in any way or use them commercially.
**Abstract Book Citations:** Authors, Title, HemaSphere, 2026;10:(S1):pages. The abstract book DOIs can be found on the "Author Information" page.
**Disclaimer:** Articles published in the journal HemaSphere exclusively reflect the opinions of the authors. The authors are responsible for all content in their abstracts including accuracy of the facts, statements, citing resources, etc.

### (PS1579) HMGN1 REGULATES THE TRANSCRIPTIONAL OUTPUT OF DIVERGENT HIF-1 REGULONS IN MLL-AF9- AND AML1-ETO-DRIVEN AML.

**Topic:** 03. Acute myeloid leukemia - Biology & translational research

Gillian Caalim*[1], Abril Arredondo-Garcia[1], David Kealy[1], William Grey[1], Katherine Bridge[1]

[1]*University of York, Centre for Blood Research, York, United Kingdom;*

### Background

The transcription factor HIF-1 coordinates survival and metabolic adaptation in the hypoxic bone marrow microenvironment. The role of HIF-1 in the pathogenesis of acute myeloid leukaemia (AML) is highly variable, and the extent to which oncogenic driver mutations alter the HIF-1 regulon remains poorly understood. As AML subtypes are defined by distinct epigenetic and chromatin states, the genetic context may influence HIF-1 chromatin occupancy and transcriptional output. The chromatin architectural protein HMGN1 is frequently overexpressed in AML and is implicated in regulating transcription, raising the possibility that chromatin context modulates HIF-1 transcriptional activity in AML. We hypothesise that AML driver mutations establish chromatin states which give rise to subtype-specific HIF-1 regulons and that HMGN1 facilitates transcription of hypoxia responsive genes.

### Aims

To characterise and compare the HIF-1 genomic binding profiles in *MLL-AF9* and *AML1-ETO* rearranged AML models, and to determine the requirement for the chromatin architectural protein HMGN1 in the transcriptional activity of HIF-1 and the downstream cellular response to hypoxia.

### Methods

Chromatin immunoprecipitation sequencing (ChIP-seq) was performed in THP-1 (MLL-AF9-driven) and Kasumi-1 (AML1-ETO-driven) AML cell lines under hypoxic conditions (1% $O_2$). To determine chromatin-dependent regulation of HIF-1 transcriptional activity, cell lines were transduced with lentiviral shRNA constructs to deplete HMGN1. The impact of HMGN1 loss on HIF-1 transcriptional output was assessed via quantitative RT-PCR across core and subtype-specific loci, while HIF-1 genomic occupancy was validated by ChIP-qPCR. Cellular responses to hypoxia were assessed by cell proliferation, apoptosis, and cell cycle analysis. To isolate HIF-specific effects independent of hypoxia, cells were treated with a PHD2 inhibitor to pharmacologically stabilise HIFs under normoxic conditions (20% $O_2$).

**Copyright Information:** (Online) ISSN: 2572-9241
©2026 The Author(s). HemaSphere is published by John Wiley & Sons Ltd on behalf of European Hematology Association. This is an open access Abstract Book distributed under the Attribution-NonCommercial-NoDerivs (CC BY-NC-ND), which allows third parties to download the articles and share them with others as long as they credit the author and the Abstract Book, but they cannot change the content in any way or use them commercially.
**Abstract Book Citations:** Authors, Title, HemaSphere, 2026;10:(S1):pages. The abstract book DOIs can be found on the "Author Information" page.
**Disclaimer:** Articles published in the journal HemaSphere exclusively reflect the opinions of the authors. The authors are responsible for all content in their abstracts including accuracy of the facts, statements, citing resources, etc.

**Results**

Differential and comparative analyses reveal substantial differences in the genomic binding profile of HIF-1 between THP-1 and Kasumi-1, with a partial overlap of binding sites indicating subtype-specific HIF-1 binding landscapes. Both cell lines shared a core HIF-1 gene signature including canonical glycolytic genes (e.g. *PGK1*, *TPI1*), consistent with established hypoxia-driven metabolic adaptation. Functional annotation of HIF-1 binding sites in THP-1 cells reveal enriched targets associated with metabolism regulation, whereas Kasumi-1 cells also exhibited enrichment in chromatin remodellers (e.g. *KDM3A*, *KDM6B*, *TET2*).

HMGN1 loss markedly reduced expression of HIF-1 regulons in both AML models, supporting a role for HMGN1 in regulating transcriptional responses to hypoxia. Phenotypically, hypoxic stress compromised proliferative capacity of AML cells, with HMGN1 loss potentiating this effect. Flow cytometric analyses suggest that reduced proliferation is primarily driven by induction of apoptosis rather than cell cycle arrest. Notably, pharmacological stabilisation of HIFs via PHD2 inhibition under normoxic conditions recapitulated the growth-suppressive effects observed in hypoxia, suggesting that hypoxia-induced growth suppression is largely HIF-dependent.

**Summary/Conclusion**

AML driver mutations establish distinct HIF-1 genomic binding profiles and downstream transcriptional responses to hypoxia. HMGN1 modulates the expression of HIF-1 regulons, highlighting the role of chromatin architecture in influencing cellular adaptation to hypoxia.

**Copyright Information:** (Online) ISSN: 2572-9241
©2026 The Author(s). HemaSphere is published by John Wiley & Sons Ltd on behalf of European Hematology Association. This is an open access Abstract Book distributed under the Attribution-NonCommercial-NoDerivs (CC BY-NC-ND), which allows third parties to download the articles and share them with others as long as they credit the author and the Abstract Book, but they cannot change the content in any way or use them commercially.
**Abstract Book Citations:** Authors, Title, HemaSphere, 2026;10:(S1):pages. The abstract book DOIs can be found on the “Author Information” page.
**Disclaimer:** Articles published in the journal HemaSphere exclusively reflect the opinions of the authors. The authors are responsible for all content in their abstracts including accuracy of the facts, statements, citing resources, etc.

### (PS1580) SYSTEMIC REPROGRAMMING OF INFLAMMATORY MICROENVIRONMENT IN AML: FROM NETWORK PATTERNS TO PROGNOSTIC PREDICTION

**Topic:** 03. Acute myeloid leukemia - Biology & translational research

Shuqing Wang*[1]

[1]*Peking Union Medical College Hospital, Beijing, China;*

### Background

Dysregulated inflammation in Acute myeloid leukemia (AML) establishes a protective, leukemia-permissive microenvironment that drives disease evolution. While it is evident that immune communication plays a critical role, current insights are predominantly derived from single-cytokine studies. This narrow perspective overlooks the systemic immune network, creating a critical gap in understanding how inflammatory shifts translate to clinical progression. Therefore, mapping this dysregulated network is essential to uncover mechanistic drivers and to identify novel prognostic markers for patient stratification.

### Aims

Here, we integrated single-cell RNA sequencing and proteomic profiling to comprehensively map the reprogramming of inflammatory networks in AML bone marrow. Given that cytokine synergies—rather than individual factors—likely dictate disease evolution, we aimed to delineate the coordinated alterations within these networks and to elucidate how such collective changes fuel leukemogenesis. This approach will not only uncover mechanistic insights but also inform future therapeutic strategies targeting the inflammatory microenvironment.

### Methods

To systematically dissect inflammatory network remodeling in AML bone marrow, we first performed single-cell RNA sequencing with flow cytometric validation of key immune subsets. We further quantified inflammatory cytokines using Luminex assays. Based on these data, we reconstructed immune communication networks through ligand-receptor interaction analysis and established an Inflammation Risk Score (IRS) via LASSO-Cox regression.

### Results

Single-cell analysis revealed three coordinated transformations: cellular communication dominance shifted from T lymphocytes to myeloid populations, signal networks reconstructed into self-amplifying circuits (CXCL8-CXCR2, CCL3-CCR5 axes), and functional outputs became cell type-dependent. Protein validation confirmed dysregulation of key mediators (CXCL8, CCL2, CCL3, IL-1A; Spearman $r > 0.7$, $p < 0.001$) with significant associations to adverse clinical features including ELN2022 risk ($p < 0.02$) and age ($r = 0.37$, $p = 0.012$). The IRS stratified patients into risk groups with markedly different survival (median >40 vs ~10 months, $p = 0.0001$), achieving sustained predictive accuracy (1-year AUC 0.776, 3-year 0.756) superior to individual biomarkers.

### Summary/Conclusion

Our findings reveal that AML orchestrates a coordinated shift in immune communication, establishing myeloid-dominated inflammatory circuits that drive disease progression. The multi-cytokine Inflammation Risk Score (IRS), derived from this network remodeling, serves as a clinically actionable prognostic tool, demonstrating clear superiority over conventional single-marker biomarkers. By positioning inflammatory network analysis as a novel dimension for risk assessment, this study provides a systems-level framework for understanding AML pathogenesis and guiding future therapeutic strategies.

**Copyright Information:** (Online) ISSN: 2572-9241
©2026 The Author(s). HemaSphere is published by John Wiley & Sons Ltd on behalf of European Hematology Association. This is an open access Abstract Book distributed under the Attribution-NonCommercial-NoDerivs (CC BY-NC-ND), which allows third parties to download the articles and share them with others as long as they credit the author and the Abstract Book, but they cannot change the content in any way or use them commercially.
**Abstract Book Citations:** Authors, Title, HemaSphere, 2026;10:(S1):pages. The abstract book DOIs can be found on the "Author Information" page.
**Disclaimer:** Articles published in the journal HemaSphere exclusively reflect the opinions of the authors. The authors are responsible for all content in their abstracts including accuracy of the facts, statements, citing resources, etc.

### (PS1581) A MULTI-OMICS PLASMACYTOID DENDRITIC CELL SIGNATURE PREDICTS PROGNOSIS AND REFINES RISK STRATIFICATION IN ACUTE MYELOID LEUKEMIA

**Topic:** 03. Acute myeloid leukemia - Biology & translational research

Xuan Wen*[1], Minming Li[1], Chengxin Deng[1], Ping Wu[1], Zesheng Lu[1], Yulian Wang[2], Suxia Geng[1], Peilong Lai[1], Xin Du[1], Jianyu Weng[1], Xin Huang[1]

*[1]Guangdong Provincial People's Hospital (Guangdong Academy of Medical Sciences), Southern Medical University, Department of Hematology, Guangzhou, China; [2]Guangdong Provincial People's Hospital (Guangdong Academy of Medical Sciences), Department of Hematology, Guangzhou, China;*

### Background

Although the European Leukemia Net (ELN) 2022 risk stratification system provides a framework for precise diagnosis and treatment of acute myeloid leukemia (AML), its genetic-based stratification still fails to fully account for the clinical heterogeneity observed among patients. The tumor immune microenvironment (TME) plays a crucial role in disease progression and treatment response, potentially harboring important complementary information. Among TME components, plasmacytoid dendritic cells (pDCs) exert dual functions in tumor immunity, yet their prognostic significance in AML remains unclear.

### Aims

Our aim is to integrate immune features to optimize the AML prognostic risk stratification system.

**Copyright Information:** (Online) ISSN: 2572-9241
©2026 The Author(s). HemaSphere is published by John Wiley & Sons Ltd on behalf of European Hematology Association. This is an open access Abstract Book distributed under the Attribution-NonCommercial-NoDerivs (CC BY-NC-ND), which allows third parties to download the articles and share them with others as long as they credit the author and the Abstract Book, but they cannot change the content in any way or use them commercially.
**Abstract Book Citations:** Authors, Title, HemaSphere, 2026;10:(S1):pages. The abstract book DOIs can be found on the "Author Information" page.
**Disclaimer:** Articles published in the journal HemaSphere exclusively reflect the opinions of the authors. The authors are responsible for all content in their abstracts including accuracy of the facts, statements, citing resources, etc.

### Methods

We collected bone marrow samples at diagnosis from 8 patients with AML who received the ABC-14 regimen (azacitidine, venetoclax, chidamide) at Guangdong Provincial People's Hospital between March 2023 and November 2024. Based on bone marrow assessment after two cycles of induction chemotherapy, patients were classified as complete remission (CR, n=3) or non-CR (n=5). All participants provided written informed consent, and the study was approved by the ethics committee (No.:KY-N-2022-103-01). We integrated our data with those of 13 AML patients from the GSE198681 dataset, who received "3+7" induction chemotherapy (Table 1). After single-cell analysis identified an adverse pDC subset (Fig.1A), we applied five machine learning algorithms to select 7 prognostic genes and constructed the GDPH score. This score was validated for its association with overall survival (OS), chemotherapy response, and immune features.

***Table1 Baseline Characteristics of AML Patients by Treatment Response***

| Characteristics | All patients (n = 21) | Non-CR group (n = 10) | CR group (n = 11) | P value |
|---|---|---|---|---|
| **Age, years [median ]** | 58 (45-63) | 60 (50-62.8) | 53 (38-61.5) | 0.585 |
| **Gender** | | | | |
| Male | 12 (57.1%) | 8 (80%) | 4 (36.4%) | 0.0805 |
| Female | 9 (42.9%) | 2 (20%) | 7 (63.6%) | |
| **Leukocytes, ×10⁹/L [median]** | 8.1 (3.2-45.3) | 6.1 (3.4-20.5) | 35.1 (4.6-69.9) | 0.379 |
| **Blasts in marrow, % [median ]** | 67.5 (45-75) | 69 (57.4-79) | 48 (44.2-71.8) | 0.409 |
| **FAB classification** | | | | |
| M0 | 2 (9.5%) | 1 (10%) | 1 (9.1%) | 1 |
| M1 | 1 (4.8%) | 0 (0%) | 1 (9.1%) | |
| M2 | 15 (71.4%) | 8 (80%) | 7 (63.6%) | |
| M4 | 1 (4.8%) | 0 (0%) | 1 (9.1%) | |
| tAML | 2 (9.5%) | 1 (10%) | 1 (9.1%) | |
| **Cytogenetics risk** | | | | |
| Adverse | 11 (52.4%) | 6 (60%) | 5 (45.5%) | 0.67 |
| Intermediate | 10 (47.6%) | 4 (40%) | 6 (54.5%) | |

### Results

The GDPH score robustly stratified patients into high- and low-risk groups with significantly distinct overall survival across training and validation cohorts(Fig.1B). The score also exhibited significant predictive value for response to induction chemotherapy (AUC > 0.7, 95% CI: 0.650–0.772, P < 0.001)(Fig.1C). Notably, this model extended beyond conventional ELN2022 stratification by uncovering a high-risk subgroup concealed within the favorable-risk category (HR = 4.15, 95% CI: 1.62–10.60, P = 0.003) and survival outcomes comparable to those of ELN2022 intermediate/adverse groups(Fig.1D). Heterogeneity in the immune microenvironment between risk groups suggested LAG3 as a potential immunotherapeutic target(Fig.1E).

### Summary/Conclusion

This study represents the first construction of a pDC-based prognostic and therapeutic response prediction model for AML, offering new insights into AML risk re-stratification and the formulation of immunotherapy strategies.

**Copyright Information:** (Online) ISSN: 2572-9241
©2026 The Author(s). HemaSphere is published by John Wiley & Sons Ltd on behalf of European Hematology Association. This is an open access Abstract Book distributed under the Attribution-NonCommercial-NoDerivs (CC BY-NC-ND), which allows third parties to download the articles and share them with others as long as they credit the author and the Abstract Book, but they cannot change the content in any way or use them commercially.
**Abstract Book Citations:** Authors, Title, HemaSphere, 2026;10:(S1):pages. The abstract book DOIs can be found on the "Author Information" page.
**Disclaimer:** Articles published in the journal HemaSphere exclusively reflect the opinions of the authors. The authors are responsible for all content in their abstracts including accuracy of the facts, statements, citing resources, etc.

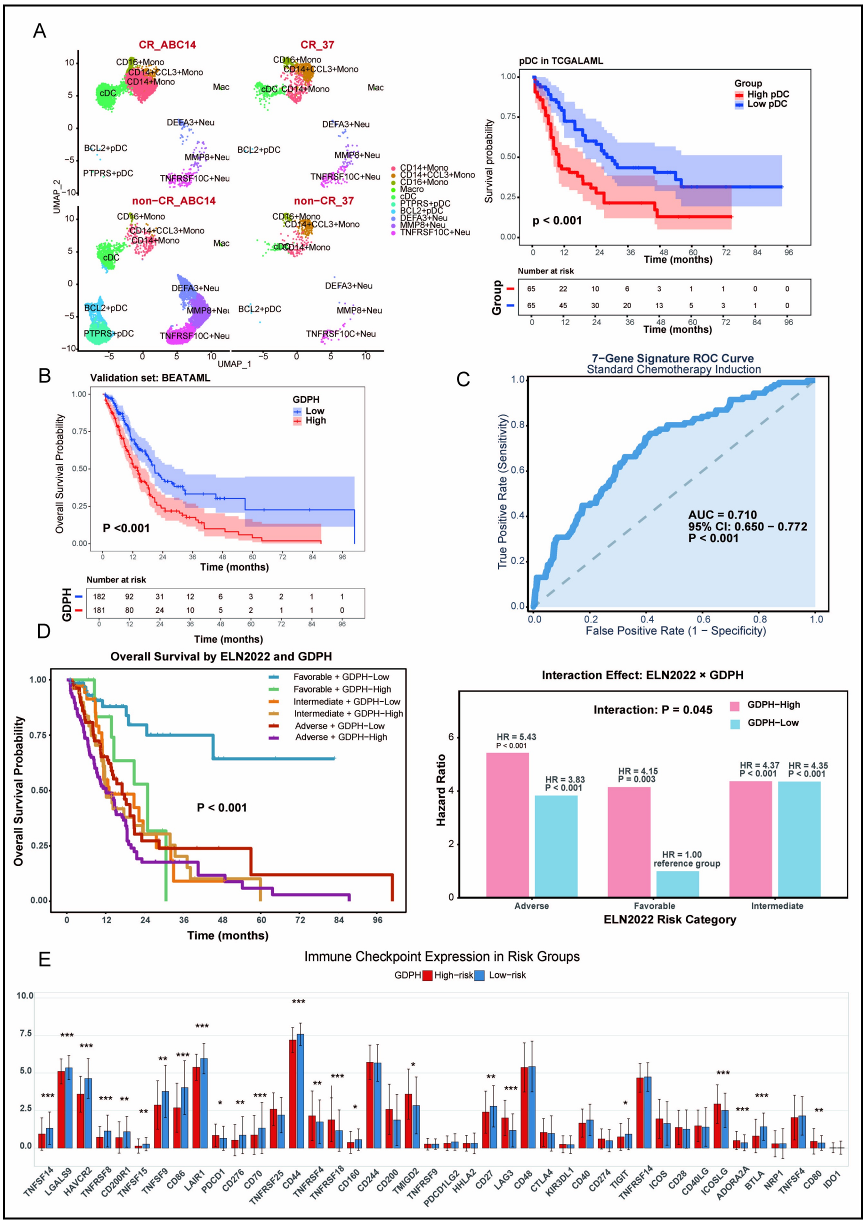

Fig. 1. Construction and validation of the pDC-based prognostic model. (A) Single-cell profiling identifies pDC subsets associated with adverse prognosis in AML. (B) Kaplan–Meier survival curves for GDPH_High vs.GDPH_Low patients in the BEAT-AML validation cohort. (C) ROC curve of the 7-gene signature for predicting response to induction chemotherapy. (D) Forest plot showing hazard ratios for mortality within each ELN2022 risk category, identifying a high-risk subgroup within the ELN2022-favorable-risk group (HR = 4.15, interaction P = 0.045). (E) Bar plot comparing expression levels of 42 immune checkpoint molecules between GDPH_high and GDPH_low subgroups.

**Copyright Information:** (Online) ISSN: 2572-9241
©2026 The Author(s). HemaSphere is published by John Wiley & Sons Ltd on behalf of European Hematology Association. This is an open access Abstract Book distributed under the Attribution-NonCommercial-NoDerivs (CC BY-NC-ND), which allows third parties to download the articles and share them with others as long as they credit the author and the Abstract Book, but they cannot change the content in any way or use them commercially.
**Abstract Book Citations:** Authors, Title, HemaSphere, 2026;10:(S1):pages. The abstract book DOIs can be found on the "Author Information" page.
**Disclaimer:** Articles published in the journal HemaSphere exclusively reflect the opinions of the authors. The authors are responsible for all content in their abstracts including accuracy of the facts, statements, citing resources, etc.

### (PS1582) SYNERGISTIC ANTI-LEUKEMIC EFFECTS OF HOMOHARRINGTONINE COMBINED WITH CLIFUTINIB IN FLT3-ITD MUTATED ACUTE MYELOID LEUKEMIA

**Topic:** 03. Acute myeloid leukemia - Biology & translational research

Wenjie Sha*[1], Haihui Zhuang[1], Fenglin Li[1], Xia Jiang[1], Ying Lu[1]

[1]*Department of Hematology, The Affiliated People's Hospital of Ningbo University, Ningbo, China;*

**Background**

FLT3-ITD mutation is one of the most aggressive molecular subtype in acute myeloid leukemia (AML) and is associated with poor prognosis and high relapse rates. Although FLT3 inhibitors have improved clinical outcomes, resistance and limited durability remain major challenges. Therefore, novel combination strategies targeting multiple oncogenic pathways are urgently needed. Homoharringtonine (HTT) and Clifutinib are both demonstrated as anti-leukemic properties through distinct mechanisms of action, making their combination a rational and promising candidate for multi-targeted therapy; however, their synergistic potential and the underlying molecular mechanisms remain unexplored.

**Aims**

This study aimed to investigate the synergistic anti-leukemic effects of HTT combined with Clifutinib in FLT3-ITD mutated AML and to elucidate the underlying molecular mechanisms both *in vitro* and *in vivo*.

**Methods**

FLT3-ITD mutated leukemia cell lines were treated with HTT and Clifutinib singly or in combination. Cell viability was assessed, and synergistic effects were evaluated using the combination index (CI) method. Apoptosis was analyzed by flow cytometry. Network pharmacology was employed to predict potential signaling pathways involved in the combined treatment. Western blotting was performed to validate the inhibition of key signaling pathways. *In vivo* efficacy was evaluated using a zebrafish xenograft model and a mouse tail-vein injection leukemia model to assess tumor burden and overall survival.

**Results**

The combination of HTT and Clifutinib demonstrated potent and synergistic anti-leukemic activity in FLT3-ITD mutated cell lines. Synergism was confirmed by combination index analysis after 48h treatment, with CI values of 0.77 at $ED_{75}$, and 0.56 at $ED_{90}$. Mechanistically, the combined treatment synergistically induced apoptosis in FLT3-ITD mutated cells. Network pharmacology analysis and subsequent western blot validation revealed that the combination concurrently inhibited multiple survival pathways, including PI3K-AKT, and JAK-STAT signaling, which are central to FLT3-ITD-driven disease progression and therapeutic resistance. Importantly, *in vivo* studies demonstrated that the combination therapy significantly reduced leukemic burden in the zebrafish model and markedly prolonged survival in leukemia-bearing mice compared with monotherapy or controls, underscoring its translational potential as a novel combinational strategy for FLT3-ITD mutated AML.

**Summary/Conclusion**

HTT combined with Clifutinib exerts synergistic anti-leukemic effects against FLT3-ITD mutated AML by inducing apoptosis and simultaneously inhibiting multiple oncogenic signaling pathways, including PI3K-AKT and JAK-STAT. These findings provide a strong preclinical rationale for further investigation of this combination strategy as a potential therapeutic approach for FLT3-ITD mutated AML.

**Copyright Information:** (Online) ISSN: 2572-9241
©2026 The Author(s). HemaSphere is published by John Wiley & Sons Ltd on behalf of European Hematology Association. This is an open access Abstract Book distributed under the Attribution-NonCommercial-NoDerivs (CC BY-NC-ND), which allows third parties to download the articles and share them with others as long as they credit the author and the Abstract Book, but they cannot change the content in any way or use them commercially.
**Abstract Book Citations:** Authors, Title, HemaSphere, 2026;10:(S1):pages. The abstract book DOIs can be found on the "Author Information" page.
**Disclaimer:** Articles published in the journal HemaSphere exclusively reflect the opinions of the authors. The authors are responsible for all content in their abstracts including accuracy of the facts, statements, citing resources, etc.

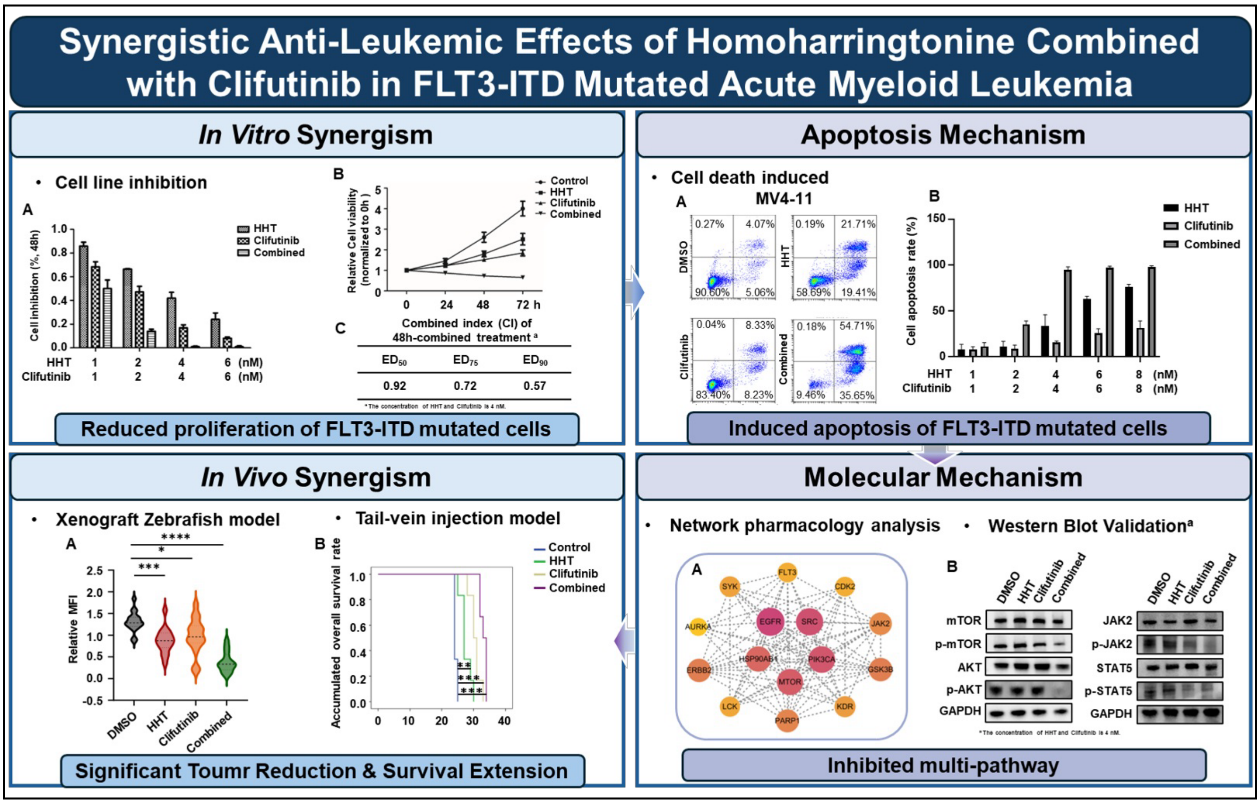

Figure 1. Synergistic efficacy and mechanisms of HHT and Clifutinib combination in FLT3-ITD AML. (A) In vitro synergistic cytotoxicity (CI < 1) in mutant cell lines. (B) Enhanced apoptosis induction by the combination treatment. (C) Mechanism: concurrent suppression of PI3K-AKT, MAPK, and JAK-STAT pathways. (D) In vivo efficacy: reduced tumor burden (zebrafish) and prolonged overall survival (mice).

Copyright Information: (Online) ISSN: 2572-9241
©2026 The Author(s). HemaSphere is published by John Wiley & Sons Ltd on behalf of European Hematology Association. This is an open access Abstract Book distributed under the Attribution-NonCommercial-NoDerivs (CC BY-NC-ND), which allows third parties to download the articles and share them with others as long as they credit the author and the Abstract Book, but they cannot change the content in any way or use them commercially.
Abstract Book Citations: Authors, Title, HemaSphere, 2026;10:(S1):pages. The abstract book DOIs can be found on the "Author Information" page.
Disclaimer: Articles published in the journal HemaSphere exclusively reflect the opinions of the authors. The authors are responsible for all content in their abstracts including accuracy of the facts, statements, citing resources, etc.

### (PS1583) DDX41-MUTATED MYELOID NEOPLASMS IN A LARGE CHINESE COHORT:DISTINCT GERMLINE HOTSPOTS, CLINICAL OUTCOMES, AND SUPERIOR RESPONSE TO HSCT

**Topic:** 03. Acute myeloid leukemia - Biology & translational research

Yueqi Sun*[1], Xiaosu Zhao[1, 2]

*[1]Peking University People's Hospital, Peking University Institute of Hematology, National Clinical Research Center for Hematologic Disease, Beijing Key Laboratory of Cell and Gene Therapy for Hematologic Malignan-cies, Peking University, Beijing, China; [2]Collaborative Innovation Center of Hematology, Peking University, Beijing, China;*

### Background

Germline and somatic DDX41 mutations are increasingly recognized in myeloid neoplasms, with variable prevalence and prognostic impact across ethnicities. Current data are predominantly derived from Western cohorts, and the comprehensive mutational spectrum, clinical characteristics, and therapeutic implications of DDX41 mutations in Chinese patients remain poorly defined.

### Aims

To characterize the clinical, molecular, and prognostic features of DDX41-mutated myeloid neoplasms in a large Chinese cohort, focusing on mutational spectrum, germline/somatic status, co-mutation patterns, and response to chemotherapy and hematopoietic stem cell transplantation (HSCT).

### Methods

We retrospectively analyzed 133 patients with DDX41-mutated acute myeloid leukemia (AML) or myelodysplastic syndromes (MDS) diagnosed between 2018 and January 2025. Clinical, laboratory, cytogenetic, and next-generation sequencing data were collected. Outcomes were evaluated using propensity score matching (PSM) to compare DDX41-mutated and wild-type cohorts.

### Results

The prevalence of DDX41 mutations was 4.13% among AML and MDS patients. Patients were predominantly male (66.9%), with a median age of 61 years and a high rate of normal karyotype (71.4%).
We identified P258L and Y259C as the two most common germline DDX41 hotspots, accounting for 34% each of all germline coding variants, representing a distinct ethnic profile compared with Western cohorts. The canonical somatic hotspot R525H remained the most prevalent somatic mutation.
Somatic DDX41 mutations were associated with more favorable overall survival (OS) and leukemia-free survival (LFS) compared with isolated germline mutations. After PSM, DDX41-mutated patients showed prolonged relapse-free survival (RFS) post-HSCT compared with wild-type patients, while no significant survival benefit was observed with chemotherapy alone.
Notably, in patients with concurrent TP53 mutations, DDX41 mutation was associated with higher complete remission rate, lower mortality, and attenuated adverse prognosis, suggesting a potential protective effect of DDX41 mutation against TP53-related dismal outcomes.

**Copyright Information:** (Online) ISSN: 2572-9241
©2026 The Author(s). HemaSphere is published by John Wiley & Sons Ltd on behalf of European Hematology Association. This is an open access Abstract Book distributed under the Attribution-NonCommercial-NoDerivs (CC BY-NC-ND), which allows third parties to download the articles and share them with others as long as they credit the author and the Abstract Book, but they cannot change the content in any way or use them commercially.
**Abstract Book Citations:** Authors, Title, HemaSphere, 2026;10:(S1):pages. The abstract book DOIs can be found on the "Author Information" page.
**Disclaimer:** Articles published in the journal HemaSphere exclusively reflect the opinions of the authors. The authors are responsible for all content in their abstracts including accuracy of the facts, statements, citing resources, etc.

### Summary/Conclusion

This large Chinese cohort reveals a unique DDX41 mutational spectrum characterized by novel germline hotspots P258L and Y259C. DDX41-mutated MN displays distinct clinical features and favorable outcomes after HSCT rather than conventional chemotherapy. DDX41 mutation may mitigate the adverse impact of TP53 abnormalities. Our findings underscore the importance of ethnic-specific genetic characterization and support early consideration of HSCT in DDX41-mutated myeloid neoplasms.

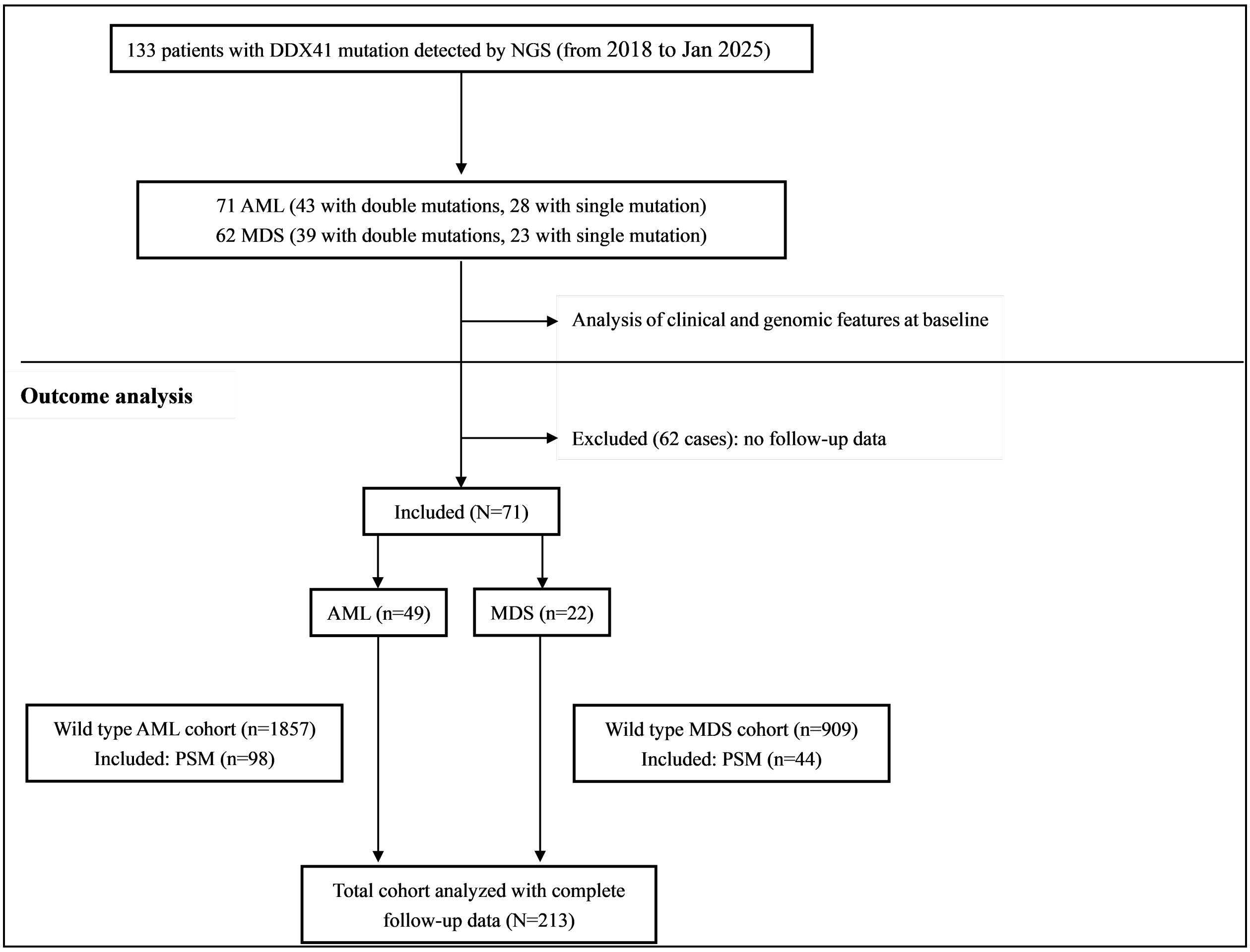

**Copyright Information:** (Online) ISSN: 2572-9241
©2026 The Author(s). HemaSphere is published by John Wiley & Sons Ltd on behalf of European Hematology Association. This is an open access Abstract Book distributed under the Attribution-NonCommercial-NoDerivs (CC BY-NC-ND), which allows third parties to download the articles and share them with others as long as they credit the author and the Abstract Book, but they cannot change the content in any way or use them commercially.
**Abstract Book Citations:** Authors, Title, HemaSphere, 2026;10:(S1):pages. The abstract book DOIs can be found on the "Author Information" page.
**Disclaimer:** Articles published in the journal HemaSphere exclusively reflect the opinions of the authors. The authors are responsible for all content in their abstracts including accuracy of the facts, statements, citing resources, etc.

### (PS1584) THE NOVEL LYSOSOME INHIBITOR LYS05 SYNERGIZES WITH VENETOCLAX TO EXERT ANTI-AML ACTIVITY BY NEGATIVELY REGULATING AUTOPHAGY

**Topic:** 03. Acute myeloid leukemia - Biology & translational research

Xia Jiang*[1], Sumeng Xiang[1], Ying Lu[1]

[1] *The Affiliated People's Hospital of Ningbo University, Department of Hematology, Ningbo, China;*

### Background

Acute myeloid leukemia (AML) is a highly aggressive hematologic malignancy with a dismal 5-year survival rate of approximately 30%. While the standard "3+7" regimen achieves complete remission in 60–80% of patients, overall prognosis remains poor. The advent of targeted therapies, such as the BCL-2 inhibitor venetoclax, has ushered in a new era for treating specific AML molecular subtypes. However, the efficacy of venetoclax-based regimens is often limited by primary and acquired resistance, underscoring an urgent need to overcome drug resistance. Lys05 is a novel lysosome-targeting agent that disrupts cellular survival mechanisms by blocking autophagy, thereby sensitizing tumor cells to chemotherapy. Nevertheless, whether Lys05 can overcome venetoclax resistance by modulating the autophagy-lysosome pathway and exert synergistic anti-AML effects, as well as the underlying molecular mechanisms, remain to be elucidated.

### Aims

To investigate the potential synergistic effect and underlying mechanism of combining the novel lysosome inhibitor Lys05 with venetoclax in AML treatment.

### Methods

Following treatment with Lys05 and venetoclax, either alone or in combination, cell viability was assessed in AML cell lines and patient-derived primary cells using proliferation assays, and combination indices were calculated. Apoptosis was evaluated by flow cytometry. RNA sequencing was performed to identify differentially expressed genes and enriched signaling pathways. Autophagy- and apoptosis-related protein expression was examined by western blotting. The in vivo anti-AML efficacy and safety of the combination were evaluated using an AML xenograft mouse model.

### Results

The combination of Lys05 and venetoclax synergistically inhibited proliferation and induced apoptosis in both AML cell lines and primary patient samples. RNA sequencing revealed that differentially expressed genes upon combination treatment were predominantly enriched in autophagy-lysosome pathways. Western blotting confirmed impaired autophagic flux, as evidenced by an increased LC3-II/LC3-I ratio and significant accumulation of the autophagy substrate p62/SQSTM1. Cross-analysis of gene sets identified five core genes spanning the autophagy-lysosome pathway: CTSB, CTSD, CTSL, LAMP2, and LAMP3. Finally, in an MV4-11-derived xenograft mouse model, the combination treatment synergistically reduced leukemia burden and significantly prolonged mouse survival.

### Summary/Conclusion

Lys05 synergizes with venetoclax to produce a potent anti-AML effect, providing a strong preclinical rationale for clinical translation and offering a promising combination strategy for AML therapy.

**Copyright Information:** (Online) ISSN: 2572-9241
©2026 The Author(s). HemaSphere is published by John Wiley & Sons Ltd on behalf of European Hematology Association. This is an open access Abstract Book distributed under the Attribution-NonCommercial-NoDerivs (CC BY-NC-ND), which allows third parties to download the articles and share them with others as long as they credit the author and the Abstract Book, but they cannot change the content in any way or use them commercially.
**Abstract Book Citations:** Authors, Title, HemaSphere, 2026;10:(S1):pages. The abstract book DOIs can be found on the "Author Information" page.
**Disclaimer:** Articles published in the journal HemaSphere exclusively reflect the opinions of the authors. The authors are responsible for all content in their abstracts including accuracy of the facts, statements, citing resources, etc.

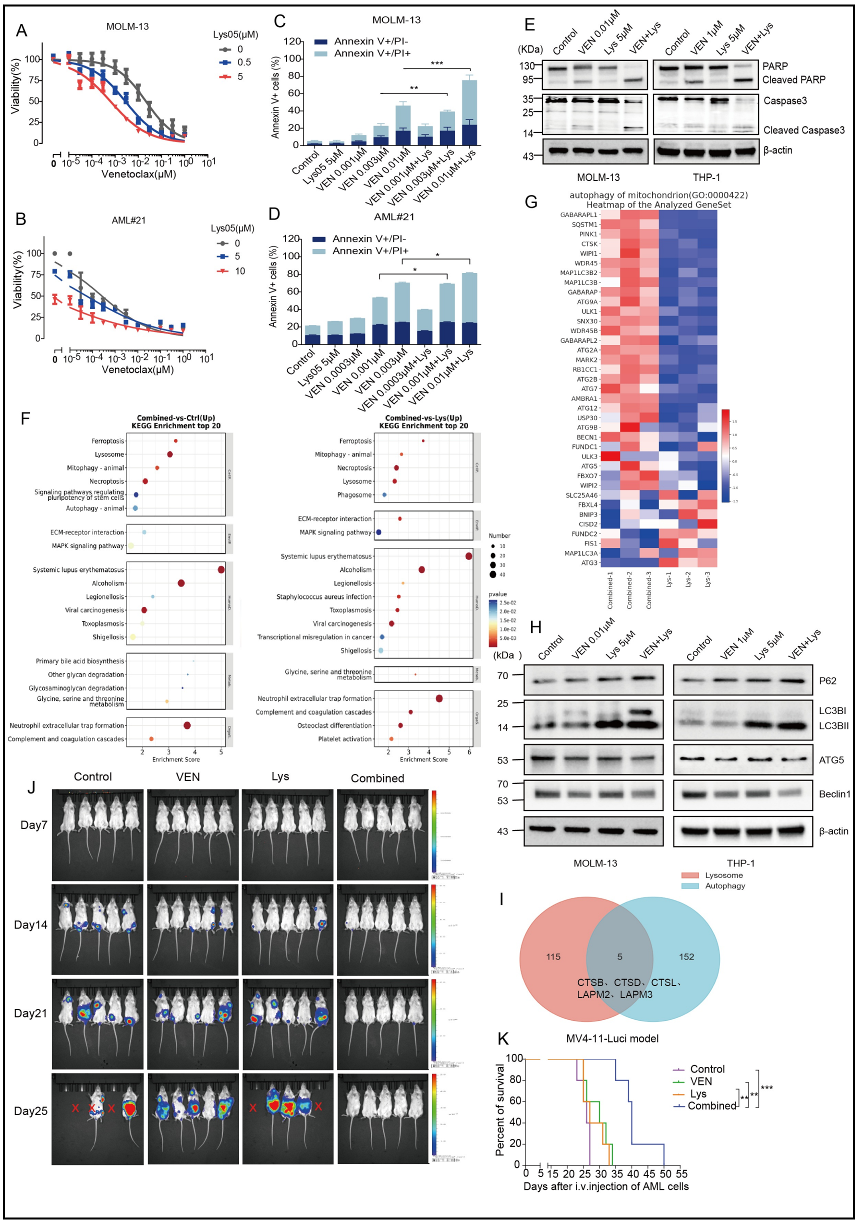

**Copyright Information:** (Online) ISSN: 2572-9241
©2026 The Author(s). HemaSphere is published by John Wiley & Sons Ltd on behalf of European Hematology Association. This is an open access Abstract Book distributed under the Attribution-NonCommercial-NoDerivs (CC BY-NC-ND), which allows third parties to download the articles and share them with others as long as they credit the author and the Abstract Book, but they cannot change the content in any way or use them commercially.
**Abstract Book Citations:** Authors, Title, HemaSphere, 2026;10:(S1):pages. The abstract book DOIs can be found on the "Author Information" page.
**Disclaimer:** Articles published in the journal HemaSphere exclusively reflect the opinions of the authors. The authors are responsible for all content in their abstracts including accuracy of the facts, statements, citing resources, etc.

### (PS1585) PATIENT-INFORMED ENDOGENOUS KMT2A::ELL AND KMT2A::MLLT1 INFANT LEUKEMIA MODELS IN HUMAN HSPCS REVEAL FUSION-DEPENDENT LINEAGE STATES AND ACTIONABLE VULNERABILITIES

**Topic:** 03. Acute myeloid leukemia - Biology & translational research

Álvaro Menchaca Muñiz*[1], Pia Radszuweit[1], Aleksei Parshenkov[1], Markus G. Manz[1], Dominik Schneidawind[1], Corina Schneidawind[1]

[1]*University Hospital Zürich, Department of Medical Oncology and Hematology, Zürich, Switzerland;*

### Background

*KMT2A* (*MLL*) rearrangements drive some of the most aggressive, chemotherapy-resistant infant acute leukemia and often arise with few cooperating mutations. Fusion-dependent characteristics and lineage switching are increasingly linked in prompting therapeutic resistance and relapse. Despite genome-scale recombinome maps and the clinical emergence of novel and promising therapeutics (i.e. menin inhibition), progress in understanding leukemogenesis and developing effective therapeutic strategies remains limited by the scarcity of fully human endogenous models that preserve leukemic state behaviour whilst enabling functional benchmarking.

### Aims

We aim to bridge this gap by establishing patient-informed endogenous *KMT2A::ELL* (AML-biased) and *KMT2A::MLLT1/ENL* (ALL-biased) leukemia model systems in primary human hematopoietic stem and progenitor cells (HSPCs) enabling full characterization of fusion states, definition and testing of actionable targets for their clinical relevance.

### Methods

Cord blood-derived HSPCs are edited with CRISPR/Cas9 RNPs targeting patient-specific breakpoints in*KMT2A* intron 9/11 and common partner loci *ELL* and *ENL/MLLT1* at intron 1. Fusion presence is confirmed by PCR and Sanger sequencing, with culture purity determined by *KMT2A* break-apart FISH analysis. Leukemic phenotypes are assessed by flow cytometry, immunohistochemistry and colony-forming assays. Bulk RNAseq and quantitative proteomics are performed and integrated to compare with public patient datasets. Therapeutic testing of a 17-compound panel designed from these RNA/protein dependencies is followed by a pre/post-treatment proteomic profiling to nominate candidate resistance pathways. *In vivo* relevance is assessed in NSG mice using serial transplantation and measurement of longitudinal peripheral blood (PB) (%hCD45$^{+}$ every 14 days) to assess leukemic development.

### Results

We have generated 15 independent fusion-positive cultures across all relevant *KMT2A::ELL and KMT2A::MLLT1/ENL* contexts. Fusions were detectable within ~3 weeks and were genetically identical across donors. By week 7, FISH demonstrated >90% purity in all cultures. Fusion-positive cultures exhibited sustained proliferation, enriched stem-like/leukemia-associated compartments (CD34$^{+}$CD38$^{-}$, CD34$^{+}$CD90$^{+}$), clonogenic growth (strongest in *KMT2A::ELL*) with activation of leukemic programs (i.e. MYC, or MEIS1). Fusion identity consistently shaped lineage state: AML-biased *KMT2A::ELL* fusions displayed immature myeloid programs (CD117/CD123$^{high}$, CD14$^{low}$) with HOXA-centred signatures, whereas ALL-biased *KMT2A::MLLT1* showed lymphoid-oriented features including upregulated CD19, CD79A & FCER2. Integrated RNA/protein profiles recapitulated patient programs and highlighted actionable nodes with existing modulators (i.e., CASP3, SNCA, NTMT1) guiding our 17-compound panel now entering screening. *In vivo* validation has begun: an age and sex randomized NSG cohort (n=28) has been injected with *KMT2Ar* lines and longitudinal PB monitoring is underway with first timepoints collected. Serial transplantation is planned subsequently.

**Copyright Information:** (Online) ISSN: 2572-9241
©2026 The Author(s). HemaSphere is published by John Wiley & Sons Ltd on behalf of European Hematology Association. This is an open access Abstract Book distributed under the Attribution-NonCommercial-NoDerivs (CC BY-NC-ND), which allows third parties to download the articles and share them with others as long as they credit the author and the Abstract Book, but they cannot change the content in any way or use them commercially.
**Abstract Book Citations:** Authors, Title, HemaSphere, 2026;10:(S1):pages. The abstract book DOIs can be found on the "Author Information" page.
**Disclaimer:** Articles published in the journal HemaSphere exclusively reflect the opinions of the authors. The authors are responsible for all content in their abstracts including accuracy of the facts, statements, citing resources, etc.

**Summary/Conclusion**

Our reproducible endogenous human HSPC platform yields high-purity *KMT2A* fusion cultures capturing fusion-dependent lineage characteristics and patient-concordant features across all molecular and cellular levels allowing for the validation of established therapeutic compounds as well as the definition of novel, precision-based approaches. Paving the way for the personalization of leukemic treatments.

**Copyright Information:** (Online) ISSN: 2572-9241
©2026 The Author(s). HemaSphere is published by John Wiley & Sons Ltd on behalf of European Hematology Association. This is an open access Abstract Book distributed under the Attribution-NonCommercial-NoDerivs (CC BY-NC-ND), which allows third parties to download the articles and share them with others as long as they credit the author and the Abstract Book, but they cannot change the content in any way or use them commercially.
**Abstract Book Citations:** Authors, Title, HemaSphere, 2026;10:(S1):pages. The abstract book DOIs can be found on the “Author Information” page.
**Disclaimer:** Articles published in the journal HemaSphere exclusively reflect the opinions of the authors. The authors are responsible for all content in their abstracts including accuracy of the facts, statements, citing resources, etc.

### (PS1586) SYNERGISTIC EFFICACY OF HDACI CHIDAMIDE WITH GL-V9 BY SUPPRESSING MYBL2 EXPRESSION IN ACUTE MYELOID LEUKEMIA

**Topic:** 03. Acute myeloid leukemia - Biology & translational research

Yazhen Bi[1], Qinglong Guo[2], Hui Hui[2], Hui Li[2], Chunhua Song[3], Zheng Ge*[1]

*[1]Department of Hematology, Zhongda Hospital, School of Medicine, Southeast University, Institute of Hematology Southeast University, Nanjing, China; [2]State Key Laboratory of Natural Medicines, Jiangsu Key Laboratory of Carcinogenesis and Intervention, School of Basic Medicine and Clinical Pharmacy, China Pharmaceutical University, Nanjing, China; [3]Division of Hematology, The Ohio State University Wexner Medical Center, The James Cancer Hospital, Columbus, United States of America;*

### Background

Traditional high-dose chemotherapy is limited to elderly and unfit patients with acute myeloid leukemia (AML). Therefore, it has significant clinical relevance to have a new targeted therapy and combined therapy for AML. Chidamide is a benzamide histone deacetylase (HDAC) inhibitor that induces growth inhibition, cell cycle arrest, and apoptosis in AML. GL-V9 is a synthesized flavonoid derived from wogonin, which is one of the main active components of Scutellarin baicalensis and exhibits anticancer activities with low toxicity as its natural features.

### Aims

This study sought to explore the synergistic efficacy and underlying mechanisms of Chidamide with GL-V9 in AML.

### Methods

Cell Counting Kit-8 was used for cell proliferation assay; CalcuSyn was applied for the synergy analysis of the two drugs. Cell cycle and apoptosis were measured by flow cytometry analysis. Cellular senescence was assessed by senescence-associated β-galactosidase (SA-β-gal) staining. The human AML cell U937-xenograft (CDX) mouse model was developed to examine the therapeutic efficacy of chidamide with GL-V9 in vivo. Transcriptome was analyzed by RNA-seq analysis in MV4-11 cells treated with chidamide or GL-V9, respectively. WB and qPCR were utilized for gene expression. Co-immunoprecipitation (Co-IP) assays were performed to verify the interaction between the two proteins in AML.

**Copyright Information:** (Online) ISSN: 2572-9241
©2026 The Author(s). HemaSphere is published by John Wiley & Sons Ltd on behalf of European Hematology Association. This is an open access Abstract Book distributed under the Attribution-NonCommercial-NoDerivs (CC BY-NC-ND), which allows third parties to download the articles and share them with others as long as they credit the author and the Abstract Book, but they cannot change the content in any way or use them commercially.
**Abstract Book Citations:** Authors, Title, HemaSphere, 2026;10:(S1):pages. The abstract book DOIs can be found on the “Author Information” page.
**Disclaimer:** Articles published in the journal HemaSphere exclusively reflect the opinions of the authors. The authors are responsible for all content in their abstracts including accuracy of the facts, statements, citing resources, etc.

### Results

GL-V9 had a dose-dependent effect on cell proliferation arrest in U937, Molm13, and MV411 cells. GL-V9 significantly sensitized this effect of chidamide vs single-drug controls, and CalcuSyn analysis showed the synergistic effect of the combination (Fig. 1A). The combination also significantly increased the % apoptotic cells vs single-drug controls (Fig. 1B). Chidamide induced G0/G1-phase arrest and GL-V9 induced G2/M arrest, whereas the combination significantly decreased the % S-phase cells (Fig. 1C). Also, combined treatment with drug chidamide and GL-V9 increased the number of SA-β-gal–positive cells (Fig. 1D). Moreover, the combination of chidamide with GL-V9 vs either single drug controls significantly extended the survival (Fig. 1F), reduced the spleen sizes and weight (Fig. 1E), % $hCD45^{+}hCD33^{+}$ leukemia cells of the spleen and bone marrow (Fig. 1G) in the U937-xengraft mouse models. The combination vs single drug controls also significantly inhibited % cell viability of the primary cells from AML patients (Fig. 1H), and CalcuSyn analysis showed the combination had a synergistic effect.

To better understand the underlying mechanism of the synergy, the transcriptome changes upon the drug treatment were analyzed, and the significantly different expression genes (DEGs) were identified upon either GL-V9 or Chidamide treatment vs vehicle control. The overlapped DEGs are mainly enriched in cell cycle and cell senescence (Fig. 1I), and MYBL2, a member of the MYB family of transcription factors, is one of the down-regulated top DEGs. MYBL2 is a central regulator of cell cycle progression, cell senescence and cell survival, dysregulated in a wide range of cancers. Combining chidamide with GL-V9 significantly down-regulated MYBL2 mRNA and protein levels compared with single drug and vehicle control (Fig. 1K). Co-IP analysis confirmed the interaction between HDAC1 and MYBL2(Fig. 1L).

### Summary/Conclusion

Combining HDAC inhibitor chidamide with GL-V9 demonstrates synergistic efficacy by suppressing MYBL2 expression in AML. Our results reveal a new potential combined therapeutic option for AML.

**Copyright Information:** (Online) ISSN: 2572-9241
©2026 The Author(s). HemaSphere is published by John Wiley & Sons Ltd on behalf of European Hematology Association. This is an open access Abstract Book distributed under the Attribution-NonCommercial-NoDerivs (CC BY-NC-ND), which allows third parties to download the articles and share them with others as long as they credit the author and the Abstract Book, but they cannot change the content in any way or use them commercially.
**Abstract Book Citations:** Authors, Title, HemaSphere, 2026;10:(S1):pages. The abstract book DOIs can be found on the "Author Information" page.
**Disclaimer:** Articles published in the journal HemaSphere exclusively reflect the opinions of the authors. The authors are responsible for all content in their abstracts including accuracy of the facts, statements, citing resources, etc.

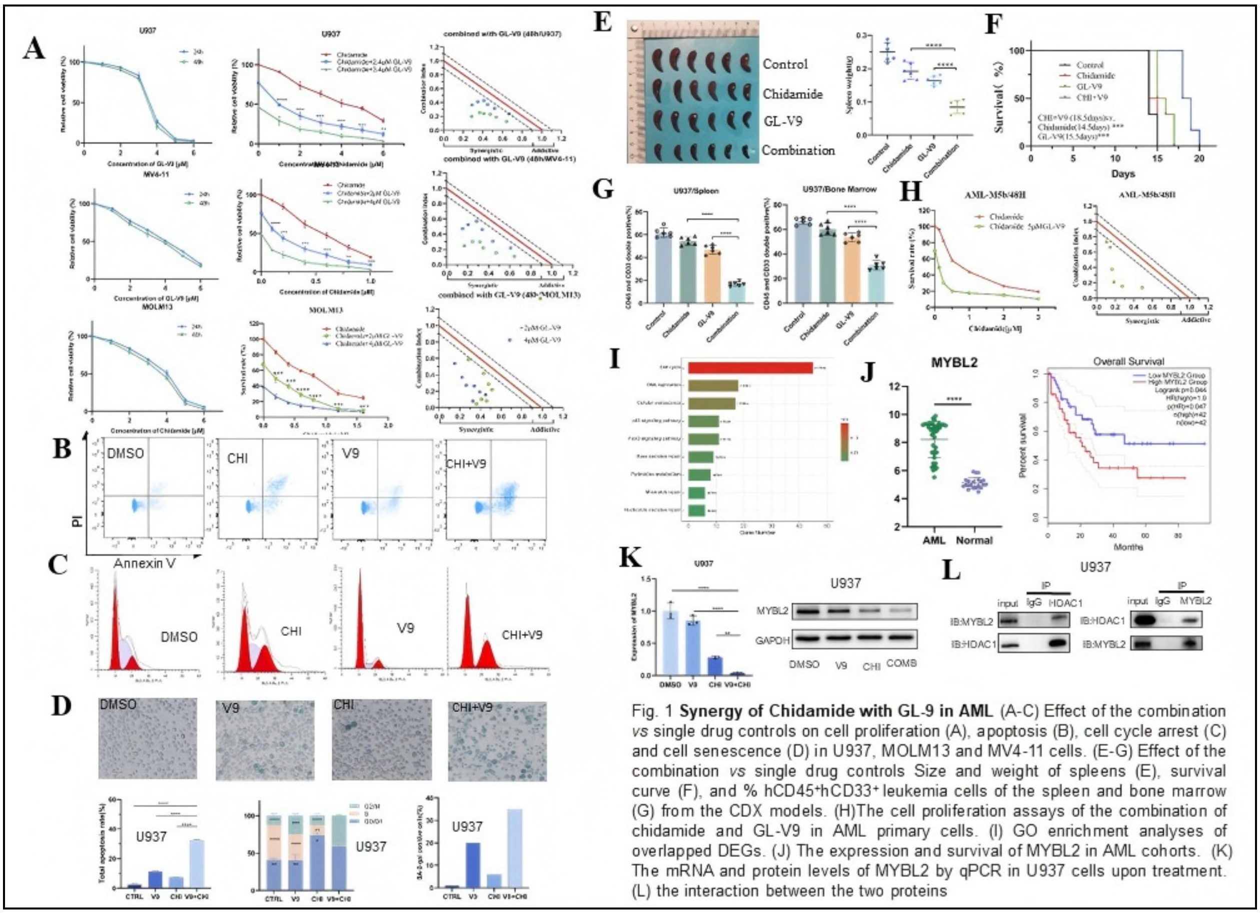

Fig. 1 **Synergy of Chidamide with GL-9 in AML** (A-C) Effect of the combination *vs* single drug controls on cell proliferation (A), apoptosis (B), cell cycle arrest (C) and cell senescence (D) in U937, MOLM13 and MV4-11 cells. (E-G) Effect of the combination *vs* single drug controls Size and weight of spleens (E), survival curve (F), and % hCD45⁺hCD33⁺ leukemia cells of the spleen and bone marrow (G) from the CDX models. (H)The cell proliferation assays of the combination of chidamide and GL-V9 in AML primary cells. (I) GO enrichment analyses of overlapped DEGs. (J) The expression and survival of MYBL2 in AML cohorts. (K) The mRNA and protein levels of MYBL2 by qPCR in U937 cells upon treatment. (L) the interaction between the two proteins

**Copyright Information:** (Online) ISSN: 2572-9241
©2026 The Author(s). HemaSphere is published by John Wiley & Sons Ltd on behalf of European Hematology Association. This is an open access Abstract Book distributed under the Attribution-NonCommercial-NoDerivs (CC BY-NC-ND), which allows third parties to download the articles and share them with others as long as they credit the author and the Abstract Book, but they cannot change the content in any way or use them commercially.
**Abstract Book Citations:** Authors, Title, HemaSphere, 2026;10:(S1):pages. The abstract book DOIs can be found on the "Author Information" page.
**Disclaimer:** Articles published in the journal HemaSphere exclusively reflect the opinions of the authors. The authors are responsible for all content in their abstracts including accuracy of the facts, statements, citing resources, etc.

### (PS1587) FINAL ANALYSIS OF THE APOLLO TRIAL FOR HIGH-RISK ACUTE PROMYELOCYTIC LEUKEMIA (APL)

**Topic:** 04. Acute myeloid leukemia - Clinical

Maria Teresa Voso*[1], Lionel Ades[2], Pau Montesinos[3], Emanuele Ammatuna[4], Pierre Fenaux[2], Claudia Baldus[5], Massimo Bernardi[6], Céline Berthon[7], Monica Bocchia[8], Caroline Bonmati[9], Erika Borlenghi[10], Martin Bornhauser[11], Diana Carp[12], Sylvain Chantepie[13], Enrico Crea[14], Fabio Efficace[15], Adriano Venditti[1], Hartmut Döhner[16], Gerhard Ehninger[11], Jordi Esteve Reyner[17], Jamilé Frayfer[18], Ana Garrido Diaz[19], Cristina Gil Cortes[20], Luca Guarnera[1], Anna Franziska Hamm[21], Mael Heiblig[22], Daniela Heidenreich[23], Alwin Johannes Krämer[24], Marie-Pierre Ledoux[25], Monia Lunghi[26], Valentina Mancini[27], Klaus Metzeler[28], Maria Cristina Miggiano[29], Carsten Müller-Tidow[24], Pierre Peterlin[30], Alfonso Piciocchi[14], Kathrin Rieger[31], Christoph Röllig[11], Giovanni Rossi[32], Miguel Sanz[3], Hubert Serve[33], Maaike Sohne[34], Karsten Spiekermann[35], Emmanuelle Tavernier Tardy[36], Christian Thiede[11], Susana Vives Polo[37], Wichard Vogel[38], Patrizia Zappasodi[39], Sven Zukunft[11], Pauline Ziller-Walter[11], Uwe Platzbecker[11]

*[1]University of Tor Vergata, Biomedicine and Prevention, Rome, Italy; [2]Université Paris Cité, Paris, France; [3]Servicio de Hematología, Hospital Universitari i Politècnic La Fe, Valencia,, Spain; [4]Afd. Hematologie, University Medical Center Groningen, Groningen, The Netherlands; [5]Department of Internal Medicine II, University Hospital Schleswig-Holstein, Kiel, Germany; [6]Ospedale San Raffaele S.r.l., U.O. Ematologia e Trapianto di Midollo, Milano, Italy;[7]Service des Maladies du sang, Le Centre Hospitalier Universitaire de Lille, Lille, France; [8]Azienda Ospedaliera Senese, Ematologia, Siena, Italy; [9]Le Centre Hospitalier Régional Universitaire de Nancy, Service d'Hématologie et Médecine Interne, Vandoeuvre-lés-Nancy, France; [10]ASST degli Spedali Civili di Brescia, Ematologia, Brescia, Italy; [11]University Hospital Carl Gustav Carus and TU Dresden - Faculty of Medicine, Medical Clinic 1, Dresden, Germany; [12]Service d'Hématologie Clinique et de Thérapie Cellulaire, Le Centre Hospitalier Régional d'Orléans, Orléans, France; [13]Service d'Hématologie Clinique, Le Centre Hospitalier Universitaire de Caen, Caen, France; [14]Fondazione GIMEMA Franco Mandelli Onlus, Roma, Italy; [15]Italian Group for Adult Hematologic Diseases, Data Center and Health Outcomes Research Unit, Rome, Italy; [16]Department of Internal Medicine III, Universitätsklinikum Ulm, Ulm, Germany; [17]Servicio de Hematología, Hospital Universitari Clínic de Barcelona, Barcelona, Spain;[18]Service d'Hématologie, Centre Hospitalier de Meaux, France, France; [19]Servicio de Hematología, Hospital de la Santa Creu i Sant Pau, Barcelona, Spain; [20]Servicio de Hematología, Hospital General Universitario de Alicante, Alicante, Spain;[21]Medical Clinic I, University Hospital Schleswig-Holstein, Lübeck, Germany; [22]Department of Hematology, Hospices Civils de Lyon-Lyon-Sud Hospital, Pierre Bénite, France; [23]Medical Clinic III, University Hospital Mannheim, Mannheim, Germany;[24]Department of Internal Medicine 5, University Hospital Heidelberg, Heidelberg, Germany; [25]Institut de cancérologie Strasbourg Europe, Strasbourg, France;[26]Division of Hematology, Department of Translational Medicine, Università del Piemonte Orientale and AOU Maggiore della Carità, Novara, Italy; [27]ASST Grande Ospedale Motropolitano Niguarda, Ematologia, Milano, Italy; [28]Department for Hematology, Cellular Therapy and Hemostaseology, University Hospital Leipzig, Leipzig, Germany; [29]Azienda ULSS 8 Bercia, Ematologia, Vicenza, Italy; [30]Service d'Hématologie Clinique, Le Centre Hospitalier Universitaire de Nantes, Nantes, France; [31]Department of Hematology, Oncology and Cancer Immunology, Campus Benjamin Franklin, Charité-Universitätsmedizin Berlin, corporate member of Freie Universität Berlin and Humboldt-Universität zu Berlin, Berlin, Germany; [32]Ospedale Casa Sollievo della Sofferenza, Ematologia, San Giovanni Rotondo, Italy; [33]Department of Medicine II, Goethe University Frankfurt, Frankfurt, Germany; [34]St Antonius Ziekenhuis, Nieuwegein, The Netherlands; [35]Department of Medicine III, University Hospital Großhadern-Ludwig-Maximilians-University, München, Germany; [36]Institut De Cancérologie Et D'hématologie Universitaire De Saint-Étienne, Saint-Priest-en-Jarez, France; [37]Servicio de Hematología, Hospital Universitari Germans Trias i Pujol, Barcelona, Spain; [38]Department of Hematology, Oncology, Clinical Immunology and Rheumatology, University Hospital Tübingen, Tübingen, Germany; [39]Fondazione IRCCS Policlinico San Matteo, S.C. Ematologia, Pavia, Italy;*

**Copyright Information:** (Online) ISSN: 2572-9241
©2026 The Author(s). HemaSphere is published by John Wiley & Sons Ltd on behalf of European Hematology Association. This is an open access Abstract Book distributed under the Attribution-NonCommercial-NoDerivs (CC BY-NC-ND), which allows third parties to download the articles and share them with others as long as they credit the author and the Abstract Book, but they cannot change the content in any way or use them commercially.
**Abstract Book Citations:** Authors, Title, HemaSphere, 2026;10:(S1):pages. The abstract book DOIs can be found on the "Author Information" page.
**Disclaimer:** Articles published in the journal HemaSphere exclusively reflect the opinions of the authors. The authors are responsible for all content in their abstracts including accuracy of the facts, statements, citing resources, etc.

**Background**

The APOLLO trial, a phase III European academic trial for high-risk APL, defined by WBC≥10 x$10^9$ /L, showed superiority of all-trans retinoic acid and arsenic trioxide (ATRA/ATO) preceded by two doses of idarubicin, and followed by four ATRA/ATO consolidation cycles (arm A), compared with the classical AIDA regimen (ATRA plus idarubicin induction followed by three chemotherapy consolidation cycles and two years of maintenance, arm B). The trial met its primary endpoint, demonstrating significantly superior 2-year event-free survival (EFS) in the ATRA/ATO arm (88% vs 71%, p=0.02) (Platzbecker et al, J Clin Oncol 2025).

**Aims**

The present analysis aimed to evaluate long-term outcomes and to assess additional prognostic factors for EFS, including short-term management measures and long-term adverse events (AEs).

**Methods**

The trial included 133 patients randomized to ATRA/ATO (n= 68) or AIDA (n= 65). Median age was 46 years (range, 18-65), with 52% males. Median body mass index (BMI) was 26.9 (range, 17.5-42.8) and was similar between treatments arms. Additional baseline patient and disease characteristics have been previously reported.

**Results**

The rate of early death during induction was similar between treatment arms, with 5 patients (7.4%) in the ATRA–ATO group and 6 patients (9.2%) in the ATRA–CHT group dying at a median of 3 days from enrollment (range, 1–26 days). During induction, 111 bleeding events were reported in Arm A and 124 in arm B (8 vs 6 CTCAE grade ≥ 3, respectively; p=0.4). Thrombosis was rarely reported, with 1 and 4 cases in arm A vs B (p=0.2). Cytoreduction with hydroxyurea (HU) was administered in 42 of 62 pts (67.7%) in arm A and 38 of 63 pts (64%) in arm B (p=0.4). Median HU duration was 3 days in both arms (IQ range, 0-6 d for both), corresponding to a median cumulative dose of 11,500 mg and 12,000 mg in arms A and B, respectively (p=0.1).
With a median follow-up of 50 months (range, 1.4-101.8), 5-year EFS was 83% (95% CI, 73-94) in arm A vs 64% (95% CI, 52-79) in arm B (Log-rank p=0.0089; Figure 1), while 5-year overall survival was 88%% (95% CI, 79-97) vs 83% (95% CI, 74-93), respectively (p=0.16). The Cox regression analysis for EFS showed that age and white blood cell count (logWBC), but not BMI, were independent predictors for inferior EFS (age: HR:1.03, CI:1.00-1.07, p=0.045; logWBC: HR:1.75, CI: 1.16-2.64, p=0.007).
The 5-year cumulative incidence of relapse (CIR) was 3.4% in the ATRA/ATO arm (1 molecular and 1 hematologic relapse) compared with 16% in the AIDA arm (7 molecular and 2 hematologic relapses), occurring at a median of 11 months (range, 6.4-33.1) from CR (HR 0.20, 95% CI: 0.04-0.9, p=0.036). Two cases of secondary acute myeloid leukemia occurred in Arm B at a median of 55 months from randomization.

**Summary/Conclusion**

The final analysis of the APOLLO trial, conducted at a median follow-up of 50 months, confirms the superiority of the nearly chemotherapy-free ATRA/ATO regimen over AIDA chemotherapy in high-risk APL, resulting in significantly longer event-free survival (EFS) and lower cumulative incidence of relapse (CIR). In the HR-APL setting, ATRA-ATO was able to early control leukocytosis similar to AIDA, as shown by similar HU total dose and duration in the two arms. Also, there were no differences in the rate of short and long-term AE, except for the previously reported reduced hematologic toxicity of the ATRA/ATO regimen.

**Copyright Information:** (Online) ISSN: 2572-9241
©2026 The Author(s). HemaSphere is published by John Wiley & Sons Ltd on behalf of European Hematology Association. This is an open access Abstract Book distributed under the Attribution-NonCommercial-NoDerivs (CC BY-NC-ND), which allows third parties to download the articles and share them with others as long as they credit the author and the Abstract Book, but they cannot change the content in any way or use them commercially.
**Abstract Book Citations:** Authors, Title, HemaSphere, 2026;10:(S1):pages. The abstract book DOIs can be found on the “Author Information” page.
**Disclaimer:** Articles published in the journal HemaSphere exclusively reflect the opinions of the authors. The authors are responsible for all content in their abstracts including accuracy of the facts, statements, citing resources, etc.

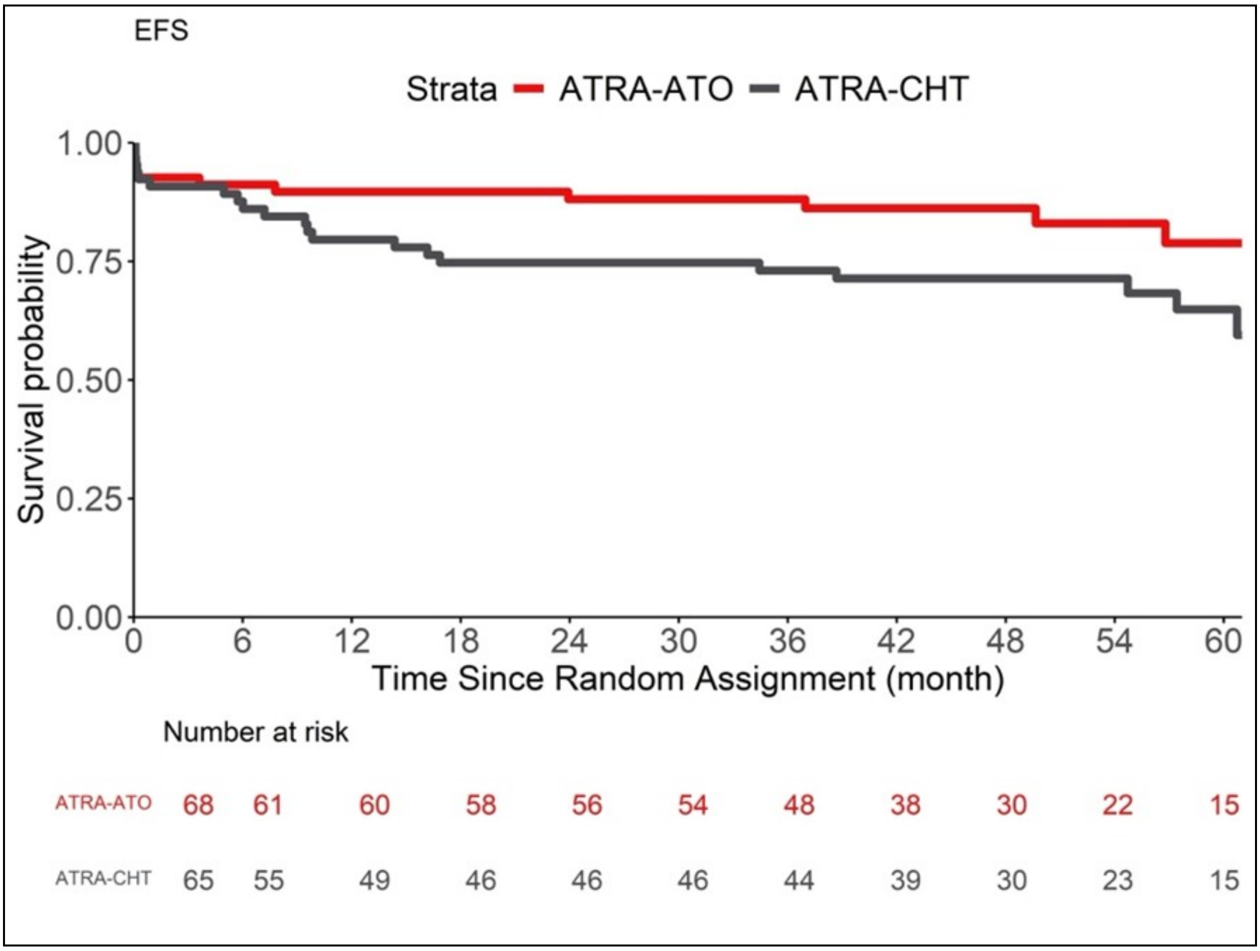

Figure 1. Event-free survival

**Copyright Information:** (Online) ISSN: 2572-9241
©2026 The Author(s). HemaSphere is published by John Wiley & Sons Ltd on behalf of European Hematology Association. This is an open access Abstract Book distributed under the Attribution-NonCommercial-NoDerivs (CC BY-NC-ND), which allows third parties to download the articles and share them with others as long as they credit the author and the Abstract Book, but they cannot change the content in any way or use them commercially.
**Abstract Book Citations:** Authors, Title, HemaSphere, 2026;10:(S1):pages. The abstract book DOIs can be found on the "Author Information" page.
**Disclaimer:** Articles published in the journal HemaSphere exclusively reflect the opinions of the authors. The authors are responsible for all content in their abstracts including accuracy of the facts, statements, citing resources, etc.

### (PS1588) CLINICAL AND MOLECULAR CHARACTERIZATION OF THERAPY-RELATED AML: INSIGHTS FROM THE HARMONY PLATFORM

**Topic:** 04. Acute myeloid leukemia - Clinical

Luca Guarnera*[1], Helena Fidalgo[2], Angela Villaverde Rimero[2], Gert Ossenkoppele[3], Marta Sobas[4], Rabea Mecklenbrauck[5], Magdalena Karasek[4], Enrico Attardi[1], Rosa María Ayala Díaz[6], Jurjen Versluis[7], Joaquín Martinez-Lopez[6], Klaus Metzeler[8], Amin Turki[9], Ian Thomas[10], Brian Huntly[11], Christian Thiede[12], Christoph Röllig[13], Ken Mills[14], Jorge Sierra Gil[15], Guadalupe Oñate[16], Anna Bohlin[17], Torsten Haferlach[18], Sören Lehmann[19], Jesus Maria Hernandez Rivas[20], Lars Bullinger[21], Konstanze Döhner[22], Hartmut Döhner[22], Maria Teresa Voso[1]

*[1]University of Tor Vergata, Biomedicine and Prevention, Rome, Italy; [2]Instituto de Investigación Biomédica de Salamanca, Instituto Universitario de Biología Molecular y Celular del Cáncer, Consejo Superior de Investigaciones Científicas, Cancer Research Center, Salamanca, Spain; [3]Department of Hematology, Amsterdam UMC location Vrije Universiteit Amsterdam, Amsterdam, The Netherlands; [4]Department of Hematology, Collegium Medicum in Bydgoszcz, Nicolaus Copernicus University in Toruń, Bydgoszcz, Poland; [5]Department of Hematology, Hemostasis, Oncology and Stem Cell Transplantation, Hannover Medical School, Hannover, Germany; [6]Hospital Universitario 12 de Octubre, National Cancer Research Center, Complutense University, Madrid, Spain; [7]Department of Hematology, Erasmus MC Cancer Institute, Erasmus University Medical Center Rotterdam, Rotterdam, The Netherlands; [8]Department for Hematology, Cellular Therapy and Hemostaseology, University Hospital Leipzig, Leipzig, Germany; [9]Computational Hematology Lab, West-German Cancer Center, University Hospital Essen, Hufelandstr. 55, 45122, Essen, Germany; [10]Cardiff University, Cardiff, United Kingdom; [11]Department of Haematology and Cambridge Stem Cell Institute, University of Cambridge, Cambridge, United Kingdom; [12]Medizinische Klinik und Poliklinik 1, Medizinische Fakultät und Universitätsklinikum Carl Gustav Carus, Technische Universität Dresden, Dresden, Germany; [13]University Hospital Carl Gustav Carus and TU Dresden - Faculty of Medicine, Medical Clinic 1, Dresden, Germany;[14]Patrick G Johnston Centre for Cancer Research, Queen's University Belfast, Belfast, United Kingdom; [15]Hematology Department, Hospital Santa Creu i Sant Pau, Barcelona, Spain; [16]Hospital de la Santa Creu i Sant Pau, Barcelona, Spain;[17]Department of Medicine, Division of Hematology, Karolinska University Hospital, Huddinge, Sweden; [18]MLL Munich Leukemia Laboratory, Munich, Germany; [19]Department of Medical Sciences, Hematology, Uppsala University, University Hospital Uppsala, Uppsala, Sweden; [20]Department of Hematology, University Hospital of Salamanca, Salamanca, Spain;[21]Department of Hematology, Oncology, and Cancer Immunology, Charité-Universitätsmedizin Berlin, corporate member of Freie Universität Berlin, Humboldt Universität zu Berlin, and Berlin Institute of Health, Berlin, Germany; [22]Department of Internal Medicine III, University Hospital of Ulm, Ulm, Germany;*

### Background

Therapy-related acute myeloid leukemias (t-AML) are characterized by high-risk karyotype abnormalities and somatic mutations, leading to an overall dismal prognosis. However, the role of "therapy-related" status has not been completely elucidated.

### Aims

We aim to investigate clinical and biological features of t-AML, as well as the impact of "therapy-related" status

### Methods

We used data on the HARMONY Platform, gathered from public and private sources, and examined genetic characteristics and outcome of a large cohort of patients with t-AML as compared to *de novo* AML.

**Copyright Information:** (Online) ISSN: 2572-9241
©2026 The Author(s). HemaSphere is published by John Wiley & Sons Ltd on behalf of European Hematology Association. This is an open access Abstract Book distributed under the Attribution-NonCommercial-NoDerivs (CC BY-NC-ND), which allows third parties to download the articles and share them with others as long as they credit the author and the Abstract Book, but they cannot change the content in any way or use them commercially.
**Abstract Book Citations:** Authors, Title, HemaSphere, 2026;10:(S1):pages. The abstract book DOIs can be found on the "Author Information" page.
**Disclaimer:** Articles published in the journal HemaSphere exclusively reflect the opinions of the authors. The authors are responsible for all content in their abstracts including accuracy of the facts, statements, citing resources, etc.

**Results**

We selected 4335 patients, 4103 *de novo* (dn-AML) and 232 t-AML, with available information on previous cytotoxic treatment, and treated with intensive chemotherapy. Patients with t-AML vs dn-AML were older (median age: 57 y vs 51 y, p<0.001), and were more frequently females (63% vs 49%, p<0.001). t-AML presented higher prevalence (p<0.05) of high-risk genetic abnormalities, including trisomy 8, del(5q), del(7), t(9;11) (11% vs 4%, 8% vs 1%, 10% vs 2%, 11% vs 1%, respectively), and *TP53*, *RUNX1* and *ASXL1* mutations (17% vs 2%, 12% vs 3%, 7% vs 3%, respectively; fig 1A). When analysing clinical outcomes, patients with t-AML presented inferior overall survival (OS) and event-free survival (EFS) when compared to dn-AML (p<0.001 for both). The ELN2022 risk classification correctly stratified both dn- and t-AML (p<0.001 for OS and EFS for both). When analysing risk subgroups, OS was similar in the favourable risk group, while it was inferior for patients with t-AML in the intermediate (p=0.008), and in the adverse groups (p<0.001; Fig. 1B-D). When investigating ELN2022 model performance, there was no difference in *dn* vs t-AML (c-index: 0.6 and 0.61 for OS, and 0.6 and 0.6 for EFS). To further assess the role of previous cytotoxic therapy, we built a multivariable model including age, ELN2022 stratification, allogenic transplantation and AML type (*dn* vs t-AML). All these variables independently correlated with OS (HR for therapy-related status: 1.3308, 95% CI: 1.1216-1.5790, p=0.001). We then investigated the role of specific mutations and karyotype abnormalities. *TP53* mutations conferred an inferior prognosis in all patients (p<0.001 for OS and EFS), with no differences between dn and t- AML. In AML with MDS-related gene mutations mutations t-AML had inferior OS and EFS as compared to *dn*-AML (both p<0.001). Karyotype abnormalities including del(7), del(5q) and t(9;11) were prognostically unfavorable in *dn*-AML (p<0.001, p<0.001, p=0.006, respectively), but not in t-AML (p=0.088, p=0.059, p=0.534, respectively). Trisomy 8 did not play a prognostic role in both disease types.

**Summary/Conclusion**

In our large cohort of intensively-treated AML patients, a therapy-related status conferred an independent, inferior prognosis. This impact was particularly evident in high-risk subsets, including the ELN22 adverse-risk subgroup and cases with mutations in MDS-related genes. *TP53* mutations were prognostically unfavourable, regardless of AML subtype

**Copyright Information:** (Online) ISSN: 2572-9241
©2026 The Author(s). HemaSphere is published by John Wiley & Sons Ltd on behalf of European Hematology Association. This is an open access Abstract Book distributed under the Attribution-NonCommercial-NoDerivs (CC BY-NC-ND), which allows third parties to download the articles and share them with others as long as they credit the author and the Abstract Book, but they cannot change the content in any way or use them commercially.
**Abstract Book Citations:** Authors, Title, HemaSphere, 2026;10:(S1):pages. The abstract book DOIs can be found on the “Author Information” page.
**Disclaimer:** Articles published in the journal HemaSphere exclusively reflect the opinions of the authors. The authors are responsible for all content in their abstracts including accuracy of the facts, statements, citing resources, etc.

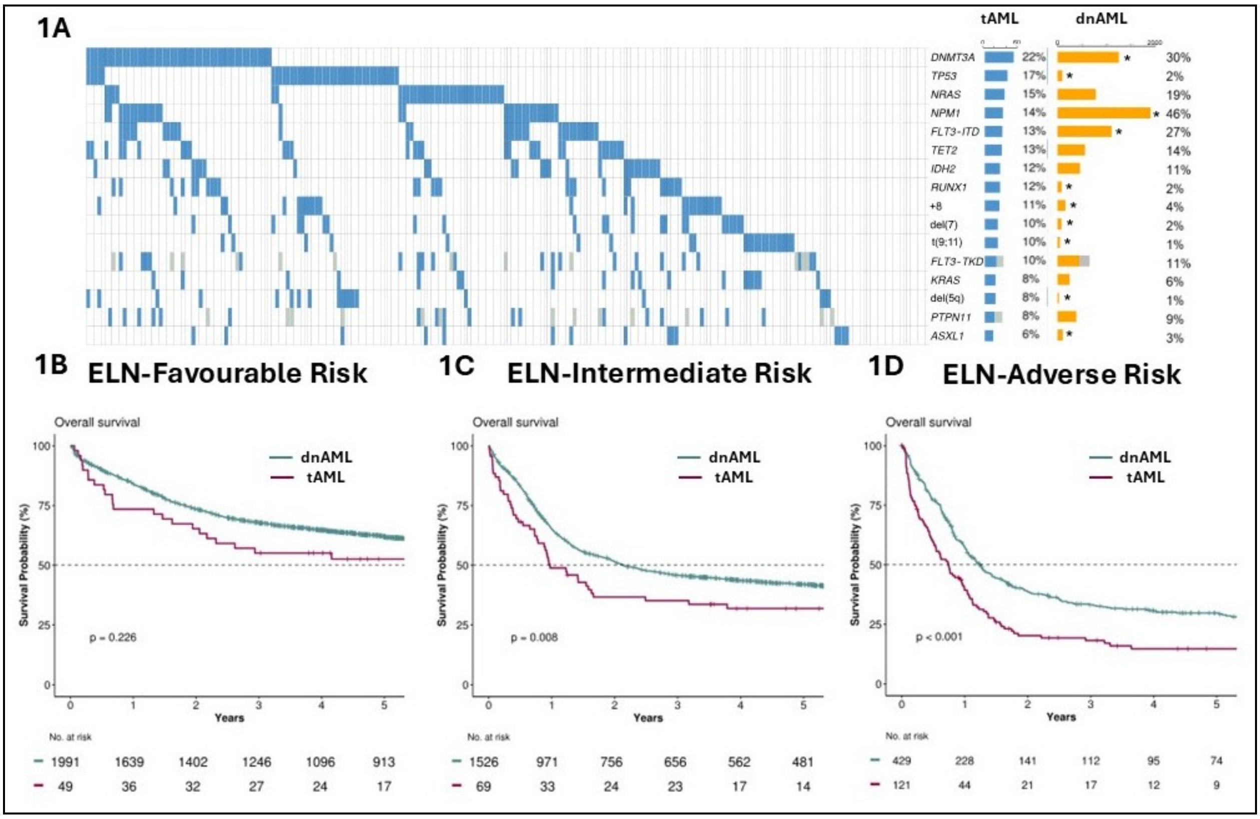

Figure 1 Oncoplot showing genetic lesions in tAML vs dnAML (1A) and Overall survival curves in tAML vs dnAML accoding to ELN risk groups (1B-D).

**Copyright Information:** (Online) ISSN: 2572-9241
©2026 The Author(s). HemaSphere is published by John Wiley & Sons Ltd on behalf of European Hematology Association. This is an open access Abstract Book distributed under the Attribution-NonCommercial-NoDerivs (CC BY-NC-ND), which allows third parties to download the articles and share them with others as long as they credit the author and the Abstract Book, but they cannot change the content in any way or use them commercially.
**Abstract Book Citations:** Authors, Title, HemaSphere, 2026;10:(S1):pages. The abstract book DOIs can be found on the "Author Information" page.
**Disclaimer:** Articles published in the journal HemaSphere exclusively reflect the opinions of the authors. The authors are responsible for all content in their abstracts including accuracy of the facts, statements, citing resources, etc.

### (PS1589) REFINING PROGNOSTIC STRATIFICATION IN ACUTE MYELOID LEUKEMIA WITH MYELODYSPLASIA-RELATED GENE MUTATIONS ACCORDING TO VARIANT ALLELE FREQUENCY

**Topic:** 04. Acute myeloid leukemia - Clinical

Yu-Sung Chang*[1, 2], Lai Wing Hei[3], Cheng-Hong Tsai[1], Feng-Ming Tien[1, 2], Sao Chih Ni[4], Min-Yen Lo[5], Wan-Hsuan Lee[1], Chien-Chin Lin[1, 6], Sze-Hwei Lee[1, 7], Kuo Yuan-Yeh[8], Yen-Ling Peng[1], Mei-Hsuan Tseng[8], Bor-Sheng Ko[1, 4], Ming Yao[1, 7], Hwei-Fang Tien[1], Wen-Chien Chou[1], Yu Hung Leung[3, 9], Hsin-An Hou[1, 2]

*[1]National Taiwan University Hospital, Department of Internal Medicine, Taipei, Taiwan;[2]National Taiwan University, Graduate Institute of Clinical Medicine, Taipei, Taiwan; [3]The University of Hong Kong, Medicine, Pok Fu Lam, Hong Kong; [4]National Taiwan University Cancer Center, Hematological Oncology, Taipei, Taiwan; [5]National Taiwan University Hospital Yunlin Branch, Internal Medicine, Yunlin, Taiwan; [6]National Taiwan University Hospital, Department of Laboratory Medicine, Taipei, Taiwan;[7]National Taiwan University Hospital, Department of Integrated Diagnostics & Therapeutics, Taipei, Taiwan; [8]Tai-Chen Cell Therapy Center, National Taiwan University, Taipei, Taiwan; [9]Hong Kong Science Park, Centre for Oncology and Immunology, New Territories, Hong Kong;*

### Background

Myelodysplasia-related gene (MRG) mutations, *ASXL1*, *BCOR*, *EZH2*, *SF3B1*, *SRSF2*, *STAG2*, *U2AF1*, *ZRSR2*, and *RUNX1*, define a distinct entity in the 2022 ICC and are categorized as adverse risk in ELN-2022. However, clinical outcomes among MRG-mutated AML are heterogeneous, suggesting that binary classification may overestimate risk in a subset of patients. Unlike *TP53*-mutated AML, no variant allele frequency (VAF) threshold currently informs risk assignment for MRG mutations.

### Aims

To assess whether incorporating VAF of MRG mutations provides incremental prognostic value beyond current ELN-2022 risk stratification.

### Methods

A total of 5,682 patients with non-M3 AML were included from four cohorts: National Taiwan University Hospital (n=1,575), University of Hong Kong (n=538), UK-NCRI (n=2,094), and AMLSG (n=1,475). Targeted next-generation sequencing identified MRG mutations, and in cases with multiple mutations, the one with the highest VAF was analyzed. Optimal VAF thresholds were derived using maximally selected rank statistics. Survival analyses were performed in intensively treated patients with multivariable Cox models adjusting for clinical covariates and transplantation in first remission as a time-dependent variable.

**Copyright Information:** (Online) ISSN: 2572-9241
©2026 The Author(s). HemaSphere is published by John Wiley & Sons Ltd on behalf of European Hematology Association. This is an open access Abstract Book distributed under the Attribution-NonCommercial-NoDerivs (CC BY-NC-ND), which allows third parties to download the articles and share them with others as long as they credit the author and the Abstract Book, but they cannot change the content in any way or use them commercially.
**Abstract Book Citations:** Authors, Title, HemaSphere, 2026;10:(S1):pages. The abstract book DOIs can be found on the "Author Information" page.
**Disclaimer:** Articles published in the journal HemaSphere exclusively reflect the opinions of the authors. The authors are responsible for all content in their abstracts including accuracy of the facts, statements, citing resources, etc.

**Results**

Among 5,682 patients, 1,800 (31.4%) had at least one MRG mutation. There was a significant co-occurrence among epigenetic regulators (e.g., *ASXL1–TET2*, *BCOR–DNMT3A*, all $p<0.05$) and mutual exclusivity between spliceosome genes (*SF3B1*, *SRSF2*, *U2AF1*, all $p<0.05$). Correlation analysis between VAF of MRG mutation and bone marrow (BM) blast percentage showed negligible associations except *RUNX1*, revealing a weak correlation (ρ=0.23, p<0.001).

Among 1,320 intensively treated patients (73.3%), patients with ≥2 MRG mutations had inferior survival compared with single mutation (median overall survival [mOS] 15 *vs.* 24 months; $p<0.001$). Among the MRG mutations, *BCOR*- and *STAG2*-mutated AML showed survival similar to ELN-intermediate risk disease (*BCOR*: 20 *vs.* 26 months, $p=0.15$; *STAG2*: 19 *vs.* 26 months, $p=0.08$).

VAF further refined risk discrimination. In the ELN-adverse risk group with MRG mutations, patients were subdivided into MRG-intermediate subgroup, defined by single MRG with VAF <40% (n=204, 12%) and MRG-adverse subgroup, defined by concurrent adverse-risk cytogenetics, a *TP53* mutation, multiple MRGs, or a single MRG with VAF ≥40% (n=823, 49%). Complete response (CR) rates were higher in MRG-intermediate than the MRG-adverse subgroup (88% *vs.* 70%, $p<0.001$). The MRG-intermediate subgroup showed similar event-free and relapse-free survival compared with the ELN-intermediate AML (EFS: 17 *vs.* 15 months, $p=0.78$; RFS, 18 *vs.* 16 months, $p=0.84$). In addition, the MRG-intermediate subgroup showed OS similar to ELN-intermediate AML (mOS 26 *vs.* 27 months; $p=0.58$) whereas the MRG-adverse group showed outcomes comparable to ELN-adverse AML excluding *TP53* mutation (mOS 17 *vs.* 15 months; $p=0.41$). In multivariable Cox models adjusted for age, sex, BM blasts, and transplantation in first CR, using the MRG-intermediate subgroup as the reference, OS was comparable in the ELN-intermediate (HR 1.04, 95% CI 0.87–1.23), but inferior in MRG-adverse (HR 1.38, 95% CI 1.16–1.64) and ELN-adverse groups (HR 1.81, 95% CI 1.49–2.20).

**Summary/Conclusion**

AML with MRG mutations is heterogeneous and not uniformly adverse. Incorporating VAF refines risk beyond ELN-2022 and reclassifies a substantial subset toward intermediate-risk behavior.

**Copyright Information:** (Online) ISSN: 2572-9241
©2026 The Author(s). HemaSphere is published by John Wiley & Sons Ltd on behalf of European Hematology Association. This is an open access Abstract Book distributed under the Attribution-NonCommercial-NoDerivs (CC BY-NC-ND), which allows third parties to download the articles and share them with others as long as they credit the author and the Abstract Book, but they cannot change the content in any way or use them commercially.
**Abstract Book Citations:** Authors, Title, HemaSphere, 2026;10:(S1):pages. The abstract book DOIs can be found on the “Author Information” page.
**Disclaimer:** Articles published in the journal HemaSphere exclusively reflect the opinions of the authors. The authors are responsible for all content in their abstracts including accuracy of the facts, statements, citing resources, etc.

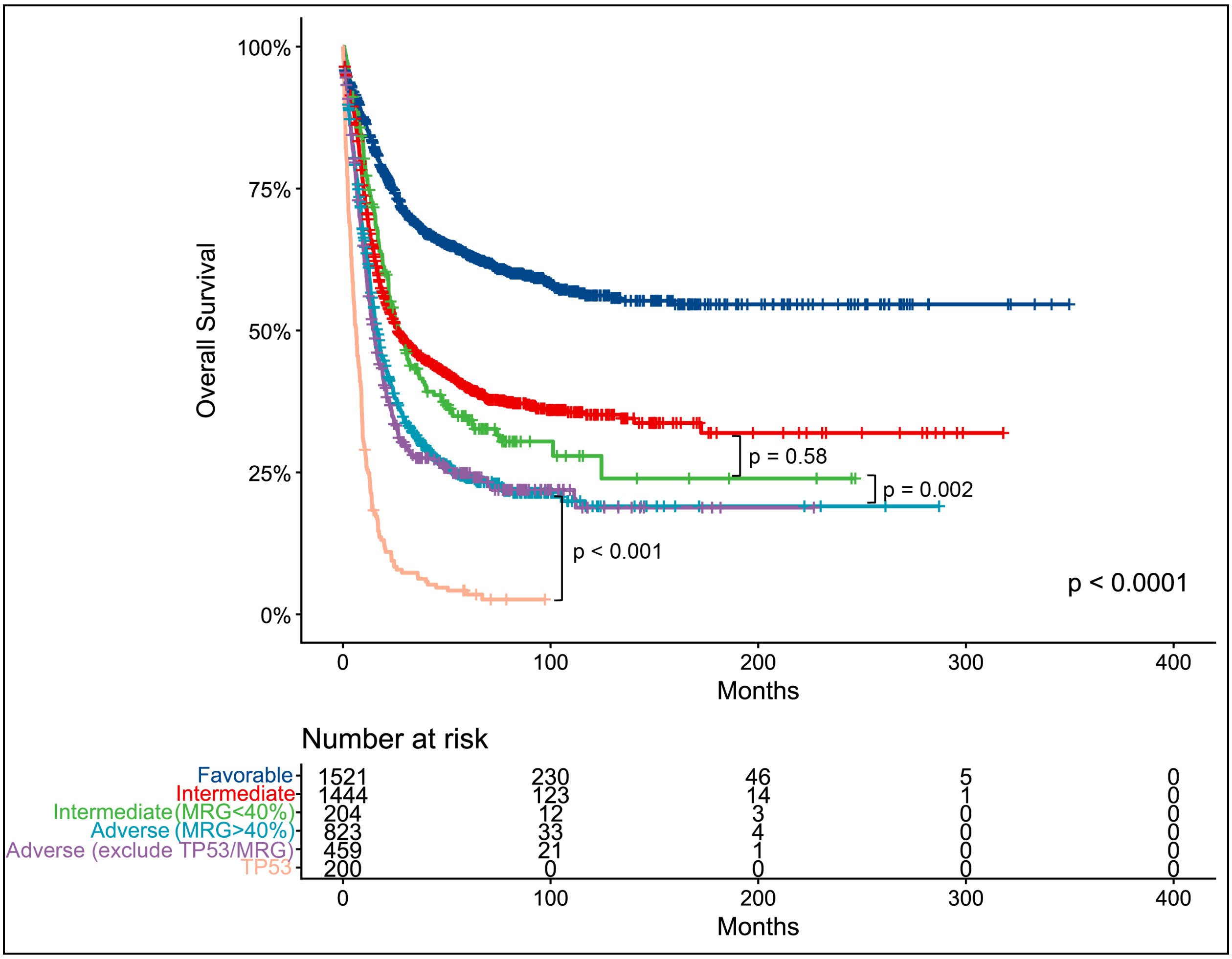

Kaplan–Meier curves of overall survival in intensively treated non-M3 AML stratified by the refined ELN classification. The MRG-intermediate subgroup showed survival comparable to ELN-intermediate risk, whereas the MRG-adverse subgroup demonstrated survival similar to ELN-adverse risk, excluding TP53 mutations.

**Copyright Information:** (Online) ISSN: 2572-9241
©2026 The Author(s). HemaSphere is published by John Wiley & Sons Ltd on behalf of European Hematology Association. This is an open access Abstract Book distributed under the Attribution-NonCommercial-NoDerivs (CC BY-NC-ND), which allows third parties to download the articles and share them with others as long as they credit the author and the Abstract Book, but they cannot change the content in any way or use them commercially.
**Abstract Book Citations:** Authors, Title, HemaSphere, 2026;10:(S1):pages. The abstract book DOIs can be found on the “Author Information” page.
**Disclaimer:** Articles published in the journal HemaSphere exclusively reflect the opinions of the authors. The authors are responsible for all content in their abstracts including accuracy of the facts, statements, citing resources, etc.

### (PS1590) VENETOCLAX-AZACITIDINE IN COMBINATION WITH CHIDAMIDE AND CAG VERSUS "3+7" REGIMEN IN FIT OLDER PATIENTS WITH NEWLY DIAGNOSED ACUTE MYELOID LEUKEMIA A MULTICENTER, RANDOMIZED, PHASE 3 TRIAL

**Topic:** 04. Acute myeloid leukemia - Clinical

Zhenyang Gu*[1], Hai Yi[2], Fang Zhou[3], Dai-Hong Liu[1], Liping Dou[1]

*[1]The Fifth Medical Center of PLA General Hospital, Beijing, China;[2]People's Liberation Army The General Hospital of Western Theater Command, Chengdu, China; [3]the 960th Hospital of the People's Liberation Army Joint Logistics Support Force, Jinan, China;*

### Background

No standard treatment has been established for elderly patients with acute myeloid leukemia (AML). Intensive chemotherapy serves as an important therapeutic option for fit elderly patients.

### Aims

This phase 3 trial aimed to evaluate the outcomes of a novel combined venetoclax-based (venetoclax-azacitidine combined with chidamide) and anthracyclines-based (aclarubicin and cytarabine) induction regimen (CACAG+VEN) versus "3+7" regimen in fit older patients aged 60-75 years with newly diagnosed AML.

### Methods

In this multicenter, randomized, phase 3 trial, newly diagnosed AML patients aged 60-75 years eligible for intensive chemotherapy, were randomly assigned (1:1) to receive either CACAG+VEN or the "3+7" regimen regimen. CACAG+VEN group: Aclarubicin at a dose of 10 mg/m² per day on days 1, 3, and 5; azacitidine at 75 mg/m² per day on days 1–7; cytarabine at 75 mg/m² per dose, twice daily on days 1–5; chidamide at 30 mg per dose, twice a week for 2 weeks (4 doses in total); venetoclax at 100 mg per day in combination with azole antifungal agents; additionally, granulocyte colony - stimulating factor (G - CSF) was administered at a dose of 5 μg/kg per day.
3+7 group: Patients received induction therapy with an anthracycline combined with cytarabine. All patients who achieved complete remission (CR) received consolidation therapy with the same induction regimen. The primary endpoint was the CR rate after one cycle of induction therapy.

### Results

Of 130 screened patients, 106 were enrolled and randomized, with 53 patients in each group. Intention-to-treat analysis showed a significantly higher complete remission rate with CACAG+VEN (89% [47/53]) compared to the "3+7" group (51% [27/53]; $P < 0.01$).
The rate of minimal residual disease (MRD) negativity was 41% in the experimental group compared with 33% in the "3+7" group ($P = 0.02$). The incidence of grade ≥ 3 infections was significantly lower in the experimental group (31% vs. 53%, $P = 0.02$). The duration of severe thrombocytopenia was shorter in the experimental group (15 days vs. 19 days, $P = 0.03$). At a median follow-up of 12 months, the overall survival rate was 75% in the experimental group and 67% in the "3+7" group, with no statistically significant difference.

**Copyright Information:** (Online) ISSN: 2572-9241
©2026 The Author(s). HemaSphere is published by John Wiley & Sons Ltd on behalf of European Hematology Association. This is an open access Abstract Book distributed under the Attribution-NonCommercial-NoDerivs (CC BY-NC-ND), which allows third parties to download the articles and share them with others as long as they credit the author and the Abstract Book, but they cannot change the content in any way or use them commercially.
**Abstract Book Citations:** Authors, Title, HemaSphere, 2026;10:(S1):pages. The abstract book DOIs can be found on the "Author Information" page.
**Disclaimer:** Articles published in the journal HemaSphere exclusively reflect the opinions of the authors. The authors are responsible for all content in their abstracts including accuracy of the facts, statements, citing resources, etc.

**Summary/Conclusion**

In conclusion, the venetoclax - combined CACAG regimen exhibits favorable efficacy and safety in fit elderly patients with newly diagnosed AML.

**Copyright Information:** (Online) ISSN: 2572-9241
©2026 The Author(s). HemaSphere is published by John Wiley & Sons Ltd on behalf of European Hematology Association. This is an open access Abstract Book distributed under the Attribution-NonCommercial-NoDerivs (CC BY-NC-ND), which allows third parties to download the articles and share them with others as long as they credit the author and the Abstract Book, but they cannot change the content in any way or use them commercially.
**Abstract Book Citations:** Authors, Title, HemaSphere, 2026;10:(S1):pages. The abstract book DOIs can be found on the "Author Information" page.
**Disclaimer:** Articles published in the journal HemaSphere exclusively reflect the opinions of the authors. The authors are responsible for all content in their abstracts including accuracy of the facts, statements, citing resources, etc.

### (PS1591) CLINICAL AND TRANSCRIPTOMIC IMPACTS OF TET2 MUTATIONS IN DE NOVO ACUTE MYELOID LEUKEMIA: A MAJOR DETERMINANT OF SURVIVAL BEYOND CURRENT RISK MODELS

**Topic:** 04. Acute myeloid leukemia - Clinical

Tsung-Han Li*[1], Cheng-Hong Tsai[1], Yu-Sung Chang[1], Chi-Yuan Yao[1, 2, 3], Min-Yen Lo[2], Chien-Chin Lin[1, 3], Feng-Ming Tien[1], Hsin-An Hou[1], Bor-Sheng Ko[1, 4], Ming Yao[1], Wen-Chien Chou[1], Hwei-Fang Tien[1, 5]

*[1]National Taiwan University Hospital, Division of Hematology and Oncology, Department of Internal Medicine, Taipei, Taiwan; [2]National Taiwan University Hospital Yunlin Branch, Division of Hematology, Department of Internal Medicine, Yunlin, Taiwan; [3]National Taiwan University Hospital, Department of Laboratory Medicine, Taipei, Taiwan; [4]National Taiwan University Cancer Center, Department of Hematological Oncology, Taipei, Taiwan; [5]Far-Eastern Memorial Hospital, Department of Internal Medicine, New Taipei city, Taiwan;*

### Background

The prognostic significance of *TET2* mutations in AML remains controversial. Early studies suggested adverse outcomes, particularly in cytogenetically intermediate-risk AML, whereas later large-scale genomic analyses indicated that prognostic impact was highly dependent on co-mutation patterns.

### Aims

To clarify the biological and prognostic implications of *TET2* mutations within the framework of contemporary AML classification.

### Methods

1,588 adult patients with de novo AML (excluding APL) were retrospectively enrolled. Clinical and biological characteristics and outcomes were collected. Next generation sequencing and bulk RNA sequencing of diagnostic bone marrow samples were performed.

**Copyright Information:** (Online) ISSN: 2572-9241
©2026 The Author(s). HemaSphere is published by John Wiley & Sons Ltd on behalf of European Hematology Association. This is an open access Abstract Book distributed under the Attribution-NonCommercial-NoDerivs (CC BY-NC-ND), which allows third parties to download the articles and share them with others as long as they credit the author and the Abstract Book, but they cannot change the content in any way or use them commercially.
**Abstract Book Citations:** Authors, Title, HemaSphere, 2026;10:(S1):pages. The abstract book DOIs can be found on the “Author Information” page.
**Disclaimer:** Articles published in the journal HemaSphere exclusively reflect the opinions of the authors. The authors are responsible for all content in their abstracts including accuracy of the facts, statements, citing resources, etc.

**Results**

*TET2* mutations were identified in 14.6% of patients. Compared with *TET2* wild-type AML, *TET2*$^{mut}$ patients were older and presented with higher WBC counts, LDH levels, and lower platelet counts.

Co-mutation analysis showed strong associations between *TET2*$^{mut}$ and *NPM1*$^{mut}$, as well as genes involved in epigenetic regulation (*ASXL1*, *EZH2*) and RNA splicing (*SRSF2*, *ZRSR2*). These findings indicate that *TET2*$^{mut}$ arises within a distinct epigenetic and splicing-related genomic context rather than in signaling-driven leukemogenesis.

Clinically, *TET2*$^{mut}$ AML was associated with significantly inferior overall survival (OS) compared with *TET2*$^{wt}$ AML (median OS 12.5 vs 20.1 months, p<0.05). When stratified by ELN 2022 risk categories, the adverse impact persisted across all groups (Figure A). Notably, patients with ELN favorable-risk AML but with concurrent *TET2*$^{mut}$ had survival similar to ELN intermediate-risk group. Similarly, those with ELN intermediate-risk AML but with *TET2*$^{mut}$ had survival comparable to ELN adverse-risk group. Although allogeneic stem cell transplantation significantly improved OS and relapse-free survival in *TET2*$^{mut}$ patients, outcomes remained inferior to those of *TET2*$^{wt}$ patients.

Integration of data from the UK-NCRI cohorts confirmed these findings. In the UK-NCRI dataset, *TET2*$^{mut}$ AML was likewise associated with worse OS (median OS 12.1 vs 21.4 months, p<0.05), supporting the robustness and generalizability of our results.

Transcriptomic profiling from 471 patients revealed that *TET2*$^{mut}$ AML didn't form a distinct gene expression cluster but instead exhibited widespread yet modest transcriptional alterations. Gene set enrichment analysis demonstrated significant negative enrichment of E2F target genes and other cell cycle–related pathways, along with suppression of metabolic programs, oncogenic signaling pathways (PI3K–AKT–mTOR, KRAS), and immune/stress response pathways. These data suggest that *TET2*$^{mut}$ AML is not characterized by a hyperproliferative phenotype but rather by altered cell cycle regulation, transcriptional instability, and clonal heterogeneity.

**Summary/Conclusion**

*TET2* mutations define a clinically aggressive and biologically distinct AML subgroup characterized by adverse clinical features, a specific co-mutation landscape, inferior survival, and broad transcriptional dysregulation. These findings support incorporating *TET2* mutational status into contemporary risk stratification models and considering early HSCT even in conventionally favorable-risk AML. Our data highlight limitations of the current ELN 2022 framework in capturing epigenetically driven risk and underscore the need to integrate *TET2* status into future AML classification and treatment strategies.

**Copyright Information:** (Online) ISSN: 2572-9241
©2026 The Author(s). HemaSphere is published by John Wiley & Sons Ltd on behalf of European Hematology Association. This is an open access Abstract Book distributed under the Attribution-NonCommercial-NoDerivs (CC BY-NC-ND), which allows third parties to download the articles and share them with others as long as they credit the author and the Abstract Book, but they cannot change the content in any way or use them commercially.
**Abstract Book Citations:** Authors, Title, HemaSphere, 2026;10:(S1):pages. The abstract book DOIs can be found on the "Author Information" page.
**Disclaimer:** Articles published in the journal HemaSphere exclusively reflect the opinions of the authors. The authors are responsible for all content in their abstracts including accuracy of the facts, statements, citing resources, etc.

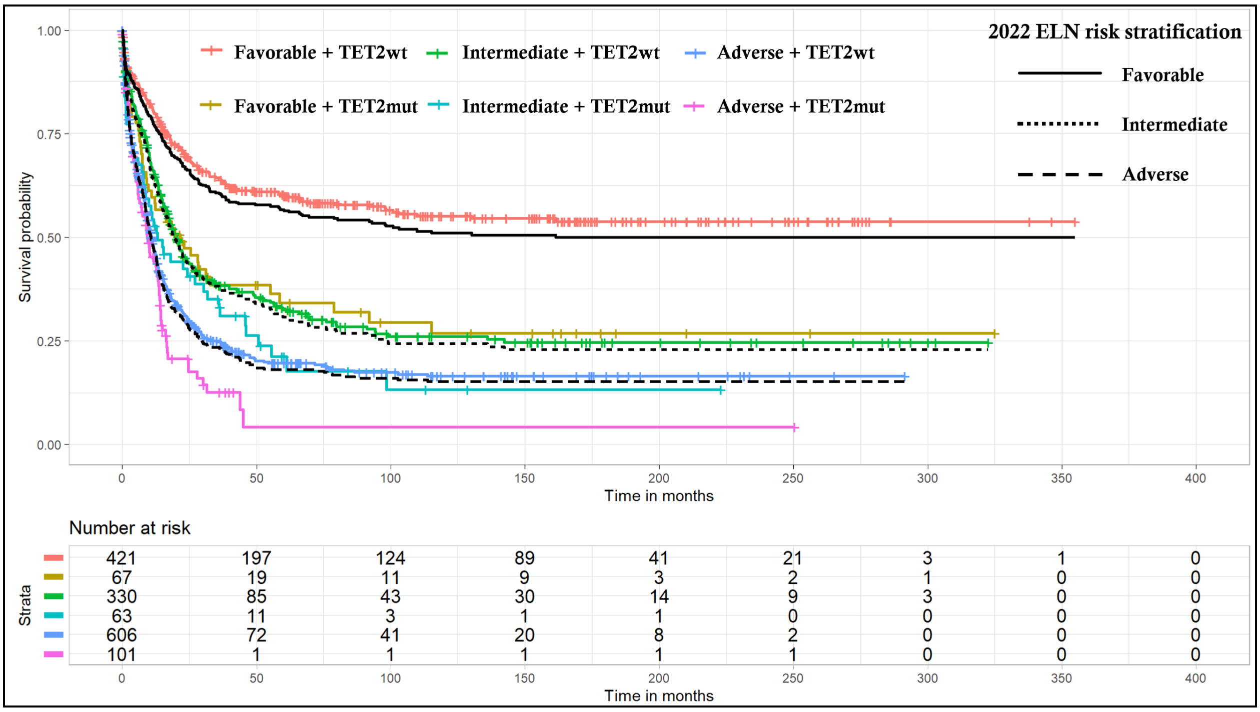

Comparison of Kaplan–Meier survival curves for overall survival among patients with AML stratified by 2022 ELN risk stratification and the combination of TET2 mutation status

Copyright Information: (Online) ISSN: 2572-9241
©2026 The Author(s). HemaSphere is published by John Wiley & Sons Ltd on behalf of European Hematology Association. This is an open access Abstract Book distributed under the Attribution-NonCommercial-NoDerivs (CC BY-NC-ND), which allows third parties to download the articles and share them with others as long as they credit the author and the Abstract Book, but they cannot change the content in any way or use them commercially.
Abstract Book Citations: Authors, Title, HemaSphere, 2026;10:(S1):pages. The abstract book DOIs can be found on the “Author Information” page.
Disclaimer: Articles published in the journal HemaSphere exclusively reflect the opinions of the authors. The authors are responsible for all content in their abstracts including accuracy of the facts, statements, citing resources, etc.

### (PS1592) RETROSPEKTIVE, MULTI-CENTER VALIDATION OF MAC-SCORING FOR PREDICTION OF HMA/VENETOCLAX RESPONSE

**Topic:** 04. Acute myeloid leukemia - Clinical

Alexander Waclawiczek*[1], Simon Renders[1, 2], Ida Vänttinen[3], Heikki Kuusanmäki[3], Ivana Jeziskova[4], Natalia Podstavkova[4], Jirina Prochazkova[4], Barbora Weinbergerová[4], Aino Maija Leppae[5], Mika Kontro[3], Michael Hundemer[2], Tim Sauer[2], Jiří Mayer[4], Carsten Müller-Tidow[2], Andreas Trumpp[1]

*[1]German Cancer Research Center, Heidelberg, Germany;[2]University Hospital Heidelberg, Heidelberg, Germany;[3]Institute for Molecular Medicine Finland (FIMM), Helsinki, Finland;[4]Masaryk University Hospital Brno, Brno, Czechia;[5]The Francis Crick Institute, London, United Kingdom;*

### Background

The combination of the BCL-2 inhibitor Venetoclax (VEN) with hypomethylating agents (HMAs) has significantly improved outcomes for older patients with acute myeloid leukemia (AML). More recently, this combination has begun to rival conventional induction chemotherapy as a first-line treatment (Lu et al.; Fathi et al., *Blood*, 2025).
Because traditional genomic risk stratification scores do not reliably predict outcomes with HMA/VEN therapy, alternative mutation-based models, such as the 4-gene score, have been introduced (Doehner et al., *Blood*, 2024). However, these mutation-driven assessments typically require turnaround times of 10 days or more.
To address this limitation, we recently introduced the “Mediators of Apoptosis Combinatorial” (MAC) score, a flow cytometry–based approach that quantifies the ratio of BCL-2 to BCL-xL plus MCL-1 in GPR56⁺ leukemic stem cells (LSCs) (Waclawiczek et al., 2023) and was predictive of VEN/HMA response in a retrospective analysis of 82 patients.

### Aims

Building on our previous findings, we sought to retrospectively validate MAC-scoring in an expanded, multi-center cohort and draw a comparison to genetic based 4-gene score prediction method.

### Methods

Cryopreserved mononuclear AML cells were stained for surface markers (CD64, CD11b, GPR56, CD45, CD3, CD235a, CD20) and intracellular proteins (BCL-2, MCL-1, BCL-xL). Protein expression levels were quantified by flow cytometry as mean fluorescence intensity (MFI) in immature GPR56⁺ LSCs. For every patient, each protein's MFI value was normalized to the median MFI of the respective protein across the cohort, as previously described. The MAC-Score was calculated using the formula: MAC-Score = normalized BCL-2 / (normalized MCL-1 + normalized BCL-xL)

**Copyright Information:** (Online) ISSN: 2572-9241
©2026 The Author(s). HemaSphere is published by John Wiley & Sons Ltd on behalf of European Hematology Association. This is an open access Abstract Book distributed under the Attribution-NonCommercial-NoDerivs (CC BY-NC-ND), which allows third parties to download the articles and share them with others as long as they credit the author and the Abstract Book, but they cannot change the content in any way or use them commercially.
**Abstract Book Citations:** Authors, Title, HemaSphere, 2026;10:(S1):pages. The abstract book DOIs can be found on the “Author Information” page.
**Disclaimer:** Articles published in the journal HemaSphere exclusively reflect the opinions of the authors. The authors are responsible for all content in their abstracts including accuracy of the facts, statements, citing resources, etc.

**Results**

The MAC-score was assessed in 228 AML patients prior to VEN/HMA treatment from University Hospital Heidelberg (N = 103), Masaryk University Hospital Brno (N = 78), and the Finnish AML Group (N = 47). VEN/HMA was administered as first-line therapy in 170 patients and as salvage therapy in 58 patients. Forty-two patients subsequently underwent allogeneic stem cell transplantation following VEN/HMA treatment. The median MAC score across the entire cohort was 0.45.

Patients who responded to VEN/HMA and achieved complete remission (CR), CR with incomplete hematologic recovery (CRi), or morphologic leukemia-free state (MLFS) had a mean MAC-score of 0.28, whereas non-responders had a mean MAC-score of 0.69. Stratification using the previously defined cut-off of 0.4 demonstrated a strong association between MAC-score and EFS as well as overall survival (OS).

Patients with MAC-scores <0.4 (N = 96) had a median EFS of 102 days and a median OS of 200 days, whereas patients with MAC-scores >0.4 (N = 132) had a median EFS of 638 days and a median OS of 862 days. In multivariable analysis, the hazard ratio for an EFS event comparing patients with MAC-scores above versus below the median was 3.3.

We next stratified the cohort according to the 4-gene score into high-benefit, intermediate-benefit, and low-benefit groups (Döhner et al. Blood, 2024). The MAC-score further discriminated EFS and OS within the high- and intermediate-benefit groups, while patients in the low-benefit group had poor outcomes irrespective of MAC-score.

**Summary/Conclusion**

Overall, MAC-Scoring is a robust and reproducible method to predict VEN/HMA response in AML using flow cytometry.

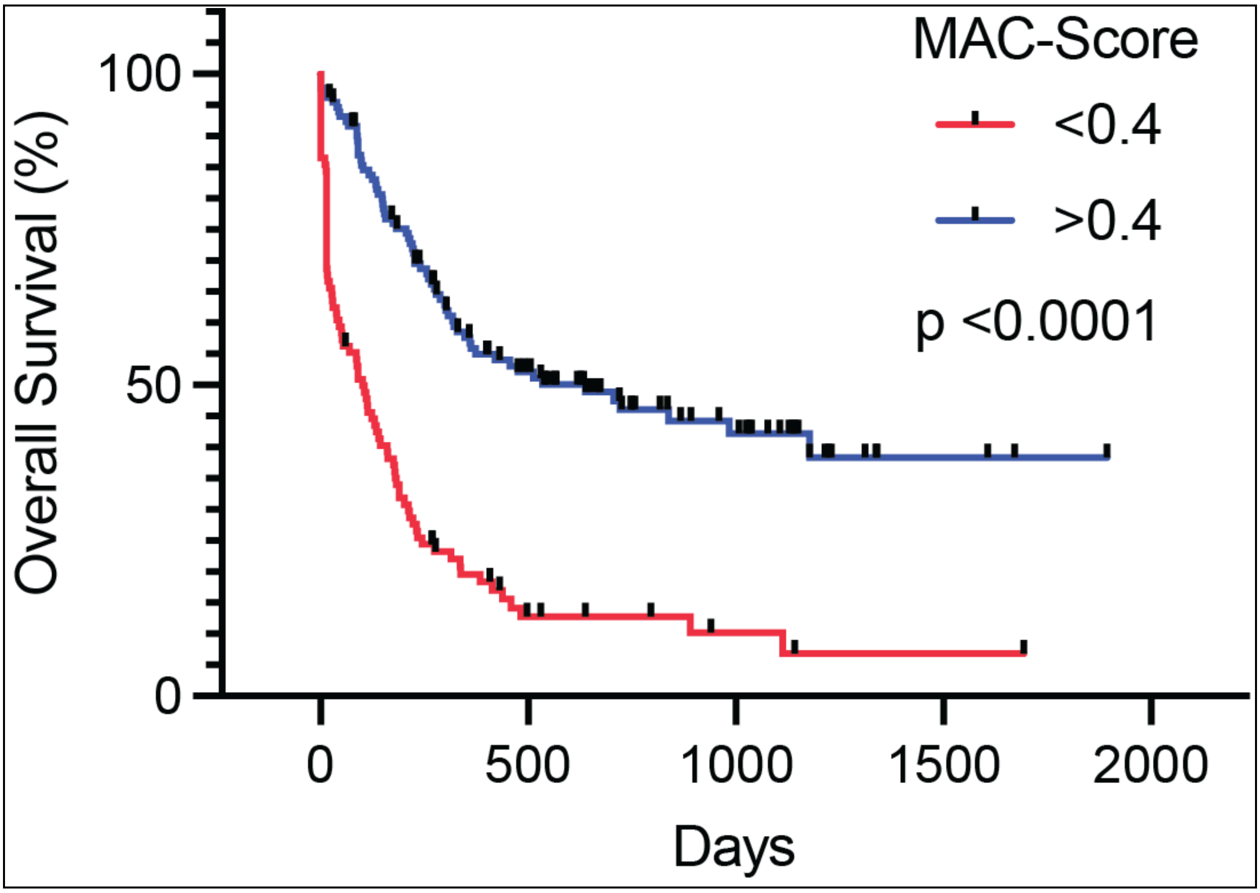

**Copyright Information:** (Online) ISSN: 2572-9241
©2026 The Author(s). HemaSphere is published by John Wiley & Sons Ltd on behalf of European Hematology Association. This is an open access Abstract Book distributed under the Attribution-NonCommercial-NoDerivs (CC BY-NC-ND), which allows third parties to download the articles and share them with others as long as they credit the author and the Abstract Book, but they cannot change the content in any way or use them commercially.
**Abstract Book Citations:** Authors, Title, HemaSphere, 2026;10:(S1):pages. The abstract book DOIs can be found on the "Author Information" page.
**Disclaimer:** Articles published in the journal HemaSphere exclusively reflect the opinions of the authors. The authors are responsible for all content in their abstracts including accuracy of the facts, statements, citing resources, etc.

### (PS1593) PROGNOSTIC RISK INTEGRATION FOR SURVIVAL MODELING IN ACUTE MYELOID LEUKEMIA (PRISM-AML): A MULITNATIONAL COHORT STUDY

**Topic:** 04. Acute myeloid leukemia - Clinical

Curtis Lachowiez*[1], Joshua Zeidner[2], Jad Othman[3], Andy Kaempf[1], Seongseok Yun[4], Mael Heiblig[5], Michael Heuser[6], Rabia Shahswar[7], Anand Patel[8], Andrius Žučenka[9], Jennifer Marvin-Peek[10], Jayastu Senapati[10], Yasmin Abaza[11], Rafael Marroquin[8], Joseph Cannova[8], Irene Zacheo[12], Federica Monaco[13], Dimitrios Drekolias[4], Fatima Tuz Zahra[4], Alok Swaroop[11], Amine Belhabri[14], Urbain Tauveron-Jalenques[15], Emmanuelle Tavernier Tardy[16], Martin Carre[17], Gaspar Aspa Requena[18], Nicola Long[1], Rachel Cook[1], Elie Traer[1], Jennifer Saultz[1], Jenny O'nions[19], Faisal Basheer[20], John Laurie[21], Shivani Handa[22], Eytan Stein[23], Maria Baer[24], Wendy Stock[8], Rebecca Olin[25], William Blum[26], Gary Schiller[27], Tara L. Lin[28], Emily Curran[29], Ying Huang[22], Rina Welkie[22], Ashley Yocum[30], Ellen Madarang[31], Jessica K. Altman[11], Naval Daver[10], Tapan Kadia[10], Giovanni Marconi[32], Yazan F. Madanat[33], Richard Dillon[34], Courtney Dinardo[10], Ronan Swords[1], Juan Arango Ossa[35], Justin Watts[31], Sanam Loghavi[36], Daniel Pollyea[37], Elsa Bernard[38]

*[1]Oregon Health & Science University, Portland, United States of America;[2]University of North Carolina, Lineberger Comprehensive Cancer Center, Medicine; Division of Hematology, Chapel Hill, United States of America; [3]Department of Medical and Molecular Genetics, King's College London, London, United Kingdom; University of Sydney, Sydney, Australia; Haematology Department, Royal North Shore Hospital, St. Leonards, Australia, Sydney, Australia; [4]Department of Malignant Hematology, H. Lee Moffitt Cancer Center & Research Institute, Tampa USA, Tampa, United States of America; [5]Hematology department, Lyon-Sud Hospital, Hospices Civils de Lyon, Pierre Bénite, France; [6]Department of Hematology, Hemostasis, Oncology and Stem Cell Transplantation, Hannover Medical School, Hannover, Germany; Department of Internal Medicine IV, University Hospital Halle (Saale), Martin-Luther-University Halle-Wittenberg, Halle, Germany, Hannover, Germany; [7]Department of Hematology, Hemostasis, Oncology and Stem Cell Transplantation, Hannover Medical School, Hannover, Germany; Molecular Pharmacology Program, Memorial Sloan Kettering Cancer Center, New York, NY, USA, Hannover, Germany; [8]Section of Hematology-Oncology, Department of Medicine, University of Chicago, Chicago, United States of America; [9]Vilnius University, Faculty of Medicine, Hematology and Oncology Department, and Hematology, Oncology, and Transfusion Medicine Center, National Cancer Center, Vilnius University Hospital Santaros Klinikos, Vilnius, Lithuania; [10]Department of Leukemia, The University of Texas MD Anderson Cancer Center, Houston, United States of America; [11]Leukemia Program, Division of Hematology and Oncology, Robert H. Lurie Comprehensive Cancer Center, Feinberg School of Medicine, Northwestern University, Chicago, United States of America; [12]Hematology Unit, IRCSS Istituto Romagnolo Per lo Studio Dei Tumori (IRST) "Dino Amadori", Meldola, United States of America; [13]U.O. Ematologia, Ospedale Infermi Rimini, AUSL ROMAGNA, Rimini, Italy; [14]Hematology Department and Centre de Recherche en Cancerologie de Lyon (CRCL) - UMR INSERM 1052 CNRS 5286, Leon Berard Cancer Center, Lyon, France; [15]Cellular Therapy and Clinical Haematology Department, Clermont-Ferrand University Hospital, Clermont-Ferrand, France; [16]University Hospital Saint Etienne, Saint Etienne, France; [17]Clinical Hematology Department, CHU Grenoble Alpes - Université Grenoble Alpes, Grenoble, France; [18]Clinical Hematology Department - Nîmes University Hospital - Gard Cancer Institute, Nîmes, France; [19]University College London Hospitals NHS Foundation Trust, London, United Kingdom;[20]Department of Haematology, Addenbrooke's Hospital, Cambridge, United Kingdom; [21]University Hospitals Sussex NHS Foundation Trust, Worthing, United Kingdom; [22]Division of Hematology, The Ohio State University Wexner Medical Center, Columbus, United States of America; [23]Leukemia Service, Department of Medicine, Memorial Sloan Kettering Cancer Center, New York, United States of America; [24]University of Maryland Greenebaum Comprehensive Cancer Center, Baltimore, United States of America;[25]University of California San Francisco, San Francisco, United States of America; [26]Winship Cancer Institute of Emory University, Atlanta, United States of America; [27]David Geffen School of Medicine at University of California, Los Angeles, United States of America; [28]Division of Hematologic Malignancies & Cellular Therapeutics, University of Kansas, Kansas City, United States of America; [29]University of Cincinnati, Cincinnati, United States of America;[30]Blood Cancer United, Washington D.C., United States of America; [31]Division of Hematology, Sylvester Comprehensive Cancer Center, University of Miami Miller School of Medicine, Miami, United States of America; [32]University of Bologna, Hematology Unit, Ospedale S. Maria delle Croci, Ravenna (RA), Italy; [33]University of Texas Southwestern Medical Center, Dallas, United States of America;[34]Department of Medical and Molecular*

**Copyright Information:** (Online) ISSN: 2572-9241
©2026 The Author(s). HemaSphere is published by John Wiley & Sons Ltd on behalf of European Hematology Association. This is an open access Abstract Book distributed under the Attribution-NonCommercial-NoDerivs (CC BY-NC-ND), which allows third parties to download the articles and share them with others as long as they credit the author and the Abstract Book, but they cannot change the content in any way or use them commercially.
**Abstract Book Citations:** Authors, Title, HemaSphere, 2026;10:(S1):pages. The abstract book DOIs can be found on the "Author Information" page.
**Disclaimer:** Articles published in the journal HemaSphere exclusively reflect the opinions of the authors. The authors are responsible for all content in their abstracts including accuracy of the facts, statements, citing resources, etc.

*Genetics, King's College, London, United Kingdom; [35]Computational Oncology Service, Department of Epidemiology & Biostatistics, Memorial Sloan Kettering Cancer Center, New York, United States of America; [36]Department of Hematopathology, MD Anderson Cancer Center, Houston, United States of America; [37]Division of Hematology, University of Colorado School of Medicine, Aurora, United States of America; [38]Paris-Saclay University, Gustave Roussy, INSERM U1361, IHU PRISM National PRecISion Medicine Center in Oncology, Villejuif, France;*

**Background**

Venetoclax (VEN) combined with hypomethylating agents (HMA) or low-dose cytarabine (LDAC) is standard treatment for older or unfit patients (pts) with newly diagnosed acute myeloid leukemia (ND-AML). The foundational 4-gene classifier (stratifying using mutations in *FLT3*-ITD, *N/KRAS*, and *TP53*) has been incorporated into ELN 2024 recommendations, however this model and others provide modest discrimination and omit clinical or cytogenetic variables.

**Aims**

We aimed to develop and externally validate a therapy-specific prognostic model—Prognostic Risk Integration for Survival Modeling (PRISM)—for pts treated with lower-intensity VEN-based therapy. We now report the final model, outcomes in key subgroups, and in expanded validation cohorts.

**Methods**

Characteristics of the internal training (n=1,385) and validation cohorts (n=587) and model generation have already been described (Lachowiez et. al. ASH 2025). Briefly, candidate variables were evaluated using elastic-net Cox regression. Reproducible features were refit using ridge regression and beta-coefficient weights were summed to generate a continuous PRISM score. For clinical interpretability, PRISM scores were categorized into tertiles (PRISM-3: low, moderate, high). Discrimination with the 4-gene classifier was assessed using Harrell's C-index.Two independent external validation cohorts are now included (EVC1, n=500; EVC2, n=222).

**Results**

Key adverse-risk features retained in PRISM included *TP53*, *KRAS*, *JAK2*, *ASXL1* mutations, *FLT3*-ITD, non-diploid cytogenetics, secondary AML, male sex, and increasing age. Favorable features included *BCOR*, *IDH1*, *IDH2*, *RUNX1*, *STAG2*, and *CEBPA* mutations.
PRISM-3 reclassified 40–50% of pts relative to the 4-gene classifier, including over half of those labeled favorable-risk using the 4-gene classifier. Complete remission/Complete remission with incomplete count recovery (CR/CRi) rates differed significantly across PRISM-3 low, moderate, and high-risk groups (training: 76% vs. 62% vs. 43%; internal validation: 74% vs. 52% vs 40%).
In the training cohort, median overall survival (mOS) across low, moderate, and high PRISM-3 groups was 23.7, 14.3, and 6.7 months ($p<0.001$); in the internal validation cohort mOS was 25.6, 13.2, and 6.1 months, respectively ($p<0.001$). Similar OS stratification was observed across PRISM-3 groups in EVC1 and EVC2 (Figure 1A-D). PRISM-3 improved OS discrimination compared with the 4-gene classifier (Harrell's C-index 0.63 vs 0.59–0.60 across training, internal validation, and EVC1 cohorts; $p<0.05$).
In pooled exploratory analyses, PRISM-3 groupings retained prognostic value compared to the 4-gene classifier among pts achieving CR/CRi (C-index 0.61 vs 0.58; p=0.012) and pts age < 65 (C-index 0.65 vs. 0.60, $p<0.001$). Superior performance was also observed with PRISM vs. Beat AML, Mayo, and revised ELN-2024 prognostic models (p-value < 0.05 for all). Allogeneic hematopoietic cell transplantation attenuated but did not eliminate risk differences across PRISM-3 groups, suggesting potential utility in guiding transplant referral and risk-adapted treatment strategies.

**Copyright Information:** (Online) ISSN: 2572-9241
©2026 The Author(s). HemaSphere is published by John Wiley & Sons Ltd on behalf of European Hematology Association. This is an open access Abstract Book distributed under the Attribution-NonCommercial-NoDerivs (CC BY-NC-ND), which allows third parties to download the articles and share them with others as long as they credit the author and the Abstract Book, but they cannot change the content in any way or use them commercially.
**Abstract Book Citations:** Authors, Title, HemaSphere, 2026;10:(S1):pages. The abstract book DOIs can be found on the "Author Information" page.
**Disclaimer:** Articles published in the journal HemaSphere exclusively reflect the opinions of the authors. The authors are responsible for all content in their abstracts including accuracy of the facts, statements, citing resources, etc.

### Summary/Conclusion

PRISM significantly improves survival stratification beyond current standards for AML treated with lower-intensity VEN-based therapy. By integrating clinical, cytogenetic, and genomic features, PRISM enables meaningful risk reclassification and supports individualized, risk-adapted clinical decision-making and trial design in the venetoclax era. A web-based risk calculator is available at prism-aml.com.

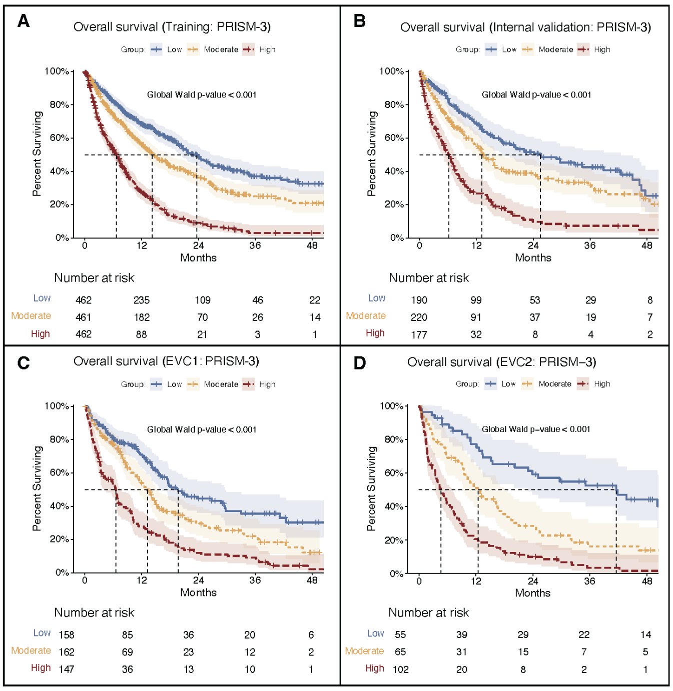

**Figure 1**: Overall survival stratified by PRISM-3 groups assessed using the Kaplan Meier method in patients with newly diagnosed AML in the (A) internal training cohort, (B) internal validation cohort, (C) external validation cohort #1, and (D) external validation cohort #2. EVC: external validation cohort. Hashed vertical lines extending down from the Kaplan-Meier curves represent the median survival in months for the respective group of interest

**Copyright Information:** (Online) ISSN: 2572-9241
©2026 The Author(s). HemaSphere is published by John Wiley & Sons Ltd on behalf of European Hematology Association. This is an open access Abstract Book distributed under the Attribution-NonCommercial-NoDerivs (CC BY-NC-ND), which allows third parties to download the articles and share them with others as long as they credit the author and the Abstract Book, but they cannot change the content in any way or use them commercially.
**Abstract Book Citations:** Authors, Title, HemaSphere, 2026;10:(S1):pages. The abstract book DOIs can be found on the “Author Information” page.
**Disclaimer:** Articles published in the journal HemaSphere exclusively reflect the opinions of the authors. The authors are responsible for all content in their abstracts including accuracy of the facts, statements, citing resources, etc.

### (PS1594) DDX41-MUTATED ACUTE MYELOID LEUKEMIA DEFINES A DISTINCT AML ENTITY

**Topic:** 04. Acute myeloid leukemia - Clinical

Carolin Seeling*[1], Maral Saadati[2], Daniela Späth[1], Axel Benner[2], Hartmut Döhner[1], Konstanze Döhner[1]

[1]*University Hospital of Ulm, Department of Internal Medicine III, Ulm, Germany;* [2]*German Cancer Research Center, Division of Biostatistics, Heidelberg, Germany;*

### Background

Pathogenic germline variants in *DDX41* are the most common inherited predisposition to adult myeloid neoplasms. *DDX41*-mutated AML typically harbors biallelic lesions, a paucity of class-defining co-mutations, and predominantly normal karyotypes. Patients present at older age, with male predominance and cytopenias. However, the molecular distinctiveness of *DDX41*-mutated AML within established AML classes remains unclear.

### Aims

Genetic and clinical characterization of *DDX41*-mutated AML and assessment of its molecular distinctiveness in independent cohorts.

### Methods

The discovery cohort included 516 intensively treated patients from AMLSG trials (*Papaemmanuil et al., N Engl J Med 2016*), selected from preestablished molecular subgroups (*No_drivers*, *No_class*, *chromatin–spliceosome*, *TP53–aneuploidy*), in which *DDX41*-mutations were anticipated based on prior reports. *DDX41* was retrospectively sequenced across the full coding region by next-generation sequencing. High-dimensional mutation profiles were projected using Uniform Manifold Approximation and Projection (UMAP). Survival outcomes were estimated with Kaplan–Meier methods and multivariable Cox regression. Validation was performed in 604 patients non-intensively treated within the ASTRAL-1 trial (*Jahn et al., Leukemia 2023*).

### Results

In the discovery cohort, 18 patients (3.5%) harbored pathogenic *DDX41* variants, most frequently in *No_drivers* (20%) vs *No_class* (3%), *chromatin–spliceosome* (2%), and *TP53–aneuploidy* (1%). Biallelic alterations were observed in 78%, with p.M1I germline and p.R525H somatic variants predominating. Co-mutations were infrequent (median 0.5, range 0-3), mainly affecting *ASXL1* (16.7%), *SRSF2* (11.1%), and *PHF6* (11.1%), and 56% had normal karyotype. UMAP projection revealed a distinct *DDX41* cluster, suggesting molecular uniqueness. Two cases, however, clustered with complex karyotype/ *TP53* or *FLT3*-ITD cases, indicating these lesions determined the primary phenotype (**Fig. 1A**). Median OS in *DDX41*-mutated patients was 37.2 vs 14.2 months for *DDX41* wild-type patients (p=0.02). Compared with established molecular subgroups, *DDX41*-mutated AML had longer OS than *No_class* (20.8 mo), *chromatin–spliceosome* (15.1 mo), and *TP53–aneuploidy* (9.1 mo), but shorter than *No_drivers* (47.5 mo; **Fig. 1B**). Median EFS was 27.3 vs 2.9 months for *DDX41* wild-type, exceeding all subgroups. In multivariable analyses, *DDX41*-mutated AML was independently associated with superior OS and EFS compared with *chromatin–spliceosome* (OS HR 0.54; p=0.04; EFS HR 0.51, p=0.02) and *TP53–aneuploidy* (OS HR 0.26, p<0.001; EFS HR 0.32, p<0.001), showed a trend toward improved outcomes relative to *No_class* (OS HR 0.67, p=0.21; EFS HR 0.56, p=0.05), and conferred no advantage compared with *No_drivers* (OS HR 1.02, p=0.96; EFS HR 1.14, p=0.7). Molecularly distinct clustering and superior prognosis of *DDX41*-mutated AML were independently validated in the ASTRAL-1 cohort.

**Copyright Information:** (Online) ISSN: 2572-9241
©2026 The Author(s). HemaSphere is published by John Wiley & Sons Ltd on behalf of European Hematology Association. This is an open access Abstract Book distributed under the Attribution-NonCommercial-NoDerivs (CC BY-NC-ND), which allows third parties to download the articles and share them with others as long as they credit the author and the Abstract Book, but they cannot change the content in any way or use them commercially.
**Abstract Book Citations:** Authors, Title, HemaSphere, 2026;10:(S1):pages. The abstract book DOIs can be found on the "Author Information" page.
**Disclaimer:** Articles published in the journal HemaSphere exclusively reflect the opinions of the authors. The authors are responsible for all content in their abstracts including accuracy of the facts, statements, citing resources, etc.

### Summary/Conclusion

Using a dedicated bioinformatic algorithm, we demonstrate that *DDX41*-mutated AML forms a distinct molecular cluster. Together with the characteristic co-mutational pattern and typical clinical presentation, these findings provide further evidence that *DDX41*-mutated AML represents a distinct entity of AML.

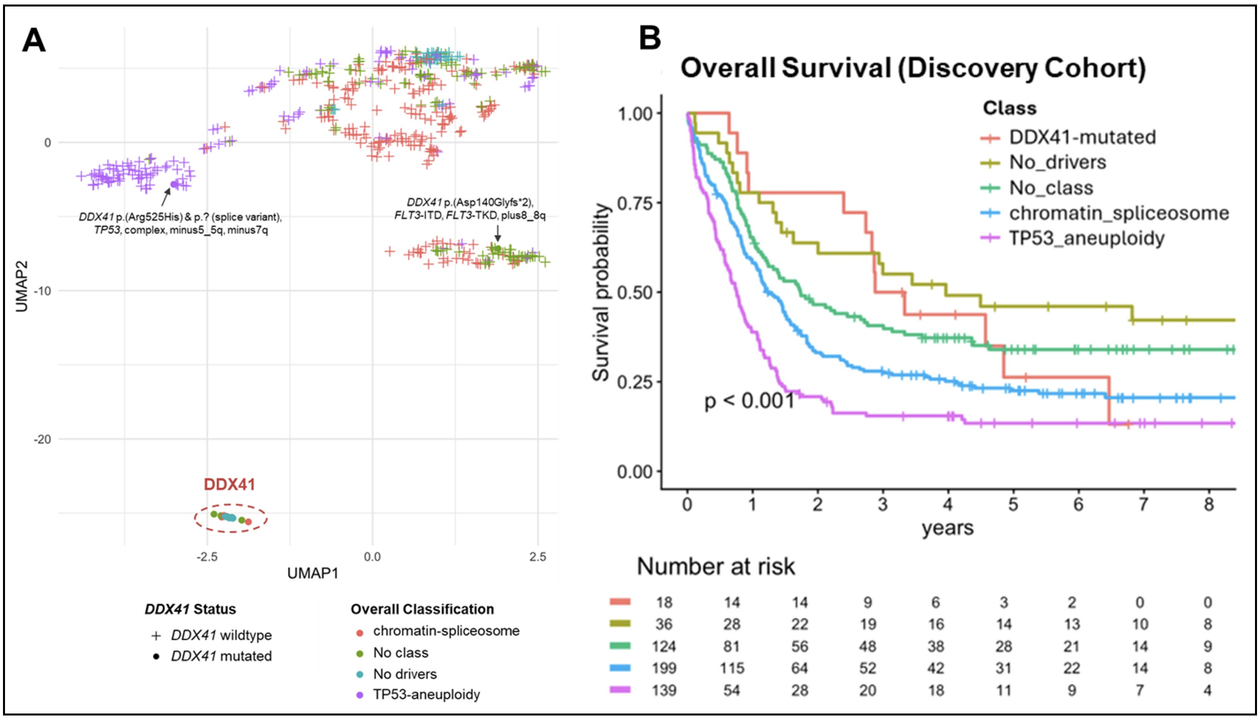

Figure 1: DDX41-mutated AML forms a distinct molecular cluster within the AML landscape. (A) UMAP clustering shows separation of DDX41-mutated AML from other subgroups. (B) Kaplan–Meier analysis reveals superior overall survival.

**Copyright Information:** (Online) ISSN: 2572-9241
©2026 The Author(s). HemaSphere is published by John Wiley & Sons Ltd on behalf of European Hematology Association. This is an open access Abstract Book distributed under the Attribution-NonCommercial-NoDerivs (CC BY-NC-ND), which allows third parties to download the articles and share them with others as long as they credit the author and the Abstract Book, but they cannot change the content in any way or use them commercially.
**Abstract Book Citations:** Authors, Title, HemaSphere, 2026;10:(S1):pages. The abstract book DOIs can be found on the "Author Information" page.
**Disclaimer:** Articles published in the journal HemaSphere exclusively reflect the opinions of the authors. The authors are responsible for all content in their abstracts including accuracy of the facts, statements, citing resources, etc.

### (PS1595) FLAG-IDA VERSUS VENETOCLAX-HMA AS SALVAGE THERAPY IN FIRST RELAPSED/REFRACTORY AML: REAL WORLD DATA

**Topic:** 04. Acute myeloid leukemia - Clinical

Gaspar Aspas Requena*[1], Pilar Lloret-Madrid[2], Carmen Botella Prieto[3], Eliana Aguiar[4], Mar Tomo[5], Maria de Las Mercedes Colorado[6], Teresa Bernal[7], Josefina Serrano Lopez[8], Maria Pilar Martinez Sanchez[9], Carlos Rodriguez Medina[10], Eduardo Rodríguez-Arbolí[11], Raimundo Garcia Boyero[12], Tamara Castaño[13], Claudia Sossa-Melo[14], Pilar Herrera[15], Susana Vives Polo[16], María José Sayas Lloris[17], Monica Alejandra Romero Riquelme[18], Olga Salamero[19], Lorenzo Algarra[20], Victor Noriega Concepción[21], Jorge Labrador[22], Maria Luz Amigo[23], Joana Infante[24], Maria Virginia Prates[25], Manuel Mateo Perez Encinas[26], Alberto Gimenez Conca[27], Marcela Espinoza[28], Beatriz De Rueda Ciller[29], Juan Miguel Bergua Burgues[30], Marta Esperanza Valero[31], Maria Marta Rivas[32], Christine Michelle Rojas Hopkins[33], Francisca Bass[34], Manuel Barrios García[35], Maria Dolores Madrigal Toscano[36], Lourdes Amador Barciela[37], Francisco Ibañez[38], Ana Alfonso Pierola[39], Esther Pérez[40], José Mario Mariz[41], Olga Arce[42], Rosalia Riaza Grau[43], Ana Basquiera[44], Maria Carmen Martinez Chamorro[45], Maria Jose Garcia Perez[46], Mayte Olave[47], Juan Antonio López[48], Maria Garcia Fortes[49], Carlos De Miguel[50], Ana Oliva Hernández[51], Maria Jose Mela Osorio[52], Leticia Rapan[53], Pau Montesinos[2]

*[1]Centre Hospitalier Universitaire de Montpellier, Service d'Hématologie Clinique, Montpellier, France;[2]Hospital Universitario y Politécnico La Fe, Servicio de Hematología, Valencia, Spain; [3]Hospital General Universitario de Alicante, Servicio de Hematología, Alicante, Spain; [4]Centro Hospitalar São João, Serviço de Hematologia, Porto, Portugal; [5]Hospital Clínico Universitario de Valencia, Instituto de Investigación Biomédica INCLIVA, Servicio de Hematología, Valencia, Spain; [6]Hospital Universitario Marqués de Valdecilla, Servicio de Hematología, Santander, Spain; [7]Hospital Universitario Central de Asturias, Servicio de Hematología, Oviedo, Spain; [8]Hospital Universitario Reina Sofía, Servicio de Hematología, Córdoba, Spain;[9]Hospital Universitario 12 de Octubre, Servicio de Hematología, Madrid, Spain; [10]Hospital Universitario de Gran Canaria Dr. Negrín, Servicio de Hematología, Las Palmas, Spain; [11]Hospital Universitario Virgen del Rocío, Servicio de Hematología, Sevilla, Spain; [12]Hospital General Universitario de Castellón, Servicio de Hematología, Castellón, Spain;[13]Hospital Universitario Fundación Jiménez Díaz, Servicio de Hematología, Madrid, Spain; [14]Clínica FOSCAL, Universidad Autónoma de Bucaramanga, Servicio de Hematología, Bucaramanga, Colombia; [15]Hospital Universitario Ramón y Cajal, Servicio de Hematología, Madrid, Spain;[16]ICO Badalona-Hospital Germans Trias i Pujol, Institut de recerca Josep Carreras, Badalona, Spain; [17]Hospital Universitario Doctor Peset, Servicio de Hematología, Valencia, Spain; [18]Hospital Guillermo Grant Benavent, Servicio de Hematología, Concepción, Chile; [19]Hospital Universitari Vall d'Hebron, Servei d'Hematologia, Barcelona, Spain; [20]Hospital General Universitario de Albacete, Servicio de Hematología, Albacete, Spain; [21]Complejo Hospitalario Universitario de A Coruña, Servicio de Hematología, A Coruña, Spain; [22]Hospital Universitario de Burgos, Servicio de Hematología, Burgos, Spain,[23]Hospital General Universitario Morales Meseguer, Servicio de Hematología, Murcia, Spain; [24]Hospital de Santa Maria, CHULN, Serviço de Hematologia, Lisbon, Portugal; [25]Hospital Italiano de La Plata,, Servicio de Hematología, La Plata, Argentina;[26]Hospital Clínico Universitario de Santiago de Compostela, Servicio de Hematología, Santiago de Compostela, Spain; [27]Hospital Italiano de Buenos Aires, Servicio de Hematología, Buenos Aires, Argentina; [28]Clínica Dávila, Servicio de Hematología, Santiago de Chile, Chile; [29]Hospital Universitario Miguel Servet, Servicio de Hematología, Zaragoza, Spain;[30]Hospital San Pedro de Alcántara, Servicio de Hematología, Cáceres, Spain; [31]Hospital Arnau de Vilanova, Servicio de Hematología, Valencia, Spain;[32]Hospital Universitario Austral, Servicio de Hematología, Buenos Aires, Argentina; [33]Hospital Gustavo Fricke, Servicio de Hematología, Viña del Mar, Chile; [34]Hospital del Salvador, Servicio de Hematología, Santiago de Chile, Chile;[35]Hospital Regional Universitario de Málaga, Servicio de Hematología, Málaga, Spain; [36]Hospital Universitario Virgen Macarena, Servicio de Hematología, Sevilla, Spain; [37]Complejo Hospitalario Universitario de Pontevedra, Servicio de Hematología, Pontevedra, Spain;[38]Hospital General Universitario de Valencia, Servicio de Hematología, Valencia, Spain; [39]Clínica Universidad de Navarra, Servicio de Hematología, Pamplona, Spain; [40]Hospital Universitario Donostia, Servicio de Hematología, San Sebastián, Spain;[41]Instituto Português de Oncologia do Porto, Serviço de Hematologia, Porto, Portugal; [42]Hospital Universitario de Basurto, Servicio de Hematología, Bilbao, Spain; [43]Hospital Universitario Severo Ochoa, Servicio de Hematología, Madrid, Spain;[44]Hospital Privado Universitario*

**Copyright Information:** (Online) ISSN: 2572-9241
©2026 The Author(s). HemaSphere is published by John Wiley & Sons Ltd on behalf of European Hematology Association. This is an open access Abstract Book distributed under the Attribution-NonCommercial-NoDerivs (CC BY-NC-ND), which allows third parties to download the articles and share them with others as long as they credit the author and the Abstract Book, but they cannot change the content in any way or use them commercially.
**Abstract Book Citations:** Authors, Title, HemaSphere, 2026;10:(S1):pages. The abstract book DOIs can be found on the "Author Information" page.
**Disclaimer:** Articles published in the journal HemaSphere exclusively reflect the opinions of the authors. The authors are responsible for all content in their abstracts including accuracy of the facts, statements, citing resources, etc.

de Córdoba, Servicio de Hematología, Córdoba, Argentina; [45]Hospital Universitario Quirón Pozuelo, Servicio de Hematología, Madrid, Spain; [46]Complejo Hospitalario Torrecárdenas, Servicio de Hematología, Almería, Spain;[47]Hospital Clínico Universitario Lozano Blesa, Servicio de Hematología, Zaragoza, Spain; [48]Hospital Universitario de Jaén, Servicio de Hematología, Jaén, Spain; [49]Hospital Universitario Virgen de la Victoria, Servicio de Hematología, Málaga, Spain;[50]Hospital Universitario Puerta de Hierro, Servicio de Hematología, Madrid, Spain; [51]Hospital Universitario Nuestra Señora de Candelaria, Servicio de Hematología, Santa Cruz de Tenerife, Spain; [52]Fundaleu, Servicio de Hematología, Buenos Aires, Argentina;[53]Sanatorio Sagrado Corazón, Servicio de Hematología, Buenos Aires, Argentina;

**Background**

The optimal salvage strategy for relapsed/refractory (R/R) acute myeloid leukemia (AML) remains undefined. FLAG-IDA is widely used for fit R/R patients, while venetoclax plus hypomethylating agents (VEN-HMA) has emerged as an alternative, particularly for older or unfit patients. Few small retrospective cohort studies have compared these approaches in the R/R setting.

**Aims**

To compare overall survival (OS), CR/CRi/MLFS rates, early mortality, and event-free survival (EFS) between FLAG-IDA and VEN-HMA as first salvage in R/R AML in adult patients reported to the multinational PETHEMA registry (Spain, Argentina-GATLA, Portugal-GPLA, Chile-GRELAM, and Colombia-RENEHOC).

**Methods**

Retrospective multicenter analysis of 1,754 adult R/R AML patients receiving first salvage with FLAG-IDA (n=1,439) or VEN-HMA (n=315) from the PETHEMA database (1998–2025). A multivariable Cox model for OS included treatment approach, R/R period (1998-2018 or 2019-2025), sex, age, prior transplant, ECOG, cytogenetic risk, WBC, and *NPM1.*

**Results**

The two cohorts differed substantially. FLAG-IDA patients were younger (median 51 vs 67 years), had higher WBC (median 12.4 vs 6.6 x$10^9$/L), higher LDH (median 498 vs 335 U/L), and more *FLT3*-ITD at diagnosis (19% vs 10%) (p<0.001). FLAG-IDA cohort more frequently received intensive first-line therapy (98% vs 68%), while allogeneic transplant in first line was lower (23% vs 30%) (p<0.001). FLAG-IDA spanned 1998–2025, whereas VEN-HMA was almost exclusively used from 2019 onward, resulting in markedly shorter follow-up for the VEN-HMA cohort. CR/CRi/MLFS rates were 54.4% for FLAG-IDA vs 40.9% for VEN-HMA (p<0.001). In unadjusted analysis, median OS was 10.4 after FLAG-IDA vs 8.2 months after VEN-HMA (HR 1.22, 95%CI 1.04–1.43, p=0.017), with same trend when restricting analyses to patients with R/R after 2019 (mOS 12.1 months after FLAG-IDA vs 8.2 months after VEN-HMA (p<0.001). Subgroup analyses showed a significant difference in refractory (HR 1.34, p=0.017) but not in relapsed patients (HR 1.13, p=0.275), and in *NPM1*-negative (HR 1.34, p=0.004) but not *NPM1*-positive (HR 1.15, p=0.40). mOS of FLAG-IDA vs VEN-HMA according to MRC2010 risk was: low- 25.7 vs 19.3 (HR 1.05, 95%CI 0.52–2.09, p=0.895); intermediate-10.9 vs 7.8 (HR 1.22, 95%CI 0.98-1.53, p=079); and high-risk 7.5 vs 7.2 (HR 1.14, 95%CI 0.85–1.55, p=0.384). Both strategies yielded comparable OS in patients ≥65 (HR 0.97, p=0.825). In the multivariable Cox model (N=525), age ≥65 at R/R, baseline WBC, ECOG 2-4, and FLAG-IDA were not independent prognostic factors (HR 0.81, 95%CI 0.55–1.2, p=0.29), while adverse cytogenetics (HR 3.33, p<0.001), *NPM1* mutation (HR 0.7, p=0.01), prior transplant (HR 0.64, p<0.001), and diagnosis of R/R ≥2019 (HR 0.72, p=0.01) retained significance.

**Copyright Information:** (Online) ISSN: 2572-9241
©2026 The Author(s). HemaSphere is published by John Wiley & Sons Ltd on behalf of European Hematology Association. This is an open access Abstract Book distributed under the Attribution-NonCommercial-NoDerivs (CC BY-NC-ND), which allows third parties to download the articles and share them with others as long as they credit the author and the Abstract Book, but they cannot change the content in any way or use them commercially.
**Abstract Book Citations:** Authors, Title, HemaSphere, 2026;10:(S1):pages. The abstract book DOIs can be found on the "Author Information" page.
**Disclaimer:** Articles published in the journal HemaSphere exclusively reflect the opinions of the authors. The authors are responsible for all content in their abstracts including accuracy of the facts, statements, citing resources, etc.

**Summary/Conclusion**

In this large retrospective and multinational cohort of adult AML patients in first R/R, FLAG-IDA was associated with higher CR/CRi/MFLS rates and OS compared with VEN-HMA. However, the baseline differences between cohorts, the substantially shorter follow-up of VEN-HMA patients, and the lack of independent prognostic impact of treatment in multivariable analysis preclude conclusions. These findings should be considered hypothesis-generating and support an individualized approach to salvage selection based on fitness, molecular profile, and transplant eligibility, pending validation in more homogeneous populations with longer follow-up.

**Copyright Information:** (Online) ISSN: 2572-9241
©2026 The Author(s). HemaSphere is published by John Wiley & Sons Ltd on behalf of European Hematology Association. This is an open access Abstract Book distributed under the Attribution-NonCommercial-NoDerivs (CC BY-NC-ND), which allows third parties to download the articles and share them with others as long as they credit the author and the Abstract Book, but they cannot change the content in any way or use them commercially.
**Abstract Book Citations:** Authors, Title, HemaSphere, 2026;10:(S1):pages. The abstract book DOIs can be found on the “Author Information” page.
**Disclaimer:** Articles published in the journal HemaSphere exclusively reflect the opinions of the authors. The authors are responsible for all content in their abstracts including accuracy of the facts, statements, citing resources, etc.

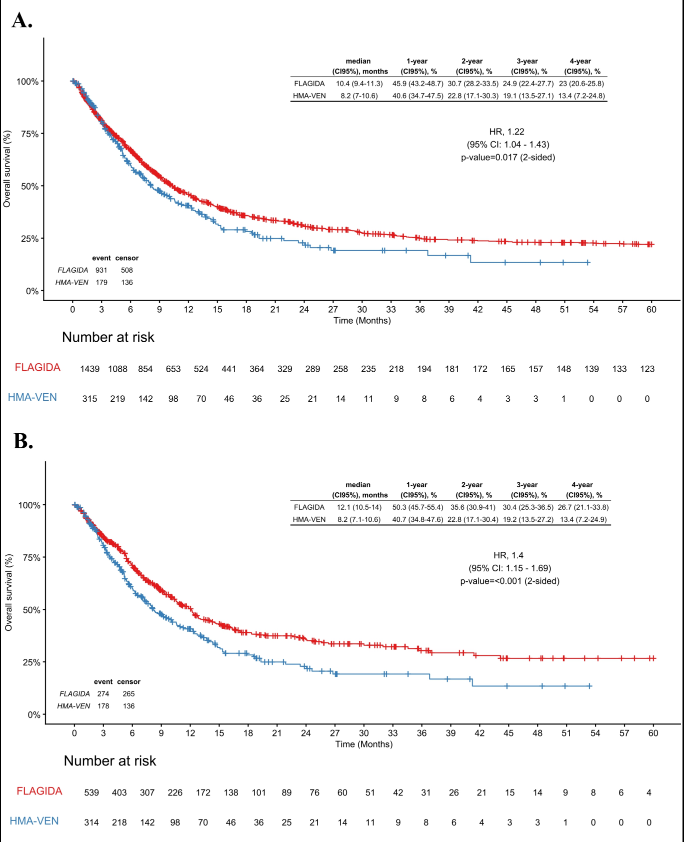

**Figure. Overall survival according to salvage regimen (FLAG-IDA vs VEN-AZA) in patients with relapsed/refractory AML from the PETHEMA registry. A.** Kaplan-Meier estimates in the overall unmatched cohort (N = 1,754; FLAG-IDA n = 1,439, VEN-AZA n = 315). **B.** Kaplan-Meier estimates restricted to patients with year of relapse or resistance from 2019 onward (N = 853; FLAG-IDA n = 539, VEN-AZA n = 314). Hazard ratios (HR) with 95% confidence intervals were estimated using Cox regression; p-values are two-sided.

Figure. Overall survival according to salvage regimen (FLAG-IDA vs VEN-AZA) in patients with relapsed/refractory AML from the PETHEMA registry. A. Kaplan-Meier estimates in the overall unmatched cohort (N = 1,754; FLAG-IDA n = 1,439, VEN-AZA n = 315). B. Kaplan-Meier estimates restricted to patients with year of relapse or resistance from 2019 onward (N = 853; FLAG-IDA n = 539, VEN-AZA n = 314). Hazard ratios (HR) with 95% confidence intervals were estimated using Cox regression; p-values are two- sided.

**Copyright Information:** (Online) ISSN: 2572-9241
©2026 The Author(s). HemaSphere is published by John Wiley & Sons Ltd on behalf of European Hematology Association. This is an open access Abstract Book distributed under the Attribution-NonCommercial-NoDerivs (CC BY-NC-ND), which allows third parties to download the articles and share them with others as long as they credit the author and the Abstract Book, but they cannot change the content in any way or use them commercially.
**Abstract Book Citations:** Authors, Title, HemaSphere, 2026;10:(S1):pages. The abstract book DOIs can be found on the "Author Information" page.
**Disclaimer:** Articles published in the journal HemaSphere exclusively reflect the opinions of the authors. The authors are responsible for all content in their abstracts including accuracy of the facts, statements, citing resources, etc.

### (PS1597) RUXOLITINIB AND DEXAMETHASONE COMBINATION THERAPY EFFECTIVELY PREVENTS DIFFERENTIATION SYNDROME IN ACUTE PROMYELOCYTIC LEUKEMIA

**Topic:** 04. Acute myeloid leukemia - Clinical

Hongming Zhu*[1], Yubing Zhao[1], Xiaojing Lin[1], Li Chen[1], Fangyi Dong[1], Junmin Li[1], Yang Shen[1]

*[1]Shanghai Institute of Hematology, State Key Laboratory of Medical Genomics, National Research Center for Translational Medicine at Shanghai, Ruijin Hospital Affiliated to Shanghai Jiao Tong University School of Medicine, Shanghai, China;*

### Background

Differentiation syndrome (DS) occurs in about 25% of patients with acute promyelocytic leukemia (APL) during all-trans retinoic acid (ATRA) and arsenic trioxide (ATO) induction therapy, particularly in intermediate-to-high-risk patients. Severe DS is closely related to early mortality. The mechanism of DS is unclear, and current prevention methods are limited and unsatisfactory.

### Aims

The study aims to optimize the prophylaxis strategy of DS among APL patients.

### Methods

The APL2022 study was a prospective, single-arm clinical trial for patients aged between 14 and 75 with newly diagnosed intermediate-to-high-risk APL (according to Sanz risk model). Patients were treated with ATRA and ATO-based induction therapy, and Ruxolitinib (Ruxo) 5mg bid plus dexamethasone (Dex) 10mg per day for prophylaxis of DS. The primary endpoint was DS rate decreased to 15% from 25% in the previous studies, which would be achieved if more than 77 out of 95 patients did not develop DS at stage 1 and over 82 out of 101 cases avoid DS at stage 2, according to Simon two-stage “minimax” method. DS score was measured according to 7 clinical features, each for 1 point: unexplained fever, dyspnea, pleural or pericardial effusion, pulmonary infiltration, renal failure, hypotension, and a weight gain ≥ 5 kg. Mild DS was measured as 1-2 points, moderate DS 3 points, severe DS ≥ 4 points. When DS score = 0 and WBC count < $5 \times 10^9$/L for more than 3 days, Ruxo could be discontinued and the dosage of Dex reduced. For safety concern, if PLT count < $10 \times 10^9$/L or any other grade III-IV adverse effects possibly related to Ruxo occurred, it should be discontinued.

### Results

From Jun 2023 to Oct 2025, 101 patients were included in the study and received Ruxo+Dex prophylaxis therapy. The median use of Ruxo was 13 days (range 3 - 23). Fifteen (14.9%) patients developed DS, all in stage 1, so that 80 patients were DS free at stage 1, and 86 at stage 2, the trial has met the primary endpoint. It was significantly reduced comparing to our historical data (26.3%, $P$ = 0.013). High-risk patients were remarkably benefited from this strategy, with the incidence of DS reduced from 30.6% to 12.2% ( $P$ = 0.023). Dyspnea, fever, pleural or pericardial effusion and weight gain were the most common symptoms of DS. Once acute renal dysfunction or hypotension occurred, patients all developed severe DS (3 cases). Seven (6.9%) patients died within 30 days from diagnosis, only 1 died from severe DS. Univariate and multivariate analyses (involving age, gender, *FLT3-ITD*, *FLT3-TKD*, *NRAS*, *WT1*, WBC, hemoglobin, PLT and fibrinogen) showed that only *NRAS* mutation was the independent risk factor for DS (OR = 5.14, 95% CI = 1.38-19.13, $P$ = 0.015). A total of 13 cases carried *NRAS* mutation, 5 (38.5%) developed DS, including 2 (15.4%) severe cases. Both overall and severe DS incidence were significantly higher than that of non *NRAS* mutant cohort. Neutrophil extracellular traps (NETs) are released by mature neutrophils after ATRA-induced differentiation, which may cause DS by damaging endothelial cells. Continuous peripheral blood NETs monitoring was conducted in 54 patients, and we found that early elevation of NETs was significantly associated with the onset of DS.

**Copyright Information:** (Online) ISSN: 2572-9241
©2026 The Author(s). HemaSphere is published by John Wiley & Sons Ltd on behalf of European Hematology Association. This is an open access Abstract Book distributed under the Attribution-NonCommercial-NoDerivs (CC BY-NC-ND), which allows third parties to download the articles and share them with others as long as they credit the author and the Abstract Book, but they cannot change the content in any way or use them commercially.
**Abstract Book Citations:** Authors, Title, HemaSphere, 2026;10:(S1):pages. The abstract book DOIs can be found on the “Author Information” page.
**Disclaimer:** Articles published in the journal HemaSphere exclusively reflect the opinions of the authors. The authors are responsible for all content in their abstracts including accuracy of the facts, statements, citing resources, etc.

### Summary/Conclusion

Ruxo and Dex combination therapy was an effective and safe strategy for preventing DS in APL patients, particularly in high-risk group. *NRAS* mutation and early elevation of NETs might serve as risk predictors for DS under this protocol. (www.chictr.org.cn, ChiCTR2300072086)

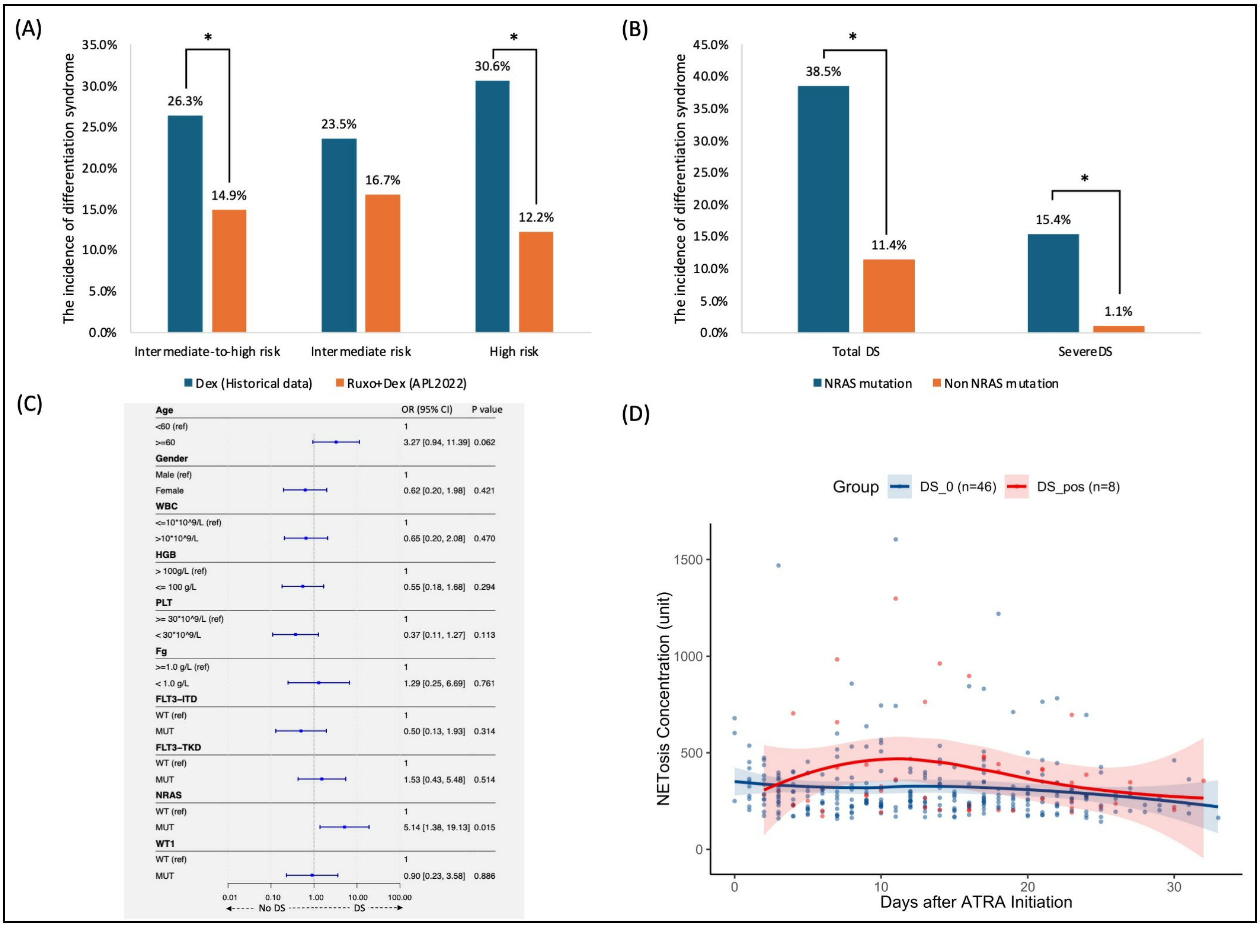

(A) The incidence of differentiation syndrome (DS) in APL2022 vs. historical data. (B) The incidence of DS in NRAS mutation vs. Non NRAS mutation patients. (C) NRAS mutation is a risk factor for DS. (D) Early elevation of NETs predicts DS.

**Copyright Information:** (Online) ISSN: 2572-9241
©2026 The Author(s). HemaSphere is published by John Wiley & Sons Ltd on behalf of European Hematology Association. This is an open access Abstract Book distributed under the Attribution-NonCommercial-NoDerivs (CC BY-NC-ND), which allows third parties to download the articles and share them with others as long as they credit the author and the Abstract Book, but they cannot change the content in any way or use them commercially.
**Abstract Book Citations:** Authors, Title, HemaSphere, 2026;10:(S1):pages. The abstract book DOIs can be found on the "Author Information" page.
**Disclaimer:** Articles published in the journal HemaSphere exclusively reflect the opinions of the authors. The authors are responsible for all content in their abstracts including accuracy of the facts, statements, citing resources, etc.

### (PS1598) REAL WORLD CHARACTERISTICS AND OUTCOMES IN A LARGE COHORT OF FIT PATIENTS WITH CBF-AML: A MULTINATIONAL PETHEMA-GPLA-ACHO-GRELAM STUDY

**Topic:** 04. Acute myeloid leukemia - Clinical

Evelyn Acuña-Cruz*[1], Rebeca Rodríguez-Veiga[2], Pilar Lloret-Madrid[1], Eva Barragán[3], Isabel Cano Ferri[1], Laura Torres Miñana[1], Delfim Duarte[4], Teresa Bernal[5], Eduardo Rodríguez-Arbolí[6], Rosa Ayala[7], Cristina Gil Cortes[8], Irene Navarro-Vicente[1], Fernanda Trigo[9], Eliana Aguiar[10], Josefina Serrano Lopez[11], Mar Tomo[12], Juan Miguel Bergua Burgues[13], Monica Alejandra Romero Riquelme[14], Francisca Bass[15], Carlos Rodriguez Medina[16], Manuel Mateo Perez Encinas[17], Olga Salamero[18], José Luis López-Lorenzo[19], Marcela Espinoza[20], Tomas Rivero Equiza[21], Lucas Cárcamo[22], Pilar Herrera-Puente[23], Maria de Las Mercedes Colorado[24], Manuel Barrios García[25], Esther Pérez[26], Lorenzo Algarra[27], Mayte Olave[28], MA. Mar Moles[28], María Belén Vidriales[29], Ana Alfonso Pierola[30], Sandra Marini[31], Torres Vivianne[32], Joana Infante[33], Maria Jose Sayas Lloris[34], Agata Almela Gallego[35], Claudia Sossa-Melo[36], María Luz Amigo[37], Raimundo Garcia Boyero[38], Mónica Lisseth Torres MacIas[38], Marta Polo[39], Rosa Fernández[40], Cristina Lemes Quintana[40], Maria Dolores Madrigal Toscano[41], Victor Noriega Concepción[42], Sofia Grille[43], Alberto Cantalapiedra[44], Natalia Carpizo Jiménez[44], Matxalen Olivares[45], Julia Moran Sanchez[46], Javier Ortiz[47], Maria Carmen Mateos Rodriguez[48], Ferran Vall-Llovera Calmet[49], Joaquín Martinez-Lopez[50], Pau Montesinos[51]

*[1]Hospital Universitari i Politècnic-IIS La Fe, Hematology Department, Valencia, Spain;[2]Hospital Universitari i Politécnic La Fe, Valencia, Spain; [3]Hospital Universitari i Politècnic-IIS La Fe, Molecular Biology Unit, Valencia, Spain; [4]Instituto Português Oncologia do Porto Francisco Gentil, IPO, Porto, Portugal; [5]Hospital Central de Asturias, Oviedo, Spain;[6]Hospital Universitario Virgen del Rocío, Instituto de Biomedicina de Sevilla (IBIS/CSIC), University of Seville, Hematology, Seville, Spain; [7]Hospital 12 de Octubre, Madrid, Spain; [8]Hospital General Universitario Doctor Balmis, Alicante, Spain;[9]Centro Hospitalar Universitário de São João, Porto, Portugal; [10]Centro Hospitalar Universitário São João, Porto, Portugal; [11]Hospital Reina Sofia, Córdoba, Spain; [12]Hospital Clínico Universitario, Instituto de investigación biomédica INCLIVA, Valencia, Spain; [13]Hospital San Pedro de Alcántara, Cáceres, Spain; [14]Hospital Guillermo Grant Benavente, Concepción, Chile; [15]Hospital del Salvador, Providencia, Chile; [16]Hospital Universitario de Gran Canaria Doctor Negrín, Las Palmas de Gran Canaria, Spain;[17]Hospital Clínico Universitario de Santiago, Santiago de Compostela, Spain; [18]Hospital U. Vall d'Hebron, Barcelona, Spain; [19]Hospital Universitario Fundación Jiménez Díaz, Madrid, Spain; [20]Clínica Dávila, Recoleta, Chile; [21]Hospital U. Germans Trias i Pujol ICO Badalona, Badalona, Spain; [22]Hospital Gustavo Fricke, Valparaiso, Chile; [23]Hospital Universitario Ramón y Cajal, Madrid, Spain; [24]Hospital U. Marqués de Valdecilla, Santander, Spain; [25]Hospital Universitario Regional Carlos Haya, Málaga, Spain;[26]Hospital Universitario Donostia, Donostia, Spain; [27]Hospital Universitario General de Albacete, Albacete, Spain;[28]Hospital Clinico Universitario Lozano Blesa, Zaragoza, Spain; [29]Hospital Universitario de Salamanca, Salamanca, Spain;[30]Clínica Universitaria de Navarra, Pamplona, Spain; [31]Centro Hospitalar e Universitário de Coimbra, EPE, Coimbra, Portugal;[32]Hospital Base de Valdivia, Valdivia, Chile; [33]Hospital de Santa Maria-Lisboa, Lisboa, Portugal;[34]Hospital Universitario Dr. Peset, Valencia, Spain; [35]Hospital Universitario de León, León, Spain;[36]Clínica FOSCAL, Universidad Autónoma de Bucaramanga-UNAB, Bucaramanga, Colombia; [37]Hospital Universitario Morales Messeguer, Murcia, Spain;[38]Hospital Universitari General de Castelló, Castellón, Spain; [39]Hospital Clínico San Carlos, Madrid, Spain; [40]Hospital Insular de Las Palmas, Las Palmas de Gran Canaria, Spain; [41]Hospital U. Virgen de la Macarena, Sevilla, Spain;[42]Hospital Universitario A Coruña, A Coruña, Spain;[43]Hospital de Clínicas, Montevideo, Uruguay; [44]Hospital Universitario Río Hortega, Valladolid, Spain; [45]Hospital Galdakao-Usansolo, Galdakao, Spain; [46]Hospital U. Puerta del Mar, Cádiz, Spain;[47]Hospital Universitario de la Princesa, Madrid, Spain;[48]Hospital Universitario de Navarra, Navarra, Spain; [49]Hospital Universitari Mútua de Terrassa, Terrassa, Spain;[50]Hospital 12 de Octubre, Universidad Complutense, CNIO, Madrid, Spain; [51]Hospital Universitari i Politècnic La Fe, CIBERONC Instituto de Salud Carlos III, Valencia, Spain;*

**Copyright Information:** (Online) ISSN: 2572-9241
©2026 The Author(s). HemaSphere is published by John Wiley & Sons Ltd on behalf of European Hematology Association. This is an open access Abstract Book distributed under the Attribution-NonCommercial-NoDerivs (CC BY-NC-ND), which allows third parties to download the articles and share them with others as long as they credit the author and the Abstract Book, but they cannot change the content in any way or use them commercially.
**Abstract Book Citations:** Authors, Title, HemaSphere, 2026;10:(S1):pages. The abstract book DOIs can be found on the "Author Information" page.
**Disclaimer:** Articles published in the journal HemaSphere exclusively reflect the opinions of the authors. The authors are responsible for all content in their abstracts including accuracy of the facts, statements, citing resources, etc.

### Background

Core-binding factor acute myeloid leukemia (CBF-AML) is characterized by the presence of inv(16)/t(16;16) or t(8;21) and is classified as a favorable risk by international AML guidelines. Despite high remission rates with intensive regimens, there is still 40% of relapses, and regimens with mylotarg (GO), among others, have emerged as potential game changers in this disease. However, real world evidence in large series is still scarce.

### Aims

To analyze clinical and biological characteristics, and to assess therapeutic patterns and outcomes of a large cohort of CBF-AML patients treated with an upfront intensive regimen.

### Methods

Patients >14 years old, treated with an intensive approach, reported to the multinational and retrospective PETHEMA database including patients from Spain, Portugal (GPLA), Colombia (ACHO), and Chile (GRELAM), diagnosed with CBF-AML from 1989 to 2025 were included in the study. Diagnosis was made by local cytogenetics and by local or central PCR and/or NGS. Disease and patient's baseline characteristics, CBF-AML mutation subtype, treatments (according to physician´s choice) and outcomes were recorded.

### Results

Among 17,748 patients with cytogenetics and/or molecular data, 1311 (7.4%) had CBF-AML at diagnosis (median age 47 years old, 54% male, 23% with extramedullary disease). Overall, 1002 patients (85%) received an intensive treatment approach. Of them, 499 (49.8%) had inv(16)/t(16;16) and 503 (50.2%) had t(8;21). Median age was 45.5 years old, similar gender distribution, 84 patients (8%) presented secondary AML (72 had therapy related AML), 85% had ECOG 0-1. Comparing inv(16)/t(16;16) vs. t(8;21) groups, M4-FAB subtype was more present in inv(16)/t(16;16) (69% vs 10%), while M2-FAB was more prevalent in t(8;21) (8% vs 65.4%, p<0.001); extramedullary disease in 34% vs 13%, respectively (p<0.001), median WBC x $10^9$/L was 30 vs. 10.5, respectively (p<0.001); FLT3-ITDmut in 6% vs. 10%, FLT3-TKDmut in 17% vs. 11%; and cKITmut 26% vs. 28%, respectively. Induction regimen consisted of idarubicin or daunorubicin plus cytarabine (3+7) in 739 (75%), 3+7+GO in 118 (12%), 3+7 plus etoposide in 62 (6.3%), 3+7 plus midostaurin in 13 (1.3%), CPX-351 in 10 (1%), FLAG-based in 9 (1%), and others in 34 (3.4%). Overall, 89% CRc was observed without significant differences between induction regimens. Median overall survival (mOS) was 181.9 months, not reached in inv(16)/t(16;16) and 145.2 months in t(8;21), p<0.001. Post-induction MRD by flow cytometry was available in 364 pts (34%), being negative in 230 (66%). Comparing 3+7 with 3+7+GO, the 5-year OS was 60.7% vs. 78.6% (p=0.03) (Figure 1-A) and the 5-year disease-free survival (DFS) was 55% vs. 70.5% (p=0.02), respectively. Stem cell transplantation (SCT) in first CRc data was available in 910 pts, 185 (18%) received an auto-SCT, 112 (12%) an allo-SCT and 633 (70%) had no SCT (p<0.001). The 5 years OS was 76.2% after auto-SCT, 71.1% after allo-SCT, and 53.8% in non-transplanted patients (p<0.001) (Figure 1-B); and the 5 years DFS was 68.1%, 56.3%, and 49.5%, respectively.

### Summary/Conclusion

When comparing CBF-AML subtypes, we found that t(8;21) had poorer prognosis. We show that intensive regimens induced very high CRc rates, with an apparent OS and DFS benefit among patients receiving 3+7+GO, but also among those receiving auto-SCT intensification. Combining both GO and auto-SCT could be explored in future well designed protocols to obtain further improvements.

**Copyright Information:** (Online) ISSN: 2572-9241
©2026 The Author(s). HemaSphere is published by John Wiley & Sons Ltd on behalf of European Hematology Association. This is an open access Abstract Book distributed under the Attribution-NonCommercial-NoDerivs (CC BY-NC-ND), which allows third parties to download the articles and share them with others as long as they credit the author and the Abstract Book, but they cannot change the content in any way or use them commercially.
**Abstract Book Citations:** Authors, Title, HemaSphere, 2026;10:(S1):pages. The abstract book DOIs can be found on the "Author Information" page.
**Disclaimer:** Articles published in the journal HemaSphere exclusively reflect the opinions of the authors. The authors are responsible for all content in their abstracts including accuracy of the facts, statements, citing resources, etc.

Figure 1-A.

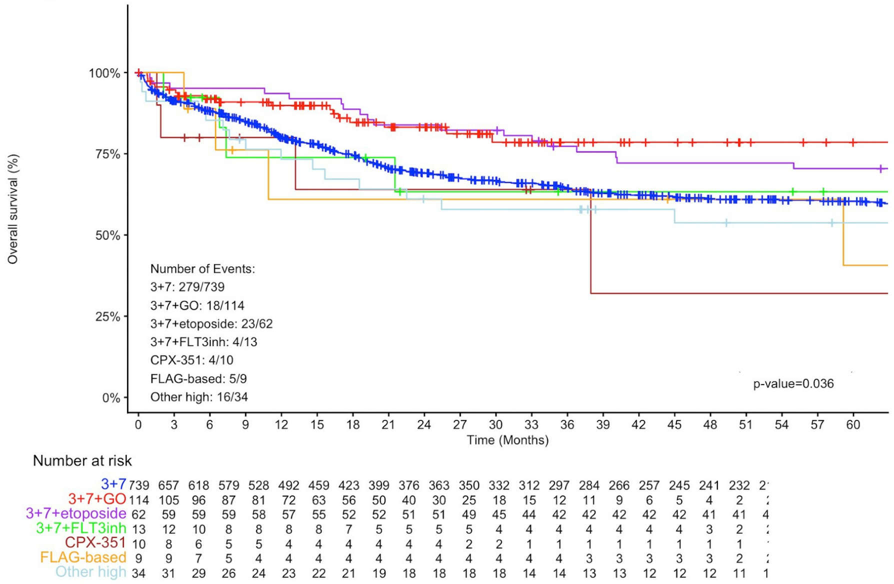

Figure 1-B.

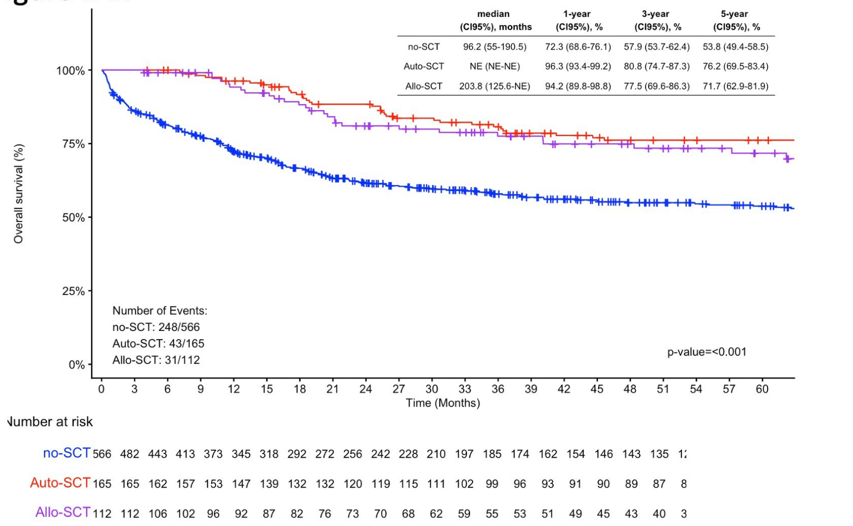

**Copyright Information:** (Online) ISSN: 2572-9241
©2026 The Author(s). HemaSphere is published by John Wiley & Sons Ltd on behalf of European Hematology Association. This is an open access Abstract Book distributed under the Attribution-NonCommercial-NoDerivs (CC BY-NC-ND), which allows third parties to download the articles and share them with others as long as they credit the author and the Abstract Book, but they cannot change the content in any way or use them commercially.
**Abstract Book Citations:** Authors, Title, HemaSphere, 2026;10:(S1):pages. The abstract book DOIs can be found on the "Author Information" page.
**Disclaimer:** Articles published in the journal HemaSphere exclusively reflect the opinions of the authors. The authors are responsible for all content in their abstracts including accuracy of the facts, statements, citing resources, etc.

### (PS1599) ALIDHE: A PHASE 3B STUDY ASSESSING IVOSIDENIB + AZACITIDINE IN ADULTS WITH NEWLY DIAGNOSED MIDH1 ACUTE MYELOID LEUKAEMIA WHO ARE INELIGIBLE FOR INTENSIVE INDUCTION CHEMOTHERAPY

**Topic:** 04. Acute myeloid leukemia - Clinical

Paresh Vyas*[1, 2], Christian Recher[3], Hartmut Döhner[4], Adriano Venditti[5], Sam Salek[6], Samantha Nier[7], Rebeca Rodríguez-Veiga[8], Sylvain Chantepie[9], Tony Marchand[10], Erika Borlenghi[11], Koen Theunissen[12], Mar Tomo[13], Antonio Curti[14], Ioana Codruta Rus[15], David de Leeuw[16, 17], Lalit Saini[18], Emanuele Ammatuna[19], Panagiotis Panagiotidis[20], Andre Schuh[21], Maria Pagoni[22], Johan Maertens[23], Bogdan Ionescu[24], Dilza Camanho[25], Adam Sullivan[26], Vanessa Lacoste[27], Mathilde Romagnoli[25], Benedicte Cognet[27], Courtney Dinardo[28], Susana Vives Polo[29]

*[1]Medical Research Council Weatherall Institute of Molecular Medicine (MRC WIMM), University of Oxford, John Radcliffe Hospital, Oxford, United Kingdom; [2]Oxford University Hospitals NHS Foundation Trust, Department of Haematology, Oxford, United Kingdom; [3]Institut Universitaire du Cancer de Toulouse Oncopole, Université Toulouse III Paul Sabatier, Toulose, France; [4]Ulm University Hospital, Department of Internal Medicine III, Ulm, Germany; [5]University Tor Vergata, Hematology, Department of Biomedicine and Prevention, Rome, Italy; [6]University of Hertfordshire, Hatfield, United Kingdom; [7]Acute Leukemia Advocates Network, Bern, Switzerland; [8]Hospital U.P. La Fe, Valencia, Spain; [9]Caen-Normandy University Hospital, Hematology Institute, Caen, France; [10]Centre Hospitalier Universitaire de Rennes, Rennes, France; [11]ASST Spedali Civili di Brescia, Brescia, Italy; [12]Jessa Hospital, Hasselt, Belgium; [13]Hospital Clinico Universitario and INCLIVA Biomedical Research Institute, Valencia, Spain; [14]IRCCS Azienda Ospedaliero-Universitaria di Bologna, Istituto di Ematologia "Seràgnoli", Bologna, Italy;[15]The Oncology Institute "Prof. Dr. Ion Chiricuta" Cluj-Napoca, Cluj, Romania; [16]Amsterdam UMC, Amsterdam, The Netherlands; [17]Cancer Center Amsterdam, Amsterdam, The Netherlands; [18]Division of Hematology, Department of Medicine, London Health Sciences Centre, London, Canada; [19]University Medical Center Groningen, Groningen, The Netherlands; [20]Hematology Clinic, National and Kapodistrian University of Athens and Laiko General Hospital, Athens, Greece; [21]Princess Margaret Cancer Centre and University of Toronto, Toronto, Canada; [22]Hematology-Lymphomas Department and BMT Unit, Athens, Greece; [23]UZ Leuven, Leuven, Belgium; [24]Clinical Institute Fundeni, Bucharest, Romania;[25]R&D Institute Servier Paris Saclay, Gif-sur Yvette, France; [26]Servier BioInnovation, Boston, United States of America; [27]Servier Medical Affairs, Suresnes, France; [28]MD Andersen Cancer Center, Houston, United States of America; [29]ICO Badalona-Hospital Germans Trias i Pujol and Institut de recerca Josep Carreras, Badalona, Spain;*

### Background

6–10% of patients with acute myeloid leukaemia (AML) have isocitrate dehydrogenase 1 (IDH1) mutations (*IDH1m*). Ivosidenib (IVO) is an oral targeted small molecule inhibitor of mutant IDH1 with activity in patients with AML. The AGILE clinical trial demonstrated that IVO + azacitidine (AZA) provides long-term clinical efficacy with a favourable safety profile in patients with newly diagnosed *IDH1m* AML who are ineligible for intensive chemotherapy (ICIE).

### Aims

To describe the effectiveness, safety, and tolerability of IVO+AZA in an international, single-arm, open-label, post-approval phase 3b study conducted in a real-world setting in ICIE patients with newly diagnosed *mIDH1* AML (NCT05907057).

**Copyright Information:** (Online) ISSN: 2572-9241
©2026 The Author(s). HemaSphere is published by John Wiley & Sons Ltd on behalf of European Hematology Association. This is an open access Abstract Book distributed under the Attribution-NonCommercial-NoDerivs (CC BY-NC-ND), which allows third parties to download the articles and share them with others as long as they credit the author and the Abstract Book, but they cannot change the content in any way or use them commercially.
**Abstract Book Citations:** Authors, Title, HemaSphere, 2026;10:(S1):pages. The abstract book DOIs can be found on the "Author Information" page.
**Disclaimer:** Articles published in the journal HemaSphere exclusively reflect the opinions of the authors. The authors are responsible for all content in their abstracts including accuracy of the facts, statements, citing resources, etc.

### Methods

ALIDHE is enrolling patients aged ≥18 years with AML and a documented *IDH1* R132 mutation. Treatment (tx) comprises IVO (500 mg) once daily and AZA (75 mg/$m^2$/day) for 7 days in 28-day cycles. The primary aim is to assess the safety of this combination. Secondary endpoints include event-free survival (EFS) and disease response. Measurable residual disease (MRD; optional consent) and biomarker correlations with clinical outcomes are exploratory endpoints. ALIDHE is a post-approval study, designed with the Acute Leukemia Advocates Network, putting patient needs, experiences, and perspectives at the forefront.

### Results

As of November 3rd 2025 (data cut off), 124 patients from 9 countries (EU and Canada) had been enrolled and treated; 84 patients remained on tx. Median age was 75.5 years; 62.1% of patients had de novo AML. Median tx duration was 6.2 months (interquartile range: 2.7; 11.3). Overall, IVO+AZA had a favourable safety profile (**Table 1**). Fifteen (12.1%) patients experienced grade ≥2 differentiation syndrome and 10 (8.1%) had grade ≥3 QT prolongation, neither of which led to tx discontinuation.
The objective response (OR) rate in the overall patient population was high (62.9%); rates of complete remission (CR) and CR with partial haematologic recovery were 46.0% and 8.1%, respectively. Responses were observed across the full range of co-occurring mutations, including CRs in 76.2% of patients with *NPM1m* and 30.3% in patients with mutations in the RTK pathway (**Figure 1**). Median durations of OR and CR were 18.2 months and 16.1 months, respectively. Over 24 cycles, haemoglobin and platelet counts increased, neutrophil counts stabilised, and mean bone marrow blasts decreased. During tx, 103 (83.1%) and 78 (62.9%) patients were red blood cell and/or platelet transfusion-free for ≥8 and ≥16 weeks, respectively. Of the 25 MRD-evaluable patients in CR, 11 (44.0%) were MRD-negative by flow cytometry, with >80% reaching MRD negativity by cycle 6.

**Table 1. Safety outcomes (N=124)**

| TEAEs | n (%) |
|---|---|
| **Any TEAE** | 118 (95.2) |
| **Grade ≥3 TEAE** | 105 (84.7) |
| **Treatment-related TEAEs (IVO and/or AZA)** | 94 (75.8) |
| **Serious TEAEs** | 72 (58.1) |
| **TEAEs leading to death[a]** | 8 (6.5)[a] |
| **TEAEs leading to discontinuation of IVO+AZA** | 8 (6.5) |
| **AESI** | 24 (19.4) |
| Grade ≥2 differentiation syndrome | 15 (12.1) |
| Grade ≥3 QT prolongation | 10 (8.1) |

[a] None were considered related to treatment.
AESI, adverse event of special interest; AZA, azacitidine; IVO, ivosidenib; TEAE, treatment-emergent adverse event.

### Summary/Conclusion

These new real-world data from the ongoing ALIDHE study in ICIE patients with newly diagnosed *IDH1m* AML confirm the IVO+AZA efficacy and safety profile seen in the pivotal AGILE study, with enhanced responses observed in the *NPM1m* molecular group. Further safety, effectiveness (including EFS), and quality-of-life data will be analysed and reported.

**Copyright Information:** (Online) ISSN: 2572-9241
©2026 The Author(s). HemaSphere is published by John Wiley & Sons Ltd on behalf of European Hematology Association. This is an open access Abstract Book distributed under the Attribution-NonCommercial-NoDerivs (CC BY-NC-ND), which allows third parties to download the articles and share them with others as long as they credit the author and the Abstract Book, but they cannot change the content in any way or use them commercially.
**Abstract Book Citations:** Authors, Title, HemaSphere, 2026;10:(S1):pages. The abstract book DOIs can be found on the "Author Information" page.
**Disclaimer:** Articles published in the journal HemaSphere exclusively reflect the opinions of the authors. The authors are responsible for all content in their abstracts including accuracy of the facts, statements, citing resources, etc.

Figure 1. Best response in the safety set population (N=124) and in those with specific co-occurring mutations when n > 20 pts

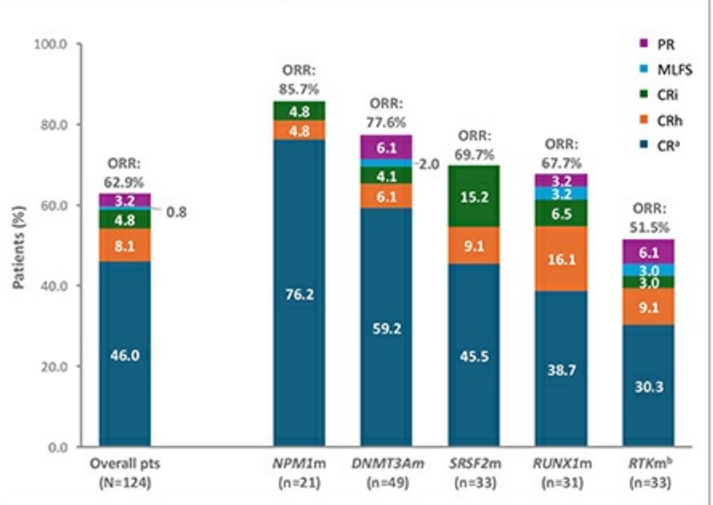

[a]The CR/CRh rate includes those patients who converted to MRD negativity; [b]RTK pathway mutations included those occurring in *FLT3, KIT, KRAS, NF1, NRAS,* and *PTPN11.*

CR, complete remission; CRh, complete remission with partial haematologic recovery; CRi, complete remission with incomplete haematologic recovery; *DNMT3Am*, DNA methyltransferase 3 alpha mutation; *FLT3*, FMS-like tyrosine kinase 3; *KRAS*, Kirsten rat sarcoma viral oncogene homologue; MLFS, morphologic leukaemia-free state; MRD, measurable residual disease; N, number of overall patients; n, number of patients in a subset; *NF1*, neurofibromin-1; *NPM1m*, nucleophosmin 1 mutation; *NRAS*; neuroblastoma RAS viral oncogene homolog; OR, objective response; PR, partial remission; *PTPN11*, protein-tyrosine phosphatase nonreceptor-type 11; pts, patients; *RTKm*, receptor tyrosine kinase pathway mutations; *RUNX1m*, Runt-related transcription factor 1 mutation; *SRSF2m*, serine/arginine-rich splicing factor 2 mutation.

**Copyright Information:** (Online) ISSN: 2572-9241
©2026 The Author(s). HemaSphere is published by John Wiley & Sons Ltd on behalf of European Hematology Association. This is an open access Abstract Book distributed under the Attribution-NonCommercial-NoDerivs (CC BY-NC-ND), which allows third parties to download the articles and share them with others as long as they credit the author and the Abstract Book, but they cannot change the content in any way or use them commercially.
**Abstract Book Citations:** Authors, Title, HemaSphere, 2026;10:(S1):pages. The abstract book DOIs can be found on the "Author Information" page.
**Disclaimer:** Articles published in the journal HemaSphere exclusively reflect the opinions of the authors. The authors are responsible for all content in their abstracts including accuracy of the facts, statements, citing resources, etc.

### (PS1600) BEYOND BCL-2: QUANTITATIVE BCL-XL/MCL-1 AND BH3 PROTEIN PROFILING IDENTIFIES VENETOCLAX RESISTANCE IN ELDERLY AML PATIENTS

**Topic:** 04. Acute myeloid leukemia - Clinical

Chia-Jen Liu*[1, 2, 3], Te-Lin Hsu[1, 3, 4, 5], Yu-Hsun Lin[6, 7], Chun-Kuang Tsai[3, 5, 8], Chiu-Mei Yeh[1]

*[1]Taipei Veterans General Hospital, Division of Transfusion Medicine, Taipei, Taiwan;[2]National Yang Ming Chiao Tung University, Institute of Emergency and Critical Care Medicine, Taipei, Taiwan; [3]National Yang Ming Chiao Tung University, School of Medicine, Taipei, Taiwan; [4]Kinmen Hospital, Ministry of Health and Welfare, Department of Medicine, Kinmen, Taiwan;[5]National Yang Ming Chiao Tung University, Institute of Clinical Medicine, Taipei, Taiwan; [6]Taipei Veterans General Hospital, Department of Pathology and Laboratory Medicine, Taipei, Taiwan; [7]National Yang Ming Chiao Tung University, Institute of Biotechnology in Medicine, Taipei, Taiwan; [8]Taipei Veterans General Hospital, Division of Hematology, Department of Medicine, Taipei, Taiwan;*

### Background

Venetoclax-based low-intensity regimens have improved outcomes for older patients with acute myeloid leukemia (AML), yet responses remain heterogeneous. Because venetoclax selectively inhibits BCL-2, we hypothesized that the broader balance of BCL-2 family proteins (pro- and anti-apoptotic) at diagnosis better captures “apoptotic priming” and may refine prognostication beyond conventional genetic risk.

### Aims

We aimed to determine whether quantitative profiling of pro- and anti-apoptotic BCL-2 family proteins in diagnostic bone marrow specimens can improve risk stratification in older AML patients treated with venetoclax-based regimens.

### Methods

We conducted a retrospective cohort study of patients aged ≥ 60 years with newly diagnosed AML treated with venetoclax-based regimens at Taipei Veterans General Hospital (January 2019–March 2023). Diagnostic bone marrow trephine biopsy specimens were formalin-fixed, paraffin-embedded and stained by automated immunohistochemistry for 11 BCL-2 family proteins (BH3-only: HRK, BAD, NOXA, PUMA, BID, BIM; anti-apoptotic: BCL-2, BCL-XL, MCL-1; effectors: BAX, BAK). Whole slide images were digitized at ×40 and quantified using QuPath (v0.5.0) with standardized tumor-region annotation and H-score computation; ≥ 10,000 cells per protein per case were analyzed. Protein expression was dichotomized using ROC-derived cutoffs (Youden’s index and closest-to-(0,1) methods). Progression-free survival (PFS) was the primary endpoint and overall survival (OS) the secondary endpoint; associations were assessed using Cox proportional hazards models adjusting for baseline clinical factors and ELN risk.

### Results

Fifty-two patients met inclusion criteria (median age 74 years; 26.9% ≥ 80; 59.6% male). Most patients had adverse ELN 2022 risk (76.9%), and 75% received venetoclax plus hypomethylating agent or low-dose cytarabine. Adverse ELN 2022 risk independently predicted shorter PFS and OS, and age ≥80 years independently predicted inferior OS. Quantitative protein profiling added prognostic information: higher expression of pro-apoptotic BH3-only sensitizers (notably BAD, HRK, and NOXA) was associated with improved outcomes, whereas higher expression of anti-apoptotic proteins BCL-XL and MCL-1 was associated with increased risk of progression and death (with MCL-1 showing borderline significance in some multivariable models). Higher BCL-2 expression itself did not correspond to superior survival. In the low-intensity subgroup, BAD remained consistently associated with longer PFS, while MCL-1 was an adverse factor for OS.

**Copyright Information:** (Online) ISSN: 2572-9241
©2026 The Author(s). HemaSphere is published by John Wiley & Sons Ltd on behalf of European Hematology Association. This is an open access Abstract Book distributed under the Attribution-NonCommercial-NoDerivs (CC BY-NC-ND), which allows third parties to download the articles and share them with others as long as they credit the author and the Abstract Book, but they cannot change the content in any way or use them commercially.
**Abstract Book Citations:** Authors, Title, HemaSphere, 2026;10:(S1):pages. The abstract book DOIs can be found on the “Author Information” page.
**Disclaimer:** Articles published in the journal HemaSphere exclusively reflect the opinions of the authors. The authors are responsible for all content in their abstracts including accuracy of the facts, statements, citing resources, etc.

### Summary/Conclusion

Digital pathology–based, quantitative IHC profiling of the BCL-2 apoptotic network at diagnosis provides clinically meaningful risk stratification in older AML treated with venetoclax. Elevated BCL-XL/MCL-1 identifies patients at risk for early failure, while higher BAD/HRK/NOXA suggests greater apoptotic priming and favorable outcomes. These findings support incorporating apoptosis biomarkers into risk models and prioritizing rational combination strategies to overcome BCL-XL/MCL-1–mediated resistance.

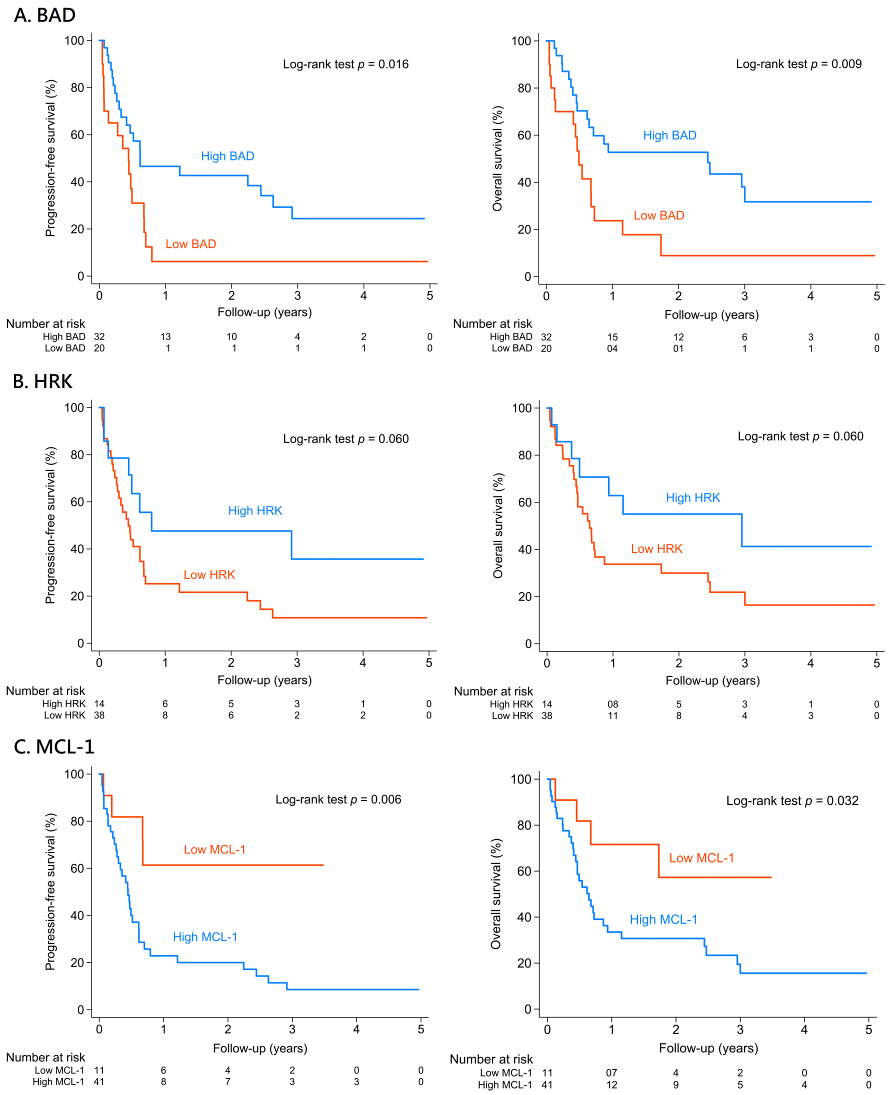

**Figure** Kaplan–Meier survival by BCL-2 family protein expression.

Progression-free survival (left) and overall survival (right) are shown for patients stratified by high vs low expression of BAD (A), HRK (B), and MCL-1 (C) using ROC-derived H-score cutoffs.

**Copyright Information:** (Online) ISSN: 2572-9241
©2026 The Author(s). HemaSphere is published by John Wiley & Sons Ltd on behalf of European Hematology Association. This is an open access Abstract Book distributed under the Attribution-NonCommercial-NoDerivs (CC BY-NC-ND), which allows third parties to download the articles and share them with others as long as they credit the author and the Abstract Book, but they cannot change the content in any way or use them commercially.
**Abstract Book Citations:** Authors, Title, HemaSphere, 2026;10:(S1):pages. The abstract book DOIs can be found on the "Author Information" page.
**Disclaimer:** Articles published in the journal HemaSphere exclusively reflect the opinions of the authors. The authors are responsible for all content in their abstracts including accuracy of the facts, statements, citing resources, etc.

### (PS1601) HMA PLUS VENETOCLAX FOR 7- VS 14- VS 21- VS 28-DAY CYCLES IN NEWLY-DIAGNOSED ACUTE MYELOID LEUKEMIA: ELN- AND MAYO GENETIC RISK–STRATIFIED ANALYSIS IN 540 PATIENTS.

**Topic:** 04. Acute myeloid leukemia - Clinical

Sudhesh Kumar[1], Momna Warraich[1], Mahnoor Fatima[1], Kristen McCullough[1], Aref Al-Kali[1], Hassan Alkhateeb[1], Kebede Begna[1], Abhishek Mangaonkar[1], Antoine Saliba[1], Mark Litzow[1], William Hogan[1], Mithun Shah[1], Mrinal Patnaik[1], Animesh Pardanani[1], Talha Badar[2], James M Foran[2], Jeanne Palmer[3], Cecilia Arana Yi[3], Ayalew Tefferi[1], Naseema Gangat*[1]

*[1]Mayo Clinic, Department of Hematology, Rochester, United States of America;[2]Mayo Clinic, Department of Hematology, Jacksonville, United States of America; [3]Mayo Clinic, Department of Hematology, Scottsdale, United States of America;*

### Background

Venetoclax plus hypomethylating agent (Ven-HMA) is the standard treatment for unfit patients with newly diagnosed acute myeloid leukemia (ND-AML), with Ven typically administered for 28 days (d) per cycle (DiNardo NEJM 2020). However, the optimal duration of Ven across risk groups remains unclear.

### Aims

We examined survival outcomes with Ven 7, 14, 21, and 28d in the context of European LeukemiaNet (ELN) 2024 (Döhner Blood 2024) and Mayo Genetic risk models (Gangat Am J Hematol 2025).

### Methods

ND-AML patients receiving Ven-HMA at the Mayo Clinic were retrospectively studied. Patients were stratified by ELN 2024 and Mayo Genetic risk and analyzed using standard statistical methods.

**Copyright Information:** (Online) ISSN: 2572-9241
©2026 The Author(s). HemaSphere is published by John Wiley & Sons Ltd on behalf of European Hematology Association. This is an open access Abstract Book distributed under the Attribution-NonCommercial-NoDerivs (CC BY-NC-ND), which allows third parties to download the articles and share them with others as long as they credit the author and the Abstract Book, but they cannot change the content in any way or use them commercially.
**Abstract Book Citations:** Authors, Title, HemaSphere, 2026;10:(S1):pages. The abstract book DOIs can be found on the “Author Information” page.
**Disclaimer:** Articles published in the journal HemaSphere exclusively reflect the opinions of the authors. The authors are responsible for all content in their abstracts including accuracy of the facts, statements, citing resources, etc.

### Results

540 patients (median age 75 years, 52% de novo) received HMA with Ven for 7d (n=33), 14d (n=117), 21d (n=96), or 28d (n=294). ELN and Mayo risk distributions were 22%/40% high, 18%/51% intermediate, 60%/9% low risk, respectively. Baseline characteristics were comparable.
CR/CRi rates were comparable across Ven durations, with the exception of lower CR/CRi with 7d vs 21d (p=0.04). Risk-stratified CR/CRi showed variations across Mayo and ELN groups but without significant differences based on Ven duration (Table).
Allogeneic transplant rates were 15%, 18%, 18%, 20% for 7, 14, 21, 28d (p=0.86).
At a median follow-up of 37.7 months, median overall survival (OS) differed by ELN risk (6.6, 11.9, 20 months) and Mayo genetic risk (7.2, 19.6, NR (Not Reached) months), for high, intermediate, low risk (p<0.01), respectively. Median OS was comparable across Ven 7, 14, 21, 28d (12.8, 12.6, 16.7, 14.3 months; p=0.84), with corresponding 1/2/3-year survival rates of 53%/32%/0%, 51%/32%/25%, 56%/35%/23%, 55%/34%/28%. Outcomes remained comparable in transplant censored survival as well (Figure).
30-/60-d mortality differed by Ven duration; 7d 10%/20%, 14d 7%/15%, 21d 4%/13%, 28d 4%/6%. Compared with 28d, 30d mortality was higher with 7d and 14d (p=0.07/0.03), and 60d mortality was higher with 7d, 14d, and 21d (p=0.02/0.01/0.05).
OS was comparable across Ven durations within ELN intermediate/low risk and all Mayo risk groups. However, among ELN high-risk patients, those receiving 14d Ven had inferior OS and transplant-censored survival compared with 21d and 28d (p<0.01 for both).
Cumulative incidence of relapse did not differ by ELN risk (p=0.61) or Mayo genetic risk (p=0.46), with 1-year rates of 19%/16%/15% for Mayo high, intermediate, and low risk and 19%/20%/16% for ELN high, intermediate, and low risk respectively.
Cumulative incidence of relapse was comparable across Ven durations, with 1-year rates of 6%/17%/19%/18% for 7, 14, 21, and 28d (p=0.65).

| | | | 7 (N=33) | 14 (N=117) | 21 (N=96) | 28 (N=294) | P-value | |
|---|---|---|---|---|---|---|---|---|
| **CR/CRi (%)** | | | 52 | 63 | 72 | 65 | 0.19 | 7≠21 |
| | **Mayo Risk** | **High** | 33 | 46 | 57 | 51 | 0.53 | - |
| | | **Intermediate** | 58 | 70 | 84 | 74 | 0.17 | 7≠21 |
| | | **Low** | 100 | 85 | 89 | 86 | 0.84 | - |
| | **ELN Risk** | **High** | 33 | 30 | 57 | 53 | 0.21 | - |
| | | **Intermediate** | 33 | 59 | 64 | 56 | 0.63 | - |
| | | **Low** | 62 | 74 | 80 | 73 | 0.46 | - |

### Summary/Conclusion

Survival outcomes were comparable across Ven 7, 14, 21, 28 d in ND-AML, with outcomes driven by Mayo Genetic and ELN risk. Despite differences by Ven duration in high-risk groups, higher early mortality with 7d and 14d likely reflects treatment selection. Prospective trials are needed to establish risk adapted Ven dosing strategies.

**Copyright Information:** (Online) ISSN: 2572-9241
©2026 The Author(s). HemaSphere is published by John Wiley & Sons Ltd on behalf of European Hematology Association. This is an open access Abstract Book distributed under the Attribution-NonCommercial-NoDerivs (CC BY-NC-ND), which allows third parties to download the articles and share them with others as long as they credit the author and the Abstract Book, but they cannot change the content in any way or use them commercially.
**Abstract Book Citations:** Authors, Title, HemaSphere, 2026;10:(S1):pages. The abstract book DOIs can be found on the "Author Information" page.
**Disclaimer:** Articles published in the journal HemaSphere exclusively reflect the opinions of the authors. The authors are responsible for all content in their abstracts including accuracy of the facts, statements, citing resources, etc.

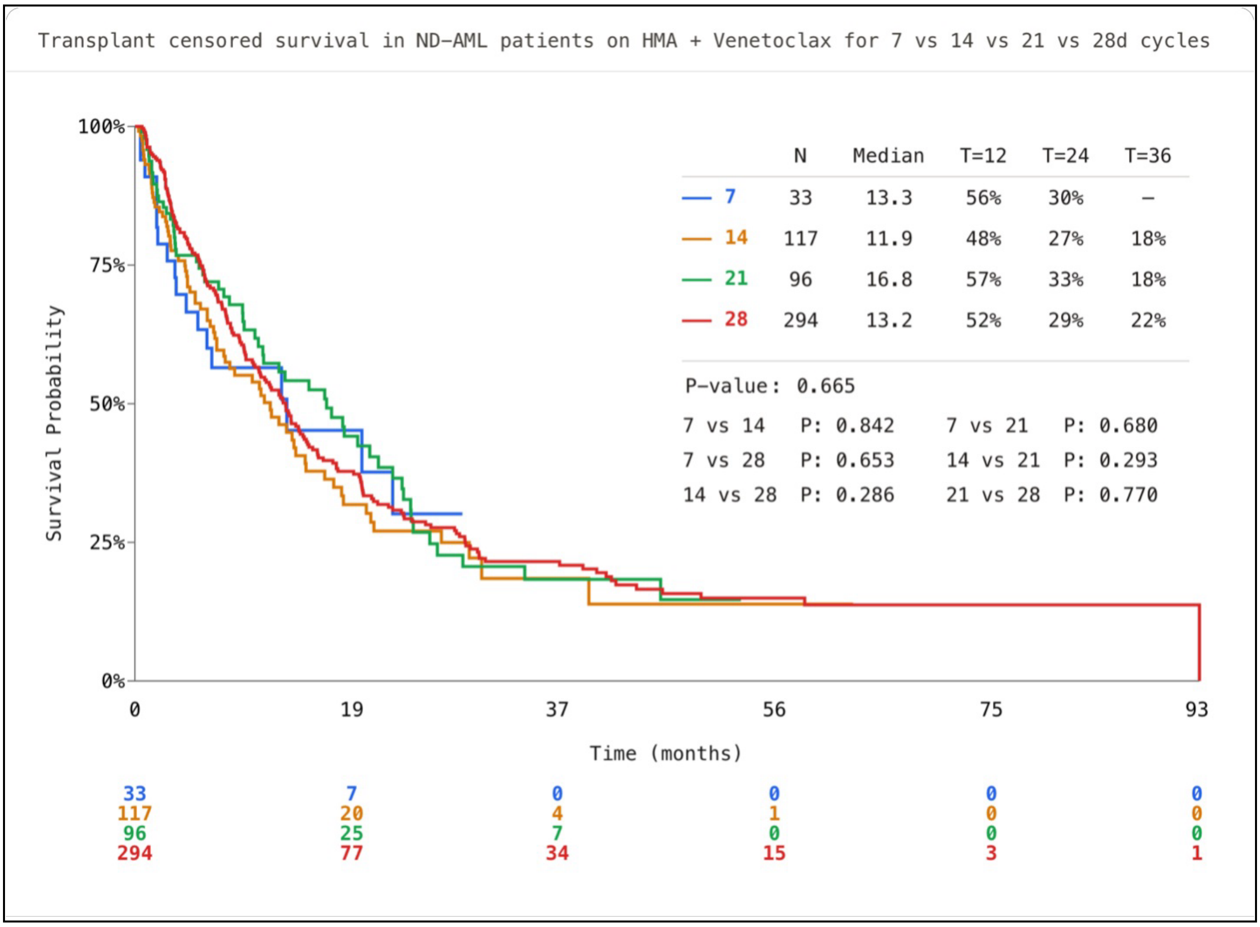

**Copyright Information:** (Online) ISSN: 2572-9241
©2026 The Author(s). HemaSphere is published by John Wiley & Sons Ltd on behalf of European Hematology Association. This is an open access Abstract Book distributed under the Attribution-NonCommercial-NoDerivs (CC BY-NC-ND), which allows third parties to download the articles and share them with others as long as they credit the author and the Abstract Book, but they cannot change the content in any way or use them commercially.
**Abstract Book Citations:** Authors, Title, HemaSphere, 2026;10:(S1):pages. The abstract book DOIs can be found on the "Author Information" page.
**Disclaimer:** Articles published in the journal HemaSphere exclusively reflect the opinions of the authors. The authors are responsible for all content in their abstracts including accuracy of the facts, statements, citing resources, etc.

### (PS1602) A PHASE 2 STUDY OF TAMIBAROTENE AND ARSENIC TRIOXIDE (ATO) FOLLOWED BY GEMTUZUMAB OZOGAMICIN (GO) FOR FIRST RELAPSED ACUTE PROMYELOCYTIC LEUKEMIA: THE JALSG APL219R STUDY

**Topic:** 04. Acute myeloid leukemia - Clinical

Hiroto Ishii*[1], Toru Kiguchi[2], Shingo Yano[3], Masashi Sawa[4], Kaichi Nishiwaki[5], Hiroyuki Fujita[6], Yuichi Ishikawa[7], Nobuhiko Kobayashi[8], Kenji Matsumoto[9], Akio Maeda[10], Masako Toyosaki[11], Eisei Kondo[12], Hisayuki Yokoyama[13], Akiko M. Saito[14], Emiko Sakaida[15], Yoshinobu Maeda[16], Takahiro Yamauchi[17], Itaru Matsumura[18], Yasushi Miyazaki[19], Hitoshi Kiyoi[20]

*[1]The Jikei University Hospital, Tokyo, Japan; [2]Dokkyo Medical University Dokkyo Medical Center, Koshigaya, Japan; [3]The Jikei University, Tokyo, Japan; [4]Anjo Kosei Hospital, Anjo, Japan; [5]The Jikei University Kashiwa Hospital, Kashiwa, Japan; [6]Saiseikai Yokohama-shi Nanbu Hospital, Yokohama, Japan; [7]Nagoya University Graduate School Of Medicine, Nagoya, Japan; [8]Gunma University, Maebashi, Japan; [9]Yokohama City University, Yokohama, Japan; [10]Hyogo Cancer Center, Akashi, Japan; [11]Tokai University, Isehara, Japan; [12]Kawasaki Medical School, Kurashiki, Japan; [13]Yamagata University, Yamagata, Japan; [14]NHO Nagoya Medical Center, Nagoya, Japan; [15]Chiba University Hospital, Chiba, Japan; [16]Okayama University Hospital, Okayama, Japan; [17]University of Fukui, Fukui, Japan; [18]Kindai University Faculty Of Medicine, Sakai, Japan; [19]Nagasaki University, Nagasaki, Japan; [20]Nagoya University Graduate School of Medicine, Nagoya, Japan;*

### Background

In Japan, the standard salvage strategy for relapsed acute promyelocytic leukemia (APL) has been established by the Japan Adult Leukemia Study Group (JALSG) APL205R trial, consisting of arsenic trioxide (ATO) reinduction and consolidation, high-dose cytarabine–based chemotherapy with peripheral blood stem cell collection, followed by autologous hematopoietic stem cell transplantation (auto-HSCT). Although favorable outcomes were reported (complete remission (CR) 81%, 1-year event-free survival (EFS) 77%, and 5-year overall survival (OS) 77%), approximately one-quarter of patients were unable to proceed to auto-HSCT due to advanced age, comorbidities, poor performance status, institutional limitations, or patient preference.

### Aims

The JALSG APL219R study excludes auto-HSCT and includes both auto-HSCT–eligible and –ineligible patients. This phase 2 study evaluates the efficacy and safety of a novel, transplant-free regimen consisting of reinduction therapy with tamibarotene, a synthetic retinoid, in combination with ATO, followed by consolidation with gemtuzumab ozogamicin (GO).

### Methods

This multicenter, single-arm, phase 2 study was conducted at JALSG-participating institutions in Japan. Eligible patients were aged ≥16 years with first relapsed of CD33-positive APL confirmed by cytogenetic or molecular analysis. The protocol comprised one reinduction course and three consolidation courses. Reinduction therapy consisted of oral tamibarotene (6 mg/m²/day) combined with intravenous ATO (0.15 mg/kg/day) until hematological remission or for a maximum of 56 days. Two subsequent consolidation courses with tamibarotene and ATO were followed by a final consolidation course with GO (3 mg/m²). The primary endpoint was 1-year EFS rate. Secondary endpoints included CR rate, OS, and safety. The study was registered with jRCT (jRCTs041190101).

**Copyright Information:** (Online) ISSN: 2572-9241
©2026 The Author(s). HemaSphere is published by John Wiley & Sons Ltd on behalf of European Hematology Association. This is an open access Abstract Book distributed under the Attribution-NonCommercial-NoDerivs (CC BY-NC-ND), which allows third parties to download the articles and share them with others as long as they credit the author and the Abstract Book, but they cannot change the content in any way or use them commercially.
**Abstract Book Citations:** Authors, Title, HemaSphere, 2026;10:(S1):pages. The abstract book DOIs can be found on the “Author Information” page.
**Disclaimer:** Articles published in the journal HemaSphere exclusively reflect the opinions of the authors. The authors are responsible for all content in their abstracts including accuracy of the facts, statements, citing resources, etc.

**Results**

Between December 2019 and December 2023, 21 of the planned 39 patients were enrolled. At the data cutoff in February 2025, the median age at relapse was 54 years (range, 35–77), and 66.7% were male. The median duration of first remission was 3.6 years. All patients were in first hematological or molecular relapse. The 1-year EFS rate was 81.0% (95% confidence interval (CI), 57.0–92.0%), exceeding the prespecified threshold of 50%. Hematological and molecular CR rates after reinduction were 95.2% and 90.5%, respectively, with a median time to hematological remission of 44 days. The 1-year OS rate was 100%. Seventeen patients (81.0%) completed the full treatment protocol. The most frequent grade ≥3 non-hematological adverse events were QTc prolongation (28.6%), febrile neutropenia (23.8%), and elevated liver enzymes (AST/ALT) (4.8/9.5%). Serious adverse events occurred in 3 patients (14.3%) and included one case each of febrile neutropenia, lower gastrointestinal hemorrhage, and COVID-19 infection; no treatment-related deaths were observed.

**Summary/Conclusion**

Despite not reaching the planned sample size, the APL219R study demonstrated a high 1-year EFS with manageable toxicity in this phase 2 cohort of patients with relapsed APL who did not undergo auto-HSCT. These results indicate that a transplant-free strategy may be a clinically meaningful treatment option, particularly for patients who are unsuitable for or lack access to auto-HSCT.

**Copyright Information:** (Online) ISSN: 2572-9241
©2026 The Author(s). HemaSphere is published by John Wiley & Sons Ltd on behalf of European Hematology Association. This is an open access Abstract Book distributed under the Attribution-NonCommercial-NoDerivs (CC BY-NC-ND), which allows third parties to download the articles and share them with others as long as they credit the author and the Abstract Book, but they cannot change the content in any way or use them commercially.
**Abstract Book Citations:** Authors, Title, HemaSphere, 2026;10:(S1):pages. The abstract book DOIs can be found on the “Author Information” page.
**Disclaimer:** Articles published in the journal HemaSphere exclusively reflect the opinions of the authors. The authors are responsible for all content in their abstracts including accuracy of the facts, statements, citing resources, etc.

### (PS1603) FLT3-ITD ALLELIC RATIO IS NO LONGER PROGNOSTIC IN AML PATIENTS TREATED WITH HIGH-DOSE ANTHRACYCLINE AND A FLT3 INHIBITOR

**Topic:** 04. Acute myeloid leukemia - Clinical

Mathieu Leclerc*[1], Etienne Audureau[2], Nicolas Duployez[3], Mathilde Hunault[4], Emmanuelle Tavernier Tardy[5], Cécile Pautas[6], Sarah Bertoli[7], Emmanuel Raffoux[8], Marie Anne Hospital[9], Tony Marchand[10], Mael Heiblig[11], Sylvain Chantepie[12], Martin Carre[13], Pierre Peterlin[14], Maria Pilar Gallego Hernanz[15], Romain Guieze[16], Célestine Simand[17], Pascal Turlure[18], Anne Huynh[19], Emilie Lemasle[20], Ludovic Gabellier[21], Juliette Lambert[22], Carlos Felipe Suarez[23], Samy Chraibi[24], Laurence Sanhes[25], Karine Celli-Lebras[26], Ariane Mineur[27], Claude Gardin[28], Jean-Valère Malfuson[29], Thomas Cluzeau[30], Norbert Vey[9], Régis Peffault de Latour[8], Lucie Rigolot[31], Isabelle Luquet[31], Dominique Penther[32], Raphael Itzykson[8], Eric Delabesse[33], Jean-François Hamel[34], Stéphane De Botton[35], Arnaud Pigneux[36], Hervé Dombret[8], Claude Preudhomme[3], Christian Recher[19], Pierre, Yves Dumas[36]

*[1]Hôpital Henri Mondor, Assistance Publique - Hôpitaux de Paris, Hematology and Cell Therapy Department, Créteil, France; [2]Clinical Research Unit (URC) Henri Mondor Hospital, Assistance Publique - Hôpitaux de Paris, IMRB U955 INSERM, Public Health Department, Créteil, France; [3]CHU de Lille, Hematology Laboratory, Lille, France; [4]CHU d'Angers, FHU GOAL- CRCINA, INSERM, Université de Nantes, Université d'Angers, Department of Hematology, Angers, France; [5]CHU de Saint-Etienne, Hematology and Cell Therapy Department, Saint-Etienne, France; [6]Hôpital Henri Mondor, Hematology and Cell Therapy Department, Créteil, France; [7]6.Université de Toulouse, CHU de Toulouse, Institut Universitaire du Cancer de Toulouse Oncopole, Service d'Hématologie, Toulouse, France; [8]Hôpital Saint-Louis, Assistance Publique - Hôpitaux de Paris, Department of Hematology, Paris, France; [9]Institut Paoli-Calmettes, Department of Hematology, Marseille, France; [10]CHU de Rennes, Department of Hematology, Rennes, France; [11]CHU Lyon Sud, Hospices Civils de Lyon, Department of Hematology, Lyon, France; [12]CHU de Caen, Department of Hematology, Caen, France; [13]CHU de Grenoble, Department of Hematology, Grenoble, France; [14]CHU de Nantes, Department of Hematology, Nantes, France; [15]CHU de Poitiers, Department of Hematology, Poitiers, France; [16]CHU Estaing, Department of Hematology, Clermont-Ferrand, France; [17]CHU de Strasbourg, Department of Hematology, Strasbourg, France; [18]CHU de Limoges, Department of Hematology, Limoges, France; [19]Université de Toulouse, CHU de Toulouse, Institut Universitaire du Cancer de Toulouse Oncopole, Service d'Hématologie, Toulouse, France; [20]Centre Henri Becquerel, Department of Hematology, Rouen, France; [21]CHU de Montpellier, Department of Clinical Hematology, Montpellier, France; [22]Hôpital Mignot, Centre Hospitalier de Versailles, Department of Hematology, Versailles, France;[23]Hôpital Necker-Enfants Malades, Assistance Publique - Hôpitaux de Paris, Department of Adult Hematology, Paris, France; [24]CHU de Nîmes, Department of Hematology, Nîmes, France; [25]Centre Hospitalier de Perpignan, Department of Hematology, Perpignan, France; [26]Acute Leukemia French Association Group Coordination, IRSL, Paris, France;[27]French Innovative Leukemia Organization Group Coordination, CHU de Bordeaux, Bordeaux, France; [28]Hôpital Avicenne, Department of Hematology, Bobigny, France; [29]Hôpital National d'Instruction des Armées Percy, Department of Hematology, Clamart, France;[30]Hôpital l'Archet, CHU de Nice, Department of Clinical Hematology, Nice, France; [31]CHU de Toulouse, Institut Universitaire du Cancer de Toulouse Oncopole, Hematology Laboratory, Toulouse, France; [32]Centre Henri Becquerel, Onco-Genetics Laboratory, Rouen, France; [33]Université de Toulouse, CHU de Toulouse, Institut Universitaire du Cancer de Toulouse Oncopole, Hematology Laboratory, Toulouse, France; [34]CHU d'Angers, Department of Biostatistics, Angers, France;[35]Institut Gustave Roussy, Department of Hematology, Villejuif, France; [36]CHU de Bordeaux, Hematology and Cell Therapy Department, Bordeaux, France;*

### Background

Internal tandem duplication of *FLT3* (*FLT3*-ITD) occurs in about 25% of acute myeloid leukemia (AML) cases and its prognostic impact has been associated with the ITD/wild-type allelic ratio (AR) and *NPM1* co-mutation status. However, *FLT3*-ITD AR was removed from the ELN-2022 classification mainly because of the lack of standardized assay and the advent of FLT3 tyrosine kinase inhibitors, even though there was no clear evidence that its prognostic value was no longer relevant.

**Copyright Information:** (Online) ISSN: 2572-9241
©2026 The Author(s). HemaSphere is published by John Wiley & Sons Ltd on behalf of European Hematology Association. This is an open access Abstract Book distributed under the Attribution-NonCommercial-NoDerivs (CC BY-NC-ND), which allows third parties to download the articles and share them with others as long as they credit the author and the Abstract Book, but they cannot change the content in any way or use them commercially.
**Abstract Book Citations:** Authors, Title, HemaSphere, 2026;10:(S1):pages. The abstract book DOIs can be found on the "Author Information" page.
**Disclaimer:** Articles published in the journal HemaSphere exclusively reflect the opinions of the authors. The authors are responsible for all content in their abstracts including accuracy of the facts, statements, citing resources, etc.

### Aims

To evaluate the prognostic impact of *FLT3*-ITD AR in a prospective cohort of younger AML patients treated with high-dose anthracycline-based induction chemotherapy and midostaurin.

### Methods

We conducted a post-hoc analysis from the BIG-1 database. The BIG-1 protocol is a large prospective multicenter study from the Acute Leukemia French Intergroup involving patients 18 to 60 years of age with newly diagnosed AML. Patients received a single high-dose anthracycline-based induction chemotherapy (either daunorubicin 90 mg/m²/d for 3 days or idarubicin 9 mg/m²/d for 5 days) followed by 3 cycles of intermediate or high-dose cytarabine consolidation cycles. High-risk patients were referred to allogeneic hematopoietic stem-cell transplantation if they had a suitable donor. After a protocol amendment in June 2018, *FLT3*-ITD patients also received midostaurin 50 mg twice daily D8-D21 in combination with chemotherapy during the induction and consolidation phases, as well as continuously for a 12-months maintenance therapy if not transplanted. *FLT3*-ITD patients were categorized as having a high (≥0.5) or low (<0.5) AR. The primary endpoint of this study is overall survival (OS) and secondary endpoints are relapse-free (RFS) and event-free survivals (EFS).

### Results

Among the 1990 patients included in the BIG-1 trial from January 2015 to July 2021 with available data concerning *FLT3* mutational status, 459 (23.1%) harbored a *FLT3*-ITD, including 186 (40.5%) with a high and 273 (59.5%) with a low AR. Median age was 49.9 years and 261 (56.9%) patients were female. Most patients were categorized as intermediate risk (79.5% of cases) according to the ELN-2022 classification and 66.2% also had a *NPM1* mutation. Patients with a high AR had a significantly higher median baseline white blood cell count. Most patients were included in the study before June 2018 and thus did not receive midostaurin (n=306, 66.7%). Composite complete remission rate after induction chemotherapy was similar between patients with a high or low AR (79.6% (n=148/186) vs 81.7% (n=223/273) respectively, P = 0.85). Median OS was 7.6 years for patients with a high AR and 6.9 years for those with a low AR (log rank test P = 0.25, Figure 1). In multivariable analysis, *FLT3*-ITD AR was not significantly and independently associated with the risk of death, even when analysis was restricted to patients that received (aHR = 1.43, 95% CI [0.83;2.44], P = 0.20) or did not receive (aHR 1.16, 95% CI [0.83;1.60], P = 0.38) midostaurin. Only midostaurin use (aHR = 0.69; 95% CI [0.5;0.95]; P = 0.024) and ELN-2022 adverse risk category (aHR = 1.66; 95% CI [1.18;2.34]; P = 0.004) independently impacted OS. Likewise, *FLT3*-ITD AR had no impact on RFS (median 2.0 versus 1.7 years, P = 0.5) nor EFS (median 1.4 versus 1.2 years, P = 0.58).

### Summary/Conclusion

In younger AML patients treated with high-dose anthracycline-based induction chemotherapy and midostaurin, *FLT3*-ITD AR has no independent prognostic value.

**Copyright Information:** (Online) ISSN: 2572-9241
©2026 The Author(s). HemaSphere is published by John Wiley & Sons Ltd on behalf of European Hematology Association. This is an open access Abstract Book distributed under the Attribution-NonCommercial-NoDerivs (CC BY-NC-ND), which allows third parties to download the articles and share them with others as long as they credit the author and the Abstract Book, but they cannot change the content in any way or use them commercially.
**Abstract Book Citations:** Authors, Title, HemaSphere, 2026;10:(S1):pages. The abstract book DOIs can be found on the "Author Information" page.
**Disclaimer:** Articles published in the journal HemaSphere exclusively reflect the opinions of the authors. The authors are responsible for all content in their abstracts including accuracy of the facts, statements, citing resources, etc.

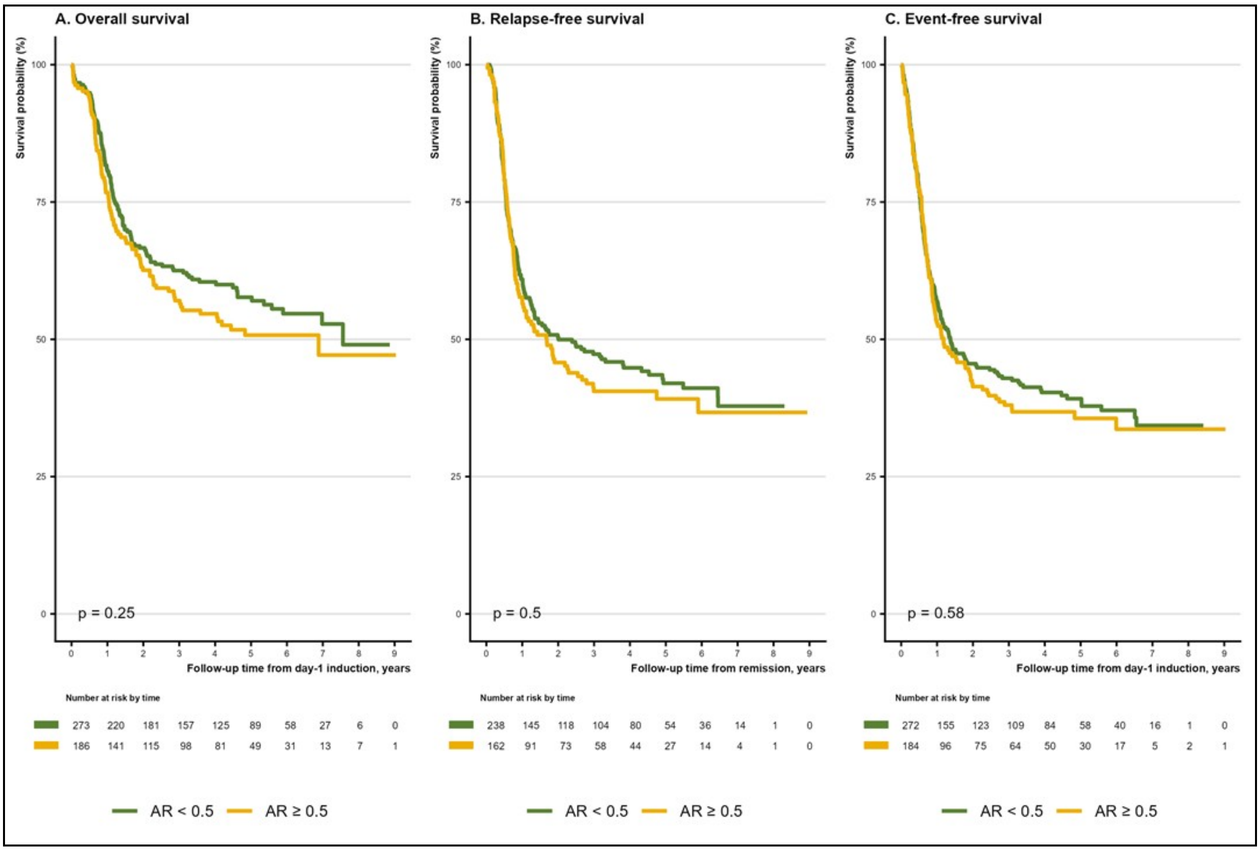

Figure 1. Survival by FLT3-ITD allelic ratio. A: Probability of overall survival according to FLT3-ITD AR (≥0.5 vs <0.5); B: Probability of relapse-free survival according to FLT3-ITD AR (≥0.5 vs <0.5); C: Probability of event-free survival according to FLT3-ITD AR (≥0.5 vs <0.5). Survival curves generated using the Kaplan-Meier method and group comparisons performed with log-rank tests.

**Copyright Information:** (Online) ISSN: 2572-9241
©2026 The Author(s). HemaSphere is published by John Wiley & Sons Ltd on behalf of European Hematology Association. This is an open access Abstract Book distributed under the Attribution-NonCommercial-NoDerivs (CC BY-NC-ND), which allows third parties to download the articles and share them with others as long as they credit the author and the Abstract Book, but they cannot change the content in any way or use them commercially.
**Abstract Book Citations:** Authors, Title, HemaSphere, 2026;10:(S1):pages. The abstract book DOIs can be found on the "Author Information" page.
**Disclaimer:** Articles published in the journal HemaSphere exclusively reflect the opinions of the authors. The authors are responsible for all content in their abstracts including accuracy of the facts, statements, citing resources, etc.

### (PS1604) A NOVEL STRATEGY TO CIRCUMVENT VENETOCLAX RESISTANCE IN RELAPSED/REFRACTORY ACUTE MYELOID LEUKEMIA : UPDATED RESULTS FROM A PHASE II TRIAL

**Topic:** 04. Acute myeloid leukemia - Clinical

Huafeng Wang*[1, 2, 3, 4], Yile Zhou[2, 5], Xiang Zhang[2, 5], Liping Mao[2, 5], Xia Li[2, 5], Xingnong Ye[2, 5], Mixue Xie[2, 5], De Zhou[2, 5], Xiudi Yang[2, 5], Jian Huang[2, 3, 4, 5], Wenjuan Yu[2, 5], Juying Wei[2, 5], Min Yang[2, 5], Yinjun Lou[2, 5], Gaixiang Xu[2, 5], Zenghua Lin[6], Hongyan Tong[2, 3, 4, 5], Jie Jin[2, 3, 4, 5]

*[1]The First Affiliated Hospital, Zhejiang University School of Medicine, Hangzhou, China;[2]Zhejiang Key Laboratory for Precision Diagnosis and Treatment of Hematological Malignancies, Hangzhou, China; [3]Zhejiang University Cancer Center, Zhejiang University, Hangzhou, China; [4]Zhejiang Provincial Clinical Research Center for Hematological disorders, Hangzhou, China; [5]Department of Hematology, the First Affiliated Hospital, Zhejiang University School of Medicine, Hangzhou, China;[6]Affiliated Hospital of Nantong University, Nantong, China;*

### Background

Venetoclax (VEN) has improved AML treatment, but options for VEN-resistant disease are limited. Mitochondrial calcium regulates VEN sensitivity, suggesting mitoxantrone, a mitochondrial calcium uniporter inhibitor, may overcome resistance. Mitoxantrone hydrochloride liposome (Lipo-MIT), a novel anthracycline nano-drug, has shown improved pharmacokinetics and activity. We initiated the MAV regimen (Lipo-MIT, cytarabine, and VEN) for R/R AML (NCT06621212). Preliminary data from 20 patients (ASH 2025, #3464) showed a 64.3% composite complete remission (CRc) rate in VEN-exposed patients, a finding that prompted expansion to a multicenter phase II trial. Updated results on 35 patients are now presented.

### Aims

To evaluate efficacy and safety of the MAV regimen in patients with R/R AML.

### Methods

This prospective study enrolled adults (≥18 years) with R/R AML and ECOG 0-2. Induction: Lipo-MIT 30 mg/m² (day 1), cytarabine 100 mg/m² (days 1-7), and venetoclax (100 mg day 2, 200 mg day 3, then 400 mg days 4-8), q4w for up to 2 cycles. Patients achieving CRc were recommended for allo-HSCT; transplant-ineligible patients received consolidation (cytarabine and venetoclax for fit patients or low-intensity therapy for unfit patients). Primary endpoint: CRc rate. Secondary endpoints: MRD negativity by flow cytometry, ORR, survival, and safety.

**Copyright Information:** (Online) ISSN: 2572-9241
©2026 The Author(s). HemaSphere is published by John Wiley & Sons Ltd on behalf of European Hematology Association. This is an open access Abstract Book distributed under the Attribution-NonCommercial-NoDerivs (CC BY-NC-ND), which allows third parties to download the articles and share them with others as long as they credit the author and the Abstract Book, but they cannot change the content in any way or use them commercially.
**Abstract Book Citations:** Authors, Title, HemaSphere, 2026;10:(S1):pages. The abstract book DOIs can be found on the “Author Information” page.
**Disclaimer:** Articles published in the journal HemaSphere exclusively reflect the opinions of the authors. The authors are responsible for all content in their abstracts including accuracy of the facts, statements, citing resources, etc.

**Results**

Between July 2024 and January 2026, 35 patients (median age 56 years; range 19-77) were enrolled. Eight (22.9%) had primary refractory AML, while 27 (77.1%) had relapsed disease. Three patients (8.6%) had secondary AML. According to the ELN 2022 criteria, the genetic risk stratification revealed favorable risk in 12 patients (34.3%), intermediate risk in 5 patients (14.3%), and adverse risk in 17 patients (48.6%). Two patients had prior allo-HSCT, and 25 patients (71.4%) had prior VEN exposure. Among these 25, 5 (20%) relapsed during VEN treatment, 10 (40%) after VEN discontinuation, and 10 (40%) were primary refractory to VEN-based therapy.
Efficacy was evaluable in all 35 patients. Following one induction cycle, CRc rates were 62.9% (22/35) in all patients, 65.6% (21/32) in de novo AML. The ORR was 68.6% (24/35). In patients achieving CRc, MRD-negative rate was 86.4% (19/22). The CRc rates were 80% (8/10) in patients without prior VEN exposure and 56% (14/25) in those with prior VEN exposure, with rates of 80% (4/5) for relapse on treatment, 60% (6/10) after discontinuation, and 40% (4/10) for primary refractory disease. The CRc rates by ELN 2022 risk were: favorable 75% (9/12), intermediate 60% (3/5), adverse 52.9% (9/17). Initial efficacy was also observed in patients with MDS-related molecular alterations. The CRc rates were 100% (2/2) in those with STAG2 mutations, 85.7% (6/7) in ASXL1, 75% (3/4) in RUNX1, and 50% (2/4) in SRSF2. With a median follow-up of 3.6 months (range 1.1-15.3), median relapse-free survival and overall survival were not reached.
Hematological toxicity were the main treatment-related adverse events (TRAEs). In patients with CRc, median durations of neutropenia (<0.5×10^9/L) and thrombocytopenia (<25×10^9/L) were 21.5 days (10-41) and 16 days (3-46). Common grade 3/4 non-hematological TRAEs included febrile neutropenia (40%), pulmonary infection (17.1%), and sepsis (8.6%).

**Summary/Conclusion**

The MAV regimen demonstrates promising efficacy and manageable toxicity in R/R AML, including particular potential to overcome VEN resistance in relapsed patients with prior venetoclax exposure, with the trial ongoing.

**Copyright Information:** (Online) ISSN: 2572-9241
©2026 The Author(s). HemaSphere is published by John Wiley & Sons Ltd on behalf of European Hematology Association. This is an open access Abstract Book distributed under the Attribution-NonCommercial-NoDerivs (CC BY-NC-ND), which allows third parties to download the articles and share them with others as long as they credit the author and the Abstract Book, but they cannot change the content in any way or use them commercially.
**Abstract Book Citations:** Authors, Title, HemaSphere, 2026;10:(S1):pages. The abstract book DOIs can be found on the "Author Information" page.
**Disclaimer:** Articles published in the journal HemaSphere exclusively reflect the opinions of the authors. The authors are responsible for all content in their abstracts including accuracy of the facts, statements, citing resources, etc.

**Figure 1. Swim lane plot**

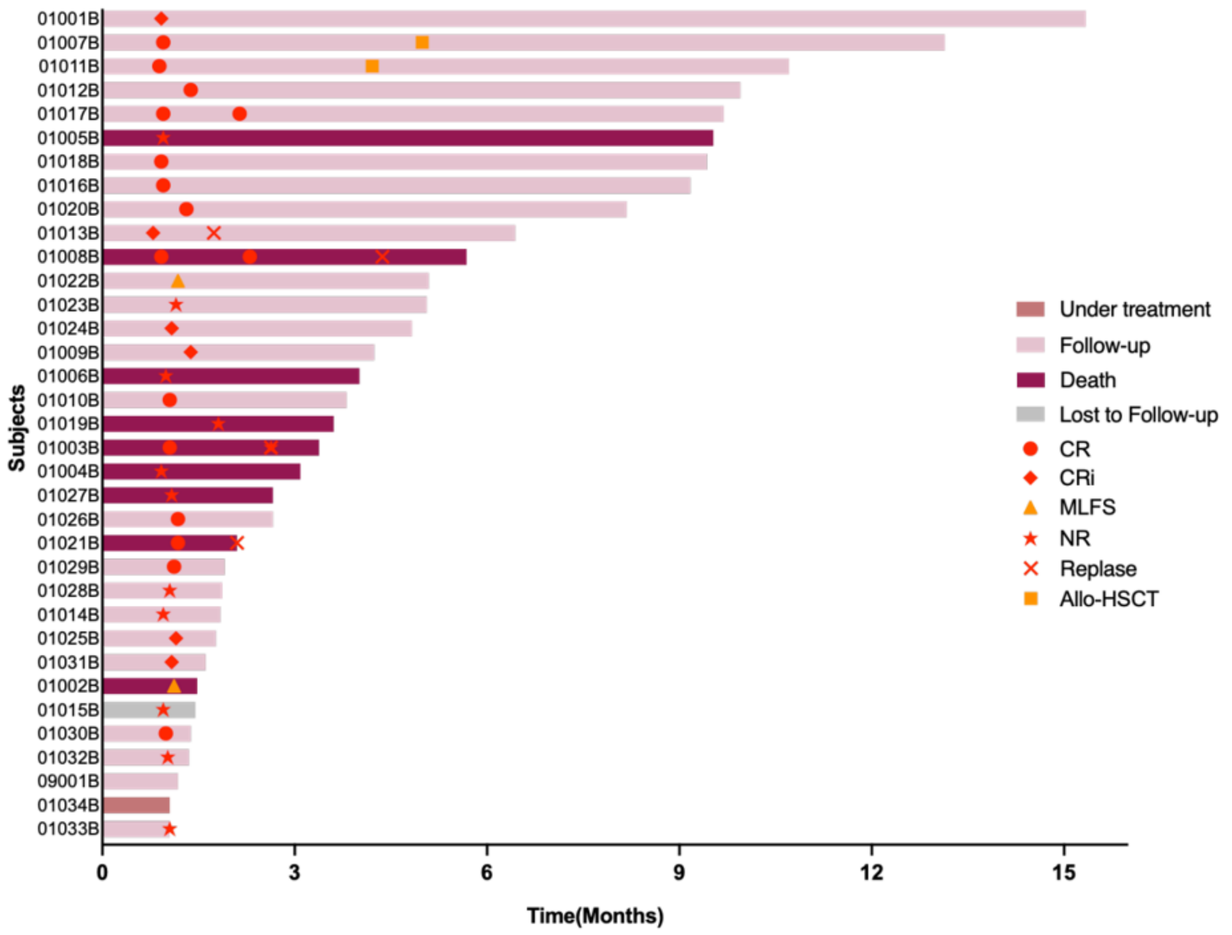

Copyright Information: (Online) ISSN: 2572-9241
©2026 The Author(s). HemaSphere is published by John Wiley & Sons Ltd on behalf of European Hematology Association. This is an open access Abstract Book distributed under the Attribution-NonCommercial-NoDerivs (CC BY-NC-ND), which allows third parties to download the articles and share them with others as long as they credit the author and the Abstract Book, but they cannot change the content in any way or use them commercially.
Abstract Book Citations: Authors, Title, HemaSphere, 2026;10:(S1):pages. The abstract book DOIs can be found on the "Author Information" page.
Disclaimer: Articles published in the journal HemaSphere exclusively reflect the opinions of the authors. The authors are responsible for all content in their abstracts including accuracy of the facts, statements, citing resources, etc.

### (PS1605) THE EFFICACY AND SAFETY OF THE DECITABINE, VENETOCLAX, AND SELINEXOR REGIMEN IN PATIENTS WITH NEWLY DIAGNOSED AML UNFIT FOR INTENSIVE CHEMOTHERAPY: A PROSPECTIVE, SINGLE-ARM CLINICAL STUDY

**Topic:** 04. Acute myeloid leukemia - Clinical

Weige Xu[1, 2], Yali Zhang[1], Tian Tian[1], Lina Xing[1], Jingnan Zhang[1], Xiaonan Guo[1], Shukai Qiao*[1]

*[1]Second Hospital of Hebei Medical University, Department of Hematology, Shijiazhuang , Hebei, China;[2]The Second Affiliated Hospital of Xingtai Medical College, Department of Hematology, Xingtai, China;*

### Background

Acute myeloid leukemia (AML) is an aggressive hematological malignancy that mainly affects older adults. Myelodysplastic syndrome (MDS) comprises a heterogeneous group of myeloid neoplasms with variable clinical courses and outcomes. Newly diagnosed elderly patients, or those with AML or MDS who are considered unfit because of significant comorbidities, often have a poor prognosis due to impaired organ function and limited tolerance to intensive chemotherapy.Venetoclax-based regimens are widely used in patients with newly diagnosed unfit acute myeloid leukemia (AML) and high-risk myelodysplastic syndrome (MDS). Resistance and suboptimal depth of response, however, remain important challenges, and prospective data on rational combination strategies are limited.

### Aims

We conducted this prospective, single-arm study to evaluate the efficacy and safety of the decitabine, venetoclax, and Selinexor (DVS) regimen in patients with newly diagnosed AML who were unfit for intensive chemotherapy and patients with high-risk MDS.

### Methods

Here, we evaluated the efficacy and safety of the decitabine, venetoclax, and Selinexor (DVS) regimen in 20 newly diagnosed unfit AML/high-risk MDS patients. This prospective, single-arm clinical study (registration number: ChiCTR230076031) was designed to evaluate the DVS regimen (decitabine, venetoclax, and selinexor) in patients with newly diagnosed AML who were unfit for intensive chemotherapy and in patients with high-risk MDS. A total of 20 evaluable patients with AML or high-risk MDS were enrolled at the Second Hospital of Hebei Medical University between October 1, 2023, and November 30, 2025. All patients were newly diagnosed, considered ineligible for intensive chemotherapy (“unfit”), and received combination treatment with decitabine, venetoclax, and selinexor, completing at least one treatment cycle. Clinical and laboratory data were collected prospectively, and patients were followed longitudinally. The primary study endpoint was the rate of complete remission (CR) or complete remission with incomplete hematologic recovery (CRi). Secondary endpoints included overall response rate (ORR), minimal residual disease (MRD) negativity rate and time to MRD negativity, as well as the incidence of grade 3–4 adverse events (AEs).

### Results

The median age was 66 years and the median follow-up was 10.5 months. After one treatment cycle, the complete remission / complete remission with incomplete hematologic recovery (CR/CRi) rate was 90.0% (18/20), and the overall response rate was 95.0%. Among patients achieving CR/CRi, 66.7% (12/18) attained minimal residual disease (MRD) negativity. The CR rate was 100% in patients with favorable- and intermediate-risk AML and MDS, whereas the CR/CRi rate was 80% in the adverse-risk group. Responses were less frequent in patients with TP53 mutations (1/3). The most frequent grade 3–4 adverse events were hematologic toxicities, with febrile neutropenia occurring in 50.0% of patients. Non-hematological adverse events were mainly infections and gastrointestinal reactions.

**Copyright Information:** (Online) ISSN: 2572-9241
©2026 The Author(s). HemaSphere is published by John Wiley & Sons Ltd on behalf of European Hematology Association. This is an open access Abstract Book distributed under the Attribution-NonCommercial-NoDerivs (CC BY-NC-ND), which allows third parties to download the articles and share them with others as long as they credit the author and the Abstract Book, but they cannot change the content in any way or use them commercially.
**Abstract Book Citations:** Authors, Title, HemaSphere, 2026;10:(S1):pages. The abstract book DOIs can be found on the “Author Information” page.
**Disclaimer:** Articles published in the journal HemaSphere exclusively reflect the opinions of the authors. The authors are responsible for all content in their abstracts including accuracy of the facts, statements, citing resources, etc.

### Summary/Conclusion

Overall, the DVS regimen achieved high remission rates with rapid MRD-negative conversion and a manageable safety profile, supporting its further investigation in newly diagnosed unfit AML/MDS patients.

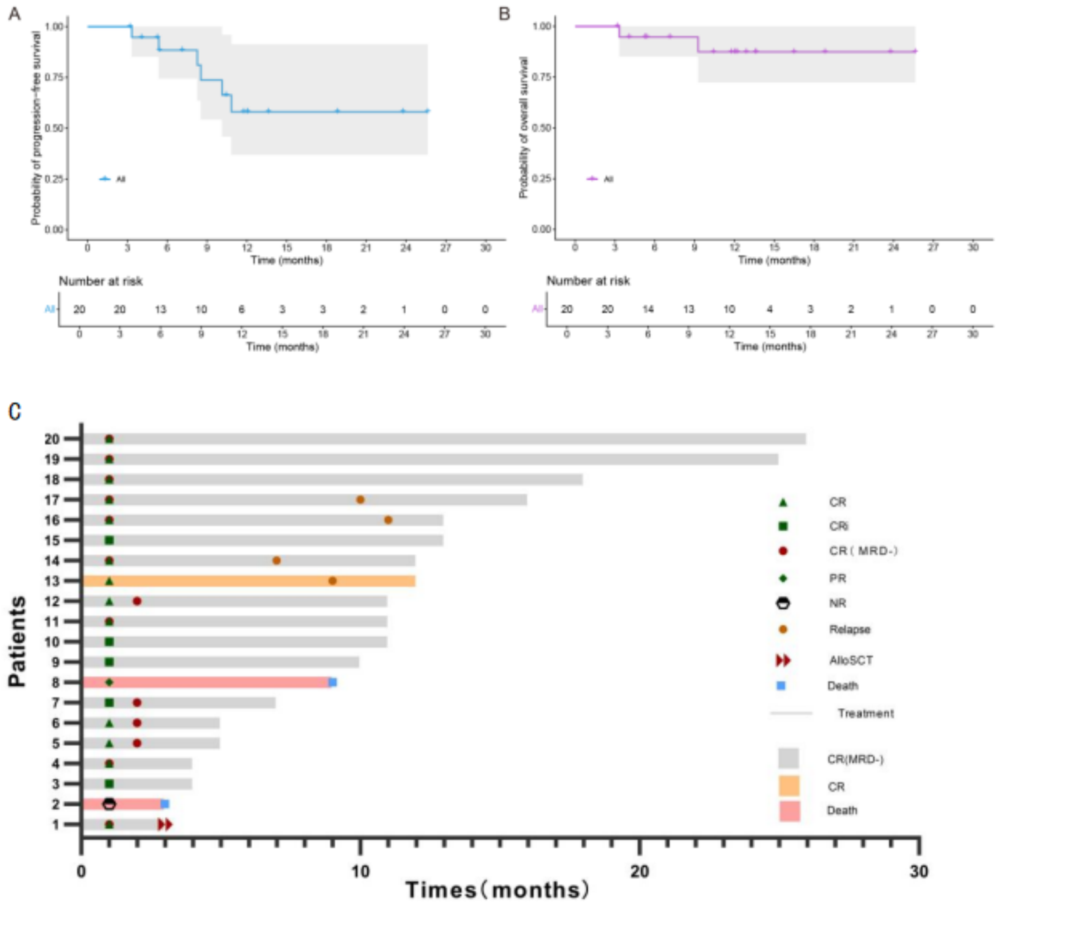

**Fig 3.** Survival outcomes in the frontline cohort. (A) Progression free survival (PFS) according to risk subgroup. (B) Overall survival (OS). （C） Swimmer's plot showing dynamic responses of patients.

**Copyright Information:** (Online) ISSN: 2572-9241
©2026 The Author(s). HemaSphere is published by John Wiley & Sons Ltd on behalf of European Hematology Association. This is an open access Abstract Book distributed under the Attribution-NonCommercial-NoDerivs (CC BY-NC-ND), which allows third parties to download the articles and share them with others as long as they credit the author and the Abstract Book, but they cannot change the content in any way or use them commercially.
**Abstract Book Citations:** Authors, Title, HemaSphere, 2026;10:(S1):pages. The abstract book DOIs can be found on the "Author Information" page.
**Disclaimer:** Articles published in the journal HemaSphere exclusively reflect the opinions of the authors. The authors are responsible for all content in their abstracts including accuracy of the facts, statements, citing resources, etc.

### (PS1606) A PHASE 2 TRIAL OF THE BCL2 INHIBITOR FCN-338 IN COMBINATION WITH AZACITIDINE OR CHEMOTHERAPY IN PATIENTS WITH RELAPSED/REFRACTORY OR NEWLY DIAGNOSED AML

**Topic:** 04. Acute myeloid leukemia - Clinical

Yu Hu*[1], Wenjuan He[1], Xudong Wei[2], Yicheng Zhang[3], Liangming MA[4], Ling Qi[5], Zunmin Zhu[6], Zeping Zhou[7], Hongyan Tong[8], Xiaoyu Zhu[9], Sujun Gao[10], Sheng Luo[11], Ruihua Mi[2], Xiaoxi Zhou[3], Xingli Wang[12], Yuchen Yang[12], Qian Xu[12]

*[1]Department of Hematology, Union Hospital, Tongji Medical College, Huazhong University of Science and Technology, Wuhan, China; [2]Department of hematology, The Affiliated Cancer Hospital of Zhengzhou University & Henan Cancer Hospital, Zhengzhou, China; [3]Department of Hematology, Tongji Hospital, Tongji Medical College, Huazhong University of Science and Technology, Wuhan, China; [4]Department of hematology，Shanxi Bethune Hospital, Taiyuan, China; [5]Center of Hematology, The First Affiliated Hospital of Nanchang University, Nanchang, China; [6]Department of Hematology, Institute of Hematology, Henan Provincial People's Hospital, Zhengzhou, China; [7]Department of Hematology, The Second Affiliated Hospital of Kunming Medical University, Kunming, China; [8]Department of Hematology, the First Affiliated Hospital, Zhejiang University School of Medicine, Hangzhou, China; [9]Department of Hematology, The First Affiliated Hospital of USTC, Division of Life Sciences and Medicine, University of Science and Technology of China, Hefei, China; [10]Department of Hematology, The First Hospital of Jilin University, Changchun, China; [11]Department of Hematology, The First Affiliated Hospital of Wenzhou Medical University, Wenzhou, China; [12]Shanghai Fosun Pharmaceutical Industrial Development Co., Ltd.,, Shanghai, China;*

### Background

"7+3" intensive chemotherapy (cytarabine and an anthracycline) is standard for acute myeloid leukemia (AML). With this treatment, only 60%–80% AML patients achieve complete remission, and 5-year overall survival is below 50%, indicating a substantial unmet clinical need. FCN-338 is a novel, orally active BCL2 inhibitor with optimized molecular design and high selectivity. Preclinical studies have demonstrated its potent antitumor activity against a broad spectrum of Bcl-2-dependent B-cell malignancies.

### Aims

To evaluate the safety and tolerability of FCN-338 combined with Azacitidine (AZA) or chemotherapy (cytarabine, daunorubicin) in AML patients. (NCT06858618)

### Methods

This open-label, multicenter, phase II study was conducted in 11 centers. In Cohort A, patients with refractory/relapsed(R/R) AML were treated with FCN-338 (400 or 600 mg, once daily, days 1–28) plus AZA (75 mg/m$^2$, days 1–7) until disease progression or unacceptable toxicity. In Cohort B, newly diagnosed, fit for intensive chemotherapy AML patients received induction therapy with FCN-338 at the dose determined from Cohort A (days 1–14), plus daunorubicin (60 mg/m$^2$, days 1–3) and cytarabine (100 mg/m$^2$, days 1–7). For patients who achieved complete remission (CR) / CR with incomplete hematologic recovery (CRi), consolidation therapy was given with medium- to high-dose cytarabine (1-3 g/m$^2$ every 12 h for 6 doses) over 3-4 cycles, followed by up to 24 cycles of maintenance (12 cycles of FCN-338 plus AZA 50 mg/m$^2$ on days 1-5, then 12 cycles of FCN-338 monotherapy). The primary endpoint were dose-limiting toxicities (DLTs) for Cohort A and CR/CRi (CR with partial hematologic recovery, (CRh) required further evaluation), measurable residual disease (MRD) negativity rate for Cohort B. Secondary endpoints included safety, time to response (TTR), duration of response (DoR), event-free survival (EFS), and overall survival (OS).

**Copyright Information:** (Online) ISSN: 2572-9241
©2026 The Author(s). HemaSphere is published by John Wiley & Sons Ltd on behalf of European Hematology Association. This is an open access Abstract Book distributed under the Attribution-NonCommercial-NoDerivs (CC BY-NC-ND), which allows third parties to download the articles and share them with others as long as they credit the author and the Abstract Book, but they cannot change the content in any way or use them commercially.
**Abstract Book Citations:** Authors, Title, HemaSphere, 2026;10:(S1):pages. The abstract book DOIs can be found on the “Author Information” page.
**Disclaimer:** Articles published in the journal HemaSphere exclusively reflect the opinions of the authors. The authors are responsible for all content in their abstracts including accuracy of the facts, statements, citing resources, etc.

### Results

As of 4 Oct 2024, 47 patients were enrolled (14 in Cohort A, 33 in Cohort B), with a median age of 52.0 years (range 19–67). Males accounted for 61.7%. In Cohort A, no DLTs were observed, and the FCN-338 maximum tolerated dose was determined to be 600 mg. In Cohort B, 27/33 reached CR, CRi or CRh (81.8%). Within 27 patients with CR, CRi, or morphologic leukemia-free state (MLFS), a total of 88.9% (24/27) reached MRD negativity. After a median follow-up of 5.5 months, median TTR was 0.8 months, whereas DoR, EFS, and OS were not yet assessable. Grade ≥3 adverse events (AEs) occurred in 13 patients (92.9%) in Cohort A and 33 patients (100%) in Cohort B. In both cohorts, the most frequent grade ≥3 AEs were platelet count decreased, neutrophil count decreased, and white-cell count decreased. In cohort B, dose interruptions due to AEs occurred in 23 patients, predominantly due to platelet count decreased. Four patients underwent dose reductions, and one patient discontinued treatment because of hypokalemia. One patient developed a laboratory TLS event, which was successfully managed without dose modification. No treatment-related fatal events occurred in Cohort B, and one treatment-related death was reported in Cohort A due to intracranial hemorrhage, infection, and acute hepatic failure.

### Summary/Conclusion

FCN-338 plus AZA or intensive chemotherapy showed promising antileukemic activity, acceptable safety and potential survival benefit in R/R and newly diagnosed fit AML patients, supporting its further exploration, especially with intensive chemotherapy for newly diagnosed fit patients.

**Copyright Information:** (Online) ISSN: 2572-9241
©2026 The Author(s). HemaSphere is published by John Wiley & Sons Ltd on behalf of European Hematology Association. This is an open access Abstract Book distributed under the Attribution-NonCommercial-NoDerivs (CC BY-NC-ND), which allows third parties to download the articles and share them with others as long as they credit the author and the Abstract Book, but they cannot change the content in any way or use them commercially.
**Abstract Book Citations:** Authors, Title, HemaSphere, 2026;10:(S1):pages. The abstract book DOIs can be found on the “Author Information” page.
**Disclaimer:** Articles published in the journal HemaSphere exclusively reflect the opinions of the authors. The authors are responsible for all content in their abstracts including accuracy of the facts, statements, citing resources, etc.

### (PS1607) CLINICAL ACTIVITY OF REVUMENIB IN PATIENTS WITH RELAPSED/REFRACTORY (R/R) NUP98-REARRANGED ACUTE LEUKEMIAS

**Topic:** 04. Acute myeloid leukemia - Clinical

Ghayas Issa*[1], Branko Cuglievan[1], John Dipersio[2], Matthew Rees[3], James S. Blachly[4], Jessica A. Pollard[5], Bill Chang[6], Sarah Tasian[7, 8], Li Yu[9], Craig W. Freyer[9], Angela Smith[9], Eytan Stein[10]

*[1]The University of Texas MD Anderson Cancer Center, Houston, TX, United States of America;[2]Washington University School of Medicine, St. Louis, MO, United States of America; [3]St. Jude Children's Research Hospital, Memphis, TN, United States of America; [4]The Ohio State University, Columbus, OH, United States of America;[5]Dana-Farber/Boston Children's Cancer and Blood Disorders Center; Harvard Medical School, Boston, MA, United States of America; [6]Knight Cancer Institute, Oregon Health and Science University, Portland, OR, United States of America; [7]Children's Hospital of Philadelphia, Philadelphia, PA, United States of America; [8]University of Pennsylvania, Philadelphia, PA, United States of America;[9]Syndax Pharmaceuticals, Inc., New York, NY, United States of America; [10]Memorial Sloan Kettering Cancer Center, New York, NY, United States of America;*

### Background

The menin-KMT2A interaction is critical for leukemogenesis in *KMT2A*-rearranged (*KMT2A*r), *NPM1*-mutated (*NPM1*m), and *NUP98*-rearranged (*NUP98*r) acute leukemias (AL). *NUP98*r AL is associated with poor prognosis, and has no approved targeted therapies. Revumenib, a first-in-class, oral, potent, and selective inhibitor of the menin-KMT2A interaction, is used for the treatment of relapsed/refractory (R/R) AL harboring a *KMT2A* translocation or R/R acute myeloid leukemia (AML) with *NPM1*m in adult and pediatric patients (pts) 1 year and older. Preclinical evidence also supports the antileukemic activity of revumenib in *NUP98*r AL.

### Aims

To describe preliminary safety and efficacy of revumenib monotherapy in pts with R/R*NUP98*r AL treated in the AUGMENT-101 phase 1 trial (NCT04065399) or via an expanded access program (EAP; NCT05918913).

### Methods

Pts from phase 1 of AUGMENT-101 received various dose levels of revumenib based on escalation rules, while pts in the EAP received the recommended phase 2 dose (160 mg with a strong CYP3A4 inhibitor [270 mg without]; 95 mg/m$^2$ if <40 kg) q12h orally in 28-day cycles or last tolerated dose received prior to transfer to EAP. Pts from AUGMENT-101 were included in the efficacy population if they had ≥1 disease response assessment while pts in the EAP were included if their first revumenib dose was ≥2 days from any previous leukemia therapy, they completed ≥1 cycle of revumenib, and they had ≥1 disease response assessment. EAP disease assessments were performed until morphologic remission was reached; no further response data were required. Responses were classified by European LeukemiaNet 2022.

**Copyright Information:** (Online) ISSN: 2572-9241
©2026 The Author(s). HemaSphere is published by John Wiley & Sons Ltd on behalf of European Hematology Association. This is an open access Abstract Book distributed under the Attribution-NonCommercial-NoDerivs (CC BY-NC-ND), which allows third parties to download the articles and share them with others as long as they credit the author and the Abstract Book, but they cannot change the content in any way or use them commercially.
**Abstract Book Citations:** Authors, Title, HemaSphere, 2026;10:(S1):pages. The abstract book DOIs can be found on the "Author Information" page.
**Disclaimer:** Articles published in the journal HemaSphere exclusively reflect the opinions of the authors. The authors are responsible for all content in their abstracts including accuracy of the facts, statements, citing resources, etc.

### Results

Twenty-six pts (AUGMENT-101: N=6, data cutoff 26Feb2025; EAP: N=20, data cutoff 25Nov2025) were included in this analysis; 23 (88%) pts had AML. Overall, 13 (50%) pts were <18 y, 11 (42%) were 18–64 y, and 2 (8%) were ≥65 y; median (range) age was 18 y (1.8–79.0). Pts received a median (range) of 3 prior lines of tx (1–7); 20 (77%) received prior venetoclax; and 13 (50%) had prior hematopoietic stem cell transplant (HSCT).

A composite complete remission (CRc) was attained in 7/25 (28.0%) pts with *NUP98*r AL from the efficacy population with a median duration of response of 6.7 mo (95% CI, 0.5–NR) and median (range) time to response of 1.9 mo (0.95–3.15). Three of 7 responders (42.9%) proceeded to HSCT while in remission and also received post-HSCT maintenance with revumenib. Of those who achieved CRc and had measurable residual disease (MRD) testing locally by flow cytometry (n=6), 1 (16.7%) pt was MRD negative. Three pts without response remained on revumenib for >4 mo (**Figure**).

Grade ≥3 tx-emergent adverse events (TEAEs) occurred in 69% of pts overall. Grade ≥3 QTcF prolongation occurred in 2 (8%) pts, each lasting 1 day. Differentiation syndrome occurred in 2 (8%) pts. No TEAEs led to discontinuation or death.

### Summary/Conclusion

Revumenib induced remission in 28% of pts with R/R *NUP98*r AL in phase 1 of AUGMENT-101 and the EAP, with responses emerging after ~2 mo, and was HSCT-enabling for some. The safety profile was manageable, with no tx-related discontinuations or deaths, and AEs consistent with the known effects of menin inhibition. These findings support the development of larger prospective studies, including frontline and combination chemotherapy regimens, to better define the clinical benefit of revumenib in this high-risk, historically undertreated population.

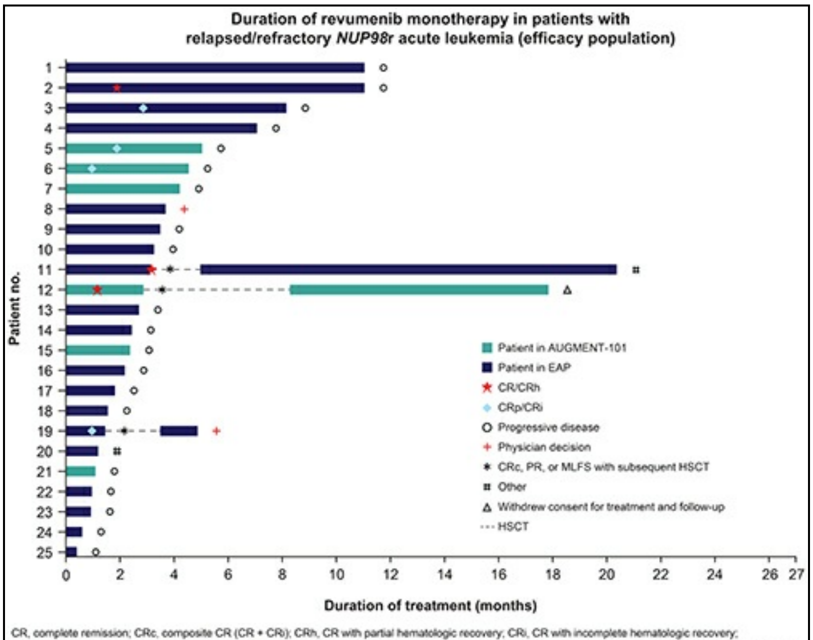

CR, complete remission; CRc, composite CR (CR + CRi); CRh, CR with partial hematologic recovery; CRi, CR with incomplete hematologic recovery; CRp, CR with incomplete platelet recovery; EAP, expanded access program; HSCT, hematopoietic stem cell transplant; MLFS, morphologic leukemia-free state; *NUP98r*, nucleoporin-98 rearrangement; PR, partial remission.

**Copyright Information:** (Online) ISSN: 2572-9241
©2026 The Author(s). HemaSphere is published by John Wiley & Sons Ltd on behalf of European Hematology Association. This is an open access Abstract Book distributed under the Attribution-NonCommercial-NoDerivs (CC BY-NC-ND), which allows third parties to download the articles and share them with others as long as they credit the author and the Abstract Book, but they cannot change the content in any way or use them commercially.
**Abstract Book Citations:** Authors, Title, HemaSphere, 2026;10:(S1):pages. The abstract book DOIs can be found on the “Author Information” page.
**Disclaimer:** Articles published in the journal HemaSphere exclusively reflect the opinions of the authors. The authors are responsible for all content in their abstracts including accuracy of the facts, statements, citing resources, etc.

### (PS1608) UPDATED SAFETY AND EFFICACY RESULTS OF A PHASE IB INVESTIGATION OF THE LSD1 INHIBITOR IADADEMSTAT (ORY-1001) IN COMBINATION WITH AZACITIDINE AND VENETOCLAX IN NEWLY DIAGNOSED AML

**Topic:** 04. Acute myeloid leukemia - Clinical

Curtis Lachowiez*[1], Andy Kaempf[1], Carolyn Lehn[1], Tara Lundberg Williams[1], Gabriella Sheldon[1], Anna Regina Samson[1], Jessica Stempel[1], Jessica Leonard[1], Gabrielle Meyers[1], Arpita Gandhi[1], Jennifer Saultz[1], Rachel Cook[1], Ronan Swords[1], Gordon Mills[1], Theodore Braun[1]

*[1]Oregon Health & Science University, Portland, United States of America;*

### Background

Azacitidine combined with venetoclax (AZA+VEN) is the standard of care treatment for older and/or unfit patients (pts) with newly diagnosed acute myeloid leukemia (ND-AML), yet most pts ultimately relapse. Novel triplet strategies are needed, particularly for those without targetable mutations.

### Aims

Herein, we report updated results of the combination therapy of Iadademstat (IADA), a lysine-specific demethylase 1 (LSD1) inhibitor in combination with AZA+VEN within an ongoing Phase 1b study in ND-AML (NCT06357182).

### Methods

Eligibility criteria have previously been reported (Lachowiez et. al. ASH 2025). Four IADA dose levels (DL) were pre-specified: DL1 (100 mcg PO daily), DL2 (150 mcg daily), and two de-escalation levels, administered 5 days on/2 days off. In 28-day cycles, IADA (Days 1–5, 8–12, 15–19) was combined with AZA (75 mg/m² IV/SubQ Days 1–7) and VEN (400 mg PO Days 1–21). The first 6 pts received a 7-day IADA lead-in (C0), later removed following a protocol amendment.
The primary endpoint was dose-limiting toxicity (DLT), defined as grade (Gr) 4–5 non-hematologic adverse events related to study drug during C0–C1, or Gr ≥4 neutropenia or thrombocytopenia persisting ≥42 days from C1D1 without disease. Secondary endpoints included composite complete remission (CRc: CR+CRh+CRi), overall response rate (ORR: CRc+MLFS+PR), and Gr ≥3 adverse events (AE).

**Copyright Information:** (Online) ISSN: 2572-9241
©2026 The Author(s). HemaSphere is published by John Wiley & Sons Ltd on behalf of European Hematology Association. This is an open access Abstract Book distributed under the Attribution-NonCommercial-NoDerivs (CC BY-NC-ND), which allows third parties to download the articles and share them with others as long as they credit the author and the Abstract Book, but they cannot change the content in any way or use them commercially.
**Abstract Book Citations:** Authors, Title, HemaSphere, 2026;10:(S1):pages. The abstract book DOIs can be found on the "Author Information" page.
**Disclaimer:** Articles published in the journal HemaSphere exclusively reflect the opinions of the authors. The authors are responsible for all content in their abstracts including accuracy of the facts, statements, citing resources, etc.

**Results**

As of February 2026, 15 patients enrolled (10 ND-AML, 2 MDS/AML, 3 sAML): 3 at DL1 and 12 at DL2. Median age was 71 years (range 64–81); 73% were male; median ECOG was 2. ELN 2022 risk was adverse in 93% and intermediate in 7%. 4-gene risk was higher-benefit in 80%, intermediate-benefit in 13%, and lower in 7%.
Among MTD-evaluable patients (n=13), 2 DLTs occurred at DL2: one Gr 4 *C. difficile* colitis and one Gr 4 neutropenia >42 days without G-CSF support.
Common Gr ≥3 AEs occurring in >1 pt were febrile neutropenia (20%) and neutropenia (20%, all Gr 4). One tumor lysis syndrome event was successfully medically managed. Three deaths occurred: two due to septic shock (both deemed unrelated to study drug) and one post–allogeneic hematopoietic cell transplantation (HCT).
Median number of treatment cycles was 2 (range: 1-4). Eleven pts discontinued protocol therapy. Discontinuation reasons include receipt of HCT (n=6), relocation/preference (n=3), and death (n=2). Four pts remain on study. Median time to ANC ≥ 500/mL and platelets ≥ 50,000/mL amongst response evaluable pts (n=14) was 32 days in C1 with no significant difference between dose levels (DL1: 31 vs. DL2: 32 days). Median number of cycles to best response was 1 (range: 1-2).
In response evaluable pts (n=14/15) the ORR was 100% with a CR rate of 79% (n=11/14) and a CRc rate of 93% (n=13/14). One pt attained MLFS as best response. After a median follow-up of 6 months, estimated 12-month OS is 74%.
RNA sequencing and reverse phase protein array following C0 of IADA monotherapy lead-in demonstrated increased expression of *GFI1*, consistent with an on-target effect of LSD1 inhibition and compensatory upregulation of bcl2, suggesting increased bcl2 dependence and a biologic rationale for synergy with VEN.

**Summary/Conclusion**

Within this expanded patient population, IADA in combination with AZA+VEN remains safe and active in ND-AML, with high response rates observed to date. Enrollment continues in this ongoing clinical trial. Additional outcomes and correlative data will be presented at the meeting.

**Copyright Information:** (Online) ISSN: 2572-9241
©2026 The Author(s). HemaSphere is published by John Wiley & Sons Ltd on behalf of European Hematology Association. This is an open access Abstract Book distributed under the Attribution-NonCommercial-NoDerivs (CC BY-NC-ND), which allows third parties to download the articles and share them with others as long as they credit the author and the Abstract Book, but they cannot change the content in any way or use them commercially.
**Abstract Book Citations:** Authors, Title, HemaSphere, 2026;10:(S1):pages. The abstract book DOIs can be found on the "Author Information" page.
**Disclaimer:** Articles published in the journal HemaSphere exclusively reflect the opinions of the authors. The authors are responsible for all content in their abstracts including accuracy of the facts, statements, citing resources, etc.

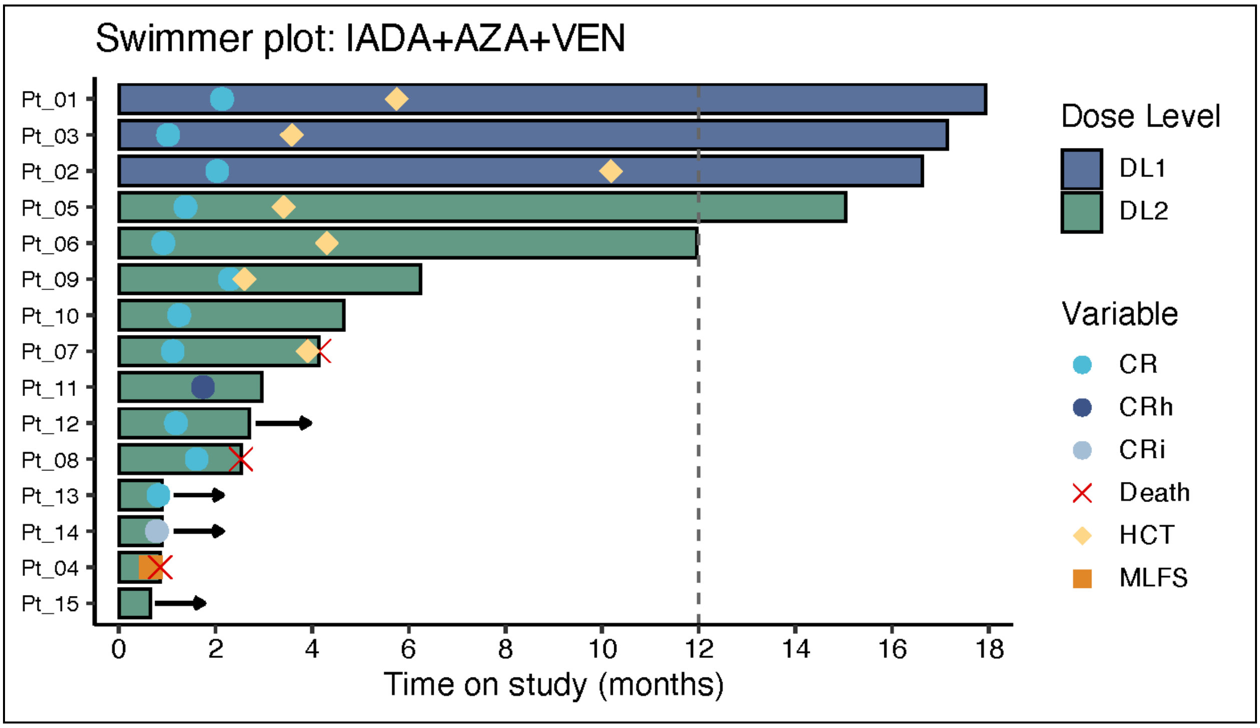

Swimmer plot depicting response and survival outcomes of patients treated on protocol through February 2026. Arrows indicate patients remaining on protocol defined therapy.

**Copyright Information:** (Online) ISSN: 2572-9241
©2026 The Author(s). HemaSphere is published by John Wiley & Sons Ltd on behalf of European Hematology Association. This is an open access Abstract Book distributed under the Attribution-NonCommercial-NoDerivs (CC BY-NC-ND), which allows third parties to download the articles and share them with others as long as they credit the author and the Abstract Book, but they cannot change the content in any way or use them commercially.
**Abstract Book Citations:** Authors, Title, HemaSphere, 2026;10:(S1):pages. The abstract book DOIs can be found on the "Author Information" page.
**Disclaimer:** Articles published in the journal HemaSphere exclusively reflect the opinions of the authors. The authors are responsible for all content in their abstracts including accuracy of the facts, statements, citing resources, etc.

### (PS1609) IMPACT OF FLOW CYTOMETRY MEASURABLE RESIDUAL DISEASE (MRD) CLEARANCE IN PATIENTS WITH ACUTE MYELOID LEUKEMIA TREATED WITH AZACITIDINE AND VENETOCLAX (AZA-VEN)

**Topic:** 04. Acute myeloid leukemia - Clinical

Aarya Murali*[1, 2], Cristiano MacHado De Freitas[1, 2], Zahra Alhaj Issa[1, 2], Aniket Bankar[1, 2], Marta Davidson[1, 2], Maria Agustina Perusini[1, 2], Dawn Maze[1, 2], Steven M Chan[1, 2], Aaron Schimmer[1, 2], Hassan, Sibai[1, 2], Karen Yee[1, 2], Andre Schuh[1, 2], Vikas Gupta[1, 2], Anne Tierens[3], Guillaume Richard-Carpentier[1, 2]

*[1]Princess Margaret Cancer Centre, Toronto, Canada;[2]University of Toronto, Toronto, Canada;[3]Toronto General Hospital, Toronto, Canada;*

### Background

The combination of Azacitidine and Venetoclax (Aza-Ven) has revolutionised the treatment of acute myeloid leukemia (AML). However, our understanding of disease response kinetics, in particular measurable residual disease (MRD) clearance with Aza-Ven is still evolving. Additionally, data is lacking on the optimal timepoint and frequency of testing as well as how we can use MRD results in real-time to adapt therapy.

### Aims

Our study aimed to assess factors associated with flow cytometry MRD clearance and characterise its prognostic impact on overall survival (OS) and event free survival (EFS).

### Methods

We performed a retrospective review of patients (pts) with AML who received front-line treatment with Aza-Ven at Princess Margaret Cancer Centre between 2017 and 2024. MRD testing was performed using multi-parametric flow cytometry with a lower limit of detection of 0.1%. The primary outcome was OS and secondary outcome was EFS both defined per ELN 2022 criteria.

**Copyright Information:** (Online) ISSN: 2572-9241
©2026 The Author(s). HemaSphere is published by John Wiley & Sons Ltd on behalf of European Hematology Association. This is an open access Abstract Book distributed under the Attribution-NonCommercial-NoDerivs (CC BY-NC-ND), which allows third parties to download the articles and share them with others as long as they credit the author and the Abstract Book, but they cannot change the content in any way or use them commercially.
**Abstract Book Citations:** Authors, Title, HemaSphere, 2026;10:(S1):pages. The abstract book DOIs can be found on the "Author Information" page.
**Disclaimer:** Articles published in the journal HemaSphere exclusively reflect the opinions of the authors. The authors are responsible for all content in their abstracts including accuracy of the facts, statements, citing resources, etc.

### Results

We analysed 175 pts with newly diagnosed AML. The median age was 74 years, 114 (65%) pts were male and 70% of pts had an ECOG 0-1. A total of 153 pts had an evaluable bone marrow (BM) at a median time of 30 days after initiation of cycle 1. At this timepoint, 104 (68%) pts had achieved morphologic complete remission (mCR, < 5% bone marrow blasts) including 33 (22%) achieving CR, 38 (25%) achieving CR with incomplete or partial hematological recovery (CRi/CRh), and 33 (22%) pts achieving morphologic leukemia free state (MLFS). Sixteen (10%) pts had partial response (PR) and 34 (22%) had no response (NR).
Among pts who achieved mCR (n=104) after cycle 1, 85 (82%) pts had flow MRD data available (not evaluable in 19 pts due to sample limitations). At the end of cycle 1, 36/85 (42%) pts had flow MRD positivity (MRD-C1pos) and 49/85 (58%) had flow MRD negativity (MRD-C1neg). No difference in baseline clinical or genetic characteristics was observed between pts achieving MRD-C1pos or MRD-C1neg. MRD-C1pos was significantly associated with increased 60-day mortality (8% vs 0%, $p$=0.04) and delayed platelet recovery >100 x $10^9$/L with a median time to recovery of 23 days vs 32 days in pts with MRD-C1neg and MRD-C1pos, respectively ($p$<0.01).
Early MRD clearance (MRD-C1neg) was associated with a trend toward improved OS (median OS 28 vs 19 months, $p$=0.24) and EFS (median EFS 18 vs 10 months, $p$=0.13). Following cycle 1, 9/36 (25%) pts within the MRD-C1pos group achieved MRD negativity. Pts who achieved MRD negativity at any time (MRD-BESTneg) had significantly improved OS (median OS 50 vs 11 months, $p$<0.01) and EFS (median EFS 24 vs 8 months, $p$<0.01) compared to those who remained MRD positive (MRD-BESTpos) (Figure 1).
Importantly, the benefit of allogeneic stem cell transplant in first remission (CR1 HSCT) was greatest in MRD-C1pos (HR 0.21, 95% CI: 0.05 – 0.97; log rank $p$=0.03) and MRD-BESTpos (HR 0.16, 95% CI 0.02 – 1.20; log rank $p$=0.04). However, this analysis was limited by the small number of CR1 HSCT in this cohort (n=13/85).

### Summary/Conclusion

Early flow MRD clearance with Aza-Ven is associated with faster platelet recovery and improved 60-day mortality, with a trend toward improved OS and EFS. Pts achieving flow MRD negativity at any time with Aza-Ven have significantly superior outcomes compared to those with persistent MRD positivity. Hence, flow MRD may assist in identifying patients at risk of early mortality as well as inferior long-term outcomes.

**Copyright Information:** (Online) ISSN: 2572-9241
©2026 The Author(s). HemaSphere is published by John Wiley & Sons Ltd on behalf of European Hematology Association. This is an open access Abstract Book distributed under the Attribution-NonCommercial-NoDerivs (CC BY-NC-ND), which allows third parties to download the articles and share them with others as long as they credit the author and the Abstract Book, but they cannot change the content in any way or use them commercially.
**Abstract Book Citations:** Authors, Title, HemaSphere, 2026;10:(S1):pages. The abstract book DOIs can be found on the “Author Information” page.
**Disclaimer:** Articles published in the journal HemaSphere exclusively reflect the opinions of the authors. The authors are responsible for all content in their abstracts including accuracy of the facts, statements, citing resources, etc.

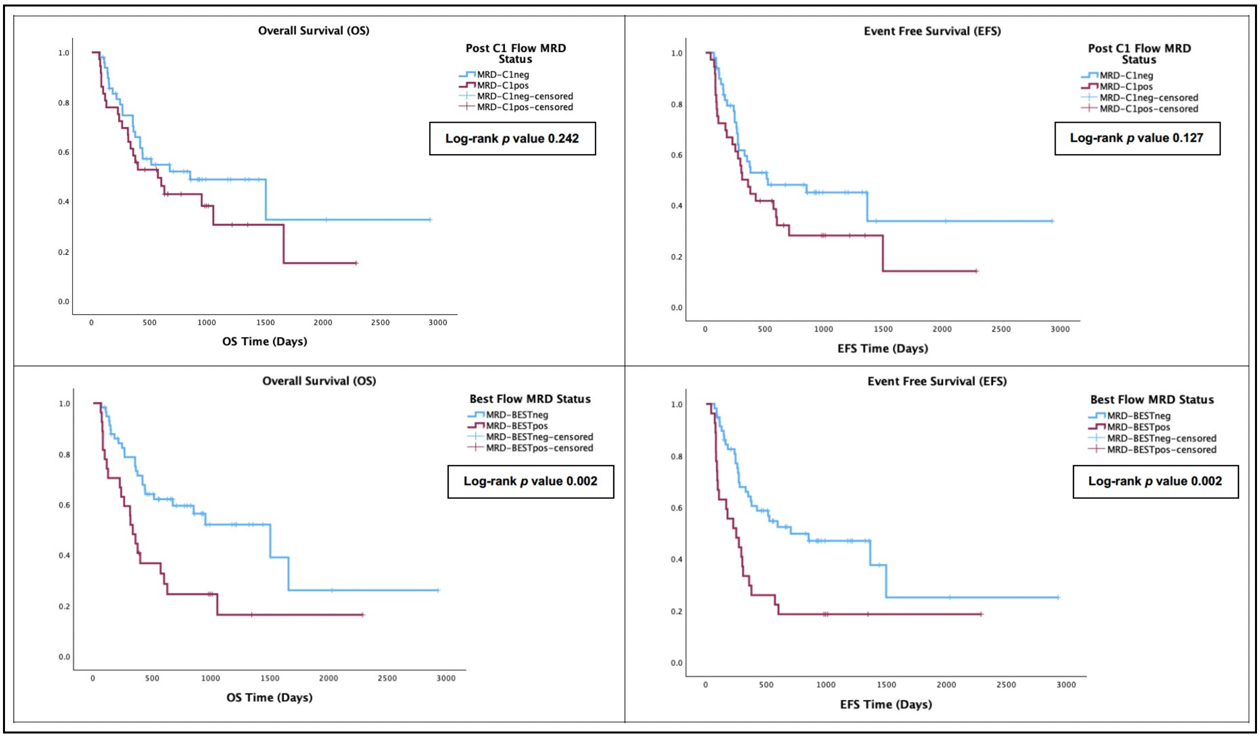

Figure 1: Overall Survival (OS) and Event Free Survival (EFS) in patients stratified by flow MRD status

**Copyright Information:** (Online) ISSN: 2572-9241
©2026 The Author(s). HemaSphere is published by John Wiley & Sons Ltd on behalf of European Hematology Association. This is an open access Abstract Book distributed under the Attribution-NonCommercial-NoDerivs (CC BY-NC-ND), which allows third parties to download the articles and share them with others as long as they credit the author and the Abstract Book, but they cannot change the content in any way or use them commercially.
**Abstract Book Citations:** Authors, Title, HemaSphere, 2026;10:(S1):pages. The abstract book DOIs can be found on the "Author Information" page.
**Disclaimer:** Articles published in the journal HemaSphere exclusively reflect the opinions of the authors. The authors are responsible for all content in their abstracts including accuracy of the facts, statements, citing resources, etc.

### (PS1610) MRD-GUIDED TREATMENT WITH PEMBROLIZUMAB AND AZACITIDINE IN NPM1-MUTATED AML PATIENTS WITH IMMINENT HEMATOLOGICAL RELAPSE – RESULTS OF THE PROSPECTIVE PEMAZA TRIAL

**Topic:** 04. Acute myeloid leukemia - Clinical

Madlen Jentzsch*[1, 2], Regina Herbst[3], Richard Schlenk[4, 5], Jan-Henrik Mikesch[6], Martin Kaufmann[7], Ullrich Graeven[8], Katharina Götze[9], Klaus Metzeler[1], Christoph Röllig[10], Sabrina Kraus[11], Sven Zukunft[10], Anna Harig[10], Christian Thiede[10, 12], Jan Moritz Middeke[10], Martin Bornhäuser[10], Uwe Platzbecker[1, 13]

*[1]University of Leipzig Medical Center, Department for Hematology, Cell Therapy, Hemostaseology, and Infectious Diseases, Leipzig, Germany; [2]Cancer Center Central Germany, Leipzig, Germany; [3]Klinikum Chemnitz, Department of Internal Medicine III, Chemnitz, Germany; [4]Heidelberg University Hospital, Department of Internal Medicine V, Hematology, Oncology, and Rheumatology, Heidelberg, Germany; [5]German Cancer Research Center, Clinical Trial Center, Heidelberg, Germany; [6]University Hospital Münster, Department of Medicine A, Hematology/Oncology, Münster, Germany; [7]Robert-Bosch-Krankenhaus, Department of Hematology, Stuttgart, Germany; [8]Kliniken Maria Hilf GmbH, Mönchengladbach, Germany; [9]Technical University of Munich School of Medicine and Health, Department of Medicine III, Munich, Germany; [10]University Hospital Carl Gustav Carus, TUD Dresden University of Technology, Department of Internal Medicine I, Dresden, Germany; [11]University Hospital Würzburg, Department of Internal Medicine II, Division of Hematology and Oncology, Würzburg, Germany; [12]Agendix GmbH, Dresden, Germany; [13]12 University Hospital Carl Gustav Carus, TUD Dresden University of Technology, Dresden, Germany;*

### Background

For acute myeloid leukemia (AML) patients (pts) in complete remission (CR) after treatment, relapse is a frequent obstacle. Evaluation of measurable residual disease (MRD) to detect imminent relapse is established, especially in patients with *NPM1* mutations. Strategies to eliminate MRD and prevent chemotherapy-resistant relapse are under investigation. Pre-emptive treatment with azacitidine (AZA) can substantially delay relapse in MRD-pos pts with AML (Platzbecker, 2018). However, immunological escape mechanisms and upregulation of checkpoint molecules following AZA treatment may induce treatment resistance and AML relapse. This suggests that combining AZA and checkpoint inhibition through the PD-1 inhibitor pembrolizumab (PEM) might improve MRD-guided treatment response, especially in immunogenic *NPM1*-mutated AML.

### Aims

PEMAZA (NCT03769532) was a single-arm multicenter phase II trial to assess the efficacy and tolerability of PEM in combination with AZA in preventing hematologic relapse in *NPM1* MRD-pos AML pts.

### Methods

Eligible adult pts were in CR after conventional cytarabine/anthracycline-based chemotherapy, not scheduled for immediate allogeneic stem cell transplantation (HSCT) and had detectable MRD > 1% *NPM1*/*ABL1* in blood or bone marrow in a central lab. Pts received up to 6 cycles AZA 75mg/m$^2$ for 7 days (optional pauses over weekends) of each 28-day cycle and PEM 200 mg starting on day 9 of the first AZA cycle, continued every 3 weeks up to 8 times. Pts were followed for 1 year after start of study treatment. The primary endpoint was proportion of event-free patients (*i.e.* without hematologic relapse, death or treatment change to anything other than AZA) after 24 weeks. Treatment efficacy was defined at response rate ≥70%, with an alpha of 5% and a power of 90%. Secondary endpoints included overall survival (OS), MRD, and safety.

**Copyright Information:** (Online) ISSN: 2572-9241
©2026 The Author(s). HemaSphere is published by John Wiley & Sons Ltd on behalf of European Hematology Association. This is an open access Abstract Book distributed under the Attribution-NonCommercial-NoDerivs (CC BY-NC-ND), which allows third parties to download the articles and share them with others as long as they credit the author and the Abstract Book, but they cannot change the content in any way or use them commercially.
**Abstract Book Citations:** Authors, Title, HemaSphere, 2026;10:(S1):pages. The abstract book DOIs can be found on the "Author Information" page.
**Disclaimer:** Articles published in the journal HemaSphere exclusively reflect the opinions of the authors. The authors are responsible for all content in their abstracts including accuracy of the facts, statements, citing resources, etc.

### Results

PEMAZA recruited between 09/2019 and 03/2025 at 9 German sites and closed prematurely due to delayed recruitment after 26/28 planned pts. One patient was excluded due to open disease relapse at study entry (intention-to-treat set), pts characteristics are shown in **Figure 1A**. Twenty-four weeks after start of MRD-guided PEMAZA, 10/25 pts (40%) were event-free, which did not exceed the prespecified benchmark of 70% (*P*>.99). Eleven pts (44%) suffered morphologic relapse after median of 3 cycles and 4 pts (16%) went off study due to treatment change; all of whom subsequently received venetoclax-based therapies. There were no treatment related deaths during the 24 weeks of study treatment. After 6 and 12 months, event-free survival (EFS) was 40% (95% CI 25%-65%), while OS was 96% (89%-100%) and 76% (61%-95%), respectively (**Figure 1B**). At end of study, 3 pts (12%) cleared their *NPM1* MRD and 3 pts (12%) showed a decline < 1%. *NPM1* MRD values during study are shown in **Figure 1C**. After the study, 7 pts (28%) proceeded to HSCT. Of all pts receiving at least 1 dose PEM (n=26), 10 pts (38%) had non-hematologic adverse events (AEs) ≥ grade 3, in 7 (27%) this was regarded as related to PEM. Grade 3 AEs observed in at least 10% of pts were febrile neutropenia (not related to PEM, 10.5%), and syncope (potentially related to PEM, 10.5%), no > grade 3 AEs were observed. A total of 9 pts (35%) experienced at least one SAE, in 5 pts (19%) this was regarded as related to study drug.

### Summary/Conclusion

While PEMAZA was safe to administer in *NPM1* mutated AML pts with impeding relapse, it appeared not more effective than AZA alone in preventing relapse.

**A Patients characteristics**

| | Intention-to-treat set, n=25 |
|---|---|
| **Age**, years (median, range) | 65 (36-79) |
| **Female Sex**, n (%) | 16 (64) |
| **ELN2017 risk at diagnosis,** n (%) | |
| Favorable | 20 (83) |
| Intermediate | 4 (17) |
| adverse | 0 (0) |
| ***FLT3*-ITD at diagnosis,** n (%) | |
| absent | 16 (64) |
| present | 8 (32) |
| **ECOG at baseline**, n (%) | |
| 0 | 20 (80) |
| 1 | 5 (20) |
| **Neutrophil count at baseline,** Gpt/l (median, range) | 2.9 (0.86-9.4) |
| **Platelets at baseline,** Gpt/l (median, range) | 196 (104-280) |
| **Blood *NPM1/ABL* at baseline**, % (median, range) | 1.3 (0.03 - 190) |
| **Bone marrow *NPM1/ABL* at baseline**, % (median, range) | 16 (1.2 - 282) |
| **Applied AZA cycles on study,** n (median, range) | 5 (1 - 6) |

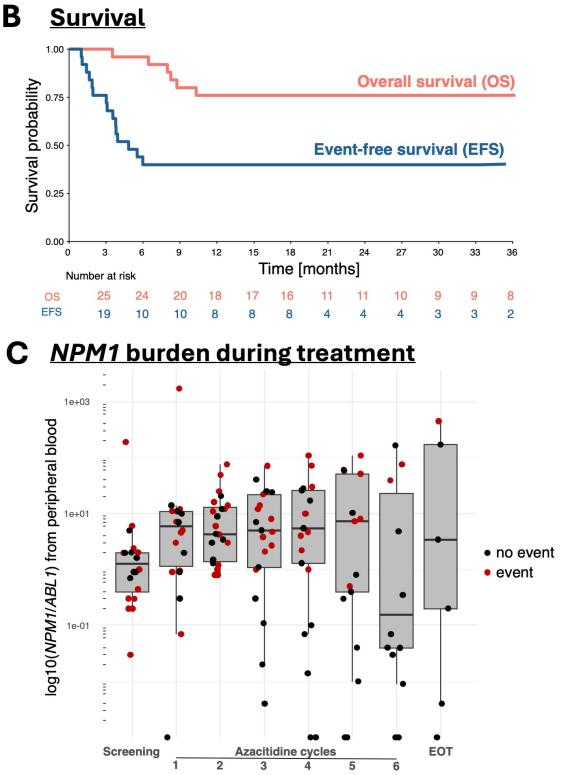

**Copyright Information:** (Online) ISSN: 2572-9241
©2026 The Author(s). HemaSphere is published by John Wiley & Sons Ltd on behalf of European Hematology Association. This is an open access Abstract Book distributed under the Attribution-NonCommercial-NoDerivs (CC BY-NC-ND), which allows third parties to download the articles and share them with others as long as they credit the author and the Abstract Book, but they cannot change the content in any way or use them commercially.
**Abstract Book Citations:** Authors, Title, HemaSphere, 2026;10:(S1):pages. The abstract book DOIs can be found on the "Author Information" page.
**Disclaimer:** Articles published in the journal HemaSphere exclusively reflect the opinions of the authors. The authors are responsible for all content in their abstracts including accuracy of the facts, statements, citing resources, etc.

### (PS1611) SAFETY, TOLERABILITY, AND PRELIMINARY ACTIVITY OF ROGOCEKIB IN PATIENTS WITH RELAPSED/REFRACTORY MYELOID MALIGNANCIES: RESULTS FROM A PHASE 1/2 STUDY (CTX-712-CL-02)

**Topic:** 04. Acute myeloid leukemia - Clinical

Guillermo Garcia-Manero*[1], Antoine Saliba[2], Cecilia Arana Yi[3], James M Foran[4], Daniel Reed[5], Anna B. Halpern[6], Jessica K. Altman[7], Yoshiyuki Tanaka[8], Yasushi Tanoue[8], Maki Yamamoto[8], Robert Oda[8], Akio Mizutani[8], Eiji Takahara[8], Hirokazu Tozaki[8], Shingo Shoji[8], Joachim Gullbo[9], Aref Al-Kali[2]

*[1]The University of Texas MD Anderson Cancer Center, Houston, United States of America;[2]Mayo Clinic Rochester, Rochester, United States of America; [3]Mayo Clinic Scottsdale, Scottsdale, United States of America;[4]Mayo Clinic Jacksonville, Jacksonville, United States of America; [5]University of Virginia Comprehensive Cancer Center, Charlottesville, United States of America; [6]University of Washington Fred Hutchinson Cancer Center, Seattle, United States of America;[7]Robert H. Lurie Comprehensive Cancer Center of Northwestern University, Chicago, United States of America; [8]Chordia Therapeutics Inc., Fujisawa, Japan; [9]Theradex Oncology, Princeton, United States of America;*

### Background

Mutations in RNA splicing factor (SF) genes are common in myeloid neoplasms, are linked to poor outcomes, and currently have no approved targeted therapies. The CDC2-like kinase (CLK) family phosphorylates SR proteins that regulate splicing, making CLK inhibition a rational therapeutic strategy in myeloid malignancies with SF-mutations (SFm). Rogocekib is a first-in-class, oral, selective small-molecule CLK inhibitor. An ongoing phase 1/2 study is evaluating its safety and efficacy in relapsed or refractory (R/R) acute myeloid leukemia (AML) and higher-risk myelodysplastic syndromes (HR-MDS).

### Aims

To assess safety, determine the maximum tolerated dose of rogocekib given once weekly (QW) or twice weekly (BIW), evaluate pharmacokinetic/pharmacodynamic (PD) and preliminary antileukemic activity.

### Methods

CTX-712-CL-02 (NCT05732103) is an open-label, multicenter phase 1/2 study enrolling adults with R/R AML, HR-MDS, or MDS/MPN (including CMML). Rogocekib is given at escalating QW doses (20-140 mg), followed by BIW (60–100 mg). Safety assessments include treatment-emergent adverse events (TEAEs), and dose-limiting toxicities (DLTs). PD activity is assessed via exon-skipping biomarkers. AML and MDS responses are assessed per ELN 2017 and IWG 2006 criteria.

**Copyright Information:** (Online) ISSN: 2572-9241
©2026 The Author(s). HemaSphere is published by John Wiley & Sons Ltd on behalf of European Hematology Association. This is an open access Abstract Book distributed under the Attribution-NonCommercial-NoDerivs (CC BY-NC-ND), which allows third parties to download the articles and share them with others as long as they credit the author and the Abstract Book, but they cannot change the content in any way or use them commercially.
**Abstract Book Citations:** Authors, Title, HemaSphere, 2026;10:(S1):pages. The abstract book DOIs can be found on the “Author Information” page.
**Disclaimer:** Articles published in the journal HemaSphere exclusively reflect the opinions of the authors. The authors are responsible for all content in their abstracts including accuracy of the facts, statements, citing resources, etc.

**Results**

To date, 41 patients (pts) have been enrolled (78% ≥65 years), including AML (53.7%), HR-MDS (39.0%), and MDS/MPN (7.3%). Pts were heavily pretreated (29.3% with ≥4 prior lines). The most common TEAEs included diarrhea (65.9%), nausea (58.5%), fatigue (39.0%), and constipation (34.1%). At QW dosing (n=25), TEAEs included nausea (48.0%), diarrhea (32.0%), ALT/AST increases (16.0%), platelet count decrease/headache (16.0%), leukopenia/fatigue (12.0%). At BIW dosing (n=16), TEAEs included nausea (62.5%), diarrhea (43.8%), constipation/vomiting (25.0% each), decreased appetite, dysgeusia, anemia, dizziness (18.8% each), worsening thrombocytopenia/neutropenia, dyspnea, hypotension, and hypokalemia (12.5% each). Grade ≥3 drug-related TEAEs occurred in 53.7%, and Grade ≥3 drug-related cytopenia in ≤15% of pts. DLTs occurred in 2 pts at 140 mg QW (pericardial/pleural effusions with dyspnea and hypotension), 1 at 80 mg BIW (esophagitis hemorrhagic), and 1 at 100 mg BIW (ALT/AST elevations). So far, the MTD for QW dosing has been determined to be 100 mg QW with BIW investigation ongoing.

Rogocekib plasma concentrations increased dose-dependently. PD analysis showed target engagement via intra-patient, baseline-normalized fold changes in THAP9-AS1 and S6K splicing ratios consistent with CLK inhibition.
Among 30 evaluable pts, 4 responses were observed in both SFm and WT pts (11.8%): 3 complete remissions with incomplete count recovery (CRi; AML) and 1 marrow CR (mCR; MDS). Stable disease (SD) was observed in 16.7% of AML and 56.3% of MDS pts. SFm were identified in 18 pts (47.4%): SRSF2 (26.3%), SF3B1 (13.2%), U2AF1/ZRSR2 (5.3%). Among SFm pts with evaluable responses (n=10), 1 CRi, 1 mCR, and 8 SD were observed. Responses were observed in both AML and MDS, including cases with co-occurring adverse-risk mutations (TP53, ASXL1, RUNX1).
Updated results will be presented at the EHA Congress.

**Summary/Conclusion**

Rogocekib demonstrated a manageable and tolerable safety profile and preliminary antileukemic activity in a heavily pretreated, high-risk population, with encouraging signals in AML (3 CRi, 16.7%). These findings support continued dose optimization and further clinical development in R/R AML and HR-MDS.

**Copyright Information:** (Online) ISSN: 2572-9241
©2026 The Author(s). HemaSphere is published by John Wiley & Sons Ltd on behalf of European Hematology Association. This is an open access Abstract Book distributed under the Attribution-NonCommercial-NoDerivs (CC BY-NC-ND), which allows third parties to download the articles and share them with others as long as they credit the author and the Abstract Book, but they cannot change the content in any way or use them commercially.
**Abstract Book Citations:** Authors, Title, HemaSphere, 2026;10:(S1):pages. The abstract book DOIs can be found on the "Author Information" page.
**Disclaimer:** Articles published in the journal HemaSphere exclusively reflect the opinions of the authors. The authors are responsible for all content in their abstracts including accuracy of the facts, statements, citing resources, etc.

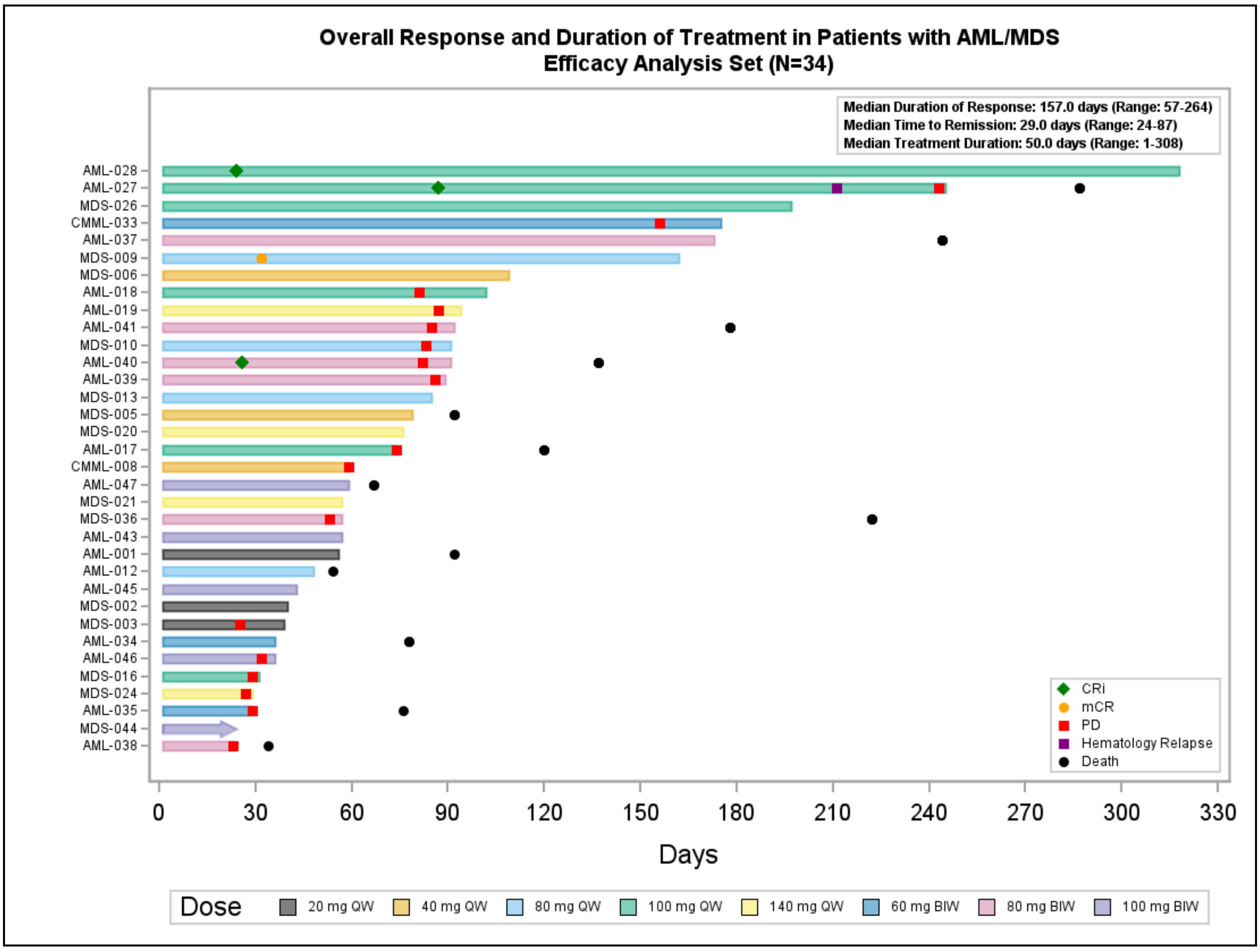

**Copyright Information:** (Online) ISSN: 2572-9241
©2026 The Author(s). HemaSphere is published by John Wiley & Sons Ltd on behalf of European Hematology Association. This is an open access Abstract Book distributed under the Attribution-NonCommercial-NoDerivs (CC BY-NC-ND), which allows third parties to download the articles and share them with others as long as they credit the author and the Abstract Book, but they cannot change the content in any way or use them commercially.
**Abstract Book Citations:** Authors, Title, HemaSphere, 2026;10:(S1):pages. The abstract book DOIs can be found on the “Author Information” page.
**Disclaimer:** Articles published in the journal HemaSphere exclusively reflect the opinions of the authors. The authors are responsible for all content in their abstracts including accuracy of the facts, statements, citing resources, etc.

### (PS1612) CLINICAL AND MOLECULAR FEATURES AND CLONAL EVOLUTION OF AML WITH KMT2A-PTD: CONCURRENT KMT2A-PTD AND FLT3-ITD MAY REFINE THE ELN 2022 RISK STRATIFICATION

**Topic:** 04. Acute myeloid leukemia - Clinical

Yi-Tsung Yang*[1, 2], Chi-Yuan Yao[1, 3], Yong-Huai Yi[1], Chein-Jun Kao[1], Yuan-Yeh Kuo[4], Ming-En Lin[1], Hsin-An Hou[1, 2], Wen-Chien Chou[1, 2], Hwei-Fang Tien[1, 5]

*[1]National Taiwan University Hospital, Division of Hematology, Department of Internal Medicine, Taipei, Taiwan;[2]College of Medicine, National Taiwan University, Department of Internal Medicine, Taipei, Taiwan; [3]National Taiwan University Hospital Yunlin Branch, Division of Hematology, Department of Internal Medicine, Yunlin, Taiwan; [4]Tai-Chen Cell Therapy Center, National Taiwan University, Taipei, Taiwan; [5]Far-Eastern Memorial Hospital, Department of Internal Medicine, New Taipei, Taiwan;*

### Background

*KMT2A* alterations occur in 5-10% of AML cases, mainly as partial tandem duplications (*KMT2A*-PTD) or rearrangements (*KMT2A*-r). While most *KMT2A*-r variants (except *KMT2A*::*MLLT3*) are considered adverse risk in the ELN 2022 classification, the prognostic impact of *KMT2A*-PTD remains to be clarified.

### Aims

We integrated clinical, genomic, transcriptomic, and clonal evolution data to assess the prognostic and therapeutic implications of *KMT2A*-PTD in AML and to compare AML with *KMT2A*-PTD vs *KMT2A*-r.

### Methods

We screened 1928 newly diagnosed non-APL AML patients from 1994 to 2025 for *KMT2A*-PTD using genomic DNA PCR and reverse transcription PCR (RT-PCR), and for *KMT2A*-r using multiplex RT-PCR; all positives were confirmed by Sanger sequencing. Targeted NGS of a 42-gene myeloid panel (excluding *KMT2A*) was performed in most patients, with bulk RNA-seq in those with available samples. Clonal evolution trees were constructed from serial samples at diagnosis, complete remission, and relapse for 21 *KMT2A*-PTD and 21 *KMT2A*-r cases. Survival analyses were stratified by ELN 2022 risk category, *FLT3*-ITD status, midostaurin use, and allogeneic hematopoietic stem cell transplantation (HSCT).

**Copyright Information:** (Online) ISSN: 2572-9241
©2026 The Author(s). HemaSphere is published by John Wiley & Sons Ltd on behalf of European Hematology Association. This is an open access Abstract Book distributed under the Attribution-NonCommercial-NoDerivs (CC BY-NC-ND), which allows third parties to download the articles and share them with others as long as they credit the author and the Abstract Book, but they cannot change the content in any way or use them commercially.
**Abstract Book Citations:** Authors, Title, HemaSphere, 2026;10:(S1):pages. The abstract book DOIs can be found on the "Author Information" page.
**Disclaimer:** Articles published in the journal HemaSphere exclusively reflect the opinions of the authors. The authors are responsible for all content in their abstracts including accuracy of the facts, statements, citing resources, etc.

**Results**

*KMT2A*-PTD and *KMT2A*-r were identified in 4.7% (90/1928) and 3.9% (76/1928) of patients, respectively. Compared with *KMT2A*-r AML, *KMT2A*-PTD AML patients were older (median age 60 vs 47 years), had more WHO-defined AML with maturation and a normal karyotype, and showed more frequent co-mutations (96% vs 65%), most commonly *FLT3*-ITD (49%), *DNMT3A* (36%), and *RUNX1* (25%). In contrast, *KMT2A*-r AML was dominated by *RAS* mutations. Transcriptomics revealed *HOXA*-cluster upregulation in both groups, but *KMT2A*-PTD AML was *HOXB*-high, *PBX3*/*MEIS1*-low, and enriched for hematopoietic stem cell signatures, whereas *KMT2A*-r AML was *HOXB*-low, *PBX3*/*MEIS1*-high, and enriched for G2/M checkpoint, MYC targets, oxidative phosphorylation, and *KMT2A*-related signatures. In serial samples, *KMT2A*-PTD and recurrent co-mutations —including CHIP-associated mutations (*DNMT3A*, *TET2*, *ASXL1*, spliceosome genes) and alterations in *IDH1/2*, *RUNX1*, or cohesin—typically persisted as stable founder events across remission and relapse. *KMT2A*-r, however, usually acted as a dominant founder event without a CHIP-like background. In both AML subtypes, signaling pathway mutations (*FLT3*, *NRAS*, *PTPN11*) were dynamic, often gained or lost at relapse. Furthermore, in intensively treated AML patients (n=960), *KMT2A*-PTD positivity (n=54) was linked to poorer survival compared with *KMT2A*-PTD negativity (n=906) (HR=1.5, p=0.01). Within *KMT2A*-PTD AML patients, concurrent *FLT3*-ITD (n=30) predicted particularly poor survival, comparable to the ELN 2022 adverse-risk group, while *FLT3*-ITD-negative cases (n=24) had outcomes closer to the intermediate-risk group (HR=3.0, p=0.002; Fig. 1). GSEA consistently showed enrichment of ERK/MAPK and oxidative phosphorylation signatures in *FLT3*-ITD-positive vs *FLT3*-ITD-negative *KMT2A*-PTD AML. In the *FLT3*-ITD-positive subgroup, midostaurin use (n=10) significantly improved survival (p=0.022). HSCT (n=32) also enhanced outcomes in *KMT2A*-PTD AML (p<0.001) and remained an independent protective factor in multivariable analyses.

**Summary/Conclusion**

*KMT2A*-PTD AML is molecularly distinct from *KMT2A*-r AML and confers poor prognosis, particularly with concurrent *FLT3*-ITD. *FLT3*-ITD status and its therapeutic targeting, together with HSCT, substantially modify prognosis and should be incorporated into risk-adapted, mutation-informed management.

**Copyright Information:** (Online) ISSN: 2572-9241
©2026 The Author(s). HemaSphere is published by John Wiley & Sons Ltd on behalf of European Hematology Association. This is an open access Abstract Book distributed under the Attribution-NonCommercial-NoDerivs (CC BY-NC-ND), which allows third parties to download the articles and share them with others as long as they credit the author and the Abstract Book, but they cannot change the content in any way or use them commercially.
**Abstract Book Citations:** Authors, Title, HemaSphere, 2026;10:(S1):pages. The abstract book DOIs can be found on the "Author Information" page.
**Disclaimer:** Articles published in the journal HemaSphere exclusively reflect the opinions of the authors. The authors are responsible for all content in their abstracts including accuracy of the facts, statements, citing resources, etc.

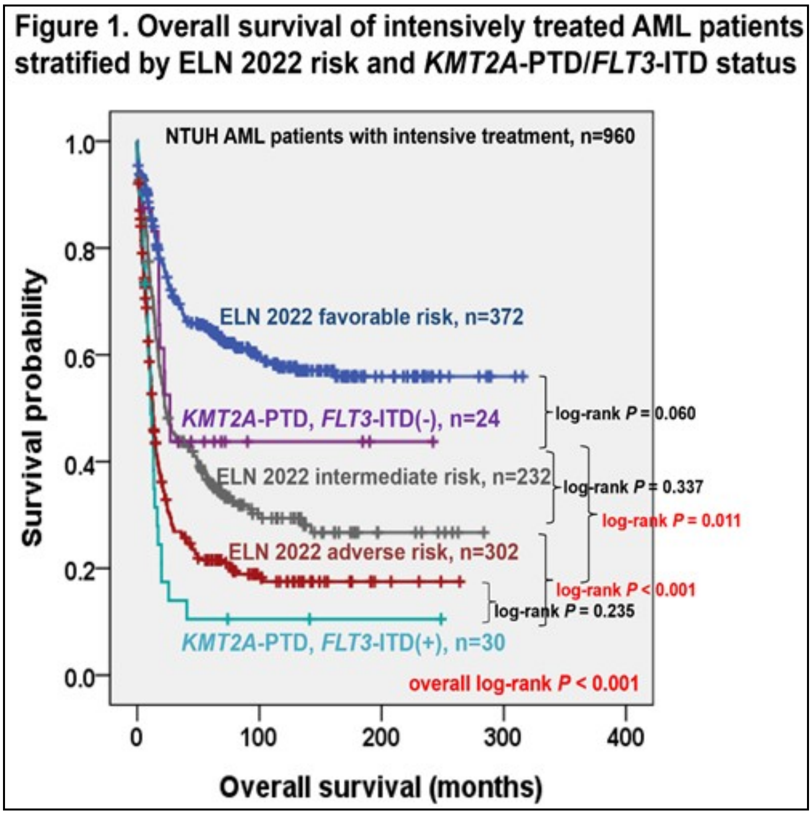

**Copyright Information:** (Online) ISSN: 2572-9241
©2026 The Author(s). HemaSphere is published by John Wiley & Sons Ltd on behalf of European Hematology Association. This is an open access Abstract Book distributed under the Attribution-NonCommercial-NoDerivs (CC BY-NC-ND), which allows third parties to download the articles and share them with others as long as they credit the author and the Abstract Book, but they cannot change the content in any way or use them commercially.
**Abstract Book Citations:** Authors, Title, HemaSphere, 2026;10:(S1):pages. The abstract book DOIs can be found on the "Author Information" page.
**Disclaimer:** Articles published in the journal HemaSphere exclusively reflect the opinions of the authors. The authors are responsible for all content in their abstracts including accuracy of the facts, statements, citing resources, etc.

### (PS1613) DISTINCT FLT3-ITD CLONAL EVOLUTION PATTERNS IN AML REFLECT PRE-EXISTING BIOLOGY AND INFLUENCE PROGNOSIS

**Topic:** 04. Acute myeloid leukemia - Clinical

Szu-Chi Yao*[1], Cheng-Hong Tsai[1], Yu-Sung Chang[1], Feng-Ming Tien[1], Yuan-Yeh Kuo[2], Mei-Hsuan Tseng[2], Yen-Ling Peng[1], Shao-Min Han[2], Bor-Sheng Ko[3], Ming Yao[1], Wen-Chien Chou[1], Hwei-Fang Tien[1], Hsin-An Hou[1]

*[1]Division of Hematology, Department of Internal Medicine, National Taiwan University Hospital, Taipei, Taiwan;[2]Tai-Chen Cell Therapy Center, National Taiwan University, Taipei, Taiwan; [3]Department of Hematological Oncology, National Taiwan University Cancer Center, National Taiwan University Hospital, Taipei, Taiwan;*

### Background

FLT3-ITD occurs in approximately 20–25% of adult acute myeloid leukemia (AML) cases and is associated with increased relapse risk and inferior survival. Although FLT3-ITD status at diagnosis is incorporated into contemporary risk stratification, emerging evidence indicates that FLT3-ITD may be acquired or lost during disease progression. The prevalence, prognostic relevance, and biological determinants of these distinct clonal evolution patterns, however, remain incompletely defined.

### Aims

To define the clinical impact of distinct FLT3-ITD clonal trajectories and determine whether they are driven by molecular programs present at diagnosis.

### Methods

Between 2000 and 2022, 808 adult patients with newly diagnosed non-M3 AML received intensive chemotherapy at National Taiwan University Hospital. Among them, 319 patients who experienced relapse and had complete *FLT3* mutation data available at both diagnosis and relapse were included in the analysis. Serial *FLT3*-ITD assessment was performed using a high-sensitivity molecular assay. Diagnostic samples underwent targeted next-generation sequencing and bulk RNA sequencing. Diagnostic transcriptomic analyses were performed for pathway characterization and predictive modeling.

**Copyright Information:** (Online) ISSN: 2572-9241
©2026 The Author(s). HemaSphere is published by John Wiley & Sons Ltd on behalf of European Hematology Association. This is an open access Abstract Book distributed under the Attribution-NonCommercial-NoDerivs (CC BY-NC-ND), which allows third parties to download the articles and share them with others as long as they credit the author and the Abstract Book, but they cannot change the content in any way or use them commercially.
**Abstract Book Citations:** Authors, Title, HemaSphere, 2026;10:(S1):pages. The abstract book DOIs can be found on the "Author Information" page.
**Disclaimer:** Articles published in the journal HemaSphere exclusively reflect the opinions of the authors. The authors are responsible for all content in their abstracts including accuracy of the facts, statements, citing resources, etc.

**Results**

Based on longitudinal *FLT3*-ITD status, patients were classified into four clonal evolution patterns: maintained negative (66.5%), loss (8.5%), acquisition (9.7%), and maintained positive (15.3%). Patients with maintained *FLT3*-ITD positivity had significantly higher white blood cell counts and peripheral blast percentages at diagnosis (both P<0.001) and were enriched for *NPM1* mutations (61.2% *vs.* 13.2% in the persistent *FLT3*-ITD–negative group, 19.4% in the acquired group, and 25.9% in the loss group; P<0.001). In contrast, AML with in-frame bZIP *CEBPA* mutations was more frequent in the maintained *FLT3*-ITD–negative group (P=0.043). ELN 2022 risk categories differed significantly across clonal evolution patterns, with higher proportions of intermediate- or adverse-risk disease observed in the maintained *FLT3*-ITD–positive and *FLT3*-ITD loss groups (P<0.001).

Distinct mutational architectures were observed across clonal trajectories. Patients remaining *FLT3*-ITD–negative exhibited a broadly distributed mutational landscape without dominant cooperating lesions, implying with relapse from persistent *FLT3*–wild-type subclones. *FLT3*-ITD acquisition at relapse occurred without enrichment of *NPM1* or *DNMT3A* mutations, indicating a mutational context distinct from the canonical FLT3-mutated genomic constellation. In contrast, maintained *FLT3*-ITD positivity was characterized by stable co-occurrence of *FLT3*-ITD, *NPM1*, and *DNMT3A* mutations, indicating strong clonal fitness.

With a median follow-up of 96.8 months, patients with *FLT3*-ITD acquisition or persistent *FLT3*-ITD had inferior overall survival and relapse-free survival compared with those who remained *FLT3*-ITD–negative (P=0.018 and 0.009, respectively). Diagnostic transcriptomic profiling revealed enrichment of immune and inflammatory Hallmark pathways in the persistent *FLT3*-ITD group, including IL6–JAK–STAT3 and TNFα signaling (Figure 1). A transcriptome-based model demonstrated robust discrimination for subsequent *FLT3*-ITD clonal loss (ROC-AUC 0.786).

**Summary/Conclusion**

Distinct *FLT3*-ITD clonal trajectories reflect pre-existing biology, influence prognosis, and can be predicted by diagnostic transcriptomic profiling, supporting refined risk stratification in AML.

**Copyright Information:** (Online) ISSN: 2572-9241
©2026 The Author(s). HemaSphere is published by John Wiley & Sons Ltd on behalf of European Hematology Association. This is an open access Abstract Book distributed under the Attribution-NonCommercial-NoDerivs (CC BY-NC-ND), which allows third parties to download the articles and share them with others as long as they credit the author and the Abstract Book, but they cannot change the content in any way or use them commercially.
**Abstract Book Citations:** Authors, Title, HemaSphere, 2026;10:(S1):pages. The abstract book DOIs can be found on the "Author Information" page.
**Disclaimer:** Articles published in the journal HemaSphere exclusively reflect the opinions of the authors. The authors are responsible for all content in their abstracts including accuracy of the facts, statements, citing resources, etc.

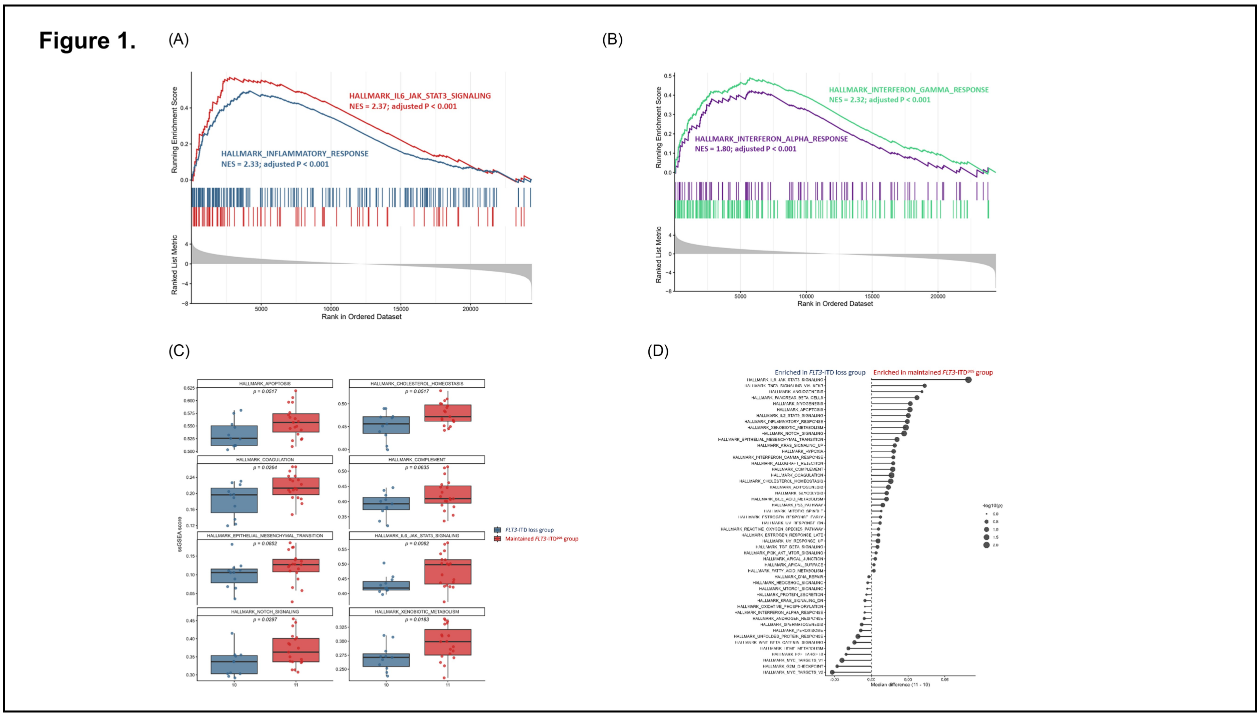

Figure 1. Transcriptomic differences associated with distinct FLT3-ITD clonal evolution patterns. (A–B) Gene set enrichment analysis (GSEA) of diagnostic RNA sequencing data demonstrating significant enrichment of inflammatory and immune-related Hallmark pathways in the maintained FLT3-ITD–positive group compared with the FLT3-ITD loss group, including IL6–JAK–STAT3 signaling, inflammatory response, interferon-α response, and interferon-γ response. Normalized enrichment scores (NES) and adjusted P values are indicated. (C) Comparison of representative Hallmark pathway enrichment scores between the FLT3-ITD loss (blue) and maintained FLT3-ITD (red) groups, including apoptosis, cholesterol homeostasis, coagulation, complement, epithelial–mesenchymal transition, IL6–JAK–STAT3 signaling, NOTCH signaling, and xenobiotic metabolism. Boxes represent interquartile ranges with median values; P values were calculated using two-sided statistical tests. (D) Summary dot plot of differentially enriched Hallmark pathways between groups, ranked by median difference in enrichment score; dot size reflects –log10(P) values. Abbreviations: GSEA, gene set enrichment analysis; NES, normalized enrichment score; ROC-AUC, area under the receiver operating characteristic curve.

**Copyright Information:** (Online) ISSN: 2572-9241
©2026 The Author(s). HemaSphere is published by John Wiley & Sons Ltd on behalf of European Hematology Association. This is an open access Abstract Book distributed under the Attribution-NonCommercial-NoDerivs (CC BY-NC-ND), which allows third parties to download the articles and share them with others as long as they credit the author and the Abstract Book, but they cannot change the content in any way or use them commercially.
**Abstract Book Citations:** Authors, Title, HemaSphere, 2026;10:(S1):pages. The abstract book DOIs can be found on the "Author Information" page.
**Disclaimer:** Articles published in the journal HemaSphere exclusively reflect the opinions of the authors. The authors are responsible for all content in their abstracts including accuracy of the facts, statements, citing resources, etc.

### (PS1614) MYELOID NEOPLASMS WITH PRDM16 VS. MECOM REARRANGEMENTS: THE FIRST AND LARGEST COMPARATIVE STUDY

**Topic:** 04. Acute myeloid leukemia - Clinical

Shaobin Yang*[1], Bingbing Han[1], Xinyue Zhang[1], Yani Lin[1], Kun Ru[2]

*[1]Sino-US Diagnostics Lab, Tianjin, China;[2]Department of Pathology and Lab Medicine,Shandong Cancer Hospital, Jinan, China;*

### Background

MECOM (PRDM3) and PRDM16 are homologous members of the PRDM family. While MECOM rearrangement (MECOMr) defines a high-risk subgroup of acute myeloid leukemia (AML) with the most adverse prognosis, PRDM16 rearrangement (PRDM16r)—approximately one-fourth as frequent—has long been undercharacterized and overlooked. Given their shared protein family origin, these entities may exhibit biological and clinical similarities, yet no systematic comparison has been conducted to date.

### Aims

To report the largest cohort to date of myeloid neoplasms with PRDM16r and to provide the first comprehensive clinicopathological and molecular comparison between PRDM16r and MECOMr neoplasms.

### Methods

We retrospectively analyzed 48 patients with PRDM16r and 185 patients with MECOMr. Baseline characteristics, cytogenetic findings, mutational profiles, and survival outcomes were systematically evaluated.

### Results

The two groups showed comparable median age (59 vs. 58 years, P=0.131) and blood counts, with anemia nearly universal in both (100% vs. 96%), though sex distribution differed (male:female 28:20 vs. 94:91, P=0.027). Cytogenetically, 87.5% of PRDM16r resulted from t(1;3)(p36;q21), while 71.9% of MECOMr were inv(3)/t(3;3). Simple translocations were more common in PRDM16r (54.2% vs. 33.5%, P=0.026), and complex karyotypes less frequent (14.6% vs. 28.6%, P=0.047). Notably, -7/7q- was enriched in MECOMr (34% vs. 6%, P<0.001), whereas -20/20q- was more frequent in PRDM16r (8.3% vs. 3.8%, P=0.01). Mutational profiling revealed striking similarities: both shared a core backbone of SF3B1, ASXL1, and GATA2 with comparable frequencies (43%/34%, 21%/15%, and 21%/10%, respectively). In PRDM16r, the most frequent mutations were RUNX1 (43%) and SF3B1 (43%), followed by ASXL1, BCOR, ETV6, and GATA2 (each 21%). In MECOMr, SF3B1 (34%), KRAS (29%), and NRAS (23%) predominated, followed by ASXL1 (15%). RUNX1 was markedly higher in PRDM16r (43% vs. 10%), while KRAS/NRAS were enriched in MECOMr (29%/23% vs. 14% each). Progression-free survival was comparable between groups (median 6.68 vs. 8.71 months, P=0.48), underscoring their equally aggressive nature.

### Summary/Conclusion

This first comprehensive comparative study demonstrates that PRDM16r and MECOMr myeloid neoplasms, despite certain cytogenetic and cooperative mutation differences, exhibit remarkable convergence in clinical presentation, core mutational architecture (SF3B1, ASXL1, GATA2), and equally dismal outcomes. These findings suggest they represent variants of a single disease entity driven by PRDM family members. We therefore propose reclassifying them as "AML with PRDMs rearrangement" to reflect their shared identity and ensure PRDM16r receives clinical attention commensurate with MECOMr.

**Copyright Information:** (Online) ISSN: 2572-9241
©2026 The Author(s). HemaSphere is published by John Wiley & Sons Ltd on behalf of European Hematology Association. This is an open access Abstract Book distributed under the Attribution-NonCommercial-NoDerivs (CC BY-NC-ND), which allows third parties to download the articles and share them with others as long as they credit the author and the Abstract Book, but they cannot change the content in any way or use them commercially.
**Abstract Book Citations:** Authors, Title, HemaSphere, 2026;10:(S1):pages. The abstract book DOIs can be found on the "Author Information" page.
**Disclaimer:** Articles published in the journal HemaSphere exclusively reflect the opinions of the authors. The authors are responsible for all content in their abstracts including accuracy of the facts, statements, citing resources, etc.

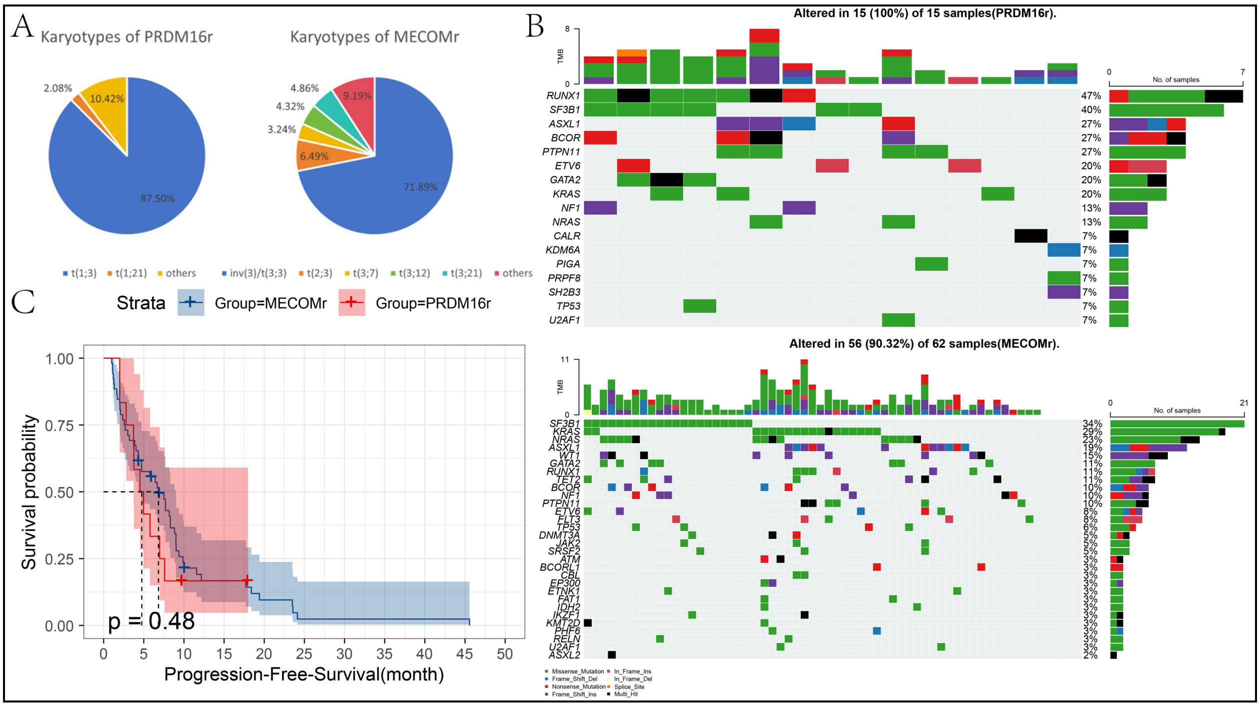

**Copyright Information:** (Online) ISSN: 2572-9241
©2026 The Author(s). HemaSphere is published by John Wiley & Sons Ltd on behalf of European Hematology Association. This is an open access Abstract Book distributed under the Attribution-NonCommercial-NoDerivs (CC BY-NC-ND), which allows third parties to download the articles and share them with others as long as they credit the author and the Abstract Book, but they cannot change the content in any way or use them commercially.
**Abstract Book Citations:** Authors, Title, HemaSphere, 2026;10:(S1):pages. The abstract book DOIs can be found on the "Author Information" page.
**Disclaimer:** Articles published in the journal HemaSphere exclusively reflect the opinions of the authors. The authors are responsible for all content in their abstracts including accuracy of the facts, statements, citing resources, etc.

### (PS1615) FIRST-STAGE RESULTS OF SELINEXOR COMBINED WITH DECITABINE AND DOSE-ADJUSTED CMG (CYTARABINEÞ+MITOXANTRONE+G-CSF) IN VENETOCLAX-EXPOSED R/R AML: A PROSPECTIVE, SINGLE-ARM, MULTICENTER STUDY

**Topic:** 04. Acute myeloid leukemia - Clinical

Zi-Yuan Nie*[1], Linhui Wang[2], Sheng-Xin Cai[1], Xiao-Lei Zhang[1], Ying Zhan[1], Jin-Ao Li[1], Yu Tao[1], Ming-Li Wang[1], Dongfeng Zeng[3], Xiao-Ling Guo[1], Jin-Hai Ren[1]

*[1]Department of Hematology, The Second Hospital of Hebei Medical University, Shijiazhuang, China;[2]Guanshanhu Campus of Guizhou Provincial People's Hospital, Guiyang, China; [3]Department of Haematology, Daping Hospital, Army Medical University, Chongqing, China;*

### Background

Relapsed/refractory (R/R )AML following venetoclax exposure represents one of the most challenging clinical scenarios.  Our previous single-center retrospective study (n=7) suggested promising anti-leukemia activity of selinexor combined with decitabine and half-dose CMG in venetoclax-exposed R/R AML, with an overall response rate (ORR) of 85.7%. Based on these encouraging preliminary data, we initiated this prospective, single-arm, multicenter clinical trial to further evaluate the efficacy and safety of this combination.

### Aims

To prospectively evaluate the efficacy and safety of selinexor combined with decitabine and dose-adjusted CMG regimen in patients with R/R AML previously exposed to venetoclax.

### Methods

This is a multicenter, prospective, single-arm clinical trial (ChiCTR2500104950). Eligible patients were aged >18 years with R/R AML following prior venetoclax exposure. Treatment regimen: decitabine (20 mg/m$^2$, d1-5), cytarabine (10 mg/m$^2$, subcutaneous BID, d4-14), mitoxantrone (5 mg, intravenous, d4-6), G-CSF (300 µg, subcutaneous, d1-14), combined with selinexor (60 mg, oral, d1,4,8,11). Cycles were repeated every 4-6 weeks. The primary endpoint was the CR/CRi/CRh/MLFS rate after the first cycle. Sample size estimation was based on Simon's two-stage design. Here we report the first-stage results.

### Results

A total of 16 patients were enrolled, with a median age of 57.5 years (range 28-68); 62.5% were male. Adverse-risk genetics were present in 75% of patients. Median number of prior lines of therapy was 2; median bone marrow blast percentage was 19%. After one cycle of treatment, 6 patients (37.5%) achieved CR, 2 (12.5%) CRi, 1 (6.25%) CRh, and 3 (18.75%) MLFS, resulting in a ORR[illegible]CR+CRi+CRh+MLFS rate[illegible] of 75.0% (12/16) and a composite complete remission (CRc) rate of 56.25%. Notably, among 4 patients with TP53 mutations, 3 achieved CR/CRi/CRh and 1 achieved MLFS, yielding a ORR of 100%.  With a median follow-up of 185 days, median OS was not reached, and the 6-month OS rate was 74%. Subgroup analysis showed that patients receiving early salvage therapy (1-2 prior lines) (n=9) had a CRc rate of 77.7% and a ORR of 88.9%, which were markedly higher compared to those receiving late salvage therapy (≥3 prior lines). The 6-month overall survival (OS) rate was also significantly prolonged [88.9% (95% CI: 43.3% - 98.4%) vs 33.3% (95% CI: 9.1% - 60.3%), *P*=0.075]. Additionally, 4 patients proceeded to allogeneic hematopoietic stem cell transplantation (including 1 non-responder), and all transplanted patients are currently alive. Grade 3/4 hematologic toxicities were universal but manageable; grade ≥ 3 infection occurred in 75% of patients, with one patient succumbing to sepsis. Non-hematologic adverse events were predominantly grade 1 or 2.

**Copyright Information:** (Online) ISSN: 2572-9241
©2026 The Author(s). HemaSphere is published by John Wiley & Sons Ltd on behalf of European Hematology Association. This is an open access Abstract Book distributed under the Attribution-NonCommercial-NoDerivs (CC BY-NC-ND), which allows third parties to download the articles and share them with others as long as they credit the author and the Abstract Book, but they cannot change the content in any way or use them commercially.
**Abstract Book Citations:** Authors, Title, HemaSphere, 2026;10:(S1):pages. The abstract book DOIs can be found on the “Author Information” page.
**Disclaimer:** Articles published in the journal HemaSphere exclusively reflect the opinions of the authors. The authors are responsible for all content in their abstracts including accuracy of the facts, statements, citing resources, etc.

### Summary/Conclusion

The combination of selinexor with decitabine and dose-adjusted CMG demonstrated promising high efficacy in patients with venetoclax-exposed R/R AML, with a ORR of 75.0% and a CRc rate of 56.25%. Notably, a 100% response rate was achieved in patients with TP53 mutations. However, efficacy and survival benefits were significantly diminished in patients who had received ≥3 prior lines of therapy, indicating that earlier application of this regimen may be more beneficial. The study is ongoing, with continued patient enrollment and extended follow-up planned to further validate the long-term efficacy and survival benefits of this regimen.

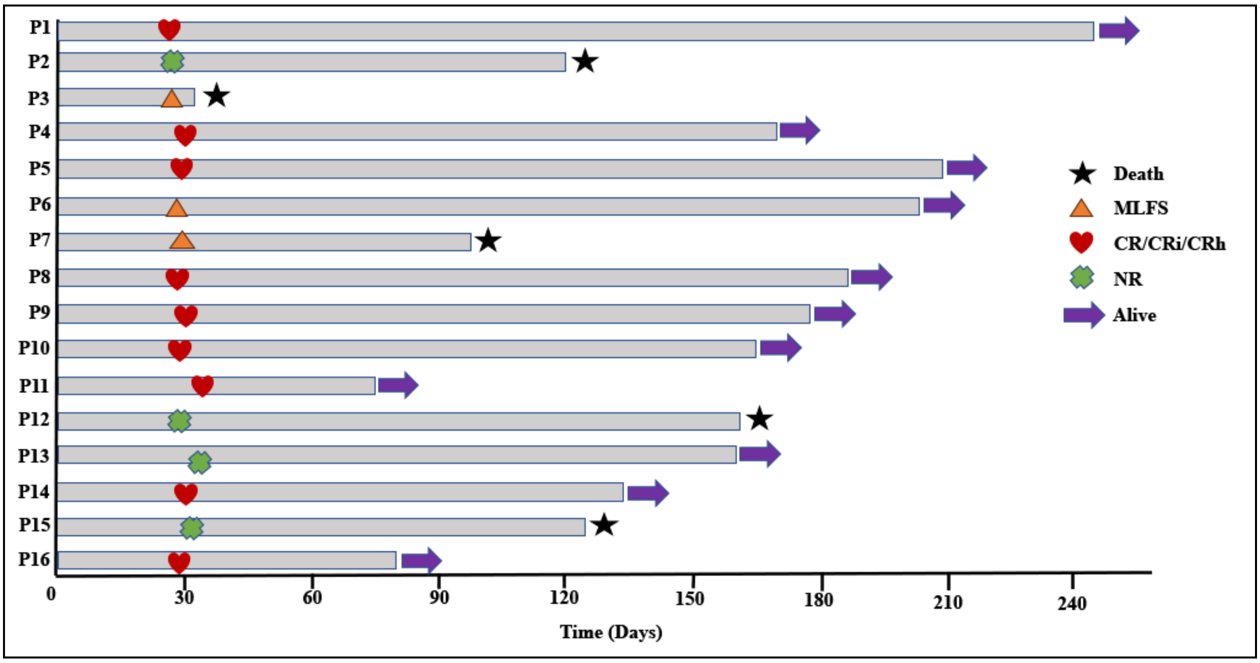

**Copyright Information:** (Online) ISSN: 2572-9241
©2026 The Author(s). HemaSphere is published by John Wiley & Sons Ltd on behalf of European Hematology Association. This is an open access Abstract Book distributed under the Attribution-NonCommercial-NoDerivs (CC BY-NC-ND), which allows third parties to download the articles and share them with others as long as they credit the author and the Abstract Book, but they cannot change the content in any way or use them commercially.
**Abstract Book Citations:** Authors, Title, HemaSphere, 2026;10:(S1):pages. The abstract book DOIs can be found on the "Author Information" page.
**Disclaimer:** Articles published in the journal HemaSphere exclusively reflect the opinions of the authors. The authors are responsible for all content in their abstracts including accuracy of the facts, statements, citing resources, etc.

### (PS1616) SPLICEOSOME MUTATIONS IDENTIFY AML PATIENTS AT HIGH RISK FOR ANTHRACYCLINE-INDUCED ATRIAL FIBRILLATION

**Topic:** 04. Acute myeloid leukemia - Clinical

Guangshuai Teng*[1], Yuhui Zhang[1], Anna Li[1], Ke Shang[1], Chenxiao Du[1], Yan Wang[1], Yanqi Li[1], Huiqin Zhang[1], Lan Peng[1], Yifan Duan[1], Weiping Yuan[1, 2], Minghui Duan[3], Jie Bai[1]

*[1]The Second Hospital of Tianjin Medical University, Department of Hematology, Tianjin, China;[2]Institute of Hematology & Blood Diseases Hospital, Chinese Academy of Medical Sciences & Peking Union Medical College, State Key Laboratory of Experimental Hematology, National Clinical Research Center for Blood Diseases, Tianjin, China; [3]Peking Union Medical College Hospital, Chinese Academy of Medical Sciences & Peking Union Medical College, Department of Hematology, Beijing, China;*

### Background

Atrial fibrillation (AF) is a life-threatening complication in patients with Acute Myeloid Leukemia (AML), but personalized risk stratification remains challenging. Spliceosome gene mutations are key drivers of myeloid malignancies. However, their specific role in the pathogenesis of AF and potential synergistic effects with anthracyclines remain unclear.

### Aims

To investigate the role of spliceosome gene mutations in new-onset AF in AML patients and their synergistic effect with anthracyclines, and to elucidate the underlying mechanisms.

### Methods

This retrospective cohort study included a discovery cohort (n=1321) and a validation cohort (n=412) of patients with myeloid malignancies (53.9% male, median age 62 years). Associations between clonal hematopoiesis-related mutations (DTA genes, spliceosome genes, DNA damage repair genes) and AF were analyzed using Firth-penalized Cox regression, competing risk models, and FDR correction. Sensitivity analyses were performed to address immortal time bias and mutation burden thresholds. Interaction analysis evaluated synergistic effects between spliceosome mutations and anthracyclines. Mechanistically, peripheral blood inflammatory profiling and human primary cell co-culture models were used to characterize the profibrotic phenotype of myeloid cells driven by spliceosome mutations.

### Results

Spliceosome gene mutations were significantly associated with an increased risk of AF in the discovery cohort (adjusted HR=1.59, P=0.030), a finding robust to sensitivity analyses and confirmed in the validation cohort (HR=2.71, P=0.013).Significant synergistic interactions between spliceosome mutations and anthracycline exposure were observed in both cohorts: discovery cohort (sHR = 2.99, P = 0.018), validation cohort (sHR = 3.78, P = 0.036).
Patients with spliceosome mutations exhibited left atrial enlargement (mediation effect: 26.6%), elevated monocyte counts, and higher levels of IL-6 and TNF-α. In vitro, monocytes from mutation carriers promoted type I collagen expression in human atrial fibroblasts.

**Copyright Information:** (Online) ISSN: 2572-9241
©2026 The Author(s). HemaSphere is published by John Wiley & Sons Ltd on behalf of European Hematology Association. This is an open access Abstract Book distributed under the Attribution-NonCommercial-NoDerivs (CC BY-NC-ND), which allows third parties to download the articles and share them with others as long as they credit the author and the Abstract Book, but they cannot change the content in any way or use them commercially.
**Abstract Book Citations:** Authors, Title, HemaSphere, 2026;10:(S1):pages. The abstract book DOIs can be found on the “Author Information” page.
**Disclaimer:** Articles published in the journal HemaSphere exclusively reflect the opinions of the authors. The authors are responsible for all content in their abstracts including accuracy of the facts, statements, citing resources, etc.

### Summary/Conclusion

Spliceosome gene mutations are a novel independent predictor of AF in AML patients, and synergistically amplify anthracycline-related AF risk. Myeloid-driven inflammation and atrial structural remodeling may represent key mediating mechanisms. These findings support intensified surveillance for AF in spliceosome-mutated patients receiving anthracyclines and highlight targeting inflammation as a potential therapeutic target for cardioprotection.

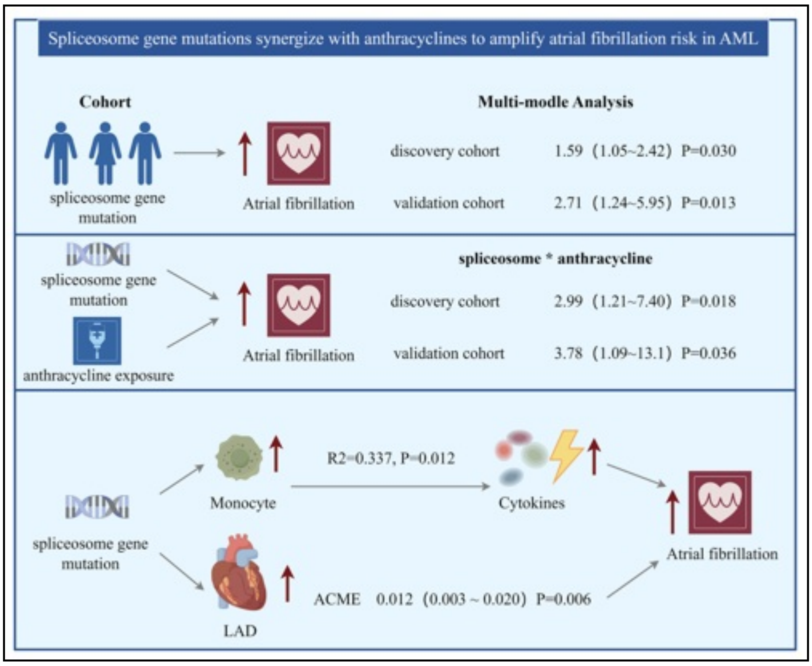

Figure. Spliceosome gene mutations synergize with anthracyclines to increase AF risk in patients with myeloid malignancies. This integrated model identifies spliceosome gene mutations as a novel independent predictor of AF in myeloid malignancies. These mutations synergize with anthracycline exposure to elevate AF risk. Exploratory analyses show that monocyte-mediated inflammation and left atrial enlargement partially mediate the link between spliceosome mutations and AF.

**Copyright Information:** (Online) ISSN: 2572-9241
©2026 The Author(s). HemaSphere is published by John Wiley & Sons Ltd on behalf of European Hematology Association. This is an open access Abstract Book distributed under the Attribution-NonCommercial-NoDerivs (CC BY-NC-ND), which allows third parties to download the articles and share them with others as long as they credit the author and the Abstract Book, but they cannot change the content in any way or use them commercially.
**Abstract Book Citations:** Authors, Title, HemaSphere, 2026;10:(S1):pages. The abstract book DOIs can be found on the "Author Information" page.
**Disclaimer:** Articles published in the journal HemaSphere exclusively reflect the opinions of the authors. The authors are responsible for all content in their abstracts including accuracy of the facts, statements, citing resources, etc.

### (PS1617) REAL-WORLD OUTCOMES IN MECOM-REARRANGED AML: THE LARGEST MULTICENTER COHORT REPORTED TO DATE

**Topic:** 04. Acute myeloid leukemia - Clinical

Cecilia Arana Yi*[1], Jesus D. Gonzalez-Lugo[2], Usman Ilyas[1], Angel Luis Orosco-Ttamina[1], David Martinez Gamboa[1], Deedra Nicolet[3], Anand Patel[4], Appoorva Ravichandran[4], Alexander Coltoff[5], Alexander Ambinder[6], Yazan F. Madanat[7], Krzysztof Mrozek[3], Michael Walker[3], Christine McMahon[8], Micahel Hochman[9], Brittany K. Ragon[10], Joshua Zeidner[11], Kieran Sahasrabudhe[12], Bianca Barredo[12], Lauren Banaszak[12], Alice Mims[3], Onyee Chan[13], Rami S. Komrokji[13], Eric Winer[14], Chenyu Lin[15], Ehab Atallah[16], Guru Subramanian Guru Murthy[16], Rory M. Shallis[13], Antoine Saliba[17], Charles Foucar[18], Anna Bode[18], Yasmin Abaza[19], Vamsi Kota[20], Annie Im[21], Tara L. Lin[2], Talha Badar[22], Ann-Kathrin Eisfeld[3]

*[1]Mayo Clinic Arizona, Internal Medicine,, Phoenix, United States of America;[2]The University of Kansas Cancer Center, Kansas City, United States of America; [3]Clara D. Bloomfield Center for Leukemia Outcomes Research, The Ohio State University Comprehensive Cancer Center, Columbus, United States of America; [4]The University of Chicago, Chicago, United States of America; [5]Medical University of South Carolina, Charleston, United States of America;[6]Sidney Kimmel Comprehensive Cancer Center at Johns Hopkins, Baltimore, United States of America; [7]UT Southwestern Medical Center, Dallas, United States of America; [8]University of Colorado Health Blood Disorders and Cell Therapies Center, Aurora, United States of America;[9]Emory Winship Cancer Institute, Atlanta, United States of America; [10]Atrium Health Levine Cancer Institute/Wake Forest University School of Medicine, Charlotte, United States of America; [11]University of North Carolina Lindeberger Comprehensive Cancer Center, Chapel Hill, United States of America; [12]University of Wisconsin, Madison, United States of America; [13]Moffitt Cancer Center, Tampa, United States of America; [14]Dana Farber Cancer Institute, Boston, United States of America; [15]Duke Blood Cancer Center, Durham, United States of America; [16]Medical College of Wisconsin, Milwaukee, United States of America; [17]Mayo Clinic, Rochester, United States of America; [18]The University of New Mexico Comprehensive Cancer Center, Albuquerque, United States of America; [19]Northwestern University Robert Lurie Cancer Center, Chicago, United States of America; [20]Medical College of Georgia, Augusta, United States of America;[21]The University of Pittsburgh Medical Center, Pittsburgh, United States of America; [22]Mayo Clinic Florida, Jacksonville, United States of America;*

### Background

MECOM-rearranged (MECOM-r) AML, associated with inv(3)(q21q26.2), t(3;3)(q21;q26.2), and other 3q26.2 abnormalities, represents a rare WHO/ICC adverse-risk subtype. Initial MARROW Consortium data were presented at ASH 2025; this expanded analysis now represents the largest real-world cohort to date.

### Aims

To characterize clinical features, molecular landscape, treatment patterns, and outcomes data in the largest US real-world MECOM-r AML population using integrated data from the COMMAND Consortium, MARROW Consortium, and Flatiron database electronic health records.

### Methods

312 newly diagnosed adults were identified (MARROW n=93; COMMAND n=88; Flatiron n=131) from 10 MARROW and 5 COMMAND institutions. Endpoints included composite CR (CRc), disease-free survival (DFS), and overall survival (OS). Logistic regression and Cox proportional hazards models evaluated predictors of response and survival.

**Copyright Information:** (Online) ISSN: 2572-9241
©2026 The Author(s). HemaSphere is published by John Wiley & Sons Ltd on behalf of European Hematology Association. This is an open access Abstract Book distributed under the Attribution-NonCommercial-NoDerivs (CC BY-NC-ND), which allows third parties to download the articles and share them with others as long as they credit the author and the Abstract Book, but they cannot change the content in any way or use them commercially.
**Abstract Book Citations:** Authors, Title, HemaSphere, 2026;10:(S1):pages. The abstract book DOIs can be found on the "Author Information" page.
**Disclaimer:** Articles published in the journal HemaSphere exclusively reflect the opinions of the authors. The authors are responsible for all content in their abstracts including accuracy of the facts, statements, citing resources, etc.

### Results

Median age was 67 years (range 19–93); 71% were ≥60 and 53% ≥70; 52% male; 86% White, 8% Black, 5% Hispanic. MECOM-r subtype distribution was inv(3)/t(3;3) 77%, t(3;21) and other variants 23%. Co-mutations included RAS-pathway 31%, TP53 17%, and AML-MR lesions 44%. Chromosome 7 abnormalities and del(5q) occurred in 44% and 14%, respectively.
Initial therapy was intensive chemotherapy (IC) for 38% of pts, hypomethylating agents + venetoclax (HMA+VEN) for (25%), and hypomethylating agent monotherapy (HMA) for (16%). Overall CRc rate was: 34%, CRc was 42% for IC, 37% for HMA+VEN, and 12% for HMA ($p < 0.001$). CRc by MECOM-r subtype was 42% for t(3;21), 28% for inv(3)/t(3;3), and 14% for other MECOM variants (p=0.06). Among pts with chromosome 7 abnormalities, CRc was 36% with IC, 31% with HMA+VEN, and 5% with HMA (p=0.01). For those with del(5q), CRc was 57% in pts who received IC, 20% with HMA+VEN, and 0% with HMA (p=0.03). Overall, the relapse rate was 50%, with no difference among treatment groups. Allogeneic stem cell transplant (AlloSCT) was performed in 28%, more often after IC (49%) than after HMA+VEN (22%) or HMA (15%) ($p<0.001$).
OS favored IC over HMA-based therapies ($p < 0.001$), with median OS of 14.8 m for IC, 7.8 m for HMA+VEN, and 6.2 m for HMA. DFS differed among treatment groups (p=0.028), with median DFS of 9.6 m for IC, 7.2 m for HMA+VEN, and 4.7 m for HMA. Pts with chromosome 7–abnormalities had mOS 12.9, 6.5, and 5.5 m with IC, HMA+VEN, and HMA, respectively ($p<0.001$). Del(5q) showed mOS 9.1 vs 5.1 m with IC vs HMA (p=0.085). MECOM-r subtypes showed mOS 14.8 m for (inv(3)/t(3;3)), 11.7 m for (t(3;21)), and 21.8 m for other MECOM-r subtypes (p=0.1938). Among AlloSCT recipients (n=52), mOS was 24.8 vs 15.8 m after IC and HMA-based therapy (p=0.4132), with overall mOS of 19.7 m.
In univariate analysis, IC was associated with higher CR (OR 1.95; p=0.02). RAS-pathway mutations did not impact CR or relapse. Older age, del(5q), and *PTPN11* mutations were adverse OS predictors. In multivariable analysis. AlloSCT was the strongest predictor of OS (HR 0.21; $p<0.001$).

### Summary/Conclusion

This largest U.S. real-world MECOM-r AML cohort confirms uniformly adverse outcomes. IC yielded the highest remission rates and, when followed by AlloSCT, the most favorable survival. HMA+VEN did not show survival benefit over HMA alone, indicating that both remain appropriate options for IC-ineligible patients. However, relapse remained frequent and survival limited even with IC and AlloSCT, underscoring the critical need for MECOM-r directed therapies.

**Copyright Information:** (Online) ISSN: 2572-9241
©2026 The Author(s). HemaSphere is published by John Wiley & Sons Ltd on behalf of European Hematology Association. This is an open access Abstract Book distributed under the Attribution-NonCommercial-NoDerivs (CC BY-NC-ND), which allows third parties to download the articles and share them with others as long as they credit the author and the Abstract Book, but they cannot change the content in any way or use them commercially.
**Abstract Book Citations:** Authors, Title, HemaSphere, 2026;10:(S1):pages. The abstract book DOIs can be found on the "Author Information" page.
**Disclaimer:** Articles published in the journal HemaSphere exclusively reflect the opinions of the authors. The authors are responsible for all content in their abstracts including accuracy of the facts, statements, citing resources, etc.

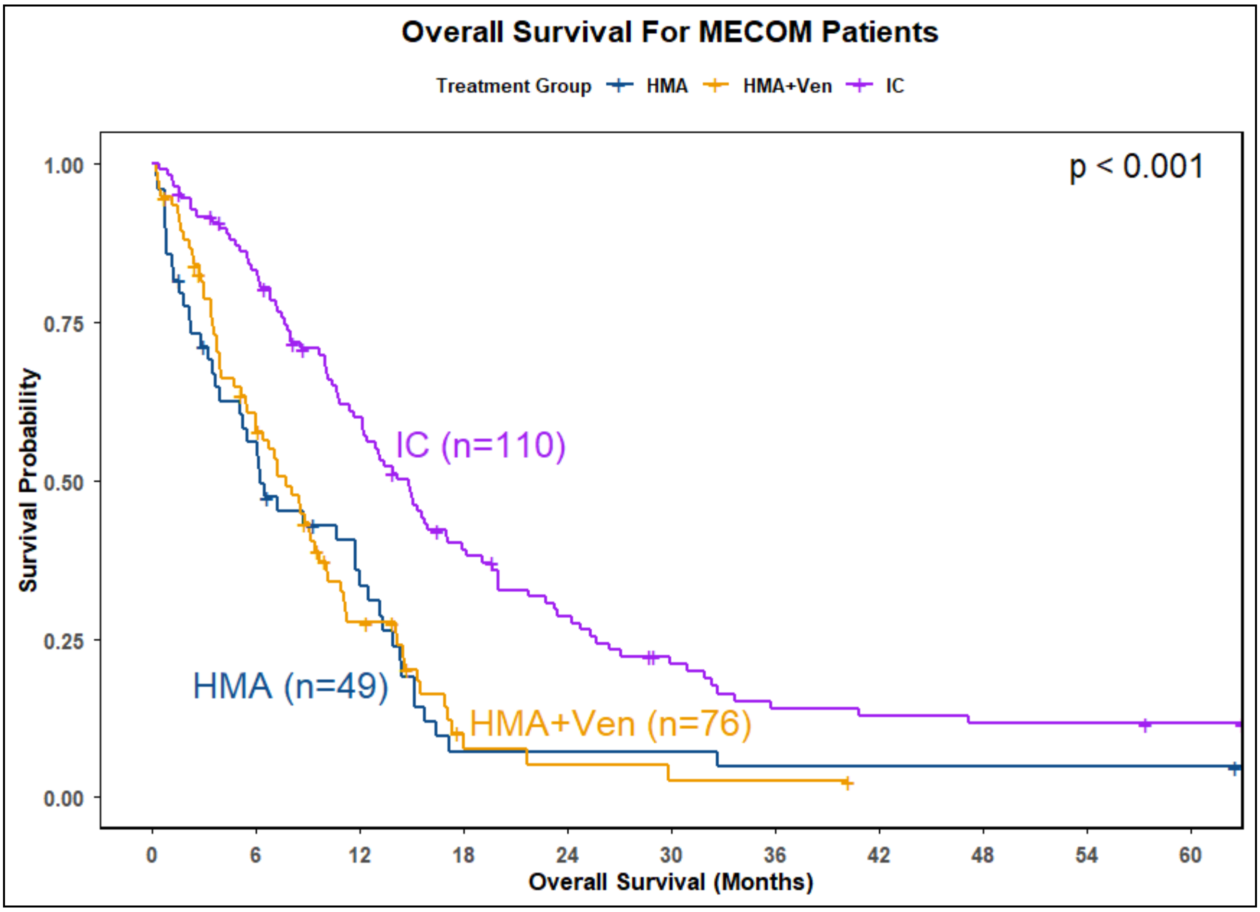

Figure 1. Overall Survival per Chemotherapy Regimen

**Copyright Information:** (Online) ISSN: 2572-9241
©2026 The Author(s). HemaSphere is published by John Wiley & Sons Ltd on behalf of European Hematology Association. This is an open access Abstract Book distributed under the Attribution-NonCommercial-NoDerivs (CC BY-NC-ND), which allows third parties to download the articles and share them with others as long as they credit the author and the Abstract Book, but they cannot change the content in any way or use them commercially.
**Abstract Book Citations:** Authors, Title, HemaSphere, 2026;10:(S1):pages. The abstract book DOIs can be found on the "Author Information" page.
**Disclaimer:** Articles published in the journal HemaSphere exclusively reflect the opinions of the authors. The authors are responsible for all content in their abstracts including accuracy of the facts, statements, citing resources, etc.

### (PS1618) GILTERITINIB RESUMPTION AFTER HEMATOPOIETIC STEM CELL TRANSPLANTATION VS BEST SUPPORTIVE CARE IN RELAPSED/REFRACTORY FLT3-MUTATED ACUTE MYELOID LEUKEMIA: A LANDMARK AND PROPENSITY-MATCHED COHORT STUDY

**Topic:** 04. Acute myeloid leukemia - Clinical

Harry Gill*[1], Erlie Jiang[2], Hsin-An Hou[3, 4], Hee-Je Kim[5], Dong-Yeop Shin[6, 7, 8], Shengli Xue[9, 10, 11], Shaun Fleming[12, 13, 14], Allesandra Paz[15], Jamie An[16], Juan Ariza[17], Jun Liu[18], Ashish Bajel[19, 20]

*[1]Department of Medicine, School of Clinical Medicine, LKS Faculty of Medicine, the University of Hong Kong, Hong Kong, China; [2]State Key Laboratory of Experimental Hematology, National Clinical Research Center for Blood Diseases, Haihe Laboratory of Cell Ecosystem, Institute of Hematology & Blood Diseases Hospital, Chinese Academy of Medical Sciences & Peking Union Medical College, Tianjin, China; [3]Division of Hematology, Department of Internal Medicine, National Taiwan University Hospital, Taipei, Taiwan; [4]Division of General Medicine, Department of Internal Medicine, National Taiwan University Hospital, Taipei, Taiwan; [5]Department of Hematology, Catholic Hematology Hospital, Seoul St. Mary's Hospital, College of Medicine, The Catholic University of Korea, Seoul, South Korea; [6]Department of Internal Medicine, Seoul National University Hospital, Seoul National University College of Medicine, Seoul, South Korea; [7]Center for Medical Innovation, Biomedical Research Institute, Seoul National University Hospital, Seoul, South Korea; [8]Cancer Research Institute, Seoul National University College of Medicine, Seoul, South Korea; [9]Department of Hematology, The First Affiliated Hospital of Soochow University, Suzhou, China;[10]Department of Blood and Marrow Transplantation, Soochow University, Suzhou, Jiangsu, China; [11]The Leukemia Working Group of National Clinical Research Center for Hematologic Diseases, Suzhou, China; [12]Alfred Pathology, The Alfred Hospital, Melbourne, Victoria, Australia; [13]Department of Malignant Haematology and Stem Cell Transplantation, The Alfred Hospital, Melbourne, Victoria, Australia; [14]Australian Centre for Blood Diseases, Monash University, Melbourne, Victoria, Australia; [15]Clinical Hospital of Porto Alegre, Porto Alegre, Brazil; [16]Astellas Pharma Singapore Pte Ltd., Singapore, Singapore; [17]Astellas Pharma INT-M Region, Bogota, Colombia; [18]Astellas Pharma Inc., Northbrook, IL, United States of America; [19]Clinical Haematology, Peter MacCallum Cancer Centre and The Royal Melbourne Hospital, Melbourne, Australia; [20]Department of Medicine, The University of Melbourne, Melbourne, Australia;*

### Background

Patients (pts) with relapsed/refractory (R/R) *FMS*-like tyrosine kinase 3-mutated (*FLT3*$^{mut+}$) acute myeloid leukemia (AML) are typically heavily pretreated, measurable residual disease-positive, experience poor outcomes, and need effective post-hematopoietic stem cell transplantation (HSCT) maintenance options.

### Aims

To evaluate efficacy outcomes in pts with R/R *FLT3*$^{mut+}$ AML who resumed gilteritinib or received best supportive care (BSC) post HSCT.

**Copyright Information:** (Online) ISSN: 2572-9241
©2026 The Author(s). HemaSphere is published by John Wiley & Sons Ltd on behalf of European Hematology Association. This is an open access Abstract Book distributed under the Attribution-NonCommercial-NoDerivs (CC BY-NC-ND), which allows third parties to download the articles and share them with others as long as they credit the author and the Abstract Book, but they cannot change the content in any way or use them commercially.
**Abstract Book Citations:** Authors, Title, HemaSphere, 2026;10:(S1):pages. The abstract book DOIs can be found on the “Author Information” page.
**Disclaimer:** Articles published in the journal HemaSphere exclusively reflect the opinions of the authors. The authors are responsible for all content in their abstracts including accuracy of the facts, statements, citing resources, etc.

### Methods

This noninterventional retrospective analysis compared pts from the ADMIRAL and COMMODORE phase 3 trials who resumed gilteritinib after on-study HSCT, and an external comparator group of real-world pts identified via chart review who received BSC (donor lymphocyte infusion, intrathecal chemotherapy, or cranial radiation) between 2015-2022, mainly before gilteritinib availability and *FLT3* mutation retesting at R/R diagnosis became common practice. Adults with R/R *FLT3*$^{mut+}$ AML who underwent HSCT, were alive at day 90 post HSCT, achieved engraftment and any complete response, and had grade ≤2 graft-vs-host disease (GvHD) were included. A 90-day post-HSCT landmark analysis was applied to minimize immortal time bias. Baseline confounding variables between the gilteritinib and BSC groups were adjusted by 1:1 propensity score matching in the per-protocol population (PPP; defined as pts meeting all protocol-specified criteria) based on prespecified covariates. Primary and secondary outcomes were relapse-free survival (RFS) and overall survival (OS), respectively.

### Results

In the intention-to-treat population (gilteritinib, n=55; BSC, n=105), age and sex distributions were similar between groups. ECOG PS score of 0 (50.9% vs 26.7%) and intermediate cytogenetic risk (74.5% vs 32.4%) were more frequent in the gilteritinib group. The PPP comprised 45 and 90 pts in the gilteritinib and BSC groups, respectively; 45/45 (100%) and 38/90 (42.2%) pts had confirmed *FLT3*$^{mut+}$ status at R/R diagnosis. Propensity score matching in the PPP yielded 40 pts per group. Post-matching balance was achieved (all absolute standardized mean differences <0.1), except for cytogenetic risk and *FLT3*$^{mut+}$ status, which were adjusted for using a multivariable Cox proportional hazards model. Median (95% CI) follow-up was 43.3 (38.5-44.2) and 52.5 (38.5-60.5) months in the gilteritinib and BSC groups, respectively. Univariate analyses of the PPP (RFS hazard ratio [HR] 0.55 [95% CI: 0.32-0.96]; OS HR 0.71 [95% CI: 0.41-1.25]) and multivariable analyses of the matched-analysis population (RFS HR 0.28 [95% CI: 0.13-0.62]; OS HR 0.53 [95% CI: 0.23-1.25]) showed that gilteritinib was associated with lower risk of relapse and death vs BSC (**Figure**). Similar results were observed in univariate analyses (RFS HR 0.38 [95% CI: 0.21-0.71]; OS HR 0.50 [95% CI: 0.27-0.95]) and multivariable analyses (RFS HR 0.36 [95% CI: 0.18-0.74]; OS HR 0.52 [95% CI: 0.25-1.08]) of the confirmed *FLT3*$^{mut+}$ status at R/R diagnosis subgroup.

### Summary/Conclusion

In pts with R/R *FLT3*$^{mut+}$ AML undergoing HSCT, resumption of gilteritinib was associated with lower risk of relapse and a trend toward improved survival vs BSC. Results may be influenced by the retrospective study design, baseline disease burden, timing of drug initiation, and HSCT-related variations. These findings address a major post-HSCT management gap and support FLT3 inhibitor resumption as a clinically meaningful strategy in this high-risk population, but further long-term studies are needed.

**Copyright Information:** (Online) ISSN: 2572-9241
©2026 The Author(s). HemaSphere is published by John Wiley & Sons Ltd on behalf of European Hematology Association. This is an open access Abstract Book distributed under the Attribution-NonCommercial-NoDerivs (CC BY-NC-ND), which allows third parties to download the articles and share them with others as long as they credit the author and the Abstract Book, but they cannot change the content in any way or use them commercially.
**Abstract Book Citations:** Authors, Title, HemaSphere, 2026;10:(S1):pages. The abstract book DOIs can be found on the “Author Information” page.
**Disclaimer:** Articles published in the journal HemaSphere exclusively reflect the opinions of the authors. The authors are responsible for all content in their abstracts including accuracy of the facts, statements, citing resources, etc.

**Figure. Kaplan–Meier plot of RFS in the overall PPP and *FLT3*$^{mut+}$ test at R/R diagnosis subgroup**

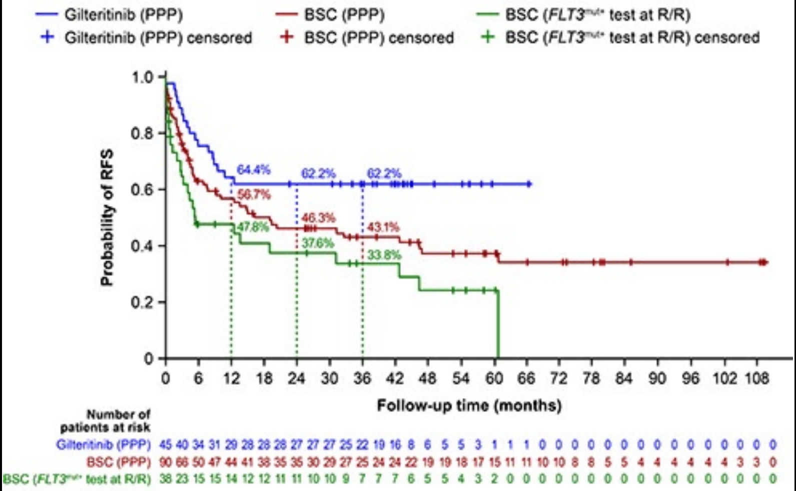

| | PPP | | *FLT3*$^{mut+}$ test at R/R |
|---|---|---|---|
| | Gilteritinib (n=45) | BSC (n=90) | BSC (n=38) |
| Events, n (%) | 17 (37.8) | 51 (56.7) | 26 (68.4) |
| Relapse | 11 (24.4) | 38 (42.2) | 18 (47.4) |
| Death | 6 (13.3) | 13 (14.4) | 8 (21.1) |
| Median RFS (95% CI), months | NE (10.8–NE) | 19.0 (7.5–46.8) | 5.4 (2.8–31.2) |

BSC, best supportive care; CI, confidence interval; *FLT3*$^{mut+}$, *FMS*-like tyrosine kinase 3-mutated; NE, not estimable; PPP, per-protocol population; RFS, relapse-free survival, R/R, relapsed/refractory.

**Copyright Information:** (Online) ISSN: 2572-9241
©2026 The Author(s). HemaSphere is published by John Wiley & Sons Ltd on behalf of European Hematology Association. This is an open access Abstract Book distributed under the Attribution-NonCommercial-NoDerivs (CC BY-NC-ND), which allows third parties to download the articles and share them with others as long as they credit the author and the Abstract Book, but they cannot change the content in any way or use them commercially.
**Abstract Book Citations:** Authors, Title, HemaSphere, 2026;10:(S1):pages. The abstract book DOIs can be found on the "Author Information" page.
**Disclaimer:** Articles published in the journal HemaSphere exclusively reflect the opinions of the authors. The authors are responsible for all content in their abstracts including accuracy of the facts, statements, citing resources, etc.

### (PS1619) REAL-WORLD TRANSFUSION INDEPENDENCE OUTCOMES WITH FRONTLINE IMETELSTAT VERSUS LUSPATERCEPT IN LOWER-RISK MYELODYSPLASTIC SYNDROMES

**Topic:** 04. Acute myeloid leukemia - Clinical

Andres Ramirez-Gamero*[1], Nehemias Guevara[2], Matthew Walter[3], Zachary Crees[3], Meagan Jacoby[3], Ramzi Abboud[3], Iskra Pusic[3], Camille Abboud[3], Giulia Petrone[3], Matthew Christopher[3], Jhon Dipersio[3], Geoffrey Uy[3], Samuel Urrutia[3]

*[1]Roger Williams Medical Center, Providence, United States of America;[2]Saint Louis University, Saint Louis, United States of America; [3]Washington University in St Louis, St. Louis, United States of America;*

### Background

Imetelstat is a telomerase inhibitor approved for patients with lower-risk myelodysplastic syndromes (LR-MDS) who have progressed after or are ineligible for erythropoiesis-stimulating agents (ESA). In a phase 3 trial, imetelstat improved durable red blood cell (RBC) transfusion independence (TI) in LR-MDS. Luspatercept, frequently used as frontline therapy, is also associated with meaningful TI rates. There are no studies comparing these drugs as frontline therapy. We evaluated transfusion outcomes of frontline imetelstat compared to frontline luspatercept using the TriNetX dataset.

### Methods

We first evaluated the real-world efficacy of imetelstat. We then compared patients who received imetelstat as frontline therapy and those who received luspatercept as frontline therapy. The primary outcome was achievement of 8- and 24-week TI, defined as absence of documented RBC transfusion within the specified time window following treatment initiation. Transfusion-free survival was estimated using Kaplan–Meier methods. As a sensitivity analysis, we performed 1:1 propensity score matching based on age and baseline hemoglobin.

### Results

In the overall imetelstat cohort (n = 107), median age was 76.5 years, and median baseline hemoglobin was 7.8 g/dL, 35 patients (32%) had ring sideroblasts. Most patients had significant prior therapy and transfusion with 74% previously exposed to luspatercept. In this population, the probabilities of achieving TI were 30.2% at 8 weeks and 26.6% at 20 weeks after imetelstat initiation.

Among 107 patients treated with imetelstat, 22 (20.5%) received it as frontline therapy, the 8-week TI probability was 44.3%, and the 24-week TI probability was 33.2%. In the frontline luspatercept cohort (n = 361), the 8- and 24-week TI probabilities were 63.2% and 53.2%, respectively. After 1:1 propensity score matching on age and baseline hemoglobin, 19 patients per cohort were retained. In the matched analysis, there was no statistically significant differences (p-value = 0.19) in 8- or 24-week TI probabilities between frontline imetelstat and frontline luspatercept (OR: 1.88, 95%CI: -0.085, 0.386) and transfusion-free survival at 8 and 24 weeks (p=0.23).

### Summary/Conclusion

In this real-world LR-MDS cohort, imetelstat achieved clinically meaningful TI, including in patients with prior luspatercept exposure and high transfusion burden. When used as frontline therapy, imetelstat produced TI rates that were not statistically different from those observed with frontline luspatercept after propensity-matched adjustment, however, frontline imetelstat use remains uncommon. Observations from this real-world cohort point toward the potential for imetelstat to be used early in treatment of LR-MDS. Studies to refine treatment sequencing are needed.

**Copyright Information:** (Online) ISSN: 2572-9241
©2026 The Author(s). HemaSphere is published by John Wiley & Sons Ltd on behalf of European Hematology Association. This is an open access Abstract Book distributed under the Attribution-NonCommercial-NoDerivs (CC BY-NC-ND), which allows third parties to download the articles and share them with others as long as they credit the author and the Abstract Book, but they cannot change the content in any way or use them commercially.
**Abstract Book Citations:** Authors, Title, HemaSphere, 2026;10:(S1):pages. The abstract book DOIs can be found on the "Author Information" page.
**Disclaimer:** Articles published in the journal HemaSphere exclusively reflect the opinions of the authors. The authors are responsible for all content in their abstracts including accuracy of the facts, statements, citing resources, etc.

### (PS1620) ASSESSING MRD BY MFC POST-SCT REFINES RISK STRATIFICATION IN AML: LONG-TERM FOLLOW-UP OF A MULTICENTER PROSPECTIVE STUDY

**Topic:** 04. Acute myeloid leukemia - Clinical

Siqi Li*[1], Fuxu Wang[2], Lanping Xu[1], Yu Wang[1], Xiaohui Zhang[1], Xiao-Dong Mo[1], Xiao-Jun Huang[1], Erlie Jiang[3], Yingjun Chang[1]

*[1]Peking University People's Hospital & Peking University Institute of Hematology, National Clinical Research Center for Hematologic Disease, Beijing Key Laboratory of Cell and Gene Therapy for Hematologic Malignancies, Beijing, China; [2]Department of Hematology, The Second Hospital of Hebei Medical University, Shijiazhuang, China;[3]State Key Laboratory of Experimental Hematology, National Clinical Research Center for Blood Diseases, Haihe Laboratory of Cell Ecosystem, Institute of Hematology & Blood Diseases Hospital, Chinese Academy of Medical Sciences & Peking Union Medical College, Tianjin, China;*

### Background

Leukemia relapse remains the leading cause of death in acute myeloid leukemia (AML) patients who receive allogeneic stem cell transplantation (allo-SCT). Measurable residual disease (MRD) is the source of leukemia relapse. Currently, the prognostic significance of post-SCT MRD has been less well investigated. In addition, there are some limitations of the available studies on the association between post-SCT MRD and clinical outcomes. For example, the retrospective nature of the studies and the small sample size of subgroup patients with short-term follow-up.

### Aims

We aime to investigat the prognostic value of posttransplantation measurable residual disease (MRD) in refining the risk stratification of patients with acute myeloid leukemia who underwent allo-SCT.

### Methods

We performed a multicenter, prospective, long-term follow-up study to evaluate the value of post-SCT MRD for predicting the outcomes of AML patients. MRD was determined using multiparameter flow cytometry.

### Results

Five hundred forty-eight AML patients were enrolled. Compared with patients with negative post-SCT MRD, patients with positive post-SCT MRD had a higher 5-year cumulative incidence of relapse (CIR, 8.1% vs. 54.5%, $P<0.001$) and a lower 5-year probability of LFS (82.4% vs. 36.4%, $P<0.001$) and OS (84.9% vs. 45.3%, $P<0.001$). Post-SCT MRD had a higher C-index (0.707 vs. 0.656) for predicting relapse than did pre-SCT tumor burden. Post-SCT MRD can be used to improve the stratification of AML patients based on other classification biomarkers, such as refined ELN 2017 risk stratification and refined pre-SCT disease status. Peri-SCT leukemia burden kinetics have more power than post-SCT MRD or pre-SCT leukemia burden in terms of predicting outcomes. We established a scoring system based on peri-SCT leukemia burden kinetics and chimerism for relapse prediction, which has a higher C-index (0.785) than do other refined stratifications.

### Summary/Conclusion

Our results reveal the predictive value of post-SCT MRD for relapse and its ability to refine the risk stratification of AML transplantation recipients. This novel risk score has the highest C-index for predicting transplant outcomes in patients with AML.

**Copyright Information:** (Online) ISSN: 2572-9241
©2026 The Author(s). HemaSphere is published by John Wiley & Sons Ltd on behalf of European Hematology Association. This is an open access Abstract Book distributed under the Attribution-NonCommercial-NoDerivs (CC BY-NC-ND), which allows third parties to download the articles and share them with others as long as they credit the author and the Abstract Book, but they cannot change the content in any way or use them commercially.
**Abstract Book Citations:** Authors, Title, HemaSphere, 2026;10:(S1):pages. The abstract book DOIs can be found on the "Author Information" page.
**Disclaimer:** Articles published in the journal HemaSphere exclusively reflect the opinions of the authors. The authors are responsible for all content in their abstracts including accuracy of the facts, statements, citing resources, etc.

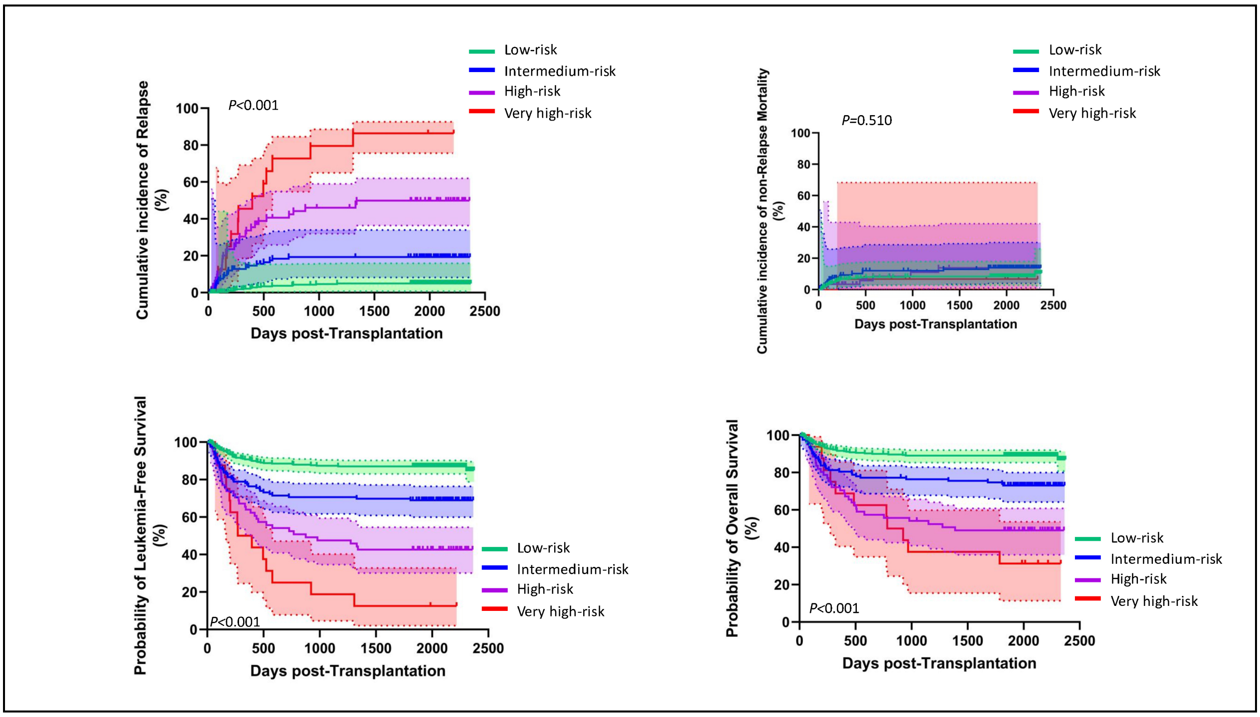

Relationships between the novel scoring system and transplant outcomes for patients with AML who underwent allo-SCT (n=548). Estimated (a) CIR, (b) cumulative incidence of NRM, (c) probability of LFS and (d) probability of OS. CIR, cumulative incidence of relapse; NRM, nonrelapse mortality; LFS, leukemia-free survival; OS, overall survival.

**Copyright Information:** (Online) ISSN: 2572-9241
©2026 The Author(s). HemaSphere is published by John Wiley & Sons Ltd on behalf of European Hematology Association. This is an open access Abstract Book distributed under the Attribution-NonCommercial-NoDerivs (CC BY-NC-ND), which allows third parties to download the articles and share them with others as long as they credit the author and the Abstract Book, but they cannot change the content in any way or use them commercially.
**Abstract Book Citations:** Authors, Title, HemaSphere, 2026;10:(S1):pages. The abstract book DOIs can be found on the "Author Information" page.
**Disclaimer:** Articles published in the journal HemaSphere exclusively reflect the opinions of the authors. The authors are responsible for all content in their abstracts including accuracy of the facts, statements, citing resources, etc.

### (PS1621) PHASE I/PHASE 2 STUDY OF QUIZARTINIB COMBINED WITH CLIA IN NEWLY DIAGNOSED AND RELAPSED/REFRACTORY FLT3-MUTATED AND WILD-TYPE ACUTE MYELOID LEUKEMIA

**Topic:** 04. Acute myeloid leukemia - Clinical

Musa Yilmaz*[1], Tapan Kadia[1], Courtney Dinardo[1], Nicholas Short[1], Cara Cooper[1], Gautam Borthakur[1], Naveen Pemmaraju[1], Yesid Alvarado[1], Fadi Haddad[1], Kelly Chien[1], Wei-Ying Jen[1], Alexandre Bazinet[1], Sanam Loghavi[1, 1], Steven Kornblau[1], Michael Andreeff[1], Elias Jabbour[1], Farhad Ravandi[1], Guillermo Garcia-Manero[1], Hagop Kantarjian[1], Naval Daver[1]

[1]*MD Anderson Cancer Center, Houston, United States of America;*

### Background

Quizartinib (QUIZ) is a potent, selective FLT3 inhibitor with demonstrated efficacy in FLT3-ITD–mutated and FLT3 wild-type AML. In QuANTUM-First and QUIWI, adding QUIZ to standard induction/consolidation improved survival in newly diagnosed FLT3-ITD–mutated and wild-type AML.

### Aims

This phase 1/2 study evaluated the safety and efficacy of QUIZ combined with cladribine, idarubicin, and cytarabine (CLIA) in newly diagnosed (ND) and relapsed/refractory (R/R) FLT3-ITD–mutated and wild-type AML.

### Methods

Patients (pts) aged <75 years with ND or R/R AML, ECOG ≤2, adequate organ function, and fit for intensive therapy were eligible. Phase 1 assessed QUIZ 17.7 mg and 26.5 mg (days 6–19 induction; days 4–28 consolidation). CLIA induction: cladribine 5 mg/m² (days 1–5), cytarabine 1.5 g/m² (days 1–5), idarubicin 10 mg/m² (days 1–3). Consolidation (≤5 cycles): cladribine 5 mg/m² (days 1–3), cytarabine 1 g/m² (days 1–3), idarubicin 8 mg/m² (days 1–2).

**Copyright Information:** (Online) ISSN: 2572-9241
©2026 The Author(s). HemaSphere is published by John Wiley & Sons Ltd on behalf of European Hematology Association. This is an open access Abstract Book distributed under the Attribution-NonCommercial-NoDerivs (CC BY-NC-ND), which allows third parties to download the articles and share them with others as long as they credit the author and the Abstract Book, but they cannot change the content in any way or use them commercially.
**Abstract Book Citations:** Authors, Title, HemaSphere, 2026;10:(S1):pages. The abstract book DOIs can be found on the "Author Information" page.
**Disclaimer:** Articles published in the journal HemaSphere exclusively reflect the opinions of the authors. The authors are responsible for all content in their abstracts including accuracy of the facts, statements, citing resources, etc.

### Results

Forty-seven pts were treated (17 phase 1, 30 phase 2). No DLTs occurred at QUIZ 17.7 mg; one myocarditis/pericarditis at 26.5 mg, which was selected as RP2D.
Thirty-two pts had FLT3-ITD–mutated AML (15ND, 17 R/R). Among 15 ND pts, median age was 38 years (range, 22–60), 12 (80%) were diploid, and median FLT3-ITD VAF was 29% (range, 1–54%). Twelve (80%) and 3 (20%) were ELN 2022 intermediate- and high-risk. Most common baseline co-mutations (>15%) were NPM1 (47%), DNMT3A (27%), IDH1/2 (20%), and WT1 (20%). CR, CRi, EOC1 MRD negativity by MFC (0.01% sensitivity), and EOC1 FLT3-ITD NGS negativity ($5\times10^{-5}$ sensitivity) were 87%, 7%, 92% (12/13), and 30% (3/10). Best MFC and FLT3-ITD NGS MRD negativity (≥2 cycles) were 100% (11/11) and 67% (6/9). Among NPM1-comutated pts, NPM1-NGS MRD negativity ($5\times10^{-5}$ sensitivity) at EOC1 and best overall were 0% (0/6) and 50% (3/6). Of 4 pts with positive pre-ASCT MRD (FLT3 and/or NPM1), all became MRD negative by post-ASCT day 100. Median cycles received were 2 (range, 1–3). Median time to ANC ≥1000 and platelets >100K in responders was 28 days (range, 22–34) and 24 days (range, 20–61). At last follow-up, 7 pts underwent ASCT, 3 completed therapy, 3 transferred care (insurance-related), 1 was lost to follow-up, and 1 remained on treatment. With median f/u of 11 months, no relapses or deaths occurred (Figure).
Among 17 R/R FLT3-ITD–mutated pts (median age 52; median 3 prior lines; 71% prior FLT3i; 67% ELN2022 adverse), CR/CRi/MLFS were each 17%. Best MRD negativity: 50% by MFC and 33% by FLT3-NGS. Of 9 responders, 7 underwent ASCT (3 relapsed, 3 ongoing CR, 1 died in CR); both non-transplanted responders relapsed. Median OS was 5.7 months.
Fifteen FLT3-wild-type pts were treated (4 ND, 11 R/R; 93% ELN2022 adverse. In ND patients, 3/4 (75%) responded (2 CR, 1 PR) and proceeded to ASCT; median OS 66 months. In R/R disease (median 3 prior lines), ORR was 27%; two responders underwent ASCT; median OS 6.2 months.
Grade ≥3 AEs were consistent with intensive chemotherapy. In ND pts, febrile neutropenia (26%), sepsis (16%), and abdominal pain (16%) were most common; grade 4 events were infrequent. In R/R patients, febrile neutropenia (57%), lung infection (50%), and sepsis (29%) predominated; grade 4/5 events were uncommon. One pt had grade 3 QTcF prolongation.

### Summary/Conclusion

QUIZ plus CLIA showed encouraging activity and manageable toxicity in FLT3-ITD–mutated and wild-type AML. ND FLT3-ITD pts achieved deep MRD-negative remissions without observed relapses at current follow-up. The study continues to accrue.

**Copyright Information:** (Online) ISSN: 2572-9241
©2026 The Author(s). HemaSphere is published by John Wiley & Sons Ltd on behalf of European Hematology Association. This is an open access Abstract Book distributed under the Attribution-NonCommercial-NoDerivs (CC BY-NC-ND), which allows third parties to download the articles and share them with others as long as they credit the author and the Abstract Book, but they cannot change the content in any way or use them commercially.
**Abstract Book Citations:** Authors, Title, HemaSphere, 2026;10:(S1):pages. The abstract book DOIs can be found on the “Author Information” page.
**Disclaimer:** Articles published in the journal HemaSphere exclusively reflect the opinions of the authors. The authors are responsible for all content in their abstracts including accuracy of the facts, statements, citing resources, etc.

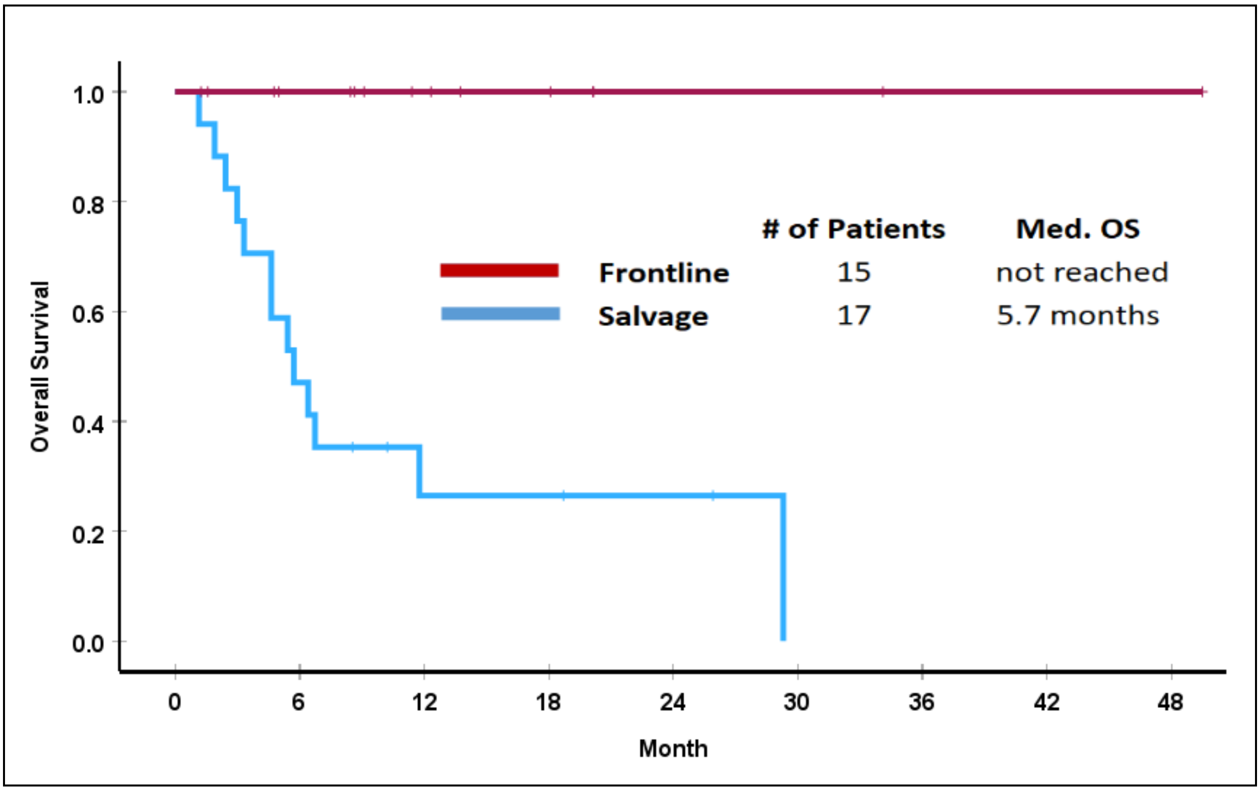

Figure. Overall Survival in FLT3-ITD Mutated Cohort (Frontline vs. Salvage)

**Copyright Information:** (Online) ISSN: 2572-9241
©2026 The Author(s). HemaSphere is published by John Wiley & Sons Ltd on behalf of European Hematology Association. This is an open access Abstract Book distributed under the Attribution-NonCommercial-NoDerivs (CC BY-NC-ND), which allows third parties to download the articles and share them with others as long as they credit the author and the Abstract Book, but they cannot change the content in any way or use them commercially.
**Abstract Book Citations:** Authors, Title, HemaSphere, 2026;10:(S1):pages. The abstract book DOIs can be found on the "Author Information" page.
**Disclaimer:** Articles published in the journal HemaSphere exclusively reflect the opinions of the authors. The authors are responsible for all content in their abstracts including accuracy of the facts, statements, citing resources, etc.

### (PS1622) CLINICAL CHARACTERISTICS AND PROGNOSTIC IMPACT OF TP53 DELETION/IDER(17)(Q10)T(15;17) IN ACUTE PROMYELOCYTIC LEUKEMIA

**Topic:** 04. Acute myeloid leukemia - Clinical

Gen Qing*[1], Yanbo Nie[1], Wen Zhao[1], Yani Lin[1], Shaobin Yang[1]

[1]*Sino-US Diagnostics Lab, Tianjin, China;*

### Background

Acute promyelocytic leukemia (APL) is highly curable with ATRA/ATO-based therapy. It is widely accepted that additional chromosomal abnormalities (ACAs) do not affect the prognosis of APL patients. However, previous large-scale ACA studies that support this conclusion rarely included or adequately represented patients with TP53 deletion. TP53 deletion is a well-established marker of poor prognosis in various cancers.

### Aims

This study aims to investigate the prognostic impact of TP53 deletion in APL patients.

### Methods

APL patients were retrospectively analyzed and divided into TP53-deleted group (with ider(17)(q10)t(15;17)) and control group. Clinical characteristics, cytogenetics, mutation profiles, and survival outcomes were compared between the two groups.

### Results

A total of 415 APL patients were enrolled, including 116 in the TP53-deleted group and 299 in the control group. Regarding baseline characteristics, the TP53-deleted group had significantly higher median WBC compared to the control group (2.9×10^9/L vs 2.27×10^9/L, P=0.013), while Hb and PLT showed no significant differences.Cytogenetically, 29.3% of patients in the TP53-deleted group had additional chromosomal abnormalities, most commonly +8 (11.2%), +ider(17)(q10)t(15;17) (11.2%), and +21 (3.4%); in the control group, 26.8% had additional abnormalities, most commonly +8 (9.0%), 7q- (3%), and 9q- (2.3%).Regarding gene mutations, 72.4% of patients in the TP53-deleted group had detectable somatic mutations, with common mutations including FLT3 (28%), WT1 (17%), NRAS (17%), and ARID1A (14%); in the control group, 72.1% had detectable mutations, with common mutations including FLT3 (47%), WT1 (24%), and NRAS (8%). FLT3 was the most frequent mutation in both groups, but was predominantly missense mutations in the TP53-deleted group versus in-frame insertions in the control group.Survival analysis showed no significant difference in progression-free survival (PFS) between the TP53-deleted group and the control group (P=0.46). Within the TP53-deleted group, no significant PFS difference was observed among different risk stratification categories; however, the intermediate-risk group exhibited a trend toward inferior PFS compared to the low-risk group. For the high-risk group, the lack of statistical significance was likely due to the limited sample size, warranting validation in larger cohorts. Univariate analysis revealed that +8, +ider(17)(q10) karyotypes, and FLT3, WT1, NRAS mutations were not associated with prognosis.

### Summary/Conclusion

This study demonstrates that although the TP53-deleted group presented with higher baseline WBC and a distinct mutation profile, TP53 deletion did not significantly affect PFS in APL patients. In the era of ATRA/ATO-based therapy, TP53 deletion may not constitute an adverse prognostic factor in APL.

**Copyright Information:** (Online) ISSN: 2572-9241
©2026 The Author(s). HemaSphere is published by John Wiley & Sons Ltd on behalf of European Hematology Association. This is an open access Abstract Book distributed under the Attribution-NonCommercial-NoDerivs (CC BY-NC-ND), which allows third parties to download the articles and share them with others as long as they credit the author and the Abstract Book, but they cannot change the content in any way or use them commercially.
**Abstract Book Citations:** Authors, Title, HemaSphere, 2026;10:(S1):pages. The abstract book DOIs can be found on the “Author Information” page.
**Disclaimer:** Articles published in the journal HemaSphere exclusively reflect the opinions of the authors. The authors are responsible for all content in their abstracts including accuracy of the facts, statements, citing resources, etc.

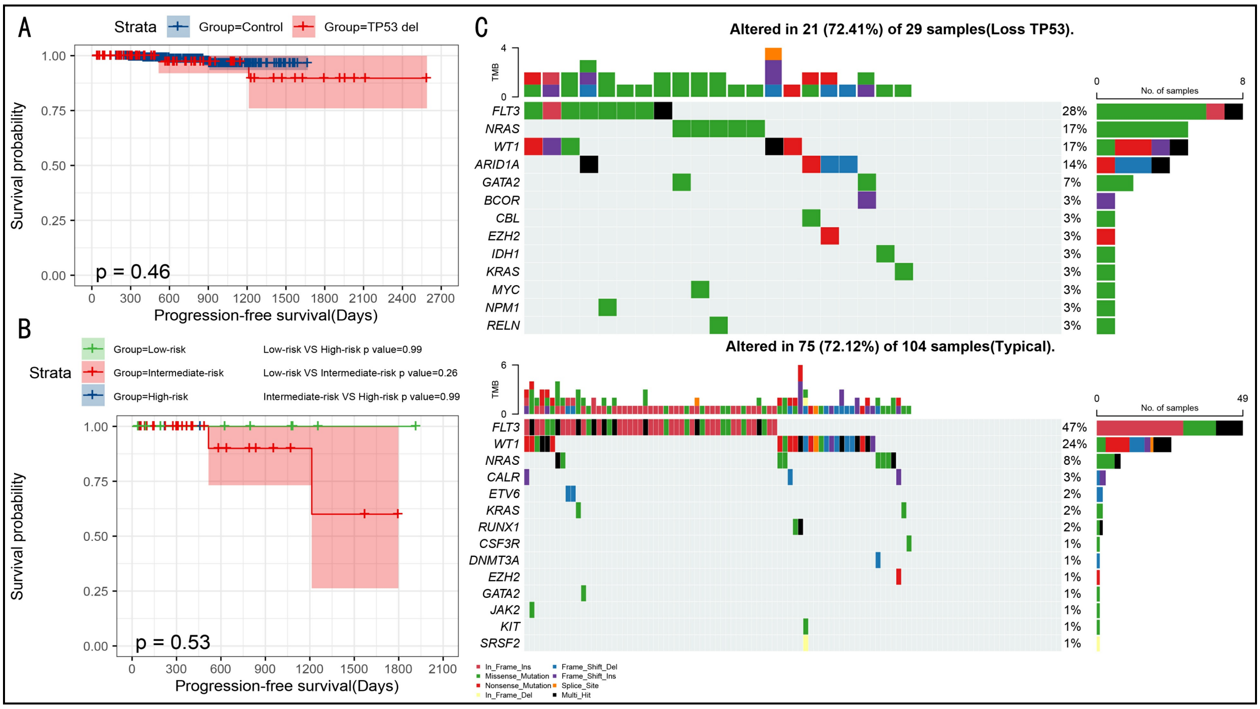

**Copyright Information:** (Online) ISSN: 2572-9241
©2026 The Author(s). HemaSphere is published by John Wiley & Sons Ltd on behalf of European Hematology Association. This is an open access Abstract Book distributed under the Attribution-NonCommercial-NoDerivs (CC BY-NC-ND), which allows third parties to download the articles and share them with others as long as they credit the author and the Abstract Book, but they cannot change the content in any way or use them commercially.
**Abstract Book Citations:** Authors, Title, HemaSphere, 2026;10:(S1):pages. The abstract book DOIs can be found on the "Author Information" page.
**Disclaimer:** Articles published in the journal HemaSphere exclusively reflect the opinions of the authors. The authors are responsible for all content in their abstracts including accuracy of the facts, statements, citing resources, etc.

### (PS1623) THE ROLE OF ALLO-HSCT IN PATIENTS WITH ACUTE MYELOID LEUKEMIA IN COMPLETE MRD-NEGATIVE REMISSION AFTER THE FIRST COURSE OF INDUCTION (RESULTS OF A RANDOMIZED STUDY)

**Topic:** 04. Acute myeloid leukemia - Clinical

Irina Lukianova*[1], Elena Parovichnikova[1], Vera Troitskaya[1], Sergey Bondarenko[2], Chabaeva Julia[1], Larisa Girshova[3], Natalia Minaeva[4], Valeriy Lapin[5], Tatyana Konstantinova[6]

*[1]National Medical Research Center for Hematology, Moscow, Russia;[2]Raisa Gorbacheva Memorial Research Institute for Pediatric Oncology, Saint-Petersburg, Russia; [3]Almazov National Medical Research Centre, Saint-Petersburg, Russia;[4]Kirov Research Institute of Hematology and Blood Transfusion, Kirov, Russia; [5]Yaroslavl Regional Clinical Hospital, Yaroslavl, Russia; [6]Sverdlovsk Regional Clinical Hospital No. 1, Ekaterinburg, Russia;*

### Background

Allogeneic hematopoietic stem cell transplantation (allo-HSCT) is a crucial step in the treatment of acute myeloid leukemia, and its timely implementation plays a crucial role in the effectiveness of AML treatment. In determining the indications for allo-HSCT, in addition to tumor clone characteristics, the minimal residual disease (MRD) status, assessed at certain key stages, also plays a key role.

### Aims

To determine the role of allo-HSCT in AML patients who achieved MRD-negative status after the first 7+3 course (excluding AML with myelodysplasia (AML-MD), *MECOM* gene involvement, and inv16).

### Methods

The Russian multicenter clinical trial AML-21 was launched in February 2021 and is ongoing (ClinTrialGov NCT05339204). At the data cutoff (October 2025), 315 patients with newly diagnosed AML aged 18 to 59 years were enrolled. The median age was 42 years (18;59), there were 177 women and 138 men. Pts with AML-MD and involving the MECOM gene (group C, n=52) received low-intensity programs (Aza/Dec+Ven or AzaIdaAraC), group A (inv16, n=25) and group B (the rest, n=235) - intensive (7+3 No. 2, FLAG No. 2, 5+5 No. 6). MRD was assessed at the central laboratory using flow cytometry (MPC) and PCR (if a marker was available). All pts in Group C were scheduled for early allo-HSCT, while for pts in Group A, allo-HSCT was considered only in the event of relapse. Pts in Group B who achieved an MRD-negative (MPC) complete response after the first 7+3 cycle were randomized to allo-HSCT or continued chemotherapy (CT).

### Results

Median follow-up was 12.7 mo (5 days - 57 months). CR was achieved in 78.8% of pts: group A - 92%, B - 80.6%, C - 63.5%. The proportion of refractory pts was 10%: patients in group C - 21%, B - 8.5%. Early mortality among all was 11.2%. Among 173 pts in group B who achieved CR after 1 course, 133 (73.6%) were MRD-negative. 120 pts were randomized: 60 - to early allo-HSCT (46.7% underwent allo-HSCT with Me from CR to allo-HSCT of 5.5 months), 60 - to CT, the results of relapse-free survival are comparable between the groups (72% and 64%, respectively, p=0,4). MRD-positive pts after the first course (n=14) and those who achieved CR after the second course were scheduled to undergo allo-HSCT. Also, all pts with CR from gr. C were scheduled to undergo allo-HSCT (performed in 69.7% of patients with a Me of 4 months). Four-year OS for the entire cohort of pts was 57%, RFS – 55%. 4-year OS for gr. A was 84%, gr. B – 51%, gr. C – 41%. 2-year RFS for gr. A was 73%, gr. B – 56%, gr. C – 35%. Relapse-free survival of patients from the randomization group was 72% and 64% for the branch with BMT and CT, respectively (p=0.4) (Fig. 1).

**Copyright Information:** (Online) ISSN: 2572-9241
©2026 The Author(s). HemaSphere is published by John Wiley & Sons Ltd on behalf of European Hematology Association. This is an open access Abstract Book distributed under the Attribution-NonCommercial-NoDerivs (CC BY-NC-ND), which allows third parties to download the articles and share them with others as long as they credit the author and the Abstract Book, but they cannot change the content in any way or use them commercially.
**Abstract Book Citations:** Authors, Title, HemaSphere, 2026;10:(S1):pages. The abstract book DOIs can be found on the “Author Information” page.
**Disclaimer:** Articles published in the journal HemaSphere exclusively reflect the opinions of the authors. The authors are responsible for all content in their abstracts including accuracy of the facts, statements, citing resources, etc.

**Summary/Conclusion**

The feasibility of performing allo-HSCT in MRD-negative pts from favorable and intermediate-risk groups remains debated. AML-21 is the only randomized study to examine the role of allo-HSCT in the first CR of AML patients who were MRD-negative after the first induction. Treatment outcomes i n randomized MRD-negative pts who underwent allo-HSCT with a median of 5.5 months from achieving remission are comparable to those in those who did not achieve remission. Early allo-HSCT in a higher proportion of MRD-positive pts improves treatment outcomes in this cohort; a similar conclusion applies to Group C.

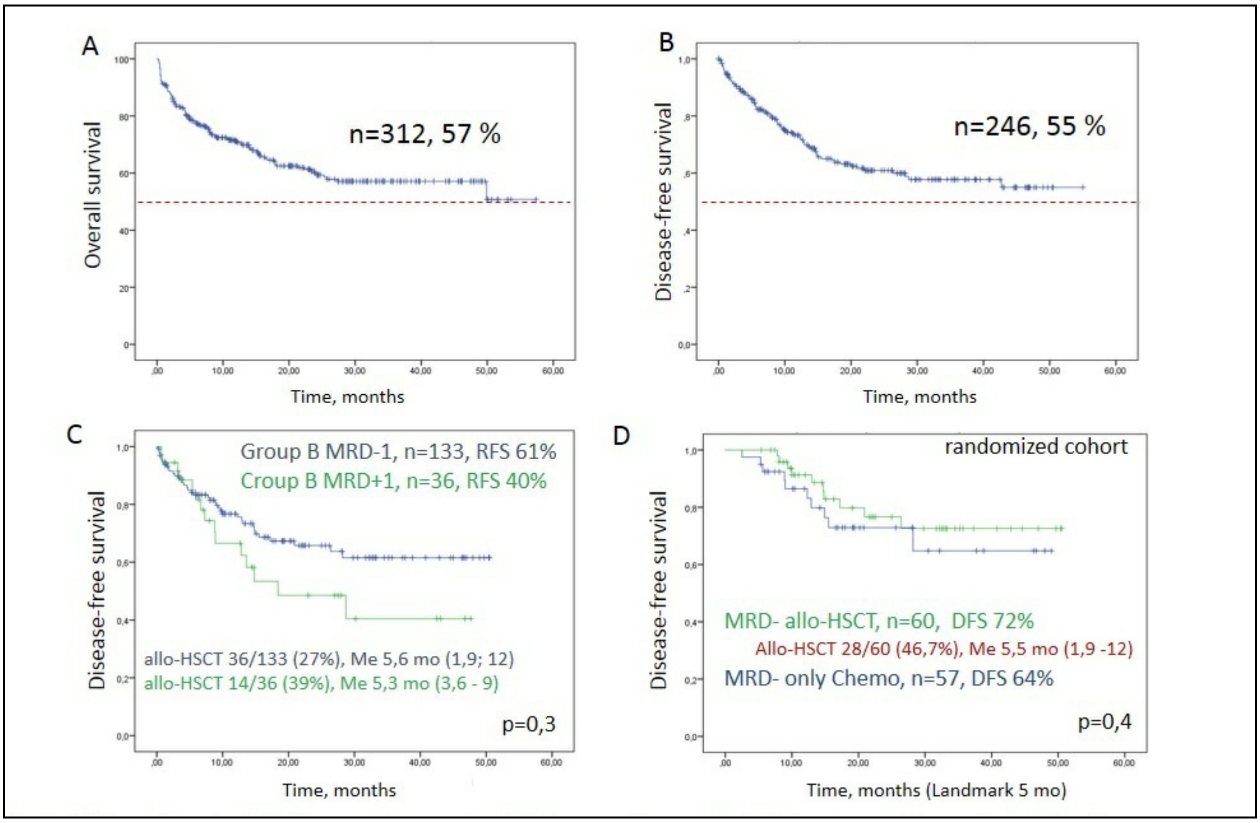

Fig. 1. (A) Overall survival; (B) Desease-free survival; (C) Desease-free survival of pts in group B in remission after 1 course depending on MRD status; (D) Desease-free survival of a randomized cohort of MRD-negative patients surviving 5 months (Landmark)

**Copyright Information:** (Online) ISSN: 2572-9241
©2026 The Author(s). HemaSphere is published by John Wiley & Sons Ltd on behalf of European Hematology Association. This is an open access Abstract Book distributed under the Attribution-NonCommercial-NoDerivs (CC BY-NC-ND), which allows third parties to download the articles and share them with others as long as they credit the author and the Abstract Book, but they cannot change the content in any way or use them commercially.
**Abstract Book Citations:** Authors, Title, HemaSphere, 2026;10:(S1):pages. The abstract book DOIs can be found on the “Author Information” page.
**Disclaimer:** Articles published in the journal HemaSphere exclusively reflect the opinions of the authors. The authors are responsible for all content in their abstracts including accuracy of the facts, statements, citing resources, etc.

### (PS1624) PROGNOSTIC MODEL FOR NEWLY DIAGNOSED M4/M5 ACUTE MYELOID LEUKEMIA TREATED WITH VENETOCLAX AND AZACITIDINE IN THE REAL-WORLD

**Topic:** 04. Acute myeloid leukemia - Clinical

Dan Wang*[1], Yuxin Li[1], Danchen Meng[1], Dinghui Zhao[1], Zhengfeng Hou[1], Jichun Yang[2], Zhenqi Huang[1], Xiaofeng Shi[3], Zhangbiao Long[1]

*[1]The First Affiliated Hospital of Anhui Medical University, Hefei, China;[2]Tianjin University of Traditional Chinese Medicine, Tianjin, China; [3]The Second Affiliated Hospital of Nanjing Medical University, Nanjing, China;*

### Background

Venetoclax combined with azacitidine (VEN+AZA) has become the standard treatment for older patients with newly diagnosed acute myeloid leukemia (AML) who are unfit for intensive chemotherapy. However, the differences in efficacy across AML subtypes remain unclear.

### Aims

This study aimed to explore independent risk factors influencing overall survival (OS) and to develop a clinical nomogram model for OS in patients with the monocytic differentiation subtype.

### Methods

Patients with newly diagnosed AML who received first-line VEN+AZA treatment were divided into a monocytic differentiation (MD) group and a non-monocytic differentiation (non-MD) group. The evaluated outcomes included composite complete remission (cCR: CR/CRi) rates, measurable residual disease (MRD) negativity rates, OS, and event-free survival (EFS). Furthermore, patients in the MD group were randomly divided into a training cohort and a validation cohort at a 7:3 ratio. In the training cohort, univariate and multivariate regression Cox analyses were performed to identify independent prognostic factors for OS and a clinical prediction model was then constructed.

### Results

A total of 262 AML patients were enrolled in this study, with 174 patients (66.4%) in the MD group and 88 patients (33.6%) in the non-MD group. The cCR rate in the MD group was significantly lower than that in the non-MD group (61.5% *vs.* 79.5%, $P$ = 0.003). The MRD negativity rate was also significantly lower (41.4% *vs.* 56.8%, $P$ = 0.018). During a median follow-up of 19.5 months, the median OS and median EFS for the entire cohort were 12.0 months and 7.0 months, respectively. The non-MD group achieved significantly longer OS and EFS compared to the MD group (18.0 months *vs.* 9.5 months, $P$ = 0.0007; 10.0 months *vs.* 5.8 months, $P$ = 0.0039). The median OS and EFS in the M4 subgroup were 12.7 months and 5.65 months, respectively, both significantly inferior to those in the non-MD group ( $P$ = 0.035 and $P$ = 0.04, respectively). Similarly, the median OS and median EFS in the M5 subgroup were 9.0 months and 6.0 months, respectively, also significantly shorter than those in the non-MD group ( $P$ < 0.0001 and $P$ = 0.0495, respectively). No significant differences in treatment response or survival were observed between the M4 and M5 subgroups. In the MD group, prior MDS/MPN, LDH level before treatment, complex karyotype, and TET2 mutation were identified as independent risk factors for EFS. In addition to these four factors, DNMT3A wild-type was associated with shorter OS. A clinical prediction model for OS was successfully developed based on these five factors. The model demonstrated good discriminative ability, calibration, and clinical net benefit, as evidenced by the concordance index (0.78 in the training cohort and 0.76 in the validation cohort), the time-dependent area under the receiver operating characteristic curve (0.827, 0.775, 0.806, and 0.751 at 6, 12, 18, and 24 months, respectively), and the plotted calibration curves and clinical decision curves.

**Copyright Information:** (Online) ISSN: 2572-9241
©2026 The Author(s). HemaSphere is published by John Wiley & Sons Ltd on behalf of European Hematology Association. This is an open access Abstract Book distributed under the Attribution-NonCommercial-NoDerivs (CC BY-NC-ND), which allows third parties to download the articles and share them with others as long as they credit the author and the Abstract Book, but they cannot change the content in any way or use them commercially.
**Abstract Book Citations:** Authors, Title, HemaSphere, 2026;10:(S1):pages. The abstract book DOIs can be found on the "Author Information" page.
**Disclaimer:** Articles published in the journal HemaSphere exclusively reflect the opinions of the authors. The authors are responsible for all content in their abstracts including accuracy of the facts, statements, citing resources, etc.

**Summary/Conclusion**

Patients with monocytic differentiation AML who received VEN+AZA treatment had significantly inferior treatment response and survival outcomes compared to those with non-monocytic differentiation. A nomogram model constructed based on the five independent risk factors mentioned above can provide reliable individualized predictions of OS for these patients. This model may assist clinicians in identifying patients with poor prognoses and facilitate timely adjustments to treatment strategies.

**Copyright Information:** (Online) ISSN: 2572-9241
©2026 The Author(s). HemaSphere is published by John Wiley & Sons Ltd on behalf of European Hematology Association. This is an open access Abstract Book distributed under the Attribution-NonCommercial-NoDerivs (CC BY-NC-ND), which allows third parties to download the articles and share them with others as long as they credit the author and the Abstract Book, but they cannot change the content in any way or use them commercially.
**Abstract Book Citations:** Authors, Title, HemaSphere, 2026;10:(S1):pages. The abstract book DOIs can be found on the "Author Information" page.
**Disclaimer:** Articles published in the journal HemaSphere exclusively reflect the opinions of the authors. The authors are responsible for all content in their abstracts including accuracy of the facts, statements, citing resources, etc.

### (PS1625) ACUTE MYELOID LEUKEMIA (AML) PATIENT, DISEASE, AND MOLECULAR CHARACTERISTICS ASSOCIATED WITH A LONG-TERM RESPONSE TO OLUTASIDENIB

**Topic:** 04. Acute myeloid leukemia - Clinical

Stéphane De Botton*[1], Brian A. Jonas[2], Arnaud Pigneux[3], Andrew H. Wei[4], Christian Recher[5], Jürgen Krauter[6], Amber Thomassen[7], Hua Tian[7], Jorge Cortes[8], Justin M. Watts[9]

*[1]Gustave Roussy Institute, Clinical Hematology, Villejuif, France; [2]University of California Davis School of Medicine, Division of Malignant Hematology/Cellular Therapy and Transplantation, Department of Internal Medicine, Sacramento, United States of America; [3]University of Bordeaux, Department of Clinical Hematology and Cell Therapy, CHU Bordeaux, Bordeaux, France;[4]The Alfred Hospital, Department of Haematology, Melbourne, Australia; [5]University of Toulouse, Department of Hematology, CHU de Toulouse, Toulouse University Cancer Institute - Oncopole, Toulouse, France; [6]Braunschweig Municipal Hospital, Medical Clinic III, Hematology and Oncology, Braunschweig, Germany; [7]Rigel Pharmaceuticals, Inc., South San Francisco, United States of America; [8]Augusta University, Department of Medicine, Georgia Cancer Center, Augusta, United States of America;[9]University of Miami Sylvester Comprehensive Cancer Center, Division of Hematology, Department of Medicine, Miami, United States of America;*

### Background

Mutations in isocitrate dehydrogenase 1 (m*IDH1*) occur in 7%-14% of patients with AML. Olutasidenib is a potent, oral small-molecule mIDH1 inhibitor that selectively inhibits IDH1 variants but preserves wild-type IDH1 function. The registrational phase 2 trial (NCT02719574) of olutasidenib in patients with relapsed/refractory (R/R) m*IDH1* AML reported a complete remission (CR)/CR with partial hematologic recovery (CRh) rate of 35% and a median CR/CRh duration of 25.3 mo.

### Aims

This analysis examines demographic, disease, and molecular characteristics of patients with a long-term CR/CRh response to olutasidenib without transplant.

### Methods

Consenting adults with R/R m*IDH1* AML treated with olutasidenib 150 mg twice daily who had a CR/CRh and a duration of response (DOR) >12 mo were included. Overall survival (OS), DOR, event-free survival (EFS), and adverse events (AEs) were assessed. Univariate and multivariate logistic regression analyses were used to examine baseline factors that predicted DOR >12 vs ≤12 mo.

**Copyright Information:** (Online) ISSN: 2572-9241
©2026 The Author(s). HemaSphere is published by John Wiley & Sons Ltd on behalf of European Hematology Association. This is an open access Abstract Book distributed under the Attribution-NonCommercial-NoDerivs (CC BY-NC-ND), which allows third parties to download the articles and share them with others as long as they credit the author and the Abstract Book, but they cannot change the content in any way or use them commercially.
**Abstract Book Citations:** Authors, Title, HemaSphere, 2026;10:(S1):pages. The abstract book DOIs can be found on the “Author Information” page.
**Disclaimer:** Articles published in the journal HemaSphere exclusively reflect the opinions of the authors. The authors are responsible for all content in their abstracts including accuracy of the facts, statements, citing resources, etc.

### Results

Of 147 evaluable patients, 51 (35%) achieved a CR/CRh; of these, 32 (63%) maintained a CR/CRh >12 mo. Excluding 6 patients who proceeded to transplant, 26 (51%) had a DOR >12 mo (DOR range, 13.5-54.6 mo) (**Table**). Among these patients at baseline, median age was 72 y; 69% were female; 73% had an R132C mutation; 12% had prior venetoclax; 88% were platelet transfusion independent (TI); 73% were red blood cell (RBC) TI. Patients had a median (range) of 2 (0, 5) comutations, most commonly *DNMT3A* (n=6) or *NPM1* (n=5); 2 patients with *NPM1* also had *FLT3* mutations. Median (95% CI) time to response was 1.9 (1.0, 1.9) mo. Median OS and EFS were not reached. Estimated 48-mo OS was 74% (95% CI: 51%, 88%). Estimated 48-mo EFS was 69% (95% CI: 47%, 83%). Five (19%) patients relapsed (including 1 after 24 mo); 10 (38%) patients were on treatment at data lock. Significant predictors of response >12 vs ≤12 mo by multivariate analysis were relapsed AML vs refractory AML (odds ratio [OR]: 0.19, *P*=0.02) and female sex (OR: 0.21; *P*=0.01). Age, number of comutations, m*IDH1* variant, RTK pathway mutations, number of prior therapies, and baseline TI were not significant predictors. Common AEs were nausea (35%), increased alanine aminotransferase (ALT), asthenia, constipation, and cough (31% each). Common grade ≥3 AEs were increased ALT, gamma glutamyltransferase increased (19% each), and RBC count decreased (15%). Nine (35%) patients experienced grade ≥3 all-cause AEs after 12 mo (only 1 AE in >1 patient [Coronavirus infection]).

### Summary/Conclusion

Olutasidenib enabled long-term CR/CRh in half of R/R m*IDH1* AML patients with CR/CRh without transplant (longest response >54 mo). Multivariate analysis identified relapsed (vs refractory) AML and female sex but not baseline TI or molecular factors as a significant predictor of long-term response with olutasidenib.

| DOR Group | Patients, n (%) |
|---|---|
| >12 mo | 26 (100) |
| >24 mo | 19 (73) |
| >36 mo | 15 (58) |
| >48 mo | 5 (19) |

**Copyright Information:** (Online) ISSN: 2572-9241
©2026 The Author(s). HemaSphere is published by John Wiley & Sons Ltd on behalf of European Hematology Association. This is an open access Abstract Book distributed under the Attribution-NonCommercial-NoDerivs (CC BY-NC-ND), which allows third parties to download the articles and share them with others as long as they credit the author and the Abstract Book, but they cannot change the content in any way or use them commercially.
**Abstract Book Citations:** Authors, Title, HemaSphere, 2026;10:(S1):pages. The abstract book DOIs can be found on the “Author Information” page.
**Disclaimer:** Articles published in the journal HemaSphere exclusively reflect the opinions of the authors. The authors are responsible for all content in their abstracts including accuracy of the facts, statements, citing resources, etc.

### (PS1626) LARGE LANGUAGE MODEL-ENHANCED CLINICAL RECORD ANNOTATION SUPPORTS RISK STRATIFICATION AND OUTCOME ANALYSES FOR AML PATIENTS TREATED WITH VENETOCLAX/AZACITIDINE FRONTLINE THERAPY

**Topic:** 04. Acute myeloid leukemia - Clinical

Ali Kamali[1], Kuo-Kai Chin[2], Baher Krayem[2], Ettaib Elmarabti[3], Alexander Chan[2], Khawaja Bilal[2], Chad Vanderbilt[2], Justin Jee[2], Ahmet Dogan[2], Eytan Stein[2], Gregory Goldgof[2], Xin Wang*[2]

*[1]Memorial Sloan Kettering Cancer Center, Pathology and Laboratory Medicine, New York, United States of America;[2]Memorial Sloan Kettering Cancer Center, Medicine, New York, United States of America; [3]Weill Cornell Medicine/NewYork-Presbyterian Hospital, New York, United States of America;*

### Background

Venetoclax/azacitidine (VEN/AZA) is increasingly used as backbone therapy for older/frail patients (pts) with AML. Although VEN/AZA-specific risk stratification tools have been developed, large-scale external validation is limited due to the labor-intensive nature of manual abstraction from unstructured records.

### Aims

We developed a large language model (LLM)-based pipeline to convert free-text clinical data into structured datasets for AML outcome analysis and comparative validation of existing VEN/AZA risk scores.

### Methods

We developed the Hematologic Multimodal Analytics Platform (HeMAP) in Python for LLM-assisted abstraction of disease trajectories in AML pts (Fig 1A). The pipeline collated demographics, pathology, molecular and cytogenetic reports, oncology notes, and HSCT records for pts with new AML treated with VEN/AZA as 1L therapy at our center (2018-2024). We used Llama 3.3 70B Instruct and Llama 4 Maverick 17B Instruct across pipeline components. Sequential LLM prompts were applied to extract clinical variables of interest. The LLM abstractions, along with available structured data, were combined with rule-based algorithms. Outputs were validated against a curated ground truth by expert clinicians.

**Copyright Information:** (Online) ISSN: 2572-9241
©2026 The Author(s). HemaSphere is published by John Wiley & Sons Ltd on behalf of European Hematology Association. This is an open access Abstract Book distributed under the Attribution-NonCommercial-NoDerivs (CC BY-NC-ND), which allows third parties to download the articles and share them with others as long as they credit the author and the Abstract Book, but they cannot change the content in any way or use them commercially.
**Abstract Book Citations:** Authors, Title, HemaSphere, 2026;10:(S1):pages. The abstract book DOIs can be found on the “Author Information” page.
**Disclaimer:** Articles published in the journal HemaSphere exclusively reflect the opinions of the authors. The authors are responsible for all content in their abstracts including accuracy of the facts, statements, citing resources, etc.

### Results

A total of 159 pts were identified. Validation (30 randomly sampled cases) showed high concordance with expert annotations: AML diagnosis date 96.7%; ELN2022 96.7%, ELN2024 100%, BEAT-AML 93.3%, Mayo 93.3% risk classifications; best ELN2022 response 70% concordant. VEN/AZA initiation date was correctly extracted within 14 days in 93.7% of pts.
Subsequent analyses were performed in the full 159-pt cohort using HeMAP-derived variables. Median age was 75 years. Median number of VEN/AZA cycles received was 2 (IQR 1-3). Composite complete remission (CRc) was achieved in 69% (n=102), flow MRD- in 33% (n=68), and HSCT in first remission in 25% of pts. The median number of cycles to CRc was 1 (IQR 1-2, range 1-6) and to flow MRD- was also 1 (IQR 1, range 1-6) across all risk models. BEAT-AML, ELN2024, and Mayo successfully stratified rate of CRc, whereas BEAT-AML, ELN2024, and ELN2022 successfully stratified rate of flow MRD-, with BEAT-AML being best for both (CRc: AUC 0.60, CI 0.51-0.69; flow MRD-: AUC 0.65, CI 0.57-0.73).
At median follow up of 44.8 months, median OS was 19.5 months. ELN2024 (C-index 0.62, p<0.001), BEAT-AML (C-index 0.60, p<0.001), and Mayo (C-index 0.57, p=0.01) models significantly stratified OS, whereas ELN2022 did not (C-index = 0.52, p=0.58) [Fig1B]. These associations persisted in analyses stratified by prior HMA exposure and by age (≥70 vs <70), except Mayo lost significance in patients ≥70.
In pts who achieved CRc, no difference in OS was noted between pts who achieved CR after 1 cycle or 2+ (HR 0.84, CI 0.47-1.44, for 2+) or those who achieved flow MRD- after 1 cycle or 2+ (HR 1.34 for, CI 0.64-2.77 for 2+). HSCT after CRc was associated with superior survival with HSCT as a time-varying covariate (HR 0.2, CI 0.07-0.34, p<0.001), including in ELN2024 low-risk pts (HR 0.13, CI 0.04-0.42).

### Summary/Conclusion

The LLM-powered pipeline accurately extracted complex clinical data and enabled robust real-world analyses of VEN/AZA treated AML. ELN2024, BEAT-AML, and Mayo models were prognostic for survival, and BEAT-AML was the best for CRc and flow MRD- stratification. HSCT was associated with improved survival in pts who achieved CRc. This AI-enabled approach may facilitate real-time, data-driven risk stratification, and evaluation of novel therapies.

**Copyright Information:** (Online) ISSN: 2572-9241
©2026 The Author(s). HemaSphere is published by John Wiley & Sons Ltd on behalf of European Hematology Association. This is an open access Abstract Book distributed under the Attribution-NonCommercial-NoDerivs (CC BY-NC-ND), which allows third parties to download the articles and share them with others as long as they credit the author and the Abstract Book, but they cannot change the content in any way or use them commercially.
**Abstract Book Citations:** Authors, Title, HemaSphere, 2026;10:(S1):pages. The abstract book DOIs can be found on the "Author Information" page.
**Disclaimer:** Articles published in the journal HemaSphere exclusively reflect the opinions of the authors. The authors are responsible for all content in their abstracts including accuracy of the facts, statements, citing resources, etc.

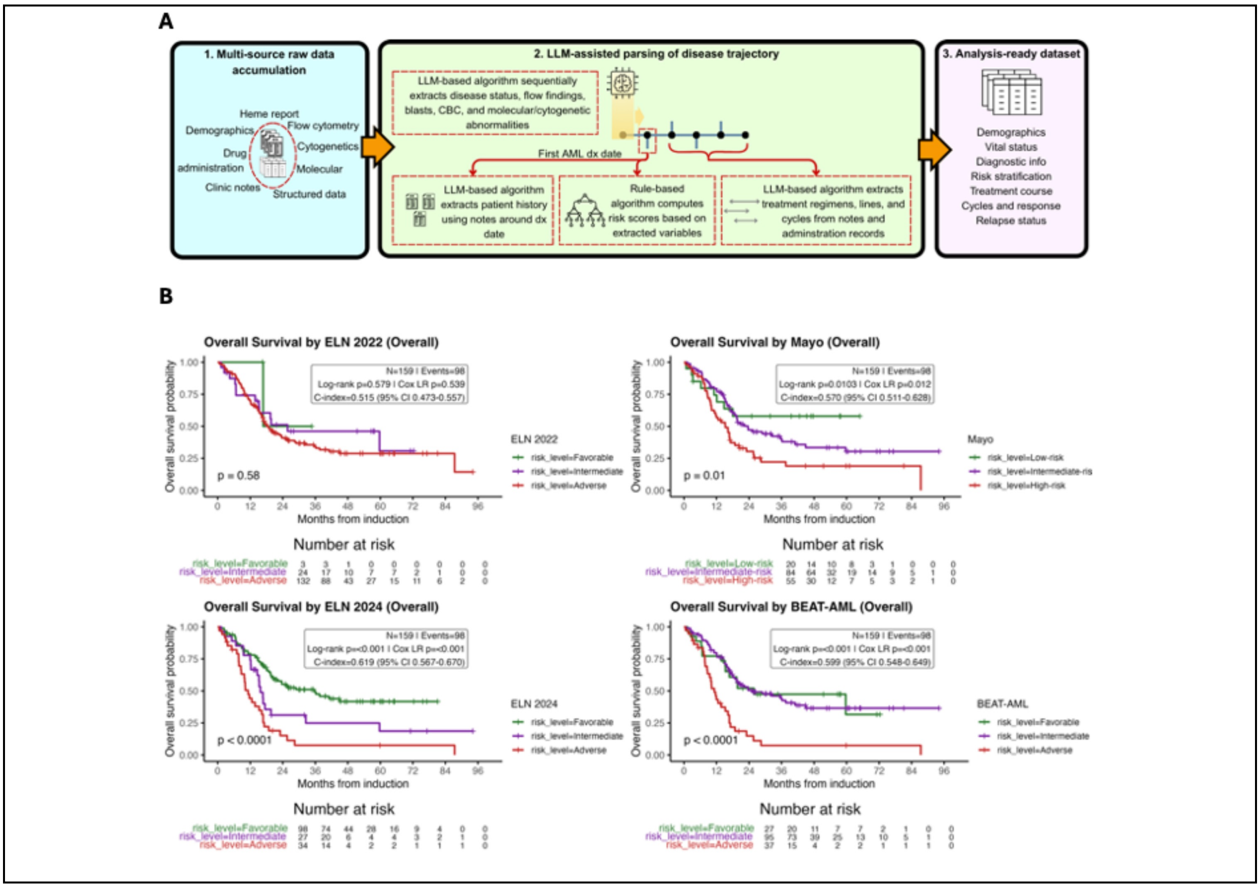

Figure 1A. HeMAP LLM-assisted abstraction algorithm. Figure 1B. Overall survival by existing risk stratification models.

**Copyright Information:** (Online) ISSN: 2572-9241
©2026 The Author(s). HemaSphere is published by John Wiley & Sons Ltd on behalf of European Hematology Association. This is an open access Abstract Book distributed under the Attribution-NonCommercial-NoDerivs (CC BY-NC-ND), which allows third parties to download the articles and share them with others as long as they credit the author and the Abstract Book, but they cannot change the content in any way or use them commercially.
**Abstract Book Citations:** Authors, Title, HemaSphere, 2026;10:(S1):pages. The abstract book DOIs can be found on the "Author Information" page.
**Disclaimer:** Articles published in the journal HemaSphere exclusively reflect the opinions of the authors. The authors are responsible for all content in their abstracts including accuracy of the facts, statements, citing resources, etc.

### (PS1627) LONG-TERM FOLLOW UP OF ORAL DECITABINE/CEDAZURIDINE (ASTX727) PLUS VENETOCLAX IN NEWLY DIAGNOSED ACUTE MYELOID LEUKEMIA: A PHASE 2 STUDY

**Topic:** 04. Acute myeloid leukemia - Clinical

Thura Win Htut*[1], Jeannot Kekedjian[1], Nicholas Short[1], Alexandre Bazinet[1], Yesid Alvarado[1], Alex Bataller[1], Ghayas Issa[1], Abhishek Maiti[1], Musa Yilmaz[1], Nitin Jain[1], Lucia Masarova[1], Steven Kornblau[1], Elias Jabbour[1], Guillermo Montalban-Bravo[1], Sherry Pierce[1], Courtney Dinardo[1], Tapan Kadia[1], Naval Daver[1], Marina Konopleva[2], Xuelin Huang[3], Guillermo Garcia-Manero[1], Farhad Ravandi[1]

*[1]The University of Texas MD Anderson Cancer Center, Department of leukemia, Houston, United States of America;[2]Albert Einstein College of Medicine, Department of oncology, New York, United States of America; [3]The University of Texas MD Anderson Cancer Center, Department of biostatistics, Houston, United States of America;*

### Background

Older or unfit patients with newly diagnosed acute myeloid leukemia (NDAML) have poor outcomes, particularly those with secondary AML (sAML).

### Aims

We report extended follow-up of a phase II study evaluating fully oral decitabine/cedazuridine plus venetoclax, focusing on outcomes by de novo vs secondary NDAML.

### Methods

A total of 68 pts with NDAML (32 de novo; 36 secondary) were included in our single-center phase II study between March 2021 and July 2025. Treatment consisted of ASTX727 ( cedazuridine 100 mg/decitabine 35 mg) orally for 5 days and venetoclax 400 mg orally for 21-28 days in 28-day cycles. Primary outcomes were overall survival (OS) and relapse-free survival (RFS), which were estimated by using the Kaplan-Meier method and compared by log-rank test. Responses were assessed per ELN (European LeukemiaNet) 2022 criteria; MRD was evaluated by multiparameter flow cytometry. Cumulative incidence of relapse (CIR), and non-relapse mortality (NRM) were analyzed and compared by Gray' test.

**Copyright Information:** (Online) ISSN: 2572-9241
©2026 The Author(s). HemaSphere is published by John Wiley & Sons Ltd on behalf of European Hematology Association. This is an open access Abstract Book distributed under the Attribution-NonCommercial-NoDerivs (CC BY-NC-ND), which allows third parties to download the articles and share them with others as long as they credit the author and the Abstract Book, but they cannot change the content in any way or use them commercially.
**Abstract Book Citations:** Authors, Title, HemaSphere, 2026;10:(S1):pages. The abstract book DOIs can be found on the "Author Information" page.
**Disclaimer:** Articles published in the journal HemaSphere exclusively reflect the opinions of the authors. The authors are responsible for all content in their abstracts including accuracy of the facts, statements, citing resources, etc.

### Results

Baseline characteristics were similar between 2 groups. 11 pts in sAML cohort had previous exposure to HMAs. Overall, median age was 80 years (76–83); 50% had ECOG performance status 2–3. TP53 mutations were detected in 12 pts (18%) and complex karyotypes in 18 pts (26%); similar incidence between 2 groups.

With median follow-up of 32 months (26–NR), overall response rate (ORR) was 75% (24/32) in de novo AML and 58% (21/36) in sAML. Composite CR (CR/CRi) and CR were observed in 75% and 56% in de novo AML vs 50% and 31% in sAML. MRD negativity among CR/CRi responders was similar (58% de novo vs 56% sAML). Median time-to-response was 41 days in both cohorts (29- 56) (Table 1).

Median OS was 12.7 months in de novo AML and 7.2 months in sAML ($p$=0.61). 1-year and 2-year OS rates were 51% and 17% in de novo AML vs 42% and 31% in sAML ($p$=0.61). Among pts achieving CR/CRi, RFS was comparable between 2 groups, with median RFS of 9.2 months in de novo AML and 11.7 months in sAML ($p$=0.56); 1 year RFS rates were 32% and 48% ($p$=0.56).

Competing-risk analysis showed that 1-year CIR was higher in de novo AML (64%) than in sAML (39%), whereas 1-year NRM was higher in sAML (19% vs 4%) (p=0.15). 4-week and 8-week mortality were 8% and 22% in sAML vs 6.2% each in de novo AML. Relapse was the predominant cause of failure in de novo responders, whereas death in remission contributed substantially in sAML.

ELN 2024 maintains strong, validated prognostic discrimination (median OS 15 vs 9 vs 2 months for favorable, intermediate, adverse; $p$<0.01). A molecular prognostic risk signature (mPRS) further refined survival (median OS 16 vs 9 vs 2 months for higher, intermediate, lower mPRS benefit groups; $p$<0.01).

Grade ≥3 AEs occurred at 66%, mainly thrombocytopenia 15% and febrile neutropenia 12%. Venetoclax dose reductions by cycle 2 were required in 59%, reflecting expected myelosuppression.

### Summary/Conclusion

Oral decitabine/cedazuridine and venetoclax achieved high remission rates in this high-risk elderly NDAML cohort. Pts with de novo AML demonstrated higher response but relapse-dominant failure, whereas those with sAML had increased early and non-relapse mortality, reflecting lower competing early mortality in de novo cohort. Molecular risk stratification remained prognostic. These data support biologically informed and toxicity-adapted optimization of fully oral ASTX727/venetoclax therapy in older AML.

**Copyright Information:** (Online) ISSN: 2572-9241
©2026 The Author(s). HemaSphere is published by John Wiley & Sons Ltd on behalf of European Hematology Association. This is an open access Abstract Book distributed under the Attribution-NonCommercial-NoDerivs (CC BY-NC-ND), which allows third parties to download the articles and share them with others as long as they credit the author and the Abstract Book, but they cannot change the content in any way or use them commercially.
**Abstract Book Citations:** Authors, Title, HemaSphere, 2026;10:(S1):pages. The abstract book DOIs can be found on the "Author Information" page.
**Disclaimer:** Articles published in the journal HemaSphere exclusively reflect the opinions of the authors. The authors are responsible for all content in their abstracts including accuracy of the facts, statements, citing resources, etc.

| | De novo NDAML (N=32) | Secondary NDAML (N=36) |
|---|---|---|
| ORR† | 24 (75%; 57–89) | 21 (58%; 41–75) |
| CRc | 24 (75%; 57–89) | 18 (50%; 33–67) |
| CR | 18 (56%; 38–74) | 11(31%; 16–48) |
| CRi | 6 (19%; 7–36) | 7 (19%; 8–36) |
| MLFS | 0 | 3 (8%; 2–23) |
| PR | 0 | 0 |
| 4-week mortality | 2 (6%; 1–21) | 3 (8%; 2–23) |
| 8-week mortality | 2 (6%; 1–21) | 8 (22%; 10–39) |
| MRD negativity (all patients) | 14 (44%; 27-61) | 10 (28%; 14-45) |
| MRD negativity (CR/CRi) | 14 (58%;37-78) | 10 (56%; 31-79) |
| Cycles to first response | 1 (1–1) | 1 (1–1) |
| Cycles to best response | 1 (1–1) | 1 (1–1) |
| Total cycles given | 4 (2–7) | 3 (1–8) |

Data are n (%; 95% CI) or median (IQR).

†Overall response rate (ORR) is the sum of complete remission (CR), complete remission with incomplete blood count recovery (CRi), partial remission (PR), and morphologic leukemia-free state (MLFS). CRc denotes composite complete remission (CR + CRi).

Table 1. Responses in evaluable patients

**Copyright Information:** (Online) ISSN: 2572-9241
©2026 The Author(s). HemaSphere is published by John Wiley & Sons Ltd on behalf of European Hematology Association. This is an open access Abstract Book distributed under the Attribution-NonCommercial-NoDerivs (CC BY-NC-ND), which allows third parties to download the articles and share them with others as long as they credit the author and the Abstract Book, but they cannot change the content in any way or use them commercially.
**Abstract Book Citations:** Authors, Title, HemaSphere, 2026;10:(S1):pages. The abstract book DOIs can be found on the "Author Information" page.
**Disclaimer:** Articles published in the journal HemaSphere exclusively reflect the opinions of the authors. The authors are responsible for all content in their abstracts including accuracy of the facts, statements, citing resources, etc.

### (PS1628) FRONTLINE LOW-INTENSITY VAH ACHIEVES HIGH MRD-NEGATIVE REMISSION RATES IN YOUNGER PATIENTS WITH NEWLY DIAGNOSED AML

**Topic:** 04. Acute myeloid leukemia - Clinical

Yannan Jia*[1], Jun Yang[1], Xinxin Xia[1], Yu Cai[1], Yin Tong[1], Baoxia Dong[1], Xianmin Song[1]

[1] *Shanghai General Hospital, Shanghai Jiaotong University School of Medicine, Shanghai, China;*

### Background

Venetoclax-based combinations have transformed AML treatment, primarily for unfit patients. However, effective frontline options for younger, fit patients remain an unmet need. Homoharringtonine (HHT) shows synergistic activity with both venetoclax and azacitidine. The triplet regimen VAH has demonstrated promise in relapsed/refractory AML, but its efficacy and safety as frontline induction in younger patients are unknown.

### Aims

To evaluate the real-world effectiveness and safety of VAH as frontline induction therapy in fit patients aged <65 years with newly diagnosed (ND) AML.

### Methods

This single-center retrospective study included 110 consecutive ND AML patients treated with VAH between August 2022 and November 2025 (Table). Eligible patients were 14–65 years with ECOG ≤2. The regimen consisted of venetoclax (100mg d1, 200mg d2, 400mg d3-28), azacitidine (75mg/m² d1-7), and HHT (1mg/m² d1-7) in 28-day cycles. Primary endpoint: composite complete remission (CRc) after cycle 1 (C1). Secondary endpoints: measurable residual disease (MRD) negativity (by MFC, 0.01% threshold), overall survival (OS), event-free survival (EFS), and safety.

### Results

The median age was 47 years (range, 14–65); 40% were ELN favorable, 20% intermediate, and 40% adverse risk, reflecting a high-risk real-world population.
After one cycle, CRc rate was 86.4% (95/110), with 69.5% of responders achieving MRD-negativity. Responses deepened after cycle 2 (C2), with CRc increasing to 97.5% (79/81) and MRD-negativity to 83.5% (**Figure**). ELN risk significantly impacted C1 CRc (95.5% favorable, 90.9% intermediate, 75.0% adverse; $P$=0.02) and C2 MRD-negativity (93.9%,76.5%,75.9%; $P$<0.001). Multivariable analysis identified ELN non-favorable risk as the sole independent predictor of C1 MRD-positivity (OR 3.21, $P$=0.021). High activity was noted in FLT3-ITD-mutated patients (94.4% C1 CRc). C2 MRD-negativity was strongly associated with superior outcomes.
With a median follow-up of 314 days, estimated 1-year OS and EFS were 86.9% and 73.4%, respectively. EFS was significantly stratified by ELN risk (1-year: 86.5% favorable, 86.4% intermediate, 53.6% adverse; $P$<0.01). Achieving C2 MRD-negativity was associated with significantly superior EFS ($P$<0.001) and OS ($P$=0.031). In multivariable analysis, C1 MRD-positivity (HR 3.90, $P$=0.001) and ELN risk (HR 2.12, $P$=0.003) were independent predictors of inferior EFS.
Grade ≥3 hematologic toxicities were frequent but manageable: neutropenia (91.8%), febrile neutropenia (36.4%), with two infection-related deaths (1.8%).

**Copyright Information:** (Online) ISSN: 2572-9241
©2026 The Author(s). HemaSphere is published by John Wiley & Sons Ltd on behalf of European Hematology Association. This is an open access Abstract Book distributed under the Attribution-NonCommercial-NoDerivs (CC BY-NC-ND), which allows third parties to download the articles and share them with others as long as they credit the author and the Abstract Book, but they cannot change the content in any way or use them commercially.
**Abstract Book Citations:** Authors, Title, HemaSphere, 2026;10:(S1):pages. The abstract book DOIs can be found on the "Author Information" page.
**Disclaimer:** Articles published in the journal HemaSphere exclusively reflect the opinions of the authors. The authors are responsible for all content in their abstracts including accuracy of the facts, statements, citing resources, etc.

**Summary/Conclusion**

Frontline VAH is a highly effective and tolerable induction regimen for fit patients with ND AML, achieving rapid and deep MRD-negative remissions across ELN risk groups, including adverse-risk and FLT3-ITD-mutated disease. Early molecular response translated into favorable 1-year survival. These real-world data support further prospective evaluation of VAH as a frontline induction strategy.

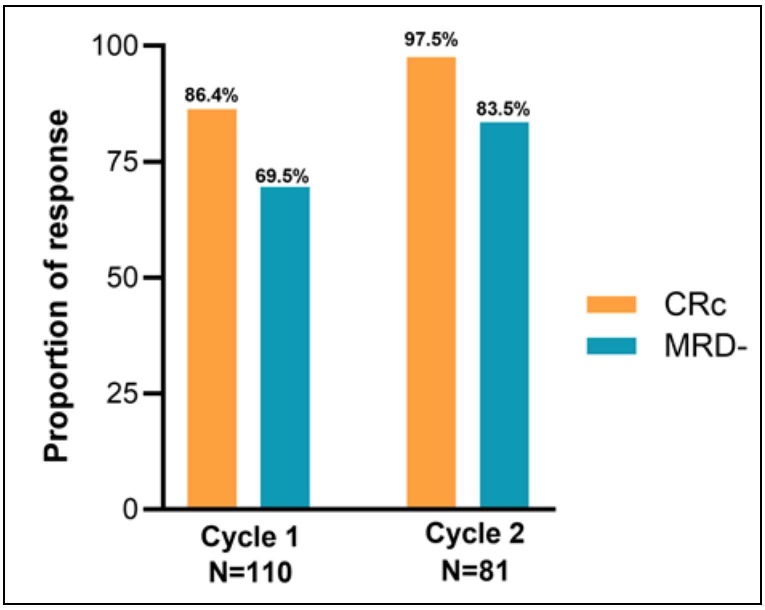

Rapid and Deep Responses Achieved with Frontline VAH in Newly Diagnosed AML

**Copyright Information:** (Online) ISSN: 2572-9241
©2026 The Author(s). HemaSphere is published by John Wiley & Sons Ltd on behalf of European Hematology Association. This is an open access Abstract Book distributed under the Attribution-NonCommercial-NoDerivs (CC BY-NC-ND), which allows third parties to download the articles and share them with others as long as they credit the author and the Abstract Book, but they cannot change the content in any way or use them commercially.
**Abstract Book Citations:** Authors, Title, HemaSphere, 2026;10:(S1):pages. The abstract book DOIs can be found on the "Author Information" page.
**Disclaimer:** Articles published in the journal HemaSphere exclusively reflect the opinions of the authors. The authors are responsible for all content in their abstracts including accuracy of the facts, statements, citing resources, etc.

### (PS1629) REVUMENIB AS MAINTENANCE FOR AML FOLLOWING ALLOGENEIC STEM CELL TRANSPLANTATION

**Topic:** 04. Acute myeloid leukemia - Clinical

Hannah Goulart*[1], Olayinka Okeleji[1], Courtney Dinardo[1], Naval Daver[1], Tapan Kadia[1], Alex Bataller[1], Hagop Kantarjian[1], Hussein Abbas[1], Wei-Ying Jen[1], Jeremy Connors[1], David McCall[1], Guillermo Garcia-Manero[1], Nicholas Short[1], Uday R. Popat[1], Demetrios Petropoulos[1], Cesar Nunez[1], Irtiza Sheikh[1], Samanta Catueno[1], Priti Tewari[1], Gheath Al-Atrash[1], Elizabeth J. Shpall[1], Branko Cuglievan[1], Ghayas Issa[1]

[1]*The University of Texas MD Anderson Cancer Center, Houston, United States of America;*

**Background**

Relapse after allogeneic stem cell transplantation (SCT) remains common in acute myeloid leukemia (AML). Revumenib is approved for relapsed/refractory (R/R) *NPM1*mt or *KMT2A*r acute leukemia; however its safety and efficacy as post-SCT maintenance has not been characterized.

**Aims**

We sought to characterize the outcomes of revumenib maintenance post-SCT in adult and pediatric patients (pts) at our institution.

**Methods**

Pts with *NPM1*mt, *KMT2A*r or *NUP98*r AML were eligible to continue post-SCT revumenib maintenance following response pre-SCT to revumenib monotherapy or combination on clinical trial. Revumenib was resumed at the pre-SCT dose with adjustment for azoles.

**Copyright Information:** (Online) ISSN: 2572-9241
©2026 The Author(s). HemaSphere is published by John Wiley & Sons Ltd on behalf of European Hematology Association. This is an open access Abstract Book distributed under the Attribution-NonCommercial-NoDerivs (CC BY-NC-ND), which allows third parties to download the articles and share them with others as long as they credit the author and the Abstract Book, but they cannot change the content in any way or use them commercially.
**Abstract Book Citations:** Authors, Title, HemaSphere, 2026;10:(S1):pages. The abstract book DOIs can be found on the “Author Information” page.
**Disclaimer:** Articles published in the journal HemaSphere exclusively reflect the opinions of the authors. The authors are responsible for all content in their abstracts including accuracy of the facts, statements, citing resources, etc.

### Results

Twenty-one pts were identified (11 adult, 10 pediatric); median age 20 years (range, 1 – 69), 62% female. Most pts (n = 16, 76%) had *KMT2A*r, 4 (19%) had *NPM1*mt, and 1 pediatric pt had *NUP98*r. Pre-SCT, revumenib was given either as monotherapy per AUGMENT-101 (NCT04065399, n = 8) or Expanded Access (NCT05918913, n = 2) or with oral decitabine and venetoclax per SAVE (NCT05360160, n = 11). Pts received a median of 3 (1 – 11) prior therapies, 12 pts (57%) had prior SCT, and 4 pts received SAVE frontline. Maintenance post-SCT on SAVE was planned for 1 year. At current SCT, 19% were in first complete remission (CR1 following SAVE), 81% were in CR2+ (SAVE or monotherapy). Median time to initiate post-SCT revumenib was 2.8 months (1.4 – 5.7) with median duration of therapy of 6.7 months (0.5 – 36).
With a median follow-up of 21.1 months (95% CI 19.3 – 46.2), median overall survival (OS) and event-free survival (EFS) from SCT have not been reached; 1- and 2-year OS rates were 89%; 1- and 2-year EFS rates were 84% and 73%, respectively (**Figure 1**). The 1-year cumulative incidence of relapse (CIR) was 11% and death was 5%. In CR2+, 2-year OS rate was 93% and 1-year CIR was 13%. For adults versus peds, the 1-year CIR and death was 25% versus 0% ($p = 0.16$) and 9% versus 0% ($p = 0.34$).
At last follow up, 24% remain on revumenib, and 76% have discontinued (relapse 14%, toxicity 19%, completion 14%, pt choice 14%, other illness 9%).
The most common any grade adverse event (AE) was thrombocytopenia (86%; grade ≥3 43%), leading to dose modification in 48% and discontinuation in 14%. Graft-versus-host-disease occurred in 14% (grade ≥3 5%), however no other AE led to dose modification. Infections occurred in 14% (grade ≥3 10%); one pt discontinued due to grade 4 septic shock. No QTc prolongation was observed.
In contrast, in a historical SCT cohort with the same genotypes treated prior to the advent of menin inhibitors (**Table 1**), pts transplanted at CR2+ had a 2-year OS of 33% and 1-year CIR of 46%.

**Table 1: Outcomes of Revumenib Versus Historical Cohorts**

| | N | 1-yr OS (%) | 2-yr OS (%) | 1-year CIR (%) | Median F/u (mos) |
|---|---|---|---|---|---|
| Rev | 21 | 89 | 89 | 11 | 21.1 |
| Rev CR2+ | 17 | 93 | 93 | 13 | 33.3 |
| Historical | 320 | 60 | 51 | 39 | 76 |
| Hist. CR2+ | 124 | 45 | 33 | 46 | 92 |

*Abbreviations: Rev: revumenib, Hist: historical, OS: overall survival, CIR: cumulative incidence of relapse, f/u: follow-up, CR: complete remission*

### Summary/Conclusion

Revumenib maintenance after SCT was feasible in this heavily pretreated cohort. Thrombocytopenia was common often requiring dose modification, but no other significant toxicities were observed. Outcomes appear favorable compared to historical cohorts, supporting prospective evaluation of menin inhibition as maintenance.

© 2026 American Society of Clinical Oncology, Inc. Reused with permission. This abstract was accepted and previously presented at the 2026 ASCO Annual Meeting. All rights reserved.

**Copyright Information:** (Online) ISSN: 2572-9241
©2026 The Author(s). HemaSphere is published by John Wiley & Sons Ltd on behalf of European Hematology Association. This is an open access Abstract Book distributed under the Attribution-NonCommercial-NoDerivs (CC BY-NC-ND), which allows third parties to download the articles and share them with others as long as they credit the author and the Abstract Book, but they cannot change the content in any way or use them commercially.
**Abstract Book Citations:** Authors, Title, HemaSphere, 2026;10:(S1):pages. The abstract book DOIs can be found on the "Author Information" page.
**Disclaimer:** Articles published in the journal HemaSphere exclusively reflect the opinions of the authors. The authors are responsible for all content in their abstracts including accuracy of the facts, statements, citing resources, etc.

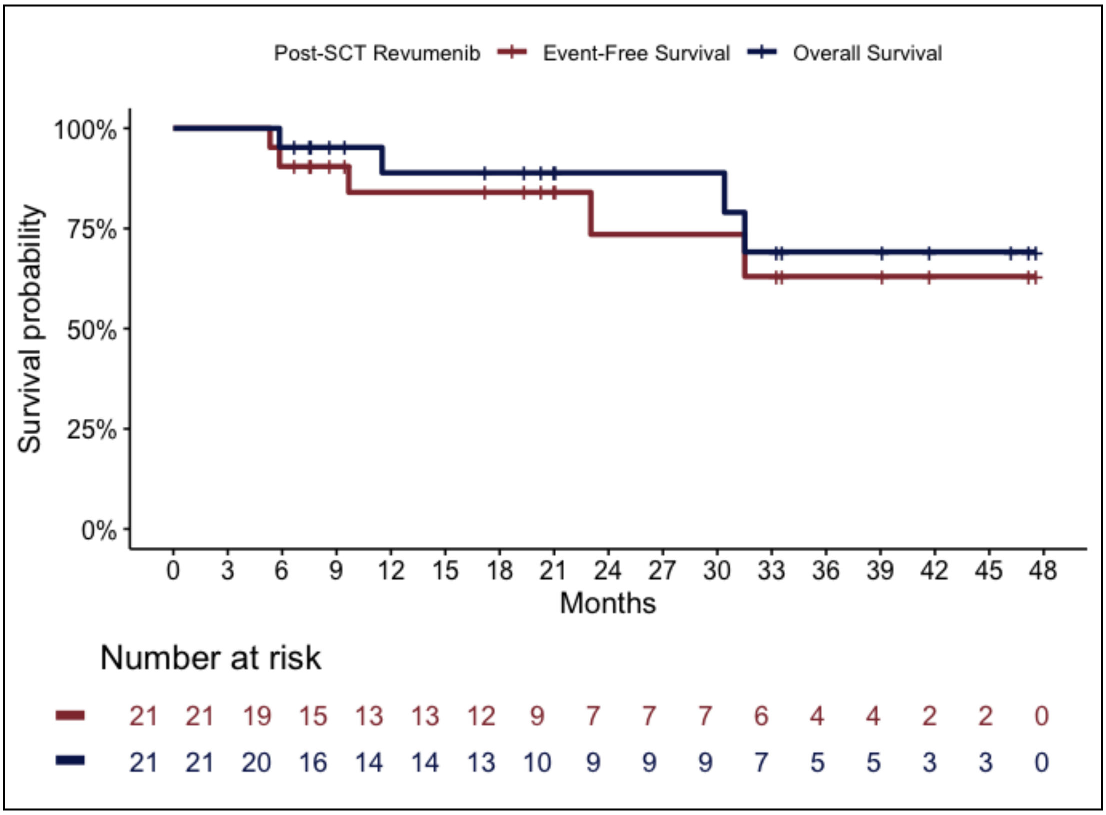

**Copyright Information:** (Online) ISSN: 2572-9241
©2026 The Author(s). HemaSphere is published by John Wiley & Sons Ltd on behalf of European Hematology Association. This is an open access Abstract Book distributed under the Attribution-NonCommercial-NoDerivs (CC BY-NC-ND), which allows third parties to download the articles and share them with others as long as they credit the author and the Abstract Book, but they cannot change the content in any way or use them commercially.
**Abstract Book Citations:** Authors, Title, HemaSphere, 2026;10:(S1):pages. The abstract book DOIs can be found on the "Author Information" page.
**Disclaimer:** Articles published in the journal HemaSphere exclusively reflect the opinions of the authors. The authors are responsible for all content in their abstracts including accuracy of the facts, statements, citing resources, etc.

### (PS1630) VENETOCLAX-BASED THERAPY OR INTENSIVE CHEMOTHERAPY FOR THE FRONTLINE TREATMENT OF FIT PATIENTS WITH IDH1/2 MUTATION ACUTE MYELOID LEUKEMIA? A MULTI-CENTER, REAL-WORLD, COHORT STUDY

**Topic:** 04. Acute myeloid leukemia - Clinical

Guopan Yu*[1, 1], Dandan Chen[1], Zhao Yin[1], Xiaoting Zhang[1], Dan Xu[1], Jing Huang[1], Qing Zhang[1]

[1]*Nanfang Hospital, Southern Medical University, Guangzhou, China;*

### Background

Intensive chemotherapy (IC) is the standard induction therapy for fit patients with IDH1/2 mutation acute myeloid leukemia (AML) according to NCCN guideline. Yet, as early molecular events, IDH1/2 mutation may not be sensitive to IC. In unfit populations, venetoclax (Ven)-based therapy (VBT) is the frontline treatment of IDH1/2 mutation AML and works well. Whether VBT could do better than IC in the frontline treatment of fit patients with IDH1/2 mutation AML remains unclear.

### Aims

To compare the efficacy and safety of venetoclax-based therapy versus intensive chemotherapy for the frontline treatment of fit patients with IDH1/2 mutation acute myeloid leukemia.

### Methods

We conducte a multi-center (the South China Hematology Alliance), real-world, cohort study to compare the outcome and safety of VBT and IC in the frontline treatment of fit patients with IDH1/2 mutation AML.

### Results

202 fit patients, including 70(34.7%) IDH1 mutation, 128(63.4%) IDH2 mutation, and 4(2.0%) harboring both mutations, with the median age of 51(17-74) years old, male and female of 108/94, were included. NPM1 was the most common co-mutation (81, 40.1%), followed by TET2 (73, 36.1%), DNMT3A (64, 31.7%), FLT3 (52, 25.7%), ASXL1 (35, 17.3%), BCOR (32, 15.8%), N/KRAS and RUNX1 (30, 14.9%), etc. MLL rearrangement was also common (80, 39.6%). There were 36(17.8%) , 60(29.7%) and 106(52.5% patients in ELN 2022 low, intermediate and high-risk group, respectively. 163(80.7%) patients received IC, and 39(19.3%) took VBT for induction. The baseline characteristics between the two treatment groups were comparable. 130(79.8%) patients in the IC group acquired response, including 111(68.1%) CR/CRi, 50(31.3%) MRD-negative, while 38(97.4%) in the VBT group obtained response (P=0.008), 34(87.2%) CR/CRi (P=0.017), and 30(76.9%) MRD-negative (P<0.001). 19/49(38.8%) patients without response in the IC group transferred to receive VBT for salvage therapy, while 1/4(25.0%) in the VBT group took IC for salvage, and finally 201(99.5%) acquired response. With a median follow-up of 16.6(2.0-74.3) months, 98(60.1%) patients in IC group, compared to 10(25.6%) in VBT group, were bridged to allo-HSCT (P<0.001). 39/162(24.1%) relapsed, 27(16.6%) died in the IC group, while 8(20.5%) relapsed, 6(25.4%) died in the VBT group. There were not significantly different in relapse and survival between the two groups, presenting as 2 year CIR 22.2%, OS 80.0%±3.8%, DFS 70.1%±4.3% in the IC group, compared to CIR 20.5% (P=0.637), OS 76.7%±8.6% (P=0.279), DFS 61.3%±11.9% (P=0.382) in the VBT group. Higher toxicity was observed in the IC than VBT, presenting as the incidence of febrile neutropenia (FN) was 84.1% in the IC group versus 45.5% in the VBT group (P=0.004), median duration of neutropenia was 23 days in the IC versus 15.5 days in the VBT (P=0.012).

**Copyright Information:** (Online) ISSN: 2572-9241
©2026 The Author(s). HemaSphere is published by John Wiley & Sons Ltd on behalf of European Hematology Association. This is an open access Abstract Book distributed under the Attribution-NonCommercial-NoDerivs (CC BY-NC-ND), which allows third parties to download the articles and share them with others as long as they credit the author and the Abstract Book, but they cannot change the content in any way or use them commercially.
**Abstract Book Citations:** Authors, Title, HemaSphere, 2026;10:(S1):pages. The abstract book DOIs can be found on the "Author Information" page.
**Disclaimer:** Articles published in the journal HemaSphere exclusively reflect the opinions of the authors. The authors are responsible for all content in their abstracts including accuracy of the facts, statements, citing resources, etc.

**Summary/Conclusion**

VBT might be a promising frontline treatment of fit patients with IDH1/2 mutation AML compared to IC, with higher response and better toleration. Further prospective studies are needed to be explored.

**Copyright Information:** (Online) ISSN: 2572-9241
©2026 The Author(s). HemaSphere is published by John Wiley & Sons Ltd on behalf of European Hematology Association. This is an open access Abstract Book distributed under the Attribution-NonCommercial-NoDerivs (CC BY-NC-ND), which allows third parties to download the articles and share them with others as long as they credit the author and the Abstract Book, but they cannot change the content in any way or use them commercially.
**Abstract Book Citations:** Authors, Title, HemaSphere, 2026;10:(S1):pages. The abstract book DOIs can be found on the "Author Information" page.
**Disclaimer:** Articles published in the journal HemaSphere exclusively reflect the opinions of the authors. The authors are responsible for all content in their abstracts including accuracy of the facts, statements, citing resources, etc.

### (PS1631) REVUMENIB THERAPY POST HEMATOPOIETIC STEM CELL TRANSPLANT FOR PATIENTS WITH RELAPSED/REFRACTORY KMT2AR, NPM1M, AND NUP98R ACUTE MYELOID LEUKEMIA: POST HOC ANALYSIS OF OUTCOMES FROM AUGMENT-101

**Topic:** 04. Acute myeloid leukemia - Clinical

Andrius Žučenka*[1], Ghayas Issa[2], Martha L Arellano[3], Wendy Stock[4], Branko Cuglievan[2], Lincy Thomas[5], Li Yu[5], Eytan Stein[6]

*[1]Vilnius University, Faculty of Medicine and Vilnius University Hospital Santaros Klinikos, National Cancer Center, Hematology, Oncology, and Transfusion Medicine Center, Vilnius, Lithuania; [2]The University of Texas MD Anderson Cancer Center, Houston, TX, United States of America; [3]Emory University School of Medicine, Atlanta, GA, United States of America; [4]University of Chicago Medicine, Chicago, IL, United States of America; [5]Syndax Pharmaceuticals, Inc., New York, NY, United States of America; [6]Memorial Sloan Kettering Cancer Center, New York, NY, United States of America;*

### Background

Hematopoietic stem cell transplant (HSCT) is a standard-of-care treatment (tx) for patients (pts) with acute myeloid leukemia (AML); however, relapse is a leading cause of HSCT failure, occurring in ~30%–40% of pts, typically within 6–7 mo after HSCT. In *KMT2A*-rearranged (*KMT2A*r) AML, median time to relapse is 4.7 mo. Revumenib is a first-in-class, oral, potent, and selective inhibitor of the menin-KMT2A interaction, which disrupts multiple key drivers of leukemogenesis (*KMT2A*r, *NPM1* mutation [*NPM1*m], and *NUP98* rearrangement [*NUP98*r]). Revumenib is used for the tx of relapsed/refractory (R/R) acute leukemias harboring a *KMT2A* translocation or R/R AML with *NPM1*m in adult and pediatric pts 1 year and older. Preclinical data indicate menin inhibition can enhance donor T-cell antitumor activity, supporting revumenib as a potential post-HSCT maintenance tx, and thus as a strategy to reduce relapse risk.

### Aims

To evaluate the efficacy and safety of revumenib as post-HSCT maintenance tx in pts with R/R*KMT2A*r, *NPM1*m, and *NUP98*r AML from pooled phase 1/2 data from AUGMENT-101 (NCT04065399).

### Methods

AUGMENT-101 is an ongoing phase 1/2, open-label, dose-escalation/-expansion study. Pts with at least partial remission could proceed to HSCT and remain on study. Revumenib tx after HSCT was allowed for pts 30–180 days post HSCT who achieved composite complete remission with no acute/chronic graft-versus-host disease (GVHD) requiring immunosuppression. Pts received revumenib at the same dose given immediately before HSCT, with adjustments for starting/stopping a strong CYP3A4 inhibitor peri HSCT. Tx was given until disease progression (PD) or unacceptable toxicity. Relapse-free survival (RFS) was defined as time from date of first dose of revumenib post HSCT to date of first PD or death, whichever occurred first. Overall survival (OS) was defined as time from date of first dose of revumenib post HSCT to date of death due to any cause.

**Copyright Information:** (Online) ISSN: 2572-9241
©2026 The Author(s). HemaSphere is published by John Wiley & Sons Ltd on behalf of European Hematology Association. This is an open access Abstract Book distributed under the Attribution-NonCommercial-NoDerivs (CC BY-NC-ND), which allows third parties to download the articles and share them with others as long as they credit the author and the Abstract Book, but they cannot change the content in any way or use them commercially.
**Abstract Book Citations:** Authors, Title, HemaSphere, 2026;10:(S1):pages. The abstract book DOIs can be found on the "Author Information" page.
**Disclaimer:** Articles published in the journal HemaSphere exclusively reflect the opinions of the authors. The authors are responsible for all content in their abstracts including accuracy of the facts, statements, citing resources, etc.

### Results

At data cutoff date (26Feb2025), 19 pts with AML had resumed revumenib tx post HSCT; 15 remained in the study, and 4 remained on revumenib tx. Majority (74%) of pts had *KMT2A*r AML; median (range) age was 39.0 y (1.3–75.0); and pts had received a median (range) of 2 prior lines of tx (1–7). Ten (53%) pts had received prior HSCT. Median (range) duration of post-HSCT revumenib tx was 27.9 wk (0–99).

Median RFS and OS were not reached (NR; 95% CI NR–NR) in pts receiving post-HSCT revumenib, regardless of genotype. Estimated 12-mo (95% CI) RFS and OS rates were 77.7% (50.7–91.1) and 94.7% (68.1–99.2), respectively. Duration of revumenib tx and pt status are shown in the **Figure**.

Grade ≥3 tx-emergent adverse events (TEAEs) occurred in 13 (68%) pts; 1 TEAE led to tx discontinuation. Most common grade ≥3 TEAEs were platelet count decreased (26% of pts), thrombocytopenia (21% of pts), and neutrophil count decreased (16% of pts). Grade ≥2 QTcF prolongation occurred in 1 (5%) pt (grade 2, lasting 1 day) and GVHD in 3 (16%) pts (2 grade 3).

### Summary/Conclusion

Revumenib demonstrated promising efficacy and a manageable safety profile as post-HSCT maintenance tx in pts with R/R *KMT2A*r, *NPM1*m, and *NUP98*r AML. Post-HSCT revumenib showed encouraging RFS and OS rates (medians NR). Low incidence of relapse and durability of remission across genotypes support the potential of menin inhibition to reduce post-HSCT relapse in this high-risk population. These findings warrant continued evaluation of revumenib as a post-HSCT maintenance tx.

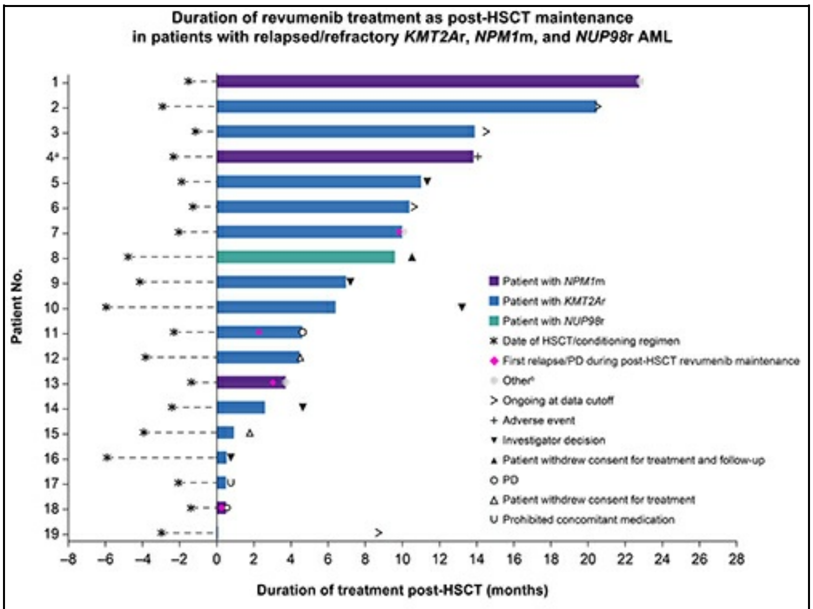

[a]Patient discontinued revumenib due to fatigue (possibly related to treatment). [b]Discontinued due to patient decision to discontinue maintenance treatment after 2 years, or due to disease relapse.
HSCT, hematopoietic stem cell transplant; PD, progressive disease.

Copyright Information: (Online) ISSN: 2572-9241
©2026 The Author(s). HemaSphere is published by John Wiley & Sons Ltd on behalf of European Hematology Association. This is an open access Abstract Book distributed under the Attribution-NonCommercial-NoDerivs (CC BY-NC-ND), which allows third parties to download the articles and share them with others as long as they credit the author and the Abstract Book, but they cannot change the content in any way or use them commercially.
Abstract Book Citations: Authors, Title, HemaSphere, 2026;10:(S1):pages. The abstract book DOIs can be found on the "Author Information" page.
Disclaimer: Articles published in the journal HemaSphere exclusively reflect the opinions of the authors. The authors are responsible for all content in their abstracts including accuracy of the facts, statements, citing resources, etc.

### (PS1633) VENETOCLAX COMBINED WITH MODIFIED INTENSIVE CHEMOTHERAPY IN NEWLY DIAGNOSED ACUTE MYELOID LEUKEMIA: A PROPENSITY SCORE-MATCHED ANALYSIS

**Topic:** 04. Acute myeloid leukemia - Clinical

Qiaoyi Zhou*[1], Hong Liu[1], Lihua Wu[1], Chunhua Liu[1], Xiyan Wang[1], Zhenzhen Wang[1], Lijun Fang[1], Runxia Gu[1], Shuning Wei[1], Ying Wang[1]

[1] *Institute of Hematology and Blood Diseases Hospital, Chinese Academy of Medical Sciences, Tianjin, China;*

### Background

Venetoclax, the B-cell lymphoma-2 (BCL-2) inhibitor, has exhibited apparent anti-leukemic effect especially for the high-risk AML and the potential for eliminating leukemic stem cells. Besides, preclinical trial supported that venetoclax could synergy with anthracycline and cytarabine to reduce AML cells and overcome resistance. Thus, several researches have tried to explore the feasibility for combination of venetoclax and the intensive chemotherapy or the modified regimen. However, few of them included direct comparisons between different regimens.

### Aims

Inspired by these, we tried to verified the superiority of the 3+7+Venetoclax regimen by performing a propensity score matched study and furthermore explored the feasibility of the 2+5+Venetoclax regimen as induction therapy to untreated AML patients.

### Methods

The study retrospectively collected the information about newly diagnosed AML patients of which 100 induced by the standard 3+7 regimen, 22 by the 3+7+ventoclax regimen and 19 by the 2+5+ventoclax regimen.This research study was approved by the Ethics Committees of the Institute of Hematology & Blood Diseases Hospital (approval number: IIT2021028-EC-1) and informed consent was obtained from all patients.Patients received the 2+5+V regimen, including idarubicin 12mg/m$^2$/day for 2 days, intravenous infusion cytarabine 100mg/m$^2$/days for 5 days and oral venetoclax escalating to 400mg for 7 days. In the 3+7+V group, owing to the serious myelosuppression causing by applying venetoclax for 14 days observed in first 3 patients, we adapted the venetoclax administration duration to 7 days. Thus, the majority of patients acquired the 3+7+V regimen including daunorubicin 60mg/m$^2$/day or idarubicin 12 mg/m$^2$/day for 3 days and cytarabine 100mg/m$^2$/days for 7 days plus venetoclax escalating to 400mg maintaining for 7 days, expect 3 applying venetoclax for 14 days. The dose of venetoclax was adjusted when confronting the usage of CYP3A4 inhibitors such as posaconazole. Moreover, the standard 3+7 group was made up of patients receiving daunorubicin 60mg/m$^2$/day or idarubicin 12mg/m$^2$/day for 3 days and cytarabine 100mg/m$^2$/days for 7 days. We performed propensity score-matched analysis to the 3+7+ventoclax group and the historical standard 3+7 group to adjust age, Charlson comorbidity index and ELN risk category. Furthermore, we tried to explored the feasibility of the 2+5+venetoclax regimen to adapt for the older or patients with more complication by comparing to the 3+7+venetoclax group.

**Copyright Information:** (Online) ISSN: 2572-9241
©2026 The Author(s). HemaSphere is published by John Wiley & Sons Ltd on behalf of European Hematology Association. This is an open access Abstract Book distributed under the Attribution-NonCommercial-NoDerivs (CC BY-NC-ND), which allows third parties to download the articles and share them with others as long as they credit the author and the Abstract Book, but they cannot change the content in any way or use them commercially.
**Abstract Book Citations:** Authors, Title, HemaSphere, 2026;10:(S1):pages. The abstract book DOIs can be found on the “Author Information” page.
**Disclaimer:** Articles published in the journal HemaSphere exclusively reflect the opinions of the authors. The authors are responsible for all content in their abstracts including accuracy of the facts, statements, citing resources, etc.

### Results

Compared with the standard 3+7 therapy, the complete remission/complete remission with incomplete hematologic recovery (CR/CRi) rate was higher in the 3+7+venetoclax group (90.91% vs 63.33%, p=0.015) and the event-free survival (EFS) was prolonged (P=0.0072). Besides, venetoclax induced deeper remission for patients at ELN favorable risk. The superiority causing by 3+7+V regimen still existed among patients classified as ELN adverse or intermediate risk group. 36.84% of the 2+5+ventoclax group aged 60 years old or older. There was no significant difference in CR/CRi rate, OS or EFS between the 2+5+venetoclax group and the 3+7+venetoclax group.

### Summary/Conclusion

The addition of venetoclax to the standard 3+7 induction therapy could notably improve the outcome of untreated AML. And the comparable efficiency with considerable security by administrating 2+5+venetoclax regimen, make it possible to substitute for the 3+7+V regimen as induction treatment, especially for elder patients.

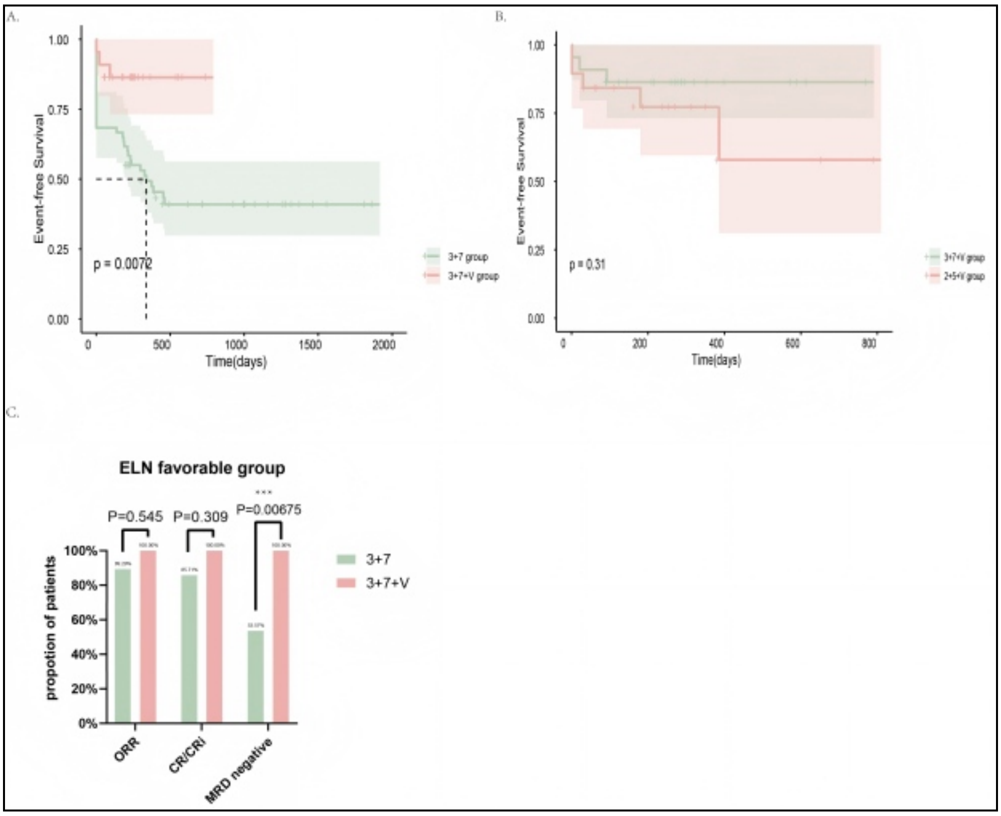

A.The EFS curve between the 3+7 group and 3+7+V group; B.The EFS curve between the 3+7+V group and 2+5+V group; C.The ORR, CR/CRi rates and the MRD negative rates among patients at favorable risk.

Copyright Information: (Online) ISSN: 2572-9241
©2026 The Author(s). HemaSphere is published by John Wiley & Sons Ltd on behalf of European Hematology Association. This is an open access Abstract Book distributed under the Attribution-NonCommercial-NoDerivs (CC BY-NC-ND), which allows third parties to download the articles and share them with others as long as they credit the author and the Abstract Book, but they cannot change the content in any way or use them commercially.
Abstract Book Citations: Authors, Title, HemaSphere, 2026;10:(S1):pages. The abstract book DOIs can be found on the "Author Information" page.
Disclaimer: Articles published in the journal HemaSphere exclusively reflect the opinions of the authors. The authors are responsible for all content in their abstracts including accuracy of the facts, statements, citing resources, etc.

### (PS1634) VENETOCLAX-BASED REGIMENS IMPROVE OUTCOMES IN ACUTE MYELOID LEUKEMIA WITH U2AF1 MUTATION: A REAL-WORLD EFFICACY COMPARATIVE ANALYSIS

**Topic:** 04. Acute myeloid leukemia - Clinical

Yaru Wang[1], Xiangli Chen*[2], Yijie Wang[1], Tiantian Dai[3], Xiaoting Xu[1], Wenyi Lu[2], Pingfan Lu[2], Fei Ge[2], Xiaona Niu[2]

*[1]Henan Provincial People's Clinical Medical School of Zhengzhou University, Department of Hematology, Zhengzhou City, China; [2]Henan Provincial People's Hospital, Department of Hematology, Zhengzhou City, China;[3]People's Hospital of Henan University, Department of Hematology, Zhengzhou City, China;*

### Background

U2AF1 is among the most frequent spliceosome mutations in acute myeloid leukemia (AML), typically associated with adverse prognosis. While venetoclax (VEN)-based combinations have transformed treatment for older/unfit AML patients, their efficacy in specific molecular subsets, particularly U2AF1-mutated AML, remains inadequately characterized, with limited data mostly from subgroup analyses of clinical trials. Large-scale real-world evidence is lacking.

### Aims

This real-world study aimed to analyze the clinical features of U2AF1-mutated AML and directly compare the efficacy and survival outcomes between VEN-based regimens and conventional intensive chemotherapy (IC) in this subset.

### Methods

We retrospectively analyzed 532 newly diagnosed AML patients who received standard induction therapy at our center (Jan 2018 - Nov 2024). Based on next-generation sequencing, patients were categorized into U2AF1-mutated (n=54) and wild-type (n=478) groups. Among the 54 U2AF1-mutated patients, two induction cohorts were defined: VEN-based group (VEN-G, n=25; combining VEN with hypomethylating agents or low-dose cytarabine) and IC group (Chemo-G, n=29; "3+7" or similar regimens). Primary endpoints were composite complete remission (CRc) and objective response rate (ORR). Secondary endpoints were progression-free survival (PFS) and overall survival (OS).

### Results

U2AF1-mutated patients had higher male proportion, lower baseline WBC, hemoglobin, and bone marrow blast percentage compared to wild-type. Trisomy 8 was more common in the mutated group. Within U2AF1-mutated patients, VEN-G achieved significantly higher CRc (72.0% vs. 37.9%, P=0.012) and ORR (96.0% vs. 62.1%, P=0.003) than Chemo-G. After a median follow-up of 17.7 months, VEN-G demonstrated significantly longer median PFS (12.6 months, 95% CI: 9.5-15.7) compared to Chemo-G (7.6 months, 95% CI: 5.1-10.1; P<0.05). Median OS was also superior in VEN-G (15.0 months, 95% CI: 7.9-22.2) vs. Chemo-G (9.0 months, 95% CI: 7.3-10.7; P<0.05). Multivariate Cox analysis confirmed that receiving a VEN-based regimen was an independent favorable prognostic factor for OS in U2AF1-mutated AML (P<0.05).

### Summary/Conclusion

This real-world study reveals distinct clinico-biological features of U2AF1-mutated AML. Venetoclax-based induction therapy yields significantly higher response rates and superior survival outcomes compared to intensive chemotherapy in this high-risk subset. These findings strongly support prioritizing VEN-based regimens as frontline induction therapy for AML patients harboring U2AF1 mutation, providing robust real-world evidence for precision treatment in this population.

**Copyright Information:** (Online) ISSN: 2572-9241
©2026 The Author(s). HemaSphere is published by John Wiley & Sons Ltd on behalf of European Hematology Association. This is an open access Abstract Book distributed under the Attribution-NonCommercial-NoDerivs (CC BY-NC-ND), which allows third parties to download the articles and share them with others as long as they credit the author and the Abstract Book, but they cannot change the content in any way or use them commercially.
**Abstract Book Citations:** Authors, Title, HemaSphere, 2026;10:(S1):pages. The abstract book DOIs can be found on the "Author Information" page.
**Disclaimer:** Articles published in the journal HemaSphere exclusively reflect the opinions of the authors. The authors are responsible for all content in their abstracts including accuracy of the facts, statements, citing resources, etc.

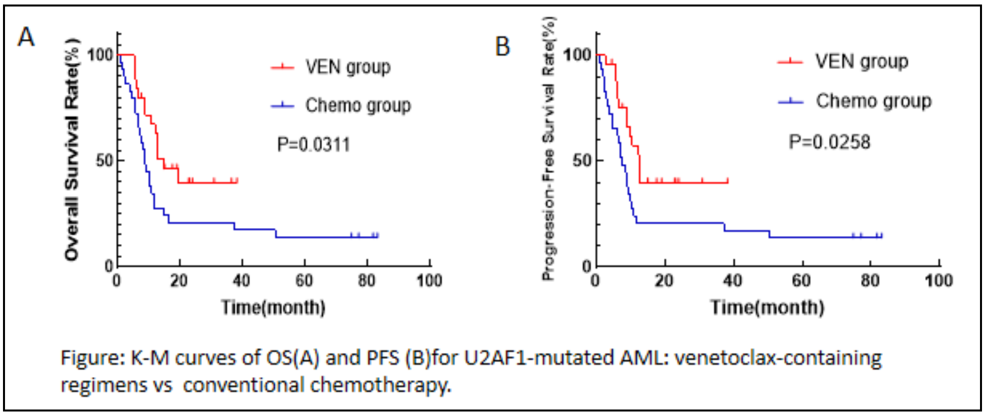

Figure:K-M curves of OS(A) and PFS(B) for U2AF1-mutated AML:venetoclax-containing regimens vs conventional chemotherapy.

**Copyright Information:** (Online) ISSN: 2572-9241
©2026 The Author(s). HemaSphere is published by John Wiley & Sons Ltd on behalf of European Hematology Association. This is an open access Abstract Book distributed under the Attribution-NonCommercial-NoDerivs (CC BY-NC-ND), which allows third parties to download the articles and share them with others as long as they credit the author and the Abstract Book, but they cannot change the content in any way or use them commercially.
**Abstract Book Citations:** Authors, Title, HemaSphere, 2026;10:(S1):pages. The abstract book DOIs can be found on the "Author Information" page.
**Disclaimer:** Articles published in the journal HemaSphere exclusively reflect the opinions of the authors. The authors are responsible for all content in their abstracts including accuracy of the facts, statements, citing resources, etc.

### (PS1635) TREATING ACUTE LEUKEMIA DURING PREGNANCY: MATERNAL AND FETAL OUTCOMES FROM A 34-YEAR EXPERIENCE

**Topic:** 04. Acute myeloid leukemia - Clinical

Fatima Alali*[1], Ahmed Abdrabou[2], Sidrah Arabi[3], Amani Zainab[3], Syed Osman Ahmed[1], Ahmad Alotaibi[1], Mansour Alfayez[1], Ayman Saad[1], Hanan Alkhaldi[1], Omar Albanyan[1], Ali Alahmari[1], Amr Suleiman Hanbali[1]

*[1]King Faisal Specialist and Research Centre, Hematology, Riyadh, Saudi Arabia;[2]King Faisal Specialist and Research Centre, Riyadh, Saudi Arabia; [3]Alfaisal University, Riyadh, Saudi Arabia;*

### Background

Acute leukemia diagnosed during pregnancy represents a rare but clinically challenging scenario, requiring a balance between optimal maternal therapy and fetal safety. Data on maternal and fetal outcomes remain limited, largely due to the rarity of the condition. This study aimed to evaluate maternal, fetal, and long-term outcomes of patients diagnosed with acute leukemia during pregnancy and managed at a single tertiary care institution.

### Aims

To assess maternal, fetal, and long-term outcomes of patients diagnosed with acute leukemia during pregnancy and managed at a tertiary care center.

### Methods

We conducted a retrospective analysis using our institutional leukemia database. All cases of acute leukemia diagnosed during pregnancy between January 1991 and October 2025 were identified. Clinical characteristics, pregnancy outcomes, treatment approaches, transplant utilization, and long-term survival were analyzed.

### Results

A total of 37 patients with acute leukemia diagnosed during pregnancy were identified. Of these, 27 patients (73%) received anti-leukemic therapy during pregnancy, resulting in 20 live births, while 10 pregnancies ended in spontaneous abortion. No cases of teratogenicity, congenital malformations, or postnatal complications were observed among live-born infants.
The remaining 10 patients did not receive chemotherapy during pregnancy; three were diagnosed after 34 weeks of gestation, delivered healthy neonates, and subsequently initiated chemotherapy postpartum, while eight patients had therapeutic abortions prior to initiating chemotherapy.
Overall, 20 patients (54%) proceeded to HLA-matched sibling allogeneic stem cell transplantation, including 5 patients with acute myeloid leukemia, 14 with acute lymphoblastic leukemia, and 1 with acute promyelocytic leukemia. After a median follow-up of 9 months, 13 patients (35%) were alive and in sustained remission, including 4 treated with chemotherapy alone and 9 who underwent allogeneic transplantation.

**Copyright Information:** (Online) ISSN: 2572-9241
©2026 The Author(s). HemaSphere is published by John Wiley & Sons Ltd on behalf of European Hematology Association. This is an open access Abstract Book distributed under the Attribution-NonCommercial-NoDerivs (CC BY-NC-ND), which allows third parties to download the articles and share them with others as long as they credit the author and the Abstract Book, but they cannot change the content in any way or use them commercially.
**Abstract Book Citations:** Authors, Title, HemaSphere, 2026;10:(S1):pages. The abstract book DOIs can be found on the "Author Information" page.
**Disclaimer:** Articles published in the journal HemaSphere exclusively reflect the opinions of the authors. The authors are responsible for all content in their abstracts including accuracy of the facts, statements, citing resources, etc.

**Summary/Conclusion**

Our findings support the feasibility and relative safety of administering anti-leukemic therapy during pregnancy, with acceptable fetal outcomes when appropriately managed. However, long-term maternal outcomes appear inferior compared with historical non-pregnant cohorts, reflecting the aggressive disease biology and complexity of treatment decisions in this unique clinical setting. These results highlight the need for individualized, multidisciplinary management and continued reporting of long-term outcomes in this rare population.

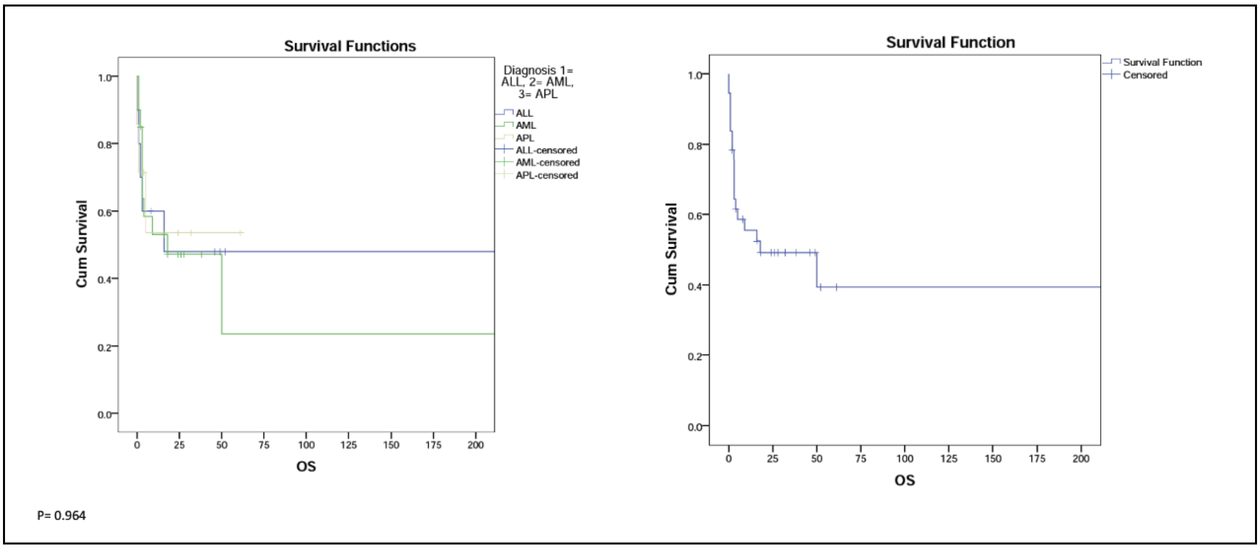

**Copyright Information:** (Online) ISSN: 2572-9241
©2026 The Author(s). HemaSphere is published by John Wiley & Sons Ltd on behalf of European Hematology Association. This is an open access Abstract Book distributed under the Attribution-NonCommercial-NoDerivs (CC BY-NC-ND), which allows third parties to download the articles and share them with others as long as they credit the author and the Abstract Book, but they cannot change the content in any way or use them commercially.
**Abstract Book Citations:** Authors, Title, HemaSphere, 2026;10:(S1):pages. The abstract book DOIs can be found on the "Author Information" page.
**Disclaimer:** Articles published in the journal HemaSphere exclusively reflect the opinions of the authors. The authors are responsible for all content in their abstracts including accuracy of the facts, statements, citing resources, etc.

### (PS1636) SWEET SYNDROME IN ACUTE MYELOID LEUKEMIA: THE THREE-SITE MAYO CLINIC EXPERIENCE

**Topic:** 04. Acute myeloid leukemia - Clinical

Noah Takacs*[1], Ali Husnain[2], Jabra Zarka[2], Oudai Alkabbani[2], Mohamad Sheikh Najeeb[2], Nathan Puwani[3], Aasiya Matin[2], Abhishek Mangaonkar[2], Mrinal Patnaik[2], Mithun Shah[2], Hemant Murthy[4], Tariq Kewan[2], Mark Litzow[2], Kebede Begna[2], Naseema Gangat[2], Nandita Khera[3], William Hogan[2], James M Foran[4], Hassan Alkhateeb[2], Aref Al-Kali[2], Antoine Saliba[2]

*[1]Mayo Clinic, Department of Medicine, Rochester, United States of America;[2]Mayo Clinic, Division of Hematology, Rochester, United States of America; [3]Mayo Clinic, Division of Hematology/Oncology, Jacksonville, United States of America;[4]Mayo Clinic, Division of Hematology/Oncology, Scottsdale, United States of America;*

### Background

Sweet syndrome, also known as acute febrile neutrophilic dermatosis, is a skin manifestation associated with various disorders including malignancy. Sweet syndrome is most often associated with underlying hematologic malignancies, particularly acute myeloid leukemia (AML). However, systematic characterization within contemporary AML cohorts remains limited.

### Aims

We sought to describe the clinical presentation, diagnostic features, treatment approaches, and outcomes of patients with Sweet syndrome occurring in the context of AML, with a focus on temporal relationships with AML therapy and disease status.

### Methods

We conducted a retrospective review of AML patients diagnosed at our institution (Minnesota, Florida, and Arizona) between 2015 and 2024. Biopsy-proven cases of Sweet syndrome were identified through clinical and dermatopathological records. Demographic data, AML subtype, timing of Sweet syndrome relative to AML diagnosis or therapy, laboratory findings, treatments administered for Sweet syndrome, and clinical outcomes were extracted. We included a control cohort of 98 consecutive AML patients diagnosed between 2022 and 2023 who did not develop Sweet syndrome, to enable comparative analyses. Descriptive statistics were generated, and associations between Sweet syndrome onset and AML features were explored in JMP 18.2.1.

**Copyright Information:** (Online) ISSN: 2572-9241
©2026 The Author(s). HemaSphere is published by John Wiley & Sons Ltd on behalf of European Hematology Association. This is an open access Abstract Book distributed under the Attribution-NonCommercial-NoDerivs (CC BY-NC-ND), which allows third parties to download the articles and share them with others as long as they credit the author and the Abstract Book, but they cannot change the content in any way or use them commercially.
**Abstract Book Citations:** Authors, Title, HemaSphere, 2026;10:(S1):pages. The abstract book DOIs can be found on the “Author Information” page.
**Disclaimer:** Articles published in the journal HemaSphere exclusively reflect the opinions of the authors. The authors are responsible for all content in their abstracts including accuracy of the facts, statements, citing resources, etc.

### Results

Patients who developed Sweet syndrome (n=32) were younger at AML diagnosis than controls (median 60 years [IQR 46–66] vs 69 years [IQR 61–75]; p<0.0001) and more likely to be female (63% vs 33%; p=0.0036). Sweet syndrome occurred at AML diagnosis (9%), during leukemia-directed therapy (66%), or post-allogeneic stem cell transplant (SCT) with (19%) or without (6%) relapse; one case preceded AML by 10 months. Excluding post-SCT cases, median time from AML diagnosis or relapse to Sweet syndrome was 21 days [IQR 13–46]. Lesions were most commonly generalized (53%) or localized to the extremities (31%), with less frequent trunk (6%) and facial involvement (3%). The predominant morphologic pattern was papular (69%), followed by nodular (25%), and vesiculobullous (6%).  Fever (59%) and painful lesions (44%) were common. Arthralgia and weight loss occurred in one patient each; ocular/auricular involvement occurred in 6%. Nearly half of patients received systemic corticosteroids (47%, 15/32). Topical steroids were used alone for 34% of patients, one patient received dapsone, and four received no therapy. No recurrences were observed. Molecular and cytogenetic characteristics are summarized in Fig. 1A. After median follow-up of 16 months, while restricting to cases at diagnosis or during initial therapy pre-SCT (n=26), overall survival (OS) was longer in the Sweet cohort (median 34 [95% CI 16–NR] vs 12 [8–20] months; log-rank p=0.0356) (Fig. 1B). Restricting to patients age<65 years, median OS remained numerically longer in the Sweet syndrome group but lost statistical significance (median 33 [12–NR] vs 17 [12–NR] months; p=0.53) (Fig. 1C). Age <65 (OR 4.5 [1.8–11.8]) and female sex (OR 3.1 [1.3–7.7]) were associated with occurrence of Sweet syndrome, whereas ELN risk, TP53, and FLT3 mutational status were not.

### Summary/Conclusion

In our cohort, Sweet syndrome occurred more frequently in younger and female AML patients and most often developed during leukemia-directed therapy. Patients with Sweet syndrome demonstrated longer OS compared with controls, suggesting it may represent a distinct clinical subset rather than an adverse prognostic marker.

**Copyright Information:** (Online) ISSN: 2572-9241
©2026 The Author(s). HemaSphere is published by John Wiley & Sons Ltd on behalf of European Hematology Association. This is an open access Abstract Book distributed under the Attribution-NonCommercial-NoDerivs (CC BY-NC-ND), which allows third parties to download the articles and share them with others as long as they credit the author and the Abstract Book, but they cannot change the content in any way or use them commercially.
**Abstract Book Citations:** Authors, Title, HemaSphere, 2026;10:(S1):pages. The abstract book DOIs can be found on the "Author Information" page.
**Disclaimer:** Articles published in the journal HemaSphere exclusively reflect the opinions of the authors. The authors are responsible for all content in their abstracts including accuracy of the facts, statements, citing resources, etc.

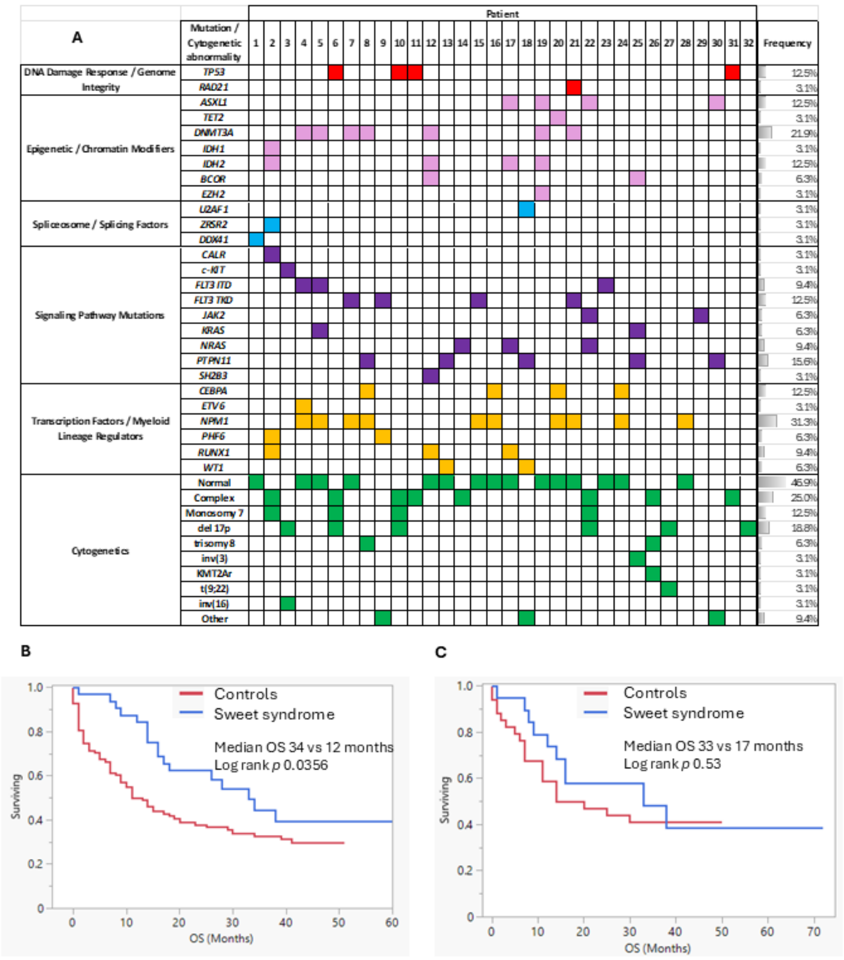

**Copyright Information:** (Online) ISSN: 2572-9241
©2026 The Author(s). HemaSphere is published by John Wiley & Sons Ltd on behalf of European Hematology Association. This is an open access Abstract Book distributed under the Attribution-NonCommercial-NoDerivs (CC BY-NC-ND), which allows third parties to download the articles and share them with others as long as they credit the author and the Abstract Book, but they cannot change the content in any way or use them commercially.
**Abstract Book Citations:** Authors, Title, HemaSphere, 2026;10:(S1):pages. The abstract book DOIs can be found on the "Author Information" page.
**Disclaimer:** Articles published in the journal HemaSphere exclusively reflect the opinions of the authors. The authors are responsible for all content in their abstracts including accuracy of the facts, statements, citing resources, etc.

### (PS1637) A PROSPECTIVE CLINICAL STUDY OF VENETOCLAX PLUS AZACITIDINE AS MAINTENANCE TREATMENT FOR POST-REMISSION ACUTE MYELOID LEUKEMIA

**Topic:** 04. Acute myeloid leukemia - Clinical

Jinqiang Guo*[1], Fang Cheng[2], Yuanyan Tang[3], Jie Tan[4], Hongbo Ren[4], Haiyan Wang[5], Lulu Zhang[6], Meifang Su[7], Zhe Zhao[8], Zhuangzhi Yang[9], Hongxiang Wang[10], Jing Zheng[5], Guolin Yuan[11], Qihuan Liu[12], Jun Qin[13], Zhengrong Liu[14], Shuai Liu[15], Xianyi Wu[16], Renying Ge[17], Rui Jiang[18], Chuancai Liu[19], Rui Qin[20], Weiming Li[1]

*[1]Union Hospital, Tongji Medical College, Huazhong University of Science and Technology, Department of Hematology, Wuhan, China; [2]Union Hospital, Tongji Medical College, Huazhong University of Science and Technology, Department of Pharmacy, Wuhan, China; [3]Jingzhou Hospital Affiliated to Yangtze University, Jingzhou, China; [4]The First People's Hospital of Jingzhou, JingZhou, China; [5]Yi Chang Central People's Hospital, The First College of Clinical Medical Science, China Three Gorges University, Department of Hematology, Yichang, China; [6]Taihe Hospital, Hubei University of Medicine, Department of Hematology, Shiyan, China; [7]Huanggang Central Hospital, Department of Hematology, Huanggang, China;[8]Minda Hospital of Hubei University for Nationalities, Department of Hematology, Enshi, China; [9]Suizhou central hospital, Hubei University of Medicine, Suizhou, China; [10]the Central Hospital of Wuhan, Wuhan, China; [11]the Central Hospital of Xiangyang, Department of Hematology, Xiangyang, China; [12]Sinopharm Dongfeng General Hospital, Hubei University of Medicine, Department of Hematology, Shiyan, China; [13]Shiyan Renmin Hospital of Hubei Medical University, Department of Hematology, Shiyan, China; [14]The People's Hospital of Anyang City, Anyang, China; [15]Dawu County People's Hospital, Department of Hematology, Xiaogan, China; [16]The First Affiliated Hospital of Guilin Medical University, Department of Hematology, guilin, China;[17]Xian Ning Center Hospital, Department of Hematology, xianning, China; [18]the Central Hospital of Jingmen, jingmen, China; [19]Ezhou Central Hospital, Department of Hematology, Ezhou, China; [20]The central hospital of enshi tujia and miao autonomous prefecture, Enshi, China;*

### Background

Most patients with acute myeloid leukemia (AML) eventually relapse despite achieving remission following induction therapy, underscoring the need for effective strategies to reduce recurrence risk. A phase 2 clinical trial (NCT0406266) has demonstrated the feasibility and tolerability of low-dose azacitidine (AZA) combined with venetoclax (VEN) as maintenance therapy in AML.

### Aims

We conducted an expanded prospective study to further evaluate the efficacy and safety of AZA plus VEN as maintenance therapy in patients with AML in remission, using an optimized treatment schedule.

### Methods

This prospective, multicenter, single-arm study enrolled adult patients (≥18 years) with non-APL AML who achieved complete remission (CR) or CR with incomplete hematologic recovery (CRi) after at least two cycles of consolidation therapy. Eligible patients were required to have confirmed measurable residual disease (MRD)-negative status by flow cytometry (threshold: 0.1%) within three months prior to enrollment. Participants were stratified into Cohort 1 (prior intensive induction chemotherapy) and Cohort 2 (prior low-intensity induction therapy). Maintenance treatment consisted of AZA 50 mg/m² intravenously or subcutaneously on Days 1–5, and VEN 400 mg orally on Days 1–14, administered in 28-day cycles. After six consecutive cycles, treatment was given every other cycle up to a total duration of two years (one year for ELN2022 low-risk patients). The primary endpoint was relapse-free survival (RFS).

**Copyright Information:** (Online) ISSN: 2572-9241
©2026 The Author(s). HemaSphere is published by John Wiley & Sons Ltd on behalf of European Hematology Association. This is an open access Abstract Book distributed under the Attribution-NonCommercial-NoDerivs (CC BY-NC-ND), which allows third parties to download the articles and share them with others as long as they credit the author and the Abstract Book, but they cannot change the content in any way or use them commercially.
**Abstract Book Citations:** Authors, Title, HemaSphere, 2026;10:(S1):pages. The abstract book DOIs can be found on the "Author Information" page.
**Disclaimer:** Articles published in the journal HemaSphere exclusively reflect the opinions of the authors. The authors are responsible for all content in their abstracts including accuracy of the facts, statements, citing resources, etc.

**Results**

As of December 2025, 78 patients were enrolled, with 42 assigned to Cohort 1 and 36 to Cohort 2. The cohort included 39 (50%) males and 39 (50%) females, with a median age of 59 years (range 18–80). The median interval from initiation of consolidation therapy to start of maintenance therapy was 7.2 months (IQR 4.6–10.4), and from completion of consolidation to maintenance initiation was 1.7 months (IQR 0.8–2.0). Patients received a median of 6 maintenance cycles (IQR 1–14). After a median follow-up of 13.4 months, the median RFS for the overall cohort was 21.5 months. Median RFS was 21.5 months (95% CI 8.8–34.3) in Cohort 2 and was not reached in Cohort 1. Univariate Cox regression analysis suggested that receipt of three cycles of consolidation therapy in Cohort 1 and four cycles in Cohort 2 were associated with improved RFS. Treatment-emergent adverse events (TEAEs) occurred in 66 patients (85%), predominantly grade 1–2. The most common grade 3–4 TEAEs were neutropenia (35%) and thrombocytopenia (15%). No treatment-related deaths or discontinuations due to serious adverse events were observed.

**Summary/Conclusion**

Maintenance therapy with VEN plus AZA demonstrated promising RFS and a manageable safety profile in patients with AML in remission.

**Copyright Information:** (Online) ISSN: 2572-9241
©2026 The Author(s). HemaSphere is published by John Wiley & Sons Ltd on behalf of European Hematology Association. This is an open access Abstract Book distributed under the Attribution-NonCommercial-NoDerivs (CC BY-NC-ND), which allows third parties to download the articles and share them with others as long as they credit the author and the Abstract Book, but they cannot change the content in any way or use them commercially.
**Abstract Book Citations:** Authors, Title, HemaSphere, 2026;10:(S1):pages. The abstract book DOIs can be found on the “Author Information” page.
**Disclaimer:** Articles published in the journal HemaSphere exclusively reflect the opinions of the authors. The authors are responsible for all content in their abstracts including accuracy of the facts, statements, citing resources, etc.

### (PS1638) ODYSSEY: A FIRST-IN-HUMAN STUDY OF THE ALDEHYDE DEHYDROGENASE (ALDH) INHIBITOR ABD-3001 IN PATIENTS WITH REFRACTORY/RELAPSED ACUTE MYELOID LEUKEMIA (AML) OR HIGH-RISK MYELODYSPLASTIC SYNDROME (MDS)

**Topic:** 04. Acute myeloid leukemia - Clinical

Mael Heiblig*[1], Lina Benajiba[2], Julien Colle[3], Sandrine Loron[1], Juliette Soret-Dulphy[2], Rafael Daltro de Oliveira[2], Geoffroy Venton[2], Marie-Virginie Larcher[1], Laure Farnault/De Lassus[3], Charlotte Gandillon[4], Muhube Yazir[4], Mileidys Perez[4], Ismail Ceylan[4], Guillaume Martin[4], Annick Pelletier[3], Jean-Jacques Kiladjian[2], Régis Costello[3]

*[1]Centre Hospitalier Lyon Sud, Service Hématologie, Centre d'Investigation des thérapeutiques en oncologie et hématologie de Lyon (CITHOL), Lyon, France; [2]Hôpital Saint-Louis, Service d'Hématologie seniors, Centre d'Investigation Clinique, 14-27 Saint-Louis, Paris, France; [3]Hôpital de la Timone, Service Hématologie et Thérapie Cellulaire, Centre d'Essais Précoces en Cancérologie de Marseille (CEPCM), Marseille, France; [4]Advanced BioDesign, Saint-Priest, France;*

### Background

Despite recent therapeutic progress in acute myeloid leukemia (AML) and high-risk myelodysplastic syndromes (HR-MDS), outcomes for patients with relapsed/refractory (R/R) disease remain poor, underscoring the need for novel targeted approaches. Aldehyde dehydrogenase 1 (ALDH1) is emerging as a promising therapeutic target, as its overexpression is associated with leukemic stemness, chemoresistance, and adverse prognosis. ABD-3001 is a first-in-class, selective ALDH1 inhibitor currently under investigation as monotherapy in R/R AML and HR-MDS (NCT05601726).

### Aims

We report results from the Dose Ranging Study (DRS) of this ongoing multicenter Phase 1 trial. The primary objective is to determine the recommended Phase 2 dose (RP2D) and evaluate safety. Secondary objectives include pharmacokinetics (PK), pharmacodynamics (PD), and preliminary clinical activity.

### Methods

Patients (pts) enrolled in the DRS received intravenous ABD-3001 on 28-day cycles for up to 3 cycles. Three regimens were assessed: 270 mg/m² once weekly (QW), 270 mg/m² twice weekly (BIW) and 405 mg/m² BIW. Adverse events (AEs) were graded by NCI CTCAE v5.0. The safety population included all pts receiving ≥1 dose. Efficacy per ELN 2017 criteria was evaluated in pts completing ≥1 cycle. Target engagement was assessed by ALDH1 activity inhibition and JNK phosphorylation (pJNK) as a molecular PD biomarker.

**Copyright Information:** (Online) ISSN: 2572-9241
©2026 The Author(s). HemaSphere is published by John Wiley & Sons Ltd on behalf of European Hematology Association. This is an open access Abstract Book distributed under the Attribution-NonCommercial-NoDerivs (CC BY-NC-ND), which allows third parties to download the articles and share them with others as long as they credit the author and the Abstract Book, but they cannot change the content in any way or use them commercially.
**Abstract Book Citations:** Authors, Title, HemaSphere, 2026;10:(S1):pages. The abstract book DOIs can be found on the "Author Information" page.
**Disclaimer:** Articles published in the journal HemaSphere exclusively reflect the opinions of the authors. The authors are responsible for all content in their abstracts including accuracy of the facts, statements, citing resources, etc.

**Results**

As of February 25, 2026, 24 pts (19 AML; 3 HR-MDS) were treated. Median age was 74 years (range 29–89); 62.5% were female; 62.5% had ECOG PS 1. ELN 2022 risk was adverse in 78.9% and intermediate in 26.3%. Pts received a median of 6 prior lines (range 2–69); 62.9% were previously exposed to venetoclax + azacitidine; 12.5% were post-HCT. Key mutations included TP53 (17%), N/KRAS (21%), and FLT3-ITD (4%).
Treatment-related AEs (TRAEs) occurred in 18/24 pts (75%). The most frequent TRAEs were fever (29.1%), infusion-related reactions (IRRs, 20.8%), catheter infection (16.6%), and diarrhea (12.5%). Grade ≥3 TRAEs occurred in 12 pts (50%), mainly IRR (8.3%), interstitial pneumonitis (8.3%), dyspnea (8.3%), and febrile neutropenia (8.3%). ABD-3001-related dose interruptions occurred in 3 pts (12.5%), one per dosing level.
Among 15 evaluable pts, 1 achieved complete remission (CR) and 1 CR with incomplete recovery (CRi). Six pts (40%) exhibited a reduction in bone marrow blasts, including three with ≥50% decrease.
PK analyses showed systemic exposure at all doses with non–dose-proportional increases and no accumulation over 3 cycles. PD data demonstrated consistent ALDH1 inhibition and pJNK activation, with 270 mg/m² showing stronger biological activity than 405 mg/m², supporting a non-linear dose–response relationship.

**Summary/Conclusion**

ABD-3001 demonstrated an acceptable and manageable safety profile across all dosing regimens in this first-in-human setting. Early signals of anti-leukemic activity were observed, with 40% of evaluable pts achieving a reduction in marrow blasts, including one CR and one CRi. PD data confirmed on-target ALDH1 inhibition and downstream pathway activation.
These findings support ongoing evaluation of ABD-3001 to determine the RP2D and guide further clinical development in R/R AML and HR-MDS.

**Copyright Information:** (Online) ISSN: 2572-9241
©2026 The Author(s). HemaSphere is published by John Wiley & Sons Ltd on behalf of European Hematology Association. This is an open access Abstract Book distributed under the Attribution-NonCommercial-NoDerivs (CC BY-NC-ND), which allows third parties to download the articles and share them with others as long as they credit the author and the Abstract Book, but they cannot change the content in any way or use them commercially.
**Abstract Book Citations:** Authors, Title, HemaSphere, 2026;10:(S1):pages. The abstract book DOIs can be found on the "Author Information" page.
**Disclaimer:** Articles published in the journal HemaSphere exclusively reflect the opinions of the authors. The authors are responsible for all content in their abstracts including accuracy of the facts, statements, citing resources, etc.

### (PS1639) EVOLVING TREATMENT PATTERNS AND SURVIVAL IN OLDER ADULTS WITH ACUTE MYELOID LEUKEMIA: A POPULATION-BASED STUDY

**Topic:** 04. Acute myeloid leukemia - Clinical

Brittany Salter*[1], Alejandro Garcia-Horton[1], Shreyash Dalmia[1], Michael Radford[1], Joseph Aziz[1], Wilson Lam[1], Kylie Lepic[1], Irwin Walker[1], Brian Leber[1], Hira Mian[1, 2], Gregory Pond[1, 2], Hsien Seow[1, 2], Tobias Berg[1, 2, 3]

*[1]McMaster University, Oncology, Hamilton, Canada;[2]Escarpment Cancer Research Institute ECRI, Hamilton, Canada;[3]Centre for Discovery in Cancer Research CDCR, Hamilton, Canada;*

### Background

Acute myeloid leukemia (AML) predominantly affects older adults, many of whom are ineligible for intensive chemotherapy. Recent advances, including hypomethylating agents (HMAs), venetoclax, and other targeted therapies, have expanded treatment options for this population.

### Aims

This study examined how access to these therapies has influenced treatment patterns and survival outcomes in AML.

### Methods

We conducted a retrospective population-based cohort study using province-wide administrative data from ICES in Ontario, Canada. Patients aged ≥60 years diagnosed with AML between January 1, 2010, and December 31, 2023, were included. Overall survival (OS) was analyzed using the Kaplan–Meier method and Cox proportional hazards models, adjusting for age, sex, comorbidity score (PopGrouper), treatment type, transplant status, and socioeconomic factors.

### Results

Among 6,161 patients, 2,708 (44%) received active therapy (19.3% intensive; 24.7% non-intensive), while 3,453 (56%) received best supportive care (BSC). In the non-intensively treated patients, 3% received low-dose cytarabine, 75% received HMA monotherapy, and 22% received venetoclax-based therapy. Use of active therapy increased over time from 29% in 2010–2014 to 46% in 2015–2019 and 56% in 2020–2023, with a corresponding decline in BSC (70%, 54%, and 44%, respectively).
Median OS improved modestly, increasing from 4.7 months in 2010-2014 to 5.8 months in 2015-2019 and 5.5 months in 2020-2023. Within the 2020-2023 interval, median OS was 15.4 months with intensive chemotherapy, 9.8 months with non-intensive therapy, and 1.4 months with BSC. Younger age, receipt of transplantation, and lower comorbidity burden were associated with improved OS, whereas greater distance to treatment center (≥50 km) was independently associated with inferior survival. Income level (except for the highest quintile) and rural residence were not associated with OS.
In the univariate analysis, both venetoclax-based therapy (HR 0.82; P=0.003) and intensive therapy (HR 0.61; P<0.0001) were associated with improved OS relative to HMA monotherapy. In the multivariable analysis, venetoclax-based therapy remained independently associated with improved OS (HR 0.82; P=0.004), whereas intensive therapy did not (HR 0.94; P=0.17).

### Summary/Conclusion

In this large population-based real-world cohort, increasing access to active therapy and transplant was associated with improved survival among older patients with AML. After adjustment for clinical factors, venetoclax-based treatment demonstrated outcomes better than HMA alone and comparable to intensive chemotherapy.

**Copyright Information:** (Online) ISSN: 2572-9241
©2026 The Author(s). HemaSphere is published by John Wiley & Sons Ltd on behalf of European Hematology Association. This is an open access Abstract Book distributed under the Attribution-NonCommercial-NoDerivs (CC BY-NC-ND), which allows third parties to download the articles and share them with others as long as they credit the author and the Abstract Book, but they cannot change the content in any way or use them commercially.
**Abstract Book Citations:** Authors, Title, HemaSphere, 2026;10:(S1):pages. The abstract book DOIs can be found on the “Author Information” page.
**Disclaimer:** Articles published in the journal HemaSphere exclusively reflect the opinions of the authors. The authors are responsible for all content in their abstracts including accuracy of the facts, statements, citing resources, etc.

**Table 1: Multivariate Analysis**

| Variable Label (Reference) | Value | Multivariate Model Results<br>Hazard Ratio<br>Estimate | Lower | Upper | P-Value |
|---|---|---|---|---|---|
| Diagnosis year (2010-2014) | 2015-2019 | 1.02 | 0.95 | 1.08 | 0.65 |
| | 2020-2023 | 1.15 | 1.07 | 1.23 | 0.00 |
| Treatment type (HMA) | Intensive | 0.94 | 0.85 | 1.03 | 0.17 |
| | Venetoclax-based | 0.82 | 0.71 | 0.94 | 0.004 |
| | BSC | 1.71 | 1.59 | 1.83 | <0.0001 |
| Transplant within 1 year of diagnosis | Yes | 0.49 | 0.43 | 0.56 | <0.0001 |
| Age category (80+) | 60-64 | 0.55 | 0.49 | 0.60 | <0.0001 |
| | 65-69 | 0.61 | 0.56 | 0.66 | <0.0001 |
| | 70-74 | 0.73 | 0.68 | 0.79 | <0.0001 |
| | 75-79 | 0.82 | 0.77 | 0.89 | <0.0001 |
| Sex (Male) | Female | 0.96 | 0.91 | 1.02 | 0.17 |
| Distance to nearest cancer centre at diagnosis (<50 km) | 50+ km | 1.16 | 1.07 | 1.25 | 0.00 |
| | Rural | 1.01 | 0.91 | 1.13 | 0.80 |
| | Urban Q2 | 1.01 | 0.93 | 1.11 | 0.78 |

**Copyright Information:** (Online) ISSN: 2572-9241
©2026 The Author(s). HemaSphere is published by John Wiley & Sons Ltd on behalf of European Hematology Association. This is an open access Abstract Book distributed under the Attribution-NonCommercial-NoDerivs (CC BY-NC-ND), which allows third parties to download the articles and share them with others as long as they credit the author and the Abstract Book, but they cannot change the content in any way or use them commercially.
**Abstract Book Citations:** Authors, Title, HemaSphere, 2026;10:(S1):pages. The abstract book DOIs can be found on the "Author Information" page.
**Disclaimer:** Articles published in the journal HemaSphere exclusively reflect the opinions of the authors. The authors are responsible for all content in their abstracts including accuracy of the facts, statements, citing resources, etc.

| | | | | | |
|---|---|---|---|---|---|
| Rural status + income quintile at diagnosis (Urban Q1 = lowest) | Urban Q3 | 1.01 | 0.93 | 1.11 | 0.78 |
| | Urban Q4 | 0.96 | 0.88 | 1.05 | 0.40 |
| | Urban Q5 | 0.91 | 0.83 | 0.99 | 0.03 |
| PopGrouper category (Major) | Moderate | 0.82 | 0.77 | 0.87 | <0.0001 |
| | Minor/non-users | 0.76 | 0.69 | 0.84 | <0.0001 |

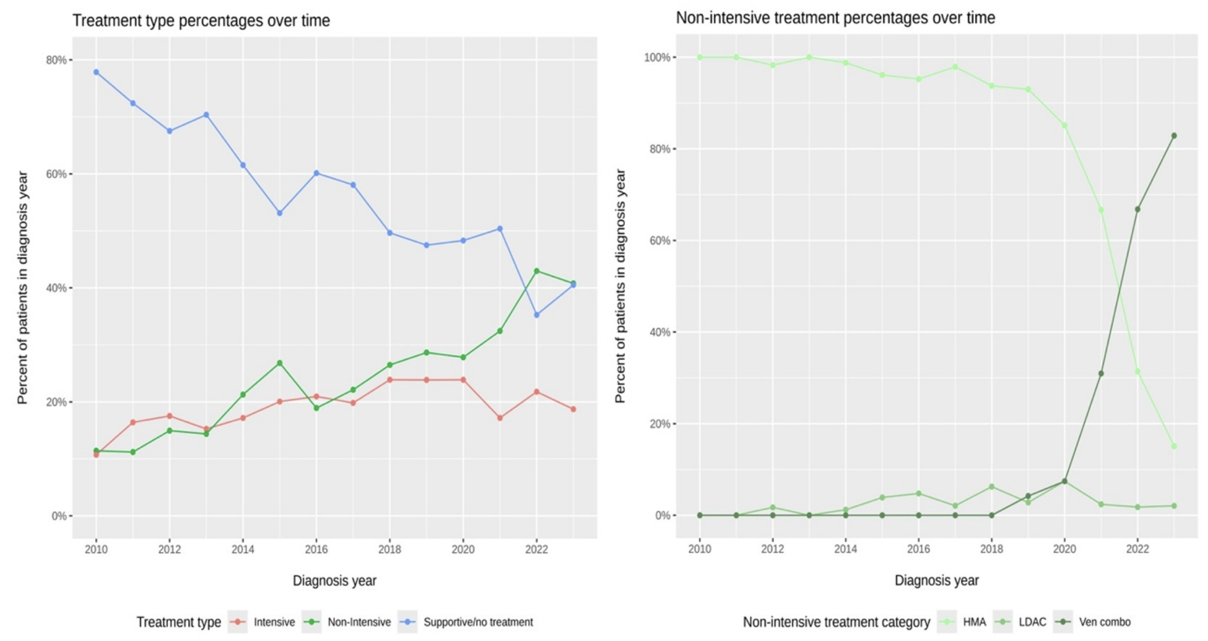

**Figure: Treatment patterns over time**

**Copyright Information:** (Online) ISSN: 2572-9241
©2026 The Author(s). HemaSphere is published by John Wiley & Sons Ltd on behalf of European Hematology Association. This is an open access Abstract Book distributed under the Attribution-NonCommercial-NoDerivs (CC BY-NC-ND), which allows third parties to download the articles and share them with others as long as they credit the author and the Abstract Book, but they cannot change the content in any way or use them commercially.
**Abstract Book Citations:** Authors, Title, HemaSphere, 2026;10:(S1):pages. The abstract book DOIs can be found on the "Author Information" page.
**Disclaimer:** Articles published in the journal HemaSphere exclusively reflect the opinions of the authors. The authors are responsible for all content in their abstracts including accuracy of the facts, statements, citing resources, etc.

### (PS1640) CPX-351 CONFERS SURVIVAL ADVANTAGE AS BRIDGE TO TRANSPLANT IN PATIENTS WITH TP53 MUTATED ACUTE MYELOID LEUKEMIAS THERAPY RELATED AND WITH MYELODISPLASTIC RELATED CHANGES ACHIEVING COMPLETE REMISSION

**Topic:** 04. Acute myeloid leukemia - Clinical

Elisabetta Todisco*[1], Ivana Lotesoriere[1], Carlos Jimenez-Chillon[2], Federico Lussana[3], Federica Gigli[4], Cristina Papayannidis[5], Roberto Cairoli[6], Massimo Bernardi[7], Michela Rondoni[8], Felicetto Ferrara[9], Annalina Piccioni[10], Michela Dargenio[11], Andrea Corbingi[12], Francesco Mannelli[13], Alessandro Cignetti[14], Patrizia Zappasodi[15], Maria Paola Martelli[16], Monia Lunghi[17], Luana Fianchi[18], Alessandra Sperotto[19], Raffaele Palmieri[20], Laura Solán[21], Olga Salamero[22], Marta Polo[23], Javier Ortiz Martin[24], Marina Diaz Beya[25], Virginia Pradillo Fernandez[26], Ana Alfonso Pierola[27], Maria Pilar Martinez Sanchez[28], Fabio Guolo[29], Gabriela Rodríguez MacÍas[2]

*[1]ASST Valle Olona, Busto Arsizio, Italy; [2]Hospital General Universitario Gregorio Marañon, Instituto de Investigación Sanitaria Gregorio Marañón (IiSGM), Madrid, Spain; [3]ASST Papa Giovanni XXIII, Bergamo, Italy; [4]IEO European Institute of Oncology, Milan, Italy; [5]University Hospital S. Orsola Malpighi, Bologna, Italy; [6]ASST Grande Ospedale Metropolitano Niguarda, Milan, Italy; [7]"San Raffaele" Scientific Institute, Milan, Italy; [8]AUSL Romagna, Ravenna, Italy; [9]A. Cardarelli Hospital, Naples, Italy; [10]San Giovanni Addolorata Hospital, Rome, Italy; [11]Vito Fazzi Hospital, Lecce, Italy; [12]Santa Maria Goretti Hospital, Latina, Italy; [13]Careggi Hospital University of Florence, Florence, Italy; [14]A.O. "Ordine Mauriziano", Turin, Italy; [15]IRCCS S. Matteo Polyclinic Foundation, Pavia, Italy; [16]Insitute of Hematology CREO University of Perugia, Perugia, Italy; [17]University of Eastern Piedmont, Novara, Italy; [18]A. Gemelli Polyclinic, Rome, Italy; [19]IOV Venetian Oncology Institute, Castelfranco Veneto, Italy; [20]Tor Vergata University, Rome, Italy; [21]Hospital Universitario Fundación Jiménez Díaz, Madrid, Spain; [22]Hospital Vall d'Hebron. Experimental del Vall d'Hebron Institut d'Oncología, Facultat de Medicina, Universitat Autònoma de Barcelona, Barcelona, Spain; [23]Hospital Clínico San Carlos de Madrid, Madrid, Spain; [24]Hospital Universitario La Princesa, Madrid, Spain; [25]Hospital Clinic Barcelona, Barcelona, Spain; [26]Hospital Universitario Quirón Pozuelo, Madrid, Spain; [27]Cancer Center Clínica Universidad de Navarra (CCUN), IdISNA, CIBERONC, Pamplona, Spain; [28]Hospital 12 Octubre, Madrid, Spain; [29]University of Genoa, Genoa, Italy;*

### Background

CPX-351 has become the standard induction therapy in Acute Myeloid Leukemias (AML) with myelodysplasia related changes (AML-MRC) and therapy-related (t-AML). TP53 mutations (TP53m) are found in about 20-35% of these AML but the assessment of their prognostic impact may be challenging considering the many variables of TP53m topography and configuration as well as the associated cytogenetic features.

### Aims

We conducted a large retrospective analysis about the outcome of TP53m AML-MRC and t-AML patients (pts) treated with CPX-351in order to evaluate the impact of biologic disease features and the role of allogeneic hematopoietic stem cell transplantation (allo-HSCT).

### Methods

This study included 51 pts treated according to the European Medicines Agency's indications for use of CPX-351 from January 2019 to December 2021 in 38 Italian centers (subanalysis of the italian real world study recently published by *Guolo F. and Todisco E., American Journal of Hematology, 2025*) and 53 pts from January 2019 to December 2025 in 20 Spanish centers.
Eligible pts proceeded to allo-HSCT as per internal standard procedures of each center.

**Copyright Information:** (Online) ISSN: 2572-9241
©2026 The Author(s). HemaSphere is published by John Wiley & Sons Ltd on behalf of European Hematology Association. This is an open access Abstract Book distributed under the Attribution-NonCommercial-NoDerivs (CC BY-NC-ND), which allows third parties to download the articles and share them with others as long as they credit the author and the Abstract Book, but they cannot change the content in any way or use them commercially.
**Abstract Book Citations:** Authors, Title, HemaSphere, 2026;10:(S1):pages. The abstract book DOIs can be found on the "Author Information" page.
**Disclaimer:** Articles published in the journal HemaSphere exclusively reflect the opinions of the authors. The authors are responsible for all content in their abstracts including accuracy of the facts, statements, citing resources, etc.

### Results

Our study population included 104 TP53m pts (76 AML-MRC and 28 t-AML) with a median age of 65 years (range 23-77); del(17p) was found in 18 pts (17%).
We restrospectively assigned TP53 allelic status according to *Grob T. et al., Blood 2022* in 94 (90%) evaluable pts (Figure 1a); 99 pts (95%) were instead evaluable for complex karyotype (CK) presence.
Forty-five pts (43%) obtained complete remission (CR) or CR with incomplete hematologic recovery (CRi) after first CPX-351 induction. In univariate analysis for CR, only del(17p) resulted having a negative prognostic impact (p < .005) while CK (p .76) and biallelic TP53 status (p .84) did not.
Thirty pts (29%) underwent allo-HSCT, 16 of them among the 51 pts of the Italian cohort (31%) and 14 (26%) in the Spanish one.
After a median follow up of 23,68 months (m) (CI95% 20,27-27,07), median overall survival (mOS) of the whole TP53m population was 7,8 m (CI95% 6,11-9,49). In univariate analysis for OS, obtaining CR was the only factor with an improving impact (mOS 16,3 m for pts in CR vs 4,8 m, p .01); instead, the significant unfavorable factor for survival was CK (6,4 vs 13,6 without CK, p .03) while biallelic TP53m status showed only a trend (6,6 m for biallelic vs 8,9 m, p .075). By including these variables into multivariate analysis for OS, only CR maintained a significant favorable impact (HR 3,79, p < .001).
In a landmark analysis of pts alive and in 1st CR at 3 m after starting induction therapy who underwent allo-HSCT (n=14), mOS for transplanted pts was 23,6 m (CI95% 16.26-not reached) vs 9,5 m (CI95%  3.75-16.53) for those not transplanted (p .04) with a 12-m survival probability of 79% vs 39% (Fig. 1b). Ten of these 14 pts (71%) had CK and 11 (79%) were biallelic while only one (7%) had del(17p). Among pts receiving allo-HSCT, there wasn't survival disadvantage for those carrying CK (mOS 20 m vs 22 m without CK, p .99).

### Summary/Conclusion

CPX-351 leads to CR 44% of TP53m pts and del(17p) seems to be the only unfavorable prognostic factor. Bridging these pts quickly to allo-HSCT provides a significant survival advantage. We are conducting an Italian retrospective and prospective expansion of the observational study about AML pts treated with CPX-351 with special attention to those carrying TP53m in order to confirm or improve these findings identifying prognostic AML features and transplant strategies.

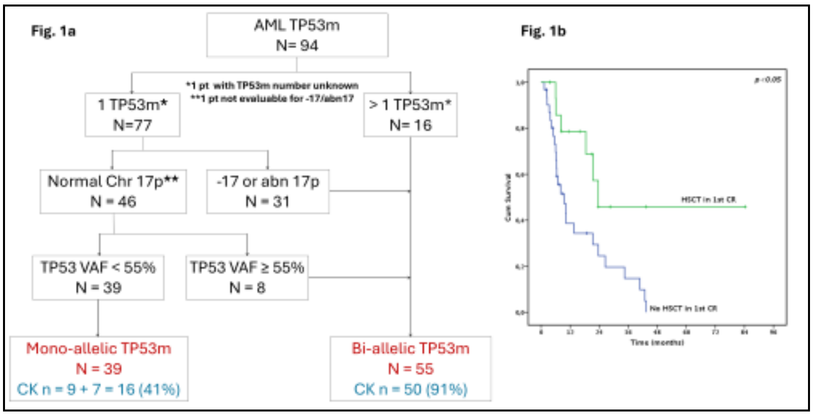

**Copyright Information:** (Online) ISSN: 2572-9241
©2026 The Author(s). HemaSphere is published by John Wiley & Sons Ltd on behalf of European Hematology Association. This is an open access Abstract Book distributed under the Attribution-NonCommercial-NoDerivs (CC BY-NC-ND), which allows third parties to download the articles and share them with others as long as they credit the author and the Abstract Book, but they cannot change the content in any way or use them commercially.
**Abstract Book Citations:** Authors, Title, HemaSphere, 2026;10:(S1):pages. The abstract book DOIs can be found on the "Author Information" page.
**Disclaimer:** Articles published in the journal HemaSphere exclusively reflect the opinions of the authors. The authors are responsible for all content in their abstracts including accuracy of the facts, statements, citing resources, etc.

### (PS1641) THE CHARACTERISTICS AND PROGNOSIS OF THERAPY-RELATED NPM1-MUTATED AML: A COMPARATIVE STUDY WITH DE NOVO NPM1-MUTATED AND THERAPY-RELATED AML IN CHINA

**Topic:** 04. Acute myeloid leukemia - Clinical

Xinyi Qi*[1], Guanghui Chang[1], Yu Zhang[2], Hongyu Zhang[3], Jinhai Ren[4], Peng-Cheng He[5], Yajing Xu[6], Hua Yan[7], Yongqian Jia[8], Liping Dou[9], Tao Wu[10], Zhi Guo[11], Xiaojun Yuan[12], Yongjun Fang[13], Xiaoyan Wu[14], Guo-Rui Ruan[1], Xiaosu Zhao[1], Qian Jiang[1], Hao Jiang[1], Lanping Xu[1], Xiaohui Zhang[1], Xiao-Jun Huang[1, 15], Feifei Tang[1]

*[1]Peking University People's Hospital, Peking University Institute of Hematology, National Clinical Research Center for Hematologic Disease, Beijing Key Laboratory of Cell and Gene Therapy for Hematologic Malignancies, Peking University, Beijing, China; [2]Nanfang Hospital, Southern Medical University, Guangzhou, China;[3]Peking University Shenzhen Hospital, Shenzhen, China; [4]Second Hospital of Hebei Medical University, Hematology Institute of Hebei Province, Shijiazhuang, China; [5]The First Affiliated Hospital of Xi'an Jiaotong University, Xi'an, China;[6]Xiangya Hospital, Central South University, Changsha, China; [7]Ruijin Hospital Affiliated to Shanghai Jiao Tong University School of Medicine, Shanghai, China;[8]West China Hospital of Sichuan University, Chengdu, China; [9]Chinese PLA General Hospital, Beijing, China; [10]The 940th Hospital of Joint Logistics Support Force of Chinese People's Liberation Army, Lanzhou, China; [11]Huazhong University of Science and Technology Union Shenzhen Hospital, Shenzhen, China; [12]Xinhua Hospital Affiliated to Shanghai Jiao Tong University School of Medicine, Shanghai, China; [13]Children's Hospital of Nanjing Medical University, Nanjing, China; [14]Union Hospital, Tongji Medical College, Huazhong University of Science and Technology, Wuhan, China; [15]Peking-Tsinghua Center for Life Sciences, Academy for Advanced Interdisciplinary Studies, Peking University, Beijing, China;*

### Background

Therapy-related AML (t-AML) typically carries poor prognosis, whereas *NPM1* mutations favor survival in de novo AML. Although 2022 ELN guidelines prioritize genetics over clinical history for risk stratification, large-scale Chinese data comparing therapy-related *NPM1*-mutated AML (t-*NPM1* AML) with de novo *NPM1*-mutated AML (dn-*NPM1* AML) and t-AML cohorts remain scarce.

### Aims

We aimed to investigate the clinical characteristics and prognosis of t-*NPM1* AML in the Chinese population by comparing the features of t-*NPM1* AML with dn-*NPM1* AML and t-AML in a multi-center cohort.

### Methods

We analyzed the baseline characteristics and prognosis of 878 patients from 14 centers across China, including 34 t-*NPM1* AML, 647 dn-*NPM1* AML, and 174 t-AML.

**Copyright Information:** (Online) ISSN: 2572-9241
©2026 The Author(s). HemaSphere is published by John Wiley & Sons Ltd on behalf of European Hematology Association. This is an open access Abstract Book distributed under the Attribution-NonCommercial-NoDerivs (CC BY-NC-ND), which allows third parties to download the articles and share them with others as long as they credit the author and the Abstract Book, but they cannot change the content in any way or use them commercially.
**Abstract Book Citations:** Authors, Title, HemaSphere, 2026;10:(S1):pages. The abstract book DOIs can be found on the "Author Information" page.
**Disclaimer:** Articles published in the journal HemaSphere exclusively reflect the opinions of the authors. The authors are responsible for all content in their abstracts including accuracy of the facts, statements, citing resources, etc.

### Results t-*NPM1* and dn-*NPM1* AML have similar cytogenetic and molecular features compared to t-AML: higher normal karyotype (58.8% vs. 71.9 % vs. 29.3%, P<0.001), *DNMT3A* (48.1% vs 56.2% vs. 16.9%, P<0.001), and *FLT3-ITD* (41% vs. 41.6% vs. 13.2%, P<0.001), but lower *TP53* (0% vs 2.9% vs. 8.4%, P=0.077) and *RUNX1* (0% vs 0.3% vs 12%, P<0.001). Clinically, t-*NPM1* AML had similar 3-year OS to dn-*NPM1* AML, and both were higher than t-AML (72.8% vs. 69.3% vs 51.6%; P<0.001). Patients with >3 mutations had inferior 3-year OS in t-*NPM1* (36.4% vs 84.6%, P=0.014) and dn-*NPM1* (74.0% vs 80.0%, P=0.046). The triple-mutated genotype predicted inferior 3-year OS in t-*NPM1* (20.0% vs 71.0%, P=0.011) and showed a trend toward inferior 3-year OS in dn-*NPM1* (67.0% vs 78.0%, P=0.074). In multivariate analysis, *TET2* mutations predicted inferior OS and DFS in both t-*NPM1* (OS: HR 39.52, P=0.003; DFS: HR 18.84, P=0.017) and dn-*NPM1* (OS: HR 1.85, P=0.017; DFS: HR 1.64, P=0.032).
Baseline characteristics, achievement of CR/CRi, and allogeneic transplant rates by disease group.

| Characteristic | t-NPM1 AML N = 34 | dn-NPM1 AML N = 647 | t-AML N = 174 | P (t-NPM1 vs dn-NPM1 AML) | P (t-NPM1 vs t-AML) |
|---|---|---|---|---|---|
| **Age, median [min-max]** | 58[37 - 71] | 49 [14 - 80] | 51 [16 - 89] | <0.001 | 0.004 |
| **Male sex (%)** | 9 (26.5%) | 291 (45.0%) | 58 (33.3%) | 0.035 | 0.622 |
| **Prior therapy** | | | | | |
| Chemotherapy alone | 19 (55.9%) | | 95 (54.6%) | | 0.999 |
| Radiotherapy alone | 6 (17.6%) | | 18 (10.3%) | | 0.241 |
| Chemotherapy + radiotherapy | 9 (26.5%) | | 61 (35.1%) | | 0.428 |
| **Latency from primary tumor (months)** | 58 [2 - 285] | | 38[1.8 - 480] | | 0.002 |
| **White cell count, $x10^9$/L** | 16[1 - 277] | 31 [1 - 558] | 8 [1 - 281] | 0.154 | 0.066 |
| **Bone marrow blasts (%)** | 57 [24 - 92] | 66 [9 - 99] | 49 [20 - 96] | 0.013 | 0.763 |
| **Cytogenetic risk group** | | | | | |
| Favorable | 3 (8.8%) | 4 (0.6%) | 38 (21.8%) | 0.003 | 0.100 |
| Normal karyotype | 20 (58.8%) | 465 (71.9%) | 51 (29.3%) | 0.121 | 0.001 |
| Other intermediate | 8 (23.5%) | 89 (13.8%) | 32 (18.4%) | 0.126 | 0.481 |
| Adverse | 1 (2.9%) | 8(1.2%) | 31 (17.8%) | 0.434 | 0.034 |
| Unknown | 2(5.9%) | 81(12.5%) | 22(12.6%) | | |
| **ELN 2022 risk group** | | | | | |
| Favorable | 17 (50.0%) | 326 (50.4%) | 48 (27.6%) | 0.999 | 0.015 |
| Intermediate | 11 (32.4%) | 226 (34.9%) | 45 (25.9%) | 0.855 | 0.526 |
| Adverse | 4 (11.8%) | 14 (2.2%) | 59 (33.9%) | 0.010 | 0.013 |
| Unknown | 2(5.9%) | 81(12.5%) | 22(12.6%) | | |
| **Intensive treatment (%)** | 19 (55.9%) | 466 (72.0%) | 108 (62.8%) | 0.051 | 0.448 |
| **Allogeneic transplant (%)** | 13 (38.2%) | 269 (41.6%) | 72 (41.4%) | 0.726 | 0.849 |
| **CR/CRi (%)** | 29 (85.3%) | 590 (91.2%) | 129 (74.1%) | 0.218 | 0.193 |

Abbreviations: AML, acute myeloid leukemia; dn-*NPM1*, de novo NPM1-mutated; t-AML, therapy-related AML with wild-type *NPM1*; t-*NPM*1, therapy-related *NPM1*-mutated AML;CR, complete remission; CRi, complete remission with incomplete hematologic recovery; ELN, European LeukemiaNet; IQR, interquartile range.

### Summary/Conclusion t-*NPM1* AML is similar to dn-*NPM1* AML in baseline demographic, cytogenetic, and molecular features, as well as clinical survival; both are superior than t-AML.

**Copyright Information:** (Online) ISSN: 2572-9241
©2026 The Author(s). HemaSphere is published by John Wiley & Sons Ltd on behalf of European Hematology Association. This is an open access Abstract Book distributed under the Attribution-NonCommercial-NoDerivs (CC BY-NC-ND), which allows third parties to download the articles and share them with others as long as they credit the author and the Abstract Book, but they cannot change the content in any way or use them commercially.
**Abstract Book Citations:** Authors, Title, HemaSphere, 2026;10:(S1):pages. The abstract book DOIs can be found on the "Author Information" page.
**Disclaimer:** Articles published in the journal HemaSphere exclusively reflect the opinions of the authors. The authors are responsible for all content in their abstracts including accuracy of the facts, statements, citing resources, etc.

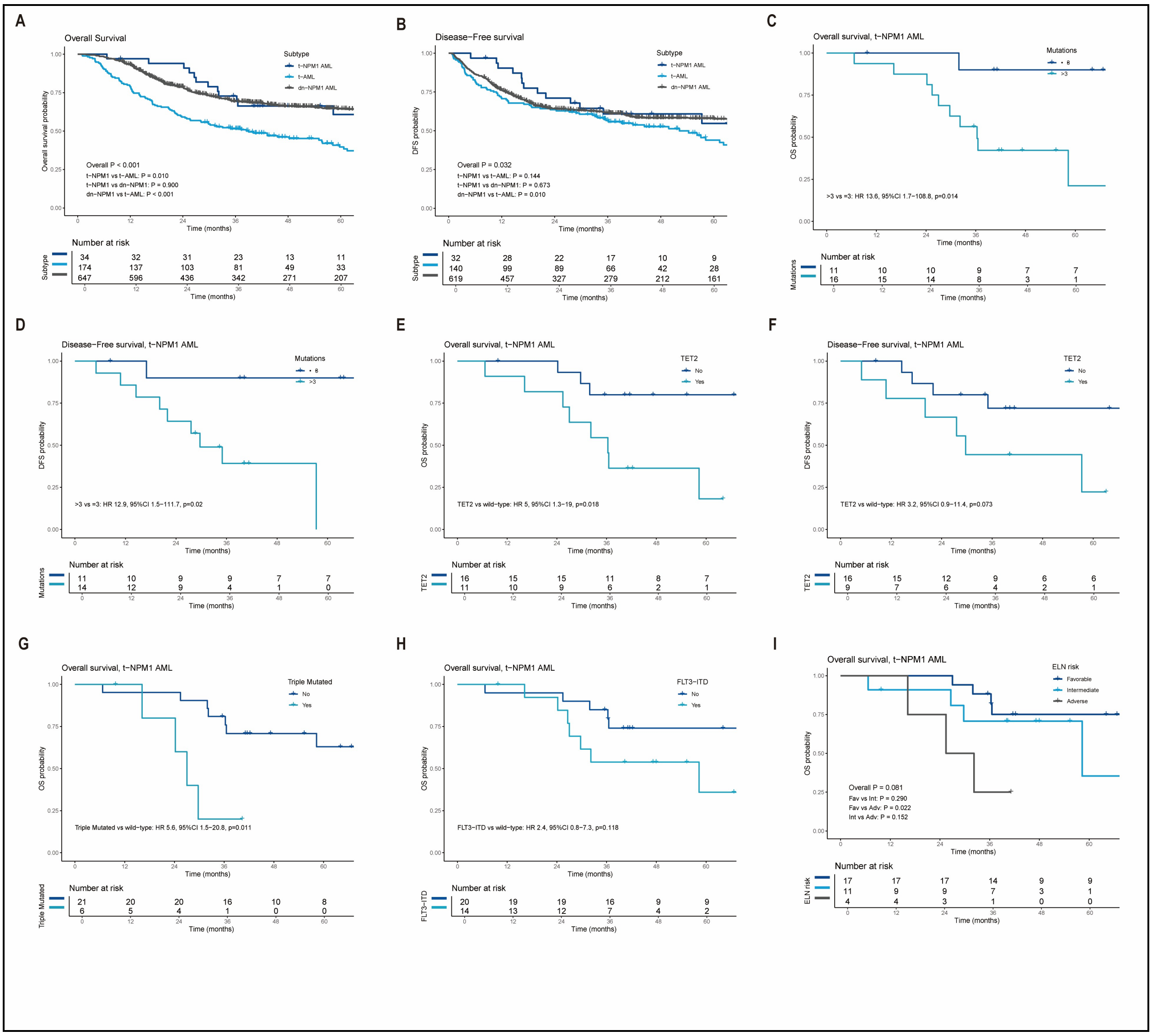

Kaplan-Meier survival curves for (A) OS and (B) DFS corresponding to the subtypes of AML (t-NPM1 AML, t-AML, and dn-NPM1 AML); (C) OS and (D) DFS of with ≤3 or >3 mutations in t-NPM1 AML patients; (E) OS and (F) DFS of with or without TET2 mutation in t-NPM1 AML patients; (G) OS of with or without triple Mutated in t-NPM1 AML patients; (H) OS of with or without FLT3-ITD mutation in t-NPM1 AML patients; (I) OS stratified by ELN risk categories (favorable, intermediate, and adverse) in t-NPM1 AML patients.

**Copyright Information:** (Online) ISSN: 2572-9241
©2026 The Author(s). HemaSphere is published by John Wiley & Sons Ltd on behalf of European Hematology Association. This is an open access Abstract Book distributed under the Attribution-NonCommercial-NoDerivs (CC BY-NC-ND), which allows third parties to download the articles and share them with others as long as they credit the author and the Abstract Book, but they cannot change the content in any way or use them commercially.
**Abstract Book Citations:** Authors, Title, HemaSphere, 2026;10:(S1):pages. The abstract book DOIs can be found on the “Author Information” page.
**Disclaimer:** Articles published in the journal HemaSphere exclusively reflect the opinions of the authors. The authors are responsible for all content in their abstracts including accuracy of the facts, statements, citing resources, etc.

### (PS1642) MOLECULAR PATTERNS AND THERAPY-SPECIFIC SURVIVAL OF PATIENTS (PTS) WITH IDH1-MUTATED ACUTE MYELOID LEUKEMIA (AML) WITH RESPECT TO SELF-REPORTED RACE-ETHNICITY

**Topic:** 04. Acute myeloid leukemia - Clinical

Jennifer Marvin-Peek*[1], Onyee Chan[2], Alexander Ambinder[3], Yazan F. Madanat[4], Kuo-Kai Chin[5], Ann Mercurio[6], Vanessa Kennedy[7], Yamuna Kondapally[7], Tapan Kadia[1], Naval Daver[1], Alice Mims[8], Ilias Oulidi[9], Praneeth Baratam[10], Deedra Nicolet[8], Krzysztof Mrozek[8], Michael Walker[8], Utsav Joshi[2], Najla Al Ali[2], Haley Roberts[8], Thomas Kehoe[11], Akriti Jain[12], Sean Ormond[13], Madelyn Burkart[14], David Swoboda[15], Elaine Mardis[16], B. Douglas Smith[3], Courtney Dinardo[1], Daniel Pollyea[17], Ann-Kathrin Eisfeld[8]

*[1]University of Texas MD Anderson Cancer Center, Leukemia, Houston, United States of America;[2]Moffitt Cancer Center, Tampa, United States of America; [3]Johns Hopkins Sidney Kimmel Comprehensive Cancer Center, Baltimore, United States of America; [4]Simmons Comprehensive Cancer Center, University of Texas Southwestern Medical Center, Dallas, United States of America; [5]Memorial Sloan Kettering Cancer Center, New York, United States of America;[6]Icahn School of Medicine at Mount Sinai Morningside/West, New York, United States of America; [7]Stanford Cancer Institute, Stanford University, Stanford, United States of America; [8]The Ohio State University Comprehensive Cancer Center, Columbus, United States of America;[9]MUSC Health Florence Medical Center, Florence, United States of America; [10]Medical University of South Carolina, Charleston, United States of America; [11]University of South Florida Morsani College of Medicine, Tampa, United States of America;[12]Taussig Cancer Institute, Cleveland Clinic, Leukemia Program, Clevelans, United States of America; [13]Atrium Health Wake Forest Baptist Medical Center, Winston-Salem, United States of America; [14]Atrium Health Wake Forest Baptist Comprehensive Cancer Center, Winston-Salem, United States of America; [15]Tampa General Hospital Cancer Institute, Tampa, United States of America; [16]Nationwide Children's Hospital, Columbus, United States of America; [17]University of Colorado, Aurora, United States of America;*

### Background

With the advent of targeted therapy, *IDH1*-mutated (*IDH1*$^{mut}$) AML pts now have several frontline treatment options, including intensive chemotherapy [(IC) ± ivosidenib (IVO)], hypomethylating agents (HMA) ± venetoclax (VEN), and HMA+IVO. However, current knowledge about the clinical efficacy of these regimens is mostly based on pts self-identified as non-Hispanic (NH) White due to underrepresentation of other populations in landmark clinical and translational studies.

### Aims

To delineate response to frontline treatments and survival outcomes of *IDH1*$^{mut}$ AML pts by treatment type and self-reported race-ethnicity (R/E).

### Methods

We identified 801 adults with newly diagnosed *IDH1*$^{mut}$ AML from a collaborative effort of 11 US-based academic institutions (n=396) and data obtained from the Flatiron Health database (n=405; AACR/Flatiron Health Real World Impact Award) between 2013 and 2025, including 198 (24.7%) pts who self-identified as not NH-White. Treatments were stratified as IC, IC+IVO, HMA+VEN, HMA+IVO, or HMA alone. Pts receiving triplet therapies were excluded. Primary outcomes included composite complete remission (CRc) rate, duration of remission (DOR), and overall survival (OS).

**Copyright Information:** (Online) ISSN: 2572-9241
©2026 The Author(s). HemaSphere is published by John Wiley & Sons Ltd on behalf of European Hematology Association. This is an open access Abstract Book distributed under the Attribution-NonCommercial-NoDerivs (CC BY-NC-ND), which allows third parties to download the articles and share them with others as long as they credit the author and the Abstract Book, but they cannot change the content in any way or use them commercially.
**Abstract Book Citations:** Authors, Title, HemaSphere, 2026;10:(S1):pages. The abstract book DOIs can be found on the “Author Information” page.
**Disclaimer:** Articles published in the journal HemaSphere exclusively reflect the opinions of the authors. The authors are responsible for all content in their abstracts including accuracy of the facts, statements, citing resources, etc.

**Results**

The median pt age was 69 years (range, 18-89) and 55% were male. Among pts not identifying as NH-White, 91 (46%) identified as NH-Black, 72 (36%) as Hispanic, and 35 (18%) as Asian. Molecular profiles did not significantly differ among R/E groups, with *DNMT3A*$^{mut}$, *NPM1*$^{mut}$, *ASXL1*$^{mut}$ and *RUNX1*$^{mut}$ being the most frequent co-occurring alterations (Fig 1A). Frontline therapy consisted of IC (n=334, 42%), IC+IVO (n=24, 3%), HMA+VEN (n=224, 28%), HMA+IVO (n=73, 9%), and HMA alone (n=102, 12%). This was different among R/E groups (p<0.001), with more non-NH-White pts receiving IC (54 vs 42%) and less receiving HMA+VEN (22 vs 32%) and HMA alone (7 vs 15%) compared to NH-White. There was no significant difference in frequency of allogeneic stem cell transplant. Among response evaluable pts other than NH-White, CRc rates were 83% for IC, 100% for IC+IVO, 62% for HMA+VEN, 44% for HMA+IVO, and 21% for HMA alone (p<0.001) (Fig 1B). CRc rates were lowest in Black pts (59%), compared to 73% and 80% in Hispanic and Asian pts (p=0.04), but the overall CRc rate in pts who identified as non-NH-white was comparable to that in White pts (68 vs 67%, p=0.73). With a median follow-up of 34 months (mo), the median OS (mOS) was 58, 41, 21, and 4 mo for non-NH-White pts treated with IC, HMA+VEN, HMA+IVO, and HMA alone, respectively (p=0.0001). In NH-Black pts age ≥60 years (yrs) (n=60), mOS was longest with HMA+VEN (27 mo), followed by IC (19 mo), HMA+IVO (9 mo) and HMA alone (3 mo). Among Hispanic pts ≥60 yrs (n=40), there were no significant differences in CRc rates (p=0.21) or mOS (p=0.79) by frontline therapy. For NH-White pts ≥60 yrs (n=479), DOR was longest with IVO-based therapy [median not reached (NR)] compared with HMA+VEN (29 mo), IC (37 mo), and HMA alone (25 mo) (p=0.04) (Fig 1C). In contrast, in non-NH-White pts ≥60 yrs (n=124), mDOR was longest with HMA+VEN (NR), compared with HMA+IVO (32 mo) and IC (18 mo).

**Summary/Conclusion**

In the largest multi-institutional analysis of *IDH1*$^{mut}$ AML pts across multiple treatment modalities and R/E groups, significant differences in treatment outcomes were observed despite similar molecular profiles. Our findings underscore the importance of proportional R/E representation in pivotal clinical trials to ensure that outcomes are accurately characterized across diverse populations.

**Copyright Information:** (Online) ISSN: 2572-9241
©2026 The Author(s). HemaSphere is published by John Wiley & Sons Ltd on behalf of European Hematology Association. This is an open access Abstract Book distributed under the Attribution-NonCommercial-NoDerivs (CC BY-NC-ND), which allows third parties to download the articles and share them with others as long as they credit the author and the Abstract Book, but they cannot change the content in any way or use them commercially.
**Abstract Book Citations:** Authors, Title, HemaSphere, 2026;10:(S1):pages. The abstract book DOIs can be found on the "Author Information" page.
**Disclaimer:** Articles published in the journal HemaSphere exclusively reflect the opinions of the authors. The authors are responsible for all content in their abstracts including accuracy of the facts, statements, citing resources, etc.

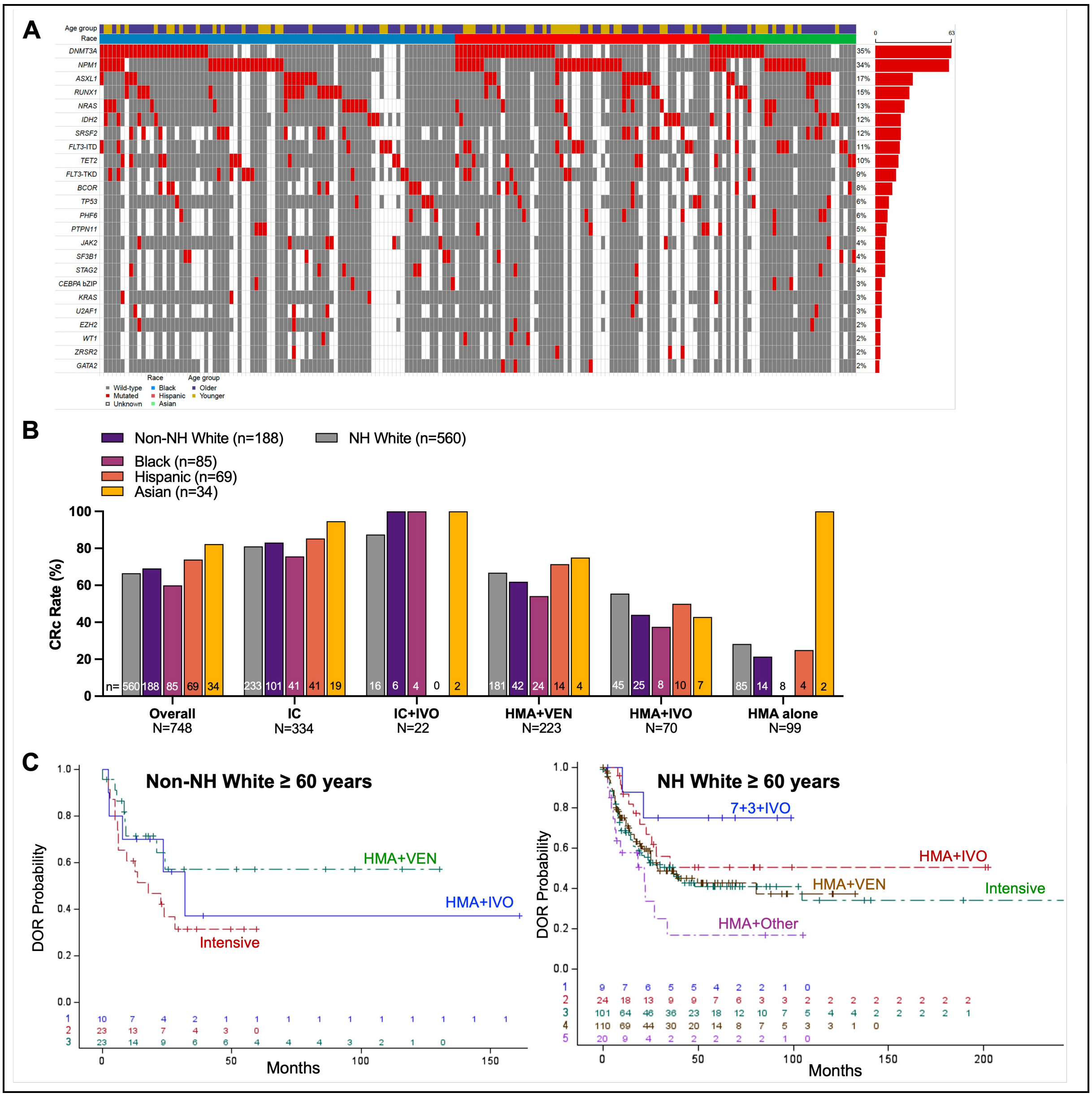

Fig 1. (A) Oncoprint depicting co-mutations in non-NH White IDH1mut AML pts separated by self-identified race-ethnicity (R/E). (B) CRc rates by treatment type and R/E in response evaluable patients. (C) Duration of remission (DOR) by on treatment type in pts ≥60 years separated by pts other than NH-White (left) or NH-White pts (right).

**Copyright Information:** (Online) ISSN: 2572-9241
©2026 The Author(s). HemaSphere is published by John Wiley & Sons Ltd on behalf of European Hematology Association. This is an open access Abstract Book distributed under the Attribution-NonCommercial-NoDerivs (CC BY-NC-ND), which allows third parties to download the articles and share them with others as long as they credit the author and the Abstract Book, but they cannot change the content in any way or use them commercially.
**Abstract Book Citations:** Authors, Title, HemaSphere, 2026;10:(S1):pages. The abstract book DOIs can be found on the "Author Information" page.
**Disclaimer:** Articles published in the journal HemaSphere exclusively reflect the opinions of the authors. The authors are responsible for all content in their abstracts including accuracy of the facts, statements, citing resources, etc.

### (PS1643) INTENSIFIED INDUCTION CHEMOTHERAPY DOES NOT APPEAR TO IMPROVE OUTCOMES FOR PATIENTS WITH UPSTREAM BINDING TRANSCRIPTION FACTOR TANDEM DUPLICATIONS (UBTF-TD) AML: RESULTS FROM THE UK NCRI AML19 TRIAL.

**Topic:** 04. Acute myeloid leukemia - Clinical

Katie Lewis*[1], Nicola Potter[1], Jad Othman[1, 2], Sylvie Freeman[3], Carlos Jimenez Vicente[1], Kezban Kaimak[1], Joanna Canham[4], Sean Johnson[4], Ian Thomas[4], Richard Whitmill[5], Edward Belsham[6], Nigel Russell[7], Richard Dillon[1, 7]

*[1]King's College London, Department of Medical and Molecular Genetics, London, United Kingdom;[2]The University of Sydney, Faculty of Medicine and Health, Sydney, Australia; [3]University of Birmingham, Institute of Immunology and Immunotherapy, Birmingham, United Kingdom; [4]Cardiff University, Centre for Trials Research, Cardiff, United Kingdom;[5]New Cross Hospital Wolverhampton, Wolverhampton, United Kingdom; [6]Portsmouth Hospitals NHS Trust, Portsmouth, United Kingdom;[7]Guy's and St Thomas' NHS Foundation Trust, Department of Haematology, London, United Kingdom;*

### Background

*UBTF*-TD is a novel recurrent genetic abnormality occurring in ~3-4% of de novo AML. Initial studies indicate that *UBTF*-TD AML has a poor prognosis and optimal treatment strategies are yet to be determined.

### Aims

To report the clinicopathological features and outcomes for patients with *UBTF*-TD AML enrolled in the UK NCRI AML19 to investigate the impact of treatment intensification.

### Methods

The UK NCRI AML19 clinical trial (ISRCTN78449203) enrolled 1695 younger adults (generally <60y) with newly-diagnosed AML, without known adverse karyotype, who were randomised between standard (DA+GO) and intensified (FLAG-Ida+GO) chemotherapy regimens. A subset of patients also received midostaurin. We retrospectively screened patients without an AML-defining genetic lesion or adverse karyotype for *UBTF*-TD by PCR and NGS and analysed patient outcomes.

**Copyright Information:** (Online) ISSN: 2572-9241
©2026 The Author(s). HemaSphere is published by John Wiley & Sons Ltd on behalf of European Hematology Association. This is an open access Abstract Book distributed under the Attribution-NonCommercial-NoDerivs (CC BY-NC-ND), which allows third parties to download the articles and share them with others as long as they credit the author and the Abstract Book, but they cannot change the content in any way or use them commercially.
**Abstract Book Citations:** Authors, Title, HemaSphere, 2026;10:(S1):pages. The abstract book DOIs can be found on the "Author Information" page.
**Disclaimer:** Articles published in the journal HemaSphere exclusively reflect the opinions of the authors. The authors are responsible for all content in their abstracts including accuracy of the facts, statements, citing resources, etc.

**Results**

A total of 683 patient's samples were screened and 43 (6.3%, accounting for 2.5% of all enrolled patients) were positive, with insertion sizes between 3-219 base pairs. There was 100% concordance between PCR and NGS results for the 15 cases where both techniques were performed. Patients had a median age of 37y (range 17-57). 33 patients (77%) had a myeloid NGS panel performed, and the most common co-mutations were *FLT3*-ITD (63%) and *WT1* (49%). Normal karyotype (20, 47%) or isolated +8 (15, 35%) were the most common cytogenetic findings.
28 patients (65%) were randomised to DA±GO and 15 (35%) to FLAG-Ida±GO for cycle 1 (C1): due to drug availability 4/28 and 6/15 patients respectively did not receive GO. 5 patients (12%) with *FLT3*-ITD received midostaurin. 5 patients (12%) who received DA±GO in C1 were randomised to receive FLAG-Ida in C2, as part of a separate "high-risk" randomisation, with the remaining patients receiving DA (17, 40%) or CPX351 (6, 14%) for C2. 31 patients (72%) underwent allogeneic stem cell transplant with the majority (28, 65%) undertaken in CR1.

The overall response rate (CR/CRi) after C1 was 77% and 93% for patients randomised to DA and FLAG-Ida respectively (p=0.38). MRD was assessed by multiparameter flow cytometry for 16 patients post-C1 and 14 post-C2. Using a threshold of 0.1%, MRD was positive in 12 (75%) and 7 (50%) respectively.

With a median follow up of 33.6 months, the 3-year OS for all patients with *UBTF*-TD AML was 56% (95% CI 0.41–0.77). There was no difference in OS according to treatment allocation (panel A): this was 63% (95% CI, 46–86) in patients treated with DA±GO and 41% (95% CI, 19–87) in those treated with FLAG-Ida±GO (HR, 1.61; 95% CI, 0.60–4.33; log-rank p = 0.34). The 3-year cumulative incidence of relapse (CIR, Figure 1. panel B) was 46% (95% CI, 23–68) in patients treated with DA±GO and 48% (95% CI, 23–73) in those treated with FLAG-Ida±GO (Gray's test p = 0.77).

For the 28 patients that received allogeneic transplant in CR1, 3y OS was 64%.

**Summary/Conclusion**

This is the largest prospective, single trial cohort of *UBTF*-TD AML reported to date, and confirms that these patients have high rates of MRD positivity and relatively poor outcomes due to a high rate of relapse. Intensification of chemotherapy did not reduce relapse or improve survival. Proactive screening and alternative therapies are required for this high-risk patient population.

**Copyright Information:** (Online) ISSN: 2572-9241
©2026 The Author(s). HemaSphere is published by John Wiley & Sons Ltd on behalf of European Hematology Association. This is an open access Abstract Book distributed under the Attribution-NonCommercial-NoDerivs (CC BY-NC-ND), which allows third parties to download the articles and share them with others as long as they credit the author and the Abstract Book, but they cannot change the content in any way or use them commercially.
**Abstract Book Citations:** Authors, Title, HemaSphere, 2026;10:(S1):pages. The abstract book DOIs can be found on the "Author Information" page.
**Disclaimer:** Articles published in the journal HemaSphere exclusively reflect the opinions of the authors. The authors are responsible for all content in their abstracts including accuracy of the facts, statements, citing resources, etc.

FIGURE 1. OUTCOMES BASED ON PRIMARY RANDOMISATION. (A) OS, (B) CIR

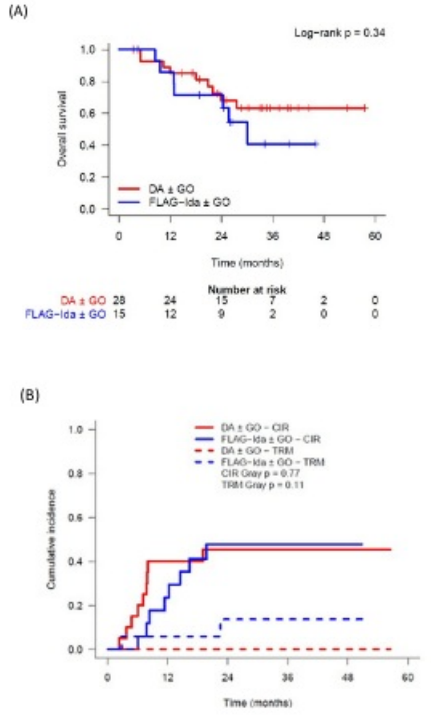

**Copyright Information:** (Online) ISSN: 2572-9241
©2026 The Author(s). HemaSphere is published by John Wiley & Sons Ltd on behalf of European Hematology Association. This is an open access Abstract Book distributed under the Attribution-NonCommercial-NoDerivs (CC BY-NC-ND), which allows third parties to download the articles and share them with others as long as they credit the author and the Abstract Book, but they cannot change the content in any way or use them commercially.
**Abstract Book Citations:** Authors, Title, HemaSphere, 2026;10:(S1):pages. The abstract book DOIs can be found on the "Author Information" page.
**Disclaimer:** Articles published in the journal HemaSphere exclusively reflect the opinions of the authors. The authors are responsible for all content in their abstracts including accuracy of the facts, statements, citing resources, etc.

### (PS1644) GENETIC CHARACTERISTICS AND PROGNOSTIC IMPACT OF MYC AND/OR KMT2A AMPLIFICATION MANIFESTING AS DOUBLE MINUTES IN ACUTE MYELOID LEUKEMIA

**Topic:** 04. Acute myeloid leukemia - Clinical

Tong Wang*[1, 2], Jingbo Ni[1], Jing Long[3], Jinan Tan[1], Xinqing Li[1], Shengxiao Gao[2], Wei Cao[1], Yan Zhang[1], Qiongjie Zhang[1], Ying Yin[2], Hongxing Liu[1, 2]

*[1]Division of Laboratory Medicine, Hebei Yanda Lu Daopei Hospital, Langfang, China;[2]Beijing Lu Daopei Institute of Hematology, Beijing, China; [3]Division of Information center, Beijing Lu Daopei Institute of Hematology, Beijing, China;*

### Background

High-level amplification of *MYC* and/or *KMT2A (MYC/KMT2A)* is a rare but highly aggressive genomic alteration in acute myeloid leukemia (AML). Double minutes (dmin), extrachromosomal circular DNA fragments, represent the most frequent cytogenetic manifestation of gene amplification in AML. However, the genetic features and prognostic significance of *MYC/KMT2A* amplification associated with dmin remain poorly defined.

### Aims

To characterize the cytogenetic, molecular features and clinical impact of *MYC/KMT2A* amplification associated with dmin in AML patients (pts).

### Methods

Among 4,200 consecutive AML pts diagnosed at Hebei Yanda Lu Daopei Hospital between 2014 and 2025, cases with dmin identified by conventional G-banding were retrospectively analyzed. Interphase FISH with dual-color break-apart probes for *MYC* and *KMT2A* was performed in all dmin-positive pts and in 24 dmin-negative controls with chromosome 8 and/or 11 abnormalities. Gene amplification was defined as >5 signals per interphase nucleus, with ≥6 signals indicating high-level amplification. Integrated analyses included karyotyping, targeted next-generation sequencing, fusion gene screening, immunophenotyping, morphology, and clinical outcomes.

### Results

Double minutes were identified in 24/4200 pts (0.57%)(Fig A, B), of whom 16/24 (66.7%) harbored high-level*MYC/KMT2A* amplification, including *MYC* amplification in 11/16 (68.8%)(Fig C), *KMT2A* amplification in 3/16 (18.8%), and concurrent amplification of both genes in 2/16 (12.5%). The median age was 61.5 years (range, 46–78), with a male-to-female ratio of 1.3:1. Complex karyotypes were frequent (68.8%). Recurrent additional chromosomal abnormalities included trisomy 6 (37.5%) and trisomy 4 (31.3%). Chromosome 17 abnormalities indicating *TP53* loss were observed in 6 pts (37.5%).
Among 15 pts with available sequencing data (Fig D), *TP53* mutations were detected in 10 (66.7%), of whom 6 showed concomitant chromosomal *TP53* loss, consistent with biallelic inactivation. *TET2* mutations were identified in 6 patients (40%).
Clinically, this subgroup demonstrated an aggressive disease course and marked treatment resistance. Among 13 evaluable pts, 9 received hypomethylating agent–based regimens combined with venetoclax or similar therapies; only 2 achieved transient responses followed by early relapse. Two pts underwent allogeneic hematopoietic stem cell transplantation (allo-HSCT): one relapsed and died within 3 months post-transplant, the other achieved durable remission after a second allo-HSCT. The median overall survival (OS) was 4.5 months, with a 6-month OS rate of 27.3% (Fig E). Pts with biallelic *TP53* inactivation experienced particularly dismal outcomes (OS range, 0.3–5 months). No significant survival difference was observed between *MYC*- and *KMT2A*- amplified pts.

**Copyright Information:** (Online) ISSN: 2572-9241
©2026 The Author(s). HemaSphere is published by John Wiley & Sons Ltd on behalf of European Hematology Association. This is an open access Abstract Book distributed under the Attribution-NonCommercial-NoDerivs (CC BY-NC-ND), which allows third parties to download the articles and share them with others as long as they credit the author and the Abstract Book, but they cannot change the content in any way or use them commercially.
**Abstract Book Citations:** Authors, Title, HemaSphere, 2026;10:(S1):pages. The abstract book DOIs can be found on the “Author Information” page.
**Disclaimer:** Articles published in the journal HemaSphere exclusively reflect the opinions of the authors. The authors are responsible for all content in their abstracts including accuracy of the facts, statements, citing resources, etc.

### Summary/Conclusion

Double minutes–associated *MYC/KMT2A* amplification in AML defines an ultra–high-risk disease entity characterized by complex karyotypes, recurrent trisomy 6/4, and a striking enrichment of *TP53* alterations with frequent biallelic inactivation. These pts exhibit extreme chemoresistance and dismal survival. FISH detection of *MYC/KMT2A* amplification may serve as a practical biomarker for risk stratification and monitoring. Novel therapeutic strategies are urgently needed, while allo-HSCT remains the only potential option for durable disease control in selected pts.

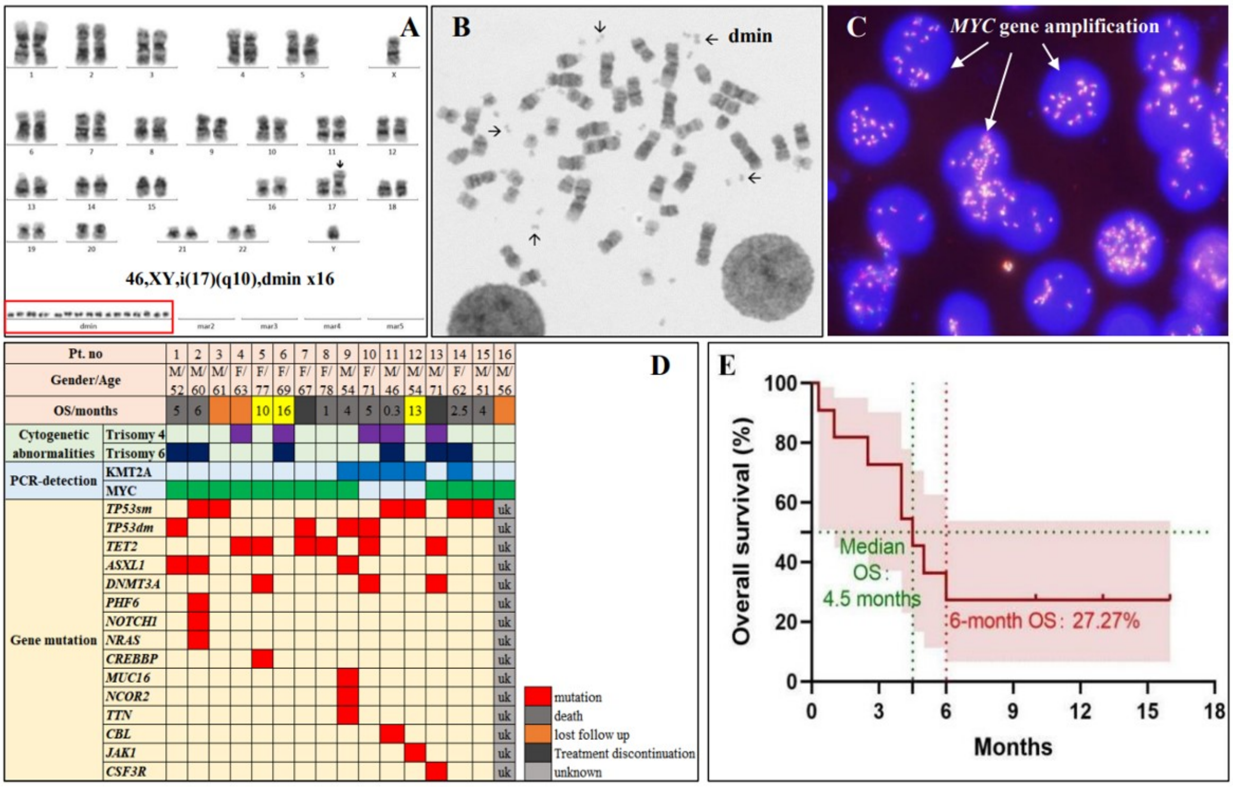

Fig: (A) Chromosome karyotype, with i(17ql0) and a large number of double minute chromosomes, i(17ql0) (indicated by the arrow) suggests loss of the *TP53* gene; (B) The arrow indicates double minutes; (C) FISH shows high amplification of the *MYC* gene in the interphase nucleus. (D) Basic Information, Karyotype, and Genetic Mutation Results of 16 Patients with Double Minutes and *MYC/KMT2A* Gene Amplification. (E) Overall survival curves of the 11 patients with complete follow-up data.

**Copyright Information:** (Online) ISSN: 2572-9241
©2026 The Author(s). HemaSphere is published by John Wiley & Sons Ltd on behalf of European Hematology Association. This is an open access Abstract Book distributed under the Attribution-NonCommercial-NoDerivs (CC BY-NC-ND), which allows third parties to download the articles and share them with others as long as they credit the author and the Abstract Book, but they cannot change the content in any way or use them commercially.
**Abstract Book Citations:** Authors, Title, HemaSphere, 2026;10:(S1):pages. The abstract book DOIs can be found on the "Author Information" page.
**Disclaimer:** Articles published in the journal HemaSphere exclusively reflect the opinions of the authors. The authors are responsible for all content in their abstracts including accuracy of the facts, statements, citing resources, etc.

### (PS1645) FRONTLINE TREATMENT INTENSITY MAY NOT SIGNIFICANTLY INFLUENCE SURVIVAL IN IDH-MUTATED AML

**Topic:** 04. Acute myeloid leukemia - Clinical

Mohamad Sheikh Najeeb[1], Oudai Alkabbani[2], Noah Takacs[1], James M Foran[3], Cecilia Arana Yi[4], Aasiya Matin[1], William Hogan[1], Mehrdad Hefazi[1], Mithun Shah[1], Kebede Begna[1], Mark Litzow[1], Naseema Gangat[1], Abhishek Mangaonkar[1], Aref Al-Kali[1], Tariq Kewan*[1]

*[1]Mayo Clinic, Hematology, Rochester, United States of America; [2]Mayo Clinic, Rochester, United States of America; [3]Mayo Clinic, Jacksonville, United States of America; [4]Mayo Clinic, Phoenix, United States of America;*

### Background

Mutations in Isocitrate dehydrogenase (*IDH*) are recurrent in acute myeloid leukemia (AML). Frontline treatment selection is typically guided by patient fitness, with intensive chemotherapy (IC) favored for fit young patients, and lower-intensity therapy (LIT), including hypomethylating agents with venetoclax and/or *IDH* inhibitors, commonly used in older or unfit patients. Comparative survival data between IC and LIT in *IDH*-mutated AML remain inconclusive with no definitive evidence establishing the superiority of one approach over the other.

### Aims

To compare clinical response and survival outcomes among patients with *IDH*-mutated AML treated with IC versus LIT.

### Methods

AML patients diagnosed at Mayo Clinic, Rochester (2016–2024) with available next-generation sequencing data were included. IRB approval was obtained. Baseline variables were recorded at time of diagnosis. Patients who received frontline *IDH*-targeted therapy were excluded. Primary outcomes were complete remission (CR), progression-free survival (PFS), and overall survival (OS) from diagnosis to death or last follow-up. Secondary outcomes included subgroup survival analyses by age, hematopoietic stem cell transplantation (HSCT) status, cytogenetic risk, and baseline *IDH* variant allele frequency (VAF). Discriptive statistics were performed using GraphPad Prism 10.0.0.

**Copyright Information:** (Online) ISSN: 2572-9241
©2026 The Author(s). HemaSphere is published by John Wiley & Sons Ltd on behalf of European Hematology Association. This is an open access Abstract Book distributed under the Attribution-NonCommercial-NoDerivs (CC BY-NC-ND), which allows third parties to download the articles and share them with others as long as they credit the author and the Abstract Book, but they cannot change the content in any way or use them commercially.
**Abstract Book Citations:** Authors, Title, HemaSphere, 2026;10:(S1):pages. The abstract book DOIs can be found on the “Author Information” page.
**Disclaimer:** Articles published in the journal HemaSphere exclusively reflect the opinions of the authors. The authors are responsible for all content in their abstracts including accuracy of the facts, statements, citing resources, etc.

**Results**

Of 625 molecularly annotated AML patients, 94 had *IDH*-mutated AML (*IDH1* 30%, *IDH2* 68%, concurrent *IDH1+IDH2* 2%); 51% received IC (n=48) and 49% LIT (n=46). The IC group was younger (median 62 vs 75 years; p=0.0001) and had more frequent normal karyotype (65% vs 35%; p=0.006), with a trend toward higher ELN 2022 adverse risk in LIT (74% vs 56%; p=0.08). IC had slightly lower hemoglobin (median 8.7 vs 9.7 g/dL; p=0.02) and lower absolute neutrophil count (median 0.30 vs 0.56 × 10^9/L; p=0.01), while platelets and marrow blasts did not differ (p=0.14 and p=0.40). Baseline *IDH* VAF was slightly higher in IC (42% vs 37%; p=0.02). Allogeneic HSCT was more common after IC (63% vs 17%; p=0.0001). CR was higher with IC vs LIT (67% vs 37%; p=0.003). Among patients with flow measurable residual disease (MRD) assessment, MRD negativity was similar (82% vs 77%; p=0.99). Relapse did not differ by treatment (30% vs 17%; p=0.32). Median PFS was similar (28.2 vs 19.3 months; p=0.31), and OS did not differ overall (IC not reached vs LIT 29.5 months; p=0.09) (Figure 1). Stratified OS was similar in patients ≥60 years (p=0.75), post-HSCT (p=0.94), and with baseline *IDH* VAF ≥40% (p=0.13), whereas IC was associated with improved OS in normal karyotype (p=0.04). In univariable Cox analysis, IC showed a nonsignificant trend toward improved OS vs LIT (HR 0.58; p=0.09), while older age was associated with worse OS (HR 1.03; p=0.03), undergoing HSCT with better OS (HR 0.39; p=0.01), and achieving CR with better OS (HR 0.50; p=0.03). In multivariable analysis adjusting for treatment type, age, complex karyotype, HSCT, baseline *IDH* VAF, and CR, IC was not independently associated with OS (aHR 1.24; p=0.63), whereas HSCT remained associated with improved OS (aHR 0.43; p=0.04); other covariates were not significant.

**Summary/Conclusion**

Among patients with *IDH*-mutated AML, the choice of frontline therapy (IC vs. LIT) did not significantly impact OS. Notably, patients treated with LIT were older and more enriched with adverse cytogenetics, yet OS outcomes remained comparable between the two groups.

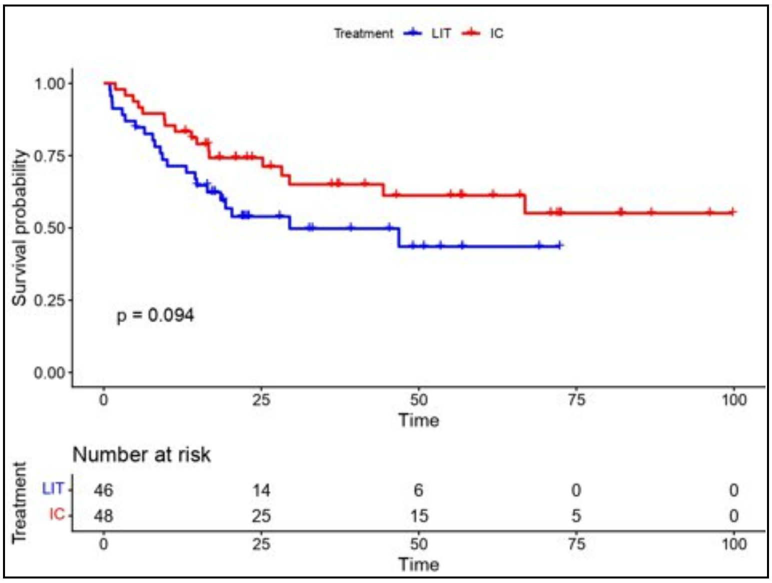

**Copyright Information:** (Online) ISSN: 2572-9241
©2026 The Author(s). HemaSphere is published by John Wiley & Sons Ltd on behalf of European Hematology Association. This is an open access Abstract Book distributed under the Attribution-NonCommercial-NoDerivs (CC BY-NC-ND), which allows third parties to download the articles and share them with others as long as they credit the author and the Abstract Book, but they cannot change the content in any way or use them commercially.
**Abstract Book Citations:** Authors, Title, HemaSphere, 2026;10:(S1):pages. The abstract book DOIs can be found on the "Author Information" page.
**Disclaimer:** Articles published in the journal HemaSphere exclusively reflect the opinions of the authors. The authors are responsible for all content in their abstracts including accuracy of the facts, statements, citing resources, etc.

### (PS1646) CLINICAL CHARACTERISTICS AND OUTCOMES OF ACUTE MYELOID LEUKEMIA WITH MECOM REARRANGEMENT: THE THREE-SITE MAYO CLINIC EXPERIENCE

**Topic:** 04. Acute myeloid leukemia - Clinical

Emma St Martin[1], Olisaemeka Ogbue[2], Ali Husnain[1], Mohamad Sheikh Najeeb[1], Oudai Alkabbani[1], Amanda Pawlenty[1], Aasiya Matin[1], Alexandra Wolanskyj-Spinner[1], Abhishek Mangaonkar[1], Mehrdad Hefazi[1], Mrinal Patnaik[1], Mithun Shah[1], Hemant Murthy[3], Mark Litzow[1], Lisa Sproat[4], Kebede Begna[1], Talha Badar[3], Rong He[1], William Hogan[1], Tariq Kewan[1], Dragan Jevremovic[1], Cecilia Arana Yi[4], Naseema Gangat[1], James M Foran[3], Hassan Alkhateeb[1], Patricia Greipp[1], Aref Al-Kali[1], Antoine Saliba*[1]

*[1]Mayo Clinic, Hematology, Rochester, United States of America;[2]Mayo Clinic, Rochester, United States of America;[3]Mayo Clinic, Jacksonville, United States of America; [4]Mayo Clinic, Phoenix, United States of America;*

### Background

Acute myeloid leukemia (AML) with MECOM rearrangement (AML-MECOM) has adverse outcomes with 3-year overall survival (OS) of <10%. Data describing real-world outcomes remain limited.

### Aims

To characterize the clinical, cytogenetic, molecular characteristics, and survival outcomes of patients (pts) with AML-MECOM treated across Mayo Clinic (Minnesota, Arizona, and Florida).

### Methods

We retrospectively identified pts diagnosed with AML-MECOM (WHO 5$^{th}$ edition) between 2014-2026. Clinical, laboratory, cytogenetic, molecular, transplant, and survival data were abstracted from the medical record. Controls consisted of consecutive AML pts without MECOM rearrangements treated during a similar period (n=196). Mutation comparisons were performed using Fisher's exact test with Bonferroni correction for multiple comparisons (adjusted $p$<0.001).

**Copyright Information:** (Online) ISSN: 2572-9241
©2026 The Author(s). HemaSphere is published by John Wiley & Sons Ltd on behalf of European Hematology Association. This is an open access Abstract Book distributed under the Attribution-NonCommercial-NoDerivs (CC BY-NC-ND), which allows third parties to download the articles and share them with others as long as they credit the author and the Abstract Book, but they cannot change the content in any way or use them commercially.
**Abstract Book Citations:** Authors, Title, HemaSphere, 2026;10:(S1):pages. The abstract book DOIs can be found on the "Author Information" page.
**Disclaimer:** Articles published in the journal HemaSphere exclusively reflect the opinions of the authors. The authors are responsible for all content in their abstracts including accuracy of the facts, statements, citing resources, etc.

### Results

41 pts (median age 65 years [IQR 53-76]; 37% female) with AML-MECOM were identified. *De novo* AML accounted for 73%, secondary AML 27%, and therapy-related AML 32%. At diagnosis, median bone marrow (BM) blasts were 20% [IQR 9-47] (9 with <10%, 4 with 10-19%, and 28 with ≥20%), median platelets 146x10⁹/L [IQR 63-256], and WBC 3.3x10⁹/L [IQR 1.8-15.6]. 36 pts received systemic therapy: 40% with intensive chemotherapy (IC) and 60% with less intensive therapy (LIT).
83% had a classic inv(3)(q21;q26.2) or t(3;3)(q21;q26.2). Monosomy 7 occurred in 32%, deletion 7q in 22%, and complex karyotype in 37%.
Among pts with available molecular data (n=29 cases, 189 controls), *SF3B1* (38% vs 3%, p<0.001) and *K/NRAS* mutations (17% vs 4%, p=0.004) were significantly more common in pts with AML-MECOM. *IDH2* mutations (0% vs 10%, p<0.001) were significantly less common in AML-MECOM.
Among 35 response evaluable pts with AML-MECOM, the disease was primary refractory in 23 pts (66%). The composite complete remission rate (CR+CRi+CRh) with first-line therapy was 8/25 (32%). Pts treated with IC demonstrated a significantly higher ORR compared with those receiving LIT (6/13, 46% vs 2/17, 12%; *p*=0.049). Median OS in patients with AML-MECOM was 12.9 months [95% CI 7.3-19.2], comparable to the control group (11.8 months) **(Fig. 1A)**.
Interestingly, pts with ≥20% BM blasts on aspirate had a higher overall response rate (ORR, including MLFS) compared with those with <20% blasts (41% vs 8%; *p*=0.047). However, this relationship was reversed with respect to OS—pts with ≥20% blasts had a median OS of 9.5 months, compared with 18.7 months for those with <20% blasts, although this difference did not reach statistical significance (log-rank *p*=0.145) **(Fig. 1B)**.
Allogeneic stem cell transplant (SCT) was performed in 32% of cases vs 28% of controls (*p*=0.23). Post-SCT relapse occurred in 85% of AML-MECOM pts compared with 16% of controls (p<0.001). One patient achieved long-term survival (18 years from diagnosis) following a second SCT. SCT was associated with longer median OS (31.2 months [95% CI 13.7-115.4] vs 6.1 months [95% CI 2.3–10.9]; log-rank *p*<0.001) **(Fig. 1C)**. In a multivariable Cox regression model including sex, age, therapy-related status, BM blasts <20%, and SCT, only SCT remained independently predictive of improved OS (HR 0.14, 95% CI 0.05-0.45; *p*<0.001).

### Summary/Conclusion

AML-MECOM portends poor response to therapy and a high risk of post-SCT relapse with infrequent long-term OS, underscoring the aggressive biology of this entity. *SF3B1* and *K/NRAS* mutations were enriched in this cohort. Novel therapeutic strategies are needed to improve outcomes and post-SCT disease control.

**Copyright Information:** (Online) ISSN: 2572-9241
©2026 The Author(s). HemaSphere is published by John Wiley & Sons Ltd on behalf of European Hematology Association. This is an open access Abstract Book distributed under the Attribution-NonCommercial-NoDerivs (CC BY-NC-ND), which allows third parties to download the articles and share them with others as long as they credit the author and the Abstract Book, but they cannot change the content in any way or use them commercially.
**Abstract Book Citations:** Authors, Title, HemaSphere, 2026;10:(S1):pages. The abstract book DOIs can be found on the “Author Information” page.
**Disclaimer:** Articles published in the journal HemaSphere exclusively reflect the opinions of the authors. The authors are responsible for all content in their abstracts including accuracy of the facts, statements, citing resources, etc.

### Figure 1

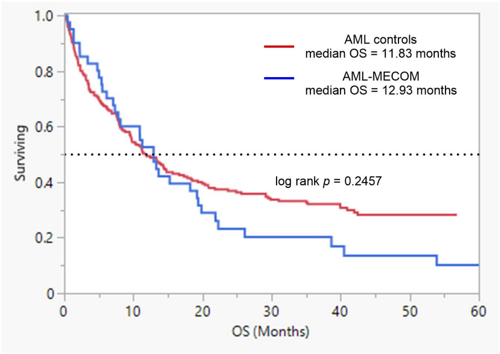

**Figure 1(A): Overall Survival in Patients with AML-MECOM Compared to AML Controls**
Kaplan-Meier analysis of overall survival (OS) in patients with AML-MECOM compared with control patients with AML without MECOM rearrangements. Survival was measured from time of diagnosis to death from any cause.

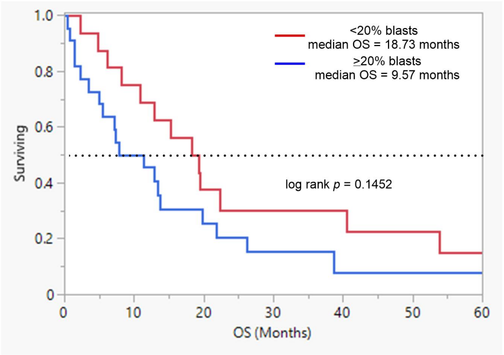

**Figure 1(B): Overall Survival in AML-MECOM Stratified by Bone Marrow Blast Percentage at Diagnosis**
Kaplan-Meier analysis of OS among patients with AML-MECOM stratified by bone marrow blast percentage at diagnosis (≥20% vs <20%). Survival was measured from time of diagnosis to death from any cause.

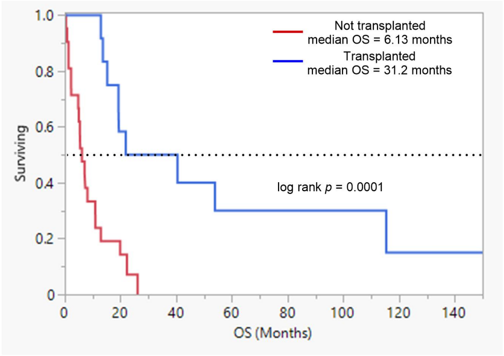

**Figure 1(C): Overall Survival in AML-MECOM Stratified by Allogeneic Stem Cell Transplant**
Kaplan-Meier analysis of OS in patients with AML-MECOM who underwent allogeneic hematopoietic cell transplantation compared with those who did not. Survival was measured from time of diagnosis to death from any cause.

**Copyright Information:** (Online) ISSN: 2572-9241
©2026 The Author(s). HemaSphere is published by John Wiley & Sons Ltd on behalf of European Hematology Association. This is an open access Abstract Book distributed under the Attribution-NonCommercial-NoDerivs (CC BY-NC-ND), which allows third parties to download the articles and share them with others as long as they credit the author and the Abstract Book, but they cannot change the content in any way or use them commercially.
**Abstract Book Citations:** Authors, Title, HemaSphere, 2026;10:(S1):pages. The abstract book DOIs can be found on the "Author Information" page.
**Disclaimer:** Articles published in the journal HemaSphere exclusively reflect the opinions of the authors. The authors are responsible for all content in their abstracts including accuracy of the facts, statements, citing resources, etc.

### (PS1647) EPIDEMIOLOGY, CLINICAL AND BIOLOGICAL CHARACTERISTICS OF UNFIT PATIENTS WITH CBF-AML: RESULTS FROM THE MULTINATIONAL PETHEMA REGISTRY

**Topic:** 04. Acute myeloid leukemia - Clinical

Evelyn Acuña-Cruz*[1], Pilar Lloret-Madrid[1], Rebeca Rodríguez-Veiga[2], Eva Barragán[3], Isabel Cano Ferri[1], Paulina Hillebrand[1], Juan Miguel Bergua Burgues[4], Francisco Ibañez[5], Josefina Serrano Lopez[6], Mar Tomo[7], Gema Miralles Albeza[1], Paula Rodríguez Gálvez[1], Olga Salamero[8], Juan Manuel Alonso Dominguez[9], Juan Antonio López[10], Cristina Gil Cortes[11], Eliana Aguiar[12], Joana Infante[13], Victor Noriega Concepción[14], Marta Valero[15], Olga Arce[16], Carlos Varón[17], Lara Maria Gomez Garcia[18], María Carmen García Garay[19], Raquel Ballester Mínguez[19], Claudia Sossa-Melo[20], Maria Carmen Martinez Chamorro[21], Maria Jose Garcia Perez[22], Azueg Hong[23], M. Soledad Casado[24], Sandra Suarez[25], Cristina Otero[26], Lissette Costilla[27], Carlos Javier Cerveró[28], Williana Torres Jiménez[28], Blanca Boluda[29], María José Cortti[29], Pedro Fernandez[30], Paola Beneit[30], Francisco-Javier Peñalver[31], Patricia García Ramírez[32], Ana Oliva Hernández[33], Maria Teresa Busnego Barreto[33], Julio Dávila-Valls[34], Jackeline Solano Tovar[35], Alba Manasa González Arias[36], Pau Montesinos[37]

*[1]Hospital Universitari i Politècnic-IIS La Fe, Hematology Department, Valencia, Spain;[2]Hospital Universitari i Politécnic La Fe, Valencia, Spain; [3]Hospital Universitari i Politècnic-IIS La Fe, Molecular Biology Unit, Valencia, Spain;[4]Hospital San Pedro de Alcántara, Cáceres, Spain; [5]Hospital General de Valencia, Valencia, Spain;[6]Hospital Reina Sofia, Córdoba, Spain;[7]Hospital Clínico Universitario, Instituto de investigación biomédica INCLIVA, Valencia, Spain; [8]Hospital U. Vall d'Hebron, Barcelona, Spain; [9]Hospital Universitario Fundación Jiménez Díaz, Madrid, Spain; [10]Hospital Universitario de Jaén, Jaén, Spain; [11]Hospital General Universitario Doctor Balmis, Alicante, Spain; [12]Centro Hospitalar Universitário de São João, Porto, Portugal; [13]Hospital de Santa Maria-Lisboa, Lisboa, Portugal; [14]Hospital Universitario A Coruña, A Coruña, Spain;[15]Hospital Arnau de Vilanova, Valencia, Spain; [16]Hospital Universitario Basurto, Bilbao, Spain; [17]Unidad Hematológica Especializada de Cúcuta, Cúcuta, Colombia; [18]Hospital Clínico de Valladolid, Valladolid, Spain;[19]Hospital U. Virgen de la Arrixaca, Murcia, Spain;[20]Clínica FOSCAL, Universidad Autónoma de Bucaramanga-UNAB, Bucaramanga, Colombia; [21]Hospital Universitario Quirón Salud Madrid, Madrid, Spain; [22]Hospital Universitario Torrecárdenas, Almería, Spain;[23]Hospital Doctor José Molina Orosa, Lanzarote, Spain; [24]Hospital Universitario de Badajoz, Badajoz, Spain;[25]Complejo Hospitalario Álvaro Cunqueiro, Vigo, Spain;[26]Hospital Maciel, Montevideo, Uruguay; [27]Hospital San Jorge, Huesca, Spain;[28]Hospital Virgen de la Luz, Cuenca, Spain;[29]Hospital Manises, Valencia, Spain; [30]H.C.U. Sant Joan d'Alacant, Alicante, Spain;[31]Hospital Universitario Fundación Alcorcón, Madrid, Spain; [32]Hospital U. Príncipe de Asturias, Madrid, Spain;[33]Hospital U. Nuestra Señora de Candelaria, Tenerife, Spain; [34]Complejo Asistencial de Ávila, Ávila, Spain;[35]Complejo Asistencial Universitario de Palencia, Palencia, Spain;[36]Hospital U. Severo Ochoa, Leganés, Spain; [37]Hospital Universitari i Politècnic La Fe, CIBERONC Instituto de Salud Carlos III, Valencia, Spain;*

### Background

Core-binding factor acute myeloid leukemia (CBF-AML) is characterized by the presence of inv(16)/t(16;16) or t(8;21), or its molecular counterparts, and is classified as a favorable risk by the 2022 ELN guidelines. Despite high remission rates and good prognosis with intensive regimens, there are no studies demonstrating favorable outcomes in unfit AML patients. Furthermore, due to its low frequency among elderly patients, there is scarce information among unfit CB-AML patients.

### Aims

To analyze clinical and biological characteristics, and to assess therapeutic patterns and outcomes of a large cohort of unfit CBF-AML patients treated with non-intensive regimens.

**Copyright Information:** (Online) ISSN: 2572-9241
©2026 The Author(s). HemaSphere is published by John Wiley & Sons Ltd on behalf of European Hematology Association. This is an open access Abstract Book distributed under the Attribution-NonCommercial-NoDerivs (CC BY-NC-ND), which allows third parties to download the articles and share them with others as long as they credit the author and the Abstract Book, but they cannot change the content in any way or use them commercially.
**Abstract Book Citations:** Authors, Title, HemaSphere, 2026;10:(S1):pages. The abstract book DOIs can be found on the “Author Information” page.
**Disclaimer:** Articles published in the journal HemaSphere exclusively reflect the opinions of the authors. The authors are responsible for all content in their abstracts including accuracy of the facts, statements, citing resources, etc.

### Methods

Adult patients diagnosed with CBF-AML and treated with a non-intensive approach were reported to the multinational and retrospective PETHEMA database, between 1989 and 2025. Diagnosis was made by local cytogenetics and by local or central PCR and/or NGS. Disease and patient's baseline characteristics, CBF-AML mutation subtype, treatments and outcomes were recorded.

### Results

Among 17,748 patients with cytogenetics and/or molecular data, 1311 (7.4%) had CBF-AML at diagnosis (median age 47 years old). Of them, 1002 (85%) received an intensive approach, 77 (6.5%) received a non-intensive approach, 46 (3.5%) only supportive care, and this information was not available in 59 (5%). Among 77 CBF-AML treated with non-intensive regimens, 48 (62%) had inv(16)/t(16;16) and 29 (38%) had t(8;21). Median age of this cohort was 73 years old (range 34 to 92 years), 57% were male, 20 (26%) had secondary AML (16 were therapy related AML), and 70% had ECOG 0-1. Comparing inv(16)/t(16;16) vs. t(8;21) groups, M4-FAB subtype was more present in inv(16)/t(16;16) (47.5% vs. 4.3%), while M2-FAB was more prevalent in t(8;21) (15% vs 43.6%, p=0.001); 9% of extramedullary disease was seen with similar distribution (8% vs 9%, respectively), median WBC x $10^9$/L was 12 vs. 13 (p=0.91); FLT3-ITDmut in 7% of inv(16)/t(16;16) vs. 20% of t(8;21) (overall 11%), FLT3-TKDmut in 9% vs. 11%, respectively; and cKITmut 23% vs. 19%. Front-line approach comprised hypomethylating agent (HMA) in 32 (42%) (median age 74, range 56 to 92), HMA plus venetoclax (VEN) in 17 (22%) (median age 76, range 42 to 83), and low-dose cytarabine-based (LDAC) regimens in 28 (36%) (median age 72, range 34 to 86). Overall, a 49% CRc was observed (75% with HMA plus VEN vs. 37% with HMA vs. 46% with LDAC-based, p=0.05). Median overall survival (mOS) was 10.4 months, 15.2 in inv(16)/t(16;16) and 6.7 months in t(8;21), p=0.009 (Figure 1-A). The mOS was 9.7 months with HMA, 8 months with LDAC, and not reached with HMA plus VEN (p=0.015) (Figure 1-B).

### Summary/Conclusion

We confirm that patients with CBF-AML are mostly treated intensively. Among patients with CBF-AML a priori non eligible for intensive chemotherapy, we found that t(8;21) had more frequently FLT3-ITD mutations, and had a poorer prognosis compared with inv(16)/t(16;16). Although numbers remain scarce and selection bias could play a role, we suggest that treatment with HMA plus VEN regimens could result in improved OS in this setting.

**Copyright Information:** (Online) ISSN: 2572-9241
©2026 The Author(s). HemaSphere is published by John Wiley & Sons Ltd on behalf of European Hematology Association. This is an open access Abstract Book distributed under the Attribution-NonCommercial-NoDerivs (CC BY-NC-ND), which allows third parties to download the articles and share them with others as long as they credit the author and the Abstract Book, but they cannot change the content in any way or use them commercially.
**Abstract Book Citations:** Authors, Title, HemaSphere, 2026;10:(S1):pages. The abstract book DOIs can be found on the "Author Information" page.
**Disclaimer:** Articles published in the journal HemaSphere exclusively reflect the opinions of the authors. The authors are responsible for all content in their abstracts including accuracy of the facts, statements, citing resources, etc.

Figure 1-A.

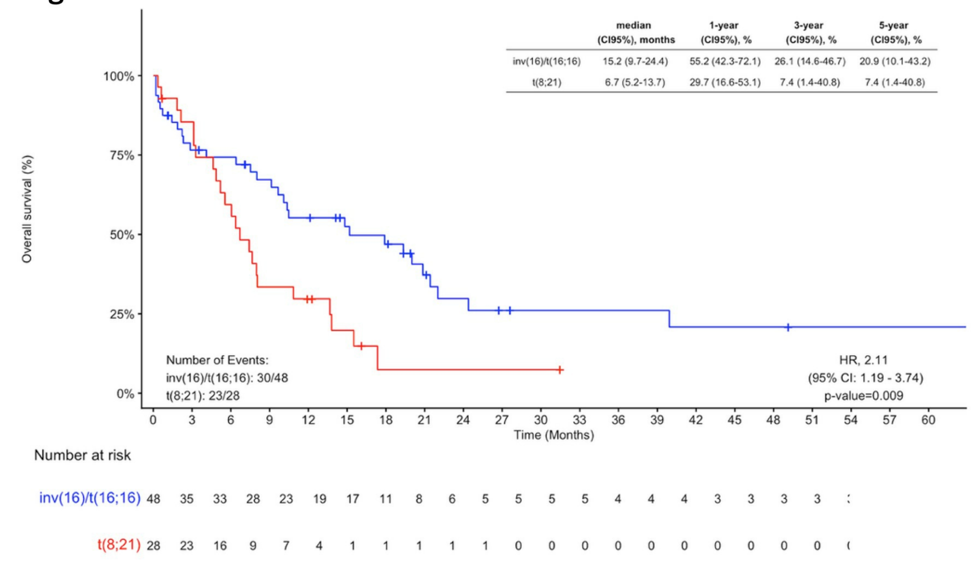

Figure 1-B.

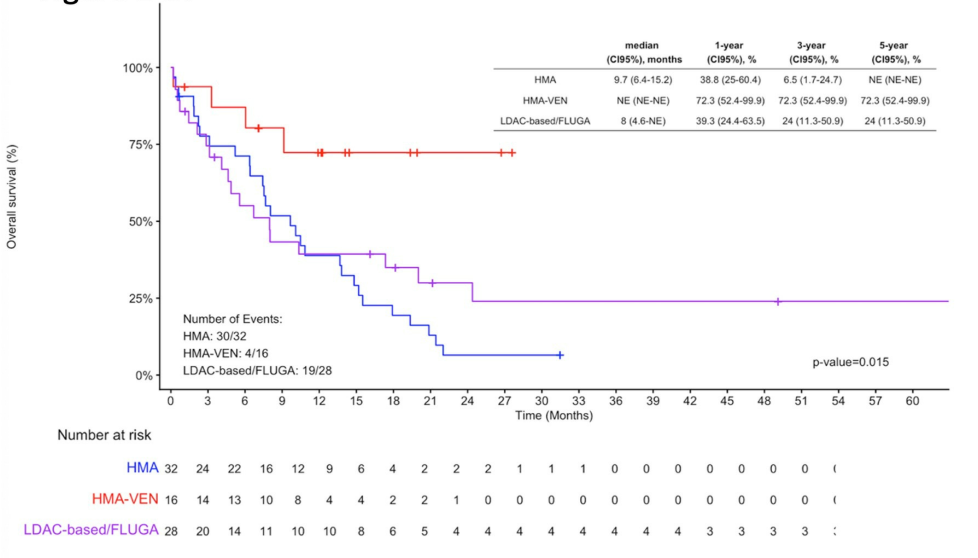

**Copyright Information:** (Online) ISSN: 2572-9241
©2026 The Author(s). HemaSphere is published by John Wiley & Sons Ltd on behalf of European Hematology Association. This is an open access Abstract Book distributed under the Attribution-NonCommercial-NoDerivs (CC BY-NC-ND), which allows third parties to download the articles and share them with others as long as they credit the author and the Abstract Book, but they cannot change the content in any way or use them commercially.
**Abstract Book Citations:** Authors, Title, HemaSphere, 2026;10:(S1):pages. The abstract book DOIs can be found on the "Author Information" page.
**Disclaimer:** Articles published in the journal HemaSphere exclusively reflect the opinions of the authors. The authors are responsible for all content in their abstracts including accuracy of the facts, statements, citing resources, etc.

### (PS1648) PERIPHERAL BLOOD FLOW CYTOMETRIC MRD MONITORING IS FEASIBLE FOR AML PATIENTS

**Topic:** 04. Acute myeloid leukemia - Clinical

Nicholas McCarthy*[1], Tatiana Raskovalova[1, 2], Eleanor Hickman[1], Vismay Deshani[3], Nicola Potter[4], Naeem Khan[1], Richard Dillon[4], John Chadwick[3], Mike Dennis[3], Vidhya Murthy[5], Iman Quereshi[6], Emma Searle[3], Sylvie Freeman[1]

*[1]School of Infection, Inflammation and Immunology, University of Birmingham College of Medicine and Health,, Birmingham, United Kingdom; [2]Laboratoire d'immunologie, Centre Hospitalier Universitaire Grenoble, Grenoble, France; [3]The Christie NHS Foundation Trust, Manchester, United Kingdom; [4]Department of Medical and Molecular Genetics, King's College, London, United Kingdom; [5]University Hospitals of Birmingham, Birmingham, United Kingdom; [6]University Hospitals Coventry and Warwickshire, Coventry, United Kingdom;*

### Background

Peripheral blood based measurable residual disease (PB MRD) testing enables less invasive serial monitoring for patients. This allows more frequent tests, mitigating the limitations from variable disease kinetics on pre-emptive relapse detection. PB MRD also avoids the pitfall of heterogenous bone marrow (BM) sampling that can reduce the accuracy of MRD quantification. In acute myeloid leukaemia (AML), BM flow cytometry (FC) is commonly used to assess MRD but PB MRD testing by this methodology requires further comparison to BM.

### Aims

To assess feasibility and potential false negative rate of FC PB MRD monitoring compared to BM.

### Methods

A 4-5ml PB sample was collected alongside routine BM MRD specimens in patients where MRD positivity was expected or BM samples likely inadequate. FC AML MRD was assessed using a validated 21-colour one-tube spectral assay incorporating all ELN-recommended MRD and LSC markers. BM MRD positivity threshold was > 0.05%.
The criterion for PB sample adequacy to report MRD negativity was a sample LOD of≤0.01%. The PB assay background was <0.005% of leukocytes and <2% of progenitors for all 30 standard DfN (LAIP-based) fixed gates across 19 control PBs (mean LOB 0.0005% of leukocytes/ 1.5% of progenitors). An average of 5 events (range 0-15) were detected in DfN gates of PB controls, allowing 20 events as the LOD threshold for detectable MRD in PB. CD33+ and LMPP-type stem cells were detectable in 4 and 1 of the 19 PB controls, respectively.

**Copyright Information:** (Online) ISSN: 2572-9241
©2026 The Author(s). HemaSphere is published by John Wiley & Sons Ltd on behalf of European Hematology Association. This is an open access Abstract Book distributed under the Attribution-NonCommercial-NoDerivs (CC BY-NC-ND), which allows third parties to download the articles and share them with others as long as they credit the author and the Abstract Book, but they cannot change the content in any way or use them commercially.
**Abstract Book Citations:** Authors, Title, HemaSphere, 2026;10:(S1):pages. The abstract book DOIs can be found on the "Author Information" page.
**Disclaimer:** Articles published in the journal HemaSphere exclusively reflect the opinions of the authors. The authors are responsible for all content in their abstracts including accuracy of the facts, statements, citing resources, etc.

### Results

In total, 86 paired BM/PB MRD samples from 57 patients (median age 61) were collected and tested March 2025 to February 2026. The majority of patients were receiving lower-intensity frontline or salvage therapy (40/57; venetoclax n=18, menin inhibitors n=14, maintenance n=3, other n=5). Of 86 sample pairs, 23 were inadequate in BM and/or PB including 10 inadequate in PB only. Median patient WBC at time of an inadequate PB sample was $0.76x10^9$/L[IQR 0.3-1.2] vs $3.10x10^9$/L[IQR 1.3-4.6] for adequate PBs, suggesting that a 10ml PB volume may be required when WBC is <1.0 $x10^9$. Inadequate PB samples were most frequent during venetoclax-based therapy.

Median MRD level was 0.32%[IQR 0.125-4.19] in the 49 MRD+ BMs and 0.081%[IQR 0.017-0.34] in the 45 MRD+ PBs. In 4 cases with inadequate BMs, PB was informative (2 MRD+, 2 MRD-). In adequate paired PB/BM samples, 5 (8%) PB results were discordant: 2 PB MRD- in patients receiving menin inhibitors (including 1 BM MRD+ but <0.1% ) and 3 PB MRD+ where the distinct aberrant cluster detected in PB could not be quantified above BM background (but BM PCR+ in the 2 cases with concurrent PCR monitoring).

Median difference in % MRD load between PB and BM paired samples was -0.626 $log_{10}$ [IQR, -0.168 to -1.255] but >-2 $log_{10}$differences were observed in some (***Figure***). Notably, the MRD proportion of progenitor compartment was similar between PB and BM (median difference 0.02 $log_{10}$[IQR, -0.13 to 0.19]).

### Summary/Conclusion

In patients receiving predominantly lower-intensity or salvage therapies, PB MRD testing by single-tube spectral FC identified 95% of BM MRD+ cases when PB sample was adequate, and detected MRD not apparent in BM in a subset of patients. These findings support the use of post-remission FC PB MRD monitoring, with BM sampling reserved for cases where PB sample sensitivity is insufficient to confirm MRD negativity, most commonly observed in patients with low WBC. The MRD+ proportion of progenitors appears to provide comparable MRD values between PB and BM.

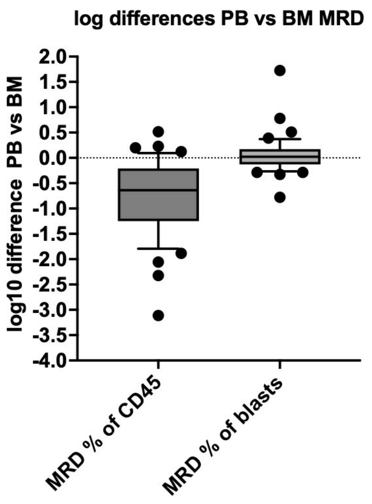

**Legend** Differences ($log_{10}$) in MRD load in PB compared to paired BM

1) % of leukocytes
2) % of progenitors (parent CD34+ or CD117+ blast gate).

Only samples with detectable MRD in both BM and PB were included (N=47, including 8 categorized as relapse /refractory by flow cytometry)

**Copyright Information:** (Online) ISSN: 2572-9241
©2026 The Author(s). HemaSphere is published by John Wiley & Sons Ltd on behalf of European Hematology Association. This is an open access Abstract Book distributed under the Attribution-NonCommercial-NoDerivs (CC BY-NC-ND), which allows third parties to download the articles and share them with others as long as they credit the author and the Abstract Book, but they cannot change the content in any way or use them commercially.
**Abstract Book Citations:** Authors, Title, HemaSphere, 2026;10:(S1):pages. The abstract book DOIs can be found on the "Author Information" page.
**Disclaimer:** Articles published in the journal HemaSphere exclusively reflect the opinions of the authors. The authors are responsible for all content in their abstracts including accuracy of the facts, statements, citing resources, etc.

### (PS1649) INTERIM RESULTS OF THE PHASE I/II STUDY INVESTIGATING KARONUDIB IN PATIENTS WITH REFRACTORY HAEMATOLOGICAL MALIGNANCIES PAIRED WITH EX-VIVO PRECISION SCREEN DRUG SENSITIVITY SCREENING

**Topic:** 04. Acute myeloid leukemia - Clinical

Austin Smith[1], Stefan Deneberg*[2], Bertil Uggla[3], Olof Breuer[2], Hans Beier Ommen[4], Claudia Schöllkopf[5], Ulrika Warpmanberglund[1], Maria Klockare[1], Thomas Helleday[2], Nona Struyf[2], Tom Erkers[2]

*[1]Oxcia, Stockholms Stad, Sweden;[2]Karolinska Institutet, Huddinge, Sweden;[3]Örebro Universitet, Grythyttan, Sweden;[4]Aarhus H, Dept Haematology, Århus C, Denmark;[5]Rigshospitalets Kirke, Department of Hematology, København Ø, Denmark;*

### Background

MutT homologue 1 (MTH1) detoxifies cells from ROS-induced DNA damage and is essential for cancer cell survival. MTH1 depletion in RAS-transformed tumour cells causes proliferative abnormalities and numerous malignancies, including haematological tumours, which overexpress MTH1. Karonudib (TH1579, OXC-101) is a mitotic MTH1 inhibitor that arrests mitosis by disrupting microtubule dynamics and chromosome alignment, and increases incorporation of oxidised nucleosides into DNA, with improved survival demonstrated across several haematological preclinical models.

### Aims

To report Part 1 and Part 2 dose-escalation interim data from the phase I/II MAATEO trial (NCT04077307) of Karonudib in adults with advanced relapsed/refractory (R/R) AML, ALL, DLBCL, MM, and high-risk MDS.

### Methods

This single-arm, dose-escalation trial enrolled patients aged 18–75 years with adequate organ function receiving escalating oral doses of Karonudib using a 3+3 design. The primary objectives were safety and tolerability, assessed by the DLT rate over the first 4 weeks. Secondary objectives included clinical benefit, pharmacokinetics, and biomarker analyses. Disease assessment was performed on Cycle 1 Day 22 and at cycle end. Part 2 evaluates Karonudib at the RP2D in combination with idarubicin in R/R AML and high-risk MDS.

**Copyright Information:** (Online) ISSN: 2572-9241
©2026 The Author(s). HemaSphere is published by John Wiley & Sons Ltd on behalf of European Hematology Association. This is an open access Abstract Book distributed under the Attribution-NonCommercial-NoDerivs (CC BY-NC-ND), which allows third parties to download the articles and share them with others as long as they credit the author and the Abstract Book, but they cannot change the content in any way or use them commercially.
**Abstract Book Citations:** Authors, Title, HemaSphere, 2026;10:(S1):pages. The abstract book DOIs can be found on the "Author Information" page.
**Disclaimer:** Articles published in the journal HemaSphere exclusively reflect the opinions of the authors. The authors are responsible for all content in their abstracts including accuracy of the facts, statements, citing resources, etc.

### Results

Fourteen patients were enrolled across 3 cohorts in the dose-escalation phase. Diagnoses included AML (n=5), MDS (n=4), DLBCL (n=3), AUL (n=1), and MM (n=1). The most common treatment-related adverse events (≥25%) were anaemia (50%), neutropenia (43%), diarrhoea (43%), nausea (36%), and thrombocytopenia (29%). One DLT of grade 5 intracranial haemorrhage (possibly related) occurred in Cohort 2, prompting enrolment of 3 additional patients and tighter entry criteria for thrombocytopenia. No grade ≥3 QTc prolongation or Hy's Law hepatotoxicity was observed. In AML (n=2 evaluable; median age 65 years; median 4 prior therapies), best responses were one partial response (133 days) and one stable disease (141 days). In MDS (n=4 evaluable; median age 72 years; median 3 prior therapies), the best response was stable disease (65 days). In DLBCL (n=2 evaluable; median age 77 years; median 4 prior therapies), the best response was stable disease (167 days). Thirteen patients were enrolled in the ongoing idarubicin combination cohort (Cohort 5), comprising 11 AML patients (all adverse risk) and 2 high-risk MDS patients (median age 68 years; range 44–81), the majority of whom were heavily pre-treated with azacitidine- and venetoclax-based regimens. Of the nine patients who discontinued, reasons included progressive disease (n=5), adverse event (n=2), patient wish (n=1), and lack of efficacy (n=1); four patients remain on treatment. One patient achieved stable disease over four cycles, with bone marrow blasts reducing from 40% to 17%; a second patient achieved stable disease at Cycle 1, with blast reduction from 17.5% to 12.4%, before progressing at Cycle 2. Drug-sensitivity scoring in AML/MDS assesses Karonudib activity and compares it to that of standard-of-care agents throughout treatment cycles.

### Summary/Conclusion

Karonudib was generally well tolerated with acceptable safety and preliminary clinical activity in R/R AML and MDS. The RP2D is now being evaluated in combination with idarubicin, with the aim of optimising delivery of this combination. The drug sensitivity screening shows strong correlation with prior and current clinical outcomes.

**Copyright Information:** (Online) ISSN: 2572-9241
©2026 The Author(s). HemaSphere is published by John Wiley & Sons Ltd on behalf of European Hematology Association. This is an open access Abstract Book distributed under the Attribution-NonCommercial-NoDerivs (CC BY-NC-ND), which allows third parties to download the articles and share them with others as long as they credit the author and the Abstract Book, but they cannot change the content in any way or use them commercially.
**Abstract Book Citations:** Authors, Title, HemaSphere, 2026;10:(S1):pages. The abstract book DOIs can be found on the "Author Information" page.
**Disclaimer:** Articles published in the journal HemaSphere exclusively reflect the opinions of the authors. The authors are responsible for all content in their abstracts including accuracy of the facts, statements, citing resources, etc.

### (PS1650) CPX-351-INDUCED DEEP MOLECULAR REMISSION (MRD-) PREDICTS EXCELLENT OUTCOMES FOLLOWING ALLOGENEIC HEMATOPOIETIC STEM CELL TRANSPLANTATION IN AML

**Topic:** 04. Acute myeloid leukemia - Clinical

Jannik Lüthje*[1, 2], Christina Ganster[2], Justin Hasenkamp[1], Miriam Leonard[2], Elzbieta Brzuszkiewicz[2], Paolo Mazzeo[1, 2], Gerald Wulf[1], Julie Schanz[1, 2, 3], Friederike Kratzsch[1, 2], Katharina Rittscher[2], Katayoon Shirneshan[2], Felicitas Thol[4], Detlef Haase[1, 2], Hannes Treiber[1, 2]

*[1]University Medical Center Göttingen, Department of Hematology and Medical Oncology, Göttingen, Germany;[2]University Medical Center Göttingen, INDIGHO laboratory, Göttingen, Germany; [3]University Medical Center Göttingen, UMG Laboratory, Göttingen, Germany; [4]Department of Hematology, Hemostasis, Oncology & Stem Cell Transplantation, Hannover Medical School, Hannover, Germany;*

### Background

CPX-351, a liposomal formulation of daunorubicin and cytarabine, has doubled long-term overall survival (OS) vs. 7+3 in patients with AML-MRC and t-AML [WHO 2016]. Response assessment is usually performed by bone marrow cytomorphology after induction. While CPX-351 achieves high complete remission (CR) rates, data on measurable residual disease (MRD) in NGS are limited, although MRD-negativity is increasingly recognized as a strong prognostic marker.

### Aims

Assess the prognostic value of post-induction/pre-HSCT MRD for OS and cumulative incidence of relapse (CIR) in pts. achieving CR after CPX-351. Additionally, we analysed genetic profiles, treatment responses to CPX-351, consolidation/salvage therapy, and the impact of further treatment in patients without CR/MRD-negativity after induction.

### Methods

All adults ≥18 years treated with CPX-351 at our tertiarycenter were screened. Inclusion criteria included initial NGS profiling (53-gene panel) with at least one non-DTA marker and ≥1 MRD assessment after induction. Remission was defined according to ELN 2022 (CR/CRi/MLFS=CR; PR/RD=PR/RD). MRD assessment included NGS (LOD 1%) for the individually mutated genes, RT-qPCR for *NPM1* (LOD 0.004%) and *KMT2A*-PTD (3.82%), and *FLT3*-ITD by fragment analysis (ratio 0.01).

**Copyright Information:** (Online) ISSN: 2572-9241
©2026 The Author(s). HemaSphere is published by John Wiley & Sons Ltd on behalf of European Hematology Association. This is an open access Abstract Book distributed under the Attribution-NonCommercial-NoDerivs (CC BY-NC-ND), which allows third parties to download the articles and share them with others as long as they credit the author and the Abstract Book, but they cannot change the content in any way or use them commercially.
**Abstract Book Citations:** Authors, Title, HemaSphere, 2026;10:(S1):pages. The abstract book DOIs can be found on the "Author Information" page.
**Disclaimer:** Articles published in the journal HemaSphere exclusively reflect the opinions of the authors. The authors are responsible for all content in their abstracts including accuracy of the facts, statements, citing resources, etc.

### Results

Among 78 treated pts., 60 were evaluable and 50 underwent HSCT (47 RIC, 3 MAC). The median age was 63.5 years (range 41–75); male n=36, female n=24. According to WHO 2022, 51 had AML-MR, 3 AML with *NPM1* mutation, 1 AML with *MECOM* rearrangement and 5 AML defined by differentiation. After induction, 15 pts. achieved CR/MRD-, 24 CR/MRD+, and 21 PR/RD. In total, 171 non-DTA and 42 DTA mutations were detected pre-induction; 38 % of non-DTA mutations became MRD-negative. 15 *TP53* mutations (11 pts.; 8 HSCT) were associated with inferior response compared to other non-DTAs (Fisher's p=0.001) and pts. had worse OS after HSCT (median OS=4m vs. 51m, p=0.031).

In pts. receiving HSCT, MRD status after induction (CR/MRD- n=12, CR/MRD+ n=21, PR/RD n=17) was predictive of superior OS for CR/MRD- patients (median OS=NA vs. 6m vs. 16m, p=0.022). Cumulative incidence of relapse and death without relapse was also lower (Gray test p=0.024). Among 38 pts. without early CR/MRD- after induction, OS was better for those receiving additional therapy prior to HSCT (consolidation n=12, salvage n=9, none n=17; median OS=NA vs. 9m vs. 5m, p=0.012).

Pre-HSCT remission data was available for 41 patients (CR/MRD- n=16, CR/MRD+ n=21, PR/RD n=4). OS was significantly higher for MRD- patients (median OS=NA vs. 9m vs. 1.5m, p<0.0001). Five patients converted to MRD- after consolidation/salvage therapy. Of four transplanted pts. with persistent blasts, three died and one relapsed after 3 months. In multivariate Cox regression (death/relapse), CR/MRD+ (HR 6.68; p=0.01) and PR/RD (HR 32.50; p<0.001) had significant impact, while *TP53* (HR 2.10; p=0.18) had no significant impact.

### Summary/Conclusion

CPX-351 can achieve deep molecular responses, with substantial MRD clearance after a single induction. Both early and pre-HSCT MRD-status strongly predict OS and CIR. Pts. failing to reach MRD-negativity benefit from additional therapy before transplant. These findings highlight the prognostic and therapeutic relevance of MRD monitoring in AML and suggest that the superior efficacy of CPX-351 may in part be explained by its capacity to induce profound molecular remissions. Achieving CR/MRD- before HSCT could be a key determinant of improved long-term outcomes in AML.

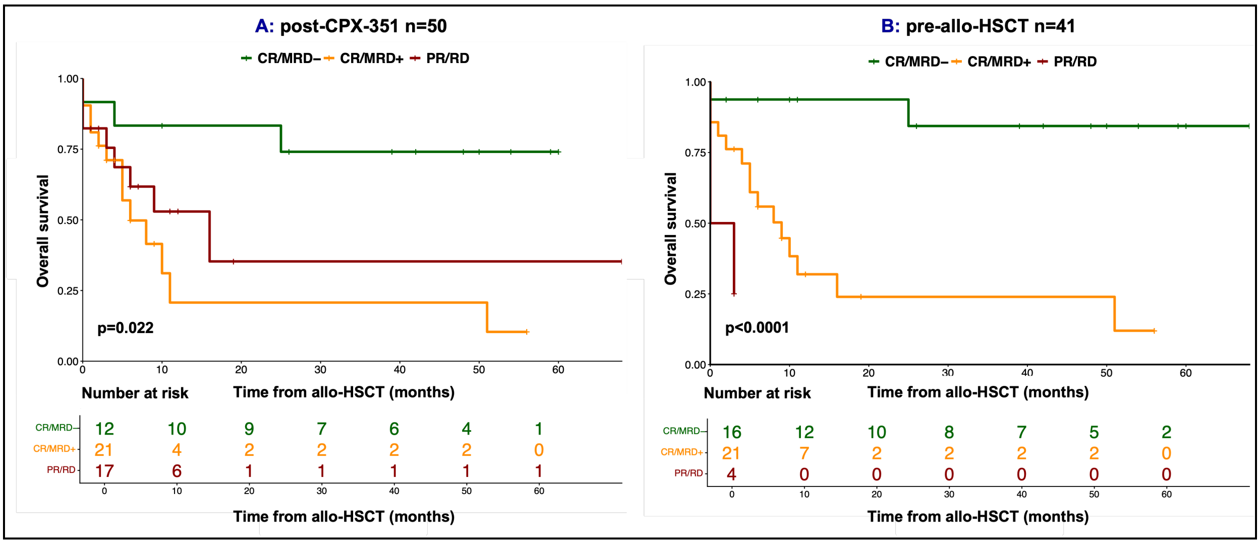

**Copyright Information:** (Online) ISSN: 2572-9241
©2026 The Author(s). HemaSphere is published by John Wiley & Sons Ltd on behalf of European Hematology Association. This is an open access Abstract Book distributed under the Attribution-NonCommercial-NoDerivs (CC BY-NC-ND), which allows third parties to download the articles and share them with others as long as they credit the author and the Abstract Book, but they cannot change the content in any way or use them commercially.
**Abstract Book Citations:** Authors, Title, HemaSphere, 2026;10:(S1):pages. The abstract book DOIs can be found on the "Author Information" page.
**Disclaimer:** Articles published in the journal HemaSphere exclusively reflect the opinions of the authors. The authors are responsible for all content in their abstracts including accuracy of the facts, statements, citing resources, etc.

### (PS1651) PATIENT TAILORED AZACITIDINE, VENETOCLAX AND GILTERITINIB TRIPLET THERAPY ACHIEVES DURABLE DISEASE CONTROL AND IMPROVES TOLERABILITY IN FLT3-MUTATED AML

**Topic:** 04. Acute myeloid leukemia - Clinical

Matilda Arvidsson Kvissberg*[1], Christer Nilsson[1], Niklas Landberg[2], Albin Osterroos[3], Aristea Papageorgiou[4], Bertil Uggla[5], Vladimir Lazarevic[6], Stefan Deneberg[1], Martin Jädersten[1]

*[1]Karolinska University Hospital/Karolinska Institutet, Hematology, Stockholm, Sweden; [2]Lund University Hospital, Division of Clinical Genetics, Lund, Sweden; [3]Uppsala University Hospital, Department of Medical Sciences, Haematology, Uppsala, Sweden; [4]Halland Hospital, Halmstad, Sweden; [5]Örebro University Hospital, Örebro, Sweden; [6]Lund University Hospital, Department of Hematology, Oncology and Radiation Physics, Lund, Sweden;*

### Background

*FLT3*-mutated acute myeloid leukemia (AML) is associated with high relapse rates and treatment resistance. Triplet therapy with azacitidine (AZA), venetoclax (VEN), and the FLT3 inhibitor gilteritinib (GILT) induces a high response rate but is frequently complicated by prolonged myelosuppression and infectious complications, limiting treatment duration. In relapse/refractory (R/R) AML, the median number of cycles is approximately two, and relapse free survival remains below 6 months. Limited tolerability of standard triplet regimens may contribute to early relapse. This retrospective study evaluated alternative GILT-based triplet schedules developed to improve tolerability while maintaining efficacy.

### Aims

To compare three GILT-based triplet regimens and identify an optimal scheduling strategy with respect to tolerability and clinical outcomes.

### Methods

We conducted a multicenter retrospective study of 29 *FLT3*-mutated AML patients treated with AZA, VEN, and GILT across five Swedish centers. The primary endpoint was overall survival (OS); secondary endpoints included event-free survival (EFS) and time to neutrophil and platelet recovery. Patients received one of three regimens: concomitant triplet therapy from day 1 (Group 1); AZA and VEN followed by concomitant VEN and GILT (Group 2); or overlapping AZA and VEN followed by GILT monotherapy (Group 3). Outcomes were also analyzed according to disease status at treatment initiation.

**Copyright Information:** (Online) ISSN: 2572-9241
©2026 The Author(s). HemaSphere is published by John Wiley & Sons Ltd on behalf of European Hematology Association. This is an open access Abstract Book distributed under the Attribution-NonCommercial-NoDerivs (CC BY-NC-ND), which allows third parties to download the articles and share them with others as long as they credit the author and the Abstract Book, but they cannot change the content in any way or use them commercially.
**Abstract Book Citations:** Authors, Title, HemaSphere, 2026;10:(S1):pages. The abstract book DOIs can be found on the “Author Information” page.
**Disclaimer:** Articles published in the journal HemaSphere exclusively reflect the opinions of the authors. The authors are responsible for all content in their abstracts including accuracy of the facts, statements, citing resources, etc.

**Results**

Patients in Group 1 (n=13) were younger, with a median age of 49 years, compared with 75 and 76 years in Group 2 (n=9) and 3 (n=7), respectively. Patients in Group 1 were generally fitter, eligible for a more myelosuppressive treatment and often continued to hematopoietic stem cell transplantation (HSCT). In contrast, the regimens used in Group 2 and 3 were selected for more frail patients, considered unfit for fully concomitant GILT triplet (Group 1) due to anticipated myelosuppression.
At 18 months, OS for the entire cohort was 78.6%, with median OS not reached after more than five years of follow-up. High response rates were observed across all schedules, with a modified composite complete remission (mCRc) rate of 94.7%. Median OS was not reached in Groups 1 and 2 after three years, whereas Group 3 had a median OS of 18 months; however, the difference did not reach statistical significance. Median EFS was 40.5 months in Group 1 and was not reached at five years in Group 2, compared with 11.5 months in Group 3 ($p$=0.04 and $p$=0.05, respectively).
Myelosuppression differed significantly between schedules. The median time to absolute neutrophil count (ANC) recovery (>0.5x$10^9$/L) after cycle 1 was 58 days in Group 1, compared to 39 days in Group 2 ($p$=0.006) and 38 days in Group 3 ($p$=0.006). The estimated median time to platelet (PLT) recovery (>50x$10^9$/L) was 105 days for Group 1, with only 11% of patients regenerating before day 60. In contrast, the median time to PLT recovery in Group 2 and 3 was 43 and 36 days, respectively (Group 1 compared to Group 2 $p$=0.004, and to Group 3 $p$=0.008).

**Summary/Conclusion**

Regimens limiting concomitant exposure to no more than two agents significantly reduced myelosuppression and infectious complications compared with fully concomitant triplet therapy. Schedules without triple-drug overlap enabled sustained treatment administration through reduced toxicity and was associated with durable disease control, including in patients ineligible for allogeneic HSCT.

**Copyright Information:** (Online) ISSN: 2572-9241
©2026 The Author(s). HemaSphere is published by John Wiley & Sons Ltd on behalf of European Hematology Association. This is an open access Abstract Book distributed under the Attribution-NonCommercial-NoDerivs (CC BY-NC-ND), which allows third parties to download the articles and share them with others as long as they credit the author and the Abstract Book, but they cannot change the content in any way or use them commercially.
**Abstract Book Citations:** Authors, Title, HemaSphere, 2026;10:(S1):pages. The abstract book DOIs can be found on the “Author Information” page.
**Disclaimer:** Articles published in the journal HemaSphere exclusively reflect the opinions of the authors. The authors are responsible for all content in their abstracts including accuracy of the facts, statements, citing resources, etc.

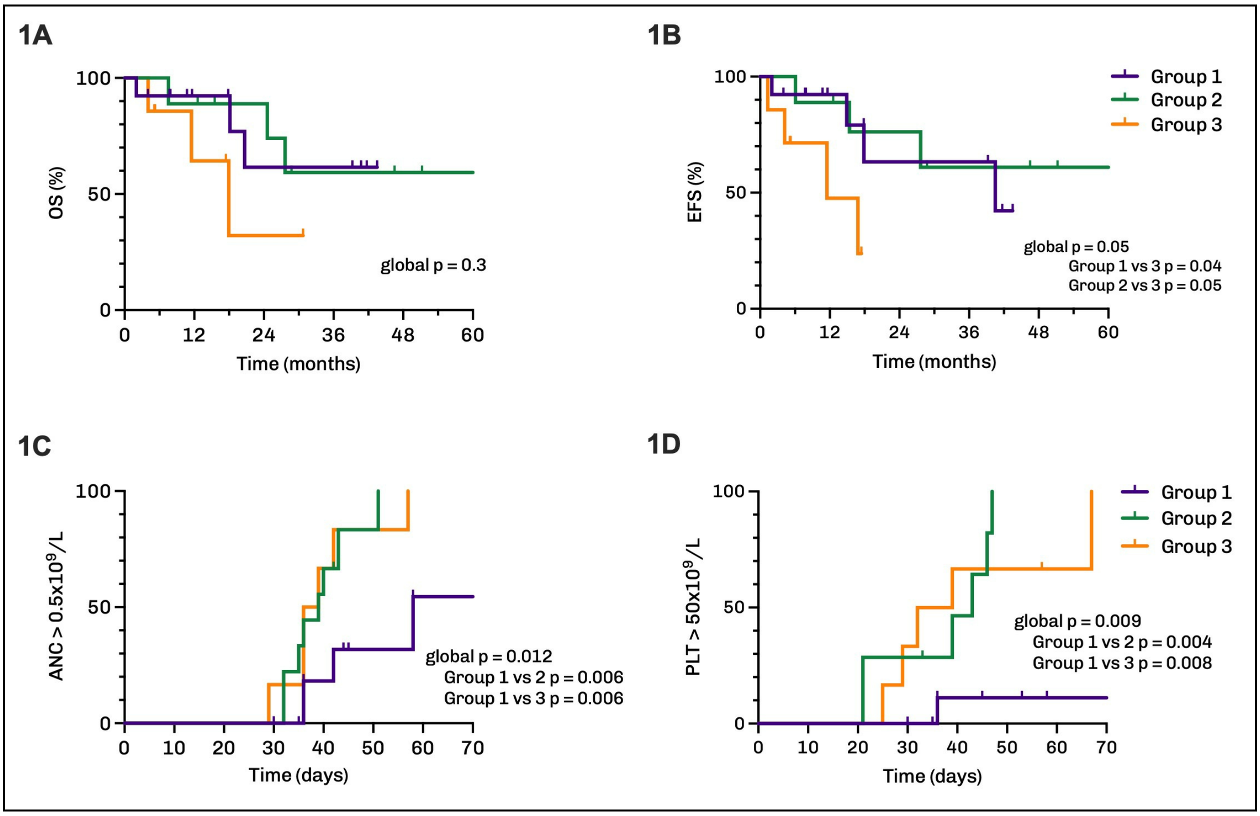

Figure 1. Outcome after GILT triplet therapy: (A) OS in the three treatment groups compared using logrank analysis. (B) EFS in the three treatment groups, compared using logrank analysis. Count regeneration after GILT triplet therapy: days until regeneration after cycle 1 of triplet therapy per group, ANC >0.5x109/L (C) PLT >50x109/L (D), data censored in patients that continued to HSCT or cycle 2 in a cytopenic state. Abbreviation: OS, overall survival; EFS, event free survival; ANC, absolute neutrophil count; PLT, platelet count; HSCT, hematopoietic stem cell transplantation.

**Copyright Information:** (Online) ISSN: 2572-9241
©2026 The Author(s). HemaSphere is published by John Wiley & Sons Ltd on behalf of European Hematology Association. This is an open access Abstract Book distributed under the Attribution-NonCommercial-NoDerivs (CC BY-NC-ND), which allows third parties to download the articles and share them with others as long as they credit the author and the Abstract Book, but they cannot change the content in any way or use them commercially.
**Abstract Book Citations:** Authors, Title, HemaSphere, 2026;10:(S1):pages. The abstract book DOIs can be found on the "Author Information" page.
**Disclaimer:** Articles published in the journal HemaSphere exclusively reflect the opinions of the authors. The authors are responsible for all content in their abstracts including accuracy of the facts, statements, citing resources, etc.

### (PS1652) ACUTE MYELOID LEUKEMIA WITH KMT2A AMPLIFICATION: A TP53-ALTERNATION ENRICHED, CHROMOANAGENESIS-DRIVEN ENTITY WITH POOR PROGNOSIS

**Topic:** 04. Acute myeloid leukemia - Clinical

Thura Win Htut*[1], Wei-Ying Jen[1], Ghayas Issa[1], Tapan Kadia[1], Sanam Loghavi[2], Abhishek Maiti[1], Alexandre Bazinet[1], Carlos Bueso Ramos[2], Qing Wei[2], Gokce A. Toruner[2], Jayastu Senapati[1], Nicholas Short[1], Farhad Ravandi[1], Naval Daver[1], Courtney Dinardo[1], Guilin Tang[2]

*[1]The University of Texas MD Anderson Cancer Center, Department of leukemia, Houston, United States of America;[2]The University of Texas MD Anderson Cancer Center, Department of hematopathology, Houston, United States of America;*

### Background

*KMT2A*, located on chromosome 11q23, encodes a histone methyltransferase essential for HOX gene regulation and hematopoiesis. *KMT2A* amplification (amp), defined as ≥5 copies of *KMT2A*, represents a distinct genomic alteration from the well-recognized *KMT2A* rearrangement in AML and has been associated with poor prognosis in small series.

### Aims

We aimed to characterize the clinicogenomic features of AML with *KMT2A*amp and evaluate outcomes in newly diagnosed (ND) and relapsed/refractory (RR) cases.

### Methods

Patients with *KMT2A*amp confirmed via fluorescence in situ hybridization (FISH) or optical genome mapping (OGM) from May 2020 to August 2025 were included. Response rates were assessed via ELN 2022 criteria. Overall survival (OS) and event-free survival (EFS) were estimated using the Kaplan–Meier method. Landmark analysis was used to assess the benefits of venetoclax and stem cell transplant (SCT).

**Copyright Information:** (Online) ISSN: 2572-9241
©2026 The Author(s). HemaSphere is published by John Wiley & Sons Ltd on behalf of European Hematology Association. This is an open access Abstract Book distributed under the Attribution-NonCommercial-NoDerivs (CC BY-NC-ND), which allows third parties to download the articles and share them with others as long as they credit the author and the Abstract Book, but they cannot change the content in any way or use them commercially.
**Abstract Book Citations:** Authors, Title, HemaSphere, 2026;10:(S1):pages. The abstract book DOIs can be found on the “Author Information” page.
**Disclaimer:** Articles published in the journal HemaSphere exclusively reflect the opinions of the authors. The authors are responsible for all content in their abstracts including accuracy of the facts, statements, citing resources, etc.

**Results**

A total of 102 pts were included, 58 ND and 44 RR with confirmed KMT2Aamp. Median age was 68 years (ND 69; RR 67). All pts had a complex karyotype. Other adverse cytogenetic abnormalities were also frequent: -5 or del(5q) in 62%, -7 in 35%, and -17/abn(17p) in 34%. No other class-defining chromosomal rearrangements were detected. *TP53* mutations were detected in 85% of patients, whereas mutations in other genes were rare. 96 (95%) cases were classified as adverse risk by ELN 2022. Genomic features were similar between the ND and RR cohorts.

Among 17 pts evaluated by OGM, all showed chromoanagenesis involving chromosome 11, suggesting that *KMT2A*amp is a manifestation of chromoanagenesis.

In ND AML, 11 pts were treated with intensive chemotherapy (IC) and 43 were treated with low intensity therapy (LIT), including 23 pts who also received venetoclax (LIT+VEN). Composite complete remission rates were 33% with IC, 23% with LIT and 54% with LIT+ven. MRD negativity among all pts was achieved at 17% with IC, 9% with LIT and 29% with LIT+ven. The median OS was 5.5 months (95% CI 4.3–7.5). OS did not differ for pts treated with IC or LIT (4.6 vs 5.8 months, *p*=0.58). However, the addition of venetoclax was associated with better OS, regardless of treatment intensity (7.1 vs 4.6 months; *p*=0.03; Fig A). Secondary AML had inferior OS compared with *de novo* disease (3.9 vs 7.5 months; *p*<0.01). SCT was associated with improved OS (14 vs 7.5 months; *p*=0.04; Fig B). Median EFS was poor at 0.8 month (95% CI 0-4.1) but venetoclax addition improved EFS (6.7 vs 0.2 months, *p*<0.01)

Pts with RR AML had a median of 1(range, 1-4) prior lines of therapy. 33 (83%) had prior HMA, 19 (48%) had prior venetoclax and 6(15%) had prior SCT. In 24 (25%) patients with prior cytogenetics data, 5 (21%) had copy number alterations of chromosome 11 before *KMT2A*amp detection; while in the remainder, *KMT2A*amp occurred with clonal evolution. The median OS was 2.4 months (95% CI 1.7–3.6) regardless of prior lines of therapy.

**Summary/Conclusion**

*KMT2A*amp in AML was invariably associated with complex karyotypes and strongly linked to *TP53* mutation and/or deletion, with few additional recurrent gene mutations. OGM findings support chromoanagenesis as the underlying mechanism for *KMT2A*amp. Patients with *KMT2A*amp often have extremely poor outcomes; however, venetoclax-based therapy appeared to confer a survival benefit.

**Copyright Information:** (Online) ISSN: 2572-9241
©2026 The Author(s). HemaSphere is published by John Wiley & Sons Ltd on behalf of European Hematology Association. This is an open access Abstract Book distributed under the Attribution-NonCommercial-NoDerivs (CC BY-NC-ND), which allows third parties to download the articles and share them with others as long as they credit the author and the Abstract Book, but they cannot change the content in any way or use them commercially.
**Abstract Book Citations:** Authors, Title, HemaSphere, 2026;10:(S1):pages. The abstract book DOIs can be found on the "Author Information" page.
**Disclaimer:** Articles published in the journal HemaSphere exclusively reflect the opinions of the authors. The authors are responsible for all content in their abstracts including accuracy of the facts, statements, citing resources, etc.

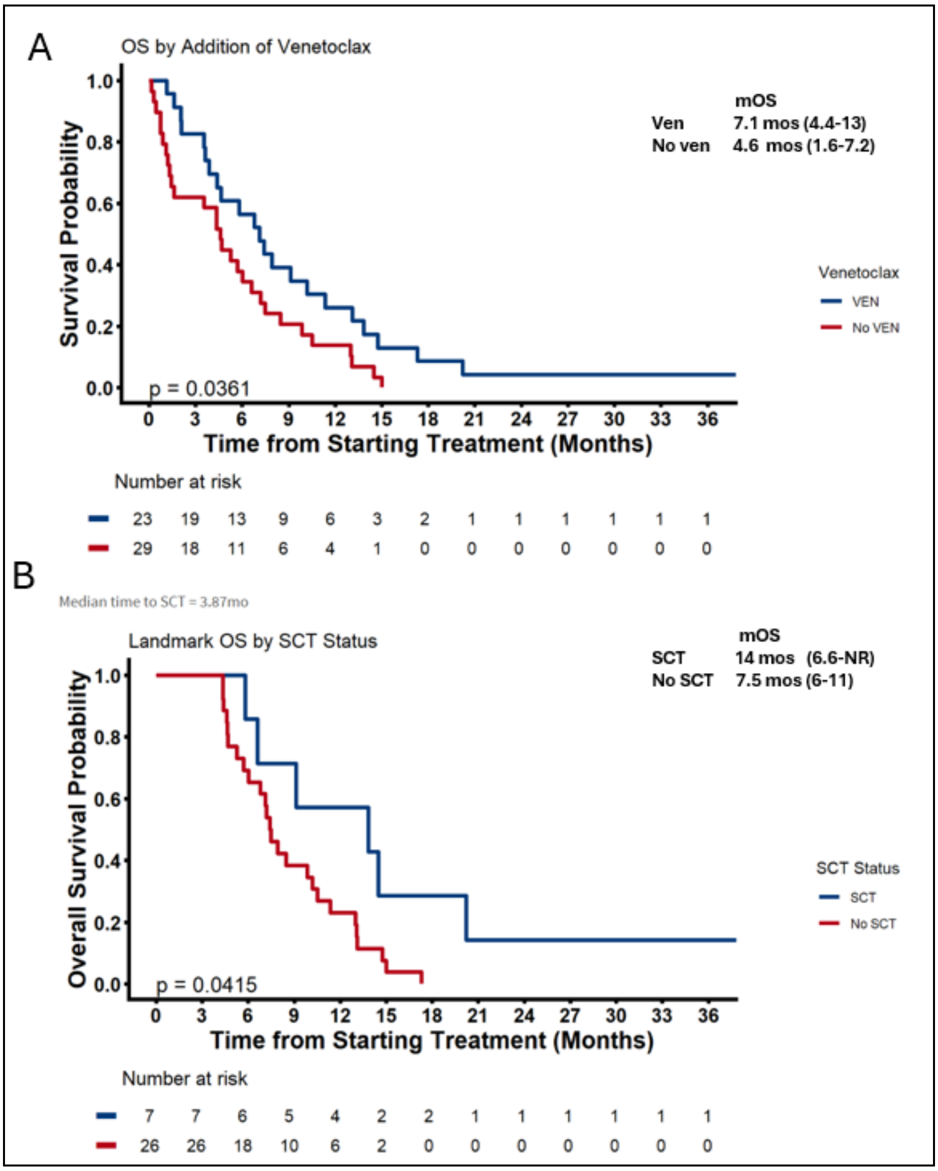

Figure A and B showing OS benefit by addition of venetoclax and SCT in NDAML with KMT2A amplification

**Copyright Information:** (Online) ISSN: 2572-9241
©2026 The Author(s). HemaSphere is published by John Wiley & Sons Ltd on behalf of European Hematology Association. This is an open access Abstract Book distributed under the Attribution-NonCommercial-NoDerivs (CC BY-NC-ND), which allows third parties to download the articles and share them with others as long as they credit the author and the Abstract Book, but they cannot change the content in any way or use them commercially.
**Abstract Book Citations:** Authors, Title, HemaSphere, 2026;10:(S1):pages. The abstract book DOIs can be found on the "Author Information" page.
**Disclaimer:** Articles published in the journal HemaSphere exclusively reflect the opinions of the authors. The authors are responsible for all content in their abstracts including accuracy of the facts, statements, citing resources, etc.

### (PS1653) OLDER AGE AND STAG2 MUTATIONS PREDICT SUPERIOR POST-TRANSPLANT SURVIVAL IN NEWLY DIAGNOSED ACUTE MYELOID LEUKEMIA TREATED WITH HMA-VENETOCLAX

**Topic:** 04. Acute myeloid leukemia - Clinical

Momna Warraich*[1], Sudhesh Kumar[1], Mahnoor Fatima[1], Kristen McCullough[1], Aref Al-Kali[1], Hassan Alkhateeb[1], Kebede Begna[1], Abhishek Mangaonkar[1], Antoine Saliba[1], Aasiya Matin[1], Mark Litzow[1], William Hogan[1], Mithun Shah[1], Mrinal Patnaik[1], Animesh Pardanani[1], James M Foran[2], Lisa Sproat[3], Nandita Khera[3], Ayalew Tefferi[1], Naseema Gangat[1]

*[1]Mayo Clinic, Hematology and Oncology, Rochester, United States of America; [2]Mayo Clinic, Jacksonville, United States of America; [3]Mayo Clinic, phoenix, United States of America;*

### Background

Venetoclax (Ven) plus hypomethylating agents (HMA) is standard frontline therapy for newly diagnosed AML (ND-AML) patients unfit for intensive chemotherapy and is also being considered in younger and fit patients (Fathi, Blood 2025). However, consolidation with allogeneic stem cell tranplant (ASCT) is often required to secure durable remission. The current study examines predictors of post-transplant survival (PTS) in the scenario.

### Methods

ND-AML patients who received upfront Ven+HMA and underwent ASCT were retrospectively studied. Clinical, cytogenetic, and molecular characteristics at diagnosis and at transplantation were collected. PTS, relapse rate, non-relapse mortality (NRM) and predictors of survival were evaluated.

### Results

111 ND-AML patients (58% male, 60% secondary/therapy-related, median age 70 [37-80 years]) received median 3 cycles of Ven-HMA. 22% had complex karyotypes. Mutations at diagnosis included *RUNX1* (18%), *SRSF2* (17%), *TP53* (16%), *ASXL1*, *IDH2*, *K/NRAS* and *TET2* (14% each), *STAG2* (11%), *DNMT3A* (8%) and at time of ASCT (N=78 evaluable);*RUNX1* (8%), *NPM1* (6%), *TP53* and *ASXL1* (5% each), and *SRSF2* and *TET2* (4% each).
At ASCT, all patients were in CR/CRi; 88% after Ven-HMA and 12% after additional bridging therapy. MRD by flow cytometry was detected in 18 (24%) of 76 evaluable patients and complex karyotype in 8 (9%) of 94. Donors were mostly HLA-matched unrelated (75%), with 51% receiving fludarabine/melphalan conditioning and 67% post-transplant cyclophosphamide (PTCy).
Af a median follow-up of 30 months, 42 patients (38%) have died, including 18 (40%) from relapse. Median PTS was not reached; 1-, 2-, and 3-year survival rates were 69%, 60%, and 57%. On multivariate analysis, age ≥65 years (NR vs 10 months; HR 0.38 p<0.01), and *STAG2* mutation (NR vs 44 months; HR 0.21p=0.04),) were independently associated with superior PTS. In analysis limited to variables at time of ASCT, age ≥65 years remained significant (p<0.01) while *TP53* mutations, donor type, and MRD status were not (p>0.1). Patients <65 years more often received bridging therapy compared to those ≥65 years (23% vs 7%; p<0.05); results were unchanged after exclusion of patients receiving bridging therapy. A risk score was developed, assigning 1 point each for age <65 years and absence of *STAG2* mutation. This model identified three prognostic groups (p<0.01): high risk (2 points), intermediate risk (1 point), and low risk (0 points), with 3-year PTS survival rates of 22%, 62%, and 91%, respectively.
18 patients (16%) experienced post-ASCT relapse with median relapse free survival (RFS) of 7 months. Cumulative incidence of relapse at 1, 2, and 3 years was 22%, 30%, and 31%; NRM was 13%, 24%, and 46%. Patients <65 years had higher 1-year relapse rates (36% vs 18%; p=0.01) with similar NRM (p=0.54). PTCy was not associated with relapse (p=0.66) but showed a borderline association with higher NRM (1-/2-/3-year 17%/31%/58% vs 4%/7%/16% in non-PTCy recipients; p=0.06). GVHD rate was 44% (50% ≥grade 2) without association with relapse (p=0.43) or NRM (p=0.99).

**Copyright Information:** (Online) ISSN: 2572-9241
©2026 The Author(s). HemaSphere is published by John Wiley & Sons Ltd on behalf of European Hematology Association. This is an open access Abstract Book distributed under the Attribution-NonCommercial-NoDerivs (CC BY-NC-ND), which allows third parties to download the articles and share them with others as long as they credit the author and the Abstract Book, but they cannot change the content in any way or use them commercially.
**Abstract Book Citations:** Authors, Title, HemaSphere, 2026;10:(S1):pages. The abstract book DOIs can be found on the "Author Information" page.
**Disclaimer:** Articles published in the journal HemaSphere exclusively reflect the opinions of the authors. The authors are responsible for all content in their abstracts including accuracy of the facts, statements, citing resources, etc.

### Summary/Conclusion

In the current study, older age (>65 years), including >75 years, and *STAG2* mutation was independently associated with superior PTS in ND-AML patients receiving Ven-HMA. By contrast, PTS was not significantly affected by genetic profile at the time of transplant, donor type, conditioning regimen, or GVHD prophylaxis.

**Figure 1. Post Transplant Survival (PTS) based on age < 65 years and absence of *STAG2* mutation ( 1 point each): low (age ≥65 years and *STAG2* mutation present; n=11), intermediate (age <65 years or *STAG2* mutation absent; n=74), high (age <65 years and *STAG2* mutation absent; n=26)**

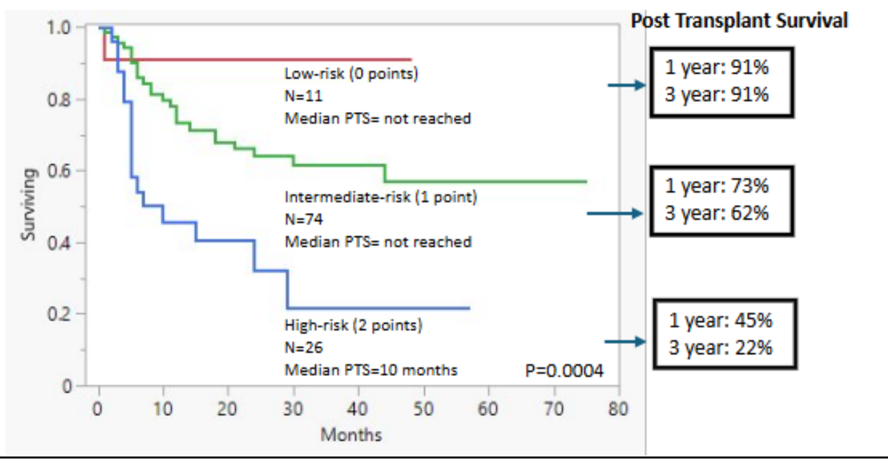

**Copyright Information:** (Online) ISSN: 2572-9241
©2026 The Author(s). HemaSphere is published by John Wiley & Sons Ltd on behalf of European Hematology Association. This is an open access Abstract Book distributed under the Attribution-NonCommercial-NoDerivs (CC BY-NC-ND), which allows third parties to download the articles and share them with others as long as they credit the author and the Abstract Book, but they cannot change the content in any way or use them commercially.
**Abstract Book Citations:** Authors, Title, HemaSphere, 2026;10:(S1):pages. The abstract book DOIs can be found on the "Author Information" page.
**Disclaimer:** Articles published in the journal HemaSphere exclusively reflect the opinions of the authors. The authors are responsible for all content in their abstracts including accuracy of the facts, statements, citing resources, etc.

### (PS1654) EFFICACY AND SAFETY ANALYSIS OF CBA CONDITIONING REGIMEN IN ALLOGENEIC HEMATOPOIETIC STEM CELL TRANSPLANTATION FOR INTERMEDIATE/HIGH-RISK ACUTE MYELOID LEUKEMIA

**Topic:** 04. Acute myeloid leukemia - Clinical

Qiuyue Wu*[1], Tao Wu[1]

[1] *The 940th Hospital of Joint Logistics Support force of Chinese People's Liberation Army, hematology, Gansu, China;*

### Background

Acute myeloid leukemia (AML) is a highly heterogeneous hematologic malignancy. For patients with intermediate/high-risk AML, allogeneic hematopoietic stem cell transplantation (allo-HSCT) remains a pivotal therapeutic modality. However, post-transplant relapse, treatment-related mortality, and non-relapse mortality (NRM) including graft-versus-host disease (GVHD) still constitute the major obstacles to successful transplantation. Exploring novel conditioning regimens that can enhance transplant efficacy while reducing treatment-related toxicity is of great clinical significance. It has been confirmed that allo-HSCT bridged by cladribine (CLAD)-based chemotherapy exerts favorable safety and remarkable efficacy in the treatment of R/R AML. Nevertheless, clinical studies on the conditioning regimen of cladribine combined with busulfan and cytarabine (CBA) for intermediate/high-risk AML remain scarce.

### Aims

To investigate the efficacy and safety of CBA conditioning regimen in patients with intermediate/high-risk AML undergoing allo-HSCT.

### Methods

A retrospective analysis was performed on 56 patients with intermediate/high-risk AML who underwent allo-HSCT in the Department of Hematology, the 940th Hospital of Joint Logistics Support Force from January 1, 2018 to December 31, 2025. Among them, 16 cases were assigned to the CBA group and 40 cases to the modified busulfan plus cyclophosphamide (BuCy) group. The efficacy and safety of the two regimens were evaluated by comparing hematopoietic reconstitution, adverse reactions and survival outcomes, and prognostic factors were further analyzed.

### Results

The CBA conditioning regimen significantly improved neutrophil engraftment (P=0.041) and accelerated hematopoietic reconstitution in AML patients. Besides, the CBA group had a lower incidence of chronic graft-versus-host disease (cGVHD) (P=0.043) and a markedly reduced incidence of nausea and vomiting (P=0.003). There were no significant differences in the 3-year progression-free survival (PFS), overall survival (OS), cumulative incidence of relapse (CIR) and NRM between the two groups. Propensity score matching (PSM) analysis revealed that the CBA conditioning regimen could significantly decrease the post-transplant incidence of cGVHD (P=0.030) and CIR (P=0.016), while the median time to relapse in the CBA group was earlier than that in the BuCy group. Logistic regression analysis identified relapsed/refractory disease as an independent risk factor for post-transplant relapse, and post-transplant maintenance therapy as an independent protective factor against post-transplant relapse. Cox regression analysis demonstrated that the grade of acute graft-versus-host disease (aGVHD) and TLS-ERG gene mutation were independent risk factors affecting OS in these patients.

**Copyright Information:** (Online) ISSN: 2572-9241
©2026 The Author(s). HemaSphere is published by John Wiley & Sons Ltd on behalf of European Hematology Association. This is an open access Abstract Book distributed under the Attribution-NonCommercial-NoDerivs (CC BY-NC-ND), which allows third parties to download the articles and share them with others as long as they credit the author and the Abstract Book, but they cannot change the content in any way or use them commercially.
**Abstract Book Citations:** Authors, Title, HemaSphere, 2026;10:(S1):pages. The abstract book DOIs can be found on the "Author Information" page.
**Disclaimer:** Articles published in the journal HemaSphere exclusively reflect the opinions of the authors. The authors are responsible for all content in their abstracts including accuracy of the facts, statements, citing resources, etc.

### Summary/Conclusion

The CBA-based conditioning regimen is safe and effective for allo-HSCT in patients with intermediate/high-risk AML, which can effectively reduce the incidence of post-transplant cGVHD, accelerate hematopoietic reconstitution and decrease the occurrence of nausea and vomiting. Relapsed/refractory disease is an independent risk factor for post-transplant relapse, and post-transplant maintenance therapy is an independent protective factor against this outcome. Additionally, the grade of aGVHD and TLS-ERG mutation are independent risk factors for poor OS in intermediate/high-risk AML patients after allo-HSCT.

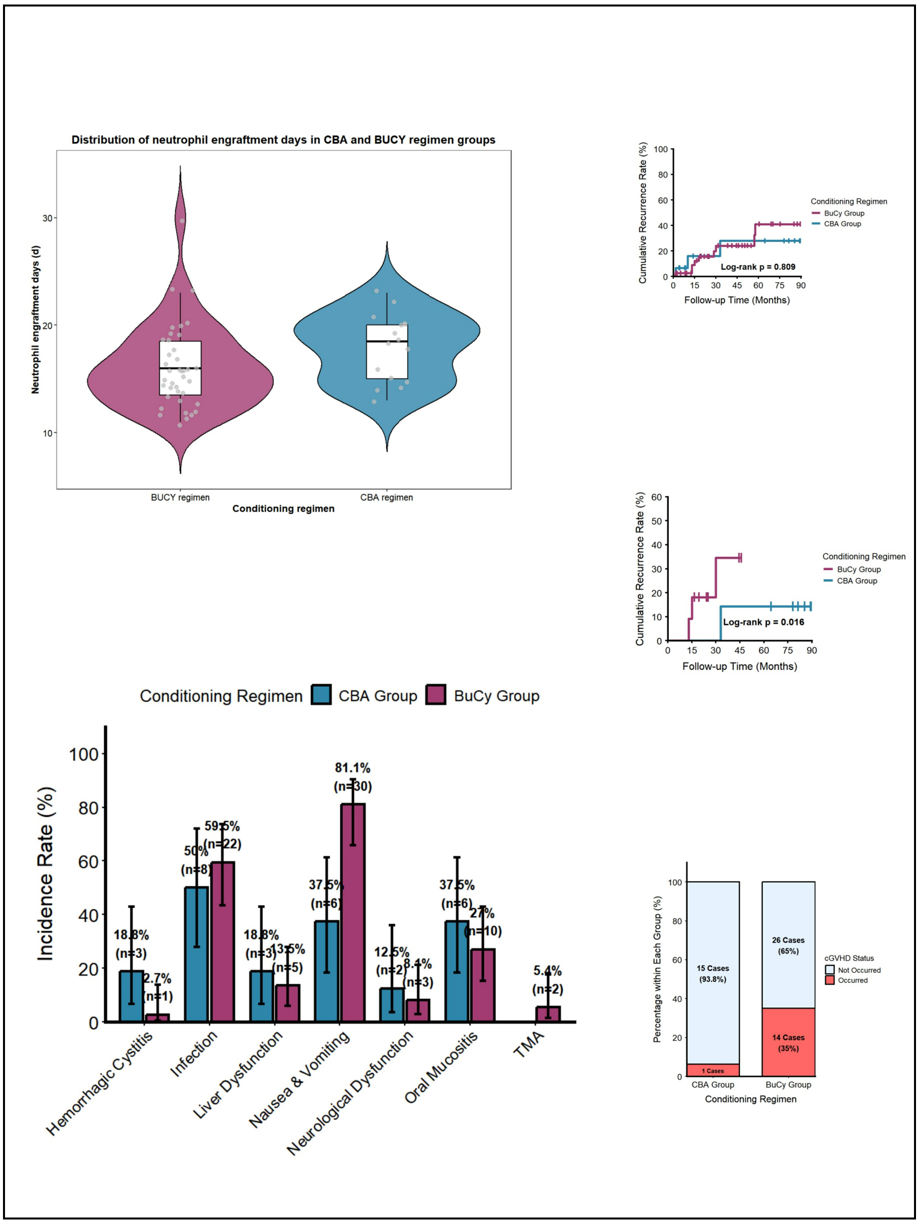

**Copyright Information:** (Online) ISSN: 2572-9241
©2026 The Author(s). HemaSphere is published by John Wiley & Sons Ltd on behalf of European Hematology Association. This is an open access Abstract Book distributed under the Attribution-NonCommercial-NoDerivs (CC BY-NC-ND), which allows third parties to download the articles and share them with others as long as they credit the author and the Abstract Book, but they cannot change the content in any way or use them commercially.
**Abstract Book Citations:** Authors, Title, HemaSphere, 2026;10:(S1):pages. The abstract book DOIs can be found on the "Author Information" page.
**Disclaimer:** Articles published in the journal HemaSphere exclusively reflect the opinions of the authors. The authors are responsible for all content in their abstracts including accuracy of the facts, statements, citing resources, etc.

### (PS1655) VENETOCLAX-BASED REGIMENS IN ELDERLY OR MEDICALLY UNFIT PATIENTS WITH NEWLY DIAGNOSED CORE BINDING FACTOR (CBF)-REARRANGED AML: UPDATED REAL-WORLD OUTCOMES BASED ON INTERNATIONAL REGISTRY DATA

**Topic:** 04. Acute myeloid leukemia - Clinical

Leo Ruhnke*[1], Christoph Röllig[1], Lydia Kretschmer[1], Jan Moritz Middeke[1], Sylvia Herold[2], David Poitz[3], Pau Montesinos[4], Maria Carmen Martinez Chamorro[5], Josefina Serrano Lopez[6], Joaquin Sanchez Garcia[6], Lara Maria Gomez Garcia[7], Francisco-Javier Peñalver[8], Guillermo Enrique Quintero Vega[9], Virginia Abello-Polo[10], Pierre, Yves Dumas[11], Arnaud Pigneux[11], Sarah Bertoli[12], Christian Recher[12], Jenny O'nions[13], Hannah Al-Yousuf[13], Jad Othman[14], Richard Dillon[14], Jiří Mayer[15], Stéphane De Botton[16], Raphael Itzykson[17], Hervé Dombret[17], Magdalena Czemerska[18], Agnieszka Wierzbowska[18], Kellen Gil[19], Andrew Kent[19], Daniel Pollyea[19], Seongseok Yun[20], Alison Walker[20], David Sallman[20], Anne Weigert[21], Madlen Jentzsch[21], Uwe Platzbecker[1, 21], Karsten Spiekermann[22], Tim Sauer[23], Carsten Müller-Tidow[23], Hubert Serve[24], Claudia Baldus[25], Martin Bornhäuser[1], Klaus Metzeler[21]

*[1]Department of Internal Medicine I, University Hospital Dresden, TU Dresden, Dresden, Germany; [2]Institute of Pathology, Molecular Pathology, University Hospital Dresden, TU Dresden, Dresden, Germany; [3]Institute of Clinical Chemistry and Laboratory Medicine, University Hospital Dresden, TU Dresden, Dresden, Germany; [4]Hematology Department, Hospital Universitario i Politècnico la Fe, Valencia, Valencia, Spain; [5]Hospital Universitario Quirónsalud Madrid and Universidad Europea de Madrid, Madrid, Spain; [6]Instituto Maimónides de Investigación Biomédica de Córdoba, Hospital Universitario Reina Sofía, Universidad de Córdoba, Córdoba, Spain; [7]Hospital Clínico de Valladolid, Valladolid, Spain; [8]Hospital Universitario Fundación Alcorcón, Madrid, Spain; [9]Fundacion Santa Fe de Bogotá, Bogotá, Colombia; [10]CTIC-Centro de tratamiento e investigacion sobre cáncer Luis Carlos Sarmiento Angulo, Bogotá, Colombia; [11]Department of Hematology, CHU de Bordeaux, Université de Bordeaux, INSERM-U1312, Bordeaux, France; [12]Université de Toulouse, Centre Hospitalier Universitaire de Toulouse, Institut Universitaire du Cancer de Toulouse-Oncopole, Service d'Hématologie, Toulouse, France; [13]Department of Hematology, University College London Hospital NHS Foundation Trust, London, United Kingdom; [14]Department of Medical and Molecular Genetics, King's College London, London, United Kingdom; [15]Department of Internal Medicine, University Hospital Brno and Masaryk University, Brno, Czechia; [16]Département d'Hématologie et Département d'Innovation Thérapeutique, Gustave Roussy, Villejuif, France; [17]Department of Hematology, Saint-Louis Institute for Research, Université Paris Cité, Hôpital Saint-Louis, Assistance Publique-Hôpitaux de Paris, Paris, France; [18]Department of Hematology, Medical University of Lodz, Multidisciplinary Provincial Centre of Oncology and Traumatology, Lodz, Poland; [19]Division of Hematology, University of Colorado School of Medicine, Aurora, Colorado, United States of America; [20]Department of Malignant Hematology, Moffitt Cancer Center, Tampa, Florida, United States of America; [21]Department of Hematology, Cellular Therapy, Hemostaseology and Infectious Disease, University of Leipzig Medical Center, Leipzig, Germany; [22]Department of Medicine III, University Hospital, LMU, Munich, Germany; [23]Department of Internal Medicine V, University of Heidelberg, Heidelberg, Germany; [24]Department of Medicine, Hematology/Oncology, Goethe University, Frankfurt, Germany; [25]Department of Hematology and Oncology, University Hospital Schleswig-Holstein, Campus Kiel, Kiel, Germany;*

### Background

Patients (pts) with CBF AML (defined by inv(16)(p13.1q22) or t(16;16)(p13.1;q22)/*CBFB::MYH11* or t(8;21)(q22;q22.1)/*RUNX1::RUNX1T1* gene fusions) are considered to have favorable risk disease when treated with intensive chemotherapy regimens. However, little is known about the efficacy of venetoclax (Ven)-based therapy in CBF AML pts ineligible for intensive treatment.

**Copyright Information:** (Online) ISSN: 2572-9241
©2026 The Author(s). HemaSphere is published by John Wiley & Sons Ltd on behalf of European Hematology Association. This is an open access Abstract Book distributed under the Attribution-NonCommercial-NoDerivs (CC BY-NC-ND), which allows third parties to download the articles and share them with others as long as they credit the author and the Abstract Book, but they cannot change the content in any way or use them commercially.
**Abstract Book Citations:** Authors, Title, HemaSphere, 2026;10:(S1):pages. The abstract book DOIs can be found on the "Author Information" page.
**Disclaimer:** Articles published in the journal HemaSphere exclusively reflect the opinions of the authors. The authors are responsible for all content in their abstracts including accuracy of the facts, statements, citing resources, etc.

### Aims

To assess the efficacy of less-intensive treatment regimens combining Ven with hypomethylating agents (HMA) or low-dose cytarabine (LDAC) in this specific patient population.

### Methods

Based on data from international study group and cancer center registries, we retrospectively analyzed adult pts with newly diagnosed CBF AML treated with Ven-based regimens. Response and survival outcomes were evaluated according the ELN 2022 criteria, best response within 180 days is reported.

### Results

We identified 66 pts with newly diagnosed CBF AML (52% with *CBFB::MYH11*, 48% with *RUNX1::RUNX1T1*) treated with Ven-based regimens between July 2019 and August 2025. The median age of the entire cohort was 76 years (range, 30-86), 41% were female, 33% of pts had ECOG PS ≥2, 23% had AML post cytotoxic therapy. According to the European LeukemiaNet (ELN) 2024 risk classification, 68%, 30%, and 2% of pts had favorable, intermediate, and adverse risk disease, respectively. The most common secondary alterations were mutations in *NRAS* (33%), *TET2* (27%), *KIT* (27%), *KRAS* (16%) and *DNMT3A* (12%). Except for a higher *KRAS* mutation prevalence in pts with *CBFB*::*MYH11* (29% vs. 4%; *p*=0.02), there were no significant differences in baseline characteristics between CBF subgroups.
Most pts (89%) received azacitidine/Ven. The median planned Ven schedule was 21 days (range, 7-28), and the median number of administered HMA/Ven or LDAC/Ven cycles was 4 (range, 1-50). Two pts underwent allogeneic HCT.
The composite complete remission rate (CRc, CR + CRi) of the entire cohort was 69%, and was similar for pts with *CBFB*::*MYH11* or *RUNX1*::*RUNX1T1* fusions (68% vs. 71%, *p*=1.00). Pts with ELN 2024 intermediate risk mutations (i.e., *FLT3*-ITD, *NRAS* and/or *KRAS*) had a CRc rate similar to those without any of these variants (70% vs. 69%, *p*=1.00). Also, the CRc rate of pts with *KIT* mutations was comparable to wild-type pts (66% vs 70%, *p*=1.00). The early mortality rate was 11%.
After a median follow-up of 21 months, the median overall survival (mOS) of the entire cohort was 14 months, with corresponding 12-month and 24-month OS rates of 55% and 40%, respectively. The mOS of pts with *RUNX1*::*RUNX1T1* was similar to those with *CBFB*::*MYH11* (13 months vs. 23 months, *p*=0.93). As compared to the corresponding wild-type pts, neither ELN 2024 intermediate risk variants nor *KIT* mutations adversely impacted mOS (*FLT3*-ITD/*NRAS*/*KRAS*, 14 vs. 18 months, *p*=0.48; *KIT*, 14 months vs. 11 months, *p*=0.91).

### Summary/Conclusion

Treatment with Ven-based regimens seems to be a valuable treatment option for CBF AML pts ineligible for intensive chemotherapy. In contrast to previous reports, outcomes did not differ between pts with *CBFB*::*MYH11*- and *RUNX1*::*RUNX1T1*-rearrangend AML. With a mOS of 14 months, outcomes appear less favorable than those previously reported for pts with ELN 2024 favorable risk disease, and similar to those observed in ELN 2024 intermediate-risk non-CBF AML pts, irrespective of *FLT3*-ITD, *NRAS* and/or *KRAS* mutations. Therefore, we propose that all pts with CBF AML treated with Ven-based less-intensive regimens should be provisionally classified as having intermediate-risk disease.

**Copyright Information:** (Online) ISSN: 2572-9241
©2026 The Author(s). HemaSphere is published by John Wiley & Sons Ltd on behalf of European Hematology Association. This is an open access Abstract Book distributed under the Attribution-NonCommercial-NoDerivs (CC BY-NC-ND), which allows third parties to download the articles and share them with others as long as they credit the author and the Abstract Book, but they cannot change the content in any way or use them commercially.
**Abstract Book Citations:** Authors, Title, HemaSphere, 2026;10:(S1):pages. The abstract book DOIs can be found on the "Author Information" page.
**Disclaimer:** Articles published in the journal HemaSphere exclusively reflect the opinions of the authors. The authors are responsible for all content in their abstracts including accuracy of the facts, statements, citing resources, etc.

### (PS1656) INTERIM BONE MARROW BIOPSY IN AML TREATED WITH VENETOCLAX PLUS “7+3” CHEMOTHERAPY IS POORLY PREDICTIVE OF END-OF-INDUCTION REMISSION

**Topic:** 04. Acute myeloid leukemia - Clinical

Anushri Soni*[1], Buse Eglenen Polat[2], Mumtu Lalla[2], Mendel Goldfinger[2], Aditi Shastri[2], Kira Gritsman[2], David Levitz[2], Noah Kornblum[2], Lauren Shapiro[2], Alejandro Sica[2], Annemarie Munoz[2], Nicole Chambers[2], Aradhika Dhawan[2], Karen Fehn[2], Davina Hoban[2], Amit Verma[2], Mimi Kim[2], Dennis Cooper[2], Marina Konopleva[2], Eric Feldman[2], Ioannis Mantzaris[2]

*[1]Jacobi Medical Center/Albert Einstein College of Medicine, New York City, United States of America;[2]Montefiore Einstein Comprehensive Cancer Center, New York City, United States of America;*

### Background

Interim bone marrow biopsy (IBMB), performed between days 14 and 21 of induction chemotherapy (IC) in patients with acute myeloid leukemia (AML), is used to assess for residual disease (≥5% blasts) and to guide decisions regarding re-induction therapy aiming for complete remission. However, the interpretation of IBMB findings is challenging during this vulnerable phase of treatment, and its clinical utility controversial, particularly with emerging highly efficacious therapies.

### Aims

To assess the predictive capacity of IBMB for end-of-induction (EOI) remission in newly diagnosed (ND) AML patients treated with venetoclax (Ven) plus “7+3” IC.

### Methods

We conducted a post-hoc analysis of an adult ND AML patient cohort treated with Ven+”7+3” between July 2022 and October 2025 on an ongoing prospective, single-center clinical trial (NCT05342584). Of 66 treated patients, 58 underwent IBMB on days 14-21 and were included. Blast percentage was assessed by morphology and immunohistochemistry (IHC) on core biopsy (n=53) and/or high-sensitivity multiparameter flow cytometry (hsMFC at Hematologics, Inc; lower limit of enumeration <0.02%) (n=48). Residual disease was defined as ≥5% blasts by IHC and/or hsMFC. EOI response (days 28-42) was assessed per ELN 2022 criteria. No patient received re-induction based on IBMB findings. We evaluated the concordance between interim and EOI assessments.

### Results

At the EOI, 52/58 patients (89.7%) achieved composite complete remission (CRc=51CR+1CRh). Among responders, IBMB showed no residual disease in 29/48 by IHC (sensitivity [Sn]: 60.4%), 34/42 by hsMFC (Sn: 81.0%), and 31/51 by either modality (Sn: 60.8%). Among non-responders, IBMB showed residual disease in 5/5 patients by IHC (specificity [Sp]: 100%), 4/6 by hsMFC (Sp: 66.7%) and 6/6 by either (Sp: 100%). Absence of residual disease on IBMB predicted CR in 34/36 (positive predictive value [PPV: 94.4%) when assessed by hsMFC and in all pts by IHC (29/29, PPV: 100%) or the two combined (31/31, PPV: 100%). On the contrary, residual disease on D14-21 had low negative predictive value (NPV) for refractory AML (IHC: 5/24; NPV: 20.8% vs hsMFC: 4/12; NPV: 33.3% vs combined: 6/26; NPV: 23.1%). Agreement between IBMB blast burden (<5% vs ≥5%) and CR status was low, with kappa values of 0.22 for IHC, 0.33 for hsMFC, and 0.25 for either modality.

**Copyright Information:** (Online) ISSN: 2572-9241
©2026 The Author(s). HemaSphere is published by John Wiley & Sons Ltd on behalf of European Hematology Association. This is an open access Abstract Book distributed under the Attribution-NonCommercial-NoDerivs (CC BY-NC-ND), which allows third parties to download the articles and share them with others as long as they credit the author and the Abstract Book, but they cannot change the content in any way or use them commercially.
**Abstract Book Citations:** Authors, Title, HemaSphere, 2026;10:(S1):pages. The abstract book DOIs can be found on the “Author Information” page.
**Disclaimer:** Articles published in the journal HemaSphere exclusively reflect the opinions of the authors. The authors are responsible for all content in their abstracts including accuracy of the facts, statements, citing resources, etc.

| **IHC** | CRc* (n=48) | No response (n=5) | % (95% CI) |
|---|---|---|---|
| Blasts <5%, n | 29 | 0 | PPV= 100 (88.1 - 100) |
| Blasts ≥5%, n | 19 | 5 | NPV= 20.8 (7.1 - 42.2) |
| % (95% CI) | Sn* = 60.4 (45.3 - 74.2) | Sp* = 100 (47.8 - 100) | |

| **hsMFC*** | CRc (n=42) | No response (n=6) | % (95% CI) |
|---|---|---|---|
| Blasts <5%, n | 34 | 2 | PPV= 94.4 (81.3 - 99.3) |
| Blasts ≥5%, n | 8 | 4 | NPV= 33.3 (9.9 - 65.1) |
| % (95% CI) | Sn= 81.0 (65.9 - 91.4) | Sp= 66.7 (22.3 - 95.7) | |

| **hsMFC+IHC** | CRc (n=51) | No response (n=6) | % (95% CI) |
|---|---|---|---|
| Blasts <5%, n | 31 | 0 | PPV= 100 (88.8 - 100) |
| Blasts ≥5%, n | 20 | 6 | NPV= 23.1 (9.0 - 43.7) |
| % (95% CI) | Sn= 60.8 (46.1 - 74.2) | Sp= 100 (54.1 - 100) | |

*IHC: immunohistochemistry, CRc: composite complete remission, Sn: sensitivity, Sp: specificity, PPV: positive predictive value, NPV: negative predictive value, hsMFC: high-resolution multiparameter flow cytometry

Table. Predictive performance of IBMB for CR

**Copyright Information:** (Online) ISSN: 2572-9241
©2026 The Author(s). HemaSphere is published by John Wiley & Sons Ltd on behalf of European Hematology Association. This is an open access Abstract Book distributed under the Attribution-NonCommercial-NoDerivs (CC BY-NC-ND), which allows third parties to download the articles and share them with others as long as they credit the author and the Abstract Book, but they cannot change the content in any way or use them commercially.
**Abstract Book Citations:** Authors, Title, HemaSphere, 2026;10:(S1):pages. The abstract book DOIs can be found on the “Author Information” page.
**Disclaimer:** Articles published in the journal HemaSphere exclusively reflect the opinions of the authors. The authors are responsible for all content in their abstracts including accuracy of the facts, statements, citing resources, etc.

**Summary/Conclusion**

Within the limitations of a single-center study and a small sample size, our results suggest that IBMB in AML induction is a poor predictor of remission when patients are treated with “7+3” plus Ven. False positive results can come at a cost of undue toxicity. With more efficacious induction strategies this practice may no longer be necessary.

**Copyright Information:** (Online) ISSN: 2572-9241
©2026 The Author(s). HemaSphere is published by John Wiley & Sons Ltd on behalf of European Hematology Association. This is an open access Abstract Book distributed under the Attribution-NonCommercial-NoDerivs (CC BY-NC-ND), which allows third parties to download the articles and share them with others as long as they credit the author and the Abstract Book, but they cannot change the content in any way or use them commercially.
**Abstract Book Citations:** Authors, Title, HemaSphere, 2026;10:(S1):pages. The abstract book DOIs can be found on the “Author Information” page.
**Disclaimer:** Articles published in the journal HemaSphere exclusively reflect the opinions of the authors. The authors are responsible for all content in their abstracts including accuracy of the facts, statements, citing resources, etc.

### (PS1657) REDUCED DOSE ALL-TRANS RETINOIC ACID AND ARSENIC TRIOXIDE TO DECREASE EARLY DEATHS IN OLDER ACUTE PROMYELOCYTIC LEUKEMIA (APL) PATIENTS. POST-HOC ANALYSIS OF PATIENTS >60 YEARS IN THE EA9131 TRIAL

**Topic:** 04. Acute myeloid leukemia - Clinical

Vamsi Kota*[1], Sandra Lee[2], Jessica Altman[3], Selina Luger[4], Martin Tallman[3], James M Foran[5], Lisa Law[6], Locke Bryan[1], Abdallah Abou Zahr[7], Kebede Begna[5], Alexander Perl[4], Joseph Vadakara[8], Rubina Qamar[9], Raymond Bergan[10], Michael Fisch[11], Ruth Carlos[12], Lynn Wagner[13], Mark Litzow[5], Anand Jillella[1]

*[1]Georgia Cancer Center at Augusta University, Augusta, United States of America;[2]Dana Farber Cancer Institute, Boston, United States of America; [3]Northwestern School of Medicine, Chicago, United States of America; [4]University of Pennsylvania, Philadelphia, United States of America; [5]Mayo Clinic, Jacksonville, United States of America; [6]Kaiser Permanente, Roseville, United States of America; [7]Sanford Health, Sanford, United States of America;[8]Geisinger Health, Danville, United States of America; [9]University of Wisconsin, Madison, United States of America; [10]University of Nebraska, Omaha, United States of America; [11]MD Anderson Cancer Center, Houston, United States of America; [12]University of Michigan, Ann Arbor, United States of America; [13]University of North Carolina, Chapel Hill, United States of America;*

### Background

Clinical trials have reported >95% durable remissions in patients with (APL). In contrast, population studies from national registries report one-year survival less than 70%. The conflicting rates are due to enrollment of younger patients in trials (median age early 40s) and exclusion of patients with co-morbid conditions. Of particular importance is the under appreciation among physicians of a high rate of Early Deaths (ED) of 17-29% in the general population and more than 50% in patients >60 years within the first 30 days of treatment. ED is the commonest cause of treatment failure in APL. Contributory factors are older age, comorbid conditions, complexity at diagnosis, lack of institutional treatment pathways and provider inexperience given the rarity of the disease. In the EA9131multi-center cooperative group prospective trial, we proposed that use of a treatment algorithm and access to APL experts at academic centers to provide remote guidance to community centers (CC) would decrease ED. Of 201 enrolled patients, there were 6 deaths with an ED rate of 3% and a 1-year survival of 94.5% (Jillella et al 2025). We herein report the care approach and exploratory analysis of outcome in patients >60 years.

### Methods

We used a treatment algorithm developed by 7 APL experts at 6 academic lead centers (LC). The trial was opened at 6 LC and 293 CC. Patients who presented to one of the 6 LC were managed with input from the local expert. Physicians at the CC contacted one of the 7 experts when a patient presented with suspected APL. Expert support was available 24/7 and the patient was eligible if the contact was made within 3 days of starting APL therapy. There were no exclusion criteria. A workup and treatment plan was provided to the CC physician based on standard of care with dose reductions for patients >60. For patients >60, ATRA at 25 mg/m2/day was suggested with addition of 50% dose reduced ATO after 10 days and patients >70 further reduction was made with ATRA at 10mg/m2/day and ATO added later or only during consolidation. Communication was maintained throughout induction. OS data was evaluated using the method of Kaplan-Meier (KM) and one-month and one-year OS rates estimated based on the KM estimate.

**Copyright Information:** (Online) ISSN: 2572-9241
©2026 The Author(s). HemaSphere is published by John Wiley & Sons Ltd on behalf of European Hematology Association. This is an open access Abstract Book distributed under the Attribution-NonCommercial-NoDerivs (CC BY-NC-ND), which allows third parties to download the articles and share them with others as long as they credit the author and the Abstract Book, but they cannot change the content in any way or use them commercially.
**Abstract Book Citations:** Authors, Title, HemaSphere, 2026;10:(S1):pages. The abstract book DOIs can be found on the "Author Information" page.
**Disclaimer:** Articles published in the journal HemaSphere exclusively reflect the opinions of the authors. The authors are responsible for all content in their abstracts including accuracy of the facts, statements, citing resources, etc.

**Results**

Of 201 patients enrolled, 72 were >60; of these 41(56.9%) were 60-69, 22 (30.6%) 70-79 and 9 (12.5%) >80. 36 (50%) were male and 62 white, 7 African American, 2 Asian, 1 unknown; 7 identified as Hispanic. 15 patients (20.8%) were high-risk, 42 (58.3) obese and 49 (68%) had a Charlson comorbid index of >3. In 5 patients the dose of ATRA was not available. In 67 with reported doses, 20 started ATRA on day 1 at 45mg/m2/day with dose reduction to 25mg/m2/day in 15 by day 5. 39 started at 25mg/m2/day on day 1 and 8 at <25mg/m2/day. In 20 patients ATRA was decreased further, held and restarted for leukocytosis or differentiation syndrome (DS). 21 patients did not receive ATO during induction and 44 started around day 10 with most receiving 50% dose reduction. There were 5 deaths (ages 70, 71, 76, 79 and 81) all from DS with a one-month survival of 93.1% and a 1-year survival of 87.5%. There were 4 relapses; 3 were non-compliant/declined consolidation.

**Summary/Conclusion**

In this elderly cohort with multiple comorbid conditions, reduced dose ATRA and ATO, late initiation of ATO, algorithm use and expert support decreased ED. Full doses of ATRA and ATO are poorly tolerated by the elderly and further studies are needed in this highly curable malignancy.

**Copyright Information:** (Online) ISSN: 2572-9241
©2026 The Author(s). HemaSphere is published by John Wiley & Sons Ltd on behalf of European Hematology Association. This is an open access Abstract Book distributed under the Attribution-NonCommercial-NoDerivs (CC BY-NC-ND), which allows third parties to download the articles and share them with others as long as they credit the author and the Abstract Book, but they cannot change the content in any way or use them commercially.
**Abstract Book Citations:** Authors, Title, HemaSphere, 2026;10:(S1):pages. The abstract book DOIs can be found on the "Author Information" page.
**Disclaimer:** Articles published in the journal HemaSphere exclusively reflect the opinions of the authors. The authors are responsible for all content in their abstracts including accuracy of the facts, statements, citing resources, etc.

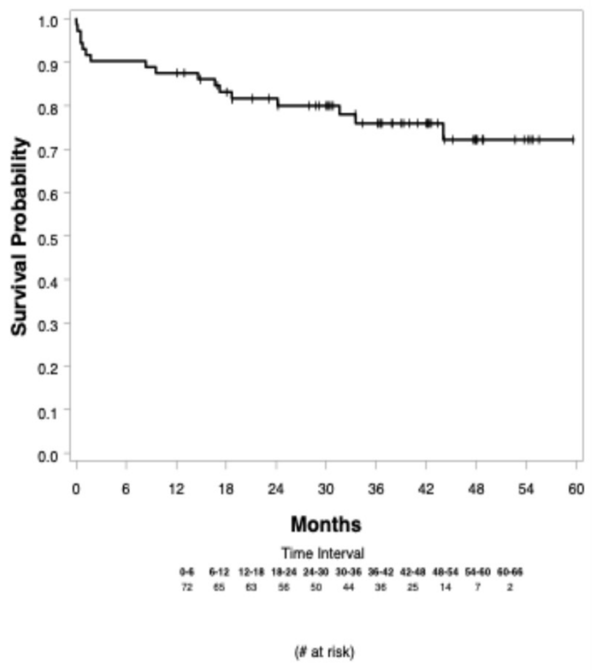

Figure 1. Survival plot for patients 60+ years of age One-month: 93.1% 95% CI (84.1%, 97.0%) One-Year:87.5%, 95% CI (77.3%, 93.3%)

**Copyright Information:** (Online) ISSN: 2572-9241
©2026 The Author(s). HemaSphere is published by John Wiley & Sons Ltd on behalf of European Hematology Association. This is an open access Abstract Book distributed under the Attribution-NonCommercial-NoDerivs (CC BY-NC-ND), which allows third parties to download the articles and share them with others as long as they credit the author and the Abstract Book, but they cannot change the content in any way or use them commercially.
**Abstract Book Citations:** Authors, Title, HemaSphere, 2026;10:(S1):pages. The abstract book DOIs can be found on the "Author Information" page.
**Disclaimer:** Articles published in the journal HemaSphere exclusively reflect the opinions of the authors. The authors are responsible for all content in their abstracts including accuracy of the facts, statements, citing resources, etc.

### (PS1658) A PHASE 1/2 STUDY OF THE MENIN INHIBITOR REVUMENIB WITH THE CELMOD MEZIGDOMIDE IN RELAPSED/REFRACTORY KMT2A-REARANGED, NPM1-MUTANT, AND NUP98-REARRANGED ACUTE LEUKEMIAS

**Topic:** 04. Acute myeloid leukemia - Clinical

Kuo-Kai Chin*[1], Sheng Cai[1], Andriy Derkach[1], Gabrielle Melamed[1], Natalie Smith[2], Neerav Shukla[3], Joshua Zeidner[4], Ibrahim Aldoss[2], Ghayas Issa[5], Eytan Stein[1]

*[1]Memorial Sloan Kettering Cancer Center, Leukemia Service, Division of Hematologic Malignancies, New York, United States of America; [2]City of Hope, Division of Leukemia, Department of Hematology & Hematopoietic Cell Transplantation, Duarte, United States of America; [3]Memorial Sloan Kettering Cancer Center, Department of Pediatrics, New York, United States of America; [4]Lineberger Comprehensive Cancer Center, University of North Carolina, Chapel Hill, United States of America;[5]The University of Texas MD Anderson Cancer Center, Department of Leukemia, Houston, United States of America;*

**Background**

Menin complexes with KMT2A and drives leukemogenesis in acute leukemias with aberrant *HOX* gene expression. Menin inhibitors (MENINi) are a new class of targeted agents effective in relapsed or refractory (R/R) *KMT2A*r and *NPM1*c acute leukemias. Other *HOX*-high acute leukemias such as *NUP98*r acute leukemias also respond to MENINi. Revumenib, the first US FDA–approved MENINi, achieved 63% ORR and 21% CR/CRh rate in R/R *KMT2A*r acute leukemias in AUGMENT-101 (NCT04065399). Despite efficacy, MENINi resistance can emerge. *MEN1* mutation emerge in approximately 40% of patients treated with revumenib, sometimes after 2 cycles. IKAROS, a critical transcription factor in lymphocyte development, is a key dependency in *KMT2A*r and *NPM1*c acute leukemias. Mezigdomide, a cereblon E3 ligase modulator (CELMoD) in phase 3 multiple myeloma trials, degrades IKAROS. Preclinical data show synergy between mezigdomide and MENINi, downregulating *HOX* genes while preventing and overcoming *MEN1* mutations.

**Aims**

We designed a phase 1/2 trial of revumenib and mezigdomide in patients with R/R *KMT2A*r, *NPM1*c, or *NUP98*r acute leukemias, including MENINi-exposed patients. This is the first clinical study to target both prevention and reversal of *MEN1*–mediated resistance to menin inhibition.

**Copyright Information:** (Online) ISSN: 2572-9241
©2026 The Author(s). HemaSphere is published by John Wiley & Sons Ltd on behalf of European Hematology Association. This is an open access Abstract Book distributed under the Attribution-NonCommercial-NoDerivs (CC BY-NC-ND), which allows third parties to download the articles and share them with others as long as they credit the author and the Abstract Book, but they cannot change the content in any way or use them commercially.
**Abstract Book Citations:** Authors, Title, HemaSphere, 2026;10:(S1):pages. The abstract book DOIs can be found on the “Author Information” page.
**Disclaimer:** Articles published in the journal HemaSphere exclusively reflect the opinions of the authors. The authors are responsible for all content in their abstracts including accuracy of the facts, statements, citing resources, etc.

### Methods

This study is a phase 1/2, multicenter, investigator-initiated study evaluating revumenib and mezigdomide in patients ≥12 years with R/R *KMT2Ar*, *NPM1*c, or *NUP98*r acute leukemias (NCT07356154). Phase 1 permits prior MENINi use. Phase 2 will include MENINi–naïve and MENINi–exposed cohorts (Figure 1). Major exclusion criteria include active use of strong or moderate CYP3A4 inhibitors due to drug-drug interactions with mezigdomide.
The trial will accrue across 4 US centers and enroll 2-18 patients in phase 1. If a recommended phase 2 dose (RP2D) is determined, up to 40 additional patients will be enrolled in phase 2 across 2 cohorts.
The phase 1 portion of the study will utilize a 3+3 dose escalation design with 3 dose levels to determine maximum tolerated dose (MTD) and RP2D and the primary endpoint will be safety and tolerability. Revumenib will be given at 270 mg every 12 hours, the FDA–approved dose. Mezigdomide doses are based on prior myeloma trials, where the RP2D was 1 mg daily on day 1-21 with dexamethasone in 28-day cycles. Selected mezigdomide dose levels (0.6 mg daily for 14 out of 28 days, 0.6 mg daily for 21 out of 28 days, and 1 mg daily for 21 out of 28 days) all showed effective degradation of AIOLOS (an IKAROS homolog) in myeloma trials.
The phase 2 portion will feature 2 cohorts and a Simon's minimax 2-stage design for each with CR/CRh within 6 cycles as primary endpoint. Cohort 1 (MENINi–naïve) will test for a CR/CRh rate of 40% vs 20% based on AUGMENT-101. Cohort 2 (MENINi–exposed) will test for a CR/CRh rate of 25% vs 5% based on retrospective data on outcomes after MENINi failure. Type-1 error is 10% and power is 80% in both cases. A stopping rule will be used to monitor toxicity. Key additional endpoints include depth and duration of response, survival, resistance mutations, and immunomodulatory effects.

### Results

The study is funded and IRB-approved, opened February 2026, and two patients have been treated at abstract submission. Dose escalation is ongoing.

### Summary/Conclusion

This ongoing phase 1/2 study evaluates the safety and preliminary efficacy of revumenib plus mezigdomide in R/R HOX-high acute leukemias. The study is designed to address an urgent unmet need: resistance to menin inhibition.

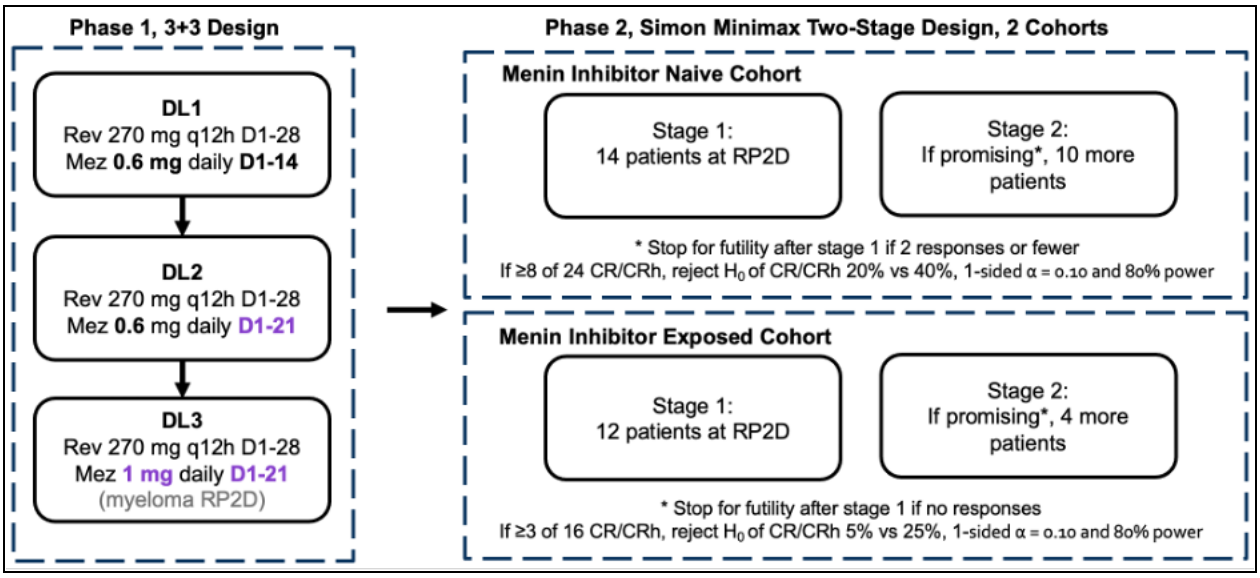

Study schema

**Copyright Information:** (Online) ISSN: 2572-9241
©2026 The Author(s). HemaSphere is published by John Wiley & Sons Ltd on behalf of European Hematology Association. This is an open access Abstract Book distributed under the Attribution-NonCommercial-NoDerivs (CC BY-NC-ND), which allows third parties to download the articles and share them with others as long as they credit the author and the Abstract Book, but they cannot change the content in any way or use them commercially.
**Abstract Book Citations:** Authors, Title, HemaSphere, 2026;10:(S1):pages. The abstract book DOIs can be found on the "Author Information" page.
**Disclaimer:** Articles published in the journal HemaSphere exclusively reflect the opinions of the authors. The authors are responsible for all content in their abstracts including accuracy of the facts, statements, citing resources, etc.

### (PS1659) EFFECT OF NPM1 MUTATION SUBTYPES AND CO-MUTATION PATTERN IN ACUTE MYELOID LEUKEMIA: A REAL-WORLD ANALYSIS FROM INTERNATIONAL COLLABORATION

**Topic:** 04. Acute myeloid leukemia - Clinical

Christopher Tham*[1], Jing Yuan Tan[1], Than Hein[1], Sarah Mott[2], Ratdanai Yodsuwan[2], Kensuke Takaoka[2], Gee Fung How[1], Kathryn Crawford[2], April Chia[1], Nicole-Ann Lim[1], Sarah Hornberg[2], Anthony Snow[2], Grerk Sutamtewagul[2], Jeffrey Quek[1], Kittika Poonsombudlert[2]

*[1]Singapore General Hospital, Singapore, Singapore;[2]University of Iowa Health Care, Iowa City, United States of America;*

**Background**

Alpermann et al. (2015) reported no difference in outcomes among *NPM1* mutation subtypes in AML. The ELN 2022 and ELN 2024 guidelines similarly classify $NPM1^{mut}$ $FLT3\text{-}ITD^{wt}$ AML in the favorable risk category regardless of subtype, based on overall treatment responsiveness. However, in clinical practice, a subset of patients is unexpectedly treatment-refractory, prompting re-evaluation of the interaction between *NPM1* subtypes and co-mutations from an international cohort.

**Aims**

To evaluate the impact of *NPM1* mutation subtypes and co-mutation patterns on complete remission (CR), relapse-free survival (RFS) after HSCT, and overall survival (OS) in AML patients treated at Singapore General Hospital (SGH) and University of Iowa Health Care (UIHC).

**Methods**

We retrospectively reviewed medical records of adult AML patients treated between 2015-2025 at SGH and UIHC.*NPM1* and co-mutations were identified using institutional molecular panels; *NPM1* subtypes were categorized per Alpermann et al. (2015). Logistic and Cox regression models were used to evaluate the association of patient, disease, and treatment characteristics with CR, RFS, and OS.

**Copyright Information:** (Online) ISSN: 2572-9241
©2026 The Author(s). HemaSphere is published by John Wiley & Sons Ltd on behalf of European Hematology Association. This is an open access Abstract Book distributed under the Attribution-NonCommercial-NoDerivs (CC BY-NC-ND), which allows third parties to download the articles and share them with others as long as they credit the author and the Abstract Book, but they cannot change the content in any way or use them commercially.
**Abstract Book Citations:** Authors, Title, HemaSphere, 2026;10:(S1):pages. The abstract book DOIs can be found on the "Author Information" page.
**Disclaimer:** Articles published in the journal HemaSphere exclusively reflect the opinions of the authors. The authors are responsible for all content in their abstracts including accuracy of the facts, statements, citing resources, etc.

**Results**

Among 229 patients (SGH N=68, UIHC N=161), median age was 63 years, and 49% were male. Racial distribution included: White (64%), Asian (30%), and Other (6%). *NPM1* subtype A was the most common (74%) followed by D (N=7%), B (5%), G/I/J/K (N=7%), and other subtypes (N=7%). Site differences included a higher percentage of non-type A (29% vs. 18%) and *FLT3-TKD* (17% vs 3%) at UIHC, whereas SGH had higher rates of *FLT3-ITD* (53% vs 27%) and *DNMT3A* (47% vs 32%).
Of 213 induction-eligible patients, 67% received high-intensity regimens (commonly anthracycline + cytarabine "7+3"), and 33% received low-intensity regimens (LIR, including hypomethylating agent + venetoclax). 78% achieved CR, with 56% also achieving measurable residual disease (MRD) negativity. On multivariable analysis, *TET2* co-mutation (OR 0.44, 95% CI 0.20-0.94) and LIR (OR 0.28, 95% CI 0.11-0.69) were associated with lower odds of CR. While significant on univariable analysis, *IDH1* co-mutation and age were no longer significantly associated with improved CR in the multivariable model.
Seventy-four patients underwent HSCT. Pre-HSCT status included CR1 (65%), CR2 (28%), ≥CR3 (1%), and active disease (6%). (67% of those in CR were MRD+ pre-HSCT). Matched unrelated donors were preferred at UIHC (69%), while haploidentical donors were more common at SGH (70%). MAC and RIC conditioning were equally used. RFS at 12 months after HSCT was 69% for subtype A patients vs 59% for non-subtype A patients.
Median follow-up was 14 months for the entire cohort, and the OS at 12 months was 73%, 79%, 62%, 52%, and 48% for subtypes A, D, B, G/I/J/K, and other, respectively. On multivariable analysis, *IDH1/2* co-mutation was associated with better OS (HR 0.46, 95% CI 0.28–0.77), whereas *TET2* co-mutation (HR 1.63, 95% CI 1.06-2.53) and LIR (HR 1.76, 95% CI 1.04-2.97) were associated with worse OS. The difference in patients' race did not translate to a difference in OS.

**Summary/Conclusion**

In this international cohort, OS did not significantly differ among *NPM1* subtypes. The limited number of non-type A cases may have been underpowered to detect the impact of NPM1 subtypes and co-mutation patterns. Additional biological drivers of adverse outcomes in some *NPM1*-mutated AML warrant further investigation.

**Copyright Information:** (Online) ISSN: 2572-9241
©2026 The Author(s). HemaSphere is published by John Wiley & Sons Ltd on behalf of European Hematology Association. This is an open access Abstract Book distributed under the Attribution-NonCommercial-NoDerivs (CC BY-NC-ND), which allows third parties to download the articles and share them with others as long as they credit the author and the Abstract Book, but they cannot change the content in any way or use them commercially.
**Abstract Book Citations:** Authors, Title, HemaSphere, 2026;10:(S1):pages. The abstract book DOIs can be found on the "Author Information" page.
**Disclaimer:** Articles published in the journal HemaSphere exclusively reflect the opinions of the authors. The authors are responsible for all content in their abstracts including accuracy of the facts, statements, citing resources, etc.

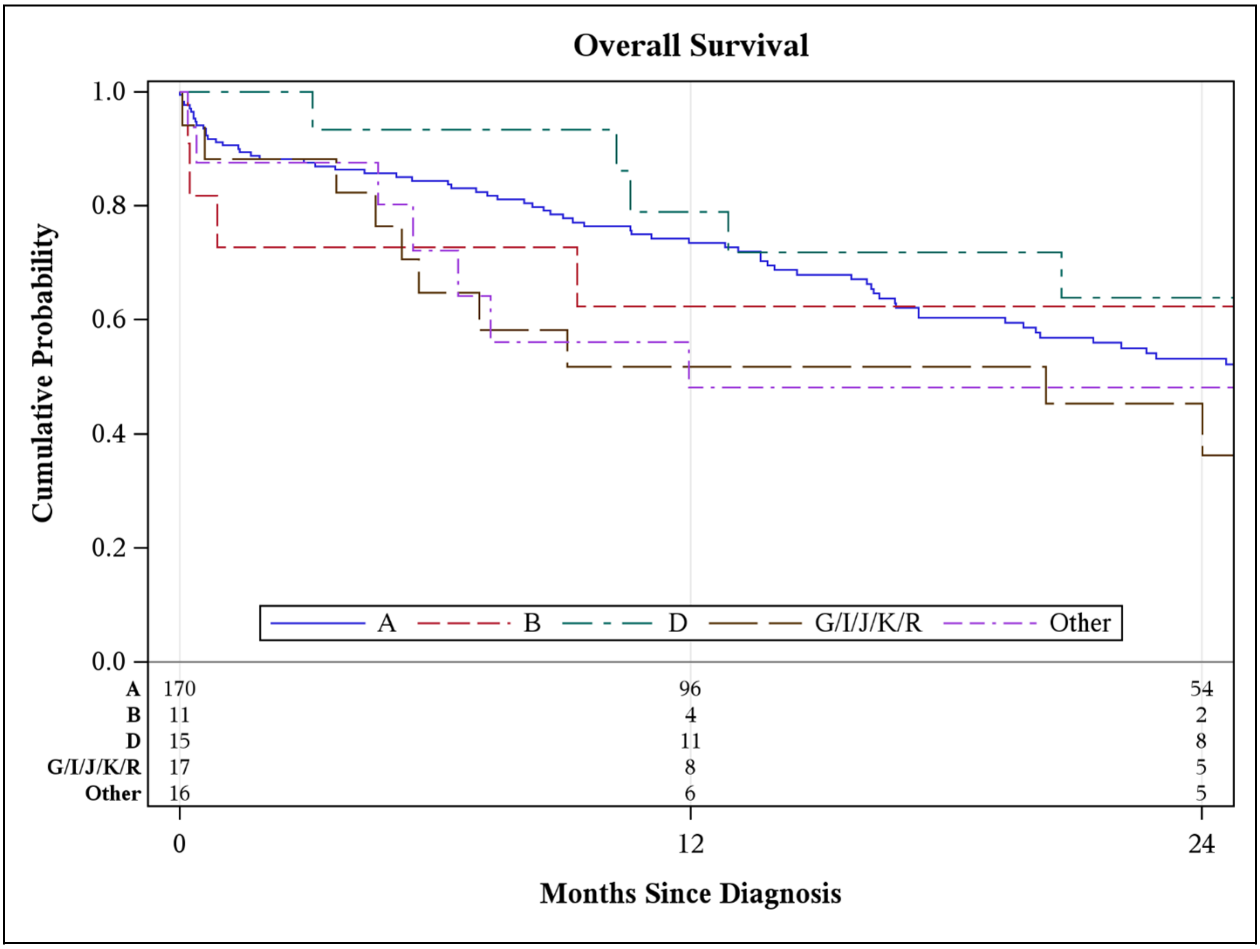

Figure 1: OS of NPM1 stratified by subtypes

**Copyright Information:** (Online) ISSN: 2572-9241
©2026 The Author(s). HemaSphere is published by John Wiley & Sons Ltd on behalf of European Hematology Association. This is an open access Abstract Book distributed under the Attribution-NonCommercial-NoDerivs (CC BY-NC-ND), which allows third parties to download the articles and share them with others as long as they credit the author and the Abstract Book, but they cannot change the content in any way or use them commercially.
**Abstract Book Citations:** Authors, Title, HemaSphere, 2026;10:(S1):pages. The abstract book DOIs can be found on the "Author Information" page.
**Disclaimer:** Articles published in the journal HemaSphere exclusively reflect the opinions of the authors. The authors are responsible for all content in their abstracts including accuracy of the facts, statements, citing resources, etc.

### (PS1660) THE GLASGOW PROGNOSTIC SCORE ADDS PROGNOSTIC INFORMATION IN ELDERLY AML PATIENTS INELIGIBLE FOR INTENSIVE TREATMENT BEYOND THE MOLECULAR RISK PROFILE – A RETROSPECTIVE MULTICENTER ANALYSIS

**Topic:** 04. Acute myeloid leukemia - Clinical

Stefan Fuchs*[1], Verena Petermichl[2], Matthias Weber[3], Charlotte Micheloud[4], Lukas Graf[5], Yannick Gerth[5], Jeroen Simon Goede[6], Thomas Lehmann[2], Christoph Driessen[2], Ulrich Jeremi Manuel Mey[1], Richard Cathomas[1], Sergio Cogliatti[3], Tobias Silzle[1, 2]

*[1]Cantonal Hospital Graubünden, Medical Oncology and Hematology, Chur, Switzerland; [2]Cantonal Hospital St. Gallen, Medical Oncology and Hematology, St. Gallen, Switzerland; [3]Cantonal Hospital St. Gallen, Institute of Pathology, St. Gallen, Switzerland; [4]Swiss Cancer Institute, Bern, Switzerland; [5]Center for Laboratory Medicine, St. Gallen, Switzerland; [6]Cantonal Hospital Winterthur, Winterthur, Switzerland;*

### Background

The Glasgow Prognostic Score (GPS) combines C-reactive protein (CRP) and albumin as biomarkers of inflammation or catabolism and malnutrition and has demonstrated prognostic value in various solid tumours. However, its prognostic impact in elderly patients with acute myeloid leukaemia (AML) or myelodysplastic neoplasms (MDS) treated with hypomethylating agents (HMAs) ± venetoclax (Ven) is less well characterised.

### Aims

To investigate the prognostic significance of the GPS within this patient population in the context of established genetic-based risk models and current treatment modalities

### Methods

We conducted a multicentric retrospective chart review of elderly patients newly diagnosed with AML or MDS/AML according to ICC2022 who were ineligible for intensive treatment. CRP and albumin levels were assessed at the time of diagnosis (±30 days) and before start of treatment. Patients were analysed according to treatment modality, the presence of infections at diagnosis and established molecular prognostic risk scores (molecular Prognostic Scoring System [mPRS] and Mayo Genetic Risk Model).

**Copyright Information:** (Online) ISSN: 2572-9241
©2026 The Author(s). HemaSphere is published by John Wiley & Sons Ltd on behalf of European Hematology Association. This is an open access Abstract Book distributed under the Attribution-NonCommercial-NoDerivs (CC BY-NC-ND), which allows third parties to download the articles and share them with others as long as they credit the author and the Abstract Book, but they cannot change the content in any way or use them commercially.
**Abstract Book Citations:** Authors, Title, HemaSphere, 2026;10:(S1):pages. The abstract book DOIs can be found on the "Author Information" page.
**Disclaimer:** Articles published in the journal HemaSphere exclusively reflect the opinions of the authors. The authors are responsible for all content in their abstracts including accuracy of the facts, statements, citing resources, etc.

**Results**

166 patients were included (female n = 72 [43.4%]; median age 78 years, range 61–91; AML n = 140 [84.3%], MDS/AML n = 26 [15.7%]).
98 patients (59.0%) received HMA+Ven, 39 patients (23.5%) received HMA alone or in combination with other agents, including hydroxyurea or ibrutinib (HOVON135/SAKK-30/15 trial) and 29 patients (17.5%) received best supportive care. An active infection at diagnosis was documented in 34 out of 163 patients (20.9%) with available baseline CRP levels. After a median follow-up of 48 months (range 0–69), 139 patients (83.7%) had died.

A GPS (available for 152 out of 166 patients) of 0 (CRP ≤10 mg/L and albumin ≥35 g/L, n=61) was associated with a significantly longer median overall survival (OS) of 13.2 months (95% confidence interval (CI): 7.2–16.8) compared with 3.9 months (95% CI: 2.1–5.4) in patients with a GPS of 1 or 2 (CRP >10 mg/L and/or albumin <35 g/L, n=91; hazard ratio (HR): 1.84 [95% CI: 1.28–2.65]; p<0.001; Figure 1A).

Similar results were observed in the subgroup of patients without infection at diagnosis treated with HMA+Ven (n = 75), with a median OS of 16.8 months (95% CI: 7.8–24.0) for GPS 0 versus 6.5 months (95% CI: 2.9–13.0) for GPS 1 or 2 (p = 0.017; HR: 1.9 [95% CI: 1.11–3.25]; Figure 1B).

In this subgroup, a GPS >0 retained its independent negative prognostic impact in bivariable Cox regression models, alongside both the mPRS (HR: 2.35; 95% CI: 1.27–4.36; p = 0.007) and the Mayo Genetic Risk Model (HR: 1.99; 95% CI: 1.03–3.83; p = 0.039).

Inclusion of the GPS resulted in higher C-indices compared to the respective molecular score alone, indicating better performance of the models combining oncogenomic information with levels of CRP and albumin:

| | mPRS | mPRS and GPS | Mayo Genetic Risk Model | Mayo Genetic Risk Model and GPS |
|---|---|---|---|---|
| C-Index | 0.62 | **0.67** | 0.64 | **0.68** |

**Summary/Conclusion**

According to this retrospective analysis, the GPS, a parameter easily accessible in daily clinical practice, provides prognostic information for elderly AML- and MDS/AML-patients independently of molecular-based risk scores. Therefore, considering CRP and albumin levels alongside oncogenomic factors may help to identify a particularly vulnerable subgroup of patients, even in the context of treatment with HMA+Ven as the current standard of care. However, our observations require validation in larger cohorts.

**Copyright Information:** (Online) ISSN: 2572-9241
©2026 The Author(s). HemaSphere is published by John Wiley & Sons Ltd on behalf of European Hematology Association. This is an open access Abstract Book distributed under the Attribution-NonCommercial-NoDerivs (CC BY-NC-ND), which allows third parties to download the articles and share them with others as long as they credit the author and the Abstract Book, but they cannot change the content in any way or use them commercially.
**Abstract Book Citations:** Authors, Title, HemaSphere, 2026;10:(S1):pages. The abstract book DOIs can be found on the "Author Information" page.
**Disclaimer:** Articles published in the journal HemaSphere exclusively reflect the opinions of the authors. The authors are responsible for all content in their abstracts including accuracy of the facts, statements, citing resources, etc.

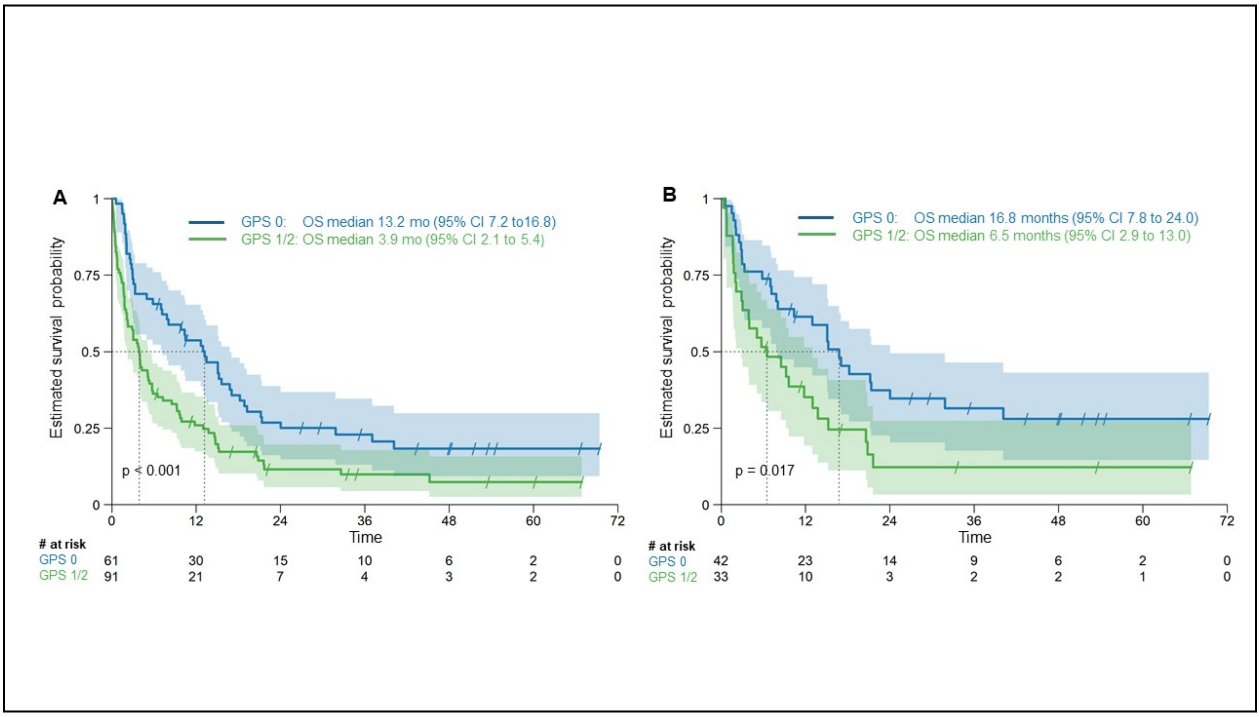

Figure 1: Kaplan Meier curves of OS by GPS (0 vs. 1/2) for the total patient cohort (A) and patients without infection at time of diagnosis treated with HMA+Ven.

**Copyright Information:** (Online) ISSN: 2572-9241
©2026 The Author(s). HemaSphere is published by John Wiley & Sons Ltd on behalf of European Hematology Association. This is an open access Abstract Book distributed under the Attribution-NonCommercial-NoDerivs (CC BY-NC-ND), which allows third parties to download the articles and share them with others as long as they credit the author and the Abstract Book, but they cannot change the content in any way or use them commercially.
**Abstract Book Citations:** Authors, Title, HemaSphere, 2026;10:(S1):pages. The abstract book DOIs can be found on the "Author Information" page.
**Disclaimer:** Articles published in the journal HemaSphere exclusively reflect the opinions of the authors. The authors are responsible for all content in their abstracts including accuracy of the facts, statements, citing resources, etc.

### (PS1661) DETERMINING PROGNOSTIC FACTORS IN PATIENTS WITH POSITIVE PRE-TRANSPLANT MRD BASED ON SPECIFIC ABNORMALITIES IN 2022 ELN

**Topic:** 04. Acute myeloid leukemia - Clinical

Ruixin Li*[1], Xiaoxiao MA[1], Aiming Pang[1], Donglin Yang[1], Yi He[1], Erlie Jiang[1]

[1] *Institute of Hematology & Blood Diseases Hospital, Chinese Academy of Medical Sciences & Peking Union Medical College, Tianjin, China;*

### Background

Pre-hematopoietic stem cell transplantation (HSCT) measurable residual disease (MRD) evaluation can be used as an indicator of relapse risk and guide therapy post-HSCT in patients with acute myeloid leukemia (AML). Risk stratifications of AML patients can be guided by genetic abnormalities at initial diagnosis. Combining specific genetic abnormalities may better determine the prognostic factors in patients with pre-transplant MRD positivity.

### Aims

To explore prognostic impact of ELN-2022-defined genetic abnormalities in AML patients with positive pre-transplant MRD.

### Methods

This single-center retrospective study included 345 AML patients in complete remission with positive MRD prior to allogeneic HSCT, enrolled from the National Longitudinal Cohort of Hematological Diseases (NCT04645199) in China. MRD was assessed using reverse transcription quantitative PCR, digital droplet PCR, and multiparametric flow cytometry. Individual molecular and cytogenetic abnormalities defined by the ELN-2022 classification were analyzed as independent prognostic variables. Optimal cutoff values for white blood cell count (WBC), infused mononuclear cells (MNC), and $CD34^{+}$ cells were determined using maximally selected log-rank statistics to achieve maximal separation of overall survival.

**Copyright Information:** (Online) ISSN: 2572-9241
©2026 The Author(s). HemaSphere is published by John Wiley & Sons Ltd on behalf of European Hematology Association. This is an open access Abstract Book distributed under the Attribution-NonCommercial-NoDerivs (CC BY-NC-ND), which allows third parties to download the articles and share them with others as long as they credit the author and the Abstract Book, but they cannot change the content in any way or use them commercially.
**Abstract Book Citations:** Authors, Title, HemaSphere, 2026;10:(S1):pages. The abstract book DOIs can be found on the "Author Information" page.
**Disclaimer:** Articles published in the journal HemaSphere exclusively reflect the opinions of the authors. The authors are responsible for all content in their abstracts including accuracy of the facts, statements, citing resources, etc.

**Results**

The median age was 37 years (interquartile range, 29-48), with a median follow-up of 1.6 years (maximum, 11.5 years). Secondary AML accounted for 11.0% of cases. Donor types included matched sibling (33.3%), matched unrelated (6.7%), and haploidentical donors (60.0%). MRD was detected by multiparametric flow cytometry in 55.1% of patients; 24.3% had core-binding factor abnormalities; 13.1% harbored gene mutations; 4.3% had adverse fusion genes; and 3.2% were WT1-positive.
The 2-year overall survival (OS), leukemia-free survival (LFS), cumulative incidence of relapse (CIR), and non-relapse mortality (NRM) were 64.0%, 60.8%, 23.1%, and 16.1%, respectively. Variables with $P < 0.05$ in univariate analyses were entered into multivariable models.
An initial WBC > 5.47 × $10^9$/L was independently associated with improved OS (HR = 0.5, 95% CI: 0.38-0.79). In contrast, infused MNC >12.8 × $10^8$/kg (HR = 1.6, 95% CI: 1.09-2.36), chromosome 5/7/17 abnormalities (HR = 2.4, 95% CI: 1.31-4.56), and wild-type NPM1 with FLT3-ITD mutation (HR = 1.8, 95% CI: 1.07-2.89) were independently associated with inferior OS (Figure 1A). Chromosome 5/7/17 abnormalities (HR = 2.5, 95% CI: 1.42-4.37) and wild-type NPM1 with FLT3-ITD mutation (HR = 1.7, 95% CI: 1.07-2.61) were also independent predictors of poorer LFS (Figure 1B).
For CIR, an initial WBC >5.5 × $10^9$/L was protective (HR = 0.6, 95% CI: 0.37-0.91), whereas refractory disease status (HR = 1.9, 95% CI: 1.08-3.48), KMT2A rearrangement (HR = 2.9, 95% CI: 1.35-6.18), chromosome 5/7/17 abnormalities (HR = 2.90, 95% CI: 1.41-5.87), and wild-type NPM1 with FLT3-ITD mutation (HR = 1.8, 95% CI: 1.04-3.20) were independent risk factors (Figure 1C). Haploidentical HSCT (HR = 3.00, 95% CI: 1.51-6.09) and infused $CD34^+$ cells > 4.2 × $10^6$/kg (HR = 0.4, 95% CI: 0.21-0.85) were independently associated with NRM (Figure 1D).

**Summary/Conclusion**

Among AML patients with positive pre-transplant MRD, specific ELN-2022-defined genetic abnormalities exert distinct and clinically meaningful effects on post-transplant survival and relapse risk. Incorporating these abnormalities may refine prognostic stratification and inform individualized post-transplant management strategies.

**Copyright Information:** (Online) ISSN: 2572-9241
©2026 The Author(s). HemaSphere is published by John Wiley & Sons Ltd on behalf of European Hematology Association. This is an open access Abstract Book distributed under the Attribution-NonCommercial-NoDerivs (CC BY-NC-ND), which allows third parties to download the articles and share them with others as long as they credit the author and the Abstract Book, but they cannot change the content in any way or use them commercially.
**Abstract Book Citations:** Authors, Title, HemaSphere, 2026;10:(S1):pages. The abstract book DOIs can be found on the "Author Information" page.
**Disclaimer:** Articles published in the journal HemaSphere exclusively reflect the opinions of the authors. The authors are responsible for all content in their abstracts including accuracy of the facts, statements, citing resources, etc.

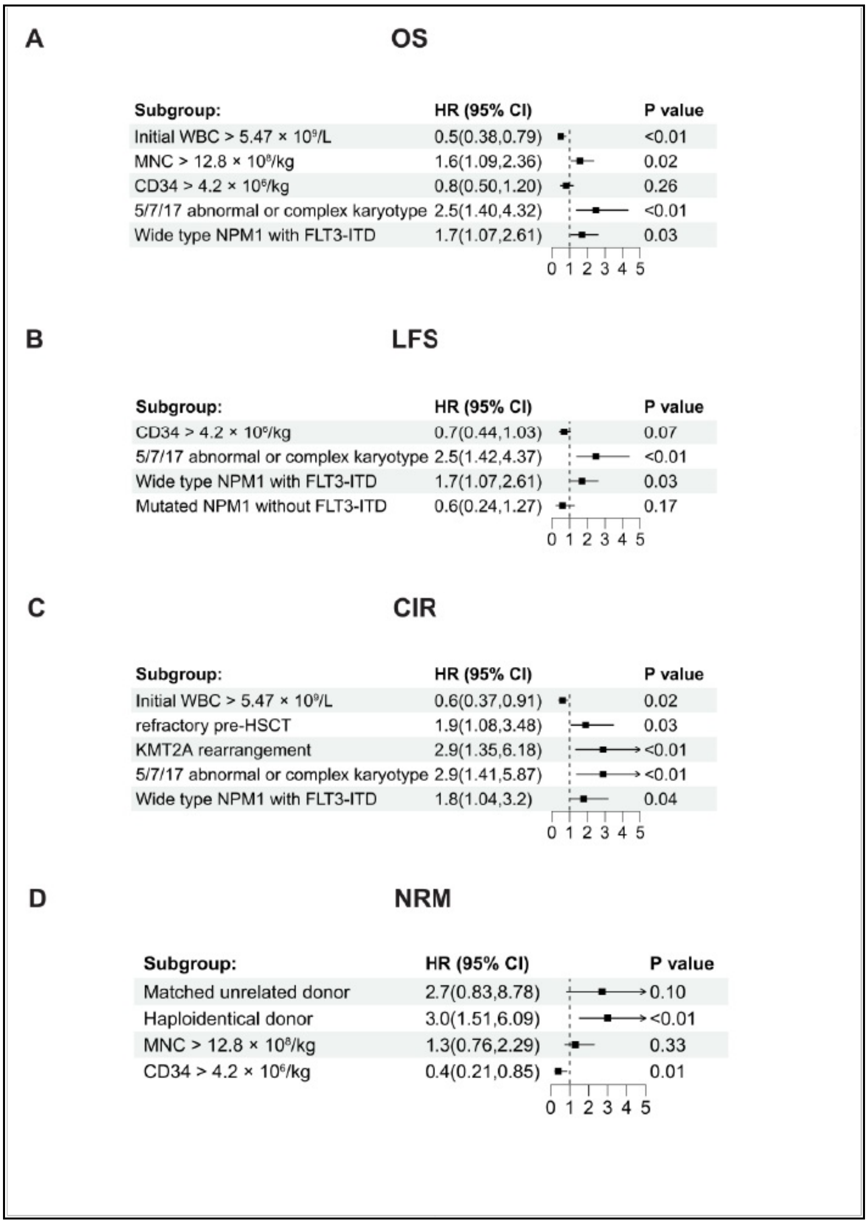

**Copyright Information:** (Online) ISSN: 2572-9241
©2026 The Author(s). HemaSphere is published by John Wiley & Sons Ltd on behalf of European Hematology Association. This is an open access Abstract Book distributed under the Attribution-NonCommercial-NoDerivs (CC BY-NC-ND), which allows third parties to download the articles and share them with others as long as they credit the author and the Abstract Book, but they cannot change the content in any way or use them commercially.
**Abstract Book Citations:** Authors, Title, HemaSphere, 2026;10:(S1):pages. The abstract book DOIs can be found on the "Author Information" page.
**Disclaimer:** Articles published in the journal HemaSphere exclusively reflect the opinions of the authors. The authors are responsible for all content in their abstracts including accuracy of the facts, statements, citing resources, etc.

### (PS1662) REAL-WORLD COMPARISON OF INTENSIVE CHEMOTHERAPY COMPARED TO NON-INTENSIVE TREATMENT OF MYELODYSPLASIA-RELATED ACUTE MYELOID LEUKAEMIA (AML-MR)

**Topic:** 04. Acute myeloid leukemia - Clinical

Trung Ngo*[1], Kavita Raj[1], Jiexin Zheng[1], Jenny O'nions[1], Rajeev Gupta[1, 2], Elspeth Payne[1, 2], Rob Sellar[1, 2], Panagiotis Kottaridis[1], Richard Burt[1, 3, 4], Marc Mansour[1, 2], Kate Stringaris[1], Samah Alimam[1], Ke Xu[1], Anish Tailor[1], Andrew Wilson[1]

*[1]University College London Hospitals NHS Trust, Haematology, London, United Kingdom;[2]UCL Cancer Institute, London, United Kingdom; [3]The Francis Crick Institute, London, United Kingdom; [4]Imperial College London, London, United Kingdom;*

### Background

Myelodysplasia-related acute myeloid leukaemia (AML-MR) accounts for one third of AML diagnoses and predominantly affects an older patient population, which limits the delivery of intensive chemotherapy. It has dismal outcomes compared to other AML subtypes. Recent Phase II clinical trials have shown that non-intensive induction treatment has comparable outcomes to intensive chemotherapy in allograft-eligible patients. Allograft consolidation remains the only therapy to achieve long-term remission in AML-MR, but it is unclear whether the intensity of induction chemotherapy impacts survival.

### Aims

To compare the efficacy of intensive chemotherapy with venetoclax-based non-intensive treatment for AML-MR with and without allograft, and to assess the impact of genomic abnormalities on treatment outcomes.

### Methods

A chart review was performed to assess patients treated for AML-MR, defined by WHO 2022 criteria, at University College London Hospitals from January 2021 to December 2025. Survival was measured by the Kaplan-Meier estimate and log-rank test. Multivariable cox regression assessed the impact of treatment intensity and allograft on genomic subgroups.

### Results

141 patients who received treatment for AML-MR were identified (Table 1). Correlation analysis illustrated that a complex/monosomal karyotype was associated with TP53 mutation (co-variance 0.52), and STAG2 was associated with ASXL1 mutation (co-variance 0.39). 46% of patients had an intention to transplant, of which 57% proceeded to transplant. Within the intention to transplant group, there was no difference in the rate of allograft between intensive and non-intensive treatment ($p = 0.999$). Patients who received an allograft had superior median overall survival than those who did not (33 vs 13 months, $p<0.0001$), which also held true with intention to transplant analysis (29 vs 13 months, $p=0.007$). Their survival curves also plateaued, indicating long-term disease control.

Of those allografted, there was no difference in survival between intensive and non-intensive chemotherapy ($p = 0.892$). This also held true for those not allografted, with no difference between intensive and non-intensive regimens ($p = 0.122$). Multivariable cox regression showed that patients with complex/monosomal karyotype (HR 3.49; 95% CI, 1.53 to 7.88) or ASXL1 mutation (HR 2.03; 95% CI, 1.05 to 3.90) had poor survival outcomes irrespective of co-mutations, treatment intensity or a consolidative allograft (Figure 1).

**Copyright Information:** (Online) ISSN: 2572-9241
©2026 The Author(s). HemaSphere is published by John Wiley & Sons Ltd on behalf of European Hematology Association. This is an open access Abstract Book distributed under the Attribution-NonCommercial-NoDerivs (CC BY-NC-ND), which allows third parties to download the articles and share them with others as long as they credit the author and the Abstract Book, but they cannot change the content in any way or use them commercially.
**Abstract Book Citations:** Authors, Title, HemaSphere, 2026;10:(S1):pages. The abstract book DOIs can be found on the "Author Information" page.
**Disclaimer:** Articles published in the journal HemaSphere exclusively reflect the opinions of the authors. The authors are responsible for all content in their abstracts including accuracy of the facts, statements, citing resources, etc.

### Summary/Conclusion

An allograft for AML-MR results in superior survival and long-term disease control, irrespective of whether an intensive or non-intensive induction regimen is used. Conversely, patients with a complex/monosomal karyotype or ASXL1 mutation have dismal outcomes regardless of treatment intensity or allograft consolidation.

**Table 1: Baseline Characteristics**

| | Intensive (%) | Non-intensive (%) |
|---|---|---|
| **Number (total 141)** | 47 | 94 |
| **Age, median years** | 58 (24 to 74) | 69 (35 to 87) |
| **Gender** | | |
| - Male | 25 (53%) | 56 (60%) |
| - Female | 22 (47%) | 38 (40%) |
| **Adverse subgroups** | | |
| - Complex/monosomal karyotype | 10 (21%) | 20 (21%) |
| - ASXL1 | 12 (26%) | 33 (35%) |
| - BCOR | 9 (19%) | 19 (20%) |
| - EZH2 | 5 (11%) | 10 (11%) |
| - RUNX1 | 12 (26%) | 37 (39%) |
| - SF3B1 | 3 (6%) | 5 (5%) |
| - SRSF2 | 9 (19%) | 27 (29%) |
| - STAG2 | 7 (15%) | 13 (14%) |
| - U2AF1 | 3 (6%) | 12 (13%) |
| - ZRSR2 | 1 (2%) | 3 (3%) |
| - TP53 | 5 (11%) | 17 (18%) |
| **Treatment:** | | |
| - Daunorubicin/Cytarabine | 14 (30%) | |
| - FLAG-Ida | 22 (47%) | |
| - CPX-351 | 11 (23%) | |
| - Venetoclax/Azacitidine | | 87 (93%) |
| - Venetoclax/Low-dose cytarabine | | 7 (7%) |
| **Allograft** | | |
| - Yes | 23 (49%) | 14 (15%) |
| - No | 24 (51%) | 80 (85%) |
| **Intention to transplant** | | |
| - Yes | 40 (85%) | 25 (27%) |
| - No | 7 (15%) | 69 (73%) |

**Copyright Information:** (Online) ISSN: 2572-9241
©2026 The Author(s). HemaSphere is published by John Wiley & Sons Ltd on behalf of European Hematology Association. This is an open access Abstract Book distributed under the Attribution-NonCommercial-NoDerivs (CC BY-NC-ND), which allows third parties to download the articles and share them with others as long as they credit the author and the Abstract Book, but they cannot change the content in any way or use them commercially.
**Abstract Book Citations:** Authors, Title, HemaSphere, 2026;10:(S1):pages. The abstract book DOIs can be found on the "Author Information" page.
**Disclaimer:** Articles published in the journal HemaSphere exclusively reflect the opinions of the authors. The authors are responsible for all content in their abstracts including accuracy of the facts, statements, citing resources, etc.

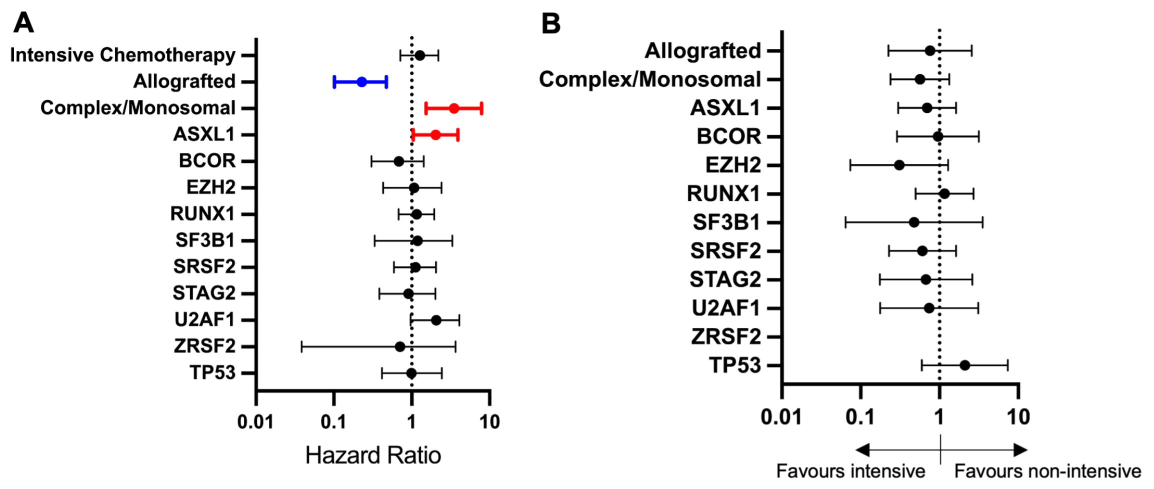

**Figure 1: Hazard ratio for overall survival.** (A) Multivariable cox regression, accounting for covariates. (B) Univariate subgroup comparison between intensive and non-intensive induction chemotherapy.

**Copyright Information:** (Online) ISSN: 2572-9241
©2026 The Author(s). HemaSphere is published by John Wiley & Sons Ltd on behalf of European Hematology Association. This is an open access Abstract Book distributed under the Attribution-NonCommercial-NoDerivs (CC BY-NC-ND), which allows third parties to download the articles and share them with others as long as they credit the author and the Abstract Book, but they cannot change the content in any way or use them commercially.
**Abstract Book Citations:** Authors, Title, HemaSphere, 2026;10:(S1):pages. The abstract book DOIs can be found on the “Author Information” page.
**Disclaimer:** Articles published in the journal HemaSphere exclusively reflect the opinions of the authors. The authors are responsible for all content in their abstracts including accuracy of the facts, statements, citing resources, etc.

### (PS1663) HIGH REMISSION RATES AND COMPARABLE POST-REMISSION OUTCOMES WITH SHORTENED VENETOCLAX SCHEDULES IN A REAL-WORLD COHORT OF AZA-VEN TREATED AML PATIENTS

**Topic:** 04. Acute myeloid leukemia - Clinical

Zeljko Prka[1], Anamarija Vrkljan Vuk*[1], Ena Soric[1], David Čičić[1], Marko Lucijanic[1, 2], Ozren Jakšić[1, 2]

*[1]University Hospital Dubrava, Zagreb, Croatia; [2]University of Zagreb School of Medicine, Zagreb, Croatia;*

### Background

Based on results of VIALE-A study combination of azacitidine and venetoclax (aza-ven) is standard of care for newly diagnosed AML patients unfit for intensive chemotherapy. Additionaly, this combination also show significant activity and meaningful responses in relapsed/refractory (RR) setting. Although generally less toxic than standard induction, it still have significant hemtologic toxicity. So, to mitigate this, in clinical practice, venetoclax duration per cycle is frequently modified due to cytopenias or pre-emptively based on patient age and comorbidities. The impact of shortened schedules on outcomes remains insufficiently characterized in real-world settings.

### Aims

To evaluate the association with venetoclax duration per cycle on remission rates and post-remission outcomes in a real-world cohort of aza-ven treated AML patients.

### Methods

We retrospectively evaluated AML patients newly diagnosed or relapsed/refractory (RR) treated with aza-ven at our institution between February 2018 and October 2025. Venetoclax duration per cycle (7, 14, 21, or 28 days) was determined by the treating physician based on disease characteristics, patient age, frailty assessment, and treatment-related toxicities, particularly cytopenias.

### Results

A total of 79 patients were evaluated; 48 (60.8%) male, median age 67 years. Of these, 58 (73.4%) had newly diagnosed and 21 (26.6%) RR AML; 23 (29.1%) had prior MDS or MPN. Remission was achieved in 54 (68.4%) patients; 21 (27.3%) proceeded to allogeneic stem cell transplantation (allo-SCT). Median follow-up was 16 months. Median overall survival (OS) was 11.1 months overall and 15.2 months among patients achieving remission.
Twenty-one patients (26.6%) received full 28-day venetoclax, while shortened 7-, 14-, and 21-day schedules were administered to 22 (27.8%), 15 (19.0%), and 21 (26.6%) patients, respectively. Patients receiving shortened schedules were significantly older (median 69 vs. 62 years, P=0.030) and less likely to have complex karyotype (12.2% vs. 41.7%, P=0.023). No significant differences were observed in sex, newly diagnosed vs. RR status, bone marrow blasts, peripheral blood counts, or FLT3, NPM1, or TP53 mutational status (P>0.05 for all).
Patients on shortened schedules achieved higher remission rates (81% vs. 33%, P<0.001, Figure 1A), with remission occurring at a similar pace regardless of schedule (median 1 cycle, range 1–4 in both groups). Cycle 1 neutropenia and thrombocytopenia duration were comparable between groups. Among patients in remission, similar proportions proceeded to allo-SCT (37% vs. 42.9%, P=0.766) and OS was comparable (P=0.349, Figure 1B).

**Copyright Information:** (Online) ISSN: 2572-9241
©2026 The Author(s). HemaSphere is published by John Wiley & Sons Ltd on behalf of European Hematology Association. This is an open access Abstract Book distributed under the Attribution-NonCommercial-NoDerivs (CC BY-NC-ND), which allows third parties to download the articles and share them with others as long as they credit the author and the Abstract Book, but they cannot change the content in any way or use them commercially.
**Abstract Book Citations:** Authors, Title, HemaSphere, 2026;10:(S1):pages. The abstract book DOIs can be found on the “Author Information” page.
**Disclaimer:** Articles published in the journal HemaSphere exclusively reflect the opinions of the authors. The authors are responsible for all content in their abstracts including accuracy of the facts, statements, citing resources, etc.

**Summary/Conclusion**

In this real-world cohort, shortened venetoclax schedules — applied pre-emptively based on age and significant comorbidities or reactively for toxicity — were associated with high remission rates and comparable post-remission outcomes relative to full 28-day dosing. The results were similar across both newly diagnosed and relapsed/refractory AML. However, these findings should be interpreted considering that patients receiving shortened schedules, while older, had more favorable cytogenetic profiles.

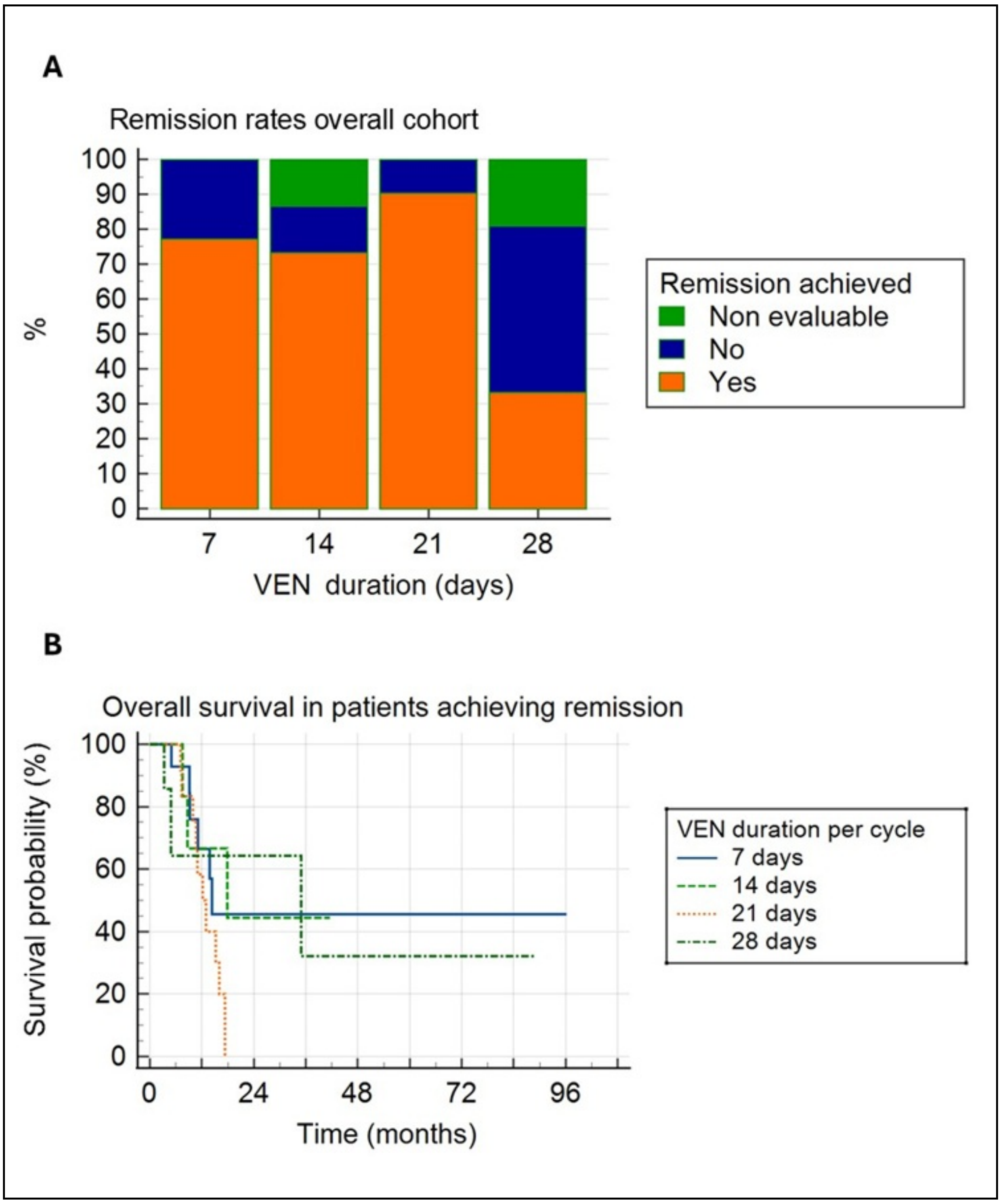

Figure 1A. Remission rates overall cohort; Figure 1B. Overall survival in patients achieving remission

**Copyright Information:** (Online) ISSN: 2572-9241
©2026 The Author(s). HemaSphere is published by John Wiley & Sons Ltd on behalf of European Hematology Association. This is an open access Abstract Book distributed under the Attribution-NonCommercial-NoDerivs (CC BY-NC-ND), which allows third parties to download the articles and share them with others as long as they credit the author and the Abstract Book, but they cannot change the content in any way or use them commercially.
**Abstract Book Citations:** Authors, Title, HemaSphere, 2026;10:(S1):pages. The abstract book DOIs can be found on the "Author Information" page.
**Disclaimer:** Articles published in the journal HemaSphere exclusively reflect the opinions of the authors. The authors are responsible for all content in their abstracts including accuracy of the facts, statements, citing resources, etc.

### (PS1664) EARLY BONE MARROW BLAST REDUCTION-GUIDED SELINEXOR-BASED REGIMEN THERAPY IN RELAPSED/REFRACTORY AND NEWLY DIAGNOSED ACUTE MYELOID LEUKEMIA

**Topic:** 04. Acute myeloid leukemia - Clinical

Yi Zhu[1], Bin Xu[1], Shuai Su[1], Xiwen Tong[1, 2], Shiyuan Zhang[1], Yuling Wan[1], Andie Fu[1], Zhuoxin Wen[1], Longlong Liu[3], Zhenqian Huang[3], Min Xiao[1], Jia Wei[1], Donghua Zhang*[1]

*[1]Tongji Hospital, Tongji Medical College, Huazhong University of Science and Technology, Hematology, Wuhan, China;[2]Sir Run Run Shaw Hospital, Zhejiang University School of Medicine, Hematology and Cell Therapy, Hangzhou, China; [3]The First Affiliated Hospital of Guangzhou Medical University, Hematology, Guangzhou, China;*

### Background

Therapeutic options for relapsed/refractory acute myeloid leukemia (R/R AML) remain limited, with poor long-term survival. Our observation of rapid blast decline kinetics in R/R AML patients (pts) treated with selinexor-based regimen predicted therapeutic efficacy. We hypothesized that these kinetics could guide therapy intensification also in newly diagnosed (ND) AML.

### Aims

This study evaluates the XAB regimen,leveraging blast-reduction patterns observed in R/R AML to optimize new diagnosed AML (ND AML) therapy.

### Methods

Pts aged 18-74 years diagnosed R/R or ND AML were included. All received the XAB regimen: selinexor (35 mg/m² twice weekly), azacitidine (75 mg/m²/day days 1–5), and venetoclax (ramp-up to 400 mg daily days 1–21). Pts who failed to achieve CR/CRi in the first week, cytarabine (50mg/day×3-5days) was added to the XAB regimen. After two weeks of treatment, pts who meet the transplantation criteria will subsequently receive alloHCT. Bone marrow assessments at week 1 and 2 were combined to classify blast reduction kinetics: Type A (sensitive): complete remission with/without count recovery (CR/CRi) at both week1 and 2; Type B (moderately sensitive): Partial Remission (PR)/No Remission(NR) at week 1 → PR/CR/CRi at week 2; Type C (low sensitivity): PR/CR/CRi at week 1 → NR at week 2; Type D (resistant): NR at both timepoints.

### Results

Among 16 R/R AML pts, blast kinetics classified 50.0% (n=8) as Type A, 6.3% (n=1) as Type B, 25.0% (n=4) as Type C, and 18.8% (n=3) as Type D. Type A/B pts continuing XAB for 2 additional weeks achieved CR/CRi in 77.8% (7/9), whereas Type C pts receiving azacitidine-intensified XAB showed no response. All Type D pts discontinued therapy without response. At median follow-up of 8.2 months, 62.5% (10/16) survived—50% (5/10) of whom were Type A responders.
Based on the above results, we applied this kinetic-guided algorithm to the newly diagnosed pts (ND pts) in the hope of obtaining a judgment on the efficacy of XAB regimen. A total of 29 ND pts were included, among whom 26 could be evaluated for 2 weeks (3 pts withdrew from the study in the second week and had no evaluation data). Among 26 ND pts, blast kinetics classified 11pts (42.3%) as Type A, 11pts (42.3%) as Type B, only 1pt (3.8%) as Type C, and 3pts (11.5%) as Type D.
In Tpye B, the initial remission status at first week consisted of 5 pts achieving PR and 6 with NR, yielding ORR rate of 45.5%. Following the introduction of cytarabine , the remission improved markedly by the second week, with 7 pts CR and 4 pts PR, resulting in ORR rate of 100%. In Tpye C, there was only 1 patient, CR in the first week and PR in the second week. 3 pts were classified as Type D, all of whom discontinued per protocol and switched to alternative intensive chemotherapy.

**Copyright Information:** (Online) ISSN: 2572-9241
©2026 The Author(s). HemaSphere is published by John Wiley & Sons Ltd on behalf of European Hematology Association. This is an open access Abstract Book distributed under the Attribution-NonCommercial-NoDerivs (CC BY-NC-ND), which allows third parties to download the articles and share them with others as long as they credit the author and the Abstract Book, but they cannot change the content in any way or use them commercially.
**Abstract Book Citations:** Authors, Title, HemaSphere, 2026;10:(S1):pages. The abstract book DOIs can be found on the "Author Information" page.
**Disclaimer:** Articles published in the journal HemaSphere exclusively reflect the opinions of the authors. The authors are responsible for all content in their abstracts including accuracy of the facts, statements, citing resources, etc.

**Summary/Conclusion**

Early bone marrow blasts reduction kinetics robustly predict therapeutic efficacy of the XAB regimen across AML disease states. Type A and B kinetics identify pts likely to benefit from continued XAB therapy, while kinetic stratification allows timely adaptation—switching non-responders (Types C and D) to intensive treatment. The current enrolled patients is predominantly classified as types A and B22/26, indicating that the XAB regimen demonstrates promising efficacy in the majority of newly diagnosed cases and supporting further exploration. This finding supports bone marrow blast kinetics as a dynamic biomarker for optimizing personalized AML therapy.

**Classification of types according to dynamic remission status**

| Type | Definition | Week 1 status | Week 2 status | Interpretation |
|---|---|---|---|---|
| A | Sensitive | CR/CRi | CR/CRi | Sensitive,durable remission |
| B | Moderately sensitive | PR/NR | PR/CR/CRi | Improved response |
| C | Low sensitivity | PR/CR/CRi | NR | Lost response |
| D | Resistant | NR | NR | Persistent non-remission |

**Copyright Information:** (Online) ISSN: 2572-9241
©2026 The Author(s). HemaSphere is published by John Wiley & Sons Ltd on behalf of European Hematology Association. This is an open access Abstract Book distributed under the Attribution-NonCommercial-NoDerivs (CC BY-NC-ND), which allows third parties to download the articles and share them with others as long as they credit the author and the Abstract Book, but they cannot change the content in any way or use them commercially.
**Abstract Book Citations:** Authors, Title, HemaSphere, 2026;10:(S1):pages. The abstract book DOIs can be found on the "Author Information" page.
**Disclaimer:** Articles published in the journal HemaSphere exclusively reflect the opinions of the authors. The authors are responsible for all content in their abstracts including accuracy of the facts, statements, citing resources, etc.

### (PS1665) RUXOLITINIB COMBINED WITH DECITABINE INTENSIFIED MODIFIED BUSULFAN/CYCLOPHOSPHAMIDE CONDITIONING REGIMEN REDUCES RELAPSE AFTER ALLO-HSCT IN AML

**Topic:** 04. Acute myeloid leukemia - Clinical

Weiqi Li[1], Liping Dou*[1]

[1]*Chinese PLA General Hospita, State Key Laboratory of Experimental Hematology, Beijing, China;*

### Background

Allogeneic hematopoietic stem cell transplantation (allo-SCT) remains the sole means of curing hematological malignancies in many cases [1], and it is the most effective treatment option for adult patients with acute myeloid leukemia (AML). However, recurrence remains the main cause of transplantation failure, with a recurrence rate of 40% - 60% [2]. Although most intensified conditioning regimens reduce recurrence, they are accompanied by an increase in non-relapse mortality (NRM). Therefore, it is crucial to explore more effective conditioning regimens to reduce recurrence.

### Aims

To analyze the impact of the ruxolitinib (Rux) and decitabine (Dec)-intensified modified busulfan/cyclophosphamide conditioning regimen (modified Bu/Cy, mBu/Cy) on the 1-year relapse rate in acute myeloid leukemia (AML) patients in first complete remission (CR1).

### Methods

This prospective single-arm cohort study enrolled patients meeting the diagnostic criteria for AML CR1. All patients received the Rux-Dec-mBu/Cy regimen (addition of ruxolitinib and decitabine to the modified busulfan/cyclophosphamide conditioning regimen). It was prospectively observed that the 1-year cumulative relapse rate (RR) in 32 AML CR1 patients who underwent allogeneic hematopoietic stem cell transplantation (allo-HSCT) with the Rux-Dec-mBu/Cy regimen at the First Medical Center of Chinese PLA General Hospital between September 2020 and June 2023 (ClinicalTrials.gov ID: NCT04582604).

### Results

The 1-year cumulative relapse rate was 9.38%. As of May 1, 2024, the median follow-up time was 1085.5 (645–1683) days, during which 5 patients experienced relapse (4 survived after relapse, and 1 died due to relapse). Seventeen patients tested positive for minimal residual disease (MRD) by flow cytometry on day +28 post-transplantation. The 1-year overall survival (OS) rate was 90.4%, disease-free survival (DFS) was 87.5%, GVHD-free relapse-free survival (GRFS) was 81.25%, and non-relapse mortality (NRM) was 9.38%. Four patients died (1 from relapse, 3 from non-relapse causes: 1 from septic shock and 2 from viral pneumonia).

### Summary/Conclusion

The ruxolitinib and decitabine-intensified modified busulfan/cyclophosphamide conditioning regimen could reduce relapse in AML CR1 patients following allo-HSCT.

**Copyright Information:** (Online) ISSN: 2572-9241
©2026 The Author(s). HemaSphere is published by John Wiley & Sons Ltd on behalf of European Hematology Association. This is an open access Abstract Book distributed under the Attribution-NonCommercial-NoDerivs (CC BY-NC-ND), which allows third parties to download the articles and share them with others as long as they credit the author and the Abstract Book, but they cannot change the content in any way or use them commercially.
**Abstract Book Citations:** Authors, Title, HemaSphere, 2026;10:(S1):pages. The abstract book DOIs can be found on the "Author Information" page.
**Disclaimer:** Articles published in the journal HemaSphere exclusively reflect the opinions of the authors. The authors are responsible for all content in their abstracts including accuracy of the facts, statements, citing resources, etc.

### (PS1666) PATIENT-SPECIFIC DIGITAL PCR MEASURABLE RESIDUAL DISEASE PREDICTS POST-TRANSPLANT OUTCOMES IN MYELODYSPLASIA-RELATED ACUTE MYELOID LEUKEMIA

**Topic:** 04. Acute myeloid leukemia - Clinical

Caroline Arvé*[1], Jonas Kassow Grønlund[1], Dorte Melsvik[1], Julia Mathews Printtans[1], Johanne Amalie Pold[1], Emma Frasez Sørensen[1], Marianne Severinsen[2], Marietta Nygaard[3], Hans Beier Ommen[1]

*[1]Aarhus University Hospital, Department of Hematology, Aarhus, Denmark;[2]Aalborg University Hospital, Department of Hematology, Aalborg, Denmark; [3]Copenhagen University Hospital – Rigshospitalet, Department of Hematology, Copenhagen, Denmark;*

### Background

In high-risk myelodysplastic syndrome (MDS) and acute myeloid leukemia with myelodysplasia-related features (AML-MR), validated molecular markers enabling highly sensitive measurable residual disease (MRD) monitoring are lacking. Moreover, various induction chemotherapy regimens are available, but studies have reported conflicting results regarding the optimal regimen in this patient population.

### Aims

To develop a target selection algorithm enabling identification of patient-specific MRD markers in patients with high-risk MDS and AML-MR, and to evaluate the prognostic impact of pre-transplant MRD levels. To compare MRD negativity rates and post-transplant outcomes according to induction therapy (CPX-351 vs standard chemotherapy [3+10±gemtuzumab ozogamicin or FLAG-Ida]) in a Danish high-risk MDS/AML-MR cohort.

### Methods

Patients diagnosed with high-risk MDS (≥10% blasts) or AML-MR who received intensive induction chemotherapy with either CPX-351 or standard regimens and underwent allogeneic hematopoietic stem cell transplantation (alloHSCT) in first complete remission between 2016 and 2024 were included (N=58). Patient-specific MRD markers were identified using NGS analyses of paired diagnostic and remission samples. Eligible variants required a diagnostic variant allele frequency (VAF) ≥10% (AML) or ≥5% (MDS) and remission VAF <5%. A gene prioritization algorithm favoring leukemia-specific alterations over clonal hematopoiesis-associated variants was used to select among multiple available variants fulfilling the VAF criteria. MRD was quantified by digital PCR (dPCR) in bone marrow (BM) and peripheral blood (PB) within 1.5 months prior to alloHSCT. MRD positivity was defined as VAF >0.1% in PB and/or BM. Overall survival (OS) and cumulative incidence of relapse (CIR), with non-relapse mortality as a competing event, were used to evaluate the prognostic impact of pre-transplant MRD and treatment effects.

**Copyright Information:** (Online) ISSN: 2572-9241
©2026 The Author(s). HemaSphere is published by John Wiley & Sons Ltd on behalf of European Hematology Association. This is an open access Abstract Book distributed under the Attribution-NonCommercial-NoDerivs (CC BY-NC-ND), which allows third parties to download the articles and share them with others as long as they credit the author and the Abstract Book, but they cannot change the content in any way or use them commercially.
**Abstract Book Citations:** Authors, Title, HemaSphere, 2026;10:(S1):pages. The abstract book DOIs can be found on the "Author Information" page.
**Disclaimer:** Articles published in the journal HemaSphere exclusively reflect the opinions of the authors. The authors are responsible for all content in their abstracts including accuracy of the facts, statements, citing resources, etc.

### Results

A molecular dPCR MRD target was identifiable in 47/58 high-risk MDS/AML-MR patients (80%). However, dPCR analysis was feasible in 40 of these 47 patients due to a lack of diagnostic material for assay validation. MRD positivity prior to alloHSCT was strongly associated with relapse (sHR 6.56, 95% CI 1.45–29.72, p=0.015). This translated into inferior OS among MRD-positive patients (HR 3.18, 95% CI 0.98–10.35; p=0.055).
MRD negativity prior to alloHSCT did not differ between induction regimens (N=40; 56% with CPX-351 vs 63% with standard chemotherapy; Fisher's exact p=0.74). Accordingly, CPX-351 was not associated with improved post-transplant outcomes compared with standard induction, with no significant difference in relapse risk (sHR 0.62, 95% CI 0.23–1.68, p=0.340) or OS (HR 1.44, 95% CI 0.56–3.67, p=0.449). There was no evidence of effect modification by MR cytogenetic status (interaction sHR p=0.501; interaction HR p=0.164), although subgroup estimates were imprecise due to limited number of events.

### Summary/Conclusion

In this molecularly homogeneous population of high-risk MDS and AML-MR, CPX-351 induction did not improve post-transplant outcomes compared with standard chemotherapy. In contrast, patient-specific dPCR-based MRD assessment prior to alloHSCT provided strong prognostic discrimination for relapse and OS. These findings support molecular MRD monitoring in a high-risk subgroup not routinely eligible for highly sensitive MRD surveillance.

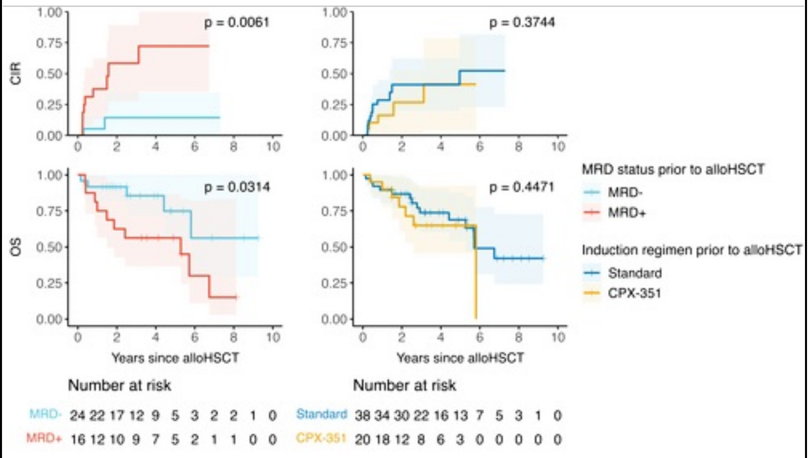

**Figure 1.** Left: Cumulative incidence of relapse (CIR; Gray's test) and overall survival (OS; log-rank) according to dPCR MRD status within 1.5 months prior to alloHSCT. Right: CIR and OS according to pre-transplant treatment (standard chemotherapy vs CPX-351). Shaded areas indicate 95% confidence intervals.

**Copyright Information:** (Online) ISSN: 2572-9241
©2026 The Author(s). HemaSphere is published by John Wiley & Sons Ltd on behalf of European Hematology Association. This is an open access Abstract Book distributed under the Attribution-NonCommercial-NoDerivs (CC BY-NC-ND), which allows third parties to download the articles and share them with others as long as they credit the author and the Abstract Book, but they cannot change the content in any way or use them commercially.
**Abstract Book Citations:** Authors, Title, HemaSphere, 2026;10:(S1):pages. The abstract book DOIs can be found on the "Author Information" page.
**Disclaimer:** Articles published in the journal HemaSphere exclusively reflect the opinions of the authors. The authors are responsible for all content in their abstracts including accuracy of the facts, statements, citing resources, etc.

### (PS1667) A RELAPSE RISK SCORING SYSTEM FOR ACUTE MYELOID LEUKEMIA PATIENTS RECEIVING VENETOCLAX-BASED THERAPY

**Topic:** 04. Acute myeloid leukemia - Clinical

Yuxin Li*[1], Dan Wang[1], Danchen Meng[1], Dinghui Zhao[1], Zhengfeng Hou[1], Jichun Yang[2], Zhenqi Huang[1], Xiaofeng Shi[3], Zhangbiao Long[1]

*[1]The First Affiliated Hospital of Anhui Medical University, Hefei, China;[2]Tianjin University of Traditional Chinese Medicine, Tianjin, China; [3]The Second Affiliated Hospital of Nanjing Medical University, Nanjing, China;*

### Background

Venetoclax-based regimens are now a first-line treatment option for elderly, unfit for intensive chemotherapy acute myeloid leukemia (AML) patients. While initial responses are promising, most patients eventually develop resistance and relapse and they often do not respond to other conventional therapies. Early detection of venetoclax resistance and timely treatment adjustment before relapse are crucial strategies. However, there is currently no effective predictive tool for assessing relapse risk after venetoclax-based regimens.

### Aims

This study aims to develop a clinical prediction model for AML relapse following venetoclax-based therapy using multicenter real-world data.

### Methods

This real-world retrospective study included patients with AML (non-M3 subtype) who had received prior venetoclax-based therapy and achieved disease remission. Patients overall survival (OS) and relapse-free survival (RFS) were compared using Kaplan-Meier survival curve analysis, with between-group differences assessed by the *log-rank* test. In the training cohort, potential influencing factors were analyzed using univariate Cox regression, followed by multivariate Cox regression. Variables were assigned scores based on their regression coefficients, and the total risk score for each patient was calculated as the sum of these individual scores. The optimal cutoff point for risk stratification was determined using the survival and survminer packages, and the surv-cutpoint function in R software, and patients were subsequently categorized into different risk groups. The model's performance was then evaluated and validated in the independent validation cohort.

### Results

We included 264 AML patients who achieved remission after venetoclax-based regimens. The median follow-up was 19.2 months. Relapse occurred in 127 patients. Patients in non-relapsed group had a significantly longer median overall survival compared to those who relapsed (Not reached *vs.* 12.8 months, $P < 0.0001$). Through univariate and multivariate Cox regression analyses, seven predictive variables were identified. These include age ≥ 65 years, presence of comorbidities, white blood cell count > $10\times10^9$/L, serum $Ca^{2+}$ < 2.1 mmol/L, total venetoclax treatment cycles < 3, MRD ≥ $1\times10^{-3}$, and *TP53*$^{mut}$. Based on their regression coefficients, a scoring system was developed to predict relapse risk (http://www.aml-relapse-risk-model.com). Depending on this system, patients were stratified into low-, intermediate-, and high-risk groups. Kaplan–Meier curves showed significant differences in relapse probability among these groups. The model demonstrated good performance with a C-index of 0.80 in the training cohort and 0.72 in the validation cohort. Time-dependent Receiver Operating Characteristic curves and calibration curves further confirmed its robust predictive ability.

**Copyright Information:** (Online) ISSN: 2572-9241
©2026 The Author(s). HemaSphere is published by John Wiley & Sons Ltd on behalf of European Hematology Association. This is an open access Abstract Book distributed under the Attribution-NonCommercial-NoDerivs (CC BY-NC-ND), which allows third parties to download the articles and share them with others as long as they credit the author and the Abstract Book, but they cannot change the content in any way or use them commercially.
**Abstract Book Citations:** Authors, Title, HemaSphere, 2026;10:(S1):pages. The abstract book DOIs can be found on the "Author Information" page.
**Disclaimer:** Articles published in the journal HemaSphere exclusively reflect the opinions of the authors. The authors are responsible for all content in their abstracts including accuracy of the facts, statements, citing resources, etc.

**Summary/Conclusion**

This model allows early prediction of secondary resistance to venetoclax-based regimens in elderly, unfit for intensive chemotherapy AML patients, indicating its significant clinical utility.

**Copyright Information:** (Online) ISSN: 2572-9241
©2026 The Author(s). HemaSphere is published by John Wiley & Sons Ltd on behalf of European Hematology Association. This is an open access Abstract Book distributed under the Attribution-NonCommercial-NoDerivs (CC BY-NC-ND), which allows third parties to download the articles and share them with others as long as they credit the author and the Abstract Book, but they cannot change the content in any way or use them commercially.
**Abstract Book Citations:** Authors, Title, HemaSphere, 2026;10:(S1):pages. The abstract book DOIs can be found on the "Author Information" page.
**Disclaimer:** Articles published in the journal HemaSphere exclusively reflect the opinions of the authors. The authors are responsible for all content in their abstracts including accuracy of the facts, statements, citing resources, etc.

### (PS1668) OUTCOMES OF ACUTE PROMYELOCYTIC LEUKEMIA IN PATIENTS AGED ≥70 YEARS: A MULTICENTER REAL-WORLD ANALYSIS OF FIRST-LINE ATO-BASED THERAPY

**Topic:** 04. Acute myeloid leukemia - Clinical

Joana Infante*[1], Eliana Aguiar[2], Ines Damasio Sa[2], Ines Dos Santos Marques[2], Miguel Neto[2], Cátia Almeida[3], Telma Nascimento[3], Marta Pereira[3], Emilia Cortesão[3], Guilherme Sapinho[1], Graca Luiza O P Neto Vasconcelos Esteves[1], Tiago Bras[4], Luísa Regadas Fernandes[4], Ana Filipa Marques Saraiva[5], Georgy Alikhanov[5], Joana Saraiva Santos[5], Patricia Ribeiro[5], Fernanda Trigo[2]

*[1]Serviço de Hematologia e Transplantação de Medula - Hospital de Santa Maria, Lisbon, Portugal;[2]Serviço de Hematologia Clínica - Hospital de São João, Porto, Portugal; [3]Serviço de Hematologia Clínica - Hospital de Coimbra, Coimbra, Portugal; [4]Serviço de Hematologia Clínica - Hospital de Santo António, Porto, Portugal;[5]Serviço de Hematologia Clínica - Hospital de Santo António dos Capuchos, Lisbon, Portugal;*

### Background

Acute promyelocytic leukemia (APL) is highly curable in the modern era, but survival of older patients remains inferior due to frailty, increased treatment-related toxicity and early death (ED), and comorbidities. Arsenic trioxide (ATO)-based regimens are better tolerated, but patients aged ≥70 years have limited data due to the rarity of APL at this age and their exclusion from the ATO clinical trials.

### Aims

In this study, we evaluated baseline characteristics and clinical outcomes of elderly patients with APL treated with ATO plus all-trans retinoic acid (ATRA) in a multicenter unselected real-world cohort.

### Methods

We retrospectively analyzed consecutive adult patients with newly diagnosed APL treated with ATRA plus ATO at five academic hospitals from the Grupo Português de Leucemias Agudas. Patients were stratified by age <70 versus ≥70 years. Clinical features, treatment-related complications, response rates, ED, and overall survival (OS) were assessed. Survival was estimated using Kaplan–Meier methods. Cox regression identified prognostic factors for OS.

**Copyright Information:** (Online) ISSN: 2572-9241
©2026 The Author(s). HemaSphere is published by John Wiley & Sons Ltd on behalf of European Hematology Association. This is an open access Abstract Book distributed under the Attribution-NonCommercial-NoDerivs (CC BY-NC-ND), which allows third parties to download the articles and share them with others as long as they credit the author and the Abstract Book, but they cannot change the content in any way or use them commercially.
**Abstract Book Citations:** Authors, Title, HemaSphere, 2026;10:(S1):pages. The abstract book DOIs can be found on the “Author Information” page.
**Disclaimer:** Articles published in the journal HemaSphere exclusively reflect the opinions of the authors. The authors are responsible for all content in their abstracts including accuracy of the facts, statements, citing resources, etc.

### Results

Among 135 patients, 23 (17%) were aged ≥70 years. Elderly patients more frequently presented with ECOG 2–3 (17.4% vs 1.8%, p=0.001), high-risk disease (26.1% vs 3.6%, p<0.001), renal dysfunction (p=0.005), higher fibrinogen levels (p=0.028), and previously diagnosed arterial hypertension (73.9% vs 17.0%, p<0.001). Time from diagnosis to treatment initiation was shorter in the elderly group (0 vs 1 day, p=0.026).
There were no significant differences between incidence of differentiation syndrome (DS), DS prophylaxis, infection rate, thrombosis rate and bleeding events during induction between age groups.
Whereas in patients <70 years a corticosteroid prophylaxis reduced the incidence of DS (14% versus 42.9% with no prophylaxis, p=0.009), in patients aged ≥70 a corticosteroid prophylaxis had no statistically significant impact on DS (15.0% versus 33.3% with no prophylaxis, p=0.435).
Despite these adverse features, hematologic complete remission after induction and molecular remission after consolidation were achieved in 100% of evaluable patients in both groups. However, 30-day ED rate and induction mortality rate were significantly higher in elderly patients (1.8% vs 13.0%, p=0.009; and 1.8% vs 17.4%, p=0.001, respectively). The causes of death during induction were intracranial bleeding (n=2), septic shock (n=1) and unspecified cardiac event (n=1).
With a median follow-up of 33.4 months, OS was inferior in patients aged ≥70 (log-rank p=0.003). 1-, 2-, and 5-year OS rates were 82.1%, 82.1% and 61.6% in elderly patients compared with 97.2% across all timepoints in younger patients (Figure 1). No relapses occurred among patients >70 years.
On univariate analysis, age ≥70 years (HR 9.1, p=0.003), high-risk disease (HR 9.3, p=0.003), and a Glasgow Coma Scale <15 at diagnosis (HR 18.1, p=0.001) predicted inferior OS. On multivariate analysis, only age ≥70 retained independent prediction of inferior OS (HR 5.5, CI95% 1.1-25.3, p=0.039).

### Summary/Conclusion

ATO-based therapy achieved excellent results in patients aged ≥70 years, with universal complete remission. Inferior survival was primarily driven by ED and baseline vulnerability rather than delays in treatment initiation, ATO/ATRA-related toxicities or therapy resistance. This study confirms ATO plus ATRA is effective in older patients, and early supportive care strategies tailored to elderly patients are needed to further improve ED in this special population.

**Copyright Information:** (Online) ISSN: 2572-9241
©2026 The Author(s). HemaSphere is published by John Wiley & Sons Ltd on behalf of European Hematology Association. This is an open access Abstract Book distributed under the Attribution-NonCommercial-NoDerivs (CC BY-NC-ND), which allows third parties to download the articles and share them with others as long as they credit the author and the Abstract Book, but they cannot change the content in any way or use them commercially.
**Abstract Book Citations:** Authors, Title, HemaSphere, 2026;10:(S1):pages. The abstract book DOIs can be found on the "Author Information" page.
**Disclaimer:** Articles published in the journal HemaSphere exclusively reflect the opinions of the authors. The authors are responsible for all content in their abstracts including accuracy of the facts, statements, citing resources, etc.

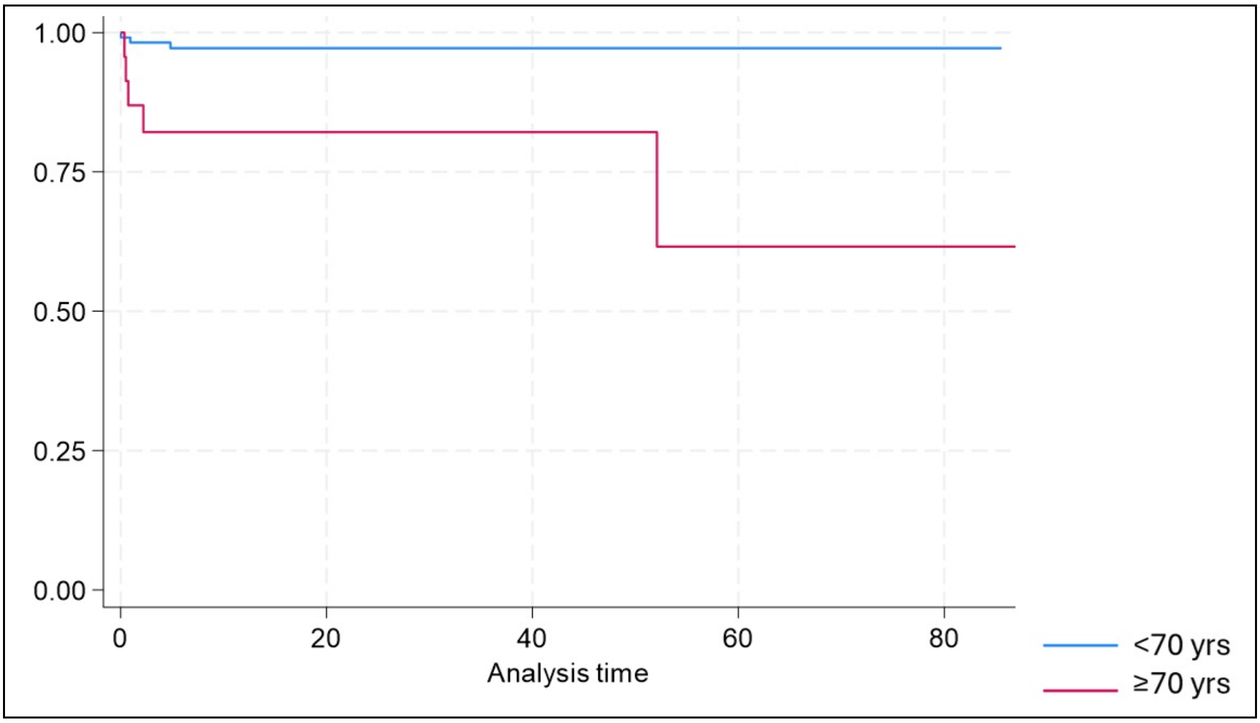

Figure 1. Overall survival of patients with APL treated with first-line ATO plus ATRA by patient age group.

**Copyright Information:** (Online) ISSN: 2572-9241
©2026 The Author(s). HemaSphere is published by John Wiley & Sons Ltd on behalf of European Hematology Association. This is an open access Abstract Book distributed under the Attribution-NonCommercial-NoDerivs (CC BY-NC-ND), which allows third parties to download the articles and share them with others as long as they credit the author and the Abstract Book, but they cannot change the content in any way or use them commercially.
**Abstract Book Citations:** Authors, Title, HemaSphere, 2026;10:(S1):pages. The abstract book DOIs can be found on the "Author Information" page.
**Disclaimer:** Articles published in the journal HemaSphere exclusively reflect the opinions of the authors. The authors are responsible for all content in their abstracts including accuracy of the facts, statements, citing resources, etc.

### (PS1669) WHOLE-GENOME PROFILING OF EARLY CLL/MBL REVEALS GENOMIC COMPLEXITY FROM DISEASE ONSET AND REFINES RISK BEYOND IPS-E

**Topic:** 05. Chronic lymphocytic leukemia and related disorders - Biology & translational research

Pauline Robbe*[1], Kate Ridout[1, 1], Grigore Gafencu[1], Niamh Appleby[1, 2], Georgeta Ciuban[1], Melinda Kormany[1], Danmei Xu[1, 2], Renata Walewska[3], Andrew Pettitt[4], Piers Patten[5], Oliver Miles[6], Helen Marr[7], Claire Hutchinson[8], Nicolas Martinez-Calle[9], Scott Marshall[10], Simon Stern[11], Toby A. Eyre[2], Murali Kesavan[2], Harriet Walter[12], Wendy Harrison[13], Charlotte Bomken[14], Yasmin Reyal[15], Savio Fernandes[16], Timothy Coutts[17], Anthony Cutts[1], Helene Dreau[1], Dimitris Vavoulis[1, 18], Anna Schuh[1, 18]

*[1]University of Oxford, Oxford, United Kingdom; [2]Oxford University Hospitals NHS Trust, Oxford, United Kingdom; [3]University Hospitals Dorset, Bournemouth, United Kingdom; [4]The Clatterbridge Cancer Centre, Liverpool, United Kingdom; [5]King's College London, London, United Kingdom; [6]Musgrove Park Hospital, Somerset, United Kingdom; [7]Freeman Hospital, Newcastle, United Kingdom; [8]University Hospitals Plymouth NHS Trust, Plymouth, United Kingdom; [9]Nottingham City Hospital, Nottingham, United Kingdom; [10]Sunderland Royal Hospital, Sunderland, United Kingdom; [11]Epsom & St Helier Hospital NHS Trust, London, United Kingdom; [12]Leicester Royal Infirmary, Leicester, United Kingdom; [13]James Paget Hospital, Great Yarmouth, United Kingdom; [14]North Tyneside General hospital, Northumbria NHS Trust, United Kingdom; [15]St George's University Hospital, London, United Kingdom; [16]Russells Hall Hospital, Dudley, United Kingdom; [17]University of Oxford, OCTO, Department of Oncology, Oxford, United Kingdom; [18]NIHR Oxford Biomedical Research Centre, Oxford, United Kingdom;*

### Background

Risk assessment of high-count monoclonal B-cell lymphocytosis (HC-MBL)/Rai stage 0–I chronic lymphocytic leukaemia (CLL) relies on the International Prognostic Score for Early-stage CLL (IPS-E). Predicting progression to symptomatic disease using the IPS-E at an individual level lacks precision. Whole-genome sequencing (WGS) may identify molecular features that improve risk assessment and guide future early intervention studies.

### Aims

To define the genomic landscape of HC-MBL/early CLL using WGS
To identify molecular features linked to time to progression (TTP) and time to first treatment (TTFT)
To develop an integrated clinico-molecular IPS-E

### Methods

We performed paired tumour–germline WGS in 320 individuals with HC-MBL/Rai stage 0–I CLL from a prospective cohort with a median follow-up of 33.4 months. Analyses included single-nucleotide variants, structural variants (including in-depth del13q characterisation), mutational signatures, driver burden, genomic complexity, telomere length, human leukocyte antigen (HLA) typing, polygenic risk scores (PRS), and immunoglobulin heavy chain variable region (IGHV) status. Subclonal structure was inferred from cancer cell fractions. Findings were compared with 473 WGS pairs from symptomatic CLL. Associations with TTP and TTFT were assessed using time-to-event analyses. Genomic features were integrated with IPS-E and internally validated using resampling.

**Copyright Information:** (Online) ISSN: 2572-9241
©2026 The Author(s). HemaSphere is published by John Wiley & Sons Ltd on behalf of European Hematology Association. This is an open access Abstract Book distributed under the Attribution-NonCommercial-NoDerivs (CC BY-NC-ND), which allows third parties to download the articles and share them with others as long as they credit the author and the Abstract Book, but they cannot change the content in any way or use them commercially.
**Abstract Book Citations:** Authors, Title, HemaSphere, 2026;10:(S1):pages. The abstract book DOIs can be found on the "Author Information" page.
**Disclaimer:** Articles published in the journal HemaSphere exclusively reflect the opinions of the authors. The authors are responsible for all content in their abstracts including accuracy of the facts, statements, citing resources, etc.

### Results

Ninety per cent of samples carried at least one recognised CLL driver, and over half had multiple drivers. Comparison with symptomatic CLL showed that canonical drivers are already present in HC-MBL, with no clear molecular boundary between HC-MBL and early-stage CLL. Subclonal diversity was detected in 96% of cases, and convergent evolution was detectable early in drivers such as ATM, indicating tumour evolution before progression. Deletion of chromosome 13q14.2 was the most frequent alteration (69%). Marked heterogeneity was observed in deletion size, structure, and clonality.
Cox regression analyses identified 19 genomic features — including driver burden, subclonal complexity, del6q, unmutated IGHV, MED12, SF3B1, and ATM/del11q — associated with early TTP and TTFT (<24 months, FDR < 0.05). IGLV3-21 R110 and subset #2 outperformed IGHV mutation status in predicting progression (P < 0.0001). Del13q14.2, as a sole event by WGS, defined the subgroup with the lowest progression risk (FDR = 0.01).
Within the high IPS-E cohort, progressors and non-progressors displayed distinct genomic landscapes: among several genomic features, 95% of progressors versus 68% of non-progressors carried high tumour burden (FDR = 0.03), while 2% of progressors versus 27% of non-progressors carried del13q as a sole event, supporting discordance between clinical and molecular risk. Finally, we incorporated the 19 progression-associated genomic features into the IPS-E to define a molecular IPS-E. This reclassified 18/103 (17%) of IPS-E high-risk and 33/126 (26%) of intermediate-risk cases into lower genomic subgroups, improving separation between high- and intermediate-risk groups (P < 0.01). Further refinement incorporating additional genomic features, longer follow-up, and immune markers is ongoing to strengthen risk classification.

### Summary/Conclusion

Our findings support the use of a combined clinico-molecular IPS-E for refined risk stratification and provide a framework to identify high-risk individuals who may benefit from early-intervention studies.

**Copyright Information:** (Online) ISSN: 2572-9241
©2026 The Author(s). HemaSphere is published by John Wiley & Sons Ltd on behalf of European Hematology Association. This is an open access Abstract Book distributed under the Attribution-NonCommercial-NoDerivs (CC BY-NC-ND), which allows third parties to download the articles and share them with others as long as they credit the author and the Abstract Book, but they cannot change the content in any way or use them commercially.
**Abstract Book Citations:** Authors, Title, HemaSphere, 2026;10:(S1):pages. The abstract book DOIs can be found on the "Author Information" page.
**Disclaimer:** Articles published in the journal HemaSphere exclusively reflect the opinions of the authors. The authors are responsible for all content in their abstracts including accuracy of the facts, statements, citing resources, etc.

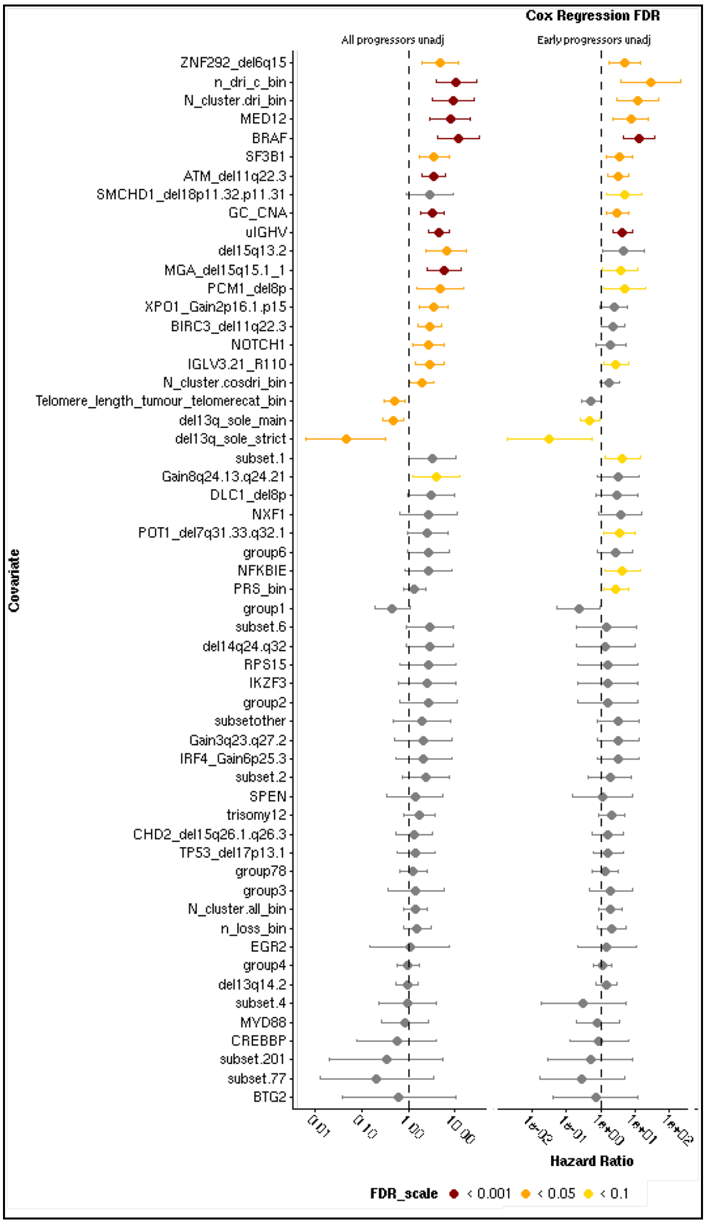

Forest plots of Cox regression analyses for progression. Hazard ratios (95% CI) are shown for all and early progressors; colour denotes FDR. Nineteen features (FDR < 0.05) were selected for further analysis. del13q_main was excluded due to high correlation with del13q_strict, which showed stronger association with lack of progression. “bin” denotes variables binarised at the median. Variables include CLL drivers, copy-number alterations, genomic complexity, telomere length, and molecular subgroups.

**Copyright Information:** (Online) ISSN: 2572-9241
©2026 The Author(s). HemaSphere is published by John Wiley & Sons Ltd on behalf of European Hematology Association. This is an open access Abstract Book distributed under the Attribution-NonCommercial-NoDerivs (CC BY-NC-ND), which allows third parties to download the articles and share them with others as long as they credit the author and the Abstract Book, but they cannot change the content in any way or use them commercially.
**Abstract Book Citations:** Authors, Title, HemaSphere, 2026;10:(S1):pages. The abstract book DOIs can be found on the “Author Information” page.
**Disclaimer:** Articles published in the journal HemaSphere exclusively reflect the opinions of the authors. The authors are responsible for all content in their abstracts including accuracy of the facts, statements, citing resources, etc.

### (PS1670) CHRONIC LYMPHOCYTIC LEUKEMIA HIJACKS THE GERMINAL CENTER-SPECIFIC TRANSCRIPTION FACTOR GFI1 TO DRIVE PROLIFERATION

**Topic:** 05. Chronic lymphocytic leukemia and related disorders - Biology & translational research

Alba Maiques-Diaz*[1, 2, 3], Liska Caviedes Cárdenas[2, 3], Anna Vidal-Crespo[2], Judith Mateos-Jaimez[2], Vicente Chapaprieta[2, 3], Víctor Jiménez-Martínez[2, 3, 4], Raúl Pérez[2, 5], Stella Charalampopoulou[2, 3], Sara Salazar Ortego[6], Ana Ortega Molina[6], Ingo Ringshausen[7], Dolors Colomer Pujol[2, 3, 4, 8], Elias Campo[2, 3, 4, 8], José Ignacio Martín-Subero[2, 3, 4, 9]

*[1]Department of Physiological Sciences,Faculty of Medicine, University of Barcelona, L'Hospitalet de Llobregat, Spain;[2]Fundació de Recerca Clínic Barcelona-Institut d'Investigacions Biomèdiques August Pi i Sunyer (FRCB-IDIBAPS), Barcelona, Spain; [3]Centro de Investigación Biomédica en Red Cáncer (CIBERONC), Madrid, Spain;[4]Department of Pathology, Faculty of Medicine, University of Barcelona, Barcelona, Spain; [5]Department of Biochemistry and Molecular Biology, Faculty of Veterinary Medicine, Complutense University of Madrid, Madrid, Spain; [6]Centro de Biología Molecular Severo Ochoa-CSIC, Madrid, Spain; [7]University College London (UCL) Cancer Institute, London, United Kingdom; [8]Hospital Clínic, Barcelona, Spain; [9]Institución Catalana de Recerca i Estudis Avançats (ICREA), Barcelona, Spain;*

### Background

Chronic lymphocytic leukemia (CLL) is clinically heterogeneous ranging from indolent to more aggressive cases that require treatment. It is known that CLL cells primarily proliferate within the lymph node (LN) microenvironment, yet the epigenetic and transcriptional rewiring taking place in LNs remains largely unexplored.

### Aims

We aimed to elucidate mechanisms underlying the proliferation of CLL cells in response to microenvironmental stimuli.

### Methods

Peripheral blood (PB) cells from treatment-naive CLL patients were co-cultured with MM1 stromal cells expressing CD40L, IL21, and BAFF to induce proliferation and a LN-like phenotype. RNA-seq and H3K27acetylation ChIP-seq were performed in five mutated IGHV (M-CLL) and five unmutated IGHV (U-CLL) cases, and CUT&RUN for GFI1 was performed in 3 primary CLLs. Data were integrated with previously published H3K27ac ChIP-seq of normal B cells; RNA-seq of a PB-LN CLL series; single cell RNA-seq from normal tonsils. GFI1 function was assessed by overexpression and CRISPR-Cas9 KO in healthy B cells, primary CLL and the HG3 CLL cell line.

**Copyright Information:** (Online) ISSN: 2572-9241
©2026 The Author(s). HemaSphere is published by John Wiley & Sons Ltd on behalf of European Hematology Association. This is an open access Abstract Book distributed under the Attribution-NonCommercial-NoDerivs (CC BY-NC-ND), which allows third parties to download the articles and share them with others as long as they credit the author and the Abstract Book, but they cannot change the content in any way or use them commercially.
**Abstract Book Citations:** Authors, Title, HemaSphere, 2026;10:(S1):pages. The abstract book DOIs can be found on the “Author Information” page.
**Disclaimer:** Articles published in the journal HemaSphere exclusively reflect the opinions of the authors. The authors are responsible for all content in their abstracts including accuracy of the facts, statements, citing resources, etc.

**Results**

LN-like stimulation triggered a convergent transcriptional and epigenetic program across CLL subtypes, upregulating cell cycle, MYC-targets, mTORC1 and OxPhos pathways. H3K27ac profiles revealed 1,168 CLL-specific active chromatin regions, and 800 shared with germinal center B cells (GCBCs) as main signatures (**panel A**). CLL-specific peaks target genes enriched in NF-kB pro-survival effectors (e.g., *IL15RA*, *TNFRSF9*) and immunomodulators (e.g., *CD70*, *IL10*), whereas CLL-GCBC shared peaks target cell cycle regulation genes (e.g., *E2F1, CDK1*) (**panel B**). Analysis of transcription factors (TF) upregulated in proliferative CLL cells including MM1-stimulated cells and PB-LN series identified 11 candidates, five of which were also upregulated in healthy GCBCs. Among them, *GFI1*, a transcriptional repressor essential in hematopoiesis, was associated with more aggressive CLL cells, as higher expression correlate with faster progression (**panel C**). GFI1 displays a primed transcriptional state in aggressive CLL subsets (U-CLL and IGLV3-21R110 M-CLL), that is selectively induced upon proliferative stimulation. Notably, GFI1 expression is induced by pro-proliferative signals (BCR stimulation, TLR activation and MM1 coculture), but not by pro-survival cues alone. scRNA-seq from healthy tonsils localized *GFI1* expression specifically to proliferative GC dark zone cells (**panel D**), suggesting a direct role in B-cell cell cycle regulation. Overexpression of *GFI1* promoted proliferation in healthy B cells and primary CLL cells (**panel E-F**), whereas CRISPR-Cas9 knockout in HG3 cells significantly impaired proliferative capacity. Finally, we identified 1,843 direct GFI1 targets in CLL primary cells including immunomodulatory proteins (e.g., *CD86, HLA-DQB1*) and negative regulators of B-cell proliferation or signaling (e.g., *CYLD, E2F5*), which were downregulated upon GFI1 overexpression, potentially explaining the more proliferative and aggressive phenotype.

**Summary/Conclusion**

The LN microenvironment induces a GC-like program in CLL cells, including GFI1 upregulation, thereby promoting proliferation while attenuating immune signaling. These findings position GFI1 as a key mediator of microenvironment-driven CLL progression.

**Copyright Information:** (Online) ISSN: 2572-9241
©2026 The Author(s). HemaSphere is published by John Wiley & Sons Ltd on behalf of European Hematology Association. This is an open access Abstract Book distributed under the Attribution-NonCommercial-NoDerivs (CC BY-NC-ND), which allows third parties to download the articles and share them with others as long as they credit the author and the Abstract Book, but they cannot change the content in any way or use them commercially.
**Abstract Book Citations:** Authors, Title, HemaSphere, 2026;10:(S1):pages. The abstract book DOIs can be found on the “Author Information” page.
**Disclaimer:** Articles published in the journal HemaSphere exclusively reflect the opinions of the authors. The authors are responsible for all content in their abstracts including accuracy of the facts, statements, citing resources, etc.

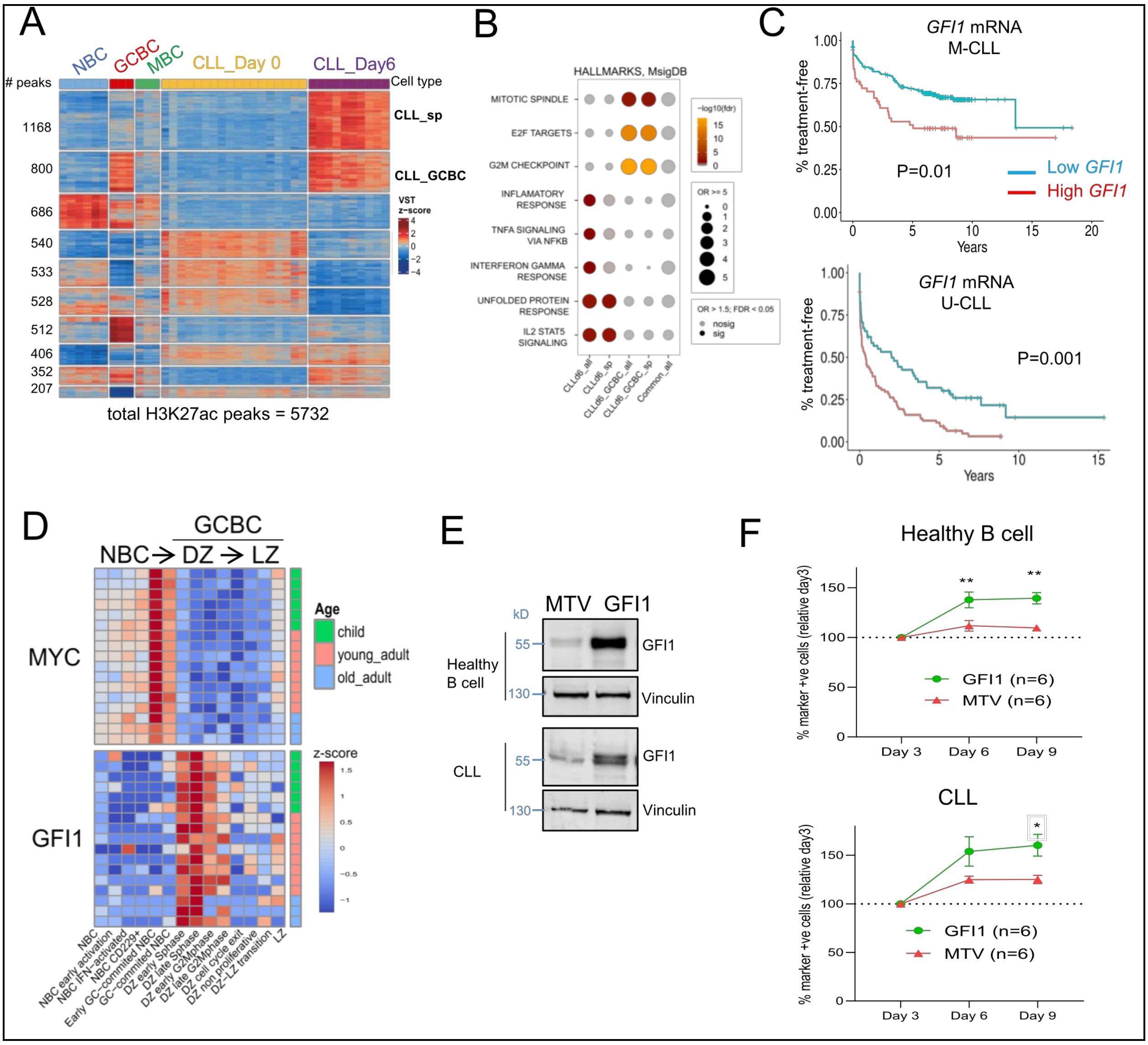

(A) Heatmap of H3K27ac signal intensities, indicated as row z-score. On the left the number of independent regions per cluster is indicated (B) Top 5 gene sets from Hallmark MSigDB identified by pathway enrichment analysis of the indicated target genes (FDR<0.05, OR>1.5). (C) Kaplan-Meier curves showing time-to-first treatment in patients with high and low GFI1 mRNA levels according to MaxStat analysis. (D) Heatmap shows scaled GFI1 expression across the indicated populations. (E) Western blot of GFI1 levels in empty-vector (MTV) or GFI1-overexpressing (GFI1) cells. (F) Mean ± SEM percentage of MTV (mCherry+) or GFI1 (GFP+) in healthy B cells and CLL cells up to 9 days post-transduction (n=6). NCB: Naive B cell; GCBC: Germinal center B cell; MBC: Memory B cell; DZ: Dark zone; LZ: Light zone; MTV: empty vector.

**Copyright Information:** (Online) ISSN: 2572-9241
©2026 The Author(s). HemaSphere is published by John Wiley & Sons Ltd on behalf of European Hematology Association. This is an open access Abstract Book distributed under the Attribution-NonCommercial-NoDerivs (CC BY-NC-ND), which allows third parties to download the articles and share them with others as long as they credit the author and the Abstract Book, but they cannot change the content in any way or use them commercially.
**Abstract Book Citations:** Authors, Title, HemaSphere, 2026;10:(S1):pages. The abstract book DOIs can be found on the “Author Information” page.
**Disclaimer:** Articles published in the journal HemaSphere exclusively reflect the opinions of the authors. The authors are responsible for all content in their abstracts including accuracy of the facts, statements, citing resources, etc.

### (PS1671) SPATIAL TRANSCRIPTOMIC PROFILING OF THE LYMPH NODE MICROENVIRONMENT IN CHRONIC LYMPHOCYTIC LEUKEMIA

**Topic:** 05. Chronic lymphocytic leukemia and related disorders - Biology & translational research

Antonella Bucci[1], Maria Carmela Vegliante[1], Giulia Notarpietro[1], Benedetta Apollonio[2], Paolo Mondelli[1], Anna Scattone[3], Sabino Ciavarella[1], Michela Frenquelli[4], Alessia Ciarrocchi[5], Benedetta Donati[5], Maurilio Ponzoni[6], Paolo Ghia[4, 7], Carla Minoia*[1]

*[1]IRCCS Istituto Tumori "Giovanni Paolo II", Hematology Unit, Bari, Italy; [2]IRCCS Istituto Tumori "Giovanni Paolo II", Rare Tumors and Melanoma Unit, Bari, Italy; [3]IRCCS Istituto Tumori "Giovanni Paolo II", Pathology Unit, Bari, Italy; [4]IRCCS Ospedale San Raffaele, B-cell Neoplasia Unit, Comprehensive Cancer Center, Milano, Italy; [5]Azienda Unità Sanitaria Locale-IRCCS di Reggio Emilia, Laboratory of Translational Research, Reggio Emilia, Italy; [6]San Raffaele Scientific Institute, Pathology Unit, Milano, Italy; [7]Università Vita-Salute San Raffaele, Medical School, Milano, Italy;*

### Background

Chronic lymphocytic leukemia (CLL) is characterized by a dependency on signals from the tumor microenvironment (TME) to survive and expand. While circulating CLL cells remain largely quiescent, active proliferation occurs within lymph nodes (LNs), in specialized niches known as proliferation centers (PCs). Deciphering spatial immune-tumor interactions within these regions is essential to understand disease progression.

### Aims

We aimed at identifying spatially resolved transcriptional profiles associated with LN PCs and transcriptomic peculiarities in CLL TME, focusing on CD3+ T lymphocytes and CD68+ macrophages both within and outside the PCs (EXTRA-PC), and to ultimately design a molecular signature predictive of clinical progression.

### Methods

17 FFPE CLL-LNs were grouped into 3 clinical subsets: indolent disease (n=6); in need of treatment (n=4); relapsed/refractory (n=7). The GeoMx® DSP platform was used to profile specific regions of interest (ROIs) corresponding to specific cytotypes segments (CD3+, CD68+ and MUM1+). Normalized matrices were generated for each cell segment. Differentially expressed genes (DEGs; $p < 0.05$, $|\log_2 \text{fold change}| > 0$) were identified through pairwise comparisons across distinct clinical subsets within the same LN compartment. Gene Ontology (GO) analysis was performed using GSEA on preranked lists of genes (FDR Q<0.05, NOM p<0.05). From the compartment-specific transcriptomic data, predictive signature of disease progression was derived using Random Forest and geNetClassifier.

**Copyright Information:** (Online) ISSN: 2572-9241
©2026 The Author(s). HemaSphere is published by John Wiley & Sons Ltd on behalf of European Hematology Association. This is an open access Abstract Book distributed under the Attribution-NonCommercial-NoDerivs (CC BY-NC-ND), which allows third parties to download the articles and share them with others as long as they credit the author and the Abstract Book, but they cannot change the content in any way or use them commercially.
**Abstract Book Citations:** Authors, Title, HemaSphere, 2026;10:(S1):pages. The abstract book DOIs can be found on the “Author Information” page.
**Disclaimer:** Articles published in the journal HemaSphere exclusively reflect the opinions of the authors. The authors are responsible for all content in their abstracts including accuracy of the facts, statements, citing resources, etc.

### Results

284 ROIs were analyzed, annotated as PC and EXTRA-PC (**Figure 1**). PCs displayed a distinct transcriptional identity compared to EXTRA-PC. PC CD3+ T cells were enriched in pro-inflammatory and activation pathways, with upregulation of *STAT1*, *NF-κB1* and *CCL19*. PC macrophages showed enhanced inflammatory signaling, chromatin remodeling, oxidative stress responses (*SOD1, TXN2*), defining a reactive immune niche. Across clinical stages, PCs underwent progressive immune remodeling. In indolent CLL, CD3+ T cells maintained an activated yet homeostatic profile (*CD247*, *STAT5B*), while macrophages exhibited surveillance and balanced inflammatory programs (*SPARCL1*, *JAK2*). In patients requiring treatment, T lymphocytes showed increased metabolic activation (*ENO1*), chromatin remodeling (*EZH2*), and amplified inflammatory pathways. Macrophages paralleled this shift with metabolic reorganization and enhanced mitochondrial translation (*NME1*). In relapsed/refractory disease, CD3+ T cells displayed impaired immune defense and altered trafficking and motility, consistent with dysfunction, whereas macrophages exhibited features of exhaustion and M2-like polarization enriched for programmed cell death pathways (*TP63*). Integration of spatial tumor and immune data led to the identification of a 50-gene tumor–immune signature that clearly distinguishes indolent from treatment-requiring CLL cases. Hierarchical clustering showed a distinct stratification of samples according to clinical status, indicating the potential prognostic relevance of this signature.

### Summary/Conclusion

Our findings indicate that CLL-LN TME is transcriptionally reprogrammed across disease evolution. PC transits from coordinated immune activation in indolent disease to dysfunctional niches in advanced relapsing CLL, highlighting spatial immune remodeling as a key player in clinical progression. The molecular predictive signature is currently under validation on an independent, multicenter cohort of 100 LN biopsies.

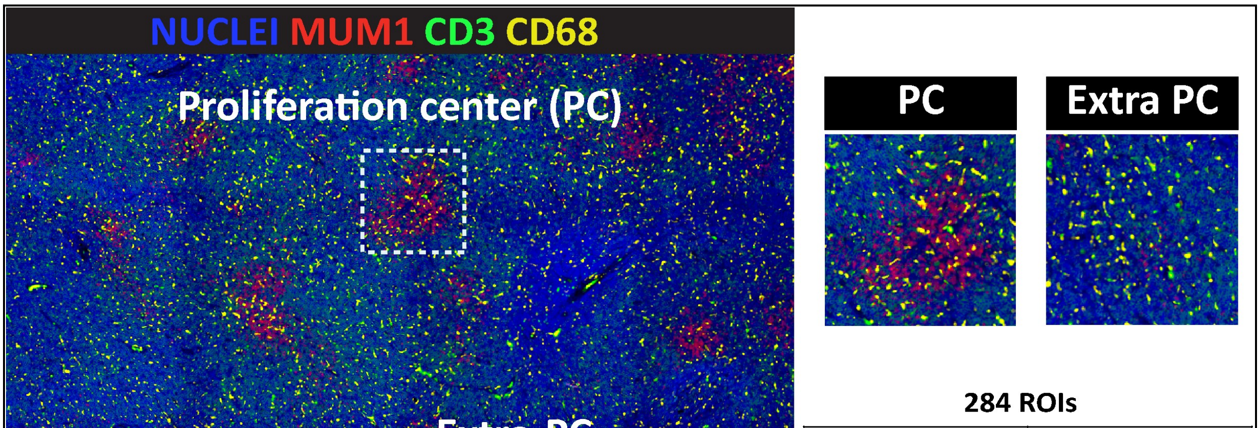

| PC (MUM1⁺) | | | Extra-PC (MUM1⁻) | | |
|---|---|---|---|---|---|
| CD3⁺ | CD68⁺ | CLL | CD3⁺ | CD68⁺ | CLL |
| 38 | 38 | 44 | 50 | 57 | 57 |

Figure 1. Digital Spatial Profiling (DSP) in CLL LNs. Immunofluorescence staining for CD3 (green), and CD68 (yellow) were used to identify T cells and macrophages respectively. MUM1 staining (red) was used to identify CLL cells in PC. Table is showing the total number of ROIs selected inside and outside PCs for each cell type.

**Copyright Information:** (Online) ISSN: 2572-9241
©2026 The Author(s). HemaSphere is published by John Wiley & Sons Ltd on behalf of European Hematology Association. This is an open access Abstract Book distributed under the Attribution-NonCommercial-NoDerivs (CC BY-NC-ND), which allows third parties to download the articles and share them with others as long as they credit the author and the Abstract Book, but they cannot change the content in any way or use them commercially.
**Abstract Book Citations:** Authors, Title, HemaSphere, 2026;10:(S1):pages. The abstract book DOIs can be found on the "Author Information" page.
**Disclaimer:** Articles published in the journal HemaSphere exclusively reflect the opinions of the authors. The authors are responsible for all content in their abstracts including accuracy of the facts, statements, citing resources, etc.

### (PF1672) DEFECTS IN MITOCHONDRIAL STRUCTURE UNDERLIE METABOLIC DYSFUNCTION IN T CELLS FROM PATIENTS WITH CHRONIC LYMPHOCYTIC LEUKEMIA

**Topic:** 05. Chronic lymphocytic leukemia and related disorders - Biology & translational research

Nienke Goedhart[1, 2], Gaspard Cretenet[1, 2], Kirill Vlaskov[1], Nicole N van der Wel[3], Anita Grootemaat[3], Frank Vrieling[4], Bob J Ignacio[5], Rinke Stienstra[4, 6], Kimberly M Bonger[5], Eric Eldering[1, 2], Arnon Philip Kater[1, 2], Helga Simon-Molas*[1, 2]

*[1]Amsterdam UMC, Departments of Experimental Immunology and Hematology, Amsterdam, The Netherlands;[2]Cancer Center Amsterdam and Amsterdam institute for Immunology and Infectious Diseases, Amsterdam, The Netherlands; [3]Amsterdam UMC, Department of Medical Biology, Electron Microscopy Center Amsterdam, The Netherlands; [4]Wageningen University and Research, Division of Human Nutrition and Health, Wageningen, The Netherlands; [5]Leiden Institute of Chemistry, Department of Chemical Biology and Immunology, Leiden, The Netherlands; [6]RadboudUMC, Department of Internal Medicine, Nijmegen, The Netherlands;*

### Background

Impaired immune responses are a hallmark of cancers, and specifically T-cell dysfunction is increasingly recognized as a major barrier to effective immunotherapies. In chronic lymphocytic leukaemia (CLL), altered T cell responses have been widely identified by us and others and low success rates of T cell-based therapies are noticeable. Our previous work identified defective metabolic programming in T cells from CLL patients and leukaemia-bearing mice (Simon-Molas, EHA Congress 2024; Goedhart, Gamal and Simon-Molas, Blood Advances, 2025). However, the mechanisms driving this dysfunction remained poorly understood.

### Aims

The main aim of this project is to identify the molecular causes of metabolic defects in T cells from patients with CLL.

### Methods

We analysed metabolic responses alongside effector function in T cells from CLL patients and age-matched healthy donors upon TCR engagement. To do so, extracellular flux analysis, metabolomics, 13C isotope tracing, confocal imaging, electron microscopy and flow cytometry were applied and integrated. All CLL samples were obtained from the B cell malignancies Biobank of Amsterdam UMC.

### Results

We demonstrate that stimulated T cells from CLL patients (CLL T cells) adopt a metabolically imbalanced state marked by persistent glycolysis and insufficient engagement of mitochondrial oxidative phosphorylation (OXPHOS). This skewed metabolic response correlates with high expression of activation markers and production of cytokines sustained too long after TCR engagement. Defective mitochondrial metabolism in CLL T cells is not caused by limitations in nutrient availability or capacity for fuel processing but related to intrinsic mitochondrial alterations. These include decreased mitochondrial network complexity (reduced area, perimeter, elongation and branching) and disrupted cristae architecture. Sustained mTORC1 signalling, maintained by increased glutamine uptake, enforces the glycolytic phenotype and is associated with aberrant proliferation. Pharmacologic mTORC1 inhibition with rapamycin reverses metabolic imbalance, reducing glucose dependence, restoring mitochondrial capacity and normalizing proliferation.

**Copyright Information:** (Online) ISSN: 2572-9241
©2026 The Author(s). HemaSphere is published by John Wiley & Sons Ltd on behalf of European Hematology Association. This is an open access Abstract Book distributed under the Attribution-NonCommercial-NoDerivs (CC BY-NC-ND), which allows third parties to download the articles and share them with others as long as they credit the author and the Abstract Book, but they cannot change the content in any way or use them commercially.
**Abstract Book Citations:** Authors, Title, HemaSphere, 2026;10:(S1):pages. The abstract book DOIs can be found on the "Author Information" page.
**Disclaimer:** Articles published in the journal HemaSphere exclusively reflect the opinions of the authors. The authors are responsible for all content in their abstracts including accuracy of the facts, statements, citing resources, etc.

**Summary/Conclusion**

Together, these findings pinpoint defects in mitochondrial structure as a central cause of T-cell metabolic impairment in CLL and identify sustained mTORC1 signalling as a key upstream driver and actionable target to counteract the imbalanced metabolic programming in these cells.

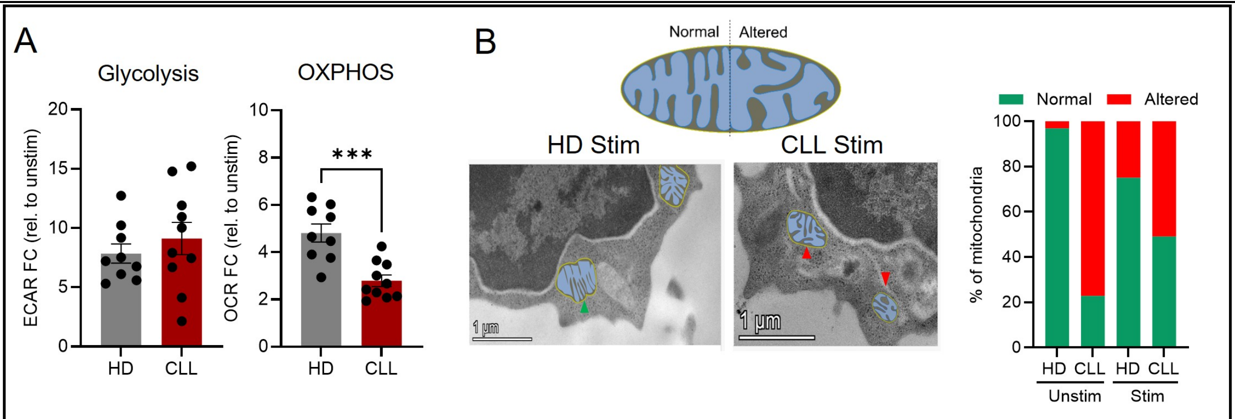

A) Fold change (FC) of basal glycolysis measured by extracellular acidification date (ECAR) and basal oxidative phosphorylation (OXPHOS) as measured by oxygen consumption rate in T cells from CLL patients or healthy donors (HD) stimulated with αCD3/28 soluble antibodies for 48h. Differences were analyzed using a two-tailed unpaired t-test, *p<0.05; **p<0.01; ****p<0.0001. Error bars represent mean ± SEM.
B) Transmission electron microscopy of T cells from HD (n=1) and CLL (n=1) unstimulated or stimulated with αCD3/28 soluble antibodies for 48h. Scoring of at least 30 mitochondria per condition was performed blindly by three independent evaluators. Mitochondria were classified as normal or altered and data are expressed as percentage of total mitochondria analyzed. Representative images from the stimulated conditions are shown.

**Copyright Information:** (Online) ISSN: 2572-9241
©2026 The Author(s). HemaSphere is published by John Wiley & Sons Ltd on behalf of European Hematology Association. This is an open access Abstract Book distributed under the Attribution-NonCommercial-NoDerivs (CC BY-NC-ND), which allows third parties to download the articles and share them with others as long as they credit the author and the Abstract Book, but they cannot change the content in any way or use them commercially.
**Abstract Book Citations:** Authors, Title, HemaSphere, 2026;10:(S1):pages. The abstract book DOIs can be found on the "Author Information" page.
**Disclaimer:** Articles published in the journal HemaSphere exclusively reflect the opinions of the authors. The authors are responsible for all content in their abstracts including accuracy of the facts, statements, citing resources, etc.

### (PS1673) INTEGRATED TRANSCRIPTOMIC AND IMMUNE PROFILING IDENTIFIES DETERMINANTS OF DEEP REMISSION WITH IBRUTINIB–VENETOCLAX COMBINATION THERAPY IN CHRONIC LYMPHOCYTIC LEUKEMIA

**Topic:** 05. Chronic lymphocytic leukemia and related disorders - Biology & translational research

Varsha Gandhi*[1], Gorkem Kismali[1], Evan N Cohen[1], Ganiraju Manyam[2], Mark Meerson[3], Anastasiia Shvyrkova[3], Ekaterina Postovalova[3], Nikita Kotlov[3], Alexander Bagaev[3], Nathan Fowler[3], William G Wierda[4], Nitin Jain[4]

*[1]Department of Translational Molecular Pathology, University of Texas MD Anderson Cancer Center, Houston, TX, United States of America; [2]Department of Bioinformatics and Computational Biology, University of Texas MD Anderson Cancer Center, Houston, TX, United States of America; [3]BostonGene, Waltham, MA, United States of America; [4]Department of Leukemia, University of Texas MD Anderson Cancer Center, Houston, TX, United States of America;*

### Background

B-cell receptor (BCR) signaling pathway and BCL-2 prosurvival proteins are established biomarkers in the pathophysiology of chronic lymphocytic leukemia (CLL). Ibrutinib, a covalent Bruton's tyrosine kinase (BTK) inhibitor blunts BCR signaling while venetoclax neutralizes BCL-2 protein. Both these drugs are effective as monotherapy; however, their synergy was transformational when combined for patients with CLL. As monotherapy, these drugs rarely result in undetectable measurable residual disease (uMRD) status. In an investigator-initiated clinical trial, we demonstrated that combined ibrutinib and venetoclax (I+V) resulted in bone marrow uMRD4 ($10^{-4}$ limit of detection) status in 60-66% of patients. This was observed in treatment-naïve (TN) disease (n=120 patients; Jain et al. NEJM 2019; Jain et al. JAMA Onc 2021) as well as relapsed-refractory (RR) CLL (n=79 patients; Swaminathan et al. Blood Cancer Discov 2026). I+V is approved in Europe based on the randomized GLOW trial (Kater et al. NEJM Evid 2022), with half of patients in the I+V arm attaining blood uMRD4 remission.

### Aims

To identify biologic and molecular mechanisms explaining the likelihood of achieving uMRD4 with I+V combination in CLL.

### Methods

In this trial, patients started with 3 cycles of ibrutinib monotherapy at 420 mg/d. On cycle 4 day 1, venetoclax (weekly ramp-up dosing reaching 400 mg/d) was added. Patients received 24 cycles of I+V. Blood samples were collected prior to therapy (C1D1), after 1 and 3 cycles of ibrutinib (C2D1 and C4D1) and after a week of I+V (C4D8). Transcriptomic data from these >200 samples (from 32 TN and 25 RR CLL patients) were analyzed. To perform differential gene expression analysis, reads were aligned using STAR (genome hg38), read counts were obtained by featurecounts, followed by limma. TPM-normalized expressions from Kallisto were used to predict cell composition using Kassandra (Zaitsev A et al. Cancer Cell 2022) algorithm.

**Copyright Information:** (Online) ISSN: 2572-9241
©2026 The Author(s). HemaSphere is published by John Wiley & Sons Ltd on behalf of European Hematology Association. This is an open access Abstract Book distributed under the Attribution-NonCommercial-NoDerivs (CC BY-NC-ND), which allows third parties to download the articles and share them with others as long as they credit the author and the Abstract Book, but they cannot change the content in any way or use them commercially.
**Abstract Book Citations:** Authors, Title, HemaSphere, 2026;10:(S1):pages. The abstract book DOIs can be found on the "Author Information" page.
**Disclaimer:** Articles published in the journal HemaSphere exclusively reflect the opinions of the authors. The authors are responsible for all content in their abstracts including accuracy of the facts, statements, citing resources, etc.

**Results**

Compared to C1D1-TN and RR samples, there was a significant decline in BCR pathway molecules (BTK, CD79A and CD79B, LYN, CCL3 and CCL4) and MCL-1 transcript levels in C2D1 and C4D1 timepoints. This was confirmed by RT-PCR assay. Corresponding protein levels also decreased. Compared to baseline, CCL3 and CCL4 plasma levels were reduced in all patient samples. In the TN cohort, MRD+ patients at the end of 24 cycles of I+V had upregulated complement components (C1QA, C1QB, $p<0.01$), lactate dehydrogenase (LDH, $p=0.02$), and cell adhesion genes (JAM3, LRP5, $p<0.01$). Results from longitudinal comprehensive immune profiling using Kassandra deconvolution demonstrated consistent increases in total T ($p=0.04$) and NK ($p=0.03$) cell populations, including subpopulations of CD4 and CD8 effector T cells in patients who did not achieve uMRD4 status. Compared to RR-uMRD4 cohort, in the MRD+ group we noted upregulation of WNT signaling (WNT5A, WNT5B, $p<0.01$) and LPL-associated genes (AKAP12, KANK2, $p<0.01$). FAM167A, a key molecule involved in non-canonical NF-κB signaling, was upregulated in patients who failed to obtain uMRD4 across both TN/RR cohorts ($p<0.01$).

**Summary/Conclusion**

Differences in peripheral immune populations and gene expression in patients who failed to achieve deep remission suggest a common resistance mechanism in both TN/RR cohorts. Targeting these universally shared pathways, especially in high-risk disease could result in improved disease control. Updated data will be presented at the meeting.

**Copyright Information:** (Online) ISSN: 2572-9241
©2026 The Author(s). HemaSphere is published by John Wiley & Sons Ltd on behalf of European Hematology Association. This is an open access Abstract Book distributed under the Attribution-NonCommercial-NoDerivs (CC BY-NC-ND), which allows third parties to download the articles and share them with others as long as they credit the author and the Abstract Book, but they cannot change the content in any way or use them commercially.
**Abstract Book Citations:** Authors, Title, HemaSphere, 2026;10:(S1):pages. The abstract book DOIs can be found on the “Author Information” page.
**Disclaimer:** Articles published in the journal HemaSphere exclusively reflect the opinions of the authors. The authors are responsible for all content in their abstracts including accuracy of the facts, statements, citing resources, etc.

### (PS1674) TRANSCRIPTIONAL AND IMMUNOGENETIC DIVERSITY IN STEREOTYPED CHRONIC LYMPHOCYTIC LEUKEMIA: INSIGHTS FROM SINGLE-CELL SEQUENCING

**Topic:** 05. Chronic lymphocytic leukemia and related disorders - Biology & translational research

Blaž Oder*[1], Leily Rabbani[1], Teresa del Peso Santos[1], Sameer Parikh[2], Karla Plevova[3, 4, 5], Larry Mansouri[1], Anastasia Chatzidimitriou[1, 6], Sarka Pospisilova[3, 4, 5], Neil Kay[2, 7], Kostas Stamatopoulos[1, 6, 8], Cecilia Österholm[1], Richard Rosenquist[1, 9]

*[1]Karolinska Institutet, Department of Molecular Medicine and Surgery, Stockholm, Sweden; [2]Mayo Clinic, Department of Internal Medicine, Division of Hematology, Rochester, MN, United States of America; [3]Masaryk University and University Hospital Brno, Medical Faculty, Department of Internal Medicine, Hematology and Oncology, Brno, Czechia; [4]Masaryk University and University Hospital Brno, Medical Faculty, Institute of Medical Genetics and Genomics, Brno, Czechia; [5]Masaryk University, Central European Institute of Technology, Brno, Czechia; [6]Centre for Research and Technology Hellas, Institute of Applied Biosciences, Thessaloniki, Greece; [7]Mayo Clinic, Department of Immunology, Rochester, MN, United States of America;[8]G. Papanicolaou Hospital, Hematology Department and HCT Unit, Thessaloniki, Greece; [9]Karolinska University Hospital, Clinical Genetics and Genomics, Stockholm, Sweden;*

### Background

Chronic lymphocytic leukemia (CLL) comprises immunogenetically defined stereotyped subsets characterized by distinct B-cell receptor immunoglobulin (BcR IG) features and divergent clinical outcomes. Subset #1 (IGHV1/5/7) is associated with unmutated IGHV genes and adverse outcome, subset #4 (IGHV4-34/IGKV2-30) represents indolent IgG-switched IGHV-mutated CLL, and subset #2 (IGHV3-21/IGLV3-21) exhibits frequent *SF3B1* mutations and poor prognosis independent of IGHV gene mutational status. Bulk transcriptomics has indicated subset-related differences, but failed to reveal intratumoral heterogeneity and rare cellular states.

### Aims

The study sought to define subset-specific transcriptional signatures and functional cellular states at single-cell resolution, and to assess how cellular heterogeneity and the tumor microenvironment contribute to the distinct disease behavior of subsets #1, #2, and #4.

### Methods

Integrated single-cell RNA and V(D)J sequencing (10x Genomics) was performed on 13 treatment-naïve primary CLL peripheral blood mononuclear cell samples representing subsets #1, #2, and #4. Analyses within the scverse framework included data integration, differential gene expression, gene set enrichment, trajectory inference, and immunogenetic analyses.

**Copyright Information:** (Online) ISSN: 2572-9241
©2026 The Author(s). HemaSphere is published by John Wiley & Sons Ltd on behalf of European Hematology Association. This is an open access Abstract Book distributed under the Attribution-NonCommercial-NoDerivs (CC BY-NC-ND), which allows third parties to download the articles and share them with others as long as they credit the author and the Abstract Book, but they cannot change the content in any way or use them commercially.
**Abstract Book Citations:** Authors, Title, HemaSphere, 2026;10:(S1):pages. The abstract book DOIs can be found on the "Author Information" page.
**Disclaimer:** Articles published in the journal HemaSphere exclusively reflect the opinions of the authors. The authors are responsible for all content in their abstracts including accuracy of the facts, statements, citing resources, etc.

**Results**

Profiling of 48,557 cells revealed a dominant leukemic B-cell compartment (91.7%), with >99.7% of B cells expressing subset-defining IG genes. Despite inter-patient variability, leukemic cells exhibited pronounced subset-specific features: subset #1 was enriched for hypoxia-related genes, subset #2 for oxidative phosphorylation (OXPHOS), MYC/E2F targets, fatty-acid metabolism, and mTORC1 signaling, whereas subset #4 showed negative enrichment of hypoxia, apoptosis, adipogenesis, and ROS pathways. Notably, a distinct metabolically active and recently emigrated/proliferative leukemic cell state, characterized by a $CXCR4^{dim}$ $CD5^{bright}$ transcriptional phenotype and coordinated enrichment of MYC targets, OXPHOS, mTORC1, and BcR signaling, was identified. "Active-state" cells were more prevalent in subsets #1 (8.7%; range, 4.2–18.3%) and #2 (14.6%; range, 9.5–28.0%) than subset #4 (2.5%; range, 0.5–6.3%). These cells displayed a conserved signature, including upregulation of metabolic and immune regulators (e.g., *ENO1*, *LDHA*, *LILRA4*, *LILRB4*). Immunogenetic analyses demonstrated dominant clonotypes in 99.7% of B cells with evidence of ongoing somatic hypermutation and intraclonal diversification in most cases (11/13). T-cell profiling revealed predominance of CD8+ effector memory (51.4%) and CD4+ central memory (30.7%) populations. CD8+ T cells formed a continuum toward terminal cytotoxic states accompanied by exhaustion-associated programs, with expanded T-cell clones largely restricted to terminal states. Ligand–receptor analyses further indicated co-stimulatory and inhibitory interactions between leukemic and immune cells.

**Summary/Conclusion**

This study identifies a metabolically and proliferatively active leukemic cell state preferentially expanded in clinically aggressive CLL subsets, suggesting a link between BcR IG features, cellular state composition, and disease behavior. Concurrent evidence of ongoing somatic hypermutation highlights continued intraclonal diversification in all subsets. Together, these findings offer an integrated view of the transcriptional and immunogenetic architecture of stereotyped CLL.

**Copyright Information:** (Online) ISSN: 2572-9241
©2026 The Author(s). HemaSphere is published by John Wiley & Sons Ltd on behalf of European Hematology Association. This is an open access Abstract Book distributed under the Attribution-NonCommercial-NoDerivs (CC BY-NC-ND), which allows third parties to download the articles and share them with others as long as they credit the author and the Abstract Book, but they cannot change the content in any way or use them commercially.
**Abstract Book Citations:** Authors, Title, HemaSphere, 2026;10:(S1):pages. The abstract book DOIs can be found on the "Author Information" page.
**Disclaimer:** Articles published in the journal HemaSphere exclusively reflect the opinions of the authors. The authors are responsible for all content in their abstracts including accuracy of the facts, statements, citing resources, etc.

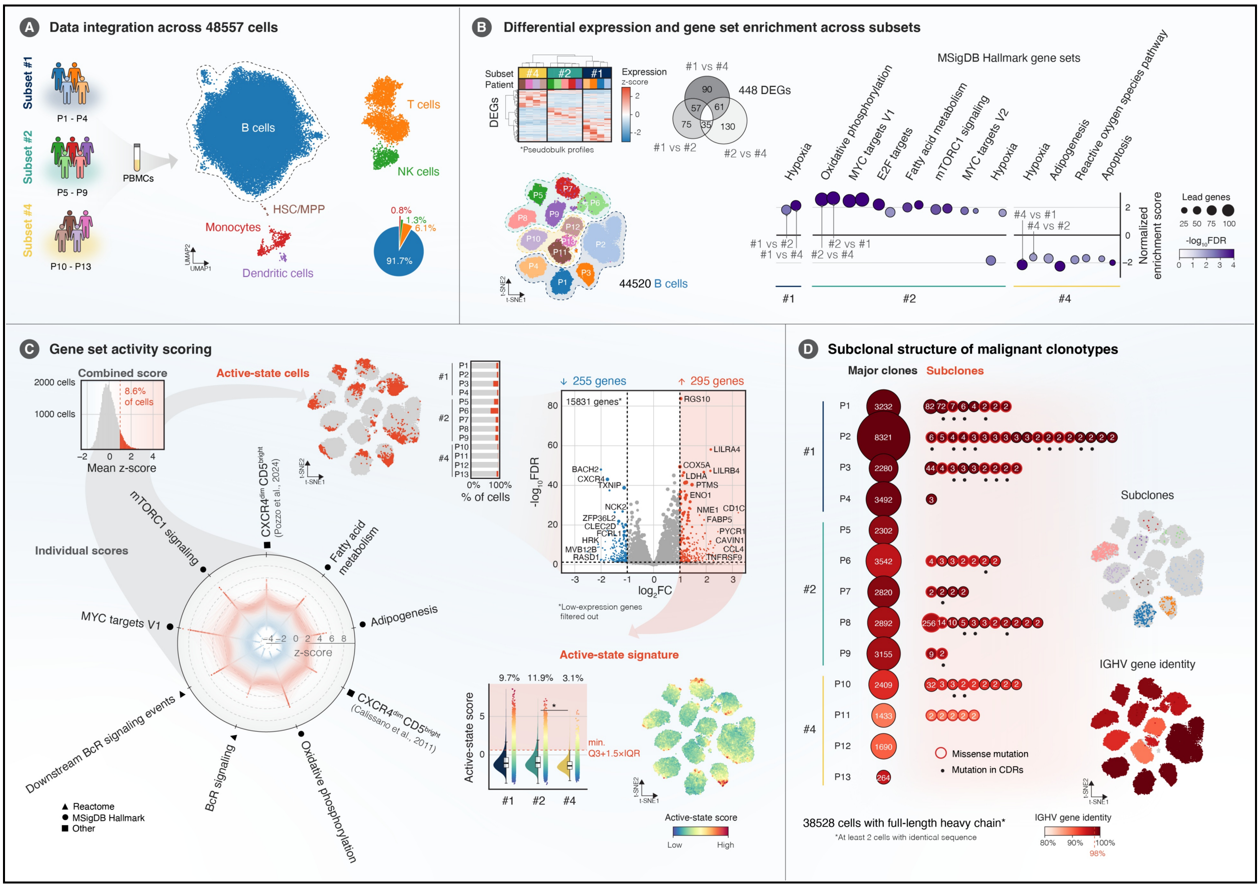

UMAP representation of 48,557 PBMCs integrated across three subsets (subset #1: P1–P4, subset #2: P5–P9, subset #4: P10–P13), with annotated immune populations (B cells, T cells, NK cells, monocytes, dendritic cells, and HSC/MPP) and their relative proportions shown in the pie chart. B. Subsetted analysis of 44,520 B cells displayed as an unintegrated t-SNE representation to retain patient-specific clustering, alongside differential gene expression (DGE) and gene set enrichment analyses (GSEA) across subsets. Hierarchical clustering of 448 unique differentially expressed genes (DEGs) identified in pairwise comparisons (#1 vs #2, #1 vs #4, and #2 vs #4) and DEG overlap are shown, with GSEA results summarized as a dot plot of normalized enrichment scores for pathways that were significant in both corresponding comparisons involving the same subset. C. Single-cell-based activity scoring for gene sets with the highest Pearson correlation, including Hallmark and Reactome pathways and recently emigrated/proliferative (CXCR4dim CD5bright) signatures (Calissano et al., Pozzo et al.). Radar plot displays z-score-normalized gene set scores, with points colored by their mean combined activity score. The distribution of combined scores is shown as a histogram, and cells with mean z-score >1 are classified as active-state cells. t-SNE representation highlights active-state cells, and the bar plot indicates their frequency per sample. DGE analysis comparing active-state versus remaining cells defines the transcriptional signature of the active-state population. Raincloud plot summarizes subset differences in active-state signature scores, with red shading denoting the proportion of cells exceeding the upper-whisker threshold (Q3+1.5×IQR) defined by the lowest such value across subsets, observed in subset #4. D. Subclonality analysis of malignant clonotypes based on full-length IGH nucleotide sequence identity. Circles represent major clones and their derived subclones, with size proportional to cell number and color indicating IGHV gene identity. Red outlines denote missense mutations, and black dots indicate mutations within CDR regions. t-SNE representations illustrate IGHV gene identity and the distribution of IGH subclones, colored by sample identity.

**Copyright Information:** (Online) ISSN: 2572-9241
©2026 The Author(s). HemaSphere is published by John Wiley & Sons Ltd on behalf of European Hematology Association. This is an open access Abstract Book distributed under the Attribution-NonCommercial-NoDerivs (CC BY-NC-ND), which allows third parties to download the articles and share them with others as long as they credit the author and the Abstract Book, but they cannot change the content in any way or use them commercially.
**Abstract Book Citations:** Authors, Title, HemaSphere, 2026;10:(S1):pages. The abstract book DOIs can be found on the "Author Information" page.
**Disclaimer:** Articles published in the journal HemaSphere exclusively reflect the opinions of the authors. The authors are responsible for all content in their abstracts including accuracy of the facts, statements, citing resources, etc.

### (PS1675) IL21-ARMORED CD72-TARGETING NANOBODY-BASED CAR-NK AS AN OFF-THE-SHELF, SAFE AND EFFECTIVE APPROACH TO ELIMINATE CHRONIC LYMPHOCYTIC LEUKEMIA AND RICHTER TRANSFORMATION CELLS

**Topic:** 05. Chronic lymphocytic leukemia and related disorders - Biology & translational research

Daniel Gil-Alós*[1, 2, 3, 4], Eva Castellano[1, 2, 3, 5], Almudena García-Ortiz[1, 2, 3, 5], Raquel Oliva[1, 2, 3, 5], Sandra López-Garcia[1, 2, 3], Rosa Mullor-Vigo[1, 2, 3], Lara Álvarez[5, 6, 7], Paula Pérez-Palomares[1, 2, 3], Fatima Miras Calvo[1, 4], Paula Río[5, 6, 7], Matthew Nix[8], Arun Wiita[8, 9, 10, 11], Joaquín Martinez-Lopez[1, 2, 3, 4, 5], Antonio Valeri[1, 2, 3, 5]

*[1]Hospital 12 de Octubre Research Institute (i+12), Department of Translational Hematology, Madrid, Spain;[2]Centro Nacional de Investigaciones Oncológicas (CNIO), Hematological Malignancies Clinical Research Unit H12O-CNIO, Madrid, Spain; [3]Instituto de Salud Carlos III, Centro de Investigación Biomédica en Red de Cáncer (CIBERONC), Madrid, Spain; [4]Hospital 12 de Octubre, Hematology Department, Madrid, Spain; [5]Instituto de Salud Carlos III, Red Española de Terapias Avanzadas (TERAV), Madrid, Spain; [6]Centro de Investigaciones Energéticas Medioambientales y Tecnológicas and Centro de Investigación Biomédica en Red de Enfermedades Raras (CIEMAT/CIBERER), Division of Hematopoietic Innovative Therapies, Madrid, Spain; [7]Instituto de Investigación Sanitaria Fundación Jiménez Díaz (IIS-FJD, UAM), Advanced Therapies Unit, Madrid, Spain; [8]University of California San Francisco (UCSF), Department of Laboratory Medicine, San Francisco (California), United States of America; [9]University of California San Francisco (UCSF), Department of Bioengineering and Therapeutic Sciences, San Francisco (California), United States of America; [10]Chan Zuckerberg Biohub, San Francisco (California), United States of America; [11]Parker Institute for Cancer Immunotherapy, San Francisco (California), United States of America;*

### Background

Double refractory Chronic Lymphocytic Leukemia (CLL) and Richter Transformation (RT) remain a major unmet clinical need. Despite the success of CAR-T therapy in other B-cell lymphoproliferative diseases, CD19 CAR-T cells have shown limited efficacy in CLL, partly due to the remarkable disease-related immune dysfunction, that also compromises product manufacturing. Allogeneic CAR-engineered natural killer (NK) cells represent an off-the-shelf alternative with preserved cytotoxic function in immunosuppressed hosts, a favorable safety profile, and improved manufacturability. CD72 expression has been consistently reported in B-ALL and other B-cell malignancies and its role as second CAR target after CD19 has been explored successfully in preclinical setting with T-cell platforms.

### Aims

To overcome the immunoeffector cell dysfunction in CLL, our aim was to develop a novel targeted allogeneic immunotherapy based on cytokine-improved CD72-CAR NK-cells combined with Antibody-Dependent Cellular Citotoxicity (ADCC)-mediating antibodies, to achieve a more cytotoxic, persistent, and safer immunotherapy.

### Methods

CD72 expression in tumor cells and healthy donor (HD) B cells was studied by flow cytometry (FCM) and plasma soluble CD72 (sCD72) was quantified by ELISA. We developed a lentiviral-transduced bicistronic anti-CD72 nanoCAR cord-blood NK product based on a VHH-derived second-generation 4-1BB CAR, co-expressing IL-21 (CAR72_21) and its monocistronic versions. Transduction efficiency was assessed by FCM and qPCR. IL21 secretion was measured by ELISA in supernatant. Cytotoxicity was evaluated by calcein-release assays at 3h after co-culture of CAR72_21 cells against tumoral cell lines and primary CLL samples, and by FCM analysis at 24h in the ADCC assay.

**Copyright Information:** (Online) ISSN: 2572-9241
©2026 The Author(s). HemaSphere is published by John Wiley & Sons Ltd on behalf of European Hematology Association. This is an open access Abstract Book distributed under the Attribution-NonCommercial-NoDerivs (CC BY-NC-ND), which allows third parties to download the articles and share them with others as long as they credit the author and the Abstract Book, but they cannot change the content in any way or use them commercially.
**Abstract Book Citations:** Authors, Title, HemaSphere, 2026;10:(S1):pages. The abstract book DOIs can be found on the "Author Information" page.
**Disclaimer:** Articles published in the journal HemaSphere exclusively reflect the opinions of the authors. The authors are responsible for all content in their abstracts including accuracy of the facts, statements, citing resources, etc.

**Results**

Seventy-three CLL patients were studied, all of them showing expression of CD72 in tumor cells and 93% of patients with >99% of cells positive for CD72, with a median RFI of 292.8 (IQR 163.7 – 421.3). There was no CD72 overexpression respect to HD B-cells. sCD72 was significantly increased in CLL patients (3.64 (±2.23) vs 0.14 (±0.07) ng/mL, p=0.0006).
CAR72_21 transduction efficiency was 38.1% (±9.9) with a vector copy number of 1.05 (±0.24) copies/cell at 7 days post-transduction. CAR72_21 supernatant analysis confirmed the presence of IL21 at 4.1 (±3) ng/ml. CAR72_21 outperformed untransduced (UT) cytotoxicity with 94.2% (±20.4) vs 65.5% (±14.5) against Raji cell line (p=0.0038, CAR72-21 vs UT, 4:1 effector:tumor (E:T) ratio) and 41.8% (±12.9) vs 22.4% (±4.6) in MEC2 cell line (p<0.0001, CAR72_21 vs UT, 4:1 E:T ratio). In addition, CAR72_21 tumor clearance against primary CLL cells was 50.8% (±7.2) vs 10.6% (±4.9) (p<0.0001, CAR72_21 vs UT, 2:1 E:T ratio) (Figure 1A). Furthermore, the combination of Obinutuzumab and CAR72_21 increased tumoral cell lysis to 91% (±4.6) compared with 41.8% (±17.2) for CAR72_21 alone and 34.8% (±17) for Obinutuzumab as single agent (Figure 1B), suggesting synergistic activity. No significant toxicity of CAR72_21 against HD PBMCs and no NK fratricide was observed.
We have developed Patient-Derived Xenograft-Human Immune System (PDX-HIS) model of CLL and a novel Cell Line-Derived Xenograft (CDX) model of RT. Safety and efficacy *in vivo* experiments are on-going.

**Summary/Conclusion**

CAR72_21 NK therapy has shown favorable in vitro data in terms of safety and efficacy to eliminate CLL cells. This off-the-shelf strategy represents a promising immunotherapeutic approach for double-refractory CLL and RT.

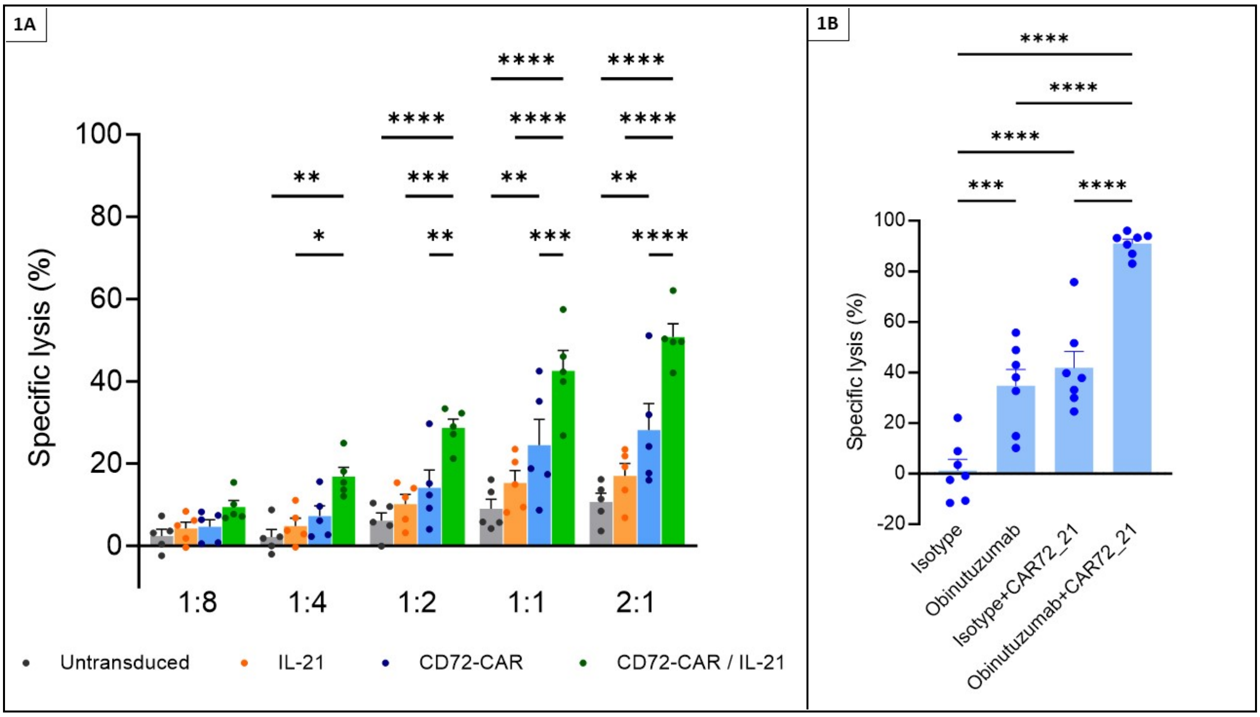

**Copyright Information:** (Online) ISSN: 2572-9241
©2026 The Author(s). HemaSphere is published by John Wiley & Sons Ltd on behalf of European Hematology Association. This is an open access Abstract Book distributed under the Attribution-NonCommercial-NoDerivs (CC BY-NC-ND), which allows third parties to download the articles and share them with others as long as they credit the author and the Abstract Book, but they cannot change the content in any way or use them commercially.
**Abstract Book Citations:** Authors, Title, HemaSphere, 2026;10:(S1):pages. The abstract book DOIs can be found on the "Author Information" page.
**Disclaimer:** Articles published in the journal HemaSphere exclusively reflect the opinions of the authors. The authors are responsible for all content in their abstracts including accuracy of the facts, statements, citing resources, etc.

### (PS1676) MECHANISMS OF RESISTANCE TO BTK INHIBITORS INDEPENDENT OF BTK AND PLCG2 MUTATIONS

**Topic:** 05. Chronic lymphocytic leukemia and related disorders - Biology & translational research

Wen Li*[1], Jialei Qi[1, 2], Rashmi Priyadharshini Dheenadayalan[1], Deyan Yosifov[1], Daniel Tews[3], Pamela Fischer-Posovszky[3], Christof Schneider[1], Eugen Tausch[1], Stephan Stilgenbauer[1], Billy Chelliah Jebaraj[1]

*[1]University Hospital Ulm, Department of Internal Medicine III, Ulm, Germany; [2]Medical School of Nanjing University, Department of Hematology, Affiliated Drum Tower Hospital, Nanjing, China; [3]University Hospital Ulm, Department of Pediatrics and Adolescent Medicine, Ulm, Germany;*

### Background

BTK inhibitor (BTKi) resistance has become a major clinical challenge, mainly due to acquired *BTK* and *PLCG2* mutations. However, clinically, some relapsed/refractory patients develop BTKi resistance without *BTK* or *PLCG2* mutations. Meanwhile, we reported REC-1 cells with acquired resistance to BTKi lacking *BTK* or *PLCG2* mutations (Qi J. et al. Blood Advance. 2023), and the underlying mechanisms are not fully resolved.

### Aims

To decipher mechanisms other than *BTK* or *PLCG2* mutations that drive resistance to BTKi, and to identify alternative treatment strategies.

### Methods

CellTiter Glo was used to assess cell viability. Western blotting (WB) was performed to evaluate protein expression. Targeted next generation sequencing (tNGS), whole-exome sequencing (WES) and RNA sequencing (RNA-seq) were used to identify genetic alterations and signaling pathway changes. Propidium iodide-based cell cycle analysis and annexin V/7-AAD analysis were performed using flow cytometry. Caspase-3/7 activity assay (Promega) was also used to measure apoptosis.

**Copyright Information:** (Online) ISSN: 2572-9241
©2026 The Author(s). HemaSphere is published by John Wiley & Sons Ltd on behalf of European Hematology Association. This is an open access Abstract Book distributed under the Attribution-NonCommercial-NoDerivs (CC BY-NC-ND), which allows third parties to download the articles and share them with others as long as they credit the author and the Abstract Book, but they cannot change the content in any way or use them commercially.
**Abstract Book Citations:** Authors, Title, HemaSphere, 2026;10:(S1):pages. The abstract book DOIs can be found on the "Author Information" page.
**Disclaimer:** Articles published in the journal HemaSphere exclusively reflect the opinions of the authors. The authors are responsible for all content in their abstracts including accuracy of the facts, statements, citing resources, etc.

**Results**

Seven lines of REC-1 cells that acquired resistance to pirtobrutinib or nemtabrutinib lacked *BTK, PLCG2* or other known resistance-associated mutations when analyzed by tNGS (Qi J. et al. Blood Advance. 2023) and WES. WB demonstrated that these cells had normal BCR signaling. Treatment with BTKis ibrutinib or nemtabrutinib, or BTK degrader (BTKd) NX-5948 resulted in suppression of BTK phosphorylation or caused BTK degradation, respectively. Both were capable of decreasing downstream PLCG2 and ERK signaling. However, these cells exhibited persistent cross-resistance to BTKi and BTKd, indicating potential BCR-independent resistance mechanisms driven by alternative pathways.
Transcriptomic analysis using RNA-seq revealed significant upregulation of OXPHOS, proteasome, and cell cycle pathways in resistant cells. Mitochondrial mass and extracellular flux assay using Agilent Seahorse showed only marginal changes in energy metabolism of BTKi resistant REC-1 cells compared to sensitive REC-1 cells. Hence, BTKi resistant REC-1 did not show significantly increased sensitivity to OXPHOS pathway inhibitor IACS-10759 either as single drug or in combination with BTKi/BTKd. The role of the proteasome pathway in BTKi resistance was previously reported by Arseni et al. (Nature Communications. 2023). Therefore, we focused on studying the role of dysregulated cell cycle pathways in BTKi resistance.
In our REC-1 BTKi resistance model, WB confirmed increased protein expression of CDK1 and cyclin A2, consistent with elevated mRNA observed in RNAseq. Flow cytometry demonstrated a distinct cell cycle distribution of BTKi resistant cells, characterized by an increased fraction of cells in the S-phase and a reduction in the G1 and G2/M phases compared to BTKi sensitive cells. In addition, the CDK inhibitor dinaciclib demonstrated efficacy as monotherapy in resistant cells, and its combination with the BTKd NX-5948 showed significant synergistic activity in both wild-type and resistant cells. caspase-3/7 activity assays and annexin V/7-AAD flow cytometry confirmed that the combination treatment of dinaciclib with BTKd resulted in synergistic increase in apoptosis.

**Summary/Conclusion**

Dysregulation of the cell cycle contributed to acquired BTKi and BTKd resistance in REC-1 cells in the absence of *BTK* or *PLCG2* mutation. Synergistic effects of CDK inhibitors with BTKd need to be further studied in patient samples and in vivo models.

**Copyright Information:** (Online) ISSN: 2572-9241
©2026 The Author(s). HemaSphere is published by John Wiley & Sons Ltd on behalf of European Hematology Association. This is an open access Abstract Book distributed under the Attribution-NonCommercial-NoDerivs (CC BY-NC-ND), which allows third parties to download the articles and share them with others as long as they credit the author and the Abstract Book, but they cannot change the content in any way or use them commercially.
**Abstract Book Citations:** Authors, Title, HemaSphere, 2026;10:(S1):pages. The abstract book DOIs can be found on the “Author Information” page.
**Disclaimer:** Articles published in the journal HemaSphere exclusively reflect the opinions of the authors. The authors are responsible for all content in their abstracts including accuracy of the facts, statements, citing resources, etc.

### (PS1677) MALT1 IS REQUIRED FOR IN VIVO CHRONIC LYMPHOCYTIC LEUKEMIA DEVELOPMENT

**Topic:** 05. Chronic lymphocytic leukemia and related disorders - Biology & translational research

Nakhle Saba*[1], Delia Carlino[2], Terri Rasmussen[2], Van Barnes[2], Jorge Belgodere[2], Matthew Burow[2], Adrian Wiestner[3]

*[1]Our Lady of the Lake Cancer Institute, School of Medicine - LSU, Baton Rouge, United States of America;[2]Tulane University, New Orleans, United States of America; [3]National Heart, Lung, and Blood Institute (NHLBI), National Institutes of Health (NIH), Bethesda, United States of America;*

### Background

Acquired resistance to Bruton's Tyrosine Kinase inhibitors (BTKi)—such as ibrutinib, acalabrutinib, and zanubrutinib—represents a significant clinical challenge in chronic lymphocytic leukemia (CLL). MALT1 connects antigen signaling to NF-κB activation. Because most known BTKi resistance mutations occur upstream of MALT1, targeting this protein offers a strategy to bypass the effects of *BTK* and *PLCG2* mutations at a critical downstream junction. Our group previously demonstrated that MALT1 targeting overcomes resistance *in vitro* in ibrutinib-resistant CLL cells, suggesting the MALT1 axis is pivotal for CLL progression and survival.

### Aims

Here, we investigated the requirement for Malt1 in CLL development by crossing the Eμ-TCL1 transgenic mouse model of spontaneous CLL with our lab generated CRISPR-mediated *Malt1* knockout mice (exon 2 deletion).

### Methods

We established four genotyped cohorts (n=25 per group): TCL1 transgenic mice with intact *Malt1* alleles (Eμ-TCL1, positive control), TCL1 mice with a single allele loss (Eμ-TCL1/*Malt1*+/-), TCL1 mice with biallelic loss (Eμ-TCL1/*Malt1*-/-), and *Malt1*-deficient mice without the TCL1 transgene (*Malt1*-/-, negative control). Ten mice per group were sacrificed at six months of age to quantify CLL burden; the remainder were monitored until the development of limb paralysis or other humane endpoints. CLL burden was assessed via spleen weight and flow cytometry (CD5+/CD19+ fraction) of bone marrow, spleen, and peritoneal fluid cells. Overall survival (OS) was defined as the time from birth to the development of limb paralysis or death.

### Results

Immunoblot analysis of cells from the peritoneum, spleen, and marrow confirmed that Malt1 protein was absent in the Eμ-TCL1/*Malt1*-/- group and significantly reduced in the Eμ-TCL1/*Malt1*+/- group compared to Eμ-TCL1 controls. Strikingly, while both Eμ-TCL1 and Eμ-TCL1/*Malt1*+/- cohorts developed CLL in all three compartments, the Eμ-TCL1/*Malt1*-/- mice showed no evidence of CLL (comparable to the negative control). These findings indicate that Malt1 is essential for leukemogenesis in this model. The Eμ-TCL1 group had the shortest median OS (266 days). This was significantly shorter than Eμ-TCL1*Malt1*-/- (310 days, $P$=0.046), and Eμ-TCL1/*Malt1*+/- (433 days, $P$<0.0001). Notably, the majority of mice Eμ-TCL1/*Malt1*-/- cohorts reached endpoints due to non-CLL complications—specifically severe dermatological lesions and ocular ulcers—rather than leukemia. Consequently, the Eμ-TCL1/*Malt1*+/- cohort achieved the longest median survival, benefiting from reduced leukemogenesis without the autoimmune-like toxicity associated with complete Malt1 ablation.
Eμ-TCL1 mice exhibited a higher burden than Eμ-TCL1/*Malt1*+/- mice at the six-month mark. However, at the time of euthanasia, the burden was lower in the Eμ-TCL1 group compared to the heterozygotes. This is likely explained by the significantly longer survival of the Eμ-TCL1/*Malt1*+/- cohort, which allowed more time for CLL cells to accumulate in tissues. This also suggests that reduced Malt1 levels result in less aggressive disease kinetics.

**Copyright Information:** (Online) ISSN: 2572-9241
©2026 The Author(s). HemaSphere is published by John Wiley & Sons Ltd on behalf of European Hematology Association. This is an open access Abstract Book distributed under the Attribution-NonCommercial-NoDerivs (CC BY-NC-ND), which allows third parties to download the articles and share them with others as long as they credit the author and the Abstract Book, but they cannot change the content in any way or use them commercially.
**Abstract Book Citations:** Authors, Title, HemaSphere, 2026;10:(S1):pages. The abstract book DOIs can be found on the "Author Information" page.
**Disclaimer:** Articles published in the journal HemaSphere exclusively reflect the opinions of the authors. The authors are responsible for all content in their abstracts including accuracy of the facts, statements, citing resources, etc.

**Summary/Conclusion**

In conclusion, Malt1 is critical for CLL leukemogenesis in Eμ-TCL1 mice. While complete *Malt1* knockout abrogates CLL development, it induces severe inflammatory complications (being currently investigated). Partial inhibition (heterozygosity) effectively delays progression and extends survival, highlighting a dose-dependent therapeutic window.

**Copyright Information:** (Online) ISSN: 2572-9241
©2026 The Author(s). HemaSphere is published by John Wiley & Sons Ltd on behalf of European Hematology Association. This is an open access Abstract Book distributed under the Attribution-NonCommercial-NoDerivs (CC BY-NC-ND), which allows third parties to download the articles and share them with others as long as they credit the author and the Abstract Book, but they cannot change the content in any way or use them commercially.
**Abstract Book Citations:** Authors, Title, HemaSphere, 2026;10:(S1):pages. The abstract book DOIs can be found on the "Author Information" page.
**Disclaimer:** Articles published in the journal HemaSphere exclusively reflect the opinions of the authors. The authors are responsible for all content in their abstracts including accuracy of the facts, statements, citing resources, etc.

### (PS1678) MATURE B-CELL NEOPLASMS ARE ASSOCIATED WITH A PROLONGED PRECURSOR PHASE CHARACTERIZED BY IGH GENE REPERTOIRE CLONALITY

**Topic:** 05. Chronic lymphocytic leukemia and related disorders - Biology & translational research

Pieter Martijn Kolijn*[1, 2], Christiane Pott[3], Florentin Späth[4], Joyce Schilperoord-Vermeulen[1], Magnus Hultdin[5], Sandrine Roulland[6], James D. McKay[7], Roel C.H. Vermeulen[2], Anton Langerak[1]

*[1]Erasmus MC, Immunology, Rotterdam, The Netherlands; [2]Utrecht University, Population Health Sciences, Utrecht, The Netherlands; [3]University Hospital Schleswig-Holstein, Second Medical Department, Kiel, Germany; [4]Umeå University, Department of Diagnostics and Intervention,, Umeå, Sweden; [5]Umeå University, Department of Medical Biosciences, Pathology, Umeå, Sweden; [6]Centre d'Immunologie de Marseille-Luminy, Aix-Marseille University, Centre National de la Recherche Scientifique, INSERM, Marseille, France; [7]International Agency for Research on Cancer (IARC) - World Health Organization, Lyon, France;*

### Background

Lymphoid malignancies comprise a heterogeneous group of cancers varying in etiology, incidence, and survival. Notably, several subtypes are preceded by precursor conditions detectable years before overt malignancy. Examples of precursor conditions include: monoclonal B cell lymphocytosis (MBL) preceding chronic lymphocytic leukemia (CLL), circulating t(14;18)-positive B-cells preceding follicular lymphoma (FL), monoclonal gammopathy of undetermined significance (MGUS) preceding multiple myeloma (MM). Studying the early development of lymphoid neoplasms holds the potential to reveal key insights into the ontogeny and etiology of these precursor conditions.
Previously, we described significant B-cell receptor immunoglobulin heavy chain (BcR IGH) gene repertoire clonality and clonotypic evolution up to 16 years before CLL diagnosis. However, it is currently unknown whether IGH gene repertoire clonality is also associated with other B-cell malignancies such as mantle cell lymphoma (MCL), FL, diffuse large B-cell lymphoma (DLBCL) and MM.

### Aims

The aim of our study was to investigate whether MCL, FL, DLCBL and MM development are associated with an extended precursor phase characterized by IGH gene repertoire clonality in the peripheral blood, as previously shown for CLL.

### Methods

In the current study, buffy coat samples from a total of 732 participants in the European Investigation into Cancer (EPIC) population cohort underwent IGH gene repertoire sequencing using a leader-based IGH assay on the Illumina MiSeq platform. The cohort included 104 controls, 135 DLBCL, 125 FL, 25 MCL and 101 MM cases. We additionally included 124 CLL cases and 118 controls from an earlier study. The timing of B-cell malignancy diagnosis ranged from 3 months to 19 years after initial blood sampling. The IGH gene repertoire was characterized using the ARResT/Interrogate immunoprofiler. Abundance ratio was calculated by dividing the abundance of the dominant clonotype by the mean of the abundance of the clonotypes ranked 3$^{rd}$-7$^{th}$. The cutoff for clonality was set at an abundance ratio of 10. We evaluated the association between clonality and lymphoid malignancy risk using Cox regression through the “survival” R package. Cox regression models were adjusted for age, sex and recruitment center.

**Copyright Information:** (Online) ISSN: 2572-9241
©2026 The Author(s). HemaSphere is published by John Wiley & Sons Ltd on behalf of European Hematology Association. This is an open access Abstract Book distributed under the Attribution-NonCommercial-NoDerivs (CC BY-NC-ND), which allows third parties to download the articles and share them with others as long as they credit the author and the Abstract Book, but they cannot change the content in any way or use them commercially.
**Abstract Book Citations:** Authors, Title, HemaSphere, 2026;10:(S1):pages. The abstract book DOIs can be found on the “Author Information” page.
**Disclaimer:** Articles published in the journal HemaSphere exclusively reflect the opinions of the authors. The authors are responsible for all content in their abstracts including accuracy of the facts, statements, citing resources, etc.

**Results**

IGH gene repertoire clonality was significantly associated with an increased risk of CLL (HR 8.7 [5.7-13.2]), MCL (HR 10 [3.5-28.1]), FL (HR 2.4 [1.4-4.2]) and DLBCL (HR 1.9 [1.04-3.3]) (Figure). IGH gene repertoire clonality was not significantly associated with an increased risk of MM (HR 1.4 [0.9-2.4]). IGH gene repertoire clonality was detected over 10 years prior to CLL, MCL, DLBCL and FL diagnosis. Notably, IGH gene repertoire clonality in the peripheral blood was more strongly associated with leukemic diseases like CLL and MCL, than nodal lymphomas such as DLBCL and FL. Half of clonal MCL cases presented with multiple dominant clonotypes with distinct IGHV/IGHJ gene usage and CDR3 regions, also known as bi- or oligoclonality, a feature that is exceedingly rare in CLL, FL and DLBCL.

**Summary/Conclusion**

IGH gene repertoire clonality is associated with an increased risk of CLL, MCL, FL and DLBCL development. Notably, IGH gene repertoire clonality in the peripheral blood is a stronger risk factor for leukemic diseases such as CLL and MCL, than for nodal lymphomas such as DLBCL and FL. Our findings suggest that, like CLL, MCL may be preceded by an extended precursor phase.

| Cancer | Clonality | N case | N control | Hazard ratio | | P-value |
|---|---|---|---|---|---|---|
| **CLL** | Not clonal | 36 | 214 | | Reference | |
| | Clonal | 88 | 8 | | 8.71 (5.73, 13.24) | <0.001 |
| **MCL** | Not clonal | 17 | 214 | | Reference | |
| | Clonal | 8 | 8 | | 9.95 (3.52, 28.14) | <0.001 |
| **FL** | Not clonal | 109 | 214 | | Reference | |
| | Clonal | 16 | 8 | | 2.43 (1.40, 4.24) | 0.002 |
| **DLBCL** | Not clonal | 118 | 214 | | Reference | |
| | Clonal | 17 | 8 | | 1.85 (1.04, 3.31) | 0.037 |
| **MM** | Not clonal | 92 | 214 | | Reference | |
| | Clonal | 9 | 8 | | 1.43 (0.86, 2.36) | 0.164 |

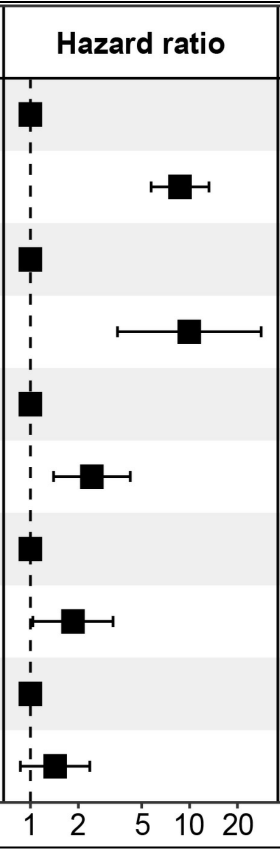

**Copyright Information:** (Online) ISSN: 2572-9241
©2026 The Author(s). HemaSphere is published by John Wiley & Sons Ltd on behalf of European Hematology Association. This is an open access Abstract Book distributed under the Attribution-NonCommercial-NoDerivs (CC BY-NC-ND), which allows third parties to download the articles and share them with others as long as they credit the author and the Abstract Book, but they cannot change the content in any way or use them commercially.
**Abstract Book Citations:** Authors, Title, HemaSphere, 2026;10:(S1):pages. The abstract book DOIs can be found on the "Author Information" page.
**Disclaimer:** Articles published in the journal HemaSphere exclusively reflect the opinions of the authors. The authors are responsible for all content in their abstracts including accuracy of the facts, statements, citing resources, etc.

### (PS1679) FIRST-IN-HUMAN IN VIVO CAR T CELL GENERATION USING A CD8-TARGETED LIPID NANOPARTICLE PLATFORM IN RELAPSED/REFRACTORY B-CELL LYMPHOMA

**Topic:** 05. Chronic lymphocytic leukemia and related disorders - Biology & translational research

Xi Zhang[1], Li Gao[1], Xixi Xiang[1], Ruihao Huang[1], Sha Zhou[1], Qu Jiang[1], Yunjing Zeng[1], Xianlan Zhao[1], Weitong Xu[2], Yuting He[2], Jun Liao[3], Hai Huang[3], Xue Li[3], Chutong Ouyang[3], Han Zheng[3], Yuquan Wei[2], Xiangrong Song*[2]

*[1]Xinqiao Hospital of Army Medical University, Chongqing, China;[2]West China Hospital, Sichuan University, Chengdu, China; [3]WestGene Biopharma Co., Ltd., Chengdu, China;*

### Background

Relapsed or refractory (R/R) B-cell lymphomas, including diffuse large B-cell lymphoma (DLBCL), mantle cell lymphoma (MCL), and follicular lymphoma (FL), remain associated with poor clinical outcomes despite advances in ex vivo–manufactured CAR T-cell therapies. However, complex manufacturing processes, treatment delays, and high costs limit the accessibility of conventional CAR T-cell therapy. In vivo generation of CAR T cells through targeted delivery platforms may provide a scalable alternative by enabling off-the-shelf and repeatable treatment approaches.

### Aims

This study aimed to evaluate the safety, pharmacodynamic activity, and preliminary clinical efficacy of a CD8-targeted lipid nanoparticle (CD8-tLNP) platform designed to generate CD19 CAR T cells directly in vivo in patients with R/R B-cell lymphoma.

### Methods

CD8-tLNPs were generated by conjugating an anti-CD8 VHH nanobody to lipid nanoparticles encapsulating mRNA encoding a CD19-directed chimeric antigen receptor.

Safety, tolerability, and pharmacodynamic activity were first evaluated in cynomolgus monkeys following intravenous administration. Subsequently, patients with R/R CD19$^+$ B-cell lymphoma received intravenous administration of CD19-CD8-tLNPs in multiple dosing cycles.

Peripheral blood samples were collected longitudinally to assess CAR expression in CD8$^+$ T cells, B-cell depletion, cytokine profiles, and laboratory safety parameters. Antitumor activity was evaluated using PET-CT imaging. Adverse events were graded according to standard clinical criteria.

**Copyright Information:** (Online) ISSN: 2572-9241
©2026 The Author(s). HemaSphere is published by John Wiley & Sons Ltd on behalf of European Hematology Association. This is an open access Abstract Book distributed under the Attribution-NonCommercial-NoDerivs (CC BY-NC-ND), which allows third parties to download the articles and share them with others as long as they credit the author and the Abstract Book, but they cannot change the content in any way or use them commercially.
**Abstract Book Citations:** Authors, Title, HemaSphere, 2026;10:(S1):pages. The abstract book DOIs can be found on the “Author Information” page.
**Disclaimer:** Articles published in the journal HemaSphere exclusively reflect the opinions of the authors. The authors are responsible for all content in their abstracts including accuracy of the facts, statements, citing resources, etc.

**Results**

Preclinical evaluation in non-human primates demonstrated favorable safety and pharmacodynamic activity, supporting clinical translation.

To date, seven patients with R/R CD19⁺ B-cell lymphoma have received treatment. Among evaluable patients, two achieved partial remission with reduction of tumor lesions, and one patient demonstrated stable disease at day 28, while four patients remain under ongoing efficacy evaluation.

Following administration, CAR expression in peripheral CD8⁺ T cells peaked within 4–6 hours and declined thereafter, consistent with transient in vivo CAR T-cell generation. Rapid and near-complete peripheral B-cell depletion was observed, with complete depletion (<1 cell/µL) occurring between 12 hours and day 3. Repeated dosing at 3-day intervals maintained sustained B-cell depletion and pharmacodynamic activity.

Treatment was generally well tolerated. No grade ≥2 cytokine release syndrome was observed. Transient increases in inflammatory markers, including C-reactive protein and ferritin, were manageable and not associated with clinically significant hepatotoxicity or prolonged cytopenias. In two patients, B-cell reconstitution with a predominantly naïve phenotype was observed by day 32 and day 47, respectively.

**Summary/Conclusion**

This first-in-human study demonstrates the feasibility of generating functional CAR T cells in vivo using a CD8-targeted lipid nanoparticle platform in patients with R/R B-cell lymphoma. The approach enables rapid and transient CAR expression, repeatable dosing, and a favorable safety profile without the need for ex vivo manufacturing or lymphodepletion. These findings provide clinical proof of concept supporting further development of scalable in vivo CAR T-cell therapies.

**Copyright Information:** (Online) ISSN: 2572-9241
©2026 The Author(s). HemaSphere is published by John Wiley & Sons Ltd on behalf of European Hematology Association. This is an open access Abstract Book distributed under the Attribution-NonCommercial-NoDerivs (CC BY-NC-ND), which allows third parties to download the articles and share them with others as long as they credit the author and the Abstract Book, but they cannot change the content in any way or use them commercially.
**Abstract Book Citations:** Authors, Title, HemaSphere, 2026;10:(S1):pages. The abstract book DOIs can be found on the "Author Information" page.
**Disclaimer:** Articles published in the journal HemaSphere exclusively reflect the opinions of the authors. The authors are responsible for all content in their abstracts including accuracy of the facts, statements, citing resources, etc.

### (PS1680) CD20×CD3 BISPECIFIC ANTIBODY ENHANCES AND EXTENDS IBRUTINIB-MEDIATED DISEASE CONTROL IN CLL IN 3D CULTURE MODELS AND IN MICE

**Topic:** 05. Chronic lymphocytic leukemia and related disorders - Biology & translational research

Cèlia Dobaño-López*[1], Annika Fitz[1], Alessia Floerchinger[1], Hannah Briesch[1], Martina Seiffert[1]

[1]*German Cancer Research Center (DKFZ), Immunology, Infection and Cancer, Heidelberg, Germany;*

### Background

Continuous ibrutinib therapy provides durable disease control in chronic lymphocytic leukemia (CLL) but rarely induces complete remissions and is limited by resistance. As ibrutinib has been shown to overcome CLL-associated T-cell dysfunction, combining it with a CD20×CD3 bispecific antibody (BsAb) may enhance anti-leukemic efficacy and prolong response.

### Aims

We therefore evaluated whether combining ibrutinib with a CD20×CD3 BsAb enhances anti-leukemic efficacy and extends disease control in CLL.

### Methods

Therapeutic efficacy was assessed in the Eμ-TCL1 mouse model across three independent experiments per treatment setting (3–10 mice per treatment arm). In parallel, patient-derived 3D lymph node–mimicking cultures (n=5 patients) were used to evaluate treatment effects on viable CLL cells and autologous T-cell–mediated cytotoxicity. Ongoing high-dimensional phenotyping using a 34-color spectral flow cytometry panel aims to further define T-cell functional states under combination therapy.

### Results

In the Eμ-TCL1 mouse model, the addition of BsAb to ibrutinib significantly improved CLL clearance and prolonged disease control compared with either monotherapy. BsAb enhanced responses both when administered concomitantly with ibrutinib and when introduced after initial ibrutinib treatment (Figure 1A), indicating extension of ibrutinib-mediated control of CLL in vivo. Ongoing studies are evaluating whether BsAb addition enables ibrutinib treatment interruptions while maintaining disease control. Consistently, in patient-derived 3D lymph node–mimicking cultures, the combination of ibrutinib with BsAb induced greater reduction of viable CLL cells and increased autologous T-cell–mediated cytotoxicity than either agent alone after 3 and 6 days (Figure 1B). Importantly, ibrutinib did not impair BsAb activity; instead, combination treatment was associated with enhanced T-cell activation and effector responses.

### Summary/Conclusion

Together, these data demonstrate that CD20×CD3 BsAb potentiates and prolongs ibrutinib responses while ibrutinib supports BsAb-driven T-cell function. The cooperative activity observed across in vivo and patient-relevant in vitro models supports combined therapy as a strategy to deepen responses and suggests the feasibility of fixed-duration ibrutinib treatment with sustained disease control in CLL.

**Copyright Information:** (Online) ISSN: 2572-9241
©2026 The Author(s). HemaSphere is published by John Wiley & Sons Ltd on behalf of European Hematology Association. This is an open access Abstract Book distributed under the Attribution-NonCommercial-NoDerivs (CC BY-NC-ND), which allows third parties to download the articles and share them with others as long as they credit the author and the Abstract Book, but they cannot change the content in any way or use them commercially.
**Abstract Book Citations:** Authors, Title, HemaSphere, 2026;10:(S1):pages. The abstract book DOIs can be found on the “Author Information” page.
**Disclaimer:** Articles published in the journal HemaSphere exclusively reflect the opinions of the authors. The authors are responsible for all content in their abstracts including accuracy of the facts, statements, citing resources, etc.

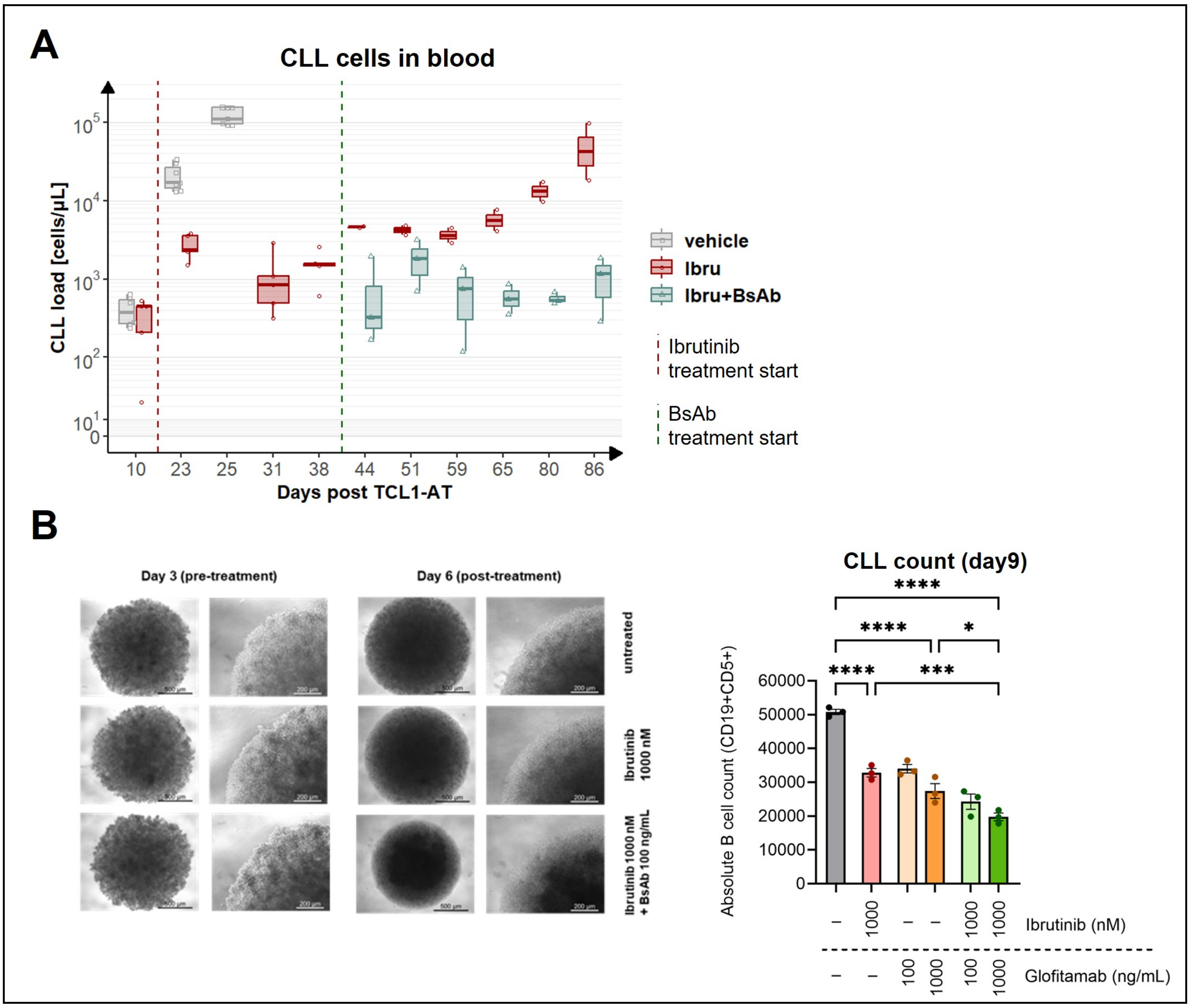

Figure 1. Ibrutinib and bsAb treatments in CLL. A) CD19+CD5+ (CLL cells) cell count/uL measured in blood overtime using the Eµ-TCL1 AT mouse model. Red line indicates the ibrutinib treatment start. Green line indicates the BsAb treatment start. B) Bright-field images of a patient-derived spheroid at day 3 (pre-treatment) and at day 6 (after 3 days of treatment) (left). CD19+CD5+ (CLL cells) absolute count under control (DMSO), ibrutinib, bsAb (Glofitamab) and the combination after 6 days of treatment (day9) (right). *p<0.05; **p<0.01; ***p<0.001; ****p<0.0001.

**Copyright Information:** (Online) ISSN: 2572-9241
©2026 The Author(s). HemaSphere is published by John Wiley & Sons Ltd on behalf of European Hematology Association. This is an open access Abstract Book distributed under the Attribution-NonCommercial-NoDerivs (CC BY-NC-ND), which allows third parties to download the articles and share them with others as long as they credit the author and the Abstract Book, but they cannot change the content in any way or use them commercially.
**Abstract Book Citations:** Authors, Title, HemaSphere, 2026;10:(S1):pages. The abstract book DOIs can be found on the “Author Information” page.
**Disclaimer:** Articles published in the journal HemaSphere exclusively reflect the opinions of the authors. The authors are responsible for all content in their abstracts including accuracy of the facts, statements, citing resources, etc.

### (PS1681) SPATIAL TRANSCRIPTOMICS UNCOVERS A BONE MARROW NICHE SUPPORTING BI-DIRECTIONAL CROSSTALK BETWEEN MONOCYTES/MACROPHAGES AND T-CELL LARGE GRANULAR LYMPHOCYTIC LEUKEMIA CELLS

**Topic:** 05. Chronic lymphocytic leukemia and related disorders - Biology & translational research

Mischa Vervoordeldonk*[1], Marnix Koops[2], Thierry van den Bosch[3], King Lam[3], Lisanne Noordam[3], Tom Cupedo[2], Mark-David Levin[4], Anton Langerak[1]

*[1]Erasmus MC, Immunology, Rotterdam, The Netherlands;[2]Erasmus MC, Hematology, Rotterdam, The Netherlands;[3]Erasmus MC, Pathology, Rotterdam, The Netherlands; [4]Albert Schweitzer Hospital, Internal Medicine, Dordrecht, The Netherlands;*

### Background

T-cell large granular lymphocytic leukemia (T-LGLL) is characterized by clonal expansion of cytotoxic T-cells. It is unknown which proliferative niche supports T-LGLL expansion before the leukemic T-cells are released into the circulation. The bone marrow (BM) is a compelling candidate for such a niche in T-LGLL, as BM infiltration is present in nearly all T-LGLL patients, and disturbance of normal blood cell production is a common clinical feature.

### Aims

Here we aimed to characterize the BM environment of T-LGLL in more detail.

### Methods

First, data was resegmented using Baysor, a tool which considers the likelihood of transcriptional composition, size and shape of a cell, to improve transcript-to-cell assignment. Next, transcripts with <99% confidence score, and all cells with ≤20 features, were removed. Finally, after quality control, 851,632 high-quality cells were retained and analyzed using a standard Seurat workflow. We were able to detect structural cell types (stromal, endothelial, and adipocytic cells) immune cell types (T-cells, B-cells, plasma cells, monocytes, macrophages, mast cells, and neutrophils), as well as erythrocytes and megakaryocytes. Ligand-receptor interactions were assessed using SpaTalk and CellChat; both methods estimate the probability of signaling between cells based on the co-expression of ligands and receptors, and in the case of SpaTalk, also intracellular propagators, while simultaneously integrating spatial distances of cells. Cellular niches were identified using BANKSY, a clustering tool that combines the features of each cell with the average of the features of its spatial neighbors.

### Results

T-LGLL samples showed a median of 6.8% T-cells (IQR; 5.4% – 9.9%). We observed an increase of monocytes/macrophages in the BM of T-LGLL patients compared to controls ($p = 0.04$; logFC = 1.8). According to spatially-aware ligand-receptor interaction analysis, interactions of T-LGLL cells were mostly with monocytes/macrophages. One of the top hits, IFNγ, produced by T-LGLL cells, may activate monocytes/macrophages, as seen by high intracellular expression of STAT1. This activation then promotes the secretion of IL-15, for which the ligand-receptor interaction was also enriched and which is a known key cytokine for T-LGLL cell survival. Of note, chemokine receptor signaling was also enriched, and the signaling through CCR1 and CCR5 suggests active attraction of these two populations. To validate this finding, we performed spatially aware clustering analysis and identified a distinct niche, termed CN9, enriched for T-LGLL cells and monocytes/macrophages, showing that these two populations do actually co-localize. This niche also captures the observed IFNγ, IL-15, and chemokine receptor signaling interactions (**Figure 1**).

**Copyright Information:** (Online) ISSN: 2572-9241
©2026 The Author(s). HemaSphere is published by John Wiley & Sons Ltd on behalf of European Hematology Association. This is an open access Abstract Book distributed under the Attribution-NonCommercial-NoDerivs (CC BY-NC-ND), which allows third parties to download the articles and share them with others as long as they credit the author and the Abstract Book, but they cannot change the content in any way or use them commercially.
**Abstract Book Citations:** Authors, Title, HemaSphere, 2026;10:(S1):pages. The abstract book DOIs can be found on the “Author Information” page.
**Disclaimer:** Articles published in the journal HemaSphere exclusively reflect the opinions of the authors. The authors are responsible for all content in their abstracts including accuracy of the facts, statements, citing resources, etc.

### Summary/Conclusion

This work identifies monocyte/macrophage–T-LGLL crosstalk in the BM as a mechanistically grounded and potentially targetable vulnerability in a disease with limited therapeutic options.

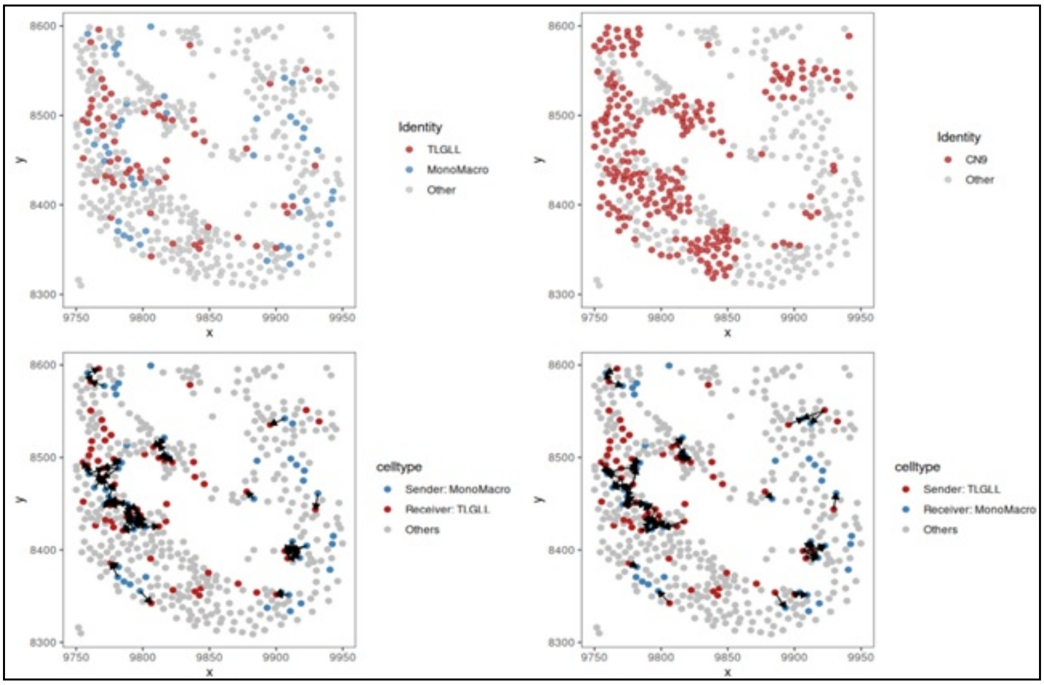

Figure 1: Co-localization of T-LGLL cells (red) and monocytes/macrophages (blue) in the BM of a representative patient (top-left). Spatial distribution of CN9, which captures the co-localization of T-LGLL cells and monocytes/macrophages (top-right). Ligand-receptor signaling between T-LGLL cells and monocytes/macrophages (bottom-left and bottom-right). Arrows indicate direction of communication between two interacting cells. Dots represent cell-centroids. X-axis and Y-axis are x- and y-coordinates in µm.

**Copyright Information:** (Online) ISSN: 2572-9241
©2026 The Author(s). HemaSphere is published by John Wiley & Sons Ltd on behalf of European Hematology Association. This is an open access Abstract Book distributed under the Attribution-NonCommercial-NoDerivs (CC BY-NC-ND), which allows third parties to download the articles and share them with others as long as they credit the author and the Abstract Book, but they cannot change the content in any way or use them commercially.
**Abstract Book Citations:** Authors, Title, HemaSphere, 2026;10:(S1):pages. The abstract book DOIs can be found on the "Author Information" page.
**Disclaimer:** Articles published in the journal HemaSphere exclusively reflect the opinions of the authors. The authors are responsible for all content in their abstracts including accuracy of the facts, statements, citing resources, etc.

### (PS1682) ZNF292 MUTATIONS IMPACT CELL CYCLE DYNAMICS AND COMPROMISE REPLICATION STRESS RESPONSE, DEFINING A POOR PROGNOSTIC SUBGROUP IN CHRONIC LYMPHOCYTIC LEUKEMIA

**Topic:** 05. Chronic lymphocytic leukemia and related disorders - Biology & translational research

Alberto Rodríguez-Sánchez*[1], María Alonso Gago[1], Claudia Perez-Carretero[2], Luis A Corchete[3], Cristina Miguel[1], Sandra Santos[1], Angela Villaverde Rimero[1], Giorgia Simonetti[4], Federico Lo Schiavo[4], Ana Rodríguez-Vicente[1], Jesus Maria Hernandez Rivas[1], Miguel Quijada-Álamo[5]

*[1] 1. University of Salamanca, IBSAL, IBMCC, CSIC, Cancer Research Center, Salamanca, Spain 2. Department of Hematology, University Hospital of Salamanca, Salamanca, Spain, SALAMANCA, Spain; [2] Translational Research Centre in Onco-hematology, Faculty of Medicine, University of Geneva, and Swiss Cancer Center Leman, Geneva, Switzerland., Geneva, Switzerland; [3] 1. Massachusetts General Hospital Center for Cancer Research, Charlestown, MA 02129 2. Harvard Medical School, Boston, MA 02215. 3. Broad Institute of MIT and Harvard, Cambridge, MA 02139., Boston, United States of America; [4] Translational Hematology Unit - Biosciences Laboratory, IRCCS Istituto Romagnolo per lo Studio dei Tumori (IRST) "Dino Amadori", Meldola, Italy; [5] 1. University of Salamanca, IBSAL, IBMCC, CSIC, Cancer Research Center, Salamanca, Spain 2. Department of Oncological Sciences, Icahn School of Medicine at Mount Sinai, New York, USA, New York, United States of America;*

### Background

The mutational landscape of chronic lymphocytic leukemia (CLL) is characterized by a “long tail” of low-frequency driver genes (<5%). *ZNF292*, a zinc-finger transcription factor, is among these recurrently mutated genes, occurring in ~3% of patients at diagnosis. However, the clinical and biological impact of this gene are not fully understood.

### Aims

To determine the prognostic relevance and functional implications of *ZNF292* alterations in CLL.

### Methods

We performed targeted Next-Generation Sequencing (54 CLL-related genes) on a cohort of 488 CLL patients. Additionally, we generated isogenic CLL cellular models (PGA1 cells) using CRISPR/Cas9 to introduce *ZNF292* mutations (*ZNF292*$^{\mathrm{MUT}}$). These cells, alongside wild-type counterparts (*ZNF292*$^{\mathrm{WT}}$), were evaluated using RNA-sequencing and subsequent functional assays.

**Copyright Information:** (Online) ISSN: 2572-9241
©2026 The Author(s). HemaSphere is published by John Wiley & Sons Ltd on behalf of European Hematology Association. This is an open access Abstract Book distributed under the Attribution-NonCommercial-NoDerivs (CC BY-NC-ND), which allows third parties to download the articles and share them with others as long as they credit the author and the Abstract Book, but they cannot change the content in any way or use them commercially.
**Abstract Book Citations:** Authors, Title, HemaSphere, 2026;10:(S1):pages. The abstract book DOIs can be found on the “Author Information” page.
**Disclaimer:** Articles published in the journal HemaSphere exclusively reflect the opinions of the authors. The authors are responsible for all content in their abstracts including accuracy of the facts, statements, citing resources, etc.

**Results**

We detected 27 *ZNF292* mutations in 27 of 488 patients (5.5%). Of these, 75% were truncating variants, primarily frameshift. Although these variants were broadly distributed throughout the coding region, a recurrent p.T2053fs variant was identified in 6 cases. *ZNF292*$^{MUT}$ cases were significantly associated with concurrent *NOTCH1* mutations (41%, p<0.001). Clinically, *ZNF292* mutations correlated with a shorter time to first treatment (TTFT, median 30 vs. 52 months, p=0.028) and reduced overall survival (median 93 vs. 150 months, p=0.016). Notably, these variants retained prognostic value in early-stage disease (Rai 0/1), exhibiting a significantly shorter TTFT (median 44 vs. 90 months; p=0.016), and refining risk stratification for these patients. In multivariate analysis adjusted for IGHV status, Rai stage and frequent co-mutations, *ZNF292* alterations (mutations and 6q deletion) constituted an independent prognostic factor for TTFT.

RNA-sequencing of cellular models revealed that *ZNF292*$^{MUT}$ cells exhibit downregulation in pathways related to protein translation, cell cycle regulation, DNA damage response and replication stress. Functional assays validated a significant reduction in protein synthesis efficiency in *ZNF292*$^{MUT}$ cells (p<0.01). Furthermore, *ZNF292* disruption altered cell cycle dynamics, leading to a higher accumulation of *ZNF292*$^{MUT}$ cells in the S-phase compared with *ZNF292*$^{WT}$ (29% vs. 26%, p<0.05). To functionally evaluate the response to replication stress, we exposed cells to camptothecin, which induces replication-dependent DNA breaks during the S-phase. Following this targeted damage, *ZNF292*$^{MUT}$ cells exhibited higher expression of phospho-RPA32 (mean *foci* 33 vs. 24, p<0.001), a hallmark of replication stress, as well as reduced CHK1 activation and increased overall DNA damage (p<0.05). Therapeutically, *ZNF292*$^{MUT}$ cells displayed heightened sensitivity to venetoclax (IC50: 0.12 vs. 0.78 µM) and to the CDK4/6 inhibitor palbociclib (IC50: 2 vs. 3.5 µM).

**Summary/Conclusion**

*ZNF292* alterations in CLL are predominantly loss-of-function variants that associate with *NOTCH1* mutations and define an independent prognostic marker for shorter TTFT. Functionally, *ZNF292* mutations alter cell cycle progression and compromise the response to replication stress. The increased *in vitro* sensitivity to venetoclax suggests that BCL2 inhibition represent a potential therapeutic strategy for *ZNF292*$^{MUT}$ patients.

**Copyright Information:** (Online) ISSN: 2572-9241
©2026 The Author(s). HemaSphere is published by John Wiley & Sons Ltd on behalf of European Hematology Association. This is an open access Abstract Book distributed under the Attribution-NonCommercial-NoDerivs (CC BY-NC-ND), which allows third parties to download the articles and share them with others as long as they credit the author and the Abstract Book, but they cannot change the content in any way or use them commercially.
**Abstract Book Citations:** Authors, Title, HemaSphere, 2026;10:(S1):pages. The abstract book DOIs can be found on the “Author Information” page.
**Disclaimer:** Articles published in the journal HemaSphere exclusively reflect the opinions of the authors. The authors are responsible for all content in their abstracts including accuracy of the facts, statements, citing resources, etc.

### (PS1683) TARGETING THE FATTY ACID TRANSPORTER SLC27A1 IMPAIRS METABOLIC FITNESS AND ENHANCES DRUG SENSITIVITY IN CHRONIC LYMPHOCYTIC LEUKEMIA

**Topic:** 05. Chronic lymphocytic leukemia and related disorders - Biology & translational research

Patrycja Tarkowska*[1, 2], Manuel Knauer[1, 2], Mohamed Elbahloul[1, 2], Wei Chen[3], Anna Herr[2, 4], Lena Osswald[1, 2, 5], Maximilian Kistner[2, 4], Alessandro Baldi[6], Torsten Haferlach[6], Marc Schmidt-Supprian[2, 5], Julia Jellusova[2, 4], Nicole Strittmatter[3], Florian Bassermann[1, 2], Maike Buchner[2, 4], Ruth Eichner[1, 2]

*[1]Department of Medicine III, TUM University Hospital, TUM School of Medicine and Health, Technical University of Munich, Munich, Germany; [2]TranslaTUM, Center for Translational Cancer Research, Technical University of Munich, Munich, Germany; [3]Department of Bioscience, TUM School of Natural Sciences, Technical University of Munich, Munich, Germany;[4]Institute of Clinical Chemistry and Pathobiochemistry, TUM School of Medicine and Health, Technical University of Munich, Munich, Germany; [5]Institute for Experimental Haematology, TUM School of Medicine and Health, Technical University of Munich, Munich, Germany; [6]MLL Munich Leukemia Laboratory, Munich, Germany;*

### Background

Chronic lymphocytic leukemia (CLL) is the most common leukemia in adults with a particularly high incidence in the Western world. Epidemiologic data indicate that CLL incidence is associated with dyslipidemia and inversely correlated with lipid-lowering therapy, implicating lipid metabolism in disease pathogenesis. Consistently, CLL cells exhibit enhanced lipid metabolic activity compared with normal B cells, suggesting metabolic dependencies that may be therapeutically exploitable. Transcriptomic analysis revealed significant overexpression of the fatty acid transporter SLC27A1 in CLL patient samples compared with healthy individuals, with the highest expression in high-risk disease subsets. We therefore hypothesized that SLC27A1, previously implicated in aggressive lymphoma and colorectal cancer, plays a critical role in CLL progression.

### Aims

This study aims to define the functional role of SLC27A1 in CLL and evaluate its therapeutic targetability as monotherapy and in combination with established CLL therapies.

### Methods

Single-cell SLC27A1 knockout clones were generated in the OSU CLL cell line by lentiviral sgRNA transduction and single-cell seeding. Pharmacological inhibition was achieved using arylpiperazine 5k. Peripheral blood mononuclear cells from CLL patients and healthy donors were co-cultured with CD40L/IL-21-expressing YK6 stromal cells to model the lymph node microenvironment. Fatty acid uptake and intracellular neutral lipids were quantified by flow cytometry using BODIPY FL C16 and BODIPY 493/503. Proliferation was assessed by CellTiter-Glo and CellTrace assays, and cell death by LIVE/DEAD staining. Mitochondrial respiration was measured using Seahorse Assays, and metabolite composition was assessed by desorption electrospray ionization mass spectrometry (DESI-MS). Finally, CLL cells were treated with arylpiperazine 5k combined with ibrutinib or venetoclax to evaluate drug responses and synergy.

**Copyright Information:** (Online) ISSN: 2572-9241
©2026 The Author(s). HemaSphere is published by John Wiley & Sons Ltd on behalf of European Hematology Association. This is an open access Abstract Book distributed under the Attribution-NonCommercial-NoDerivs (CC BY-NC-ND), which allows third parties to download the articles and share them with others as long as they credit the author and the Abstract Book, but they cannot change the content in any way or use them commercially.
**Abstract Book Citations:** Authors, Title, HemaSphere, 2026;10:(S1):pages. The abstract book DOIs can be found on the "Author Information" page.
**Disclaimer:** Articles published in the journal HemaSphere exclusively reflect the opinions of the authors. The authors are responsible for all content in their abstracts including accuracy of the facts, statements, citing resources, etc.

**Results**

SLC27A1 knockout in the OSU CLL cell line reduced fatty acid uptake and neutral lipid storage, resulting in a marked proliferative deficit. Similar effects were observed upon pharmacological inhibition with arylpiperazine 5k. SLC27A1-deficient cells showed decreased oxygen consumption and ATP production, indicating reduced metabolic activity. DESI-MS metabolomic analysis further demonstrated a broad reduction in intracellular fatty acids as well as tricarboxylic acid cycle intermediates, consistent with impaired oxidative metabolism. In primary cell models, arylpiperazine 5k significantly impaired primary CLL cell proliferation compared with healthy B cells in YK6 co-culture mimicking the lymph node niche, demonstrating a CLL-specific metabolic dependency. Finally, arylpiperazine 5k synergized with ibrutinib and venetoclax in CLL cell lines and primary cells.

**Summary/Conclusion**

In line with transcriptomic data, we identify SLC27A1 as a key mediator of fatty acid uptake and metabolic fitness in CLL. Targeting SLC27A1 disrupts lipid utilization, impairs leukemic cell proliferation, and enhances sensitivity to ibrutinib and venetoclax. These findings position SLC27A1 as a promising metabolic vulnerability and therapeutic target in CLL.

**Copyright Information:** (Online) ISSN: 2572-9241
©2026 The Author(s). HemaSphere is published by John Wiley & Sons Ltd on behalf of European Hematology Association. This is an open access Abstract Book distributed under the Attribution-NonCommercial-NoDerivs (CC BY-NC-ND), which allows third parties to download the articles and share them with others as long as they credit the author and the Abstract Book, but they cannot change the content in any way or use them commercially.
**Abstract Book Citations:** Authors, Title, HemaSphere, 2026;10:(S1):pages. The abstract book DOIs can be found on the "Author Information" page.
**Disclaimer:** Articles published in the journal HemaSphere exclusively reflect the opinions of the authors. The authors are responsible for all content in their abstracts including accuracy of the facts, statements, citing resources, etc.

### (PS1684) RESTORATION OF MIR-146B IN T-CELL LARGE GRANULAR LYMPHOCYTE LEUKEMIA (T-LGLL): SELECTIVE MOLECULAR REPROGRAMMING TOWARD AN RNA-BASED TARGETED THERAPEUTIC STRATEGY

**Topic:** 05. Chronic lymphocytic leukemia and related disorders - Biology & translational research

Elisa Rampazzo*[1, 2], Antonella Teramo[1, 2], Eleonora Roncaglia[3], Barbara Mariotti[4], Giulia Calabretto[1, 2], Elena Buson[1, 2], Enrico Gaffo[5], Silvia Orsi[3], Rebecca Crivellari[1, 2], Giulia Pertile[1, 2], Valentina Trimarco[1], Monica Facco[1], Flavia Bazzoni[4], Francesca Nonne[6], Antonio Palomba[6], Vittorio Tedde[6], Daniela Pagnozzi[6], Salvatore Pisanu[6], Livio Trentin[1], Gianpietro Semenzato[2], Stefania Bortoluzzi[5], Renato Zambello[1, 2]

*[1]Department of Medicine, Hematology Branch, University of Padova, Padova, Italy;[2]Veneto Institute of Molecular Medicine (VIMM), Padova, Italy; [3]Department of Molecular Medicine, University of Padova, Padova, Italy; [4]Department of Medicine, Division of General Pathology, University of Verona, Verona, Italy; [5]Department of Surgery, Oncology and Gastroenterology, University of Padova, Padova, Italy; [6]Proteomics and Immunodiagnostics Laboratory, Porto Conte Ricerche Srl, Alghero, Italy;*

### Background

Despite advances in the understanding of its molecular basis, T-cell Large Granular Lymphocyte Leukemia (T-LGLL) remains a rare, chronic lymphoproliferative disorder lacking effective targeted therapies, representing a major unmet clinical need. Current immunosuppressive treatments offer limited benefits, with frequent relapses and therapy-related toxicities. The disease is driven by recurrent activating mutations, mainly in *STAT3* and *STAT5B*, within a pro-inflammatory microenvironment. *STAT3* mutations, common in CD8+ T-LGLL, are strongly linked to symptomatic disease and severe neutropenia, adversely affecting patient outcome. We recently showed that miR-146b downregulation promotes neutropenia via the STAT3–miR-146b–ELAVL1–FasL axis, increasing FasL production and neutrophil apoptosis.

### Aims

This project investigates the molecular effects of miR-146b restoration in patient-derived cells and its potential as an RNA-based targeted therapy for T-LGLL.

### Methods

LGLs from six CD8+ STAT3-mutated T-LGLL patients and CTLs from six healthy donors (HD) were purified and transfected with a miR-146b mimic or scramble control. After 24h, viability and transfection efficiency were assessed by flow cytometry and RT-qPCR. RNA-seq (24h) and mass spectrometry (24, 48h) were then performed. Gene expression was quantified with CircComPara2; DESeq2 (adj-P≤0.05) was used for differential expression analysis and functional enrichment was assessed with clusterProfiler and enrichplot. Proteomic analysis (Proteome Discoverer) was performed after Tandem Mass Tag labeling for multiplexed quantification. Differentially expressed proteins were identified by t-test (adj-P<0.05, LFC≥0.5).

**Copyright Information:** (Online) ISSN: 2572-9241
©2026 The Author(s). HemaSphere is published by John Wiley & Sons Ltd on behalf of European Hematology Association. This is an open access Abstract Book distributed under the Attribution-NonCommercial-NoDerivs (CC BY-NC-ND), which allows third parties to download the articles and share them with others as long as they credit the author and the Abstract Book, but they cannot change the content in any way or use them commercially.
**Abstract Book Citations:** Authors, Title, HemaSphere, 2026;10:(S1):pages. The abstract book DOIs can be found on the "Author Information" page.
**Disclaimer:** Articles published in the journal HemaSphere exclusively reflect the opinions of the authors. The authors are responsible for all content in their abstracts including accuracy of the facts, statements, citing resources, etc.

### Results

MiR-146b transfection was successful in both HD and T-LGLL samples compared to scramble control ($p<0.01$), with ≥60% cell viability at 24h. Transcriptomic profiling showed minimal impact of miR-146b restoration in normal CTLs (154 genes, 1.2% differentially expressed), whereas patient-derived cells showed profound transcriptional reprogramming (2,404 genes). Restoration normalized key pathogenic targets, notably *ELAVL1*, reducing *FasL* expression and reverting the STAT3-driven neutropenia axis. Moreover, miR-146b transfection fully or partially corrected nearly half of the genes dysregulated in patients, while inducing only mild changes in genes unaffected by the disease. Direct oncogenic targets such as *IRAK1* were downregulated, and disease-related genes including *CCL5* ($p<0.01$) decreased to levels comparable to HD. Pathway analysis showed suppression of TNFα/NF-kB, IL-6/JAK/STAT3 and inflammatory response. Proteomics further strengthened these results, demonstrating significant downregulation of MCL1, a critical STAT3-dependent anti-apoptotic regulator of LGLs survival, together with modulation of MAPK signalling components, including reduced MAPK8 expression. Notably, CD28 expression remained unchanged and undetectable, confirming that miR-146b restoration selectively targets pathogenic pathways without altering the differentiated LGL phenotype. Overall, transcriptomic and proteomic data support the disease-specific therapeutic potential of miR-146b restoration.

### Summary/Conclusion

MiR-146b restoration induces selective molecular reprogramming in STAT3-mutated LGLs with minimal effects on healthy CTLs. By normalizing the STAT3–miR-146b–ELAVL1–FasL axis and suppressing key inflammatory and oncogenic pathways, it mitigates pathogenic signalling and supports the development of a CD57-targeted RNA-based therapy for T-LGLL.

**Copyright Information:** (Online) ISSN: 2572-9241
©2026 The Author(s). HemaSphere is published by John Wiley & Sons Ltd on behalf of European Hematology Association. This is an open access Abstract Book distributed under the Attribution-NonCommercial-NoDerivs (CC BY-NC-ND), which allows third parties to download the articles and share them with others as long as they credit the author and the Abstract Book, but they cannot change the content in any way or use them commercially.
**Abstract Book Citations:** Authors, Title, HemaSphere, 2026;10:(S1):pages. The abstract book DOIs can be found on the "Author Information" page.
**Disclaimer:** Articles published in the journal HemaSphere exclusively reflect the opinions of the authors. The authors are responsible for all content in their abstracts including accuracy of the facts, statements, citing resources, etc.

### (PS1685) CLINICAL AND TRANSCRIPTOMIC INSIGHTS INTO THE NPM1-XPO1 AXIS IN CHRONIC LYMPHOCYTIC LEUKEMIA AND ITS IMPLICATIONS FOR TARGETED THERAPY

**Topic:** 05. Chronic lymphocytic leukemia and related disorders - Biology & translational research

Monika Szelest*[1], Magdalena Paziewska[1], Michal Kielbus[1], Agnieszka Karczmarczyk[1], Joanna Zaleska[1], Marta Masternak[1], Marta Morawska[1], Krzysztof Giannopoulos[1]

[1]*Medical University of Lublin, Department of Experimental Hematooncology, Lublin, Poland;*

### Background

Despite major advances in targeted therapy, durable responses in chronic lymphocytic leukemia (CLL) are not achieved in all patients. Resistance to Bruton tyrosine kinase inhibitors or BCL2 antagonists is not exclusively mutation-driven and may reflect dysregulation of pro-survival signaling and microenvironmental interactions. Nucleophosmin-1 (NPM1), a multifunctional nucleolar protein involved in ribosome biogenesis and nucleocytoplasmic trafficking, directly interacts with exportin-1 (XPO1), a key regulator of nuclear export.

### Aims

To investigate the molecular and prognostic significance of *NPM1* expression in CLL and to explore the functional relevance of the NPM1-XPO1 axis in the context of targeted therapy.

### Methods

A total of 309 treatment-naïve CLL patients were included in the study. NPM1 expression was quantified by RT-qPCR (n=309) and *XPO1* transcript levels by ddPCR (n=79). RNA-seq (n=24, Illumina approach) was performed to characterize transcriptomic profiles and alternative splicing according to *NPM1* and *XPO1* levels. To evaluate whether pharmacologic inhibition of XPO1 modulates *NPM1* expression and influences response to targeted agents, primary CLL cells were exposed to selinexor alone and in combination with ibrutinib or venetoclax.

### Results

*NPM1* expression was significantly higher in patients with advanced-stage disease (Rai III-IV vs Rai 0 and I-II; p=0.0142 and p=0.0064, respectively), in CD38-positive cases (p=0.0126), and in del(11q) patients (p =0.0006), whereas trisomy 12 was associated with lower *NPM1* levels (p=0.0465). In multivariable analysis, the prognostic impact of *NPM1* expression was dependent on IGHV mutation status, with higher *NPM1* levels associated with longer TTFT in IGHV-mutated cases (interaction p=0.044). *NPM1* expression negatively correlated with *XPO1* transcript levels (r =-0.4297, p=0.0001, Fig. 1A). Increased *XPO1* expression was observed in patients with *TP53* mutation and del(17p) (p=0.0035 and p=0.0016, respectively), while trisomy 12 was associated with lower *XPO1* levels (p=0.0201). Transcriptomic profiling revealed limited gene expression differences according to *NPM1* expression. In contrast, high *XPO1* expression was associated with 48 differentially expressed genes (FDR<0.05), including those involved in cell cycle regulation, immune signaling, and metabolism. Differential splicing analysis identified widespread isoform alterations associated with both *NPM1* and *XPO1* levels, predominantly affecting genes involved in RNA metabolism, immune regulation, and cellular stress responses. Several shared alternatively spliced genes (e. g. *TLR1* and *TBC1D5*) displayed opposite inclusion patterns depending on *NPM1* or *XPO1* expression status, suggesting differential regulation of common targets. In primary CLL cells, selinexor demonstrated concentration-dependent synergistic interactions with venetoclax and ibrutinib (ZIP scores +13.1 and +10.2, respectively, Fig. 1BC), resulting in significant reduction in cell viability compared with single-agent treatment. At the protein level, selinexor-based combinations were associated with modulation of *NPM1* expression.

**Copyright Information:** (Online) ISSN: 2572-9241
©2026 The Author(s). HemaSphere is published by John Wiley & Sons Ltd on behalf of European Hematology Association. This is an open access Abstract Book distributed under the Attribution-NonCommercial-NoDerivs (CC BY-NC-ND), which allows third parties to download the articles and share them with others as long as they credit the author and the Abstract Book, but they cannot change the content in any way or use them commercially.
**Abstract Book Citations:** Authors, Title, HemaSphere, 2026;10:(S1):pages. The abstract book DOIs can be found on the "Author Information" page.
**Disclaimer:** Articles published in the journal HemaSphere exclusively reflect the opinions of the authors. The authors are responsible for all content in their abstracts including accuracy of the facts, statements, citing resources, etc.

### Summary/Conclusion

Our study integrates clinical, transcriptomic, and functional data to identify the NPM1-XPO1 axis as a biologically relevant and molecular context-dependent pathway in CLL. These findings suggest that dysregulation of nuclear export may influence response to targeted therapy and support further investigation of the NPM1-XPO1 axis in CLL.

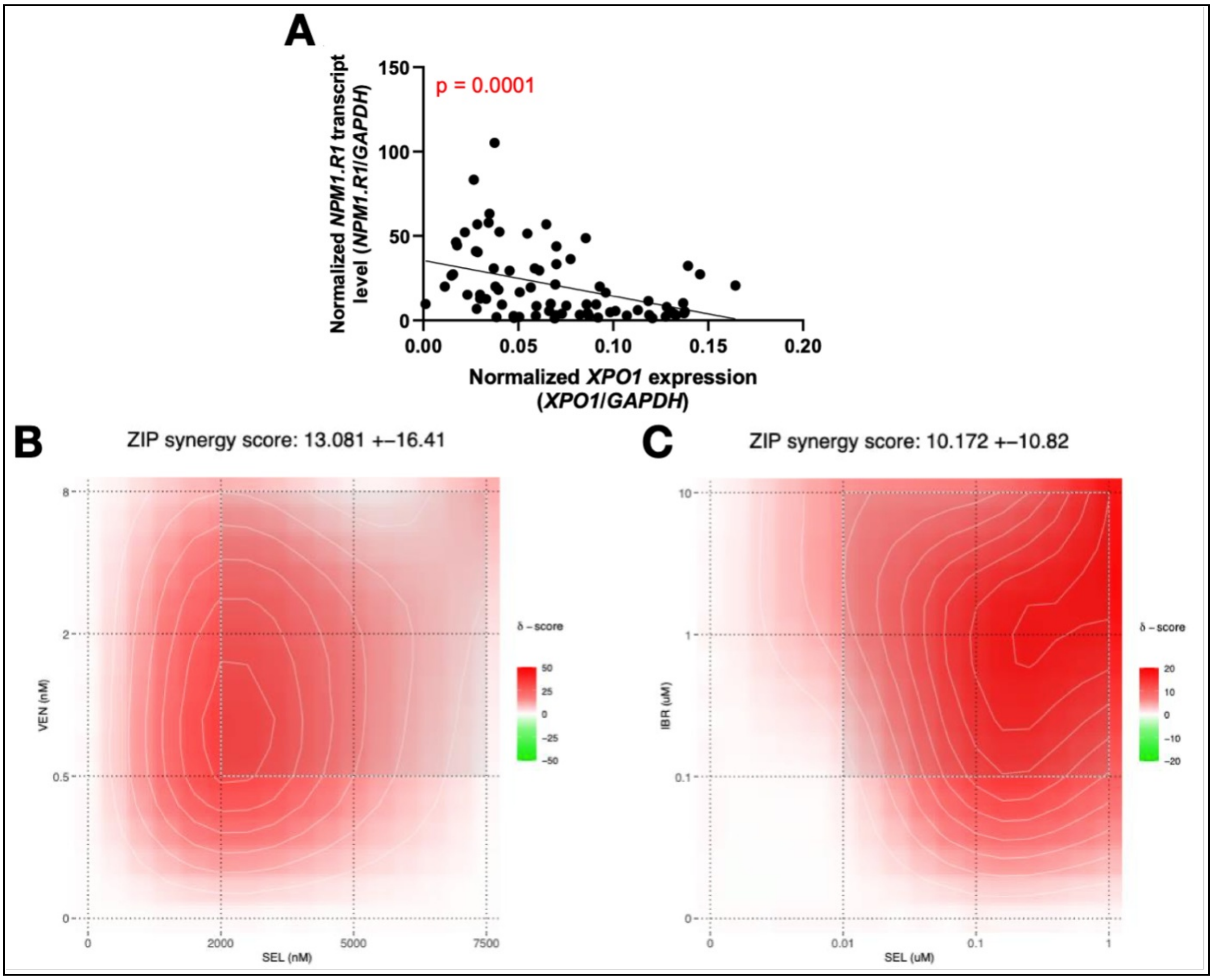

Expression correlation between main NPM1 transcript (NPM1.R1) and XPO1 (A) and functional effects of XPO1 inhibition in primary CLL cells (B, C).

**Copyright Information:** (Online) ISSN: 2572-9241
©2026 The Author(s). HemaSphere is published by John Wiley & Sons Ltd on behalf of European Hematology Association. This is an open access Abstract Book distributed under the Attribution-NonCommercial-NoDerivs (CC BY-NC-ND), which allows third parties to download the articles and share them with others as long as they credit the author and the Abstract Book, but they cannot change the content in any way or use them commercially.
**Abstract Book Citations:** Authors, Title, HemaSphere, 2026;10:(S1):pages. The abstract book DOIs can be found on the "Author Information" page.
**Disclaimer:** Articles published in the journal HemaSphere exclusively reflect the opinions of the authors. The authors are responsible for all content in their abstracts including accuracy of the facts, statements, citing resources, etc.

### (PS1686) DISTINCTIVE IN VIVO CLASS-SWITCH RECOMBINATION PATTERNS IN CHRONIC LYMPHOCYTIC LEUKEMIA STEREOTYPED SUBSET #1: MOLECULAR EVIDENCE FOR TUMOR MICROENVIRONMENT-DRIVEN ACTIVATION

**Topic:** 05. Chronic lymphocytic leukemia and related disorders - Biology & translational research

Aikaterini Mallou*[1, 2], Uday Aditya Sarkar[3, 4], Anastasia Anastasiadou[1], Nikolaos Pechlivanis[1], Blaž Oder[5], Ferran Nadeu[6, 7], Konstantina Zampouridou[1], Athina Tziogkidou[1], Konstantinos Kardamiliotis[1, 2], Anastasia Iatrou[1], Cecilia Österholm[5], Leily Rabbani[5], Daniel Hägerstrand[5], Ian Márquez-López[6], Marek Mraz[8, 9], Fotis Psomopoulos[1], Andreas Agathangelidis[2], Richard Rosenquist[5], Elias Campo[6, 7], Anastasia Chatzidimitriou[1, 5], Paolo Ghia[3, 4, 10, 11], Kostas Stamatopoulos[1, 5]

*[1]Institute of Applied Biosciences, Centre for Research and Technology Hellas, Thessaloniki, Greece;[2]Department of Biology, School of Science, National and Kapodistrian University of Athens, Athens, Greece; [3]Department of Medical Oncology, Dana-Farber Cancer Institute, Boston, United States of America; [4]Department of Medicine, Harvard Medical School, Boston, United States of America; [5]Department of Molecular Medicine and Surgery, Karolinska Institutet, Stockholm, Sweden; [6]Fundació de Recerca Clínic Barcelona-Institut d'Investigacions Biomèdiques August Pi i Sunyer (FRCB-IDIBAPS), Barcelona, Spain; [7]Centro de Investigación Biomédica en Red de Cáncer (CIBERONC), Madrid, Spain; [8]Department of Internal Medicine, Hematology and Oncology, University Hospital Brno and Faculty of Medicine, Masaryk University, Brno, Czechia; [9]CEITEC, Masaryk University, Brno, Czechia; [10]Medical School, Università Vita-Salute San Raffaele, Milano, Italy; [11]Comprehensive Cancer Center, IRCCS Ospedale San Raffaele, Milano, Italy;*

### Background

CLL stereotyped subset #1 is an adverse-prognostic disease variant, defined by the expression of unmutated immunoglobulin of the μ isotype utilizing clan I IGHV (IGHV1, 5 and 7) genes paired with the IGKV1-39/1D-39 gene. We have preliminarily reported that a fraction of the malignant clone undergoes *in vivo* class-switch recombination (CSR) at a significantly higher frequency compared to other CLL subgroups. The underlying biological basis remains unknown.

### Aims

We aimed to obtain insight into the enrichment of CSR in CLL subset #1 by comprehensive transcriptomic profiling and comparisons with other CLL subgroups.

### Methods

We studied two independent RNA-seq datasets: the first (cohort 1) comprised only CLL stereotyped subset cases (n=91), while the second (cohort 2) included both subset and non-subset CLL cases (n=289). Immune repertoires were reconstructed using the TRUST4 pipeline. Differential gene expression analysis was performed with DESeq2, followed by gene set enrichment (GSEA) and over-representation analysis (ORA). Peripheral blood mononuclear cells of 11 subset #1 patients were stimulated *in vitro* with CD40L/IL-4, and the resulting transcriptional changes were profiled as above.

**Copyright Information:** (Online) ISSN: 2572-9241
©2026 The Author(s). HemaSphere is published by John Wiley & Sons Ltd on behalf of European Hematology Association. This is an open access Abstract Book distributed under the Attribution-NonCommercial-NoDerivs (CC BY-NC-ND), which allows third parties to download the articles and share them with others as long as they credit the author and the Abstract Book, but they cannot change the content in any way or use them commercially.
**Abstract Book Citations:** Authors, Title, HemaSphere, 2026;10:(S1):pages. The abstract book DOIs can be found on the "Author Information" page.
**Disclaimer:** Articles published in the journal HemaSphere exclusively reflect the opinions of the authors. The authors are responsible for all content in their abstracts including accuracy of the facts, statements, citing resources, etc.

**Results**

TRUST4-based analysis demonstrated a significantly ($p<0.05$) higher prevalence of CSR events, evidenced by the presence of clonally related γ and α transcripts, in subset #1 vs any other CLL subgroup (stereotyped or not). In cohort 1, CSR events were detected in 26/36 (72.2%) subset #1 cases vs 11/33 (33.3%) other cases with unmutated IGHV genes (U-CLL) and 10/22 (45.5%) cases with mutated IGHV genes (M-CLL). In cohort 2, CSR was observed in 8/12 (66.7%) subset #1 cases vs 32/96 (33.3%) other U-CLL cases and 49/181 (27.1%) M-CLL cases. Differential expression analysis of both cohorts indicated that while subset #1 retains core transcriptional features of U-CLL, it segregates as a distinct transcriptomic entity. GSEA supported a phenotype characterized by enhanced extracellular matrix remodeling (e.g. *MMP14*, *BSG*, *COL8A2*), increased capacity for tumor microenvironmental (TME) interactions (e.g. *ITGA6*, *IQGAP1*), together with broad cytokine responsiveness (e.g. *EGR1*, *IL10/IL10R*, *IFNGR2*, *IL13*, *IL3RA*) and signatures consistent with intensified crosstalk with both adaptive and innate immune compartments (e.g. *ICOS*, *CCL3*, *TLR7*, *TYROBP*). Several lncRNAs (e.g. *ENSG00000285810*, *ENSG00000288771*) were also differentially expressed, alluding to additional layers of transcriptional regulation. CD40L/IL-4 stimulation elicited a significant upregulation of *AICDA* in all samples, accompanied by reinforced B-cell receptor responsiveness and intensified downstream signaling cascades (e.g. *CD79B*, *BTK*, *PLCG2*, *GRB2)*; as well as enhanced cytoskeletal remodeling, adhesion and migratory capacity (e.g. *RAC2*, *VAV1* , *ACTB*, *ACTG1*), consistent with intensified microenvironmental crosstalk and a survival-optimized activation phenotype (e.g. *CCL22, CISH*, *NFKBIZ*).

**Summary/Conclusion**

Our findings highlight subset #1 as a biologically distinct CLL variant characterized by enriched CSR and a transcriptomic profile suggestive of intensified engagement with the TME. The responses obtained by CD40L/IL-4 activation support that induction of AID by antigen engagement and T-cell mediated stimulation may underlie the extensive *in vivo* CSR characterizing CLL subset #1.

**Copyright Information:** (Online) ISSN: 2572-9241
©2026 The Author(s). HemaSphere is published by John Wiley & Sons Ltd on behalf of European Hematology Association. This is an open access Abstract Book distributed under the Attribution-NonCommercial-NoDerivs (CC BY-NC-ND), which allows third parties to download the articles and share them with others as long as they credit the author and the Abstract Book, but they cannot change the content in any way or use them commercially.
**Abstract Book Citations:** Authors, Title, HemaSphere, 2026;10:(S1):pages. The abstract book DOIs can be found on the “Author Information” page.
**Disclaimer:** Articles published in the journal HemaSphere exclusively reflect the opinions of the authors. The authors are responsible for all content in their abstracts including accuracy of the facts, statements, citing resources, etc.

### (PS1687) INCREASED SMU-3′RR RECOMBINATION RESULTING FROM MYC OVEREXPRESSION AFTER HEIGHTENED ACTIVATION IN CLL FURTHER SUPPORTS A ROLE FOR C-MYC AS A DRIVER OF GENOMIC INSTABILITY

**Topic:** 05. Chronic lymphocytic leukemia and related disorders - Biology & translational research

Milène Parquet[1], Kenza Guiyedi[1], Justine Pollet[1], Hugo Bonnemain-Pascual[1], Israa Al Jamal[1], Maxime Roubinet[1], Jasmine Chauzeix[1], David Rizzo[1], Jean Feuillard[1], Nathalie Gachard[1], Sophie Peron*[1]

[1]*CRIBL INSERM, Limoges, France;*

### Background

Being still incurable, chronic lymphocytic leukemia (CLL) is an indolent non-Hodgkin B-cell lymphoma of the elderly, characterized by the expansion of malignant $CD5^+$ B-cells. A hallmark of this cancer is its relatively clinical homogeneity at diagnosis which contrasts with a wide range of genetic abnormalities and equally diverse disease progressions. This heterogeneity raises questions whether CLL may encompass a spectrum of resembling related disease subtypes rather than a single uniform entity.

### Aims

In a previous work (Al Jamal et al., Haematologica, 2024), we separated CLL patients based on the number of recombination between the switch µ (Sµ) and the 3' regulatory (3'RR) regions (Sµ-3'RRrec) detected in their peripheral blood mononuclear cells (PBMCs). We found that patients, designated as Sµ-3'RRrec$^{High}$, with a high count of junctions, consistently exhibited elevated *MYC* expression compared the Sµ-3'RRrec$^{Low}$ patients. To further elucidate the biological basis of this phenomenon, we aimed to characterize the cellular and molecular features that distinguish Sµ-3'RRrec$^{High}$ patients, who are associated with *MYC* overexpression, from the Sµ-3'RRrec$^{Low}$ CLLs.

### Methods

Samples from CLL patients, PBMCs and lymph node biopsies, were used accordingly to the Declaration of Helsinki. We quantified *MYC* and *BCL2* mRNA levels by qRT-PCR. To evaluate clonal evolution dynamics, we performed IGHV-based lineage tree analysis. We conducted total RNA sequencing on PBMCs to identify transcriptomic signatures. Flow cytometry analysis of CXCR4 and CD5 expression in $CD19^+CD5^+$ PBMCs was used to quantify resting versus recently SLO-emerging CLL cells. BCR signaling was examined by western blot analysis of P-BTK and P-ERK before and after BCR stimulation. Proliferation and apoptosis in lymph nodes were assessed by Ki67 immunofluorescence staining and TUNEL assay.

Copyright Information: (Online) ISSN: 2572-9241
©2026 The Author(s). HemaSphere is published by John Wiley & Sons Ltd on behalf of European Hematology Association. This is an open access Abstract Book distributed under the Attribution-NonCommercial-NoDerivs (CC BY-NC-ND), which allows third parties to download the articles and share them with others as long as they credit the author and the Abstract Book, but they cannot change the content in any way or use them commercially.
**Abstract Book Citations:** Authors, Title, HemaSphere, 2026;10:(S1):pages. The abstract book DOIs can be found on the "Author Information" page.
**Disclaimer:** Articles published in the journal HemaSphere exclusively reflect the opinions of the authors. The authors are responsible for all content in their abstracts including accuracy of the facts, statements, citing resources, etc.

### Results

We demonstrated that Sμ-3′RRrec$^{High}$ CLL PBMCs are characterized by significant *MYC* overexpression, whereas BCL2 levels tend to be higher in Sμ-3′RRrec$^{Low}$ cases, leading to a discriminative BCL2/MYC ratio between the groups. This exclusive expression pattern is maintained in lymph node samples. Clonal evolution analyses based on IGHV lineage trees revealed no major differences indicating that expansion dynamics are similar. In contrast, transcriptomic profiling uncovered clearly distinct gene expression signatures, with Sμ-3′RRrec$^{High}$ samples showing downregulation of genes involved in cellular signaling and immune regulation. Phenotypically, Sμ-3′RRrec$^{High}$ PBMCs displayed an enrichment of recently SLO-emerging cells, consistent with sustained activation. Functionally, these cases remained responsive to BCR stimulation with increased c-MYC protein expression upon activation, whereas Sμ-3′RRrec$^{Low}$ cases exhibited BCR unresponsiveness. Finally, although overall proliferation rates in lymph nodes were comparable, Sμ-3′RRrec$^{High}$ samples showed higher Ki67 staining intensity, suggesting reduced quiescence and enhanced cell cycle engagement.

### Summary/Conclusion

Collectively, these findings identify *MYC*-driven Sμ-3′RR recombination as a marker of genomic instability and delineate Sμ-3′RRrec$^{High}$ CLL as a distinct *MYC*-driven entity marked by sustained activation and proliferative readiness. They further underscore the role of *MYC* as a potential driver of disease aggressiveness and suggest that *MYC* expression may contribute to complement the biological assessment of CLL.

**Copyright Information:** (Online) ISSN: 2572-9241
©2026 The Author(s). HemaSphere is published by John Wiley & Sons Ltd on behalf of European Hematology Association. This is an open access Abstract Book distributed under the Attribution-NonCommercial-NoDerivs (CC BY-NC-ND), which allows third parties to download the articles and share them with others as long as they credit the author and the Abstract Book, but they cannot change the content in any way or use them commercially.
**Abstract Book Citations:** Authors, Title, HemaSphere, 2026;10:(S1):pages. The abstract book DOIs can be found on the “Author Information” page.
**Disclaimer:** Articles published in the journal HemaSphere exclusively reflect the opinions of the authors. The authors are responsible for all content in their abstracts including accuracy of the facts, statements, citing resources, etc.

### (PS1688) ANALYSIS OF CHRONIC LYMPHOCYTIC LEUKEMIA (CLL) PATIENTS WITH 11Q DELETION: CLINICOBIOLOGICAL PROFILE AND GENOMIC CHARACTERIZATION BY OGM

**Topic:** 05. Chronic lymphocytic leukemia and related disorders - Biology & translational research

Joanna Kamaso*[1], Silvia Ramos-Campoy[1], Carme Melero[1], Marta Lorenzo[1], María Rodríguez-Rivera[1], Diego Quimis[1], Eva Gimeno[2], Alicia Serrano[3], Blanca Ferrer[3], Miguel Alcoceba Sánchez[4], Miguel Bastos Boente[4], Angel Ramirez Payer[5], Alicia Rodriguez Fernandez[6], Ana Carla De Oliveira[7], Raquel Santiago Alonso[8], Angel Serna Pareja[9], Pau Abrisqueta[9], Marta Crespo Maull[9], Alba Cabirta[10], Maria Dolores Garcia Malo[11], Miguel Ángel Perera Álvarez[12], Alexia Teresa Suarez Cabrera[12], MacArena Ortiz Pareja[13], María Teresa Villalobos Prego[14], Rosa Collado Nieto[15], Rocío García-Serra[15], Marta Julia Arenas[16], Rocio Salgado[17], Sergio Ramos Cillan[17], Gemma Azaceta[18], Javier De La Serna[19], Xabier Gutierrez Lopez De Ocariz[20], Ainara Ferrero[21], Janilson Do Nascimento Ferreira[22], Iñigo Olazabal Eizaguirre[23], Rafael Andreu[24], Eduardo Rios Herranz[25], Miguel Angel Rodriguez Gil[26], José María Arguiñano[27], Lucrecia Yáñez[28], Isabel Teresa González Gascón Y Marín[29], Emil De Leon Marcano[30], Ana Muntañola Prat[31], Miguel Argüello-Tomás[31], Javier Loscertales Pueyo[32], Paloma García Martín[33], Ángeles Medina[34], Maria Victoria Calle Gordo[35], Marta Salido[1], Ana Ferrer[1], Christelle Ferrà[2], Francisco Bosch Albareda[9], Anna Puiggros[1], Blanca Espinet[1]

*[1]Laboratori de Citogenètica Molecular, Laboratori de Citologia Hematològica, Servei de Patologia, Grup de Recerca Translacional en Neoplàsies Hematològiques, Programa de Recerca en Càncer, Hospital del Mar Research Institute, Barcelona, Spain; [2]Servei d'Hematologia Clínica, Hospital del Mar, Barcelona, Spain;[3]Hospital Clínico Universitario de Valencia y INCLIVA, Instituto de Investigación Sanitaria, Valencia, Spain; [4]Hospital Universitario de Salamanca (HUS-IBSAL), CIBERONC y Centro de Investigación del Cáncer (CIC-IBMCC, USAL-CSIC), Salamanca, Spain; [5]Hospital Universitario Central de Asturias, Oviedo, Spain; [6]Hospital Universitario Virgen de la Macarena, Sevilla, Spain;[7]Institut Català d'Oncologia - Hospital Duran i Reynals, IDIBELL, Hospitalet de Llobregat, Spain; [8]Hospital Sant Joan de Déu de Manresa, Manresa, Spain;[9]Servicio de Hematología, H.U. Vall d'Hebron/Vall d'Hebron Instituto de Oncología (VHIO), Barcelona, Spain; [10]Hospital Universitario de A Coruña, A Coruña, Spain; [11]Hospital General Universitario Morales Meseguer, Murcia, Spain; [12]Hospital Universitario de Gran Canaria Doctor Negrín, Las Palmas de Gran Canarias, Spain; [13]Hospital Regional Universitario de Málaga, Málaga, Spain; [14]Hospital Universitari Mútua Terrassa, Terrassa, Spain; [15]Consorcio Hospital General Universitario de Valencia, Valencia, Spain; [16]Hospital Universitari Germans Trias i Pujol, Badalona, Spain; [17]Hospital Universitario Fundación Jiménez Díaz, Madrid, Spain; [18]Hospital Clínico Universitario Lozano Blesa, Zaragoza, Spain; [19]Hospital Universitario 12 de Octubre, Madrid, Spain; [20]Hospital Universitario Araba – Txagorritxu, Vitoria, Spain; [21]Hospital Universitari Arnau de Vilanova, Lleida, Spain;[22]Hospital Universitari Joan XXIII, Tarragona, Spain; [23]Hospital Universitario Donostia, San Sebastián, Spain;[24]Hospital Universitari i Politècnic La Fe, Valencia, Spain; [25]Hospital Universitario Virgen de Valme, Sevilla, Spain;[26]Hospital Universitario Virgen de las Nieves, Granada, Spain; [27]Hospital Universitario de Navarra, Pamplona, Spain;[28]Hospital Universitario Marqués de Valdecilla, Santander, Spain; [29]Hospital Universitario Infanta Leonor, Madrid, Spain; [30]Hospital Médico-Quirúrgico de Jaén, Jaén, Spain; [31]Hospital de la Santa Creu i Sant Pau, Barcelona, Spain; [32]Hospital Universitario de La Princesa, Madrid, Spain;[33]Hospital San Cecilio de Granada, Granada, Spain; [34]Hospital Universitario Costa del Sol, Marbella, Spain;[35]Hospital Universitario Torrecárdenas, Almería, Spain;*

### Background

In chronic lymphocytic leukemia (CLL), 11q22q23 deletion [del(11q)] is associated with B symptoms, lymphadenopathy, and shorter time to first treatment. The minimally deleted region includes *ATM*, essential for DNA repair, and coexists with *BIRC3* deletion in 80-85% of cases. Although del(11q) was considered an adverse prognostic marker in the chemoimmunotherapy (CIT) era, its impact appears attenuated in patients treated with BTK or BCL2 inhibitors (BTKi or BCL2i). However, the genomic context of this deletion and its relationship with genomic complexity remain poorly understood.

**Copyright Information:** (Online) ISSN: 2572-9241
©2026 The Author(s). HemaSphere is published by John Wiley & Sons Ltd on behalf of European Hematology Association. This is an open access Abstract Book distributed under the Attribution-NonCommercial-NoDerivs (CC BY-NC-ND), which allows third parties to download the articles and share them with others as long as they credit the author and the Abstract Book, but they cannot change the content in any way or use them commercially.
**Abstract Book Citations:** Authors, Title, HemaSphere, 2026;10:(S1):pages. The abstract book DOIs can be found on the "Author Information" page.
**Disclaimer:** Articles published in the journal HemaSphere exclusively reflect the opinions of the authors. The authors are responsible for all content in their abstracts including accuracy of the facts, statements, citing resources, etc.

### Aims

(1) To define the clinicobiological characteristics of CLL patients with del(11q) in a Spanish real-world cohort treated with targeted agents and compare them with other high-risk genetic alterations. (2) To describe alterations within the 11q region and assess genomic complexity by Optical Genome Mapping (OGM).

### Methods

We included 434 patients from 35 Spanish centers treated with BTKi/BCL2i (±anti-CD20) from November 2021-November 2025. Among them, 85 cases with del(11q) detected by FISH were selected. Patients were characterized by clinicobiological features and high-risk biomarkers [*TP53* status, IGHV mutational status, and complex karyotype (CK)]. Peripheral blood samples from 55/85 patients were analyzed by OGM to detect numerical/structural alterations in 11q and assess genomic complexity (GC; ≥5 alterations ≥5Mb and/or rearrangements).

### Results

Patients with del(11q) represented 20% (85/434) of the cohort (Figure 1A), of whom 81% were in Binet stage B/C. Regarding treatment, 62% received BTKi, 28% BCL2i, and 10% both. Overall, 38% (32/85) had ≥2 treatment lines and 34% (29/84) prior CIT exposure. A high frequency of unmutated IGHV (UM-IGH, 89%), CK (34%), and concomitant *TP53* abnormalities (19%) was observed in del(11q) patients. Compared with *TP53*-altered cases, they showed higher frequency of UM-IGHV and a lower CK rates (Figure 1B). Among the 55 OGM-analyzed cases, the median size of del(11q) was 33.6 Mb (range: 422 Kb–42.6 Mb). All cases included *ATM* and 87% *BIRC3*, without clinicobiological differences by *BIRC3* status. In 19 (35%) patients, del(11q) was associated with additional structural events (simple translocations (n=6); multiple involving 11q (n=5) and/or other chromosomes (n=8)). In two cases, the deletion was part of a chromothripsis (CHT) event. Multiple 11q deletions were detected in 7 cases (two deletions in six cases and three in one), suggesting distinct clones. The median number of alterations per case was 6 (range: 1–46), and 58% showed GC. Chromoanagenesis (CAG) events were identified in 18% of cases, most frequently chromoplexy (n=5), involving chromosomes 2q, 11q, 13q, and 17q with no recurrent breakpoints. CHT was observed in 4 cases and one chromoanasynthesis event affecting 17q. While *BIRC3* involvement within the 11q deletion did not impact GC or CAG frequency, patients with concomitant *TP53* alterations and del(11q) showed higher genomic complexity compared to those with isolated del(11q) (Figure 1C).

### Summary/Conclusion

(1) Del(11q) in CLL is associated with an adverse biological profile and a high frequency of UM-IGHV. (2) OGM reveals wide structural variability at 11q22q23 and high GC in these patients. (3) CAG events are common, particularly chromoplexy. (4) Concomitant *TP53* alterations significantly increase GC, highlighting their biological relevance.

ACKNOWLEDGMENTS. 2021SGR25, PI21/00022, PI24/00229, FEHH-GELLC.

**Copyright Information:** (Online) ISSN: 2572-9241
©2026 The Author(s). HemaSphere is published by John Wiley & Sons Ltd on behalf of European Hematology Association. This is an open access Abstract Book distributed under the Attribution-NonCommercial-NoDerivs (CC BY-NC-ND), which allows third parties to download the articles and share them with others as long as they credit the author and the Abstract Book, but they cannot change the content in any way or use them commercially.
**Abstract Book Citations:** Authors, Title, HemaSphere, 2026;10:(S1):pages. The abstract book DOIs can be found on the "Author Information" page.
**Disclaimer:** Articles published in the journal HemaSphere exclusively reflect the opinions of the authors. The authors are responsible for all content in their abstracts including accuracy of the facts, statements, citing resources, etc.

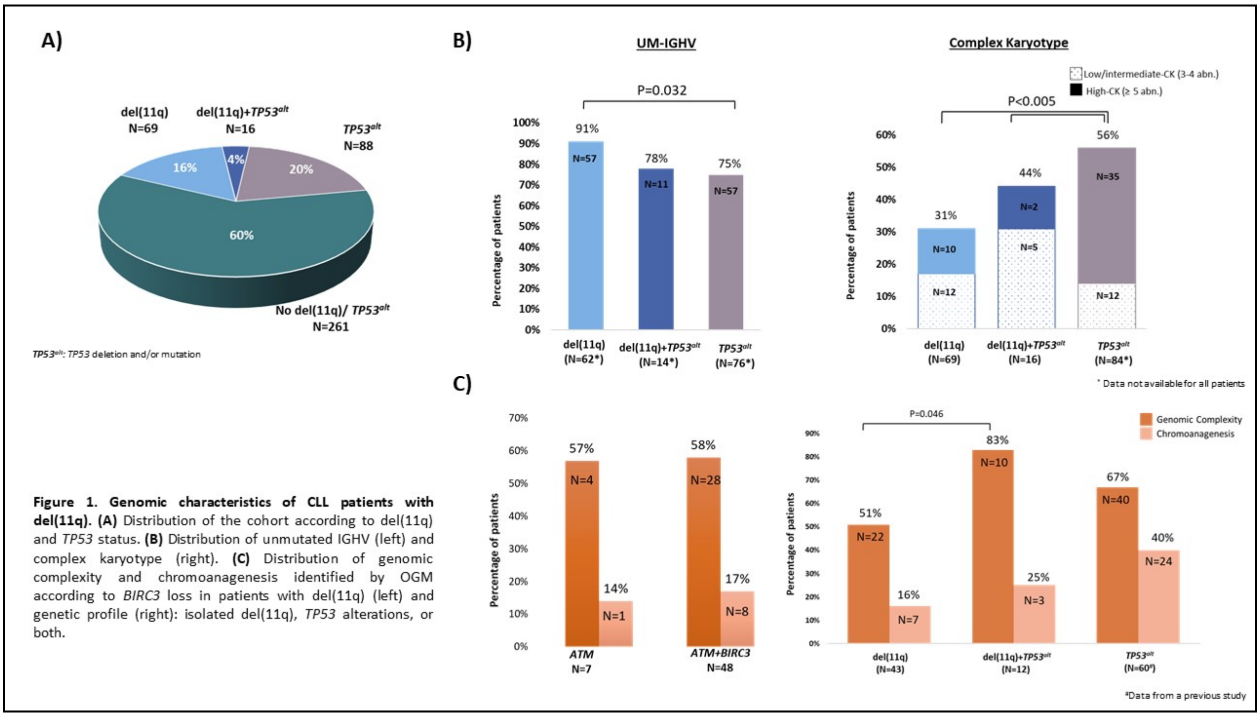

**Figure 1. Genomic characteristics of CLL patients with del(11q). (A)** Distribution of the cohort according to del(11q) and *TP53* status. **(B)** Distribution of unmutated IGHV (left) and complex karyotype (right). **(C)** Distribution of genomic complexity and chromoanagenesis identified by OGM according to *BIRC3* loss in patients with del(11q) (left) and genetic profile (right): isolated del(11q), *TP53* alterations, or both.

**Copyright Information:** (Online) ISSN: 2572-9241
©2026 The Author(s). HemaSphere is published by John Wiley & Sons Ltd on behalf of European Hematology Association. This is an open access Abstract Book distributed under the Attribution-NonCommercial-NoDerivs (CC BY-NC-ND), which allows third parties to download the articles and share them with others as long as they credit the author and the Abstract Book, but they cannot change the content in any way or use them commercially.
**Abstract Book Citations:** Authors, Title, HemaSphere, 2026;10:(S1):pages. The abstract book DOIs can be found on the "Author Information" page.
**Disclaimer:** Articles published in the journal HemaSphere exclusively reflect the opinions of the authors. The authors are responsible for all content in their abstracts including accuracy of the facts, statements, citing resources, etc.

### (PS1689) INITIAL RESULTS OF MULTICENTER PHASE 2 BOVEN-ΔMRD400 TRIAL OF ZANUBRUTINIB, OBINUTUZUMAB, AND VENETOCLAX (BOVEN) WITH TREATMENT DURATION GUIDED BY MRD KINETICS (ΔMRD400) IN TREATMENT-NAÏVE (TN) CLL

**Topic:** 06. Chronic lymphocytic leukemia and related disorders - Clinical

Jacob Soumerai*[1], Meghan C. Thompson[2], Shuo MA[3], Venkatraman Seshan[2], Natalie Slupe[2], Jeremy S. Abramson[1], Prioty Islam[2], Jeffrey Barnes[1], J. Erika Haydu[1], P. Connor Johnson[1], Jane Winter[3], Ephraim Hochberg[1], Philip Caron[2], Efrat Luttwak[2], Jae H. Park[2], Anita Kumar[2], Pallawi Torka[2], Ariela Noy[2], M. Lia Palomba[2], Robert Stuver[2], Tamanna Haque[2], Raphael Steiner[2], Kevin David[2], Paul Hamlin[2], Steven Horwitz[2], Lindsey Roeker*[4], Ahmet Dogan[2], Omar Abdel-Wahab[2], Gilles Salles[2], Joanna Mi[2], Neena Mahajan[2], Jessica Pendleton[2], Alyssa Labarre[2], Clare Grieve[2], Ashlee Joseph[2], Colleen Dorsey[2], Jamila Brutus[2], Rachel Nazzaro[1], Jayne Coyle[1], Rosalba Martignetti[1], Olivia Maas[1], Elizabeth Simkins[1], Akshata Kakolu[1], Cameron Gordon[1], Eshani Baez[1], Bernadette Beatty[1], Michelle Gulloto[1], Sherry Ratcliff[1], Grace Bir[1], Robert A Redd[5], Jamie Hirata[6], Mina Shahkarami[7], Allison Jacob[8], Andrew Zelenetz[2]

*[1]Mass General Brigham Cancer Institute; Harvard Medical School, Boston, Massachusetts, United States of America;[2]Memorial Sloan Kettering Cancer Center, New York, NY, United States of America; [3]Robert H. Lurie Comprehensive Cancer Center of Northwestern University, Chicago, IL, United States of America; [4]Mayo Clinic, Rochester, MN, United States of America;[5]Dana-Farber Cancer Institute, Boston, United States of America; [6]BeOne Medicines, Ltd., San Mateo, CA, United States of America; [7]Genentech Inc., South San Francisco, CA, United States of America;[8]Adaptive Biotechnologies, Seattle, WA, United States of America;*

### Background

In the initial phase II trial of BOVen in TN CLL, treatment (Tx) duration was based on uMRD4 status and 92% achieved both peripheral blood (PB) and bone marrow (BM) uMRD4 with a median Tx duration of 10 months (range, 8-24). Response kinetics defined as ΔMRD400 (≥400-fold reduction in PB MRD by clonoSEQ [Adaptive] at C5D1) predicted more durable uMRD4 despite less time on Tx. We initiated the BOVen-ΔMRD400 phase 2 trial to evaluate if ΔMRD400 can be used to guide triplet therapy duration.

### Aims

Determine the rate of uMRD5, prospectively validate ΔMRD400 for predicting MRD outcomes, and evaluate its potential as a biomarker to guide triplet therapy duration.

### Methods

In this multicenter, phase 2 trial (NCT03824483), eligible pts had TN CLL/SLL requiring Tx per iwCLL 2018 and were enriched for TP53 aberrancy. Informed consent was obtained from all pts. BOVen was administered in 28-day cycles: Zanubrutinib 160mg PO BID started D1; Obinutuzumab 1000mg IV on D1 (or split D1-2), 8, 15 of C1, and D1 of C2-8; Venetoclax ramp up started C3D1 (target 400mg PO QD). Tx duration was guided by ΔMRD400: 10 vs 24 cycles for ΔMRD400 achievers vs non-achievers. MRD and ΔMRD400 were assessed with clonoSEQ testing. Response was assessed per iwCLL 2018. AEs was assessed per CTCAE v5.

**Copyright Information:** (Online) ISSN: 2572-9241
©2026 The Author(s). HemaSphere is published by John Wiley & Sons Ltd on behalf of European Hematology Association. This is an open access Abstract Book distributed under the Attribution-NonCommercial-NoDerivs (CC BY-NC-ND), which allows third parties to download the articles and share them with others as long as they credit the author and the Abstract Book, but they cannot change the content in any way or use them commercially.
**Abstract Book Citations:** Authors, Title, HemaSphere, 2026;10:(S1):pages. The abstract book DOIs can be found on the "Author Information" page.
**Disclaimer:** Articles published in the journal HemaSphere exclusively reflect the opinions of the authors. The authors are responsible for all content in their abstracts including accuracy of the facts, statements, citing resources, etc.

### Results

The study accrued 79 pts (2023-2025): median age 67 (35-87), male 76%, TP53 aberrant 37% (N=29; del17p only [N=18], TP53 mut only [N=4], both [N=7]), IGHV-UM 54% (N=43), 79 evaluable for safety and 78 for efficacy.
ΔMRD400 was achieved by 60% (N=47); all ΔMRD400 achievers completed Tx and all ΔMRD400 non-achievers completed ≥10 cycles (8 completed 24 cycles, all others on ongoing Tx). Of 72 pts with EOT/C13D1 MRD results (EOT after 10 cycles for ΔMRD400 achievers; C13D1 for ΔMRD400 non-achievers continuing on Tx), PB uMRD4 and uMRD5 rates were 83% (N=60) and 74% (N=53). ΔMRD400 was strongly associated with EOT/C13D1 PB uMRD4/uMRD5 overall and irrespective of IGHV or del(17p)/*TP53* mutation status (all p<0.01). All pts achieved iwCLL response (PR or CR).
Among ΔMRD400 achievers (60%; N=47), PB uMRD4 and uMRD5 rates at EOT were 100% (N=47) and 94% (44/47) and BM uMRD4 and uMRD5 rates at C10D28 were 93% (39/42) and 69% (29/42). Among ΔMRD400 non-achievers (40%; N=31), PB uMRD4 and uMRD5 rates at C13D1 were 52% (13/25) and 36% (9/25) and BM uMRD4 and uMRD5 rates at C10D28 were 30% (9/30) and 17% (5/30). Additional MRD data including EOT/2-yr for non-achievers will be presented.
We performed univariate analyses to identify factors associated with ΔMRD400. Higher risk CLL-IPI was associated with ΔMRD400 failure (p=0.025); Rai, LN diameter, ALC, B2M, IGHV status, TP53 aberrancy, and hierarchical FISH were not.
The most common AEs (all-cause) were neutropenia (58%), diarrhea (54%), thrombocytopenia (48%), bruising (46%), nausea (41%). The most common grade ≥3 AEs were neutropenia (42%), thrombocytopenia (14%), hypertension (8.9%), anemia (5.1%). No grade 5 AEs. One lab TLS on C1D1. All COVID-19 infections were grade 1-2 (N=11).

### Summary/Conclusion

BOVen with Tx duration guided by MRD kinetics is well-tolerated with high rates of uMRD4/uMRD5 within 10-12 mo. In this prospective validation, ΔMRD400 identified pts with higher rates of early PB uMRD4 (100% vs 52%) and uMRD5 (94% vs 36%). Additional follow-up in ΔMRD400 non-achievers is needed to learn if extended Tx improves outcomes. Traditional factors do not appear to be associated with ΔMRD400. Correlative investigations are underway to identify the biology underlying ΔMRD400.

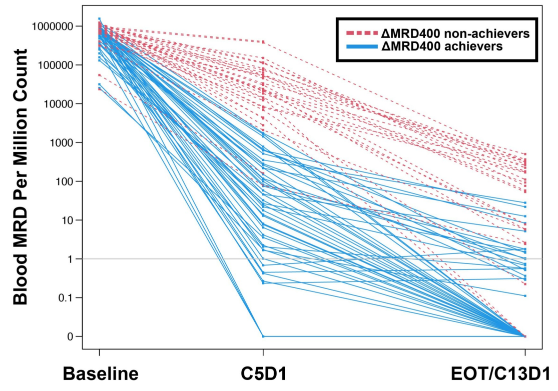

| | ΔMRD400 Achievers (N=47) |
|---|---|
| TP53 mutation or deletion, % (N) | 35% (N=16) |
| IGHV unmutated, % (N) | 55% (N=26) |
| ΔMRD (Pre to C5), median (IQR) | 12,008 (3,039-16,0349) |
| EOT (10-mo) Blood uMRD4, % (N) | 100%: (47/47) |
| EOT (10-mo) Blood uMRD5, % (N) | 94% (44/47) |
| | **ΔMRD400 Non-Achievers (N=31)** |
| TP53 mutation or deletion, % (N) | 42% (N=13) |
| IGHV unmutated, % (N) | 55% (N=17) |
| ΔMRD (Pre to C5), median (IQR) | 34 (12-102) |
| C13D1 Blood uMRD4, % (N) | 52% (13/25) |
| C13D1 Blood uMRD5, % (N) | 36% (9/25) |

ΔMRD = Baseline MRD level ÷ C5D1 MRD level measured by clonoSEQ testing.
ΔMRD400 achievers demonstrate a ΔMRD of ≥400, i.e., a ≥400-fold MRD reduction.

Copyright Information: (Online) ISSN: 2572-9241
©2026 The Author(s). HemaSphere is published by John Wiley & Sons Ltd on behalf of European Hematology Association. This is an open access Abstract Book distributed under the Attribution-NonCommercial-NoDerivs (CC BY-NC-ND), which allows third parties to download the articles and share them with others as long as they credit the author and the Abstract Book, but they cannot change the content in any way or use them commercially.
Abstract Book Citations: Authors, Title, HemaSphere, 2026;10:(S1):pages. The abstract book DOIs can be found on the "Author Information" page.
Disclaimer: Articles published in the journal HemaSphere exclusively reflect the opinions of the authors. The authors are responsible for all content in their abstracts including accuracy of the facts, statements, citing resources, etc.

### (PS1690) UPDATED RESULTS FROM A PHASE 1B STUDY OF NON-COVALENT PAN-MUTANT BTK INHIBITOR DOCIRBRUTINIB (AS-1763) IN PATIENTS WITH PREVIOUSLY TREATED B-CELL MALIGNANCIES

**Topic:** 06. Chronic lymphocytic leukemia and related disorders - Clinical

Nitin Jain*[1], Catherine Coombs[2], Nirav N Shah[3], Jonathan S. Goldberg[4], Seung Tae Lee[5], Jacqueline Barrientos[6], Sunil Babu[7], John Nemunaitis[8], Shuo MA[9], Andrew Gillis-Smith[10], Danielle M. Brander[11], Julio Peguero[12], Shirou Kirita[13], Koji Yoshida[13], Masaaki Sawa[13], Kyoko Miyamoto[14], Akinori Arimura[14], William Wierda[1], Varsha Gandhi[15], Javier Pinilla[16]

*[1]The University of Texas MD Anderson Cancer Center, Department of Leukemia, Houston, TX, United States of America; [2]University of California Irvine, Division of Hematology/Oncology, Irvine, CA, United States of America;[3]Medical College of Wisconsin, Division of Hematology/Oncology, Milwaukee, WI, United States of America; [4]Optum Medical Care, Department of Hematology and Oncology, Mount Kisco, NY, United States of America; [5]University of Maryland, Greenebaum Comprehensive Cancer, Baltimore, MD, United States of America; [6]Mount Sinai Medical Center, Miami Beach, FL, United States of America; [7]American Oncology Partners, Fort Wayne Medical Oncology and Hematology, Fort Wayne, IN, United States of America;[8]Taylor Cancer Research Center, Maumee, OH, United States of America; [9]Northwestern Memorial Hospital, Robert H. Lurie Comprehensive Cancer Center, Division of Hematology-Oncology, Chicago, IL, United States of America; [10]University of Massachusetts Memorial Medical Center, Hematology/Oncology, Worcester, MA, United States of America; [11]Duke Cancer Institute, Division of Hematologic Malignancies and Cellular Therapy, Durham, NC, United States of America; [12]Oncology Consultants, Houston, TX, United States of America; [13]Carna Biosciences, Inc., Kobe, Japan; [14]CarnaBio USA, Inc., South San Francisco, CA, United States of America; [15]The University of Texas MD Anderson Cancer Center, Department of Translational Molecular Pathology, Houston, TX, United States of America; [16]Moffitt Cancer Center, H Lee Moffitt Cancer Center and Research Institute, Tampa, FL, United States of America;*

### Background

Docirbrutinib is a highly selective, pan-mutant non-covalent BTK inhibitor (ncBTKi), which inhibits both wild-type and various c/ncBTKi-resistant mutations including C481x, T474x and L528x with $IC_{50}$ values of <10 nM and shows strong anti-tumor activity in B-cell lymphoma cell lines harboring resistant BTK mutations including a kinase-dead BTK L528W. Here we present updated results from the ongoing Phase 1b study of docirbrutinib in B-cell malignancies including chronic lymphocytic leukemia/small lymphocytic lymphoma (CLL/SLL), follicular lymphoma (FL), mantle cell lymphoma (MCL), Waldenström macroglobulinemia (WM), and marginal zone lymphoma (MZL) (NCT05602363).

### Aims

To investigate the safety, efficacy and pharmacokinetics of docirbrutinib in Phase 1b study in B-cell malignancies.

### Methods

This is a multicenter, open-label, Phase 1b study in patients (pts) with CLL/SLL or B-cell NHL who received ≥2 prior therapies including c/ncBTKis. The study includes dose escalation and expansion parts. The dose expansion part consists of three cohorts; Cohorts 1 and 2 include CLL/SLL and NHL pts, respectively, for determination of the recommended Phase 2 dose, and Cohort 3 includes CLL/SLL/MCL pts previously treated with a ncBTKi, pirtobrutinib, for exploratory efficacy evaluation.

**Copyright Information:** (Online) ISSN: 2572-9241
©2026 The Author(s). HemaSphere is published by John Wiley & Sons Ltd on behalf of European Hematology Association. This is an open access Abstract Book distributed under the Attribution-NonCommercial-NoDerivs (CC BY-NC-ND), which allows third parties to download the articles and share them with others as long as they credit the author and the Abstract Book, but they cannot change the content in any way or use them commercially.
**Abstract Book Citations:** Authors, Title, HemaSphere, 2026;10:(S1):pages. The abstract book DOIs can be found on the “Author Information” page.
**Disclaimer:** Articles published in the journal HemaSphere exclusively reflect the opinions of the authors. The authors are responsible for all content in their abstracts including accuracy of the facts, statements, citing resources, etc.

**Results**

As of 9 Feb 2026, 53 pts (30 CLL/SLL, 8 MCL, 6 FL, 5 MZL, 4 WM) were assigned to 5 dose levels: 100 (n=3), 200 (n=3), 300 (n=23), 400 (n=21), and 500 mg twice daily (BID) (n=3). Median age was 69 years (range, 46-86). The median number of prior lines of therapy for CLL/SLL was 4 (range, 2-13) including cBTKi (30/30 [100%]), BCL2i (18/30 [60%]) and pirtobrutinib (2/30 [7%]), and that for NHL was 4 (range, 2-8) including cBTKi (3/8 [38%] for MCL, 1/6 [17%] for FL, 2/5 [40%] for MZL, 2/4 [50%] for WM) and pirtobrutinib (2/8 [25%] for MCL). For 30 CLL/SLL pts, baseline genetic features included IGHV-unmutated (29/29, 100%), del(17p)/TP53-mutated (10/29, 34%), del(11q) (4/27, 15%), BTK C481x (9/25, 36%), A428D (1/25, 4%), and L528W mutations (1/25, 4%), and PLCG2 mutation (1/25, 4%). Median treatment duration was 7.4 (range, 0.4-30.3) months for CLL/SLL and 3.5 (range, 0.4-22.4) months for NHL. Among all 53 pts, no drug-related atrial fibrillation or hypertension was reported. Drug-related ≥G3 adverse events (AEs) were reported in 5 pts (9%): neutropenia in 3 pts, ALT/AST increased in 1 pt, hematuria in 1 pt, hematoma on concomitant anticoagulation, anemia, and hemorrhagic shock in 1 pt. Due to drug-related AEs, 2 pts experienced dose interruption/reduction (ALT/AST elevation and fatigue), and 1 pt discontinued (hematoma). In the efficacy evaluable pts, excluding Cohort 3, overall response rate (ORR) was 56% (14/25; 12 partial response [PR] and 2 PR with lymphocytosis) for CLL/SLL, 75% (3/4; 2 complete response and 1 PR) for MCL, 100% (4/4; 4 PR) for WM, 50% (1/2; 1 PR) for MZL, and 0% (0/5) for FL. ORR for CLL/SLL pts assigned to the 400 mg BID dose level was 88% (7/8), including 5 pts double-exposed to cBTKi and BCL2i. The median progression free survival for CLL/SLL, MCL, WM and MZL have not yet been reached. In Cohort 3, the efficacy evaluation remains immature, as 3 of 4 pts have not yet reached the first post-dose assessment point.

**Summary/Conclusion**

Docirbrutinib demonstrated a favorable safety profile and robust efficacy in pts with CLL/SLL including those double-exposed to cBTKi and BCL2i and showed promising responses in pts with MCL and WM. Updated data will be presented at the annual meeting.

**Copyright Information:** (Online) ISSN: 2572-9241
©2026 The Author(s). HemaSphere is published by John Wiley & Sons Ltd on behalf of European Hematology Association. This is an open access Abstract Book distributed under the Attribution-NonCommercial-NoDerivs (CC BY-NC-ND), which allows third parties to download the articles and share them with others as long as they credit the author and the Abstract Book, but they cannot change the content in any way or use them commercially.
**Abstract Book Citations:** Authors, Title, HemaSphere, 2026;10:(S1):pages. The abstract book DOIs can be found on the “Author Information” page.
**Disclaimer:** Articles published in the journal HemaSphere exclusively reflect the opinions of the authors. The authors are responsible for all content in their abstracts including accuracy of the facts, statements, citing resources, etc.

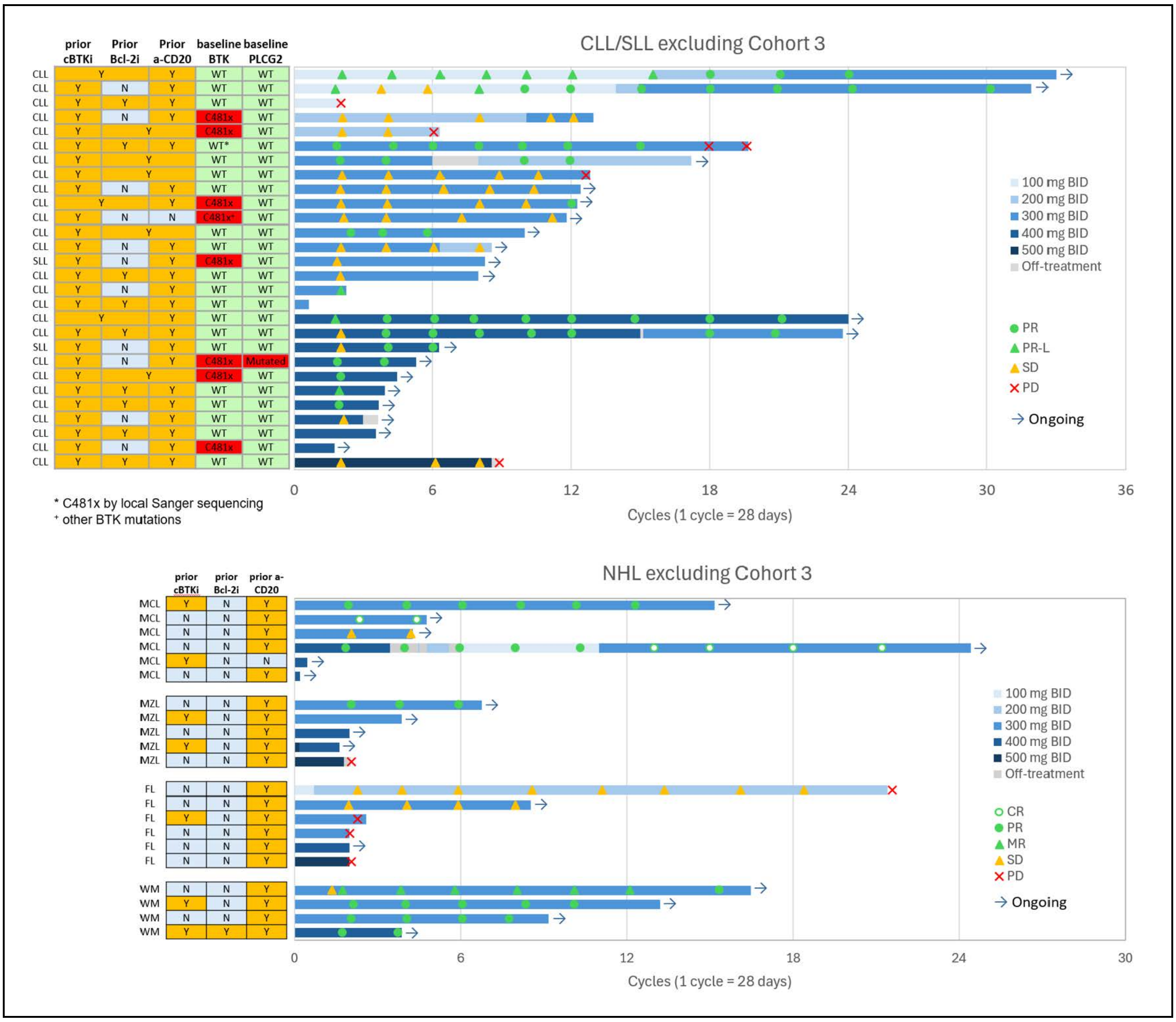

**Copyright Information:** (Online) ISSN: 2572-9241
©2026 The Author(s). HemaSphere is published by John Wiley & Sons Ltd on behalf of European Hematology Association. This is an open access Abstract Book distributed under the Attribution-NonCommercial-NoDerivs (CC BY-NC-ND), which allows third parties to download the articles and share them with others as long as they credit the author and the Abstract Book, but they cannot change the content in any way or use them commercially.
**Abstract Book Citations:** Authors, Title, HemaSphere, 2026;10:(S1):pages. The abstract book DOIs can be found on the "Author Information" page.
**Disclaimer:** Articles published in the journal HemaSphere exclusively reflect the opinions of the authors. The authors are responsible for all content in their abstracts including accuracy of the facts, statements, citing resources, etc.

### (PS1691) ERIC REAL-WORLD RETROSPECTIVE ANALYSIS OF OUTCOMES IN CLL PATIENTS TREATED WITH CHEMOTHERAPY-FREE FRONTLINE TARGETED THERAPIES DEMONSTRATES THE IMPACT OF TP53 DISRUPTION IN THE ERA OF NOVEL THERAPY.

**Topic:** 06. Chronic lymphocytic leukemia and related disorders - Clinical

Sunil Iyengar*[1], Li Yuan Chan[1], Dima El-Sharkawi[1], Persefoni Talimtzi[2], Sneha Abburu[3], Bhawana Adhikari[4], Alyssa Adlaon[5], Ahmad Alhuraiji[6], Salem Alshemmari[7], Mary Anderson[8], Maria Angelopoulou[9], Miguel Argüello-Tomás[10], Celso Arrais-Rodrigues[11], Fanny Baran Marszak[12], Aisling Barrett[13], Leonidas Benetatos[14], Ruben Blommers[15], Ana Manuel Cano[16], Aina Chang[17], Evdoxia Chatzicharisi[18], Anastasia Chatzidimitriou[2], Sofia Chatzileontiadou[18], Eliza Chung[19], Marta Coscia[20], Marta Crespo Maull[21], Gemma Cutting[1], Zadie Davis[22], Filomena De Falco[23], Maria Dimou[24], Michael Doubek[25], Maria Efstathopoulou[26], Blanca Espinet Sola[27], Toby A. Eyre[28], Gianmarco Favrin[29], Concepción Fernández-Rodriguez[27], Francesco Forconi[5], Gianluca Gaidano[30], José Antonio García Vela[31], Eva Gimeno[27], Satyen Gohil[32], Isabel Teresa González Gascón Y Marín[33], Sabrina Habib[3], Yair Herishanu[34], Eleftheria Hatzimichael[35], Kevin Joyce[36], Elżbieta Kalicińska[37], Sofia Kazmi[38], Adam Kittai[39], Andreas-Epameinondas Koumpis[35], Ioannis Kyriakou[40], Denise Lee[41], Mark-David Levin[15], Alexandros MacHairas[9], Jitka Malcikova[25], Helen Marr[42], Nicolas Martinez-Calle[38], Francesca Martini[43], Sean McKeague[8], Eva Minga[2], Riccardo Moia[30], Marta Morawska[44], Roberta Murru[45], Iliana Konstantinou[46], Uttam Kumar Nath[4], Chin Neoh[47], Maura Nicolosi[48], Ana Carla De Oliveira[49], Stephen Opat[50], MacArena Ortiz Pareja[51], Gerassimos Pangalis[26], Papageorgiou Sotirios[52], Vassiliki Pappa[52], Piers Patten[17], Verena Pfister[53], Viola Maria Popov[54], Edward Poynton[55], Anna Puiggros[27], John Riches[56], Fazla Saheed[6], Jawad Saeed[32], Benjamin Sale[5], Alessandro Sanna[57], Lydia Scarfò[58], Andrea Serafin[59], Angel Serna Pareja[21], Matevz Skerget[60], Joao Tadeu Souto Filho[61], Paolo Sportoletti[23], Niki Stavrogianni[62], Tamar Tadmor[63], Livio Trentin[59], Marzia Varettoni[29], Theodoros Vassilakopoulos[9], Candida Vitale[64], Andrea Visentin[59], Ewa Wasik-Szczepanek[65], Graham Wehmeyer[39], Cameron Wellard[19], Stella Williams[36], Claire Dearden[1], Kostas Stamatopoulos[2], Paolo Ghia[66], Sarka Pavlova[25], Thomas Chatzikonstantinou[2]

*[1]The Royal Marsden Hospital NHS Foundation Trust, Haemato-Oncology, London, United Kingdom;[2]Institute of Applied Biosciences, Centre for Research and Technology Hellas, Thessaloniki, Greece; [3]Barts Health NHS Trust, St. Bartholomew's Hospital, London, United Kingdom; [4]Department of Medical Oncology Haematology, All India Institute of Medical Sciences, Rishikesh, India; [5]Cancer Sciences, Faculty of Medicine, University of Southampton, Southampton, United Kingdom;[6]Kuwait Cancer Control Center, Kuwait city, Kuwait; [7]Faculty of Medicine, Department of Medicine, Kuwait University, Kuwait city, Kuwait; [8]Peter MacCallum Cancer Centre and Royal Melbourne Hospital, Melbourne, Australia;[9]Laikon General Hospital, Athens, Greece; [10]Hospital de la Santa Creu i Sant Pau, Barcelona, Spain;[11]Universidade Federal de São Paulo/Escola Paulista de Medicina, Department of Hematology, São Paulo, Brazil; [12]Hôpital Avicenne Assistance Publique des Hôpitaux de Paris, Service d'hématologie biologique, Bobigny, France; [13]Nuffield Department of Population Health, University of Oxford, Oxford, United Kingdom; [14]Department of Hematology, Faculty of Medicine, (School of Health Sciences), University of Ioannina, Ioannina, Greece; [15]Department of Internal Medicine, Albert Schweitzer Hospital, Dordrecht, The Netherlands; [16]Hospital Regional Universitario de Málaga, Malaga,, Spain; [17]Department of Haematology, King's College Hospital, Denmark Hill UK and Department of Haematology, Comprehensive Cancer Centre, King's College London, London, United Kingdom; [18]Aristotle University of Thessaloniki, AHEPA Hospital, Thessaloniki, Greece; [19]School of Public Health and Preventive Medicine, Monash University, Melbourne, Australia; [20]Department of Medicine and Surgery, University of Insubria, Varese, Italy;[21]Department of Medicine, Universitat Autònoma de Barcelona, Bellaterra, Spain; Servei d'Hematologia, Vall d'Hebron Hospital Universitari, Experimental Hematology, Vall d'Hebron Institute of Oncology (VHIO), Vall d'Hebron Barcelona Hospital Campus, Barcelona, Spain; [22]Royal Bournemouth Hospital, Bournemouth, United Kingdom;[23]University of Perugia, Perugia, Italy; [24]Department of Haematology and Bone Marrow Transplantation Unit, National and Kapodistrian University of Athens, Laiko, Athens, Greece; [25]Department of Internal Medicine – Hematology and Oncology, Faculty of Medicine, Masaryk university and University Hospital Brno, Brno, Czechia; [26]Iatriko Medical Center, Athens, Greece; [27]Hospital del Mar, Barcelona, Spain;[28]Department of Clinical Haematology, Oxford University Hospitals NHS Trust, Oxford, United Kingdom; [29]Fondazione IRCCS Policlinico, San Matteo, Italy; [30]Università del Piemonte Orientale, Novara, Italy; [31]Hospital Universitario Puerta de Hierro, Madrid, Spain, Madrid, Spain;*

**Copyright Information:** (Online) ISSN: 2572-9241
©2026 The Author(s). HemaSphere is published by John Wiley & Sons Ltd on behalf of European Hematology Association. This is an open access Abstract Book distributed under the Attribution-NonCommercial-NoDerivs (CC BY-NC-ND), which allows third parties to download the articles and share them with others as long as they credit the author and the Abstract Book, but they cannot change the content in any way or use them commercially.
**Abstract Book Citations:** Authors, Title, HemaSphere, 2026;10:(S1):pages. The abstract book DOIs can be found on the "Author Information" page.
**Disclaimer:** Articles published in the journal HemaSphere exclusively reflect the opinions of the authors. The authors are responsible for all content in their abstracts including accuracy of the facts, statements, citing resources, etc.

[32]Department of Haematology, University College Hospital, London, United Kingdom;[33]Hospital Universitario Infanta Leonor, Madrid, Spain; [34]Tel Aviv Sourasky Medical Center, Tel Aviv, Israel, Tel Aviv, Israel;[35]Department of Hematology, Faculty of Medicine, (School of Health Sciences), University of Ioannina., Ioannina, Greece; [36]Clatterbridge Cancer Centre NHS Foundation Trust, Liverpool, United Kingdom; [37]Department of Hematology, Cell Therapies and Internal Diseases, Wrocław Medical University, Wroclaw, Poland; [38]Department of Haematology, Nottingham University Hospitals NHS Trust, Nottingham, United Kingdom; [39]Icahn School of Medicine at Mount Sinai, New York, United States of America;[40]G. Papanicolaou Hospital, Thessaloniki, Greece; [41]Olivia Newton-John Cancer Research Institute at Austin Health, Heidelberg, Australia;[42]Department of Haematology, The Newcastle Upon Tyne Hospitals NHS Foundation Trust, Newcastle Upon Tyne, United Kingdom; [43]Strategic Research Program on CLL, Comprehensive Cancer Center, IRCCS Ospedale San Raffaele, Milano, Italy; [44]Experimental Hemato-Oncology Department, Medical University of Lublin, Lublin, Poland; [45]Hematology and Stem Cell Transplantation Unit, Ospedale Oncologico A. Businco, ARNAS G. Brotzu, Cagliari, Italy; [46]National and Kapodistrian University of Athens, Laiko Hospital, Athens, Greece; [47]Department of Haematology, Royal Surrey County Hospital, Royal Surrey NHS Trust, Guildford, United Kingdom; [48]Hematology Division A.O.U. Città della Salute e della Scienza di Torino, Torino, Italy;[49]Department of Clinical Hematology, Catalan Institute of Oncology (ICO)-Hospital Duran i Reynals, IDIBELL, Barcelona, Spain; [50]Department of Medicine, School of Clinical Sciences at Monash Health, Monash University, Clayton, Australia; [51]Hospital Regional Universitario de Málaga, Malaga, Spain; [52]2nd Department of Internal Medicine, Propaedeutic Hematology Unit, U.G.H "ATTIKON", Athens, Greece; [53]Department of Hematology, Universidade Federal de São Paulo/Escola Paulista de Medicina, São Paulo, Brazil; [54]Colentina Clinical Hospital, 1st Hematology Department, Bucharest, Romania;[55]Department of Haematology, Northwick Park Hospital, London North West University Healthcare NHS Trust, London, United Kingdom; [56]Barts Health NHS Trust, St. Bartholomew's Hospital, West Smithfield, London, United Kingdom; [57]AOU Careggi, Firenze,, Italy;[58]Università Vita-Salute San Raffaele, Milano, Italy; [59]Hematology Unit, Department of Medicine DIMED, University of Padua, Padova, Italy;[60]Department of Hematology, University Medical Centre Ljubljana, Ljubljana, Slovenia; [61]Faculdade de Medicina de Campos, Campos dos Goytacazes & Instituto Federal de Educação, Ciência e Tecnologia Fluminense, Campos dos Goytacazes, Rio de Janeiro, Brazil; [62]G. Papanicolaou Hospital, Thessaloniki, United Kingdom; [63]Bnai-Zion Medical Center, and the Ruth and Bruce Rappaport Faculty of Medicine, Technion, Haifa, Israel; [64]University of Turin, Torino, Italy; [65]Department of Hemato-Oncology and Bone Marrow Transplantation, Lublin, Poland; [66]Università Vita-Salute San Raffaele and IRCCS Ospedale San Raffaele, Milano, Italy;

**Background**

*TP53* testing is widely employed in the pre-treatment investigation of CLL. Yet the impact of*TP53* disruption on prognosis and treatment selection in the context of chemotherapy-free, frontline targeted therapy is not fully understood. Although not compared head-to-head in the context of *TP53* disruption, clinical trial data suggest that continuous therapy provides longer remissions than time-limited therapy (TLT). On the other hand, TLT has the advantage of providing treatment-free intervals while potentially limiting development of resistance to subsequent therapies.

**Aims**

We aimed to describe the clinico-biological characteristics and outcomes in a real-world cohort of CLL patients treated with frontline targeted therapies to better understand the relevance of *TP53* disruption in this setting.

**Methods**

A retrospective, multicentre analysis was conducted by the European Research Initiative on CLL across 55 centres internationally. All patients treated with frontline targeted therapy with prior testing for *TP53* disruption [*TP53* mutation, del(17p) or both] were included, irrespective of *TP53* status. Data gathered included *TP53* variant allele frequency, types and number of mutations,*IGHV* status as well as types of treatment. *TP53* variants were checked and curated centrally. Time to next treatment or death (TTNT-D) and overall survival (OS) were analysed using the Kaplan-Meier method.

**Copyright Information:** (Online) ISSN: 2572-9241
©2026 The Author(s). HemaSphere is published by John Wiley & Sons Ltd on behalf of European Hematology Association. This is an open access Abstract Book distributed under the Attribution-NonCommercial-NoDerivs (CC BY-NC-ND), which allows third parties to download the articles and share them with others as long as they credit the author and the Abstract Book, but they cannot change the content in any way or use them commercially.
**Abstract Book Citations:** Authors, Title, HemaSphere, 2026;10:(S1):pages. The abstract book DOIs can be found on the "Author Information" page.
**Disclaimer:** Articles published in the journal HemaSphere exclusively reflect the opinions of the authors. The authors are responsible for all content in their abstracts including accuracy of the facts, statements, citing resources, etc.

**Results**

Data from 1633 patients were available for analysis. Most patients were male (1041, 63.7%) with median age 68yrs (IQR=59,75yrs) at the time of frontline treatment. Patients with *TP53* disruption [*TP53* mutation and/or del(17p)], accounted for 31% of cases. *IGHV* mutation status was available in 1409 cases. Unmutated *IGHV* constituted 65.4% of total and 72.5% of *TP53* disrupted cases. Median follow up on treatment was 37.2mths (IQR=20.9, 58.8mths). Comparing patients harbouring both del(17p) and *TP53* mutation to patients with del(17p) without *TP53* mutation, median TTNT-D was 55.7mths vs 71.7mths (P=0.06) and median OS 76.7mths vs NR (p=0.012). Patients with 2 or more *TP53* mutations did not appear to have a worse outcome when compared to those with single *TP53* mutations.
In total, 1072 patients received continuous therapy including 434 *TP53* disrupted cases. *TP53* disruption conferred an inferior TTNT-D (median 56mths vs 104.3mths, p<0.001) and OS (99.6mths vs 137.6mths, p<0.001). 561 patients received TLT, including 73 cases of *TP53* disrupted CLL. Only a small minority received MRD-directed therapy within this group. In comparison to the continuous therapy cohort, median follow-up for the TLT cohort was shorter (32.8mths vs 41.3mths) with a lower median age (64yrs vs 70yrs), and a higher percentage of *IGHV* mutated cases (37.8% vs 32.9%). Median TTNT-D and OS were not reached in both *TP53* disrupted and non-disrupted groups with TLT. *TP53* disruption adversely affected TTNT-D (p=0.04) but there was no significant OS difference between *TP53* disrupted and non-disrupted cases (p=0.5) with TLT.

**Summary/Conclusion**

This is the largest study of *TP53* disrupted CLL treated with targeted therapy and provides useful insights into the impact of *TP53* disruptions in this setting. Although targeted therapy has markedly improved outcomes in *TP53* disrupted CLL, this study suggests outcomes are still inferior compared to *TP53* non-disrupted CLL. Extended follow-up is needed to fully understand the impact of frontline therapy choice in *TP53* disrupted CLL, particularly with TLT.

**Copyright Information:** (Online) ISSN: 2572-9241
©2026 The Author(s). HemaSphere is published by John Wiley & Sons Ltd on behalf of European Hematology Association. This is an open access Abstract Book distributed under the Attribution-NonCommercial-NoDerivs (CC BY-NC-ND), which allows third parties to download the articles and share them with others as long as they credit the author and the Abstract Book, but they cannot change the content in any way or use them commercially.
**Abstract Book Citations:** Authors, Title, HemaSphere, 2026;10:(S1):pages. The abstract book DOIs can be found on the “Author Information” page.
**Disclaimer:** Articles published in the journal HemaSphere exclusively reflect the opinions of the authors. The authors are responsible for all content in their abstracts including accuracy of the facts, statements, citing resources, etc.

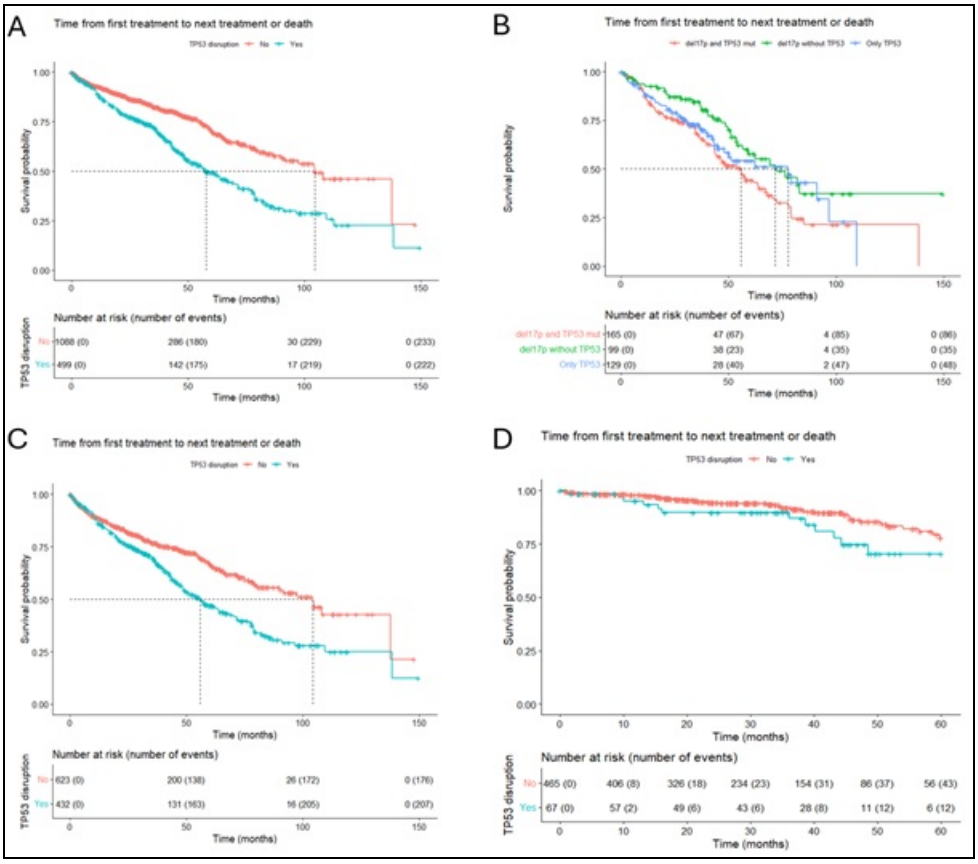

Figure 1. TTNT-D analyses (A) for overall cohort by TP53 status, (B) by TP53 mutation and 17p deletion status where both tests were performed, (C) with continuous therapy by TP53 status and (D) with time-limited therapy by TP53 status.

**Copyright Information:** (Online) ISSN: 2572-9241
©2026 The Author(s). HemaSphere is published by John Wiley & Sons Ltd on behalf of European Hematology Association. This is an open access Abstract Book distributed under the Attribution-NonCommercial-NoDerivs (CC BY-NC-ND), which allows third parties to download the articles and share them with others as long as they credit the author and the Abstract Book, but they cannot change the content in any way or use them commercially.
**Abstract Book Citations:** Authors, Title, HemaSphere, 2026;10:(S1):pages. The abstract book DOIs can be found on the "Author Information" page.
**Disclaimer:** Articles published in the journal HemaSphere exclusively reflect the opinions of the authors. The authors are responsible for all content in their abstracts including accuracy of the facts, statements, citing resources, etc.

### (PS1692) UPDATED CLINICAL DATA FROM THE PHASE 1 STUDY OF DIBOTATUG (DR-01), A NON-FUCOSYLATED ANTI-CD94 ANTIBODY, IN PATIENTS WITH LARGE GRANULAR LYMPHOCYTIC LEUKEMIA

**Topic:** 06. Chronic lymphocytic leukemia and related disorders - Clinical

Tapan Kadia*[1], Enrica Marchi[2], Ryotaro Nakamura[3], Christina Poh[3], Mengyang Di[4], Costas K. Yannakou[5, 6], Mitul Gandhi[7], Jasmine Zain[8], Steven Horwitz[8], Yumeng Zhang[9], Lubomir Sokol[9], Lauren Shea[10], Jenna Reed[11], Jennifer Richardson[11], Ruoshi Shi[11], Alissa Keegan[11], Wan-Jen Hong[11], Nenad Tomasevic[11], Natali Pflug[12], Therry Lamy[13], Marco Herling[14], Jonathan Brammer[15], Thomas Loughran Jr[2]

*[1]MD Anderson Cancer Center, Houston, United States of America;[2]University of Virginia Comprehensive Cancer Center, Charlottesville, United States of America; [3]City of Hope, Duarte, United States of America;[4]Fred Hutchinson Cancer Center, Seattle, United States of America; [5]Epworth HealthCare, Melbourne, Australia; [6]University of Melbourne, Melbourne, Australia; [7]NEXT Oncology, Fairfax, United States of America;[8]Memorial Sloan Kettering Cancer Center, New York, United States of America; [9]H. Lee Moffitt Cancer Center, Tampa, United States of America; [10]University of Alabama Birmingham, Birmingham, United States of America; [11]Dren Bio, San Carlos, United States of America;[12]University of Cologne, Cologne, Germany; [13]Pontchaillou University Hospital, Rennes, France; [14]University of Leipzig, Leipzig, Germany; [15]The Ohio State University Comprehensive Cancer Center, Columbus, United States of America;*

### Background

Large granular lymphocytic leukemia (LGLL) is a chronic, incurable lymphoproliferative disorder of cytotoxic T (γδ and CD8) and natural killer (NK) cells that leads to neutropenia, recurrent infections, and transfusion-dependent anemia. No approved therapies exist, and chronic immunosuppression with available therapy is poorly tolerated and modestly effective. Dibotatug (DR-01), a non-fucosylated human IgG antibody targeting CD94, which is expressed on NK cells, γδ T cells, and terminal effector $CD8^+$ T cells, leads to their depletion predominantly by antibody-dependent cellular cytotoxicity and is a potential new targeted therapy for patients with LGLL.

### Aims

Assess safety, PK, pharmacodynamics, and efficacy in patients with LGLL.

### Methods

A Phase 1/2 open-label, dose-escalation study (NCT05475925) assessing dibotatug enrolled LGLL patients who failed ≥1 prior line of therapy and cytotoxic lymphoma patients. Here we summarize outcomes in LGLL All patients provided informed consent.  Patients received intravenous (IV) dibotatug (0.3–10 mg/kg) followed by monthly dosing during dose escalation, followed by dose optimization was then explored at 1 or 6 mg/kg (induction) on C1D1/D8/D15 followed by once monthly thereafter. Efficacy was assessed using ECOG 5998 response criteria (Loughran 2015).

**Copyright Information:** (Online) ISSN: 2572-9241
©2026 The Author(s). HemaSphere is published by John Wiley & Sons Ltd on behalf of European Hematology Association. This is an open access Abstract Book distributed under the Attribution-NonCommercial-NoDerivs (CC BY-NC-ND), which allows third parties to download the articles and share them with others as long as they credit the author and the Abstract Book, but they cannot change the content in any way or use them commercially.
**Abstract Book Citations:** Authors, Title, HemaSphere, 2026;10:(S1):pages. The abstract book DOIs can be found on the "Author Information" page.
**Disclaimer:** Articles published in the journal HemaSphere exclusively reflect the opinions of the authors. The authors are responsible for all content in their abstracts including accuracy of the facts, statements, citing resources, etc.

**Results**

As of October 2025, 52 LGLL patients (48 [92%] T cell LGLL; 4 [8%] NK cell LGLL) were enrolled, 35 [67%] male; median age, 64 years [range, 24–86]). ECOG performance status was: 0 (33%), 1 (60%), or 2 (8%) at study entry. Primary indications for treatment were severe neutropenia in 19 (37%), transfusion-dependent anemia in 18 (35%), symptomatic anemia in 10 (19%), and neutropenia with recurrent infections in 5 (10%) patients. Median number of prior lines of therapy was 2 (range, 1–10). Prior therapies included methotrexate (n=46 [89%]), cyclophosphamide (25 [48%]), cyclosporine (23 [44%]), and alemtuzumab (6 [12%)). Time on treatment ranged from 1–24 months; 38 patients (73%) remain on study treatment.The most frequent treatment-related adverse event was infusion-related reactions (IRR; n=10 [17%]) with only 1 grade 3 event in the first patient who did not receive IRR prophylaxis. No IRR led to dose reduction or discontinuation except in the first patient before IRR prophylaxis was optimized. No deaths or dose-limiting toxicities occurred.

In 43 response-evaluable patients, the overall response rate (ORR) was 47% with (11 [26%] complete responses [CR]; 9 [21%] partial responses [PR]). Time to first response was 0.9–3.75 months; 8/19 (42%) responders reached at least PR by C2D1. Among those with CR, primary indications for treatment were neutropenia (n=6) and transfusion-dependent anemia (n=4). All responders remain in response on study treatment except for 1 patient who went on to allogeneic stem cell transplant. The longest duration of response (21+ months) was observed in a heavily pretreated patient who achieved a best response of CR. In 35 patients receiving C1D1/D8/D15 induction, ORR was 54% (29% CR; 26% PR). Dibotatug PK was linear within the dose range tested. Peripheral blood LGL count reduction and dominant TCR clonotype eradication and reduction of serum granzyme B, a cytotoxic marker, accompanied responses.

**Summary/Conclusion**

Preliminary safety and efficacy data show that dibotatug is well-tolerated. Response rates are promising with deep and durable responses which support continued dibotatug development as a novel therapy for LGLL.

**Copyright Information:** (Online) ISSN: 2572-9241
©2026 The Author(s). HemaSphere is published by John Wiley & Sons Ltd on behalf of European Hematology Association. This is an open access Abstract Book distributed under the Attribution-NonCommercial-NoDerivs (CC BY-NC-ND), which allows third parties to download the articles and share them with others as long as they credit the author and the Abstract Book, but they cannot change the content in any way or use them commercially.
**Abstract Book Citations:** Authors, Title, HemaSphere, 2026;10:(S1):pages. The abstract book DOIs can be found on the “Author Information” page.
**Disclaimer:** Articles published in the journal HemaSphere exclusively reflect the opinions of the authors. The authors are responsible for all content in their abstracts including accuracy of the facts, statements, citing resources, etc.

### (PS1693) BRUTON TYROSINE KINASE (BTK) DEGRADER BGB-16673 IN BTK INHIBITOR–NAIVE PATIENTS WITH CLL/SLL AND OTHER B-CELL MALIGNANCIES: RESULTS FROM THE PHASE 1 CADANCE-101 STUDY

**Topic:** 06. Chronic lymphocytic leukemia and related disorders - Clinical

Irina Mocanu*[1], Anna Maria Frustaci[2], Abel Costa[3], Masa Lasica[4], Christof Schneider[5], Chan Y. Cheah[6, 7, 8], Eric Mou[9], Franck Morschhauser[10], Mariana Bastos-Oreiro[11], Nikesh Shah[12], Yucai Wang[13], Yanan Zhang[14], Linlin Xu[14], Kunthel By[14], Shannon Fabre[14], Shelonitda Rose[14], Levani Makhaldiani[15]

*[1]The Institute of Oncology, ARENSIA Exploratory Medicine, Chisinau, Moldova;[2]ASST Grande Ospedale Metropolitano Niguarda, Milano, Italy; [3]D'Or Institute for Research and Education, São Paulo, Brazil; [4]St Vincent's Hospital Melbourne, Melbourne, VIC, Australia; [5]Ulm University, Department of Internal Medicine 3, Division of CLL, Ulm, Germany;[6]Sir Charles Gairdner Hospital, Nedlands, WA, Australia; [7]Medical School, University of Western Australia, Crawley, WA, Australia;[8]Linear Clinical Research, Nedlands, WA, Australia; [9]University of Iowa Hospitals and Clinics, Iowa City, IA, United States of America; [10]CHU de Lille, Lille, France; [11]Gregorio Marañón University General Hospital, Madrid, Spain; [12]Tampa General Hospital Cancer Institute, Tampa, FL, United States of America; [13]Mayo Clinic, Rochester, MN, United States of America; [14]BeOne Medicines, Ltd, San Carlos, CA, United States of America; [15]ARENSIA Exploratory Medicine, Tbilisi, Georgia;*

### Background

BGB-16673 is a BTK degrader that blocks signaling by tagging BTK for degradation through the cell's proteasome pathway, leading to tumor regression. CaDAnCe-101 (BGB-16673-101; NCT05006716) is an ongoing open-label, phase 1/2 study of BGB-16673 monotherapy in patients (pts) with B-cell malignancies. Previous reports have shown promising activity of BGB-16673 in heavily pretreated pts exposed to BTK inhibitors. This is the first report on the safety/efficacy of a BTK degrader in BTK inhibitor-naive pts.

### Aims

To report safety and efficacy results for phase 1 of the CaDAnCe-101 trial in BTK inhibitor–naive pts with B-cell malignancies.

### Methods

Eligible pts had a confirmed diagnosis of chronic lymphocytic leukemia/small lymphocytic lymphoma (CLL/SLL), mantle cell lymphoma (MCL), marginal zone lymphoma (MZL), Waldenström macroglobulinemia (WM), or Richter transformation (RT); an ECOG performance status of 0-2 (0/1 in the EU); and no prior treatment with a BTK inhibitor. BGB-16673 was administered orally at 200 mg once daily. The primary objective was to evaluate safety; secondary objectives included assessing overall response rate (ORR), with the first assessment occurring after 12 weeks of treatment (4 weeks for WM).

**Copyright Information:** (Online) ISSN: 2572-9241
©2026 The Author(s). HemaSphere is published by John Wiley & Sons Ltd on behalf of European Hematology Association. This is an open access Abstract Book distributed under the Attribution-NonCommercial-NoDerivs (CC BY-NC-ND), which allows third parties to download the articles and share them with others as long as they credit the author and the Abstract Book, but they cannot change the content in any way or use them commercially.
**Abstract Book Citations:** Authors, Title, HemaSphere, 2026;10:(S1):pages. The abstract book DOIs can be found on the "Author Information" page.
**Disclaimer:** Articles published in the journal HemaSphere exclusively reflect the opinions of the authors. The authors are responsible for all content in their abstracts including accuracy of the facts, statements, citing resources, etc.

**Results**

As of Dec 15, 2025, 54 pts (CLL/SLL, n=29; MCL, n=8; MZL, n=10; RT, n=2; WM, n=5) were enrolled and treated with BGB-16673. Median age was 68 (range, 42-81) years and pts had a median of 2 (range, 0-9) prior lines of therapy. Median follow-up was 8.3 (range, 0.4-12.8) months and 43 pts (79.6%) remained on treatment at data cutoff.
Any-grade treatment-emergent adverse events (TEAEs) occurred in 83.3% of pts; grade ≥3 TEAEs occurred in 33.3%. Any-grade TEAEs in ≥15% of pts were contusion (bruising; 27.8%) and petechiae (18.5%). Grade ≥3 TEAEs in ≥2 pts were neutropenia/neutrophil count decreased (14.8%) and anemia, hypertension, lower respiratory tract infection, pneumonia, and thrombocytopenia (each 3.7%). No cases of major hemorrhage, opportunistic infections or febrile neutropenia occurred. TEAEs led to treatment discontinuation in 2 pts (3.7%; metastatic neoplasm, n=1; back pain, n=1; neither considered treatment related) and death in 1 pt (1.9%; metastatic neoplasm in the brain; not considered treatment related); all had CLL/SLL.
In pts with CLL/SLL (n=29), the most common grade ≥3 TEAE was neutropenia/neutrophil count decreased (20.7%).
In 22 evaluable pts with CLL/SLL, the ORR was 86.4% (partial response [PR], n=15; PR with lymphocytosis, n=4) with median follow-up of 8.2 months. At data cutoff, all responses were ongoing (n=19). Median time to first response was 2.8 (range, 2.7-5.6) months, around the time of first disease assessment. Responses occurred in all 4 pts with known unmutated IGHV and in 4 of 6 pts with known del(17p) and/or *TP53* mutation. At 6 months, no progression-free survival events were observed. Efficacy is not reported at this time in other cohorts due to small sample sizes.
BTK degradation data were available for 44 pts in peripheral blood; all showed complete BTK degradation at steady-state.

**Summary/Conclusion**

In a first report of BTK degrader clinical activity in a BTK inhibitor–naive population, BGB-16673 was well tolerated. Relative to BTK inhibitor–exposed pts, BTK inhibitor–naive pts had fewer grade ≥3 TEAEs and no reported opportunistic infections or major hemorrhage. BGB-16673 shows promising antitumor activity in BTK inhibitor–naive pts with CLL/SLL. This encouraging safety and efficacy could inform future clinical trials.

**Copyright Information:** (Online) ISSN: 2572-9241
©2026 The Author(s). HemaSphere is published by John Wiley & Sons Ltd on behalf of European Hematology Association. This is an open access Abstract Book distributed under the Attribution-NonCommercial-NoDerivs (CC BY-NC-ND), which allows third parties to download the articles and share them with others as long as they credit the author and the Abstract Book, but they cannot change the content in any way or use them commercially.
**Abstract Book Citations:** Authors, Title, HemaSphere, 2026;10:(S1):pages. The abstract book DOIs can be found on the “Author Information” page.
**Disclaimer:** Articles published in the journal HemaSphere exclusively reflect the opinions of the authors. The authors are responsible for all content in their abstracts including accuracy of the facts, statements, citing resources, etc.

### (PS1694) CLINICAL, CELLULAR AND MOLECULAR EFFECTS OF SUSPENDED AND REPEATED (ON-OFF) IBRUTINIB TREATMENT IN PATIENTS WITH CHRONIC LYMPHOCYTIC LEUKEMIA IN SUSTAINED PARTIAL REMISSION.

**Topic:** 06. Chronic lymphocytic leukemia and related disorders - Clinical

Marzia Palma*[1, 2], Maria Andersson[1, 2], Julia Hauenstein[3], Nicolai Frengen[3], Lucía Peña-Pérez[4, 5], Anna Berglöf[3], Olina Lind[1], Kia Heimersson[1], Qing Wang[3], Maja Cidh Ronge[3], Tom Aadrian Mulder[1, 2], Rula Zain[3, 6, 7], Lotta Hansson[1, 2], Magdalena Kättström[8], Anders Uddevik[9], Max Flogegård[10], Teresa del Peso Santos[4], Cecilia Österholm[4], Richard Rosenquist[4, 7], C.I. Edvard Smith[3, 6], Robert Månsson[3, 11, 12], Anders Osterborg[1, 2], Jeanette Lundin[1, 2]

*[1]Karolinska Institutet, Department of Oncology-pathology, Stockholm, Sweden;[2]Karolinska University Hospital, Department of Hematology, Stockholm, Sweden; [3]Karolinska Institutet, Department of Laboratory Medicine, Stockholm, Sweden;[4]Karolinska Institutet, Department of Molecular Medicine and Surgery, Stockholm, Sweden; [5]Karolinska University Hospital, Centre for Inherited Metabolic Diseases, Stockholm, Sweden; [6]Karolinska Institutet, Karolinska ATMP Center, Stockholm, Sweden; [7]Karolinska University Hospital, Department of Clinical Genetics and Genomics, Stockholm, Sweden;[8]Örebro University Hospital, Department of Medicine, Section of Hematology, Örebro, Sweden; [9]Gävle Hospital, Department of Medicine, Section of Hematology, Gävle, Sweden; [10]Falu lasarett, Department of Medicine, Falun, Sweden;[11]Royal Institute of Technology, National Genomics Infrastructure, Science for Life Laboratory, Stockholm, Sweden; [12]Karolinska University Hospital, Department of Clinical Immunology and Transfusion Medicine, Stockholm, Sweden;*

### Background

In chronic lymphocytic leukemia (CLL), Bruton's tyrosine kinase inhibitors (BTKi) monotherapy is usually given continuously until progressive disease (PD) or intolerance, but long-term use can cause cumulative toxicity and resistance. Beyond B-cell receptor (BCR) inhibition, BTKi induce broad immunologic and molecular changes, whose reversibility after treatment interruption remains unclear.

### Aims

This investigator-initiated, multicenter phase 1b/2 study evaluated repeated response-guided interruptions of ibrutinib (ibru) in patients (pts) with CLL who achieved at least partial remission after ≥6 months (mo) of therapy.

### Methods

Treatment was discontinued and reinitiated at early asymptomatic PD defined by prespecified criteria. Peripheral blood (PB) samples were collected before treatment interruption (baseline (BL)/pre-OFF1), during the first off-treatment phase (OFF1), and before restart (pre-ON1), as well as at later interruptions and restarts (pre-OFF2, pre-ON2 etc.). When available, pre-ibru PB samples were also analyzed. Immune cell subsets were analyzed by flow cytometry (FC), plasma proteins by proximity extension assay, hotspot *BTK* p.C481 c.1442G>C and c.1441T>A mutations by droplet digital PCR (ddPCR), gene-regulatory changes (gene expression and chromatin accessibility) by RNA- and ATAC-sequencing on FC-sorted CLL cells.

**Copyright Information:** (Online) ISSN: 2572-9241
©2026 The Author(s). HemaSphere is published by John Wiley & Sons Ltd on behalf of European Hematology Association. This is an open access Abstract Book distributed under the Attribution-NonCommercial-NoDerivs (CC BY-NC-ND), which allows third parties to download the articles and share them with others as long as they credit the author and the Abstract Book, but they cannot change the content in any way or use them commercially.
**Abstract Book Citations:** Authors, Title, HemaSphere, 2026;10:(S1):pages. The abstract book DOIs can be found on the "Author Information" page.
**Disclaimer:** Articles published in the journal HemaSphere exclusively reflect the opinions of the authors. The authors are responsible for all content in their abstracts including accuracy of the facts, statements, citing resources, etc.

### Results

Forty-nine pts were enrolled; median OFF1 was 17 mo, with 73% off >12 mo, 37% >24 mo, and 5 pts >3 years. Of the 40 pts who restarted BTKi (ON1), 36 received ibru and 4 zanubrutinib (due to changed standard of care); 39 responded. Longer OFF1 was associated with mutated immunoglobulin heavy variable (IGHV) genes (median 5.3 vs 1.2 years; HR 0.24), complete response at entry, and longer prior ibru exposure (Fig.1). ddPCR detected *BTK* mutations in 7/36 pts (19%) at pre-ON1 (5 with VAF 0.03–1.1; 2 with VAF 20-94). At BL/pre-OFF1, mutations were detected in 2/7 pts.
Immune profiling at pre-OFF1, pre-ON1, pre-OFF2 and pre-ON2 showed phase-dependent changes paralleling disease burden. At pre-ON1, $CD4^+$ and $CD8^+$ T-cell counts, memory subsets, and immune checkpoint expression increased significantly vs. BL/pre-OFF1, partially normalizing after treatment resumed.
Plasma proteomic profiling performed at pre-ibru (n=11), at BL/pre-OFF1, at 2 wks, 3 mo, 6 mo, 12 mo during OFF1 and at pre-ON1 confirmed suppression of canonical BCR-responsive chemokines (e.g. CCL3 and CCL4) during ibru therapy. During OFF1, most treatment-associated protein changes reverted toward pre-ibru levels at pre-ON1, though some remained persistently altered.
For 6 selected pts with sustained remission, FC-sorted CLL cells from samples taken at pre-ibru, BL/pre-OFF1, 3 and 6 mo during OFF1 and at pre-ON1 were analyzed by ATAC-seq and RNA-seq. Differential expression/chromatin accessibility analysis identified 151 genes and 258 chromatin regions altered on-treatment; although many reverted after discontinuation, not all changes fully returned to the pre-ibru state during OFF1.

### Summary/Conclusion

Response-guided repeated interruption of ibru is feasible and safe. Treatment duration before interruption, depth of remission and IGHV mutational status may influence the length of the 1$^{st}$ off-phase. Immunologic and plasma biomarkers are mostly treatment-dependent — normalized at discontinuation, shifting with PD during the off-phase, and reverting after BTKi restart — while some transcriptional and chromatin accessibility changes in CLL cells persist even after prolonged OFF-periods.

**Copyright Information:** (Online) ISSN: 2572-9241
©2026 The Author(s). HemaSphere is published by John Wiley & Sons Ltd on behalf of European Hematology Association. This is an open access Abstract Book distributed under the Attribution-NonCommercial-NoDerivs (CC BY-NC-ND), which allows third parties to download the articles and share them with others as long as they credit the author and the Abstract Book, but they cannot change the content in any way or use them commercially.
**Abstract Book Citations:** Authors, Title, HemaSphere, 2026;10:(S1):pages. The abstract book DOIs can be found on the "Author Information" page.
**Disclaimer:** Articles published in the journal HemaSphere exclusively reflect the opinions of the authors. The authors are responsible for all content in their abstracts including accuracy of the facts, statements, citing resources, etc.

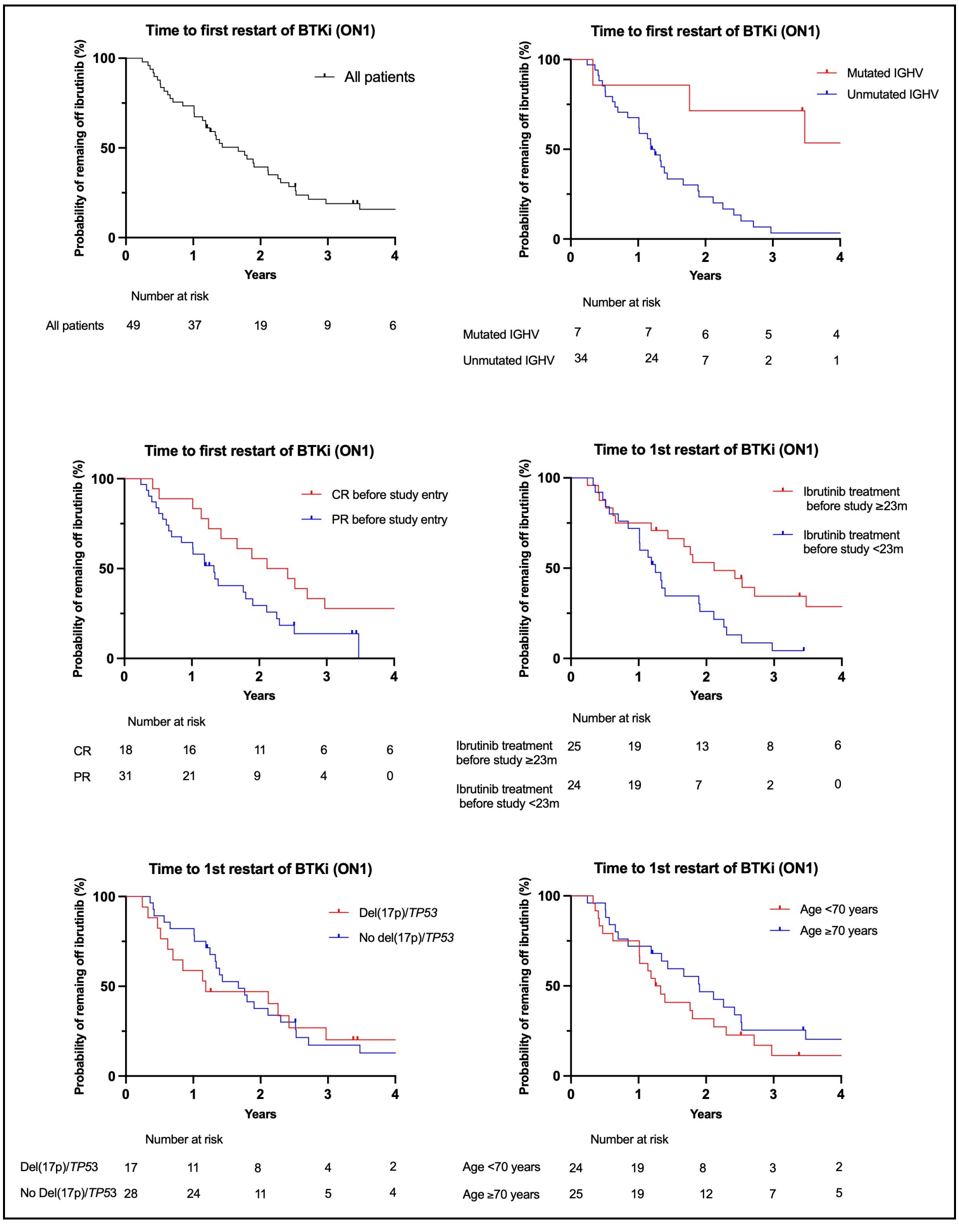

Figure 1. Kaplan–Meier estimates of time to first BTKi re-initiation stratified by age, IGHV mutational status, response status at study entry (CR vs PR), prior ibrutinib exposure (<23 vs ≥23 months), del(17p)/TP53 aberration status and age.

**Copyright Information:** (Online) ISSN: 2572-9241
©2026 The Author(s). HemaSphere is published by John Wiley & Sons Ltd on behalf of European Hematology Association. This is an open access Abstract Book distributed under the Attribution-NonCommercial-NoDerivs (CC BY-NC-ND), which allows third parties to download the articles and share them with others as long as they credit the author and the Abstract Book, but they cannot change the content in any way or use them commercially.
**Abstract Book Citations:** Authors, Title, HemaSphere, 2026;10:(S1):pages. The abstract book DOIs can be found on the "Author Information" page.
**Disclaimer:** Articles published in the journal HemaSphere exclusively reflect the opinions of the authors. The authors are responsible for all content in their abstracts including accuracy of the facts, statements, citing resources, etc.

### (PS1695) ACALABRUTINIB TREATMENT ACHIEVES DURABLE RESPONSES AND QUALITY OF LIFE IMPROVEMENTS IN OLDER (≥80Y) AND/OR FRAIL PATIENTS WITH CLL – FINAL ANALYSIS OF THE CLL-FRAIL TRIAL

**Topic:** 06. Chronic lymphocytic leukemia and related disorders - Clinical

Florian Simon*[1], Jan-Paul Bohn[2], Rudy Ligtvoet[1], Thomas Noesslinger[3], Julia von Tresckow[4], Rüdiger Liersch[5], Tobias Gaska[6], Kathleen Jentsch-Ullrich[7], Michael Gärtner[8], Thomas Wolff[9], Ingo Schwaner[10], Dominik Wolf[2], Christof Schneider[11], Ursula Vehling-Kaiser[12], Matthias Ritgen[13], Christian Spoer[14], Michael Eckart[15], Thomas Decker[16], Geothy Chakupurakal[17], Björn Schöttker[18], Jens Kisro[19], Karl-Anton Kreuzer[1], Eugen Tausch[11], Stephan Stilgenbauer[11], Christina Paulitschek[1], Katharina Löhers[1], Sandra Robrecht[1], Janina Stumpf[1], Moritz Fürstenau[1], Kirsten Fischer[1], Valentin Goede[20], Petra Langerbeins[1], Michael Hallek[1], Barbara Eichhorst[1]

*[1]Department I of Internal Medicine and Center of Integrated Oncology Aachen, Bonn, Cologne, Düsseldorf, Faculty of Medicine and University Hospital of Cologne, University of Cologne, Cologne, Germany; [2]Internal Medicine V, Department of Hematology and Oncology, Comprehensive Cancer Center Innsbruck (CCCI), Medical University of Innsbruck, Innsbruck, Germany; [3]Hanusch Krankenhaus Wien, 3rd Medical Department for Hematology and Oncology, Vienna, Austria;[4]Clinic for Hematology and Stem Cell Transplantation, West German Cancer Center, University Hospital Essen, University of Duisburg-Essen, Essen, Germany; [5]Gemeinschaftspraxis für Hämatologie und Onkologie, Clemenshospital Münster, Münster, Germany; [6]Brüderkrankenhaus St. Josef Paderborn, Klinik für Hämatologie und Onkologie, Paderborn, Germany;[7]Gemeinschaftspraxis für Hämatologie und Onkologie, Magdeburg, Germany; [8]Onkologisches Ambulanzzentrum Hannover, Hannover, Germany; [9]OncoResearch Lerchenfeld, Hamburg, Germany; [10]Onkologische Schwerpunktpraxis Kurfürstendamm, Berlin, Germany; [11]Department of Internal Medicine III, Division of CLL, Ulm University, Ulm, Germany; [12]Outpatient Clinic, Landshut, Germany; [13]Department II of Internal Medicine, University of Schleswig-Holstein, Kiel, Germany;[14]MVZ Klinikum Deggendorf, Deggendorf, Germany; [15]Practice for Oncology and Hematology, Erlangen, Germany; [16]Oncological Practice, Ravensburg, Germany; [17]Praxis für Hämatologie und Onkologie, Koblenz, Germany; [18]Practice for Oncology and Hematology, Würzburg, Germany; [19]Lübecker Onkologische Schwerpunktpraxis, Lübeck, Germany; [20]Department of Oncogeriatrics, St. Marien-Hospital, Cologne, Germany;*

### Background

The CLL-Frail trial is a prospective study specifically designed for older and/or frail patients (pts) with chronic lymphocytic leukemia (CLL). The primary endpoint analysis (Simon et. al, *Blood*, 2025) demonstrated an overall response rate (ORR) of 81.1% and 53.5% of pts reporting improvement in frailty symptoms. Adverse events (AEs), however, were common, and approximately one third of pts discontinued therapy prematurely.

### Aims

The final analysis presented here provides new efficacy and safety data after 3 years of follow-up, as well as the first comprehensive report of quality of life (QoL) outcomes.

### Methods

CLL-Frail is an investigator-initiated, prospective, multicenter phase II trial enrolling pts with active CLL aged ≥80 years and/or considered frail based on self-
assessment (via FRAIL-scale). One
prior line of treatment was allowed. Pts received acalabrutinib monotherapy until intolerance, disease progression or death. QoL was assessed via EORTC-QLQ30 and CLL17 questionnaires three-monthly until final restaging.

**Copyright Information:** (Online) ISSN: 2572-9241
©2026 The Author(s). HemaSphere is published by John Wiley & Sons Ltd on behalf of European Hematology Association. This is an open access Abstract Book distributed under the Attribution-NonCommercial-NoDerivs (CC BY-NC-ND), which allows third parties to download the articles and share them with others as long as they credit the author and the Abstract Book, but they cannot change the content in any way or use them commercially.
**Abstract Book Citations:** Authors, Title, HemaSphere, 2026;10:(S1):pages. The abstract book DOIs can be found on the "Author Information" page.
**Disclaimer:** Articles published in the journal HemaSphere exclusively reflect the opinions of the authors. The authors are responsible for all content in their abstracts including accuracy of the facts, statements, citing resources, etc.

**Results**

Between 2021 and 2023, 53 pts were enrolled and 52 received at least one dose of treatment. Median age was 81, median CIRS was 8 and 73.6% of patients had a creatinin-clearance <70ml/min. After a median follow-up of 34 months, five additional pts discontinued treatment after the primary endpoint analysis, resulting in 29 of 52 pts (55.8%) remaining on therapy until the per protocol defined end of trial in May 2025. Sixteen of 23 (69.7%) early treatment discontinuations occurred before cycle 18.
A total of seven progressive disease events were observed, all occurring in pts who had previously discontinued treatment. Median progression-free survival (PFS) was not reached, with a 3-year PFS rate of 70.1% (95% CI: 56.7 - 83.5**) (Figure 1A**). Seven deaths occurred overall; infections, including COVID-19, accounted for 57.1% of all deaths, while one was attributed to CLL. Median overall survival (OS) was not reached, with a 3-year OS rate of 85.5% (75.6 - 95.5) (Figure 1B).
Atrial fibrillation and hypertension/hypertensive crises each occurred in 4 pts (7.7%). Infections remained a concern, with 34.6% of all pts experiencing ≥°3 infections. However, adverse event trajectories from month 3 to month 24 showed that the severe and related (as assessed by investigator) incidence rates of adverse events decreased from 204.4 (102.1 – 365.8) to 46 (5.6 – 166.2) and 408 (256.2 – 619) to 69 (14.2 – 201.6), respectively, per 1000 patient months.
Improvements in self-assessed frailty were durable at final restaging (cycle 24, day 1), with 18.9% of pts considering themselves frail, compared to 47.8% at screening and 20.9% at interim staging (cycle 7, day 1). Patient-reported QoL improved across multiple domains, with frail pts showing larger gains compared to non-frail pts, particularly in global health status, physical functioning, and role functioning (mean increases from baseline to peak of 45.5→74.2, 43.6→66.4, and 39.6→74.2, respectively), followed by partial attenuation at month 24 (final restaging) (**Figure 1C and D**).

**Summary/Conclusion**

Acalabrutinib treatment in old and/or frail pts with CLL results in durable responses and sustained improvements in frailty and quality of life. These findings underscore the potential of targeted therapies to provide meaningful clinical benefit across age and frailty groups.

**Copyright Information:** (Online) ISSN: 2572-9241
©2026 The Author(s). HemaSphere is published by John Wiley & Sons Ltd on behalf of European Hematology Association. This is an open access Abstract Book distributed under the Attribution-NonCommercial-NoDerivs (CC BY-NC-ND), which allows third parties to download the articles and share them with others as long as they credit the author and the Abstract Book, but they cannot change the content in any way or use them commercially.
**Abstract Book Citations:** Authors, Title, HemaSphere, 2026;10:(S1):pages. The abstract book DOIs can be found on the "Author Information" page.
**Disclaimer:** Articles published in the journal HemaSphere exclusively reflect the opinions of the authors. The authors are responsible for all content in their abstracts including accuracy of the facts, statements, citing resources, etc.

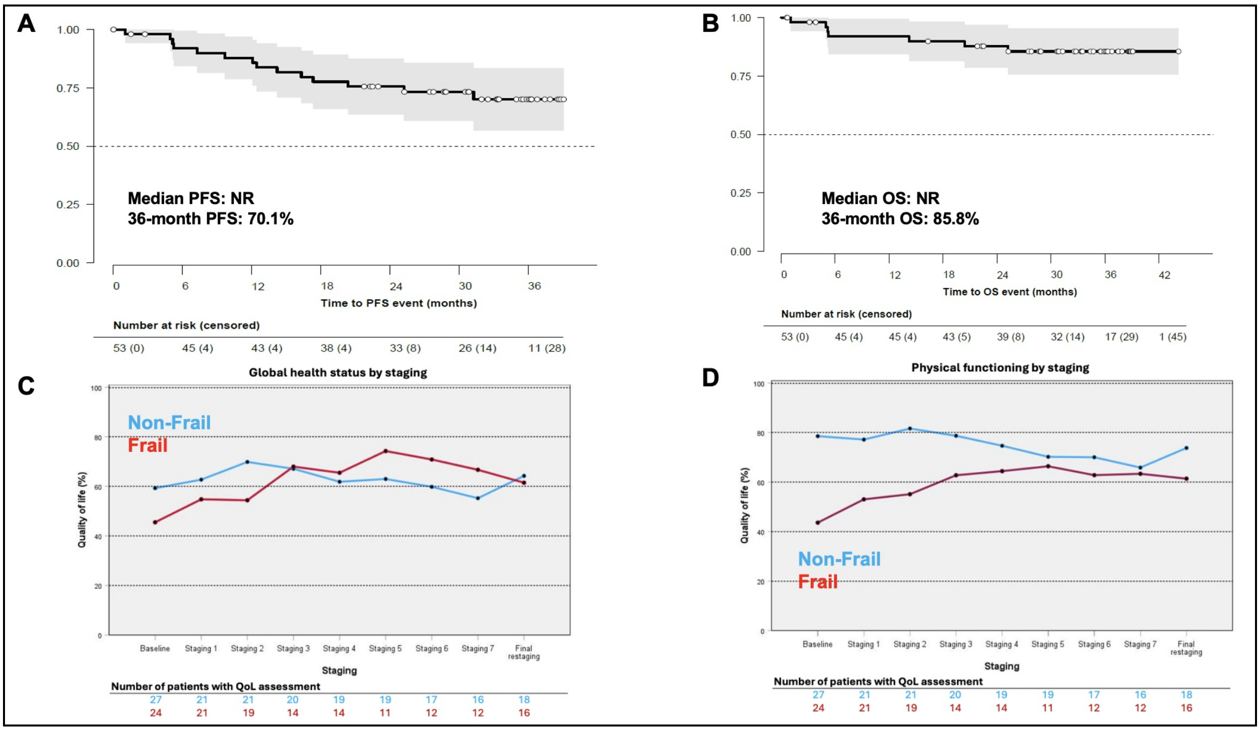

Figure 1: Survival and QoL gains during acalabrutinib treatment. A: Kaplan-Meier curves for Progression-Free Survival (PFS). B: Kaplan-Meier Curves for Overall Survival (OS). C: Patient reported quality of life for global health status. D: Patient-reported quality of life for physical functioning. Light blue: FRAIL scale score <3; red: FRAIL Scale score ≥3.

**Copyright Information:** (Online) ISSN: 2572-9241
©2026 The Author(s). HemaSphere is published by John Wiley & Sons Ltd on behalf of European Hematology Association. This is an open access Abstract Book distributed under the Attribution-NonCommercial-NoDerivs (CC BY-NC-ND), which allows third parties to download the articles and share them with others as long as they credit the author and the Abstract Book, but they cannot change the content in any way or use them commercially.
**Abstract Book Citations:** Authors, Title, HemaSphere, 2026;10:(S1):pages. The abstract book DOIs can be found on the "Author Information" page.
**Disclaimer:** Articles published in the journal HemaSphere exclusively reflect the opinions of the authors. The authors are responsible for all content in their abstracts including accuracy of the facts, statements, citing resources, etc.

### (PS1696) INCIDENCE OF REAL-WORLD CARDIOVASCULAR (CV) EVENTS IN PATIENTS INITIATING COVALENT BTK INHIBITORS (CBTKI) IN 1L CHRONIC LYMPHOCYTIC LEUKEMIA (CLL) IN THE UNITED STATES

**Topic:** 06. Chronic lymphocytic leukemia and related disorders - Clinical

David Dingli*[1], Siyang Leng[2], Mohammed Farooqui[2], Halit Yapici[3], Jonathan Lilley[3], Weiqi Jiao[3], Hayden Hyatt[3], Xuan Zhang[3], Kunal Lodaya[3], Mavis Obeng-Kusi[2], Enrico de Nigris[4]

*[1]Mayo Clinic, Rochester, United States of America; [2]Merck & Co., Inc., Rahway, United States of America; [3]Boston Strategic Partners, Boston, United States of America; [4]MSD (UK) Limited, London, United Kingdom;*

### Background cBTKis have become a standard of care in CLL/small lymphocytic lymphoma (SLL) patients. However, cBTKis have been linked to an increased risk of CV disease. While 2$^{nd}$-generation cBTKis (acalabrutinib and zanubrutinib) may offer improved safety profiles compared to 1$^{st}$-generation ibrutinib, it is crucial to evaluate the occurrence of cardiovascular toxicities in real-world practice.

### Aims

This study evaluated the occurrence of incident cardiovascular events in CLL/SLL patients during first-line (1L) treatment with cBTKis in the real-world setting.

### Methods

Patients diagnosed with CLL/SLL who initiated cBTKi treatment (index date) as 1L between 2020-2025 were identified using claims data (Optum's de-identified Clinformatics® Data Mart Database). Patient demographics were assessed. The occurrence of incident (not present pre-index) CV events was assessed during the treatment-specific observation period. Incident CV event rates were reported per 1,000 patient-months and compared among cBTKi treatment groups using incidence rate ratios, with p-values derived from two-tailed Z-tests based on a normal approximation of the log-transformed rates.

### Results

A total of 3,370 patients (40.4% female) were identified with a mean (SD) age of 71.9 (8.7) years and a treatment-specific observation period of 16.8 (13.1) months. Of all patients, 1,122 (33.3%) initiated ibrutinib, 1,480 (43.9%) initiated acalabrutinib, and 768 (22.8%) initiated zanubrutinib.

Overall, 32.7% of patients receiving 1$^{st}$-generation cBTKis and 18.8% for those receiving 2$^{nd}$-generation cBTKis developed at least one incident CVAE. On a per-1000 patient month basis, the occurrence rate for specific CV events in patients treated with 1$^{st}$-generation cBTKis compared to those treated with 2$^{nd}$-generation cBTKis were as follows (rates shown first, followed by p-value; % of patients with events): 22.9 vs 13.0 (p<0.001; 36.7% vs 18.9%) for hypertension; 8.8 vs 4.6 (p<0.001; 16.6% vs 6.9%) for atrial flutter (AF); 8.3 vs 4.4 (p<0.001; 15.7% vs 6.6%) for atrial fibrillation (AFib); 7.0 vs 6.1 (p=0.205; 13.8% vs 8.9%) for heart failure; 3.1 vs 2.2 (p=0.039; 6.3% vs 3.2%) for myocardial infarction; 1.9 vs 1.9 (p=0.875; 3.9% vs 2.8%) for cardiomyopathy; 1.6 vs 0.6 (p<0.001; 3.2% vs 1.0%) for ventricular arrhythmias; and 0.7 vs 0.5 (p=0.258; 1.4% vs 0.7%) for cardiac-related death. For those treated with 2$^{nd}$-generation cBTKis, the rate of developing at least one CV event (of all types assessed) was similar between acalabrutinib vs zanubrutinib treated patients (14.2 vs 13.4 per-1000 patient months, p=0.565; 21.0% vs 14.5%, Figure 1).

**Copyright Information:** (Online) ISSN: 2572-9241
©2026 The Author(s). HemaSphere is published by John Wiley & Sons Ltd on behalf of European Hematology Association. This is an open access Abstract Book distributed under the Attribution-NonCommercial-NoDerivs (CC BY-NC-ND), which allows third parties to download the articles and share them with others as long as they credit the author and the Abstract Book, but they cannot change the content in any way or use them commercially.
**Abstract Book Citations:** Authors, Title, HemaSphere, 2026;10:(S1):pages. The abstract book DOIs can be found on the "Author Information" page.
**Disclaimer:** Articles published in the journal HemaSphere exclusively reflect the opinions of the authors. The authors are responsible for all content in their abstracts including accuracy of the facts, statements, citing resources, etc.

### Summary/Conclusion

While 2nd-generation cBTKis demonstrate improved CV safety compared to 1st-generation cBTKis, the incidence of CV events– such as hypertension, AF, and AFib– remains substantial among cBTKi treated patients. Acalabrutinib and zanubrutinib demonstrated similar CV profiles; however, CV rates, particularly hypertension, remain high across 2nd-generation agents. Although often asymptomatic, hypertension significantly increases the long-term risk for major CV events, such as myocardial infarction and stroke. Collectively, these results underscore the critical unmet need for management strategies and novel therapies that both improve cardiovascular tolerability and efficacy.

**Figure 1. Cardiovascular Event Rates (PPPM) in 1L Patients with CLL/SLL Treated with cBTKis**

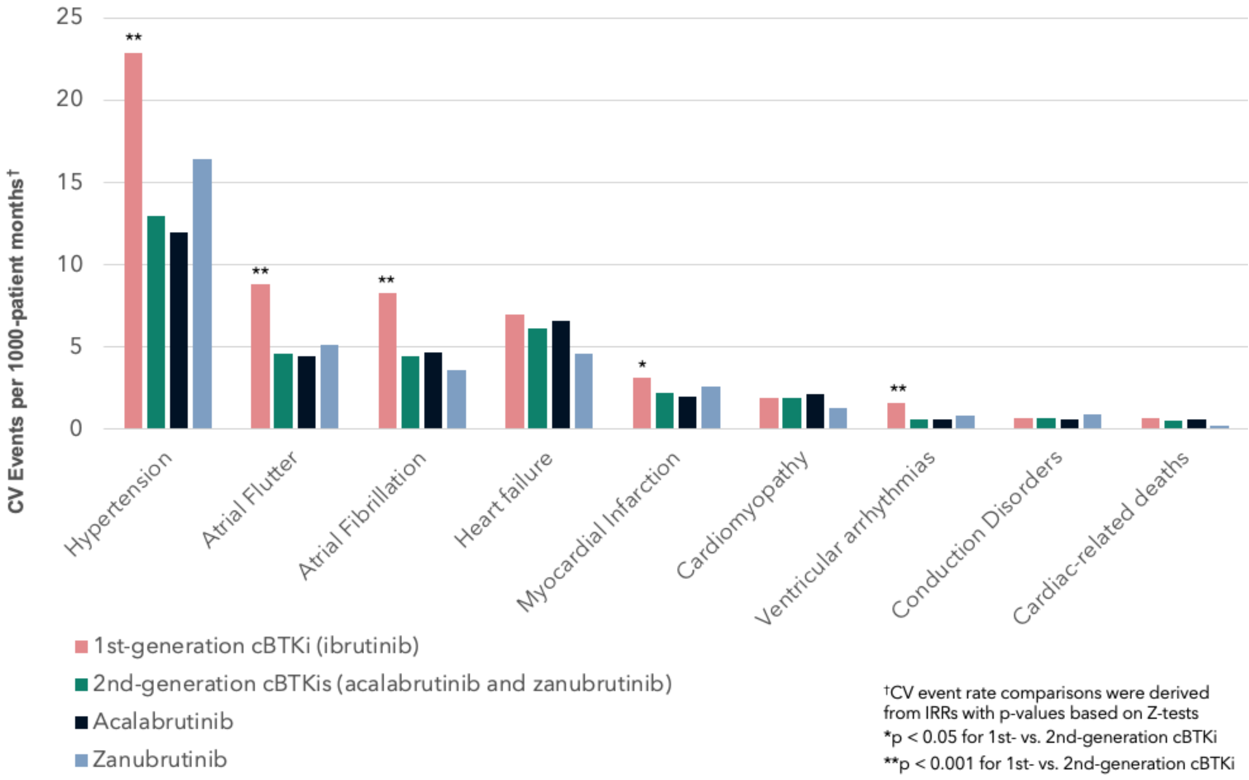

**Copyright Information:** (Online) ISSN: 2572-9241
©2026 The Author(s). HemaSphere is published by John Wiley & Sons Ltd on behalf of European Hematology Association. This is an open access Abstract Book distributed under the Attribution-NonCommercial-NoDerivs (CC BY-NC-ND), which allows third parties to download the articles and share them with others as long as they credit the author and the Abstract Book, but they cannot change the content in any way or use them commercially.
**Abstract Book Citations:** Authors, Title, HemaSphere, 2026;10:(S1):pages. The abstract book DOIs can be found on the "Author Information" page.
**Disclaimer:** Articles published in the journal HemaSphere exclusively reflect the opinions of the authors. The authors are responsible for all content in their abstracts including accuracy of the facts, statements, citing resources, etc.

### (PS1697) UPDATED SAFETY AND EFFICACY OF ALL-ORAL SONROTOCLAX + ZANUBRUTINIB IN RELAPSED/REFRACTORY (R/R) CHRONIC LYMPHOCYTIC LEUKEMIA/SMALL LYMPHOCYTIC LYMPHOMA (CLL/SLL), INCLUDING PATIENTS WITH DEL(17P)/TP53

**Topic:** 06. Chronic lymphocytic leukemia and related disorders - Clinical

Stephen Opat*[1], Constantine S. Tam[2], Mary Anderson[3, 4], Alessandra Tedeschi[5], Emma Verner[6, 7], Masa Lasica[8], Alejandro Arbelaez[9], Stephan Stilgenbauer[10], Peter Browett[11], Sophie Leitch[12], Eva Gonzalez Barca[13], Mazyar Shadman[14, 15], Jing-Zhou Hou[16], Herbert Eradat[17], David Westerman[18, 19], Binghao Wu[20], Gabriel Man[21], Yiqian Fang[20], Sheel Patel[21], Remus Vezan[21], Chan Y. Cheah[22, 23, 24]

*[1]Lymphoma Research Group, School of Clinical Sciences at Monash Health, Monash University, Clayton, VIC, Australia;[2]Alfred Hospital and Monash University, Melbourne, VIC, Australia; [3]Royal Melbourne Hospital and Peter MacCallum Cancer Centre, Melbourne, VIC, Australia; [4]The Walter and Eliza Hall Institute, Melbourne, VIC, Australia; [5]ASST Grande Ospedale Metropolitano Niguarda, Milano, Italy; [6]Concord Repatriation General Hospital, Concord, NSW, Australia;[7]University of Sydney, Sydney, NSW, Australia; [8]St Vincent's Hospital Melbourne, Melbourne, VIC, Australia;[9]Pindara Private Hospital, Benowa, QLD, Australia; [10]Ulm University, Ulm, Germany; [11]Auckland City Hospital, Grafton, Auckland, New Zealand; [12]Te Whatu Ora, Health New Zealand-Waitemata, Auckland, New Zealand; [13]Institut Català d'Oncologia Hospitalet, Universitat de Barcelona, IDIBELL, Barcelona, Spain; [14]Fred Hutchinson Cancer Center, Seattle, WA, United States of America; [15]University of Washington, Seattle, WA, United States of America; [16]University of Pittsburgh Medical Center, Pittsburgh, PA, United States of America;[17]David Geffen School of Medicine at UCLA, Los Angeles, CA, United States of America; [18]Peter MacCallum Cancer Centre, Melbourne, VIC, Australia; [19]University of Melbourne, Melbourne, VIC, Australia;[20]BeOne Medicines, Ltd, Shanghai, China;[21]BeOne Medicines, Ltd, San Carlos, CA, United States of America; [22]Sir Charles Gairdner Hospital, Nedlands, WA, Australia;[23]Medical School, University of Western Australia, Crawley, WA, Australia; [24]Linear Clinical Research, Nedlands, WA, Australia;*

### Background

BCL2 + BTK inhibition is an effective 1L treatment (tx) strategy for CLL/SLL but undetectable minimal residual disease (uMRD) rates are suboptimal. Sonrotoclax (BGB-11417; sonro), a next-generation BCL2 inhibitor, is a more selective and pharmacologically potent inhibitor of BCL2 than venetoclax, with a shorter half-life and no drug accumulation. Zanubrutinib (zanu), a next-generation BTK inhibitor, has shown superior progression-free survival (PFS) with fewer cardiac adverse events vs ibrutinib in R/R CLL/SLL.

### Aims

To report updated safety/efficacy for sonro + zanu (SZ) in patients (pts) with R/R CLL/SLL from BGB-11417-101 (NCT04277637), a phase 1/1b global study.

### Methods

Pts with R/R CLL/SLL (≥1 prior tx; no progressive disease [PD] on prior BTK inhibitor) received zanu (320 mg QD or 160 mg BID) for 8-12 wk before sonro was added by ramp-up to target dose (40/80/160/320/640 mg QD). Tx continued until PD, unacceptable toxicity, or protocol-defined elective discontinuation after 96 wk. The primary endpoint was safety/tolerability. Secondary/exploratory endpoints included overall response rate (ORR; partial response [PR] with lymphocytosis or better per iwCLL 2018) and peripheral blood (PB) uMRD4 by flow cytometry, respectively.

**Copyright Information:** (Online) ISSN: 2572-9241
©2026 The Author(s). HemaSphere is published by John Wiley & Sons Ltd on behalf of European Hematology Association. This is an open access Abstract Book distributed under the Attribution-NonCommercial-NoDerivs (CC BY-NC-ND), which allows third parties to download the articles and share them with others as long as they credit the author and the Abstract Book, but they cannot change the content in any way or use them commercially.
**Abstract Book Citations:** Authors, Title, HemaSphere, 2026;10:(S1):pages. The abstract book DOIs can be found on the "Author Information" page.
**Disclaimer:** Articles published in the journal HemaSphere exclusively reflect the opinions of the authors. The authors are responsible for all content in their abstracts including accuracy of the facts, statements, citing resources, etc.

**Results**

As of 06Dec2025, 47 pts with R/R CLL/SLL were enrolled, including 22 to the sonro 320-mg cohort. Median age was 65 y (range, 36-76); 37% (16/43) had *TP53* mutation or del(17p) and 77% (34/44) had unmutated IGHV. Pts had a median of 1 prior tx (range, 1-3); median duration of last systemic tx was 5.0 mo (range, 0.1-86.6). Seven pts had a BTK inhibitor as a last prior tx. Overall, median study follow-up was 37.8 mo (range, 10.2-57.8). Of 47 pts, 16 (34%) remained on tx and 31 (66%) discontinued SZ, most due to protocol-defined elective discontinuation (51%); 3 pts (2%) discontinued due to PD.

TEAEs led to discontinuation of SZ in 4 pts (8%; arthralgia, myelodysplastic syndrome, meningococcal sepsis, and multiple myeloma) and zanu alone in 1 pt (2%; intracranial hemorrhage). Grade ≥3 TEAEs occurred in 72%, most commonly neutropenia (28%), hypertension (11%), and pneumonia (11%). Serious TEAEs occurred in 47%; pneumonia (11%), cellulitis (9%), and pyrexia (4%) were those seen in >1 pt. No TLS occurred, and no TEAEs led to death.

In 46 efficacy-evaluable pts, ORR was 98% and complete response (CR) rate was 54%. Median time to response was 2.6 mo (range, 1.5-24.6). Overall, 36-mo PFS rate was 95% (95% CI, 83%-99%). In 21 efficacy-evaluable pts in the sonro 320-mg cohort, ORR was 100% (52% CR) and median time to response was 2.5 mo (range, 1.6-6.9). Of 3 evaluable BTK inhibitor-pretreated pts, 3 achieved PR. For the sonro 320-mg cohort (median follow-up, 28.7 mo [range, 20.1-48.9]), the 24-month PFS rate was 94% (95% CI, 67%-99%). Only 1 PD was observed, in a pt with *TP53* mutation/del(17p)/mutated IGHV. In 24 pts who electively discontinued, median time off tx was 11.3 mo (range, 0.2-21.5).

In 21 MRD-evaluable pts in the sonro 320-mg cohort, the best PB uMRD4 rate was 86% (18/21). Median time from reaching sonro target dose to uMRD4 was 5.3 mo (range, 2.5-16.3). Best uMRD4 rates by wk 24, 48, and 96 were 52% (11/21), 81% (17/21) and 82% (14/17), respectively. No pt with uMRD4 reverted to MRD4+.

**Summary/Conclusion**

In pts with R/R CLL/SLL, sonro 320 mg + zanu was well tolerated, with low rates of tx discontinuation due to TEAEs, and continued to demonstrate substantial antitumor activity (ORR 100%; CR 52%; uMRD4 86%), including in BTK inhibitor-pretreated pts.

**Copyright Information:** (Online) ISSN: 2572-9241
©2026 The Author(s). HemaSphere is published by John Wiley & Sons Ltd on behalf of European Hematology Association. This is an open access Abstract Book distributed under the Attribution-NonCommercial-NoDerivs (CC BY-NC-ND), which allows third parties to download the articles and share them with others as long as they credit the author and the Abstract Book, but they cannot change the content in any way or use them commercially.
**Abstract Book Citations:** Authors, Title, HemaSphere, 2026;10:(S1):pages. The abstract book DOIs can be found on the "Author Information" page.
**Disclaimer:** Articles published in the journal HemaSphere exclusively reflect the opinions of the authors. The authors are responsible for all content in their abstracts including accuracy of the facts, statements, citing resources, etc.

### (PS1698) REAL-WORLD CARDIOVASCULAR BURDEN, TREATMENT (TX) SEQUENCES, AND CLINICAL OUTCOMES IN PATIENTS INITIATING 1L COVALENT BRUTON'S TYROSINE KINASE INHIBITORS (CBTKIS) IN CHRONIC LYMPHOCYTIC LEUKEMIA (CLL)

**Topic:** 06. Chronic lymphocytic leukemia and related disorders - Clinical

Enrico de Nigris[1], Siyang Leng[2], Mohammed Farooqui[2], Halit Yapici[3], Jonathan Lilley[3], Weiqi Jiao[3], Hayden Hyatt[3], Xuan Zhang[3], Kunal Lodaya[3], Mavis Obeng-Kusi[2], David Dingli*[4]

*[1]MSD (UK) Limited, London, United Kingdom;[2]Merck & Co., Inc., Rahway, United States of America;[3]Boston Strategic Partners, Boston, United States of America; [4]Mayo Clinic, Rochester, United States of America;*

### Background cBTKis, including 1$^{st}$-generation (1$^{st}$-gen) ibrutinib (ibru) and 2$^{nd}$-generation (2$^{nd}$-gen) acalabrutinib (acala) and zanubrutinib (zanu), are a mainstay of tx for CLL/Small lymphocytic lymphoma (SLL). While randomized trials have demonstrated improved cardiovascular (CV) tolerability with 2nd-gen cBTKis compared with ibru, real-world (RW) data describing CV burden, tx patterns (utilization and sequencing) and clinical outcomes remain limited.

### Aims

This study evaluated the RW pre-tx CV disease burden, tx patterns, and clinical outcomes in patients (pts) initiating cBTKis.

### Methods

Adult pts diagnosed with CLL/SLL who initiated cBTKi (ibru, acala, or zanu) as first-line (1L) therapy between 2020-2025 (index date) were identified using claims data (Optum's de-identified Clinformatics® Data Mart Database). Pt demographics, pre-tx CV disease prevalence, and tx sequences including re-treatments and switch to a sequential cBTKi were assessed. Clinical outcomes of time to CV event (TTCE) and time to next-tx (NT) or death (TTNT+D) were estimated with Kaplan-Meier (KM) methods and compared via log-rank tests; hazard ratios (HRs) and 95% confidence intervals (CIs) were evaluated with Cox proportional hazards models. Analyses compared 1$^{st}$- vs. 2$^{nd}$-gen cBTKis and between 2$^{nd}$-gen cohorts.

### Results

Overall, 3,370 pts (40.4% female) were identified with a mean (SD) age of 71.9 (8.7) years. At baseline, 65.0% of pts had hypertension, 13.3% had heart failure, 11.4% had atrial fibrillation, and 10.4% had atrial flutter. Acala was the most prominent tx (43.9%), followed by ibru (33.3%), and zanu (22.8%). Uptake of 2$^{nd}$-gen cBTKis was higher in recent years: 58.1% of zanu and 25.8% of acala pts initiated therapy in 2024 vs. 8.2% for ibru. Overall, 835 pts received NT; this included 36.2% (406/1,122) of ibru and 19.1% (429/2,248) of 2$^{nd}$-gen cBTKi pts. Among pts with NT, 17.6% and 24.3% received B-cell lymphoma 2 inhibitor (BCL2i) and CD20±chemotherapy (CIT), respectively. Of ibru pts receiving NT, 28.3% underwent re-treatment and 36.0% switched to an alternative cBTKi tx. For 2$^{nd}$-gen cBTKi pts receiving NT, 37.1% were re-treated and 15.4% switched to an alternative cBTKi. Additionally, the KM estimate for remaining free of incident CV events at 24 months was 73.9% with 2$^{nd}$-gen cBTKis vs 63.1% with ibru ($p<0.001$; HR [CI]: 0.65 [0.56–0.75]; Fig. 1); median TTCE not reached (NR). Similarly, 2nd-gen cBTKis resulted in higher probability of not receiving NT or dying at 24 months compared to ibru (62.5% vs. 55.4%; $p < 0.001$; HR [CI]: 0.79 [0.71–0.88]), with a median TTNT+D of 2.7 years vs. 2.3 years. No differences were detected between the 2nd-gen cBTKis acala and zanu for TTCE and TTNT+D (log rank test $p > 0.05$).

**Copyright Information:** (Online) ISSN: 2572-9241
©2026 The Author(s). HemaSphere is published by John Wiley & Sons Ltd on behalf of European Hematology Association. This is an open access Abstract Book distributed under the Attribution-NonCommercial-NoDerivs (CC BY-NC-ND), which allows third parties to download the articles and share them with others as long as they credit the author and the Abstract Book, but they cannot change the content in any way or use them commercially.
**Abstract Book Citations:** Authors, Title, HemaSphere, 2026;10:(S1):pages. The abstract book DOIs can be found on the "Author Information" page.
**Disclaimer:** Articles published in the journal HemaSphere exclusively reflect the opinions of the authors. The authors are responsible for all content in their abstracts including accuracy of the facts, statements, citing resources, etc.

### Summary/Conclusion

Our findings show the high and increasing uptake of $2^{nd}$-gen cBTKis over time as 1L tx for CLL/SLL. Notably, the data reveal a considerable pre-treatment CV burden among pts treated in the RW setting, with ~two-thirds of pts having hypertension. Clinically, $2^{nd}$-gen cBTKis showed improvements in TTCE and TTNT+D over ibru, with similar rates observed between acala and zanu. Nevertheless, the high rates of cBTKi switching/restart following 1L therapy alongside the frequent use of CIT as NT, which deviates from current standards of care, emphasize an ongoing unmet need in these pts. RW studies with longer follow-up are crucial to evaluate CV risks, clinical outcomes with $2^{nd}$-gen cBTKis, and to guide development of novel therapies, particularly for patients with a high baseline CV burden.

**Figure 1. Kaplan-Meier Estimates for TTCE and TTNT+D in Patients with CLL/SLL Treated with 1st vs. $2^{nd}$-Generation cBTKis**

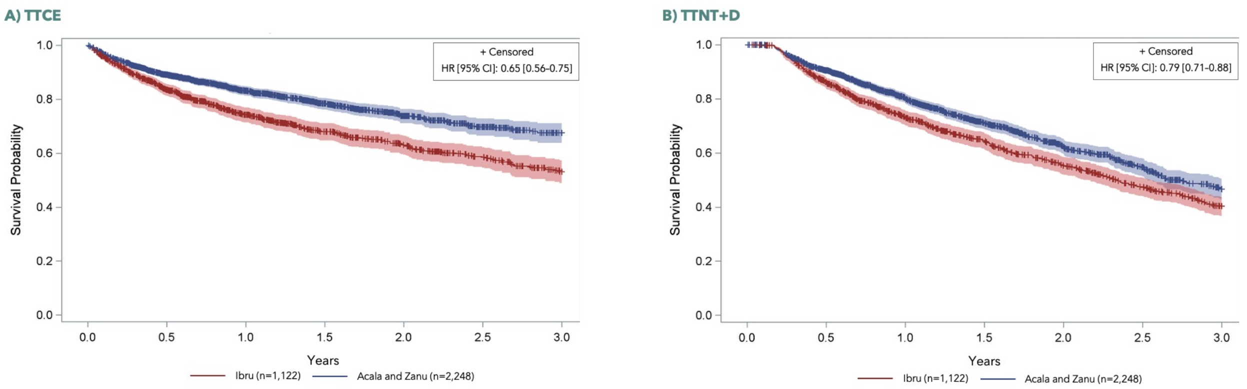

**Copyright Information:** (Online) ISSN: 2572-9241
©2026 The Author(s). HemaSphere is published by John Wiley & Sons Ltd on behalf of European Hematology Association. This is an open access Abstract Book distributed under the Attribution-NonCommercial-NoDerivs (CC BY-NC-ND), which allows third parties to download the articles and share them with others as long as they credit the author and the Abstract Book, but they cannot change the content in any way or use them commercially.
**Abstract Book Citations:** Authors, Title, HemaSphere, 2026;10:(S1):pages. The abstract book DOIs can be found on the "Author Information" page.
**Disclaimer:** Articles published in the journal HemaSphere exclusively reflect the opinions of the authors. The authors are responsible for all content in their abstracts including accuracy of the facts, statements, citing resources, etc.

### (PS1699) SECONDARY CANCERS IN CLL PATIENTS TREATED WITH TARGETED THERAPIES AT FIRST LINE: A REAL-WORLD COMPARATIVE STUDY

**Topic:** 06. Chronic lymphocytic leukemia and related disorders - Clinical

Liron Hofstetter*[1, 2], Ofir Wolach[1, 2], Pia Raanani[1, 2, 3], Emma Hajaj[4, 5]

*[1]Davidoff Cancer Centre, Rabin Medical Centre, Institute of Hematology, Petah Tikva, Israel;[2]Tel Aviv University, The Gray Faculty of Medical and Health Sciences, Tel Aviv, Israel; [3]Felsenstein Medical Research centre, Rabin Medical Centre, Petah Tikva, Israel; [4]Rabin Medical centre, Department of Internal Medicine DI, Petah Tikva, Israel;[5]The Weizmann Institute of Science, . Department of Molecular Cell Biology, Rehovot, Israel;*

### Background

Patients with chronic lymphocytic leukemia (CLL) exhibit impaired innate and adaptive immunity, predisposing them to secondary primary malignancies (SPMs). While historical chemotherapy increased this risk, real-world data regarding SPMs in the era of targeted therapies remain limited. Understanding if these agents alter the natural history of SPM development compared to observation is critical for clinical management

### Aims

We aimed to evaluate the risk of secondary cancer in CLL patients treated with targeted agents at first line and to compare them to the risk of CLL patients in observation.

### Methods

De-identified patient-level data were retrieved from Clalit Health Services (CHS). Patients aged 40–90 years diagnosed with CLL (ICD9: 204.1) between 01.01.2004- 01.03.2024, were extracted. Three groups were formed: (1) CLL patients without treatment; (2) First-line CLL patients treated with BTKi (Ibrutinib or Acalabrutinib); and (3) Patients treated with VO. Patients with a diagnosis of malignancy prior to CLL diagnosis or treatment were excluded.

**Copyright Information:** (Online) ISSN: 2572-9241
©2026 The Author(s). HemaSphere is published by John Wiley & Sons Ltd on behalf of European Hematology Association. This is an open access Abstract Book distributed under the Attribution-NonCommercial-NoDerivs (CC BY-NC-ND), which allows third parties to download the articles and share them with others as long as they credit the author and the Abstract Book, but they cannot change the content in any way or use them commercially.
**Abstract Book Citations:** Authors, Title, HemaSphere, 2026;10:(S1):pages. The abstract book DOIs can be found on the "Author Information" page.
**Disclaimer:** Articles published in the journal HemaSphere exclusively reflect the opinions of the authors. The authors are responsible for all content in their abstracts including accuracy of the facts, statements, citing resources, etc.

### Results

6084 patients with CLL were extracted from the database, The final analysis included 2,517 untreated patients, 153 BTKi-treated patients, and 73 VO-treated patients. The VO group was younger compared to the BTKi group and had a lower lymphocyte count and higher GFR (table1).
At 2 years of follow-up, the cumulative incidence (CI) of hematological malignancies (HM) in males was 8.2% in the observation cohort, 2.9% in the BTKi group, and 2.2% in the VO group, with no statistically significant differences between treatments. In females, CI were 7.0%, 4.6%, and 0%, respectively, likewise without significant differences. By 4 years, HM CI in males increased to 10.7%, 13.1%, and 2.2%, respectively, and in females to 9.9%, 4.6%, and 0%, with all comparisons remaining non-significant.
For skin malignancies (SKM), the 2-year CI in males was 7.5% with no treatment, 6.7% with BTKi, and 12.0% with VO. In females, 2-year SKM CI was 4.7%, 1.9%, and 6.3%, respectively, without statistically significant differences. At 4 years, SGM CI rose to 12.4%, 6.7%, and 12.0% in males and to 8.7%, 1.9%, and 14.1% in females, with no significant pairwise differences observed.
The CI for solid non-skin malignancies in males at 2 years were 6.6% with no treatment, 4.4% with BTKi, and 4.9% with VO; in females, corresponding rates were 7.2%, 6.5%, and 8.4%, with no significant differences. By 4 years, male estimates converged at 10% across all therapies (9.7–9.9%; all $p$=1.0). In females, 4-year CI ranged from 6.5% with BTKi to 14.9% with VG, but differences were not statistically significant ($p \geq 0.5$).

Table 1 Patient's characteristics

| Feature | no T (n=2517) | BTKI (n=153) | VO (n=73) |
|---|---|---|---|
| Age (years) | 71.48±11.1 | 69.11±10.4 | 65.39±9.8 |
| Male (N (%)) | 1293 (51.4) | 93 (60.8) | 48 (65.7) |
| WBC (x10^9/L) | 22.25±26.3 | 111.03±83.7 | 86.01±92.5 |
| HGB (g/dL) | 13.11±1.9 | 11.14±1.8 | 11.39±2.2 |
| PLT (x10^3/µL) | 222.31±86.4 | 154.51±61.0 | 157.48±76.2 |
| LYMP.abs (x10^3/µL) | 13.02±16.1 | 85.54±76.4 | 62.13±70.4 |
| GLOBULIN (g/dL) | 2.68±0.5 | 2.37±0.7 | 2.3±0.6 |
| LACTATE DEHYDROGENASE (LDH) (IU/L) | 386.32±181.8 | 378.56±177.3 | 465.12±379.0 |
| ALBUMIN (g/dL) | 4.18±0.4 | 4.31±0.4 | 4.13±0.5 |
| GLUCOSE- BLOOD (mg/dL) | 114.79±43.3 | 109.65±37.4 | 112.31±39.4 |
| GFR_CKDEPII | 74.11±20.5 | 70.54±18.7 | 76.66±20.2 |

### Summary/Conclusion

Targeted treatment does not increase the risk for secondary malignancies in comparison to CLL in observation. Time limited treatment with VO confers the same risk as BTKi for development of secondary malignancies.

**Copyright Information:** (Online) ISSN: 2572-9241
©2026 The Author(s). HemaSphere is published by John Wiley & Sons Ltd on behalf of European Hematology Association. This is an open access Abstract Book distributed under the Attribution-NonCommercial-NoDerivs (CC BY-NC-ND), which allows third parties to download the articles and share them with others as long as they credit the author and the Abstract Book, but they cannot change the content in any way or use them commercially.
**Abstract Book Citations:** Authors, Title, HemaSphere, 2026;10:(S1):pages. The abstract book DOIs can be found on the "Author Information" page.
**Disclaimer:** Articles published in the journal HemaSphere exclusively reflect the opinions of the authors. The authors are responsible for all content in their abstracts including accuracy of the facts, statements, citing resources, etc.

### (PS1700) TREATMENT AND OUTCOMES OF ACCELERATED CHRONIC LYMPHOCYTIC LEUKEMIA/ SMALL LYMPHOCYTIC LYMPHOMA IN THE TARGETED THERAPY ERA

**Topic:** 06. Chronic lymphocytic leukemia and related disorders - Clinical

Rafal Asmaro[1], Emma Holmes[1], Russell Sterrett[2], Amelie Fontaine[2], Lesley Street[1], Sarah Perry[1], Nanette Cox-Kennett[1], Anthea Peters[2], Etienne Mahe[1], Robert Puckrin*[1]

*[1]Arthur Child Comprehensive Cancer Centre and University of Calgary, Calgary, Canada; [2]Cross Cancer Institute and University of Alberta, Edmonton, Canada;*

### Background

Accelerated chronic lymphocytic leukemia/small lymphocytic lymphoma (CLL/SLL) is an uncommon but clinically aggressive histologic variant for which limited data exist in the era of targeted therapies.

### Aims

To describe the clinical characteristics, treatment patterns, and outcomes of accelerated CLL/SLL.

### Methods

This retrospective, population-based study included consecutive patients with accelerated CLL/SLL in Alberta, Canada between 2015 and 2023. Tissue biopsies were independently reviewed by expert hematopathologists to confirm the presence of ≥1 diagnostic criterion: expanded proliferation centers (>20× field) or high proliferation rate (>2.4 mitoses/proliferation center or Ki-67 >40%).

**Copyright Information:** (Online) ISSN: 2572-9241
©2026 The Author(s). HemaSphere is published by John Wiley & Sons Ltd on behalf of European Hematology Association. This is an open access Abstract Book distributed under the Attribution-NonCommercial-NoDerivs (CC BY-NC-ND), which allows third parties to download the articles and share them with others as long as they credit the author and the Abstract Book, but they cannot change the content in any way or use them commercially.
**Abstract Book Citations:** Authors, Title, HemaSphere, 2026;10:(S1):pages. The abstract book DOIs can be found on the "Author Information" page.
**Disclaimer:** Articles published in the journal HemaSphere exclusively reflect the opinions of the authors. The authors are responsible for all content in their abstracts including accuracy of the facts, statements, citing resources, etc.

**Results**

Accelerated CLL/SLL was identified in 58 of 2,284 patients diagnosed with CLL/SLL, corresponding to a prevalence of 2.5%. Median age was 69 years (range 51-95), and 35 (60%) were male. An antecedent history of CLL/SLL was present in 35 (60%), while 3 (5%) had prior Richter transformation (RT); 13 (22%) had received prior therapy for CLL/SLL or RT.
Diagnosis of accelerated CLL/SLL was established on biopsy of lymph node (n=54), nasopharynx/tonsil (n=2), bowel (n=1), or bone (n=1), which was performed due to clinical suspicion for RT in 36 (62%). IGHV was mutated in 5 (9%), unmutated in 20 (34%), indeterminate in 7 (12%), and untested in 26 (45%). Deletion(17p)/TP53 mutation was identified in 15/42 (36%) evaluable cases.
At diagnosis, peripheral blood lymphocytosis >5 was present in 44 (76%) and serum LDH was elevated in 28/54 (52%). Hypercalcemia developed in 10 (17%). Involved disease sites at diagnosis included peripheral blood (n=49), lymph nodes (n=56), spleen (n=32), marrow (n= 9), and other extranodal sites (n=15).
Median number of systemic therapies following diagnosis was 1 (range 0-6). Forty-seven (81%) patients received systemic therapy, initiated at diagnosis in 40 (69%) or after a period of active surveillance in 7 (12%). Nine (16%) were managed with observation alone and never required systemic treatment, while 1 (2%) died of disease and 1 (2%) was lost to follow-up before treatment initiation.
First-line therapy included a covalent BTK inhibitor (cBTKi; n=17), chemoimmunotherapy (n=15), BCL2 inhibitor (BCLi; n=13), cBTKi+BCL2i (n=1), and anti-CD20 monoclonal antibody (n=1). Second-line therapy consisted of a BCL2i (n=7), chemoimmunotherapy (n=5), cBTKi (n=4), and non-covalent BTKi (ncBTKi; n=1). Subsequent therapies included a cBTKi (n=3), ncBTKi (n=3),
chemoimmunotherapy (n=3), BCL2i (n=2), and BTKi + allotransplant (n=2).
Median progression-free survival (PFS) following first-line therapy was 27.8 months and numerically favored targeted therapy over chemoimmunotherapy (median PFS 65.2 versus 27.7 months, p=0.29). Median PFS was 26.6 months after second-line and 7.1 months after third-line therapy. Biopsy-confirmed RT developed in 7 (12%).
With a median follow-up of 4.4 years, median overall survival was 6.2 years from diagnosis. Causes of death included CLL/SLL or RT (n=12), infection (n=5), other malignancies (n=4), and other/unknown causes (n=5).

**Summary/Conclusion**

Although targeted therapies may partially improve outcomes, accelerated CLL/SLL remains associated with aggressive clinical behavior, a relatively high risk of transformation, and suboptimal outcomes, underscoring the need for further research into understanding the adverse disease biology and optimizing treatment strategies for this high-risk subgroup.

**Copyright Information:** (Online) ISSN: 2572-9241
©2026 The Author(s). HemaSphere is published by John Wiley & Sons Ltd on behalf of European Hematology Association. This is an open access Abstract Book distributed under the Attribution-NonCommercial-NoDerivs (CC BY-NC-ND), which allows third parties to download the articles and share them with others as long as they credit the author and the Abstract Book, but they cannot change the content in any way or use them commercially.
**Abstract Book Citations:** Authors, Title, HemaSphere, 2026;10:(S1):pages. The abstract book DOIs can be found on the “Author Information” page.
**Disclaimer:** Articles published in the journal HemaSphere exclusively reflect the opinions of the authors. The authors are responsible for all content in their abstracts including accuracy of the facts, statements, citing resources, etc.

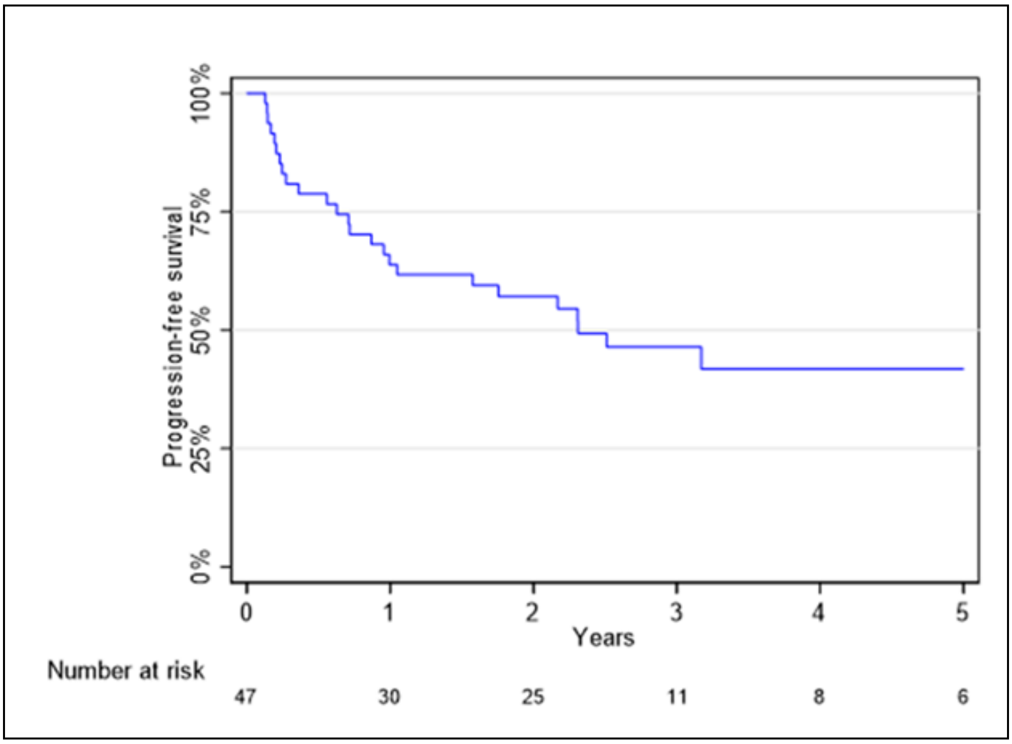

Figure 1: Progression-free survival from start of first-line therapy

**Copyright Information:** (Online) ISSN: 2572-9241
©2026 The Author(s). HemaSphere is published by John Wiley & Sons Ltd on behalf of European Hematology Association. This is an open access Abstract Book distributed under the Attribution-NonCommercial-NoDerivs (CC BY-NC-ND), which allows third parties to download the articles and share them with others as long as they credit the author and the Abstract Book, but they cannot change the content in any way or use them commercially.
**Abstract Book Citations:** Authors, Title, HemaSphere, 2026;10:(S1):pages. The abstract book DOIs can be found on the "Author Information" page.
**Disclaimer:** Articles published in the journal HemaSphere exclusively reflect the opinions of the authors. The authors are responsible for all content in their abstracts including accuracy of the facts, statements, citing resources, etc.

### (PS1701) PIRTOBRUTINIB IN TREATMENT-NAIVE PATIENTS WITH CLL/SLL: POOLED RESULTS FROM BRUIN CLL-313 AND BRUIN CLL-314

**Topic:** 06. Chronic lymphocytic leukemia and related disorders - Clinical

Jennifer Woyach*[1], William Wierda[2], José Antonio García Vela[3], Marcelo Capra[4], Gerardo Musuraca[5], Michal Kwiatek[6], Lugui Qiu[7], Jianyong Li[8], Hiro Tatetsu[9], Shinsuke Iida[10], Kamel Laribi[11], Lutz Jacobasch[12], Catherine Coombs[13], Katharine Lewis[14], Katherine Bao[15], Alejandro Levy[15], Yuanyuan Bian[15], Weiji Su[15], Marisa Hill[16], Wojciech Jurczak[17]

*[1]The Ohio State University Comprehensive Cancer Center, Columbus, OH, United States of America;[2]University of Texas MD Anderson Cancer Center, Houston, TX, United States of America; [3]Department of Haematology, Hospital Universitario Puerta de Hierro, Madrid, Spain; [4]Centro Integrado de Hematologia e Oncologia, Hospital Mãe de Deus, Porto Alegre, Brazil;[5]Hematology Unit, IRCCS Istituto Romagnolo per lo Studio dei Tumori (IRST) “Dino Amadori”, Mendola, Italy; [6]AIDPORT Clinical Trials Hospital, Skorzewo, Poland; [7]Institute of Hematology & Blood Diseases Hospital, Chinese Academy of Medical Sciences, Tianjin, China; [8]Department of Hematology, Key Laboratory of Nanjing Medical University, The First Affiliated Hospital of Nanjing Medical University, Jiangsu Province Hospital, Collaborative Innovation Center for Cancer Personalized Medicine, Nanjing, China; [9]Department of Hematology, Rheumatology and Infectious Diseases, Kumamoto University Hospital, Kuma moto, Japan; [10]Department of Hematology and Oncology, Nagoya City University Graduate School of Medical Sciences, Nagoya, Japan; [11]CH Le Mans, Le Mans, France; [12]Praxis of Haematology and Oncology, Dresden, Germany; [13]University of California Irvine, California, United States of America; [14]Division of Haematology, Sir Charles Gairdner Hospital, Nedlands, WA, Australia;[15]Eli Lilly and Company, Indianapolis, IN, United States of America; [16]Eli Lilly and Company, Clinical Research, Indianapolis, IN, United States of America; [17]National Research Institute of Oncology, Krakow, Poland;*

### Background

Pirtobrutinib, a highly selective, non-covalent Bruton tyrosine kinase inhibitor (BTKi), is approved globally for patients (pts) with chronic lymphocytic leukemia/small lymphocytic lymphoma (CLL/SLL) previously treated with a covalent BTKi.

### Aims

BRUIN CLL-313 and BRUIN CLL-314 are open-label, randomized phase 3 studies evaluating pirtobrutinib for therapy of pts with CLL/SLL, including in the treatment-naïve (TN) setting [versus bendamustine plus rituximab (BendaR; CLL-313, NCT05023980) and versus ibrutinib (CLL-314, NCT05254743)].

### Methods

Pooled data from TN CLL pts randomized to receive pirtobrutinib from CLL-313 and CLL-314 were used to assess efficacy [overall response rate (ORR), progression-free survival (PFS) and overall survival (OS)] and safety. PFS and OS estimates were calculated by the Kaplan-Meier method. All pts (253) randomized within each study to receive pirtobrutinib are included in efficacy analyses and all pts (252) who received pirtobrutinib treatment are included in safety analyses.

**Copyright Information:** (Online) ISSN: 2572-9241
©2026 The Author(s). HemaSphere is published by John Wiley & Sons Ltd on behalf of European Hematology Association. This is an open access Abstract Book distributed under the Attribution-NonCommercial-NoDerivs (CC BY-NC-ND), which allows third parties to download the articles and share them with others as long as they credit the author and the Abstract Book, but they cannot change the content in any way or use them commercially.
**Abstract Book Citations:** Authors, Title, HemaSphere, 2026;10:(S1):pages. The abstract book DOIs can be found on the “Author Information” page.
**Disclaimer:** Articles published in the journal HemaSphere exclusively reflect the opinions of the authors. The authors are responsible for all content in their abstracts including accuracy of the facts, statements, citing resources, etc.

**Results**

A total of 253 TN pts, median age of 66 yrs, were assigned to receive pirtobrutinib (CLL-313 n=141; CLL-314 n=112); all but one received treatment. Among pts with evaluable samples 134/233 (58%) had unmutated IGHV, 14/253 (6%) had del(17p) and 21/220 (10%) had mutated TP53. Efficacy was consistent between the two studies (Table). Overall, ORR was 93.3%. Median PFS was not reached; the 24-month PFS rate was 93.2% (95%CI, 89.1-95.8). Median OS was also not reached; the 24-month OS rate was 97.5% (95%CI, 94.6-98.9). Median duration of therapy was 29.1 months. Treatment discontinuation due to TEAEs occurred in 4.8% of pts with 1.6% treatment related. Any-grade infection occurred in 59.9% of pts; neutropenia in 18.3% and grade ≥3 bleeding in 2.4%. Any-grade hypertension occurred in 10.7% of pts and atrial fibrillation/flutter was reported in 2.8%.

**Copyright Information:** (Online) ISSN: 2572-9241
©2026 The Author(s). HemaSphere is published by John Wiley & Sons Ltd on behalf of European Hematology Association. This is an open access Abstract Book distributed under the Attribution-NonCommercial-NoDerivs (CC BY-NC-ND), which allows third parties to download the articles and share them with others as long as they credit the author and the Abstract Book, but they cannot change the content in any way or use them commercially.
**Abstract Book Citations:** Authors, Title, HemaSphere, 2026;10:(S1):pages. The abstract book DOIs can be found on the “Author Information” page.
**Disclaimer:** Articles published in the journal HemaSphere exclusively reflect the opinions of the authors. The authors are responsible for all content in their abstracts including accuracy of the facts, statements, citing resources, etc.

**Summary/Conclusion**

In 253 TN pts with CLL randomized on two phase 3 studies, pirtobrutinib showed a high ORR and favorable 24-month PFS and OS rates. The safety profile was consistent with prior studies using pirtobrutinib in more heavily treated pts, with low rates of atrial fibrillation/flutter, hypertension and TEAE-related discontinuations. These findings reinforce the potential of pirtobrutinib as a meaningful and clinically relevant treatment option for pts with TN disease.

**Table. Efficacy of Pirtobrutinib in TN patients with CLL/SLL**

| | Parameter | CLL-313<br>N = 141 | CLL-314<br>N = 112 | Pooled TN pts<br>N=253 |
|---|---|---|---|---|
| ORR | ORR, % (95% CI) | 94.3 (89.1, 97.5) | 92.0 (85.3, 96.3) | 93.3 (89.5, 96.0) |
| PFS | 24-month PFS rates, % (95% CI) | 92.7 (86.8, 96.0) | 94.3 (87.8, 97.4) | 93.2 (89.1-95.8) |
| | Median FU, months | 28.1 | 22.4 | 27.7 |
| OS | 24-month OS rates, % (95% CI) | 97.8 (93.3, 99.3) | 97.2 (91.5, 99.1) | 97.5 (94.6-98.9) |
| | Median FU, months | 32.7 | 26.7 | 29.5 |

ORR includes a best response of partial response or better

© 2026 American Society of Clinical Oncology, Inc. Reused with permission. This abstract was accepted and previously presented at the 2026 ASCO Annual Meeting. All rights reserved.

**Copyright Information:** (Online) ISSN: 2572-9241
©2026 The Author(s). HemaSphere is published by John Wiley & Sons Ltd on behalf of European Hematology Association. This is an open access Abstract Book distributed under the Attribution-NonCommercial-NoDerivs (CC BY-NC-ND), which allows third parties to download the articles and share them with others as long as they credit the author and the Abstract Book, but they cannot change the content in any way or use them commercially.
**Abstract Book Citations:** Authors, Title, HemaSphere, 2026;10:(S1):pages. The abstract book DOIs can be found on the “Author Information” page.
**Disclaimer:** Articles published in the journal HemaSphere exclusively reflect the opinions of the authors. The authors are responsible for all content in their abstracts including accuracy of the facts, statements, citing resources, etc.

### (PS1702) UNDETECTABLE MINIMAL RESIDUAL DISEASE AND SURVIVAL ASSOCIATION IN OVER 4000 FIRST-LINE CHRONIC LYMPHOCYTIC LEUKEMIA PATIENTS: ARTIFICIAL INTELLIGENCE–ASSISTED PATIENT-LEVEL PATIENT ANALYSES

**Topic:** 06. Chronic lymphocytic leukemia and related disorders - Clinical

Anthony Mato*[1, 2], Nicola Rath[3], Florencia Tettamanti[3], Azadeh Shohoudi[4], Sonia Rodrigues[3], Ioannis Kagiampakis[5], Mishka Gidwani[4], Carol Moreno[6], Judy Li[5], Simon Rule[3], Mauricio Oberti[4], Veerendra Mungalavadla[5], Sashka Dimitrievska[4]

*[1]AstraZeneca, New York, United States of America;[2]Cayuga Medical Center, Ithaca, United States of America;[3]AstraZeneca, Cambridge, United Kingdom;[4]AstraZeneca, Gaithersburg, United States of America;[5]AstraZeneca, South San Francisco, United States of America;[6]AstraZeneca, Barcelona, Spain;*

### Background

Minimal residual disease (MRD) assessment in chronic lymphocytic leukemia (CLL) may inform clinical decision making and accelerate therapy development. However, heterogeneity in treatment regimens and methodologies challenges validation of MRD as a surrogate for survival, especially given recent data (AMPLIFY, CLL17) showing Bruton tyrosine kinase and B-cell lymphoma 2 inhibitor doublets (BTKi–BCL2i) achieve longer progression-free survival (PFS) despite lower undetectable MRD (uMRD) rates.

### Aims

Leveraging artificial intelligence (AI)-supported approaches, we built a framework for literature review, data extraction, and cross-trial harmonization enabling evaluation of MRD and survival. We tested MRD–survival associations using trial and patient (pt)-level data from 4337 first-line (1L) CLL pts.

### Methods

An AI-supported literature review identified time-limited 1L CLL trials reporting MRD and Kaplan-Meier (KM) plots of PFS/overall survival (OS) stratified by MRD status. MRD was quantified by flow cytometry, next-generation sequencing, or allele-specific oligonucleotide polymerase chain reaction (ASO-PCR) within 6 months post-end of treatment (EOT). Sample size, median age, follow-up, regimen, MRD methods, and outcomes were extracted for harmonization. If needed, individual survival data were reconstructed from digitized KM curves with validated AI and quality control. uMRD was defined at $10^{-4}$ sensitivity in the peripheral blood. Cox models estimated hazard ratios (HR) and 95% confidence intervals (CIs).

### Results

In total, 4337 pts from 12 global 1L studies were included (2937 uMRD pts). Baseline: 61 y median age, 61% unmutated immunoglobulin heavy-chain variable region gene (uIGHV), 10% del17p, 53.5% chemoimmunotherapy (CIT)-treated, 44.6% venetoclax (Ven)-based treated. Across the entire study cohort, uMRD was associated with longer PFS (HR 0.26; 95% CI 0.23–0.29 [n=4337]) and OS (HR 0.35; 95% CI 0.29–0.42 [n=3136]). In subgroup analyses, uMRD was associated with improved PFS in pts treated with CIT, Ven-based combinations, and BTKi–BCL2i doublets (**Table**). Further, in the BTKi–BCL2i subgroup, uMRD was associated with significantly longer PFS in pts with uIGHV but not mutated IGHV (**Table**). Notably, detectable MRD (dMRD) pts on BTKi–BCL2i doublets had longer PFS vs dMRD pts on CIT/Ven–obinutuzumab (HR 0.43; 95% CI 0.34–0.54). uMRD's prognostic value was not impacted by MRD methods within the confinement of $10^{-4}$ assay sensitivity.

**Copyright Information:** (Online) ISSN: 2572-9241
©2026 The Author(s). HemaSphere is published by John Wiley & Sons Ltd on behalf of European Hematology Association. This is an open access Abstract Book distributed under the Attribution-NonCommercial-NoDerivs (CC BY-NC-ND), which allows third parties to download the articles and share them with others as long as they credit the author and the Abstract Book, but they cannot change the content in any way or use them commercially.
**Abstract Book Citations:** Authors, Title, HemaSphere, 2026;10:(S1):pages. The abstract book DOIs can be found on the "Author Information" page.
**Disclaimer:** Articles published in the journal HemaSphere exclusively reflect the opinions of the authors. The authors are responsible for all content in their abstracts including accuracy of the facts, statements, citing resources, etc.

### Summary/Conclusion

Across >4000 1L CLL pts, uMRD was associated with longer PFS and OS. Similar to CLL17 and AMPLIFY, these data indicate that EOT MRD status is informative within a given treatment approach, but less so for cross-regimen comparisons. This analysis supports further exploration of EOT MRD as a potential intermediate endpoint for survival in 1L CLL. AI was instrumental in conducting this work efficiently, and further work will provide regulatory-grade evidence.

**Table. Survival Endpoint Analysis by MRD Status**

| | **PFS HR [95% CI] (n)** |
|---|---|
| **Overall cohort** | 0.26 [0.23–0.29]* (4337) |
| **CIT** | 0.18 [0.16–0.21]* (2402) |
| **Ven-based regimens** | 0.5 [0.42–0.60]* (1935) |
| **BTKi–BCL2i doublets** | 0.38 [0.23–0.65]* (379) |
| **BTKi–BCL2i doublets uIGHV** | 0.21 [0.09–0.46]* (167) |
| **BTKi–BCL2i doublets mIGHV** | 0.87 [0.37–2.08]$^{ns}$ (124) |

*$P$<0.01; ns: $P$>0.05. HR display uMRD vs dMRD.

**Copyright Information:** (Online) ISSN: 2572-9241
©2026 The Author(s). HemaSphere is published by John Wiley & Sons Ltd on behalf of European Hematology Association. This is an open access Abstract Book distributed under the Attribution-NonCommercial-NoDerivs (CC BY-NC-ND), which allows third parties to download the articles and share them with others as long as they credit the author and the Abstract Book, but they cannot change the content in any way or use them commercially.
**Abstract Book Citations:** Authors, Title, HemaSphere, 2026;10:(S1):pages. The abstract book DOIs can be found on the "Author Information" page.
**Disclaimer:** Articles published in the journal HemaSphere exclusively reflect the opinions of the authors. The authors are responsible for all content in their abstracts including accuracy of the facts, statements, citing resources, etc.

### (PS1703) LONG-TERM FOLLOW-UP FOR SAFETY AND EFFICACY OF ZANUBRUTINIB IN ELDERLY (≥80 YEARS) TREATMENT-NAÏVE CLL/SLL PATIENTS, INCLUDING THOSE WITH DEL(17P): SUBGROUP ANALYSIS FROM THE SEQUOIA TRIAL

**Topic:** 06. Chronic lymphocytic leukemia and related disorders - Clinical

Alessandra Tedeschi*[1], Anders Osterborg[2], Talha Munir[3], Marcus Lefebure[4], Jiayi Shen[5], Wassim Aldairy[5], Jamie Hirata[5], Constantine S. Tam[6]

*[1]ASST Grande Ospedale Metropolitano Niguarda, Milan, Italy; [2]Karolinska University Hospital, Stockholm, Sweden; [3]Leeds Teaching Hospitals NHS Trust, Leeds, United Kingdom; [4]BeOne Medicines Ltd, London, United Kingdom; [5]BeOne Medicines, Ltd, San Carlos, United States of America; [6]6Alfred Health and Monash University, Melbourne, Australia;*

### Background

Chronic lymphocytic leukemia (CLL) and small lymphocytic lymphoma (SLL) predominantly affects older adults, with a median age at diagnosis of 72 years (yrs). Advancing age, comorbidities, and poorer performance status can limit the tolerability and effectiveness of therapeutic interventions. However, clinical data in an advanced-age population remain limited and with short follow-up. In the phase 3 SEQUOIA study (NCT03336333), zanubrutinib (zanu), a potent and selective next-generation BTKi, demonstrated superiority over bendamustine + rituximab (BR) in pts with treatment-naïve (TN) CLL/SLL without del(17p), with an estimated 6yr PFS rate of 74% vs 32% (COVID-adjusted: 77% vs 33%), respectively, and also showed robust efficacy in pts with del(17p).

### Aims

With a median follow-up of over 6.5yrs, this SEQUOIA subgroup analysis assessed the efficacy and safety of zanu in pts aged ≥80yrs with CLL/SLL, including those with del(17p).

### Methods

SEQUOIA enrolled TN pts with CLL/SLL considered unsuitable for FCR, defined as age ≥65yrs and/or CIRS >6, creatinine clearance <70 mL/min, and/or a recent history of serious or multiple infections. In Cohort 1/1a, pts without del(17p) were randomized 1:1 to receive continuous zanu (Arm A) or six cycles of BR (Arm B). Cohort 2 (Arm C) included pts with del(17p), all of whom received continuous zanu. This post hoc subgroup analysis included pts aged ≥80yrs from both cohorts who received zanu.

### Results

A total of 38 pts treated with zanu (Arm A: 28 pts; Arm C: 10 pts) were aged ≥80yrs at study entry. Median age was 81yrs (range; 80–87) and 23 (60.5%) were male. Del(17p) and/or *TP53* mutations were present in 14 (36.8%) pts, complex karyotype (≥3 copy-number aberrations) was detected in 4/24 (16.7%) evaluable pts, and 14 (36.8%) and 22 (57.9%) pts had mutated or unmutated IGHV, respectively. As of 31 Oct 2025, the median follow-up was 78.8mo (range; 5.0–92.1). The overall response rate was 100%, with a complete response rate of 18.4%. Estimated investigator-assessed progression free survival (PFS) rate (95% CI) at 72mo was 63.8% (44.6–77.8) (Figure). Estimated overall survival (OS) rate (95% CI) at 72mo was 75.8% (58.6–86.6).
Grade ≥3 treatment-emergent adverse events (TEAEs) occurred in 78.9% of pts. TEAE of interest (any grade/grade ≥3) included atrial fibrillation/flutter (15.8%/0%), major hemorrhage (21.1%/13.2%), hypertension (28.9%/18.4%) and infections (76.3%/36.8%). TEAEs leading to treatment discontinuation occurred in 39.5% of pts, and TEAEs leading to death were observed in 13.2% of pts; infection (two cases; pneumonia and meningitis cryptococcal) was the only TEAE leading to death in >1 pt. In total, 36.8% pts remain on zanu.

**Copyright Information:** (Online) ISSN: 2572-9241
©2026 The Author(s). HemaSphere is published by John Wiley & Sons Ltd on behalf of European Hematology Association. This is an open access Abstract Book distributed under the Attribution-NonCommercial-NoDerivs (CC BY-NC-ND), which allows third parties to download the articles and share them with others as long as they credit the author and the Abstract Book, but they cannot change the content in any way or use them commercially.
**Abstract Book Citations:** Authors, Title, HemaSphere, 2026;10:(S1):pages. The abstract book DOIs can be found on the "Author Information" page.
**Disclaimer:** Articles published in the journal HemaSphere exclusively reflect the opinions of the authors. The authors are responsible for all content in their abstracts including accuracy of the facts, statements, citing resources, etc.

### Summary/Conclusion

Very elderly pts remain underrepresented in CLL trials despite the disease predominantly affecting older adults, making dedicated evaluation in this age group essential. SEQUOIA includes one of the largest populations of patients aged ≥80 years in a ph3 setting and provides the longest follow-up in this subgroup. Pts aged ≥80 years achieved durable benefit from zanu, with 64% of pts alive and progression free at 6yrs despite advanced age and high-risk characteristics. The tolerability profile observed in this subgroup is as expected for a very elderly population and is comparable to the established safety profile reported for zanu. These results support the use of zanu as an effective first-line treatment for pts with TN CLL/SLL, including those aged ≥80yrs.

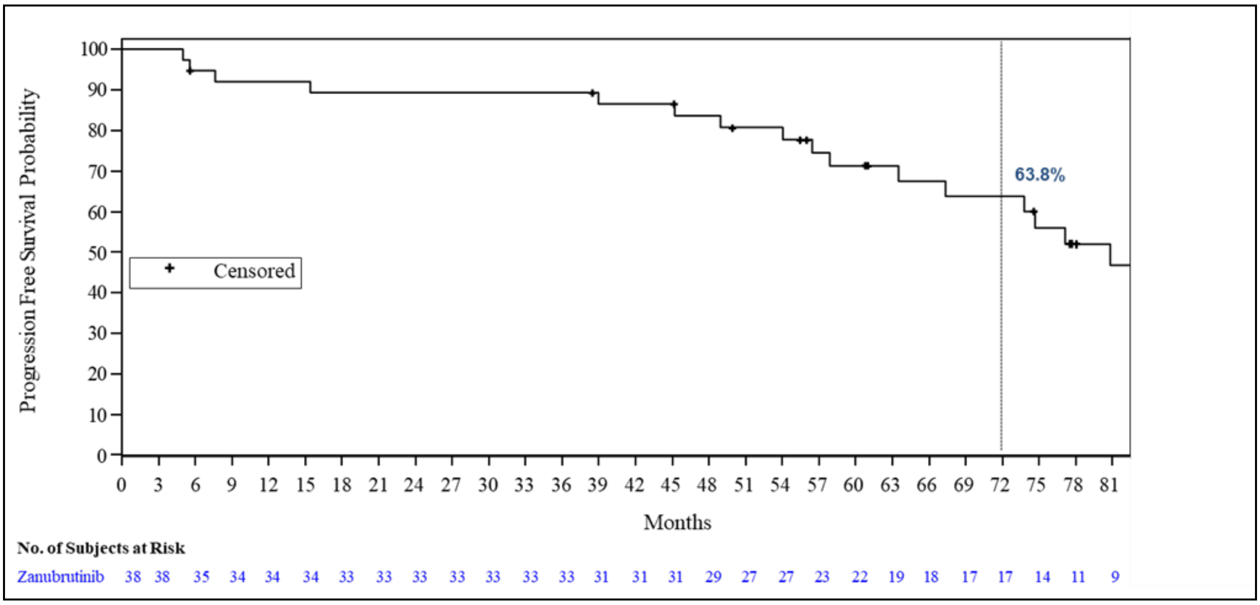

Figure: PFS

Copyright Information: (Online) ISSN: 2572-9241
©2026 The Author(s). HemaSphere is published by John Wiley & Sons Ltd on behalf of European Hematology Association. This is an open access Abstract Book distributed under the Attribution-NonCommercial-NoDerivs (CC BY-NC-ND), which allows third parties to download the articles and share them with others as long as they credit the author and the Abstract Book, but they cannot change the content in any way or use them commercially.
Abstract Book Citations: Authors, Title, HemaSphere, 2026;10:(S1):pages. The abstract book DOIs can be found on the "Author Information" page.
Disclaimer: Articles published in the journal HemaSphere exclusively reflect the opinions of the authors. The authors are responsible for all content in their abstracts including accuracy of the facts, statements, citing resources, etc.

### (PS1704) STAT3-MUTATED LARGE GRANULAR LYMPHOCYTIC LEUKEMIA: A RETROSPECTIVE ANALYSIS OF AN ITALIAN REFERRAL CENTRE

**Topic:** 06. Chronic lymphocytic leukemia and related disorders - Clinical

Luca Massarotti*[1], Antonella Teramo[1, 2], Elisa Rampazzo[1, 2], Laura Pavan[1], Giulia Calabretto[1, 2], Elena Buson[1, 2], Rebecca Crivellari[1, 2], Giulia Pertile[1, 2], Gregorio Barila'[3], Valentina Trimarco[1], Livio Trentin[1], Monica Facco[1, 2], Gianpietro Semenzato[2], Renato Zambello[1, 2]

*[1]Hematology Unit, University of Padova, Department of Medicine, Padova, Italy; [2]Veneto Institute of Molecular Medicine (VIMM), Padova, Italy; [3]Hematology Unit, San Bortolo Hospital, Vicenza, Italy;*

### Background

Large Granular Lymphocyte Leukemia (LGLL) is a rare and chronic lymphoproliferative disorder characterized by LGLs expansion. The disease displays a remarkable clinical heterogeneity, with patients being asymptomatic or developing cytopenia, mostly neutropenia. STAT3 activation promotes leukemic lymphocytes survival and proliferation, and its mutation is associated with higher rates of neutropenia and autoimmune disease (AD), increased need for therapy, and inferior overall survival (OS). The therapy is based on immunosuppressive drugs, such as Methotrexate (MTX), Cyclophosphamide (CTX) and Cyclosporin A (CyA). However, due to the paucity of data in phenotypic or genetic subsets, the therapeutic approach is the same for all types of LGLL, including those with STAT3 mutation.

### Aims

The aim of this study is to analyze clinical and biological features of a cohort of STAT3-mutated LGLL patients to identify prognostic markers affecting patients' outcome and to explore efficacy of first-line therapies.

### Methods

Data of 73 LGLL patients carrying STAT3 mutation, followed at our centre between 1993 and 2025, were retrospectively collected. Comparisons of biological parameters were performed using Fisher's exact test. Survival outcomes were estimated using the Kaplan–Meier method, with differences assessed by the log-rank test and Cox model.

**Copyright Information:** (Online) ISSN: 2572-9241
©2026 The Author(s). HemaSphere is published by John Wiley & Sons Ltd on behalf of European Hematology Association. This is an open access Abstract Book distributed under the Attribution-NonCommercial-NoDerivs (CC BY-NC-ND), which allows third parties to download the articles and share them with others as long as they credit the author and the Abstract Book, but they cannot change the content in any way or use them commercially.
**Abstract Book Citations:** Authors, Title, HemaSphere, 2026;10:(S1):pages. The abstract book DOIs can be found on the "Author Information" page.
**Disclaimer:** Articles published in the journal HemaSphere exclusively reflect the opinions of the authors. The authors are responsible for all content in their abstracts including accuracy of the facts, statements, citing resources, etc.

### Results

64 patients had Tα/β-LGLL (87%), 7 had a TCRγ/δ subtype (10%) and 2 belonged to NK-LGLL. The most frequent variant of STAT3 mutation was Y640F (59%), followed by D661Y (29%) and other less common genetic alterations. At baseline, neutropenia (ANC<1500/μL) was the main feature (70%), with severe neutropenia (ANC<500/μL) being present in 25% of patients; 15% of patients had significant anemia (Hb<10g/dL) and 36% of patients were "high burden", defined as >3.0x109/L circulating LGLs. 23 patients (31%) had a concomitant AD, of which about half (n=12) with rheumatoid arthritis and 5 with autoimmune cytopenias. On univariate analysis, baseline Hb<10 g/dL (p=0.0015) and need for treatment less than 6 months from diagnosis (p=0.0075) were associated with worse OS. The presence of D661Y appears to be associated with less requirement for treatment, although non statistically significant (p=0.06).

Only 32 patients (44%) required immunosuppressive therapy, with a median interval from diagnosis of 7.5 months. The main treatment indication was severe neutropenia (59%), followed by transfusion-dependent anemia (22%), symptomatic anemia and moderate neutropenia with recurrent infections (9% each). MTX was used in the majority of patients as a first-line option (56%), whereas CTX and CyA were used in 22% and 9% of cases, respectively. The overall response rate (ORR) was 78% (higher than the median ORRs reported in literature in LGLL), with a median time to the best response of 7 months, and a complete response rate of 41%. No biological or clinical factor influencing ORR has been identified, including the immunosuppressive agent used, STAT3 mutation type or TCR phenotype. Median progression-free survival (PFS) after first-line therapy was 34 months (Figure) and having a concomitant AD was linked with worse PFS (p=0.0159).

### Summary/Conclusion

Our study confirms that STAT3 mutated LGLL cases represent a subset with high frequency of neutropenia and associated AD. Our results highlight that this group can be characterized by higher ORR regardless of the chosen therapy, although the duration of response remains unsatisfactory, especially in those with ADs.

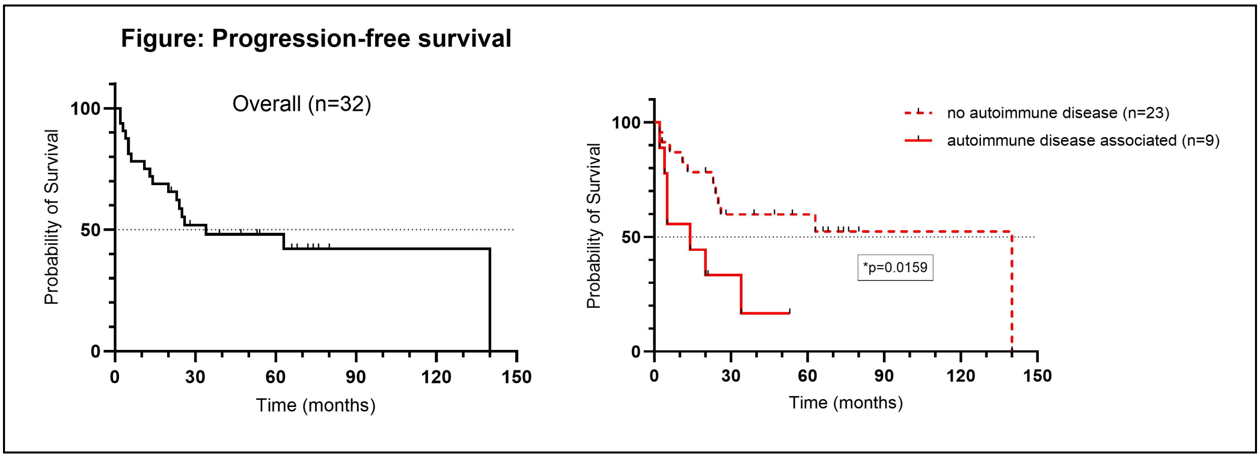

**Copyright Information:** (Online) ISSN: 2572-9241
©2026 The Author(s). HemaSphere is published by John Wiley & Sons Ltd on behalf of European Hematology Association. This is an open access Abstract Book distributed under the Attribution-NonCommercial-NoDerivs (CC BY-NC-ND), which allows third parties to download the articles and share them with others as long as they credit the author and the Abstract Book, but they cannot change the content in any way or use them commercially.
**Abstract Book Citations:** Authors, Title, HemaSphere, 2026;10:(S1):pages. The abstract book DOIs can be found on the "Author Information" page.
**Disclaimer:** Articles published in the journal HemaSphere exclusively reflect the opinions of the authors. The authors are responsible for all content in their abstracts including accuracy of the facts, statements, citing resources, etc.

### (PS1705) CARDIOVASCULAR TOXICITY OF CONTEMPORARY FIRST-LINE CLL TREATMENT REGIMENS: A POOLED ANALYSIS OF THE GERMAN CLL STUDY GROUP

**Topic:** 06. Chronic lymphocytic leukemia and related disorders - Clinical

Florian Simon*[1], Rudy Ligtvoet[1], Moritz Fürstenau[1], Othman Al-Sawaf[1], Henriette Huber[2], Julia von Tresckow[3], Thomas Nösslinger[4], Christian Bjørn Poulsen[5], Thomas Illmer[6], Lothar Müller[7], Ursula Vehling-Kaiser[8], Liliya Sivcheva[9], Javier Loscertales Pueyo[10], Kathleen Jentsch-Ullrich[11], Holger Hebart[12], Christof Schneider[13], Eugen Tausch[13], Stephan Stilgenbauer[13], Sandra Robrecht[1], Anna-Maria Fink[1], Kirsten Fischer[1], Arnon Philip Kater[14], Carsten Niemann[15], Michael Hallek[1], Barbara Eichhorst[1], Petra Langerbeins[1]

*[1]Department I of Internal Medicine and Center of Integrated Oncology Aachen, Bonn, Cologne, Düsseldorf, Faculty of Medicine and University Hospital of Cologne, University of Cologne, Cologne, Germany; [2]Städtisches Klinikum Karlsruhe, Karlsruhe, Germany; [3]Department of Hematology, Oncology and Palliative Care, Katholisches Klinikum Bochum, Ruhr University Bochum, Bochum, Germany; [4]Hanusch Krankenhaus Wien, 3rd Medical Department for Hematology and Oncology, Vienna, Austria; [5]Department of hematology, Zealand University Hospital, Roskilde, Denmark; [6]Hematology Private Practice, Dresden, Germany; [7]Study Centrum Unter Ems, Practice for Oncology and Hematology, Leer, Germany; [8]Outpatient Clinic, Landshut, Germany; [9]First Department of Internal Medicine, Multiprofile Hospital for Active Treatment – HristoBotev, Vratsa, Bulgaria; [10]Hematology Department, Hospital Universitario de La Princesa, Madrid, Spain; [11]Gemeinschaftspraxis für Hämatologie und Onkologie, Magdeburg, Germany; [12]Department of Internal Medicine, Hematology and Oncology, Stauferklinikum, Mutlangen, Germany; [13]Department of Internal Medicine III, Division of CLL, Ulm University, Ulm, Germany; [14]Department of Hematology and Cancer Center Amsterdam, Amsterdam, The Netherlands; [15]Department of Hematology, Rigshospitalet, Copenhagen University Hospital, Copenhagen, Denmark;*

### Background

First-line treatment of chronic lymphocytic leukemia (CLL) includes targeted approaches such as BTK-inhibitor or venetoclax-based regimens which differ in their cardiovascular risk profiles. Comparative cardiotoxicity data across treatment classes, including chemoimmunotherapy (CIT) and untreated patients (pts), remain limited.

### Aims

To evaluate cardiovascular toxicity during different therapy regimens in patients with CLL treated in four prospective trials of the German CLL study group (GCLLSG).

### Methods

Data from the CLL12, CLL13, CLL14 and CLL2-GIVe trials were pooled. Cardiac, vascular, bleeding, and thrombotic events were assessed separately as well as combined under the term cardiovascular (CV) events, assessed from the first dose of any study medication until the end of treatment plus 28 days. Time to onset of first CV event was analyzed using Kaplan-Meier methodology. Univariate logistic regression analyses for associations of baseline factors and development of CV events were performed.

**Copyright Information:** (Online) ISSN: 2572-9241
©2026 The Author(s). HemaSphere is published by John Wiley & Sons Ltd on behalf of European Hematology Association. This is an open access Abstract Book distributed under the Attribution-NonCommercial-NoDerivs (CC BY-NC-ND), which allows third parties to download the articles and share them with others as long as they credit the author and the Abstract Book, but they cannot change the content in any way or use them commercially.
**Abstract Book Citations:** Authors, Title, HemaSphere, 2026;10:(S1):pages. The abstract book DOIs can be found on the “Author Information” page.
**Disclaimer:** Articles published in the journal HemaSphere exclusively reflect the opinions of the authors. The authors are responsible for all content in their abstracts including accuracy of the facts, statements, citing resources, etc.

**Results**

1,717 pts (median age 65 years, IQR 56-72) were allocated to: ibrutinib continuous monotherapy: 170 pts (9.9%), triplet (ibrutinib, venetoclax + obinutuzumab): 272 pts (15.8%), placebo: 168 pts (9.8%), venetoclax+CD20 antibody (venetoclax+CD20ab): 677 pts (39.4%), and CIT: 430 pts (25%). Median treatment duration was 36.1 (range 0.2-91.1), 12.2 (1.0-37.2), 25.0 (0.1-88.8), 11.2 (0.1-14.3) and 5.5 (0-13.6) months, respectively. Baseline cardiac comorbidity (CIRS Heart ≥1) and hypertension (CIRS Blood pressure ≥1) was present in 15.3% and 56.5% for ibrutinib, 14.3 and 37.9% for ibrutinib-venetoclax-based, 10.1% and 53% for placebo, 25.5% and 45.6% for venetoclax+CD20ab and 24.7% and 37.9% for CIT, respectively.
A total of 912 CV events were reported in 566 (33.0%) pts. The proportion of pts with CV events was 62.4% (cardiac events 29.4%, grade ≥3 cardiac events 12.9%) in the ibrutinib group, 43.4% (17.3%, 6.3%) in the triplet group, 38.1% (17.3%, 6.5%) in the placebo group, and 27.0% (8.0%, 1.8%) in the venetoclax+CD20ab cohort, and 22.1% (6.7%, 2.3%) in the CIT group. Exposure-adjusted incidence rates were 27.7 (95% CI 23.9-31.8), 54.9 (47.7-62.6), 20.4 (16.8-24.3), 32.9 (29.0-37.1), and 41.0 (34.6-48.0) CV events per 1,000 patient-months, respectively.
Median CV event-free survival was 17.0 months in the ibrutinib group (12-month rate 58.0%; 95% CI 50.2% – 65.8%), 18.7 months in the triplet group (57.8%; 51.9% – 63.8%), 58.1 months in the placebo group (79.7%; 73.4% – 86.0%), and not reached in both venetoclax+CD20ab (72.5%; 69.1% – 76.0%) and CIT (74.7%; 69.6% – 79.5%) group.
Factors associated with CV event occurrence were baseline hypertension (CIRS blood pressure ≥1; odds ratio [OR] 2.56; 95% CI 1.35 – 4.83, p=0.004) for the ibrutinib group, and CIRS overall >3 (1.78; 1.07 – 2.95, p=0.026) as well as CIRS heart ≥1 (3.07; 1.50 – 6.27, p=0.002) for the triplet group. In contrast, the placebo, CIT and venetoclax+CD20ab groups showed no associations between developing a CV event and baseline cardiovascular comorbidities, including cardiac disease or hypertension.

**Summary/Conclusion**

This pooled analysis of four prospective GCLLSG trials confirms the class-associated CV toxicity of ibrutinib-containing regimens, particularly in patients with baseline hypertension and cardiac comorbidity. These data underscore the importance of cardiovascular risk assessment in treatment selection and support venetoclax-based regimens as a preferred option for CLL patients with elevated CV risk.

**Copyright Information:** (Online) ISSN: 2572-9241
©2026 The Author(s). HemaSphere is published by John Wiley & Sons Ltd on behalf of European Hematology Association. This is an open access Abstract Book distributed under the Attribution-NonCommercial-NoDerivs (CC BY-NC-ND), which allows third parties to download the articles and share them with others as long as they credit the author and the Abstract Book, but they cannot change the content in any way or use them commercially.
**Abstract Book Citations:** Authors, Title, HemaSphere, 2026;10:(S1):pages. The abstract book DOIs can be found on the “Author Information” page.
**Disclaimer:** Articles published in the journal HemaSphere exclusively reflect the opinions of the authors. The authors are responsible for all content in their abstracts including accuracy of the facts, statements, citing resources, etc.

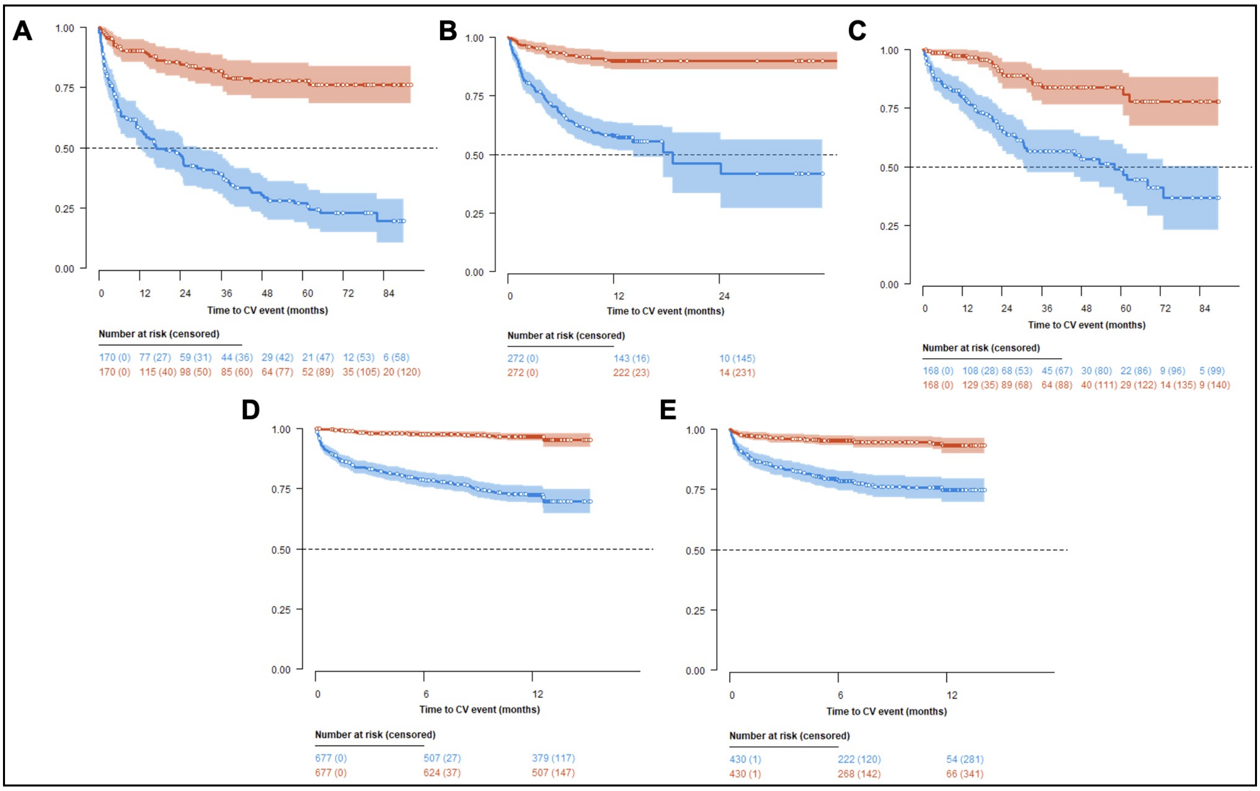

Figure 1: Cardiovascular Event-Free Survival. X-Axis scale reflects the respective treatment/exposure duration. Blue: Any CV AE, Red: Any CV SAE. A: Ibrutinib cohort, B: Triplet cohort; C Placebo cohort, D: Venetoclax-based cohort, E: CIT cohort.

**Copyright Information:** (Online) ISSN: 2572-9241
©2026 The Author(s). HemaSphere is published by John Wiley & Sons Ltd on behalf of European Hematology Association. This is an open access Abstract Book distributed under the Attribution-NonCommercial-NoDerivs (CC BY-NC-ND), which allows third parties to download the articles and share them with others as long as they credit the author and the Abstract Book, but they cannot change the content in any way or use them commercially.
**Abstract Book Citations:** Authors, Title, HemaSphere, 2026;10:(S1):pages. The abstract book DOIs can be found on the “Author Information” page.
**Disclaimer:** Articles published in the journal HemaSphere exclusively reflect the opinions of the authors. The authors are responsible for all content in their abstracts including accuracy of the facts, statements, citing resources, etc.

### (PS1706) QUALITY OF LIFE AND SYMPTOMS WITH FIXED-DURATION ACALABRUTINIB + VENETOCLAX ± OBINUTUZUMAB VS CHEMOIMMUNOTHERAPY IN TREATMENT-NAIVE CHRONIC LYMPHOCYTIC LEUKEMIA: PATIENT-REPORTED OUTCOMES FROM AMPLIFY

**Topic:** 06. Chronic lymphocytic leukemia and related disorders - Clinical

Arnon Philip Kater*[1], Jennifer R. Brown[2], Paolo Ghia[3, 4], Barbara Eichhorst[5], Wojciech Jurczak[6], Leif Simmatis[7], Cara Arizmendi[8], Lysbeth Floden[9], Batul Suterwala[10], Alen Ostojic[11], John F. Seymour[12]

*[1]Department of Hematology, Cancer Center Amsterdam, Amsterdam University Medical Centers, University of Amsterdam, Amsterdam, The Netherlands; [2]Dana-Farber Cancer Institute, Boston, United States of America; [3]Università Vita-Salute San Raffaele, Milano, Italy; [4]Comprehensive Cancer Center, IRCCS Ospedale San Raffaele, Milano, Italy; [5]University Hospital Cologne, Department I of Internal Medicine and Center for Integrated Oncology Aachen Bonn Cologne Duesseldorf, Cologne, Germany; [6]Department of Clinical Oncology, Maria Sklodowska-Curie National Research Institute of Oncology, Kraków, Poland; [7]Evinova, Toronto, Canada; [8]Evinova, Durham, United States of America; [9]Evinova, Waltham, United States of America; [10]AstraZeneca, South San Francisco, United States of America; [11]AstraZeneca, Barcelona, Spain; [12]Peter MacCallum Cancer Centre, Royal Melbourne Hospital, The University of Melbourne, Melbourne, Australia;*

### Background

The phase 3 AMPLIFY trial evaluated fixed-duration (FD) acalabrutinib + venetoclax ± obinutuzumab (AV/AVO) vs chemoimmunotherapy (CIT; fludarabine + cyclophosphamide + rituximab or bendamustine + rituximab [FCR/BR]) in patients (pts) with treatment-naive (TN) chronic lymphocytic leukemia (CLL). In the interim analysis, FD AV and AVO significantly prolonged progression-free survival compared with CIT (Brown JR et al. *NEJM* 2025), but the impact of these different regimens on pts is not well studied.

### Aims

To evaluate differential treatment (tx) effects on pt-reported outcomes, with a focus on quality of life (QoL) and disease- and tx-related symptoms in pts treated with FD AV and AVO compared with CIT.

### Methods

The AMPLIFY (NCT03836261) trial is an ongoing, randomized, open-label, phase 3 trial in pts with TN CLL aged ≥18 y with ECOG performance status ≤2 and without del(17p) or *TP53* mutation. Pts were randomized 1:1:1 to receive AV (A: cycles [C] 1–14; V: C3–14), AVO (AV as described; O: C2–7), or investigator's choice of CIT (FCR or BR; C1–6) in 28-day Cs. Pt-reported outcomes were assessed before (baseline [BL]), during (monthly), and after tx using the European Organisation for Research and Treatment of Cancer (EORTC) Quality of Life Questionnaire Core 30 (performed through 1 y following end of tx [1 y post-tx]) and Item Library 27 (performed through 12 wk after the 30-d safety follow-up if no disease progression [first post-tx follow-up]).

**Copyright Information:** (Online) ISSN: 2572-9241
©2026 The Author(s). HemaSphere is published by John Wiley & Sons Ltd on behalf of European Hematology Association. This is an open access Abstract Book distributed under the Attribution-NonCommercial-NoDerivs (CC BY-NC-ND), which allows third parties to download the articles and share them with others as long as they credit the author and the Abstract Book, but they cannot change the content in any way or use them commercially.
**Abstract Book Citations:** Authors, Title, HemaSphere, 2026;10:(S1):pages. The abstract book DOIs can be found on the "Author Information" page.
**Disclaimer:** Articles published in the journal HemaSphere exclusively reflect the opinions of the authors. The authors are responsible for all content in their abstracts including accuracy of the facts, statements, citing resources, etc.

### Results

In total, 834 pts received ≥1 dose of AV (n=291), AVO (n=284), or CIT (n=259). BL QoL scores were comparable across tx arms. Global health status (GHS)/QoL and physical functioning improved from BL across all regimens, with numerically greater and more sustained improvements observed with AV and AVO compared with CIT. The proportion of pts with a clinically meaningfully improvement in GHS/QoL score (≥10-point increase) increased from BL to C6 day (D) 1 (AV 38.4%, AVO 34.1%, CIT 34.9%), increased further from BL to 1 y post-tx (AV 47.8%, AVO 43.5%, CIT 39.0%), and was higher with AV and AVO vs CIT. Mean change (standard deviation [SD]) in GHS/QoL score from BL to C6D1 was 6.47 (19.95) for AV, 4.91 (20.33) for AVO, and 6.54 (20.24) for CIT; mean change from BL to 1 y post-tx was clinically meaningful for AV (11.73 [SD 21.36]) and AVO (10.84 [19.08]) but not for CIT (7.81 [22.20]). Similar patterns were observed for physical functioning, with greater improvement rates from BL to 1 y post-tx for AV (33.8%) and AVO (30.1%) vs CIT (23.9%), and larger mean changes from BL to 1 y post-tx for AV and AVO vs CIT (AV 5.93 [SD 14.63], AVO 4.55 [15.64], CIT 1.59 [16.23]). Outcomes were broadly similar between AV and AVO. Improvements in role functioning, fatigue, and nausea/vomiting were generally comparable across tx arms. Among tx- and disease-related symptoms, night sweats showed the largest early improvement, with greater reductions from BL by C2D1 in AV and AVO compared with CIT (**Figure**). Fever incidence remained low (>80% reporting no symptoms) and similar across tx arms throughout follow-up. Muscle or joint aches or pains and bruising showed limited change from BL. Additional time points will be presented.

### Summary/Conclusion

FD AV and AVO were associated with earlier symptom relief and more durable improvements in pt-reported QoL and physical functioning compared with CIT, without increased tx-related symptom burden.

**Figure. Proportion of patients reporting symptoms in IL27 questionnaire over time**

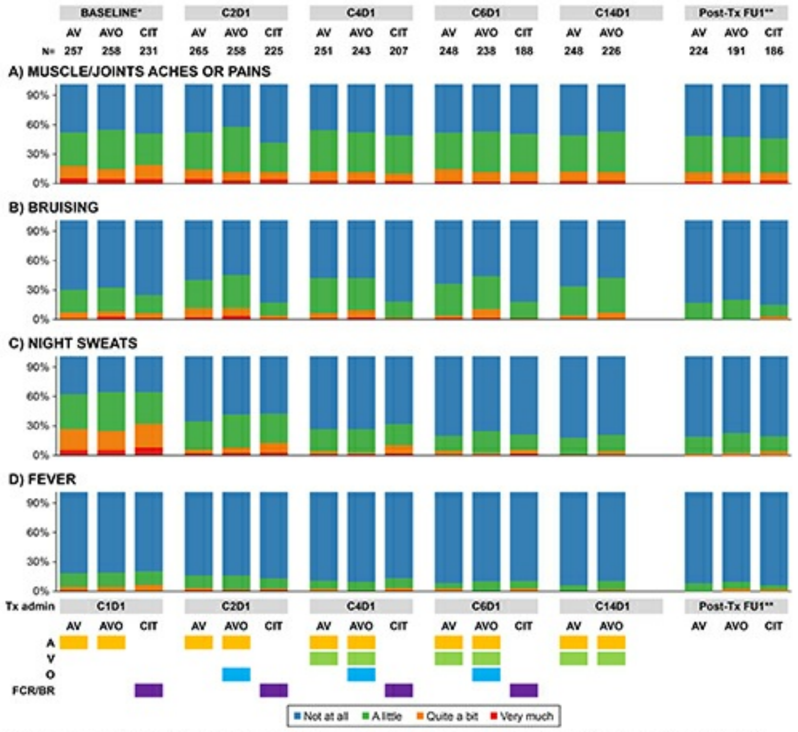

*Prior to treatment start. **First post-tx FU after 30-day safety FU, ~12 weeks later if no progression; approx. from treatment start: for AV/AVO ~17 mo; for CIT ~9 mo.
AV, acalabrutinib + venetoclax; AVO, acalabrutinib + venetoclax + obinutuzumab; C, cycle; CIT, chemoimmunotherapy; D, day; FCR/BR, fludarabine + cyclophosphamide + rituximab/bendamustine + rituximab; FU, follow-up; Tx, treatment.

**Copyright Information:** (Online) ISSN: 2572-9241
©2026 The Author(s). HemaSphere is published by John Wiley & Sons Ltd on behalf of European Hematology Association. This is an open access Abstract Book distributed under the Attribution-NonCommercial-NoDerivs (CC BY-NC-ND), which allows third parties to download the articles and share them with others as long as they credit the author and the Abstract Book, but they cannot change the content in any way or use them commercially.
**Abstract Book Citations:** Authors, Title, HemaSphere, 2026;10:(S1):pages. The abstract book DOIs can be found on the “Author Information” page.
**Disclaimer:** Articles published in the journal HemaSphere exclusively reflect the opinions of the authors. The authors are responsible for all content in their abstracts including accuracy of the facts, statements, citing resources, etc.

### (PS1707) NEMTABRUTINIB PLUS VENETOCLAX IN PARTICIPANTS WITH RELAPSED OR REFRACTORY CHRONIC LYMPHOCYTIC LEUKEMIA/SMALL LYMPHOCYTIC LYMPHOMA: PART 1 OF THE PHASE 3 BELLWAVE-010 STUDY

**Topic:** 06. Chronic lymphocytic leukemia and related disorders - Clinical

Paolo Ghia*[1, 2], Mauricio Chandía[3], Vernon Louw[4, 5], Maria Carmen Martinez Chamorro[6, 7], Gonzalo Garate[8], Valeria Buccheri[9], Raimundo Gazitua[10], Alejandro Berkovits[11], Michael Africa-Cass[12], Eva Gonzalez Barca[13], Jose Sandoval-Sus[14], Muhit Ozcan[15], Sven Erik Ojavee[16], Ima Paydar[16], Mohammed Farooqui[16], Ohad Benjamini[17]

*[1]Università Vita-Salute San Raffaele, Milan, Italy; [2]Comprehensive Cancer Center, IRCCS Ospedale San Raffaele, Milan, Italy; [3]Biocenter, Concepción, Chile; [4]Stellenbosch University, Cape Town, South Africa; [5]University of Cape Town and Groote Schuur Hospital, Cape Town, South Africa; [6]Hospital Universitario Quironsalud Madrid, Madrid, Spain; [7]Universidad Europea de Madrid, Madrid, Spain; [8]Hospital Aleman-Oncohematologic Diseases, Buenos Aires, Argentina; [9]ICESP – Instituto do Câncer do Estado de São Paulo, Faculdade de Medicina da Universidade de Sao Paulo, São Paulo, Brazil; [10]Instituto Oncológico Fundación Arturo López Pérez (FALP), Providencia, Chile; [11]Inmunocel Chile, Providencia, Chile; [12]Haemalife, Kuils River, Cape Town, South Africa; [13]Instituto Catalan de Oncologia - Hospital Duran i Reynals-DIBELL, Universitat de Barcelona, Barcelona, Spain;[14]Moffitt Cancer Center at Memorial Healthcare System, Pembroke Pines, United States of America; [15]Ankara University School of Medicine, Ankara, Türkiye; [16]Merck & Co., Inc., Rahway, United States of America;[17]Chaim Sheba Medical Center, Ramat Gan, Israel;*

### Background

Venetoclax plus rituximab (VR) is a standard-of-care regimen for patients with relapsed or refractory (R/R) chronic lymphocytic leukemia/small lymphocytic lymphoma (CLL/SLL). Nemtabrutinib, a reversible Bruton tyrosine kinase inhibitor, showed durable antitumor activity in participants with CLL/SLL in the phase 1/2 BELLWAVE-001 study. BELLWAVE-010 (NCT05947851) is an open-label, randomized, phase 3 study evaluating the safety and efficacy of nemtabrutinib plus venetoclax versus VR in R/R CLL/SLL. Part 1 is a nonrandomized dose-escalation and dose-confirmation phase designed to assess the safety and tolerability, and to establish the recommended dose, of nemtabrutinib plus venetoclax. Part 2 is a randomized phase evaluating efficacy and safety of nemtabrutinib plus venetoclax versus VR.

### Aims

To present initial results from part 1 of the BELLWAVE-010 study.

### Methods

Participants ≥18 years of age with R/R CLL/SLL refractory to ≥1 prior therapy per International Workshop on CLL 2018 criteria and an Eastern Cooperative Oncology Group performance status (ECOG PS) of 0 to 2 were eligible. Participants received nemtabrutinib at 2 dose levels (45 mg escalating to 65 mg by mouth once daily) for 4 weeks, followed by venetoclax with a standard 28-day ramp-up (20-400 mg by mouth once daily). The primary end points were safety and tolerability, dose-limiting toxicity (DLT), and establishment of the recommended phase 2 dose. Efficacy end points for part 1 included objective response rate and duration of response. The data cutoff date was April 9, 2025.

**Copyright Information:** (Online) ISSN: 2572-9241
©2026 The Author(s). HemaSphere is published by John Wiley & Sons Ltd on behalf of European Hematology Association. This is an open access Abstract Book distributed under the Attribution-NonCommercial-NoDerivs (CC BY-NC-ND), which allows third parties to download the articles and share them with others as long as they credit the author and the Abstract Book, but they cannot change the content in any way or use them commercially.
**Abstract Book Citations:** Authors, Title, HemaSphere, 2026;10:(S1):pages. The abstract book DOIs can be found on the “Author Information” page.
**Disclaimer:** Articles published in the journal HemaSphere exclusively reflect the opinions of the authors. The authors are responsible for all content in their abstracts including accuracy of the facts, statements, citing resources, etc.

**Results**

A total of 31 participants were enrolled in the DLT-evaluable population; 15 received nemtabrutinib 45 mg plus venetoclax and 16 received nemtabrutinib 65 mg plus venetoclax. Median study follow-up was 10.3 months (range, 1.2-19.4). Median age was 70 years (range, 39-89), 22 participants (71%) were male, 5 (16%) had received ≥3 prior lines of therapy, and 3 (10%) had an ECOG PS of 2. At the data cutoff date, 2 participants (6%) had discontinued treatment due to an adverse event (AE). DLTs occurred in 2 participants receiving nemtabrutinib 65 mg, both due to pneumonia (1 grade 3 [resolved] and 1 grade 5). Treatment-related AEs of any grade were reported in all participants (100%) in both arms. Grade 3-5 treatment-related AEs occurred in 11 participants (73%) in the nemtabrutinib 45-mg arm and 12 participants (75%) in the nemtabrutinib 65-mg arm. One treatment-related death occurred in the nemtabrutinib 65-mg arm (pneumonia); no treatment-related deaths occurred in the nemtabrutinib 45-mg arm. Objective response rate was 100% (95% CI, 78%-100%; complete response [CR], 13%; partial response [PR], 87%) in the nemtabrutinib 45-mg arm and 88% (95% CI, 62%-98%; CR, 13%; PR, 75%) in the nemtabrutinib 65-mg arm. Median duration of response was not reached in either arm.

**Summary/Conclusion**

In part 1 of BELLWAVE-010, nemtabrutinib 45 mg plus venetoclax showed promising antitumor activity with a manageable safety profile. These findings support continued evaluation of this combination in participants with R/R CLL/SLL.

**Copyright Information:** (Online) ISSN: 2572-9241
©2026 The Author(s). HemaSphere is published by John Wiley & Sons Ltd on behalf of European Hematology Association. This is an open access Abstract Book distributed under the Attribution-NonCommercial-NoDerivs (CC BY-NC-ND), which allows third parties to download the articles and share them with others as long as they credit the author and the Abstract Book, but they cannot change the content in any way or use them commercially.
**Abstract Book Citations:** Authors, Title, HemaSphere, 2026;10:(S1):pages. The abstract book DOIs can be found on the "Author Information" page.
**Disclaimer:** Articles published in the journal HemaSphere exclusively reflect the opinions of the authors. The authors are responsible for all content in their abstracts including accuracy of the facts, statements, citing resources, etc.

### (PS1708) BRUTON TYROSINE KINASE INHIBITORS NEUTRALIZE THE ADVERSE PROGNOSIS IMPACT OF THE IGLV3-21R110 MUTATION IN PATIENTS WITH CHRONIC LYMPHOCYTIC LEUKEMIA

**Topic:** 06. Chronic lymphocytic leukemia and related disorders - Clinical

Juan A. Piñeyroa*[1, 2, 3], Charlotte Syrykh[4], Irene López-Oreja[1, 2, 5], Miguel Argüello de Tomás[6], Sonia Piernas[7], María Teresa Villalobos Prego[8], Montse García-Caro[9], Marta Garcia Pintos[10], José Rodruiguez[11], Loic Ysebaert[4], Laetitia Largeaud[4], Ares Martínez-Farran[12], Aleix Real[12], Eric Delabesse[13], Monica Lopez-Guerra[1], Ferran Nadeu[2, 14], Elias Campo[1, 2, 3], Pablo Javier Mozas Fernandez[1, 12], Dolors Colomer Pujol[1, 3, 12, 14]

*[1]Hospital Clínic De Barcelona, Barcelona, Spain;[2]Institut d'Investigacions Biomèdiques August Pi i Sunyer, Barcelona, Spain; [3]Universitat de Barcelona, Barcelona, Spain;[4]Institut Universitaire du Cancer de Toulouse Oncopole, Toulouse, France;[5]Centro de Investigación Biomédica en Red de Cáncer, Madrid, Spain; [6]Hospital De La Santa Creu I Sant Pau, Barcelona, Spain; [7]Hospital Parc Taulí De Sabadell, Sabadell, Spain;[8]Hospital Universitari MutuaTerrassa, Terrassa, Spain;[9]Hospital Universitari De Vic, Vic, Spain; [10]Consorci Sanitari De Terrassa, Terrassa, Spain;[11]Hospital De Mollet, Mollet del Vallès, Spain;[12]Institut d'Investigacions Biomèdiques August Pi i Sunyer (IDIBAPS), Barcelona, Spain; [13]Centre hospitalier universitaire (CHU) de Toulouse, Toulouse, France; [14]Centro de Investigación Biomédica en Red de Cáncer (CIBERONC), Madrid, Spain;*

### Background

The IGLV3-21$^{R110}$ mutation defines a Chronic lymphocytic leukemia subgroup with specific biological features. The mutation is a single G>C substitution at the immnoglobulin lambda light chain, changing the glycine at position 110 to arginine triggering cell-autonomous B-cell receptor signaling. Clinically, it has been associated with shorter time to first treatment (TTFT) and inferior overall survival in the chemotherapy era. However, evidence regarding its impact under targeted therapies remains limited.

### Aims

To describe the clinical, genomic, and treatment outcomes of CLL patients with the IGLV3-21$^{R110}$ mutation treated with BTK inhibitors (BTKi).

### Methods

We performed an international, multicenter retrospective study including 399 CLL patients treated with BTKi between 2013–2025 across 8 hospitals. IGLV3-21$^{R110}$ was identified using the SOPHiA GENETICS "all-CLL" NGS panel and identifided with the Igcaller software. Clinical, biochemical, and genomic features were collected with the NGS data obtained before BTKi initiation or at relapse. Sequential NGS data was available in 167 patients.

### Results

The prevalence of IGLV3-21$^{R110}$ was 9%. This subgroup showed a significantly higher frequency of borderline IGHV mutational status (97–98%), subset #2 assignment, *SF3B1* mutations, and absence of trisomy 12 and del(17p). No significant differences were observed in baseline clinical characteristics, laboratory parameters, complex karyotype, or other driver mutations [Table 1].
In paired samples, we observed significant acquisition of *TP53* (14% to 29%, P=0.0001), *NOTCH1* (18% to 24%, P=0.004), and *BTK/PLCG2* mutations (0 to 44%, P=0.003). No patient gained or lost the IGLV3-21$^{R110}$ mutation over time.
Overall, 59% received BTKi in first line, with no differences in BTKi choice by IGLV3-21$^{R110}$ status. Stratified by IGHV status, IGLV3-21$^{R110}$ cases showed a shorter TTFT than IGHV mutated patients (HR=2.0 [1.4–3], P<0.001) and a similar interval to

**Copyright Information:** (Online) ISSN: 2572-9241
©2026 The Author(s). HemaSphere is published by John Wiley & Sons Ltd on behalf of European Hematology Association. This is an open access Abstract Book distributed under the Attribution-NonCommercial-NoDerivs (CC BY-NC-ND), which allows third parties to download the articles and share them with others as long as they credit the author and the Abstract Book, but they cannot change the content in any way or use them commercially.
**Abstract Book Citations:** Authors, Title, HemaSphere, 2026;10:(S1):pages. The abstract book DOIs can be found on the "Author Information" page.
**Disclaimer:** Articles published in the journal HemaSphere exclusively reflect the opinions of the authors. The authors are responsible for all content in their abstracts including accuracy of the facts, statements, citing resources, etc.

IGHV unmutated cases. The median follow-up of the entire cohort was 4 years (range 0.1–9.5). There were no significant differences in progression free survival and survival from BTKi initiation according to IGLV3-21$^{R110}$ status in either first line or later line settings, although IGLV3-21$^{R110}$ patients showed a trend toward better outcomes with BTKi in first line [Figure 1]. No differences were found in the incidence of Richter transformation.

**Table 1**

| | **IGLV3-21$^{R110}$** | | ***P*** |
|---|---|---|---|
| | **No** | **Yes** | |
| Age | 70y | 72y | NS |
| Male sex | 61% | 59% | NS |
| ECOG≥2 | 11% | 13% | NS |
| Rai III-IV | 39% | 42% | NS |
| Complex karyotype (≥3) | 27% | 13% | NS |
| FISH | | | |
| del(13q) | 40% | 59% | NS |
| Trisomy 12 | 21% | 0 | 0.01 |
| del(11q) | 18% | 21% | NS |
| del(17p) | 14% | 0 | 0.04 |
| Subset #2 | 1% | 32% | <0.01 |
| Mutated gene | | | |
| *ATM* | 15% | 17% | NS |
| *BIRC3* | 9% | 3% | NS |

**Copyright Information:** (Online) ISSN: 2572-9241
©2026 The Author(s). HemaSphere is published by John Wiley & Sons Ltd on behalf of European Hematology Association. This is an open access Abstract Book distributed under the Attribution-NonCommercial-NoDerivs (CC BY-NC-ND), which allows third parties to download the articles and share them with others as long as they credit the author and the Abstract Book, but they cannot change the content in any way or use them commercially.
**Abstract Book Citations:** Authors, Title, HemaSphere, 2026;10:(S1):pages. The abstract book DOIs can be found on the "Author Information" page.
**Disclaimer:** Articles published in the journal HemaSphere exclusively reflect the opinions of the authors. The authors are responsible for all content in their abstracts including accuracy of the facts, statements, citing resources, etc.

| | | | |
|---|---|---|---|
| *EGR2* | 7% | 0 | NS |
| *FBXW7* | 3% | 0 | NS |
| *KRAS* | 6% | 0 | NS |
| *NFKBIE* | 8% | 13% | NS |
| *NOTCH1* | 23% | 13% | NS |
| *POT1* | 3% | 10% | NS |
| *SF3B1* | 15% | 53% | <0.001 |
| *TP53* | 25% | 23% | NS |
| BTKi | | | |
| Ibrutinib | 68% | 56% | NS |
| Ibrutinib+Venetoclax | 7% | 12% | |
| Acalabrutinib | 16% | 24% | |
| Zanubrutinib | 9% | 9% | |

**Summary/Conclusion**

Despite requiring earlier treatment initiation, CLL patients with IGLV3-21$^{R110}$ did not show poorer outcomes when treated with BTKi.

**Copyright Information:** (Online) ISSN: 2572-9241
©2026 The Author(s). HemaSphere is published by John Wiley & Sons Ltd on behalf of European Hematology Association. This is an open access Abstract Book distributed under the Attribution-NonCommercial-NoDerivs (CC BY-NC-ND), which allows third parties to download the articles and share them with others as long as they credit the author and the Abstract Book, but they cannot change the content in any way or use them commercially.
**Abstract Book Citations:** Authors, Title, HemaSphere, 2026;10:(S1):pages. The abstract book DOIs can be found on the "Author Information" page.
**Disclaimer:** Articles published in the journal HemaSphere exclusively reflect the opinions of the authors. The authors are responsible for all content in their abstracts including accuracy of the facts, statements, citing resources, etc.

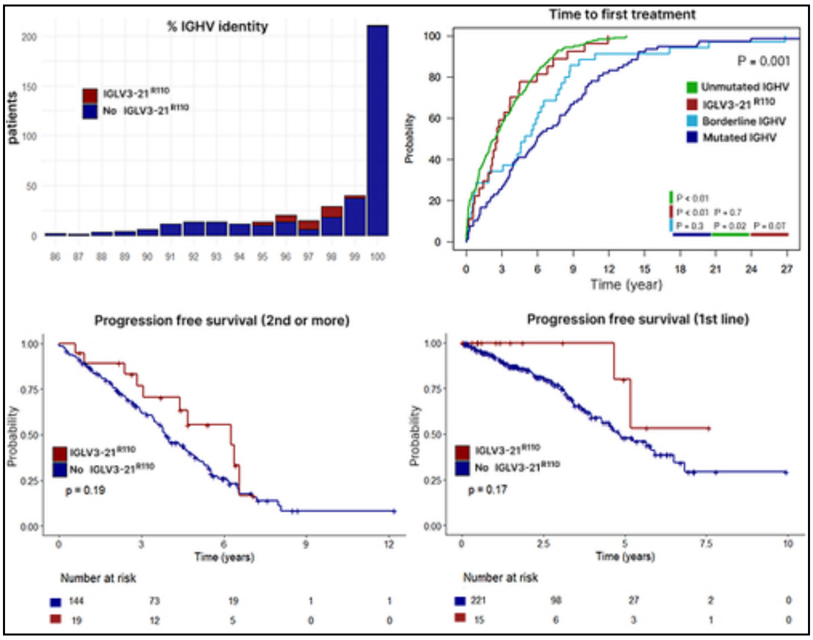

IGHV identity percentage, TTFT, and PFS according to the presence or absence of the IGLVR110 mutation.

**Copyright Information:** (Online) ISSN: 2572-9241
©2026 The Author(s). HemaSphere is published by John Wiley & Sons Ltd on behalf of European Hematology Association. This is an open access Abstract Book distributed under the Attribution-NonCommercial-NoDerivs (CC BY-NC-ND), which allows third parties to download the articles and share them with others as long as they credit the author and the Abstract Book, but they cannot change the content in any way or use them commercially.
**Abstract Book Citations:** Authors, Title, HemaSphere, 2026;10:(S1):pages. The abstract book DOIs can be found on the "Author Information" page.
**Disclaimer:** Articles published in the journal HemaSphere exclusively reflect the opinions of the authors. The authors are responsible for all content in their abstracts including accuracy of the facts, statements, citing resources, etc.

### (PS1709) MATURE LYMPHOID NEOPLASMS IN PATIENTS WITH CHRONIC LYMPHOCYTIC LEUKEMIA: RESULTS FROM THE SPANISH MULTICENTER GELLC STUDY

**Topic:** 06. Chronic lymphocytic leukemia and related disorders - Clinical

Miguel Argüello de Tomás*[1], Juan A. Piñeyroa[2], Pablo Javier Mozas Fernandez[2], Marta Crespo Maull[3], Angel Serna Pareja[3], Ángeles Medina[4], Almudena Navarro[5], Blanca Espinet[6], Anna Puiggros[6], Eva Gimeno[6], Fatima Miras Calvo[7], José Antonio García Vela[8], María Teresa Villalobos Prego[9], Ana Carla De Oliveira[10], Marta Julia Arenas[11], Javier Loscertales Pueyo[12], Francisco Bosch Albareda[3], Ana Muntañola Prat[1]

*[1]Hospital de la Santa Creu i Sant Pau, Hematology, Barcelona, Spain;[2]Hospital Clínic de Barcelona, Hematology, Barcelona, Spain; [3]Hospital Universitari Vall d'Hebron - VHIO, Hematology, Barcelona, Spain;[4]Hospital Universitario Costa del Sol, Hematology, Marbella, Spain; [5]Complejo Asistencial Universitario de Salamanca, Salamanca, Spain;[6]Hospital del Mar, Barcelona, Spain; [7]Hospital Universitario 12 de Octubre, Hematology, Madrid, Spain;[8]Hospital Universitario Puerta de Hierro, Majadahonda, Spain; [9]Hospital Universitari Mutua Terrassa, Hematology, Terrassa, Spain; [10]Institut Català d'Oncologia (ICO) Hospital Duran i Reynals, Hematology, L'Hospital de Llobregat, Spain; [11]ICO Badalona, Hematology, Badalona, Spain; [12]Hospital Universitario La Princesa, Hematology, Madrid, Spain;*

### Background

Chronic lymphocytic leukemia (CLL) is frequently associated with secondary neoplasms, including solid and hematologic malignancies. These events are related to a reduced overall survival (OS), especially in cases of myeloid neoplasia or Richter transformation (RT).

### Aims

To describe the frequency and characteristics of patients with CLL and other mature lymphoid neoplasms (MLN).

### Methods

Patients were identified from the multicentric Registry of the Spanish CLL Group (GELLC), comprising patients with CLL or small lymphocytic lymphoma (SLL) diagnosed since 2010. Clinical and biological parameters (FISH, IGHV, and *TP53* mutational status) were collected. Additional cases with CLL and MLN not captured by the GELLC registry were also submitted from collaborating centers. RT cases were not considered as MLN.

**Copyright Information:** (Online) ISSN: 2572-9241
©2026 The Author(s). HemaSphere is published by John Wiley & Sons Ltd on behalf of European Hematology Association. This is an open access Abstract Book distributed under the Attribution-NonCommercial-NoDerivs (CC BY-NC-ND), which allows third parties to download the articles and share them with others as long as they credit the author and the Abstract Book, but they cannot change the content in any way or use them commercially.
**Abstract Book Citations:** Authors, Title, HemaSphere, 2026;10:(S1):pages. The abstract book DOIs can be found on the "Author Information" page.
**Disclaimer:** Articles published in the journal HemaSphere exclusively reflect the opinions of the authors. The authors are responsible for all content in their abstracts including accuracy of the facts, statements, citing resources, etc.

### Results

We included 2,350 patients (93.3% CLL, 6.7% SLL) from the GELLC Registry. At diagnosis, median age was 71 years (19–99), 53.7% were male, 60.5% were IGHV-mutated (49.6% missing), and 13.4% had *TP53* abnormalities (55.3% missing). After a median follow-up of 4.3 years (0.1–16.2), 450 patients (19.1%) developed a secondary neoplasm: 318 solid tumors (13.5%), 19 myeloid neoplasms (0.8%), 63 RT (2.7%), and 50 MLN (2.1%). Compared with patients without MLN, patients were more frequently SLL (14.8% vs 6.7%, p=0.015), male (68.6% vs 50.8%, p=0.03), Binet B stage (19.9% vs 9.5%, p=0.04), producers of a monoclonal component (27.3% vs 12.1%, p=0.001), and less frequently required CLL treatment (33.2% vs 49.1%, p=0.04). No other significant differences were found.

Twenty-one additional patients with MLN and CLL not recorded in the GELLC Registry were included, bringing the total to 71 MLN cases. The most frequent MLNs were marginal zone lymphoma (n=18) and follicular lymphoma (n=12). Coexistence of both entities was demonstrated in at least one anatomical site in 38 patients (53.5%): peripheral blood n=22, bone marrow n=14, lymph node n=11, skin n=2, spleen n=1. Forty-one patients required treatment for the MLN, most frequently chemoimmunotherapy regimens (n=17). In six patients with CLL and B lymphomas, a treatment directed at one entity induced remission of both, using targeted therapy in 5 cases. Patients without other neoplasms had a median OS of 14.4 years, comparable to patients with MLN (12 years, p=0.34), but significantly longer than patients with solid tumors (9.3 years, p=0.03), RT (8.1 years, p<0.01), or myeloid neoplasms (7.5 years, p<0.01) (Figure).

**Table. Characteristics of Patients with CLL and MLN (n=71)**

| Characteristic | N (%) |
|---|---|
| Lineage | B: 57 (80.3%), T: 14 (19.7%) |
| MLN Diagnosis | |
| · Follicular lymphoma | 12 (16.9%) |
| · Mantle cell lymphoma | 5 (7%) |
| · Marginal zone lymphoma | 18 (25.4%) |
| · Lymphoplasmacytic lymphoma | 3 (4.2%) |
| · Multiple myeloma | 4 (5.6%) |
| · T-cell prolymphocytic leukemia | 4 (5.6%) |
| · Other indolent B-cell NHL | 11 (15.5%) |
| · T-cell lymphoma and others | 8 (11.3%) |
| · Mycosis fungoides/Sézary syndrome | 6 (8.5%) |
| Diagnosis MLN compared to CLL | |
| Preceding | 23 (32.4%) |
| Simultaneous | 12 (16.9%) |
| Subsequent | 36 (50.7%) |
| Median age at CLL diagnosis | 71 years |
| Median age at MLN diagnosis | 71 years |
| IGHV mutated (CLL) | 23/39 (59%) |
| *TP53* altered (CLL) | 7/50 (14%) |

### Summary/Conclusion

In our series, the prevalence of MLN among patients with CLL was 2.1%. These patients showed a predominance of nodal disease and an indolent course requiring less frequent CLL treatment and without a negative impact on OS. MLN and CLL can coexist in the same anatomical territories and respond to the same agents.

**Copyright Information:** (Online) ISSN: 2572-9241
©2026 The Author(s). HemaSphere is published by John Wiley & Sons Ltd on behalf of European Hematology Association. This is an open access Abstract Book distributed under the Attribution-NonCommercial-NoDerivs (CC BY-NC-ND), which allows third parties to download the articles and share them with others as long as they credit the author and the Abstract Book, but they cannot change the content in any way or use them commercially.
**Abstract Book Citations:** Authors, Title, HemaSphere, 2026;10:(S1):pages. The abstract book DOIs can be found on the “Author Information” page.
**Disclaimer:** Articles published in the journal HemaSphere exclusively reflect the opinions of the authors. The authors are responsible for all content in their abstracts including accuracy of the facts, statements, citing resources, etc.

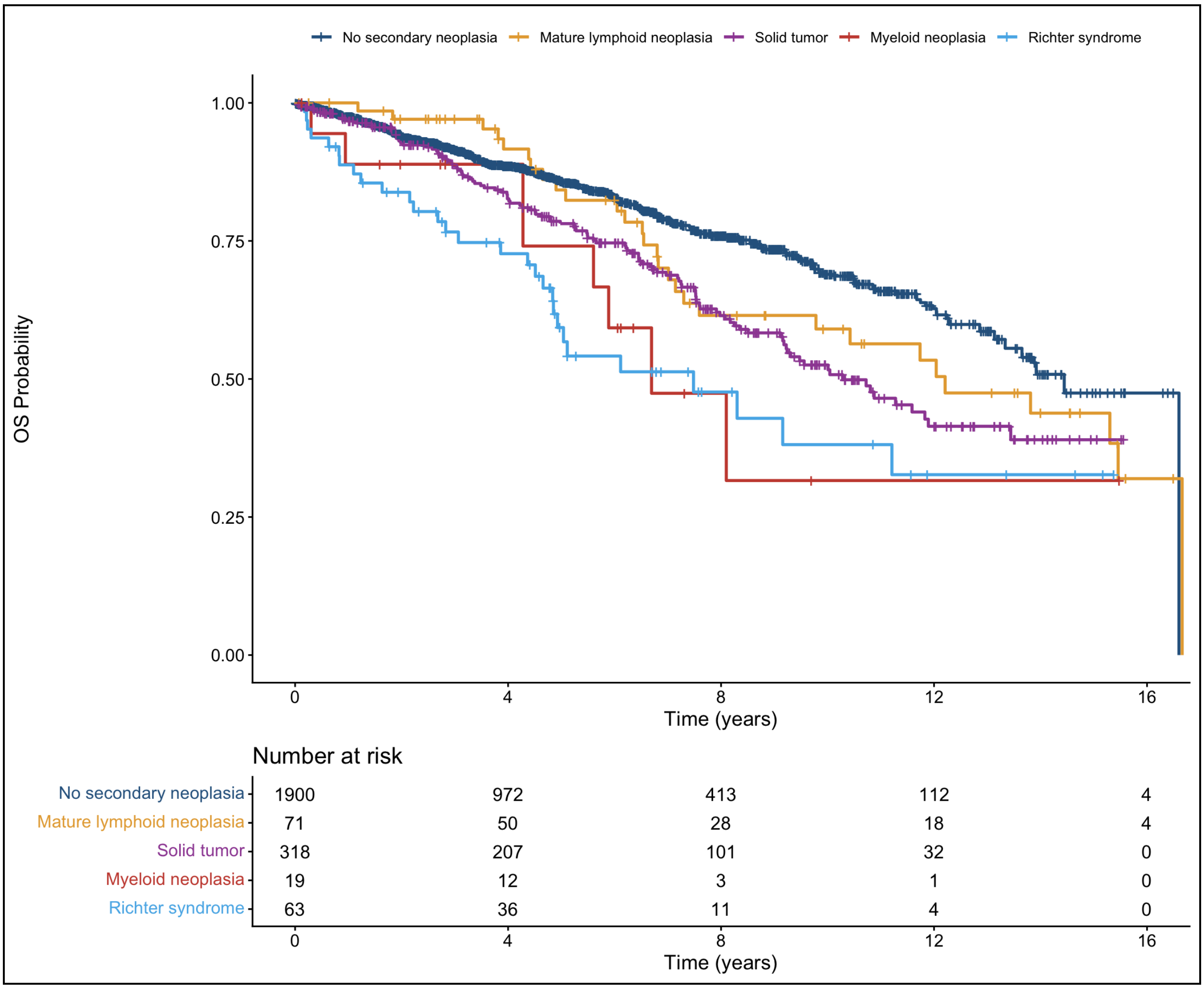

Figure. Overall survival from chronic lymphocytic leukemia (CLL) according to the presence and characteristics of associated primary neoplasms. OS was considered from the CLL diagnosis.

**Copyright Information:** (Online) ISSN: 2572-9241
©2026 The Author(s). HemaSphere is published by John Wiley & Sons Ltd on behalf of European Hematology Association. This is an open access Abstract Book distributed under the Attribution-NonCommercial-NoDerivs (CC BY-NC-ND), which allows third parties to download the articles and share them with others as long as they credit the author and the Abstract Book, but they cannot change the content in any way or use them commercially.
**Abstract Book Citations:** Authors, Title, HemaSphere, 2026;10:(S1):pages. The abstract book DOIs can be found on the "Author Information" page.
**Disclaimer:** Articles published in the journal HemaSphere exclusively reflect the opinions of the authors. The authors are responsible for all content in their abstracts including accuracy of the facts, statements, citing resources, etc.

### (PS1710) REAL-WORLD COMPARATIVE ANALYSIS OF TREATMENT DISCONTINUATION WITH COVALENT BRUTON TYROSINE KINASE INHIBITORS IN FIRST-LINE CHRONIC LYMPHOCYTIC LEUKEMIA (CLL)

**Topic:** 06. Chronic lymphocytic leukemia and related disorders - Clinical

Nakhle S. Saba*[1], Erin Mulvey[2], Mei Xue[3], Keri Yang[3], Catherine Fu[3], Dong Yuan[3], Derrick Van Beuge[3], Sikander Ailawadhi[4]

*[1]Our Lady of the Lake Cancer Institute, Baton Rouge, LA, United States of America;[2]Weill Cornell Medicine, New York, NY, United States of America; [3]BeOne Medicines Ltd, San Carlos, CA, United States of America; [4]Mayo Clinic, Jacksonville, FL, United States of America;*

### Background

While covalent Bruton tyrosine kinase inhibitors (cBTKis) are commonly used first-line (1L) therapies for CLL/small lymphocytic lymphoma (SLL), real-world data on treatment patterns remain limited.

### Aims

To evaluate real-world treatment persistence and treatment discontinuation associated with 1L cBTKi use in CLL.

### Methods

This retrospective observational study used the US IQVIA PharMetrics® closed claims database to identify adult patients (pts) with ≥2 entries of ICD codes for CLL/SLL diagnoses who initiated 1L cBTKi monotherapy (ibrutinib [ibru], acalabrutinib [acala], or zanubrutinib [zanu]) from 01/1/2022 to 02/28/2025; 1L start date was the index date. Eligible pts had continuous enrollment in health insurance for ≥3 months before the start of each cBTKi treatment and were followed until the end of the study period or censoring due to loss to follow-up, whichever occurred first. Baseline pt characteristics and outcomes were examined by each cBTKi. Outcomes included treatment discontinuation (defined as time from index to regimen end date if there was a subsequent therapy, or a gap of ≥120 days between regimen end date and last activity/enrollment date) and treatment persistence (defined as the probability of a pt continuing on treatment). Time to treatment discontinuation (TTD) was analyzed using Kaplan-Meier (KM) estimates. A multivariable Cox proportional hazards model adjusted for age, sex, race, ethnicity, payer type, geographic region, and Charlson Comorbidity Index and was used to estimate the hazard ratio of discontinuation between zanu and acala or ibru. A subgroup analysis was conducted in elderly pts aged ≥65 years.

### Results

A total of 1850 pts were included (zanu=608; acala=826; ibru=416). Baseline pt demographic and clinical characteristics were generally comparable between cBTKis, though median age was higher with zanu than acala or ibru (69.0 vs 68.0 vs 67.0 years; *P*=.0007), with a greater proportion of pts aged ≥65 years (73.9% vs 68.9% vs 65.9%;*P*=.0229). Median follow-up periods were 12 months for zanu, 14 months for acala, and 17 months for ibru. Zanu demonstrated significantly longer TTD (**Figure**) and higher treatment persistence at 6, 12, 18, 24, and 30 months than acala and ibru. At 12 months, treatment persistence was highest with zanu (82.8%; 95% CI, 79.1-85.9), followed by acala (78.7%; 95% CI, 75.4-81.7) and ibru (68.1%; 95% CI, 62.8-72.9; *P*<.0001). Similarly, at 24 months, treatment persistence was highest with zanu (74.7%; 95% CI, 69.3-79.3) than with acala (66.3%; 95% CI, 61.6-70.6) or ibru (58.7%; 95% CI, 52.7-64.3; *P*=.0002). Similar results were observed in the elderly pt subgroup. Multivariable analysis showed that the risk of treatment discontinuation was significantly lower with zanu than acala (adjusted hazard ratio [aHR]=0.76; 95% CI, 0.59-0.97; *P*=.0257) or ibru (aHR=0.51; 95% CI, 0.39-0.66; 66; *P*<.0001). Similar results were seen in pts ≥65 years (zanu vs acala: aHR=0.74; 95% CI, 0.55-0.98; *P*=.0364; zanu vs ibru: aHR=0.48; 95% CI, 0.36-0.66; *P*<.0001).

**Copyright Information:** (Online) ISSN: 2572-9241
©2026 The Author(s). HemaSphere is published by John Wiley & Sons Ltd on behalf of European Hematology Association. This is an open access Abstract Book distributed under the Attribution-NonCommercial-NoDerivs (CC BY-NC-ND), which allows third parties to download the articles and share them with others as long as they credit the author and the Abstract Book, but they cannot change the content in any way or use them commercially.
**Abstract Book Citations:** Authors, Title, HemaSphere, 2026;10:(S1):pages. The abstract book DOIs can be found on the "Author Information" page.
**Disclaimer:** Articles published in the journal HemaSphere exclusively reflect the opinions of the authors. The authors are responsible for all content in their abstracts including accuracy of the facts, statements, citing resources, etc.

### Summary/Conclusion

In this real-world study, zanu demonstrated significantly lower risk of treatment discontinuation and greater treatment persistence than acala or ibru in 1L CLL, especially in pts aged ≥65 years. While this study provides comparative insights into real-world treatment discontinuation, additional studies with longer follow-up are needed to better understand the reasons for and consequences of treatment discontinuation in 1L CLL.

**Figure.** Kaplan-Meier Curve of TTD

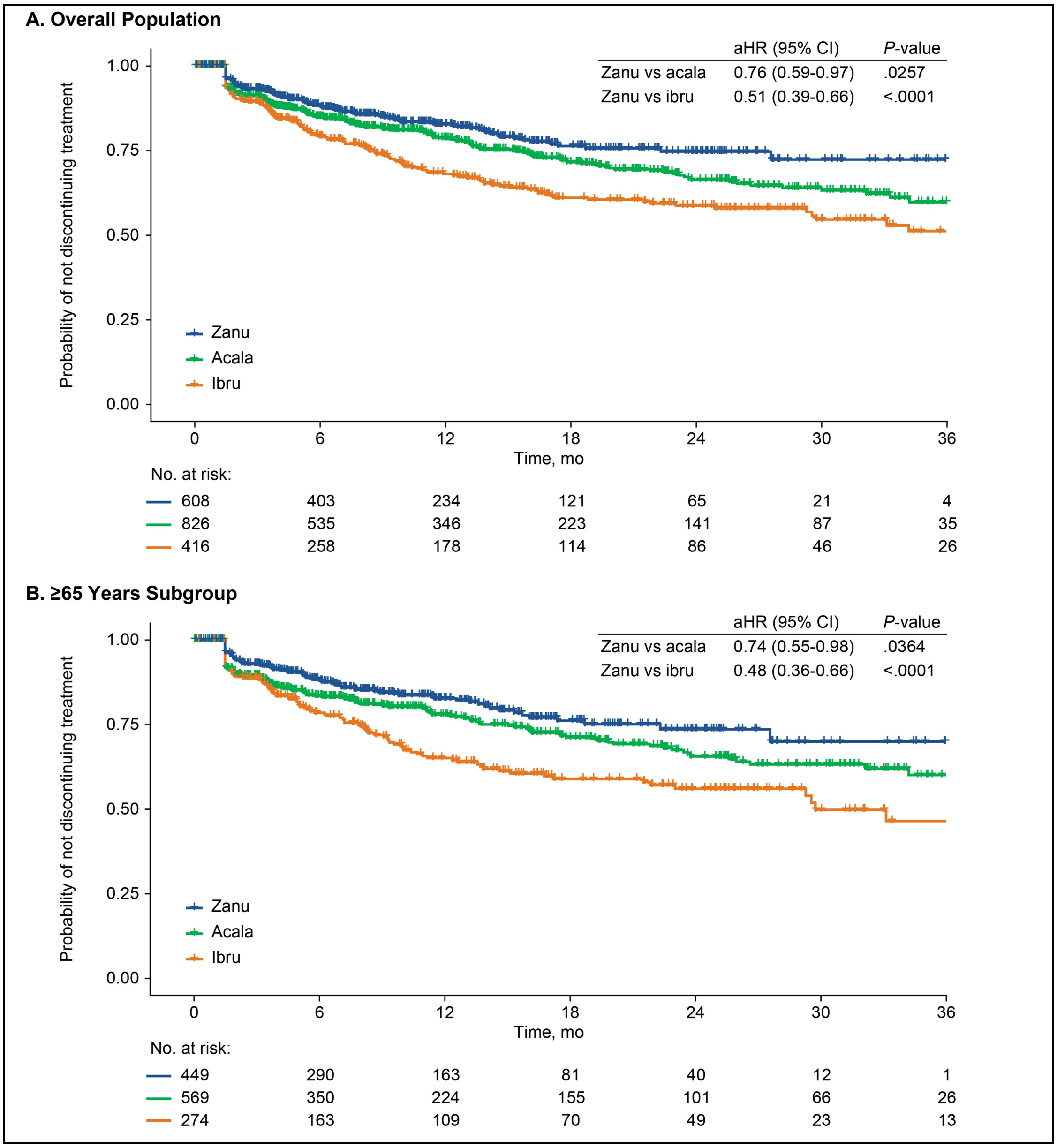

**Copyright Information:** (Online) ISSN: 2572-9241
©2026 The Author(s). HemaSphere is published by John Wiley & Sons Ltd on behalf of European Hematology Association. This is an open access Abstract Book distributed under the Attribution-NonCommercial-NoDerivs (CC BY-NC-ND), which allows third parties to download the articles and share them with others as long as they credit the author and the Abstract Book, but they cannot change the content in any way or use them commercially.
**Abstract Book Citations:** Authors, Title, HemaSphere, 2026;10:(S1):pages. The abstract book DOIs can be found on the "Author Information" page.
**Disclaimer:** Articles published in the journal HemaSphere exclusively reflect the opinions of the authors. The authors are responsible for all content in their abstracts including accuracy of the facts, statements, citing resources, etc.

### (PS1711) BGB-16673, A BRUTON TYROSINE KINASE (BTK) DEGRADER, HAS LOW RISK OF CYP3A-MEDIATED DRUG-DRUG INTERACTION (DDI): PHASE 1 ABSORPTION, DISTRIBUTION, METABOLISM, AND EXCRETION AND DDI STUDY RESULTS

**Topic:** 06. Chronic lymphocytic leukemia and related disorders - Clinical

Bilal Tariq*[1], Heather Zhang[1], Motohisa Takai[1], Prasanna Kumar[1], Teresa Zhang[1], Yanan Zhang[1], Tingting Cai[2], Fan Wang[1], Shelonitda Rose[1], William Hanley[1], Ying Ou[1]

*[1]BeOne Medicines, Ltd, San Carlos, CA, United States of America;[2]BeOne Medicines, Ltd, Shanghai, China;*

### Background

BGB-16673 is a BTK degrader that blocks signaling by tagging BTK for degradation through the cell's proteasome pathway, leading to tumor regression. Real-world data indicate that cytochrome P450 3A (CYP3A) inhibitors (eg, azole antifungals) are used by 15%-30% of patients with B-cell malignancies. Currently available covalent and noncovalent BTK inhibitors are primarily metabolized by CYP3A and require dose modification or caution when coadministered with CYP3A modulators due to exposure changes.

### Aims

To characterize the human absorption, distribution, metabolism, and excretion (ADME) profile and to evaluate the impact of strong CYP3A modulators on BGB-16673 pharmacokinetics (PK).

### Methods

BGB-16673-106 (NCT06776679) was a phase 1 human ADME study in healthy male participants (n=8) receiving a single oral dose of [$^{14}$C]-BGB-16673 (200 mg). Parent drug concentration and total radioactivity were measured in the plasma, urine, and feces; metabolite profiling was conducted using radiometric and mass spectrometric methods.
BGB-16673-105 (NCT06906809) was a phase 1, fixed-sequence, DDI study in healthy participants (n=37). Part A evaluated the strong CYP3A inducer phenytoin (100 mg three times daily) with a single-dose of BGB-16673 (200 mg). Part B evaluated the strong CYP3A inhibitor itraconazole (200 mg once or twice daily) with a single-dose of BGB-16673 (50 mg). Primary PK endpoints were area under the plasma concentration–time curve (AUC) and maximum observed plasma concentration ($C_{max}$), which were analyzed using mixed-effects models to derive geometric mean ratios (GMRs) and 90% CIs.
Both studies were conducted per the Declaration of Helsinki; all participants provided written informed consent.

Copyright Information: (Online) ISSN: 2572-9241
©2026 The Author(s). HemaSphere is published by John Wiley & Sons Ltd on behalf of European Hematology Association. This is an open access Abstract Book distributed under the Attribution-NonCommercial-NoDerivs (CC BY-NC-ND), which allows third parties to download the articles and share them with others as long as they credit the author and the Abstract Book, but they cannot change the content in any way or use them commercially.
**Abstract Book Citations:** Authors, Title, HemaSphere, 2026;10:(S1):pages. The abstract book DOIs can be found on the “Author Information” page.
**Disclaimer:** Articles published in the journal HemaSphere exclusively reflect the opinions of the authors. The authors are responsible for all content in their abstracts including accuracy of the facts, statements, citing resources, etc.

**Results**

Following a single oral dose of [$^{14}C$]-BGB-16673, mean total radioactivity recovery was 88.5%, with the vast majority recovered in feces and minimal recovery in urine. Unchanged BGB-16673 was the predominant circulating drug-related component, with no significant plasma metabolites detected. Oxadiazole ring-cleaved metabolites generated by gut microbiota predominated in feces, whereas oxidative and amide hydrolysis pathways were minimal, indicating limited contribution of hepatic metabolism to systemic clearance. These findings are consistent with metabolic pathways observed in nonclinical species and support predominant elimination via intestinal or biliary secretion of the unchanged parent drug, with negligible renal contribution. The median time to the maximum observed concentration was 12 hours, and the geometric mean terminal half-life of BGB-16673 was ≈82 hours.
In the DDI study, phenytoin coadministration resulted in no clinically meaningful exposure change in BGB-16673 (AUC GMR [coadministration vs alone], ≈0.9-1.0; $C_{max}$ GMR, ≈1.2) (Table). Similarly, itraconazole did not meaningfully increase exposure (AUC GMR, ≈1.1), with a modest reduction in $C_{max}$ (GMR, ≈0.77). Despite moderate variability, these changes were not clinically relevant.

**Summary/Conclusion**

BGB-16673 is primarily eliminated via intestinal or biliary excretion of the unchanged parent drug, with negligible renal clearance and minimal contribution of hepatic metabolism. Consistent with this profile, strong CYP3A induction and inhibition had no clinically meaningful impact on systemic exposure. These findings suggest a low risk of clinically relevant CYP3A-mediated DDIs and support greater dosing flexibility with BGB-16673.

**Copyright Information:** (Online) ISSN: 2572-9241
©2026 The Author(s). HemaSphere is published by John Wiley & Sons Ltd on behalf of European Hematology Association. This is an open access Abstract Book distributed under the Attribution-NonCommercial-NoDerivs (CC BY-NC-ND), which allows third parties to download the articles and share them with others as long as they credit the author and the Abstract Book, but they cannot change the content in any way or use them commercially.
**Abstract Book Citations:** Authors, Title, HemaSphere, 2026;10:(S1):pages. The abstract book DOIs can be found on the “Author Information” page.
**Disclaimer:** Articles published in the journal HemaSphere exclusively reflect the opinions of the authors. The authors are responsible for all content in their abstracts including accuracy of the facts, statements, citing resources, etc.

**Table. Statistical Analysis of Pharmacokinetic Parameters From BGB-16673-105**

| Parameter | Treatment | n | GLSM | Test vs reference: GLSM ratio (90% CI) | Test vs reference: Intrapatient CV (%) |
|---|---|---|---|---|---|
| **BGB-16673-105 part A: BGB-16673/phenytoin Interaction** | | | | | |
| **$AUC_{0\text{-tlast}}$, h × ng/mL** | BGB-16673 200 mg (reference) | 19 | 2640 | — | — |
| | BGB-16673 200 mg + phenytoin 100 mg (test) | 14 | 2880 | 1.09 (0.84-1.42) | 44.7 |
| **$AUC_{0\text{-}\infty}$, h × ng/mL** | BGB-16673 200 mg (reference) | 17 | 3130 | — | — |
| | BGB-16673 200 mg + phenytoin 100 mg (test) | 16 | 2870 | 0.92 (0.70-1.21) | 49.9 |
| **$C_{max}$, ng/mL** | BGB-16673 200 mg (reference) | 19 | 31.7 | — | — |
| | BGB-16673 200 mg + phenytoin 100 mg (test) | 16 | 38.6 | 1.22 (0.95-1.56) | 42.6 |
| **BGB-16673-105 part B: BGB-16673/itraconazole Interaction** | | | | | |
| **$AUC_{0\text{-tlast}}$, h × ng/mL** | BGB-16673 50 mg (reference) | 18 | 1270 | — | — |
| | BGB-16673 50 mg + itraconazole 200 mg (test) | 14 | 1330 | 1.05 (0.84-1.33) | 37.5 |
| **$C_{max}$, ng/mL** | BGB-16673 50 mg (reference) | 18 | 15.6 | — | — |
| | BGB-16673 50 mg + itraconazole 200 mg (test) | 14 | 12.0 | 0.77 (0.58-1.02) | 45.5 |

$AUC_{0\text{-}\infty}$, area under the plasma concentration–time curve from time 0 to infinity; $AUC_{0\text{-tlast}}$, area under the plasma concentration–time curve from time 0 to last quantifiable concentration; $C_{max}$, maximum observed plasma concentration; CV, coefficient of variation; GLSM, geometric least-squares mean.

**Copyright Information:** (Online) ISSN: 2572-9241
©2026 The Author(s). HemaSphere is published by John Wiley & Sons Ltd on behalf of European Hematology Association. This is an open access Abstract Book distributed under the Attribution-NonCommercial-NoDerivs (CC BY-NC-ND), which allows third parties to download the articles and share them with others as long as they credit the author and the Abstract Book, but they cannot change the content in any way or use them commercially.
**Abstract Book Citations:** Authors, Title, HemaSphere, 2026;10:(S1):pages. The abstract book DOIs can be found on the "Author Information" page.
**Disclaimer:** Articles published in the journal HemaSphere exclusively reflect the opinions of the authors. The authors are responsible for all content in their abstracts including accuracy of the facts, statements, citing resources, etc.

### (PS1712) REAL WORLD OUTCOMES IN OF 50 PATIENTS WITH CHRONIC LYMPHOCYTIC LEUKEMIA (CLL) TREATED WITH PIRTOBRUTINIB AFTER A COVALENT BRUTON TYROSINE KINASE INHIBITOR AND A BCL2 INHIBITOR: A GIMEMA STUDY

**Topic:** 06. Chronic lymphocytic leukemia and related disorders - Clinical

Gian Matteo Rigolin[1], Andrea Visentin[2], Maura Nicolosi[3], Annamaria Tomasso[4], Roberta Murru[5], Sara Pepe[6], Roberto Marasca[7], Lucia Farina[8], Alberto Fresa[9], Annalisa Arcari[10], Vanessa Innao[11], Elisa Albi[12], Corrado Benevolo Savelli[13], Claudia Giordano[14], Elsa Pennese[15], Candida Vitale[16], Jacopo Olivieri[17], Isacco Ferrarini[18], Chiara Salvetti[19], Myriam Foglietta[20], Laura Arletti[21], Maurizio Cavallari[22], Nicola Franzoni[23], Riccardo Moia[24], Esmeralda Conte[25], Arcangelo Liso[26], Martina Bullo[27], Maria Ilaria DEL Principe[28], Valentina Arena[29], Francesca Paoloni[29], Alfonso Piciocchi[29], Lydia Scarfò[30], Paolo Ghia[30], Antonio Cuneo*[1]

*[1]Hematology Section, Department of Medical Sciences, Arcispedale S. Anna, Azienda Ospedaliero-Universitaria, University of Ferrara, Ferrara, Italy; [2]Haematology Unit, Department of Medicine, University of Padova, Padova, Italy; [3]Hematology Division, A.O.U. Città della Salute e della Scienza di Torino, C.so Bramante 88, 10126, Torino, Italy; [4]Hematology Unit, Fondazione Policlinico Gemelli, IRCCS, Catholic University of Sacred Hearth, 00168, Rome, Italy; [5]Hematology and Stem Cell Transplantation Unit, Ospedale Oncologico A. Businco, ARNAS "G. Brotzu", Cagliari, Italy; [6]Hematology, Department of Translational and Precision Medicine, Sapienza University of Rome, Rome, Italy; [7]Hematology Unit, University of Modena and Reggio Emilia, Modena, Italy; [8]Hematology, Fondazione IRCCS Istituto Nazionale dei Tumori, Milano, Italy; [9]Hematology-Oncology and Stem-Cell Transplantation Unit, National Cancer Institute - IRCCS - Fondazione "G. Pascale", Naples, Italy; [10]Hematology Unit, Ospedale Guglielmo da Saliceto, Piacenza, Italy; [11]UOC Ematologia, ARNAS Garibaldi, Catania, Italy; [12]Comprehensive Cancer Center, IRCCS San Raffaele Hospital, Milano, Italy; [13]Candiolo Cancer Institute, FPO-IRCCS, Candiolo, (Turin), Italy; [14]Federico II University, Naples, Italy; [15]Ospedale Civile S. Spirito, Pescara, Italy; [16]Department of Molecular Biotechnology and Health Sciences, University of Turin, Turin, Italy And University Division of Hematology, A.O.U. Città della Salute e della Scienza di Torino, Turin, Italy; [17]Clinica Ematologica, Azienda Sanitaria Universitaria Friuli Centrale, Udine, Italy; [18]Department of Engineering for Innovation Medicine, Section of Hematology, University of Verona, Verona, Italy; [19]Clinic of Hematology, IRCCS Ospedale Policlinico San Martino, Genoa, Italy; [20]Hematology Unit, Santa Croce e Carle General Hospital, Cuneo, Italy; [21]Hematology unit, AUSL-IRCCS Reggio Emilia, Reggio Emilia, Italy;[22]Hematology Section, Ospedale dell'Angelo, Mestre, Italy; [23]Azienda Ulss 5 Polesana, Rovigo, Italy; [24]Division of Hematology, Department of Translational Medicine, University of Eastern Piedmont, Novara, Italy; [25]Hematology, Department of Clinical and Molecular Medicine, Sant'Andrea University Hospital, Sapienza University of Rome, Rome, Italy; [26]Department of Medicine and Surgery University of Perugia, Perugia Italy and Hematology/Oncology Unit Terni Hospital, Terni, Italy; [27]Hematology Unit, Azienda Ospedaliera Ordine Mauriziano di Torino, Turin, Italy; [28]Haematology, Department of Biomedicine and Prevention, University of Rome Tor Vergata, Rome, Italy;[29]GIMEMA, Rome, Italy; [30]Comprehensive Cancer Center, IRCCS San Raffaele Hospital, Milan, Italy And Medical School, Università Vita-Salute San Raffaele, Milan, Italy;*

### Background

Pirtobrutinib is a non-covalent Bruton tyrosine kinase inhibitor (ncBTKi) with documented efficacy in CLL previously exposed to covalent BTKi (cBTKi). To date, it is the only agent approved in the post-BTKi setting with evidence derived from dedicated clinical trials, including patients with double exposure (DE) to a cBTKi and to a BCL2 inhibitor. As of today, efficacy and safety data in a real-world setting are lacking.

**Copyright Information:** (Online) ISSN: 2572-9241
©2026 The Author(s). HemaSphere is published by John Wiley & Sons Ltd on behalf of European Hematology Association. This is an open access Abstract Book distributed under the Attribution-NonCommercial-NoDerivs (CC BY-NC-ND), which allows third parties to download the articles and share them with others as long as they credit the author and the Abstract Book, but they cannot change the content in any way or use them commercially.
**Abstract Book Citations:** Authors, Title, HemaSphere, 2026;10:(S1):pages. The abstract book DOIs can be found on the "Author Information" page.
**Disclaimer:** Articles published in the journal HemaSphere exclusively reflect the opinions of the authors. The authors are responsible for all content in their abstracts including accuracy of the facts, statements, citing resources, etc.

**Aims**

To analyze the efficacy of pirtobrutinib in a nationwide named-patient program for DE patients with CLL.

**Methods**

The data were obtained from the GIMEMA CLL2121 study, an observational study aimed at collecting clinicobiologic data of CLL patients diagnosed in Italy. The study was approved by the EC and all patients signed a written informed consent.

The endpoints of this analysis were overall response rate (ORR), time to discontinuation for progression, death and toxicity (TPDT), obtained by censoring all patients who discontinued the study drug for reasons other than progression, death or toxicity, time to treatment discontinuation (TTD), defined as the time occurring between the first administration of pirtobrutinib and treatment discontinuation for any cause, including death or lost to follow-up and overall survival (OS).

**Results**

Fifty patients with DE CLL were treated with pirtobrutinib at 28 centers. Median age was 70y (range 51- 87). Median time from diagnosis to first dose of pirtobrutinib was 9.6 years (IQ: 7.5-14.22). Other baseline characteristics are shown in table 1.

ORR was 57%. With a median follow-up of 9.7 months (IQR 3.39;24.38) the estimated 12-month probability to be on treatment, using TPTD as endpoint, was 66% (95% CI: 50 - 87), (Figure 1). The median TTD was 13.3 months (95% CI: 10.1 - NA), with a 58% probability of being on treatment at 12 months (95% CI: 42 - 79). The reasons of discontinuation in 16 patients were failure in 5, death in 6, toxicity in 1 and other causes in 4 patients. An estimated 80% probability of being alive at 12 months was observed (95% CI: 68 - 94). ORR, TPDT, TTD and OS were not influenced by baseline characteristics, including age and *TP53* aberrations. Median OS in 10 patients who discontinued pirtobrutinib for reasons other than death was 3.68 months (95% CI: 0.329 – NA). Causes of death were CLL progression in 6 patients, Richter transformation in 4, second primary malignancy in 1, unknown or not reported in 4.

Grade 3-4 adverse events were reported in 11 patients: 3 had an infection, 3 had neutropenia; febrile neutropenia, diarrhea, chest pain, pulmonary toxicity, and increased transaminase levels were recorded in 1 patient each. One patient had fungal pneumonia, and 1 patient had an episode of atrial fibrillation (grade not reported).

**Copyright Information:** (Online) ISSN: 2572-9241
©2026 The Author(s). HemaSphere is published by John Wiley & Sons Ltd on behalf of European Hematology Association. This is an open access Abstract Book distributed under the Attribution-NonCommercial-NoDerivs (CC BY-NC-ND), which allows third parties to download the articles and share them with others as long as they credit the author and the Abstract Book, but they cannot change the content in any way or use them commercially.
**Abstract Book Citations:** Authors, Title, HemaSphere, 2026;10:(S1):pages. The abstract book DOIs can be found on the “Author Information” page.
**Disclaimer:** Articles published in the journal HemaSphere exclusively reflect the opinions of the authors. The authors are responsible for all content in their abstracts including accuracy of the facts, statements, citing resources, etc.

### Summary/Conclusion

In this real-world analysis we adopted objective endpoints to assess the effectiveness of pirtobrutinib in DE CLL. Our findings show that pirtobrutinib proved to be efficacious and safe in a predominantly elderly and heavily pre-treated patient population. Though the number of patients is relatively small the effectiveness was not influenced by unfavorable clinicobiologic characteristics. The outcome after pirtobrutinib discontinuation was dismal and the causes of death were mostly related to disease progression and Richter transformation.

**Table 1**

| Characteristic | % of pts |
|---|---|
| Sex *(M/F)* | 60/40 |
| uIGHV/mIGHV | 74/26 |
| *TP53* mutated (yes/no) | 50/50 |
| 17p- (yes/no) | 30/70 |
| Binet stage A or B/C | 62/38 |
| N. of previous therapies *(2/3/4+)* | 14/42/44 |

**Copyright Information:** (Online) ISSN: 2572-9241
©2026 The Author(s). HemaSphere is published by John Wiley & Sons Ltd on behalf of European Hematology Association. This is an open access Abstract Book distributed under the Attribution-NonCommercial-NoDerivs (CC BY-NC-ND), which allows third parties to download the articles and share them with others as long as they credit the author and the Abstract Book, but they cannot change the content in any way or use them commercially.
**Abstract Book Citations:** Authors, Title, HemaSphere, 2026;10:(S1):pages. The abstract book DOIs can be found on the "Author Information" page.
**Disclaimer:** Articles published in the journal HemaSphere exclusively reflect the opinions of the authors. The authors are responsible for all content in their abstracts including accuracy of the facts, statements, citing resources, etc.

### (PS1713) CORRELATION OF BASELINE CHARACTERISTICS WITH PROGNOSIS IN PATIENTS WITH CHRONIC LYMPHOCYTIC LEUKEMIA/SMALL LYMPHOCYTIC LYMPHOMA (CLL/SLL) TREATED WITH LISAFTOCLAX (APG-2575) IN A PIVOTAL PHASE 2 STUDY

**Topic:** 06. Chronic lymphocytic leukemia and related disorders - Clinical

Keshu Zhou*[1], Tingyu Wang[2], Ting Niu[3], Meifang Zheng[4], Jie Jin[5], Eric Liang[6], Min Yu[7], Lei Wang[7], Fang Lu[7], Yan Xiong[7], Zi Chen[7], Dajun Yang[6, 7, 8], Jianyong Li[9], Yifan Zhai[6, 7]

*[1]Henan Cancer Hospital, Zhengzhou, China;[2]Institute of Hematology & Blood Diseases Hospital, Chinese Academy of Medical Sciences, Tianjin, China; [3]West China Hospital of Sichuan University, Chengdu, China;[4]Linyi Cancer Hospital, Linyi, China;[5]The First Affiliated Hospital of Zhejiang University School of Medicine, Hangzhou, China; [6]Ascentage Pharma Group Inc., Rockville, MD, United States of America; [7]Ascentage Pharma (Suzhou) Co., Ltd., Suzhou, China;[8]Department of Experimental Research, State Key Laboratory of Oncology in South China Collaborative Innovation Center for Cancer Medicine, Sun Yat-sen University Cancer Center, Guangzhou, China; [9]Jiangsu Provincial People's Hospital, Nanjing, China;*

### Background

BCL-2 inhibitor monotherapy or combination with BTK inhibitors (BTKis) or anti-CD20 antibodies has demonstrated efficacy in CLL/SLL. However, prognostic factors for patients with CLL/SLL treated with BCL-2 inhibitors, particularly in the post-BTKi setting, remain poorly defined because of disease heterogeneity. Lisaftoclax is a novel, oral BCL-2 inhibitor evaluated in a pivotal phase 2 study (NCT05147467) in patients with R/R CLL/SLL previously treated with BTKis.

### Aims

We conducted a correlative analysis to identify prognostic markers associated with clinical outcomes in patients receiving lisaftoclax.

### Methods

Patients with R/R CLL/SLL refractory to BTKis (n = 77) received lisaftoclax 600 mg QD until disease progression or unacceptable toxicity. ORR was assessed per IRC and investigators, and PFS per the Kaplan-Meier method. ORR and PFS were assessed at a median follow-up of 22.0 months. Baseline cytogenetics were assessed by conventional karyotyping and fluorescence in situ hybridization. Gene mutations were assessed by next-generation sequencing (NGS) using a 175-gene panel in bone marrow aspirate (BMA) obtained before treatment. MRD testing in peripheral blood (PB) and/or BMA was performed using 8-color flow cytometry. Undetectable MRD (uMRD) was defined as < $10^{-4}$ (<1 CLL cell per $10^4$ leukocytes). Fisher's exact test, Kaplan-Meier survival analyses, log-rank test, and multivariate Cox or logistic regression analyses were used to assess biomarker associations with clinical outcomes (ORR, PFS, uMRD).

**Copyright Information:** (Online) ISSN: 2572-9241
©2026 The Author(s). HemaSphere is published by John Wiley & Sons Ltd on behalf of European Hematology Association. This is an open access Abstract Book distributed under the Attribution-NonCommercial-NoDerivs (CC BY-NC-ND), which allows third parties to download the articles and share them with others as long as they credit the author and the Abstract Book, but they cannot change the content in any way or use them commercially.
**Abstract Book Citations:** Authors, Title, HemaSphere, 2026;10:(S1):pages. The abstract book DOIs can be found on the "Author Information" page.
**Disclaimer:** Articles published in the journal HemaSphere exclusively reflect the opinions of the authors. The authors are responsible for all content in their abstracts including accuracy of the facts, statements, citing resources, etc.

### Results

Among 72 evaluable patients, median PFS was 23.9 months and ORRs were 62.5% (per IRC) and 58.3% (per investigators). Among 55 patients who underwent MRD testing, 12 (21.8%) achieved uMRD in PB. Of 11 patients evaluable for BMA MRD, 6 (54.5%) achieved uMRD. According to NGS, *BTK* and *TP53* were the most frequently mutated genes in all patients, with mutation frequencies of 44.1% and 39.7%, respectively, followed by *ATM*, *SF3B1*, and *NOTCH1* (each > 20%). Univariate analyses revealed that *TP53*/del(17p) (in 39.7%), complex karyotype (CK; ≥ 3 chromosomal abnormalities; in 47.7%) and*SF3B1*, *KIT*, *BLM*, or *SETD2* mutations (in 25%, 4.4%, 7.4%, 7.4%) were associated with significantly shorter PFS (each p < 0.05) as shown in Figure 1; multivariate Cox regression revealed that baseline CK and tumor size were independent risk factors for inferior PFS (adjusted hazard ratio, 4.50 [95% CI, 1.50-13.5]; p = 0.0073 for baseline CK and 4.57 [95% CI, 1.11–18.8]; p = 0.0350 for tumor size). *SF3B1* mutation (in 25%) was also associated with inferior ORR (p < 0.05). Baseline absolute lymphocyte count was independently associated with uMRD by multivariate logistic regression (adjusted odds ratio, 0.372 [95% CI, 0.123-0.759]; p = 0.0314).

### Summary/Conclusion

Baseline *TP53*/del(17p); CK; tumor size; and *SF3B1, KIT, BLM,* and *SETD2* mutations are high-risk factors in patients with CLL/SLL receiving lisaftoclax. These results may refine risk stratification in the post-BTKi setting and support development of risk-adapted combination therapeutic strategies.

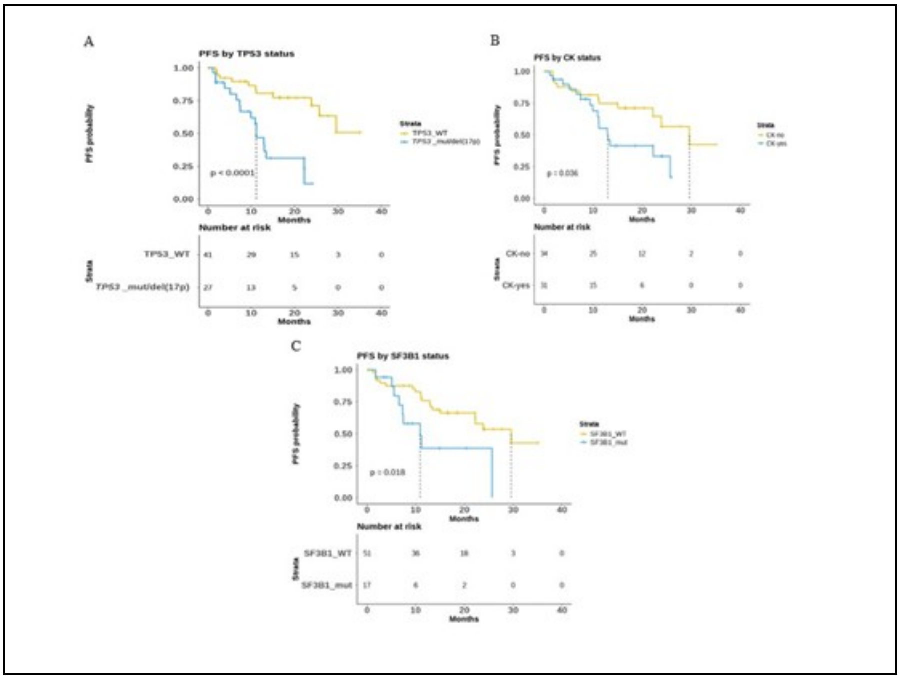

Figure 1. PFS according to baseline characteristics. A. PFS is shorter in patients with TP53 mutations or del(17p) (TP53_mut/del(17p); blue) than in those without (TP53_WT; yellow). B. PFS is shorter in patients with CK (blue) than in those without CK (yellow). C. PFS is shorter in patients with SF3B1 mutations than in those without (SF3B1_WT; yellow). Statistical significance was determined by log-rank test with a p-value threshold of 0.05.

**Copyright Information:** (Online) ISSN: 2572-9241
©2026 The Author(s). HemaSphere is published by John Wiley & Sons Ltd on behalf of European Hematology Association. This is an open access Abstract Book distributed under the Attribution-NonCommercial-NoDerivs (CC BY-NC-ND), which allows third parties to download the articles and share them with others as long as they credit the author and the Abstract Book, but they cannot change the content in any way or use them commercially.
**Abstract Book Citations:** Authors, Title, HemaSphere, 2026;10:(S1):pages. The abstract book DOIs can be found on the "Author Information" page.
**Disclaimer:** Articles published in the journal HemaSphere exclusively reflect the opinions of the authors. The authors are responsible for all content in their abstracts including accuracy of the facts, statements, citing resources, etc.

### (PS1714) BTKI-FIRST VERSUS VENETOCLAX-OBINUTUZUMAB-FIRST SEQUENCING IN CLL: A PROPENSITY SCORE-MATCHED TOTAL DISEASE MANAGEMENT ANALYSIS

**Topic:** 06. Chronic lymphocytic leukemia and related disorders - Clinical

Shankar Biswas*[1], Yashasvi Srivastava[1], Kridhay Jindal[2, 2], Raghabendra Kumar Mahato[3, 3]

*[1]Ivano-Frankivsk National Medical University, Internal Medicine, Ivano-Frankivsk, Ukraine; [2]Government Medical College, Internal Medicine, Patiala, India; [3]Gandaki Medical College Teaching Hospital and Research Center, Internal Medicine, Pokhara, Nepal;*

### Background

BTK inhibitors (BTKi) and venetoclax-obinutuzumab (VO) are established first-line CLL therapies. The CLL17 trial showed non-inferior PFS with fixed-duration VO versus continuous ibrutinib at 3 years (Al-Sawaf et al., Blood 2025), but follow-up is insufficient for second-line outcomes. As most patients eventually require subsequent therapy, the optimal sequence of BTKi-first then venetoclax (BTKi→Ven) versus VO-first then BTKi (VO→BTKi) still remains unknown. Evidence is limited to small single-arm series (n=23–85) evaluating individual lines rather than complete sequences (Hampel et al., ASH 2024; Soumerai et al., Blood Adv 2025).

### Aims

To compare total disease management outcomes across first-two-line treatment sequences (BTKi→Ven vs VO→BTKi) in CLL using a large real-world dataset.

### Methods

Retrospective cohort study using the TriNetX Global Collaborative Network (171 healthcare organizations). Cohort A (BTKi→Ven): CLL patients receiving ibrutinib, acalabrutinib, or zanubrutinib first-line followed by venetoclax ≥90 days later. Cohort B (VO→BTKi): patients receiving VO first-line followed by a BTKi ≥90 days later. Index was first-line initiation. Propensity score matching (1:1) balanced age, sex, race, comorbidities (AF, hypertension, heart failure, CKD, diabetes), CLL features (lymphadenopathy, splenomegaly, autoimmune cytopenias), and labs (hemoglobin, platelets, WBC, creatinine, LDH). Primary outcome: all-cause mortality from first-line start. Secondary: new-onset AF, sepsis, HSCT/CAR-T receipt, hospitalization, ED visits, and pancytopenia.

### Results

After matching, 1,776 patients per cohort were analyzed (median age 68–69 years; 64% male; 79% White; all standardized differences <0.10). Median follow-up: 720 days (VO→BTKi) versus 686 days (BTKi→Ven). Mortality was 24.9% versus 26.7% (HR 0.917; 95% CI 0.805–1.044; p=0.188); median survival 2,823 versus 2,787 days. No significant differences were found for AF (HR 0.960; p=0.747), sepsis (HR 0.985; p=0.872), HSCT/CAR-T (HR 0.870; p=0.394), hospitalization (HR 1.001; p=0.987), ED visits (HR 1.076; p=0.492), or pancytopenia (HR 1.004; p=0.961). Proportional hazards assumptions held for all endpoints.

### Summary/Conclusion

In this large propensity score-matched analysis, total disease management outcomes were comparable between BTKi-first and VO-first sequences. Point estimates for mortality and HSCT/CAR-T numerically favored VO→BTKi, though not significantly. Clinicians may choose either sequence based on patient-specific factors. Planned sensitivity analyses including restriction by BTKi generation and landmark analysis from second-line start will address residual survivorship bias.

**Copyright Information:** (Online) ISSN: 2572-9241
©2026 The Author(s). HemaSphere is published by John Wiley & Sons Ltd on behalf of European Hematology Association. This is an open access Abstract Book distributed under the Attribution-NonCommercial-NoDerivs (CC BY-NC-ND), which allows third parties to download the articles and share them with others as long as they credit the author and the Abstract Book, but they cannot change the content in any way or use them commercially.
**Abstract Book Citations:** Authors, Title, HemaSphere, 2026;10:(S1):pages. The abstract book DOIs can be found on the "Author Information" page.
**Disclaimer:** Articles published in the journal HemaSphere exclusively reflect the opinions of the authors. The authors are responsible for all content in their abstracts including accuracy of the facts, statements, citing resources, etc.

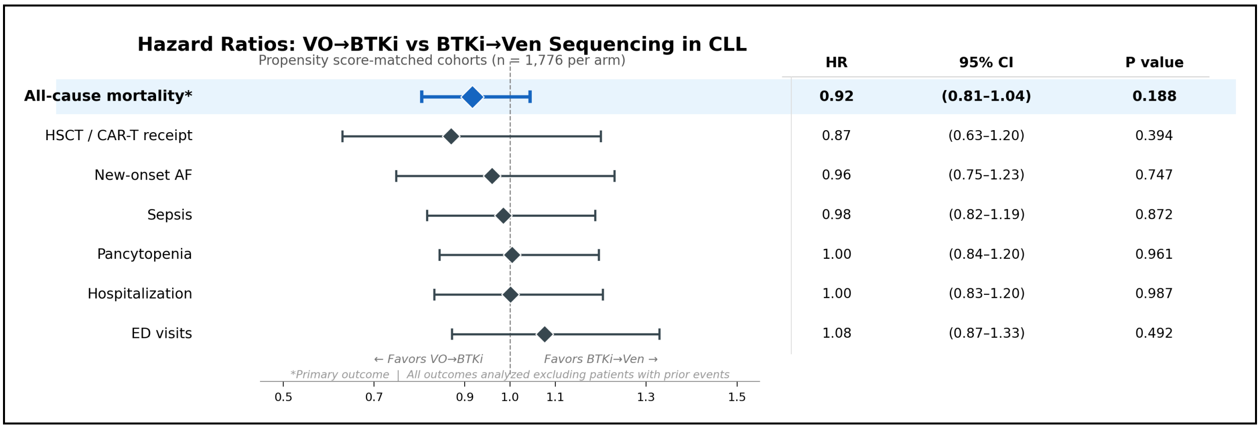

Abbreviations: AF, atrial fibrillation; BTKi, Bruton's tyrosine kinase inhibitor; CAR-T, chimeric antigen receptor T-cell therapy; CI, confidence interval; CLL, chronic lymphocytic leukemia; ED, emergency department; HR, hazard ratio; HSCT, hematopoietic stem cell transplantation; Ven, venetoclax; VO, venetoclax-obinutuzumab. All outcomes were analyzed using propensity score-matched cohorts (n=1,776 per arm) excluding patients with prior events; asterisk () denotes the primary outcome. HR <1.0 favors VO→BTKi sequencing; no statistically significant difference was observed across any outcome.*

Copyright Information: (Online) ISSN: 2572-9241
©2026 The Author(s). HemaSphere is published by John Wiley & Sons Ltd on behalf of European Hematology Association. This is an open access Abstract Book distributed under the Attribution-NonCommercial-NoDerivs (CC BY-NC-ND), which allows third parties to download the articles and share them with others as long as they credit the author and the Abstract Book, but they cannot change the content in any way or use them commercially.
Abstract Book Citations: Authors, Title, HemaSphere, 2026;10:(S1):pages. The abstract book DOIs can be found on the “Author Information” page.
Disclaimer: Articles published in the journal HemaSphere exclusively reflect the opinions of the authors. The authors are responsible for all content in their abstracts including accuracy of the facts, statements, citing resources, etc.

### (PS1715) REAL-WORLD DATA OF FIRST-LINE FIXED-DURATION WITH IBRUTINIB-VENETOCLAX (IV) OR VENETOCLAX-OBINUTUZUMAB (VO) IN CHRONIC LYMPHOCYTIC LEUKEMIA: A MULTICENTER STUDY FROM THE GELLC REGISTRY

**Topic:** 06. Chronic lymphocytic leukemia and related disorders - Clinical

Lucrecia Yáñez*[1, 2], Almudena Navarro[3], Ana Muntañola Prat[4], Angel Ramirez Payer[5], Tycho Stephan Baumann[6], Ángeles Medina[7], José-Angel Hernández-Rivas[8], Pau Abrisqueta[9, 10], Pablo Javier Mozas Fernandez[11], María José Terol[12], Eva Gimeno[13], Javier Loscertales Pueyo[14], Alexia Teresa Suarez Cabrera[15], Sergio Ramos Cillan[16], Miguel Argüello-Tomás[4], Cristina De Ramón[3], Diana Sánchez-Argüello[17], Luis Ignacio Sancho Val[18], María Argüello Marina[19], Amalia Cuesta[20], Martin Cabero[21], Arantxa García-Raso[16], Javier De La Serna[6], Marta Crespo Maull[10], Francisco Bosch Albareda[9, 10]

*[1]Hospital Universitario Marqués de Valdecilla, Hematologia, Santander, Spain;[2]University of Cantabria, Santander, Spain; [3]Hospital Universitario de Salamanca, Salamanca, Spain;[4]Hospital de la Santa Creu i Sant Pau, Hematologia, Barcelona, Spain; [5]Hospital Universitario Central de Asturias, Hema, Oviedo, Spain;[6]Hospital Universitario 12 de Octubre, Madrid, Spain;[7]Hospital Universitario Costa del Sol, Marbella, Spain; [8]Hospital Universitario Infanta Leonor, Madrid, Spain; [9]Hospital Universitario Vall d'Hebron, Barcelona, Spain; [10]Vall D'hebron Institute Of Oncology, Barcelona, Spain; [11]Hospital Clinic de Barcelona, Hematologia, Barcelona, Spain; [12]Hospital Clinico de Valencia, Valencia, Spain; [13]Hospital del Mar, Barcelona, Spain; [14]Hospital Universitario de La Princesa, Madrid, Spain; [15]Hospital Universitario De Gran Canaria, Las Palmas de Gran Canaria, Spain; [16]Fundación Jiménez Díaz, Madrid, Spain; [17]Hospital de Laredo, Laredo, Spain; [18]Hospital Lozano Blesa, Zaragoza, Spain; [19]Hospital Universitario de Guadalajara, Guadalajara, Spain;[20]Hospital Sierrallana, Torrelavega, Spain; [21]Hospital General La Mancha Centro, Ciudad Real, Spain;*

### Background

The therapeutic paradigm in Chronic Lymphocytic Leukemia (CLL) has shifted from continuous BTK inhibition to fixed-duration (finite) regimens, aiming to minimize cumulative toxicity and avoid clonal resistance. While recent clinical trials have established high efficacy for Venetoclax-Obinutuzumab (VO) and Ibrutinib-Venetoclax (IV), head-to-head data in unselected, real-world populations remain scarce.

### Aims

The GELLC-RWE3 study aims to analyze treatment compliance and clinical outcomes of finite first-line therapies in a multicenter, unselected cohort from the Spanish Group of CLL (GELLC).

### Methods

We conducted a multicenter, retrospective, observational study using data from the GELLC registry. We identified patients (pts) diagnosed with CLL or SLL according to iwCLL criteria who received IV or VO as first-line therapy in routine clinical practice. To ensure a strictly real-world evidence cohort, exclusion criteria included participation in interventional clinical trials. This interim analysis reports baseline characteristics, molecular/genetic risk profiles [del(17p), *TP53*, and IGHV status], and early efficacy data.

**Copyright Information:** (Online) ISSN: 2572-9241
©2026 The Author(s). HemaSphere is published by John Wiley & Sons Ltd on behalf of European Hematology Association. This is an open access Abstract Book distributed under the Attribution-NonCommercial-NoDerivs (CC BY-NC-ND), which allows third parties to download the articles and share them with others as long as they credit the author and the Abstract Book, but they cannot change the content in any way or use them commercially.
**Abstract Book Citations:** Authors, Title, HemaSphere, 2026;10:(S1):pages. The abstract book DOIs can be found on the "Author Information" page.
**Disclaimer:** Articles published in the journal HemaSphere exclusively reflect the opinions of the authors. The authors are responsible for all content in their abstracts including accuracy of the facts, statements, citing resources, etc.

**Results**

As of February 18, 2026, 144 pts were evaluable (IV: n=59; VO: n=85). The IV cohort was younger (mean 61 vs. 65 years; p=0.05). The treatment period per cohort was similar: VO 2021–2025; IV 2023–2025. Table 1 shows clinical and biological data in each group.
The comorbidity analysis revealed that VO was preferred in patients with pre-existing cardiovascular risk : atrial fibrillation (19.5% in VO vs. 0% in IV; p<0.01) and other cardiopathies (39% in VO vs. 13.3% in IV; p<0.05). No significant differences were observed regarding hypertension, diabetes, or renal failure.
High-risk CLL [del(17p) and/or *TP53* mutation] were identified in 12.9% of the total population, with a higher prevalence in the VO group (20.4%) compared to IV (8.6%). IGHV status was unmutated in 82.4% of VO pts and 57.1% of IV pts.
Efficacy (n=90) showed a high overall response rate for both cohorts: 96.9% for IV and 98.3% for VO. Peripheral blood minimal residual disease (PB-MRD) at EOT was assessed in 44 pts by flow cytometry; PB-MRD $\leq 10^{-2}$ was significantly more frequent in the VO cohort (90.9%) than in the IV cohort (45.5%; p<0.01). Data for undetectable MRD ($<10^{-4}$) is not available at this moment.

**Summary/Conclusion**

In this real-world cohort, both IV and VO demonstrate high efficacy in treatment-naïve CLL. Current clinical practice in Spain appears to favor the VO regimen for elderly patients and those with higher cardiovascular comorbidity, while IV appears the preferred fixed duration combination for younger CLL.
Recruitment in this study is ongoing, and updated data will be presented. These findings will contribute to guide treatment selection in the finite-duration era.

| Characteristic | I+V (N=59) | V+O (N=85) |
|---|---|---|
| **Age, years, mean (±SD)** | 61.17 (11.351) | 64.74 (10.109) |
| **Male sex, n/N (%)** | 38/59 (64.4%) | 60/85 (70.6%) |
| **Comorbidities, patients with data** | 30 | 41 |
| Hypertension, n/N (%) | 13/30 (43.3%) | 25/41 (61.0%) |
| Atrial fibrillation, n/N (%) | 0/30 (0.0%) | 8/41 (19.5%) |
| Other cardiac disease, n/N (%) | 4/30 (13.3%) | 16/41 (39.0%) |
| Diabetes mellitus, n/N (%) | 5/30 (16.7%) | 10/41 (24.4%) |
| Renal insufficiency, n/N (%) | 1/30 (3.3%) | 2/41 (4.9%) |
| **Genetic and molecular features** | | |
| Unmutated IGHV, n/N with data (%) | 8/14 (57.1%) | 14/17 (82.4%) |
| Del(17p13), n/N with data (%) | 1/36 (2.8%) | 10/49 (20.4%) |
| Del(11q23), n/N with data (%) | 8/36 (22.2%) | 7/49 (14.3%) |
| TP53 mutation, n/N with data (%) | 3/35 (8.6%) | 3/43 (7.0%) |

Table 1. Patients characteristics

**Copyright Information:** (Online) ISSN: 2572-9241
©2026 The Author(s). HemaSphere is published by John Wiley & Sons Ltd on behalf of European Hematology Association. This is an open access Abstract Book distributed under the Attribution-NonCommercial-NoDerivs (CC BY-NC-ND), which allows third parties to download the articles and share them with others as long as they credit the author and the Abstract Book, but they cannot change the content in any way or use them commercially.
**Abstract Book Citations:** Authors, Title, HemaSphere, 2026;10:(S1):pages. The abstract book DOIs can be found on the “Author Information” page.
**Disclaimer:** Articles published in the journal HemaSphere exclusively reflect the opinions of the authors. The authors are responsible for all content in their abstracts including accuracy of the facts, statements, citing resources, etc.

### (PS1716) YIELD AND APPROPRIATENESS OF IMMUNOPHENOTYPING IN MILD LYMPHOCYTOSIS: A 6-YEAR SINGLE-CENTRE ANALYSIS OF OVER 1000 TESTS

**Topic:** 06. Chronic lymphocytic leukemia and related disorders - Clinical

Firinna McKenna Beattie*[1], Maureen O' Donnell[1], Amjad Hayat[1]

[1] *Galway University Hospital, Galway, Ireland;*

### Background

Mild lymphocytosis is a common encounter in haematology outpatient settings. As life expectancy continues to increase, the number of patients living with small asymptomatic B-cell clonal populations will continue to grow exponentially. Immunophenotyping can establish whether a clonal population is present, but it is costly and the benefit of diagnosing asymptomatic patients is unclear.

### Aims

To establish further data to guide the use of routine immunophenotyping in patients with mild lymphocytosis. To describe the relationship between lymphocyte count and the presence of a B-cell clone. To audit practice within our lab by reviewing thresholds for lymphocyte panel requests.

### Methods

We reviewed all chronic lymphocyte panels carried out on peripheral blood in Galway University Hospital from January 2020 to December 2025. Data collected included indication for test, age, gender, full blood count and immunophenotype determined by flow cytometry. The presence of a B-cell clone was classified as either Chronic Lymphocytic Leukaemia (CLL), Monoclonal B-cell Lymphocytosis (MBL) or other Clonal B-cell Population (all phenotypes other than CLL). In addition, a subset of patients with MBL or early CLL from 2020-2021 were followed retrospectively to determine outcomes at mean follow up of 5 years.

**Copyright Information:** (Online) ISSN: 2572-9241
©2026 The Author(s). HemaSphere is published by John Wiley & Sons Ltd on behalf of European Hematology Association. This is an open access Abstract Book distributed under the Attribution-NonCommercial-NoDerivs (CC BY-NC-ND), which allows third parties to download the articles and share them with others as long as they credit the author and the Abstract Book, but they cannot change the content in any way or use them commercially.
**Abstract Book Citations:** Authors, Title, HemaSphere, 2026;10:(S1):pages. The abstract book DOIs can be found on the "Author Information" page.
**Disclaimer:** Articles published in the journal HemaSphere exclusively reflect the opinions of the authors. The authors are responsible for all content in their abstracts including accuracy of the facts, statements, citing resources, etc.

**Results**

1048 lymphocyte panels were performed on peripheral blood samples in Galway University Hospital from 2020 to 2025. 81% of requests were for lymphocytosis (n = 852) with a median age of 67 years (range 16 to 96) and slight male predominance (58.7%). Other recurring indications for request included suspected lymphoproliferative disorders with a normal lymphocyte count (n = 112) and cytopenias (n = 70).

Of the 852 samples with lymphocytosis, median lymphocyte count was 7.0 x $10^9$/L (IQR 4.6 to 11.9, range 3.0 to 311). A B-cell clone was detected in 64.6% of samples (n = 550), compromising of 280 cases of CLL (median lymphocyte count 15.3 x $10^9$/L), 127 cases of MBL (median lymphocyte count of 6.5 x $10^9$/L) and 143 clonal B-cell populations with a non-CLL phenotype (median lymphocyte count of 8.1 x $10^9$/L). Clonality was strongly associated with male gender and increased age. There was no B-cell clone detected in 35.4% of tests for lymphocytosis (n = 302) with a median lymphocyte count of 4.3 x $10^9$/L in this cohort (IQR 3.7 to 5.5, range 3.0 to 22).

30% of tests (n = 259) were for lymphocyte counts < 5 x $10^9$/L, of which 25% had a small clonal population (n = 65). In samples with a lymphocyte count ≥ 10 × $10^9$/L (n = 288, 34%), a clone was detected in 95% of cases (n = 274). No patient with MBL or CLL was symptomatic or required treatment at lymphocyte counts <10 × $10^9$/L.

We identified 56 patients who were diagnosed with either MBL or early CLL at lymphocyte counts of < 10 x $10^9$/L in 2020-2021. At a mean follow up of 5 years, 9% have required treatment for symptomatic CLL (n = 5). 9 patients died (16%) of age related causes not known to be linked to CLL.

**Summary/Conclusion**

Almost one third of tests were on samples with lymphocyte counts of < 5 x $10^9$/L, highlighting the need for more careful consideration among doctors requesting the test and stricter lab criteria. Progression of small clones to treatment was low at 5 years in our study. In an effort to balance cost with uncertainty in the benefit of following small B-cell clones in asymptomatic patients, it may be useful to consider a higher threshold than 5 x $10^9$/L for referral to haematology in select patients.

**Copyright Information:** (Online) ISSN: 2572-9241
©2026 The Author(s). HemaSphere is published by John Wiley & Sons Ltd on behalf of European Hematology Association. This is an open access Abstract Book distributed under the Attribution-NonCommercial-NoDerivs (CC BY-NC-ND), which allows third parties to download the articles and share them with others as long as they credit the author and the Abstract Book, but they cannot change the content in any way or use them commercially.
**Abstract Book Citations:** Authors, Title, HemaSphere, 2026;10:(S1):pages. The abstract book DOIs can be found on the "Author Information" page.
**Disclaimer:** Articles published in the journal HemaSphere exclusively reflect the opinions of the authors. The authors are responsible for all content in their abstracts including accuracy of the facts, statements, citing resources, etc.

### (PS1717) TREATMENT OUTCOMES WITH FIRST-LINE VENETOCLAX+OBINUTUZUMAB THERAPY IN YOUNGER ADULTS WITH CHRONIC LYMPHOCYTIC LEUKEMIA/SMALL LYMPHOCYTIC LYMPHOMA: A REAL-WORLD, INTERNATIONAL STUDY

**Topic:** 06. Chronic lymphocytic leukemia and related disorders - Clinical

Andres Chang*[1], Lori Leslie[2], Nicolas Martinez-Calle[3], Lindsey Roeker*[4], Beenish Manzoor[5], Chaitra Ujjani[6], Deborah Stephens[7], Brian Hill[8], Nicole Lamanna[9], Jennifer R. Brown[10], Nilanjan Ghosh[11], Hande Tuncer[10], Matthew Davids[10], Toby A. Eyre[12], Paul Barr[13], Christopher Jensen[7], Catherine Coombs[14], Nnadozie Emechebe[5], Alan Skarbnik[15], Bita Fakhri[16], Meghan C. Thompson[17], Wendy Sinai[5], Joanna Rhodes[18], Isabelle Fleury[19], Frederick Lansigan[20], Michael Coyle[21], Samin Houshyar[13], Laurie Pearson[22], Jennifer Huang[6], Nancy Kaddis[10], Stephen Schuster[23], Irina Pivneva[24], Henrietta Blinder[24], Annie Guerin[24], Mazyar Shadman[6]

*[1]Winship Cancer Institute of Emory University, Atlanta, GA, United States of America;[2]John Theurer Cancer Center, Hackensack Meridian Health, Hackensack, NJ, United States of America; [3]Nottingham University Hospitals, NHS Trust, Nottingham, United Kingdom; [4]Mayo Clinic, Rochester, MN, United States of America;[5]AbbVie Inc., North Chicago, IL, United States of America; [6]Fred Hutchinson Cancer Research Center, Seattle, WA, United States of America;[7]Lineberger Comprehensive Cancer Center, University of North Carolina at Chapel Hill, Chapel Hill, NC, United States of America; [8]Cleveland Clinic, Cleveland, OH, United States of America; [9]Columbia University, New York, NY, United States of America; [10]Dana-Farber Cancer Institute, Boston, MA, United States of America; [11]Levine Cancer Institute/Atrium Health, Wake Forest University School of Medicine, Charlotte, NC, United States of America; [12]Churchill Hospital, Oxford University, Oxford, United Kingdom; [13]Wilmot Cancer Institute, University of Rochester Medical Center, Rochester, NY, United States of America; [14]University of California, Irvine, CA, United States of America; [15]Lymphoma and CLL Program, Novant Health Cancer Institute, Charlotte, NC, United States of America;[16]Stanford Cancer Institute, Stanford University, Stanford, CA, United States of America; [17]CLL Program, Leukemia Service, Division of Hematologic Malignancies, Memorial Sloan Kettering Cancer Center, New York, NY, United States of America; [18]Rutgers Cancer Institute, New Brunswick, NJ, United States of America; [19]CHU de Québec, Université Laval, Quebec, Canada;[20]Dartmouth-Hitchcock Medical Center, Lebanon, NH, United States of America; [21]Lowell General Hospital, Lowell, MA, United States of America; [22]University of Massachusetts, Worcester, MA, United States of America;[23]Lymphoma Program, Abramson Cancer Center, University of Pennsylvania, Philadelphia, PA, United States of America; [24]Analysis Group, Inc., Montreal, QC, Canada;*

**Background**

Given the chronicity of chronic lymphocytic leukemia/small lymphocytic lymphoma (CLL/SLL), younger adults represent a distinct clinical subgroup with unique long-term treatment considerations, particularly those with high-risk disease features. Venetoclax+obinutuzumab (VO) is a fixed-duration, chemotherapy-free regimen approved for first line (1L) use. The CLL13 trial of VO reported durable outcomes in a fit, younger population, including those with unmutated IGHV (uIGHV). However, there is a paucity of real-world evidence for VO use in younger adults.

**Aims**

Assess clinical outcomes in younger adults (<55 years) with CLL treated with VO as 1L, in the overall sample and for those with uIGHV.

**Copyright Information:** (Online) ISSN: 2572-9241
©2026 The Author(s). HemaSphere is published by John Wiley & Sons Ltd on behalf of European Hematology Association. This is an open access Abstract Book distributed under the Attribution-NonCommercial-NoDerivs (CC BY-NC-ND), which allows third parties to download the articles and share them with others as long as they credit the author and the Abstract Book, but they cannot change the content in any way or use them commercially.
**Abstract Book Citations:** Authors, Title, HemaSphere, 2026;10:(S1):pages. The abstract book DOIs can be found on the “Author Information” page.
**Disclaimer:** Articles published in the journal HemaSphere exclusively reflect the opinions of the authors. The authors are responsible for all content in their abstracts including accuracy of the facts, statements, citing resources, etc.

### Methods

Data were analyzed from the retrospective, international, CLL Collaborative Study of Real-World Evidence (CORE). Adults with CLL/SLL were eligible if they started VO as 1L and were <55 years at VO initiation (i.e., index date). Outcomes included: overall response rate (ORR: physician-assessed clinical complete/partial response [CR/PR], as documented in the patient chart); progression-free survival (PFS: time from index to disease progression/death or end of follow-up); time to next treatment or death (TTNT-D: time from index to next treatment/death or end of follow-up); and overall survival (OS: time from index to death or end of follow-up). Kaplan-Meier methods were used to assess 24-month rates for time-to-event data. Outcomes were reported overall and for those with uIGHV (i.e., uIGHV subgroup; those with a confirmed del(17p)/*TP53* mutation or unknown/not tested del(17p)/*TP53* mutation status were excluded).

### Results

Of 2,822 patients in CORE, 85 adults <55 years initiated VO as 1L (outside of a clinical trial: 57.6%). At index, patients had a median age of 49.6 years (IQR: 45.1, 52.7), with 68.2% male, 95.3% with ECOG grade 0-1, and 29.4% with cardiovascular conditions (e.g., hypertension). Among all tested patients, 5/80 (6.3%) had a del(17p)/*TP53* mutation; 52/72 (72.2%) patients had uIGHV, of whom 45 (86.5%) had uIGHV without del(17p)/*TP53* mutation for inclusion in the uIGHV subgroup.
Over a median follow-up of 22.7 months (IQR: 8.6, 31.6), median VO treatment duration was 11.0 months (IQR: 8.1, 13.0), with 25 (29.4%) patients still on VO at the end of follow-up. The most common reasons for ending VO (non-mutually exclusive) were due to completion of fixed-duration therapy (35 [58.3%]), based on available minimal residual disease status information (21 [35.0%]), and watchful waiting (8 [13.3%]). At the end of follow-up, 3 (3.5%) patients initiated a subsequent regimen (covalent Bruton tyrosine kinase inhibitor: 2; cyclophosphamide: 1).
For the overall sample, the ORR in the 70 patients with available response data was 94.3% (CR: 53 [75.7%]; PR: 13 [18.6%]). The 24-month rate for PFS was 98.3%; TTNT-D and OS were both 100.0%.
Among the 45 patients in the uIGHV subgroup, the ORR for the 41 patients with available response data was 95.1% (CR: 31 [75.6%]; PR: 8 [19.5%]). The 24-month rates for PFS, TTNT-D, and OS were all 100.0%.

### Summary/Conclusion

The results of this real-world study demonstrate that younger adults with CLL/SLL treated with fixed-duration VO as 1L, including those with higher-risk uIGHV, achieved high response rates and 24-month PFS, TTNT-D, and OS, supporting the effectiveness of VO. Longer follow up in this cohort is needed to confirm response durability, as well as in a larger sample of patients with other high-risk features.

**Copyright Information:** (Online) ISSN: 2572-9241
©2026 The Author(s). HemaSphere is published by John Wiley & Sons Ltd on behalf of European Hematology Association. This is an open access Abstract Book distributed under the Attribution-NonCommercial-NoDerivs (CC BY-NC-ND), which allows third parties to download the articles and share them with others as long as they credit the author and the Abstract Book, but they cannot change the content in any way or use them commercially.
**Abstract Book Citations:** Authors, Title, HemaSphere, 2026;10:(S1):pages. The abstract book DOIs can be found on the “Author Information” page.
**Disclaimer:** Articles published in the journal HemaSphere exclusively reflect the opinions of the authors. The authors are responsible for all content in their abstracts including accuracy of the facts, statements, citing resources, etc.

**Figure 1.** Clinical outcomes in younger adults treated with VO as 1L, overall and for those with uIGHV[1].

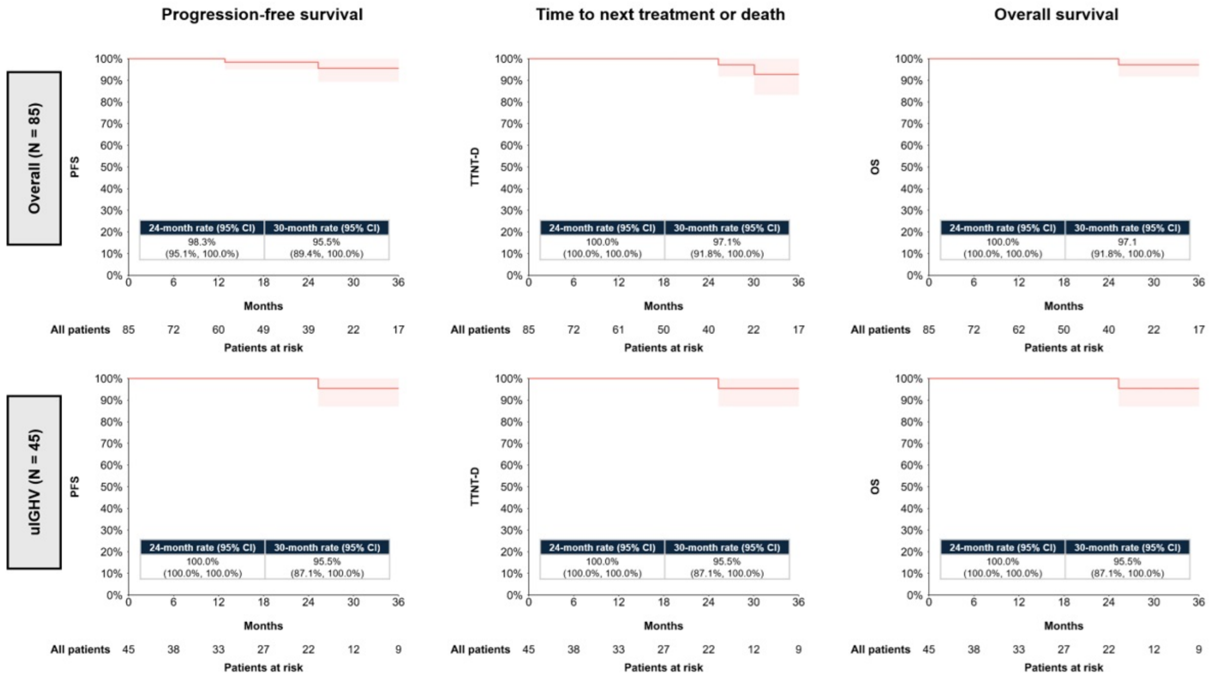

**Abbreviations:** 1L: first line; CI: confidence interval; OS, overall survival; PFS: progression-free survival; TTNT-D: time to next treatment or death; uIGHV: unmutated IGHV; VO: venetoclax+obinutuzumab.

**Note:**

[1] Patients with uIGHV were excluded if they also had a confirmed del(17p)/TP53 mutation or unknown/not tested del(17p)/TP53 mutation status.

**Copyright Information:** (Online) ISSN: 2572-9241
©2026 The Author(s). HemaSphere is published by John Wiley & Sons Ltd on behalf of European Hematology Association. This is an open access Abstract Book distributed under the Attribution-NonCommercial-NoDerivs (CC BY-NC-ND), which allows third parties to download the articles and share them with others as long as they credit the author and the Abstract Book, but they cannot change the content in any way or use them commercially.
**Abstract Book Citations:** Authors, Title, HemaSphere, 2026;10:(S1):pages. The abstract book DOIs can be found on the "Author Information" page.
**Disclaimer:** Articles published in the journal HemaSphere exclusively reflect the opinions of the authors. The authors are responsible for all content in their abstracts including accuracy of the facts, statements, citing resources, etc.

### (PS1718) ZANUBRUTINIB VS IBRUTINIB IN TREATMENT-NAIVE CHRONIC LYMPHOCYTIC LEUKEMIA (CLL): IMPLICATIONS FOR INTERPRETING FIXED-DURATION TREATMENT OUTCOMES FROM CLL17

**Topic:** 06. Chronic lymphocytic leukemia and related disorders - Clinical

Talha Munir*[1], Wojciech Jurczak[2], Leyla Mohseninejad[3], Sheng Xu[4], Marcus Lefebure[5], Keri Yang[6], Binod P. Neupane[7], Balazs Dobi[8], G. Rhys Williams[6], Alessandra Tedeschi[9]

*[1]Leeds Teaching Hospital NHS Trust, Leeds, United Kingdom;[2]Maria Sklodowska-Curie National Research Institute of Oncology, Kraków, Poland; [3]BeOne Medicines, Ltd, Schipol, The Netherlands; [4]BeOne Medicines, Ltd, Shanghai, China; [5]BeOne Medicines, Ltd, London, United Kingdom; [6]BeOne Medicines, Ltd, San Carlos, CA, United States of America; [7]PPD Evidera, Montreal, QC, Canada; [8]PPD Evidera, Budapest, Hungary; [9]ASST Grande Ospedale Metropolitano Niguarda, Milano, Italy;*

### Background

In the phase 3 CLL17 trial (NCT04608318), fixed-duration (FD) venetoclax + obinutuzumab (V+O) and FD venetoclax + ibrutinib (V+I) demonstrated noninferiority to continuous ibrutinib in patients with treatment-naive (TN) CLL. However, as ibrutinib is a first-generation Bruton tyrosine kinase inhibitor (BTKi), the extent to which these findings can be extrapolated to newer BTKis, such as zanubrutinib, remains uncertain. In the phase 3 SEQUOIA study (NCT03336333), continuous zanubrutinib (arm A) demonstrated superior progression-free survival (PFS) compared with bendamustine + rituximab (arm B) in patients with TN CLL without del(17p), while PFS outcomes in zanubrutinib-treated patients with del(17p) (arm C) were comparable to those observed in patients without del(17p).

### Aims

To compare the efficacy of continuous zanubrutinib with treatments evaluated in the CLL17 study and assess whether conclusions for ibrutinib are generalizable to zanubrutinib.

### Methods

In the absence of head-to-head trials, unanchored matching-adjusted indirect comparisons (MAICs) were conducted using individual patient data from SEQUOIA (median follow-up, 58.0 months) and published aggregate data from CLL17 (median follow-up, 34.2 months). Feasibility assessment confirmed alignment in study design and eligibility criteria. However, owing to the limited follow-up for FD regimens in CLL17 and the absence of COVID-19–adjusted overall survival (OS) data, continuous ibrutinib was the only methodologically feasible comparator. Zanubrutinib-treated patients were reweighted to match the CLL17 ibrutinib intent-to-treat population based on age, sex, Binet stage, ECOG performance status, presence of bulky disease, cancer type, and genomic risk factors. Alternative matching factor sets were tested in sensitivity analysis. Investigator-assessed PFS (PFS-INV) and OS were evaluated. As no COVID-19–related deaths were reported in the CLL17 ibrutinib arm, COVID-19–adjusted analyses were performed for SEQUOIA to improve comparability.

**Copyright Information:** (Online) ISSN: 2572-9241
©2026 The Author(s). HemaSphere is published by John Wiley & Sons Ltd on behalf of European Hematology Association. This is an open access Abstract Book distributed under the Attribution-NonCommercial-NoDerivs (CC BY-NC-ND), which allows third parties to download the articles and share them with others as long as they credit the author and the Abstract Book, but they cannot change the content in any way or use them commercially.
**Abstract Book Citations:** Authors, Title, HemaSphere, 2026;10:(S1):pages. The abstract book DOIs can be found on the “Author Information” page.
**Disclaimer:** Articles published in the journal HemaSphere exclusively reflect the opinions of the authors. The authors are responsible for all content in their abstracts including accuracy of the facts, statements, citing resources, etc.

### Results

After weighting, baseline characteristics were balanced, with an effective sample size of 91 for zanubrutinib. COVID-19–adjusted PFS-INV significantly favored zanubrutinib vs ibrutinib after matching (hazard ratio, 0.23; 95% CI, 0.12-0.42) (**Table**). COVID-19–adjusted analyses for OS numerically favored zanubrutinib. Several sensitivity analyses using alternative matching variables yielded consistent results.

**Table. COVID-19–Adjusted Comparative Efficacy of Zanubrutinib (SEQUOIA) vs Ibrutinib (CLL17)**

| Outcomes | Naive HR (95% CI) | MAIC-weighted HR (95% CI) |
|---|---|---|
| PFS-INV | 0.44 (0.30-0.64) | 0.23 (0.12-0.42) |
| OS | 1.17 (0.61-2.25) | 0.70 (0.29-1.73) |

**Abbreviations:** HR, hazard ratio; MAIC, matching-adjusted indirect comparison; OS, overall survival; PFS-INV, investigator-assessed progression-free survival.

### Summary/Conclusion

This MAIC demonstrates substantially improved PFS and numerically favorable OS with zanubrutinib compared with ibrutinib in TN CLL. These findings suggest that efficacy observed with ibrutinib in CLL17 may not be representative of the whole BTKi class. Therefore, any conclusions supporting broad use of FD regimens should not be extrapolated beyond the specific comparator studied, given differences in efficacy across continuous BTKis. Real-world data or indirect comparisons using similar follow-up duration for FD treatments vs continuous zanubrutinib are warranted to define the optimal frontline approach for patients with CLL.

**Copyright Information:** (Online) ISSN: 2572-9241
©2026 The Author(s). HemaSphere is published by John Wiley & Sons Ltd on behalf of European Hematology Association. This is an open access Abstract Book distributed under the Attribution-NonCommercial-NoDerivs (CC BY-NC-ND), which allows third parties to download the articles and share them with others as long as they credit the author and the Abstract Book, but they cannot change the content in any way or use them commercially.
**Abstract Book Citations:** Authors, Title, HemaSphere, 2026;10:(S1):pages. The abstract book DOIs can be found on the "Author Information" page.
**Disclaimer:** Articles published in the journal HemaSphere exclusively reflect the opinions of the authors. The authors are responsible for all content in their abstracts including accuracy of the facts, statements, citing resources, etc.

### (PS1719) MORPHOLOGY-BASED CELL SORTING USING AI-DRIVEN GHOST CYTOMETRY COMBINED WITH SINGLE-CELL RNA SEQUENCING REVEALS POTENTIAL THERAPEUTIC TARGETS IN CML LEUKEMIC STEM CELLS

**Topic:** 07. Chronic myeloid leukemia - Biology & translational research

Tomoiku Takaku*[1, 2], Kohjin Suzuki[1, 2, 3], Hiroko Nomaru[4], Naoki Watanabe[2], Kazuhiro Yamada[3], Miki Ando[2], Sadao Ota[4, 5]

*[1]Saitama Medical University Hospital, Hematology, Saitama, Japan;[2]Juntendo University Graduate School of Medicine, Hematology, Tokyo, Japan;[3]Sysmex Corporation, Hyogo, Japan;[4]ThinkCyte K.K., Tokyo, Japan;[5]The University of Tokyo, Research Center for Advanced Science and Technology, Tokyo, Japan;*

### Background

The achievement rate of treatment-free remission (TFR), one of the therapeutic goals in chronic myeloid leukemia (CML), remains at approximately 50%. The persistence of CML leukemic stem cells (LSCs) after treatment has been identified as a major cause of relapse. Although the development of novel therapeutic strategies targeting LSCs is highly desired, none have yet been implemented in clinical practice. Previously, we have successfully detected CML cells using ghost cytometry (GC) (Ota S, et al. *Science* 2018), a technology that enables label-free acquisition of fine cellular morphological information and cell discrimination by artificial intelligence (AI) (Suzuki K, et al. *Cancer Sci.* 2024; Suzuki K, et al. *Sci. Rep.* 2025).
In this study, we aimed to employ GC to reveal previously unrecognized morphological phenotypes within CML-LSCs and to integrate these findings with single-cell RNA sequencing (scRNA-seq) to discover novel therapeutic targets.

### Methods

Bone marrow mononuclear cells (BM-MNCs) derived from newly diagnosed chronic phase CML patients (n = 3) and healthy donors (n = 3) were mixed, and multidimensional waveform signals including morphological information were obtained using GC. The acquired signals were subjected to dimensionality reduction and visualization by uniform manifold approximation and projection (UMAP), and cell populations characteristic of CML patients were extracted. Using GC trained by AI to recognize these populations, specific cell populations were sorted from CML BM-MNCs (n = 3) and analyzed by scRNA-seq using the 10X Genomics platform (Figure A).

### Results

We successfully identified CML-associated cell populations via GC analysis, sorted these cells for high-purity isolation, and applied scRNA-seq to reveal their transcriptional profiles. UMAP analysis of the scRNA-seq data revealed that the GC-sorted cells were classified into multiple clusters, each exhibiting characteristic gene expression profiles (Figure B). Notably, a hematopoietic stem cell cluster enriched for $CD34^{+}/38^{-}$ cells was identified (Figure C), which included LSC-like cell populations expressing known LSC markers such as *DPP4 (CD26), IL1RAP,* and *IL2RA (CD25)*, with significant activation of signals such as *PROM1, BAALC,* and *HLF.* Furthermore, this cell population exhibited myeloid-primed features with upregulation of *CTSG, MPO*, and *PRTN3*, as well as activation of negative regulators of inflammatory signaling such as *NFKBIA, TNFAIP3*, and *SOCS3.* In addition, increased expressions of known therapeutic target candidates such as *SRC, BCL2*, and *FLT3*, as well as novel candidates including *CXCR4* and *CALCRL*, were also observed (Figure D, E, $p < 0.0001$ for all).

**Copyright Information:** (Online) ISSN: 2572-9241
©2026 The Author(s). HemaSphere is published by John Wiley & Sons Ltd on behalf of European Hematology Association. This is an open access Abstract Book distributed under the Attribution-NonCommercial-NoDerivs (CC BY-NC-ND), which allows third parties to download the articles and share them with others as long as they credit the author and the Abstract Book, but they cannot change the content in any way or use them commercially.
**Abstract Book Citations:** Authors, Title, HemaSphere, 2026;10:(S1):pages. The abstract book DOIs can be found on the "Author Information" page.
**Disclaimer:** Articles published in the journal HemaSphere exclusively reflect the opinions of the authors. The authors are responsible for all content in their abstracts including accuracy of the facts, statements, citing resources, etc.

### Summary/Conclusion

By combining morphology-based cell sorting using GC and scRNA-seq analysis, we identified a myeloid-primed leukemia stem/progenitor cell population. This population shares features with known LSCs, while also exhibiting a novel LSC state that actively utilizes inflammatory signaling. The observed upregulation of candidate therapeutic target molecules suggests the utility of this approach for the discovery of new therapeutic targets for CML-LSCs.
In conclusion, we established a novel morphology-based approach for discovering therapeutic candidates targeting CML-LSCs. Further studies are warranted to clarify the clinical significance of this cell population.

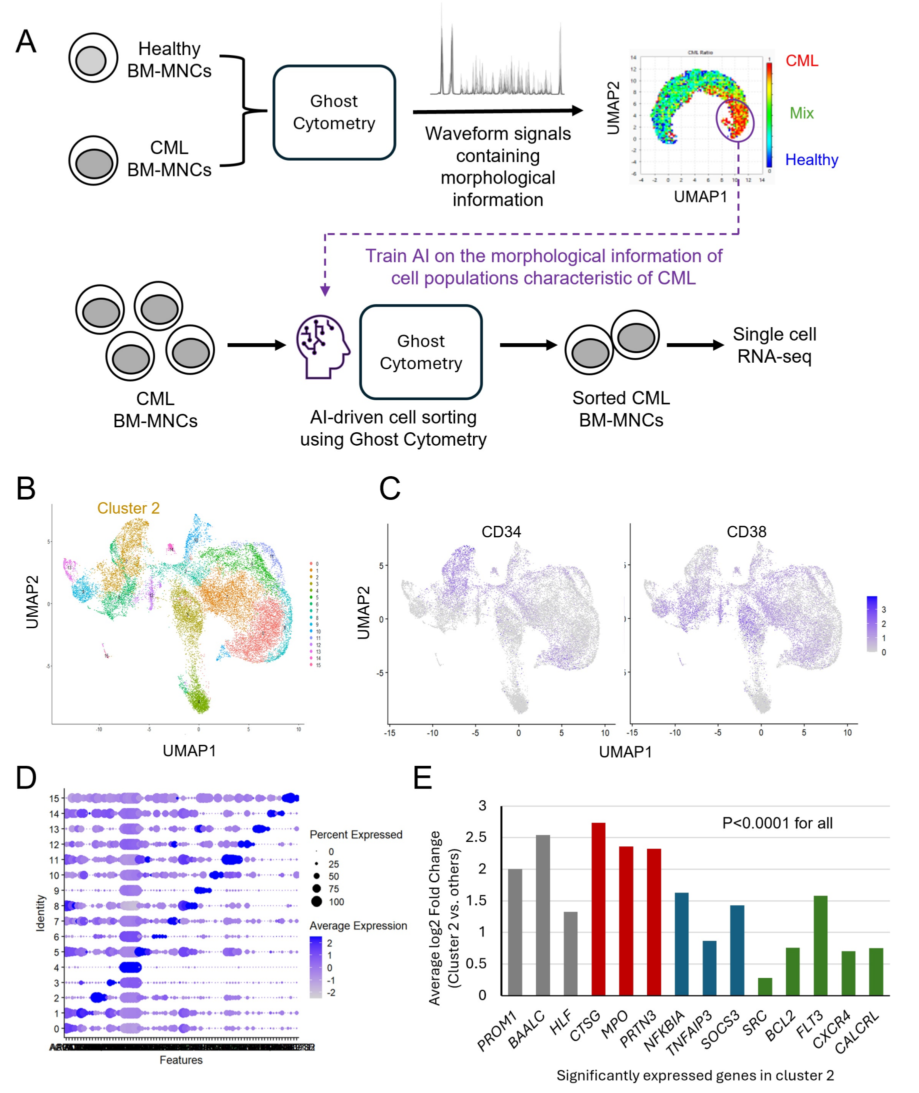

Figures. **A.** Schematic overview of the experimental procedure. **B.** UMAP clustering analysis of scRNA-seq data. **C.** Expression of CD34 and CD38 within the clusters. **D.** Dot plot showing differentially expressed genes. **E.** Genes significantly upregulated in cluster 2.

**Copyright Information:** (Online) ISSN: 2572-9241
©2026 The Author(s). HemaSphere is published by John Wiley & Sons Ltd on behalf of European Hematology Association. This is an open access Abstract Book distributed under the Attribution-NonCommercial-NoDerivs (CC BY-NC-ND), which allows third parties to download the articles and share them with others as long as they credit the author and the Abstract Book, but they cannot change the content in any way or use them commercially.
**Abstract Book Citations:** Authors, Title, HemaSphere, 2026;10:(S1):pages. The abstract book DOIs can be found on the “Author Information” page.
**Disclaimer:** Articles published in the journal HemaSphere exclusively reflect the opinions of the authors. The authors are responsible for all content in their abstracts including accuracy of the facts, statements, citing resources, etc.

### (PS1720) CITE-SEQ RESOLVES THE LEUKEMIC STEM CELL HIERARCHY AND IMMUNE MICROENVIRONMENT IN CML DISEASE PROGRESSION VIA MACHINE LEARNING MODELS

**Topic:** 07. Chronic myeloid leukemia - Biology & translational research

Kristina Kirschner*[1], Hira Jabeen[2], Ya-Ching Hsieh[2], Ross Laidlaw[2], Helen Wheadon[2], Thomas Otto[3], Gillian Horne[2], Tamir Chandra[1], Mhairi Copland[2], Neil Robertson[1]

*[1]Mayo Clinic, Hematology, Rochester, United States of America;[2]University of Glasgow, Glasgow, United Kingdom;[3]Bernhard Nocht Institute for Tropical Medicine, Hamburg, United Kingdom;*

### Background

Chronic myeloid leukemia (CML) is a myeloproliferative disorder driven by the BCR-ABL1 oncogenic tyrosine kinase, arising from the t(9;22)(q34;q11.2) translocation in myeloid stem cells. While tyrosine kinase inhibitors (TKIs) have normalised life expectancy for many patients, a subset of TKI-insensitive leukemic stem cells (LSCs) can drive progression from chronic phase (CP) to lethal blast phase (BP) CML. LSCs are also implicated in immune evasion through suppression of T cells, NK cells, and dendritic cells. However, the molecular features governing LSC hierarchy and their role in immune-mediated disease progression remain poorly understood.

### Aims

To resolve the LSC hierarchy and identify key pathways driving disease progression by combining CITE-Seq with machine learning approaches.

.

### Methods

CITE-Seq was performed on PBMCs from 3 CP-CML and 3 BP-CML patients, profiling 50,851 cells to simultaneously quantify transcriptome and surface proteins. Unsupervised clustering with reference-based annotation characterised cellular composition across disease phases. An ensemble classifier integrating protein and transcriptomic data was developed to identify putative BCR-ABL1+ LSCs, with training labels defined by CD26 ADT expression and CD25/CD123 as co-markers following the Warfvinge framework. Gaussian mixture modelling separated CD26-high from CD26-low progenitors, and elastic net logistic regression and random forest classifiers were trained using 5-fold cross-validation. Pathway enrichment was assessed using AUCell, and cell-cell communication analysis evaluated ligand-receptor interactions between LSC-enriched progenitors and immune populations.

### Results

BP-CML was enriched for HSPCs with elevated LSC markers CD26 (DPP4) and CD93 relative to CP-CML. The ensemble classifier achieved AUC = 0.89 and 0.88 with 87.5% concordance, revealing marked enrichment of predicted BCR-ABL1+ cells in BP-CML (21.4–96.9%) versus CP-CML (13.9–20.8%). Top features included cytokine genes (IL5, IL32, CCL1) and microenvironment molecules (LGALS3, VCAN). BCR-ABL1+ progenitors were enriched for inflammatory, KRAS, TNFα/NFκB, and JAK-STAT3 pathways with downregulation of haem metabolism (Cohen's d = −0.80), consistent with a differentiation block at the MEP stage. Differential ligand-receptor interactions between LSC-enriched progenitors and B cells, T cells, and monocytes in BP-CML suggest active immune microenvironment remodelling to facilitate immune evasion.

**Copyright Information:** (Online) ISSN: 2572-9241
©2026 The Author(s). HemaSphere is published by John Wiley & Sons Ltd on behalf of European Hematology Association. This is an open access Abstract Book distributed under the Attribution-NonCommercial-NoDerivs (CC BY-NC-ND), which allows third parties to download the articles and share them with others as long as they credit the author and the Abstract Book, but they cannot change the content in any way or use them commercially.
**Abstract Book Citations:** Authors, Title, HemaSphere, 2026;10:(S1):pages. The abstract book DOIs can be found on the "Author Information" page.
**Disclaimer:** Articles published in the journal HemaSphere exclusively reflect the opinions of the authors. The authors are responsible for all content in their abstracts including accuracy of the facts, statements, citing resources, etc.

**Summary/Conclusion**

CITE-Seq combined with ensemble machine learning resolves the LSC hierarchy in CML peripheral blood, identifying a transcriptional programme linked to LSC surface phenotype independent of classification markers. LSC expansion in BP-CML, alongside its distinct signalling profile, provides new insight into disease progression and highlights potential therapeutic vulnerabilities in the LSC compartment.

**Copyright Information:** (Online) ISSN: 2572-9241
©2026 The Author(s). HemaSphere is published by John Wiley & Sons Ltd on behalf of European Hematology Association. This is an open access Abstract Book distributed under the Attribution-NonCommercial-NoDerivs (CC BY-NC-ND), which allows third parties to download the articles and share them with others as long as they credit the author and the Abstract Book, but they cannot change the content in any way or use them commercially.
**Abstract Book Citations:** Authors, Title, HemaSphere, 2026;10:(S1):pages. The abstract book DOIs can be found on the "Author Information" page.
**Disclaimer:** Articles published in the journal HemaSphere exclusively reflect the opinions of the authors. The authors are responsible for all content in their abstracts including accuracy of the facts, statements, citing resources, etc.

### (PS1721) DUAL TARGETING WITH PONATINIB AND CRIZOTINIB TO OVERCOME ASCIMINIB RESISTANCE IN CHRONIC MYELOID LEUKEMIA

**Topic:** 07. Chronic myeloid leukemia - Biology & translational research

Seiichi Okabe*[1], Seiichiro Yoshizawa[1], Daigo Akahane[1]

[1]*Tokyo Medical University, Department of Hematology, Tokyo, Japan;*

### Background

Chronic myeloid leukemia (CML) is driven by the BCR::ABL1 fusion gene, which encodes a constitutively active tyrosine kinase that promotes aberrant signaling and uncontrolled myeloid proliferation. The development of ABL1 tyrosine kinase inhibitors (TKIs) has markedly improved survival outcomes. However, resistance remains a significant clinical issue, particularly in patients harboring BCR::ABL1 compound mutations. Asciminib is a first-in-class allosteric ABL1 inhibitor targeting the myristoyl pocket and is approved for patients with chronic-phase CML, including use in the frontline setting and in those previously treated with multiple TKIs. Although asciminib has shown substantial clinical benefit, the molecular mechanisms underlying resistance are not fully elucidated.

### Aims

This study aimed to establish and comprehensively characterize in vitro models of asciminib-resistant CML, to identify and molecularly define resistance-associated BCR::ABL1 mutations, and to elucidate the biological mechanisms underlying resistance. Furthermore, we sought to systematically evaluate potential therapeutic strategies using clinically available targeted agents through high-throughput drug screening and functional validation assays.

### Methods

Asciminib-resistant Ba/F3 cells (Ba/F3 asc-R: Y139D and T315I) were established and characterized. Ba/F3 V468D mutant exhibiting asciminib resistance was also generated. A high-throughput screen of 1700 clinically available compounds was conducted to identify candidate therapeutic agents. Selected compounds were subsequently evaluated in multiple CML models, including Ba/F3 parental BCR::ABL, Ba/F3 T315I, K562, and ABL TKI–resistant K562 cells.

### Results

Ba/F3 asc-R cells exhibited marked resistance to asciminib. Ba/F3 V468D mutant demonstrated robust resistance to asciminib. High-throughput screening of 1700 clinically available compounds identified ponatinib and crizotinib as candidate therapeutic agents with activity against asciminib-resistant cells. Ponatinib monotherapy induced dose-dependent inhibition of cell proliferation. Crizotinib also suppressed cell proliferation in a dose-dependent manner, accompanied by increased cytotoxicity and significantly elevated caspase-3/7 activity. These effects were observed not only in asciminib-resistant Ba/F3 models but also in Ba/F3 T315I and ABL TKI–resistant K562 cells. Combination treatment with ponatinib and crizotinib produced significantly enhanced anti-leukemic effects compared with either agent alone and was associated with augmented apoptotic signaling. Cytotoxicity and caspase-3/7 activity were further increased in the combination group. Colony formation assays confirmed a marked reduction in colony numbers following dual treatment. Moreover, mitochondrial membrane potential was significantly decreased, accompanied by reduced intracellular ATP levels, indicating mitochondrial dysfunction associated with the combination strategy.

**Copyright Information:** (Online) ISSN: 2572-9241
©2026 The Author(s). HemaSphere is published by John Wiley & Sons Ltd on behalf of European Hematology Association. This is an open access Abstract Book distributed under the Attribution-NonCommercial-NoDerivs (CC BY-NC-ND), which allows third parties to download the articles and share them with others as long as they credit the author and the Abstract Book, but they cannot change the content in any way or use them commercially.
**Abstract Book Citations:** Authors, Title, HemaSphere, 2026;10:(S1):pages. The abstract book DOIs can be found on the “Author Information” page.
**Disclaimer:** Articles published in the journal HemaSphere exclusively reflect the opinions of the authors. The authors are responsible for all content in their abstracts including accuracy of the facts, statements, citing resources, etc.

**Summary/Conclusion**

The T315I, Y139D, and V468D mutations conferred marked resistance to asciminib. Crizotinib exhibited significant anti-leukemic activity, inducing dose-dependent growth inhibition and apoptosis in asciminib-resistant Ba/F3 models, as well as in Ba/F3 T315I and ABL TKI–resistant K562 cells. These findings identify crizotinib as a potential therapeutic option for asciminib-resistant and TKI-resistant chronic myeloid leukemia, warranting further preclinical and clinical evaluation.

**Copyright Information:** (Online) ISSN: 2572-9241
©2026 The Author(s). HemaSphere is published by John Wiley & Sons Ltd on behalf of European Hematology Association. This is an open access Abstract Book distributed under the Attribution-NonCommercial-NoDerivs (CC BY-NC-ND), which allows third parties to download the articles and share them with others as long as they credit the author and the Abstract Book, but they cannot change the content in any way or use them commercially.
**Abstract Book Citations:** Authors, Title, HemaSphere, 2026;10:(S1):pages. The abstract book DOIs can be found on the "Author Information" page.
**Disclaimer:** Articles published in the journal HemaSphere exclusively reflect the opinions of the authors. The authors are responsible for all content in their abstracts including accuracy of the facts, statements, citing resources, etc.

### (PS1722) METABOLOMIC PROFILING REVEALS SUBTYPES WITH DISTINCT METABOLIC FEATURES AND PROGNOSIS IN CLL PATIENTS

**Topic:** 07. Chronic myeloid leukemia - Biology & translational research

Luomengjia Dai[1], Qian Wang[2], Ziyuan Zhou[1], Dan Yang[1], Tonglu Qiu[1], Shuchao Qin[1], Yi-Lin Kong[1], Hui Jin[1], Lei Fan[1], Juncheng Dai[2], Jianyong Li[1], Huayuan Zhu*[1]

*[1]The First Affiliated Hospital with Nanjing Medical University, Department of Hematology, Nanjing, China;[2]Center for Global Health, School of Public Health, Nanjing Medical University, Epidemiology, Nanjing, China;*

### Background

Metabolic reprogramming is a hallmark of cancer and play a key role in driving disease progression. However, the characteristics of metabolic reprogramming of CLL remain unclear, nor did its clinical relevance.

### Aims

To employ untargeted metabolomics to depict the metabolic reprogramming landscape and investigate heterogenous metabolic subtype within CLL patients and investigate its prognostic potential for predicting time to first treatment (TTFT).

### Methods

Between Jan 2022 and Nov 2024, patients newly diagnosed with CLL/SLL in Jiangsu Province Hospital with available serum samples were enrolled (N=149). LC-MS based untargeted metabolomics analysis was performed. Distance matrix-based variance estimation was used to quantify the contribution of each factor to inter-individual variations in the serum metabolome. Non-negative matrix factorization (NMF) was used to identify underlying metabolic subtypes. Metabolite Set Enrichment Analysis(MSEA) and Pathway Enrichment based on KEGG database were performed in MetaboAnalyst. Clinical and biological characteristics and time to first treatment (TTFT) were compared.

**Copyright Information:** (Online) ISSN: 2572-9241
©2026 The Author(s). HemaSphere is published by John Wiley & Sons Ltd on behalf of European Hematology Association. This is an open access Abstract Book distributed under the Attribution-NonCommercial-NoDerivs (CC BY-NC-ND), which allows third parties to download the articles and share them with others as long as they credit the author and the Abstract Book, but they cannot change the content in any way or use them commercially.
**Abstract Book Citations:** Authors, Title, HemaSphere, 2026;10:(S1):pages. The abstract book DOIs can be found on the “Author Information” page.
**Disclaimer:** Articles published in the journal HemaSphere exclusively reflect the opinions of the authors. The authors are responsible for all content in their abstracts including accuracy of the facts, statements, citing resources, etc.

### Results

The cohort included 149 patients(aged 24-87 years), 82 patients were asymptomatic and 67 patients met the criteria of treatment according to iwCLL 2018. Serum untargeted metabolomics based on LC-MS totally identified 1157 metabolites. Multivariate PERMANOVA analysis showed that clinical or biological factors explained 0.7–1.9% of the inter-individual variance in the serum metabolome. EGR2 mutation accounted for the largest proportion ($R2 = 1.9\%$, $p = 0.056$). Totally, these factors explained 34.2% of the variance($R2 = 34.2\%$ , $p = 0.23$). NMF analysis identified two distinct metabolic clusters. Top 50-ranked characteristic metabolites in each cluster were visualized to highlight differential metabolic features. Quantitative comparison showed that Cluster 1 exhibited specific elevations in amino acids, including L-phenylalanine, 4-Oxoproline, L-valine, L-histidine, L-tyrosine, taurine, and L-Lysine, suggesting perturbations in amino acid metabolism. In contrast, Cluster 2 was predominantly characterized by increased levels of phosphatidylethanolamine(PE), phosphatidylcholines (PC), phosphatidylinositols (PIs), 1-hexadecanoyl-sn-glycero-3-phospho-D-myo-inositol (LPIs), and free fatty acids (Linoleate, Hexadecanoic acid, Octadecanoic acid), underscoring substantial lipid remodeling. Pathway enrichment analysis revealed that Cluster 1 showed significant enrichment in phenylalanine, valine, and tyrosine metabolism pathways while Cluster 2 exhibited marked enrichment in biosynthesis of unsaturated fatty acids, Linoleic acid metabolism, and glycosylphosphatidylinositol (GPI)-anchor biosynthesis pathways. Clinical relevance analysis showed that patients in Cluster 2 were younger ($p = 0.037$), with EGR2 mutations exclusively observed in Cluster 2 ($p = 0.002$), whereas disease status, comorbidities, and other biological features were comparable between clusters. Survival analysis further demonstrated a significantly higher risk of requiring first treatment in Cluster 2 (HR = 1.463, 95% CI: 1.004–2.12, $p = 0.048$), with median TTFT of 26.3 months compared to 34.5 months in Cluster 1.

### Summary/Conclusion

Herein, we investigate serum metabolic profiles of CLL patients and revealed the existence of two clusters with distinct metabolic profiles and prognosis.

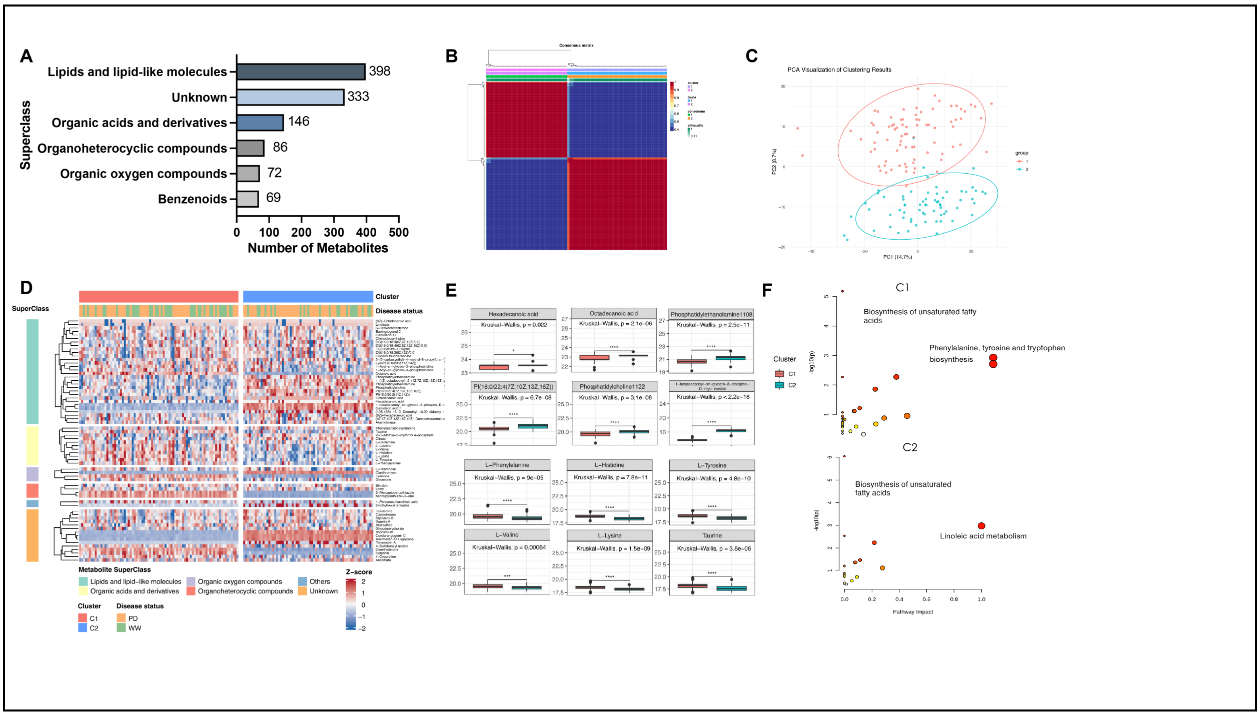

**Copyright Information:** (Online) ISSN: 2572-9241
©2026 The Author(s). HemaSphere is published by John Wiley & Sons Ltd on behalf of European Hematology Association. This is an open access Abstract Book distributed under the Attribution-NonCommercial-NoDerivs (CC BY-NC-ND), which allows third parties to download the articles and share them with others as long as they credit the author and the Abstract Book, but they cannot change the content in any way or use them commercially.
**Abstract Book Citations:** Authors, Title, HemaSphere, 2026;10:(S1):pages. The abstract book DOIs can be found on the "Author Information" page.
**Disclaimer:** Articles published in the journal HemaSphere exclusively reflect the opinions of the authors. The authors are responsible for all content in their abstracts including accuracy of the facts, statements, citing resources, etc.

### (PS1723) NEW 2-AMINOPYRIMIDINE PONATINIB DERIVATIVES AS POTENTIAL BCR-ABL1 TYROSINE KINASE INHIBITORS IN CHRONIC MYELOID LEUKEMIA

**Topic:** 07. Chronic myeloid leukemia - Biology & translational research

Stefany Moura[1, 2, 3, 4, 5], Raquel Alves[1, 2, 3], Ana Cristina Gonçalves[1, 2, 3], Joana Jorge[1, 2, 3], Beatriz Lapa[1, 2, 3], Tânia Gaspar[1, 2, 3], João Silva[1, 2, 3], Luiz Claudio Pimentel[5], Monica Bastos[4, 5], Núbia Boechat[4, 5], Ana Ribeiro*[1, 2, 3, 6]

*[1]University of Coimbra, Faculty of Medicine (FMUC), Coimbra, Portugal; [2]University of Coimbra, Coimbra Institute for Clinical and Biomedical Research (iCBR) – Environment, Genetics, and Oncobiology Group (CIMAGO) – FMUC, Center for Innovative Biomedicine and Biotechnology (CIBB), Coimbra, Portugal, Coimbra, Portugal; [3]University of Coimbra, Laboratory of Oncobiology and Hematology (LOH), Coimbra, Portugal; [4]University of Rio de Janeiro (UFRJ), Graduate Program in Pharmacology and Medicinal Chemistry, Institute of Biomedical Sciences (ICB), Rio de Janeiro, Brazil; [5]Oswaldo Cruz Foundation, Institute of Pharmaceutical Technology, Farmanguinhos, Rio de Janeiro, Brazil; [6]Coimbra University Hospitals (CHUC), Hematology Department, Coimbra Local Health Unit (ULS Coimbra), Coimbra, Portugal;*

### Background

The dysregulation of protein kinases is associated with several types of cancer. In chronic myeloid leukemia (CML), the t(9;22) reciprocal translocation generates the *BCR::ABL1* fusion gene, which encodes a constitutively active tyrosine kinase. Imatinib (IMA) is the first-line therapy; however, resistance remains a major clinical challenge. Ponatinib (PNT), a third-generation TKI, overcomes several ABL1 kinase domain mutations, including T315I, but its clinical use is limited by severe vascular toxicity and cardiovascular adverse events. Therefore, the development of new BCR-ABL1 TKIs with improved safety profiles is urgently needed.

### Aims

This study aimed to design and biologically evaluate 13 PNT analogs (**C-9** and 12 novel: **B1–B6**, **T1–T6**) with improved selectivity and safety profiles using CML cell lines sensitive to IMA (K562) and resistant, the K562-RC (continuous resistant model) and K562-RD cells (discontinuous resistant model) and in a non-tumoral human B-lymphocyte cell line immortalized by the Epstein–Barr virus (IMC cells).

### Methods

The synthesis of 13 PNT analogs was carried out at the Laboratory of Drug Synthesis (LASFAR), Institute of Pharmaceutical Technology (Farmanguinhos/Fiocruz, Brazil). he compounds, together with PNT, were evaluated *in vitro* in monotherapy for 48 hours at concentrations ranging from 10 to 100,000 nM in CML cell lines sensitive to IMA (K562) and resistant (K562-RC, a continuous resistance model with constant supplementation of 250 nM imatinib mesylate, and K562-RD, a discontinuous resistance model with 15-day cycles with and without supplementation), developed at the Laboratory of Oncobiology and Hematology (LOH). Metabolic viability was determined by the resazurin assay, enabling the generation of concentration–response curves and $IC_{50}$ values, with statistical analysis performed at a 95% significance level. For SI calculation, the IMC cell line, a non-tumoral human B-lymphocyte cell line immortalized by the Epstein–Barr virus, developed at the Laboratory of Cytogenetics and Genomics of the Coimbra Institute for Clinical and Biomedical Research (iCBR), was included.

**Copyright Information:** (Online) ISSN: 2572-9241
©2026 The Author(s). HemaSphere is published by John Wiley & Sons Ltd on behalf of European Hematology Association. This is an open access Abstract Book distributed under the Attribution-NonCommercial-NoDerivs (CC BY-NC-ND), which allows third parties to download the articles and share them with others as long as they credit the author and the Abstract Book, but they cannot change the content in any way or use them commercially.
**Abstract Book Citations:** Authors, Title, HemaSphere, 2026;10:(S1):pages. The abstract book DOIs can be found on the "Author Information" page.
**Disclaimer:** Articles published in the journal HemaSphere exclusively reflect the opinions of the authors. The authors are responsible for all content in their abstracts including accuracy of the facts, statements, citing resources, etc.

**Results**

T2 and T5 were the most potent among the 12 analogs. In parental K562 cells, both exhibited high potency ($IC_{50}$: T5 = 19.24 nM; T2 = 26.34 nM), although lower than PNT ($IC_{50}$ = 3.92 nM). Activity was maintained in the discontinuous resistance model (K562-RD: T2 = 11.66 nM; T5 = 12.23 nM); however, only T5 remained effective in the continuous resistance model (K562-RC = 63.14 nM), whereas T2 showed a marked loss of potency ($IC_{50}$ = 603.7 nM).
In non-tumoral IMC cells, most compounds did not reach 50% inhibition up to 100000 nM. T5 was an exception ($IC_{50}$ = 69.9 µM), compared with PNT ($IC_{50}$ = 727.1 nM). This disparity resulted in a Selectivity Index (SI) of 3630 for T5, surpassing PNT (SI = 177.8) and indicating greater specificity toward CML cell lines and an improved therapeutic window.

**Summary/Conclusion**

Our preliminary findings indicate that the newly developed PNT analogs exhibit promising antileukemic activity. Although less potent than PNT in parental K562 cells, T5 maintained activity in resistant models and demonstrated markedly improved tumor selectivity. Overall, these findings support the effectiveness of the molecular design strategy, demonstrating enhanced selectivity toward tumor cells and reduced cytotoxicity in non-tumoral cells. Future studies will focus on elucidating cell death mechanisms and cell cycle effects by flow cytometry.

**Copyright Information:** (Online) ISSN: 2572-9241
©2026 The Author(s). HemaSphere is published by John Wiley & Sons Ltd on behalf of European Hematology Association. This is an open access Abstract Book distributed under the Attribution-NonCommercial-NoDerivs (CC BY-NC-ND), which allows third parties to download the articles and share them with others as long as they credit the author and the Abstract Book, but they cannot change the content in any way or use them commercially.
**Abstract Book Citations:** Authors, Title, HemaSphere, 2026;10:(S1):pages. The abstract book DOIs can be found on the "Author Information" page.
**Disclaimer:** Articles published in the journal HemaSphere exclusively reflect the opinions of the authors. The authors are responsible for all content in their abstracts including accuracy of the facts, statements, citing resources, etc.

### (PS1725) CHRONIC MYELOID LEUKEMIA (CML) TREATMENT DISCONTINUATION AFTER A TWO-STEP DOSE REDUCTION. FINAL RESULTS OF THE PHASE 2 STUDY HALF.

**Topic:** 08. Chronic myeloid leukemia - Clinical

Jiří Mayer*[1], Katerina MacHova Polakova[2], Edgar Faber[3], Hana Klamová[2], Petra Belohlavkova[4], Lukas Stejskal[5], Michal Karas[6], Eduard Cmunt[7], Olga Cerna[8], Anežka Hronská[1], Petra Cicatkova[1], Ivana Jeziskova[1], Tomas Jurcek[1], Marie Jarosova[1], Marek Borsky[1], Hana Zizkova[2], Romana Vajnerová[2], Dana Srbová[2], Cyril Salek[2], Tomas Papajik[3], Jaroslava Voglova[4], Pavel Jindra[6], Žák Pavel[4], Ondřej Wiewiorka[9], Regina Demlová[10], Adéla Bártová[10], Jitka Rychlíčková[10], Andrea Gottschalk[11], Ingo Roeder[11], Ingmar Glauche[11], Jirina Prochazkova[1], Adam Svobodník[12], Radka Stepanova[10], Daniela Žáčková[1]

*[1]University Hospital Brno, Brno, Czechia; [2]Institute of Hematology and Blood Transfusion, Prague, Czechia;[3]University Hospital Olomouc, Olomouc, Czechia; [4]University Hospital Hradec Králové, Hradec Králové, Czechia; [5]University Hospital Ostrava, Ostrava, Czechia; [6]University Hospital Plzeň, Plzeň, Czechia; [7]General Faculty Hospital, Praha, Czechia;[8]University Hospital Královské Vinohrady, Praha, Czechia; [9]St. Anne's University Hospital, Brno, Czechia; [10]Masaryk University, Brno, Czechia; [11]TU Dresden, Dresden, Germany; [12]Anova CRO, Brno, Czechia;*

### Background

Discontinuation of tyrosine kinase inhibitor (TKI) therapy has become an established management in clinical practice for eligible patients. However, molecular recurrences and TKI withdrawal syndrome remain relatively common.

### Aims

The objectives of the HALF clinical trial were to evaluate the proportion of patients achieving treatment-free remission (TFR), to identify predictive factors for molecular relapse, and to characterize the dynamics of adverse events following TKI discontinuation.

### Methods

Between 2020 and 2023, the HALF trial was offered to eligible patients in the Czech Republic. Eligibility criteria included a TKI treatment duration for at least 4 years and a MR4 duration at least 2 years. After enrollment, the TKI dose was reduced to half of the standard dose; after 6 months, this reduced dose was administered every other day for an additional 6 months. TKI therapy was then discontinued. Multiple clinical and laboratory parameters were collected before and after discontinuation. Reinitiation of TKI therapy was triggered by confirmed loss of major molecular response (MMR).

**Copyright Information:** (Online) ISSN: 2572-9241
©2026 The Author(s). HemaSphere is published by John Wiley & Sons Ltd on behalf of European Hematology Association. This is an open access Abstract Book distributed under the Attribution-NonCommercial-NoDerivs (CC BY-NC-ND), which allows third parties to download the articles and share them with others as long as they credit the author and the Abstract Book, but they cannot change the content in any way or use them commercially.
**Abstract Book Citations:** Authors, Title, HemaSphere, 2026;10:(S1):pages. The abstract book DOIs can be found on the "Author Information" page.
**Disclaimer:** Articles published in the journal HemaSphere exclusively reflect the opinions of the authors. The authors are responsible for all content in their abstracts including accuracy of the facts, statements, citing resources, etc.

**Results**

A total of 207 patients were enrolled (median age 61.8 years; 24–86), with a median TKI treatment duration of 8.6 years (4–20). Imatinib was the most common first-line therapy (79%). The TFR rate at 36 months was 63.9%.
With this dose-reduction approach, none of the parameters analyzed to date showed a statistically significant association ($p<0.05$) with TFR: age at diagnosis; age at enrollment; sex ($p=0.06$); basophil count ($p=0.06$), eosinophils, blasts, and platelet count at diagnosis; risk scores; transcript type; early molecular response ($p=0.08$); duration of TKI therapy or MR4; type of TKI; CD4+, CD8+, regulatory T cells, B cells, and NK cells; or the presence of clonal hematopoiesis at enrollment.
At month 24, the following laboratory parameters significantly ($p<0.05$) decreased: creatinine, Na, Cl, AST, creatine kinase, amylase, lipase, and adiponectin; whereas leukocytes, platelets, hemoglobin, urea, K, Ca, P, and ALP increased. Total cholesterol, LDL, and HDL cholesterol also increased. These changes differed according to the TKI discontinued.
Among adverse events present at enrollment with a frequency ≥5%, the following significantly decreased ($p<0.05$): arthralgia, myalgia, skin rash, nausea, peripheral edema, diarrhea, muscle cramps, fatigue, and conjunctivitis. However, new adverse events developed in some patients, most commonly arthralgia, myalgia, and fatigue. Withdrawal-related symptoms were generally mild and transient: three patients received corticosteroids; one resumed TKI therapy; other patients received oral magnesium supplementation (n=9) or analgesics (n=7).
Assessment of *BCR::ABL1* kinetics during both dose-reduction phases showed that linear trend analysis may help identify patients at risk of TFR failure. Patients with increasing *BCR::ABL1* levels during either phase had a higher likelihood of subsequent loss of MMR.

**Summary/Conclusion**

A two-step TKI dose-reduction strategy prior to discontinuation appears to be safe and effective and results in a relatively high TFR rate. *BCR::ABL1* kinetics during dose de-escalation may predict subsequent loss of MMR. In addition, the incidence of withdrawal syndrome appears to be reduced. However, TKI discontinuation does not eliminate all treatment-related adverse effects, and some new mild symptoms may emerge. This two-step de-escalation approach could become a routine strategy in appropriately selected patients.
Supported by AZV and CELL

**Copyright Information:** (Online) ISSN: 2572-9241
©2026 The Author(s). HemaSphere is published by John Wiley & Sons Ltd on behalf of European Hematology Association. This is an open access Abstract Book distributed under the Attribution-NonCommercial-NoDerivs (CC BY-NC-ND), which allows third parties to download the articles and share them with others as long as they credit the author and the Abstract Book, but they cannot change the content in any way or use them commercially.
**Abstract Book Citations:** Authors, Title, HemaSphere, 2026;10:(S1):pages. The abstract book DOIs can be found on the "Author Information" page.
**Disclaimer:** Articles published in the journal HemaSphere exclusively reflect the opinions of the authors. The authors are responsible for all content in their abstracts including accuracy of the facts, statements, citing resources, etc.

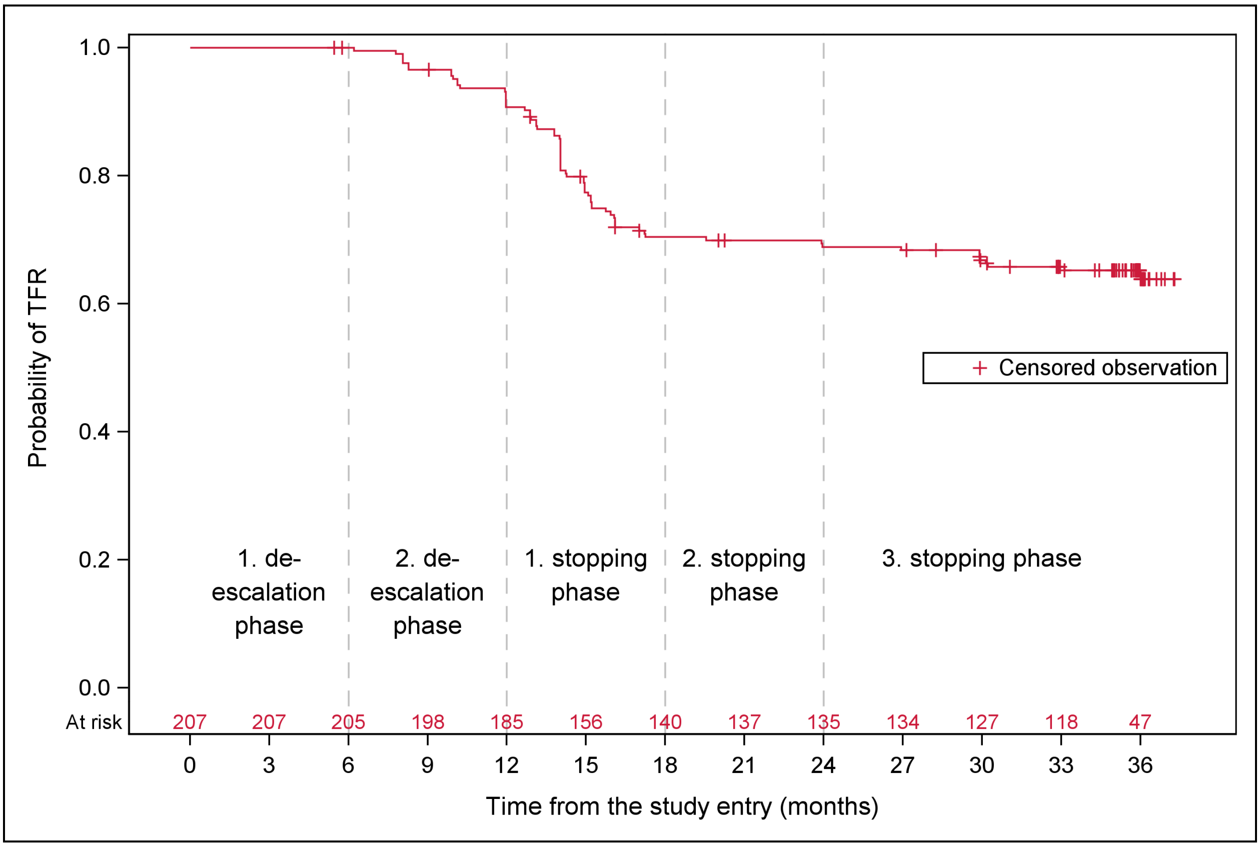

Treatment-free remission after a two-step TKI deescalations and stopping

**Copyright Information:** (Online) ISSN: 2572-9241
©2026 The Author(s). HemaSphere is published by John Wiley & Sons Ltd on behalf of European Hematology Association. This is an open access Abstract Book distributed under the Attribution-NonCommercial-NoDerivs (CC BY-NC-ND), which allows third parties to download the articles and share them with others as long as they credit the author and the Abstract Book, but they cannot change the content in any way or use them commercially.
**Abstract Book Citations:** Authors, Title, HemaSphere, 2026;10:(S1):pages. The abstract book DOIs can be found on the "Author Information" page.
**Disclaimer:** Articles published in the journal HemaSphere exclusively reflect the opinions of the authors. The authors are responsible for all content in their abstracts including accuracy of the facts, statements, citing resources, etc.

### (PS1727) EFFICACY OF OLVEREMBATINIB IN PATIENTS WITH CHRONIC-PHASE CHRONIC MYELOID LEUKEMIA (CP-CML) WITH PRIOR RESISTANCE TO PONATINIB OR ASCIMINIB AND ASXL1 MUTATIONS

**Topic:** 08. Chronic myeloid leukemia - Clinical

Elias Jabbour*[1], Paul Koller[2], Dennis Kim[3], Jorge Cortes[4], Omer Jamy[5], Vivian G. Oehler[6], Maria Baer[7], Anthony M. Hunter[8], Sudipto Mukherjee[9], Huanshan Guo[10], Dengkun Xiong[11], Eric Liang[12], Zhihong Yang[12], Zi Chen[11], Hagop Kantarjian[1], Yifan Zhai[10, 11, 12]

*[1]Department of Leukemia, The University of Texas MD Anderson Cancer Center, Houston, TX, United States of America; [2]Department of Hematology and Hematopoietic Cell Transplantation in the Division of Leukemia, City of Hope National Medical Center, Duarte, CA, United States of America; [3]Princess Margaret Cancer Centre, University Health Network, University of Toronto, Toronto, Canada; [4]Georgia Cancer Center, Augusta University, Augusta, GA, United States of America; [5]Division of Hematology and Oncology, Department of Medicine, University of Alabama at Birmingham, Birmingham, AL, United States of America; [6]Translational Science and Therapeutics, Fred Hutchinson Cancer Center, Seattle, WA, United States of America; [7]University of Maryland Greenebaum Comprehensive Cancer Center, Baltimore, United States of America; [8]Department of Hematology and Medical Oncology, Winship Cancer Institute, Emory University School of Medicine, Atlanta, GA, United States of America; [9]Department of Hematology and Medical Oncology, Leukemia Program, Taussig Cancer Institute, Cleveland Clinic, Cleveland, OH, United States of America; [10]Guangzhou Healthquest Pharma Co., Ltd., Guangzhou, China; [11]Ascentage Pharma (Suzhou) Co., Ltd., Suzhou, China; [12]Ascentage Pharma Group Inc., Rockville, MD, United States of America;*

### Background

Patients with CP-CML resistant to multiple BCR::ABL1 TKIs have limited therapeutic options. Mutations in *ASXL1* are associated with unfavorable prognosis in patients with CP-CML, who experience inferior response rates and failure-free survival, with elevated risk of disease progression and secondary *ABL1* kinase domain (KD) mutations. Outcomes of patients with *ASXL1* mutations at diagnosis are equally poor, regardless of TKI used. Olverembatinib, a third-generation BCR::ABL1 TKI, exhibits antileukemic activity in a range of *BCR::ABL1* mutations, including T315I and compound mutations. In a phase 1b trial (Jabbour E et al. *JAMA Oncol* 2025;11:28-35), 43.0% and 33.3% of patients with ponatinib and asciminib resistance, respectively, achieved major molecular response (MMR) with olverembatinib. However, olverembatinib's activity across different mutational backgrounds remains unclear.

### Aims

We characterized the baseline mutational spectrum of patients with ponatinib/asciminib-resistant CP-CML treated with olverembatinib in the phase 1b study and evaluated olverembatinib's antileukemic activity in the context of different mutations.

### Methods

Genetic profiling using a 529-gene pan-cancer panel (including *ASXL1*) characterized the baseline mutational profiles of patients with CP-CML. Peripheral blood samples from 22 patients collected at screening were analyzed. Of these, 16 had ponatinib-resistant (10 ponatinib only, 6 dual-TKI-resistant) and 12 had asciminib-resistant disease (6 asciminib only, 6 dual-TKI-resistant). The therapeutic responses of these patients to olverembatinib were analyzed with their mutational spectrum. The cutoff date for efficacy data was January 2026.

**Copyright Information:** (Online) ISSN: 2572-9241
©2026 The Author(s). HemaSphere is published by John Wiley & Sons Ltd on behalf of European Hematology Association. This is an open access Abstract Book distributed under the Attribution-NonCommercial-NoDerivs (CC BY-NC-ND), which allows third parties to download the articles and share them with others as long as they credit the author and the Abstract Book, but they cannot change the content in any way or use them commercially.
**Abstract Book Citations:** Authors, Title, HemaSphere, 2026;10:(S1):pages. The abstract book DOIs can be found on the "Author Information" page.
**Disclaimer:** Articles published in the journal HemaSphere exclusively reflect the opinions of the authors. The authors are responsible for all content in their abstracts including accuracy of the facts, statements, citing resources, etc.

### Results

Among 22 patients with mutational analysis results (median 3.5 [range 2-6] prior therapies), 10 and 8 experienced lack of efficacy on ponatinib and asciminib, respectively, as their immediate prior TKI. Baseline *BCR::ABL* transcript levels ranged between 0.4% and 417%. Seven out of 22 patients had *ABL1* KD mutations, including T315I (in 5), F359I, and the E255V/F317L multiple mutations (both in 1 each). One of the 2 patients with concurrent *ASXL1* mutations and T315I achieved MMR; another with concurrent *ASXL1* mutations and E255V/F317L achieved partial cytogenetic response (PCyR). *ASXL1* mutations were present in 40.9% (9/22) of patients, including in 5, 3, and 1 patient with ponatinib-, asciminib-, and dual-TKI–resistant disease, respectively. After olverembatinib treatment, 44.4% (4/9) of patients with *ASXL1* mutations achieved clinical responses, including 22.2% (2/9) of patients with MMR (one MR4.5); one each achieved complete cytogenetic response and PCyR. The median duration of cytogenetic response was 12.6 months (range 0.03-19.6) for the 4 cytogenetic responders and the duration of MMR was 2.8 and 22.1 months, respectively, for the 2 patients who achieved MMR and above; the shorter duration (2.8 months) reflected early withdrawal for transplantation.

### Summary/Conclusion

To our knowledge, these data provide the first evidence of responses to olverembatinib in patients with ponatinib/asciminib-resistant CP-CML, including those with *ASXL1* mutations. Olverembatinib may offer an effective new treatment option for patients with relapsed/refractory CP-CML that exhibits challenging genotypes and has progressed through multiple TKIs, including other third-generation agents.

**Copyright Information:** (Online) ISSN: 2572-9241
©2026 The Author(s). HemaSphere is published by John Wiley & Sons Ltd on behalf of European Hematology Association. This is an open access Abstract Book distributed under the Attribution-NonCommercial-NoDerivs (CC BY-NC-ND), which allows third parties to download the articles and share them with others as long as they credit the author and the Abstract Book, but they cannot change the content in any way or use them commercially.
**Abstract Book Citations:** Authors, Title, HemaSphere, 2026;10:(S1):pages. The abstract book DOIs can be found on the “Author Information” page.
**Disclaimer:** Articles published in the journal HemaSphere exclusively reflect the opinions of the authors. The authors are responsible for all content in their abstracts including accuracy of the facts, statements, citing resources, etc.

### (PS1728) THE EFFICACY AND SAFETY OF SWITCHING TO OLVEREMBATINIB OR CONTINUING ORIGINAL TKI THERAPY IN CML-CP PATIENTS TREATED WITH AT LEAST TWO PRIOR TKIS: A PROSPECTIVE, MULTICENTER, CONTROLLED TRIAL

**Topic:** 08. Chronic myeloid leukemia - Clinical

Bingbing Wen*[1, 2], Junchong Peng[1], Jixian Huang[3], Mingjie Tang[3], Fei Wang[4], Liling Zheng[5], Chan Long[6], Zan Li[7], Xiaojing Hu[8], Xiaoliang Li[9], Haifei Chen[10], Huiqing He[11], Guoshu Chen[12], Bingcheng Liu[13], Jian Huang[2], Xin Du[1]

*[1]The Second People's Hospital of Shenzhen, The First Affiliated Hospital of Shenzhen University, Shenzhen, China;[2]The First Affiliated Hospital, Zhejiang University School of Medicine, Hangzhou, China; [3]Yuebei People's hospital, Shaoguan, China; [4]Central People's Hospital of Zhanjiang, Zhanjiang, China;[5]Guangdong Second People's Hospital, Guangzhou, China;[6]Huizhou First People's Hospital, Huizhou, China; [7]Maoming People's Hospital, Maoming, China;[8]Dongguan People's Hospital, Dongguan, China; [9]Zhuhai People's Hospital, Zhuhai, China; [10]Shenzhen Luohu People's Hospital, Shenzhen, China; [11]Zhongshan City People's Hospital, Zhongshan, China; [12]Huizhou Central People's Hospital, Huizhou, China; [13]Institute of Hematology and Blood Diseases Hospital, Tianjian, Tianjin, China;*

### Background

Olverembatinib (HQP1351) is a novel third-generation BCR-ABL1 tyrosine kinase inhibitor (TKI) in chronic myeloid leukemia-chronic phase (CML-CP). However, patients with CML-CP who failed to achieve MMR were aproved with shortened PFS and overall survival (OS).

### Aims

This study evaluated the efficacy and safety of switching to olverembatinib in patients with CML-CP who failed to achieve MMR for more than 18 months and were treated at least two prior TKIs.

### Methods

In this multicenter, prospective controlled trial (CTR2300072680), patients with CML-CP who had failed for treatment with ≥2 prior TKIs for ≥18 months were enrolled. Patients were assigned at 1:2 ratio to receive either olverembatinib (40 mg orally every other day) or continue their most recent TKI therapy (control group). The primary endpoint was the major molecular response (MMR) rate at 6 months. Secondary endpoints included deep molecular responses (DMR), cumulative response rates, OS, and safety.

**Copyright Information:** (Online) ISSN: 2572-9241
©2026 The Author(s). HemaSphere is published by John Wiley & Sons Ltd on behalf of European Hematology Association. This is an open access Abstract Book distributed under the Attribution-NonCommercial-NoDerivs (CC BY-NC-ND), which allows third parties to download the articles and share them with others as long as they credit the author and the Abstract Book, but they cannot change the content in any way or use them commercially.
**Abstract Book Citations:** Authors, Title, HemaSphere, 2026;10:(S1):pages. The abstract book DOIs can be found on the “Author Information” page.
**Disclaimer:** Articles published in the journal HemaSphere exclusively reflect the opinions of the authors. The authors are responsible for all content in their abstracts including accuracy of the facts, statements, citing resources, etc.

**Results**

Between August 1, 2023 and January 1, 2026,120 patients were assessed for eligibility, 105 patients were screened and enrolled in this prospective, multicenter, controlled trial, with 35 patients assigned to olverembatinib group and 70 patients to control group (**Fig. A**). The mean age was 48.0794 (SD 14.7422) years, and males comprised 81/105 (77.1%) totally. The median disease duration at baseline was 9 years (range, 2-25 years). All patients expressed P210 transcript type. 40% (14/35) of patients in the olverembatinib group and 31.43% (22/70) in the control group were recorded with 3/4 lines of prior TKI therapy (**Fig. B**). After 6 months switching to olverembatinib treatment, the 6 months-MMR rate in olverembatinib group was significantly higher than those in control group (olverembatinib group, 19/35 (54.3%) versus control group 7/70 (10.0%); P<0.001) (**Fig. C**). At 12 months, the cumulative incidence of MMR was 57.14% (20/35) in the olverembatinib group compared to 21.43% (15/70) in the control group (P<0.0001) (**Fig. D**). The most common treatment-emergent adverse events (TEAEs) of olverembatinib were primarily grade 1-2 and manageable. The most common grade 3/4 hematological TEAEs included thrombocytopenia (15/35, 42.86%) and anemia (6/35, 17.14%), followed by leukopenia (6/35, 17.14%) and neutropenia (2/35, 5.71%). Grade 3/4 non-hematological events were rare (**Fig. E**). Interestingly, 78.57% of AEs relatived to prior TKI therapy in CML-CP patients has been improved after patients switched to olverembatinib treatment (**Fig. F**).

**Summary/Conclusion**

A higher proportion of efficacy in patients switching to olverembatinib treatment than those remained on treatment and continued to derive benefit over time, supporting olverembatinib may as a standard of care for patients with CML-CP previously treated with ≥2 TKIs. Olverembatinib also can improve the AEs relatived to prior TKI therapy and maybe a valuable therapeutic option for CML-CP patients.

**Copyright Information:** (Online) ISSN: 2572-9241
©2026 The Author(s). HemaSphere is published by John Wiley & Sons Ltd on behalf of European Hematology Association. This is an open access Abstract Book distributed under the Attribution-NonCommercial-NoDerivs (CC BY-NC-ND), which allows third parties to download the articles and share them with others as long as they credit the author and the Abstract Book, but they cannot change the content in any way or use them commercially.
**Abstract Book Citations:** Authors, Title, HemaSphere, 2026;10:(S1):pages. The abstract book DOIs can be found on the "Author Information" page.
**Disclaimer:** Articles published in the journal HemaSphere exclusively reflect the opinions of the authors. The authors are responsible for all content in their abstracts including accuracy of the facts, statements, citing resources, etc.

A

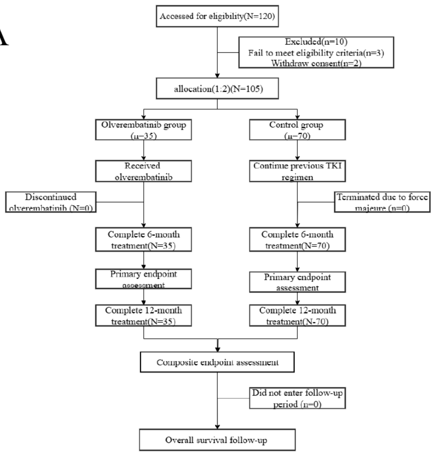

B

| | Olverembatinib group | Control group | Total | P |
|---|---|---|---|---|
| **Patient numbers** | **35** | **70** | **105** | |
| Age,Mean±SD,year | 46.0286±12.2198 | 47.6286±15.1305 | 48.0794± 14.7422 | 0.588 |
| Disease course,median(range),year | 5(2-25) | 9(2-20) | 9(2-25) | 0.004 |
| Gender,n(%) | | | | 1.000 |
| Male | 27(77.1) | 54(77.1) | 81(77.1) | |
| Female | 8(22.9) | 16(22.9) | 24(22.9) | |
| Transcript type,n | | | | |
| P210 | 35 | 70 | 105 | |
| BCR-ABL1 mutation status by next-generation sequencing,n(%) | | | | 1.000 |
| No mutation | 31(88.6) | 63(90.0) | 94(89.5) | |
| Other mutations+ | 4(11.4) | 7(10.0) | 11(10.5) | |
| Comorbidity,n(%) | | | | |
| Hypertension | 4(11.4) | 7(10.0) | 11(10.5) | 1.000 |
| Diabetes mellitus | 3(8.6) | 1(1.4) | 4(3.8) | 0.299 |
| Chronic kidney disease | 0 | 4(5.7) | 4(3.8) | 0.203 |
| Cardiac insufficiency | 0 | 4(5.7) | 4(3.8) | 0.203 |
| Viral hepatitis | 2(5.7) | 7(10.0) | 9(8.6) | 0.714 |
| Hyperlipidemia | 1(2.9) | 4(5.7) | 5(4.8) | 0.663 |
| Prior TKIs,n(%) | | | | |
| Imatinib | 30(85.7) | 65(92.9) | 95(90.5) | 0.295 |
| Dasatinib | 13(37.1) | 35(50.0) | 48(45.7) | 0.299 |
| Nilotinib | 23(65.7) | 42(60.0) | 65(61.9) | 0.671 |
| Flumatinib | 15(42.9) | 19(27.1) | 34(32.4) | 0.124 |
| Ponatinib | 0 | 6(8.6) | 6(5.7) | 0.097 |
| Number of lines of prior TKI therapy,n(%) | | | | |
| 2 | 21(60.0) | 47(67.1) | 68(64.8) | 0.519 |
| 3 | 13(41.9) | 18(25.7) | 31(29.5) | 0.260 |
| 4 | 1(2.9) | 4(5.7) | 5(4.8) | 0.663 |

C

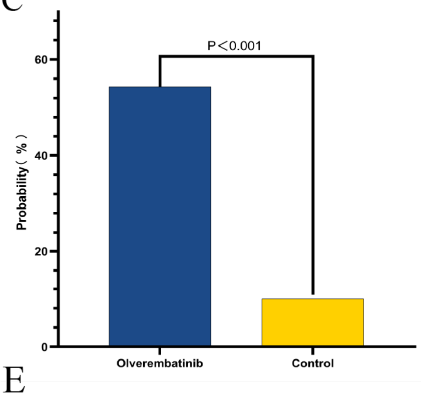

D

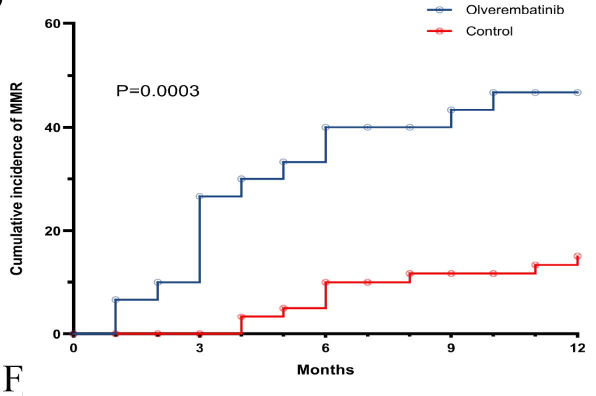

E

| | Any grade | 3/4 grade |
|---|---|---|
| **Hematological** | | |
| Anemia | 18(51.43) | 6(17.14) |
| Thrombocytopenia | 25(71.43) | 15(42.86) |
| Neutropenia | 4(11.43) | 2(5.71) |
| Leukopenia | 8(22.86) | 6(17.14) |
| **Non-hematological** | | |
| Rash | 4(11.43) | 0 |
| Pyrexia | 6(17.14) | 0 |
| Asthenia | 2(5.71) | 0 |
| Arthralgia | 2(5.71) | 0 |
| Proteinuria | 16(45.71) | 0 |
| Hypokalemia | 8(22.86) | 0 |
| Hyperuricemia | 4(11.43) | 0 |
| Hyperglycemia | 7(20.00) | 0 |
| Sinus tachycardia | 6(17.14) | 0 |
| Skin pigmentation | 25(71.43) | 0 |
| Hyperbilirubinemia | 15(42.86) | 0 |
| Hypertriglyceridemia | 18(51.43) | 2(5.71) |
| Increased γ-glutamyl transferase | 9(25.71) | 0 |
| Increased creatine phosphokinase | 12(34.29) | 2(5.71) |
| Increased alanine aminotransferase | 13(37.14) | 0 |
| Increased aspartate aminotransferase | 13(37.14) | 0 |

F

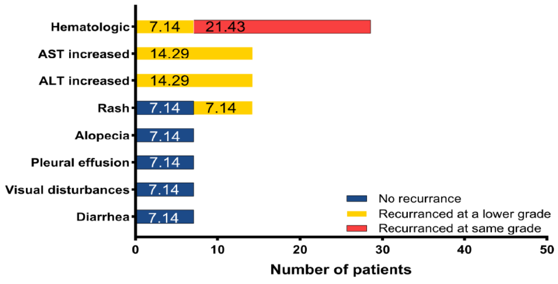

**Copyright Information:** (Online) ISSN: 2572-9241
©2026 The Author(s). HemaSphere is published by John Wiley & Sons Ltd on behalf of European Hematology Association. This is an open access Abstract Book distributed under the Attribution-NonCommercial-NoDerivs (CC BY-NC-ND), which allows third parties to download the articles and share them with others as long as they credit the author and the Abstract Book, but they cannot change the content in any way or use them commercially.
**Abstract Book Citations:** Authors, Title, HemaSphere, 2026;10:(S1):pages. The abstract book DOIs can be found on the "Author Information" page.
**Disclaimer:** Articles published in the journal HemaSphere exclusively reflect the opinions of the authors. The authors are responsible for all content in their abstracts including accuracy of the facts, statements, citing resources, etc.

### (PS1729) RISK OF PERMANENT FRONTLINE DISCONTINUATION DURING THE FIRST 36 MONTHS OF THERAPY WITH TYROSINE KINASE INHIBITORS IN CHRONIC MYELOID LEUKEMIA: A “CAMPUS CML” STUDY WITH A NEW “AD HOC” SCORE PROPOSAL

**Topic:** 08. Chronic myeloid leukemia - Clinical

Roberto Latagliata*[1], Matteo Caridi[1, 2], Isabella Capodanno[3], Maria Cristina Miggiano[4], Alessandra Iurlo[5], Francesco Cavazzini[6], Sabrina Leonetti Crescenzi[7], Sabina Russo[8], Giuseppina Loglisci[9], Ida Carmosino[10], Andrea Bernardelli[11], Luigiana Luciano[12], Olga Mulas[13], Chiara Elena[14], Antonella Russo Rossi[15], Gianni Binotto[16], Elena Crisà[17], Atelda Romano[18], Ambra Di Veroli[1], Anna Rita Scortechini[19], Anna Paola Leporace[20], Maria Basile[21], Monica Crugnola[22], Pamela Murgano[23], Davide Rapezzi[24], Debora Luzi[25], Daniele Cattaneo[5], Monica Bocchia[26], Carmen Fava[27], Alessandra Malato[28], Sara Galimberti[29], Iolanda Donatella Vincelli[30], Malgorzata Monica Trawinska[31], Michele Pizzuti[32], Francesca Lunghi[33], Fabio Stagno[34], Giovanni Caocci[13], Massimiliano Bonifacio[11], Federica Sora[35], Giuseppe Saglio[36], Giorgina Specchia[15], Massimo Breccia[10], Mario Tiribelli[37]

*[1]UOC Hematology - Santa Rosa Hospital, Viterbo, Italy;[2]Department of Physiology and Pharmacology, Sapienza University, Rome, Italy, Rome, Italy; [3]Hematology, Azienda USL - IRCCS di Reggio Emilia, Reggio Emilia, Italy;[4]Hematology Department, San Bortolo Hospital, Azienda ULSS8 "Berica" di Vicenza, Vicenza, Italy; [5]Hematology Division, Foundation IRCCS Ca' Granda Ospedale Maggiore Policlinico, Milan, Italy; [6]Hematology Section, Department of Medical Sciences, Azienda Ospedaliero-Universitaria, Arcispedale S. Anna, University of Ferrara, Ferrara, Italy; [7]UOC Hematology - San Giovanni Hospital, Rome, Italy; [8]Hematology Section, AOU Policlinico "G. Martino", Messina, Italy;[9]UOC Hematology, Vito Fazzi Hospital, Lecce, Italy; [10]Hematology, Department of Translational and Precision Medicine, Sapienza University of Rome, Rome, Italy; [11]Department of Engineering for Innovation Medicine, Section of Innovation Biomedicine, Hematology Area - University of Verona, Verona, Italy; [12]Hematology, University Federico II of Neaples, Neaples, Italy; [13]Department of Medical Sciences and Public Health, University of Cagliari, Cagliari, Italy; [14]Hematology, Policlinico San Matteo, University of Pavia, Pavia, Italy; [15]Hematology and Transplantation Unit, University of Bari, Bari, Italy; [16]Department of Medicine, Hematology and Clinical Immunology, University of Padua, Padua, Italy; [17]UOC Hematology, Ospedale Maggiore, Novara, Italy; [18]Hematology, IRCCS Regina Elena National Cancer Institute, Rome, Italy; [19]Hematology, Department of Clinical and Molecular Sciences, Università Politecnica delle Marche, Ancona, Italy; [20]Hematology - Sant'Andrea Hospital, Rome, Italy; [21]UOC Hematology, San Giuseppe Moscati Hospital, Taranto, Italy; [22]Hematology Unit, University of Parma, Parma, Italy;[23]Division of Hematology, Sant'Elia Hospital, Caltanissetta, Italy; [24]Hematology, S. Croce e Carle Hospital, Cuneo, Italy;[25]Onco-Hematology Division, Santa Maria Hospital, Terni, Italy; [26]Hematology Unit, Azienda Ospedaliero Universitaria Senese, Siena, Italy; [27]Hematology, Mauriziano Hospital, Turin, Italy; [28]UOC Hematology, Cervello Hospital, Palermo, Italy; [29]Division of Hematology, University of Pisa, Pisa, Italy;[30]UOC Hematology - Bianchi Melacrino Morelli Hospital, Reggio Calabria, Italy; [31]Division of Hematology, Ospedale S. Eugenio, Rome, Italy;[32]UOC Hematology - San Carlo Hospital, Potenza, Italy; [33]Hematology, San Raffaele Hospital, Milan, Italy; [34]Division of Hematology, Department of Human Pathology in Adulthood and Childhood 'Gaetano Barresi', University of Messina, Messina, Italy; [35]Hematology Division, Policlinico Gemelli, Catholic University of Sacred Hearth of Rome, Rome, Italy;[36]Clinical and Biological Sciences, University of Turin, Turin, Italy; [37]Division of Hematology and BMT, Department of Medical Area, University of Udine, Udine, Italy;*

### Background

The first 3 years of frontline therapy with Tyrosine Kynase Inhibitors (TKI) are crucial to achieve optimal response in patients (pts) with Chronic Myeloid Leukemia (CML): in this early phase, however, different toxicities or suboptimal response/resistance may occur, leading to permanent frontline TKI discontinuation and need for a 2nd-line switch.

**Copyright Information:** (Online) ISSN: 2572-9241
©2026 The Author(s). HemaSphere is published by John Wiley & Sons Ltd on behalf of European Hematology Association. This is an open access Abstract Book distributed under the Attribution-NonCommercial-NoDerivs (CC BY-NC-ND), which allows third parties to download the articles and share them with others as long as they credit the author and the Abstract Book, but they cannot change the content in any way or use them commercially.
**Abstract Book Citations:** Authors, Title, HemaSphere, 2026;10:(S1):pages. The abstract book DOIs can be found on the “Author Information” page.
**Disclaimer:** Articles published in the journal HemaSphere exclusively reflect the opinions of the authors. The authors are responsible for all content in their abstracts including accuracy of the facts, statements, citing resources, etc.

### Aims

To evaluate in a large real-life cohort of CML pts all types of events leading to a permanent frontline TKI discontinuation during the first 3 years and to propose a new “ad hoc” score.

### Methods

1713 CML pts diagnosed from 1/2012 and 12/2019 at 35 Hematology Centres were retrospectively analysed. To derive a clinical score, a stratified data partition was performed, splitting pts into a training cohort (1/3, n = 573) and an independent validation cohort (2/3, n = 1140) using stratified sampling based on the joint distribution of treatment discontinuation status at 36 months. Variables showing a significant association by univariate analysis were considered for multivariable modeling. To enhance model stability and explore data augmentation strategies, a synthetic expansion of the training dataset was performed. Several algorithms were applied to generate a 3-fold synthetic expansion of the training dataset (n = 1719): the synthetic dataset with the lowest propensity score Mean Squared Error (pMSE) and a standardized pMSE value closest to 1 was selected for further analysis. A data-driven grid search approach was applied to determine optimal cut-off values for these variables. The resulting risk score was finally tested in the independent validation cohort to evaluate its discriminative performance.

### Results

Frontline TKI was IM in 976 (56.9%) and 2G-TKIs in 737 (43.1%) pts. Total number of pts who had discontinued frontline TKI at the 36th month of observation was 536/1713 (31.3%), being higher with IM (366/976, 37.5%) than with 2G-TKIs (170/737, 23.0%) ($p<0.001$). Among the 536 pts who discontinued frontline TKI, the main causes were hematologic toxicity in 34 (6.4%), extra-hematologic toxicity in 171 (31.9%), primary resistance in 228 (42.5%), secondary resistance in 27 (5.0%), blastic phase evolution in 23 (4.3%), unrelated deaths in 42 (7.8%) and other causes in 11 (2.1%). At multivariate analysis, age (OR 1.01, 95% CI 1.00–1.02; $p = 0.002$), hemoglobin (OR 0.87, 95% CI 0.82–0.92; $p < 0.001$) and spleen size (OR 1.07, 95% CI 1.03–1.11; $p < 0.001$) retained an independent predictive role for TKI discontinuation. The optimal combined cut-offs identified by the grid-search procedure were age ≥ 63.5 years, hemoglobin ≤ 10.2 g/dL, and the presence of any degree of splenomegaly. An aggregated score based on these 3 cut-offs (low-risk 0-1 variables, high risk 2-3 variables) enabled a strong risk stratification of pts, effectively predicting the likelihood of treatment discontinuation. This discriminative ability was consistently observed in both the training and validation cohorts and was maintained across subgroup analyses stratified by frontline imatinib versus 2G-TKI treatment (Figure).

### Summary/Conclusion

By integrating conventional statistical methods with advanced machine learning approaches, we developed a clinically applicable risk score based on simple baseline parameters. This score enables effective stratification of the risk of TKI early discontinuation, facilitating practical bedside implementation and supporting a more personalized therapeutic strategy.

**Copyright Information:** (Online) ISSN: 2572-9241
©2026 The Author(s). HemaSphere is published by John Wiley & Sons Ltd on behalf of European Hematology Association. This is an open access Abstract Book distributed under the Attribution-NonCommercial-NoDerivs (CC BY-NC-ND), which allows third parties to download the articles and share them with others as long as they credit the author and the Abstract Book, but they cannot change the content in any way or use them commercially.
**Abstract Book Citations:** Authors, Title, HemaSphere, 2026;10:(S1):pages. The abstract book DOIs can be found on the “Author Information” page.
**Disclaimer:** Articles published in the journal HemaSphere exclusively reflect the opinions of the authors. The authors are responsible for all content in their abstracts including accuracy of the facts, statements, citing resources, etc.

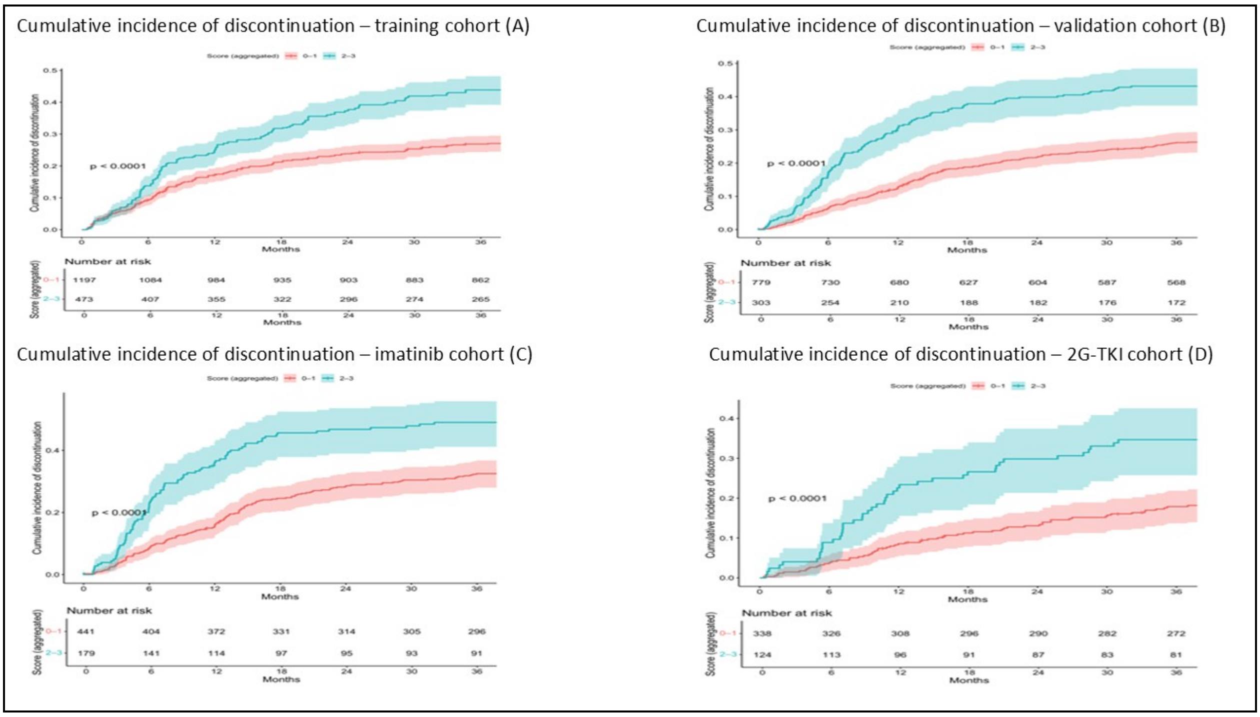

Cumulative incidence of permanent frontline TKI discontinuation in the training cohort (A), vakidation cohort (B), imatinib cohort (C) and 2G-TKI cohort (D)

**Copyright Information:** (Online) ISSN: 2572-9241
©2026 The Author(s). HemaSphere is published by John Wiley & Sons Ltd on behalf of European Hematology Association. This is an open access Abstract Book distributed under the Attribution-NonCommercial-NoDerivs (CC BY-NC-ND), which allows third parties to download the articles and share them with others as long as they credit the author and the Abstract Book, but they cannot change the content in any way or use them commercially.
**Abstract Book Citations:** Authors, Title, HemaSphere, 2026;10:(S1):pages. The abstract book DOIs can be found on the "Author Information" page.
**Disclaimer:** Articles published in the journal HemaSphere exclusively reflect the opinions of the authors. The authors are responsible for all content in their abstracts including accuracy of the facts, statements, citing resources, etc.

### (PS1730) OPTIMAL TIMING AND PARAMETERS FOR TYROSINE KINASE INHIBITOR DOSE DE-ESCALATION AFTER ACHIEVING MAJOR MOLECULAR RESPONSE IN CHRONIC MYELOID LEUKEMIA

**Topic:** 08. Chronic myeloid leukemia - Clinical

Sewon Lee*[1], Yeung-Chul Mun[1], Hongtae Kim[2], Semin Lee[2], Sung-Hyun Kim[3], Young Rok Do[4], Eunjung Jang[5], Sahee Park[5], Dw Kim[5, 6]

*[1]Ewha Womans University Mokdong Hospital, Seoul, South Korea; [2]Ulsan National Institute of Science & Technology, Department of Biological Science, Ulsan, South Korea; [3]Dong-A University Medical Center, Busan, South Korea; [4]Dongsan Medical Center, Keimyung University, Daegu, South Korea; [5]Leukemia Omics Research Institute, Eulji University, Uijeongbu, South Korea; [6]Uijeongbu Eulji Medical Center, Uijeongbu, South Korea;*

### Background

Tyrosine kinase inhibitors (TKIs) are effective across all phases of chronic myeloid leukemia (CML). However, long-term treatment is frequently complicated by TKI-related adverse events (AEs), which negatively impact quality of life. In clinical practice, dose reduction or discontinuation is often required to mitigate AEs; however, the impact of such modifications on long-term molecular responses remains uncertain.

### Aims

This study aimed to evaluate the clinical outcomes of TKI dose reduction in CML patients who achieved a major molecular response (MMR) following standard-dose therapy. Specifically, we sought to determine whether dose reduction compromises molecular response, assess the incidence of molecular relapse, and identify the optimal timing of dose reduction and potential predictors of maintenance of molecular response in a real-world setting.

### Methods

We retrospectively analyzed 193 patients with CML who achieved a MMR or deeper responses after TKI therapy and subsequently underwent dose reduction due to several reasons, including AEs. Patients were initially treated with imatinib (400–800 mg once daily), nilotinib (150–300 mg twice daily), radotinib (200–400 mg twice daily), dasatinib (50–140 mg once daily), or ponatinib (45 mg once daily). Dose reduction was implemented after achieving MMR and molecular monitoring was performed using quantitative PCR every three months.

**Copyright Information:** (Online) ISSN: 2572-9241
©2026 The Author(s). HemaSphere is published by John Wiley & Sons Ltd on behalf of European Hematology Association. This is an open access Abstract Book distributed under the Attribution-NonCommercial-NoDerivs (CC BY-NC-ND), which allows third parties to download the articles and share them with others as long as they credit the author and the Abstract Book, but they cannot change the content in any way or use them commercially.
**Abstract Book Citations:** Authors, Title, HemaSphere, 2026;10:(S1):pages. The abstract book DOIs can be found on the "Author Information" page.
**Disclaimer:** Articles published in the journal HemaSphere exclusively reflect the opinions of the authors. The authors are responsible for all content in their abstracts including accuracy of the facts, statements, citing resources, etc.

**Results**

Among 3,204 CML patient registry pool, a total of 193 patients (183 in CP and 10 in AP) were included in de-escalation analysis. The median time to MMR was 6.1 months (range: 1.8–145.7), and the median time from TKI initiation to the first dose reduction was 82.7 months (range: 1.6–274.1). At the time of dose reduction, patients were receiving imatinib 200 mg (n=65), nilotinib 200 mg (n=25), radotinib 200 mg (n=64), dasatinib 20 mg (n=35), or ponatinib 15 mg every other day (n=4). Additionally, 127 (65.8%) patients were receiving first-line TKI therapy, 40 (20.7%) second-line, 14 (7.3%) third-line, and 12 (6.2%) fourth-line or later TKIs. Dose reductions were primarily due to typical TKI-related AEs, and all patients experienced improvement following dose adjustment. During a median follow-up of 30.7 months (range, 2.8–92.2 months), only two (1.0%) patients lost MMR. Thirty-two (16.6%) patients experienced fluctuating molecular responses but maintained MMR, while 31 (16.1%) achieved deeper molecular responses. The remaining 128 (66.3%) patients maintained a stable response after dose reduction. During the first 12 months after frontline TKI therapy,  64 patients had not achieved optimal molecular response, and 17 patients switched to another TKI due to refractory or inadequate molecular response to prior therapy. Despite these results, subsequent dose reduction did not appear to negatively affect molecular outcomes among these patients. In addition, second-generation TKIs showed a trend toward a lower rate of molecular relapse compared to imatinib; however, further analysis is required. Treatment-free remission (TFR) was attempted in 26 patients who maintained MR5.0 after dose reduction, and 19 of 26 (73.1%) successfully sustained TFR at 12 months.

**Summary/Conclusion**

In this real-world cohort, TKI dose reduction after achieving MMR did not adversely affect subsequent molecular outcomes in CML patients. Dose reduction effectively alleviates AEs while maintaining durable molecular responses. Further studies are warranted to identify predictors of molecular relapse following TKI dose de-escalation.

**Copyright Information:** (Online) ISSN: 2572-9241
©2026 The Author(s). HemaSphere is published by John Wiley & Sons Ltd on behalf of European Hematology Association. This is an open access Abstract Book distributed under the Attribution-NonCommercial-NoDerivs (CC BY-NC-ND), which allows third parties to download the articles and share them with others as long as they credit the author and the Abstract Book, but they cannot change the content in any way or use them commercially.
**Abstract Book Citations:** Authors, Title, HemaSphere, 2026;10:(S1):pages. The abstract book DOIs can be found on the “Author Information” page.
**Disclaimer:** Articles published in the journal HemaSphere exclusively reflect the opinions of the authors. The authors are responsible for all content in their abstracts including accuracy of the facts, statements, citing resources, etc.

### (PS1731) CHARACTERISTICS, TREATMENT AND OUTCOME OF 15 TO 18-YEAR-OLD ADOLESCENTS WITH CHRONIC MYELOID LEUKEMIA (CML): THE EXPERIENCE OF THE INTERNATIONAL REGISTRY OF CHILDHOOD CML (I-CML-PED STUDY)

**Topic:** 08. Chronic myeloid leukemia - Clinical

Frédéric Millot*[1], Morgane Froment[2], Lisa Durocher[1], Markus Metzler[3], Barbara De Moerloose[4], Silvia Regina Brandalise[5], Marina Borisevich[6], Petr Sedlacek[7], Adalet Meral Güneş[8], Birgitte Lausen[9], Antonio Molines Honrubia[10], Gordana Jakovljevic[11], Maaike Luesink[12], Krzysztof Kałwak[13], Alexandra Kolenova[14], Meinolf Suttorp[15]

*[1]Inserm CIC 1402, POITIERS, France; [2]Robert Debré Hospital, Paris, France; [3]Department of Pediatrics and Adolescent Medicine, University Hospital, Erlangen, Germany; [4]Ghent University Hospital., Ghent, Belgium; [5]Centro Infantil Boldrini, Sao Paulo, Brazil; [6]Belarusian Research Centre for Pediatric Oncology, Haematology and Immunology, Minsk, Belarus; [7]Department of Pediatric Hematology-Oncology, University Hospital Motol, Charles University, Prague, Czechia; [8]Pediatric Hematology and Oncology Hospital Görükle, Nilüfer/Bursa, Türkiye; [9]Department of Paediatrics and Adolescent Medicine, Rigshospitalet, University of Copenhagen, Copenhagen, Denmark; [10]Department of Pediatric Hematology, CHU insular Materno-infantil, Las Palmas de Gran Canaria, Spain; [11]Department of Oncology and Hematology, Children's Hospital Zagreb, Zagreb, Croatia; [12]Princess Máxima Center for Pediatric Oncology, Utrecht, The Netherlands; [13]Department of Pediatric Hematology, Oncology and Bone Marrow Transplantation, Wroclaw Medical University, Wroclaw, Poland; [14]Department of Pediatric Hematology and Oncology, National Institute of Children's Diseases and Comenius University, Bratislava, Slovakia; [15]Pediatric Hematology and Oncology, Medical Faculty, Technical University, Dresden, Germany;*

### Background

**Chronic Myeloid Leukemia (CML) is a rare disease in children and adolescents and data regarding adolescents with CML are limited. The International Registry of Pediatric CML (I-CML-Ped Study, Poitiers, France, registered at www.clinicaltrials.gov NCT01281735) enabled the retrospective analysis of a large cohort of adolescents treated upfront with tyrosine kinase inhibitor (TKI). Chronic Myeloid Leukemia (CML) is a rare disease in children and adolescents and data regarding adolescents with CML are limited. The International Registry of Pediatric CML (I-CML-Ped Study, Poitiers, France, registered at www.clinicaltrials.gov NCT01281735) enabled the retrospective analysis of a large cohort of adolescents treated upfront with tyrosine kinase inhibitor (TKI)**

### Aims

**To report on features at diagnosis and response to TKI treatment of adolescents (15-18 years of age at diagnosis) with CML in chronic phase (CML-CP) and to compare their outcome with younger children.**

### Methods

**The I-CML-Ped Study enrolled patients (pts) less than 18 years of age at diagnosis of CML. Data from adolescents with CML-CP enrolled from 2011 to 2021 were analyzed and compared to data from children less than 15 years with CML-CP enrolled during the same period of time.**

**Copyright Information:** (Online) ISSN: 2572-9241
©2026 The Author(s). HemaSphere is published by John Wiley & Sons Ltd on behalf of European Hematology Association. This is an open access Abstract Book distributed under the Attribution-NonCommercial-NoDerivs (CC BY-NC-ND), which allows third parties to download the articles and share them with others as long as they credit the author and the Abstract Book, but they cannot change the content in any way or use them commercially.
**Abstract Book Citations:** Authors, Title, HemaSphere, 2026;10:(S1):pages. The abstract book DOIs can be found on the "Author Information" page.
**Disclaimer:** Articles published in the journal HemaSphere exclusively reflect the opinions of the authors. The authors are responsible for all content in their abstracts including accuracy of the facts, statements, citing resources, etc.

Results

Among the 614 pts registered, 144 (23.4%) adolescents (15-18 years of age at diagnosis) with sufficient data were identified. Among them, 132 pts presented with CML-CP according to the European Leukemia Net criteria : ratio male/female was 1.75, papable spleen was reported in 67% of the pts (median spleen size of 10 cm below the costal margin [interquartile range, 5-18]). Pain, asthenia and weight loss were the most frequent symptoms at diagnosis. At diagnosis, median leukocyte count was 181 G/L [range, 70-327]; median platelet count was 516 G/L [range, 287-721] and mean hemoglobin level was 11 g/dL (± 2 standard deviations). BCR::ABL1 transcript type b3a2 was present in 55% of the assessable pts. Overall, 9% of the pts were allocated to the high-risk group according to the ELTS score. Among the 132 adolescents with CML-CP, 124 (94%) were treated with imatinib as first-line treatment. In these 124 pts, the ratio BCR::ABL1/ABL1 after 3 months of imatinib was < 10% in 54% of 91 pts with available data; the cumulative incidence of major molecular responses (MMR) at month 12 and month 36 were 33.4% (95% confidence interval CI, 24.9-41.9) and 79.8% (95% CI, 71.8-87.7), respectively, with a median time to achieve MMR of 16 months (95% CI, 14-19.6). With a median follow-up of 4 years (95% CI : 3.6-4.9), 49 (40%) of these 124 pts receiving imatinib as first-line therapy were switched to another treatment and 11 (9%) pts progressed to the advanced phase. Death occurred in 5 (4%) pts including 4 deaths related to complications associated with hematopoietic stem cell transplantation. Overall survival (OS) rate of these 124 adolescents was 95.2% (95% CI, 90.8-99.9%) at 5 years. We did not find a significant statistical difference in terms of cumulative responses (complete cytogenetic response and MMR) to treatment and outcome (progression-free survival, OS) between these adolescents and children less than 15 years treated with imatinib front-line.

Summary/Conclusion

Response to treatment and outcome in adolescents with CML-CP are satisfying in the tyrosine kinase inhibitor era and are similar to results in children less than 15 years. Futher studies comparing characteristics and outcomes of adolescents to young adults are needed.

Copyright Information: (Online) ISSN: 2572-9241
©2026 The Author(s). HemaSphere is published by John Wiley & Sons Ltd on behalf of European Hematology Association. This is an open access Abstract Book distributed under the Attribution-NonCommercial-NoDerivs (CC BY-NC-ND), which allows third parties to download the articles and share them with others as long as they credit the author and the Abstract Book, but they cannot change the content in any way or use them commercially.
Abstract Book Citations: Authors, Title, HemaSphere, 2026;10:(S1):pages. The abstract book DOIs can be found on the “Author Information” page.
Disclaimer: Articles published in the journal HemaSphere exclusively reflect the opinions of the authors. The authors are responsible for all content in their abstracts including accuracy of the facts, statements, citing resources, etc.

### (PS1732) KINETICS AND DURABILITY OF MOLECULAR RESPONSE AS PREDICTORS OF TREATMENT-FREE REMISSION IN CHRONIC MYELOID LEUKEMIA

**Topic:** 08. Chronic myeloid leukemia - Clinical

Sewon Lee*[1], Yeung-Chul Mun[1], Hongtae Kim[2], Semin Lee[3], Sung-Ho Park[2], Sung-Hyun Kim[4], Young Rok Do[5], Soo-Hyun Kim[6], Kyung-Mi Kee[6], Dw Kim[6, 7]

*[1]Ewha Womans University Mokdong Hospital, Seoul, South Korea; [2]Ulsan National Institute of Science & Technology, Department of Biological Science, Ulsan, South Korea; [3]Ulsan National Institute of Science and Technology, Department of Biomedical Engineering, Ulsan, South Korea; [4]Dong-A University Medical Center, Busan, South Korea; [5]Dongsan Medical Center, Keimyung University, Daegu, South Korea; [6]Leukemia Omics Research Institute, Eulji University, Uijeongbu, South Korea; [7]Uijeongbu Eulji Medical Center, Uijeongbu, South Korea;*

**Background**

Tyrosine kinase inhibitor (TKI) discontinuation, or treatment-free remission (TFR), has become an important therapeutic goal in chronic-phase chronic myeloid leukemia (CP-CML). Although nearly half of patients may meet eligibility criteria for TFR, only approximately 50% maintain durable remission after discontinuation.

**Aims**

While TKI duration, molecular response depth, and baseline disease risk have been associated with successful TFR, further real-world validation of predictive factors remains necessary.

**Methods**

We conducted a combined retrospective and prospective observational study of Korean CP-CML patients who discontinued TKI therapy after sustaining MR4.5 (BCR::ABL1 IS ≤0.0032%) for at least four years. Molecular monitoring was performed using RT-qPCR. Upon loss of major molecular response (MMR), the prior TKI was resumed at the same dose, with monthly monitoring until MMR recovery.

**Copyright Information:** (Online) ISSN: 2572-9241
©2026 The Author(s). HemaSphere is published by John Wiley & Sons Ltd on behalf of European Hematology Association. This is an open access Abstract Book distributed under the Attribution-NonCommercial-NoDerivs (CC BY-NC-ND), which allows third parties to download the articles and share them with others as long as they credit the author and the Abstract Book, but they cannot change the content in any way or use them commercially.
**Abstract Book Citations:** Authors, Title, HemaSphere, 2026;10:(S1):pages. The abstract book DOIs can be found on the "Author Information" page.
**Disclaimer:** Articles published in the journal HemaSphere exclusively reflect the opinions of the authors. The authors are responsible for all content in their abstracts including accuracy of the facts, statements, citing resources, etc.

### Results

A total of 306 patients discontinued TKI therapy. Median age at diagnosis was 44.1 years (range 15–79). EURO risk scores were low (43.1%), intermediate (34.3%), high (8.5%), or unknown (14.1%). Before discontinuation, patients had received TKI therapy for a median of 98.1 months and maintained MR4.5 for 53 months. At discontinuation, 71.1% were on first-line TKI therapy.
After a median follow-up of 49.4 months, TFR rates at 12, 24, and 48 months were 77.6%, 72.2%, and 70.3%, respectively. Among all patients, 88 (28.8%) lost MMR at a median of 3.7 months after discontinuation, with most relapses occurring within six months. All patients who lost MMR resumed the same TKI, and 94.3% regained MMR within a median of 4.5 months.
Late molecular relapse occurred in six patients, including two cases beyond four years and one at ten years after discontinuation. Notably, the two latest relapses progressed to blast phase following loss of undetectable minimal residual disease (UMRD). One patient remains in complete molecular response after allogeneic stem cell transplantation, while the other died during treatment. These findings underscore the need for long-term molecular monitoring, even in patients with prolonged TFR.
Among patients who restarted TKI after an initial unsuccessful TFR attempt and subsequently maintained MR4.5 for at least two years, 26 (29.5%) proceeded to a second TFR attempt. At data cutoff, 11 patients who underwent multiple discontinuation attempts remained off TKIs and maintained remission. The success rates after each attempt were 26.9% for the second, 16.6% for the third, and 33.3% for the fourth attempt. Although the probability of sustained remission may decline with repeated attempts, a subset of patients can still achieve durable TFR after multiple discontinuations.
Multivariate analysis identified significant predictors of molecular relapse, including slower BCR::ABL1 halving time (≥13.6 days), shorter duration of complete molecular response (<24.8 months), higher EURO risk score, absence of withdrawal syndrome, and younger age at discontinuation. Patients who received approximately nine years of TKI therapy and sustained deep molecular response for more than five years had the highest probability of durable TFR, regardless of TKI type.

### Summary/Conclusion

In conclusion, longer TKI exposure and sustained deep molecular response significantly improve the likelihood of successful TFR. However, late relapse remains possible, emphasizing the importance of prolonged molecular surveillance and individualized decision-making in TKI discontinuation strategies.

**Copyright Information:** (Online) ISSN: 2572-9241
©2026 The Author(s). HemaSphere is published by John Wiley & Sons Ltd on behalf of European Hematology Association. This is an open access Abstract Book distributed under the Attribution-NonCommercial-NoDerivs (CC BY-NC-ND), which allows third parties to download the articles and share them with others as long as they credit the author and the Abstract Book, but they cannot change the content in any way or use them commercially.
**Abstract Book Citations:** Authors, Title, HemaSphere, 2026;10:(S1):pages. The abstract book DOIs can be found on the “Author Information” page.
**Disclaimer:** Articles published in the journal HemaSphere exclusively reflect the opinions of the authors. The authors are responsible for all content in their abstracts including accuracy of the facts, statements, citing resources, etc.

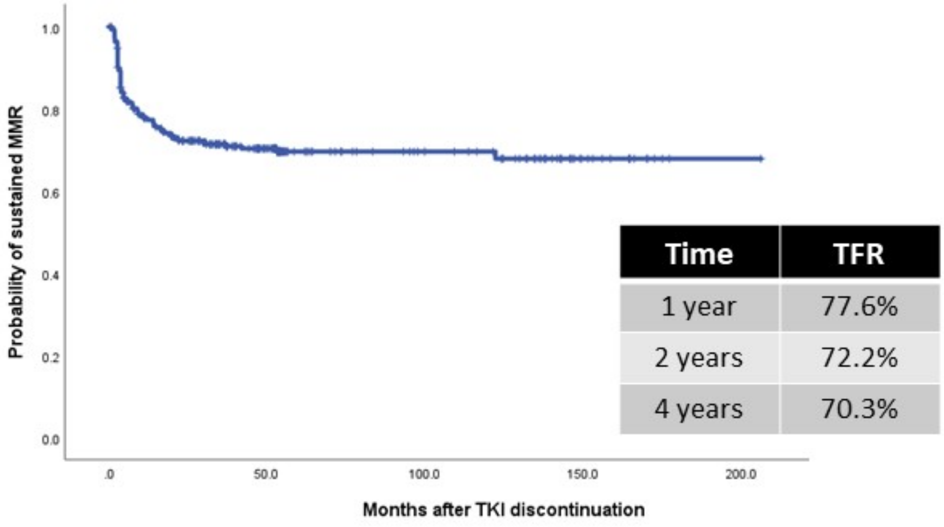

| Time | TFR |
|---|---|
| 1 year | 77.6% |
| 2 years | 72.2% |
| 4 years | 70.3% |

**Figure 1. Cumulative rate of overall TFR**

**Table1. Univariate analysis with significant factors**

| | HR | 95% CI | P |
|---|---|---|---|
| **Halving time, 11.9 days≤** | 2.395 | 1.068-5.370 | 0.034 |
| **CMR duration, 24.9 months≤** | 0.262 | 0.109-0.628 | 0.003 |
| **EURO risk score** | | | |
| Intermediate | 9.900 | 2.695-36.360 | <0.001 |
| High | 21.854 | 2.124-224.874 | 0.010 |
| **Withdrawal Symptoms** | 0.262 | 0.109-0.628 | 0.003 |
| **Age at discontinuation, 52.4years≤** | 0.107 | 0.220-0.514 | 0.005 |

**Copyright Information:** (Online) ISSN: 2572-9241
©2026 The Author(s). HemaSphere is published by John Wiley & Sons Ltd on behalf of European Hematology Association. This is an open access Abstract Book distributed under the Attribution-NonCommercial-NoDerivs (CC BY-NC-ND), which allows third parties to download the articles and share them with others as long as they credit the author and the Abstract Book, but they cannot change the content in any way or use them commercially.
**Abstract Book Citations:** Authors, Title, HemaSphere, 2026;10:(S1):pages. The abstract book DOIs can be found on the "Author Information" page.
**Disclaimer:** Articles published in the journal HemaSphere exclusively reflect the opinions of the authors. The authors are responsible for all content in their abstracts including accuracy of the facts, statements, citing resources, etc.

### (PS1733) UPDATED EFFICACY AND SAFETY OF OLVEREMBATINIB (HQP1351) AS SECOND-LINE THERAPY IN PATIENTS WITH CHRONIC-PHASE CHRONIC MYELOID LEUKEMIA (CP-CML)

**Topic:** 08. Chronic myeloid leukemia - Clinical

Weiming Li*[1], Yanli Zhang[2], Huanling Zhu[3], Yunfan Yang[3], Na Xu[4], Bingcheng Liu[5], Zi Chen[6], Wei Chu[6], Yifan Zhai[6, 7, 8], Yu Hu[1]

*[1]Union Hospital, Tongji Medical College, Huazhong University of Science and Technology, Department of Hematology, Wuhan, China; [2]Affiliated Cancer Hospital of Zhengzhou University, Department of Hematology, Zhengzhou, China;[3]West China Hospital of Sichuan University, Department of Hematology, Chengdu, China; [4]Nanfang Hospital, Southern Medical University, Department of Hematology, Guangzhou, China; [5]Institute of Hematology and Blood Diseases Hospital, Chinese Academy of Medical Sciences and Peking Union Medical College, Tianjin, China; [6]Ascentage Pharma (Suzhou) Co., Ltd., Suzhou, China;[7]Ascentage Pharma Group Inc., Rockville, MD, United States of America; [8]Guangzhou Healthquest Pharma Co., Ltd., Guangzhou, China;*

### Background

Olverembatinib is a third-generation BCR::ABL1 tyrosine kinase inhibitor (TKI) approved in China for chronic myeloid leukemia (CML) resistant/intolerant to ≥ 2 TKIs or with the T315I mutation.

### Aims

This study assessed olverembatinib as second-line treatment in patients with CP-CML resistant/intolerant to first-line TKIs without the T315I mutation.

### Methods

This single-arm, multicenter, open-label phase 2 study (ChiCTR2200061655) enrolled patients (ECOG PS 0-2, adequate hepatic/renal function) who receive olverembatinib 40 mg orally every other day in 28-day cycles. Efficacy and safety were assessed every 3 cycles. The primary endpoint was complete cytogenetic response (CCyR) rate.

### Results

From August 4, 2022, through January 14, 2026, 47 patients were enrolled (91.5% first-line TKI resistant, 8.5% intolerant). The median age was 42.0 (19-70) years; 66.0% were male. Twelve (25.5%) received first-line imatinib, and 35 (74.5%) received first-line second-generation TKIs. Thirty-six (76.6%) had no baseline *BCR::ABL1* kinase domain mutations, and 11 (23.4%) had non-T315I mutations. At data cutoff, 33 (70.2%) continued treatment, while 14 (29.8%) discontinued because of adverse events (n = 3), treatment failure (n = 4), progression (n = 2), or other reasons (n = 5). Among 42 evaluable patients, 76.2% achieved CCyR and 47.6% major molecular response (MMR). CCyR/MMR rates increased progressively: cycle 6 (57.5%/25.0%), cycle 9 (60.4%/33.3%), cycle 12 (75.0%/37.5%), cycle 15 (83.3%/46.7%), cycle 18 (89.3%/50.0%), cycle 21 (92.0%/64.0%), and cycle 24 (91.3%/60.9%). Complete hematologic response was achieved in 83.3% (15/18) and major cytogenetic response in 83.3% (35/42). Among 32 patients pretreated with first-line second-generation TKIs, 81.3% achieved CCyR and 50% MMR. The median treatment duration was 16.0 (6-38) cycles. Any-grade treatment-related adverse events (TRAEs) occurred in 42 (89.4%) patients; 21 (44.7%) had grade ≥ 3 TRAEs; and 6 (12.8%) had serious adverse events (SAEs). Nonhematologic TRAEs (mainly grade 1-2) included skin hyperpigmentation (51.1%), hyperuricemia (31.9%), and creatine phosphokinase increased (27.7%). Grade ≥ 3 hematologic toxicities included platelet count decreased (42.6%), neutropenia (25.5%), and anemia (8.5%). Olverembatinib-related SAEs included platelet count decreased (6.4%), anemia, myelosuppression, and pyrexia (2.1% each). Most hematologic TRAEs were manageable with supportive care. Two cardiovascular events (grade 1 hypertension) were possibly treatment-related. No deaths occurred.

**Copyright Information:** (Online) ISSN: 2572-9241
©2026 The Author(s). HemaSphere is published by John Wiley & Sons Ltd on behalf of European Hematology Association. This is an open access Abstract Book distributed under the Attribution-NonCommercial-NoDerivs (CC BY-NC-ND), which allows third parties to download the articles and share them with others as long as they credit the author and the Abstract Book, but they cannot change the content in any way or use them commercially.
**Abstract Book Citations:** Authors, Title, HemaSphere, 2026;10:(S1):pages. The abstract book DOIs can be found on the “Author Information” page.
**Disclaimer:** Articles published in the journal HemaSphere exclusively reflect the opinions of the authors. The authors are responsible for all content in their abstracts including accuracy of the facts, statements, citing resources, etc.

**Summary/Conclusion**

Olverembatinib shows good tolerability and leads to high MMR and CCyR in patients with CP-CML without T315I mutation that is resistant/intolerant to first-line TKIs.

**Copyright Information:** (Online) ISSN: 2572-9241
©2026 The Author(s). HemaSphere is published by John Wiley & Sons Ltd on behalf of European Hematology Association. This is an open access Abstract Book distributed under the Attribution-NonCommercial-NoDerivs (CC BY-NC-ND), which allows third parties to download the articles and share them with others as long as they credit the author and the Abstract Book, but they cannot change the content in any way or use them commercially.
**Abstract Book Citations:** Authors, Title, HemaSphere, 2026;10:(S1):pages. The abstract book DOIs can be found on the "Author Information" page.
**Disclaimer:** Articles published in the journal HemaSphere exclusively reflect the opinions of the authors. The authors are responsible for all content in their abstracts including accuracy of the facts, statements, citing resources, etc.

### (PS1734) EARLY MOLECULAR KINETICS PREDICT DEEP MOLECULAR RESPONSE AND INFORM FRONTLINE TKI SELECTION IN PEDIATRIC CHRONIC MYELOID LEUKEMIA: A REAL-WORLD MULTICENTER STUDY

**Topic:** 08. Chronic myeloid leukemia - Clinical

Wenbin An*[1], Xingchen Wang[1], Fenying Zhao[2], Xue Li[3], Lingzhen Wang[4], Ninning Wang[5], Fu Li[6], Futian MA[7], Xiangling He[8], Wenpeng Hao[9], Xue Han[10], Yan Gu[11], Jianping Liu[12], Xiaofan Zhu[1], Wenyu Yang[1]

*[1]State Key Laboratory of Experimental Hematology, National Clinical Research Center for Blood Diseases, Haihe Laboratory of Cell Ecosystem, Institute of Hematology & Blood Diseases Hospital, Chinese Academy of Medical Sciences & Peking Union Medical College, Pediatric Hematology and Oncology, Tianjin, China; [2]Children's Hospital, Zhejiang University School of Medicine, National Clinical Research Center for Children and Adolescents' Health and Diseases,, Pediatric Hematology Oncology, Hangzhou, China; [3]Qilu Hospital of Shandong University, Pediatrics, Jinan,, China; [4]the affiliated hospital of Qingdao University, Qingdao, China; [5]The Second Affiliated Hospital of Anhui Medical University, Pediatric Hematology and Oncology, Hefei, China; [6]Children's Hospital Affiliated to Shandong University, Hematology and Oncology, Jinan, China; [7]Department of Hematology, Hebei Children's Hospital, Hematology, Shijiazhuang, China; [8]Children's Medical Center, Hunan Provincial People's Hospital/First Hospital of Hunan Normal University, Hematology and Oncology, Changsha, China; [9]Hematology and Tumor Research Center of Harbin First Hospital, Harbin, China; [10]Department of Hematology, Kaifeng Children's Hospital, kaifeng, China; [11]Pediatric Hematology Department, The First Affiliated Hospital of Shandong First Medical University (Shandong Qianfoshan Hospital), Jinan, China; [12]Department of Pediatrics, Inner Mongolia Autonomous Region Maternal and Child Health Hospital, Hohhot, China;*

### Background

The prognostic utility of adult-derived scoring systems and early molecular response (EMR, defined as *BCR*::*ABL1* level ≤10% at 3 months) in pediatric chronic myeloid leukemia (CML) requires further validation.

### Aims

This study aimed to evaluate the EUTOS Long-Term Survival (ELTS) score and 3-month molecular response in a real-world pediatric cohort and compare frontline imatinib (IM) versus second-generation tyrosine kinase inhibitors (2G-TKIs).

### Methods

This multicenter, retrospective study analyzed 248 pediatric CML patients in chronic phase from 13 Chinese hospitals. Molecular responses were assessed per ELN recommendations. The majority (89.9%) received first-line imatinib therapy. The impact of the ELTS risk , 3-month *BCR*::*ABL1* level, TKI switching, age, baseline bone marrow fibrosis, and spleen size on outcomes was evaluated using Kaplan-Meier and Cox regression analyses.

**Copyright Information:** (Online) ISSN: 2572-9241
©2026 The Author(s). HemaSphere is published by John Wiley & Sons Ltd on behalf of European Hematology Association. This is an open access Abstract Book distributed under the Attribution-NonCommercial-NoDerivs (CC BY-NC-ND), which allows third parties to download the articles and share them with others as long as they credit the author and the Abstract Book, but they cannot change the content in any way or use them commercially.
**Abstract Book Citations:** Authors, Title, HemaSphere, 2026;10:(S1):pages. The abstract book DOIs can be found on the "Author Information" page.
**Disclaimer:** Articles published in the journal HemaSphere exclusively reflect the opinions of the authors. The authors are responsible for all content in their abstracts including accuracy of the facts, statements, citing resources, etc.

### Results

With a median follow-up of 45.3 months, the 5-year overall and progression-free survival rates were 98.9% and 96.2%, respectively. The EMR group had faster rates of achieving major molecular response (MMR: median time 9 vs. 24 months, p≤0.001) and deep molecular response (DMR: median time 18 vs. 50 months, p≤0.001). Cumulative incidences were significantly higher for the EMR group (12-month MMR: 71.4% vs. 21.2%; HR 2.50; p<0.001; 24-month DMR: 64.7% vs. 27.4%; HR 3.26;p<0.001)(Figure 1). Multivariable analysis confirmed 3-month *BCR*::*ABL1* >10% was independent predictors of longer time to MMR and DMR. Furthermore, the EMR attained group also demonstrated superior 5-year event-free survival (74.5% vs. 49.7%; HR 2.89, p=0.0006) and failure-free survival (73.6% vs. 47.8%; HR 2.92, p=0.004). Of the patients who remained on their front-line therapy, front-line 2G-TKIs (n=18) led to a significantly shorter median time to DMR (median time15 vs. 24 months, p=0.0067) and higher 24-month DMR rate (100% vs. 52.5%, p=0.00666) compared to IM (n=168), though 12-month MMR rates were similar. However, baseline ELTS risk stratification (low- vs. intermediate/high-risk) showed no prognostic value for PFS, EFS and FFS.

### Summary/Conclusion

In pediatric CML, early molecular kinetics at 3 months are strong predictors of long-term outcomes. When the treatment goal is rapid and deep molecular response (e.g., aiming for future TFR), frontline 2G-TKIs may offer a significant benefit over IM. This decision must be balanced against their distinct toxicity profiles and cost. The prognostic value of the ELTS score in pediatric CML needs further assessment.

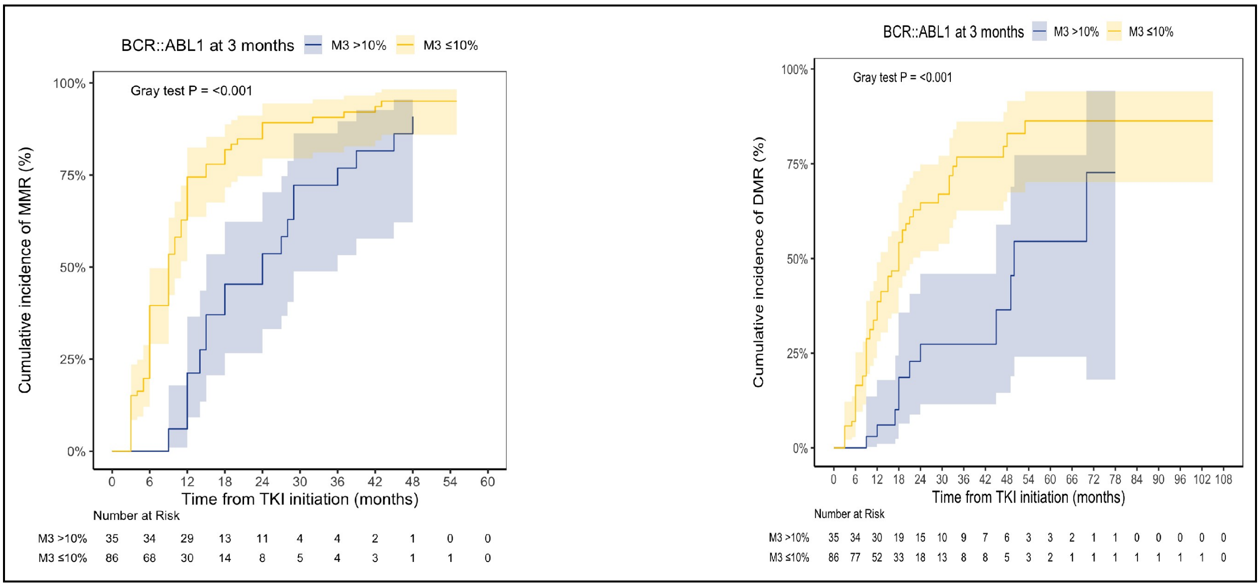

The cumulative incidences of MMR and DMR in the patients with first-line IM treatment. In the EMR group, patients achieved MMR median at 9 months (95% CI, 9–11) and achieved DMR median at 18 months (95% CI, 13–22), which was 24 (15-29) months and 50 (45-NA) months in the patients without EMR, respectively.

**Copyright Information:** (Online) ISSN: 2572-9241
©2026 The Author(s). HemaSphere is published by John Wiley & Sons Ltd on behalf of European Hematology Association. This is an open access Abstract Book distributed under the Attribution-NonCommercial-NoDerivs (CC BY-NC-ND), which allows third parties to download the articles and share them with others as long as they credit the author and the Abstract Book, but they cannot change the content in any way or use them commercially.
**Abstract Book Citations:** Authors, Title, HemaSphere, 2026;10:(S1):pages. The abstract book DOIs can be found on the "Author Information" page.
**Disclaimer:** Articles published in the journal HemaSphere exclusively reflect the opinions of the authors. The authors are responsible for all content in their abstracts including accuracy of the facts, statements, citing resources, etc.

### (PS1735) FRONT-LINE SECOND-GENERATION TKI THERAPY SIGNIFICANTLY IMPROVES THE SUCCESS RATE OF TREATMENT-FREE REMISSION IN CHRONIC MYELOID LEUKEMIA: A MULTICENTER RETROSPECTIVE STUDY OF THE REAL WORLD

**Topic:** 08. Chronic myeloid leukemia - Clinical

Yue Chen[1], Lisi Huang[2], Yanli Zhang[3], Li Zhou[4], Huangling Zhu[5], Yunfan Yang[5], Yu Zhu[6], Na Xu[7], Xin Du[8], Weiming Li[9], Zhenfang Liu[10], Bingcheng Liu[11], Weng Jianyu[2], Qian Jiang*[1]

*[1]Peking University People's Hospital, Beijing, China;[2]Guangdong Provincial People's Hospital, Guangzhou, China;[3]Henan Cancer Hospital, Zhengzhou, China; [4]State Key Laboratory of Medical Genomics, National Research Center for Translational Medicine at Shanghai, Ruijin Hospital Affiliated to Shanghai Jiao Tong University School of Medicine, Shanghai, China; [5]West China Hospital, Sichuan University, Chengdu, China; [6]The First Affiliated Hospital of Nanjing Medical University, Jiangsu Province Hospital, Nanjing, China; [7]Nanfang Hospital, Southern Medical University, Guangzhou, China;[8]The Second People's Hospital of Shenzhen, Shenzhen, China; [9]Union Hospital, Tongji Medical College. Huazhong University of Science and Technology, Wuhan, China; [10]The First Affiliated Hospital of Guangxi Medical University, Guangxi, China;[11]Institute Of Hematology & Blood Diseases Hospital, Chinese Academy Of Medical Sciences & Peking Union Medical College, Tianjin, China;*

### Background

Treatment-free remission (TFR) after tyrosine kinase inhibitor (TKI) discontinuation is an emerging goal in patients with chronic myeloid leukemia (CML). Whether the initial use of a second-generation (2G) TKI can improve the success rate of TFR remains an unanswered question.

### Aims

To identify covariates associated with TFR success following TKI discontinuation.

### Methods

In the multicenter, retrospective, real-word study, data on the outcomes of TKI discontinuation in the adults with chronic-phase CML (CML-CP) who had a imatinib therapy duration ≥5 years or a 2G-TKI [nilotinib, dasatinib, or flumatinib] therapy duration ≥3 years and a sustained deep molecular response (DMR) ≥2 years from 17 Chinese hospitals were analyzed. Cox regression model was utilized to identify covariates associated with loss of MMR. Propensity score matching (PSM) analysis was used to adjust for differences in baseline covariates including gender, age at diagnosis, interval from starting TKI therapy to achieving DMR and DMR duration between initial TKI therapy groups. X-tile plots were constructed to determine the optimal cut-off values of continuous covariates for predicting MMR loss.

**Copyright Information:** (Online) ISSN: 2572-9241
©2026 The Author(s). HemaSphere is published by John Wiley & Sons Ltd on behalf of European Hematology Association. This is an open access Abstract Book distributed under the Attribution-NonCommercial-NoDerivs (CC BY-NC-ND), which allows third parties to download the articles and share them with others as long as they credit the author and the Abstract Book, but they cannot change the content in any way or use them commercially.
**Abstract Book Citations:** Authors, Title, HemaSphere, 2026;10:(S1):pages. The abstract book DOIs can be found on the "Author Information" page.
**Disclaimer:** Articles published in the journal HemaSphere exclusively reflect the opinions of the authors. The authors are responsible for all content in their abstracts including accuracy of the facts, statements, citing resources, etc.

### Results

940 CML-CP patients receiving initial imatinib (n = 740) or 2G-TKIs (n=200) therapy were included in this study. Median TKI treatment duration and DMR duration before TKI discontinuation were 93 months (IQR, 73 - 121 months) and 70 months (IQR, 53 - 95 months), respectively. With a median follow-up of 41 months (IQR, 20 - 65 months) after TKI discontinuation, 261 (28%) patients lost MMR at a median of 4 months (IQR, 3 - 11 months). The 5-year probability of a sustained MMR was 68% (95% confidence interval [CI], [64%, 72%]). In the multivariate analyses the front-line 2G-TKIs (reference, imatinib; HR = 0.6 [0.4, 0.8], $p = 0.002$) and longer DMR duration prior to discontinuation (HR = 0.4 [0.3, 0.6], $p < 0.001$) were significantly-associated with a higher success rate of TFR. After PSM at a 1:1 ratio in patients without TKI switch during TKI therapy, 166 pairs of patients receiving imatinib or a 2G-TKI were included. Patients receiving an initial 2G-TKI had significantly higher rates of 5 years sustained MMR than those receiving initial imatinib (81% versus 65%, $p = 0.004$). By X-tile plots, the optimal cut-off values of DMR duration were 94 months for the imatinib group and 61 months for the 2G-TKI group, respectively. The longer DMR subgroup had higher rates of 5 years sustained MMR than the short DMR subgroup in both imatinib (78% versus 61%, $p < 0.001$) and 2G-TKI (89% versus 69%, $p = 0.038$) groups.

### Summary/Conclusion

The real-world multicenter retrospective study revealed that the front-line treatment with a 2G-TKI improved the success rate of TFR in CML-CP patients beyond achieving a longer DMR.

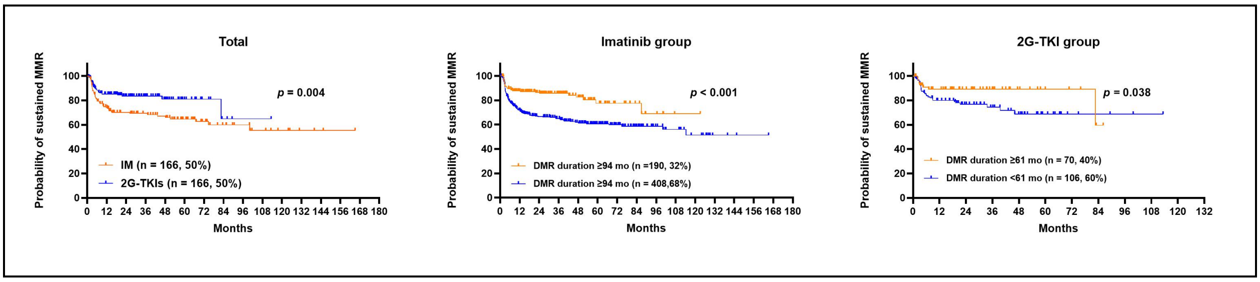

**Copyright Information:** (Online) ISSN: 2572-9241
©2026 The Author(s). HemaSphere is published by John Wiley & Sons Ltd on behalf of European Hematology Association. This is an open access Abstract Book distributed under the Attribution-NonCommercial-NoDerivs (CC BY-NC-ND), which allows third parties to download the articles and share them with others as long as they credit the author and the Abstract Book, but they cannot change the content in any way or use them commercially.
**Abstract Book Citations:** Authors, Title, HemaSphere, 2026;10:(S1):pages. The abstract book DOIs can be found on the "Author Information" page.
**Disclaimer:** Articles published in the journal HemaSphere exclusively reflect the opinions of the authors. The authors are responsible for all content in their abstracts including accuracy of the facts, statements, citing resources, etc.

### (PS1736) AGE-STRATIFIED ANALYSIS OF CML-CP IN ADOLESCENT AND YOUNG ADULT (AYA) PATIENTS: A MULTICENTER RETROSPECTIVE STUDY

**Topic:** 08. Chronic myeloid leukemia - Clinical

Xiaoyan MA[1], Tian Zeng[1], Qinmei Han[1], Zhikang Zheng[1], Rumeng Li[1], Bingbing Wen[1], Jia Li[1], Xiaojing Bao[1], Hongyan Tong[1], Jie Jin[1], Jian Huang*[1]

[1] *The First Affiliated Hospital, Zhejiang University School of Medicine, Hangzhou, China;*

### Background

Chronic myeloid leukemia (CML) is a myeloproliferative neoplasm driven by the BCR-ABL1 fusion gene. Adolescent and young adult (AYA) patients, typically defined as those aged 15–39 years, represent a distinct subgroup within CML who face unique biological characteristics and clinical challenges.  Therefore, age-specific management strategies are warranted in this population.

### Aims

This study aims to systematically compare the differences between AYA and non-AYA patients with CML in terms of baseline characteristics, gene mutation profiles, and responses to TKI therapy, in order to better optimize clinical management strategies for different age-stratified populations.

### Methods

This multicenter retrospective study enrolled CML patients with complete medical records from multiple centers in Zhejiang Province. Patients were stratified by age at diagnosis into AYA and non-AYA groups. Baseline demographic and clinical characteristics were collected and compared. The primary efficacy endpoints were time to major molecular response and overall survival (OS). Molecular responses were assessed according to standardized criteria. Next-generation sequencing was used to analyze the frequency of cancer-related gene (CRG) mutations. The Kaplan-Meier method and Cox regression analysis were employed to evaluate the impact of age and treatment regimens on clinical outcomes.

### Results

A total of 3251 patients with CML were enrolled in this study, including 1010 in the AYA group and 2241 in the non-AYA group. Baseline comparisons showed that the AYA group had higher white blood cell counts P<0.001at diagnosis than the non-AYA group. In contrast, non-AYA patients had a higher proportion of splenomegaly P<0.001 and more severe bone marrow fibrosis P=0.018). Next-generation sequencing analysis performed in 98 patients revealed that AYA patients had a lower frequency of ASXL1 mutations 13.51% vs 22.41%and ABL kinase domain mutations 2.70% vs 10.34% than the non-AYA group. NOTCH2 ( 8.11% vs 0%) and CSMD10% vs 12.07% were identified as relatively specific mutations in the AYA and non-AYA groups respectively. Under first-line TKI therapy, no statistically significant differences were observed between AYA and non-AYA patients in major molecular response rates (including MMR, MR4, and MR4.5) nor in OS. Cox proportional hazards regression analysis demonstrated that first-line treatment with second-generation TKIs significantly shortened the time to MMR compared with first-generation TKIs HR=1.82195% CI 1.535-2.160 P<0.001.And a significant age-treatment interaction was observed HR 1.45495% CI  1.036-2.042P=0.031, indicating that the efficacy advantage of second-generation TKIs was more pronounced in AYA patients. Among second-generation TKIs (dasatinib, nilotinib, and flumatinib), no significant differences were observed in the median time to achieve MMR.

**Copyright Information:** (Online) ISSN: 2572-9241
©2026 The Author(s). HemaSphere is published by John Wiley & Sons Ltd on behalf of European Hematology Association. This is an open access Abstract Book distributed under the Attribution-NonCommercial-NoDerivs (CC BY-NC-ND), which allows third parties to download the articles and share them with others as long as they credit the author and the Abstract Book, but they cannot change the content in any way or use them commercially.
**Abstract Book Citations:** Authors, Title, HemaSphere, 2026;10:(S1):pages. The abstract book DOIs can be found on the “Author Information” page.
**Disclaimer:** Articles published in the journal HemaSphere exclusively reflect the opinions of the authors. The authors are responsible for all content in their abstracts including accuracy of the facts, statements, citing resources, etc.

**Summary/Conclusion**

AYA patients with CML presented with a higher tumor burden at diagnosis, yet achieved rates of major molecular response and OS comparable to non-AYA patients after TKI therapy. The CRG mutation profiles differed between AYA and non-AYA populations. Notably, first-line treatment with second-generation TKIs conferred a greater efficacy advantage in AYA patients with comparable median times to MMR among dasatinib, nilotinib, and flumatinib.
This research was supported by the National Natural Science Foundation of China (No. 82570191) and the Zhejiang Provincial Health Highlevel Innovative Talent Project (2022-2026).

**Copyright Information:** (Online) ISSN: 2572-9241
©2026 The Author(s). HemaSphere is published by John Wiley & Sons Ltd on behalf of European Hematology Association. This is an open access Abstract Book distributed under the Attribution-NonCommercial-NoDerivs (CC BY-NC-ND), which allows third parties to download the articles and share them with others as long as they credit the author and the Abstract Book, but they cannot change the content in any way or use them commercially.
**Abstract Book Citations:** Authors, Title, HemaSphere, 2026;10:(S1):pages. The abstract book DOIs can be found on the "Author Information" page.
**Disclaimer:** Articles published in the journal HemaSphere exclusively reflect the opinions of the authors. The authors are responsible for all content in their abstracts including accuracy of the facts, statements, citing resources, etc.

### (PS1737) HOW MANY CHRONIC-PHASE CML PATIENTS STARTING TYROSINE KINASE INHIBITORS CAN ACHIEVE A PERSISTENT TREATMENT FREE REMISSION AND BE POTENTIALLY CURED? A SINGLE CENTER REAL-LIFE EXPERIENCE.

**Topic:** 08. Chronic myeloid leukemia - Clinical

Mariella D'adda*[1, 2], Valeria Cancelli[1, 2], Giulia Vittoria Facchetti[1, 2], Samantha Ferrari[1, 2], Nicola Bianchetti[1, 2], Chiara Bottelli[1, 2], Riccardo Panebianco[1, 2], Simone Maifredi[1, 2], Ilaria Accarino[1, 2], Alessandra Tucci[1, 2]

*[1]S.C. Ematologia, ASST Spedali Civili, Brescia, Italy; [2]ERN/EuroBloodNet, Brescia, Italy;*

### Background

Treatment free remission (TFR) is currently one of the main goals of chronic myeloid leukemia (CML) therapy and consolidated data of literature show that 40-60% of patients (pts) that discontinue tyrosine kinase inhibitors (TKIs) after a sustained deep molecular response (sDMR) of at least 2 years remain in TFR. Conversely, few data are available about how many pts with newly diagnosed CML starting TKIs will achieve a durable TFR.

### Aims

To evaluate how many chronic-phase chronic myeloid leukemia (CP-CML) pts starting TKIs in our center achieve durable TFR and are potentially cured.

### Methods

We evaluated all consecutive CP-CML pts treated with TKIs in our center in TKIs era, from 2000 to the end of 2025. The main criteria for 1st TKIs discontinuation were at least 5 years of TKIs therapy and at least 2 years of sustained deep molecular response (sDMR). Even for 2$^{nd}$ TKIs discontinuation a sDMR of at least 2 years was required.

**Copyright Information:** (Online) ISSN: 2572-9241
©2026 The Author(s). HemaSphere is published by John Wiley & Sons Ltd on behalf of European Hematology Association. This is an open access Abstract Book distributed under the Attribution-NonCommercial-NoDerivs (CC BY-NC-ND), which allows third parties to download the articles and share them with others as long as they credit the author and the Abstract Book, but they cannot change the content in any way or use them commercially.
**Abstract Book Citations:** Authors, Title, HemaSphere, 2026;10:(S1):pages. The abstract book DOIs can be found on the "Author Information" page.
**Disclaimer:** Articles published in the journal HemaSphere exclusively reflect the opinions of the authors. The authors are responsible for all content in their abstracts including accuracy of the facts, statements, citing resources, etc.

### Results

The total number of CP-CML patients treated with TKIs was 332. Of them 19 have received Interferon before. Sixty-two (19 %) pts died, 4 for progression to blast phase and 58 (whose median age at death was 83 years, range 58-95) for causes not related to CML; 6 pts were lost at follow up; 264 pts are presently in follow up (in TKIs treatment or in TFR). Among the 222 pts with a follow up of 5 years or more, focus of our analysis, 145 (65%) reached TFR criteria and discontinued TKIs (TFR group), while the remaining 77 pts (35%) didn't reach TFR criteria and continued treatment (no-TFR group). The characteristics of the 2 groups are summarized in table 1. In univariate analysis, they showed statistically different features, in detail: pts in TFR group had a longer follow up, better overall survival, a higher percentage of e14a2 transcript type compared to e13a2, a higher percentage of frontline second generation TKIs (2GTKIs). In the no-TFR group more pts needed TKIs beyond 1$^{st}$ line therapy and more pts were treated with reduced TKIs dosage. No patients with follow up $\geq$ 5 years died for progression to blast phase. Between "TFR group" and "no-TFR group" there were no significant differences of age at the moment of CML diagnosis and of the 3 different Sokal risk score category. At the last contact, 99/145 pts (68%) in TFR group remain in TFR, that is 99/222 (45%) of the pts with a follow up of at least 5 years. Fourteen out of 99 pts reached a persistent TFR after a 2$^{nd}$ TKIs discontinuation attempt. In the TFR group, median follow up from CML diagnosis to the last contact was 157 months (64-460), median follow up from the start of TKIs treatment to TKIs discontinuation 100 months (60-266), median follow up from TKIs discontinuation 45 months (4-138). The impact of different variables on the TFR maintenance was analyzed: the duration of TKIs therapy $\geq$ 10 years resulted a statistically significant favorable factor (p 0.05) while only a trend in favor of e14a2 transcript type and full dosage TKIs therapy was observed. None of the pts in TFR group experienced disease progression to blast phase. Pts failing TFR attempt resumed therapy with rapid recovery of an optimal molecular response.

### Summary/Conclusion

According to our experience, more than 40% of CP-CML pts starting TKIs can reach and maintain a durable TFR: therefore, these pts can be considered potentially cured. Frontline 2GTKIs, full dosage TKIs treatment and the presence of the e14a2 transcript type are associated to a higher probability of achieving this goal.

**Copyright Information:** (Online) ISSN: 2572-9241
©2026 The Author(s). HemaSphere is published by John Wiley & Sons Ltd on behalf of European Hematology Association. This is an open access Abstract Book distributed under the Attribution-NonCommercial-NoDerivs (CC BY-NC-ND), which allows third parties to download the articles and share them with others as long as they credit the author and the Abstract Book, but they cannot change the content in any way or use them commercially.
**Abstract Book Citations:** Authors, Title, HemaSphere, 2026;10:(S1):pages. The abstract book DOIs can be found on the "Author Information" page.
**Disclaimer:** Articles published in the journal HemaSphere exclusively reflect the opinions of the authors. The authors are responsible for all content in their abstracts including accuracy of the facts, statements, citing resources, etc.

| | TFR group | no TFR group | p |
|---|---|---|---|
| **Pts, n** | 145 | 77 | |
| **M/F** | 66/79 | 42/35 | 0.2 |
| **Age at CML diagnosis, years: median (range)** | 52 (13-80) | 58 (27-82) | 0.09 |
| **Follow up from CML diagnosis to the last contact, months: median (range)** | 157 (60-460) | 109 (37-322 | 0.00021 |
| **Died pts, n.** | 17 | 20 | 0.0084 |
| **Sokal score risk**<br>• Low, n<br>• Intermediate, n<br>• High, n<br>• Not available, n | <br>50<br>41<br>23<br>31 | <br>16<br>22<br>13<br>26 | <br>0.7 |
| **Type of transcript**<br>• e13a2, n<br>• e14a2, n<br>• e14a2+a13a2, n<br>• Atypical/not available, n | <br>42<br>97<br>2<br>4 | <br>39<br>36<br>1<br>1 | <br>0.0020 |
| **Frontline TKI**<br>• Imatinib, n<br>• 2GTKI, n | <br>93<br>52 | <br>61<br>16 | <br>0.022 |
| **Interferon before TKI, n** | 16 | 3 | 0.081 |
| **Pts treated with ≥2nd line TKI** | 50 | 39 | 0.0021 |
| **TKI dosage** (before TKI discontinuation or at last follow up for pts in no TFR group)<br>• full dosage<br>• reduced dosage | <br>73<br>71 | <br>15<br>62 | <br>0.0001 |

Table 1

**Copyright Information:** (Online) ISSN: 2572-9241
©2026 The Author(s). HemaSphere is published by John Wiley & Sons Ltd on behalf of European Hematology Association. This is an open access Abstract Book distributed under the Attribution-NonCommercial-NoDerivs (CC BY-NC-ND), which allows third parties to download the articles and share them with others as long as they credit the author and the Abstract Book, but they cannot change the content in any way or use them commercially.
**Abstract Book Citations:** Authors, Title, HemaSphere, 2026;10:(S1):pages. The abstract book DOIs can be found on the "Author Information" page.
**Disclaimer:** Articles published in the journal HemaSphere exclusively reflect the opinions of the authors. The authors are responsible for all content in their abstracts including accuracy of the facts, statements, citing resources, etc.

### (PS1738) THERAPEUTIC STRATEGIES IN ADVANCED OR TKI-REFRACTORY CHRONIC MYELOID LEUKEMIA IN THE ERA OF TYROSINE KINASE INHIBITORS: THE ITALIAN EXPERIENCE

**Topic:** 08. Chronic myeloid leukemia - Clinical

Elisa Quinti*[1], Barbara Scappini[2], Francesca Crupi[3], Chiara Lucarelli[2], Elisabetta Abruzzese[4], Malgorzata Monica Trawinska[4], Francesca Lunghi[5], Daniele Sannipoli[6], Alessandra Iurlo[7], Chiara Elena[8], Uros Markovic[9], Davide Rapezzi[10], Isabella Capodanno[11], Carmen Fava[12], Massimiliano Bonifacio[13], Giuseppina Loglisci[14], Sara Galimberti[15], Daniele Cattaneo[16], Simona Bernardi[17], Maria Cristina Miggiano[18], Michele Pizzuti[19], Monica Crugnola[20], Francesco Muriano[21], Gianni Binotto[22], Antonella Vita Russo Rossi[23], Raffaele Palmieri[24], Rosaria Sancetta[25], Antonella Gozzini[26]

*[1]Azienda Ospedaliera Universitaria Senese, Hematology, Siena, Italy; [2]Azienda Ospedaliera Universitaria Careggi, Hematology, Florence, Italy; [3]Azienda Ospedaliera Universitaria Careggi, Florence, Italy; [4]Ospedale S. Eugenio, Tor Vergata University, ASL Roma2, Roma, Italy; [5]Centro Trasfusionale SIMT Policlinico San Matteo di Pavia, Pavia, Italy; [6]IRCCS San Raffaele Scientific Institute, Milan, Italy; [7]Fondazione IRCCS Cá Granda – Ospedale Maggiore Policlinico, Milan, Italy; [8]Foundation IRCCS Policlinico San Matteo, University of Pavia, Pavia, Italy; [9]A.O.U. Policlinico "G.Rodolico-San Marco", Catania, Italy; [10]Azienda Ospedaliera S. Croce e Carle di Cuneo, Cuneo, Italy; [11]Struttura Complessa di Ematologia, Azienda USL – IRCCS di Reggio Emilia, Reggio Emilia, Italy; [12]AO Ordine Mauriziano di Torino, Hematology, Torino, Italy; [13]Department of Engineering for Innovation Medicine, Section of Innovation Biomedicine - University of Verona, Hematology Area, Verona, Italy; [14]Ospedale Vito Fazzi, U.O.C. Ematologia con Trapianto, Lecce, Italy; [15]Department of Clinical and Experimental Medicine, University of Pisa, Hematology Unit, Pisa, Italy; [16]Policlinico Di Milano Ospedale Maggiore Fondazione Irccs Ca' Granda Italy, Milan, Italy; [17]Department of Clinical and Experimental Sciences, University of Brescia, Brescia, Italy; [18]San Bortolo Hospital, Hematology Department, Vicenza, Italy; [19]Ospedale San Carlo, ematologia con trapianto, Potenza, Italy; [20]University Hospital of Parma, Hematology Unit, Parma, Italy; [21]Department of Clinical Medicine and Surgery, Hematology Section, University of Naples, Napoli, Italy; [22]Padua School of Medicine, Department of Medicine Hematology and Clinical Immunology Unit, Padova, Italy; [23]AOU Consorziale Policlinico, Hematology and Stem Cell Transplantation Unit, Bari, Italy; [24]Università degli studi Tor Vergata, Ematologia, Dipartimento di Biomedicina e Prevenzione, Roma, Italy; [25]Dell'Angelo Hospital, ULSS 3, Hematology Unit, Venezia-Mestre, Italy; [26]Azienda Ospedaliera Universitaria Careggi, Transplant unit, Firenze, Italy;*

### Background

Despite major advances achieved with tyrosine kinase inhibitors (TKIs), a subset of patients with chronic myeloid leukemia (CML) remains resistant to all available agents resulting in probable progression to blast phase (BP). A treatment strategy has not yet been standardized for patients presenting with BP-CML and available evidence is largely derived from small and single-center studies. Allogeneic hematopoietic stem cell transplantation (HSCT) remains the only curative option in this setting.

### Aims

To evaluate real-world clinical and therapeutic management strategies for advanced-phase CML in the TKI-era across major Italian hematology centers.

### Methods

The clinical data were collected through a multicenter retrospective survey. The study included new cases of CML in BP (according to the WHO 2022 classification) and patients in chronic phase (CP) resistant to available TKIs.

**Copyright Information:** (Online) ISSN: 2572-9241
©2026 The Author(s). HemaSphere is published by John Wiley & Sons Ltd on behalf of European Hematology Association. This is an open access Abstract Book distributed under the Attribution-NonCommercial-NoDerivs (CC BY-NC-ND), which allows third parties to download the articles and share them with others as long as they credit the author and the Abstract Book, but they cannot change the content in any way or use them commercially.
**Abstract Book Citations:** Authors, Title, HemaSphere, 2026;10:(S1):pages. The abstract book DOIs can be found on the "Author Information" page.
**Disclaimer:** Articles published in the journal HemaSphere exclusively reflect the opinions of the authors. The authors are responsible for all content in their abstracts including accuracy of the facts, statements, citing resources, etc.

**Results**

From January 2000 to June 2025 a total of 58 patients were analyzed, including 24 with BP-CML and 34 CP-CML (Figure 1A). Factors associated with lower overall survival (OS) were age over 60 years and BP disease (median OS of 28 months for age over 60 years compared to 223.4 months for age ≤60 years, $p<0.001$; median OS of 209.1 months for CP compared with 80.4 months for BP, $p=0.017$; Figure 1B-C). During follow-up, 19% of patients with CP-CML progressed to BP, a higher incidence than expected in the TKI era, likely reflecting limited access to TKIs at diagnosis and unfavorable biological characteristics of the disease.
From comparative analyses between CP-CML and BP-CML at diagnosis, we observed a significantly longer median time from diagnosis to HSCT and a mayor number of treatment lines prior to transplantation in CP-CML (21.2 months vs. 7 months, $p<0.001$; 3 treatment lines in CP-CML vs. 2 lines in BP-CML, $p=0.027$). The proportion of patients undergoing HSCT did not significantly differ between CP- and BP-CML (82.4% vs. 70.8%).
Although information on genetic mutations, including BCR::ABL1 T315I, was available for only a minority of patients, it was comparable between groups, with no statistically significant differences. However, all cases with ASXL1 mutations were diagnosed in CP-CML.
Regarding bridging strategies to HSCT, achievement of major molecular response (MMR) prior to transplantation did not appear to be mandatory. In CP-CML, patients treated with TKIs alone had significantly superior OS compared with those receiving TKI plus chemotherapy (median OS 221 vs. 45.3 months, respectively, $p<0.001$). In contrast, among patients with BP-CML at diagnosis, OS was comparable between those treated with TKIs alone and those receiving combined TKI and chemotherapy (median OS 81 vs. 71 months, respectively, $p=0.916$; figure 1D), suggesting no clear survival benefit from chemotherapy in this setting. In both CP- and BP-CML patients undergoing HSCT, post-transplant TKI maintenance was started in patients with relapse or residual disease positivity, with a trend toward poorer outcomes, likely due to underlying resistance to TKIs, higher-risk disease profiles, and limited sample size.

**Summary/Conclusion**

This multicenter real-world study provides an overview of therapeutic strategies currently adopted in Italy for advanced-phase of CML over the past 25 years. While highlighting progress achieved in the TKI era, our findings underscore persistent unmet clinical needs and identify key areas for optimization in the future management of advanced and TKI-refractory CML.

**Copyright Information:** (Online) ISSN: 2572-9241
©2026 The Author(s). HemaSphere is published by John Wiley & Sons Ltd on behalf of European Hematology Association. This is an open access Abstract Book distributed under the Attribution-NonCommercial-NoDerivs (CC BY-NC-ND), which allows third parties to download the articles and share them with others as long as they credit the author and the Abstract Book, but they cannot change the content in any way or use them commercially.
**Abstract Book Citations:** Authors, Title, HemaSphere, 2026;10:(S1):pages. The abstract book DOIs can be found on the "Author Information" page.
**Disclaimer:** Articles published in the journal HemaSphere exclusively reflect the opinions of the authors. The authors are responsible for all content in their abstracts including accuracy of the facts, statements, citing resources, etc.

| Patients | Chronic Phase 34 | | Blast phase 24 | | p |
|---|---|---|---|---|---|
| Age | 48 y | 23-74 | 44 y | 26-85 | 0.943 |
| Age >60y | 3/34 | 8.8% | 6/24 | 25% | 0.142 |
| Mutation BCR::ABL | 10/34 | 29.4% | 4/24 | 16.6% | |
| T315I | 6/34 | 17.6% | 6/24 | 25% | 0.498 |
| ASXL1 | 5/34 | 14.7% | 0/24 | 0% | 0.070 |
| Miscellaneous | 15/34 | 44.1% | 6/24 | 25% | 0.171 |
| N. lines pre-HSCT (median) | 3 | 1-3 | 2 | 1-3 | **0.027** |
| Median time from diagnosis to HSCT | 21.2 | 15.1-43.2 | 7 | 5.9-16.8 | **0.000** |
| HSCT | 28/34 | 82.4% | 17/24 | 70.8% | 0.304 |
| Response pre HSCT | | | | | |
| Not response | 6/28 | 21.4% | 0/17 | 0% | |
| CHR | 9/28 | 32.1% | 3/17 | 17.6% | **0.030** |
| CCyR | 9/28 | 32.1% | 6/17 | 35.3% | |
| MMR | 4/28 | 14.3% | 8/17 | 47.1% | |
| Only 1 TKI pre-HSCT | 3/34 | 8.8% | 5/24 | 20.8% | |
| >1 TKI pre-HSCT | 26/34 | 76.5% | 9/24 | 37.5% | **0.010** |
| TKI+CHT pre-HSCT | 5/34 | 14.7% | 10/24 | 41.7% | |
| MMR post TKI+CHT | 1/4 | 25% | 6/8 | 75% | 0.222 |
| MMR in only TKI | 3/4 | 75% | 2/8 | 25% | |
| TKI post HSCT | 11/28 | 39.3% | 11/17 | 65% | 0.130 |
| Relapse | 4/11 | 36.4% | 5/11 | 45.5% | |
| Preemptive | 4/11 | 36.4% | 2/11 | 18.2% | 0.750 |
| Prophylaxis | 3/11 | 27.3% | 4/11 | 36.4% | |

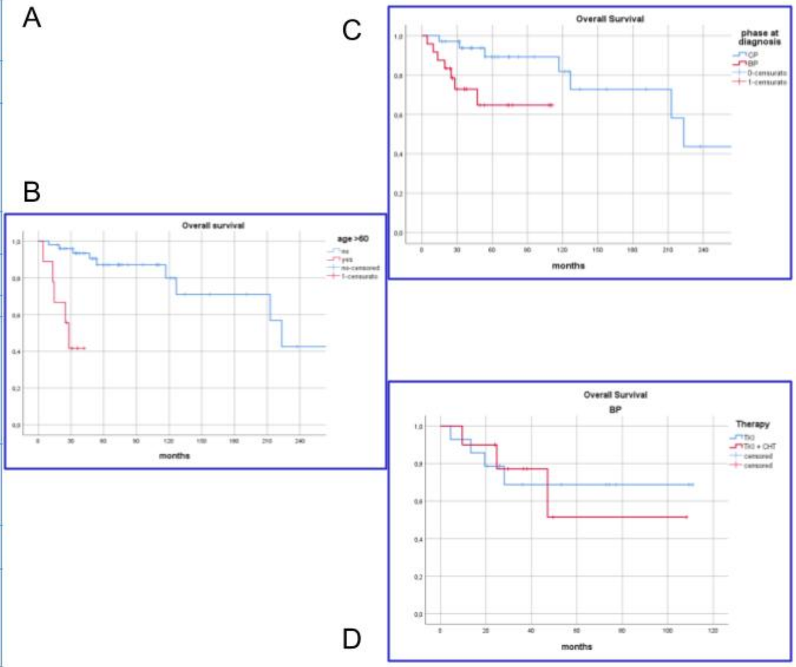

**Copyright Information:** (Online) ISSN: 2572-9241
©2026 The Author(s). HemaSphere is published by John Wiley & Sons Ltd on behalf of European Hematology Association. This is an open access Abstract Book distributed under the Attribution-NonCommercial-NoDerivs (CC BY-NC-ND), which allows third parties to download the articles and share them with others as long as they credit the author and the Abstract Book, but they cannot change the content in any way or use them commercially.
**Abstract Book Citations:** Authors, Title, HemaSphere, 2026;10:(S1):pages. The abstract book DOIs can be found on the "Author Information" page.
**Disclaimer:** Articles published in the journal HemaSphere exclusively reflect the opinions of the authors. The authors are responsible for all content in their abstracts including accuracy of the facts, statements, citing resources, etc.

### (PS1739) MRD-TAILORED TREATMENT WITH DECITABINE COMBINED WITH DASATINIB IN MYELOID BLAST PHASE CHRONIC MYELOID LEUKEMIA INELIGIBLE FOR INTENSIVE INDUCTION THERAPY: REAL-WORLD DATA IN RESOURCE-LIMITED SETTING

**Topic:** 08. Chronic myeloid leukemia - Clinical

Paras Satadeve[1], Adamya Gupta[1], Reshma Benson[1], Arjun Kachhwaha[1], Bibhant Shah[1], Prisla Dalton[1], Kavya Ronanki[1], Nikhil Nagpal[1], Karthik Kumar[1], Uttam Kumar Nath*[1]

*[1]All India Institute of Medical Sciences, Rishikesh, Department of Medical Oncology Hematology, Rishikesh, India;*

### Background

Treatment of myeloid blast phase chronic myeloid leukemia (CML) consists of tyrosine kinase inhibitor (TKI) plus intensive induction chemotherapy, followed by allogeneic hematopoietic cell transplantation (HCT). Patients ineligible for intensive induction and treated with single-agent TKI have poor survival outcomes.

### Aims

To determine efficacy & safety of decitabine with dasatinib in myeloid blast phase CML patients ineligible/unfit for intensive induction chemotherapy.

### Methods

Patients with myeloid blast phase CML who were either unfit for intensive induction chemotherapy or ineligible due to financial constraints were treated with dasatinib & decitabine induction therapy. The dose of decitabine was 20 mg/m² daily for 5 days per 28 days' cycle, administered by one hour' intravenous infusion. The starting dose of dasatinib was 140 mg daily, which was reduced to 100 mg for grade 4 neutropenia/ thrombocytopenia, together with prophylactic filgrastim & romiplostim. Dasatinib dose was also reduced for any grade 3-4 non-hematological toxicity, and temporarily withheld during febrile neutropenia. Bone marrow morphological & flowcytometric minimal residual disease (FCM-MRD) response were assessed after 1$^{st}$ cycle and repeated after 2$^{nd}$ cycle if morphological complete response (CR) was not achieved after 1$^{st}$ cycle. Patients who achieved CR were considered for post-remission therapy with high-dose cytarabine (HiDAC) followed by HCT, if feasible. Patients who were ineligible for intensive post-remission chemotherapy or HCT, either due to age/comorbidities or due to financial constrains were continued on decitabine plus dasatinib therapy, with monitoring of molecular response.

**Copyright Information:** (Online) ISSN: 2572-9241
©2026 The Author(s). HemaSphere is published by John Wiley & Sons Ltd on behalf of European Hematology Association. This is an open access Abstract Book distributed under the Attribution-NonCommercial-NoDerivs (CC BY-NC-ND), which allows third parties to download the articles and share them with others as long as they credit the author and the Abstract Book, but they cannot change the content in any way or use them commercially.
**Abstract Book Citations:** Authors, Title, HemaSphere, 2026;10:(S1):pages. The abstract book DOIs can be found on the "Author Information" page.
**Disclaimer:** Articles published in the journal HemaSphere exclusively reflect the opinions of the authors. The authors are responsible for all content in their abstracts including accuracy of the facts, statements, citing resources, etc.

**Results**

Data of 16 patients was available for analysis. Median age of patients was 39 (IQR 35-49.5, range 25-64) years, with male: female ratio of 1.3:1. Median number of decitabine cycles received was 4 (IQR 4-6.5, range 2-34).Fourteen patients (87.5%) achieved morphological complete response (CR), 12/16 patients (75%) achieved negative FCM-MRD, 11/16 (69%) achieved major molecular response (MMR), and 6/16 (37.5%) achieved complete molecular response (CMR). Four patients received HiDAC, but none had allogeneic HCT due to financial constraints. Median overall survival (OS) was 16 months (IQR 11.5-27, range 6-40), with significantly higher OS among patients who achieved negative FCM-MRD compared to non-responders (not reached vs 10 months; $P < 0.05$). F317L mutation was detected in two patients who did not achieve CR. Only one of 14 patients who achieved CR, had very early CNS relapse after 5 months and was detected to have T315I mutation despite being FCM-MRD negative. Another patient had loss of MMR due to T315I mutation while on maintenance dasatinib but retained CR & negative flow-MRD status and was switched to ponatinib. Grade 3-4 hematological AEs during first cycle decitabine+dasatinib induction were thrombocytopenia (50%), neutropenia (38%), anemia (38%), & febrile neutropenia (31%). Commonest non-hematological AEs were diarrhea and nausea/vomiting. Three patients required dasatinib dose reduction/interruption in $1^{st}$ cycle due to febrile neutropenia, and one patient required dasatinib interruption due to grade 3 pleural effusion.

**Summary/Conclusion**

Decitabine plus dasatinib was highly effective and safe induction regimen in myeloid blast phase CML patients ineligible/unfit for intensive induction chemotherapy, and was associated with excellent CR, MRD negativity & long-term survival in resource-limited settings.

**Copyright Information:** (Online) ISSN: 2572-9241
©2026 The Author(s). HemaSphere is published by John Wiley & Sons Ltd on behalf of European Hematology Association. This is an open access Abstract Book distributed under the Attribution-NonCommercial-NoDerivs (CC BY-NC-ND), which allows third parties to download the articles and share them with others as long as they credit the author and the Abstract Book, but they cannot change the content in any way or use them commercially.
**Abstract Book Citations:** Authors, Title, HemaSphere, 2026;10:(S1):pages. The abstract book DOIs can be found on the "Author Information" page.
**Disclaimer:** Articles published in the journal HemaSphere exclusively reflect the opinions of the authors. The authors are responsible for all content in their abstracts including accuracy of the facts, statements, citing resources, etc.

### (PS1740) LUSPATERCEPT REDUCES CARDIAC STRESS AND IMPROVES CARDIAC FUNCTION IN TET2-DEFICIENT HFPEF-LIKE MOUSE MODEL: TRANSLATIONAL EVIDENCE FROM PRECLINICAL STUDIES AND PHASE 3 COMMANDS TRIAL

**Topic:** 09. Myelodysplastic syndromes - Biology & translational research

Manuel Ugidos Guerrero[1], Sarita Nehra[2], Manoj Dokania[2], Gobinathan Rengarasu[2], Guillermo Garcia-Manero*[3], Uwe Platzbecker[4], Anita K. Gandhi[1], Ching-Pin Chang[1], Ricardo Garcia[5], Jyoti Gulia[2], Rajasekhar N.V.S. Suragani[1], Maroof Hasan[1]

*[1]Bristol Myers Squibb, Princeton, NJ, United States of America;[2]Bristol Myers Squibb, Mumbai, India;[3]University of Texas MD Anderson Cancer Center, Houston, TX, United States of America;[4]University Hospital Leipzig, Leipzig, Germany;[5]GeneToBe, Ann Arbor, MI, United States of America;*

### Background

Luspatercept (inhibitor of activin receptor–Smad2/3 signaling) is approved as first-line treatment of anemia in adult patients with lower-risk myelodysplastic syndromes (LR-MDS) based on the COMMANDS trial (NCT03682536). Most frequent MDS-related somatic mutations (e.g., *SF3B1, TET2, DNMT3A, ASXL1*) can lead to dysregulated immune signaling which may drive both hematological malignancies and cardiovascular complications. Recent evidence also indicates a higher prevalence of *TET2* and *SF3B1* mutations in patients with heart failure with preserved ejection fraction (HFpEF). TET2 loss in murine studies can induce anemia and drive HFpEF pathogenesis with enhanced pro-inflammatory signaling. Despite the heightened cardiovascular risk in MDS, no therapies currently target the underlying mechanistic drivers linked to cardiac dysfunction.

### Aims

To integrate clinical observations with a preclinical model exhibiting HFpEF-like features to identify potential novel mechanisms of luspatercept that may modulate cellular signaling pathways and have impact on cardiac function.

### Methods

In the COMMANDS trial, the primary endpoint was red blood cell-transfusion independence (RBC-TI) for ≥12 weeks with concurrent mean hemoglobin increase ≥1.5 g/dL (Weeks 1–24). Baseline and Week 24 serum and bone marrow were used for transcriptomics/proteomics analyses. Baseline bone marrow mononuclear cell DNA (n=350) underwent targeted next-generation sequencing of 82 myeloid genes (NovaSeq 6000, Illumina). Multivariable Cox regression analyses were performed for survival outcomes. The murine analogue of luspatercept (RAP-536) was tested in an experimental HFpEF model in wild-type and TET2-knockout C57BL/6 mice. Echocardiographic assessment was done for cardiac hypertrophy and function, and blood parameters were measured after 4 weeks of RAP-536 treatment.

**Copyright Information:** (Online) ISSN: 2572-9241
©2026 The Author(s). HemaSphere is published by John Wiley & Sons Ltd on behalf of European Hematology Association. This is an open access Abstract Book distributed under the Attribution-NonCommercial-NoDerivs (CC BY-NC-ND), which allows third parties to download the articles and share them with others as long as they credit the author and the Abstract Book, but they cannot change the content in any way or use them commercially.
**Abstract Book Citations:** Authors, Title, HemaSphere, 2026;10:(S1):pages. The abstract book DOIs can be found on the "Author Information" page.
**Disclaimer:** Articles published in the journal HemaSphere exclusively reflect the opinions of the authors. The authors are responsible for all content in their abstracts including accuracy of the facts, statements, citing resources, etc.

### Results

At baseline, 87% (306/350) of patients had ≥1 of the most frequent MDS-related mutations with median variant allele frequency ranging from 3%–50%. Patients with common MDS-related mutations had higher cardiac stress marker, NT-proBNP levels (median 2200 pg/mL vs 1725 pg/mL). Cardiac disorders reported as adverse events and/or cause of death occurred in 20% of patients, of which 89% were predominantly associated with common MDS-related mutations. Among responders, luspatercept reduced inflammatory signaling and NT-proBNP levels (Δ median −620 pg/mL; $P$=0.002) and lowered hepcidin levels (Δ median −44 ng/mL; $P$≤0.0001). In multivariable Cox models, elevated NT-proBNP and hepcidin were associated with higher mortality risk (HR 1.83, $P$=0.003; HR 2.13, $P$≤0.001); luspatercept reduced this risk (HR 0.78, $P$=0.2), likely via reductions in NT-proBNP, hepcidin, and inflammatory signaling. In a TET2-deficient mice with features of HFpEF and anemia, RAP-536 significantly reduced cardiac hypertrophy ($P$<0.01), improved diastolic dysfunction ($P$≤0.0001), and improved anemia ($P$<0.0001).

### Summary/Conclusion

Our integrated translational approach demonstrates the potential beneficial effects of luspatercept in patients with anemia and HFpEF harboring frequent MDS-related somatic mutations. These findings reveal a previously unrecognized, cardioprotective action of luspatercept, highlighting its potential to address the substantial cardiovascular morbidity in LR-MDS and establishing a new paradigm for therapeutic benefit beyond hematologic improvement.

**Copyright Information:** (Online) ISSN: 2572-9241
©2026 The Author(s). HemaSphere is published by John Wiley & Sons Ltd on behalf of European Hematology Association. This is an open access Abstract Book distributed under the Attribution-NonCommercial-NoDerivs (CC BY-NC-ND), which allows third parties to download the articles and share them with others as long as they credit the author and the Abstract Book, but they cannot change the content in any way or use them commercially.
**Abstract Book Citations:** Authors, Title, HemaSphere, 2026;10:(S1):pages. The abstract book DOIs can be found on the “Author Information” page.
**Disclaimer:** Articles published in the journal HemaSphere exclusively reflect the opinions of the authors. The authors are responsible for all content in their abstracts including accuracy of the facts, statements, citing resources, etc.

### (PS1741) DISTINCT IMMUNE AND TRANSCRIPTOMIC PROGRAMS UNDERLIE ESA RESPONSE ACCORDING TO TIMING OF INITIATION IN LOWER-RISK MDS: CORRELATIVE BIOLOGICAL RESULTS OF THE EPO-PRETAR TRIAL

**Topic:** 09. Myelodysplastic syndromes - Biology & translational research

Sophie Park*[1], David Rombaut[2], Yannick Simoni[2], Borhane Slama[3], Marius Moldovan[4], Chloe Friedrich[2], Ismael Boussaid[2], Aurore Tourville[2], Mathieu Meunier[1], Aspasia Stamatoullas Bastard[5], Maud D Aveni[6], Stephane Cheze[7], Sylvan Thepot[8], Miguel Torregrosa-Diaz[9], Sophie Dimicoli Salazar[10], Kamel Laribi[11], Reza Tabrizi[12], Anne Banos[13], Alice Garnier[14], Celia Salanoubat[15], Laurent Pascal[16], Clemence Santana[17], Ali Arar[18], Gaelle Fossard[19], Adrien Contejean[20], Ramdane Tekrane[21], Fatiha Chermat[21], Pierre Fenaux[21], Oliver Kosmider[22], Michaela Fontenay[22]

*[1]Université Grenoble Alpes, CHU Grenoble Alpes, INSERM U1209, CNRS 5309, Hematology, GRENOBLE, France;[2]Université Paris Cité, Institut Cochin, INSERM U1016, CNRSUMR8104; Assistance Publique-Hôpitaux de Paris Centre, Hôpital Cochin, Paris, Laboratory of Hematology, Paris, France; [3]CH Avignon, Hematology, Avignon, France; [4]CH Perigueux, Hematology, Perigueux, France; [5]Unicancer, Hematology, Rouen, France; [6]CHU Nancy, Hematology, Nancy, France;[7]Institut Basse Normandie, Hematology, Caen, France; [8]CHU Angers, Hematology, Angers, France;[9]CHU Poitiers, Hematology, Poitiers, France; [10]Hopital De Haut Leveque-hematologie Clinique, Hematology, Bordeaux, France; [11]Centre Hospitalier du Mans, Hematology, Le Mans, France; [12]Centre Hospitalier de Mont de Marsan, Hematology, Le Mont de Marsan, France;[13]C.H. Côte Basque, Hematology, Bayonne, France; [14]Hôpital Hôtel-Dieu, Hematology, Nantes, France; [15]Sud Francilien Hospital, Hematology, Corbeil Essones, France; [16]Groupement des Hôpitaux de l'Institut Catholique de Lille - Hôpital Saint Vincent de Paul, Hematology, Lille, France; [17]Gh De Valence, Valence, France; [18]CH Orleans, Hematology, Orleans, France; [19]Lyon University Hospital, Lyon, France, Hematology, Lyon, France; [20]CH Annecy, Hematology, Annecy, France; [21]Service d'Hématologie Séniors, Hôpital Saint-Louis, Université Paris 7, Hematology, Paris, France; [22]Assistance Publique-Hôpitaux de Paris. Centre-Université Paris Cité, Service d'hématologie biologique, Hôpital Cochin, Laboratory of Hematology, Paris, France;*

### Background

Beyond clonal hematopoiesis, myelodysplastic syndromes (MDS) arise within a chronically dysregulated immune microenvironment that shapes hematopoietic failure. While predictors of erythropoiesis-stimulating agent (ESA) response have mainly focused on erythropoietic and iron metabolism pathways (Park S, Hematologica 2019), increasing evidence suggests that adaptive and inflammatory immune signals influence MDS bone marrow niche and evolution (Prummel KD, Nat Com 2025; Chen L,Nat Com 2025). The timing of ESA introduction may therefore interact with distinct immune landscapes, yet the biological consequences of early versus delayed initiation remain poorly understood. This is the first randomized study linking ESA timing and response to distinct immune trajectories and transfusion dependence in LR-MDS.

### Aims

To characterize immune cytokine patterns and bone marrow transcriptomic programs associated with early or delayed ESA initiation in lower-risk MDS (LR-MDS) and to identify immune determinants of response and transfusion dependence.

### Methods

The phase III EPO-PRETAR trial randomized 84 non–transfusion-dependent LR-MDS patients to early (arm A) or delayed (arm B) epoetin-alfa treatment. Peripheral cytokines were quantified at baseline and longitudinally. Bone marrow mononuclear cell comprehensive RNA-sequencing was performed in 44 baseline samples. Differential expression (DESeq2, $|\log_2 FC| \geq 1$, adj. $p<0.05$) and Reactome pathway enrichment were analyzed according to ESA response, treatment arm, and transfusion status.

**Copyright Information:** (Online) ISSN: 2572-9241
©2026 The Author(s). HemaSphere is published by John Wiley & Sons Ltd on behalf of European Hematology Association. This is an open access Abstract Book distributed under the Attribution-NonCommercial-NoDerivs (CC BY-NC-ND), which allows third parties to download the articles and share them with others as long as they credit the author and the Abstract Book, but they cannot change the content in any way or use them commercially.
**Abstract Book Citations:** Authors, Title, HemaSphere, 2026;10:(S1):pages. The abstract book DOIs can be found on the "Author Information" page.
**Disclaimer:** Articles published in the journal HemaSphere exclusively reflect the opinions of the authors. The authors are responsible for all content in their abstracts including accuracy of the facts, statements, citing resources, etc.

**Results**

Baseline clinical characteristics, mutation profiles, monocyte counts, CRP, and cytokine levels were comparable between arms. However, transcriptomic profiling revealed a distinct immune activation pattern in armA, enriched for IL-10, IFNγ, and T-cell receptor signaling pathways, together with relative depletion of DNA replication, repair, cell cycle checkpoints and ribosome-biogenesis programs—consistent with T-cell–driven proliferation stress. Within armA, responders displayed an adaptive immune orientation marked by increased IFNγ and IL-4 levels and enrichment of IFNα/β, IFNγ, IL-4, and IL-12 pathways, with upregulation of CD8A, ZAP70, IFNG, PD-L1, and epigenetic chromatin regulatory genes. In armB, responders demonstrated preserved proliferative and stress-response pathways, whereas non-responders showed inflammatory activation with elevated IL-6 and IL-10 and enrichment of IL-6, IL-10, TGFβ, and NFκB signaling.

Longitudinally, early-arm non-transfused patients sustained adaptive immune signatures and high IL-4, while late-arm non-responders maintained IL-6/IL-10 elevation up to 24months. Across both arms, baseline high IL-6 combined with low IL-4 predicted transfusion dependence. Integrated analyses showed that early-arm, non-transfused responders activated immune-response and chromatin-modifying programs, whereas delayed-arm responders retained proliferation/DNA-repair signatures with reduced inflammatory signaling.

**Summary/Conclusion**

The timing of ESA exposure delineates divergent immune trajectories in LR-MDS. Early initiation occurs within a T-cell–activated niche where responders engage adaptive rather than inflammatory immunity, while delayed initiation associates with sustained inflammatory reprogramming. Overall, inflammation appears to drive transfusion requirement. These data provide a biological rationale to prioritize early ESA initiation in LR-MDS patients and to explore immune-modulating combinations in late inflammatory disease stages.

**Copyright Information:** (Online) ISSN: 2572-9241
©2026 The Author(s). HemaSphere is published by John Wiley & Sons Ltd on behalf of European Hematology Association. This is an open access Abstract Book distributed under the Attribution-NonCommercial-NoDerivs (CC BY-NC-ND), which allows third parties to download the articles and share them with others as long as they credit the author and the Abstract Book, but they cannot change the content in any way or use them commercially.
**Abstract Book Citations:** Authors, Title, HemaSphere, 2026;10:(S1):pages. The abstract book DOIs can be found on the "Author Information" page.
**Disclaimer:** Articles published in the journal HemaSphere exclusively reflect the opinions of the authors. The authors are responsible for all content in their abstracts including accuracy of the facts, statements, citing resources, etc.

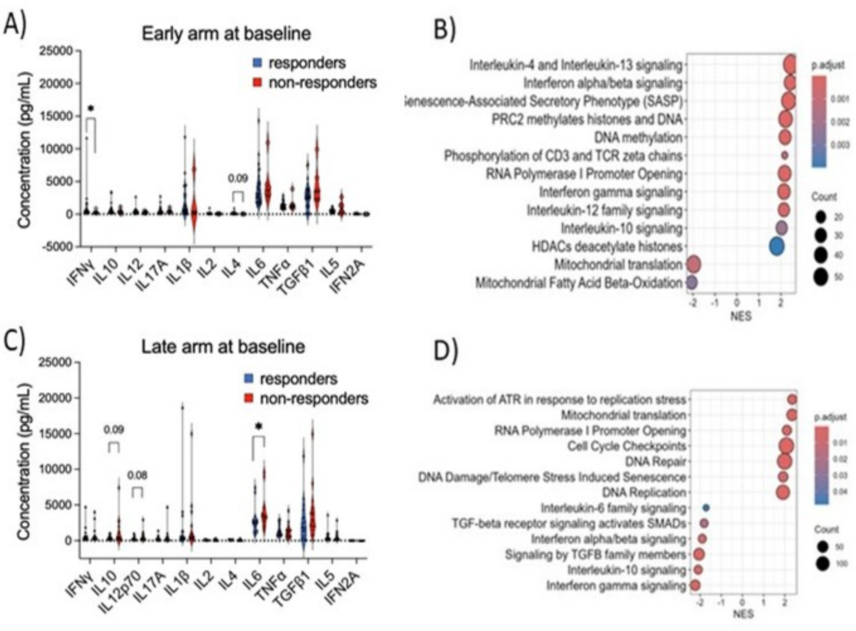

A. Cytokine multiplex profiling according to the response to ESA Baseline profile according to response in the early arm
B. Enrichment analysis of Reactome pathways. Pathways enriched in the comparison of responders to non-responders of the early arm with adjusted P-values ordered by normalized enrichment scores or NES.
C. Cytokine multiplex profiling according to the response to ESA in the late arm
D. Enrichment analysis of Reactome pathways. Pathways enriched in the comparison of responders to non-responders of the late arm with adjusted P-values ordered by normalized enrichment scores or NES.

**Copyright Information:** (Online) ISSN: 2572-9241
©2026 The Author(s). HemaSphere is published by John Wiley & Sons Ltd on behalf of European Hematology Association. This is an open access Abstract Book distributed under the Attribution-NonCommercial-NoDerivs (CC BY-NC-ND), which allows third parties to download the articles and share them with others as long as they credit the author and the Abstract Book, but they cannot change the content in any way or use them commercially.
**Abstract Book Citations:** Authors, Title, HemaSphere, 2026;10:(S1):pages. The abstract book DOIs can be found on the "Author Information" page.
**Disclaimer:** Articles published in the journal HemaSphere exclusively reflect the opinions of the authors. The authors are responsible for all content in their abstracts including accuracy of the facts, statements, citing resources, etc.

### (PS1742) ORAL HYPOMETHYLATING AGENT NTX-301 DEMONSTRATES POTENT ANTI-LEUKEMIC ACTIVITY AND ENHANCES RESPONSE TO VENETOCLAX IN CHRONIC MYELOMONOCYTIC LEUKEMIA (CMML)

**Topic:** 09. Myelodysplastic syndromes - Biology & translational research

Arnold Kloos*[1], Nadine Kattre[2], Isabell Arnhardt[2], Jyoti Kandarp[2], Michael Heuser[1]

*[1]University Hospital Halle, Department of Internal Medicine IV, Halle, Germany;[2]Hannover Medical School, Hematology, Hemostasis, Oncology, and Stem Cell Transplantation, Hannover, Germany;*

### Background

Chronic myelomonocytic leukemia (CMML) is a clonal hematologic malignancy characterized by limited treatment options and suboptimal responses to currently available therapies, including hypomethylating agents and cytoreductive drugs. Novel therapeutic approaches are needed to improve disease control and reduce leukemic burden. NTX-301 is a novel oral hypomethylating agent with potential anti-leukemic activity, but its efficacy in CMML has not been characterized.

### Aims

To evaluate the therapeutic activity of NTX-301 in primary CMML samples and in vivo CMML patient-derived xenograft (PDX) models, and to assess potential synergistic effects in combined drug therapy.

### Methods

Primary CMML cells were cultured in cytokine-supplemented suspension assays and treated with NTX-301 alone or in comparison with clinically relevant agents including venetoclax, trametinib, decitabine, azacitidine, and hydroxyurea. Combination experiments were performed with NTX-301 and venetoclax. Cell viability, apoptosis, and differentiation were assessed by flow cytometry. $IC_{50}$ values were calculated, and colony-forming cell (CFC) assays evaluated progenitor activity.
In vivo efficacy was investigated in two CMML-PDX models (Kloos et al. Leukemia 2020). Mice received vehicle, NTX-301, venetoclax, azacitidine, or NTX-301 plus venetoclax. Leukemic engraftment was monitored by serial measurement of human $CD45^+$ cells in peripheral blood and hematopoietic tissues. Survival, hematologic parameters, and differentiation marker expression were analyzed.

### Results

NTX-301 inhibited primary CMML cell viability with $IC_{50}$ values in the nanomolar to low micromolar range (23 nM to 1497 nM) and demonstrated greater potency than decitabine, venetoclax, azacitidine, trametinib and hydroxyurea, tested in parallel in 14 patient samples. Combination treatment with venetoclax resulted in synergistic effects in multiple patient samples, reflected by reduced $IC_{50}$ values and enhanced inhibition in dose–response analyses.
NTX-301 induced apoptosis and promoted partial myeloid differentiation, as indicated by increased CD14 expression, while CD15 expression remained largely unchanged. In colony-forming cell (CFC) assays, NTX-301 significantly reduced colony formation across three independent primary CMML samples. In one CMML sample, combination treatment with venetoclax or trametinib further reduced progenitor growth compared to NTX-301 alone, whereas venetoclax or trametinib monotherapy showed no relevant effect.
In both CMML-PDX models, NTX-301 significantly reduced leukemic engraftment and prolonged survival compared with vehicle controls (median survival 118 vs. 241 days in model 1 and 122.5 vs. 216 days in model 2, vehicle vs NTX-301, respectively).
Combination therapy with venetoclax enhanced inhibition of leukemic burden in peripheral blood, spleen and bone marrow, without further prolongation of overall survival compared with NTX-301 or azacitidine monotherapy or azacitidine plus venetoclax combination therapy. Increased CD14 expression during therapy suggested induction of differentiation in vivo.

**Copyright Information:** (Online) ISSN: 2572-9241
©2026 The Author(s). HemaSphere is published by John Wiley & Sons Ltd on behalf of European Hematology Association. This is an open access Abstract Book distributed under the Attribution-NonCommercial-NoDerivs (CC BY-NC-ND), which allows third parties to download the articles and share them with others as long as they credit the author and the Abstract Book, but they cannot change the content in any way or use them commercially.
**Abstract Book Citations:** Authors, Title, HemaSphere, 2026;10:(S1):pages. The abstract book DOIs can be found on the “Author Information” page.
**Disclaimer:** Articles published in the journal HemaSphere exclusively reflect the opinions of the authors. The authors are responsible for all content in their abstracts including accuracy of the facts, statements, citing resources, etc.

**Summary/Conclusion**

NTX-301 demonstrates potent anti-leukemic activity in primary CMML cells and robust efficacy in two CMML-PDX models. The observed synergy with venetoclax and the consistent reduction of leukemic burden support further clinical development of NTX-301 as a promising therapeutic strategy for CMML.

**Copyright Information:** (Online) ISSN: 2572-9241
©2026 The Author(s). HemaSphere is published by John Wiley & Sons Ltd on behalf of European Hematology Association. This is an open access Abstract Book distributed under the Attribution-NonCommercial-NoDerivs (CC BY-NC-ND), which allows third parties to download the articles and share them with others as long as they credit the author and the Abstract Book, but they cannot change the content in any way or use them commercially.
**Abstract Book Citations:** Authors, Title, HemaSphere, 2026;10:(S1):pages. The abstract book DOIs can be found on the "Author Information" page.
**Disclaimer:** Articles published in the journal HemaSphere exclusively reflect the opinions of the authors. The authors are responsible for all content in their abstracts including accuracy of the facts, statements, citing resources, etc.

### (PS1743) CHARACTERIZATION AND MECHANISM OF T CELL EXHAUSTION IN THE BONE MARROW IMMUNE MICROENVIRONMENT OF MYELODYSPLASTIC NEOPLASMS PATIENTS WITH TP53 MUTATIONS

**Topic:** 09. Myelodysplastic syndromes - Biology & translational research

Lingxu Jiang*[1], Li Ye[1], Xin Huang[1], Yu Xu[1], Qiaoli Li[1], Wei Wang[1], Xinping Zhou[1], Liya MA[1], Gaixiang Xu[1], Liangyi Liu[1], Jingjing Zhu[1], Lu Wang[1], Yanling Ren[1], Yudi Zhang[1], Jiejin Qian[1], Wei Lang[1], Chen Mei[1], Xianbo Huang[1], Jie Sun[1], Hongyan Tong[1]

[1]*The First Affiliated Hospital, Zhejiang University School of Medicine, Department of Hematology, Hangzhou, China;*

### Background

TP53 mutations is one of the most common genomic alterations in myelodysplastic neoplasms (MDS), associated with adverse prognosis. While recent studies suggested that T cell immune escape participates in the pathogenesis of TP53-mutant neoplasms, the role of T cell exhaustion in the bone marrow immune microenvironment of MDS with TP53 mutation remain poorly characterized.

### Aims

To characterize T cell exhaustion in TP53-mutant MDS and investigate the potential molecular mechanism.

### Methods

Fresh bone marrow samples from MDS patients and healthy donors were collected. Immune checkpoint expression on T cells was detected by flow cytometry. Single-cell transcriptome sequencing was performed on $CD3^+$ T cells sorted by magnetic bead separation. A p53-knockout MOLM-13 cell model was established using CRISPR/Cas9 and co-cultured with T cells.

### Results

Flow cytometric analysis showed that the expression levels of PD-1, CTLA-4, TIM-3, LAG-3, and TIGIT on bone marrow $CD8^+$ T cells were significantly higher in TP53-mutant patients (N=21) than those in TP53-wildtype patients (N=71) ($P<0.05$). In $CD4^+$ T cells, the expression levels of CTLA-4, TIM-3 , and LAG-3 were also significantly elevated in TP53-mutant patients compared to wildtype patients ($P<0.05$) (Figure A). Other common gene mutations and clinical variables almostly showed no obviously correlation with immune checkpoint expression on T cells. These findings reveal the presence of an immunosuppressive T cell phenotype in the bone marrow microenvironment of TP53-mutant MDS patients.

Single-cell transcriptome sequencing was then performed on bone marrow $CD3^+$ T cells from 6 TP53-mutant MDS patients, 12 TP53-wildtype MDS patients, and 4 healthy donors (Figure B). A total of 128,998 $CD3^+$ T cells were obtained. In both $CD8^+$ and $CD4^+$ T cell subsets, the immune checkpoint expression and exhaustion scores were significantly higher in TP53-mutant patients than in healthy donors and TP53-wildtype patients (Figure C-E) . The proportions of $CD8^+$ exhausted T cells (CD8_Tex) and $CD4^+$ exhausted T cells (CD4_Tex) were significantly increased in the TP53-mutant group compared to healthy donors and TP53-wildtype patients ($P < 0.05$) (Figure F, G) . Chemokine signaling pathway was enriched in exhausted T cells, and chemokine receptor CCR5 expression was significantly upregulated and positively correlated with multiple immune checkpoints. These results suggest that the CCR5 signaling pathway may play a key role in T cell exhaustion in TP53-mutant MDS patients.

In vitro, p53-knockout MOLM-13 cells significantly upregulated immune checkpoints on co-cultured T cells, compared to the control MOLM-13 cells. p53 knockout markedly increased the expression and secretion of CCR5 ligands (CCL2, CCL3, CCL4, CCL5) in MOLM-13 cells. Knockdown of CCR5 in T cells significantly downregulated immune checkpoint expression, partially reversing T cell exhaustion.

**Copyright Information:** (Online) ISSN: 2572-9241
©2026 The Author(s). HemaSphere is published by John Wiley & Sons Ltd on behalf of European Hematology Association. This is an open access Abstract Book distributed under the Attribution-NonCommercial-NoDerivs (CC BY-NC-ND), which allows third parties to download the articles and share them with others as long as they credit the author and the Abstract Book, but they cannot change the content in any way or use them commercially.
**Abstract Book Citations:** Authors, Title, HemaSphere, 2026;10:(S1):pages. The abstract book DOIs can be found on the "Author Information" page.
**Disclaimer:** Articles published in the journal HemaSphere exclusively reflect the opinions of the authors. The authors are responsible for all content in their abstracts including accuracy of the facts, statements, citing resources, etc.

### Summary/Conclusion

TP53-mutant MDS exhibits characteristic T cell exhaustion with upregulated immune checkpoints and increased exhausted subsets. Mechanistically, TP53 mutation enhances CCR5 ligand expression and secretion in MDS cells, activating CCR5 signaling on T cells to induce exhaustion. Blocking CCR5 signaling partially reverses this immunosuppressive phenotype. This study reveals a novel mechanism of immune escape via the "CCR5 ligand/CCR5 axis" in TP53-mutant MDS, providing a rationale for targeting CCR5 to improve immunotherapy in these high-risk patients.

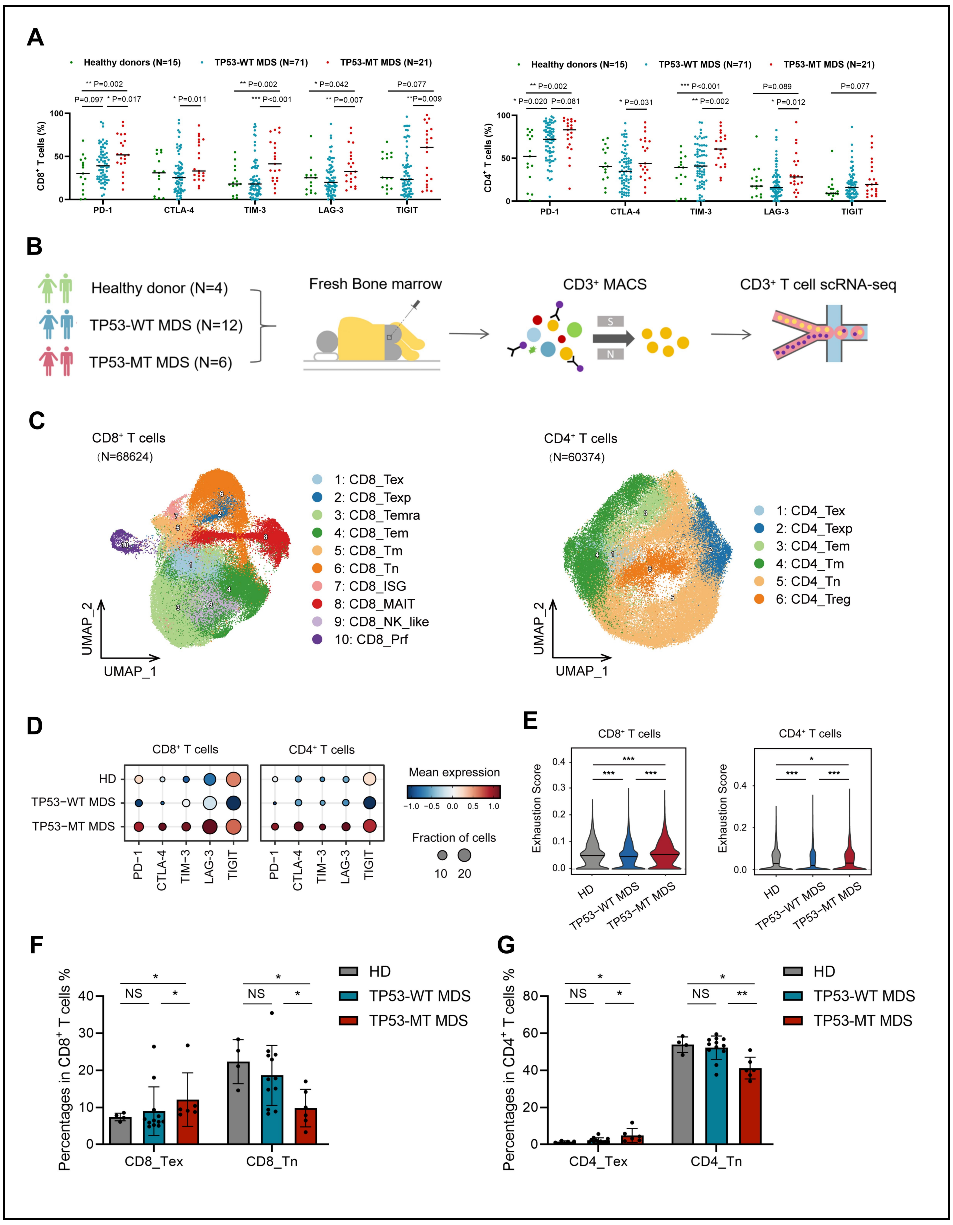

Characterization of T cell exhaustion in the bone marrow immune microenvironment of myelodysplastic neoplasms patients with TP53 mutations

**Copyright Information:** (Online) ISSN: 2572-9241
©2026 The Author(s). HemaSphere is published by John Wiley & Sons Ltd on behalf of European Hematology Association. This is an open access Abstract Book distributed under the Attribution-NonCommercial-NoDerivs (CC BY-NC-ND), which allows third parties to download the articles and share them with others as long as they credit the author and the Abstract Book, but they cannot change the content in any way or use them commercially.
**Abstract Book Citations:** Authors, Title, HemaSphere, 2026;10:(S1):pages. The abstract book DOIs can be found on the “Author Information” page.
**Disclaimer:** Articles published in the journal HemaSphere exclusively reflect the opinions of the authors. The authors are responsible for all content in their abstracts including accuracy of the facts, statements, citing resources, etc.

### (PS1744) DISTINCT PROGENITOR PROGRAMS DRIVE SUBGROUP-SPECIFIC MECHANISMS OF ANEMIA IN MYELODYSPLASTIC SYNDROMES

**Topic:** 09. Myelodysplastic syndromes - Biology & translational research

Carmen Rodriguez-Res*[1], Aintzane Díaz-Mazkiaran[1, 2, 3], Paula García Olloqui[2, 3], Nerea Berastegui Zufiaurre[2, 3], Guillermo Serrano[1, 4], Idoia Recalde[2], Gabriela Fernández-Ruz[2], Patxi San Martín[2], Paula Aguirre[2], Amaia Vilas Zornoza[2, 3], Marina Ainciburu[2, 3], Sofia Huerga-Dominguez[5], Jose Maria Lamo de Espinosa[6], Pamela Acha[7, 8], Maria Julia Montoro Gomez[8], Tamara Jiménez Solas[3, 9], Francesc Sole Ristol[7], David Valcárcel[8], María Díez Campelo[9], Idoia Ochoa[10, 11], Irene Gañán Gómez[2, 3], Ana Alfonso Pierola[3, 5], Felipe Prosper[2, 3, 5], Mikel Hernaez[1, 3, 11], Teresa Ezponda[2, 3]

*[1]Computational Biology Program CIMA-Universidad de Navarra, Cancer Center Clínica Universidad de Navarra (CCUN), , IdISNA, Pamplona, Spain; [2]Hematology-Oncology Program, CIMA, Cancer Center Clínica Universidad de Navarra (CCUN), IdiSNA, Pamplona, Spain; [3]Centro de Investigación Biomédica en Red de Cáncer, CIBERONC, Pamplona, Spain;[4]Biological and Environmental Science and Engineering Division, King Abdullah University of Science and Technology (KAUST), Saudi Arabia; [5]Hematology and Cell Therapy Service, Cancer Center Clínica Universidad de Navarra (CCUN), IdISNA, Pamplona, Spain; [6]Department of Orthopedics, Clínica Universidad de Navarra, Pamplona, Spain;[7]MDS Research Group, Josep Carreras Leukaemia Research Institut, Universitat Autònoma de Barcelona, Barcelona, Spain; [8]Service of Hematology, Hospital Universitari Vall d'Hebron, Barcelona; Vall d'Hebron Instituto de Oncología (VHIO), Barcelona, Spain; [9]Department of Hematology, Hospital Universitario de Salamanca-IBSAL, Salamanca, Salamanca, Spain; [10]Department of Electrical and Electronics engineering, School of Engineering (Tecnun), University of Navarra, San Sebastian, Spain; [11]Instituto de Ciencia de los Datos e Inteligencia Artificial (DATAI), University of Navarra, Pamplona, Spain;*

### Background

The molecular basis of ineffective hematopoiesis in myelodysplastic syndromes (MDS) remains incompletely understood.

### Aims

To explore transcriptional and regulatory alterations driving MDS pathogenesis, we profiled hematopoietic stem and progenitor cells ($CD34^+$) from 15 newly diagnosed MDS patients and 3 age-matched healthy donors by single-cell RNA-sequencing (10x Genomics).

### Methods

MiloR identified compositional shifts and aberrant progenitor subsets. Four independent bulk $CD34^+$ cohorts were deconvoluted with Sweetwater for clinical validation. Differential expression and GRN analyses (SimiC) identified dysregulated genes and transcription factors.

**Copyright Information:** (Online) ISSN: 2572-9241
©2026 The Author(s). HemaSphere is published by John Wiley & Sons Ltd on behalf of European Hematology Association. This is an open access Abstract Book distributed under the Attribution-NonCommercial-NoDerivs (CC BY-NC-ND), which allows third parties to download the articles and share them with others as long as they credit the author and the Abstract Book, but they cannot change the content in any way or use them commercially.
**Abstract Book Citations:** Authors, Title, HemaSphere, 2026;10:(S1):pages. The abstract book DOIs can be found on the "Author Information" page.
**Disclaimer:** Articles published in the journal HemaSphere exclusively reflect the opinions of the authors. The authors are responsible for all content in their abstracts including accuracy of the facts, statements, citing resources, etc.

### Results

We identified 13 progenitor populations spanning HSCs to lineage-committed cells (Fig 1A). All populations were detected across samples, but MDS showed marked compositional shifts (Fig. 1B). A subset of patients displayed enrichment of early/late erythroid progenitors, whereas others showed myeloid expansion (Fig 1C). Erythroid enrichment associated with severe anemia, low blast counts, and lower IPSS-R risk and was reproduced in four public bulk cohorts of CD34+ cells after deconvolution-based inference of erythroid proportions (Fig D). No clinical associations were observed in the myeloid-enriched group.
We then analyzed cell compositional changes along the erythroid trajectory in erythroid-enriched MDS. When focusing on HSCs, we uncover two distinct subpopulations: 1) a stem, quiescent population more prevalent in healthy donors (Fig1 E, HSC-red), and 2) a less stem population with similar healthy/MDS proportions (Fig1 E, HSC-grey). Examination of these subpopulations revealed that n MDS, the stem HSC population was markedly reduced relative to its proportion in healthy donors. Transcriptional profiling showed that HSCs from these patients exhibit increased early erythroid priming, elevated cytokine expression, and reduced inflammatory response. MEPs displayed a similar pattern, with reduced stem-like fractions and enhanced erythroid gene expression, consistent with early erythroid priming (Fig 1G, H).
Among early and late erythroid progenitors, two MDS populations emerged (Fig 1E): one with similar proportion of healthy and MDS cells, and an MDS-specific subset absent in healthy controls that preferentially expanded within the late erythroid compartment (Fig 1I). MDS-specific cells showed the highest proliferation, lowest EPO responsiveness, and elevated HMGB1, consistent with impaired EPO receptor signaling (Fig 1J). Gene regulatory analysis revealed increased erythroid regulator activity but reduced STAT5/STAT3 signaling, particularly in MDS-specific cells. Altered ZEB2 activity further supports defective maturation despite progenitor expansion.
When MDS patients without erythroid enrichment were analyzed, this erythroid-specific population was absent indicating distinct mechanisms of anemia. Ongoing analyses are uncovering distinct transcriptional and regulatory alterations in these two types of patients.

### Summary/Conclusion

Erythroid-skewed MDS at the CD34+ progenitor level defines a biologically and clinically distinct subgroup, with depletion of stem-like cells, premature erythroid commitment, and expansion of a hyperproliferative yet EPO-unresponsive population. These features link erythroid expansion to ineffective erythropoiesis and anemia. Anemic MDS patients without erythroid enrichment show no expansion of erythroid progenitors, suggesting partially distinct mechanisms underlying their anemia

**Copyright Information:** (Online) ISSN: 2572-9241
©2026 The Author(s). HemaSphere is published by John Wiley & Sons Ltd on behalf of European Hematology Association. This is an open access Abstract Book distributed under the Attribution-NonCommercial-NoDerivs (CC BY-NC-ND), which allows third parties to download the articles and share them with others as long as they credit the author and the Abstract Book, but they cannot change the content in any way or use them commercially.
**Abstract Book Citations:** Authors, Title, HemaSphere, 2026;10:(S1):pages. The abstract book DOIs can be found on the “Author Information” page.
**Disclaimer:** Articles published in the journal HemaSphere exclusively reflect the opinions of the authors. The authors are responsible for all content in their abstracts including accuracy of the facts, statements, citing resources, etc.

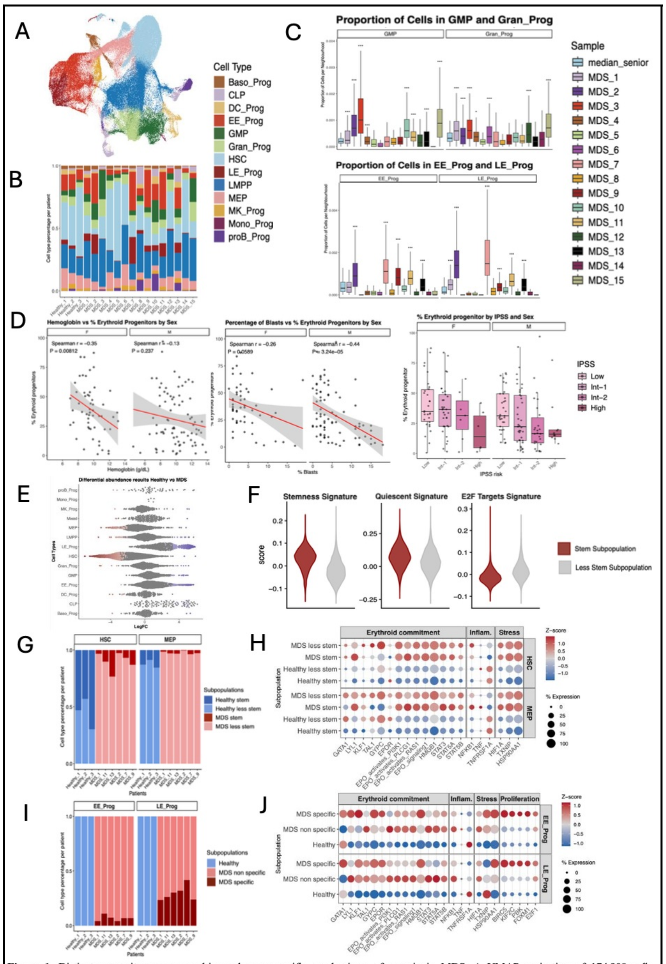

**Figure 1**. Distinct progenitor programs drive subgroup-specific mechanisms of anemia in MDS. **A** UMAP projection of 174,908 cells corresponding to the 13 expected hematopoietic progenitor populations after integration of samples from healthy donors (n=3) and MDS patients (n=15): HSC, LMPP, GMP, Gran_Prog, Mono_Prog, DC_Prog, CLP, B-cell progenitors, MEP, MK_Prog, EE_Prog, LE_Prog, and Baso_Prog. **B** Bar plot illustrating the relative contribution of each patient and healthy donor to the different progenitor populations. **C** Boxplot showing the abundance of erythroid progenitors (EE_Prog and LE_Prog) and myeloid progenitors (GMP and Gran_Prog) as determined by MiloR. Six patients exhibit a statistically significant enrichment in erythroid progenitors, whereas nine show enrichment in myeloid progenitors. P-values were calculated using a two-sided Wilcoxon signed-rank test (* $p < 0.05$; ** $p < 0.01$; *** $p < 0.001$). **D** Correlation between the erythroid proportion inferred by Sweetwater after deconvolution and clinical parameters, including hemoglobin levels and bone marrow blast percentage, assessed by Spearman correlation. Boxplot showing erythroid proportion stratified by IPSS risk categories. Each point represents an individual patient. **E** Violin plots showing MiloR differential abundance comparing MDS patients with erythroid enrichment to healthy donors. Each dot represents a transcriptionally defined cell subpopulation. Subpopulations more abundant in MDS (blue) correspond to MDS-specific early (EE) and late (LE) erythroid progenitors, subpopulations more prevalent in healthy donors (red) are observed in HSC and MEP compartments, and subpopulations with similar proportions (grey) show no differential abundance. **F** Violin plots showing stemness, quiescence, and proliferation scores for HSC cells from panel E, with the red group of cells forming the Stem population and the grey group forming the Less Stem population. **G** Bar plot showing the relative proportions of Stem and Less Stem subpopulations in HSC and MEP compartments of healthy donors and MDS patients. **H** Dot plot showing the top differentially expressed genes identified by DEG analysis between MDS and healthy cells within Stem and Less Stem HSC subpopulations. **I** Bar plot showing the contribution of early (EE) and late (LE) erythroid subpopulations, defined by MiloR, in healthy donors and MDS patients. **J** Dot plot showing the top differentially expressed genes between MDS-specific, MDS non-specific, and healthy erythroid subpopulations, highlighting transcriptional differences across these groups.

**Copyright Information:** (Online) ISSN: 2572-9241
©2026 The Author(s). HemaSphere is published by John Wiley & Sons Ltd on behalf of European Hematology Association. This is an open access Abstract Book distributed under the Attribution-NonCommercial-NoDerivs (CC BY-NC-ND), which allows third parties to download the articles and share them with others as long as they credit the author and the Abstract Book, but they cannot change the content in any way or use them commercially.
**Abstract Book Citations:** Authors, Title, HemaSphere, 2026;10:(S1):pages. The abstract book DOIs can be found on the "Author Information" page.
**Disclaimer:** Articles published in the journal HemaSphere exclusively reflect the opinions of the authors. The authors are responsible for all content in their abstracts including accuracy of the facts, statements, citing resources, etc.

### (PS1745) MACHINE LEARNING UNCOVERS INVARIANT EVOLUTIONARY MOLECULAR TRAJECTORIES IN MDS.

**Topic:** 09. Myelodysplastic syndromes - Biology & translational research

Matteo D'addona*[1], Arda Durmaz[2], Carlos Bravo Pérez[2], Luca Guarnera[2], Simona Pagliuca[2], Carmelo Gurnari[2], Valeria Visconte[2], Jaroslaw P MacIejewski[2]

*[1]Scuola Medica Salernitana, University of Salerno, Department of Medicine, Surgery, and Dentistry, Salerno, Italy, Salerno, Italy; [2]Taussig Cancer Institute, Department of Translational Hematology and Oncology Research, Cleveland, United States, Cleveland, United States of America;*

### Background

Myelodysplastic syndromes (MDS) display marked genetic heterogeneity, where distinct molecular drivers may converge into similar prognostic groups, limiting the ability of outcome-supervised systems to reflect shared pathogenesis. We previously developed an unbiased autoencoder-based machine-learning (ML) approach defining 14 molecular clusters (MCs) based on combinatorial genetic similarity ("molecular nosology" of MDS). While MCs capture convergent leukemogenic pathways at diagnosis, clonal architecture is dynamic; thus, stationary MCs may encode not only disease stage but also direction and velocity of progression.

### Aims

To determine whether serial application of molecular nosology reveals invariant patterns of molecular evolution in MDS and secondary AML (sAML), and to define the clinical significance of molecular cluster transitions.

### Methods

The molecular nosology algorithm, originally trained and validated in 3,588 patients, was applied to an expanded cohort of 3,810 MDS patients. Serial molecular profiling was available for 320 patients, including 210 clinical progressors and 110 non-progressors. MC distribution, stability, transitions (MC switching), and intra-cluster evolution were analyzed longitudinally to define evolutionary trajectories and ontogenetic relationships among MCs.

### Results

MC composition in the expanded cohort mirrored the original dataset. At baseline, MC2 (22%), MC13 (20%), and MC7 (11%) were most frequent. At progression, MC distribution shifted toward advanced-risk clusters, particularly MC13 and MC7. Among progressors, 40% underwent MC reassignment while 60% retained their original MC, indicating non-random clonal reorganization. MC13 emerged as the dominant progression node, increasing from 20% to 34%, with high internal stability. MC7 expanded (11→19%) and frequently transitioned to MC13, functioning as a gateway cluster. MC2 declined markedly (22→11%) and often transitioned to MC7 or MC13 despite association with normal karyotype and DNMT3A/RAS mutations. MC8 (del5q, DNMT3A, early TP53) was unstable, with over half progressing to MC13. MC10 disappeared entirely at progression. MC6 showed intrinsic instability independent of overt progression, suggesting a transient molecular state. In contrast, MC3 remained stable, while MC9, MC11, and MC12 rarely transitioned to MC13. MC transitions followed invariant temporal patterns, allowing inference of founder MCs preceding diagnosis.

**Copyright Information:** (Online) ISSN: 2572-9241
©2026 The Author(s). HemaSphere is published by John Wiley & Sons Ltd on behalf of European Hematology Association. This is an open access Abstract Book distributed under the Attribution-NonCommercial-NoDerivs (CC BY-NC-ND), which allows third parties to download the articles and share them with others as long as they credit the author and the Abstract Book, but they cannot change the content in any way or use them commercially.
**Abstract Book Citations:** Authors, Title, HemaSphere, 2026;10:(S1):pages. The abstract book DOIs can be found on the "Author Information" page.
**Disclaimer:** Articles published in the journal HemaSphere exclusively reflect the opinions of the authors. The authors are responsible for all content in their abstracts including accuracy of the facts, statements, citing resources, etc.

**Summary/Conclusion**

Molecular nosology defines a dynamic yet constrained evolutionary framework in MDS, with invariant molecular trajectories governing progression. Advanced MCs represent evolutionary destinations arising from specific founder states, with MC13 as a central malignant node. Serial MC analysis captures disease kinetics beyond static prognostic models and may enable early identification of aggressive progression patterns.

**Copyright Information:** (Online) ISSN: 2572-9241
©2026 The Author(s). HemaSphere is published by John Wiley & Sons Ltd on behalf of European Hematology Association. This is an open access Abstract Book distributed under the Attribution-NonCommercial-NoDerivs (CC BY-NC-ND), which allows third parties to download the articles and share them with others as long as they credit the author and the Abstract Book, but they cannot change the content in any way or use them commercially.
**Abstract Book Citations:** Authors, Title, HemaSphere, 2026;10:(S1):pages. The abstract book DOIs can be found on the "Author Information" page.
**Disclaimer:** Articles published in the journal HemaSphere exclusively reflect the opinions of the authors. The authors are responsible for all content in their abstracts including accuracy of the facts, statements, citing resources, etc.

### (PS1746) SINGLE-CELL JOINT PROFILING OF GENOTYPES, EPIGENOME, AND TRANSCRIPTOMES IN TET2-MUTANT CLONAL PRE-LEUKEMIC LESIONS

**Topic:** 09. Myelodysplastic syndromes - Biology & translational research

Masanori Motomura*[1], Yotaro Ochi[1], Yasuhito Nannya[1, 2], Nobuyuki Kakiuchi[1, 3], Nobuo Sezaki[4], Senji Kasahara[5], Tomoe Nakagawa[1], Tomonori Hirano[1], Zhao Lanying[1, 6], Shuichi Matsuda[7], Masahiro Nakagawa[1], Seishi Ogawa[1, 6, 8]

*[1]Kyoto University, Department of Pathology and Tumor Biology, Graduate School of Medicine, Kyoto, Japan;[2]The University of Tokyo, Division of Hematopoietic Disease Control, Institute of Medical Science, Tokyo, Japan; [3]Kyoto University, The Hakubi Center for Advanced Research, Kyoto, Japan; [4]Chugoku Central Hospital, Department of Hematology, Hiroshima, Japan; [5]Gifu Municipal Hospital, Department of Hematology, Gifu, Japan; [6]Kyoto University, Institute for the Advanced Study of Human Biology (WPI-ASHBi), Kyoto, Japan; [7]Kyoto University, Department of Orthopedic Surgery, Graduate School of Medicine, Kyoto, Japan; [8]Kindai University, Department of Innovative Medicine, Faculty of Medicine, Osaka, Japan;*

### Background

Age-related clonal hematopoiesis (CH) and clonal cytopenia of undetermined significance (CCUS) frequently harbor mutations in epigenetic regulators, including *TET2*. However, it is poorly understood how epigenomic alterations induced by these mutations dysregulate gene expression and lead to aberrant hematopoiesis, thereby contributing to CH/CCUS development. This is largely due to technical limitations in investigating epigenomes and gene expression in *TET2*-mutated (*TET2*-mut) cells that typically represent a small fraction of patient samples.

### Aims

We aimed to establish a novel platform that enables simultaneous measurements of DNA mutations, chromatin accessibility, and gene expression, which was used to understand how *TET2*-mut cells contribute to CH/CCUS.

### Methods

We developed a novel platform enabling simultaneous single-cell ATAC-seq and genotyping by modifying the Mission Bio Tapestri platform, which was applied to 11 *TET2*-mut CH/CCUS bone marrow samples. Among these, three samples were also analyzed using 10x Chromium single-cell ATAC+RNA-seq ('Multiome'). By combining the paired Tapestri and Multiome data via ATAC profiles, we performed a joint analysis of genotypes, chromatin accessibility, and gene expression at the single-cell level. Briefly, we generated a model to impute single-cell *TET2* genotypes in the Multiome data. To correlate chromatin and transcriptional alterations in *TET2*-mut cells with DNA methylation changes, we performed EM-seq analysis of *TET2*-mut CCUS cases (n = 4) and wild-type subjects (n = 5).

**Copyright Information:** (Online) ISSN: 2572-9241
©2026 The Author(s). HemaSphere is published by John Wiley & Sons Ltd on behalf of European Hematology Association. This is an open access Abstract Book distributed under the Attribution-NonCommercial-NoDerivs (CC BY-NC-ND), which allows third parties to download the articles and share them with others as long as they credit the author and the Abstract Book, but they cannot change the content in any way or use them commercially.
**Abstract Book Citations:** Authors, Title, HemaSphere, 2026;10:(S1):pages. The abstract book DOIs can be found on the "Author Information" page.
**Disclaimer:** Articles published in the journal HemaSphere exclusively reflect the opinions of the authors. The authors are responsible for all content in their abstracts including accuracy of the facts, statements, citing resources, etc.

**Results**

Using the Tapestri-based platform, we analyzed a total of 37,190 cells from 11 patients with *TET2*-mut CH/CCUS. *TET2*-mut cells exhibited biased differentiation toward myeloid and plasmacytoid dendritic cells (pDCs), with reduced representation in mature lymphoid cells. In line with this, *TET2*-mut hematopoietic stem/progenitor cells showed increased accessibility to the motifs of transcription factors (TFs) related to myeloid/pDC differentiation, including CEBPE, RUNX1, and IKZF1, while *TET2*-mut B cells had reduced accessibility to the motifs of key TFs for B cell maturation, such as BACH2, IRF4, and IRF8.
Based on the Multiome data with predicted *TET2* genotypes, *TET2*-mut cells were shown to be depleted from the most mature CD16(+) subset, suggesting impaired monocyte maturation of *TET2*-mut progenitors. To delineate the underlying mechanisms, we assessed the impact of *TET2* mutations on TF activity, inferred from both accessibility at TF-targeting enhancers and expression of their targeted genes. Consistent with the monocyte maturation impairment, *TET2*-mut monocytes exhibited reduced activity of key monocyte maturation-associated TFs, such as CEBPB and PU.1. Furthermore, bulk EM-seq analysis of bone marrowmonocytes identified widespread hypermethylation in *TET2*-mut CCUS cases, preferentially affecting enhancers targeted by the TFs with reduced activity, supporting a model in which enhancer hypermethylation by *TET2* mutations contributes to the attenuation of enhancer-mediated TF activity.

**Summary/Conclusion**

We established single-cell multimodal frameworks linking somatic genotypes with gene regulation and transcriptional outputs. Our findings provide mechanistic insights into the epigenetic and transcriptional basis of dysregulated hematopoietic differentiation and maturation in *TET2*-mutant CH/CCUS.

**Copyright Information:** (Online) ISSN: 2572-9241
©2026 The Author(s). HemaSphere is published by John Wiley & Sons Ltd on behalf of European Hematology Association. This is an open access Abstract Book distributed under the Attribution-NonCommercial-NoDerivs (CC BY-NC-ND), which allows third parties to download the articles and share them with others as long as they credit the author and the Abstract Book, but they cannot change the content in any way or use them commercially.
**Abstract Book Citations:** Authors, Title, HemaSphere, 2026;10:(S1):pages. The abstract book DOIs can be found on the “Author Information” page.
**Disclaimer:** Articles published in the journal HemaSphere exclusively reflect the opinions of the authors. The authors are responsible for all content in their abstracts including accuracy of the facts, statements, citing resources, etc.

### (PS1747) DOWN-REGULATION OF THE NFAT/TOX PATHWAY IMPROVES THE T-CELL EXHAUSTION STATE IN THE BONE MARROW OF PATIENTS WITH MYELODYSPLASTIC SYNDROME ACCOMPANIED BY T LARGE GRANULAR LYMPHOCYTE PROLIFERATION

**Topic:** 09. Myelodysplastic syndromes - Biology & translational research

Chenxiao Du*[1], Shiqin Luo[1], Yuhui Zhang[1], Guangshuai Teng[1], Yifan Duan[1], Yan Wang[1], Yanqi Li[1], Huiqin Zhang[1], Yuan Zhou[2], Yajing Chu[2], Weiping Yuan[1, 2], Jie Bai[1]

*[1]The Second Hospital of Tianjin Medical University, Department of Hematology, Tianjin, China;[2]Institute of Hematology & Blood Diseases Hospital, Chinese Academy of Medical Sciences & Peking Union Medical College, State Key Laboratory of Experimental Hematology, National Clinical Research Center for Blood Diseases, Tianjin, China;*

### Background

Myelodysplastic syndrome with increased blasts (MDS-IB) is a group of high-risk myeloid malignancies that are prone to transformation into acute myeloid leukemia. At the initial diagnosis, 5% of patients with MDS-IB are accompanied by T large granular lymphocyte proliferation (MDS-IB-LGLP), and its pathogenesis may be distinct from that of primary T-LGLL driven by STAT3.

### Aims

To elucidate the pathogenesis of large granular lymphoproliferation in MDS-IB patients and identify potential novel therapeutic directions, we conducted a comprehensive investigation through three components: clinical data analysis, single-cell sequencing, and cellular experiments.

### Methods

We first conducted a retrospective analysis of the clinical and laboratory data of 100 patients (40 cases of MDS-IB-LGLP and 60 cases of MDS-IB). Then, we analyzed the differences in T-cell function at the single-cell sequencing level between MDS-IB-LGLP and MDS-IB patients (3 cases of MDS-IB-LGLP and 3 cases of MDS-IB). To investigate the differences in $CD8^+$ T cell function between MDS-IB-LGLP and MDS-IB patients, and to clarify the interaction of transcription factor TOX, NFAT signaling pathway, and TIM-3, we performed immunophenotyping and Western blot analysis by sorting $CD8^+$ T lymphocytes from both groups of patients, measuring the expression of TOX, NFAT, and TIM-3, and analyzing their correlations. Two groups of patients were selected to co-culture $CD8^+$ T lymphocytes with the MOLM-13 cell line for 48 hours, followed by observation of tumor cell proliferation. Immunophenotyping and Western blot were used to detect the expression of the tumor surface ligand GAL-9, while ELISA was employed to measure the expression levels of cytokines on the surface of co-cultured cells.

**Copyright Information:** (Online) ISSN: 2572-9241
©2026 The Author(s). HemaSphere is published by John Wiley & Sons Ltd on behalf of European Hematology Association. This is an open access Abstract Book distributed under the Attribution-NonCommercial-NoDerivs (CC BY-NC-ND), which allows third parties to download the articles and share them with others as long as they credit the author and the Abstract Book, but they cannot change the content in any way or use them commercially.
**Abstract Book Citations:** Authors, Title, HemaSphere, 2026;10:(S1):pages. The abstract book DOIs can be found on the "Author Information" page.
**Disclaimer:** Articles published in the journal HemaSphere exclusively reflect the opinions of the authors. The authors are responsible for all content in their abstracts including accuracy of the facts, statements, citing resources, etc.

### Results

Clinical data analysis shows that compared to MDS-IB patients, MDS-IB-LGLP patients have less T cell depletion and better survival (P=0.003, P=0.02). The bone marrow single-cell sequencing analysis of MDS-IB-LGLP patients showed that the transcription factors *NFATc1* and *TOX* expression in the *NFAT* signaling pathway of $CD8^{+}$ T cells were downregulated (P<0.001, P<0.001), and positively correlated with the expression of immune suppressive genes such as PD-1 and TIM-3 (P<0.001, P<0.001). After co culturing lymphocytes with tumor cells, it was found that the MDS-LGLP $CD8^{+}$ T cell group had a greater advantage in inhibiting tumor growth than the MDS $CD8^{+}$ T group. The expression of GAL-9 on the surface of tumor cells in the MDS-LGLP $CD8^{+}$ T group decreased (P<0.05), while the expression of IFN - γ and TNF - α increased (P<0.05, P<0.05).

### Summary/Conclusion

These data suggest that in MDS-LGLP patients, TOX may be crucial for NFAT regulated depletion of $CD8^{+}$ T cells. Downregulation of TOX affects the expression of TIM-3 receptors on T cell surfaces and GAL-9 ligands on tumor cell surfaces, relieving $CD8^{+}$ T cell immune tolerance and enhancing anti-tumor immunity. This study provides evidence for the regulation of NFAT by TOX in MDS-IB-LGLP patients and proposes for the first time that downregulating the expression or activity of TOX and NFAT may be a promising new strategy for MDS immunotherapy. Currently, further experiments are underway to comprehensively understand the potential mechanisms and possible clinical treatment methods of the disease.

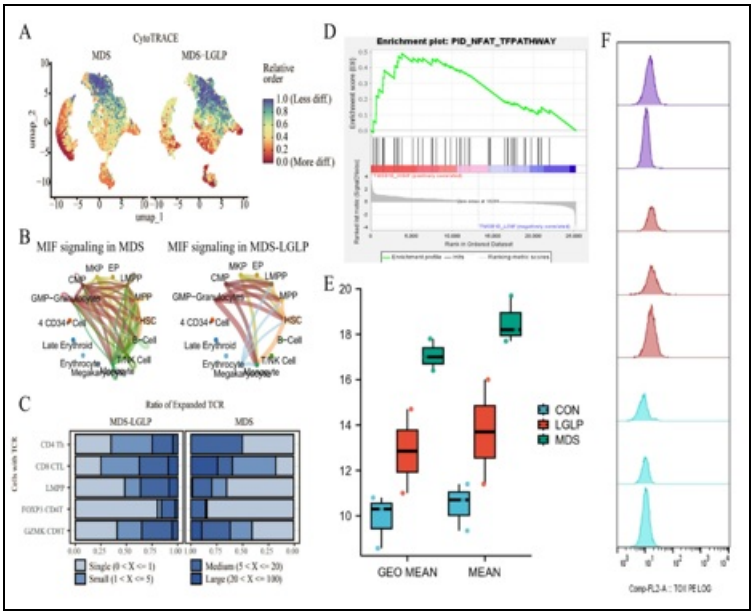

A.CytoTrace analysis of CD34+/CD117+ BMMNC in MDS-IB (Left) and MDS-IB-LGLP (Right) groups reveals differentiation trends. B.MIF signaling pathway network between MDS-IB and MDS-LGLP-IB-LGLP group. C.UMAP of T cells their phenotypes show the size of cell clones. Cells with detected TCR were divided into single, small, medium, large. D.GSEA analysis showed that NFAT pathway were upregulated in the T cells in MDS-IB paitent. E-F. Compared to MDS-IB patients, the transcription factor tox has decreased T cell expression levels in MDS-IB-LGLP patients, but is higher than in healthy controls.

**Copyright Information:** (Online) ISSN: 2572-9241
©2026 The Author(s). HemaSphere is published by John Wiley & Sons Ltd on behalf of European Hematology Association. This is an open access Abstract Book distributed under the Attribution-NonCommercial-NoDerivs (CC BY-NC-ND), which allows third parties to download the articles and share them with others as long as they credit the author and the Abstract Book, but they cannot change the content in any way or use them commercially.
**Abstract Book Citations:** Authors, Title, HemaSphere, 2026;10:(S1):pages. The abstract book DOIs can be found on the "Author Information" page.
**Disclaimer:** Articles published in the journal HemaSphere exclusively reflect the opinions of the authors. The authors are responsible for all content in their abstracts including accuracy of the facts, statements, citing resources, etc.

### (PS1748) MAMMALIAN DIAPHANOUS-RELATED FORMIN 1 IS REQUIRED FOR MAINTAINING HEMATOPOIETIC STEM CELL QUIESCENCE AND SURVIVAL VIA REGULATING MITOCHONDRIA OXIDATIVE METABOLIC PATHWAY

**Topic:** 09. Myelodysplastic syndromes - Biology & translational research

Baoxia Dong*[1], Ming Liu[2], Ben Pinder[2], Katherine Siminovitch[2], Jinyi Zhang[3]

*[1]Shanghai General Hospital, Shanghai Jiao Tong University, School of Medicine, Department of Hematology, Shanghai, China; [2]Lunenfeld-Tanenbaum Research Institute, Toronto, Canada; [3]Wenzhou Medical University, Oujiang Laboratory, Zhejiang Lab for Regenerative Medicine, Vision and Brain Health, Wenzhou, China;*

### Background

Myelo*dys*plastic neoplasms (MDS) is a malignant disease mainly originating from Hematopoietic stem cells(HSCs) in the elderly characterized by hyperproliferation in Bone Marrow (BM) and morphological dysplasia, with genetic instability and easy to transform into acute myeloid leukemia (AML) . The defective hematopoiesis shows as peripheral pancytopenia, but associated with enhanced proliferation and, paradoxically, increased apoptosis of hematopoietic progenitors. While MDS is a highly heterogeneous disease with many factors affecting the pathogenesis, However, it is believed that MDS is a disease process in which HSCs aging and clonal hematopoiesis are intertwined and overlapped . The underlying mechanism of the HSC defects engendering MDS has not been fully understood.

### Aims

HSCs exist in a relatively quiescent state in bone marrow (BM) niche to maintain the balance between self-renewal and multi-lineage differentiation. Rho family GTPases have been shown to play critical roles in HSCs maintenance and functions, even aging. The mammalian diaphanous-related formin 1 (mDia1), a cytoskeleton regulatory protein, as a downstream effector of RhoA, has been implicated in hematopoiesis and its deficiency is associated with MDS like phenotypes. This study aim to investigate the underlying mechanism of how mDia1 regulates HSCs functions and malignant transformation to MDS.

### Methods

Fluorescence-activated cell sorting were used to purify HSCs. Clonogenic assays were used for committed hematopoietic progenitor growth. DNA dye Hoechst33342 and RNA dye Pyronin Y were used for the cell cycle analysis. Competitive reconstitution assay were used to detect the mDia1$^{-/-}$HSCs reconstitution ability. Mitochondrial membrane potential, Mitochondrial mass and ROS Level were detected based on the immuno-fluorescence staining and FACS analysis.Seahorse XF96 for OCR and ECAR measurement for ROS detection. RNA Sequencing was used to explore the underlying signal pathway and underlying mechanism.

**Copyright Information:** (Online) ISSN: 2572-9241
©2026 The Author(s). HemaSphere is published by John Wiley & Sons Ltd on behalf of European Hematology Association. This is an open access Abstract Book distributed under the Attribution-NonCommercial-NoDerivs (CC BY-NC-ND), which allows third parties to download the articles and share them with others as long as they credit the author and the Abstract Book, but they cannot change the content in any way or use them commercially.
**Abstract Book Citations:** Authors, Title, HemaSphere, 2026;10:(S1):pages. The abstract book DOIs can be found on the "Author Information" page.
**Disclaimer:** Articles published in the journal HemaSphere exclusively reflect the opinions of the authors. The authors are responsible for all content in their abstracts including accuracy of the facts, statements, citing resources, etc.

**Results**

We found that the deficiency of mDia1 leads to anemia/thrombocytopenia in knockout mice, and the deletion of mDia1 causes decreased HSC pool with increased cell cycle entry and apoptotic death of mDia1$^{-/-}$ HSCs, and skewed myeloid-biased differentiation. The impaired reconstituting capability of mDia1$^{-/-}$ HSCs is cell-autonomous. These phenotypes were consistent with the HSCs aging when mDia1 deficiency.mDia1$^{-/-}$ HSCs exhibited lower self-renewal ability and aberrantly exit from quiescence and enter active cell cycle. The mDia1$^{-/-}$ HSCs also exhibit increased DNA damage and programmed cell death, resulting in decreased frequency of HSCs and skewed differentiation towards myeloid lineages, with elevated higher level of oxygen phosphorylation and reactive oxygen species (ROS). Furthermore, single cell sequence analysis of mDia1$^{-/-}$ HSCs showed impaired mitochondrial function and over-activated phosphorylation pathway. Taken together, our finding identifies an essential role for mDia1 as a key regulator for HSCs homeostasis.

**Summary/Conclusion**

This finding establish a link between the cytoskeletal regulatory protein mDia1 and the function of HSCs, and provide the explanation about the mechanism of mDia1 playing a role in regulating the mitochondrial function and ROS production in HSCs aging and malignant transformation of MDS.

**Copyright Information:** (Online) ISSN: 2572-9241
©2026 The Author(s). HemaSphere is published by John Wiley & Sons Ltd on behalf of European Hematology Association. This is an open access Abstract Book distributed under the Attribution-NonCommercial-NoDerivs (CC BY-NC-ND), which allows third parties to download the articles and share them with others as long as they credit the author and the Abstract Book, but they cannot change the content in any way or use them commercially.
**Abstract Book Citations:** Authors, Title, HemaSphere, 2026;10:(S1):pages. The abstract book DOIs can be found on the “Author Information” page.
**Disclaimer:** Articles published in the journal HemaSphere exclusively reflect the opinions of the authors. The authors are responsible for all content in their abstracts including accuracy of the facts, statements, citing resources, etc.

### (PS1749) AN INTEGRATIVE APPROACH COMBINING SINGLE-CELL DNA-SEQ AND SURFACEOME REVEALS DISTINCT EVOLUTIONARY TRAJECTORIES SPECIFIC TO SOMATIC ABNORMALITIES IN GATA2 DEFICIENCY

**Topic:** 09. Myelodysplastic syndromes - Biology & translational research

Hedi Somai[1], Pauline Enfedaque*[1], Simon Boutinaud[2], Camille Hamelle[3], Quentin Rigaud[1], Stéphanie Dufrechou[3], Naïs Prade[3], Mony Fahd[4], Flore Sicre de Fontbrune[5], Claire Fieschi[5], Christine Bellanne Chantelot[4], Jean Donadieu[6], Christine Didier[1], Bastien Gerby[1], Cyril Broccardo[1], Eric Delabesse[1, 3], Marlène Pasquet[2], Laetitia Largeaud[1, 3]

*[1]Research Center in Cancer de Toulouse, Toulouse, France; [2]CHU Purpan, Toulouse, France; [3]IUCT Oncopole, Toulouse, France; [4]Hôpital Universitaire Robert Debré, Paris, France; [5]Hôpital Saint-Louis, Paris, France; [6]Hôpital Armand Trousseau, Paris, France;*

### Background

GATA2 deficiency is a hereditary predisposition syndrome characterized by a high risk of myelodysplastic syndromes (MDS) and acute myeloid leukemia (AML), with nearly 80% of patients developing hematologic malignancies before the age of 40. Leukemia transformation is commonly associated with somatic alterations such as STAG2, ASXL1 and SETBP1 mutations, as well as monosomy 7 and trisomy 8. However, bulk sequencing approaches cannot resolve their clonal architecture and define the specific cell types in which they arise.

### Aims

Deciphering the hierarchy and timing of these events is critical to refine risk stratification and optimize transplantation timing. To address these issues, we implemented an integrated single-cell strategy combining DNA sequencing and surfaceome profiling. This approach enabled us to delineate the clonal architecture of somatic alterations and assess their impact on cellular differentiation trajectories.

### Methods

We analyzed the clonal architecture of five bone marrow samples from GATA2-deficient patients using single-cell DNA sequencing coupled with surface protein profiling (Tapestri® technology and TotalSeq-D antibody panel (BioLegend®)).

### Results

In spectrum 1 patients (hypoplastic bone marrow with dysmyelopoiesis), we found several clones coexisting, dominated by molecular abnormalities such as STAG2 and ASXL1 mutations or by cytogenetic abnormalities such as trisomy 8. STAG2 mutations are strictly restricted to the erythroblastic compartment, while ASXL1 mutations affect both granulocytic and erythroblastic lineages, indicating distinct consequences on differentiation trajectories.
In spectrum 2 patients (excess of blasts), original events substantially influence cell progression. Monosomy 7 appears to be an initiating event detectable across multiple hematopoietic compartments, including lymphoid cells, consistent with acquisition in a multipotent stem cell. A subclone subsequently acquired a SETBP1 mutation, which is associated with blast expansion and monocytic differentiation features, despite the characteristic monocytopenia observed in GATA2 deficiency. These findings identify monosomy 7 as a leukemogenic initiating event, with SETBP1 acting as a progression driver reshaping lineage output. Our clonal architecture analysis shows that STAG2 mutations can sometimes constitute the initiating event in leukemia transformation through sequential acquisition of additional alterations including EZH2 or RUNX1 mutations or partial chromosome 7 deletion. This progression is associated with early erythroid differentiation blockage at the megakaryocyte-erythroid progenitor (MEP) stage, and an excess of blasts visible in cytology.

**Copyright Information:** (Online) ISSN: 2572-9241
©2026 The Author(s). HemaSphere is published by John Wiley & Sons Ltd on behalf of European Hematology Association. This is an open access Abstract Book distributed under the Attribution-NonCommercial-NoDerivs (CC BY-NC-ND), which allows third parties to download the articles and share them with others as long as they credit the author and the Abstract Book, but they cannot change the content in any way or use them commercially.
**Abstract Book Citations:** Authors, Title, HemaSphere, 2026;10:(S1):pages. The abstract book DOIs can be found on the "Author Information" page.
**Disclaimer:** Articles published in the journal HemaSphere exclusively reflect the opinions of the authors. The authors are responsible for all content in their abstracts including accuracy of the facts, statements, citing resources, etc.

**Summary/Conclusion**

Overall, our study highlights distinct evolutionary trajectories depending on the founding events in GATA2 deficiency, emphasizing the importance of longitudinal follow-up of these clones. STAG2-mutated clones are restricted to the erythroblastic population and display a lineage-restricted expansion without immediate transformation, whereas acquisition of monosomy 7 confer a higher leukemic potential, particularly upon acquisition of SETBP1, which redirect differentiation towards the monocyte lineage. By integrating clonal architecture with cellular phenotype, this approach provides a framework to anticipate blast progression and refine therapeutic decision, including optimal timing of hematopoietic stem cell transplantation.

**Copyright Information:** (Online) ISSN: 2572-9241
©2026 The Author(s). HemaSphere is published by John Wiley & Sons Ltd on behalf of European Hematology Association. This is an open access Abstract Book distributed under the Attribution-NonCommercial-NoDerivs (CC BY-NC-ND), which allows third parties to download the articles and share them with others as long as they credit the author and the Abstract Book, but they cannot change the content in any way or use them commercially.
**Abstract Book Citations:** Authors, Title, HemaSphere, 2026;10:(S1):pages. The abstract book DOIs can be found on the "Author Information" page.
**Disclaimer:** Articles published in the journal HemaSphere exclusively reflect the opinions of the authors. The authors are responsible for all content in their abstracts including accuracy of the facts, statements, citing resources, etc.

### (PS1750) ASXL1 MUTATIONS ARE A NOVEL RISK FACTOR FOR MAJOR BLEEDING IN PATIENTS WITH MYELODYSPLASTIC SYNDROMES

**Topic:** 09. Myelodysplastic syndromes - Biology & translational research

Elay Amidor*[1], Romi Kazoom[1], Vladimir Vainstein[2], Revital Saban[2], Ela Shai[2], Batia Roth Jelinek[2], Ariela Arad[2], Noa Buchman[2], Yossi Kalish[2], Eran Zimran[2]

*[1] 1. Faculty of Medicine, Hebrew University of Jerusalem, Jerusalem, Israel;[2] 2. Hadassah Medical Organization and Faculty of Medicine, Hebrew University of Jerusalem, Hematology, Jerusalem, Israel;*

### Background

Cardiovascular disease is a major cause of morbidity and mortality in patients with myelodysplastic syndromes (MDS), with a relative risk of approximately twofold compared with age-matched individuals. Bleeding events, usually attributed to thrombocytopenia, also contribute significantly to morbidity and mortality in this frail patient population. Myeloid gene mutations detected by next-generation sequencing (NGS) confer a higher risk of thrombosis in individuals with clonal hematopoiesis and patients with myeloproliferative neoplasms; however, their impact in MDS has not been studied.

### Aims

To evaluate the association between myeloid gene mutations, especially in *DNMT3A*, *TET2,* and *ASXL1* (collectively: DTA) and thrombosis and bleeding events in patients with MDS.

### Methods

We prospectively followed 191 consecutive patients with MDS evaluated at our center between 2020 to 2025 who had underwent NGS panel testing using Archer® VariantPlex® Myeloid Panel. Arterial and venous thrombotic events, as well as major bleeding events, were documented. All patients provided written informed consent.

**Copyright Information:** (Online) ISSN: 2572-9241
©2026 The Author(s). HemaSphere is published by John Wiley & Sons Ltd on behalf of European Hematology Association. This is an open access Abstract Book distributed under the Attribution-NonCommercial-NoDerivs (CC BY-NC-ND), which allows third parties to download the articles and share them with others as long as they credit the author and the Abstract Book, but they cannot change the content in any way or use them commercially.
**Abstract Book Citations:** Authors, Title, HemaSphere, 2026;10:(S1):pages. The abstract book DOIs can be found on the “Author Information” page.
**Disclaimer:** Articles published in the journal HemaSphere exclusively reflect the opinions of the authors. The authors are responsible for all content in their abstracts including accuracy of the facts, statements, citing resources, etc.

**Results**

Median age was 69 years and 56% of the patients were male. Seventy percent of the patients had at least one cardiovascular comorbidity, most commonly hypertension. Seventeen percent had high-risk MDS according to IPSS-R, 10% had adverse cytogenetics, 14% were treated with hypomethylating agents and 21% were RBC transfusion dependent. Ninety percent harbored at least one myeloid gene mutation, and >70% had at least one DTA mutation (Fig 1A). Within a median follow-up of 2.8 years, 23 patients (12%) experienced thrombotic events, most commonly arterial including coronary events and strokes, and 16 patients (8%) experienced major bleeding including gastrointestinal bleeding (n=6), epistaxis (n=5) and intracranial or subdural hematomas (n=5). Age, cardiovascular comorbidities, and male sex were significantly associated with arterial thrombosis, while an association between myeloid gene mutations (including DTA) and either arterial or venous thrombosis was not observed. In univariate analysis, anemia, ≥2 cytopenias, and *ASXL1* mutations (HR: 3.5, CI: 2.1-6.5, p<0.001) were significantly associated with major bleeding, while thrombocytopenia alone and higher-risk MDS were not (Fig 1B). In multivariate analysis, only *ASXL1* mutations remained significant and were independent of age, hemoglobin or platelet counts, disease risk, leukemic transformation, and exposure to anticoagulation (Fig 1C).
To validate this finding, we used TriNetX, a global network that aggregates electronic health records from multiple healthcare organizations (HCOs). At the time of analysis (1/2026) this network encompassed 171 HCOs comprising 183,351,845 individuals across 20 countries. Among 7,522 MDS patients tested for *ASXL1* mutations, excluding those with leukemic transformation or referral for allogeneic transplantation, a significant association between *ASXL1* mutations and major bleeding was confirmed (HR: 1.55, CI: 1.3-1.82, p<0.001) and was independent of age, cytopenias, and exposure to anticoagulation (Fig 1D).

**Summary/Conclusion**

*ASXL1* mutations were associated with a higher risk of major bleeding events in patients with MDS, independent of age, blood counts, and disease-risk. This association was confirmed in a large international healthcare database. Further investigation is warranted to elucidate the mechanisms and clinical implications of this association.

**Copyright Information:** (Online) ISSN: 2572-9241
©2026 The Author(s). HemaSphere is published by John Wiley & Sons Ltd on behalf of European Hematology Association. This is an open access Abstract Book distributed under the Attribution-NonCommercial-NoDerivs (CC BY-NC-ND), which allows third parties to download the articles and share them with others as long as they credit the author and the Abstract Book, but they cannot change the content in any way or use them commercially.
**Abstract Book Citations:** Authors, Title, HemaSphere, 2026;10:(S1):pages. The abstract book DOIs can be found on the “Author Information” page.
**Disclaimer:** Articles published in the journal HemaSphere exclusively reflect the opinions of the authors. The authors are responsible for all content in their abstracts including accuracy of the facts, statements, citing resources, etc.

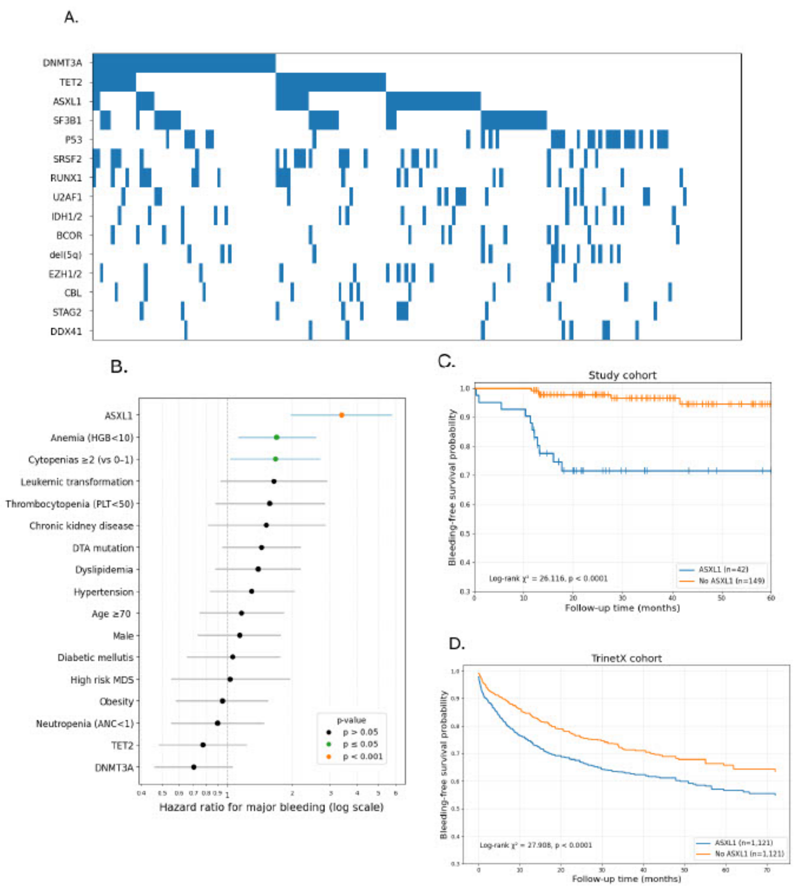

**Figure 1. A)** Distribution of myeloid gene mutations in 191 consecutive patients with MDS between 2020-2025, by order of prevalence. **B)** Univariate analysis of the association between various factors and major bleeding events. **C)** Kaplan Meier analysis of major bleeding free survival in MDS patients with and without an *ASXL1* mutation in the Hadassah cohort. **D)** Kaplan Meier analysis of bleeding free survival in MDS patients with and without an *ASXL1* mutation in the TriNetX network.

**Copyright Information:** (Online) ISSN: 2572-9241
©2026 The Author(s). HemaSphere is published by John Wiley & Sons Ltd on behalf of European Hematology Association. This is an open access Abstract Book distributed under the Attribution-NonCommercial-NoDerivs (CC BY-NC-ND), which allows third parties to download the articles and share them with others as long as they credit the author and the Abstract Book, but they cannot change the content in any way or use them commercially.
**Abstract Book Citations:** Authors, Title, HemaSphere, 2026;10:(S1):pages. The abstract book DOIs can be found on the “Author Information” page.
**Disclaimer:** Articles published in the journal HemaSphere exclusively reflect the opinions of the authors. The authors are responsible for all content in their abstracts including accuracy of the facts, statements, citing resources, etc.

### (PS1751) A NEW MURINE CMML-LIKE MODEL: INDUCIBLE HSC-SPECIFIC UTX DELETION MIMICS KEY DISEASE FEATURES

**Topic:** 09. Myelodysplastic syndromes - Biology & translational research

Iryna Kovtun*[1, 2], Ekaterina Balaian[1], Katja Sockel[1], Anupam Sinha[2], Martin Bornhäuser[1], Ben Wielockx[2], Tatyana Grinenko[2, 3]

*[1]Universitätsklinikum Carl Gustav Carus Dresden, Medizinische Klinik und Poliklinik I, Dresden, Germany;[2]Technische Universität Dresden, Institut für Klinische Chemie und Laboratoriumsmedizin, Dresden, Germany; [3]Ruijin Hospital Affiliated to Shanghai Jiao Tong University School of Medicine, Institute of Hematology, National Research Center for Translational Medicine at Shanghai, Shanghai, China;*

### Background

Chronic myelomonocytic leukemia (CMML) is a clonal hematopoietic disorder characterized by persistent monocytosis, dysplastic hematopoiesis, frequently splenomegaly, and a substantial risk of progression to acute myeloid leukemia. Despite advances in molecular characterization and supportive care, treatment options remain limited, and median overall survival is still under three years. A major limitation in the field is the lack of faithful mouse models that recapitulate key CMML features. Most available models involving UTX deletion rely on broad or pan-hematopoietic gene inactivation, lack true clonal hematopoiesis arising from a defined hematopoietic stem cell (HSC) clone, or often result in aggressive disease with rapid lethality. Therefore, establishing a novel *in vivo* model that overcomes these limitations is essential for advancing both fundamental and translational research.

### Aims

We aimed to develop a physiologically relevant CMML-like mouse model by inducing an HSC-restricted deletion of the histone demethylase UTX (KDM6A), a gene recurrently mutated in a subset of CMML patients.

### Methods

Serial line breeding established a new HSC-Scl:cre-ER$^{T2}$;UTX$^{f/f}$ mouse strain with tamoxifen-inducible UTX deletion restricted to HSCs. Deletion was induced at 12 weeks of age, and bone marrow (BM), spleen, and peripheral blood (pB) were analyzed by flow cytometry at 3, 9, and 18 months post-induction, and bulk RNA-sequencing of HSCs at 4 months.

**Copyright Information:** (Online) ISSN: 2572-9241
©2026 The Author(s). HemaSphere is published by John Wiley & Sons Ltd on behalf of European Hematology Association. This is an open access Abstract Book distributed under the Attribution-NonCommercial-NoDerivs (CC BY-NC-ND), which allows third parties to download the articles and share them with others as long as they credit the author and the Abstract Book, but they cannot change the content in any way or use them commercially.
**Abstract Book Citations:** Authors, Title, HemaSphere, 2026;10:(S1):pages. The abstract book DOIs can be found on the “Author Information” page.
**Disclaimer:** Articles published in the journal HemaSphere exclusively reflect the opinions of the authors. The authors are responsible for all content in their abstracts including accuracy of the facts, statements, citing resources, etc.

**Results**

Efficient UTX deletion was confirmed by marked protein loss in HSCs four weeks after induction. Importantly, mice were followed for extended periods (up to 18 months) without leukemic transformation or disease-related lethality.
Progressive hematologic changes were observed in pB over time. Red blood cell counts declined from three months post-induction and continued to decrease. Hemoglobin values were similar at baseline, declined by 12 months after deletion (9.53 vs 8.48 mmol/L, p<0.001) and further at 18 months (8.74 vs 6.64 mmol/L, p<0.001). Absolute monocyte counts were normal at baseline, elevated two months after induction (0.33 vs 0.88 x$10^3$/ul, p<0.05), and showed a sustained increase up to 18 months (0.55 vs 1.93 x$10^3$/ul, p<0.001).
In the BM, HSCs markedly expanded across all time points (3 months: 0.08 vs 0.25; 18 months: 0.14 vs 1.48 % in BM, p<0.0001), while multipotent progenitors decreased. Myeloid progenitors were transiently expanded at 3 and 9 months but declined by 18 months after UTX deletion, while erythroid progenitors were consistently reduced. Consistently, HSCs from UTX-deficient mice upregulated genes involved in myeloid differentiation (such as *Clu, Vwf, Neo1, Itgb3*) and downregulated erythroid associated genes (*Klf1, Bpgm, Slc40a1*).
Splenomegaly appeared early and progressed over time, with significantly higher spleen weights at 3 months (99 vs 118 mg) and 18 months (92 vs 235 mg; p<0.05).
Notably, macrophages and classical inflammatory monocytes were persistently elevated in pB, BM, and spleen, suggesting chronic inflammation characteristic of CMML.

**Summary/Conclusion**

We established a novel mouse model enabling HSC-specific UTX deletion, thereby restricting the genetic alteration to a stem cell compartment rather than the entire hematopoietic system. This model more accurately recapitulates the cellular origin and progressive clonal evolution characteristic of CMML and offers a robust platform for mechanistic and preclinical studies.

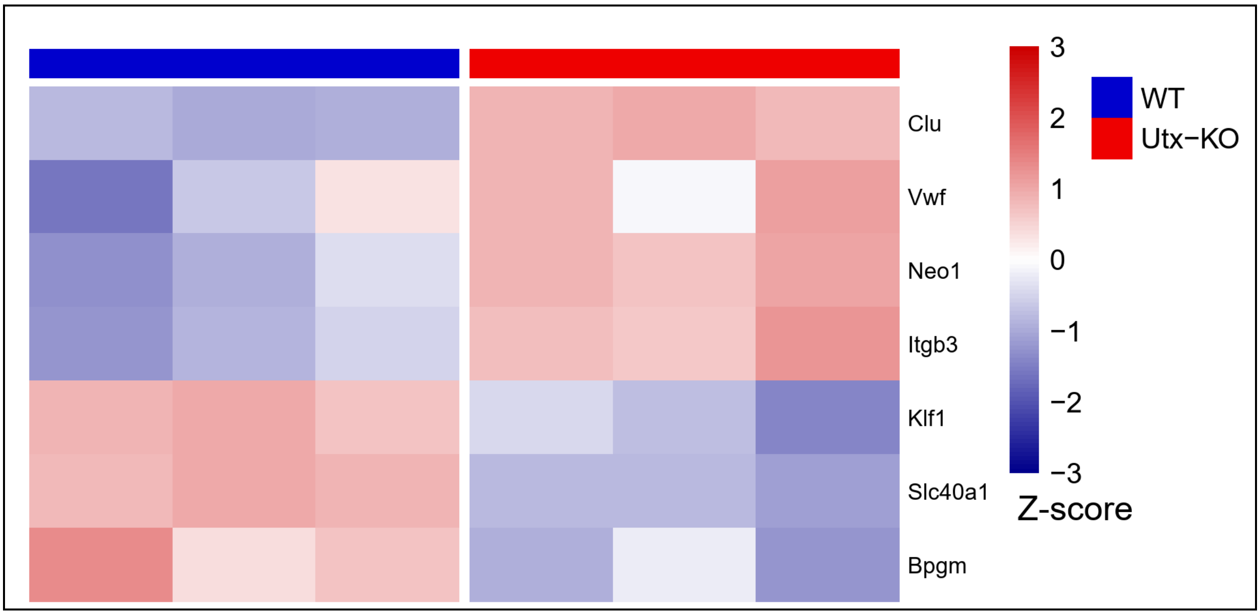

Heatmap representation of transcriptional differences in HSCs from WT and UTX-deficient mice, showing upregulation of myeloid-associated and downregulation of erythroid-associated genes

**Copyright Information:** (Online) ISSN: 2572-9241
©2026 The Author(s). HemaSphere is published by John Wiley & Sons Ltd on behalf of European Hematology Association. This is an open access Abstract Book distributed under the Attribution-NonCommercial-NoDerivs (CC BY-NC-ND), which allows third parties to download the articles and share them with others as long as they credit the author and the Abstract Book, but they cannot change the content in any way or use them commercially.
**Abstract Book Citations:** Authors, Title, HemaSphere, 2026;10:(S1):pages. The abstract book DOIs can be found on the "Author Information" page.
**Disclaimer:** Articles published in the journal HemaSphere exclusively reflect the opinions of the authors. The authors are responsible for all content in their abstracts including accuracy of the facts, statements, citing resources, etc.

### (PS1752) DIAGNOSTIC UTILITY OF B-CELL IMMUNOPHENOTYPING IN UNEXPLAINED CYTOPENIA

**Topic:** 09. Myelodysplastic syndromes - Biology & translational research

Andy Rawstron[1], Richard Leach[1], Rukhsaar Ali[1], Paul Glover[1], Helen Warren[1], Suzan Van Hoppe[1], Alex Smith[2], Ruth Detute[1], Catherine Cargo*[1]

*[1]Haematological Malignancy Diagnostic Service, Leeds Cancer Centre, Leeds Teaching Hospitals NHS Trust, Leeds, United Kingdom; [2]University of York, Epidemiology and Cancer Statistics Group, York, United Kingdom;*

### Background

Depletion of normal B-progenitors is a characteristic feature of myelodysplastic neoplasms (MDS) and there is increasing recognition that immune dysregulation may play a role in the evolution of myeloid disorders. Clonal B-cell expansions can also be associated with cytopenia even when the level of abnormal B-cells is low, and diagnostic work-up of unspecified cytopenia frequently identifies abnormal B-lineage populations as a potential underlying cause.

### Aims

To characterise B-cell subset distribution and clonality across diagnostic groups in a large, prospective, population-based cytopenia cohort, and to assess the diagnostic utility of B-cell immunophenotyping in routine workup.

### Methods

Patients aged >18 referred for cytopenia investigation with no history of myeloid malignancy or lymphadenopathy were eligible; patients with a B-lymphoproliferative disorder identified at screening were excluded. Targeted sequencing was conducted alongside routine clinical testing. B-cell subsets were assessed by 8-colour flow cytometry. Clonality was defined as κ:λ ratio <0.43:1 or >4:1 (calculated to identify a clonal population representing >=50% of B-cells in the subset) or if >=20% are sIg-negative within a subset.

### Results

Data were available for 646 cases of idiopathic cytopenia of unknown significance (ICUS); 243 cases of clonal cytopenia of unknown significance (CCUS); 128 MDS cases (41/128 with increased blasts, 87 with low blasts of which 5/87 have 5q deletion and 23/87 have *SF3B1* mutation) and 20 other Myeloid disorders (incl. 8 AML, 7 MPN, 2 CMML, 1 MDS/MPN and 2 other).
Pro-B CD10++CD34+/CD45(wk) progenitors cell numbers were significantly decreased in CCUS/MDS/Myeloid disorders at a median (95% CI) of 0.04% (0.03-0.05), 0.02%(0.01-0.03) and 0.01%(0.01-0.02) respectively compared to 0.09% (0.08-0.11) in ICUS. Pre-B CD10+CD34-CD45(int) B-cell precursors and naïve B-cell numbers showed a similar pattern with significant decreases in CCUS/MDS/Myeloid disorders compared to ICUS. Overall maturation appeared relatively normal indicating a decrease across the spectrum of immature B-cells in patients with CCUS/MDS/myeloid disorders.
Non-naïve B-cell numbers were broadly similar across diagnostic groups however clonal B-cell expansions were more common in MDS/Myeloid disorders (25.9%) compared to ICUS/CCUS (14.7%/15.2% respectively, Chi-square P=0.004). Similarly low-level CLL-phenotype populations were more common in MDS/Myeloid disorders (23.6%) compared to ICUS/CCUS (14.6%/13.6% respectively, Chi-square P=0.014).

**Copyright Information:** (Online) ISSN: 2572-9241
©2026 The Author(s). HemaSphere is published by John Wiley & Sons Ltd on behalf of European Hematology Association. This is an open access Abstract Book distributed under the Attribution-NonCommercial-NoDerivs (CC BY-NC-ND), which allows third parties to download the articles and share them with others as long as they credit the author and the Abstract Book, but they cannot change the content in any way or use them commercially.
**Abstract Book Citations:** Authors, Title, HemaSphere, 2026;10:(S1):pages. The abstract book DOIs can be found on the "Author Information" page.
**Disclaimer:** Articles published in the journal HemaSphere exclusively reflect the opinions of the authors. The authors are responsible for all content in their abstracts including accuracy of the facts, statements, citing resources, etc.

**Summary/Conclusion**

B-progenitor and naïve B-cell numbers are decreased in CCUS, MDS and Myeloid disorders compared to ICUS. This suggests that impaired B-lymphopoiesis may be an early event during the evolution of MDS. In contrast, light-chain restricted (clonal) B-cell expansions including CLL-type MBL, are more common in MDS/myeloid disorders compared to ICUS/CCUS. The relationship between B-cell clonal expansions / immune defects and clinical outcome in CCUS vs. MDS/MPN will be reported. However, there is a clear indication to investigate B-cell subsets in work-up of cytopenia to exclude B-cell malignancies as an underlying cause of the cytopenia. The i4MDS consortium is working towards developing standardised panels for this purpose.

**Copyright Information:** (Online) ISSN: 2572-9241
©2026 The Author(s). HemaSphere is published by John Wiley & Sons Ltd on behalf of European Hematology Association. This is an open access Abstract Book distributed under the Attribution-NonCommercial-NoDerivs (CC BY-NC-ND), which allows third parties to download the articles and share them with others as long as they credit the author and the Abstract Book, but they cannot change the content in any way or use them commercially.
**Abstract Book Citations:** Authors, Title, HemaSphere, 2026;10:(S1):pages. The abstract book DOIs can be found on the “Author Information” page.
**Disclaimer:** Articles published in the journal HemaSphere exclusively reflect the opinions of the authors. The authors are responsible for all content in their abstracts including accuracy of the facts, statements, citing resources, etc.

### (PS1754) ASSOCIATION OF MHC CLASS I POLYPEPTIDE-RELATED SEQUENCE A (MICA) AND B (MICB) ALLELE POLYMORPHISMS WITH SUSCEPTIBILITY TO MYELODYSPLASTIC SYNDROMES (MDS)

**Topic:** 09. Myelodysplastic syndromes - Biology & translational research

Sotirios Bristogiannis[1], Michalis Panousieris[1], Kyriaki Kontitsi[1], Antonis Patrinos[1], Eleni Porfyriou*[1], Alexandra Kontoula[1], Diamanto Kouniaki[2], Theofilos Athanasiadis[2], Maria Garofalaki[3], Tatiana Tzenou[1], Ifigeneia Tzannou[1], Georgia Kourti[1], Ioannis Tsonis[1], Stavros Gigantes[1], Vasiliki Kitsiou[2], Aikaterini Tarassi[2], Ioannis Baltadakis[1], Alexandra Tsirogianni[2]

*[1]"Evangelismos" General Hospital, Department of Haematology and Lymphomas and Bone Marrow Transplantation Unit, Athens, Greece; [2]"Evangelismos" General Hospital, Immunology and Histocompatibility Department, Athens, Greece; [3]"Evangelismos" General Hospital, Molecular Haematology Lab, Athens, Greece;*

### Background

MDS constitutes a clonal hematopoietic disorder characterized by ineffective hematopoiesis, cytopenias, dysplasia, and transformation risk to acute myeloid leukemia (AML). Pathogenesis integrates somatic mutations with immune dysregulation, including defective NK-cell surveillance of dysplastic clones. MICA and MICB are higly polymorphic, non-classical MHC class I genes, encoding stress-induced NKG2D ligands on NK, CD8+ and γδ T-cells. Thus, their expression and polymorphisms influence immune recognition and evasion mechanisms relevant to MDS pathogenesis and progression. No prior germline genetic studies have systematically investigated MICA/MICB polymorphisms in MDS susceptibility.

### Aims

Identify MICA/MICB alleles associated with MDS susceptibility via case-control frequency analysis.

### Methods

We performed NGS-based HLA typing including MICA/B alleles in 125 consecutive MDS patients and compared allele frequencies to 95 healthy controls from registry (PMID:32153595) using chi-square/Fisher's exact test with False Discovery Correction Rate (FDR) correction. Overall distribution rates were evaluated using chi-square tests of homogeneity ($\alpha$=0.05).

### Results

MICA locus showed profound skewing: 7/23 alleles differed significantly between the two cohorts (FDR<0.05) [Image 1A]. The MICA*008:01 allele was significantly less frequent in MDS patients, occurring in 15.6% versus 58.4% of healthy controls ($\chi^2$=42.8, p=1.62e-20), conferring strong protection to MDS. MICA*010:01 was, also, underrepresented in patients with MDS (2.4% vs 7.9% in controls; p=0.019) Conversely, MDS patients were enriched for MICA*008:04, *009:01, *016:01, and *018:01 alleles (all p<0.05), indicating that these alleles confer susceptibility.
MICB analysis revealed significant differences between the two groups in 4/16 alleles at FDR<0.05 (Image 1B). MICB*004:01 was 3.1-fold rarer in MDS patients ($\chi^2$=30.71, p<1e-16); whereas MICB*008:01 was 4.8-fold rarer ($\chi^2$=18.86, p<10e-10), both proving to have a protective role. Conversely, the MICB*002:01 allele was significantly more prevalent in MDS patients ($\chi^2$=12.8, p<1e-16), marking susceptibility.
Overall MICA and MICB distribution differed dramatically between cohorts [ ($\chi^2$=∞, p<1e-16) ($\chi^2$=101.4, p=7.07×10e-15), respectively]. These non-random patterns confirm the role of NKG2D-axis to MDS predisposition.

**Copyright Information:** (Online) ISSN: 2572-9241
©2026 The Author(s). HemaSphere is published by John Wiley & Sons Ltd on behalf of European Hematology Association. This is an open access Abstract Book distributed under the Attribution-NonCommercial-NoDerivs (CC BY-NC-ND), which allows third parties to download the articles and share them with others as long as they credit the author and the Abstract Book, but they cannot change the content in any way or use them commercially.
**Abstract Book Citations:** Authors, Title, HemaSphere, 2026;10:(S1):pages. The abstract book DOIs can be found on the "Author Information" page.
**Disclaimer:** Articles published in the journal HemaSphere exclusively reflect the opinions of the authors. The authors are responsible for all content in their abstracts including accuracy of the facts, statements, citing resources, etc.

### Summary/Conclusion

This pioneering NGS study demonstrates genome-wide significant germline MICA/MICB associations with MDS susceptibility. Protective alleles (MICA*008:01/010:01; MICB*004:01/008:01) likely confer NKG2D-mediated immunosurveillance, substantially reducing MDS risk. Susceptibility alleles (MICA*008:04, *009:01, *016:01, *018:01; MICB*002:01) promote ligand evasion, enhancing disease penetrance. These findings position MICA/MICB as novel immunogenetic risk modifiers that complement somatic mutational profiling for personalized MDS risk stratification. Preclinical evidence suggests hypomethylating agents upregulate MICA/MICB surface expression, identifying these loci as promising therapeutic targets to restore NKG2D-axis immunosurveillance in high-risk MDS.

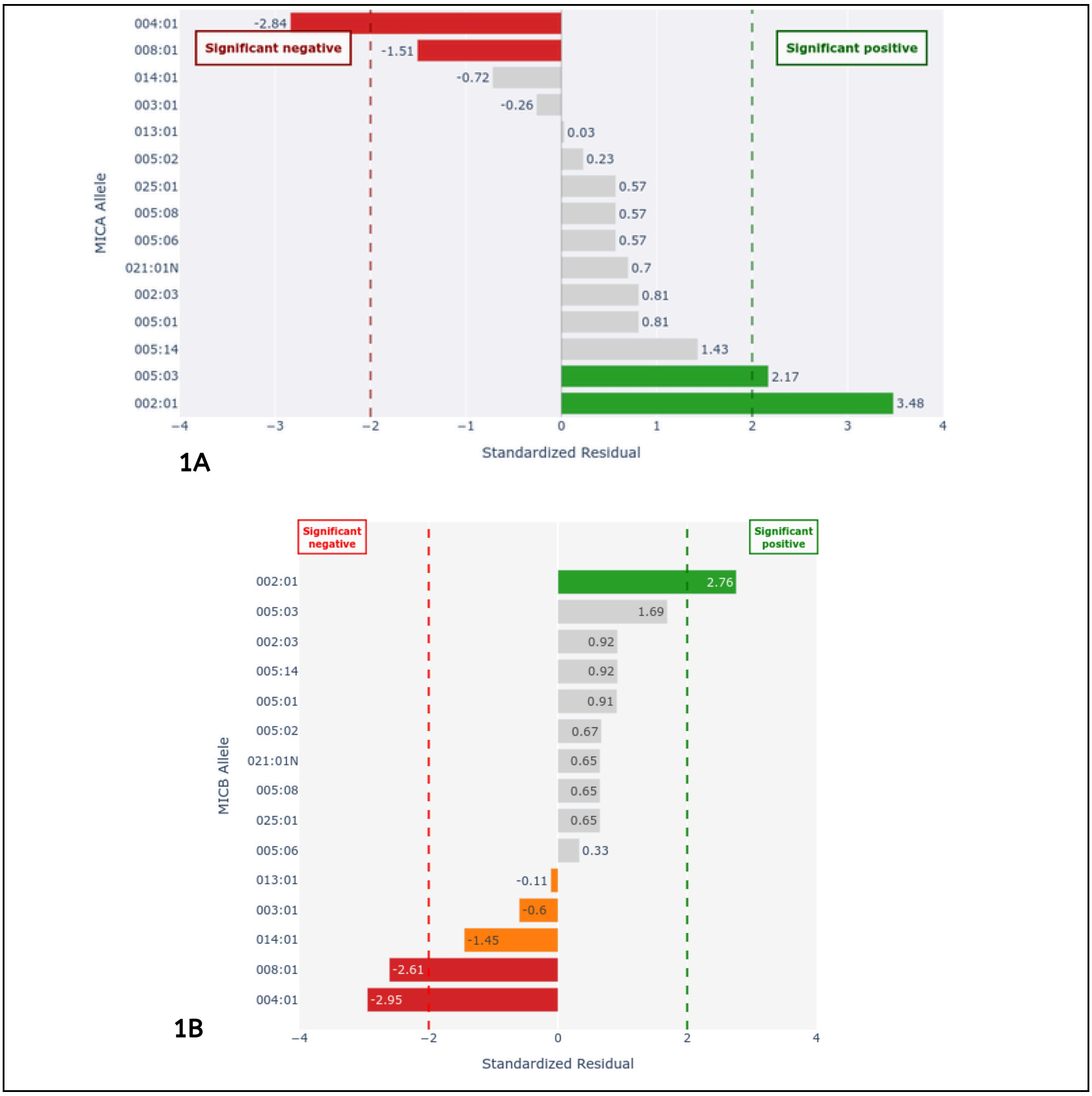

1A: Association of MICA allele polymorphisms with MDS susceptibility; 1B: Association of MICB allele polymorphisms with MDS susceptibility

**Copyright Information:** (Online) ISSN: 2572-9241
©2026 The Author(s). HemaSphere is published by John Wiley & Sons Ltd on behalf of European Hematology Association. This is an open access Abstract Book distributed under the Attribution-NonCommercial-NoDerivs (CC BY-NC-ND), which allows third parties to download the articles and share them with others as long as they credit the author and the Abstract Book, but they cannot change the content in any way or use them commercially.
**Abstract Book Citations:** Authors, Title, HemaSphere, 2026;10:(S1):pages. The abstract book DOIs can be found on the "Author Information" page.
**Disclaimer:** Articles published in the journal HemaSphere exclusively reflect the opinions of the authors. The authors are responsible for all content in their abstracts including accuracy of the facts, statements, citing resources, etc.

### (PS1755) LONGITUDINAL EVALUATION OF CLONAL HEMATOPOIESIS DYNAMICS IN OVARIAN CANCER PATIENTS DURING PARP INHIBITORS TREATMENT

**Topic:** 09. Myelodysplastic syndromes - Biology & translational research

Elisabetta Todisco*[1], Martina Fontanini[2], Chiara Ronchini[2], Raffaella Furgi[2], Yinxiu Zhan[2], Viviana Amato[2], Alessandro Davini[2], Ramadan Safaa Mahmoud[2], Federica Gigli[2], Fabio Giglio[2], Gabriella Parma[2], Ivana Lotesoriere[1], Francesco Ballardini[1], Nicoletta Colombo[2], Enrico Derenzini[2], Corrado Tarella[2], Pier Giuseppe Pelicci[2], Myriam Alcalay[2]

*[1]ASST Valle Olona, Busto Arsizio, Italy; [2]European Institute of Oncology, Milan, Italy;*

### Background

PARP inhibitors (PARPi) are used as maintenance therapy in platinum-sensitive epithelial ovarian cancer (EOC) patients. Prolonged exposures in combination with chemotherapy may increase the risk of hematological complications. We recently reported an 8.7% incidence of therapy related myeloid neoplasms (t-MNs) in EOC patients after a median PARPi exposure of 18.5 months and identified actionable mutations in cases with clonal cytopenia of undetermined significance (CCUS) after PARPi discontinuation. Chemotherapy may also promote clonal hematopoiesis (CH), an age-related condition defined by hematopoietic cells with somatic mutations in myeloid-associated genes without evident disease. Identifying genetic alterations associated with t-MN risk in PARPi-treated patients is warranted.

### Aims

We aimed to assess CH dynamics in samples collected longitudinally during PARPi exposure from the same patient and to identify genetic alterations associated with increased risk of t-MN in this clinical context.

### Methods

Genomic profiling was performed using custom gene panels and validated workflows for detection of germline and subclonal CH-associated variants. Germline analyses were conducted on buccal DNA at enrollment, while CH analyses on peripheral blood DNA pre and every six months during PARPi treatment. We employed a high-sensitivity sequencing approach with a variant allele frequency (VAF) detection limit of 0.1%, enabling detection of CH variants below the 2% clinical threshold of CHIP.

### Results

We analyzed 58 EOC patients (median age 60.5 years, range 38-85): 52% received PARPi as first-line maintenance therapy and 48% in later lines after platinum-based chemotherapy; 53% received Olaparib, 31% Niraparib and 16% Rucaparib. We identified 23 pathogenic germline mutations, 87% of which were heterozygous mutations in *BRCA1/2* genes, in 22 (38%) patients. CH analyses were performed on 58 patients pre-PARPi and in 56, 31, 22, 14 and 7 patients after 6, 12, 18, 24 and 30 months of treatment, respectively. Across all timepoints, we identified 600 CH mutations, 70% of which defined as potential driver-CH (PD-CH). PD-CH burden increased significantly with age, and the most frequently mutated genes were *DNMT3A*, *TP53* and *PPM1D*. 91% of mutations had VAF <2%, confirming the sensitivity of the approach. Pre-PARPi, 91% of patients had CH mutations. No associations were observed between numbers of PD-CH mutations and patients' *BRCA* status, number of cycles of platinum-based chemotherapy, or line of PARPi treatment. Longitudinal analyses revealed complex CH dynamics including acquisition, loss and persistence of CH mutated clones. VAF increased significantly during PARPi treatment (up to 2 years), with the most pronounced and persistent expansions observed in *PPM1D*-mutated clones. CHIP/CCUS cases nearly doubled during PARPi, from 16 to 30 patients within 2 years; although not significant, multiple *PPM1D* and *TP53* mutations were associated with higher likelihood of CCUS.

**Copyright Information:** (Online) ISSN: 2572-9241
©2026 The Author(s). HemaSphere is published by John Wiley & Sons Ltd on behalf of European Hematology Association. This is an open access Abstract Book distributed under the Attribution-NonCommercial-NoDerivs (CC BY-NC-ND), which allows third parties to download the articles and share them with others as long as they credit the author and the Abstract Book, but they cannot change the content in any way or use them commercially.
**Abstract Book Citations:** Authors, Title, HemaSphere, 2026;10:(S1):pages. The abstract book DOIs can be found on the "Author Information" page.
**Disclaimer:** Articles published in the journal HemaSphere exclusively reflect the opinions of the authors. The authors are responsible for all content in their abstracts including accuracy of the facts, statements, citing resources, etc.

**Summary/Conclusion**

Using a high-sensitivity approach, we observed high prevalence of CH mutations in PARPi treated EOC patients, mainly at low VAF. Longitudinal expansion of PD-CH clones, particularly those with *PPM1D* mutations, suggests a role of PARPi exposure in clonal selection. These findings support screening for CH pre-PARPi treatment and longitudinal clonal monitoring to identify patients at increased risk of t-MNs.

**Copyright Information:** (Online) ISSN: 2572-9241
©2026 The Author(s). HemaSphere is published by John Wiley & Sons Ltd on behalf of European Hematology Association. This is an open access Abstract Book distributed under the Attribution-NonCommercial-NoDerivs (CC BY-NC-ND), which allows third parties to download the articles and share them with others as long as they credit the author and the Abstract Book, but they cannot change the content in any way or use them commercially.
**Abstract Book Citations:** Authors, Title, HemaSphere, 2026;10:(S1):pages. The abstract book DOIs can be found on the "Author Information" page.
**Disclaimer:** Articles published in the journal HemaSphere exclusively reflect the opinions of the authors. The authors are responsible for all content in their abstracts including accuracy of the facts, statements, citing resources, etc.

### (PS1756) SINGLE-CELL INSIGHTS INTO THE DIFFERENTIATION SKEWING IN AML USING BLOOD-FORMING GASTRULOIDS.

**Topic:** 09. Myelodysplastic syndromes - Biology & translational research

Robel Tesfaye*[1], Pavan Kumar Mysuru Shivalingappa[1], Anuntxi Monsalve-Iguiniz[1], Anna Hogmalm[1], Elin Hilpold-Berntsson[1], Pekka Jaako[1], Lars Palmqvist[2], Carolina Guibentif[1]

*[1]Institute of Biomedicine, Sahlgrenska Center for Cancer Research, University of Gothenburg, Department of Microbiology and Immunology, Gothenburg, Sweden; [2]Institute of Biomedicine, University of Gothenburg, Department of Clinical Chemistry, Gothenburg, Sweden;*

### Background

Both leukemia and hematopoiesis are believed to follow a hierarchical model of development. At the apex, cancer stem cells and hematopoietic stem cells (HSCs), respectively, differentiate into downstream cell types. While mutations leading to clonal hematopoiesis give a subset of HSCs a fitness advantage, other mutations could lead to inefficient hematopoiesis characteristic of myelodysplastic syndromes and potentially leukemia. The t(7;12)(q36;p13) cryptic translocation between chromosomes 7 and 12 results in a differentiation blockade at myeloid progenitors' stages. This translocation is estimated to account for 18 to 30% of infant AML cases, with a median age of patients of about 6 months. Even though fusion transcripts can be detected in patients, a critical factor for leukemogenesis seems to be *MNX1* overexpression. While the transcriptomic and epigenomic consequences of *MNX1* overexpression were described recently, the factors underlying the differentiation blockade remain unclear.

### Aims

The main objective of the study is to characterize the differentiation trajectories occurring within a human pluripotent stem cell (hPSCs)-based gastruloid model of t(7;12) AML. The study seeks to identify mechanisms preceding the observed hematopoietic differentiation skewing and reveal potential therapeutic targets.

### Methods

Two clones of hPSC lines bearing the t(7;12) translocation and the parental wild-type hPSCs were differentiated into blood-forming human gastruloids (BFhG) in vitro. Cells were then harvested at different time points to evaluate cell-type heterogeneity, hematopoietic, and stromal output, through flow cytometry. Single-cell RNA sequencing was performed to compare cellular composition and differentiation dynamics between wild-type and mutant cells.

### Results

Flow cytometry data indicate that mutant cells generate a higher proportion of hematopoietic cells and lower mesenchymal/stromal cell populations relative to wild-type cells. While endothelial populations show comparable proportions between mutant and wild-type cells, a higher proportion of hemogenic endothelium is observed in mutant cells, potentially supporting more efficiently hematopoietic differentiation. Single-cell RNA sequencing analysis indicates that *MNX1* expression is exclusive to mutant cells. *MNX1* expression is induced in endothelial precursor cells, suggesting that *MNX1* could drive the higher hematopoietic output in mutant cells. Mutant cells also show cell-type compositional alterations within the remaining mesodermal populations.

**Copyright Information:** (Online) ISSN: 2572-9241
©2026 The Author(s). HemaSphere is published by John Wiley & Sons Ltd on behalf of European Hematology Association. This is an open access Abstract Book distributed under the Attribution-NonCommercial-NoDerivs (CC BY-NC-ND), which allows third parties to download the articles and share them with others as long as they credit the author and the Abstract Book, but they cannot change the content in any way or use them commercially.
**Abstract Book Citations:** Authors, Title, HemaSphere, 2026;10:(S1):pages. The abstract book DOIs can be found on the "Author Information" page.
**Disclaimer:** Articles published in the journal HemaSphere exclusively reflect the opinions of the authors. The authors are responsible for all content in their abstracts including accuracy of the facts, statements, citing resources, etc.

**Summary/Conclusion**

A comparative analysis of the differentiation trajectories taking place in mutant cells relative to parental wild-type cells suggests that a unique mesodermal composition, combined with overexpression of *MNX1*, underlies the differentiation skewing observed in t(7;12) AML models. The BFhG models offer a unique insight into mechanisms preceding tumor initiation and the environment supporting tumoral development.

**Copyright Information:** (Online) ISSN: 2572-9241
©2026 The Author(s). HemaSphere is published by John Wiley & Sons Ltd on behalf of European Hematology Association. This is an open access Abstract Book distributed under the Attribution-NonCommercial-NoDerivs (CC BY-NC-ND), which allows third parties to download the articles and share them with others as long as they credit the author and the Abstract Book, but they cannot change the content in any way or use them commercially.
**Abstract Book Citations:** Authors, Title, HemaSphere, 2026;10:(S1):pages. The abstract book DOIs can be found on the "Author Information" page.
**Disclaimer:** Articles published in the journal HemaSphere exclusively reflect the opinions of the authors. The authors are responsible for all content in their abstracts including accuracy of the facts, statements, citing resources, etc.

### (PS1757) COEXISTING BUT GENETICALLY DISTINCT HEMATOPOIETIC AND STROMAL ALTERATIONS IN MYELODYSPLASTIC SYNDROMES

**Topic:** 09. Myelodysplastic syndromes - Biology & translational research

Andrés Durán Adame[1, 2], Carlos Puerta[3], Mayte García Antúnez[1], Raúl Azibeiro-Melchor[3], Eva Lumbreras[2], Irene Rodríguez Iglesias[2], Sara González Briones[2], Cristina Díaz Pescador[1], Marta Fonseca Santos[3], Araceli Sama-Barroso[1, 2], Teresa Bernal[4], Juan Carlos Caballero[5], Almudena Fernandez Gonzalez[6], Jesus Maria Hernandez Rivas[1, 2, 3], María Díez Campelo*[1, 3], Sandra Muntion[1], Mónica del Rey[1, 2]

*[1]Instituto de Investigación Biomédica de Salamanca (IBSAL), Universidad de Salamanca, Departamento de Medicina, Salamanca, Spain; [2]IBMCC, Centro de Investigación del Cáncer (CIC), Universidad de Salamanca, CSIC, Salamanca, Spain; [3]Hospital Universitario de Salamanca, Salamanca, Spain;[4]Hospital Universitario Central de Asturias, Oviedo, Spain;[5]Hospital Clínico Universitario de Valladolid, Valladolid, Spain; [6]Hospital Universitario de Cabueñes, Gijón, Spain;*

### Background

The somatic mutational landscape of hematopoietic cells (HSC) in Myelodysplastic Syndromes (MDS) has been extensively characterized. However, whether mesenchymal stromal cells (MSC)—key regulators of hematopoietic stem cell maintenance—harbor somatic mutations and, in particular, whether they share genetic alterations with the hematopoietic compartment remains unclear.

### Aims

To compare the mutational profiles of MSC and HSC in MDS, determine whether alterations are shared or compartment-specific, and evaluate their potential biological relevance.

### Methods

A total of 154 samples were obtained from 72 MDS patients. These included 72 mononuclear cells (MNC) and 72 MSC samples isolated using Ficoll density-gradient centrifugation. MSCs were expanded to passage 3 under standard culture conditions. In addition, $CD3^+$ cells were isolated by magnetic separation from peripheral blood samples collected from 10 patients (autoMACS, Miltenyi Biotec) and used as germline controls. Targeted NGS was performed in MNC and MSC of 62 patients using a custom panel of 108 MDS-related genes (Agilent SureSelect; sequenced on NextSeq, Illumina). Whole Exome Sequencing (WES) was conducted in MNC, MSC and $CD3^+$ cells of 10 patients (Twist Exome 2.0; NovaSeq X Plus, Illumina). Only high-quality exonic or splice-site protein-altering variants were retained for analysis.

**Copyright Information:** (Online) ISSN: 2572-9241
©2026 The Author(s). HemaSphere is published by John Wiley & Sons Ltd on behalf of European Hematology Association. This is an open access Abstract Book distributed under the Attribution-NonCommercial-NoDerivs (CC BY-NC-ND), which allows third parties to download the articles and share them with others as long as they credit the author and the Abstract Book, but they cannot change the content in any way or use them commercially.
**Abstract Book Citations:** Authors, Title, HemaSphere, 2026;10:(S1):pages. The abstract book DOIs can be found on the "Author Information" page.
**Disclaimer:** Articles published in the journal HemaSphere exclusively reflect the opinions of the authors. The authors are responsible for all content in their abstracts including accuracy of the facts, statements, citing resources, etc.

**Results**

The cohort was representative of patients with MDS. According to the WHO-2017 classification, the most frequent subtypes were MDS with multilineage dysplasia (37%), MDS with isolated del(5q) (22%) and MDS with multilineage dysplasia and ring sideroblasts (13%). In MNC, 93 pathogenic variants were identified, affecting 46 of 72 patients (63.9%). In contrast, MSC harbored 11 pathogenic variants, detected in 8 of the 72 patients (11.1%) (**Fig. 1**). Importantly, none of the pathogenic variants found in MSC were shared with their paired MNC samples.
Five out of eleven pathogenic variants identified in MSC corresponded to MDS-associated genes and were detected across the 72-patient cohort: *DNMT3A* (p.S160fs, VAF=12.7%; p.G354C, VAF=4.5%) ( **Fig. 1: P1, P2** ), *KRAS* (p.G12D, VAF=20.4%; p.Q61H, VAF=8.5%) (**Fig. 1: P3, P4** ), and *STAG2* (p.W1038*, VAF=4.1%) ( **Fig. 1: P4**). The remaining six variants affected non-MDS related genes ( *UGT1A1*, *CA10*, *NEB*, *GABRA5*, *BACH2*, and *PPP2R1A*) and were identified within the subset of ten patients analyzed by WES.
Among the eight patients harboring MSC mutations, three of them harbored two variants. One patient presented both an MDS-associated and a non–MDS-associated alteration (*KRAS* and *GABRA5*; **Fig. 1: P3**), another harbored two MDS-associated variants (*KRAS* and *STAG2*; **Fig. 1: P4**) and a third displayed two non–MDS-associated variants ( *UGT1A1* and *CA10*; **Fig. 1: P5**).
Regarding cytogenetics, 3/8 patients with MSC mutations presented complex karyotypes, representing 50% of all complex karyotypes identified in the 72-patient cohort. Two of these harbored *TP53* mutations in MNC, including one multi-hit case with two *TP53* variants and one mono-hit case (**Fig. 1: P2, P3** ), whereas the remaining one did not present any mutations in MNC (**Fig. 1: P1** ). The other five patients showed normal (n=3), −Y, or +8 karyotypes, all of whom harbored *SF3B1* mutations in MNC.

**Summary/Conclusion**

Altogether, our results demonstrate that stromal niches can acquire pathogenic alterations independent of hematopoietic mutations, highlighting the microenvironment as an active and genetically altered component in MDS pathogenesis.
**Funding:** PI23/01103, PMP 24/00025

**Copyright Information:** (Online) ISSN: 2572-9241
©2026 The Author(s). HemaSphere is published by John Wiley & Sons Ltd on behalf of European Hematology Association. This is an open access Abstract Book distributed under the Attribution-NonCommercial-NoDerivs (CC BY-NC-ND), which allows third parties to download the articles and share them with others as long as they credit the author and the Abstract Book, but they cannot change the content in any way or use them commercially.
**Abstract Book Citations:** Authors, Title, HemaSphere, 2026;10:(S1):pages. The abstract book DOIs can be found on the "Author Information" page.
**Disclaimer:** Articles published in the journal HemaSphere exclusively reflect the opinions of the authors. The authors are responsible for all content in their abstracts including accuracy of the facts, statements, citing resources, etc.

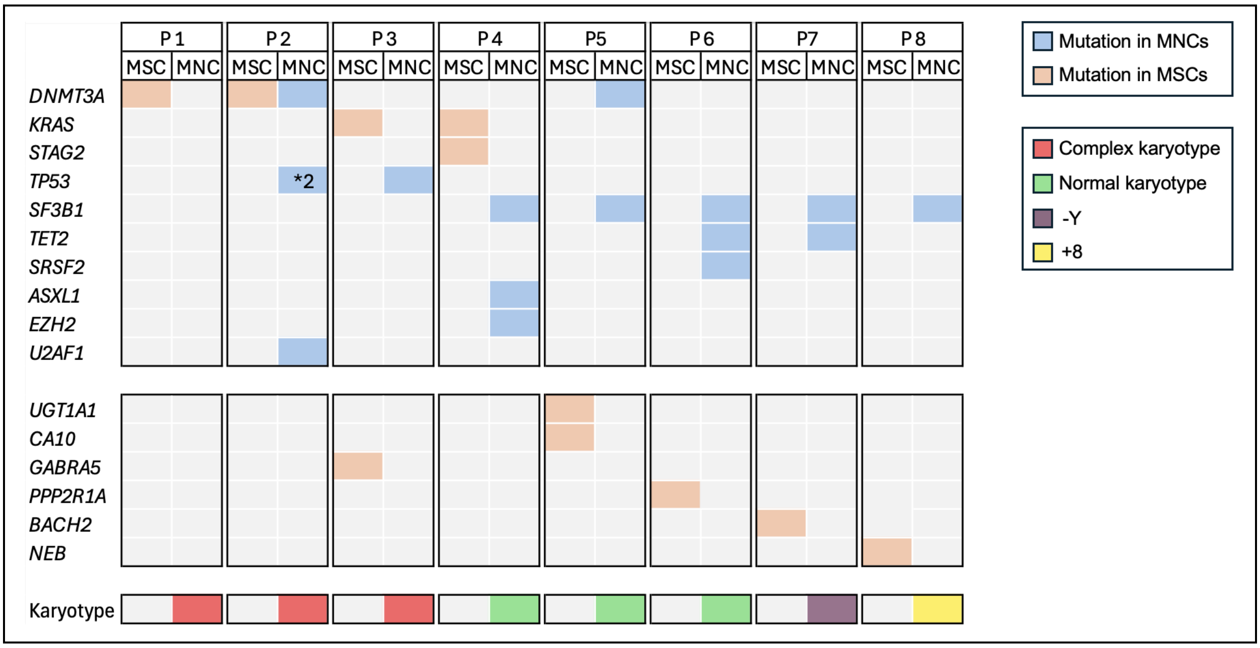

Figure 1. Distribution of pathogenic variants in 8 MDS patients with MSC somatic alterations. *2: Patient 2 (P2) harbored two independent TP53 mutations (p.E153K, VAF=25.1% and p.S88F, VAF=26.4%), consistent with a multi-hit TP53 status.

**Copyright Information:** (Online) ISSN: 2572-9241
©2026 The Author(s). HemaSphere is published by John Wiley & Sons Ltd on behalf of European Hematology Association. This is an open access Abstract Book distributed under the Attribution-NonCommercial-NoDerivs (CC BY-NC-ND), which allows third parties to download the articles and share them with others as long as they credit the author and the Abstract Book, but they cannot change the content in any way or use them commercially.
**Abstract Book Citations:** Authors, Title, HemaSphere, 2026;10:(S1):pages. The abstract book DOIs can be found on the "Author Information" page.
**Disclaimer:** Articles published in the journal HemaSphere exclusively reflect the opinions of the authors. The authors are responsible for all content in their abstracts including accuracy of the facts, statements, citing resources, etc.

### (PS1758) VEXAS PROGNOSTIC SCORE: PERSONALIZED MACHINE LEARNING DERIVED RISK STRATIFICATION MODEL

**Topic:** 10. Myelodysplastic syndromes - Clinical

Tariq Kewan*[1], Yael Kusne[2], Tian Shulan[1], Ryan Iverson[1], Aubrey Pinteric[2], Terra Lasho[1], Jenna Fernandez[1], Kaaren Reichard[1], Horatiu Olteanu[1], Aasiya Matin[1], Mehrdad Hefazi[1], Mithun Shah[1], Kebede Begna[1], Talha Badar[3], William Hogan[1], James M Foran[3], Cecilia Arana Yi[2], Patricia Greipp[1], Antoine Saliba[1], Naseema Gangat[1], Mark Litzow[1], David Dingli[1], Taxiarchis Kourelis[1], Rong He[1], Dragan Jevremovic[1], Hassan Alkhateeb[1], Megan Sullivan[1], Kenneth Warrington[1], Aref Al-Kali[1], Mrinal Patnaik[1], Matthew Koster[1], Abhishek Mangaonkar[1]

*[1]Mayo Clinic, Department of Internal Medicine, Rochester, United States of America;[2]Mayo Clinic Hospital, Phoenix, United States of America; [3]Mayo Clinic, Jacksonville, United States of America;*

### Background

VEXAS (vacuoles, E1 enzyme, X-linked, autoinflammatory, somatic) syndrome is a systemic auto inflammatory disease secondary to acquired somatic mutations in UBA1 ($UBA1^{mut}$). Factors associated with favorable or adverse prognosis are not well established.

### Aims

To develop a risk score to guide risk stratification and treatment decisions for patients (pts) with VEXAS syndrome.

### Methods

Pts with VEXAS syndrome diagnosed at Mayo Clinic between 2015 and 2025 were included. Gradient boosting machine (GBM) Cox model using clinical, bone marrow biopsy, and molecular data developed to predict overall survival (OS). Pts who received allogenic hematopoietic stem cell transplant (allo-HCT) were censored at the time of transplant. The model was adjusted for the use of hypomethylating agents (HMA) or disease modifying antirheumatic drugs. Model performance optimized using hyperparameter tuning with 5-fold cross-validation and evaluated using Harrell's C-index. SHAP analysis used to identify significant predictors for OS. Predicted risk scores by the GBM model used to define risk groups.

**Copyright Information:** (Online) ISSN: 2572-9241
©2026 The Author(s). HemaSphere is published by John Wiley & Sons Ltd on behalf of European Hematology Association. This is an open access Abstract Book distributed under the Attribution-NonCommercial-NoDerivs (CC BY-NC-ND), which allows third parties to download the articles and share them with others as long as they credit the author and the Abstract Book, but they cannot change the content in any way or use them commercially.
**Abstract Book Citations:** Authors, Title, HemaSphere, 2026;10:(S1):pages. The abstract book DOIs can be found on the "Author Information" page.
**Disclaimer:** Articles published in the journal HemaSphere exclusively reflect the opinions of the authors. The authors are responsible for all content in their abstracts including accuracy of the facts, statements, citing resources, etc.

### Results

142 pts (median age of 71 [IQR: 65-77] years) included. All the pts were male except one female pt. Thirty (21%) had concurrent myelodysplastic syndrome (MDS). *UBA1* p.Met41Thr (44%), p.Met41Leu (25%), and p.Met41Val (22%) were the most common mutations. Only 10% had splice site variants. The median *UBA1*$^{mut}$ VAF was 65 (43–81)%. Skin rash (82%), peripheral arthritis (47%), and recurrent fever (46%) were the most common symptoms. *DNMT3A* (20%), *SF3B1* (4%), and *ASXL1* (2%) were the most common co-mutations. For treatment, only 34 (24%) pts received HMA and 25 (18%) underwent allo-HCT.
SHAP analysis identified weight loss, anti-nuclear antibodies, absolute monocyte count, chondritis, and hypercellular bone marrow as the most influential predictors of survival in the GBM-Cox model. Co-MDS contributed minimally to risk prediction (Figure 1A). Three risk groups (low = 48, intermediate = 47, and high = 47) identified. The high-risk group (vs. low-risk) was characterized by a higher frequency of hypercellular bone marrow (87% vs. 69%, p=0.029), bone marrow vacuoles (85% vs. 67%, p=0.039), and peripheral arthritis (51% vs. 31%, p=0.018). High-risk pts had higher rates of RBC transfusion (51% vs. 6%, p<0.001) and platelet transfusions (19% vs. 0%, p<0.001). Laboratory differences included higher median MCV (111 vs. 103 fL, p<0.001) and UBA1$^{mut}$ VAF (75% vs. 56%, p=0.036), lower hemoglobin (9.3 vs. 11.5 g/dL, p<0.001) and platelet counts (124 vs.193 $\times 10^9$/L, p<0.001) in the high-risk group. The median glucocorticoid dose was also higher in the high-risk group (40 mg vs. 30 mg, p=0.022).
The median follow up from diagnosis was 27 (24-31) months (mo). The median OS (mo) was significantly different between the pts assigned to low-risk (69, 95% CI: NA-NA), intermediate-risk (29, 95%CI: 26-NA), and high-risk (14, 95%CI 9-16) groups, p<0.001 (Figure 1B). Significant OS differences between the risk groups observed among pts treated with HMA (p<0.001) and pts received allo-HCT (p = 0.01).

### Summary/Conclusion

We developed a machine learning risk score that stratifies pts with VEXAS syndrome into three prognostic groups with distinct OS. This risk model will provide a practical framework to guide risk-adapted treatment strategies, including consideration of early allo-HCT in high-risk pts and enrollment in clinical trials. Validation in an external cohort is currently ongoing.

**Copyright Information:** (Online) ISSN: 2572-9241
©2026 The Author(s). HemaSphere is published by John Wiley & Sons Ltd on behalf of European Hematology Association. This is an open access Abstract Book distributed under the Attribution-NonCommercial-NoDerivs (CC BY-NC-ND), which allows third parties to download the articles and share them with others as long as they credit the author and the Abstract Book, but they cannot change the content in any way or use them commercially.
**Abstract Book Citations:** Authors, Title, HemaSphere, 2026;10:(S1):pages. The abstract book DOIs can be found on the “Author Information” page.
**Disclaimer:** Articles published in the journal HemaSphere exclusively reflect the opinions of the authors. The authors are responsible for all content in their abstracts including accuracy of the facts, statements, citing resources, etc.

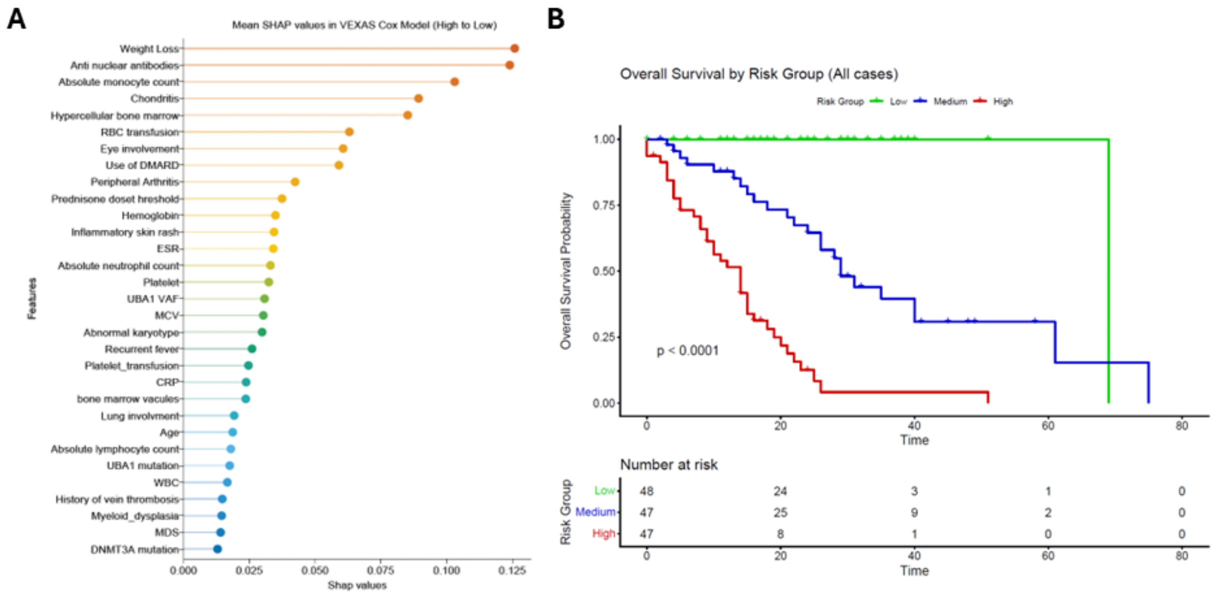

**Figure 1. A:** Mean SHAP values for the most significant variables included in the mayo Clinic VEXAS score. **B.** Kaplan–Meier analysis of overall survival (OS) among patients with VEXAS syndrome stratified into low, intermediate, and high risk groups according to the Mayo Clinic VEXAS Score.

**Copyright Information:** (Online) ISSN: 2572-9241
©2026 The Author(s). HemaSphere is published by John Wiley & Sons Ltd on behalf of European Hematology Association. This is an open access Abstract Book distributed under the Attribution-NonCommercial-NoDerivs (CC BY-NC-ND), which allows third parties to download the articles and share them with others as long as they credit the author and the Abstract Book, but they cannot change the content in any way or use them commercially.
**Abstract Book Citations:** Authors, Title, HemaSphere, 2026;10:(S1):pages. The abstract book DOIs can be found on the “Author Information” page.
**Disclaimer:** Articles published in the journal HemaSphere exclusively reflect the opinions of the authors. The authors are responsible for all content in their abstracts including accuracy of the facts, statements, citing resources, etc.

### (PS1759) PHASE II STUDY OF CLADRIBINE, LOW DOSE CYTARABINE AND VENETOCLAX, ALTERNATING WITH AZACITIDINE AND VENETOCLAX, IN HIGHER RISK MYELODYSPLASTIC SYNDROMES

**Topic:** 10. Myelodysplastic syndromes - Clinical

Guillermo Montalban-Bravo*[1], Naval Daver[1], Nicholas Short[1], Yesid Alvarado[1], Kelly Chien[1], Gautam Borthakur[1], Ghayas Issa[1], Elias Jabbour[1], Courtney Dinardo[1], Mahesh Swaminathan[1], Abhishek Maiti[1], Danielle Hammond[1], Jo Ishizawa[1], Maro Ohanian[1], Lucia Masarova[1], Steven Kornblau[1], Prithviraj Bose[1], Alex Bataller[1], Fadi Haddad[1], Alexandre Bazinet[1], Xuelin Huang[2], Sanam Loghavi[1], Heather Schneider[1], Stephany Hendrickson[1], Tapan Kadia[1], Guillermo Garcia-Manero[1]

*[1]The University of Texas MD Anderson Cancer Center, Hematopathology, Houston, United States of America;[2]The University of Texas MD Anderson Cancer Center, Biostatistics, Houston, United States of America;*

### Background

Responses to hypomethylating agents (HMAs) in patients (pts) with higher-risk myelodysplastic syndromes (HR-MDS) are short-lived, with high risk of transformation to acute myeloid leukemia (AML). Cladribine (CDA) is active in AML when combined with low dose cytarabine (LDAC) and venetoclax (VEN).

### Aims

To evaluate the safety and activity of CDA, LDAC and VEN in HR-MDS.

### Methods

We designed a phase II clinical trial (NCT05365035) for newly diagnosed (ND) or relapsed/refractory (R/R) HR-MDS and other chronic myeloid neoplasms. Results for HR-MDS pts are reported here. Induction consisted of CDA 5mg/m$^2$ daily IV for 3-4 days, LDAC 20mg s.c bid for 5-7 days and VEN 400mg daily for 7-10 days followed by azacitidine 75mg/m$^2$ daily days 1-7 with venetoclax 400mg daily days 1-7 or CDA 5mg/m$^2$ days 1-3, LDAC 20mg s.c bid days 1-5 and VEN 400mg days 1-7 alternating every 2 cycles. Primary end points included safety and efficacy.

**Copyright Information:** (Online) ISSN: 2572-9241
©2026 The Author(s). HemaSphere is published by John Wiley & Sons Ltd on behalf of European Hematology Association. This is an open access Abstract Book distributed under the Attribution-NonCommercial-NoDerivs (CC BY-NC-ND), which allows third parties to download the articles and share them with others as long as they credit the author and the Abstract Book, but they cannot change the content in any way or use them commercially.
**Abstract Book Citations:** Authors, Title, HemaSphere, 2026;10:(S1):pages. The abstract book DOIs can be found on the "Author Information" page.
**Disclaimer:** Articles published in the journal HemaSphere exclusively reflect the opinions of the authors. The authors are responsible for all content in their abstracts including accuracy of the facts, statements, citing resources, etc.

### Results

As of 02/2026, 41 pts have been treated (16 [%] ND and 25 [%] R/R) including 5 (12%) with high and 31 (76%) with very high IPSS Molecular risk. The median age was 71 years (range 52-83). Median bone marrow blasts were 13% (range 6-19). Eighteen (44%) pts had complex karyotype and 8 (20%) had multihit *TP53* (*multiTP53*). Among R/R pts: the median number of prior therapies was 1 (range 1-4); the median number of prior HMA cycles was 9 (range 2-60); 7 (28%) pts had received prior VEN and 1 (4%) prior allogeneic stem-cell transplant (SCT).

The median number of cycles received was 2 (range 1-7). The overall response rates (ORR) by IWG 2006 response criteria were 52% (n=13, complete response [CR]: 4 [16%]; marrow complete response [mCR]: 6 [24%]; mCR + hematological improvement [HI]: 3 [12%]) and 73% (n=11, CR: 7 [44%]; mCR: 4 [25%]) in R/R and ND pts, respectively. By IWG 2023 response criteria, ORR were 48% (n=12, CR: 5 [20%]; CR bilineage [$CR_{bi}$]: 4 [16%], CR unilineage [$CR_{uni}$]: 2 [8%]; CR with partial hematologic recovery [$CR_h$]: 1 [4%]) and 56% (n=9, CR: 8 [50%]; $CR_{bi}$: 1 [6%]) in R/R or ND pts, respectively. Among ND patients who achieved CR 2 (25%) had complete cytogenetic responses. Among pts with *multiTP53* only 2 (25%) achieved response to therapy including 1 (13%) CR by both IWG 2006 and 2023, and 1 (13%) mCR by IWG 2006. Median number of cycles to best response was 1 (range 1-3). At time of data cut-off 3 (7%) pts remain on study, 13 (32%) came off study to undergo SCT, 7 (17%) due to AML progression, 5 (12%) due to no response, 4 (10%) due to physician choice, 4 (10%) due to patient choice, 4 (10%) due to relapse, 1 (2%) due to death.

The 4- and 8-week mortality were 4% and 4% for R/R, and 0% and 6% for ND pts, respectively. Most common grade ³3 treatment-emergent adverse events were thrombocytopenia (n=13, 32%), febrile neutropenia (n=6, 15%) and neutropenia (n=6, 15%). Among responders, 71% and 75% of pts achieved neutrophil (>1x$10^9$/L) and platelet (>100x$10^9$/L) recovery after cycle 1 after a median of 27 (range 24-38) and 21 (range 15-43) days, respectively.

With a median follow up of 19.8 months, the median overall survival (OS) is 17.7 and 5.8 months for ND and R/R pts, respectively. Among R/R pts, *multiTP53* or multiple prior lines of therapy/prior VEN were associated with numerically shorter OS compared to that of patients with isolated prior HMA exposure (median OS 4.0 months, 5.8 months, 27.4 months, respectively).

### Summary/Conclusion

CDA, LDAC and VEN is safe in pts with HR-MDS with promising results in ND pts with wild-type*TP53* gene status.

**Copyright Information:** (Online) ISSN: 2572-9241
©2026 The Author(s). HemaSphere is published by John Wiley & Sons Ltd on behalf of European Hematology Association. This is an open access Abstract Book distributed under the Attribution-NonCommercial-NoDerivs (CC BY-NC-ND), which allows third parties to download the articles and share them with others as long as they credit the author and the Abstract Book, but they cannot change the content in any way or use them commercially.
**Abstract Book Citations:** Authors, Title, HemaSphere, 2026;10:(S1):pages. The abstract book DOIs can be found on the “Author Information” page.
**Disclaimer:** Articles published in the journal HemaSphere exclusively reflect the opinions of the authors. The authors are responsible for all content in their abstracts including accuracy of the facts, statements, citing resources, etc.

### (PS1760) HYPOMETHYLATING AGENTS WITH OR WITHOUT VENETOCLAX IN TREATMENT-NAIVE MYELODYSPLASTIC SYNDROMES WITH EXCESS BLASTS: RESULTS FROM A LARGE RETROSPECTIVE COHORT

**Topic:** 10. Myelodysplastic syndromes - Clinical

Alex Bataller*[1], Alexandre Bazinet[1], Ian Bouligny[1], Guillermo Montalban-Bravo[1], Kelly Chien[1], Danielle Hammond[1], Nicholas Short[1], Ghayas Issa[1], Tapan Kadia[1], Farhad Ravandi[1], Elias Jabbour[1], Naval Daver[1], Courtney Dinardo[1], Sanam Loghavi[1], Harold Keer[2], Uday R. Popat[3], Guillermo Garcia-Manero[1]

*[1]The University of Texas MD Anderson Cancer Center, Leukemia Department, Houston, United States of America;[2]Taiho Oncology Inc, Princeton, New Jersey, United States of America; [3]The University of Texas MD Anderson Cancer Center, Department of Stem Cell Transplantation and Cellular Therapy, Houston, United States of America;*

### Background

There is rationale for combining Venetoclax (Ven) with hypomethylating agents (HMAs) in higher-risk myelodysplastic syndromes (HR-MDS). The Phase 3 randomized VERONA trial (Azacitidine [Aza] ± Ven) did not improve overall survival (OS). Debate remains about which HR-MDS subgroups may benefit from Ven.

### Aims

To analyze and compare a large cohort of patients with MDS with excess-blasts (MDS-EB) treated with HMA± Ven, focusing on the outcomes in specific groups and clinical scenarios.

### Methods

This retrospective single-center study included treatment-naïve MDS-EB (≥5% blasts in the bone marrow [BM]) diagnosed from 2010 to 2025. Patients were grouped by frontline therapy received (trial or non-trial): HMAs alone (HMA) or HMAs with Ven (HMAVen). Outcomes were analyzed using Kaplan Meier, Cox regression and multi-state Markov multi-state models.

**Copyright Information:** (Online) ISSN: 2572-9241
©2026 The Author(s). HemaSphere is published by John Wiley & Sons Ltd on behalf of European Hematology Association. This is an open access Abstract Book distributed under the Attribution-NonCommercial-NoDerivs (CC BY-NC-ND), which allows third parties to download the articles and share them with others as long as they credit the author and the Abstract Book, but they cannot change the content in any way or use them commercially.
**Abstract Book Citations:** Authors, Title, HemaSphere, 2026;10:(S1):pages. The abstract book DOIs can be found on the "Author Information" page.
**Disclaimer:** Articles published in the journal HemaSphere exclusively reflect the opinions of the authors. The authors are responsible for all content in their abstracts including accuracy of the facts, statements, citing resources, etc.

**Results**

A total of 358 patients were included: 245 (HMA) and 113 (HMAVen). Median age was similar (69 vs 70 years, *P*=ns). Median BM blast percentage was higher in the HMAVen cohort (12% vs 8%, *P*<0.001). IPSS-R risk distribution was comparable between both cohorts with high-risk disease in 29% of both cohorts and very high-risk disease in 47 vs 60% (*P*=ns). Mutations were balanced across cohorts; the most frequent mutations were *TP53* (HMA 35% vs HMAVen 32%), *ASXL1* (HMA 23% vs HMAVen 32%) and *DNMT3A* (HMA 17% vs HMAVen 14%). Treatment included Aza (HMA 28% vs HMA-Ven 41%, P=ns), decitabine (HMA 47% vs HMAVen 59%, P=ns) and guadecitabine (HMA 25% vs HMAVen 0%, P<0.001). In the HMAVen cohort, 93% of patients received Ven 8-14 days, 4% for 1-17 days, and 3% >15 days during cycle 1.
By IWG 2006 criteria, the overall response rate (ORR) was 62% (HMA) vs 89% (HMAVen, *P*<0.001), including complete response rates of 34% and 35%, respectively. By IWG 2023 criteria, the ORR was 51% (HMA) vs 77% (HMAVen) (P<0.001). HSCT rates were 26% (HMA) and 43% (HMAVen) (P<0.001).
Median OS and 2-yr OS was 19 months/41% vs 18 months/46% for the HMA vs HMAVen (P=ns), with no OS differences across HMA types. Univariate subgroup analysis showed improved OS with HMAVen in patients with *ASXL1* mutation (*ASXL1mut*; HR 0.45 [0.24-0.83], P=0.01), IPSS-R very high (HR 0.65 [0.45-0.93], P=0.03), IPSS-M very high (HR 0.65 [0.44-0.96], P=0.03) and BM blasts ≥10% (HR 0.69 [0.49-0.99], P=0.04, Figure 1A). Adjustment for HSCT attenuated significance: *ASXL1*mut (HR 0.59 [0.31-1.11], P=0.09), IPSS-R very high (HR 0.71 [0.5-1.03] P=0.07), IPSS-M very high (HR 0.77 [0.52-1.14], P=0.19) and BM blasts ≥10% (HR 0.81 [0.56-1.16], 0.25), whereas HSCT remained strongly protective (HR <1, P <0.001). Markov multistate modeling showed HMAVen significantly increased the hazard of transition from treatment to HSCT (HR of 2.27 [ASXL1mut, Figure 1B], 1.81 [IPSS-R very high], 1.83 [IPSS-M very high] and 2.33 [BM blasts ≥10%]; all P<0.05), without increasing pre- or post-HSCT mortality versus HMA.

**Summary/Conclusion**

Specific MDS-EB subgroups benefited from adding Ven to HMA (i.e. high-risk MDS, *ASXL1*mut, BM blasts ≥10%). Ven was associated with an increased transition to HSCT, likely contributing to improved survival. Larger cohorts with longer follow-up are needed to further define Ven role in HR-MDS.

**Copyright Information:** (Online) ISSN: 2572-9241
©2026 The Author(s). HemaSphere is published by John Wiley & Sons Ltd on behalf of European Hematology Association. This is an open access Abstract Book distributed under the Attribution-NonCommercial-NoDerivs (CC BY-NC-ND), which allows third parties to download the articles and share them with others as long as they credit the author and the Abstract Book, but they cannot change the content in any way or use them commercially.
**Abstract Book Citations:** Authors, Title, HemaSphere, 2026;10:(S1):pages. The abstract book DOIs can be found on the “Author Information” page.
**Disclaimer:** Articles published in the journal HemaSphere exclusively reflect the opinions of the authors. The authors are responsible for all content in their abstracts including accuracy of the facts, statements, citing resources, etc.

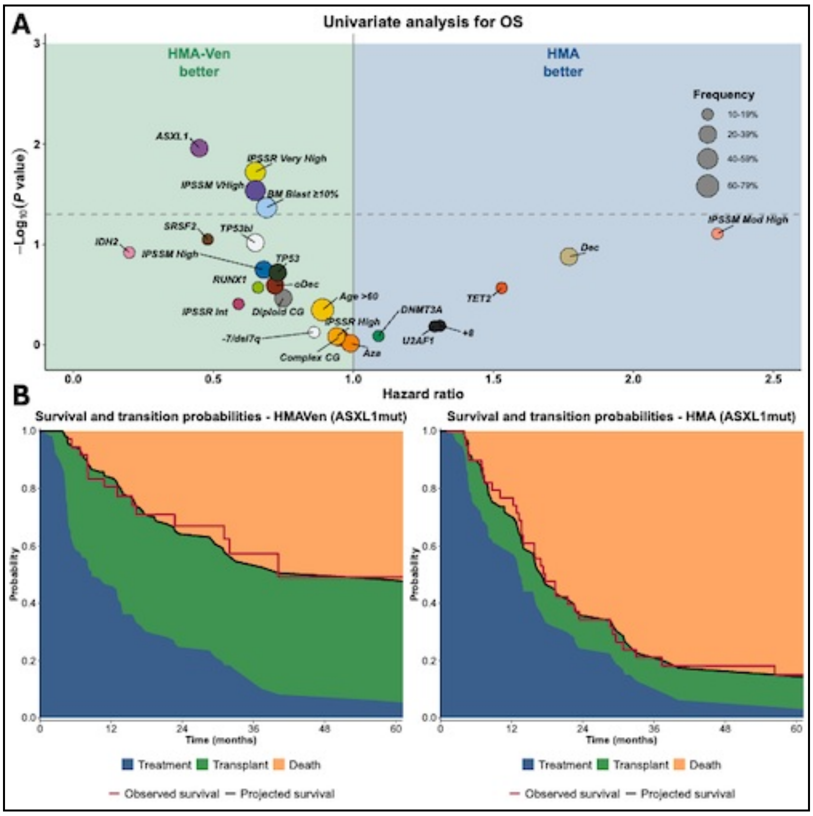

A. Univariate analysis for OS. B. OS (red line) and stacked transition probabilities in ASXL1mut patients HMA vs HMA Ven (blue: HMA/HMA Ven treatment, green: HSCT, yellow: death)

**Copyright Information:** (Online) ISSN: 2572-9241
©2026 The Author(s). HemaSphere is published by John Wiley & Sons Ltd on behalf of European Hematology Association. This is an open access Abstract Book distributed under the Attribution-NonCommercial-NoDerivs (CC BY-NC-ND), which allows third parties to download the articles and share them with others as long as they credit the author and the Abstract Book, but they cannot change the content in any way or use them commercially.
**Abstract Book Citations:** Authors, Title, HemaSphere, 2026;10:(S1):pages. The abstract book DOIs can be found on the "Author Information" page.
**Disclaimer:** Articles published in the journal HemaSphere exclusively reflect the opinions of the authors. The authors are responsible for all content in their abstracts including accuracy of the facts, statements, citing resources, etc.

### (PS1761) UPDATED PHASE 2 RESULTS OF ELRITERCEPT IN PATIENTS (PTS) WITH TRANSFUSION-DEPENDENT (TD) LOWER-RISK MYELODYSPLASTIC NEOPLASMS (LR-MDS)

**Topic:** 10. Myelodysplastic syndromes - Clinical

María Díez Campelo*[1], Shuh Ying Tan[2], David Valcarcel Ferreiras[3], Anish Puliyayil[4], Alejandro Arbelaez[5], Montserrat Arnan Sangerman[6], David Ross[7], Devendra Hiwase[8], Teresa Bernal[9], Thomas Cluzeau[10], Aristoteles Giagounidis[11], Radha Ramesh[12], Camden Bay[12], Kevin Galinsky[12], Leopold Sellner[12], Kaveri Suryanarayan[12], Lynette Chee[13]

*[1]Servicio de Hematología, Hospital Universitario de Salamanca, IBSAL, Salamanca, Spain;[2]St. Vincent's Hospital Melbourne, Fitzroy, Australia; [3]Vall d'Hebron University Hospital, Barcelona, Spain;[4]Border Medical Oncology and Haematology, Albury, Australia; [5]Tweed Valley Hospital, Cancer Care Services, Tweed Heads, Australia;[6]Institut Català d'Oncologia, Hospitalet de Llobregat, Barcelona, Spain; [7]Flinders Medical Centre, Adelaide, Australia;[8]The Royal Adelaide Hospital, Adelaide, Australia; [9]Hospital Universitario Central de Asturias, ISPA, IUOPA, Oviedo, Asturias, Spain;[10]CHU de Nice, Nice, France; [11]Marien Hospital Düsseldorf, Düsseldorf, Germany; [12]Takeda Development Center Americas, Inc. (TDCA), Cambridge, United States of America; [13]The Royal Melbourne Hospital/Peter MacCallum Cancer Centre, Department of Clinical Haematology, Melbourne, Australia;*

### Background

Anemia is common in pts with LR-MDS, and transfusion dependency can be a substantial burden. Over time, all pts may become resistant to first-line erythroid-stimulating agents (ESAs), requiring ongoing red blood cell (RBC) transfusions. Elritercept (TAK-226), an investigational modified activin receptor type IIA ligand trap, rebalances TFG-β superfamily signaling, which may lead to durable transfusion independence (TI) and improve quality of life.

### Aims

We report updated safety and efficacy data from a phase 2 trial of elritercept in pts with LR-MDS (NCT04419649).

### Methods

TD pts with International Prognostic Scoring System-Revised (IPSS-R) Very Low-, Low-, or Intermediate-risk MDS were enrolled. During dose escalation, 31 pts received treatment every 4 weeks (wks; Q4W) for ≤24 cycles to establish the recommended part 2 dose (RP2D). The expansion phase (n=71) evaluated the RP2D of 3.75 mg/kg Q4W, with potential for up-titration to 5.0 mg/kg. Pts deriving clinical benefit continued in a long-term study. Endpoints included safety (assessed in all pts who received ≥1 RP2D), RBC TI ≥8 wks and hematologic improvement-erythroid (HI-E) per Modified International Working Group 2006 criteria. TI rates (≥8 wks and ≥24 wks) were analysed over the first 24 and 48 wks of treatment, respectively, in a modified intent-to-treat-24 ($mITT_{24}$) population (all pts who received ≥3.75 mg/kg Q4W and had 24 wks of treatment or discontinued before reaching Wk 24 at data cutoff, and received ≥2 RBC units [U] at baseline). Pts in the HI-E analysis were defined similarly but did not require ≥2 RBC U; response was defined as a mean increase in hemoglobin (Hgb) ≥1.5 g/dL (in low transfusion burden [LTB] pts) or reduction in transfusion of ≥4 RBC U/8 wks (high transfusion burden [HTB]) on treatment vs 8-Wk pretreatment period. Overall erythroid response was defined as HI-E or TI over any 8-wk period.

**Copyright Information:** (Online) ISSN: 2572-9241
©2026 The Author(s). HemaSphere is published by John Wiley & Sons Ltd on behalf of European Hematology Association. This is an open access Abstract Book distributed under the Attribution-NonCommercial-NoDerivs (CC BY-NC-ND), which allows third parties to download the articles and share them with others as long as they credit the author and the Abstract Book, but they cannot change the content in any way or use them commercially.
**Abstract Book Citations:** Authors, Title, HemaSphere, 2026;10:(S1):pages. The abstract book DOIs can be found on the "Author Information" page.
**Disclaimer:** Articles published in the journal HemaSphere exclusively reflect the opinions of the authors. The authors are responsible for all content in their abstracts including accuracy of the facts, statements, citing resources, etc.

### Results

As of April 3, 2025, 96 pts were enrolled and treated with the RP2D. Median treatment exposure was 13.8 months. Drug-related treatment-emergent adverse events (TEAEs) occurred in 44 (45.8%) pts [grade ≥3 in 5 (5.2%)], with nausea (7.3%) and diarrhea (6.3%) reported most commonly. Overall erythroid response and TI rates are shown in Figure 1. By Wk 24, TI ≥8 wks was achieved by 38.5% of all evaluable pts, 29.3% of HTB pts, 44.2% of ring sideroblast (RS)+ pts, 28.0% of non-RS pts, 37.2% of pts with erythropoietin (EPO)<500 U/L HTB, 48.9% of RS+ pts with EPO<500 U/L, and 43.8% of non-RS pts with EPO<500 U/L (Figure 1). By Wk 48, 26.9% of pts achieved TI ≥24 wks (HTB: 15.5%) with a median time to first TI of 0.4 wks (95% confidence interval [CI]: 0.3–5.3). Sustained TI ≥24 wks was seen in 67.3% of pts with TI ≥8 wks. Median TI duration was 110.9 wks (95% CI: 24.3–not estimable) in pts who achieved TI ≥8 wks by Wk 24. In pts with *SF3B1* mutation, TI ≥8 wks occurred in 51.2% pts at Wk 24, 56.1% at Wk 48, and 63.4% overall. By Wk 24, HI-E response was achieved by 49.0% of evaluable pts, 48.3% of HTB pts, 51.9% of pts with EPO<500 U/L, 53.1% of RS+ pts, and 38.7% of non-RS pts, with a median time to response of 3.1 wks (95% CI: 1.1–8.1). Median duration of HI-E was 43.9 wks (95% CI: 24.6–59.1). Sustained increases for ≥8 wks in absolute neutrophil and platelets counts were seen in 31.8% and 33.3% of pts, respectively.

### Summary/Conclusion

Elritercept was well tolerated and led to rapid, durable responses in pts with LR-MDS, including non-RS and HTB pts.
*©American Society of Hematology (2025). Reused with permission.*

**Figure 1: Transfusion independence rates for ≥8 weeks by Week 24 in the $mITT_{24}$ population and across subgroups**

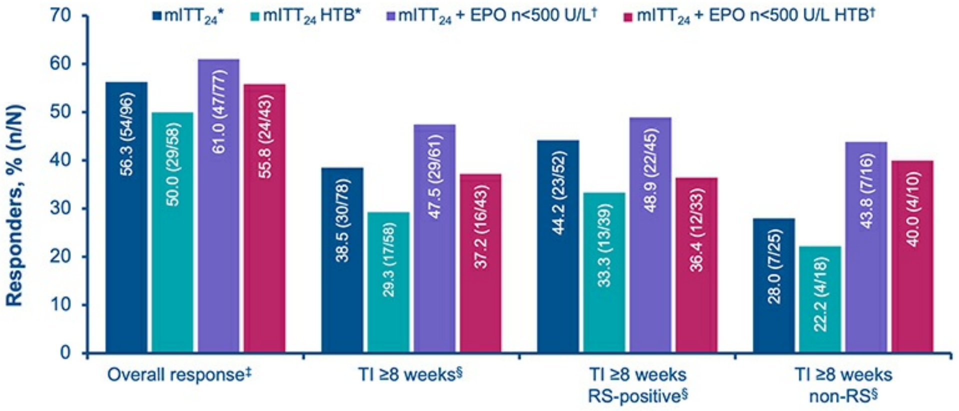

*Includes data for Weeks 0–24 for $mITT_{24}$ patients. †Includes data for Weeks 0–24 in $mITT_{24}$ patients with baseline EPO <500 U/L, excluding one patient with del5q MDS. ‡Defined as achieving modified IWG 2006 HI-E and/or TI. §Includes all MDS RP2D patients who have had 24 weeks of treatment or have discontinued before reaching week 24 at the data cutoff date in addition to having ≥2 units of RBC transfusion at baseline. EPO, erythropoietin; HI-E, hematologic improvement-erythroid; HTB, high transfusion burden; IWG, International Working Group; MDS, myelodysplastic neoplasms; $mITT_{24}$, modified intent-to-treat-24 includes all MDS RP2D patients who have had 24 weeks of treatment or have discontinued before reaching week 24 at the data cutoff date; RP2D, recommended part 2 dose; RS, ring sideroblast; TI, transfusion independence; U/L, units per liter.

**Copyright Information:** (Online) ISSN: 2572-9241
©2026 The Author(s). HemaSphere is published by John Wiley & Sons Ltd on behalf of European Hematology Association. This is an open access Abstract Book distributed under the Attribution-NonCommercial-NoDerivs (CC BY-NC-ND), which allows third parties to download the articles and share them with others as long as they credit the author and the Abstract Book, but they cannot change the content in any way or use them commercially.
**Abstract Book Citations:** Authors, Title, HemaSphere, 2026;10:(S1):pages. The abstract book DOIs can be found on the "Author Information" page.
**Disclaimer:** Articles published in the journal HemaSphere exclusively reflect the opinions of the authors. The authors are responsible for all content in their abstracts including accuracy of the facts, statements, citing resources, etc.

### (PS1762) A FOUR-GENE STEMNESS SCORE PREDICTS HMA RESISTANCE AND IDENTIFIES RATIONAL 5-AZACITIDINE COMBINATION STRATEGIES IN MYELODYSPLASTIC SYNDROMES

**Topic:** 10. Myelodysplastic syndromes - Clinical

Daniel Stowitschek[1], Chien-Chin Lin[2], Yu-Hung Wang*[3], Carmelo Gurnari[4], Pascal Krezer[1], Wan-Hsuan Lee[2], Sze-Hwei Lee[2], Yuan-Yeh Kuo[5], Bor-Sheng Ko[3], Hsin-An Hou[2], Jaroslaw P MacIejewski[6], Wen-Chien Chou[2], Maya Andre[1], Hwei-Fang Tien[2]

*[1]Universität Tübingen, Tübingen, Germany; [2]National Taiwan University Hospital, Taipei, Taiwan; [3]National Taiwan University Cancer Center, Taipei, Taiwan; [4]Tor Vergata University, Rome, Italy; [5]Tai-Chen Cell Therapy Center, National Taiwan University, Taipei, Taiwan; [6]Taussig Cancer Institute, Cleveland Clinic, Cleveland, United States of America;*

### Background

Myelodysplastic syndromes (MDS) are clinically and molecularly heterogeneous malignant hematopoietic stem cell disorders. Hitherto, hypomethylating agents (HMAs) remain the only disease-modifying therapy for higher-risk disease; however, responses are suboptimal (~50%), unpredictable, and typically transient. Robust biomarkers capable of predicting HMA response are still lacking. Combination strategies to improve HMA efficacy have shown limited benefit, as shown in recent negative high profile trials. Leukemic stemness cell (LSC) scores are strongly prognostic in both acute myeloid leukemia (AML) and MDS, but their relevance to HMA resistance remains undefined.

### Aims

We aim to identify LSC-associated signatures linked to HMA resistance and clinical outcomes, and to nominate/validate candidate therapeutic agents to overcome HMA resistance in MDS.

### Methods

The primary cohort comprised 101 cases treated with 5-azacitidine (5-AzaC) monotherapy at National Taiwan University Hospital, with whole-marrow RNA-seq data available at diagnosis. Publicly available $CD34^+$ RNA-seq datasets were merged to form Validation Cohort 1 (VC1). Prognostic relevance was assessed in the Cleveland Clinic cohort and 3 additional public MDS cohorts (VC2–5). Patient stratification was performed using maximally selected rank statistics independently in each cohort. Candidate agents identified via oncoPredict were tested in vitro, including 5-AzaC combination assays in various MDS/AML cell lines.

**Copyright Information:** (Online) ISSN: 2572-9241
©2026 The Author(s). HemaSphere is published by John Wiley & Sons Ltd on behalf of European Hematology Association. This is an open access Abstract Book distributed under the Attribution-NonCommercial-NoDerivs (CC BY-NC-ND), which allows third parties to download the articles and share them with others as long as they credit the author and the Abstract Book, but they cannot change the content in any way or use them commercially.
**Abstract Book Citations:** Authors, Title, HemaSphere, 2026;10:(S1):pages. The abstract book DOIs can be found on the “Author Information” page.
**Disclaimer:** Articles published in the journal HemaSphere exclusively reflect the opinions of the authors. The authors are responsible for all content in their abstracts including accuracy of the facts, statements, citing resources, etc.

### Results

The median age of 5-AzaC-treated patients was 65 years, with a male predominance; 58/101 (57.4%) achieved initial complete or partial response to 5-AzaC. A backward conditional multivariable Cox model identified four stemness-related genes ( *LAPTM4B*, *SOCS2*, *ZBTB46*, *MMRN1*) independently associated with overall survival (OS), which were integrated into a weighted HMA-LSC4 score.

High HMA-LSC4 scores correlated with adverse clinical and genetic features, including older age, higher marrow blasts, and *ASXL1*, *SRSF2*, and *TP53* mutations. The score strongly predicted 5-AzaC response (low vs high HMA-LSC4: 77% vs 38%; $p<0.001$, Fig 1A) and significantly stratified both AML-free and OS (both $p<0.001$) independent of IPSS-R and abovementioned mutations. VC1 independently validated prediction of HMA response and OS, with prognostic relevance reproduced across VC2–5. GSEA demonstrated enrichment of LSC pathways in high-score patients across all cohorts.

The HMA-LSC4 score correlated strongly with ssGSEA-derived HMA resistance signatures across four datasets (Fig 1B), supporting its biological coherence. Drug-response modelling identified compounds for which high HMA-LSC4 scores predicted increased sensitivity, notably an inhibitor of apoptosis protein (IAP) antagonist and a myeloid cell leukemia-1 (MCL-1) inhibitor, implicating potential add-on candidates to overcome HMA resistance. *In vitro* testing confirmed robust synergy between each agent and azacitidine in MDS/AML cell lines (all synergy scores >30; p<0.001, Fig 1C). *In vivo* studies are ongoing, with results to be presented.

### Summary/Conclusion

We developed and validated a concise HMA-LSC4 score that robustly predicts 5-AzaC resistance and clinical outcomes in MDS. Beyond prognostication, computational drug-response modelling identified biologically coherent and highly active combination partners, with substantial and consistent synergy between 5-AzaC and both IAP and MCL-1 inhibitors, presenting a novel targeted approach to improve HMA response for this unmet clinical need.

**Copyright Information:** (Online) ISSN: 2572-9241
©2026 The Author(s). HemaSphere is published by John Wiley & Sons Ltd on behalf of European Hematology Association. This is an open access Abstract Book distributed under the Attribution-NonCommercial-NoDerivs (CC BY-NC-ND), which allows third parties to download the articles and share them with others as long as they credit the author and the Abstract Book, but they cannot change the content in any way or use them commercially.
**Abstract Book Citations:** Authors, Title, HemaSphere, 2026;10:(S1):pages. The abstract book DOIs can be found on the "Author Information" page.
**Disclaimer:** Articles published in the journal HemaSphere exclusively reflect the opinions of the authors. The authors are responsible for all content in their abstracts including accuracy of the facts, statements, citing resources, etc.

**Fig 1A. The HMA-LSC4 score significantly stratifies HMA response and overall survival in MDS patients, with validation across multiple independent cohorts.**

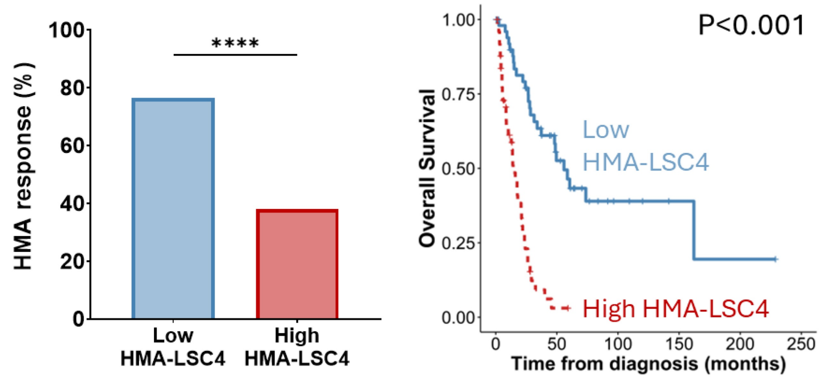

**Fig 1B. The HMA-LSC4 score shows a strong positive correlation with published HMA-resistance signatures**

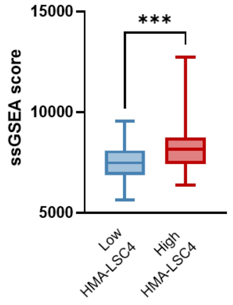

**Fig 1C. In vitro assays demonstrated marked synergy between the IAP antagonist and 5-AzaC in the HEL cell line. *In vivo* work is ongoing and will be presented.**

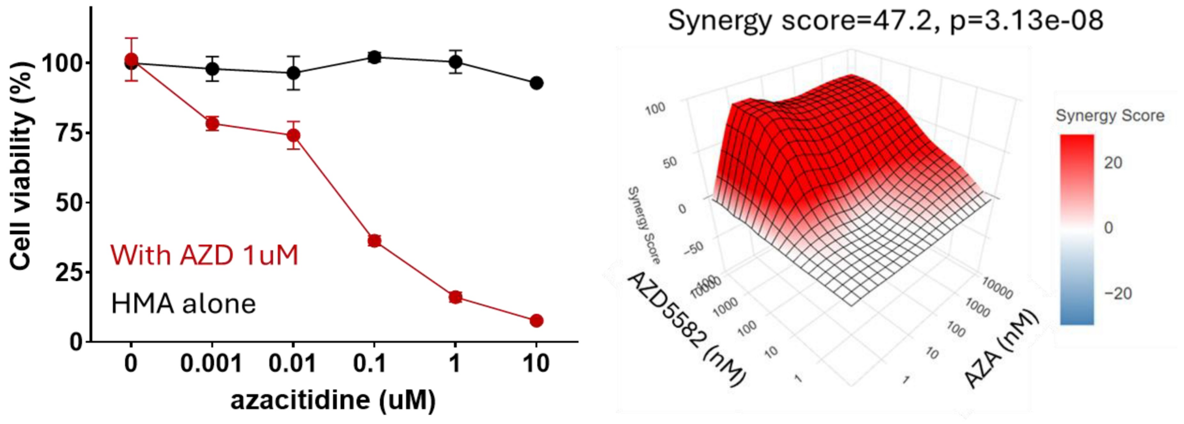

**Copyright Information:** (Online) ISSN: 2572-9241
©2026 The Author(s). HemaSphere is published by John Wiley & Sons Ltd on behalf of European Hematology Association. This is an open access Abstract Book distributed under the Attribution-NonCommercial-NoDerivs (CC BY-NC-ND), which allows third parties to download the articles and share them with others as long as they credit the author and the Abstract Book, but they cannot change the content in any way or use them commercially.
**Abstract Book Citations:** Authors, Title, HemaSphere, 2026;10:(S1):pages. The abstract book DOIs can be found on the "Author Information" page.
**Disclaimer:** Articles published in the journal HemaSphere exclusively reflect the opinions of the authors. The authors are responsible for all content in their abstracts including accuracy of the facts, statements, citing resources, etc.

### (PS1763) CLINICAL, GENETIC, AND TRANSCRIPTOMIC CHARACTERIZATION OF MYELODYSPLASTIC SYNDROME/NEOPLASMS WITH AUTOIMMUNE DISEASES

**Topic:** 10. Myelodysplastic syndromes - Clinical

Tai-Chung Tseng*[1], Wan-Hsuan Lee[1], Yu-Sung Chang[1], Jui-Che Chen[2], Sao Chih Ni[2], Feng-Ming Tien[1], Cheng-Hong Tsai[1], Chien-Chin Lin[1], Chi-Yuan Yao[1], Min-Yen Lo[3], Yuan-Yeh Kuo[4], Mei-Hsuan Tseng[4], Yen-Ling Peng[5], Bor-Sheng Ko[2], Ming Yao[1], Hwei-Fang Tien[1, 6], Hsin-An Hou[1], Wen-Chien Chou[1]

*[1]National Taiwan University Hospital, Internal Medicine, Taipei, Taiwan; [2]National Taiwan University Cancer Center, Taipei, Taiwan; [3]National Taiwan University Hospital Yunlin Branch, Internal Medicine, Yunlin, Taiwan; [4]Tai-Chen Cell Therapy Center, National Taiwan University, Taipei, Taiwan; [5]National Taiwan University Hospital, Taipei, Taiwan; [6]Far-Eastern Memorial Hospital, Internal Medicine, New Taipei City, Taiwan;*

### Background

Autoimmune disease (AID) occurs in a subset of patients with myelodysplastic syndrome/neoplasms (MDS), yet its biological significance and prognostic impact remain unclear.

### Aims

We examined whether AID defines a distinct clinical and molecular MDS subtype and whether temporal relationship between AID and MDS influences outcomes.

### Methods

We retrospectively reviewed 1,196 consecutive patients diagnosed with MDS at National Taiwan University Hospital between 1981 and 2025. Clinical features, AID status, treatments, and outcomes were collected. Targeted myeloid sequencing and RNA sequencing were performed in available samples. Survival was analyzed using multivariate Cox proportional hazards models adjusting for clinical covariates.

**Copyright Information:** (Online) ISSN: 2572-9241
©2026 The Author(s). HemaSphere is published by John Wiley & Sons Ltd on behalf of European Hematology Association. This is an open access Abstract Book distributed under the Attribution-NonCommercial-NoDerivs (CC BY-NC-ND), which allows third parties to download the articles and share them with others as long as they credit the author and the Abstract Book, but they cannot change the content in any way or use them commercially.
**Abstract Book Citations:** Authors, Title, HemaSphere, 2026;10:(S1):pages. The abstract book DOIs can be found on the “Author Information” page.
**Disclaimer:** Articles published in the journal HemaSphere exclusively reflect the opinions of the authors. The authors are responsible for all content in their abstracts including accuracy of the facts, statements, citing resources, etc.

### Results

Among 1,196 MDS patients, 137 (11.5%) had AID. The most common AIDs were systemic lupus erythematosus (n=21), rheumatoid arthritis (n=16), Sjögren syndrome (n=12), autoimmune hemolytic anemia (n=12), and autoimmune thyroiditis (n=11). Compared to non-AID patients, those with AID had higher female proportion, higher leukocyte and platelet counts, lower lymphocyte-to-monocyte ratios, lower bone marrow blasts, and lower IPSS-R risk. Transcriptomic data were available for 221 patients (31 AID, 190 non-AID). Differentially expressed gene analysis identified *FCGR1A, SOCS3, NLRP3, IFI30, IL10, and IL13RA1* among most upregulated genes in patients with AID. Gene Set Enrichment Analysis showed enrichment of interferon-α response, IL6–JAK–STAT3 signaling, unfolded protein response, PI3K–AKT–mTOR signaling, protein secretion, and DNA repair pathways, with suppressed heme metabolism. Reactome inflammasome, pyroptosis, and Toll-like receptor signaling were also enriched. These findings imply a distinct proinflammatory and innate immune–activated transcriptional state. After adjustment for age, sex, IPSS-R, hypomethylating agent use, and hematopoietic stem cell transplantation, AID was independently associated with inferior overall survival (OS; adjusted hazard ratio [aHR] 1.362, p=0.028) and leukemia-free survival (LFS; aHR 1.403, p=0.012).
Patients with AID were stratified by disease chronology. 106 patients had preceding or concurrent AID (diagnosed before or within 60 days of MDS diagnosis), and 31 had successive AID (>60 days after MDS diagnosis). Preceding or concurrent AID conferred worse OS (aHR 1.532, p=0.008) and LFS (aHR 1.585, p=0.003) compared to non-AID, whereas successive AID had no survival impact. Preceding or concurrent AID showed higher mutational burden involving transcriptional regulation, splicing machinery, signaling pathways, and DNA methylation linked to adverse-risk MDS biology (such as *RUNX1, TP53, EZH2, IDH2, SRSF2, U2AF1, ETV6, DNMT3A, FLT3-ITD/TKD, KMT2A/PTD, NRAS*) than successive AID. Exploratory transcriptomic analyses suggested distinct immune programs by disease chronology. Preceding or concurrent AID enriched adaptive immune signatures (including immunoglobulin- and T cell receptor–related transcripts), whereas successive AID showed inflammatory alarmin pathways activation, notably *S100A8/A9*.

### Summary/Conclusion

AID in MDS independently associates with inferior survival, particularly when preceding or concurrent with MDS that is accompanied by high-risk mutations. Transcriptomic analyses reveal proinflammatory, innate immune–activated signature, supporting immune dysregulation in driving adverse outcomes. Chronology-specific immune patterns imply distinct underlying pathobiology.

**Copyright Information:** (Online) ISSN: 2572-9241
©2026 The Author(s). HemaSphere is published by John Wiley & Sons Ltd on behalf of European Hematology Association. This is an open access Abstract Book distributed under the Attribution-NonCommercial-NoDerivs (CC BY-NC-ND), which allows third parties to download the articles and share them with others as long as they credit the author and the Abstract Book, but they cannot change the content in any way or use them commercially.
**Abstract Book Citations:** Authors, Title, HemaSphere, 2026;10:(S1):pages. The abstract book DOIs can be found on the "Author Information" page.
**Disclaimer:** Articles published in the journal HemaSphere exclusively reflect the opinions of the authors. The authors are responsible for all content in their abstracts including accuracy of the facts, statements, citing resources, etc.

### (PS1764) SEQUENTIAL THERAPY WITH DECITABINE AND AN XPO-1 INHIBITOR FOLLOWED BY ALLO-HSCT IN PATIENTS WITH MYELODYSPLASTIC NEOPLASM WITH BIALLELIC TP53 INACTIVATION : A PRELIMINARY CASE SERIES.

**Topic:** 10. Myelodysplastic syndromes - Clinical

Yanling Ren*[1], Chen Mei[1], Yu Xu[1], Xianbo Huang[1], Shasha Wang[1], Jiejin Qian[1], Hongyan Tong[1]

*[1]The First Affiliated Hospital of Zhejiang University, College of Medicine, Hangzhou, China;*

### Background

Patients with Myelodysplastic neoplasm with biallelic TP53 inactivation (MDS-biTP53) represent an ultra-high-risk subgroup with a dire prognosis. They respond poorly to conventional therapies and experience extremely high relapse rates even after allogeneic hematopoietic stem cell transplantation (allo-HSCT), underscoring a critical need for novel therapeutic strategies.

### Aims

We conducted this retrospective analysis to evaluate the outcomes of a small case series from our center, in which patients with MDS-biTP53 were treated with a novel sequential protocol of decitabine (DAC) plus the XPO-1 inhibitor selinexor, followed immediately by allo-HSCT.

### Methods

This single-center, retrospective study included 8 consecutive adults with MDS-biTP53 who received the sequential protocol from September 2024 to December 2025. Key data on patient demographics, pre-transplant disease characteristics, and transplant details were collected. Treatment involved a 2-week induction with decitabine (20 mg/m²/day, days 1–5) and selinexor (20 mg, twice weekly), followed directly by a decitabine-based myeloablative conditioning regimen (DAC-BuCy or Dec/Flu/Bu) and allo-HSCT. Key endpoints analyzed were neutrophil/platelet engraftment rates, cumulative incidence of relapse (CIR), relapse-free survival (RFS), overall survival (OS), incidences of acute graft-versus-host disease (GVHD) and transplant-related serious adverse events.

### Results

At a median follow-up of 6.4 months (range, 2.8-16.7 months), promising efficacy was observed. The OS was 87.5% (7/8), and the RFS was 75% (6/8). Notably, the CIR was low at 12.5% (1/8). This single relapsed patient successfully achieved a second complete remission following preemptive therapy with low-dose decitabine combined with donor lymphocyte infusion and remained in remission at the last follow-up. The incidence of grade III-IV acute GVHD was 25% (2/8). Two patients developed serious infections during the peritransplant period. One patient, who had a pre-transplant intestinal colonization of carbapenem-resistant Enterobacteriaceae (CRE), ultimately died due to CRE bloodstream infection followed by intestinal GVHD. The overall treatment-related toxicity was deemed manageable. All patients achieved prompt neutrophil engraftment(Table 1 and Fig 1).

Table 1 Clinical characteristics and transplant outcomes

| | Patient 1 | Patient 2 | Patient 3 | Patient 4 | Patient 5 | Patient 6 | Patient 7 | Patient 8 |
|---|---|---|---|---|---|---|---|---|
| Age(years) | 61 | 59 | 68 | 52 | 64 | 58 | 55 | 63 |
| WHO-type | IB2 | IB1 | IB2 | IB2 | IB1 | IB2 | IB1 | IB2 |
| TP53 mutation | c.700T>C;p.Y234H, VAF:69.67% | c.422G>A p.Cys141Try VAF:7.19% | c.587G>C p.R196P�VAF: 26.69%�c.503A>T p.H168L�VAF:1.35%�c.473G>A p.R158H� VAF:28.50% | c.916C>T,p.R306X, VAF,82.45%, | c.659A>G VAF:85.15% | c.1009C>T,p.R337C, VAF:79% | c.824G>T,p.C275F�VAF:66.25%� c.555delC,p.S185fs� VAF:5% | c.375+1G>T VAF: 80.85% |
| Karyotype | CK(-17) | CK(-17) | CK | CK(-17) | CK(-17) | CK(-17P) | CK(-17) | CK(-17) |
| IPSS-R | 8 | 7.5 | 7.5 | 8 | 8.5 | 6.5 | 7 | 8 |
| IPSS-M | 3.4 | 2.7 | 3.36 | 3.07 | 3.5 | 2.6 | 3.6 | 2.76 |
| Initial treatment | AZA+CD47 | AZA+XPO-1 | AZA | AZA+XPO-1 | DEC | support care | support care | support care |
| Status at transplant | PD | mCR | SD | PD | PD | SD | SD | SD |
| Grade III–IV aGvHD | no | yes | no | no | yes | no | no | yes |
| OS�Months) | 16.7 | 11.6 | 10.6 | 6.4 | 5.1 | 4.2 | 3.8 | 2.8 |
| NRM | no | no | no | no | no | no | no | GVHD |

**Copyright Information:** (Online) ISSN: 2572-9241
©2026 The Author(s). HemaSphere is published by John Wiley & Sons Ltd on behalf of European Hematology Association. This is an open access Abstract Book distributed under the Attribution-NonCommercial-NoDerivs (CC BY-NC-ND), which allows third parties to download the articles and share them with others as long as they credit the author and the Abstract Book, but they cannot change the content in any way or use them commercially.
**Abstract Book Citations:** Authors, Title, HemaSphere, 2026;10:(S1):pages. The abstract book DOIs can be found on the “Author Information” page.
**Disclaimer:** Articles published in the journal HemaSphere exclusively reflect the opinions of the authors. The authors are responsible for all content in their abstracts including accuracy of the facts, statements, citing resources, etc.

**Summary/Conclusion**

This preliminary study provides encouraging evidence that sequential DAC and selinexor therapy followed by allo-HSCT may improve outcomes with acceptable toxicity in patients with ultra-high-risk MDS-biTP53. However, these findings are limited by the small sample size, relatively short follow-up, and retrospective, single-center design. Confirmation through larger prospective trials is warranted.

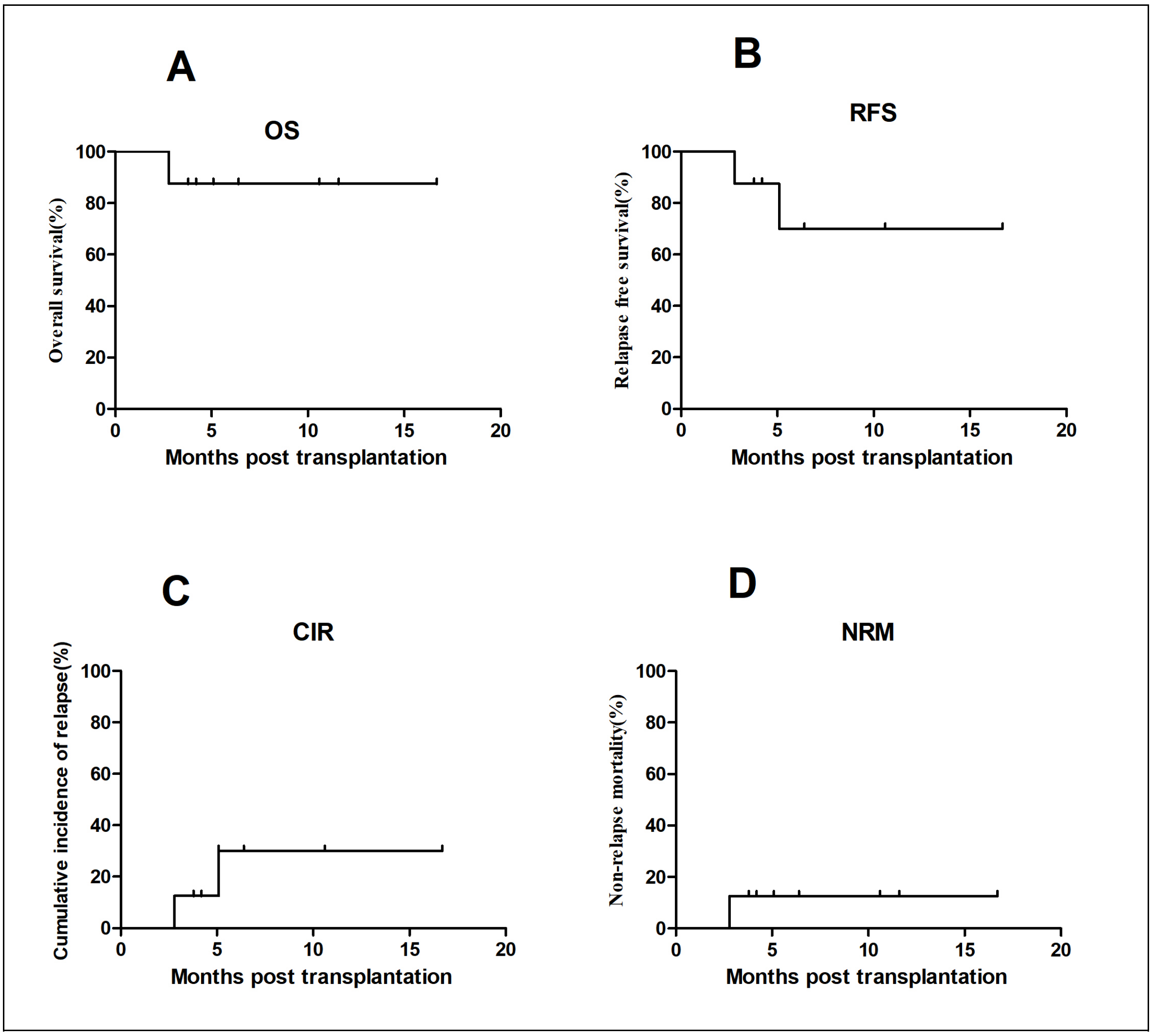

**Copyright Information:** (Online) ISSN: 2572-9241
©2026 The Author(s). HemaSphere is published by John Wiley & Sons Ltd on behalf of European Hematology Association. This is an open access Abstract Book distributed under the Attribution-NonCommercial-NoDerivs (CC BY-NC-ND), which allows third parties to download the articles and share them with others as long as they credit the author and the Abstract Book, but they cannot change the content in any way or use them commercially.
**Abstract Book Citations:** Authors, Title, HemaSphere, 2026;10:(S1):pages. The abstract book DOIs can be found on the "Author Information" page.
**Disclaimer:** Articles published in the journal HemaSphere exclusively reflect the opinions of the authors. The authors are responsible for all content in their abstracts including accuracy of the facts, statements, citing resources, etc.

### (PS1765) IMPACT OF VENETOCLAX BRIDGING ON HEMATOPOIETIC STEM CELL TRANSPLANTATION OUTCOMES IN ADVANCED MYELODYSPLASTIC NEOPLASMS

**Topic:** 10. Myelodysplastic syndromes - Clinical

Zhong-Shi Lyu[1], Yuqian Sun[1], Xiao-Dong Mo[1], Meng Lv[1], Yi-Fei Cheng[1], Lanping Xu[1], Xiaohui Zhang[1], Xiao-Jun Huang[1], Yu Wang*[1]

*[1]Peking University People's Hospital, Peking University Institute of Hematology,, Beijing, China;*

### Background

Allogeneic hematopoietic stem cell transplantation (allo-HSCT) is the only curative option for advanced myelodysplastic neoplasms (MDS), but relapse and non-relapse mortality (NRM) remain major causes of treatment failure. While venetoclax combined with hypomethylating agents (VEN-HMA) improves response rates in the non-transplant setting, its role as a bridge to allo-HSCT remains unclear.

### Aims

To evaluate the impact of venetoclax-based bridging therapy on post-transplant outcomes in patients with advanced myelodysplastic neoplasms undergoing allogeneic hematopoietic stem cell transplantation.

### Methods

We retrospectively analyzed 240 advanced MDS patients undergoing allo-HSCT, divided into three groups based on pre-transplant strategy: best supportive care (BSC, n=124), VEN-HMA bridging (VEN, n=37), or induction chemotherapy bridging (IC, n=79). Multivariate Cox regression and propensity score matching (PSM) were used to adjust for confounders, particularly IPSS-M risk.

### Results

In this study, unadjusted Cox models showed that VEN bridging was associated with inferior OS (HR 3.09, P=0.043) and inferior RFS (HR 2.84, P=0.0097) versus BSC. In multivariable Cox models adjusting for age, sex, and IPSS-M risk, VEN remained independently associated with inferior RFS (adjusted HR 2.51, P=0.030), while OS did not reach statistical significance (adjusted HR 2.86, P=0.074). In PSM analyses, 32 VEN patients were matched to 56 BSC patients with substantially improved balance. In the matched cohort, OS was not significantly different and RFS showed a borderline difference (P=0.052). Notably, in a responder-restricted PSM analysis, VEN responders had significantly inferior RFS (P=0.028) despite no significant OS difference. Among 13 NRM deaths, infection was the leading cause.

### Summary/Conclusion

Neither VEN-HMA nor IC demonstrated a statistically significant impact on post-transplant survival compared to BSC. Bridging therapy pre-HSCT did not necessarily improve post-HSCT prognosis.

**Copyright Information:** (Online) ISSN: 2572-9241
©2026 The Author(s). HemaSphere is published by John Wiley & Sons Ltd on behalf of European Hematology Association. This is an open access Abstract Book distributed under the Attribution-NonCommercial-NoDerivs (CC BY-NC-ND), which allows third parties to download the articles and share them with others as long as they credit the author and the Abstract Book, but they cannot change the content in any way or use them commercially.
**Abstract Book Citations:** Authors, Title, HemaSphere, 2026;10:(S1):pages. The abstract book DOIs can be found on the "Author Information" page.
**Disclaimer:** Articles published in the journal HemaSphere exclusively reflect the opinions of the authors. The authors are responsible for all content in their abstracts including accuracy of the facts, statements, citing resources, etc.

### (PS1766) HETROMBOPAG FOR THROMBOCYTOPENIA IN LOWER-RISK MYELODYSPLASTIC SYNDROMES: A PROSPECTIVE SINGLE-CENTER STUDY

**Topic:** 10. Myelodysplastic syndromes - Clinical

Yumei Liu[1], Yingao Liang[1], Huaquan Wang[1], Rong Fu*[1]

[1] *Tianjin Medical University General Hospital, Tianjin, China;*

### Background

Thrombocytopenia occurs in up to 65% of patients with lower-risk myelodysplastic syndromes (MDS) and is associated with bleeding complications and inferior outcomes. Therapeutic options remain limited, and platelet transfusion is often required. Hetrombopag is an oral thrombopoietin receptor agonist (TPO-RA) with demonstrated efficacy in immune thrombocytopenia and aplastic anemia; however, data in MDS patients are limited.

### Aims

To evaluate the efficacy and safety of hetrombopag in adult patients with lower-risk MDS and thrombocytopenia.

### Methods

Adult patients diagnosed with MDS according to the WHO 2022 classification, with IPSS low or intermediate-1 risk, bone marrow blasts <5%, and platelet counts $<30\times10^9/L$ or $30–100\times10^9/L$ accompanied by grade ≥1 bleeding were prospectively enrolled. Hetrombopag was initiated at 5 mg/day and titrated up to 15 mg/day according to response. Supportive care was permitted except for recombinant human TPO or other TPO-RAs. Platelet response (PLT-R) was assessed according to International Working Group (IWG 2006) criteria. The primary endpoints were PLT-R within 24 weeks and safety.

### Results

Fifty-six patients who received at least 12 weeks of treatment were included in the efficacy analysis (median age 55 years; 50% female). The median follow-up was 61 weeks (IQR 29–108). Overall, PLT-R was achieved in 33 patients (58.9%), including 10 complete responses. 30 patients (53.6%) achieved PLT-R within 24 weeks. The median time to response was 6 weeks (IQR 3–12), and the median duration of response was not reached. Baseline platelet count $<30\times10^9/L$ and IPSS-M score >0 were associated with lower PLT-R rates (Figure 1).
Bleeding events decreased during treatment. Among 21 patients with platelet transfusion dependence, 12 (57.1%) achieved transfusion independence. Of 30 patients with red blood cell transfusion dependence, 9 (30.0%) became transfusion-independent. Erythroid improvement was observed in 23 of 46 anemic patients (50.0%).
Adverse events occurred in 15 patients, and no arterial or venous thrombotic events were observed. Grade 3–4 non-hematologic adverse events included pulmonary infection (n=3), angina (n=1), heart failure (n=1) and sepsis (n=1); none were considered treatment-related by investigators. During follow-up, one patient progressed to IB2 and one transformed to acute myeloid leukemia. Six patients died due to severe infection, cerebral hemorrhage or severe anemia.

### Summary/Conclusion

Hetrombopag demonstrated clinically meaningful platelet responses and reduced transfusion burden in patients with lower-risk MDS and thrombocytopenia, with manageable toxicity and no apparent signal of increased leukemic progression during follow-up. These findings support further evaluation of hetrombopag in this patient population.

**Copyright Information:** (Online) ISSN: 2572-9241
©2026 The Author(s). HemaSphere is published by John Wiley & Sons Ltd on behalf of European Hematology Association. This is an open access Abstract Book distributed under the Attribution-NonCommercial-NoDerivs (CC BY-NC-ND), which allows third parties to download the articles and share them with others as long as they credit the author and the Abstract Book, but they cannot change the content in any way or use them commercially.
**Abstract Book Citations:** Authors, Title, HemaSphere, 2026;10:(S1):pages. The abstract book DOIs can be found on the "Author Information" page.
**Disclaimer:** Articles published in the journal HemaSphere exclusively reflect the opinions of the authors. The authors are responsible for all content in their abstracts including accuracy of the facts, statements, citing resources, etc.

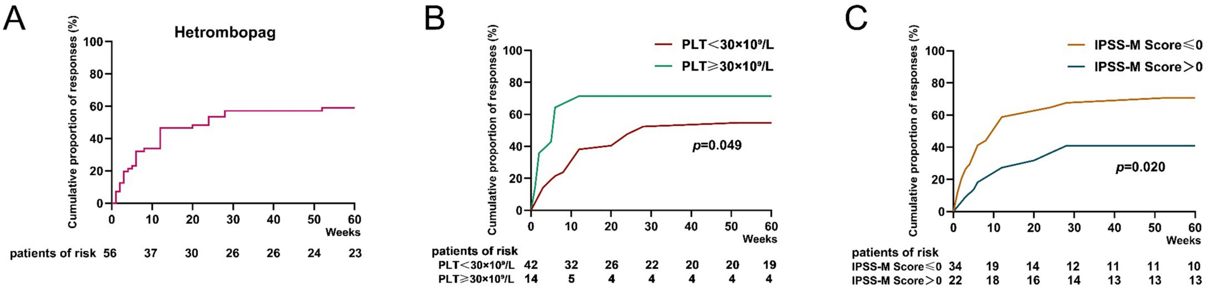

**Figure 1. Platelet response to hetrombopag in lower-risk MDS.** (A) Time to platelet response according to IWG 2006 criteria. (B) Platelet response rate stratified by baseline platelet count. (C) Platelet response rate stratified by IPSS-M score.

**Copyright Information:** (Online) ISSN: 2572-9241
©2026 The Author(s). HemaSphere is published by John Wiley & Sons Ltd on behalf of European Hematology Association. This is an open access Abstract Book distributed under the Attribution-NonCommercial-NoDerivs (CC BY-NC-ND), which allows third parties to download the articles and share them with others as long as they credit the author and the Abstract Book, but they cannot change the content in any way or use them commercially.
**Abstract Book Citations:** Authors, Title, HemaSphere, 2026;10:(S1):pages. The abstract book DOIs can be found on the “Author Information” page.
**Disclaimer:** Articles published in the journal HemaSphere exclusively reflect the opinions of the authors. The authors are responsible for all content in their abstracts including accuracy of the facts, statements, citing resources, etc.

### (PS1767) CLINICAL AND SURVIVAL IMPACT OF PRIOR IMMUNE AND ONCOLOGIC EXPOSURES IN ZRSR2-MUTATED MYELOID NEOPLASMS AND CCUS

**Topic:** 10. Myelodysplastic syndromes - Clinical

Mahmoud Yacout[1], Bahga Katamesh[1], Yazan Jabban[1], Rong He[1], David Viswanatha[1], Dragan Jevremovic[1], Patricia Greipp[1], Kurt Bessonen[1], Jeanne Palmer[1], James M Foran[1], Antoine Saliba[1], Mehrdad Hefazi[1], Kebede Begna[1], William Hogan[1], Mrinal Patnaik[1], Mithun Shah[1], Hassan Alkhateeb[1], Aref Al-Kali*[1]

[1]*Mayo Clinic, Hematology, Rochester, United States of America;*

### Background

*ZRSR2* mutations are spliceosome alterations observed across myeloid neoplasms and clonal cytopenia of undetermined significance (CCUS). Yet, the clinical impact of prior immune and oncologic exposures on disease phenotype and outcomes remains poorly defined. Immune dysregulation and prior cancer-directed therapies may influence disease evolution, but their relationship with clinical presentation and survival in ZRSR2-mutated disease has not been systematically evaluated.

### Aims

To investigate the pattern of prior immunotherapy, chemo-/radiotherapy, prior neoplasms, and autoimmune conditions on clinical phenotype, molecular characteristics, and survival outcomes in patients with *ZRSR2*-mutated myeloid neoplasms and CCUS.

### Methods

After obtaining IRB approval, we performed a retrospective cohort analysis of patients harboring *ZRSR2* mutations identified by next-generation sequencing (NGS). Clinical, laboratory, and molecular variables were compared between exposure groups using independent-sample t-tests or Welch correction for continuous variables and Fisher's exact tests for categorical variables. Overall survival (OS) was assessed using Kaplan–Meier analysis and log-rank testing. Multivariable linear regression models were used to evaluate independent associations with peripheral blast burden after adjustment for coexisting exposures.

### Results

Our *ZRSR2m* cohort included 168 patients diagnosed with CCUS (23.8%) or myeloid neoplasm (76.2%). Prior immunotherapy was present in 19%, autoimmune conditions in 9.5%, and prior neoplasms in 11.3%. The most frequent prior neoplasms were lymphoma (52.6%) and multiple myeloma (26.3%), while autoimmune conditions were primarily inflammatory arthritis and rheumatoid arthritis. Rituximab (7.7%) and hydroxychloroquine (4.8%) were the most common prior immunotherapies.
Baseline disease characteristics at NGS were comparable across exposure groups. However, diagnostic classification differed significantly. Patients with prior immunotherapy were more likely to have MDS than AML(78.1% vs 45.9%, p=0.001), whereas patients with prior cancers more frequently had AML(84.2% vs 48.0%, p=0.003). Prior immunotherapy was associated with reduced disease burden, including lower frequency of bone marrow blasts >5% (9.4% vs 30.6%, p=0.014) and absence of peripheral circulating blasts (0% vs 12.8%, p=0.046). Molecular features, including isolated *ZRSR2* mutations and *TET2* co-mutation frequency, were similar across groups.
Despite lower blast burden, Patients with prior immune or oncologic exposure demonstrated numerically inferior survival (median OS: prior immunotherapy 27 vs 52 months, p=0.69; prior neoplasm 25 vs 51 months, p=0.32; prior chemo/radiotherapy 23 vs 52 months, p=0.08). Survival differed significantly according to exposure to prior neoplasm subtype (p=0.048). In multivariable models, exposure variables were not independently associated with blast burden after adjustment.

**Copyright Information:** (Online) ISSN: 2572-9241
©2026 The Author(s). HemaSphere is published by John Wiley & Sons Ltd on behalf of European Hematology Association. This is an open access Abstract Book distributed under the Attribution-NonCommercial-NoDerivs (CC BY-NC-ND), which allows third parties to download the articles and share them with others as long as they credit the author and the Abstract Book, but they cannot change the content in any way or use them commercially.
**Abstract Book Citations:** Authors, Title, HemaSphere, 2026;10:(S1):pages. The abstract book DOIs can be found on the "Author Information" page.
**Disclaimer:** Articles published in the journal HemaSphere exclusively reflect the opinions of the authors. The authors are responsible for all content in their abstracts including accuracy of the facts, statements, citing resources, etc.

### Summary/Conclusion

Prior immune and/or chemo-radiotherapy exposures in *ZRSR2*-mutated myeloid neoplasms are associated with altered clinical phenotypes characterized by lower blast burden and distinct diagnostic classification without major molecular differences. Despite this less proliferative phenotype, exposed patients demonstrated numerically worse survival, with significant heterogeneity by prior neoplasm subtype, suggesting that exposure history may modify disease biology beyond baseline disease burden.

| Domain | Variable / Comparison | Key result | p value | Interpretation |
|---|---|---|---|---|
| **Baseline diagnosis** | Prior immunotherapy vs none (AML vs MDS) | MDS more frequent (78.1% vs 45.9%) | **0.001** | Different diagnostic trajectory |
| | Prior neoplasm vs none (AML vs MDS) | AML more frequent (84.2% vs 48.0%) | **0.003** | Different disease history |
| **Blast burden** | BM blasts >5% (prior immunotherapy vs none) | Lower frequency (9.4% vs 30.6%) | **0.014** | Lower marrow blast burden |
| | Peripheral blasts present (prior immunotherapy vs none) | 0% vs 12.8% | **0.046** | Reduced peripheral blasts |
| **Survival (exposure)** | Prior immunotherapy (Yes vs No) | Median OS 27 vs 52 months | 0.69 | Numerically worse survival |
| | Prior neoplasm (Yes vs No) | Median OS 25 vs 51 months | 0.32 | Numerically worse survival |
| | Prior chemo/radiotherapy (Yes vs No) | Median OS 23 vs 52 months | 0.08 | Trend toward worse survival |
| **Survival (subgroup)** | Prior neoplasm subtype | Survival differed by subtype | **0.048** | Significant survival heterogeneity |
| | • Lymphoma | Median OS 25 months | — | |
| | • BPDCN | Median OS 0 months | — | |
| | • CLL | Median OS 15 months | — | |
| | • Bladder cancer | Median OS 22 months | — | |

**Copyright Information:** (Online) ISSN: 2572-9241
©2026 The Author(s). HemaSphere is published by John Wiley & Sons Ltd on behalf of European Hematology Association. This is an open access Abstract Book distributed under the Attribution-NonCommercial-NoDerivs (CC BY-NC-ND), which allows third parties to download the articles and share them with others as long as they credit the author and the Abstract Book, but they cannot change the content in any way or use them commercially.
**Abstract Book Citations:** Authors, Title, HemaSphere, 2026;10:(S1):pages. The abstract book DOIs can be found on the "Author Information" page.
**Disclaimer:** Articles published in the journal HemaSphere exclusively reflect the opinions of the authors. The authors are responsible for all content in their abstracts including accuracy of the facts, statements, citing resources, etc.

### (PS1768) LONG-TERM RISK OF HEMATOLOGIC MALIGNANCIES IN PATIENTS WITH UNEXPLAINED LEUKOPENIA: A 10-YEAR RETROSPECTIVE GLOBAL COHORT STUDY

**Topic:** 10. Myelodysplastic syndromes - Clinical

Huei-Ying Lee[1], Yi-Tsung Yang[2, 3], Ruyi Huang*[4, 5, 6], Wen-Chien Chou[2, 3]

*[1]Taipei Tzuchi Hospital, Department of Internal Medicine, New Taipei, Taiwan;[2]National Taiwan University Hospital, Division of Hematology, Department of Internal Medicine, Taipei, Taiwan; [3]College of Medicine, National Taiwan University, Department of Internal Medicine, Taipei, Taiwan; [4]Taipei Tzuchi Hospital, Department of Family Medicine, Taipei, Taiwan;[5]College of Medicine, Tzu Chi University, Department of Family Medicine, Hualien, Taiwan; [6]National Taiwan University and Academia Sinica, Data Science Degree Program, Taipei, Taiwan;*

### Background

Leukopenia is common in routine care and may reflect subclinical marrow dysfunction that predisposes to hematologic malignancies. However, large-scale longitudinal studies examining this association are limited.

### Aims

This study aimed to investigate the long-term risk of hematologic malignancies among adults with unexplained leukopenia compared with individuals with normal white blood cell (WBC) counts.

### Methods

We conducted a multinational, multicenter retrospective cohort study using the TriNetX Global Collaborative Network, which contains de-identified electronic health records from 148 healthcare organizations in 19 countries. Adult patients with leukopenia (WBC < 4000/μL) at the index health examination were propensity score–matched 1:1 to controls (WBC 4000-10000/μL). We excluded patients with infection within 14 days before the index exam, potential drug-induced leukopenia, prior chemo/radiotherapy, autoimmune diseases, or hematologic malignancies diagnosed within 6 months after the index exam. The primary outcomes were incidence of hematologic malignancies, specifically acute myeloid leukemia (AML), acute lymphoblastic leukemia (ALL), chronic lymphocytic leukemia (CLL), myelodysplastic syndrome (MDS), myelofibrosis (MF), hairy cell leukemia (HCL), and multiple myeloma (MM), identified by ICD-10-CM codes over up to 10 years of follow-up. Hazard ratios (HR) with 95% confidence intervals (CI) were calculated. Sensitivity analyses used alternative WBC cutoffs, and additional analyses assessed differences by age, sex, hemoglobin (Hb), and platelet (Plt) levels.

**Copyright Information:** (Online) ISSN: 2572-9241
©2026 The Author(s). HemaSphere is published by John Wiley & Sons Ltd on behalf of European Hematology Association. This is an open access Abstract Book distributed under the Attribution-NonCommercial-NoDerivs (CC BY-NC-ND), which allows third parties to download the articles and share them with others as long as they credit the author and the Abstract Book, but they cannot change the content in any way or use them commercially.
**Abstract Book Citations:** Authors, Title, HemaSphere, 2026;10:(S1):pages. The abstract book DOIs can be found on the "Author Information" page.
**Disclaimer:** Articles published in the journal HemaSphere exclusively reflect the opinions of the authors. The authors are responsible for all content in their abstracts including accuracy of the facts, statements, citing resources, etc.

### Results

We identified 30510 patients with leukopenia and 450984 controls. After 1:1 matching, two equal-sized cohorts had well-balanced baseline covariates. Median follow-up was 3558 days in the leukopenia cohort and 3650 days in the control cohort. Leukopenia was associated with increased risks of MDS (HR 3.70, 95% CI 1.94-7.44; $p < 0.001$; absolute risk difference 0.28%; Figure 1A) and MM (HR 2.62, 95% CI 1.38-4.96; $p = 0.002$; absolute risk difference 0.16%; Figure 1B), but not AML, ALL, CLL, HCL, or MF. In sensitivity analyses, stricter leukopenia definitions strengthened these associations. The highest risks were observed with WBC < 3500/μL vs 4000-10000/μL for both MDS (HR 4.57, 95% CI 2.82-7.41) and MM (HR 5.50, 95% CI 2.85-10.78). Furthermore, in additional analyses within the conventional leukopenia cohort (WBC < 4000/μL), MDS risk was higher in older adults (≥ 65 vs 18-64 years: HR 3.86, 95% CI 2.00-7.45). MM risk was also higher in those ≥ 65 years (vs 18-64 years: HR 4.27, 95% CI 2.22-8.23) and in males (vs females HR 1.95, 95% CI 1.15-3.31). Concurrent cytopenias further increased MM risk: leukopenia with anemia (Hb < 12 g/dL) had an HR of 3.01 (95% CI 1.47-6.13) and leukopenia with thrombocytopenia (Plt < 150000/μL) had an HR of 2.70 (95% CI 1.31-5.57), compared with leukopenia without additional cytopenias.

### Summary/Conclusion

In this large multinational cohort, leukopenia identified on health examinations was associated with a substantially increased 10-year risk of MDS and MM, especially in older patients and those with more severe leukopenia; MM risk was further amplified by male sex and additional cytopenias. These findings support the notion that unexplained leukopenia may serve as an early marker of marrow dysfunction and may warrant closer hematologic evaluation and longitudinal surveillance in high-risk subgroups.

Figure 1. Ten-Year Cumulative Incidence of Myelodysplastic Syndrome and Multiple Myeloma in Leukopenia and Normal WBC Cohorts

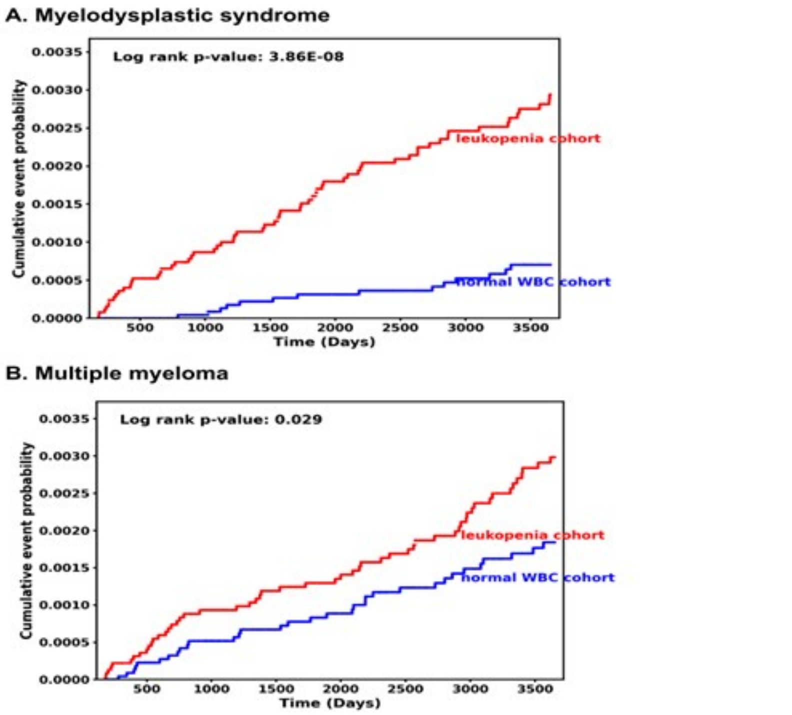

**Copyright Information:** (Online) ISSN: 2572-9241
©2026 The Author(s). HemaSphere is published by John Wiley & Sons Ltd on behalf of European Hematology Association. This is an open access Abstract Book distributed under the Attribution-NonCommercial-NoDerivs (CC BY-NC-ND), which allows third parties to download the articles and share them with others as long as they credit the author and the Abstract Book, but they cannot change the content in any way or use them commercially.
**Abstract Book Citations:** Authors, Title, HemaSphere, 2026;10:(S1):pages. The abstract book DOIs can be found on the "Author Information" page.
**Disclaimer:** Articles published in the journal HemaSphere exclusively reflect the opinions of the authors. The authors are responsible for all content in their abstracts including accuracy of the facts, statements, citing resources, etc.

### (PS1769) COMPARATIVE PROGNOSTIC PERFORMANCE OF SIX RISK SCORES AND EXPLORATORY INFLAMMATORY INDICES IN A REAL-WORLD CHRONIC MYELOMONOCYTIC LEUKEMIA COHORT

**Topic:** 10. Myelodysplastic syndromes - Clinical

Tomas Rivero Equiza*[1, 2], Laura Palomo[1, 2], Pablo Castillo Jimenez[1, 2], Helena Pomares[3], Montserrat Arnan Sangerman[3], Lisbeth Cifuentes Saenz[3], Lucia Moreno[4], Maria Raquel De Paz Arias[4], Marta Gomez Nuñez[5], Laura Segura De La Torre[5], Maria del Carme Talarn Forcadell[6], Jordi Vila Bou[7], Mireia Morgades[1, 2], Isabel Granada[1, 2], Anna Arrufat[1, 2], Alba Mesa[1, 2], Elisa Orna Montero[1, 2], Lurdes Zamora Plana[1, 2], Juan-Manuel Sancho Cia[1, 2], Blanca Xicoy Cirici[1, 2]

*[1]Institut Català d'Oncologia Badalona- Hospital Germans Trias i Pujol, Badalona, Spain;[2]Josep Carreras Research Institute., Badalona, Spain; [3]Institut Català d'Oncologia Hospitalet, Barcelona, Spain; [4]Hospital Universitario La Paz, Madrid, Spain; [5]Hospital Universitario Parc Taulí de Sabadell, Sabadell, Spain;[6]Institut Català d'Oncologia Tarragona, Tarragona, Spain; [7]Institut Català d'Oncologia Girona, Girona, Spain;*

### Background

Prognostication in chronic myelomonocytic leukemia (CMML) remains challenging due to marked biological heterogeneity, particularly between myelodysplastic (MD) and myeloproliferative (MP) subtypes. Multiple risk scores have been developed; however, their comparative performance within the same real-world cohort is not well established. In addition, simple inflammatory indexes derived from routine blood counts have emerged as potential surrogate prognostic markers.

### Aims

To compare the prognostic performance of established CMML risk scores for overall survival (OS), evaluate the applicability of the Triple-A score in this setting, and explore the discriminatory capacity of readily available inflammatory indexes.

### Methods

Adult patients (≥18 years) diagnosed with CMML according to WHO and ICC 2022 criteria and with NGS data at diagnosis were included. The following scores were assessed: IPSS-M, CPSS, CPSS-Mol, BLAST, BLAST-Mol and Triple-A. Inflammatory indexes including neutrophil/lymphocyte ratio (NLR), neutrophil/monocyte ratio (NMR), monocyte/lymphocyte ratio (MLR) and platelet/lymphocyte ratio (PLR) were also analyzed. Discriminatory performance was evaluated using Harrell's C-index. Optimal cut-offs for continuous inflammatory variables were determined using maximally selected rank statistics with Monte Carlo resampling.

**Copyright Information:** (Online) ISSN: 2572-9241
©2026 The Author(s). HemaSphere is published by John Wiley & Sons Ltd on behalf of European Hematology Association. This is an open access Abstract Book distributed under the Attribution-NonCommercial-NoDerivs (CC BY-NC-ND), which allows third parties to download the articles and share them with others as long as they credit the author and the Abstract Book, but they cannot change the content in any way or use them commercially.
**Abstract Book Citations:** Authors, Title, HemaSphere, 2026;10:(S1):pages. The abstract book DOIs can be found on the "Author Information" page.
**Disclaimer:** Articles published in the journal HemaSphere exclusively reflect the opinions of the authors. The authors are responsible for all content in their abstracts including accuracy of the facts, statements, citing resources, etc.

### Results

A total of 239 patients (pts) were included between December 2007 and October 2025. Median age was 71 years (41- 89); 67% were male. CMML-1 and MD subtype accounted for 85% and 79% of cases, respectively, and 20% had oligomonocytic CMML. *TET2, SRSF2* and *ASXL1* mutations were present in 69%, 36% and 38%, respectively. Eleven percent underwent allo-HSCT. Median follow-up among alive pts was 2.15 years; median OS was 4.5 years (95% CI 3.8–6.7) and 5-year cumulative incidence of AML progression was 21% (95% CI 15–28%).
Marked biological differences were observed between MD and MP subtypes, including higher leukocyte counts and inflammatory ratios, as well as increased *ASXL1* frequency in MP disease. Survival differed significantly, with median OS of 5.6 years in MD versus 1.6 years in MP pts (p<0.001) (Figure 1A).
All six scores significantly stratified OS. BLAST-Mol (C-index 0.678) and BLAST (0.671) showed the highest discriminatory performance (Figure 1B). Notably, although Triple-A showed modest global performance, it identified a higher-risk subgroup within MD disease (Figure 1C), while failing to clearly stratify MP cases.
In exploratory univariable analyses, neutrophil count, NLR and MLR (Figure 1D) consistently discriminated OS in the global cohort and within MD, suggesting potential prognostic relevance of inflammatory burden.

### Summary/Conclusion

In this large real-world CMML cohort, established risk scores significantly stratified survival, although overall discriminatory performance was moderate. BLAST and BLAST-Mol showed the highest prognostic discrimination. The marked survival difference between MD and MP subtypes underscores the biological heterogeneity of CMML.
Although global performance of Triple-A was modest, subtype-specific analyses identified a higher-risk subgroup within the MD subtype. Exploratory inflammatory indexes showed significant univariable discrimination and may represent accessible surrogate markers; however, further analysis is required to establish their independent prognostic value.

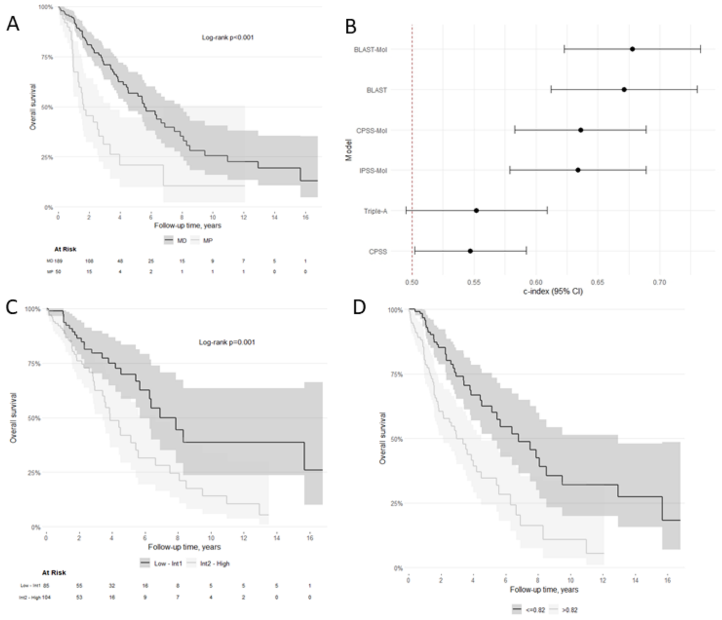

**Figure 1. OS according to CMML subtype (A); according to triple A in MD subgroup (C) and according to MLR in the entire cohort (D). Forest plot of Harrel´s C-index for OS across prognostic models (B).**

**Copyright Information:** (Online) ISSN: 2572-9241
©2026 The Author(s). HemaSphere is published by John Wiley & Sons Ltd on behalf of European Hematology Association. This is an open access Abstract Book distributed under the Attribution-NonCommercial-NoDerivs (CC BY-NC-ND), which allows third parties to download the articles and share them with others as long as they credit the author and the Abstract Book, but they cannot change the content in any way or use them commercially.
**Abstract Book Citations:** Authors, Title, HemaSphere, 2026;10:(S1):pages. The abstract book DOIs can be found on the “Author Information” page.
**Disclaimer:** Articles published in the journal HemaSphere exclusively reflect the opinions of the authors. The authors are responsible for all content in their abstracts including accuracy of the facts, statements, citing resources, etc.

### (PS1770) REAL-WORLD EFFECTIVENESS OF LUSPATERCEPT IN TRANSFUSION-DEPENDENT LOWER-RISK MYELODYSPLASTIC SYNDROMES: RESULTS FROM THE CZECH MYDYS REGISTRY

**Topic:** 10. Myelodysplastic syndromes - Clinical

Anna Jonášová*[1], Libor Cervinek[2], Petra Belohlavkova[3], Tomas Prochazka[4], Markéta Šťastná Marková[5], Jaroslav Cermak[5], Renata MacHová[6], Barbora Dluhosova[7], Olga Cerna[8]

*[1]Charles University General Hospital Prague, Hematology, Prague, Czechia;[2]IHOK, Hematology, Brno, Czechia; [3]Faculty hospital Charles University Hradec Kralove, Hematology, Hradec Kralove, Czechia; [4]Faculty hospital Plzen, Hematology, Plzen, Czechia; [5]Institute of hematology and blood transfusion, Prague, Czechia;[6]FN Olomouc, Olomouc, Czechia; [7]FN Ostrava, Ostrava, Czechia; [8]Faculty hospital Kralovske Vinohrady, Prague, Czechia;*

### Background

Luspatercept improves anemia in transfusion-dependent lower-risk myelodysplastic syndromes (LR-MDS), particularly in patients with ring sideroblasts (RS). Robust real-world data on baseline transfusion burden, prior therapy, concomitant erythropoietin (EPO) use, subgroup responses, and survival remain limited.

### Aims

To evaluate patient characteristics, transfusion dependence, treatment exposure, concomitant therapy, effectiveness, and survival of luspatercept in routine clinical practice.

### Methods

We performed a retrospective analysis of LR-MDS patients treated with luspatercept within the Czech MYDYS registry. The most recent treatment course was evaluated. Endpoints included transfusion independence (TI), hematologic improvement–erythroid (HI-E), treatment duration, and overall survival (OS), estimated using the Kaplan–Meier method.

### Results

Among 2,685 registry patients, 150 received luspatercept. Median age at diagnosis was 71 years (range 45–89); 88 patients were male and 62 female. Median time from diagnosis to treatment initiation was 27.6 months (range 0.4–181.7). MDS-RS was present in 77.9% of patients, and SF3B1 mutation in 72.1% of those tested. IPSS-R risk was predominantly very low/low (76.3%).
Prior erythropoiesis-stimulating agents were used in 75%, steroids in 12.0%, hypomethylating or other disease-modifying agents (including azacitidine, lenalidomide, and imetelstat) in 7.8%, and G-CSF in 7.1%.
Before luspatercept initiation, patients had a median transfusion burden of 6 RBC units over 8 weeks (range 1–23), with 63.4% exhibiting high transfusion burden (>4 units).
Median duration of completed therapy was 12.1 months (range 0.6–46.2). Concomitant EPO was administered at treatment initiation in 36.2% of patients, and overall 54.5% received EPO at some point during therapy. Among patients receiving EPO, TI ≥8 weeks during weeks 1–24 was achieved in 56.7%, and HI-E during weeks 1–48 in 52.1%.
In all evaluable patients, TI ≥8 weeks during weeks 1–24 occurred in 53.9%, TI ≥12 weeks in 42.3%, and TI ≥16 weeks in 38.2%; during weeks 1–48, TI ≥12 weeks occurred in 54.3% and TI ≥16 weeks in 45.5%. HI-E was observed in 48.5% (weeks 1–24) and 54.1% (weeks 1–48). In MDS-RS patients, TI ≥8 weeks (1–24) was 54.2% and HI-E (1–48) 60.0%; in non-RS patients, 58.8% and 38.7%, respectively. Among SF3B1-mutated patients, TI ≥8 weeks (1–24) was 54.7% and HI-E (1–48) 50.9%.
Median OS from diagnosis was 123.9 months (95% CI 79.4–NA), and median OS from luspatercept initiation was 45.9 months (95% CI 20.4–NA).

**Copyright Information:** (Online) ISSN: 2572-9241
©2026 The Author(s). HemaSphere is published by John Wiley & Sons Ltd on behalf of European Hematology Association. This is an open access Abstract Book distributed under the Attribution-NonCommercial-NoDerivs (CC BY-NC-ND), which allows third parties to download the articles and share them with others as long as they credit the author and the Abstract Book, but they cannot change the content in any way or use them commercially.
**Abstract Book Citations:** Authors, Title, HemaSphere, 2026;10:(S1):pages. The abstract book DOIs can be found on the "Author Information" page.
**Disclaimer:** Articles published in the journal HemaSphere exclusively reflect the opinions of the authors. The authors are responsible for all content in their abstracts including accuracy of the facts, statements, citing resources, etc.

**Summary/Conclusion**

This large real-world analysis demonstrates substantial and durable erythroid responses to luspatercept in heavily transfusion-dependent LR-MDS, including key biological subgroups and patients receiving concomitant EPO, supporting its effectiveness in routine clinical practice.

**Copyright Information:** (Online) ISSN: 2572-9241
©2026 The Author(s). HemaSphere is published by John Wiley & Sons Ltd on behalf of European Hematology Association. This is an open access Abstract Book distributed under the Attribution-NonCommercial-NoDerivs (CC BY-NC-ND), which allows third parties to download the articles and share them with others as long as they credit the author and the Abstract Book, but they cannot change the content in any way or use them commercially.
**Abstract Book Citations:** Authors, Title, HemaSphere, 2026;10:(S1):pages. The abstract book DOIs can be found on the "Author Information" page.
**Disclaimer:** Articles published in the journal HemaSphere exclusively reflect the opinions of the authors. The authors are responsible for all content in their abstracts including accuracy of the facts, statements, citing resources, etc.

### (PS1771) EVALUATING THE PROGNOSTIC SIGNIFICANCE OF MARROW COMPLETE REMISSION (MCR) IN HIGHER-RISK MDS: A META-REGRESSIVE ASSESSMENT OF SURROGATE ENDPOINT VALIDITY

**Topic:** 10. Myelodysplastic syndromes - Clinical

Luis E. Aguirre*[1], Jenn Beardsley-Kooyoumjian[2], Melanie Castro-Mollo[3], Nuttavut Sumransub[3], Jonathan Fang[2], Alain Mina[1], Jan Bewersdorf[1], Lourdes Mendez[1], Nikolai Podoltsev[1], Stephanie Halene[1], Amer M. Zeidan[1], Richard Stone[3], David Sallman[4], Rami S. Komrokji[4], Maximilian Stahl[1]

*[1]Yale School of Medicine, Section of Medical and Oncology and Hematology, New Haven, United States of America;[2]Harvard T.H. Chan School of Public Health, Boston, United States of America; [3]Dana-Farber Cancer Institute, Department of Medical Oncology, Boston, United States of America; [4]H. Lee Moffitt Cancer Center and Research Institute, Department of Malignant Hematology, Tampa, United States of America;*

### Background

Marrow complete remission (mCR) is frequently used in trials evaluating therapies for higher-risk MDS (HR-MDS) as an indicator of treatment efficacy and is often incorporated into composite response endpoints. Although mCR increases reported response rates, evidence suggests that patients achieving mCR derive inconsistent benefit, and its association with improved overall survival (OS) remains unclear. If mCR lacks prognostic value, its inclusion in composite endpoints may overestimate treatment efficacy and misguide clinical interpretation, trial design, and regulatory decisions.

### Aims

To evaluate whether reliance on mCR-based composite endpoints is justified by assessing the trial-arm relationship between mCR rate and median OS in prospective HMA trials of HR-MDS.

### Methods

PubMed and Embase were systematically searched from July 2006 through August 2025. We identified prospective phase I–III trials of HMA enrolling adults with HR-MDS per IPSS/IPSS-R that reported mCR (IWG 2006) and survival outcomes. Retrospective studies, case series, reviews, conference abstracts, duplicate datasets, and post-transplant trials were excluded. Five reviewers independently screened 2,273 records in Covidence, with inclusion determined by consensus of ≥2 reviewers. Data were abstracted in accordance with PRISMA guidelines. For studies lacking 95% CIs for mOS (n=2), individual patient data were reconstructed from Kaplan–Meier curves using the Guyot algorithm to derive CIs. Quantitative synthesis included random-effects meta-analysis (REma) of mOS and a primary mixed-effects meta-regression with random intercepts for study and treatment arm. The primary endpoint was the arm-level association between mCR proportion and mOS. An unadjusted random-effects meta-regression was performed as a sensitivity analysis.

**Copyright Information:** (Online) ISSN: 2572-9241
©2026 The Author(s). HemaSphere is published by John Wiley & Sons Ltd on behalf of European Hematology Association. This is an open access Abstract Book distributed under the Attribution-NonCommercial-NoDerivs (CC BY-NC-ND), which allows third parties to download the articles and share them with others as long as they credit the author and the Abstract Book, but they cannot change the content in any way or use them commercially.
**Abstract Book Citations:** Authors, Title, HemaSphere, 2026;10:(S1):pages. The abstract book DOIs can be found on the “Author Information” page.
**Disclaimer:** Articles published in the journal HemaSphere exclusively reflect the opinions of the authors. The authors are responsible for all content in their abstracts including accuracy of the facts, statements, citing resources, etc.

### Results

Twelve trials (16 arms; 859 patients) were included (1,562 unique records screened; 105 full texts assessed). Across arms, mean mCR was 23.5% (SD, 12.5%). REma of 11 arms showed an estimated pooled mOS of 12.8 months ($I^2$=95.0%). Knapp–Hartung–adjusted, disease-setting estimates were 16.1 months (frontline), 12.1 months (mixed), and 8.9 months (relapsed/refractory, R/R). In the primary mixed-effects meta-regression (MR, final analytic dataset: 9 arms with complete mCR/mOS data), higher arm-level mCR was not associated with longer survival: each 10%-point increase in mCR corresponded to a 1.02-fold higher mOS (95% CI, 0.30–2.16; P=.60), while heterogeneity remained high ($I^2$=97.7%). Findings were concordant in unadjusted MR, with each 10%-point increase in mCR corresponding to a 1.25-fold higher mOS (95% CI, 0.61–2.53; P=.55; $I^2$=98.2%). Covariate adjustment (R/R status, *TP53*mt) did not materially reduce heterogeneity or strengthen the association.

### Summary/Conclusion

Across prospective HMA trials in HR-MDS,arm-level mCR rates did not reliably track with median OS, providing no evidence that mCR functions as a robust surrogate for survival at the trial-arm level. Any apparent association was attenuated after adjustment for adverse biology and R/R status, suggesting that the relationship may reflect confounding rather than true surrogacy. These data caution against reliance on mCR-driven composite response endpoints, which may inflate apparent efficacy and misdirect late-phase development and interpretation.

.

**Copyright Information:** (Online) ISSN: 2572-9241
©2026 The Author(s). HemaSphere is published by John Wiley & Sons Ltd on behalf of European Hematology Association. This is an open access Abstract Book distributed under the Attribution-NonCommercial-NoDerivs (CC BY-NC-ND), which allows third parties to download the articles and share them with others as long as they credit the author and the Abstract Book, but they cannot change the content in any way or use them commercially.
**Abstract Book Citations:** Authors, Title, HemaSphere, 2026;10:(S1):pages. The abstract book DOIs can be found on the “Author Information” page.
**Disclaimer:** Articles published in the journal HemaSphere exclusively reflect the opinions of the authors. The authors are responsible for all content in their abstracts including accuracy of the facts, statements, citing resources, etc.

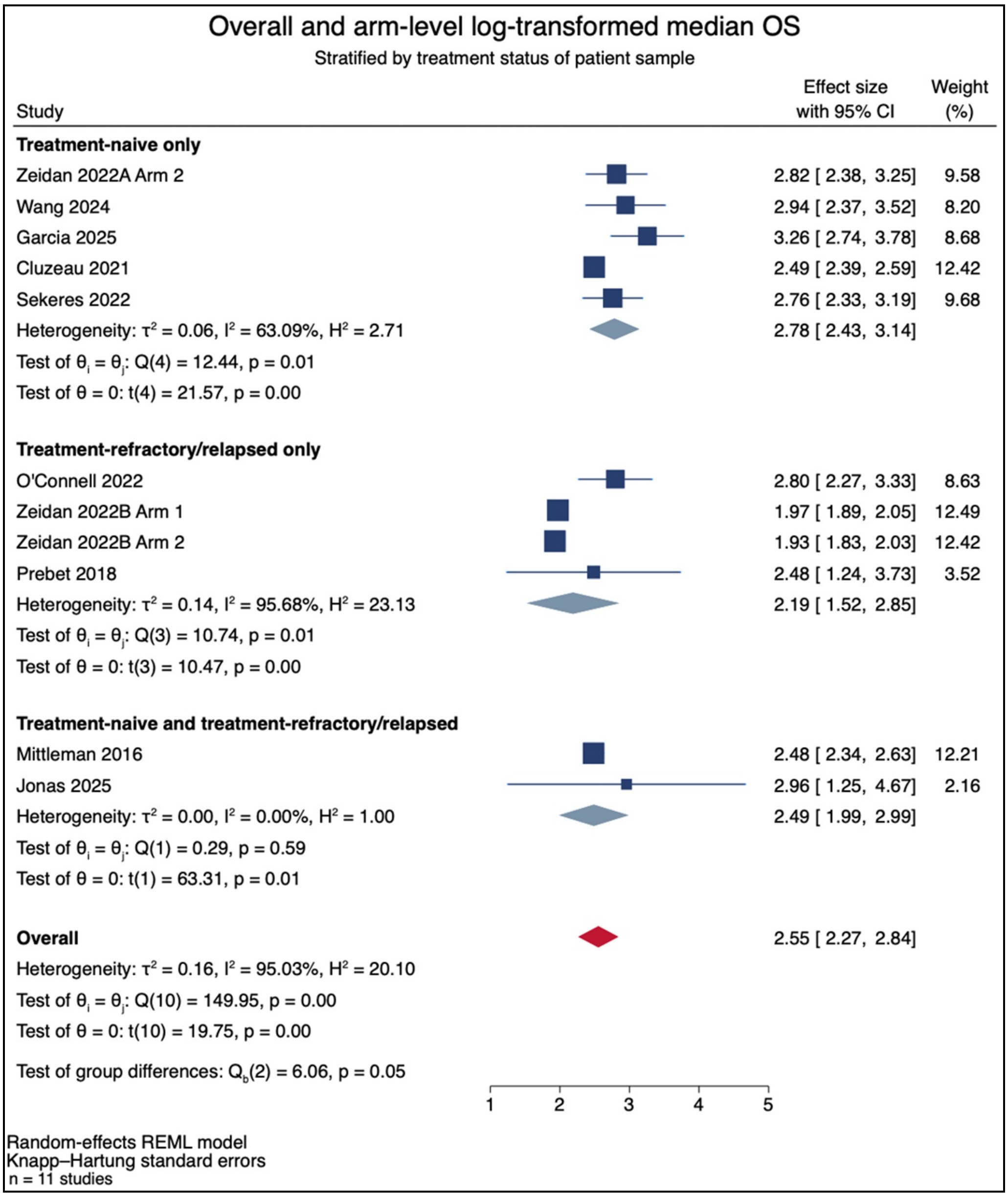

Figure 1.- Forest plot summarizing log-transformed median overall survival (OS) across 11 eligible study arms, stratified by patient treatment status (treatment-naïve, mixed, or refractory).

**Copyright Information:** (Online) ISSN: 2572-9241
©2026 The Author(s). HemaSphere is published by John Wiley & Sons Ltd on behalf of European Hematology Association. This is an open access Abstract Book distributed under the Attribution-NonCommercial-NoDerivs (CC BY-NC-ND), which allows third parties to download the articles and share them with others as long as they credit the author and the Abstract Book, but they cannot change the content in any way or use them commercially.
**Abstract Book Citations:** Authors, Title, HemaSphere, 2026;10:(S1):pages. The abstract book DOIs can be found on the "Author Information" page.
**Disclaimer:** Articles published in the journal HemaSphere exclusively reflect the opinions of the authors. The authors are responsible for all content in their abstracts including accuracy of the facts, statements, citing resources, etc.

### (PS1772) IPSS-M OUTPERFORMS IPSS-R IN PREDICTING TIME TO TRANSFUSION DEPENDENCY IN PATIENTS WITH LOW RISK MYELODYSPLASTIC SYNDROMES

**Topic:** 10. Myelodysplastic syndromes - Clinical

Federica Pilo*[1], Alessandro Costa[2], Arianna Basciu[2], Martina Pettinau[1], Marianna Greco[1], Olga Mulas[2], Giovanni Caocci[2]

*[1]Hematology and BMT Unit, "A. Businco" Hospital, ARNAS Brotzu, Cagliari, Cagliari, Italy; [2]Hematology, Department of Medical Sciences and Public Health, University of Cagliari, 09121 Cagliari, Italy, Cagliari, Italy;*

### Background

Transfusion dependence (TD) is a clinically significant endpoint in myelodysplastic syndromes (MDS) that affects quality of life and prognosis. Although the International Prognostic Scoring System Revised (IPSS-R) and the Molecular IPSS (IPSS-M) are validated prognostic tools for survival, they have not been compared in terms of their ability to predict the time to acquire TD. This aspect becomes even more important with the advent of new drugs for ineffective erythropoiesis.

### Aims

To evaluate the ability of IPSS-M to predict incident TD in comparison with low and intermediate IPSS-R, and to assess whether molecular stratification, including SF3B1 mutational status, provides additional prognostic information beyond clinical risk scores.

### Methods

We retrospectively analyzed 70 consecutive patients with low or intermediate-I IPSS-R MDS for whom comprehensive NGS profiling was available at diagnosis. Patients with high IPSS-R and other causes of anemia were excluded. The primary endpoint was time from diagnosis to transfusion dependence. Time-to-event analyses were performed using Kaplan–Meier estimates and Cox proportional hazards models. Model discrimination was assessed by time-dependent AUC at 12 months and concordance index (C-index). Interaction with ESA therapy was formally tested. Molecular analyses included SF3B1 mutational status, co-mutation patterns, and multivariable models adjusted for IPSS-M. The **table 1** shows patients' characteristics.

### Results

A total of 70 patients were included in the study. At the time of diagnosis, 23 patients (32%) were already TD. Of the remaining 47 patients who were transfusion-independent at diagnosis, six (12,7%) developed incident TD during follow-up, with a median time to TD of 13.9 months (interquartile range [IQR], 2.2–24.0). In the incident TD cohort, the IPSS-M was significantly associated with a shorter time to TD (hazard ratio [HR], 3.67; 95% confidence interval [CI], 1.40–9.60; p = 0.008), whereas the IPSS-R showed only a non-significant trend (HR, 2.31; 95% CI, 0.91–5.90; p = 0.080). In a model that compared both scores, IPSS-M was independently predictive (HR 3.69, 95% CI 1.08–12.6; p = 0.037), while IPSS-R was not (p = 0.989). Specifically, patients with low or intermediate-I IPSS-R scores who were reclassified as high IPSS-M showed the shortest time to TD. SF3B1 mutations were present in 18 out of 47 patients (38,3%) in the incident cohort. SF3B1 single mutations showed a trend toward a shorter time to TD (HR, 4.47; P = 0.085), whereas no events occurred among SF3B1 co-mutated cases. After adjusting for IPSS-M, the association between SF3B1 single mutations and TD remained significant (HR 6.68; p = 0.037).

**Copyright Information:** (Online) ISSN: 2572-9241
©2026 The Author(s). HemaSphere is published by John Wiley & Sons Ltd on behalf of European Hematology Association. This is an open access Abstract Book distributed under the Attribution-NonCommercial-NoDerivs (CC BY-NC-ND), which allows third parties to download the articles and share them with others as long as they credit the author and the Abstract Book, but they cannot change the content in any way or use them commercially.
**Abstract Book Citations:** Authors, Title, HemaSphere, 2026;10:(S1):pages. The abstract book DOIs can be found on the "Author Information" page.
**Disclaimer:** Articles published in the journal HemaSphere exclusively reflect the opinions of the authors. The authors are responsible for all content in their abstracts including accuracy of the facts, statements, citing resources, etc.

### Summary/Conclusion

In our study, IPSS-M outperformed IPSS-R in predicting TD incident.
Patients with low or intermediate-I IPSS-R scores who were reclassified as high IPSS-M showed the shortest time to TD.Data support the added clinical utility of molecular risk stratification for anticipating the introduction of new drugs that target ineffective erythropoiesis in MDS. Exploratory analyses suggest a potential additional effect of SF3B1 single mutations, as expected considering its known interaction with iron homeostasis and reduced response to ESA. Validation in larger case studies is warranted.

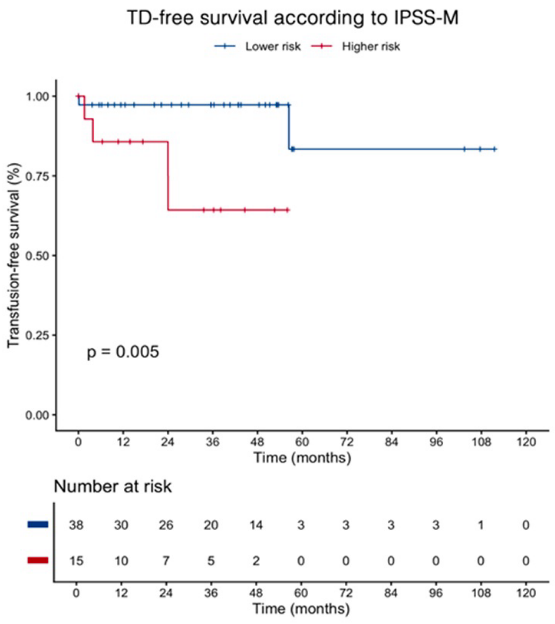

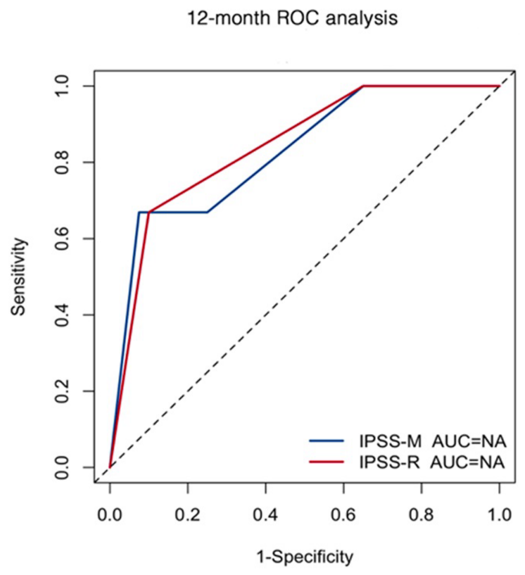

TAB 1: Patients' characteristics

| | Transfusion dependency at diagnosis | No transfusion dependency at diagnosis |
|---|---|---|
| Number (%) | 23 (32%) | 47 (67%) |
| Sex | M 13; F 10 | M 24; F 23 |
| Median age at diagnosis | 65 | 64 |
| Median Hb ad diagnosis gr/dL | 7,5 | 12,3 |
| Median Blasts at diagnosis | 3 | 2 |
| Very low IPSS-R | 4 | 15 |
| Low IPSS-R | 14 | 24 |
| Intermediate IPSS-R | 5 | 7 |
| Very low IPSS-M | 2 | 14 |
| Low IPSS-M | 2 | 6 |
| Moderate low IPSS-M | 11 | 23 |
| Moderate high IPSS-M | 3 | 2 |
| High IPSS-M | 4 | 2 |
| Very high IPSS-M | 1 | 0 |
| SF3B1 wild type | 10 | 29 |
| SF3B1 single mutation | 2 | 7 |
| SF3B1 co-mutation | 11 | 11 |
| ESA treatment | 0 | 23 |

**Copyright Information:** (Online) ISSN: 2572-9241
©2026 The Author(s). HemaSphere is published by John Wiley & Sons Ltd on behalf of European Hematology Association. This is an open access Abstract Book distributed under the Attribution-NonCommercial-NoDerivs (CC BY-NC-ND), which allows third parties to download the articles and share them with others as long as they credit the author and the Abstract Book, but they cannot change the content in any way or use them commercially.
**Abstract Book Citations:** Authors, Title, HemaSphere, 2026;10:(S1):pages. The abstract book DOIs can be found on the "Author Information" page.
**Disclaimer:** Articles published in the journal HemaSphere exclusively reflect the opinions of the authors. The authors are responsible for all content in their abstracts including accuracy of the facts, statements, citing resources, etc.

### (PS1773) OUTCOMES OF PATIENTS DIAGNOSED WITH MYELODYSPLASTIC SYNDROME OR ACUTE MYELOID LEUKEMIA FOLLOWING SOLID ORGAN TRANSPLANTATION

**Topic:** 10. Myelodysplastic syndromes - Clinical

Charan Vegivinti*[1], Guillermo Montalban-Bravo[2], Kelly Chien[2], Danielle Hammond[2], Koji Sasaki[2], Alex Bataller[2], Ian Bouligny[2], Wei-Ying Jen[2], Mahesh Swaminathan[2], Sanam Loghavi[3], Uday R. Popat[4], Guillermo Garcia-Manero[2], Alexandre Bazinet[2]

*[1]MD Anderson Cancer Center, Department of Cancer Medicine, Houston, United States of America;[2]MD Anderson Cancer Center, Department of Leukemia, Houston, United States of America; [3]MD Anderson Cancer Center, Department of Hematopathology, Houston, United States of America; [4]MD Anderson Cancer Center, Department of Stem Cell Transplantation and Cellular Therapy, Houston, United States of America;*

### Background

The rate of solid organ transplantation (SOT) has increased in recent years. Long-term immunosuppression, while essential for graft maintenance, increases secondary malignancy risk, with two-fold higher cancer mortality versus the general population. Skin cancers and post-transplantation lymphoproliferative disorders (PTLDs) are well described after SOT. Myeloid neoplasms are less common and remain poorly characterized.

### Aims

To evaluate the characteristics and outcomes of patients developing myelodysplastic syndrome (MDS) or acute myeloid leukemia (AML) after SOT.

### Methods

This was a single-center retrospective study (2015-2025) including adult SOT recipients who developed MDS or AML per WHO 2016 criteria. Risk was stratified using IPSS-R/IPSS-M for MDS and ELN 2022 for AML. Treatment responses were evaluated per IWG 2023 (MDS) and ELN 2022 (AML) criteria. Higher risk MDS (HR-MDS) was defined as IPSS-R >3.5. NGS using 28- or 81-gene panels was carried out when accessible. Biallelic *TP53* status was defined as a single *TP53* mutation with VAF >50%, two *TP53* mutations, or a *TP53* mutation with concurrent deletion of chromosome 17p. Overall survival (OS) was calculated from diagnosis to death from any cause and estimated using the Kaplan-Meier method.

**Copyright Information:** (Online) ISSN: 2572-9241
©2026 The Author(s). HemaSphere is published by John Wiley & Sons Ltd on behalf of European Hematology Association. This is an open access Abstract Book distributed under the Attribution-NonCommercial-NoDerivs (CC BY-NC-ND), which allows third parties to download the articles and share them with others as long as they credit the author and the Abstract Book, but they cannot change the content in any way or use them commercially.
**Abstract Book Citations:** Authors, Title, HemaSphere, 2026;10:(S1):pages. The abstract book DOIs can be found on the “Author Information” page.
**Disclaimer:** Articles published in the journal HemaSphere exclusively reflect the opinions of the authors. The authors are responsible for all content in their abstracts including accuracy of the facts, statements, citing resources, etc.

**Results**

We identified 18 patients diagnosed with myeloid neoplasm following SOT (12 MDS, 6 AML). The median age at diagnosis was 62 years. The median time from SOT to diagnosis was 8.6 years in MDS, 8.3 years in AML. Kidney transplants predominated (78%), followed by liver (17%), and heart (6%). The most common indications for SOT were glomerulonephritis (n=5) and renal cell carcinoma (n=3) for kidney, MASH-cirrhosis for liver (n=2), and non-ischemic cardiomyopathy for heart transplants. Among MDS patients, 75% were HR-MDS by IPSS-R, and 86% were high/very-high risk by IPSS-M. Most AML patients (83%) were adverse-risk by ELN 2022. The most common mutations were *TP53* (47%), *NF1* (27%), *ASXL1* (24%), and *RUNX1* (24%). 88% of *TP53* mutations were biallelic. Complex cytogenetics occurred in 33% of MDS and 83% of AML cases. 5/6 (83%) AML cases were biallelic *TP53*-mutated (Fig 1A).

MDS patients were treated with hypomethylating agent (HMA)-based combinations in 58%, erythroid-stimulating agents (ESAs) in 17%, luspatercept in 8%, lenalidomide in 8%, and observation alone in 8%. Of the 4 patients who were IWG 2023 response evaluable, 2 (50%) responded (1 CR, 1 $CR_L$). In the AML patients, 83% received low intensity HMA or low-dose cytarabine-based therapy and 17% received intensive chemotherapy (7+3). The overall response rate was 33% (1 CR, 1 CRp) in AML. Three patients (2 MDS, 1 AML) underwent allogeneic SCT.

The median OS was 15.7 months for the full cohort, 26 months for MDS, and 9.7 months for AML (Fig 1B). HR-MDS had significantly worse survival than low-risk MDS (19.6 vs 74.5 months, p=0.03). Infections were responsible for 8/12 (67%) of deaths with known cause. Among renal transplant recipients, 2 (17%) patients experienced ≥30% creatinine elevation (both on HMA therapy), and one required initiation of dialysis (treated with ESA alone). No heart/liver graft failures occurred. All three SCT recipients died, two from graft failure and one from pneumonia.

**Summary/Conclusion**

Post-SOT myeloid neoplasms represent generally high-risk entities with poor survival. Treatment approaches must carefully consider toxicities in this population.

**Copyright Information:** (Online) ISSN: 2572-9241
©2026 The Author(s). HemaSphere is published by John Wiley & Sons Ltd on behalf of European Hematology Association. This is an open access Abstract Book distributed under the Attribution-NonCommercial-NoDerivs (CC BY-NC-ND), which allows third parties to download the articles and share them with others as long as they credit the author and the Abstract Book, but they cannot change the content in any way or use them commercially.
**Abstract Book Citations:** Authors, Title, HemaSphere, 2026;10:(S1):pages. The abstract book DOIs can be found on the "Author Information" page.
**Disclaimer:** Articles published in the journal HemaSphere exclusively reflect the opinions of the authors. The authors are responsible for all content in their abstracts including accuracy of the facts, statements, citing resources, etc.

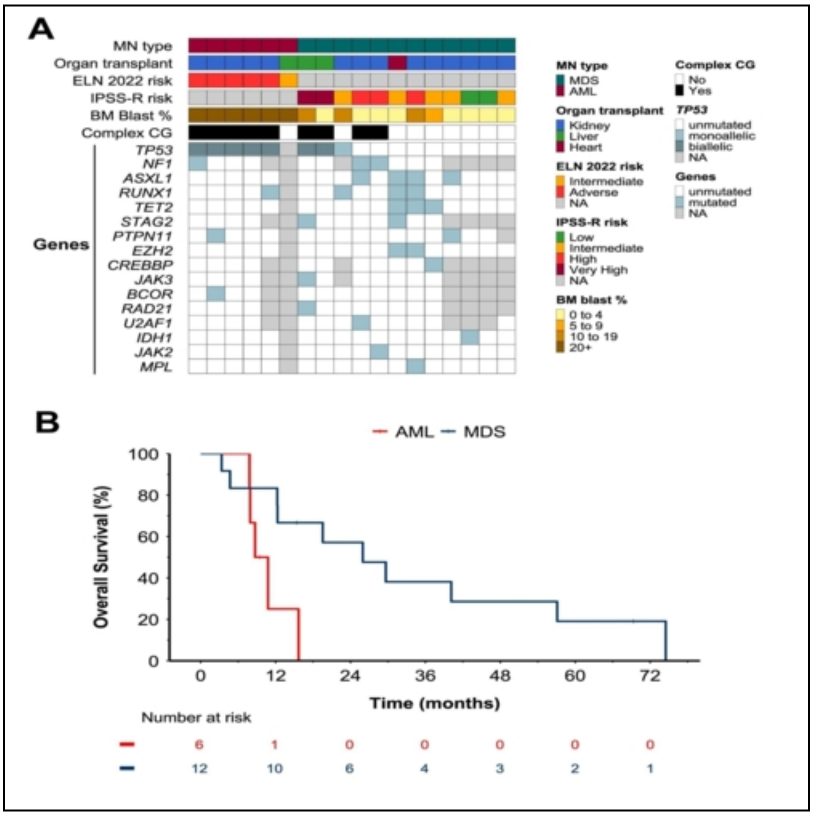

Figure 1A: Oncoprint of baseline characteristics and molecular cytogenetics Figure 1B: Overall survival in MDS and AML

**Copyright Information:** (Online) ISSN: 2572-9241
©2026 The Author(s). HemaSphere is published by John Wiley & Sons Ltd on behalf of European Hematology Association. This is an open access Abstract Book distributed under the Attribution-NonCommercial-NoDerivs (CC BY-NC-ND), which allows third parties to download the articles and share them with others as long as they credit the author and the Abstract Book, but they cannot change the content in any way or use them commercially.
**Abstract Book Citations:** Authors, Title, HemaSphere, 2026;10:(S1):pages. The abstract book DOIs can be found on the “Author Information” page.
**Disclaimer:** Articles published in the journal HemaSphere exclusively reflect the opinions of the authors. The authors are responsible for all content in their abstracts including accuracy of the facts, statements, citing resources, etc.

### (PS1774) BORTEZOMIB COMBINED WITH AZACITIDINE AND VENETOCLAX IN HIGH-RISK MYELODYSPLASTIC NEOPLASMS: A SINGLE-CENTER RETROSPECTIVE STUDY

**Topic:** 10. Myelodysplastic syndromes - Clinical

Zi-Yuan Nie*[1], Yajuan Cui[2], Liwei Fang[3]

*[1]Department of Hematology, The Second Hospital of Hebei Medical University, Shijiazhuang, China;[2]Department of Hematology，The second xiangya Hospital，Central South University, changsha, China; [3]State Key Laboratory of Experimental Hematology, National Clinical Research Center for Blood Diseases, Haihe Laboratory of Cell Ecosystem, Institute of Hematology & Blood Diseases Hospital, Chinese Academy of Medical Sciences & Peking Union Medical College, Tianjin, China;*

### Background

High-risk myelodysplastic neoplasms (HR-MDS) are associated with a poor prognosis, and the efficacy of current therapies, such as azacitidine plus venetoclax (VA regimen), remains suboptimal. Preclinical and clinical studies have demonstrated the activity of bortezomib, either alone or in combination with hypomethylating agents, in MDS. However, no study to date has evaluated the combination of bortezomib with azacitidine and venetoclax (VAB regimen).

### Aims

This study aimed to assess the efficacy and safety of this triplet regimen in treatment-naïve HR-MDS patients.

### Methods

This single-center retrospective study enrolled 20 treatment-naïve HR-MDS patients with an IPSS-R score >4.5, treated between June 2021 and June 2024. All patients received the VAB regimen: azacitidine 75 mg/m²/day on days 1–7, venetoclax 400 mg/day on days 1–14, and bortezomib 1.3 mg/m²/dose on days 1, 8, 15, and 22 in cycle 1, and on days 1 and 8 in subsequent cycles. Cycle length was 28–42 days.

### Results

A total of 20 patients with HR-MDS were enrolled, with a median age of 51.5 years (range, 18–66 years) and a male-to-female ratio of 1:1 (10 males, 10 females). According to the WHO 2016 classification, 9 patients (45%) had EB1, 9 (45%) had EB2, and 2 (10%) had MLD. IPSS-R stratification classified 9 patients (45%) as high-risk and 11 (55%) as very high-risk. The median number of gene mutations was 3 (range, 0–7). Cytogenetic analysis revealed normal karyotypes in 9 patients (45%) and abnormal karyotypes in 11 (55%), including complex karyotypes (≥3 abnormalities) in 5 patients (25%). Eight patients (40%) underwent hematopoietic stem cell transplantation. The overall best composite remission rate (CRc) ( including CR, CRi, mCR+HI, and CRbi), was 75%. The high-risk group had a significantly higher best CRc rate than the very high-risk group (100% vs. 54.5%, p=0.03). After the first treatment cycle, the CRc rate was 60% (12/20) (CR in 3, CRi in 2, mCR+HI in 4, and CRbi in 3). The first-cycle CRc rate was 77.8%  in the high-risk group and 45.5%in the very high-risk group (p=0.21). With a median follow-up of 17.4 months, 4 patients died and 2 progressed to AML. The median overall survival (mOS) for the entire cohort was not reached. mOS was not reached in the high-risk group  and was 11.0 months in the very high-risk group (p=0.34). . The 1-year and 2-year OS rates were both 78.6%. Grade ≥3 hematological toxicity was the most common adverse event but was manageable.

**Copyright Information:** (Online) ISSN: 2572-9241
©2026 The Author(s). HemaSphere is published by John Wiley & Sons Ltd on behalf of European Hematology Association. This is an open access Abstract Book distributed under the Attribution-NonCommercial-NoDerivs (CC BY-NC-ND), which allows third parties to download the articles and share them with others as long as they credit the author and the Abstract Book, but they cannot change the content in any way or use them commercially.
**Abstract Book Citations:** Authors, Title, HemaSphere, 2026;10:(S1):pages. The abstract book DOIs can be found on the "Author Information" page.
**Disclaimer:** Articles published in the journal HemaSphere exclusively reflect the opinions of the authors. The authors are responsible for all content in their abstracts including accuracy of the facts, statements, citing resources, etc.

### Summary/Conclusion

This retrospective study is the first to report the efficacy and safety of the VAB triplet regimen in treatment-naïve patients with HR-MDS. The regimen induced a high early CRc rate and was associated with prolonged OS, including in the very high-risk subgroup. The safety profile was manageable. These findings provide a novel perspective for optimizing first-line treatment strategies in HR-MDS.

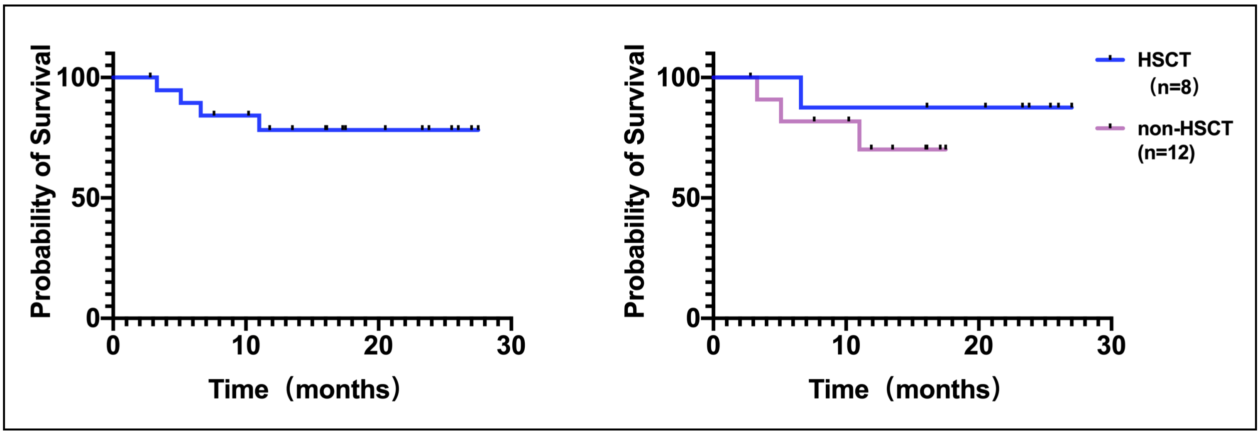

**Copyright Information:** (Online) ISSN: 2572-9241
©2026 The Author(s). HemaSphere is published by John Wiley & Sons Ltd on behalf of European Hematology Association. This is an open access Abstract Book distributed under the Attribution-NonCommercial-NoDerivs (CC BY-NC-ND), which allows third parties to download the articles and share them with others as long as they credit the author and the Abstract Book, but they cannot change the content in any way or use them commercially.
**Abstract Book Citations:** Authors, Title, HemaSphere, 2026;10:(S1):pages. The abstract book DOIs can be found on the "Author Information" page.
**Disclaimer:** Articles published in the journal HemaSphere exclusively reflect the opinions of the authors. The authors are responsible for all content in their abstracts including accuracy of the facts, statements, citing resources, etc.

### (PS1775) LONGITUDINAL IPSS-M RISK EVALUATION IN 205 MDS PATIENTS: IS REASSESSMENT BEYOND INITIAL DIAGNOSIS NECESSARY?

**Topic:** 10. Myelodysplastic syndromes - Clinical

Anetta Marcinek*[1], Stephan Hutter[1], Sandra Huber[1], Gregor Hörmann[1], Franziska Schneider[1], Saskia Kolb[1], Rabea Ritter[1], Manja Meggendorfer[1], Torsten Haferlach[1], Constance Baer[1]

[1]*MLL Munich Leukemia Laboratory, Munich, Germany;*

### Background

Since 2022, the molecular International Prognostic Scoring System (IPSS-M) has refined risk assessment in myelodysplastic neoplasms (MDS) by integrating recurrent somatic mutations with hematologic and cytogenetic parameters. The IPSS-M was primarily developed for use at initial diagnosis (ID) and does not account for disease dynamics. Patients diagnosed before 2022 or without baseline molecular profiling at ID often lack IPSS-M-based risk assessment. In addition, the value of repeated IPSS-M evaluations during follow-up (FU) remains unclear.

### Aims

Through a longitudinal study, we aimed to elucidate the impact of prognostic restratification on MDS patients to answer: (1) whether an IPSS-M assessment is reasonable at FU in patients lacking IPSS-M evaluation at ID, and (2) whether disease dynamics are reflected in a shift to a higher (upstaging) or lower (downstaging) IPSS-M risk category.

### Methods

We analyzed 205 paired bone marrow (BM) samples from MDS patients, all at ID and at different FU timepoints, by morphology, cyto-, and molecular genetics. Clinical (% BM blasts, hemoglobin (Hb), and platelet (PLT) count) and genetic (IPSS-R cytogenetic risk and count of mutated genes) parameters were analyzed for each timepoint. The IPSS-M was then calculated for both timepoints, and the minimal time difference between ID and FU was one year (median: 2.1 years [range: 1.01-14.9 years]). Patients with allogeneic SCT were excluded.

**Copyright Information:** (Online) ISSN: 2572-9241
©2026 The Author(s). HemaSphere is published by John Wiley & Sons Ltd on behalf of European Hematology Association. This is an open access Abstract Book distributed under the Attribution-NonCommercial-NoDerivs (CC BY-NC-ND), which allows third parties to download the articles and share them with others as long as they credit the author and the Abstract Book, but they cannot change the content in any way or use them commercially.
**Abstract Book Citations:** Authors, Title, HemaSphere, 2026;10:(S1):pages. The abstract book DOIs can be found on the "Author Information" page.
**Disclaimer:** Articles published in the journal HemaSphere exclusively reflect the opinions of the authors. The authors are responsible for all content in their abstracts including accuracy of the facts, statements, citing resources, etc.

**Results**

About half of the patients changed to another IPSS-M, mostly by upstaging, which was particularly seen in cases with an initially very low to moderate low IPSS-M. In detail, for each group the following changes were observed: Very low and low (up: 66/143 (46%), down: 4/143 (3%), no: 73/143(51%)), moderate low and moderate high (up: 22/46 (48%), down: 11/46 (24%), no: 13/46 (28%)), high and very high (up: 5/16 (31%), down: 3/16 (19%), no: 8/16 (50%); Fig. 1). Significant changes in clinical parameters from ID to FU were observed in patients who were up- or downstaged compared to those with unchanged IPSS-M (details are depicted in the table).

| | Changes from ID to FU (median) | | |
|---|---|---|---|
| | Upstaged (n=93; 45%) | Downstaged (n=18; 8%) | Stable (n=94; 46%) |
| BM blasts (%) | 3.5 to 5 [p <0.0001] | 4.5 to 1.75 [p=0.0041] | 3.5 to 2.5 [ns] |
| Hb (g/dL) | 10.5 to 8.6 [p <0.0001] | 8.5 to 10.6 [p=0.0002] | 10.1 to 9.8 [ns] |
| PLT count (G/l) | 124 to 73 [p <0.0001] | 117.5 to 80 [p=0.002] | 150 to 114 [ns] |

Progression in upstaged patients was accompanied by significant changes in cytogenetic and molecular parameters: a gain of alterations leading to a higher IPSS-R cytogenetic risk like +8, -7 or gain of a complex karyotype and an increase in the count of mutated genes relevant for IPSS-M calculation. Mutations were acquired in several high-risk genes including *TP53*, *CBL*, *EZH2*, *NRAS*, and *RUNX1*.

**Summary/Conclusion**

Approximately half of the patients experienced a change in their IPSS-M status throughout the follow-up, suggesting a high need to determine all parameters at ID to apply the IPSS-M correctly. For cases with stable disease and absent molecular genetic screening at ID, later scoring during FU may be a justified alternative. Conversely, disease progression, indicated by clinical parameters (i.e., blood counts), was associated with a higher likelihood of clonal evolution and altered IPSS-M risk grouping. Further studies are needed to determine if longitudinal monitoring of IPSS-M with repeated cytogenetic and molecular screening should be included in any clinical decision-making.

**Copyright Information:** (Online) ISSN: 2572-9241
©2026 The Author(s). HemaSphere is published by John Wiley & Sons Ltd on behalf of European Hematology Association. This is an open access Abstract Book distributed under the Attribution-NonCommercial-NoDerivs (CC BY-NC-ND), which allows third parties to download the articles and share them with others as long as they credit the author and the Abstract Book, but they cannot change the content in any way or use them commercially.
**Abstract Book Citations:** Authors, Title, HemaSphere, 2026;10:(S1):pages. The abstract book DOIs can be found on the "Author Information" page.
**Disclaimer:** Articles published in the journal HemaSphere exclusively reflect the opinions of the authors. The authors are responsible for all content in their abstracts including accuracy of the facts, statements, citing resources, etc.

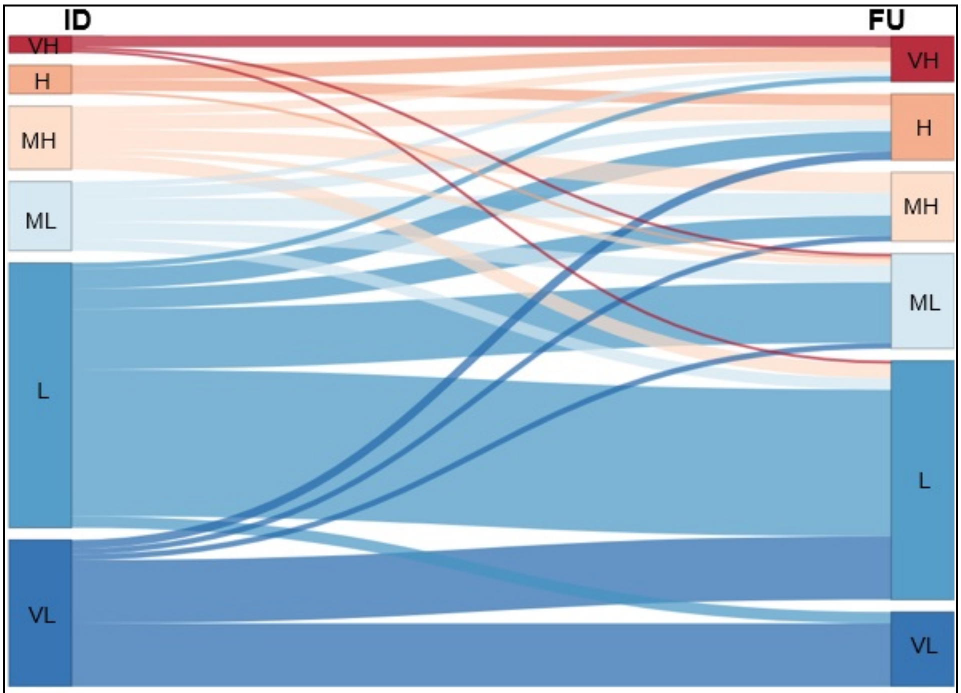

Figure 1: Changes of the IPSS-M risk category from initial diagnosis (ID) to follow-up (FU) of 205 MDS patients. VH: Very High, H: High, MH: Moderate High, ML: Moderate Low, L: Low, VL: Very Low

**Copyright Information:** (Online) ISSN: 2572-9241
©2026 The Author(s). HemaSphere is published by John Wiley & Sons Ltd on behalf of European Hematology Association. This is an open access Abstract Book distributed under the Attribution-NonCommercial-NoDerivs (CC BY-NC-ND), which allows third parties to download the articles and share them with others as long as they credit the author and the Abstract Book, but they cannot change the content in any way or use them commercially.
**Abstract Book Citations:** Authors, Title, HemaSphere, 2026;10:(S1):pages. The abstract book DOIs can be found on the "Author Information" page.
**Disclaimer:** Articles published in the journal HemaSphere exclusively reflect the opinions of the authors. The authors are responsible for all content in their abstracts including accuracy of the facts, statements, citing resources, etc.

### (PS1776) GENOMIC LANDSCAPE AND SURVIVAL OUTCOMES IN MYELODYSPLASTIC SYNDROMES WITH FIBROSIS: A REAL-WORLD ANALYSIS FOR REFINING THE HIGH-RISK PHENOTYPE IN THE IPSS-M ERA

**Topic:** 10. Myelodysplastic syndromes - Clinical

Arda Bayar*[1], Muhammed Fatih Kırcalı[2], Semih Özerol[1], Rana Ozince[1], Ceren Uzunoğlu Güren[1], Fatma Temiz[1], Elif Sakcı[1], Fatma Arikan[1], Asu Fergün Aydın[1], Isik Atagunduz[1], Tayfur Toptas[1]

*[1]Marmara University, Hematology Department, Istanbul, Türkiye;[2]Marmara University, Internal Medicine Department, Istanbul, Türkiye;*

### Background

Integration of molecular genetics through IPSS-M has transformed prognostication in myelodysplastic syndromes (MDS), yet the role of the marrow microenvironment remains incompletely captured. Although WHO 2022 recognizes bone marrow fibrosis as an adverse feature, it is not routinely incorporated into current risk models. Whether fibrosis adds independent prognostic value beyond IPSS-R and IPSS-M in real-world cohorts remains uncertain in contemporary real-world cohorts.

### Aims

This study characterizes the clinical and genomic phenotype of fibrotic MDS, evaluates its independent association with overall survival beyond IPSS-R and IPSS-M, and assesses whether incorporating fibrosis improves prognostic score discrimination.

### Methods

In this single-center retrospective cohort, we analyzed adults with primary MDS diagnosed between 2015–2025. Fibrosis was graded (MF0–MF3) using the European Consensus scale and dichotomized as MF0/1 vs MF2/3. Overall survival was estimated by Kaplan–Meier and multivariable Cox models adjusting for IPSS-R/IPSS-M (HSCT-censored OS). Model discrimination was evaluated using Harrell's C-index with bootstrap correction.

### Results

Among 130 patients (median age 67 years), 49 (37.6%) had MF2/3. MDS-MF2/3 showed male predominance (73.5% vs 44.4%, p=0.002), higher median cellularity (70% vs 55%, p=0.013), and excess blasts (38.8% vs 9.9%, p<0.001). Targeted sequencing was available in 57 patients; within this subset, signal-transduction pathway mutations (e.g., SH2B3, SETBP1) were enriched in MF2/3 (37% vs 7.4%, p=0.011). Median OS was markedly inferior for MDS-MF2/3 (19 vs 72 months, p<0.001). In multivariable Cox models, MDS-MF2/3 remained an independent predictor of OS when adjusted for IPSS-R (HR 2.14, p=0.015) and for IPSS-M (HR 3.37, p=0.021). Adding fibrosis to prognostic models improved discrimination (IPSS-R C-index 0.734→0.785, LRT p=0.013; IPSS-M C-index 0.767→0.810, LRT p=0.009). The adverse impact persisted in patients with <5% blasts (median OS 101 months vs. 57 months, p=0.010) and within lower-risk scoring strata (for IPSS-R subset, HR 3.55, p<0.001; for IPSS-M subset, HR 4.73, p=0.011). Fibrosis showed a trend toward higher risk and shorter time to transfusion dependence (multivariable HR 2.56, p=0.056).

### Summary/Conclusion

Moderate-to-severe fibrosis identifies a biologically aggressive MDS phenotype with inferior survival independent of molecular risk. Incorporation of fibrosis improves IPSS-R/IPSS-M performance, suggesting that stromal remodeling may represent a prognostic dimension not captured by genomics alone. Routine integration of fibrosis grading may refine risk stratification and guide earlier therapeutic decision-making.

**Copyright Information:** (Online) ISSN: 2572-9241
©2026 The Author(s). HemaSphere is published by John Wiley & Sons Ltd on behalf of European Hematology Association. This is an open access Abstract Book distributed under the Attribution-NonCommercial-NoDerivs (CC BY-NC-ND), which allows third parties to download the articles and share them with others as long as they credit the author and the Abstract Book, but they cannot change the content in any way or use them commercially.
**Abstract Book Citations:** Authors, Title, HemaSphere, 2026;10:(S1):pages. The abstract book DOIs can be found on the "Author Information" page.
**Disclaimer:** Articles published in the journal HemaSphere exclusively reflect the opinions of the authors. The authors are responsible for all content in their abstracts including accuracy of the facts, statements, citing resources, etc.

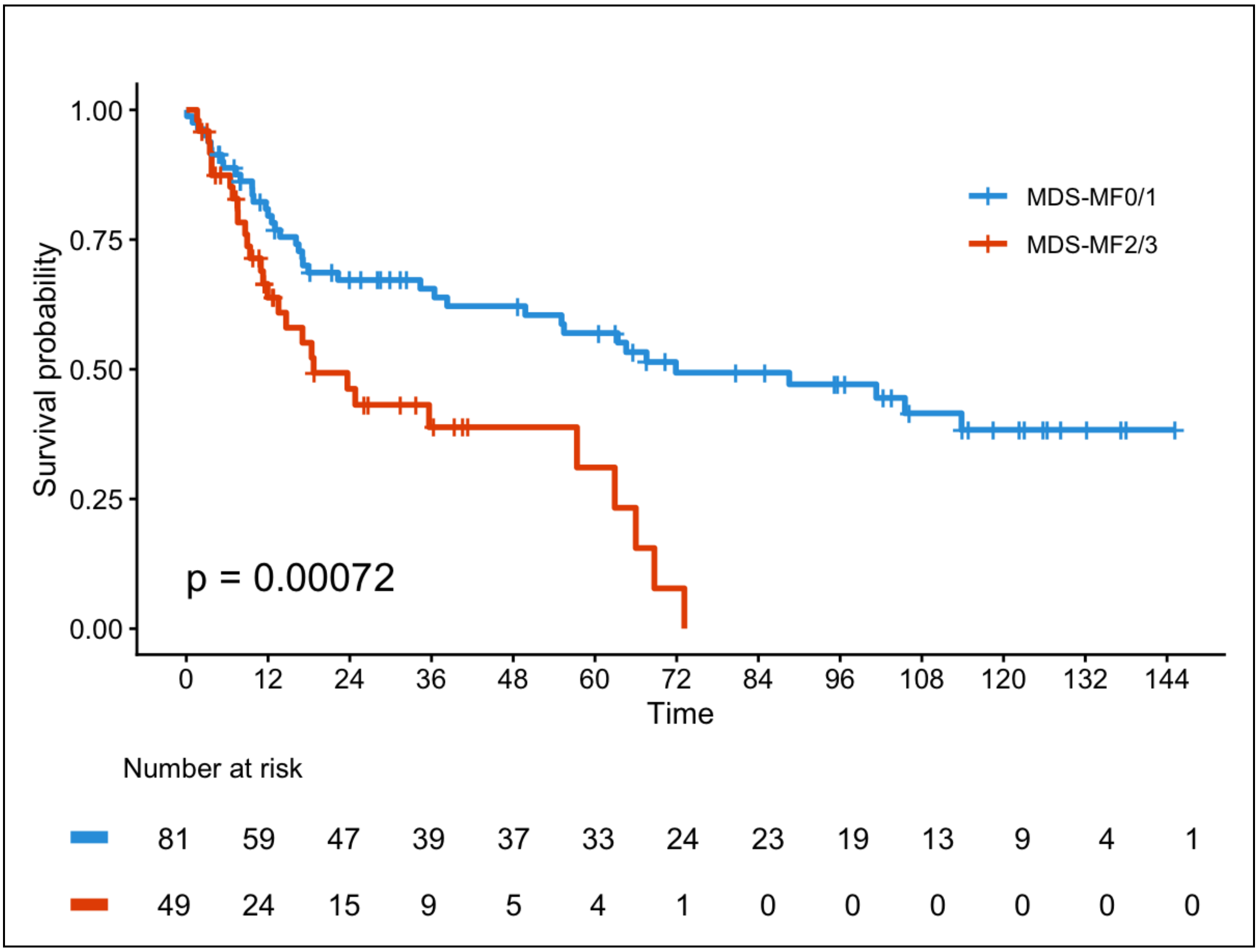

**Copyright Information:** (Online) ISSN: 2572-9241
©2026 The Author(s). HemaSphere is published by John Wiley & Sons Ltd on behalf of European Hematology Association. This is an open access Abstract Book distributed under the Attribution-NonCommercial-NoDerivs (CC BY-NC-ND), which allows third parties to download the articles and share them with others as long as they credit the author and the Abstract Book, but they cannot change the content in any way or use them commercially.
**Abstract Book Citations:** Authors, Title, HemaSphere, 2026;10:(S1):pages. The abstract book DOIs can be found on the "Author Information" page.
**Disclaimer:** Articles published in the journal HemaSphere exclusively reflect the opinions of the authors. The authors are responsible for all content in their abstracts including accuracy of the facts, statements, citing resources, etc.

### (PS1777) THERAPY-RELATED MYELOID NEOPLASMS FOLLOWING TREATMENT FOR PRIMARY HEMATOLOGIC MALIGNANCIES: A MULTI-CENTER RETROSPECTIVE ANALYSIS

**Topic:** 10. Myelodysplastic syndromes - Clinical

Andrea Lattanzi*[1, 2], Elisa Diral[1], Camilla Pacchioni[2], Lorenzo Rizzo[3], Marta Riva.[3], Gianluca Scorpio[1], Sara Mastaglio[1], Luca Vago[1, 2], Massimo Bernardi[1], Fabio Giglio[1], Andrea Assanelli[1], Annalisa Ruggeri[1], Lorenzo Lazzari[1], Simona Piemontese[1], Alessandro Bruno[1], Anna Francesio[1, 2], Maria Teresa Lupo Stanghellini[1], Fabio Ciceri[1, 2]

*[1]IRCCS San Raffaele Scientific Institute, Unit of Hematology and Bone Marrow Transplantation, Milano, Italy;[2]Vita-Salute San Raffaele University, Milano, Italy; [3]ASST Grande Ospedale Metropolitano Niguarda, S.C. EMATOLOGIA Dipartimento di Ematologia, Oncologia e Medicina Molecolare Niguarda Cancer Center, Milano, Italy;*

### Background

Therapy-related myeloid neoplasms (t-MNs) represent a subgroup of myeloid malignancies arising after exposure to chemotherapy (CT) and/or radiotherapy (RT) for prior malignancies. t-MNs include therapy related myelodysplasia (t-MDS) and acute myeloid leukemia (t-AML) and are associated with poor prognosis. Notably, treatment for hematologic malignancies, including lymphoma and multiple myeloma, also confers a significant risk of developing t-MNs.

### Aims

To analyze clinical characteristics, risk factors, and outcomes of patients who developed t-MN following treatment for primary hematological malignancies (pHM).

### Methods

We conducted a retrospective analysis on 64 t-MN cases diagnosed at IRCCS Ospedale San Raffaele and ASST Grande Ospedale Metropolitano Niguarda (2009-2025) after CT/RT for pHM. Collected variables included for both pHM and t-MN: type, cytogenetics, molecular data, treatment, and survival.

**Copyright Information:** (Online) ISSN: 2572-9241
©2026 The Author(s). HemaSphere is published by John Wiley & Sons Ltd on behalf of European Hematology Association. This is an open access Abstract Book distributed under the Attribution-NonCommercial-NoDerivs (CC BY-NC-ND), which allows third parties to download the articles and share them with others as long as they credit the author and the Abstract Book, but they cannot change the content in any way or use them commercially.
**Abstract Book Citations:** Authors, Title, HemaSphere, 2026;10:(S1):pages. The abstract book DOIs can be found on the "Author Information" page.
**Disclaimer:** Articles published in the journal HemaSphere exclusively reflect the opinions of the authors. The authors are responsible for all content in their abstracts including accuracy of the facts, statements, citing resources, etc.

**Results**

The cohort included 43 males (67%) and 21 females (33%) with a median age at t-MN diagnosis of 68 years (IQR 13 years). Median latency of t-MN onset from completion of pHM therapy was 37 months (IQR 105 months). pHM were mainly lymphomas (40, 62%), with 31 non-Hodgkin lymphoma and 9 Hodgkin disease, multiple myeloma and AML (8, 12% each, ***figure 1A***). All patients except two received cytotoxic CT for pHM, mainly in association with other therapies: 18 patients received RT, 37 immunotherapy, 25 underwent hematopoietic cell transplant (HCT), of which 18 autologous and 7 allogeneic. One patient received CAR-T cell therapy.

CT regimens used for pHM included: alkylating agents (46, 72%), anthracyclines (38, 59%), antimetabolites (36, 56%), vinca alkaloids (31, 48%), topoisomerase inhibitors (19, 30%), platinum compounds (13, 20%), bendamustine or similar (8, 13%) and tyrosine kinases inhibitors (6, 9%).

At t-MN diagnosis, 44 patients (72%) had MDS, 18 (25%) AML and 2 (3%) MDS/MPN overlap syndrome; 9 MDS patients (14% of total cohort) progressed to AML during follow up. Of note, 7 of the 64 t-MN patients developed donor derived cell leukemia after allo-HCT.

Conventional cytogenetic analysis, performed in 61 patients, revealed a complex karyotype in 18 cases (30%). NGS data (n=40) showed recurrent mutations in TP53 (30%), DNMT3A and TET2 (17.5% each), ASXL1 and RUNX1 (15% each), and SF3B1 and NRAS (10% each).

High-risk t-MN was observed in 40 patients: 24 with MDS (59%, IPSS-R ≥3.5), 15 with AML (83%, ELN 2017 intermediate or adverse risk), and one patient with MDS/MPN. IPSS-M was assessed in 14 patients, with 50% classified as high-risk. Twenty-three patients (36%) underwent HCT. Median overall survival (mOS) for the whole cohort was 15.5 months; mOS in high-risk patients was longer in allo-HCT recipients (42 vs 8 months, ***figure 1B***); low risk patients had a mOS of 31 months.

At latest follow-up, 23 patients (36%) were alive. The main causes of death were disease progression (19 patients, 53%) and infections (10, 28%).

We conducted multivariate analysis investigating the prognostic role of *TP53* mutations, complex karyotype, and the use of hypomethylating agents in high-risk patients. No statistically significant association with overall survival was observed.

**Summary/Conclusion**

Our findings confirm the high-risk biological profile and poor outcomes of t-MNs following pHM. Allo-HCT remains the only potentially curative approach in eligible patients with t-MN. The analysis of larger cohorts might further elucidate the prognostic impact of disease characteristics on clinical outcomes.

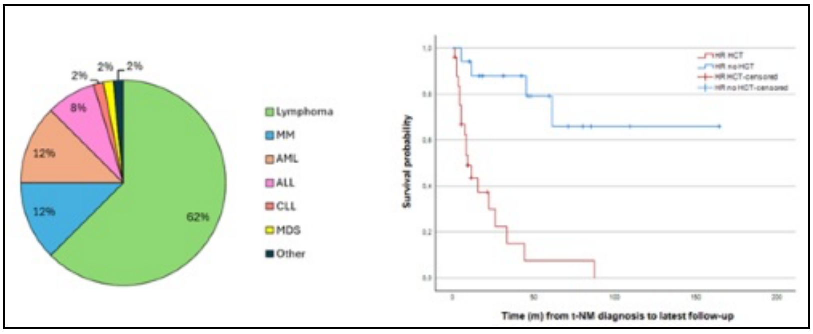

Figure A. Distribution of patients according to type of pHM; Figure B. Kaplan–Meier estimates of OS in patients with high-risk t-NM (blue line: HCT recipient; red line: did not receive HCT)

**Copyright Information:** (Online) ISSN: 2572-9241
©2026 The Author(s). HemaSphere is published by John Wiley & Sons Ltd on behalf of European Hematology Association. This is an open access Abstract Book distributed under the Attribution-NonCommercial-NoDerivs (CC BY-NC-ND), which allows third parties to download the articles and share them with others as long as they credit the author and the Abstract Book, but they cannot change the content in any way or use them commercially.
**Abstract Book Citations:** Authors, Title, HemaSphere, 2026;10:(S1):pages. The abstract book DOIs can be found on the "Author Information" page.
**Disclaimer:** Articles published in the journal HemaSphere exclusively reflect the opinions of the authors. The authors are responsible for all content in their abstracts including accuracy of the facts, statements, citing resources, etc.

### (PS1778) CHARACTERIZATION OF ALN-CFB, AN INVESTIGATIONAL SIRNA TARGETING HEPATIC COMPLEMENT FACTOR B, IN NON-HUMAN PRIMATES AND CFB-HUMANIZED MICE

**Topic:** 11. Bone marrow failure syndromes incl. PNH - Biology & translational research

Kishor Devalaraja-Narashimha*[1], Sripathi Sureban[1], Kristina Yucius[2], Nikhil Singh[1], Marc Cao[1], Huey Ling Liew[1], John O'brien[1], Yifan Luo[1], Marc Retter[1], Lori Morton[1]

*[1]Regeneron Pharmaceuticals, Inc., Tarrytown, United States of America;[2]Alnylam Pharmaceutical, Inc., Cambridge, United States of America;*

### Background

The alternative pathway (AP) of the complement system is a key component of innate immunity; its dysregulation contributes to paroxysmal nocturnal hemoglobinuria (PNH), rare kidney diseases, and dry age-related macular degeneration. Approved AP-directed therapies can be limited by suboptimal blockade or frequent dosing. ALN-CFB is an investigational RNA interference therapeutic; this GalNAc-conjugated siRNA reduces complement factor B (CFB) messenger RNA (mRNA), inhibiting production of CFB protein, an indispensable AP serine protease. The ALN-CFB target sequence is conserved between humans and monkeys.

### Aims

To evaluate ALN-CFB in preclinical studies in monkeys and CFB-humanized mice.

### Methods

A pharmacokinetic (PK) study was conducted in female cynomolgus monkeys receiving a single subcutaneous (SC) dose of 0.6, 2, or 6 mg/kg (n=5/group) on Day 1, followed to Day 116. Pharmacodynamic studies were conducted in monkeys (SC 2 or 6 mg/kg; n=3/group; to Day 113) and in CFB-humanized mice ($CFB^{Hu/Hu}$; SC 0.3, 1, or 10 mg/kg; n=5/group; to Day 70). Concentrations of ALN-CFB and its active metabolites were measured in monkey plasma and liver using a qualified high-performance liquid chromatography (LC) coupled with high-resolution mass spectrometry (MS) method. Serum CFB protein was quantified by LC/MS. AP activity was assessed using both the traditional AP hemolysis (AH) assay and the Wieslab complement AP (CAP) assay. Circulating Ba and Bb, CFB activation split products, were measured with a Quidel multiplex enzyme-linked immunosorbent assay. Hepatic *CFB* mRNA was evaluated by reverse-transcription quantitative real-time polymerase chain reaction.

### Results

After SC administration in monkeys, ALN-CFB was rapidly absorbed and cleared from plasma, with predominantly hepatic distribution. Plasma PK was linear over 1 day, and hepatic PK was generally linear throughout the study across all doses, with minimal renal excretion. Drug and active metabolite levels persisted longer in the liver than in plasma, with a hepatic ALN-CFB half-life ranging between 18–22 days. ALN-CFB led to a potent, dose-dependent reduction of CFB protein in monkeys; at 6 mg/kg, suppression exceeded 95% by Day 15, was sustained through Day 64, and remained generally linear throughout the study across all doses. AP activity was inhibited in a dose-dependent manner, with the 6 mg/kg dose reducing CAP activity by ≥99% from Day 15 through Day 92 and AH activity by >95% from Day 15 through Day 64. CFB protein reduction strongly correlated with AP inhibition. At Days 22 and 43, ALN-CFB achieved >90% knockdown of hepatic *CFB* mRNA and reductions in circulating Bb, and >80% reductions in circulating Ba. These pharmacologic effects were reversible, with partial recovery by the end of the study. Aligning with monkey studies, ALN-CFB yielded >95% reductions in circulating CFB from Day 7, sustained to Day 42 in $CFB^{Hu/Hu}$ mice.

**Copyright Information:** (Online) ISSN: 2572-9241
©2026 The Author(s). HemaSphere is published by John Wiley & Sons Ltd on behalf of European Hematology Association. This is an open access Abstract Book distributed under the Attribution-NonCommercial-NoDerivs (CC BY-NC-ND), which allows third parties to download the articles and share them with others as long as they credit the author and the Abstract Book, but they cannot change the content in any way or use them commercially.
**Abstract Book Citations:** Authors, Title, HemaSphere, 2026;10:(S1):pages. The abstract book DOIs can be found on the "Author Information" page.
**Disclaimer:** Articles published in the journal HemaSphere exclusively reflect the opinions of the authors. The authors are responsible for all content in their abstracts including accuracy of the facts, statements, citing resources, etc.

**Summary/Conclusion**

Single SC doses of ALN-CFB produced robust, durable CFB protein reduction and near-complete AP inhibition in non-human primates, with marked suppression of hepatic *CFB* mRNA and prolonged hepatic drug/metabolite presence. Effects were consistent in $CFB^{Hu/Hu}$ mice. If translated to humans, these data suggest the potential for robust AP inhibition with convenient, infrequent SC dosing. ALN-CFB is under investigation in a phase 1/2 study for safety and efficacy in participants with PNH and persistent anemia on a C5 inhibitor (NCT07187401).

**Copyright Information:** (Online) ISSN: 2572-9241
©2026 The Author(s). HemaSphere is published by John Wiley & Sons Ltd on behalf of European Hematology Association. This is an open access Abstract Book distributed under the Attribution-NonCommercial-NoDerivs (CC BY-NC-ND), which allows third parties to download the articles and share them with others as long as they credit the author and the Abstract Book, but they cannot change the content in any way or use them commercially.
**Abstract Book Citations:** Authors, Title, HemaSphere, 2026;10:(S1):pages. The abstract book DOIs can be found on the "Author Information" page.
**Disclaimer:** Articles published in the journal HemaSphere exclusively reflect the opinions of the authors. The authors are responsible for all content in their abstracts including accuracy of the facts, statements, citing resources, etc.

### (PS1779) REAL-WORLD OUTCOMES OF IPTACOPAN IN PAROXYSMAL NOCTURNAL HEMOGLOBINURIA (PNH) PATIENTS: INSIGHTS FROM THE FRENCH EARLY ACCESS PROGRAM.

**Topic:** 11. Bone marrow failure syndromes incl. PNH - Biology & translational research

Edouard Forcade[1], Nicolas Blin[2], Michael Loschi[3], Vincent Alcazer[4], Virginie Savary de Beauregard[5], Claire Perrin[5], Pauline Mechali[5], Régis Peffault de Latour*[6], Flore Sicre de Fontbrune[6]

*[1]CeRAMIC, CHU Bordeaux, Hopital Haut-Leveque, Pessac, France;[2]CeRAMIC ,Hôpital Universitaire de Nantes, Université de Nantes, Nantes, France; [3]CeRAMIC, Hôpital Universitaire de Nice, Université Côte d'Azur, Nice, France;[4]CeRAMIC, Service d'Hématologie Clinique, Hospices Civils de Lyon &Université Lyon 1, Lyon, France; [5]Novartis, Rueil Malmaison, France; [6]Centre de Référence Aplasie Médullaire, Service d'Hématologie Greffe, Assistance Publique des Hôpitaux de Paris, Hôpital Saint-Louis, Paris, France;*

### Background

Iptacopan is the 1st oral monotherapy targeting Factor B to inhibit the proximal alternative complement pathway, effectively controlling both intra and extravascular hemolysis in paroxysmal nocturnal hemoglobinuria (PNH). In the APPLY-PNH randomized trial, iptacopan was superior to antiC5 therapies (C5i) in improving hemoglobin (Hb) and reducing red blood cell transfusion (RBCT) in patients with persistent anemia. Since May 2, 2024, an Early Access Program (EAP) have enabled iptacopan access in France for adults with PNH with anemia despite C5i.

### Aims

To report the real-world effectiveness and safety of iptacopan in France.

### Methods

Eligibility criteria were adult patients with PNH clone size ≥10% and Hb <10 g/dL despite 6 months of C5i. Patients intolerant to 2nd line proximal inhibitor (C3i) were eligible if previously received ≥6 months of C5i with Hb <10 g/dL at switch. Treatment initiation required vaccination and validation by the French reference center multidisciplinary team. Clinical and biological data are collected at treatment request, initiation and subsequently at day 15 (D15), month 2 (M2), and every 2 months during year 1, then every 4 months thereafter.

**Copyright Information:** (Online) ISSN: 2572-9241
©2026 The Author(s). HemaSphere is published by John Wiley & Sons Ltd on behalf of European Hematology Association. This is an open access Abstract Book distributed under the Attribution-NonCommercial-NoDerivs (CC BY-NC-ND), which allows third parties to download the articles and share them with others as long as they credit the author and the Abstract Book, but they cannot change the content in any way or use them commercially.
**Abstract Book Citations:** Authors, Title, HemaSphere, 2026;10:(S1):pages. The abstract book DOIs can be found on the "Author Information" page.
**Disclaimer:** Articles published in the journal HemaSphere exclusively reflect the opinions of the authors. The authors are responsible for all content in their abstracts including accuracy of the facts, statements, citing resources, etc.

**Results**

Overall, 51 initiated iptacopan and were included by 22 physicians (mean age 47.9 ± 17.9 years; 64.7% women). Prior thrombotic events were reported for 15 patients (29.4%). Among these patients, 4 had previously started iptacopan through compassionate access and 7 initiated treatment following intolerance to a 2$^{nd}$ line C3i. These 11 patients had Hb >10 g/dL at treatment request. Among the other 40 patients previously treated by C5i, RBCT in the 12 months preceding treatment was required for 22 patients (55.5%) (mean number transfusions per patient: 10.6 ± 10.1). Mean exposure to iptacopan was 7.6 ± 4.5 months (min: 0; max: 17.4 months).

In the Hb ≤10 g/dL subgroup (N=40), mean (±SD) Hb increased significantly and rapidly from baseline (8.65 ± 0.97 g/dL) by 3.01 ± 1.79 g/dL (11.66 ± 1.53 g/dL) at D15, followed by progressive increases at subsequent visits:+4.44 ± 1.40 g/dL at M6 (13.07 ± 1.11 g/dL), and +4.60 ± 1.92 at M12 (13.26 ± 1.44 g/dL). Transfusion independence increased from 50% at baseline to 95% at D15 and 100% at M12. In the Hb >10 g/dL subgroup (N=11), mean Hb (baseline: 12.30 ± 0.95 g/dL) increased by 0.56 ± 0.81 at D15, and by 0.99 ± 1.24 at M2 and then remained stable, exceeding 13g/dL during follow-up, demonstrating durable long-term control in this previously treated population. All patients were transfusion independent from D15.

No death, thromboembolic events or breakthrough hemolysis (BTH) were reported. Overall, 6 patients experienced a total of 16 serious adverse drug reactions (ADR) and 9 non-serious. Two serious infections (pneumonia and cystitis) were reported. Of the 4 patients experiencing serious ADRs two achieved full recovery, one was reported as condition improving, and the remaining case had no documented outcome. None of the ADR resulted in modification or discontinuation of iptacopan.

**Summary/Conclusion**

The EAP confirms the real-world effectiveness and safety of iptacopan demonstrated in the clinical trials. Patients with Hb <10 g/dL showed rapid and sustained Hb improvement with markedly reduced transfusion needs from D15, while patients with prior iptacopan or C3i treatment with Hb >10 g/dL remained stable without BTH reported. Overall, no new safety signals were identified.

**Copyright Information:** (Online) ISSN: 2572-9241
©2026 The Author(s). HemaSphere is published by John Wiley & Sons Ltd on behalf of European Hematology Association. This is an open access Abstract Book distributed under the Attribution-NonCommercial-NoDerivs (CC BY-NC-ND), which allows third parties to download the articles and share them with others as long as they credit the author and the Abstract Book, but they cannot change the content in any way or use them commercially.
**Abstract Book Citations:** Authors, Title, HemaSphere, 2026;10:(S1):pages. The abstract book DOIs can be found on the "Author Information" page.
**Disclaimer:** Articles published in the journal HemaSphere exclusively reflect the opinions of the authors. The authors are responsible for all content in their abstracts including accuracy of the facts, statements, citing resources, etc.

### (PS1780) IMMUNE REPERTOIRE SIGNATURE LINKS HEPATITIS AND THE DESTRUCTION OF HAEMATOPOIETIC PRECURSORS IN HEPATITIS-ASSOCIATED APLASTIC ANAEMIA

**Topic:** 11. Bone marrow failure syndromes incl. PNH - Biology & translational research

Daniel Dulla*[1, 2], Michaela Reiterova[1, 2], Michael Svaton[1], Martina Vášková[1], Andrea Rennerova[1, 2], Ema Joudalova[1], Ondřej Fabián[3], Markéta Kalinová[4], Veronika Kanderová[1], Vit Campr[5], Tomáš Votava[6], Pavel Timr[7], Dagmar Pospíšilová[8], Ondřej Zapletal[9], Tomáš Kuhn[10], Iveta Janotova[2], Ondrej Hrusak[1], Jan Trka[1], Lucie Sramkova[2], Jan Stary[2], Martina Suková[2], Ester Mejstrikova[1, 2], Eva Fronkova[1, 2]

*[1]CLIP, Second Faculty of Medicine, Charles University and Motol and Homolka University Hospital, Department of Paediatric Haematology and Oncology, Prague, Czechia; [2]Department of Paediatric Haematology and Oncology, Motol and Homolka University Hospital, Prague, Czechia; [3]University Thomayer Hospital and Third Faculty of Medicine, Charles University, Department of Pathology and Molecular Medicine, Prague, Czechia; [4]University Hospital Kralovske Vinohrady, Central Laboratories, Prague, Czechia; [5]Department of Pathology and Molecular Medicine, Second Faculty of Medicine, Charles University, and Motol and Homolka University Hospital, Prague, Czechia; [6]University Hospital Pilsen, Department of Paediatrics, Pilsen, Czechia; [7]České Budějovice Hospital, Department of Paediatrics, České Budějovice, Czechia;[8]Palacky University and University Hospital, Department of Pediatrics, Olomouc, Czechia; [9]University Hospital and Masaryk University Brno, Brno, Czechia; [10]University Hospital Ostrava, Department of Paediatric Haematology and Haematooncology, Ostrava, Czechia;*

### Background

Hepatitis-associated aplastic anaemia (HA-AA) is a rare variant of aplastic anaemia preceded by an attack of acute non-A-E hepatitis. Histologically, some patients are classified as refractory cytopenia of childhood (RCC). The mechanistic link between the hepatitis and bone marrow destruction, and the extent to which antigen-driven T-cell responses contribute, remain incompletely defined.

### Aims

This study sought to describe the T-cell receptor beta (TRB) repertoire of HA-AA in a longitudinal and cross-compartment fashion, and to assess the plausibility of antigen-driven T cell recruitment. Additionally, we assessed potential germline predispositions to abnormal immune response and bone marrow failure (BMF).

### Methods

We evaluated a national cohort of 43 idiopathic AA (IAA) and 29 HA-AA patients treated between 2004 and 2026. Among HA-AA patients, 21 were histologically AA, and 8 as RCC. Two patients required liver transplantation. All samples were evaluated by flow cytometry. For HA-AA, longitudinal peripheral blood and bone marrow samples underwent TRB sequencing. Whole exome sequencing (WES) was performed to screen for predispositions to immune dysregulation or BMF. In IAA patients with suspected germline predisposition, WES or panel sequencing was performed to exclude inherited BMF. In two patients with available explanted liver tissue, TRB sequencing of liver-infiltrating T cells was performed. Clonotype motif clustering was performed using turboGliph. Age-matched peripheral blood samples were used for healthy controls.

**Copyright Information:** (Online) ISSN: 2572-9241
©2026 The Author(s). HemaSphere is published by John Wiley & Sons Ltd on behalf of European Hematology Association. This is an open access Abstract Book distributed under the Attribution-NonCommercial-NoDerivs (CC BY-NC-ND), which allows third parties to download the articles and share them with others as long as they credit the author and the Abstract Book, but they cannot change the content in any way or use them commercially.
**Abstract Book Citations:** Authors, Title, HemaSphere, 2026;10:(S1):pages. The abstract book DOIs can be found on the “Author Information” page.
**Disclaimer:** Articles published in the journal HemaSphere exclusively reflect the opinions of the authors. The authors are responsible for all content in their abstracts including accuracy of the facts, statements, citing resources, etc.

### Results

Immunophenotyping distinguished HA-AA from IAA by a reduced CD4/CD8 ratio ($p = 0.0007$) and increased $CD8^{+}$ T cell activation based on HLA-DR expression ($p < 0.0001$). TRB repertoire diversity at diagnosis of HA-AA was significantly decreased compared with healthy controls ($p = 0.0003$). Diversity at diagnosis did not correlate with bone marrow histology or predict success of immunosuppressive therapy (IST). Clones above the contamination threshold were private with occasional matches to viral sequences, such as CMV. Clonal expansion at day 120 of IST was common and did not indicate poor treatment response. Notably, in 2 patients with available liver samples, expanded clonotypes spanning the hepatic, peripheral and bone marrow compartments could be traced across timepoints. These contracted on IST. TurboGliph clustering revealed enriched patient-specific CDR3aa k-mer motif clusters. Further, in a representative patient with multi-compartment data, motif-associated clonotypes at the time of diagnosis disappeared upon remission while immunodominant clonotypes contracted but persisted. WES revealed no monogenic cause in HA-AA but detected variants of unknown significance in genes associated with BMF or immune dysregulation, including *DNAJC21*, *WRAP53*, *RAG1*, *ADA2* and *IFIH1*.

### Summary/Conclusion

HA-AA is associated with a distinct immunophenotypic pattern irrespective of histology. Its TRB repertoire is skewed and exhibits a variable degree of private-clonotype expansion. Longitudinal cross-compartment tracing of clonotypes across the liver, peripheral blood and bone marrow establishes a molecular link between the hepatitis and the destruction of bone marrow precursors. The presence of enriched CDR3aa k-mer clusters points towards antigen-driven convergent TCR recruitment. The selective disappearance of motif-associated clonotypes suggests pathogenicity is confined to the most antigen-adapted, rather than the most expanded clones.

*E.M. and E.F. contributed equally*

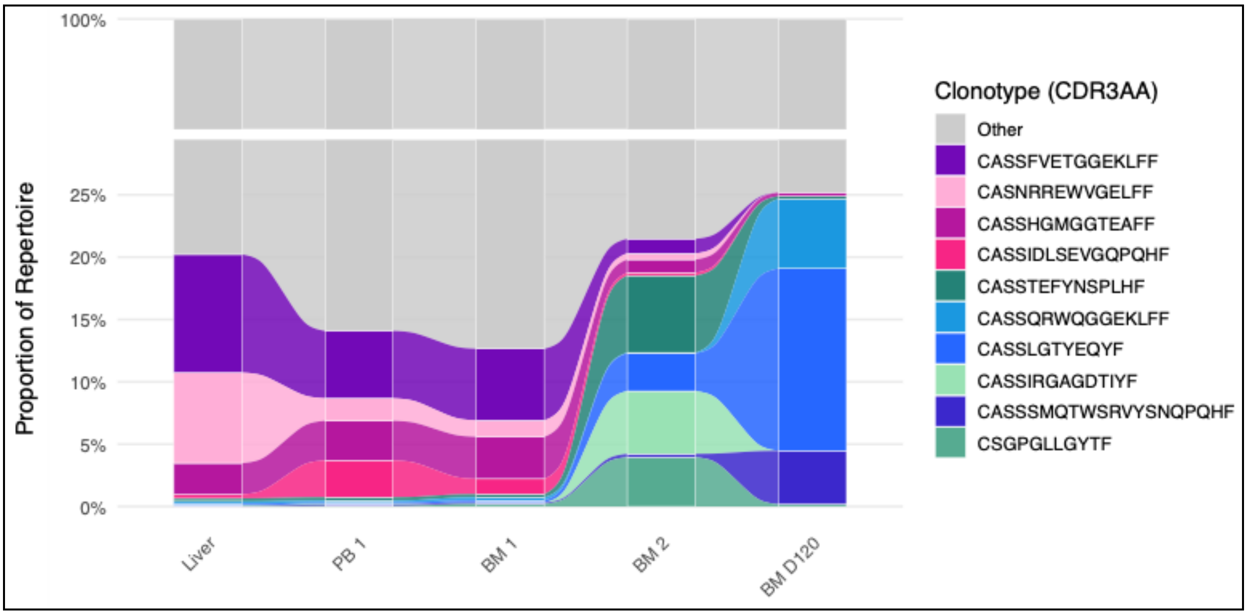

Longitudinal cross-compartment clonotype tracing in a representative HA-AA patient.

**Copyright Information:** (Online) ISSN: 2572-9241
©2026 The Author(s). HemaSphere is published by John Wiley & Sons Ltd on behalf of European Hematology Association. This is an open access Abstract Book distributed under the Attribution-NonCommercial-NoDerivs (CC BY-NC-ND), which allows third parties to download the articles and share them with others as long as they credit the author and the Abstract Book, but they cannot change the content in any way or use them commercially.
**Abstract Book Citations:** Authors, Title, HemaSphere, 2026;10:(S1):pages. The abstract book DOIs can be found on the "Author Information" page.
**Disclaimer:** Articles published in the journal HemaSphere exclusively reflect the opinions of the authors. The authors are responsible for all content in their abstracts including accuracy of the facts, statements, citing resources, etc.

### (PS1781) DIAMOND BLACKFAN ANEMIA SYNDROME ERYTHROID PROGENITORS DISPLAY ABNORMAL METABOLOMIC PROFILE

**Topic:** 11. Bone marrow failure syndromes incl. PNH - Biology & translational research

Veronica Riccardi*[1], Jonathan de Wilde[2], Julie Reisz[3], Angelo D'alessandro[3], Richard Pozzetto Huot[1], Yuefeng Tang[4], Alexis Bertrand[5], Naomi Taylor[6], Vincent Schulz[7], Enrica Federti[1], Patrick Gallagher[8], Jeffrey Lipton[2], Roberta Russo[9], Achille Iolascon[9], Leonardo Rivadeneyra[10], Megan Wind-Rotolo[10], Mohandas Narla[11], Julien Papoin[12], Lucia De Franceschi[1], Lionel Blanc[2]

*[1]university of verona, Verona, Italy; [2]Feinstein Institute for Medical Research, manhasset, United States of America;[3]university of colorado, aurora, United States of America; [4]University of Massachusetts Chan Medical School, worcester, United States of America; [5]northwell health, new york, United States of America;[6]Center for Cancer Research, bethesda, United States of America; [7]university of yale, west haven, United States of America;[8]Ohio State University, ohio, United States of America; [9]università di napoli federico II, napoli, Italy; [10]agios pharmaceutical, Inc, cambridge, United States of America; [11]new york blood center, new york, United States of America; [12]northwell, manhasset, United States of America;*

### Background

Diamond-Blackfan anemia syndrome (DBAS) is a rare, hereditary bone marrow failure syndrome, primarily characterized by erythroid aplasia, congenital anomalies, and an increased risk to develop cancer. Recently, we generated two conditional models of *Rps19* and *Rpl5* haploinsufficiency under a Vav-iCre promoter, representing two of the most commonly affected genes. In these models, we observed that in fetal hematopoiesis the *Rpl5* haploinsufficient erythroid progenitors failed to progress to the late progenitor/early precursors stage. This was associated with increased oxidative stress (as evidenced by increased ROS levels) and lipid peroxidation. Ferroptosis is an autophagy related cell death event due to sustained protein and lipid oxidation, linked to iron homeostasis and integrated with cell metabolism. We therefore hypothesized that ferroptosis may be involved in the premature cell death in *Rpl5* haploinsufficiency. This was strengthened by the transcriptional upregulation of pro-ferroptotic genes, alongside a decreased transcription of anti-ferroptotic genes in erythroid progenitors from *Rpl5* haploinsufficiency mice. The metabolic profile of these *Rpl5* haploinsufficient progenitors and its relation to ferroptosis remains to be elucidated.

### Aims

To understand the impact of metabolome of erythroid progenitors from DBA, we profiled the metabolome of*RPL5* haploinsufficient human progenitors compared to healthy controls.

### Methods we performed a metabolomic analysis of erythroid progenitors derived from CD34+ cells of DBAS patients with*RPL5* haploinsufficiency (n=3) compared to healthy controls (n=5).

**Copyright Information:** (Online) ISSN: 2572-9241
©2026 The Author(s). HemaSphere is published by John Wiley & Sons Ltd on behalf of European Hematology Association. This is an open access Abstract Book distributed under the Attribution-NonCommercial-NoDerivs (CC BY-NC-ND), which allows third parties to download the articles and share them with others as long as they credit the author and the Abstract Book, but they cannot change the content in any way or use them commercially.
**Abstract Book Citations:** Authors, Title, HemaSphere, 2026;10:(S1):pages. The abstract book DOIs can be found on the "Author Information" page.
**Disclaimer:** Articles published in the journal HemaSphere exclusively reflect the opinions of the authors. The authors are responsible for all content in their abstracts including accuracy of the facts, statements, citing resources, etc.

### Results

DBAS cells' differentiation and growth was significantly delayed when compared to healthy controls. The metabolomic profile revealed numerous fatty acids and acylcarnitines elevated in DBA relative to control cells. In contrast, levels of redox metabolites, like reduced and oxidized glutathione (GSH and GSSG, respectively), and nucleotides were lower in DBAS cells when compared to healthy controls. Metabolites in central energy pathways downstream of glucose were significantly altered. DBAS cells showed lower levels of ATP, pentose phosphate pathway intermediates, Krebs cycle metabolites, and later glycolytic metabolites but higher pyruvate and lactate content. Taken together these data illustrate metabolic defects in energy and redox metabolism that may be suggestive of dysfunctional cellular energy. As the enzyme pyruvate kinase (PK) plays a vital role in glycolysis, we further investigated the two isoforms of PK (i.e., PKM2 and PKR) taking advantage of the *RPL5* haploinsufficient mice. We found increased phospho-PKM2 displaying non-glycolytic function in mouse *Rpl5* haploinsufficient progenitors when compared to healthy controls, suggesting a possible link with modulation of genes involved in ferroptosis.

### Summary/Conclusion

In conclusion, our data indicate a deep perturbation of cellular energy in*RPL5* haploinsufficient cells from DBAS patients when compared to healthy controls. Thus, the disturbed metabolism may also be reflected in a non-glycolytic function of PKM2, which could play a role in the failure of erythroid progenitors to progress to the late progenitor/early precursors stage.

**Copyright Information:** (Online) ISSN: 2572-9241
©2026 The Author(s). HemaSphere is published by John Wiley & Sons Ltd on behalf of European Hematology Association. This is an open access Abstract Book distributed under the Attribution-NonCommercial-NoDerivs (CC BY-NC-ND), which allows third parties to download the articles and share them with others as long as they credit the author and the Abstract Book, but they cannot change the content in any way or use them commercially.
**Abstract Book Citations:** Authors, Title, HemaSphere, 2026;10:(S1):pages. The abstract book DOIs can be found on the "Author Information" page.
**Disclaimer:** Articles published in the journal HemaSphere exclusively reflect the opinions of the authors. The authors are responsible for all content in their abstracts including accuracy of the facts, statements, citing resources, etc.

### (PS1782) THE ROLE OF NEUTROPHIL EXTRACELLULAR TRAPS IN THROMBOSIS OF PAROXYSMAL NOCTURNAL HEMOGLOBINURIA

**Topic:** 11. Bone marrow failure syndromes incl. PNH - Biology & translational research

Mengting Che[1], Hui Liu[1], Lijuan Li[1], Rong Fu*[1]

[1] *Tianjin Medical University General Hospital, Tianjin, China;*

### Background

Thrombosis is the leading cause of disability and death in patients with paroxysmal nocturnal hemoglobinuria (PNH), but its pathogenesis is complex and incompletely understood. Neutrophil extracellular traps (NETs) link immune activation and the coagulation cascade, and play important roles in thrombotic diseases. However, the exact function and regulatory mechanism of NETs in PNH-associated thrombosis remain unclear.

### Aims

This study aimed to examine NETs expression and clinical relevance in PNH patients, characterize NETs forming ability and the plasma microenvironment's regulatory role, identify key NETs-regulating molecules via transcriptomic analysis, and evaluate NETs' prothrombotic effects and targeted inhibitors' therapeutic efficacy in animal models. The results will provide new experimental evidence and potential targets for PNH thrombosis prevention and management.

### Methods

Serum levels of NETs markers, complement components, inflammatory chemokines and thrombosis-related factors in PNH patients were determined by ELISA and Luminex xMAP assays. Platelet-neutrophil aggregates (PNAs) were quantified via flow cytometry, with correlations among parameters analyzed. Neutrophils from PNH patients were used to compare NETs formation under different stimulatory conditions, and cross-incubation assays evaluated the plasma microenvironment's regulatory role. Transcriptome sequencing identified differentially expressed genes, with CXCR4 selected as a key candidate. CXCR4 expression was verified by qPCR and flow cytometry, and its role in mediating NETs formation was confirmed via ligand stimulation and inhibitor blockade. Additionally, venous and arterial thrombosis models were established in PNH mice to assess in vivo NETs deposition and CXCR4 inhibitors' inhibitory effects on thrombosis progression.

### Results

Serum NETs markers (MPO-DNA, CitH3) were significantly higher in PNH patients than in normal controls, and further elevated in PNH patients with thrombosis, especially the new thrombosis subgroup. These markers correlated positively with granulocyte clone load, complement CH50, inflammatory factors, thrombosis-related factors, and PNAs.In vitro, PNH neutrophils showed enhanced basal NETs formation and higher sensitivity to inducers (C5a, PMA, G-CSF, C3a). Cross-incubation assays confirmed that inherent PNH neutrophil dysfunction and abnormal plasma microenvironment synergistically promoted excessive NETs release.Transcriptome sequencing showed significant up-regulation of CXCR4, a core chemokine signaling molecule; qPCR and flow cytometry confirmed increased CXCR4 mRNA and protein expression in PNH neutrophils. CXCL12 stimulation promoted PNH neutrophil NETs formation, which was reversed by CXCR4 inhibitors.In vivo, PNH mouse thrombosis models had enhanced thrombotic capacity, increased NETs deposition in thrombi, and a positive correlation between NETs levels and thrombosis severity. CXCR4 inhibitor intervention significantly reduced NETs levels, decreased thrombus weight, and prolonged vascular occlusion time in PNH mice.

**Copyright Information:** (Online) ISSN: 2572-9241
©2026 The Author(s). HemaSphere is published by John Wiley & Sons Ltd on behalf of European Hematology Association. This is an open access Abstract Book distributed under the Attribution-NonCommercial-NoDerivs (CC BY-NC-ND), which allows third parties to download the articles and share them with others as long as they credit the author and the Abstract Book, but they cannot change the content in any way or use them commercially.
**Abstract Book Citations:** Authors, Title, HemaSphere, 2026;10:(S1):pages. The abstract book DOIs can be found on the "Author Information" page.
**Disclaimer:** Articles published in the journal HemaSphere exclusively reflect the opinions of the authors. The authors are responsible for all content in their abstracts including accuracy of the facts, statements, citing resources, etc.

**Summary/Conclusion**

NETs contribute to thrombosis in PNH through the combined effects of intrinsic neutrophil dysregulation and an abnormal plasma microenvironment. CXCR4 is a critical mediator of NETs formation and represents a potential therapeutic target for preventing thrombosis in PNH.

**Copyright Information:** (Online) ISSN: 2572-9241
©2026 The Author(s). HemaSphere is published by John Wiley & Sons Ltd on behalf of European Hematology Association. This is an open access Abstract Book distributed under the Attribution-NonCommercial-NoDerivs (CC BY-NC-ND), which allows third parties to download the articles and share them with others as long as they credit the author and the Abstract Book, but they cannot change the content in any way or use them commercially.
**Abstract Book Citations:** Authors, Title, HemaSphere, 2026;10:(S1):pages. The abstract book DOIs can be found on the "Author Information" page.
**Disclaimer:** Articles published in the journal HemaSphere exclusively reflect the opinions of the authors. The authors are responsible for all content in their abstracts including accuracy of the facts, statements, citing resources, etc.

### (PS1783) BONE MARROW BIOPSIES AND PLASMA REVEAL A DISRUPTED ANGIOGENIC LANDSCAPE IN SHWACHMAN–DIAMOND SYNDROME PATIENTS

**Topic:** 11. Bone marrow failure syndromes incl. PNH - Biology & translational research

Jennifer Romagnoli*[1], Clarissa Gervasoni[2], Alice Martina Giussani[2], Erica Dander[2], Mara Riminucci[3], Samantha Donsante[2], Biagio Palmisano[3], Valentino Bezzerri[4], Francesco Pegoraro[5], Andrea Biondi[6], Paola Corti[7], Fabiola Guerra[7], Piero Farruggia[8], Giovanna Giagnuolo[9], Anna Pegoraro[4], Simone Cesaro[10], Marco Cipolli[4], Giovanna D'amico[2]

*[1]University of Milano Bicocca, Milano, Italy; [2]Fondazione Tettamanti, Fondazione IRCCS San Gerardo dei Tintori, Monza, Italy; [3]Sapienza University of Rome, Roma, Italy; [4]Cystic Fibrosis Center, Azienda Ospedaliera Universitaria Integrata,, Verona, Italy; [5]AOU Meyer IRCCS, Firenze, Italy; [6]School of Medicine and Surgery, University of Milano-Bicocca, Monza, Italy; [7]Pediatrics, Fondazione IRCCS San Gerardo dei Tintori,, Monza, Italy; [8]Ospedali "Civico Di Cristina Benfratelli",, Palermo, Italy; [9]Pediatric Hemato-Oncology Unit, Santobono-Pausilipon Hospital,, Napoli, Italy; [10]Pediatric Hematology-Oncology, Azienda Ospedaliera Universitaria Integrata, Verona, Italy;*

### Background

Shwachman-Diamond syndrome (SDS) is a rare inherited bone marrow failure disorder characterized by cytopenias and an increased risk of myeloid malignancies. Increasing evidence suggests that, beyond intrinsic hematopoietic defects, dysfunction of the bone marrow (BM) microenvironment contributes to disease pathogenesis, including stromal abnormalities and impaired angiogenesis. We previously demonstrated that SDS BM mesenchymal stromal cells exhibit reduced angiogenic potential both *in vitro* and *in vivo* compared with healthy donor (HD)–MSCs, supporting the presence of structural and functional alterations within the vascular–stromal niche.

### Aims

To comprehensively characterize the angiogenic landscape of SDS bone marrow by integrating morphologic and immunohistochemical analyses of bone marrow biopsies (BMBs) with quantitative profiling of angiogenic mediators in BM plasma from SDS patients, in order to define structural and molecular alterations of the vascular niche and clarify their contribution to disease pathogenesis.

### Methods

BM biopsies from SDS patients were fixed, decalcified, and paraffin-embedded. Four-µm sections were stained with hematoxylin and eosin for histologic and histomorphometric evaluation. $CD34^+$ vessels were assessed by immunohistochemistry using a monoclonal anti-CD34 antibody with peroxidase–DAB detection and quantified in terms of vessel density, area fraction, lumen size, perimeter, and total vascular area. BM aspirates from SDS patients (n=19) and HDs (n=19) were collected in EDTA tubes. Plasma was isolated by centrifugation, and angiogenesis-related factors were quantified using a bead-based multiplex assay.

**Copyright Information:** (Online) ISSN: 2572-9241
©2026 The Author(s). HemaSphere is published by John Wiley & Sons Ltd on behalf of European Hematology Association. This is an open access Abstract Book distributed under the Attribution-NonCommercial-NoDerivs (CC BY-NC-ND), which allows third parties to download the articles and share them with others as long as they credit the author and the Abstract Book, but they cannot change the content in any way or use them commercially.
**Abstract Book Citations:** Authors, Title, HemaSphere, 2026;10:(S1):pages. The abstract book DOIs can be found on the "Author Information" page.
**Disclaimer:** Articles published in the journal HemaSphere exclusively reflect the opinions of the authors. The authors are responsible for all content in their abstracts including accuracy of the facts, statements, citing resources, etc.

**Results**

Morphologic and immunohistochemical analyses of SDS BMBs revealed markedly increased marrow adiposity at the expense of hematopoietic tissue, consistent with a compromised hematopoietic microenvironment. CD34 staining demonstrated altered vascular architecture in SDS samples. Quantitative analysis demonstrated a 30% increase in vessel number (p=0.014), a 35% increase in vessel area, and a 46% increase in lumen size compared with HD controls, whereas vessel perimeter and total vascular area were not significantly different. Multiplex analysis of BM plasma showed significantly reduced levels of key pro-angiogenic and vascular homeostasis factors in SDS patients, including BMP9 (p=0.0071), endothelin-1 (p=0.0077), HB-EGF (p=0.0446), and VEGF-C (p=0.0008). These mediators are critical for vessel maturation, stabilization, and stromal–endothelial cross-talk; their reduction suggests an impaired capacity to maintain physiological vascular remodeling and niche integrity. In contrast, SDS plasma displayed significantly increased levels of G-CSF (p=0.0012) and IL-8 (p=0.008), both known to promote endothelial activation and angiogenic sprouting, possibly reflecting a compensatory response to vascular dysfunction.

**Summary/Conclusion**

SDS bone marrow biopsies demonstrate increased vessel density and vascular alterations. Concomitantly, BM plasma from SDS patients reveals an imbalance in angiogenic mediators essential for vascular niche homeostasis. Although no malignant transformation was observed at biopsy, the vascular features resemble patterns reported in pre-malignant conditions. Collectively, these findings indicate that SDS bone marrow harbors early microenvironmental and vascular alterations, underscoring the need for mechanistic studies and targeted therapeutic strategies.

**Copyright Information:** (Online) ISSN: 2572-9241
©2026 The Author(s). HemaSphere is published by John Wiley & Sons Ltd on behalf of European Hematology Association. This is an open access Abstract Book distributed under the Attribution-NonCommercial-NoDerivs (CC BY-NC-ND), which allows third parties to download the articles and share them with others as long as they credit the author and the Abstract Book, but they cannot change the content in any way or use them commercially.
**Abstract Book Citations:** Authors, Title, HemaSphere, 2026;10:(S1):pages. The abstract book DOIs can be found on the “Author Information” page.
**Disclaimer:** Articles published in the journal HemaSphere exclusively reflect the opinions of the authors. The authors are responsible for all content in their abstracts including accuracy of the facts, statements, citing resources, etc.

### (PS1784) DISSOCIATION BETWEEN VISCERAL AND SKELETAL DISEASE IN MILD GENOTYPES OF GAUCHER DISEASE TYPE 1

**Topic:** 11. Bone marrow failure syndromes incl. PNH - Biology & translational research

Irene Serrano-Gonzalo*[1, 2], Sonia Roca Esteve[1, 2], Isidro Arévalo-Vargas[1, 2], Mercedes Roca-Espiau[1], Esther Valero-Tena[1, 3], Marcio Andrade-Campos[1], Pilar Giraldo[1, 4]

*[1]Fundación Española para el Estudio y Terapéutica de la Enfermedad de Gaucher y otras lisosomales (FEETEG), Zaragoza, Spain; [2]University of Zaragoza, Zaragoza, Spain;[3]MAZ hospital, Intern medicine, Zaragoza, Spain; [4]QuironSalud Hospital, Hematology, Zaragoza, Spain;*

### Background

One of the most prevalent manifestations in Gaucher disease type 1 (GD1) is at the bone level, where the bone marrow is displaced by Gaucher cells, causing various bone complications. Although the c.[1226A>G] (N370S) variant is classically associated with a mild phenotype, bone disease remains highly heterogeneous, even among genotypes considered "mild". Direct comparisons between N370S homozygotes and N370S/mild compound heterozygotes are limited.

### Aims

We conducted a retrospective study using the Spanish Gaucher Disease Registry to compare skeletal disease at diagnosis in GD1 patients homozygous for c.[1226A>G] versus c.[1226A>G]/mild genotypes, and to identify potential clinical and biochemical modifiers.

### Methods

Genotypes were classified as c.[1226A>G]; c.[1226A>G] or c.[1226A>G]/mild, with "mild" defined by functional severity. Skeletal involvement was assessed by the Spanish MRI scale (S-MRI) for bone crises, osteonecrosis, vertebral deformities and cell infiltration and DXA for the loss of bone mineral density. Biomarkers (glucosylsphingosine, chitotriosidase activity and CCL18/PARC), hematological, and visceral parameters were recorded. Analyses were adjusted for age, sex, splenectomy, and treatment exposure.

### Results

Among 436 GD1 patients, 56 were c.[1226A>G] homozygous and 39 c.[1226A>G]/mild. Clinically relevant skeletal disease (S-MRI ≥11) affected half of c.[1226A>G]/mild patients versus one third of homozygotes. Glucosylsphingosine levels were over twice as high in c.[1226A>G]/mild patients (median 98.3 vs 44.8 ng/mL), indicating higher metabolic burden. Osteonecrosis prevalence was similar (10.7% vs 10.2%), while bone crises were slightly less frequent in homozygotes (5.5% vs 7.1%). Osteopenia/osteoporosis at diagnosis was low and comparable (13.3% vs 17.9%). Visceral and hematological parameters did not differ. After ~20 years of follow-up, new bone crises and joint replacement were rare and similar between groups.

### Summary/Conclusion

Despite being considered "mild," c.[1226A>G] homozygotes exhibit a more favorable skeletal profile than c.[1226A>G]/mild patients. These results highlight the dissociation between visceral and skeletal disease in GD1 and support the need for genotype-informed, individualized skeletal monitoring, even in clinically mild cases.

**Copyright Information:** (Online) ISSN: 2572-9241
©2026 The Author(s). HemaSphere is published by John Wiley & Sons Ltd on behalf of European Hematology Association. This is an open access Abstract Book distributed under the Attribution-NonCommercial-NoDerivs (CC BY-NC-ND), which allows third parties to download the articles and share them with others as long as they credit the author and the Abstract Book, but they cannot change the content in any way or use them commercially.
**Abstract Book Citations:** Authors, Title, HemaSphere, 2026;10:(S1):pages. The abstract book DOIs can be found on the "Author Information" page.
**Disclaimer:** Articles published in the journal HemaSphere exclusively reflect the opinions of the authors. The authors are responsible for all content in their abstracts including accuracy of the facts, statements, citing resources, etc.

### (PS1785) A HOMOZYGOUS LOSS-OF-FUNCTION MUTATION IN ARHGAP25 AS A NOVEL MECHANISM FOR CONGENITAL NEUTROPENIA

**Topic:** 11. Bone marrow failure syndromes incl. PNH - Biology & translational research

Maksim Klimiankou*[1], Jannik Herold[1], Larissa Doll[1], Patrick Münzer[2], Ivan Tesakov[1], Cornelia Zeidler[3], Sevda Kadah[3], Natalia Borbaran Bravo[1], Olga Klimenkova[1], Betül Findik[1], Karl Welte[1], Julia Skokowa[1]

*[1]University Hospital Tübingen, Internal Medicine II, Tübingen, Germany;[2]University Hospital Tübingen, Internal Medicine III, Tübingen, Germany; [3]Hannover Medical School, Severe Chronic Neutropenia International Registry, Hannover, Germany;*

### Background

Severe congenital neutropenia (CN) is characterized by a primary maturation arrest of myeloid progenitors, resulting in a low number of mature neutrophils in the peripheral blood (PB). Clinically, this manifests as a high susceptibility to severe and recurrent bacterial infections.

### Aims

To elucidate the genetic etiology and molecular pathogenesis in a CN patient presenting with atypical clinical presentations, and to functionally validate the identified candidate mutations using comprehensive *in vivo* and *in vitro* models.

### Methods

Whole-exome sequencing (WES) and Sanger sequencing were utilized to identify the candidate genetic variants. Protein expression was evaluated via Western blot analysis. *In vivo* validation of variant pathogenicity was performed by introduction of *arhgap25* knockout in zebrafish neutrophil reporter lines (mpo:*gfp*+). We applied CRISPR/Cas9 RNP nucleofection to knockout *ARHGAP25* in human cord blood CD34$^{+}$ cells, followed by liquid culture differentiation (LCD). Functional assays evaluated filamentous actin (F-actin) content, reactive oxygen species (ROS) generation upon N-formyl-methionyl-leucyl-phenylalanine (fMLP) stimulation, and chemotaxis assay toward CXCL8 and CXCL12.

**Copyright Information:** (Online) ISSN: 2572-9241
©2026 The Author(s). HemaSphere is published by John Wiley & Sons Ltd on behalf of European Hematology Association. This is an open access Abstract Book distributed under the Attribution-NonCommercial-NoDerivs (CC BY-NC-ND), which allows third parties to download the articles and share them with others as long as they credit the author and the Abstract Book, but they cannot change the content in any way or use them commercially.
**Abstract Book Citations:** Authors, Title, HemaSphere, 2026;10:(S1):pages. The abstract book DOIs can be found on the "Author Information" page.
**Disclaimer:** Articles published in the journal HemaSphere exclusively reflect the opinions of the authors. The authors are responsible for all content in their abstracts including accuracy of the facts, statements, citing resources, etc.

**Results**

The patient was admitted to the hospital with bone marrow hypocellularity, almost complete absence of granulopoiesis, life-threatening opportunistic infections, relative increase in erythroid and megakaryocytic precursors, and multilineage dysplasia. The absolute neutrophil counts (ANC) spiked in response to G-CSF (300µg) from 0 to 7,7x$10^3$ cells/µl. Lab tests for CMV, ANA, ANCA, rheumatoid factor, vitamin B12/folate deficiency, hemophagocytosis, paroxysmal nocturnal hemoglobinuria, Fanconi anemia, dyskeratosis congenita, telomeropathies, AML/MDS sequencing panel were all negative.
WES revealed a homozygous stop-gain mutation in the ARHGAP25 gene (NM_001007231.3:c.1132C>T, p.Arg378X). No homozygous carriers of *ARHGAP25* loss-of-function variants are observed in large population databases. The presence of *ARHGAP25* p.Arg378X was further confirmed by Sanger sequencing. Western blotting revealed the complete absence of ARHGAP25 protein in patient PB mononuclear cells. ARHGAP25 is a hematopoietic-specific GTPase-activating protein (GAP) that negatively regulates the Rho-family small GTPases Rac1 and Rac2. These GTPases are essential for controlling cytoskeletal actin dynamics, directional chemotaxis, and ROS production.
Patient neutrophils demonstrated severely impaired migration toward CXCL8 but intact chemotaxis toward CXCL12. LCD-derived *ARHGAP25* knockout human neutrophils differentiated normally, demonstrated production of ROS after stimulation with 1µM fMLP similar to healthy donors, normal level of chemotactic response to CXCL12 but exhibited increased F-actin accumulation. Zebrafish Arhgap25 KO and truncation mutants displayed reduced basal trunk neutrophil numbers but paradoxically elevated recruitment to wound sites. The number of neutrophils was fully rescued by *csf3a* overexpression.

**Summary/Conclusion**

A homozygous p.Arg378X mutation in *ARHGAP25* represents a novel genetic etiology for CN. The lack of ARHGAP25 leads to a neutrophil migration defect driven by unbalanced Rac signaling and CXCL8 hypo-responsiveness. Based on our earlier publication (Kasper B, et al. Blood 2000), G-CSF treatment can bypass the GAP deficiency by shifting the balance of Rac2 regulation via decreasing Rac2 expression and increasing its sequestration by the guanosine diphosphate (GDP)-dissociation inhibitor.

**Copyright Information:** (Online) ISSN: 2572-9241
©2026 The Author(s). HemaSphere is published by John Wiley & Sons Ltd on behalf of European Hematology Association. This is an open access Abstract Book distributed under the Attribution-NonCommercial-NoDerivs (CC BY-NC-ND), which allows third parties to download the articles and share them with others as long as they credit the author and the Abstract Book, but they cannot change the content in any way or use them commercially.
**Abstract Book Citations:** Authors, Title, HemaSphere, 2026;10:(S1):pages. The abstract book DOIs can be found on the “Author Information” page.
**Disclaimer:** Articles published in the journal HemaSphere exclusively reflect the opinions of the authors. The authors are responsible for all content in their abstracts including accuracy of the facts, statements, citing resources, etc.

### (PS1786) EFFICACY AND SAFETY OF SWITCHING TO ORAL HSK39297 MONOTHERAPY IN PAROXYSMAL NOCTURNAL HEMOGLOBINURIA PATIENTS WITH PERSISTENT ANEMIA DESPITE ANTI-C5 THERAPY: A PHASE 3 STUDY (HSK39297-302)

**Topic:** 12. Bone marrow failure syndromes incl. PNH - Clinical

Gaixiang Xu*[1], Guangsheng He[2], Lina Zhang[3], Shunqing Wang[4], Zijing Guo[5], Jie Bai[6], Zenghua Lin[7], Xizhe Guo[8], Xiaobing Huang[9], Hongyan Tong[1]

*[1]the First Affiliated Hospital,Zhejiang University School of Medicine, Hangzhou, China;[2]The First Affiliated Hospital of Nanjing Medical University, Department of Hematology, Nanjing, China; [3]Henan Cancer Hospital, Department of Hematology, Zhengzhou, China; [4]Guangzhou First People's Hospital, Department of Hematology, Guangzhou, China;[5]Hebei Provincial Hospital of Traditional Chinese Medicine, Department of Hematology, Shijiazhuang, China; [6]The Second Hospital of Tianjin Medical University, Department of Hematology, Tianjin, China; [7]Affiliated Hospital of Nantong University, Nantong, China;[8]The Second Affiliated Hospital of Fujian Medical University, Fujian, China; [9]Sichuan Academy of Medical Sciences Sichuan Provincial People's Hospital, Chengdu, China;*

### Background

A significant proportion of patients with paroxysmal nocturnal hemoglobinuria (PNH) remain anemic despite standard-of-care anti-C5 therapy due to C3-mediated extravascular hemolysis. HSK39297 is a novel, oral, once-daily factor B inhibitor targeting proximal complement activation.

### Aims

To evaluate the efficacy and safety of switching to HSK39297 monotherapy in PNH patients with persistent anemia after anti-C5 therapy.

### Methods

This was a Phase 3, open-label, single-arm, multicenter study (NCT07052838). Thirty-six adult PNH patients (hemoglobin [Hb] < 100 g/L) who had received stable anti-C5 therapy (eculizumab/crovalimab) for at least 6 months were switched to oral HSK39297 200 mg once daily for 24 weeks. The primary endpoint was the proportion of patients achieving Hb ≥ 120 g/L in ≥ 3 of 4 measurements between Weeks 18-24, without red blood cell (RBC) transfusions after Week 2. Secondary endpoints included transfusion avoidance, changes in Hb, lactate dehydrogenase (LDH), absolute reticulocyte count (ARC), C3 fragment deposition, FACIT-Fatigue score, and safety.

**Copyright Information:** (Online) ISSN: 2572-9241
©2026 The Author(s). HemaSphere is published by John Wiley & Sons Ltd on behalf of European Hematology Association. This is an open access Abstract Book distributed under the Attribution-NonCommercial-NoDerivs (CC BY-NC-ND), which allows third parties to download the articles and share them with others as long as they credit the author and the Abstract Book, but they cannot change the content in any way or use them commercially.
**Abstract Book Citations:** Authors, Title, HemaSphere, 2026;10:(S1):pages. The abstract book DOIs can be found on the "Author Information" page.
**Disclaimer:** Articles published in the journal HemaSphere exclusively reflect the opinions of the authors. The authors are responsible for all content in their abstracts including accuracy of the facts, statements, citing resources, etc.

### Results

All 36 patients completed the 24-week treatment. The study met its prespecified success criterion for the primary endpoint. The target hemoglobin response  was achieved by 52.8% (19/36) of patients (95% CI: 35.5, 69.6)(Figure1). Additionally, 91.7% (33/36) of patients (95% CI: 77.5, 98.2) achieved the clinically meaningful threshold of a ≥ 20 g/L increase in Hb from baseline. Mean Hb increased from 81.04 g/L at baseline to 123.58 g/L at Week 24, with a least squares mean (LSM) change from baseline of +42.08 g/L (95% CI: 37.21, 46.94) during Weeks 18-24. Transfusion avoidance (Weeks 2-24) was achieved in 97.2% (35/36) of patients. Markers of hemolysis improved: mean ARC decreased by an LSM of -166.29 x10$^{9}$/L (95% CI: -179.45, -153.12), and mean C3 fragment deposition on RBCs decreased from 9.31% to 0.19% at Week 24 (LSM change: -9.08%). FACIT-Fatigue scores improved by an LSM of +9.6 points (95% CI: 8.4, 10.9). Adverse events (AE) occurred in 80.6% (29/36) of patients, mostly Common Terminology Criteria for Adverse Events (CTCAE) Grade 1-2. The most common AEs were platelet count decreased (22.2%), neutrophil count decreased (16.7%), and hypertriglyceridaemia (16.7%). One patient (2.8%) experienced breakthrough hemolysis, no major adverse vascular events occurred. No AEs led to treatment discontinuation or death.

### Summary/Conclusion

Switching to oral HSK39297 monotherapy resulted in significant and sustained hemoglobin improvement, near-complete elimination of transfusion need, effective control of hemolysis (including resolution of C3-mediated extravascular hemolysis), and reduced fatigue in PNH patients with persistent anemia on anti-C5 therapy, with a favorable safety profile. HSK39297 represents a promising once-daily oral treatment option for this patient population.

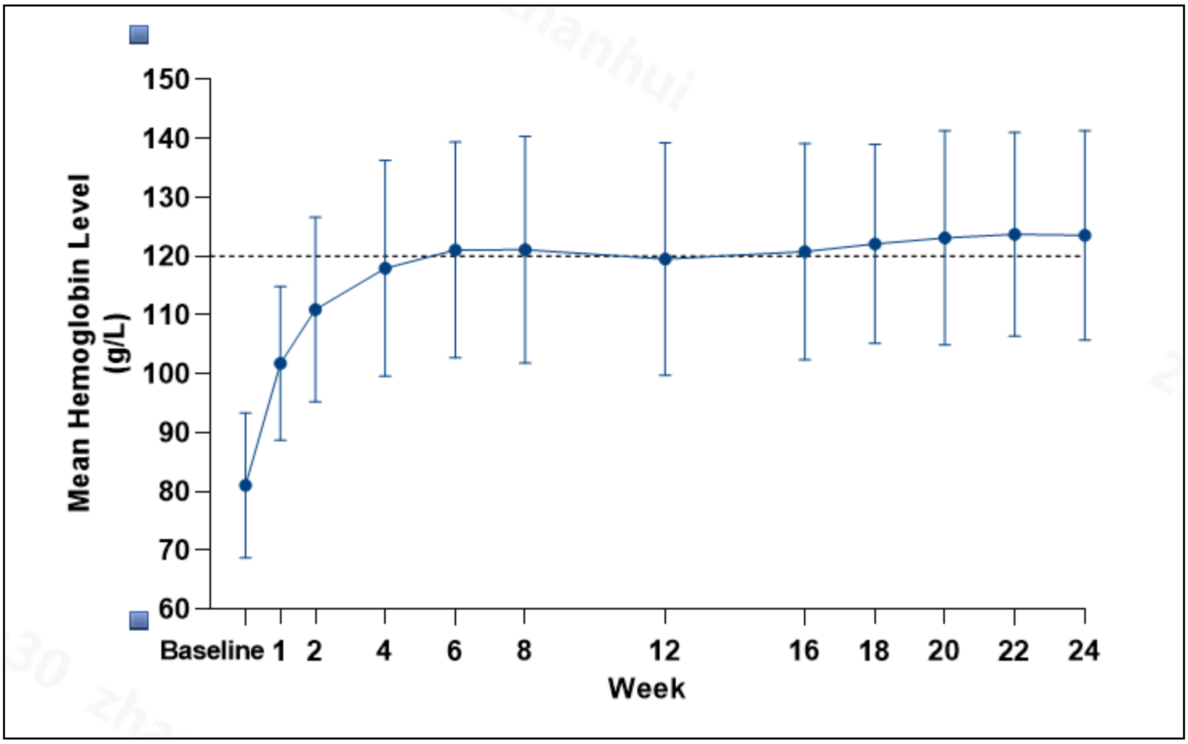

**Copyright Information:** (Online) ISSN: 2572-9241
©2026 The Author(s). HemaSphere is published by John Wiley & Sons Ltd on behalf of European Hematology Association. This is an open access Abstract Book distributed under the Attribution-NonCommercial-NoDerivs (CC BY-NC-ND), which allows third parties to download the articles and share them with others as long as they credit the author and the Abstract Book, but they cannot change the content in any way or use them commercially.
**Abstract Book Citations:** Authors, Title, HemaSphere, 2026;10:(S1):pages. The abstract book DOIs can be found on the "Author Information" page.
**Disclaimer:** Articles published in the journal HemaSphere exclusively reflect the opinions of the authors. The authors are responsible for all content in their abstracts including accuracy of the facts, statements, citing resources, etc.

### (PS1787) ORAL MY008211A DEMONSTRATES SUPERIOR EFFICACY VERSUS ECULIZUMAB IN COMPLEMENT INHIBITOR–NAÏVE CHINESE PATIENTS WITH PAROXYSMAL NOCTURNAL HEMOGLOBINURIA (PNH)

**Topic:** 12. Bone marrow failure syndromes incl. PNH - Clinical

Fengkui Zhang*[1], Bing Han[2], Hongyan Tong[3], Hong Chang[4], Linghui Xia[5], Zengmei Sheng[6], Weng Jianyu[7], Minkai Song[8], Rongjun Liu[8], Rui Xia[8], Qin Xu[8], Yongkai Chen[8], Wilson Chunlin Wang[8], Geoffrey Ross[9], Chuanrui Xu[9]

*[1]Chinese Academy of Medical Sciences & Peking Union Medical College, Tianjin, China;[2]Peking Union Medical College Hospital (PUMCH), Chinese Academy of Medical Science, Department of Hematology, Beijing, China; [3]First Affiliated Hospital, Zhejiang University School of Medicine, Department of Hematology, Hangzhou, China; [4]West China Hospital, Sichuan University, Department of Hematology, Chengdu, China; [5]Tongji Medical College, Huazhong University of Science and Technology, Wuhan, China; [6]The Third Hospital of Changsha, Changsha, China;[7]Guangdong Provincial People's Hospital, Guangzhou, China; [8]Wuhan Createrna Science and Technology Co., Ltd., Wuhan, China;[9]Wuhan Createrna Science and Technology Co., Ltd., Cornwall, United Kingdom;*

### Background

Eculizumab, a C5 inhibitor, is standard therapy for paroxysmal nocturnal hemoglobinuria (PNH), but its use requires intravenous infusions every 2 weeks, and many patients maintain persistent anemia from residual hemolysis. MY008211A is an oral, selective complement factor B inhibitor that blocks alternative complement pathway activation.

### Aims

To investigate the efficacy and safety of oral MY008211A versus intravenous eculizumab in achieving transfusion independence with hemoglobin (Hb) normalization in complement inhibitor–naïve Chinese patients with PNH.

### Methods

This multicenter, randomized, open-label Phase III trial (NCT06932744) enrolled 68 adult Chinese patients with PNH who were naïve to complement inhibitors and had active hemolysis (lactate dehydrogenase ≥1.5 × upper limit of normal; PNH clone ≥10%). Patients were randomized 1:1 to receive MY008211A 400 mg orally twice daily (n=34) or intravenous eculizumab administered according to the approved dosing regimen (n=34, 33 treated) for 24 weeks. The primary endpoint was the proportion of patients achieving transfusion avoidance from Day 14 to Day 168, together with Hb ≥120 g/L, at ≥3 of 4 assessments during Weeks 18–24. Secondary endpoints included transfusion avoidance with Hb increase ≥20 g/L, control of hemolysis, changes in Hb and reticulocyte count from baseline, and proportion of patients with transfusion avoidance. All patients provided informed consent.

**Copyright Information:** (Online) ISSN: 2572-9241
©2026 The Author(s). HemaSphere is published by John Wiley & Sons Ltd on behalf of European Hematology Association. This is an open access Abstract Book distributed under the Attribution-NonCommercial-NoDerivs (CC BY-NC-ND), which allows third parties to download the articles and share them with others as long as they credit the author and the Abstract Book, but they cannot change the content in any way or use them commercially.
**Abstract Book Citations:** Authors, Title, HemaSphere, 2026;10:(S1):pages. The abstract book DOIs can be found on the "Author Information" page.
**Disclaimer:** Articles published in the journal HemaSphere exclusively reflect the opinions of the authors. The authors are responsible for all content in their abstracts including accuracy of the facts, statements, citing resources, etc.

**Results**

A significantly greater proportion of patients in the MY008211A arm achieved the primary endpoint compared with the eculizumab arm (50.0% versus 9.1%; odds ratio, 19.0; 95% CI, 3.62–99.67; *P*=0.0005 for superiority). Rates of transfusion avoidance with an increase in Hb ≥20 g/L were 91.2% for MY008211A versus 63.6% for eculizumab (*P*=0.038). Hemolysis control was achieved in 91.2% of patients treated with MY008211A versus 81.8% who received eculizumab (*P*=0.2475), and breakthrough hemolysis rates were similar (5.9% versus 6.1%). Mean Hb increase from baseline was significantly greater with MY008211A versus eculizumab (least squares [LS] mean 52.3 versus 25.6 g/L; difference, 26.7 g/L; 95% CI, 17.52–35.77; *P*<0.0001). Reduction in reticulocyte count was significantly greater in the MY008211A arm (LS mean difference, −0.17 × $10^{12}$/L; *P*<0.0001). Rates of transfusion avoidance were 100% versus 93.9% (*P*=0.2388). Both treatments were generally well tolerated. The most common adverse events with MY008211A were neutrophil count decreased (29.4%), upper respiratory infection (26.5%), and white blood cell count decreased (23.5%). No major adverse vascular events occurred in either arm.

**Summary/Conclusion**

MY008211A met its primary endpoint and demonstrated superiority over eculizumab, with a greater proportion of patients achieving transfusion avoidance with Hb normalization. Oral MY008211A resulted in significant improvements in Hb and superior reduction in reticulocyte counts compared with eculizumab while maintaining a favorable safety profile. These results support MY008211A as a potentially practice-changing oral therapy for patients with PNH.

**Copyright Information:** (Online) ISSN: 2572-9241
©2026 The Author(s). HemaSphere is published by John Wiley & Sons Ltd on behalf of European Hematology Association. This is an open access Abstract Book distributed under the Attribution-NonCommercial-NoDerivs (CC BY-NC-ND), which allows third parties to download the articles and share them with others as long as they credit the author and the Abstract Book, but they cannot change the content in any way or use them commercially.
**Abstract Book Citations:** Authors, Title, HemaSphere, 2026;10:(S1):pages. The abstract book DOIs can be found on the “Author Information” page.
**Disclaimer:** Articles published in the journal HemaSphere exclusively reflect the opinions of the authors. The authors are responsible for all content in their abstracts including accuracy of the facts, statements, citing resources, etc.

### (PS1788) IPTACOPAN DEMONSTRATES SUSTAINED EFFICACY AND SAFETY IN PAROXYSMAL NOCTURNAL HEMOGLOBINURIA: UP TO 4 YEARS OF FOLLOW-UP IN PATIENTS FROM APPLY, APPOINT AND ROLL-OVER EXTENSION PROGRAM

**Topic:** 12. Bone marrow failure syndromes incl. PNH - Clinical

Régis Peffault de Latour*[1, 2, 3], Austin G. Kulasekararaj[3, 4, 5, 6], Phillip Scheinberg[7], Yasutaka Ueda[8], Alexander Röth[9], Bing Han[10], Li Zhang[11], Eros Di Bona[12], Rong Fu[13], Morag Griffin[14], Richard J. Kelly[14], Jens Panse[15, 16], Hubert Schrezenmeier[17, 18], Philippe Ferber[19], Rajeshri Karki[20], Silvia Sanz[21], Olivier Somenzi[19], Luca Monaco[19], Erik Houtsma[22], Antonio M. Risitano[3, 23, 24]

*[1]French Reference Center for Aplastic Anemia and Paroxysmal Nocturnal Hemoglobinuria, Saint-Louis Hospital, Paris, France; [2]Assistance Publique Hôpitaux de Paris, Université Paris Cité, Paris, France;[3]The Severe Aplastic Anemia Working Party of the European Society for Blood and Marrow Transplantation, Barcelona, Spain; [4]King's College Hospital NHS, London, United Kingdom; [5]National Institute for Health and Care Research and Wellcome King's Research Facility, London, United Kingdom; [6]King's College London, London, United Kingdom; [7]Hospital A Beneficência Portuguesa, São Paolo, Brazil; [8]The University of Osaka Graduate School of Medicine, Suita, Japan; [9]Department of Hematology and Stem Cell Transplantation, West German Cancer Center, University Hospital Essen, University Duisburg-Essen, Essen, Germany; [10]Peking Union Medical College Hospital, Chinese Academy of Medical Science and Peking Union Medical College, Beijing, China; [11]Anemia Therapeutic Center, Institute of Hematology and Blood Diseases Hospital, Chinese Academy of Medical Sciences and Peking Union Medical College, Tianjin, China; [12]UOC Oncoematologia, AULSS7 Pedemontana, Bassano del Grappa, Vicenza, Italy; [13]Tianjin Medical University General Hospital, Tianjin, China; [14]St James's University Hospital, Leeds, United Kingdom; [15]University Hospital RWTH Aachen, Aachen, Germany; [16]Center for Integrated Oncology Aachen Bonn Cologne Düsseldorf, Aachen, Germany; [17]University of Ulm, Institute of Transfusion Medicine and Immunogenetics, Ulm, Germany; [18]German Red Cross Blood Transfusion Service Baden-Württemberg-Hessen and University Hospital Ulm, Ulm, Germany; [19]Novartis Pharma AG, Basel, Switzerland; [20]Novartis Pharmaceuticals Corporation, East Hanover, NJ, United States of America; [21]Novartis Farmacéutica, S.A., Barcelona, Spain; [22]Novartis Ireland Ltd., Dublin, Ireland; [23]U.O.C Ematologia e Terapie cellulari avanzate, AORN San Giuseppe Moscati, Avellino, Italy; [24]University of Naples Federico II, Naples, Italy;*

### Background

Iptacopan demonstrated sustained hematologic control and a favorable safety profile in patients with paroxysmal nocturnal hemoglobinuria (PNH) in phase 3 studies APPLY-PNH (NCT04558918) and APPOINT-PNH (NCT04820530), with efficacy and safety maintained from 24-48 weeks in the parent studies to 2 years in the roll-over extension program (PNH-REP; NCT04747613).

### Aims

To assess the long-term safety and efficacy of iptacopan monotherapy in patients with PNH who initiated treatment with iptacopan in APPLY-PNH and APPOINT-PNH with up to 4 years' follow-up.

### Methods

All patients received iptacopan monotherapy 200 mg twice daily across parent studies and PNH-REP; the first dose in parent study was considered as baseline (BL). Efficacy assessments included hemoglobin (Hb), lactate dehydrogenase (LDH), absolute reticulocyte count (ARC), transfusion avoidance, breakthrough hemolysis (BTH) rates and prevention of major adverse vascular events (MAVEs). Safety assessments included treatment-emergent adverse events and serious adverse events (SAEs).

**Copyright Information:** (Online) ISSN: 2572-9241
©2026 The Author(s). HemaSphere is published by John Wiley & Sons Ltd on behalf of European Hematology Association. This is an open access Abstract Book distributed under the Attribution-NonCommercial-NoDerivs (CC BY-NC-ND), which allows third parties to download the articles and share them with others as long as they credit the author and the Abstract Book, but they cannot change the content in any way or use them commercially.
**Abstract Book Citations:** Authors, Title, HemaSphere, 2026;10:(S1):pages. The abstract book DOIs can be found on the "Author Information" page.
**Disclaimer:** Articles published in the journal HemaSphere exclusively reflect the opinions of the authors. The authors are responsible for all content in their abstracts including accuracy of the facts, statements, citing resources, etc.

**Results**

At data cut-off, study participation was completed or ongoing in 122/136 patients (89.7%). Hematologic efficacy was sustained, with results comparable across 2, 3 and 4 years (Figure). At 3 and 4 years, mean (SD) Hb was 12.9 g/dL (1.8) and 12.3 g/dL (1.5), respectively, with Hb ≥12.0 g/dL achieved in 76/104 (73.1%) and 14/20 patients (70.0%), irrespective of blood transfusion. Control of intravascular hemolysis was maintained, with LDH levels <1.5×ULN in 95/105 patients (90.5%) at 3 years and 18/19 (94.7%) at 4 years. Markers of extravascular hemolysis showed improvement; mean (SD) ARC decreased by −100.9 (69.1) and $-114.7\times10^{9}$/L (55.8) at 3 and 4 years, with ARC normalization observed in 96/104 (92.3%) and 18/19 patients (94.7%), respectively. Mean (SD) changes from BL for bilirubin at 3 and 4 years were −18.3 µmol/L (23.4) and −15.1 µmol/L (9.8), respectively. Mean (SD) changes from BL in platelet count were $-10.3\times10^{9}$/L (52.8) and $-11.2\times10^{9}$/L (46.1), respectively. Overall, 118/136 patients (86.8%) remained transfusion-free throughout the treatment period. Mean (SD) FACIT-F score improvements from BL were 9.3 (10.8) and 8.2 (9.1) at 3 and 4 years, respectively.

There were 37 BTH events in 23 patients (8.3 events/100 patient years [py]), including 6 serious events (1.3 events/100 py); all events resolved, and no BTH led to treatment discontinuation. Six MAVEs occurred in 5 pts (1.3 events/100 py), including 3 severe events in 2 patients; 1 event of intestinal infarction resulted in death.

SAEs occurred in 50/136 patients (36.8%), including serious infections and infestations in 24/136 patients (17.6%; 8.7/100 py). Five deaths (3.7%) occurred due to cardiopulmonary failure following sepsis, gastrointestinal hemorrhage, metastatic colorectal cancer, pneumonia, and infection with ascites followed by intestinal infarction. No death was related to iptacopan treatment. Nine pregnancies were reported; of these, 4 resulted in elective abortions, 2 in spontaneous abortions, 1 in ectopic pregnancy and 2 in healthy live births.

**Summary/Conclusion**

With up to 4 years of follow-up, iptacopan monotherapy demonstrated sustained efficacy, with hemolysis control, high transfusion avoidance, and improved fatigue in patients with PNH. Long-term treatment was well tolerated, with no new safety findings.

**Copyright Information:** (Online) ISSN: 2572-9241
©2026 The Author(s). HemaSphere is published by John Wiley & Sons Ltd on behalf of European Hematology Association. This is an open access Abstract Book distributed under the Attribution-NonCommercial-NoDerivs (CC BY-NC-ND), which allows third parties to download the articles and share them with others as long as they credit the author and the Abstract Book, but they cannot change the content in any way or use them commercially.
**Abstract Book Citations:** Authors, Title, HemaSphere, 2026;10:(S1):pages. The abstract book DOIs can be found on the “Author Information” page.
**Disclaimer:** Articles published in the journal HemaSphere exclusively reflect the opinions of the authors. The authors are responsible for all content in their abstracts including accuracy of the facts, statements, citing resources, etc.

**Figure.** Sustained Hematologic and Biomarker Responses Through 2-4 years in Patients With PNH from APPLY-PNH, APPOINT-PNH, and PNH-REP

| | 2 Years | 3 Years | 4 Years |
|---|---|---|---|
| Mean (SD) Hb levels, g/dL | 12.7 (2.0), n=124 | 12.9 (1.8), n=104 | 12.3 (1.5), n=20 |
| Hb ≥12.0 g/dL*, n/N (%) | 89/124 (71.8) | 76/104 (73.1) | 14/20 (70.0) |
| Mean (SD) LDH levels, U/L | 300.4 (196.6), n=124 | 273.1 (87.8), n=105 | 272.1 (100.0), n=19 |
| Patients with no RBC transfusions, n/N (%) | 123/136 (90.4) | 120/136 (88.2) | 118/136 (86.8)† |
| Mean (SD) platelet counts, $x10^9$/L | 136.3 (54.8), n=122 | 142.1 (66.0), n=102 | 132.3 (59.8), n=18 |
| Mean (SD) bilirubin, µmol/L | 14.4 (11.9), n=123 | 13.5 (11.6), n=104 | 11.5 (6.6), n=19 |

*Irrespective of RBC transfusion.
†During the entire study.
Hb, hemoglobin; LDH, lactate dehydrogenase; RBC, red blood cell; SD, standard deviation.

**Copyright Information:** (Online) ISSN: 2572-9241
©2026 The Author(s). HemaSphere is published by John Wiley & Sons Ltd on behalf of European Hematology Association. This is an open access Abstract Book distributed under the Attribution-NonCommercial-NoDerivs (CC BY-NC-ND), which allows third parties to download the articles and share them with others as long as they credit the author and the Abstract Book, but they cannot change the content in any way or use them commercially.
**Abstract Book Citations:** Authors, Title, HemaSphere, 2026;10:(S1):pages. The abstract book DOIs can be found on the "Author Information" page.
**Disclaimer:** Articles published in the journal HemaSphere exclusively reflect the opinions of the authors. The authors are responsible for all content in their abstracts including accuracy of the facts, statements, citing resources, etc.

### (PS1789) HEMATOLOGIC MANIFESTATIONS OF NON-TINF2 AUTOSOMAL DOMINANT TELOMERE BIOLOGY DISORDERS

**Topic:** 12. Bone marrow failure syndromes incl. PNH - Clinical

Emma Groarke*[1], Fernanda Gutierrez-Rodrigues[1], Fieke Hoff[1], Luiz Fernando Bazzo Catto[1], Nicholas Lee[1], Jennifer Lotter[1], Tania MacHado[1], Colin Wu[1], Ruba Shalhoub[1], Neal Young[1], Bhavisha Patel[1]

[1]*National Heart, Lung and Blood Institute, Hematology Branch, Bethesda, United States of America;*

### Background

Telomere biology disorders (TBDs) comprise a group of syndromes caused by germline mutations in telomere-maintenance genes. Typical clinical manifestations include bone marrow failure (BMF), pulmonary fibrosis (PF), liver disease, and cancer predisposition. Genetic inheritance can be autosomal or x-linked recessive (AR/XLR), autosomal dominant (AD), most commonly due to heterozygous *TERT*, *TERC*, *RTEL1,* and *TINF2* pathogenic germline variants (PGVs). Non-*TINF2* AD-inheritance (AD-TBD) has been associated with adult disease onset, milder phenotype, and better overall survival, compared to other TBD genotypes.

### Aims

Characterize the hematologic manifestations of AD-TBDs in a large cohort.

### Methods

Patients with AD-TBD were enrolled and assessed. Gene specific analysis included *TERT*, *TERC*, *PARN* and *RTEL1* variants. We assessed life-time cumulative incidence (CI) of cytopenia, documented transfusion, and myeloid malignancy, and looked for gene-specific differences. All time to event analyses were censored for hematopoietic cell transplant (HCT).

**Copyright Information:** (Online) ISSN: 2572-9241
©2026 The Author(s). HemaSphere is published by John Wiley & Sons Ltd on behalf of European Hematology Association. This is an open access Abstract Book distributed under the Attribution-NonCommercial-NoDerivs (CC BY-NC-ND), which allows third parties to download the articles and share them with others as long as they credit the author and the Abstract Book, but they cannot change the content in any way or use them commercially.
**Abstract Book Citations:** Authors, Title, HemaSphere, 2026;10:(S1):pages. The abstract book DOIs can be found on the "Author Information" page.
**Disclaimer:** Articles published in the journal HemaSphere exclusively reflect the opinions of the authors. The authors are responsible for all content in their abstracts including accuracy of the facts, statements, citing resources, etc.

**Results**

Of a total 110 TBD patients, 91 were AD-TBDs due to PGVs in *TERT* (n=49; 54%), *TERC* (n=32; 35%), *RTEL1* (n=7; 8%), and *PARN* (n=3; 3%). Cytopenia occurred in the majority (n=74; 81%): Pancytopenia was the first documented cytopenia in 27 (39%) patients, followed by isolated thrombocytopenia (n=17; 24%), anemia (n=8; 11%), or neutropenia (n=2; 3%). Others presented with bi-cytopenia (n=20), with anemia/thrombocytopenia (n=8) being the commonest.
The median age at first documented cytopenia was 22 years. CI of cytopenia was 36% by age 20 and 64% by age 40. Genotype correlations showed that patients with *TERT* had a CI of cytopenia of 28% by age 20 and 56% by age 40; in contrast, patients with *TERC* had a younger age at cytopenia onset, with 51% CI by age 20 and 86% by age 40 (Figure 1A).
One-third (n=30; 33%) required a lifetime RBC transfusion. Median time to first RBC transfusion was 29 years. By age 20, 8% required a RBC transfusion, which rose to 26% by age 40 and 44% by age 60. Of those who required RBCs, in 5 were temporary during childbirth. CI of RBC transfusion was higher in *TERC* than in *TERT*; 11% by age 20 and 42% by age 40, compared to 6% by age 20 and 26% by age 40%, respectively.
Over a quarter of patients (n=25; 28%) also required a lifetime platelet transfusion. Median time to first platelet transfusion was 28 years. By age 20, 7% required transfusion, rising to 28% by age 40. Again, some patients required transfusion only during childbirth (n=4) or peri-procedure (n=1). Patients with *TERC* had higher CI of platelet transfusion of 11% by age 20 and 42% by age 40 compared to *TERT* at 6% by age 20 and 26% by age 40.
Median time to myeloid malignancy was 45 years and did not occur in patients <20 years of age. CI of myeloid malignancy by age of 60 was higher in patients with *TERC* v *TERT*- PGVs (54% vs. 9.5%, respectively (P=0.04; Figure 1B).
Patients with AD-*RTEL1* (n=7) presented at older age (all >60 years) and only 3 manifested hematologic disease. One presented with acute myeloid leukemia, one severe aplastic anemia, and one had mild cytopenia with prior diagnosis of solid malignancy. In patients with *PARN* PGVs (n=3), 2 had BMF and one had PF.
Eight patients underwent HCT, 2 for MDS and the rest for progressive BMF.

**Summary/Conclusion**

Hematologic manifestations in AD-TBDs are common, increase with age, and correlate with genotype. Patients with *TERC* PGVs have earlier onset of BMF and higher CI of transfusion than those with *TERT*.

**Copyright Information:** (Online) ISSN: 2572-9241
©2026 The Author(s). HemaSphere is published by John Wiley & Sons Ltd on behalf of European Hematology Association. This is an open access Abstract Book distributed under the Attribution-NonCommercial-NoDerivs (CC BY-NC-ND), which allows third parties to download the articles and share them with others as long as they credit the author and the Abstract Book, but they cannot change the content in any way or use them commercially.
**Abstract Book Citations:** Authors, Title, HemaSphere, 2026;10:(S1):pages. The abstract book DOIs can be found on the "Author Information" page.
**Disclaimer:** Articles published in the journal HemaSphere exclusively reflect the opinions of the authors. The authors are responsible for all content in their abstracts including accuracy of the facts, statements, citing resources, etc.

### (PS1790) ROMIPLOSTIM IN CHEMORADIOTHERAPY-ASSOCIATED APLASTIC ANAEMIA: A PROSPECTIVE SINGLE-ARM STUDY

**Topic:** 12. Bone marrow failure syndromes incl. PNH - Clinical

Qi Wang*[1], Ziwei Liu[1], Chen Yang[1], Jian Li[1], Miao Chen[1], Bing Han[1]

*[1]Peking Union Medical College Hospital and Chinese Academy of Medical Sciences,, Department of Hematology, Beijing, China;*

### Background

Chemoradiotherapy-associated aplastic anemia (AA) is a severe and potentially life-threatening complication that may compromise continuation of anticancer therapy. Effective treatment options for this condition remain undefined.

### Aims

To evaluate the efficacy and safety of romiplostim in patients with chemoradiotherapy-associated aplastic anaemia (AA) in a prospective single-arm study.

### Methods

In this single-center, prospective, open-label, single-arm study (ClinicalTrials.gov identifier: NCT06516484), adult patients who developed AA at least 3 months after completion of chemoradiotherapy were enrolled. Diagnosis of AA was established according to standard criteria, including bone marrow hypocellularity and peripheral cytopenias, after exclusion of other causes. The primary endpoint was overall response rate (ORR; complete response [CR]+partial response [PR]) at 6 months. Secondary endpoints included ORR at 3 months and the incidence of relapse, clonal evolution, and adverse events during follow-up.

### Results

Sixty-nine patients were enrolled and followed for a median of 16 months [IQR, 10–22]. The study included 22 males (31.9%) and 47 females (68.1%) with a median age of 61 years [IQR, 54–70]; 35 (51%) patients had severe AA, and 34 (49%) had non-severe AA (NSAA). At 3 months, 84.1% (58/69) patients achieved ORR, including 23.2% CR. At 6 months, ORR reached 90.9% (60/66), with 37.9% achieving CR. Median times to overall and complete response were 2.0 months (IQR, 1.8–5.5) and 3.6 months (IQR, 2.3–8.0), respectively. During a median follow-up of 16 months [IQR, 10–22], relapse occurred in three (4.3%) patients, and one patient developed del(7q) with subsequent progression to myelodysplastic syndrome. Treatment was well tolerated, adverse events were predominantly grade 1–2, most commonly arthralgia (42%), fatigue (33%), and myalgia (30%). No thromboembolic events or treatment discontinuations due to adverse events were observed.

### Summary/Conclusion

Romiplostim achieved high response rates with rapid hematologic recovery and an acceptable safety profile in patients with chemoradiotherapy-associated AA, supporting its consideration as a safe and effective therapeutic option in this clinically challenging setting.

**Copyright Information:** (Online) ISSN: 2572-9241
©2026 The Author(s). HemaSphere is published by John Wiley & Sons Ltd on behalf of European Hematology Association. This is an open access Abstract Book distributed under the Attribution-NonCommercial-NoDerivs (CC BY-NC-ND), which allows third parties to download the articles and share them with others as long as they credit the author and the Abstract Book, but they cannot change the content in any way or use them commercially.
**Abstract Book Citations:** Authors, Title, HemaSphere, 2026;10:(S1):pages. The abstract book DOIs can be found on the "Author Information" page.
**Disclaimer:** Articles published in the journal HemaSphere exclusively reflect the opinions of the authors. The authors are responsible for all content in their abstracts including accuracy of the facts, statements, citing resources, etc.

### (PS1791) BEYOND HEMATOLOGIC RESPONSE: SUSTAINED DEEP REMISSIONS AND FAVORABLE SAFETY WITH EXTENDED TPO-RA PLUS ANTI-THYMOCYTE GLOBULIN AND CYCLOSPORINE AS FIRST-LINE THERAPY FOR SEVERE APLASTIC ANEMIA

**Topic:** 12. Bone marrow failure syndromes incl. PNH - Clinical

Jingnan Sun*[1], Dexiang Ji[2], Yuemin Gong[3], Jiaqi Wang[1], Ye Baodong[4], Jinsong Jia[5], Shuqian Xu[6], Chuanming Lin[7], Chengtao Qin[8], Yehui Tan[1], Fei Li[2], Guangsheng He[3]

*[1]Hematology Department，The First Hospital of Jilin University, Changchun, China;[2]Hematology Center of the First Affiliated Hospital of Nanchang University, Nanchang, China; [3]The First Affiliated Hospital of Nanjing Medical University,Jiangsu Province Hospital, Nanjing, China; [4]Hematology Department of Zhejiang Provincial Hospital of Traditional Chinese Medicine, Hangzhou, China; [5]Peking University People's Hospital,Peking University Institute of Hematology, Beijing, China;[6]Hematology Department, Qilu hospital of Shandong University, Jinan, China; [7]Department of Hematology, First Affiliated Hospital of Gannan Medical University, Ganzhou, China; [8]The First Affiliated Hospital of Nanjing MedicalUniversity.Jiangsu Provincial People's Hospital, Nanjing, China;*

### Background

Anti-thymocyte globulin（ATG）and cyclosporine combined with TPO-RA is the first-line recommendation for patients with severe aplastic anemia (SAA) who are not eligible for hematopoietic stem cell transplantation.The addition of TPO-RA to immunosuppressive therapy significantly improves the depth of hematological response and early response speed in SAA patients.Previous trials from NIH and EBMT limited TPO-RA to a maximum of 6 months. To date, rare study has investigated whether extended TPO-RA therapy results in favorable efficacy and safety outcomes in patients with SAA, particularly in Chinese populations.

### Methods

Between Jan.1, 2015 and Dec. 31, 2024, 262 new diagnosed adult SAA from 6 centers in Chinese Eastern Collaboration Group of Anemia(CECGA)were enrolled in this prospective multicenter registry study(ChiCTR2100045895). Patients received ATG and CsA without TPO-RA(AC cohort n=86),combined with extended TPO-RA(ACHE cohort n=176)including hetrombopag (ACH cohort n=98) or eltrombopag (ACE cohort n=78). Hetrombopag 15 mg/d or eltrobopag 75mg mg/d was administered for at least 6 months. Subsequent duration of TPO-RA depends the depth of remission and acceptability of pharmacoeconomics. For patients achieving CR, TPO-RA were maintained for 3 months before gradual tapering to discontinuation. For patients with PR, treatment continued to response plateau, then maintained and gradually taperred. ATG was administered intravenously for 5 consecutive days, and CsA 3~5mg/kg/d was given for at least 12 months according to plasma concentration.

**Copyright Information:** (Online) ISSN: 2572-9241
©2026 The Author(s). HemaSphere is published by John Wiley & Sons Ltd on behalf of European Hematology Association. This is an open access Abstract Book distributed under the Attribution-NonCommercial-NoDerivs (CC BY-NC-ND), which allows third parties to download the articles and share them with others as long as they credit the author and the Abstract Book, but they cannot change the content in any way or use them commercially.
**Abstract Book Citations:** Authors, Title, HemaSphere, 2026;10:(S1):pages. The abstract book DOIs can be found on the “Author Information” page.
**Disclaimer:** Articles published in the journal HemaSphere exclusively reflect the opinions of the authors. The authors are responsible for all content in their abstracts including accuracy of the facts, statements, citing resources, etc.

### Results

Regarding baseline demographics and clinical characteristics, no significant differences were observed between the two cohorts in terms of age, disease severity, PNH clone, HGB, RDW-CV, PLT, absolute reticulocyte count , or ferritin levels. However, patients in the ACHE cohort did present with significantly higher baseline levels of Tregs and absolute neutrophils, as well as a higher rate of pre-ATG infections.. Comparing to AC cohort, ACHE resulted in progressively improvement of both CR and OR at 3 6 9 12 and 24 months(Figure1A). No differences were observed in OR rates between ACH and ACE, but CR rates demonstrated significant higher trend for ACH cohort at 24 months post-ATG(Figure1B) . Liver function impairment occurred in 38.7% of AC cohort and 34.1% of ACHE cohort(*P*=0.062), while renal function impairment was observed in 9.7% of AC cohort and 10.6% of ACHE cohort (*P*=0.089). After a median follow-up of 42.1 months (median TPO-RA exposure: 16.9 months), the ACHE group showed no increase in clonal evolution.No unexpected safety events observed 1 year post-ATG.

### Summary/Conclusion

Extended TPO-RA stratedy provides a time-dependent, sustained deep remission without additional AE concerns including clonal evolution.OR obtains a plateau after 12 months, CR demonstrates progressive benefits over time, extending up to 24 months post-ATG.

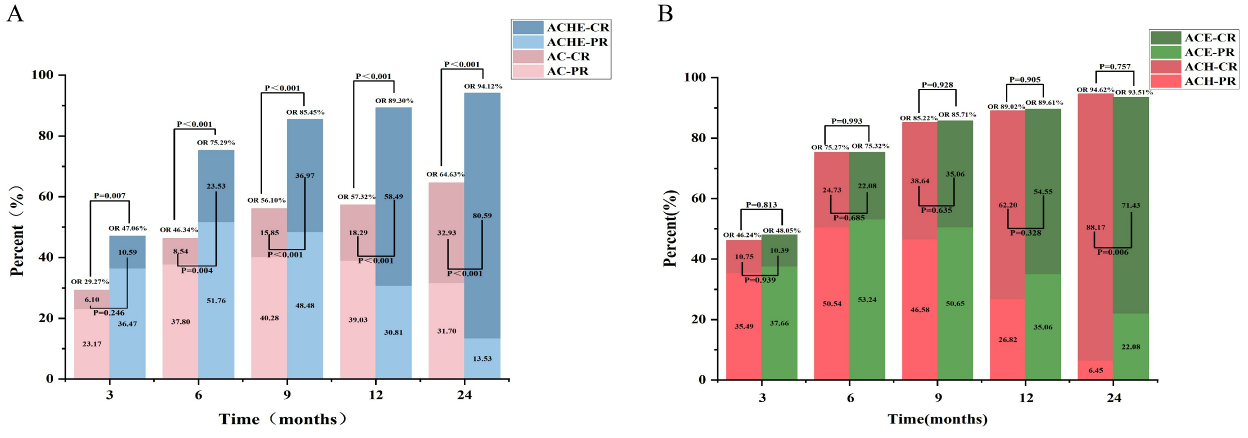

**Figure1. Hematological response to first-line treatment at different months comparing ATG plus cyclosporine with or without Extended TPO-RAs.**
**A. Cumulative CR and OR at 3,6,9,12,24months post-ATG for AC and ACHE cohort. B. Cumulative CR and OR at 3,6,9,12,24months post-ATG for ACH and ACE cohort.**

**Copyright Information:** (Online) ISSN: 2572-9241
©2026 The Author(s). HemaSphere is published by John Wiley & Sons Ltd on behalf of European Hematology Association. This is an open access Abstract Book distributed under the Attribution-NonCommercial-NoDerivs (CC BY-NC-ND), which allows third parties to download the articles and share them with others as long as they credit the author and the Abstract Book, but they cannot change the content in any way or use them commercially.
**Abstract Book Citations:** Authors, Title, HemaSphere, 2026;10:(S1):pages. The abstract book DOIs can be found on the “Author Information” page.
**Disclaimer:** Articles published in the journal HemaSphere exclusively reflect the opinions of the authors. The authors are responsible for all content in their abstracts including accuracy of the facts, statements, citing resources, etc.

### (PS1792) BONE MARROW FEATURES AND CLINICAL OUTCOMES IN ELANE-ASSOCIATED CONGENITAL NEUTROPENIA: A NAPAAC/SCNIR STUDY

**Topic:** 12. Bone marrow failure syndromes incl. PNH - Clinical

Helen Reed*[1], Ilonze Chibuzo[2], Frederick Goldman[2], Nicholas Gloude[3], Helena Yu[3], Tamanna Shamrin[4], Jessica Uhrich[4], Marcin Wlodarski[4], Irem Eldem[5], Melanie Fields[5], Sei-Gyung Sze[6], Taizo Nakano[7], Lauri Burroughs[8], Amy Geddis[8], Kathryn Dickerson[9], Kathleen Overholt[10], Jessica Seaborn[10], Melissa Rose[11], Jordan Milner[12], Thomas D. Coates[13], Hyojeong Han[14], Sandy Mostaert[15], Anjali Sharathkumar[16], Gloria Contreras Yametti[17], Kirsty Hiller[17], Larisa Broglie[18], Alejandra Escobar Vasco[18], Julie Waldhart[18], David C. Dale[19], Peter Newburger[20], Kasiani C. Myers[21], Akiko Shimamura[1], Jane Koo[21]

*[1]Boston Children's Hospital, Dana-Farber Cancer Institute, Harvard Medical School, Division of Hematology/Oncology, Department of Pediatrics, Boston, United States of America; [2]University of Alabama at Birmingham, Division of Pediatric Hematology Oncology, Birmingham, United States of America; [3]Rady Children's Hospital San Diego, University of California San Diego, Division of Pediatric Hematology/Oncology, Department of Pediatrics, San Diego, United States of America; [4]St. Jude Children's Research Hospital, Department of Hematology, Memphis, United States of America; [5]Washington University School of Medicine, Division of Hematology and Oncology, Department of Pediatrics, St. Louis, United States of America; [6]Tufts University School of Medicine, Division of Pediatric Hematology and Oncology, Department of Pediatrics, Portland, United States of America; [7]Children's Hospital Colorado, University of Colorado School of Medicine,, Center for Cancer and Blood Disorders, Aurora, United States of America; [8]Seattle Children's Hospital, Cancer and Blood Disorders Center, Department of Pediatrics, Seattle, United States of America; [9]UT Southwestern Medical Center, Division of Pediatric Hematology and Oncology, Department of Pediatrics, Dallas, United States of America; [10]Riley Children's Hospital at Indiana University, Division of Pediatric Hematology, Oncology and Stem Cell Transplant, Indianapolis, United States of America; [11]Nationwide Children's Hospital, The Ohio State University College of Medicine, Division of Pediatric Hematology Oncology, Department of Pediatrics, Columbus, United States of America; [12]University of Florida, Division of Pediatric Hematology Oncology, and Stem Cell Transplantation, Department of Pediatrics,, Gainesville, United States of America; [13]Children's Hospital Los Angeles, University of Southern California Keck School of Medicine, Cancer and Blood Disease Institute,, Los Angeles, United States of America; [14]Children's National Hospital, Division of Hematology, Washington DC, United States of America; [15]University of Iowa Hospital and Clinics, ICTS-Clinical Research Unit, Iowa City, United States of America; [16]University of Iowa Carver College of Medicine, Stead Family Department of Pediatrics, Iowa City, United States of America; [17]Hassenfield Children's Hospital at New York University Langone Health, Division of Pediatric Hematology Oncology, New YOrk, United States of America; [18]Medical College of Wisconsin, Division of Pediatric Hematology, Oncology, Blood and Marrow Transplant, Milwaukee, United States of America; [19]University of Washington, Department of Medicine, Seattle, United States of America; [20]University of Massachusetts Chan Medical School, Departments of Pediatrics and Molecular, Cell and Cancer Biology,, Worcester, United States of America; [21]Cincinnati Children's Hospital Medical Center, Division of Bone Marrow Transplantation and Immune Deficiency, Cincinnati, United States of America;*

### Background

Congenital neutropenia (CN) due to germline *ELANE* mutations is associated with an increased risk of myeloid malignancy. The diagnostic criteria for MDS and AML have evolved, so updated data are needed to inform treatment decisions.

### Aims

To characterize the genetic, clinical, and pathologic features of *ELANE* CN, including BM morphology and somatic mutational profiles, and to evaluate myeloid malignancy incidence and outcomes.

**Copyright Information:** (Online) ISSN: 2572-9241
©2026 The Author(s). HemaSphere is published by John Wiley & Sons Ltd on behalf of European Hematology Association. This is an open access Abstract Book distributed under the Attribution-NonCommercial-NoDerivs (CC BY-NC-ND), which allows third parties to download the articles and share them with others as long as they credit the author and the Abstract Book, but they cannot change the content in any way or use them commercially.
**Abstract Book Citations:** Authors, Title, HemaSphere, 2026;10:(S1):pages. The abstract book DOIs can be found on the "Author Information" page.
**Disclaimer:** Articles published in the journal HemaSphere exclusively reflect the opinions of the authors. The authors are responsible for all content in their abstracts including accuracy of the facts, statements, citing resources, etc.

### Methods

We conducted a retrospective, multicenter cohort study using data from 379 pediatric and adult patients with genetically confirmed *ELANE* CN enrolled through the North American Pediatric Aplastic Anemia Consortium (NAPAAC) and the Severe Chronic Neutropenia International Registry (SCNIR). Clinical, laboratory, genetic, and pathology data were abstracted from medical records. BM features, including somatic mutation testing where available, and G-CSF exposure were characterized. Central review was conducted to apply the 2022 WHO and ICC diagnostic criteria for myelodysplastic syndrome (MDS) and acute myeloid leukemia (AML). Overall survival (OS) was estimated using the Kaplan-Meier method and compared using the log-rank (Mantel-Cox) test.

### Results

Demographic and clinical features are summarized in Table 1. The estimated OS at 50 years was 72.4% (95% CI: 63.5% - 84.27%). Thirteen (3.4%) and 23 (6.1%) patients were diagnosed with MDS and AML, respectively. Median OS following MDS and AML diagnosis were 5.5 years (95% CI: 0.8 years – NR) and 2.7 years (95% CI: 0.7 – 6.2 years), respectively. A total of 65 (17.2%) patients had undergone HSCT. 53.5% (95% CI: 36.5% - 74.3%) were alive at 5 years following HSCT for malignancy vs. 80.4% (95% CI: 69.9% - 100.0%) following HSCT for non-malignant indications ($p = 0.008$).
Among 309 BM procedures, 227 (73.5%) were performed for hematologic surveillance. Among available marrow pathology reports without a myeloid malignancy diagnosis, left-shifted granulopoiesis was frequent (179/272, 65.8%), while myeloid maturation arrest was explicitly noted in 40 (14.7%) and eosinophilia in 116 (42.6%) examinations. Erythroid abnormalities were present in 55/268 (20.5%) marrows and were typically mild, consisting of preserved maturation with megaloblastoid changes; marked erythroid dysplasia or severe hypoplasia was rare (3.4%). Dysmegakaryopoiesis was identified in 41/263 (15.5%) marrows. Monosomy 7/del(7q) was detected in 16 BM procedures from 3 patients, including 7 surveillance marrows without other features of dysplasia or malignancy. One patient had monosomy 7 detected on an earlier bone marrow evaluation and subsequently progressed to AML. Another patient demonstrated monosomy 7 only at the time of AML diagnosis. A third patient had monosomy 7 (0.4% detected by FISH) and did not progress to AML. Somatic variants were identified in 19/50 (38%) procedures, most commonly involving *CSF3R* and *RUNX1*.

### Summary/Conclusion

In this large, multicenter cohort of patients with genetically confirmed *ELANE* CN, myeloid malignancy risk was high, and OS following MDS/AML using current diagnostic criteria was poor. Among those who underwent HSCT, survival was superior among those transplanted for a non-malignant indication compared to those transplanted for MDS/AML. This supports the need for further investigation into the utility of surveillance and distinguishing high-risk bone marrow features indicative of impending malignant transformation from common baseline features in *ELANE* CN.

**Copyright Information:** (Online) ISSN: 2572-9241
©2026 The Author(s). HemaSphere is published by John Wiley & Sons Ltd on behalf of European Hematology Association. This is an open access Abstract Book distributed under the Attribution-NonCommercial-NoDerivs (CC BY-NC-ND), which allows third parties to download the articles and share them with others as long as they credit the author and the Abstract Book, but they cannot change the content in any way or use them commercially.
**Abstract Book Citations:** Authors, Title, HemaSphere, 2026;10:(S1):pages. The abstract book DOIs can be found on the “Author Information” page.
**Disclaimer:** Articles published in the journal HemaSphere exclusively reflect the opinions of the authors. The authors are responsible for all content in their abstracts including accuracy of the facts, statements, citing resources, etc.

**Table 1. Demographics and Clinical Features of *ELANE* Congenital Neutropenia Cohort**

| | n = 379 |
|---|---|
| **Median age (range) in years** | 17.5 (0.1 – 84.7) |
| **Sex, n (%)** | Female = 193 (50.9%)<br>Male = 186 (49.1%) |
| **Race, n (%)** | White = 310 (81.8%)<br>Black = 16 (4.2%)<br>Asian = 16 (4.2%)<br>Other/Unknown = 37 (9.8%) |
| **Vital status at last follow-up, n (%)** | Alive = 337 (88.9%)<br>Dead = 42 (11.1%) |
| **MDS diagnosis, n (%)** | 13 (3.4%) |
| **Median age at diagnosis (range) in years** | 16.9 (2.1 – 42.0) |
| **AML diagnosis, n (%)** | 23 (6.1%) |
| **Median age at diagnosis (range) in years** | 19.5 (4.2 – 55.5) |
| **History of HSCT (n, %)** | 65 (17.2%) |
| **Age at HSCT (range), years** | 7.2 (0.3 – 56.0) |

Table 1. Cohort characteristics

**Copyright Information:** (Online) ISSN: 2572-9241
©2026 The Author(s). HemaSphere is published by John Wiley & Sons Ltd on behalf of European Hematology Association. This is an open access Abstract Book distributed under the Attribution-NonCommercial-NoDerivs (CC BY-NC-ND), which allows third parties to download the articles and share them with others as long as they credit the author and the Abstract Book, but they cannot change the content in any way or use them commercially.
**Abstract Book Citations:** Authors, Title, HemaSphere, 2026;10:(S1):pages. The abstract book DOIs can be found on the "Author Information" page.
**Disclaimer:** Articles published in the journal HemaSphere exclusively reflect the opinions of the authors. The authors are responsible for all content in their abstracts including accuracy of the facts, statements, citing resources, etc.

### (PS1793) MOMELOTINIB IN VEXAS SYNDROME: A PRELIMINARY REPORT EVALUATION OF SAFETY AND EFFECTIVENESS

**Topic:** 12. Bone marrow failure syndromes incl. PNH - Clinical

Elisa Diral*[1], Gregorio Maria Bergonzi[1, 2], Costanza Piccolo[2, 3], Corrado Campochiaro[2, 3], Alessandro Tomelleri[2, 3], Enrico Cozzo[1, 2], Carmelo Gurnari[4, 5], Francesca Romano[5], Rossella Puzziferri[6], Anna Baldisserotto[6], Luca Vago[1, 2], Marco Matucci-Cerinic[2, 3], Lorenzo Dagna[2, 3], Fabio Ciceri[1, 2]

*[1]IRCCS San Raffaele Hospital, Unit of Hematology and Bone Marrow Transplantation, Milan, Italy; [2]Vita-Salute San Raffaele University, Milan, Italy, Milan, Italy; [3]IRCCS San Raffaele Hospital, Unit of Immunology, Rheumatology, Allergy and Rare Diseases (UnIRAR), Milan, Italy; [4]Taussig Cancer Institute, Cleveland Clinic, Cleveland, Ohio, USA, Department of Translational Hematology and Oncology Research, Cleveland, United States of America; [5]University of Rome Tor Vergata, Department of Biomedicine and Prevention, Rome, Italy; [6]IRCCS San Raffaele Hospital, Pharmacy Unit, Italy;*

### Background

VEXAS (Vacuoles, E1 enzyme, X-linked, Autoinflammatory, Somatic) syndrome is an acquired autoinflammatory disease caused by somatic mutations in the *UBA1* gene. Janus kinase (JAK) inhibitors, particularly ruxolitinib (RUXO), have proven their efficacy in VEXAS syndrome. However, RUXO-associated anemia frequently leads to dose reduction or treatment discontinuation. Momelotinib (MMB), a JAK1/JAK2 and activin A receptor type 1 inhibitor, has been demonstrated to improve anemia in myelofibrosis and has been suggested as a potential therapeutic option in VEXAS syndrome.

### Aims

To evaluate the effectiveness and safety of MMB in a single-center cohort of patients with VEXAS syndrome.

### Methods

Patients with genetically confirmed VEXAS syndrome were identified. MMB treatment was authorized by the Regional Rare Disease Fund, in the absence of approved therapies and clinical trials. Data collected included: disease and clinical features at baseline; variant allele frequency (VAF) of *UBA1* mutations by means of droplet digital PCR (ddPCR) at baseline and at 3 months since MMB introduction; hemoglobin (Hb) levels, C-reactive protein (CRP), and glucocorticoid dose at baseline and at one, two and three months since MMB initiation.

**Copyright Information:** (Online) ISSN: 2572-9241
©2026 The Author(s). HemaSphere is published by John Wiley & Sons Ltd on behalf of European Hematology Association. This is an open access Abstract Book distributed under the Attribution-NonCommercial-NoDerivs (CC BY-NC-ND), which allows third parties to download the articles and share them with others as long as they credit the author and the Abstract Book, but they cannot change the content in any way or use them commercially.
**Abstract Book Citations:** Authors, Title, HemaSphere, 2026;10:(S1):pages. The abstract book DOIs can be found on the "Author Information" page.
**Disclaimer:** Articles published in the journal HemaSphere exclusively reflect the opinions of the authors. The authors are responsible for all content in their abstracts including accuracy of the facts, statements, citing resources, etc.

### Results

Six male patients with VEXAS, with a median age of 67 years [IQR 66–70], were identified. All patients had concomitant lower risk myelodysplastic syndrome. Four (67%) patients had been previously treated with RUXO, that was discontinued for worsening anemia (n = 3) or for skin rash (n = 1). At baseline, all patients were on glucocorticoids (median daily dose 15 mg [IQR 10-23.75] of prednisone) and 4 (67%) patients were on erythropoiesis-stimulating agents (ESA) (median weekly dose 40000 IU [IQR 40000-50000]. All patients were started on MMB 200 mg daily. MMB was discontinued in one patient for subjective intolerance by day 30, whereas in another patient the dose was reduced to 100 mg daily for interactions with concomitant medications. Three patients (50%) experienced respiratory tract infections requiring admission in two cases. One patient experienced a disease flare in the first month since MMB start, requiring an increase in glucocorticoid dose, while no patient experienced thrombotic events. An increase in Hb levels of a median 1 g/dl [IQR 0.5-2] in the first month and a median of 2 g/dl [IQR 0-3] at three months (Figure 1A), compared to baseline was observed. In one patient ESA was discontinued at 3 months from MMB start. CRP levels decreased from a median of 25 mg/l [4.6-48.6] at baseline to a median of 16 mg/l [8-27.6] at 3 months (Figure 1B). Moreover, glucocorticoid dose was reduced from a median of 20 mg [IQR 15-25] daily at baseline to 15 mg [IQR 10-17.5] daily at 3 months (Figure 1C) since MMB initiation. Furthermore, we observed an overall decrease of *UBA1* VAF from a median of 67% [62.7-67.9] at baseline to a median of 29.8% [23.8-40.5] at three months (Figure 1D).

### Summary/Conclusion

Our study represents one of the first reports evaluating MMB in patients with VEXAS syndrome and shows encouraging results, with an acceptable safety profile and no life-threatening adverse events. Treatment was associated with improvements in Hb levels, reductions in median CRP values, and a steroid-sparing effect. Of note, a reduction in UBA1 VAF was achieved. These findings highlight the potential effectiveness and tolerability of MMB in VEXAS syndrome and support further investigation in larger, prospective studies.

**Copyright Information:** (Online) ISSN: 2572-9241
©2026 The Author(s). HemaSphere is published by John Wiley & Sons Ltd on behalf of European Hematology Association. This is an open access Abstract Book distributed under the Attribution-NonCommercial-NoDerivs (CC BY-NC-ND), which allows third parties to download the articles and share them with others as long as they credit the author and the Abstract Book, but they cannot change the content in any way or use them commercially.
**Abstract Book Citations:** Authors, Title, HemaSphere, 2026;10:(S1):pages. The abstract book DOIs can be found on the “Author Information” page.
**Disclaimer:** Articles published in the journal HemaSphere exclusively reflect the opinions of the authors. The authors are responsible for all content in their abstracts including accuracy of the facts, statements, citing resources, etc.

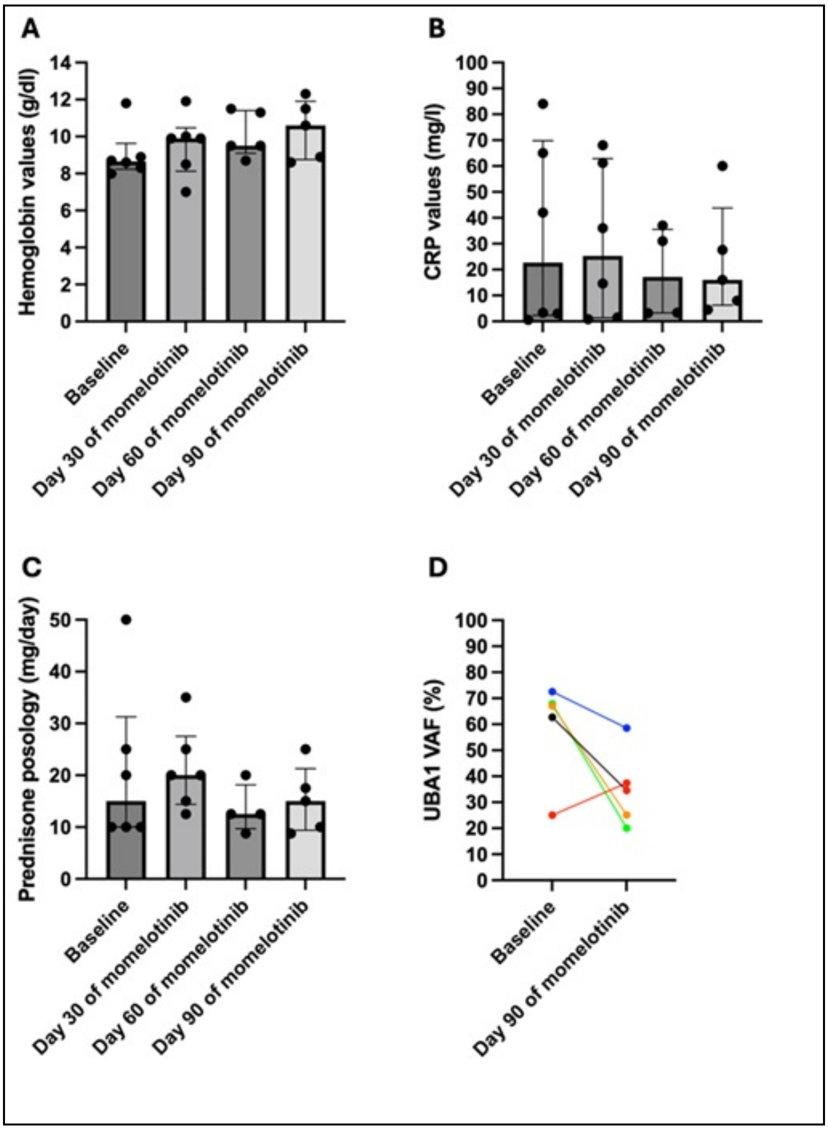

**Copyright Information:** (Online) ISSN: 2572-9241
©2026 The Author(s). HemaSphere is published by John Wiley & Sons Ltd on behalf of European Hematology Association. This is an open access Abstract Book distributed under the Attribution-NonCommercial-NoDerivs (CC BY-NC-ND), which allows third parties to download the articles and share them with others as long as they credit the author and the Abstract Book, but they cannot change the content in any way or use them commercially.
**Abstract Book Citations:** Authors, Title, HemaSphere, 2026;10:(S1):pages. The abstract book DOIs can be found on the "Author Information" page.
**Disclaimer:** Articles published in the journal HemaSphere exclusively reflect the opinions of the authors. The authors are responsible for all content in their abstracts including accuracy of the facts, statements, citing resources, etc.

### (PS1794) SUSTAINED LONG-TERM REAL-WORLD EFFECTIVENESS AND SAFETY OF PEGCETACOPLAN IN PATIENTS WITH PNH: ONGOING COMPLETE PHASE 4 STUDY

**Topic:** 12. Bone marrow failure syndromes incl. PNH - Clinical

Lenka Mastíková*[1], Carmen González[2], Anna-Elina Lehtinen[3], Roochi Trikha[4], Håkan Malmström[5], Luis López Lázaro[5], Michael O'malley[5], Benjamin Chin-Yee[6], Danny Hsu[7], Régis Peffault de Latour[8]

*[1]Institute of Hematology and Blood Transfusion, Prague, Czechia;[2]Donostiako Unibertsitate Ospitalea, Donostialdeko ESIa, San Sebastián, Spain; [3]Helsinki University Hospital, Comprehensive Cancer Centre and University of Helsinki, Coagulation Disorders Unit, Department of Hematology,, Helsinki, Finland; [4]King's College Hospital NHS, London, United Kingdom; [5]Swedish Orphan Biovitrum AB, Stockholm, Sweden; [6]Western University, London Health Sciences Centre, London, Canada; [7]Liverpool Hospital, Sydney, Australia; [8]French Reference Center for Aplastic Anemia and Paroxysmal Nocturnal Hemoglobinuria; Université Paris Cité, Paris, France;*

### Background

Paroxysmal nocturnal haemoglobinuria (PNH) is characterised by complement-mediated intravascular haemolysis (IVH) and an increased risk of thrombosis, which manifest with various symptoms. Complement 5 inhibitors (C5is) control IVH, but residual IVH and/or emerging extravascular haemolysis (EVH) and persistent anaemia may be experienced by most patients (pts), potentially causing transfusion dependence and compromised quality of life. Pegcetacoplan (PEG) is a complement 3 protein (C3)/C3b inhibitor approved for the treatment of adults with PNH, targeting both IVH and EVH. Phase 3 trials in C5i-experienced (PEGASUS) and complement inhibitor-naïve (PRINCE) pts with PNH have shown that PEG significantly improved haematological and other clinical outcomes. Emerging real-world evidence confirms the efficacy and safety of PEG to treat PNH observed in clinical trials.

### Aims

To present interim analysis results from the ongoing Phase 4 COMPLETE (NCT05776472) study to describe the long-term real-world effectiveness of PEG for the treatment of adults (≥18 years) with PNH.

### Methods

COMPLETE is a long-term, observational study enrolling pts in Europe, the Middle East, Canada, and Australia. Adults with PNH who initiate routine PEG treatment before or at enrolment are included. Key data are collected from PEG initiation and pts are followed prospectively for 36 months. Retrospective data ≤12 months before PEG initiation are also captured. The primary endpoint is change in haemoglobin (Hb) at 6 months after PEG initiation. Secondary endpoints include Hb improvement (Hb ≥12 g/dl or a ≥2 g/dl increase), and changes in lactate dehydrogenase, absolute reticulocyte count, bilirubin, and ferritin, assessed every 6 months after PEG initiation. The annualised number and units of red blood cell transfusions (RBCTs) are also evaluated. Adverse events (AEs) are recorded from enrolment.

**Copyright Information:** (Online) ISSN: 2572-9241
©2026 The Author(s). HemaSphere is published by John Wiley & Sons Ltd on behalf of European Hematology Association. This is an open access Abstract Book distributed under the Attribution-NonCommercial-NoDerivs (CC BY-NC-ND), which allows third parties to download the articles and share them with others as long as they credit the author and the Abstract Book, but they cannot change the content in any way or use them commercially.
**Abstract Book Citations:** Authors, Title, HemaSphere, 2026;10:(S1):pages. The abstract book DOIs can be found on the "Author Information" page.
**Disclaimer:** Articles published in the journal HemaSphere exclusively reflect the opinions of the authors. The authors are responsible for all content in their abstracts including accuracy of the facts, statements, citing resources, etc.

### Results

As of 5 January 2026, 125 eligible pts were enrolled across 14 countries. At baseline, median age was 53.0 years, 64 (51.2%) pts were female, 42 (33.6%) pts had a history of aplastic anaemia, and 100 (80.0%) pts had recorded previous C5is. Median time from PNH diagnosis to PEG initiation was 49.5 months and median duration of PEG treatment was 21.8 months. Outcomes after PEG initiation at 6-month intervals up to 30 months are shown in **Table 1**. From baseline to 6 months, the median (interquartile range [IQR]) absolute change in Hb was 2.2 (0.8, 3.4) g/dl and the median (IQR) change in Hb was 24.1% (8.0, 39.5). At 6 months, median Hb was 11.4 g/dl and 42 (39.3%) pts had Hb levels ≥12 g/dl; at 30 months, the corresponding values were 11.2 g/dl and 11 (33.3%) pts. The mean number of annualised RBCTs and units of annualised RBCTs decreased from ≤12 months before PEG initiation (2.8 and 4.5; n=125) to 0–12 months after initiation (1.2 and 1.8; n=113) and remained low up to 24–36 months after initiation (1.7 and 2.6; n=34). Haemolytic events requiring additional intervention occurred in 34 (27.2%) pts. AEs occurred in 61 (48.8%) pts. Serious AEs occurred in 30 (24.0%) pts and AEs leading to treatment discontinuation were reported for 1 (0.8%) pt.

### Summary/Conclusion

The COMPLETE study includes a broad and representative population of pts with PNH, mirroring real-world clinical practice. Treatment with PEG led to marked improvements in haematological and other clinical parameters by 6 months, with these benefits maintained through 30 months of follow-up. No new safety signals were observed, supporting the long-term use of PEG.

**Table 1. Outcomes after treatment initiation at 6-month intervals up to 30 months for the FAS (n=125)**

| Outcome | n | Baseline | n | 6 months | n | 12 months | n | 18 months | n | 24 months | n | 30 months |
|---|---|---|---|---|---|---|---|---|---|---|---|---|
| Hb, median (IQR), g/dl | 118 | 9.5 (8.6, 10.6) | 107 | 11.4 (10.3, 12.7) | 63 | 11.5 (9.8, 12.7) | 64 | 11.2 (10.4, 12.4) | 44 | 11.1 (9.9, 12.5) | 33 | 11.2 (10.3, 12.2) |
| Absolute Hb CFB, median (IQR),[a] g/dl | - | - | 102 | 2.2 (0.8, 3.4) | - | - | - | - | - | - | - | - |
| Hb CFB, median (IQR),[a] % | - | - | 102 | 24.1 (8.0, 39.5) | - | - | - | - | - | - | - | - |
| Hb ≥12 g/dl, n (%) | 118 | 5 (4.2) | 107 | 42 (39.3) | 63 | 23 (36.5) | 64 | 19 (29.7) | 44 | 17 (38.6) | 33 | 11 (33.3) |
| Hb increase of ≥2 g/dl achieved vs baseline,[b] n (%) | - | - | 107 | 55 (51.4) | 63 | 31 (49.2) | 64 | 30 (46.9) | 44 | 20 (45.5) | 33 | 15 (45.5) |
| LDH, median (IQR), U/l | 105 | 310.0 (233.0, 396.0) | 98 | 223.5 (175.0, 258.0) | 59 | 213.0 (178.0, 312.0) | 60 | 202.5 (167.0, 307.5) | 42 | 222.5 (164.0, 342.0) | 29 | 188.0 (150.0, 217.0) |
| ARC, median (IQR), $\times 10^9$ cells/l | 88 | 190.5 (134.9, 258.2) | 85 | 98.0 (73.3, 126.0) | 50 | 106.0 (81.0, 122.1) | 49 | 107.0 (90.0, 138.0) | 37 | 113.0 (92.0, 144.0) | 27 | 99.6 (64.0, 139.6) |
| Total bilirubin, median (IQR), µmol/l | 112 | 25.5 (16.6, 41.3) | 100 | 13.1 (9.2, 19.9) | 60 | 13.0 (9.0, 15.7) | 60 | 11.8 (8.9, 16.3) | 43 | 10.0 (8.6, 15.4) | 30 | 11.4 (8.0, 16.0) |
| Ferritin, median (IQR), µg/l | 57 | 534.0 (191.9, 1231.0) | 64 | 473.7 (122.5, 1025.1) | 39 | 415.9 (84.0, 1660.0) | 42 | 224.0 (43.0, 1170.0) | 27 | 270.0 (125.0, 670.0) | 20 | 168.5 (56.5, 293.7) |

[a]Absolute and percentage Hb CFB reported for FAS: 6 months (n=107) for patients with values available at both baseline and 6 months; [b]relative to pegcetacoplan treatment initiation.
ARC, absolute reticulocyte count; CFB, change from baseline; FAS, full analysis set; Hb, haemoglobin; IQR, interquartile range; LDH, lactate dehydrogenase.

Copyright Information: (Online) ISSN: 2572-9241
©2026 The Author(s). HemaSphere is published by John Wiley & Sons Ltd on behalf of European Hematology Association. This is an open access Abstract Book distributed under the Attribution-NonCommercial-NoDerivs (CC BY-NC-ND), which allows third parties to download the articles and share them with others as long as they credit the author and the Abstract Book, but they cannot change the content in any way or use them commercially.
Abstract Book Citations: Authors, Title, HemaSphere, 2026;10:(S1):pages. The abstract book DOIs can be found on the "Author Information" page.
Disclaimer: Articles published in the journal HemaSphere exclusively reflect the opinions of the authors. The authors are responsible for all content in their abstracts including accuracy of the facts, statements, citing resources, etc.

### (PS1795) TRANSPLANT OUTCOMES FOR PATIENTS WITH SEVERE ACQUIRED APLASTIC ANEMIA OVER THE AGE OF 40 USING BUSULFAN, FLUDARABINE, REDUCED-DOSE CYCLOPHOSPHAMIDE, AND ANTI-THYMOCYTE GLOBULIN CONDITIONING REGIMEN.

**Topic:** 12. Bone marrow failure syndromes incl. PNH - Clinical

Fan Lin*[1], Tingting Han[1], Yun He[1], Zheng-Li Xu[1], Yuan-Yuan Zhang[1], Yu-Hong Chen[1], Jing-Zhi Wang[1], Fengrong Wang[1], Wei Han[1], Yao Chen[1], Yuqian Sun[1], Xiao-Dong Mo[1], Yiheng Zhao[1], Yu Wang[1], Xiaohui Zhang[1], Xiao-Jun Huang[1], Lanping Xu[1]

*[1]Peking University People's Hospital, Peking University Institute of Hematology, Beijing, China;*

### Background

Allogeneic hematopoietic stem cell transplantation (HCT) is well established as curative treatment for severe acquired aplastic anemia (SAA). However, the transplant outcomes remain unsatisfactory in patients over the age of 40. EBMT data suggests the lack of further improvements in survival in 2010 to 2015, as compared with 2001 to 2009, in patients aged over 40. Hence there is an unmet need to improve the management of this older group of SAA patients. Given their higher comorbidity indexes and poorer physical condition, older patients are exposed to a higher risk of organ toxicities. Fludarabine (Flu)-based conditioning regimens are the most prevalent option for mitigating regimen-related toxicities (RRT) and could be an effective way to improve outcomes for this group of patients.

### Aims

We aimed to determine the safety and efficacy of a fludarabine-based regimen comprising low-dose busulfan, fludarabine, reduced-dose cyclophosphamide, and anti-thymocyte globulin (BFCA regimen) for older SAA patients.

### Methods

HCT conducted between July 2018 and June 2025 were reviewed. Patients received transplants from available MRD, 10/10 HLA-matched unrelated donors (MUD), or haploidentical donors (HID). We have identified and analyzed 56 consecutive cases of SAA patients over the age of 40 who received the BFCA regimen prior to HCT. This is a retrospective analysis of a prospectively maintained and updated database, and data were analyzed in January 2026.

**Copyright Information:** (Online) ISSN: 2572-9241
©2026 The Author(s). HemaSphere is published by John Wiley & Sons Ltd on behalf of European Hematology Association. This is an open access Abstract Book distributed under the Attribution-NonCommercial-NoDerivs (CC BY-NC-ND), which allows third parties to download the articles and share them with others as long as they credit the author and the Abstract Book, but they cannot change the content in any way or use them commercially.
**Abstract Book Citations:** Authors, Title, HemaSphere, 2026;10:(S1):pages. The abstract book DOIs can be found on the “Author Information” page.
**Disclaimer:** Articles published in the journal HemaSphere exclusively reflect the opinions of the authors. The authors are responsible for all content in their abstracts including accuracy of the facts, statements, citing resources, etc.

**Results**

Regimen-related toxicities of the BFCA regimen were acceptable. Severe RRTs (grade 3-4) occurred in 5 patients (8.9%). The cumulative incidence of primary engraftment was 92.9% (81.3%-97.4%). No graft failure occurred. During the follow-up, 48 patients achieved platelet engraftment at a median time of 15 days (range, 7-589). The cumulative incidence of 100-day platelet engraftment was 83.9% (70.8%-91.5%). The 100-day cumulative incidences of grade 2-4 and grade 3-4 acute graft-versus-host disease (aGVHD) were 25.0% (14.5%-36.9%) and 7.1% (2.3%-15.9%), respectively. The 3-year cumulative incidences of chronic graft-versus-host disease (cGVHD) and moderate-to-severe cGVHD were 33.7% (20.5%-47.4%) and 10.8% (3.9%-21.9%), respectively. The favorable 3-year overall survival (OS) of 71.4%, failure-free survival (FFS) of 71.4%, and GVHD/failure-free survival (GFFS) of 61.4% were achieved. Recipient age of ≥ 50 years and ECOG score of ≥ 2 were independent risk factor for OS and FFS, as well as GFFS.
Patients were divided into two group for subgroup analysis: aged 40-50 years (n = 35) and aged over 50 years (n = 21). The BFCA regimen yielded comparable engraftment and GVHD in the two age groups. However, patients aged over 50 years achieved significantly poorer OS (85.7% (74.9%-98.1%) vs. 46.2% (28.7%-74.2%), p = 0.003), FFS (85.7% (74.9%-98.1%) vs. 46.2% (28.7%-74.2%), p = 0.003), and GFFS (74.2% (61.0%-90.2%) vs. 40.4% (23.5%-69.5%), p = 0.016) compared to those under age 50 when receiving the BFCA regimen. Older patients were more likely to had infection-related mortality (p = 0.042). The BFCA regimen resulted in delayed immune reconstitution in patients aged over 50.

**Summary/Conclusion**

In conclusion, the BFCA regimen is feasible for conditioning SAA patients over the age of 40 and leads to promising outcomes. Patients who aged 40-50 years or maintained a good performance status can be more likely to benefit from HCT with the BFCA regimen.

**Copyright Information:** (Online) ISSN: 2572-9241
©2026 The Author(s). HemaSphere is published by John Wiley & Sons Ltd on behalf of European Hematology Association. This is an open access Abstract Book distributed under the Attribution-NonCommercial-NoDerivs (CC BY-NC-ND), which allows third parties to download the articles and share them with others as long as they credit the author and the Abstract Book, but they cannot change the content in any way or use them commercially.
**Abstract Book Citations:** Authors, Title, HemaSphere, 2026;10:(S1):pages. The abstract book DOIs can be found on the "Author Information" page.
**Disclaimer:** Articles published in the journal HemaSphere exclusively reflect the opinions of the authors. The authors are responsible for all content in their abstracts including accuracy of the facts, statements, citing resources, etc.

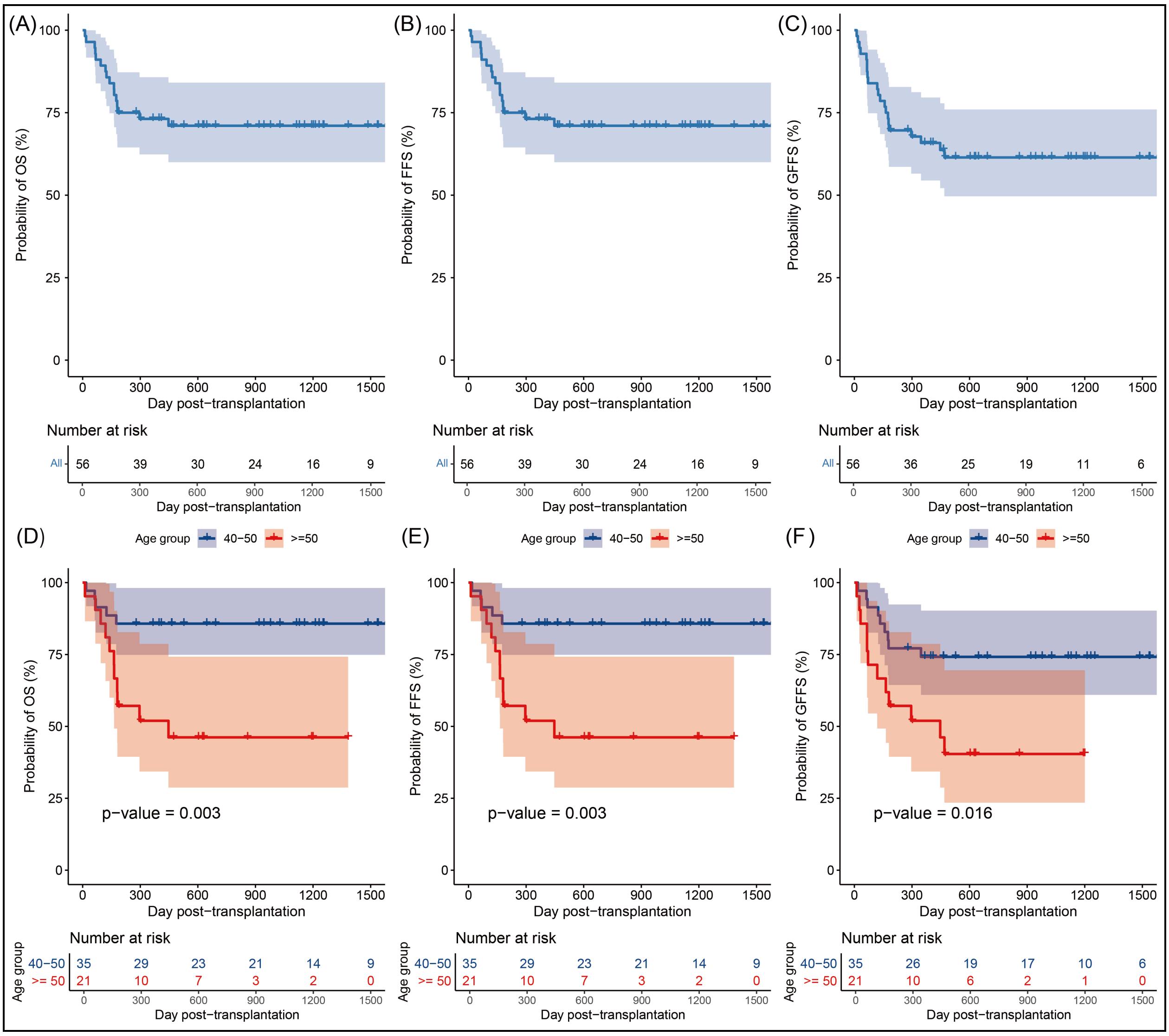

**Copyright Information:** (Online) ISSN: 2572-9241
©2026 The Author(s). HemaSphere is published by John Wiley & Sons Ltd on behalf of European Hematology Association. This is an open access Abstract Book distributed under the Attribution-NonCommercial-NoDerivs (CC BY-NC-ND), which allows third parties to download the articles and share them with others as long as they credit the author and the Abstract Book, but they cannot change the content in any way or use them commercially.
**Abstract Book Citations:** Authors, Title, HemaSphere, 2026;10:(S1):pages. The abstract book DOIs can be found on the "Author Information" page.
**Disclaimer:** Articles published in the journal HemaSphere exclusively reflect the opinions of the authors. The authors are responsible for all content in their abstracts including accuracy of the facts, statements, citing resources, etc.

### (PS1796) REAL-WORLD HEMOGLOBIN PROFILES AND TRANSFUSION BURDEN IN PATIENTS WITH PAROXYSMAL NOCTURNAL HEMOGLOBINURIA RECEIVING COMPLEMENT INHIBITORS: A US CLAIMS ANALYSIS

**Topic:** 12. Bone marrow failure syndromes incl. PNH - Clinical

Carlos de Castro*[1], Peter Blandino[2], Fiston Vuvu[2], Enrico Zanardo[3], Lynn Huynh[3], Robert Schell[3], Nathaniel Downes[3], Yixin Yang[3], Mei Sheng Duh[3], Peter Hillmen[2]

*[1]Duke University, Durham, United States of America;[2]Apellis Pharmaceuticals, Inc, Boston, United States of America;[3]Analysis Group Inc, Boston, United States of America;*

### Background

Anemia and transfusion dependence are common in patients with paroxysmal nocturnal hemoglobinuria (PNH). Terminal inhibitors target intravascular hemolysis (IVH), but residual IVH and the development of extravascular hemolysis can contribute to ongoing anemia. Randomized trials show that proximal complement inhibition improves hemoglobin (Hb) concentrations and reduces transfusion dependence, but real-world evidence is limited.

### Aims

To evaluate real-world Hb concentrations and transfusion burden among patients with PNH treated with terminal or proximal complement inhibitors.

### Methods

We conducted a retrospective cohort study using the Komodo Research Database (January 1, 2016 to April 30, 2025), a large, nationally representative US claims database with linked Hb data. Adults with PNH initiating terminal (eculizumab, ravulizumab) or proximal complement inhibitors (pegcetacoplan, iptacopan) were categorized into three cohorts: terminal inhibitor only (no switch to proximal inhibitor), terminal-to-proximal switchers, and proximal inhibitor at index (initiated proximal inhibitor as first treatment). Transfusion burden (transfusion rates per patient per year [PPPY] and transfusion dependence [≥4 transfusions/year]) was assessed at baseline (3 months before first treatment) and follow-up (≥1 month). For switchers, follow-up was divided into pre- and post-switch periods. Hb levels and proportions of patients with Hb <12 g/dL were assessed for patients with available Hb data during baseline or follow-up (pre-/post-switch for switchers).

### Results

We identified 972 patients (**Table**): 783 (80.6%) terminal inhibitor only, 147 (15.1%) terminal-to-proximal switchers, 42 (4.3%) proximal inhibitor at index. Among terminal-only patients, mean (SD) Hb was 9.3 (2.2) g/dL at baseline and 10.8 (2.4) g/dL at follow-up. The proportion of patients with Hb <12 g/dL was 85.1% at baseline and 65.8% at follow-up. Mean (SD) transfusion rates were 4.99 (12.78) PPPY at baseline and 2.59 (7.97) PPPY at follow-up, and 29.4% were transfusion dependent at baseline and 13.8% at follow-up. Terminal-to-proximal switchers had Hb levels of 8.1 (2.2) g/dL at baseline, 9.5 (2.0) g/dL after terminal initiation before switch to proximal, and 11.0 (2.4) g/dL after switch to proximal. The proportion with Hb <12 g/dL was 100% at baseline, 87.0% pre-switch, and 58.3% post-switch. Mean (SD) transfusion rates were 3.97 (12.57) PPPY at baseline, 4.63 (11.85) PPPY pre-switch, and 2.77 (10.40) PPPY post-switch. The proportion with transfusion dependence was 29.3% at baseline, 22.4% pre-switch, and 10.9% post-switch. Among patients with proximal inhibitor at index, mean (SD) transfusion rates were 2.95 (8.20) PPPY at baseline and 1.30 (2.94) PPPY at follow-up. Transfusion dependence was 19.0% at baseline and 9.5% at follow-up. Few patients had Hb data (n=2 at baseline; n=6 at follow-up).

**Copyright Information:** (Online) ISSN: 2572-9241
©2026 The Author(s). HemaSphere is published by John Wiley & Sons Ltd on behalf of European Hematology Association. This is an open access Abstract Book distributed under the Attribution-NonCommercial-NoDerivs (CC BY-NC-ND), which allows third parties to download the articles and share them with others as long as they credit the author and the Abstract Book, but they cannot change the content in any way or use them commercially.
**Abstract Book Citations:** Authors, Title, HemaSphere, 2026;10:(S1):pages. The abstract book DOIs can be found on the "Author Information" page.
**Disclaimer:** Articles published in the journal HemaSphere exclusively reflect the opinions of the authors. The authors are responsible for all content in their abstracts including accuracy of the facts, statements, citing resources, etc.

### Summary/Conclusion

Patients with PNH treated with terminal inhibitors may experience persistent anemia and transfusion burden. Hb-based metrics provide clinically meaningful insight into disease control beyond IVH alone. In this study, patients switching from terminal to proximal inhibitors had mean Hb levels of 9.5 g/dL before and 11.0 g/dL after switching, and the proportion with transfusion dependence was 22.4% before and 10.9% after switching. These findings highlight an ongoing unmet need in PNH and support treatment strategies addressing the full spectrum of complement-mediated hemolysis.

**Table 1. Baseline characteristics, transfusion burden, and hemoglobin concentration among patients with PNH initiating complement inhibitors**

| | Terminal inhibitor at index, no switch to proximal n = 783 | | Terminal inhibitor at index, switch to proximal n = 147 | | | Proximal inhibitor at index n = 42 | |
|---|---|---|---|---|---|---|---|
| **Patient characteristics** | | | | | | | |
| **Age at index, years, mean (SD)** | 46.4 (18.3) | | 45.2 (18.3) | | | 52.4 (17.4) | |
| **Female, n (%)** | 423 (54.0) | | 76 (51.7) | | | 25 (59.5) | |
| **Complement inhibitor agent,[a] n (%)** | | | *Terminal inhibitor (pre-switch)* | | *Proximal inhibitor (post-switch)* | | |
| **Eculizumab** | 459 (58.6) | | 63 (42.9) | | – | – | |
| **Ravulizumab** | 325 (41.5) | | 84 (57.1) | | – | – | |
| **Pegcetacoplan** | – | | – | | 88 (59.9) | 25 (59.5) | |
| **Iptacopan** | – | | – | | 59 (40.1) | 17 (40.5) | |
| **Transfusion burden** | | | | | | | |
| | *Baseline* | *Follow-up* | *Baseline* | *Pre-switch* | *Post-switch* | *Baseline* | *Follow-up* |
| **Transfusions PPPY, mean (SD)** | 4.99 (12.78) | 2.59 (7.97) | 3.97 (12.57) | 4.63 (11.85) | 2.77 (10.40) | 2.95 (8.20) | 1.30 (2.94) |
| **Transfusion dependence, n (%)** | 230 (29.4) | 108 (13.8) | 43 (29.3) | 33 (22.4) | 16 (10.9) | 8 (19.1) | 4 (9.5) |
| **Hemoglobin concentration** | | | | | | | |
| | *Baseline* | *Follow-up* | *Baseline* | *Pre-switch* | *Post-switch* | *Baseline* | *Follow-up* |
| **Patients with hemoglobin availability, n** | 74 | 158 | 11 | 23 | 24 | 2 | 6 |
| **Hemoglobin levels[b], mean (SD), g/ dL** | 9.3 (2.2) | 10.8 (2.4) | 8.1 (2.2) | 9.5 (2.0) | 11.0 (2.4) | 10.4 (0.8) | 11.4 (1.6) |
| **Patients with average Hb <12 g/dL,[b] n (%)** | 63 (85.1) | 104 (65.8) | 11 (100.0) | 20 (87.0) | 14 (58.3) | 2 (100.0) | 4 (66.7) |

Abbreviations: Hb, hemoglobin; PNH, paroxysmal nocturnal hemoglobinuria; PPPY: per patient per year.

[a] One patient in the *terminal inhibitor, no switch* cohort initiated on eculizumab and ravulizumab simultaneously. Crovalimab was included in the original search criteria, but no patients were identified as having initiated crovalimab during the study period.

[b] Values reflect means across patients with available data.

Copyright Information: (Online) ISSN: 2572-9241
©2026 The Author(s). HemaSphere is published by John Wiley & Sons Ltd on behalf of European Hematology Association. This is an open access Abstract Book distributed under the Attribution-NonCommercial-NoDerivs (CC BY-NC-ND), which allows third parties to download the articles and share them with others as long as they credit the author and the Abstract Book, but they cannot change the content in any way or use them commercially.
Abstract Book Citations: Authors, Title, HemaSphere, 2026;10:(S1):pages. The abstract book DOIs can be found on the "Author Information" page.
Disclaimer: Articles published in the journal HemaSphere exclusively reflect the opinions of the authors. The authors are responsible for all content in their abstracts including accuracy of the facts, statements, citing resources, etc.

### (PS1797) LONG-TERM HEMATOLOGIC CONTROL AND SAFETY IN PATIENTS WITH PAROXYSMAL NOCTURNAL HEMOGLOBINURIA TREATED WITH IPTACOPAN: 6-YEAR FOLLOW-UP FROM PHASE 2 STUDIES AND ROLL-OVER EXTENSION PROGRAM

**Topic:** 12. Bone marrow failure syndromes incl. PNH - Clinical

Antonio M. Risitano*[1, 2, 3], Camilla Frieri[4], Bor-Sheng Ko[5], Lily Wong Lee Lee[6], Alexander Röth[7], Flore Sicre de Fontbrune[2, 8], Eng-Soo Yap[9], Sung-Soo Yoon[10], Philippe Ferber[11], Aurelie Verles[11], Silvia Sanz[12], Luca Monaco[11], Darlington Mapiye[13], Régis Peffault de Latour[2, 8, 14]

*[1]AORN Moscati, Avellino, Italy; [2]The Severe Aplastic Anemia Working Party of the European Society for Blood and Marrow Transplantation, Barcelona, Spain; [3]University of Naples Federico II, Naples, Italy; [4]U.O.C Ematologia e Terapie cellulari avanzate, AORN San Giuseppe Moscati, Avellino, Italy; [5]National Taiwan University Cancer Center, Taipei, Taiwan; [6]Queen Elizabeth Hospital, Kota Kinabalu, Malaysia; [7]Department of Hematology and Stem Cell Transplantation, West German Cancer Center, University Hospital Essen, University Duisburg-Essen, Essen, Germany; [8]French Reference Center for Aplastic Anemia and Paroxysmal Nocturnal Hemoglobinuria, Saint-Louis Hospital, Paris, France; [9]University Hospital Singapore, Singapore, Singapore; [10]Seoul National University Hospital, Seoul, South Korea; [11]Novartis Pharma AG, Basel, Switzerland; [12]Novartis Farmacéutica, S.A., Barcelona, Spain; [13]IQVIA, Reading, United Kingdom; [14]Assistance Publique Hôpitaux de Paris, Université Paris Cité, Paris, France;*

### Background

Iptacopan has shown sustained hematologic control and a favorable safety profile in patients with paroxysmal nocturnal hemoglobinuria (PNH).

### Aims

To report long-term safety and efficacy outcomes with up to 6 years of follow-up from patients with PNH who received iptacopan 200 mg twice daily (BID) in two phase 2 studies (NCT03439839 and NCT03896152) and an ongoing roll-over extension program (PNH-REP; NCT04747613).

### Methods

Long-term data were reported for patients from the phase 2 studies and PNH-REP. Baseline was defined as the first day of receiving iptacopan 200 mg BID. Efficacy outcomes included mean hemoglobin (Hb) levels and Hb ≥12.0 g/dL, mean lactate dehydrogenase (LDH) levels and LDH <1.5×upper limit of normal (ULN), mean absolute reticulocyte count (ARC) and ARC normalization, transfusion independence, hematologic parameters, Functional Assessment of Chronic Illness Therapy (FACIT)-Fatigue, breakthrough hemolysis (BTH) rates, and prevention of major adverse vascular events (MAVEs). Safety assessments included frequency and number of events per 100 patient years (py) of treatment-emergent adverse events (TEAEs) and serious TEAEs.

**Copyright Information:** (Online) ISSN: 2572-9241
©2026 The Author(s). HemaSphere is published by John Wiley & Sons Ltd on behalf of European Hematology Association. This is an open access Abstract Book distributed under the Attribution-NonCommercial-NoDerivs (CC BY-NC-ND), which allows third parties to download the articles and share them with others as long as they credit the author and the Abstract Book, but they cannot change the content in any way or use them commercially.
**Abstract Book Citations:** Authors, Title, HemaSphere, 2026;10:(S1):pages. The abstract book DOIs can be found on the "Author Information" page.
**Disclaimer:** Articles published in the journal HemaSphere exclusively reflect the opinions of the authors. The authors are responsible for all content in their abstracts including accuracy of the facts, statements, citing resources, etc.

### Results

Of the 26 patients treated with iptacopan 200 mg BID in the phase 2 studies, 22 entered the PNH-REP; at data cut-off, 21/26 (80.8%) patients were ongoing, 1 (3.8%) had completed the study, and 4 (15.4%) had discontinued. Sixteen of 26 (61.5%) patients had ≥5 years and 8/26 (30.8%) had ≥6 years of exposure; median duration of exposure was 66.1 months. Hematologic response was sustained from 6 months to 6 years (Figure). At 5 and 6 years, Hb ≥12.0 g/dL irrespective of red blood cell (RBC) transfusion was achieved by 7/12 (58.3%) and 6/7 patients (85.7%) with available data, respectively, and LDH <1.5×ULN was achieved by 10/12 (83.3) and 6/7 patients (85.7%), respectively. ARC normalization at 5 and 6 years was achieved by 12/12 (100%) and 6/7 patients (85.7%), respectively, with mean (SD) being 73.5 (16.8) and $81.4 \times 10^9$/L (36.1). Most patients (23/26 [88.5%] at 5 and 6 years) remained RBC transfusion-free. Bilirubin and haptoglobin levels, platelet and neutrophil counts, and FACIT-Fatigue scores observed at 6 months were sustained to 6 years. Overall, 6 BTH events (none serious) occurred in 5/26 patients (19.2%; 4.7/100 py): 2 mild, 3 moderate, and 1 severe event (deemed study drug-related by investigators). A MAVE (unstable angina) was reported in 1/26 patients (3.8%; 0.8/100 py) who had pre-existing hypertension, left ventricular hypertrophy, and nephropathy.
In total, 23/26 patients (88.5%) reported ≥1 TEAE, most frequently COVID-19 and pyrexia (30.8% each). Serious TEAEs occurred in 11/26 patients (42.3%; 13.2/100 py), with serious infections in 3/26 patients (11.5%; 3.9/100 py). Three of 26 (11.5%) patients died during phase 2 studies; no events leading to deaths were deemed study drug-related.

### Summary/Conclusion

Over a 6-year follow-up, iptacopan treatment was associated with sustained hematologic control, including maintenance of Hb levels and transfusion independence. In most of the patients, LDH <1.5×ULN and ARC normalization were achieved at 6 months through to 6 years, showing control of intravascular hemolysis and absence of treatment-emergent extravascular hemolysis. Low rates of BTH and MAVEs were observed. A consistent safety profile was maintained, with no new safety findings compared with previous reports. These findings further characterize the long-term effects of iptacopan treatment in patients with PNH.

**Copyright Information:** (Online) ISSN: 2572-9241
©2026 The Author(s). HemaSphere is published by John Wiley & Sons Ltd on behalf of European Hematology Association. This is an open access Abstract Book distributed under the Attribution-NonCommercial-NoDerivs (CC BY-NC-ND), which allows third parties to download the articles and share them with others as long as they credit the author and the Abstract Book, but they cannot change the content in any way or use them commercially.
**Abstract Book Citations:** Authors, Title, HemaSphere, 2026;10:(S1):pages. The abstract book DOIs can be found on the “Author Information” page.
**Disclaimer:** Articles published in the journal HemaSphere exclusively reflect the opinions of the authors. The authors are responsible for all content in their abstracts including accuracy of the facts, statements, citing resources, etc.

**Figure.** Long-Term Consistency of Key Hematologic Responses With up to 6 Years of Iptacopan Treatment

| | **6 Months n=26** | **5 Years n=16** | **6 Years n=8** | **Last available N=26** |
|---|---|---|---|---|
| Hb ≥12.0 g/dL, n/N (%)* | 14/20 (70.0) | 7/12 (58.3) | 6 /7 (85.7) | 17/26 (65.4) |
| Hb (g/dL), mean (SD) | 12.8 (1.9) | 12.4 (1.6) | 13.0 (1.5) | 12.4 (1.7) |
| LDH <1.5xULN, n/N (%) | 18/19 (94.7) | 10/12 (83.3) | 6/7 (85.7) | 23/26 (88.5) |
| LDH (U/L), mean (SD) | 246.1 (59.9) | 272.8 (81.0) | 309.1 (70.6) | 274.9 (87.3) |
| ARC ($\times 10^9$/L), mean (SD) | 94.8 (39.5) | 73.5 (16.8) | 81.4 (36.1) | 91.4 (92.2) |
| No RBC transfusions, n/N (%) | 26/26 (100) | 23/26 (88.5)† | 23/26 (88.5)‡ | 23/26 (88.5) |
| Bilirubin (μmol/L), mean (SD), | 13.1 (8.1) | 11.2 (5.8) | 13.4 (8.5) | 14.6 (7.8) |
| Haptoglobin (g/L), mean (SD) | 0.2 (0.3) | 0.5 (0.6) | 0.3 (0.4) | 0.3 (0.3) |
| Platelets ($x10^9$/L), mean (SD) | 113.4 (55.2) | 133.4 (59.4) | 123.1 (61.7) | 137.2 (53.7) |
| Neutrophils ($x10^9$/L), mean (SD) | 2.5 (1.8) | 3.1 (1.4) | 2.3 (0.6) | 2.8 (1.2) |
| FACIT-Fatigue score, mean (SD) | 44.3 (8.6) | 45.4 (5.3) | 45.3 (7.6) | 44.3 (10.1) |

*Irrespective of RBC transfusion; †During Years 1–5; ‡During Years 1–6.
ARC, absolute reticulocyte count; FACIT, Functional Assessment of Chronic Illness Therapy; Hb, hemoglobin; LDH, lactate dehydrogenase; NR, not reported; RBC, red blood cell; SD, standard deviation; ULN, upper limit of normal.

Copyright Information: (Online) ISSN: 2572-9241
©2026 The Author(s). HemaSphere is published by John Wiley & Sons Ltd on behalf of European Hematology Association. This is an open access Abstract Book distributed under the Attribution-NonCommercial-NoDerivs (CC BY-NC-ND), which allows third parties to download the articles and share them with others as long as they credit the author and the Abstract Book, but they cannot change the content in any way or use them commercially.
Abstract Book Citations: Authors, Title, HemaSphere, 2026;10:(S1):pages. The abstract book DOIs can be found on the "Author Information" page.
Disclaimer: Articles published in the journal HemaSphere exclusively reflect the opinions of the authors. The authors are responsible for all content in their abstracts including accuracy of the facts, statements, citing resources, etc.

### (PS1798) ANDROGEN TREATMENT FOR TELOMERE BIOLOGY DISORDERS: LONG-TERM RESULTS FROM A RETROSPECTIVE REGISTRY ANALYSIS

**Topic:** 12. Bone marrow failure syndromes incl. PNH - Clinical

Nergis Güzel[1, 2], Mareike Tometten[2, 3], Jana Freudenberg[1, 2], Kim Kricheldorf[2, 3], Lucia Vankann[2, 3], Martin Kirschner[2, 3], Jens Panse[2, 3], Marcelo A. Szymanski de Toledo[2, 3], Marcin Wlodarski[4], Miriam Erlacher[5], Florian Kraft[1, 2], Miriam Elbracht[1, 2], Ingo Kurth[1, 2], Tim Henrik Brümmendorf[2, 3], Robert Meyer[1, 2], Fabian Beier*[2, 3]

*[1]Center for Human Genetics and Genomic Medicine, Faculty of Medicine, RWTH Aachen University, Aachen, Germany;[2]Center for Integrated Oncology Aachen Bonn Cologne Düsseldorf (CIO ABCD), Aachen, Germany; [3]Department of Hematology, Oncology, Hemostaseology and Stem Cell Transplantation, Medical Faculty, RWTH Aachen University, Aachen, Germany; [4]Department of Hematology, St. Jude Children´s Research Hospital, Memphis, United States of America;[5]Department of Pediatrics and Adolescent Medicine, University Medical Center Ulm, Ulm, Germany;*

### Background

Telomere biology disorders (TBD) comprise a spectrum of rare, inherited conditions caused by pathogenic variants in genes essential for telomere maintenance. TBDs often manifest as bone marrow failure (BMF), but may also present with multisystem involvement, including pulmonary fibrosis and hepatic cirrhosis. Treatment options for BMF in TBD include androgens or hematopoietic stem cell transplantation (HSCT). Androgen therapy has been shown to improve hematopoiesis. However, comprehensive long-term outcome data remain limited.

### Aims

Here, we report on the clinical course, hematologic response, telomere dynamics, and hematopoietic clonal evolution in a cohort of 19 TBD patients receiving androgen therapy for BMF.

### Methods

TBD diagnosis was established based on the presence of a pathogenic or likely pathogenic germline variant, lymphocyte telomere length (TL) below the 1st percentile and compatible clinical features such as BMF, pulmonary or hepatic fibrosis. Nineteen patients with a confirmed diagnosis of TBD and a history of androgen therapy were identified from the German Aplastic Anemia and Bone Marrow Failure Registry. All patients received danazol (n=18) or oxymetholone (n=1) for treatment of BMF. Serial telomere length was assessed by flow-FISH and Telo-Seq, a nanopore based long-read sequencing technology. Clonal hematopoiesis (CH, VAF > 4 %) was assessed using whole exome sequencing focusing on known recurrently mutated genes in myeloid malignancies.

**Copyright Information:** (Online) ISSN: 2572-9241
©2026 The Author(s). HemaSphere is published by John Wiley & Sons Ltd on behalf of European Hematology Association. This is an open access Abstract Book distributed under the Attribution-NonCommercial-NoDerivs (CC BY-NC-ND), which allows third parties to download the articles and share them with others as long as they credit the author and the Abstract Book, but they cannot change the content in any way or use them commercially.
**Abstract Book Citations:** Authors, Title, HemaSphere, 2026;10:(S1):pages. The abstract book DOIs can be found on the "Author Information" page.
**Disclaimer:** Articles published in the journal HemaSphere exclusively reflect the opinions of the authors. The authors are responsible for all content in their abstracts including accuracy of the facts, statements, citing resources, etc.

**Results**

The median age at initiation of androgen therapy was 37.3 years (range, 9–72 years); 12 patients were male and 7 were female. Disease-causing variants were identified in TERC (n = 8), TERT (n = 9), RTEL1 (n = 2), and NAF1 (n = 1), with one patient carrying two pathogenic variants. Pulmonary fibrosis was present in 8 patients (42%) and liver fibrosis in 10 patients (53%). Three patients had a history of solid malignancy or lymphoma prior to androgen initiation.
Danazol was administered at daily doses ranging from 200 to 600 mg. A hematologic response, defined as achieving transfusion independence or improvement in at least one hematopoietic lineage, was observed in 79% (15/19) of patients. The median duration of therapy was 1.2 years (range, 0.1–10.1 years). Treatment discontinuation due to adverse events occurred in 4 patients due to acne, virilization, reduced libido, and sinus vein thrombosis. Median event-free survival (events defined as HSCT, solid organ transplantation, or death) was 6.8 years after androgen initiation. During follow-up, three patients underwent HSCT. Eight patients in our cohort died during follow-up, with a median overall survival after androgen initiation of 7.1 years.
Serial telomere length measurements were available in 12 patients. Five of twelve exhibited relevant telomere elongation (>0.5 kb) in granulocytes and lymphocytes during treatment. In two cases, telomere elongation was confirmed using Telo-Seq at the single-telomere level.
Cytogenetics were normal in all patients, and CH was present at baseline in three patients, indicated by mutations in PPM1D, ASXL1, and U2AF1. Longitudinal analysis in nine patients with a median follow-up of 1.8 years (range, 0.3–8 years) revealed no relevant clonal expansion, acquisition of new driver mutations, or new cytogenetic aberrations during treatment.

**Summary/Conclusion**

Androgen therapy is associated with hematologic improvement in TBD patients and transient telomere elongation. Our data support the use of androgens as a safe therapeutic strategy in TBD.

**Copyright Information:** (Online) ISSN: 2572-9241
©2026 The Author(s). HemaSphere is published by John Wiley & Sons Ltd on behalf of European Hematology Association. This is an open access Abstract Book distributed under the Attribution-NonCommercial-NoDerivs (CC BY-NC-ND), which allows third parties to download the articles and share them with others as long as they credit the author and the Abstract Book, but they cannot change the content in any way or use them commercially.
**Abstract Book Citations:** Authors, Title, HemaSphere, 2026;10:(S1):pages. The abstract book DOIs can be found on the "Author Information" page.
**Disclaimer:** Articles published in the journal HemaSphere exclusively reflect the opinions of the authors. The authors are responsible for all content in their abstracts including accuracy of the facts, statements, citing resources, etc.

### (PS1799) COMPARISON OF EFFICACY BETWEEN INTENSIFIED IMMUNOSUPPRESSION COMBINED WITH UMBILICAL CORD BLOOD THERAPY AND ALLOGENEIC HEMATOPOIETIC STEM CELL TRANSPLANTATION IN PATIENTS WITH SAA AGED OVER 40 YEARS

**Topic:** 12. Bone marrow failure syndromes incl. PNH - Clinical

Jijia Li[1], Xiaolin Yu[2], Xiaochen Song[2], Qianqian Xiao[2], Lei Deng[2], Jing Wang[2], Fang Zhou*[2]

*[1]The 960th Hospital of The People's Liberation Army (PLA) Joint Logistics Support Force; Shandong Second Medical University, Jinan, China; [2]The 960th Hospital of The People's Liberation Army (PLA) Joint Logistics Support Force, Jinan, China;*

### Background

Immunosuppressive therapy(IST) and allogeneic hematopoietic stem cell transplantation(allo-HSCT) represent the two main effective treatment modalities for patients with severe aplastic anemia(SAA). Age is one of the strongest predictors of survival. In the past, patients with SAA over the age of 40 often deferred allo-HSCT until after the failure of at least one course of IST. IST combined with umbilical cord blood infusion(IIST-UCB), as an alternative approach, remains an effective treatment option for SAA patients aged over 40. IIST-UCB is increasingly being adopted as an alternative treatment for adult patients lacking a matched related donor, owing to its lower incidence of graft-versus-host disease(GVHD) and less stringent HLA matching requirements. In contrast, although allo-HSCT offers a shorter time to hematopoietic engraftment, it requires stricter HLA matching and is associated with a higher incidence of GVHD. This article aims to provide a basis for treatment selection in elderly patients by retrospectively analyzing the efficacy of these two therapeutic strategies in SAA patients aged 40 years and older.

### Aims

Comparison of the efficacy of allo-HSCT and IIST-UCB in the treatment of SAA in patients aged 40 years and older.

### Methods

A retrospective analysis was conducted on the clinical data and prognosis of 34 patients aged 40 years and older with SAA who received different treatment regimens between 2004 and 2024. Among them, 21 patients received allo-HSCT and 13 patients received IIST-UCB. The primary study objective was to compare the overall survival(OS) of SAA patients aged 40 years and older treated with different therapeutic regimens. The secondary study objectives included clinical outcomes such as the incidence of infection, time to engraftment, and GVHD-free, failure-free survival(GFFS).

### Results

The study results demonstrated that patients in the allo-HSCT group achieved significantly faster platelet engraftment compared to those in the IIST-UCB group ($P$=0.002). The incidence of GVHD in the allo-HSCT group was 42.9%. IIST-UCB group was not associated with complications such as GVHD. Compared with allo-HSCT, IIST-UCB had an equivalent incidence of cytomegalovirus(28.6% vs. 30.8%, $P$>0.05), incidence of epstein-Barr virus viremia(28.6% vs. 30.8%, $P$>0.05), hemorrhagic cystitis(19.0% vs. 15.4%, $P$>0.05) and pulmonary infection(61.9% vs. 92.3%, $P$=0.107). No statistically significant differences were observed between the two groups in terms of time to neutrophil engraftment ($P$=0.1). The median follow-up duration was 9 months (range, 0-149 months) for the allo-HSCT group and 8 months (range, 0-91 months) for the IIST-UCB group. The estimated 3-year OS was 80.1% in the allo-HSCT group compared to 84.6% in the IIST-UCB group, with no statistically significant difference between the groups. The 3-year GFFS was 41.6% in the allo-HSCT group and 26.9% in the IIST-UCB group, also showing no significant difference ($P$=0.955).

**Copyright Information:** (Online) ISSN: 2572-9241
©2026 The Author(s). HemaSphere is published by John Wiley & Sons Ltd on behalf of European Hematology Association. This is an open access Abstract Book distributed under the Attribution-NonCommercial-NoDerivs (CC BY-NC-ND), which allows third parties to download the articles and share them with others as long as they credit the author and the Abstract Book, but they cannot change the content in any way or use them commercially.
**Abstract Book Citations:** Authors, Title, HemaSphere, 2026;10:(S1):pages. The abstract book DOIs can be found on the “Author Information” page.
**Disclaimer:** Articles published in the journal HemaSphere exclusively reflect the opinions of the authors. The authors are responsible for all content in their abstracts including accuracy of the facts, statements, citing resources, etc.

**Summary/Conclusion**

Our study found that for SAA patients aged ≥40 years, the 3-year OS was comparable between the allo-HSCT group and the IIST-UCB group. allo-HSCT was associated with faster platelet engraftment, whereas the IIST-UCB group was not associated with complications such as GVHD. Therefore, IIST-UCB represents an effective alternative treatment option for SAA patients aged ≥40 years who have failed for IST and lack an HLA-matched donor for allo-HSCT.

**Copyright Information:** (Online) ISSN: 2572-9241
©2026 The Author(s). HemaSphere is published by John Wiley & Sons Ltd on behalf of European Hematology Association. This is an open access Abstract Book distributed under the Attribution-NonCommercial-NoDerivs (CC BY-NC-ND), which allows third parties to download the articles and share them with others as long as they credit the author and the Abstract Book, but they cannot change the content in any way or use them commercially.
**Abstract Book Citations:** Authors, Title, HemaSphere, 2026;10:(S1):pages. The abstract book DOIs can be found on the "Author Information" page.
**Disclaimer:** Articles published in the journal HemaSphere exclusively reflect the opinions of the authors. The authors are responsible for all content in their abstracts including accuracy of the facts, statements, citing resources, etc.

### (PS1800) IRON OVERLOAD IN PAROXYSMAL NOCTURNAL HEMOGLOBINURIA (PNH) PATIENTS ON C5 - INHIBITORS MAY RESULT FROM ABNORMAL IRON REGULATION IN ADDITION TO INCREASED EXTRAVASCULAR HEMOLYSIS.

**Topic:** 12. Bone marrow failure syndromes incl. PNH - Clinical

Mira Jani*[1], Alexandra Misura[1], Yuxuan (Tina) Luo[1], Mehak Mahajan[1], Noor Kundu[1], Thibika Gunalingam[1], Winston Ukpong[1], Katarina Czibere[1], Tina Khazaee[2], David Spaner[3], Graeme Quest[4], Hubert Tsui[2], Signy Chow[5]

*[1]Sunnybrook Research Institute, University of Toronto, Toronto, Canada;[2]Sunnybrook Health Sciences Centre, University of Toronto, Laboratory Medicine and Molecular Diagnostics, Toronto, Canada;[3]Odette Cancer Centre and Sunnybrook Health Sciences Centre, University of Toronto, Toronto, Canada;[4]Queens University, Kingston Health Sciences Centre, Kingston, Canada;[5]Odette Cancer Centre and Sunnybrook Health Sciences Centre, University of Toronto, Medical Oncology and Hematology, Toronto, Canada;*

### Background

Paroxysmal Nocturnal Hemoglobinuria (PNH) is a clonal hematologic disorder caused by a loss-of-function mutation in the PIG-A gene, leading to an absence of GPI anchored complement-regulatory proteins, rendering red blood cells (RBCs) susceptible to complement-mediated intravascular hemolysis (IVH). This results in a plethora of clinical manifestations, including fatigue, thrombosis, smooth muscle spasm, hemoglobuinuria and iron deficiency. C5 complement inhibition (C5i) is first-line therapy for PNH, substantially reducing IVH. Patients on C5i may subsequently develop RBC C3d opsonization and resultant extravascular hemolysis (EVH), which is associated with clinical iron overload.

### Aims

We aimed to: (1) to characterize the incidence and degree of iron overload in PNH patients receiving C5i, and (2) to explore abnormalities in iron metabolism in PNH.

### Methods

PNH patients participating in the Sunnybrook Hematology Biobank at Sunnybrook Health Sciences Centre (SHSC, Toronto, ON, Canada) were included in the study. Peripheral blood samples were available from these patients as well as patients consenting separately to a prospective observational study looking at iron overload. The study was approved by the local ethics board (REB #3359, #6176).

Clinical charts were reviewed for diagnostic, laboratory and treatment history. Liver iron concentrations (LICs) were measured by abdominal MRI for patients with ferritin >600 μg/L. Hepcidin was quantified using the Intrinsic Hepcidin IDx™ ELISA (Intrinsic LifeSciences, La Jolla, CA, USA) from biobanked samples. Hepcidin analyses were performed at multiple timepoints if available. Flow cytometry for C3d deposition was performed at Kingston Health Sciences Centre (Kingston, ON, Canada) or SHSC core clinical laboratory. MRIs were performed on a 1.5T magnet using a routine Siemens standard LiverLab Multi-echo Dixon VIBE sequence and analyzed on post-processing software. LICs were calculated in mg/g dry weight from R2*. Statistical analyses were performed with GraphPad Prism.

Copyright Information: (Online) ISSN: 2572-9241
©2026 The Author(s). HemaSphere is published by John Wiley & Sons Ltd on behalf of European Hematology Association. This is an open access Abstract Book distributed under the Attribution-NonCommercial-NoDerivs (CC BY-NC-ND), which allows third parties to download the articles and share them with others as long as they credit the author and the Abstract Book, but they cannot change the content in any way or use them commercially.
Abstract Book Citations: Authors, Title, HemaSphere, 2026;10:(S1):pages. The abstract book DOIs can be found on the "Author Information" page.
Disclaimer: Articles published in the journal HemaSphere exclusively reflect the opinions of the authors. The authors are responsible for all content in their abstracts including accuracy of the facts, statements, citing resources, etc.

### Results

24 patients were included in the study: 19 on active therapy, 3 with PNH clones >10% who did not meet criteria for treatment and were not on any anticomplement therapy and 2 patients with small clones (<10%) that were included as controls. 3/19 were on proximal complement inhibitors, including one who switched from C5i to C3i. 2/19 donated both pre-treatment and on-treatment samples. 4/19 patients had current or historical iron overload with a LIC > 6mg/g. For patients on C5i, those with iron overload had higher reticulocytes (293 vs. 139 x$10^9$ cells/L), ferritin levels (1507 vs. 401 ug/L) and higher rates of C3d loading (23.8% vs. 3.72%) measured by flow cytometry, consistent with increased EVH. Patients with high LIC had inappropriately low hepcidin levels relative to ferritin, with partial resolution after starting iron chelation (Figure 1).

### Summary/Conclusion

Iron overload in PNH in patients on C5i is strongly associated with ongoing hemolysis/reticulocytosis and specifically with EVH, as measured by the extent of C3d loading. Surprisingly, we found inappropriately low levels of hepcidin in patients with iron overload, measured on multiple occasions in these patients, suggesting that additional abnormalities in iron and hepcidin regulation may contribute to iron overload in PNH patients on C5i.

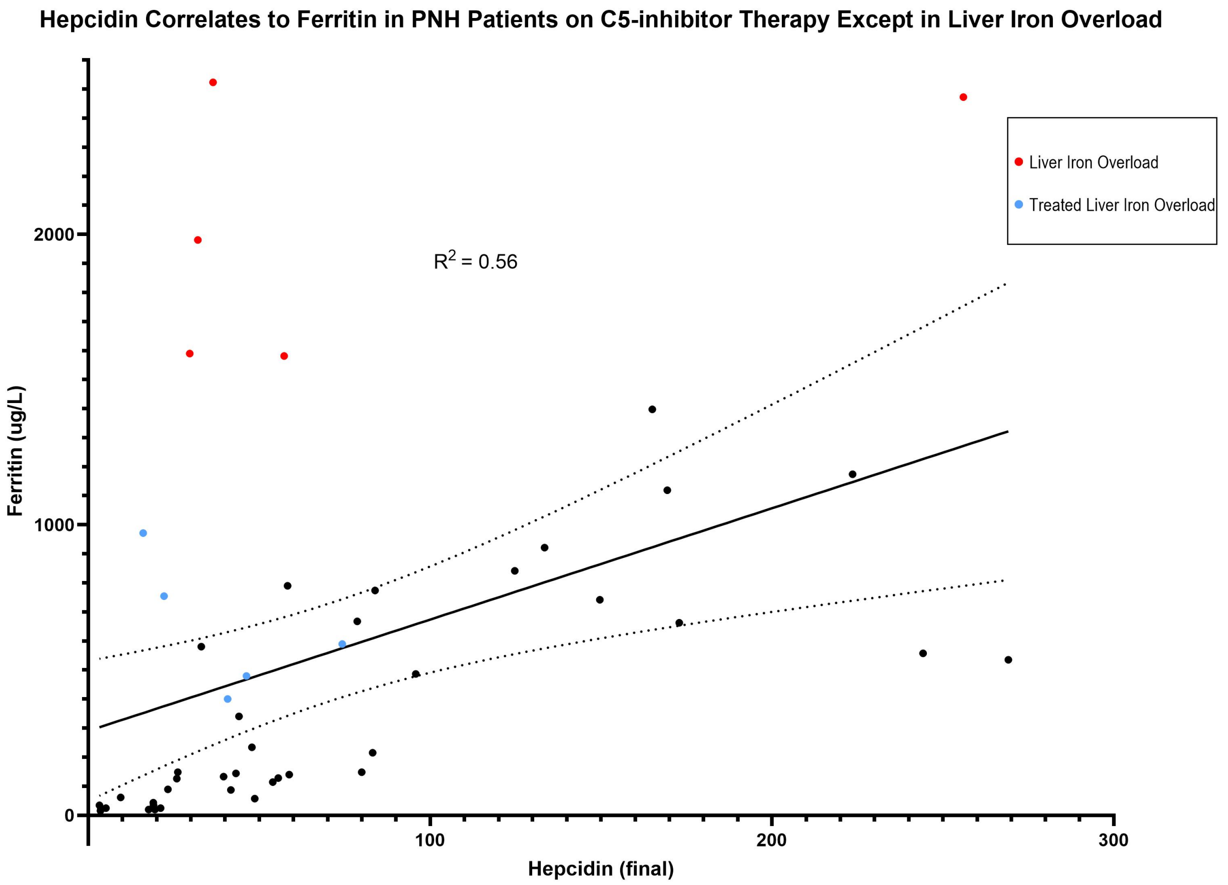

**Figure 1: Hepcidin Correlates to Ferritin in PNH Patients on C5-inhibitor Therapy Except in Liver Iron Overload.** Hepcidin correlates closely to ferritin as expected for iron homeostasis and regulation except in patient with liver iron overload. $R^2$ excludes patients with current or treated liver iron overload. Iron overload is defined as liver iron concentration> 6mg/g.

**Copyright Information:** (Online) ISSN: 2572-9241
©2026 The Author(s). HemaSphere is published by John Wiley & Sons Ltd on behalf of European Hematology Association. This is an open access Abstract Book distributed under the Attribution-NonCommercial-NoDerivs (CC BY-NC-ND), which allows third parties to download the articles and share them with others as long as they credit the author and the Abstract Book, but they cannot change the content in any way or use them commercially.
**Abstract Book Citations:** Authors, Title, HemaSphere, 2026;10:(S1):pages. The abstract book DOIs can be found on the "Author Information" page.
**Disclaimer:** Articles published in the journal HemaSphere exclusively reflect the opinions of the authors. The authors are responsible for all content in their abstracts including accuracy of the facts, statements, citing resources, etc.

### (PS1801) CLINICAL CHARACTERISTICS AND HEMATOPOIETIC STEM CELL TRANSPLANTATION PROGNOSIS IN SHWACHMAN-DIAMOND SYNDROME: A REPORT OF 116 CASES FROM A SINGLE CHINISE CENTER

**Topic:** 12. Bone marrow failure syndromes incl. PNH - Clinical

Jiao Chen*[1], Zhouyang Liu[1], Zhende Hou[1], Xiaoxia Li[1], Shifen Fan[1], Fan Jiang[1], Yuan Sun[1]

[1]*Beijing Jingdu Children's Hospital, Beijing, China;*

### Background

Shwachman-Diamond syndrome (SDS) is a rare hereditary bone marrow failure syndrome,characterized by pancreatic exocrine insufficiency,bone marrow dysfunction, bone malformation. Allogeneic hematopoietic stem cell transplantation (allo-HSCT) is required when the patienets complicated with severe hemocytopenia,myelodysplastic syndrome (MDS) or leukemia.

### Aims

To Summarize the clinical presentation, molecular genetics, and explore the safety and efficacy of HSCT in SDS.

### Methods

A total of 116 SDS patients who were managed at Beijing Jingdu Children's Hospital from July 2015 to February 2026 were enrolled.The clinical manifestations,gene analysis and details about HSCT were summarized.

### Results

There were 64 males and 52 females. Gene analysis revealed compound heterozygous SBDS mutations in 92 cases, homozygous SBDS mutations in 23 cases, and compound heterozygous EFL1 mutations in 1 case.Median age at onset and diagnosis was <1 month (<1-96 months) and 14 months (1 day–15 years).Chronic diarrhoea was present in 86.2%, cytopenia in 97.4%. One patient (0.9%) progressed to AML, and 11 (9.5%) progressed to MDS. Hepatic dysfunction occurred in 86.2%. Other manifestations included neurological abnormalities (50%), dental abnormalities (41.4%), skeletal abnormalities (37.9%), cardiac abnormalities (36.0%), and eczema with allergies (50%). 28 patients underwent allo-HSCT at Beijing Jingdu Children's Hospital . The median age at transplant was 26.5 months (range, 7–192 months), median weight was 9.6 kg (range, 5.0–38.2 kg), and median height was 80 cm (range, 60–160cm). All patients had neutropenia,1 patient was diagnosed with AML, 5 with MDS, 2 had complex karyotypes, and 5 harbored TP53 mutations. The donors consisted of 16 unrelated cord blood(UCB),7 mismatched related donors(MMRD) and 6 unrelated donors(UD).All patients used myeloablating conditioning.GVHD prophylaxis consisted of cyclosporine and mycophenolate mofetil with balliximab or methotrexate. Successful engraftment was achieved in 27 cases (96.3%) after the primary transplant. 1 patient with failed cord blood engraftment subsequently received haploid HSCT with successful engraftment.The median time for neutrophil engraftment was 14(range,10-22) days in UCB group and 11 (range,9-12) days in UD and MMRD group.The median time for platelet engraftment was 20(range, 15-66) days in UCB group and 14 (range, 11-17) days in UD and MMRD group.There were 0/15, 3/7, 2/6 cases with acuteGVHD(grades II to IV) and 2/15, 2/7, 1/6 mild chronic GVHD in UCB,MMRD and UD groups.There was no middle and severe chronic GVHD.Within the follow-up of 16.5(ranging from 0.5 to 126) months after HSCT, 2 patients died.One died of multiple organ failure due to sepsis and another died of respiratory failure due to pneumonia.A patient diagnosed as MDS-LB accompanied with complex karyotype and Tp53 mutation relapsed after UCBT,then he underwent haploid HSCT, He relapsed repeatedly post the second transplant and developed AML, he is currently under palliative treatment.The overall survival and event-free survival rate was 92.9% and 89.3%.

**Copyright Information:** (Online) ISSN: 2572-9241
©2026 The Author(s). HemaSphere is published by John Wiley & Sons Ltd on behalf of European Hematology Association. This is an open access Abstract Book distributed under the Attribution-NonCommercial-NoDerivs (CC BY-NC-ND), which allows third parties to download the articles and share them with others as long as they credit the author and the Abstract Book, but they cannot change the content in any way or use them commercially.
**Abstract Book Citations:** Authors, Title, HemaSphere, 2026;10:(S1):pages. The abstract book DOIs can be found on the "Author Information" page.
**Disclaimer:** Articles published in the journal HemaSphere exclusively reflect the opinions of the authors. The authors are responsible for all content in their abstracts including accuracy of the facts, statements, citing resources, etc.

**Summary/Conclusion**

The manifestations mainly include cytopenia, diarrhoea and hepatic dysfunction. When SDS is complicated by severe cytopenia, MDS or AML, allo-HSCT is a safe and effective treatment. Compared with MMRD and UD, UCB transplantation is associated with a lower incidence of GVHD. Patients with recurrent karyotypic abnormalities combined with TP53 mutations have a extremely poor prognosis.

**Copyright Information:** (Online) ISSN: 2572-9241
©2026 The Author(s). HemaSphere is published by John Wiley & Sons Ltd on behalf of European Hematology Association. This is an open access Abstract Book distributed under the Attribution-NonCommercial-NoDerivs (CC BY-NC-ND), which allows third parties to download the articles and share them with others as long as they credit the author and the Abstract Book, but they cannot change the content in any way or use them commercially.
**Abstract Book Citations:** Authors, Title, HemaSphere, 2026;10:(S1):pages. The abstract book DOIs can be found on the "Author Information" page.
**Disclaimer:** Articles published in the journal HemaSphere exclusively reflect the opinions of the authors. The authors are responsible for all content in their abstracts including accuracy of the facts, statements, citing resources, etc.

### (PS1802) THROMBOEMBOLISM IN PATIENTS WITH PAROXYSMAL NOCTURNAL HEMOGLOBINURIA IN THE ERA OF COMPLEMENT INHIBITORS – MULTICENTER ANALYSIS BY THE POLISH ADULT LEUKEMIA GROUP

**Topic:** 12. Bone marrow failure syndromes incl. PNH - Clinical

Joanna Drozd-Sokolowska[1], Patrycja Mensah-Glanowska[2], Katarzyna Budziszewska[3], Justyna Kozińska[4], Michal Kurlapski[5], Anna Szmigielska-Kapłon[6], Joana Rupa Matysek[7], Łukasz Bołkun[8], Łukasz Szukalski[9], Anna Kopinska[10], Bogusław MacHaliński[11], Adrianna Wlodyka-Pekosz[12], Martyna Krzyzewska[13], Aleksandra Butrym[14], Justyna Rybka[15], Magdalena Partyka[16], Elzbieta Sienkiewicz[17], Piotr Kacprzyk[1], Paweł Bernatowicz[8], Krzysztof Madry[1], Jadwiga Dwilewicz-Trojaczek[1], Marek Hus[4], Jarosław Czyż[9], Grzegorz Helbig[10], Mirosław Markiewicz[12], Jan MacIej Zaucha[5], Grzegorz Basak[1], Jerzy Windyga[3], Agnieszka Piekarska*[5]

*[1]Medical University of Warsaw, Warsaw, Poland;[2]Jagiellonian University Collegium Medicum, University Hospital in Cracow, Kraków, Poland; [3]Institute of Hematology and Transfusion Medicine, Warsaw, Poland;[4]Medical University of Lublin, Lublin, Poland; [5]Medical University of Gdansk, Gdansk, Poland; [6]Medical University of Lodz, Lodz, Poland;[7]Poznan University of Medical Sciences, Poznan, Poland; [8]Medical University of Bialystok, Bialystok, Poland;[9]Collegium Medicum in Bydgoszcz, Nicolaus Copernicus University in Toruń, Bydgoszcz, Poland; [10]Medical University of Silesia, Katowice, Poland; [11]Pomeranian Medical University, Szczecin, Poland; [12]Faculty of Medicine, Collegium Medicum, University of Rzeszów, Rzeszow, Poland; [13]Department of Hematooncology in Brzozow, Brzozow, Poland; [14]Wroclaw Medical University, Branch in Walbrzych, Walbrzych, Poland; [15]Wroclaw Medical University, Wroclaw, Poland; [16]Department of Hematology, Biala Podlaska, Biala Podlaska, Poland; [17]Warsaw University of Technology, Department of Mathematics and Information Science, Warsaw, Poland;*

### Background

Both arterial and venous thromboses are recognized clinical manifestations of paroxysmal nocturnal hemoglobinuria (PNH). Prior to the advent of complement inhibition therapy, thrombotic events accounted for 40–67% of PNH-related mortality. Although the incidence of thrombosis has markedly declined in the era of complement inhibitors (CIs), epidemiological data remain limited.

### Aims

To determine the incidence of thromboembolic events (TE) in patients with PNH exposed to CIs
.

### Methods

A retrospective analysis of patients with PNH who received at least one dose of any CI. Patients were evaluated for the occurrence of TEs both before and during CI therapy, as well as anticoagulation used.

**Copyright Information:** (Online) ISSN: 2572-9241
©2026 The Author(s). HemaSphere is published by John Wiley & Sons Ltd on behalf of European Hematology Association. This is an open access Abstract Book distributed under the Attribution-NonCommercial-NoDerivs (CC BY-NC-ND), which allows third parties to download the articles and share them with others as long as they credit the author and the Abstract Book, but they cannot change the content in any way or use them commercially.
**Abstract Book Citations:** Authors, Title, HemaSphere, 2026;10:(S1):pages. The abstract book DOIs can be found on the "Author Information" page.
**Disclaimer:** Articles published in the journal HemaSphere exclusively reflect the opinions of the authors. The authors are responsible for all content in their abstracts including accuracy of the facts, statements, citing resources, etc.

**Results**

Among 137 patients included in the study, 69 (50.4%) were male, the median age at PNH diagnosis was 39 years (range, 15-84). Seventy-one (52%) patients did have anamnesis of either previous or concomitant bone marrow failure, most frequently aplastic anemia (46/71; 64.8%). The median age at first CI initiation was 40 years (15-84). All patients received at least one dose of CI, with the first anti-complement treatment being eculizumab in 85 (62%) or ravulizumab in 42 (30.7%) patients. In total, 72 patients underwent at least one treatment modification, consisting of either a switch to another inhibitor (including proximal inhibitors) or the addition of danicopan.
Forty-two patients (34.3%) received antithrombotic treatment of any duration because of PNH [low-molecular-weight heparin (LMWH) in 20 cases, direct oral anticoagulants (DOAC) in 10 cases, vitamin K antagonist (VKA) in 6 cases]. Thirteen additional patients received antithrombotic prophylaxis due to causes unrelated to PNH (LMWH 6, DOAC 6, VKA 1).
Twenty-five (18.2%) patients did have a positive history of TE before PNH diagnosis, with 19 cases being venous, 5 arterial, and 1 mixed (both venous and arterial) thrombotic episodes. The TEs were considered associated with PNH in 23 cases. There were 12 cases of intraabdominal TEs, including 5 cases of Budd-Chiari syndrome (BCS). An additional seven TEs were diagnosed in five patients with an established diagnosis of PNH. The first episode in those patients consisted of: BCS (n = 1), deep vein thrombosis of the lower extremity (n = 1), intramuscular vein thrombosis of the lower extremity (n = 1), cerebral venous sinus thrombosis (CVST; n = 1), and coronary artery thrombosis (n = 1). Only 4 out of those 7 episodes were diagnosed in patients on CIs (all on eculizumab), translating into the incidence of TEs of 0.8 events per 100 patient-years while on CIs. One TE episode (CVST ), diagnosed concomitantly with PNH contributed to the death of the affected patient, as judged by the investigator. For the 7 episodes diagnosed after the diagnosis of PNH the treatment comprised LMWH in 6 cases, DOAC in 2.

**Summary/Conclusion**

The incidence of TE in PNH patients is substantial, with nearly 20% of them experiencing a thrombotic event (frequently intraabdominal), which may finally lead to the diagnosis of PNH. CIs significantly reduce the frequency of TEs, however, even in the era of complement inhibition, TE attributable to PNH remains a serious complication and may still result in patients’ death.

**Copyright Information:** (Online) ISSN: 2572-9241
©2026 The Author(s). HemaSphere is published by John Wiley & Sons Ltd on behalf of European Hematology Association. This is an open access Abstract Book distributed under the Attribution-NonCommercial-NoDerivs (CC BY-NC-ND), which allows third parties to download the articles and share them with others as long as they credit the author and the Abstract Book, but they cannot change the content in any way or use them commercially.
**Abstract Book Citations:** Authors, Title, HemaSphere, 2026;10:(S1):pages. The abstract book DOIs can be found on the “Author Information” page.
**Disclaimer:** Articles published in the journal HemaSphere exclusively reflect the opinions of the authors. The authors are responsible for all content in their abstracts including accuracy of the facts, statements, citing resources, etc.

### (PS1803) MANAGEMENT OF CARDIOVASCULAR RISK IN PATIENTS WITH PAROXYSMAL NOCTURNAL HEMOGLOBINURIA TREATED WITH IPTACOPAN

**Topic:** 12. Bone marrow failure syndromes incl. PNH - Clinical

Isla Storey*[1], Louise Arnold[1], Pooja Mahapatra[1], Briony Robinson[1], Matthew Holt[1], Nora Youngs[1], Sateesh Nagumantry[2], Katharine Lowndes[3], Lindsay Mitchell[4], Malinthi Adikarama[1], Jivendra Gosai[5], Alexandra Pike[1], Talha Munir[1], Petra Muus[1], Morag Griffin[1], Richard J. Kelly[1]

*[1]Leeds Teaching Hospitals NHS Trust, Leeds, United Kingdom;[2]Peterborough District General Hospital, Peterborough, United Kingdom; [3]Royal Hampshire County Hospital, Winchester, United Kingdom;[4]Univeristy Hospital Monklands, Alrdire, United Kingdom; [5]Bradford Teaching Hospitals, Bradford, United Kingdom;*

### Background

Paroxysmal Nocturnal Hemoglobinuria (PNH) is a rare blood disorder characterized by intravascular hemolysis and an increased risk of thrombosis. Iptacopan (ipta) is an oral complement factor B inhibitor shown to be an effective therapy for adult patients with PNH. Clinical trial data in patients with PNH treated with ipta indicated it can cause an increase in total and LDL-cholesterol. QRISK is a calculator that estimates the 10-year risk of developing cardiovascular (CV) disease. It includes age, ethnicity, smoking, diabetes, blood pressure, cholesterol, body mass index, family history of heart disease, some medications, medical conditions and socioeconomic factors. A score of 10-20% is deemed moderate risk and greater than 20% indicates a high CV risk. Guidelines recommend offering lipid modification at a score of greater than or equal to 10%.

### Aims

To evaluate changes in total and LDL-cholesterol and QRISK score on ipta to identify individuals at higher risk.

### Methods

All patients receiving ipta at the Leeds National PNH center were evaluated at initiation of ipta and at 3, 6, 12, 24 and 36 month time points. At these time points a QRISK score was calculated and blood tests including total and LDL-cholesterol were performed.

**Copyright Information:** (Online) ISSN: 2572-9241
©2026 The Author(s). HemaSphere is published by John Wiley & Sons Ltd on behalf of European Hematology Association. This is an open access Abstract Book distributed under the Attribution-NonCommercial-NoDerivs (CC BY-NC-ND), which allows third parties to download the articles and share them with others as long as they credit the author and the Abstract Book, but they cannot change the content in any way or use them commercially.
**Abstract Book Citations:** Authors, Title, HemaSphere, 2026;10:(S1):pages. The abstract book DOIs can be found on the "Author Information" page.
**Disclaimer:** Articles published in the journal HemaSphere exclusively reflect the opinions of the authors. The authors are responsible for all content in their abstracts including accuracy of the facts, statements, citing resources, etc.

**Results**

This was a retrospective analysis of 85 patients (40 male and 45 female) receiving ipta between March 2022 and January 2026. Four received ipta as the initial therapy and 81 after changing from an alternative anti-complement drug. The mean duration of ipta therapy was 11.4 months (range 1-42 months) with 4 patients discontinuing treatment (1 non-compliant, 1 death, 1 deranged liver function, 1 uncontrolled lactate dehydrogenase).
At commencement of ipta, the mean QRISK score was found to be 9.87%. Twenty-nine patients (34%) had a QRISK score >10% with 11/29 having a moderate risk score of 10-20% and 18/29 having a high risk score of greater than 20%. The mean total cholesterol at ipta initiation was 4.31mmol/L (n=56) and LDL-cholesterol of 2.1 mmol/L (n=47).
At 3 months on ipta, the mean QRISK score was 9.9% (n=72). Thirty-one patients had a QRISK score >10% (43%) with 16/31 having a moderate risk score of 10-20% and 15/31 having a high risk score of greater than 20%. In 6 cases the QRISK score had increased from low to moderate risk and in 2 cases from moderate to high risk.
The mean total cholesterol increased by 0.9 mmol/L from baseline, n=44) and LDL-cholesterol increased by 0.7 mmol/L from baseline, n=41).
At 6 months on ipta, the mean QRISK score was 8.4% (n=60). Twenty-two patients had a QRISK score >10% (37%) with 12/22 having moderate risk and 10/22 having high risk. No patients had a further change in their QRISK category.
At 12 months on ipta, the mean QRISK score was 7.9% (n=33). Eleven patients had a QRISK score >10% (33%) with 5/11 having a moderate risk and 6/11 having a high risk. One patient had a QRISK increase from low to moderate risk.
At 12 months the mean total cholesterol increased by 0.8 mmol/L from baseline, n=25) and LDL-cholesterol increased by 0.6 mmol/L from baseline, n=23).
Only 7 patients had received ipta for over 24 months and 2 for over 36 months.

**Summary/Conclusion**

The QRISK category increased in 8 cases (11%) after 3 months on ipta and in a further case after 12 months on ipta. This score can be used to identify the small proportion of patients whose CV risk increases and should be considered for interventions to reduce this risk. Moderate increases in total and LDL-cholesterol were also observed.

**Copyright Information:** (Online) ISSN: 2572-9241
©2026 The Author(s). HemaSphere is published by John Wiley & Sons Ltd on behalf of European Hematology Association. This is an open access Abstract Book distributed under the Attribution-NonCommercial-NoDerivs (CC BY-NC-ND), which allows third parties to download the articles and share them with others as long as they credit the author and the Abstract Book, but they cannot change the content in any way or use them commercially.
**Abstract Book Citations:** Authors, Title, HemaSphere, 2026;10:(S1):pages. The abstract book DOIs can be found on the “Author Information” page.
**Disclaimer:** Articles published in the journal HemaSphere exclusively reflect the opinions of the authors. The authors are responsible for all content in their abstracts including accuracy of the facts, statements, citing resources, etc.

### (PS1804) CLINICAL HETEROGENEITY AND LONG-TERM OUTCOMES OF CHILDHOOD AUTOIMMUNE NEUTROPENIA: A 20-YEAR COHORT STUDY

**Topic:** 12. Bone marrow failure syndromes incl. PNH - Clinical

Ioanna Saougou[1], Chrysoula Kosmeri[1], Nikolaos Chaliasos[1], Irene Mavroudi[2, 3], Charalampos Pontikoglou[2, 3], Alexandros Makis*[1]

*[1]Department of Pediatrics, University Hospital of Ioannina, Faculty of Medicine, School of Health Sciences, University of Ioannina, Ioannina, Greece; [2]Department of Hematology, University Hospital of Heraklion, Heraklion, Greece;[3]Haemopoiesis Research Laboratory, School of Medicine, University of Crete, Heraklion, Greece;*

### Background

Autoimmune neutropenia (AIN) in childhood comprises heterogeneous clinical forms that extend beyond the classic primary AIN of infancy. However, comprehensive evaluation of phenotype-specific immune characteristics and infectious outcomes remains limited.

### Aims

To describe the clinical presentation, immunohematological features, and long-term outcomes of three distinct childhood AIN phenotypes within a 20-year single-centre cohort.

### Methods

Retrospective cohort study of 112 children with confirmed AIN (granulocyte immunofluorescence test and/or granulocyte agglutination test positivity) at the Pediatric Hematology Unit of the University Hospital of Ioannina, Northwestern Greece (2005–2025). Three mutually exclusive phenotypes were defined per Fioredda et al. consensus criteria: primary AIN (pAIN), long-lasting neutropenia (LL-Np), and late-onset neutropenia (LO-Np). Outcomes included immunoglobulin levels and lymphocyte subset counts assessed against age-adjusted reference intervals, infection-related hospitalisation incidence rates per 100 person-years (exact Poisson 95% CI), and time-to-resolution by Kaplan-Meier analysis.

### Results

The cohort consisted of 90 pAIN (80.4%), 10 LL-Np (8.9%), and 12 LO-Np (10.7%) patients. Significant demographic differences were observed, particularly age at diagnosis, which was youngest in pAIN and markedly older in LO-Np. Family history of autoimmunity was uncommon in pAIN but highly prevalent in LL-Np and LO-Np. Agranulocytosis (ANC $<0.2 \times 10^9$/L) was observed in 45.6% of pAIN and 60.0% of LL-Np but no LO-Np patient. LO-Np was uniquely characterized by leukopenia (median WBC $3.85 \times 10^9$/L), CD3+ lymphopenia (33.3%), CD4+ lymphopenia (33.3%), CD19+ lymphopenia (50.0%), ANA positivity (50.0%), and systemic autoimmune diseases (25.0%), none of which occurred in pAIN or LL-Np. Diagnostic work-up was more extensive in LO-Np, with more frequent bone marrow biopsies and genetic testing, all supporting an autoimmune rather than congenital etiology. Despite the most severe immune phenotype, LO-Np had zero infection-related hospitalizations across 160.3 person-years (0.00/100 PY, 95% CI 0.00–2.30), compared with 1.38/100 PY in pAIN and 5.58/100 person-years in LL-Np. Immunoglobulin levels were within age-adjusted normal ranges in all 112 patients. Overall, 78.6% of patients achieved resolution of neutropenia. Median time to resolution was 19.0 months (pAIN), 47.0 months (LO-Np), and 67.0 months (LL-Np; $p<0.001$ vs pAIN) (Figure).

**Copyright Information:** (Online) ISSN: 2572-9241
©2026 The Author(s). HemaSphere is published by John Wiley & Sons Ltd on behalf of European Hematology Association. This is an open access Abstract Book distributed under the Attribution-NonCommercial-NoDerivs (CC BY-NC-ND), which allows third parties to download the articles and share them with others as long as they credit the author and the Abstract Book, but they cannot change the content in any way or use them commercially.
**Abstract Book Citations:** Authors, Title, HemaSphere, 2026;10:(S1):pages. The abstract book DOIs can be found on the "Author Information" page.
**Disclaimer:** Articles published in the journal HemaSphere exclusively reflect the opinions of the authors. The authors are responsible for all content in their abstracts including accuracy of the facts, statements, citing resources, etc.

**Summary/Conclusion**

Childhood AIN comprises three clinically and immunologically distinct phenotypes. LO-Np appears to represent a separate entity, marked by leukopenia, multi-lineage lymphopenia, and associations with systemic autoimmunity, yet paradoxically demonstrating no infection-related hospitalisation risk, possibly due to preserved humoral immunity. Identification of these phenotypes may support risk-adapted prognostic counselling and more individualized immunological follow-up.

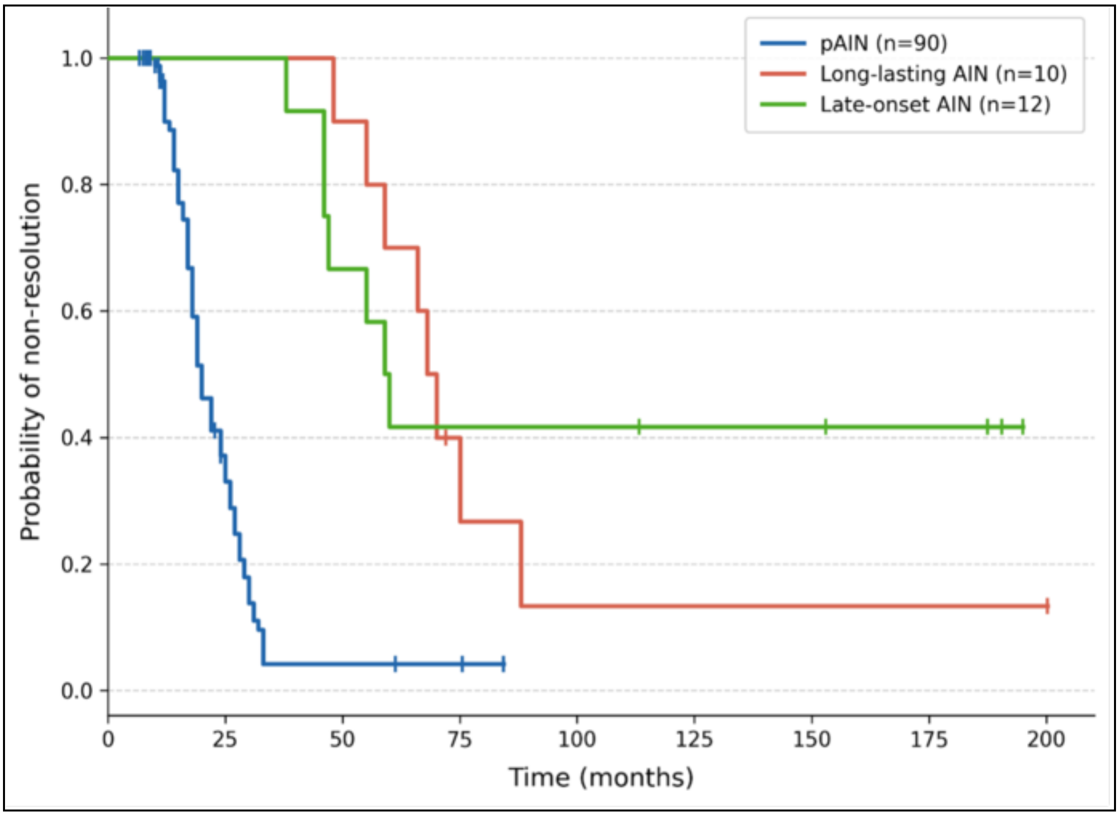

Kaplan-Meier curves for time to resolution of neutropenia by phenotype group.

**Copyright Information:** (Online) ISSN: 2572-9241
©2026 The Author(s). HemaSphere is published by John Wiley & Sons Ltd on behalf of European Hematology Association. This is an open access Abstract Book distributed under the Attribution-NonCommercial-NoDerivs (CC BY-NC-ND), which allows third parties to download the articles and share them with others as long as they credit the author and the Abstract Book, but they cannot change the content in any way or use them commercially.
**Abstract Book Citations:** Authors, Title, HemaSphere, 2026;10:(S1):pages. The abstract book DOIs can be found on the "Author Information" page.
**Disclaimer:** Articles published in the journal HemaSphere exclusively reflect the opinions of the authors. The authors are responsible for all content in their abstracts including accuracy of the facts, statements, citing resources, etc.

### (PS1805) INDIVIDUALS HETEROZYGOUS FOR TERT P.ASP684GLY OR P.ARG774* ARE AT RISK OF TELOMERE BIOLOGY DISORDER

**Topic:** 12. Bone marrow failure syndromes incl. PNH - Clinical

Tuulia Räisänen*[1, 2], Laura Tommila[3], Suvi Douglas[1, 2], Riitta Kaarteenaho[4, 5], Outi Kuismin[6, 7], Jukka Moilanen[6, 7], Anna Hakonen[3], Outi Kilpivaara[1, 2, 8], Ulla Wartiovaara-Kautto[1, 9]

*[1]University of Helsinki, Applied Tumor Genomics Research Program, Faculty of Medicine, Helsinki, Finland;[2]University of Helsinki, Department of Medical and Clinical Genetics, Medicum, Faculty of Medicine, Helsinki, Finland; [3]Helsinki University Hospital and University of Helsinki, Department of Clinical Genetics, HUS Diagnostic Center, Helsinki, Finland; [4]University of Oulu, Research Unit of Biomedicine and Internal Medicine, Oulu, Finland; [5]Oulu University Hospital, Center of Internal Medicine and Respiratory Medicine, and Medical Research Center Oulu, Oulu, Finland; [6]Oulu University Hospital, Department of Clinical Genetics, and Medical Research Center Oulu, Oulu, Finland; [7]University of Oulu, Research Unit of Clinical Medicine, Oulu, Finland; [8]Foundation for the Finnish Cancer Institute, K. Albin Johansson Cancer Researcher Fellow, Helsinki, Finland;[9]Helsinki University Hospital Comprehensive Cancer Center and University of Helsinki, Department of Hematology, Helsinki, Finland;*

### Background

Germline variants in *TERT* are the most prevalent findings in patients with telomere biology disorders (TBD). Heterozygous carriers of pathogenic or likely pathogenic variants in *TERT* may cause later onset and milder phenotype of the disease compared to biallelic variants. This, as well as the wide spectrum of distinct variants, including variants of uncertain significance, challenges clinical care and standardized recommendations for surveillance.
*TERT* variants c.2051A>G, p.Asp684Gly and c.2320C>T, p.Arg774* are enriched in Finland compared to the rest of the world with a combined carrier frequency of 1/210 in the Finnish population (gnomAD Finns minor allele frequency (MAF) 0.001753 vs gnomAD all 0.00007146 and 0.0006665 vs 0.00002790, respectively). Due to this, we had a unique opportunity to study these variants and their effects.

### Aims

We aimed to explore the clinical significance of rare germline *TERT* p.Asp684Gly and p.Arg774* variants in heterozygous carriers.

### Methods

We developed an extensive questionnaire focusing on symptoms and findings typical of TBDs, as well as family history. We interviewed 35 Finnish individuals with *TERT* founder variants from eight families in the Helsinki and Oulu University Hospitals. Additionally, we collected relevant clinical data from patient medical records. Written informed consent was obtained from all study individuals.

### Results

Our study included thirty individuals heterozygous for either *TERT* p.Asp684Gly or p.Arg774*, two were compound heterozygous (p.Asp684Gly;Arg774* and p.Asp684Gly;Glu1068Lys) and three homozygous for p.Asp684Gly. Out of all individuals 18/35 (51%) were male), 17/35 (49%) were index patients and 18/35 (51%) relatives. The median age for heterozygous variant carriers was 50 (range 22-72) and 26 for biallelic carriers (range 20-52).
Telomeres were measured in 20/30 heterozygous individuals. Short telomeres were detected in 70% of these (median length -2.10, range -3.00 to +3.50) (Table 1). Among heterozygous individuals, malignancies and hematological disturbances were prevalent and more than 10% had other TBD-associated clinical features (Table 1).

**Copyright Information:** (Online) ISSN: 2572-9241
©2026 The Author(s). HemaSphere is published by John Wiley & Sons Ltd on behalf of European Hematology Association. This is an open access Abstract Book distributed under the Attribution-NonCommercial-NoDerivs (CC BY-NC-ND), which allows third parties to download the articles and share them with others as long as they credit the author and the Abstract Book, but they cannot change the content in any way or use them commercially.
**Abstract Book Citations:** Authors, Title, HemaSphere, 2026;10:(S1):pages. The abstract book DOIs can be found on the "Author Information" page.
**Disclaimer:** Articles published in the journal HemaSphere exclusively reflect the opinions of the authors. The authors are responsible for all content in their abstracts including accuracy of the facts, statements, citing resources, etc.

**Table 1.** Major TBD -related findings in patients with germline *TERT* variants.

| **Findings** | p.Asp684Gly (n=21) | p.Arg774* (n=9) | Total heterozygous (n=30) | Biallelic (n=5) |
|---|---|---|---|---|
| short telomeres | 12/15 (80%) | 2/5 (40%) | 14/20 (70%) | 3/3 (100%) |
| any cancer | 5 (24%) | 2 (22%) | 7 (23%) | 1 (20%) |
| myelodysplastic syndrome | 2 (9.5%) | 0 | 2 (6.7%) | 1 (20%) |
| abnormal blood count | 11 (52%) | 5 (56%) | 16 (53%) | 5 (100%) |
| diagnosed with dyskeratosis congenita | 3 (14%) | 0 | 3 (10%) | 4 (80%) |
| pulmonary fibrosis | 3 (14%) | 1 (11%) | 4 (13%) | 0 |
| hepatic condition | 2 (9,5%) | 3 (33%) | 5 (17%) | 2 (40%) |
| acid reflux/dysphagia | 11 (52%) | 5 (56%) | 16 (53%) | 3 (60%) |
| osteoporosis | 2 (9,5%) | 1 (11%) | 3 (10%) | 2 (40%) |
| infertility | 3 (14%) | 0 | 3 (10%) | 0 |
| oral leukoplakia | 2 (9,5%) | 1 (11%) | 3 (10%) | 3 (60%) |
| nail dysplasia | 8 (38%) | 4 (44%) | 12 (30%) | 3 (60%) |
| abnormal skin pigmentation | 9 (43%) | 3 (33%) | 12 (40%) | 1 (20%) |

**Copyright Information:** (Online) ISSN: 2572-9241
©2026 The Author(s). HemaSphere is published by John Wiley & Sons Ltd on behalf of European Hematology Association. This is an open access Abstract Book distributed under the Attribution-NonCommercial-NoDerivs (CC BY-NC-ND), which allows third parties to download the articles and share them with others as long as they credit the author and the Abstract Book, but they cannot change the content in any way or use them commercially.
**Abstract Book Citations:** Authors, Title, HemaSphere, 2026;10:(S1):pages. The abstract book DOIs can be found on the "Author Information" page.
**Disclaimer:** Articles published in the journal HemaSphere exclusively reflect the opinions of the authors. The authors are responsible for all content in their abstracts including accuracy of the facts, statements, citing resources, etc.

### Summary/Conclusion

Our study demonstrates that despite fairly high MAF, individuals with heterozygous *TERT* p.Asp684Gly or p.Arg774* may carry a significant risk for end organ damages. The exact penetrance of distinct symptoms calls for further studies. However, the findings indicate the need for careful clinical examination of all identified heterozygous carriers, testing of family members, and surveillance.

**Copyright Information:** (Online) ISSN: 2572-9241
©2026 The Author(s). HemaSphere is published by John Wiley & Sons Ltd on behalf of European Hematology Association. This is an open access Abstract Book distributed under the Attribution-NonCommercial-NoDerivs (CC BY-NC-ND), which allows third parties to download the articles and share them with others as long as they credit the author and the Abstract Book, but they cannot change the content in any way or use them commercially.
**Abstract Book Citations:** Authors, Title, HemaSphere, 2026;10:(S1):pages. The abstract book DOIs can be found on the "Author Information" page.
**Disclaimer:** Articles published in the journal HemaSphere exclusively reflect the opinions of the authors. The authors are responsible for all content in their abstracts including accuracy of the facts, statements, citing resources, etc.

### (PS1806) REAL-WORLD BASELINE CHARACTERISTICS OF PATIENTS INITIATING RAVULIZUMAB FOR PAROXYSMAL NOCTURNAL HEMOGLOBINURIA: INSIGHTS FROM THE POLISH MULTICENTER OBSERVATIONAL STUDY (PNH RECORD)

**Topic:** 12. Bone marrow failure syndromes incl. PNH - Clinical

Patrycja Mensah-Glanowska[1], Sławomir Jurek[2], Bernadeta Ceglarek[2], Adela Gwozdowska[2], Justyna Kozińska[3], Marek Hus[3], Joanna Drozd-Sokołowska[4], Piotr Kacprzyk[4], Bogusław MacHaliński[5], Piotr Kulig[5], Anna Szmigielska-Kapłon[6], Jacek Krzanowski[7], Martyna Krzyzewska[7], Jarosław Czyż[8], Łukasz Szukalski[8], Michał Kurlapski[9], Aleksandra Butrym[10, 11], Paweł Chrząstek[12], Jarosław Piszcz[13], Szymon Drygała[14], Pawel Dubiela[14, 15], Agnieszka Piekarska*[9]

*[1]Department of Hematology, Collegium Medicum Jagiellonian University, Cracow, Poland;[2]Department of Hemostasis Disorders and Internal Medicine, Laboratory of Hemostasis and Metabolic Diseases, Institute of Hematology and Transfusion Medicine, Warsaw, Poland; [3]Department of Hematooncology and Bone Marrow Transplantation, Medical University in Lublin, Lublin, Poland; [4]Department of Hematology, Transplantation and Internal Medicine, Medical University of Warsaw, Warsaw, Poland; [5]Department of Hematology and Transplantology, Pomeranian Medical University, Szczecin, Poland; [6]Department of Hematology, Medical University in Lodz, Copernicus Hospital, Lodz, Poland; [7]Brzozow Oncology Center, Brzozow, Poland; [8]Department of Hematology, Collegium Medicum in Bydgoszcz, Nicolaus Copernicus University in Toruń, Bydgoszcz, Poland; [9]Department of Hematology, Transplantalogy and Cellular Therapies, Medical University of Gdansk, Gdansk, Poland; [10]Department of Cancer Prevention and Therapy, Wroclaw Medical University, Wroclaw, Poland; [11]Alfred Sokolowski Specialist Hospital in Walbrzych Oncology Support Centre for Clinical Trials, Walbrzych, Poland; [12]Department of Hematology, Institute of Medical Sciences, University of Opole, Opole, Poland; [13]Department of Hematology, Medical University of Bialystok, Bialystok, Poland; [14]AstraZeneca Pharma Poland, Warsaw, Poland; [15]Centre of Regenerative Medicine, Medical University of Bialystok, Bialystok, Poland;*

### Background

**Background:** Paroxysmal nocturnal hemoglobinuria (PNH) is a rare, life-threatening hematologic disorder characterized by uncontrolled terminal complement-mediated hemolysis, thrombotic complications, and bone marrow failure. Ravulizumab, a second-generation C5i, engineered from eculizumab, offers immediate, complete and sustained terminal complement inhibition with extended dosing interval (8-weeks), resulting in effective IVH control, reduced morbidity (thrombosis rate), and mortality (improved survival). However, comprehensive real-world data on baseline (BL) characteristics of patients initiating ravulizumab is limited for certain European countries.

### Aims

To describe BL demographic, clinical and laboratory characteristics of adults initiating ravulizumab in routine Polish practice, and to compare complement inhibitor (Ci)–naïve and Ci-experienced (exp) patients.

### Methods

PNH RECORD is a multicenter, longitudinal, observational study across Polish hematology centers. BL data were collected at ravulizumab initiation (Index Date) and summarized descriptively overall and by prior Ci exposure. Medical history and concomitant medications are reported at BL.

**Copyright Information:** (Online) ISSN: 2572-9241
©2026 The Author(s). HemaSphere is published by John Wiley & Sons Ltd on behalf of European Hematology Association. This is an open access Abstract Book distributed under the Attribution-NonCommercial-NoDerivs (CC BY-NC-ND), which allows third parties to download the articles and share them with others as long as they credit the author and the Abstract Book, but they cannot change the content in any way or use them commercially.
**Abstract Book Citations:** Authors, Title, HemaSphere, 2026;10:(S1):pages. The abstract book DOIs can be found on the "Author Information" page.
**Disclaimer:** Articles published in the journal HemaSphere exclusively reflect the opinions of the authors. The authors are responsible for all content in their abstracts including accuracy of the facts, statements, citing resources, etc.

**Results**

The interim analysis set included 53 patients; 40 (75.5%) were Ci-exp and 13 (24.5%) were Ci-naïve. Median age was 43 years (Q1–Q3: 31–62) and 26/53 (49.1%) were female. Ci-naïve patients were younger than Ci-exp patients (median 35 vs 44 years) and had a shorter median PNH duration (3.0 vs 50.1 months). BL medical history (events occurring at any time prior to the Index Date) included thrombosis or other serious vascular events in 15/53 (28.3%) patients (23.1% Ci-naïve; 30.0% Ci-exp) and aplastic anemia in 10/53 (18.9%) (38.5% vs 12.5%, respectively); consistent with the longer disease duration in the Ci-exp cohort. In the 12 months before ravulizumab initiation, 15/53 (28.3%) patients required transfusions (38.5% Ci-naïve; 25.0% Ci-exp); the corresponding transfusion rate was 3.4 blood units per patient-year overall (8.1 Ci-naïve; 1.9 Ci-exp). Marked differences in hemolysis were observed at BL: mean LDH was 877.8 U/L in Ci-naïve patients versus 257.5 U/L in Ci-exp patients, and LDH <1.5×ULN was present in 0% vs 95.0%, respectively. Comorbidities were reported in 77.4% (41/53) of patients, particularly vascular (30.2%), gastrointestinal (22.6%) and metabolic/nutritional disorders (22.6%), and were more frequent in Ci-exp than Ci-naïve patients (82.5% vs 61.5%). Concomitant medications were used by 45/53 (84.9%), most often reparations (41.5%), antithrombotic agents (24.5%) and immunosuppressants (18.9%); beta-blockers (18.9%) and renin–angiotensin system agents (15.1%) were also frequently recorded.

**Summary/Conclusion**

In Polish routine practice, patients initiating ravulizumab showed distinct BL profiles by treatment history; had higher hemolytic activity, greater pre-index transfusion burden, and higher prevalence of aplastic anemia compared with Ci-exp patients. These BL data may provide important context for interpreting longitudinal outcomes with ravulizumab treatment in this study.

**Copyright Information:** (Online) ISSN: 2572-9241
©2026 The Author(s). HemaSphere is published by John Wiley & Sons Ltd on behalf of European Hematology Association. This is an open access Abstract Book distributed under the Attribution-NonCommercial-NoDerivs (CC BY-NC-ND), which allows third parties to download the articles and share them with others as long as they credit the author and the Abstract Book, but they cannot change the content in any way or use them commercially.
**Abstract Book Citations:** Authors, Title, HemaSphere, 2026;10:(S1):pages. The abstract book DOIs can be found on the "Author Information" page.
**Disclaimer:** Articles published in the journal HemaSphere exclusively reflect the opinions of the authors. The authors are responsible for all content in their abstracts including accuracy of the facts, statements, citing resources, etc.

### (PS1807) A PROSPECTIVE RANDOMIZED CONTROLLED STUDY OF RAPID OR SLOW CYCLOSPORIN A TAPERING REGIMENS IN ACQUIRED PURE RED CELL APLASIA

**Topic:** 12. Bone marrow failure syndromes incl. PNH - Clinical

Xiaoqing Liu[1], Guangsheng He*[2]

*[1]the Second Hospital of Nanjing, Hematology, Nanjing, China; [2]The First Affiliated Hospital with Nanjing Medical University, Nanjing, China;*

### Background

Cyclosporin A (CsA) effectively improves bone marrow hematopoietic function and induces hematological remission in patients with acquired pure red cell aplasia (aPRCA). However, long-term CsA administration is associated with potential adverse reactions, and improper tapering often leads to disease recurrence. Currently, there is no unified clinical standard for the optimal CsA tapering regimen after remission induction in aPRCA patients to balance efficacy and recurrence risk.

### Aims

This study aimed to explore the differences in efficacy and recurrence risk between rapid and slow CsA tapering regimens after remission induction in aPRCA patients, thereby providing evidence-based support for optimizing clinical CsA tapering strategies.

### Methods

This was a prospective randomized controlled trial designed to enroll 30 aPRCA patients who achieved hematological remission with CsA treatment and maintained remission for ≥12 months. Patients were randomly divided into the rapid tapering group and the slow tapering group (15 cases each) using a random number table. The rapid group received a tapering regimen of 0.8 mg/kg/month, while the slow tapering group received 0.3–0.7 mg/kg/month. The primary endpoint was the recurrence rate (defined as a decrease in hemoglobin level of ≥20 g/L requiring resumption of full-dose CsA treatment), and the secondary endpoint was the duration of remission. Data from 28 enrolled patients were analyzed. This study was conducted in accordance with the Helsinki Declaration. All patients had given informed consent with a clinical trial registration number of ChiCTR2100043485.

### Results

Among the 28 patients, 13 had primary aPRCA and 15 had large granular lymphocytic leukemia (LGLL)-related aPRCA. In the rapid tapering group, 10 cases relapsed, with a recurrence rate of 66.7% (10/15); in the slow tapering group, 8 cases relapsed, with a recurrence rate of 61.5% (8/13). There was no statistically significant difference in the recurrence rate between the two groups ($P$=1.0). The median duration of remission after tapering was 9 (2–41) months in the rapid tapering group and 17 (2–45) months in the slow tapering group, but this difference was not statistically significant ($P$=0.558) (Fig. 1). No deaths occurred in either group during the follow-up period.

### Summary/Conclusion

The recurrence risks of the rapid and slow CsA tapering regimens are comparable, while the slow tapering regimen tends to prolong the duration of remission. The slow tapering regimen is more suitable for patients requiring long-term maintenance of remission, whereas the rapid tapering regimen can be used for patients intolerant to CsA. However, further studies with larger sample sizes are needed to validate these findings, so as to provide references for the individualized formulation of clinical CsA tapering strategies.

**Copyright Information:** (Online) ISSN: 2572-9241
©2026 The Author(s). HemaSphere is published by John Wiley & Sons Ltd on behalf of European Hematology Association. This is an open access Abstract Book distributed under the Attribution-NonCommercial-NoDerivs (CC BY-NC-ND), which allows third parties to download the articles and share them with others as long as they credit the author and the Abstract Book, but they cannot change the content in any way or use them commercially.
**Abstract Book Citations:** Authors, Title, HemaSphere, 2026;10:(S1):pages. The abstract book DOIs can be found on the “Author Information” page.
**Disclaimer:** Articles published in the journal HemaSphere exclusively reflect the opinions of the authors. The authors are responsible for all content in their abstracts including accuracy of the facts, statements, citing resources, etc.

### (PS1808) ORAL MY008211A MONOTHERAPY ACHIEVES TRANSFUSION AVOIDANCE AND HEMOGLOBIN NORMALIZATION IN CHINESE PNH PATIENTS WITH RESIDUAL ANEMIA DESPITE C5 INHIBITION

**Topic:** 12. Bone marrow failure syndromes incl. PNH - Clinical

Bing Han*[1], Fengkui Zhang[1], Hongyan Tong[2], Minkai Song[3], Rongjun Liu[3], Rui Xia[3], Xuguang Guo[3], Yihan Zhang[3], Wilson Chunlin Wang[3], Geoffrey Ross[4], Chuanrui Xu[3]

*[1]Peking Union Medical College Hospital (PUMCH), Chinese Academy of Medical Science, Department of Hematology, Beijing, China; [2]First Affiliated Hospital, Zhejiang University School of Medicine, Department of Hematology, Hangzhou, China;[3]Wuhan Createrna Science and Technology Co., Ltd., Wuhan, China; [4]Wuhan Createrna Science and Technology Co., Ltd., Cornwall, United Kingdom;*

### Background

Paroxysmal nocturnal hemoglobinuria (PNH) is a rare acquired hematopoietic disorder characterized by complement-mediated intravascular hemolysis. While C5 monoclonal antibodies (mAbs) reduce intravascular hemolysis, many patients retain persistent anemia from residual hemolysis within and outside vasculature. MY008211A is an oral complement factor B inhibitor that blocks proximal complement activation.

### Aims

To evaluate efficacy and safety of MY008211A monotherapy in Chinese patients with PNH and persistent anemia (hemoglobin [Hb] <100 g/L) despite receiving stable C5 mAb treatment for ≥6 months.

### Methods

This multicenter, single-arm, open-label Phase III study (NCT06932471) enrolled 20 adults with PNH and Hb <100 g/L despite stable C5 mAb therapy. Patients received oral MY008211A 400 mg twice daily for 24 weeks after discontinuing C5 mAb. The primary endpoint was the proportion of patients achieving transfusion avoidance (Days 14–168) alongside sustained Hb normalization (Hb ≥120 g/L in ≥3 of 4 assessments during Weeks 18–24). Secondary endpoints included transfusion avoidance with a sustained Hb improvement of ≥20 g/L and changes from baseline in Hb, lactate dehydrogenase (LDH), reticulocyte count, and fatigue scores (FACIT-Fatigue). All patients provided informed consent.

### Results

Twenty patients were enrolled (mean age, 45.4 years; 55% female). At baseline, mean Hb was 79.5 g/L, with 60% having received prior transfusions. The primary endpoint of sustained transfusion avoidance with Hb normalization was achieved by 70.0% (14 of 20) of patients. Furthermore, 95.0% (19 of 20) of patients achieved transfusion avoidance combined with a sustained Hb improvement of ≥20 g/L. Treatment with MY008211A increased Hb levels by 48.0 g/L from baseline to Weeks 18–24 (127.5 g/L). Hemolysis was effectively controlled: 85.0% of patients achieved sustained LDH levels <1.5 × the upper limit of normal. Reticulocyte counts normalized (mean decrease, 141.4 × $10^9$/L). FACIT-Fatigue scores improved (+9.6 points). No breakthrough hemolysis or major adverse vascular events occurred. Treatment was well tolerated; the most common treatment-emergent adverse events were hyperlipidemia (30.0%) and platelet count decreased (20.0%). No serious adverse events were treatment-related, and no patients discontinued due to adverse events.

**Copyright Information:** (Online) ISSN: 2572-9241
©2026 The Author(s). HemaSphere is published by John Wiley & Sons Ltd on behalf of European Hematology Association. This is an open access Abstract Book distributed under the Attribution-NonCommercial-NoDerivs (CC BY-NC-ND), which allows third parties to download the articles and share them with others as long as they credit the author and the Abstract Book, but they cannot change the content in any way or use them commercially.
**Abstract Book Citations:** Authors, Title, HemaSphere, 2026;10:(S1):pages. The abstract book DOIs can be found on the "Author Information" page.
**Disclaimer:** Articles published in the journal HemaSphere exclusively reflect the opinions of the authors. The authors are responsible for all content in their abstracts including accuracy of the facts, statements, citing resources, etc.

**Summary/Conclusion**

MY008211A monotherapy demonstrated robust efficacy in Chinese patients with PNH and residual anemia despite prior C5 mAb therapy. Notably, 95.0% of patients achieved continuous transfusion avoidance, with 70% reaching complete hemoglobin normalization, accompanied by remarkable improvements in fatigue-related quality of life. The favorable safety profile and convenient twice-daily oral dosing support MY008211A as a promising alternative to address the unmet need of persistent anemia in patients with PNH who have inadequate response to C5 inhibition.

**Copyright Information:** (Online) ISSN: 2572-9241
©2026 The Author(s). HemaSphere is published by John Wiley & Sons Ltd on behalf of European Hematology Association. This is an open access Abstract Book distributed under the Attribution-NonCommercial-NoDerivs (CC BY-NC-ND), which allows third parties to download the articles and share them with others as long as they credit the author and the Abstract Book, but they cannot change the content in any way or use them commercially.
**Abstract Book Citations:** Authors, Title, HemaSphere, 2026;10:(S1):pages. The abstract book DOIs can be found on the "Author Information" page.
**Disclaimer:** Articles published in the journal HemaSphere exclusively reflect the opinions of the authors. The authors are responsible for all content in their abstracts including accuracy of the facts, statements, citing resources, etc.

### (PS1809) EFFICACY AND SAFETY OF LUSUTROMBOPAG IN PATIENTS WITH NON-SEVERE APLASTIC ANEMIA: RESULTS FROM A PROSPECTIVE, SINGLE-ARM, OPEN-LABEL, EXPLORATORY STUDY

**Topic:** 12. Bone marrow failure syndromes incl. PNH - Clinical

Ting Wang*[1], Rong Fu[1]

[1]*Tianjin Medical University General Hospital, Tianjin, China;*

### Background

Non-severe aplastic anemia (NSAA) is characterized by bone marrow failure and peripheral is cytopenia. While immunosuppressive therapy (IST) is the standard of care, its efficacy is limited by suboptimal response rates and potential toxicity. Lusutrombopag, a second-generation thrombopoietin receptor agonist (TPO-RA), promotes platelet production with a favorable safety profile and no dietary restrictions. However, its therapeutic potential in NSAA has not yet been systematically evaluated.

### Aims

This study (LUS-AA-2024, ChiCTR2400084012) aimed to evaluate the preliminary efficacy and safety of Lusutrombopag in patients with NSAA.

### Methods

In this single-center, single-arm, open-label study, 15 patients with NSAA (including treatment-naïve and those non-responsive to prior IST) were enrolled. A dose-titration regimen was employed, with lusutrombopag initiated at 3 mg once daily. The dose could be increased by 3 mg every two weeks based on platelet count and safety profile, up to a maximum of 12 mg/day.  The total treatment duration was 8 weeks. The primary endpoint was the proportion of patients achieving hematologic response (HR, defined as response in at least one lineage) at Week 8. Secondary endpoints included time to response, multilineage response, overall response rate (ORR), and safety.

### Results

A total of 15 patients were enrolled, with a mean age of 44.1 years and a mean baseline platelet count of $25.9\times10^9$/L. At Week 8, the hematologic response (HR) rate was 63.6% (95% CI: 30.8%, 89.1%). The ORR was consistent at 63.6%. Among responders, hematologic improvement was rapid, with a median time to response of 20.0 days (range: 6–31 days). Regarding multilineage recovery, 18.2% achieved a bilineage response, and 9.1% achieved a trilineage response. Mean platelet counts increased from $25.9\times10^9$/L at baseline to $51.2\times10^9$/L at Week 8. Lusutrombopag demonstrated a good safety profile. Treatment-emergent adverse events (TEAEs) occurred in 26.7% of patients, while no treatment-related adverse events (TRAEs) were reported. One patient experienced a serious TEAE (pancreatitis) leading to discontinuation, which was assessed as unrelated to the study drug. No Grade 4-5 adverse events or deaths were reported.

### Summary/Conclusion

In this exploratory study, Lusutrombopag demonstrated promising efficacy in NSAA patients with a rapid onset of action (median 20 days). The drug was well-tolerated with no treatment-related adverse events reported. These findings support Lusutrombopag is a potential therapeutic option for NSAA and warrant further investigation in larger, randomized controlled trials.

**Copyright Information:** (Online) ISSN: 2572-9241
©2026 The Author(s). HemaSphere is published by John Wiley & Sons Ltd on behalf of European Hematology Association. This is an open access Abstract Book distributed under the Attribution-NonCommercial-NoDerivs (CC BY-NC-ND), which allows third parties to download the articles and share them with others as long as they credit the author and the Abstract Book, but they cannot change the content in any way or use them commercially.
**Abstract Book Citations:** Authors, Title, HemaSphere, 2026;10:(S1):pages. The abstract book DOIs can be found on the “Author Information” page.
**Disclaimer:** Articles published in the journal HemaSphere exclusively reflect the opinions of the authors. The authors are responsible for all content in their abstracts including accuracy of the facts, statements, citing resources, etc.

### (PS1810) A REAL-WORLD STUDY: EFFICACY AND SAFETY OF COMPLEMENT INHIBITORS (ECULIZUMAB OR IPTACOPAN) COMBINED WITH CYCLOSPORINE IN PATIENTS WITH APLASTIC ANEMIA-PAROXYSMAL NOCTURNAL HEMOGLOBINURIA SYNDROME

**Topic:** 12. Bone marrow failure syndromes incl. PNH - Clinical

Chuan He[1], Yuemin Gong[2], Shuqian Xu[3], Xinmer Yu[1], Puzhou Wang[3], Yawen Zhang[2], Guangsheng He*[2]

*[1]Institute of Hematology, West China Hospital, Sichuan University, Chengdu, China;[2]The First Affiliated Hospital of Nanjing Medical University, Nanjing, China; [3]Qilu Hospital of Shandong University, Jinan, China;*

### Background

Aplastic anemia (AA) often occurs concurrently with paroxysmal nocturnal hemoglobinuria (PNH), representing a bone marrow failure disorder characterized by both immune-mediated hematopoietic hypoplasia and complement-mediated intravascular hemolysis. Controlling hemolysis and improving hematopoiesis must be addressed simultaneously in the treatment. In general, cyclosporine combined with complement inhibitors is recommended. However, data on the safety and efficacy of proximal complement inhibitors combined with cyclosporine in real-world practice remain lacking.

### Aims

To evaluate the efficacy and safety of complement inhibitors (eculizumab and iptacopan) combined with cyclosporine in Chinese adult patients with AA and PNH for reference of subsequent clinical use.

### Methods

Enrolled adult patients had a confirmed diagnosis of AA and PNH. Patients received standard-dose eculizumab or iptacopan combined with cyclosporine. The observation period was 24 weeks. Primary endpoints included the proportion of patients with an increase in hemoglobin (Hb) level ≥2 g/dL from baseline, the proportion of patients with Hb normalization (Hb ≥12 g/dL), and safety at Week 24. Secondary endpoints included the proportion of transfusion independence at Week 24, changes from baseline in Hb and lactate dehydrogenase (LDH) levels, breakthrough hemolysis, and major adverse vascular events (MAVEs).

### Results

A total of 38 patients were enrolled between January 2024 and October 2025. Baseline characteristics were balanced between the two groups (Table 1). At Week 24, the proportion of patients achieving the primary endpoint was 28.6% in the eculizumab group and 52.9% in the iptacopan group (P=0.185). Kaplan-Meier analysis showed that the time to achieve a Hb increase ≥2 g/dL was significantly shorter in the iptacopan group (P=0.014), with a median time to response of 12 weeks, compared with >24 weeks in the eculizumab group. The proportion of patients with normalized Hb was 14.3% and 47.1%, respectively (P=0.037). The mean Hb increase from baseline was +1.1 g/dL and +3.7 g/dL in the two groups (P=0.01); the median Hb increase was +1.0 g/dL and +4.2 g/dL, respectively (P=0.044). The proportion of patients achieving transfusion independence was 46.2% and 100%, respectively (P=0.007) (Table 2). At Week 24, LDH levels were significantly and rapidly controlled in both groups, with mean reductions from baseline of 74.8% and 68.9%, respectively, and mean LDH levels remained below 1.5×ULN in both groups.No treatment-related adverse events of grade ≥3, breakthrough hemolysis or MAVEs were observed. Among evaluable patients (eculizumab, n=8; iptacopan, n=14), D-dimer levels decreased by 24.4% and 81.0%, respectively. During the 24-week treatment period, serum creatinine and estimated glomerular filtration rate (eGFR) remained stable in both groups, with eGFR improved in 6 and 8 patients, respectively. AST/ALT levels also showed a downward trend, with favorable hepatic and renal safety profiles.

**Copyright Information:** (Online) ISSN: 2572-9241
©2026 The Author(s). HemaSphere is published by John Wiley & Sons Ltd on behalf of European Hematology Association. This is an open access Abstract Book distributed under the Attribution-NonCommercial-NoDerivs (CC BY-NC-ND), which allows third parties to download the articles and share them with others as long as they credit the author and the Abstract Book, but they cannot change the content in any way or use them commercially.
**Abstract Book Citations:** Authors, Title, HemaSphere, 2026;10:(S1):pages. The abstract book DOIs can be found on the "Author Information" page.
**Disclaimer:** Articles published in the journal HemaSphere exclusively reflect the opinions of the authors. The authors are responsible for all content in their abstracts including accuracy of the facts, statements, citing resources, etc.

### Summary/Conclusion

This real-world study demonstrates that eculizumab or iptacopan combined with cyclosporine can rapidly and effectively control hemolysis and significantly improve anemia in Chinese patients with AA and PNH. Both regimens are well tolerated. Iptacopan shows a superior trend in increasing hemoglobin and achieving transfusion independence. Further studies with larger sample sizes and longer follow-up are warranted to evaluate efficacy, impact on disease progression, and comprehensive benefits on patient outcomes.

**Table 1. Patient Baseline Characteristics**

| Characteristic | Eculizumab Group (N=21) | Iptacopan Group (N=17) |
|---|---|---|
| **Demographics** | | |
| Age，years，median (range) | 43(16-70) | 38(19-68) |
| Female，n(%) | 14(66.7) | 10(58.8) |
| **Disease characteristics** | | |
| History of pre-existing AA，n(%) | 16(76.2) | 15(88.2) |
| Disease duration，years，median (range) | 2.5(1-15) | 2(0.5-16) |
| **Treatment history** | | |
| Baseline cyclosporine treatment，n(%) | 17(81) | 12(70.6) |
| **Laboratory parameters** | | |
| Transfusion dependence，n(%) | 13(61.9) | 10(58.8) |
| Hb(g/dL)，mean±SD | 7.1±2.4 | 7.4±2.6 |
| LDH (U/L)，mean±SD | 1234.8±1109.4 | 816.9±788.0 |
| **PNH clone (granulocytes)** | | |
| ≥50%, n(%) | 16(76.2) | 13(76.5) |
| 10%—50%，n(%) | 5(23.8) | 4(23.5) |

**Table 2. Efficacy Parameters at Week 24**

| Efficacy Parameter | Eculizumab Group (N=21) | Iptacopan Group (N=17) | Method | P-value |
|---|---|---|---|---|
| **Hemoglobin (Hb)** | | | | |
| Mean Hb (g/dL)，Mean±SD | 8.1±2.7 | 11.1±2.4 | | |
| Mean change from baseline (g/dL，%) | +1.1(+15.4%) | +3.7(+49.6%) | Independent samples t-test | 0.01 |
| Median change in Hb from baseline (g/dL) | +1.0 | +4.2 | Wilcoxon rank-sum test | 0.044 |
| Patients with Hb increase ≥2 g/dL，n（%） | 6(28.6) | 9(52.9) | Fisher's exact test | 0.185 |
| Patients with Hb ≥12 g/dL，n（%） | 3(14.3) | 8(47.1) | Fisher's exact test | 0.037 |
| **Lactate dehydrogenase (LDH)** | | | | |
| Mean LDH (U/L)，Mean±SD | 310.9±150.3 | 253.8±92.7 | | |
| Mean change from baseline (%) | -74.8 | -68.9 | | |
| **Clinical outcome** | | | | |
| Patients achieving transfusion independence*，n/N(%) | 6/13(46.2) | 10/10(100) | Fisher's exact test | 0.007 |

*Note: Calculated only for patients with baseline transfusion dependence

Copyright Information: (Online) ISSN: 2572-9241
©2026 The Author(s). HemaSphere is published by John Wiley & Sons Ltd on behalf of European Hematology Association. This is an open access Abstract Book distributed under the Attribution-NonCommercial-NoDerivs (CC BY-NC-ND), which allows third parties to download the articles and share them with others as long as they credit the author and the Abstract Book, but they cannot change the content in any way or use them commercially.
Abstract Book Citations: Authors, Title, HemaSphere, 2026;10:(S1):pages. The abstract book DOIs can be found on the "Author Information" page.
Disclaimer: Articles published in the journal HemaSphere exclusively reflect the opinions of the authors. The authors are responsible for all content in their abstracts including accuracy of the facts, statements, citing resources, etc.

### (PS1811) REDUCED DOSE OF GLUCOCORTICOIDS FOR PROPHYLAXIS OF SERUM SICKNESS IN APLASTIC ANEMIA POST ANTI-HUMAN T-LYMPHOCYTE GLOBULIN (ATG) TREATMENT: A RANDOMIZED TRIAL

**Topic:** 12. Bone marrow failure syndromes incl. PNH - Clinical

Ling Gao[1], Yuemin Gong[2], Jingnan Sun[3], Guangsheng He*[2]

*[1]Jiangsu Province (Suqian) Hospital, Suqian, China; [2]The First Affiliated Hospital with Nanjing Medical University, Nanjing, China; [3]The First Bethune Hospital of Jilin University, Changchun, China;*

### Background

ATG is first-line for transfusion-dependent non-severe and severe apastic anemia (TD-NSAA/SAA) who is ineligible for transplant. Glucocorticoids are co-administered to prevent infusion reactions and serum sickness, but optimal dosing is not confirmed. Given adverse effects, we attempted reduced-dose prophylaxis in elderly/diabetic patients, finding no significant difference in serum sickness (9.5% vs 17.1%). Thus, we conducted this multicenter, open-label, randomized trial comparing reduced- vs full-dose glucocorticoids for serum sickness prevention post-ATG (ChiCTR2400087735).

### Aims

To evaluate non-inferiority of reduced-dose glucocorticoids in preventing serum sickness following ATG in AA.

### Methods

This trial plans to enroll 80 patients (15-64y, no cardiovascular/endocrine comorbidities) randomized 1:1 to reduced- or full-dose groups. All receive methylprednisolone 1 mg/kg/d during ATG infusion (Day 1-5). Post-ATG, reduced-dose group receives 0.5 mg/kg/d tapered by 20% q3d; full-dose group continues 1 mg/kg/d to Day 14 then tapered by 20% q3d. Primary endpoint: incidence of serum sickness within 28 days. Secondary: severity/duration of serum sickness, glucocorticoid-related adverse events (AEs), hematologic response rate (HRR).

### Results

This interim analysis included 46 patients (23 per group) with balanced baseline characteristics (Table 1). In the reduced-dose, serum sickness occurred in 6 (26.1%) cases (2 grade1 and 4 grade 2), with a median onset time of Day 12.5 (range: 10-20), and a median duration of 3.5 (range: 3-8) days. In the full-dose, only 1 case (4.3%) developed grade 1 serum sickness on Day 13, which lasted for 1 day. There was a difference in the incidence of serum sickness between groups [p=0.10; odds ratio [OR], 7.8; 95% confidence interval (CI), 0.8-70.8]. Common ≥ grade 3 glucocorticoid-related AEs: in reduced-dose, infection (69.6%), hypertension (13.0%), and hypokalemia (13.0%); in full-dose, infection (43.5%) and edema (17.4%). Overall, ≥ grade 3 AEs did not differ significantly, but 4 full-dose patients required dose reduction/cessation due to serious AEs (2 for edema, 1 for GI bleeding, 1 for septic shock), whereas no such cases occurred in the reduced-dose. 3-month HRR: 68.2% (15/22) in the reduced-dose vs 70.0% (14/20) in the full-dose (p=0.90).

**Copyright Information:** (Online) ISSN: 2572-9241
©2026 The Author(s). HemaSphere is published by John Wiley & Sons Ltd on behalf of European Hematology Association. This is an open access Abstract Book distributed under the Attribution-NonCommercial-NoDerivs (CC BY-NC-ND), which allows third parties to download the articles and share them with others as long as they credit the author and the Abstract Book, but they cannot change the content in any way or use them commercially.
**Abstract Book Citations:** Authors, Title, HemaSphere, 2026;10:(S1):pages. The abstract book DOIs can be found on the “Author Information” page.
**Disclaimer:** Articles published in the journal HemaSphere exclusively reflect the opinions of the authors. The authors are responsible for all content in their abstracts including accuracy of the facts, statements, citing resources, etc.

Table 1. Baseline Characteristics

| Characteristics | Reduced-dose (n=23) | Full-dose (n=23) | P-value |
|---|---|---|---|
| Gender (female, %) | 69.6 | 65.2 | 0.75 |
| Age (y, mean±SD) | 42.4±14.8 | 35.6±13.0 | 0.10 |
| Body Mass Index (kg/m$^2$, mean±SD) | 24.6±4.7 | 23.5±3.3 | 0.36 |
| ATG species (n) | | | |
| Rabbit | 15 | 13 | 0.55 |
| Porcine | 8 | 10 | |
| Severity (n) | | | |
| TD-NSAA | 1 | 1 | |
| SAA | 12 | 14 | 0.76 |
| VSAA | 10 | 8 | |
| Follow-up [weeks, median (range)] | 33.4 (5.0-69.3) | 32.3 (4.1-72.9) | 0.95 |

Table 2. Endpoints Assessment

| Endpoint | Reduced-dose (n=23) | Full-dose (n=23) | P-value |
|---|---|---|---|
| Serum Sickness (n) | 6 | 1 | 0.10 |
| Grade 1 | 2 | 1 | |

Copyright Information: (Online) ISSN: 2572-9241
©2026 The Author(s). HemaSphere is published by John Wiley & Sons Ltd on behalf of European Hematology Association. This is an open access Abstract Book distributed under the Attribution-NonCommercial-NoDerivs (CC BY-NC-ND), which allows third parties to download the articles and share them with others as long as they credit the author and the Abstract Book, but they cannot change the content in any way or use them commercially.
Abstract Book Citations: Authors, Title, HemaSphere, 2026;10:(S1):pages. The abstract book DOIs can be found on the "Author Information" page.
Disclaimer: Articles published in the journal HemaSphere exclusively reflect the opinions of the authors. The authors are responsible for all content in their abstracts including accuracy of the facts, statements, citing resources, etc.

| | | | |
|---|---|---|---|
| | | | 0.21 |
| Grade 2 | 4 | 0 | |
| Duration [days, median (range)] | 3.5 (3-8) | 1 | 0.29 |
| ≥ Grade 3 AEs Related to Glucocorticoids (n) | | | |
| Infection | 16 | 10 | 0.07 |
| Hypertension | 3 | 2 | 1.00 |
| Edema | 0 | 4 | 0.11 |
| Hypertriglyceridemia | 0 | 2 | 0.49 |
| Myalgia | 1 | 1 | 1.00 |
| Hypokalaemia | 3 | 0 | 0.23 |
| GI bleeding | 0 | 1 | 1.00 |
| Nausea | 1 | 0 | 1.00 |
| Elevated transaminases | 0 | 1 | 1.00 |
| Heart failure | 1 | 0 | 1.00 |
| 3-month Hematologic Response (%) | | | |
| Overall | 68.2 | 70.0 | 0.90 |

**Copyright Information:** (Online) ISSN: 2572-9241
©2026 The Author(s). HemaSphere is published by John Wiley & Sons Ltd on behalf of European Hematology Association. This is an open access Abstract Book distributed under the Attribution-NonCommercial-NoDerivs (CC BY-NC-ND), which allows third parties to download the articles and share them with others as long as they credit the author and the Abstract Book, but they cannot change the content in any way or use them commercially.
**Abstract Book Citations:** Authors, Title, HemaSphere, 2026;10:(S1):pages. The abstract book DOIs can be found on the “Author Information” page.
**Disclaimer:** Articles published in the journal HemaSphere exclusively reflect the opinions of the authors. The authors are responsible for all content in their abstracts including accuracy of the facts, statements, citing resources, etc.

| | | | |
|---|---|---|---|
| Complete | 13.6 | 10.0 | 0.72 |

**Summary/Conclusion**

Reduced-dose methylprednisolone (<0.5 mg/kg/d) post-ATG increased serum sickness but eliminated dose reduction/cessation due to severe AEs. Future optimization of dose/duration is needed to balance efficacy and safety.

**Copyright Information:** (Online) ISSN: 2572-9241
©2026 The Author(s). HemaSphere is published by John Wiley & Sons Ltd on behalf of European Hematology Association. This is an open access Abstract Book distributed under the Attribution-NonCommercial-NoDerivs (CC BY-NC-ND), which allows third parties to download the articles and share them with others as long as they credit the author and the Abstract Book, but they cannot change the content in any way or use them commercially.
**Abstract Book Citations:** Authors, Title, HemaSphere, 2026;10:(S1):pages. The abstract book DOIs can be found on the "Author Information" page.
**Disclaimer:** Articles published in the journal HemaSphere exclusively reflect the opinions of the authors. The authors are responsible for all content in their abstracts including accuracy of the facts, statements, citing resources, etc.

### (PS1812) LATE AND SUSTAINED RESPONSES TO IMMUNOSUPPRESSIVE THERAPY PLUS ROMIPLOSTIM IN TREATMENT-NAÏVE APLASTIC ANEMIA

**Topic:** 12. Bone marrow failure syndromes incl. PNH - Clinical

Kohei Hosokawa*[1], Jong Wook Lee[2], Jun Ho Jang[3], Naoshi Obara[4], Masashi Sawa[5], Kensuke Usuki[6, 7], Gaku Oshikawa[8], Masahiro Kizaki[9], Koji Nagafuji[10], Ting-An Lin[11], Hitoshi Hanamoto[12], Yeung-Chul Mun[13], Toshiro Kawakita[14], Kazunori Imada[15], June-Won Cheong[16], Sung-Soo Park[17], Takayuki Tabayashi[9], Masaru Gamou[18], Kenta Murotani[19], Hirohito Yamazaki[20]

*[1]Kanazawa University Hospital, Department of Hematology, Kanazawa, Japan;[2]Hanyang University Seoul Hospital, Division of Hematology-Oncology, Seoul, South Korea; [3]Sungkyunkwan University Samsung Medical Center, Department of Hematology, Seoul, South Korea; [4]University of Tsukuba Hospital, Department of Hematology, Ibaraki, Japan;[5]Anjo Kosei Hospital, Department of Hematology and Oncology, Aichi, Japan; [6]NTT Medical Center Tokyo, Department of Hematology, Tokyo, Japan; [7]Mizonokuchi Hospital, Teikyo University School of Medicine, Forth Department of Internal Medicine, Kanagawa, Japan; [8]Japanese Red Cross Musashino Hospital, Department of Hematology, Tokyo, Japan;[9]Saitama Medical Center, Saitama Medical University, Department of Hematology, Saitama, Japan; [10]Kurume University Hospital, Department of Hematology, Kurume, Japan; [11]Taipei Veterans General Hospital, Department of Medicine, Taipei, Taiwan; [12]Kindai University Nara Hospital, Department of Hematology, Nara, Japan; [13]Ewha Womans' University College of Medicine, Department of Internal Medicine, Seoul, South Korea; [14]National Hospital Organization Kumamoto Medical Center, Department of Hematology, Kumamoto, Japan; [15]Japanese Red Cross Osaka Hospital, Department of Hematology, Osaka, Japan;[16]Yonsei University College of Medicine, Severance Hospital, Division of Hematology, Department of Internal Medicine, Seoul, South Korea; [17]Seoul St. Mary's Hospital, The Catholic University of Korea, Division of Hematology, Seoul, South Korea; [18]Kyowa Kirin Co., Ltd., Tokyo, Japan; [19]Kurume University, Biostatistics Center, Kurume, Japan; [20]Kanazawa University Hospital, Division of Medical Safety, Kanazawa, Japan;*

### Background

Results of phase II/III trials of rabbit antithymocyte globulin (ATG) + cyclosporine (CsA) + romiplostim (ROMI) (531-003; NCT03957694) and CsA + ROMI (531-004; NCT04095936) for treatment-naïve patients (pts) with aplastic anemia (AA) have previously been reported (*Blood Adv* 2025, *BJH* 2025). Long-term efficacy and safety data (up to 5 years) have been reported for the prospective observational study 531-005 (NCT04870346). Although efficacy was evaluated at weeks (wk) 27 and 53 in the trials, the effects of treatment continuation and discontinuation after (beyond) 27 wks have been insufficiently investigated. Therefore, we conducted an additional analysis of data from 531-005 to identify pts who achieved a response with ROMI treatment after wk 27 who had no response at wk 27 (late response) and those who sustained complete or partial responses (sustained CR/PR) after ROMI discontinuation, and to explore factors associated with these outcomes.

### Aims

To explore factors associated with late response and sustained CR/PR in treatment-naïve AA pts treated with immunosuppressive therapy (IST) + ROMI.

### Methods

We analyzed pts who achieved a late response or sustained CR/PR in 531-005 and explored associations with baseline characteristics.

**Copyright Information:** (Online) ISSN: 2572-9241
©2026 The Author(s). HemaSphere is published by John Wiley & Sons Ltd on behalf of European Hematology Association. This is an open access Abstract Book distributed under the Attribution-NonCommercial-NoDerivs (CC BY-NC-ND), which allows third parties to download the articles and share them with others as long as they credit the author and the Abstract Book, but they cannot change the content in any way or use them commercially.
**Abstract Book Citations:** Authors, Title, HemaSphere, 2026;10:(S1):pages. The abstract book DOIs can be found on the "Author Information" page.
**Disclaimer:** Articles published in the journal HemaSphere exclusively reflect the opinions of the authors. The authors are responsible for all content in their abstracts including accuracy of the facts, statements, citing resources, etc.

### Results

A total of 36 pts were enrolled in 531-005 (531-003, n=15; 531-004, n=21) (median observation period, 4.1 years [range, 0.1–5.0]). Of 13 pts who did not achieve a response at wk 27, six continued ROMI. Of these 6 pts, 4 and 2 pts achieved responses within 1 year (wk 53) and after 1 year, respectively (wks 31, 34, 40, 41, 57 and 126, respectively). All 4 pts who responded by wk 53 maintained their response through the final observation (wks 176, 186, 201, and 236), with 2 continuing ROMI, 1 switching to eltrombopag (EPAG) in CR, and 1 discontinuing ROMI. The remaining two patients had transient no response, but achieved a response at the final observation point with ROMI treatment. In late responders who achieved a response by wk 53, there was a numerical increase in reticulocyte and neutrophil counts from baseline (Figure). Although these increases did not reach statistical significance, the slope of the change from baseline to wks 27 was positive.
ROMI was discontinued by or after wk 27 in 11 of 13 pts who responded to ATG + CsA + ROMI. Excluding one patient treated with EPAG during the response period, 5 pts had sustained CR/PR, and achieved treatment-free remission with discontinuation of CsA by the last observation point. Sustained CR/PR was more frequently observed in patients with higher baseline reticulocyte counts (≥20,000/μL; 3/5 [60.0%] vs 1/6 [16.7%]), higher platelet peaks and speed of increase during treatment. Most pts with platelet counts ≥100,000/μL within 1 or 2 years (5/8, 62.5%) and 2 of 3 pts with counts ≥200,000/μL by wk 27 achieved sustained CR/PR.

### Summary/Conclusion

In treatment-naïve pts with AA treated with IST+ROMI, factors potentially associated with late response and sustained CR/PR were investigated in an exploratory analysis. The findings suggest that ROMI continued beyond wk 27 may have clinically meaningful effects. Additionally, treatment strategies aimed at achieving greater or earlier increases in platelet counts with ROMI may facilitate sustained CR/PR and treatment-free remission. Future therapeutic options therefore warrant further investigation.

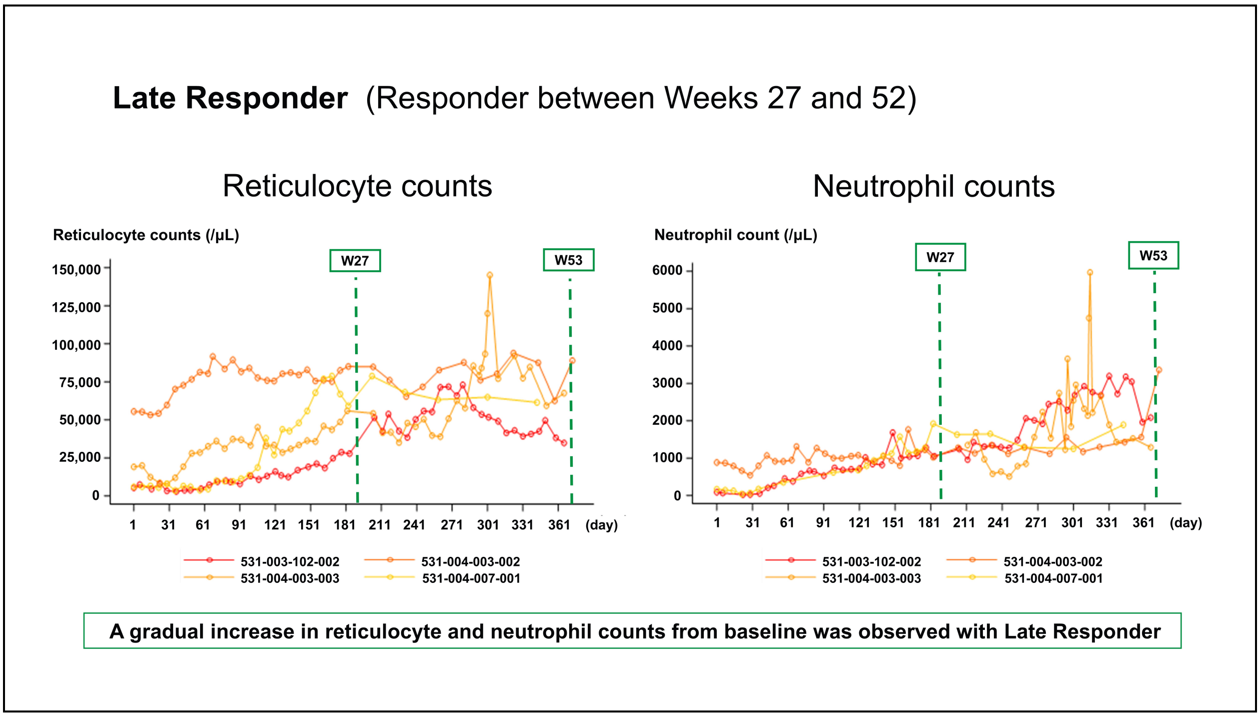

**Copyright Information:** (Online) ISSN: 2572-9241
©2026 The Author(s). HemaSphere is published by John Wiley & Sons Ltd on behalf of European Hematology Association. This is an open access Abstract Book distributed under the Attribution-NonCommercial-NoDerivs (CC BY-NC-ND), which allows third parties to download the articles and share them with others as long as they credit the author and the Abstract Book, but they cannot change the content in any way or use them commercially.
**Abstract Book Citations:** Authors, Title, HemaSphere, 2026;10:(S1):pages. The abstract book DOIs can be found on the "Author Information" page.
**Disclaimer:** Articles published in the journal HemaSphere exclusively reflect the opinions of the authors. The authors are responsible for all content in their abstracts including accuracy of the facts, statements, citing resources, etc.

### (PS1813) CLINICAL EFFICACY AND SAFETY OF COMPLEMENT INHIBITOR MONOTHERAPY AND COMBINED IMMUNOTHERAPY IN PATIENTS WITH PAROXYSMAL NOCTURNAL HEMOGLOBINURIA-APLASTIC ANEMIA (PNH-AA): A SINGLE-CENTER COHORT STUDY

**Topic:** 12. Bone marrow failure syndromes incl. PNH - Clinical

Xiaoqi Pang[1], Hui Liu[1], Rong Fu*[1]

[1] *Tianjin Medical University General Hospital, Department of Hematology, Tianjin, China;*

### Background

Paroxysmal nocturnal hemoglobinuria-aplastic anemia (PNH-AA) patients present with concurrent hemolysis and bone marrow failure, which complicates clinical management. During treatment, some aplastic anemia (AA) patients develop a high proportion of detectable PNH clones accompanied by overt hemolytic features. Patients with recovered bone marrow function may benefit from complement inhibitor monotherapy, whereas those with persistent bone marrow failure may require combination therapy with complement inhibitors and immunotherapy. However, clinical evidence supporting these two therapeutic strategies in PNH-AA remains limited.

### Aims

To clarify the differences in clinical characteristics between two subtypes of PNH-AA, namely classic PNH and PNH with bone marrow failure, and to investigate the clinical efficacy and safety of complement inhibitor monotherapy and combined immunotherapy in PNH-AA patients, respectively.

### Methods

A retrospective analysis was conducted on 37 patients diagnosed with PNH-AA who were treated at our center from 2021 to 2025. Among them, 17 patients progressed from AA to classic PNH and received complement inhibitor monotherapy, while 19 patients developed AA-related PNH with bone marrow failure and received combination therapy with complement inhibitors and immunotherapy. The median follow-up duration was 1.25 (1–4) years. Parameters monitored included hemoglobin levels, lactate dehydrogenase levels, PNH clone size, bone marrow hematopoietic function (absolute neutrophil count, reticulocytes, platelets). Adverse events were also recorded.

**Copyright Information:** (Online) ISSN: 2572-9241
©2026 The Author(s). HemaSphere is published by John Wiley & Sons Ltd on behalf of European Hematology Association. This is an open access Abstract Book distributed under the Attribution-NonCommercial-NoDerivs (CC BY-NC-ND), which allows third parties to download the articles and share them with others as long as they credit the author and the Abstract Book, but they cannot change the content in any way or use them commercially.
**Abstract Book Citations:** Authors, Title, HemaSphere, 2026;10:(S1):pages. The abstract book DOIs can be found on the "Author Information" page.
**Disclaimer:** Articles published in the journal HemaSphere exclusively reflect the opinions of the authors. The authors are responsible for all content in their abstracts including accuracy of the facts, statements, citing resources, etc.

### Results

1. Compared with patients with bone marrow failure, those with classic PNH-AA exhibited more severe fatigue and hemoglobinuria. Laboratory examinations revealed larger PNH clones and higher levels of lactate dehydrogenase, total bilirubin and direct bilirubin. Patients with bone marrow failure had lower absolute neutrophil and platelet counts, whereas hemoglobin levels were comparable between the two groups.
2. Complement inhibitor monotherapy effectively controlled hemolysis in classic subtype PNH-AA. Within one year of treatment, 33.3% (6/18) of patients achieved complete response (CR), with no breakthrough hemolysis or secondary bone marrow failure occurred.
3. Combination therapy effectively controlled hemolysis and improved bone marrow hematopoietic function in bone marrow failure subtype PNH-AA. Within one year of treatment, 15.79% (3/19) of patients achieved significant response (SR), while 26.32% (5/19) achieved good response (GR), with no breakthrough hemolysis or progression of bone marrow failure occurred.
4. Combination therapy suppressed T-cell cytotoxicity in the bone marrow failure subtype PNH-AA. The proportion of CD8+ T cells was significantly decreased, accompanied by an increased proportion of CD4+ T cells. The proportions of Treg cells and B cells were increased, and the levels of inflammatory cytokines such as IL-6 and TNF-α were decreased. No significant differences were observed in NK cells after treatment.
5. The major adverse events included infection, abnormal liver function, leukopenia, and neutropenia. No significant differences in these adverse events were observed between the two groups, and all symptoms could be relieved after symptomatic treatment.

### Summary/Conclusion

Complement inhibitors effectively control hemolysis in all PNH-AA subtypes, and their combination with immunotherapy to improve bone marrow hematopoietic function is clinically feasible.

**Copyright Information:** (Online) ISSN: 2572-9241
©2026 The Author(s). HemaSphere is published by John Wiley & Sons Ltd on behalf of European Hematology Association. This is an open access Abstract Book distributed under the Attribution-NonCommercial-NoDerivs (CC BY-NC-ND), which allows third parties to download the articles and share them with others as long as they credit the author and the Abstract Book, but they cannot change the content in any way or use them commercially.
**Abstract Book Citations:** Authors, Title, HemaSphere, 2026;10:(S1):pages. The abstract book DOIs can be found on the "Author Information" page.
**Disclaimer:** Articles published in the journal HemaSphere exclusively reflect the opinions of the authors. The authors are responsible for all content in their abstracts including accuracy of the facts, statements, citing resources, etc.

### (PS1814) IPTACOPAN TREATMENT FOR CHINESE PATIENTS WITH HEMOLYTIC PAROXYSMAL NOCTURNAL HEMOGLOBINURIA (PNH): A MULTICENTER RETROSPECTIVE STUDY

**Topic:** 12. Bone marrow failure syndromes incl. PNH - Clinical

Leyu Wang[1], Min Zhang[2], Hong Chang[3], Hu Zhou[4], Shiyu MA[5], Yanping MA[6], Dong Miao[7], Zeping Zhou[8], Jinsong Jia[9], Ruijuan Zhang[10], Guoxiang Zhang[11], Jie Liu[12], Qiujuan Zhu[13], Bin Yu[14], Hongjuan Wang[15], Ruihong Hou[16], Wenjun Ge[17], Lixin Wang[18], Junyuan Lang[19], Hongling MA[20], Yuan Yang[21], Bing Han*[1]

*[1]Peking Union Medical College Hospital, Chinese Academy of Medical Science and Peking Union Medical College, Hematology, Beijing, China; [2]Union Hospital, Tongji Medical College, Huazhong University of Science and Technology, wuhan, China; [3]Department of Hematology, Institute of Hematology, West China Hospital, Sichuan University, 37 GuoXueXiang Street,, Chengdu, China; [4]The Affiliated Cancer Hospital of Zhengzhou University & Henan Cancer Hospital, Zhengzhou, China;[5]The First Hospital of China Medical University, Beijing, China; [6]The Second Hospital of Shanxi Medical University, shanxi, China; [7]Huaihe Hospital of Henan University, kaifeng, China;[8]The Second Affiliated Hospital of Kunming Medical University, kunming, China; [9]Peking University People's Hospital, Peking University Institute of Hematology, National Clinical Research Center for Hematologic Disease, Beijing, China; [10]The First Hospital of Shanxi Medical University, shanxi, China;[11]Heping Hospital Affiliated to Changzhi Medical College, changzhi, China; [12]Sinopharm Tongmei General Hospital, Datong, China;[13]Shanxi Bethune Hospital, shanxi, China; [14]Shanxi Traditional Chinese Medical Hospital, shanxi, China; [15]Yuncheng Traditional Chinese Medicine Hospital, China,, yuncheng, China; [16]Linfen People's Hospital, China,, Linfen, China; [17]The Fifth People's Hospital of Datong, China,, Datong, China; [18]The First People's Hospital of Jinzhong,, Jinzhong, China;[19]Jincheng People's Hospital, Jincheng, China; [20]The Second People's Hospital of Datong (Datong Cancer Hospital), China,, Datong, China;[21]Department of Hematology, Peking University Third Hospital, Beijing, China;*

### Background

Paroxysmal Nocturnal Hemoglobinuria (PNH) is a rare, acquired clonal disorder of hematopoietic stem cells. Iptacopan is an oral, proximal complement inhibitor that targets factor B, which is a key protease in alternative complement pathway initiation. Moreover, iptacopan is more effective in obviating the need for red-cell transfusions, elevating hemoglobin levels, and reducing the frequency of clinical breakthrough hemolysis compared with anti-C5 complement inhibitors.

### Aims

This study aims to evaluate the real-world efficacy and safety of iptacopan in complement inhibitor-naïve patients with PNH in China.

### Methods

This retrospective study included data collected from patients diagnosed with PNH at Peking Union Medical College Hospital and other hospitals all over China between June 2024 and May 2025, who were consistently treated with iptacopan for a minimum of 6 months. Changes in clinical and laboratory indicators were examined after 1, 3, and 6 months of iptacopan therapy and during the subsequent follow-up. The incidence rates of clinical breakthrough hemolysis (BTH), extravascular hemolysis (EVH), and adverse events were also recorded.

**Copyright Information:** (Online) ISSN: 2572-9241
©2026 The Author(s). HemaSphere is published by John Wiley & Sons Ltd on behalf of European Hematology Association. This is an open access Abstract Book distributed under the Attribution-NonCommercial-NoDerivs (CC BY-NC-ND), which allows third parties to download the articles and share them with others as long as they credit the author and the Abstract Book, but they cannot change the content in any way or use them commercially.
**Abstract Book Citations:** Authors, Title, HemaSphere, 2026;10:(S1):pages. The abstract book DOIs can be found on the "Author Information" page.
**Disclaimer:** Articles published in the journal HemaSphere exclusively reflect the opinions of the authors. The authors are responsible for all content in their abstracts including accuracy of the facts, statements, citing resources, etc.

### Results

Totally 54 patients were enrolled, including 17 (31.5%) females, with a median age of 38 years (range, 21–82). Twenty-six (48.1%) patients had classic PNH and 28 (51.9%) had aplastic anemia (AA)/PNH. Before iptacopan treatment, 20 cases (37%) were transfusion-dependent. The median duration of follow-up was 10 months (range, 6–18). During the entire follow-up period, among the 54 patients, 53 (98.1%) had LDH ≤ 1.5 ULN at least once and 50 (92.6%) displayed LDH ≤ 1.0 ULN at least once, with median times to first attainment of 1 month (range, 1–3) and 1 month (range, 1–6), respectively.At 1, 3, 6, 12 and 18 months, 94.4% (51/54), 96.3% (52/54), 94.4% (51/54), 95% (19/20) and 84.6% (11/13) patients achieved LDH ≤ 1.5 ULN, respectively, whereas 79.6% (43/54), 68.5% (37/54), 70.4% (38/54), 60% (12/20) and 53.8% (7/13) patients achieved LDH ≤ 1.0 ULN (Figure 1A-B), respectively. During the entire follow-up period, 49 (90.7%) patients exhibited hemoglobin level increases from baseline of ≥ 20 g/L, at a median time of 1 month (range, 1–6). On the other hand, 46 (85.2%) patients had hemoglobin levels ≥ 120 g/L, at a median of 3 months (range, 1–6, Figure 1C-D). All those who were transfusion dependent at baseline did not need transfusion 14 days after iptacopan treatment and maintained transfusion independence until the end of follow-up. No significant differences were noted in hemolytic parameters between patients with classic PNH and those with AA/PNH. BTH in 4 (7.4%) patients, with a median time of onset at 10 months (range, 3.5–18). Adverse events were reported in 20 (37.0%) patients, all of which were mild in severity. The most common adverse events were headache (13.0%), upper respiratory tract infection (13.0%), skin rash (5.6%) and transient elevation of liver enzymes (5.6%). No new thrombosis or microvascular events, EVH, and breakthrough infections by Neisseria meningitidis or Streptococcus pneumoniae were noticed during the observation period. No deaths or adverse events leading to treatment discontinuation were recorded.

### Summary/Conclusion

Iptacopan showed sustained hemolytic control, significant hemoglobin improvement and favorable safety in Chinese PNH patients.

**Copyright Information:** (Online) ISSN: 2572-9241
©2026 The Author(s). HemaSphere is published by John Wiley & Sons Ltd on behalf of European Hematology Association. This is an open access Abstract Book distributed under the Attribution-NonCommercial-NoDerivs (CC BY-NC-ND), which allows third parties to download the articles and share them with others as long as they credit the author and the Abstract Book, but they cannot change the content in any way or use them commercially.
**Abstract Book Citations:** Authors, Title, HemaSphere, 2026;10:(S1):pages. The abstract book DOIs can be found on the "Author Information" page.
**Disclaimer:** Articles published in the journal HemaSphere exclusively reflect the opinions of the authors. The authors are responsible for all content in their abstracts including accuracy of the facts, statements, citing resources, etc.

### (PS1815) 3-YEAR EFFICACY AND SAFETY OF CROVALIMAB IN PATIENTS WITH PAROXYSMAL NOCTURNAL HEMOGLOBINURIA (PNH) WHO SWITCHED FROM RAVULIZUMAB: LONG-TERM DATA FROM THE PHASE III COMMODORE 1 TRIAL

**Topic:** 12. Bone marrow failure syndromes incl. PNH - Clinical

Yasutaka Ueda*[1], Hazzaa Alzahrani[2], Alejandro Contento Gonzalo[3], Martin Höglund[4], Jin Seok Kim[5], Austin G. Kulasekararaj[6], Jens Panse[7], Melissa Rose[8], Jörg Schubert[9], Eng-Soo Yap[10], Sammy Chebon[11], Siobhan Connor-Ahmad[12], Sai Li[11], Sasha Sreckovic[13], Phillip Scheinberg[14]

*[1]Department of Hematology and Oncology, Graduate School of Medicine, Faculty of Medicine, Osaka University, Suita, Japan; [2]Department of Hematology, Stem Cell Transplantation and Cellular Therapy, King Faisal Specialist Hospital & Research Center, Riyadh, Saudi Arabia; [3]Regional University Hospital of Málaga, Málaga, Spain;[4]Department of Medical Sciences, Uppsala University, Uppsala, Sweden; [5]Yonsei University College of Medicine, Severance Hospital, Seoul, South Korea; [6]Department of Haematological Medicine, King's College Hospital; National Institute for Health Research and Wellcome King's Clinical Research Facility and King's College London, London, United Kingdom; [7]Department of Oncology, Hematology, Hemostaseology and Stem Cell Transplantation, University Hospital RWTH Aachen; Centre for Integrated Oncology (CIO), Aachen, Bonn, Cologne, Düsseldorf (ABCD), London, United Kingdom; [8]Division of Pediatric Hematology Oncology, Nationwide Children's Hospital, Department of Pediatrics, The Ohio State University, Columbus, United States of America; [9]Elblandklinikum, Riesa, Germany; [10]Department of Laboratory Medicine, National University Hospital, Singapore, Singapore; [11]F. Hoffmann-La Roche Ltd, Basel, Switzerland; [12]Roche Products Ltd, Welwyn Garden City, United Kingdom; [13]Genentech, Inc South, San Francisco, United States of America; [14]Division of Hematology, Hospital A Beneficencia Portuguesa de São Paulo, Sao Paulo, Brazil;*

### Background

Crovalimab (crova; PiaSky®), a novel C5 inhibitor (C5i), enables maintenance subcutaneous injections every 4 weeks (wks) in patients (pts) with PNH. In COMMODORE 2 (NCT04434092), crova demonstrated non-inferior efficacy vs eculizumab (ecu) in C5i-naive pts (Röth 2024). In the global COMMODORE 1 trial (NCT04432584), crova showed similar safety and efficacy vs ecu in randomized pts who switched from ecu (Scheinberg 2024); this study also included a non-randomized cohort of pts who switched from ravulizumab (ravu; a C5i given intravenously every 8 wks) to crova.

### Aims

Report 3-year follow-up ravu switch data from COMMODORE 1.

### Methods

The ravu-pretreated cohort enrolled pts with PNH who received ravu for ≥16 wks and had lactate dehydrogenase (LDH) ≤2×the upper limit of normal (ULN) at screening. Pts received crova (weight-based tiered dosing) for a 24-wk primary treatment period, followed by an extension period.

Efficacy was evaluated from baseline (BL) to Week (W)25 (primary treatment period) and from W25–169 (extension period). Exploratory efficacy endpoints included: the proportions of pts with hemolysis control (LDH ≤1.5×ULN) at each visit; and with transfusion avoidance (TA), breakthrough hemolysis (BTH), and hemoglobin (Hb) stabilization over consecutive 24-wk intervals. Fatigue was assessed by visit using the Functional Assessment of Chronic Illness Therapy (FACIT)-Fatigue scale. Safety was assessed from BL to the clinical cutoff date (Nov 3, 2025).

**Copyright Information:** (Online) ISSN: 2572-9241
©2026 The Author(s). HemaSphere is published by John Wiley & Sons Ltd on behalf of European Hematology Association. This is an open access Abstract Book distributed under the Attribution-NonCommercial-NoDerivs (CC BY-NC-ND), which allows third parties to download the articles and share them with others as long as they credit the author and the Abstract Book, but they cannot change the content in any way or use them commercially.
**Abstract Book Citations:** Authors, Title, HemaSphere, 2026;10:(S1):pages. The abstract book DOIs can be found on the "Author Information" page.
**Disclaimer:** Articles published in the journal HemaSphere exclusively reflect the opinions of the authors. The authors are responsible for all content in their abstracts including accuracy of the facts, statements, citing resources, etc.

### Results

28 pts were enrolled (11 with a history of aplastic anemia; median treatment duration, 152.2 wks [range 4.1–224.0]).
Mean normalized LDH remained ≤1.5×ULN at each visit; hemolysis control was achieved in 82–100% of pts from BL–W25 and 89%–100% of pts from W25–169. TA, Hb stabilization, and BTH rates were consistent with the 2-year follow-up data (**Table**). Most BTH events were singular events due to a concurrent complement-activating condition. Mean FACIT-Fatigue scores ranged from 30.9 to 36.9 from BL–W25 and 32.8 to 37.8 from W25–169.
Adverse events (AEs) with incidence ≥5 per 100 pt-years (PY) were summarized for crova (prior ravu) (28 pts; 71.5 PY). The incidence per 100 PY of serious AEs (SAEs) was 50.4 (95% CI 35.3, 69.7), most commonly type III immune complex mediated reactions (7.0 [2.3, 16.3]) and COVID-19, hemolysis, and cholangitis (4.2 [0.9, 12.3] each); incidence per 100 PY of infection SAEs was 16.8 (8.7, 29.3). No deaths occurred.
Transient immune complex mediated reactions (TICRs; one-time events that some pts may experience only when switching between crova and another C5i) occurred in 9 of 28 pts. Common symptoms were pyrexia (n=4), rash (n=3), and arthralgia (n=3); there were no renal manifestations. Median time to 1st TICR onset was 2.0 wks (range 1.3–4.3) and median TICR duration was 1.7 wks (0.6–11.9). No life-threatening/fatal events occurred and most TICRs were mild/moderate. No meningococcal infections occurred.

### Summary/Conclusion

Long-term follow-up data from COMMODORE 1 showed maintained disease control in pts who switched from ravu to crova. Long-term sustained efficacy and tolerable safety were achieved in pts who switch from ravu to crova. These exploratory data warrant cautious interpretation due to the small sample size and disease variability.

| Endpoint | BL–W25 | W25–W169 |
|---|---|---|
| TA | 67% | 78%–95% |
| Hb stabilization | 54% | 78%–95% |
| BTH | 29% | 0%–11% |

BTH, breakthrough hemolysis; Hb, hemoglobin; TA, transfusion avoidance.

**Copyright Information:** (Online) ISSN: 2572-9241
©2026 The Author(s). HemaSphere is published by John Wiley & Sons Ltd on behalf of European Hematology Association. This is an open access Abstract Book distributed under the Attribution-NonCommercial-NoDerivs (CC BY-NC-ND), which allows third parties to download the articles and share them with others as long as they credit the author and the Abstract Book, but they cannot change the content in any way or use them commercially.
**Abstract Book Citations:** Authors, Title, HemaSphere, 2026;10:(S1):pages. The abstract book DOIs can be found on the "Author Information" page.
**Disclaimer:** Articles published in the journal HemaSphere exclusively reflect the opinions of the authors. The authors are responsible for all content in their abstracts including accuracy of the facts, statements, citing resources, etc.

### (PS1816) TEMPI SYNDROME PATIENT MONOCLONAL ANTIBODIES ARE PATHOGENIC DRIVERS OF ERYTHROPOIETIN PRODUCTION

**Topic:** 13. Myeloma and other monoclonal gammopathies - Biology & translational research

David Sykes*[1], Jens Wrammert[2], Wilfried Schroyens[3]

*[1]Massachusetts General Hospital, Hematology, Boston, United States of America;[2]Emory University, Atlanta, United States of America;[3]University of Antwerp, Antwerp, Belgium;*

### Background

We discovered the TEMPI syndrome in 2011. TEMPI is a rare monoclonal gammopathy of clinical significance (MGCS) where patients commonly display five hallmark features: (1) Telangiectasias (involving the upper torso and face), (2) elevated serum Erythropoietin (EPO) levels and erythrocytosis, (3) Monoclonal gammopathy, (4) Perinephric fluid collections, and (5) microscopic Intrapulmonary shunting (Fig 1A). To date, we have identified 53 patients across the world. TEMPI is an acquired syndrome without any specific geographic, racial, or gender predisposition.
TEMPI patients have a strikingly elevated serum EPO, with EPO often >500-times the upper limit of normal. This suggests a severe dysregulation of hypoxia sensing and of EPO production. As expected, elevated serum EPO leads to uncontrolled red blood cell production and marked erythrocytosis. Patients are commonly misdiagnosed with polycythemia vera and started on programs of phlebotomy.
In some TEMPI patients, plasma cell directed therapies (e.g., bortezomib, lenalidomide, daratumumab, teclistamab, autologous transplant) have been used to eradicate the TEMPI MGUS. Remarkably, elimination of the MGUS leads to complete resolution of the other TEMPI syndrome symptoms, implicating the monoclonal antibody as the pathologic driver of the disease.

### Aims

We hypothesize that TEMPI syndrome is driven by the auto-reactive monoclonal gammopathy (Fig 1B). Using a toolkit of TEMPI syndrome patient monoclonal antibodies, we sought to identify the antigenic target that leads to dysregulated EPO production.

### Methods

Bone marrow aspirates were collected from 13 TEMPI syndrome patients. Plasma cells were isolated, RNA was purified and long read RNA sequencing was performed to identify the exact heavy and light chain sequences of the monoclonal antibody (mAb). This permitted the cloning and the *in vitro* production of unique, pure, recombinant, TEMPI patient mAbs.

### Results

(1) TEMPI mAbs were injected into wild-type mice at a dose mimicking the serum monoclonal antibody concentration as seen in patients. The TEMPI mAbs increased the serum EPO concentration as well as increasing the hemoglobin and hematocrit. (2) TEMPI mAbs were used to screen a focused pathogen array. This implicated a potential previous exposure to toxoplasmosis as a trigger in two TEMPI syndrome patients. (3) Two TEMPI mAbs were used for flow cytometric screening of a membrane proteome array of ~6000 human membrane proteins. This has generated a candidate list of potential TEMPI syndrome membrane protein auto-antigens, currently awaiting experimental validation.

**Copyright Information:** (Online) ISSN: 2572-9241
©2026 The Author(s). HemaSphere is published by John Wiley & Sons Ltd on behalf of European Hematology Association. This is an open access Abstract Book distributed under the Attribution-NonCommercial-NoDerivs (CC BY-NC-ND), which allows third parties to download the articles and share them with others as long as they credit the author and the Abstract Book, but they cannot change the content in any way or use them commercially.
**Abstract Book Citations:** Authors, Title, HemaSphere, 2026;10:(S1):pages. The abstract book DOIs can be found on the “Author Information” page.
**Disclaimer:** Articles published in the journal HemaSphere exclusively reflect the opinions of the authors. The authors are responsible for all content in their abstracts including accuracy of the facts, statements, citing resources, etc.

**Summary/Conclusion**

Validation experiments are underway to confirm the target of the TEMPI syndrome monoclonal antibodies. We hope to understand how an extracellular monoclonal antibody can mimic hypoxia and lead to dysregulated EPO production. This has the potential to reveal a previously unknown antibody-antigen interaction capable of driving hypoxia-independent erythropoietin production.
TEMPI syndrome is a model MGCS, where antibody target identification may shed light on disease pathogenesis. We present straightforward workflow from bone marrow biopsy to recombinant antibody production where patient-specific mAbs can be readily generated for *in vitro* diagnostic and research approaches.
TEMPI syndrome is also a model of international hematology collaboration. In a disease with ~50 patients world-wide, sample collection and research has depended on the generosity and thoughtfulness of colleagues and patients across the globe.

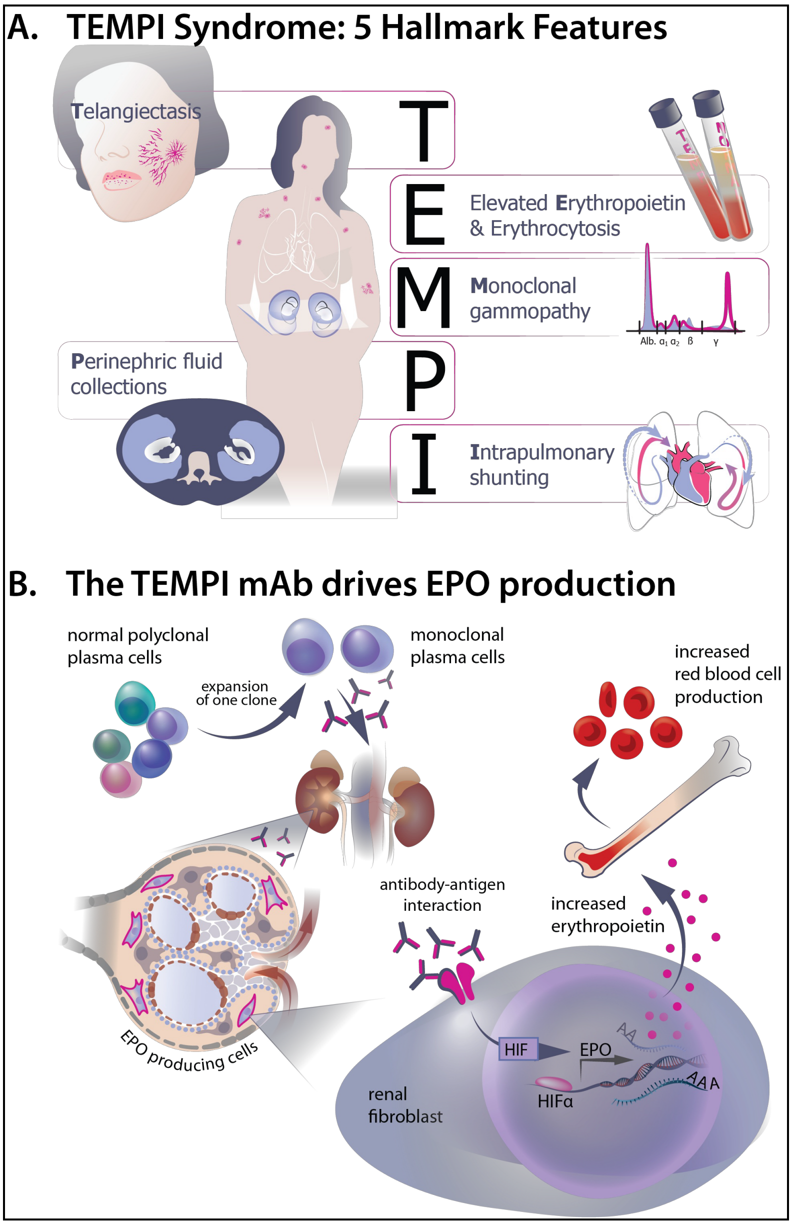

**Copyright Information:** (Online) ISSN: 2572-9241
©2026 The Author(s). HemaSphere is published by John Wiley & Sons Ltd on behalf of European Hematology Association. This is an open access Abstract Book distributed under the Attribution-NonCommercial-NoDerivs (CC BY-NC-ND), which allows third parties to download the articles and share them with others as long as they credit the author and the Abstract Book, but they cannot change the content in any way or use them commercially.
**Abstract Book Citations:** Authors, Title, HemaSphere, 2026;10:(S1):pages. The abstract book DOIs can be found on the "Author Information" page.
**Disclaimer:** Articles published in the journal HemaSphere exclusively reflect the opinions of the authors. The authors are responsible for all content in their abstracts including accuracy of the facts, statements, citing resources, etc.

### (PS1817) DUAL BTK AND BCL-2 INHIBITION DISMANTLES CXCR4 MUTATION–DRIVEN RESISTANCE IN WALDENSTROM MACROGLOBULINEMIA

**Topic:** 13. Myeloma and other monoclonal gammopathies - Biology & translational research

Eleni Kalafati*[1, 2], Nefeli Mavrianou Koutsoukou[1], Ioannis Kollias[1], Ioanna Galani[3], Christine Liacos[1], Alexandra Papadimou[1], Konstantina Taouxi[1], Katerina Chrisostomidou[1], Meletios A. Dimopoulos[1], Evangelos Terpos[1], Nicholas Anagnou[2], Efstathios Kastritis[1], Tina Bagratuni[1]

*[1]Department of Clinical Therapeutics, School of Medicine, National and Kapodistrian University of Athens, Athens, Greece; [2]Laboratory of Cell and Gene Therapy, Centre of Basic Research, Biomedical Research Foundation of the Academy of Athens (BRFAA), Athens, Greece; [3]Laboratory of Immunobiology, Centre for Clinical, Experimental Surgery and Translational Research, Biomedical Research Foundation of the Academy of Athens, Athens, Greece;*

### Background

In Waldenström macroglobulinemia (WM), somatic *CXCR4* mutations (CXCR4$^{MUT}$) are detected in 20-40% of patients and promote enhanced survival signaling, retention of malignant cells within the bone marrow niche, and attenuated responses to Bruton's tyrosine kinase inhibitors (BTKi), especially to the 1$^{st}$ generation BTKi, ibrutinib (IBR).

### Aims

To directly interrogate the mechanism of BTKi resistance, the most prevalent *CXCR4*$^{MUT}$ were introduced into experimental models *in vitro* and *in vivo*. We then evaluated the efficacy of IBR or the 2$^{nd}$ generation BTKi zanubrutinib (ZAN) in combination with the BCL-2 inhibitor venetoclax (VEN), to delineate the molecular, functional, and microenvironmental mechanisms underlying their synergistic activity.

### Methods

MWCL-1, BCWM-1 and OCI-LY3 cells were transfected with CXCR4$^{S338X}$, CXCR4$^{R334X}$, CXCR4$^{G336X}$, CXCR4$^{E343K}$ or CXCR4$^{E343X}$ plasmids using Lipofectamine$^{TM}$ 3000. CXCR4 expression was determined using qPCR and flow cytometry (FC). Transfected cells were cultured in the presence of IBR (5μM), ZAN (5μM), VEN (1μM) or BTKIs/VEN combination for 48 h. Proliferation was estimated using CCK-8. Cell cycle and apoptosis were evaluated by propidium iodide and Annexin V/7-AAD staining (FC), respectively. RNA-Seq analysis was performed in MWCL-1 and BCWM-1 cells transfected with CXCR4$^{MUT}$ or wild-type plasmids with or without BTKIs, VEN or their combination. Cytokine and growth-factor dynamics were profiled using Milliplex assays. Therapeutic efficacy and tolerability of ZAN/VEN combination (25mg/kg each) were validated in a *CXCR4*$^{S338X}$ xenograft NOD/SCID mouse model. Tumor burden, angiogenesis, circulating tumor cells, IgM secretion, and systemic cytokine secretion were analyzed using confocal microscopy, FC and ELISA, respectively. Patient serum cytokine profiles before and after BTKi therapy were also measured by Milliplex assay.

**Copyright Information:** (Online) ISSN: 2572-9241
©2026 The Author(s). HemaSphere is published by John Wiley & Sons Ltd on behalf of European Hematology Association. This is an open access Abstract Book distributed under the Attribution-NonCommercial-NoDerivs (CC BY-NC-ND), which allows third parties to download the articles and share them with others as long as they credit the author and the Abstract Book, but they cannot change the content in any way or use them commercially.
**Abstract Book Citations:** Authors, Title, HemaSphere, 2026;10:(S1):pages. The abstract book DOIs can be found on the "Author Information" page.
**Disclaimer:** Articles published in the journal HemaSphere exclusively reflect the opinions of the authors. The authors are responsible for all content in their abstracts including accuracy of the facts, statements, citing resources, etc.

**Results**

*$CXCR4^{MUT}$* conferred marked resistance to single-agent BTKi and limited sensitivity to VEN. In contrast, combined BTK and BCL-2 inhibition profoundly reduced proliferation, enforced $G_1/G_0$ arrest, and triggered robust apoptosis across all $CXCR4^{MUT/WT}$ cells. ZAN consistently outperformed IBR both alone and in combination with VEN. RNA-Seq revealed that $CXCR4^{MUT}$-mediated resistance was driven by sustained AKT, RAS, and NFκB signaling. Combination treatment collapsed BCR/NFκB signaling and activated apoptotic transcriptional networks in both $CXCR4^{WT}$ and $CXCR4^{MUT}$ cells. Cytokine profiling showed that dual inhibition also reshaped the cytokine milieu, suppressing inflammatory and microenvironment tumor-supportive factors. *In vivo*, ZAN/VEN administration was well tolerated and induced tumor regression up to 95.39 ± 0.64% ($p<0.0001$, n=4 *vs.* untreated), eliminated circulating tumor cells, reduced angiogenesis and IgM secretion, accompanied by favorable systemic cytokine modulation. Furthermore, analysis in serum samples from WM patients (n=6) undergoing therapy with the above agents mirrored preclinical findings, demonstrating decreased levels of IL-6, IL-10, GM-CSF, and TNFα following ZAN-based therapy.

**Summary/Conclusion**

Dual BTK and BCL-2 targeting disrupts the molecular axis of $CXCR4^{MUT}$ driven resistance in WM by simultaneously silencing survival signaling and unlocking apoptosis. The ZAN/VEN combination delivers deep antitumor activity that neither agent can achieve alone. These findings support the exploration of combination strategies in WM patients harboring an unfavorable *CXCR4* genotype.

**Copyright Information:** (Online) ISSN: 2572-9241
©2026 The Author(s). HemaSphere is published by John Wiley & Sons Ltd on behalf of European Hematology Association. This is an open access Abstract Book distributed under the Attribution-NonCommercial-NoDerivs (CC BY-NC-ND), which allows third parties to download the articles and share them with others as long as they credit the author and the Abstract Book, but they cannot change the content in any way or use them commercially.
**Abstract Book Citations:** Authors, Title, HemaSphere, 2026;10:(S1):pages. The abstract book DOIs can be found on the “Author Information” page.
**Disclaimer:** Articles published in the journal HemaSphere exclusively reflect the opinions of the authors. The authors are responsible for all content in their abstracts including accuracy of the facts, statements, citing resources, etc.

### (PS1818) IRF2 IS AN ESSENTIAL TRANSCRIPTION FACTOR WITH PATHOGENIC AND PROGNOSTIC IMPACT IN MULTIPLE MYELOMA

**Topic:** 13. Myeloma and other monoclonal gammopathies - Biology & translational research

Nahia Gomez-Echarte*[1], Arantxa Carrasco-León[2], Alba Maiques Diaz[3], Naroa Barrena[4], Estibaliz Miranda[2], Leire Garate[5], Ane Amundarain[2], Patxi San Martín[1], Stella Charalampopoulou[3], Luis V. Valcárcel[4], Benat Ariceta[1], Paula Rodriguez-Marquez[2], Juan Roberto Rodriguez-Madoz[2], Kazuya Ishiguro[6], Francisco Planes[4], Paula Rodríguez-Otero[7], Constantine Mitsiades[6], José Ignacio Martín-Subero[8], Edurne San José Enériz[2], Felipe Prosper[9], Xabier Agirre[2]

*[1]Centre for Applied Medical Research (CIMA), Navarre Medical Research Institute (idiSNA), Cancer Centre Clínica Universidad de Navarra (CCUN)., Pamplona, Spain; [2]Centre for Applied Medical Research (CIMA), Navarre Medical Research Institute (idiSNA), Cancer Centre Clínica Universidad de Navarra (CCUN), Pamplona, Navarre, Spain. Cancer Networking Biomedical Research Centre (CIBERONC), Madrid, Spain., Pamplona, Spain; [3]Fundació Clínic per la Recerca Biomèdica-Institut d'Investigacions Biomèdiques August Pi i Sunyer (FCRB-IDIBAPS), Barcelona, Spain; [4]Tecnun School of Engineering, University of Navarre, San Sebastián, Spain; [5]Cancer Centre Clínica Universidad de Navarra (CCUN), Servicio de Hematología y Terapia Celular, Clínica Universidad de Navarra (CUN), Pamplona, Navarra, Spain. Cancer Networking Biomedical Research Centre (CIBERONC), Madrid, Spain., Pamplona, Spain; [6]Department of Medical Oncology, Dana-Farber Cancer Institute., Boston, United States of America; [7]Servicio de Hematología y Terapia Celular, Clínica Universidad de Navarra (CUN)., Pamplona, Spain; [8]Institució Catalana de Recerca i Estudis Avançats (ICREA), Fundació Clínic per la Recerca Biomèdica-Institut d'Investigacions Biomèdiques August Pi i Sunyer (FCRB-IDIBAPS), Barcelona, Spain. Cancer Networking Biomedical Research Centre (CIBERONC), Madrid, Spain., Barcelona, Spain; [9]Centre for Applied Medical Research (CIMA), Navarre Medical Research Institute (idiSNA), Cancer Centre Clínica Universidad de Navarra (CCUN), Servicio de Hematología y Terapia Celular, Clínica Universidad de Navarra (CUN), Pamplona, Navarra, Spain. Cancer Networking Biomedical Research Centre (CIBERONC), Madrid, Spain., Pamplona, Spain;*

### Background

Multiple Myeloma (MM), the second most prevalent hematologic malignancy, remains incurable, highlighting the need to identify the molecular drivers of disease resistance. A comprehensive understanding of the molecular mechanisms underlying MM pathogenesis is essential for developing therapeutic strategies capable of overcoming disease heterogeneity and treatment relapses.
Aberrant activity of key Transcription Factors (TFs) can rewire gene expression programs, promoting uncontrolled proliferation, survival, and therapeutic resistance. Although TFs were long considered "undruggable", recent advances in therapeutic modalities have reshaped this view and opened new opportunities for TF-directed interventions in MM.

### Aims

Here, we sought to identify essential TFs and to define their functional role in MM pathogenesis.

### Methods

Leveraging the power of a custom CRISPR-Cas9 screening in MM cell lines, we systematically identified TF dependencies. The role of TF candidates was then interrogated by CUT&RUN and confirmed by transcriptomic and functional analyses in different MM cell lines. Besides, subcutaneous and intravenous mouse models were established using TF-depleted MM cell lines to determine the role of the TF in tumor growth and overall survival, respectively.

**Copyright Information:** (Online) ISSN: 2572-9241
©2026 The Author(s). HemaSphere is published by John Wiley & Sons Ltd on behalf of European Hematology Association. This is an open access Abstract Book distributed under the Attribution-NonCommercial-NoDerivs (CC BY-NC-ND), which allows third parties to download the articles and share them with others as long as they credit the author and the Abstract Book, but they cannot change the content in any way or use them commercially.
**Abstract Book Citations:** Authors, Title, HemaSphere, 2026;10:(S1):pages. The abstract book DOIs can be found on the "Author Information" page.
**Disclaimer:** Articles published in the journal HemaSphere exclusively reflect the opinions of the authors. The authors are responsible for all content in their abstracts including accuracy of the facts, statements, citing resources, etc.

### Results

We identified 22 essential TFs, including members of the Interferon Regulatory Factor (IRF) family. Remarkably, in addition to the well-known IRF4, IRF2 emerged as an underappreciated TF in MM. The essential role of IRF2 was further validated *in vitro* and *in vivo,* where a subcutaneous mouse model carrying IRF2 knockout (KO) MM cells demonstrated its impact in tumor growth (**Figure 1A**), and intravenous mouse model with IRF2 KO MM cells led to increased survival rates correlated with a less infiltration of MM cells in the bone marrow (**Figure 1B**). Also, the exogenous expression of *IRF2* upon its silencing rescued cell viability loss in MM cell lines (**Figure 1C**).
CUT&RUN experiments revealed that IRF2 binds extensively to chromatin, both independently and in cooperation with IRF1 and IRF4. While IRF2-unique regions were predominantly associated with active promoters, regions bound by IRF2/1/4 were biased towards introns (**Figure 1D**). These IRF2-bound regions indicate its role in key pathways related to MM pathobiology, further validated by RNA-seq and functional assays after *IRF2* silencing. IRF2 directly regulates specific genes, and its inhibition results in the dysregulation of key pathways. Functionally, IRF2 supports MM cell survival by suppressing necroptosis, as shown by propidium iodide staining and western blot analysis of phosphorylated MLKL (**Figure 1E**). Moreover, IRF2 promotes MM cell migration, demonstrated by wound-healing and transwell assays (**Figure 1F**).
Notably, IRF2-dependent transcriptional dysregulation was evident in precursor conditions such as MGUS and SMM, suggesting a role in early disease evolution. In addition to its role as early factor, *IRF2* levels also seem to influence disease progression, as MMs with higher expression showed worse Progression-Free Survival and Overall Survival in both univariate and multivariate analyses, even after adjusting for common MM genetic risk factors (**Figure 1G**).

### Summary/Conclusion

IRF2 constitutes an underappreciated essential TF involved in the pathogenesis and clinical behavior of MM. Its inhibition leads to dysregulation of key signaling pathways in disease progression, highlighting its potential as a therapeutic target.

**Copyright Information:** (Online) ISSN: 2572-9241
©2026 The Author(s). HemaSphere is published by John Wiley & Sons Ltd on behalf of European Hematology Association. This is an open access Abstract Book distributed under the Attribution-NonCommercial-NoDerivs (CC BY-NC-ND), which allows third parties to download the articles and share them with others as long as they credit the author and the Abstract Book, but they cannot change the content in any way or use them commercially.
**Abstract Book Citations:** Authors, Title, HemaSphere, 2026;10:(S1):pages. The abstract book DOIs can be found on the "Author Information" page.
**Disclaimer:** Articles published in the journal HemaSphere exclusively reflect the opinions of the authors. The authors are responsible for all content in their abstracts including accuracy of the facts, statements, citing resources, etc.

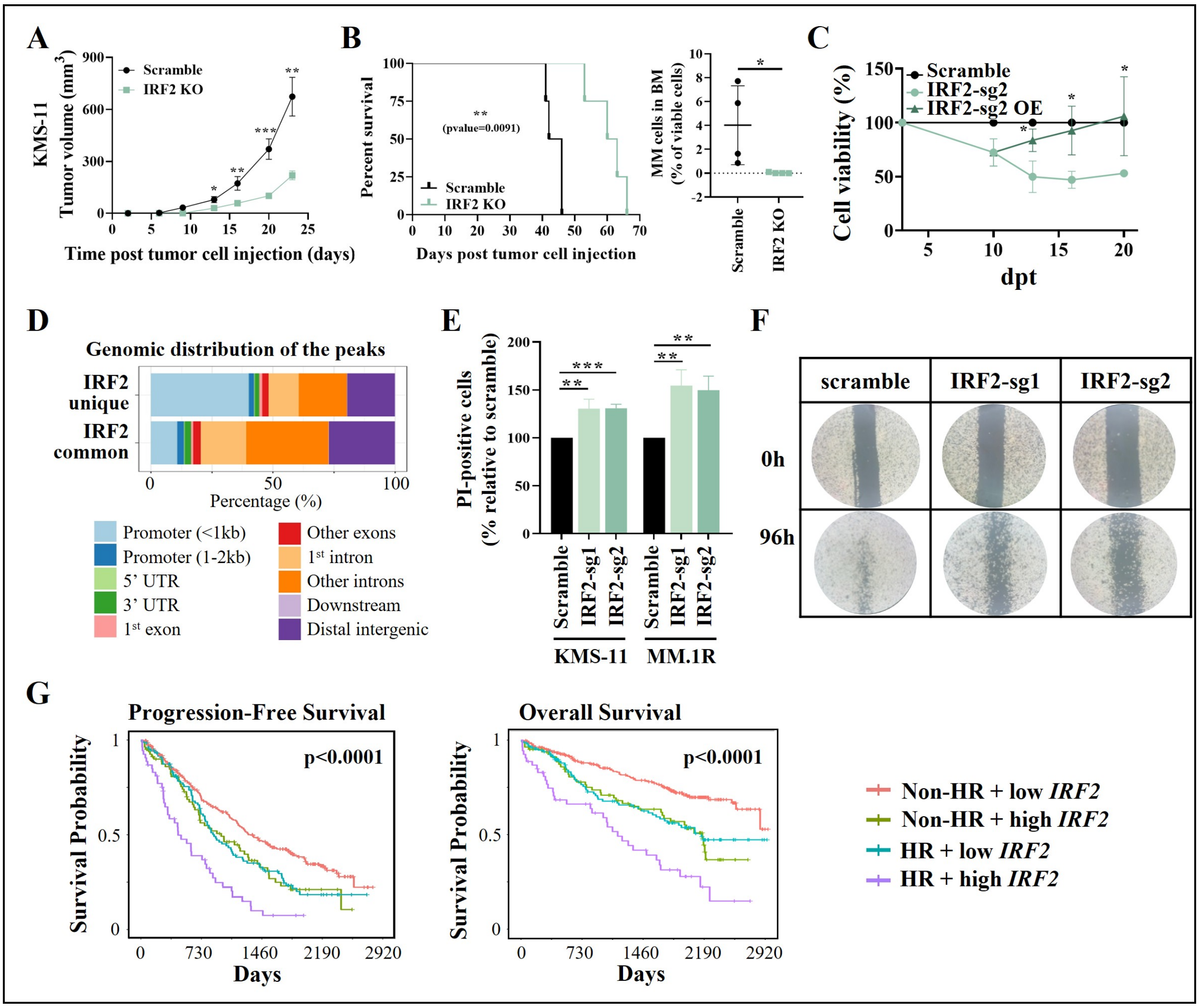

Figure 1. IRF2 is an underappreciated TF in MM. A) Tumor growth monitoring from the subcutaneous mouse model using KMS-11 cell line carrying IRF2 knockout (KO) or not (scramble) (error bars show SEM); B) Kaplan–Meier survival curves of mice injected with control MM cells (scramble) or IRF2 KO MM cells (left) and percentage of MM cells in Bone Marrow (BM) of scramble and IRF2 KO groups (right); C) Cell viability following IRF2 CRISPR/Cas9 inhibition, with or without subsequent IRF2 overexpression (OE); D) Bar plot showing the genomic distribution of IRF2 unique and IRF2 common peaks obtained by CUT&RUN; E) Percentage of propidium iodide (PI)-positive cells showing a significant increase after IRF2 inhibition using two CRISPR/Cas9 sgRNAs (IRF2-sg1 and IRF2-sg2) in KMS-11 and MM.1R cell lines; F) Wound-healing migration assay after the inhibition of IRF2 using IRF2-sg1 and IRF2-sg2; and G) Kaplan-Meier curves of the survival analysis regarding IRF2 expression in patients with High-Risk (HR) and non-HR genetic alterations, showing the results for Progression-Free Survival (PFS; left) and Overall Survival (OS; right).

Copyright Information: (Online) ISSN: 2572-9241
©2026 The Author(s). HemaSphere is published by John Wiley & Sons Ltd on behalf of European Hematology Association. This is an open access Abstract Book distributed under the Attribution-NonCommercial-NoDerivs (CC BY-NC-ND), which allows third parties to download the articles and share them with others as long as they credit the author and the Abstract Book, but they cannot change the content in any way or use them commercially.
Abstract Book Citations: Authors, Title, HemaSphere, 2026;10:(S1):pages. The abstract book DOIs can be found on the "Author Information" page.
Disclaimer: Articles published in the journal HemaSphere exclusively reflect the opinions of the authors. The authors are responsible for all content in their abstracts including accuracy of the facts, statements, citing resources, etc.

### (PS1819) CIRCULATING TUMOR CELLS AND PLASMA MYD88 MAF AS PROGNOSTIC MARKERS FOR STAGING AND IBRUTINIB RESPONSE IN WALDENSTRÖM MACROGLOBULINEMIA

**Topic:** 13. Myeloma and other monoclonal gammopathies - Biology & translational research

Tina Bagratuni*[1], Ioannis Kollias[1], Stamatia Skourti[1], Christos Vlachos[1], Nefeli Mavrianou Koutsoukou[1], Eleni Kalafati[1], Maria Sakkou[1], Alexandra Papadimou[1, 1], Konstantina Taouxi[1], Katerina Chrisostomidou[1], Ioannis Kostopoulos[1], Agisilaos Papadopoulos[1], Eleni Thanou[1], Christine Liacos[1], Eirini Solia[1], Ourania Tsitsiloni[1], Athina Markou[1], Maria Gavriatopoulou[1], Evangelos Terpos[1], Meletios A. Dimopoulos[1, 1], Efstathios Kastritis[1]

[1]*National and Kapodistrian University of Athens, Clinical Therapeutics, Athens, Greece;*

### Background

Characterization of liquid biopsy in Waldenström's macroglobulinemia (WM) as represented by the presence of circulating tumor cells (CTCs) and cell-free tumor DNA (ctDNA), may provide a powerful tool for disease burden estimation taking advantage of the non-invasive approach and the ability to capture the spatial heterogeneity of the bone marrow (BM) that a single BM biopsy misses.

### Aims

In this study, we evaluated the feasibility of detecting CTCs in patients with IgM monoclonal gammopathies and quantified the *MYD88*$^{L265}$ mutation allele frequency (MAF) in matched ctDNA. Our aim was to provide a more comprehensive assessment of disease burden and to determine whether these noninvasive markers could offer independent prognostic information beyond current clinical evaluation strategies.

### Methods

We performed multiparameter flow cytometry to detect CTCs as determined by clonal B cells (CD19$^+$ IgM$^+$ with light chain restriction) at giga per liter (10^9/L) in the peripheral blood of patients with IgM monoclonal gammopathy. All samples were stained with established phenotypic markers of the WM clone including positive expression of CD19, CD20, IgM, CD22, CD25, CD27 and exclusion of CD5 and CD23. Light-chain restriction was evaluated with surface kappa and lambda antibodies. Data were acquired on Accuri™ II cytometer.  In parallel, we performed ddPCR assay on Absolute Quantstudio for the detection and quantification of *MYD88*$^{L265P}$ mutation in ctDNA with a detection sensitivity of
$5\times10^{-3}$.

**Copyright Information:** (Online) ISSN: 2572-9241
©2026 The Author(s). HemaSphere is published by John Wiley & Sons Ltd on behalf of European Hematology Association. This is an open access Abstract Book distributed under the Attribution-NonCommercial-NoDerivs (CC BY-NC-ND), which allows third parties to download the articles and share them with others as long as they credit the author and the Abstract Book, but they cannot change the content in any way or use them commercially.
**Abstract Book Citations:** Authors, Title, HemaSphere, 2026;10:(S1):pages. The abstract book DOIs can be found on the "Author Information" page.
**Disclaimer:** Articles published in the journal HemaSphere exclusively reflect the opinions of the authors. The authors are responsible for all content in their abstracts including accuracy of the facts, statements, citing resources, etc.

**Results**

Among the 196 patients with IgM monoclonal gammopathies, CTCs were detected in 70%. Specifically, CTCs were detected in 22 out of 66 IgM-MGUS (35%, median:0.001, range: 0.0004- 0.029 x10^9/L), in 33 out of 43 patients with asymptomatic WM (76%, median: 0.0031, range: 0.001-0.3 x10^9/L) and in 81 out of 91 symptomatic WM patients (89%, median: 0.05 x10^9/L, range: 0.002-1.7 x10^9/L). There was a significant difference in the CTC distributions among the disease status groups (Kruskal-Wallis, $p<10^{-6}$). Concurrently, *MYD88*$^{L265P}$ MAF was evaluated in plasma samples available from the same patient cohort (n = 116). Similarly, *MYD88*$^{L265P}$ MAF (in patients harboring the mutation) was higher in symptomatic WM with a median of 4.6% (range:0.62 – 86.41%) compared to asymptomatic WM (median: 1.33%; range: 0.12 – 19.91%) and IgM-MGUS patients (median: 0.53%; range: 0.1 – 8.59%) indicating its potential use for monitoring tumor burden in WM (Kruskal-Wallis, $p<0.001$). No significant correlation was observed between CTC counts and *MYD88*$^{L265P}$ MAF.
Furthermore, we analyzed 37 sequential samples of WM patients at diagnosis and 6 months post-ibrutinib (IBR) therapy. Patients achieving partial response (PR) or better after 6 months of therapy were defined as responders (RG, n=24) while patients with less than PR were defined as non-responder (NRG, n=13). CTCs counts pre- and post-IBR were higher in the NRG compared to the RG ($p$= 0.0002) at the time of diagnosis but decreased significantly in the RG post therapy ($p$=0.025) but not in the NRG and was also associated with shorter progression-free survival ($p$=0.01). Concurrently, *MYD88*$^{L265P}$ MAF in ctDNA significantly decreased in RG ($p$=0.0076), while slightly increased in the NRG patients with most cases having a stable MAF pre and post treatment ($p$=0.6).

**Summary/Conclusion**

Our findings support the potential of liquid biopsy with CTCs and *MYD88*$^{L265P}$ MAF as a potential tool for assessing dynamic changes in disease status and treatment efficacy.

**Copyright Information:** (Online) ISSN: 2572-9241
©2026 The Author(s). HemaSphere is published by John Wiley & Sons Ltd on behalf of European Hematology Association. This is an open access Abstract Book distributed under the Attribution-NonCommercial-NoDerivs (CC BY-NC-ND), which allows third parties to download the articles and share them with others as long as they credit the author and the Abstract Book, but they cannot change the content in any way or use them commercially.
**Abstract Book Citations:** Authors, Title, HemaSphere, 2026;10:(S1):pages. The abstract book DOIs can be found on the "Author Information" page.
**Disclaimer:** Articles published in the journal HemaSphere exclusively reflect the opinions of the authors. The authors are responsible for all content in their abstracts including accuracy of the facts, statements, citing resources, etc.

### (PS1820) IMMUNE EXCLUSION IN EXTRAMEDULLARY MYELOMA VERSUS SOLITARY BONE PLASMACYTOMA DEPICTED VIA IMAGING MASS CYTOMETRY

**Topic:** 13. Myeloma and other monoclonal gammopathies - Biology & translational research

Maximilian Steinhardt*[1], William Sherman[2], Mariam Stein[2], Taxiarchis Kourelis[1], Dragan Jevremovic[3], Angela Dispenzieri[1], Morie Gertz[1], Karen Rech[3], Surendra Dasari[2], Jansen Seheult[3], Kevin Pavelko[4], Michael Strausbauch[4], Megan Weivoda[5], Wilson Gonsalves[1], Prashant Kapoor[1], Vincent Rajkumar[1], Shaji Kumar[1], Saurabh Zanwar[1]

*[1]Mayo Clinic, Department of Hematology, Rochester, United States of America;[2]Mayo Clinic, Department of Biomedical Informatics, Rochester, United States of America; [3]Mayo Clinic, Department of Laboratory Medicine and Pathology, Rochester, United States of America; [4]Mayo Clinic, Immune Monitoring Core, Rochester, United States of America; [5]Mayo Clinic, Division of Hematology Research, Rochester, United States of America;*

### Background

Extramedullary myeloma (EMD) is associated with aggressive clinical behavior and inferior responses to therapy, including novel T cell-redirecting approaches. Solitary plasmacytoma (SPM), which shares histological features but has favorable outcomes, can serve as a valid comparator to define immune architectures linked to tumor control.

### Aims

To characterize cellular composition and spatial single–cell organization in EMD compared with SPM.

### Methods

Imaging mass cytometry was performed on 8 regions of interest from 2 EMD and 2 SPM biopsies. A total of 101,311 cells underwent single-cell phenotyping and spatial mapping. Cells were classified into broad immunologic subsets including plasma cells, B cells, helper/cytotoxic/regulatory T cells, monocytes/macrophages, dendritic cells, and matrix and epithelial cells. Cell composition, nearest-neighbor distances, cell–cell interaction networks, and checkpoint receptor expression were compared between entities. Nonparametric testing and Bonferroni correction for multiple comparisons was applied for statistical analysis.

**Copyright Information:** (Online) ISSN: 2572-9241
©2026 The Author(s). HemaSphere is published by John Wiley & Sons Ltd on behalf of European Hematology Association. This is an open access Abstract Book distributed under the Attribution-NonCommercial-NoDerivs (CC BY-NC-ND), which allows third parties to download the articles and share them with others as long as they credit the author and the Abstract Book, but they cannot change the content in any way or use them commercially.
**Abstract Book Citations:** Authors, Title, HemaSphere, 2026;10:(S1):pages. The abstract book DOIs can be found on the "Author Information" page.
**Disclaimer:** Articles published in the journal HemaSphere exclusively reflect the opinions of the authors. The authors are responsible for all content in their abstracts including accuracy of the facts, statements, citing resources, etc.

**Results**

Plasma cells dominated both tissues (71% overall) with comparable density (plasma cell-plasma cell distance 7.1 µm vs 8.4 µm in EMD and SPM, respectively). EMD samples from lymph node samples showed a significantly higher relative abundance of B cells compared with SPM ($p = 0.019$), and T helper cells proportion was lower in EMD ($p=0.025$).  Other cell population percentages were comparable between EMD and SPM.
Nearest-neighbor analysis demonstrated preserved immune effector abundance and infiltration in both EMD and SPM, but all types of immune cells showed increased distances to plasma cells in EMD compared to SPM (EMD/SPM distance ratio 1.3-4.5, $p < 0.001$ for all absolute distance differences). Specifically, immune effector cells were spatially more separated in EMD compared to SPM (mean distance of cytotoxic T cells 68.3 µm in EMD vs 41.4 µm in SPM, macrophages 26.9 µm in EMD vs 20.8 µm in SPM; $p < 0.001$, respectively). Helper T cells and regulatory T cells were closer to cytotoxic T cells in EMD compared to SPM (EMD/SPM distance ratio 0.6 and 0.8; $p < 0.001$ for all absolute distance differences), whereas dendritic cells were significantly more distanced (EMD/SPM distance ratio 2.1; $p < 0.001$ for absolute distance differences, figure 1).
Permutation tests for cell/cell interactions based on occurrence of cell types within a 40 µm radius suggested reorganization of immune cells into condensed niches in EMD, with increased separation of plasma cells from both effector and antigen-presenting cells, whereas plasma cell- regulatory T cell associations were enriched. In contrast, SPM retained a network in which effector T cells were in proximity to both plasma cells and antigen-presenting cells. These quantitative differences are mirrored by the histologic appearance with secluded immune areas within EMD vs evenly distributed immune cells throughout SPM samples.
Helper and cytotoxic T cells in EMD displayed higher TIGIT, PD-1, PD-L1, and TIM-3 expression compared to SPMs, consistent with an exhausted phenotype ($p < 0.001$).

**Summary/Conclusion**

This analysis demonstrates that EMD is characterized by spatial decoupling of effector cells cells from antigen presentation and malignant plasma cells together with enhanced regulatory interactions compared to SPM, rather than absence of immune infiltration. This immune-excluding spatial organization may contribute to the limited responsiveness of EMD to current immunotherapies.

**Copyright Information:** (Online) ISSN: 2572-9241
©2026 The Author(s). HemaSphere is published by John Wiley & Sons Ltd on behalf of European Hematology Association. This is an open access Abstract Book distributed under the Attribution-NonCommercial-NoDerivs (CC BY-NC-ND), which allows third parties to download the articles and share them with others as long as they credit the author and the Abstract Book, but they cannot change the content in any way or use them commercially.
**Abstract Book Citations:** Authors, Title, HemaSphere, 2026;10:(S1):pages. The abstract book DOIs can be found on the "Author Information" page.
**Disclaimer:** Articles published in the journal HemaSphere exclusively reflect the opinions of the authors. The authors are responsible for all content in their abstracts including accuracy of the facts, statements, citing resources, etc.

**Figure 1: Relative cell distances, EMD / SPM**

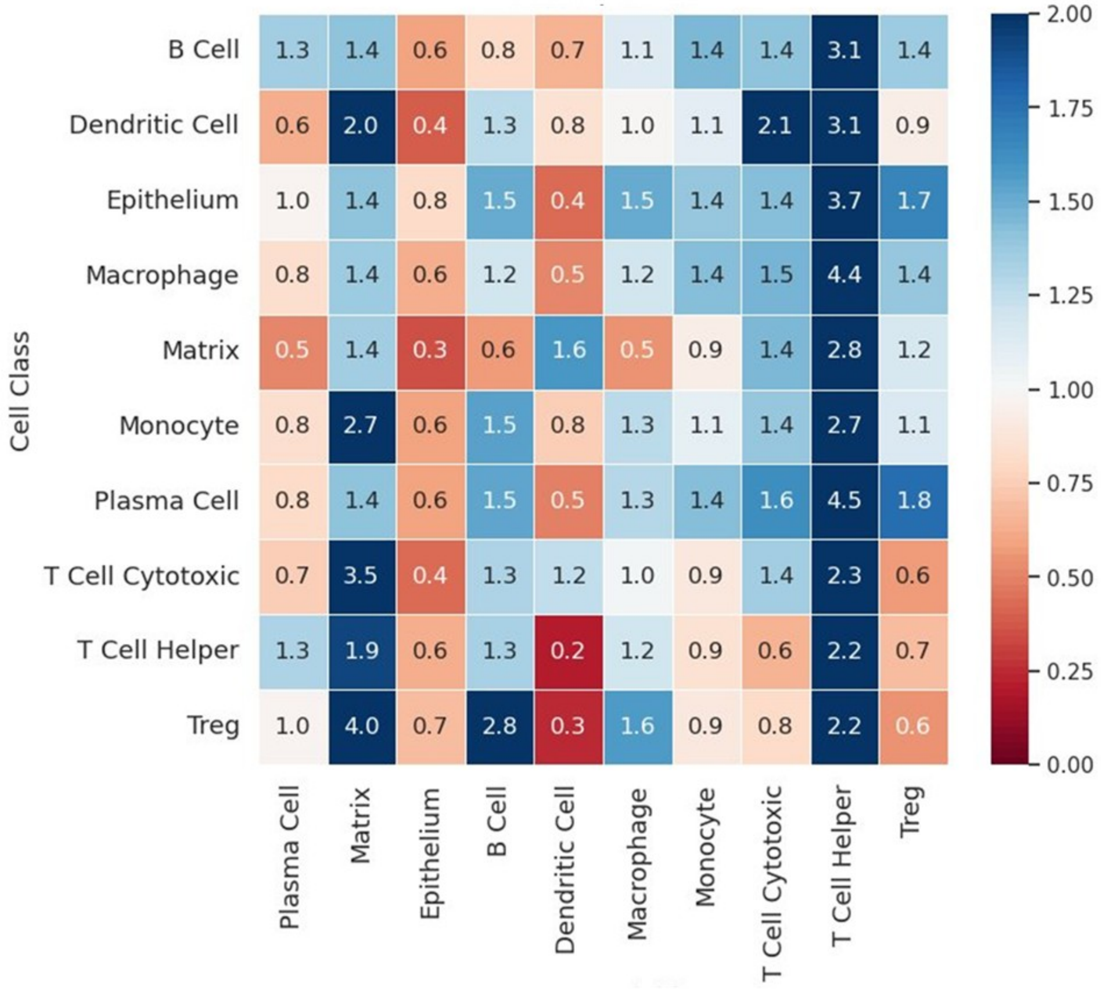

EMD/SPM ratio of mean nearest-neighbor distances between cell classes. Rows denote the index cell class and columns indicate the corresponding nearest-neighbor cell type. Values <1 (red) indicate shorter average distances in EMD relative to SPM, values >1 (blue) indicate larger distances.

**Copyright Information:** (Online) ISSN: 2572-9241
©2026 The Author(s). HemaSphere is published by John Wiley & Sons Ltd on behalf of European Hematology Association. This is an open access Abstract Book distributed under the Attribution-NonCommercial-NoDerivs (CC BY-NC-ND), which allows third parties to download the articles and share them with others as long as they credit the author and the Abstract Book, but they cannot change the content in any way or use them commercially.
**Abstract Book Citations:** Authors, Title, HemaSphere, 2026;10:(S1):pages. The abstract book DOIs can be found on the “Author Information” page.
**Disclaimer:** Articles published in the journal HemaSphere exclusively reflect the opinions of the authors. The authors are responsible for all content in their abstracts including accuracy of the facts, statements, citing resources, etc.

### (PS1821) CHROMATIN-DRIVEN TRANSCRIPTIONAL REPROGRAMMING SHAPES DISEASE EVOLUTION FROM MGUS TO MULTIPLE MYELOMA

**Topic:** 13. Myeloma and other monoclonal gammopathies - Biology & translational research

Raghad Shuwaikan*[1], Aleksandra Kurowska[2], Azari Bantan[2], Nahia Gomez-Echarte[3], Itziar Cenzano Armendariz[2], Paula Aguirre[3], Estibaliz Miranda[3], Leire Garate[3], Amaia Vilas Zornoza[3], Patxi San Martín[3], Marta Larrayoz Ilundain[3], Benat Ariceta[3], Daniel Soto Ortiz[2], Turki Sobahy[2], José Ignacio Martín-Subero[4, 5], Vincenzo Lagani[2], Jesper Tegner[2], Jose A. Martinez-Climent[3], Mikel Hernaez[1, 6, 7], Nuria Planell[1], Felipe Prosper[3, 6, 8], Xabier Agirre[3], David Gomez Cabrero[2, 9]

*[1]Centre for Applied Medical Research (CIMA), Instituto de Investigaciones Sanitarias de Navarra (IdiSNA), Cancer Center Clinica Universidad de Navarra (CCUN), Computational Biology Program, Pamplona, Spain; [2]King Abdullah University of Science and Technology, Biomedical Sciences Division, Thuwal, Saudi Arabia; [3]Centre for Applied Medical Research (CIMA), Instituto de Investigaciones Sanitarias de Navarra (IdiSNA), Cancer Center Clinica Universidad de Navarra (CCUN), Hematology and Oncology Program, Pamplona, Spain; [4]Institució Catalana de Recerca i Estudis Avançats (ICREA), Barcelona, Spain;[5]Institut d'Investigacions Biomèdiques August Pi i Sunyer (IDIBAPS), Barcelona, Spain; [6]Centro de Investigacion Biomedica en Red de Cancer (CIBERONC), Madrid, Spain; [7]Center for Data Science and Artificial Intelligence (DATAI), University of Navarra, Pamplona, Spain; [8]Clinica Universidad de Navarra, IdiSNA, Hematology and Cell Therapy Department, Pamplona, Spain; [9]Navarrabiomed, Universidad Pública de Navarra (UPNA), Translational Bioinformatics Unit, Pamplona, Spain;*

### Background

Multiple myeloma (MM) is a heterogeneous malignancy characterized by the clonal proliferation of plasma cells (PCs) within the bone marrow (BM). Despite therapeutic advances, MM remains incurable due to inevitable relapses and the transition to a refractory state. MM evolves along a clinical continuum from asymptomatic precursor stages: monoclonal gammopathy of undetermined significance (MGUS) and smoldering multiple myeloma (SMM). While the genomic drivers of this disease trajectory are well characterized, the contribution of epigenetic regulation remains less defined.

### Aims

We leveraged a bulk multiomic cohort to systematically map the epigenetic landscape of PCs across precursor to symptomatic MM. Our aim was to dissect chromatin changes underlying disease evolution and identify biological programs that may serve as novel targets for early therapeutic intervention.

### Methods

We employed an integrative approach involving 197 individuals across all disease stages and healthy donors. The cohort encompasses a broad spectrum of primary genetic aberrations, ensuring comprehensive coverage of molecular subtypes across precursor and malignant stages. Using BM-isolated CD138+ PCs, we generated paired ATAC-seq and RNA-seq profiles. To maximize analytical power, we performed H3K27ac ChIP-seq over a subset of individuals (n=128) to identify active chromatin regulatory regions. These data were integrated to define the epigenetic regulatory landscape of PCs across the MGUS-SMM-MM transition.

**Copyright Information:** (Online) ISSN: 2572-9241
©2026 The Author(s). HemaSphere is published by John Wiley & Sons Ltd on behalf of European Hematology Association. This is an open access Abstract Book distributed under the Attribution-NonCommercial-NoDerivs (CC BY-NC-ND), which allows third parties to download the articles and share them with others as long as they credit the author and the Abstract Book, but they cannot change the content in any way or use them commercially.
**Abstract Book Citations:** Authors, Title, HemaSphere, 2026;10:(S1):pages. The abstract book DOIs can be found on the "Author Information" page.
**Disclaimer:** Articles published in the journal HemaSphere exclusively reflect the opinions of the authors. The authors are responsible for all content in their abstracts including accuracy of the facts, statements, citing resources, etc.

### Results

Differential accessibility of open chromatin regions (OCRs) revealed a stage-dependent accumulation of candidate regulatory elements with progressively increasing accessibility during disease progression. Notably, 96.8% of upregulated OCRs identified at the MGUS stage were retained in SMM, and 93.6% of those persisted in MM. Transcriptomic analysis mirrored this pattern. We hypothesized that transcription factors (TFs) exploit the permissive chromatin landscape of MGUS to drive PCs toward a myeloma phenotype through opportunistic binding at newly accessible regulatory elements. To investigate disease-associated perturbations, we constructed a gene regulatory network linking active and differential OCRs to upregulated genes. Genes associated with these OCRs were enriched in key myeloma pathways, including early activation of migration programs, followed by cellular remodeling and MM-specific growth and bone pathways. Meanwhile, core signaling pathways were established in early stages of the disease and reinforced throughout disease progression. TF binding enrichment analysis identified several regulators of these pathways, including known and novel candidates, with expanding predicted target repertoires across disease stages. For example, IRF1 targets linked to the chemotaxis pathway increased from 9 regulated genes in MGUS to 14 in SMM and 24 in MM. These novel TFs could have a previously undescribed functional role in malignant transformation.

### Summary/Conclusion

Our findings demonstrate that epigenetic remodeling at precursor stages primes the genome for progression to symptomatic MM. This high-resolution multiomic atlas delineates the regulatory circuitry underlying malignant transformation and the opportunistic engagement of TFs, providing a framework for novel epigenetic-based strategies to intercept MM at early stages.

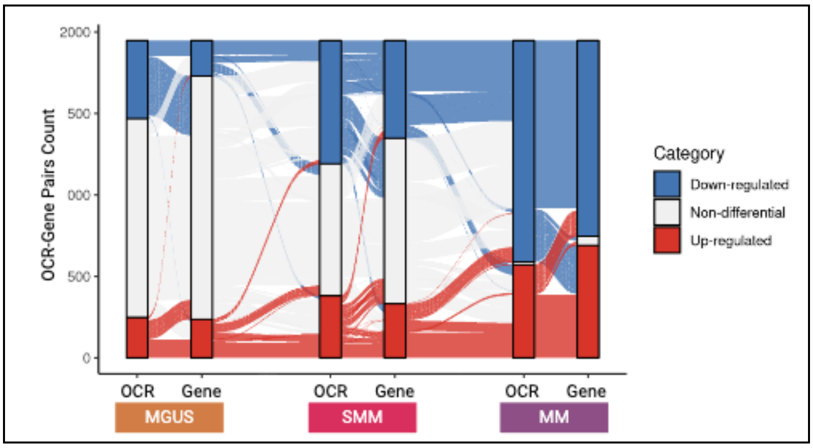

Stepwise expansion of GRNs during myeloma progression, showing accumulation of active OCRs and their linked upregulated genes.

**Copyright Information:** (Online) ISSN: 2572-9241
©2026 The Author(s). HemaSphere is published by John Wiley & Sons Ltd on behalf of European Hematology Association. This is an open access Abstract Book distributed under the Attribution-NonCommercial-NoDerivs (CC BY-NC-ND), which allows third parties to download the articles and share them with others as long as they credit the author and the Abstract Book, but they cannot change the content in any way or use them commercially.
**Abstract Book Citations:** Authors, Title, HemaSphere, 2026;10:(S1):pages. The abstract book DOIs can be found on the “Author Information” page.
**Disclaimer:** Articles published in the journal HemaSphere exclusively reflect the opinions of the authors. The authors are responsible for all content in their abstracts including accuracy of the facts, statements, citing resources, etc.

### (PS1822) TUMOR-SPECIFIC IFNΒ GENE THERAPY ENABLES SYSTEMIC ERADICATION OF MULTIPLE MYELOMA

**Topic:** 13. Myeloma and other monoclonal gammopathies - Biology & translational research

Eleni Kalafati*[1, 2], Evangelos Balafas[3], Anastasios Gaitanis[3], Aggeliki Tsalta[1], Nikolaos Kostomitsopoulos[3], Anna Agapaki[1], Konstantinos Konstantopoulos[4], Els Verhoeyen[5], Evangelos Terpos[2], Efstathios Kastritis[2], Nicholas Anagnou[1]

*[1]Laboratory of Cell and Gene Therapy, Centre of Basic Research, Biomedical Research Foundation of the Academy of Athens (BRFAA), Athens, Greece; [2]Department of Clinical Therapeutics, School of Medicine, National and Kapodistrian University of Athens, Athens, Greece; [3]Laboratory Animal Facilities, Centre of Clinical, Experimental Surgery and Translational Research, Biomedical Research Foundation of the Academy of Athens, Athens, Greece; [4]Department of Hematology and Bone Marrow Transplantation, National and Kapodistrian University of Athens, Laikon General Hospital, Athens, Greece; [5]CIRI-International Center for Infectiology Research, Inserm, U1111, Université Claude Bernard Lyon 1, CNRS, UMR5308, Ecole Normale Supérieure de Lyon, Université Lyon, Lyon and Université Côte d'Azur, INSERM, Nice, France;*

### Background

Multiple myeloma (MM) is a plasma cell malignancy characterized by systemic dissemination, monoclonal immunoglobulin secretion, and recurrent cycles of remission and relapse, despite considerable advances in treatment strategies. Therefore, innovative therapeutic approaches are required to improve treatment efficacy while maintaining an acceptable safety profile. Gene delivery using viral vectors provides a platform for tumor-directed therapeutic intervention. In this context, we developed a lentiviral vector pseudotyped with engineered measles virus HF glycoproteins to deliver the interferon-β (IFNβ) transgene, a cytokine with antitumor and immunomodulatory activity.

### Aims

The aim was to evaluate the *in vivo* therapeutic efficacy, biodistribution, and safety of a tumor-targeted IFNβ-expressing vector (IFNβ/HF) in a systemic xenograft model of MM.

### Methods

Systemic MM was established in NSG mice by intravenous injection of H929 cells. Four weeks later, following confirmation of disease engraftment by microPET/CT, mice received intravenous administration of either IFNβ/HF ($10^7$ transduction units, n = 10), or the reference vector GFP/HF ($10^7$ transduction units, n = 10), or PBS (n = 5). Clinical status was monitored longitudinally by assessing body weight, locomotor activity, grooming behavior, pain scoring, and hematological parameters. Circulating tumor cells were quantified by flow cytometry. Disease burden was evaluated by microPET/CT. Serum paraprotein and cytokine levels were measured by ELISA. Vector biodistribution was assessed by in vivo fluorescence imaging (Xenogen®) and vector copy number (VCN) analysis in several key tissues.

**Copyright Information:** (Online) ISSN: 2572-9241
©2026 The Author(s). HemaSphere is published by John Wiley & Sons Ltd on behalf of European Hematology Association. This is an open access Abstract Book distributed under the Attribution-NonCommercial-NoDerivs (CC BY-NC-ND), which allows third parties to download the articles and share them with others as long as they credit the author and the Abstract Book, but they cannot change the content in any way or use them commercially.
**Abstract Book Citations:** Authors, Title, HemaSphere, 2026;10:(S1):pages. The abstract book DOIs can be found on the “Author Information” page.
**Disclaimer:** Articles published in the journal HemaSphere exclusively reflect the opinions of the authors. The authors are responsible for all content in their abstracts including accuracy of the facts, statements, citing resources, etc.

**Results**

IFNβ/HF administration resulted in marked systemic disease control compared to GFP/HF and PBS groups. IFNβ/HF-treated mice maintained stable body weight, preserved activity, and low pain scores, while hematological parameters remained within physiological ranges, indicating good tolerability. Circulating myeloma cells were significantly reduced, reaching near-background levels at days 7 and 21 post-treatment ($p \leq 0.0001$). MicroPET/CT demonstrated regression of myeloma lesions with no evidence of new infiltrates. Survival was substantially prolonged, with 80% (95% CI: 55–100%) of IFNβ/HF-treated mice alive at the end of the 5-month follow-up. Moreover, serum IgA paraprotein levels were profoundly reduced, decreasing from 466.5 ± 90.8 ng/mL in GFP/HF-treated mice to 15.6 ± 41.8 ng/mL in IFNβ/HF-treated mice by day 21 ($p \leq 0.0001$). Cytokine profiling revealed decreased IL-6, MCP-1, GM-CSF, and TNFα, alongside increased IFNγ, IP-10, and MIG, consistent with interferon pathway activation and microenvironmental reprogramming. Histological analysis of tissues obtained from treated mice revealed no evidence of treatment-related toxicity. Biodistribution analyses integrating in vivo imaging and VCN quantification demonstrated preferential bone marrow tropism with minimal detection in non-target organs, supporting a favorable safety profile.

**Summary/Conclusion**

Tumor-targeted IFNβ gene therapy mediated by the novel vector, reduced tumor burden and paraprotein levels, reshaped the cytokine milieu, and prolonged survival in a systemic xenograft model. To our knowledge, this is the first demonstration of durable systemic MM control, employing a self-inactivating lentiviral vector, supported by concordant imaging and serological measurements. These findings support the therapeutic potential of IFNβ/HF as a promising novel therapeutic strategy for MM.

**Copyright Information:** (Online) ISSN: 2572-9241
©2026 The Author(s). HemaSphere is published by John Wiley & Sons Ltd on behalf of European Hematology Association. This is an open access Abstract Book distributed under the Attribution-NonCommercial-NoDerivs (CC BY-NC-ND), which allows third parties to download the articles and share them with others as long as they credit the author and the Abstract Book, but they cannot change the content in any way or use them commercially.
**Abstract Book Citations:** Authors, Title, HemaSphere, 2026;10:(S1):pages. The abstract book DOIs can be found on the "Author Information" page.
**Disclaimer:** Articles published in the journal HemaSphere exclusively reflect the opinions of the authors. The authors are responsible for all content in their abstracts including accuracy of the facts, statements, citing resources, etc.

### (PS1823) IDENTIFICATION OF PRECURSOR CELLS OF EXTRAMEDULLARY DISEASE IN MULTIPLE MYELOMA AND THEIR THERAPEUTIC TARGETS BY INTEGRATED OMICS ANALYSIS

**Topic:** 13. Myeloma and other monoclonal gammopathies - Biology & translational research

Shojiro Haji*[1], Hiroki Muta[1], Motoaki Shiratsuchi[2], Youko Suehiro[3], Yoshihiro Ogawa[1]

*[1]Kyushu University, Department of Medicine and Bioregulatory Science, Fukuoka, Japan;[2]Iizuka hospital, Department of hematology, Iizuka, Japan; [3]Kyushu Cancer Center, Department of Blood and Cell Therapy, Fukuoka, Japan;*

### Background

Despite the advent of novel therapies, most patients with multiple myeloma (MM) experience relapse due to residual disease that persists even during clinical remission. Extramedullary disease (EMD) is an aggressive manifestation of MM, and relapse with EMD is associated with dismal outcomes. Therefore, EMD represents a major unmet medical need. However, the myeloma subpopulations in the bone marrow (BM) that serve as precursor cells of EMD, as well as the molecular mechanisms required for their survival, are not fully understood, hindering the development of effective strategies to prevent and treat EMD.

### Aims

To identify EMD-precursor cells and their therapeutic targets.

### Methods

We conducted longitudinal single-cell RNA sequencing (scRNA-seq) of three sequential BM samples from a patient with EMD relapse, identifying putative EMD-precursor cells that persisted during remission. To evaluate the generalizability of this population, we analyzed samples from 98 patients from eight publicly scRNA-seq studies of MM cells in the BM. To elucidate the interactions of EMD-precursor cells with the tumor microenvironment, spatial transcriptomics using the Xenium platform was performed on two sequential BM biopsies from a second patient with EMD relapse. To characterize EMD-precursor cells within established EMD, we analyzed scRNA-seq data from five EMD samples, comprising three pleural effusions from registry cases and two institutional samples (one pleura from a second patient and one lymph node from a third patient), along with Xenium data from two institutional EMD samples. Therapeutic targets, identified through scRNA-seq and Xenium analysis for BM and EMD, were assessed by exposing primary MM cells to inhibitors and measuring cytotoxicity against EMD precursor cells using an MTS viability assay.

**Copyright Information:** (Online) ISSN: 2572-9241
©2026 The Author(s). HemaSphere is published by John Wiley & Sons Ltd on behalf of European Hematology Association. This is an open access Abstract Book distributed under the Attribution-NonCommercial-NoDerivs (CC BY-NC-ND), which allows third parties to download the articles and share them with others as long as they credit the author and the Abstract Book, but they cannot change the content in any way or use them commercially.
**Abstract Book Citations:** Authors, Title, HemaSphere, 2026;10:(S1):pages. The abstract book DOIs can be found on the “Author Information” page.
**Disclaimer:** Articles published in the journal HemaSphere exclusively reflect the opinions of the authors. The authors are responsible for all content in their abstracts including accuracy of the facts, statements, citing resources, etc.

### Results

We identified undifferentiated residual cells (URC) that were present even during remission. Gene set enrichment analysis of differentially expressed genes (DEGs) in URC revealed the upregulation of pathways related to epithelial-mesenchymal transition and metastasis. Notably, 96% of the DEGs in URC were associated with poor survival outcomes. Analysis of public scRNA-seq datasets confirmed the existence of URC in BM across all 98 patients, with increased proportions in patients with EMD relapse. Gene expression profiles of URC were resembled those of the bulk EMD data reported previously. Next, we analyzed public scRNA-seq data from paired BM and pleural effusion samples from the same patient and conducted a pseudotime trajectory analysis. The trajectories indicated a continuous progression from the URC in BM toward EMD cells along a single pseudotemporal axis, suggesting that the URC possesses an EMD-precursor transcriptional state. Among the 12 DEGs shared by URC across all MM patients (n=99), we identified BIRC5 as a potential therapeutic target based on DepMap CRISPR/Cas9 loss-of-function screening data and its specific expression profile. In addition, integrated spatial proximity and cell–cell interaction analyses using scRNA-seq and Xenium data from BM and EMD suggested that URC rely on autocrine signaling via PPIA–BSG for survival rather than paracrine signaling from surrounding non-tumor cells. Finally, we confirmed that the inhibitors of BIRC5 and PPIA suppressed URC growth in a dose-dependent manner *in vitro*.

### Summary/Conclusion

We identified putative EMD–precursor cells and their therapeutic targets, which may offer novel strategies for the prevention and treatment of EMD.

**Copyright Information:** (Online) ISSN: 2572-9241
©2026 The Author(s). HemaSphere is published by John Wiley & Sons Ltd on behalf of European Hematology Association. This is an open access Abstract Book distributed under the Attribution-NonCommercial-NoDerivs (CC BY-NC-ND), which allows third parties to download the articles and share them with others as long as they credit the author and the Abstract Book, but they cannot change the content in any way or use them commercially.
**Abstract Book Citations:** Authors, Title, HemaSphere, 2026;10:(S1):pages. The abstract book DOIs can be found on the "Author Information" page.
**Disclaimer:** Articles published in the journal HemaSphere exclusively reflect the opinions of the authors. The authors are responsible for all content in their abstracts including accuracy of the facts, statements, citing resources, etc.

### (PS1824) HIGH-RISK GENOMIC FEATURES PREDICTING EXTRAMEDULLARY PROGRESSION IN MULTIPLE MYELOMA WITH PARASKELETAL PLASMACYTOMAS

**Topic:** 13. Myeloma and other monoclonal gammopathies - Biology & translational research

Martin Stork*[1], Eva Ondrouskova[1], Johana Mayerova[1], Andrea Mareckova[1], Jakub Pawel Porc[2, 3], Tomas Reigl[2, 3], Klara Mensikova[1], Nela Sendlerova[1], Ivanna Biochuk[1], Jakub Cieślar[1], Michaela Bilcikova[1], Zdenek Adam[1], Marta Krejčí[1], Viera Sandecká[1], Zdenka Knechtova[1], Zuzana Jelinkova[4], Milan Krticka[5], Vladimir Nekuda[5], Tomas Rohan[6], Viera Hrabcakova[1], Lenka Janska-Vojtkova[1], Marek Borsky[1], Lenka Radova[2, 3], Marie Jarosova[1, 2, 3], Ludek Pour[1], Jana Kotaskova[1, 3]

*[1]Department of Internal Medicine, Hematology and Oncology, University Hospital Brno and Faculty of Medicine, Masaryk University, Brno, Czechia; [2]Central European Institute of Technology, Masaryk University, Brno, Czechia;[3]Institute of Medical Genetics and Genomics, Faculty of Medicine, Masaryk University and University Hospital Brno, Brno, Czechia; [4]Department of Burns and Plastic Surgery, University Hospital Brno and Faculty of Medicine, Masaryk University, Brno, Czechia; [5]Trauma Surgery Department, University Hospital Brno and Faculty of Medicine, Masaryk University, Brno, Czechia; [6]Department of Radiology and Nuclear Medicine, University Hospital Brno and Faculty of Medicine, Masaryk University, Brno, Czechia;*

### Background

Bone-related paraskeletal (PS) plasmacytomas, represent a frequent manifestation of multiple myeloma (MM), but only a subset exhibits an aggressive clinical course. Their biological heterogeneity and risk of progression to secondary extramedullary disease (EMD) remain incompletely understood. High-risk MM is currently defined by the International Myeloma Society/International Myeloma Working Group (IMS/IMWG) criteria based on specific cytogenetic and molecular abnormalities.

### Aims

To identify IMS/IMWG-defined high-risk genomic features among newly diagnosed MM (NDMM) patients with PS plasmacytomas associated with subsequent development of secondary EMD.

### Methods

We retrospectively analyzed 888 NDMM patients treated between 2015 and 2025 with systematic imaging-based assessment. Genomic profiling of CD138⁺ bone marrow plasma cells (BMPCs), performed using an in-house targeted next-generation sequencing (NGS) panel and interphase fluorescence in situ hybridization (I-FISH), was available for 167 patients. In addition, plasmacytoma tissue profiling was conducted in 46 patients, including samples obtained at NDMM (2 EMD and 14 PS) and at relapsed/refractory multiple myeloma (RRMM) (19 EMD and 11 PS).

### Results

Among patients without baseline EMD (n = 872), secondary EMD developed more frequently in patients with PS lesions present at diagnosis (n=226) than in those without baseline PS involvement (OR 2.24; 95% CI 1.19–4.22; p = 0.017). In BMPC-based profiling of NDMM patients, concurrent del(1p32) and gain(1q21) (OR 8.85; 95% CI 2.17–36.09) and rare biallelic activating MAPK pathway mutations (OR 13.45; 95% CI 2.96–61.2) were strongly associated with secondary EMD development.

PS plasmacytoma tissue in NDMM patients showed marked heterogeneity, with biallelic alterations (*TP53, TENT5C, NRAS, RB1*) predominantly restricted to aggressive cases, while paired BMPCs typically harbored only monoallelic hits.
In EMD tissue from RRMM patients, concurrent del(1p32) and gain(1q21) was detected in 21.1% (4/19) of cases. Similarly, biallelic *TP53* inactivation was present in 21.1% (4/19), while biallelic MAPK pathway alterations were observed in 15.8% (3/19) of samples.

**Copyright Information:** (Online) ISSN: 2572-9241
©2026 The Author(s). HemaSphere is published by John Wiley & Sons Ltd on behalf of European Hematology Association. This is an open access Abstract Book distributed under the Attribution-NonCommercial-NoDerivs (CC BY-NC-ND), which allows third parties to download the articles and share them with others as long as they credit the author and the Abstract Book, but they cannot change the content in any way or use them commercially.
**Abstract Book Citations:** Authors, Title, HemaSphere, 2026;10:(S1):pages. The abstract book DOIs can be found on the “Author Information” page.
**Disclaimer:** Articles published in the journal HemaSphere exclusively reflect the opinions of the authors. The authors are responsible for all content in their abstracts including accuracy of the facts, statements, citing resources, etc.

**Summary/Conclusion**

Our study identifies a high-risk subset of NDMM patients with PS lesions defined by the combination of del(1p32) and gain(1q21), which in the context of the current IMS/IMWG prognostic framework should be understood as an EMD-specific high-risk feature. Biallelic inactivation of tumor suppressor genes and rare activating *MAPK* pathway alterations represent additional hallmarks of aggressive disease evolution, regardless of whether they are detected in BMPCs or plasmacytoma tissue.
This work was supported by conceptual development of research organization (FNBr 65269705) and Brno Ph.D. Talent

**Copyright Information:** (Online) ISSN: 2572-9241
©2026 The Author(s). HemaSphere is published by John Wiley & Sons Ltd on behalf of European Hematology Association. This is an open access Abstract Book distributed under the Attribution-NonCommercial-NoDerivs (CC BY-NC-ND), which allows third parties to download the articles and share them with others as long as they credit the author and the Abstract Book, but they cannot change the content in any way or use them commercially.
**Abstract Book Citations:** Authors, Title, HemaSphere, 2026;10:(S1):pages. The abstract book DOIs can be found on the "Author Information" page.
**Disclaimer:** Articles published in the journal HemaSphere exclusively reflect the opinions of the authors. The authors are responsible for all content in their abstracts including accuracy of the facts, statements, citing resources, etc.

### (PS1825) STROMAL NANOTUBE NETWORKS PROMOTE TRANSMITOPHAGY AND METABOLIC ADAPTATION IN MULTIPLE MYELOMA

**Topic:** 13. Myeloma and other monoclonal gammopathies - Biology & translational research

Antonio Giovanni Solimando*[1], Francesco Di Palma[1], Vanessa Desantis[1], Angelo Vacca[1], Maria Svelto[1], Francesco Pisani[1]

[1]*Bari University, Department of Precision and Regenerative Medicine and Ionian Area (DiMePRe-J), Bari, Italy;*

**Background**

Multiple myeloma (MM) evolves within a hypoxic and metabolically heterogeneous bone marrow microenvironment that promotes tumor adaptation and therapeutic resistance. Although stromal cells are known to support MM survival, the mechanisms through which the niche actively mitigates metabolic stress remain incompletely understood. Emerging evidence suggests that intercellular mitochondrial and membrane trafficking contributes to tumour plasticity, yet its organisation and functional relevance under marrow-like stress conditions remain unclear.

**Aims**

We aimed to determine whether stromal intercellular connectivity enables cooperative mitochondrial and mebrane processing through tunneling nanotubes (TNTs) and transmitophagy, thereby sustaining MM survival under metabolic stress conditions mimicking the bone marrow niche.

**Methods**

MM cells and bone marrow stromal cells (BMSCs) were cultured under oxygen–glucose deprivation (OGD) to reproduce hypoxic metabolic stress. Mitochondrial membrane potential and apoptosis were assessed using fluorescence-based assays. TNT formation and dynamics were analyzed by F-actin staining, live-cell imaging, and confocal microscopy. Mitochondrial transfer was traced using fluorescent labeling, and lysosomal colocalization analyses were performed to identify transmitophagy. Functional relevance was evaluated by pharmacologic disruption of TNTs followed by survival assays.

**Results**

OGD induced mitochondrial dysfunction and apoptosis in MM cells, whereas BMSCs maintained metabolic stability and rescued MM viability in co-culture. Metabolic stress promoted formation of an extensive homotypic TNT network between BMSCs. MM-derived mitochondria and plasma membrane were transferred into stromal cells, where they colocalized with lysosomal compartments and exhibited features consistent with transmitophagy. TNTs further enabled redistribution of tumor-derived mitochondria between stromal cells, indicating cooperative mitochondrial processing within the stromal compartment. Disruption of TNTs abolished stromal rescue and restored apoptosis under stress conditions, demonstrating the functional requirement of stromal networking.

**Copyright Information:** (Online) ISSN: 2572-9241
©2026 The Author(s). HemaSphere is published by John Wiley & Sons Ltd on behalf of European Hematology Association. This is an open access Abstract Book distributed under the Attribution-NonCommercial-NoDerivs (CC BY-NC-ND), which allows third parties to download the articles and share them with others as long as they credit the author and the Abstract Book, but they cannot change the content in any way or use them commercially.
**Abstract Book Citations:** Authors, Title, HemaSphere, 2026;10:(S1):pages. The abstract book DOIs can be found on the “Author Information” page.
**Disclaimer:** Articles published in the journal HemaSphere exclusively reflect the opinions of the authors. The authors are responsible for all content in their abstracts including accuracy of the facts, statements, citing resources, etc.

**Summary/Conclusion**

These findings identify TNT-mediated transmitophagy as a novel mechanism linking microenvironmental architecture to metabolic adaptation in MM. Stromal cells form cooperative networks that collectively buffer tumor metabolic damage rather than acting as passive supporters. Targeting stromal connectivity may therefore represent a therapeutic strategy to disrupt niche-dependent resistance. More broadly, the study supports a model in which treatment response is shaped by the spatial and functional organization of the tumor microenvironment, providing a framework for microenvironment-directed precision approaches in multiple myeloma.

**Copyright Information:** (Online) ISSN: 2572-9241
©2026 The Author(s). HemaSphere is published by John Wiley & Sons Ltd on behalf of European Hematology Association. This is an open access Abstract Book distributed under the Attribution-NonCommercial-NoDerivs (CC BY-NC-ND), which allows third parties to download the articles and share them with others as long as they credit the author and the Abstract Book, but they cannot change the content in any way or use them commercially.
**Abstract Book Citations:** Authors, Title, HemaSphere, 2026;10:(S1):pages. The abstract book DOIs can be found on the "Author Information" page.
**Disclaimer:** Articles published in the journal HemaSphere exclusively reflect the opinions of the authors. The authors are responsible for all content in their abstracts including accuracy of the facts, statements, citing resources, etc.

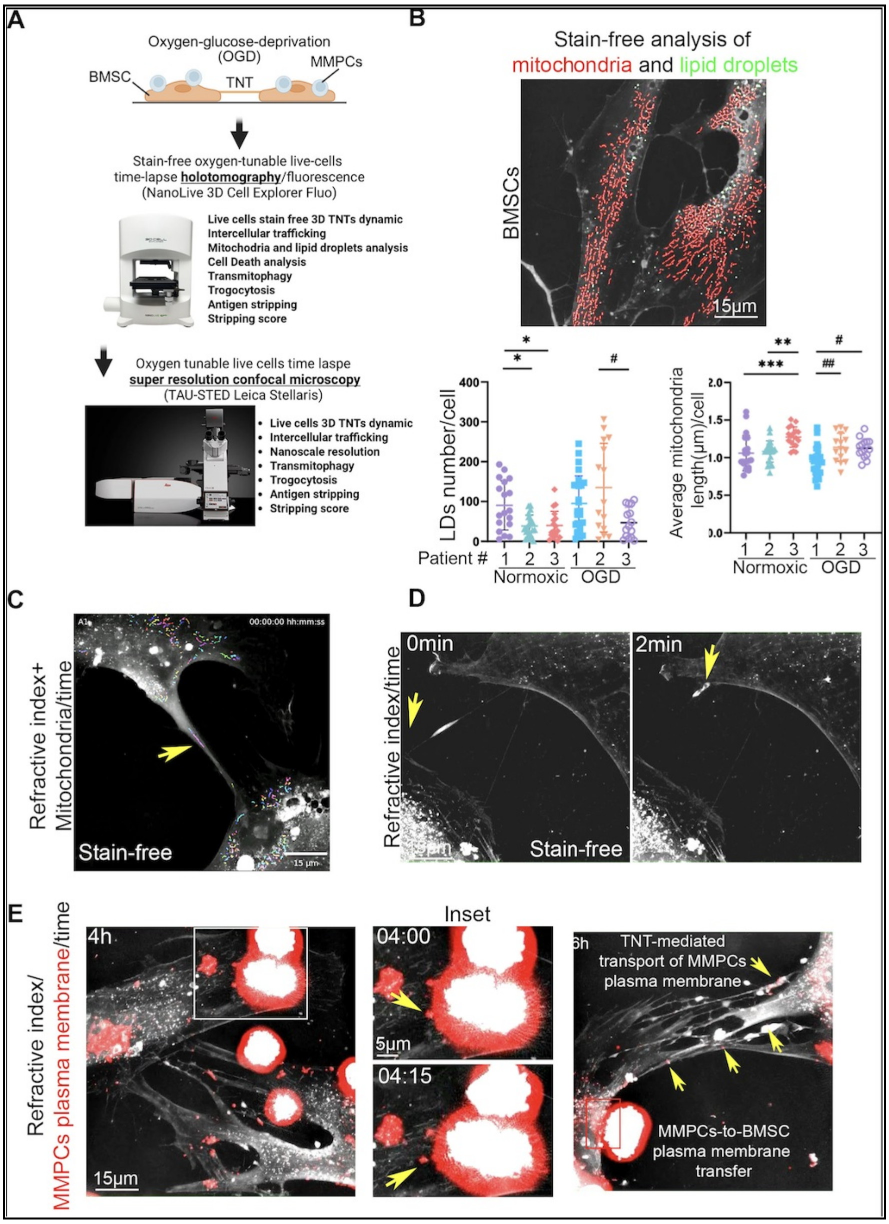

Image. (A) Schematic representation of the co-culture model developed under oxygen–glucose deprivation (OGD), used to analyze metabolic reprogramming and intercellular crosstalk. (B) Live-cell, stain-free holotomographic analysis of mitochondria (red) and lipid droplets (LDs, green) in primary BMSC cultures. (C–D) Live-cell, stain-free analysis of tunneling nanotube (TNT) dynamics between BMSCs under OGD, revealing TNT-mediated mitochondrial transport (C) and cytoplasmic mass transfer (D) between BMSCs. (E) Transfer of plasma membrane from MMPCs to BMSCs, including internalization of MMPC-derived plasma membrane within BMSCs and its subsequent transfer between BMSCs via TNTs.

**Copyright Information:** (Online) ISSN: 2572-9241
©2026 The Author(s). HemaSphere is published by John Wiley & Sons Ltd on behalf of European Hematology Association. This is an open access Abstract Book distributed under the Attribution-NonCommercial-NoDerivs (CC BY-NC-ND), which allows third parties to download the articles and share them with others as long as they credit the author and the Abstract Book, but they cannot change the content in any way or use them commercially.
**Abstract Book Citations:** Authors, Title, HemaSphere, 2026;10:(S1):pages. The abstract book DOIs can be found on the "Author Information" page.
**Disclaimer:** Articles published in the journal HemaSphere exclusively reflect the opinions of the authors. The authors are responsible for all content in their abstracts including accuracy of the facts, statements, citing resources, etc.

### (PS1826) LOSS OF LET-7C INCURRED BY PROMOTER HYPERMETHYLATION DRIVES MM DEVELOPMENT

**Topic:** 13. Myeloma and other monoclonal gammopathies - Biology & translational research

Nana Ren[1], Yonghuai Feng*[1, 2]

*[1]affiliated hospital of Zunyi medical university, hematology department, Zunyi, China;[2]Dongguan People's Hospital, hematology department, Dongguan, China;*

### Background

The pathogenesis of multiple myeloma (MM) remains to be fully elucidated. Let-7c, a tumor-suppressive miRNA, is thought to inhibit MM proliferation by targeting HMGA2 - a gene frequently overexpressed in tumors. However, the specific role and molecular mechanisms through which the let-7c/HMGA2 axis promotes malignant progression in MM are still unclear.

### Aims

This study aims to (i) investigate how the let-7c/HMGA2 axis regulates the proliferation of MM cells and (ii) define the contribution of DNA methylation, particularly promoter hypermethylation, to the aberrant expression of let-7c in MM.

### Methods

Bone marrow specimens were collected from 40 newly diagnosed MM patients and 10 healthy donors at the Affiliated Hospital of Zunyi Medical University between January 2024 and June 2025. The mRNA expression of let-7c and HMGA2 were measured by RT-qPCR. The targeting relationship between let-7c and HMGA2 was validated using a dual-luciferase reporter assay. To manipulate let-7c expression, MM cell lines were transfected with let-7c mimic or inhibitor, and cell viability was detected by CCK-8 assay and Hoechst 33342 staining. A cell line-derived xenografts(CDX) model was established in nude mice to evaluate the effect of let-7c agomir on subcutaneous tumor growth. Western blotting (WB) was performed to detect HMGA2 protein levels as well as two epithelial-mesenchymal transition (EMT)-related markers, E-cadherin and N-Cadherin. Furthermore, MM cells were treated with decitabine, followed by re-examination of let-7c and HMGA2 transcript levels and cell proliferation. A dCas9-TET1CD-sgRNA based targeted demethylation system was employed to specifically demethylate the let-7c promoter region, after which transcriptional changes of let-7c and alterations in cell proliferation were analyzed.

### Results

Both MM patients and cell lines exhibited significantly lower expression of let-7c and higher expression of HMGA2 compared to controls ($P < 0.05$). The dual-luciferase reporter assay confirmed that let-7c directly binds to the 3'-UTR of HMGA2, resulting in a marked decrease in luciferase activity ($P < 0.05$). Overexpression of let-7c suppressed MM cell proliferation ($P < 0.05$), while inhibition of let-7c enhanced malignant phenotypes. In vivo, administration of the let-7c agomir significantly attenuated the growth of xenografted tumors in nude mice ($P < 0.05$). corresponding tumor tissues showed down-regulated protein levels of HMGA2 and N-cadherin, and up-regulated E-cadherin, indicating inhibition of EMT. Furthermore, both treatment with the broad-spectrum demethylating agent decitabine and targeted demethylation of the let-7c promoter using dCas9-TET1CD-sgRNA system effectively elevated let -7c expression and inhibited MM cell proliferation ($P < 0.05$).

**Copyright Information:** (Online) ISSN: 2572-9241
©2026 The Author(s). HemaSphere is published by John Wiley & Sons Ltd on behalf of European Hematology Association. This is an open access Abstract Book distributed under the Attribution-NonCommercial-NoDerivs (CC BY-NC-ND), which allows third parties to download the articles and share them with others as long as they credit the author and the Abstract Book, but they cannot change the content in any way or use them commercially.
**Abstract Book Citations:** Authors, Title, HemaSphere, 2026;10:(S1):pages. The abstract book DOIs can be found on the "Author Information" page.
**Disclaimer:** Articles published in the journal HemaSphere exclusively reflect the opinions of the authors. The authors are responsible for all content in their abstracts including accuracy of the facts, statements, citing resources, etc.

**Summary/Conclusion**

In summary, this study demonstrates that promoter hypermethylation-mediated loss of let-7c promotes MM progression via derepression of HMGA2. The let-7c/HMGA2 axis regulates MM cell proliferation and tumor growth, partially through modulating EMT markers. Importantly, both pharmacological and targeted epigenetic reactivation of let-7c effectively restore its expression and suppress MM cell proliferation. This findings reveal a DNA methylation-dependent mechanism underlying let-7c silencing in MM, and suggest that epigenetic editing of the let-7c promoter represents a potential therapeutic strategy for MM.

**Copyright Information:** (Online) ISSN: 2572-9241
©2026 The Author(s). HemaSphere is published by John Wiley & Sons Ltd on behalf of European Hematology Association. This is an open access Abstract Book distributed under the Attribution-NonCommercial-NoDerivs (CC BY-NC-ND), which allows third parties to download the articles and share them with others as long as they credit the author and the Abstract Book, but they cannot change the content in any way or use them commercially.
**Abstract Book Citations:** Authors, Title, HemaSphere, 2026;10:(S1):pages. The abstract book DOIs can be found on the "Author Information" page.
**Disclaimer:** Articles published in the journal HemaSphere exclusively reflect the opinions of the authors. The authors are responsible for all content in their abstracts including accuracy of the facts, statements, citing resources, etc.

### (PS1827) INTEGRATED SINGLE-CELL AND IMMUNE PROFILING IDENTIFIES DETERMINANTS OF MRD OUTCOME IN MULTIPLE MYELOMA

**Topic:** 13. Myeloma and other monoclonal gammopathies - Biology & translational research

Anna Maria Corsale*[1], Maria Speciale[2], Cristina Aquilina[1], Emilia Gigliotta[1], Andrea Rizzuto[1], Marta Biondo[3], Elena Tofacchi[1], Andrea Romano[2], Marta Di Simone[1], Alessia Gallo[4], Nadia Caccamo[5], Francesco Dieli[5], Serena Meraviglia[5], Sergio Siragusa[1], Cirino Botta[1]

*[1]Department of Health Promotion, Mother and Child Care, Internal Medicine and Medical Specialties (ProMISE), University of Palermo, Palermo, Italy; [2]Department of Surgical, Oncological and Stomatological Disciplines (DICHIRONS), University of Palermo, Palermo, Italy; [3]Department of General Surgery and Medical-Surgical Specialties, Hematology Section, University of Catania, Catania, Italy; [4]IRCCS-ISMETT, Palermo, Italy; [5]Department of Biomedicine, Neuroscience and Advanced Diagnosis (Bi.N.D.), University of Palermo, palermo, Italy;*

### Background

Minimal residual disease (MRD) status represents one of the most robust prognostic indicators across all treatment settings in multiple myeloma (MM). Nonetheless, the immune mechanisms responsible for MRD eradication or persistence are still not fully elucidated.

### Aims

To explore this issue, we analyzed the immunological determinants associated with MRD status through an integrated multi-omic strategy aimed at characterizing both the cellular landscape and the functional remodeling of the bone marrow (BM) microenvironment following therapy.

### Methods

Multiparametric flow cytometry was performed on BM aspirates obtained from 23 consecutively enrolled, ASCT-eligible, newly diagnosed multiple myeloma (NDMM) patients (14 with undetectable MRD [uMRD] and 9 with persistent MRD [pMRD]), both at diagnosis and at day +100 after autologous stem cell transplantation. In a subgroup of eight patients (4 uMRD and 4 pMRD) with available baseline samples, four additional 10-color panels were applied to enable a more comprehensive immune characterization of BM and peripheral blood compartments. Furthermore, baseline samples from seven patients (4 uMRD and 3 pMRD) underwent single-cell RNA sequencing combined with TCR/BCR profiling and 30-antibody CITE-seq, allowing an in-depth characterization of the BM immune ecosystem.

**Copyright Information:** (Online) ISSN: 2572-9241
©2026 The Author(s). HemaSphere is published by John Wiley & Sons Ltd on behalf of European Hematology Association. This is an open access Abstract Book distributed under the Attribution-NonCommercial-NoDerivs (CC BY-NC-ND), which allows third parties to download the articles and share them with others as long as they credit the author and the Abstract Book, but they cannot change the content in any way or use them commercially.
**Abstract Book Citations:** Authors, Title, HemaSphere, 2026;10:(S1):pages. The abstract book DOIs can be found on the "Author Information" page.
**Disclaimer:** Articles published in the journal HemaSphere exclusively reflect the opinions of the authors. The authors are responsible for all content in their abstracts including accuracy of the facts, statements, citing resources, etc.

**Results**

At baseline, pMRD patients showed reduced frequencies of $CD27^{-}/CD28^{-}/CD38^{-}$ T/NK cells compared with uMRD ($p<0.05$). At day +100, pMRD cases exhibited increased granulocytes ($p<0.01$), decreased lymphocytes ($p<0.01$), and higher granulocyte-to-lymphocyte ratio ($p<0.05$), together with expansion of $CD27^{+}/CD28^{-}/CD38^{-}$ T/NK cells ($p<0.05$) (Figure 1A,B). Extended profiling revealed that, despite lower total T-cell frequencies, pMRD patients displayed an altered effector-to-naïve balance, with enrichment of BM $CD8^{+}$ effector memory T cells (p=0.05) and depletion of $CD8^{+}$ naïve cells (p=0.0075), a pattern mirrored in peripheral blood (Figure 1C). As no additional quantitative differences in immune subset distribution emerged, we subsequently focused on qualitative transcriptional features using single-cell RNA sequencing. Baseline transcriptomic analysis identified distinct immune signatures associated with MRD status. In uMRD patients, $CD8^{+}$ effector T cells were enriched in gene programs related to adaptive immunity and memory maintenance pathways—including T-cell activation and differentiation, antigen processing and presentation, and memory development—suggesting a BM microenvironment conducive to durable immune surveillance and effective B–T cell crosstalk (Figure 1D). Similarly, $CD14^{+}$ monocytes from uMRD cases showed enrichment in activation and chemotactic pathways, indicative of a supportive role in T-cell–mediated anti-myeloma responses.In contrast, $CD14^{+}$ monocytes from pMRD patients preferentially upregulated genes associated with innate and humoral immune pathways, including antibacterial responses, antimicrobial peptide production, B-cell activation and isotype switching, and toll-like receptor signaling (Figure 1E).

**Summary/Conclusion**

Integrated immunophenotypic and transcriptomic profiling indicates that uMRD patients harbor, already at diagnosis, a functionally coordinated immune microenvironment that is reinforced by therapy. In contrast, MRD persistence associates with immune dysregulation and ineffective anti-tumor responses. These findings support the development of strategies aimed at restoring immune competence in immune-dysfunctional MM patients.

**Copyright Information:** (Online) ISSN: 2572-9241
©2026 The Author(s). HemaSphere is published by John Wiley & Sons Ltd on behalf of European Hematology Association. This is an open access Abstract Book distributed under the Attribution-NonCommercial-NoDerivs (CC BY-NC-ND), which allows third parties to download the articles and share them with others as long as they credit the author and the Abstract Book, but they cannot change the content in any way or use them commercially.
**Abstract Book Citations:** Authors, Title, HemaSphere, 2026;10:(S1):pages. The abstract book DOIs can be found on the "Author Information" page.
**Disclaimer:** Articles published in the journal HemaSphere exclusively reflect the opinions of the authors. The authors are responsible for all content in their abstracts including accuracy of the facts, statements, citing resources, etc.

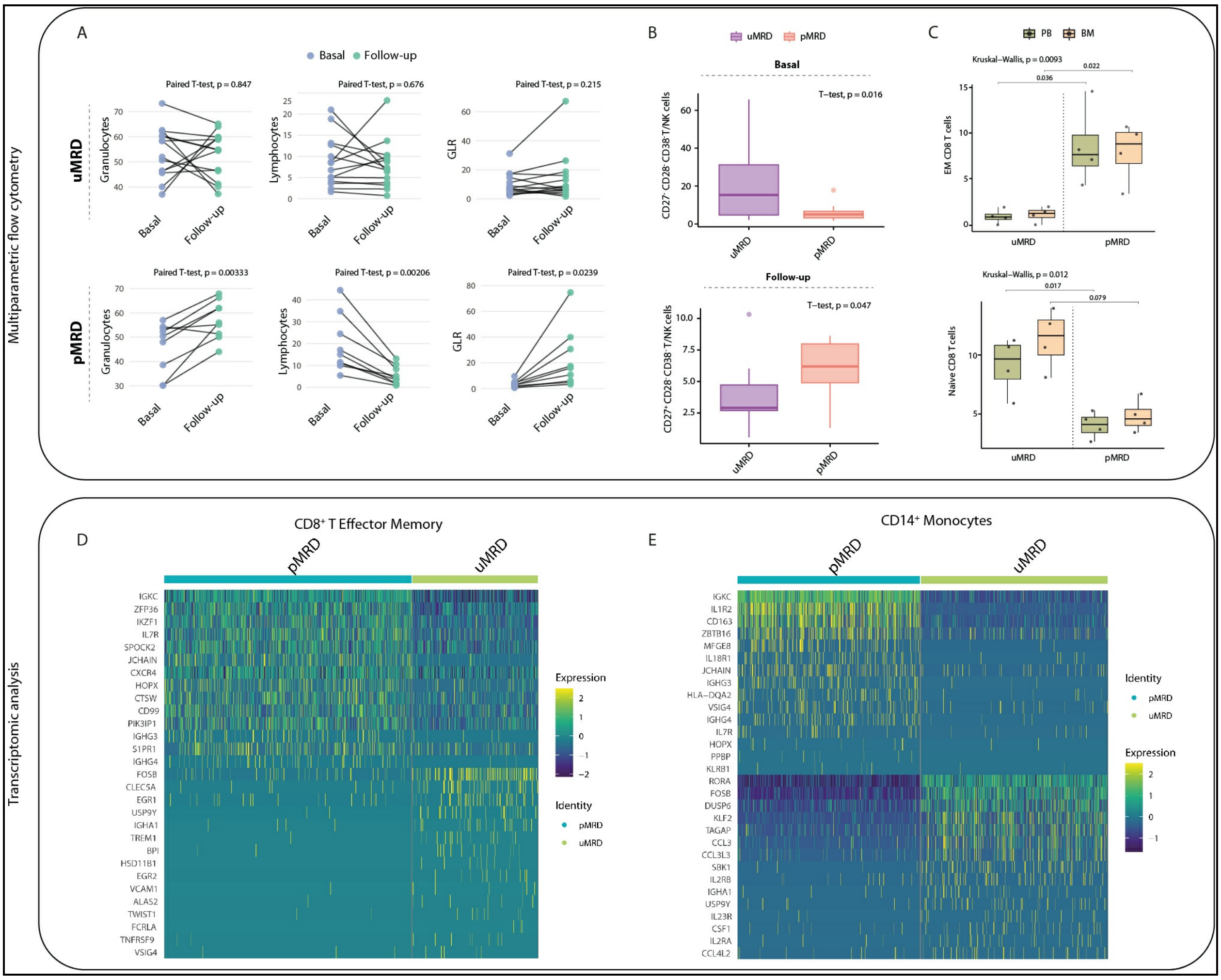

Multiparametric flow cytometry and transcriptomic profiling according to MRD status.

Copyright Information: (Online) ISSN: 2572-9241
©2026 The Author(s). HemaSphere is published by John Wiley & Sons Ltd on behalf of European Hematology Association. This is an open access Abstract Book distributed under the Attribution-NonCommercial-NoDerivs (CC BY-NC-ND), which allows third parties to download the articles and share them with others as long as they credit the author and the Abstract Book, but they cannot change the content in any way or use them commercially.
Abstract Book Citations: Authors, Title, HemaSphere, 2026;10:(S1):pages. The abstract book DOIs can be found on the "Author Information" page.
Disclaimer: Articles published in the journal HemaSphere exclusively reflect the opinions of the authors. The authors are responsible for all content in their abstracts including accuracy of the facts, statements, citing resources, etc.

### (PS1828) AN NGS LIQUID BIOPSY APPROACH FOR GENOMIC CHARACTERIZATION IN MULTIPLE MYELOMA: HEAD-TO-HEAD COMPARISON WITH BONE MARROW FISH

**Topic:** 13. Myeloma and other monoclonal gammopathies - Biology & translational research

Paola Tononi[1], Alberto Ferrarini[1], Marianna Garonzi[1], Federica Sola[1], Giuseppe Giacomo Montefrancesco[1], Aliki Ntzifa[2], Helen Theochari[2], Delphine Chabut[1], Evi Lianidou[2], Efstathios Kastritis*[2], Nicolò Manaresi[1]

*[1]Menarini Silicon Biosystems SpA, Castel Maggiore (BO), Italy;[2]National and Kapodistrian University of Athens, Dept of Chemistry, Athens, Greece;*

### Background

Multiple myeloma (MM) is a genetically complex and spatially heterogeneous disease characterized by significant variability in clinical outcomes. While bone marrow (BM) aspirates are the gold standard for genomic risk stratification, its invasiveness and inability to capture spatial heterogeneity represent significant drawbacks. We present a peripheral blood-based NGS panel targeting genomic alterations for risk stratification, as well as recurrent alterations with prognostic or potential therapeutic value, enabling detection of IGH translocations, aneuploidies, focal copy number alterations (CNAs) and mutations across 147 genes.

### Aims

The aim was to evaluate the assay's performance in accurately reflecting the tumor genomic landscape in comparison with conventional cytogenetics.

### Methods

Pools of 125 to 300 circulating MM cells from 50 MM patients (36 at diagnosis, 4 at relapse, 10 from precursor stages), out of which 43 had matched BM FISH data at the same timepoint, were FACS-sorted after enrichment of peripheral blood using the CELLSEARCH® System. After lysis, samples underwent whole-genome DNA library preparation using the DEPArray LibPrep kit and targeted enrichment using a custom-designed panel (Twist Bioscience). Sequencing was performed on the Illumina NextSeq 1000. Results were compared with paired BM-FISH data. To further evaluate analytical performance in detecting CNAs and to assess the analytical performance in detecting sequence variants, pools of sorted cells from 38 patients were also analyzed using the orthogonal methods Ampli1 LowPass and Ampli1 OncoSeek.

### Results

All patient samples showed at least one alteration across the three categories analyzed: IGH translocations (18 patients), recurrent CNAs (45 patients), and mutations (35 patients). Notably, two out of five MGUS (Monoclonal Gammopathy of Undetermined Significance) patients, who showed no mutations or CNAs, still harbored high-risk translocations. The NGS panel demonstrated strong concordance with the amplicon-based Ampli1 OncoSeek panel. All 31 variants in shared target regions were detected by both platforms, showing an excellent variant allele frequency (VAF) correlation (97%). For copy number alterations, the average positive percent agreement (PPA) with Ampli1 Lowpass was greater than 92%, and exceeded 96% for alterations that were ≥15 Mbp. By using the BM-FISH as a comparator a PPA of 96.4% and a Positive Predictive Value (PPV) of 98.8% were achieved including alterations with a Cancer Cell Fraction (CCF) ranging from 4.4% to 100% (median: 74.6%). Notably the targeted NGS panel also identified a focal del(17p) that was undetected by conventional BM-FISH, suggesting superior resolution for localized genomic events. Six patients exhibited *TP53* mutations, now included in the definition of high-risk MM, which are undetectable by FISH. Four of these six mutations were part of double-hit biallelic *TP53* events, which significantly affects patient prognosis.

**Copyright Information:** (Online) ISSN: 2572-9241
©2026 The Author(s). HemaSphere is published by John Wiley & Sons Ltd on behalf of European Hematology Association. This is an open access Abstract Book distributed under the Attribution-NonCommercial-NoDerivs (CC BY-NC-ND), which allows third parties to download the articles and share them with others as long as they credit the author and the Abstract Book, but they cannot change the content in any way or use them commercially.
**Abstract Book Citations:** Authors, Title, HemaSphere, 2026;10:(S1):pages. The abstract book DOIs can be found on the "Author Information" page.
**Disclaimer:** Articles published in the journal HemaSphere exclusively reflect the opinions of the authors. The authors are responsible for all content in their abstracts including accuracy of the facts, statements, citing resources, etc.

**Summary/Conclusion**

We present a robust NGS liquid biopsy assay for the genomic risk stratification of MM patients. This approach overcomes FISH limitations, such as the inherent difficulty in detecting focal CNAs, while enabling the detection of point mutations, including those currently included into MM risk-stratification guidelines. Overall, this assay provides a more comprehensive molecular profile than cytogenetics alone, offering a minimally invasive tool for enhanced risk assessment.

**Copyright Information:** (Online) ISSN: 2572-9241
©2026 The Author(s). HemaSphere is published by John Wiley & Sons Ltd on behalf of European Hematology Association. This is an open access Abstract Book distributed under the Attribution-NonCommercial-NoDerivs (CC BY-NC-ND), which allows third parties to download the articles and share them with others as long as they credit the author and the Abstract Book, but they cannot change the content in any way or use them commercially.
**Abstract Book Citations:** Authors, Title, HemaSphere, 2026;10:(S1):pages. The abstract book DOIs can be found on the "Author Information" page.
**Disclaimer:** Articles published in the journal HemaSphere exclusively reflect the opinions of the authors. The authors are responsible for all content in their abstracts including accuracy of the facts, statements, citing resources, etc.

### (PS1829) RISK-STRATIFIED PATIENT SELECTION IN MULTIPLE MYELOMA FOR FRONTLINE TREATMENT DECISION-MAKING BASED ON A TRANSCRIPTOMIC CLASSIFIER

**Topic:** 13. Myeloma and other monoclonal gammopathies - Biology & translational research

Maria Kuzmicheva[1], Karen Baghdasaryan[1], Stanislav Kurpe[1], Andrey Kravets[1], Evgenia Alekseeva[1], Nazar Aryutyunian[1], Oleg Baranov[1], Eduardo Shugaev-Mendosa[1], Konstantin Chernyshov[1], Nikita Kotlov*[1]

*[1]BostonGene Corporation, Waltham, MA, United States of America;*

### Background

The treatment paradigm for newly diagnosed multiple myeloma (NDMM) is rapidly evolving, with daratumumab-based quadruplet regimen established as standard of care and CAR-T and BiTEs therapies moving into the frontline setting. Current clinical risk assessment tools—including the International Staging System (ISS) based on biochemical and cytogenetic markers, as well as transcriptome-based classifiers such as SKY92 and CoMMpass—identify high-risk populations but lack a unified framework to guide risk-stratified frontline treatment.

### Aims

To address this gap, we integrated SKY92 and CoMMpass classifications using NGS, to develop a consensus model for risk stratification. This model refines risk categories and identifies patients who may benefit from intensive frontline strategies, including BiTE combinations or CAR-T therapies.

### Methods

We used the CoMMpass (N=715) and SKY92 (N=282) cohorts to develop transcriptomic signatures that identify tumors with a highly aggressive proliferative phenotype. Genes strongly associated with poor survival and high inter-gene correlation were selected. Signature scores were computed using a modified single-sample Gene Set Enrichment Analysis (ssGSEA) approach and integrated into a consensus logistic regression model to yield a proliferative risk (PR) score. This PR-based model stratifies patients into three groups: high risk (worst survival), intermediate risk, and low risk (best survival). Validation was performed using the GSE136324 and GSE2658 cohorts. Progression-free survival (PFS) and overall survival (OS) were assessed with Kaplan–Meier estimates.

### Results

Compared with conventional classifiers (SKY92 and CoMMpass), our consensus PR-based model provides more granular risk stratification, identifying both high-risk and intermediate-risk patients. While CoMMpass and SKY92 classified 8% and 20% of patients as high-risk, respectively, our integrated model identified 10.8% (n=76) as high-risk and 20.1% (n=142) as intermediate-risk. These subgroups were significantly associated with inferior PFS and OS ($p<0.001$). On the test dataset, the model achieved ROC > 0.9 for predicting OS and PFS. Validation on an independent dataset confirmed platform-independence, with risk classification remaining significantly prognostic for OS and PFS. Combining the PR-based model with ISS further enhanced risk stratification, identifying distinct intermediate-risk subgroups within ISS standard-risk categories. Furthermore, we identified an adaptive Unfolded Protein Response (UPR) signature in the high-risk subgroup, derived from co-expressed genes involved in selective transcription and RNA quality control. Notably, the UPR score increased progressively across risk groups ($p<0.001$), linking high proliferative risk with an adaptive stress response that promotes proteasome inhibitor resistance.

**Copyright Information:** (Online) ISSN: 2572-9241
©2026 The Author(s). HemaSphere is published by John Wiley & Sons Ltd on behalf of European Hematology Association. This is an open access Abstract Book distributed under the Attribution-NonCommercial-NoDerivs (CC BY-NC-ND), which allows third parties to download the articles and share them with others as long as they credit the author and the Abstract Book, but they cannot change the content in any way or use them commercially.
**Abstract Book Citations:** Authors, Title, HemaSphere, 2026;10:(S1):pages. The abstract book DOIs can be found on the "Author Information" page.
**Disclaimer:** Articles published in the journal HemaSphere exclusively reflect the opinions of the authors. The authors are responsible for all content in their abstracts including accuracy of the facts, statements, citing resources, etc.

**Summary/Conclusion**

Our consensus PR-based model, combined with ISS, outperforms conventional classifiers by precisely defining high- and intermediate-risk NDMM subgroups. The identification of an adaptive UPR signature in high-risk patients highlights a biological mechanism linked to treatment resistance. These findings support evaluation of the PR-based model in patients receiving daratumumab-based regimens and suggest that high-risk patients may benefit from more intensive frontline strategies, such as BiTE combinations or CAR-T therapies.

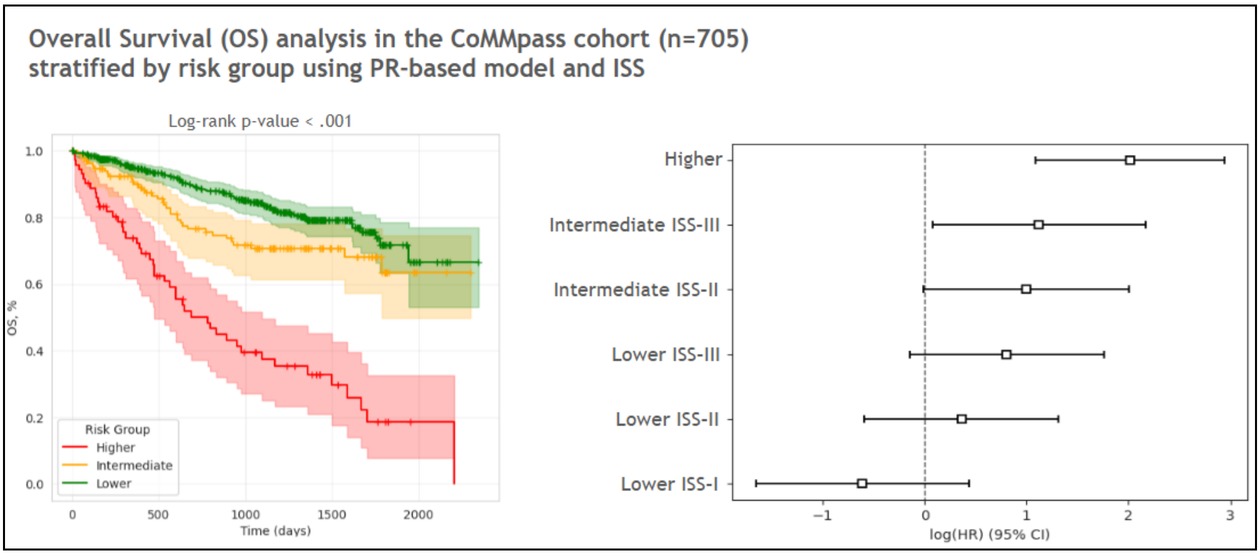

**Copyright Information:** (Online) ISSN: 2572-9241
©2026 The Author(s). HemaSphere is published by John Wiley & Sons Ltd on behalf of European Hematology Association. This is an open access Abstract Book distributed under the Attribution-NonCommercial-NoDerivs (CC BY-NC-ND), which allows third parties to download the articles and share them with others as long as they credit the author and the Abstract Book, but they cannot change the content in any way or use them commercially.
**Abstract Book Citations:** Authors, Title, HemaSphere, 2026;10:(S1):pages. The abstract book DOIs can be found on the "Author Information" page.
**Disclaimer:** Articles published in the journal HemaSphere exclusively reflect the opinions of the authors. The authors are responsible for all content in their abstracts including accuracy of the facts, statements, citing resources, etc.

### (PS1830) ACUTE DEGRADATION OF ONCOGENIC MAF IN MYELOMA CELLS REVEALS MAF-DEPENDENT IRF4 CO-BINDING AND REGULATION OF ONCOGENIC TRANSCRIPTIONAL PROGRAMMES.

**Topic:** 13. Myeloma and other monoclonal gammopathies - Biology & translational research

Qizhen Jia*[1], Pierangela Sabbattini[1], Katrina Fordwor-Hepburn[1], Nikolaos Trasanidis[1], Sophie Ball[1], Yiming Huang[1], Wenqi Gong[1], Harry Whitwell[2], Alexia Katsarou[1], Aristeidis Chaidos[1], Nicholas T Crump[1], Anastasios Karadimitris[1]

*[1]Imperial College London, Hugh and Josseline Langmuir Centre for Myeloma Research, Centre for Haematology, Department of Immunology and Inflammation, London, United Kingdom; [2]Imperial College London, National Phenome Centre and Imperial Clinical Phenotyping Centre, Department of Metabolism, Digestion and Reproduction, London, United Kingdom;*

**Background**

MAF is recurrently activated by chromosomal rearrangements in 5% of cases of multiple myeloma (MM). Oncogenic MAF activates transcriptional programs that promote plasma cell proliferation and adhesion. Although MAF overexpression is implicated in MM pathogenesis through its chromatin co-binding with IRF4, the functional and chromatin-level consequences of acute MAF loss on IRF4 co-occupancy are incompletely characterised.

**Aims**

To define the chromatin state and transcriptional consequences of oncogenic MAF activation and its co-operation with IRF4, as revealed by MAF protein depletion in myeloma cells

**Methods**

An FKBP-HA degron tag was introduced at the endogenous MAF locus in the MM.1S cells using CRISPR/Cas9-mediated genome editing. Correct integration and allelic zygosity were confirmed by diagnostic PCR, and protein expression and degradation kinetics were validated by immunoblotting and intracellular flow cytometry. Acute MAF degradation was induced using the VHL-recruiting degrader dTAGv1. RNA-seq, ChIP-seq (MAF, IRF4, H3K27ac, H3K4me1, H3K4me3), and ATAC-seq were performed following 24h MAF degradation across three degron clones. GSEA enrichment analyses were performed to characterise functional programs. Integration of chromatin binding and differential gene expression was performed using BETA.

**Copyright Information:** (Online) ISSN: 2572-9241
©2026 The Author(s). HemaSphere is published by John Wiley & Sons Ltd on behalf of European Hematology Association. This is an open access Abstract Book distributed under the Attribution-NonCommercial-NoDerivs (CC BY-NC-ND), which allows third parties to download the articles and share them with others as long as they credit the author and the Abstract Book, but they cannot change the content in any way or use them commercially.
**Abstract Book Citations:** Authors, Title, HemaSphere, 2026;10:(S1):pages. The abstract book DOIs can be found on the “Author Information” page.
**Disclaimer:** Articles published in the journal HemaSphere exclusively reflect the opinions of the authors. The authors are responsible for all content in their abstracts including accuracy of the facts, statements, citing resources, etc.

**Results**

dTAGv1 treatment induced rapid and sustained depletion of MAF protein within 2 to 6 hours, persisting beyond 120 hours, and consistently impaired cellular proliferation across multiple independent MM.1S degron clones.
RNA-seq analysis revealed consistent transcriptional reprogramming across three clones, identifying a shared MAF-responsive gene signature enriched for cell-cycle progression, DNA replication, mitotic control, and DNA repair pathways. Comparison with a published shRNA-mediated *MAF* silencing dataset identified 878 shared differentially expressed genes, demonstrating convergence across MAF perturbation approaches and reinforcing the robustness of the MAF-dependent transcriptional network.
MAF ChIP-seq and chromatin state annotation identified promoter and enhancer-associated binding sites that substantially overlapped with previously published wild-type MAF occupancy, indicating preservation of genomic binding specificity following FKBP–HA tagging. Integration of ChIP-seq and RNA-seq data demonstrated significant enrichment of downregulated genes proximal to MAF binding sites (BETA, $P < 0.001$), consistent with a predominantly activating transcriptional role.
IRF4 ChIP-seq following MAF depletion revealed 1,272 lost and 3,612 gained binding sites, respectively. Notably, among the 1,272 IRF4 binding sites lost upon MAF depletion, 858 overlapped with MAF binding peaks (70% of them at enhancers), whereas only one gained IRF4 site that overlapped with MAF, indicating strong MAF-dependent IRF4 binding to chromatin. These 858 MAF–IRF4 co-binding sites are predicted to regulate genes involved in immune cell differentiation, activation, and cell adhesion.
Histone mark profiling revealed loss of H3K27ac peaks at regions significantly enriched for MAF and IRF4 co-binding sites, commensurate with reductions in chromatin accessibility. In contrast, only a small number of H3K4me1/3 changes occurred upon MAF depletion.

**Summary/Conclusion**

Oncogenic MAF sustains IRF4 binding in MAF-translocated myeloma cells, and the two transcription factors cooperate in the context of H3K27ac-marked chromatin at promoters and enhancers to activate cell adhesion transcriptional programs.

**Copyright Information:** (Online) ISSN: 2572-9241
©2026 The Author(s). HemaSphere is published by John Wiley & Sons Ltd on behalf of European Hematology Association. This is an open access Abstract Book distributed under the Attribution-NonCommercial-NoDerivs (CC BY-NC-ND), which allows third parties to download the articles and share them with others as long as they credit the author and the Abstract Book, but they cannot change the content in any way or use them commercially.
**Abstract Book Citations:** Authors, Title, HemaSphere, 2026;10:(S1):pages. The abstract book DOIs can be found on the "Author Information" page.
**Disclaimer:** Articles published in the journal HemaSphere exclusively reflect the opinions of the authors. The authors are responsible for all content in their abstracts including accuracy of the facts, statements, citing resources, etc.

### (PS1831) PERIPHERAL BLOOD CIRCULATING MYELOMA CELLS AS A REAL-TIME, NONINVASIVE SURROGATE FOR BONE MARROW MRD AFTER CELLULAR THERAPY AND AUTOLOGOUS TRANSPLANT

**Topic:** 13. Myeloma and other monoclonal gammopathies - Biology & translational research

Ehsan Malek*[1], Rahul Thakur[1], Marco Davila[1], Shernan Holtan[1, 1], Brian Betts[1], James Driscoll[2]

[1]*Roswell Park Cancer Center, Buffalo, United States of America;* [2]*University Hospital Cleveland Medical Center, Cleveland, United States of America;*

### Background

Minimal residual disease (MRD) negativity has become a central therapeutic goal in multiple myeloma (MM) and is strongly associated with prolonged progression-free and overall survival. Bone marrow (BM) MRD assessment by next-generation sequencing (NGS) remains the reference standard but is constrained by procedural invasiveness, spatial heterogeneity, and limited sampling frequency. In the era of highly active therapies—including CAR T-cell therapy and autologous stem cell transplant (ASCT)—more dynamic and less invasive disease monitoring strategies are needed.
Circulating multiple myeloma cells (CMMCs), defined as $CD138^+CD38^+CD19^-CD45^-$ malignant plasma cells detectable in peripheral blood, represent a potential liquid biomarker of systemic tumor burden. While CMMCs have been explored in other treatment settings, their performance relative to deep BM MRD assessment in patients receiving cellular therapy or ASCT remains incompletely defined.

### Aims

To define CMMC performance relative to deep BM MRD assessment in patients receiving cellular therapy or ASCT remains incompletely defined.

### Methods

We prospectively evaluated 51 patients with MM undergoing CAR T-cell therapy or ASCT at Roswell Park Comprehensive Cancer Center. Paired peripheral blood and BM aspirates were obtained at the time of response assessment.
BM MRD was assessed using NGS (ClonoSEQ) with a sensitivity of $10^{-6}$. Peripheral blood (4 mL) was analyzed using the CELLSEARCH® platform following CD138-based ferrofluid enrichment and fluorescent labeling (CD38-PE, CD19/CD45-APC, DAPI). CMMC positivity was defined as >2 cells per 4 mL of blood.
Diagnostic performance metrics—including sensitivity, specificity, overall concordance, and Cohen's kappa—were calculated. Quantitative association between CMMC counts and BM MRD burden was assessed using Spearman correlation.

**Copyright Information:** (Online) ISSN: 2572-9241
©2026 The Author(s). HemaSphere is published by John Wiley & Sons Ltd on behalf of European Hematology Association. This is an open access Abstract Book distributed under the Attribution-NonCommercial-NoDerivs (CC BY-NC-ND), which allows third parties to download the articles and share them with others as long as they credit the author and the Abstract Book, but they cannot change the content in any way or use them commercially.
**Abstract Book Citations:** Authors, Title, HemaSphere, 2026;10:(S1):pages. The abstract book DOIs can be found on the "Author Information" page.
**Disclaimer:** Articles published in the journal HemaSphere exclusively reflect the opinions of the authors. The authors are responsible for all content in their abstracts including accuracy of the facts, statements, citing resources, etc.

### Results

The median age was 66 years (range 45–77), with 39% harboring high-risk cytogenetic features. At sampling, 53% were in VGPR or better, and 65% had received daratumumab-based induction.
CMMCs were detected in 28 of 51 patients (54.9%). Among these, 25 were BM MRD-positive. Peripheral blood CMMC detection demonstrated:

- Sensitivity: 89.3%
- Specificity: 91.3%
- Overall concordance: 90.2%
- Cohen's $\kappa = 0.79$

CMMC enumeration strongly correlated with quantitative BM MRD levels ($\rho = 0.72$, $p < 0.001$). Performance was consistent across therapeutic modalities, with concordance of 88.2% in the CAR T cohort and 90.9% in the ASCT cohort.

### Summary/Conclusion

Peripheral blood CMMC enumeration provides a highly concordant and minimally invasive surrogate for deep BM MRD assessment following CAR T-cell therapy and ASCT. This liquid-based strategy enables scalable, real-time disease monitoring and may allow earlier identification of molecular relapse or MRD conversion. Integration of CMMC-based monitoring into post–cellular therapy surveillance strategies warrants prospective evaluation.

**Copyright Information:** (Online) ISSN: 2572-9241
©2026 The Author(s). HemaSphere is published by John Wiley & Sons Ltd on behalf of European Hematology Association. This is an open access Abstract Book distributed under the Attribution-NonCommercial-NoDerivs (CC BY-NC-ND), which allows third parties to download the articles and share them with others as long as they credit the author and the Abstract Book, but they cannot change the content in any way or use them commercially.
**Abstract Book Citations:** Authors, Title, HemaSphere, 2026;10:(S1):pages. The abstract book DOIs can be found on the "Author Information" page.
**Disclaimer:** Articles published in the journal HemaSphere exclusively reflect the opinions of the authors. The authors are responsible for all content in their abstracts including accuracy of the facts, statements, citing resources, etc.

### (PS1832) PROGNOSTIC VALUE OF STANDARDIZED QUANTITATIVE FDG PET/CT BIOMARKERS IN NEWLY DIAGNOSED MULTIPLE MYELOMA

**Topic:** 13. Myeloma and other monoclonal gammopathies - Biology & translational research

Georg-Michael Krause*[1], Sanne Wiegers[1], Gerben Zwezerijnen[1], Sandeep Golla[1], Martijn Heymans[1], Kazimierz Groen[2], Sonja Zweegman[2], Josée Zijlstra[2], Ronald Boellaard[1]

*[1]Amsterdam UMC, Department of Radiology & Nuclear Medicine, Amsterdam, The Netherlands;[2]Amsterdam UMC, Department of Hematology, Amsterdam, The Netherlands;*

### Background

18F-FDG PET/CT holds information on glucose metabolism, tumor spread and tumor volume in Multiple Myeloma (MM). However, risk stratification based on FDG PET/CT imaging remains challenging, mainly due to the heterogeneity of disease manifestations found in newly diagnosed and relapsed patients. As a result, risk stratification of MM patients in current clinical practice is based solely on serum markers and cytogenetics. Nevertheless, several recent studies have indicated prognostic value of FDG PET/CT, internally validated on small patient cohorts. For clinical application of PET-based risk stratification, a standardized and widely available segmentation method for lesion delineation in MM is required. Furthermore, prognostic models based on FDG PET/CT need extensive external validation, given the small numerical size of most FDG PET/CT-trials in MM.

### Aims

In this analysis, we developed prognostic models to predict event-free survival (EFS) based on single-threshold tumor delineation of baseline FDG PET/CT scans in newly diagnosed MM patients. Lesion segmentation at an absolute SUV threshold was aimed to address the need for a standardized, widely available lesion segmentation method. We further demonstrated the robustness of our prognostic models by external validation on an independent cohort of patients.

### Methods

Following a recently published lesion segmentation benchmark for FDG PET/CT based prognosis in diffuse large B-cell, follicular and Hodgkin lymphoma (Boellaard et al, 2025), we applied the same benchmark (lesion delineation at SUV>4 threshold) in baseline FDG PET/CT studies of newly diagnosed MM patients. SUV-peak (peak standardized uptake value), Dmax (maximum distance between two lesions), and TMTV (total metabolic tumor volume) were derived and used as quantitative imaging biomarkers. Using univariate and multivariate logistic regression, we trained models on these imaging biomarkers to predict EFS. The prediction models were internally and externally validated on two comparable, but fully independent study cohorts of elderly, non-transplant eligible NDMM patients undergoing different induction regimens (HOVON-126 trial: N=47, ITd induction; HOVON-87 trial: N=36, MPT/R induction).

### Results

SUV-peak, Dmax and TMTV were consistently negatively correlated with EFS at short-term (2 year EFS), intermediate (3-4 year EFS) and long-term survival (5 year EFS). Internal cross-validation on the HOVON-126 trial yielded an area-under-the-curve of AUC=0.69 for log(TMTV), AUC=0.65 for SUV-peak and AUC=0.73 for Dmax at 5-year EFS. In external validation on the HOVON-87 trial, we retrieved prognostic powers of AUC=0.84, AUC=0.83 and AUC=0.77 for log(TMTV), SUV-peak and Dmax, respectively. To demonstrate the survival benefit of SUV>4 biomarkers in NDMM, we split both study cohorts using the median biomarker values of the HOVON-126 cohort and plotted Kaplan-Meier survival curves (Figure 1). Figure 1 shows that all three PET-biomarkers effectively split the study cohorts into low and high-survival sub-groups.

**Copyright Information:** (Online) ISSN: 2572-9241
©2026 The Author(s). HemaSphere is published by John Wiley & Sons Ltd on behalf of European Hematology Association. This is an open access Abstract Book distributed under the Attribution-NonCommercial-NoDerivs (CC BY-NC-ND), which allows third parties to download the articles and share them with others as long as they credit the author and the Abstract Book, but they cannot change the content in any way or use them commercially.
**Abstract Book Citations:** Authors, Title, HemaSphere, 2026;10:(S1):pages. The abstract book DOIs can be found on the “Author Information” page.
**Disclaimer:** Articles published in the journal HemaSphere exclusively reflect the opinions of the authors. The authors are responsible for all content in their abstracts including accuracy of the facts, statements, citing resources, etc.

### Summary/Conclusion

To our knowledge, this is the first publication to validate quantitative FDG-PET biomarkers on an external cohort in newly diagnosed MM. We demonstrated the feasibility of the SUV4 benchmark segmentation method on FDG-PET in MM and found consistent prognostic value of SUV-peak, Dmax and TMTV in both cohorts. The results promise clinical application and added value complementary to biomarkers currently used for risk stratification.

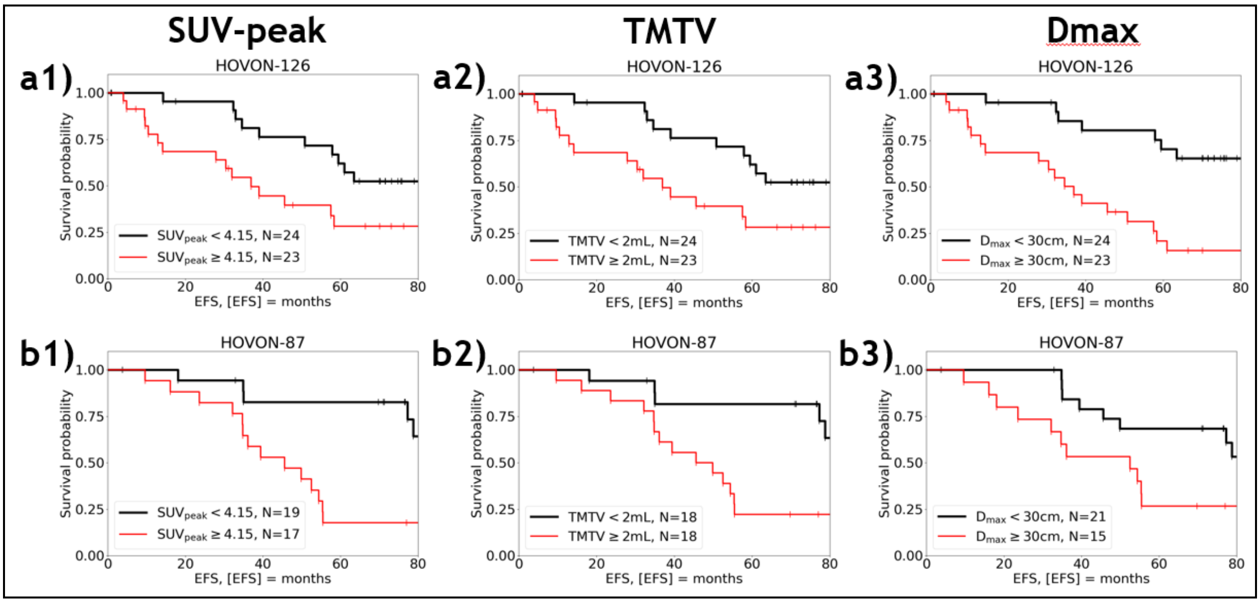

Figure 1: Predictive value of SUV4 PET-biomarkers for event-free survival in newly diagnosed Multiple Myeloma.

**Copyright Information:** (Online) ISSN: 2572-9241
©2026 The Author(s). HemaSphere is published by John Wiley & Sons Ltd on behalf of European Hematology Association. This is an open access Abstract Book distributed under the Attribution-NonCommercial-NoDerivs (CC BY-NC-ND), which allows third parties to download the articles and share them with others as long as they credit the author and the Abstract Book, but they cannot change the content in any way or use them commercially.
**Abstract Book Citations:** Authors, Title, HemaSphere, 2026;10:(S1):pages. The abstract book DOIs can be found on the "Author Information" page.
**Disclaimer:** Articles published in the journal HemaSphere exclusively reflect the opinions of the authors. The authors are responsible for all content in their abstracts including accuracy of the facts, statements, citing resources, etc.

### (PS1833) THERAPY-ASSOCIATED IMMUNE SENESCENCE IN MULTIPLE MYELOMA: A QUANTITATIVE MODEL TO INFORM TIMING OF CELLULAR THERAPY

**Topic:** 13. Myeloma and other monoclonal gammopathies - Biology & translational research

Ehsan Malek*[1], Marco Davila[1], Shernan Holtan[1], Brian Betts[1], James Driscoll[1]

[1]*Roswell Park Cancer Center, Buffalo, United States of America;*

### Background

Successful outcomes following autologous stem cell transplant (ASCT) and CAR T-cell therapy depend on functional immune reserve. While chronological age is commonly used in clinical decision-making, it incompletely captures adaptive immune competence. Multiple myeloma (MM) and its frontline therapies may induce accelerated immune senescence prior to transplant conditioning or lymphodepletion, potentially compromising cellular therapy efficacy.
We applied donor-referenced immune modeling to quantify therapy-associated immune aging in MM patients completing induction therapy and to evaluate its relevance as a functional biomarker of cellular therapy readiness.

### Aims

To assess immune age of Myeloma patient

### Methods

Peripheral blood samples from 219 MM patients following a median of four cycles of proteasome inhibitor + IMiD–based induction (pre-HCT timepoint) and 150 healthy related donors (pre-apheresis) were analyzed between 2020–2024. Multiparameter flow cytometry quantified naïve T-cell ($CD4^+$ and $CD8^+$ $CD45RA^+CCR7^+$) and naïve B-cell ($CD19^+CD45RA^+$) compartments. Generalized additive models constructed from donor data defined age-associated immune trajectories. These models were inverted to estimate "immune age" for each MM patient. The immune-age gap was defined as estimated immune age minus chronological age. Cohort-specific slope differences were evaluated using interaction terms.

### Results

The MM cohort (median chronological age 62 years) demonstrated significant contraction of naïve $CD4^+$ T cells compared with age-matched donors ($p = 0.006$). This corresponded to a median immune-age acceleration of approximately +15 to +18 years.
Naïve $CD8^+$ T-cell and $CD19^+$ B-cell compartments showed similar but less pronounced declines. Notably, MM patients under age 45 frequently exhibited immune profiles comparable to donors aged ≥65 years, indicating substantial therapy-associated immune remodeling early in the disease course.
These findings demonstrate that clinically meaningful immune aging occurs prior to transplant conditioning or CAR T lymphodepletion.

**Copyright Information:** (Online) ISSN: 2572-9241
©2026 The Author(s). HemaSphere is published by John Wiley & Sons Ltd on behalf of European Hematology Association. This is an open access Abstract Book distributed under the Attribution-NonCommercial-NoDerivs (CC BY-NC-ND), which allows third parties to download the articles and share them with others as long as they credit the author and the Abstract Book, but they cannot change the content in any way or use them commercially.
**Abstract Book Citations:** Authors, Title, HemaSphere, 2026;10:(S1):pages. The abstract book DOIs can be found on the "Author Information" page.
**Disclaimer:** Articles published in the journal HemaSphere exclusively reflect the opinions of the authors. The authors are responsible for all content in their abstracts including accuracy of the facts, statements, citing resources, etc.

**Summary/Conclusion**

Induction therapy in MM is associated with measurable acceleration of adaptive immune aging, particularly within the $CD4^+$ naïve T-cell compartment. Quantitative immune-age modeling provides a functional metric of immune reserve beyond chronological age. Incorporation of immune-age assessment into pre-ASCT or pre-CAR T evaluation may:

- Inform optimal timing of leukapheresis
- Guide conditioning or lymphodepletion intensity
- Identify candidates for immune-rejuvenation strategies
- Improve stratification in cellular therapy trials

Prospective validation is warranted to determine whether immune-age metrics predict CAR T expansion, immune reconstitution, and long-term clinical outcomes.

**Copyright Information:** (Online) ISSN: 2572-9241
©2026 The Author(s). HemaSphere is published by John Wiley & Sons Ltd on behalf of European Hematology Association. This is an open access Abstract Book distributed under the Attribution-NonCommercial-NoDerivs (CC BY-NC-ND), which allows third parties to download the articles and share them with others as long as they credit the author and the Abstract Book, but they cannot change the content in any way or use them commercially.
**Abstract Book Citations:** Authors, Title, HemaSphere, 2026;10:(S1):pages. The abstract book DOIs can be found on the "Author Information" page.
**Disclaimer:** Articles published in the journal HemaSphere exclusively reflect the opinions of the authors. The authors are responsible for all content in their abstracts including accuracy of the facts, statements, citing resources, etc.

### (PS1834) TARGETING THE DNA DAMAGE RESPONSE IN DORMANT MYELOMA CELLS

**Topic:** 13. Myeloma and other monoclonal gammopathies - Biology & translational research

Abigail Foston*[1], Haider Al-Janabi[1], Alexandria Sprules[1], Victor Canuas-Landero[1], Helen Bryant[1], Alanna Green[1]

[1]*University of Sheffield, Clinical Medicine, Sheffield, United Kingdom;*

### Background

Multiple myeloma is the second most common haematological cancer in Europe, but it remains incurable due to the prevalence of relapse following therapeutic treatment. Currently approved standard of care therapies often lead to a state of 'minimal residual disease' (MRD), as cancer cells can evade the treatment, causing inevitable relapse. This can be initiated by reactivation of dormant, chemo-resistant myeloma cells. Entry into dormancy is considered a protective mechanism to prolong cellular survival, which may be triggered by DNA damage in myeloma.
No treatments effectively kill dormant cells, so new therapeutics are needed to fill this gap, either to prevent reactivation or to cause complete removal of MRD. Taking advantage of increased levels of DNA damage in dormant myeloma cells may be an unexplored new treatment avenue, with a possibility to exploit changes in the DNA damage response (DDR).

### Aims

To determine whether DNA damage levels differ in proliferating and dormant myeloma cells using *in vitro* and *in vivo* (mouse) models.

### Methods

Two mouse models were used to measure DNA damage levels. In the first model, ~8-week-old C57BL/KaLwRij immunocompetent mice were i.v. injected with 2x10$^{6}$ DiD-labelled 5TGM1-luc cells and culled 21 days post-injection. In the second model, ~8-week-old NSG immunocompromised mice were i.v. injected with 1x10$^{6}$ DiD-labelled human U266-GFP-Luc cells. When tumour burden was high mice were treated with chemotherapy 50mg/kg lenalidomide and 0.75mg/kg bortezomib. Tumour was monitored via bioluminescent imaging using an IVIS imaging system. Flow cytometry was used to identify dormant myeloma cells by DiD-retention, AXL and p21 expression, and DNA damage was measured by γH2AX staining of bone marrow samples.
*In vitro* comparisons were made between dormant and proliferating cells cultured for 7 days using flow cytometry, and immunofluorescence staining for γH2AX was performed after FACS sorting for DiD$^{Hi}$ (dormant) and DiD$^{Low}$ (proliferating) cell populations. DNA damage foci were quantified using ImageJ. Activity of DNA damage response inhibitor drugs (olaparib, abemaciclib, berzosertib and Ku55933) on dormant and proliferating myeloma cells was assessed using resazurin viability assay after 1 week of treatment.
Expression of DDR genes were analysed using publicly available single cell RNA seq data (SRA: PRJNA453652).

**Copyright Information:** (Online) ISSN: 2572-9241
©2026 The Author(s). HemaSphere is published by John Wiley & Sons Ltd on behalf of European Hematology Association. This is an open access Abstract Book distributed under the Attribution-NonCommercial-NoDerivs (CC BY-NC-ND), which allows third parties to download the articles and share them with others as long as they credit the author and the Abstract Book, but they cannot change the content in any way or use them commercially.
**Abstract Book Citations:** Authors, Title, HemaSphere, 2026;10:(S1):pages. The abstract book DOIs can be found on the "Author Information" page.
**Disclaimer:** Articles published in the journal HemaSphere exclusively reflect the opinions of the authors. The authors are responsible for all content in their abstracts including accuracy of the facts, statements, citing resources, etc.

**Results**

NSG and C57BL/KaLwRij models exhibited significantly increased levels of DNA damage (γH2AX+) in dormant compared to proliferating populations. This was supported by *in vitro* experiments where γH2AX foci were significantly higher in the DiD-high (dormant) groups compared to DiD-low (proliferative) in both human and mouse cell lines, indicating dormant myeloma cells have higher levels of DNA damage.
Abemeciclib (CDK4/6 inhibitor), berzosertib (ATR inhibitor) and Ku55933 (ATM inihibitor) dose-dependently inhibited viability in proliferating cells. PARP inhibitor olaparib reduced viability in BRCA deficient 5TGM1 cells, but not U266 cells.
DDR gene expression profiles between dormant and proliferative myeloma cells were comparable. However, gene expression does not represent potential activity levels and low input phospho-proteomics is being used to observe changes in activation between different cellular states.

**Summary/Conclusion**

Dormant myeloma cells exhibit higher levels of DNA damage across modelling approaches. Thus, targeting the DNA damage response is a promising approach to target chemo-resistant dormant myeloma cells.

**Copyright Information:** (Online) ISSN: 2572-9241
©2026 The Author(s). HemaSphere is published by John Wiley & Sons Ltd on behalf of European Hematology Association. This is an open access Abstract Book distributed under the Attribution-NonCommercial-NoDerivs (CC BY-NC-ND), which allows third parties to download the articles and share them with others as long as they credit the author and the Abstract Book, but they cannot change the content in any way or use them commercially.
**Abstract Book Citations:** Authors, Title, HemaSphere, 2026;10:(S1):pages. The abstract book DOIs can be found on the "Author Information" page.
**Disclaimer:** Articles published in the journal HemaSphere exclusively reflect the opinions of the authors. The authors are responsible for all content in their abstracts including accuracy of the facts, statements, citing resources, etc.

### (PS1835) TRANSCRIPTOMIC PROFILING OF MULTIPLE MYELOMA REVEALS COMMON AND TREATMENT SPECIFIC MOLECULAR SIGNATURES OF RELAPSE AND DRUG RESISTANCE FOLLOWING DARATUMUMAB, PI AND IMID THERAPY

**Topic:** 13. Myeloma and other monoclonal gammopathies - Biology & translational research

Eleni Kalafati*[1], Christine Liacos[1], Ourania Theologi[1], Alexandra Papadimou[1], Konstantina Taouxi[1], Katerina Chrisostomidou[1], Tina Bagratuni[1], Foteini Theodorakakou[1], Despina Fotiou[1], Evangelos Eleutherakis-Papaiakovou[1], Magda Migkou[1], Ioannis Ntanasis-Stathopoulos[1], Panagiotis Malandrakis[1], Vassiliki Spiliopoulou[1], Maria Gavriatopoulou[1], Evangelos Terpos[1], Meletios A. Dimopoulos[1], Efstathios Kastritis[1]

*[1]Department of Clinical Therapeutics, School of Medicine, National and Kapodistrian University of Athens, Athens, Greece;*

### Background

Relapse in multiple myeloma (MM) after immunotherapeutic and targeted treatment reflects clonal disease escape which may also be the result of profound biological reprogramming. Monoclonal antibodies against CD38 such as Daratumumab (DARA) now constitute the backbone of current regimens; however, the transcriptional landscape underlying treatment-specific relapse remains incompletely characterized.

### Aims

To delineate the transcriptomic evolution of MM from diagnosis to relapse in order to identify resistance-associated molecular programs across distinct therapy-specific transcriptional patterns, particularly following DARA, immunomodulators (IMiDs), and proteasome inhibitors (PIs)-based therapies.

### Methods

Bulk RNA sequencing was performed on CD138$^+$ clonal bone marrow plasma cells from 114 MM patients, including newly diagnosed, previously untreated (ND; n = 32) and relapsed cases following DARA-based therapy (n = 23), IMiDs (n = 18), PIs (n = 18), or combined PIs and IMiDs (n = 23). Differentially expressed genes (DEGs) were identified using DESeq2, with all relapse cohorts compared to ND samples. Functional annotation included Gene Ontology (GO) and pathway enrichment analyses. Interaction network analysis was performed using PANTHER and ReactomeFI.

**Copyright Information:** (Online) ISSN: 2572-9241
©2026 The Author(s). HemaSphere is published by John Wiley & Sons Ltd on behalf of European Hematology Association. This is an open access Abstract Book distributed under the Attribution-NonCommercial-NoDerivs (CC BY-NC-ND), which allows third parties to download the articles and share them with others as long as they credit the author and the Abstract Book, but they cannot change the content in any way or use them commercially.
**Abstract Book Citations:** Authors, Title, HemaSphere, 2026;10:(S1):pages. The abstract book DOIs can be found on the “Author Information” page.
**Disclaimer:** Articles published in the journal HemaSphere exclusively reflect the opinions of the authors. The authors are responsible for all content in their abstracts including accuracy of the facts, statements, citing resources, etc.

**Results**

DEGs analysis (padj < 0.05; |fold change| ≥ 1.5) showed that relapse after DARA was characterized by coordinated upregulation of genes involved in chromatin remodeling (PHF13, EZH2, DNMT3B), ER stress signaling (CREB3L4), and survival pathways (NFKB1, DUSP10, MIF), alongside downregulation of genes mediating immune activation, including IL-1 signaling (IL1RAP, IL1B), complement activation (C1QA–C), and T-cell engagement markers (HLA-DQA1, LAG3, TAGAP). This concomitant induction of immunosuppressive mediators (CXCL8, MIF, and EZH2) may contribute to the establishment of an immune-cold tumor microenvironment. Gene set enrichment analysis (GSEA) revealed enrichment of E2F, MYC, and G2M checkpoint molecular pathways, coupled with suppression of interferon pathways. Moreover, interaction network analysis identified a central axis of mitotic cell cycle activation (fold enrichment = 4.11; p < 0.0001). MM relapse following PIs and IMiDs shared transcriptional features characterized by immune attenuation and enhanced MYC signaling. Notably, relapse following PI treatment was characterized by selective enrichment of oxidative phosphorylation and mTORC1 signaling, indicating a metabolically reprogrammed and energetically adaptive phenotype. Conversely, the transcriptome profile after IMiD therapy exhibited an enhanced cytokine-driven and immune-modulated shift. The combination of PIs/IMiDs defined a relapse transcriptome dominated by cell cycle acceleration and genomic stress adaptation, while lacking the distinct metabolic or cytokine-centered pathway enrichment pattern observed in relapse after single-agent treatment. Comparative analysis across all relapse cohorts identified a DARA-associated immunosuppressive and cell survival expression pattern. Lastly, all relapse samples shared a common 8-gene expression signature (upregulated: INAVA, MIF; downregulated: A2M, LILRB5, SLC40A1, SDC3, CD72, ADAP2), revealing a conserved core relapse-associated transcriptional program.

**Summary/Conclusion**

Our study identified a conserved core relapse-associated transcriptional backbone alongside therapy-specific adaptive programs in MM, providing a molecular framework to further investigate mechanisms of resistance and rational therapeutic tailoring.

**Copyright Information:** (Online) ISSN: 2572-9241
©2026 The Author(s). HemaSphere is published by John Wiley & Sons Ltd on behalf of European Hematology Association. This is an open access Abstract Book distributed under the Attribution-NonCommercial-NoDerivs (CC BY-NC-ND), which allows third parties to download the articles and share them with others as long as they credit the author and the Abstract Book, but they cannot change the content in any way or use them commercially.
**Abstract Book Citations:** Authors, Title, HemaSphere, 2026;10:(S1):pages. The abstract book DOIs can be found on the "Author Information" page.
**Disclaimer:** Articles published in the journal HemaSphere exclusively reflect the opinions of the authors. The authors are responsible for all content in their abstracts including accuracy of the facts, statements, citing resources, etc.

### (PS1836) EXPLORING THE CELL-FREE TRANSCRIPTOME AS A BIOMARKER SOURCE IN IGM GAMMOPATHIES: AN ANALYSIS FROM THE "BIO-WM" TRIAL OF THE FONDAZIONE ITALIANA LINFOMI (FIL)

**Topic:** 13. Myeloma and other monoclonal gammopathies - Biology & translational research

Philippe Decruyenaere*[1, 2, 3], Daniela Drandi[4], Kimberly Verniers[2, 3], Simone Ragaini[4], Jasper Anckaert[2, 3], Enrico Amaducci[4], Elisa Genuardi[4], Federica Cavallo[4, 5], Luigi Marcheselli[6], Irene Dogliotti[5], Emanuele Cencini[7], Filomena Russo[8], Angela Ferrari[9], Luca Laurenti[10], Giacomo Loseto[11], Monia Marchetti[12, 13], Annelien Morlion[2, 3], Ramón García Sanz[14], Fritz C. Offner[1], Benedetto Bruno[4, 5], Marzia Varettoni[15], Jo Vandesompele[2, 3], Simone Ferrero[4, 5]

*[1]Ghent University Hospital, Hematology, Ghent, Belgium; [2]Cancer Research Institute Ghent (CRIG), OncoRNALab, Ghent, Belgium; [3]Ghent University, Biomolecular Medicine, Ghent, Belgium; [4]University of Torino, Molecular Biotechnology and health sciences, Hematology Division, Torino, Italy; [5]A.O.U. Città della Salute e della Scienza di Torino, Hematology, Torino, Italy; [6]Fondazione Italiana Linfomi (FIL), Alessandria, Italy; [7]AOU Senese, U.O.C. Ematologia, Siena, Italy; [8]Azienda Ospedaliera Universitaria di Parma, UO Ematologia e CTMO, Parma, Italy; [9]AO Arcispedale Santa Maria Nuova - IRCCS, Ematologia, Reggio Emilia, Italy; [10]Università Cattolica S. Cuore, Ematologia, Roma, Italy; [11]IRCCS Istituto Tumori Giovanni Paolo II, U.O.C Ematologia, Bari, Italy; [12]Ospedale Cardinal Massaia, S.O.S. Ematologia, Asti, Italy; [13]University Hospital SS Antonio e Biagio e cesare Arrigo, Hematology unit, Alessandria, Italy; [14]University Hospital of Salamanca, Research Biomedical Institute of Salamanca (IBSAL), CIBERONC and Center for Cancer Research-IBMCC (USAL-CSIC), Haematology Department, Salamanca, Spain; [15]Fondazione IRCCS Policlinico San Matteo, SC Ematologia 1, Pavia, Italy;*

### Background

IgM gammopathies encompass a spectrum of lymphoplasmacytic disorders ranging from IgM-MGUS to asymptomatic (aWM) and symptomatic Waldenström Macroglobulinemia (sWM). While genomic profiling of bone marrow (BM) and plasma cell-free DNA (cfDNA) has established recurrent mutations such as *MYD88* L265P in 90–95% of WM cases, the potential of circulating cell-free RNA (cfRNA) as a non-invasive biomarker source remains largely unexplored.

### Aims

To characterize plasma cfRNA profiles across the IgM gammopathy spectrum and to evaluate their potential for disease classification and prognostication.

### Methods

Plasma samples from 60 patients enrolled in the Fondazione Italiana Linfomi (FIL)-BIO-WM trial (NCT03521516) and 28 healthy controls were analyzed. CfRNA was isolated from 200 µL of blood plasma and sequenced on a NovaSeq 6000 instrument using SMARTer Stranded Total RNA-seq Pico v3. Due to markedly superior cfRNA quality from EDTA plasma, downstream analyses included 49 patients (12 IgM-MGUS, 17 aWM, 20 sWM) and 14 healthy controls. Differential expression, pathway enrichment, and survival analyses were performed, integrating *MYD88* L265P cfDNA and clinical data.

**Copyright Information:** (Online) ISSN: 2572-9241
©2026 The Author(s). HemaSphere is published by John Wiley & Sons Ltd on behalf of European Hematology Association. This is an open access Abstract Book distributed under the Attribution-NonCommercial-NoDerivs (CC BY-NC-ND), which allows third parties to download the articles and share them with others as long as they credit the author and the Abstract Book, but they cannot change the content in any way or use them commercially.
**Abstract Book Citations:** Authors, Title, HemaSphere, 2026;10:(S1):pages. The abstract book DOIs can be found on the "Author Information" page.
**Disclaimer:** Articles published in the journal HemaSphere exclusively reflect the opinions of the authors. The authors are responsible for all content in their abstracts including accuracy of the facts, statements, citing resources, etc.

### Results

CfRNA concentrations were significantly higher in sWM versus aWM ($p=0.017$), IgM-MGUS ($p<0.001$), and healthy controls ($p<0.001$), correlating with serum IgM levels ($\rho=0.48$, $p<0.001$) and BM infiltration ($\rho=0.43$, $p=0.0022$). Immune cell deconvolution of cfRNA demonstrated expanding plasma cell fractions in sWM compared with aWM ($p=0.017$), IgM-MGUS ($p=0.0042$), and healthy controls ($p<0.001$). A total of 2107, 72, and 727 differentially abundant genes were identified in the comparisons of WM versus healthy controls, IgM-MGUS versus healthy controls, and WM versus IgM-MGUS, respectively. Multiclass cfRNA profiling identified high-performing classifiers, and attenuation of platelet-driven variance refined these signatures to a smaller, biologically distinct gene set with significantly improved discriminative performance. A five-gene signature (*LINC02609*, *PPP1R3C*, *PPIL6*, *RPS2P32*, *HSALNG0126719*) predicted a shorter time to progression after first-line therapy in sWM patients ($p<0.001$).

### Summary/Conclusion

Plasma cfRNA profiling captures biologically and clinically relevant features across the IgM gammopathy spectrum, enabling disease classification and risk stratification. These findings position cfRNA as a promising, minimally invasive biomarker capable of enhancing disease diagnosis, monitoring and prognostication in WM.

**Copyright Information:** (Online) ISSN: 2572-9241
©2026 The Author(s). HemaSphere is published by John Wiley & Sons Ltd on behalf of European Hematology Association. This is an open access Abstract Book distributed under the Attribution-NonCommercial-NoDerivs (CC BY-NC-ND), which allows third parties to download the articles and share them with others as long as they credit the author and the Abstract Book, but they cannot change the content in any way or use them commercially.
**Abstract Book Citations:** Authors, Title, HemaSphere, 2026;10:(S1):pages. The abstract book DOIs can be found on the "Author Information" page.
**Disclaimer:** Articles published in the journal HemaSphere exclusively reflect the opinions of the authors. The authors are responsible for all content in their abstracts including accuracy of the facts, statements, citing resources, etc.

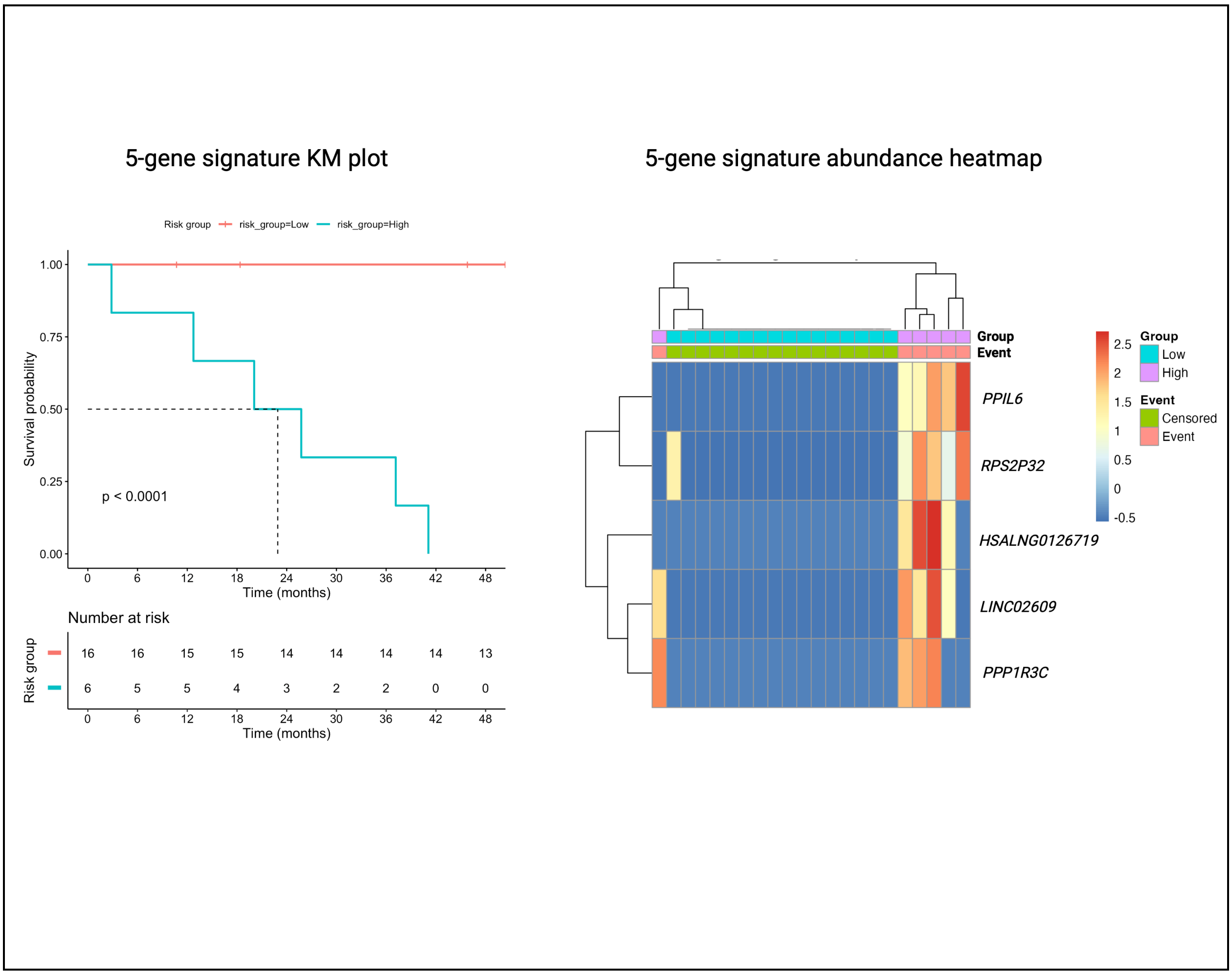

Prognostic value of a 5-gene signature for PFS in treated WM patients. Kaplan–Meier curves showing overall PFS stratified by a multigene Cox-derived risk score using a median-based cutoff (left). Heatmap of z-scored expression of the five signature genes across all samples, clustered by expression similarity (right). PFS: progression-free survival; WM: Waldenström Macroglobulinemia.

**Copyright Information:** (Online) ISSN: 2572-9241
©2026 The Author(s). HemaSphere is published by John Wiley & Sons Ltd on behalf of European Hematology Association. This is an open access Abstract Book distributed under the Attribution-NonCommercial-NoDerivs (CC BY-NC-ND), which allows third parties to download the articles and share them with others as long as they credit the author and the Abstract Book, but they cannot change the content in any way or use them commercially.
**Abstract Book Citations:** Authors, Title, HemaSphere, 2026;10:(S1):pages. The abstract book DOIs can be found on the “Author Information” page.
**Disclaimer:** Articles published in the journal HemaSphere exclusively reflect the opinions of the authors. The authors are responsible for all content in their abstracts including accuracy of the facts, statements, citing resources, etc.

### (PS1837) GENOMIC PROFILING UNVEILS ACTIONABLE VULNERABILITIES AND CLONAL EVOLUTION IN RELAPSED/REFRACTORY MULTIPLE MYELOMA

**Topic:** 13. Myeloma and other monoclonal gammopathies - Biology & translational research

Ruijie He[1], Qinlong Zheng[1], Yajing Zhang*[1]

[1]*Beijing GoBroad Boren Hospital, Beijing, China;*

### Background

Relapsed/refractory multiple myeloma (R/R MM) remains a formidable challenge, especially for patients with extramedullary disease (EMD) or resistance to immunotherapies. R/R MM undergoes complex clonal evolution under therapeutic pressure, acquiring genomic aberrations that confer aggressive phenotypes and multidrug resistance. Deciphering this genomic complexity is critical for identifying novel actionable targets and developing personalized salvage strategies.

### Aims

To delineate the comprehensive genomic landscape of a real-world R/R MM cohort using ultra-deep targeted next-generation sequencing (NGS) and to evaluate the feasibility of genome-guided targeted interventions.

### Methods

We retrospectively analyzed clinical and genomic data in R/R MM patients from 2024.04 to 2026.02. Bone marrow aspirates and EMD biopsies underwent customized targeted NGS (mean depth >1500x) encompassing up to 172 hematologic malignancy-related genes. Single nucleotide variants (SNVs) and small insertions/deletions (InDels) were functionally annotated. Therapeutic actionability was systematically assessed by mapping pathogenic variants to FDA/NMPA-approved targeted agents based on preclinical and clinical evidence.

### Results

Ultra-deep sequencing identified a highly heterogeneous mutational landscape, revealing at least one actionable somatic alteration in a significant proportion of heavily pretreated patients. The genomic architecture driving clonal evolution was predominantly characterized by aberrations in cell cycle regulation, epigenetic modification, and PI3K/AKT/DNA damage repair (DDR) pathways:
1.Cell Cycle Dysregulation: Activating mutations in the PEST domain of *CCND3* (e.g., p.Q280X, p.P284S) and loss-of-function truncations in the tumor suppressor *CDKN2A* (e.g., p.R80X) were prevalent. These alterations stabilize Cyclin D3 and bypass the p16INK4a-Rb checkpoint, providing a strong mechanistic rationale for repurposing CDK4/6 inhibitors (e.g., palbociclib, dalpiciclib).
2.Epigenetic Reprogamming: Recurrent variants in histone modifiers, including frameshifts in CREBBP (p.E1061Rfs38) and KMT2D (p.E1207Gfs5, p.M1379Vfs*52), alongside DNMT3A missense mutations (p.P904L), were enriched in patients with EMD. These mutations drive clonal stemness but confer synthetic lethality to HDAC inhibitors (e.g., chidamide) and hypomethylating agents.
3.Signaling & DDR Defects: We identified actionable activating mutations in the PI3K/AKT pathway, such as *PIK3R1* frameshifts (p.D68Afs*7) that disrupt PTEN stability, and DDR defects including ATM truncations (p.Q2280X) conferring susceptibility to PARP inhibitors. Furthermore, NOTCH1 truncations (p.P2514Rfs*4) and *SAMHD1* variants (p.R366H) were observed in aggressive clones. Clinically, identifying these intrinsic vulnerabilities enabled the deployment of genome-guided off-label targeted combinations as salvage or bridging therapies prior to cellular immunotherapy.

**Copyright Information:** (Online) ISSN: 2572-9241
©2026 The Author(s). HemaSphere is published by John Wiley & Sons Ltd on behalf of European Hematology Association. This is an open access Abstract Book distributed under the Attribution-NonCommercial-NoDerivs (CC BY-NC-ND), which allows third parties to download the articles and share them with others as long as they credit the author and the Abstract Book, but they cannot change the content in any way or use them commercially.
**Abstract Book Citations:** Authors, Title, HemaSphere, 2026;10:(S1):pages. The abstract book DOIs can be found on the "Author Information" page.
**Disclaimer:** Articles published in the journal HemaSphere exclusively reflect the opinions of the authors. The authors are responsible for all content in their abstracts including accuracy of the facts, statements, citing resources, etc.

**Summary/Conclusion**

Ultra-deep targeted NGS unveils a complex, actionable molecular architecture in R/R MM, particularly in cases with EMD or aggressive clonal evolution. The enrichment of specific cell cycle (*CCND3/CDKN2A*), epigenetic (*CREBBP/KMT2D/DNMT3A*), and kinase (*PIK3R1*) mutations presents profound therapeutic opportunities. Integrating genome-driven precision medicine—such as repurposing CDK4/6, HDAC, and PI3K/mTOR inhibitors—offers a promising, mechanistically sound salvage strategy for R/R MM patients who have exhausted standard options.

| Pathway | Key Mutated Genes | Representative Variants | Potential Targeted Agents |
|---|---|---|---|
| Cell Cycle | CCND3, CDKN2A | p.Q280X, p.P284S, p.R80X | CDK4/6 inhibitors |
| Epigenetic | CREBBP, KMT2D, DNMT3A | p.E1061Rfs*38, p.E1207Gfs*5, p.P904L | HDAC inhibitors, HMAs |
| PI3K/AKT | PIK3R1 | p.D68Afs*7 | PI3K/mTOR inhibitors |
| DNA Repair | ATM | p.Q2280X | PARP inhibitors |

Actionable Genomic Alterations and Genome-Guided Strategies

**Copyright Information:** (Online) ISSN: 2572-9241
©2026 The Author(s). HemaSphere is published by John Wiley & Sons Ltd on behalf of European Hematology Association. This is an open access Abstract Book distributed under the Attribution-NonCommercial-NoDerivs (CC BY-NC-ND), which allows third parties to download the articles and share them with others as long as they credit the author and the Abstract Book, but they cannot change the content in any way or use them commercially.
**Abstract Book Citations:** Authors, Title, HemaSphere, 2026;10:(S1):pages. The abstract book DOIs can be found on the "Author Information" page.
**Disclaimer:** Articles published in the journal HemaSphere exclusively reflect the opinions of the authors. The authors are responsible for all content in their abstracts including accuracy of the facts, statements, citing resources, etc.

### (PS1838) PSMD10 AS A SELECTIVE REGULATOR OF MHC CLASS II EXPRESSION IN MULTIPLE MYELOMA

**Topic:** 13. Myeloma and other monoclonal gammopathies - Biology & translational research

Zuzanna Tracz-Gaszewska*[1], Patryk Krzemiński[2], Filip Garbicz[1], Luis A. Corchete[3, 4], Agnieszka Druzd-Sitek[5], Agnieszka Końska[6], Agata Malinowska[7], Agnieszka Stewart[7], Marta Pelon[1], Maria Czyżewska[8], Marcin Rymko[8], Isabel Isidro[3, 4], Elizabeta A. Rojas[3, 4], Ewa Lech-Marańda[6], Przemyslaw Juszczynski[1], Norma Gutiérrez[3, 4], Irena Misiewicz-Krzemińska[1]

*[1]Institute of Hematology and Transfusion Medicine, Department of Experimental Hematology, Warsaw, Poland;[2]Warsaw University of Life Sciences, Biology Institute, Department of Nanobiotechnology, Warsaw, Poland; [3]University Hospital of Salamanca, Institute of Biomedical Research of Salamanca, Hematology Department, Salamanca, Spain; [4]University of Salamanca–National Research Council (USAL-CSIC), Institute of Cancer Molecular and Cellular Biology, Cancer Research Center, Salamanca, Spain; [5]Maria Sklodowska-Curie National Research Institute of Oncology, Warsaw, Poland;[6]Institute of Hematology and Transfusion Medicine, Hematology Clinic, Warsaw, Poland; [7]Polish Academy of Sciences, Institute of Biochemistry and Biophysics, Mass Spectrometry Laboratory, Warsaw, Poland; [8]The Ludwik Rydygier Regional Hospital in Toruń, Toruń, Poland;*

### Background

Multiple myeloma (MM) remains incurable in part due to a highly immunosuppressive tumor microenvironment (TME). Malignant plasma cells typically display low MHC class II (MHC-II) expression and, together with dysfunctional antigen-presenting cells, impair CD4⁺ T-cell priming and antitumor immunity. Recognized mechanisms of MHC-II downregulation, such as epigenetic silencing of the MHC-II master transactivator CIITA and its ubiquitin-dependent proteasomal degradation by E3 ligases, do not fully account for the persistently low MHC-II levels in MM, suggesting additional regulation. Our studies identify PSMD10 as a novel, highly selective suppressor of MHC-II expression, providing new insights into TME immunosuppression in MM.

### Aims

To elucidate the role of PSMD10 in the regulation of MHC-II expression in MM, and to identify molecular mechanisms of selective MHC-II suppression that can be exploited as entry points for immune-modulating therapies.

### Methods

Kaplan–Meier progression-free survival (PFS) analysis of newly diagnosed MM (NDMM) patients from the MMRF CoMMpass cohort assessed the prognostic impact of PSMD10 expression in MM cells, stratifying PI+IMiD versus non-PI therapies. PSMD10 protein levels were analysed in paired diagnosis–relapse bone marrow samples. PSMD10 inhibition or loss was achieved using CRISPR/Cas9 knockout (KO), Cjoc42, and inducible shRNA knockdown (KD) to examine effects on MHC-I and MHC-II (HLA-DR) expression at mRNA and protein levels by multi-omics and flow cytometry. RNA-seq with gene set enrichment analysis (GSEA) was performed in two MM lines after PSMD10 shRNA KD to identify enriched pathways. Mass spectrometry-based proteomics and Western blotting in PSMD10-KO MM cells validated changes in MHC-I/II components, CD74, and immunoproteasome subunits.

**Copyright Information:** (Online) ISSN: 2572-9241
©2026 The Author(s). HemaSphere is published by John Wiley & Sons Ltd on behalf of European Hematology Association. This is an open access Abstract Book distributed under the Attribution-NonCommercial-NoDerivs (CC BY-NC-ND), which allows third parties to download the articles and share them with others as long as they credit the author and the Abstract Book, but they cannot change the content in any way or use them commercially.
**Abstract Book Citations:** Authors, Title, HemaSphere, 2026;10:(S1):pages. The abstract book DOIs can be found on the "Author Information" page.
**Disclaimer:** Articles published in the journal HemaSphere exclusively reflect the opinions of the authors. The authors are responsible for all content in their abstracts including accuracy of the facts, statements, citing resources, etc.

### Results

High PSMD10 expression in MM was associated with significantly shorter PFS in NDMM patients treated with PI+IMiD regimens, but not in those receiving non-PI therapies (Fig. 1A). PSMD10 protein levels increased at relapse compared with diagnosis, consistent with the selection of PSMD10-high clones and acquired resistance. PSMD10 inhibition or loss robustly upregulated HLA-DR without affecting MHC-I. RNA-seq showed enrichment of signatures related to antigen processing and presentation, IFN-γ/IFN-α responses, and IL-6/JAK/STAT3 signaling, with upregulation of MHC-II components, CD74, and peptide-processing cathepsins (Fig. 1B), indicating an immune-permissive state. Unchanged immunoproteasome subunits point to a selective effect of PSMD10 depletion on MHC-II antigen presentation rather than global proteasome remodeling, while proteomics confirmed increased MHC-II and reduced MHC-I in PSMD10-KO cells, potentially shifting antigen presentation toward CD4⁺ T-cell activation. Planned MHC-II-immunopeptidomics will define PSMD10-dependent surface peptide repertoires and candidate neoantigens.

### Summary/Conclusion

PSMD10 emerges as a key regulator of MHC-II suppression in MM, with elevated expression strongly associated with unfavorable prognosis and disease progression. Its inhibition selectively enhances MHC-II–restricted antigen expression and activates immune response pathways, potentially overcoming resistance mechanisms within the immunosuppressive TME. These findings support PSMD10 as a promising target for immune-modulatory strategies to restore anti-tumor immunity in MM. Funded by National Science Center 2019/35/B/NZ5/02824 and IHIT grant B5-T3-2026.

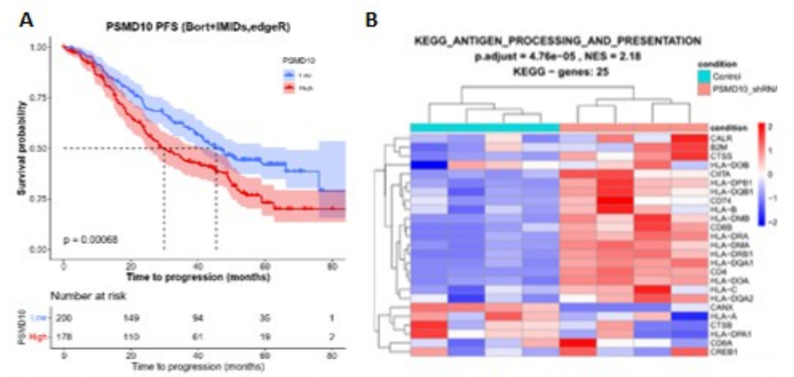

Figure 1. A) Kaplan-Meier analysis of newly diagnosed MM patients from the MMRF CoMMpass cohort treated with bortezomib + IMiD regimens showing that high PSMD10 expression in MM cells is associated with significantly shorter progression-free survival (PFS). B) RNA-seq-based analysis after two weeks of PSMD10 shRNA knockdown in two MM cell lines (JJN3, U266) shows strong enrichment of the KEGG "Antigen Processing and Presentation" pathway.

Copyright Information: (Online) ISSN: 2572-9241
©2026 The Author(s). HemaSphere is published by John Wiley & Sons Ltd on behalf of European Hematology Association. This is an open access Abstract Book distributed under the Attribution-NonCommercial-NoDerivs (CC BY-NC-ND), which allows third parties to download the articles and share them with others as long as they credit the author and the Abstract Book, but they cannot change the content in any way or use them commercially.
Abstract Book Citations: Authors, Title, HemaSphere, 2026;10:(S1):pages. The abstract book DOIs can be found on the "Author Information" page.
Disclaimer: Articles published in the journal HemaSphere exclusively reflect the opinions of the authors. The authors are responsible for all content in their abstracts including accuracy of the facts, statements, citing resources, etc.

### (PS1839) INVESTIGATION ON THE EFFECT AND MOLECULAR MECHANISM OF THE ATP1B4 ON THE RESISTANCE TO PROTEASOME INHIBITORS IN MULTIPLE MYELOMA

**Topic:** 13. Myeloma and other monoclonal gammopathies - Biology & translational research

Xueming Jia*[1], Huihan Wang[1]

[1]*Shengjing Hospital of China Medical University, Department of Hematology, Liaoning Province, China;*

### Background

Multiple myeloma (MM) is a common hematological malignancy, accounting for approximately 10% of all hematological malignancies. Its annual incidence exceeds 3 per 100,000 individuals and continues to rise with the aging of the population. The 5-year survival rate of MM is 59.8%. Although proteasome inhibitors (PIs), such as carfilzomib, have significantly improved patient outcomes in clinical practice, the emergence and progression of drug resistance have become an increasingly prominent challenge. Studies have shown that extrachromosomal circular DNA (eccDNA), the mTOR signaling pathway, and fatty acid oxidation (FAO) are involved in tumor drug resistance.

### Aims

The precise mechanisms linking eccDNA, mTOR, and FAO remain unclear. Exploring these molecular connections and PI resistance mechanisms is valuable for finding new treatments, reversing MM cell drug resistance, and prolonging patient survival.

### Methods

1. RNA sequencing identified the PI resistance hub genes in MM cells. qRT-PCR verified ATP1B4 mRNA and eccDNA abundance, and western blotting verified protein expression. EccDNA transfection was used to examine the effect of ATP1B4 overexpression on PI resistance.
2. The ATP1B4 gene was silenced using shRNA. CCK-8 and flow cytometry were used to assess cell viability and CFZ-induced apoptosis. Bone marrow organoids were used to test CFZ's pro-apoptotic effect after ATP1B4 silencing.
3. A subcutaneous xenograft tumor model of PI-resistant MM cells was developed in nude mice using CFZ injections with ATP1B4-targeting shRNA lentivirus. In vivo imaging was used to monitor tumor growth.
4. LC/MS was used to screen ATP1B4 upstream regulatory proteins, and western blotting was used to verify PPARγ expression. CRISPRi was used to silence PPARγ and assess ATP1B4 expression and eccDNA abundance, followed by evaluation of viability and apoptosis.
5. CRISPRi silenced LIG3 to determine ATP1B4 and eccDNA abundance. ChIP confirmed LIG3-PPARγ binding to the ATP1B4 promoter. Co-IP and IF analyses were used to analyze their binding and PPRE elements.
6. LC/MS analysis verified CPT1A expression. CRISPRi silenced CPT1A to assess ATP1B4 expression. GSVA-enriched pathways and detected FAO rates for CPT1A-PPARγ regulation.
7. Western blotting was used to detect mTOR in the resistant cells. CRISPRi silenced mTOR to assess ATP1B4 expression. IF observed CPT1A-LAMP1 co-localization. Autophagy inhibitors verified mTOR-mediated CPT1A regulation.

**Copyright Information:** (Online) ISSN: 2572-9241
©2026 The Author(s). HemaSphere is published by John Wiley & Sons Ltd on behalf of European Hematology Association. This is an open access Abstract Book distributed under the Attribution-NonCommercial-NoDerivs (CC BY-NC-ND), which allows third parties to download the articles and share them with others as long as they credit the author and the Abstract Book, but they cannot change the content in any way or use them commercially.
**Abstract Book Citations:** Authors, Title, HemaSphere, 2026;10:(S1):pages. The abstract book DOIs can be found on the "Author Information" page.
**Disclaimer:** Articles published in the journal HemaSphere exclusively reflect the opinions of the authors. The authors are responsible for all content in their abstracts including accuracy of the facts, statements, citing resources, etc.

### Results

1. ATP1B4 exists in PI-resistant MM cell (PR) eccDNA, with higher levels than sensitive cells (PS).eccDNA transfection increases ATP1B4 in PS cells. CFZ ineffective on PR cells until ATP1B4 silencing. ATP1B4 downregulation promoted CFZ-induced apoptosis in organoids.In xenografts, CFZ with ATP1B4-targeting shRNA reduced tumors, reversing PI resistance. PPARγ regulates ATP1B4, showing increased PR expression. PPARγ-LIG3 complex enables ATP1B4 transcription. Their inhibition reduces ATP1B4 and eccDNA. CPT1A increases in PR cells; its silencing reduced ATP1B4, PPARγ, and enhanced CFZ effect. mTOR upregulation maintains CPT1A by inhibiting degradation, activating FAO pathway.

### Summary/Conclusion

ATP1B4 is key for PI resistance in MM cells, existing in PR cell eccDNA. ATP1B4 silencing restores PI sensitivity. PPARγ-LIG3 complex activates ATP1B4 transcription on eccDNA. CPT1A regulates PPARγ activation and PI resistance. mTOR affects resistance by regulating CPT1A degradation, forming core regulatory axis in MM cells.

**Copyright Information:** (Online) ISSN: 2572-9241
©2026 The Author(s). HemaSphere is published by John Wiley & Sons Ltd on behalf of European Hematology Association. This is an open access Abstract Book distributed under the Attribution-NonCommercial-NoDerivs (CC BY-NC-ND), which allows third parties to download the articles and share them with others as long as they credit the author and the Abstract Book, but they cannot change the content in any way or use them commercially.
**Abstract Book Citations:** Authors, Title, HemaSphere, 2026;10:(S1):pages. The abstract book DOIs can be found on the "Author Information" page.
**Disclaimer:** Articles published in the journal HemaSphere exclusively reflect the opinions of the authors. The authors are responsible for all content in their abstracts including accuracy of the facts, statements, citing resources, etc.

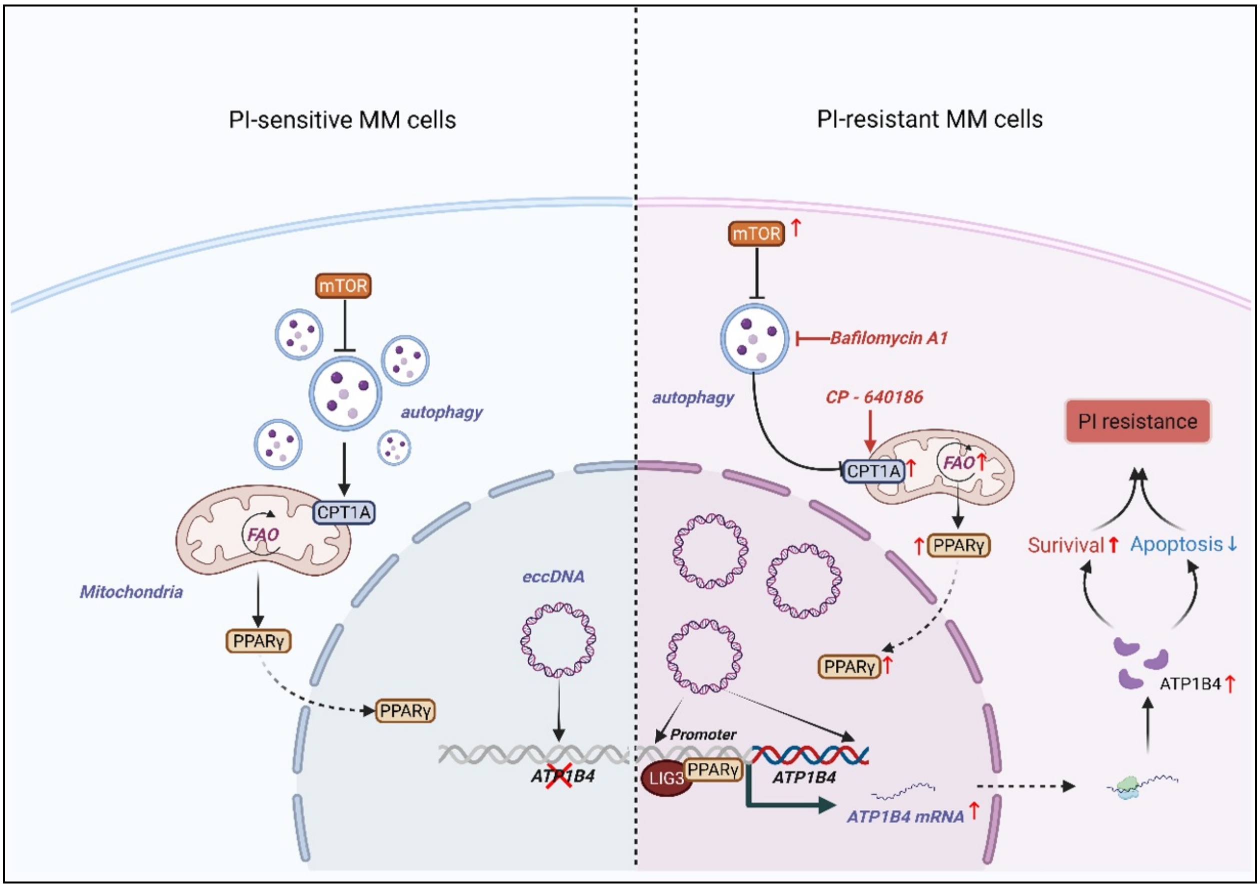

A schematic illustration of how ATP1B4 affects the sensitivity and resistance of multiple myeloma (MM) cells to proteasome inhibitors (PIs) is as follows: In the left panel (PI-sensitive MM cells), the ATP1B4 gene is not present in the extrachromosomal circular DNA (eccDNA, a form of DNA found outside chromosomes), and no molecular cascade that promotes resistance is formed, so the cells remain sensitive to PI treatment. In the right panel (PI-resistant MM cells), the expression level of the protein kinase mTOR (mechanistic target of rapamycin) is increased, which continuously supports the autophagy (cellular self-digestion) process; this process is coupled with fatty acid oxidation (FAO, a metabolic process dependent on the enzyme carnitine palmitoyltransferase 1A, or CPT1A) and is closely related to the upregulation of peroxisome proliferator-activated receptor gamma (PPARγ, a protein that regulates gene expression). Meanwhile, eccDNA accumulates in large amounts in resistant cells, driving increased ATP1B4 transcription (seen as increased ATP1B4 mRNA levels) through interactions with DNA ligase 3 (LIG3, an enzyme involved in DNA repair) and PPARγ. These molecular events ultimately enhance MM cell survival, inhibit their programmed cell death (apoptosis), and thereby cause resistance to PI treatment.

**Copyright Information:** (Online) ISSN: 2572-9241
©2026 The Author(s). HemaSphere is published by John Wiley & Sons Ltd on behalf of European Hematology Association. This is an open access Abstract Book distributed under the Attribution-NonCommercial-NoDerivs (CC BY-NC-ND), which allows third parties to download the articles and share them with others as long as they credit the author and the Abstract Book, but they cannot change the content in any way or use them commercially.
**Abstract Book Citations:** Authors, Title, HemaSphere, 2026;10:(S1):pages. The abstract book DOIs can be found on the "Author Information" page.
**Disclaimer:** Articles published in the journal HemaSphere exclusively reflect the opinions of the authors. The authors are responsible for all content in their abstracts including accuracy of the facts, statements, citing resources, etc.

### (PS1840) THE NOVEL RETINOID WYC-209 SENSITIZES MULTIPLE MYELOMA TO CARFILZOMIB VIA EPIGENETICALLY UPREGULATING ZMYND8

**Topic:** 13. Myeloma and other monoclonal gammopathies - Biology & translational research

Jiaxuan Xu[1], Jie Yan[2], Xiaoqing Dong[1], Bing Chen*[1, 2]

*[1]Nanjing Drum Tower Hospital Clinical College of Nanjing University of Chinese Medicine, Department of Hematology, Nanjing, China; [2]Nanjing Drum Tower Hospital, Affiliated Hospital of Medical School, Nanjing University, Department of Hematology, Nanjing, China;*

### Background

Carfilzomib (CFZ) is a cornerstone therapy for multiple myeloma (MM), yet clinical efficacy is often limited by inadequate response. We previously identified ZMYND8 as a key determinant of CFZ sensitivity in MM cells, suggesting that pharmacological activation of ZMYND8 represents a promising strategy to enhance CFZ efficacy.

### Aims

This study aimed to identify novel retinoid compounds capable of epigenetically activating ZMYND8 expression and to investigate whether such compounds could synergize with CFZ to improve anti-MM efficacy in a ZMYND8-dependent manner.

### Methods

Transcriptomic sequencing, dual-luciferase reporter assays, and bioinformatic analysis were employed to screen retinoid compounds for ZMYND8 activation. Epigenetic mechanisms were investigated using CUT&Tag and ChIP-qPCR analyses of histone modifications at the ZMYND8 promoter. The synergistic effects of WYC-209 with CFZ were evaluated in MM cell lines, patient-derived primary cells, and ZMYND8-knockout models through cell viability assays, apoptosis analysis, transmission electron microscopy, and drug synergy modeling. In vivo therapeutic synergy was assessed in NSG mouse xenograft models by monitoring tumor burden, survival, and bone marrow infiltration.

### Results

Both ATRA and the novel synthetic retinoid WYC-209 significantly upregulated ZMYND8 transcription by remodeling the epigenetic landscape, particularly through increased H3K27ac enrichment at predicted retinoic acid response elements within the ZMYND8 promoter. WYC-209 exhibited superior ZMYND8 transcriptional activation compared to other retinoid analogs. Combined treatment with WYC-209 and CFZ demonstrated pronounced synergistic cytotoxicity in MM cells, with ZIP synergy scores exceeding 10. This synergy was characterized by enhanced apoptosis and exacerbated endoplasmic reticulum dilation, effects that were markedly attenuated upon ZMYND8 knockout. Notably, the optimal synergistic concentration window for WYC-209+CFZ occurred at lower CFZ concentrations (1–10 nM) compared to ATRA+CFZ (10–30 nM). In vivo, WYC-209 significantly potentiated the antitumor efficacy of CFZ, improving mouse survival, reducing tumor burden, and decreasing MM cell infiltration in bone marrow—all in a ZMYND8-dependent manner.

**Copyright Information:** (Online) ISSN: 2572-9241
©2026 The Author(s). HemaSphere is published by John Wiley & Sons Ltd on behalf of European Hematology Association. This is an open access Abstract Book distributed under the Attribution-NonCommercial-NoDerivs (CC BY-NC-ND), which allows third parties to download the articles and share them with others as long as they credit the author and the Abstract Book, but they cannot change the content in any way or use them commercially.
**Abstract Book Citations:** Authors, Title, HemaSphere, 2026;10:(S1):pages. The abstract book DOIs can be found on the “Author Information” page.
**Disclaimer:** Articles published in the journal HemaSphere exclusively reflect the opinions of the authors. The authors are responsible for all content in their abstracts including accuracy of the facts, statements, citing resources, etc.

### Summary/Conclusion

This study establishes ZMYND8 as a pivotal effector mediating retinoid-induced sensitization of MM cells to CFZ. We identify WYC-209 as a novel epigenetic activator of ZMYND8 that synergizes potently with CFZ through exacerbating ER stress in a ZMYND8-dependent manner. These findings provide a mechanistic rationale for a combination strategy using WYC-209 to augment CFZ efficacy, offering translational potential for improving outcomes in high-risk or relapsed MM patients.

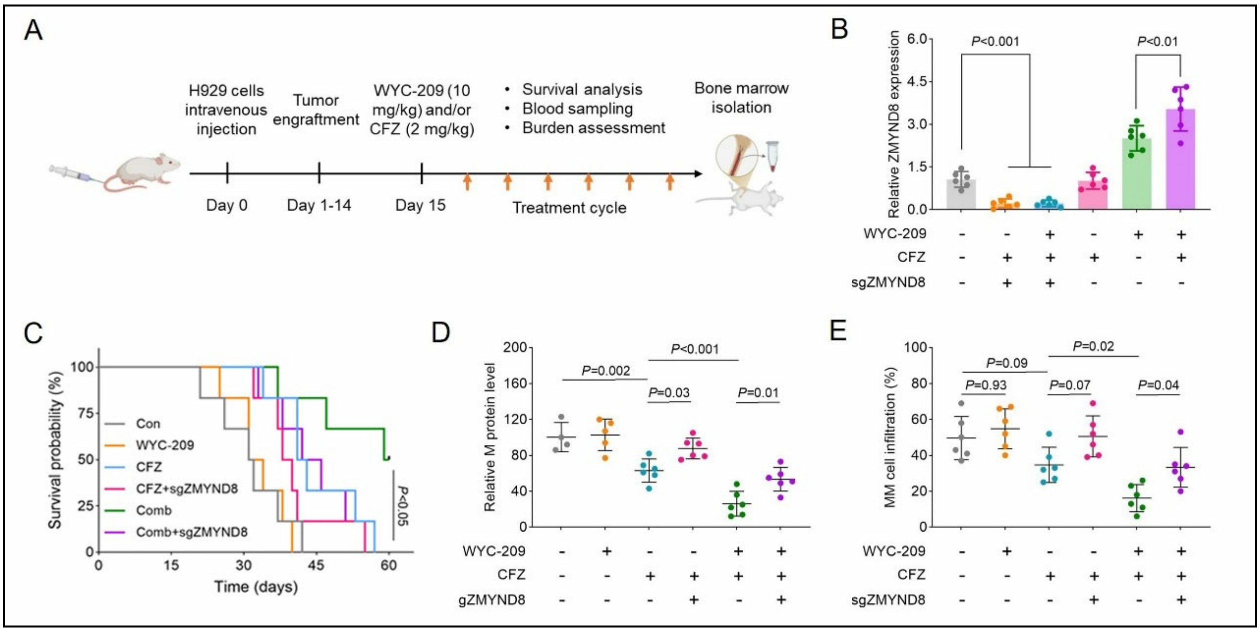

Figure 1. Combined treatment with WYC-209 and CFZ exhibits pronounced synergistic effects on MM in a ZMYND8-dependent manner. (A) Schematic representation of the experimental timeline for tumor cell inoculation and drug administration in NSG mice. (B) Relative ZMYND8 mRNA expression in CD138+ MM cells isolated from murine bone marrow (n = 6). (C) Kaplan-Meier survival analysis of tumor-bearing mice across treatment groups (n = 6). (D) Serum levels of human immunoglobulin light chain, quantified by ELISA 30 days post-tumor injection. (E) Proportion of MM cell infiltration in murine bone marrow across experimental groups (n = 6). Data are presented as mean±SD.

**Copyright Information:** (Online) ISSN: 2572-9241
©2026 The Author(s). HemaSphere is published by John Wiley & Sons Ltd on behalf of European Hematology Association. This is an open access Abstract Book distributed under the Attribution-NonCommercial-NoDerivs (CC BY-NC-ND), which allows third parties to download the articles and share them with others as long as they credit the author and the Abstract Book, but they cannot change the content in any way or use them commercially.
**Abstract Book Citations:** Authors, Title, HemaSphere, 2026;10:(S1):pages. The abstract book DOIs can be found on the "Author Information" page.
**Disclaimer:** Articles published in the journal HemaSphere exclusively reflect the opinions of the authors. The authors are responsible for all content in their abstracts including accuracy of the facts, statements, citing resources, etc.

### (PS1841) CHROMOANAGENESIS DETECTED BY OPTICAL GENOMIC MAPPING PREDICTS ADVERSE OUTCOMES IN MULTIPLE MYELOMA

**Topic:** 13. Myeloma and other monoclonal gammopathies - Biology & translational research

Lorenzo Gensini[1], Charan Vegivinti*[2], Ruxandra Irimia[3], Lei Feng[4], Sheeba K. Thomas[1], Lu Dai[1], Oren Pasvolsky[1], Mahmoud R. Gaballa[1], Melody R. Becnel[1], Neeraj Saini[5], Donna Weber[1], Hans C. Lee[6], Krina Patel[1], Robert Z Orlowski[1], Pei Lin[7], Ying Zou[8], Guilin Tang[7], Christian Gocke[9], Jing Christine Ye[1]

*[1]MD Anderson Cancer Center, Department of Lymphoma/Myeloma, Houston, United States of America;[2]MD Anderson Cancer Center, Department of Cancer Medicine, Houston, United States of America; [3]Carol Davila University of Medicine and Pharmacy, Department of Hematology, Bucharest, Romania; [4]MD Anderson Cancer Center, Department of Biostatistics, Houston, United States of America; [5]MD Anderson Cancer Center, Department of Stem Cell Transplantation and Cellular Therapy, Houston, United States of America; [6]Sarah Cannon Research Institute, Department of Myeloma, Nashville, United States of America;[7]MD Anderson Cancer Center, Department of Hematopathology, Houston, United States of America; [8]Johns Hopkins University School of Medicine, Department of Pathology, Baltimore, United States of America; [9]Johns Hopkins Sidney Kimmel Comprehensive Cancer Center., Department of Oncology, Baltimore, United States of America;*

### Background

Multiple Myeloma (MM) is a heterogeneous disease. The role of chromoanagenesis (ChrAG) and MYC rearrangements in disease biology and clinical outcomes remains unclear in newly diagnosed multiple myeloma (NDMM) and relapsed refractory multiple myeloma (RRMM). Optical genome mapping (OGM) identifies intricate genome-wide rearrangements, offering opportunities to comprehensively characterize ChrAG and MYC rearrangements that are not covered by routine FISH testing.

### Aims

To evaluate the clinical utility of ChrAG and MYC rearrangements detected by OGM in both NDMM and RRMM patients.

### Methods

Both NDMM and RRMM patients from two major cancer centers in the US were included in this study. Plasma cells from bone marrow were isolated for OGM analysis.

**Copyright Information:** (Online) ISSN: 2572-9241
©2026 The Author(s). HemaSphere is published by John Wiley & Sons Ltd on behalf of European Hematology Association. This is an open access Abstract Book distributed under the Attribution-NonCommercial-NoDerivs (CC BY-NC-ND), which allows third parties to download the articles and share them with others as long as they credit the author and the Abstract Book, but they cannot change the content in any way or use them commercially.
**Abstract Book Citations:** Authors, Title, HemaSphere, 2026;10:(S1):pages. The abstract book DOIs can be found on the “Author Information” page.
**Disclaimer:** Articles published in the journal HemaSphere exclusively reflect the opinions of the authors. The authors are responsible for all content in their abstracts including accuracy of the facts, statements, citing resources, etc.

**Results**

A total of 142 patients were enrolled with OGM performed between March 2023 and September 2025.
In the NDMM cohort (n=89) median age was 67 years, R-ISS staging showed 16 (18.4%) patients with stage I, 48 (55.2%) with stage II, 23 (26.4%) with stage III disease. Triplet induction was administered in 16 patients, bispecific in 3 patients and quadruplet in 67 patients. ChrAG was detected in 22 cases (25%): 11 cases (12%) involved one chromosome and another 11 cases (12%) involved two or more chromosomes. There was a trend toward inferior OS with ChrAG involving >=2 chromosomes (n=11, 12.4%) compared to those with ChrAG involving 0-1 chromosomes (n=78, 87.6%) (p=0.09). The difference in PFS and TTNT between these two groups was statistically significant (p=0.003).
MYC gene rearrangement was detected in 24 (27%) NDMM patients, involving 9 different partner chromosomes, the most common rearrangements were t(8;22) in 8 cases and t(8;14) in 5 cases. There was a trend toward worse OS in patients with MYC gene rearrangement compared to those without (p=0.26).
In the RRMM cohort (n=53), median age was 67 years. ChrAG was detected in 19 patients (36%): 6 cases (11%) involved 0-1 chromosomes and 13 cases (25%) involved >=2 chromosomes. RRMM demonstrated a higher prevalence of ≥2 ChrAG events compared with NDMM (26% vs 13%; p=0.045). Among the 13 cases with ChrAG involving >2 chromosomes, 3 presented with EMD, another 3 presented with secondary PCL. OS was inferior in the group with ChrAG involving two or more chromosomes (p=0.06) although PFS was not significantly different.

**Summary/Conclusion**

ChrAG, characterized by complex rearrangements of one or more chromosomes, can be detected with high sensitivity using OGM. Our data demonstrate that in NDMM, the presence of ChrAG involving more than one chromosome is associated with significant worse PFS and TTNT and a trend toward worse OS. The frequency of ChrAG is substantially higher in RRMM. ChrAG involving more than one chromosome is associated with a trend toward a shorter OS in RRMM. Larger cohorts may further enhance the prognostic value of ChrAG.

**Copyright Information:** (Online) ISSN: 2572-9241
©2026 The Author(s). HemaSphere is published by John Wiley & Sons Ltd on behalf of European Hematology Association. This is an open access Abstract Book distributed under the Attribution-NonCommercial-NoDerivs (CC BY-NC-ND), which allows third parties to download the articles and share them with others as long as they credit the author and the Abstract Book, but they cannot change the content in any way or use them commercially.
**Abstract Book Citations:** Authors, Title, HemaSphere, 2026;10:(S1):pages. The abstract book DOIs can be found on the “Author Information” page.
**Disclaimer:** Articles published in the journal HemaSphere exclusively reflect the opinions of the authors. The authors are responsible for all content in their abstracts including accuracy of the facts, statements, citing resources, etc.

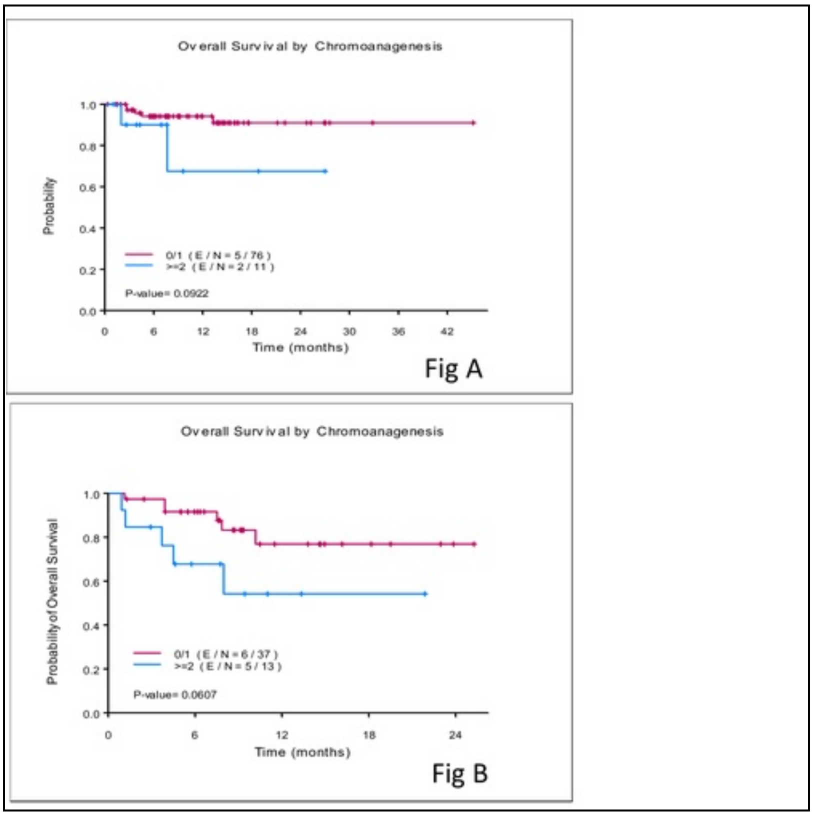

Association of ChrAG with overall survival in (Figure 1A) NDMM and (Figure 1B) RRMM cohorts.

**Copyright Information:** (Online) ISSN: 2572-9241
©2026 The Author(s). HemaSphere is published by John Wiley & Sons Ltd on behalf of European Hematology Association. This is an open access Abstract Book distributed under the Attribution-NonCommercial-NoDerivs (CC BY-NC-ND), which allows third parties to download the articles and share them with others as long as they credit the author and the Abstract Book, but they cannot change the content in any way or use them commercially.
**Abstract Book Citations:** Authors, Title, HemaSphere, 2026;10:(S1):pages. The abstract book DOIs can be found on the "Author Information" page.
**Disclaimer:** Articles published in the journal HemaSphere exclusively reflect the opinions of the authors. The authors are responsible for all content in their abstracts including accuracy of the facts, statements, citing resources, etc.

### (PS1842) MDSCS DRIVE T CELL SENESCENCE TO FACILITATE TUMOR GROWTH AND STEM CELL-LIKE PROPERTIES IN MULTIPLE MYELOMA

**Topic:** 13. Myeloma and other monoclonal gammopathies - Biology & translational research

Lisha Ai*[1]

[1] *Institute of Hematology, Union Hospital,Tongji Medical College, Huazhong University of Science and Technology, Wuhan, China;*

### Background

Multiple myeloma (MM) is a hematologic malignancy characterized by clonal proliferation of plasma cells in the bone marrow. Despite extended overall survival through proteasome inhibitors, immunomodulatory drugs, CD38 monoclonal antibodies, stem cell transplantation, and CAR-T therapy, drug resistance/relapse remains challenging.

### Aims

Myelomagenesis and progression are closely linked to the immunosuppressive tumor microenvironment (TME). This study investigates the functional impact of $CD3^+CD8^+CD28^-CD57^+$ senescent T cells in MM patients, alongside exploring potential mechanisms of T-cell senescence induction within the MM TME.

### Methods

Flow cytometry quantified $CD3^+CD8^+CD28^-CD57^+$ senescent T cells in bone marrow and peripheral blood from MM patients and healthy donors, correlating levels with the Revised International Staging System (R-ISS). Immunosuppressive cells—regulatory T cells, myeloid-derived suppressor cells, tumor-associated macrophages, and regulatory B cells —were isolated and co-cultured with naïve T cells. T cell senescence was assessed via SA-β-gal staining and downregulation of costimulatory molecules CD27/CD28. MDSCs were pretreated with a TLR8 agonist to evaluate its impact on T cell senescence induction. Then senescent T cells were co-cultured with MM cell lines. Tumor growth kinetics were analyzed via cell counting and MTT assays, stemness maintenance was evaluated by tumor sphere formation assay and stemness-related proteins (Nanog, Oct-4, Sox-2) expression level.

### Results

Patients with multiple myeloma (MM) exhibited a significantly higher proportion of $CD3^+CD8^+CD28^-CD57^+$ senescent T cells compared to healthy controls, and this proportion positively correlated with the Revised International Staging System (R-ISS) stage. Furthermore, the frequency of monocytic myeloid-derived suppressor cells (M-MDSCs) in MM patients positively correlated with the proportion of senescent T cells. MM patient-derived M-MDSCs were capable of inducing T cell senescence in vitro, as manifested by increased SA-β-gal-positive staining and significantly decreased expression of the co-stimulatory molecules CD27 and CD28. Pretreatment of M-MDSCs with the TLR8 agonist polyG3 resulted in reduced SA-β-gal-positive T cells compared to co-culture with untreated M-MDSCs. Moreover, co-culture with MM-derived senescent T cells promoted accelerated proliferation, increased tumor sphere formation, and upregulated expression of stemness-associated proteins in MM cell lines.

### Summary/Conclusion

This study demonstrates that $CD3^+CD8^+CD28^-CD57^+$ senescent T cells expand in MM patients, and positively correlate with R-ISS stage. MM-derived M-MDSCs drive T-cell senescence in vitro, potentially via TLR8 signaling. Critically, senescent T cells functionally promote tumor growth and stemness maintenance in MM. These findings provide novel insights into MM relapse/resistance mechanisms and reveal senescent T cells maybe a new target for MM therapy.

**Copyright Information:** (Online) ISSN: 2572-9241
©2026 The Author(s). HemaSphere is published by John Wiley & Sons Ltd on behalf of European Hematology Association. This is an open access Abstract Book distributed under the Attribution-NonCommercial-NoDerivs (CC BY-NC-ND), which allows third parties to download the articles and share them with others as long as they credit the author and the Abstract Book, but they cannot change the content in any way or use them commercially.
**Abstract Book Citations:** Authors, Title, HemaSphere, 2026;10:(S1):pages. The abstract book DOIs can be found on the "Author Information" page.
**Disclaimer:** Articles published in the journal HemaSphere exclusively reflect the opinions of the authors. The authors are responsible for all content in their abstracts including accuracy of the facts, statements, citing resources, etc.

### (PS1843) ARGINASE INHIBITION ENABLES NEUTROPHIL GRANULOCYTE-DRIVEN HYPERACTIVATION OF T CELLS FROM PATIENTS WITH MULTIPLE MYELOMA

**Topic:** 13. Myeloma and other monoclonal gammopathies - Biology & translational research

Ann-Katrin Gebel*[1], Stephanie Reichert[1], David Charalambous[1], Matthias Bros[2], Matthias Theobald[1, 3, 4], Hakim Echchannaoui[1, 3], Markus Munder[1, 4, 5]

*[1]Department of Internal Medicine III, University Medical Center Mainz, Mainz, Germany;[2]Department of Dermatology, University Medical Center Mainz, Mainz, Germany; [3]German Cancer Consortium (DKTK), Partner Site Frankfurt/Mainz, Germany;[4]Research Center for Immunotherapy (FZI), University Medical Center Mainz, Mainz, Germany; [5]Department of Hematology and Medical Oncology, Deaconess Foundation Hospital Speyer, Speyer, Germany;*

### Background

T cells in the tumor microenvironment of multiple myeloma frequently become functionally impaired. A key mechanism of this tumor-promoting immune suppression is the arginase-mediated depletion of arginine, predominantly mediated by polymorphonuclear neutrophils (PMNs). We previously observed that pharmacological arginase inhibition can restore activation even in severely dysfunctional patient-derived T cells.

### Aims

This study aims to assess whether anergic T cells from multiple myeloma patients can be converted into a non-anergic, fully activated state through inhibition of PMN-derived arginase and to characterize the resulting activation profile.

### Methods

Human $CD3^+$ T cells from patients diagnosed with multiple myeloma (MM; either newly diagnosed = nd or relapsed/refractory = rr) isolated from peripheral blood (PB) or bone marrow (BM) were activated using anti-CD3/anti-CD28–coated beads and cultured for 48 h or 96 h in PMN-derived supernatants supplemented with the arginase inhibitor nor-NOHA. PMNs were obtained either from healthy donors or from MM patients. As a control, T cells were cultured in medium containing physiological 150 µM arginine. Experimental readouts were mainly eFluor450 dye dilution for proliferation, flow cytometric surface marker profiling to determine differentiation, MitoTracker®- and Seahorse XF analysis based assessment of mitochondrial and overall cellular metabolic activity, as well as multiplex cytokine bead arrays to characterize secretory patterns.

### Results

Upon arginase inhibition, T cells cultured in the PMN-derived supernatant showed hyperproliferation (eFluor450 mean MFI 18,1 ±9,96) compared to control conditions (mean MFI 37,4 ± 7,04). This effect was consistently observed in both, BM- and PB-derived T cells from ndMM as well as rrMM patients. In addition to enhanced proliferation, T cells displayed altered differentiation profiles. In ndMM PB T cells, the frequency of early differentiated subsets increased by 54%, whereas terminally differentiated EMRA T cells as well as senescent $CD28^-CD57^+$ T cells both decreased significantly (by 68% and 60%, respectively). Mitochondrial fitness was assessed by MitoTracker® staining and revealed increased levels of functional mitochondria. Enhanced metabolic activity was detectable, with increased glycolysis and oxidative phosphorylation including a 78% increase in maximal respiration. Finally, T cells isolated from MM patients demonstrated significantly elevated cytokine secretion under arginase inhibition, most notably a 4-fold increase in IFN-γ and a 17-fold increase in TNF-α relative to control conditions.

**Copyright Information:** (Online) ISSN: 2572-9241
©2026 The Author(s). HemaSphere is published by John Wiley & Sons Ltd on behalf of European Hematology Association. This is an open access Abstract Book distributed under the Attribution-NonCommercial-NoDerivs (CC BY-NC-ND), which allows third parties to download the articles and share them with others as long as they credit the author and the Abstract Book, but they cannot change the content in any way or use them commercially.
**Abstract Book Citations:** Authors, Title, HemaSphere, 2026;10:(S1):pages. The abstract book DOIs can be found on the “Author Information” page.
**Disclaimer:** Articles published in the journal HemaSphere exclusively reflect the opinions of the authors. The authors are responsible for all content in their abstracts including accuracy of the facts, statements, citing resources, etc.

### Summary/Conclusion

In summary, our findings demonstrate that arginase inhibition in human PMN promotes a distinct hyperactivated state in patient-derived T cells from multiple myeloma patients. Arginase inhibition reverses PMN-mediated T cell immunosuppression by promoting proliferation, shifting differentiation towards less terminally differentiated subsets, enhancing mitochondrial fitness, and increasing pro-inflammatory cytokine secretion. Importantly, the use of primary patient-derived T cells underscores the translational and clinical relevance of our findings, as T cell dysfunction is a central limitation in multiple myeloma immunotherapy.

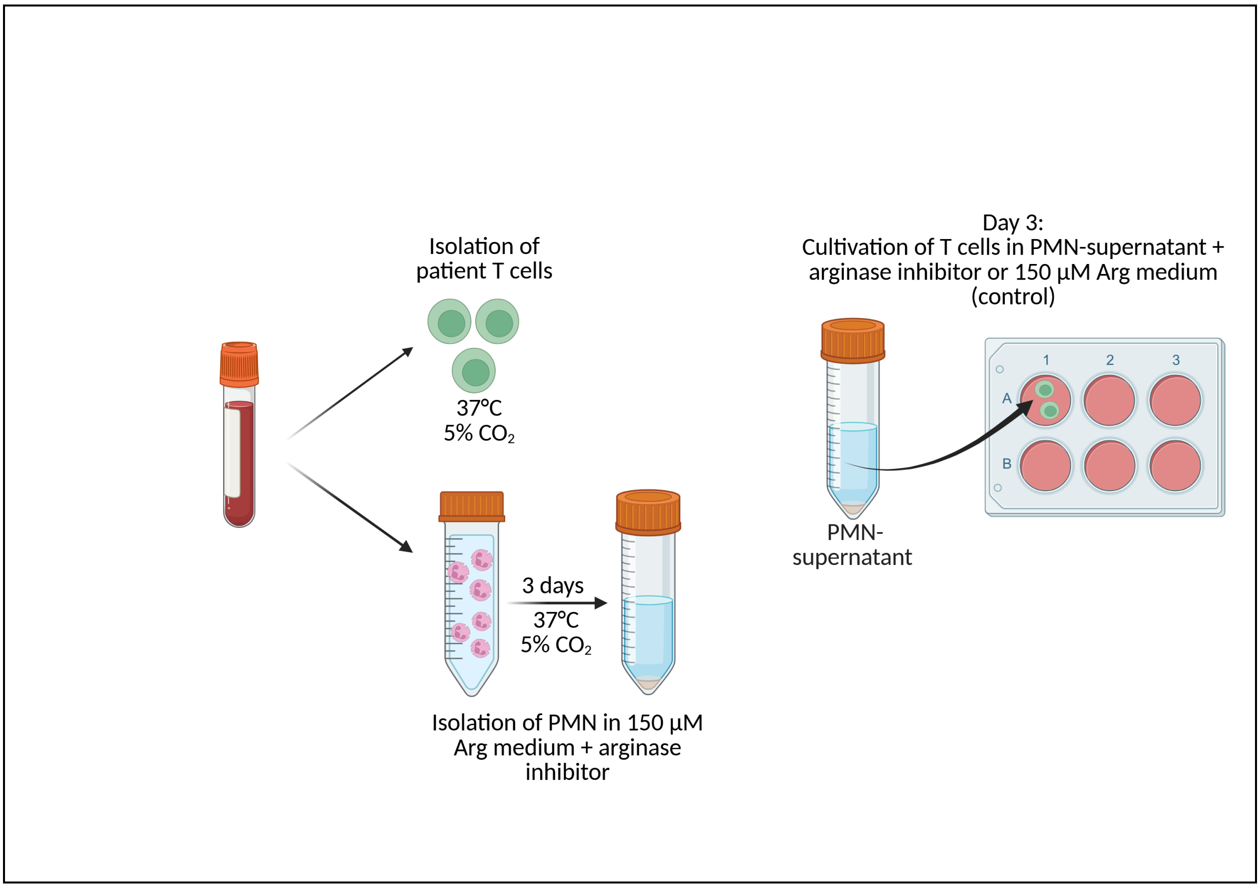

Experimental workflow for the isolation and cultivation of T cells and PMNs (Created with BioRender.com).

**Copyright Information:** (Online) ISSN: 2572-9241
©2026 The Author(s). HemaSphere is published by John Wiley & Sons Ltd on behalf of European Hematology Association. This is an open access Abstract Book distributed under the Attribution-NonCommercial-NoDerivs (CC BY-NC-ND), which allows third parties to download the articles and share them with others as long as they credit the author and the Abstract Book, but they cannot change the content in any way or use them commercially.
**Abstract Book Citations:** Authors, Title, HemaSphere, 2026;10:(S1):pages. The abstract book DOIs can be found on the "Author Information" page.
**Disclaimer:** Articles published in the journal HemaSphere exclusively reflect the opinions of the authors. The authors are responsible for all content in their abstracts including accuracy of the facts, statements, citing resources, etc.

### (PS1844) DR4/DR5 AGONISTS SYNERGIZE WITH BH3 MIMETICS VENETOCLAX TO PROMOTE APOPTOSIS IN MULTIPLE MYELOMA

**Topic:** 13. Myeloma and other monoclonal gammopathies - Biology & translational research

Yiwen Jiang*[1], Qingyu Xu[1], Jingjing Deng[1], Huixing Zhou[1], Zhiyao Zhang[1], Menghan Liu[1], Wenjing Li[1], Ying Tian[1], Guangzhong Yang[1], Yin Wu[1], Yun Leng[1], Ai-Jun Liu[1], Wen Gao[1], Honghu Zhu[1], Wenming Chen[1]

[1]*Beijing Chao-Yang Hospital, Capital Medical University, Beijing, China;*

### Background

Multiple myeloma (MM) remains incurable despite treatment advances, with most patients developing relapsed or refractory disease. Circularly Permuted TRAIL (CPT, Aponermin) activates the extrinsic apoptotic pathway via death receptors DR4 and DR5, whereas Venetoclax targets the intrinsic BCL-2–dependent pathway. We therefore hypothesized that dual targeting of these complementary apoptotic mechanisms could overcome resistance and produce synergistic antitumor efficacy.

### Aims

To evaluate the synergistic effects of CPT and Venetoclax in myeloma cells by dissecting DR4/DR5- and BCL-2–mediated apoptotic pathways, and to identify predictive biomarkers that could inform patient selection for combination therapy.

### Methods

Cell viability was measured using CellTiter-Glo, apoptosis by flow cytometry and caspase-8, -9, and -3/7 activity, and cell cycle by DAPI staining. Dose–response relationships were characterized for both single-agent and combination treatments, and caspase dependency was examined using selective inhibitors. RNA sequencing followed by gene set enrichment analysis was performed to evaluate pathway activation and mechanisms. Primary patient-derived plasma cells were isolated from bone marrow aspirates via $CD138^+$ MACS. Purified primary cells were used for ex vivo drug treatment across dose–response matrices to assess synergy using the Bliss model, and for flow cytometry analysis of DR4/DR5 and key BCL2 family proteins (BCL2, MCL1, BCL-xL).

### Results

CPT synergized with Venetoclax in killing both dexamethasone-resistant MM1R and bortezomib-resistant KMS11/BTZ cells (Bliss synergy scores = 10.88 and 20.29, respectively). Venetoclax activity was also enhanced in combination with DR4/DR5 agonists in KMS12 PE cells (Bliss synergy score = 6.75). RNA-seq analysis of KMS11/BTZ cells demonstrated that combination treatment further enriched the apoptosis and TNF signaling pathway compared with either agent alone (NES = 1.506 and 2.11, respectively; nominal p-value $< 0.0001$), indicating stronger apoptotic induction. Caspase-3/7, -8, and -9 activities increased and G1-phase cell cycle arrest was observed after combined treatment. Then the analysis of 20 $CD138^+$ primary myeloma bone marrow samples showed that mean Bliss synergy scores positively correlated with DR4 and DR5 expression (Spearman r = 0.610 and 0.497, respectively; $p < 0.05$), but not with BCL2 expression. ROC analysis indicated that DR4 and DR5 effectively discriminated synergistic from non-synergistic samples (AUCs = 0.898 and 0.778, respectively), with combined analysis increasing the AUC to 0.955. Based on ex vivo synergy profiling of primary myeloma cells, several patients subsequently demonstrated improved clinical responses following CPT–Venetoclax regimen.

**Copyright Information:** (Online) ISSN: 2572-9241
©2026 The Author(s). HemaSphere is published by John Wiley & Sons Ltd on behalf of European Hematology Association. This is an open access Abstract Book distributed under the Attribution-NonCommercial-NoDerivs (CC BY-NC-ND), which allows third parties to download the articles and share them with others as long as they credit the author and the Abstract Book, but they cannot change the content in any way or use them commercially.
**Abstract Book Citations:** Authors, Title, HemaSphere, 2026;10:(S1):pages. The abstract book DOIs can be found on the “Author Information” page.
**Disclaimer:** Articles published in the journal HemaSphere exclusively reflect the opinions of the authors. The authors are responsible for all content in their abstracts including accuracy of the facts, statements, citing resources, etc.

**Summary/Conclusion**

CPT synergizes with Venetoclax to induce caspase-dependent apoptosis in multiple myeloma, and DR4/DR5 expression levels predict the magnitude of synergy, highlighting their potential as biomarkers for guiding combination therapy.

**Copyright Information:** (Online) ISSN: 2572-9241
©2026 The Author(s). HemaSphere is published by John Wiley & Sons Ltd on behalf of European Hematology Association. This is an open access Abstract Book distributed under the Attribution-NonCommercial-NoDerivs (CC BY-NC-ND), which allows third parties to download the articles and share them with others as long as they credit the author and the Abstract Book, but they cannot change the content in any way or use them commercially.
**Abstract Book Citations:** Authors, Title, HemaSphere, 2026;10:(S1):pages. The abstract book DOIs can be found on the "Author Information" page.
**Disclaimer:** Articles published in the journal HemaSphere exclusively reflect the opinions of the authors. The authors are responsible for all content in their abstracts including accuracy of the facts, statements, citing resources, etc.

### (PS1845) POLB 001, AN ORAL P38 MAPK INHIBITOR FOR THE PREVENTION OF CYTOKINE RELEASE SYNDROME

**Topic:** 13. Myeloma and other monoclonal gammopathies - Biology & translational research

Liam Tremble[1], Paula Maguire[1], Meena Arora[1], Joe Froggatt[2], Jeremy Skillington[1], Brendan Buckley[1], Rakesh Popat*[3], Emma Searle[2, 4]

*[1]Poolbeg Pharma, London, United Kingdom; [2]The Christie NHS Foundation Trust University of Manchester, Manchester, United Kingdom; [3]University College London Hospitals Nhs Foundation Trust, London, United Kingdom; [4]University of Manchester, Manchester, United Kingdom;*

### Background

Cytokine Release Syndrome (CRS) is a potentially severe adverse effect of cancer immunotherapies including CAR T cell therapies and T cell-engaging bispecific antibodies (BsAbs) which carry black box warnings for CRS, and management of CRS requires close monitoring and can be severe if not recognized and treated promptly. Monitoring & management of patients with CRS places significant strain on the healthcare infrastructure. Initial dosing typically requires inpatient admission, or outpatient care only where patients can remain near the clinical setting with a carer. Availability of a convenient and effective prophylactic could enable an expansion in outpatient delivery. As use of CRS-inducing immunotherapies expands across more indications and earlier lines of therapy improved management strategies are needed.
POLB 001 is an oral p38 MAPK inhibitor in development for prevention of immunotherapy-induced CRS. POLB 001 has previously been evaluated in two Phase 1 trials: a first-in-human SAD/MAD PK/PD study and an LPS challenge study, both in healthy subjects.

### Aims

To evaluate the ability of POLB 001 to prevent CRS without compromising anti-tumour efficacy.

### Methods

MHC I/II double knockout NSG mice were treated with a CD28 agonist or CD3xCD19 bispecific antibody to induce CRS; POLB 001 was dosed prophylactically. The effects of POLB 001 on T cell killing, proliferation and phenotype were investigated in in vitro models of BCMAxCD3 bispecific antibody induced tumour cell killing using human PBMCs. POLB 001 is being investigated in a non-randomised, single-arm, multi-centre, open label Phase I/II trial in patients with multiple myeloma eligible to receive the bispecific t-cell engager antibody, teclistamab, within its licensed indication (Figure 1).

### Results

In an *in vivo* CD3xCD19 BsAb-induced CRS model, POLB 001 reduced levels of all cytokines tested (TNF, IFNγ, IL-6, IL-10) at all timepoints, with significant decreases of peak levels ($p \leq 0.05$). In an *in vivo* CD28-induced CRS model, peak levels of all cytokines tested were reduced ;TNF, IFNγ, MIP1A, IL-2, IL-4, IL-6, IL-8, IL-10 (IL-6 & IL-4, $p<0.05$, IL-8 & MIP1A, $p<0.01$, IL-2, IL-10, TNF & IFNγ, non-significant). In *in vitro* models of CD3xBCMA BsAb-induced tumour cell death with human PBMCs, POLB 001 had no significant effect on tumour cell death T cell surface markers (CCR7, 4-1BB, CD25 or CD45RO) or intracellular granzyme B;, a mild increase in CD45RA+ cells was observed in some donors.

**Copyright Information:** (Online) ISSN: 2572-9241
©2026 The Author(s). HemaSphere is published by John Wiley & Sons Ltd on behalf of European Hematology Association. This is an open access Abstract Book distributed under the Attribution-NonCommercial-NoDerivs (CC BY-NC-ND), which allows third parties to download the articles and share them with others as long as they credit the author and the Abstract Book, but they cannot change the content in any way or use them commercially.
**Abstract Book Citations:** Authors, Title, HemaSphere, 2026;10:(S1):pages. The abstract book DOIs can be found on the "Author Information" page.
**Disclaimer:** Articles published in the journal HemaSphere exclusively reflect the opinions of the authors. The authors are responsible for all content in their abstracts including accuracy of the facts, statements, citing resources, etc.

### Summary/Conclusion

POLB 001 reduced key CRS-associated cytokines and clinical CRS scores in humanised mouse models without impairing BsAb anti-tumour efficacy in in vitro models and had no meaningful impact on T cell phenotype. A Phase I/II trial of POLB 001 in teclistamab-treated patients with multiple myeloma is now commencing.

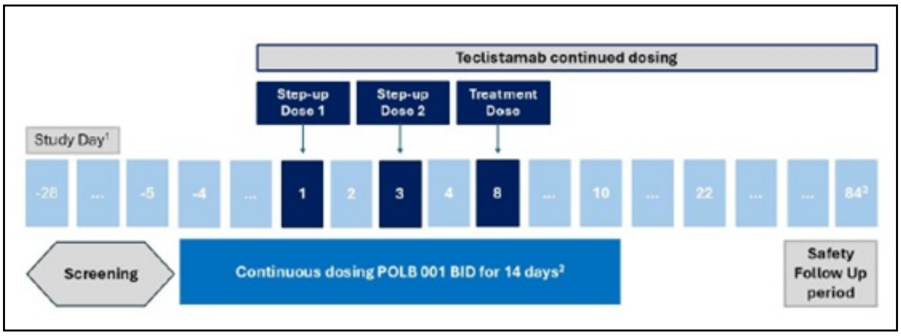

Figure 1. Dosing schedule in single arm, multi-centre, open label Phase I/II trial in patients with multiple myeloma

**Copyright Information:** (Online) ISSN: 2572-9241
©2026 The Author(s). HemaSphere is published by John Wiley & Sons Ltd on behalf of European Hematology Association. This is an open access Abstract Book distributed under the Attribution-NonCommercial-NoDerivs (CC BY-NC-ND), which allows third parties to download the articles and share them with others as long as they credit the author and the Abstract Book, but they cannot change the content in any way or use them commercially.
**Abstract Book Citations:** Authors, Title, HemaSphere, 2026;10:(S1):pages. The abstract book DOIs can be found on the "Author Information" page.
**Disclaimer:** Articles published in the journal HemaSphere exclusively reflect the opinions of the authors. The authors are responsible for all content in their abstracts including accuracy of the facts, statements, citing resources, etc.

### (PS1846) MONOCLONAL LIGHT CHAIN N-GLYCOSYLATION IS A RISK FACTOR FOR NEWLY DIAGNOSED MULTIPLE MYELOMA PROGRESSION

**Topic:** 13. Myeloma and other monoclonal gammopathies - Biology & translational research

Yu Chen[1, 2], Liangshun You[1, 3], Li Zhu[1, 3], Xuejiao Yin[1, 3], Qiumei Yao[1, 3], Shengnan Ding[1, 3], Yi Liu[1, 3], Hongyan Tong[1, 3], Jie Jin[1, 2, 3], Haitao Meng[1, 3], Min Yang*[1, 3]

*[1]Department of Hematology, The First Affiliated Hospital, College of Medicine, Zhejiang University, Hangzhou, Zhejiang, China; [2]Zhejiang Province Key Laboratory of Hematology Oncology Diagnosis and Treatment, Hangzhou, Zhejiang, China;[3]Zhejiang Provincial Clinical Research Center for Hematologic Diseases, Hangzhou, Zhejiang, China;*

### Background

The light chain N-glycosylation of monoclonal immunoglobulins represents an emerging post-translational modification that can be identified through mass spectrometry. Our study provides evidence indicating a link between this modification and more severe disease phenotypes in plasma cell disorders. In summary, monoclonal light chain glycosylation constitutes a potent risk factor for progression to newly diagnosed multiple myeloma in the new era of R2-ISS staging. We propose that monoclonal light chain N-glycosylation may enable earlier prognosis, potentially leading to reduced morbidity and mortality.

### Aims

To assess the prevalence and the independent prognostic significance of serum M-protein light chain N-glycosylation in a real-world, consecutive cohort of newly diagnosed MM (NDMM) patients.

### Methods

We conducted a retrospective, single-center cohort study of consecutive patients with newly diagnosed multiple myeloma (NDMM) diagnosed between January 2023 and January 2026. Baseline serum samples were analyzed using MALDI-TOF mass spectrometry (iMS-IP Assay) to detect N-glycosylation of the primary paraprotein (Gly+). Patients were categorized into Gly+ and Gly- groups. The primary clinical endpoints included overall response rate (ORR), complete response (CR) rate, and progression-free survival (PFS). High-risk (HR) disease was defined as R2-ISS stage III-IV. Statistical analysis comprised chi-square tests to assess response rates and Kaplan-Meier curves with log-rank tests to compare PFS. A multivariable Cox regression model was employed, adjusting for key prognostic factors including R2-ISS stage, treatment regimen, transplant status, age, and light chain isotype.

### Results

N-glycosylation of the M-protein light chain emerged as a strong, independent predictor of disease progression to newly diagnosed multiple myeloma in the new era of R2-ISS staging. In a multivariable Cox model adjusted for R2-ISS stage, treatment intensity, transplant status, age, and light-chain isotype, Gly+ status was associated with a hazard ratio of 3.58 (95% CI 1.79–7.16, $p<0.001$). Glycosylation was detected in 11.5% (33/287) of patients. Gly+ status was independently associated with significantly inferior outcomes: lower overall response rate (57.6% vs. 85.4%, $p<0.001$) and complete response rate (24.2% vs. 55.5%, $p=0.001$), and a >3.5-fold higher rate of early disease progression (33.3% vs. 9.1%, $p<0.001$).
This adverse prognostic association remained consistent across different risk groups. Notably, in the high-risk (R2-ISS III-IV) subgroup (n=140), Gly+ patients who experienced disease progression had a significantly shorter median progression-free survival of 3 months (95% CI, 2.5–12) compared with 15 months (95% CI, 1–24) in Gly- patients, indicating an high-risk clinical course.

**Copyright Information:** (Online) ISSN: 2572-9241
©2026 The Author(s). HemaSphere is published by John Wiley & Sons Ltd on behalf of European Hematology Association. This is an open access Abstract Book distributed under the Attribution-NonCommercial-NoDerivs (CC BY-NC-ND), which allows third parties to download the articles and share them with others as long as they credit the author and the Abstract Book, but they cannot change the content in any way or use them commercially.
**Abstract Book Citations:** Authors, Title, HemaSphere, 2026;10:(S1):pages. The abstract book DOIs can be found on the "Author Information" page.
**Disclaimer:** Articles published in the journal HemaSphere exclusively reflect the opinions of the authors. The authors are responsible for all content in their abstracts including accuracy of the facts, statements, citing resources, etc.

**Summary/Conclusion**

Our real-world study confirms that M-protein light chain N-glycosylation serves as a robust, independent biomarker identifying an high-risk NDMM subgroup characterized by inherent treatment resistance and rapid disease progression. This finding offers critical prognostic evidence for R2-ISS staging, supporting its immediate adoption in risk-adapted management strategies while underscoring the need for prospective validation.

**Copyright Information:** (Online) ISSN: 2572-9241
©2026 The Author(s). HemaSphere is published by John Wiley & Sons Ltd on behalf of European Hematology Association. This is an open access Abstract Book distributed under the Attribution-NonCommercial-NoDerivs (CC BY-NC-ND), which allows third parties to download the articles and share them with others as long as they credit the author and the Abstract Book, but they cannot change the content in any way or use them commercially.
**Abstract Book Citations:** Authors, Title, HemaSphere, 2026;10:(S1):pages. The abstract book DOIs can be found on the "Author Information" page.
**Disclaimer:** Articles published in the journal HemaSphere exclusively reflect the opinions of the authors. The authors are responsible for all content in their abstracts including accuracy of the facts, statements, citing resources, etc.

### (PS1848) BIODEGRADABLE ALBUMIN-BASED NANOCAPSULES CO-DELIVERING ANGIOSTATIN AND THROMBOSPONDIN-1 INHIBIT PRO-ANGIOGENIC SIGNALING AND PROLIFERATION IN MULTIPLE MYELOMA.

**Topic:** 13. Myeloma and other monoclonal gammopathies - Biology & translational research

Karolina Łuczkowska[1], Maria Dąbkowska[2], Patrycja Stodolak*[1], Alicja Szatanik[2], Dawid Tabaka[2], Ewa Pius-Sadowska[1], Bogusław MacHaliński[1]

*[1]Pomeranian Medical University in Szczecin, Department of General Pathology, Szczecin, Poland;[2]Pomeranian Medical University in Szczecin, Department of Pharmacokinetics, Biopharmacy and Drug Delivery Systems, Szczecin, Poland;*

### Background

Angiogenesis is a key process supporting tumor growth and invasion, regulated by a balance of pro- and anti-angiogenic factors. Anti-angiogenic therapy aims to normalize tumor vasculature, reduce hypoxia, and improve drug delivery; however, strategies such as anti-VEGF antibodies show limited efficacy due to compensatory angiogenesis. To overcome this limitation, we developed biodegradable nanocapsules composed of albumin and hyaluronic acid for sustained release of angiostatin and thrombospondin-1, selected to inhibit alternative angiogenic pathways in multiple myeloma (MM) cells.

### Aims

The aim of the study was to synthesize innovative biodegradable nanoparticles with a human serum albumin core for the prolonged release of anti-angiogenic molecules and to investigate their inhibitory role in the formation and function of the pro-angiogenic microenvironment in multiple myeloma.

### Methods

U266 and MM1S cells were incubated for 72 h in six experimental groups: (a) control (untreated cells), (b) albumin (HSA), (c) HSA + hyaluronic acid (HA), (d) HSA + angiostatin + thrombospondin-1, (e) HSA + HA + angiostatin + thrombospondin-1, and (f) free proteins without a carrier. Nanocapsules contained angiostatin and thrombospondin-1 at a concentration of 24 ng/mL each. Identical concentrations were used in the free-protein group to allow for a direct comparison between the nanocapsule formulation and carrier-free proteins. Thirty-five proteins in supernatants and lysates were analyzed using Luminex xMAP (Luminex 200), and 15 proteins by automated Western blot (Jess). Proliferation was assessed after 8 days with alamarBlue (Varioskan Lux). Statistics: one-way ANOVA with FDR correction and Tukey’s post hoc test ($p.adj < 0.05$).

### Results

Full nanocapsules (HSA + HA + angiostatin + thrombospondin-1) significantly reduced pro-angiogenic proteins ($p.adj < 0.05$) vs. control, HSA, HSA + HA, and free proteins. In U266 supernatants, VEGF-A, CCL2, and TGF-α decreased significantly; in MM1S, MMP-9, CXCL10, and Angiopoietin-1 were reduced. CXCL10 also decreased in MM1S lysates ($p.adj < 0.05$). Western blot showed increased phospho-MAPK in U266 and decreased PCNA in MM1S; other proteins were unchanged. After 8 days, proliferation was inhibited by 47% in U266 and 32% in MM1S vs. control and free proteins ($p.adj < 0.05$). Effects were strongest with full nanocapsules, underscoring the importance of the carrier structure for efficient delivery and activity.

**Copyright Information:** (Online) ISSN: 2572-9241
©2026 The Author(s). HemaSphere is published by John Wiley & Sons Ltd on behalf of European Hematology Association. This is an open access Abstract Book distributed under the Attribution-NonCommercial-NoDerivs (CC BY-NC-ND), which allows third parties to download the articles and share them with others as long as they credit the author and the Abstract Book, but they cannot change the content in any way or use them commercially.
**Abstract Book Citations:** Authors, Title, HemaSphere, 2026;10:(S1):pages. The abstract book DOIs can be found on the “Author Information” page.
**Disclaimer:** Articles published in the journal HemaSphere exclusively reflect the opinions of the authors. The authors are responsible for all content in their abstracts including accuracy of the facts, statements, citing resources, etc.

**Summary/Conclusion**

The nanocapsules effectively deliver angiostatin and thrombospondin-1, suppress key pro-angiogenic factors, and significantly inhibit MM cell proliferation. Dual targeting of alternative angiogenic and proliferative pathways may overcome limitations of anti-VEGF therapy. This system represents a promising multi-target anti-angiogenic strategy with high therapeutic potential in MM.

Funding: This research was founded by the Medical Research Agency-Poland, grant number KPOD.07.07-IW.07-0026/24 by B.M.

**Copyright Information:** (Online) ISSN: 2572-9241
©2026 The Author(s). HemaSphere is published by John Wiley & Sons Ltd on behalf of European Hematology Association. This is an open access Abstract Book distributed under the Attribution-NonCommercial-NoDerivs (CC BY-NC-ND), which allows third parties to download the articles and share them with others as long as they credit the author and the Abstract Book, but they cannot change the content in any way or use them commercially.
**Abstract Book Citations:** Authors, Title, HemaSphere, 2026;10:(S1):pages. The abstract book DOIs can be found on the "Author Information" page.
**Disclaimer:** Articles published in the journal HemaSphere exclusively reflect the opinions of the authors. The authors are responsible for all content in their abstracts including accuracy of the facts, statements, citing resources, etc.

### (PS1849) UROLITHIN A AS A DUAL-ACTION ENHANCER OF CAR-T THERAPY IN MULTIPLE MYELOMA

**Topic:** 13. Myeloma and other monoclonal gammopathies - Biology & translational research

Raquel Ancos-Pintado*[1, 2], Roberto Garcia-Vicente[1], Alba Rodríguez-García[1], Andrea Adrados[1], Raquel Oliva[1], Antonio Valeri[1], María Hernández-Sánchez[1, 2, 3], Joaquín Martinez-Lopez[1, 3, 4], María Linares[1, 2, 3]

*[1]Research Institute Hospital 12 de Octubre (i+12), Department of Translational Hematology, Hematological Malignancies Clinical Research Unit H120-CNIO, Madrid, Spain; [2]Pharmacy School, Universidad Complutense de Madrid, Department of Biochemistry and Molecular Biology, Madrid, Spain; [3]Co-senior author, Madrid, Spain; [4]Medicine School, Universidad Complutense de Madrid, Department of Medicine, Madrid, Spain;*

### Background

Gut microbiota has a significant role in modulating the response of different neoplasms to immunotherapy. Our group has recently described that short chain fatty acids (SCFAs) such as the microbiota metabolite butyrate enhances CAR-T cytotoxicity in lymphoma cells (García-Vicente *et al.* Clinical Cancer Research, 2026). Concerning Multiple Myeloma (MM), we have recently discovered a correlation between SCFAs with progression and response to treatment (Rodríguez-García *et al.* Clinical Cancer Research, 2024). Additionally, Urolithin A (UroA), another microbiota metabolite, presence in stool in MM patients has been associated with higher progression-free survival (Rodríguez-García *et al.* Microbiome, 2024). Furthermore, UroA has shown antitumoral effect *in vivo* and *in vitro*, and has exhibited a synergistic effect with first line therapies, which arises the interest in studying its role in immunotherapy.

### Aims

The objective of this project is to analyse the dual effect of UroA, in monotherapy and in combination with BCMA CAR-T therapy in MM.

### Methods

To analyse the effect of UroA in monotherapy, *in vitro* IC50 analyses were conducted in MM lines such as JJN3, MM.1S, ARP-1, XG-1 and RPMI-8226.
To test the toxicity of UroA, cytotoxicity studies were performed in PBMCs and T cells from healthy donors. Then, early and late activation of T-cells in presence of UroA was tested. For cytotoxicity experiments, T-cells from healthy donors were transduced by spinoculation with a lentiviral vector. Robust expression of the BCMA-CAR was checked by flow cytometry. CAR-T cells were co-cultured with JJN3 cell line in presence or absence of UroA at different E:T ratios. UroA 15µM was added during T-cells expansion and cytotoxicity assay.

**Copyright Information:** (Online) ISSN: 2572-9241
©2026 The Author(s). HemaSphere is published by John Wiley & Sons Ltd on behalf of European Hematology Association. This is an open access Abstract Book distributed under the Attribution-NonCommercial-NoDerivs (CC BY-NC-ND), which allows third parties to download the articles and share them with others as long as they credit the author and the Abstract Book, but they cannot change the content in any way or use them commercially.
**Abstract Book Citations:** Authors, Title, HemaSphere, 2026;10:(S1):pages. The abstract book DOIs can be found on the “Author Information” page.
**Disclaimer:** Articles published in the journal HemaSphere exclusively reflect the opinions of the authors. The authors are responsible for all content in their abstracts including accuracy of the facts, statements, citing resources, etc.

**Results**

An antiproliferative effect of this metabolite was observed in different MM cell lines with a half maximal inhibitory concentration in the micromolar range (Fig. 1A).
Interestingly, UroA did not show antiproliferative effect on either T-cells or PBMCs at micromolar range, making it eligible for combination with immunotherapy (Fig. 1B). Moreover, treatment with this metabolite in T cells caused an increase in the expression of CD69, a rapid-acting marker of lymphocyte activation after 24h of stimulation, and an increase in the expression of CD25 marker, associated with late activation after 12 days of stimulation (Fig. 1C).
Subsequently, we attempted to demonstrate the effect of UroA when applied to CAR-T therapy. The antiproliferative effect of CAR-T cells in MM cells with a E:T ratio of 1:4, 1:8 and 1:16 was significantly increased when UroA was added not only during cytotoxicity with the MM cell line, but also during prior expansion of T-cell (Fig. 1D). Based on these results, we focused on the 1:8 ratio to show that treatment with UroA during the cytotoxicity assay also caused a significantly greater antitumor effect. (Fig. 1E).

**Summary/Conclusion**

The antiproliferative effect on MM lines confirms the therapeutic potential of this metabolite individually. Furthermore, the absence of toxicity in T-cells and the enhancement of CAR-T cells effect after stimulation and during the co-culture support the dual potential of UroA to activate T-cells and its antiproliferative effect in MM. These findings unlock new therapeutic opportunities based on the combination of this metabolite with immunotherapy.

This work was supported by grant PID2024-155654OB-I00 funded by MCIN/AEI/10.13039/501100011033 and ERDF A way of making Europe

**Copyright Information:** (Online) ISSN: 2572-9241
©2026 The Author(s). HemaSphere is published by John Wiley & Sons Ltd on behalf of European Hematology Association. This is an open access Abstract Book distributed under the Attribution-NonCommercial-NoDerivs (CC BY-NC-ND), which allows third parties to download the articles and share them with others as long as they credit the author and the Abstract Book, but they cannot change the content in any way or use them commercially.
**Abstract Book Citations:** Authors, Title, HemaSphere, 2026;10:(S1):pages. The abstract book DOIs can be found on the "Author Information" page.
**Disclaimer:** Articles published in the journal HemaSphere exclusively reflect the opinions of the authors. The authors are responsible for all content in their abstracts including accuracy of the facts, statements, citing resources, etc.

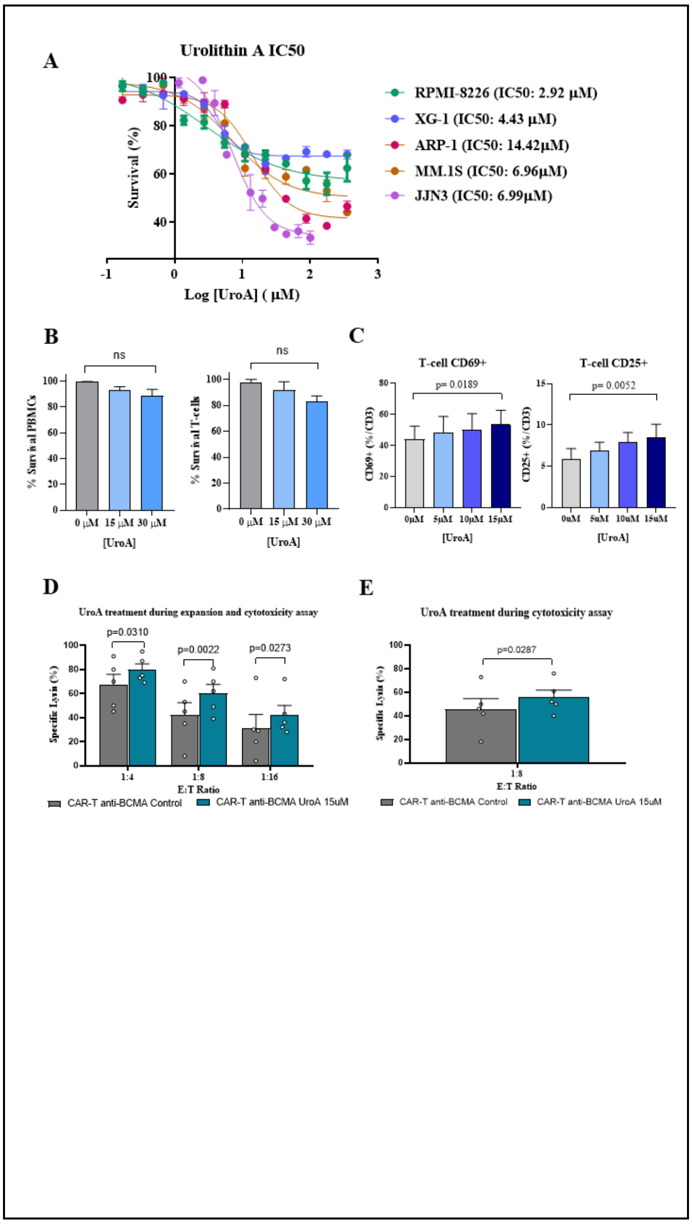

Figure 1. A) Dose-response curves after 48 hours of exposure to urolithin A in RPMI-8226, XG-1, ARP-1, MM.1S and JJN3 cell lines. Mean ± SEM of data normalized to the control condition. B) Bar chart shows the mean ± SEM of survival percentage in PBMCs and T-cells from healthy donors upon 48 h exposure to urolithin A (UroA) at 15 µM (n=3) or 30 µM (n=3). No significant differences were observed. C) Analysis of CD69 (rapid-acting marker of activation) and CD25 (late activation) surface expression by flow cytometry in T-cells (n=6) after 24 hours and 12 days respectively of UroA treatment with increasing concentrations. D) Antiproliferative effect of anti-BCMA CAR-T cells treated for 24h during expansion with UroA 15 µM against JJN3 cell line at different E:T ratios of 1:4, 1:8 and 1:16 over a 24h co-culture period in presence of UroA 15 µM (n=5). E) Antiproliferative effect of anti-BCMA CAR-T cells against JJN3 cell line at 1:8 E:T ratio over a 24h co-culture period in presence of UroA 15 µM (n=5).

**Copyright Information:** (Online) ISSN: 2572-9241
©2026 The Author(s). HemaSphere is published by John Wiley & Sons Ltd on behalf of European Hematology Association. This is an open access Abstract Book distributed under the Attribution-NonCommercial-NoDerivs (CC BY-NC-ND), which allows third parties to download the articles and share them with others as long as they credit the author and the Abstract Book, but they cannot change the content in any way or use them commercially.
**Abstract Book Citations:** Authors, Title, HemaSphere, 2026;10:(S1):pages. The abstract book DOIs can be found on the "Author Information" page.
**Disclaimer:** Articles published in the journal HemaSphere exclusively reflect the opinions of the authors. The authors are responsible for all content in their abstracts including accuracy of the facts, statements, citing resources, etc.

### (PS1850) SINGLE-CELL PROTEIN AND TRANSCRIPT PROFILING OF BONE MARROW IDENTIFIES IMMUNE FEATURES ASSOCIATED WITH RESPONSE AND PROGRESSION FOLLOWING TALQUETAMAB IN MULTIPLE MYELOMA.

**Topic:** 13. Myeloma and other monoclonal gammopathies - Biology & translational research

Selma Bekri*[1, 2, 3, 4], Tarek Mouhieddine[5], Adolfo Aleman[1, 2, 3, 4], Gargi Damle[1, 2, 6], Deniz Demircioglu[2, 6, 7], Zhihong Chen[1, 8], Darwin D'souza[1, 8], Seunghee Kim-Schulze[1, 8], Shambavi Richard[1, 2, 3], Cesar Rodriguez[1, 2, 3], Gurbakhash Kaur[1, 2, 3], Larysa Sanchez[1, 2, 3], Abhinav Rohatgi[1, 2, 3], Joshua Richter[1, 2, 3], Adriana Rossi[1, 2, 3], Santiago Thibaud[1, 2, 3], Samir Parekh[1, 2, 3, 4], Sundar Jagannath[1, 2, 3], Michael Nixon[9], Cedric Dos Santos[10], Hearn Jay Cho[1, 2, 3, 4]

*[1]Icahn School of Medicine at Mount Sinai, Hematology and Medical Oncology, New York, United States of America;[2]Tisch Cancer Center at Mount Sinai, New York, United States of America; [3]Multiple Myeloma Center of Excellence at Mount Sinai, New York, United States of America; [4]Precision Immunology Institute at Mount Sinai, New York, United States of America;[5]Dana-Farber Cancer Institute, Boston, United States of America; [6]Mount Sinai Bioinformatics for Next Generation Sequencing (BiNGS) Core, New York, United States of America; [7]Icahn School of Medicine at Mount Sinai, New York, United States of America; [8]Human Immune Monitoring Center at Mount Sinai, New York, United States of America;[9]Genentech, South San Francisco, United States of America; [10]AstraZeneca, South San Francisco, United States of America;*

### Background

Talquetamab is a GPRC5D-targeting T-cell–redirecting bispecific antibody with established clinical activity in relapsed/refractory multiple myeloma (MM). However, immune features that distinguish response from progression following GPRC5D-directed therapy remain poorly defined. Longitudinal single-cell profiling of the bone marrow microenvironment enables high-resolution characterization of immune states associated with clinical outcome.

### Aims

To characterize bone marrow immune features observed at baseline (BL), complete response (CR), and progressive disease (PD) in patients treated with talquetamab using complementary single-cell protein and transcriptomic approaches.

### Methods

Bone marrow mononuclear cells from 14 patients treated with single-agent talquetamab were analyzed longitudinally at BL and at CR or PD when available. Spectral flow cytometry (Cytek Aurora) was performed on 28 samples (14 BL, 9 CR, 5 PD). Single-cell RNA sequencing (10x Genomics) was performed on 23 samples meeting quality criteria (10 BL, 9 CR, 4 PD).

**Copyright Information:** (Online) ISSN: 2572-9241
©2026 The Author(s). HemaSphere is published by John Wiley & Sons Ltd on behalf of European Hematology Association. This is an open access Abstract Book distributed under the Attribution-NonCommercial-NoDerivs (CC BY-NC-ND), which allows third parties to download the articles and share them with others as long as they credit the author and the Abstract Book, but they cannot change the content in any way or use them commercially.
**Abstract Book Citations:** Authors, Title, HemaSphere, 2026;10:(S1):pages. The abstract book DOIs can be found on the “Author Information” page.
**Disclaimer:** Articles published in the journal HemaSphere exclusively reflect the opinions of the authors. The authors are responsible for all content in their abstracts including accuracy of the facts, statements, citing resources, etc.

### Results

Spectral flow cytometry identified differences in immune composition and inhibitory receptor expression across clinical timepoints. CR samples were associated with increased representation of $CD16^+$ NK cell populations and reduced frequency of myeloid-derived suppressor cells (MDSCs) compared with BL and PD, consistent with a less suppressive bone marrow microenvironment. In contrast, PD samples exhibited higher expression of checkpoint inhibitors including LAG-3, TIGIT, PD-1, and TIM-3 across T-cell and NK-cell compartments. The coordinated upregulation of these inhibitory receptors is consistent with previously described dysfunctional or exhaustion-associated immune phenotypes in cancer.
In scRNA-seq analyses, large shifts in major immune cell frequencies were not observed. However, neighborhood-based differential abundance analysis within T and NK cell compartments identified relative enrichment of $CD16^+$ and $CD56^+$ NK cell clusters in CR. Conversely, $GZMK^+$ CD8 effector memory (TEM) and HLA-$DR^+$ CD8 TEM cells, which have been described in chronic antigen exposure and exhaustion in cancer, were relatively reduced in CR compared with BL and PD.
Across both modalities, NK cell enrichment at CR and increased inhibitory receptor expression at PD were directionally concordant, reinforcing the consistency of these immune patterns within this cohort.

### Summary/Conclusion

This longitudinal single-cell analysis defines immune features observed at response and progression following talquetamab therapy in MM. Complete response was characterized by enrichment of NK cell populations and relative reduction of chronically stimulated and pre-exhausted CD8 effector-memory cells, suggesting a shift toward NK-dominant cytotoxic control. In contrast, progression was associated with increased inhibitory receptor expression across immune compartments and persistence of chronically stimulated CD8 subsets, consistent with activation of exhaustion-associated inhibitory programs. Despite the limited cohort size, the concordance of findings across orthogonal protein and transcriptomic platforms strengthens confidence in these observed immune patterns. These findings provide a biologically coherent framework for future validation of immune correlates of outcome in GPRC5D-treated cohorts and may inform rational combination strategies aimed at overcoming resistance to bispecific antibodies including talquetamab.

**Copyright Information:** (Online) ISSN: 2572-9241
©2026 The Author(s). HemaSphere is published by John Wiley & Sons Ltd on behalf of European Hematology Association. This is an open access Abstract Book distributed under the Attribution-NonCommercial-NoDerivs (CC BY-NC-ND), which allows third parties to download the articles and share them with others as long as they credit the author and the Abstract Book, but they cannot change the content in any way or use them commercially.
**Abstract Book Citations:** Authors, Title, HemaSphere, 2026;10:(S1):pages. The abstract book DOIs can be found on the "Author Information" page.
**Disclaimer:** Articles published in the journal HemaSphere exclusively reflect the opinions of the authors. The authors are responsible for all content in their abstracts including accuracy of the facts, statements, citing resources, etc.

### (PS1851) APOBEC3B COUPLES MITOCHONDRIAL RESPIRATORY ADAPTATION AND INFLAMMATORY REMODELLING IN MULTIPLE MYELOMA

**Topic:** 13. Myeloma and other monoclonal gammopathies - Biology & translational research

Faezeh Borzooee*[1], Afsaneh Panahi[1], Hamidreza Galavi[1, 2], Mani Larijani[3], Stephane Flibotte[4], Gerald Krystal[1, 5], Ingrid Elisia[1], Kevin Song[6], Christopher Venner[6, 7], Arefeh Rouhi[1, 2], Florian Kuchenbauer[1, 6]

*[1]BC Cancer Research Institute, Terry Fox Laboratory, Vancouver, BC, Canada; [2]University of British Columbia,, Department of Medicine, Vancouver, BC, Canada; [3]Faculty of Science, Simon Fraser University, Department of Molecular Biology and Biochemistry, Burnaby, Canada; [4]University of British Columbia, Life Sciences Institute, Vancouver, BC, Canada; [5]University of British Columbia, Department of Pathology & Laboratory Medicine, Vancouver, BC, Canada; [6]Vancouver General Hospital, Leukemia/Bone Marrow Transplant Program of British Columbia, Vancouver, BC, Canada; [7]Cross Cancer Institute, Department of Medicine, Edmonton, Canada;*

### Background

APOBEC mutational activity drives genomic instability in multiple myeloma (MM) and is associated with disease progression. While APOBEC3B (A3B) is established as a DNA cytosine deaminase, whether it functionally contributes to myeloma cell stress adaptation beyond mutagenesis remains unclear.

### Aims

To determine whether A3B regulates mitochondrial respiratory capacity under proteasome stress and whether A3B expression in MM patient cells associates with metabolic programs.

### Methods

CRISPR-Cas9 A3B knockout (KO), rescue, and lentiviral overexpression (OE) models were generated in human MM cell lines. Proteotoxic stress was induced using bortezomib (BTZ), with or without the mitochondrial complex I inhibitor metformin. Mitochondrial respiration was assessed by Seahorse XF extracellular flux analysis, measuring oxygen consumption rate (OCR). Cytokines were analyzed using 15-plex Luminex multiplex assays. Transcriptomic analyses were performed in newly diagnosed MM patient cells from the MMRF CoMMpass cohort (RNA-seq n=921; WGS n=700).

**Copyright Information:** (Online) ISSN: 2572-9241
©2026 The Author(s). HemaSphere is published by John Wiley & Sons Ltd on behalf of European Hematology Association. This is an open access Abstract Book distributed under the Attribution-NonCommercial-NoDerivs (CC BY-NC-ND), which allows third parties to download the articles and share them with others as long as they credit the author and the Abstract Book, but they cannot change the content in any way or use them commercially.
**Abstract Book Citations:** Authors, Title, HemaSphere, 2026;10:(S1):pages. The abstract book DOIs can be found on the "Author Information" page.
**Disclaimer:** Articles published in the journal HemaSphere exclusively reflect the opinions of the authors. The authors are responsible for all content in their abstracts including accuracy of the facts, statements, citing resources, etc.

**Results**

BTZ exposure increased APOBEC3A/B expression, indicating induction under therapeutic stress. To test whether A3B contributes to metabolic adaptation, mitochondrial respiration was assessed in engineered models. A3B KO reduced basal and maximal respiration and spare respiratory capacity by 30-60% versus controls (all $p \leq 0.01$), demonstrating impaired oxidative phosphorylation (OXPHOS) capacity and reduced metabolic flexibility.
Under BTZ, OCR declined in both groups; however, KO cells maintained lower maximal respiration and spare capacity ($p \leq 0.01$), indicating diminished stress adaptability. Combined BTZ and metformin caused near-complete collapse of respiratory reserve in KO cells ($p<0.01$). Conversely, A3B OE enhanced basal, ATP-linked, and maximal respiration and increased spare capacity by 40-60% ($p \leq 0.001$). During BTZ±metformin exposure, A3B OE cells preserved mitochondrial reserve ($p \leq 0.01$), supporting a role for A3B in maintaining respiratory resilience under energetic stress. These findings suggest A3B promotes adaptive mitochondrial reprogramming, potentially via modulation of nuclear-encoded electron transport chain components.
Despite these metabolic differences, A3B status did not alter acute BTZ $IC_{50}$ values, indicating A3B supports adaptive fitness rather than immediate cytotoxic resistance. Cytokine profiling revealed A3B-dependent modulation of inflammatory mediators, including increased IL-6 and MIP-1α (CCL3) secretion under stress ($p<0.05$), consistent with coordinated metabolic, inflammatory remodelling.
To assess clinical relevance, A3B expression was examined in primary MM patient cells. A3B-high MM patient cells were enriched for proliferative programs (FDR<0.001). Although canonical OXPHOS pathways were not globally enriched, targeted analysis identified coordinated modulation of nuclear-encoded electron transport chain components, aligning with the in vitro mitochondrial phenotype.

**Summary/Conclusion**

Beyond mutagenesis, A3B enhances mitochondrial respiratory reserve and stress adaptation in MM cells under proteasome inhibition. A3B-high MM patient cells display proliferative and mitochondrial transcriptional features consistent with enhanced metabolic fitness, suggesting potential mitochondrial vulnerabilities in A3B-high disease.

**Copyright Information:** (Online) ISSN: 2572-9241
©2026 The Author(s). HemaSphere is published by John Wiley & Sons Ltd on behalf of European Hematology Association. This is an open access Abstract Book distributed under the Attribution-NonCommercial-NoDerivs (CC BY-NC-ND), which allows third parties to download the articles and share them with others as long as they credit the author and the Abstract Book, but they cannot change the content in any way or use them commercially.
**Abstract Book Citations:** Authors, Title, HemaSphere, 2026;10:(S1):pages. The abstract book DOIs can be found on the “Author Information” page.
**Disclaimer:** Articles published in the journal HemaSphere exclusively reflect the opinions of the authors. The authors are responsible for all content in their abstracts including accuracy of the facts, statements, citing resources, etc.

### (PS1852) TURNING IMMUNOSUPPRESSION INTO THERAPY: LIPOSOMAL DEXAMETHASONE WITH ANTI-CD47 IN MULTIPLE MYELOMA

**Topic:** 13. Myeloma and other monoclonal gammopathies - Biology & translational research

Jelena Lazarevic[1, 2], Fabio Testaquadra[1, 2], Margherita Vieri[1, 2], Marcelo A. Szymanski de Toledo[1, 2], Mirna Barsoum[3], Julian Baumeister[1, 2], Federica De Lorenzi[2, 4, 5], Sara El Safy[4], Katja Klausz[6], Alessia Nucci[4], Anita Schulz[1, 2], Nathalie Steinke[7], Siddharth Gupta[1, 2], Nicolas Chatain[1, 2], Milena Kalmer[1, 2], Maria Jimena Rodriguez[1, 2], Henrike Jacobi[1, 2], Dorothee Winterberg[6], Svenja Andreas[6], Patrick Weiand[1, 2], Jochen Maurer[8], Christian Preisinger[3], Fabian Beier[1, 2], Deniz Nogueira Gezer[1, 2], Julia Franzen[9], Steffen Koschmieder[1, 2], Josbert Metselaar[4], Fabian Kiessling[4], Wolfgang Wagner[10, 11], Twan Lammers[4], Matthias Peipp[6], Tim Brümmendorf[1, 2], Alexandros Marios Sofias[4], Mirle Schemionek*[1, 2, 12]

*[1]Department of Hematology, Oncology, Hemostaseology and Stem Cell Transplantation, RWTH Aachen University, Aachen, Germany; [2]Center for Integrated Oncology Aachen Bonn Cologne Düsseldorf (CIO ABCD), Aachen, Germany; [3]Proteomics Facility, Interdisciplinary Centre for Clinical Research, RWTH Aachen University Medical School, Aachen, Germany; [4]Institute for Experimental Molecular Imaging, Medical Faculty, RWTH Aachen University, Aachen, Germany; [5]Department of Medical Biosciences, Radboud University Medical Centre, Nijmegen, The Netherlands, Aachen, Germany; [6]Division of Antibody-Based Immunotherapy, Department of Internal Medicine II, University Medical Center Schleswig-Holstein, Campus Kiel, Kiel, Germany; [7]Flow Cytometry Facility, Interdisciplinary Centre for Clinical Research, University Hospital RWTH Aachen, Aachen, Germany, Aachen, Germany; [8]Department of Obstetrics and Gynecology, RWTH Aachen University Hospital, 52074 Aachen, Germany, Aachen, Germany; [9]Genomics Facility, Interdisciplinary Centre for Clinical Research, University Hospital RWTH Aachen, Aachen, Germany, Aachen, Germany; [10]Helmholtz-Institute for Biomedical Engineering, Stem Cell Biology and Cellular Engineering, RWTH Aachen University, Aachen, Germany., Aachen, Germany; [11]Institute for Biomedical Engineering - Cell Biology, RWTH Aachen University Medical School, Aachen, Germany, Aachen, Germany; [12]Institute for Functional Gene Analytics (IFGA), Bonn-Rhein-Sieg University of Applied Sciences, Aachen, Germany;*

### Background

Multiple myeloma (MM) is an incurable clonal plasma cell malignancy driven by immune dysregulation, tumor microenvironmental support, and therapy-resistant stem-like cell populations. Tumor-associated macrophages (TAMs) play a central role in disease progression and drug resistance by promoting MM immune evasion.

### Aims

To develop a MM mouse model with bone marrow (BM) stem cell-like support and to subsequently evaluate macrophage-mediated MM cell clearance of disease-initiating and therapy-resistant cell populations by combining anti-CD47 checkpoint blockade with free or liposomal dexamethasone (dex/lipodex).

**Copyright Information:** (Online) ISSN: 2572-9241
©2026 The Author(s). HemaSphere is published by John Wiley & Sons Ltd on behalf of European Hematology Association. This is an open access Abstract Book distributed under the Attribution-NonCommercial-NoDerivs (CC BY-NC-ND), which allows third parties to download the articles and share them with others as long as they credit the author and the Abstract Book, but they cannot change the content in any way or use them commercially.
**Abstract Book Citations:** Authors, Title, HemaSphere, 2026;10:(S1):pages. The abstract book DOIs can be found on the “Author Information” page.
**Disclaimer:** Articles published in the journal HemaSphere exclusively reflect the opinions of the authors. The authors are responsible for all content in their abstracts including accuracy of the facts, statements, citing resources, etc.

### Methods

*In vitro*, MM1.S cells were used in mono- or co-culture with macrophages, differentiated from healthy donor-derived monocytes using M-CSF. Dex (2 µM) treated cells were analysed for CD47, calreticulin (CALR), and CD138 by flow cytometry. $CD138^+$ and $CD138^-$ RPMI 8667 cells were FACS-sorted, treated with dex (3 µM), and evaluated in colony-forming unit assays. Phagocytosis of MM1.S wild-type (WT) and CD47-knockout (KO) cells was measured by live-cell fluorescence imaging upon dex monotherapy or combined with anti-CD47 antibody (Hu5F9-IgG2σ, 10 µg/mL). CD47 KO was generated using CRISPR–Cas9. CD47 and CALR expression was also analysed in primary cells.
NSG mice were transplanted with $GFP^+/luc^+$ MM1.S cells +/- HS27a fibroblasts. Tumor burden was monitored by µCT-FLT/BLT imaging and validated via flow cytometry, BM immunohistochemistry, and serum Igλ ELISA. Mice were treated with dex, lipodex, anti-CD47, or combinations. Sorted MM cells and macrophages underwent bulk RNA sequencing.

### Results

Patient-derived BM and peripheral blood MM cells showed higher CD47 and CALR expression compared to non-MM cells (**$p < 0.01$). Dex enriched $CD138^-$ MM cells increased CALR while maintaining high CD47 surface expression *in vitro* (*$p < 0.05$–***$p < 0.001$). Combined stromal and MM cell transplantation increased stem-cell like cells *in vivo* (1.6-fold, *$p < 0.05$). Cy7-labeled liposomes showed significant BM uptake (5% ID/g), and lipodex outperformed free dex by reducing serum Igλ levels (*$p < 0.05$) with lower toxicity. Lipodex treatment modulated mTOR and interferon-related pathways, downregulated immune evasion genes, and impacted therapeutic resistance. Enhanced macrophage phagocytosis occurred upon dex plus CD47 blockade in WT MM cells (*$p < 0.05$). *In vivo*, anti-CD47 reduced BM tumor burden and Igλ, while combination with lipodex selectively targeted $CD138^-$ stem-like cells, suppressed JAK2/HOXA9 signaling, and enhanced macrophage phagocytic pathways.

### Summary/Conclusion

Combined transplantation of stromal and MM cells inceased stem-cell like cells*in vivo*. Anti-CD47 checkpoint inhibition not only restored macrophage phagocytosis but also effectively targeted stem-like MM cells upon lipodex combination, thereby transforming the immunosuppressive liability of conventional therapy into a rational combination strategy that overcomes resistance and enhance immunotherapy.

**Copyright Information:** (Online) ISSN: 2572-9241
©2026 The Author(s). HemaSphere is published by John Wiley & Sons Ltd on behalf of European Hematology Association. This is an open access Abstract Book distributed under the Attribution-NonCommercial-NoDerivs (CC BY-NC-ND), which allows third parties to download the articles and share them with others as long as they credit the author and the Abstract Book, but they cannot change the content in any way or use them commercially.
**Abstract Book Citations:** Authors, Title, HemaSphere, 2026;10:(S1):pages. The abstract book DOIs can be found on the "Author Information" page.
**Disclaimer:** Articles published in the journal HemaSphere exclusively reflect the opinions of the authors. The authors are responsible for all content in their abstracts including accuracy of the facts, statements, citing resources, etc.

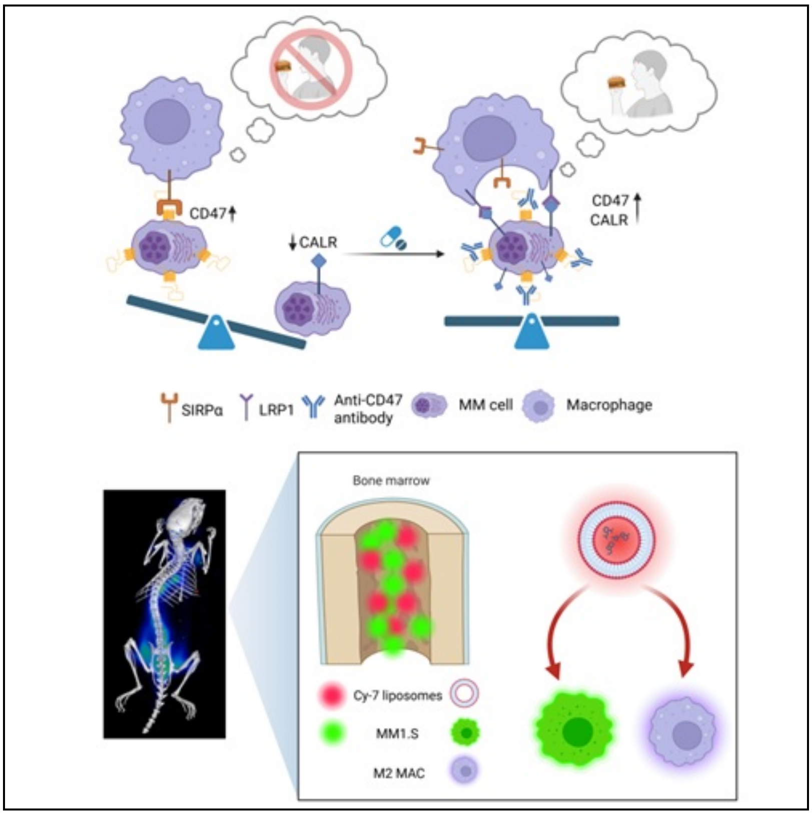

Graphical abstract

**Copyright Information:** (Online) ISSN: 2572-9241
©2026 The Author(s). HemaSphere is published by John Wiley & Sons Ltd on behalf of European Hematology Association. This is an open access Abstract Book distributed under the Attribution-NonCommercial-NoDerivs (CC BY-NC-ND), which allows third parties to download the articles and share them with others as long as they credit the author and the Abstract Book, but they cannot change the content in any way or use them commercially.
**Abstract Book Citations:** Authors, Title, HemaSphere, 2026;10:(S1):pages. The abstract book DOIs can be found on the "Author Information" page.
**Disclaimer:** Articles published in the journal HemaSphere exclusively reflect the opinions of the authors. The authors are responsible for all content in their abstracts including accuracy of the facts, statements, citing resources, etc.

### (PS1853) CD38 EXPRESSION IN PARASKELETAL AND EXTRAMEDULLARY PLASMACYTOMAS IN MULTIPLE MYELOMA: ASSESSMENT IN RELATION TO ANTI-CD38 THERAPY

**Topic:** 13. Myeloma and other monoclonal gammopathies - Biology & translational research

Klara Mensikova[1], Jakub Cieślar[1], Marek Borsky[1], Viera Hrabcakova[1], Lenka Janska-Vojtkova[1], Nela Sendlerova[1], Ivanna Biochuk[1], Viera Sandecká[1], Zdenek Adam[1], Marta Krejčí[1], Zuzana Jelinkova[2], Milan Krticka[3], Vladimir Nekuda[3], Tomas Rohan[4], Martin Stork*[1], Ludek Pour[1]

*[1]Department of Internal Medicine, Hematology and Oncology, University Hospital Brno and Faculty of Medicine, Masaryk University, Brno, Czech Republic, Brno, Czechia; [2]Department of Burns and Plastic Surgery, Faculty of Medicine, Masaryk University Brno, Czech Republic, Brno, Czechia; [3]Trauma Surgery Department, University Hospital Brno and Faculty of Medicine, Masaryk University, Brno, Czech Republic, Brno, Czechia; [4]Department of Radiology and Nuclear Medicine, University Hospital Brno and Masaryk University Brno, Brno, Czechia;*

### Background

Multiple myeloma (MM) is characterized by clonal plasma cell (cPC) proliferation. A subset of patients develops extraosseous MM (EMM), either as paraskeletal plasmacytomas (PS) or true extramedullary plasmacytomas (EMD). Although anti-CD38 monoclonal antibodies (mAbs) significantly improved outcomes, CD38 expression on cPCs may vary across spatial and temporal disease compartments within the same individual, potentially influencing personalized therapeutic decision-making.

### Aims

To evaluate CD38 antigen expression by flow cytometry in cPCs derived from both bone marrow and plasmacytoma lesions in the context of anti-CD38 therapy.

### Methods

We analyzed 37 plasmacytomas: 20 anti-CD38-naive, 12 historically exposed, and 5 refractory in the last treatment line. Subsets included 19 PS and 18 EMD tissues. Parallel bone marrow plasma cells (BMPCs) and tumor plasma cells (TPCs) were available in 89% (33/37) of patients. CD38 expression was quantified as median fluorescence intensity (MFI) in TPCs and BMPCs on the same flow cytometer using identical settings and anti-human CD38 multi-epitope probe that allows the identification of CD38 molecule on plasma cells, even in patients under anti-CD38 therapy.

**Copyright Information:** (Online) ISSN: 2572-9241
©2026 The Author(s). HemaSphere is published by John Wiley & Sons Ltd on behalf of European Hematology Association. This is an open access Abstract Book distributed under the Attribution-NonCommercial-NoDerivs (CC BY-NC-ND), which allows third parties to download the articles and share them with others as long as they credit the author and the Abstract Book, but they cannot change the content in any way or use them commercially.
**Abstract Book Citations:** Authors, Title, HemaSphere, 2026;10:(S1):pages. The abstract book DOIs can be found on the "Author Information" page.
**Disclaimer:** Articles published in the journal HemaSphere exclusively reflect the opinions of the authors. The authors are responsible for all content in their abstracts including accuracy of the facts, statements, citing resources, etc.

**Results**

In the anti-CD38–naive cohort, EMD-derived TPCs showed significantly lower CD38 expression vs. PS lesions (median MFI 64,299 [IQR 34,942–87,293] vs. 141,458 [IQR 101,437–200,021]; p<0.001). Conversely, bone marrow (BM) CD38 expression did not differ between EMD and PS patients (BMPCs: median 154,048 [IQR 88,580–207,841] vs. 190,275 [IQR 161,937–294,751]; p=0.261). Importantly, patient-paired tumor-vs-BM analysis demonstrated significant CD38 downregulation in the EMD tumor compartment relative to matched BMPCs (median 64,299 [IQR 34,942–87,293] vs. 154,048 [IQR 81,327–228,857]; p=0.011). In contrast, this tumor-to-BM difference was not observed in PS patients (median 141,458 [IQR 101,437–200,021] vs. 190,275 [IQR 155,568–310,933]; p=0.052).

In the anti-CD38–exposed cohort, TPC CD38 expression was low in both EMD and PS, with no significant difference (median MFI 40,802 [IQR 17,102–98,344] vs. 43,257 [IQR 25,164–66,055]; p=1.000). MFI did not differ between patients refractory to anti-CD38 therapy and those who were not (median 33,835 [IQR 12,430–64,731] vs. 40,802 [IQR 19,774–66,121]; p=0.721). In the exposed cohort, TPCs demonstrated downregulation in CD38 expression compared with BMPCs (median 40,802 [IQR 20,830–53,482] vs. 147,934 [IQR 64,892–549,250]; p=0.011).

**Summary/Conclusion**

In naive patients, EMD-derived TPCs exhibit significantly lower CD38 density compared to PS. Paired analysis revealed a tumor-to-BM CD38 downregulation specific to EMD, suggesting that EMD tissue promotes a phenotypic shift characterized by CD38 loss independent of therapeutic pressure. This phenomenon was absent in PS cohorts.

Anti-CD38 exposure eventually leads to a convergent low-CD38 phenotype across all tissues, irrespective of whether the treatment was administered in the distant past or has only recently failed. In patient-paired analysis, anti-CD38–exposed PS and EMD tissues exhibited reduced CD38 expression compared with BMPCs.

Baseline CD38 deficiency in EMD lesions likely underlies the reduced efficacy of anti-CD38 mAbs. Similarly, low CD38 expression in exposed PS should be considered in further therapeutic selection.

Supported by Conceptual development of research organization (FNBr 65269705).

**Copyright Information:** (Online) ISSN: 2572-9241
©2026 The Author(s). HemaSphere is published by John Wiley & Sons Ltd on behalf of European Hematology Association. This is an open access Abstract Book distributed under the Attribution-NonCommercial-NoDerivs (CC BY-NC-ND), which allows third parties to download the articles and share them with others as long as they credit the author and the Abstract Book, but they cannot change the content in any way or use them commercially.
**Abstract Book Citations:** Authors, Title, HemaSphere, 2026;10:(S1):pages. The abstract book DOIs can be found on the "Author Information" page.
**Disclaimer:** Articles published in the journal HemaSphere exclusively reflect the opinions of the authors. The authors are responsible for all content in their abstracts including accuracy of the facts, statements, citing resources, etc.

### (PS1854) PERIPHERAL BLOOD IMMUNOPROFILING DEFINES THREE DISTINCT IMMUNE STATES ASSOCIATED WITH CLINICAL AND MOLECULAR AXES IN MULTIPLE MYELOMA

**Topic:** 13. Myeloma and other monoclonal gammopathies - Biology & translational research

David G. Coffey[1], Anastasia Radko*[2], Ivan Zubarev[2], Diana Lupova[2], Elina Ohanjanyan[2], Julia Alesse[2], Noel English[2], Roman Movsisyan[2], Ksenia Markova[2], Michael Goldberg[2], C. Ola Landgren[1]

*[1]University of Miami, Sylvester Comprehensive Cancer Center, Division of Myeloma, Miami, United States of America;*
*[2]BostonGene Corporation, Waltham, United States of America;*

### Background

Multiple myeloma (MM) cases are clinically and biologically heterogeneous. While immune dysregulation in the bone marrow has been extensively studied in MM, the architecture of peripheral blood immune compartments is less clearly defined. Given the increasing therapeutic complexity of MM, it is important to characterize the circulating immune states to gain insights into MM disease biology and clinical risk stratification.

### Aims

We aim to define distinct blood immune states in MM and smoldering multiple myeloma (SMM) and to assess their association with clinical parameters, cytogenetic risk, and therapy exposure.

### Methods

Cryopreserved peripheral blood mononuclear cells (PBMCs) from patients with MM (n = 56) and SMM (n = 5) were profiled using high-dimensional multiparameter flow cytometry. Immune cell subsets were quantified as percentages of their parent populations. Association with clinical features, cytogenetic subtypes, therapy exposure, and clinical risk variables was assessed using non-parametric statistical testing. To capture coordinated immune variation, we aggregated biologically related subsets into composite immune state scores using z-scored frequencies. Pairwise Spearman correlation analysis was used to evaluate independence between these scores. Statistical comparisons were then performed across clinical and molecular subgroups.

### Results

Differential immune profiling across SMM/MM status, cytogenetic abnormalities, and therapy exposure revealed a significant association between multiple T-cell and myeloid populations with clinical and molecular variables ($p<0.05$). Three distinct composite immune states with low inter-score correlations ($|\rho|\leq0.25$) emerged, each representing coordinated variation across biologically related subsets.

Immune state 1 was enriched in naïve T-cells, CD31+ CD4+ T cells, and CXCR5+ T-follicular helper cells. It was significantly more prominent in patients with SMM than those with MM (Δ median=0.86, p=0.003), in those with ≤4 prior lines of therapy (Δ median=0.43, p=0.008), and in patients last treated with lenalidomide (Δ median=0.53, p=0.005).

Immune state 2 was associated with high-risk cytogenetic abnormalities, including 1q amplification (Δ median=0.80, p=0.010) and 13q deletion (Δ median=0.78, p=0.016). It showed increased proportions of monocytes and proliferating Ki-67+ CD8+ T cells.

Immune state 3 was defined by skewing of non-naïve CD4+ T cells toward greater Th2 polarization, with elevated PD-1 and TIGIT expression on T cells. It was linked to high-risk cytogenetics: 11;14 translocations (Δ median=0.49, p=0.028), 17p deletions (Δ median=0.37, p=0.019), and >4 prior lines of therapy (Δ median=0.40, p=0.011).

**Copyright Information:** (Online) ISSN: 2572-9241
©2026 The Author(s). HemaSphere is published by John Wiley & Sons Ltd on behalf of European Hematology Association. This is an open access Abstract Book distributed under the Attribution-NonCommercial-NoDerivs (CC BY-NC-ND), which allows third parties to download the articles and share them with others as long as they credit the author and the Abstract Book, but they cannot change the content in any way or use them commercially.
**Abstract Book Citations:** Authors, Title, HemaSphere, 2026;10:(S1):pages. The abstract book DOIs can be found on the "Author Information" page.
**Disclaimer:** Articles published in the journal HemaSphere exclusively reflect the opinions of the authors. The authors are responsible for all content in their abstracts including accuracy of the facts, statements, citing resources, etc.

**Summary/Conclusion**

Peripheral blood immunoprofiling revealed three distinct immune states, each aligned with plasma cell dyscrasia subtypes, cytogenetic risks, and prior treatment history. These profiles reflect coordinated patterns of T-cell differentiation, polarization, and proliferation along with checkpoint enrichment and monocyte expansion across disease and treatment groups, rather than isolated immune alterations. Our findings show that peripheral immune architecture captures biologically and clinically meaningful heterogeneity in MM and supports blood-based immunoprofiling. Further analysis in expanded cohorts is required to better define its clinical utility and advance biological insight into disease evolution.

**Copyright Information:** (Online) ISSN: 2572-9241
©2026 The Author(s). HemaSphere is published by John Wiley & Sons Ltd on behalf of European Hematology Association. This is an open access Abstract Book distributed under the Attribution-NonCommercial-NoDerivs (CC BY-NC-ND), which allows third parties to download the articles and share them with others as long as they credit the author and the Abstract Book, but they cannot change the content in any way or use them commercially.
**Abstract Book Citations:** Authors, Title, HemaSphere, 2026;10:(S1):pages. The abstract book DOIs can be found on the "Author Information" page.
**Disclaimer:** Articles published in the journal HemaSphere exclusively reflect the opinions of the authors. The authors are responsible for all content in their abstracts including accuracy of the facts, statements, citing resources, etc.

### (PS1855) NEUTROPHIL DYNAMICS AND NEUTROPHIL EXTRACELLULAR TRAPS (NETS) IN MULTIPLE MYELOMA

**Topic:** 13. Myeloma and other monoclonal gammopathies - Biology & translational research

Alessandra Romano*[1, 2], Ilaria Dulcamare[1], Daniela Cambria[3], Deborah Calvo[1], Mohammadreza Khosropoor[2], Concetta Conticello[2], Rossella Cacciola[1], Emma Cacciola[1], Francesco Di Raimondo[1, 2], Mariarosaria Galdiero[4]

*[1]Università degli Studi di Catania, Dipartimento di Chirurgia e Specialità Medico Chirurgiche, Catania, Italy;[2]AOU Policlinico Rodolico San Marco, UOC Ematologia, Catania, Italy; [3]University of Palermo, Department of Health Promotion, Mother and Child Care, Internal Medicine and Medical Specialties, Palermo, Italy; [4]Federico II University of Naples, Department of Translational Medical Sciences (DiSMeT), Napoli, Italy;*

**Background**

In multiple myeloma (MM), a hematological malignancy characterized by accumulating tumor plasma cells in the bone marrow (BM), the bidirectional interaction between MM cells and surrounding cells regulates immune editing, transforming the microenvironment into a tumor-promoting and immune-suppressive milieu mediating tumor growth, progression, and cell adhesion. Neutrophil extracellular traps (NETs) are extracellular decondensed chromatin released from activated neutrophils that are beneficial in the context of infection. However, when excessive release occurs, NETs contribute to various pathological conditions, including cancer.

**Aims**

Since the ability of MM cells to induce NET formation has not been studied or associated with clinical outcome, we aimed to evaluate NETs as biomarker of clinical outcome in myeloma patients treated upfront with immunotherapy including daratumumab, IMiDs (thalidomide, lenalidomide, pomalidomide) and proteasome inhibitors (bortezomib, carfilzomib).

**Methods**

We simultaneously measured neutrophil extracellular trap (NET) NET-osis area and neutrophil-related mediators and biomarkers in the sera of 67 consecutive newly diagnosed MM and 36 healthy subjects patients using commercial ELISA kits. Key inflammatory markers, including MMP-9, MPO, neutrophil elastase (NE), CXCL8/IL-8, GM-CSF, IL-32, tryptase, and Arginase 1 were assessed. Additionally, NET formation was specifically identified by detecting MPO-DNA complexes and Citrullinated Histone H3 (CitH3) using high- sensitivity monoclonal antibodies.

**Copyright Information:** (Online) ISSN: 2572-9241
©2026 The Author(s). HemaSphere is published by John Wiley & Sons Ltd on behalf of European Hematology Association. This is an open access Abstract Book distributed under the Attribution-NonCommercial-NoDerivs (CC BY-NC-ND), which allows third parties to download the articles and share them with others as long as they credit the author and the Abstract Book, but they cannot change the content in any way or use them commercially.
**Abstract Book Citations:** Authors, Title, HemaSphere, 2026;10:(S1):pages. The abstract book DOIs can be found on the “Author Information” page.
**Disclaimer:** Articles published in the journal HemaSphere exclusively reflect the opinions of the authors. The authors are responsible for all content in their abstracts including accuracy of the facts, statements, citing resources, etc.

**Results**

Baseline characteristics are summarized in Table 1: median age 68 (IQR 45-87), 41 males( 62%); high-risk cytogenetics 21%. In MM patients, NET-osis area was significant higher than in healthy subjects.
To establish if NET-osis was increased as consequence of soluble factors present in MM sera, we evaluated the effects of patient sera on neutrophils from healthy donors .
After incubation with MM sera for 24 h, donor neutrophils showed significantly higher NET-osis area levels than those cultured with sera from healthy donors (516 $uM^2$ vs 806 $uM^2$, p<0.007). If exposed to autologous serum or PMA, a NETosis inducer, healthy HDNs made shorter NETs than those induced by MM sera, suggesting that soluble factors in MM are involved in NET-osis.
MM patients showed significantly higher serum levels of neutrophil-related mediators compared to healthy controls. Specifically, CitH3, MMP-9, and MPO levels increased steadily over time. While IL-32 and Arginase 1 were elevated, MCT levels were notably decreased. Correlation analyses indicated a complex inflammatory network where NET biomarkers positively correlated with key cytokines. Interestingly, IL-32 consistently exhibited a negative correlation with multiple neutrophil activation markers. During treatment with daratumumab some patients exhibited a strong reduction of CXCL8, however neither baseline levels nor paired quantifications could predict clinical outcome or early response to daratumumab.

**Summary/Conclusion**

These findings show that MM patients are in a state of ongoing neutrophil activation and increased NETosis, which worsens as the disease advances. The difference between elevated Arginase 1 and decreased MCT indicates a functional reprogramming of the myeloid cells. This self-sustaining inflammatory network likely influences the tumor microenvironment, providing new insights into the immune-crosstalk sustaining MM onset.

**Copyright Information:** (Online) ISSN: 2572-9241
©2026 The Author(s). HemaSphere is published by John Wiley & Sons Ltd on behalf of European Hematology Association. This is an open access Abstract Book distributed under the Attribution-NonCommercial-NoDerivs (CC BY-NC-ND), which allows third parties to download the articles and share them with others as long as they credit the author and the Abstract Book, but they cannot change the content in any way or use them commercially.
**Abstract Book Citations:** Authors, Title, HemaSphere, 2026;10:(S1):pages. The abstract book DOIs can be found on the "Author Information" page.
**Disclaimer:** Articles published in the journal HemaSphere exclusively reflect the opinions of the authors. The authors are responsible for all content in their abstracts including accuracy of the facts, statements, citing resources, etc.

### (PS1856) PREDICTIVE IMMUNE BIOMARKERS OF RESPONSE TO BISPECIFIC ANTIBODIES IN TRIPLE-CLASS REFRACTORY MULTIPLE MYELOMA PATIENTS

**Topic:** 13. Myeloma and other monoclonal gammopathies - Biology & translational research

Elena Tofacchi*[1], Anna Maria Corsale[1], Cristina Aquilina[1], Maria Speciale[2], Andrea Romano[2], Marta Biondo[3], Marta Di Simone[1], Emilia Gigliotta[1], Alessia Gallo[4], Fabrizio Accardi[5], Francesco Dieli[6], Serena Meraviglia[6], Stefania Grimaudo[1], Sergio Siragusa[1], Cirino Botta[1]

*[1]Department of Health Promotion, Mother and Child Care, Internal Medicine and Medical Specialties ProMISE.."G. D'Alessandro", University of Palermo,, Palermo, Italy; [2]Department of Surgical, Oncological and Stomatological Disciplines (DICHIRONS), University of Palermo,, Palermo, Italy; [3]Hematology Section, Department of General Surgery and Medical-Surgical Specialties, University of Catania,, Catania, Italy; [4]Department of Research, IRCCS ISMETT (Istituto Mediterraneo per i Trapianti e Terapie ad alta Specializzazione), UPMC, Palermo, Italy; [5]U.O.C di Oncoematologia, Azienda Ospedaliera Ospedali Riuniti Villa Sofia - Cervello,, Palermo,, Italy; [6]Central Laboratory of Advanced Diagnosis and Biomedical Research, University of Palermo, Palermo,, Italy;*

### Background

Patients with triple-class relapsed/refractory multiple myeloma (RRMM) have extremely poor outcomes. Although bispecific antibodies (BsAbs) have significantly improved survival, up to 40% of patients exhibit primary resistance. The identification of predictive biomarkers remains an unmet clinical need.

### Aims

This study aimed to identify immunological and transcriptional determinants of response and resistance to novel anti-MM BsAbs by integrating clinical, laboratory and single-cell data in a cohort of triple-class RRMM.

### Methods

Clinical data were collected and analysed from a cohort of 18 triple-class–exposed/refractory multiple myeloma patients (RRMM) (7 treated with Teclistamab, 7 with Elranatamab, and 4 with Talquetamab). Flow cytometry and single-cell RNA-seq/CITE-seq analyses were performed in a subset of patients for whom high-quality samples were available (n=11). Immunophenotyping was conducted on bone marrow cells (BMMCs) using standardized 8-color flow cytometry panels and analysed with the FlowCT semi-automated pipeline. Subsequently, single-cell transcriptomic analyses were performed to characterize immune cell states and pathway-level differences associated with treatment response. All patients provided written informed consent, and the study was approved by the institutional ethics committee (approval numbers 05/2021 and 02/2022; protocol codename: MMVision).

**Copyright Information:** (Online) ISSN: 2572-9241
©2026 The Author(s). HemaSphere is published by John Wiley & Sons Ltd on behalf of European Hematology Association. This is an open access Abstract Book distributed under the Attribution-NonCommercial-NoDerivs (CC BY-NC-ND), which allows third parties to download the articles and share them with others as long as they credit the author and the Abstract Book, but they cannot change the content in any way or use them commercially.
**Abstract Book Citations:** Authors, Title, HemaSphere, 2026;10:(S1):pages. The abstract book DOIs can be found on the "Author Information" page.
**Disclaimer:** Articles published in the journal HemaSphere exclusively reflect the opinions of the authors. The authors are responsible for all content in their abstracts including accuracy of the facts, statements, citing resources, etc.

### Results

Non-responders patients (n=7) showed higher ISS stage (p=0.001), increased monoclonal component (p=0.039), and lower haemoglobin levels (p=0.03) compared with responders (n=11). Responding patients experienced a significant reduction in lymphocyte counts following step-up dosing (p<0.001). *Fig.1(a)* Flow cytometry analysis revealed that non-responding patients exhibit marked alterations in the lymphocyte compartment. Specifically, we observed that non-responding patients showed a significant reduction in the percentage of B cells compared with their responding counterpart (p =0.032). *Fig.(1b*) Single-cell RNA-seq analysis of 39,195 cells revealed little to no differences in term of population abundances, but confirmed the flow cytometry findings, showing a marked reduction of B cells in non-responding patients (p = 0.0092). *Fig.(1c)* Subsequentially, transcriptomic profiling demonstrated an immunosuppressive and tolerogenic signature in non-responders, characterized by monocytes overexpressing innate immune and TLR-related genes (e.g. *IRAK1*) and inflammatory/chemotactic mediators such as *CXCR4*. In contrast, responders exhibited coordinated activation of cytotoxic programs in CD8 effector memory T cells and NK cells, with increased expression of *GNLY*, *GZMA/GZMH* and *NKG7*. Non-responders were enriched in exhausted T cells expressing inhibitory receptors, including *HAVCR2/TIM-3*. *Fig.(1d)*

### Summary/Conclusion

By integrating clinical, single-cell, and flow cytometry data, our study shows that bispecific antibody efficacy in RRMM is driven by tumor burden and baseline immune competence. Higher baseline non-malignant B cells in responders likely reflect a preserved microenvironment permissive to T-cell redirection. In contrast, primary resistance is linked to immunosuppressive and exhausted states. These findings support baseline immune profiling and pre-treatment strategies to reduce tumor burden and optimize effector-to-target ratios.

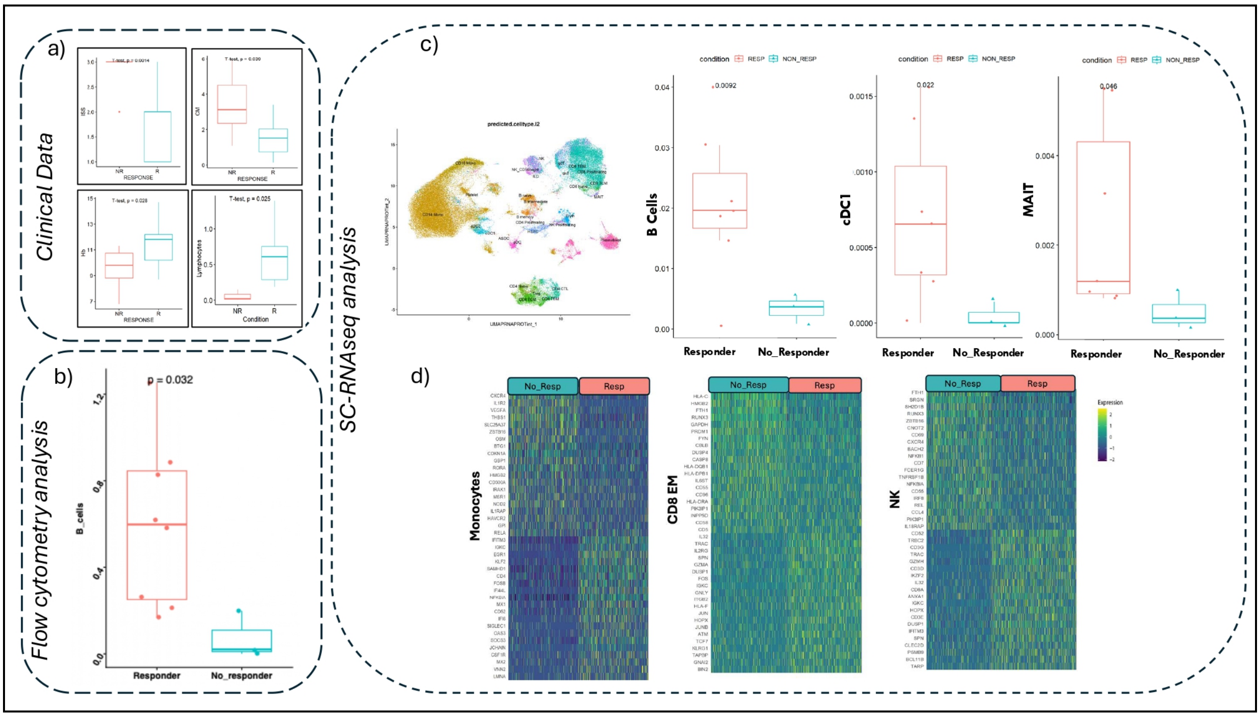

**Copyright Information:** (Online) ISSN: 2572-9241
©2026 The Author(s). HemaSphere is published by John Wiley & Sons Ltd on behalf of European Hematology Association. This is an open access Abstract Book distributed under the Attribution-NonCommercial-NoDerivs (CC BY-NC-ND), which allows third parties to download the articles and share them with others as long as they credit the author and the Abstract Book, but they cannot change the content in any way or use them commercially.
**Abstract Book Citations:** Authors, Title, HemaSphere, 2026;10:(S1):pages. The abstract book DOIs can be found on the "Author Information" page.
**Disclaimer:** Articles published in the journal HemaSphere exclusively reflect the opinions of the authors. The authors are responsible for all content in their abstracts including accuracy of the facts, statements, citing resources, etc.

### (PS1858) THE NOVEL INDIRUBIN DERIVATIVE I3MV-8B AUGMENTS ANTI-CD38 MONOCLONAL ANTIBODY THERAPY IN MULTIPLE MYELOMA: A DUAL MECHANISM INVOLVING NK CELL IMMUNOMODULATION AND INCREASED CD38 EXPRESSION

**Topic:** 13. Myeloma and other monoclonal gammopathies - Biology & translational research

Xiaoyu Zhang*[1], Xiyue Sun[1], Yijie Wang[1], Lanting Liu[1], Zhen Yu[1], Mu Hao[1], Lugui Qiu[1]

*[1]State Key Laboratory of Experimental Hematology, National Clinical Research Center for Blood Diseases, Haihe Laboratory of Cell Ecosystem, Institute of Hematology & Blood Diseases Hospital, Chinese Academy of Medical Sciences & Peking Union Medical College, Tianjin, China;*

### Background

Multiple myeloma (MM) is a currently incurable hematologic malignancy whose therapy is significantly hindered by genetic heterogeneity, clonal evolution, and the consequent development of treatment resistance and disease relapse. Previously, we identified a novel I3MO derivative, I3MV-8b, which functions as a dual inhibitor of the proteasome and HDAC6 and demonstrates promising anti-myeloma activity. Here, we report that I3MV-8b significantly restores immune cell function and upregulates CD38 expression on MM cells, thereby enhancing the therapeutic efficacy of anti-CD38 monoclonal antibodies and offering a novel strategy to overcome treatment resistance.

### Aims

This study aimed to evaluate the ability of 8b to facilitate the therapeutic effect of anti-CD38 monoclonal antibodies against MM and to elucidate the underlying mechanisms.

### Methods

The immunomodulatory effects of I3MV-8b were assessed via spectral flow cytometry in C57BL/KaLwRij mouse models. In vitro, its impact on NK cell function was examined, and differential gene expression was analyzed by RNA-seq and confirmed with flow cytometry. Additionally, the combination of I3MV-8b with anti-CD38 antibodies was evaluated using an NK-MM co-culture cytotoxicity assay in vitro and in an NK-humanized NSG mouse model in vivo.

**Copyright Information:** (Online) ISSN: 2572-9241
©2026 The Author(s). HemaSphere is published by John Wiley & Sons Ltd on behalf of European Hematology Association. This is an open access Abstract Book distributed under the Attribution-NonCommercial-NoDerivs (CC BY-NC-ND), which allows third parties to download the articles and share them with others as long as they credit the author and the Abstract Book, but they cannot change the content in any way or use them commercially.
**Abstract Book Citations:** Authors, Title, HemaSphere, 2026;10:(S1):pages. The abstract book DOIs can be found on the “Author Information” page.
**Disclaimer:** Articles published in the journal HemaSphere exclusively reflect the opinions of the authors. The authors are responsible for all content in their abstracts including accuracy of the facts, statements, citing resources, etc.

### Results

8b demonstrated potent antitumor and immunomodulatory effects in the C57BL/KaLwRij myeloma mouse model. Spectral flow cytometry analysis revealed that 8b treatment significantly increased the proportion of NK cells and enhanced the production of the activation marker IFN-γ within this population, while concurrently downregulating the expression of NK cell exhaustion markers, including TIGIT. Further in vitro co-culture experiments of NK cells and MM cells confirmed that 8b treatment inhibited the expression of exhaustion markers TIM-3 and TIGIT. RNA-seq analysis indicated that the differentially expressed genes following 8b treatment were enriched in autophagy-related pathways. Subsequent validation demonstrated that 8b suppressed NK cell exhaustion in the co-culture system by promoting autophagy via the STING signaling pathway. Given the crucial role of NK cell function in anti-CD38 antibody therapy, we evaluated the potential synergy between 8b and anti-CD38 antibodies. In a co-culture system of human NK cells and MM cells, 8b treatment enhanced the cytotoxicity mediated by daratumumab (Dara) against MM cells in vitro. This synergistic effect was corroborated in an NK-humanized NSG mouse model, where 8b significantly improved the in vivo efficacy of Dara. Furthermore, 8b was found to increase CD38 expression on MM cells. Flow cytometry confirmed that 8b significantly upregulated CD38 expression in both MM cell lines and primary patient cells. Mechanistically, 8b-mediated HDAC6 inhibition elevated the levels of active histone marks, H3K27ac and H3K9ac. ChIP-seq analysis demonstrated the specific enrichment of these acetylated histones at the enhancer and promotor regions of the CD38 gene, and this epigenetic modification was confirmed to facilitate CD38 transcription through integrated assay for ATAC-seq and ChIP-qPCR validation.

### Summary/Conclusion

I3MV-8b enhances the efficacy of anti-CD38 antibody therapy via a dual mechanism: first, by modulating NK cell activity, and second, through an epigenetic pathway that upregulates CD38 expression. This combination represents a promising strategy to overcome therapy resistance in multiple myeloma.

**Copyright Information:** (Online) ISSN: 2572-9241
©2026 The Author(s). HemaSphere is published by John Wiley & Sons Ltd on behalf of European Hematology Association. This is an open access Abstract Book distributed under the Attribution-NonCommercial-NoDerivs (CC BY-NC-ND), which allows third parties to download the articles and share them with others as long as they credit the author and the Abstract Book, but they cannot change the content in any way or use them commercially.
**Abstract Book Citations:** Authors, Title, HemaSphere, 2026;10:(S1):pages. The abstract book DOIs can be found on the "Author Information" page.
**Disclaimer:** Articles published in the journal HemaSphere exclusively reflect the opinions of the authors. The authors are responsible for all content in their abstracts including accuracy of the facts, statements, citing resources, etc.

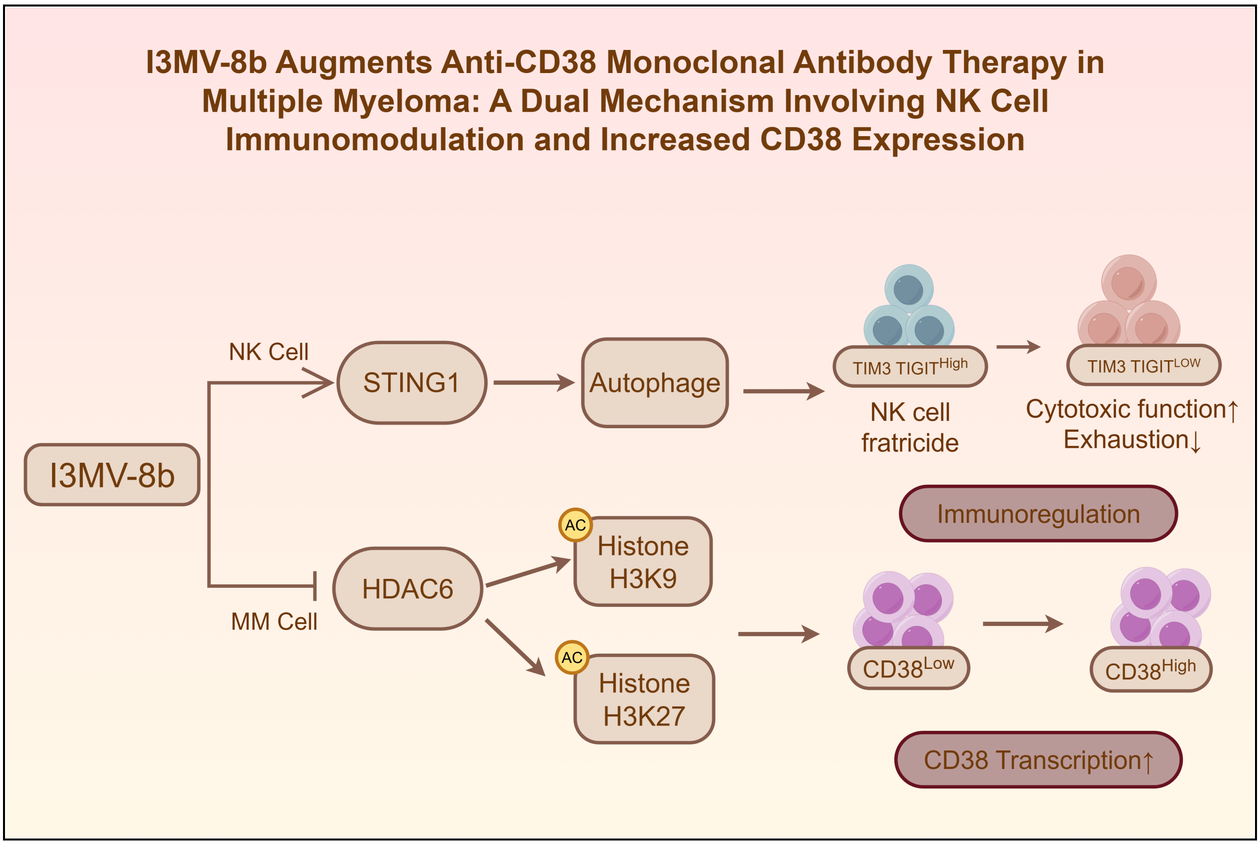

**Copyright Information:** (Online) ISSN: 2572-9241
©2026 The Author(s). HemaSphere is published by John Wiley & Sons Ltd on behalf of European Hematology Association. This is an open access Abstract Book distributed under the Attribution-NonCommercial-NoDerivs (CC BY-NC-ND), which allows third parties to download the articles and share them with others as long as they credit the author and the Abstract Book, but they cannot change the content in any way or use them commercially.
**Abstract Book Citations:** Authors, Title, HemaSphere, 2026;10:(S1):pages. The abstract book DOIs can be found on the "Author Information" page.
**Disclaimer:** Articles published in the journal HemaSphere exclusively reflect the opinions of the authors. The authors are responsible for all content in their abstracts including accuracy of the facts, statements, citing resources, etc.

### (PS1859) REVERTING OF MICROENVIRONMENTAL INVASIVENESS AND IMMUNESUPPRESSION THROUGH A NOVEL ANTI-CD138 ANTIBODY NANOPLATFORM FOR TARGETING OF MULTIPLE MYELOMA

**Topic:** 13. Myeloma and other monoclonal gammopathies - Biology & translational research

Carmela Tangredi*[1], Monica Argenziano[2], Davide Busato[3], Sara Capolla[3], Antonella Zucchetto[3], Valter Gattei[3], Roberta Cavalli[2], Paolo MacOr[4], Giuseppe Toffoli[5], Michele Dal Bo[3]

*[1]Centro di Riferimento Oncologico (CRO), IRCCS - University of Trieste, Clinical and Experimental Onco-Hematology, Aviano (PN), Italy; [2]University of Torino, Department of drug science and technology, Torino (TO), Italy;[3]Centro di Riferimento Oncologico (CRO), IRCCS, Clinical and Experimental Onco-Hematology, Aviano (PN), Italy; [4]University of Trieste, Department of Life Science, Trieste (TS), Italy; [5]Centro di Riferimento Oncologico (CRO), IRCCS, Experimental and clinical Pharmacology, Aviano (PN), Italy;*

### Background

CD138 (i.e. Syndecan-1) may be a useful immunotherapeutic target for multiple myeloma (MM) treatment being highly and specifically expressed on MM cell surfaces (Riccardi et al, Front Oncol 2024). We recently developed a mouse monoclonal antibody of the IgG class directed against a CD138 epitope in close proximity to the cell membrane (AT801, submitted patent n 102025000010005, Ministero delle Imprese e del Made in Italy). Anti-CD138 antibodies such as AT801 can be conjugated with chitosan nanobubbles (CS-NBs) loaded with chemotherapeutics, to increase their payload amount reaching tumor site. Due to chitosan composition, CS-NBs can revert immunological crosstalk within tumor niche and shift different immune cell type, like macrophages, towards an inflammatory phenotype (Reichel et al, Nanotheranostics 2019, Vol.3). Thus, they might contribute to reverting the immunosuppressive tumor microenvironment (TME).

### Aims

To investigate the cytotoxic effects and capability to modify TME interactions of CS-NBs conjugated with AT801 for MM treatment.

### Methods

AT801 was developed by in-vivo electroporation protocol and hybridoma production. CS-NBs were conjugated with AT801 or left unconjugated as control.

**Copyright Information:** (Online) ISSN: 2572-9241
©2026 The Author(s). HemaSphere is published by John Wiley & Sons Ltd on behalf of European Hematology Association. This is an open access Abstract Book distributed under the Attribution-NonCommercial-NoDerivs (CC BY-NC-ND), which allows third parties to download the articles and share them with others as long as they credit the author and the Abstract Book, but they cannot change the content in any way or use them commercially.
**Abstract Book Citations:** Authors, Title, HemaSphere, 2026;10:(S1):pages. The abstract book DOIs can be found on the "Author Information" page.
**Disclaimer:** Articles published in the journal HemaSphere exclusively reflect the opinions of the authors. The authors are responsible for all content in their abstracts including accuracy of the facts, statements, citing resources, etc.

**Results**

The ability of AT801 to bind CD138 was demonstrated by flow cytometry (FC) (more than 95% of CD138+ cells) and immunofluorescence (IF) in both CD138-expressing MM-like RPMI 8226 cell line and in human MM samples. A siRNA-mediated knockdown was used to investigate AT801 specificity for CD138. FC showed more than 97-98 % of positive cells upon transfection with control siRNA; this fluorescent signal drops by 60-70% upon transfection with the pull siRNA against CD138. When cross reactivity was evaluated by FC on both mouse bone marrow and human blood sample, AT801 showed high specificity for the human samples whereas it did not bind murine samples. Synthesized CS-NBs had a positive charge and a diameter of ~360nm by DLS analysis. In-vivo biodistribution in RPMI 8226 tumor bearing xenograft mice demonstrated that CS-NBs can accumulate in the tumor mass. This was enhanced by AT801 conjugation (p=0.0015) on CS-NB surface. The CS-NBs effects on TME were also evaluated ex-vivo in extracted tumor masses from biodistribution studies by investigating the M2:M1 ratio of macrophages in PBS, CS-NBs and AT801-CS-NBs treated groups. IF performed using specific markers such as CD206 (M2) and iNOS (M1) showed no differences in the recruitment of M2 macrophages but revealed an increase of M1 population in the tumor masses of CS-NBs and AT801-CS-NBs treated mice compared to the PBS treated group. To evaluate whether this increase was due to a repolarization effect, RAW 264.7 cell lines were treated in vitro with CS-NBs after being polarized into M1 and M2 phenotype. FC results showed no effect in M1-like cell lines while in M2-like cells, CS-NBs significantly reduced CD206 (p<0,0001) and increased CD86 (M1) expression marker. ELISA assay in M2-like cells showed that CS-NBs did not affect IL-10 production suggesting that CS-NBs could reprogram M2 macrophages toward M1 phenotype.

**Summary/Conclusion**

AT801 is a useful targeting agent for nanoplatforms to revert microenvironmental invasiveness and immunesuppression for MM treatment.

**Copyright Information:** (Online) ISSN: 2572-9241
©2026 The Author(s). HemaSphere is published by John Wiley & Sons Ltd on behalf of European Hematology Association. This is an open access Abstract Book distributed under the Attribution-NonCommercial-NoDerivs (CC BY-NC-ND), which allows third parties to download the articles and share them with others as long as they credit the author and the Abstract Book, but they cannot change the content in any way or use them commercially.
**Abstract Book Citations:** Authors, Title, HemaSphere, 2026;10:(S1):pages. The abstract book DOIs can be found on the “Author Information” page.
**Disclaimer:** Articles published in the journal HemaSphere exclusively reflect the opinions of the authors. The authors are responsible for all content in their abstracts including accuracy of the facts, statements, citing resources, etc.

### (PS1860) FCГRIIIA GENOTYPE IN MULTIPLE MYELOMA AND ITS PRECURSORS IS ASSOCIATED WITH BONE DISEASE EXTENSION, ADVERSE CLINICAL PRESENTATION AND IMMUNE DYSFUNCTION

**Topic:** 13. Myeloma and other monoclonal gammopathies - Biology & translational research

Daniela Cambria[1], Mariateresa Cannizzaro[2], Nunziatina Laura Parrinello[3], Sara Marino[4], Ilaria Dulcamare[4], Deborah Calvo[4], Mohammadreza Khosropoor[3], Noemi Puccio[5], Concetta Conticello[3], Francesco Di Raimondo[3, 4], Lavinia Raimondi[6], Gianluca Giavaresi[6], Cirino Botta[1], Antonino Neri[5], Alessandra Romano*[3, 4]

*[1]University of Palermo, Department of Health Promotion, Mother and Child Care, Internal Medicine and Medical Specialties, Palermo, Italy; [2]AOU Policlinico Rodolico San Marco, UO Radiologia, Catania, Italy; [3]AOU Policlinico Rodolico San Marco, UOC Ematologia, Catania, Italy; [4]Università degli Studi di Catania, Dipartimento di Chirurgia e Specialità Medico Chirurgiche, Catania, Italy; [5]Azienda USL-IRCCS di Reggio Emilia, Reggio Emilia, Italy; [6]IRCCS Istituto Ortopedico Rizzoli, SS Piattaforma Scienze Omiche per Ortopedia Personalizzata (SOP), Palermo, Italy;*

### Background

Polymorphic variants of the constant fraction region of immunoglobulins FcγRIIIA presents a single nucleotide polymorphism at location 158 (V/F), which affects its binding to the fragment crystallizable (Fc) of antibodies (Abs).

### Aims

Since in presence of immune complexes FcγRIIIA can mediate the severity of bone disease and osteoclastogenic activity, we compared the allele frequency of FcγRIIIA-F158V polymorphism in patients affected by multiple myeloma and its precursors, namely monoclonal gammopathy of uncertain significance (MGUS) and smoldering myeloma.

### Methods

Between October 2023 and May 2025, 72 consecutive newly diagnosed MM, 31 SMM and 42 MGUS patients have been included in this study. Bone osteolytic lesions were addressed using whole-body low-dose CT. The criteria for the identification of MM-related bone disease was the presence of at least one well-defined lytic lesion of the trabecular bone (≥5 mm) with no sclerotic margins, in line with established guidelines.

**Copyright Information:** (Online) ISSN: 2572-9241
©2026 The Author(s). HemaSphere is published by John Wiley & Sons Ltd on behalf of European Hematology Association. This is an open access Abstract Book distributed under the Attribution-NonCommercial-NoDerivs (CC BY-NC-ND), which allows third parties to download the articles and share them with others as long as they credit the author and the Abstract Book, but they cannot change the content in any way or use them commercially.
**Abstract Book Citations:** Authors, Title, HemaSphere, 2026;10:(S1):pages. The abstract book DOIs can be found on the “Author Information” page.
**Disclaimer:** Articles published in the journal HemaSphere exclusively reflect the opinions of the authors. The authors are responsible for all content in their abstracts including accuracy of the facts, statements, citing resources, etc.

### Results

FcγR3AV158F polymorphisms frequencies were 4.8% for FcγR3A-158 V/V homozygous genotype, 54.8% for Fcγ3A-158 F/V heterozygous genotype and 40.5% for FcγR3A-158 F/F homozygous genotype. Among SMM patients (N=31), FcγR3AV158F polymorphisms frequencies were 6.5% for FcγR3A-158 V/V homozygous genotype, 80.0% for Fcγ3A-158 F/V heterozygous genotype and 12.9% for FcγR3A-158 F/F homozygous genotype. Among MM patients (N=72), FcγR3AV158F polymorphisms frequencies were 30.6% for FcγR3A-158 V/V homozygous genotype, 50.0% for Fcγ3A-158 F/V heterozygous genotype and 19.4% for FcγR3A-158 F/F homozygous genotype.
The frequency of FcγR3A genotypes was like those published in prior studies with predominantly Caucasian populations only in MGUS cohort, since SMM cohort was significantly enriched of F/V heterozygous genotype ($\chi^2$ 23.28, p=0.0001). FcγR3A-158 V/V homozygous genotype was associated with high-risk cytogenetics, anemia, high beta-2 microglobulin levels, and more than 10 osteolytic lesions.
In 41 cases (12 MGUS, 9 SMM and 20 MM) ultra-low-pass whole-genome sequencing was applied to investigate key mutations in a targeted panel. We identified a median of 0 mutations per patient in MGUS (range: 0–2), 0.5 in SMM (range: 0–2) and 2 in MM (range: 0–3). Overall, we found at least one mutation in 41%, 77% and 78% of MGUS, SMM and MM patients, respectively, supporting the notion that mutations are present throughout the natural history of plasma-cells dyscrasias. Carrying VV homozygous genotype was associated to carrying at least one mutation in N-RAS, K-RAS or B-RAF (p<0.0001), despite we failed to identify an association between any specific mutation and FcγR3A genotype.

### Summary/Conclusion

Taken together, our data highlight the interactions between genetic variants, tumor microenvironment, and therapeutic response, with the aim of identifying molecular biomarkers useful for precision medicine in MM and its precursors MGUS and SMM.

Copyright Information: (Online) ISSN: 2572-9241
©2026 The Author(s). HemaSphere is published by John Wiley & Sons Ltd on behalf of European Hematology Association. This is an open access Abstract Book distributed under the Attribution-NonCommercial-NoDerivs (CC BY-NC-ND), which allows third parties to download the articles and share them with others as long as they credit the author and the Abstract Book, but they cannot change the content in any way or use them commercially.
Abstract Book Citations: Authors, Title, HemaSphere, 2026;10:(S1):pages. The abstract book DOIs can be found on the "Author Information" page.
Disclaimer: Articles published in the journal HemaSphere exclusively reflect the opinions of the authors. The authors are responsible for all content in their abstracts including accuracy of the facts, statements, citing resources, etc.

### (PS1861) EXPLORING THE EPIGENETIC ROLE OF PRDM1: A TARGET FOR IMID-RESISTANT MULTIPLE MYELOMA?

**Topic:** 13. Myeloma and other monoclonal gammopathies - Biology & translational research

Sophie Ball*[1], Jason Taslim[1], Katrina Fordwor-Hepburn[1], Zuzanna Krause[1], Aristeidis Chaidos[1], Niklas Feldhahn[1], Nicholas T Crump[1]

[1]*Imperial College London, Department of Immunology and Inflammation, London, United Kingdom;*

### Background

Multiple myeloma (MM) is an incurable plasma cell malignancy that accounts for 15% of all blood cancers. Immunomodulatory drugs (IMiDs) are a first-line treatment for MM and patients have often developed resistance to them at relapse. IMiDs operate by degrading the key transcription factors (TFs) IKZF1/Ikaros and IKZF3/Aiolos, which play a pivotal role in transforming terminally differentiated plasma cells into actively dividing myeloma cells. The degradation of these TFs is commonly blocked in IMiD-resistant patients, meaning relapse myeloma cells remain dependent on their IKZF1/3. PRDM1 is another key TF, which is known to heterodimerise with IKZF1/3, suggesting that it may provide an alternative therapeutic target to disrupt their activity in IMiD-resistant patients. PRDM1 has counterintuitive roles in both plasma cell differentiation and MM proliferation, and it is unclear how its behaviour is altered in MM to promote myelomagenesis.

### Aims

To investigate the role of PRDM1 alongside IKZF1/3 in MM to assess its potential as a novel therapeutic target in IMiD-resistant patients.

### Methods

We used ChIP-seq to map PRDM1 and IKZF1/3 binding in MM cell lines. ChIP-seq for histone H3K27 acetylation (H3K27ac) was used to identify active enhancers and promoters. We introduced an $FKBP12^{F36V}$ degron tag to the endogenous PRDM1 in MM1S cells, allowing rapid, inducible degradation, followed by transient transcriptome sequencing (TT-seq) to look at immediate effects on transcription, and ChIP-seq for TFs and histone acetylation (H3K27ac). This was compared to the effects of IKZF1/3 degradation with the IMiD pomalidomide.

### Results

PRDM1 displayed widespread enhancer and promoter binding, including at key oncogenes, and degradation of PRDM1 resulted in both up- and downregulated transcription. Upregulated genes, especially those bound by IKZF1/3, were strongly enriched for PRDM1 binding, supporting a direct repressive role at these targets. In support of this, H3K27ac, a mark of activation, increased dramatically at both enhancers and promoters of the upregulated genes, following PRDM1 degradation. Downregulated genes were also bound by PRDM1, although with bias towards enhancer binding, arguing for a parallel role in gene activation. ChIP-seq showed co-occupancy with IKZF1/3 at many loci, including differentially expressed genes. Overlap of differential genes following PRDM1 or IKZF1/3 degradation identified genes that were dependent on the continued presence of both factors. Interestingly, IKZF3 binding was seen to decrease following PRDM1 degradation, arguing that IKZF3 may be dependent on PRDM1 for continued chromatin binding. Notably, combined treatment with dTAG to induce PRDM1 degradation and the IMiD pomalidomide resulted in a greater reduction in cell growth compared to either treatment alone.

**Copyright Information:** (Online) ISSN: 2572-9241
©2026 The Author(s). HemaSphere is published by John Wiley & Sons Ltd on behalf of European Hematology Association. This is an open access Abstract Book distributed under the Attribution-NonCommercial-NoDerivs (CC BY-NC-ND), which allows third parties to download the articles and share them with others as long as they credit the author and the Abstract Book, but they cannot change the content in any way or use them commercially.
**Abstract Book Citations:** Authors, Title, HemaSphere, 2026;10:(S1):pages. The abstract book DOIs can be found on the "Author Information" page.
**Disclaimer:** Articles published in the journal HemaSphere exclusively reflect the opinions of the authors. The authors are responsible for all content in their abstracts including accuracy of the facts, statements, citing resources, etc.

**Summary/Conclusion**

This work suggests a MM gene regulatory network coordinated by PRDM1 and IKZF1/3, with the activity of PRDM1 influenced by binding to IKZF1/3 and vice versa. This argues that targeting PRDM1 in IMiD-resistant patients could disrupt the same transcriptional networks that IMiDs successfully block, providing alternative treatment options for relapse patients.

**Copyright Information:** (Online) ISSN: 2572-9241
©2026 The Author(s). HemaSphere is published by John Wiley & Sons Ltd on behalf of European Hematology Association. This is an open access Abstract Book distributed under the Attribution-NonCommercial-NoDerivs (CC BY-NC-ND), which allows third parties to download the articles and share them with others as long as they credit the author and the Abstract Book, but they cannot change the content in any way or use them commercially.
**Abstract Book Citations:** Authors, Title, HemaSphere, 2026;10:(S1):pages. The abstract book DOIs can be found on the "Author Information" page.
**Disclaimer:** Articles published in the journal HemaSphere exclusively reflect the opinions of the authors. The authors are responsible for all content in their abstracts including accuracy of the facts, statements, citing resources, etc.

### (PS1862) BELANTAMAB MAFODOTIN, BORTEZOMIB, AND DEXAMETHASONE VS DARATUMUMAB, BORTEZOMIB, AND DEXAMETHASONE IN RELAPSED/REFRACTORY MULTIPLE MYELOMA: UPDATED 4-YEAR RESULTS OF THE PHASE 3 DREAMM-7 TRIAL

**Topic:** 14. Myeloma and other monoclonal gammopathies - Clinical

Vania Hungria*[1], Marek Hus[2], Vera Zherebtsova[3], Christopher Ward[4], P. Joy Ho[5], Angelo Maiolino[6], Roman Hájek[7], Kihyun Kim[8], Hanlon Sia[9], Adam Bryant[10], Marcelo Pitombeira De Lacerda[11, 12], Anna Sureda[13, 14], Benga Kazeem[15], Hena Baig[16], Nick Pirooz[17], Sybil Varghese[17], Joe Lee[18], Paweł Robak[19], María-Victoria Mateos[20]

*[1]Clinica São Germano, São Paulo, Brazil; [2]Medical University of Lublin, Lublin, Poland; [3]Gorodskaya Klinicheskaya Bol'nitsa Im. S.p. Botkina, Moscow, Russia; [4]Royal North Shore Hospital, Sydney, Australia; [5]Royal Prince Alfred Hospital, and University of Sydney, Camperdown, NSW, Australia; [6]Instituto Américas, Rio de Janeiro, Brazil; [7]Department of Hematooncology, University Hospital Ostrava, Ostrava and Department of Hematooncology, University of Ostrava, Ostrava, Czechia; [8]Sungkyunkwan University, Samsung Medical Center, Seoul, South Korea; [9]Pindara Private Hospital, Gold Coast, QLD, Australia; [10]Liverpool Hospital, Sydney, Australia; [11]Universidade da Região de Joinville, Santa Catarina, Brazil; [12]Centro de Hematologia e Oncologia, Joinville, Santa Catarina, Brazil; [13]Institut Català d'Oncologia - L'Hospitalet, Barcelona, Spain; [14]Institut de Ciències Biomèdiques de Bellvitge (IDIBELL), Barcelona, Spain; [15]GSK, Stevenage, United Kingdom; [16]GSK, Mississauga, ON, Canada; [17]GSK, Collegeville, United States of America; [18]GSK, London, United Kingdom; [19]Medical University of Łódź, Łódź, Poland; [20]University Hospital of Salamanca/IBSAL/Cancer Research Center-IBMCC (USAL-CSIC), Salamanca, Spain;*

### Background

Targeting B-cell maturation antigen (BCMA) has transformed the relapsed/refractory multiple myeloma (RRMM) landscape with unprecedented overall survival (OS) outcomes vs standard-of-care (SOC) daratumumab triplet regimens. Belantamab mafodotin (belamaf) is a BCMA-targeting antibody-drug conjugate with a multimodal mechanism of action that delivers superior progression-free survival (PFS) and OS vs daratumumab, bortezomib, and dexamethasone (DVd) with minimal immunosuppression and no evidence of inducing T-cell exhaustion.

### Aims

To assess the continued long-term treatment benefit of belamaf, bortezomib, and dexamethasone (BVd) vs an anti-CD38–containing triplet regimen in patients with RRMM with ≥1 prior line of therapy (LOT).

### Methods

DREAMM-7 (NCT04246047) is a phase 3, open-label, randomized trial evaluating the efficacy and safety of belamaf vs daratumumab with Vd backbone (BVd vs DVd) in patients with RRMM and ≥1 prior LOT. Patients underwent 8 cycles (21 days per cycle) of BVd or DVd followed by belamaf or daratumumab monotherapy, respectively, in cycles 9 and beyond. The primary endpoint was investigator-assessed PFS. Secondary endpoints included OS, response rates, and safety. The data cutoff for this descriptive analysis was Jan 9, 2026.

**Copyright Information:** (Online) ISSN: 2572-9241
©2026 The Author(s). HemaSphere is published by John Wiley & Sons Ltd on behalf of European Hematology Association. This is an open access Abstract Book distributed under the Attribution-NonCommercial-NoDerivs (CC BY-NC-ND), which allows third parties to download the articles and share them with others as long as they credit the author and the Abstract Book, but they cannot change the content in any way or use them commercially.
**Abstract Book Citations:** Authors, Title, HemaSphere, 2026;10:(S1):pages. The abstract book DOIs can be found on the "Author Information" page.
**Disclaimer:** Articles published in the journal HemaSphere exclusively reflect the opinions of the authors. The authors are responsible for all content in their abstracts including accuracy of the facts, statements, citing resources, etc.

**Results**

At >4 years of follow-up (median, 52.9 months), BVd (n=243) treatment maintained continued OS benefit vs DVd (n=251). In the intention-to-treat (ITT) population, median OS in the BVd arm was not reached (NR; 95% CI, 63.0 months-NR) and was 58.3 months (95% CI, 41.4 months-NR) in the DVd arm (HR, 0.62; 95% CI, 0.46-0.82). PFS benefit was maintained in the ITT population, with durable outcomes in PFS and OS also observed across response categories. A total of 38% of patients achieved a stringent complete response (sCR) or CR with BVd treatment vs 21% with DVd. At the 4-year landmark, OS rates in the BVd arm were 98% in patients with sCR and 82% in those with CR, indicating sustained OS. Further, median PFS was not reached in patients with sCR or CR treated with BVd and was 56.2 and 45.0 months, respectively, in similar responding patients treated with DVd. No new safety concerns emerged with >4 years of follow-up. Adverse event (AE) incidence remained consistent with previous analyses, and no new fatal AEs were observed. Infection rates were unchanged with no new safety concerns. HRQOL remained stable over time, underscoring the long-term tolerability of belamaf monotherapy.

**Summary/Conclusion**

With >4 years of follow-up for BVd, OS outcomes remain notably superior to those with DVd. A substantial proportion of patients achieved deep remission and PFS benefit. The overall safety profile remained consistent with the known profile of belamaf. These results, along with the convenience of outpatient administration, further support BVd as a potential new SOC therapy in RRMM at first relapse or later.

**Funding statement:** This study was sponsored by GSK. Editorial support was provided by Nucleus Global, an Inizio company, and funded by GSK (study ID 207503). Drug-linker technology was licensed from Seagen Inc.; monoclonal antibody produced using POTELLIGENT Technology licensed from BioWa.

**Copyright Information:** (Online) ISSN: 2572-9241
©2026 The Author(s). HemaSphere is published by John Wiley & Sons Ltd on behalf of European Hematology Association. This is an open access Abstract Book distributed under the Attribution-NonCommercial-NoDerivs (CC BY-NC-ND), which allows third parties to download the articles and share them with others as long as they credit the author and the Abstract Book, but they cannot change the content in any way or use them commercially.
**Abstract Book Citations:** Authors, Title, HemaSphere, 2026;10:(S1):pages. The abstract book DOIs can be found on the “Author Information” page.
**Disclaimer:** Articles published in the journal HemaSphere exclusively reflect the opinions of the authors. The authors are responsible for all content in their abstracts including accuracy of the facts, statements, citing resources, etc.

### (PS1864) NON-RELAPSE MORTALITY AFTER CAR T-CELL THERAPY FOR RELAPSED/REFRACTORY MULTIPLE MYELOMA: AN IFM STUDY FROM THE DESCAR-T REGISTRY

**Topic:** 14. Myeloma and other monoclonal gammopathies - Clinical

Jeanne Loiseau*[1], Alexis Talbot[2], Lionel Karlin[3], Olivier Decaux[4], Cyrille Touzeau[5], Jean Noël Bastie[6], Salomon Manier[7], Jean Marc Schiano De Colella[8], Aurore Perrot[9], Mohamad Mohty[10], Karim Belhadj Merzoug[11], Xavier Leleu[12], Titouan Cazaubiel[13], Thomas Chalopin[14], Carine Chateleix[15], Michael Loschi[16], Camille Bigenwald[17], Jean-Richard Eveillard[18], Fabrice Jardin[19], Pierre Morel[20], Laurent Garderet[21], Phillipe Rey[22], Caroline Jacquet[23], Cécile Sonntag[24], Adeline Pierache[25], Bertrand Arnulf[2], Laure Vincent[1]

*[1]CHU de Montpellier, Clinical hematology, Montpellier, France;[2]Hôpital Saint Louis, Paris, France;[3]CHU de LYON, Pierre-Bénite, France; [4]CHU de Rennes, Rennes, France;[5]CHU de Nantes, Nantes, France;[6]CHU de Dijon Bourgogne, Dijon, France;[7]CHU de Lille, Lille, France; [8]Institut Paoli Calmettes, Marseille, France; [9]IUCT Oncopole, Toulouse, France; [10]Hôpital Saint Antoine, Paris, France; [11]Hopital Henri Mondor, Créteil, France; [12]CHU de Poitiers, Poitiers, France; [13]CHU de Bordeaux, Bordeaux, France; [14]CHU de Tours, Tours, France; [15]CHU de Clermont Ferrand, Clermont Ferrand, France; [16]CHU de Nice, Nice, France; [17]Institut Gustave Roussy, Villejuif, France; [18]CHU de Brest, Brest, France; [19]CENTRE HENRI BECQUEREL, Rouen, France; [20]CHU d'Amiens, Amiens, France; [21]HOPITAL DE LA PITIE SALPETRIERE, Paris, France;[22]CENTRE LEON BERARD, Lyon, France; [23]CHU de Nancy, Nancy, France;[24]Institut de cancérologie de Strasbourg, Strasbourg, France;[25]Exystat, Malakoff, France;*

### Background

Idecabtagene vicleucel (Ide-cel), an autologous B-cell maturation antigen (BCMA) chimeric antigen receptor (CAR) T-cell therapy, has transformed outcomes for patients with relapsed/refractory multiple myeloma (RRMM) achieving a median overall survival (OS) of 19.4 months in the pivotal trial (1). In France, Ide-cel has been available since 2021 for patients who have received at least three prior lines of therapy, including an immunomodulatory agent, a proteasome inhibitor, and an anti-CD38 monoclonal antibody with disease progression on the last line of therapy.

### Aims

While disease progression remains the leading cause of death following Ide-cel, approximately 6% of patients in real-world experience (2) die from non-relapse causes. To date, the causes and risk factors of non-relapse mortality (NRM) remain poorly described.

### Methods

Using data from the French DESCAR-T registry, we assessed the incidence, timing, and causes of NRM in patients treated with Ide-cel under standard of care conditions.

**Copyright Information:** (Online) ISSN: 2572-9241
©2026 The Author(s). HemaSphere is published by John Wiley & Sons Ltd on behalf of European Hematology Association. This is an open access Abstract Book distributed under the Attribution-NonCommercial-NoDerivs (CC BY-NC-ND), which allows third parties to download the articles and share them with others as long as they credit the author and the Abstract Book, but they cannot change the content in any way or use them commercially.
**Abstract Book Citations:** Authors, Title, HemaSphere, 2026;10:(S1):pages. The abstract book DOIs can be found on the “Author Information” page.
**Disclaimer:** Articles published in the journal HemaSphere exclusively reflect the opinions of the authors. The authors are responsible for all content in their abstracts including accuracy of the facts, statements, citing resources, etc.

**Results**

Between August 2021 and December 2025, 805 patients received Ide-cel. At data cut-of, 170 patients (18.8%) had died including 39 (4.8%) from NRM. Early NRM (<28 days post-infusion) occurred in 9 patients, and late NRM (≥ 28 days) occurred in 30 patients. The main causes of NRM were infections (*33.3%, including COVID-19 23%)* second primary malignancies (*SPM) (23.1%),* heart and hemodynamic failure (*15.4%*), severe bleeding (*12.8%, including 50% of intracerebral haemorrhage*), immune effector cell–associated toxicity (IEC); equally split between cytokine release syndrome and neurotoxicity (*12.8%).* Early NRM was only due to IEC toxicity (56%), severe bleeding (22%) or heart and hemodynamic failure (22%). Late NRM was largely dominated by infections (43.3%) occurring for the majority during the first year after CAR-T infusion.  Univariate analysis identified several biological and one clinical risk factors at the time of CAR-T cell infusion that were significantly associated with early NRM. These include elevated lactate dehydrogenase (LDH) levels (p=0.02), increased ferritin levels (p<0.01), cytopenias — anemia (p<0.01), neutropenia (p=0.04), thrombocytopenia (p=0.02) — and the presence of extramedullary disease (EMD) (p=0.03). Because of the limited number of early NRM events, multivariate analysis was not performed. Regarding late NRM, multivariate analysis identified three significant risk factors: thrombopenia at time of infusion (p=0.03), CRS grade >2 (p<0.01) and ICANS (p=0.02).
38 patients developed a SPM (4.7%). Hematologic malignancies were the most common with 15 cases (11 myelodysplastic syndromes, 4 acute leukemias, and 1 diffuse large B-cell lymphoma). SPMs were responsible for 9 deaths.

**Summary/Conclusion**

This study is the first to specifically focus on NRM following CAR T-cell in patients with RRMM within a national real-world cohort. The overall NRM observed in our cohort was comparable to rates reported in real-world studies of diffuse large B-cell lymphoma (3). Early and late NRM were associated with some similar initial risk factors but late NRM was also largely influenced by IEC, underscoring the need for dynamic risk stratification and tailored surveillance and preventive strategies, particularly for bleeding and infections which should be maintained several months after infusion.

**References:**

1. Munshi NC  et al. N Engl J Med. 2021 Feb 25;384(8):705–16.
2. Sidana S et al. Blood. 2025 Jul 10;146(2):167-177.
3. Lemoine J et al. Blood Adv. 2023 Nov 14;7(21):6589–98.

**Copyright Information:** (Online) ISSN: 2572-9241
©2026 The Author(s). HemaSphere is published by John Wiley & Sons Ltd on behalf of European Hematology Association. This is an open access Abstract Book distributed under the Attribution-NonCommercial-NoDerivs (CC BY-NC-ND), which allows third parties to download the articles and share them with others as long as they credit the author and the Abstract Book, but they cannot change the content in any way or use them commercially.
**Abstract Book Citations:** Authors, Title, HemaSphere, 2026;10:(S1):pages. The abstract book DOIs can be found on the “Author Information” page.
**Disclaimer:** Articles published in the journal HemaSphere exclusively reflect the opinions of the authors. The authors are responsible for all content in their abstracts including accuracy of the facts, statements, citing resources, etc.

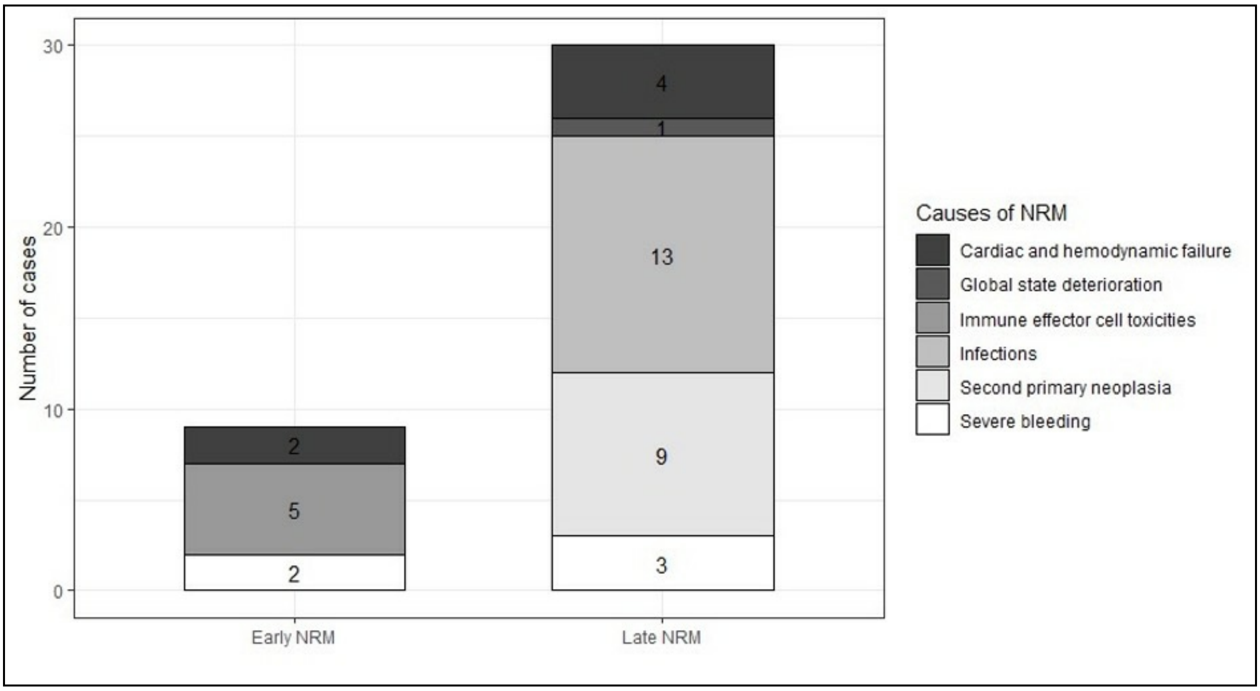

**Copyright Information:** (Online) ISSN: 2572-9241
©2026 The Author(s). HemaSphere is published by John Wiley & Sons Ltd on behalf of European Hematology Association. This is an open access Abstract Book distributed under the Attribution-NonCommercial-NoDerivs (CC BY-NC-ND), which allows third parties to download the articles and share them with others as long as they credit the author and the Abstract Book, but they cannot change the content in any way or use them commercially.
**Abstract Book Citations:** Authors, Title, HemaSphere, 2026;10:(S1):pages. The abstract book DOIs can be found on the "Author Information" page.
**Disclaimer:** Articles published in the journal HemaSphere exclusively reflect the opinions of the authors. The authors are responsible for all content in their abstracts including accuracy of the facts, statements, citing resources, etc.

### (PS1865) CEVOSTAMAB (FCRH5XCD3 BISPECIFIC ANTIBODY) INDUCES DURABLE RESPONSES IN PATIENTS WITH LATE-LINE RRMM WHO HAVE RECEIVED PRIOR BCMA-TARGETED CAR T-CELL THERAPY: EXPANSION RESULTS FROM THE CAMMA 2 STUDY

**Topic:** 14. Myeloma and other monoclonal gammopathies - Clinical

Shaji Kumar*[1], Alexander Lesokhin[2], Ashraf Badros[3], Laura Rosiñol Dachs[4], Douglas Sborov[5], Bertrand Arnulf[6], Yael Cohen[7], Paolo Corradini[8], Michel Delforge[9], Xavier Leleu[10], Christoph Scheid[11], Ameet Mishra[12], Kerstin Trunzer[13], Charlotte Vignal[13], Maureen Cannon[12], Allen Yang[14], Elisabeth Wassner Fritsch[13], Joshua Richter[15]

*[1]Mayo Clinic, Rochester, United States of America; [2]Memorial Sloan Kettering Cancer Center, New York, United States of America; [3]Greenebaum Comprehensive Cancer Center, University of Maryland, Baltimore, United States of America;[4]Hospital Clinic de Barcelona and Insitut d'Investigacions Biomèdiques August Pi i Sunyer (IDIBAPS), Barcelona, Spain; [5]Huntsman Cancer Institute, University of Utah, Salt Lake City, United States of America; [6]Hôpital Saint-Louis, Paris, France; [7]Sourasky Medical Center and Gray Faculty of Health and Medical Sciences, Tel Aviv University, Tel Aviv, Israel; [8]Fondazione IRCCS Istituto Nazionale dei Tumori, University of Milan, Milan, Italy; [9]Universitair Ziekenhuis (UZ) Leuven, Leuven, Belgium; [10]CHU de Poitiers, Poitiers, France; [11]Uniklinik Köln, Cologne, Germany; [12]Genentech, Inc., South San Francisco, United States of America; [13]F. Hoffmann-La Roche Ltd, Basel, Switzerland; [14]Roche (China) Holding Ltd, Shanghai, China; [15]Tisch Cancer Institute, Icahn School of Medicine at Mount Sinai, New York, United States of America;*

### Background

Proven salvage therapies for patients (pts) with triple-class refractory multiple myeloma (MM) who progress on B-cell maturation antigen (BCMA)-targeted chimeric antigen receptor T-cell (CAR-T) therapies are lacking. Fc receptor-homolog 5 (FcRH5) is a type I membrane protein that is ubiquitously expressed on MM cells. Cevostamab is a first-in-class FcRH5xCD3 bispecific antibody. In the initial Phase I study, cevostamab monotherapy was highly active and had a manageable safety profile in pts with late-line MM (Richter et al. ASH 2024). In the subsequent Phase I/II CAMMA 2 study (NCT05535244), cevostamab induced deep and durable responses and had manageable safety in an exploratory cohort of pts with triple-class refractory MM who had received prior BCMA-targeted antibody–drug conjugate (n=10) or CAR-T therapy (n=11) (Kumar et al. EHA 2024).

### Aims

We present pooled data from the exploratory and expansion cohorts of CAMMA 2 for pts who had received prior BCMA-targeted CAR-T therapy.

### Methods

Eligible pts were those who were triple-class relapsed or refractory and had received a prior BCMA-targeted CAR-T. Cevostamab was initiated with step-up dosing (0.3 mg on Cycle [C] 1 Day [D] 1 and 3.3 mg on C1D2, D3 or D4 depending on the emergence of cytokine release syndrome [CRS] after initial administration) and continued at target dose (160 mg) on C1D8 and D1 of each subsequent 21-day cycle until disease progression or unacceptable toxicity. Primary objectives were evaluation of safety and efficacy (primary endpoint: investigator-assessed objective response rate [ORR] per IMWG). Informed consent was provided.

**Copyright Information:** (Online) ISSN: 2572-9241
©2026 The Author(s). HemaSphere is published by John Wiley & Sons Ltd on behalf of European Hematology Association. This is an open access Abstract Book distributed under the Attribution-NonCommercial-NoDerivs (CC BY-NC-ND), which allows third parties to download the articles and share them with others as long as they credit the author and the Abstract Book, but they cannot change the content in any way or use them commercially.
**Abstract Book Citations:** Authors, Title, HemaSphere, 2026;10:(S1):pages. The abstract book DOIs can be found on the “Author Information” page.
**Disclaimer:** Articles published in the journal HemaSphere exclusively reflect the opinions of the authors. The authors are responsible for all content in their abstracts including accuracy of the facts, statements, citing resources, etc.

### Results

As of 15 September 2025, 41 pts had been enrolled across the exploratory and expansion cohorts (median age: 66 years [range: 36–81]; male: 65.9%). Eight pts (19.5%) had extramedullary disease and 16/26 pts (61.5%) who were evaluable had high-risk cytogenetics (t(4;14), t(14;16) or del(17p)). Median number of prior lines was 5 (range: 3–15). All pts were triple-class refractory and 31.7% were refractory to their last line.
Median time on study was 12.9 months (range: 1.1–34.2). ORR was 43.9% and the very good partial response (VGPR) or better rate was 34.1%; 19.5% had a stringent complete response, 2.4% a complete response, 12.2% a VGPR and 9.8% a partial response. The median duration of response (DOR) was 17.4 months (95% confidence interval: 9.0–not estimable), with 44.4% (8/18) of responders still in remission at cut-off and a DOR rate at 15 months of 65.8% (**Figure**).
Grade (Gr) 3–4 adverse events (AEs) occurred in 75.6% of pts; Gr 3–4 AEs in ≥10% were neutropenia (41.5%), anemia (12.2%) and thrombocytopenia (12.2%). Gr 3–4 infections occurred in 39.0%. Twenty-one pts (51.2%) received immunoglobulin supplementation. CRS occurred in 75.6%. CRS events were all Gr 1 (36.6%) or Gr 2 (39.0%) and 76.9% occurred within 24 hours of the end of infusion. CRS was frequently managed with tocilizumab (51.6%) or steroids (51.6%); all events resolved. Gr 5 (fatal) AEs occurred in 2 pts (4.9%); both events (pneumonia, myelodysplastic syndrome [MDS]) were considered unrelated to treatment. AEs leading to cevostamab discontinuation occurred in 1 patient (Gr 5 MDS).

### Summary/Conclusion

Cevostamab induces deep and durable responses in pts with late-line, triple-class refractory MM who have received prior BCMA-targeted CAR-T therapy. AEs are generally manageable, with a low rate of AEs leading to cevostamab discontinuation.

**Figure**: Kaplan-Meier curve of DOR among all patients who had received prior BCMA-targeted CAR-T therapy in the CAMMA 2 study.

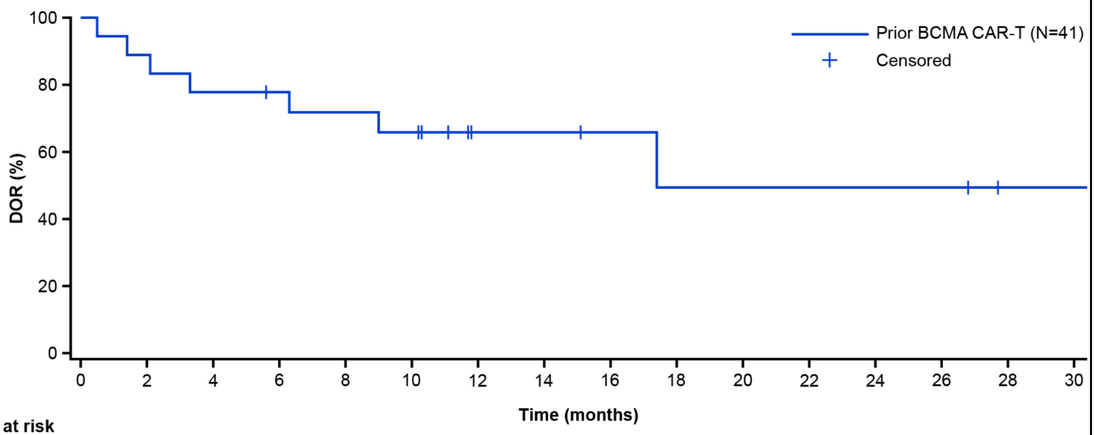

Patients remaining at risk

| | | | | | | | | | | | | | | | | |
|---|---|---|---|---|---|---|---|---|---|---|---|---|---|---|---|---|
| Prior BCMA CAR-T | 18 | 16 | 14 | 13 | 12 | 11 | 5 | 5 | 4 | 3 | 3 | 3 | 3 | 3 | 1 | 1 |

BCMA, B-cell maturation antigen; CAR-T, chimeric antigen receptor T-cell; DOR, duration of response

Copyright Information: (Online) ISSN: 2572-9241
©2026 The Author(s). HemaSphere is published by John Wiley & Sons Ltd on behalf of European Hematology Association. This is an open access Abstract Book distributed under the Attribution-NonCommercial-NoDerivs (CC BY-NC-ND), which allows third parties to download the articles and share them with others as long as they credit the author and the Abstract Book, but they cannot change the content in any way or use them commercially.
Abstract Book Citations: Authors, Title, HemaSphere, 2026;10:(S1):pages. The abstract book DOIs can be found on the “Author Information” page.
Disclaimer: Articles published in the journal HemaSphere exclusively reflect the opinions of the authors. The authors are responsible for all content in their abstracts including accuracy of the facts, statements, citing resources, etc.

### (PS1866) FIRST INTERIM ANALYSIS OF DOMAIN 1 – TECLISTAMAB (TEC) AND TALQUETAMAB (TAL) OF THE CENTRALIZED PLATFORM FOR FUNCTIONAL HIGH-RISK MULTIPLE MYELOMA — ZEPFHR STUDY AMARC 24-01.

**Topic:** 14. Myeloma and other monoclonal gammopathies - Clinical

Sueh-Li Lim*[1, 2], John Reynolds[1, 2], Yujin Kim[1, 2], Nirija Ranjit Anderson[3, 4], Nicole Wong Doo[5, 6], David Routledge[7, 8], Olga Motorna[9, 10], Jay Hocking[11], P. Joy Ho[6, 12], Nicholas Weber[13], Yachao Deng[14], Khoa Le[14], Andrew Spencer[1, 2]

*[1]Alfred Hospital Bayside Health, Department of Malignant Haematology, Transplantation and Cellular Therapy Services, Melbourne, Australia; [2]Monash University, Australian Centre for Blood Diseases, Melbourne, Australia; [3]Princess Alexandra Hospital, Department of Haematology, Brisbane, Australia; [4]University of Queensland, Brisbane, Australia; [5]Concord Hospital, Department of Haematology, Sydney, Australia; [6]University of Sydney, Sydney, Australia; [7]Peter MacCallum and Royal Melbourne Hospital, Department of Clinical Haematology, Melbourne, Australia; [8]University of Melbourne, Sir Peter MacCallum Department of Oncology, Melbourne, Australia; [9]Eastern Health, Department of Haematology, Melbourne, Australia; [10]Monash University, Eastern Clinical School, Melbourne, Australia; [11]Austin Hospital, Department of Haematology, Melbourne, Australia; [12]Royal Prince Alfred Hospital, Department of Haematology, Sydney, Australia; [13]Royal Brisbane and Women's Hospital, Department of Haematology, Brisbane, Australia; [14]Alfred Hospital Bayside Health, Australasian Myeloma Research Consortium, Melbourne, Australia;*

### Background

Functional high-risk (FHR) MM patients, identified by progressive disease ≤12 months from commencement of 1$^{st}$-line (1L) therapy, were shown in an Australian and New Zealand Myeloma and Related Diseases Registry landmark survival analysis to have a median overall survival of 20.2 compared to 60.7 months for those progressing >12 months (p<0.001).

### Methods

The Australasian Myeloma Research Consortium (AMaRC) ZePFHR study (ACTRN12625000429459) is a multi-domain platform study evaluating novel therapies in patients with early disease progression, inclusive of primary refractory disease and early relapse, within 18 months of initiation of 1L therapy. Domain 1 evaluated combination therapy with TEC+TAL in 20 patients. During C1, priming doses TEC (60µg/kg) and TAL (10µg/kg) were administered prior to step-up doses 1 and 2 (TEC: 300/1500µg/kg; TAL: 60/400 µg/kg), followed by TEC (1500µg/kg) and TAL(800µg/kg) on C1D15 and fortnightly thereafter at target dose TEC (3000µg/kg) and TAL (800µg/kg) during C2-4. C5+ TAL continued Q2W, switching to Q4W upon achievement of VGPR; TEC continues Q4W irrespective, for a total of 24 months of therapy. Primary objective is to establish within C1-4, the overall response (ORR), defined as ≥PR and safety. Protocol defined proof of concept (PoC) criteria were an observed ORR ≥70% and posterior probability that the true ORR is ≥60% is ≥0.8. We present the planned interim analysis of the first 15 evaluable patients.

**Copyright Information:** (Online) ISSN: 2572-9241
©2026 The Author(s). HemaSphere is published by John Wiley & Sons Ltd on behalf of European Hematology Association. This is an open access Abstract Book distributed under the Attribution-NonCommercial-NoDerivs (CC BY-NC-ND), which allows third parties to download the articles and share them with others as long as they credit the author and the Abstract Book, but they cannot change the content in any way or use them commercially.
**Abstract Book Citations:** Authors, Title, HemaSphere, 2026;10:(S1):pages. The abstract book DOIs can be found on the "Author Information" page.
**Disclaimer:** Articles published in the journal HemaSphere exclusively reflect the opinions of the authors. The authors are responsible for all content in their abstracts including accuracy of the facts, statements, citing resources, etc.

**Results**

The first patient was enrolled 22MAY2025. As of 11FEB2026, 15 participants are evaluable – of these, 66.7% males, median age 62.0 years (range,37-76), 6.7% were R-ISS III at diagnosis, 7/15 had EMD at screening, 12/15 patients had diagnostic cytogenetics/FISH – 50%, 33.3%, 8.3%, 16.7% had 0, 1, 2, 3 high-risk cytogenetic abnormalities (HRCAs), only 3 (25%) fulfilled 2025 IMS/IMWG genomic high-risk criteria. 14/15 patients received PI-IMID 1L (VRd 73.3%, VRd+Selinexor 6.7%, VRd+DCEP 6.7%, DARA-VRd 6.7%), 1/15 (6.7%) VCd, 8/15 autologous stem cell transplant, 2/15 primary refractory, 13/15 relapsed after initial response to 1L, of these 9 (69.2%) had achieved ≥VGPR prior to relapse. Median time from initiation of 1L to TEC+TAL was 9.3 (3.8-21.8) months. At data lock, 10/15 (66.6%), 2 (13.3%), 2 (13.3%) and 1 (6.7%) had received 4, 3, 2 and 1 cycles of TEC+TAL. Regarding the primary end-point, all patients achieved ≥PR within C1-4, ORR 100% (95%CrI79.13-99.84%). The posterior probability that the true ORR rate>60% is 99.9% and thus protocol-specific PoC criteria were met. Observed CR/sCR, VGPR and PR were 13.3, 46.67 and 40.0% (n=15), respectively.

Response was rapid, median time ≥PR 1.2 months. 14/15 remain on study, 1 genomic standard-risk pt with EMD at study entry demonstrated EMD relapse despite PR at C2D1 and subsequently died from progressive myeloma. Survival analyses are planned when all patients remaining on study have 12 months follow-up. Grd 2/3 CRS occurred in 4 patients, Grd 1 ICANs in 2 patients, AEs were consistent with established side effect profile for TAL and TEC, with cytopenias being the only Grd 4 AEs reported. There were 3 reported Grd 3 viral infections.

**Summary/Conclusion**

Domain 1 TEC+TAL of ZePFHR, the first study globally to assess bispecifics in FHR-MM, demonstrates unprecedented efficacy with an ORR 100%, fulfilling PoC criteria with manageable CRS/ICANs with standard therapies and safety profile consistent with previous experience with TEC+TAL.

**Copyright Information:** (Online) ISSN: 2572-9241
©2026 The Author(s). HemaSphere is published by John Wiley & Sons Ltd on behalf of European Hematology Association. This is an open access Abstract Book distributed under the Attribution-NonCommercial-NoDerivs (CC BY-NC-ND), which allows third parties to download the articles and share them with others as long as they credit the author and the Abstract Book, but they cannot change the content in any way or use them commercially.
**Abstract Book Citations:** Authors, Title, HemaSphere, 2026;10:(S1):pages. The abstract book DOIs can be found on the “Author Information” page.
**Disclaimer:** Articles published in the journal HemaSphere exclusively reflect the opinions of the authors. The authors are responsible for all content in their abstracts including accuracy of the facts, statements, citing resources, etc.

### (PS1867) DISTINCT MUTATIONAL NETWORK ARCHITECTURE DRIVES RACIAL GENOMIC DISPARITIES IN MULTIPLE MYELOMA RATHER THAN INDIVIDUAL MUTATIONS

**Topic:** 14. Myeloma and other monoclonal gammopathies - Clinical

Chidiebube Ugwu[1], Amin Turki*[2]

*[1]Jefferson University Hospital, Philadelphia, United States of America; [2]University Hospital Bochum/Essen, Bochum, Germany;*

### Background

Multiple myeloma (MM) disproportionately affects Black Americans, as compared to White Americans (14.4 vs 6.4 per 100,000) (Waxman, *Blood*, 2010). Paradoxically, Black American patients equally achieve comparable or superior progression-free survival rates (Dong, *Blood Cancer J*, 2022; Fillmore, *Blood*, 2019). Prior genomic studies highlighted the impact of the mutational landscape on outcome (Maura F et al. *JCO* 2024, Schavgoulidze A et al. *Blood* 2024), however co-mutational network architecture is unexplored as a source of disparity.

### Aims

We aimed to decipher the impact of the co-mutational network architecture as source of racial disparities in MM.

### Methods

We analyzed 1,020 MM patients (Black American n=181; White American n=839) from AACR Project GENIE v18 (AACR Project GENIE Consortium, *Cancer Discov*, 2017). Pairwise co-occurrence rates were computed with Fisher's exact tests and false detection rate (FDR) correction. Mutational co-occurrence networks were constructed with genes as nodes and statistically significant co-occurrence rates as edges. Co-occurrence networks were analyzed using graph topology metrics, with hub genes defined by co-occurrence degree and betweenness centrality. Global co-occurrence distributions were compared by Mann-Whitney U test. Survival analyses used Kaplan-Meier estimation, Cox proportional hazards models, and propensity score matching.

### Results

Mutation burden (median 3 per group; p=0.162) and Fraction of Genome altered (FGA) (p=0.877) were comparable. No gene reached significance after FDR correction (*TP53*: 13.9% White American vs 7.7% Black American, FDR=0.21; *KRAS*: 16.3% vs 9.9%, FDR=0.22). Global co-occurrence distributions differed markedly (p=0.001). White American patients harbored dense *TP53*-anchored networks: *TP53-TET2* 11.1% vs 0%, *TP53-FAT1* 10.2% vs 0%, *TP53-KRAS* 8.3% vs 0%, with hubs at *SETD2* (degree=12, betweenness=53) and *RB1* (degree=10). Black American patients showed sparser *NRAS*-centered networks: *KRAS-NRAS* 7.41% vs 1.35%, *NRAS-CYLD* 6.17% vs 1.57%, with hubs at *KRAS* (degree=9) and *DIS3* (degree=7). Non-Hispanic subgroup replication excluded ethnic admixture as the driver.
Established high-risk features (*TP53* mutation, FGA 20% or greater) and recurrent driver mutations (*RAS/MAPK* alterations) were similarly prevalent between groups, yet cumulative hazards in Cox models were higher in Black Americans: HR 3.99 (95% CI 1.76 to 9.05) for two or more high-risk lesions vs 2.47 (95% CI 1.72 to 3.55) in White Americans. Propensity-matched five-year survival was 57.1% vs 74.9% (difference 17.7%), with race remaining independently prognostic (HR 1.35 to 1.45) after adjustment for age, sex, and genomic risk burden.

**Copyright Information:** (Online) ISSN: 2572-9241
©2026 The Author(s). HemaSphere is published by John Wiley & Sons Ltd on behalf of European Hematology Association. This is an open access Abstract Book distributed under the Attribution-NonCommercial-NoDerivs (CC BY-NC-ND), which allows third parties to download the articles and share them with others as long as they credit the author and the Abstract Book, but they cannot change the content in any way or use them commercially.
**Abstract Book Citations:** Authors, Title, HemaSphere, 2026;10:(S1):pages. The abstract book DOIs can be found on the "Author Information" page.
**Disclaimer:** Articles published in the journal HemaSphere exclusively reflect the opinions of the authors. The authors are responsible for all content in their abstracts including accuracy of the facts, statements, citing resources, etc.

### Summary/Conclusion

Genomic sources of racial disparities in MM are driven by differences in mutational network organization, not individual gene frequencies. White American patients harbor *TP53*-anchored co-mutation networks; Black American patients show *NRAS*-centered architectures with amplified hazards due to cumulative genomic risk. Network-aware approaches to MM risk stratification refine race-aware risk assessment that complements current IMWG risk classifications.

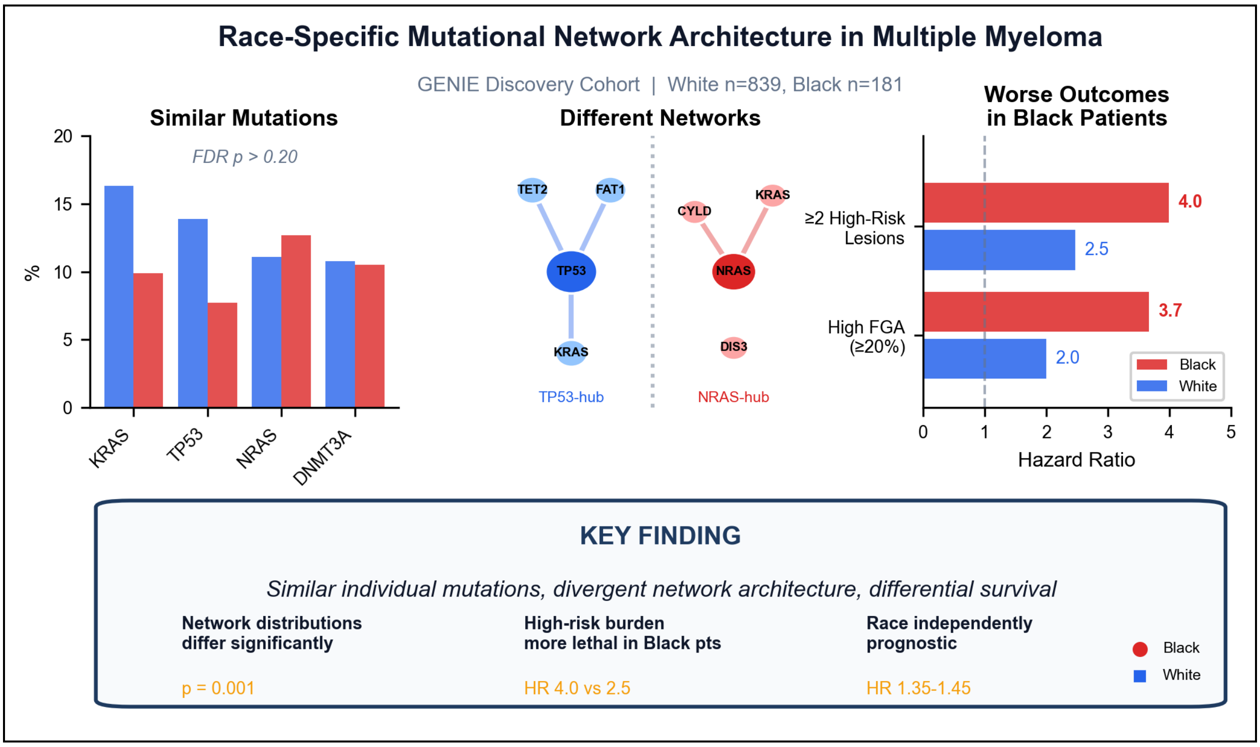

**Copyright Information:** (Online) ISSN: 2572-9241
©2026 The Author(s). HemaSphere is published by John Wiley & Sons Ltd on behalf of European Hematology Association. This is an open access Abstract Book distributed under the Attribution-NonCommercial-NoDerivs (CC BY-NC-ND), which allows third parties to download the articles and share them with others as long as they credit the author and the Abstract Book, but they cannot change the content in any way or use them commercially.
**Abstract Book Citations:** Authors, Title, HemaSphere, 2026;10:(S1):pages. The abstract book DOIs can be found on the "Author Information" page.
**Disclaimer:** Articles published in the journal HemaSphere exclusively reflect the opinions of the authors. The authors are responsible for all content in their abstracts including accuracy of the facts, statements, citing resources, etc.

### (PS1868) REALITEC-2 SUBGROUP ANALYSIS: AN INTERNATIONAL OBSERVATIONAL RETROSPECTIVE STUDY OF TECLISTAMAB IN PATIENTS WITH RELAPSED/REFRACTORY MULTIPLE MYELOMA IN THE REAL-WORLD

**Topic:** 14. Myeloma and other monoclonal gammopathies - Clinical

K Martin Kortum*[1], Katarina Uttervall[2], Rakesh Popat[3], Hila Magen[4], Markus Hansson[5], Tamir Shragai[6], Evangelos Terpos[7], Charlotte Toftmann Hansen[8], Salomon Manier[9, 10], Henrik Gregersen[11], Øyvind Ottestad[12], Cyrille Touzeau[13], Evdoxia Chatzicharisi[14, 15], Sarah Leeth Farmer[16], Eirini Katodritou[17], Emmanuil Spanoudakis[18], Maja Ølholm Vase[19], Carmine Liberatore[20, 21], Maria Papathanasiou[22], Maria Angelopoulou[7, 23], Maria Esther Clavero Sanchez[24], Raivo Kittus[25], Pavel Smirnov[26], Peter Hu[27], Guntug Gungor[28], Diptendu Santra[29], Eva Rubio Azpeitia[30], Aurore Perrot[31]

*[1]University Hospital of Würzburg, Würzburg, Germany; [2]Karolinska University Hospital, Stockholm, Sweden; [3]University College London Hospitals NHS Foundation Trust, London, United Kingdom; [4]Chaim Sheba Medical Center, Ramat Gan and Tel-Aviv University, Tel-Aviv, Israel; [5]Sahlgrenska University Hospital, Gothenburg, Sweden; [6]Tel-Aviv Sourasky Medical Center, Tel-Aviv, Israel; [7]National and Kapodistrian University of Athens, Athens, Greece; [8]Odense University Hospital, Odense, Denmark; [9]University of Lille, Lille, France; [10]Centre Hospitalier Universitaire de Lille, Lille, France; [11]Aalborg University Hospital, Aalborg, Denmark; [12]Akershus University Hospital, Lorenskog, Norway; [13]University Hospital Hôtel-Dieu, Nantes, France; [14]AHEPA University Hospital, Thessaloniki, Greece; [15]Aristotle University of Thessaloniki, Thessaloniki, Greece; [16]Vejle Hospital, Vejle, Denmark; [17]Theagenio Cancer Hospital, Thessaloniki, Greece; [18]Democritus University of Thrace, Medical School, Alexandroupolis, Greece; [19]Aarhus University Hospital, Aarhus, Denmark; [20]Santo Spirito Hospital, Pescara, Italy; [21]"G. d'Annunzio" University, Chieti, Italy; [22]G Papanicolaou Hospital, Thessaloniki, Greece; [23]Laikon General Hospital, Athens, Greece; [24]Hospital Universitario Virgen De Las Nieves, Granada, Spain; [25]Johnson & Johnson, London, United Kingdom; [26]Johnson & Johnson, Milan, Italy; [27]Johnson & Johnson, Raritan, United States of America; [28]Johnson & Johnson, Breda, The Netherlands; [29]Parexel International, Sheffield, United Kingdom; [30]Johnson & Johnson, Madrid, Spain; [31]Université de Toulouse, Toulouse, France;*

### Background

Teclistamab is the most widely used BCMA bispecific antibody for triple-class-exposed relapsed/refractory multiple myeloma (TCE RRMM). In the MajesTEC-1 trial, with a median follow-up of 30.4 months, teclistamab induced deep and durable responses across all patients, including those more difficult-to-treat such as the penta-refractory, with high-risk cytogenetics, and older than 75 years. The first REALiTEC cohort, an international observational study including patients treated mainly through pre-approval access programs, showed response rates and clinical outcomes consistent with MajesTEC-1, with no reduction in effectiveness in subgroups typically associated with poor prognosis. These findings further established teclistamab as a standard-of-care option in a real-world TCE RRMM population.

### Aims

Here, we report the subgroup analysis of the second cohort of REALiTEC, REALiTEC-2, involving patients starting commercial teclistamab in 2023 and 2024.

### Methods

REALiTEC is a retrospective, multicohort, non-interventional study based on clinical data extracted from patient medical records. Treatment effectiveness was assessed using standard endpoints, including response rates, duration of response (DOR), progression-free survival (PFS), and overall survival (OS). Safety was also evaluated.

**Copyright Information:** (Online) ISSN: 2572-9241
©2026 The Author(s). HemaSphere is published by John Wiley & Sons Ltd on behalf of European Hematology Association. This is an open access Abstract Book distributed under the Attribution-NonCommercial-NoDerivs (CC BY-NC-ND), which allows third parties to download the articles and share them with others as long as they credit the author and the Abstract Book, but they cannot change the content in any way or use them commercially.
**Abstract Book Citations:** Authors, Title, HemaSphere, 2026;10:(S1):pages. The abstract book DOIs can be found on the "Author Information" page.
**Disclaimer:** Articles published in the journal HemaSphere exclusively reflect the opinions of the authors. The authors are responsible for all content in their abstracts including accuracy of the facts, statements, citing resources, etc.

**Results**

REALiTEC-2 included 260 patients from 60 sites across 11 countries in Europe and Israel. Median age was 68 years and 67.6% of patients had documented high-risk cytogenetic abnormalities. Extramedullary disease (EMD) excluding bone-related soft tissue plasmacytomas was reported in 19.1% of cases, and 60% of patients would not have met eligibility criteria for MajesTEC-1. Patients had received a median of four prior lines of therapy (pLOTs). Overall response rate (ORR) was 66.5%, with 54.6% being very good partial response or better (≥VGPR). With a median follow-up of 12.7 months, median DOR was not reached (NR), with a 12-month estimate of 64.4%, median PFS was 15.2 months, and median OS was NR, with a 12-month estimate of 69.8%. No significant differences were found in ORR and ≥VGPR rates in clinically relevant subgroups such as penta-refractory patients, high-risk cytogenetics, ≥75 years, International Staging System (ISS) III or EMD patients as compared to the overall cohort (**Figure**). DOR, PFS and OS were also similar between these subgroups and the overall study population with significantly better outcomes observed in the elderly patients in DOR and PFS ($p=0.026$ for both); and in patients with ISS I-II as compared to ISS III in PFS and OS ($p=0.022$ and $p \leq 0.001$, respectively). Furthermore, patients achieving deep responses (≥VGPR) had significantly longer DOR, PFS, and OS ($p<0.001$).

**Summary/Conclusion**

REALiTEC-2 showed comparable outcomes to MajesTEC-1 in TCE RRMM patients in the real world in a broader patient population. No significant differences in effectiveness were observed across subgroups typically associated with poorer prognosis, indicating consistent treatment outcomes across heterogeneous patient populations. Collectively, these results further substantiate the role of teclistamab as an effective standard of care for TCE RRMM in the real-world.

**Copyright Information:** (Online) ISSN: 2572-9241
©2026 The Author(s). HemaSphere is published by John Wiley & Sons Ltd on behalf of European Hematology Association. This is an open access Abstract Book distributed under the Attribution-NonCommercial-NoDerivs (CC BY-NC-ND), which allows third parties to download the articles and share them with others as long as they credit the author and the Abstract Book, but they cannot change the content in any way or use them commercially.
**Abstract Book Citations:** Authors, Title, HemaSphere, 2026;10:(S1):pages. The abstract book DOIs can be found on the "Author Information" page.
**Disclaimer:** Articles published in the journal HemaSphere exclusively reflect the opinions of the authors. The authors are responsible for all content in their abstracts including accuracy of the facts, statements, citing resources, etc.

Figure: Response rates by subgroups

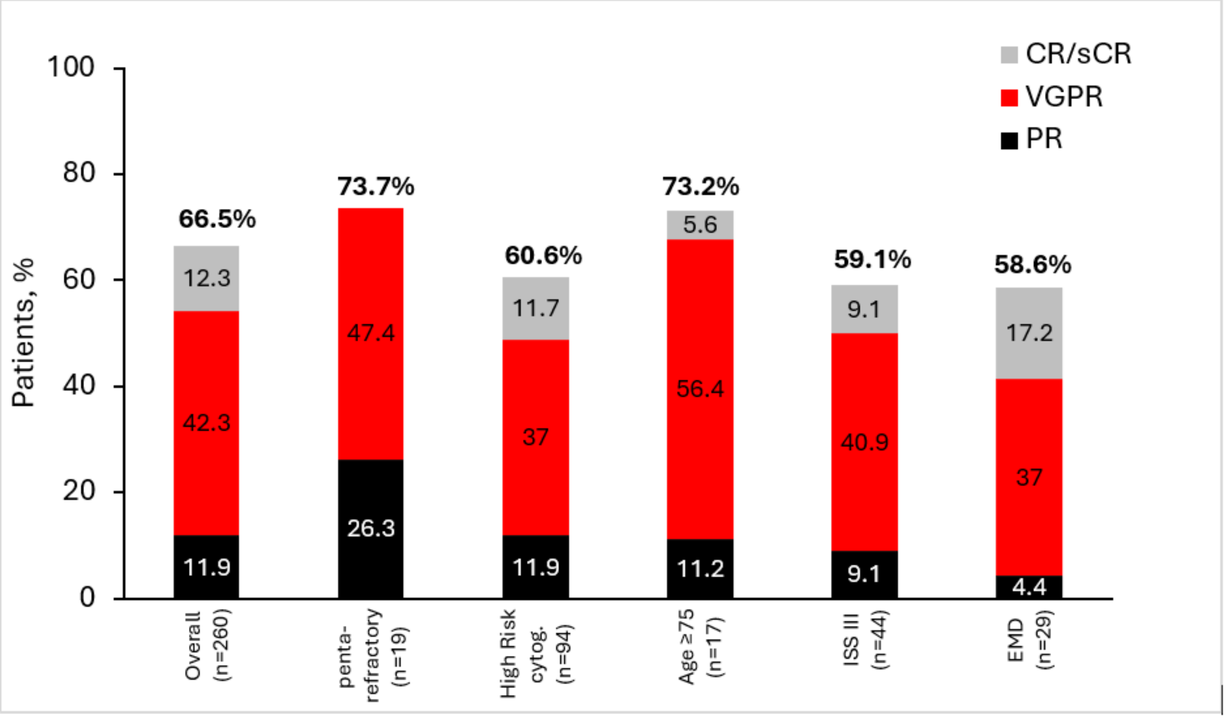

EMD, extramedullary disease; CR, complete response; ISS, international staging system; PR, partial response; sCR, stringent complete response; VGPR, very good partial response

**Copyright Information:** (Online) ISSN: 2572-9241
©2026 The Author(s). HemaSphere is published by John Wiley & Sons Ltd on behalf of European Hematology Association. This is an open access Abstract Book distributed under the Attribution-NonCommercial-NoDerivs (CC BY-NC-ND), which allows third parties to download the articles and share them with others as long as they credit the author and the Abstract Book, but they cannot change the content in any way or use them commercially.
**Abstract Book Citations:** Authors, Title, HemaSphere, 2026;10:(S1):pages. The abstract book DOIs can be found on the "Author Information" page.
**Disclaimer:** Articles published in the journal HemaSphere exclusively reflect the opinions of the authors. The authors are responsible for all content in their abstracts including accuracy of the facts, statements, citing resources, etc.

### (PS1869) NEW IMS/IMWG RISK CRITERIA BY NEXT-GENERATION SEQUENCING: ANALYSIS OF DARATUMUMAB BENEFIT IN BOTH HIGH- AND STANDARD-RISK PATIENTS IN THE PERSEUS STUDY

**Topic:** 14. Myeloma and other monoclonal gammopathies - Clinical

Luca Bertamini*[1], Carolina Terragna[2], Niccolò Bolli[3], Philippe Moreau[4], Paula Rodríguez-Otero[5], Mario Boccadoro[6], Joan Blade[7], Niels W C J van de Donk[8], Francesca Gay[9], Roberto Mina[10], Aurore Perrot[11], P. Joy Ho[12], Annemiek Broijl[1], Meral Beksac[13], Mark van Duin[1], Diego Vieyra[14], Fredrik Borgsten[15], Melissa Rowe[16], Meletios A. Dimopoulos[17, 18], Pieter Sonneveld[1]

*[1]Department of Hematology, Erasmus MC Cancer Institute, Rotterdam, The Netherlands;[2]IRCCS Azienda Ospedaliero-Universitaria di Bologna, Istituto di Ematologia "Seràgnoli", Bologna, Italy; [3]Hematology Unit, Foundation IRCCS Ca' Granda Ospedale Maggiore Policlinico; Department of Oncology and Hemato-Oncology, University of Milan, Milan, Italy; [4]Hematology Department at the University Hospital of Nantes, Nantes, France; [5]Cancer Center Clinica Universidad de Navarra, University of Navarra, Pamplona, Spain; [6]European Myeloma Network, EMN, Torino, Italy; [7]Amyloidosis and Multiple Myeloma Unit, Hematology, Hospital Clinic of Barcelona August Pi i Sunyer Biomedical Research Institute (IDIBAPS), Barcelona, Spain; [8]Department of Hematology, Cancer Center Amsterdam, Amsterdam UMC, Vrije Universiteit Amsterdam, Amsterdam, The Netherlands; [9]Division of Hematology, AOU Città della Salute e della Scienza di Torino, University of Torino and Department of Molecular Biotechnology and Health Sciences, University of Torino, Torino, Italy; [10]Department of Hematology and Medical Oncology, Winship Cancer Institute, Emory University, Atlanta, United States of America; [11]Université de Toulouse, CHU de Toulouse Hématologie, IUCT-Oncopole, Toulouse, France; [12]Multiple Myeloma Research Unit and Thalassemia/Haemoglobinopathy Unit at the Institute of Haematology, Royal Prince Alfred Hospital, Camperdown, Australia; [13]Department of Hematology, Ankara Liv Hospital, Istinye University, Ankara, Türkiye;[14]Johnson & Johnson, Spring House, United States of America; [15]Johnson & Johnson, Raritan, United States of America; [16]Johnson & Johnson, High Wycombe, United Kingdom; [17]Department of Clinical Therapeutics, School of Medicine, National and Kapodistrian University of Athens, Athens, Greece; [18]Department of Medicine, Korea University, Seoul, South Korea;*

### Background

IMS/IMWG recently proposed novel Consensus Genomic Staging (CGS) high-risk criteria for newly diagnosed multiple myeloma (NDMM), including genetic alterations not identifiable by fluorescence in situ hybridization (FISH) but by next-generation sequencing (NGS; eg, *TP53* mutations). PERSEUS/EMN017 (NCT03710603) evaluated the addition of daratumumab to bortezomib, lenalidomide, and dexamethasone (VRd) induction/consolidation and to R maintenance (DVRd arm) vs VRd+R (VRd arm) in transplant-eligible (TE) patients. PERSEUS previously reported that DVRd significantly improved progression-free survival (PFS) regardless of cytogenetic risk as defined by previous criteria.

### Aims

To assess daratumumab benefit in both high- and standard-risk patients in the PERSEUS study using CGS high-risk criteria.

### Methods

709 patients with NDMM were randomized 1:1 to DVRd vs VRd. Unique molecular assay (UMA)-NGS target panel was used on DNA extracted from CD138+ bone marrow (BM) cells. Of 664 patients from whom BM was collected, 434 patient DNA samples were available for NGS testing. Per the new IMS/IMWG CGS criteria, high risk was defined post hoc by the presence of del17p/*TP53* mutations; t(4;14) or t(14;16) or t(14;20) with additional amp1q or del1p; amp1q and del1p; biallelic del1p; and high β2 microglobulin with normal renal function. Classic FISH high risk was defined per-protocol by the presence of del17p, t(14;16), and/or t(4;14). Minimal residual disease negativity (MRD neg) was assessed by NGS (Adaptive) in patients with complete response or better.

**Copyright Information:** (Online) ISSN: 2572-9241
©2026 The Author(s). HemaSphere is published by John Wiley & Sons Ltd on behalf of European Hematology Association. This is an open access Abstract Book distributed under the Attribution-NonCommercial-NoDerivs (CC BY-NC-ND), which allows third parties to download the articles and share them with others as long as they credit the author and the Abstract Book, but they cannot change the content in any way or use them commercially.
**Abstract Book Citations:** Authors, Title, HemaSphere, 2026;10:(S1):pages. The abstract book DOIs can be found on the “Author Information” page.
**Disclaimer:** Articles published in the journal HemaSphere exclusively reflect the opinions of the authors. The authors are responsible for all content in their abstracts including accuracy of the facts, statements, citing resources, etc.

**Results**

Of 434 patients evaluated, 152 (35%) were high risk by CGS. The most common genetic lesions were del17p/*TP53* (n=59, 14%), IgH translocation with amp1q/del1p (12%), and del1p with amp1q (9%); 2% had biallelic focal del1p. Using CGS criteria, 17% of patients were reclassified from standard risk to high risk; additionally, 4% of patients previously classified as high risk were reclassified as standard risk. DVRd was associated with increased MRD neg rates at $10^{-6}$ compared with VRd in both CGS standard-risk (67% vs 39%, $P$<0.0001) and high-risk patients (56% vs 26%, $P$=0.0003). Sustained MRD neg ($10^{-6}$) rates for ≥12 months (+/-1 mo) were higher with DVRd vs VRd for both CGS standard-risk (53% vs 22%; $P$<0.0001) and high-risk patients (37% vs 17%; $P$=0.0004), and patients who achieved sustained MRD negativity ($10^{-6}$) had superior PFS outcomes versus those who did not, regardless of CGS risk status ($P$<0.0001). DVRd led to improved PFS in both CGS standard-risk (HR 0.38, 95% CI 0.20–0.74; $P$=0.005) and high-risk patients (HR 0.54, 95% CI 0.31–0.95; $P$=0.03), adjusting for ISS, lactate dehydrogenase, and age (>65 years).

**Summary/Conclusion**

DVRd improved MRD negativity and PFS over VRd in both the new IMS/IMWG CGS high-risk and standard-risk subgroups. UMA-NGS panel successfully stratified risk according to CGS criteria, identifying nearly twice as many high-risk patients in the PERSEUS study. These data further support DVRd induction/consolidation and DR maintenance as standard of care for TE-NDMM, regardless of cytogenetic risk.
© 2026 American Society of Clinical Oncology, Inc. Reused with permission. This abstract was accepted and previously presented at the 2026 ASCO Annual Meeting. All rights reserved.

**Copyright Information:** (Online) ISSN: 2572-9241
©2026 The Author(s). HemaSphere is published by John Wiley & Sons Ltd on behalf of European Hematology Association. This is an open access Abstract Book distributed under the Attribution-NonCommercial-NoDerivs (CC BY-NC-ND), which allows third parties to download the articles and share them with others as long as they credit the author and the Abstract Book, but they cannot change the content in any way or use them commercially.
**Abstract Book Citations:** Authors, Title, HemaSphere, 2026;10:(S1):pages. The abstract book DOIs can be found on the "Author Information" page.
**Disclaimer:** Articles published in the journal HemaSphere exclusively reflect the opinions of the authors. The authors are responsible for all content in their abstracts including accuracy of the facts, statements, citing resources, etc.

### (PS1870) DE-ESCALATED DOSING OF BELANTAMAB MAFODOTIN PLUS VD REDUCES THE INCIDENCE OF OCULAR EVENTS WHILE MAINTAINING EFFICACY IN RELAPSED/REFRACTORY MULTIPLE MYELOMA: A CZECH MULTICENTER PHASE 2 STUDY

**Topic:** 14. Myeloma and other monoclonal gammopathies - Clinical

Tereza Popková*[1, 2], Jakub Radocha[3], Martin Stork[4], Jiri Minarik[5], Jan Straub[6], Alexandra Jungová[7], Petr Pavlíček[8], Tomas Jelinek[1, 2], Vladimir Maisnar[3], Ludek Pour[4], Tomáš Pika[5], František Sedlák[6], Jan Soukup[8], Hana Plonková[1], Ludmila Muronova[1, 2], Viera Sandecká[4], Petra Krhovská[5], ,David Žihala[1, 2], Roman Hájek[1, 2]

*[1]University Hospital Ostrava, Department of Haematooncology, Ostrava, Czechia; [2]University of Ostrava, Faculty of Medicine, Department of Haematooncology, Ostrava, Czechia; [3]University Hospital Hradec Králové, Charles University, Faculty of Medicine in Hradec Králové, 4th Department of Internal Medicine – Hematology, Hradec Kralove, Czechia; [4]University Hospital Brno and Faculty of Medicine Masaryk University, Department of Internal Medicine, Hematology and Oncology, Brno, Czechia; [5]University Hospital Olomouc and Faculty of Medicine and Dentistry, Palacky University Olomouc, Department of Hemato-oncology, Olomouc, Czechia; [6]First Faculty of Medicine and General Teaching Hospital Charles University, 1st Medical Department – Clinical Department of Haematology, Prague, Czechia; [7]Charles University Hospital Pilsen, Hematology and Oncology Department, Pilsen, Czechia; [8]University Hospital Kralovske Vinohrady, Department of Internal Medicine and Hematology, Prague, Czechia;*

### Background

Belantamab mafodotin (belamaf)–containing regimens have demonstrated substantial efficacy and a favorable benefit–risk profile within the anti-myeloma therapeutic armamentarium. However, ocular adverse events represent a known risk; therefore, dose and schedule optimization are warranted. We designed an optimization study to reduce grade (gr.) ≥3 ocular findings by at least 75% while preserving efficacy, defined as maintaining ≥80% of the ORR observed in DREAMM-6. Accordingly, the primary endpoints were to decrease gr.≥3 ocular findings from 55% to <14% and to maintain an ORR ≥63% (80% of 78%).

### Aims

This phase 2 study (EudraCT 2022-002515-34) was conducted across 7 Czech university hemato-oncology centers. The aim was to evaluate whether a lower dose of belamaf (1.9 mg/kg) administered less frequently (Q8W) in combination with weekly bortezomib and dexamethasone in patients (pts) with multiple myeloma after ≥1 line of therapy could achieve comparable efficacy with improved toxicity compared with the standard dosing regimen (2.5 mg/kg Q3W).

### Methods

The inclusion/exclusion criteria mirrored DREAMM-7 study. Treatment consisted of eight 28-day cycles of bortezomib (1.3 mg/m² SC days 1, 8, 15, and 22) and dexamethasone (20 mg PO days 1, 2, 8, 9, 15, 16, 22, and 23), plus belamaf (1.9 mg/kg IV Q8W) until disease progression or unacceptable toxicity. The data cut-off was December 31, 2025. All patients provided informed consent.

**Copyright Information:** (Online) ISSN: 2572-9241
©2026 The Author(s). HemaSphere is published by John Wiley & Sons Ltd on behalf of European Hematology Association. This is an open access Abstract Book distributed under the Attribution-NonCommercial-NoDerivs (CC BY-NC-ND), which allows third parties to download the articles and share them with others as long as they credit the author and the Abstract Book, but they cannot change the content in any way or use them commercially.
**Abstract Book Citations:** Authors, Title, HemaSphere, 2026;10:(S1):pages. The abstract book DOIs can be found on the "Author Information" page.
**Disclaimer:** Articles published in the journal HemaSphere exclusively reflect the opinions of the authors. The authors are responsible for all content in their abstracts including accuracy of the facts, statements, citing resources, etc.

**Results**

A total of 45 pts after a median of 3 (1-7) prior lines of therapy (LOT) initiated treatment. Median age was 71 (44-89) years. High-risk cytogenetics [t(4;14), t(14;16), del(17p)] was found in 35% (15/43) of pts; 9% (4/45) and 31% (14/45) of pts were double and triple-class refractory, respectively. Response was evaluable in 41 pts who reached cycle 2 (excluding 4: 2 pts died due to infection after 20 and 37 days of the treatment course, respectively; 1 patient withdrew consent after 8 days; 1 patient discontinued treatment due to gr.3 hepatopathy after 18 days): ORR was 68% (28/41), including CR in 22% (9/41), VGPR in 24% (10/41), and PR in 22% (9/41) of pts. One patient (2%; 1/41) achieved MR, 27% (11/41) of pts had SD, and 1 patient (2%; 1/41) had progressive disease. The MRD negativity rate was 14.6% (6/41). The median time to response was 28 days.
Ocular findings were evaluable in 40 pts with ≥2 ophthalmologic assessments (excluding 5: 4 pts described above, 1 died due to postoperative cardiac failure after 44 days of the treatment course): treatment-related ocular findings developed in 65% (26/40) of pts, including maximum keratopathy visual acuity (KVA) gr.1 in 20% (8/40), gr.2 in 37.5% (15/40), and gr.3 in 7.5% (3/40) of pts. KVA gr.3 developed after 2nd, 3rd and 3rd dose of belamaf.
After a median follow-up of 24.3 months (IQR 20.7;28.1), 27% (12/45) of pts remain on treatment. Among patients receiving ≥3 belamaf infusions, mPFS was 23.7 months (14.3;NA), ORR was 82% (23/28), and MRD negativity rate was 21% (6/28).

**Summary/Conclusion**

The primary endpoints were successfully achieved: the incidence of KVA gr.3 was substantially reduced, while efficacy was maintained. These findings support modification of the original belantamab dosing schedule to mitigate ocular adverse events without compromising clinical efficacy. Despite the inclusion/exclusion criteria mirrored DREAMM-7 study, direct comparison with any DREAMM study is limited by an older, more heavily pretreated cohort (median of 3 prior LOT).
Funding and product were provided by GSK.

**Copyright Information:** (Online) ISSN: 2572-9241
©2026 The Author(s). HemaSphere is published by John Wiley & Sons Ltd on behalf of European Hematology Association. This is an open access Abstract Book distributed under the Attribution-NonCommercial-NoDerivs (CC BY-NC-ND), which allows third parties to download the articles and share them with others as long as they credit the author and the Abstract Book, but they cannot change the content in any way or use them commercially.
**Abstract Book Citations:** Authors, Title, HemaSphere, 2026;10:(S1):pages. The abstract book DOIs can be found on the “Author Information” page.
**Disclaimer:** Articles published in the journal HemaSphere exclusively reflect the opinions of the authors. The authors are responsible for all content in their abstracts including accuracy of the facts, statements, citing resources, etc.

### (PS1871) REAL-WORLD BISPECIFIC ANTIBODIES IN MULTIPLE MYELOMA IN THE NETHERLANDS (T-REX): DOSING INTERVALS, INFECTION RISK AND QUALITY OF LIFE.

**Topic:** 14. Myeloma and other monoclonal gammopathies - Clinical

Marjolein Appelman*[1], Rimke Oostvogels[2], Paulus A.F. Geerts[3], Laurens Franssen[4], Ellen van der Spek[5], Heleen De Lil[6], Cleo van Rooijen[7], Paula Ypma[8], Monique Minnema[2], Wilfried Roeloffzen[9], Wouter Plattel[9], Kazimierz Groen[10], Catharina Van Elssen[11], Elizabeth De Kort[12], Inger Nijhof[13], Ruth Wester[1]

*[1]Erasmus MC Cancer Institute, Hematology, Rotterdam, The Netherlands; [2]UMC Utrecht, Hematology, Utrecht, The Netherlands; [3]Isala, Hematology, Zwolle, The Netherlands; [4]Jeroen Bosch Hospital, Hematology, 's-Hertogenbosch, The Netherlands; [5]Rijnstate, Arnhem, The Netherlands; [6]Máxima MC, Eindhoven, The Netherlands; [7]Medisch Spectrum Twente, Enschede, The Netherlands; [8]Haga Hospital, Den Haag, The Netherlands; [9]University Medical Center Groningen, Groningen, The Netherlands; [10]Amsterdam University Medical Center, Amsterdam, The Netherlands; [11]MUMC+, Maastricht, The Netherlands; [12]Radboud University Medical Center, Nijmegen, The Netherlands; [13]St. Antonius Hospital, Nieuwegein, The Netherlands;*

### Background

Bispecific antibodies (BsAbs) targeting BCMA and GPRC5D are approved for relapsed/refractory multiple myeloma (RRMM) after ≥3 prior lines of therapy (LOT) in triple-class–exposed (TCE) patients. Real-world data are essential to evaluate effectiveness, safety, and treatment patterns in broader patient populations.

### Methods

This nationwide, multicenter observational registry captures outcomes of teclistamab (TEC) and talquetamab (TAL) with focus on infections, infection prophylaxis, dosing modifications, and quality of life (QoL; EORTC QLQ-C30 and QLQ-MY20).

**Copyright Information:** (Online) ISSN: 2572-9241
©2026 The Author(s). HemaSphere is published by John Wiley & Sons Ltd on behalf of European Hematology Association. This is an open access Abstract Book distributed under the Attribution-NonCommercial-NoDerivs (CC BY-NC-ND), which allows third parties to download the articles and share them with others as long as they credit the author and the Abstract Book, but they cannot change the content in any way or use them commercially.
**Abstract Book Citations:** Authors, Title, HemaSphere, 2026;10:(S1):pages. The abstract book DOIs can be found on the "Author Information" page.
**Disclaimer:** Articles published in the journal HemaSphere exclusively reflect the opinions of the authors. The authors are responsible for all content in their abstracts including accuracy of the facts, statements, citing resources, etc.

### Results

A total of 301 patients from 13 hospitals were included (median 4 prior LOT; all TCE; 72% triple-class refractory). Notably, 58% would not have met MajesTEC-1 or MonumenTAL-1 eligibility criteria, mainly due to poor performance status, cytopenia, or renal impairment. TEC was administered to 261 patients. Response rates for TEC and TAL, including relevant subgroups, are shown in Table 1. Responses were consistently high across groups, with favorable outcomes in patients with AL amyloidosis and lower responses in MM with extramedullary disease. For TEC-treated patients, median progression-free survival (mPFS) was 11.9 months and median overall survival (mOS) was 33.2 months. mPFS was 10.5 months in trial-ineligible vs. 22.8 months in eligible patients ($p = 0.045$). In multivariate Cox analysis, poor performance status (HR 2.05, $p<0.001$) and cytopenia (HR 1.96, $p<0.001$) at start of BsAb were independently associated with shorter PFS. At 14.3 months median follow-up (mFU), median duration of response (mDoR) was not reached; 62.0% of responders remained in remission at 24 months.
Cytokine release syndrome (CRS) and ICANS occurred in 51% and 6% of patients, respectively, predominantly grade 1–2; 45% received tocilizumab. Infections occurred in 70% (grade ≥3 in 31%); prophylaxis was widely implemented (IVIG 82%, valaciclovir 95%, cotrimoxazole 94%). Infection rates varied by TEC dosing interval, with weekly dosing showing significantly higher infection rates in comparison to extended intervals (2–3-weekly and >4-weekly) (Table 2). Patients who switched to an extended dosing interval before 4 months showed no inferior PFS compared with those who remained on weekly dosing (Fig. 1).
Forty-three patients received TAL, 80% with prior TEC exposure, mPFS was 5.3 months, mDoR 4.4 months, and mOS 15.7 months. A preliminary analysis of QoL in 27 patients showed that the median physical functioning score was 33 (SD 21) at baseline, increasing slightly to 37 (SD 21) at mFU of 75 days.
Table 1

| | Patients (n) | ORR (%) | ≥VGPR (%) | Median follow-up (months) |
|---|---|---|---|---|
| **Teclistamab** | 261 | 69 | 63 | 11.5 |
| Prior talquetamab | 13 | 77 | 69 | 12.0 |
| Prior CAR-T therapy | 5 | 60 | 60 | 23.9 |
| AL amyloidosis | 8 | 88 | 88 | 19.5 |
| Extramedullary disease | 65 | 54 | 46 | 6.5 |
| **Talquetamab** | 43 | 67 | 49 | 7.6 |
| Prior teclistamab | 34 | 62 | 50 | 7.5 |
| No prior teclistamab | 9 | 89 | 44 | 9.6 |

**Table 2**

| Dosinginterval | Patient months | Infections (n), all grade | Infection rate (per 100 pm), all grade | Infections (n), grade ≥3 | Infection rate (per 100 pm), grade ≥3 |
|---|---|---|---|---|---|
| **Weekly** | 1114 | 257 | 23.1 | 85 | 7.6 |
| **2-3 weekly** | 753 | 112 | 14.9* | 15 | 2.0* |
| **>4 weekly** | 709 | 110 | 15.5* | 13 | 1.8* |

*P = <0.001

### Summary/Conclusion

Real-world response rates and PFS were consistent with clinical trials, despite a substantial part of patients that would have been trial-ineligible. Infectious complications were frequent but appeared manageable with prophylaxis and dosing adjustments. QoL results may suggest a modest improvement in physical functioning over the early follow-up period; further analyses are currently ongoing.

**Copyright Information:** (Online) ISSN: 2572-9241
©2026 The Author(s). HemaSphere is published by John Wiley & Sons Ltd on behalf of European Hematology Association. This is an open access Abstract Book distributed under the Attribution-NonCommercial-NoDerivs (CC BY-NC-ND), which allows third parties to download the articles and share them with others as long as they credit the author and the Abstract Book, but they cannot change the content in any way or use them commercially.
**Abstract Book Citations:** Authors, Title, HemaSphere, 2026;10:(S1):pages. The abstract book DOIs can be found on the "Author Information" page.
**Disclaimer:** Articles published in the journal HemaSphere exclusively reflect the opinions of the authors. The authors are responsible for all content in their abstracts including accuracy of the facts, statements, citing resources, etc.

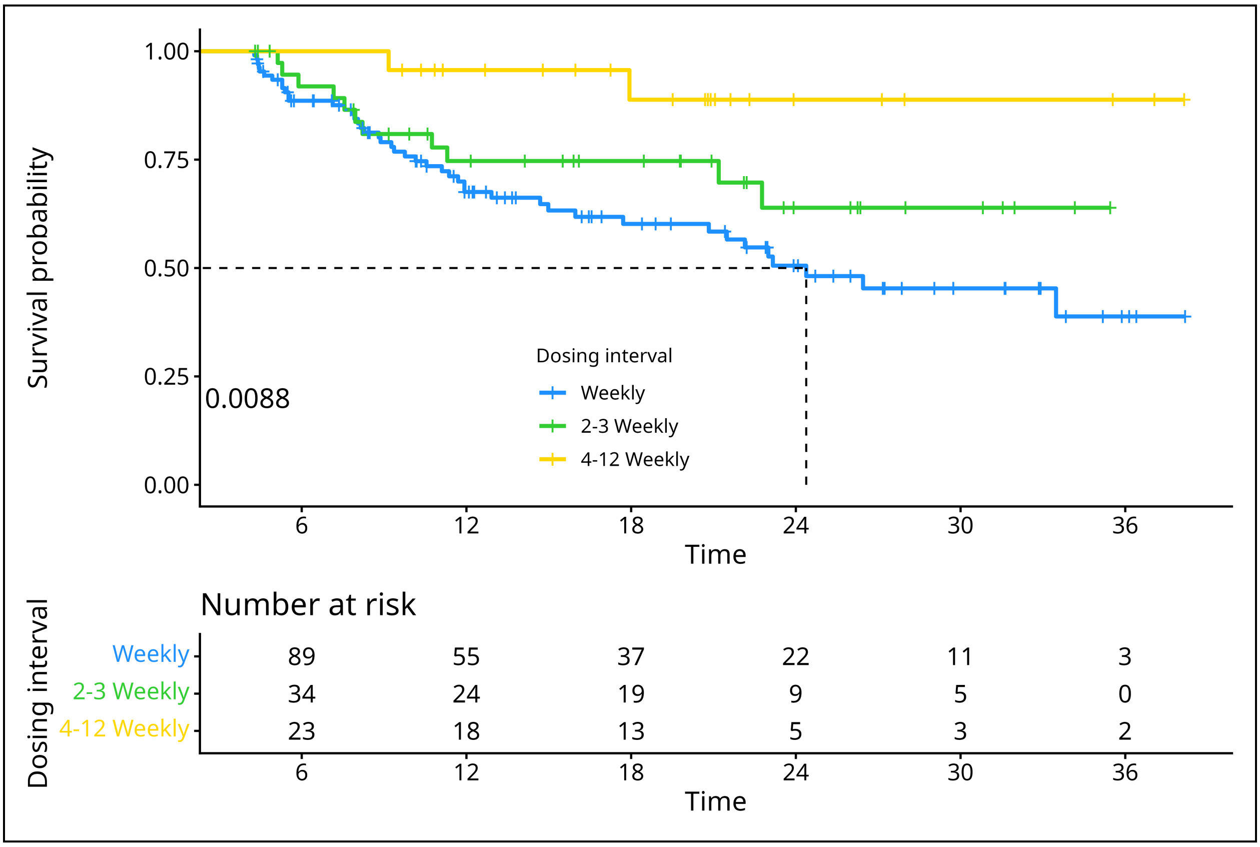

Figure 1: PFS by dosing interval

**Copyright Information:** (Online) ISSN: 2572-9241
©2026 The Author(s). HemaSphere is published by John Wiley & Sons Ltd on behalf of European Hematology Association. This is an open access Abstract Book distributed under the Attribution-NonCommercial-NoDerivs (CC BY-NC-ND), which allows third parties to download the articles and share them with others as long as they credit the author and the Abstract Book, but they cannot change the content in any way or use them commercially.
**Abstract Book Citations:** Authors, Title, HemaSphere, 2026;10:(S1):pages. The abstract book DOIs can be found on the "Author Information" page.
**Disclaimer:** Articles published in the journal HemaSphere exclusively reflect the opinions of the authors. The authors are responsible for all content in their abstracts including accuracy of the facts, statements, citing resources, etc.

### (PS1872) SERIAL CIRCULATING TUMOR PLASMA CELL MONITORING DYNAMICALLY STRATIFIES PROGRESSION RISK IN A SCREENED SMOLDERING MULTIPLE MYELOMA COHORT

**Topic:** 14. Myeloma and other monoclonal gammopathies - Clinical

Sigrún Thorsteinsdóttir*[1, 2], Sæmundur Rögnvaldsson[1, 3], Andri Ólafsson[1], Ásdís Rósa Þórðardóttir[1], Brynjar Vidarsson[4], Páll Torfi Önundarson[4], Bjarni Agnar Agnarsson[4], Margrét Sigurðardóttir[4], Ingunn Þorsteinsdóttir[4], Ísleifur Ólafsson[4], Elías Eyþórsson[4], Ásbjörn Jónsson[5], Thorvardur Jon Love[1, 4], Sigurdur Yngvi Kristinsson[1, 4], Jón Þórir Óskarsson[1, 4]

*[1]University of Iceland, Faculty of Medicine, Reykjavik, Iceland;[2]Rigshospitalet, Department of Hematology, Copenhagen, Denmark; [3]Sahlgrenska University Hospital, Gothenburg, Sweden; [4]Landspitali University Hospital, Reykjavik, Iceland; [5]Akureyri Hospital, Akureyri, Iceland;*

### Background

Smoldering multiple myeloma (SMM) is an asymptomatic precursor to multiple myeloma (MM) and related plasma cell disorders, including amyloid light-chain amyloidosis (AL). As early intervention is increasingly considered for selected high-risk SMM, biomarkers are needed that can dynamically refine progression risk to guide clinical decision-making while limiting overtreatment of indolent disease. Monitoring circulating tumor plasma cells (CTPC) in peripheral blood (PB) by next-generation flow cytometry (NGF) offers a minimally invasive approach to longitudinal risk assessment.

### Aims

To evaluate baseline and serial CTPC assessment by NGF in relation to progression risk in a screened SMM cohort.

### Methods

Individuals diagnosed with SMM in the iStopMM population-based screening study who underwent NGF analysis of PB were included. Samples were stained with the EuroFlow MM-MRD panel and processed according to EuroFlow NGF protocols. CTPC detection was defined as ≥20 events, and the median limit of detection (LOD) was 0.00023% (range 0.00010-0.00064%). Progression analyses used a predefined CTPC threshold of ≥0.001%, selected to exceed the least sensitive LOD and limit between-sample variability in assay sensitivity. Progression hazard was evaluated using Cox regression at baseline and Cox regression for serial CTPC measurements with CTPC status updated over time, modeled as an absorbing state (participants remained classified as ≥0.001% after first crossing), including one-interval lag sensitivity analysis.

**Copyright Information:** (Online) ISSN: 2572-9241
©2026 The Author(s). HemaSphere is published by John Wiley & Sons Ltd on behalf of European Hematology Association. This is an open access Abstract Book distributed under the Attribution-NonCommercial-NoDerivs (CC BY-NC-ND), which allows third parties to download the articles and share them with others as long as they credit the author and the Abstract Book, but they cannot change the content in any way or use them commercially.
**Abstract Book Citations:** Authors, Title, HemaSphere, 2026;10:(S1):pages. The abstract book DOIs can be found on the “Author Information” page.
**Disclaimer:** Articles published in the journal HemaSphere exclusively reflect the opinions of the authors. The authors are responsible for all content in their abstracts including accuracy of the facts, statements, citing resources, etc.

**Results**

In total, 133 individuals with SMM (373 PB samples) underwent NGF. Median age was 68 years (range 44-88) and 63.2% were male. Median serum M-protein was 6.0 g/L (range 0.1-35.0 g/L), and median involved/uninvolved FLC ratio was 5.82 (range 1.01-70.8). Mayo 2/20/20 risk distribution was 58.6% low-, 28.6% intermediate-, and 12.8% high-risk.
At baseline, CTPC were detected in 90/133 (67.7%), with a median CTPC level of 0.00047% (range <LOD-0.059%). Among 107 individuals with follow-up samples (median 3.1 years), 14 progressed (12 MM and 2 AL), 30 were censored because of treatment trial inclusion and 8 died without progression. Among 14 progressors, CTPC were detected at diagnosis in 12/14 and prior to progression in 13/14, the only exception progressed to AL.
Baseline CTPC ≥0.001% was significantly associated with progression to MM (HR 3.93: 95% CI 1.06-14.55; p=0.040) and showed a similar, though non-significant trend when progression to both MM and AL were combined (HR 3.05: 95% CI 0.90-10.33; p=0.073). Using serial PB NGF, 50/107 (46.7%) reached CTPC ≥0.001% during follow-up, including 14/71 (19.7%) below the threshold at baseline. Progression hazard was higher during CTPC ≥0.001% intervals (HR 6.43: 95% CI 1.74-23.75; p=0.0052) and remained elevated with a one-interval lag (HR 4.02: 95% CI 1.38-11.72; p=0.011).

**Summary/Conclusion**

Serial CTPC monitoring provided dynamic progression risk information using a predefined CTPC threshold of ≥0.001%, with substantially stronger association with progression than at baseline assessment alone. CTPC levels were particularly predictive of progression to MM, whereas their predictive value for AL was more limited, although this observation was limited by small number of AL cases. These findings support CTPC monitoring as a minimally invasive tool to complement existing SMM risk stratification and enable longitudinal risk-adapted surveillance.

**Copyright Information:** (Online) ISSN: 2572-9241
©2026 The Author(s). HemaSphere is published by John Wiley & Sons Ltd on behalf of European Hematology Association. This is an open access Abstract Book distributed under the Attribution-NonCommercial-NoDerivs (CC BY-NC-ND), which allows third parties to download the articles and share them with others as long as they credit the author and the Abstract Book, but they cannot change the content in any way or use them commercially.
**Abstract Book Citations:** Authors, Title, HemaSphere, 2026;10:(S1):pages. The abstract book DOIs can be found on the "Author Information" page.
**Disclaimer:** Articles published in the journal HemaSphere exclusively reflect the opinions of the authors. The authors are responsible for all content in their abstracts including accuracy of the facts, statements, citing resources, etc.

### (PS1873) TECLISTAMAB IN RELAPSED/REFRACTORY LIGHT CHAIN AMYLOIDOSIS: A RETROSPECTIVE MULTICENTER STUDY BY THE GERMAN SOCIETY FOR AMYLOID DISEASES

**Topic:** 14. Myeloma and other monoclonal gammopathies - Clinical

Alexander Carpinteiro*[1], Christoph Kimmich[2], Despina Trajanova[3], Ute Hegenbart[3], Timon Hansen[4], Udo Holtick[5], Vera von Landenberg-Roberg[6], Stephan Bohl[6], Raphael Teipel[7], Ivana von Metzler[8], Monika Engelhardt[9], Evgenii Shumilov[10], Maximilian Steinhardt[11], Hans C. Reinhardt[12], Sara Oubari[12], Stefan Schoenland[3]

*[1]University Hospital of Essen, Hämatology and Stem Cell Transplantation, Essen, Germany;[2]University Clini Oldenburg, Department of Oncology and Hematology, Oldenburg, Germany; [3]University Hospital Heidelberg, Department of Internal Medicine V, Heidelberg, Germany; [4]Onkologicum HOPA, Hamburg, Germany; [5]University Hospital of Cologne, Cologne, Germany; [6]Charité - University Medicine Berlin, Department of Hematology and Cancer Immunology, Berlin, Germany; [7]University Hospital Carl Gustav Carus, Dresden, Germany; [8]University Hospital Frankfurt, Frankfurt, Germany; [9]University Hospital Freiburg, Department of Hematology and Oncology, Freiburg, Germany; [10]University Hospital Muenster, Department of Hematology and Oncology, Muenster, Germany; [11]University Hospital Wuerzburg, Medizinische Klinik und Poliklinik 2, Wuerzburg, Germany; [12]University Hospital Essen, Department of Hematology and Stem Cell Transplantation, Essen, Germany;*

### Background

Systemic light chain (AL) amyloidosis is a rare, acquired protein misfolding disorder characterized by extracellular deposition of misfolded immunoglobulin light chain fibrils, resulting in organ damage. Treatment is based on anti-plasma cell regimens derived from multiple myeloma therapy. To date, no approved regimens exist for relapsed/refractory cases. Teclistamab is a bispecific antibody, which targets the CD3 receptor complex on T-cells and the B-cell maturation antigen (BCMA) receptor on plasma cells. In AL amyloidosis, only a limited number of reports describing teclistamab efficacy have been published, mostly involving small patient cohorts and brief follow-up periods. The populations published to date were mainly treated within the scope of the approval for multiple myeloma, which does not correspond to the actual AL patient population and therefore essentially represent a biased subpopulation.

### Aims

To evaluate the efficacy and tolerability of the treatment with teclistamab in 52 patients with relapsed/refractory AL amyloidosis.

### Methods

A retrospective multicenter study performed at 11 centers across Germany.

**Copyright Information:** (Online) ISSN: 2572-9241
©2026 The Author(s). HemaSphere is published by John Wiley & Sons Ltd on behalf of European Hematology Association. This is an open access Abstract Book distributed under the Attribution-NonCommercial-NoDerivs (CC BY-NC-ND), which allows third parties to download the articles and share them with others as long as they credit the author and the Abstract Book, but they cannot change the content in any way or use them commercially.
**Abstract Book Citations:** Authors, Title, HemaSphere, 2026;10:(S1):pages. The abstract book DOIs can be found on the "Author Information" page.
**Disclaimer:** Articles published in the journal HemaSphere exclusively reflect the opinions of the authors. The authors are responsible for all content in their abstracts including accuracy of the facts, statements, citing resources, etc.

**Results**

The median age of the cohort was 66.5 years. 19% of patients had concomitant multiple myeloma at diagnosis. Cardiac involvement was observed in 83% of patients, 29% of patients were in Mayo stage IIIa and 25% in Mayo stage IIIb. Renal involvement was present in 77% of patients with 19% in renal stage III and 12% requiring dialysis. Patients received a median of two prior lines of therapy. The median duration of treatment was 5.3 cycles (range 0.25 - 42.5), with a median of 11.5 full doses of teclistamab administered (range 0 - 54). Cytokine release syndrome occurred in 37% of cases, with two classified as grade 3 and none as grade 4. ICANS were not observed in any case. Neutropenia grade 3 or 4 occurred in 10% of patients. All but two patients responded to therapy, both non-responders had multiple myeloma. Hematologic response (≥VGPR) was achieved in 81% of patients at day 15, which increased to 95% at 3 months. Flow-MRD was negative in 96% (23/24) of cases. Cardiac and renal responses (≥PR) at 6 months were 65% and 78%, respectively. The one-year overall survival was 83% and the median overall survival was not reached after a median follow-up time of 8.8 months. Factors including dFLC ≥180 mg/L, NTproBNP ≥8500 pg/mL, GFR <20 mL/min/1.73 $m^2$ and dialysis did not significantly impact overall survival. In all 34 patients, in whom therapy was discontinued in hematologic remission, no cases of hematologic or organ progression was observed until data cut-off after a median treatment-free survival period of 5.7 months (range 0.5 – 20.8 months).
All patients developed a secondary immunoglobulin deficiency due to B-cell depletion during treatment. Substitution with intravenous or subcutaneous immunoglobulins (IRT) was performed in 83% (43/52) of patients. In nine patients, in whom IRT was not given, four died of bacterial sepsis, whereas in the 43 patients with IRT, two patients died of bacterial sepsis. Patients receiving immunoglobulin replacement therapy had a 2.01 lower risk of infections grade 3 or 4 and a 6.14 lower risk of death.

**Summary/Conclusion**

Our study reports, for the first time, the use of teclistamab in a classic AL population. We demonstrate high efficacy of teclistamab in a heavily diseased and pretreated cohort with AL amyloidosis and highlight the necessity of a concomitant immunoglobulin replacement therapy.

**Copyright Information:** (Online) ISSN: 2572-9241
©2026 The Author(s). HemaSphere is published by John Wiley & Sons Ltd on behalf of European Hematology Association. This is an open access Abstract Book distributed under the Attribution-NonCommercial-NoDerivs (CC BY-NC-ND), which allows third parties to download the articles and share them with others as long as they credit the author and the Abstract Book, but they cannot change the content in any way or use them commercially.
**Abstract Book Citations:** Authors, Title, HemaSphere, 2026;10:(S1):pages. The abstract book DOIs can be found on the "Author Information" page.
**Disclaimer:** Articles published in the journal HemaSphere exclusively reflect the opinions of the authors. The authors are responsible for all content in their abstracts including accuracy of the facts, statements, citing resources, etc.

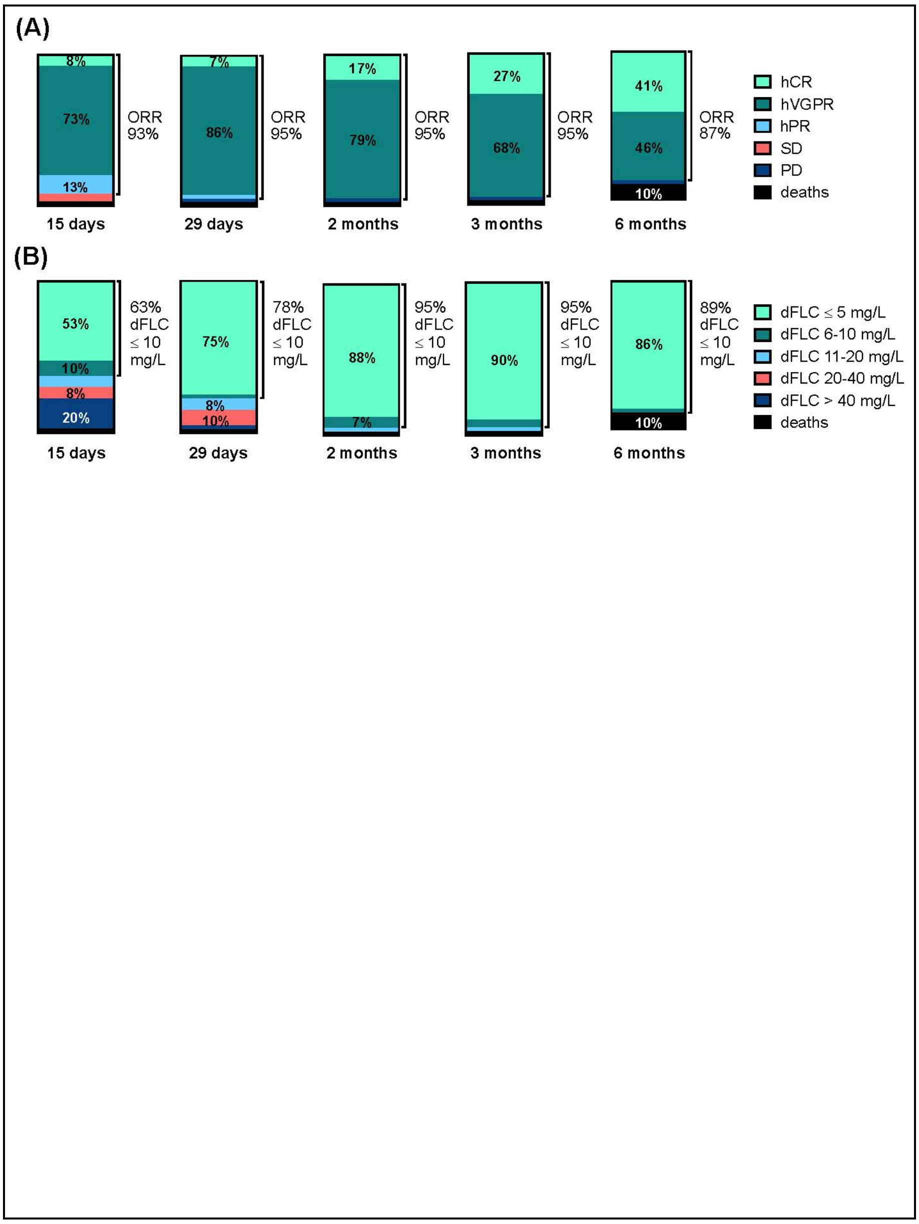

FIGURE 1 (A) hematologic response regarding ISA response criteria, (B) depth of dFLC reduction in relapsed/refractory systemic AL amyloidosis treated with teclistamab. Evaluable patients: 40, 42, 42, 41 and 39 at days 15, 29 as well as at 2, 3 and 6 months, respectively. Hematologic response was not evaluable due to low dFLC<20 mg/L in 6 patients; time point of analyses not reached in 2, 5, 6 patients at day 57, and at 3, 6 months, respectively; missing data in 6, 4, 2, 1 patients at days 15, 29, and at 2, 6 months, respectively. hCR, complete hematologic response; dFLC, difference between involved and non-involved light chains. PD, progressive disease; ORR, overall response rate defined as hPR or better; hPR, partial response; SD, stable disease; and hVGPR, very good partial response.

**Copyright Information:** (Online) ISSN: 2572-9241
©2026 The Author(s). HemaSphere is published by John Wiley & Sons Ltd on behalf of European Hematology Association. This is an open access Abstract Book distributed under the Attribution-NonCommercial-NoDerivs (CC BY-NC-ND), which allows third parties to download the articles and share them with others as long as they credit the author and the Abstract Book, but they cannot change the content in any way or use them commercially.
**Abstract Book Citations:** Authors, Title, HemaSphere, 2026;10:(S1):pages. The abstract book DOIs can be found on the "Author Information" page.
**Disclaimer:** Articles published in the journal HemaSphere exclusively reflect the opinions of the authors. The authors are responsible for all content in their abstracts including accuracy of the facts, statements, citing resources, etc.

### (PS1874) INTERIM REPORT OF PHASE I RESULTS OF A PHASE I/II, OPEN LABEL STUDY TO EVALUATE SAFETY, TOLERABILITY, AND EFFICACY OF ELRANATAMAB IN PATIENTS WITH RELAPSED AL AMYLOIDOSIS

**Topic:** 14. Myeloma and other monoclonal gammopathies - Clinical

Shahrier Hossain[1], Jacob P. Laubach[1], Shannon Miller[1], Adam S. Sperling[2], Pedro Vianna[3], Emily A. Simpson[1], Madison P. Chuma[1], Brendan S. Fink[1], Samantha L. Geberth[1], Emily Magnavita[1], Jack Khouri[4], James E. Hoffman[5], Jonathan Kaufman[6], Raymond Comenzo[7], Giada Bianchi*[2]

*[1]Dana Farber Cancer Institute, Boston, United States of America; [2]Dana Farber Cancer Institute/Brigham and Women's Hospital, Medicine, Boston, United States of America; [3]Brigham and Women's Hospital, Boston, United States of America; [4]Cleveland Clinic, Cleveland, United States of America; [5]University of Miami, Milami, United States of America;[6]Winship Cancer Institute, Atlanta, United States of America; [7]Tufts Medical Center, Boston, United States of America;*

### Background

AL amyloidosis is a rare and incurable plasma cell cancer that causes progressive organ failure due to deposition of involved free light chain (iFLC) amyloid fibrils. A deep and rapid normalization of iFLC is necessary for overall survival. However, there are no FDA-approved therapies for patients with relapsed AL amyloidosis. Elranatamab is a bispecific T-cell redirecting antibody targeting B-cell maturation antigen (BCMA) expressed by clonal plasma cells and CD3 on T cells. Elranatamab is FDA-approved for multiple myeloma patients but has not been prospectively evaluated in AL amyloidosis patients.

### Aims

The primary objective of Phase I is to determine the recommended phase II dosing (RP2D).

### Methods

Adult patients with AL amyloidosis who had progressed after at least 1 prior line of therapy, inclusive of daratumumab and/or cyclophosphamide/bortezomib/dexamethasone were eligible. Patients were excluded for overlapping multiple myeloma or previous BCMA exposure, with the exception of belanatamab mafodotin if it was not the last line of therapy before enrollment. Disease progression was defined as a difference between involved and uninvolved free light chain (dFLC) of at least 20 mg/L after at least 2 cycles of therapy. In cycle 1, Elranatamab was administed in two step-up doses at 12 mg SQ on Day 1 and 32 mg SQ on Day 4, followed by weekly treatment at 76 mg SQ (Dose Level 0). If patients achieved a very good partial response or better (≥VGPR) after 2 cycles, treatment was modified to every other week (Day 1 and Day 15) starting Cycle 3. All patients received a fixed duration of 6 cycles.

**Copyright Information:** (Online) ISSN: 2572-9241
©2026 The Author(s). HemaSphere is published by John Wiley & Sons Ltd on behalf of European Hematology Association. This is an open access Abstract Book distributed under the Attribution-NonCommercial-NoDerivs (CC BY-NC-ND), which allows third parties to download the articles and share them with others as long as they credit the author and the Abstract Book, but they cannot change the content in any way or use them commercially.
**Abstract Book Citations:** Authors, Title, HemaSphere, 2026;10:(S1):pages. The abstract book DOIs can be found on the “Author Information” page.
**Disclaimer:** Articles published in the journal HemaSphere exclusively reflect the opinions of the authors. The authors are responsible for all content in their abstracts including accuracy of the facts, statements, citing resources, etc.

### Results

At the data cut off, eight patients were enrolled in Phase I and received at least one full dose of elranatamab (range: 3-16). The median age was 67 years old (range 54-69) with a median time since diagnosis of 33.5 months (5 – 120). The median prior lines of therapy were 2 (range: 1-3) with all patients exposed to daratumumab and bortezomib. At screening, median NT-proBNP was 575 ng/mL (308-3512) and median hs-troponin T was 21 pg/mL (range:10-130). Cytokine release syndrome occurred in 5 patients (grade 1 = 25% and grade 2 = 37.5%). Tocilizumab was used in 3 patients (37.5%), additional steroids used in 2 patients (25%), and supplemental oxygen in 2 patients (37.5%). There were no neurotoxicity events. After a median follow-up of 5.7 months (1.8 – 10.6), all (100%) patients achieved a VGPR or better with 6 (75%) patients achieving a complete response. All (100%) patients achieved iFLC less than 10 mg/L after first cycle of treatment. In five patients for whom MRD testing via clonoseq was available at the end of 6 cycles, 4 were MRD negative at $10^{-6}$ and one below limit of detection. Patient reported outcome as measured via SF-36 scale showed no significant decline across the study population in physical and mental component score (PCS and MCS, respectively, Figure 1). At the time of EHA meeting, updated data comprising the entire phase 1 cohort of the trial (10 patients) will be presented. The phase II of the study is in the process of opening to external sites with a RP2D of 76 mg.

### Summary/Conclusion

Elranatamab demonstrated no new safety signals and rapid, deep and frequent hematological response in patients with relapsed, AL amyloidosis, representing a promising therapeutic option to address this critical unmet need.

**Copyright Information:** (Online) ISSN: 2572-9241
©2026 The Author(s). HemaSphere is published by John Wiley & Sons Ltd on behalf of European Hematology Association. This is an open access Abstract Book distributed under the Attribution-NonCommercial-NoDerivs (CC BY-NC-ND), which allows third parties to download the articles and share them with others as long as they credit the author and the Abstract Book, but they cannot change the content in any way or use them commercially.
**Abstract Book Citations:** Authors, Title, HemaSphere, 2026;10:(S1):pages. The abstract book DOIs can be found on the “Author Information” page.
**Disclaimer:** Articles published in the journal HemaSphere exclusively reflect the opinions of the authors. The authors are responsible for all content in their abstracts including accuracy of the facts, statements, citing resources, etc.

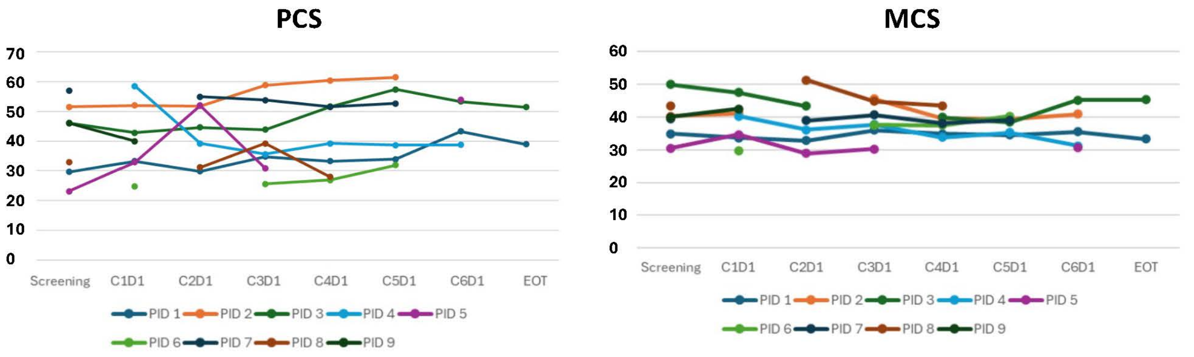

**Figure 1.** Individual patient reported outcome measured according to the SF-36 scale for 9 patients included in the phase 1 of this study showed no significant decline in physical or mental component score over time.

Figure 1. Individual patient reported outcome measured according to the SF-36 scale for 9 patients included in the phase 1 of this study showed no significant decline in physical or mental component score over time.

**Copyright Information:** (Online) ISSN: 2572-9241
©2026 The Author(s). HemaSphere is published by John Wiley & Sons Ltd on behalf of European Hematology Association. This is an open access Abstract Book distributed under the Attribution-NonCommercial-NoDerivs (CC BY-NC-ND), which allows third parties to download the articles and share them with others as long as they credit the author and the Abstract Book, but they cannot change the content in any way or use them commercially.
**Abstract Book Citations:** Authors, Title, HemaSphere, 2026;10:(S1):pages. The abstract book DOIs can be found on the "Author Information" page.
**Disclaimer:** Articles published in the journal HemaSphere exclusively reflect the opinions of the authors. The authors are responsible for all content in their abstracts including accuracy of the facts, statements, citing resources, etc.

### (PS1875) IBERDOMIDE PLUS DARATUMUMAB AND DEXAMETHASONE IN NEWLY DIAGNOSED MULTIPLE MYELOMA (NDMM) BY RENAL FUNCTION: SUBGROUP ANALYSIS OF THE CC-220-MM-001 TRIAL

**Topic:** 14. Myeloma and other monoclonal gammopathies - Clinical

Anna Sureda*[1], Abdullah M. Khan[2], Albert Oriol[3], Mercedes Gironella Mesa[4], Faiz Anwer[5], Cristina Encinas[6], Brea Lipe[7], Paula Rodríguez-Otero[8], Ping Chen[9], Hongxia Lin[9], Kexin Jin[9], Thomas Solomon[9], Lilia Taningco[9], Olumoroti Aina[9], Paulo Maclag[9], Sagar Lonial[10]

*[1]Hematology Department, Institut Català d'Oncologia – Hospitalet, IDIBELL, University of Barcelona, Barcelona, Spain;[2]The James Cancer Hospital and Solove Research Institute, The Ohio State University Comprehensive Cancer Center, Columbus, OH, United States of America; [3]Catalan Institute of Oncology and Josep Carreras Institute, Hospital Germans Trias i Pujol, Badalona, Spain; [4]Hematology Department, Vall d'Hebron Hospital, Barcelona, Spain;[5]Department of Hematology and Medical Oncology, Taussig Cancer Institute, Cleveland Clinic, Cleveland, OH, United States of America; [6]Hospital General Universitario Gregorio Marañón (HGUGM), IiSGM, Madrid, Spain; [7]The Department of Medicine, UR Medicine, Rochester, NY, United States of America; [8]Clínica Universidad de Navarra, CIMA, IDISNA, CIBERONC, Pamplona, Spain;[9]Bristol Myers Squibb, Princeton, NJ, United States of America; [10]Winship Cancer Institute, Emory University, Atlanta, GA, United States of America;*

### Background

Lenalidomide (LEN) plus daratumumab (DARA) and dexamethasone (DEX) is a standard regimen for transplant-ineligible (TNE) NDMM. Iberdomide (IBER), an oral cereblon E3 ligase modulator (CELMoD), binds cereblon with unique interactions and higher affinity than LEN, resulting in enhanced antitumor and immunostimulatory effects.
The ongoing phase 1/2 CC-220-MM-001 trial (NCT02773030) has shown the combination IBER plus DARA and DEX (IberDd) to be effective and well tolerated in patients (pts) with TNE NDMM. Prior analyses in relapsed/refractory MM showed that renal impairment (RI) did not affect clinical outcomes of pts treated with IBER plus DEX (van de Donk NWCJ, et al. *Clin Lymphoma Myeloma Leuk* 2022;22[suppl]:S191–S192).

### Aims

To assess whether RI impacts efficacy, safety, and pharmacokinetics (PK) of IberDd in pts with untreated symptomatic NDMM ineligible or not planned to receive transplant.

### Methods

Pts were assigned sequentially to each cohort and received 1:1:1 of oral IBER (1.0, 1.3, or 1.6 mg; days 1–21 of each 28-day cycle [C]) plus subcutaneous DARA (1800 mg: weekly in C1–2, every 2 weeks in C3–6, every 4 weeks in ≥C7), and weekly DEX (40 mg; 20 mg if >75 years of age). Baseline creatinine clearance (CrCl) defined RI categories (none: ≥90 mL/min, mild: 60–<90 mL/min, moderate: 30–<60 mL/min). IBER dosing was not adjusted for RI. Primary endpoint was overall response rate (ORR); duration of response (DOR) and safety by RI were also assessed. Oral IBER clearance (CL/F) was estimated from an integrated population PK (popPK) model. Logistic regression assessed correlations between RI and efficacy/safety outcomes.

Copyright Information: (Online) ISSN: 2572-9241
©2026 The Author(s). HemaSphere is published by John Wiley & Sons Ltd on behalf of European Hematology Association. This is an open access Abstract Book distributed under the Attribution-NonCommercial-NoDerivs (CC BY-NC-ND), which allows third parties to download the articles and share them with others as long as they credit the author and the Abstract Book, but they cannot change the content in any way or use them commercially.
Abstract Book Citations: Authors, Title, HemaSphere, 2026;10:(S1):pages. The abstract book DOIs can be found on the “Author Information” page.
Disclaimer: Articles published in the journal HemaSphere exclusively reflect the opinions of the authors. The authors are responsible for all content in their abstracts including accuracy of the facts, statements, citing resources, etc.

**Results**

Seventy-five pts received IberDd; 44 (58.7%) were ≥75 years of age, 42 (56.0%) were male, and 31 (41.3%) had high-risk cytogenetics. At data cutoff (March 3, 2025), median follow-up was 22.3 months; 50 (66.7%) pts remained on therapy, with a median treatment duration of 22.4 months (median 23 cycles). Of 74 evaluable pts, 10 (13.5%) had no RI, 29 (39.2%) had mild RI, and 35 (47.3%) had moderate RI. Median treatment duration was similar across subgroups. Patients across all IBER dose levels were evenly distributed per subgroup.
Overall, ORR was 94.7% (90.0% no RI; 93.1% mild RI; 97.1% moderate RI), with a complete response or better (≥CR) achieved in 67.6% (80.0% no RI; 55.2% mild RI; 74.3% moderate RI) of patients (Figure). Minimal residual disease-negativity at any time was 80.0% (no RI), 48.3% (mild RI), and 71.4% (moderate RI). Median DOR was not reached in any subgroup.
Grade (Gr) 3/4 treatment-emergent adverse events (TEAEs) occurred in 97.3% of pts, and incidence was comparable across subgroups. Gr 3/4 neutropenia occurred in 78.4% of pts (60.0% no RI; 75.9% mild RI; 85.7% moderate RI). The proportion of pts experiencing Gr 3/4 infections was similar across groups (52.7% overall; 50.0% no RI; 58.6% mild RI; 48.6% moderate RI). Dose reductions and median dose intensity were comparable across subgroups.
PopPK analysis showed no effect of baseline CrCl and RI category on oral IBER CL/F. Logistic regression suggested no correlation between baseline CrCl and ≥CR, nor trends between baseline CrCl and Gr 3/4 neutropenia or TEAEs with ≥1 dose reduction.

**Summary/Conclusion**

RI did not impact clinical outcomes of pts with TNE NDMM receiving IberDd, suggesting that IBER dose modifications are not required for pts with mild to moderate RI. ©ASH (2025). Reused with permission.

**Copyright Information:** (Online) ISSN: 2572-9241
©2026 The Author(s). HemaSphere is published by John Wiley & Sons Ltd on behalf of European Hematology Association. This is an open access Abstract Book distributed under the Attribution-NonCommercial-NoDerivs (CC BY-NC-ND), which allows third parties to download the articles and share them with others as long as they credit the author and the Abstract Book, but they cannot change the content in any way or use them commercially.
**Abstract Book Citations:** Authors, Title, HemaSphere, 2026;10:(S1):pages. The abstract book DOIs can be found on the "Author Information" page.
**Disclaimer:** Articles published in the journal HemaSphere exclusively reflect the opinions of the authors. The authors are responsible for all content in their abstracts including accuracy of the facts, statements, citing resources, etc.

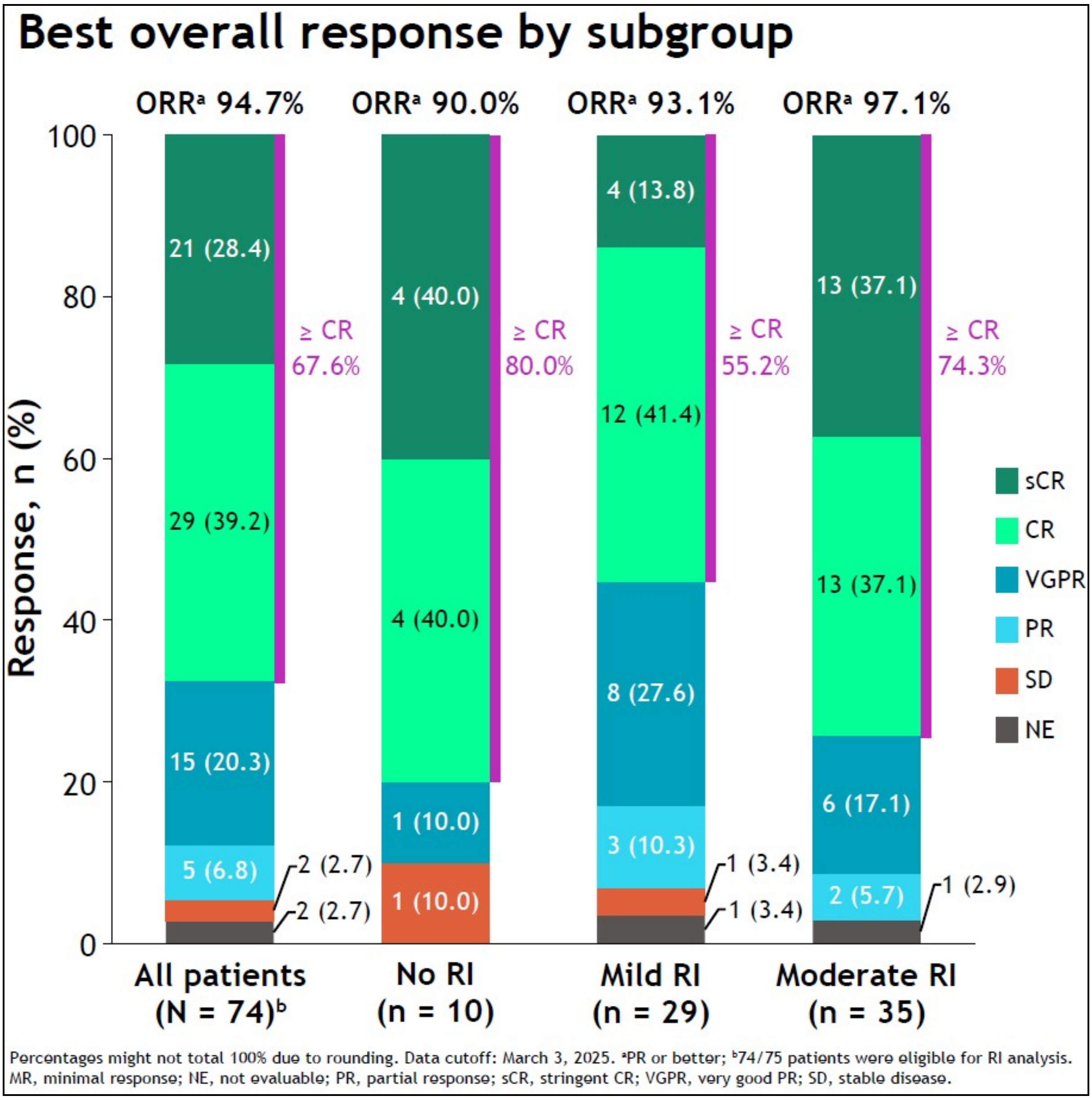

**Copyright Information:** (Online) ISSN: 2572-9241
©2026 The Author(s). HemaSphere is published by John Wiley & Sons Ltd on behalf of European Hematology Association. This is an open access Abstract Book distributed under the Attribution-NonCommercial-NoDerivs (CC BY-NC-ND), which allows third parties to download the articles and share them with others as long as they credit the author and the Abstract Book, but they cannot change the content in any way or use them commercially.
**Abstract Book Citations:** Authors, Title, HemaSphere, 2026;10:(S1):pages. The abstract book DOIs can be found on the "Author Information" page.
**Disclaimer:** Articles published in the journal HemaSphere exclusively reflect the opinions of the authors. The authors are responsible for all content in their abstracts including accuracy of the facts, statements, citing resources, etc.

### (PS1876) CIBMTR REAL-WORLD OUTCOMES OF FRAIL PATIENTS TREATED WITH IDECABTAGENE VICLEUCEL

**Topic:** 14. Myeloma and other monoclonal gammopathies - Clinical

Omar Nadeem*[1], Othman Salim Akhtar[2, 3], Ajay Nooka[4], Surbhi Sidana[5], Krina Patel[6], Marcello Pasquini[2, 3], Sophie Hello[7], Maryna Levikova-Denzler[7], Romain Piault[7], Devender Dhanda[7], Doris Hansen[8]

*[1]Dana-Farber Cancer Institute, Boston, Massachusetts, United States of America; [2]Medical College of Wisconsin, Milwaukee, Wisconsin, United States of America; [3]Center of Internal Blood & Marrow Transplant Research, Milwaukee, Wisconsin, United States of America; [4]Winship Cancer Institute, Atlanta, Georgia, United States of America; [5]Stanford University School of Medicine, Stanford, California, United States of America; [6]The University of Texas MD Anderson Cancer Center, Houston, Texas, United States of America; [7]Bristol Myers Squibb, Princeton, New Jersey, United States of America; [8]Moffitt Cancer Center, Tampa, Florida, United States of America;*

### Background

Idecabtagene vicleucel (ide-cel) is approved for patients (pts) with relapsed/refractory multiple myeloma (RRMM) after ≥ 2 prior lines of therapy. A prior real-world study (Akhtar et al. *Blood Adv.* 2025;54:212) evaluated outcomes by frailty with a median follow-up of 11.6 mo.

### Aims

Using data with longer follow-up and a higher number of pts, this analysis further assesses ide-cel effectiveness and safety in frail pts.

### Methods

This retrospective study used real-world data from the CIBMTR registry and included pts who received commercial ide-cel for RRMM between 12 May 2021 and
10 August 2023 and had the 100-day post infusion form completed. Outcomes included overall response rate (ORR), progression-free survival (PFS), overall survival (OS), and safety. Frailty was determined based on age, hematopoietic cell transplantation-specific comorbidity index, and Eastern Cooperative Oncology Group performance status (ECOG PS; score ≥ 2: frail). This same frailty definition was used by Akhtar et al, which is an adapted version of the simplified frailty index (Facon et al. *Leukemia.* 2020;34:224; Yohay et al. *Br J Haematol.* 2024;205:2370). Frailty status could not be determined for 56 of 996 pts (5.6%) as ≥ 1 components of the frailty index were missing.

**Copyright Information:** (Online) ISSN: 2572-9241
©2026 The Author(s). HemaSphere is published by John Wiley & Sons Ltd on behalf of European Hematology Association. This is an open access Abstract Book distributed under the Attribution-NonCommercial-NoDerivs (CC BY-NC-ND), which allows third parties to download the articles and share them with others as long as they credit the author and the Abstract Book, but they cannot change the content in any way or use them commercially.
**Abstract Book Citations:** Authors, Title, HemaSphere, 2026;10:(S1):pages. The abstract book DOIs can be found on the "Author Information" page.
**Disclaimer:** Articles published in the journal HemaSphere exclusively reflect the opinions of the authors. The authors are responsible for all content in their abstracts including accuracy of the facts, statements, citing resources, etc.

**Results**

Of 996 pts, 461 (46.3%) were frail. Median follow-up was 24.4 mo. Compared with non-frail pts, frail pts were older (median: 68.1 yrs vs 64.5 yrs), had higher prevalence of comorbidities (95.9% vs 61.8%), high-risk cytogenetics (26.5% vs 19.2%), and ECOG PS ≥ 2 (11.1% vs 0%). Time from diagnosis to infusion was similar between frail and non-frail pts; however, frail pts were significantly older at diagnosis than non-frail pts.
ORR (95% CI) was comparable in frail and non-frail pts (77.0% [72.9–80.8] vs 78.5% [74.5–82.1]), including VGPR+ rates of 62.3% vs 63.7%. Median PFS (95% CI) was 10.7 (9.1–12.1) mo and 11.5 (9.5–12.5) mo, respectively. At 2 yrs, 25.6% (21.2–30.1) of frail pts and 26.7% (22.5–31.1) of non-frail pts were alive and progression free. OS (95% CI) at 24 mo was 59.1% (54.1–63.7) vs 64.0% (59.2–68.4).
Grade ≥ 3 cytokine release syndrome occurred in 4.3% of frail vs 2.3% of non-frail pts. Frail pts had higher rates of grade ≥ 3 immune cell–associated neurotoxicity syndrome (ICANS; 6.7% vs 3.8%; $P$=0.04). Treatment-related mortality (95% CI) was comparable in frail vs non-frail pts at 12 mo (4.9% [3.2–7.2] vs 3.2% [1.9–5.1]) and 24 mo (6.4% [4.3–9.0] vs 5.0% [3.2–7.3]), with no evidence of a difference over the entire follow-up by Gray's test ($P$=0.29). Relapse rates were similar at both timepoints with no difference over time by Gray's test ($P$=0.94). Prolonged cytopenia occurred in 32.1% vs 25.1% ($P$=0.023) and infections in 64.9% vs 60.8% ($P$=0.22). Main causes of death in frail pts were myeloma progression (30.2%), bacterial infection (2.4%), and pulmonary failure (1.3%).

**Summary/Conclusion**

With extended follow-up, ide-cel demonstrated comparable effectiveness and broadly similar safety, except for higher ICANS, in frail and non-frail pts, supporting its use across a wide RRMM population.

**Copyright Information:** (Online) ISSN: 2572-9241
©2026 The Author(s). HemaSphere is published by John Wiley & Sons Ltd on behalf of European Hematology Association. This is an open access Abstract Book distributed under the Attribution-NonCommercial-NoDerivs (CC BY-NC-ND), which allows third parties to download the articles and share them with others as long as they credit the author and the Abstract Book, but they cannot change the content in any way or use them commercially.
**Abstract Book Citations:** Authors, Title, HemaSphere, 2026;10:(S1):pages. The abstract book DOIs can be found on the "Author Information" page.
**Disclaimer:** Articles published in the journal HemaSphere exclusively reflect the opinions of the authors. The authors are responsible for all content in their abstracts including accuracy of the facts, statements, citing resources, etc.

### (PS1877) BELANTAMAB MAFODOTIN, POMALIDOMIDE, AND DEXAMETHASONE (BPD) DEMONSTRATED IMPROVED OUTCOMES AS SECOND-LINE THERAPY VS POMALIDOMIDE, BORTEZOMIB, AND DEXAMETHASONE (PVD) IN PATIENTS WITH MULTIPLE MYELOMA

**Topic:** 14. Myeloma and other monoclonal gammopathies - Clinical

Meral Beksaç*[1], Esther González García[2], Sosana Delimpasi[3], Paweł Robak[4], Kamaraj Karunanithi[5], Felipe de Arriba de la Fuente[6], Hang Quach[7], Jakub Radocha[8], Kihyun Kim[9], Sergey Voloshin[10], Vera Zherebtsova[11], Yurii Osipov[12], Syed F. Zafar[13], Ivan Špička[14], Kazuhito Suzuki[15], Xin Du[16], Ainslee Moore[17], Eric Lewis[18], Margaret Polinkovsky[18], Kelly Brock[18], Joanna Grams[19], Elisabet E. Manasanch[18], María-Victoria Mateos[20], Suzanne Trudel[21], Meletios A. Dimopoulos[22, 23]

*[1]Department of Hematology, Adana Baskent University, Adana, Türkiye;[2]Jefa de Hematologia, Hospital Universitario Cabueñes, Gijón, Spain; [3]General Hospital Evangelismos, Athens, Greece;[4]Department of Hematology, Medical University of Łódź, Łódź, Poland; [5]Royal Stoke University Hospital, Stoke-on-Trent, United Kingdom;[6]Hematology Department, Hospital General Universitario Morales Meseguer, IMIB-Pascual Parrilla, University of Murcia, Murcia, Spain; [7]University of Melbourne, St Vincent's Hospital Melbourne, Melbourne, VIC, Australia; [8]4th Department of Internal Medicine – Hematology, University Hospital Hradec Králové, Faculty of Medicine in Hradec Králové, Charles University, Hradec Králové, Czechia; [9]Sungkyunkwan University, Samsung Medical Center, Seoul, South Korea; [10]Leningrad Regional Clinical Hospital, Saint Petersburg, Russia; [11]Gorodskaya Klinicheskaya Bol'nitsa Im. S.p. Botkina, Moscow, Russia; [12]V.A. Almazov National Medical Research Centre, Saint Petersburg, Russia; [13]Florida Cancer Specialists and Research Institute, Fort Myers, FL, United States of America; [14]First Faculty of Medicine, Charles University and General Hospital, Prague, Czechia; [15]Division of Clinical Oncology/Hematology, Department of Internal Medicine, The Jikei University School of Medicine, Tokyo, Japan; [16]Shenzhen Second People's Hospital, Shenzhen, China; [17]GSK, Stevenage, United Kingdom; [18]GSK, Collegeville, PA, United States of America; [19]GSK, Warsaw, Poland; [20]Hematology Department, University Hospital of Salamanca/IBSAL/Cancer Research Center-IBMCC (USAL-CSIC), Salamanca, Spain; [21]Department of Medical Oncology and Hematology, Princess Margaret Cancer Centre, Toronto, ON, Canada;[22]Department of Clinical Therapeutics, School of Medicine, National and Kapodistrian University of Athens, Athens, Greece; [23]Department of Medicine, Korea University, Department of Medicine, Seoul, South Korea;*

### Background

Targeting B-cell maturation antigen (BCMA) has transformed the relapsed/refractory multiple myeloma (RRMM) treatment landscape. Belantamab mafodotin represents an accessible and unique BCMA-targeting modality as an antibody-drug conjugate and was recently approved as part of a triplet therapy for RRMM. At the primary analysis of DREAMM-8 (median follow-up, 21.8 months; data cutoff [DCO]: January 29, 2024), BPd demonstrated significant improvement in progression-free survival (PFS) vs PVd in patients with RRMM who had ≥1 prior line of therapy (LOT) (hazard ratio [HR], 0.52; 95% CI 0.37-0.73, $P$<0.001). Longer-term follow-up (median, 35.8 months; DCO: July 7, 2025) found a 2.5-fold higher median PFS benefit with BPd vs PVd (32.6 vs 12.5 months; HR, 0.49; 95% CI 0.36-0.67). Herein, we assess the magnitude of BPd treatment benefit vs PVd in a subgroup of patients who only received 1 prior LOT.

### Aims

To evaluate the efficacy and safety of second-line (2L) BPd vs 2L PVd treatment in patients with RRMM.

**Copyright Information:** (Online) ISSN: 2572-9241
©2026 The Author(s). HemaSphere is published by John Wiley & Sons Ltd on behalf of European Hematology Association. This is an open access Abstract Book distributed under the Attribution-NonCommercial-NoDerivs (CC BY-NC-ND), which allows third parties to download the articles and share them with others as long as they credit the author and the Abstract Book, but they cannot change the content in any way or use them commercially.
**Abstract Book Citations:** Authors, Title, HemaSphere, 2026;10:(S1):pages. The abstract book DOIs can be found on the "Author Information" page.
**Disclaimer:** Articles published in the journal HemaSphere exclusively reflect the opinions of the authors. The authors are responsible for all content in their abstracts including accuracy of the facts, statements, citing resources, etc.

### Methods

DREAMM-8 (NCT04484623) is a phase 3, open-label, randomized trial evaluating the efficacy and safety of BPd vs PVd in patients with RRMM with ≥1 prior LOT. All patients were lenalidomide exposed and had not received prior BCMA-targeted therapy. The primary endpoint was PFS. Secondary endpoints included overall survival (OS), PFS2, duration of response (DOR), response rates, and minimal residual disease (MRD) negativity (next generation sequencing threshold of $10^{-5}$). This post hoc analysis assessed efficacy in a subgroup of patients with only 1 prior LOT.

### Results

Of the 302 patients in DREAMM-8, 159 (BPd, n=82; PVd, n=77) had received only 1 prior LOT (2L) and had a median follow-up of 36.8 months (DCO: July 7, 2025). PFS in 2L favored BPd vs PVd treatment (24-month PFS: 65% vs 36%, respectively; median not reached vs 18.4 months; HR, 0.46; 95% CI 0.29-0.72). PFS benefit was also trending in favor of BPd vs PVd across key subgroups, including patients who were lenalidomide refractory, were anti-CD38 exposed, and had high-risk cytogenetics. BPd exhibited deeper and more durable response vs PVd in 2L: complete response or better (≥ CR) rate was 51% (42/82) vs 23% (18/77), respectively; the ≥ CR-based MRD-negativity rate was 39% (32/82) vs 7% (5/77); and among patients with ≥ CR, the MRD-negativity rate was 76% (32/42) vs 28% (5/18). DOR at 24 months was 77% vs 39%, respectively. BPd benefit was also maintained following subsequent therapies (PFS2 at 24 months: 74% vs 58%; HR, 0.48; 95% CI 0.30-0.78). OS remains immature.

### Summary/Conclusion

BPd as 2L treatment in patients with RRMM demonstrated durable benefits in PFS, DOR, and PFS2, in addition to deep responses. This analysis further supports the use of BPd as early as first relapse. With BCMA-targeted treatments making notable advancements in outcomes for patients with RRMM, belamaf combinations offer both broad accessibility to durable outcomes as well as a patient-centric profile, with extended dosing intervals and outpatient administration.

**Funding statement:** This study was sponsored by GSK. Editorial support was provided by Nucleus Global, an Inizio company, and funded by GSK (study ID 207499). Drug linker technology licensed from Seagen Inc.; monoclonal antibody produced using POTELLIGENT Technology licensed from BioWa.

**Copyright Information:** (Online) ISSN: 2572-9241
©2026 The Author(s). HemaSphere is published by John Wiley & Sons Ltd on behalf of European Hematology Association. This is an open access Abstract Book distributed under the Attribution-NonCommercial-NoDerivs (CC BY-NC-ND), which allows third parties to download the articles and share them with others as long as they credit the author and the Abstract Book, but they cannot change the content in any way or use them commercially.
**Abstract Book Citations:** Authors, Title, HemaSphere, 2026;10:(S1):pages. The abstract book DOIs can be found on the “Author Information” page.
**Disclaimer:** Articles published in the journal HemaSphere exclusively reflect the opinions of the authors. The authors are responsible for all content in their abstracts including accuracy of the facts, statements, citing resources, etc.

### (PS1878) MRD-GUIDED MAINTENANCE THERAPY WITH BELANTAMAB MAFODOTIN AND LENALIDOMIDE AFTER AUTO-HCT IN NEWLY DIAGNOSED MULTIPLE MYELOMA: INTERIM ANALYSIS

**Topic:** 14. Myeloma and other monoclonal gammopathies - Clinical

Yosra Aljawai*[1], Nimisha Patel[1], Azadeh Razmandi[1], Cristina Knape[1], Ruby Delgado[1], Yago Nieto[1], Neeraj Saini[1], Samer A. Srour[1], Jeremy L. Ramdial[1], Chitra Hosing[1], Uday R. Popat[1], Betul Oran[1], Partow Kebriaei[1], Sairah Ahmed[1], Oren Pasvolsky[1], Krina Patel[1], Sheeba K. Thomas[1], Mahmoud R. Gaballa[1], Jing Christine Ye[1], Robert Z Orlowski[1], Shaheer Mannan[1], Richard E. Champlin[1], Elizabeth J. Shpall[1], Muzaffar H. Qazilbash[1], Qaiser Bashir[1]

*[1]The University of Texas MD Anderson Cancer Center, Houston, United States of America;*

### Background

Minimal residual disease (MRD) negativity predicts longer progression-free survival (PFS) in multiple myeloma (MM). Although lenalidomide maintenance improves PFS, prolonged therapy increases cumulative toxicity. MRD can guide personalized maintenance duration. Belantamab mafodotin, an anti-BCMA antibody-drug conjugate, is highly active in relapsed/refractory MM. This study evaluates MRD-guided maintenance with belantamab mafodotin plus lenalidomide after autologous hematopoietic cell transplantation (auto-HCT) in newly diagnosed MM.

### Aims

To evaluate safety and summarize early MRD-related efficacy outcomes in patients receiving belantamab mafodotin + lenalidomide as maintenance.

### Methods

This ongoing single-arm phase II study (NCT05091372) enrolls NDMM patients achieving ≥PR after auto-HCT. Patients receive belantamab mafodotin IV every 12 weeks (1.0 mg/kg with protocol-directed escalation to 1.4 mg/kg or de-escalation to 0.75 mg/kg) plus daily lenalidomide 10 mg. MRD is assessed by NGS (clonoSEQ®, sensitivity $10^{-6}$) and NGF (sensitivity $10^{-5}$). Patients with sustained MRD-negative CR by NGS after 2 years discontinue maintenance. The primary endpoint is the MRD-negative CR rate by NGS.

**Copyright Information:** (Online) ISSN: 2572-9241
©2026 The Author(s). HemaSphere is published by John Wiley & Sons Ltd on behalf of European Hematology Association. This is an open access Abstract Book distributed under the Attribution-NonCommercial-NoDerivs (CC BY-NC-ND), which allows third parties to download the articles and share them with others as long as they credit the author and the Abstract Book, but they cannot change the content in any way or use them commercially.
**Abstract Book Citations:** Authors, Title, HemaSphere, 2026;10:(S1):pages. The abstract book DOIs can be found on the "Author Information" page.
**Disclaimer:** Articles published in the journal HemaSphere exclusively reflect the opinions of the authors. The authors are responsible for all content in their abstracts including accuracy of the facts, statements, citing resources, etc.

### Results

Nineteen patients (median age 64) started maintenance; 56% (10/18) had high-risk cytogenetics (Table). No grade ≥IV AEs occurred and no patients discontinued for toxicity. Grade III AEs occurred in 47% (n=9), most commonly neutropenia (32%; n=6) and pneumonia (16%; n=3). Ocular AEs (KVA-graded) occurred in 89% (n=17): 42% (n=8) grade I and 47% (n=9) grade II; none were grade ≥III. Common grade II ocular events included blurred vision (n=6), eye pain (n=1), dry eyes (n=1), and asymptomatic keratopathy (n=1). Median time to grade II ocular events was 74 days (24–380), and median resolution to ≤grade I was 29 days (27–52). Belantamab mafodotin was held for grade II events; all but one patient resumed at a reduced dose. One patient remains on hold due to grade II BCVA decline after 114 days.

Before maintenance, MRD-negative CR rates were 37% (7/19) by NGF and 18% (3/17) by NGS. Fourteen patients (74%) completed 1 year of maintenance, with MRD-negative CR improving to 67% (8/12) by NGF and 60% (6/10) by NGS. One patient was removed after 5 cycles due to breast cancer; all others remain on study. No progression or deaths have occurred.

**Table. Patient Characteristics**

| **Characterisitcs** | **Variable** |
|---|---|
| Total (N) | 19 |
| Median age, (range) – Yr | 64 (47-75) |
| Male sex, no. (%) | 13 (68) |
| Race | |
| African American, no. (%) | 4 (21) |
| Caucasian, no (%) | 10 (53) |
| Hispanic, no (%) | 4 (21) |
| Other, no (%) | 1 (5) |
| R-ISS, no (%) | |
| Stage I | 3 (16) |
| Stage II | 11 (58) |
| Stage III | 3 (16) |
| Unknown | 2 (11) |
| Cytogenetic risk category, no (%) | |
| Standard-risk | 8 (42) |
| High-risk | 10 (53) |
| Unknown | 1 (5) |
| Induction therapy, no (%) | |
| D-VRd | 9 (47) |
| D-KRd | 4 (21) |
| D-Rd | 1 (5) |
| D-Vd | 1 (5) |
| VRd | 4 (21) |

R-ISS, Revised International Staging System; D-VRd, daratumumab, bortezomib, lenalidomide, dexamethasone; D-KRd, daratumumab, carfilzomib, lenalidomide, dexamethasone; D-Rd, daratumumab, lenalidomide, dexamethasone; D-Vd, daratumumab, bortezomib, dexamethasone; VRd, bortezomib, lenalidomide, dexamethasone

### Summary/Conclusion

Belantamab mafodotin plus lenalidomide maintenance shows promising MRD-negative rates with predictable, reversible ocular toxicity. Ocular events were frequent but limited to grade 1–2. The every-12-week belantamab schedule offers an effective, lower-burden dosing approach for patients and providers.

**Copyright Information:** (Online) ISSN: 2572-9241
©2026 The Author(s). HemaSphere is published by John Wiley & Sons Ltd on behalf of European Hematology Association. This is an open access Abstract Book distributed under the Attribution-NonCommercial-NoDerivs (CC BY-NC-ND), which allows third parties to download the articles and share them with others as long as they credit the author and the Abstract Book, but they cannot change the content in any way or use them commercially.
**Abstract Book Citations:** Authors, Title, HemaSphere, 2026;10:(S1):pages. The abstract book DOIs can be found on the “Author Information” page.
**Disclaimer:** Articles published in the journal HemaSphere exclusively reflect the opinions of the authors. The authors are responsible for all content in their abstracts including accuracy of the facts, statements, citing resources, etc.

### (PS1879) A REAL-WORLD STAGING SYSTEM FOR MYELOMA INTEGRATING AGE, PS, RENAL FUNCTION AND THE NEW HIGH-RISK IMS/IMWG SYSTEM OUTPERFORMS EXISTING MODELS: A MACHINE LEARNING STUDY BY THE GREEK MYELOMA STUDY GROUP

**Topic:** 14. Myeloma and other monoclonal gammopathies - Clinical

Eirini Katodritou*[1], Efstathios Kastritis[2], Dimitra Dalampira[1], Emmanouil Spanoudakis[3], Ioannis Ntanasis-Stathopoulos[2], Vasiliki Douka[4], Aikaterini Daioy[1], Nikolaos Kanellias[2], Elisabeth Karadrakonti[1], Panagiotis Oikonomopoulos[5], Theodosia Papadopoulou[1], Dionysios Stoumpos[6], Xanthippi Dimitriadou[1], Despina Fotiou[2], Vasiliki Tara[1], Elpiniki Rentzeperi[1], Theodora Triantafyllou[1], Deniz Tachir Oglou[1], Chrysavgi Lalagianni[4], Kyriaki Tsirou[1], Sosana Delimpasi[7], Maria Tsirogianni[5], Maria Kotsopoulou[8], Konstantinos Kostikas[9], Maria Gavriatopoulou[2], Meletios A. Dimopoulos[2], Evangelos Terpos[2]

*[1]Theagenio Cancer Hospital, Hematology-Oncology, Thessaloniki, Greece; [2]National and Kapodistrian University, School of Medicine, Athens, Clinical Therapeutics, Athens, Greece; [3]Democritus University, Alexandroupolis, Hematology, Alexandroupolis, Greece; [4]"G. Papanikolaou" General Hospital, Department of Hematology and Bone Marrow transplantation Unit, Thessaloniki, Greece; [5]Agios Savvas Cancer Hospital, Hematology, Athens, Greece; [6]Metaxa Cancer Hospital, Hematology, Athens, Greece; [7]Evangelismos General Hospital, Hematology and Bone Marrow transplantation Unit, Athens, Greece; [8]Metaxa Cancer Hospital, Hematology, Pireaus, Greece; [9]HIPA (Health Information Platform Services), Thessaloniki, Greece;*

### Background

Prognostication in multiple myeloma (MM) has improved with biologically driven models like the Revised International Staging System (R-ISS), R2-ISS and the recently proposed IMS/IMWG Consensus Genomic Staging (CGS) for High-risk (HR) MM. However, these systems often underrepresent elderly patients or patients with comorbidities (i.e. renal impairment), thereby limiting their applicability and generalizability to real-world (RW) clinical practice.

### Aims

We aimed to develop and validate a robust Real-World Staging System (RWSS) utilizing conventional statistics and machine learning (ML), explicitly accounting for patient vulnerability and tumor biology.

### Methods

We analyzed 1538 newly diagnosed MM patients from the Greek Myeloma Study Group registry (2003-2024). Traditional statistical analyses were performed to identify prognostic factors associated with progression-free survival (PFS) and overall survival (OS). The factors demonstrating the strongest prognostic impact were each assigned one point, thereby forming a Real-World Staging System (RWSS). To rigorously validate the RWSS and estimate the predictive ceiling of our dataset, we applied Random Survival Forests (RSF) across 10 repeated seed runs (70/30 train/test splits). Two ML models were predefined: (1) a strict baseline model (*RSF_10_Pred*) using only diagnosis-available clinical and cytogenetic features to avoid immortal time bias; and (2) a treatment-adjusted explanatory model (*RSF_16_Exp*) integrating clinically relevant markers, biology, and treatment/response dynamics.

**Copyright Information:** (Online) ISSN: 2572-9241
©2026 The Author(s). HemaSphere is published by John Wiley & Sons Ltd on behalf of European Hematology Association. This is an open access Abstract Book distributed under the Attribution-NonCommercial-NoDerivs (CC BY-NC-ND), which allows third parties to download the articles and share them with others as long as they credit the author and the Abstract Book, but they cannot change the content in any way or use them commercially.
**Abstract Book Citations:** Authors, Title, HemaSphere, 2026;10:(S1):pages. The abstract book DOIs can be found on the “Author Information” page.
**Disclaimer:** Articles published in the journal HemaSphere exclusively reflect the opinions of the authors. The authors are responsible for all content in their abstracts including accuracy of the facts, statements, citing resources, etc.

**Results**

The cohort's median age was 66 years (range 33-91) [M/F: 773/765; IgG: 923, IgA: 399, Light Chain: 188, IgD: 11, non-secretory: 15, IgM: 2)]. Overall, 88% received novel agents, 38% lenalidomide plus PI or daratumumab-based regimens, and 32% underwent autologous transplantation (ASCT). Over a median follow-up of 64 months, 694 (45%) patients died; median PFS and OS were 31 and 79 months, respectively. Multivariate cox regression analysis confirmed CGS-HR cytogenetics, age, eGFR, and ECOG as the strongest independent predictors. The proposed RWSS, assigning one point each to age ≥75, ECOG ≥2, eGFR <40ml/min/1.72m2, and CGS-defined HR cytogenetics, identified four distinct risk groups: low (0), low-intermediate (1), intermediate-high (2), and high (3), with superior discrimination for OS (HzRs: 0.17, 0.32, 0.55; p<0.001) and PFS (HzRs: 0.37, 0.49, 0.73; p<0.001). Compared to the established R2-ISS (OS C-index: 0.643±0.018; PFS: 0.589±0.015), the 4-point RWSS displayed significantly superior discrimination (OS C-index: 0.701±0.013; PFS: 0.631±0.020; OS AUC-12m/60m: 0.777/0.726) (Figure 1). The baseline ML model (*RSF_10_Pred*) yielded an OS C-index of 0.720±0.016, confirming that the simple RWSS captures most of the prognostic signal at diagnosis. The explanatory model (*RSF_16_Exp*) achieved the absolute highest performance (OS C-index: 0.745±0.015; OS AUC-12/36/60m: 0.804/0.776/0.771), highlighting the added predictive value of treatment and response.

**Summary/Conclusion**

Integrating CGS-defined cytogenetics with clinically relevant markers enables the development of a pragmatic staging system (RWSS) that significantly outperforms existing models in RW settings. Validated using advanced ML, the RWSS represents a robust, ready-to-implement tool for individualized risk stratification. These findings underscore that the concurrent assessment of disease biology and patient-specific characteristics is essential for accurate prognostication.

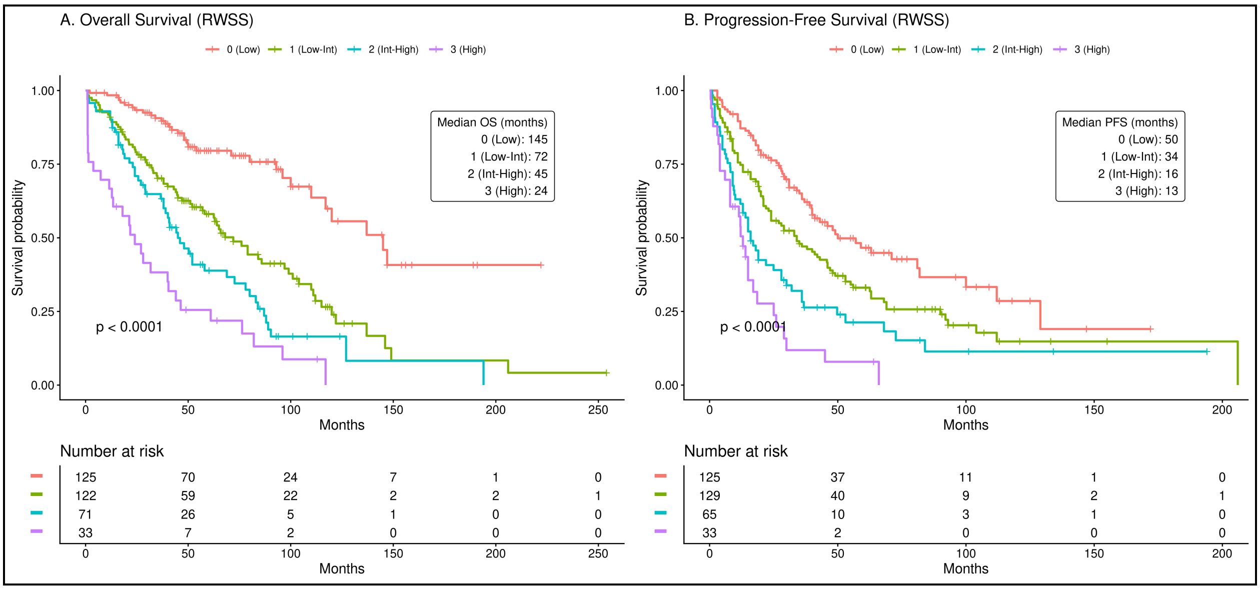

Kaplan–Meier estimates were generated for OS and PFS according to RWSS, a four-level additive score (age ≥75, ECOG ≥2, eGFR <40 mL/min/1.73m², high-risk cytogenetics; 0–3 points). Median survival was reported per stratum.

**Copyright Information:** (Online) ISSN: 2572-9241
©2026 The Author(s). HemaSphere is published by John Wiley & Sons Ltd on behalf of European Hematology Association. This is an open access Abstract Book distributed under the Attribution-NonCommercial-NoDerivs (CC BY-NC-ND), which allows third parties to download the articles and share them with others as long as they credit the author and the Abstract Book, but they cannot change the content in any way or use them commercially.
**Abstract Book Citations:** Authors, Title, HemaSphere, 2026;10:(S1):pages. The abstract book DOIs can be found on the "Author Information" page.
**Disclaimer:** Articles published in the journal HemaSphere exclusively reflect the opinions of the authors. The authors are responsible for all content in their abstracts including accuracy of the facts, statements, citing resources, etc.

### (PS1880) REAL-WORLD OUTCOMES OF ZEVORCABTAGENE AUTOLEUCEL, A FULLY HUMAN BCMA-TARGETED CAR-T THERAPY, IN 136 PATIENTS WITH MULTIPLE MYELOMA (MM): A MULTICENTER EXPERIENCE FROM CHINA

**Topic:** 14. Myeloma and other monoclonal gammopathies - Clinical

Wanyan Ouyang*[1], Zexing Fu[1], Weiping Zhang[1], Jie Xu[1], Yan Wang[1], Yi Tao[1], Yuanfang Liu[1], Yang Shen[1], Jiong Hu[1], Zhiyong Zeng[2], Bing Chen[3], Yanchao Duan[4], Rong Fu[5], Pengcheng He[6], Hongmei Jing[7], Jianping Lan[8], Yuhua Li[9], Zhenling Li[10], Guoqiang Liu[11], Yao Liu[12], Jun Luo[13], Guifang Ouyang[14], Hongling Peng[15], Wanling Sun[16], Yamin Tan[17], Jiesong Wang[18], Jing Wang[19, 19], Li Wang[20], Zheng Xu[21], Xiaojing Yan[22], Rong Zhan[23], Chunkang Chang[24], Siguo Hao[25], Songfu Jiang[26], Jian Li[27], Yonghua Li[28], Zengjun Li[29], Dai-Hong Liu[30], Ying Lu[31], Jin Lu[32], Ling Qin[33], Bing Xu[34], Lingzhi Yan[35], Ying Zhao[36], Xingbing Wang[37], Xiaoyu Zhu[37], Baijun Fang[38], Jian Hou[39], Shunqing Wang[40], Liye Zhong[41], Wenjun Zhang[42], Aibin Liang[43], Hua Wang[44, 44], Su Li[45], Chunrui Li[46], Xiaoqiang Fan[47], Juan Du[47], Guangxun Gao[48], Chong Wang[49], Zhongxing Jiang[49], Wenbin Qian[50], Heng Mei[51], Jian-qing Mi[1]

*[1]Ruijin Hospital-Shanghai Jiao Tong University School of Medicine, shanghai, China;[2]The First Affiliated Hospital of Fujian Medical University, Fujian, China; [3]Nanjing Drum Tower Hospital, The Affiliated Hospital of Nanjing University Medical School, Nanjing, China; [4]The Second Affiliated Hospital of Shandong First Medical University, Tai'an, China;[5]Tianjin Medical University General Hospital, Tianjin, China; [6]The First Affiliated Hospital of Xi'an Jiaotong University, Xi'an, China;[7]Peking University Third Hospital, Beijing, China; [8]Zhejiang Provincial People's Hospital, Hangzhou, China;[9]Zhujiang Hospital, Southern Medical University, Guangzhou, China; [10]China-Japan Friendship Hospital, Beijing, China; [11]Shengli Oilfield Central Hospital, Dongying, China; [12]Chongqing University Cancer Hospital, Chongqing, China; [13]The First Affiliated Hospital of Guangxi Medical University, Nanning, China; [14]The First Affiliated Hospital of Ningbo University, Ningbo, China;[15]The Second Xiangya Hospital of Central South University, Changsha, China; [16]Xuanwu Hospital Capital Medical University, Beijing, China; [17]Cancer Hospital of the University of Chinese Academy of Sciences (Zhejiang Cancer Hospital), Hangzhou, China; [18]FUJIAN CANCER HOSPITAL, Fuzhou, China; [19]Yangpu Hospital, Tongji University School of Medicine, Shanghai, China;[20]The First Affiliated Hospital of Chongqing Medical University, Chongqing, China; [21]Xinyang Central Hospital, Xinyang, China;[22]First Hospital of China Medical University, Shenyang, China; [23]Fujian Medical University Union Hospital, Fuzhou, China;[24]Shanghai Sixth People's Hospital Affiliated to Shanghai Jiao Tong University School of Medicine, Shanghai, China; [25]Xinhua Hospital Affiliated to Shanghai Jiaotong University School of Medicine, Shanghai, China; [26]The First Affiliated Hospital of Wenzhou Medical University, Wenzhou, China; [27]Peking Union Medical College Hospital, Chinese Academy of Medical Sciences and Peking Union Medical College, Beijing, China; [28]PLA General Hospital of Southern Theater Command, Guangzhou, China;[29]Shandong Cancer Hospital and Institute, Shandong First Medical University and Shandong Academy of Medical Sciences, Jinan, China; [30]The Fifth Medical Center, Chinese PLA General Hospital, Beijing, China; [31]Yinzhou Hospital, Affiliated to College of Medicine, Ningbo University, Ningbo, China; [32]Peking University People's Hospital, Beijing, China;[33]The First Affiliated Hospital of Henan University of Science and Technology, Luoyang, China; [34]The First Affiliated Hospital of Xiamen University, Xiamen, China;[35]The First Affiliated Hospital of Soochow University, Suzhou, China; [36]The First People's Hospital of Foshan, Foshan, China;[37]The First Affiliated Hospital of University of Science and Technology of China (Anhui Provincial Hospital), Hefei, China; [38]Henan Cancer Hospital, Zhezhou, China; [39]Renji Hospital Affiliated With Shanghai Jiao Tong University School of Medicine, Shanghai, China; [40]Guangzhou First People's Hospital, Guangzhou, China;[41]The First Affiliated Hospital of Jinan University, Guangzhou, China; [42]Tongji Hospital Affiliated to Tongji University, Shanghai, China;[43]Tongji Hospital of Tongji University, Shanghai, China;[44]Sun Yat-sen university cancer center, Guangzhou, China; [45]Gaobo Medical Group Shanghai Liquan Hospital, Shanghai, China; [46]Tongji Hospital of Tongji Medical College, Huazhong University of Science and Technology, Wuhan, China;[47]Shanghai Changzheng Hospital, Shanghai, China; [48]xijing Hospital, Shanghai, China;[49]The First Affiliated Hospital of Zhengzhou University, Zhezhou, China; [50]The Second Affiliated Hospital Zhejiang University School of Medicine, Hangzhou, China;[51]Union Hospital Tongji Medical College, Huazhong University of Science and Technology, Wuhan, China;*

**Copyright Information:** (Online) ISSN: 2572-9241
©2026 The Author(s). HemaSphere is published by John Wiley & Sons Ltd on behalf of European Hematology Association. This is an open access Abstract Book distributed under the Attribution-NonCommercial-NoDerivs (CC BY-NC-ND), which allows third parties to download the articles and share them with others as long as they credit the author and the Abstract Book, but they cannot change the content in any way or use them commercially.
**Abstract Book Citations:** Authors, Title, HemaSphere, 2026;10:(S1):pages. The abstract book DOIs can be found on the "Author Information" page.
**Disclaimer:** Articles published in the journal HemaSphere exclusively reflect the opinions of the authors. The authors are responsible for all content in their abstracts including accuracy of the facts, statements, citing resources, etc.

### Background

Zevorcabtagene Autoleucel (Zevor-cel) is a fully human BCMA-targeted CAR-T therapy. Based on its pivotal registrational clinical study (LUMMICAR-1), it received NMPA approval in February 2024 for adults with relapsed/refractory multiple myeloma (RRMM) after ≥3 prior therapies.

### Aims

This study presents, for the first time, the efficacy and safety of Zevor-cel in a real-world study in China, providing evidence-based support for its application in clinical practice.

### Methods

We conducted analysis on data from 136 patients who received infusion therapy across 51 centers between April 1, 2024 and December 31, 2025. Median follow-up was 7.5 months. All patients received a single infusion of Zevor-cel at the dose of $1.5\times10^8$ CAR-T cells/kg. Responses were evaluated per IMWG criteria, and the overall response rate (ORR) was defined as at least ≥PR.

### Results

All 136 patients underwent leukapheresis followed by Zevor-cel infusion. Median age was 63.5 years (range: 22–83), with 30.1% aged ≥70. Disease characteristics included high-risk cytogenetics (58.3%, 67/115; 21 of 136 patients had no available FISH results), extramedullary myeloma (EMM) (60.3%, 82/136), plasma cell leukemia (PCL) (14.7%, 20/136), and central nervous system (CNS) involvement (5.1%, 7/136). The median prior therapy lines were 3 (range:1–11).
In the efficacy-evaluable population (n=120; excluding 6 deaths and 1 lost to follow-up), the ORR was 90.8% (109/120), with 68.3% (82/120) achieving complete response (CR) or better. Among these patients, 8 had prior exposure to BCMA-targeted therapies, including CAR-T, BiTE, or ADC. One patient relapsed after GPRC5D-directed CAR-T therapy. Among these 9 patients, the ORR also reached 77.8% (7/9). In the EMM subgroup, ORR was 90.5% vs 95.7% in the non-EMM subgroup, with ≥CR rates 60.8% and 80.4% respectively. The ORR and CR rates varied between treatment lines, with early-line therapy (1–3 prior lines) achieving an ORR of 97.9% and a CR of 72.9%, compared to 86.1% ORR and 65.3% CR in late-line settings (3rd line or later). Patients without high-risk cytogenetics had a higher ORR rate (94% vs 87.8%).
The most common zevor-cel-related treatment-emergent adverse events (TEAEs) were cytopenias, with at least 1 grade ≥3 haematological toxicity in all patients. Hematotoxicity resolved within 30 days in most patients. Regarding the incidence of prolonged hematotoxicity, the proportions of grade 3/4 neutropenia, lymphopenia, anemia, and thrombocytopenia were 38.2%, 27.6%, 33.3%, and 34.1%, respectively. CRS of any grade was reported in 88 (73.3%) patients, with the majority classified as grade 1 and grade 2. Only 1 patient experienced grade 3 cytokine release syndrome (CRS). Immune effector cell-associated neurotoxicity syndrome (ICANS) was 5.8% (7/120), with 6 patients classified as grade 1 and 1 patient as grade 3.

### Summary/Conclusion

Zevor-cel, as a fully human BCMA-targeted CAR-T therapy, demonstrated high efficacy in 120 evaluable patients. Despite high-risk features including high-risk cytogenetics (58.3%)EMM (60.3%), PCL (14.7%), and CNS infiltration (5.1%), outcomes aligned with the pivotal LUMMICAR STUDY1(NCT03975907) trial. Safety profiles showed predominantly low-grade CRS and an extremely low incidence of ICANS. These results validate Zevor-cel as an effective therapeutic option with a potential superior safety profile, although longer follow-up is required to evaluate survival outcomes and long-term safety profiles.

**Copyright Information:** (Online) ISSN: 2572-9241
©2026 The Author(s). HemaSphere is published by John Wiley & Sons Ltd on behalf of European Hematology Association. This is an open access Abstract Book distributed under the Attribution-NonCommercial-NoDerivs (CC BY-NC-ND), which allows third parties to download the articles and share them with others as long as they credit the author and the Abstract Book, but they cannot change the content in any way or use them commercially.
**Abstract Book Citations:** Authors, Title, HemaSphere, 2026;10:(S1):pages. The abstract book DOIs can be found on the “Author Information” page.
**Disclaimer:** Articles published in the journal HemaSphere exclusively reflect the opinions of the authors. The authors are responsible for all content in their abstracts including accuracy of the facts, statements, citing resources, etc.

### (PS1881) SAFETY AND EFFICACY OF APONERMIN-BASED CHEMOTHERAPY AS BRIDGING THERAPY BEFORE CAR-T CELL TREATMENT IN RELAPSED/REFRACTORY MULTIPLE MYELOMA

**Topic:** 14. Myeloma and other monoclonal gammopathies - Clinical

Huanxin Zhang[1], Yi Wang*[2], Zhenyu Li[1], Yanli Feng[2], Yang Liu[1]

*[1]The Affiliated Hospital of Xuzhou Medical University, Xuzhou, China;[2]Shaanxi Provincial People's Hospital, Shaanxi, China;*

### Background

Chimeric antigen receptor (CAR) T-cell therapy represents a breakthrough in the treatment of relapsed or refractory (R/R) multiple myeloma (MM), however, severe cytokine release syndrome and neurotoxicity may compromise outcomes. A well-tolerated bridging therapy that controls disease without additional toxicities could improve the safety and efficacy of subsequent CAR-T infusion. Aponermin, a novel circularly permuted TRAIL ligand, induces selective apoptosis in malignant plasma cells through death receptors DR4 and DR5. The safety and efficacy of Aponermin-based regimens as bridging therapy before CAR-T treatment remain to be explored.

### Aims

The primary endpoint is overall response rate (ORR). The secondary endpoints.include-ORR.after Aponermin-based-bridging-therapy and-adverse-events.

### Methods

We performed a retrospective, three-centre cohort study of consecutive RRMM patients who received Aponermin-based chemotherapy after leukapheresis and before lymphodepletion (fludarabine 30 mg/m²/day and cyclophosphamide 300 mg/m²/day × 3 days) followed by a single BCMA- based CAR-T infusion. Safety and efficacy were assessed from the start of bridging therapy until 30 days after CAR-T administration.

### Results

A total of 30 patients were included. Median age was 55 years (range 44–81); median prior lines of therapy were 4 (range 2–7). Thirteen patients (43.3%) had prior autologous HSCT. Nineteen (63.3%) had high-risk cytogenetics and 27 (90%) had extramedullary disease (EMD). All patients were refractory to proteasome inhibitors, immunomodulatory drugs, and CD38 monoclonal antibodies. Sixteen patients received anti-BCMA CAR-T, and fourteen received BCMA/GPRC5D dual-target CAR-T. All patients received Aponermin-based bridging regimens, most commonly Apo-Td (n=12) and Apo-KTd (n=5), with other combinations administered according to prior exposure and disease characteristics.

Of the 30 patients, 25 received one cycle of bridging therapy, 3 received two cycles, and 2 patient received three cycles. After Aponermin-based bridging therapy, ORR was 26.7% (8/30), including a complete response (CR) in 6.7 % (2/30) patients and a partial response (PR) in 20.0 % (6/30) patients. Stable disease (SD) was observed in 18 (60.0%) patients, and 2 (6.7%) patients experienced disease progression after bridging therapy.

The safety profile of bridging therapy was favorable, with only one case of hypersensitivity reaction and two cases of elevated transaminases.Hematologic toxicity was manageable: grade 3–4 cytopenias occurred mainly in combination-intensive regimens (notably Apo-KTd and Apo-DECP). No grade ≥3 hepatotoxicity was observed. Importantly, 6 patients with SD after Apo-Td bridging experienced significant platelet recovery, potentially reducing bleeding risk during subsequent CAR-T therapy.

Among 27 evaluable patients after CAR-T infusion, ORR reached 85.7%, including sCR 7.4%, CR 18.5%, VGPR 7.4%, and PR 55.6%. CRS occurred in 23 patients (85.2%), with grade 3–4 CRS in 4 patients. Median PFS was 8.0 months.

**Copyright Information:** (Online) ISSN: 2572-9241
©2026 The Author(s). HemaSphere is published by John Wiley & Sons Ltd on behalf of European Hematology Association. This is an open access Abstract Book distributed under the Attribution-NonCommercial-NoDerivs (CC BY-NC-ND), which allows third parties to download the articles and share them with others as long as they credit the author and the Abstract Book, but they cannot change the content in any way or use them commercially.
**Abstract Book Citations:** Authors, Title, HemaSphere, 2026;10:(S1):pages. The abstract book DOIs can be found on the "Author Information" page.
**Disclaimer:** Articles published in the journal HemaSphere exclusively reflect the opinions of the authors. The authors are responsible for all content in their abstracts including accuracy of the facts, statements, citing resources, etc.

### Summary/Conclusion

Aponermin-based bridging therapy is well tolerated, does not exacerbate organ dysfunction or marrow suppression, and provides significant disease control or platelet recovery. These findings support prospective evaluation of Aponermin-based regimens as an effective bridge to BCMA-based CAR-T therapy in R/R MM.

**Copyright Information:** (Online) ISSN: 2572-9241
©2026 The Author(s). HemaSphere is published by John Wiley & Sons Ltd on behalf of European Hematology Association. This is an open access Abstract Book distributed under the Attribution-NonCommercial-NoDerivs (CC BY-NC-ND), which allows third parties to download the articles and share them with others as long as they credit the author and the Abstract Book, but they cannot change the content in any way or use them commercially.
**Abstract Book Citations:** Authors, Title, HemaSphere, 2026;10:(S1):pages. The abstract book DOIs can be found on the "Author Information" page.
**Disclaimer:** Articles published in the journal HemaSphere exclusively reflect the opinions of the authors. The authors are responsible for all content in their abstracts including accuracy of the facts, statements, citing resources, etc.

### (PS1882) ESTABLISHING FULLY OUTPATIENT CILTACABTAGENE AUTOLEUCEL FOR RELAPSED/REFRACTORY MULTIPLE MYELOMA: FIRST CLINICAL DATA FROM EUROPE

**Topic:** 14. Myeloma and other monoclonal gammopathies - Clinical

Tim Richardson*[1], Philipp Gödel[1], Udo Holtick[1], Christoph Scheid[1]

[1]*University hospital of cologne, Cologne, Germany;*

### Background

BCMA-directed CAR-T therapy with ciltacabtagene autoleucel (cilta-cel) is highly effective in relapsed/refractory multiple myeloma (MM) but is commonly delivered with prolonged inpatient monitoring due to concerns about cytokine release syndrome (CRS) and reimbursement issues. In Germany and most European healthcare systems, inpatient care remains standard. With increasing CAR-T volumes and pressure on hospital resources, outpatient treatment concepts are of growing interest. Data on fully outpatient CAR-T programs in Europe are limited.

### Aims

To assess feasibility, safety, and early clinical outcomes of a structured fully outpatient cilta-cel program in selected MM patients at a tertiary care center in Germany.

### Methods

Between August 2023 and June 2025, 12 patients with relapsed/refractory MM were treated with cilta-cel in a predefined outpatient pathway. Eligibility criteria included ECOG 0–1, adequate organ function, low tumor burden or good response to bridging therapy, travel time <60 minutes, and availability of a dedicated caregiver. Lymphodepleting chemotherapy and CAR-T infusion were administered outpatient. Post-infusion monitoring included in-person visits three times weekly, daily telephone contact, home monitoring of vital signs and ICE scores, and 24/7 access to CAR-T–experienced physicians. Predefined triggers for hospitalization included CRS ≥ grade 2, any ICANS, persistent fever, or absolute lymphocyte count peak $\geq 3 \times 10^9$/L. CRS and ICANS were graded per ASTCT criteria.

### Results

Median age was 61 years (range 47–73), median ECOG 0 (0–1), and patients had received a median of 2 prior lines of therapy (range 1–9). CRS occurred in 9/12 patients and was grade 1 in 8 and grade 2 in 1. No ICANS was observed. Four patients (33%) required short inpatient admission within 30 days, mainly for persistent fever or grade 2 CRS. Median length of stay was 3 days (range 1–5), resulting in 12 inpatient days in total. This compares with an estimated 192 inpatient days if routine inpatient monitoring had been applied to all patients. Tocilizumab was administered in all hospitalized patients with rapid symptom resolution. No patient required ICU care, and non-relapse mortality was 0%. Best overall response was complete response in 11 patients and stable disease in 1 patient with prior CAR-T exposure. After a median follow-up of 9.9 months, no deaths were observed.

### Summary/Conclusion

Fully outpatient administration of cilta-cel is feasible and safe in carefully selected MM patients when embedded in a structured monitoring and safety framework with a low threshold for hospitalization. This approach substantially reduced inpatient bed utilization without compromising patient safety. Outpatient CAR-T programs may represent a viable model for experienced European centers as CAR-T therapy expands.

**Copyright Information:** (Online) ISSN: 2572-9241
©2026 The Author(s). HemaSphere is published by John Wiley & Sons Ltd on behalf of European Hematology Association. This is an open access Abstract Book distributed under the Attribution-NonCommercial-NoDerivs (CC BY-NC-ND), which allows third parties to download the articles and share them with others as long as they credit the author and the Abstract Book, but they cannot change the content in any way or use them commercially.
**Abstract Book Citations:** Authors, Title, HemaSphere, 2026;10:(S1):pages. The abstract book DOIs can be found on the “Author Information” page.
**Disclaimer:** Articles published in the journal HemaSphere exclusively reflect the opinions of the authors. The authors are responsible for all content in their abstracts including accuracy of the facts, statements, citing resources, etc.

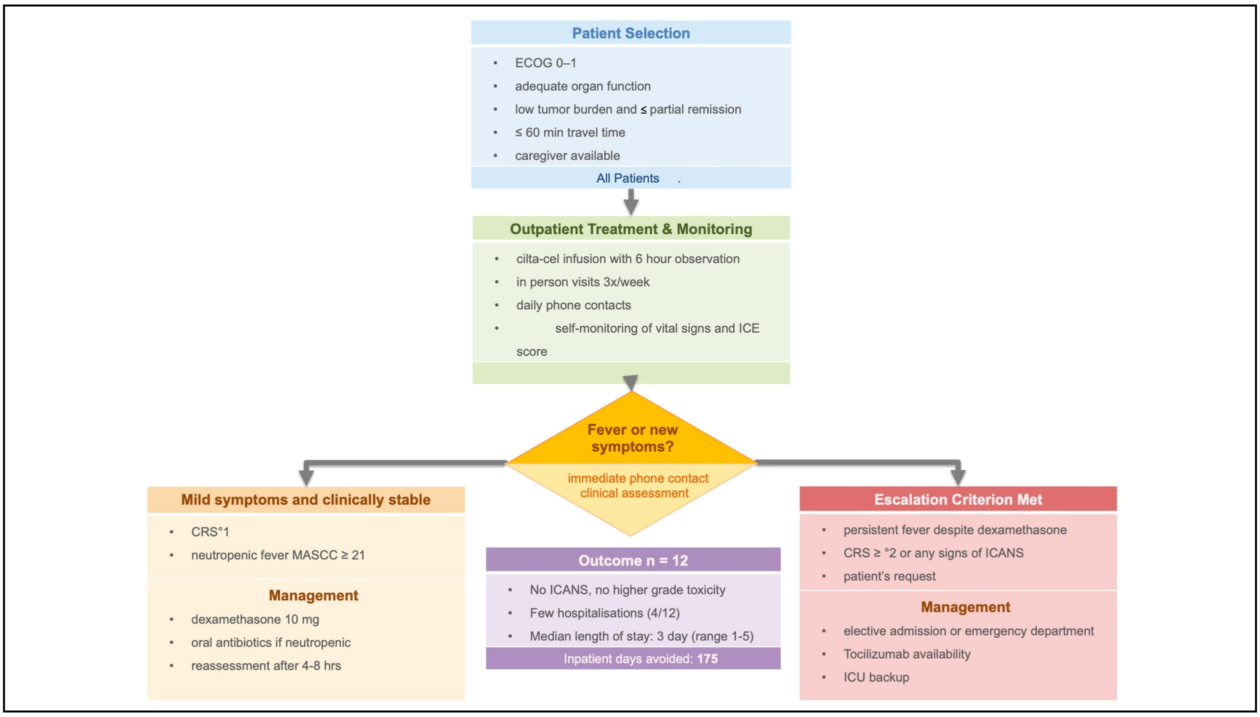

**Copyright Information:** (Online) ISSN: 2572-9241
©2026 The Author(s). HemaSphere is published by John Wiley & Sons Ltd on behalf of European Hematology Association. This is an open access Abstract Book distributed under the Attribution-NonCommercial-NoDerivs (CC BY-NC-ND), which allows third parties to download the articles and share them with others as long as they credit the author and the Abstract Book, but they cannot change the content in any way or use them commercially.
**Abstract Book Citations:** Authors, Title, HemaSphere, 2026;10:(S1):pages. The abstract book DOIs can be found on the "Author Information" page.
**Disclaimer:** Articles published in the journal HemaSphere exclusively reflect the opinions of the authors. The authors are responsible for all content in their abstracts including accuracy of the facts, statements, citing resources, etc.

### (PS1883) UPDATED RESULTS FROM RANDOMIZED PHASE II DOSE OPTIMIZATION STUDY OF INOBRODIB (CCS1477), IN COMBINATION WITH POMALIDOMIDE AND DEXAMETHASONE IN RELAPSED/REFRACTORY MULTIPLE MYELOMA (RRMM)

**Topic:** 14. Myeloma and other monoclonal gammopathies - Clinical

Nisha Joseph*[1], Victoria Louise Campbell[2], Sarah A. Holstein[3], Dan Vogl[4], Sarah Gooding[5], Heather Okervee[6], Paula Rodríguez-Otero[7], Matthew Jenner[8], Jenny O'nions[9], Thomas Creasey[10], Aristeidis Chaidos[11], Dean Edward Smith[12], Albert Oriol[13], Ceri Bygrave[14], Charlotte Pawlyn[15], Firas Al-Kaisi[16], Sally Moore[17], Gillian Brearton[18], Pablo Bedano[19], Victor Priego[20], Tim Somervaille[21], Tomasz Knurowski[22], Karen Clegg[22], Sofia Paul[22], Kristopher Frese[22], Emma Searle[21]

*[1]Emory Winship Cancer Institute, Atlanta, United States of America;[2]Western General Hospital, Edinburgh, United Kingdom; [3]University of Nebraska Medical Center, Omaha, United States of America;[4]University of Pennsylvania, Philadelphia, United States of America; [5]Oxford University Hospitals, Oxford, United Kingdom; [6]St. Barts Health NHS Trust, London, United Kingdom; [7]Universidad de Navarra, Pamplona, Spain;[8]University Hospital Southampton, Southampton, United Kingdom;[9]University College London Hospitals, London, United Kingdom; [10]Newcastle-Upon-Tyne Hospitals, Newcastle-Upon-Tyne, United Kingdom; [11]Imperial College Healthcare NHS Trust, London, United Kingdom; [12]Nottingham University Hospitals, Nottingham, United Kingdom; [13]Josep Carreras Leukaemia Research Institute, Badalona, Spain; [14]University Hospital Wales, Cardiff, United Kingdom; [15]The Royal Marsden NHS Foundation Trust, Sutton, United Kingdom; [16]Royal Derby Hospital, Derby, United Kingdom; [17]University Hospitals Bristol & Weston NHS Trust, Bristol, United Kingdom; [18]Clatterbridge Cancer Cantre, Liverpool, United Kingdom; [19]Community Health Network, Indianapolis, United States of America;[20]Center for Cancer and Blood Disorders, Bethesda, United States of America; [21]The Christie NHS Foundation Trust, Manchester, United Kingdom;[22]CellCentric Ltd., Cambridge, United Kingdom;*

### Background

Inobrodib (CCS1477) is a first-in-class, oral inhibitor of the p300/CBP bromodomains with demonstrated anti-myeloma activity and synergy with pomalidomide (Pom). In combination with Pom and dexamethasone (dex) (InoPd), it has previously shown manageable safety profile and clinical activity in heavily pretreated relapsed/refractory multiple myeloma (RRMM).

### Aims

To determine the optimal dose (RP2D) of InoPd in patients with RRMM (NCT04068597).

### Methods

Eligible RRMM patients had exhausted standard therapies or received ≥2 prior lines including lenalidomide and a proteasome inhibitor (if Pom-naïve). Patients were randomized to inobrodib 20, 30, or 40 mg taken twice daily, irrespective of food intake (4 days on/3 days off) plus Pom 4 mg (days 1–21) and age-adjusted dex (20–40 mg) in 28-day cycles. Randomization was stratified by prior Pom esposure. Adverse events (AEs) were graded per CTCAE v5.0; responses were assessed by IMWG criteria.

**Copyright Information:** (Online) ISSN: 2572-9241
©2026 The Author(s). HemaSphere is published by John Wiley & Sons Ltd on behalf of European Hematology Association. This is an open access Abstract Book distributed under the Attribution-NonCommercial-NoDerivs (CC BY-NC-ND), which allows third parties to download the articles and share them with others as long as they credit the author and the Abstract Book, but they cannot change the content in any way or use them commercially.
**Abstract Book Citations:** Authors, Title, HemaSphere, 2026;10:(S1):pages. The abstract book DOIs can be found on the "Author Information" page.
**Disclaimer:** Articles published in the journal HemaSphere exclusively reflect the opinions of the authors. The authors are responsible for all content in their abstracts including accuracy of the facts, statements, citing resources, etc.

**Results**

Enrolment has completed (63 pts total) and as of 16Feb2026 median follow-up was 217 day (range 11-427). Median age was 70 years (44–83); 78% patients had ECOG 1. Poor prognostic features, such as high risk by the IMWG Consensus Genomic Staging (37%), were noted in significant proportion of patients. Median number of prior therapies was 5 (2–17); 60% had 1-2 prior transplants, 54% were Pom-refractory, 73% triple-class refractory, 25% penta-drug refractory, and 65% had prior BCMA- and/or T-cell engager–directed therapy.
Grade 3/4 treatment-emergent AEs occurred in 71% of patients, mainly hematologic (thrombocytopenia 36%, neutropenia 34%, anemia 20%). Thrombocytopenia was the primary overlapping toxicity for inobrodib and pomalidomide. At the 20-mg dose, the frequency of G3/4 thrombocytopenia 29%, neutropenia 24% and anaemia 24% was comparable with published Pom/dex data. Non-haematological events were mostly mild or moderate, with fatigue being most frequent at 51%. Two patients (3%) discontinued treatment due to adverse event and 52% due to progressive disease.
Overall response rates (ORR) were 67%, 60%, and 41% in the 20-, 30-, and 40-mg cohorts, respectively. The lower ORR in the 40mg cohort was likely due to a higher interruption rate in the initial cycles which resulted in early discontinuations due to progression. In high unmet-need subgroup (Pom-refractory and prior BCMA/TCE), ORR was 67% at 20 and 30 mg. Median time to response was ~1 month, with deepening over time; deep responses (CR with MRD negativity) were noted in each dose level; and VGPR+ rates were 33% and 35% at 20 and 30 mg. In non–Pom-refractory patients (n=11, of which 55% were also post BCMA/TCE) at 20 mg, ORR was 91%. DOR and PFS are maturing.

**Summary/Conclusion**

InoPd shows manageable safety and encouraging activity in heavily pretreated RRMM, including patients previously treated with anti-BCMA therapy and/or T-cell engagers. The 20-mg dose provided the most favorable balance of safety and efficacy and was selected for the pivotal trials DOMMINO-1 and DOMMINO-2.

**Copyright Information:** (Online) ISSN: 2572-9241
©2026 The Author(s). HemaSphere is published by John Wiley & Sons Ltd on behalf of European Hematology Association. This is an open access Abstract Book distributed under the Attribution-NonCommercial-NoDerivs (CC BY-NC-ND), which allows third parties to download the articles and share them with others as long as they credit the author and the Abstract Book, but they cannot change the content in any way or use them commercially.
**Abstract Book Citations:** Authors, Title, HemaSphere, 2026;10:(S1):pages. The abstract book DOIs can be found on the "Author Information" page.
**Disclaimer:** Articles published in the journal HemaSphere exclusively reflect the opinions of the authors. The authors are responsible for all content in their abstracts including accuracy of the facts, statements, citing resources, etc.

### (PS1884) IMPACT OF IMMUNOMODULATORY CONTAINING HOLDING THERAPY REGIMENS ON OUTCOMES IN RELAPSED/REFRACTORY MULTIPLE MYELOMA PATIENTS RECEIVING CHIMERIC ANTIGEN RECEPTOR T-CELL THERAPY

**Topic:** 14. Myeloma and other monoclonal gammopathies - Clinical

Veena Krishnan*[1], Rishil Chopra[2], Hira Shaikh[2], Abhiraj Saxena[3], Mansi R. Shah[4], Jivtesh Singh[5], Prerna Mewawalla[5], Al-Ola Abdallah[6], Jordan Snyder[6], Zahra Mahmoudjafar[6], Jianzheng Wu[6], Emily Casteen[6], Benjamin Hane[6], William Muehlebach[6], Murtaza Shoaib[6], Lauren Scott[6], Dinesh Mudaranthakam[6], Isuru Ratnayake[6], Shebli Atrash[7], Cindy Varga[7]

*[1]Wake Forest Baptist Medical Center, Internal Medicine, Winston-Salem, United States of America;[2]University of Iowa, Hospital Medicine, Iowa City, United States of America; [3]Rutgers Health, Internal Medicine, Newark, United States of America;[4]Rutgers Cancer Institute, Hematology/Oncology, Newark, United States of America; [5]Allegheny Health Network, Internal Medicine, Pittsburgh, United States of America; [6]University of Kansas Medical Center, Department of Biostatics & Data Science, Westwood, United States of America; [7]Atrium Health Levine Cancer Institute, Cancer Medicine, Charlotte, United States of America;*

### Background

Chimeric Antigen Receptor T-Cell Therapy (CAR-T) is an effective treatment strategy for relapsed/refractory multiple myeloma (RRMM). Many patients require holding therapy (HT) prior to T-cell apheresis to maintain disease control. Immunomodulatory agents (IMiDs) have been shown to modulate the T-cell phenotype and reduce regulatory T cells, potentially affecting CAR-T efficacy. However, the impact of IMiD-containing HT on CAR-T outcomes has not been well defined.

### Aims

This retrospective study aimed to assess the impact of IMiD-containing HT regimens on Progression Free Survival (PFS), Overall Survival (OS), response to CAR-T and CAR-T associated toxicities in patients with RRMM.

### Methods

We retrospectively analyzed data from five centers participating in the U.S. Myeloma Innovations Research Collaborative (USMIRC). This study examined the records of 334 patients who received holding therapy prior to commercial CAR-T from 2021 to 2024 for RRMM. Baseline demographic and disease characteristics were collected. The primary outcome was PFS, and secondary outcomes included OS, the incidence of CRS/ICANS, ICAHT, IEC-HS and infectious complications. Statistical significance was set at $p < 0.05$. OS and PFS distributions were estimated using the Kaplan–Meier method.

**Copyright Information:** (Online) ISSN: 2572-9241
©2026 The Author(s). HemaSphere is published by John Wiley & Sons Ltd on behalf of European Hematology Association. This is an open access Abstract Book distributed under the Attribution-NonCommercial-NoDerivs (CC BY-NC-ND), which allows third parties to download the articles and share them with others as long as they credit the author and the Abstract Book, but they cannot change the content in any way or use them commercially.
**Abstract Book Citations:** Authors, Title, HemaSphere, 2026;10:(S1):pages. The abstract book DOIs can be found on the “Author Information” page.
**Disclaimer:** Articles published in the journal HemaSphere exclusively reflect the opinions of the authors. The authors are responsible for all content in their abstracts including accuracy of the facts, statements, citing resources, etc.

### Results

Among the 334 patients who received HT prior to CAR-T, 135 received an IMiD-containing regimen and 199 did not. The IMiD and non IMiD cohorts were similar in age (median: 68 years vs 66 years) and had similar mean number of prior lines of therapy (5 vs 4). The number of patients with high-risk cytogenetics was 44 (41.9%) in the IMiD cohort and 61 (58.0%) in the non-IMiD cohort. Sixty-eight (50.3%) patients in the IMiD cohort versus 86 (43.2%) in the non-IMiD cohort were penta-refractory. IMiD use was associated with a statistically significant improvement in PFS according to the restricted mean survival time. At 24 months, the IMiD group had a 3.10-month longer PFS compared to the non-IMiD group (17.05 vs 13.95 months; **p = 0.0049**). By 45 months, this difference increased to 6.44 months (25.7 vs19.26 months; **p = 0.0139**). OS differences were not statistically significant at 24 months (20.33 vs 18.86 months; **p = 0.1155**); however, at 48 months, the IMiD cohort showed a statistically significant 6.48-month OS advantage (36.24 vs 29.76 months; **p = 0.0119**). Between the IMiD and non-IMiD cohorts, rates of CRS (80% vs 75%, p = 0.3), ICANS (18% vs 21%, p = 0.4), ICAHT (14% vs 16%, p=0.6), IEC-HS (1.5% vs 3.6%, p = 0.3), infections (27% vs 30%, p = 0.6), CR/sCR rates (68% vs 70%, p = 0.2), and post-CAR-T MRD negativity status (58% vs 62%, p = 0.7) did not differ significantly.

### Summary/Conclusion

IMiD-containing HT regimens were associated with a superior PFS and OS despite comparable CR and MRD negativity rates, suggesting IMiDs may enhance T-cell quality at collection and improve durability of CAR-T outcomes. There were also no differences in CAR-T associated toxicities between the IMiD and non-IMiD cohorts.  As such, IMiD-containing HT regimens are safe and effective options associated with improved survival in RRMM patients, supporting prospective validation.

**Copyright Information:** (Online) ISSN: 2572-9241
©2026 The Author(s). HemaSphere is published by John Wiley & Sons Ltd on behalf of European Hematology Association. This is an open access Abstract Book distributed under the Attribution-NonCommercial-NoDerivs (CC BY-NC-ND), which allows third parties to download the articles and share them with others as long as they credit the author and the Abstract Book, but they cannot change the content in any way or use them commercially.
**Abstract Book Citations:** Authors, Title, HemaSphere, 2026;10:(S1):pages. The abstract book DOIs can be found on the "Author Information" page.
**Disclaimer:** Articles published in the journal HemaSphere exclusively reflect the opinions of the authors. The authors are responsible for all content in their abstracts including accuracy of the facts, statements, citing resources, etc.

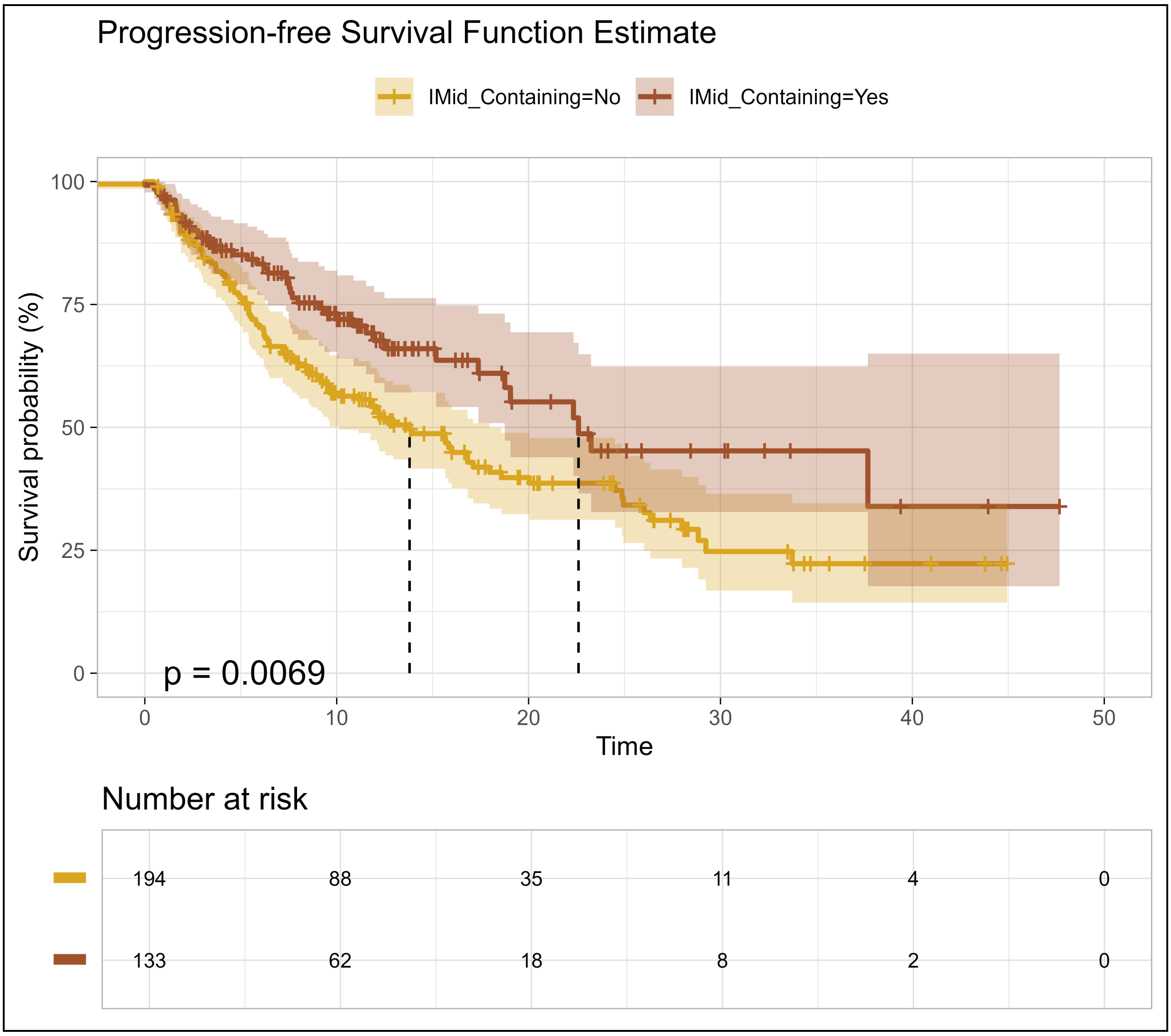

Kaplan-Meier Curves for PFS in IMiD vs Non-IMiD HT in RRMM Patients Receiving CAR-T

**Copyright Information:** (Online) ISSN: 2572-9241
©2026 The Author(s). HemaSphere is published by John Wiley & Sons Ltd on behalf of European Hematology Association. This is an open access Abstract Book distributed under the Attribution-NonCommercial-NoDerivs (CC BY-NC-ND), which allows third parties to download the articles and share them with others as long as they credit the author and the Abstract Book, but they cannot change the content in any way or use them commercially.
**Abstract Book Citations:** Authors, Title, HemaSphere, 2026;10:(S1):pages. The abstract book DOIs can be found on the "Author Information" page.
**Disclaimer:** Articles published in the journal HemaSphere exclusively reflect the opinions of the authors. The authors are responsible for all content in their abstracts including accuracy of the facts, statements, citing resources, etc.

### (PS1885) OBESITY IS AN INDEPENDENT AND MODIFIABLE RISK FACTOR FOR MONOCLONAL GAMMOPATHY OF UNDETERMINED SIGNIFICANCE AND ITS PROGRESSION TO MULTIPLE MYELOMA: A PROSPECTIVE POPULATION-BASED SCREENING STUDY

**Topic:** 14. Myeloma and other monoclonal gammopathies - Clinical

Kjartan Kristjánsson[1], Sigrún Thorsteinsdóttir[1, 2], Elías Eyþórsson[1, 3], Jón Þórir Óskarsson[1, 3], Brynjar Viðarsson[3], Páll Torfi Önundarson[1], Bjarni Agnar Agnarsson[1], Margrét Sigurðardóttir[3], Ísleifur Ólafsson[3], Ingunn Þorsteinsdóttir[3], Andri Ólafsson[1], Ásbjörn Jónsson[4], Thorvardur Jon Love[1, 3], Sigurdur Yngvi Kristinsson[1, 3], Sæmundur Rögnvaldsson*[1, 5]

*[1]University of Iceland, Faculty of Medicine, Reykjavik, Iceland;[2]Rigshospitalet, Department of Haematology, Copenhagen, Denmark; [3]Landspítali University Hospital, Reykjavik, Iceland;[4]Akureyri Hospital, Department of Radiology, Akureyri, Iceland; [5]Sahlgrenska University Hospital, Department of Haematology and Coagulation, Gothenburg, Sweden;*

### Background

Obesity and metabolic derangement have been identified as risk factors for multiple myeloma (MM). All cases of MM are preceded by the asymptomatic precursors monoclonal gammopathy of undetermined significance (MGUS) and smoldering MM (SMM). Studying these precursors is key to understanding how MM develops. In this study we present, to our knowledge the largest study to date in a screened cohort, on the association of obesity with the prevalence of these precursors and their progression, including the effect of weight loss.

### Aims

We aimed to determine the relationship between obesity and MGUS development and progression in a population-based screening study with longitudinal follow-up.

### Methods

Participants were acquired from the Iceland Screens, Treats, or Prevents Multiple Myeloma, a nationwide screening study for MGUS in Iceland which included 75,422 Icelanders over the age of 40. Two thirds of those with MGUS were randomized to active follow-up including recordings of body mass index (BMI). All participants were sent a questionnaire about their height and weight. For this study we assessed the association between BMI and the risk of MGUS using logistic regression adjusting for age and sex and other features of metabolic syndrome. Second, we assessed the association of obesity and the risk for MGUS progression using a Cox model with adjustment for age, sex, and isotype. Finally, we assessed whether changes in BMI over time affected M protein trajectory using a linear mixed effects model.

**Copyright Information:** (Online) ISSN: 2572-9241
©2026 The Author(s). HemaSphere is published by John Wiley & Sons Ltd on behalf of European Hematology Association. This is an open access Abstract Book distributed under the Attribution-NonCommercial-NoDerivs (CC BY-NC-ND), which allows third parties to download the articles and share them with others as long as they credit the author and the Abstract Book, but they cannot change the content in any way or use them commercially.
**Abstract Book Citations:** Authors, Title, HemaSphere, 2026;10:(S1):pages. The abstract book DOIs can be found on the "Author Information" page.
**Disclaimer:** Articles published in the journal HemaSphere exclusively reflect the opinions of the authors. The authors are responsible for all content in their abstracts including accuracy of the facts, statements, citing resources, etc.

### Results

Of the 75 422 participants screened in the iStopMM study, 39 633 participants had at least one recorded BMI, of whom 2,616 (6.6%) participants had an M-protein. Of these participants, 1,767 had longitudinal diagnostic data and were included in progression analysis. A total of 10,170 participants were obese (BMI≥30). Obesity was associated with a 1.33 fold increased odds of having MGUS (odds ratio (OR)=1.33; 95% confidence interval (CI): 1.21-1.45; p<0.001) after adjusting for age, sex, and other features of metabolic syndrome. This relationship was dose dependent and was seen for IgG and IgA MGUS but not IgM MGUS. Other features of metabolic syndrome, including diabetes mellitus were not associated with MGUS after adjustment for obesity. For those with MGUS, obesity at baseline was associated with an increased risk of progression to MM or other active malignancy (HR=2.59; 95%CI: 1.0-6.6; p=0.046). A total of 5,647 paired measures of M protein and BMI for 1,832 participants were available of whom 224 were diagnosed with SMM. A decrease in BMI over time was associated with a lower expected M protein concentration at 5 years (Difference of -0.29 g/L 95%CI: -0.49, -0.09; p=0.008 with a baseline BMI of 30 and ΔBMI=5). This effect was even more pronounced among those with SMM (Difference of -1.18 g/L 95%CI: -1.71, -0.66 with a baseline BMI of 30 and ΔBMI=5).

### Summary/Conclusion

In this large population-based screening study we identified obesity as a risk factor of MGUS independent of other metabolic derangement. The subsequent prospective longitudinal follow-up study identified obesity as a risk factor for MGUS progression. Finally, we showed how decreasing BMI was associated with a change in M protein trajectory. These findings indicate that obesity is an independent and, importantly, a modifiable risk factor for MGUS and its development into MM. Utilizing these findings in practice and future trials could enable disease prevention in the field of MM.

**Copyright Information:** (Online) ISSN: 2572-9241
©2026 The Author(s). HemaSphere is published by John Wiley & Sons Ltd on behalf of European Hematology Association. This is an open access Abstract Book distributed under the Attribution-NonCommercial-NoDerivs (CC BY-NC-ND), which allows third parties to download the articles and share them with others as long as they credit the author and the Abstract Book, but they cannot change the content in any way or use them commercially.
**Abstract Book Citations:** Authors, Title, HemaSphere, 2026;10:(S1):pages. The abstract book DOIs can be found on the "Author Information" page.
**Disclaimer:** Articles published in the journal HemaSphere exclusively reflect the opinions of the authors. The authors are responsible for all content in their abstracts including accuracy of the facts, statements, citing resources, etc.

**Figure: Effect of obesity, diabetes and dyslipidaemia on odds of positive screening for MGUS.**

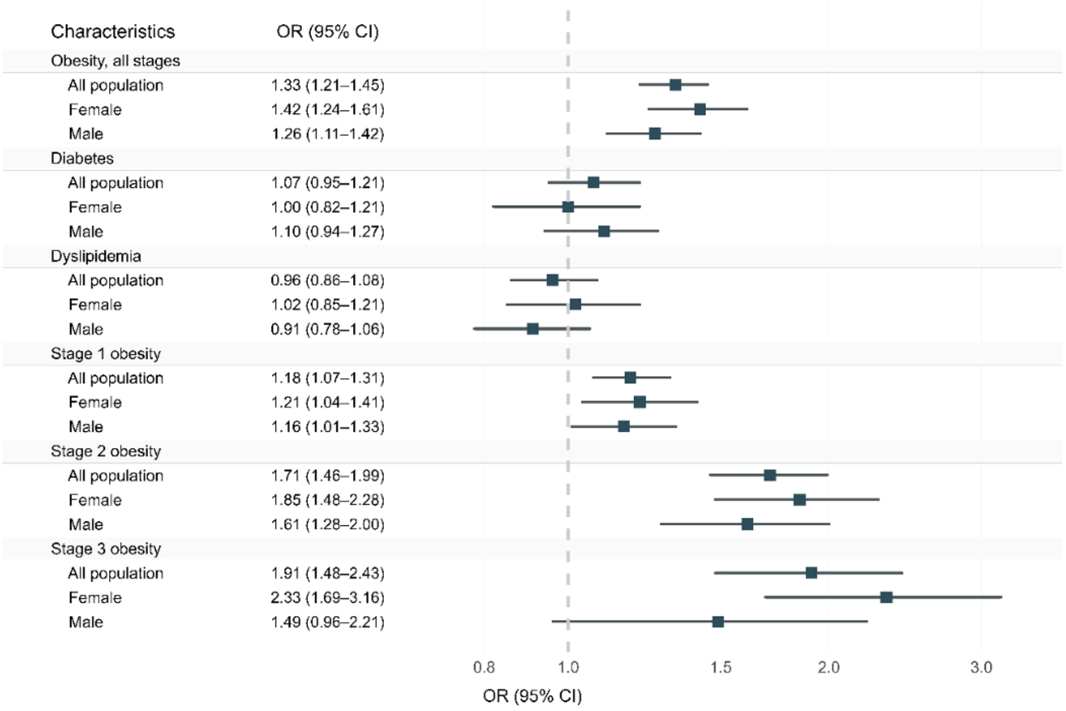

*Odds-ratios (ORs) of positive screening for MGUS, with 95% confidence intervals. Effects were calculated using a logistic-regression model with age, obesity, diabetes, and dyslipidaemia as covariates.*

**Copyright Information:** (Online) ISSN: 2572-9241
©2026 The Author(s). HemaSphere is published by John Wiley & Sons Ltd on behalf of European Hematology Association. This is an open access Abstract Book distributed under the Attribution-NonCommercial-NoDerivs (CC BY-NC-ND), which allows third parties to download the articles and share them with others as long as they credit the author and the Abstract Book, but they cannot change the content in any way or use them commercially.
**Abstract Book Citations:** Authors, Title, HemaSphere, 2026;10:(S1):pages. The abstract book DOIs can be found on the "Author Information" page.
**Disclaimer:** Articles published in the journal HemaSphere exclusively reflect the opinions of the authors. The authors are responsible for all content in their abstracts including accuracy of the facts, statements, citing resources, etc.

### (PS1886) AGE AND FRAILTY ANALYSES OF TRANSPLANT-INELIGIBLE PATIENTS WITH NEWLY DIAGNOSED MULTIPLE MYELOMA IN PHASE 3 MAIA/CEPHEUS TRIALS OF DARATUMUMAB + (BORTEZOMIB)-LENALIDOMIDE-DEXAMETHASONE

**Topic:** 14. Myeloma and other monoclonal gammopathies - Clinical

Christopher Venner[1], Sonja Zweegman[2], Shaji Kumar[3], Thierry Facon[4], Aurore Perrot[5], Philippe Moreau[6], Noopur Raje[7], Cyrille Hulin[8], Supratik Basu[9], Vania Hungria[10], Yael Cohen[11], Hartmut Goldschmidt[12], George Wang[13], Kasey Bolyard[14], Lorena Lopez-Masi[14], Matteo Loi[15], Fredrik Borgsten[14], Melissa Rowe[16], Nizar J Bahlis[17], Saad Z Usmani*[18]

*[1]Cross Cancer Institute, University of Alberta, Edmonton, and BC Cancer – Vancouver Centre, University of British Columbia, Vancouver, Canada; [2]Amsterdam UMC, Vrije Universiteit Amsterdam, Cancer Center Amsterdam, Amsterdam, The Netherlands; [3]Mayo Clinic Rochester, Rochester, United States of America;[4]University of Lille, CHU Lille, Service des Maladies du Sang, Lille, France; [5]CHU de Toulouse, IUCT-O, Université de Toulouse, UPS, Service d'Hématologie, Toulouse, France; [6]University Hospital Hôtel-Dieu, Nantes, France; [7]Massachusetts General Hospital, Harvard Medical School, Boston, United States of America; [8]Hôpital Haut Lévêque, University Hospital, Pessac, France;[9]Royal Wolverhampton NHS Trust and University of Wolverhampton, CRN West Midlands, NIHR, Wolverhampton, United Kingdom; [10]Clínica Médica São Germano, São Paulo, Brazil; [11]Tel Aviv Sourasky (Ichilov) Medical Center; Gray Faculty of Medical and Health Sciences, Tel Aviv University, Tel Aviv, Israel; [12]GMMG-Study Group at University Hospital Heidelberg, Internal Medicine, Heidelberg, Germany; [13]Johnson & Johnson, Spring House, United States of America; [14]Johnson & Johnson, Raritan, United States of America; [15]Johnson & Johnson, Leiden, The Netherlands; [16]Johnson & Johnson, High Wycombe, United Kingdom; [17]Arnie Charbonneau Cancer Research Institute, University of Calgary, Calgary, Canada; [18]Memorial Sloan Kettering Cancer Center, New York, United States of America;*

### Background

MAIA and CEPHEUS trials demonstrated that daratumumab (D) with standard of care (SOC) lenalidomide-dexamethasone (Rd) or bortezomib-Rd (VRd) significantly improved clinical outcomes versus SOC in transplant-ineligible (TIE) newly diagnosed multiple myeloma (NDMM).

### Aims

Here we describe efficacy and safety in TIE patients from both trials by age and baseline (BL) frailty using the latest data cuts.

### Methods

Patients were randomized 1:1 to DRd (n=368) or Rd (n=369) in MAIA and 1:1 to DVRd (n=144) or VRd (n=145) in CEPHEUS. Progression-free survival (PFS), complete response or better (≥CR), overall minimal residual disease (MRD) negativity (neg) at $10^{-5}$ with ≥CR, sustained MRD neg with ≥CR (confirmed MRD neg ≥12 ± 1 months [mo] apart without MRD positivity in between), and safety were assessed by age (<70, 70–<75, or ≥75 years [y]) and BL frailty (assessed by Intergroupe Francophone du Myélome [IFM] simplified frailty score: 0–1, non-frail; ≥2, frail).

**Copyright Information:** (Online) ISSN: 2572-9241
©2026 The Author(s). HemaSphere is published by John Wiley & Sons Ltd on behalf of European Hematology Association. This is an open access Abstract Book distributed under the Attribution-NonCommercial-NoDerivs (CC BY-NC-ND), which allows third parties to download the articles and share them with others as long as they credit the author and the Abstract Book, but they cannot change the content in any way or use them commercially.
**Abstract Book Citations:** Authors, Title, HemaSphere, 2026;10:(S1):pages. The abstract book DOIs can be found on the "Author Information" page.
**Disclaimer:** Articles published in the journal HemaSphere exclusively reflect the opinions of the authors. The authors are responsible for all content in their abstracts including accuracy of the facts, statements, citing resources, etc.

**Results**

Median follow-up was 64.5 mo in MAIA and 76.0 mo in CEPHEUS. In MAIA, 155 (21%) patients were <70 y, 261 (35%) 70–<75 y, and 321 (44%) ≥75 y; 341 (46%) patients were frail, 88.0% of whom were ≥70 y. In CEPHEUS TIE patients, 70 (24%) patients were <70 y, 133 (46%) 70–<75 y, and 86 (30%) ≥75 y; 83 (29%) patients were frail, 85.5% of whom were ≥70 y.
DRd or DVRd improved PFS, MRD neg rates, sustained MRD neg rates, and ≥CR rates vs Rd or VRd arms across age and frailty subgroups in both trials (Table).
Safety was consistent with established individual drug profiles, and adverse event rates were generally comparable across treatment arms and subgroups within both trials. Additional data on patient-specific factors relevant to these subgroups will be presented.

**Summary/Conclusion**

Daratumumab-based regimens improved efficacy outcomes across subgroups in MAIA and CEPHEUS trials, reinforcing daratumumab-based regimens as SOC in TIE NDMM regardless of age or frailty. These data offer clinically relevant insights to help guide treatment selection for TIE patients.
© 2026 American Society of Clinical Oncology, Inc. Reused with permission. This abstract was accepted and previously presented at the 2026 ASCO Annual Meeting. All rights reserved.

| | PFS, hazard ratio (95% CI) | | ≥CR rate, % | | MRD neg with ≥CR ($10^{-5}$) rate, % | | Sustained MRD neg with ≥CR ($10^{-5}$) rate, % | |
|---|---|---|---|---|---|---|---|---|
| **Age / Frailty status** | **MAIA*** | **CEPHEUS**** | **MAIA*** | **CEPHEUS**** | **MAIA*** | **CEPHEUS**** | **MAIA*** | **CEPHEUS**** |
| <70 y | 0.35 (0.21–0.56) | 0.60 (0.31–1.18) | 56.4 vs 31.2 | 85.7 vs 68.6 | 35.9 vs 11.7 | 74.3 vs 42.9 | 25.6 vs 3.9 | 62.9 vs 28.6 |
| 70–<75 y | 0.64 (0.45–0.89) | 0.60 (0.37–0.99) | 56.2 vs 31.3 | 77.9 vs 69.2 | 36.2 vs 12.2 | 55.9 vs 47.7 | 20.8 vs 5.3 | 45.6 vs 38.5 |
| ≥75 y | 0.59 (0.44–0.79) | 0.46 (0.22–0.94) | 44.4 vs 28.6 | 80.5 vs 44.4 | 26.9 vs 9.9 | 58.5 vs 26.7 | 13.8 vs 3.1 | 43.9 vs 15.6 |
| Frail | 0.64 (0.48–0.85) | 0.62 (0.32–1.18) | 44.8 vs 33.1 | 72.9 vs 54.3 | 25.6 vs 13.0 | 56.3 vs 34.3 | 15.7 vs 4.1 | 39.6 vs 17.1 |
| Non-frail | 0.48 (0.36–0.64) | 0.51 (0.34–0.78) | 56.5 vs 27.5 | 84.4 vs 63.6 | 37.8 vs 9.5 | 63.5 vs 41.8 | 21.4 vs 4.0 | 54.2 vs 32.7 |

*MAIA: DRd vs Rd; **CEPHEUS: DVRd vs VRd

Copyright Information: (Online) ISSN: 2572-9241
©2026 The Author(s). HemaSphere is published by John Wiley & Sons Ltd on behalf of European Hematology Association. This is an open access Abstract Book distributed under the Attribution-NonCommercial-NoDerivs (CC BY-NC-ND), which allows third parties to download the articles and share them with others as long as they credit the author and the Abstract Book, but they cannot change the content in any way or use them commercially.
Abstract Book Citations: Authors, Title, HemaSphere, 2026;10:(S1):pages. The abstract book DOIs can be found on the “Author Information” page.
Disclaimer: Articles published in the journal HemaSphere exclusively reflect the opinions of the authors. The authors are responsible for all content in their abstracts including accuracy of the facts, statements, citing resources, etc.

### (PS1887) A PROSPECTIVE STUDY OF OPTIMIZING THERAPY BASED ON DFLC REDUCTION AT WEEK 1 FOR IMPROVED OUTCOMES IN PATIENTS WITH T(11;14) AMYLOIDOSIS

**Topic:** 14. Myeloma and other monoclonal gammopathies - Clinical

Yang Liu*[1], Jin Lu[1]

*[1]Peking University People's Hospital, Hematology, Beijing, China;*

### Background

Systemic AL amyloidosis is a multi-organ damage disease caused by the deposition of misfolded proteins produced by abnormal plasma cells in the bone marrow, with the t(11;14) translocation accounting for 40%-60% of cases. Although first-line therapy has been upgraded from the CyborD regimen to the Dara-CyborD regimen and the therapeutic goal has been raised to pursuing complete response (CR), some patients still have an inadequate response to standard treatment. Moreover, early CR is crucial for reducing light chain toxicity and delaying organ failure. Previous studies by our team have confirmed that the degree of decrease in difference free light chains (dFLC) one week after treatment can predict the 6-month CR rate, and numerous studies have shown that the Bcl-2 inhibitor, venetoclax exerts a favorable efficacy in AL amyloidosis patients with the t(11;14) translocation.

### Aims

This prospective study aimed to evaluate the efficacy and safety of switching to the Daratumumab combined with Venetoclax (Dara+Vene) regimen in AL amyloidosis patients with the t(11;14) translocation who failed to reach the preset cutoff value of dFLC reduction one week after first-line Dara-CyborD/DaraBD treatment. The core objective was to improve the CR rate and long-term survival.

### Methods

A total of 22 pathologically confirmed patients with systemic AL amyloidosis were enrolled in the study, all of whom had the t(11;14) translocation, received first-line Dara-CyborD/DaraBD treatment and met the preset inclusion and exclusion criteria. The dFLC reduction was evaluated in patients one week after treatment, and those with a dFLC reduction of less than 67% or failing to achieve very good partial response (VGPR) were switched to the Dara+Vene regimen. Data including demographic characteristics, efficacy and adverse events were collected.

### Results

The median age of the patients was 66.0 years (range: 41-83 years), with males accounting for 63.6% (14/22). According to Mayo 2004 staging: stage 1, 2, 3a, 3b included 5, 9, 7, 1 cases, respectively. For Mayo 2012 staging, there were 2, 10, 5, and 5 cases in stages 1, 2, 3, and 4, respectively. Cardiac involvement was present in 77.3% of the patients (median NT-proBNP: 2441 pg/mL), and renal involvement was observed in 95.5% of the patients (median 24-hour urinary protein: 2.17 g/24h). At one week after treatment, 45.5% (10/22) of the patients were rapid responders and 54.5% (12/22) were slow responders; the median overall dFLC reduction rate at one week was 63.5% (range: -8.9% to 98.2%). The overall hematological complete response (CHR) rate reached 90.9% (20/22); the median number of cycles to achieve CHR was only 1.5. With a median follow-up of 15.9 months, one patient died of the disease and the others survived. Treatment-related adverse reactions were generally mild. Grade 3 hematological toxicities included 1 case of grade 3 lymphopenia, 1 case of anemia and 1 case of neutropenia; grade 3 non-hematological toxicities included 1 case of pneumonia.

**Copyright Information:** (Online) ISSN: 2572-9241
©2026 The Author(s). HemaSphere is published by John Wiley & Sons Ltd on behalf of European Hematology Association. This is an open access Abstract Book distributed under the Attribution-NonCommercial-NoDerivs (CC BY-NC-ND), which allows third parties to download the articles and share them with others as long as they credit the author and the Abstract Book, but they cannot change the content in any way or use them commercially.
**Abstract Book Citations:** Authors, Title, HemaSphere, 2026;10:(S1):pages. The abstract book DOIs can be found on the "Author Information" page.
**Disclaimer:** Articles published in the journal HemaSphere exclusively reflect the opinions of the authors. The authors are responsible for all content in their abstracts including accuracy of the facts, statements, citing resources, etc.

**Summary/Conclusion**

For AL amyloidosis patients with the t(11;14) translocation who have an inadequate response to first-line Dara-CyborD/DaraBD treatment at one week, timely switching to the Dara+Vene regimen can achieve a high CR rate of 90.9%. This regimen provides an effective and safe therapeutic option for AL amyloidosis patients with the t(11;14) translocation and is worthy of clinical promotion and application.

**Copyright Information:** (Online) ISSN: 2572-9241
©2026 The Author(s). HemaSphere is published by John Wiley & Sons Ltd on behalf of European Hematology Association. This is an open access Abstract Book distributed under the Attribution-NonCommercial-NoDerivs (CC BY-NC-ND), which allows third parties to download the articles and share them with others as long as they credit the author and the Abstract Book, but they cannot change the content in any way or use them commercially.
**Abstract Book Citations:** Authors, Title, HemaSphere, 2026;10:(S1):pages. The abstract book DOIs can be found on the "Author Information" page.
**Disclaimer:** Articles published in the journal HemaSphere exclusively reflect the opinions of the authors. The authors are responsible for all content in their abstracts including accuracy of the facts, statements, citing resources, etc.

### (PS1888) REAL-WORLD TREATMENT PATTERNS AND SEQUENCING OF BCMA-TARGETED THERAPIES IN RELAPSED/REFRACTORY MULTIPLE MYELOMA: A GLOBAL MULTI-CENTER ANALYSIS

**Topic:** 14. Myeloma and other monoclonal gammopathies - Clinical

Nicolas Blin*[1], Christine Maï[2], Elodie Schneider[3], Marine Leberre[3], Alexandre Raffy[3], Emma Pedrot[3], Melanie Oliveira[3]

*[1]Hôtel-Dieu, Loire Atlantique, Nantes, France; [2]APLUSA REAL WORLD, Lyon, France;[3]APLUSA, Lyon, France;*

### Background

Despite expanding therapeutic options, most patients with multiple myeloma (MM) eventually relapse. B-cell maturation antigen (BCMA)-targeted therapies—specifically chimeric antigen receptor (CAR) T-cell therapies and bispecific T-cell engagers (TCEs)—have revolutionized the treatment landscape. However, real-world data on the optimal sequencing and clinical drivers for selecting between these modalities remain limited.

### Aims

This large-scale study characterized real-world treatment patterns, sequencing, and primary determinants influencing the selection of anti-BCMA CAR T-cells versus TCEs in relapsed/refractory MM (RRMM).

### Methods

A retrospective analysis of 1,942 unique patient charts was conducted across the EU5 (France, Germany, Italy, Spain, UK), Japan, and China during 2025. The cohort included 460 patients receiving CAR T-cell products (ide-cel, cilta-cel) and 1,482 patients receiving TCEs (teclistamab, elranatamab, talquetamab). Comparative analyses focused on treatment lines, disease biology (ISS, cytogenetics), and patient fitness (age, ECOG PS, comorbidities).

### Results

The CAR T-cell cohort (n=460) had a median age of 63.6 years. Notably, 40% received CAR T-cells in early lines (2L/3L), reflecting a trend toward earlier intervention. This group was characterized by high-risk features, including high-risk cytogenetics (44%) and ISS stage III (32%), yet maintained a favorable fitness profile (85% ECOG 0-1; 66% "fit" status). Renal function was relatively preserved, with only a small minority exhibiting moderate-to-severe impairment.
In contrast, the TCE cohort (n=1,482) was significantly older (median age 68.8 years) and primarily treated in later lines (82% in 4L/5L). While ISS stage III prevalence was comparable to the CAR-T group, the TCE population included more patients with comorbidities and a higher ECOG PS (≥2). Direct comparison confirmed that CAR-T patients were significantly younger and treated earlier ($p < 0.05$ for all parameters). No major disparities were observed between the three TCEs (BCMA vs. GPRC5D) or across EU5 regions, though a trend toward older age and increased cytogenetic risk was noted in Japan.
Notably, 111 patients (6% of the total cohort) received a TCE following CAR T-cell failure, predominantly in 4L (39%) and 5L (59%). This "post-CAR T" subgroup maintained a relatively favorable performance status (78% ECOG 0-1) and a "fit" status in 54% of cases, suggesting that TCEs are a viable salvage strategy even in heavily pretreated, fit patients who have exhausted cellular therapies.

**Copyright Information:** (Online) ISSN: 2572-9241
©2026 The Author(s). HemaSphere is published by John Wiley & Sons Ltd on behalf of European Hematology Association. This is an open access Abstract Book distributed under the Attribution-NonCommercial-NoDerivs (CC BY-NC-ND), which allows third parties to download the articles and share them with others as long as they credit the author and the Abstract Book, but they cannot change the content in any way or use them commercially.
**Abstract Book Citations:** Authors, Title, HemaSphere, 2026;10:(S1):pages. The abstract book DOIs can be found on the “Author Information” page.
**Disclaimer:** Articles published in the journal HemaSphere exclusively reflect the opinions of the authors. The authors are responsible for all content in their abstracts including accuracy of the facts, statements, citing resources, etc.

**Summary/Conclusion**

These real-world data indicate that CAR T-cell therapy is preferentially allocated to younger, fitter patients in earlier lines, while TCEs serve as a versatile option for older populations and as a critical salvage strategy post-CAR T-cell exhaustion. Age, fitness, and prior exposure remain the definitive drivers of therapeutic selection in global clinical practice.

**Copyright Information:** (Online) ISSN: 2572-9241
©2026 The Author(s). HemaSphere is published by John Wiley & Sons Ltd on behalf of European Hematology Association. This is an open access Abstract Book distributed under the Attribution-NonCommercial-NoDerivs (CC BY-NC-ND), which allows third parties to download the articles and share them with others as long as they credit the author and the Abstract Book, but they cannot change the content in any way or use them commercially.
**Abstract Book Citations:** Authors, Title, HemaSphere, 2026;10:(S1):pages. The abstract book DOIs can be found on the "Author Information" page.
**Disclaimer:** Articles published in the journal HemaSphere exclusively reflect the opinions of the authors. The authors are responsible for all content in their abstracts including accuracy of the facts, statements, citing resources, etc.

### (PS1889) APHERESIS CD4/CD8 RATIO PREDICTS MOVEMENT AND NEUROCOGNITIVE TOXICITY (MNT) AFTER BCMA CAR-T CELL THERAPY IN MULTIPLE MYELOMA

**Topic:** 14. Myeloma and other monoclonal gammopathies - Clinical

Theresia Akhlaghi*[1], Mateo Mejia Saldarriaga[1], Joshua Kassner[1], David Jayabalan[1], Frank Costanzo[2], Kameisha Gulgar[2], Christian Gordillo[3], Sarah Wesley[3], Jorge Monge[4], Roger Pearse[1], Giorgio Inghirami[2], Ran Reshef[3], Ruben Niesvizky[1], Mark Bustoros[1]

*[1]Weill Cornell Medicine Hematology & Oncology, New York, United States of America;[2]Weill Cornell Medicine Pathology & Laboratory Medicine, New York, United States of America; [3]Columbia University Irving Medical Center, New York, United States of America; [4]University of Colorado Cancer Center, Aurora, United States of America;*

### Background

Movement and neurocognitive toxicity (MNT) is a serious complication of B-cell maturation antigen (BCMA)-directed chimeric antigen receptor T cell (CAR-T) therapy, manifesting as motor, cognitive, and cranial nerve deficits weeks to months after infusion. We and others have shown that maximum absolute lymphocyte count (ALC max) $\geq 3 \times 10^9$/L within the first two weeks post-infusion is associated with MNT risk, but this marker is only available after treatment. Pre-treatment predictive biomarkers are urgently needed to guide treatment decisions and enable prophylactic interventions.

### Aims

To identify predictive biomarkers at the time of apheresis and characterize the underlying immune phenotypes that correlate with the incidence of MNT.

### Methods

We retrospectively analyzed 105 patients with relapsed/refractory multiple myeloma (RRMM) treated with cilta-cel (n=87) or ide-cel (n=18) at two institutions. CD4, CD8, and NK cell counts were measured by flow cytometry at apheresis. We performed single-cell RNA sequencing in a subset of 17 patients enriched for MNT (n=6 with MNT, n=11 without) on blood mononuclear cells at apheresis. Cell type annotation was performed using Azimuth with the PBMC reference.

We used univariable logistic regression to identify risk factors for MNT. Variables with $p \leq 0.1$ were included in a multivariable model. ROC analysis identified optimal cutoffs using the Youden Index. Pseudobulk differential expression analysis was conducted using DESeq2.

**Copyright Information:** (Online) ISSN: 2572-9241
©2026 The Author(s). HemaSphere is published by John Wiley & Sons Ltd on behalf of European Hematology Association. This is an open access Abstract Book distributed under the Attribution-NonCommercial-NoDerivs (CC BY-NC-ND), which allows third parties to download the articles and share them with others as long as they credit the author and the Abstract Book, but they cannot change the content in any way or use them commercially.
**Abstract Book Citations:** Authors, Title, HemaSphere, 2026;10:(S1):pages. The abstract book DOIs can be found on the "Author Information" page.
**Disclaimer:** Articles published in the journal HemaSphere exclusively reflect the opinions of the authors. The authors are responsible for all content in their abstracts including accuracy of the facts, statements, citing resources, etc.

**Results**

Thirteen patients (12.4%) developed MNT, of whom 10 had motor involvement. Patients who developed MNT had significantly lower CD8 counts at apheresis (median 148 vs 495 cells/µL, p=0.003) and higher CD4/CD8 ratios (median 1.8 vs 0.8, p=0.0005), while CD4 counts were similar (p=0.45). ROC analysis for the CD4/CD8 ratio yielded an AUC of 0.801 with an optimal cutoff of 1.61 (sensitivity 69.2%, specificity 85.1%). This pre-infusion biomarker performed comparably to post-infusion ALC max (AUC 0.784, p=0.87). In multivariable analysis adjusting for age, prior lines of therapy, and extramedullary disease, CD4/CD8 ratio >1.6 and ALC max ≥3 x$10^9$/L were the only independent predictors of MNT with OR 7.8 (95% CI 1.66-36.54, p=0.009) and OR 6.6 (95% CI 1.35-32.4, p=0.02), respectively.

ScRNAseq analysis confirmed these findings, with MNT patients having depleted CD8 effector memory T cells (TEM: 22% vs 49% of T cells, p=0.003) and enrichment of CD4 central memory T cells (TCM: 60% vs 33% of T cells, p=0.005). Pseudobulk differential expression of CD8 TEM revealed nominally reduced expression of cytotoxic effector genes (FCRL6, CCL4L2, NKG7) and upregulation of interferon-stimulated genes (IFI44L, MX1) in MNT patients. After correction for multiple testing, CYSLTR1 (cysteinyl leukotriene receptor 1, log2FC=1.68, padj=0.01), a receptor implicated in neuroinflammation, and TMIGD2 (log2FC=2.76, padj=0.04), a T cell co-stimulatory receptor, were significantly upregulated in MNT patients.

**Summary/Conclusion**

Elevated apheresis CD4/CD8 ratio, driven by CD8 T cell depletion rather than CD4 enrichment, is a novel pre-infusion biomarker for MNT after BCMA CAR-T therapy. Notably, its predictive performance is comparable to ALC max, with the advantage of being available at the time of apheresis, rather than after infusion, which may be too late to prevent MNT. These findings may guide patient selection for CAR-T therapy and, in those who proceed, identify a high-risk population in whom prophylactic intervention for MNT can be explored.

**Copyright Information:** (Online) ISSN: 2572-9241
©2026 The Author(s). HemaSphere is published by John Wiley & Sons Ltd on behalf of European Hematology Association. This is an open access Abstract Book distributed under the Attribution-NonCommercial-NoDerivs (CC BY-NC-ND), which allows third parties to download the articles and share them with others as long as they credit the author and the Abstract Book, but they cannot change the content in any way or use them commercially.
**Abstract Book Citations:** Authors, Title, HemaSphere, 2026;10:(S1):pages. The abstract book DOIs can be found on the "Author Information" page.
**Disclaimer:** Articles published in the journal HemaSphere exclusively reflect the opinions of the authors. The authors are responsible for all content in their abstracts including accuracy of the facts, statements, citing resources, etc.

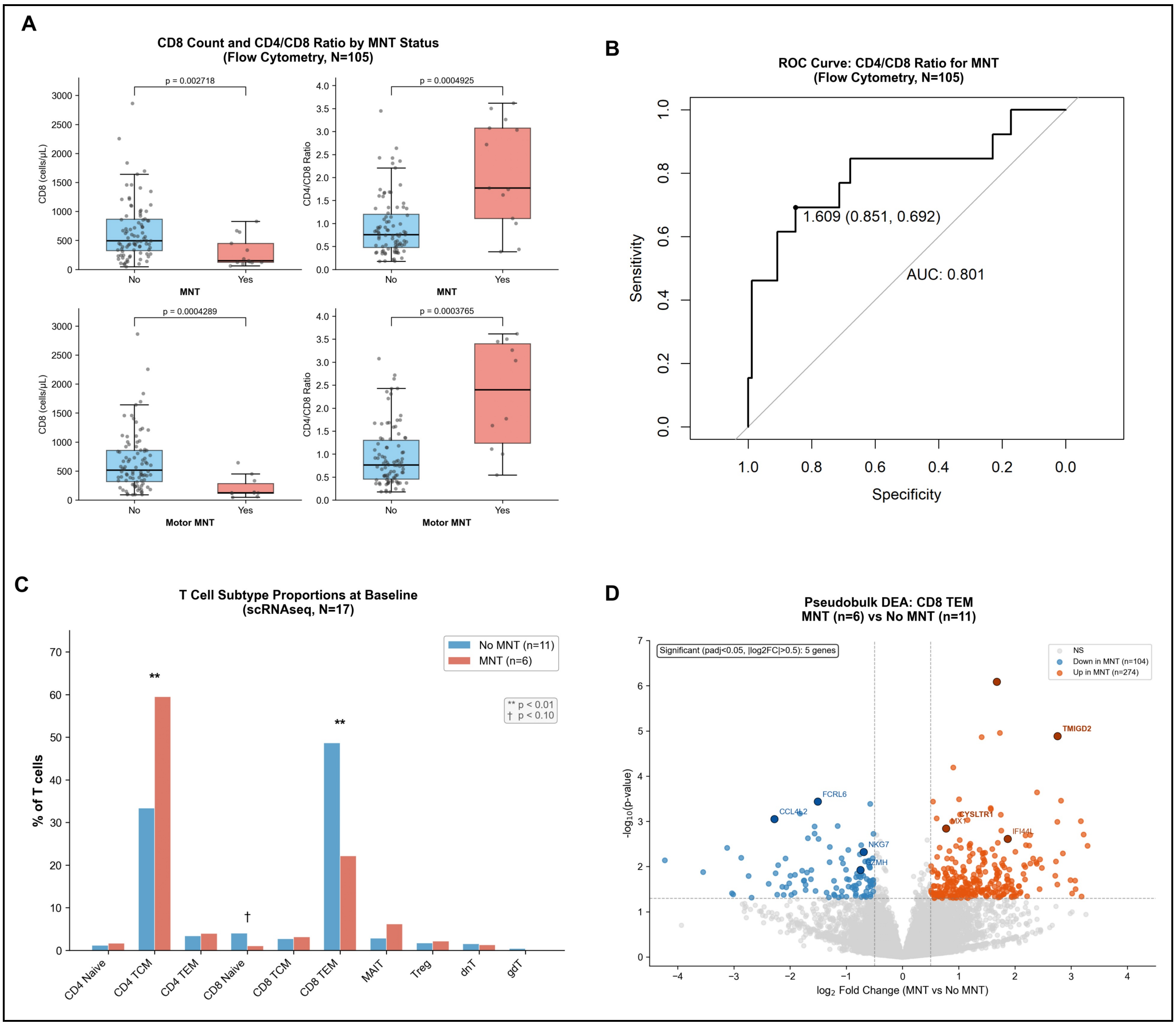

**Copyright Information:** (Online) ISSN: 2572-9241
©2026 The Author(s). HemaSphere is published by John Wiley & Sons Ltd on behalf of European Hematology Association. This is an open access Abstract Book distributed under the Attribution-NonCommercial-NoDerivs (CC BY-NC-ND), which allows third parties to download the articles and share them with others as long as they credit the author and the Abstract Book, but they cannot change the content in any way or use them commercially.
**Abstract Book Citations:** Authors, Title, HemaSphere, 2026;10:(S1):pages. The abstract book DOIs can be found on the "Author Information" page.
**Disclaimer:** Articles published in the journal HemaSphere exclusively reflect the opinions of the authors. The authors are responsible for all content in their abstracts including accuracy of the facts, statements, citing resources, etc.

### (PS1890) MITIGATING THE SEVERITY OF CILTA-CEL ASSOCIATED DELAYED NEUROTOXICITY: A COMPARATIVE ANALYSIS OF PROPHYLACTIC CYCLOPHOSPHAMIDE VERSUS DEXAMETHASONE IN HIGH-EXPANSION PATIENTS

**Topic:** 14. Myeloma and other monoclonal gammopathies - Clinical

Christoph Schaefers*[1], Melinda Tan[1], Kenneth Jin Chang Lim[1], Darin Carabenciov[1], Katharine Dooley[1], Nadine Abdallah[1], Saurabh Chhabra[2], Ricardo D. Parrondo[3], Morie Gertz[1], Taxiarchis Kourelis[1], Rahma Warsame[1], Joselle Cook[1], Moritz Binder[1], Chen Wu[1], Andre Demenezes Silva Corraes[1], Malvika Gupta[1], Zuoyi Shao[1], Kevin Regan[1], Rayaan Kamal[1], Wilson Gonsalves[1], Yadav Udit[2], P. Leif Bergsagel[2], Julia Wiedmeier-Nutor[2], Saurabh Zanwar[1], Rafael Fonseca[2], Prashant Kapoor[1], Susan Geyer[1], Sikander Ailawadhi[1], Shaji Kumar[1], Anastasia Zekeridou[1], Yi Lin[1]

*[1]Mayo Clinic, Rochester, United States of America; [2]Mayo Clinic, Phoenix, United States of America; [3]Mayo Clinic, Jacksonville, United States of America;*

### Background

Delayed neurotoxicity (DNT)—including movement and neurocognitive treatment-emergent adverse events like Parkinsonism (IEC-PKS), and cranial nerve palsies (IEC-CNP)—occurs in approximately 10–15% of myeloma patients (pts) treated with ciltacabtagene autoleucel (cilta-cel). A high post-infusion peak absolute lymphocyte count ($ALC_{peak}$ ≥3x$10^9$/L; $ALC_{hi}$) is a known biomarker for increased DNT risk (25–30%).

### Aims

While prophylactic dexamethasone (PPX DEX) does not significantly reduce DNT incidence, we evaluated whether prophylactic cyclophosphamide (PPX CTX) at $ALC_{peak}$ could mitigate expansion kinetics and improve clinical outcomes.

### Methods

We conducted a retrospective analysis of 407 patients treated with cilta-cel at Mayo Clinic. The study compared 3 cohorts: historical (n=230) with no prophylactic treatment for $ALC_{hi}$ (n=98, 43%); PPX DEX cohort (n=120) where pts with $ALC_{hi}$ received DEX 10mg twice a day for at least three days (n=54, 45%), and a PPX CTX cohort (n=57) where pts with $ALC_{hi}$ received 500 mg IV/PO of cyclophosphamide once (n=23, 40%). Clinical outcomes and ALC kinetics—including $ALC_{peak}$, Day 28 ALC ($ALC_{D28}$), and Area Under the Curve ($ALC_{AUC}$) from days 0–28—were evaluated and compared among the 3 cohorts. Whole blood cellular immune composition were assessed by flow.

**Copyright Information:** (Online) ISSN: 2572-9241
©2026 The Author(s). HemaSphere is published by John Wiley & Sons Ltd on behalf of European Hematology Association. This is an open access Abstract Book distributed under the Attribution-NonCommercial-NoDerivs (CC BY-NC-ND), which allows third parties to download the articles and share them with others as long as they credit the author and the Abstract Book, but they cannot change the content in any way or use them commercially.
**Abstract Book Citations:** Authors, Title, HemaSphere, 2026;10:(S1):pages. The abstract book DOIs can be found on the "Author Information" page.
**Disclaimer:** Articles published in the journal HemaSphere exclusively reflect the opinions of the authors. The authors are responsible for all content in their abstracts including accuracy of the facts, statements, citing resources, etc.

**Results**

Pre-LD (lymphodepletion) characteristics including disease burden were across cohorts, although the PPX DEX cohort was slightly older (p=0.01). CRS and ICANS incidence and severity were similar among cohorts. PPX CTX was well-tolerated. Among $ALC_{hi}$ pts, there were no statistically significant differences among cohorts in median $ALC_{peak}$ (p=0.6), $ALC_{D28}$ (p=0.2) and $ALC_{AUC}$ (p=0.2). No significant differences in CD4/CD8 ratio or CAR-T were observed among the historical, PPX DEX, and PPX CTX cohorts. $ALC_{peak}$ correlated with CAR-T expansion; however, CAR-T contraction was pronounced by month 1 in no DNT pts, whereas CAR-T persisted or continued to expand in the DNT pts. Among the DNT pts, 63% (5/8) of IEC-CNP had CD4 expansion (CD4:CD8>1); 89% (8/9) of IEC-PKS had CD4 expansion. Among $ALC_{hi}$ cohorts, DNT rate was comparable among the groups (historical cohort: 25%, 14 IEC-CNP, 10 IEC-PKS; PPX DEX: 19%, 8 IEC-CNP, 2 IEC-PKS; PPX CTX: 22%, 1 IEC-CNP, 4 IEC-PKS). However, IEC-PKS presentations in the PPX CTX group were milder: 3/4 pts presented word finding difficulties and flat affect without other features of IEC-PKS. Notably, all 4 pts with IEC-PKS remained alive to date and had symptom improvement or resolution with treatment, including 2/4 (50%) treated with steroid and IVIG only. This is in contrast with 50-60% mortality from IEC-PKS in other cohorts.

**Summary/Conclusion**

Prophylactic CTX is a safe strategy that does not appear to reduce ALC expansion or the incidence of DNT. However, the initial presentation of IEC-PKS appears milder and more responsive to treatment with reduced mortality with PPX CTX.

**Copyright Information:** (Online) ISSN: 2572-9241
©2026 The Author(s). HemaSphere is published by John Wiley & Sons Ltd on behalf of European Hematology Association. This is an open access Abstract Book distributed under the Attribution-NonCommercial-NoDerivs (CC BY-NC-ND), which allows third parties to download the articles and share them with others as long as they credit the author and the Abstract Book, but they cannot change the content in any way or use them commercially.
**Abstract Book Citations:** Authors, Title, HemaSphere, 2026;10:(S1):pages. The abstract book DOIs can be found on the “Author Information” page.
**Disclaimer:** Articles published in the journal HemaSphere exclusively reflect the opinions of the authors. The authors are responsible for all content in their abstracts including accuracy of the facts, statements, citing resources, etc.

### (PS1891) FIRST INDEPENDENT REAL-WORLD EVIDENCE FROM TWO NATIONWIDE COMPASSIONATE-USE PROGRAMS FOR BELANTAMAB MAFODOTIN-BASED COMBINATION THERAPY IN MULTIPLE MYELOMA

**Topic:** 14. Myeloma and other monoclonal gammopathies - Clinical

Julia Mersi[1], Natalie Schub[2], Maximilian Alexander Funk[3], Romans Zukovs[4], Marion Hoegner[5], Bernhard Heilmeier[6], Zuzana Rethmann[7], Udo Holtick[8], Britta Besemer[9], Julia Meyne[10], Andreas Hilbig-Cordes[11], Karin Schmitz[12], Giulia Graziani[13], Susanne Strifler[14], Anca-Maria Albici[2], Maximilian Al-Bazaz[15], Kerstin Brinkert[16], Margarethe Schmier[17], Angeliki Giesecke[4], Moritz Fürstenau[8], Lydia Bussemer[1], Carolin Blasberg[1], Julia Noderer[1], Yoko Tamamushi[1], Torsten Steinbrunn[1], Lisa Leypoldt[15], Katja C. Weisel[15], Hermann Einsele[1], Leo Rasche[1], K Martin Kortum[1], Sebastian Theurich[3], Stefanie Lemnitz[17], Christoph Schaefers[15], Annamaria Brioli[16], Johannes Waldschmidt*[1]

*[1]University of Würzburg Medical Center, Würzburg, Germany; [2]University Hospital of Schleswig-Holstein Campus Kiel, Kiel, Germany; [3]LMU University Hospital, Munich, Germany; [4]Universitätsklinikum Düsseldorf, Düsseldorf, Germany; [5]TUM Klinikum Rechts der Isar, Munich, Germany; [6]Krankenhaus Barmherzige Brüder, Regensburg, Germany; [7]MVZ Westerwald, Dernbach, Germany; [8]Universitaetsklinikum Koeln, Köln, Germany; [9]Universitaetsklinikum Tübingen, Tübingen, Germany; [10]Asklepios Klinik St. Georg, Hamburg, Germany; [11]St. Barbara- Klinik, Hamm, Germany; [12]Ev. Diakonie-Krankenhaus, Bremen, Germany; [13]Robert-Bosch-Krankenhaus, Stuttgart, Germany; [14]Klinikum Nürnberg, Nürnberg, Germany; [15]Universitätsklinikum Hamburg-Eppendorf, Hamburg, Germany; [16]Medizinische Hochschule Hannover, Hannover, Germany; [17]Städtisches Klinikum Karlsruhe, Karlsruhe, Germany;*

### Background

Belantamab mafodotin (belamaf)- based combination therapy has shown promising efficacy in relapsed/refractory multiple myeloma (RRMM), but its feasibility remains uncertain due to a lack of real-world data on optimal sequencing, dosing and toxicity management.

### Aims

This multicenter observational study investigated n=60 RRMM patients (pts) treated as part of two nationwide compassionate-use programs at 17 academic centers and local practices in Germany between 05-09/2020 and 01/2025-02/2026.

### Methods

Pts were eligible irrespective of prior BCMA exposure. Treatment followed DREAMM-2/7/8 trial regimens (belamaf mono, belamaf-bortezomib-dexamethasone (BVd), belamaf-pomalidomide-dexamethasone (BPd)) with modifications according to institutional practice. Responses were assessed by IMWG criteria and adverse events were graded per CTCAE v5.0.

**Copyright Information:** (Online) ISSN: 2572-9241
©2026 The Author(s). HemaSphere is published by John Wiley & Sons Ltd on behalf of European Hematology Association. This is an open access Abstract Book distributed under the Attribution-NonCommercial-NoDerivs (CC BY-NC-ND), which allows third parties to download the articles and share them with others as long as they credit the author and the Abstract Book, but they cannot change the content in any way or use them commercially.
**Abstract Book Citations:** Authors, Title, HemaSphere, 2026;10:(S1):pages. The abstract book DOIs can be found on the "Author Information" page.
**Disclaimer:** Articles published in the journal HemaSphere exclusively reflect the opinions of the authors. The authors are responsible for all content in their abstracts including accuracy of the facts, statements, citing resources, etc.

**Results**

Median age was 68.7 (range 39.4–88.1) years. High disease burden was common, including extramedullary disease (36.7%), high-risk cytogenetics (36.7%), advanced R-ISS stage (≥II in 85.0%) and a median number of prior lines of therapy (LOT) of 5 (1-15) across the entire cohort. Pts were grouped into i) a focus population of BVd/ BPd treated pts with prior LOT ≤5 (n=31; median 3 (1-5)), ii) BVd/ BPd treated end-stage pts defined by prior LOT ≥6 (n=14; median 9 (6-15)) and iii) belamaf monotherapy treated pts (n=15, median prior LOT 6 (3-11)). Triple-class refractory status and prior exposure to anti-BCMA therapy were observed in 64.5% and 9.7% of pts in the focus group vs. 100% and 92.9% in end-stage pts. The median number of applied belamaf doses was 3 (1-21), 2 (1-7) and 2 (1-18) for focus, end-stage and monotherapy pts, respectively. Combination therapy enabled longer treatment spacing with median intervals between consecutive cycles of 43.5 (28-62) in focus vs. 28 (27-29) in end-stage and 27 (21-35) days in monotherapy pts. Grade 3/4 treatment-related neutropenia, anemia and thrombocytopenia were noted in 21.7%, 23.3% and 36.7% of all pts, respectively, with no particular safety signals for BVd/ BPd-treated focus pts (19.4%, 25.8%, 35.5%), despite the longest treatment exposure across all subgroups. Grade 3/4 infections were observed in 16.7% and were highest in monotherapy pts (26.7%). IVIG use was required in 11/14 (78.6%) end-stage pts, but only in 7/31 (22.6%) focus pts, consistent with prior exposure to anti-BCMA bsAb or CAR-T as main drivers of hypogammaglobulinemia. Clinically significant keratopathy occurred in 27/60 (45%) pts, and was lowest in end-stage pts (14.3%), likely reflecting shorter exposure and underreporting in this heavily pre-treated subcohort. No pt had to stop therapy due to ocular events. Across the entire program, the overall response rate (ORR) and clinical benefit rate (CBR) were 51.7% and 63.3%, respectively, with highest efficacy in the focus subgroup (ORR 71.0%, CR 6.5%, unconfirmed ≥VGPR 32.3%, *Fig. 1*). Median progression-free survival (PFS) was 2.3 mos for end-stage and 1.4 mos for monotherapy pts compared to a 72.1% 6-mos PFS rate in the focus pt subgroup (*P*=0.0006). Subsequent therapy was given in 21/60 (35.0%) of pts, mostly consisting of anti-GPRC5D/ BCMA combinatorial approaches.

**Summary/Conclusion**

This first real-world analysis demonstrates that the EMA-approved triplet therapies BVd and BPd are feasible and effective in moderately pretreated MM. Our data support early use, extended dosing intervals and low need for IVIG in BCMA-naïve pts.

**Copyright Information:** (Online) ISSN: 2572-9241
©2026 The Author(s). HemaSphere is published by John Wiley & Sons Ltd on behalf of European Hematology Association. This is an open access Abstract Book distributed under the Attribution-NonCommercial-NoDerivs (CC BY-NC-ND), which allows third parties to download the articles and share them with others as long as they credit the author and the Abstract Book, but they cannot change the content in any way or use them commercially.
**Abstract Book Citations:** Authors, Title, HemaSphere, 2026;10:(S1):pages. The abstract book DOIs can be found on the “Author Information” page.
**Disclaimer:** Articles published in the journal HemaSphere exclusively reflect the opinions of the authors. The authors are responsible for all content in their abstracts including accuracy of the facts, statements, citing resources, etc.

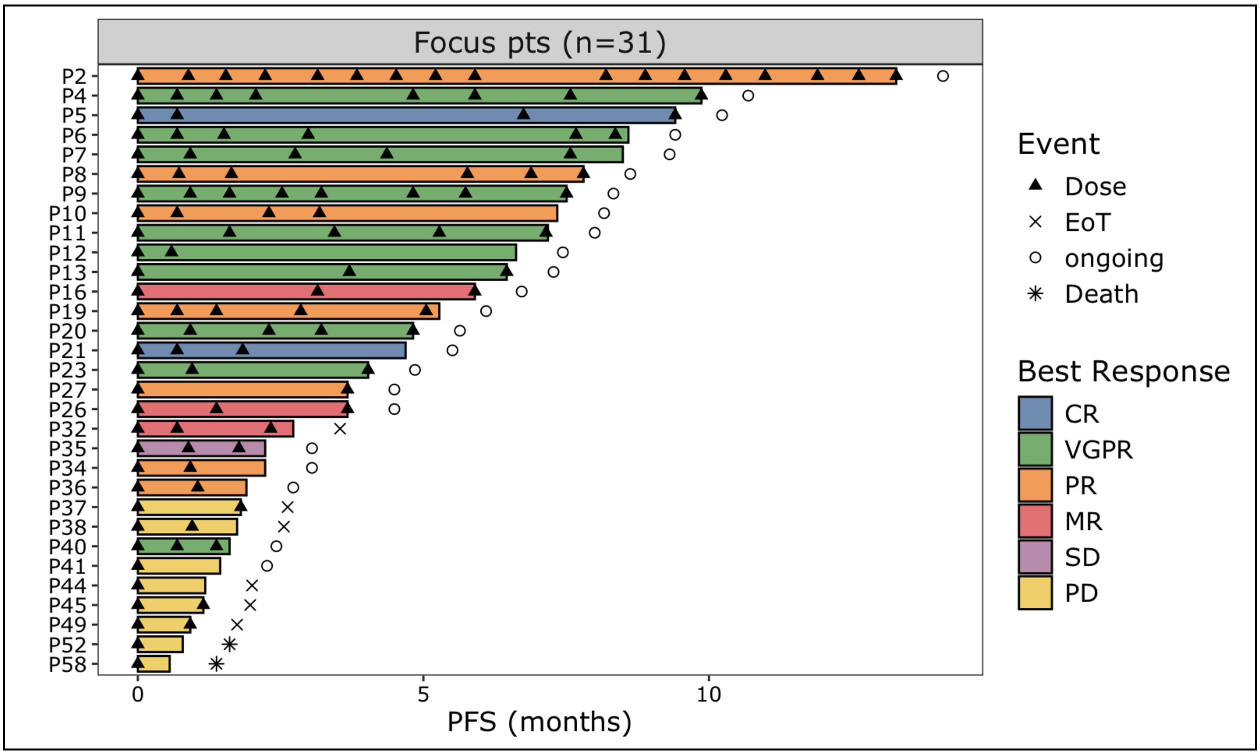

Figure 1. Progression-free survival and dosing intervals in BVd/ BPd focus patient subgroup (n=31)

**Copyright Information:** (Online) ISSN: 2572-9241
©2026 The Author(s). HemaSphere is published by John Wiley & Sons Ltd on behalf of European Hematology Association. This is an open access Abstract Book distributed under the Attribution-NonCommercial-NoDerivs (CC BY-NC-ND), which allows third parties to download the articles and share them with others as long as they credit the author and the Abstract Book, but they cannot change the content in any way or use them commercially.
**Abstract Book Citations:** Authors, Title, HemaSphere, 2026;10:(S1):pages. The abstract book DOIs can be found on the “Author Information” page.
**Disclaimer:** Articles published in the journal HemaSphere exclusively reflect the opinions of the authors. The authors are responsible for all content in their abstracts including accuracy of the facts, statements, citing resources, etc.

### (PS1892) SAFETY AND EFFICACY OF FIRST-LINE SELINEXOR PLUS BORTEZOMIB, LENALIDOMIDE AND DEXAMETHASONE (XVRD) IN HIGH RISK NEWLY DIAGNOSED MULTIPLE MYELOMA: A MULTI-CENTER, SINGLE-ARM, PHASE IB/II TRIAL

**Topic:** 14. Myeloma and other monoclonal gammopathies - Clinical

Yun Wang[1], Xiaoqin Chen[1], Zhijian Liang[1], Weida Wang[1], Hua Wang[1], Bingyi Wu[1], Yang Liang[1], Zhongjun Xia*[1]

*[1]State Key Laboratory of Oncology in South China, Guangdong Provincial Clinical Research Center for Cancer, Sun Yat-Sen University Cancer Center, Department of Hematological Oncology, Guangzhou, China;*

### Background

High-risk (HR) factors for multiple myeloma (MM) include del(17p), t (4; 14), t (14; 16), p53 mutation, gain/amp 1q, R-ISS stage 3, High Plasma Cell S-phase and high-risk GEP. Newly diagnosed MM (NDMM) harboring HR factors often presents with with characteristics of early relapse or primary refractoriness, and there is no unified treatment standard yet. XPO1 protein overexpression in MM leads to nuclear export of tumor suppressor proteins and glucocorticoid receptors, facilitating oncogene translation. Selinexor, a selective XPO1 inhibitor, exerts anti-myeloma effects.

### Aims

This study aimed to evaluate the safety and efficacy of selinexor plus VRd as a first-line treatment for NDMM patients with HR factors (NCT05422027).

### Methods

Phase Ib objectives included determining the recommended phase II dose (RP2D) and maximum tolerated dose (MTD) of selinexor. Dose expansion at the RP2D were designed to evaluate efficacy, including complete response (CR) rate, overall response rate (ORR), very good partial response (VGPR) rate, minimal residual disease (MRD) negativity rate, safety and tolerability. Patients received the XVRd regimen (selinexor 40/60 mg on d1,8,15, bortezomib 1.3mg/m$^2$ on d1,4,8,11, lenalidomide 25 mg on d1-14, dexamethasone 20 mg/d on d1,2,8,9,15,16 or 40 mg on d1,8,15) as induction therapy for 4 cycles (21 days/cycle). Following induction, patients undergoing autologous stem cell transplantation (ASCT) received 2-4 cycles of XVRd consolidation; whlie non-ASCT patients received total 8 cycles of XVRd consolidation. All patients received XR as maintenance therapy until disease progression, death, intolerance or other discontinuation reasons.

**Copyright Information:** (Online) ISSN: 2572-9241
©2026 The Author(s). HemaSphere is published by John Wiley & Sons Ltd on behalf of European Hematology Association. This is an open access Abstract Book distributed under the Attribution-NonCommercial-NoDerivs (CC BY-NC-ND), which allows third parties to download the articles and share them with others as long as they credit the author and the Abstract Book, but they cannot change the content in any way or use them commercially.
**Abstract Book Citations:** Authors, Title, HemaSphere, 2026;10:(S1):pages. The abstract book DOIs can be found on the “Author Information” page.
**Disclaimer:** Articles published in the journal HemaSphere exclusively reflect the opinions of the authors. The authors are responsible for all content in their abstracts including accuracy of the facts, statements, citing resources, etc.

### Results

Between July 2022 and February 2026, 30 NDMM patients were enrolled, with a median age of 55.5 years (range:40-71). All patients had HR cytogenetic abnormalities, with 83.3% exhibiting 1q21 gain/amp,10.0% exhibiting P53 deficiency, 23.3% exhibiting t(4;14) variant, 20.0% exhibiting 2 cytogenetic abnormalities. The distribution of free light chain (FLC) was 73.3% κ-type and the 26.7% λ-type. Extramedullary diseases (EMD) were present in 33.3% of patients.
In the phase Ib study, the following selinexor dosages were administered: 40 mg qw (n=4) and 60 mg qw (n=3). No dose-limiting toxicities (DLTs) occurred. The MTD and RP2D were established as selinexor 60 mg qw.
By February 2026, 30 patients were evaluable for response. The ORR was 96.7%, with CR/sCR, ≥VGPR, and PR rates of 46.7%, 90.0%, and 6.7%, respectively. The 12-month PFS and OS rate was 96.0% and 100%, respectively. Among MRD evaluable patients, the MRD negativity rate was 63.6%. The most common grade 3/4 treatment-related AE (TRAE) were thrombocytopenia (70.0%), neutropenia (53.3%), and peripheral neuropathy (30.0%). All TRAEs were manageable with supportive care and dose modifications.

| Baseline characteristics | All patients (n=30) |
|---|---|
| Median age, years (range) | 55.5 (40-71) |
| Sex, no.(%) | |
| Male | 17 (56.7%) |
| Female | 13 (43.3%) |
| R-ISS III, no.(%) | 6 (20.0%) |
| High-risk cytogenetic, no. (%) | |
| 1q21 gain/amp | 25 (83.3%) |
| P53 deficiency | 3 (10%) |
| t(4;14) variant | 7 (23.3%) |
| t(14;16) variant | 1 (3.3%) |
| ≥2 cytogenetic abnormalities | 6 (20.0%) |
| Extramedullary diseases(EMD), no.(%) | 10 (33.3%) |
| Free light chain (FLC), no.(%) | |
| κ | 22 (73.3%) |
| λ | 8 (26.7%) |

### Summary/Conclusion

The combination of selinexor with VRd demonstrates encouraging efficacy and a manageable safety profile in patients with HR NDMM.

**Copyright Information:** (Online) ISSN: 2572-9241
©2026 The Author(s). HemaSphere is published by John Wiley & Sons Ltd on behalf of European Hematology Association. This is an open access Abstract Book distributed under the Attribution-NonCommercial-NoDerivs (CC BY-NC-ND), which allows third parties to download the articles and share them with others as long as they credit the author and the Abstract Book, but they cannot change the content in any way or use them commercially.
**Abstract Book Citations:** Authors, Title, HemaSphere, 2026;10:(S1):pages. The abstract book DOIs can be found on the “Author Information” page.
**Disclaimer:** Articles published in the journal HemaSphere exclusively reflect the opinions of the authors. The authors are responsible for all content in their abstracts including accuracy of the facts, statements, citing resources, etc.

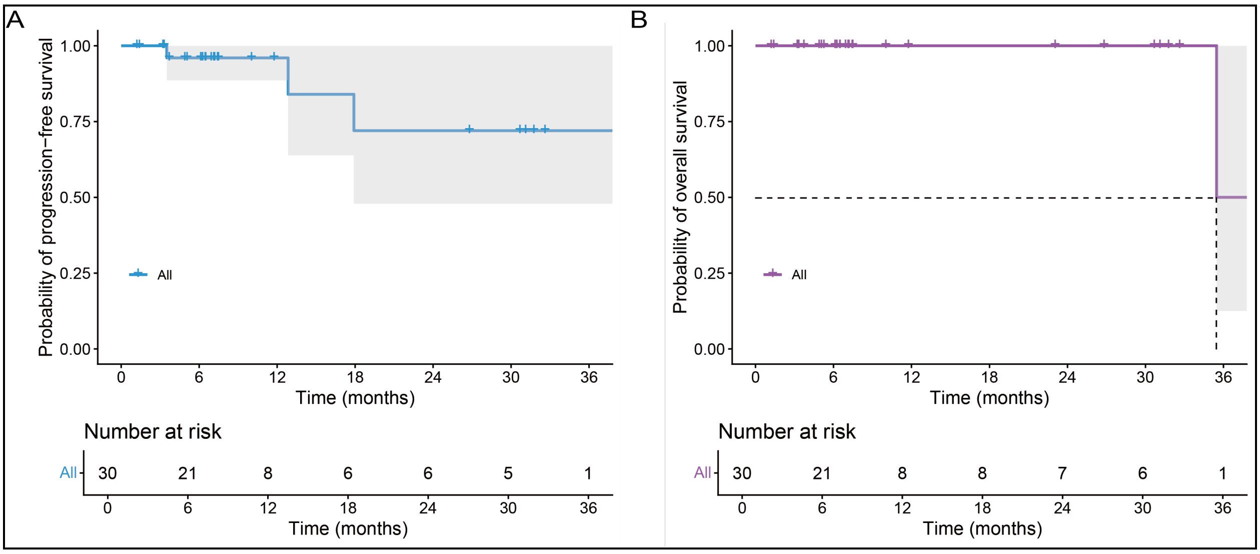

Figure 1 Survival curves of PFS (A) and OS (B).

**Copyright Information:** (Online) ISSN: 2572-9241
©2026 The Author(s). HemaSphere is published by John Wiley & Sons Ltd on behalf of European Hematology Association. This is an open access Abstract Book distributed under the Attribution-NonCommercial-NoDerivs (CC BY-NC-ND), which allows third parties to download the articles and share them with others as long as they credit the author and the Abstract Book, but they cannot change the content in any way or use them commercially.
**Abstract Book Citations:** Authors, Title, HemaSphere, 2026;10:(S1):pages. The abstract book DOIs can be found on the "Author Information" page.
**Disclaimer:** Articles published in the journal HemaSphere exclusively reflect the opinions of the authors. The authors are responsible for all content in their abstracts including accuracy of the facts, statements, citing resources, etc.

### (PS1893) PHASE 2 STUDY OF APONERMIN + BISPECIFIC ANTIBODIES IN PATIENTS(PTS) WITH RELAPSED/REFRACTORY MULTIPLE : A PROSPECTIVE SINGLE-ARM STUDY

**Topic:** 14. Myeloma and other monoclonal gammopathies - Clinical

Lefu Huang[1], Ying Tian[1], Rui Zhang[2], Tiejun Gong[3], Huijuan Wang[1], Jingrong Wang[4], Zunmin Zhu[5], Liping Su[4], Xiaojing Yan[2], MA Jun[3], Honghu Zhu[1], Wenming Chen[1], Aijun Liu*[1]

*[1]Beijing Chaoyang Hospital, affiliated to Capital Medical University, Beijing, China;[2]The First Affiliated Hospital of China Medical University, Shenyang, China; [3]Harbin Institute of Hematology and Oncology, Harbin, China;[4]Shanxi Cancer Hospital (Shanxi Cancer Center, Taiyuan, China; [5]Henan Provincial People's Hospital, Zhengzhou, China;*

### Background

Although bispecific antibodies have shown promising efficacy with high infection in pts, relapsed/ refractory multiple myeloma (RRMM) remains incurable, particularly in pts exposed to multiple-lines of therapy and those with high-risk(HR) disease features. Aponermin (Apo), the first approved DR4/DR5 agonist, triggering the extrinsic apoptotic pathway through caspase cascade activation, presenting high anti-myeloma activity with low toxicity.

### Aims

This study was to evaluate the efficacy and safety of Apo+ Bispecific antibodies(BsAbs)+Thalidomide and Dexamethasone(Td) regimen* in pts of RRMM.

### Methods

This multicenter, single-arm, phase 2 study (ChiCTR2500106279) aimed to enroll 31 pts with RRMM according to IMWG criteria, RR to the last line of therapy (LOT).  Pts received Apo+ BsAbs+Td regimen* for at least 4-6 cycles. The primary endpoint was overall response rate (ORR) after two cycles of treatment. AEs were graded per CTCAE v5.0. Cytokine release syndrome (CRS) and immune effector cell-associated neurotoxicity syndrome (ICANS) were graded per ASTCT criteria.

**Copyright Information:** (Online) ISSN: 2572-9241
©2026 The Author(s). HemaSphere is published by John Wiley & Sons Ltd on behalf of European Hematology Association. This is an open access Abstract Book distributed under the Attribution-NonCommercial-NoDerivs (CC BY-NC-ND), which allows third parties to download the articles and share them with others as long as they credit the author and the Abstract Book, but they cannot change the content in any way or use them commercially.
**Abstract Book Citations:** Authors, Title, HemaSphere, 2026;10:(S1):pages. The abstract book DOIs can be found on the “Author Information” page.
**Disclaimer:** Articles published in the journal HemaSphere exclusively reflect the opinions of the authors. The authors are responsible for all content in their abstracts including accuracy of the facts, statements, citing resources, etc.

**Results**

As of February 28, 2026, 22 patients were enrolled. The median age was 66 years (range, 50–79), and the median number of prior lines of therapy was 4 (range, 2–11). High-risk cytogenetics were present in 59.1%, 68.2%(15/22) of pts with extramedullary disease (EMD), including 66.7%(10/15) of pts with big EMD(diameter≥5cm), 60.0% (9/15) with both HR cytogenetics and big EMD. 90.9% (20/22) were triple- class exposed, two patients had undergone BCMA-CART cell infusion, and 31.8% (7/22) had previously received Apo. Most of pts received BCMA-targeted bites, except 3 of GPRC5D -targeted bites.
The ORR of one and two months was 82.4% (14/17) and 86.6% (13/15), A ≥VGPR of two months was achieved in 66.7% (10/15), including a complete remission (CR) in 46.7% (7/15). Notably, the responses were rapid: 47.1% (8/17) achieving ≥VGPR, including 3 of pts with severe renal impairment(RI) at one month.
With a median follow-up of 5 months (range, 0.8–9.0), the median PFS and overall survival were not reached. Six months PFS rate was 100%(8/8).
The most common treatment-emergent adverse events (TEAEs) were CRS (85.0%; grade [gr] 3, 1, 5%, no gr 4), neutropenia (60%, gr≥3, 4, 23.5%); infections ( 41.2%, gr≥3, 4, 23.5%) , 3 pts received immunoglobulin Infusion; deep vein thrombosis(DVT) (4, 23.5%); 3 pts changed to pomalidomide cause of DVT or thalidomide intolerance. Severe TEAEs occurred in 3 of pts with severe AI. Overall, toxicities were manageable with supportive care and dose adjustments.

**Summary/Conclusion**

The aponermin–bispecific antibody–IMiDs regimen demonstrated rapid and meaningful clinical activity with manageable toxicities in heavily pretreated and high-risk RRMM, including patients with extramedullary disease, triple-class exposure, high-risk cytogenetics and severe renal impairment.
Clinical trial information: ChiCTR2500106279.

**Copyright Information:** (Online) ISSN: 2572-9241
©2026 The Author(s). HemaSphere is published by John Wiley & Sons Ltd on behalf of European Hematology Association. This is an open access Abstract Book distributed under the Attribution-NonCommercial-NoDerivs (CC BY-NC-ND), which allows third parties to download the articles and share them with others as long as they credit the author and the Abstract Book, but they cannot change the content in any way or use them commercially.
**Abstract Book Citations:** Authors, Title, HemaSphere, 2026;10:(S1):pages. The abstract book DOIs can be found on the "Author Information" page.
**Disclaimer:** Articles published in the journal HemaSphere exclusively reflect the opinions of the authors. The authors are responsible for all content in their abstracts including accuracy of the facts, statements, citing resources, etc.

### (PS1894) RISKFACTORS OF INFECTIONS IN TRANPLANT-INELIGBLE MULTIPLE MYELOMA PATIENTS. A POPULATION-BASED STUDY ON 6,456 PATIENTS DIAGNOSED 2008-2021 FROM THE SWEDISH MYELOMA REGISTRY

**Topic:** 14. Myeloma and other monoclonal gammopathies - Clinical

Cecilie Hveding Blimark*[1], Moshtaak Karma[2], Mariana Villegas- Scivetti[1], Gunnar Larfors[3], Sigrun Einarsdottir[1], Karin Larsson[3], Ingigerdur Sverrisdottir[1]

*[1]Sahlgrenska University Hospital, Dep. of Hematology, Göteborg, Sweden;[2]Södra Älvsborg Hospital, Borås, Sweden;[3]Uppsala University Hospital, Uppsala, Sweden;*

### Background

Infections still represent a major cause of morbidity and mortality in patients with multiple myeloma (MM). Previous studies have shown an up to 5-fold increase in infections in MM patients compared to controls, and one-third of patients die as a result of a serious infection. Vulnerable patients may need closer infection surveillance and more targeted prophylaxis.

### Aims

To identify risk factors for infections among Swedish newly diagnosed (ND) transplant-ineligible (TIE) MM patients and to compare overall survival between the groups with and without infections after diagnosis.

### Methods

All TIE MM patients diagnosed between 2008 and 2021 were identified through the Swedish population-based Myeloma Registry. This registry provided blood values, cytogenetics, staging, and radiological findings at diagnosis. Prior comorbidities and cancer diagnoses were obtained from national patient registries, using the International Classification of Diseases (ICD)-10 codes. Risk factors for infections and overall survival were assessed using a multi-state Cox analysis, adjusted for age, year of diagnosis, and sex. Results were presented as hazard ratios (HR) with 95% Confidence intervals (Ci). Patients were followed until death or until 1$^{st}$ January 2023.

**Copyright Information:** (Online) ISSN: 2572-9241
©2026 The Author(s). HemaSphere is published by John Wiley & Sons Ltd on behalf of European Hematology Association. This is an open access Abstract Book distributed under the Attribution-NonCommercial-NoDerivs (CC BY-NC-ND), which allows third parties to download the articles and share them with others as long as they credit the author and the Abstract Book, but they cannot change the content in any way or use them commercially.
**Abstract Book Citations:** Authors, Title, HemaSphere, 2026;10:(S1):pages. The abstract book DOIs can be found on the “Author Information” page.
**Disclaimer:** Articles published in the journal HemaSphere exclusively reflect the opinions of the authors. The authors are responsible for all content in their abstracts including accuracy of the facts, statements, citing resources, etc.

### Results

A total of 6,456 TIE MM patients were included, of whom 4,216 had at least 1 infection after diagnosis. Patients with infections had signs of more advanced disease, see Table 1. No differences were found in the Charlson comorbidity index, bone disease, or hypercalcemia. Analyzing specific risk factors in the patient's history, heart failure (HR=1.71; 95%CI 1.45-2.03), chronic lung disease (HR=1.65; 95%CI 1.35-2.01) and chronic renal disease (HR=1.63; 95%CI 1.46-1.81) were associated with an increased risk of infections. Further, characteristics of high tumor load, such as elevated LDH, ISS/R-ISS stage and the cytogenetic deletion 17p, were identified as risk factors. Previous cancer or autoimmune disease did not increase the risk of infections after MM diagnosis. Overall survival was decreased for patients with infections compared to those without (HR=4.15; 95%CI (3.76-4.57)).

| **Characteristics** | **No infections** | **Infections** | **p-value**[1] |
|---|---|---|---|
| | N = 2,240 | N = 4,216[1] | |
| **Age** | | | <0.001 |
| Median (Q1, Q3) | 77.0 (71.0, 83.0) | 75.0 (70.0, 81.0) | |
| **Heart failure, n (%)** | 322 (14%) | 492 (12%) | 0.001 |
| **Chronic Lung Disease, n (%)** | 32 (1.4%) | 116 (2.8%) | <0.001 |
| **Chronic renal disease, n (%)** | 383 (17%) | 915 (22%) | <0.001 |
| **Hemoglobin** | 108.1 (18.7) | 106.6 (18.4) | 0.003 |
| **Creatinin** | | | <0.001 |
| Median (Q1, Q3) | 91.0 (71.0, 133.0) | 96.0 (73.0, 149.0) | |
| **b2 microglobulin*** | | | <0.001 |
| Median (Q1, Q3) | 4.2 (3.0, 7.0) | 4.7 (3.1, 7.6) | |
| **ISS, n(%)** | | | <0.001 |
| I | 3131 (19%) | 474 (16%) | |
| II | 722 (44%) | 1,295 (43%) | |
| III | 583 (36%) | 1,232 (41%) | |
| **R ISS** | | | 0.008 |
| I | 43 (8%) | 14 (3%) | |
| II | 483 (84%) | 365 (86%) | |
| III | 46 (8.3%) | 45 (11%) | |

### Summary/Conclusion

In this large population-based study of 6,456 Swedish TIE NDMM patients, individuals who developed infections were primarily characterized by indicators of high tumor burden at diagnosis. Furthermore, specific risk factors for infection were identified as elevated disease stage at diagnosis, heart failure, and chronic renal or lung disease in the patient´s prior history. Interestingly, the Charlson comorbidity index could not predict an increased risk of infections in TIE MM patients. The identified risk factors can facilitate early recognition of patients at higher risk of infection and guide personalized prophylactic strategies in MM patients, potentially improving survival in this patient group.

**Copyright Information:** (Online) ISSN: 2572-9241
©2026 The Author(s). HemaSphere is published by John Wiley & Sons Ltd on behalf of European Hematology Association. This is an open access Abstract Book distributed under the Attribution-NonCommercial-NoDerivs (CC BY-NC-ND), which allows third parties to download the articles and share them with others as long as they credit the author and the Abstract Book, but they cannot change the content in any way or use them commercially.
**Abstract Book Citations:** Authors, Title, HemaSphere, 2026;10:(S1):pages. The abstract book DOIs can be found on the "Author Information" page.
**Disclaimer:** Articles published in the journal HemaSphere exclusively reflect the opinions of the authors. The authors are responsible for all content in their abstracts including accuracy of the facts, statements, citing resources, etc.

### (PS1895) NEXT-GENERATION FLOW CYTOMETRY-BASED DETECTION OF CIRCULATING PLASMA CELLS PREDICTS CYTOGENETIC HIGH-RISK DISEASE AND DEPTH OF RESPONSE IN NEWLY DIAGNOSED MULTIPLE MYELOMA

**Topic:** 14. Myeloma and other monoclonal gammopathies - Clinical

Neha Singh*[1, 2], Pradeep Arumugam[1, 2], Deepali Saxena[1, 2], Anil Singh[2, 3], Ankita Pal[2, 4], Anil Yadav[2, 5], Nilesh Dhole[1, 2], Rohit Kori[1, 2], Snehal Jaiswar[1, 2], Seema Biswas[1, 2], Bal Mishra[2, 3]

*[1]TATA MEMORIAL CENTRE (HBCH & MPMMCC), HEMATOPATHOLOGY, VARANASI, India;[2]HOMI BHABHA NATIONAL INSTITUTE, MUMBAI, Mumbai, India; [3]TATA MEMORIAL CENTRE (HBCH & MPMMCC), ADULT HEMATOLYMPHOID UNIT, VARANASI, India; [4]TATA MEMORIAL CENTRE (HBCH & MPMMCC), BIOSTATISTICS AND EPIDEMIOLOGY, VARANASI, India; [5]TATA MEMORIAL CENTRE (HBCH & MPMMCC), CANCER CYTOGENETICS, VARANASI, India;*

### Background

Circulating plasma cells (CPCs) have emerged as clinically meaningful entities beyond the confines of the bone marrow microenvironment. Advanced next-generation flow cytometry (NGF) now enables sensitive and standardised detection of CPCs at remarkably low thresholds, facilitating their integration into routine prognostic workup. The interplay between CPC burden, cytogenetic risk, and treatment response in non-transplant patients warrants prospective evaluation.

### Aims

This study aimed to determine the prevalence of CPCs in a cohort of newly diagnosed MM patients and to examine their association with FISH-defined cytogenetic risk profiles and depth of response following frontline bortezomib-based chemotherapy. A secondary aim was to assess the impact of CPC positivity on relapse-free survival (RFS) and overall survival (OS).

### Methods

This prospective study enrolled 137 newly diagnosed multiple myeloma patients over a period of two years. Next-generation flowcytometry was performed on peripheral blood using a customized ten-color myeloma panel. Concurrent cytogenetic risk stratification was conducted using fluorescence in-situ hybridisation (FISH) targeting key MM-associated abnormalities, including deletion 17p, t(4;14), t(14;16), t(14;20), IgH::FGFR3, hyperdiploidy, and 1q21 gain.  Treatment responses were classified per International Myeloma Working Group criteria. Survival analyses utilised Kaplan-Meier methodology with log-rank testing.

### Results

CPCs (≥0.001%) were detected in 64.96% (89/137) of patients at baseline, ranging from 0.0037-41.07%. Four patients had plasma cell leukemia at presentation. Median age was 59.5 years with M:F ratio of 2.3:1. Patients with lower normal to total plasma cells ratio in bone marrow had strong correlation with presence of circulating plasma cells (p= <0.001). The commonest cytogenetic abnormalities were 1q21 gain (33.6%) followed by hyperdiploidy (25.5%) and IgH::FGFR3 (9.5%). No FISH abnormalities were observed in 29.2% patients. One or more poor-risk cytogenetic features i.e del(17p), t(4;14), t(14;16), t(14;20) and 1q21 gain, were present in 42.3% patients.  CPC positivity ≥0.001% as well as ≥0.01% showed significant correlation with poor-risk cytogenetics i.e p = 0.0026 and p=0.0027 respectively. 27.0% patients belonged to stage III as per the Revised ISS staging for myeloma. However, no significant correlation could be established between staging and CPCs (p=0.42), attributable possibly to non-inclusion of 1q21 gain in the high risk cytogenetics of the R-ISS staging system. CR or better response rates were achieved in 31.25% and 52.63% of patients in the CPC-positive and CPC-negative subgroups, and the difference was statistically significant (p=0.032). 2-year RFS and 2-year OS rates were 93.8% and 95.6% respectively for the entire cohort. However, the difference was not significant in the CPC-positive versus the CPC-negative subgroups (p=0.24).

**Copyright Information:** (Online) ISSN: 2572-9241
©2026 The Author(s). HemaSphere is published by John Wiley & Sons Ltd on behalf of European Hematology Association. This is an open access Abstract Book distributed under the Attribution-NonCommercial-NoDerivs (CC BY-NC-ND), which allows third parties to download the articles and share them with others as long as they credit the author and the Abstract Book, but they cannot change the content in any way or use them commercially.
**Abstract Book Citations:** Authors, Title, HemaSphere, 2026;10:(S1):pages. The abstract book DOIs can be found on the "Author Information" page.
**Disclaimer:** Articles published in the journal HemaSphere exclusively reflect the opinions of the authors. The authors are responsible for all content in their abstracts including accuracy of the facts, statements, citing resources, etc.

**Summary/Conclusion**

CPC quantification by NGF is a clinically accessible prognostic tool in newly diagnosed MM, with demonstrated utility in predicting early treatment response and identifying high-risk cytogenetic disease. CPC–R-ISS discordance supports incorporating 1q21 gain into future staging revisions. The absence of significant survival separation likely reflects limited follow-up; prospective multicentre studies with serial CPC monitoring are needed to validate CPC kinetics as dynamic response biomarkers.

**Copyright Information:** (Online) ISSN: 2572-9241
©2026 The Author(s). HemaSphere is published by John Wiley & Sons Ltd on behalf of European Hematology Association. This is an open access Abstract Book distributed under the Attribution-NonCommercial-NoDerivs (CC BY-NC-ND), which allows third parties to download the articles and share them with others as long as they credit the author and the Abstract Book, but they cannot change the content in any way or use them commercially.
**Abstract Book Citations:** Authors, Title, HemaSphere, 2026;10:(S1):pages. The abstract book DOIs can be found on the "Author Information" page.
**Disclaimer:** Articles published in the journal HemaSphere exclusively reflect the opinions of the authors. The authors are responsible for all content in their abstracts including accuracy of the facts, statements, citing resources, etc.

### (PS1896) ELRANATAMAB IN COMBINATION WITH CARFILZOMIB AND DEXAMETHASONE IN PATIENTS WITH RELAPSED OR REFRACTORY MULTIPLE MYELOMA: RESULTS FROM THE PHASE 1B MAGNETISMM-20 TRIAL

**Topic:** 14. Myeloma and other monoclonal gammopathies - Clinical

C. Ola Landgren*[1], Muhamed Baljević[2], Hira Shaikh[3], Syed Abbas Ali[4], Eli Gabayan[5], Craig Hofmeister[6], Imad Din[7], Kunhwa Kim[8], Jay Cheng[9], Erik Vandendries[10], Alexander Lesokhin[11]

*[1]Sylvester Comprehensive Cancer Center, Miami, FL, United States of America;[2]Vanderbilt-Ingram Cancer Center, Vanderbilt University Medical Center, Nashville, United States of America; [3]University of Iowa, Division of Hematology, Oncology and Blood & Marrow Transplantation, Iowa, United States of America; [4]The Sydney Kimmel Comprehensive Cancer Center, Johns Hopkins University School of Medicine, Baltimore, United States of America; [5]Beverley Hills Cancer Center, Beverley Hills, United Kingdom; [6]Winship Cancer Institute, Emory University, Atlanta, United States of America;[7]Pfizer Inc, San Francisco, United States of America; [8]Pfizer Inc, New York, United States of America;[9]Pfizer Inc, Bothell, United States of America; [10]Pfizer Inc, Cambridge, United States of America; [11]Memorial Sloan Kettering Cancer Center, New York, United States of America;*

### Background

Elranatamab (ELRA), a B-cell maturation antigen (BCMA)-CD3 bispecific antibody, demonstrated deep, durable responses with manageable safety in BCMA-naive patients (pts) with relapsed or refractory multiple myeloma (RRMM) in the phase 2 MagnetisMM-3 study (NCT04649359). Combining ELRA with carfilzomib (CFZ) may enhance immune-mediated anti-myeloma activity through complementary mechanisms. MagnetisMM-20 (NCT05675449) Part 1 is a phase 1b, open-label, multicenter, non-randomized study of ELRA + CFZ + dexamethasone (EKd) in pts with RRMM. Previous results from Part 1 demonstrated predictable safety signals and clinical efficacy with EKd (Tomasson, ASH 2024).

### Aims

To present updated safety and efficacy results of EKd in pts with RRMM from Part 1 dose escalation of MagnetisMM-20.

### Methods

Eligible pts were aged ≥18 y with RRMM, 1-3 prior lines of therapy, ECOG performance status ≤1, and no prior BCMA-directed therapy or CFZ. Three dose levels (DL) were evaluated: DL1, DL2, and DL1A. In cycle (C) 0, ELRA step-up doses (12/32 mg on day [D]1/D4) preceded the first full dose on D8 (44 mg weekly [QW] in DL1; 76 mg QW in DL2 and every 2 weeks [Q2W] in DL1A). In DL1 and DL2, ELRA dosing frequency was reduced to Q2W after 6 mo in pts who achieved ≥ partial response (PR) for ≥2 consecutive cycles; in DL2, further reduction to every 4 weeks (Q4W) was permitted based on response. In DL1A, ELRA dosing frequency was reduced to Q4W at protocol-defined timepoints in pts who achieved ≥PR for ≥2 cycles. CFZ (20 mg/m$^2$ on C1D1, then 70 mg/m$^2$ thereafter) was given on D1, D8, and D15 of each 28-day cycle; dexamethasone 40 mg QW C1-C3.
The primary endpoint was dose limiting toxicities (DLTs) from C0D1 through end of C1. Secondary endpoints included safety, objective response rate (ORR), complete response rate (CRR), duration of response (DOR) and time to response (TTR) per IMWG criteria.

**Copyright Information:** (Online) ISSN: 2572-9241
©2026 The Author(s). HemaSphere is published by John Wiley & Sons Ltd on behalf of European Hematology Association. This is an open access Abstract Book distributed under the Attribution-NonCommercial-NoDerivs (CC BY-NC-ND), which allows third parties to download the articles and share them with others as long as they credit the author and the Abstract Book, but they cannot change the content in any way or use them commercially.
**Abstract Book Citations:** Authors, Title, HemaSphere, 2026;10:(S1):pages. The abstract book DOIs can be found on the "Author Information" page.
**Disclaimer:** Articles published in the journal HemaSphere exclusively reflect the opinions of the authors. The authors are responsible for all content in their abstracts including accuracy of the facts, statements, citing resources, etc.

**Results**

Among 21 pts (DL1: 4, DL2: 8, DL1A: 9), median age was 68.0 y (range, 45-84), 66.7% were male. 19.0% had high-risk cytogenetics, 23.8% had ≥30% baseline bone marrow plasma cells, and 42.9% had triple-class refractory disease. Median prior lines of therapy was 2 (range, 1-3).
At data cutoff (December 15, 2025), median treatment duration was 8.8 mo (range, 0.1-35.0); 38.1% of pts remained on ELRA. No DLTs were reported among 19 evaluable pts. Treatment-emergent adverse events (TEAEs) occurred in 100% (Grade [G]≥3 90.5%) of pts and hematologic TEAEs in 85.7% (71.4% G≥3). G≥3 hematologic TEAEs occurred in 75.0%, 87.5%, and 55.6% of pts in DL1, DL2, and DL1A, respectively. Using a consistent 120-day assessment window from C0D1, G≥3 hematologic TEAEs occurred in 33.3% in DL1A versus 75.0% in DL1 and 62.5% in DL2. Overall, infections occurred in 85.7% (G≥3 19.0%), cytokine release syndrome in 57.1% (all G1/2); no ICANS was reported. Common TEAEs are in Table 1.
At a median duration of follow-up of 14.3 mo (range, 0.5-35.2), confirmed ORR by investigator was 95.2% (95% CI, 76.2-99.9); confirmed and unconfirmed CRR were 66.7% (95% CI, 43.0-85.4) and 81.0% (95% CI, 58.1-94.6), respectively. Median DOR was not reached; probability of maintaining response at 21 mo was 82.6% (95% CI, 54.6-94.1). Median TTR was 1.4 mo (range, 0.5-4.5).

**Summary/Conclusion**

In BCMA-naive pts with RRMM, EKd demonstrated a predictable safety profile with encouraging clinical efficacy. Dose expansion is ongoing at DL1A, selected for a favorable exposure–tolerability balance, dosing convenience, and manageable safety profile.

**Table 1. Most frequent (any grade in ≥40% or grade ≥3 in ≥10% of pts) TEAEs[a]**

| TEAEs, % | Total (N=21) | |
|---|---|---|
| | Any grade | Grade ≥3[b] |
| Hematologic | | |
| Neutropenia | 71.4 | 66.7 |
| Thrombocytopenia | 52.4 | 23.8 |
| Anemia | 47.6 | 19.0 |
| Leukopenia | 42.9 | 23.8 |
| Lymphopenia | 33.3 | 23.8 |
| Nonhematologic | | |
| Infections[c] | 85.7 | 19.0 |
| Fatigue | 76.2 | 14.3 |
| Cough | 61.9 | 9.5 |
| Cytokine release syndrome | 57.1 | 0 |
| Diarrhea | 47.6 | 4.8 |
| Nausea | 47.6 | 0 |
| Injection site reaction | 47.6 | 0 |
| Insomnia | 42.9 | 14.3 |
| Acute kidney injury | 14.3 | 14.3 |

[a] Preferred terms were based on MedDRA v28.1 coding dictionary; [b] There were 3 grade 5 TEAEs (sudden death, COVID-19 pneumonia, and cardiorespiratory arrest); [c] System organ class of infections and infestations

**Copyright Information:** (Online) ISSN: 2572-9241
©2026 The Author(s). HemaSphere is published by John Wiley & Sons Ltd on behalf of European Hematology Association. This is an open access Abstract Book distributed under the Attribution-NonCommercial-NoDerivs (CC BY-NC-ND), which allows third parties to download the articles and share them with others as long as they credit the author and the Abstract Book, but they cannot change the content in any way or use them commercially.
**Abstract Book Citations:** Authors, Title, HemaSphere, 2026;10:(S1):pages. The abstract book DOIs can be found on the "Author Information" page.
**Disclaimer:** Articles published in the journal HemaSphere exclusively reflect the opinions of the authors. The authors are responsible for all content in their abstracts including accuracy of the facts, statements, citing resources, etc.

### (PS1897) DEVELOPMENT AND VALIDATION OF A NOVEL SOCIAL FRAILTY–INTEGRATED STAGING SYSTEM FOR PREDICTING SURVIVAL IN MULTIPLE MYELOMA

**Topic:** 14. Myeloma and other monoclonal gammopathies - Clinical

Kosuke Shima*[1, 2], Daichi Terunuma[1], Kazuki Nagao[1], Naoya Fukuda[1], Hajime Sakuma[1], Masanori Toho[1], Atsushi Uehara[1], Masami Takeuchi[1], Kosei Matsue[1]

*[1]Kameda Medical Center, Chiba, Japan; [2]Graduate School of Medicine, The University of Tokyo, Department of Hematology and Oncology, Tokyo, Japan;*

### Background

Most cases of multiple myeloma (MM) progress over several years from precursor conditions such as smoldering multiple myeloma (SMM) or monoclonal gammopathy of undetermined significance (MGUS). Typical MM related organ damage includes hypercalcemia, renal impairment, anemia, and bone lesions (CRAB), which manifest as fatigue, appetite loss, dyspnea, and bone pain. Progression of these symptoms may reduce opportunities for going out and interacting with others, leading to diminished social connectedness. Thus, MM patients may experience unrecognized declines in social activity, suggesting a close association between MM and social frailty, defined as loss of social roles and social networks. Because MM treatment often requires frequent outpatient visits, maintaining sufficient social function is clinically important. However, reports examining the relationship between MM and social frailty are scarce, and the IMWG frailty score does not directly assess social frailty.

### Aims

This study aimed to evaluate social frailty as a prognostic indicator in MM and develop a novel staging system incorporating social frailty.

### Methods

We retrospectively analyzed 387 MM patients diagnosed at our institution (Kameda Medical Center) between 2009 and 2025. Social frailty was assessed at diagnosis or first admission using the Makizako Social Frailty Index by hematologists, nurses, and physical therapists. Five items—reduced frequency of going out, decreased interaction with acquaintances/friends, loss of self-perceived social usefulness, living alone, and absence of someone to talk with daily—were evaluated, and ≥2 points indicated social frailty. We constructed a novel staging system, the Kameda MM Frailty Score (KFS), using ISS stage III, IMWG frailty (≥2 points), and social frailty (≥2 points), and classified patients into three groups (0 = Stage 1, 1–2 = Stage 2, 3 = Stage 3). Progression-free survival (PFS) and overall survival (OS) were compared. Cox proportional hazards models were performed, including the representative physical frailty scores—Fried frailty phenotype and SPPB.

### Results

Median age was 73 years; 56% were male. Median follow-up was 41 months, median PFS was 42 months, and median OS was 94 months. IMWG frailty was present in 51.4% and social frailty in 34.1%. IMWG-frail patients had significantly shorter PFS (30 months vs. 68 months, $P = 5.74\times10^{-9}$) and OS (53 months vs. 186 months, $P = 4.42\times10^{-11}$). Socially frail patients had inferior PFS (34 months vs. 56 months, $P = 0.00139$) and OS (53 months vs. 119 months , $P = 7.26\times10^{-6}$).KFS distribution was: 0 points 20%; 1 point 25%; 2 points 26%; 3 points 17%. Median PFS decreased across groups (119 months, 60 months, 32 months, 21 months; $P = 2.08\times10^{-10}$), as did OS (168 months, 186 months, 53 months, 39 months; $P = 1.54\times10^{-12}$).Cox analysis identified KFS and R-ISS as independent risk factors for PFS, while KFS and Age (≥75) independently predicted OS. Physical frailty scores (Fried, SPPB) were not significant.

**Copyright Information:** (Online) ISSN: 2572-9241
©2026 The Author(s). HemaSphere is published by John Wiley & Sons Ltd on behalf of European Hematology Association. This is an open access Abstract Book distributed under the Attribution-NonCommercial-NoDerivs (CC BY-NC-ND), which allows third parties to download the articles and share them with others as long as they credit the author and the Abstract Book, but they cannot change the content in any way or use them commercially.
**Abstract Book Citations:** Authors, Title, HemaSphere, 2026;10:(S1):pages. The abstract book DOIs can be found on the “Author Information” page.
**Disclaimer:** Articles published in the journal HemaSphere exclusively reflect the opinions of the authors. The authors are responsible for all content in their abstracts including accuracy of the facts, statements, citing resources, etc.

### Summary/Conclusion

Social frailty was significantly associated with prognosis in MM. Our proposed KFS, integrating ISS stage III, IMWG frailty, and social frailty, may serve as a simple and accurate predictor of PFS and OS. This scoring system introduces social frailty as a novel perspective complementary to conventional prognostic factors and may have prognostic value comparable to or exceeding that of physical frailty indices.

**The Kameda MM Frail Score (KFS) is a potential novel indicator of social frailty**

KFS=the Makizako Social Frailty Index + ISS stage III + IMWG frailty
Stage Ⅰ :KFS 0 points, Stage Ⅱ :KFS 1 or 2 points, Stage Ⅲ :KFS 3 points

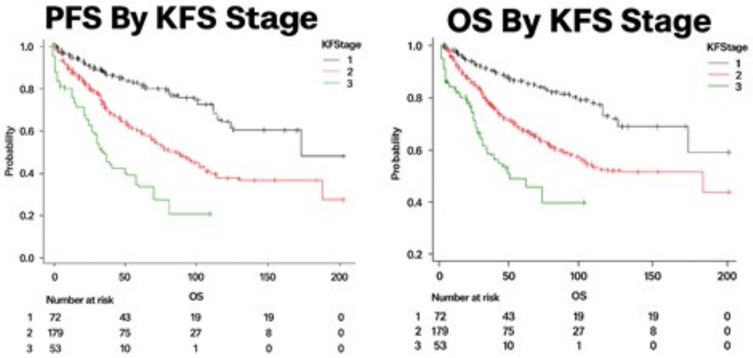

| Hazard Ratio | | | | |
|---|---|---|---|---|
| | PFS | | OS | |
| Factor | Hazard.ratio | p.value | Hazard.ratio | p.value |
| Age(≧75) | 1.46 (0.89-2.39) | 0.14 | **1.76 (0.94-3.31)** | **0.078** |
| Fried.Frailty.Phenotype(≧3) | 0.85 (0.44-1.66) | 0.64 | 1.59 (0.63-4.01) | 0.32 |
| IMWG.frailty.score(≧2) | 0.94 (0.47-1.87) | 0.86 | 1.30 (0.53-3.15) | 0.57 |
| **KFS (score : 0-3)** | **1.42 (0.98-2.05)** | **0.062** | **1.54 (0.98-2.42)** | **0.059** |
| **R.ISS** | **1.97 (1.30-2.98)** | **0.0014** | 1.23 (0.74-2.05) | 0.43 |
| SPPBscore(≦9) | 1.39 (0.71-2.71) | 0.34 | 0.96 (0.39-2.34) | 0.92 |

**Copyright Information:** (Online) ISSN: 2572-9241
©2026 The Author(s). HemaSphere is published by John Wiley & Sons Ltd on behalf of European Hematology Association. This is an open access Abstract Book distributed under the Attribution-NonCommercial-NoDerivs (CC BY-NC-ND), which allows third parties to download the articles and share them with others as long as they credit the author and the Abstract Book, but they cannot change the content in any way or use them commercially.
**Abstract Book Citations:** Authors, Title, HemaSphere, 2026;10:(S1):pages. The abstract book DOIs can be found on the “Author Information” page.
**Disclaimer:** Articles published in the journal HemaSphere exclusively reflect the opinions of the authors. The authors are responsible for all content in their abstracts including accuracy of the facts, statements, citing resources, etc.

### (PS1898) LOW-DOSE VENETOCLAX PLUS WEEKLY DEXAMETHASONE IN T(11;14)-POSITIVE RELAPSED/REFRACTORY MULTIPLE MYELOMA: FINAL EFFICACY AND SAFETY RESULTS FROM THE PHASE II VICTORIA STUDY

**Topic:** 14. Myeloma and other monoclonal gammopathies - Clinical

Astrid Hundebøll Torpe*[1, 2], Sarah Leeth Farmer[3], Mette Niemann Christoffersen[4], Jonathan Thorsen[2, 3, 5], Thomas Lund[3, 4, 6], Niels Abildgaard[4], Torben Plesner[3], Agoston Szabo[1, 2, 3]

*[1]Department of Hematology, Copenhagen University Hospital, Rigshospitalet, Copenhagen, Denmark;[2]Dept. of Clinical Medicine, University of Copenhagen, Copenhagen, Denmark; [3]Dept. of Hematology, Vejle Hospital, Vejle, Denmark;[4]Dept. of Hematology, Odense University Hospital, Odense, Denmark; [5]COPSAC, Herlev and Gentofte Hospital, University of Copenhagen, Copenhagen Prospective Studies on Asthma in Childhood, Copenhagen, Denmark; [6]Centre for Innovative Medical Technology, Odense University Hospital, Odense, Denmark;*

### Background

Translocation t(11;14), present in ~15–20% of multiple myeloma cases, is associated with high BCL-2 expression and sensitivity to BCL-2 inhibition. Early-phase studies of venetoclax (800–1200 mg daily) demonstrated ORRs of 40–60% in t(11;14)-positive relapsed/refractory disease. In BELLINI, venetoclax improved PFS, with the greatest benefit observed in the t(11;14) subgroup, although infection-related safety concerns emerged. In CANOVA, venetoclax–dexamethasone (800 mg daily) achieved higher response rates than pomalidomide–dexamethasone but did not meet its primary PFS endpoint.

### Aims

The aim of the VICTORIA study was to evaluate low-dose venetoclax (400 mg once daily) plus weekly dexamethasone in patients with t(11;14)-positive RRMM.

### Methods

VICTORIA is a Danish, investigator-initiated, open-label, multicenter phase II study in patients with t(11;14)-positive RRMM after ≥1 prior line of therapy. Venetoclax 400 mg once daily plus weekly dexamethasone (20 mg) was administered in 28-day cycles until progression or unacceptable toxicity. The primary endpoint was ORR per 2011 IMWG criteria. Secondary endpoints included PFS, OS, DoR, and safety (CTCAE v5.0). Between July 2020 - April 2025, 50 patients were enrolled over 58 months (0.9 patients/month).

**Copyright Information:** (Online) ISSN: 2572-9241
©2026 The Author(s). HemaSphere is published by John Wiley & Sons Ltd on behalf of European Hematology Association. This is an open access Abstract Book distributed under the Attribution-NonCommercial-NoDerivs (CC BY-NC-ND), which allows third parties to download the articles and share them with others as long as they credit the author and the Abstract Book, but they cannot change the content in any way or use them commercially.
**Abstract Book Citations:** Authors, Title, HemaSphere, 2026;10:(S1):pages. The abstract book DOIs can be found on the “Author Information” page.
**Disclaimer:** Articles published in the journal HemaSphere exclusively reflect the opinions of the authors. The authors are responsible for all content in their abstracts including accuracy of the facts, statements, citing resources, etc.

**Results**

Fifty patients received treatment. Median (range) age was 74.6 years (45.7–88.2); median time since diagnosis was 4.5 years (0.2–11.1); median number of prior lines was 3 (1–9). Fifty-eight percent were male; ECOG PS was 1/2/3 in 26/20/4 patients, respectively. High-risk cytogenetics included del(17p) in 10% and gain(1q) in 16%. Prior exposure rates were PI 92%, IMiD 92%, CD38 antibody 70%, and alkylator 62%. In total, 64% (n=32) were triple-class exposed and 34% (n=17) were triple-class refractory.
ORR was 60% (n=30), including sCR 6% (n=3), CR 6% (n=3), VGPR 22% (n=11), and PR 26% (n=13). Stable disease occurred in 22% (n=11) and PD in 10% (n=5); one patient discontinued without response assessment.
Median follow-up, calculated by reverse Kaplan–Meier, was 23.2 months (IQR 14.5–29.7). Thirty-five PFS events occurred; median PFS was 7.4 months, and 12-month PFS rate was 50%. Among responders, median DoR was 19.2 months, with 12-month DoR rate of 63%. Seventeen deaths were recorded; median OS was not reached; 12- and 24-month OS rate were 73% and 64%, respectively.

Grade ≥3 AEs occurred in 25 patients (50%; 49 events), 71% were non-hematologic and 29% hematologic. Most frequent were upper respiratory infection (n=7), lung infection (n=5), thrombocytopenia (n=5), and neutropenia (n=5). Twelve patients (24%) had ≥Grade 3 infectious AEs and 6 (12%) ≥Grade 3 hematologic AEs. Three patients (6%) experienced Grade 4–5 events, including one Grade 5 sepsis. Discontinuation was primarily due to progressive disease (n=37); 1 patient discontinued due to treatment-related toxicity, 1 due to death, and 3 due to other reasons.

**Summary/Conclusion**

Venetoclax 400 mg once daily combined with weekly dexamethasone demonstrates compelling and durable efficacy with a generally mild toxicity profile in t(11;14)-positive RRMM.
Achieving these outcomes at a lower dose of venetoclax than used in prior randomized studies suggests that effective BCL-2 inhibition may be attainable without higher-dose exposure. As a simple and accessible all-oral regimen, this approach offers a practical targeted strategy. Despite the absence of regulatory approval of venetoclax for RRMM, the observed efficacy and safety provide a strong rationale for off-label use.

**Copyright Information:** (Online) ISSN: 2572-9241
©2026 The Author(s). HemaSphere is published by John Wiley & Sons Ltd on behalf of European Hematology Association. This is an open access Abstract Book distributed under the Attribution-NonCommercial-NoDerivs (CC BY-NC-ND), which allows third parties to download the articles and share them with others as long as they credit the author and the Abstract Book, but they cannot change the content in any way or use them commercially.
**Abstract Book Citations:** Authors, Title, HemaSphere, 2026;10:(S1):pages. The abstract book DOIs can be found on the “Author Information” page.
**Disclaimer:** Articles published in the journal HemaSphere exclusively reflect the opinions of the authors. The authors are responsible for all content in their abstracts including accuracy of the facts, statements, citing resources, etc.

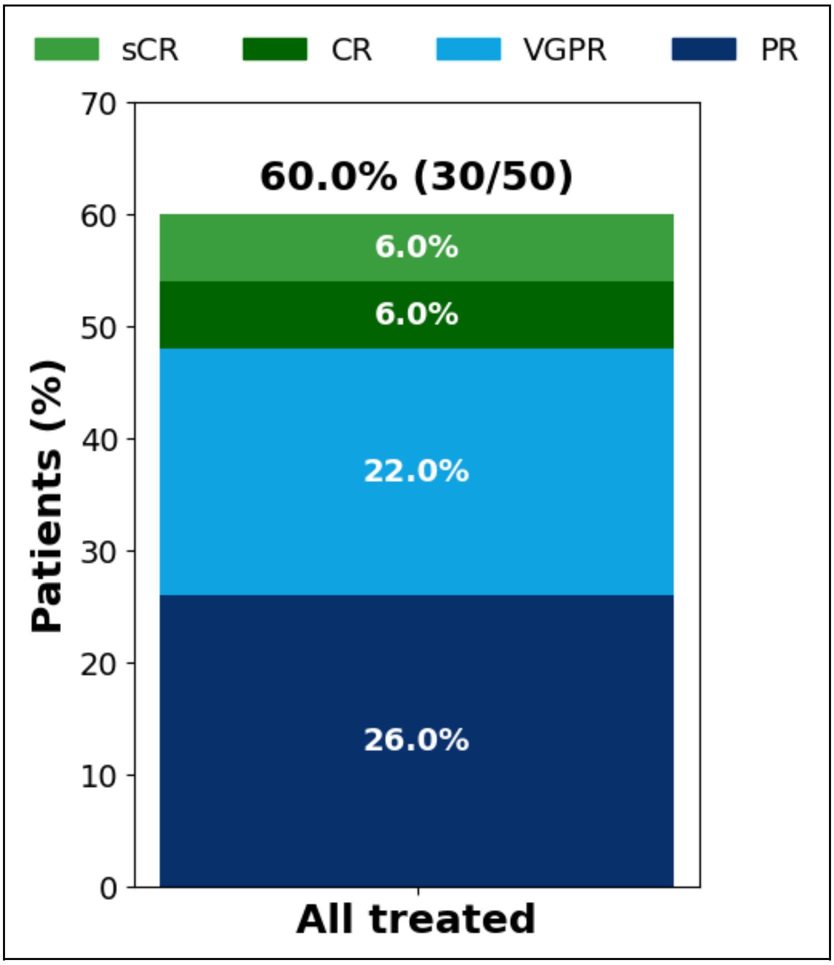

Overall response rate (ORR) with low-dose venetoclax (400 mg once daily) plus weekly dexamethasone in patients with t(11;14)-positive RRMM.

**Copyright Information:** (Online) ISSN: 2572-9241
©2026 The Author(s). HemaSphere is published by John Wiley & Sons Ltd on behalf of European Hematology Association. This is an open access Abstract Book distributed under the Attribution-NonCommercial-NoDerivs (CC BY-NC-ND), which allows third parties to download the articles and share them with others as long as they credit the author and the Abstract Book, but they cannot change the content in any way or use them commercially.
**Abstract Book Citations:** Authors, Title, HemaSphere, 2026;10:(S1):pages. The abstract book DOIs can be found on the "Author Information" page.
**Disclaimer:** Articles published in the journal HemaSphere exclusively reflect the opinions of the authors. The authors are responsible for all content in their abstracts including accuracy of the facts, statements, citing resources, etc.

### (PS1899) REAL-WORLD EFFECTIVENESS AND SAFETY OF ELRANATAMAB IN PATIENTS WITH RELAPSED/REFRACTORY MULTIPLE MYELOMA: PRELIMINARY RESULTS FROM THE PROSPECTIVE, NON-INTERVENTIONAL MAGNETISMM-16 STUDY

**Topic:** 14. Myeloma and other monoclonal gammopathies - Clinical

Jindriska Lindsay*[1], Werner Freier[2], John Jones[3], Supratik Basu[4], Firas Al-Kaisi[5], Bhuvan Kishore[6], Arief Gunawan[7], Pratap Neelakantan[8], Dean Edward Smith[9], Sunil Lakhwani[10], Alexander Pohlmann[11], Steffen Dörfel[12], Jay Cheng[13], Araceli Loredo[14], Rebecca Levin[15], Didem Aydın[16], Kamaraj Karunanithi[17]

*[1]East Kent Hospital University Foundation Trust, Canterbury, United Kingdom;[2]Medicinum Hildesheim, Hildesheim, Germany; [3]Brighton and Sussex Medical School, University of Brighton and University of Sussex, Brighton, United Kingdom;[4]Royal Wolverhampton NHS Trust and University, Wolverhampton, United Kingdom; [5]Royal Derby Hospital, Derby, United Kingdom; [6]Birmingham Heartlands Hospital, Birmingham, United Kingdom; [7]King's College Hospital, London, United Kingdom; [8]Royal Berkshire Hospital, Berkshire, United Kingdom; [9]Nottingham University Hospital NHS Trust, Nottingham, United Kingdom; [10]Hospital Universitario de Canarias, La Laguna, Spain; [11]University Hospital Muenster, Muenster, Germany; [12]Center of Oncology Dresden, Dresden, Germany; [13]Pfizer Inc, Bothell, WA, United States of America; [14]Pfizer Inc, Mexico City, Mexico; [15]Pfizer Inc, New York, United States of America; [16]Pfizer Inc, Istanbul, Türkiye; [17]University Hospitals of North Midlands NHS Trust, Stoke-on-Trent, United Kingdom;*

### Background

Elranatamab is a B-cell maturation antigen-CD3 bispecific antibody approved for the treatment of adult patients with relapsed or refractory multiple myeloma (RRMM) in several countries. The efficacy and safety of elranatamab were demonstrated in previous clinical trials, and real-world data for elranatamab are currently being accumulated.

### Aims

This interim analysis provides an early look into the results from a real-world primary data collection study of elranatamab in patients with RRMM.

### Methods

MagnetisMM-16 (EUPAS106401) is a prospective, international, multicenter, non-interventional post-authorization safety study evaluating the effectiveness and safety of elranatamab in patients aged ≥18 years with RRMM treated in real-world settings. Patients are being recruited from hematology/oncology clinics, primary care settings, and academic centers in the United Kingdom, Germany, Spain, and Italy. Investigators were asked in the study protocol to follow recommendations in the local health authority–approved product label, including infection precautions and antimicrobial prophylaxis. This interim analysis is based on a September 15, 2025 data cut and describes preliminary results.

**Copyright Information:** (Online) ISSN: 2572-9241
©2026 The Author(s). HemaSphere is published by John Wiley & Sons Ltd on behalf of European Hematology Association. This is an open access Abstract Book distributed under the Attribution-NonCommercial-NoDerivs (CC BY-NC-ND), which allows third parties to download the articles and share them with others as long as they credit the author and the Abstract Book, but they cannot change the content in any way or use them commercially.
**Abstract Book Citations:** Authors, Title, HemaSphere, 2026;10:(S1):pages. The abstract book DOIs can be found on the "Author Information" page.
**Disclaimer:** Articles published in the journal HemaSphere exclusively reflect the opinions of the authors. The authors are responsible for all content in their abstracts including accuracy of the facts, statements, citing resources, etc.

**Results**

A total of 52 patients were included in the analysis. The median age of patients was 70.0 years (range, 48.0-87.0), 59.6% were male, and 88.5% were white. The patients were heavily pre-treated with more than half (61.5%) receiving ≥4 prior lines of therapy, 94.2% were double-class exposed to a prior anti-CD38 monoclonal antibody and proteasome inhibitor, and 84.6% were triple-class exposed to a prior anti-CD38 monoclonal antibody, proteasome inhibitor, and immunomodulatory therapy. International Staging System disease stage at baseline was reported for 32 patients, 46.9% (n=15) had stage III and 34.4% (n=11) had stage II disease. The most common (≥10%) comorbidities were hypertension (32.6%), atrial fibrillation (16.3%), type 2 diabetes mellitus (14.0%), and chronic kidney disease (11.6%).

**Summary/Conclusion**

Preliminary real-world effectiveness and safety data for elranatamab will be presented for this heavily pre-treated RRMM patient population at the congress.

**Copyright Information:** (Online) ISSN: 2572-9241
©2026 The Author(s). HemaSphere is published by John Wiley & Sons Ltd on behalf of European Hematology Association. This is an open access Abstract Book distributed under the Attribution-NonCommercial-NoDerivs (CC BY-NC-ND), which allows third parties to download the articles and share them with others as long as they credit the author and the Abstract Book, but they cannot change the content in any way or use them commercially.
**Abstract Book Citations:** Authors, Title, HemaSphere, 2026;10:(S1):pages. The abstract book DOIs can be found on the "Author Information" page.
**Disclaimer:** Articles published in the journal HemaSphere exclusively reflect the opinions of the authors. The authors are responsible for all content in their abstracts including accuracy of the facts, statements, citing resources, etc.

### (PS1900) PATTERNS OF MGUS IN MEN AND WOMEN PARTICIPATING IN THE SCREENED POPULATION-BASED ISTOPMM STUDY: EARLIER ONSET OR ACCELERATED M PROTEIN INCREASE IN MEN?

**Topic:** 14. Myeloma and other monoclonal gammopathies - Clinical

Anna Richardson*[1], Ingigerdur Sverrisdottir[1, 2], Sæmundur Rögnvaldsson[1], Sara Ekberg[3], Baojing Li[3], Ingunn Þorsteinsdóttir[4], Ísleifur Ólafsson[4], Ásdís Rósa Þórðardóttir[1], Andri Ólafsson[1], Þórir Einarsson Long[1, 5], Malin Hultcrantz[6], C. Ola Landgren[7], Thorvardur Jon Love[1, 3], Sigurdur Yngvi Kristinsson[1, 3], Sigrún Thorsteinsdóttir[1, 8]

*[1]University of Iceland, Reykjavik, Iceland;[2]Sahlgrenska University Hospital, Department of Haematology, Gothenburg, Sweden;[3]Red Door Analytics AB, Stockholm, Sweden;[4]Landspítali - The National University Hospital of Iceland, Reykjavík, Iceland;[5]Skane University Hospital, Lund, Sweden;[6]Memorial Sloan Kettering Cancer Center, New York, United States of America;[7]University of Miami, Sylvester Comprehensive Cancer Center, Miami, United States of America;[8]Rigshospitalet, Department of Hematology, Copenhagen, Denmark;*

### Background

Multiple myeloma (MM) and its precursor, monoclonal gammopathy of undetermined significance (MGUS), are consistently found to be more common in men than in women. We previously demonstrated that men are 1.35 times more likely to have MGUS and have an 11% higher M protein concentration at screening compared to women in the screened Iceland screens, treats, or prevents multiple myeloma (iStopMM) cohort. The disease trajectory of MGUS between the sexes has not been reported previously.

### Aims

The aim of the current study was to evaluate sex-specific differences in longitudinal M protein trajectories among individuals diagnosed with MGUS.

### Methods

All residents of Iceland aged 40 or older were offered screening with serum protein electrophoresis (SPEP) and a serum free light chain (FLC) assay in the iStopMM study. All individuals with abnormal screening results were included as cases (MGUS). Clinical characteristics in MGUS, M protein isotype and concentration, presence of immunoparesis, and abnormal FLC ratio, were compared between the sexes. To assess the change in M protein concentration, we used linear mixed effect models with log-transformed M protein concentration and random intercepts and slopes for attained age. The model assumed a linear effect of age on the log-transformed outcome. The model was adjusted for isotype, with attained age as the timescale. In a secondary analysis restricted to participants with available BMI data, we additionally adjusted for BMI at screening. For graphical displays, age was modelled using restricted cubic splines with 5 knots.

### Results

The study included 1,926 individuals with MGUS. Men accounted for 53% of individuals with MGUS. The median age at screening was 69 years (interquartile range (IQR) = 62-77). The rate of increase in M protein concentration over time was higher in men with a 6% average annual increase in M protein level (exp(β) = 1.06, 95% CI: [1.05, 1.07]) compared to 4% in women (exp(β) = 1.04, 95% CI: [1.03, 1.05]) (Figure). The sex-age interaction was statistically significant ($p = 0.004$). Adjustment for BMI did not attenuate the association. Stratified analyses (BMI <25 vs ≥25) suggested a more pronounced sex difference among individuals with BMI ≥25. When stratified by isotype, the sex difference was observed only in IgG and biclonal MGUS (Figure).

**Copyright Information:** (Online) ISSN: 2572-9241
©2026 The Author(s). HemaSphere is published by John Wiley & Sons Ltd on behalf of European Hematology Association. This is an open access Abstract Book distributed under the Attribution-NonCommercial-NoDerivs (CC BY-NC-ND), which allows third parties to download the articles and share them with others as long as they credit the author and the Abstract Book, but they cannot change the content in any way or use them commercially.
**Abstract Book Citations:** Authors, Title, HemaSphere, 2026;10:(S1):pages. The abstract book DOIs can be found on the "Author Information" page.
**Disclaimer:** Articles published in the journal HemaSphere exclusively reflect the opinions of the authors. The authors are responsible for all content in their abstracts including accuracy of the facts, statements, citing resources, etc.

### Summary/Conclusion

In the large population-based screening study, we found that men have a higher prevalence of MGUS, higher M protein levels at diagnosis, and a more rapid increase in M protein over time than women. Furthermore, we observed that the sex difference seems to be confined to IgG and biclonal MGUS. These findings indicate that sex is a key factor influencing MGUS biology, possibly due to earlier disease onset or a more aggressive disease course in men. Further studies should clarify the underlying mechanisms, determine whether these differences translate into higher rates of progression to treatment-requiring disease, and explore the potential for more individualised monitoring strategies.

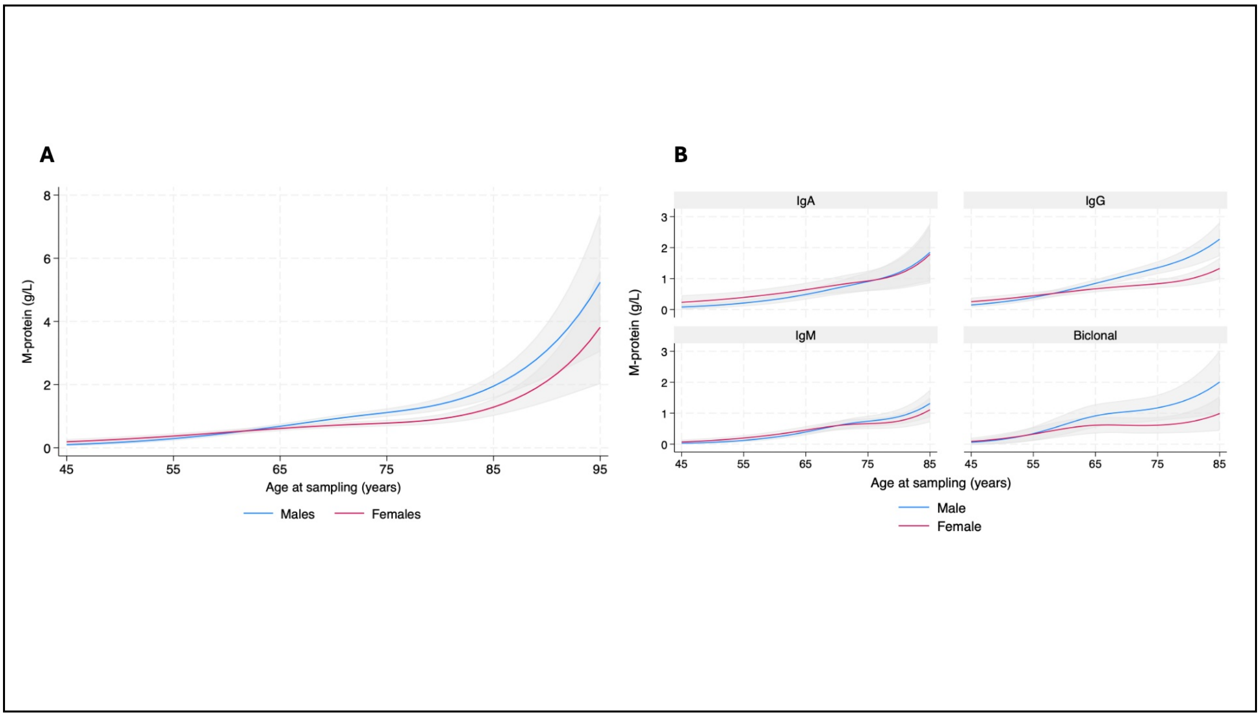

Figure. A) Predicted M protein (g/L) over age at sampling by sex from linear mixed models with random intercept and slope for attained age. B) Predicted M protein (g/L) over age at sampling (45–85) by sex and isotype category from linear mixed models with random intercept and slope for attained age. Age was modelled as restricted cubic splines with 5 knots, including sex by age interaction. Lines represent males (blue) and females (red); shaded areas indicate 95% confidence intervals.

**Copyright Information:** (Online) ISSN: 2572-9241
©2026 The Author(s). HemaSphere is published by John Wiley & Sons Ltd on behalf of European Hematology Association. This is an open access Abstract Book distributed under the Attribution-NonCommercial-NoDerivs (CC BY-NC-ND), which allows third parties to download the articles and share them with others as long as they credit the author and the Abstract Book, but they cannot change the content in any way or use them commercially.
**Abstract Book Citations:** Authors, Title, HemaSphere, 2026;10:(S1):pages. The abstract book DOIs can be found on the “Author Information” page.
**Disclaimer:** Articles published in the journal HemaSphere exclusively reflect the opinions of the authors. The authors are responsible for all content in their abstracts including accuracy of the facts, statements, citing resources, etc.

### (PS1901) TRENDS IN HOSPITALIZATION FOR MULTIPLE MYELOMA IN SPAIN (2016–2023): RISING HEALTHCARE BURDEN AND DECLINING IN-HOSPITAL MORTALITY

**Topic:** 14. Myeloma and other monoclonal gammopathies - Clinical

Ane Brieva Lopez*[1], Maria Ordoñez Garcia[1], Rafael Garcia Carretero[2], Pavel Manuel Acevedo Pérez[1], Mariana Cerrato Salas[1], Juan Sastre Ortega[1], Esther Jaro Arias[1], Alicia Lorenzo Jambrina[1], Maria Angeles Andreu Costa[1]

*[1]Hospital Universitario de Móstoles, Madrid, Spain;[2]Hospital Universitario Severo Ochoa, Madrid, Spain;*

### Background

Multiple myeloma (MM) represents the second most common hematological cancer in Europe. Analysis of hospitalization patterns provides valuable insight into disease burden and patient outcomes. Since population-based data on admissions due to MM remain limited, a detailed analysis is therefore needed to quantify healthcare demand and temporal trends at a national level.

### Aims

The primary objective of this study was to evaluate trends in MM admissions and mortality over a 8-year study period in Spain.

### Methods

We performed a nationwide, retrospective study from the Spanish National Health System. All hospital admissions with a diagnosis of MM between 2016 and 2023 were included. Demographic characteristics, comorbidities, and in-hospital mortality were analyzed. Hospitalization rates per 100,000 population were calculated, and temporal trends were assessed using Poisson regression models.

**Copyright Information:** (Online) ISSN: 2572-9241
©2026 The Author(s). HemaSphere is published by John Wiley & Sons Ltd on behalf of European Hematology Association. This is an open access Abstract Book distributed under the Attribution-NonCommercial-NoDerivs (CC BY-NC-ND), which allows third parties to download the articles and share them with others as long as they credit the author and the Abstract Book, but they cannot change the content in any way or use them commercially.
**Abstract Book Citations:** Authors, Title, HemaSphere, 2026;10:(S1):pages. The abstract book DOIs can be found on the “Author Information” page.
**Disclaimer:** Articles published in the journal HemaSphere exclusively reflect the opinions of the authors. The authors are responsible for all content in their abstracts including accuracy of the facts, statements, citing resources, etc.

### Results

A total of 107,496 MM-related admissions were recorded, corresponding to 43,383 unique patients and an average of 2.5 admissions per patient. The median age was 73 years (interquartile range 63–81). Women accounted for 47% of hospitalizations, with no significant change in sex distribution (p=0.81). The annual number of admissions rose from 10,939 in 2016 to 15,641 in 2022 (Figure 1A). Similarly, the hospitalization rates increased from 25.1 to 34.5 per 100,000 population (p<0.001). A total of 5,107 patients died during an episode of hospitalization, corresponding to an overall in-hospital mortality rate of 11% of unique patients (Table 1). The in-hospital mortality rate showed a significant, non-linear trend over the 8-year period (Figure 1B), showing its maximum of 11.9% in 2020, corresponding to the peak of the COVID-19 pandemic. Post-2020, the rate declined consistently, up to 9.6% in 2023.

*Table 1. Characteristics and hospitalization trends in multiple myeloma, Spain 2016–2023.*

| | **Total Patients** | **2016** | **2017** | **2018** | **2019** | **2020** | **2021** | **2022** | **2023** | **p-value** |
|---|---|---|---|---|---|---|---|---|---|---|
| | 43,383 | 6,857 | 5,464 | 5,582 | 5,231 | 4,677 | 4,991 | 5,221 | 5,360 | |
| **Admissions (% per patient)** | 107,496 (2.4%) | 10,939 (1.6%) | 11,901 (2.1%) | 13,466 (2.4%) | 14,505 (2.7%) | 12,744 (2.7%) | 13,172 (2.6%) | 15,128 (2.9%) | 15,641 (2.9%) | <0.001 |
| **Stage** | | | | | | | | | | |
| No remission | 36,462 (84%) | 5,612 (82%) | 4,597 (84%) | 4,561 (82%) | 4,352 (83%) | 3,961 (85%) | 4,234 (85%) | 4,547 (87%) | 4,598 (86%) | <0.001 |
| Complete remission | 3,012 (6.9%) | 537 (7.8%) | 371 (6.8%) | 450 (8.1%) | 408 (7.8%) | 311 (6.6%) | 318 (6.4%) | 282 (5.4%) | 335 (6.3%) | <0.001 |
| Relapses | 1,591 (3.7%) | 393 (5.7%) | 252 (4.6%) | 278 (5.0%) | 182 (3.5%) | 142 (3.0%) | 143 (2.9%) | 107 (2.0%) | 94 (1.8%) | <0.001 |
| **Age** | 73 (63, 81) | 72 (63, 81) | 72 (63, 81) | 73 (64, 82) | 73 (64, 81) | 72 (62, 81) | 73 (63, 81) | 73 (63, 81) | 73 (63, 81) | 0.002 |
| **Sex (women)** | 20,179 (47%) | 3,161 (46%) | 2,587 (47%) | 2,589 (46%) | 2,463 (47%) | 2,145 (46%) | 2,305 (46%) | 2,431 (47%) | 2,498 (47%) | 0.81 |
| **Hematopoietic stem cell transplantation** | 5,608 (13%) | 904 (13%) | 677 (12%) | 667 (12%) | 652 (12%) | 634 (14%) | 668 (13%) | 689 (13%) | 717 (13%) | 0.12 |
| **Deaths** | 5,107 | 894 | 645 | 624 | 569 | 606 | 576 | 586 | 607 | <0.001 |

### Summary/Conclusion

The 43% increase in MM-related admissions highlights a rising healthcare burden despite advances in disease management. Improved diagnostic techniques and the introduction of new therapeutic options lead to extended patient survival. The average of 2.5 hospitalizations per patient suggests that MM is being managed as a chronic condition requiring repeated hospital care, driven by disease progression, treatment-related toxicity, and comorbid conditions. These population-based data emphasise the need for strategic healthcare planning to address the growing demand for inpatient care among patients with MM.

**Copyright Information:** (Online) ISSN: 2572-9241
©2026 The Author(s). HemaSphere is published by John Wiley & Sons Ltd on behalf of European Hematology Association. This is an open access Abstract Book distributed under the Attribution-NonCommercial-NoDerivs (CC BY-NC-ND), which allows third parties to download the articles and share them with others as long as they credit the author and the Abstract Book, but they cannot change the content in any way or use them commercially.
**Abstract Book Citations:** Authors, Title, HemaSphere, 2026;10:(S1):pages. The abstract book DOIs can be found on the "Author Information" page.
**Disclaimer:** Articles published in the journal HemaSphere exclusively reflect the opinions of the authors. The authors are responsible for all content in their abstracts including accuracy of the facts, statements, citing resources, etc.

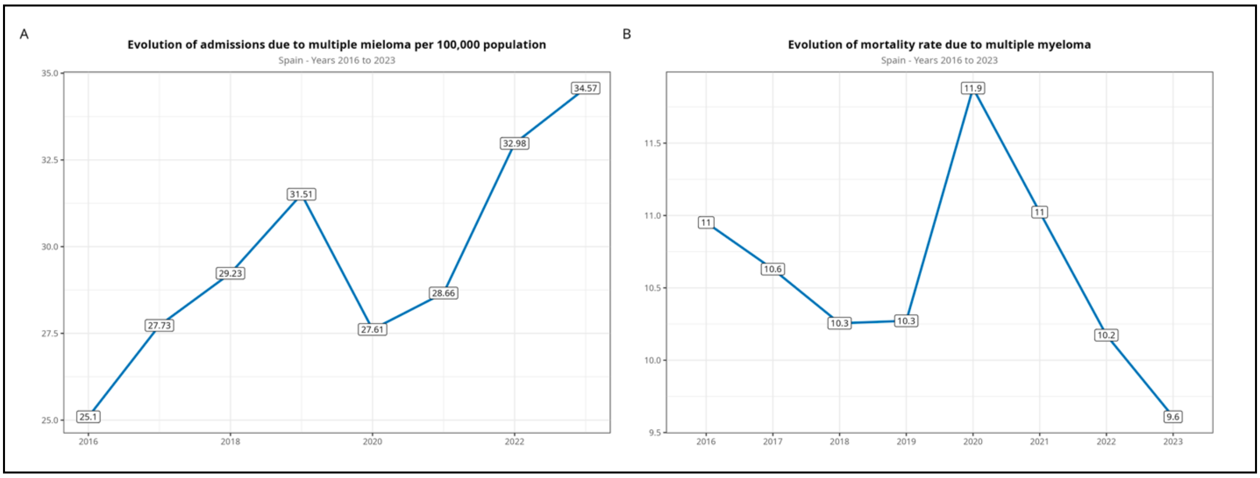

Figure 1. Trends in hospitalization and mortality rates in multiple myeloma, Spain 2016-2023.

**Copyright Information:** (Online) ISSN: 2572-9241
©2026 The Author(s). HemaSphere is published by John Wiley & Sons Ltd on behalf of European Hematology Association. This is an open access Abstract Book distributed under the Attribution-NonCommercial-NoDerivs (CC BY-NC-ND), which allows third parties to download the articles and share them with others as long as they credit the author and the Abstract Book, but they cannot change the content in any way or use them commercially.
**Abstract Book Citations:** Authors, Title, HemaSphere, 2026;10:(S1):pages. The abstract book DOIs can be found on the "Author Information" page.
**Disclaimer:** Articles published in the journal HemaSphere exclusively reflect the opinions of the authors. The authors are responsible for all content in their abstracts including accuracy of the facts, statements, citing resources, etc.

### (PS1902) COMMITMENTT-TEC: A GLOBAL REAL-WORLD (RW) STUDY OF TECLISTAMAB IN RELAPSED/REFRACTORY MULTIPLE MYELOMA (RRMM)

**Topic:** 14. Myeloma and other monoclonal gammopathies - Clinical

Guido Lancman*[1], Gloria Huei-Jong Graf[2], Naama Kipperman[2], Sikander Ailawadhi[3], Rahul Banerjee[4], Vania Hungria[5], Jian Li[6], Joaquín Martinez-Lopez[7], Kazuhito Suzuki[8], Johannes Waldschmidt[9], Jill Zitzewitz[10], Rakesh Popat[11], Niodita Gupta-Werner[12], Shuchita Kaila[12], Eva Rubio Azpeitia[13], Guntug Gungor[14], Lori Parisi[2], Mark Wildgust[2], Bingcao Wu[2], Mariana Fernandez[13]

*[1]Princess Margaret Cancer Centre, Toronto, Canada; [2]Johnson & Johnson, Raritan, United States of America; [3]Mayo Clinic, Jacksonville, United States of America; [4]Fred Hutchinson Cancer Center, Seattle, United States of America; [5]Clínica São Germano, São Paulo, Brazil; [6]Peking Union Medical College Hospital (PUMCH), Beijing, China; [7]Hospital Universitario 12 de Octubre, Madrid, Spain; [8]The Jikei University School of Medicine, Tokyo, Japan; [9]Würzburg University Hospital, Würzburg, Germany; [10]UMass Chan Medical School, Worcester, United States of America; [11]University College London Hospitals NHS Foundation Trust, London, United Kingdom; [12]Johnson & Johnson, Horsham, United States of America; [13]Johnson & Johnson, Madrid, Spain; [14]Johnson & Johnson, Breda, The Netherlands;*

### Background

Teclistamab is the most widely used bispecific antibody worldwide for the treatment of triple-class-exposed RRMM. While multiple single-center or single-country RW studies have reported safety and effectiveness outcomes consistent with the MajesTEC-1 trial, large global RW studies are needed to characterize treatment strategies and clinical outcomes across multiple regions and in clinically challenging populations.

### Aims

CoMMitmenTT is a global, chart-review-based platform study pooling data from multiple sources to evaluate real-world treatment patterns and outcomes among patients receiving teclistamab or talquetamab commercially or through expanded-access programs. Here, we report initial pooled results from three teclistamab-treated patient cohorts in the United States (US), Europe/Middle East/Africa (EMEA), and Canada along with subgroup analyses of patients with renal impairment, high-risk cytogenetic abnormalities (HRCA), and elderly patients (≥75 years).

### Methods

We pooled patient-level data from two retrospective, company-sponsored chart reviews in the US (data collection March 2024–May 2025) and EMEA (Dec 2023–Aug 2024), and aggregate data from the Canadian Myeloma Research Group (CMRG) registry (May 2023–Dec 2025). Eligibility criteria included confirmed RRMM diagnosis, initiation of teclistamab monotherapy outside of clinical trial, adult age (≥18 years), and ≥1 month follow-up. Renal impairment was defined as recorded and collected within each cohort. HRCA was defined as presence of at least one of the following: del(17p), t(4;14), t(14;16), amp(1q), del(1p). Naïve pooling methods were used to combine individual patient data from chart reviews with aggregate CMRG data, using weighted averages and summed counts as appropriate.

**Copyright Information:** (Online) ISSN: 2572-9241
©2026 The Author(s). HemaSphere is published by John Wiley & Sons Ltd on behalf of European Hematology Association. This is an open access Abstract Book distributed under the Attribution-NonCommercial-NoDerivs (CC BY-NC-ND), which allows third parties to download the articles and share them with others as long as they credit the author and the Abstract Book, but they cannot change the content in any way or use them commercially.
**Abstract Book Citations:** Authors, Title, HemaSphere, 2026;10:(S1):pages. The abstract book DOIs can be found on the "Author Information" page.
**Disclaimer:** Articles published in the journal HemaSphere exclusively reflect the opinions of the authors. The authors are responsible for all content in their abstracts including accuracy of the facts, statements, citing resources, etc.

**Results**

A total of 356 patients were eligible for study inclusion (mean age 67 years, mean prior lines of therapy (PL) 5.2 [range 1-13], 66% triple-class refractory (TCR), 15% prior BCMA exposure; 19% with renal impairment, 29% HRCA, 17% aged ≥75 years). Median follow-up was 13.8 months in the US cohort, 15.1 in the EMEA cohort, and 9.9 in the CMRG cohort. Subgroup patient characteristics were comparable to the overall cohort (Renal impairment: mean age 71, mean PL 5.8, 68% TCR, 18% prior BCMA. HRCA: mean age 66, mean PL 5, 75% TCR, 17% prior BCMA. Elderly: mean age 80, mean PL 5, 71% TCR, 12% prior BCMA). ORR in the overall cohort was 73% (57% ≥VGPR); 12-mo PFS and OS estimates were 54% and 68%. Results were comparable across subgroups (renal impairment, HRCA, elderly: ORR/≥VGPR 68%/57%, 75%/65%, 80%/74%; 12-mo PFS 45%, 55%, 59%; 12-mo OS 60%, 63%, 62%). Incidence of all-grade CRS was 40% (10% Gr2, 0.9% Gr3-4) in the overall cohort; incidence of all-grade ICANS was 6% (0.6% Gr≥3). Incidence and severity of CRS and ICANS did not differ substantially across subgroups. In the overall cohort, 15% of patients experienced at least one severe (Gr≥3) infection, compared to 23%, 20%, and 12% in the renal impairment, HRCA, and elderly subgroups. Across groups, most severe infections occurred within 6 months of teclistamab initiation.

**Summary/Conclusion**

In this global cohort spanning 10 countries, real-world effectiveness outcomes were consistent with MajesTEC-1 across a range of treatment contexts and clinically challenging patient profiles. Safety outcomes were numerically favorable compared with MajesTEC-1, potentially reflecting evolution of prophylaxis and management strategies over time.

**Copyright Information:** (Online) ISSN: 2572-9241
©2026 The Author(s). HemaSphere is published by John Wiley & Sons Ltd on behalf of European Hematology Association. This is an open access Abstract Book distributed under the Attribution-NonCommercial-NoDerivs (CC BY-NC-ND), which allows third parties to download the articles and share them with others as long as they credit the author and the Abstract Book, but they cannot change the content in any way or use them commercially.
**Abstract Book Citations:** Authors, Title, HemaSphere, 2026;10:(S1):pages. The abstract book DOIs can be found on the "Author Information" page.
**Disclaimer:** Articles published in the journal HemaSphere exclusively reflect the opinions of the authors. The authors are responsible for all content in their abstracts including accuracy of the facts, statements, citing resources, etc.

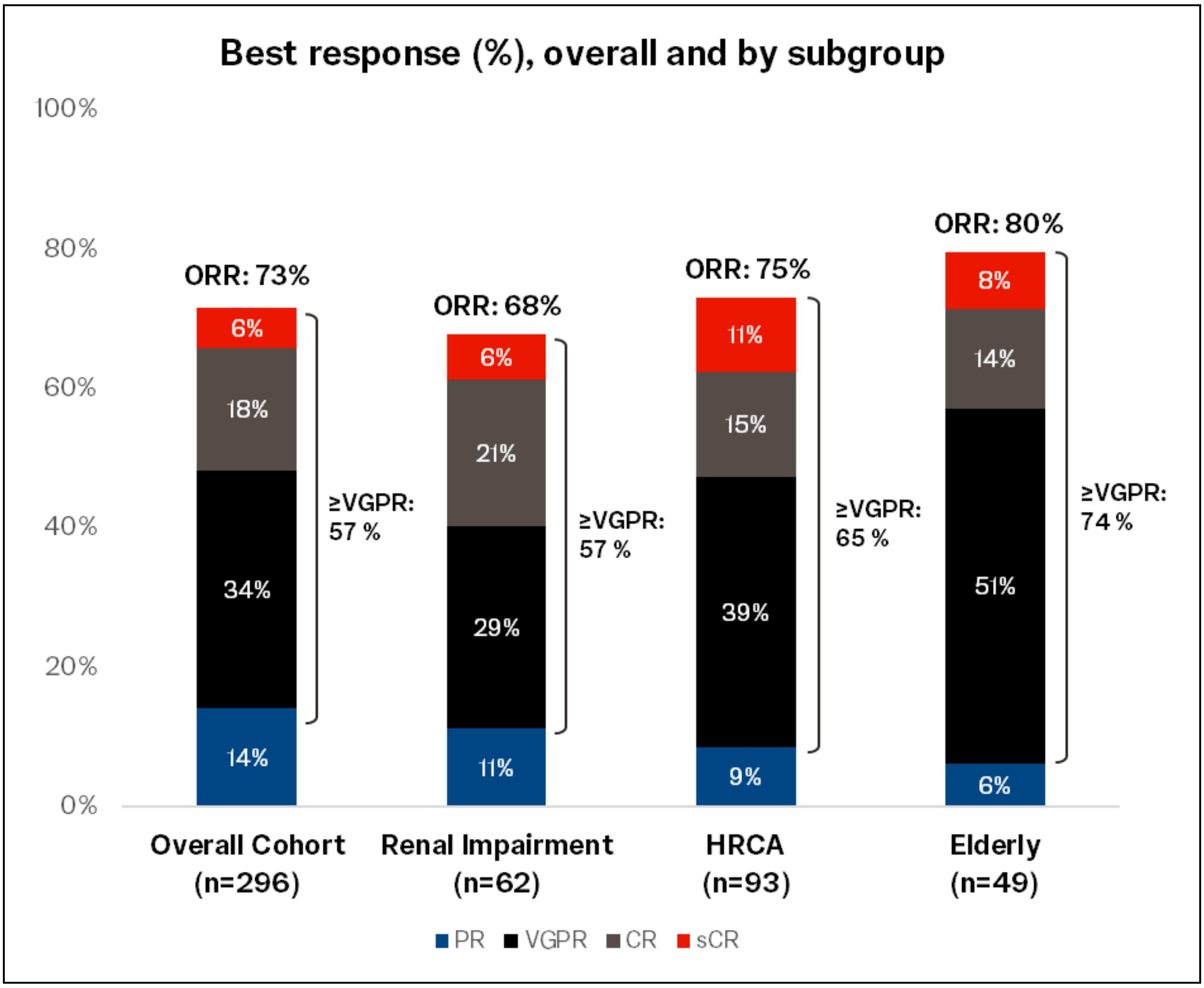

Copyright Information: (Online) ISSN: 2572-9241
©2026 The Author(s). HemaSphere is published by John Wiley & Sons Ltd on behalf of European Hematology Association. This is an open access Abstract Book distributed under the Attribution-NonCommercial-NoDerivs (CC BY-NC-ND), which allows third parties to download the articles and share them with others as long as they credit the author and the Abstract Book, but they cannot change the content in any way or use them commercially.
Abstract Book Citations: Authors, Title, HemaSphere, 2026;10:(S1):pages. The abstract book DOIs can be found on the "Author Information" page.
Disclaimer: Articles published in the journal HemaSphere exclusively reflect the opinions of the authors. The authors are responsible for all content in their abstracts including accuracy of the facts, statements, citing resources, etc.

### (PS1903) DYNAMICS OF SARCOPENIA PARAMETERS PREDICT SURVIVAL IN OLDER PATIENTS WITH MULTIPLE MYELOMA

**Topic:** 14. Myeloma and other monoclonal gammopathies - Clinical

Febe Smits*[1, 2, 3], Kazem Nasserinejad[4, 5], Gerben Zwezerijnen[6], Kazimierz Groen[1, 2], Mark-David Levin[7], Claudia Stege[5], Rjw Van Kampen[8], Ellen van der Spek[9], Yavuz M. Bilgin[10], Noortje Thielen[11], Inger Nijhof[12], Inge Ludwig[13], Esther de Waal[14], Yorick Sandberg[15], Alain Kentos[16], Gert Jan Timmers[17], Josien Regelink[18], Matthijs Westerman[19], Koen de Heer[20], Marie-Christiane Vekemans[21], Nazik Durdu-Rayman[22], Nicole de Graauw[23], Maarten Seefat[1, 2], Paula Ypma[3], Ronald Boellaard[6], Niels W C J van de Donk[1, 2], Sonja Zweegman[1, 2]

*[1]Amsterdam UMC, Hematology, Amsterdam, The Netherlands; [2]Cancer Center Amsterdam, Treatment and Quality of Life, Amsterdam, The Netherlands; [3]Haga Hospital, Hematology, The Hague, The Netherlands; [4]HOVON Data Center, Rotterdam, The Netherlands; [5]Erasmus Medical Center, Hematology, Rotterdam, The Netherlands; [6]Amsterdam UMC, Radiology and Nuclear Medicine, Amsterdam, The Netherlands; [7]Albert Schweitzer Hospital, Internal Medicine, Dordrecht, The Netherlands; [8]Zuyderland Hospital, Internal Medicine, Sittard, The Netherlands; [9]Rijnstate Hospital, Internal Medicine, Arnhem, The Netherlands; [10]Adrz Hospital, Internal Medicine, Goes, The Netherlands; [11]Diakonessenhuis, Internal Medicine, Utrecht, The Netherlands; [12]Antonius Hospital, Internal Medicine, Nieuwegein, The Netherlands; [13]Bernhoven Hospital, Hematology, Uden, The Netherlands; [14]Frisius Medical Center, Internal Medicine, Leeuwarden, The Netherlands; [15]Maasstad Hospital, Internal Medicine, Rotterdam, The Netherlands; [16]Centre Hospitalier Jolimont, Hematology, Haine-Saint-Paul, Belgium; [17]Amstelland Hospital, Internal Medicine, Amstelveen, The Netherlands; [18]Meander Medical Center, Internal Medicine, Amersfoort, The Netherlands; [19]Northwest Clinices, Internal Medicine, Alkmaar, The Netherlands; [20]Flevoziekenhuis, Internal Medicine, Almere, The Netherlands; [21]St. Luc Hospital, Hematology, Brussels, Belgium; [22]Franciscus Hospital, Internal Medicine, Schiedam, The Netherlands; [23]Bravis Hospital, Internal Medicine, Roosendaal, The Netherlands;*

### Background

Clinical outcomes of older patients with newly-diagnosed multiple myeloma (NDMM) are largely dependent on the level of frailty, assessed by the International Myeloma Working Group Frailty Index (IMWG-FI). Sarcopenia constitutes a key biological substrate of frailty; however, data on its incidence, prognostic relevance and temporal dynamics in frail populations remain limited.

### Aims

To evaluate the incidence, longitudinal evolution and predictive value of sarcopenia for clinical outcomes in intermediate-fit and frail patients treated within the HOVON 143 trial.

### Methods

The HOVON 143 study included NDMM classified as intermediate-fit or frail by the IMWG-FI. Patients were treated with ixazomib, daratumumab and low dose dexamethasone. Gait speed and grip strength were assessed at baseline, and after 3, 9, 15, 21, and 33 cycles. Skeletal muscle index (SMI, $cm^2/m^2$) at L3 was quantified from CT-scans obtained at baseline and after 9 cycles, using a machine learning-based segmentation tool (Mosamatic). Sarcopenia was defined according to EWGSOP2 criteria based on grip strength, gait speed, and SMI. Joint models were employed to model longitudinal sarcopenia data and survival outcomes (R, package JM).

**Copyright Information:** (Online) ISSN: 2572-9241
©2026 The Author(s). HemaSphere is published by John Wiley & Sons Ltd on behalf of European Hematology Association. This is an open access Abstract Book distributed under the Attribution-NonCommercial-NoDerivs (CC BY-NC-ND), which allows third parties to download the articles and share them with others as long as they credit the author and the Abstract Book, but they cannot change the content in any way or use them commercially.
**Abstract Book Citations:** Authors, Title, HemaSphere, 2026;10:(S1):pages. The abstract book DOIs can be found on the "Author Information" page.
**Disclaimer:** Articles published in the journal HemaSphere exclusively reflect the opinions of the authors. The authors are responsible for all content in their abstracts including accuracy of the facts, statements, citing resources, etc.

**Results**

A total of 130 patients were included, of whom 65 were frail and 65 were intermediate-fit. Baseline sarcopenia data were available for 41/65 and 42/65, respectively. At diagnosis, 4/41 (10%) frail patients had probable sarcopenia and 10 (24%) sarcopenia, compared with 1 (2%) and 5 (12%) in intermediate-fit patients (p=0.11). Frail patients had significantly lower baseline gait speed (β = -0.12 m/s, p=0.008) and grip strength (β = -4.05 kg, p=0.004) as compared to the intermediate-fit.
Mean gait speed and grip strength did not significantly change over time, nor between intermediate-fit and frail (**Figure 1A-C**) and frail subgroups **(Figure 1B-D)**. However, both measures showed substantial inter- and intra-individual variability over time.
After 9 cycles, sarcopenia data were available in 20/41 (49%) of frail patients who were still on protocol, and in 27/41 (59%) of intermediate-fit patients. Four/10 (40%) frail patients with sarcopenia at baseline improved to no sarcopenia, 1 to 'probable sarcopenia'. Three/27 (11%) frail patients without sarcopenia at baseline developed sarcopenia. In intermediate-fit patients, 1/5 patients with sarcopenia improved to no sarcopenia. Two/37 (5%) intermediate-fit patients deteriorated to 'probable sarcopenia' and 1/37 patient to having sarcopenia.
In univariate cox regression, baseline gait speed was significantly associated with overall survival (HR 0.36, 95%CI 0.16 – 0.81, p=0.01), whereas grip strength, SMI and sarcopenia were not. Improvements in gait speed over time were also significantly associated with overall survival (HR 0.77, 95% CI 0.62 – 0.96, p=0.02), with each increase of 0.1 m/s corresponding to a 23% reduced hazard of death. Increased grip strength was associated with an 18% reduction of mortality risk per 5 kg, although not statistically significant (HR 0.82, 95% CI 0.60 – 1.13, p=0.23).

**Summary/Conclusion**

This is the first study to describe the long-term dynamics of sarcopenia-related parameters in non-fit NDMM patients. Improvement of grip strength and gait speed were associated with a reduced risk of mortality. Repeated functional assessments, particularly of gait speed, may more accurately reflect functional frailty and contribute to guide personalized interventions to improve survival in older patients with multiple myeloma.

**Copyright Information:** (Online) ISSN: 2572-9241
©2026 The Author(s). HemaSphere is published by John Wiley & Sons Ltd on behalf of European Hematology Association. This is an open access Abstract Book distributed under the Attribution-NonCommercial-NoDerivs (CC BY-NC-ND), which allows third parties to download the articles and share them with others as long as they credit the author and the Abstract Book, but they cannot change the content in any way or use them commercially.
**Abstract Book Citations:** Authors, Title, HemaSphere, 2026;10:(S1):pages. The abstract book DOIs can be found on the "Author Information" page.
**Disclaimer:** Articles published in the journal HemaSphere exclusively reflect the opinions of the authors. The authors are responsible for all content in their abstracts including accuracy of the facts, statements, citing resources, etc.

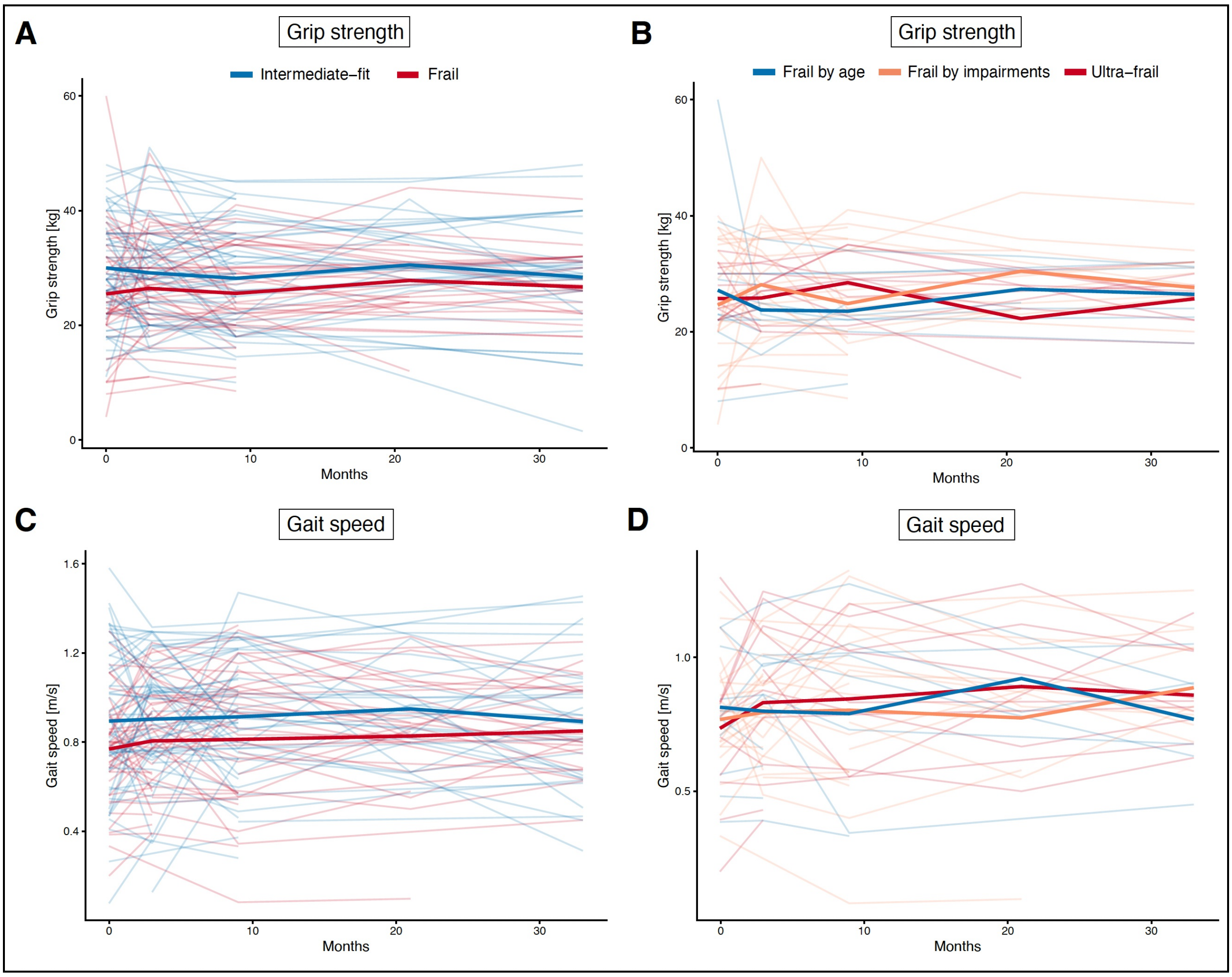

Figure 1. Dynamics of grip strength (A-B) and gait speed (C-D) in intermediate-fit and frail patients and across frail subgroups (B-C) over the course of 33 treatment cycles.

**Copyright Information:** (Online) ISSN: 2572-9241
©2026 The Author(s). HemaSphere is published by John Wiley & Sons Ltd on behalf of European Hematology Association. This is an open access Abstract Book distributed under the Attribution-NonCommercial-NoDerivs (CC BY-NC-ND), which allows third parties to download the articles and share them with others as long as they credit the author and the Abstract Book, but they cannot change the content in any way or use them commercially.
**Abstract Book Citations:** Authors, Title, HemaSphere, 2026;10:(S1):pages. The abstract book DOIs can be found on the "Author Information" page.
**Disclaimer:** Articles published in the journal HemaSphere exclusively reflect the opinions of the authors. The authors are responsible for all content in their abstracts including accuracy of the facts, statements, citing resources, etc.

### (PS1904) EVALUATION OF EARLY M-PROTEIN KINETICS POST-CAR T CELL THERAPY WITH CLONOTYPIC MASS SPECTROMETRY AND COMPARISON WITH BONE MARROW-BASED MEASURABLE RESIDUAL DISEASE.

**Topic:** 14. Myeloma and other monoclonal gammopathies - Clinical

Michael Slade[1], Abir Khaled[2], Julie Fortier[1], Zac McDonald[2], Sarah Kelley[1], Mark Fiala[1], Mark Schroeder[1], Keith Stockerl-Goldstein[1], Liqiang Yang[2], Ravi Vij*[1]

*[1]Washington University School of Medicine, Department of Medicine, Division of Oncology, Blood Cancer Section, Saint Louis, United States of America; [2]Rapid Novor, Kitchener, Canada;*

### Background

Monitoring response following chimeric antigen receptor (CAR) T cell therapy for relapsed multiple myeloma (MM) remains a challenge. Standard-of-care (SoC) assays lack specificity and have well-established limits of detection. Individualized M-protein measurement by clonotypic mass spectrometry (CT-MS) may allow for better understanding of disease kinetics.

### Aims

We aim to describe post-CAR T M-protein kinetics using CT-MS and compare this to SoC clonoSEQ measurable residual disease (MRD).

### Methods

Patients receiving CAR T cells for relapsed MM consented to serial serum collection for CT-MS analysis using the EasyM assay (Rapid Novor, Kitchener, ON). Clonotype generation generally requires a sample with M-protein ≥0.2 g/dL by serum protein electrophoresis (SPEP) or >20 mg/dL (light chain). Patients were included regardless of M-protein isotype. Baseline and follow up samples were analyzed by Rapid Novor, who were blinded to clinical data. Percent residual CT-MS was used as a measure of dynamic M-protein response. Bone marrow-based MRD was analyzed using clonoSEQ at a threshold of $>10^{-6}$ MM cells/cells measured.

### Results

Baseline clonotype by CT-MS was established in all 51 patients who met the study criteria, including 4 patients with M-protein <0.2 g/dL. The cohort was 57% male and 88% White. Median age was 67 years (range: 42 – 80). 94% of patients received cilta-cel. Median time from diagnosis to CAR T cell was 7.2 years. M-protein isotypes were IgG (n = 33), IgA (9), FLC (8) and IgD (1). Post-CAR samples were available on day +30, +100 and +365 in 92%, 73% and 37% of patients. M-protein kinetics by CT-MS varied significantly by isotype; short half-life (SHL) M-proteins (IgA/IgD/FLC) were more likely to be undetectable than long half-life (LHL) M-proteins (IgG) at day +30 (17% vs. 0%, $p = 0.02$) and day +100 (45% vs. 0%, $p < 0.001$). This difference persisted in the small cohort of patients (n = 18) with sample at 1-year post-CAR (80% vs. 7%, $p = 0.001$). Median percent residual M-protein versus pre-LDC baseline was higher in LHL versus SHL M-proteins at day +30 (median: 41.07% vs. 4.26%, $p < 0.001$) and day +100 (16.28% vs. 0.052%, $p < 0.001$). Similarly, absolute M-protein was lower for IgA/IgD versus IgG at day +30 (median: 0.011 vs. 0.323 g/dL, $p = 0.001$) and day +100 (0.001 vs. 0.154, $p = 0.002$).
43 (84%) and 42 (82%) patients had clonoSEQ MRD testing at days +30 and +100. Of these, 6 (14%) and 4 (10%) were MRD+ at day +30 and +100, with 2 persistently positive patients. MRD+ patients had numerically higher residual M-protein than MRD- patients at day +30 (median: 62.94% vs. 19.07%, $p = 0.09$) and day +100 (36.26% vs. 16.28%, $p = 0.40$). All but one clonoSEQ MRD+ samples were also positive by CT-MS ; one MRD+ day +100 sample was with baseline FLC was negative by CT-MS. The patient relapsed with non-secretory extramedullary disease at day +181.

**Copyright Information:** (Online) ISSN: 2572-9241
©2026 The Author(s). HemaSphere is published by John Wiley & Sons Ltd on behalf of European Hematology Association. This is an open access Abstract Book distributed under the Attribution-NonCommercial-NoDerivs (CC BY-NC-ND), which allows third parties to download the articles and share them with others as long as they credit the author and the Abstract Book, but they cannot change the content in any way or use them commercially.
**Abstract Book Citations:** Authors, Title, HemaSphere, 2026;10:(S1):pages. The abstract book DOIs can be found on the "Author Information" page.
**Disclaimer:** Articles published in the journal HemaSphere exclusively reflect the opinions of the authors. The authors are responsible for all content in their abstracts including accuracy of the facts, statements, citing resources, etc.

**Summary/Conclusion**

Our analysis highlights the potential utility and challenges of highly sensitive CT-MS for MRD monitoring post-CAR T cell therapy for MM. Given the high frequency of MRD-negativity by clonoSEQ, CT-MS may be a better dynamic measure of disease activity. However, our analysis suggests that characterizing CT-MS MRD status based solely on a “detectable” result is misguided due to varying half-lives. Isotype-specific, kinetic thresholds are needed. Collection of additional samples and disease outcomes are ongoing and will evaluate the prognostic value of CT-MS in this setting.

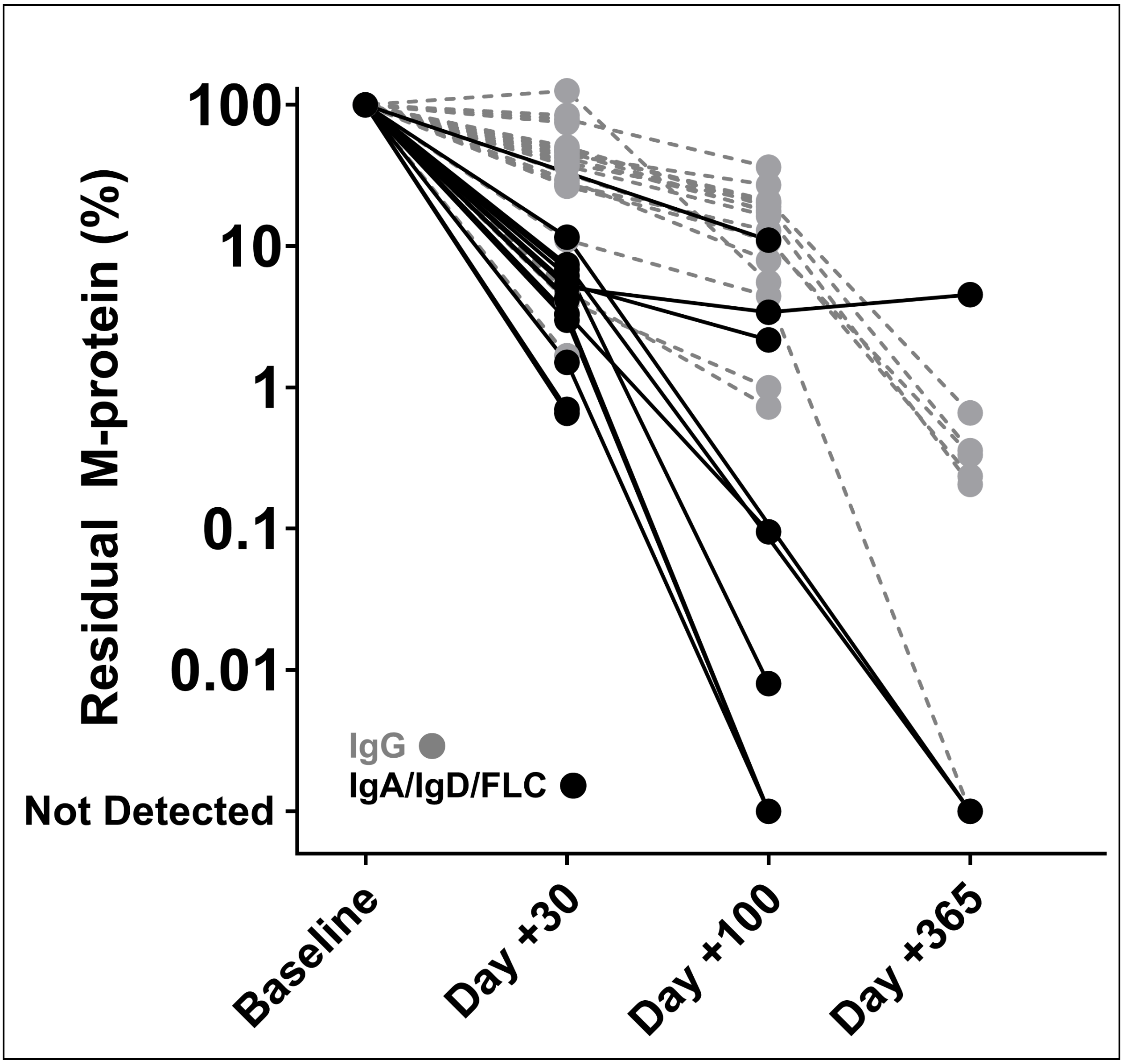

Figure 1: CT-MS M-protein kinetics post-CAR T for relapsed multiple myeloma.

**Copyright Information:** (Online) ISSN: 2572-9241
©2026 The Author(s). HemaSphere is published by John Wiley & Sons Ltd on behalf of European Hematology Association. This is an open access Abstract Book distributed under the Attribution-NonCommercial-NoDerivs (CC BY-NC-ND), which allows third parties to download the articles and share them with others as long as they credit the author and the Abstract Book, but they cannot change the content in any way or use them commercially.
**Abstract Book Citations:** Authors, Title, HemaSphere, 2026;10:(S1):pages. The abstract book DOIs can be found on the “Author Information” page.
**Disclaimer:** Articles published in the journal HemaSphere exclusively reflect the opinions of the authors. The authors are responsible for all content in their abstracts including accuracy of the facts, statements, citing resources, etc.

### (PS1905) MRD-NEGATIVE RESPONSE AFTER VRD INDUCTION, HD-ASCT AND VRD CONSOLIDATION IN TRANSPLANT-ELIGIBLE NEWLY DIAGNOSED MULTIPLE MYELOMA: PHASE 2 REMNANT STUDY WITH BROAD INCLUSION CRITERIA

**Topic:** 14. Myeloma and other monoclonal gammopathies - Clinical

Frida Bugge Askeland*[1, 2], Anne-Marie Rasmussen[1], Anna Lysén[1], Pegah Abdollahi[1], Einar Haukås[3], Magnus Moksnes[4], Anette L. Eilertsen[5], Galina Tsykunova[6], Birgitte Dahl Eiken[7], Nils Morten Leknes[8], Jürgen Rolke[9], Vidar Stavseth[10], Eivind Samstad[11, 12], Randi Fykse Hallstensen[13], Damian Szatkowski[14], Liv Osnes[15], Mona Høysæter Fenstad[16], Anna Parente-Ribes[17], Barbora Jacobsen[16], Ariane Aasbø Hansen[1], Anita Smith Nilsen[1], Tobias S. Slørdahl[18, 19], Fredrik Schjesvold[1]

*[1]Oslo Myeloma Center, Oslo University Hospital, Department of Hematology, Oslo, Norway;[2]Institute of Clinical Medicine, University of Oslo, Oslo, Norway; [3]Stavanger University Hospital, Department of Hematology and Oncology, Stavanger, Norway; [4]Vestfold Hospital Trust, Cancer and Hematology Center, Tønsberg, Norway;[5]Akershus University Hospital, Department of Hematology, Lørenskog, Norway; [6]Haukeland University Hospital, Department of Medicine, Bergen, Norway;[7]Hospital Oestfold, Department of Oncology and Hematology, Kalnes, Norway; [8]University Hospital of North Norway, Department of Hematology, Tromsø, Norway; [9]Sørlandet Hospital, Department of Hematology, Kristiansand, Norway; [10]Levanger Hospital, Medical Department, Levanger, Norway; [11]Ålesund Hospital, Department of Medicine, Ålesund, Norway; [12]Norwegian University of Science and Technology, Department of Health Sciences in Ålesund, Ålesund, Norway; [13]Nordland Hospital, Department of Hematology, Bodø, Norway; [14]Førde Central Hospital, Department of Hematology and Oncology, Førde, Norway; [15]Oslo University Hospital, Department of immunology, Oslo, Norway; [16]St. Olavs Hospital, Department of immunology and transfusion medicine, Trondheim, Norway; [17]BCNpeptides, Barcelona, Spain; [18]St. Olavs Hospital, Department of Hematology, Trondheim, Norway; [19]Norwegian University of Science and Technology, Department of clinical and molecular medicine, Trondheim, Norway;*

### Background

High-dose melphalan followed by autologous stem-cell transplantation (HD-ASCT) improves responses and prolongs progression-free survival (PFS) in transplant-eligible newly diagnosed multiple myeloma (TE NDMM). However, most transplant trials exclude patients > 65 years or those with renal impairment (RI), poor performance status, or significant comorbidities, limiting data on minimal residual disease (MRD)–negative complete response (CR) in broader TE NDMM populations. The REMNANT study was designed to evaluate outcomes in a cohort with wide inclusion criteria reflective of routine clinical practice.

### Aims

To determine the rate of MRD-negative CR after induction, single or tandem HD-ASCT, and consolidation in an unselected TE NDMM population.

### Methods

REMNANT is an academic, multicenter phase 2/3 study of TE NDMM patients aged 18–75 years with minimal exclusions. No restrictions were applied for bone marrow, renal, or liver function, and concurrent plasma cell malignancies were allowed. One prior induction cycle was permitted. In the non-randomized phase 2 component, all patients received four 21-day cycles of bortezomib, lenalidomide, and dexamethasone (VRd) induction, single or tandem HD-ASCT, and four 21-day cycles of VRd consolidation. Lenalidomide dosing was higher-than-label for patients with RI, and tandem transplantation was not limited to high-risk cytogenetics. MRD testing was performed in all patients achieving ≥very good partial response (VGPR) after consolidation. The primary endpoint was MRD-negative CR (NGF $10^{-5}$) post-consolidation; key secondary endpoints included PFS, overall survival and safety.

**Copyright Information:** (Online) ISSN: 2572-9241
©2026 The Author(s). HemaSphere is published by John Wiley & Sons Ltd on behalf of European Hematology Association. This is an open access Abstract Book distributed under the Attribution-NonCommercial-NoDerivs (CC BY-NC-ND), which allows third parties to download the articles and share them with others as long as they credit the author and the Abstract Book, but they cannot change the content in any way or use them commercially.
**Abstract Book Citations:** Authors, Title, HemaSphere, 2026;10:(S1):pages. The abstract book DOIs can be found on the "Author Information" page.
**Disclaimer:** Articles published in the journal HemaSphere exclusively reflect the opinions of the authors. The authors are responsible for all content in their abstracts including accuracy of the facts, statements, citing resources, etc.

**Results**

From August 2020 to September 2024, 382 patients were enrolled. Median age was 63 years; 40% were ≥65, 10% were ≥70, and 26% had started induction therapy before inclusion. Osteolytic lesions were present in 79%, anemia in 52%, renal insufficiency in 19%, and hypercalcemia in 11%. ECOG performance status ≥2 occured in 15%, and 12% had significant comorbidities, including prior invasive cancer (11%) or AL amyloidosis (1%). High-risk features included ISS III (26%), high-risk cytogenetics (21%), soft-tissue plasmacytomas (11%), and plasma cell leukemia (0.3%).

Post-consolidation, 33% achieved MRD-negative CR and 86% ≥VGPR (Table 1). At 34.7 months median follow-up, 12- and 24-month PFS rates were 88% and 79% (Figure 1A). Responses and PFS were similar in patients ≥65 and <65 years (Table 1, Figure 1B). Grade 3–4 adverse events were mainly infections (44%) and cytopenias (18%). Seven on-study deaths occurred (four infections, one cardiac arrest, one interstitial lung disease, one flail chest secondary to myeloma).

**Table 1: Best overall response by investigator assessment**

| | **All patients (n=382)** | **<65 years (n=229)** | **≥65 years (n=153)** |
|---|---|---|---|
| Overall response | 359 (94%) | 212 (93%) | 147 (96%) |
| MRD-negative CR* | 127 (33%) | 73 (32%) | 54 (35%) |
| CR or better | 165 (43%) | 90 (39%) | 75 (49%) |
| VGPR | 162 (42%) | 102 (45%) | 60 (39%) |
| VGPR or better | 327 (86%) | 192 (84%) | 135 (88%) |
| PR | 32 (8%) | 20 (9%) | 12 (8%) |
| SD | 17 (4%) | 13 (6%) | 4 (3%) |
| PD | 2 (1%) | 2 (1%) | 0 (0%) |
| Not evaluable | 4 (1%) | 2 (1%) | 2 (1%) |

Data are n (%). *$<1.0 \times 10^{-5}$
MRD=minimal residual disease. CR=complete response. VGPR=very good partial response. PR=partial response. SD=stable disease. PD=progressive disease.

**Copyright Information:** (Online) ISSN: 2572-9241
©2026 The Author(s). HemaSphere is published by John Wiley & Sons Ltd on behalf of European Hematology Association. This is an open access Abstract Book distributed under the Attribution-NonCommercial-NoDerivs (CC BY-NC-ND), which allows third parties to download the articles and share them with others as long as they credit the author and the Abstract Book, but they cannot change the content in any way or use them commercially.
**Abstract Book Citations:** Authors, Title, HemaSphere, 2026;10:(S1):pages. The abstract book DOIs can be found on the "Author Information" page.
**Disclaimer:** Articles published in the journal HemaSphere exclusively reflect the opinions of the authors. The authors are responsible for all content in their abstracts including accuracy of the facts, statements, citing resources, etc.

### Summary/Conclusion

VRd induction, single or tandem HD-ASCT, and VRd consolidation achieves notable MRD-negative CR rates in a clinically diverse NDMM population whose baseline characteristics mirror Norwegian real-world data from 2008–2020, supporting the broad applicability of the findings.

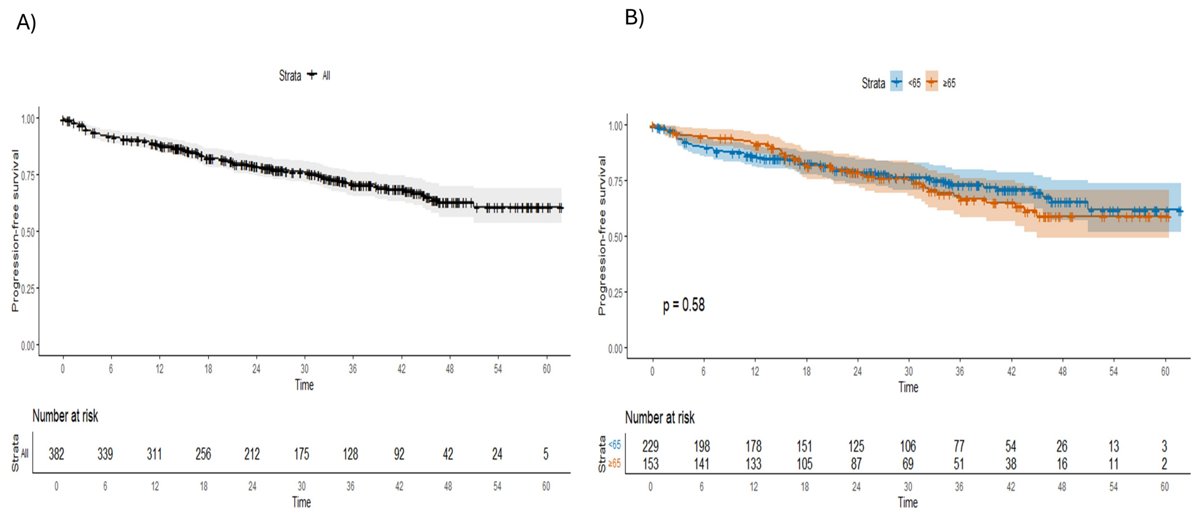

**Kaplan–Meier plots of progression-free survival. A) all patients and B) stratified by age group**

Shaded area represents 95% confidence intervals. Time 0 corresponds to the time of study inclusion

**Copyright Information:** (Online) ISSN: 2572-9241
©2026 The Author(s). HemaSphere is published by John Wiley & Sons Ltd on behalf of European Hematology Association. This is an open access Abstract Book distributed under the Attribution-NonCommercial-NoDerivs (CC BY-NC-ND), which allows third parties to download the articles and share them with others as long as they credit the author and the Abstract Book, but they cannot change the content in any way or use them commercially.
**Abstract Book Citations:** Authors, Title, HemaSphere, 2026;10:(S1):pages. The abstract book DOIs can be found on the "Author Information" page.
**Disclaimer:** Articles published in the journal HemaSphere exclusively reflect the opinions of the authors. The authors are responsible for all content in their abstracts including accuracy of the facts, statements, citing resources, etc.

### (PS1906) IMPACT OF BASELINE BONE MARROW IMMUNE CELL SUBSETS ON OUTCOMES IN MULTIPLE MYELOMA TREATED WITH DARATUMUMAB

**Topic:** 14. Myeloma and other monoclonal gammopathies - Clinical

Jianlin Hu[1], Ning An[1], Yuhan Bao[1], Xinran Wang[1], Qiuxia Yu[1], Yang Gao[1], Peiling Zhang[1], Di Wang[1], Chunrui Li*[1]

[1]*Department of Hematology, Tongji Hospital, Tongji Medical College, Huazhong University of Science and Technology, Wuhan, China;*

### Background

Multiple myeloma (MM) is a plasma cell malignancy. The bone marrow tumor–immune ecosystem is highly heterogeneous. Anti-CD38 antibodies, particularly daratumumab, are integral to MM therapy from frontline to later lines. Yet responses vary widely between patients. We hypothesized that baseline marrow immune composition influences sensitivity to daratumumab-based therapy and the risk of progression.

### Aims

To profile baseline bone marrow immune cell subsets in MM patients treated with daratumumab and to evaluate their associations with response and progression-free survival (PFS).

### Methods

We retrospectively reviewed 158 consecutive MM patients who received standard daratumumab-based regimens and completed ≥4 cycles between January 2018 and May 2025. Baseline bone marrow aspirates were obtained before daratumumab initiation and analyzed by multiparameter flow cytometry. Lymphocytes were gated by CD45 expression and side scatter. T cells were defined as CD3+ lymphocytes, and NK cells as CD56+CD3− lymphocytes within the lymphocyte gate. We quantified the proportions of NK cells, T lymphocytes, and aberrant monoclonal plasma cells. Clinical variables were recorded, including extramedullary disease and baseline blood counts. PFS was defined from daratumumab start to progression or death; overall survival (OS) was tracked. Associations with outcomes were assessed using univariate and multivariable Cox models. The optimal NK-cell cut-off was derived by ROC analysis.

### Results

Median age was 58.5 years, and 57.0% were male. Daratumumab was used in early lines (≤1 prior line) in 77.8% of patients. Median baseline proportions within bone marrow lymphocytes were 17.2% for NK cells, 70.65% for T lymphocytes, and 3.05% for aberrant monoclonal plasma cells. Median follow-up was 20.8 months. Median PFS was 48.8 months, while median OS was not reached. Early-line daratumumab was associated with superior PFS compared with later-line use (median PFS not reached vs 22.77 months; P=0.0041). Patients who experienced disease progression had higher baseline NK-cell and aberrant monoclonal plasma cell proportions within bone marrow lymphocytes. ROC analysis identified 17.25% as the optimal cut-off for NK-cell proportion. On univariate analysis, PFS was significantly associated with NK-cell proportion, T-cell proportion, aberrant plasma cell proportion, extramedullary disease, platelet count, hemoglobin level, and early-line daratumumab use (all P<0.05). In multivariable analysis, high NK-cell proportion (≥17.25%), extramedullary disease, and thrombocytopenia remained independent adverse factors for PFS.

**Copyright Information:** (Online) ISSN: 2572-9241
©2026 The Author(s). HemaSphere is published by John Wiley & Sons Ltd on behalf of European Hematology Association. This is an open access Abstract Book distributed under the Attribution-NonCommercial-NoDerivs (CC BY-NC-ND), which allows third parties to download the articles and share them with others as long as they credit the author and the Abstract Book, but they cannot change the content in any way or use them commercially.
**Abstract Book Citations:** Authors, Title, HemaSphere, 2026;10:(S1):pages. The abstract book DOIs can be found on the "Author Information" page.
**Disclaimer:** Articles published in the journal HemaSphere exclusively reflect the opinions of the authors. The authors are responsible for all content in their abstracts including accuracy of the facts, statements, citing resources, etc.

**Summary/Conclusion**

Baseline bone marrow immune features correlate with response and prognosis in MM patients treated with daratumumab. A high NK-cell proportion (≥17.25%), extramedullary disease, and thrombocytopenia independently predicted shorter PFS. Baseline immune profiling may complement standard risk assessment and help refine patient stratification and monitoring in the daratumumab era.

**Copyright Information:** (Online) ISSN: 2572-9241
©2026 The Author(s). HemaSphere is published by John Wiley & Sons Ltd on behalf of European Hematology Association. This is an open access Abstract Book distributed under the Attribution-NonCommercial-NoDerivs (CC BY-NC-ND), which allows third parties to download the articles and share them with others as long as they credit the author and the Abstract Book, but they cannot change the content in any way or use them commercially.
**Abstract Book Citations:** Authors, Title, HemaSphere, 2026;10:(S1):pages. The abstract book DOIs can be found on the “Author Information” page.
**Disclaimer:** Articles published in the journal HemaSphere exclusively reflect the opinions of the authors. The authors are responsible for all content in their abstracts including accuracy of the facts, statements, citing resources, etc.

### (PS1907) TWO STAGE AI UNMASKS IMMUNE DRIVEN FUNCTIONAL HIGH-RISK IN CYTOGENETIC STANDARD RISK MYELOMA: GUIDING DARATUMUMAB AND ASCT THERAPY

**Topic:** 14. Myeloma and other monoclonal gammopathies - Clinical

Arjun Raj Rajanna[1], Jorge Arturo Hurtado[2], David G. Coffey[1], Dickran Kazandjian[1], Benjamin Diamond[1], Michael Durante[1], Brian Walker[1], James E. Hoffman[1], Abhishek Pandey[1], Emilia Mason[1], Stephanie Fernandes[1], Shaivi Manish Shah[1], Michelle Armogan[1], Maria Laura Pascual[1], Jennifer Ahlstrom[2], Samuel Bennion[2], Laura Priscila Ochoa Loza[2], Ana Echenique Alcázar[2], Patricia Flores Pérez[2], Magaly Valeria Escobedo Cruz[2], Eduardo Franco Hernandez[2], Andrea Robles Espinoza[2], Karla Franco[2], Pamela Hugues[2], Danielle Smith[2], Daniel Bilbao[1], Yi Zhou[1], Jennifer Chapman[1], Rafat Abonour[1], Jay R. Hydren[2], C. Ola Landgren*[1]

*[1]Sylvester Comprehensive Cancer Center, Myeloma Institute, University of Miami, Miami, United States of America;[2]HealthTree Foundation, Lehi, United States of America;*

### Background

Cytogenetic Standard Risk (SR) in newly diagnosed multiple myeloma (NDMM) represents a heterogeneous population. A subset experience early relapse, termed functional high-risk, despite lacking canonical FISH markers. As frontline daratumumab (Dara) expands, identifying SR patients that require monoclonal antibody therapy versus those achieving durable responses on VRd remains critical. Because Dara relies on CD16 expressing NK cells for ADCC, we hypothesized that endogenous deficient CD16-mediated immunity, not undetected genomics alone, drives functional high-risk outcomes in SR NDMM, and that spatial AI profiling could guide therapeutic rescue.

### Aims

To deploy two stage AI spatial profiling on routine hematoxylin and eosin (H&E) bone marrow biopsy slides to identify CD16 deficiency within SR myeloma and assess its prognostic impact across Dara and ASCT treatment subgroups.

### Methods

We analyzed digitized baseline H&E slides from 211 NDMM patients (HealthTree registry) treated with VRd (n=135; median follow up 24.5 months) and Dara-VRd (n=76; 16.7 months). A two stage AI pipeline was applied: First, the CORAL histomorph attention-based model (Rajanna et al., ASH 2025) predicted cytogenetic status from H&E, excluding high-risk markers, yielding 157 SR patients (VRd n=101, Dara-VRd n=56). Second, the GigaTIME foundation model quantified spatial CD16 (High vs Low) via zero shot inference, bypassing expensive multiplex assays. Primary endpoint: time to next treatment (TTNT).

**Copyright Information:** (Online) ISSN: 2572-9241
©2026 The Author(s). HemaSphere is published by John Wiley & Sons Ltd on behalf of European Hematology Association. This is an open access Abstract Book distributed under the Attribution-NonCommercial-NoDerivs (CC BY-NC-ND), which allows third parties to download the articles and share them with others as long as they credit the author and the Abstract Book, but they cannot change the content in any way or use them commercially.
**Abstract Book Citations:** Authors, Title, HemaSphere, 2026;10:(S1):pages. The abstract book DOIs can be found on the “Author Information” page.
**Disclaimer:** Articles published in the journal HemaSphere exclusively reflect the opinions of the authors. The authors are responsible for all content in their abstracts including accuracy of the facts, statements, citing resources, etc.

**Results**

CORAL identified high-risk cytogenetics in 25.6% of patients (1q gain 20.4%, t(4;14) 7.1%, del(17p) 4.7%). Inference of CD16 from routine H&E using our two stage model unveiled functional high-risk disease masked by standard cytogenetics. In VRd, immune fitness stratified outcomes (log rank p=0.0003) into low CD16 tumor microenvironment (TME) patients deferring ASCT and subsequently experienced relapses (93.8% events; median TTNT 4.6 months), with 84.2% carrying exclusively standard risk FISH. ASCT rescued this phenotype, extending the median to 26.7 months (p=0.003). Conversely, high CD16 patients achieved excellent outcomes with no ASCT benefit (p=0.79). In Dara-VRd, outcomes converged across all CD16/ASCT groups (p=0.19), rescuing low CD16 without ASCT patients to median 41.0 months (8.3% events vs 93.8% on VRd). High CD16 patients on Dara-VRd deferring ASCT achieved median not reached (13.3% events), indistinguishable from ASCT recipients (14.3% events; p=0.98). Multivariate Cox confirmed high CD16 as an independent predictor (HR 0.28, p=0.003). In VRd, low CD16 conferred 2-fold increased risk (HR 1.72, p=0.019). ASCT rescued low CD16 (HR 0.35, p=0.005) but not high CD16 (p=0.79). CD16 classification achieved 76% NPV for functional high-risk.

**Summary/Conclusion**

Cytogenetic risk stratification in NDMM ignores TME. Two stage AI profiling of routine H&E identifies functional high-risk patients independent of FISH status. This is achieved by capturing CD16 biology across NK cells, macrophages, and neutrophils, supporting a mechanism in which Dara overcomes this deficient phenotype, enabling ASCT deferral in immune-fit patients. This scalable H&E based platform makes precision oncology broadly available, extracting genomic risk and immune status from baseline bone marrow slides to optimize treatment for individual patients.

**Copyright Information:** (Online) ISSN: 2572-9241
©2026 The Author(s). HemaSphere is published by John Wiley & Sons Ltd on behalf of European Hematology Association. This is an open access Abstract Book distributed under the Attribution-NonCommercial-NoDerivs (CC BY-NC-ND), which allows third parties to download the articles and share them with others as long as they credit the author and the Abstract Book, but they cannot change the content in any way or use them commercially.
**Abstract Book Citations:** Authors, Title, HemaSphere, 2026;10:(S1):pages. The abstract book DOIs can be found on the "Author Information" page.
**Disclaimer:** Articles published in the journal HemaSphere exclusively reflect the opinions of the authors. The authors are responsible for all content in their abstracts including accuracy of the facts, statements, citing resources, etc.

### (PS1908) MRD–PET DISCORDANCE REVEALS COMPARTMENTALIZED DISEASE IN MULTIPLE MYELOMA: PROGNOSTIC IMPLICATIONS FROM 1138 PAIRED ASSESSMENTS

**Topic:** 14. Myeloma and other monoclonal gammopathies - Clinical

Max Mendez-Lopez*[1], Marco Talarico[2], Thomas Lehmann[1], Christoph Driessen[1]

*[1]HOCH Ostschweiz, Department of Oncology and Hematology, St. Gallen, Switzerland;[2]IRCCS Azienda Ospedaliero – Universitaria di Bologna, Bologna, Italy;*

### Background

Bone marrow minimal residual disease (MRD) negativity and PET–CT negativity are widely accepted response endpoints and increasingly guide therapeutic decisions. Whether these modalities identify the same patients when assessed simultaneously remains unresolved.

### Aims

To quantify simultaneous MRD–PET discordance and evaluate the prognostic stability of dual-modality negativity as a high-confidence response phenotype.

### Methods

We conducted a systematic review and random-effects meta-analysis (2015–2026) of studies reporting paired bone marrow MRD and PET–CT assessments performed at the same clinical landmark with all four joint response categories available. Concordance was summarized using pooled concordant proportion and Cohen's κ. Directional discordance was modeled using study-level log-odds. The prognostic impact of dual MRD/PET negativity on progression-free survival (PFS) was synthesized using restricted maximum likelihood with Hartung–Knapp adjustment, with 95% prediction intervals. Risk of bias was assessed using QUADAS-2 and QUIPS.

### Results

Ten cohorts comprising 1138 paired assessments met strict inclusion criteria. The pooled joint distribution was MRD–/PET+ 12.7%, MRD–/PET– 43.8%, MRD+/PET– 27.2%, and MRD+/PET+ 16.2%, corresponding to observed agreement of 60.0%. However, chance-corrected agreement was limited (pooled κ 0.14; 95% CI 0.033–0.25), and discordance predominantly reflected MRD+/PET– classifications (pooled directional log-odds −0.83), indicating systematic biological non-overlap rather than random measurement variability. In five studies reporting PFS, dual MRD/PET negativity reproducibly identified a favorable-risk subgroup (pooled HR 0.34; 95% CI 0.22–0.51; 95% prediction interval 0.18–0.65), with consistent estimates across sensitivity analyses.

### Summary/Conclusion

Simultaneous MRD and PET–CT do not define equivalent response states in multiple myeloma. Discordant phenotypes are common and reflect compartment-specific residual disease, whereas dual negativity delineates a robust, high-confidence remission phenotype. As deeper responses are achieved with next-generation and cellular therapies, integrated multimodal response are required to replace single-modality response paradigms.

**Copyright Information:** (Online) ISSN: 2572-9241
©2026 The Author(s). HemaSphere is published by John Wiley & Sons Ltd on behalf of European Hematology Association. This is an open access Abstract Book distributed under the Attribution-NonCommercial-NoDerivs (CC BY-NC-ND), which allows third parties to download the articles and share them with others as long as they credit the author and the Abstract Book, but they cannot change the content in any way or use them commercially.
**Abstract Book Citations:** Authors, Title, HemaSphere, 2026;10:(S1):pages. The abstract book DOIs can be found on the "Author Information" page.
**Disclaimer:** Articles published in the journal HemaSphere exclusively reflect the opinions of the authors. The authors are responsible for all content in their abstracts including accuracy of the facts, statements, citing resources, etc.

### (PS1909) BLEEDING EVENTS IN PATIENTS WITH MULTIPLE MYELOMA TREATED WITH BCMA-DIRECTED CAR T-CELLTHERAPY: A SYSTEMATIC REVIEW OF CLINICAL TRIALS

**Topic:** 14. Myeloma and other monoclonal gammopathies - Clinical

Pedro Henrique Fernandes Do Carmo Las Casas*[1], Javier Marco[2, 3], Thamiris Silva Soares[4, 5], Eleftheria Lefkou[6], Mohammed A. Baghdadi[1, 7], Prakasha Kempaiah[8], Jawed Fareed[8], Patrick Van Dreden[1, 9], Vania Hungria[10], Marina Marchetti[11], Anna Falanga[11], Laurent Garderet[12], Grigorios Gerotziafas[1, 8]

*[1]Saint Antoine University Hospital Assistance Publique Hôpitaux de Paris (AP-HP), Sorbonne University, INSERM UMR_S_938, CRSA, Research Department "Cancer, Vessels, Biology and Therapeutics" (CaVITE), Research Group "Cancer-Angiogenesis-Thrombosis and Haemostasis," Thrombosis Center, University Institute of Cancerology (UIC),, Paris, France; [2]University Hospital Morales Meseguer, Hematology Department,, Murcia, Spain; [3]Biomedical Research Institute of Murcia Pascual Parrilla-IMIB, CIBERER, Murcia, Spain; [4]Federal University of Minas Gerais, Faculty of Medicine, Belo Horizonte, Brazil;[5]Luxemburgo Hospital, Instituto Mario Penna, Belo Horizonte, Brazil; [6]Larissa University Hospital, Faculty of Medicine, University of Thessaly, Hematology-Transfusion Medicine Department,, Larissa, Greece; [7]King Faisal Specialist Hospital and Research Center, Research Center, Jeddah, Saudi Arabia; [8]Loyola University Stritch School of Medicine, Maywood, United States of America; [9]Diagnostica Stago, Department of Clinical Research, Gennevilliers, France; [10]Clinica São Germano, São Paulo, Brazil; [11]Hospital Papa Giovanni XXIII, Department of Immunohematology and Transfusion Medicine, Bergamo, Italy; [12]Hôpital Universitaire Pitié-Salpêtrière, Assistance Publique Hôpitaux de Paris (AP-HP), Service Hématologie, Paris, France;*

### Background

BCMA-directed chimeric antigen receptor T-cell (CAR-T) therapy has substantially improved outcomes in relapsed/refractory multiple myeloma (MM). In our previous systematic review focusing on venous thromboembolism (VTE), thrombotic events were infrequent, suggesting that classical hypercoagulability may not represent the dominant hemostatic phenotype in this setting. However, cytokine release syndrome (CRS), a frequent complication of CAR-T therapy, is characterized by intense systemic inflammation, endothelial activation, capillary leak, and coagulation disturbances. Laboratory abnormalities during CRS often include hypofibrinogenemia, platelet consumption, and increased von Willebrand factor levels, raising concern for a potential bleeding-prone phenotype in this population.

### Aims

To evaluate the incidence, severity, anatomical distribution, and clinical relevance of bleeding events in patients with MM treated with BCMA-directed CAR-T therapy.

### Methods

This systematic review was conducted according to PRISMA guidelines and prospectively registered in PROSPERO (CRD420251105194). The methodology mirrored our previously published VTE analysis. PubMed, Embase, and Scopus were systematically searched using predefined strategies over the same study period. Among 542 records identified, 41 studies underwent full-text review. Clinical trials reporting bleeding outcomes in adult MM patients treated with idecabtagene vicleucel or ciltacabtagene autoleucel were included. Studies without bleeding data were excluded. Bleeding events were extracted as reported in the original trials and categorized as major or minor according to study-specific definitions. Data were synthesized descriptively. All included studies reported informed consent and local ethics committee approval.

**Copyright Information:** (Online) ISSN: 2572-9241
©2026 The Author(s). HemaSphere is published by John Wiley & Sons Ltd on behalf of European Hematology Association. This is an open access Abstract Book distributed under the Attribution-NonCommercial-NoDerivs (CC BY-NC-ND), which allows third parties to download the articles and share them with others as long as they credit the author and the Abstract Book, but they cannot change the content in any way or use them commercially.
**Abstract Book Citations:** Authors, Title, HemaSphere, 2026;10:(S1):pages. The abstract book DOIs can be found on the "Author Information" page.
**Disclaimer:** Articles published in the journal HemaSphere exclusively reflect the opinions of the authors. The authors are responsible for all content in their abstracts including accuracy of the facts, statements, citing resources, etc.

### Results

Five studies met inclusion criteria, comprising 442 patients treated with BCMA-directed CAR-T therapy. Overall, 31 patients (7%) experienced bleeding events. Major bleeding was uncommon, occurring in 9 patients (2%), but involved clinically significant sites such as central nervous system, gastrointestinal, and pulmonary bleeding. Minor bleeding occurred in 22 patients (5%), most frequently epistaxis. In the randomized CARTITUDE-4 trial, bleeding incidence was low and comparable between arms, occurring in 3.3% of patients in the CAR-T arm and 5.6% in the standard-of-care arm. No major bleeding events were reported in the control group.

### Summary/Conclusion

Bleeding complications following BCMA-directed CAR-T therapy in MM appear infrequent but can be clinically meaningful when major events occur. The inflammatory and endothelial perturbations associated with CRS may contribute to a transient hemostatic imbalance. Careful monitoring of coagulation parameters, fibrinogen levels, and platelet counts during the early post-infusion period is warranted. Larger prospective studies are needed to better define bleeding risk, identify predictive biomarkers, and clarify the safety of anticoagulation strategies in this population.

**Table 1.** Incidence and localisation of Bleeding in MM patients treated with ide-cel or cilta-cel in key clinical trials, and in randomised controlled trials compared with Standard-of-Care (NR: not reported, SoC – Standard of care – DaraPd e PVd)

| Trial | Type of Trial | n | Therapy | Thrombocytopenia (any grade) | All Bleeding Events (%) | Major Bleeding Events (%) | Major Bleeding Type | Minor Bleeding Events | Minor Bleeding Types (%) | RNI abnormalities | TTPa abnormalties | Median follow up (months) |
|---|---|---|---|---|---|---|---|---|---|---|---|---|
| KarMMa-1 | Phase I/IIb | 128 | Ide-Cel | 95 | 17 (13.3%) | 6 (4.6%) | 2 Gastrointestinal<br>3 CNS<br>1 Pos-procedural | 11 (8.5%) | 11 Epistaxis | 7 | 7 | 13.3 |
| KarMMa-3 | RCT | 254 | Ide-Cel | NR | NR | NR | NR | NR | NR | NR | NR | 18.6 |
| | | 132 | SoC* | NR | NR | NR | -NR | NR | NR | NR | NR | 19.6 |
| CARTITUDE-1 | Phase I/IIb | 97 | Cilta-cel | 76 | 7 (7,2%) | 2 (2%) | 1 Pulmonary<br>1 Gastrointestinal | 5 (5,1%) | 5 Epistaxis | 4 | 9 | 12.4 |
| CARTITUDE-1 Japan | Phase I/IIb | 9 | Cilta-cel | 7 | 0 | 0 | 0 | 0 | 0 | 0 | 0 | 8.5 |
| CARTITUDE-4 | RCT | 208 | Cilta-cel | 113 | 7 (3.3%) | 1 (0,5%) | 1 CNS | 6 (2.8%) | 6 Epistaxis | NR | NR | 15.9 |
| | | 211 | SoC | 67 | 12 (5.6%) | 0 | 0 | 12.(5.6%) | 12 Epistaxis | NR | NR | 15.9 |
| **TOTAL CART (excluding KarMMa-3)** | RCTs | 442 | Cilta-cel and Ida-cel | - | 31(7%) | 9 (2%) | - | 22(5%) | - | - | - | - |

Table 1: Incidence and localisation of Bleeding in MM patients treated with ide-cel or cilta-cel in key clinical trials, and in randomised controlled trials compared with Standard-of-Care (NR: not reported, SoC – Standard of care – DaraPd e PVd)

**Copyright Information:** (Online) ISSN: 2572-9241
©2026 The Author(s). HemaSphere is published by John Wiley & Sons Ltd on behalf of European Hematology Association. This is an open access Abstract Book distributed under the Attribution-NonCommercial-NoDerivs (CC BY-NC-ND), which allows third parties to download the articles and share them with others as long as they credit the author and the Abstract Book, but they cannot change the content in any way or use them commercially.
**Abstract Book Citations:** Authors, Title, HemaSphere, 2026;10:(S1):pages. The abstract book DOIs can be found on the "Author Information" page.
**Disclaimer:** Articles published in the journal HemaSphere exclusively reflect the opinions of the authors. The authors are responsible for all content in their abstracts including accuracy of the facts, statements, citing resources, etc.

### (PS1910) DYNAMIC MONITORING OF CIRCULATING PLASMA CELLS IN NEWLY DIAGNOSED MULTIPLE MYELOMA: UPDATED RESULTS FROM A PROSPECTIVE MULTICENTER STUDY IN CHINA

**Topic:** 14. Myeloma and other monoclonal gammopathies - Clinical

Qun Li*[1], Xiaojing Yan[2], Jian-qing Mi[3], Aijun Liao[4], Bing-Zong Li[5], Huihan Wang[4], Huili Cai[6], Xin Li[7], Yafei Wang[8], Li Cai[1], Yu Hu[1], Chunyan Sun[1]

*[1]Union Hospital, Tongji Medical College, Huazhong University of Science and Technology, Institute of Hematology, Wuhan, China; [2]The First Affiliated Hospital of China Medical University, Institute of Hematology, Shenyang, China;[3]Ruijin Hospital, Shanghai Jiao Tong University School of Medicine, Institute of Hematology, Shanghai, China; [4]Shengjing Hospital of China Medical University, Institute of Hematology, Shenyang, China; [5]The Second Affiliated Hospital of Soochow University, Institute of Hematology, Soochow, China; [6]Yichang Central People's Hospital, Institute of Hematology, Yichang, China;[7]The Third Xiangya Hospital of Central South University, Institute of Hematology, Changsha, China; [8]Tianjin Medical University Cancer Institute and Hospital, Tianjin, China;*

### Background

The minimally invasive nature of circulating plasma cells (CPCs) assessment provides a valuable strategy for real-time tumor burden evaluation. However, their clinical significance has not been validated in large-scale prospective studies in Chinese multiple myeloma (MM) patients. In addition, the lack of standardized detection protocols and clinically applicable cut-off values limits the implementation of CPCs monitoring in routine practice.

### Aims

In this prospective multicenter study, we adopted a standardized eight-color flow cytometry protocol to dynamically evaluate the clinical utility of serial CPCs monitoring in the Chinese myeloma population.

### Methods

Newly diagnosed MM patients have been continuously enrolled from eight medical centers since April 1, 2024. EDTA-anticoagulated peripheral blood samples were analyzed using standardized eight-color flow cytometry. Antibodies included CD38, CD138, CD45, CD56, CD19, CD27, and cytoplasmic kappa and lambda immunoglobulin light chains. CPCs monitoring was conducted every two cycles during induction therapy. The study remains ongoing with continuous patient enrollment, and the current report presents the latest available analysis.

**Copyright Information:** (Online) ISSN: 2572-9241
©2026 The Author(s). HemaSphere is published by John Wiley & Sons Ltd on behalf of European Hematology Association. This is an open access Abstract Book distributed under the Attribution-NonCommercial-NoDerivs (CC BY-NC-ND), which allows third parties to download the articles and share them with others as long as they credit the author and the Abstract Book, but they cannot change the content in any way or use them commercially.
**Abstract Book Citations:** Authors, Title, HemaSphere, 2026;10:(S1):pages. The abstract book DOIs can be found on the "Author Information" page.
**Disclaimer:** Articles published in the journal HemaSphere exclusively reflect the opinions of the authors. The authors are responsible for all content in their abstracts including accuracy of the facts, statements, citing resources, etc.

**Results**

As of November 2025, a total of 631 newly diagnosed MM patients underwent baseline CPCs assessment. CPCs were detected in 532 patients, yielding a positivity rate of 84.3%. Among patients with detectable CPCs, higher baseline CPCs levels were significantly associated with advanced ISS, R-ISS, and R2-ISS stages (all $P<0.001$) and high-risk cytogenetic abnormalities (HRCAs) ($P=0.008$), but not with extramedullary disease. CPCs levels showed a modest correlation with bone marrow plasma cell (BMPC) infiltration(r=0.237). Compared to the baseline CPCs-negative group (n=99), the baseline CPCs-positive group (n=532) exhibited more advanced disease stages (ISS-III: 49.3% vs. 32.6%, $P=0.003$; R-ISS-III: 26.4% vs. 6.8%, $P<0.001$; R2-ISS-III/IV: 54.0%/15.7% vs. 43.4%/5.3%, $P<0.001$), higher tumor burden (BMPC ≥30%: 49.6% vs. 27.2%, $P<0.001$; β2-MG≥5.5mg/L: 48.0% vs. 30.4%, $P=0.002$; LDH≥ULN: 19.9% vs. 9.3%, $P=0.013$), an increased tendency toward anemia (Hb<100g/L: 63.3% vs. 45.4%, $P<0.001$), and unfavorable cytogenetic abnormalities (55.8% vs. 32.3%, $P<0.001$). To date, 279 patients have completed CPCs assessment after two induction cycles. Patients with persistent CPCs positivity (n=30) demonstrated significantly inferior treatment responses compared with those achieving CPCs clearance (n=249) (≥CR: 7.7% vs. 9.3%; VGPR: 7.7% vs. 40.4%; ≤PR: 84.6% vs. 50.2%; $P<0.001$).

**Summary/Conclusion**

This ongoing prospective multicenter study provides the first large-scale validation of CPCs monitoring in Chinese MM patients. Updated results demonstrate that elevated baseline CPCs levels are associated with more aggressive disease characteristics. Early clearance of CPCs is closely linked to superior treatment response. With ongoing enrollment and extended follow-up, the study is expected to further elucidate the clinical implications of dynamic CPCs monitoring in MM.

**Copyright Information:** (Online) ISSN: 2572-9241
©2026 The Author(s). HemaSphere is published by John Wiley & Sons Ltd on behalf of European Hematology Association. This is an open access Abstract Book distributed under the Attribution-NonCommercial-NoDerivs (CC BY-NC-ND), which allows third parties to download the articles and share them with others as long as they credit the author and the Abstract Book, but they cannot change the content in any way or use them commercially.
**Abstract Book Citations:** Authors, Title, HemaSphere, 2026;10:(S1):pages. The abstract book DOIs can be found on the "Author Information" page.
**Disclaimer:** Articles published in the journal HemaSphere exclusively reflect the opinions of the authors. The authors are responsible for all content in their abstracts including accuracy of the facts, statements, citing resources, etc.

### (PS1911) OUTCOMES OF THERAPY AFTER PROGRESSION ON TALQUETAMAB IN PATIENTS WITH HEAVILY PRETREATED RELAPSED REFRACTORY MULTIPLE MYELOMA

**Topic:** 14. Myeloma and other monoclonal gammopathies - Clinical

Gannon Ray*[1], Omar Alkharabsheh[2], Hira Shaikh[3], Zoe Ryan[3], Jonathan Lochner[3], Shebli Atrash[4], Barry Paul[4], Donald Moore[4], Manisha Bhutani[4], Prerna Mewawalla[5], Mansi R. Shah[6], Zahra Mahmoudjafar[7], Jordan Snyder[7], Muhammad Umair Mushtaq[7], Alma Habib[7], Al-Ola Abdallah[7], Abdullah M. Khan[8], Kimberly Green[9], Muhammad Faisal[10], Anita Mazloom[1]

*[1]University of South Alabama Mitchell Cancer Institute, Division of Hematology and Oncology, Mobile, AL, United States of America; [2]University of Cincinnati, Division of Hematology and Oncology, Cincinnati, OH, United States of America;[3]University of Iowa, Division of Hematology, Oncology, and Blood & Marrow Transplantation, Iowa City, IA, United States of America; [4]Levine Cancer Institute, Atrium Health, Wake Forest University School of Medicine, Charlotte, NC, United States of America; [5]Allegheny Health Network Cancer Institute, Division of Hematology and Cellular Therapy, Pittsburgh, PA, United States of America; [6]Rutgers Cancer Institute of New Jersey, Division of Hematology, New Brunswick, NJ, United States of America; [7]University of Kansas Medical Center, Division of Hematologic Malignancies & Cellular Therapeutics, Westwood, KS, United States of America; [8]The Ohio State University, Division of Hematology, Columbus, OH, United States of America; [9]Medical University of South Carolina, Division of Hematology and Oncology, Charleston, SC, United States of America; [10]Stephenson Cancer Center at the University of Oklahoma, Division of Hematology and Oncology, Oklahoma City, OK, United States of America;*

### Background

Talquetamab is a G protein-coupled receptor, class C, group 5, member D targeting bispecific T-cell engager approved in August 2023 for patients with relapsed-refractory multiple myeloma (RRMM) who have received at least four prior lines of therapy (LOT). Optimal treatment after progression on talquetamab remains unclear. We present real world outcomes for various treatments post-talquetamab in RRMM.

### Methods

We conducted a multicenter retrospective analysis study across nine academic centers from October 2022 to January 2025 through the US Myeloma Innovations Research Collaborative. RRMM patients who progressed after talquetamab monotherapy were included. Response to therapy was evaluated using the International Myeloma Working Group criteria. Comparisons based on patient characteristics and therapy type were performed using the Kruskal–Wallis rank-sum test and Fisher's exact test.

**Copyright Information:** (Online) ISSN: 2572-9241
©2026 The Author(s). HemaSphere is published by John Wiley & Sons Ltd on behalf of European Hematology Association. This is an open access Abstract Book distributed under the Attribution-NonCommercial-NoDerivs (CC BY-NC-ND), which allows third parties to download the articles and share them with others as long as they credit the author and the Abstract Book, but they cannot change the content in any way or use them commercially.
**Abstract Book Citations:** Authors, Title, HemaSphere, 2026;10:(S1):pages. The abstract book DOIs can be found on the "Author Information" page.
**Disclaimer:** Articles published in the journal HemaSphere exclusively reflect the opinions of the authors. The authors are responsible for all content in their abstracts including accuracy of the facts, statements, citing resources, etc.

**Results**

45 patients receiving therapy after talquetamab progression were analyzed across four groups: chemotherapy-based (n=16), proteasome inhibitor (PI)/immunomodulatory drug (IMiD)-based (n=12), cellular therapy (n=9), and non-cellular/non-PI/IMiD (n=8). The cellular therapy group was primarily comprised of teclistamab and allo-BCMA CAR-T therapy. Of note, 78% (7/9) of the cellular therapy group had prior BCMA exposure. Baseline characteristics including age (median 66; IQR 59–71), gender (62% male), race (89% White), ECOG PS (83% PS 1), R-ISS stage (70% stage II/III), cytogenetic risk (52% high-risk), prior ASCT (82%), and extramedullary disease (44%) were balanced across groups (all p>0.2). Median prior LOT differed significantly across groups (p=0.045): chemotherapy-based 8.5 (IQR 6.5–10.0), PI/IMiD-based 7.5 (IQR 5.5–9.0), cellular therapy 7.0 (IQR 6.0–8.0), and non-cellular/non-PI/IMiD 6.0 (IQR 5.0–6.5).
Overall, 23 of 41 evaluable patients (56%) achieved a response to therapy post-talquetamab. Response rates differed significantly across therapy groups (p=0.005). Chemotherapy-based therapy demonstrated the highest overall response rate at 80% (12/15), followed by cellular therapy at 78% (7/9), PI/IMiD-based therapy at 27% (3/11), and non-cellular, non-PI/IMiD therapies at 17% (1/6). When depth of response was evaluated, significant differences were also observed across groups (p=0.021). Chemotherapy-based therapy achieved the highest rate of VGPR or better at 27% (4/15), followed by cellular therapy at 11% (1/9). Notably, none of the patients in the PI/IMiD-based therapy group nor the non-cellular, non-PI/IMiD therapy group achieved VGPR or better.

**Summary/Conclusion**

After progression on talquetamab, chemotherapy-based regimens and cellular therapies demonstrated the highest rate and depth of response in RRMM patients, despite these patients having been exposed to the highest median number of prior LOT. In contrast, PI/IMiD-based and non-cellular, non-PI/IMiD therapies showed limited efficacy in this setting. These findings suggest that chemotherapy-based regimens and cellular therapies may represent effective treatment strategies for patients with RRMM following talquetamab progression. Prospective studies with larger cohorts are warranted to identify optimal sequencing strategies in this heavily pretreated population. In addition, further investigation of emerging treatment options is needed to improve outcomes in this setting.

**Copyright Information:** (Online) ISSN: 2572-9241
©2026 The Author(s). HemaSphere is published by John Wiley & Sons Ltd on behalf of European Hematology Association. This is an open access Abstract Book distributed under the Attribution-NonCommercial-NoDerivs (CC BY-NC-ND), which allows third parties to download the articles and share them with others as long as they credit the author and the Abstract Book, but they cannot change the content in any way or use them commercially.
**Abstract Book Citations:** Authors, Title, HemaSphere, 2026;10:(S1):pages. The abstract book DOIs can be found on the "Author Information" page.
**Disclaimer:** Articles published in the journal HemaSphere exclusively reflect the opinions of the authors. The authors are responsible for all content in their abstracts including accuracy of the facts, statements, citing resources, etc.

**Table 1.** Patient Characteristics and Response Rate and Depth by Therapy Type after Progression on Talquetamab

| Characteristic | Overall (N=45) | Cellular Therapy (n=9) | Chemo-Based Therapy (n=16) | Non-Cell, Non-PI/IMiD (n=8) | PI/IMiD-Based (n=12) | *p-value* |
|---|---|---|---|---|---|---|
| **Age, median (IQR)** | 66 (59, 71) | 62 (44, 77) | 70 (63, 72) | 66 (62, 72) | 66 (59, 69) | 0.9 |
| **Gender, n (%)** | | | | | | 0.2 |
| Female | 17 (38) | 2 (22) | 7 (44) | 1 (13) | 7 (58) | |
| Male | 28 (62) | 7 (78) | 9 (56) | 7 (88) | 5 (42) | |
| **Race, n (%)** | | | | | | >0.9 |
| White | 40 (89) | 8 (89) | 13 (81) | 8 (100) | 11 (92) | |
| African American | 4 (8.9) | 1 (11) | 2 (13) | 0 (0) | 1 (8.3) | |
| Other | 1 (2.2) | 0 (0) | 1 (6.3) | 0 (0) | 0 (0) | |
| **ECOG Performance Status, n (%)** | | | | | | 0.4 |
| 0 | 4 (9.5) | 2 (22) | 0 (0) | 0 (0) | 2 (17) | |
| 1 | 35 (83) | 7 (78) | 14 (88) | 5 (100) | 9 (75) | |
| 2 | 3 (7.1) | 0 (0) | 2 (13) | 0 (0) | 1 (8.3) | |
| **R-ISS Stage at Diagnosis, n (%)** | | | | | | 0.3 |
| I | 11 (30) | 4 (50) | 5 (36) | 0 (0) | 2 (29) | |
| II | 20 (54) | 4 (50) | 7 (50) | 6 (75) | 3 (43) | |
| III | 6 (16) | 0 (0) | 2 (14) | 2 (25) | 2 (29) | |
| **Myeloma Subtype, n (%)** | | | | | | 0.4 |
| IgG Kappa | 10 (22) | 3 (33) | 4 (25) | 2 (25) | 1 (8.3) | |
| IgG Lambda | 12 (27) | 1 (11) | 5 (31) | 1 (13) | 5 (42) | |
| IgA Kappa | 11 (24) | 1 (11) | 3 (19) | 4 (50) | 3 (25) | |
| IgA Lambda | 3 (6.7) | 1 (11) | 1 (6.3) | 1 (13) | 0 (0) | |
| Free Kappa LC | 3 (6.7) | 0 (0) | 2 (13) | 0 (0) | 1 (8.3) | |
| Free Lambda LC | 4 (8.9) | 3 (33) | 0 (0) | 0 (0) | 1 (8.3) | |
| PCL | 2 (4.4) | 0 (0) | 1 (6.3) | 0 (0) | 1 (8.3) | |
| **Cytogenetic Risk, n (%)** | | | | | | 0.2 |
| High | 23 (52) | 4 (50) | 7 (44) | 7 (88) | 5 (42) | |
| Standard | 21 (48) | 4 (50) | 9 (56) | 1 (13) | 7 (58) | |
| **Prior ASCT, n (%)** | | | | | | 0.2 |
| Yes | 37 (82) | 8 (89) | 15 (94) | 6 (75) | 8 (67) | |
| No | 8 (18) | 1 (11) | 1 (6.3) | 2 (25) | 4 (33) | |
| **Extramedullary Disease, n (%)** | | | | | | 0.7 |
| Yes | 20 (44) | 4 (44) | 9 (56) | 3 (38) | 4 (33) | |
| No | 25 (56) | 5 (56) | 7 (44) | 5 (63) | 8 (67) | |
| **Prior LOT, median (IQR)** | 7 (6, 9) | 7 (6, 8) | 8.5 (6.5, 10) | 6 (5, 6.5) | 7.5 (5.5, 9) | **0.045** |
| **Response Post-Talquetamab, n (%)** | | | | | | **0.005** |
| Yes | 23 (56) | 7 (78) | 12 (80) | 1 (17) | 3 (27) | |
| No | 18 (44) | 2 (22) | 3 (20) | 5 (83) | 8 (73) | |
| **Depth of Response, n (%)** | | | | | | **0.021** |
| VGPR or Better | 5 (12) | 1 (11) | 4 (27) | 0 (0) | 0 (0) | |
| Partial Response | 18 (44) | 6 (67) | 8 (53) | 1 (17) | 3 (27) | |

*Abbreviations: ASCT, autologous stem cell transplant; ECOG, Eastern Cooperative Oncology Group; IQR, interquartile range; LC, light chain; LOT, lines of therapy; PCL, plasma cell leukemia; PI/IMiD, proteasome inhibitor/immunomodulatory drug; R-ISS, Revised International Staging System; SD, stable disease; PD, progressive disease; VGPR, very good partial response.*

*P-values by Kruskal-Wallis rank sum test (continuous variables) and Fisher's exact test (categorical variables). Bold p-values indicate statistical significance ($p<0.05$). Shaded rows denote primary outcome measures. Unknown values excluded from percentage calculations.*

Copyright Information: (Online) ISSN: 2572-9241
©2026 The Author(s). HemaSphere is published by John Wiley & Sons Ltd on behalf of European Hematology Association. This is an open access Abstract Book distributed under the Attribution-NonCommercial-NoDerivs (CC BY-NC-ND), which allows third parties to download the articles and share them with others as long as they credit the author and the Abstract Book, but they cannot change the content in any way or use them commercially.
Abstract Book Citations: Authors, Title, HemaSphere, 2026;10:(S1):pages. The abstract book DOIs can be found on the "Author Information" page.
Disclaimer: Articles published in the journal HemaSphere exclusively reflect the opinions of the authors. The authors are responsible for all content in their abstracts including accuracy of the facts, statements, citing resources, etc.

### (PS1912) HIGHER INCIDENCE OF SECONDARY EXTRAMEDULLARY DISEASE AFTER ANTI-CD38 THERAPY IN MULTIPLE MYELOMA: A CZECH NATIONAL REGISTRY REAL-WORLD MATCHED STUDY

**Topic:** 14. Myeloma and other monoclonal gammopathies - Clinical

Martin Stork*[1], Klara Mensikova[1], Jakub Radocha[2], Tomas Stopka[3], Tereza Popková[4, 5], Jiri Minarik[6], Alexandra Jungová[7], Petr Pavlíček[8], Denisa Novakova[2], František Sedlák[3], Ivan Špička[3], Ivanna Biochuk[1], Vojtech Latal[6], Nela Sendlerova[1], Jakub Cieślar[1], Tereza Dekojová[7], Jan Soukup[8], Vladimir Maisnar[2], Tomas Jelínek[4, 5], Roman Hájek[4, 5], Ludek Pour[1]

*[1]Department of Internal Medicine, Hematology and Oncology, University Hospital Brno and Faculty of Medicine, Masaryk University, Brno, Czechia; [2]4th Department of Internal Medicine – Hematology, University Hospital Hradec Kralove and Faculty of Medicine in Hradec Kralove, Charles University, Hradeck Kralove, Czechia; [3]1st Medical Department – Clinical Department of Hematology of the First Faculty of Medicine and General Teaching Hospital Charles University, Prague, Czechia; [4]Department of Hematooncology, University Hospital Ostrava, Ostrava, Czechia; [5]Department of Hematooncology, Faculty of Medicine University of Ostrava, Ostrava, Czechia; [6]Department of Hemato-Oncology, University Hospital Olomouc and Faculty of Medicine and Dentistry, Palacky University Olomouc, Olomouc, Czechia; [7]Hematology and Oncology Department, Charles University Hospital, Pilsen, Czechia; [8]Department of Internal Medicine and Haematology 3rd Faculty of Medicine, Charles University and University Hospital Kralovske Vinohrady, Prague, Czechia;*

### Background

The incidence of extramedullary disease (EMD) in multiple myeloma (MM) appears to be increasing in the era of novel agents. EMD is characterized by reduced CD38 expression and altered adhesion properties. CD38 interacts with its endothelial ligand CD31 (PECAM-1), and this interaction may contribute to retention of plasma cells within the bone marrow niche. This raises the possibility that anti-CD38 therapy, while generally improving bone marrow disease control, could contribute to selective outgrowth of immune-evasive EMD clones.

### Aims

To evaluate the population-level association between anti-CD38 monoclonal antibody exposure and the risk of EMD development.

### Methods

We conducted a national retrospective real-world analysis using the Registry of Monoclonal Gammopathies (RMG) of the Czech Myeloma Group (CMG). MM patients treated with anti-CD38 antibodies (daratumumab and isatuximab) within their first four treatment lines were compared to control cohort never exposed to anti-CD38 therapy. Patients without high-sensitivity imaging were excluded. Line-by-line matching was performed to exclude patients with EMD prior to the analyzed treatment line in both analyzed groups. Within each treatment line, patients were matched 1:1 using propensity score matching based on age, sex, paraskeletal plasmacytoma at diagnosis, 1q21 gains, del(17p), and follow-up duration. Secondary EMD was defined as true soft-tissue extramedullary plasmacytoma. A Cox proportional hazards regression model was calculated for matched patients.

**Copyright Information:** (Online) ISSN: 2572-9241
©2026 The Author(s). HemaSphere is published by John Wiley & Sons Ltd on behalf of European Hematology Association. This is an open access Abstract Book distributed under the Attribution-NonCommercial-NoDerivs (CC BY-NC-ND), which allows third parties to download the articles and share them with others as long as they credit the author and the Abstract Book, but they cannot change the content in any way or use them commercially.
**Abstract Book Citations:** Authors, Title, HemaSphere, 2026;10:(S1):pages. The abstract book DOIs can be found on the “Author Information” page.
**Disclaimer:** Articles published in the journal HemaSphere exclusively reflect the opinions of the authors. The authors are responsible for all content in their abstracts including accuracy of the facts, statements, citing resources, etc.

**Results**

From 9,298 MM patients (21,808 treatment lines), 374 anti-CD38–treated patients were matched to 374 controls. Baseline characteristics and predefined EMD-related risk factors were balanced between cohorts. Imaging strategies differed after matching: the anti-CD38 group more frequently underwent PET/CT (31.6% vs. 13.9%, p<0.001) and LDCT (61.8% vs. 27.3%, p<0.001), whereas the control group more often underwent CT (38.2% vs. 28.9%, p=0.007) and MRI (37.2% vs. 27.5%, p=0.004), reflecting a more symptom-focused CT/MRI approach. Secondary EMD developed in 36 patients from anti-CD38 group versus 15 patients in control group (HR 2.52, 95% CI 1.38–4.60; p=0.003). Median time-to-EMD (TTEMD) from the matched treatment line was comparable between both groups (26.0 vs. 28.0 months; p=0.149). Competing-risk analysis confirmed significantly higher cumulative incidence of EMD in the anti-CD38 group (Gray's test p<0.001), reaching 4.1% vs. 2.4% at 1 year, 8.0% vs. 3.2% at 2 years, and 10.0% vs. 4.1% at 4 years (Figure 1). Overall survival was longer in the anti-CD38 cohort (median 82.6 vs. 64.7 months; p<0.001).

**Summary/Conclusion**

In this national real-world matched cohort, anti-CD38 exposure was associated with a higher incidence of secondary EMD. Although imaging modalities differed between groups, the comparable TTEMD and persistent separation of cumulative incidence curves argue against a pure lead-time effect driven solely by more sensitive imaging. Despite of clear great clinical benefit of anti-CD38 therapy in MM patients, these findings suggest possible link to secondary EMD development and warrant further prospective a causal investigation.

**Copyright Information:** (Online) ISSN: 2572-9241
©2026 The Author(s). HemaSphere is published by John Wiley & Sons Ltd on behalf of European Hematology Association. This is an open access Abstract Book distributed under the Attribution-NonCommercial-NoDerivs (CC BY-NC-ND), which allows third parties to download the articles and share them with others as long as they credit the author and the Abstract Book, but they cannot change the content in any way or use them commercially.
**Abstract Book Citations:** Authors, Title, HemaSphere, 2026;10:(S1):pages. The abstract book DOIs can be found on the "Author Information" page.
**Disclaimer:** Articles published in the journal HemaSphere exclusively reflect the opinions of the authors. The authors are responsible for all content in their abstracts including accuracy of the facts, statements, citing resources, etc.

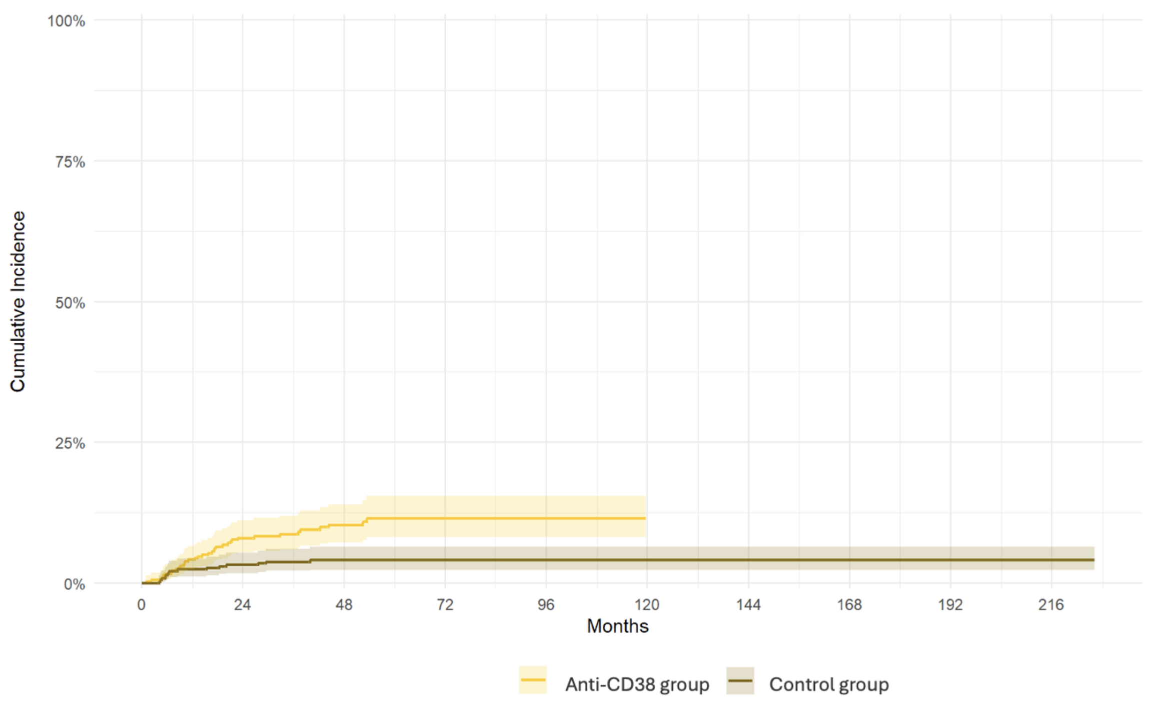

| | 0 | 24 | 48 | 72 | 96 | 120 | 144 | 168 | 192 | 216 |
|---|---|---|---|---|---|---|---|---|---|---|
| **Anti-CD38 group** | | | | | | | | | | |
| At Risk | 374 | 198 | 81 | 25 | 3 | 0 | 0 | 0 | 0 | 0 |
| Events | 0 | 28 | 34 | 36 | 36 | 36 | 36 | 36 | 36 | 36 |
| **Control group** | | | | | | | | | | |
| At Risk | 374 | 217 | 86 | 40 | 15 | 8 | 3 | 2 | 2 | 1 |
| Events | 0 | 12 | 15 | 15 | 15 | 15 | 15 | 15 | 15 | 15 |

Figure 1.: Cumulative incidence of secondary EMD events

**Copyright Information:** (Online) ISSN: 2572-9241
©2026 The Author(s). HemaSphere is published by John Wiley & Sons Ltd on behalf of European Hematology Association. This is an open access Abstract Book distributed under the Attribution-NonCommercial-NoDerivs (CC BY-NC-ND), which allows third parties to download the articles and share them with others as long as they credit the author and the Abstract Book, but they cannot change the content in any way or use them commercially.
**Abstract Book Citations:** Authors, Title, HemaSphere, 2026;10:(S1):pages. The abstract book DOIs can be found on the "Author Information" page.
**Disclaimer:** Articles published in the journal HemaSphere exclusively reflect the opinions of the authors. The authors are responsible for all content in their abstracts including accuracy of the facts, statements, citing resources, etc.

### (PS1913) ETENTAMIG IN PATIENTS (PTS) WITH RELAPSED/REFRACTORY MULTIPLE MYELOMA (RRMM) WITH PRIOR EXPOSURE TO B-CELL MATURATION ANTIGEN (BCMA)-TARGETED THERAPY

**Topic:** 14. Myeloma and other monoclonal gammopathies - Clinical

Saurabh Chhabra*[1], Emma Searle[2], Rakesh Popat[3], Hila Magen[4], Moshe Gatt[5], Irit Avivi Mazza[6], Muhamed Baljević[7], Neha Korde[8], Cesar Rodriguez[9], Eben Lichtman[10], Cyrille Touzeau[11], Xavier Leleu[12], Anders Svensson[13], Thomas Doerr[13], Jovian Yu[13], Shane Lee[13], Aarif Ahsan[13], Tanya Rosenberg[13], Chetasi Talati[13], Shaji Kumar[14]

*[1]Mayo Clinic Arizona, Phoenix, United States of America;[2]The Christie NHS Foundation Trust, Manchester, United Kingdom; [3]University College London Hospitals NHS Foundation Trust, London, United Kingdom;[4]Tel Aviv University, Department of Hematology, Tel Aviv Sourasky Medical Center & Gray Faculty of Medical and Health Sciences, Tel Aviv, Israel; [5]Hadassah Medical Center and Hebrew University of Jerusalem, Jerusalem, Israel; [6]Tel Aviv Sourasky Medical Center, Tel Aviv, Israel; [7]Vanderbilt University Medical Center, Nashville, United States of America;[8]Memorial Sloan Kettering Cancer Center, New York, United States of America; [9]Mount Sinai, New York, United States of America; [10]UNC Lineberger Comprehensive Cancer Center, Chapel Hill, United States of America; [11]University Hospital Hôtel-Dieu, Nantes, France; [12]Service d'Hématologie et Thérapie Cellulaire, Poitiers Cedex, France; [13]AbbVie, Inc, North Chicago, United States of America; [14]Mayo Clinic, Rochester, United States of America;*

### Background

Data on response to BCMA-targeted therapies among BCMA-exposed patients with RRMM are limited. Outcomes after sequential/subsequent anti-BCMA bispecific T-cell engagers (Bs-TCE) are vital to evaluate. Etentamig is being developed as a next-generation BCMA x CD3 Bs-TCE composed of a bivalent BCMA-binding domain with a high-avidity, low-affinity CD3-binding domain potentially reducing cytokine release syndrome (CRS), and a silenced Fc tail for extended half-life and convenient monthly (Q4W) dosing.

### Aims

Here we present phase 1 results of etentamig in RRMM showing robust efficacy and potential best-in-class safety.

### Methods

Arm B of the open-label, Phase 1b MOVISO study (NCT05650632) evaluated etentamig in patients with RRMM with ≥2 prior lines of therapy (PLT), including triple-class and prior BCMA exposure (antibody-drug conjugate [ADC] >30 days or CAR-T >6 months [mo] after last exposure). Primary safety endpoints were CRS and immune effector cell-associated neurotoxicity syndrome (ICANS); efficacy endpoints included objective response rate (ORR; IMWG 2016), duration of response (DoR), and progression-free survival (PFS). Patients received etentamig (60 mg IV; no step-up dosing [SUD]) on Cycle 1 Day 1 and Q4W thereafter.

**Copyright Information:** (Online) ISSN: 2572-9241
©2026 The Author(s). HemaSphere is published by John Wiley & Sons Ltd on behalf of European Hematology Association. This is an open access Abstract Book distributed under the Attribution-NonCommercial-NoDerivs (CC BY-NC-ND), which allows third parties to download the articles and share them with others as long as they credit the author and the Abstract Book, but they cannot change the content in any way or use them commercially.
**Abstract Book Citations:** Authors, Title, HemaSphere, 2026;10:(S1):pages. The abstract book DOIs can be found on the "Author Information" page.
**Disclaimer:** Articles published in the journal HemaSphere exclusively reflect the opinions of the authors. The authors are responsible for all content in their abstracts including accuracy of the facts, statements, citing resources, etc.

### Results

At cutoff (June 11, 2025), 41 prior BCMA-exposed patients (CAR-T, n=24; ADC, n=17) had been treated; 24 (57%) were male, 38 (93%) were White, median (mdn) (range) age was 68 (43–89) years, 37 (88%) had ECOG PS ≤1, and 34 (81%) were triple-class refractory. Mdn (range) PLT was 6 (3–15). For 21 (51%) patients, the last PLT was BCMA directed; 23 (56%) had previously responded to anti-BCMA therapy (17/24 [71%] for CAR-T and 6/17 [35%] for ADC). Mdn (range) time from last prior CAR-T or ADC to first dose of etentamig was 15 (1–30) and 4 (1–45) mo, respectively. Mdn (range) follow-up was 5.5 (0.2–17.1) mo. Despite no SUD, CRS and ICANS were reported in 24 (57%) and 3 (7%) patients, respectively; all G1/2 events, except 1 G3 ICANS. G3/4 neutropenia and infections were reported in 15 (36%) and 17 (41%) patients, respectively. Efficacy outcomes are detailed in the **Table** (NE, not estimable). For patients whose last PLT was BCMA directed, MRD negativity (clonoSEQ®, $10^{-5}$) was seen in 2/3 (67%) evaluable patients, along with robust peak activation and proliferation of CD8+ T cells.

### Summary/Conclusion

Patients treated with etentamig achieved durable responses; rates were higher in patients who received CAR-T as last line. No new safety signals were observed; only low-grade CRS was seen despite no SUD.

**Table**

| | All prior BCMA (N=41) | Prior BCMA CAR-T (N=24) |
|---|---|---|
| **ORR**, n/n (%) [95% CI] | | |
| Overall | 17/36 (47) [30–65] | 11/22 (50) [28–72] |
| BCMA-directed last PLT | 11/19 (58) [34–80] | 7/11 (64) [31–89] |
| **Mdn DoR**, mo [95% CI], No. of pts | | |
| Overall | 13 [7–NE], N=17 | 13 [7–NE], N=11 |
| BCMA-directed last PLT | 13 [7–NE], N=11 | 13 [7–NE], N=7 |
| **Mdn PFS**, mo [95% CI], No. of pts | | |
| Overall | 3.4 [3–11], N=41 | 8.3 [2–NE], N=24 |
| BCMA-directed last PLT | 9.4 [2–NE], N=21 | 9.4 [2–NE], N=12 |

**Copyright Information:** (Online) ISSN: 2572-9241
©2026 The Author(s). HemaSphere is published by John Wiley & Sons Ltd on behalf of European Hematology Association. This is an open access Abstract Book distributed under the Attribution-NonCommercial-NoDerivs (CC BY-NC-ND), which allows third parties to download the articles and share them with others as long as they credit the author and the Abstract Book, but they cannot change the content in any way or use them commercially.
**Abstract Book Citations:** Authors, Title, HemaSphere, 2026;10:(S1):pages. The abstract book DOIs can be found on the "Author Information" page.
**Disclaimer:** Articles published in the journal HemaSphere exclusively reflect the opinions of the authors. The authors are responsible for all content in their abstracts including accuracy of the facts, statements, citing resources, etc.

### (PS1914) 1Q GAINS DETECTED BY OPTICAL GENOME MAPPING (OGM) ARE MORE PREDICTIVE OF PROGNOSIS THAN THOSE DETECTED BY FISH IN NEWLY DIAGNOSED MULTIPLE MYELOMA

**Topic:** 14. Myeloma and other monoclonal gammopathies - Clinical

Adela Cisneros[1, 2], Anna Moreno*[1], Mar Mallo[3], Laura Abril Sabater[1], Gladys Virginia Ibarra Fernandez[1], Neus Ruiz-Xivillé[1], Aleix Mendez-Lopez[1], Marisol Xandri[1], Carmen Villena[1], Encarnación Santafé[1], Alex Méndez[1], Jessica Tijero[3], Itziar Carro Arostegui[4], Maria Lourdes Escoda Teigell[5], Yolanda Gonzalez Montes[6], Xavier Ortín Font[7], Rosa Collado Nieto[8], Mariam Ibáñez Company[8], Dolors Costa[9], Silvia Bea[9, 10], Cristina Lopez[9, 10], Maria Laura Blanco[11], Miguel Ángel Perera Álvarez[12], Carlos Javier Armas[12, 13], Sara Medina[12, 13], Josgrey Del Valle[14], Cristina De Ramón Sánchez[14], Norma Gutierrez Gutierrez[14], Cristina Baca[15], María del Mar Rodríguez Rivera[16], Marta Lorenzo[16], Marta Salido[16], Anna Puiggros[16], Blanca Espinet[16], Jose Tomás Navarro Ferrando[1, 2], Isabel Granada[1], Albert Oriol[1, 2]

*[1]Hematology Department, Institut Catala D'oncologia (ICO Badalona) –Institut de Recerca Contra la Leucemia Josep Carreras (IJC), Hospital Germans Trias i Pujol, Barcelona, Spain., Hematology, Badalona, Spain; [2]Medicine Department, Universitat Autonoma de Barcelona, Barcelona, Spain., Medicine, Badalona, Spain; [3]Microarrays Unit and MDS Research Group, Institut de Recerca Contra la Leucemia Josep Carreras (IJC), ICO-Hospital Germans Trias i Pujol, Universitat Autonoma de Barcelona, Badalona, Spain., Badalona, Spain; [4]Clinical Hematology Department, Institut Catala d'Oncologia (ICO), Hospitalet de Llobregat, Spain., Hematology, Barcelona, Spain; [5]Hematology Department, Institut Catala d'Oncologia (ICO)-hospital Joan XXIII, Tarragona, Spain., Hematology, Tarragona, Spain; [6]Hematology Department, Institut Catala d'Oncologia (ICO)-Hospital Josep Trueta, Girona, Spain., Hematology, Girona, Spain; [7]Hematology Department, Hospital Verge de la Cinta, Tortosa, Spain., Hematology, Tortosa, Spain; [8]Hematology Department. HospitaGeneral Universitario de Valencia. Fundación de Investigación Hospital General Universitario de Valencia. Valencia, Spain., Hematology, Valencia, Spain; [9]Hematopathology Unit, Department of Pathology, Hospital Clínic de Barcelona, Barcelona, Spain, Hematology, Barcelona, Spain; [10]Barcelona Clinical Research Foundation-August Pi i Sunyer Biomedical Research Institute (FRCB-IDIBAPS), Barcelona, Spain. Cancer Network Biomedical Research Center (CIBERONC), Madrid, Spain. University of Barcelona, Spain., Hematology, Barcelona, Spain; [11]Hematology Department, Hospital de la Santa Creu i Sant Pau Barcelona, Spain., Hematology, Barcelona, Spain; [12]Hospital Universitario de Gran Canaria Doctor Negrín, Las Palmas, Spain., Hematology, Las Palmas de Gran Canaria, Spain; [13]Instituto de Investigación Sanitaria de Canarias (IISC), Las Palmas, Spain, Hematology, Las Palmas de Gran Canaria, Spain; [14]Hematology Department, University Hospital of Salamanca, IBSAL, Cancer Research Center-IBMCC, Salamanca, Spain., Hematology, Salamanca, Spain; [15]Hematology Service, Hospital del Mar, Barcelona, Spain, Hematology, Barcelona, Spain; [16]Molecular Cytogenetics Laboratory, Pathology Service, Hospital del Mar, Barcelona, Spain Hospital del Mar Research Institute, Barcelona, Spain., Pathology, Barcelona, Spain;*

### Background

Gain of 1q is an adverse prognostic factor in multiple myeloma (MM) and is considered high-risk in both the Revised International Staging System (R-ISS) and the R2-ISS. Fluorescence in situ hybridization (FISH) is currently the standard technique for its detection. Optical genome mapping (OGM) has recently emerged as a novel technology that overcomes several technical limitations of FISH.

### Aims

The aim of this study was to compare the clinical impact of 1q gains detected by FISH with those identified by OGM.

**Copyright Information:** (Online) ISSN: 2572-9241
©2026 The Author(s). HemaSphere is published by John Wiley & Sons Ltd on behalf of European Hematology Association. This is an open access Abstract Book distributed under the Attribution-NonCommercial-NoDerivs (CC BY-NC-ND), which allows third parties to download the articles and share them with others as long as they credit the author and the Abstract Book, but they cannot change the content in any way or use them commercially.
**Abstract Book Citations:** Authors, Title, HemaSphere, 2026;10:(S1):pages. The abstract book DOIs can be found on the "Author Information" page.
**Disclaimer:** Articles published in the journal HemaSphere exclusively reflect the opinions of the authors. The authors are responsible for all content in their abstracts including accuracy of the facts, statements, citing resources, etc.

**Methods**

A total of 148 newly diagnosed MM patients from 19 different centers were prospectively analyzed by both FISH and OGM. The cohort included 84 males and 64 females, with a mean age of 67.5 years (SD 8.3). All patients received uniform treatment.
According to the Revised International Staging System (R2-ISS), 22 (15%) patients were classified as stage I (low risk), 39 (28%) as stage II (intermediate-low), 68 (48%) as stage III (intermediate-high), and 13 (9%) as stage IV (high risk); staging data were unavailable in 3 cases.
FISH was performed on CD138+ selected plasma cells in all cases. The cut-off values were >10% for rearrangements and >20% for gains and losses, using the following probes: CDKN2C (1p32.3)/CKS1B (1q21–22), TP53/17cen, t(4;14) FGFR3/IGH, t(11;14) CCND1/IGH, and t(14;16) MAF/IGH.
OGM was performed on CD138+ selected cells in 60 patients, on whole bone marrow samples in 85 patients, on peripheral blood in 2 patients, and using both positive selection and negative fraction analysis in 1 patient. Analysis was conducted according to the criteria of the Spanish OGM Working Group in Hematology, using a specific BED file for MM and a ≥200 kb filter for additional alterations.
Progression-free survival (PFS) was defined as the time from MM diagnosis to disease progression or death, according to the criteria of the International Myeloma Working Group.

**Results**

Based on the combined FISH and OGM data, the following abnormalities were detected: deletion of 1p32 in 19 cases (20%), gain of 1q in 55 cases (37%), TP53 loss in 16 cases (11%), IGH::FGFR3 in 13 cases (9%), IGH::CCND1 in 30 cases (20%), IGH::CCND3 in 2 cases (1.5%), IGH::MAF in 4 cases (3%), IGH::MAFB in 1 case (0.7%), and MYC rearrangements in 26 cases (18%).
A complete response (CR) or better was achieved in 49% of patients. Median PFS for the whole cohort was 3.7 years, and median overall survival (OS) was not reached.
When assessed by OGM, 1q gain was the strongest predictor of CR achievement among cytogenetic markers in both univariate and multivariate analyses (odds ratio 0.38; 95% CI 0.17–0.83; p = 0.016). In contrast, 1q gain assessed by FISH did not retain statistical significance in multivariate analysis.
1q gains detected by either method were also associated with shorter PFS. Median PFS was 4.3 years (95% CI 3.7–not reached) in patients without 1q gain and 2.7 years (95% CI 1.6–not reached) in those with 1q gain when assessed by OGM (p = 0.02) (Figure 1).

**Summary/Conclusion**

1q gains detected by OGM were superior to those detected by FISH in predicting achievement of CR in newly diagnosed MM patients. The adverse prognostic impact of 1q gain was also confirmed in terms of PFS.

**Copyright Information:** (Online) ISSN: 2572-9241
©2026 The Author(s). HemaSphere is published by John Wiley & Sons Ltd on behalf of European Hematology Association. This is an open access Abstract Book distributed under the Attribution-NonCommercial-NoDerivs (CC BY-NC-ND), which allows third parties to download the articles and share them with others as long as they credit the author and the Abstract Book, but they cannot change the content in any way or use them commercially.
**Abstract Book Citations:** Authors, Title, HemaSphere, 2026;10:(S1):pages. The abstract book DOIs can be found on the "Author Information" page.
**Disclaimer:** Articles published in the journal HemaSphere exclusively reflect the opinions of the authors. The authors are responsible for all content in their abstracts including accuracy of the facts, statements, citing resources, etc.

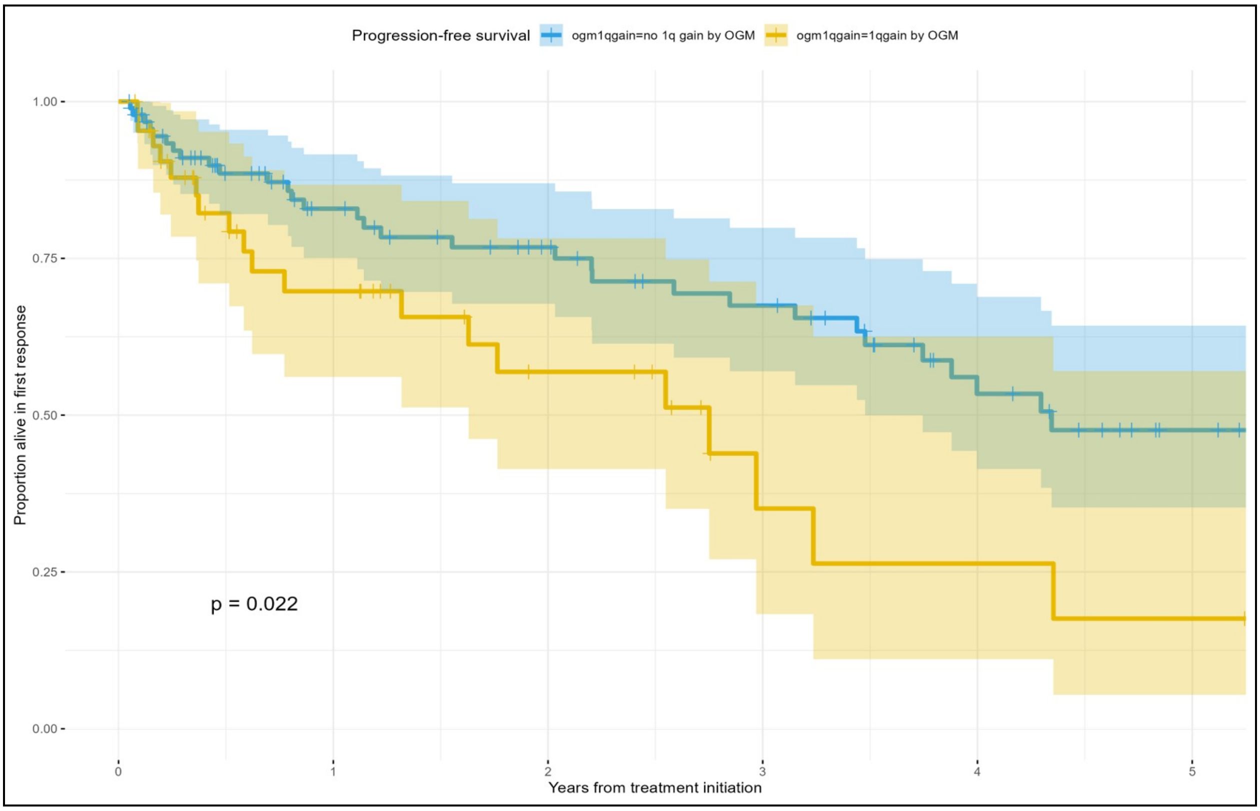

Figure 1. Kaplan Meier curves for progression-free survival (PFS) according to 1q gain status assessed by optical genome mapping (OGM). Patients with 1q gain had significantly shorter PFS compared to those without 1q gain (median 2.7 vs 4.3 years; log-rank p = 0.022).

**Copyright Information:** (Online) ISSN: 2572-9241
©2026 The Author(s). HemaSphere is published by John Wiley & Sons Ltd on behalf of European Hematology Association. This is an open access Abstract Book distributed under the Attribution-NonCommercial-NoDerivs (CC BY-NC-ND), which allows third parties to download the articles and share them with others as long as they credit the author and the Abstract Book, but they cannot change the content in any way or use them commercially.
**Abstract Book Citations:** Authors, Title, HemaSphere, 2026;10:(S1):pages. The abstract book DOIs can be found on the "Author Information" page.
**Disclaimer:** Articles published in the journal HemaSphere exclusively reflect the opinions of the authors. The authors are responsible for all content in their abstracts including accuracy of the facts, statements, citing resources, etc.

### (PS1915) STAGE-ADAPTED TREATMENT STRATEGY IN NON-AMYLOID MONOCLONAL GAMMOPATHY OF RENAL SIGNIFICANCE: A REAL-WORLD COHORT ANALYSIS

**Topic:** 14. Myeloma and other monoclonal gammopathies - Clinical

Xianghong Jin[1], Yongrui Zhang[2], Wenling Ye[3], Yuhang Song[4], Fujing Zhang[2], Limeng Chen[3], Junling Zhuang*[2]

*[1]Peking Union Medical College Hospital, Chinese Academy of Medical Science & Peking Union Medical College, Rare Diseases, BeiJing, China; [2]Peking Union Medical College Hospital, Chinese Academy of Medical Science & Peking Union Medical College, Hematology, BeiJing, China; [3]Peking Union Medical College Hospital, Chinese Academy of Medical Science & Peking Union Medical College, Nephrology, BeiJing, China; [4]Sichuan Provincial People's Hospital, University of Electronic Science and Technology of China, Rheumatology, Chengdu, China;*

### Background

Non-amyloid monoclonal gammopathy of renal significance (MGRS-NA) comprises a heterogeneous group of clonal disorders characterized by nephrotoxic monoclonal immunoglobulin–mediated injury. Although clone-directed therapy is recommended, treatment timing and intensity across chronic kidney disease (CKD) stages remain poorly defined.

### Aims

To evaluate stage-specific clinical characteristics and outcomes in MGRS-NA and explore whether renal function–based stratification may inform treatment strategies.

### Methods

We conducted a single-center ambidirectional cohort study including 64 biopsy-proven MGRS-NA patients between 2012 and 2025. Patients were stratified by baseline CKD stage using CKD-EPI formula: CKD1–2 (eGFR ≥60 ml/min/1.73m², n=13), CKD3 (30–59, n=34), and CKD4–5 (<30, n=17). Primary endpoints included renal response (≥25% improvement in eGFR or reduction in 24-hour proteinuria), hematological response (≥PR according to IMWG-adapted criteria), and survival outcomes. Median follow-up was 32.2 months.

### Results

CKD3 represented the largest subgroup (53.1%). Light chain deposit disease (LCDD) accounted for the majority in CKD4–5 (64.7%), whereas Proliferative glomerulonephritis with monoclonal immunoglobulin deposit (PGNMID) and cryoglobulinemic glomerulonephritis (CG) were more evenly distributed in early CKD stages. Bortezomib-based regimens were predominant in front-line treatment (53.1%). Four patients underwent autologous stem cell transplantation (ASCT), all in CKD1–3. Renal responses (CR+PR) were achieved depending different stages (50.0% in CKD3, 46.2% in CKD1–2, and 29.4% in CKD4–5; p=0.038). Hematologic response rates of PR or better were similar among groups. Five patients died (7.8%), with mortality highest in CKD4–5 (17.6%). Dialysis dependence at or shortly after diagnosis did not reversed exclusively in CKD4–5 patients.

### Summary/Conclusion

MGRS-NA demonstrates stage-dependent heterogeneity in pathological subtypes and clinical outcomes. Advanced CKD4–5 was associated with inferior renal response and survival. These findings suggest that mild renal damage provides a greater opportunity for therapeutic intervention, deserving prospective validation in further large cohorts.

**Copyright Information:** (Online) ISSN: 2572-9241
©2026 The Author(s). HemaSphere is published by John Wiley & Sons Ltd on behalf of European Hematology Association. This is an open access Abstract Book distributed under the Attribution-NonCommercial-NoDerivs (CC BY-NC-ND), which allows third parties to download the articles and share them with others as long as they credit the author and the Abstract Book, but they cannot change the content in any way or use them commercially.
**Abstract Book Citations:** Authors, Title, HemaSphere, 2026;10:(S1):pages. The abstract book DOIs can be found on the "Author Information" page.
**Disclaimer:** Articles published in the journal HemaSphere exclusively reflect the opinions of the authors. The authors are responsible for all content in their abstracts including accuracy of the facts, statements, citing resources, etc.

### (PS1916) SEX DIFFERENCES IN PROGRESSION TO MALIGNANCY FROM MONOCLONAL GAMMOPATHY OF UNDETERMINED SIGNIFICANCE

**Topic:** 14. Myeloma and other monoclonal gammopathies - Clinical

Ingigerdur Sverrisdottir*[1], Lærke Sloth Andersen[2], Cecilie Maeng[2, 3, 4], Christian Brieghel[2, 4, 5], Ida Kristensen[6, 7], Carsten Niemann[2, 4], Sigrún Thorsteinsdóttir[2, 8]

*[1]Sahlgrenska University Hospital, Gothenburg, Sweden; [2]Rigshospitalet, Copenhagen University Hospital, Department of Hematology, Copenhagen, Denmark; [3]Biotech Research and Innovation Center, University of Copenhagen, Cophenhagen, Denmark; [4]Danish Cancer Institute, Cophenhagen, Denmark; [5]Zealand University Hospital, Department of Hematology, Roskilde, Denmark; [6]Odense University Hospital, Department of Hematology, Odense, Denmark; [7]University of Southern Denmark, Department of Clinical Research, Odense, Denmark; [8]University of Iceland, Faculti of Medicine, Reykjavik, Iceland;*

### Background

Gender discrepancies in monoclonal gammopathy of undetermined significance (MGUS) and multiple myeloma (MM) are well known. MM incidence is 1.5 times higher in men and 1.3-1.8 in MGUS, respectively. The causes of these differences remain unclear. In general, hematological malignancies are more common in men, possibly due to differences in genetics, immune function, sex hormones, environmental factors, and risk behaviours. However, sex differences in progression from MGUS to MM or amyloid light chain (AL) amyloidosis have not been studied. Given the higher MM incidence in men, a greater progression risk from MGUS could partly explain this disparity.

### Aims

To investigate the risk of progression from MGUS to MM and AL amyloidosis in women and men with MGUS and evaluate risk factors for progression.

### Methods

*The cohort*
Participants were identified using the Danish Lymphoid Cancer Research (DALY-CARE) data resource, which contains data on all Danish adults registered with a lymphoid-lineage cancer or its precursor from 2002 to 2024. All non-IgM MGUS, MM, and AL-amyloidosis diagnoses in the DALY-CARE database were identified using the International Classification of Diseases (ICD-10). Individuals with IgG, IgA, IgD, or IgE type of M protein, as well as light-chain MGUS, were included in the study. The date of MGUS diagnosis was defined as the date of the first detectable M protein/FLC measurement. Progression was defined as a diagnosis of MM or AL-amyloidosis >90 days after MGUS diagnosis.
*Statistical analysis*
Cumulative incidence of progression was calculated using Aalen-Johanson estimator, accounting for death as a competing risk and compared using Gray´s test. Hazard ratio for progression risk was assessed using a cause-specific Cox regression model, adjusted for age.

**Copyright Information:** (Online) ISSN: 2572-9241
©2026 The Author(s). HemaSphere is published by John Wiley & Sons Ltd on behalf of European Hematology Association. This is an open access Abstract Book distributed under the Attribution-NonCommercial-NoDerivs (CC BY-NC-ND), which allows third parties to download the articles and share them with others as long as they credit the author and the Abstract Book, but they cannot change the content in any way or use them commercially.
**Abstract Book Citations:** Authors, Title, HemaSphere, 2026;10:(S1):pages. The abstract book DOIs can be found on the "Author Information" page.
**Disclaimer:** Articles published in the journal HemaSphere exclusively reflect the opinions of the authors. The authors are responsible for all content in their abstracts including accuracy of the facts, statements, citing resources, etc.

### Results

In total, 10,523 individuals with MGUS were included; 5,412 (51.4%) were men, with a median age of 72 years. The most common M protein isotype was IgG. Men had a statistically significantly higher M protein level (2.4 g/L) than women (1.9 g/L) (Wilcoxon rank-sum test; p-value < 0.001). A total of 28% of males and 20% of females died before progression. The cumulative incidence of progression to MM or AL amyloidosis was 3.3% and 3.4% at 5 years, 4.6% and 4.9% at 10 years for men and women, respectively (Grays test, p=0.24) (Figure). In both univariate and multivariable analyses, male sex was not associated with an increased risk of progression. However, both higher M protein level and an abnormal FLC ratio were associated with an increased risk of progression (Table).

| Variables | Age adjusted univariate analysis HR (95% CI) | Multivarible analysis HR (95% CI) |
|---|---|---|
| Male sex | 1.02 (0.86-1.21) | 1.01 (0.80-1.28) |
| IgA isotype (IgG ref.) | 1.28 (1.01-1-60) | 1.34 (0.98-1.83) |
| M protein concentration | 1.13 (1.11-1.15) | 1.11 (1.09-1.14) |
| Abnormal FLC-ratio | 3.37 (2.65-4.30) | 2.23 (1.73-2.90) |

**Table**. Risk factors of progression to MM or AL amyloidosis. HR: Hazard ratio, CI: Confidence interval, FLC: Free light chain.

### Summary/Conclusion

In this large population-based study of >10,000 individuals with MGUS, we did not find a difference in risk of progression to overt malignancy between the sexes. Males have higher MGUS prevalence and seem to be more likely to die of other causes. These results indicate that the higher incidence of MM in males stems from a higher incidence of MGUS rather than an increased risk of progression from MGUS.

**Copyright Information:** (Online) ISSN: 2572-9241
©2026 The Author(s). HemaSphere is published by John Wiley & Sons Ltd on behalf of European Hematology Association. This is an open access Abstract Book distributed under the Attribution-NonCommercial-NoDerivs (CC BY-NC-ND), which allows third parties to download the articles and share them with others as long as they credit the author and the Abstract Book, but they cannot change the content in any way or use them commercially.
**Abstract Book Citations:** Authors, Title, HemaSphere, 2026;10:(S1):pages. The abstract book DOIs can be found on the “Author Information” page.
**Disclaimer:** Articles published in the journal HemaSphere exclusively reflect the opinions of the authors. The authors are responsible for all content in their abstracts including accuracy of the facts, statements, citing resources, etc.

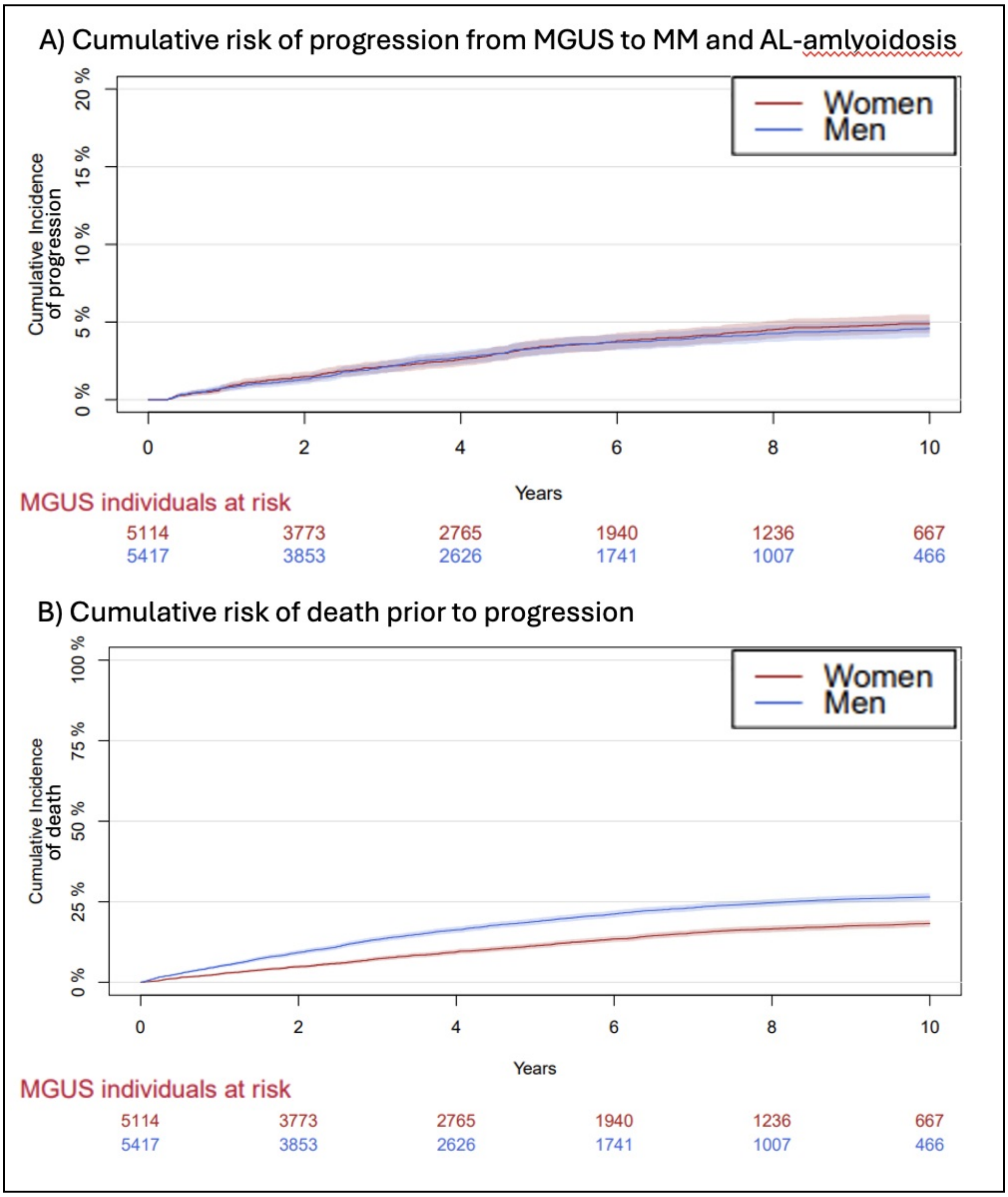

**Copyright Information:** (Online) ISSN: 2572-9241
©2026 The Author(s). HemaSphere is published by John Wiley & Sons Ltd on behalf of European Hematology Association. This is an open access Abstract Book distributed under the Attribution-NonCommercial-NoDerivs (CC BY-NC-ND), which allows third parties to download the articles and share them with others as long as they credit the author and the Abstract Book, but they cannot change the content in any way or use them commercially.
**Abstract Book Citations:** Authors, Title, HemaSphere, 2026;10:(S1):pages. The abstract book DOIs can be found on the "Author Information" page.
**Disclaimer:** Articles published in the journal HemaSphere exclusively reflect the opinions of the authors. The authors are responsible for all content in their abstracts including accuracy of the facts, statements, citing resources, etc.

### (PS1917) REAL-WORLD TREATMENT SEQUENCING AFTER CAR-T VS BISPECIFIC ANTIBODIES IN MULTIPLE MYELOMA: NEXT-LINE PATTERNS AND TIME-TO-NEXT-LINE INITIATION IN THE HEALTHTREE REGISTRY

**Topic:** 14. Myeloma and other monoclonal gammopathies - Clinical

Jorge Arturo Hurtado Martinez*[1], Jennifer Ahlstrom[1], Karla Mariana Castro Bórquez[1], Patricia Flores Pérez[1], Ana Echenique Alcázar[1], Martha Paola Anchondo Commesse[1], Samuel Bennion[1], Jay R. Hydren[1]

[1]*HealthTree Foundation, South Jordan, United States of America;*

### Background

CAR-T and bispecific antibodies (BsAb) have transformed relapsed/refractory multiple myeloma (MM), yet real-world treatment sequencing after these immune therapies lacks characterization, particularly by line of therapy and by product.

### Aims

To describe subsequent therapy patterns after index CAR-T versus BsAb and to estimate time-to-next line-of-therapy (LOT) initiation using an EHR-derived patient registry.

### Methods

We conducted a retrospective cohort study in the HealthTree Registry. Immune therapy exposures were classified as CAR-T or BsAb. Analyses were performed across all qualifying immune-therapy LOTs and in a patient-level index cohort by first observed immune therapy per patient. Observed subsequent LOT required a documented next LOT start date. Follow-up was assessed from index immune therapy date to censoring date. TTNT was analyzed using Kaplan–Meier (Greenwood 95% CI), log-rank testing, and Cox proportional hazards models adjusted for age, sex, and late line (≥5). Sequencing patterns among patients with an observed subsequent LOT were summarized by next-LOT therapy class and visualized using a Sankey diagram (index immune therapy → dominant next-LOT class). Post-LOT regimen complexity was summarized by the number of MM drugs/classes after excluding supportive/conditioning/bridging medications.

### Results

We identified 206 patients contributing 262 qualifying immune-therapy LOT exposures (CAR-T: 146 patients/151 LOTs; BsAb: 96 patients/111 LOTs). In the index cohort, 141 patients received CAR-T and 65 received BsAb. Index product distribution was dominated by ciltacabtagene autoleucel (62%) and idecabtagene vicleucel (25%) among CAR-T, and teclistamab (65%) and talquetamab (23%) among BsAb.
A documented subsequent LOT was observed for 41/141 (29%) CAR-T and 17/65 (26%) BsAb index exposures; therefore, sequencing summaries are conditional on next-line ascertainment. Among those with an observed subsequent LOT, dominant next-LOT classes after CAR-T most commonly included BsAb (LOT 1-4: 46, LOT ≥5: 62%), while after BsAb, proteasome inhibitor including regimens were the most frequently used (42%), with smaller proportions receiving anti-CD38, additional BsAb, or CAR-T (16.6% each). Early next-LOT initiation was more frequent after BsAb than CAR-T among patients with an observed subsequent LOT: ≤90 days 41.2% vs 7.3% (Fisher p=0.004) and ≤180 days 58.8% vs 24.4% (p=0.017). In TTNT adjusted analyses, no statistically significant difference was detected between cohorts (log-rank p=0.50). Six- and 12-month probabilities of no subsequent LOT were 0.918 (95% CI 0.871–0.968) and 0.816 (0.746–0.894) after CAR-T, and 0.839 (0.753–0.936) and 0.760 (0.656–0.880) after BsAb. In adjusted Cox analysis, BsAb vs CAR-T was not associated with TTNT (HR 0.87, 95% CI 0.49–1.55).

**Copyright Information:** (Online) ISSN: 2572-9241
©2026 The Author(s). HemaSphere is published by John Wiley & Sons Ltd on behalf of European Hematology Association. This is an open access Abstract Book distributed under the Attribution-NonCommercial-NoDerivs (CC BY-NC-ND), which allows third parties to download the articles and share them with others as long as they credit the author and the Abstract Book, but they cannot change the content in any way or use them commercially.
**Abstract Book Citations:** Authors, Title, HemaSphere, 2026;10:(S1):pages. The abstract book DOIs can be found on the "Author Information" page.
**Disclaimer:** Articles published in the journal HemaSphere exclusively reflect the opinions of the authors. The authors are responsible for all content in their abstracts including accuracy of the facts, statements, citing resources, etc.

### Summary/Conclusion

In the HealthTree Registry, real-world sequencing after immune therapies differed by index modality and line of therapy: subsequent treatment after CAR-T frequently incorporated BsAb, whereas post BsAb fell more frequently into proteasome inhibitor including regimens. Although overall TTNT did not significantly differ, early next-LOT initiation was more frequent after BsAb among patients with an observed subsequent LOT. However, these findings describe contemporary sequencing patterns that should not be interpreted as comparative effectiveness due to differences in therapy response monitoring and commercial access.

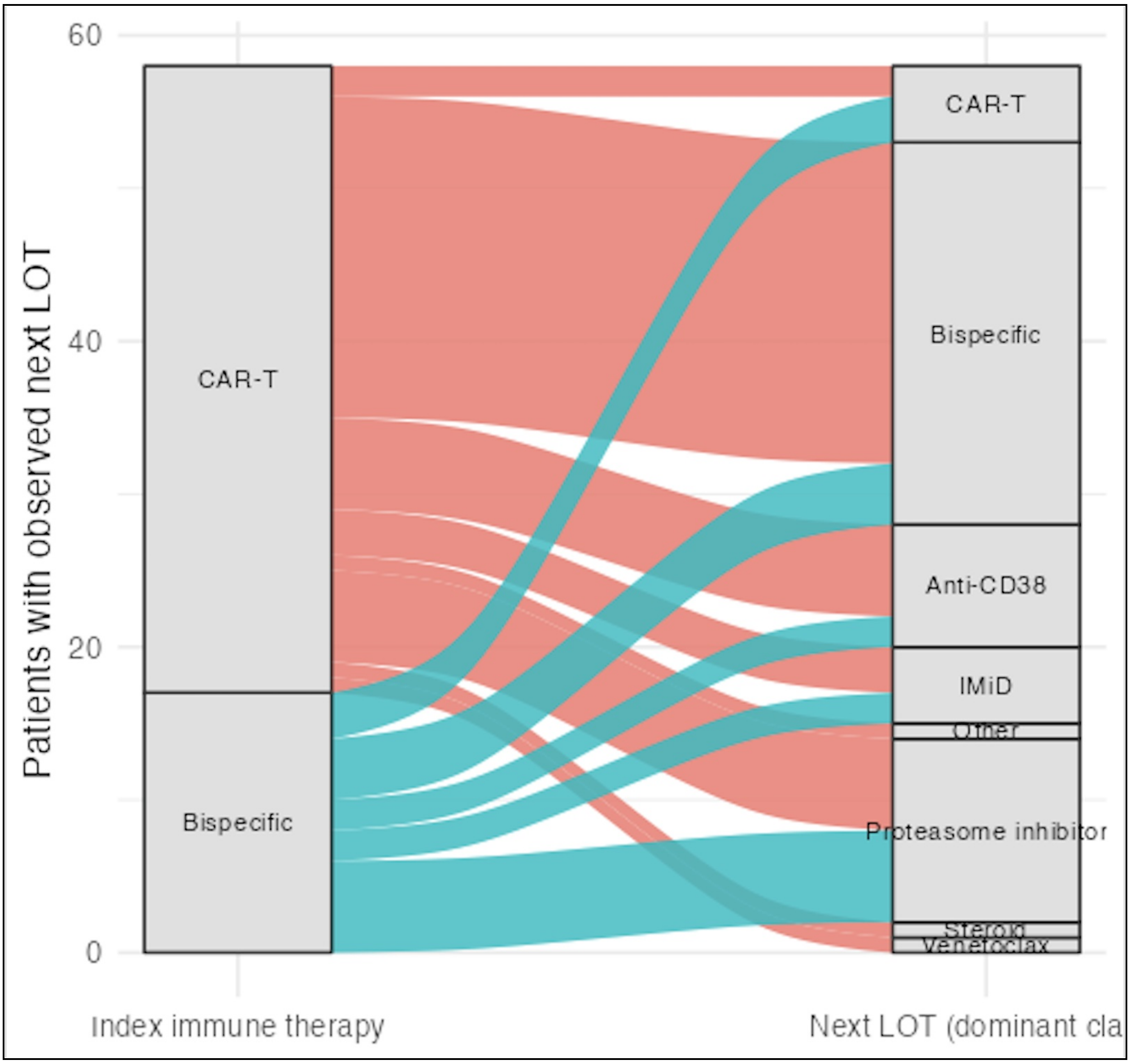

Figure 1. Subsequent therapy after CAR-T vs bispecific antibodies in MM (HealthTree Registry). Sankey diagram depicting sequencing from index immune therapy (first observed CAR-T or bispecific antibody per patient) to the dominant class of the next documented line of therapy (LOT) in the HealthTree Registry. Analysis is restricted to patients with an observed subsequent LOT. Dominant class was assigned based on a rule-based hierarchy when multiple agents were present; supportive, conditioning, and non–myeloma-directed medications were excluded from regimen classification. Flow widths are proportional to patient counts.

**Copyright Information:** (Online) ISSN: 2572-9241
©2026 The Author(s). HemaSphere is published by John Wiley & Sons Ltd on behalf of European Hematology Association. This is an open access Abstract Book distributed under the Attribution-NonCommercial-NoDerivs (CC BY-NC-ND), which allows third parties to download the articles and share them with others as long as they credit the author and the Abstract Book, but they cannot change the content in any way or use them commercially.
**Abstract Book Citations:** Authors, Title, HemaSphere, 2026;10:(S1):pages. The abstract book DOIs can be found on the "Author Information" page.
**Disclaimer:** Articles published in the journal HemaSphere exclusively reflect the opinions of the authors. The authors are responsible for all content in their abstracts including accuracy of the facts, statements, citing resources, etc.

### (PS1918) EFFICACY AND SAFETY OF ABECMA® CAR T-CELL THERAPY IN RELAPSED/REFRACTORY MULTIPLE MYELOMA PATIENTS AGED ≥75 YEARS: REAL-WORLD DATA FROM THE FRENCH DESCAR-T REGISTRY

**Topic:** 14. Myeloma and other monoclonal gammopathies - Clinical

Jihane Hssein*[1], Olivier Decaux[2], Lionel Karlin[3], Stephanie Harel[4], Karim Belhadj Merzoug[5], Andrea Pieragostini[6], Arthur Bobin[7], Salomon Manier[8], Jean Marc Schiano De Colella[9], Cyrille Touzeau[10], Michael Loschi[11], Florent Malard[12], Carine Chateleix[13], Aurore Perrot[14], Laurent Garderet[15], Cécile Sonntag[16], Camille Bigenwald[17], Phillipe Rey[18], Jean-Richard Eveillard[19], Thomas Chalopin[20], Adeline Pierache[21], Marie-Odile Petillon[22], Bertrand Arnulf[4], Laure Vincent[1]

*[1]CHU de Montpellier, Clinical hematology, Montpellier, France;[2]CHU de Rennes, Rennes, France;[3]CHU de LYON, Pierre-Bénite, France; [4]Hôpital Saint Louis, Paris, France;[5]Hopital Henri Mondor, Créteil, France; [6]CHU de Dijon Bourgogne, Dijon, France; [7]CHU de Poitiers, Poitiers, France;[8]CHU de Lille, Lille, France;[9]Institut Paoli Calmettes, Marseille, France; [10]CHU de Nantes, Nantes, France; [11]CHU de Nice, Nice, France; [12]Hôpital Saint Antoine, Paris, France; [13]CHU de Clermont Ferrand, Clermont Ferrand, France; [14]IUCT Oncopole, Toulouse, France; [15]Hôpital Pitié Salpêtrière, Paris, France; [16]Institut de cancérologie de Strasbourg, Strasbourg, France; [17]Institut Gustave Roussy, Villejuif, France; [18]Centre Léon Berard, Lyon, France; [19]CHU de Brest, Brest, France; [20]CHU de Tours, Tours, France; [21]Exystat, Malakoff, France; [22]Institut francophone du myélome, Paris, France;*

### Background

Multiple myeloma (MM) predominantly affects older adults, with a median age at diagnosis of 69 years and one-third of cases diagnosed in patients aged ≥75 years. Despite this, elderly patients remain underrepresented in clinical trials, leaving evidence gaps for CAR T-cell therapy targeting BCMA, in relapsed/refractory MM after ≥2 prior lines and triple class exposure.
This retrospective analysis from the French DESCAR-T registry (NCT06304636) evaluates idecabtagene vicleucel (Abecma®) in patients aged ≥75 years.

### Aims

The primary objective was to assess efficacy through overall response rate (ORR), progression-free survival (PFS), and overall survival (OS). Secondary objectives included safety evaluations of cytokine release syndrome (CRS), immune effector cell-associated neurotoxicity syndrome (ICANS) per ASTCT criteria, cytopenias (CTCAE v5.0) and infections.

### Methods

We analyzed the 93 patients ≥75 years (median 76, range 75-85) treated in France with commercial Abecma® prior October 2024 (median follow-up 12.6 months). Baseline characteristics included ECOG PS (94% ≤1), comorbidities (42%), high-risk cytogenetics (61%), extramedullary disease (9%), and prior therapies (median 3 prior lines, triple class exposed (98%), prior anti-BCMA therapy (5%)). Bridging therapy was used in 73%. 83% of scored patients (33/40) had low-risk CAR-HEMATOTOX.

### Results

Post-infusion ORR (best response) was 95% (4% of stringent complete response [sCR]; 56% of CR; 23% of very good partial response [VGPR]). Median time to best response was 2.8 months. 59% of patients were in sCR/CR and 19.6% VGPR at 1 year. Median PFS was 16.1 months. Median OS was 42.9 months (21 deaths, 19% due to toxicity, 57% due to progression). Safety profiles showed CRS in 100% (grade 1 (69%), grade 2 (28%), grade ≥3 (3%), median duration 3 days) and ICANS in 23% (grade 1 (10%), grade 2 (9%), grade≥3 (4%), median duration 4 days). Grade≥3 cytopenia at 1 month included neutropenia (91%), thrombocytopenia (86%), and anemia (85%). Infections occurred in 16% (47% bacterial). ICU admissions affected 6% (median stay, 5 days).

**Copyright Information:** (Online) ISSN: 2572-9241
©2026 The Author(s). HemaSphere is published by John Wiley & Sons Ltd on behalf of European Hematology Association. This is an open access Abstract Book distributed under the Attribution-NonCommercial-NoDerivs (CC BY-NC-ND), which allows third parties to download the articles and share them with others as long as they credit the author and the Abstract Book, but they cannot change the content in any way or use them commercially.
**Abstract Book Citations:** Authors, Title, HemaSphere, 2026;10:(S1):pages. The abstract book DOIs can be found on the “Author Information” page.
**Disclaimer:** Articles published in the journal HemaSphere exclusively reflect the opinions of the authors. The authors are responsible for all content in their abstracts including accuracy of the facts, statements, citing resources, etc.

**Summary/Conclusion**

Abecma® demonstrated high efficacy with deep and durable responses and manageable toxicity in this cohort of selected fit patients aged ≥75 years with RRMM—outcomes appear comparable to those in younger pivotal trial cohorts. For comparison, median PFS on overall DESCAR-T cohort for Abecma® was 17,8 months (range 15,8 – 18,9). These real-world data support broader use in selected older adults, emphasizing frailty assessment over chronologic age. Prospective studies are warranted.

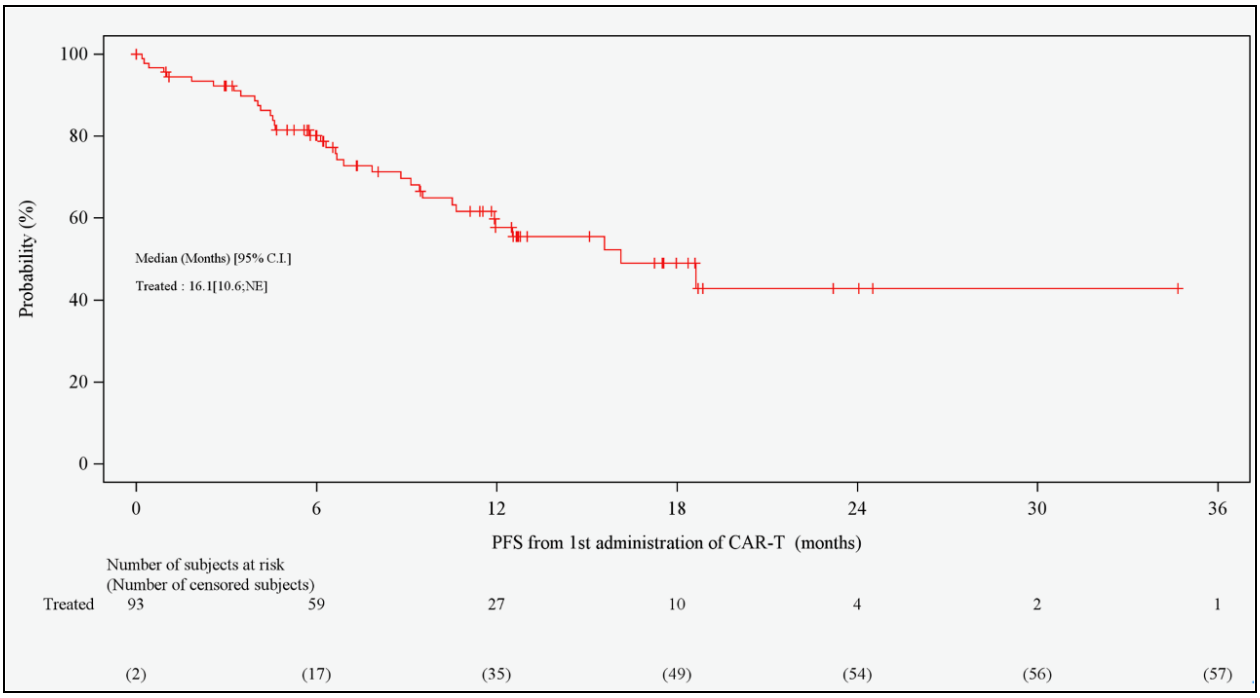

PFS from CAR-T injection

**Copyright Information:** (Online) ISSN: 2572-9241
©2026 The Author(s). HemaSphere is published by John Wiley & Sons Ltd on behalf of European Hematology Association. This is an open access Abstract Book distributed under the Attribution-NonCommercial-NoDerivs (CC BY-NC-ND), which allows third parties to download the articles and share them with others as long as they credit the author and the Abstract Book, but they cannot change the content in any way or use them commercially.
**Abstract Book Citations:** Authors, Title, HemaSphere, 2026;10:(S1):pages. The abstract book DOIs can be found on the "Author Information" page.
**Disclaimer:** Articles published in the journal HemaSphere exclusively reflect the opinions of the authors. The authors are responsible for all content in their abstracts including accuracy of the facts, statements, citing resources, etc.

### (PS1919) OUTPATIENT STEP-UP DOSING OF TECLISTAMAB OR TALQUETAMAB WITH PROPHYLACTIC TOCILIZUMAB IN PATIENTS WITH RELAPSED/REFRACTORY MULTIPLE MYELOMA: REAL-WORLD EVIDENCE FROM A LARGE US CANCER CENTER

**Topic:** 14. Myeloma and other monoclonal gammopathies - Clinical

Cesar Rodriguez*[1], Mohammad Rattu[1], Alex Lieberman-Cribbin[1], Allison Lilley[1], Tianxiang Sheng[1], Maria Bodea[1], Sundar Jagannath[1], Adriana Rossi[1], Shambavi Richard[1], Joshua Richter[1], Hearn Jay Cho[1], Gurbakhash Kaur[1], Larysa Sanchez[1], Santiago Thibaud[1], Maithili Deshpande[2], Niodita Gupta-Werner[2], Ming-Hui Tai[2], Jessica Fowler[2], Tonya Le Blanc[2], Shuchita Kaila[2], Samir Parekh[1]

*[1]The Tisch Cancer Institute, Icahn School of Medicine at Mount Sinai, New York, United States of America;[2]Johnson & Johnson, Horsham, United States of America;*

### Background

Teclistamab (Tec) and talquetamab (Tal) are first-in-class and widely used T-cell-engaging bispecific antibodies (BsAbs) approved for relapsed/refractory multiple myeloma (RRMM) worldwide. The USPI recommends inpatient (IP) step-up dosing (SUD) to manage cytokine release syndrome (CRS) and immune effector cell-associated neurotoxicity syndrome (ICANS) for both Tec and Tal. To expand patient (pt) access and reduce resource use, a large U.S. academic center implemented: fully outpatient SUD with prophylactic tocilizumab (OP-toci), and IP SUD with CRS managed with toci and early discharge to OP care (Hybrid–HY).

### Aims

This real-world study describes pt characteristics, 14-day CRS and ICANS rates, and 14- and 30-day CRS, ICANS, or infection-related hospitalization or readmission rates, among pts with RRMM receiving Tec or Tal SUD in OP-toci or HY settings at this large U.S. academic center.

### Methods

This retrospective chart review included adults with RRMM who initiated Tec after October 25, 2022 or Tal after August 9, 2023. In the OP-toci cohort, pts received prophylactic toci [8mg/kg] with Tec or Tal SUD1 within 10 hours of SUD1. In the HY cohort, SUD was initiated IP; pts who developed CRS received toci and were discharged after resolution to complete SUD OP. OP eligibility for both cohorts required a caregiver, absence of cytopenias requiring transfusion or growth factor, and no significant co-morbidities. Descriptive results are presented.

**Copyright Information:** (Online) ISSN: 2572-9241
©2026 The Author(s). HemaSphere is published by John Wiley & Sons Ltd on behalf of European Hematology Association. This is an open access Abstract Book distributed under the Attribution-NonCommercial-NoDerivs (CC BY-NC-ND), which allows third parties to download the articles and share them with others as long as they credit the author and the Abstract Book, but they cannot change the content in any way or use them commercially.
**Abstract Book Citations:** Authors, Title, HemaSphere, 2026;10:(S1):pages. The abstract book DOIs can be found on the "Author Information" page.
**Disclaimer:** Articles published in the journal HemaSphere exclusively reflect the opinions of the authors. The authors are responsible for all content in their abstracts including accuracy of the facts, statements, citing resources, etc.

**Results**

This study included 45 RRMM pts: 23 in OP-toci cohort (Tec=7, Tal=16) and 22 in HY cohort (Tec=4, Tal=18). Across OP-toci and HY cohorts, 34.8% and 36.4% were ≥75 years, 13% and 18.2% were Black, 61%, and 59% had high-risk cytogenetics, 95.6% and 100% were triple class refractory, 73.9% and 77.3% were penta-drug exposed, and 30.4% and 18.2% had extramedullary disease, respectively. The median prior lines of therapy were 4 (Tec) and 4.5 (Tal) in the OP-toci cohort, and 4.5 (Tec) and 5 (Tal) in the HY cohort.

In the OP-toci cohort, no CRS occurred within 14 days of Tec or Tal initiation. Only one Tal pt experienced Grade (G) 1 ICANS within 14-days that resolved with pocket dexamethasone; no recurrent ICANS occurred. Two Tal pts were hospitalized within 30 days: one for dysgeusia-related failure to thrive; one for renal failure. All OP-toci pts successfully completed SUD without Tec or Tal discontinuations.

In the HY cohort, 1 Tec pt developed G1 ICANS with concurrent CRS and later had recurrent G1 ICANS, and 1 Tal pt developed G2 ICANS. G1 ICANS was managed with dexamethasone, while G2 was treated with dexamethasone and anakinra per institutional SOP. All CRS and ICANS resolved prior to discharge, and all but one Tec HY pt successfully completed SUD OP. One Tec HY pt was readmitted within 30-days for infection; no 14- or 30-day readmissions occurred in the Tal HY cohort. Interpretation of the Tec HY may be limited due to the small sample size.

**Summary/Conclusion**

OP SUD can broaden pt access to Tec and Tal and reduce healthcare resource utilization. OP SUD using prophylactic toci can reduce hospitalization with Tec and Tal while a hybrid model facilitates rapid discharge of pts to complete their treatment in the OP setting. Both strategies facilitate broad medical adoption of Tec and Tal across practice settings.

**Copyright Information:** (Online) ISSN: 2572-9241
©2026 The Author(s). HemaSphere is published by John Wiley & Sons Ltd on behalf of European Hematology Association. This is an open access Abstract Book distributed under the Attribution-NonCommercial-NoDerivs (CC BY-NC-ND), which allows third parties to download the articles and share them with others as long as they credit the author and the Abstract Book, but they cannot change the content in any way or use them commercially.
**Abstract Book Citations:** Authors, Title, HemaSphere, 2026;10:(S1):pages. The abstract book DOIs can be found on the “Author Information” page.
**Disclaimer:** Articles published in the journal HemaSphere exclusively reflect the opinions of the authors. The authors are responsible for all content in their abstracts including accuracy of the facts, statements, citing resources, etc.

### (PS1920) REAL-WORLD OUTCOMES IN SMOLDERING MULTIPLE MYELOMA IN EUROPE: FIRST RESULTS FROM THE SPARK STUDY

**Topic:** 14. Myeloma and other monoclonal gammopathies - Clinical

María-Victoria Mateos*[1], Mamta Garg[2], Sandra Ketzler-Henkel[3], Thomas Chalopin[4], Ido Lapidot[5], Julien Thevenon[6], Lada Mitchell[7], Nichola Erler-Yates[8], Francesca Gay[9]

*[1]Departamento de Hematología, Hospital Universitario de Salamanca, (HUSA/IBSAL), Centro de Investigación del Cáncer-IBMCC (CSIC/USAL), CIBERONC, Salamanca, Spain; [2]University Hospitals of Leicester NHS Trust, Leicester, United Kingdom; [3]Practice for Hematology and Oncology, Dortmund, Germany; [4]Hematology, CHU, Tours, France; [5]J-C Health Care Ltd., Kibbutz Shefayim, Israel; [6]Medical Affairs, Janssen Pharmaceuticals, Issy-les-Moulineaux, France; [7]J&J, Allschwil, Switzerland; [8]Janssen-Cilag GmbH, Neuss, Germany; [9]Myeloma Unit, Division of Hematology, University of Torino, Azienda Ospedaliero-Universitaria Città della Salute e della Scienza, Torino, Italy;*

### Background

Smoldering multiple myeloma (SMM) is a precursor plasma cell disorder with variable risk of progression to symptomatic multiple myeloma (MM). Accurate risk stratification is essential to identify patients at highest risk of progression and to address the unmet need for early intervention. However, real-world data on European SMM patient characteristics, progression rates, overall-survival (OS), organ damage at MM diagnosis, and treatment patterns by risk category remain limited.

### Aims

The SPARK study aims to provide a comprehensive description of these parameters using different risk models, including Mayo 20-2-20, IMWG 2020 scoring system incorporating cytogenetics (IMWG), and the AQUILA criteria.

### Methods

SPARK is a real-world retrospective chart review study involving hematology centers across Europe. Adult patients (≥18 years) diagnosed with SMM between January 2016 and December 2021 were included if they had at least 2 years of follow-up data available or died within 2 years of diagnosis.
Categorical data was expressed as frequencies and percentages, and continuous data was summarized using median, mean, standard deviation, and range. Time-to-event outcomes, including Time to MM progression, PFS and OS, were analyzed using Kaplan-Meier methods. This first SPARK publication includes all available data collected up to the cut-off date of October 2025.

**Copyright Information:** (Online) ISSN: 2572-9241
©2026 The Author(s). HemaSphere is published by John Wiley & Sons Ltd on behalf of European Hematology Association. This is an open access Abstract Book distributed under the Attribution-NonCommercial-NoDerivs (CC BY-NC-ND), which allows third parties to download the articles and share them with others as long as they credit the author and the Abstract Book, but they cannot change the content in any way or use them commercially.
**Abstract Book Citations:** Authors, Title, HemaSphere, 2026;10:(S1):pages. The abstract book DOIs can be found on the "Author Information" page.
**Disclaimer:** Articles published in the journal HemaSphere exclusively reflect the opinions of the authors. The authors are responsible for all content in their abstracts including accuracy of the facts, statements, citing resources, etc.

**Results**

A total of 373 patients with SMM from UK, Germany, Italy, Spain and France were included, of whom 78 (20.9 %) were classified as high-risk according to the Mayo 20-2-20 criteria (Mayo HR). In the Mayo HR group, median follow-up was 53.1 months (range 1-95.4 months), median age at SMM diagnosis was 69 years (range 41-92 years), 37.2 % were female, and 78.9% had BMPC >20%. The majority (84.4%) had serum M-protein >2 g/dL, and 65.6% had an involved/uninvolved FLCr >20.
Median time to progression to MM or death was 27.4 months (95% CI: 17.08, 41.17), 30.8 months (95% CI: 16.7, 44.2), and 47.7 months (95% CI: 40.5, 65.7) for Mayo HR, IMWG HR and AQUILA HR, respectively. Out of 45 Mayo HR patients who progressed to MM, 29 (64.4%) progressed to MM within 2 years. The 2-year PFS rate was 55.6 % ("free of MM or death"), (95% CI:44.08, 67.03).
Out of 45 Mayo HR patients that progressed to MM, 64.4% met CRAB criteria, 24.4% met SLiM at MM diagnosis, and the rest with unknown type of progression. 26 (57.8%) patients had organ damage at MM diagnosis per physician assessment, most commonly bone pain (26.9%), bone fracture (19.2%), anemia symptoms (50%) and other (23.1%). Similar patterns were observed in IMWG HR and AQUILA HR.
SMM treatment was initiated in 9/373 (2.4%) patients, 56% were Mayo HR, with regimens including proteasome inhibitors, immunomodulatory drugs, corticosteroids, and monoclonal antibodies.

**Summary/Conclusion**

In this large real-world European dataset, despite active monitoring, nearly half of HR SMM patients who progressed to MM had major organ damage, most commonly bone pain, bone fractures and anemia. These data show that active monitoring alone might be insufficient for avoiding organ damage in many high-risk patients. These findings reinforce the need for enhanced monitoring strategies and improved risk stratification in order to identify patients that could benefit from early intervention. Full baseline characteristics, time to MM, PFS and OS in high-risk and non high-risk patients according to Mayo 20-2-20, IMWG and AQUILA will be presented in the final presentation.

**Copyright Information:** (Online) ISSN: 2572-9241
©2026 The Author(s). HemaSphere is published by John Wiley & Sons Ltd on behalf of European Hematology Association. This is an open access Abstract Book distributed under the Attribution-NonCommercial-NoDerivs (CC BY-NC-ND), which allows third parties to download the articles and share them with others as long as they credit the author and the Abstract Book, but they cannot change the content in any way or use them commercially.
**Abstract Book Citations:** Authors, Title, HemaSphere, 2026;10:(S1):pages. The abstract book DOIs can be found on the "Author Information" page.
**Disclaimer:** Articles published in the journal HemaSphere exclusively reflect the opinions of the authors. The authors are responsible for all content in their abstracts including accuracy of the facts, statements, citing resources, etc.

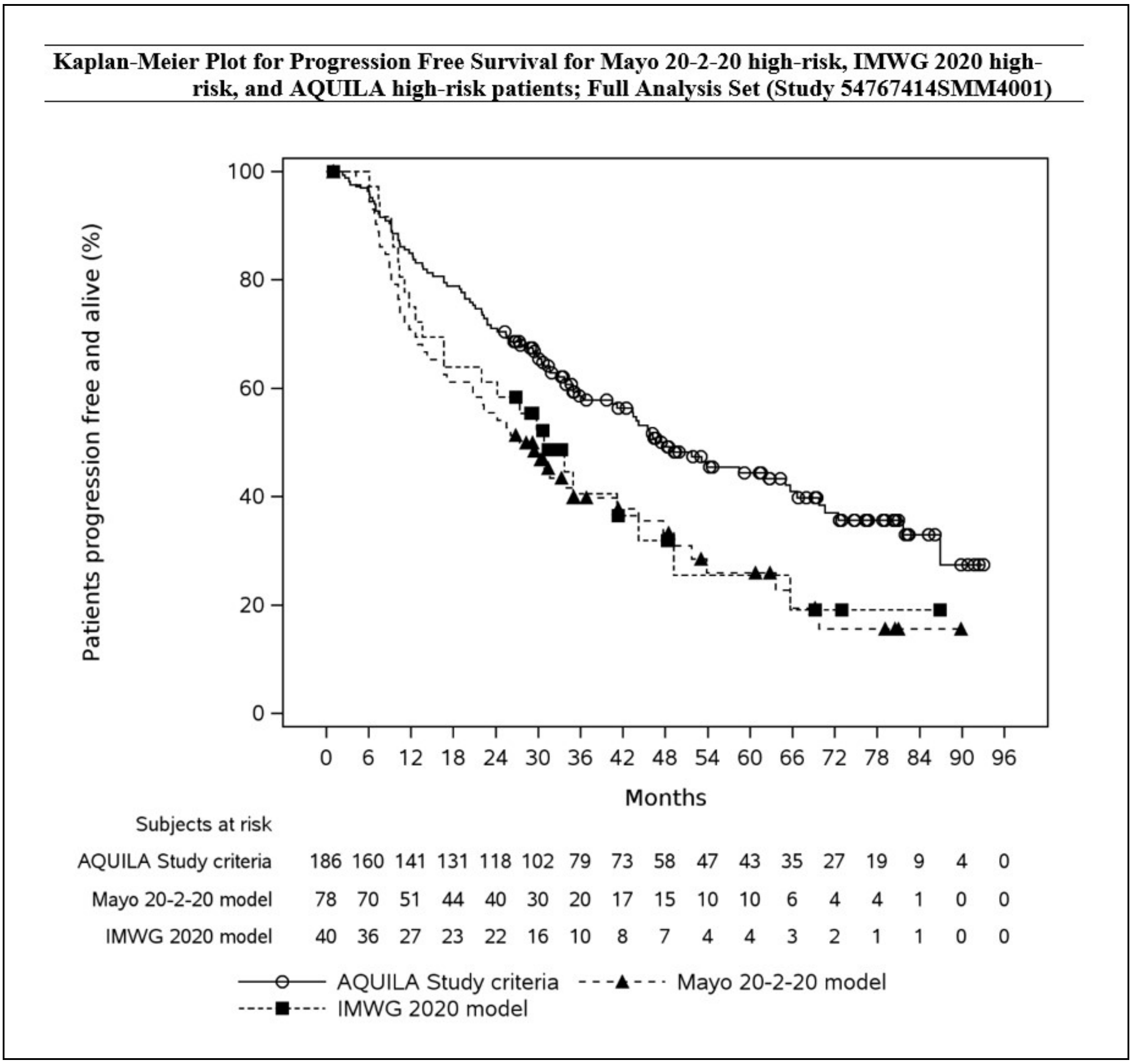

Kaplan-Meier Plot for Progression Free Survival for Mayo 20-2-20 high-risk, IMWG 2020 high-risk, and AQUILA high-risk patients; Full Analysis Set (Study 54767414SMM4001)

**Copyright Information:** (Online) ISSN: 2572-9241
©2026 The Author(s). HemaSphere is published by John Wiley & Sons Ltd on behalf of European Hematology Association. This is an open access Abstract Book distributed under the Attribution-NonCommercial-NoDerivs (CC BY-NC-ND), which allows third parties to download the articles and share them with others as long as they credit the author and the Abstract Book, but they cannot change the content in any way or use them commercially.
**Abstract Book Citations:** Authors, Title, HemaSphere, 2026;10:(S1):pages. The abstract book DOIs can be found on the "Author Information" page.
**Disclaimer:** Articles published in the journal HemaSphere exclusively reflect the opinions of the authors. The authors are responsible for all content in their abstracts including accuracy of the facts, statements, citing resources, etc.

### (PS1921) AI-BASED MODEL FOR IDENTIFICATION FUNCTIONAL HIGH-RISK MULTIPLE MYELOMA

**Topic:** 14. Myeloma and other monoclonal gammopathies - Clinical

Sonia More'*[1, 2], Massimo Offidani[1], Laura Corvatta[3], Tommaso Za[4], Francesca Fazio[5], Martina Gherardini[5], Velia Bongarzoni[6], Barbara Anaclerico[6], Luca Franceschini[7], Silvia Ferraro[8], Luca Cupelli[8], Carmine Liberatore[9, 10], Laura De Padua[11], Angela Rago[12], Silvia Gentili[13], Roberto Latagliata[14], Mariagrazia Garzia[15], Iole Cordone[16], Giulia Orlandi[16], Roberta Merola[16], Valeria Mezzanotte[7], Elena Rossi[4], Francesca Di Landro[4], Maria Zaira Limongi[5], Erika Morsia[1, 2], Antonella Poloni[1, 2], Maria Teresa Petrucci[5], Lucia Mannucci[2]

*[1]AOU delle Marche, Ancona, Italy; [2]Dipartimento di Scienze Cliniche e Molecolari, UNIVPM, Ancona, Italy; [3]Ospedale Profili, Fabriano, Italy; [4]Policlinico Universitario Agostino Gemelli, Roma, Italy; [5]Ematologia, Dipartimento di Medicina Traslazionale e di Precisione, Sapienza Università di Roma, Roma, Italy; [6]Department of Hematology San Giovanni-Addolorata Hospital, Roma, Italy; [7]Policlinico Tor Vergata, Roma, Italy; [8]UOC Hematology, Hospital S. Eugenio, Roma, Italy; [9]Santo Spirito Civil Hospital, Pescara, Italy; [10]Department of Medicine and Aging Sciences, "G d'Annunzio" University, Chieti, Italy; [11]UOC di Ematologia, Trapianto di Cellule Staminali e Terapia Genica, Frosinone, Frosinone, Italy; [12]UOSD Ematologia ASL Roma1, Roma, Italy; [13]AST Macerata, Civitanova Marche, Italy; [14]Hematology - Santa Rosa Hospital, Viterbo, Italy; [15]San Camillo Forlanini Hospital, Roma, Italy; [16]IRCCS Regina Elena National Cancer Institute, Roma, Italy;*

### Background

Functional high-risk multiple myeloma (FHR MM) includes 10–20% of patients who relapse early despite lacking conventional high-risk features and modern frontline therapies, revealing limits of current staging. The growing availability of multicenter datasets and advanced analytical methods, including machine learning, (ML) offers an opportunity to improve FHR identification through standardized preprocessing, rigorous validation, and interpretable models for risk-adapted treatment.

### Aims

We retrospectively analyzed a large multicenter Italian cohort of newly diagnosed MM patients treated with upfront therapy, including anti-CD38–based regimens in both transplant-eligible (TE) and ineligible (NTE) settings to enhance FHR prediction using routinely available clinical parameters through ML approaches.

### Methods

A total of 1,011 patients were included after duplicate removal and data cleaning. Clinical, laboratory, cytogenetic and treatment variables were processed through standardized pipelines, including imputation of missing data and class-imbalance correction. PFS and OS were assessed by Kaplan–Meier and Cox models, including fixed-time PFS at 12, 18, 24 and 36 months. Eligible patients had experienced first relapse or, if not relapsed, had ≥18 months (TE) or ≥12 months (NTE) follow-up. Three ML algorithms—logistic regression (LogReg), random forest (RF) and generalized additive models (pyGAM)—were trained and evaluated using a nested cross-validation framework. A reduced model was derived using SHAP-based feature selection and coefficient stability.

**Copyright Information:** (Online) ISSN: 2572-9241
©2026 The Author(s). HemaSphere is published by John Wiley & Sons Ltd on behalf of European Hematology Association. This is an open access Abstract Book distributed under the Attribution-NonCommercial-NoDerivs (CC BY-NC-ND), which allows third parties to download the articles and share them with others as long as they credit the author and the Abstract Book, but they cannot change the content in any way or use them commercially.
**Abstract Book Citations:** Authors, Title, HemaSphere, 2026;10:(S1):pages. The abstract book DOIs can be found on the "Author Information" page.
**Disclaimer:** Articles published in the journal HemaSphere exclusively reflect the opinions of the authors. The authors are responsible for all content in their abstracts including accuracy of the facts, statements, citing resources, etc.

### Results

Overall, 43% of patients received CD38-based regimens, 56% PI and/or IMiD combinations and 226 patients (22%) met FHR criteria. After a median follow-up of 41 months, median PFS for the cohort was 45.5 months, while FHR patients showed markedly inferior outcomes (median PFS 7 *vs* 57.5 months; p<0.001). Median OS was not reached overall, but was 19 months in FHR *vs* not reached in non-FHR (p<0.001). Among the evaluated algorithms, logistic regression trained on the computed dataset achieved the best balance of discrimination and calibration (ROC-AUC 0.71; PR-AUC 0.43; accuracy 0.69; recall 0.72). Random forest achieved AUCs of 0.60–0.61, and pyGAM reached AUC 0.66 with lower precision. The reduced 21-feature LogReg model preserved performance (AUC 0.71; PR-AUC 0.42) and improved calibration. Retained predictors included hemoglobin, age, platelets, albumin, performance status, cytogenetics, LDH, renal insufficiency and ISS stage. Predicted FHR probabilities showed strong prognostic separation for PFS across tertiles and binary thresholds (log-rank p<0.001; HR 52.6) (Fig). Time-dependent AUCs remained robust at early horizons. Associations with OS were weaker but still significant for high- *vs* low-risk groups. Overall, the nested-CV logistic model demonstrated solid discrimination and interpretability, and the reduced version maintained equivalent performance with a simplified structure. Online calculator available for baseline risk-adapted decisions: https://github.com/mannuccilucia/FHR-Predictor/releases.

### Summary/Conclusion

ML applied to a large real-world cohort enabled accurate identification of FHR MM using routine clinical variables. Logistic regression with nested validation provided the best balance of performance and interpretability, and a reduced model preserved accuracy. These findings support ML-based tools as a promising approach for risk-adapted strategies in MM.

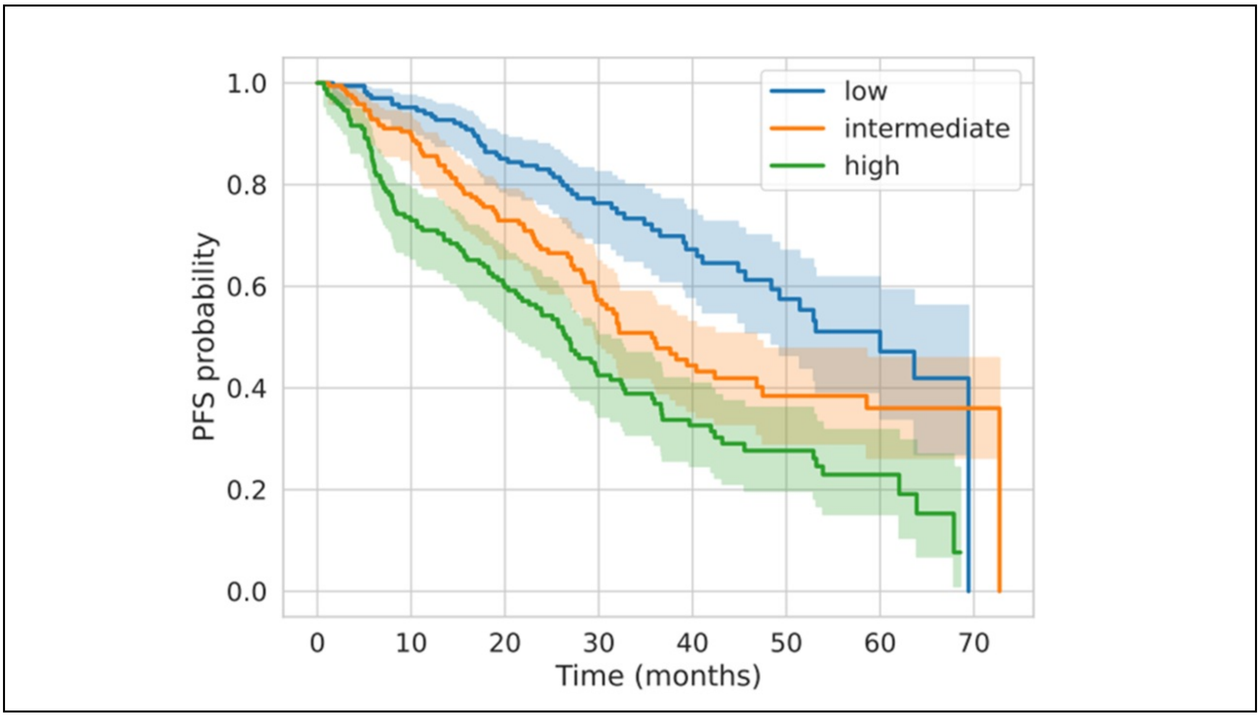

PFS across tertiles (low, intermediate and high risk groups)

Copyright Information: (Online) ISSN: 2572-9241
©2026 The Author(s). HemaSphere is published by John Wiley & Sons Ltd on behalf of European Hematology Association. This is an open access Abstract Book distributed under the Attribution-NonCommercial-NoDerivs (CC BY-NC-ND), which allows third parties to download the articles and share them with others as long as they credit the author and the Abstract Book, but they cannot change the content in any way or use them commercially.
**Abstract Book Citations:** Authors, Title, HemaSphere, 2026;10:(S1):pages. The abstract book DOIs can be found on the "Author Information" page.
**Disclaimer:** Articles published in the journal HemaSphere exclusively reflect the opinions of the authors. The authors are responsible for all content in their abstracts including accuracy of the facts, statements, citing resources, etc.

### (PS1922) THE IMS-IMWG 2025 GENOMIC CLASSIFICATION IDENTIFIES HIGH-RISK MULTIPLE MYELOMA PATIENTS IN THE ERA OF BCMA-DIRECTED CAR-T CELL THERAPY.

**Topic:** 14. Myeloma and other monoclonal gammopathies - Clinical

Almudena Zaratiegui*[1], Magdalena Corona[1], Natalia Vega-Larburu[1], María Sánchez Tabernero[1], Rafael Alonso[1], Alberto Blanco[1], M Nieves Lopez[1], Rodrigo Íñiguez[1], Alicia Giménez Sánchez[2], Laura Moreno[2], Inmaculada Rapado[1], Manuela Fernández Guijarro[1], Rosa María Ayala Diaz[1, 3], Maria Calbacho[1], Joaquín Martinez-Lopez[1, 2, 4], Jose María Sánchez-Pina[1]

*[1]Hospital 12 de Octubre, Haematology and Hemotherapy, Madrid, Spain;[2]Foundation for Biomedical Research of Hospital 12 de Octubre, Department of Translational Haematology, Madrid, Spain; [3]Complutense University of Madrid, Madrid, Spain; [4]National Cancer Research Centre (CNIO), Madrid, Spain;*

### Background

The IMS-IMWG 2025 Consensus Genomic Staging system redefines high-risk Multiple Myeloma (MM) incorporating co-occurring genomic abnormalities in patients treated with conventional therapies. Although BCMA-directed CAR-T cell therapy is highly effective in patients with MM, the prognostic relevance of this integrated genomic framework in this therapeutic context remains uncertain.

### Aims

To assess the prognostic significance of the new IMS-IMWG 2025 genomic staging system in patients with MM receiving BCMA-directed CAR-T cell therapy.

### Methods

This single-centre retrospective study included patients with MM who received BCMA-directed CAR-T cell therapy between 2020 and 2025. Cytogenetic and genomic abnormalities were assessed using FISH and NGS on CD138-selected bone marrow plasma cells prior to CAR-T infusion. According to the IMS-IMWG 2025, high-risk genomic alterations included: (1) del(17p) with a clonal fraction cutoff >20% and/or TP53 mutation; (2) IgH translocations [t(4;14), t(14;16), t(14;20)] co-occurring with 1q gain or amplification (1q+) and/or del(1p32); (3) monoallelic del(1p32) with 1q+ or biallelic del(1p32). R-ISS high-risk cytogenetic abnormalities included del(17p), t(4;14), or t(14;16). Only the genomic and cytogenetic criteria of both classifications were compared. Overall survival (OS) and progression-free
survival (PFS) were estimated using the Kaplan-Meier method, compared using the log-rank test, and modeled using Cox proportional hazards regression models.

**Copyright Information:** (Online) ISSN: 2572-9241
©2026 The Author(s). HemaSphere is published by John Wiley & Sons Ltd on behalf of European Hematology Association. This is an open access Abstract Book distributed under the Attribution-NonCommercial-NoDerivs (CC BY-NC-ND), which allows third parties to download the articles and share them with others as long as they credit the author and the Abstract Book, but they cannot change the content in any way or use them commercially.
**Abstract Book Citations:** Authors, Title, HemaSphere, 2026;10:(S1):pages. The abstract book DOIs can be found on the "Author Information" page.
**Disclaimer:** Articles published in the journal HemaSphere exclusively reflect the opinions of the authors. The authors are responsible for all content in their abstracts including accuracy of the facts, statements, citing resources, etc.

### Results

We analysed 71 patients with MM, 87% with relapsed/refractory disease. The median age at infusion was 62 years (IQR 51-69), and 53.5% were male. CAR-T cell products included Cilta-cel (63.4%), academic BCMA-directed CAR-T (25.4%), and Ide-cel (11.3%). The most frequent IMS-IMWG 2025 high-risk abnormality was del(17p) (14%), followed by IgH translocations combined with 1q+ (9.8%), and del(1p32) combined with 1q+ (4.2%). TP53 mutations were identified in 18.5% of patients (n=5/27). A total of 25.4% were classified as high-risk using the R-ISS cytogenetic criteria and 28.2% according to the IMS-IMWG 2025 genomic features. Under the updated system, 4.2% of patients were reclassified from high to standard genomic risk, and 7% from standard to high genomic risk.

At a median follow-up of 9 months (IQR 3-23), the estimated 1-year OS was 81.7% [95% CI: 71.1-92.3], and the 1-year PFS was 68.2% [95% CI: 55.1-81.3]. According to the IMS-IMWG 2025 classification, 1-year PFS was lower across genomic high-risk patients (51.3% [95% CI: 26.4–76.2]) compared with standard-risk patients (74.6% [95% CI: 59.3–89.9]), corresponding HR 2.48 [95% CI: 1.07–5.73] (p=0.034). High-risk patients also exhibited numerically lower 1-year OS: 70.5% [95% CI: 48.6–92.4] vs 86.5% [95% CI: 75.1–97.9]; corresponding HR 2.57 [95% CI: 0.86–7.67] (p=0.091). (Figure 1A, 1B)

In contrast, classic R-ISS high-risk cytogenetic criteria failed to discriminate outcomes. At 1-year, PFS (50.7% vs 73.9%, HR 1.44 [95% CI: 0.59–3.54], p=0.427) and OS (73.8% vs 84.4%, HR 1.70 [95% CI: 0.56–5.22], p=0.350) were comparable across the high and standard risk groups respectively. (Figure 1C, 1D)

### Summary/Conclusion

Our findings suggest that the IMS-IMWG 2025 classification improves genomic high-risk stratification in patients receiving BCMA-directed CAR-T cell therapy relative to conventional cytogenetic criteria. This highlights the importance of integrating multimodal platforms, such as NGS-based TP53 mutation analysis, also within novel immunotherapies.

**Copyright Information:** (Online) ISSN: 2572-9241
©2026 The Author(s). HemaSphere is published by John Wiley & Sons Ltd on behalf of European Hematology Association. This is an open access Abstract Book distributed under the Attribution-NonCommercial-NoDerivs (CC BY-NC-ND), which allows third parties to download the articles and share them with others as long as they credit the author and the Abstract Book, but they cannot change the content in any way or use them commercially.
**Abstract Book Citations:** Authors, Title, HemaSphere, 2026;10:(S1):pages. The abstract book DOIs can be found on the “Author Information” page.
**Disclaimer:** Articles published in the journal HemaSphere exclusively reflect the opinions of the authors. The authors are responsible for all content in their abstracts including accuracy of the facts, statements, citing resources, etc.

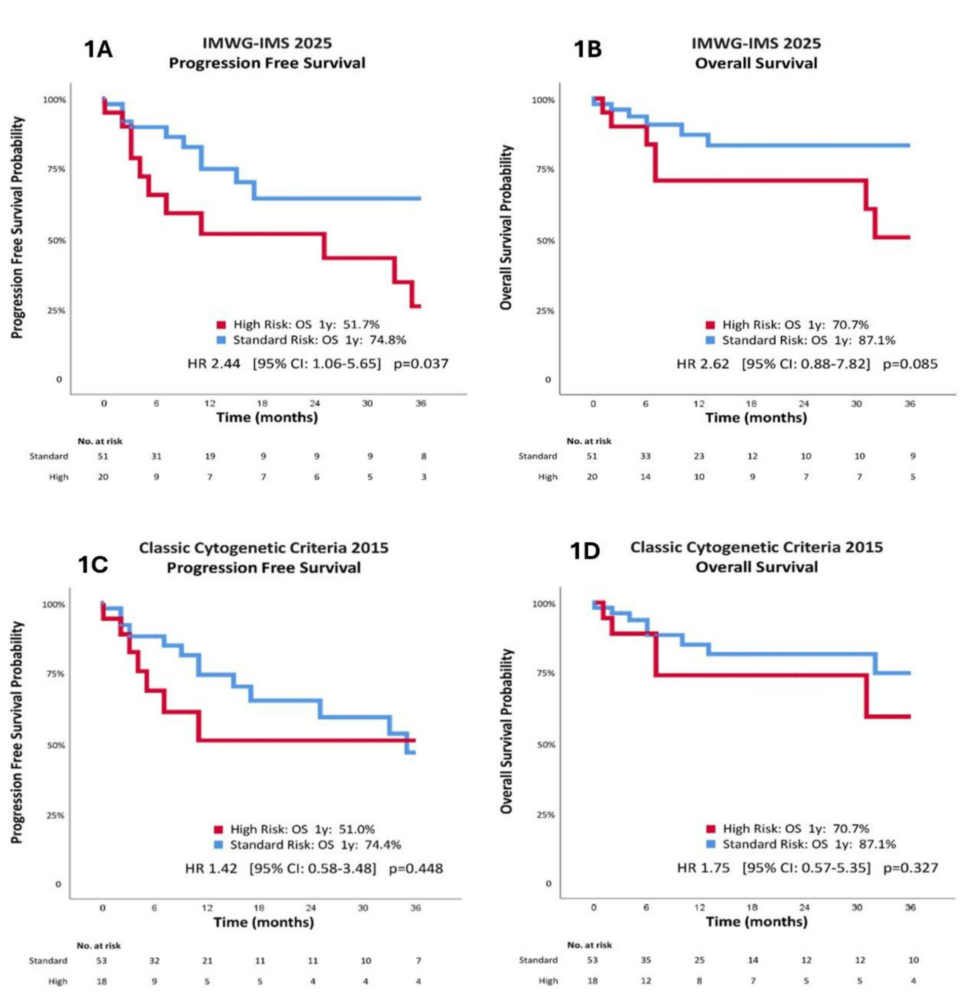

**Figure 1. Outcomes Stratified in high and standard-risk patients by the IMS-IMWG 2025 Genomic Classification and Classic Cytogenetic Criteria.** (A) Progression free survival by IMS-IMWG 2025 Genomic Classification. (B) Overall survival by IMS-IMWG 2025 Genomic Classification. (C) Progression free survival by classic cytogenetic criteria. (D) Overall survival by classic cytogenetic criteria.

**Copyright Information:** (Online) ISSN: 2572-9241
©2026 The Author(s). HemaSphere is published by John Wiley & Sons Ltd on behalf of European Hematology Association. This is an open access Abstract Book distributed under the Attribution-NonCommercial-NoDerivs (CC BY-NC-ND), which allows third parties to download the articles and share them with others as long as they credit the author and the Abstract Book, but they cannot change the content in any way or use them commercially.
**Abstract Book Citations:** Authors, Title, HemaSphere, 2026;10:(S1):pages. The abstract book DOIs can be found on the "Author Information" page.
**Disclaimer:** Articles published in the journal HemaSphere exclusively reflect the opinions of the authors. The authors are responsible for all content in their abstracts including accuracy of the facts, statements, citing resources, etc.

### (PS1923) DOES DOSE FRACTIONATION IMPROVE TOLERABILITY IN AUTOLOGOUS STEM CELL TRANSPLANTATION FOR MULTIPLE MYELOMA? A PROPENSITY SCORE–MATCHED ANALYSIS

**Topic:** 14. Myeloma and other monoclonal gammopathies - Clinical

Ceren Uzunoğlu Güren*[1], Derya Demirtas[2], Ezel Elgün[3], Tuğçe Bulun[1], Elif Suyani[2], Bilal Aygün[2], Didar Yanardag Acik[2], Vildan Özkocaman[3], Asu Fergün Aydın[1], Ayse Tulin Tuglular[4], Isik Atagunduz[1], Tayfur Toptas[1]

*[1]Marmara University, Department of Hematology, İstanbul, Türkiye;[2]Adana City Training and Research Hospital, Department of Hematology, Adana, Türkiye; [3]Uludağ University, Department of Hematology, Bursa, Türkiye;[4]Fulya Acıbadem Hospital, İstanbul, Türkiye;*

### Background

The ideal administration schedule of high-dose melphalan (HDM) preceding autologous stem cell transplantation (ASCT) in multiple myeloma is still ambiguous. Dose fractionation has been suggested to reduce toxicity; nevertheless, comparison data are scarce and conflicting.

### Aims

We aimed to compare toxicity, engraftment kinetics, and survival outcomes between single-day and split-dose HDM in a propensity score–matched cohort.

### Methods

This multicenter retrospective study included consecutive patients undergoing ASCT between 2016 and 2026. After 1:1 propensity score matching for age, sex, ECOG performance status, ISS stage, and renal function, 202 patients were analyzed (101 single-day; 101 split-dose). The primary endpoint was severe gastrointestinal (GI) toxicity, defined as grade ≥3 mucositis and/or diarrhea. Secondary endpoints included organ toxicities, engraftment, Day +90 response, progression-free survival (PFS), and overall survival (OS).

### Results

Severe GI toxicity was significantly less frequent in the single-day group (19.8% vs 36.6%; OR 0.43, 95% CI 0.21–0.84; p=0.012), largely driven by lower rates of grade ≥3 diarrhea. Creatinine and hepatic transaminase elevations were also reduced with single-day administration. Split-dose conditioning was associated with earlier neutrophil (median 11 vs 12 days; p<0.001) and platelet engraftment (11 vs 13 days; p<0.001); however, multivariable analyses demonstrated that this effect was confined to patients receiving stem cell infusion 48 hours after melphalan. Day +90 response rates were comparable between groups. After a median follow-up of 36.5 months, no significant differences were observed in OS (HR 0.84; p=0.598) or PFS (HR 0.88; p=0.552).

### Summary/Conclusion

Single-day HDM was associated with reduced severe toxicity without compromising disease control. Differences in engraftment kinetics appeared largely driven by the melphalan–infusion interval, highlighting timing as a potentially modifiable component of conditioning strategy.

**Copyright Information:** (Online) ISSN: 2572-9241
©2026 The Author(s). HemaSphere is published by John Wiley & Sons Ltd on behalf of European Hematology Association. This is an open access Abstract Book distributed under the Attribution-NonCommercial-NoDerivs (CC BY-NC-ND), which allows third parties to download the articles and share them with others as long as they credit the author and the Abstract Book, but they cannot change the content in any way or use them commercially.
**Abstract Book Citations:** Authors, Title, HemaSphere, 2026;10:(S1):pages. The abstract book DOIs can be found on the “Author Information” page.
**Disclaimer:** Articles published in the journal HemaSphere exclusively reflect the opinions of the authors. The authors are responsible for all content in their abstracts including accuracy of the facts, statements, citing resources, etc.

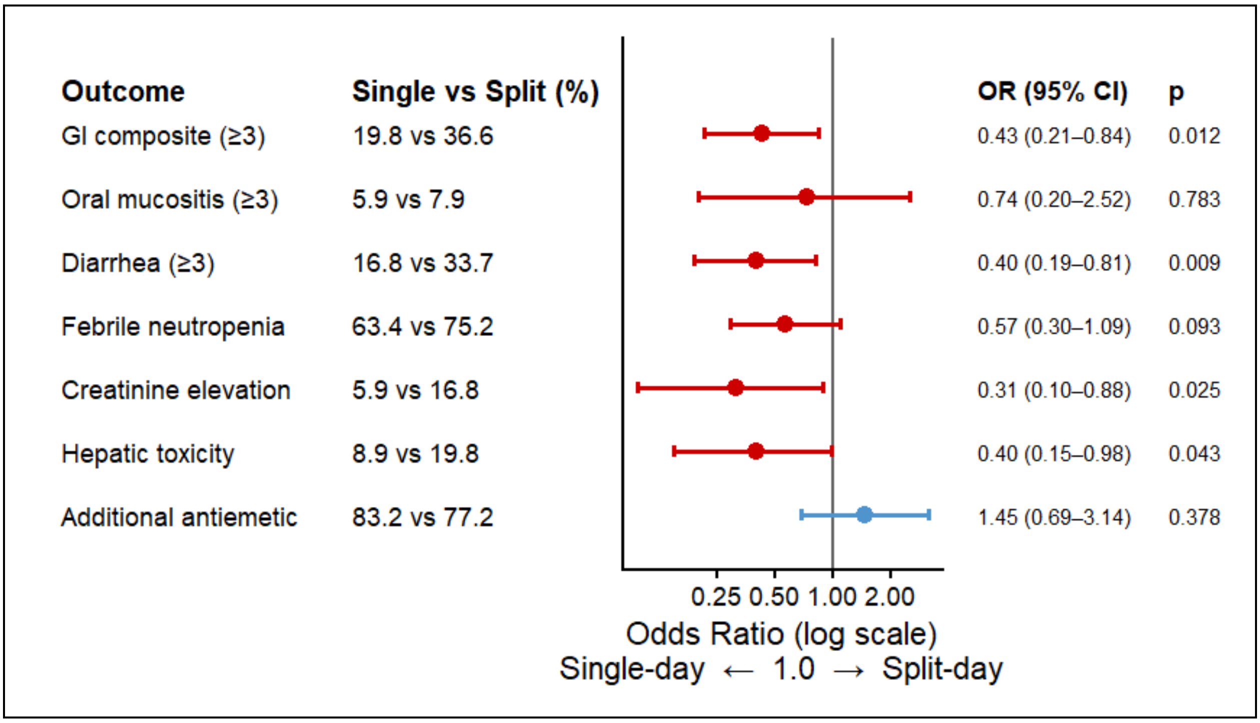

**Copyright Information:** (Online) ISSN: 2572-9241
©2026 The Author(s). HemaSphere is published by John Wiley & Sons Ltd on behalf of European Hematology Association. This is an open access Abstract Book distributed under the Attribution-NonCommercial-NoDerivs (CC BY-NC-ND), which allows third parties to download the articles and share them with others as long as they credit the author and the Abstract Book, but they cannot change the content in any way or use them commercially.
**Abstract Book Citations:** Authors, Title, HemaSphere, 2026;10:(S1):pages. The abstract book DOIs can be found on the "Author Information" page.
**Disclaimer:** Articles published in the journal HemaSphere exclusively reflect the opinions of the authors. The authors are responsible for all content in their abstracts including accuracy of the facts, statements, citing resources, etc.

### (PS1924) SEQUENTIAL TRANSITION TO ALL-ORAL IRD THERAPY FOLLOWING DARA-BASED INDUCTION IN TRANSPLANT-INELIGIBLE NDMM: DEEPENING RESPONSE AND MRD NEGATIVITY

**Topic:** 14. Myeloma and other monoclonal gammopathies - Clinical

Xibin Xiao*[1], Teng Yu[1], Panpan Chen[1], Jing Xie[1], Yongsheng Xie[1], Huyi Lei[2], Wenbin Qian[1]

*[1]The Second Affifiliated Hospital, College of Medicine, Zhejiang University, Department of Hematology, Hangzhou, China;*
*[2]Affiliated Hospital of Shaoxing University(Shaoxing Municipal Hospital), Department of Hematology, Shaoxing, China;*

### Background

Daratumumab-based (Dara-based) induction has significantly improved outcomes in newly diagnosed multiple myeloma (NDMM). For transplant-ineligible (TIE) or elderly patients, parenteral administration of CD38 monoclonal antibodies may pose logistical burdens. Transitioning to an all-oral regimen, such as Ixazomib, Lenalidomide, and Dexamethasone (IRd), after initial induction may sustain and deepen efficacy while enhancing treatment convenience and patient quality of life.

### Aims

To evaluate the efficacy and safety of a sequential therapeutic strategy transitioning to IRd therapy in TIE-NDMM patients who achieved at least a partial response (PR) after 4 cycles of Dara-based induction.

### Methods

This is a single-center, open-label, prospective Phase II clinical study. TIE-NDMM patients received 4 cycles of Dara-based induction. Those achieving ≥ PR were transitioned to the all-oral IRd regimen (ixazomib 4mg, Days 1, 8, 15; lenalidomide 25mg days 1-14; dexamethasone 20mg days 1,8,15,22) until disease progression or intolerable toxicity. The primary endpoint was 24-month progression-free survival (PFS) from the start of IRd. Secondary endpoints included response kinetics, MRD negativity, and safety.

**Copyright Information:** (Online) ISSN: 2572-9241
©2026 The Author(s). HemaSphere is published by John Wiley & Sons Ltd on behalf of European Hematology Association. This is an open access Abstract Book distributed under the Attribution-NonCommercial-NoDerivs (CC BY-NC-ND), which allows third parties to download the articles and share them with others as long as they credit the author and the Abstract Book, but they cannot change the content in any way or use them commercially.
**Abstract Book Citations:** Authors, Title, HemaSphere, 2026;10:(S1):pages. The abstract book DOIs can be found on the "Author Information" page.
**Disclaimer:** Articles published in the journal HemaSphere exclusively reflect the opinions of the authors. The authors are responsible for all content in their abstracts including accuracy of the facts, statements, citing resources, etc.

### Results

Between August 2024 and January 2026, 20 patients were enrolled with a median age of 71 years (range: 63–85); 25% were R-ISS stage III. Cytogenetic abnormalities observed in the cohort included 1q21 amplification (20%), del(17p) (10%), t(4;14) (10%), t(14;20) (5%), and t(14;16) (5%). Extramedullary disease was present in 5% and plasma cell leukemia features (≥5% circulating plasma cells) in 10% of patients.

All 20 patients completed 4 cycles of Dara-based induction, achieved ≥PR (CR 40%, VGPR 30%, PR 30%), and transitioned to IRd. At the data cutoff, the median number of IRd cycles was 9.0 (range: 3–12).
Response kinetics demonstrated significant deepening following the transition to IRd:

- Response Deepening: The rate of ≥CR increased from 40% (8/20) at the start of IRd to 60% (sCR 25%, CR 35%) as the best response during the sequential IRd phase. Overall, 9 patients (45%) achieved a deeper response level after switching to IRd.
- MRD Status: The MRD negativity rate ($10^{-5}$sensitivity) improved from 35% (7/20) at the end of Dara-induction to 55% (11/20) after 4 cycles of IRd therapy.

Survival: With a median follow-up of 9.9 (95% CI: 8.7-12.1) months, only 1 patient experienced disease progression. The estimated 1-year PFS rate was 94.7% (95% CI: 85.2%–100%). Median PFS and OS have not yet been reached.
Safety: All patients experienced at least one adverse event (AE), with 123 events reported in total. The majority (96.7%) were Grade 1–2. No Grade 4–5 AEs occurred. Common AEs included infusion-related fever (16.3%, mainly during Dara induction), nausea (13.0%), diarrhea (13.0%), and fatigue (12.2%). Grade 3 AEs were rare (n=4, 3.2%), including diarrhea (n=2), thrombocytopenia (n=1), and infusion-related fever (n=1).

### Summary/Conclusion

A sequential transition to all-oral IRd therapy after 4 cycles of Dara-based induction is feasible and effective for TIE-NDMM patients. This approach significantly deepens clinical responses and increases MRD negativity with a manageable safety profile. These findings suggest that the IRd regimen provides a potent and convenient therapeutic option to optimize outcomes in elderly population following abbreviated CD38-targeted induction.

**Copyright Information:** (Online) ISSN: 2572-9241
©2026 The Author(s). HemaSphere is published by John Wiley & Sons Ltd on behalf of European Hematology Association. This is an open access Abstract Book distributed under the Attribution-NonCommercial-NoDerivs (CC BY-NC-ND), which allows third parties to download the articles and share them with others as long as they credit the author and the Abstract Book, but they cannot change the content in any way or use them commercially.
**Abstract Book Citations:** Authors, Title, HemaSphere, 2026;10:(S1):pages. The abstract book DOIs can be found on the "Author Information" page.
**Disclaimer:** Articles published in the journal HemaSphere exclusively reflect the opinions of the authors. The authors are responsible for all content in their abstracts including accuracy of the facts, statements, citing resources, etc.

### (PS1925) COMPARATIVE ANALYSIS OF SUBCUTANEOUS ISA VRD (ISASOCUT) AND INTRAVENOUS ISA VRD (IMROZ) IN TRANSPLANT-INELIGIBLE NEWLY DIAGNOSED MULTIPLE MYELOMA ACROSS AGE GROUPS

**Topic:** 14. Myeloma and other monoclonal gammopathies - Clinical

Arthur Bobin*[1], Mohamad Mohty[2], Philippe Moreau[3], Hartmut Goldschmidt[4], Meletios A. Dimopoulos[5], Robert Z Orlowski[6], Cyrille Touzeau[3], Aurore Perrot[7], Thomas Chalopin[8], Mamoun Dib[9], Lydia Montes[10], Umer Khan[11], Mony Morisse[11], Rick Zhang[12], Corina Oprea[13], Florence Suzan[14], Thierry Facon[15], Xavier Leleu[1]

*[1]Centre Hospitalier Universitaire de Poitiers, Poitiers, France;[2]Sorbonne University, Hôpital Saint-Antoine, INSERM UMRs 938, Paris, France; [3]Centre Hospitalier Universitaire de Nantes, Nantes, France; [4]Internal Medicine V, Hematology, Oncology and Rheumatology, German-speaking Myeloma Multicenter Group (GMMG) at the Heidelberg University Hospital, Heidelberg, Germany; [5]National and Kapodistrian University of Athens, Athens, Greece; [6]The University of Texas MD Anderson Cancer Center, Houston, United States of America; [7]Hôpital Universitaire de Toulouse, Toulouse, France;[8]Hôpital Universitaire de Tours, Tours, France; [9]Hôpital Vendée, La Roche-sur-Yon, France; [10]Hôpital Universitaire d'Amiens, Amiens, France; [11]Sanofi, Cambridge, MA, United States of America; [12]Sanofi, Morristown, United States of America; [13]Sanofi, Paris, France; [14]Sanofi, R&D, Vitry-sur-Seine, France; [15]Department of Haematology, Lille University Hospital, Lille, and French National Academy of Medicine, Paris, France;*

### Background

Intravenous (IV) isatuximab (Isa) plus bortezomib, lenalidomide, and dexamethasone (VRd) significantly improved progression free survival in patients with transplant-ineligible newly diagnosed multiple myeloma (Ti NDMM) in the IMROZ randomized Phase 3 study (NCT03319667). Efficacy and safety of subcutaneous (SC) Isa VRd, using an innovative on-body injector (OBI), has been demonstrated previously, including in Ti NDMM patients in the ISASOCUT Phase 2 study (NCT05889221). Bortezomib was administered twice weekly (BIW) in IMROZ vs weekly in ISASOCUT.

### Aims

To evaluate the safety and efficacy of Isa OBI VRd (ISASOCUT) and Isa IV VRd (IMROZ) at 8 months follow-up.

### Methods

This analysis included 74 patients from ISASOCUT (Isa OBI VRd) and 265 from IMROZ (Isa IV VRd). Isa OBI VRd patients received Isa 1400 mg (weekly cycle [C]1, then every 2 weeks [Q2W] up to C12, and Q4W thereafter); each cycle lasted 28 days. SC bortezomib (1.3 mg/m$^2$) was given BIW in C1 and weekly thereafter. Isa IV VRd patients received Isa 10 mg/kg weekly in C1, then Q2W, followed by Q4W from C18 onwards. SC bortezomib (1.3 mg/m$^2$) was given BIW at C1-4. C1-4 lasted 42 days, with the remainder cycles lasting 28 days each. All patients provided informed consent. This comparative analysis evaluated safety and efficacy at 8 months follow-up by baseline age group (<75 yrs, ≥75 yrs).

**Copyright Information:** (Online) ISSN: 2572-9241
©2026 The Author(s). HemaSphere is published by John Wiley & Sons Ltd on behalf of European Hematology Association. This is an open access Abstract Book distributed under the Attribution-NonCommercial-NoDerivs (CC BY-NC-ND), which allows third parties to download the articles and share them with others as long as they credit the author and the Abstract Book, but they cannot change the content in any way or use them commercially.
**Abstract Book Citations:** Authors, Title, HemaSphere, 2026;10:(S1):pages. The abstract book DOIs can be found on the "Author Information" page.
**Disclaimer:** Articles published in the journal HemaSphere exclusively reflect the opinions of the authors. The authors are responsible for all content in their abstracts including accuracy of the facts, statements, citing resources, etc.

**Results**

Baseline demographic and disease characteristics were similar between Isa OBI VRd and Isa IV VRd patients. Incidence of serious treatment emergent adverse events (TEAEs) were similar between groups (35.1% and 42.2% for Isa OBI VRd and Isa IV VRd, respectively) and across age groups. The rate of systemic infusion reaction was lower with Isa OBI VRd vs Isa IV VRd (4 [5%] vs 60 [22.8%]). Grade ≥3 neutropenic complications occurred in 2.7% Isa SC OBI patients and 3.8% Isa IV patients and were similar across age groups (<75 yrs, 2% vs 4.1%; ≥75 yrs, 4% vs 2.9%). Study discontinuation due to TEAEs occurred in 2.7% of Isa SC OBI patients and 8.7% of Isa IV patients, with lower rates observed in Isa SC OBI treated patients aged <75 yrs (0% vs 8.2%). Among patients aged ≥75 yrs, the rates were 8% for Isa SC OBI VRd and 10.3% for Isa IV VRd. Across both age groups, a lower incidence of Grade 2 peripheral neuropathy (PN) was observed with Isa OBI VRd (weekly V) vs Isa IV VRd (BIW V) (<75 yrs, 20.4% vs 28.7%; ≥75 yrs, 20% vs 35.3%) as well as Grade ≥3 PN (<75 yrs, 2% vs 9.2%; ≥75 yrs, 0% vs 7.4%). Isa OBI VRd (weekly V) was associated with less bortezomib dose reduction due to nervous system disorders (Isa OBI VRd [weekly V] vs Isa IV VRd [BIW V]: <75 yrs, 14.3% vs 37.4%; ≥75 yrs, 28.0% vs 35.3%). In patients aged <75 yrs, very good partial response or better (≥VGPR) rates for Isa OBI VRd and Isa IV VRd were 87.8% and 83.2%, respectively (RR: 1.055; 95% CI: 0.934-1.192), whereas rates in patients aged ≥75 yrs were 80.0% and 82.6%, respectively (RR: 0.968; 95% CI: 0.774-1.211).

**Summary/Conclusion**

In this comparative analysis, Isa OBI VRd with weekly bortezomib (ISASOCUT) is more tolerable for patients regarding PN than the BIW schedule (IMROZ). Both groups maintained similar ≥VGPR rates across age groups. These findings support the efficacy and safety of the Isa SC OBI with weekly bortezomib.

© 2026 American Society of Clinical Oncology, Inc. Reused with permission. This abstract was accepted and previously presented at the 2026 ASCO Annual Meeting. All rights reserved.

**Copyright Information:** (Online) ISSN: 2572-9241
©2026 The Author(s). HemaSphere is published by John Wiley & Sons Ltd on behalf of European Hematology Association. This is an open access Abstract Book distributed under the Attribution-NonCommercial-NoDerivs (CC BY-NC-ND), which allows third parties to download the articles and share them with others as long as they credit the author and the Abstract Book, but they cannot change the content in any way or use them commercially.
**Abstract Book Citations:** Authors, Title, HemaSphere, 2026;10:(S1):pages. The abstract book DOIs can be found on the "Author Information" page.
**Disclaimer:** Articles published in the journal HemaSphere exclusively reflect the opinions of the authors. The authors are responsible for all content in their abstracts including accuracy of the facts, statements, citing resources, etc.

### (PS1926) RENAL OUTCOMES BY PALLADINI STAGE AND RESPONSE CRITERIA IN SYSTEMIC AL AMYLOIDOSIS TREATED WITH FIRST-LINE DARA-CYBORD: REAL-WORLD EXPERIENCE FROM A SINGLE AMYLOIDOSIS REFERRAL CENTER

**Topic:** 14. Myeloma and other monoclonal gammopathies - Clinical

James Di Palma-Grisi*[1], Alexander Cardiero[1], Noa Biran[2], David H. Vesole[2], Pooja Phull[2], Joseph Franz[2], Zalman Suldan[1, 1], Dominic Santoriello[3], David Siegel[2], Harsh Parmar[2]

*[1]Hackensack University Medical Center, Hackensack, United States of America;[2]John Theurer Cancer Center, Hackensack, United States of America; [3]Columbia University Irving Medical Center, Pathology, New York, United States of America;*

### Background

Immunoglobulin light-chain (AL) amyloidosis is a rare plasma cell dyscrasia characterised by misfolding and extracellular deposition of monoclonal light chains in vital organs, causing organ dysfunction and mortality. Cardiac involvement is the most common and the principal determinant of prognosis, but the kidneys can also be affected, driving renal failure and haemodialysis (HD) (Sanchorawala, 2024). Cyclophosphamide, bortezomib and dexamethasone (CyBorD) was the mainstay of treatment until daratumumab, an anti-CD38 monoclonal antibody, emerged as a potent therapy in plasma cell disorders. The ANDROMEDA trial demonstrated that daratumumab with the CyBorD backbone (Dara-CyBorD) achieved partial responses or better in 78.5% of patients with newly diagnosed AL amyloidosis, compared to 49.2% with CyBorD alone (Kastritis et. al., 2021).

### Aims

To assess whether the criteria described by Palladini et. al. are predictive of renal outcomes in our cohort of patients with newly diagnosed AL amyloidosis treated with first-line Dara-CyBorD.

### Methods

Retrospective chart review was conducted under an IRB-approved protocol among all visits between November 1, 2019 to December 31, 2025 to our amyloidosis referral center. We identified 142 patients with biopsy-confirmed systemic AL amyloidosis, 52 of whom had received upfront Dara-CyBorD. We excluded two patients who had already started on haemodialysis prior to initial therapy. Renal involvement was staged by proteinuria and estimated glomerular filtration rate (eGFR), with proteinuria > 5g/24h and eGFR < 50 mL/min corresponding to stage III, one but not both corresponding to stage II, and the absence of both corresponding to stage I. Six months post-treatment, progression was defined as an increase in eGFR ≥ 25% and response is defined as a reduction of proteinuria ≥ 30% or drop below 0.5g/24h in the absence of progression (Palladini et. al., 2014).

**Copyright Information:** (Online) ISSN: 2572-9241
©2026 The Author(s). HemaSphere is published by John Wiley & Sons Ltd on behalf of European Hematology Association. This is an open access Abstract Book distributed under the Attribution-NonCommercial-NoDerivs (CC BY-NC-ND), which allows third parties to download the articles and share them with others as long as they credit the author and the Abstract Book, but they cannot change the content in any way or use them commercially.
**Abstract Book Citations:** Authors, Title, HemaSphere, 2026;10:(S1):pages. The abstract book DOIs can be found on the “Author Information” page.
**Disclaimer:** Articles published in the journal HemaSphere exclusively reflect the opinions of the authors. The authors are responsible for all content in their abstracts including accuracy of the facts, statements, citing resources, etc.

**Results**

Median age at diagnosis was 67.6 years [IQR 58.1 - 76.3], 33/50 were Caucasian, 30/50 were male. Baseline characteristics were comparable within each group. 33/50 had cardiac involvement at diagnosis. Median follow-up was 41.2 months. Median overall survival (OS) for the entire cohort was 100.6 months. Median OS was met in the Stage III cohort and was 44.1 months (Figure 1). Median OS was not reached in the Stage II or Stage I cohorts. Median OS in the progression group was 29.7 months, in the responders group was 100.6 months and in the nonresponders group it was not reached (p=0.009). Hazard ratios (HR) were calculated for each renal stage and response group in terms of progression to HD (Table 1).

| Stage or Response Categories | Hazard Ratio [95% Confidence Interval] | p-value |
|---|---|---|
| Stage II vs I | 2.3 [0.37 - 13.7] | 0.37 |
| Stage III vs II | 1.91 [0.32 - 11.47] | 0.48 |
| Stage III vs I | 4.3 [0.6 - 31] | 0.14 |
| Progression vs Response | 9.5 [1.1, 81.9] | 0.04 |

Table 1. Hazard ratios by renal stage or response group. There were no haemodialysis events in the non-response group.

**Summary/Conclusion**

Stage at diagnosis was not significant in our single-center cohort treated with up-front Dara-CyBorD. In view of our relatively small numnber of events (progression to haemodialysis), this finding would need to be validated in larger, adequately powered and prospective studies. There was a statistically significant HR (9.5, p=0.04) between the renal response and renal progression groups, suggesting that this component of the risk stratification criteria remains highly predictive even in our relatively small sample.

**Copyright Information:** (Online) ISSN: 2572-9241
©2026 The Author(s). HemaSphere is published by John Wiley & Sons Ltd on behalf of European Hematology Association. This is an open access Abstract Book distributed under the Attribution-NonCommercial-NoDerivs (CC BY-NC-ND), which allows third parties to download the articles and share them with others as long as they credit the author and the Abstract Book, but they cannot change the content in any way or use them commercially.
**Abstract Book Citations:** Authors, Title, HemaSphere, 2026;10:(S1):pages. The abstract book DOIs can be found on the "Author Information" page.
**Disclaimer:** Articles published in the journal HemaSphere exclusively reflect the opinions of the authors. The authors are responsible for all content in their abstracts including accuracy of the facts, statements, citing resources, etc.

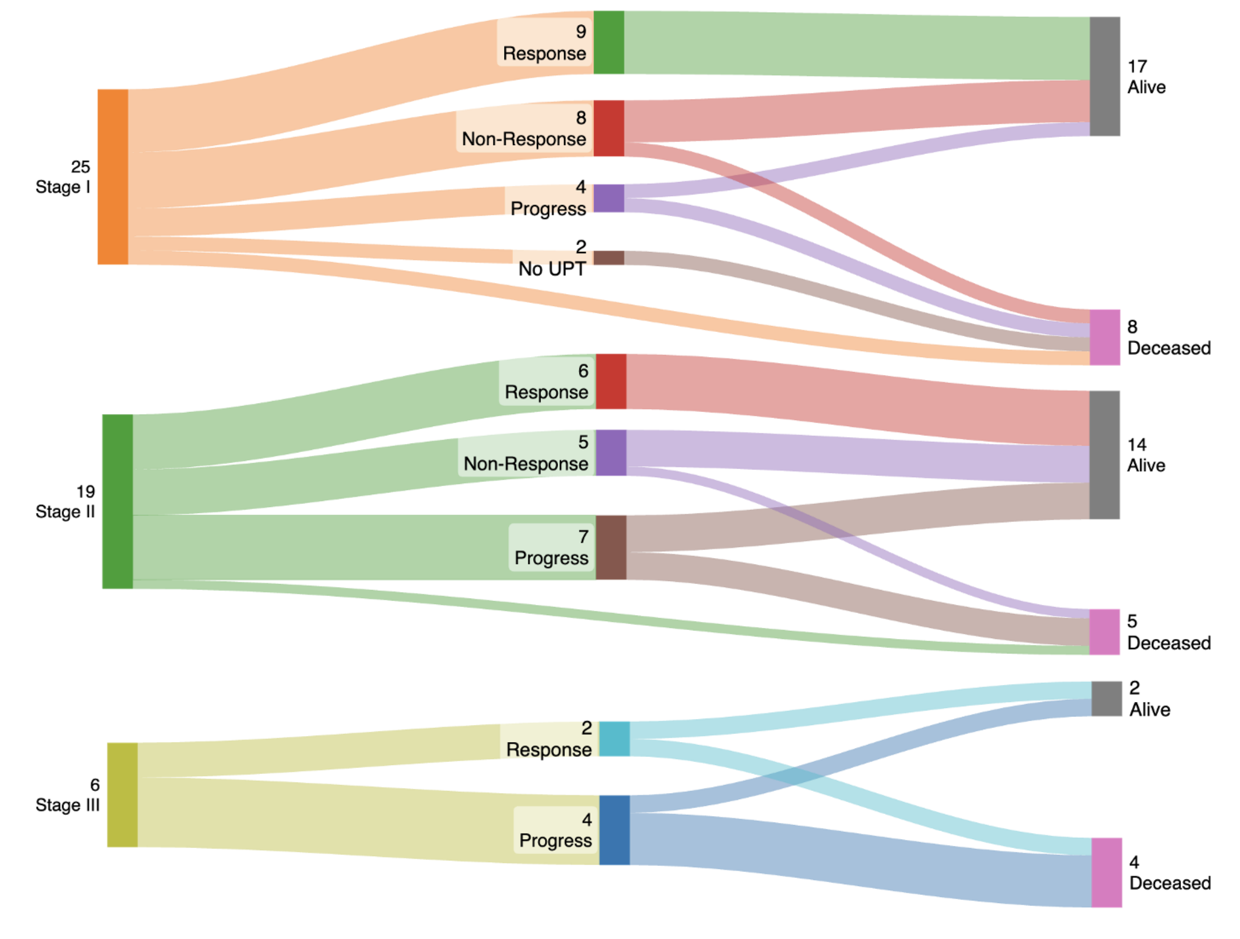

Figure 1. Sankey diagram demonstrating outcomes from each stage-response grouping.

**Copyright Information:** (Online) ISSN: 2572-9241
©2026 The Author(s). HemaSphere is published by John Wiley & Sons Ltd on behalf of European Hematology Association. This is an open access Abstract Book distributed under the Attribution-NonCommercial-NoDerivs (CC BY-NC-ND), which allows third parties to download the articles and share them with others as long as they credit the author and the Abstract Book, but they cannot change the content in any way or use them commercially.
**Abstract Book Citations:** Authors, Title, HemaSphere, 2026;10:(S1):pages. The abstract book DOIs can be found on the "Author Information" page.
**Disclaimer:** Articles published in the journal HemaSphere exclusively reflect the opinions of the authors. The authors are responsible for all content in their abstracts including accuracy of the facts, statements, citing resources, etc.

### (PS1927) DIAGNOSTIC VALUE OF TONGUE BIOPSY IN PATIENTS WITH LIGHT CHAIN AMYLOIDOSIS

**Topic:** 14. Myeloma and other monoclonal gammopathies - Clinical

Yuping Zhong*[1], Shuyuan Yu[1]

[1]*University of Health and Rehabilitation Science, Qingdao Municipal Hospital, Qingdao, China;*

### Background

The diagnosis of light chain amyloidosis relies on pathological examination, but in clinical practice, it is often difficult to obtain a pathological sample. There is an urgent need to explore simple and feasible biopsy sites.

### Aims

Amyloidosis is a rare systemic disorder characterized by the extracellular deposition of misfolded proteins as insoluble β-sheet-rich amyloid fibrils, leading to progressive organ dysfunction. if untreated, mortality. This study systematically investigates the clinical phenotypes, patterns of multi-organ involvement, and diagnostic utility of tongue biopsy in the pathological confirmation of light chain (AL) amyloidosis among patients managed at our center, thereby contributing evidence to support timely diagnosis and histopathological validation.

### Methods

We conducted a retrospective cohort analysis of 44 consecutive patients diagnosed with AL amyloidosis at our center between January 2021 and December 2025. Clinical variables—including sex, age at diagnosis, etiologic subtype (primary vs. secondary), spectrum and frequency of organ involvement, median diagnostic delay (defined as time from symptom onset to definitive diagnosis), and histopathological findings from multi-site biopsies—were extracted from electronic medical records and analyzed descriptively and comparatively.

### Results

Of the 44 patients, 26 (59.1%) were male and 18 (40.9%) were female; median age at diagnosis was 64.5 years (range: 42–83). Etiologically, 19 cases (43.2%) were classified as primary AL amyloidosis and 25 (56.8%) as secondary AL amyloidosis. Organ involvement was observed in 15 distinct sites: tongue, bone marrow, heart, kidney, gingiva, stomach, small intestine, peripheral nerve, skin, lung, abdominal wall fat, liver, thyroid, lymph nodes, and parotid gland. Median diagnostic delay was 9 months (IQR: 5–14). Tongue biopsy was performed in 18 patients, all Congo red–positive amyloid deposits (100% sensitivity)among these, bone marrow biopsy was concurrently performed in 17 patients, demonstrating Congo red positivity in only 2 cases (11.8% sensitivity); gastric and intestinal biopsies were each performed once and both yielded negative results.

### Summary/Conclusion

AL amyloidosis exhibits heterogeneous and widespread organ tropism, accompanied by substantial diagnostic delays. Tongue biopsy demonstrates exceptional diagnostic sensitivity, safety, and procedural simplicity for histopathological confirmation of AL amyloidosis, positioning it as a highly valuable first-line invasive diagnostic tool in suspected cases. In contrast, the markedly lower sensitivity of bone marrow, gastric, and intestinal biopsies—even when performed in patients with confirmed tongue amyloidosis—suggests that tongue biopsy may offer superior diagnostic method for systemic AL amyloidosis.

**Copyright Information:** (Online) ISSN: 2572-9241
©2026 The Author(s). HemaSphere is published by John Wiley & Sons Ltd on behalf of European Hematology Association. This is an open access Abstract Book distributed under the Attribution-NonCommercial-NoDerivs (CC BY-NC-ND), which allows third parties to download the articles and share them with others as long as they credit the author and the Abstract Book, but they cannot change the content in any way or use them commercially.
**Abstract Book Citations:** Authors, Title, HemaSphere, 2026;10:(S1):pages. The abstract book DOIs can be found on the "Author Information" page.
**Disclaimer:** Articles published in the journal HemaSphere exclusively reflect the opinions of the authors. The authors are responsible for all content in their abstracts including accuracy of the facts, statements, citing resources, etc.

### (PS1928) LENALIDOMIDE-BASED THERAPY IN TRANSPLANT-INELIGIBLE PATIENTS WITH NEWLY DIAGNOSED MULTIPLE MYELOMA AND RENAL IMPAIRMENT: SAFETY OF HIGHER-THAN-LABEL DOSING FROM THE REST TRIAL

**Topic:** 14. Myeloma and other monoclonal gammopathies - Clinical

Hedda Berg Monsen*[1, 2], Einar Haukås[3], Tobias S. Slørdahl[4, 5], Anja Klostergaard[6], Tatjana Alexandersen[1], Anna Lysén[1], Emil Hermansen[7], Fredrik Schjesvold[1], Frida Bugge Askeland[1, 2]

*[1]Oslo Myeloma center, Department of Hematology, Oslo University Hospital, Oslo, Norway; [2]University of Oslo, Institute of Clinical Medicine, Oslo, Norway; [3]Stavanger University Hospital, Stavanger, Department of Hematology and Oncology, Stavanger, Norway; [4]St. Olavs Hospital, Trondheim, Department of Hematology, Trondheim, Norway; [5]Norwegian University of Science and Technology (NTNU), Department of clinical and molecular medicine, Trondheim, Norway; [6]Aarhus University Hospital, Department of Hematology, Aarhus, Denmark; [7]Copenhagen University Hospital Rigshospitalet, Department of Hematology, Copenhagen, Denmark;*

### Background

Renal impairment (RI) is frequently present at diagnosis and a clinically important complication of multiple myeloma (MM) associated with inferior outcomes. Lenalidomide-based regimens are standard of care in first-line therapy for newly diagnosed (ND) MM patients. According to the prescribing information, lenalidomide doses should be substantially reduced in patients with RI, based primarily on data from a pharmacokinetic study. Recent results from clinical studies have questioned this cautionary approach.

### Aims

To evaluate safety and renal outcomes in NDMM patients with baseline RI (eGFR <50) treated with higher-than-label lenalidomide dosing.

### Methods

The REST trial is an academic, multicenter, open-label, single-arm phase 2 study conducted in Norway and Denmark. Transplant-ineligible patients with NDMM received isatuximab–bortezomib–lenalidomide–dexamethasone. Lenalidomide was dosed higher-than-label: 25 mg/day for eGFR ≥30 mL/min/1.73 m² and 15 mg/day for eGFR <30 mL/min/1.73 m². This subanalysis compared patients with baseline eGFR <50 versus ≥50 mL/min/1.73 m². Renal function was assessed from cycle 1 to cycle 7 and renal response was assessed using a creatinine-based eGFR formula. Lenalidomide-related adverse events and best overall response were evaluated.

**Copyright Information:** (Online) ISSN: 2572-9241
©2026 The Author(s). HemaSphere is published by John Wiley & Sons Ltd on behalf of European Hematology Association. This is an open access Abstract Book distributed under the Attribution-NonCommercial-NoDerivs (CC BY-NC-ND), which allows third parties to download the articles and share them with others as long as they credit the author and the Abstract Book, but they cannot change the content in any way or use them commercially.
**Abstract Book Citations:** Authors, Title, HemaSphere, 2026;10:(S1):pages. The abstract book DOIs can be found on the "Author Information" page.
**Disclaimer:** Articles published in the journal HemaSphere exclusively reflect the opinions of the authors. The authors are responsible for all content in their abstracts including accuracy of the facts, statements, citing resources, etc.

### Results

Between 30 June 2021 and 19 January 2023, 51 patients were included; 39 (76%) had preserved renal function and 12 (24%) had RI. Median age was higher in patients with RI (80 vs 76 years). Hypertension was more common in patients with RI (83% vs 46%), and non-myeloma kidney disease was observed exclusively in these patients (17%). Median baseline eGFR at cycle 1 day 1 was 70 mL/min/1.73 m² (IQR 60.5–83.5) in patients with preserved renal function and 42 mL/min/1.73 m² (IQR 38.0–46.2) in patients with RI. Four patients with preserved renal function discontinued all study treatment before cycle 7, none with baseline RI discontinued before cycle 7.

In patients with preserved renal function, eGFR remained stable (median change +1 mL/min/1.73 m²), whereas in patients with RI, eGFR increased by a median of 11 mL/min/1.73 m² (Figure 1). Renal response was observed in 58% of patients with RI.

Adverse events were largely comparable, except for skin rash, which occurred only in patients with preserved renal function (28%), and constipation, which was more frequent in patients with RI (58% vs 21%). One event of worsening renal function (grade 3) occurred in the preserved group. Lenalidomide was temporarily discontinued and resumed at a reduced dose. No worsening in renal function was observed in patients with baseline RI.

Best overall response rates are shown in Table 1. All patients achieved at least partial response.

**Table 1. Best overall response by investigator assessment**

| **Response category** | **eGFR ≥50 (n=39)** | **eGFR <50 (n=12)** | **P** |
|---|---|---|---|
| Overall response | 39 (100%) | 12 (100%) | - |
| Stringent complete response | 16 (41%) | 6 (50%) | 0.741 |
| Complete response | 2 (5%) | 1 (8%) | 0.561 |
| Very good partial response (VGPR) | 12 (31%) | 5 (42%) | 0.503 |
| VGPR or better | 30 (77%) | 12 (100%) | 0.094 |
| Partial response | 9 (23%) | 0 (0%) | 0.094 |

Data are n (%).P-values compare eGFR ≥50 vs eGFR <50 using two-sided Fisher's exact test. Data cutoff sep 2025. eGFR, estimated glomerular filtration rate.

### Summary/Conclusion

Higher-than-label lenalidomide dosing within quadruplet induction was tolerated in patients with baseline RI, without excess toxicity and without apparent compromise in response rates compared with preserved renal function. Renal function improved in most patients with baseline RI.

**Copyright Information:** (Online) ISSN: 2572-9241
©2026 The Author(s). HemaSphere is published by John Wiley & Sons Ltd on behalf of European Hematology Association. This is an open access Abstract Book distributed under the Attribution-NonCommercial-NoDerivs (CC BY-NC-ND), which allows third parties to download the articles and share them with others as long as they credit the author and the Abstract Book, but they cannot change the content in any way or use them commercially.
**Abstract Book Citations:** Authors, Title, HemaSphere, 2026;10:(S1):pages. The abstract book DOIs can be found on the "Author Information" page.
**Disclaimer:** Articles published in the journal HemaSphere exclusively reflect the opinions of the authors. The authors are responsible for all content in their abstracts including accuracy of the facts, statements, citing resources, etc.

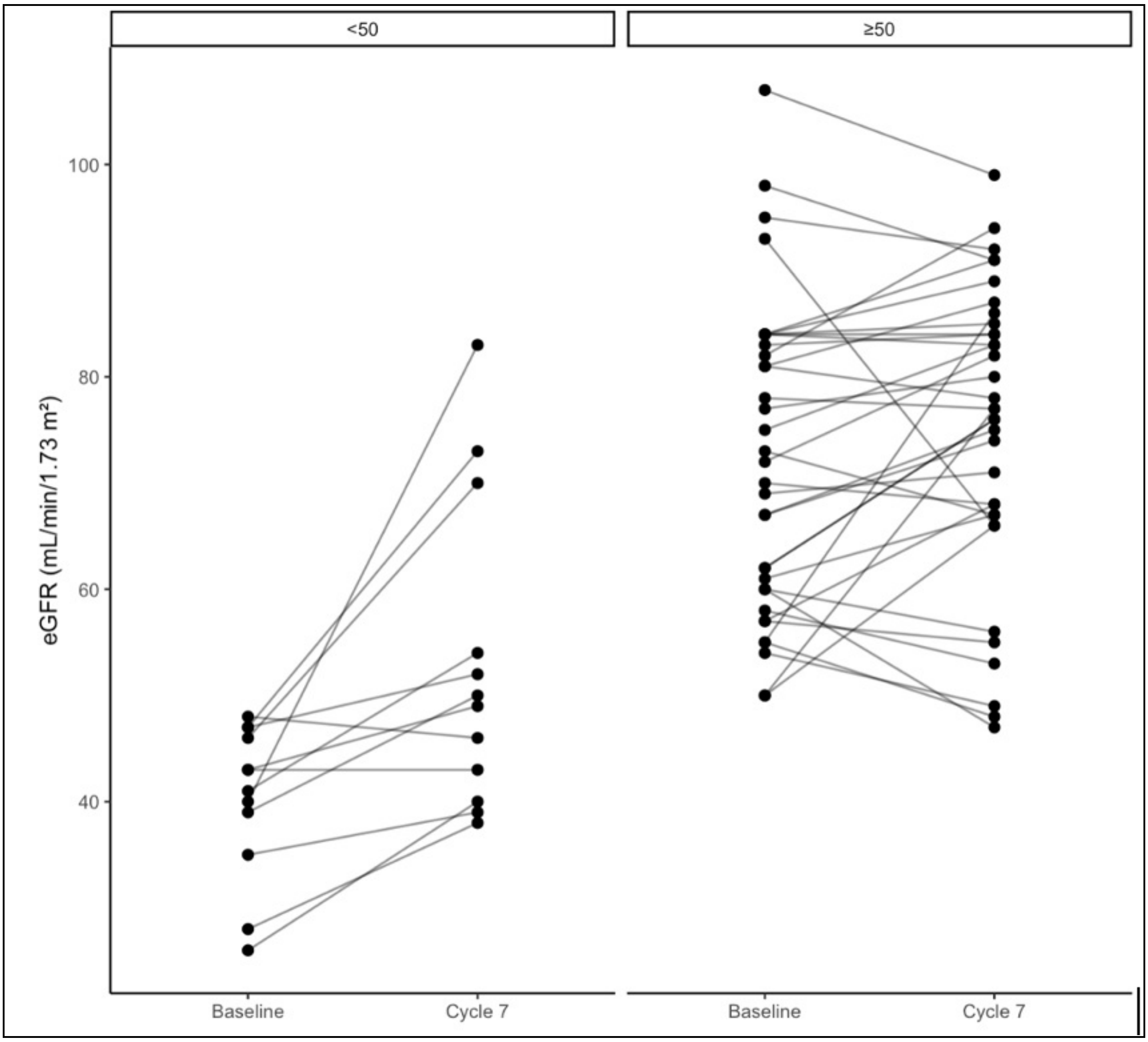

Figure 1: Individual changes in estimated glomerular filtration rate (eGFR) from baseline (Cycle 1) to Cycle 7 are shown for patients stratified by baseline eGFR <50 and ≥50 mL/min/1.73 m2. Each line represents an individual patient.

**Copyright Information:** (Online) ISSN: 2572-9241
©2026 The Author(s). HemaSphere is published by John Wiley & Sons Ltd on behalf of European Hematology Association. This is an open access Abstract Book distributed under the Attribution-NonCommercial-NoDerivs (CC BY-NC-ND), which allows third parties to download the articles and share them with others as long as they credit the author and the Abstract Book, but they cannot change the content in any way or use them commercially.
**Abstract Book Citations:** Authors, Title, HemaSphere, 2026;10:(S1):pages. The abstract book DOIs can be found on the “Author Information” page.
**Disclaimer:** Articles published in the journal HemaSphere exclusively reflect the opinions of the authors. The authors are responsible for all content in their abstracts including accuracy of the facts, statements, citing resources, etc.

### (PS1929) RENAL RECOVERY AND DEPTH OF RESPONSE WITH DARATUMUMAB-BASED QUADRUPLET VERSUS TRIPLET INDUCTION THERAPY IN TRANSPLANT-ELIGIBLE NDMM WITH RENAL IMPAIRMENT: A MULTICENTER KMMWP2309 STUDY

**Topic:** 14. Myeloma and other monoclonal gammopathies - Clinical

Ji Hyun Lee*[1], Kihyun Kim[2], Ja Min Byun[3], Sung-Soo Park[4], Dok Hyun Yoon[5], Hyeong-Woo Cho[5], Joon Ho Moon[6], Hee Jeong Cho[6], Ho-Young Yhim[7], Ho-Jin Shin[8], Do Young Kim[8], Myung-Won Lee[9], Min Kyoung Kim[10], Ka-Won Kang[11], Jae-Cheol Jo[12], Sung-Hyun Kim[1], Ho Sup Lee[13], Kyoung Ha Kim[14], Ji Yun Lee[15], Hyeon-Seok Eom[16], Jongheon Jung[16], Cheongin Yang[17], Sungnam Lim[18], Young Rok Do[19], Hyo Jung Kim[20], Ji Min Choi[21], Eunseok Lee[22], Chang-Ki Min[4], Sung-Hoon Jung[23]

*[1]Dong-A University College of Medicine, Busan, Republic of Korea, Division of Hematology-Oncology, Department of Internal Medicine, Busan, South Korea; [2]Samsung Medical Center, Sungkyunkwan University School of Medicine, Division of Hematology-Oncology, Department of Medicine, Seoul, South Korea; [3]Seoul National University Hospital, Seoul National University College of Medicine, Division of Hematology-Oncology, Department of Internal Medicine, Seoul, South Korea; [4]Seoul St. Mary's Hospital, College of Medicine, The Catholic University of Korea, Department of Hematology, Seoul, South Korea; [5]Asan Medical Center, University of Ulsan College of Medicine, Department of Oncology, Seoul, South Korea; [6]Kyungpook National University Hospital, School of Medicine, Kyungpook National University, Department of Hematology/Oncology, Daegu, South Korea; [7]Jeonbuk National University Medical School, Department of Internal Medicine, Jeonju, South Korea;[8]Pusan National University Hospital, Division of Hematology-Oncology, Department of Internal Medicine, Busan, South Korea; [9]Chungnam National University Hospital, Division of Hematology and Oncology, Department of Internal Medicine, Daejeon, South Korea; [10]Yeungnam University College of Medicine, Division of Hemato-Oncology, Department of Internal Medicine, Daegu, South Korea; [11]Korea University College of Medicine, Division of Hematology Oncology, Department of Internal Medicine, Seoul, South Korea; [12]Ulsan University Hospital, University of Ulsan College of Medicine, Department of Internal Medicine, Ulsan, South Korea; [13]Kosin University College of Medicine, Department of Internal Medicine, Busan, South Korea; [14]Soonchunhyang University Hospital, Seoul, Republic of Korea, Department of Internal Medicine, Seoul, South Korea; [15]Seoul National University Bundang Hospital, Seoul National University College of Medicine, Department of Internal Medicine, Seongnam, South Korea; [16]Center for Hematologic Malignancy, National Cancer Center, Department of Hematology-Oncology, Goyang, South Korea; [17]Ajou University Hospital, Department of Internal Medicine, Suwon, South Korea; [18]Inje University Haeundae Paik Hospital, Department of Internal Medicine, Busan, South Korea; [19]Keimyung University, Dongsan Medical Center, Department of Hemato-Oncology, Daegu, South Korea; [20]Hallym University Medical Center, Hallym University College of Medicine, Department of Hematology-Oncology, Anyang, South Korea; [21]College of Business Administration, Dong-A University, Department of Management Information Systems, Busan, South Korea; [22]The State University of New Jersey, Rutgers, New Jersey, United States of America;[23]Chonnam National University Hwasun Hospital, Chonnam National University Medical School, Department of Hematology-Oncology, Hwasun, South Korea;*

### Background

Renal impairment (RI) affects approximately one-third of patients with newly diagnosed multiple myeloma (NDMM) and remains a major adverse prognostic factor. Although daratumumab-based quadruplet induction improves depth of response in transplant-eligible NDMM, comparative real-world data on renal recovery remain limited.

### Aims

To compare hematologic response, renal recovery, and survival outcomes among D-VTd, RVd, and VTd induction regimens in transplant-eligible NDMM patients presenting with RI.

**Copyright Information:** (Online) ISSN: 2572-9241
©2026 The Author(s). HemaSphere is published by John Wiley & Sons Ltd on behalf of European Hematology Association. This is an open access Abstract Book distributed under the Attribution-NonCommercial-NoDerivs (CC BY-NC-ND), which allows third parties to download the articles and share them with others as long as they credit the author and the Abstract Book, but they cannot change the content in any way or use them commercially.
**Abstract Book Citations:** Authors, Title, HemaSphere, 2026;10:(S1):pages. The abstract book DOIs can be found on the "Author Information" page.
**Disclaimer:** Articles published in the journal HemaSphere exclusively reflect the opinions of the authors. The authors are responsible for all content in their abstracts including accuracy of the facts, statements, citing resources, etc.

### Methods

This nationwide multicenter retrospective study (KMMWP2309) included transplant-eligible NDMM patients aged ≥19 years with baseline estimated glomerular filtration rate (eGFR) <50 mL/min/1.73m² treated between May 2021 and May 2023. Patients received D-VTd, RVd, or VTd. Renal response was assessed using IMWG renal criteria. Overall response rate (ORR) was defined as PR or better; and patients with negative serum and urine immunofixation but without bone marrow confirmation were classified as near complete response (nCR) and included within the CR category for analysis. Continuous ΔeGFR was compared using Kruskal–Wallis testing with Dunn's post hoc adjustment. Survival outcomes were estimated using Kaplan–Meier methods and compared by log-rank test; Cox regression was performed for hazard ratio (HR) estimation.

### Results

A total of 230 patients were analyzed (D-VTd n=64; RVd n=101; VTd n=75). Depth of hematologic response favored D-VTd compared with triplets. In pairwise comparison, VGPR or better was significantly higher with D-VTd versus RVd (92.2% vs 80.4%, p=0.040), whereas CR rates were comparable. ORR did not differ significantly across all three regimens (Table 1). Categorical renal response showed borderline global difference (p=0.050), with numerically higher PR renal or better rates in D-VTd (60.9%) compared with RVd (55.4%) and VTd (45.3%) (Table 2). Renal function improvement differed among groups (Kruskal–Wallis p=0.033). Mean rank of ΔeGFR was highest in D-VTd (133.8), followed by VTd (110.7) and RVd (106.7). Post hoc analysis demonstrated significantly greater ΔeGFR improvement with D-VTd compared with RVd (adjusted p=0.036) (Figure 1). With median follow-up of 29.5 months, 79 PFS events occurred. Kaplan–Meier analysis demonstrated improved PFS with D-VTd (log-rank p=0.05). Compared with D-VTd, RVd (HR 1.95, p=0.034) and VTd (HR 2.01, p=0.026) were associated with significantly inferior PFS (Figure 2A). Overall survival also favored D-VTd (log-rank p=0.02), with higher mortality risk observed in RVd (HR 3.13, p=0.009) and VTd (HR 2.38, p=0.049) compared with D-VTd (Figure 2B). Grade ≥3 adverse events were comparable, though thrombocytopenia and neutropenia were more frequent in RVd. Autologous transplantation rates were higher in the D-VTd cohort.

### Summary/Conclusion

In transplant-eligible NDMM patients with renal impairment, daratumumab-based quadruplet induction was associated with deeper hematologic response, greater improvement in renal function, and superior PFS and OS compared with triplet regimens in a real-world nationwide cohort. These findings support the preferential use of D-VTd induction regimen compared with bortezomib-based triplet regimens in this group of patients. This work was supported by J&J Innovative Medicine (Reference number: 54767414MMY4067).

**Copyright Information:** (Online) ISSN: 2572-9241
©2026 The Author(s). HemaSphere is published by John Wiley & Sons Ltd on behalf of European Hematology Association. This is an open access Abstract Book distributed under the Attribution-NonCommercial-NoDerivs (CC BY-NC-ND), which allows third parties to download the articles and share them with others as long as they credit the author and the Abstract Book, but they cannot change the content in any way or use them commercially.
**Abstract Book Citations:** Authors, Title, HemaSphere, 2026;10:(S1):pages. The abstract book DOIs can be found on the "Author Information" page.
**Disclaimer:** Articles published in the journal HemaSphere exclusively reflect the opinions of the authors. The authors are responsible for all content in their abstracts including accuracy of the facts, statements, citing resources, etc.

**Table 1. Hematologic response after induction**

| Response | D-VTd | RVd | VTd |
|---|---|---|---|
| Response evaluable | 64 | 97 | 73 |
| sCR | 10.9% | 8.2% | 6.8% |
| CR / near CR | 23.4% | 24.7% | 19.2% |
| VGPR | 57.8% | 47.4% | 56.2% |
| PR | 7.8% | 13.4% | 15.1% |
| MR | 0.0% | 3.1% | 1.4% |
| SD | 0.0% | 2.1% | 1.4% |
| PD | 0.0% | 1.0% | 0.0% |

**Table 2. Renal response after induction**

| Renal response | D-VTd | RVd | VTd |
|---|---|---|---|
| Response evaluable | 64 | 99 | 73 |
| CR renal | 54.7% | 49.5% | 43.8% |
| PR renal | 6.3% | 7.1% | 2.7% |
| MR renal | 21.9% | 15.2% | 20.5% |
| No response | 17.2% | 22.2% | 32.9% |
| Renal progression | 0.0% | 6.1% | 0.0% |

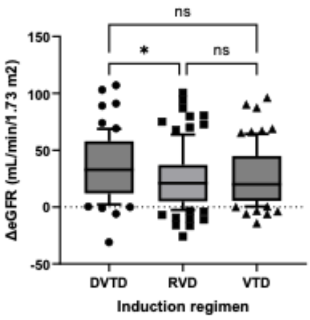

**Figure 1. Differences of eGFR after induction therapy**

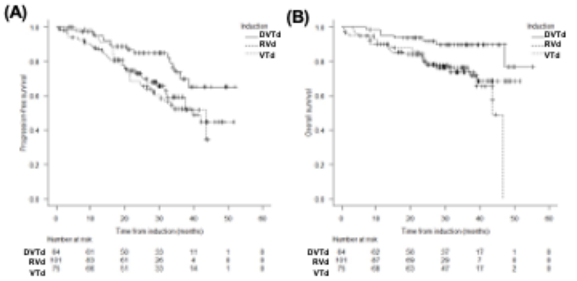

**Figure 2. (A) Progression-free, and (B) overall survival after induction therapy**

**Copyright Information:** (Online) ISSN: 2572-9241
©2026 The Author(s). HemaSphere is published by John Wiley & Sons Ltd on behalf of European Hematology Association. This is an open access Abstract Book distributed under the Attribution-NonCommercial-NoDerivs (CC BY-NC-ND), which allows third parties to download the articles and share them with others as long as they credit the author and the Abstract Book, but they cannot change the content in any way or use them commercially.
**Abstract Book Citations:** Authors, Title, HemaSphere, 2026;10:(S1):pages. The abstract book DOIs can be found on the "Author Information" page.
**Disclaimer:** Articles published in the journal HemaSphere exclusively reflect the opinions of the authors. The authors are responsible for all content in their abstracts including accuracy of the facts, statements, citing resources, etc.

### (PS1930) WHOLE-BODY DIFFUSION-WEIGHTED MRI VERSUS FDG PET/CT IN MULTIPLE MYELOMA: A PRISMA-BASED SYSTEMATIC REVIEW HIGHLIGHTING DISEASE STAGING AND CLINICAL VALUE IN GLOBAL HEMATOLOGY AND LOW-RESOURCE SETTINGS

**Topic:** 14. Myeloma and other monoclonal gammopathies - Clinical

Kalysta Borges*[1], Felipe Costa[2], Giulia Manuella Almeida[3], Bianca Victória Almeida[1], Sandrea Queiroz[4], Cairo Borges Junior[1], Dahra Teles Soares Cruz[5]

*[1]ONCOLÓGICA DO BRASIL, Santarém, Brazil; [2]SANCLIN, Santarém, Brazil; [3]SANTA CASA DE RIBEIRÃO PRETO, RIBEIRÃO PRETO, Brazil; [4]ULBRA, Santarém, Brazil; [5]HOSPITAL SANTA JOANA RECIFE, RECIFE, Brazil;*

### Background

Accurate whole-body imaging is central to the diagnosis, staging, and treatment response assessment in multiple myeloma (MM). FDG PET/CT and whole-body magnetic resonance imaging (WB-MRI), particularly with diffusion-weighted imaging (DWI), are recommended imaging modalities by international guidelines. However, PET/CT is limited by ionizing radiation exposure, false-negative results in low-hexokinase–expressing disease, high costs, and restricted availability in many regions worldwide. WB-MRI with DWI represents a radiation-free alternative with high sensitivity for bone marrow infiltration. This study aimed to systematically compare the diagnostic performance of FDG PET/CT and WB-MRI/DWI in MM, with emphasis on applicability in global hematology settings.

### Aims

To evaluate WB-MRI/DWI versus FDG PET/CT for myeloma staging, highlighting applicability in low-resource and limited-access settings.

### Methods

A systematic review was conducted in accordance with PRISMA guidelines.  MEDLINE, Embase, and the Cochrane Library were searched for studies evaluating FDG PET/CT and whole-body MRI with or without diffusion-weighted imaging (DWI) for multiple myeloma staging, detection of focal bone marrow lesions, and assessment of overall disease burden. Studies providing sufficient data to construct 2×2 contingency tables were included. Methodological quality was assessed using the QUADAS-2 tool. Pooled sensitivity, specificity, diagnostic odds ratio (DOR), and corresponding 95% confidence intervals (CI) were estimated using random-effects models. Pearson correlation coefficients (r), with 95% CI and associated p-values, were calculated to assess agreement between imaging modalities and reference standards, while summary receiver operating characteristic (SROC) curves and area under the curve (AUC) values were generated to evaluate overall diagnostic performance.

### Results

Across included studies, WB-MRI with diffusion-weighted imaging (DWI) demonstrated higher sensitivity for detecting bone marrow involvement compared with FDG PET/CT, particularly in diffuse and early infiltrative disease patterns. Pooled sensitivity for lesion detection ranged from 0.90–0.96 for WB-MRI/DWI versus 0.75–0.85 for FDG PET/CT, while specificity was comparable between modalities (0.88–0.94 vs 0.89–0.95, respectively). Agreement between WB-MRI/DWI and reference standards showed a strong positive correlation (Pearson $r = 0.81$; 95% CI 0.73–0.88; $p < 0.001$), exceeding that observed for FDG PET/CT ($r = 0.67$; 95% CI 0.54–0.77; $p < 0.001$). WB-MRI/DWI also demonstrated superior detection of focal marrow lesions ≤5 mm and higher lesion counts per patient. In treatment-naïve patients, WB-MRI/DWI identified occult marrow disease in up to 20–30% of cases with negative PET/CT findings. No significant publication bias was detected.

**Copyright Information:** (Online) ISSN: 2572-9241
©2026 The Author(s). HemaSphere is published by John Wiley & Sons Ltd on behalf of European Hematology Association. This is an open access Abstract Book distributed under the Attribution-NonCommercial-NoDerivs (CC BY-NC-ND), which allows third parties to download the articles and share them with others as long as they credit the author and the Abstract Book, but they cannot change the content in any way or use them commercially.
**Abstract Book Citations:** Authors, Title, HemaSphere, 2026;10:(S1):pages. The abstract book DOIs can be found on the "Author Information" page.
**Disclaimer:** Articles published in the journal HemaSphere exclusively reflect the opinions of the authors. The authors are responsible for all content in their abstracts including accuracy of the facts, statements, citing resources, etc.

**Summary/Conclusion**

In multiple myeloma, whole-body MRI combined with diffusion-weighted imaging (WB-MRI/DWI) demonstrates non-inferior and frequently superior diagnostic performance compared with FDG PET/CT for whole-body disease assessment, with particular strength in detecting diffuse and early marrow infiltration. As a radiation-free modality with high sensitivity for marrow-based disease, WB-MRI/DWI constitutes a high-value alternative and complementary strategy to PET/CT, supporting repeated longitudinal assessments and offering substantial clinical and implementation advantages for hematology programs in low- and middle-income settings where PET access is limited.

**Copyright Information:** (Online) ISSN: 2572-9241
©2026 The Author(s). HemaSphere is published by John Wiley & Sons Ltd on behalf of European Hematology Association. This is an open access Abstract Book distributed under the Attribution-NonCommercial-NoDerivs (CC BY-NC-ND), which allows third parties to download the articles and share them with others as long as they credit the author and the Abstract Book, but they cannot change the content in any way or use them commercially.
**Abstract Book Citations:** Authors, Title, HemaSphere, 2026;10:(S1):pages. The abstract book DOIs can be found on the “Author Information” page.
**Disclaimer:** Articles published in the journal HemaSphere exclusively reflect the opinions of the authors. The authors are responsible for all content in their abstracts including accuracy of the facts, statements, citing resources, etc.

### (PS1931) EFFICACY AND SAFETY OF IXAZOMIB PLUS REDUCED-DOSE LENALIDOMIDE VERSUS IXAZOMIB MONOTHERAPY AS MAINTENANCE IN HIGH-RISK NEWLY DIAGNOSED MULTIPLE MYELOMA: AN INTERIM ANALYSIS OF A PROSPECTIVE STUDY

**Topic:** 14. Myeloma and other monoclonal gammopathies - Clinical

Xuzhao Zhang*[1], Jingcheng Zhang[2], Qunyi Guo[3], Hui Zeng[4], Gongqiang Wu[5], Wei Jiang[6], Minchao Yan[4], Xia-Yin Zhu[3], Huiqing Qiu[7], Yang Xu[1], Wenbin Qian[1]

*[1]The Second Affiliated Hospital of Zhejiang University School of Medicine, Hangzhou, China;[2]Affiliated Jinhua Hospital, Zhejiang University School of Medicine, Hematology, Jinhua, China; [3]Taizhou Hospital of Zhejiang Province, Hematology, Taizhou, China; [4]The Affiliated Hospital of Jiaxing University, Hematology, Jiaxing, China;[5]Dongyang Hospital Affiliated with Wenzhou Medical University, Hematology, Jinhua, China; [6]Shangyu People's Hospital of Shaoxing, Hematology, Shaoxing, China;[7]Quzhou Second People's Hospital, Hematology and Medical Oncology, Quzhou, China;*

### Background

High-risk (HR) cytogenetic abnormalities confer poor prognosis in multiple myeloma (MM). Maintenance with single-agent proteasome inhibitors or immunomodulatory drugs provides only limited benefit in HR patients. Combining agents with synergistic mechanisms may improve outcomes.

### Aims

To compare the efficacy and safety of ixazomib plus reduced-dose lenalidomide (Ir) versus ixazomib monotherapy (I) as maintenance in transplant-eligible or -ineligible HR newly diagnosed MM (NDMM) patients after VRD/VCD induction/consolidation.

### Methods

This ongoing open-label, multicenter, prospective randomized study (NCT05722405) enrolls NDMM patients with at least one HR cytogenetic abnormality [t(4;14), t(14;16), del(17p)]. All patients receive 6 cycles of VRD/VCD-based induction/consolidation (with ASCT if eligible). Responding patients are randomized 1:1 to 28-day cycles of oral I (4 mg days 1,8,15) or Ir (I 4 mg days 1,8,15 + lenalidomide 10 mg days 1–21). Primary endpoints are progression-free survival (PFS). Here we report an interim analysis of the first 41 patients (data cutoff Feb 10, 2026).

### Results

41 patients were randomized (Ir, n=19; I, n=22). Baseline characteristics were well balanced (Table 1). At a median follow-up of 22 months, the median PFS was 36 months (95% CI 18.2-53.8) in the Ir arm versus 19 months (95% CI 10.7-27.3) in the I arm (log-rank p=0.102). Due to the small sample size not reaching the planned enrollment (target n=50 per arm), the study was underpowered to detect a statistically significant difference. No unexpected safety signals were observed; grade ≥3 adverse events were comparable between groups.

### Summary/Conclusion

In this interim analysis of high-risk NDMM patients, combination maintenance with Ir showed a numerically longer median PFS (36 vs 19 months) compared with I monotherapy, although the difference did not reach statistical significance owing to limited sample size. Updated results with a larger cohort and longer follow-up are required. The study continues as planned.

**Copyright Information:** (Online) ISSN: 2572-9241
©2026 The Author(s). HemaSphere is published by John Wiley & Sons Ltd on behalf of European Hematology Association. This is an open access Abstract Book distributed under the Attribution-NonCommercial-NoDerivs (CC BY-NC-ND), which allows third parties to download the articles and share them with others as long as they credit the author and the Abstract Book, but they cannot change the content in any way or use them commercially.
**Abstract Book Citations:** Authors, Title, HemaSphere, 2026;10:(S1):pages. The abstract book DOIs can be found on the "Author Information" page.
**Disclaimer:** Articles published in the journal HemaSphere exclusively reflect the opinions of the authors. The authors are responsible for all content in their abstracts including accuracy of the facts, statements, citing resources, etc.

### (PS1932) PROGNOSTIC VALUE OF MINIMAL RESIDUAL DISEASE STATUS FOR SURVIVAL OUTCOMES IN TRANSPLANT-ELIGIBLE PATIENTS WITH NEWLY DIAGNOSED MULTIPLE MYELOMA: A META-ANALYSIS

**Topic:** 14. Myeloma and other monoclonal gammopathies - Clinical

Hira Mian*[1], Saad Z Usmani[2], Elias K. Mai[3], C. Ola Landgren[4], Hartmut Goldschmidt[5], Meletios A. Dimopoulos[6], Erin Comerford[7], Chinelo Morah[7], Gaye Siliman[8], Marroon Thabane[8], Kyle Fahrbach[9], Sohan Deshpande[10], Van Nguyen[11], Iwona Pustulka[12], Nizar J Bahlis[13]

*[1]McMaster University, Hamilton, Ontario, Canada;[2]Memorial Sloan Kettering Cancer Center (MSKCC), New York, NY, United States of America; [3]Heidelberg Myeloma Center and German-speaking Myeloma Multicenter Group (GMMG), Department of Internal Medicine V, Heidelberg University Hospital and Medical Faculty Heidelberg, Heidelberg University, Heidelberg, Germany; [4]Sylvester Comprehensive Cancer Center, University of Miami; Don Soffer Clinical Research Center, Miami, Florida, United States of America; [5]Internal Medicine V, Hematology, Oncology and Rheumatology, German-speaking Myeloma Multicenter Group (GMMG) at the Heidelberg University Hospital, Heidelberg, Germany; [6]Plasma Cell Dyscrasias Unit, Section of Hematology and Medical Oncology, Department of Clinical Therapeutics, School of Medicine, National and Kapodistrian University of Athens; Alexandra Hospital, Athens, Greece; [7]Sanofi, Cambridge, MA, United States of America; [8]Sanofi, Toronto, Ontario, Canada; [9]Thermo Fisher Scientific, Waltham, United States of America; [10]Thermo Fisher Scientific, London, United Kingdom; [11]Thermo Fisher Scientific, Paris, France; [12]Thermo Fisher Scientific, Warsaw, Poland; [13]Arthur Child Comprehensive Cancer Center; Arnie Charbonneau Cancer Research Institute; University of Calgary, Calgary, Alberta, Canada;*

### Background

Therapeutic advances in multiple myeloma (MM) have led to deeper responses and prolonged survival. Consequently, the evaluation of traditional survival endpoints in transplant-eligible (TE) newly diagnosed multiple myeloma (NDMM) requires long-term follow-up, potentially delaying access to effective therapies. Minimal residual disease (MRD) has emerged as a promising prognostic biomarker and is recognized by the FDA as an endpoint to support accelerated approvals. As health agencies continue to evaluate MRD's prognostic value, it is critical to update the available evidence with a focus on contemporary TE NDMM studies given the recent approvals of novel aCD38 combinations in the frontline setting.

### Aims

To estimate the association between MRD and survival in patients with TE NDMM, namely progression-free survival (PFS) and overall survival (OS), using systematic literature review (SLR) and meta-analysis.

### Methods

Medline and EMBASE databases were searched for articles published between June 2019 and August 2024, while earlier studies were sourced from Munshi et al. (2020). Grey literature was also searched to identify relevant research. Randomized and non-randomized studies were considered eligible for the SLR if they enrolled patients with TE NDMM and reported PFS and/or OS stratified by MRD status.

A random-effects meta-analysis was conducted to estimate pooled hazard ratios (HRs) and 95% confidence intervals (CIs) comparing PFS and OS between MRD-negative versus MRD-positive patients. The primary analysis included TE NDMM studies with MRD measured 100 days post-transplant, post-consolidation, or at the start of maintenance using an MRD threshold of $10^{-5}$. Fixed-effects models were used as sensitivity analyses, and one study using a $10^{-6}$ threshold was assessed in a scenario analysis.

**Copyright Information:** (Online) ISSN: 2572-9241
©2026 The Author(s). HemaSphere is published by John Wiley & Sons Ltd on behalf of European Hematology Association. This is an open access Abstract Book distributed under the Attribution-NonCommercial-NoDerivs (CC BY-NC-ND), which allows third parties to download the articles and share them with others as long as they credit the author and the Abstract Book, but they cannot change the content in any way or use them commercially.
**Abstract Book Citations:** Authors, Title, HemaSphere, 2026;10:(S1):pages. The abstract book DOIs can be found on the "Author Information" page.
**Disclaimer:** Articles published in the journal HemaSphere exclusively reflect the opinions of the authors. The authors are responsible for all content in their abstracts including accuracy of the facts, statements, citing resources, etc.

**Results**

A total of 1,537 records (*n=1,492* from electronic database and *n=45* from relevant SLRs) were identified, with 48 records representing 47 unique studies: 41 peer-reviewed journal articles, 5 conference abstracts, and 2 letters to the editor. Twelve PFS and six OS studies were included in the primary analysis. Multiparameter flow cytometry (MFC; n=6) and next-generation sequencing (NGS; n=3) were the most common MRD assays. Most studies applied a sensitivity threshold of $10^{-5}$ as recommended in the current IMWG response criteria. MRD-negativity post-transplant, post-consolidation, or at the start of maintenance ranged from 23.0% to 87.5% across studies. In the primary meta-analysis, MRD negativity was associated with significantly improved PFS (HR 0.34; 95% CI 0.25–0.46; p<0.0001) and OS (HR 0.48; 95% CI 0.32–0.72, p=0.0003) in TE patients with NDMM (**Figure**). Across individual studies, HRs for PFS ranged from 0.12 to 0.61 and from 0.26 to 0.71 for OS. Scenario analyses incorporating MRD sensitivity thresholds of $10^{-5}$ and $10^{-6}$ showed a consistent OS benefit for MRD-negative TE patients with NDMM (HR: 0.49; 95% CI: 0.34–0.71, p=0.0001).

**Summary/Conclusion**

Achieving MRD negativity is increasingly recognized as an important goal in NDMM due to its ability to predict long-term outcomes. Using a more recent evidence base, this meta-analysis demonstrated that MRD negativity is associated with improved PFS and OS in TE patients with NDMM. These findings further support the role of MRD as a surrogate endpoint for PFS and OS.

**Copyright Information:** (Online) ISSN: 2572-9241
©2026 The Author(s). HemaSphere is published by John Wiley & Sons Ltd on behalf of European Hematology Association. This is an open access Abstract Book distributed under the Attribution-NonCommercial-NoDerivs (CC BY-NC-ND), which allows third parties to download the articles and share them with others as long as they credit the author and the Abstract Book, but they cannot change the content in any way or use them commercially.
**Abstract Book Citations:** Authors, Title, HemaSphere, 2026;10:(S1):pages. The abstract book DOIs can be found on the "Author Information" page.
**Disclaimer:** Articles published in the journal HemaSphere exclusively reflect the opinions of the authors. The authors are responsible for all content in their abstracts including accuracy of the facts, statements, citing resources, etc.

**Figure. Forest plot association of (A) PFS and (B) OS with MRD negativity — base-case analysis**

**(A)**

| Study | logHR | SE(logHR) | Number Analyzed | HR | 95% CI |
|---|---|---|---|---|---|
| Turner, 2022 | -0.8109 | 0.3684 | 112 | 0.44 | [0.22–0.91] |
| CARDAMON | -0.6419 | 0.2100 | 181 | 0.53 | [0.35–0.79] |
| Tao, 2024 | -1.7699 | 0.3545 | 168 | 0.17 | [0.09–0.34] |
| Martinez-Lopez, 2020 | -1.2641 | 0.3065 | 159 | 0.28 | [0.15–0.52] |
| GMMG-CONCEPT | -2.1371 | 0.4527 | 93 | 0.12 | [0.05–0.29] |
| FORTE | -1.3093 | 0.1972 | 331 | 0.27 | [0.18–0.40] |
| Pasvolsky, 2024 | -0.4943 | 0.0865 | * | 0.61 | [0.51–0.72] |
| Wang, 2024 | -0.9650 | 0.4385 | 104 | 0.38 | [0.16–0.90] |
| TT3b-TT5a | -0.5978 | 0.4666 | 72 | 0.55 | [0.22–1.37] |
| Jasielec, 2020 | -1.0906 | 0.4698 | 76 | 0.34 | [0.13–0.84] |
| Mori, 2024 | -1.7487 | 0.4925 | 71 | 0.17 | [0.07–0.46] |
| GRIFFIN-D-Rvd | -1.7661 | 0.7815 | 207 | 0.17 | [0.04–0.79] |
| GRIFFIN-RVd | -0.2433 | 0.5689 | 207 | 0.78 | [0.26–2.39] |
| **Fixed effects model** | | | | **0.46** | **[0.41–0.53]** |
| **Random effects model** | | | | **0.34** | **[0.25–0.46]** |

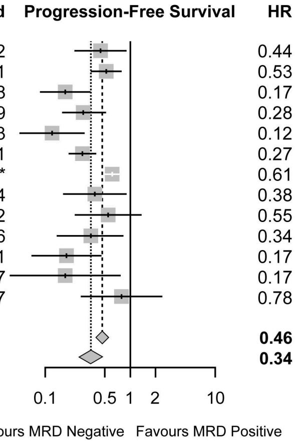

Hetrogeneity: $I^2 = 73.3\%$, $\tau^2 = 0.1745$, $p < 0.0001$
Test for overall effect (fixed effects): $p < 0.0001$
Test for overall effect (random effects): $p < 0.0001$
PFS, Basecase

**(B)**

| Study | logHR | SE(logHR) | Number Analyzed | HR | 95% CI |
|---|---|---|---|---|---|
| Tao, 2024 | -0.3429 | 0.6770 | 168 | 0.71 | [0.19–2.68] |
| FORTE | -1.1712 | 0.2948 | 361 | 0.31 | [0.17–0.55] |
| Pasvolsky, 2024 | -0.4943 | 0.1443 | * | 0.61 | [0.46–0.81] |
| Wang, 2024 | -1.4106 | 0.7663 | 104 | 0.24 | [0.05–1.10] |
| TT3b-TT5a | -0.1744 | 0.5294 | 72 | 0.84 | [0.30–2.37] |
| Jasielec, 2020 | -1.3394 | 0.6973 | 76 | 0.26 | [0.07–1.03] |
| **Fixed effects model** | | | | **0.53** | **[0.42–0.67]** |
| **Random effects model** | | | | **0.48** | **[0.32–0.72]** |

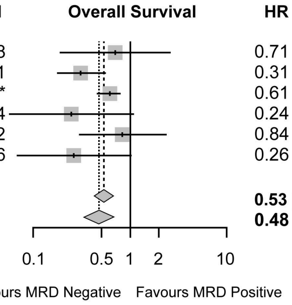

Hetrogeneity: $I^2 = 31.0\%$, $\tau^2 = 0.0816$, $p = 0.2030$
Test for overall effect (fixed effects): $p < 0.0001$
Test for overall effect (random effects): $p = 0.0003$
OS, Basecase

* Indicates that the study did not report the number of patients analyzed.
CI, confidence interval; D-RVd, daratumumab in combination with lenalidomide, bortezomib, dexamethasone; HR, hazard ratio; MRD, Minimal residual disease; OS, overall survival; PFS, progression-free survival; RVd, lenalidomide, bortezomib, dexamethasone; SE, standard error; logHR, logarithm of hazard ratio; $I^2$, heterogeneity statistic (percentage of variation across studies).

**Copyright Information:** (Online) ISSN: 2572-9241
©2026 The Author(s). HemaSphere is published by John Wiley & Sons Ltd on behalf of European Hematology Association. This is an open access Abstract Book distributed under the Attribution-NonCommercial-NoDerivs (CC BY-NC-ND), which allows third parties to download the articles and share them with others as long as they credit the author and the Abstract Book, but they cannot change the content in any way or use them commercially.
**Abstract Book Citations:** Authors, Title, HemaSphere, 2026;10:(S1):pages. The abstract book DOIs can be found on the "Author Information" page.
**Disclaimer:** Articles published in the journal HemaSphere exclusively reflect the opinions of the authors. The authors are responsible for all content in their abstracts including accuracy of the facts, statements, citing resources, etc.

### (PS1933) OVERALL SURVIVAL OF ANTI-BCMA THERAPIES: INDIRECT COMPARISON OF BELANTAMAB MAFODOTIN/BORTEZOMIB/DEXAMETHASONE (BVD) VS TECLISTAMAB/DARATUMUMAB (TEC-DARA) IN RELAPSED/REFRACTORY MULTIPLE MYELOMA (RRMM)

**Topic:** 14. Myeloma and other monoclonal gammopathies - Clinical

Joshua Richter*[1], Venediktos Kapetanakis[2], Nicholas Meader[3], Santosh Kumar[2], James Truscott[2], Balazs Dobi[4], Molly Purser[5], Omar Ladhani[5], Moshe Y. Levy[6], Douglas Sborov[7], Rahul Banerjee[8], Nick Ballew[5]

*[1]Mount Sinai, New York, NY, United States of America; [2]Evidera, a business unit of PPD, part of Thermo Fisher Scientific, London, United Kingdom; [3]GSK, London, United Kingdom; [4]Evidera, a business unit of PPD, part of Thermo Fisher Scientific, Budapest, Hungary; [5]GSK, Upper Providence, PA, United States of America; [6]Baylor University Medical Center, Department of Hematology and Medical Oncology, Dallas, TX, United States of America; [7]Huntsman Cancer Institute, University of Utah School of Medicine, Salt Lake City, UT, United States of America; [8]Fred Hutchinson Cancer Center, Seattle, WA, United States of America;*

### Background

Targeting B-cell maturation antigen (BCMA) has transformed outcomes in RRMM, with novel mechanisms of action (MoA) including antibody-drug conjugates (ADCs) and bispecifics (BsAb). Both BsAb combinations and ADC combinations have demonstrated overall survival (OS) improvements against conventional anti-CD38-based treatments in randomized trials; however, infection risks and treatment access can vary significantly between the two. Given a lack of head-to-head data, we performed an indirect treatment comparison (ITC) to assess OS and infections across matched patients (pts) from the phase 3 DREAMM-7 (BVd vs daratumumab [dara]-bortezomib-dexamethasone [DVd]) and MajesTEC-3 (tec-dara vs DVd or dara-pomalidomide-dexamethasone [DPd]) trials.

### Aims

Compare OS and infections between BCMA-targeting ADC- and BsAb-based regimens by evaluating outcomes in matched pts from DREAMM-7 (BVd) and MajesTEC-3 (tec-dara) in pts with RRMM using an ITC.

### Methods

An unanchored matching-adjusted indirect comparison (MAIC) of BVd vs tec-dara was performed, as DREAMM-7 and MajesTEC-3 lacked a common comparator arm due to DVd + DPd being analyzed collectively in MajesTEC-3. Outcomes were evaluated using data from MajesTEC-3 pts and the subgroup from DREAMM-7 meeting MajesTEC-3 eligibility (1–3 prior lines of therapy [LOT] including a proteasome inhibitor [PI] and lenalidomide [len], and len refractory if only 1 prior LOT). Individual pt data (IPD) were available for BVd-treated pts (n=79); only published summary data were available for tec-dara-treated pts (n=291), with reconstructed IPD (RIPD) derived from published Kaplan-Meier (KM) curves. BVd-treated pts were weighted using IPD to match tec-dara-treated pts in terms of the following baseline characteristics: refractoriness to len, cytogenetic risk, number of prior LOT, age group, prior PI exposure, prior dara exposure, and extramedullary disease. Outcomes for weighted BVd-treated pts were compared to those for tec-dara-treated pts. Primary objective was comparison of OS (measured as restricted mean survival time [RMST]) at Years 1/2/3 (estimated using weighted KM estimator applied to IPD and incorporating MAIC weights for BVd, and using RIPD for tec-dara). Secondary objective was comparison of infection rates (using logistic regression).

**Copyright Information:** (Online) ISSN: 2572-9241
©2026 The Author(s). HemaSphere is published by John Wiley & Sons Ltd on behalf of European Hematology Association. This is an open access Abstract Book distributed under the Attribution-NonCommercial-NoDerivs (CC BY-NC-ND), which allows third parties to download the articles and share them with others as long as they credit the author and the Abstract Book, but they cannot change the content in any way or use them commercially.
**Abstract Book Citations:** Authors, Title, HemaSphere, 2026;10:(S1):pages. The abstract book DOIs can be found on the “Author Information” page.
**Disclaimer:** Articles published in the journal HemaSphere exclusively reflect the opinions of the authors. The authors are responsible for all content in their abstracts including accuracy of the facts, statements, citing resources, etc.

### Results

After matching, equal proportions in both arms were ≥75 years (10. 7%), with median age of 64 years; had 2–3 prior LOT (62.8%); were refractory to len (82.5%); had prior PI (99.7%); had prior dara (5.2%); had high cytogenetic risk (45.2%); and had extramedullary disease (4.8%). Effective sample size of weighted BVd-treated pts was 47. OS RMST at 1, 2, and 3 years was generally comparable between treatments, with no statistical difference in the RMST ratio at any timepoint (RMST ratio 1.04 at years 1 and 2, and 1.01 at year 3) (**Table**). Odds of infection were substantially lower with BVd than tec-dara (odds ratio 0.24 [95% confidence interval 0.10, 0.61]) (**Table**).

### Summary/Conclusion

BVd demonstrated similar OS and lower odds of infection vs tec-dara when adjusting for differences in key baseline pt characteristics, within the assumptions/limitations of unanchored MAICs. These results highlight the transformative benefit of targeting BCMA while offering clinically meaningful insights to inform benefit-risk considerations and broaden access to BCMA-directed therapies, particularly in healthcare settings and pt populations where intensive infection management/hospitalization are impractical.
**Funding:** GSK

**Table. OS and infections**

| | BVd (ESS=47) | Tec-dara (N=291) |
|---|---|---|
| **OS** | | |
| RMST (months) at 1 year (SE) | 11.6 (0.25) | 11.2 (0.15) |
| RMST ratio (95% CI) | 1.04 (0.99, 1.09); p-value 0.141 | |
| RMST (months) at 2 years (SE) | 22.5 (0.67) | 21.7 (0.37) |
| RMST ratio (95% CI) | 1.04 (0.97, 1.11); p-value 0.277 | |
| RMST (months) at 3 years (SE) | 32.2 (1.3) | 31.9 (0.60) |
| RMST ratio (95% CI) | 1.01 (0.93, 1.10); p-value 0.801 | |
| **Infections, %** | 86.8 | 96.5 |
| Infections, odds ratio (95% CI) | 0.24 (0.10, 0.61); p-value 0.0026 | |

CI, confidence interval; ESS, effective sample size; SE, standard error.

**Copyright Information:** (Online) ISSN: 2572-9241
©2026 The Author(s). HemaSphere is published by John Wiley & Sons Ltd on behalf of European Hematology Association. This is an open access Abstract Book distributed under the Attribution-NonCommercial-NoDerivs (CC BY-NC-ND), which allows third parties to download the articles and share them with others as long as they credit the author and the Abstract Book, but they cannot change the content in any way or use them commercially.
**Abstract Book Citations:** Authors, Title, HemaSphere, 2026;10:(S1):pages. The abstract book DOIs can be found on the "Author Information" page.
**Disclaimer:** Articles published in the journal HemaSphere exclusively reflect the opinions of the authors. The authors are responsible for all content in their abstracts including accuracy of the facts, statements, citing resources, etc.

### (PS1934) PROGRESSION OF DISEASE WITHIN 24 MONTHS (POD24) IS AN INDEPENDENT PROGNOSTIC FACTOR FOR SURVIVAL IN NEWLY DIAGNOSED PATIENTS WITH WALDENSTRÖM MACROGLOBULINEMIA TREATED WITH DRC

**Topic:** 14. Myeloma and other monoclonal gammopathies - Clinical

Maria Gavriatopoulou*[1], Ioannis Ntanasis-Stathopoulos[1], Charalampos Filippatos[1], Panagiotis Malandrakis[1], Ilias Katsadouros[1], Panagiotis Boutsikos[1], Magdalini Migkou[1], Nikolaos Kanellias[1], Evangelos Eleutherakis-Papaiakovou[1], Foteini Theodorakakou[1], Eirini Solia[1], Evangelos Terpos[1], Efstathios Kastritis[1], Meletios A. Dimopoulos[1]

*[1]National and Kapodistrian University of Athens, Department of Clinical Therapeutics, Athens, Greece;*

### Background

The prognostic significance of progression of disease within 24 months (POD24) is well-established in many lymphomas, but data in Waldenström Macroglobulinemia (WM) remain limited and affected by therapeutic decisions in frontline setting.

### Aims

In this study we evaluated the impact of POD24 on the survival of patients treated with frontline dexamethasone-rituximab-cyclophosphamide (DRC) in a tertiary academic center in Greece.

### Methods

This retrospective study enrolled consecutive, unselected newly diagnosed WM (NDWM) patients who received frontline treatment with DRC. Patients treated primarily for WM related neuropathy were excluded from the analysis. Individuals without a progression event and/or a follow-up period of less than 24 months were also excluded from the analysis. Follow-up was calculated from the 24-month mark for the POD24 and non-POD24 cohorts. Moreover, POD24 was utilized as a time-dependent covariate in order to evaluate its prognostic impact in subsequent OS. In this context, survival endpoints were calculated from the time of progression for the POD24 subgroup and from the 24-month timepoint for the non-POD24 subgroup.

### Results

A total of 206 NDWM patients were enrolled; median age was 70 years (range 35-86) and 123 (59.7%) were male. After a median follow-up of 7.6 years, 5-year PFS and OS was 49.0% and 80.3%, respectively. Among patients with available MYD88 mutational status (30.0%), $MYD^{L265P}$ was associated with worse PFS compared to $MYD^{WT}$ (5-year PFS 60.0% vs 45.8%, respectively), but results were not statistically significant (HR=1.81, 95%CI: 0.64-5.14, p=0.264). CXCR4 status was available in a small subset of patients (23.2%) and $CXCR4^{mutated}$ was observed only in 9 patients, thus no reliable analysis could be performed. After treatment with DRC 58 patients (28.2%) achieved minor response, 141 (68.5%) partial response and 7 (3.4%) complete remission.
POD24 occurred in 47 patients (22.7%); median follow-up was 5.2 years for POD24 patients and 5.7 years for the non-POD24 subgroup. POD24 was associated with inferior subsequent OS (5-year OS 62.8% vs 82.1% in non-POD24, p=0.001), despite the follow-up starting at the 24-months timepoint for non-POD24 individuals. Specifically, POD24 patients demonstrated a two-fold increase in the risk of death (HR=2.14, 95%CI: 1.33-3.45, p=0.018) compared to non-POD24. This increased risk of death among POD24 patients was further observed when adjusting for R-IPSSWM (aHR=1.65, 95%CI: 1.38-2.14, p=0.044).
In a sensitivity analysis accounting for non-WM related death as a competing event, POD24 remained a prognostic factor for survival (SHR=2.30, 95%CI: 1.17-4.52, p=0.016); the cumulative incidence of WM-related death at 5 years between POD24 and non-POD24, was 24.0% and 7.1%, respectively. Additionally, POD24 maintained its prognostic significance after adjusting for patient scores per R-IPSSWM (aSHR= 1.88, 95%CI: 1.01-3.75, p=0.048).

**Copyright Information:** (Online) ISSN: 2572-9241
©2026 The Author(s). HemaSphere is published by John Wiley & Sons Ltd on behalf of European Hematology Association. This is an open access Abstract Book distributed under the Attribution-NonCommercial-NoDerivs (CC BY-NC-ND), which allows third parties to download the articles and share them with others as long as they credit the author and the Abstract Book, but they cannot change the content in any way or use them commercially.
**Abstract Book Citations:** Authors, Title, HemaSphere, 2026;10:(S1):pages. The abstract book DOIs can be found on the “Author Information” page.
**Disclaimer:** Articles published in the journal HemaSphere exclusively reflect the opinions of the authors. The authors are responsible for all content in their abstracts including accuracy of the facts, statements, citing resources, etc.

### Summary/Conclusion

POD24 seems to be an independent predictor of inferior survival in patients with newly diagnosed Waldenström Macroglobulinemia treated with frontline DRC in a real-world cohort with prolonged follow up. Even when accounting for immortal time bias through landmark analysis and adjusting for baseline risk via the R-IPSSWM and competing risks, patients experiencing POD24 demonstrated significantly higher risk of mortality. These findings suggest that the POD24 subgroup represents potentially a high-risk phenotype that may require more intensified and tailored management.

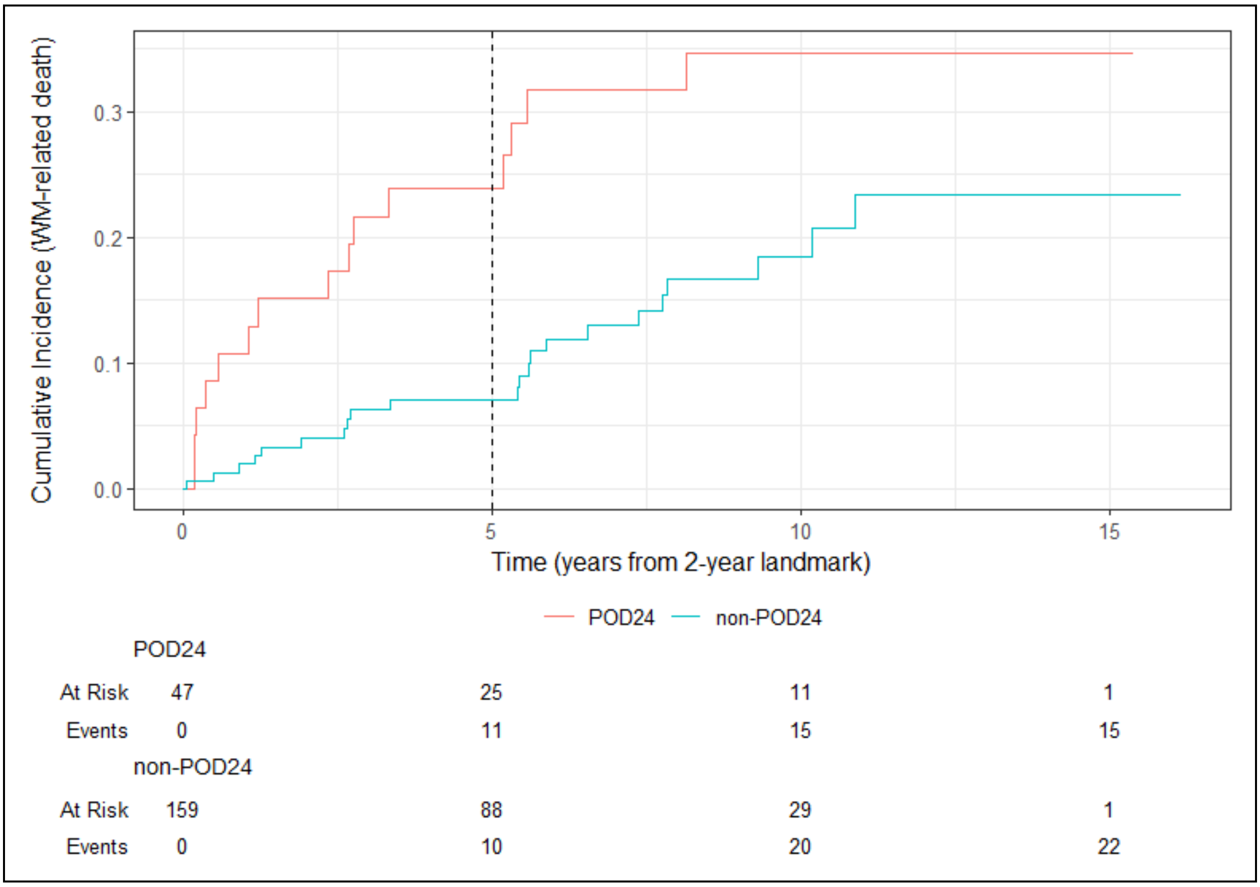

**Copyright Information:** (Online) ISSN: 2572-9241
©2026 The Author(s). HemaSphere is published by John Wiley & Sons Ltd on behalf of European Hematology Association. This is an open access Abstract Book distributed under the Attribution-NonCommercial-NoDerivs (CC BY-NC-ND), which allows third parties to download the articles and share them with others as long as they credit the author and the Abstract Book, but they cannot change the content in any way or use them commercially.
**Abstract Book Citations:** Authors, Title, HemaSphere, 2026;10:(S1):pages. The abstract book DOIs can be found on the "Author Information" page.
**Disclaimer:** Articles published in the journal HemaSphere exclusively reflect the opinions of the authors. The authors are responsible for all content in their abstracts including accuracy of the facts, statements, citing resources, etc.

### (PS1935) DUFFY NULL GENOTYPE MODIFIES TREATMENT OUTCOMES IN NEWLY DIAGNOSED MULTIPLE MYELOMA (NDMM): EVIDENCE FROM THE COMMPASS STUDY

**Topic:** 14. Myeloma and other monoclonal gammopathies - Clinical

Chaitanya Acharya*[1], Lauren Merz[2], Steven Foltz[1], Rebecca Zon[3], Matthew J. Pianko[2], Jeffrey A. Zonder[4], Craig Cole[4], Hearn Cho[1], Paul Richardson[3], John Carpten[5], George Mulligan[1]

*[1]Multiple Myeloma Research Foundation, Translational Research, Norwalk, United States of America;[2]University of Michigan, Division of Hematology/Oncology, Ann Arbor, United States of America; [3]Dana-Farber Cancer Institute, Harvard Medical School, Medical Oncology, Boston, United States of America; [4]Barbara Ann Karmanos Cancer Institute, School of Medicine, Detroit, United States of America; [5]Beckman Research Institute, Department of Integrative Translational Sciences, City of Hope, United States of America;*

### Background

The ACKR1 (atypical chemokine receptor 1) promoter variant rs2814778 (c.-67T>C) disrupts erythroid Duffy antigen expression, resulting in the Duffy null phenotype. This genotype is common in individuals of African or Middle Eastern descent (~66% of those self-identifying as these groups in the United States) and is associated with 40–50% lower baseline absolute neutrophil counts (ANC) without increased infection risk. While the presence of this genotype has also been linked to altered leukocyte trafficking and inflammatory pathways, its clinical relevance in myeloma and interaction with standard-of-care frontline treatment strategies is an area of active research.

### Aims

To determine whether Duffy C/C genotype independently predicts progression-free survival (PFS) and whether it modifies benefit from frontline treatment strategies.

### Methods

NDMM patients from the CoMMpass study with rs2814778 genotype data were classified as Duffy C/C (null; N=99) or non-C/C (N=825). PFS was assessed using Kaplan-Meier survival curves and log-rank tests. Multivariable Cox proportional hazards models adjusted for age, self-reported race, IMS-IMWG 2025 genomic risk score, treatment received, ANC, and beta-2 microglobulin. A prespecified genotype-by-treatment interaction term was evaluated using likelihood ratio testing. The following treatments were assessed: transplant (ASCT) alone, RVd alone, RVd+ASCT, and neither RVd nor ASCT (reference group).

### Results

Among evaluable patients (n=824), 10.4% (86/824) were Duffy null; 72% (85/118) of African American patients were Duffy null. Duffy null patients had lower ANC (median = 3.2 X $10^9$/L) compared with non-null (median = 3.6 X $10^9$/L) patients (p<0.001). Median follow-up was 883 days (69 events) for Duffy null and 1,290 days (532 events) for non-null patients. Kaplan-Meier analysis demonstrated inferior PFS among Duffy null patients (HR 1.33, 95% CI 1.04–1.71, p=0.025). In a multivariable analysis, Duffy null status remained independently associated with inferior PFS (HR 1.43, 95% CI 1.07–1.92, p=0.02). However, adjustment for self-reported race attenuated this association (HR 0.80), suggesting partial confounding by population structure. A significant genotype-by-treatment interaction was observed (p=0.049) in multivariable analysis which accounted for self-reported race. While no significant interaction was observed among Duffy null patients treated with RVd alone or ASCT alone, an ASCT-based triplet strategy significantly reduced risk of inferior PFS among Duffy null patients (HR 0.29, 95% CI 0.15–0.56, p<0.001).

**Copyright Information:** (Online) ISSN: 2572-9241
©2026 The Author(s). HemaSphere is published by John Wiley & Sons Ltd on behalf of European Hematology Association. This is an open access Abstract Book distributed under the Attribution-NonCommercial-NoDerivs (CC BY-NC-ND), which allows third parties to download the articles and share them with others as long as they credit the author and the Abstract Book, but they cannot change the content in any way or use them commercially.
**Abstract Book Citations:** Authors, Title, HemaSphere, 2026;10:(S1):pages. The abstract book DOIs can be found on the "Author Information" page.
**Disclaimer:** Articles published in the journal HemaSphere exclusively reflect the opinions of the authors. The authors are responsible for all content in their abstracts including accuracy of the facts, statements, citing resources, etc.

**Summary/Conclusion**

In this exploratory CoMMpass analysis, the Duffy rs2814778 C/C (null) genotype was associated with inferior PFS and modified frontline treatment benefit. Although the association attenuated after adjustment for self-reported race, findings suggest ASCT-based triplet therapy may mitigate adverse outcomes among Duffy null patients. This contrasts with the sub-analysis of the randomized phase 3 DETERMINATION trial. Differences may reflect clinical trial versus real-world settings, where access, socioeconomic factors, and patient preferences exert greater influence. While interpretation is limited by collinearity between Duffy genotype and self-reported race, these results identify Duffy status as a potentially actionable host genetic determinant of treatment response and support prospective validation in MM, particularly in the era of quadruplet induction therapy.

**Copyright Information:** (Online) ISSN: 2572-9241
©2026 The Author(s). HemaSphere is published by John Wiley & Sons Ltd on behalf of European Hematology Association. This is an open access Abstract Book distributed under the Attribution-NonCommercial-NoDerivs (CC BY-NC-ND), which allows third parties to download the articles and share them with others as long as they credit the author and the Abstract Book, but they cannot change the content in any way or use them commercially.
**Abstract Book Citations:** Authors, Title, HemaSphere, 2026;10:(S1):pages. The abstract book DOIs can be found on the “Author Information” page.
**Disclaimer:** Articles published in the journal HemaSphere exclusively reflect the opinions of the authors. The authors are responsible for all content in their abstracts including accuracy of the facts, statements, citing resources, etc.

### (PS1936) HYPERDIPLOID MULTIPLE MYELOMA: GENETIC HETEROGENEITY

**Topic:** 14. Myeloma and other monoclonal gammopathies - Clinical

Anikó Ujfalusi*[1], Anna Szilágyi[1], Beata Bessenyei[1], Katalin Koczok[1], István Balogh[1]

[1] *University of Debrecen, Faculty of Medicine, Department of Medical Genetics, Debrecen, Hungary;*

### Background

Based on the primary chromosomal abnormalities involved in the pathogenesis of multiple myeloma (MM), two major cytogenetic subgroups can be distinguished: hyperdiploid (HMM) and non-hyperdiploid (NHMM) myeloma. HMM is characterized by trisomies of ≥2 chromosomes, predominantly odd-numbered chromosomes (3, 5, 7, 9, 11, 15, 19, and 21). HMM is a common subtype of MM (approximately 50%) but is frequently under-recognized. It represents a distinct biological and clinical entity generally associated with a favorable prognosis; however, it remains genetically heterogeneous. It is still unclear whether hyperdiploidy can mitigate the adverse prognostic impact of coexisting high-risk cytogenetic abnormalities.

### Aims

Our aim was to evaluate the effectiveness of the 5p15/9q22/15q22 FISH probe-mix in identifying true HMM cases. In addition, we retrospectively analyzed the associated cytogenetic abnormalities in HMM patients and correlated these findings with other clinical data.

### Methods

Between 2021 and 2025, bone marrow samples obtained at diagnosis from 353 MM patients underwent CD138+ plasma cell enrichment using the EasySep™ Human CD138 Positive Selection Kit (Miltenyi Biotec). Fluorescence in situ hybridization (FISH) was performed according to the manufacturer's instructions using the following probe panel: 1p32/1q21, TP53/17cen, t(11;14), 5p15/9q22/15q22, t(14;16), and t(4;14) (MetaSystems). Two hundred interphase nuclei were analyzed per probe.

### Results

Positive FISH findings were detected in 283/353 cases (80%), revealing one or more genetic abnormalities. Based on the detected alterations, 140/283 cases (49%) were classified as NHMM and 143/283 (51%) as HMM. In HMM cases, aneuploidy of chromosomes 5, 9, and 15 demonstrated marked heterogeneity. The most commonly observed aneuploidy affected chromosome 9 (67%; 96/143), followed by chromosome 15 (57%; 81/143) and chromosome 5 (43%; 62/143). In 35 cases (24%), trisomy of all three chromosomes was detected. An additional copy of the *CCND*1 locus (corresponding to trisomy 11) was identified in 74/143 (52%) cases, further supporting hyperdiploid status. The most frequent associated abnormalities in HMM patients were +1q (43%; 62/143), +17 (21%; 30/143), del(17p) (10%; 15/143), and del(1p) (10%; 15/143). *IGH* translocations were identified concomitantly in 14% (20/143) of HMM cases; among these, t(11;14) was the most common (n = 5).

### Summary/Conclusion

The applied 5p15/9q22/15q22 FISH probe-mix reliably identified the HMM subgroup, detecting aneuploidy in approximately 50% of analyzed cases. The most frequently identified supernumerary chromosome was chromosome 9, followed by chromosome 15. High-risk cytogenetic abnormalities, including dup/amp(1q), del(1p), and del(17p), were frequently observed in association with hyperdiploidy. In a minority of cases, *IGH* gene translocations co-occurred with hyperdiploidy. Further analysis is warranted to clarify the prognostic interaction between hyperdiploidy and high-risk cytogenetic alterations.

**Copyright Information:** (Online) ISSN: 2572-9241
©2026 The Author(s). HemaSphere is published by John Wiley & Sons Ltd on behalf of European Hematology Association. This is an open access Abstract Book distributed under the Attribution-NonCommercial-NoDerivs (CC BY-NC-ND), which allows third parties to download the articles and share them with others as long as they credit the author and the Abstract Book, but they cannot change the content in any way or use them commercially.
**Abstract Book Citations:** Authors, Title, HemaSphere, 2026;10:(S1):pages. The abstract book DOIs can be found on the "Author Information" page.
**Disclaimer:** Articles published in the journal HemaSphere exclusively reflect the opinions of the authors. The authors are responsible for all content in their abstracts including accuracy of the facts, statements, citing resources, etc.

### (PS1937) GAPS IN ACCESS TO CHIMERIC ANTIGEN RECEPTOR T-CELL THERAPY POST LEUKAPHERESIS: WAITING TIME AND TREATMENT PATTERNS IN RELAPSED/REFRACTORY MULTIPLE MYELOMA: REAL-WORLD EVIDENCE FROM US CLAIMS

**Topic:** 14. Myeloma and other monoclonal gammopathies - Clinical

Sikander Ailawadhi[1], Natalie Boytsov[2], Jasjit Multani[3], Zifan Zhou[3], Nicole Niehoff[4], Molly Purser[2], John Hartman*[2], Teresa Palumbo[5], Chi-Chang Chen[6]

*[1]Division of Hematology, Mayo Clinic, Jacksonville, FL, United States of America;[2]RWE & HO Research, GSK, Upper Providence, PA, United States of America; [3]Health Economics and Outcomes Research, IQVIA, Falls Church, VA, United States of America; [4]RWE & HO Research, GSK, Durham, NC, United States of America;[5]Medical Affairs, GSK, New York, NY, United States of America; [6]Health Economics and Outcomes Research, IQVIA, Wayne, PA, United States of America;*

### Background

Targeting B-cell maturation antigen (BCMA) has transformed outcomes in relapsed/refractory multiple myeloma (RRMM) with three different commercially available mechanisms of action (MoA): chimeric antigen receptor T-cells (CAR-T), bispecific antibodies and antibody-drug conjugates. While each MoA and molecule has a different profile, the overall survival benefit over important standards of care has been consistently demonstrated. The suitability of BCMA-targeting therapies varies per patient, with CAR-T suitability limited by delays/manufacturing failure and changes in pt condition, financial situation, or access to specialized treatment centers.

### Aims

We characterized pts with RRMM who underwent leukapheresis to prepare for CAR-T and pt treatment waiting time, to understand the delays incurred prior to CAR-T infusion.

### Methods

A real-world study in the US using the PharMetrics Plus claims database (July 2020–September 2024) was performed. Eligible pts were aged ≥18 years with RRMM, had their first leukapheresis claim (index date) during the index period (March 2021–August 2024), were continuously enrolled in medical/pharmacy benefits for ≥180 days prior to and ≥30 days after index date, and were not clinical trial participants. Pt characteristics, rate of CAR-T receipt, and time to CAR-T receipt were assessed during the variable follow-up.

**Copyright Information:** (Online) ISSN: 2572-9241
©2026 The Author(s). HemaSphere is published by John Wiley & Sons Ltd on behalf of European Hematology Association. This is an open access Abstract Book distributed under the Attribution-NonCommercial-NoDerivs (CC BY-NC-ND), which allows third parties to download the articles and share them with others as long as they credit the author and the Abstract Book, but they cannot change the content in any way or use them commercially.
**Abstract Book Citations:** Authors, Title, HemaSphere, 2026;10:(S1):pages. The abstract book DOIs can be found on the "Author Information" page.
**Disclaimer:** Articles published in the journal HemaSphere exclusively reflect the opinions of the authors. The authors are responsible for all content in their abstracts including accuracy of the facts, statements, citing resources, etc.

**Results**

Overall, 337 pts who received leukapheresis were identified. Median (interquartile range) age was 62 (55–67) years, and 59.1% of pts were male. Mean National Cancer Institute (NCI) comorbidity index was 0.52. Most pts received care in the Midwest (31.8%) or Northeast (24.6%) regions. Most pts (95.9%) received leukapheresis in an outpatient setting. Median available follow-up time was 255 days (mean 337 days). During follow-up, 183 pts (54.3%) did not receive CAR-T after leukapheresis. Among pts who had ≥12 months of follow-up (n=143), the proportion of pts who did not receive CAR-T was similar at 53.1% (n=76). Among pts who received CAR-T during the follow-up (n=154; 45.7%), the median waiting time was 54.0 days (mean 56.7 days). The cohort of pts who did not receive CAR-T had a higher proportion of females (48.6%/31.8%), were more likely to have Medicaid (29.5%/7.8%), had higher NCI comorbidity index (scores 0.55/0.47), and most commonly received care in the Northeast (35.5%/11.7%) while pts who received CAR-T most commonly received care in the Midwest (23.0%/42.2%).
The most common first classes of multiple myeloma medications after leukapheresis but prior to CAR-T infusion in pts who did/did not receive CAR-T were an alkylating agent (65.6%/36.1%), proteasome inhibitor (40.3%/30.6%), steroid (38.3%/39.3%), immunomodulatory drug (22.7%/27.3%), anti-CD38 (20.8%/20.8%), and stem cell transplant (0%/17.5%).

**Summary/Conclusion**

Over half of pts receiving leukapheresis did not receive CAR-T infusion, highlighting a persistent gap between eligibility and treatment delivery. These data emphasize an urgent need for broadly accessible BCMA-targeted therapies that provide deep and durable responses.

**Funding:** GSK

This abstract was previously submitted to the American Society of Clinical Oncology (ASCO) Annual Meeting, June 11–14, 2026, and is submitted on behalf of the original authors with their permission. ©2026 American Society of Clinical Oncology, Inc. Reused with permission. All rights reserved.

**Copyright Information:** (Online) ISSN: 2572-9241
©2026 The Author(s). HemaSphere is published by John Wiley & Sons Ltd on behalf of European Hematology Association. This is an open access Abstract Book distributed under the Attribution-NonCommercial-NoDerivs (CC BY-NC-ND), which allows third parties to download the articles and share them with others as long as they credit the author and the Abstract Book, but they cannot change the content in any way or use them commercially.
**Abstract Book Citations:** Authors, Title, HemaSphere, 2026;10:(S1):pages. The abstract book DOIs can be found on the “Author Information” page.
**Disclaimer:** Articles published in the journal HemaSphere exclusively reflect the opinions of the authors. The authors are responsible for all content in their abstracts including accuracy of the facts, statements, citing resources, etc.

### (PS1938) DEVELOPMENT AND PRELIMINARY CONTENT VALIDATION OF THE PROSIM-Q: A PATIENT-REPORTED OCULAR SYMPTOM AND IMPACT QUESTIONNAIRE FOR ONCOLOGY TRIALS

**Topic:** 14. Myeloma and other monoclonal gammopathies - Clinical

Farrah Pompilus*[1], Noam Livnat[2], Jean Jacobs Bothwell[3], Ana Popielnicki[1], Christos Nikolaidis[4], Elisabet E. Manasanch[3], Peter Barth[1], Tom Ling[5], Omar Ladhani[3], Julia R. Correll[6], Mona Martin[6]

*[1]GSK, Boston, MA, United States of America; [2]GSK, Tel-Aviv, Israel; [3]GSK, Collegeville, PA, United States of America; [4]GSK, Baar, Switzerland; [5]GSK, Stevenage, United Kingdom; [6]PPD Evidera Patient-Centered Research Thermo Fisher Scientific, Waltham, MA, United States of America;*

### Background

Many antimyeloma therapies may cause ocular symptoms ranging from mild dryness to serious ocular adverse events (AEs). These ocular symptoms/AEs may impact patients' ability to carry out their daily activities and are important safety considerations as patients receive treatment. Patient-reported outcome (PRO) questionnaires provide the patient perspective on symptom experience and functional impact.

### Aims

Develop the Patient-Reported Ocular Symptom and Impact Monitoring-Questionnaire (PROSIM-Q) to assess treatment-related ocular AEs from the patient's perspective for use in oncology trials.

### Methods

Development followed best-practice mixed-methods PRO methodology. Evidence sources included a targeted literature review, qualitative patient interviews, and clinician interviews. Qualitative interviews were conducted with trial participants (n=153) in DREAMM-1, DREAMM-2, DREAMM-4, and DREAMM-5 receiving belantamab mafodotin as monotherapy or in combination with pembrolizumab, pomalidomide, lenalidomide, daratumumab, or other antimyeloma agents. Concepts were mapped by frequency, severity, and level of bother. Draft items were generated using evidence from literature and patient interviews. Rationale for the final list of items was confirmed through consultations with 4 multidisciplinary clinicians.

### Results

Concepts were identified in the literature and confirmed by patients. Core symptom domains included visual effects (eg, blurred vision, double vision, seeing halos) and physical sensations (eg, foreign-body sensation, dryness, tearing, irritation), while core impact domains included reading, driving, mobility, work, emotional well-being, and self-care concepts. Interviews with clinicians led to the initial development of the draft PROSIM-Q items, covering 9 symptoms with a 24-hour recall, 5 impacts with a 7-day recall, and 5-point Likert scales designed to capture ocular AEs associated with antimyeloma agents from the patient perspective.

**Copyright Information:** (Online) ISSN: 2572-9241
©2026 The Author(s). HemaSphere is published by John Wiley & Sons Ltd on behalf of European Hematology Association. This is an open access Abstract Book distributed under the Attribution-NonCommercial-NoDerivs (CC BY-NC-ND), which allows third parties to download the articles and share them with others as long as they credit the author and the Abstract Book, but they cannot change the content in any way or use them commercially.
**Abstract Book Citations:** Authors, Title, HemaSphere, 2026;10:(S1):pages. The abstract book DOIs can be found on the "Author Information" page.
**Disclaimer:** Articles published in the journal HemaSphere exclusively reflect the opinions of the authors. The authors are responsible for all content in their abstracts including accuracy of the facts, statements, citing resources, etc.

**Summary/Conclusion**

PROSIM-Q demonstrated initial content validity as a brief, patient-centered instrument to monitor ocular symptoms and impacts in oncology populations. Concept validation interviews will be conducted to confirm the content and usability of the draft PROSIM-Q. Lastly, ongoing longitudinal psychometric validation and scoring development are underway to support clinical implementation.
**Funding statement:** This study was sponsored by GSK. Editorial support was provided by Nucleus Global, an Inizio company, and funded by GSK. Drug linker technology licensed from Seagen Inc.; monoclonal antibody produced using POTELLIGENT Technology licensed from BioWa.

**Copyright Information:** (Online) ISSN: 2572-9241
©2026 The Author(s). HemaSphere is published by John Wiley & Sons Ltd on behalf of European Hematology Association. This is an open access Abstract Book distributed under the Attribution-NonCommercial-NoDerivs (CC BY-NC-ND), which allows third parties to download the articles and share them with others as long as they credit the author and the Abstract Book, but they cannot change the content in any way or use them commercially.
**Abstract Book Citations:** Authors, Title, HemaSphere, 2026;10:(S1):pages. The abstract book DOIs can be found on the "Author Information" page.
**Disclaimer:** Articles published in the journal HemaSphere exclusively reflect the opinions of the authors. The authors are responsible for all content in their abstracts including accuracy of the facts, statements, citing resources, etc.

### (PS1939) SUSTAINED MRD WITH OR WITHOUT ASCT IN THE ERA OF NEW REGIMENS FOR MULTIPLE MYELOMA: A SYSTEMATIC REVIEW AND NETWORK ANALYSIS

**Topic:** 14. Myeloma and other monoclonal gammopathies - Clinical

Giulio Caridà[1], Luca Scalise[1], Antonio Mosca[1], Eugenio Piro[1], Laura De Fazio[1], Marco Rossi*[1]

*[1]AOU Renato Dulbecco, Haematology Unit, Catanzaro, Italy;*

### Background

Minimal residual disease (MRD) negativity and sustained Minimal residual disease (sMRD) are established surrogates of deep response and are strongly associated with improved progression-free survival and overall survival in multiple myeloma. The introduction of innovative therapeutic options in first line raised the question whether the role of upfront autologous stem cell transplantation (ASCT) can be challenged by these highly active regimens capable of inducing profound responses in both transplant-eligible and transplant-ineligible populations.

### Methods

No randomized trials directly compare ASCT-based approaches with novel not transplant based combinations across heterogeneous patient populations or systematically assess their relative capacity to
achieve MRD negativity. Consequently, a network meta-analysis represents the only available methodological framework to enable indirect comparisons across regimens and treatment strategies and to generate a comprehensive estimate of their relative effectiveness in inducing MRD negativity, considered as first MRD analysis, and sMRD, considered after 12 months, even if its core assumption of transitivity may be violated. We performed a systematic review and Bayesian network meta-analysis with MRD negativity achievement as primary endpoint, preferentially extracted according to each study's definition and assay threshold (e.g., $10^{-5}$ or $10^{-6}$).

### Results

We performed a systematic review, evaluating 3,458 records, out of which 12 clinical trials were ultimately included: PERSEUS, IMROZ, MAIA, CEPHEUS, GMMG-HD7, IsKia, DETERMINATION, IFM 2009, COBRA, FORTE, BENEFIT, and ADVANCE. Treatment strategies were categorized as follows: anti-CD38-KRd, anti-CD38-Rd, anti-CD38-VRd, anti-CD38-VRd plus ASCT, KCd plus ASCT, KRd, KRd plus ASCT, Rd, VRd, and VRd plus ASCT. Ranking analyses based on SUCRA values indicated that anti-CD38–containing quadruplet approaches were most likely to induce MRD negativity. Specifically, anti-CD38-KRd and anti-CD38-VRd plus ASCT showed the highest probability of being the best treatment, with SUCRA values of 94.3 and 86.2, and probabilities of being the best treatment of 65.1% and 20.7%, respectively. Among non–anti-CD38 strategies, KRd plus ASCT (SUCRA 66.7) and KRd (SUCRA 58.0) ranked next, followed by anti-CD38-VRd (SUCRA 55.9). Poorest performance were observed with anti-CD38-Rd (SUCRA 16.4) and Rd (SUCRA 0). When considering sustained MRD (sMRD) at 12 months, ASCT-based strategies showed a higher probability of being the best treatment. The top three positions were occupied by anti-CD38-VRd plus ASCT (SUCRA 100%), anti-CD38-KRd plus ASCT (SUCRA 88.9%), and KRd plus ASCT (SUCRA 77.8), suggesting a consistent contribution of ASCT to achieving sustained MRD negativity.

**Copyright Information:** (Online) ISSN: 2572-9241
©2026 The Author(s). HemaSphere is published by John Wiley & Sons Ltd on behalf of European Hematology Association. This is an open access Abstract Book distributed under the Attribution-NonCommercial-NoDerivs (CC BY-NC-ND), which allows third parties to download the articles and share them with others as long as they credit the author and the Abstract Book, but they cannot change the content in any way or use them commercially.
**Abstract Book Citations:** Authors, Title, HemaSphere, 2026;10:(S1):pages. The abstract book DOIs can be found on the “Author Information” page.
**Disclaimer:** Articles published in the journal HemaSphere exclusively reflect the opinions of the authors. The authors are responsible for all content in their abstracts including accuracy of the facts, statements, citing resources, etc.

**Summary/Conclusion**

Comparisons between transplant-based and non-transplant strategies suggest that highly active quadruplet regimens without ASCT achieve MRD negativity rates comparable to, and in some cases numerically similar to ASCT-containing approaches. However, when sustained MRD (sMRD) is considered, ASCT-based strategies appear to confer a greater probability of achieving deeper and more durable MRD negativity.

**Copyright Information:** (Online) ISSN: 2572-9241
©2026 The Author(s). HemaSphere is published by John Wiley & Sons Ltd on behalf of European Hematology Association. This is an open access Abstract Book distributed under the Attribution-NonCommercial-NoDerivs (CC BY-NC-ND), which allows third parties to download the articles and share them with others as long as they credit the author and the Abstract Book, but they cannot change the content in any way or use them commercially.
**Abstract Book Citations:** Authors, Title, HemaSphere, 2026;10:(S1):pages. The abstract book DOIs can be found on the "Author Information" page.
**Disclaimer:** Articles published in the journal HemaSphere exclusively reflect the opinions of the authors. The authors are responsible for all content in their abstracts including accuracy of the facts, statements, citing resources, etc.

### (PS1940) BCMA-DIRECTED CAR-T CELL THERAPY IN MULTIPLE MYELOMA PATIENTS REQUIRING HEMODIALYSIS (HD): A MULTICENTER REAL-WORLD ANALYSIS

**Topic:** 14. Myeloma and other monoclonal gammopathies - Clinical

Eli Zolotov[1], Reema Mody[2], Kimberley Doucette[3], Chappell Aimee[3], Chantal Accolatse-Mati[4], James Di Palma-Grisi*[2], Harsh Parmar[1], Noa Biran[1], Pooja Phull[1], David S. Siegel[1], David H. Vesole[1]

*[1]Hackensack University Medical Center, John Theurer Cancer Center, Division of Myeloma, Hackensack, United States of America; [2]Hackensack University Medical Center, Hackensack, United States of America;[3]Georgetown University Hospital, Washington, United States of America; [4]Georgetown University Hospital, Hematology-Oncology, Washington, United States of America;*

### Background

Patients (pts) with end-stage renal disease requiring hemodialysis (HD) have been systematically excluded from pivotal CAR-T cell therapy trials in multiple myeloma. Real-world data on the optimal lymphodepletion strategies, safety, and efficacy for BCMA-directed CAR-T cell (BCMA-CAR-T) therapy in this vulnerable population remain limited, representing a critical knowledge gap in clinical practice.

### Aims

To evaluate the safety, efficacy, and clinical outcomes of BCMA-CAR-T therapy in pts with relapsed/refractory multiple myeloma (RRMM) requiring hemodialysis, with particular focus on lymphodepletion regimen modification, toxicity profiles and clinical outcomes.

### Methods

We conducted a multicenter retrospective analysis of all pts with multiple myeloma who received CAR-T therapy at Hackensack University Medical Center and Georgetown University Hospital between June 2021 to December 2025. Data were collected on patient demographics, disease characteristics, lymphodepletion regimens, CAR-T product type, toxicity profiles including cytokine release syndrome (CRS) and immune effector cell-associated neurotoxicity syndrome (ICANS), hematologic toxicities, response rates and survival outcomes.

**Copyright Information:** (Online) ISSN: 2572-9241
©2026 The Author(s). HemaSphere is published by John Wiley & Sons Ltd on behalf of European Hematology Association. This is an open access Abstract Book distributed under the Attribution-NonCommercial-NoDerivs (CC BY-NC-ND), which allows third parties to download the articles and share them with others as long as they credit the author and the Abstract Book, but they cannot change the content in any way or use them commercially.
**Abstract Book Citations:** Authors, Title, HemaSphere, 2026;10:(S1):pages. The abstract book DOIs can be found on the "Author Information" page.
**Disclaimer:** Articles published in the journal HemaSphere exclusively reflect the opinions of the authors. The authors are responsible for all content in their abstracts including accuracy of the facts, statements, citing resources, etc.

**Results**

Among 284 pts who received CAR-T therapy during the study period,2.8% pts required HD and comprised the study cohort. Of pts requiring dialysis, 89% who were receiving HD both prior to and during CAR-T infusion were included in the final analysis.
The cohort was heavily pretreated (median 6 prior lines), with a median age of 63 years. Most pts were female (62.5%), Black (75%), and non-Hispanic/Latino (87.5%); 5 received cilta-cel and 3 ide-cel. Lymphodepletion consisted of 95-175 mg bendamustine (Bend) or fludarabine/cyclophosphamide (Flu/Cy) over 3 days; fludarabine was dose-reduced by 50% due to renal impairment, with HD performed 12 hours after each dose.
CRS occurred in 37.5% of pts (all Grade 1), and ICANS occurred in 12.5% of pts (Grade 4).
With respect to hematologic safety, all pts achieved neutrophil recovery (ANC ≥1,000/µL) by Day 30 following CAR-T infusion and at day 90 post-CAR-T infusion, platelet counts ranged from 84-337×10³/µL. One patient who developed Grade 4 ICANS died in the ICU from invasive fungal pneumonia complicated by pulmonary hemorrhage in the setting of prolonged pre-existing cytopenias.
With respect to efficacy, median progression-free survival (PFS) was 13.6 months. Median overall survival (OS) was not reached. There was no statistically significant difference in outcomes between pts treated with ide-cel and those treated with cilta-cel regarding PFS or OS.

**Summary/Conclusion**

This multicenter analysis demonstrates that BCMA-CAR-T therapy is feasible and effective in carefully selected RRMM pts requiring hemodialysis. Despite heavy pretreatment and systematic exclusion from pivotal trials, HD-dependent pts achieved encouraging efficacy outcomes with median PFS of 13.6 months and OS not yet reached, comparable to landmark trial results. The toxicity profile was favorable, with only low-grade CRS and ICANS. Both lymphodepletion strategies-bendamustine and dose-reduced fludarabine/cyclophosphamide-appear safe and effective. These real-world data support the inclusion of dialysis-dependent pts in CAR-T treatment algorithms, provided appropriate dose modifications for renal impairment are implemented. Prospective studies are needed to optimize management strategies and establish evidence-based guidelines for this underrepresented population.

**Copyright Information:** (Online) ISSN: 2572-9241
©2026 The Author(s). HemaSphere is published by John Wiley & Sons Ltd on behalf of European Hematology Association. This is an open access Abstract Book distributed under the Attribution-NonCommercial-NoDerivs (CC BY-NC-ND), which allows third parties to download the articles and share them with others as long as they credit the author and the Abstract Book, but they cannot change the content in any way or use them commercially.
**Abstract Book Citations:** Authors, Title, HemaSphere, 2026;10:(S1):pages. The abstract book DOIs can be found on the "Author Information" page.
**Disclaimer:** Articles published in the journal HemaSphere exclusively reflect the opinions of the authors. The authors are responsible for all content in their abstracts including accuracy of the facts, statements, citing resources, etc.

### (PS1941) REAL-WORLD TREATMENT PATTERNS AND SURVIVAL OUTCOMES IN PATIENTS WITH MULTIPLE MYELOMA AFTER 1–2 PRIOR LINES OF THERAPY FROM THE HONEUR FEDERATED DATA NETWORK

**Topic:** 14. Myeloma and other monoclonal gammopathies - Clinical

Markus Rückert*[1], Guillaume Azarias[1], Jiri Minarik[2], Martin Stork[3], Alexandra Jungová[4], Jakub Radocha[5, 6], Michel Delforge[7], Jolien Raddoux[7], Javier De La Rubia Comos[8], Mario Arnao Herraiz[8], Giselle Lostaunau Costa[9], Yuwei Wang[10], Wout Vekemans[11], Peter Moorthamer[11], Nolen Perualila[12], Blanca Gros Otero[9], Roman Hájek[13, 14]

*[1]TriNetX Oncology GmbH, Freiburg, Germany; [2]University Hospital Olomouc and Palacky University, Olomouc, Czechia; [3]Hematology and Oncology, University Hospital Brno and Masaryk University, Brno, Czechia; [4]Charles University Hospital Pilsen, Pilsen, Czechia; [5]University Hospital Hradec Kralove, Hradec Kralove, Czechia; [6]Charles University, Hradec Kralove, Czechia; [7]University Hospitals Leuven, Leuven, Belgium; [8]Hospital Universitario y Politécnico La Fe, Valencia, Spain; [9]Johnson & Johnson, Madrid, Spain; [10]Johnson & Johnson, Breda, The Netherlands; [11]Johnson & Johnson, Beerse, Belgium; [12]Johnson & Johnson, Allschwil, Switzerland; [13]University Hospital Ostrava, Ostrava, Czechia; [14]University of Ostrava, Ostrava, Czechia;*

### Background

Treatment decisions following relapse in multiple myeloma (MM) have become increasingly complex due to a rapidly evolving therapeutic landscape and changing dynamics based on prior exposure. Understanding real-world outcomes is essential to identify target populations for novel combination therapies that may increase survival.

### Aims

To describe the baseline characteristics, prior treatment exposure, treatment patterns, and survival outcomes of patients with MM identified at second line (2L) or third line (3L) of treatment.

### Methods

From 2016–2025, we analyzed patients with MM from 3 registries (IUCT, France; TriNetX Oncology, Germany; and RMG, Czech Republic). Locally collected patient-level data were analyzed uniformly, and site-specific results were aggregated centrally using the HONEUR federated data network. The study population included patients at 2L and 3L with the index date defined as the treatment initiation date. Baseline characteristics and treatment patterns were descriptively analyzed. High-risk cytogenetics was defined as any presence of del(17p), and/or t(4;14), and/or t(14;16). Time to next treatment (TTNT), as a proxy for progression-free survival, and overall survival (OS) were assessed using Kaplan-Meier.

**Copyright Information:** (Online) ISSN: 2572-9241
©2026 The Author(s). HemaSphere is published by John Wiley & Sons Ltd on behalf of European Hematology Association. This is an open access Abstract Book distributed under the Attribution-NonCommercial-NoDerivs (CC BY-NC-ND), which allows third parties to download the articles and share them with others as long as they credit the author and the Abstract Book, but they cannot change the content in any way or use them commercially.
**Abstract Book Citations:** Authors, Title, HemaSphere, 2026;10:(S1):pages. The abstract book DOIs can be found on the "Author Information" page.
**Disclaimer:** Articles published in the journal HemaSphere exclusively reflect the opinions of the authors. The authors are responsible for all content in their abstracts including accuracy of the facts, statements, citing resources, etc.

**Results**

We examined 13,720 treatment lines in patients with 1 prior LOT (2L) and 8558 treatment lines in patients with 2 prior LOT (3L). In the 2L cohort, 31.8% of patients were ≥75 years old, 62.7% had ISS stage 2–3, 17.7% had high-risk cytogenetics, and 30.6% had received stem cell transplantation (SCT). Baseline characteristics were similar in the 3L cohort: 33.7% were ≥75 years old, 63.6% had ISS stage 2–3, 18.1% had high-risk cytogenetics, and 32.8% had received SCT.
The proportion of patients without prior exposure to CD38 (CD38 naïve) before starting 2L remained substantial over time from 99.6% in 2016 to 73.7% in 2024. The proportion of CD38-naïve patients starting 3L decreased from 99.3% in 2016 to 37.0% in 2024.
At 2L, 44.6% of patients received immunomodulatory drug (IMiD)-based regimens, 37.1% received CD38-based regimens, and 12.4% received proteasome inhibitor (PI)-only regimens (remaining 5.9% others/unknown). At 3L, 42.8% received IMiD-based regimens, 29.1% CD38-based regimens, and 14.7% PI-only regimens (remaining 13.4% others/unknown).
Median follow-up for the 2L cohort was 36.5 months and 31.0 months for the 3L cohort. Median OS was 56.6 months in the 2L cohort and 34.9 months in the 3L cohort (Figure). Median TTNT was 18.7 months in the 2L cohort and 11.9 months in the 3L cohort.

**Summary/Conclusion**

This real-world, multi-registry study indicates suboptimal outcomes in earlier lines of therapy and underscores the need to adopt novel combination regimens. Notably, a substantial proportion of patients starting 2L remains CD38 naïve, potentially indicating that earlier use of CD38-containing novel combinations could improve survival.

**Copyright Information:** (Online) ISSN: 2572-9241
©2026 The Author(s). HemaSphere is published by John Wiley & Sons Ltd on behalf of European Hematology Association. This is an open access Abstract Book distributed under the Attribution-NonCommercial-NoDerivs (CC BY-NC-ND), which allows third parties to download the articles and share them with others as long as they credit the author and the Abstract Book, but they cannot change the content in any way or use them commercially.
**Abstract Book Citations:** Authors, Title, HemaSphere, 2026;10:(S1):pages. The abstract book DOIs can be found on the “Author Information” page.
**Disclaimer:** Articles published in the journal HemaSphere exclusively reflect the opinions of the authors. The authors are responsible for all content in their abstracts including accuracy of the facts, statements, citing resources, etc.

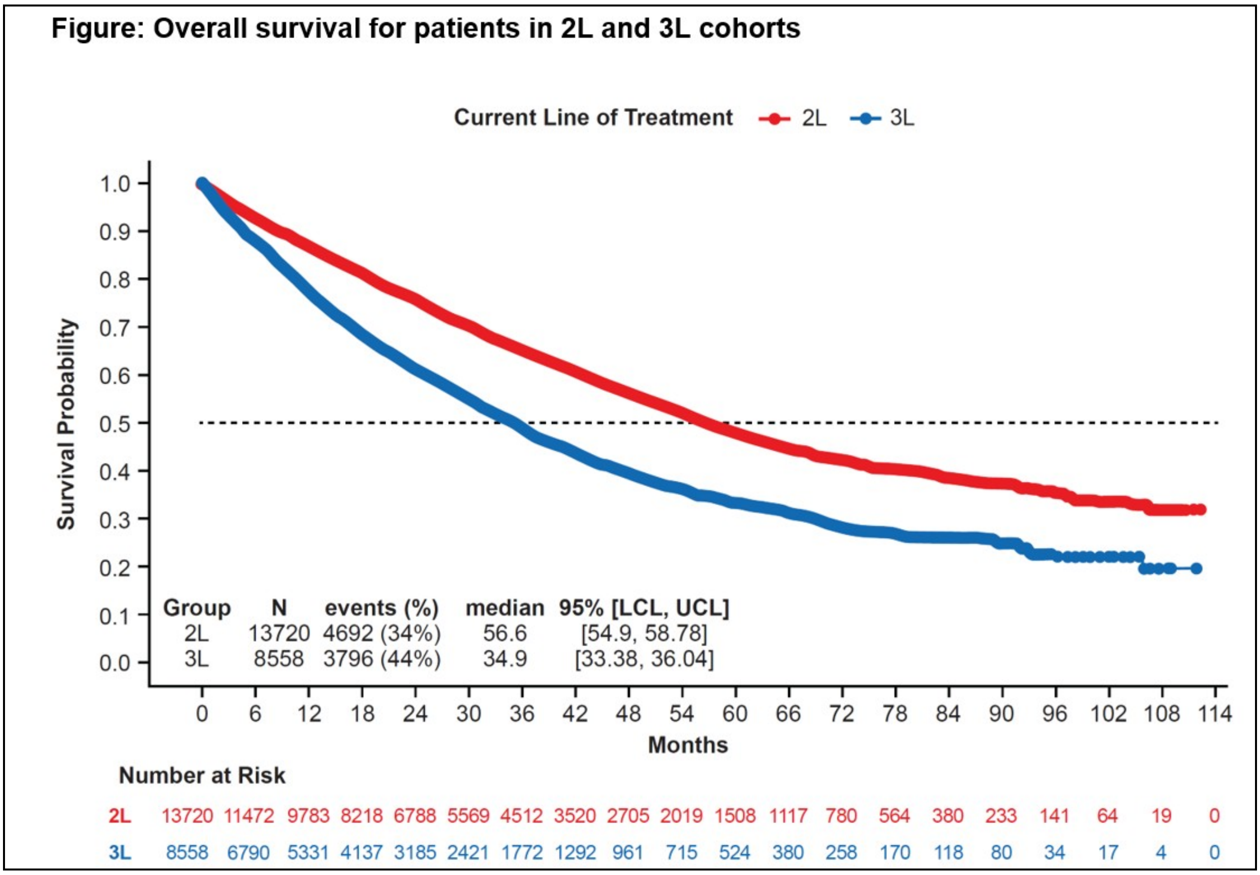

2L, second line; 3L, third line.

**Copyright Information:** (Online) ISSN: 2572-9241
©2026 The Author(s). HemaSphere is published by John Wiley & Sons Ltd on behalf of European Hematology Association. This is an open access Abstract Book distributed under the Attribution-NonCommercial-NoDerivs (CC BY-NC-ND), which allows third parties to download the articles and share them with others as long as they credit the author and the Abstract Book, but they cannot change the content in any way or use them commercially.
**Abstract Book Citations:** Authors, Title, HemaSphere, 2026;10:(S1):pages. The abstract book DOIs can be found on the "Author Information" page.
**Disclaimer:** Articles published in the journal HemaSphere exclusively reflect the opinions of the authors. The authors are responsible for all content in their abstracts including accuracy of the facts, statements, citing resources, etc.

### (PS1942) DUAL-RISK ADJUSTED THERAPY FOR ELDERLY PATIENTS WITH NEWLY DIAGNOSED MULTIPLE MYELOMA BASED ON DYNAMIC FRAILTY ASSESSMENT AND R-ISS: A PROSPECTIVE CLINICAL TRIAL

**Topic:** 14. Myeloma and other monoclonal gammopathies - Clinical

Jianmei Xu[1], Hua Xue*[1], Jie Li[2], Juan Wang[3], Suo Xiaohui[4], Haisheng Liu[5], Songying Zhao[1], Jing Wang[1], Huimei Guo[1], Jiangbo Zhang[1]

*[1]Affiliated Hospital of Hebei University, Baoding, China;[2]Hebei Provincial People's Hospital, Shijiazhuang, China;[3]Central Hospital of Cangzhou City, Cangzhou, China; [4]Handan Central Hospital, Handan, China;[5]The Fourth Hospital of Hebei Medical University, Shijiazhuang, China;*

### Background

Elderly patients with multiple myeloma (MM) are heterogeneous, facing challenges from both frailty and high-risk cytogenetics. Dynamic frailty assessment offers better prognostic value than baseline evaluation. Incorporating R-ISS and sequential therapy based on frailty improvement may better capture the disease course in elderly NDMM. All-oral regimens may improve adherence and reduce burden.

### Aims

We initiated a prospective multicenter trial to evaluate dual-risk adjusted therapy in elderly NDMM based on dynamic frailty and R-ISS (ChiCTR2300078876).

### Methods

In this prospective trial treatment consisted of initial 28 day-induction cycles consisting of ixazomib (I) 4 mg (days 1, 8, 15) and dexamethasone (D) 40mg (days 1, 8, 15, 22) for intermediate-fit patients, dexamethasone (D) 20mg (days 1, 8, 15, 22) for frail patients. Frailty was reassessed at cycles 3, 6, 12 using gait speed, grip strength, and IMWG-FI. Upon frailty improvement, a third drug was added (lenalidomide for intermediate-fit; cyclophosphamide for frail) in: 1[illegible]R-ISS high-risk group, or 2) R-ISS intermediate-low risk group with efficacy < PR.Others continued ID. Patients achieving VGPR or higher at the 12th cycle proceeded to ixazomib or lenalidomide maintenance therapy. Inclusion: NDMM ≥60 years, IMWG-FI intermediate-fit or frail.

### Results

**Copyright Information:** (Online) ISSN: 2572-9241
©2026 The Author(s). HemaSphere is published by John Wiley & Sons Ltd on behalf of European Hematology Association. This is an open access Abstract Book distributed under the Attribution-NonCommercial-NoDerivs (CC BY-NC-ND), which allows third parties to download the articles and share them with others as long as they credit the author and the Abstract Book, but they cannot change the content in any way or use them commercially.
**Abstract Book Citations:** Authors, Title, HemaSphere, 2026;10:(S1):pages. The abstract book DOIs can be found on the "Author Information" page.
**Disclaimer:** Articles published in the journal HemaSphere exclusively reflect the opinions of the authors. The authors are responsible for all content in their abstracts including accuracy of the facts, statements, citing resources, etc.

| Characteristics | N=51 |
|---|---|
| Median age, years | 74⌧60-86⌧ |
| Age ≥75 years | 48⌧94.1%⌧ |
| Male⌧% | 35⌧68.6%⌧ |
| Renal insufficiency,% | 13⌧25.5%⌧ |
| CCI | |
| ≤1 | 35⌧68.6%⌧ |
| ≥2 | 16⌧31.4%⌧ |
| IMWG-GA | |
| 1 | 9⌧17.6%⌧ |
| 2 | 18⌧35.3%⌧ |
| ≥3 | 24⌧47.1%⌧ |
| ECOG | |
| 1 | 8⌧15.7%⌧ |
| 2 | 18⌧35.3%⌧ |
| ≥3 | 25⌧49.0%⌧ |
| ISS(stage at diagnosis) | |
| Ⅰ⌧% | 5⌧9.8%⌧ |
| Ⅱ⌧% | 17⌧33.3%⌧ |
| Ⅲ⌧% | 29⌧56.9%⌧ |
| FISH⌧n=36⌧ | |
| t(4;14) | 6⌧16.7%⌧ |
| t(11;14) | 5⌧13.9%⌧ |
| t(14;20) | 1⌧2.8%⌧ |
| 1q+ | 21⌧58.3%⌧ |
| 13q- | 12⌧33.3%⌧ |
| TP53- | 3⌧9.1%⌧ |
| Negative for all FISH markers | 7⌧19.4%⌧ |

As of August 1, 2025, a total of 82 patients underwent screening, with 55 meeting the inclusion criteria. Of the enrolled patients, 4 discontinuedtreatment and withdrew after receiving only 1–2 courses. There were 8 cases in the intermediate-fit group and 43 cases in the frail group based on IMWG-FI. The characteristics of the 51 patients are shown in Table 1.The preliminary efficacy was satisfactory, with an ORR of 88.2% (45/51), of which 7 CR, 21 VGPR, 17 PR. The 32 patients exhibited improvement in their frailty state, while 26 patients were administered a third drug (IRD, n=5; ICD, n=21) . 14 patients achieved a VGPR or CR without treatment adjustments. 9 patients experienced disease progression and were subsequently switched therapy.

Two patients died early, one died due to incorrect oral medication at the 6th month, and the other one suffered sudden cardiac death at the 3rd month. Two patients experienced disease progression at 7 and 12 months, respectively, and subsequently discontinued treatment and died. No patient discontinued treatment owing to intolerable adverse reactions. Due to the short follow-up period, the median PFS and OS were not reached.All patients exhibited grade 1-2 adverse events. One patient developed grade 4 pulmonary infection and required admission to the ICU.

### Summary/Conclusion

The preliminary results indicate that the dual-risk adjusted therapy demonstrates reduced treatment-related toxicity, discontinuation, and early mortality. Furthermore, it indicates satisfactory initial response rates and confirms the benefits and the sustainable application of an all-oral drug regimen in elderly patients. This treatment may be able to alleviate the current treatment dilemma for frail elderly patients with NDMM.

**Copyright Information:** (Online) ISSN: 2572-9241
©2026 The Author(s). HemaSphere is published by John Wiley & Sons Ltd on behalf of European Hematology Association. This is an open access Abstract Book distributed under the Attribution-NonCommercial-NoDerivs (CC BY-NC-ND), which allows third parties to download the articles and share them with others as long as they credit the author and the Abstract Book, but they cannot change the content in any way or use them commercially.
**Abstract Book Citations:** Authors, Title, HemaSphere, 2026;10:(S1):pages. The abstract book DOIs can be found on the “Author Information” page.
**Disclaimer:** Articles published in the journal HemaSphere exclusively reflect the opinions of the authors. The authors are responsible for all content in their abstracts including accuracy of the facts, statements, citing resources, etc.

### (PS1943) EFFICACY OF VENETOCLAX-BASED THERAPY IN T(11;14)–POSITIVE RELAPSED/REFRACTORY MULTIPLE MYELOMA: A SYSTEMATIC REVIEW AND META-ANALYSIS.

**Topic:** 14. Myeloma and other monoclonal gammopathies - Clinical

Vasu Malhotra*[1], Shreya Patel[1], Marialaina Carter[2], Sravani Bhavanam[3], Muhammad Farooq[4], Ken Shain[5]

*[1]Lakeland Regional Health, Internal Medicine, Lakeland, United States of America;[2]USA Health University Hospital, Mobile, United States of America; [3]Brookdale University Hospital & Medical Center, Brooklyn, United States of America;[4]Lakeland Regional Health, Lakeland, United States of America; [5]Moffitt Cancer Center, Tampa, United States of America;*

### Background

Translocation between chromosomes 11 and 14 [t(11;14)] is one of the most common primary cytogenetic abnormalities in multiple myeloma. Venetoclax is a highly selective, oral BH3 mimetic that binds B-cell lymphoma-2 (BCL-2), inducing apoptosis in BCL-2–dependent plasma cells. Early phase studies suggested promising activity of venetoclax-based regimens, particularly in t(11;14) relapsed/refractory multiple myeloma (RRMM). However, venetoclax has not received FDA approval for the treatment of RRMM, in part due to results from two phase III randomized trials.

### Aims

Venetoclax demonstrated significant activity in phase I and II studies of t(11;14)-RRMM, including monotherapy and bortezomib-based combinations, with responses enriched in this cytogenetic subgroup. However, subsequent phase III trials have produced discordant outcomes. The primary aim of this study was to synthesize available phase III randomized evidence to estimate the pooled effect of venetoclax-based therapy on progression-free survival (PFS) in patients with t(11;14)-positive RRMM. Secondary aims were to evaluate pooled effects on overall survival (OS) and time to deterioration in disease-related symptoms (TTDDS), and to assess between-trial heterogeneity to better contextualize differences in trial design and comparator regimens.

### Methods

A systematic literature search was performed across PubMed-MEDLINE, Embase-OVID, CENTRAL, ClinicalTrials.gov, and Web of Science databases up to January 1, 2025. Randomized phase III trials evaluating venetoclax-based therapy versus standard-of-care regimens in patients with t(11;14)-positive RRMM were included. Progression-free survival (PFS) was the primary outcome. Overall survival (OS) and time to deterioration in disease-related symptoms (TTDDS) were secondary outcomes. Hazard ratios (HRs) were pooled using a random-effects inverse-variance model with heterogeneity assessed using the $I^2$ statistic.

### Results

A total of 298 patients from two phase III randomized controlled trials (CANOVA and BELLINI) were included. Venetoclax-based therapy was associated with a directionally favorable improvement in PFS (pooled HR 0.40; 95% CI, 0.08–1.91). No pooled OS benefit was observed (HR 1.15; 95% CI, 0.78–1.71). Venetoclax-based regimens demonstrated a trend toward prolonged TTDDS (TTDDS HR 0.70; 95% CI, 0.46–1.05). Substantial heterogeneity was observed for PFS ($I^2$ = 88.8%), while heterogeneity was minimal for OS and TTDDS ($I^2$ = 0%). None of the pooled efficacy outcomes reached statistical significance.

**Copyright Information:** (Online) ISSN: 2572-9241
©2026 The Author(s). HemaSphere is published by John Wiley & Sons Ltd on behalf of European Hematology Association. This is an open access Abstract Book distributed under the Attribution-NonCommercial-NoDerivs (CC BY-NC-ND), which allows third parties to download the articles and share them with others as long as they credit the author and the Abstract Book, but they cannot change the content in any way or use them commercially.
**Abstract Book Citations:** Authors, Title, HemaSphere, 2026;10:(S1):pages. The abstract book DOIs can be found on the "Author Information" page.
**Disclaimer:** Articles published in the journal HemaSphere exclusively reflect the opinions of the authors. The authors are responsible for all content in their abstracts including accuracy of the facts, statements, citing resources, etc.

**Summary/Conclusion**

Venetoclax-based therapy demonstrates a directionally favorable effect on PFS and TTDDS in t(11;14)-positive RRMM. In BELLINI, despite a small subgroup sample size, venetoclax demonstrated an effect size rarely observed in RRMM, whereas CANOVA was conducted against a highly active comparator. Future trials should prioritize t(11;14) populations and consider non-inferiority or combination-based comparator designs. The lack of statistical significance in pooled outcomes likely reflects differences in trial design and treatment backbone rather than absence of biological activity.

© 2026 American Society of Clinical Oncology, Inc. Reused with permission. This abstract was accepted and previously presented at the 2026 ASCO Annual Meeting. All rights reserved.

| Study | Venetoclax-Based Regimen | Control Regimen | PFS* HR (95% CI*) | OS* HR (95% CI*) | TTDDS* HR (95% CI*) |
|---|---|---|---|---|---|
| CANOVA | Venetoclax + Dexamethasone | Pomalidomide + Dexamethasone | 0.82 (0.60–1.14) | 1.19 (0.80–1.77) | 0.77 (0.49–1.21) |
| BELLINI | Venetoclax + Bortezomib + Dexamethasone | Placebo + Bortezomib + Dexamethasone | 0.16 (0.06–0.45) | 0.35 (0.03–3.84) | 0.46 (0.18–1.18) |
| | | Pooled Estimates | 0.40 (0.08–1.91) | 1.15 (0.78–1.71) | 0.70 (0.46–1.05) |

Table 1. Phase III randomized trial outcomes and pooled efficacy estimates of venetoclax-based therapy in t(11;14)-positive RRMM

**Copyright Information:** (Online) ISSN: 2572-9241
©2026 The Author(s). HemaSphere is published by John Wiley & Sons Ltd on behalf of European Hematology Association. This is an open access Abstract Book distributed under the Attribution-NonCommercial-NoDerivs (CC BY-NC-ND), which allows third parties to download the articles and share them with others as long as they credit the author and the Abstract Book, but they cannot change the content in any way or use them commercially.
**Abstract Book Citations:** Authors, Title, HemaSphere, 2026;10:(S1):pages. The abstract book DOIs can be found on the "Author Information" page.
**Disclaimer:** Articles published in the journal HemaSphere exclusively reflect the opinions of the authors. The authors are responsible for all content in their abstracts including accuracy of the facts, statements, citing resources, etc.

### (PS1944) REAL-WORLD IMPACT OF ANTI-CD38 MONOCLONAL ANTIBODY THERAPY AND HEMATOPOIETIC STEM CELL TRANSPLANTATION IN PRIMARY PLASMA CELL LEUKEMIA: A SINGLE-CENTER STUDY

**Topic:** 14. Myeloma and other monoclonal gammopathies - Clinical

Vincent Luong*[1, 2], Karin Larsson[2, 3], Katarina Uttervall[1, 2], Monika Klimkowska[4, 5], Hazel Reilly[1], Nursen Cetrez[1], Gösta Gahrton[1], Evren Alici[1, 2], Piotr Kozlowski[6], Johan Lund[1, 2]

*[1]Karolinska Institutet, Department of Medicine, Center for Hematology and Regenerative Medicine, Stockholm, Sweden; [2]Karolinska University Hospital, Department of Hematology, Stockholm, Sweden;[3]Uppsala University Hospital, Department of Medical Sciences, Division of Hematology, Uppsala, Sweden; [4]Karolinska Institutet, Department of Laboratory Medicine, Division of Pathology, Stockholm, Sweden; [5]Karolinska University Hospital, Department of Clinical Pathology and Cancer Diagnostics, Stockholm, Sweden; [6]Karolinska University Hospital, Center for Cell Therapy and Allogeneic Stem Cell Transplantation, Stockholm, Sweden;*

### Background

Primary plasma cell leukemia (pPCL) is associated with poor outcomes. Hematopoietic stem cell transplantation (HSCT), whether autologous or allogeneic, is crucial in achieving lasting remissions. Recent addition of anti-CD38 monoclonal antibodies (mAb) has markedly improved survival in multiple myeloma, supporting its use in pPCL. We have previously reported outcomes after first-line (1L) treatment at our center (IMS 2025, abstract PA-355). Here we provide updated analyses, including second-line (2L) therapy outcomes.

### Aims

To investigate 1L and 2L outcomes in pPCL patients treated with and without anti-CD38 mAb and HSCT.

### Methods

Our single-center study explored outcomes in pPCL patients diagnosed between 2002 and 2025. PCL was defined using the 2021 criteria by the International Myeloma Working Group, and applied retrospectively to subjects before 2021. We evaluated progression-free survival (PFS) and overall survival (OS) from start of 1L and 2L therapies. Survival was assessed for the full cohort, and stratified by anti-CD38 mAb exposure and by HSCT status.

**Copyright Information:** (Online) ISSN: 2572-9241
©2026 The Author(s). HemaSphere is published by John Wiley & Sons Ltd on behalf of European Hematology Association. This is an open access Abstract Book distributed under the Attribution-NonCommercial-NoDerivs (CC BY-NC-ND), which allows third parties to download the articles and share them with others as long as they credit the author and the Abstract Book, but they cannot change the content in any way or use them commercially.
**Abstract Book Citations:** Authors, Title, HemaSphere, 2026;10:(S1):pages. The abstract book DOIs can be found on the "Author Information" page.
**Disclaimer:** Articles published in the journal HemaSphere exclusively reflect the opinions of the authors. The authors are responsible for all content in their abstracts including accuracy of the facts, statements, citing resources, etc.

### Results

Fifty-three patients were included, 49% were male and median age at pPCL diagnosis was 70 years (range 40-94). Median duration of follow-up from pPCL diagnosis was 18.3 months (IQR 9.6-46.5), and 57% of the patients had ISS stage III. Cytogenetic risk profiles were available for 28 patients, of whom 23 were high-risk (82% of available). The 1L median PFS (mPFS) for the full cohort was 11.2 months (95%CI: 8.0-25.1), median OS (mOS) was 25.3 months (95%CI: 12.7-50.1).
Anti-CD38 mAb exposed patients during 1L (n=21), had a mPFS of 27.9 months (95%CI: 8.5-NA) and mOS 38.6 months (95%CI: 17.4-NA). Among patients not exposed (n=32), mPFS was 10.9 months (95%CI: 6.4-14.7) and mOS 16.4 months (95%CI: 10.9-49.8). From the 6-month landmark, patients alive and progression-free who proceeded to HSCT (n=18) had a mPFS of 21.9 months (95%CI: 8.7-NA) and mOS of 50.7 months (95%CI: 32.6-NA). In contrast, patients not undergoing HSCT at the 6-month landmark (n=13) had a mPFS and mOS of 7.5 months (95%CI: 4.9-NA) and 43.8 months (95%CI: 9.0-NA), respectively. Allogeneic HSCT patients (n=7) had a mPFS of 21.3 months (95%CI: 4.3-NA), while mOS was not reached (95%CI: 4.3-NA) from the 6-month landmark.
An overall response rate of 85% was observed on 1L therapy. Patients receiving doublet had a mPFS of 7.7 months (95%CI: 1.0-NA) and mOS of 17.4 months (95%CI: 8.8-NA). Patients receiving triplet had a mPFS 10.9 months (95%CI: 5.0-25.1) and mOS of 16.2 months (95%CI: 11.9-50.1). In patients receiving quadruplet, mPFS was 32.1 months (95%CI: 10.3-NA) and mOS was not reached (35.4-NA). Univariate analysis of 1L indicates circulating plasma cells ≥20% and renal failure were associated with poor PFS, while diagnosis after 2018, HSCT, allogeneic HSCT, anti-CD38 mAb, IMiD and quadruplet regimen were associated with improved PFS. A multivariable model suggests IMiD therapy and HSCT are independent prognostic markers.
Analysis of 2L outcomes in patients who progressed on 1L therapy (n=34), showed a mPFS of 8.0 months (95% CI: 2.7-11.4) and mOS of 15.7 months (95%CI: 6.5-34.2).

### Summary/Conclusion

Our results indicate anti-CD38 mAb therapy improves outcomes in pPCL, while HSCT (including allogeneic) and IMiD therapy remain important therapeutic considerations. Study limitations are the single-center design and small patient cohort, which highlights the need for larger, multicenter studies to validate findings.

**Copyright Information:** (Online) ISSN: 2572-9241
©2026 The Author(s). HemaSphere is published by John Wiley & Sons Ltd on behalf of European Hematology Association. This is an open access Abstract Book distributed under the Attribution-NonCommercial-NoDerivs (CC BY-NC-ND), which allows third parties to download the articles and share them with others as long as they credit the author and the Abstract Book, but they cannot change the content in any way or use them commercially.
**Abstract Book Citations:** Authors, Title, HemaSphere, 2026;10:(S1):pages. The abstract book DOIs can be found on the “Author Information” page.
**Disclaimer:** Articles published in the journal HemaSphere exclusively reflect the opinions of the authors. The authors are responsible for all content in their abstracts including accuracy of the facts, statements, citing resources, etc.

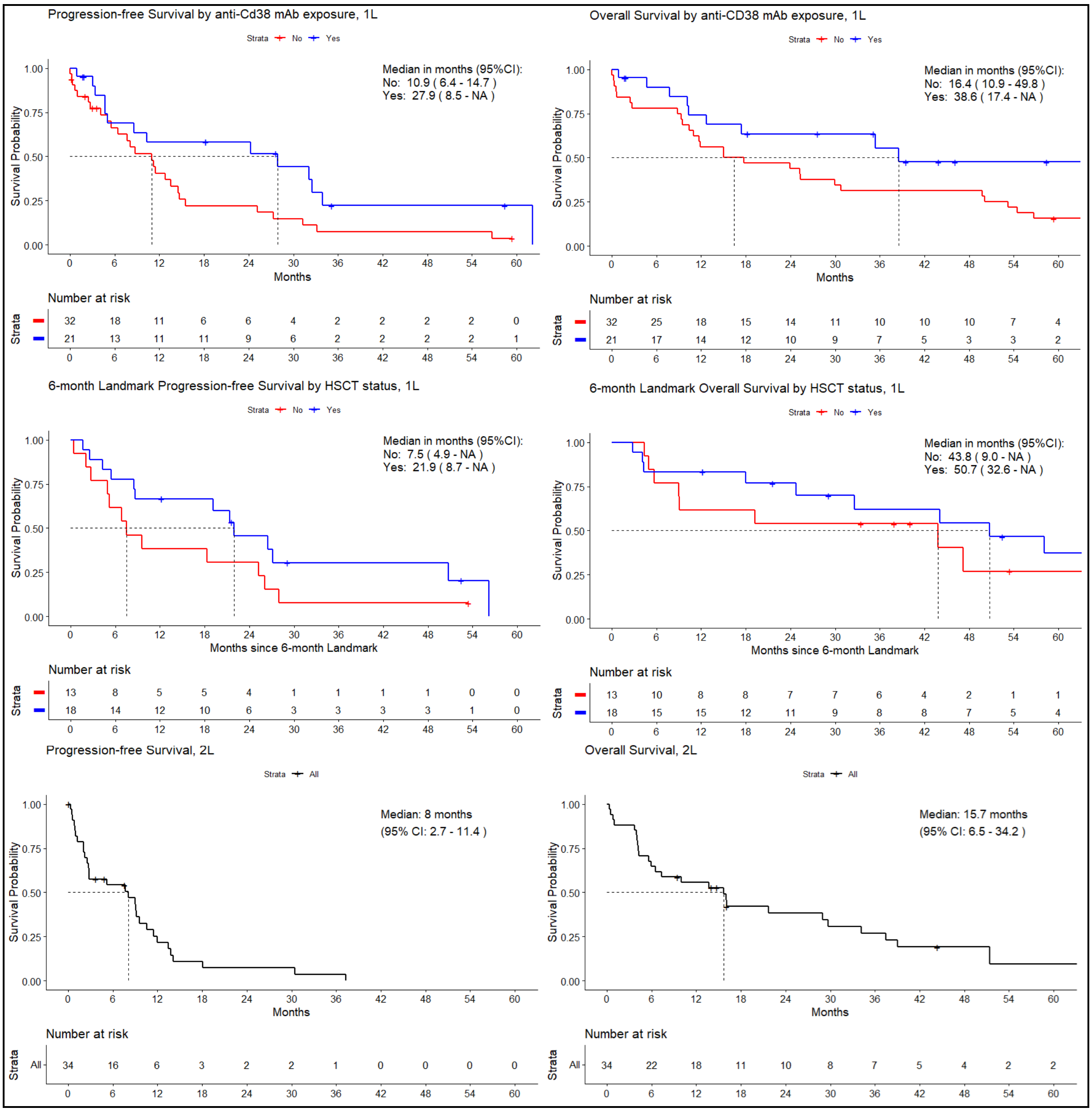

**Copyright Information:** (Online) ISSN: 2572-9241
©2026 The Author(s). HemaSphere is published by John Wiley & Sons Ltd on behalf of European Hematology Association. This is an open access Abstract Book distributed under the Attribution-NonCommercial-NoDerivs (CC BY-NC-ND), which allows third parties to download the articles and share them with others as long as they credit the author and the Abstract Book, but they cannot change the content in any way or use them commercially.
**Abstract Book Citations:** Authors, Title, HemaSphere, 2026;10:(S1):pages. The abstract book DOIs can be found on the "Author Information" page.
**Disclaimer:** Articles published in the journal HemaSphere exclusively reflect the opinions of the authors. The authors are responsible for all content in their abstracts including accuracy of the facts, statements, citing resources, etc.

### (PS1945) REAL-WORLD SAFETY OF ELRANATAMAB IN PATIENTS WITH RENAL IMPAIRMENT: FINDINGS FROM THE SUMMIT STUDY

**Topic:** 14. Myeloma and other monoclonal gammopathies - Clinical

Satoshi Yoshihara*[1], Hsu-Wen Chou[2], Charlotte Aubrac[3], Aster Meche[4], Sarasa Johnson[3], Guido Nador[5], Shohei Ikoma[6], Patrick Hlavacek[4], Chandra Prakash Yadav[7], Hiroki Kawano[6], Chenan Zhang[8], Marco Dibonaventura[4], Yong Chen[9]

*[1] 1Department of Hematology, Hyogo Medical University Hospital, Nishinomiya, Japan;[2]Pfizer Ltd., Taipei, Taiwan; [3]Statlog Inc., Quebec, Canada; [4]Pfizer Inc, New York, United States of America;[5]Pfizer Ltd., Surrey, United Kingdom;[6]Pfizer Japan Inc., Tokyo, Japan; [7]Pfizer Ltd., Chennai, India;[8]Pfizer Inc., South San Francisco, CA, United States of America;[9]Pfizer Inc., Collegeville, PA, United States of America;*

### Background

Elranatamab is a BCMAxCD3 bispecific antibody approved for heavily pre-treated multiple myeloma (MM) in Japan. Renal impairment (RI) is a major complication of MM associated with poor prognosis and limited treatment options. It is important to characterize the real-world (RW) use, safety and tolerability of elranatamab in patients with RI in Japan.

### Aims

To characterize the administration patterns and safety of elranatamab among RW Japanese MM patients by RI status.

### Methods

SUMMIT is a cohort study using the Japan Medical Data Vision administrative claims database. MM patients ≥18 years old were included if they first received elranatamab (index date) outside of clinical trials from March 26, 2024 to March 31, 2025, and had available renal function data. Patients were categorized as having either “normal to moderate RI” (estimated glomerular filtration rate (eGFR) ≥ 30mL/min/1.73m$^2$ within 90 days [d] pre-index date) or “severe RI” (eGFR < 30 mL/min/1.73m$^2$ within 90d or dialysis within 30d pre-index date). Administration patterns and baseline characteristics were descriptively reported. Relative administration intensity (RAI) was calculated as the frequency of administration received over expected administration within 30d post-index date. The incidence of adverse events (AEs) was estimated using disease codes following elranatamab initiation among patients with no history of the respective AEs within the preceding 30d. AEs included cytokine release syndrome (CRS) and immune effector cell-associated neurotoxicity syndrome (ICANS) within 14d, and cytopenia within 8d, 14d, and 30d.

### Results

Among 269 patients treated with elranatamab, 48 had available renal function data and were included in this analysis (normal to moderate RI [13.4%]; severe RI [4.5%]) (Table 1). Among patients with severe RI, 6 (50.0%) were on dialysis. Elranatamab administration patterns were comparable across RI groups (median time to first full dose: 7.0d vs. 9.0d; 30d RAI: 100.0% in both). ICANS was numerically less frequent in the severe RI group (8.3% vs 11.1%), whereas other AEs were numerically more frequent (66.7% vs 55.6% for CRS and 9.1-18.2% vs 6.5-11.1% for cytopenia 8d-30d).

**Copyright Information:** (Online) ISSN: 2572-9241
©2026 The Author(s). HemaSphere is published by John Wiley & Sons Ltd on behalf of European Hematology Association. This is an open access Abstract Book distributed under the Attribution-NonCommercial-NoDerivs (CC BY-NC-ND), which allows third parties to download the articles and share them with others as long as they credit the author and the Abstract Book, but they cannot change the content in any way or use them commercially.
**Abstract Book Citations:** Authors, Title, HemaSphere, 2026;10:(S1):pages. The abstract book DOIs can be found on the “Author Information” page.
**Disclaimer:** Articles published in the journal HemaSphere exclusively reflect the opinions of the authors. The authors are responsible for all content in their abstracts including accuracy of the facts, statements, citing resources, etc.

**Summary/Conclusion**

Across RI categories, the observed incidences of CRS, ICANS, and cytopenias were broadly consistent with the known safety profile of elranatamab. Numerical differences in the frequency of certain safety events were observed between RI groups, including slightly lower ICANS rates and higher CRS and cytopenia rates in patients with severe RI; however, these findings should be interpreted with caution given the limited sample size, particularly in the severe RI subgroup.

Table 1. Baseline characteristics and safety outcomes by RI status

| | Normal to moderate RI n=36 | Severe RI n=12 |
|---|---|---|
| Age (median [IQR]) | 74.5 (67.0-79.0) | 75.0 (68.0-81.5) |
| Female, n (%) | 18 (50.0) | 7 (58.3) |
| Time to first full dose, d (median [IQR]) | 7.0 (7.0-9.0) | 9.0 (7.0-13.0) |
| RAI, % (median [IQR]) | 100.0 (73.3-100.0) | 100.0 (83.3-100.0) |
| CRS, n (%*) | 20 (55.6) | 8 (66.7) |
| ICANS, n (%*) | 4 (11.1) | 1 (8.3) |
| Cytopenia (8d), n (%*) | 2 (6.5) | 1 (9.1) |
| Cytopenia (14d), n (%*) | 3 (10.7) | 2 (18.2) |
| Cytopenia (30d), n (%*) | 3 (11.1) | 2 (18.2) |

* Percentages were calculated based on the at-risk population which defined as patients with no history of the respective events within the preceding 30 days.

**Copyright Information:** (Online) ISSN: 2572-9241
©2026 The Author(s). HemaSphere is published by John Wiley & Sons Ltd on behalf of European Hematology Association. This is an open access Abstract Book distributed under the Attribution-NonCommercial-NoDerivs (CC BY-NC-ND), which allows third parties to download the articles and share them with others as long as they credit the author and the Abstract Book, but they cannot change the content in any way or use them commercially.
**Abstract Book Citations:** Authors, Title, HemaSphere, 2026;10:(S1):pages. The abstract book DOIs can be found on the "Author Information" page.
**Disclaimer:** Articles published in the journal HemaSphere exclusively reflect the opinions of the authors. The authors are responsible for all content in their abstracts including accuracy of the facts, statements, citing resources, etc.

### (PS1946) REAL-WORLD SAFETY OF ELRANATAMAB IN LOW-WEIGHT PATIENTS: FINDINGS FROM THE SUMMIT STUDY

**Topic:** 14. Myeloma and other monoclonal gammopathies - Clinical

Satoshi Yoshihara*[1], Aster Meche[2], Ariane Faucher[3], Hsu-Wen Chou[4], Sarasa Johnson[3], Guido Nador[5], Shohei Ikoma[6], Patrick Hlavacek[2], Chandra Prakash Yadav[7], Chenan Zhang[8], Marco Dibonaventura[2], Erman Guler[6], Yong Chen[9]

*[1]Hyogo Medical University Hospital, Department of Hematology, Nishinomiya, Japan;[2]Pfizer Inc, New York, United States of America; [3]Statlog Inc., Quebec, Canada; [4]Pfizer Ltd., Taipei, Taiwan; [5]Pfizer Ltd., Surrey, United Kingdom;[6]Pfizer Japan Inc., Tokyo, Japan; [7]Pfizer Ltd., Chennai, India;[8]Pfizer Inc., South San Francisco, CA, United States of America;[9]Pfizer Inc., Collegeville, PA, United States of America;*

### Background

Elranatamab is the first B-cell maturation antigen bispecific antibody approved in Japan for treatment of patients with heavily pre-treated multiple myeloma (MM). Although prior clinical work supports the safety of elranatamab's fixed dose formulation (Elmeliegy 2025), assessing its tolerability across different patient body weights in a real-world (RW) context is important, especially in typically lower weight Asian populations.

### Aims

This study aimed to characterize the safety of elranatamab among RW patients with MM by body weight in Japan.

### Methods

SUMMIT is a retrospective cohort study using deidentified administrative claims data from the Japan Medical Data Vision database. Adult patients with a diagnosis of MM who first received elranatamab from March 26, 2024, to March 31, 2025, outside of clinical trials, were included in the study. Five body weight subgroups were defined: four based on baseline body weight quartiles and one very low-weight (VLW) subgroup comprising patients weighing ≤40 kg. Baseline characteristics and elranatamab administration patterns were reported descriptively. The incidence of adverse events, including cytokine release syndrome (CRS), immune effector cell-associated neurotoxicity syndrome (ICANS), and cytopenia (encompassing anemia, leukopenia, lymphopenia, neutropenia, pancytopenia, and thrombocytopenia), was estimated among at-risk patients who had no history of the respective events within the 30 days (d) preceding elranatamab initiation. Events in the 14d following initiation were identified using disease codes (for cytopenia only: 8d and 30d).

### Results

Results for patients who received elranatamab (n=258) are summarized in Table 1. Safety outcomes were similar across all body weight groups, with CRS (14d) ranging from 45.5% to 50.8%. Cytopenia (30d) was similar across quartiles (23.2-28.3%) but differed in the VLW group (43.8%).

**Copyright Information:** (Online) ISSN: 2572-9241
©2026 The Author(s). HemaSphere is published by John Wiley & Sons Ltd on behalf of European Hematology Association. This is an open access Abstract Book distributed under the Attribution-NonCommercial-NoDerivs (CC BY-NC-ND), which allows third parties to download the articles and share them with others as long as they credit the author and the Abstract Book, but they cannot change the content in any way or use them commercially.
**Abstract Book Citations:** Authors, Title, HemaSphere, 2026;10:(S1):pages. The abstract book DOIs can be found on the "Author Information" page.
**Disclaimer:** Articles published in the journal HemaSphere exclusively reflect the opinions of the authors. The authors are responsible for all content in their abstracts including accuracy of the facts, statements, citing resources, etc.

### Summary/Conclusion

Safety outcomes and elranatamab administration patterns were comparable for Japanese patients by weight. Further investigation in larger samples of this population is warranted.

**Table 1. Baseline characteristics, elranatamab administration and incidence of safety outcomes by body weight for RW Japanese patients**

| | **VLW ≤40.0 kg (n=22)** | **Quartile 1 ≤48.0 kg (n=66)** | **Quartile 2 >48.0-54.6 kg (n=63)** | **Quartile 3 >54.6-62.0 kg (n=66)** | **Quartile 4 >62.0-96.1 kg (n=63)** |
|---|---|---|---|---|---|
| Age, median (IQR) | 76.5 (69.0-82.0) | 74.0 (70.0-80.0) | 76.0 (69.0-79.0) | 74.5 (68.0-78.0) | 72.0 (64.0-76.0) |
| Female, n (%) | 22 (100.0) | 59 (89.4) | 43 (68.3) | 24 (36.4) | 12 (19.0) |
| Time to first full dose, median (IQR), d | 9.0 (7.0-14.0) | 8.0 (7.0-14.0) | 10.0 (7.0-13.0) | 7.0 (7.0-10.0) | 7.0 (7.0-8.0) |
| CRS (14d), n (%*) | 10 (45.5) | 30 (45.5) | 32 (50.8) | 32 (49.2) | 29 (46.0) |
| ICANS (14d), n (%*) | 0 (0.0) | 0 (0.0) | 4 (6.3) | 3 (4.5) | 2 (3.2) |
| Cytopenia (8d), n (%*) | 3 (16.7) | 6 (10.5) | 13 (22.8) | 10 (16.7) | 11 (19.3) |
| Cytopenia (14d), n (%*) | 4 (23.5) | 8 (14.5) | 14 (25.0) | 12 (20.7) | 11 (20.0) |
| Cytopenia (30d), n (%*) | 7 (43.8) | 15 (28.3) | 14 (25.9) | 13 (23.2) | 13 (24.5) |

* Percentages are calculated based on the at-risk population, not the total cohort.

**Copyright Information:** (Online) ISSN: 2572-9241
©2026 The Author(s). HemaSphere is published by John Wiley & Sons Ltd on behalf of European Hematology Association. This is an open access Abstract Book distributed under the Attribution-NonCommercial-NoDerivs (CC BY-NC-ND), which allows third parties to download the articles and share them with others as long as they credit the author and the Abstract Book, but they cannot change the content in any way or use them commercially.
**Abstract Book Citations:** Authors, Title, HemaSphere, 2026;10:(S1):pages. The abstract book DOIs can be found on the "Author Information" page.
**Disclaimer:** Articles published in the journal HemaSphere exclusively reflect the opinions of the authors. The authors are responsible for all content in their abstracts including accuracy of the facts, statements, citing resources, etc.

### (PS1947) EFFICACY AND SAFETY OF REDUCED-DOSE DARATUMUMAB PLUS BORTEZOMIB AND DEXAMETHASONE (DVD-LITE) IN NEWLY DIAGNOSED MGRS PATIENTS

**Topic:** 14. Myeloma and other monoclonal gammopathies - Clinical

Huan Xie*[1], Maoyuan Xiang[1], Dongfeng Zeng[1]

*[1]Daping Hospital, Army Medical University, Department of Hematology, Chongqing, China;*

**Background**

Clone-directed therapy is essential for managing monoclonal gammopathy of renal significance (MGRS), as it reduces the nephrotoxic monoclonal protein burden and preserves renal function. However, due to the rarity and frequent misdiagnosis of MGRS, there is no evidence-based standard first-line treatment for MGRS.

**Aims**

This open-label, prospective clinical trial aimed to assess the efficacy and safety of the combination of reduced-dose daratumumab (Dara), bortezomib, and dexamethasone (DVd-lite) on ten newly diagnosed MGRS patients.

**Methods**

The treatment protocol consisted of six cycles of the DVd-lite regimen, which included Dara (8-10 mg biweekly), bortezomib (1.3 mg/m$^2$ weekly) and dexamethasone (5-20 mg weekly).

**Results**

The median follow-up duration of this cohort was 13.1 months (range: 4.7-17.6 months). The results showed a hematological overall response rate (ORR) of 80% and a kidney ORR of 90%. Moreover, the complete response rates in hematology and kidney were both 60%. In addition, only one patient experienced a grade 3 treatment-related adverse event.

**Summary/Conclusion**

These findings demonstrated that the DVd-lite regimen is an effective and well-tolerated first-line treatment option for newly diagnosed MGRS patients.

**Copyright Information:** (Online) ISSN: 2572-9241
©2026 The Author(s). HemaSphere is published by John Wiley & Sons Ltd on behalf of European Hematology Association. This is an open access Abstract Book distributed under the Attribution-NonCommercial-NoDerivs (CC BY-NC-ND), which allows third parties to download the articles and share them with others as long as they credit the author and the Abstract Book, but they cannot change the content in any way or use them commercially.
**Abstract Book Citations:** Authors, Title, HemaSphere, 2026;10:(S1):pages. The abstract book DOIs can be found on the "Author Information" page.
**Disclaimer:** Articles published in the journal HemaSphere exclusively reflect the opinions of the authors. The authors are responsible for all content in their abstracts including accuracy of the facts, statements, citing resources, etc.

### (PS1948) CO-OCCURRENCE, TIMING, AND SURVIVAL IN COMPOSITE HEMATOLOGIC MALIGNANCIES: SPOTLIGHT ON MULTIPLE MYELOMA

**Topic:** 14. Myeloma and other monoclonal gammopathies - Clinical

Benedikt Pelzer*[1], Sara Jennrich[2], Tudor Magdas[3], Hans C. Reinhardt[2], Bastian von Tresckow[2], Julia von Tresckow[4]

*[1]Weill Cornell Medical College, Hematology, New York City, United States of America;[2]University Hospital Essen, Department of Hematology, Essen, Germany; [3]Iuliu Hatieganu University of Medicine and Pharmacy, Cluj-Napoca, Romania;[4]Katholisches Klinikum Bochum, Ruhr University Bochum, Department of Hematology, Oncology and Palliative Care, Bochum, Germany;*

### Background

The coexistence of two distinct lymphoma subtypes within a single patient, either at the same anatomical site (composite lymphoma, CL) or at different sites (discordant lymphoma, DL), represents a rare entity. Both terms are frequently used interchangeably, leading to conflicting terminology in clinical practice. These lymphomas may arise simultaneously (synchronous lymphoma, SyncL) or sequentially (sequential lymphoma, SeqL), with timing potentially reflecting distinct biological mechanisms and carrying different prognostic implications.

### Aims

To characterize the clinical features, co-occurrence patterns, and survival outcomes of CL and DL, as well as SyncL and SeqL, with a focused analysis on Multiple Myeloma (MM) in the setting of dual hematologic malignancies.

### Methods

We conducted a comprehensive literature review via PubMed and Web of Science through December 2025. Inclusion criteria required two lymphomas diagnosed synchronously or within 6 months of each other; cases of indolent lymphomas were included if a sequential lymphoma arose. Cases showing histological transformation were excluded. Survival analyses were restricted to cases diagnosed after 1990.

### Results

A total of 1,410 cases were included, with a median age of 65 years and a male predominance (64%). The most common pairings were MM/CLL and MM/B-ALL, followed by FL/HL, DLBCL/HL, and AITL/DLBCL (Fig. A). SeqL demonstrated significantly inferior overall survival compared to SyncL (p=0.0017, HR 1.5, CI95 1.17–1.92; Fig. B), while no survival difference was observed between CL and DL. This survival disadvantage was confined to B-cell lymphoma combinations (p=0.0003, HR 1.78, CI95 1.31–2.42; Fig. C), with no significant timing-related difference observed in cases involving T-cell lymphomas (p=0.74; Fig. D). Given the unexpectedly high prevalence of MM in dual lymphoma cases, we further characterized this subgroup (Fig. E). The co-occurring lymphoma subtype had a striking influence on survival outcomes within MM-containing cases (Fig. F): while favorable outcomes in MM/T-large granulocyte leukemia and poor outcomes in MM/plasmablastic lymphoma (PBL) combinations were anticipated, the near-identical survival of MM/CLL and MM/B-ALL cases was unexpected.

**Copyright Information:** (Online) ISSN: 2572-9241
©2026 The Author(s). HemaSphere is published by John Wiley & Sons Ltd on behalf of European Hematology Association. This is an open access Abstract Book distributed under the Attribution-NonCommercial-NoDerivs (CC BY-NC-ND), which allows third parties to download the articles and share them with others as long as they credit the author and the Abstract Book, but they cannot change the content in any way or use them commercially.
**Abstract Book Citations:** Authors, Title, HemaSphere, 2026;10:(S1):pages. The abstract book DOIs can be found on the "Author Information" page.
**Disclaimer:** Articles published in the journal HemaSphere exclusively reflect the opinions of the authors. The authors are responsible for all content in their abstracts including accuracy of the facts, statements, citing resources, etc.

### Summary/Conclusion

This large comprehensive analysis underscores the clinical and biological relevance of distinguishing SyncL from SeqL, with sequential presentation conferring inferior survival driven by B-cell lymphoma combinations. MM emerges as a biologically distinct entity within dual hematologic malignancies, characterized by a unique co-occurrence landscape and unexpected survival patterns. These findings warrant further investigation and may have implications for therapeutic decision-making.

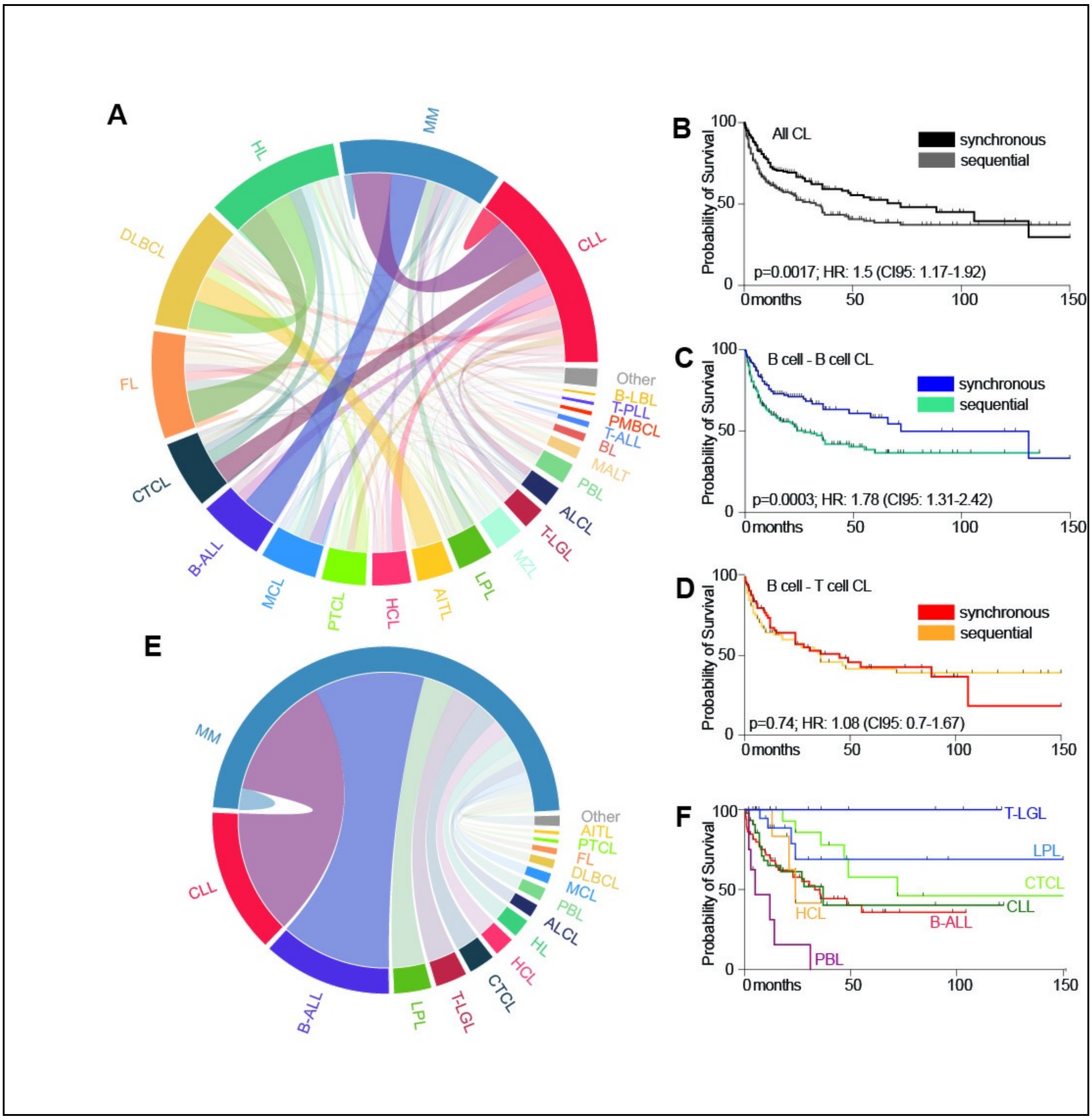

Figure legend: A) Circos plot illustrating all combinations of dual lymphomas. (B) Overall survival (OS) of patients with synchronous lymphomas (SyncL) versus sequential lymphomas (SeqL). (C) OS of SyncL versus SeqL in dual B-cell lymphomas. (D) OS of SyncL versus SeqL in dual lymphomas comprising both B-cell and T-cell neoplasms. (B–D) Survival curves were generated using the Kaplan–Meier method and compared with the log-rank test; follow-up was censored at 150 months. (E) Circos plot showing combinations of dual lymphomas that include multiple myeloma (MM). (F) OS of MM-containing dual lymphomas stratified by the histologic subtype of the second lymphoma.

**Copyright Information:** (Online) ISSN: 2572-9241
©2026 The Author(s). HemaSphere is published by John Wiley & Sons Ltd on behalf of European Hematology Association. This is an open access Abstract Book distributed under the Attribution-NonCommercial-NoDerivs (CC BY-NC-ND), which allows third parties to download the articles and share them with others as long as they credit the author and the Abstract Book, but they cannot change the content in any way or use them commercially.
**Abstract Book Citations:** Authors, Title, HemaSphere, 2026;10:(S1):pages. The abstract book DOIs can be found on the “Author Information” page.
**Disclaimer:** Articles published in the journal HemaSphere exclusively reflect the opinions of the authors. The authors are responsible for all content in their abstracts including accuracy of the facts, statements, citing resources, etc.

### (PS1949) REAL-WORLD (RW) ELRANATAMAB DOSING PATTERNS AND TIMES TO NEXT TREATMENT (TTNT) AMONG UNITED STATES (US) PATIENTS WITH MULTIPLE MYELOMA (MM): RESULTS FROM THE ALTITUDE-1 & 2 STUDIES

**Topic:** 14. Myeloma and other monoclonal gammopathies - Clinical

Meera Mohan*[1], Rahul Banerjee[2], Bhavesh Shah[3], Patricia Prince[4], Nileesa Gautam[4], Elisha Beebee[4], Cera Cantu[5], Sapna Prasad[5], Pooja Patel[5], David Hughes[6], Guido Nador[7], Patrick Hlavacek[8], Rickard Sandin[9], Benjamin Li[8], Aster Meche[8], Chai Kim[8], Isabel Perez Cruz[8], Mohsena Sumaya[8], Marco Dibonaventura[8]

*[1]Medical College of Wisconsin, Milwaukee, United States of America;[2]Fred Hutchinson Cancer Center, Seattle, United States of America; [3]Boston Medical Center, Boston, United States of America; [4]Aetion, New York, United States of America; [5]Clarify Health, San Francisco, United States of America; [6]Pfizer Inc, Cambridge, MA, United States of America; [7]Pfizer Ltd., Surrey, United Kingdom; [8]Pfizer Inc, New York, NY, United States of America;[9]Pfizer AB, Stockholm, Sweden;*

### Background

Elranatamab (ELRA) is a bispecific antibody that targets both B-cell maturation antigen (BCMA)-expressing cells and CD3-expressing T lymphocytes. ELRA is approved for the treatment of relapsed/refractory MM in several countries; however, RW data for US patients remain limited.

### Aims

To describe patient characteristics, treatment patterns, and outcomes associated with ELRA using 2 US RW databases.

### Methods

ALTITUDE-1 (EUPAS1000000229) and ALTITUDE-2 (EUPAS1000000293) are ongoing, non-interventional database studies designed to capture RW ELRA use among US patients with MM. Data sources included administrative claims from Komodo Health's Healthcare Map™ (ALTITUDE-1; October 28, 2025 data cut) and Medicare Fee-For-Service data (ALTITUDE-2; September 2025 data cut). Adult patients with MM with ≥1 claim for ELRA and ≥180 days of pre-index enrollment were included. ALTITUDE-2 also required ≥30 days of post-index enrollment. The first ELRA claim represented the index date. ELRA dosing patterns were reported separately for the step-up dosing period ("SUD period"; Index to Day 8), Cycles 1-6 ("C1-6"; months 1-6, where per label QW is expected), and Cycles 7+ ("C7+"; month 7+, where per label Q2W is expected, depending on patient response). QW and Q2W dosing were calculated based on average day intervals between doses.  Median TTNT, a commonly used RW proxy for progression-free survival in claims datasets, and overall survival (OS) were reported based on Kaplan-Meier methods.

**Copyright Information:** (Online) ISSN: 2572-9241
©2026 The Author(s). HemaSphere is published by John Wiley & Sons Ltd on behalf of European Hematology Association. This is an open access Abstract Book distributed under the Attribution-NonCommercial-NoDerivs (CC BY-NC-ND), which allows third parties to download the articles and share them with others as long as they credit the author and the Abstract Book, but they cannot change the content in any way or use them commercially.
**Abstract Book Citations:** Authors, Title, HemaSphere, 2026;10:(S1):pages. The abstract book DOIs can be found on the "Author Information" page.
**Disclaimer:** Articles published in the journal HemaSphere exclusively reflect the opinions of the authors. The authors are responsible for all content in their abstracts including accuracy of the facts, statements, citing resources, etc.

### Results

181 patients (ALTITUDE-1) and 391 patients (ALTITUDE-2) were included, with median follow-up of 5.8 months and 8.0 months, respectively. In ALTITUDE-1, the median (interquartile range [IQR]) age was 73 years (65-78 years), 44.3% female, 47.5% White, and 26.8% Black. 43.2% were penta-drug exposed and 17.5% had a prior BCMA-directed therapy (including 9.3% with prior CAR T therapy). Demographic and clinical characteristics in ALTITUDE-2 were similar. During the SUD period in ALTITUDE-1, administrations occurred at a mean of 5.2 days apart (median: 6, IQR: 3-7). During C1-6, the mean number of days between administrations was similar across studies (ALTITUDE-1: 10.4 days (median:7, IQR:7-14); ALTITUDE-2: 10.8 days (median:7, IQR: 7-14 days)), with a quarter of administrations already occurring on a Q2W cadence during C1-6. During C7+, the mean number of days between administrations were also similar (ALTITUDE 1: 20.6 days (median: 14, IQR: 14-28); ALTITUDE-2: 20.9 days (median: 14 (IQR: 14-28)), with a quarter of administrations occurring on a Q4W cadence. Median TTNT was not reached in either study (ALTITUDE-1: [IQR: 6.4 months, NR]; ALTITUDE-2: [95% CI: 18.7 months, NR]). Median OS was also not reached in either study (ALTITUDE-1 [IQR: 12.6 months, NR]; ALTITUDE-2 [95% CI: 21.0 months, NR]). In ALTITUDE-2, 62.0% (95%CI: 55.4%, 66.8%) and 57.8% (95%CI: 51.2%, 63.6%) of patients remained without an event at 12 and 18 months. Additionally, the estimated OS at 12 and 18 months was 71.5% (95%CI: 66.2%, 76.1%) and 67.2% (95%CI: 60.1%, 73.0%), respectively.

### Summary/Conclusion

Across 2 US RW databases, patients treated with ELRA were heavily pre-treated, and prior exposure to BCMA-directed therapy was common. During C1-6 and C7+, real-world treatment patterns suggest less frequent administration of ELRA compared with the label. TTNT and OS were not reached with longer follow-up required to assess RW effectiveness.

**Copyright Information:** (Online) ISSN: 2572-9241
©2026 The Author(s). HemaSphere is published by John Wiley & Sons Ltd on behalf of European Hematology Association. This is an open access Abstract Book distributed under the Attribution-NonCommercial-NoDerivs (CC BY-NC-ND), which allows third parties to download the articles and share them with others as long as they credit the author and the Abstract Book, but they cannot change the content in any way or use them commercially.
**Abstract Book Citations:** Authors, Title, HemaSphere, 2026;10:(S1):pages. The abstract book DOIs can be found on the "Author Information" page.
**Disclaimer:** Articles published in the journal HemaSphere exclusively reflect the opinions of the authors. The authors are responsible for all content in their abstracts including accuracy of the facts, statements, citing resources, etc.

### (PS1950) REAL-WORLD OUTCOMES OF MULTIPLE MYELOMA IN PATIENTS ≥80 YEARS: A BICENTRIC ITALIAN RETROSPECTIVE STUDY AND THE IMPACT OF NOVEL THERAPIES.

**Topic:** 14. Myeloma and other monoclonal gammopathies - Clinical

Daniele Derudas[1], Luca Deiana*[2], Cristina Depau[1], Sabrina Chiriu[2], Annamaria Liguori[1], Marco Murineddu[2], Rita Floris[1], Angelo Domenico Palmas[2]

*[1]S.C. di Ematologia e C.T.M.O. - Ospedale Oncologico di Riferimento Regionale "A. Businco", Cagliari, Italy;[2]S.C. di Ematologia - Ospedale "San Francesco", Nuoro, Italy;*

### Background

Population aging has resulted in an increasing number of patients aged ≥80 years with multiple myeloma (MM), a group that is under-represented in registrational trials and often presents with multiple comorbidities and associated frailty, complicating treatment selection and outcome analysis in routine clinical practice.

### Aims

To describe clinical features, treatments, responses, toxicity, and survival of MM patients diagnosed at age ≥80 years in a real-world setting, with particular focus on therapeutic evolution and the introduction of novel therapies particularly anti-CD38–containing regimens in clinical practice.

### Methods

We performed a retrospective observational study at two Italian hematology institutions (Ospedale Oncologico “A. Businco” in Cagliari and Ospedale “S. Francesco” in Nuoro). We identified a total of 111 patients diagnosed with MM at age ≥80 years between January 2010 and January 2025. After exclusions for incomplete documentation or very early death, 104 patients were evaluable. We collected variables including CRAB features, ISS/R-ISS, comorbidities, frailty according to simplified IMWG frailty score, therapeutic regimens, and response per IMWG criteria. Primary endpoint was overall survival (OS) estimated by Kaplan–Meier; secondary endpoints included overall response rates and toxicity.

### Results

Median age at diagnosis was 82 years (range 80–91), with balanced sex distribution. Disease burden at diagnosis was considerable: anemia 63.1%, renal impairment 29.7%, hypercalcemia 15.3%, and skeletal lesions 77.2%; 51.9% had ISS stage III. Frailty was prominent (the vast majority classified as frail by simplified IMWG), and >50% had ≥3 comorbidities. Overall, 90/111 patients (81.1%) received at least one anti-myeloma treatment; common frontline regimens were VMP, Rd, Dara-Rd, and Dara-VMP. Frontline overall response rate (≥PR) was 83.3%, with >1/3 achieving ≥VGPR. Anti-CD38–based regimens showed higher ORR than other regimens (95.2% vs 79.7%). Median follow-up (reverse Kaplan–Meier) was 55.7 months. Median OS for the whole cohort was 41.0 months; OS at 12, 24, and 60 months was 87.9%, 67.9%, and 33.8%, respectively. Renal impairment at diagnosis was associated with significantly worse survival and was the only CRAB component clearly linked to inferior outcome; extramedullary/paraosseous disease also identified a poor-prognosis subgroup. Toxicity was overall manageable: hematologic toxicity occurred in 41.1% (grade 3–4: 16.7%), non-hematologic toxicity in 44.4% (grade 3–4: 1.1%); the most frequent events were gastrointestinal, infectious, neuropathic, and cutaneous.

**Copyright Information:** (Online) ISSN: 2572-9241
©2026 The Author(s). HemaSphere is published by John Wiley & Sons Ltd on behalf of European Hematology Association. This is an open access Abstract Book distributed under the Attribution-NonCommercial-NoDerivs (CC BY-NC-ND), which allows third parties to download the articles and share them with others as long as they credit the author and the Abstract Book, but they cannot change the content in any way or use them commercially.
**Abstract Book Citations:** Authors, Title, HemaSphere, 2026;10:(S1):pages. The abstract book DOIs can be found on the “Author Information” page.
**Disclaimer:** Articles published in the journal HemaSphere exclusively reflect the opinions of the authors. The authors are responsible for all content in their abstracts including accuracy of the facts, statements, citing resources, etc.

### Summary/Conclusion

In this real-world bicentric cohort of ultra-octogenarians with MM, most patients could receive disease-directed therapy and achieved high response rates with a median OS of 41 months despite marked frailty and comorbidity. The progressive adoption of modern triplets and anti-CD38–containing regimens was associated with deeper responses and a favorable survival gradient, supporting that chronological age ≥80 years alone should not preclude contemporary therapy when adapted to frailty, comorbidities, and when effective toxicity management is provided.

**Real-world outcomes of multiple myeloma in patients ≥80 years: a bicentric Italian retrospective study and the impact of novel therapies.**

**Background:** Population aging has resulted in an increasing number of patients aged ≥80 years with multiple myeloma (MM), a group that is under-represented in registrational trials and often presents with multiple comorbidities and associated frailty, complicating treatment selection and outcome analysis in routine clinical practice.

**Aims:** To describe clinical features, treatments, responses, toxicity, and survival of MM patients diagnosed at age ≥80 years in a real-world setting, with particular focus on therapeutic evolution and the introduction of novel therapies particularly anti-CD38–containing regimens in clinical practice.

**Methods:** We performed a retrospective observational study at two Italian hematology institutions (Ospedale Oncologico "A. Businco" in Cagliari and Ospedale "S. Francesco" in Nuoro). We identified a total of 111 patients diagnosed with MM at age ≥80 years between January 2010 and January 2025. After exclusions for incomplete documentation or very early death, 104 patients were evaluable. We collected variables including CRAB features, ISS/R-ISS, comorbidities, frailty according to simplified IMWG frailty score, therapeutic regimens, and response per IMWG criteria. Primary endpoint was overall survival (OS) estimated by Kaplan–Meier; secondary endpoints included overall response rates and toxicity.

**Results:** Median age at diagnosis was 82 years (range 80–91), with balanced sex distribution. Disease burden at diagnosis was considerable: anemia 63.1%, renal impairment 29.7%, hypercalcemia 15.3%, and skeletal lesions 77.2%; 51.9% had ISS stage III. Frailty was prominent (the vast majority classified as frail by simplified IMWG), and >50% had ≥3 comorbidities. Overall, 90/111 patients (81.1%) received at least one anti-myeloma treatment; common frontline regimens were VMP, Rd, Dara-Rd, and Dara-VMP. Frontline overall response rate (≥PR) was 83.3%, with >1/3 achieving ≥VGPR. Anti-CD38–based regimens showed higher ORR than other regimens (95.2% vs 79.7%). Median follow-up (reverse Kaplan–Meier) was 55.7 months. Median OS for the whole cohort was 41.0 months; OS at 12, 24, and 60 months was 87.9%, 67.9%, and 33.8%, respectively. Renal impairment at diagnosis was associated with significantly worse survival and was the only CRAB component clearly linked to inferior outcome; extramedullary/paraosseous disease also identified a poor-prognosis subgroup. Toxicity was overall manageable: hematologic toxicity occurred in 41.1% (grade 3–4: 16.7%), non-hematologic toxicity in 44.4% (grade 3–4: 1.1%); the most frequent events were gastrointestinal, infectious, neuropathic, and cutaneous.

**Summary/Conclusion:** In this real-world bicentric cohort of ultra-octogenarians with MM, most patients could receive disease-directed therapy and achieved high response rates with a median OS of 41 months despite marked frailty and comorbidity. The progressive adoption of modern triplets and anti-CD38–containing regimens was associated with deeper responses and a favorable survival gradient, supporting that chronological age ≥80 years alone should not preclude contemporary therapy when adapted to frailty, comorbidities, and when effective toxicity management is provided.

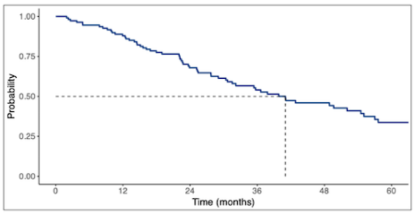

Figure 1. OS Kaplan-Meier of whole cohort.

**Copyright Information:** (Online) ISSN: 2572-9241
©2026 The Author(s). HemaSphere is published by John Wiley & Sons Ltd on behalf of European Hematology Association. This is an open access Abstract Book distributed under the Attribution-NonCommercial-NoDerivs (CC BY-NC-ND), which allows third parties to download the articles and share them with others as long as they credit the author and the Abstract Book, but they cannot change the content in any way or use them commercially.
**Abstract Book Citations:** Authors, Title, HemaSphere, 2026;10:(S1):pages. The abstract book DOIs can be found on the "Author Information" page.
**Disclaimer:** Articles published in the journal HemaSphere exclusively reflect the opinions of the authors. The authors are responsible for all content in their abstracts including accuracy of the facts, statements, citing resources, etc.

### (PS1951) STAGE ALONE IS NOT ENOUGH: INTEGRATING CLONAL BURDEN AND TREATMENT INTENSITY REDEFINES SURVIVAL IN SYSTEMIC AL AMYLOIDOSIS

**Topic:** 14. Myeloma and other monoclonal gammopathies - Clinical

Kubra Aslantas[1], Samil Ozlu[2], Isınsu Kuzu[2], Pervin Topcuoglu[3], Selami Toprak[3], Meral Beksac[4], Güldane Cengiz Seval*[3]

*[1]Ankara University Faculty of Medicine, Department of Internal Medicine, Ankara, Türkiye;[2]Ankara University Faculty of Medicine, Department of Pathology, Ankara, Türkiye; [3]Ankara University Faculty of Medicine, Department of Hematology, Ankara, Türkiye; [4]Istinye University, Department of Hematology, Ankara, Türkiye;*

### Background

Prognosis in systemic light-chain (AL) amyloidosis is traditionally determined by cardiac stage. However, marked survival heterogeneity persists within stage categories, suggesting additional determinants of outcome. Bone marrow plasma cell (BMPC) infiltration reflects clonal tumor burden, yet its independent prognostic significance remains debated. Concurrently, the role of autologous stem-cell transplantation (ASCT) in the daratumumab era continues to be questioned.

### Aims

We hypothesized that survival in AL amyloidosis is driven not solely by stage, but by the interaction between biological aggressiveness and therapeutic intensity.

### Methods

We retrospectively analyzed 116 consecutive patients diagnosed with systemic AL amyloidosis between 2010 and 2024. BMPC infiltration at diagnosis was dichotomized at 10%. Treatment was initiated in 104 patients, in whom hematologic response, overall survival (OS), and progression-free survival (PFS) were assessed. Clinical characteristics, dFLC levels, Revised Mayo 2012 stage, organ involvement, and treatment modalities—including ASCT and daratumumab-based therapy—were evaluated. Survival analyses were performed using Kaplan–Meier estimates and multivariable Cox regression adjusted for age, cardiac stage, and number of involved organs. Logistic regression identified predictors of early mortality.

### Results

Of 116 patients, 104 received treatment and were included in response and survival analyses.Patients with BMPC >10% had higher dFLC levels (p=0.0004), more advanced Revised Mayo stage (p=0.039), and accounted for 82.8% of stage IV disease (p=0.0004). Among treated patients, BMPC >10% was associated with inferior hematologic response and increased early mortality; in multivariable analysis, it independently predicted early death (p=0.031).Median OS was 56 months in the BMPC ≤10% group versus 16 months in the BMPC >10% group. In multivariable Cox models restricted to treated patients, BMPC >10% remained independently associated with inferior OS (p=0.03) and PFS (p=0.013). Among treated patients, 37 (35.6%) underwent ASCT and 67 (64.4%) received chemoimmunotherapy without ASCT. Median OS was significantly longer in the ASCT group compared with the non-ASCT group (153.1 vs. 15.9 months; p=0.000008), with similarly prolonged PFS (96.4 vs. 12.7 months; p=0.0001). Both ASCT and daratumumab were independently associated with improved OS and PFS. Stratification by ASCT and daratumumab exposure revealed pronounced survival gradients: the poorest outcomes occurred in ASCT(−)/Dara(−), whereas the most favorable were seen in ASCT(+)/Dara(+), in whom median OS was not reached. Three-year OS rates were 21%, 60%, 69.5%, and 100%, respectively. Notably, the adverse impact of high BMPC infiltration was attenuated in patients receiving integrative or intensive therapy.

**Copyright Information:** (Online) ISSN: 2572-9241
©2026 The Author(s). HemaSphere is published by John Wiley & Sons Ltd on behalf of European Hematology Association. This is an open access Abstract Book distributed under the Attribution-NonCommercial-NoDerivs (CC BY-NC-ND), which allows third parties to download the articles and share them with others as long as they credit the author and the Abstract Book, but they cannot change the content in any way or use them commercially.
**Abstract Book Citations:** Authors, Title, HemaSphere, 2026;10:(S1):pages. The abstract book DOIs can be found on the "Author Information" page.
**Disclaimer:** Articles published in the journal HemaSphere exclusively reflect the opinions of the authors. The authors are responsible for all content in their abstracts including accuracy of the facts, statements, citing resources, etc.

**Summary/Conclusion**

Stage alone is insufficient to explain survival heterogeneity in systemic AL amyloidosis. Prognosis reflects the interaction between clonal burden and therapeutic intensity. BMPC infiltration >10% identifies a biologically high-risk subset characterized by early mortality and inferior survival, yet this risk can be independently and additively modified by effective therapies, including ASCT and daratumumab. These findings support a shift toward biology-driven and treatment-adapted management strategies that may meaningfully alter the survival trajectory of AL amyloidosis.

**Copyright Information:** (Online) ISSN: 2572-9241
©2026 The Author(s). HemaSphere is published by John Wiley & Sons Ltd on behalf of European Hematology Association. This is an open access Abstract Book distributed under the Attribution-NonCommercial-NoDerivs (CC BY-NC-ND), which allows third parties to download the articles and share them with others as long as they credit the author and the Abstract Book, but they cannot change the content in any way or use them commercially.
**Abstract Book Citations:** Authors, Title, HemaSphere, 2026;10:(S1):pages. The abstract book DOIs can be found on the "Author Information" page.
**Disclaimer:** Articles published in the journal HemaSphere exclusively reflect the opinions of the authors. The authors are responsible for all content in their abstracts including accuracy of the facts, statements, citing resources, etc.

### (PS1952) SUTIMLIMAB IN PATIENTS WITH COLD AGGLUTININ DISEASE AND PRIOR RITUXIMAB EXPOSURE: A POST-HOC ANALYSIS OF THE PHASE 3 CADENZA STUDY

**Topic:** 14. Myeloma and other monoclonal gammopathies - Clinical

Marc Michel*[1], Hind El Amiri[2], Andrea Piacentini[3], Francis Shupo[4], Mounia Beloueche[4], Alexander Röth[5]

*[1]Hôpital Henri-Mondor, Université Paris-Est Créteil, Unit of Internal Medicine, Créteil, France;[2]Recordati Rare Diseases, Lyon, France; [3]Recordati HQ, Milan, Italy; [4]Recordati Rare Diseases, Hemel Hempstead, United Kingdom;[5]West German Cancer Center, University Hospital Essen, University of Duisburg-Essen, Department of Hematology and Stem Cell Transplantation, Essen, Germany;*

### Background

Sutimlimab (SUT) is a humanized monoclonal antibody that inhibits the classical complement pathway by targeting C1s and is the first approved treatment for cold agglutinin disease (CAD). Rituximab (RTX) remains a commonly used off-label frontline treatment for CAD. The Phase 3 randomized, double-blind placebo (PBO)-controlled CADENZA study (NCT03347422) compared SUT with placebo in patients with CAD without recent transfusion history.

### Aims

To describe the efficacy of SUT vs PBO in patients with prior RTX exposure in the CADENZA study.

### Methods

This *post-hoc* analysis of CADENZA included patients with prior RTX exposure (all participants provided informed consent). The primary outcome was responder rate at treatment assessment timepoint (TAT; mean value from Weeks 23, 25, and 26); i.e., the proportion of patients meeting 3 response criteria: (i) hemoglobin (Hb) increase from baseline ≥1.5 g/dL at TAT; [ii] absence of blood transfusions from Week 5–26; [iii] avoidance of protocol-prohibited CAD medications from Week 5–26. Secondary outcomes included changes from baseline to TAT in Hb, bilirubin and lactate dehydrogenase (LDH) levels; changes in the Functional Assessment of Chronic Illness Therapy-Fatigue scale (FACIT-Fatigue) score; and incidence of solicited symptomatic anemia and improvement by visit. Change from baseline to TAT in haptoglobin levels was included as an additional evaluation. All secondary and additional outcomes were analyzed without multiplicity adjustment.

### Results

Twenty-five patients with prior RTX exposure were included (SUT, n=12; PBO, n=13). Mean (standard deviation) age was 65.2 (9.6) years, and 22/25 (88.0%) patients were women. Demographic and baseline characteristics were generally well balanced between arms. Responder rate at TAT was 83.3% (SUT) vs 7.7% (PBO) (odds ratio [95% confidence interval], 26.98 [2.92, 249.64]; p<0.001; **Figure**). Statistically significant improvements were observed for change-from-baseline in Hb and bilirubin, while directional improvements were observed in LDH and FACIT-Fatigue scores (**Table**). Improvements in most anemia symptoms and reductions in symptom severity were more frequent at Week 26 in patients treated with SUT vs PBO.

**Copyright Information:** (Online) ISSN: 2572-9241
©2026 The Author(s). HemaSphere is published by John Wiley & Sons Ltd on behalf of European Hematology Association. This is an open access Abstract Book distributed under the Attribution-NonCommercial-NoDerivs (CC BY-NC-ND), which allows third parties to download the articles and share them with others as long as they credit the author and the Abstract Book, but they cannot change the content in any way or use them commercially.
**Abstract Book Citations:** Authors, Title, HemaSphere, 2026;10:(S1):pages. The abstract book DOIs can be found on the “Author Information” page.
**Disclaimer:** Articles published in the journal HemaSphere exclusively reflect the opinions of the authors. The authors are responsible for all content in their abstracts including accuracy of the facts, statements, citing resources, etc.

### Summary/Conclusion

In this exploratory *post-hoc* subgroup analysis, the clinical efficacy of SUT vs PBO in patients with prior RTX exposure was consistent with the overall CADENZA population across key endpoints. The findings suggest that prior RTX treatment does not negatively impact response to SUT in patients with CAD without recent transfusion history, warranting further prospective validation in a larger study.

**Table. Summary of secondary and additional outcomes: changes from baseline to TAT**

| Outcome | LS mean change from baseline to TAT (95% CI) | | | p-value |
|---|---|---|---|---|
| | SUT (n=12) | PBO (n=13) | Difference SUT versus PBO | |
| Hb (g/dL) | 2.81 (2.21, 3.41) | -0.10 (-0.66, 0.47) | 2.90 (2.08, 3.73) | <0.001 |
| Bilirubin (µmol/L) | -22.70 (-28.30, -17.10) | -0.43 (-5.95, 5.08) | -22.27 (-30.14, -14.39) | <0.001 |
| LDH (U/L) | -154.59 (-303.56, -5.61) | 25.44 (-117.53, 168.41) | -180.03 (-386.53, 26.47) | 0.083 |
| FACIT-Fatigue score | 9.10 (-5.49, 23.69) | 1.05 (-12.33, 14.43) | 8.05 (-11.72, 27.82) | 0.248 |
| Haptoglobin (g/L) | 0.15 (0.00, 0.30) | 0.04 (-0.11, 0.19) | 0.11 (-0.10, 0.32) | 0.295 |

CI, confidence interval; FACIT-Fatigue, Functional Assessment of Chronic Illness Therapy-Fatigue scale; Hb, hemoglobin; LDH, lactate dehydrogenase; LS, least squares; PBO, placebo; RTX, rituximab; SUT, sutimlimab; TAT, treatment assessment timepoint.

**Figure. Composite primary outcome comprising Hb levels, transfusions, and need for CAD medications in patients with CAD and with prior RTX use by treatment group.**
[a]Two patients had missing Hb values at all visits of Week 23, 25 and 26 and their status was therefore considered to be 'unknown'. [b]Two patients discontinued prior to Week 23 without an event and their status was therefore considered to be 'unknown'. CAD, cold agglutinin disease; Hb, hemoglobin

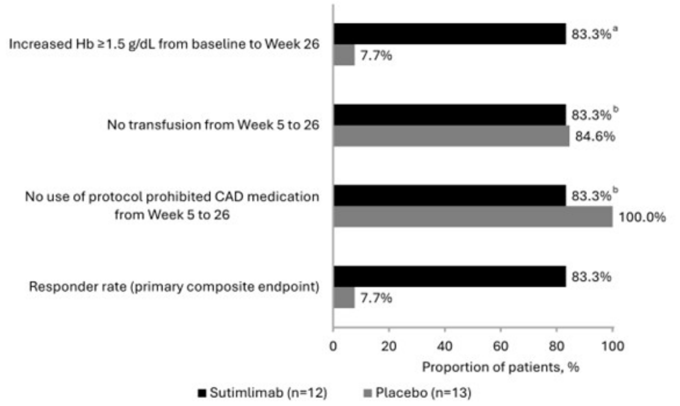

**Copyright Information:** (Online) ISSN: 2572-9241
©2026 The Author(s). HemaSphere is published by John Wiley & Sons Ltd on behalf of European Hematology Association. This is an open access Abstract Book distributed under the Attribution-NonCommercial-NoDerivs (CC BY-NC-ND), which allows third parties to download the articles and share them with others as long as they credit the author and the Abstract Book, but they cannot change the content in any way or use them commercially.
**Abstract Book Citations:** Authors, Title, HemaSphere, 2026;10:(S1):pages. The abstract book DOIs can be found on the "Author Information" page.
**Disclaimer:** Articles published in the journal HemaSphere exclusively reflect the opinions of the authors. The authors are responsible for all content in their abstracts including accuracy of the facts, statements, citing resources, etc.

### (PS1953) MULTI-OMIC CHARACTERIZATION OF INTERFERON-Α DIFFERENTIAL EFFECT ON JAK2V617F MPN AND WILD-TYPE STEM CELLS

**Topic:** 15. Myeloproliferative neoplasms - Biology & translational research

Duanya Liu*[1], Stephane Giraudier[2], Nabih Maslah[2], Bruno Cassinat[2], Jean-Jacques Kiladjian[2]

[1]*INSERM, Paris, France;* [2]*AP-HP, PARIS, France;*

### Background

Interferon-α (IFNα) is the only disease-modifying therapy in myeloproliferative neoplasms (MPN), capable of inducing durable hematologic and molecular remissions in patients with JAK2V617F mutations. Unlike JAK inhibitors, IFNα preferentially targets malignant hematopoietic stem cells (HSCs), yet its clinical use is limited by toxicity and an incomplete understanding of its selective mechanisms of action.

### Aims

To define the cellular and molecular mechanisms underlying the differential sensitivity of JAK2V617F versus wild-type (WT) hematopoietic stem and progenitor cells to IFNα.

### Methods

We used a competitive bone marrow transplantation model combining WT and JAK2V617F cells followed by pegylated IFNα treatment. Hematopoietic stem and progenitor compartments were analyzed by flow cytometry, proliferation and apoptosis assays, and secondary transplantation. Transcriptomic profiling was performed on sorted LSK cells. Parallel transcriptomic and proteomic analyses were conducted in isogenic UT7 cell lines expressing JAK2WT or JAK2V617F.

### Results

PegIFNα treatment significantly reduced JAK2V617F chimerism, normalized peripheral blood counts, and resolved splenomegaly. These findings confirm a selective disease-modifying effect rather than nonspecific hematopoietic toxicity.
Transcriptomic profiling of LSK cells showed that IFNα activated largely overlapping interferon- and cell cycle–associated programs in WT and JAK2V617F cells. However, JAK2V617F HSCs consistently displayed a greater magnitude of transcriptional remodeling, reflecting an exaggerated response to IFNα signaling. Functionally, IFNα induced cell-cycle entry in both genotypes but resulted in disproportionately increased apoptosis and loss of long-term repopulating capacity in JAK2V617F HSCs, leading to stem cell exhaustion.
Multi-omic integration identified selective suppression of EGR1, a key regulator of hematopoietic stem cell quiescence, in JAK2V617F cells following IFNα exposure, accompanied by activation of pro-apoptotic programs. At the pathway level, both in vitro and in vivo models showed conserved induction of MYC/E2F-driven cell-cycle programs together with attenuation of inflammatory signaling, including TNFα pathways. Consistent with these molecular changes, pegIFNα remodeled the systemic cytokine milieu, reducing TNFα, CCL4, and CXCL10 levels and suppressing inflammation-dependent splenic extramedullary hematopoiesis.

**Copyright Information:** (Online) ISSN: 2572-9241
©2026 The Author(s). HemaSphere is published by John Wiley & Sons Ltd on behalf of European Hematology Association. This is an open access Abstract Book distributed under the Attribution-NonCommercial-NoDerivs (CC BY-NC-ND), which allows third parties to download the articles and share them with others as long as they credit the author and the Abstract Book, but they cannot change the content in any way or use them commercially.
**Abstract Book Citations:** Authors, Title, HemaSphere, 2026;10:(S1):pages. The abstract book DOIs can be found on the “Author Information” page.
**Disclaimer:** Articles published in the journal HemaSphere exclusively reflect the opinions of the authors. The authors are responsible for all content in their abstracts including accuracy of the facts, statements, citing resources, etc.

### Summary/Conclusion

IFNα exerts its disease-modifying effects in MPN through a dual mechanism combining intrinsic exhaustion of JAK2V617F HSCs and extrinsic suppression of inflammatory niches. Rather than inducing mutation-specific toxicity, IFNα amplifies a pre-existing inflammatory and stress-primed state in JAK2V617F cells, leading to EGR1 repression, loss of quiescence, and apoptotic attrition. These findings provide a mechanistic framework for the selective clinical efficacy of IFNα and highlight potential targets for developing better-tolerated IFN-mimetic therapies in MPN.

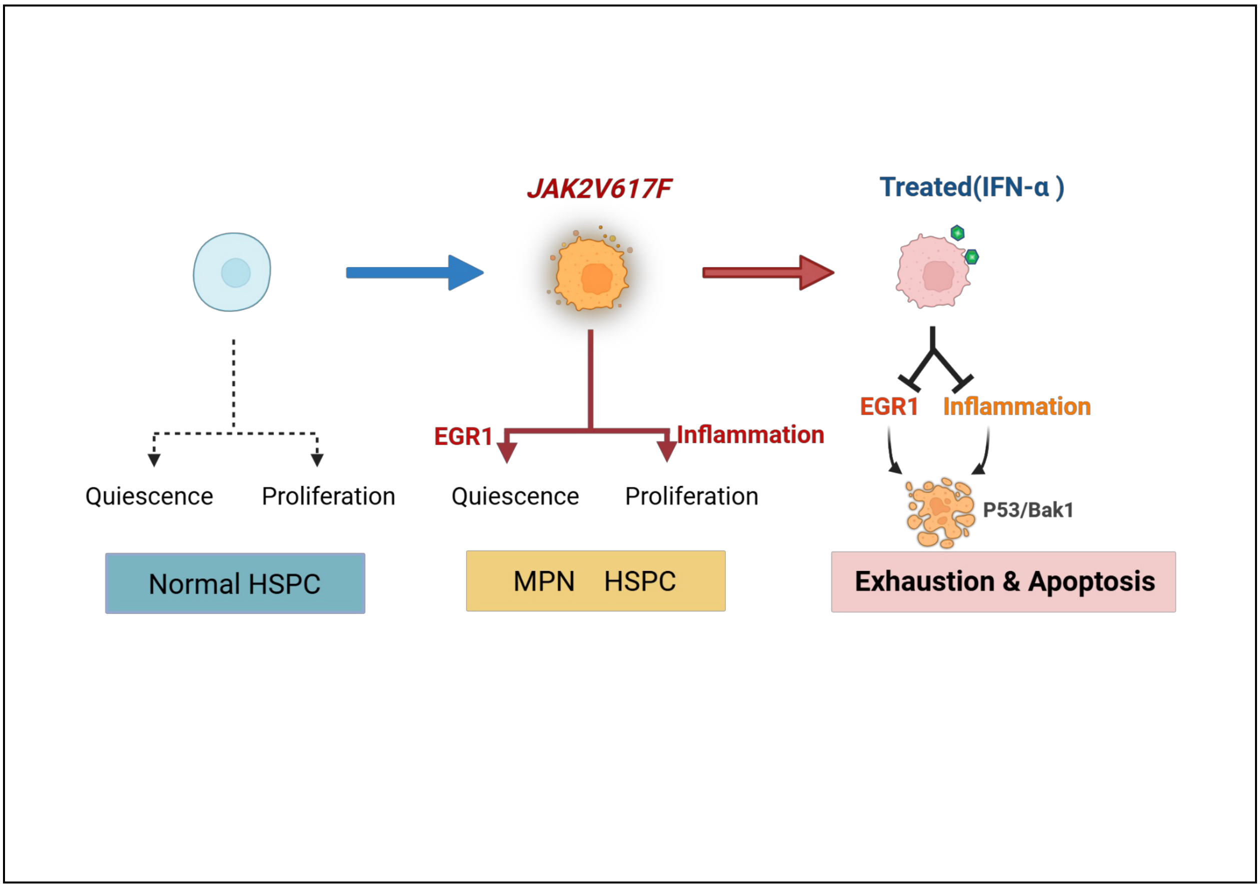

**Copyright Information:** (Online) ISSN: 2572-9241
©2026 The Author(s). HemaSphere is published by John Wiley & Sons Ltd on behalf of European Hematology Association. This is an open access Abstract Book distributed under the Attribution-NonCommercial-NoDerivs (CC BY-NC-ND), which allows third parties to download the articles and share them with others as long as they credit the author and the Abstract Book, but they cannot change the content in any way or use them commercially.
**Abstract Book Citations:** Authors, Title, HemaSphere, 2026;10:(S1):pages. The abstract book DOIs can be found on the "Author Information" page.
**Disclaimer:** Articles published in the journal HemaSphere exclusively reflect the opinions of the authors. The authors are responsible for all content in their abstracts including accuracy of the facts, statements, citing resources, etc.

### (PS1954) EXPRESSION OF JAK2V617F IN ERYTHROBLASTIC ISLAND MACROPHAGES CONTRIBUTES TO THE PATHOGENESIS OF POLYCYTHEMIA VERA

**Topic:** 15. Myeloproliferative neoplasms - Biology & translational research

Xinhua Guo[1], Donghao Liu[1], Huan Zhang[1], Yongshuai Han[1], Qianqian Yang[1], Anqi Guo[1], Xiuli An*[1]

[1]*New York Blood Center, Rye, United States of America;*

### Background

Polycythemia vera (PV) represents a classic Philadelphia chromosome-negative myeloproliferative neoplasm, characterized by microcytic erythrocytosis. Pathogenetically, approximately 95% of PV cases are attributable to the somatic JAK2V617F (Jak2$^{V/F}$) gain-of-function mutation. The overproduction of red blood cells has been thought to be caused mainly by constitutive activation of EPO/EPOR signaling in erythroid progenitors. Notably, it has been documented that erythroblastic island macrophages (EBI macrophages) contributed to the pathogenesis of PV. However, the mechanisms by which EBI macrophages support the pathological red cell production in PV remain unclear. We recently discovered that in addition to be expressed in erythroid cells, EPOR is also expressed in EBI macrophages. Based on these findings, we reasoned that Jak2$^{V/F}$ mutation in EBI macrophages may lead to activation of EPO/EPOR signaling in EBI macrophages, contributing to the pathogenesis of PV.

### Aims

To investigate the contribution of Jak2$^{V/F}$ mutation in EBI macrophages to the pathogenesis of PV and its underlying mechanisms.

### Methods

We generated mouse model in which Jak2$^{V/F}$ mutation was introduced not only in erythroid cells but also in EBI macrophages (Epor-Jak2$^{V/F}$ mice) by crossing the floxed Jak2$^{V/F}$ mouse with the Epor-tdTomato-cre mouse. We also generated mouse model in which Jak2$^{V/F}$ mutation was only introduced in erythroid cells (Gypa-Jak2$^{V/F}$ mice) by crossing the floxed Jak2$^{V/F}$ mouse with Gypa-eGFP-cre mouse. We measured peripheral blood red cell parameters using Advia. We assessed erythroid progenitors by erythroid colony assays. We quantified erythroblasts and analyzed EBI macrophages by flow cytometry. We performed both bulk RNA- and scRNA-seq analyses on EBI macrophages.

**Copyright Information:** (Online) ISSN: 2572-9241
©2026 The Author(s). HemaSphere is published by John Wiley & Sons Ltd on behalf of European Hematology Association. This is an open access Abstract Book distributed under the Attribution-NonCommercial-NoDerivs (CC BY-NC-ND), which allows third parties to download the articles and share them with others as long as they credit the author and the Abstract Book, but they cannot change the content in any way or use them commercially.
**Abstract Book Citations:** Authors, Title, HemaSphere, 2026;10:(S1):pages. The abstract book DOIs can be found on the "Author Information" page.
**Disclaimer:** Articles published in the journal HemaSphere exclusively reflect the opinions of the authors. The authors are responsible for all content in their abstracts including accuracy of the facts, statements, citing resources, etc.

**Results**

We found that while most Epor-Jak2$^{V/F}$ mice died before 4 months old, Gypa-Jak2$^{V/F}$ mice had a normal lifespan. We also found that while Epor-Jak2$^{V/F}$ mice exhibited the characteristic microcytic erythrocytosis of PV, Gypa-Jak2$^{V/F}$ mice were macrocytic. Furthermore, although both Epor-Jak2$^{V/F}$ mice and Gypa-Jak2$^{V/F}$ mice exhibited erythrocytosis, the increase of red cell count was significantly higher in Epor-Jak2$^{V/F}$ mice than that in Gypa-Jak2$^{V/F}$ mice. Importantly, EPO/EPOR signaling in EBI macrophages of the Epor-Jak2$^{V/F}$ mice was constitutively activated as demonstrated by constitutive phosphorylation of Stat5, leading to significant increases in the BM and spleen EBI macrophages along with increased percentage of Ki67$^{+}$ macrophages, indicating hyperproliferation of EBI macrophages. Quantification of erythropoiesis revealed increases in erythroid progenitors and erythroblasts in both BM and spleen with much more increases in Epor-Jak2$^{V/F}$ mice than that in Gypa-Jak2$^{V/F}$ mice. At the molecular level, RNA-Seq analyses revealed that pathways involved in DNA replication and cell cycle are significantly upregulated in the Jak2$^{V/F}$-mutant macrophages. Additionally, the expression levels of growth factors Igf1 and Scf were also increased in the Jak2$^{V/F}$-mutant macrophages.

**Summary/Conclusion**

Our findings demonstrate that Jak2$^{V/F}$ mutation in Epor$^{+}$ macrophages led to hyperproliferation of the Epor$^{+}$ macrophages along with increased expression of growth factors which in turn further enhanced production of red blood cells, evenly leading to extreme erythrocytosis. These findings underscore the critical role of Jak2$^{V/F}$-mutant EBI macrophages in PV pathology, offering potential new avenues for microenvironment targeted therapies.

**Copyright Information:** (Online) ISSN: 2572-9241
©2026 The Author(s). HemaSphere is published by John Wiley & Sons Ltd on behalf of European Hematology Association. This is an open access Abstract Book distributed under the Attribution-NonCommercial-NoDerivs (CC BY-NC-ND), which allows third parties to download the articles and share them with others as long as they credit the author and the Abstract Book, but they cannot change the content in any way or use them commercially.
**Abstract Book Citations:** Authors, Title, HemaSphere, 2026;10:(S1):pages. The abstract book DOIs can be found on the "Author Information" page.
**Disclaimer:** Articles published in the journal HemaSphere exclusively reflect the opinions of the authors. The authors are responsible for all content in their abstracts including accuracy of the facts, statements, citing resources, etc.

### (PF1956) DISCOVERY OF A FIRST-IN-CLASS ALLELE-SELECTIVE XNAZYME THAT KNOCKS DOWN MUTANT JAK2 V617F MRNA IN A MOUSE MODEL OF POLYCYTHEMIA VERA

**Topic:** 15. Myeloproliferative neoplasms - Biology & translational research

Vadim Fedyuk*[1], Alex Rosenberg[1], Einat Amit Romach[1], Rotem Volkman[1], Dmitry Torchinsky[1], Gur Roshwalb[1], Joel Kaye[1]

*[1] 1E Therapeutics, R&D, Rehovot, Israel;*

### Background

Polycythemia vera (PV) is a clonal myeloproliferative neoplasm driven by the somatic JAK2 V617F mutation in 95% of patients. This results in dysregulated erythroid progenitor proliferation and subsequent erythrocytosis with increased blood viscosity and thrombotic risk. Current JAK2 inhibitors lack selectivity for the V617F mutant allele, thereby interfering with WT JAK2-dependent hematopoiesis and leading to cytopenias. In contrast, allele specific knockdown of JAK2 V617F has the potential to be disease-modifying by preferentially suppressing the mutant clone and reducing variant allele burden while sparing WT JAK2 dependent hematopoiesis.
Therapeutic oligonucleotide modalities such as siRNAs and antisense oligonucleotides are powerful tools for targeting the fundamental genetic drivers of disease on the RNA level, providing opportunity to modify disease at its root, however achieving single nucleotide precision with existing technologies remains extremely challenging.
Here, we present 'X-Zymes', chemically evolved catalytic xeno-nucleotide enzymes (XNAzymes) engineered to enable potent, durable and single base allele-selective RNA silencing in vivo.

### Aims

To evaluate the allele selectivity and target engagement of X-Zyme targeting mutant JAK2 V617F in preclinical models of PV.

### Methods

Fully modified X-Zymes were designed to selectively cleave JAK2 V617F transcripts while sparing wild-type JAK2. Potency and selectivity were assessed using kinetic cleavage assays and allele-specific knockdown in cellular reporter systems. For in vivo proof of concept, the candidate X-Zymes were formulated with NOV340 liposomes and evaluated in a bone marrow transplantation model of PV (SclCre;JAK2-V617F;GFP). Mice were treated with a single IV dose and target engagement was assessed by RT-qPCR quantification of mutant-selective JAK2 V617F knockdown in sorted erythroid progenitors (BFU-E and CFU-E).

### Results

X-Zymes demonstrated potent, allele-selective catalytic cleavage of JAK2 V617F with discrimination against wild-type JAK2 and achieved mutant-selective knockdown in cellular assays. NOV340 encapsulation enabled efficient, dose-dependent delivery of the X-Zyme payload to erythroid populations in both bone marrow and spleen. In the PV mouse model, X-Zyme administration resulted in selective knockdown of the V617F mutant allele, and reduction in the frequency of erythroid progenitor cells (CFU-E/BFU-E).

### Summary/Conclusion

X-Zymes enable allele-selective degradation of the mutant JAK2 V617F transcript, a mechanistically distinct approach from JAK inhibitors. X-Zymes were delivered to erythroid progenitors and demonstrated median knockdown of mutant JAK2 V617F mRNA by 70%. These data support a potentially disease-modifying strategy for V617F-driven myeloproliferative neoplasms.

**Copyright Information:** (Online) ISSN: 2572-9241
©2026 The Author(s). HemaSphere is published by John Wiley & Sons Ltd on behalf of European Hematology Association. This is an open access Abstract Book distributed under the Attribution-NonCommercial-NoDerivs (CC BY-NC-ND), which allows third parties to download the articles and share them with others as long as they credit the author and the Abstract Book, but they cannot change the content in any way or use them commercially.
**Abstract Book Citations:** Authors, Title, HemaSphere, 2026;10:(S1):pages. The abstract book DOIs can be found on the "Author Information" page.
**Disclaimer:** Articles published in the journal HemaSphere exclusively reflect the opinions of the authors. The authors are responsible for all content in their abstracts including accuracy of the facts, statements, citing resources, etc.

### (PS1957) METABOLIC REWIRING BY ONCOGENIC JAK2V617F CREATES A GLUCOSE TRANSPORT VULNERABILITY IN MYELOPROLIFERATIVE NEOPLASMS

**Topic:** 15. Myeloproliferative neoplasms - Biology & translational research

Patrick Weiand[1, 2], Nicolas Chatain[1, 2], Marcelo A. Szymanski de Toledo[1, 2], Julia Moellmann[3], Tabea Pirker[3], Siddharth Gupta[1, 2], Henrike Jacobi[1, 2], Jelena Lazarevic[1, 2], Margherita Vieri[1, 2], Maria Jimena Rodriguez[1, 2], Steffen Koschmieder[1, 2], Deniz Nogueira Gezer[1, 2, 4], Julian Baumeister*[1, 2, 4]

*[1]Department of Hematology, Oncology, Hemostaseology, and Stem Cell Transplantation, Faculty of Medicine, RWTH Aachen University, Aachen, Germany; [2]Center for Integrated Oncology Aachen Bonn Cologne Düsseldorf (CIO ABCD), Aachen, Germany; [3]Department of Internal Medicine I, Cardiology, University Hospital RWTH Aachen, Aachen, Germany;[4]Contributed equally, Aachen, Germany;*

### Background

Myeloproliferative neoplasms (MPN) are clonal hematologic malignancies driven by mutations in *JAK2*, *CALR*, or *MPL*. Although these mutations result in persistent JAK-STAT activation and dysregulated hematopoiesis, current therapies fail to eliminate the malignant clone. We previously identified hypoxia–inducible factor 1 (HIF-1) as a selective vulnerability in JAK2V617F–mutant cells. However, the downstream metabolic dependencies sustaining this oncogenic state remain poorly defined.

### Aims

To characterize the metabolic landscape governed by the JAK2–HIF-1 axis and evaluate glucose transport as a therapeutically exploitable vulnerability in MPN.

### Methods

*Slc2a1* (GLUT1), *Slc2a3* (GLUT3), or both glucose transporters were knocked out using CRISPR–Cas9 in *Jak2*V617F and *Jak2*WT 32D MPL cells, and the resulting phenotypes were assessed by RNA sequencing, glucose uptake assays, Seahorse extracellular flux analysis, and proliferation and apoptosis assays.
Metabolic dependencies were further investigated in vivo using a murine *Jak2*V617F knock-in model to evaluate the effects of HIF-1 inhibition with acriflavine (ACF) and GLUT inhibition with Glutor and KL-11743 on disease burden and hematopoiesis. The translational relevance of GLUT1/3 targeting was validated ex vivo using primary peripheral blood mononuclear cells from a clinically diverse cohort of MPN patients harboring *JAK2*, *CALR*, or *MPL* mutations and healthy donors.

**Copyright Information:** (Online) ISSN: 2572-9241
©2026 The Author(s). HemaSphere is published by John Wiley & Sons Ltd on behalf of European Hematology Association. This is an open access Abstract Book distributed under the Attribution-NonCommercial-NoDerivs (CC BY-NC-ND), which allows third parties to download the articles and share them with others as long as they credit the author and the Abstract Book, but they cannot change the content in any way or use them commercially.
**Abstract Book Citations:** Authors, Title, HemaSphere, 2026;10:(S1):pages. The abstract book DOIs can be found on the "Author Information" page.
**Disclaimer:** Articles published in the journal HemaSphere exclusively reflect the opinions of the authors. The authors are responsible for all content in their abstracts including accuracy of the facts, statements, citing resources, etc.

**Results**

Metabolic profiling revealed that JAK2V617F forces a hypermetabolic state with increased glycolytic and oxidative flux, heavily dependent on a functional redundancy between GLUT1 and GLUT3. While single–transporter loss was well–tolerated, dual genetic pharmacological ablation collapsed glucose uptake and glycolytic capacity. This metabolic crisis selectively induced apoptosis in *Jak2*V617F cells, whereas *Jak2*WT cells demonstrated superior metabolic resilience and maintained higher viability (57.8 ± 10.8% (*Jak2*WT) vs 10.6 ± 6.4% (*Jak2*V617F) viable cells, mean ± SD, $p < 0.0001$, Fig. A).
Transcriptomic analysis demonstrated that *Jak2*V617F cells lack the metabolic plasticity required to adapt to glucose deprivation. In contrast to *Jak2*WT controls, *Jak2*V617F cells failed to engage compensatory pathways, instead activating DNA repair and cell cycle stress programs.
In vivo, HIF-1/GLUT inhibition modulated erythroid niche distribution but was insufficient to reverse the MPN phenotype. Importantly, primary MPN patient samples across all three mutation subtypes exhibited significantly greater sensitivity to these single-agent treatments than healthy donor cells yielding highly selective suppression of proliferation (ACF/Glutor: $p < 0.0001$; KL-11743: $p = 0.0002$, Fig. B), viability (ACF/Glutor: $p = 0.0002$; KL-11743: $p = 0.0045$, Fig. C), and clonogenicity (Glutor/KL-11743: $p = 0.0238$, Fig. D). Interestingly, co-treatment with the glutaminase inhibitor CB-839 provided no further benefit.

**Summary/Conclusion**

Our results define the HIF-1–GLUT1/3 axis as a central, targetable metabolic bottleneck in MPN. The selective exhaustion of patient–derived clones, regardless of the specific driver mutation, establishes glucose transport as a shared therapeutic vulnerability. These data provide a mechanistic rationale for further investigation of HIF-1 or GLUT inhibitors. By targeting this fundamental metabolic requirement, such agents may help overcome clinical limitations to achieve disease modification and eradicate the malignant MPN clone.

**Copyright Information:** (Online) ISSN: 2572-9241
©2026 The Author(s). HemaSphere is published by John Wiley & Sons Ltd on behalf of European Hematology Association. This is an open access Abstract Book distributed under the Attribution-NonCommercial-NoDerivs (CC BY-NC-ND), which allows third parties to download the articles and share them with others as long as they credit the author and the Abstract Book, but they cannot change the content in any way or use them commercially.
**Abstract Book Citations:** Authors, Title, HemaSphere, 2026;10:(S1):pages. The abstract book DOIs can be found on the "Author Information" page.
**Disclaimer:** Articles published in the journal HemaSphere exclusively reflect the opinions of the authors. The authors are responsible for all content in their abstracts including accuracy of the facts, statements, citing resources, etc.

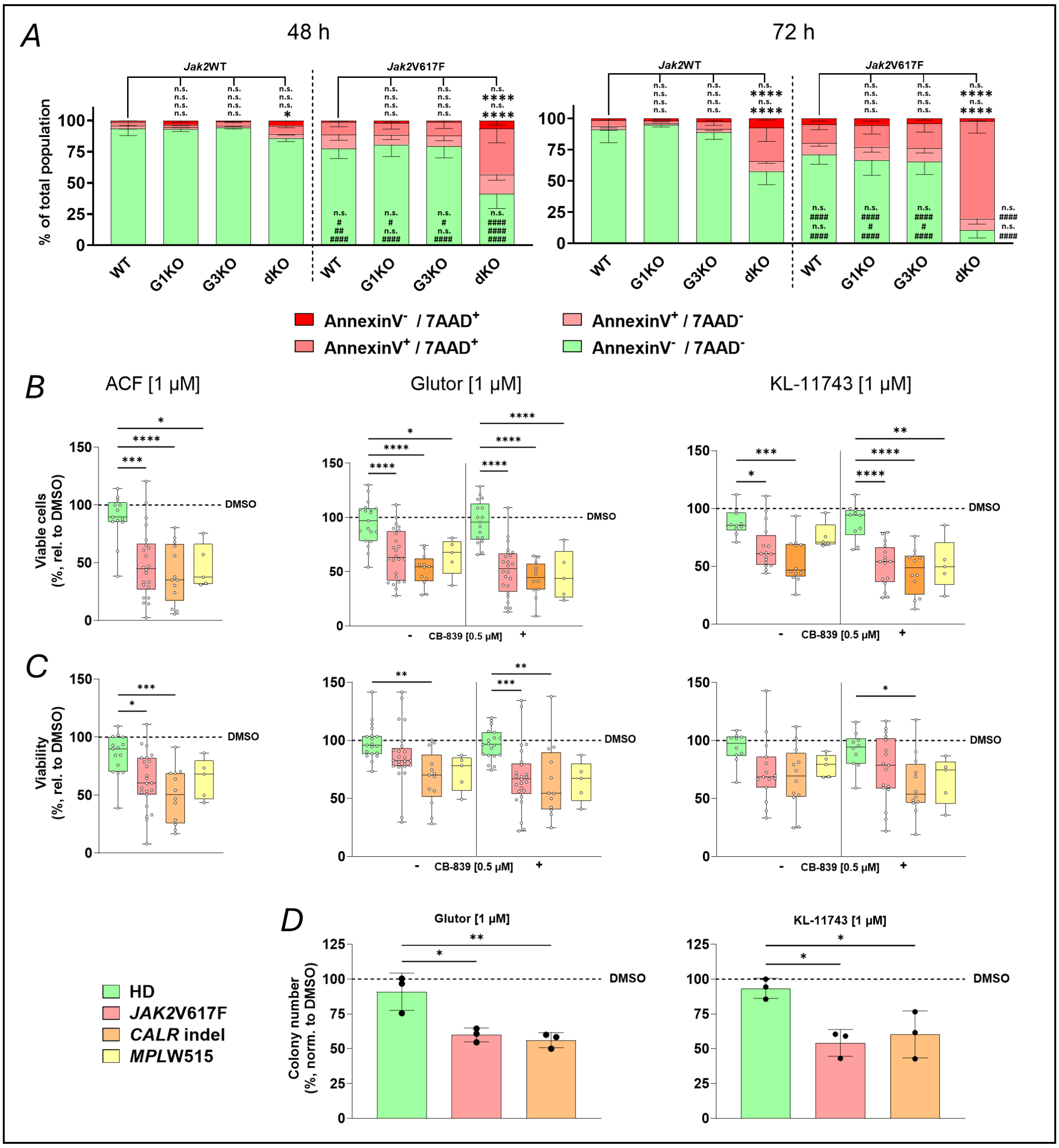

**Copyright Information:** (Online) ISSN: 2572-9241
©2026 The Author(s). HemaSphere is published by John Wiley & Sons Ltd on behalf of European Hematology Association. This is an open access Abstract Book distributed under the Attribution-NonCommercial-NoDerivs (CC BY-NC-ND), which allows third parties to download the articles and share them with others as long as they credit the author and the Abstract Book, but they cannot change the content in any way or use them commercially.
**Abstract Book Citations:** Authors, Title, HemaSphere, 2026;10:(S1):pages. The abstract book DOIs can be found on the "Author Information" page.
**Disclaimer:** Articles published in the journal HemaSphere exclusively reflect the opinions of the authors. The authors are responsible for all content in their abstracts including accuracy of the facts, statements, citing resources, etc.

### (PS1958) STAT5B INDUCES SEX-SPECIFIC PHENOTYPES IN JAK2 V617F-POSITIVE MYELOPROLIFERATIVE NEOPLASMS

**Topic:** 15. Myeloproliferative neoplasms - Biology & translational research

Marie Ploderer[1], Gabriela Feurstein[1], Barbara Maurer[1], Lena Schreiberhuber[1], Andrea Müllebner[1], Abhishek Sawalkar[2], Anandi Krishnan[2, 3], Wencke Walter[4], Gregor Hörmann[4], Emir Hadzijusufovic[5, 6], Peter Valent[5, 6], Thorsten Klampfl[7], Richard Moriggl[8], Heidi A. Neubauer*[1]

*[1]Unit for Medical Biochemistry, University of Veterinary Medicine Vienna, Vienna, Austria;[2]Rutgers Cancer Institute of New Jersey and Department of Biomedical Engineering, Rutgers University, New Brunswick, United States of America; [3]Stanford Cancer Institute, Stanford University School of Medicine, Stanford, United States of America; [4]MLL Munich Leukemia Laboratory, Munich, Germany; [5]Department of Internal Medicine I, Division of Hematology and Hemostaseology, Medical University of Vienna, Vienna, Austria; [6]Ludwig Boltzmann Institute for Hematology and Oncology, Medical University of Vienna, Vienna, Austria; [7]Unit for Pharmacology and Toxicology, University of Veterinary Medicine Vienna, Vienna, Austria; [8]Department of Biosciences and Medical Biology, Paris Lodron University Salzburg, Salzburg, Austria;*

### Background

Myeloproliferative neoplasms (MPNs) are a group of malignant diseases marked by the expansion of myeloid cells originating from a mutated hematopoietic stem cell (HSC). There are clear sex-related differences across MPN disease phenotypes and outcomes, including overall increased mortality and worse disease phenotypes in male MPN patients. However, little is known about the underlying causes. The most frequent driver mutation in MPN is JAK2$^{V617F}$, which activates the transcription factors STAT5A and STAT5B, known to be essential for MPN disease manifestation. STAT5B has well known sex-specific roles in the liver but was more recently predicted to mediate sex-specific gene transcription across a wide range of tissues. A sex-specific role for STAT5B in the hematopoietic compartment and in MPN has not been previously investigated.

### Aims

The aim of this study was to explore a potential sex-specific role of STAT5B in the context of JAK2$^{V617F}$ mutated MPN, using a novel mouse model as well as human MPN patient data.

### Methods

We generated a transgenic mouse model with a hematopoietic-restricted JAK2$^{V617F}$ mutation and increased STAT5B levels. We profiled disease parameters and quantified cells of the HSC and myeloid compartment in these mice (and littermate control mice) using flow cytometry. STAT5B-regulated sex-specific gene expression profiles were assessed in the MPN mice using RNA-seq of peripheral blood (PB)-derived neutrophils. RNA-seq data from PB cells of polycythemia vera (PV) patients were analysed for sex-specific gene expression patterns correlating with *STAT5B* expression, and Gene Set Enrichment Analysis (GSEA) was performed.

**Copyright Information:** (Online) ISSN: 2572-9241
©2026 The Author(s). HemaSphere is published by John Wiley & Sons Ltd on behalf of European Hematology Association. This is an open access Abstract Book distributed under the Attribution-NonCommercial-NoDerivs (CC BY-NC-ND), which allows third parties to download the articles and share them with others as long as they credit the author and the Abstract Book, but they cannot change the content in any way or use them commercially.
**Abstract Book Citations:** Authors, Title, HemaSphere, 2026;10:(S1):pages. The abstract book DOIs can be found on the “Author Information” page.
**Disclaimer:** Articles published in the journal HemaSphere exclusively reflect the opinions of the authors. The authors are responsible for all content in their abstracts including accuracy of the facts, statements, citing resources, etc.

**Results**

We assessed *STAT5B* mRNA levels in JAK2$^{V617F}$-positive MPN patients, revealing significantly higher *STAT5B* expression in both male and female MPN patients compared to healthy controls. To explore the impact of elevated STAT5B levels in MPN, we generated a mouse model harbouring hematopoietic-restricted JAK2$^{V617F}$ with enhanced STAT5B expression. Using this model, we observed that increased STAT5B levels resulted in significantly worsened disease in male mice but not in female mice. Specifically, STAT5B caused increased splenomegaly, a further expansion of HSCs and mature myeloid cells, and overall decreased survival only in male MPN mice. RNA-seq of PB-derived neutrophils from the MPN mice identified a set of STAT5B-regulated genes with sex biased expression patterns, a majority of which were more highly expressed in males compared with females. In line, RNA-seq data from PB cells of PV patients revealed sex biased genes whose expression positively correlated with *STAT5B* expression in a largely male-dominant manner. Importantly, the STAT5B-regulated, male-dominant genes identified in our MPN mouse model were significantly enriched in the human PV gene set. The core enriched genes were associated with biological processes such as cell cycle and metabolism.

**Summary/Conclusion**

Our data suggest that sex biases in MPN could be driven, at least in part, by a sex-specific role of STAT5B, a central transcription factor in MPN pathogenesis. In a JAK2$^{V617F}$ context, we demonstrate the adverse impact of STAT5B signaling on the survival and disease phenotypes of male mice, in line with sex biases observed in MPN patients. Mechanistically, we identify a set of genes that appear to be sex-specifically regulated by STAT5B in both the mouse model and in MPN patients. Overall, our study reveals a novel role for STAT5B as a sex-specific disease-driver in MPN.

**Copyright Information:** (Online) ISSN: 2572-9241
©2026 The Author(s). HemaSphere is published by John Wiley & Sons Ltd on behalf of European Hematology Association. This is an open access Abstract Book distributed under the Attribution-NonCommercial-NoDerivs (CC BY-NC-ND), which allows third parties to download the articles and share them with others as long as they credit the author and the Abstract Book, but they cannot change the content in any way or use them commercially.
**Abstract Book Citations:** Authors, Title, HemaSphere, 2026;10:(S1):pages. The abstract book DOIs can be found on the "Author Information" page.
**Disclaimer:** Articles published in the journal HemaSphere exclusively reflect the opinions of the authors. The authors are responsible for all content in their abstracts including accuracy of the facts, statements, citing resources, etc.

### (PS1959) DRP1-DEPENDENT MITOCHONDRIAL REPROGRAMMING DRIVES TGF-Β1–INDUCED STROMAL ACTIVATION IN MYELOFIBROSIS

**Topic:** 15. Myeloproliferative neoplasms - Biology & translational research

Rose Guignard*[1], Delphine Muller[1], Hélène Duparc[1], Merwan Saddar[1], Monika Wittner[1], William Vainchenker[1], Iléana Antony Debré[1], Sarah Hassan[2], Michelangelo Campanella[2], Antonin Marchais[3], Gael Moquin-Beaudry[3], Guillemette Fouquet[1], Caroline Marty[1], Isabelle Plo[1], Hana Raslova[1]

*[1]INSERM UMR 1287, Equipe labellisée Ligue Nationale Contre le Cancer, Villejuif, France;[2]METSY-UMR 9018, Gustave Roussy, Villejuif, France; [3]INSERM U1015, Gustave roussy, Villejuif, France;*

### Background

Myelofibrosis (MF) is a clonal hematologic malignancy belonging to BCR::ABL-negative myeloproliferative neoplasms (MPNs), alongside essential thrombocythemia and polycythemia vera. It is driven by acquired mutations in hematopoietic stem cells, primarily affecting JAK2 (60%), CALR (30%), or MPL (5%), resulting in constitutive activation of the JAK2/STAT pathway, splenomegaly, and progressive bone marrow fibrosis induced by pathological megakaryocytes (MKs). Current therapeutic options remain largely symptomatic, and allogeneic stem cell transplantation is the only curative treatment associated with significant mortality.

### Aims

The aim of this study was to elucidate the mechanisms underlying bone marrow fibrosis in myelofibrosis, with a particular focus on the role of Drp1-dependent mitochondrial dynamics and oxidative stress in TGF-β1–mediated stromal cell activation driven by pathological megakaryocytes.

### Methods

We used WT, Jak2V617F knock-in (KI), and p19 knockout/Jak2V617F knock-in (KOKI) mouse models, the latter developing a homogeneous myelofibrosis phenotype three weeks after induction of the Jak2 mutation. Spatial transcriptomics was performed on tibial sections. KOKI mice were treated with Mdivi-1 (Drp1 inhibitor) or N-acetyl-L-cysteine (NAC) to evaluate the effect of mitochondrial fission and oxidative stress inhibition on fibrosis. In vitro, TGF-β1–stimulated MS-5 stromal cells and co-culture with pathological megakaryocytes were used to assess mitochondrial remodeling, ROS production, and profibrotic gene expression.

### Results

Spatial transcriptomic analyses of tibial sections in KI and KOKI mice revealed a stromal compartment enriched in megakaryocytes, fibroblasts, and myofibroblasts, associated with platelet activation, fibrosis, and altered oxidative metabolism. Treatment of KOKI mice with Mdivi-1, an inhibitor of mitochondrial fission, abolished this transcriptional program, highlighting a central role for oxidative stress and mitochondrial dynamics in stromal activation. Consistently, Mdivi-1 treatment reduced bone marrow fibrosis, partially restored cellularity, and lowered oxidative stress in stromal cells, supporting the role of altered metabolism in disease progression. In parallel, in vitro TGF-β1 stimulation of stromal cells induced mitochondrial remodeling, increased metabolic activity, and promoted oxidative stress, highlighting the contribution of mitochondrial dynamics to stromal activation. This mitochondrial remodeling was accompanied by increased oxidative phosphorylation and glycolytic capacity, elevated ROS production, and induction of profibrotic genes (Col1α2, Fn1, α-SMA). These effects were markedly attenuated by Mdivi-1 or by the ROS inhibitor NAC. Treatment of KOKI mice with NAC also led to a significant decrease in MF. Furthermore, co-culture experiments confirmed that TGF-β1 secreted by pathological MKs from KOKI mice activates stromal cells. This activation was partially reversed by Mdivi-1 through reduction of ROS levels and decreased expression of TGF-β1 target genes.

**Copyright Information:** (Online) ISSN: 2572-9241
©2026 The Author(s). HemaSphere is published by John Wiley & Sons Ltd on behalf of European Hematology Association. This is an open access Abstract Book distributed under the Attribution-NonCommercial-NoDerivs (CC BY-NC-ND), which allows third parties to download the articles and share them with others as long as they credit the author and the Abstract Book, but they cannot change the content in any way or use them commercially.
**Abstract Book Citations:** Authors, Title, HemaSphere, 2026;10:(S1):pages. The abstract book DOIs can be found on the "Author Information" page.
**Disclaimer:** Articles published in the journal HemaSphere exclusively reflect the opinions of the authors. The authors are responsible for all content in their abstracts including accuracy of the facts, statements, citing resources, etc.

**Summary/Conclusion**

In conclusion, this study identifies a key mechanism of bone marrow fibrosis in which MK-derived TGF-β1 reprograms stromal metabolism via mitochondrial dynamics and ROS. In vivo inhibition of mitochondrial fission with Mdivi-1 reduces oxidative stress, limits fibroblast activation, and attenuates fibrosis, highlighting mitochondrial metabolism as a potential therapeutic target in myelofibrosis..

**Copyright Information:** (Online) ISSN: 2572-9241
©2026 The Author(s). HemaSphere is published by John Wiley & Sons Ltd on behalf of European Hematology Association. This is an open access Abstract Book distributed under the Attribution-NonCommercial-NoDerivs (CC BY-NC-ND), which allows third parties to download the articles and share them with others as long as they credit the author and the Abstract Book, but they cannot change the content in any way or use them commercially.
**Abstract Book Citations:** Authors, Title, HemaSphere, 2026;10:(S1):pages. The abstract book DOIs can be found on the "Author Information" page.
**Disclaimer:** Articles published in the journal HemaSphere exclusively reflect the opinions of the authors. The authors are responsible for all content in their abstracts including accuracy of the facts, statements, citing resources, etc.

### (PS1960) RUXOLITINIB COMBINED WITH HOMOHARRINGTONINE SYNERGISTICALLY TARGETS CDK2 IN RUXOLITINIB-RESISTANT MYELOFIBROSIS AND SECONDARY ACUTE MYELOID LEUKEMIA

**Topic:** 15. Myeloproliferative neoplasms - Biology & translational research

Rumeng Li[1], Qingmei Han[1], Tian Zeng[1], Xiudi Yang[1], Jian Huang*[1]

[1] *The First Affiliated Hospital, Zhejiang University School of Medicine, Hangzhou, China;*

### Background

Myeloproliferative neoplasms (MPNs) frequently develop secondary resistance and progress to secondary acute myeloid leukemia (sAML), which demonstrates poor response to JAK inhibitors and confers extremely unfavorable prognosis.

### Aims

This study aims to investigate the efficacy and mechanism of ruxolitinib combined with homoharringtonine (HHT) in the treatment of JAK-mutant drug-resistant MPNs and sAML.

### Methods

We performed single-cell RNA sequencing in ruxolitinib-resistant and -sensitive MPN patients and established drug-resistant JAK2-mutant cell lines. CDK2 expression was examined in clinical samples and cell lines. Drug combination effects were evaluated by CellTiter-Lumi assay and validated in patient samples. Apoptosis and cell cycle were analyzed by flow cytometry. Key pathways were identified by RNA-seq and proteomics and validated by RT-qPCR and Western blot. A xenograft model was used to assess tumor burden, survival, and toxicity.

### Results

Single-cell sequencing revealed a significantly higher proportion of hematopoietic stem cells (HSCs) in resistant patients, whereas B cells and T cells were more abundant in sensitive patients. Additionally, CDK2 expression was upregulated in resistant patients and exhibited high expression in established resistant cell lines. Further studies demonstrated that CDK2 was universally overexpressed in MPNs, with expression levels further elevated in patients with leukemic transformation; notably, high CDK2 expression was closely associated with increased sensitivity to HHT. Based on these findings, we employed a ruxolitinib combined with homoharringtonine regimen. Cell viability assays indicated that combination therapy significantly inhibited cell proliferation, induced apoptosis, and produced stronger G1-phase arrest compared with monotherapy. RNA-seq, proteomics, and GSEA analyses showed that combination treatment specifically suppressed E2F, PI3K-AKT, mTORC1, c-Myc, and JAK-STAT signaling pathways; meanwhile, both single-cell sequencing and resistant cell RNA-seq data confirmed significant activation of the PI3K/AKT pathway in resistant cells. Western blot and qPCR verified that the two-drug combination exerted synergistic effects through the AKT/mTOR/MYC/CDK2/RB axis to promote cell apoptosis. In NSG mouse models, combination therapy significantly reduced leukemia burden, markedly prolonged mouse survival, and did not increase toxicity.

**Copyright Information:** (Online) ISSN: 2572-9241
©2026 The Author(s). HemaSphere is published by John Wiley & Sons Ltd on behalf of European Hematology Association. This is an open access Abstract Book distributed under the Attribution-NonCommercial-NoDerivs (CC BY-NC-ND), which allows third parties to download the articles and share them with others as long as they credit the author and the Abstract Book, but they cannot change the content in any way or use them commercially.
**Abstract Book Citations:** Authors, Title, HemaSphere, 2026;10:(S1):pages. The abstract book DOIs can be found on the "Author Information" page.
**Disclaimer:** Articles published in the journal HemaSphere exclusively reflect the opinions of the authors. The authors are responsible for all content in their abstracts including accuracy of the facts, statements, citing resources, etc.

**Summary/Conclusion**

This study demonstrates that CDK2 is highly expressed in MPNs, with expression levels further elevated in patients with leukemic transformation; high CDK2 expression is closely associated with increased sensitivity to HHT. Ruxolitinib combined with homoharringtonine synergistically induces cell cycle arrest, inhibits pro-survival signaling, and activates apoptosis through the AKT/mTOR/MYC/CDK2/Cyclin A2/pRB axis, providing a novel therapeutic strategy for MPN patients with leukemic transformation and high CDK2 expression, with promising clinical translational potential.

This research was supported by the National Natural Science Foundation of China (No. 82570191) and the Zhejiang Provincial Health Highlevel Innovative Talent Project (2022-2026).* Correspondence to:Jian Huang, email: househuang@zju.edu.cn

**Copyright Information:** (Online) ISSN: 2572-9241
©2026 The Author(s). HemaSphere is published by John Wiley & Sons Ltd on behalf of European Hematology Association. This is an open access Abstract Book distributed under the Attribution-NonCommercial-NoDerivs (CC BY-NC-ND), which allows third parties to download the articles and share them with others as long as they credit the author and the Abstract Book, but they cannot change the content in any way or use them commercially.
**Abstract Book Citations:** Authors, Title, HemaSphere, 2026;10:(S1):pages. The abstract book DOIs can be found on the “Author Information” page.
**Disclaimer:** Articles published in the journal HemaSphere exclusively reflect the opinions of the authors. The authors are responsible for all content in their abstracts including accuracy of the facts, statements, citing resources, etc.

### (PS1961) AI-QUANTIFIED BONE MARROW FIBROSIS AND MEGAKARYOCYTE FEATURES CORRELATE WITH DRIVER VAF AND OUTCOMES IN THE MOST MPN STUDY

**Topic:** 15. Myeloproliferative neoplasms - Biology & translational research

Len Brandes*[1], Ruoyu Wang[1], Thomas Fletcher[1], Sharon Ruane[1], Alan Aberdeen[1], Daniel Royston[1, 2], Korsuk Sirinukunwattana[1], Cynthia Timmers[3], Evan Braunstein[3], Erin Crowgey[3]

*[1]Ground Truth Labs, Oxford, United Kingdom; [2]Radcliffe Department of Medicine, University of Oxford, Oxford, United Kingdom; [3]Incyte Corporation, Wilmington, DE, United States of America;*

### Background

Normalization of bone marrow (BM) pathology is needed for complete remission per European LeukemiaNet criteria for essential thrombocythemia (ET) and myelofibrosis (MF). However, manual histopathology review is subjective and lacks empirical standards. AI-powered BM analysis can provide objective and quantitative morphologic characterization, scaled to analyze large multicenter cohorts such as the MOST observational study (NCT02953704).

### Aims

1. Quantitatively characterize core BM morphology features of MOST ET and MF patient (pt) samples using previously developed Continuous Indexing of Fibrosis (CIF) and Megakaryocyte Characterization (MC) algorithms
2. Contextualize MOST study cohort against a large multicenter reference cohort
3. Explore the correlation of CIF- and MC-derived features with molecular burden and pt outcomes

### Methods

MOST enrollment criteria included primary and secondary MF and low risk by DIPSS (except age >65 y). ET pts were high risk or treated with ET-directed therapy. Of ~1200 enrolled pts, whole slide images from 136 reticulin (89 ET, 47 MF) and 148 H&E stained (98 ET, 50 MF) biopsies were available for histologic analysis. CIF (Ryou et al. *Leukemia* 2023) was applied to reticulin stains and reported as mean fibrosis score above the 70th percentile (CIF score). MC (Sirinukunwattana et al. *Blood Adv* 2020) was applied to H&E stains to quantify megakaryocyte (Mk) pleomorphism (standard deviation of major axis length) and Mk clustering (≥3 Mks). The large, independent reference cohort comprised BM biopsies from >10 referral centers. The CIF (reticulin) reference comprised normal (n=62), ET (n=575), and MF (n=144) biopsies. The MC (H&E) reference comprised normal (n=119), ET (n=1108) and MF (n=154). Reported values are median [IQR].

### Results

MOST MF median CIF was significantly lower vs reference MF cohort (0.40 [0.26–0.56] vs 0.74 [0.57–0.85];$P$<0.001), reflecting DIPSS low-risk enrollment criteria (**Figure 1A**). Among MF pts, higher CIF score was predictive of disease progression (0.51 [0.37–0.65] vs 0.32 [0.15–0.41]; $P$=0.007). CIF also correlated positively with variant allele frequency (VAF) for *JAK2* ($\rho$=0.557; $P$<0.001) and *CALR* ($\rho$=0.540; $P$<0.001) across the MOST cohort. CIF was lower in MOST ET vs reference ET cohort (0.20 [0.11–0.37] vs 0.30 [0.17–0.49]; $P$<0.001) but lacked statistical significance vs normal reference pts (0.20 [0.14–0.26]; $P$=0.61).
Mk pleomorphism and clustering in MOST ET and MF pts were similar to respective reference cohorts ($P$>0.2 for all) but elevated vs reference normal samples ($P$<0.001 for all; **Figure 1B**). Notably, clustering proportion correlated with VAF for *JAK2* ($\rho$=0.304; $P$=0.008) and *CALR* ($\rho$=0.387; $P$=0.024) across the MOST cohort.

**Copyright Information:** (Online) ISSN: 2572-9241
©2026 The Author(s). HemaSphere is published by John Wiley & Sons Ltd on behalf of European Hematology Association. This is an open access Abstract Book distributed under the Attribution-NonCommercial-NoDerivs (CC BY-NC-ND), which allows third parties to download the articles and share them with others as long as they credit the author and the Abstract Book, but they cannot change the content in any way or use them commercially.
**Abstract Book Citations:** Authors, Title, HemaSphere, 2026;10:(S1):pages. The abstract book DOIs can be found on the “Author Information” page.
**Disclaimer:** Articles published in the journal HemaSphere exclusively reflect the opinions of the authors. The authors are responsible for all content in their abstracts including accuracy of the facts, statements, citing resources, etc.

### Summary/Conclusion

Digital pathology combined with a reference cohort enables a consistent data analysis approach for clinical trial cohorts that improves upon manual curation methods. MOST samples followed the expected distribution of the reference myeloproliferative neoplasm (MPN) cohort given the study's recruitment criteria, consistent across both fibrosis- and Mk-based metrics. CIF showed predictive capacity for MF progression and correlated with driver mutation VAF. Mk clustering proportion also correlated with molecular driver burden. BM features in the MOST cohorts were statistically different than healthy controls. Collectively, these digital pathology techniques can improve quantitative interpretation of shifts in BM features post–intervention.

**Figure 1. MOST Samples Compared to a Large Reference Cohort**

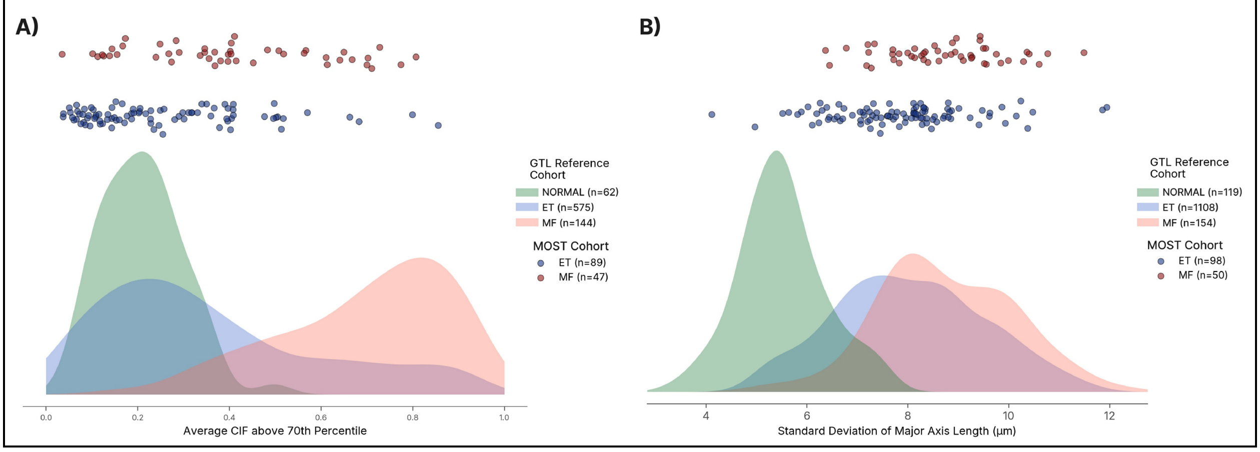

ET, essential thrombocythemia; GTL, Ground Truth Labs; MF, myelofibrosis.

**Copyright Information:** (Online) ISSN: 2572-9241
©2026 The Author(s). HemaSphere is published by John Wiley & Sons Ltd on behalf of European Hematology Association. This is an open access Abstract Book distributed under the Attribution-NonCommercial-NoDerivs (CC BY-NC-ND), which allows third parties to download the articles and share them with others as long as they credit the author and the Abstract Book, but they cannot change the content in any way or use them commercially.
**Abstract Book Citations:** Authors, Title, HemaSphere, 2026;10:(S1):pages. The abstract book DOIs can be found on the "Author Information" page.
**Disclaimer:** Articles published in the journal HemaSphere exclusively reflect the opinions of the authors. The authors are responsible for all content in their abstracts including accuracy of the facts, statements, citing resources, etc.

### (PS1962) COMBINED TARGETING OF KDM4 HISTONE DEMETHYLASES REDUCES JAK2V617F-DRIVEN MYELOPROLIFERATION

**Topic:** 15. Myeloproliferative neoplasms - Biology & translational research

Jana Schulze[1], Christoph Köllerer[1], Anja Mueller[1], Annika Lutz[1], Heike Pahl[1], Hans Staehle*[1]

[1] *University Medical Center Freiburg, Freiburg, Germany;*

### Background

Expression of the histone demethylase KDM4C is increased in patients with myeloproliferative neoplasms (MPN) and acute myeloid leukemia (AML). KDM4C belongs to the large KDM4 protein family and catalyzes the demethylation of mono-, di-, and trimethylated histones H3 at lysine 9 and 36 (H3K9 and H3K36). Although KDM4C has been shown to mediate a functional dependency in JAK2-mutant neoplasms, redundancy among KDM4 family members limits the therapeutic potential of targeting KDM4C alone.

### Aims

In this study we tested the hypothesis that combined targeting of two KDM4 histone demethylases attenuates $JAK2^{V617F}$-driven myeloproliferation.

### Methods

We lentivirally depleted KDM4A and KDM4C, individually and in combination, in three $JAK2^{V617F}$-mutant cell lines UKE1, SET2, and HEL, and assessed cell proliferation and apoptosis. In addition, we knocked down KDM4A and KDM4C in $JAK2^{V617F}$ knock-in mice and evaluated disease progression by peripheral blood counts, spleen weight, and flow-cytometric analysis. Finally, we tested pharmacological KDM4 inhibition, both *in vitro* and *in vivo*. To gain mechanistic insight into KDM4A and KDM4C function, we performed ChIP-seq, RNA-seq, and ATAC-seq in KDM4A- and KDM4C-depleted HEL cells, enabling integrated multi-omics analyses.

### Results

Combined knockdown of KDM4A and KDM4C effectively reduced proliferation of $JAK2^{V617F}$-mutant UKE1, SET2, and HEL cells compared to control cells by increasing apoptosis. Similar results were observed in the $JAK2^{V617F}$ knock-in mouse model, in which combined knockdown of KDM4A and KDM4C significantly reduced leukocytosis, thrombocytosis, and splenomegaly. Pharmacological inhibition with the KDM4C-specific inhibitor SD70 was less effective than treatment with the KDM4 pan-inhibitor TACH101. The latter both effectively attenuated $JAK2^{V617F}$-driven myeloproliferation *in vitro*, and reduced leukemic burden in a HEL $Rag2^{-/-}$ $\gamma c^{-/-}$ xenograft mouse model. RNA-seq and ATAC-seq analyses in HEL cells revealed that combined knockdown of KDM4A and KDM4C synergistically reduced gene expression of proliferation-associated genes, including those involved in STAT, BCL6 and PAX3 transcription factor signaling. Moreover, integration of the RNA- and ATAC-seq data with our ChIP-seq analyses showed that KDM4A and KDM4C synergistically mediate their pro-leukemic effects through regulation of PIM1, NQO2, and PTP4A3.

**Copyright Information:** (Online) ISSN: 2572-9241
©2026 The Author(s). HemaSphere is published by John Wiley & Sons Ltd on behalf of European Hematology Association. This is an open access Abstract Book distributed under the Attribution-NonCommercial-NoDerivs (CC BY-NC-ND), which allows third parties to download the articles and share them with others as long as they credit the author and the Abstract Book, but they cannot change the content in any way or use them commercially.
**Abstract Book Citations:** Authors, Title, HemaSphere, 2026;10:(S1):pages. The abstract book DOIs can be found on the “Author Information” page.
**Disclaimer:** Articles published in the journal HemaSphere exclusively reflect the opinions of the authors. The authors are responsible for all content in their abstracts including accuracy of the facts, statements, citing resources, etc.

**Summary/Conclusion**

Combined targeting of KDM4 histone demethylases effectively reduced JAK2$^{V617F}$-driven myeloproliferation *in vitro* and *in vivo*. Therefore, pharmacological inhibition with the KDM4 pan-inhibitor TACH101, which is currently being tested in a phase II clinical trial for advanced solid tumors, represents a promising therapeutic approach for JAK2$^{V617F}$-mutated myeloid malignancies.

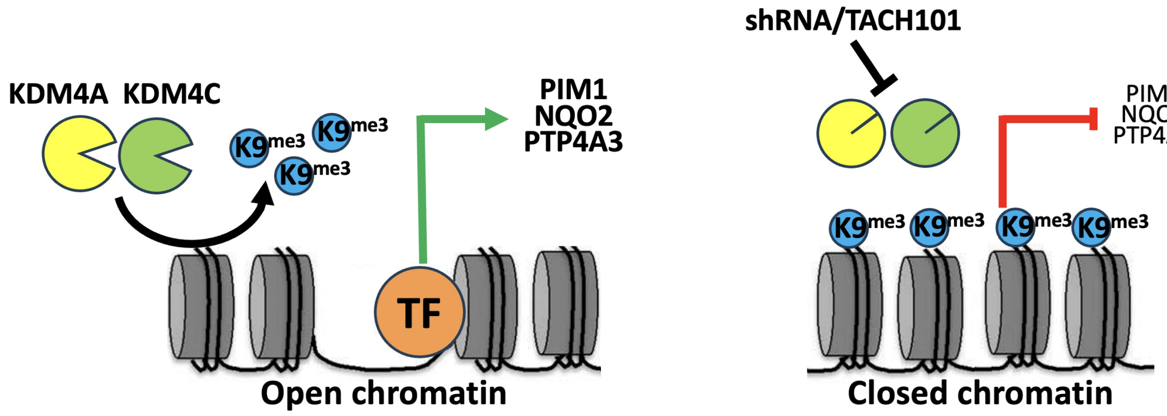

**Figure 1. Targeting KDM4 histone demethylases reduces leukemic signaling in the JAK2$^{V617F}$ context.** KDM4 histone demethylases promote chromatin opening at the PIM1, NQO2, and PTP4A3 loci thereby contributing to pro-leukemic signaling. In contrast, knockdown or pharmacologic inhibition of KDM4 demethylases lead to chromatin closure and reduced expression of these pro-leukemic genes, ultimately resulting in increased apoptosis of malignant cells.

**Copyright Information:** (Online) ISSN: 2572-9241
©2026 The Author(s). HemaSphere is published by John Wiley & Sons Ltd on behalf of European Hematology Association. This is an open access Abstract Book distributed under the Attribution-NonCommercial-NoDerivs (CC BY-NC-ND), which allows third parties to download the articles and share them with others as long as they credit the author and the Abstract Book, but they cannot change the content in any way or use them commercially.
**Abstract Book Citations:** Authors, Title, HemaSphere, 2026;10:(S1):pages. The abstract book DOIs can be found on the "Author Information" page.
**Disclaimer:** Articles published in the journal HemaSphere exclusively reflect the opinions of the authors. The authors are responsible for all content in their abstracts including accuracy of the facts, statements, citing resources, etc.

### (PS1963) SILK-BASED BONE MARROW NICHES ENABLE MECHANISTIC DISSECTION OF STROMAL-REGULATED THROMBOPOIESIS IN HEALTH AND FIBROSIS

**Topic:** 15. Myeloproliferative neoplasms - Biology & translational research

Christian Di Buduo*[1], Carolina Miguel[1], Delfina Tosi[2], Vittorio Abbonante[1], Daniele Cattaneo[3, 3], Alessandra Iurlo[3], Umberto Gianelli[2], Alessandra Balduini[1]

*[1]University of Pavia, Pavia, Italy; [2]University of Milan, Milan, Italy; [3]Foundation Irccs Ca' Granda Ospedale Maggiore Policlinico, Milan, Italy;*

### Background

Thrombopoiesis in the bone marrow (BM) is regulated by dynamic interactions among hematopoietic stem and progenitor cells (HSPCs), mesenchymal stromal cells (MSCs), and extracellular matrix (ECM) components, integrating biochemical and mechanical cues that govern megakaryocyte development. In myeloproliferative neoplasms (MPNs), elevated TGF-β1 promotes MSC reprogramming into ECM-producing myofibroblasts, driving fibrosis and megakaryocyte dysfunction. Although MSC involvement is well recognized, MSC–HSPC crosstalk within the BM niche remains incompletely understood in both physiological and pathological contexts.

### Aims

To determine how silk-based BM niches preserve and spatially organize MSC–HSPC interactions through material-driven control of architecture, mechanics, and functionalization, enabling mechanistic dissection of stromal regulation of human thrombopoiesis.

### Methods

3D scaffolds were fabricated from silk fibroin derived from *Bombyx mori* cocoons and engineered to recapitulate native BM properties. Primary human $CD34^+$ HSPCs and BM-MSCs were co-cultured within the scaffold, which reproduced key marrow features, including ECM remodeling and perfusion flow, allowing direct evaluation of thrombopoiesis. Scaffold functionalization with TGF-β1 induced MSC transition toward a myofibroblast phenotype. HSPCs from MPN patients were analyzed under physiological and fibrotic niche conditions.

**Copyright Information:** (Online) ISSN: 2572-9241
©2026 The Author(s). HemaSphere is published by John Wiley & Sons Ltd on behalf of European Hematology Association. This is an open access Abstract Book distributed under the Attribution-NonCommercial-NoDerivs (CC BY-NC-ND), which allows third parties to download the articles and share them with others as long as they credit the author and the Abstract Book, but they cannot change the content in any way or use them commercially.
**Abstract Book Citations:** Authors, Title, HemaSphere, 2026;10:(S1):pages. The abstract book DOIs can be found on the “Author Information” page.
**Disclaimer:** Articles published in the journal HemaSphere exclusively reflect the opinions of the authors. The authors are responsible for all content in their abstracts including accuracy of the facts, statements, citing resources, etc.

**Results**

3D silk scaffolds supported stable MSC engraftment. Transcriptional profiling showed that MSCs within the compliant 3D microenvironment adopted a gene expression program distinct from 2D substrates. In 2D, MSCs upregulated genes involved in collagen biosynthesis and fibril organization (e.g., COL1A1, COL4A1, COL4A2, COL8A2), matrix crosslinking and ECM remodeling (e.g., LOX, SPARC, PXDN). In contrast, the 3D silk niche promoted a bone marrow–like matrix signature with enhanced deposition of hematopoietic-supportive components such as fibronectin and laminins.
Human HSPCs co-cultured with compliant MSCs in the 3D scaffold efficiently differentiated into β1-tubulin$^{+}$CD41$^{+}$CD42b$^{+}$ megakaryocytes and displayed superior maturation compared with 2D cultures, with increased proplatelet formation and platelet production. Released platelets showed morphology and functional competence comparable to peripheral blood platelets, including discoid forms with defined β1-tubulin marginal bands and preserved granules.
TGF-β1 functionalization of the 3D silk BM model converted MSCs into α-SMA$^{+}$ myofibroblast-like cells with smooth muscle gene upregulation, fibrotic ECM signatures, increased reticulin deposition, and EDA-fibronectin splicing, recapitulating BM fibrosis. In this fibrotic niche, healthy HSPCs generated smaller, dysmorphic megakaryocytes with impaired proplatelet formation and reduced platelet release under flow.
When MPN patient–derived HSPCs were introduced into the 3D system, the fibrotic niche exacerbated intrinsic megakaryocyte defects, whereas the physiological silk niche partially restored maturation.

**Summary/Conclusion**

The silk-based bone marrow niche establishes a tunable and physiologically faithful 3D platform that preserves stromal identity and function while enabling precise manipulation of niche architecture and mechanics. The platform offers a robust translational tool to unravel microenvironment-driven thrombopoietic dysfunction and to test therapeutic strategies targeting both malignant cells and their niche.

**Copyright Information:** (Online) ISSN: 2572-9241
©2026 The Author(s). HemaSphere is published by John Wiley & Sons Ltd on behalf of European Hematology Association. This is an open access Abstract Book distributed under the Attribution-NonCommercial-NoDerivs (CC BY-NC-ND), which allows third parties to download the articles and share them with others as long as they credit the author and the Abstract Book, but they cannot change the content in any way or use them commercially.
**Abstract Book Citations:** Authors, Title, HemaSphere, 2026;10:(S1):pages. The abstract book DOIs can be found on the "Author Information" page.
**Disclaimer:** Articles published in the journal HemaSphere exclusively reflect the opinions of the authors. The authors are responsible for all content in their abstracts including accuracy of the facts, statements, citing resources, etc.

### (PS1964) THE ORDER OF MYELOID MUTATION ACQUISITION INFLUENCES OUTCOMES IN A LARGE POLYCYTHEMIA VERA COHORT

**Topic:** 15. Myeloproliferative neoplasms - Biology & translational research

Paola Guglielmelli*[1], Elena Nacca[1], Camilla Ghetti[1], Valentina Boldrini[1], Lorenzo Fagiolo[1], Giuseppe Gaetano Loscocco[1], Giada Rotunno[1], Leonardo Signori[1], Francesca Gesullo[1], Alessio Enderti[1], Claudia Bindi[1], Irene Marri[1], Alessandro Maria Vannucchi[1]

*[1]Center for Research and Innovation in Myeloproliferative Diseases (CRIMM) , Careggi University Hospital, University of Florence, Department of Experimental and Clinical Medicine, Florence, Italy;*

### Background

Polycythemia vera (PV) is a chronic myeloproliferative neoplasm mainly driven by *JAK2* mutations. Approx 40-50% of the patients (pts) also harbor additional myeloid gene mutations (MyMut) with potential prognostic impact, but ~~their~~ prevalence and contribution to phenotype, clonal architecture, and outcomes remain incompletely defined.

### Aims

To evaluate impact of clonal heterogeneity and hierarchy on clinical outcomes in pts with PV

### Methods

Patients fulfilling 2022 WHO/ICC criteria for PV with available DNA (at diagnosis or chronic phase without progression) and complete clinical/outcome data were genotyped using Targeted NGS (SOPHiA DDM Myeloid Solution). Co-mutations were grouped by functional class (epigenetic modifiers, RAS/MAPK pathway, splicing factors), and VAFs were compared with the co-occurring driver mutation to infer mutation order (driver-first vs functional-first) by Pyclone. Clonal architecture was quantified using the Shannon Diversity Index (SDI) as a measure of clonal diversity (Cerrano M, Leukemia 2021). SDI was normalized by each patient's mutation count, and the median normalized SDI was used to classify patients as clonal equilibrium (SDI ≥ median) or clonal dominance (SDI < median), within mutation-burden strata.

**Copyright Information:** (Online) ISSN: 2572-9241
©2026 The Author(s). HemaSphere is published by John Wiley & Sons Ltd on behalf of European Hematology Association. This is an open access Abstract Book distributed under the Attribution-NonCommercial-NoDerivs (CC BY-NC-ND), which allows third parties to download the articles and share them with others as long as they credit the author and the Abstract Book, but they cannot change the content in any way or use them commercially.
**Abstract Book Citations:** Authors, Title, HemaSphere, 2026;10:(S1):pages. The abstract book DOIs can be found on the "Author Information" page.
**Disclaimer:** Articles published in the journal HemaSphere exclusively reflect the opinions of the authors. The authors are responsible for all content in their abstracts including accuracy of the facts, statements, citing resources, etc.

### Results

A total of 637 PV pts were analyzed (55.7% male; median age 61.3 years, range 14.4–91.8). During follow-up, 138 (21.7%) progressed to post-PV myelofibrosis and 15 (2.4%) to blast phase; 109 patients (17.1%) died. Overall, 222 (34.9%) had a history of thrombosis (arterial (n=121), venous (n=125), 174 (27.3%) at diagnosis or within 5 years and 111 (17.4%) during follow-up; of these, 61 were thrombotic recurrences. *JAK2* mutation was detected in 99.4% of cases: V617F in 625 (98.1%) and exon 12 variants in 8 (1.3%); in four pts non-canonical *JAK2* variants were identified. MyMut were present in 283 (44.4%) patients, with 33.0%, 8.9%, and 2.4% harboring 1, 2, or ≥3 mutations, respectively. The most frequently mutated genes were *TET2* (21.4%, mean VAF 23.9%), *ASXL1* (12.0%, mean VAF 24.4%), and *DNMT3A* (9.9%, mean VAF 17.8%). The presence of at least one MyMut was associated with poorer prognosis. In particular, *ASXL1* (p<0.001) and *EZH2* (p=0.002) were significantly associated with progression to post-PV myelofibrosis, whereas *DNMT3A* mutations were inversely correlated with MF progression (p<0.001). With respect to overall survival, mutations in *DNMT3A* (p=0.012), *TET2* (p<0.001), and *U2AF1* (p=0.002), as well as the presence of ≥2 variants (p<0.001), were significantly associated with inferior outcomes. Within pts with at least 1 MyMut stratified by mutation acquisition order, epigenetic-first (32.9%) and spliceosome-first (28.6%) group had reduced OS compared Driver-first pts (log rank p=0.012, p=0.0089). Patients in the clonal equilibrium group (n=43; 15.2%) showed a trend toward reduced overall survival compared with those with clonal dominance (n=239; 84.8%) during follow-up (log-rank p=0.12), suggesting that clinical phenotype and outcomes may be driven more by the specific mutations involved (qualitative effects) and their clonal architecture (e.g., founder vs subclonal events) than by overall clonal diversity.

### Summary/Conclusion

These findings confirm the prognostic relevance of selected mutated myeloid genes in PV and, for the first time, suggest that the longitudinal sequence of mutation acquisition, rather than overall clonal complexity, may be a key determinant of clinical outcomes.

**Copyright Information:** (Online) ISSN: 2572-9241
©2026 The Author(s). HemaSphere is published by John Wiley & Sons Ltd on behalf of European Hematology Association. This is an open access Abstract Book distributed under the Attribution-NonCommercial-NoDerivs (CC BY-NC-ND), which allows third parties to download the articles and share them with others as long as they credit the author and the Abstract Book, but they cannot change the content in any way or use them commercially.
**Abstract Book Citations:** Authors, Title, HemaSphere, 2026;10:(S1):pages. The abstract book DOIs can be found on the “Author Information” page.
**Disclaimer:** Articles published in the journal HemaSphere exclusively reflect the opinions of the authors. The authors are responsible for all content in their abstracts including accuracy of the facts, statements, citing resources, etc.

### (PS1965) JAK2 E837K. FIRST CHARACTERIZATION OF A NOVEL JH2-JH1 INTERDOMAIN LINKER MUTATION DRIVING STAT ACTIVATION AND METABOLIC REPROGRAMMING IN POST-POLYCYTHEMIA VERA MYELOFIBROSIS

**Topic:** 15. Myeloproliferative neoplasms - Biology & translational research

Gloria Pasqualini*[1, 2], Mariana Gallo[3], Marco Giovannini[4], Elisabetta De Marinis[5, 6], Pasquale Niscola[4], Alessandra Zaza[1], Eliezer Espina Barroso[1], Elisabetta Abruzzese[4], Malgorzata Monica Trawinska[4], Maria Ilaria DEL Principe[4, 7], Gianfranco Catalano[1, 7], Clara Nervi[5], Nelida I. Noguera[1, 7]

*[1]I.R.C.C.S Santa Lucia Foundation, Rome, Italy; [2]University of Rome La Sapienza, Department of Biology and Biotechnology "Charles Darwin", Rome, Italy; [3]University of Parma, Department of Medicine and Surgery, Parma, Italy; [4]Hematology Unit, S. Eugenio Hospital (ASL Roma 2), Rome, Italy; [5]University of Rome La Sapienza, Department of Medical and Surgical Sciences and Biotechnologies, Rome, Italy; [6]European University of Rome, Department of Health and Life Sciences, Rome, Italy; [7]Tor Vergata University, Department of Biomedicine and Prevention, Rome, Italy;*

### Background

JAK2 mutations are key drivers of myeloproliferative neoplasms (MPNs), inducing constitutive kinase activation by disrupting JH2-JH1 autoinhibition or stabilizing active dimers, leading to aberrant JAK2-STAT signaling (Glassman et al., 2022; Abraham et al., 2024). Aberrant JAK2-STAT activation is also associated with metabolic reprogramming in MPNs, including coordinated increases in glycolysis and oxidative phosphorylation (Rao et al., 2019; Ma et al., 2025). To date, activating mutations have been reported exclusively in functional domains and regulatory interfaces of JAK2, but not in the JH2-JH1 interdomain linker. Here, we describe E837K, a novel pathogenic mutation within the JH2-JH1 interdomain linker.

### Aims

We aimed to characterize the structural, functional, and metabolic consequences of the noncanonical JAK2 E837K mutation identified in post-polycythemia vera myelofibrosis (PPV-MF), localized in the JH2-JH1 linker, to define its pathogenic role.

### Methods

The JAK2 E837K mutation was identified by NGS performed on the NextSeq 550 platform using the Archer VariantPlex panel. Wild-type and E837K JAK2 structures were modeled using AlphaFold2 via ColabFold-AF2. A colony-forming unit (CFU) assay was performed on peripheral blood mononuclear cells (PBMCs) from the JAK2 E837K PPV-MF patient, 4 healthy donors (HDs) and 8 JAK2 V617F PV patients, cultured with increasing erythropoietin (EPO) concentrations. STAT3/5 and AKT activation were assessed by Western blot. Mitochondrial respiration and glycolytic activity were analyzed using a Seahorse XFe96 analyzer.

**Copyright Information:** (Online) ISSN: 2572-9241
©2026 The Author(s). HemaSphere is published by John Wiley & Sons Ltd on behalf of European Hematology Association. This is an open access Abstract Book distributed under the Attribution-NonCommercial-NoDerivs (CC BY-NC-ND), which allows third parties to download the articles and share them with others as long as they credit the author and the Abstract Book, but they cannot change the content in any way or use them commercially.
**Abstract Book Citations:** Authors, Title, HemaSphere, 2026;10:(S1):pages. The abstract book DOIs can be found on the “Author Information” page.
**Disclaimer:** Articles published in the journal HemaSphere exclusively reflect the opinions of the authors. The authors are responsible for all content in their abstracts including accuracy of the facts, statements, citing resources, etc.

### Results

According to NCBI Genome Data Viewer and UniProt, the E837K mutation lies in exon 19 within the JH2-JH1 interdomain linker, a highly conserved region across species (Fig. 1A-B). Using AlphaFold2, five high-confidence structural models were generated for wild-type and E837K JAK2. Wild-type JAK2 samples both the closed (autoinhibited) and elongated (active) conformations, with preference for the closed state, whereas E837K adopts exclusively the elongated conformation (Fig. 1C-D). The mutation disrupts a salt-bridge network in the JH2-JH1 linker (E837-R839-E845-R847), introducing electrostatic repulsion between K837 and R839 and causing rotation of the K837 side chain. This rearrangement promotes further repulsive interactions with basic residues in the adjacent FERM domain, stabilizing JAK2 in the open conformation, promoting dimerization and constitutive activation (Fig. 1E-F). Functionally, this structural shift is reflected in enhanced cellular responsiveness as indicated by the pronounced hypersensitivity to EPO of JAK2 E837K-derived colonies, comparable to that of JAK2 V617F-derived colonies (Fig. 1G). Consistent with this, JAK2 E837K colonies showed increased STAT5 and AKT phosphorylation, whereas STAT3 activation remained unchanged (1H). Moreover, metabolic profiling revealed that JAK2 E837K-derived colonies possess elevated mitochondrial respiration and glycolytic activity compared to healthy donors (Fig. 1I-L), linking the conformational change directly to both signaling and metabolic rewiring.

### Summary/Conclusion

In conclusion, we provide the first evidence that a mutation located within the JH2-JH1 interdomain linker can act as a driver mutation, as demonstrated by E837K in a PPV-MF patient lacking other known mutations. The E837K mutation locks the protein in the open conformation, inducing EPO hypersensitivity, constitutive STAT5 and AKT activation and metabolic reprogramming, supporting its pathogenic role.

**Copyright Information:** (Online) ISSN: 2572-9241
©2026 The Author(s). HemaSphere is published by John Wiley & Sons Ltd on behalf of European Hematology Association. This is an open access Abstract Book distributed under the Attribution-NonCommercial-NoDerivs (CC BY-NC-ND), which allows third parties to download the articles and share them with others as long as they credit the author and the Abstract Book, but they cannot change the content in any way or use them commercially.
**Abstract Book Citations:** Authors, Title, HemaSphere, 2026;10:(S1):pages. The abstract book DOIs can be found on the "Author Information" page.
**Disclaimer:** Articles published in the journal HemaSphere exclusively reflect the opinions of the authors. The authors are responsible for all content in their abstracts including accuracy of the facts, statements, citing resources, etc.

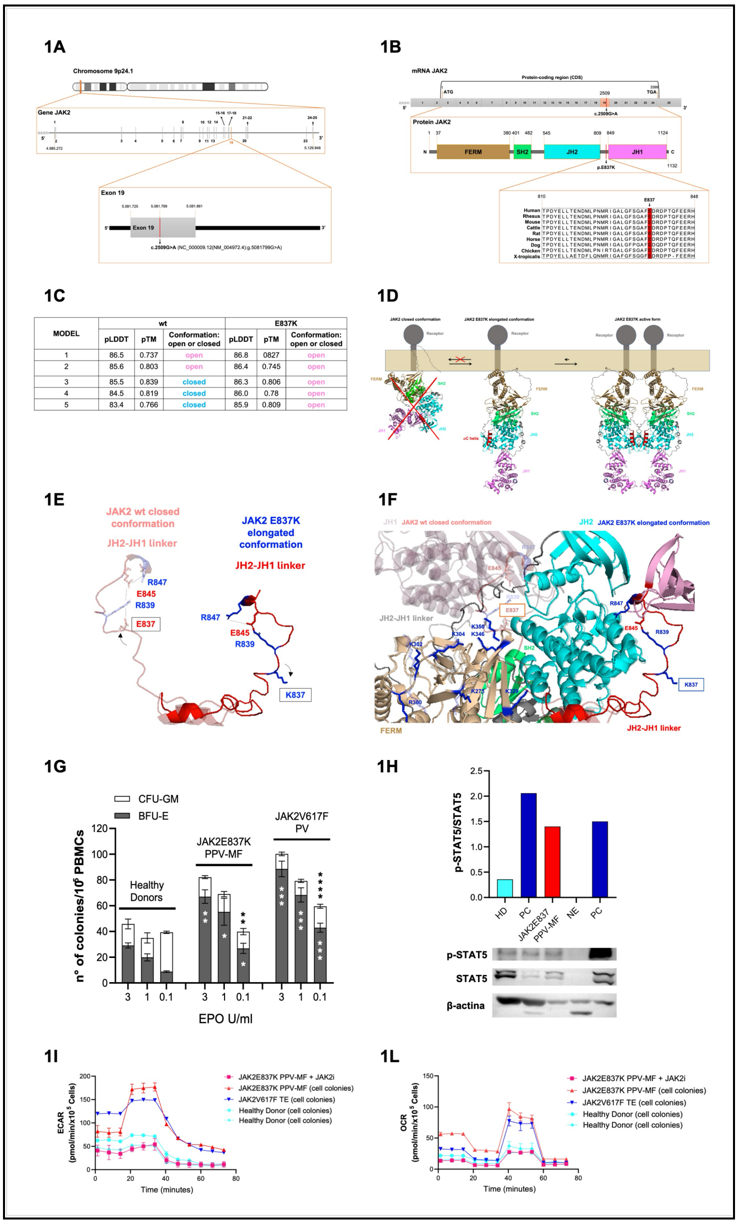

| MODEL | wt | | | E837K | | |
|---|---|---|---|---|---|---|
| | pLDDT | pTM | Conformation: open or closed | pLDDT | pTM | Conformation: open or closed |
| 1 | 86.5 | 0.737 | open | 86.8 | 0827 | open |
| 2 | 85.6 | 0.803 | open | 86.4 | 0.745 | open |
| 3 | 85.5 | 0.839 | closed | 86.3 | 0.806 | open |
| 4 | 84.5 | 0.819 | closed | 86.0 | 0.78 | open |
| 5 | 83.4 | 0.766 | closed | 85.9 | 0.809 | open |

Fig. 1A Schematic representation of the chromosomal localization and genomic structure of the human JAK2 gene, including exon 19, according to the NCBI Genome Data Viewer (GRCh38.p14; Chr 9: NC_000009.12). Fig. 1B Schematic representation of JAK2 mRNA and protein domains, including evolutionary conservation analysis. Fig. 1C AlphaFold2 confidence scores (pLDDT, pTM) and predicted conformations of WT and JAK2 E837K. Fig. 1D Proposed structural model of JAK2 E837K. Fig. 1E-F Superposition of the E837K mutant protein in its open conformation with the wild-type protein in the closed conformation (displayed with transparency), illustrating how structural differences in the JH1-JH2 linker govern the relative positioning of the JH1 domain. Fig. 1G Colony-forming capacity of PBMCs isolated from four healthy donors, one JAK2 E837K-positive PPV MF case, and eight JAK2 V617F-positive PV cases, cultured with 3, 1, and 0.1 U/mL EPO. Fig. 1H Western blot analysis of proteins from colonies of the JAK2 E837K PPV-MF patient, from a healthy donor (HD), positive controls (PC), and negative controls (NC); NE, not evaluable. Fig. 1I-L Extracellular acidification rate (ECAR, left) and mitochondrial oxygen consumption rate (OCR, right) measured in erythroid colonies from the JAK2 E837K PPV-MF patient (red), PBMCs from the same JAK2 E837K PPV-MF patient undergoing ruxolitinib treatment (pink), erythroid colonies from a JAK2 V617F patient (blue) and erythroid colonies from two healthy donors (light blue).

**Copyright Information:** (Online) ISSN: 2572-9241
©2026 The Author(s). HemaSphere is published by John Wiley & Sons Ltd on behalf of European Hematology Association. This is an open access Abstract Book distributed under the Attribution-NonCommercial-NoDerivs (CC BY-NC-ND), which allows third parties to download the articles and share them with others as long as they credit the author and the Abstract Book, but they cannot change the content in any way or use them commercially.
**Abstract Book Citations:** Authors, Title, HemaSphere, 2026;10:(S1):pages. The abstract book DOIs can be found on the “Author Information” page.
**Disclaimer:** Articles published in the journal HemaSphere exclusively reflect the opinions of the authors. The authors are responsible for all content in their abstracts including accuracy of the facts, statements, citing resources, etc.

### (PS1966) DECIPHERING HEMATOPOIETIC STRESS RESPONSES IN JAK2V617F MYELOPROLIFERATIVE NEOPLASMS: A COMBINED IN VIVO AND MATHEMATICAL MODELING APPROACH

**Topic:** 15. Myeloproliferative neoplasms - Biology & translational research

Duanya Liu*[1], Stephane Giraudier[2], Nabih Maslah[3], Panhong Gou[4], Bruno Cassinat[5]

*[1]INSERM, Paris, France; [2]AP-HP, PARIS, France; [3]INSERM, PARIS, France; [4]University Paris Cite, Paris, France; [5]AP-HP, Paris, France;*

### Background

Hematopoietic stem cells (HSCs) are continuously exposed to proliferative and inflammatory stress, which must be tightly regulated to preserve long-term function. In myeloproliferative neoplasms (MPN), the JAK2V617F mutation drives chronic signaling proliferation and inflammation; however, how mutant HSCs adapt to sustained hematopoietic stress remains incompletely understood.

### Aims

To determine whether JAK2V617F steady-state hematopoiesis recapitulates normal acute stress hematopoiesis, to characterize the responsiveness of JAK2V617F cells to additional acute stress, and assess whether stress accelerates JAK2V617F clonal dominance.

### Methods

Using JAK2V617F knock-in mouse models and wild-type controls, hematopoietic stress was induced by phenylhydrazine (PHZ)–mediated hemolytic anemia. Dynamic changes in hematopoietic stem and progenitor compartments from wild-type and JAK2V617F mice were assessed during stress induction and recovery. HSC function was evaluated by flow cytometry, proliferation and apoptosis assays. Finally, competitive transplantation and transcriptomic analyses were performed to define stress-induced pathological advantage and stress-associated molecular programs.

### Results

At steady state, JAK2V617F mice exhibited 2-fold expansion of LT-HSCs and MPPs, 4-fold expansion of MEPs and GMPs, a 5-fold increase in LT-HSC proliferation rate, 5-fold greater spleen mass and cellularity versus wild-type (WT). Serum levels of IL-1α, IL-1β, IL-2, IL-6, MCP-1, and TNF-α in JAK2V617F mice at baseline equaled or exceeded peak concentrations observed in PHZ-stressed WT animals. Wnt, Hedgehog, and TNF-α stress pathways were constitutively activated in JAK2V617F LSK cells. Mathematical modeling confirmed that steady-state division kinetics in JAK2V617F HSPCs matched those of acutely stressed WT cells. Upon PHZ challenge, WT LT-HSC proliferation increased 20-fold, whereas JAK2V617F showed only a mild increase; WT splenic MEPs expanded 100-fold but JAK2V617F-MEP only 6-fold demonstrating a relative resistance to acute stress of JAK2V617F cells. EPO levels remained undetectable in JAK2V617F animals both before and after stress when it drastically increased in WT animals. In competitive chimeras, PHZ induced a 4-fold decrease in WT MEP chimerism but a 2-fold increase in JAK2V617F MEP by day 3, with concordant shifts across LT-HSC, MPP, and CMP compartments.

**Copyright Information:** (Online) ISSN: 2572-9241
©2026 The Author(s). HemaSphere is published by John Wiley & Sons Ltd on behalf of European Hematology Association. This is an open access Abstract Book distributed under the Attribution-NonCommercial-NoDerivs (CC BY-NC-ND), which allows third parties to download the articles and share them with others as long as they credit the author and the Abstract Book, but they cannot change the content in any way or use them commercially.
**Abstract Book Citations:** Authors, Title, HemaSphere, 2026;10:(S1):pages. The abstract book DOIs can be found on the "Author Information" page.
**Disclaimer:** Articles published in the journal HemaSphere exclusively reflect the opinions of the authors. The authors are responsible for all content in their abstracts including accuracy of the facts, statements, citing resources, etc.

**Summary/Conclusion**

JAK2V617F intrinsically rewires hematopoiesis into a constitutive stress-like state, recapitulating the cytokine profile, proliferative kinetics, and pathway activation of normal acute stress erythropoiesis. Because JAK2V617F HSPCs are already maximally engaged in a stress program, they are refractory to additional acute stress. In a competitive hematopoietic environment, acute stress selectively suppresses WT hematopoiesis while sparing JAK2V617F progenitors, rapidly amplifying clonal dominance. These findings identify chronic inflammatory stress-erythropoiesis as a core pathogenic mechanism in MPN, and provide acute stress as a mechanistic basis for clonal advantage and disease progression.

**Copyright Information:** (Online) ISSN: 2572-9241
©2026 The Author(s). HemaSphere is published by John Wiley & Sons Ltd on behalf of European Hematology Association. This is an open access Abstract Book distributed under the Attribution-NonCommercial-NoDerivs (CC BY-NC-ND), which allows third parties to download the articles and share them with others as long as they credit the author and the Abstract Book, but they cannot change the content in any way or use them commercially.
**Abstract Book Citations:** Authors, Title, HemaSphere, 2026;10:(S1):pages. The abstract book DOIs can be found on the "Author Information" page.
**Disclaimer:** Articles published in the journal HemaSphere exclusively reflect the opinions of the authors. The authors are responsible for all content in their abstracts including accuracy of the facts, statements, citing resources, etc.

### (PS1967) CRISPR-BASED MODELING OF VARIANTS OF UNCERTAIN SIGNIFICANCE IN MYELOPROLIFERATIVE NEOPLASMS REVEALS REDUCED CLONOGENICITY AND MYELOID-SKEWED DIFFERENTIATION

**Topic:** 15. Myeloproliferative neoplasms - Biology & translational research

Antonia-Zoe Kouroutzidou*[1], Stamatia Laidou[1], Anna C Navis[2], Paraskevi Terzidou[1], Konstantinos I Delakis[1], Anna Christoforidou[3], Angeliki Xagorari[4], Sofia Papadaki[5], Konstantia Skarmoutsou[3], Pavlina Marinaki[6], Damianos Sotiropoulos[4], Anastasia Chatzidimitriou[1, 7], Bernhard Schmierer[2], Kostas Stamatopoulos[1, 7]

*[1]Institute of Applied Biosciences, Centre for Research and Technology Hellas, Thessaloniki, Greece;[2]CRISPR Functional Genomics, SciLifeLab and Department of Medical Biochemistry and Biophysics, Karolinska Institutet, Stockholm, Sweden; [3]Private Consultant Hematology Services, Thessaloniki, Greece; [4]Public Cord Blood Bank, Department of Hematology, "G. Papanicolaou" Hospital, Thessaloniki, Greece; [5]Aimagenesis Medical Office, Hematology Practice, Thessaloniki, Greece; [6]Department of Hematology, Interbalkan Medical Center, Thessaloniki, Greece;[7]Department of Molecular Medicine and Surgery, Karolinska Institutet, Stockholm, Sweden;*

### Background

Next-generation sequencing (NGS) has revolutionized the molecular characterization of myeloid malignancies, enabling precision-based diagnosis, risk stratification and treatment selection. However, a substantial proportion of detected variants are classified as Variants of Uncertain Significance (VUS), even in key driver genes. The lack of functional evidence limits their clinical interpretation, especially when a VUS represents the sole genomic finding.

### Aims

To assess the impact of 3 VUS recurrently identified in patients with myeloproliferative neoplasm (MPNs) on the clonogenic capacity and differentiation dynamics of CD34⁺ hematopoietic stem and progenitor cells (HSPCs).

### Methods

Three recurrent VUS detected in patients with MPN lacking established driver mutations -*MPL* p.Pro70Leu (identified in 2 cases and segregating within one family), *EZH2* p.Asn322Ser (4 cases) and *JAK2* p.Gly571Ser (2 cases)- were selected from institutional diagnostic NGS data. CD34⁺ HSPCs were isolated from healthy donor umbilical cord blood via immunomagnetic isolation (>95% purity) and cultured in expansion medium supplemented with SCF, Flt3L and TPO. Variants were introduced by CRISPR/Cas9-mediated homology-directed repair. sgRNAs were designed using CRISPOR and their cutting efficiency was validated in hTERT-RPE1 cells followed by Sanger sequencing and deconvolution by ICE tool. Ultramer™ ssDNA templates (~200bp homology arms) carried the desired substitutions and a PAM-disrupting synonymous change. Cas9-sgRNA RNPs were electroporated with HDR templates using the Neon system in the presence of Alt-R™ HDR Enhancer. Editing was confirmed by Oxford Nanopore sequencing. Edited cells were plated in MethoCult® H4434 medium ($1\times10^3$ cells/mL, triplicates) for colony-forming unit assays. After 14 days, colonies were counted, morphologically classified and pooled for FACS analysis of lineage distribution and maturation status.

**Copyright Information:** (Online) ISSN: 2572-9241
©2026 The Author(s). HemaSphere is published by John Wiley & Sons Ltd on behalf of European Hematology Association. This is an open access Abstract Book distributed under the Attribution-NonCommercial-NoDerivs (CC BY-NC-ND), which allows third parties to download the articles and share them with others as long as they credit the author and the Abstract Book, but they cannot change the content in any way or use them commercially.
**Abstract Book Citations:** Authors, Title, HemaSphere, 2026;10:(S1):pages. The abstract book DOIs can be found on the “Author Information” page.
**Disclaimer:** Articles published in the journal HemaSphere exclusively reflect the opinions of the authors. The authors are responsible for all content in their abstracts including accuracy of the facts, statements, citing resources, etc.

**Results**

sgRNA cutting efficiency showed indel frequencies of 55% for *MPL* VUS, 87% for *EZH2* VUS and 90% for *JAK2* VUS. Precise editing resulted in variant allele frequencies (VAF) of 55.7% (*EZH2* VUS) and 54.2% (*JAK2* VUS), whereas *MPL* VUS editing efficiency was lower (VAF 6.2%). Control cells (water-electroporated) generated 165 colonies/1000 plated cells (16.5% clonogenicity), while all VUS-edited samples showed reduced clonogenic capacity: 132 colonies (13.2%) for *MPL* VUS, 88 (8.8%) for *EZH2* VUS and 75 (7.5%) for *JAK2* VUS. Importantly, no significant differences were observed in the relative distribution of colony subtypes, indicating absence of skewing at the progenitor colony level. In contrast, FACS analysis revealed significant lineage alterations. Both *EZH2* and *JAK2* VUS were associated with a significant reduction in erythroid cells (85.4% in control vs. 75.3%, in *EZH2*, p=0.027; 80.5% in *JAK2*, p=0.039) and a concomitant increase in $CD11b^+$ myeloid cells (6.9% in control vs. 12.4% in *EZH2*, p=0.045; 10.9% in *JAK2*, p=0.036). Additionally, *EZH2* VUS-edited cells showed a modest but significant decrease in $CD117^+$ progenitor cells (85% to 80.9%, p=0.025), suggesting diminished progenitor maintenance and enhanced progression toward differentiated myeloid states.

**Summary/Conclusion**

Functional modeling in primary human $CD34^+$ HSPCs showed that *EZH2* and *JAK2* VUS variants cause early differentiation defects and myeloid skewing, indicating that they are not biologically neutral. Our approach establishes a novel framework for investigating the role of VUS in MPNs.

**Copyright Information:** (Online) ISSN: 2572-9241
©2026 The Author(s). HemaSphere is published by John Wiley & Sons Ltd on behalf of European Hematology Association. This is an open access Abstract Book distributed under the Attribution-NonCommercial-NoDerivs (CC BY-NC-ND), which allows third parties to download the articles and share them with others as long as they credit the author and the Abstract Book, but they cannot change the content in any way or use them commercially.
**Abstract Book Citations:** Authors, Title, HemaSphere, 2026;10:(S1):pages. The abstract book DOIs can be found on the "Author Information" page.
**Disclaimer:** Articles published in the journal HemaSphere exclusively reflect the opinions of the authors. The authors are responsible for all content in their abstracts including accuracy of the facts, statements, citing resources, etc.

### (PS1968) MACHINE LEARNING-DERIVED CIRCULATING INFLAMMATORY PROTEIN SIGNATURE FOR RISK STRATIFICATION IN SYSTEMIC MASTOCYTOSIS

**Topic:** 15. Myeloproliferative neoplasms - Biology & translational research

Wei Xiao[1], Jennifer Marvin-Peek*[1], Annie Hoai Nguyen[1], Elisabeth Rebboah[1], Yu-An Kuo[1], Leticia Campoverde[1], Maria Hachem[1], Ivo Veletic[1], Taghi Manshouri[1], Naveen Pemmaraju[1], Lucia Masarova[1], Prithviraj Bose[1], Hussein Abbas[1]

[1]*University of Texas MD Anderson Cancer Center, Leukemia, Houston, United States of America;*

### Background

Systemic mastocytosis (SM) is a clonal mast cell disorder characterized by aberrant mast cell accumulation in bone marrow and extracutaneous organs, leading to chronic inflammation and a wide spectrum of clinical manifestations. Although inflammatory signaling is central to SM biology, circulating proteomic biomarkers that reflect disease burden and clinical outcomes remain poorly defined. High-dimensional plasma proteomics combined with machine learning may enable objective, biology-driven risk stratification.

### Aims

To evaluate whether SM is characterized by a disease-specific inflammatory signature that distinguishes advanced versus non-advanced SM beyond conventional clinical criteria.

### Methods

Baseline plasma from 82 adults with WHO-confirmed SM (2007–2024) was profiled using the NULISAseq Inflammation Panel (250 proteins). After excluding one outlier, 81 patients were included for modeling. To reduce overfitting and assess robustness, we performed 100 random 80/20 train-test splits. Within each training set, differential expression and Random Forest with Boruta were applied for feature selection. The 10 most frequently selected proteins across iterations defined a fixed proteomic signature. We then trained 3 parallel classifiers using: the proteomic signature alone, available baseline clinical variables alone, or a combination of both, and evaluated them on held-out test sets. Performance was assessed by ROC analysis and accuracy across iterations, with feature contributions examined using SHAP (Fig 1A).

### Results

The cohort had a median age of 50.5 years and was 62% female, including ISM (74%), SSM (2%), ASM (11%), and SM-AHN (12%). Across iterations, our machine learning feature selection pipeline consistently identified a stable subset of discriminatory markers. The random forest classifier built on the top 10 inflammatory biomarkers demonstrated strong discrimination between advanced and non-advanced SM (85.4% accuracy) and outperformed the model trained with clinical variables only (75.8% accuracy, $p < 0.001$). The integrated model combining proteomic and clinical variables achieved the highest overall performance, supporting complementary prognostic value from circulating inflammatory proteins (Fig 1B). Key features, including IL1R1, FASLG, LDH, VCAM1, and IL1RL1, reflect inflammatory signaling, apoptosis pathways, metabolic activity, and endothelial activation, underscoring biological relevance to SM pathobiology (Fig 1C). Validation of these proteomic signatures in independent cohorts and on orthogonal platforms is ongoing.

**Copyright Information:** (Online) ISSN: 2572-9241
©2026 The Author(s). HemaSphere is published by John Wiley & Sons Ltd on behalf of European Hematology Association. This is an open access Abstract Book distributed under the Attribution-NonCommercial-NoDerivs (CC BY-NC-ND), which allows third parties to download the articles and share them with others as long as they credit the author and the Abstract Book, but they cannot change the content in any way or use them commercially.
**Abstract Book Citations:** Authors, Title, HemaSphere, 2026;10:(S1):pages. The abstract book DOIs can be found on the “Author Information” page.
**Disclaimer:** Articles published in the journal HemaSphere exclusively reflect the opinions of the authors. The authors are responsible for all content in their abstracts including accuracy of the facts, statements, citing resources, etc.

### Summary/Conclusion

High-throughput inflammatory proteomics integrated with machine learning models identifies a stable circulating protein signature that distinguishes advanced from non-advanced SM beyond conventional clinical features, supporting development of inflammatory proteomic biomarkers for risk stratification and disease monitoring in systemic mastocytosis.

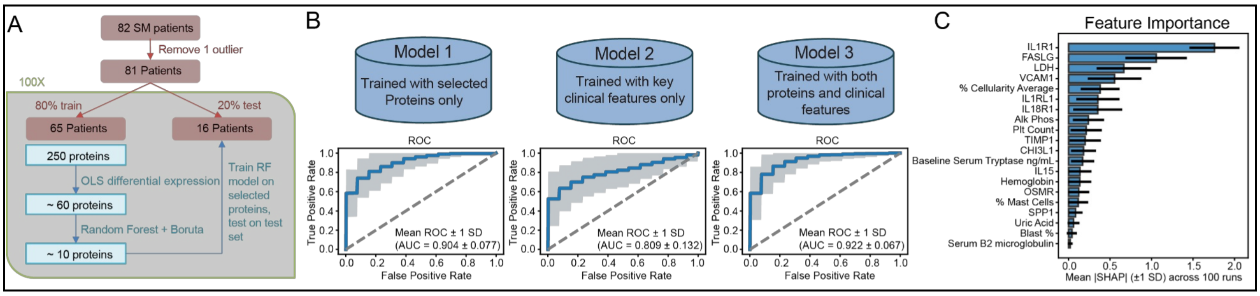

Figure 1. A) Overview of the machine learning pipeline used to identify protein signatures for stratification of SM. B) Comparison of three predictive models trained using selected proteomic features alone (Model 1), key clinical features alone (Model 2), or an integrated set of both (Model 3). Integrated models achieved the highest discriminatory performance. C) Mean SHAP feature importance scores from the integrated model identify key inflammatory, hematologic, and mast cell-related contributors driving classification.

**Copyright Information:** (Online) ISSN: 2572-9241
©2026 The Author(s). HemaSphere is published by John Wiley & Sons Ltd on behalf of European Hematology Association. This is an open access Abstract Book distributed under the Attribution-NonCommercial-NoDerivs (CC BY-NC-ND), which allows third parties to download the articles and share them with others as long as they credit the author and the Abstract Book, but they cannot change the content in any way or use them commercially.
**Abstract Book Citations:** Authors, Title, HemaSphere, 2026;10:(S1):pages. The abstract book DOIs can be found on the "Author Information" page.
**Disclaimer:** Articles published in the journal HemaSphere exclusively reflect the opinions of the authors. The authors are responsible for all content in their abstracts including accuracy of the facts, statements, citing resources, etc.

### (PS1969) MECHANISTIC CHARACTERIZATION OF PRURITUS IN MYELOPROLIFERATIVE NEOPLASMS IDENTIFIES C-FIBER SENSORY DYSFUNCTION

**Topic:** 15. Myeloproliferative neoplasms - Biology & translational research

Madeline Caduc*[1, 2, 3], Saskia Steinhoff[3, 4], Konstantin Agelopoulos[5], Marcelo A. Szymanski de Toledo[1, 2], Ng Yin Fong[1, 2], Kim Kricheldorf[1, 2], Tim Brümmendorf[1, 2], Martin Schmelz[6], Sonja Ständer[5], Barbara Namer[4, 7, 8], Steffen Koschmieder[1, 2, 8]

*[1]Department of Hematology, Oncology, Hemostaseology, and SCT, Faculty of Medicine, RWTH Aachen University, Aachen, Germany; [2]Center for Integrated Oncology Aachen Bonn Cologne Düsseldorf (CIO ABCD), Aachen, Germany; [3]shared first authorship, Aachen, Germany; [4]Institute of Neurophysiology, Uniklinik RWTH Aachen University, Aachen, Germany; [5]Pruritus Medicine Section, Department of Dermatology and Center for Chronic Pruritus (KCP), University Hospital Münster, Münster, Germany; [6]Department of Experimental Pain Research, MCTN, Medical Faculty Mannheim, Heidelberg University, Mannheim, Germany; [7]Department of Anaesthesiology, Intensive Care, Emergency and Pain Medicine, Zentrum Operative Medizin (ZOM), Würzburg, Germany; [8]shared senior authorship, Aachen, Germany;*

### Background

Myeloproliferative neoplasms (MPN) are characterized by constitutive JAK–STAT activation and chronic inflammation, resulting in a high symptom burden including chronic pruritus, particularly aquagenic pruritus. Although mast cells and cytokine dysregulation have been implicated, the pathomechanisms of MPN-associated pruritus remain unclear.

### Aims

We performed integrated clinical and molecular profiling of MPN patients with high versus low pruritus burden to delineate disease-specific itch mechanisms and identify shared features with other chronic pruritus conditions.

### Methods

32 MPN patients (polycythemia vera n=20, essential thrombocythemia n=7, myelofibrosis n=5) were prospectively enrolled and stratified by pruritus severity using a numeric rating scale (NRS 0-10; high pruritus [HP] ≥3, n=15; low pruritus [LP] <3, n=17). Comprehensive profiling included validated patient-reported outcomes (MPN-SAF, NeuroDerm, 5PLQ, SF-12, HADS, STAI-G), serum markers (IL-6, tryptase), and electrostimulation evoked itch/pain assessment in a pruritus-relevant test area (forearm) and control area (dorsum pedis). Quantitative sensory testing (QST) was performed to assess peripheral sensory function. Intraepidermal nerve fiber density (IENFD) was assessed in skin biopsies.

**Copyright Information:** (Online) ISSN: 2572-9241
©2026 The Author(s). HemaSphere is published by John Wiley & Sons Ltd on behalf of European Hematology Association. This is an open access Abstract Book distributed under the Attribution-NonCommercial-NoDerivs (CC BY-NC-ND), which allows third parties to download the articles and share them with others as long as they credit the author and the Abstract Book, but they cannot change the content in any way or use them commercially.
**Abstract Book Citations:** Authors, Title, HemaSphere, 2026;10:(S1):pages. The abstract book DOIs can be found on the "Author Information" page.
**Disclaimer:** Articles published in the journal HemaSphere exclusively reflect the opinions of the authors. The authors are responsible for all content in their abstracts including accuracy of the facts, statements, citing resources, etc.

### Results

Age, MPN subtype distribution, and driver mutation profiles were comparable between groups. Serum tryptase levels were significantly elevated in HP versus LP patients (p=0.02), independent of MPN subtype or mutational status. IL-6 showed a trend toward upregulation (p=0.10). Compared to LP, treatment with (ro)pegylated interferon-alpha (pegIFNa) was more frequent in the HP group (54% vs. 24%), while fewer had received ruxolitinib (13% vs. 30%). This distribution is aligning with the reported antipruritic effects of JAK1/2 inhibition and pruritogenic potential of pegIFNa.
Electrostimulation elicited significantly higher, intensity-dependent itch perception in HP compared with LP patients at both control and pruritus-relevant sites (Figure 1). At the control site, this was paralleled by increased pain ratings in HP versus LP patients, whereas stimulation at the pruritus-relevant site predominantly enhanced itch without relevant differences in pain, supporting specific sensitization of pruriceptive signaling. QST profiles demonstrated a mixed loss- and gain-of-function sensory phenotype in HP patients, characterized by impaired thermal detection, indicating small-fiber hypofunction, alongside increased mechanical pain sensitivity consistent with central sensitization. This pattern supports the hypothesis of peripheral C-fiber dysfunction with concomitant central amplification of sensory signaling in pruritic MPN. PGP9.5 staining revealed a significant reduction in IENFD and nerve fiber branching in HP and LP patients versus age- and sex-matched controls (p<0.001); no significant differences were observed between HP and LP patients.

### Summary/Conclusion

MPN-associated pruritus is characterized by a distinct neurosensory phenotype with gain- and loss-of-function and alterations in peripheral and central processing. Impaired thermal perception consistent with small-fiber neuropathy, together with enhanced itch and mechanical pain hypersensitivity, indicates complex sensory dysregulation in pruritic MPN. Reduced IENFD across MPN cohorts identifies peripheral nerve fiber degeneration as pruritus intensity-independent MPN-associated feature.

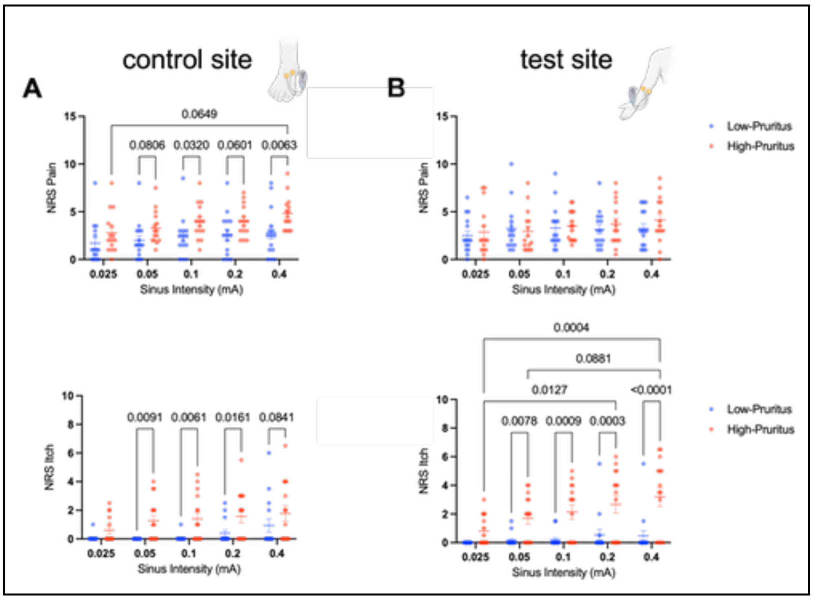

Figure 1. Electrostimulation-induced itch and pain responses in MPN patients with high (HP) versus low pruritus (LP). (A) Pain and itch ratings at the control site (dorsum pedis) across increasing stimulation intensities. (B) Pain and itch ratings at the test site. Data are shown as individual values with mean ± SEM. Statistical analysis was performed using two-way ANOVA with Sidak's multiple comparisons test.

**Copyright Information:** (Online) ISSN: 2572-9241
©2026 The Author(s). HemaSphere is published by John Wiley & Sons Ltd on behalf of European Hematology Association. This is an open access Abstract Book distributed under the Attribution-NonCommercial-NoDerivs (CC BY-NC-ND), which allows third parties to download the articles and share them with others as long as they credit the author and the Abstract Book, but they cannot change the content in any way or use them commercially.
**Abstract Book Citations:** Authors, Title, HemaSphere, 2026;10:(S1):pages. The abstract book DOIs can be found on the "Author Information" page.
**Disclaimer:** Articles published in the journal HemaSphere exclusively reflect the opinions of the authors. The authors are responsible for all content in their abstracts including accuracy of the facts, statements, citing resources, etc.

### (PS1970) IDENTIFICATION OF BIOMARKERS TO PREDICT DISEASE PROGRESSION VIA MOLECULAR ANALYSIS OF PATIENTS (PTS) WITH LOW-RISK MYELOFIBROSIS (MF) ENROLLED IN THE MOST STUDY

**Topic:** 15. Myeloproliferative neoplasms - Biology & translational research

Naveen Pemmaraju*[1], Ellen Ritchie[2, 3], John Mascarenhas[4], Salman Fazal[5], Erin Crowgey[6], Taehyung (Simon) Kim[6], Jennifer Hamer-Maansson[6], Tricia Kalafut[6], Evan Braunstein[6], Abdulraheem Yacoub[7]

*[1]The University of Texas MD Anderson Cancer Center, Houston, TX, United States of America;[2]Weill Cornell Medical College, Department of Medicine, New York, NY, United States of America; [3]The New York Presbyterian Hospital, New York, NY, United States of America; [4]Icahn School of Medicine at Mount Sinai, New York, NY, United States of America;[5]AHN Cancer Institute, Pittsburgh, PA, United States of America; [6]Incyte Corporation, Wilmington, DE, United States of America;[7]University of Kansas Medical Center, Westwood, KS, United States of America;*

### Background

The Myelofibrosis and Essential Thrombocythemia Observational Study (MOST; NCT02953704) was a prospective observational study of pts with low-risk MF defined by Dynamic International Prognostic Scoring System (DIPSS) criteria. A previous analysis of data from MOST showed that 61% of pts with low-risk MF progressed to higher-risk disease over a median 54 months of follow-up (Yacoub A, et al. *Blood*. 2024;144[suppl 1]:3136).

### Aims

To investigate the correlation between molecular gene signatures at enrollment and MF disease progression of pts in MOST.

### Methods

MOST included pts with a physician-reported MF diagnosis (primary MF, post-polycythemia vera, or post-essential thrombocythemia) and low-risk status by DIPSS (except age >65 y). Optional biospecimens were collected at usual care visits. MF progression was defined previously (Yacoub A, et al. *Blood*. 2024;144[suppl 1]:3136). Of 232 pts with MF enrolled, 140 (60%) had biospecimens available for RNA sequencing (85 pts with and 55 pts without progression), performed using Illumina NovaSeq™ X Plus. Age-matched samples from healthy donors (n=98) served as the controls (Discovery Life Sciences, AL, USA). RNAseq data were analyzed by differential gene expression (DGE) analysis via limma; pathway enrichment analyses utilized gene set variation analysis and gene set enrichment analysis (GSEA).

### Results

The MF cohort (n=140) consisted of 81 pts with a driver mutation in *JAK2*, 35 in *CALR*, 8 in *MPL*; 4 pts had 2 driver mutations; 12 were triple-negative. DGE analysis of pts in MF vs control samples identified 560 downregulated and 1524 upregulated genes (fold change >1.5; adjusted $P$<0.05). *CREB3L1*, previously correlated with increased risk of MF progression, was among the most upregulated genes in the MF cohort. Enrichment analysis highlighted heme metabolism, cell cycle checkpoint (G2M), coagulation, DNA replication (E2F targets), epithelial mesenchymal transition, and allograft rejection as differentially expressed pathways in the MF cohort.
*JAK2*V617F variant allele frequency (VAF) at enrollment was predictive of progression (with vs without progression, mean 0.48 vs 0.37; $P$=0.026). Comparison with the control samples showed more genes were differentially expressed in pts with (n=2836) than without (n=1347) MF progression. These GSEA patterns reflected those seen for total MF vs the control samples; however, the magnitude of change trended higher in pts with progression **(Figure)**.

**Copyright Information:** (Online) ISSN: 2572-9241
©2026 The Author(s). HemaSphere is published by John Wiley & Sons Ltd on behalf of European Hematology Association. This is an open access Abstract Book distributed under the Attribution-NonCommercial-NoDerivs (CC BY-NC-ND), which allows third parties to download the articles and share them with others as long as they credit the author and the Abstract Book, but they cannot change the content in any way or use them commercially.
**Abstract Book Citations:** Authors, Title, HemaSphere, 2026;10:(S1):pages. The abstract book DOIs can be found on the "Author Information" page.
**Disclaimer:** Articles published in the journal HemaSphere exclusively reflect the opinions of the authors. The authors are responsible for all content in their abstracts including accuracy of the facts, statements, citing resources, etc.

**Summary/Conclusion**

This analysis of molecular markers of MF progression identified *JAK2* V617F VAF as predictive of progression, with significantly higher VAF at enrollment seen in pts with vs without progression. DGE analysis identified other biomarkers of interest, including *CREB3L1,* previously associated with progression in myeloproliferative neoplasms and other cancers, and involved in cellular response to endoplasmic reticulum stress and DNA damage. DGE analysis also identified several pathways differentially expressed in pts with MF vs controls, with trends consistent in pts with and without progression. These differences vs controls were greater in pts with vs without MF progression, suggesting progression involves worsening of already dysregulated oncogenic pathways. Genes differentially expressed in pts with MF progression may be used for prognostic stratification and disease monitoring, while also revealing therapeutic vulnerabilities. Additional analyses are ongoing to validate biomarkers of MF progression and identify targets for disease monitoring.

**Copyright Information:** (Online) ISSN: 2572-9241
©2026 The Author(s). HemaSphere is published by John Wiley & Sons Ltd on behalf of European Hematology Association. This is an open access Abstract Book distributed under the Attribution-NonCommercial-NoDerivs (CC BY-NC-ND), which allows third parties to download the articles and share them with others as long as they credit the author and the Abstract Book, but they cannot change the content in any way or use them commercially.
**Abstract Book Citations:** Authors, Title, HemaSphere, 2026;10:(S1):pages. The abstract book DOIs can be found on the “Author Information” page.
**Disclaimer:** Articles published in the journal HemaSphere exclusively reflect the opinions of the authors. The authors are responsible for all content in their abstracts including accuracy of the facts, statements, citing resources, etc.

**Figure. Correlation Between Disease Progression Status and Fold Change in Gene Expression and Pathway Signatures**

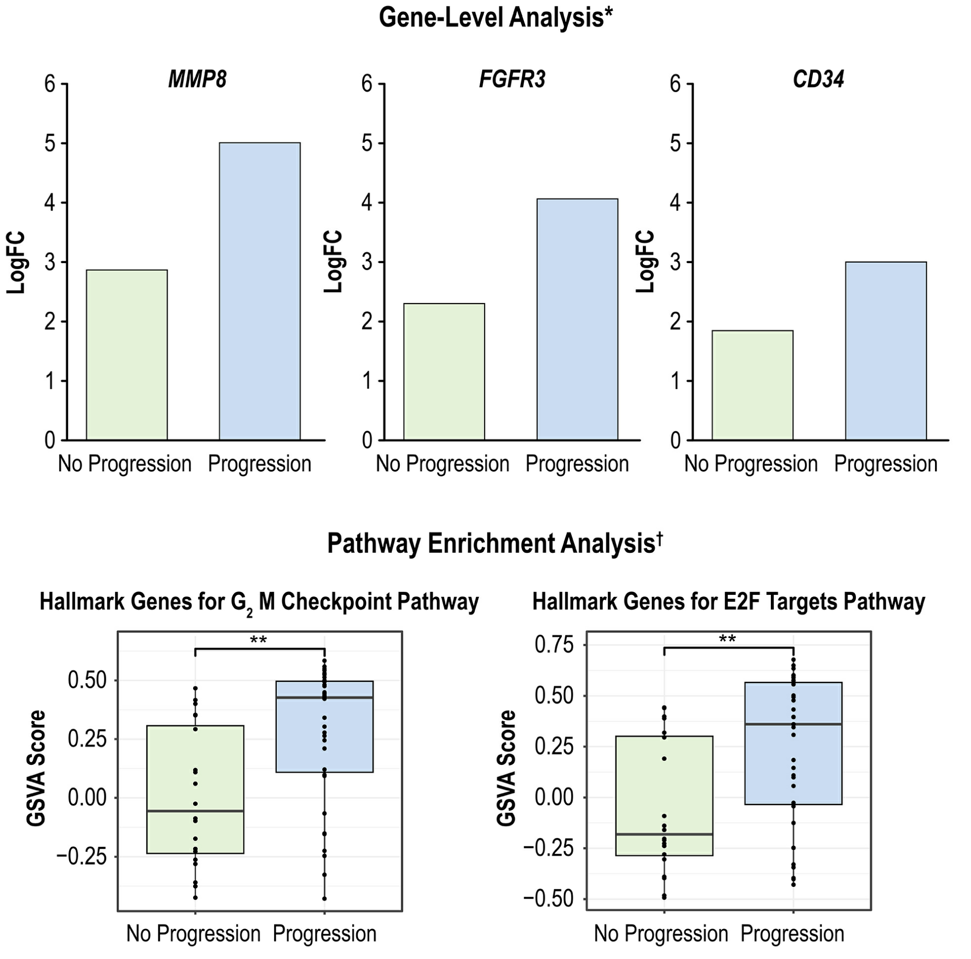

*Genes displayed had a significant *P* value (<0.05). †A pathway score was determined for each patient using GSVA and KEGG pathways and plotted per cohort. ***P*<0.01.
E2F, E2 promoter-binding factor; FC, fold change; GSVA, gene set variation analysis; KEGG, Kyoto Encyclopedia of Genes and Genomes.

**Copyright Information:** (Online) ISSN: 2572-9241
©2026 The Author(s). HemaSphere is published by John Wiley & Sons Ltd on behalf of European Hematology Association. This is an open access Abstract Book distributed under the Attribution-NonCommercial-NoDerivs (CC BY-NC-ND), which allows third parties to download the articles and share them with others as long as they credit the author and the Abstract Book, but they cannot change the content in any way or use them commercially.
**Abstract Book Citations:** Authors, Title, HemaSphere, 2026;10:(S1):pages. The abstract book DOIs can be found on the "Author Information" page.
**Disclaimer:** Articles published in the journal HemaSphere exclusively reflect the opinions of the authors. The authors are responsible for all content in their abstracts including accuracy of the facts, statements, citing resources, etc.

### (PS1971) A NOVEL GERMLINE JAK2 MUTATION JAK2G127D COOPERATION WITH JAK2V617F TO ACCELERATE MYELOFIBROSIS PROGRESSION IN MPNS

**Topic:** 15. Myeloproliferative neoplasms - Biology & translational research

Yanjie Lan*[1], Yuhui Zhang[1], Guangshuai Teng[1], Yifan Duan[1], Yan Wang[1], Huiqin Zhang[1], Yanqi Li[1], Lan Peng[1], Xiaojing Wei[1], Yuan Zhou[2], Weiping Yuan[1, 2], Jie Bai[1]

*[1]The Second Hospital of Tianjin Medical University, Department of Hematology, Tianjin, China;[2]Institute of Hematology & Blood Diseases Hospital, Chinese Academy of Medical Sciences & Peking Union Medical College, State Key Laboratory of Experimental Hematology, National Clinical Research Center for Blood Diseases, Tianjin, China;*

### Background

The disease course of myeloproliferative neoplasms (MPN) is characterized by significant heterogeneity, among which progression to myelofibrosis is a critical determinant of poor patient outcomes[1]. Although the concept of somatic driver mutations in MPNs is well established, emerging evidence indicates that germline JAK2 mutations play an active regulatory role in modulating disease pathogenesis[2,3]. Germline mutation *JAK2*$^{G127D}$ has been reported as one of the most common atypical JAK2 variants, witch was found to exclusively co-occur with *JAK2*$^{V617F}$ and was associated with higher grades of myelofibrosis[4]. However, the underlying mechanisms remain incompletely understand.

### Aims

This study aimed to elucidate the presence of *JAK2*$^{G127D}$ and *JAK2*$^{V617F}$ mutations in a cohort of MPN patients to characterize the double-mutation carriers and gain insight into the potential molecular mechanisms of coexisting mutations in driving myelofibrosis progression in MPNs.

### Methods

The clinical characteristics and survival outcomes of 290 patients with MPN who were first diagnosed in our center from 2018 to 2025 were collected. Next-generation sequencing was used to detect gene mutation. Survival analysis was calculated from the data of diagnosis to the date of death or the last date of follow-up. Furthermore, cellular models were established to investigate the underlying mechanisms.

**Copyright Information:** (Online) ISSN: 2572-9241
©2026 The Author(s). HemaSphere is published by John Wiley & Sons Ltd on behalf of European Hematology Association. This is an open access Abstract Book distributed under the Attribution-NonCommercial-NoDerivs (CC BY-NC-ND), which allows third parties to download the articles and share them with others as long as they credit the author and the Abstract Book, but they cannot change the content in any way or use them commercially.
**Abstract Book Citations:** Authors, Title, HemaSphere, 2026;10:(S1):pages. The abstract book DOIs can be found on the “Author Information” page.
**Disclaimer:** Articles published in the journal HemaSphere exclusively reflect the opinions of the authors. The authors are responsible for all content in their abstracts including accuracy of the facts, statements, citing resources, etc.

**Results**

Samples from 249 MPN patients positive for *JAK2*$^{V617F}$ were enrolled (Figure A). Fifteen of 249 *JAK2*$^{V617F}$ MPN patients were found to carry concurrent *JAK2*$^{V617F}$ and *JAK2*$^{G127D}$ mutations (6.17%, 15/243) (Figure B). Notably, *JAK2*$^{G127D}$ mutations only co-occurred with *JAK2*$^{V617F}$ (Figure C). Variant allele frequency (VAF) analysis revealed that *JAK2*$^{G127D}$ exhibited VAFs of approximately 50% or 100%, strongly suggesting a germline origin. Then, all *JAK2*$^{G127D}$ mutations were confirmed its germline origin through hair follicle (Figure D). Moreover, patients harboring the *JAK2*$^{G127D}$ and *JAK2*$^{V617F}$ mutations exhibited significantly shortened myelofibrosis-free survival ( *P*=0.0062) (Figure E), and those in advanced disease stages presented with higher grades of myelofibrosis and higher VAF of *JAK2*$^{V617F}$ compared to patients harbor only *JAK2*$^{V617F}$ mutations (Figure F). Correspondingly, a significantly higher level of TGF-β were observed in double-mutation carriers compared with *JAK2*$^{V617F+}$ patients (Figure F). These findings suggested that germline mutation *JAK2*$^{G127D}$ was associated with myelofibrosis progression in *JAK2*$^{V617F+}$ MPNs.
Cellular models were used to assess the functional consequences of the coexisting mutations. We modeled JAK2 mutants in Ba/F3 and 32D cells expressing EPOR. Ba/F3-EPOR or 32D-EPOR expressing *JAK2*$^{WT}$, *JAK2*$^{V617F}$, *JAK2*$^{G127D}$, and *JAK2*$^{V617F+G127D}$. We measured cellular proliferation in response to cytokines and found that *JAK2*$^{G127D}$ co-expression with *JAK2*$^{V617F}$ synergistically enhances cytokine-independent proliferation and markedly activates the JAK2-STAT5 signaling pathway (Figure G-H).

**Summary/Conclusion**

Here, we provided insight into the role of the novel germline *JAK2*$^{G127D}$ mutations, demonstrating the *JAK2*$^{G127D}$ and *JAK2*$^{V617F}$ mutations synergistically drive myelofibrosis progression in MPN. We also demonstrated the evidence that the germline *JAK2*$^{G127D}$ mutation synergizes with *JAK2*$^{V617F}$ to significantly potentiate the activation of the JAK-STAT signaling pathway *in vitro*. To further elucidate more detailed functional role of *JAK2*$^{G127D}$, mouse model will be pursued.

**Copyright Information:** (Online) ISSN: 2572-9241
©2026 The Author(s). HemaSphere is published by John Wiley & Sons Ltd on behalf of European Hematology Association. This is an open access Abstract Book distributed under the Attribution-NonCommercial-NoDerivs (CC BY-NC-ND), which allows third parties to download the articles and share them with others as long as they credit the author and the Abstract Book, but they cannot change the content in any way or use them commercially.
**Abstract Book Citations:** Authors, Title, HemaSphere, 2026;10:(S1):pages. The abstract book DOIs can be found on the “Author Information” page.
**Disclaimer:** Articles published in the journal HemaSphere exclusively reflect the opinions of the authors. The authors are responsible for all content in their abstracts including accuracy of the facts, statements, citing resources, etc.

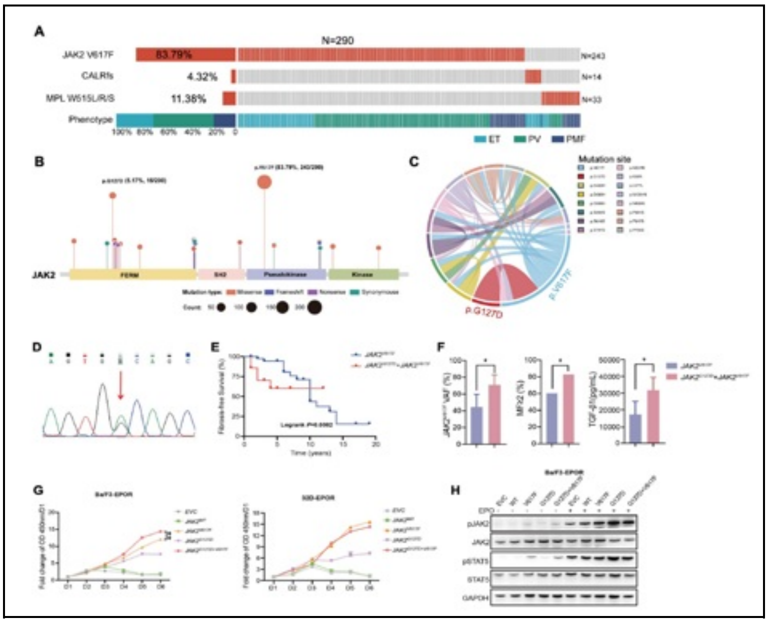

A. Driver mutation spectrum and phenotype distribution in 290 MPN patients; B. Distribution of JAK2 mutations across protein domains; C. Co-occurrence analysis of JAK2 mutations; D. Sanger sequencing validation of the germline JAK2G127D mutation; E. Myelofibrosis-free survival curves of the two groups; F. Among the subgroup of patients who progressed to myelofibrosis: JAK2V617F variant allele frequency (left), myelofibrosis grade (middle), and plasma TGF-β levels (right); G. Proliferation of cells from each group under IL-3-free conditions; H. Western blot analysis of pJAK2, total JAK2, pSTAT5, and total STAT5 protein levels in each group with (+) or without (-) EPO stimulation.

**Copyright Information:** (Online) ISSN: 2572-9241
©2026 The Author(s). HemaSphere is published by John Wiley & Sons Ltd on behalf of European Hematology Association. This is an open access Abstract Book distributed under the Attribution-NonCommercial-NoDerivs (CC BY-NC-ND), which allows third parties to download the articles and share them with others as long as they credit the author and the Abstract Book, but they cannot change the content in any way or use them commercially.
**Abstract Book Citations:** Authors, Title, HemaSphere, 2026;10:(S1):pages. The abstract book DOIs can be found on the "Author Information" page.
**Disclaimer:** Articles published in the journal HemaSphere exclusively reflect the opinions of the authors. The authors are responsible for all content in their abstracts including accuracy of the facts, statements, citing resources, etc.

### (PS1972) PHYLOGENY, ORDER OF MUTATIONS, AND CELLULAR ORIGIN OF INDOLENT AND ADVANCED SYSTEMIC MASTOCYTOSIS

**Topic:** 15. Myeloproliferative neoplasms - Biology & translational research

Tetsuichi Yoshizato*[1], Petter Woll[1], Kajsa Ax[1], Cecilia Karlström[1, 2], Joakim Dahlin[3], Sten Eirik Jacobsen[1, 2, 4], Johanna Ungerstedt[1, 5, 6]

*[1]Karolinska Institutet, Department of Medicine Huddinge, Center for Hematology and Regenerative Medicine, Stockholm, Sweden; [2]Karolinska University Hospital, ME Hematology, Stockholm, Sweden; [3]Karolinska Institutet, Center for Molecular Medicine, Stockholm, Sweden; [4]Karolinska Institutet, Department of Cell and Molecular Biology, Stockholm, Sweden; [5]Linköping University, Department of Biomedical and Clinical Sciences, Linköping, Sweden; [6]Linköping University Hospital, Hematology Clinic, Linköping, Sweden;*

### Background

Systemic mastocytosis (SM) is a myeloid neoplasm characterized by the expansion of neoplastic mast cells (MCs), primarily driven by the *KIT* D816V mutation. Clinical presentation ranges from indolent SM (ISM), associated with near-normal life expectancy, to advanced SM (AdvSM), which carries a poor prognosis. However, the molecular and cellular determinants underlying these distinct disease phenotypes remain incompletely understood.

### Aims

We aimed to elucidate the cellular origin and the temporal order of somatic mutation acquisition in SM, and to determine how these factors contribute to the clinical divergence between ISM and AdvSM.

### Methods

Whole-exome sequencing (WES) was performed on bone marrow mononuclear cells (BMMNCs), purified MCs, and T cells from 29 patients (17 ISM, 12 AdvSM including 3 aggressive SM and 7 SM with an associated other neoplasm (SMAHN). Hematopoietic stem and progenitor cell (HSPC) fractions—including hematopoietic stem cells (HSCs), granulocyte-monocyte progenitors, and erythroid progenitors—were isolated by flow cytometry. Mutation frequencies of *KIT* D816V and patient-specific variants were assessed using droplet digital PCR (ddPCR) to track clonal involvement across hematopoiesis at high resolution.

**Copyright Information:** (Online) ISSN: 2572-9241
©2026 The Author(s). HemaSphere is published by John Wiley & Sons Ltd on behalf of European Hematology Association. This is an open access Abstract Book distributed under the Attribution-NonCommercial-NoDerivs (CC BY-NC-ND), which allows third parties to download the articles and share them with others as long as they credit the author and the Abstract Book, but they cannot change the content in any way or use them commercially.
**Abstract Book Citations:** Authors, Title, HemaSphere, 2026;10:(S1):pages. The abstract book DOIs can be found on the "Author Information" page.
**Disclaimer:** Articles published in the journal HemaSphere exclusively reflect the opinions of the authors. The authors are responsible for all content in their abstracts including accuracy of the facts, statements, citing resources, etc.

**Results**

WES comprehensively characterized the mutational landscape of MCs and, apart from *KIT* and other known myeloid driver genes, did not identify additional recurrent driver mutations in either ISM or AdvSM. AdvSM harbored significantly more nonsynonymous mutations than ISM and demonstrated greater overall clonal involvement.
Phylogenetic analysis based on WES data in MCs, BMMNCs, and T cells showed that *KIT* mutations were largely restricted to MCs in ISM, whereas AdvSM patients exhibited extensive sharing of mutations between MCs and BMMNCs, consistent with a common ancestral clone. Using sensitive ddPCR in purified HSPC fractions, *KIT* D816V was detected in HSPCs in all AdvSM cases and approximately one-third of ISM patients, indicating that in AdvSM and at least a subset of ISM, the disease originates in the HSPC compartment. *KIT* D816V represented the initiating driver in 73% of ISM cases. In AdvSM,*KIT* D816V was frequently acquired as a secondary event following other myeloid driver mutations as previously described (Jahwar et al. Leukemia. 2015;29(5):1115-2), although it was identified as the initial hit in one case, indicating that mutation order alone does not determine the disease phenotype of ISM or AdvSM.
Notably, the key determinant of disease progression was the presence of additional myeloid driver mutations in genes beyond those commonly associated with clonal hematopoiesis (*DNMT3A* or *TET2*). Significant expansion of *KIT* D816V clones within the HSPC compartment was observed only in cases harboring such additional driver mutations. In contrast, *KIT* D816V alone promoted MC expansion but failed to confer clonal dominance at the HSPC level.

**Summary/Conclusion**

We demonstrate that both ISM and AdvSM can originate in the HSPC compartment, and that disease severity is not determined by the temporal order of *KIT* D816V acquisition but by the presence of additional driver mutations that confer proliferative advantage within HSPCs. *KIT D816V* alone is sufficient to drive MC expansion but, as an isolated mutation, is insufficient to induce HSPC clonal dominance. These findings provide a biological framework explaining the divergent clinical courses of ISM and AdvSM and identify HSPC-level clonal expansion as a key determinant of advanced disease.

**Copyright Information:** (Online) ISSN: 2572-9241
©2026 The Author(s). HemaSphere is published by John Wiley & Sons Ltd on behalf of European Hematology Association. This is an open access Abstract Book distributed under the Attribution-NonCommercial-NoDerivs (CC BY-NC-ND), which allows third parties to download the articles and share them with others as long as they credit the author and the Abstract Book, but they cannot change the content in any way or use them commercially.
**Abstract Book Citations:** Authors, Title, HemaSphere, 2026;10:(S1):pages. The abstract book DOIs can be found on the “Author Information” page.
**Disclaimer:** Articles published in the journal HemaSphere exclusively reflect the opinions of the authors. The authors are responsible for all content in their abstracts including accuracy of the facts, statements, citing resources, etc.

### (PS1973) BEYOND JAK INHIBITION: WT1 AS A BIOMARKER AND THERAPEUTIC TARGET IN HIGH-RISK PRIMARY MYELOFIBROSIS

**Topic:** 15. Myeloproliferative neoplasms - Biology & translational research

Qingmei Han[1], Rumeng Li[1], Bingbing Wen[2], Tian Zeng[1], Zhikang Zheng[3], Xiaojing Bao[3], Xiaoyan MA[1], Jia Li[1], Jian Huang*[1]

*[1]The First Affiliated Hospital, Zhejiang University School of Medicine, Hangzhou, China;[2]The second hospital of shenzhen, Shenzhen, China; [3]The Fourth Affiliated Hospital, Zhejiang University School of Medicine, Hangzhou, China;*

### Background

Philadelphia-negative myeloproliferative neoplasms (MPNs), particularly primary myelofibrosis (PMF), carry a risk of acute myeloid leukemia transformation. The JAK1/2 inhibitor ruxolitinib alleviates symptoms in MPN patients but rarely induces complete remission. WT1, a marker of clonal hematopoiesis, overexpressed in acute myeloid leukemia and myelodysplastic syndromes has a poorly characterized role in MPNs. Decitabine (DAC), is used in higher-risk myelodysplastic syndromes and elderly acute myeloid leukemia. We investigated whether WT1 expression correlates with disease risk in MPN and whether targeting WT1 could enhance ruxolitinib efficacy.

### Aims

This study evaluated WT1 expression in MPN patients and examined whether combining DAC with ruxolitinib exerts synergistic effects through WT1 modulation.

### Methods

Bone marrow WT1 expression was measured by RT-qPCR (normalized to ABL) in 105 newly diagnosed MPN patients (49 ET, 18 PV, 38 PMF). Correlations with MIPSS70 score, lactate dehydrogenase (LDH), and blast percentage were analyzed.This was a retrospective analysis. In vitro, SET-2 and HEL cells were treated with decitabine (DAC) and/or ruxolitinib. Viability (ATP assay), apoptosis (flow cytometry), colony formation (soft agar), and protein expression (western blot) were assessed.In vivo, NSG mice xenografted with SET2luc cells received vehicle, DAC, ruxolitinib, or the combination; tumour burden was monitored by bioluminescence.

### Results

Median WT1 expression in the MPN cohort was 0.099% (0.044%–0.225%). PMF patients exhibited higher WT1 levels (0.135%, 0.05%–0.42%) than ET (0.063%, 0.025%–0.11%) or PV (0.10%, 0.046%–0.21%). In PMF patients with WT1 >0.075%, WT1 correlated positively with MIPSS70 and MIPSS70+ score (r=0.648, P=0.0008), LDH (r=0.550, P=0.0066) and bone marrow blast percentage (r=0.547, P=0.0021). The Western blotting also revealed that the expression of WT1 in PBMCs of PMF patients was higher than that in healthy individuals. As elevated WT1 identified higher-risk PMF, we examined whether targeting WT1 could enhance ruxolitinib response. In SET-2 and HEL cells, the combination of DAC and ruxolitinib treatment produced greater inhibition, with combination index <1 indicating synergy. Apoptosis was higher with combination than with monotherapy. Ruxolitinib induced G1 arrest, while DAC did not affect cell cycle. Colony formation was nearly abolished by the combination. Mechanistically, the combination leads to the downregulation of WT1, which subsequently reduces Bcl-2 levels, concomitantly activating both Caspase-8 and Caspase-9. This activation ultimately results in a marked increase in cleaved Caspase-3 and cleaved PARP, thereby promoting apoptosis. In vivo, combination therapy reduced tumor burden and prolonged median survival.

**Copyright Information:** (Online) ISSN: 2572-9241
©2026 The Author(s). HemaSphere is published by John Wiley & Sons Ltd on behalf of European Hematology Association. This is an open access Abstract Book distributed under the Attribution-NonCommercial-NoDerivs (CC BY-NC-ND), which allows third parties to download the articles and share them with others as long as they credit the author and the Abstract Book, but they cannot change the content in any way or use them commercially.
**Abstract Book Citations:** Authors, Title, HemaSphere, 2026;10:(S1):pages. The abstract book DOIs can be found on the “Author Information” page.
**Disclaimer:** Articles published in the journal HemaSphere exclusively reflect the opinions of the authors. The authors are responsible for all content in their abstracts including accuracy of the facts, statements, citing resources, etc.

**Summary/Conclusion**

WT1 is elevated in PMF and correlates with established risk parameters, suggesting utility as a biomarker for high-risk disease. Combining DAC with ruxolitinib synergistically suppresses MPN cell growth in vitro and in vivo, an effect associated with WT1 downregulation and modulation of the Bcl-2/caspase pathway. These findings identify WT1 as a potential therapeutic target beyond JAK inhibition in high-risk primary myelofibrosis.
This research was supported by the National Natural Science Foundation of China (No. 82570191) and the Zhejiang Provincial Health Highlevel Innovative Talent Project (2022-2026).
*Correspondence to: Prof Jian Huang, E-mail: househuang@zju.edu.cn.

**Copyright Information:** (Online) ISSN: 2572-9241
©2026 The Author(s). HemaSphere is published by John Wiley & Sons Ltd on behalf of European Hematology Association. This is an open access Abstract Book distributed under the Attribution-NonCommercial-NoDerivs (CC BY-NC-ND), which allows third parties to download the articles and share them with others as long as they credit the author and the Abstract Book, but they cannot change the content in any way or use them commercially.
**Abstract Book Citations:** Authors, Title, HemaSphere, 2026;10:(S1):pages. The abstract book DOIs can be found on the "Author Information" page.
**Disclaimer:** Articles published in the journal HemaSphere exclusively reflect the opinions of the authors. The authors are responsible for all content in their abstracts including accuracy of the facts, statements, citing resources, etc.

### (PS1974) THE PROGENITOR CELLS AND THE MEGAKARYOCYTES FROM THE BONE MARROW OF THE GATA1LOW MOUSE MODEL OF MYELOFIBROSIS ACQUIRE A STRONG RIBOSOMAL SIGNATURE WITH DISEASE PROGRESSION

**Topic:** 15. Myeloproliferative neoplasms - Biology & translational research

Allison Cote*[1], Jeff Viestra[1], Anna Rita Migliaccio[1]

[1]*Altius Institute For Biomedical Sciences, Seattle, United States of America;*

### Background

Myelofibrosis (MF), the most severe of the Myeloproliferative Neoplasms, is characterized by fibrosis and hematopoietic failure in the bone marrow (BM), possibly induced by the malignant megakaryocytes corrupted by a ribosomal signature which impairs their maturation by decreasing GATA1. The disease is recapitulated by mice carrying the Gata1low mutation which develop myelofibrosis with age.

### Aims

Our aim is to characterize the unique abnormalities acquired by BM cells from Gata1low mice with age.

### Methods

We performed scRNAseq of CD1 (young, Y, n = 4, 7,314 cells, old, O, n = 6, 29,391 cells) and Gata1low (Y n = 4, 19,076 cells, O n = 6, 49,199 cells) mice. Data were aligned with STARsolo, processed for quality, clustering, and differential expression (DE) with scanpy, and pathway enrichment of DE genes was assessed with GSEApy. High quality cells were defined by QC with >500 counts/cell, >100 genes/cell, and mito reads<20%. To avoid bias, each group was clustered individually and then related clusters from each combination identified via pseudobulk comparison.

### Results

Y-CD1 BM cells exhibited 9 distinct cell clusters consisting of multiple progenitor cell types (CMP, CLP, B cell progenitors, T cell progenitors, and MK/HSPC) and differentiated cell clusters (erythroid cells, macrophages, and two neutrophil subsets).
O-CD1 mice exhibited several of the clusters found in Y-CD1 plus three new distinct cell types (an HSPC specific cluster, MEP, and a distinct macrophage subset). In CD1 mice, the frequency of cells in the CMP and macrophage clusters decreased with age, and the O-CD1 lost one of the two neutrophil subsets.
Y-Gata1low mice exhibited many of the cell types identified in Y-CD1. However, Y-Gata1low mice exhibited a distinct MEP subset and increased numbers of MK/HSPC.
O-Gata1low mice retained the Gata1low specific MEP, as well as an HSPC cluster similar to that of O-CD1. They also exhibited increased numbers in one macrophage subset and in B cell progenitors.
Large differences in gene expression were identified in the clusters according to age and genotype. There were 14,280 unique genes across cell type, age, and genotypes with significant (adjusted p-value < 0.05) and large fold change (log2(FoldChange) >1 or <-1) differences. There were many (average 68) significantly enriched pathways within each differentially enriched gene set, notably many of them were related to changes in ribogenesis/translation and oxidative stress that increased with both age and genotype (greater in O-Gata1low) (in CMP, CLP, T cell progenitors, and macrophages for Y- vs O-CD1, Y- vs O-Gata1low, and O-CD1 vs O-Gata1low) such as Cytoplasmic Translation, Regulation of Translation, Cytoplasmic Ribosomal Proteins, Oxidative Phosphorylation, Oxidative Damage Response. Additional pathways enriched in O-Gata1low mice over O-CD1 include IL17 signaling and apoptosis in macrophages; proliferation in HSPC, HIF-1 signaling/platelet activation in MK; and type II interferon, IL3, and IL2 signaling in T cell progenitors.

**Copyright Information:** (Online) ISSN: 2572-9241
©2026 The Author(s). HemaSphere is published by John Wiley & Sons Ltd on behalf of European Hematology Association. This is an open access Abstract Book distributed under the Attribution-NonCommercial-NoDerivs (CC BY-NC-ND), which allows third parties to download the articles and share them with others as long as they credit the author and the Abstract Book, but they cannot change the content in any way or use them commercially.
**Abstract Book Citations:** Authors, Title, HemaSphere, 2026;10:(S1):pages. The abstract book DOIs can be found on the "Author Information" page.
**Disclaimer:** Articles published in the journal HemaSphere exclusively reflect the opinions of the authors. The authors are responsible for all content in their abstracts including accuracy of the facts, statements, citing resources, etc.

**Summary/Conclusion**

These data provide mechanistic insights on the factors driving myelofibrosis in Gata1low mice: 1) The increased ribosomal signature with age in CD1, which is further increased in O-Gata1low mice, suggests that hypomorphic Gata1 exacerbates age related changes that misregulate the functions of multiple progenitor cell types, leading to BM failure; 2) the increased inflammatory signature in O-Gata1low mice may be responsible for the inflammatory milieu driving fibrosis.

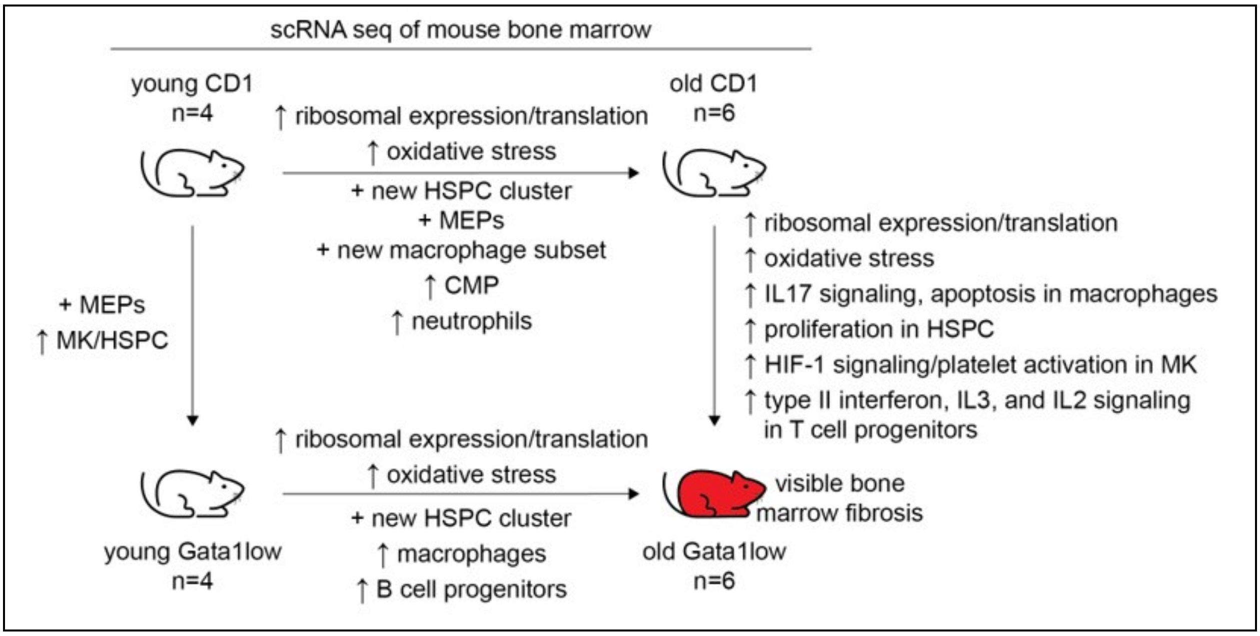

Summary of the Results

**Copyright Information:** (Online) ISSN: 2572-9241
©2026 The Author(s). HemaSphere is published by John Wiley & Sons Ltd on behalf of European Hematology Association. This is an open access Abstract Book distributed under the Attribution-NonCommercial-NoDerivs (CC BY-NC-ND), which allows third parties to download the articles and share them with others as long as they credit the author and the Abstract Book, but they cannot change the content in any way or use them commercially.
**Abstract Book Citations:** Authors, Title, HemaSphere, 2026;10:(S1):pages. The abstract book DOIs can be found on the "Author Information" page.
**Disclaimer:** Articles published in the journal HemaSphere exclusively reflect the opinions of the authors. The authors are responsible for all content in their abstracts including accuracy of the facts, statements, citing resources, etc.

### (PS1975) TGF-BETA SIGNALING IS DEREGULATED BY TYPE I CALRETICULIN MUTATIONS IN MYELOPROLIFERATIVE NEOPLASM

**Topic:** 15. Myeloproliferative neoplasms - Biology & translational research

Aditi Singh*[1], Saadia Naseer[1], Shayeri Chowdhury[1], Anita Roy[1]

[1]*Indian Institute of Technology Delhi, Kusuma School of Biological Sciences, New Delhi, India;*

### Background

Myeloproliferative neoplasm (MPN) is a clonal disorder associated with aberrant proliferation of hematopoietic stem cells and myeloid progenitors. 30% of all MPN patients carry gene-encoding calreticulin (*CALR*) mutations. The two most frequent CALR exon 9 mutations detected in patients, *CALR* del52 (type I) and ins5 (type II), show varying prognosis. Moreover, calreticulin mutants show differences in the phenotypes in mice models where type I shows bone marrow fibrosis while type II does not. Since TGF-beta signaling pathway is responsible for tissue fibrosis in most cases, our objective was to study the effect of calreticulin mutation on TGF-beta signaling pathway.

### Aims

To elucidate the role of calreticulin mutations in TGFBRIII pathway-mediated progression of myeloproliferative neoplasm.

### Methods

We used a cell-based model in which we overexpressed HEK cells with calreticulin (CALR) WT/del52/ins5, and checked their interaction with TGFβ receptors. We also checked the phosphorylation levels of SMAD2/3, which are direct effectors of TGF-β signaling. To further assess the effect of secreted TGFβ receptor, we cultured cells expressing CALR WT or mutants and checked the cell media for secreted TGFβ receptors by immunoprecipitation followed by western blotting.

### Results

Our results showed that TGFBRIII, which is a co-receptor of TGF-beta signaling interacts with wild-type and mutant CALR. This interaction was specific for TGBRIII and absent for the main receptors, TGFBRI and TGFBRII. We also observed decreased TGBRIII protein levels in the presence of CALR mutants compared to wild type. This was not due to an impaired CALR chaperone activity in the two mutants since no significant difference was found between the melting temperature of TGBRIII in the presence of CALR WT and mutants. Instead, we observed an increased SMAD2/3 phosphorylation in the presence of CALR del52, indicating increased activation of TGF-β signaling in these cells. We detected secreted TGFBRIII in the supernatant of cells expressing CALR mutants but not in the wild-type condition. When incubated with conditioned media from HEK cells expressing CALR del52 cells, we observed trans-activation of SMAD2/3 in HepG2 cells.

### Summary/Conclusion

Although the role of calreticulin mutants in driving cytokine-independent megakaryopoiesis is well-established, their role in the TGF-β pathway is unknown. Our results showed that type I CALR mutant (CALR del52) activates TGF-β signaling through direct interaction and enhanced secretion of TGFBRIII. We hypothesize that such interaction may lead to bone marrow fibrosis as observed in the patients.

**Copyright Information:** (Online) ISSN: 2572-9241
©2026 The Author(s). HemaSphere is published by John Wiley & Sons Ltd on behalf of European Hematology Association. This is an open access Abstract Book distributed under the Attribution-NonCommercial-NoDerivs (CC BY-NC-ND), which allows third parties to download the articles and share them with others as long as they credit the author and the Abstract Book, but they cannot change the content in any way or use them commercially.
**Abstract Book Citations:** Authors, Title, HemaSphere, 2026;10:(S1):pages. The abstract book DOIs can be found on the "Author Information" page.
**Disclaimer:** Articles published in the journal HemaSphere exclusively reflect the opinions of the authors. The authors are responsible for all content in their abstracts including accuracy of the facts, statements, citing resources, etc.

### (PS1976) FUNCTIONAL ANALYSIS OF A NOVEL GERMLINE JAK2 VARIANT ASSOCIATED WITH THROMBOCYTOSIS

**Topic:** 15. Myeloproliferative neoplasms - Biology & translational research

Kateřina Kuželová*[1], Lukas Dvoracek[1], Jana Markova[1], Dana Grebeňová[1], Jana Sopkova de Oliveira Santos[2], Marie Lauermannova[1], Jiri Schwarz[1]

[1]*Institute of Hematology and Blood Transfusion, Prague, Czechia;*[2]*University of Normandy, Caen, France;*

### Background

Janus kinase 2 (JAK2) mediates signaling from cytokine receptors, particularly MPL (the thrombopoietin receptor) and EPOR (the erythropoietin receptor), in hematopoietic cells. Alterations in JAK2 activity significantly influence hematopoiesis through effects on cell proliferation and differentiation. While the somatic JAK2 p.Val617Phe mutation is a well-established driver of myeloproliferative neoplasms (MPN), the functional impact of germline *JAK2* variants is largely unknown. JAK2 activation requires dimerization of pre-associated JAK2–receptor monomers, (trans)autophosphorylation of JAK2, and subsequent receptor phosphorylation. Activated JAK2 then phosphorylates downstream signaling molecules, such as STAT transcription factors.

### Aims

We identified a previously undescribed germline *JAK2* variant, p.Val1043Phe (V1043F), in a patient with thrombocytosis and elevated erythropoietin (EPO) levels during next-generation sequencing for MPN diagnostics. The second *JAK2* allele carried a frameshift mutation likely resulting in complete loss of function. This study aimed to characterize the biological impact of the V1043F variant within this hemizygote-like context.

### Methods

The effect of V1043F on downstream JAK2 signaling was assessed in HEK293T and HeLa cells transiently transfected with plasmids encoding wild-type (WT) JAK2 or JAK2 V1043F together with MPL or EPOR. STAT1, STAT3, and STAT5 phosphorylation was analyzed by western blot, with band intensities normalized to transfection efficiency determined by flow cytometry. The open dimer of human JAK2 was modeled by sequence homology modeling, and both the wild-type (WT) and mutated structures were subsequently subjected to molecular dynamics simulations using NAMD.

### Results

When co-expressed with MPL, JAK2 V1043F activated STAT5 and STAT3 to a similar extent as JAK2 WT. In contrast, EPOR-mediated signaling of JAK2 V1043F was markedly impaired for both STAT5 and STAT3, even in the presence of physiological EPO concentrations (0.01–0.03 U/mL). STAT1 phosphorylation downstream of JAK2 V1043F was largely reduced compared to JAK2 WT, irrespective of the co-transfected receptor. Overall, the V1043F variant exhibited a substrate-specific loss of function, with EPOR signaling substantially more affected than MPL signaling, and STAT1 more affected than STAT3/STAT5.Structural modeling revealed that Val1043, located within the kinase domain, contributes to the catalytic cleft important for substrate binding. The Val→Phe substitution induced a twist in the amino-acid chain that altered the spatial orientation of Asp976, which interacts directly with JAK2 substrates. While Asp976 is exposed towards the substrate-accomodating area in the WT protein, it becomes buried in the V1043F variant. This might result in altered substrate binding and coordination.

**Copyright Information:** (Online) ISSN: 2572-9241
©2026 The Author(s). HemaSphere is published by John Wiley & Sons Ltd on behalf of European Hematology Association. This is an open access Abstract Book distributed under the Attribution-NonCommercial-NoDerivs (CC BY-NC-ND), which allows third parties to download the articles and share them with others as long as they credit the author and the Abstract Book, but they cannot change the content in any way or use them commercially.
**Abstract Book Citations:** Authors, Title, HemaSphere, 2026;10:(S1):pages. The abstract book DOIs can be found on the “Author Information” page.
**Disclaimer:** Articles published in the journal HemaSphere exclusively reflect the opinions of the authors. The authors are responsible for all content in their abstracts including accuracy of the facts, statements, citing resources, etc.

**Summary/Conclusion**

The JAK2 V1043F variant reduces JAK2 kinase activity in a substrate-specific manner, likely due to altered conformation of the catalytic cleft. Impared signaling from EPOR may shift differentiation of bipotent megakaryocyte-erythroid progenitors towards thrombopoiesis. Increased EPO levels observed in the V1043F proband likely represent a compensatory response to ineffective erythropoiesis and helps restore erythroid output.

**Copyright Information:** (Online) ISSN: 2572-9241
©2026 The Author(s). HemaSphere is published by John Wiley & Sons Ltd on behalf of European Hematology Association. This is an open access Abstract Book distributed under the Attribution-NonCommercial-NoDerivs (CC BY-NC-ND), which allows third parties to download the articles and share them with others as long as they credit the author and the Abstract Book, but they cannot change the content in any way or use them commercially.
**Abstract Book Citations:** Authors, Title, HemaSphere, 2026;10:(S1):pages. The abstract book DOIs can be found on the “Author Information” page.
**Disclaimer:** Articles published in the journal HemaSphere exclusively reflect the opinions of the authors. The authors are responsible for all content in their abstracts including accuracy of the facts, statements, citing resources, etc.

### (PS1977) HETEROGENEOUS CYTOKINE EXPRESSION IN CHRONIC MYELOMONOCYTIC LEUKEMIA: ADVANCES IN DEMETHYLATION THERAPY

**Topic:** 15. Myeloproliferative neoplasms - Biology & translational research

Yan Wang*[1], Xi Luo[1], Yifan Duan[1], Guangshuai Teng[1], Chenxiao Du[1], Yanqi Li[1], Huiqin Zhang[1], Yuhui Zhang[1], Lan Peng[1], Xiaojing Wei[1], Ke Shang[1], Yanjie Lan[1], Zonghong Shao[1], Yuan Zhou[2], Weiping Yuan[1, 2], Jie Bai[1]

*[1]The Second Hospital of Tianjin Medical University, Department of Hematology, Tianjin, China;[2]Institute of Hematology & Blood Diseases Hospital, Chinese Academy of Medical Sciences & Peking Union Medical College, State Key Laboratory of Experimental Hematology, National Clinical Research Center for Blood Diseases, Tianjin, China;*

### Background

Chronic myelomonocytic leukemia (CMML) is a clonal malignant disease of hematopoietic stem cells, it is characterized by marked monocytosis and a risk of leukemic transformation. Multiple pro-inflammatory cytokines are elevated in CMML, which contribute to disease progression. hypomethylation agent(HMA) may affect the changes in inflammatory cytokines, providing early warning markers for disease progression and even potential opportunities for the development of targeted therapies.

### Aims

This study aims to analyze the clinical characteristics of patients with CMML under different treatment regimens, clonal evolution during disease progression, and changes in inflammatory factors, so as to provide clinical decision-making evidence for the early identification of patients at high risk of disease progression and the early initiation of treatment.

### Methods

A total of 24 CMML(intermediate and high-risk groups) patients admitted to the Department of Hematology, The Second Hospital of Tianjin Medical University from September 2017 to December 2025 were retrospectively enrolled.A panel of 325 mutated genes associated was detected by NGS.We analyzed the clinical characteristics between HMA group and hydroxycarbamide/best supportive care (HC/BSC) group.and molecularbiological features at various disease stages.Enzymelinked immunosorbent assay (ELISA) was performed to measure and compare the levels of inflammatory factors including interleukin (IL)-10 and interferon (IFN)-γ, et al in bone marrow supernatant.

### Results

① In the HMA group, the initial white blood cell count ,absolute monocyte count were significantly higher than those in the HC/BSC group(P[illegible]0.05). ② All 14 patients with CMML NGS after disease progression. Compared with samples obtained before HMA among patients with progressive CMML post-treatment, 3 cases (37.5%) had newly acquired mutations in the RAS signaling pathway, and 1 case (12.5%) showed an increase in variant allele frequency (VAF) ≥10%. In the HC/BSC group, 1 case (16.7%) had newly acquired mutations, and 1 case (16.7%) had an increase in VAF ≥10%.③Compared with the untreated group, the levels of CXCL2, CXCL3,TNF-α,IL-1β,IL-18,IL-10 were significantly increased in the HMA-resistant group(all $P < 0.05$). Compared with the HMA-responsive group, the HMAresistant group showed significantly higher levels of IL1β($P < 0.05$).Compared with the untreated group, CCL2,IFN-γ,IL-1β,IL-8 were significantly decreased in the the HMA-resistant group.

**Copyright Information:** (Online) ISSN: 2572-9241
©2026 The Author(s). HemaSphere is published by John Wiley & Sons Ltd on behalf of European Hematology Association. This is an open access Abstract Book distributed under the Attribution-NonCommercial-NoDerivs (CC BY-NC-ND), which allows third parties to download the articles and share them with others as long as they credit the author and the Abstract Book, but they cannot change the content in any way or use them commercially.
**Abstract Book Citations:** Authors, Title, HemaSphere, 2026;10:(S1):pages. The abstract book DOIs can be found on the "Author Information" page.
**Disclaimer:** Articles published in the journal HemaSphere exclusively reflect the opinions of the authors. The authors are responsible for all content in their abstracts including accuracy of the facts, statements, citing resources, etc.

### Summary/Conclusion

In CMML patients , the risk of RAS signaling pathway mutations increases after HMA resistance. Elevated IL-1β is associated with HMA resistance, whereas reduced IL-1β correlates with a favorable response.

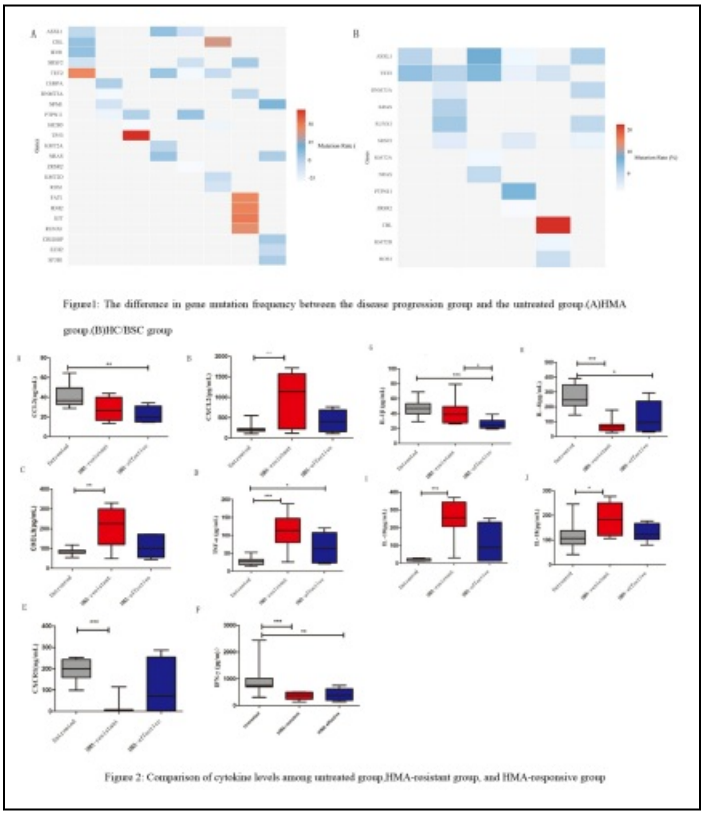

Figure1: The difference in gene mutation frequency between the disease progression group and the untreated group (A)HMA group (B)HC/BSC group

Figure 2: Comparison of cytokine levels among untreated group,HMA-resistant group, and HMA-responsive group

**Copyright Information:** (Online) ISSN: 2572-9241
©2026 The Author(s). HemaSphere is published by John Wiley & Sons Ltd on behalf of European Hematology Association. This is an open access Abstract Book distributed under the Attribution-NonCommercial-NoDerivs (CC BY-NC-ND), which allows third parties to download the articles and share them with others as long as they credit the author and the Abstract Book, but they cannot change the content in any way or use them commercially.
**Abstract Book Citations:** Authors, Title, HemaSphere, 2026;10:(S1):pages. The abstract book DOIs can be found on the “Author Information” page.
**Disclaimer:** Articles published in the journal HemaSphere exclusively reflect the opinions of the authors. The authors are responsible for all content in their abstracts including accuracy of the facts, statements, citing resources, etc.

### (PS1978) PROTEOMIC PROFILING FOR NOVEL BIOMARKERS OF THE PROGRESSION FROM ESSENTIAL THROMBOCYTHEMIA TO POST-ET MYELOFIBROSIS: A RETROSPECTIVE COHORT STUDY

**Topic:** 15. Myeloproliferative neoplasms - Biology & translational research

Mingyu Jin*[1], Ting Sun[2], Renchi Yang[2], Lei Zhang[2]

*[1]Tianjin Medical University, Tianjin, China;[2]Thrombosis and Hemostasis Center, Institute of Hematology & Blood Diseases Hospital, Chinese Academy of Medical Sciences & Peking Union Medical College,, Tianjin, China;*

### Background

Essential thrombocythemia (ET) is a chronic BCR/ABL1-negative myeloproliferative neoplasm that may evolve into post-ET myelofibrosis (MF), an irreversible condition associated with poor prognosis.Early identification of patients at risk for disease progression is therefore critical for improving outcomes.Most existing predictive models focus on the genetic level, with limited research targeting fibrotic progression at that protein translation level.

### Aims

In this study, we applied deep proteomic profiling on data from bone marrow biopsy samples from patients with ET and post-ET MF to identify protein signatures associated with the fibrotic transformation.
By integrating proteomic, genomic, and clinical features, we developed a protein-based prediction model to identify associated biomarkers.

### Methods

This retrospective cohort study included patients with ET and post-ET MF diagnosed between December 1, 2020, and December 1, 2024.
Cohort 1 (370 ET, 65 post-ET MF) was used to identify diagnostic clinical variables via logistic regression. Cohort 2(64 ET, 24 post-ET MF) with well-preserved bone marrow pathology specimens, underwent DIA-based proteomic differential profiling using formalin-fixed, paraffin-embedded (FFPE) bone marrow samples.
For data analysis, Random Forest and LASSO regression were applied to differentially expressed proteins to select robust biomarker candidates.

**Copyright Information:** (Online) ISSN: 2572-9241
©2026 The Author(s). HemaSphere is published by John Wiley & Sons Ltd on behalf of European Hematology Association. This is an open access Abstract Book distributed under the Attribution-NonCommercial-NoDerivs (CC BY-NC-ND), which allows third parties to download the articles and share them with others as long as they credit the author and the Abstract Book, but they cannot change the content in any way or use them commercially.
**Abstract Book Citations:** Authors, Title, HemaSphere, 2026;10:(S1):pages. The abstract book DOIs can be found on the "Author Information" page.
**Disclaimer:** Articles published in the journal HemaSphere exclusively reflect the opinions of the authors. The authors are responsible for all content in their abstracts including accuracy of the facts, statements, citing resources, etc.

### Results

LC-MS/MS analysis of bone marrow samples from 64 ET and 24 post-ET MF patients identified 8370 proteins. PCA revealed clear proteomic separation between the two groups, indicating distinct protein expression profiles. Totally 1000 differentially expressed proteins (DEPs) were detected in the post-ET MF group(609 upregulated, 391 downregulated).The upregulated proteins in the post-ET MF group were enriched in extracellular matrix organization, cell adhesion, and inflammatory response pathways, while the downregulated proteins were related to T cell signaling and DNA repair processes.
Subsequently, we performed Random Forest and LASSO regression to identify nine significant DEPs in the post-ET MF group. Among the nine DEPs, von Willebrand factor A domain-containing protein 1 (VWA1), interferon-induced transmembrane protein 3 (IFITM3) and cadherin-11 (CDH11) were upregulated in post-ET MF, mainly participating in ECM organization, immune regulation, and cell adhesion. Conversely, alpha-hemoglobin-stabilizing protein (AHSP), and DNA replication ATP-dependent helicase (DNA2) were downregulated, functions linked to erythroid differentiation, and DNA repair.
CDH11, VWA1, and AHSP demonstrated the highest individual predictive power and the expression levels are shown. A model combining these three markers yielded an improved AUC of 0.947. We have constructed a clinical model in cohort 1(LDH, HDL-C, Hb, and CALR mutations, AUC of 0. 902). When applied to Cohort 2, the previously established model showed excellent performance with an AUC of 0.973 (95% CI: 0.934–1.000, $p < 0.0001$).We developed a protein-based prediction model including CDH11 and LDH, showing excellent predictive performance with an AUC of 0.997,significantly outperforming both the three-protein model (AUC = 0.947)and the clinical model (AUC = 0.973).

### Summary/Conclusion

The proteomics-based predictive model offers higher diagnostic accuracy and holds significant potential for personalized monitoring and early detection of ET-to-MF progression.

**Copyright Information:** (Online) ISSN: 2572-9241
©2026 The Author(s). HemaSphere is published by John Wiley & Sons Ltd on behalf of European Hematology Association. This is an open access Abstract Book distributed under the Attribution-NonCommercial-NoDerivs (CC BY-NC-ND), which allows third parties to download the articles and share them with others as long as they credit the author and the Abstract Book, but they cannot change the content in any way or use them commercially.
**Abstract Book Citations:** Authors, Title, HemaSphere, 2026;10:(S1):pages. The abstract book DOIs can be found on the "Author Information" page.
**Disclaimer:** Articles published in the journal HemaSphere exclusively reflect the opinions of the authors. The authors are responsible for all content in their abstracts including accuracy of the facts, statements, citing resources, etc.

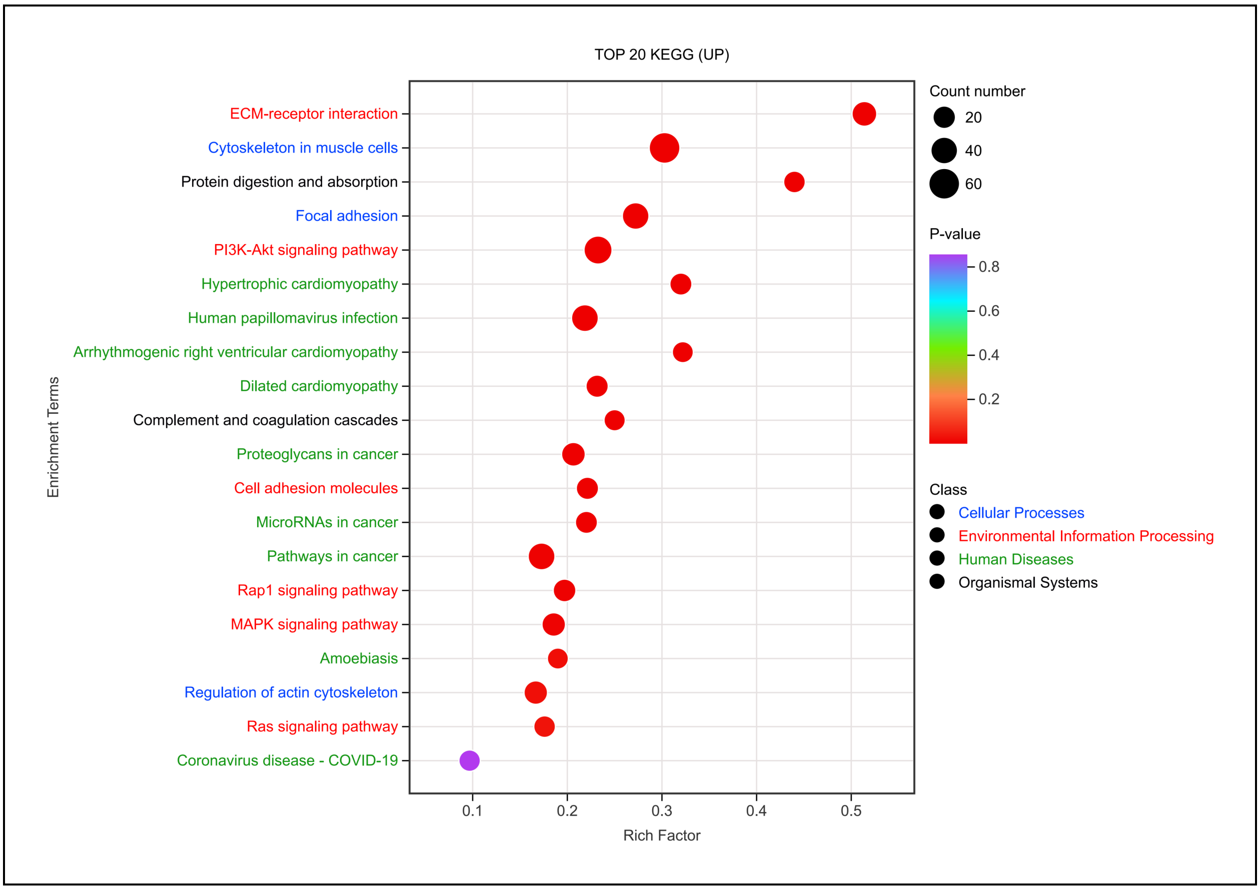

**Copyright Information:** (Online) ISSN: 2572-9241
©2026 The Author(s). HemaSphere is published by John Wiley & Sons Ltd on behalf of European Hematology Association. This is an open access Abstract Book distributed under the Attribution-NonCommercial-NoDerivs (CC BY-NC-ND), which allows third parties to download the articles and share them with others as long as they credit the author and the Abstract Book, but they cannot change the content in any way or use them commercially.
**Abstract Book Citations:** Authors, Title, HemaSphere, 2026;10:(S1):pages. The abstract book DOIs can be found on the "Author Information" page.
**Disclaimer:** Articles published in the journal HemaSphere exclusively reflect the opinions of the authors. The authors are responsible for all content in their abstracts including accuracy of the facts, statements, citing resources, etc.

### (PS1979) THE GENOMIC LANDSCAPE OF MYELOPROLIFERATIVE NEOPLASMS (MPNS) BY DISEASE SUBTYPE: BEYOND JAK2 V617F, CALR, AND MPL

**Topic:** 15. Myeloproliferative neoplasms - Biology & translational research

Kirti Arora[1], Jessica Lee[2], Chelsea Marcus[2], Lincoln Pasquina[2], Abhay Singh*[1]

*[1]Cleveland Clinic, Cleveland, United States of America; [2]Foundation Medicine, Inc., Boston, United States of America;*

### Background

Alterations (alts), particularly *JAK2* V617F, *CALR*, and *MPL* have been well characterized in MPNs. However, there is still no comprehensive description of a broad genomic panel stratified by disease subtype, i.e., primary myelofibrosis [PMF], essential thrombocytothemia [ET], polycythemia vera [PV], other rare entities such as atypical chronic myelogenous leukemia (aCML), and myelodysplastic syndrome (MDS)/MPN overlap syndromes including chronic myelomonocytic leukemia (CMML) and MDS/MPN with ring sideroblasts and thrombosis (RS-T).

### Aims

To define the spectrum of genomic alts across MPN subtypes in a contemporary cohort undergoing broad genomic profiling.

### Methods

Cases of suspected and confirmed MPN were analyzed by clinical next generation sequencing (NGS) on FoundationOneHeme, a laboratory developed test that analyzes 406 genes by DNA for short variants, indels, copy number alts (CNAs), and rearrangements (REs) and 265 by RNA for REs.
MPN subtype classification was based on documentation submitted by the provider at time of analysis. Analyses of ET, PV, and PMF were limited to cases with diagnostic alts or, for PMF, myeloid alts.

**Copyright Information:** (Online) ISSN: 2572-9241
©2026 The Author(s). HemaSphere is published by John Wiley & Sons Ltd on behalf of European Hematology Association. This is an open access Abstract Book distributed under the Attribution-NonCommercial-NoDerivs (CC BY-NC-ND), which allows third parties to download the articles and share them with others as long as they credit the author and the Abstract Book, but they cannot change the content in any way or use them commercially.
**Abstract Book Citations:** Authors, Title, HemaSphere, 2026;10:(S1):pages. The abstract book DOIs can be found on the "Author Information" page.
**Disclaimer:** Articles published in the journal HemaSphere exclusively reflect the opinions of the authors. The authors are responsible for all content in their abstracts including accuracy of the facts, statements, citing resources, etc.

### Results

Across 2,371 evaluable cases, the distribution of "driver" alts differed markedly by subtype (**Table**). PV was almost uniformly *JAK2* V617F positive. PMF was enriched for JAK2 V617F with lower *CALR* and *MPL*, while ET showed a mixed driver profile. Overlap/atypical myeloid categories had higher rates of MDS-related, signaling, or spliceosome alts. CMML demonstrated high frequencies of *TET2*, *ASXL1*, *SRSF2*, and RAS-pathway alts and aCML (n=11) showed very high*ASXL1* with enrichment of *SRSF2* and *SETBP1*, aligning with contemporary reports. MDS/MPN NOS (n=175) was enriched for *ASXL1* and RAS-pathway alts. RS-T (n=11) showed no putative *SF3B1* alts despite the expected ~90% prevalence, suggesting likely misclassification, though limited clinician-submitted data prevented confirmation in this dataset.
Other potentially actionable alts were infrequent as expected but were clinically notable. *FLT3*-ITD was present in MDS/MPN NOS (2.9%), with *FLT3*-TKD likewise seen in CMML (2.2%) and MDS/MPN NOS (3.4%). Additional targetable biology included recurrent *EZH2* and *IDH1*/*IDH2*. *TP53* alts were observed across multiple diagnoses, highest in RS-T (18.2%), ET (11.2%), and MDS/MPN NOS (8.0%), defining a high-risk subset. Finally, non-V617F *JAK2* alts were detected (PV 1.9%; MPN NOS and CMML 0.4%), supporting their diagnostic value in triple negative cases and the importance of considering germline predisposition in select patients (PMID: 39657124).

| | ET | PV | PMF | MPN | CMML | aCML | RS-T | MDS-MPN NOS |
|---|---|---|---|---|---|---|---|---|
| N | 249 | 154 | 31 | 1118 | 231 | 11 | 11 | 175 |
| *JAK2* V617F (% for all alts) | 67.5 | 98.7 | 71 | 41.8 | 11.7 | 9.1 | - | 16.6 |
| *JAK2* non-V617F | - | 1.9 | - | 0.4 | 0.4 | - | - | - |
| *JAK2* CNA/RE | 0.4 | - | - | 0.4 | - | - | - | - |
| *MPL* | 11.6 | 1.3 | 3.2 | 4.4 | 3 | - | - | 4.6 |
| *CALR* | 24.1 | - | 12.9 | 10.5 | - | - | - | 1.1 |
| *FLT3* ITD | - | - | - | 0.3 | - | 9.1 | - | 2.9 |
| *FLT3* TKD | - | - | - | 0.4 | 2.2 | 9.1 | - | 3.4 |
| *ASXL1* | 16.9 | 9.1 | 32.3 | 19.9 | 42.4 | 81.8 | - | 42.3 |
| *EZH2* | 2.4 | 1.3 | 9.7 | 3 | 5.6 | 9.1 | - | 7.4 |
| *TET2* | 17.7 | 10.4 | 19.4 | 14.4 | 55.4 | 27.3 | - | 37.1 |
| *IDH1* | 2.8 | 1.9 | 3.2 | 1.0 | 1.3 | - | - | 1.1 |
| *IDH2* | 1.6 | 3.9 | - | 2.2 | 5.2 | - | 9.1 | 3.4 |
| *TP53* | 11.2 | 5.2 | - | 5.8 | 6.5 | 9.1 | 18.2 | 8 |
| *SETBP1* | 0.8 | 0.6 | - | 3 | 6.5 | 27.3 | - | 10.9 |
| *SF3B1* | 5.6 | 5.8 | 3.2 | 5.6 | 4.3 | - | - | 13.1 |
| *SRSF2* | 4 | 2.6 | 16.1 | 8.3 | 42 | 45.5 | 18.2 | 29.1 |
| *KRAS* | 1.2 | 1.9 | 3.2 | 3.3 | 18.6 | 9.1 | - | 13.1 |
| *NRAS* | 1.2 | 1.3 | 3.2 | 4.5 | 20.3 | 18.2 | - | 14.9 |

### Summary/Conclusion

Genomic alts beyond canonical *JAK2* V617F, *CALR*, and *MPL* drivers were identified. Within smaller subgroups, actionable driver alts emerged that may inform future clinical trials in select MPN and overlap patients. Our findings show that broad-panel NGS enables comprehensive detection of alts with diagnostic, prognostic, and therapeutic relevance.

**Copyright Information:** (Online) ISSN: 2572-9241
©2026 The Author(s). HemaSphere is published by John Wiley & Sons Ltd on behalf of European Hematology Association. This is an open access Abstract Book distributed under the Attribution-NonCommercial-NoDerivs (CC BY-NC-ND), which allows third parties to download the articles and share them with others as long as they credit the author and the Abstract Book, but they cannot change the content in any way or use them commercially.
**Abstract Book Citations:** Authors, Title, HemaSphere, 2026;10:(S1):pages. The abstract book DOIs can be found on the "Author Information" page.
**Disclaimer:** Articles published in the journal HemaSphere exclusively reflect the opinions of the authors. The authors are responsible for all content in their abstracts including accuracy of the facts, statements, citing resources, etc.

### (PS1980) THE TUMOR-ASSOCIATED FIBROBLAST BIOMARKER PRO-C6 IS ELEVATED IN PATIENTS WITH MYELOPROLIFERATIVE NEOPLASMS

**Topic:** 15. Myeloproliferative neoplasms - Biology & translational research

Caroline Norup Bistrup*[1], Morten Kranker Larsen[2], Peter Junker[3], Vibe Skov[2], Lasse Kjær[2], Trine Alma Knudsen[2], Morten Karsdal[1], Nicholas Willumsen[1], Hans Hasselbalch[2]

[1]*Nordic Bioscience, Oncology, Herlev, Denmark;* [2]*Zealand University Hospital, Department of Hematology, Roskilde, Denmark;* [3]*Odense University Hospital, Department of Rheumatology, Odense, Denmark;*

### Background

Myeloproliferative neoplasms (MPNs) are associated with an increased risk of cardiovascular diseases (CVDs) and thrombotic complications, mainly driven by chronic inflammation, fibrosis and are closely linked to the acquisition of the *JAK2V617F* mutation. As CVD contributes to the morbidity and mortality of patients with MPN, it is of high interest to improve early detection of inflammation-driven CVD in MPNs. Activated fibroblasts produce the extracellular matrix (ECM) -derived signaling molecule endotrophin, released during type VI collagen formation, and involved in fibrotic tissue remodeling. Endotrophin is measured by circulating levels of PRO-C6, which is a serological biomarker, reflecting tumor-associated fibroblast activity. PRO-C6 has previously been associated with an increased risk of mortality across multiple chronic diseases such as chronic kidney disease (CKD), biliary tract cancer, and cardiovascular diseases (CVDs). Furthermore, often endotrophin was also associated with degree of disease severity in these chronic diseases.

### Aims

The aim of this study was to evaluate the PRO-C6 biomarker in patients with MPN and investigate possible associations with disease phenotypes, bone marrow fibrosis, disease activity and hematological variables.

### Methods

PRO-C6 was measured by ELISA in pre-treatment serum from patients enrolled in the DALIAH trial (NCT01387763), a randomized phase III trial of hydroxyurea (HU) vs. pegylated interferon-α2 (IFN-α2) in newly diagnosed or untreated patients with MPN (n=130, subtypes: ET=49, PV=60, pre-MF=8, PMF=13). Biomarker levels in patients with MPN were compared to age- and sex-matched healthy individuals (n=32) by using an unpaired t-test, and were assessed according to disease subtypes, somatic driver mutation status, and *JAK2V617F* allele burden using ordinary one-way ANOVA. Furthermore, biomarker levels were correlated with hematological variables including platelet count, hemoglobin, hematocrit, white blood cells, and lactase dehydrogenase (LDH).

### Results

PRO-C6 was significantly elevated in patients with MPN (mean=7.6 ng/ml) compared to healthy individuals (mean(95%CI) of 5.8 ng/ml, (0.78 – 2.78), P=0.0005). Stratifying into disease subtypes, PRO-C6 was significantly elevated in pre-MF patients (mean=11.0 ng/ml) compared with ET and PV (mean(95%CI) of 6.9 ng/ml, (-6.63 - -1.58), P=0.0003 and mean(95%CI) of 7.2 ng/ml, (-6.27 - -1.27), P=0.0008, respectively) and in patients with PMF (mean=9.4 ng/ml) compared with ET and PV (mean(95%CI) of 6.9 ng/ml, (-4.57 - -0.44), P=0.01and mean(95%CI) of 7.2 ng/ml, (-4.2 - -0.14), P=0.03, respectively). These changes in PRO-C6 levels were independent of mutation status and *JAK2V617F* allele burden. PRO-C6 biomarker levels negatively correlated with platelet count (P=0.02) and positively correlated with leukocytes (P=0.0133) and LDH (P<0.0001). PRO-C6 did not correlate with hemoglobin or hematocrit levels.

**Copyright Information:** (Online) ISSN: 2572-9241
©2026 The Author(s). HemaSphere is published by John Wiley & Sons Ltd on behalf of European Hematology Association. This is an open access Abstract Book distributed under the Attribution-NonCommercial-NoDerivs (CC BY-NC-ND), which allows third parties to download the articles and share them with others as long as they credit the author and the Abstract Book, but they cannot change the content in any way or use them commercially.
**Abstract Book Citations:** Authors, Title, HemaSphere, 2026;10:(S1):pages. The abstract book DOIs can be found on the “Author Information” page.
**Disclaimer:** Articles published in the journal HemaSphere exclusively reflect the opinions of the authors. The authors are responsible for all content in their abstracts including accuracy of the facts, statements, citing resources, etc.

**Summary/Conclusion**

The tumor-associated fibroblast biomarker PRO-C6 is significantly elevated in patients with MPN, particularly in fibrotic subtypes (pre-MF and PMF), suggesting enhanced fibroblast driven extracellular matrix remodeling in fibrotic disease phenotypes. Importantly, PRO-C6 levels were independent of driver mutation status and *JAK2V617F* allele burden. These findings support further investigation of PRO-C6 as a potential biomarker of fibrotic remodeling and inflammation in patients with MPN, likely detecting the patients with CVD risk earlier.

**Copyright Information:** (Online) ISSN: 2572-9241
©2026 The Author(s). HemaSphere is published by John Wiley & Sons Ltd on behalf of European Hematology Association. This is an open access Abstract Book distributed under the Attribution-NonCommercial-NoDerivs (CC BY-NC-ND), which allows third parties to download the articles and share them with others as long as they credit the author and the Abstract Book, but they cannot change the content in any way or use them commercially.
**Abstract Book Citations:** Authors, Title, HemaSphere, 2026;10:(S1):pages. The abstract book DOIs can be found on the "Author Information" page.
**Disclaimer:** Articles published in the journal HemaSphere exclusively reflect the opinions of the authors. The authors are responsible for all content in their abstracts including accuracy of the facts, statements, citing resources, etc.

### (PS1981) PLASMA METABOLOMIC LANDSCAPE REVEALS A PROTHROMBOTIC MICROENVIRONMENT AND PREDICTIVE BIOMARKERS IN ESSENTIAL THROMBOCYTHEMIA

**Topic:** 15. Myeloproliferative neoplasms - Biology & translational research

Mingyu Jin*[1], Ting Sun[2], Lei Zhang[2], Renchi Yang[2]

*[1]Tianjin Medical University, Haihe Laboratory of Cell Ecosystem, Tianjin, China;[2]Thrombosis and Hemostasis Center, Institute of Hematology & Blood Diseases Hospital, Chinese Academy of Medical Sciences & Peking Union Medical College, Tianjin, China;*

### Background

ET is a myeloproliferative neoplasm characterized by clonal proliferation of hematopoietic progenitor cells and is associated with an increased risk of thrombotic events (TEs). Earlier detection of plasma biomarkers especially associated with thrombosis events is significant for the prognostic evaluation and prompt intervention in patients with ET.

### Aims

This study aims to characterize the serum metabolome of ET and compare it with that of thrombosis history patients, thereby identifying metabolic biomarkers in the thrombosis process of ET. By integrating metabolic, genomic, and clinical features, we developed a metabolomics-based prediction model to identify associated biomarkers.

### Methods

We retrospectively included 392 consecutive patients diagnosed with essential thrombocythemia (ET) from December 2020 to December 2024. Patients with a history of thrombosis prior to diagnosis were classified as the ET with TEs group. Major TEs comprise arterial thrombosis (ischemic stroke/TIA, myocardial infarction, peripheral/visceral infarction) and venous thrombosis, including both common (deep vein thrombosis, pulmonary embolism) and rare-site (splenic/cerebral venous) events.
The study population was divided into two cohorts: Cohort 1 (n=229;69 with TEs) was used to identify initial clinical predictors via logistic regression. Cohort 2 (n=70;24 with TEs) underwent LC–MS–based non-targeted metabolomic profiling of plasma samples. Machine learning algorithms were then employed to select the most informative metabolite features for the construction of a prediction model.

**Copyright Information:** (Online) ISSN: 2572-9241
©2026 The Author(s). HemaSphere is published by John Wiley & Sons Ltd on behalf of European Hematology Association. This is an open access Abstract Book distributed under the Attribution-NonCommercial-NoDerivs (CC BY-NC-ND), which allows third parties to download the articles and share them with others as long as they credit the author and the Abstract Book, but they cannot change the content in any way or use them commercially.
**Abstract Book Citations:** Authors, Title, HemaSphere, 2026;10:(S1):pages. The abstract book DOIs can be found on the "Author Information" page.
**Disclaimer:** Articles published in the journal HemaSphere exclusively reflect the opinions of the authors. The authors are responsible for all content in their abstracts including accuracy of the facts, statements, citing resources, etc.

**Results**

ET Patients with thrombosis were more often male and older at diagnosis, with a higher proportion aged ≥60 years ($P<0.001$). Cardiovascular risk factors more prevalent and RDW-SD increased ($P=0.016$). The VIP score of the OPLS-DA model was used to identify key metabolites. The screening criteria for differentially expressed metabolites (DEMs) are metabolites with VIP > 1 and P - value < 0.05 (Student's t test).

Among the 83 DEMs (35 upregulated and 48 downregulated) in the ET-thrombosis group, SVM and Boruta further identified 22 significant metabolites.RF ranking selected three key metabolites—3-amino-2,3-dihydrobenzoic acid, sulfated prenylated polyphenol, and pyridine-3,4-dicarboxylic acid for prediction model construction.A clinical prediction model (AUC=0.79) for thrombosis in ET patients was first developed in the full cohort (n = 392) using multivariable logistic regression, including Age>60 , CVF, JAK2V617F , and Crea .The clinical model in the metabolomics sub-cohort (n=94;OOF AUC=0.73) had an adequate covariate balance after IPW adjustment (all SMDs <0.1).For seven candidate predictors,LASSO (λmin) selected four clinical variables plus two metabolites for the joint model.
In the metabolomics sub-cohort (n = 94), 5-fold OOF cross-validation showed an AUC of 0.73 (95% CI 0.60–0.84) for the clinical model, 0.77 (0.65–0.88) for the metabolite model, and 0.79 (0.66–0.89) for the joint model. DCA further demonstrated that the joint model provided a higher net clinical benefit than the clinical model.

**Summary/Conclusion**

Integrated clinical and metabolomic analyses suggest that inflammation, oxidative stress, metabolic reprogramming, platelet hyperreactivity, and endothelial dysfunction contribute to thrombosis. The metabolomics-based predictive model offers higher diagnostic accuracy and holds significant potential for personalized monitoring and early detection of high risk of thrombosis progression.

**Copyright Information:** (Online) ISSN: 2572-9241
©2026 The Author(s). HemaSphere is published by John Wiley & Sons Ltd on behalf of European Hematology Association. This is an open access Abstract Book distributed under the Attribution-NonCommercial-NoDerivs (CC BY-NC-ND), which allows third parties to download the articles and share them with others as long as they credit the author and the Abstract Book, but they cannot change the content in any way or use them commercially.
**Abstract Book Citations:** Authors, Title, HemaSphere, 2026;10:(S1):pages. The abstract book DOIs can be found on the "Author Information" page.
**Disclaimer:** Articles published in the journal HemaSphere exclusively reflect the opinions of the authors. The authors are responsible for all content in their abstracts including accuracy of the facts, statements, citing resources, etc.

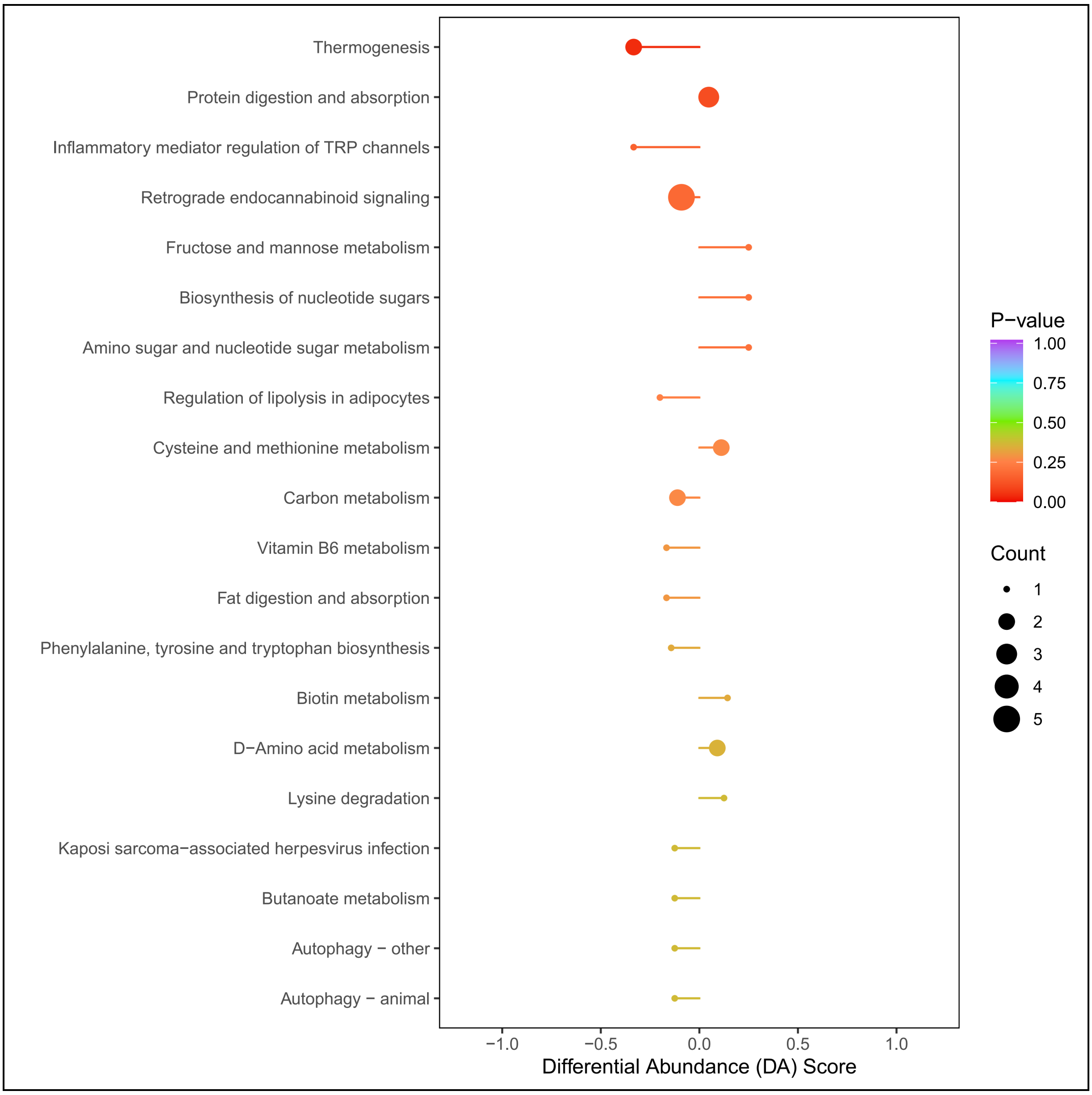

**Copyright Information:** (Online) ISSN: 2572-9241
©2026 The Author(s). HemaSphere is published by John Wiley & Sons Ltd on behalf of European Hematology Association. This is an open access Abstract Book distributed under the Attribution-NonCommercial-NoDerivs (CC BY-NC-ND), which allows third parties to download the articles and share them with others as long as they credit the author and the Abstract Book, but they cannot change the content in any way or use them commercially.
**Abstract Book Citations:** Authors, Title, HemaSphere, 2026;10:(S1):pages. The abstract book DOIs can be found on the "Author Information" page.
**Disclaimer:** Articles published in the journal HemaSphere exclusively reflect the opinions of the authors. The authors are responsible for all content in their abstracts including accuracy of the facts, statements, citing resources, etc.

### (PS1982) COEXISTENCE OF SF3B1 AND DRIVER MUTATIONS IN MYELOID NEOPLASMS: A CONTINUOUS CLINICAL-MOLECULAR SPECTRUM

**Topic:** 15. Myeloproliferative neoplasms - Biology & translational research

Jose Antonio Farah Gamarra*[1], Fernando Vázquez Lagos[2], Jose Angel Diaz Arias[2], Javier Alberto Rojas Martinez[2], Ana Fernández[1], Beatriz Quintans Castro[3], Celsa Quinteiro Garcia[3], Manuel Mateo Perez Encinas[1]

*[1]University Hospital of Santiago de Compostela, Department of Hematology, Myeloid Neoplasms Unit., Santiago de Compostela, Spain; [2]University Hospital of Santiago de Compostela, Department of Hematology, Santiago de Compostela, Spain;[3]University Hospital of Santiago de Compostela, Galician Public Foundation of Xenomics Medicine., Santiago de Compostela, Spain;*

### Background

The SF3B1 mutation defines a subtype of MDS with ring sideroblasts and favorable prognosis. Its coexistence with driver mutations (JAK2, CALR, MPL) creates a diagnostic grey zone between MDS, classical MPN, and MDS/MPN overlap syndromes, with phenotypic heterogeneity and uncertain clinical outcomes. These cases may be classified into different entities by morphological criteria, despite sharing a common molecular profile.

### Aims

To characterize the clinical, hematological, and molecular profile of patients with myeloid neoplasms harboring SF3B1 and concomitant driver mutations, and to identify differences between diagnostic subgroups within the MDS-MPN overlap spectrum.

### Methods

Retrospective single-center study of 25 consecutive patients (≥18 years) with SF3B1-mutated myeloid neoplasms and a driver mutation (JAK2/CALR/MPL), diagnosed between 2009–2025, classified by WHO 2022 into: MDS-SF3B1, classical MPN, and MDS/MPN-SF3B1 with thrombocytosis. Hematological, morphological, and molecular variables were analyzed at diagnosis. Mutational detection by NGS and/or PCR, determining VAF of all variants. Comparisons: Kruskal-Wallis/Mann-Whitney U (continuous), Chi-square/Fisher (categorical), Bonferroni correction ($p<0.016$). Survival: Kaplan-Meier and log-rank. Median follow-up: 102 months.

**Copyright Information:** (Online) ISSN: 2572-9241
©2026 The Author(s). HemaSphere is published by John Wiley & Sons Ltd on behalf of European Hematology Association. This is an open access Abstract Book distributed under the Attribution-NonCommercial-NoDerivs (CC BY-NC-ND), which allows third parties to download the articles and share them with others as long as they credit the author and the Abstract Book, but they cannot change the content in any way or use them commercially.
**Abstract Book Citations:** Authors, Title, HemaSphere, 2026;10:(S1):pages. The abstract book DOIs can be found on the “Author Information” page.
**Disclaimer:** Articles published in the journal HemaSphere exclusively reflect the opinions of the authors. The authors are responsible for all content in their abstracts including accuracy of the facts, statements, citing resources, etc.

**Results**

The cohort included 14 MPN (56%), 5 MDS-SF3B1 (20%), and 6 MDS/MPN (24%), median age 71 years. JAK2V617F was the predominant driver (68%), followed by CALR (20%) and MPL (12%). Sixty percent had additional mutations: TET2 (32%), TP53 (12%), ASXL1 (8%), DNMT3A (8%). SF3B1 was first mutation in 36% and coexistent in 64%; dominant clone in 48%, though with ΔVAF >10% only 24% showed clear dominance, suggesting frequent allelic co-dominance.

SF3B1 VAF was higher in MDS-SF3B1 (43.5%) and MDS/MPN (41.8%) versus MPN (19.25%; p=0.029), suggesting SF3B1 clonal burden drives the dysplastic phenotype. Hematological parameters revealed a gradient: platelets in MPN (736×10⁹/L) and MDS/MPN (672×10⁹/L) versus MDS-SF3B1 (239×10⁹/L; p=0.003); hemoglobin in MPN (13.0 g/dL) vs MDS/MPN (11.5) and MDS-SF3B1 (10.3; p=0.004); leukocytes: MDS/MPN (10.56×10⁹/L) > MPN (8.47×10⁹/L) > MDS-SF3B1 (4.50×10⁹/L; p=0.035).

Morphological findings: ring sideroblasts in MDS/MPN (36%) and MDS-SF3B1 (15%) with absence in MPN (p<0.001); erythroid dysplasia in 100% of MDS/MPN and 80% of MDS-SF3B1 versus 21% in MPN (p<0.001); anemia in MDS-SF3B1 (100%) and MDS/MPN (83%) versus MPN (21%; p=0.002); fibrosis ≥G1 in MDS/MPN (33%) and MPN (29%) versus MDS-SF3B1 (0%; p=0.003).

First-line treatment mirrored this duality: hydroxyurea (64%) for proliferative features and erythropoietin (28%) for dysplastic ones. Estimated median OS was 149 months; only MPN reached a group-specific median (141 months), while MDS-SF3B1 and MDS/MPN had not reached median OS at last follow-up (log-rank p=0.846). AML progression occurred in 20% (MPN: 4 cases, MDS-SF3B1: 1, MDS/MPN: 0); overall mortality 28%.

**Summary/Conclusion**

Our data demonstrate a continuous clinical-molecular spectrum in myeloid neoplasms with SF3B1/driver coexistence. MDS/MPN-SF3B1 with thrombocytosis occupies an intermediate position between MDS-SF3B1 (higher SF3B1 clonal burden, cytopenias, ring sideroblasts, low AML risk) and classical MPN (thrombocytosis, lower VAF SF3B1, absence of ring sideroblasts, high AML risk). The SF3B1/driver VAF ratio emerges as a promising phenotypic determinant, with implications for classification, prognosis, and treatment requiring multicenter prospective validation.

**Copyright Information:** (Online) ISSN: 2572-9241
©2026 The Author(s). HemaSphere is published by John Wiley & Sons Ltd on behalf of European Hematology Association. This is an open access Abstract Book distributed under the Attribution-NonCommercial-NoDerivs (CC BY-NC-ND), which allows third parties to download the articles and share them with others as long as they credit the author and the Abstract Book, but they cannot change the content in any way or use them commercially.
**Abstract Book Citations:** Authors, Title, HemaSphere, 2026;10:(S1):pages. The abstract book DOIs can be found on the "Author Information" page.
**Disclaimer:** Articles published in the journal HemaSphere exclusively reflect the opinions of the authors. The authors are responsible for all content in their abstracts including accuracy of the facts, statements, citing resources, etc.

**Table 1.** Clinical, analytical and molecular characteristics of the global cohort (N = 25)

| Variable | Result | Variable | Result |
|---|---|---|---|
| **Demographics and neoplasm type** | | | |
| Age (years), median (range) | 71 (58–84) | Classical MPN, N (%) | 14 (56%) |
| Sex F/M | 12/13 (48/52%) | MDS with SF3B1, N (%) | 5 (20%) |
| | | MDS/MPN-SF3B1 with thrombocytosis, N (%) | 6 (24%) |
| **Hematological parameters and bone marrow** | | | |
| Hb (g/dL), median (range) | 12.2 (8.2–15.8) | BM blasts (%), median | 0.5% |
| Platelets (×10⁹/L), median | 633 (81–1,410) | Ring sideroblasts >15%, N (%) | 9 (36%) |
| Leukocytes (×10⁹/L), median | 8.24 (2.35–21.51) | Hypercellular BM, N (%) | 20 (80%) |
| Hb <12.2 g/dL, N (%) | 13 (52%) | Fibrosis ≥G1, N (%) | 6 (24%) |
| Erythroid dysplasia, N (%) | 13 (52%) | Megakaryocytic dysp., N (%) | 19 (76%) |
| **Molecular profile and clonal architecture** | | | |
| **Driver mutation, N (%)** | | **VAF SF3B1 (%), median (range)** | 35.1 (3–50) |
| JAK2V617F | 17 (68%) | **VAF driver (%), median** | |
| CALR | 5 (20%) | JAK2 | 26% |
| MPL | 3 (12%) | CALR / MPL | 42% / 19% |
| **SF3B1/driver VAF ratio, median (range)†** | **1.03 (0.07–16.10)** | *MDS-SF3B1:* **2.38** \| *MDS/MPN:* **1.04** \| *MPN:* **1.00** | — |
| **Acquisition order, N (%)** | | **Clonal dominance (highest VAF), N (%)** | |
| SF3B1 first (1st mutation) | 9 (36%) | SF3B1 dominant | 12 (48%) |
| Coexistent (simultaneous) | 16 (64%) | JAK2 dominant | 5 (20%) |
| **Additional mutations, N (%)** | 15 (60%) | CALR dominant | 2 (8%) |
| TET2 / TP53 / ASXL1 | 8 / 3 / 2 | | |
| **Treatment, outcomes and survival** | | | |
| **1st-line treatment, N (%)** | | **Survival and outcomes** | |
| Hydroxyurea | 16 (64%) | Follow-up (months), median | 102 |
| Erythropoietin (EPO) | 7 (28%) | Estimated OS (months), median | 149 |
| | | AML progression, N (%) | 5 (20%) |
| | | Mortality, N (%) | 7 (28%) |

*Med: median. BM: bone marrow. VAF: variant allele frequency. OS: overall survival.*

*AML: acute myeloid leukemia. EPO: erythropoietin. F/M: female/male.*

*†SF3B1/driver VAF ratio by diagnostic group: MDS-SF3B1: 2.38, MDS/MPN: 1.04, MPN: 1.00.*

**Table 2.** Comparative analysis between MDS with SF3B1, MDS/MPN with SF3B1 and thrombocytosis, and classical MPN

| Variable | MDS-SF3B1 | MDS/MPN SF3B1+Thrombocytosis | Classical MPN | p value | trend |
|---|---|---|---|---|---|
| | (n = 5) | (n = 6) | (n = 14) | global | |
| | ◄ Dysplasia | — *grey zone* — | Proliferation ► | | |
| **Hematological parameters (median)** | | | | | |
| Age (years) | 75 | 78 | 69.5 | **.043** | |
| Hb (g/dL) | 10.30 | 11.50 | 13.00 | **.004** | |
| Platelets (×10⁹/L) | 239 | 672 | 736 | **.003** | |
| Leukocytes (×10⁹/L) | 4.50 | 10.56 | 8.47 | **.035** | |
| Anemia (<12.2 g/dL), N (%) | 5 (100%) | 5 (83%) | 3 (21%) | **.002** | |
| **Bone marrow** | | | | | |
| Ring sideroblasts (%), median | 15 | 36 | 0 | **<.001** | |
| Erythroid dysplasia, N (%) | 4 (80%) | 6 (100%) | 3 (21%) | **<.001** | |
| Fibrosis ≥G1 at dx, N (%) | 0 | 2 (33%) | 4 (29%) | **.003** | |
| **Molecular profile (median)** | | | | | |
| VAF SF3B1 (%) | 43.5 | 41.8 | 19.25 | **.029** | |
| **VAF driver (%)** | 8 | 36.75 | 28 | .096 | |

*p value global: Kruskal-Wallis (quantitative) / Chi-square/Fisher (qualitative).*

*Bonferroni correction: p<0.016 (bold values). dx: diagnosis.*

*Trend column: gradient bars show direction of values across MDS-SF3B1 → MDS/MPN → MPN*

*(◄ red = higher in MDS-SF3B1, ► blue = higher in MPN, purple peak = higher in MDS/MPN).*

**Copyright Information:** (Online) ISSN: 2572-9241
©2026 The Author(s). HemaSphere is published by John Wiley & Sons Ltd on behalf of European Hematology Association. This is an open access Abstract Book distributed under the Attribution-NonCommercial-NoDerivs (CC BY-NC-ND), which allows third parties to download the articles and share them with others as long as they credit the author and the Abstract Book, but they cannot change the content in any way or use them commercially.
**Abstract Book Citations:** Authors, Title, HemaSphere, 2026;10:(S1):pages. The abstract book DOIs can be found on the "Author Information" page.
**Disclaimer:** Articles published in the journal HemaSphere exclusively reflect the opinions of the authors. The authors are responsible for all content in their abstracts including accuracy of the facts, statements, citing resources, etc.

### (PS1983) MUTANT CALRETICULIN–SPECIFIC MONOCLONAL ANTIBODY, INCA033989, IS WELL TOLERATED AND ACHIEVES RAPID AND SUSTAINED HEMATOLOGIC AND MOLECULAR RESPONSES IN PATIENTS WITH ESSENTIAL THROMBOCYTHEMIA (ET)

**Topic:** 16. Myeloproliferative neoplasms - Clinical

John Mascarenhas*[1], Tania Jain[2], Aaron T. Gerds[3], Vikas Gupta[4], Claire Harrison[5], Chi-Joan How[6], David Ross[7], Lucia Masarova[8], Bethan Psaila[9], Abdulraheem Yacoub[10], Terrence Bradley[11], Caroline McNamara[12], Andrew Perkins[13], William Shomali[14], Makoto Yoshimitsu[15], Junichiro Yuda[16], Haifa Kathrin Al-Ali[17], Lynette Chee[18], Jean-Jacques Kiladjian[19], Ruben Mesa[20], Francesco Passamonti[21], Raajit K Rampal[22], Tim Somervaille[23], Alessandro Maria Vannucchi[24], Hiroki Yamaguchi[25], Steffen Koschmieder[26], Jason Gotlib[14], Jyoti Nangalia[27], Chenwei Tian[28], Betty Lamothe[28], Erin Crowgey[28], Tatiana Zinger[28], Evan Braunstein[28], Haris Ali[29]

*[1]Icahn School of Medicine at Mount Sinai, New York, NY, United States of America;[2]Sidney Kimmel Comprehensive Cancer Center, Johns Hopkins University, Baltimore, MD, United States of America; [3]Cleveland Clinic, Cleveland, OH, United States of America; [4]Princess Margaret Cancer Centre, Toronto, ON, Canada;[5]Guy's and St Thomas' NHS Foundation Trust, London, United Kingdom; [6]Brigham and Women's Hospital and Dana-Farber Cancer Institute, Boston, MA, United States of America; [7]Royal Adelaide Hospital, Adelaide, SA, Australia;[8]MD Anderson Cancer Center, Houston, TX, United States of America; [9]University of Oxford, Oxford, United Kingdom; [10]The University of Kansas Cancer Center, Kansas City, KS, United States of America; [11]University of Miami Sylvester Comprehensive Cancer Center, Division of Hematology, Miami, FL, United States of America; [12]Royal Brisbane and Women's Hospital, Brisbane, QLD, Australia; [13]The Alfred Hospital, Melbourne, VIC, Australia; [14]Stanford Cancer Institute/Stanford University School of Medicine, Stanford, CA, United States of America; [15]Kagoshima University Hospital, Kagoshima, Japan; [16]Department of Hematology, National Cancer Center Hospital East, Chiba, Japan; [17]University Medicine Halle, Saale, Germany; [18]Peter MacCallum Cancer Centre and The Royal Melbourne Hospital, Melbourne, VIC, Australia; [19]Saint-Louis Hospital, Paris Cité University, INSERM, Paris, France;[20]Wake Forest University Baptist Medical Center, Winston-Salem, NC, United States of America; [21]Fondazione IRCCS Ca Ganda Ospedale Maggiore, Milan, Italy; [22]Memorial Sloan Kettering Cancer Center, New York, NY, United States of America;[23]Leukaemia Biology Laboratory, Cancer Research UK Manchester Institute, The University of Manchester, Manchester, United Kingdom; [24]Azienda Ospedaliero-Universitaria Careggi, Florence, Italy; [25]Nippon Medical School Hospital, Tokyo, Japan;[26]RWTH Aachen University, Faculty of Medicine, and Center for Integrated Oncology (CIO-ABCD), Aachen, Germany; [27]The Sanger Institute, Cambridge, United Kingdom; [28]Incyte Corporation, Wilmington, DE, United States of America;[29]City of Hope Medical Center, Duarte, CA, United States of America;*

### Background

ET is a myeloproliferative neoplasm characterized by megakaryocytic hyperplasia and clustering, thrombocytosis, and elevated risk of thrombosis, hemorrhage, and transformation to myelofibrosis (MF). About 25% of patients (pts) with ET harbor calreticulin mutations (mut*CALR*). INCA033989 is a novel, fully human, Fc-silenced, IgG1 monoclonal antibody that selectively targets mut*CALR* in complex with thrombopoietin receptor and inhibits oncogenic signaling and cell proliferation. INCA033989-101 (NCT05936359) and -102 (NCT06034002) are phase 1, first-in-human, multicenter, open-label studies evaluating INCA033989 in pts with ET or MF.

### Aims

To evaluate safety and efficacy of INCA033989 in pts with ET and mut*CALR*.

**Copyright Information:** (Online) ISSN: 2572-9241
©2026 The Author(s). HemaSphere is published by John Wiley & Sons Ltd on behalf of European Hematology Association. This is an open access Abstract Book distributed under the Attribution-NonCommercial-NoDerivs (CC BY-NC-ND), which allows third parties to download the articles and share them with others as long as they credit the author and the Abstract Book, but they cannot change the content in any way or use them commercially.
**Abstract Book Citations:** Authors, Title, HemaSphere, 2026;10:(S1):pages. The abstract book DOIs can be found on the "Author Information" page.
**Disclaimer:** Articles published in the journal HemaSphere exclusively reflect the opinions of the authors. The authors are responsible for all content in their abstracts including accuracy of the facts, statements, citing resources, etc.

### Methods

Pts enrolled had ET with mut*CALR*; high risk disease (age ≥60 y; history of thrombosis, major bleeding, or extreme thrombocytosis); resistance/intolerance to ≥1 prior line of cytoreductive treatment (tx); and a platelet count (PLT) >450 G/L. The primary endpoint was safety and tolerability. Efficacy was evaluated by hematologic response: PLT ≤400 G/L (complete hematologic response [CHR]) or ≤600 G/L (partial hematologic response [PHR]); a leukocyte count of <10 G/L. Reduction in mut*CALR* variant allele frequency (VAF) was also evaluated.

### Results

As of Jan 5, 2026, 110 pts were enrolled as part of dose escalation (24-2500 mg) and dose expansion; no DLTs were observed; MTD was not reached. The median (range) age was 61.5 y (23, 89); 60% of pts were female. The most frequent prior cytoreductive tx were hydroxycarbamide (84%), anagrelide (31%), and interferons (32%). Fifty-one (46%), 40 (36%), and 19 pts (17%) had Type 1, Type 2, and other mut*CALR*, respectively. Baseline median (Q1, Q3) PLT was 913 G/L (657, 1214); median (Q1, Q3) mut*CALR* VAF was 33% (28, 37).
INCA033989 was well tolerated with 106/110 pts (96.4%) continuing tx. The median (range) exposure was 28 weeks (4, 109). Tx-emergent adverse events (TEAEs) occurred in 94 pts (85%); most frequently (≥20%) fatigue (24%) and headache (20%). In 84% of pts, all TEAEs were grade 1-2 and most resolved during tx; 18 pts (16%) had grade ≥3 TEAEs. 7 serious AEs were reported in 3 pts (all grade 3; max duration, 12 days); 1 event (venous thrombosis, 24 mg) led to discontinuation, 1 event (diverticulitis, 400mg) led to dose reduction (pt remained on study and later dose escalated). Infusion reactions were rare (6%), with interruptions occurring in only 5 pts (5%).
Across all doses, 75 (68%) pts had a CHR and 93 (85%) had a CHR/PHR, with a median (range) duration of CHR/PHR of 15 (0.14, 95) weeks. The median (range) time to onset of durable (≥12 weeks) CHR was 2.1 (1.1, 64) weeks. At C7D1 (~24 weeks), doses of ≥750 mg resulted in 100% of Type 1 and 47% of non-Type 1 pts having PLT <600 G/L, and 92% of Type 1 and 33% of non-Type 1 pts having PLT <400 G/L. A ≥25% VAF reduction correlated with durable CHR (nominal $P<0.0001$); among 38 pts who achieved durable CHR and have ≥1 post-baseline VAF assessment, 71% (27/38) achieved a ≥25% reduction in VAF (**Figure**). Histopathology analysis of bone marrow showed reduction in megakaryocyte cell clustering from baseline to C7D1, correlating with a ≥25% reduction in VAF (nominal $P=0.02$).

### Summary/Conclusion

INCA033989 continues to demonstrate favorable safety and efficacy in pts with ET and resistance/intolerance to prior cytoreductive tx. Most pts had a rapid, durable CHR that correlated with a reduction in mut*CALR* VAF. These data support the planned initiation of phase 3 trials in pts with ET.

**Copyright Information:** (Online) ISSN: 2572-9241
©2026 The Author(s). HemaSphere is published by John Wiley & Sons Ltd on behalf of European Hematology Association. This is an open access Abstract Book distributed under the Attribution-NonCommercial-NoDerivs (CC BY-NC-ND), which allows third parties to download the articles and share them with others as long as they credit the author and the Abstract Book, but they cannot change the content in any way or use them commercially.
**Abstract Book Citations:** Authors, Title, HemaSphere, 2026;10:(S1):pages. The abstract book DOIs can be found on the "Author Information" page.
**Disclaimer:** Articles published in the journal HemaSphere exclusively reflect the opinions of the authors. The authors are responsible for all content in their abstracts including accuracy of the facts, statements, citing resources, etc.

**Figure. In Patients with ET Treated with INCA033989, Greater Improvement in VAF from Baseline is Associated with Durable Hematologic Response**

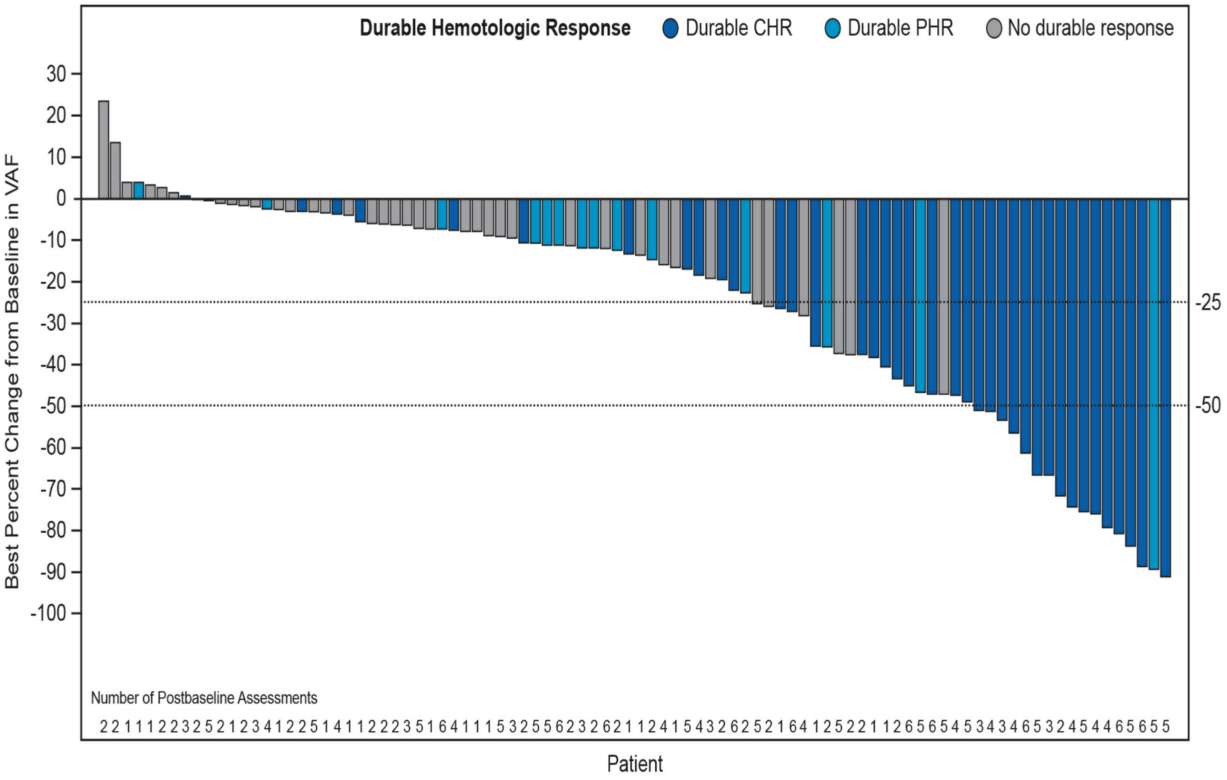

CHR defined as platelet count ≤400 G/L and leukocytes <10 G/L, PHR defined as platelet count ≤600 G/L and leukocytes <10 G/L (baseline platelet count >600 G/L).
Durable response defined as maintaining for ≥12 weeks.
CHR, complete hematologic response; PHR, partial hematologic response; VAF, variant allele frequency.

**Copyright Information:** (Online) ISSN: 2572-9241
©2026 The Author(s). HemaSphere is published by John Wiley & Sons Ltd on behalf of European Hematology Association. This is an open access Abstract Book distributed under the Attribution-NonCommercial-NoDerivs (CC BY-NC-ND), which allows third parties to download the articles and share them with others as long as they credit the author and the Abstract Book, but they cannot change the content in any way or use them commercially.
**Abstract Book Citations:** Authors, Title, HemaSphere, 2026;10:(S1):pages. The abstract book DOIs can be found on the "Author Information" page.
**Disclaimer:** Articles published in the journal HemaSphere exclusively reflect the opinions of the authors. The authors are responsible for all content in their abstracts including accuracy of the facts, statements, citing resources, etc.

### (PS1984) FEDRATINIB + NIVOLUMAB COMBINATION IMPROVES OUTCOMES IN MYELOFIBROSIS PATIENTS RESISTANT OR SUBOPTIMALLY RESPONDING TO JAK-INHIBITOR THERAPY – RESULTS FROM THE FRACTION TRIAL (IKF051)

**Topic:** 16. Myeloproliferative neoplasms - Clinical

Susanne Isfort*[1], Nikolaschristiancornelius Bubnoff[2], Nadja Jäkel[3], Heiko Becker[4], Frank Stegelmann[5], Thorsten Götze[6], Philipp Le Coutre[7], Martin Griesshammer[8], Adrian Schwarzer[9], Luisa Wohn[6], Salah-Eddin Al-Batran[6], Johanna Riedel[6], Francesca Palandri[10], Kirsi Manz[1], Manuela Neubert[9], Yankai Xiao[1], Christian Schultze-Florey[1], Andreas Hochhaus[11], Konstanze Döhner[5], Florian Heidel[1]

*[1]Hannover Medical School (MHH), Hematology, Hemostasis, Oncology and Stem Cell Transplantation, Comprehensive Cancer Center Lower Saxony (CCC-N), Hannover, Germany; [2]University Medical Center and University Cancer Center Schleswig-Holstein Campus Lübeck, Department of Hematology and Oncology, Lübeck, Germany; [3]University Hospital Halle, Halle/Saale, Germany; [4]Medical Center - University of Freiburg, Faculty of Medicine, Department of Medicine I, Freiburg, Germany; [5]University Hospital Ulm, Department of Internal Medicine III, Ulm, Germany; [6]Frankfurter Institut für Klinische Krebsforschung IKF, Frankfurt, Germany; [7]Charite Berlin, Department of Hematology, Oncology and Stem Cell Transplantation, Berlin, Germany; [8]Johannes Wesling Medical Center Minden, University Clinic for Hematology, Oncology, Haemostaseology and Palliative Care, Minden, Germany; [9]University Medicine Greifswald, Internal Medicine C, Hematology, Oncology, Stem Cell Transplantation and Palliative Care, Greifswald, Germany; [10]IRCCS Azienda Ospedaliero-Universitaria di Bologna, Istituto di Ematologia "Seràgnoli", Bologna, Italy; [11]Jena University Hospital, Hematology/Oncology, Jena, Germany;*

### Background

Checkpoint inhibitors have been tested as monotherapy or in combination with ruxolitinib so far with rather unsatisfying results, most likely due to short exposure time or potential antagonistic effects. Fedratinib is JAK2 specific and permissive for immune functions. Here, nivolumab, an established checkpoint inhibitor with favorable safety profile was studied as combinatorial therapy with fedratinib.

### Aims

Efficacy and safety of the combination of fedratinib and nivolumab in myelofibrosis (MF) patients (pts) with suboptimal response to prior JAK-inhibitor treatment was evaluated.

### Methods

FRACTION is a single arm, open label Phase II trial investigating the combination of fedratinib with nivolumab in patients with MF and suboptimal or lack of response to JAK-inhibitor therapy. The primary efficacy endpoint is the response rate defined as the proportion of pts with a best response within 12 treatment cycles acc. to the IWG-MRT criteria of complete remission (CR), partial remission (PR), clinical improvement (CI) or red blood cell transfusion independency (RBC-TI). Additional details on study design were published previously (Isfort, S. et al. PMID: 38967662). Experimental analysis included sequential assessment of T cell characteristics by spectral flow and molecular analysis by targeted Next-Generation Sequencing.

**Copyright Information:** (Online) ISSN: 2572-9241
©2026 The Author(s). HemaSphere is published by John Wiley & Sons Ltd on behalf of European Hematology Association. This is an open access Abstract Book distributed under the Attribution-NonCommercial-NoDerivs (CC BY-NC-ND), which allows third parties to download the articles and share them with others as long as they credit the author and the Abstract Book, but they cannot change the content in any way or use them commercially.
**Abstract Book Citations:** Authors, Title, HemaSphere, 2026;10:(S1):pages. The abstract book DOIs can be found on the "Author Information" page.
**Disclaimer:** Articles published in the journal HemaSphere exclusively reflect the opinions of the authors. The authors are responsible for all content in their abstracts including accuracy of the facts, statements, citing resources, etc.

**Results**

30 of all 31 enrolled patients were included in the full analysis set. Median age was 66.5y, 70 % were male. 17 (57%) were high or very high risk according to MIPSS70+2.0 (Mutation profiles in Figure 1). Median time from diagnosis to treatment was 64.1 mo. 9 pts were transfusion-dependent at baseline. 27% of the pts received more than one prior JAK2-inhibitor.
The median duration for the combinatorial treatment was 4.2 mo (range 0.5-24.4). The most common reasons for treatment discontinuation included lack of response/ end of study (each n=5), death (n=4) and toxicity/patient wish/unrelated medical illness or complication (each n=3).
Acc. to the study´s A'Hern design, the treatment is considered promising if at least five pts achieve a response as defined by the primary endpoint. 12/30 (40%) evaluable pts responded, with best responses being CI in 5 pts (17%) and RBC-TI in 7 pts (23%). 43% of pts showed clinical benefit incl. 7 pts (23%) with stable disease (SD) and symptom response and 1 pt with SD and anemia response. Among 21 pts with palpable splenomegaly, 7 (33%) pts achieved a spleen response. Pts with JAK2 vs. CALR mutations tended to show better response patterns (50% vs. 29%) as well as pts with RBC-transfusion dependence at baseline (67% vs. 29%). Progression as defined by the IWG-MRT criteria occurred in 1 pt (progressive splenomegaly). With a median follow-up of 16.5 months, median overall survival (OS) is estimated at 31.8 mo, which is above the expected median OS according to MIPSS70+2.0. 5 pts became eligible for allogeneic stem cell transplantation.
30 pts were evaluable for safety. Adverse events (AEs) occurring in >25% of pts were dyspnea (57%), diarrhea (43%), anemia (40%), nausea (40%), fatigue (37%), edema limbs (27%) and creatinine increase (27%). AEs with a grade ≥ 3 occurred in 83% of pts. Treatment-related AEs (TRAE) occurred in 28 pts with 60% of pts had a TRAE grade ≥3.

**Summary/Conclusion**

Combination of fedratinib with nivolumab demonstrates promising clinical efficacy by successfully achieving the primary endpoint. Furthermore, transfusion-dependent pts showed a higher response rate. The safety profile raised no relevant concerns.

**Copyright Information:** (Online) ISSN: 2572-9241
©2026 The Author(s). HemaSphere is published by John Wiley & Sons Ltd on behalf of European Hematology Association. This is an open access Abstract Book distributed under the Attribution-NonCommercial-NoDerivs (CC BY-NC-ND), which allows third parties to download the articles and share them with others as long as they credit the author and the Abstract Book, but they cannot change the content in any way or use them commercially.
**Abstract Book Citations:** Authors, Title, HemaSphere, 2026;10:(S1):pages. The abstract book DOIs can be found on the "Author Information" page.
**Disclaimer:** Articles published in the journal HemaSphere exclusively reflect the opinions of the authors. The authors are responsible for all content in their abstracts including accuracy of the facts, statements, citing resources, etc.

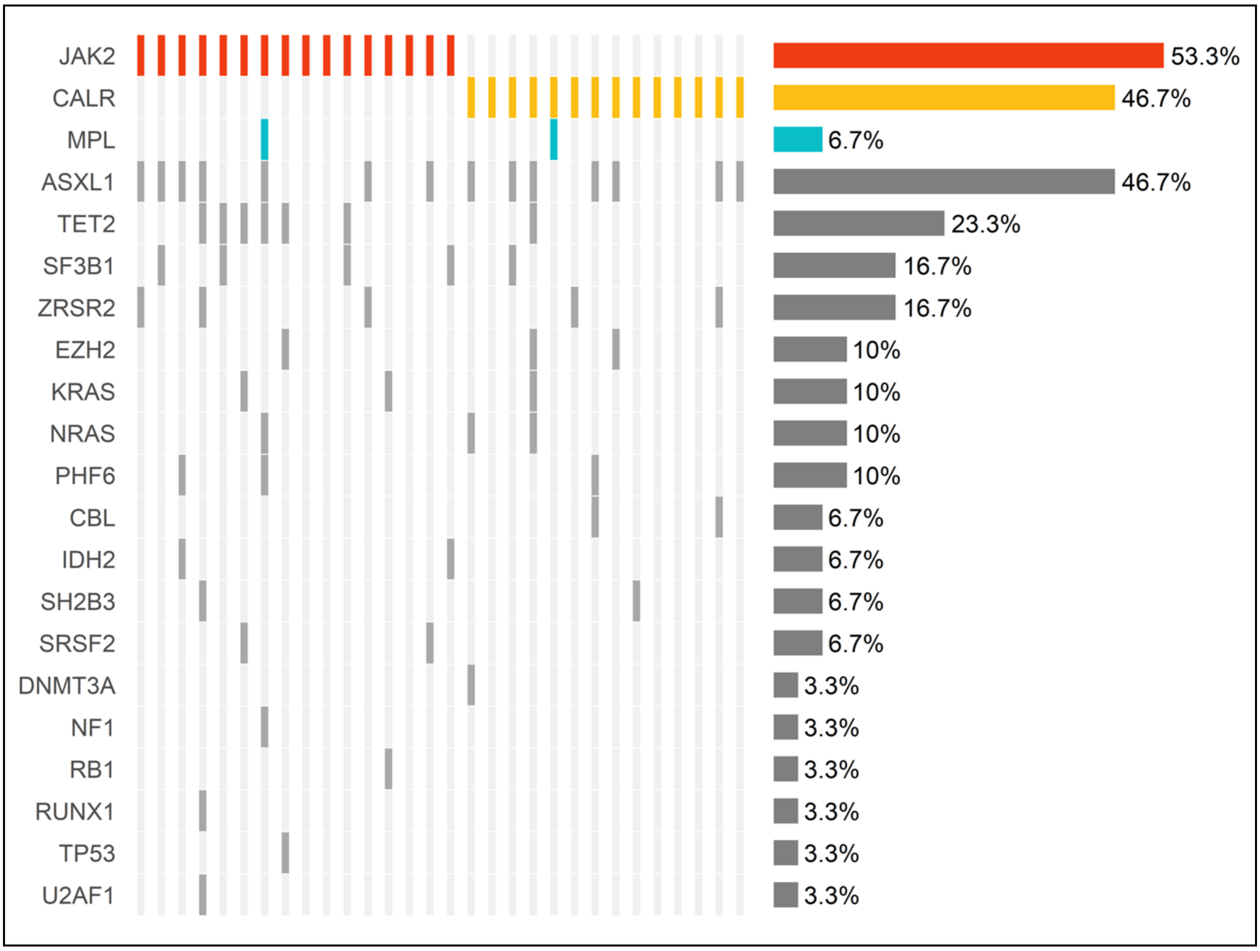

Fig. 1: Mutational profile at baseline. Driver mutations are highlighted in a distinct color relative to additional tested genes (in grey). 3 patients acquired ≥1 additional mutation over the course of the trial, 7 pts had at one point in time over the course of the trial fewer mutations compared to baseline, and 2 showed an unchanged number of mutations with a switch in the affected genes.

**Copyright Information:** (Online) ISSN: 2572-9241
©2026 The Author(s). HemaSphere is published by John Wiley & Sons Ltd on behalf of European Hematology Association. This is an open access Abstract Book distributed under the Attribution-NonCommercial-NoDerivs (CC BY-NC-ND), which allows third parties to download the articles and share them with others as long as they credit the author and the Abstract Book, but they cannot change the content in any way or use them commercially.
**Abstract Book Citations:** Authors, Title, HemaSphere, 2026;10:(S1):pages. The abstract book DOIs can be found on the "Author Information" page.
**Disclaimer:** Articles published in the journal HemaSphere exclusively reflect the opinions of the authors. The authors are responsible for all content in their abstracts including accuracy of the facts, statements, citing resources, etc.

### (PS1985) INVESTIGATIONAL PIM1 INHIBITOR NUVISERTIB IN COMBINATION WITH MOMELOTINIB SHOWED PROMISING CLINICAL ACTIVITY IN PATIENTS WITH MYELOFIBROSIS AND ANEMIA: DATA FROM AN ONGOING GLOBAL PHASE 1/2 STUDY

**Topic:** 16. Myeloproliferative neoplasms - Clinical

James McCloskey*[1], Lindsay Rein[2], Andrew Srisuwananukorn[3], John Mascarenhas[4], Prithviraj Bose[5], Vikas Gupta[6], Raajit K Rampal[7], Joseph M Scandura[8], Kazuya Shimoda[9], Timothy Devos[10], Alessandro Lucchesi[11], Firas El Chaer[12], Srinivas Tantravahi[13], Noboru Asada[14], Noriko Fukuhara[15], Junichiro Yuda[16], Michiko Ichii[17], Marcello Rotta[18], Anthony M. Hunter[19], David Ross[20], Ashwin Kishtagari[21], Vincent Ribrag[22], Marina Kremyanskaya[4], Moshe Talpaz[23], Dominiek Mazure[24], Alessandra Iurlo[25], Ciro Rinaldi[26], Florian Heidel[27], Ronald Hoffman[4], Ruben Mesa[28], Masataka Seki[29], Prasanthi Pellakuru[29], Zhonggai Li[29], Hongliang Cai[29], Jinny Lee[29], Eric I Sbar[29], Sujan Kabir[29], Claire Harrison[30], Francesco Passamonti[25]

*[1]John Theurer Cancer Center, Hackensack University Medical Center, Hackensack, United States of America;[2]Duke University Medical Center, Durham, United States of America; [3]The Ohio State University Comprehensive Cancer Center, Columbus, United States of America; [4]Icahn School of Medicine at Mount Sinai, New York, United States of America;[5]The University of Texas MD Anderson Cancer Center, Houston, United States of America; [6]Princess Margaret Cancer Centre, Toronto, Canada; [7]Memorial Sloan Kettering Cancer Center, New York, United States of America; [8]Weill Cornell Medicine, New York, United States of America; [9]University of Miyazaki, Miyazaki, Japan; [10]Department of Hematology, University Hospitals Leuven and Department of Microbiology and Immunology, Laboratory of Molecular Immunology (Rega Institute), KU Leuven, Belgium; [11]IRCCS Istituto Romagnolo per lo Studio e la Cura dei Tumori (IRST) "DinoAmadori", Hematology Unit, Meldola, Italy; [12]Miami Cancer Institute, Baptist Health, Miami, United States of America; [13]Huntsman Cancer Institute, University of Utah, Salt lake city, United States of America; [14]Okayama University Hospital, Okayama, Japan; [15]Tohoku University Hospital, Sendai, Japan; [16]National Cancer Center Hospital East, Kashiwa, Japan; [17]The University of Osaka Graduate School of Medicine, Suita, Japan;[18]Colorado Blood Cancer Institute, Denver, United States of America; [19]Emory University School of Medicine, Atlanta, United States of America; [20]Royal Adelaide Hospital, Adelaide, Australia;[21]Vanderbilt University Medical Center, Nashville, United States of America; [22]Institut Gustave Roussy, Villejuif, France; [23]University of Michigan, Ann Arbor, United States of America; [24]Universitair Ziekenhuis Gent, Gent, Belgium; [25]Foundation IRCCS Ca' Granda Ospedale Maggiore Policlinico, Milan, Italy; [26]United Lincolnshire Hospitals NHS Trust, Lincoln, United Kingdom; [27]Hannover Medical School (MHH), Hematology, Hemostasis, Oncology and Stem Cell Transplantation, Hannover, Germany; [28]Atrium Health Levine Cancer Institute, Charlotte, United States of America; [29]Sumitomo Pharma America, Inc., Marlborough, United States of America; [30]Guy's and St Thomas' NHS Foundation Trust, London, United Kingdom;*

### Background

PIM1 is downstream of JAK/STAT signaling leading to its upregulation in myelofibrosis (MF). Additionally, PIM1 expression can be upregulated by NF-kB and ERG, which function as JAK-independent compensatory pathways that drive MF pathogenesis. Dual inhibition of JAK and PIM1 may provide more comprehensive suppression of these combined disease-relevant pathways. Momelotinib (MMB), a JAK/ACVR1 inhibitor, showed ≥50% reduction in total symptom score (TSS50) of 25%, ≥25% spleen volume reduction (SVR25) of 39% and anemia improvement in patients (pts) with relapsed/refractory (R/R) MF. Nuvisertib (nuvi), an oral investigational selective PIM1 inhibitor, monotherapy was well tolerated with stable hemoglobin (Hgb) and platelets (PLT) and showed TSS50 and SVR25 responses correlating with cytokine modulation in pts with R/R MF. Higher plasma exposure of nuvi was observed under fed conditions (fed) vs. fasted in normal healthy volunteers. The combination of nuvi with MMB may enhance clinical activity while preserving anemia response advantage of MMB. Here, we present preliminary data of nuvi + MMB combination (fed) for the first time.

**Copyright Information:** (Online) ISSN: 2572-9241
©2026 The Author(s). HemaSphere is published by John Wiley & Sons Ltd on behalf of European Hematology Association. This is an open access Abstract Book distributed under the Attribution-NonCommercial-NoDerivs (CC BY-NC-ND), which allows third parties to download the articles and share them with others as long as they credit the author and the Abstract Book, but they cannot change the content in any way or use them commercially.
**Abstract Book Citations:** Authors, Title, HemaSphere, 2026;10:(S1):pages. The abstract book DOIs can be found on the "Author Information" page.
**Disclaimer:** Articles published in the journal HemaSphere exclusively reflect the opinions of the authors. The authors are responsible for all content in their abstracts including accuracy of the facts, statements, citing resources, etc.

### Aims

This global Phase 1/2 study (NCT04176198, Arm 3) aims to identify the RP2D of nuvi + MMB (fed) in pts with R/R MF, and to assess the safety, clinical activity, and PK/PD.

### Methods

Key eligibility includes primary or secondary MF, previously treated with a JAK inhibitor (JAKi, except MMB), DIPSS intermediate or high-risk, Hgb <10 g/dL, PLT ≥50 x $10^9$/L, splenomegaly (≥450 $cm^3$), and ≥2 symptoms with each score ≥3 or TSS of ≥10 per MFSAF v4.

### Results

As of 06 Dec 2025, total 26 pts enrolled in 4 dose levels of nuvi (fed) from 240, 360, 480 and 720 mg BID + MMB 200 mg QD. At baseline, median age 71 years (56, 86); TSS 23 (11, 57); spleen volume 1386 $cm^3$ (484, 5428); Hgb 8.9 g/dL (4.5, 10.6; 46% pts required transfusion); and PLT 140 x $10^9$/L (61, 488; 35% pts with PLT <100 x $10^9$/L). All pts received prior JAKi, and 41% pts had high molecular risk mutations. Median treatment duration was 14 weeks (2, 37), and 92% (24/26) pts remained on treatment. No DLTs occurred. Treatment-related adverse events (TRAEs) occurring in ≥20% pts were diarrhea, nausea, and thrombocytopenia (TCP). Grade 3 TRAE occurring in ≥2 pts included TCP without bleeding (n=3); no Grade 4/5 event. 23% (6/26) pts required dose modification, no discontinuation due to TRAE. Mean Hgb and PLT remained stable throughout the 24-week treatment. 15 pts completed ≥12 weeks of treatment and were efficacy evaluable. TSS reduction was observed in 11 pts (median -53%, 11% to -98%); TSS50 at Wk12 was 53% (8/15) pts and at Wk24 was 60% (3/5) pts (Fig 1A). Absolute reduction was observed in all 7 symptoms, including 50% reduction in mean fatigue score. SVR was achieved in 14 pts (median –35%, 43% to –77%); SVR25 at Wk12 was 73% (11/15) pts and at Wk24 was 100% (5/5) pts (Fig 1B). Anemia response by IWG ELN2024 was observed in 50% (6/12) pts at any time. At Wk24, 60% (3/5) pts achieved all three responses (TSS50, SVR25 and anemia). Decreased IL-18 and EN-RAGE and increased adiponectin were observed in 64% (9/14) pts. Dose escalation is ongoing.

### Summary/Conclusion

Preliminary data from the ongoing Phase 1 dose escalation study in pts with R/R MF and anemia, nuvi + MMB (fed) appeared well tolerated and showed promising clinical activity including symptom and spleen responses, anemia improvement and cytokine modulation. Emerging data supports further development of nuvi + MMB combination (fed) for pts with MF.

**Copyright Information:** (Online) ISSN: 2572-9241
©2026 The Author(s). HemaSphere is published by John Wiley & Sons Ltd on behalf of European Hematology Association. This is an open access Abstract Book distributed under the Attribution-NonCommercial-NoDerivs (CC BY-NC-ND), which allows third parties to download the articles and share them with others as long as they credit the author and the Abstract Book, but they cannot change the content in any way or use them commercially.
**Abstract Book Citations:** Authors, Title, HemaSphere, 2026;10:(S1):pages. The abstract book DOIs can be found on the “Author Information” page.
**Disclaimer:** Articles published in the journal HemaSphere exclusively reflect the opinions of the authors. The authors are responsible for all content in their abstracts including accuracy of the facts, statements, citing resources, etc.

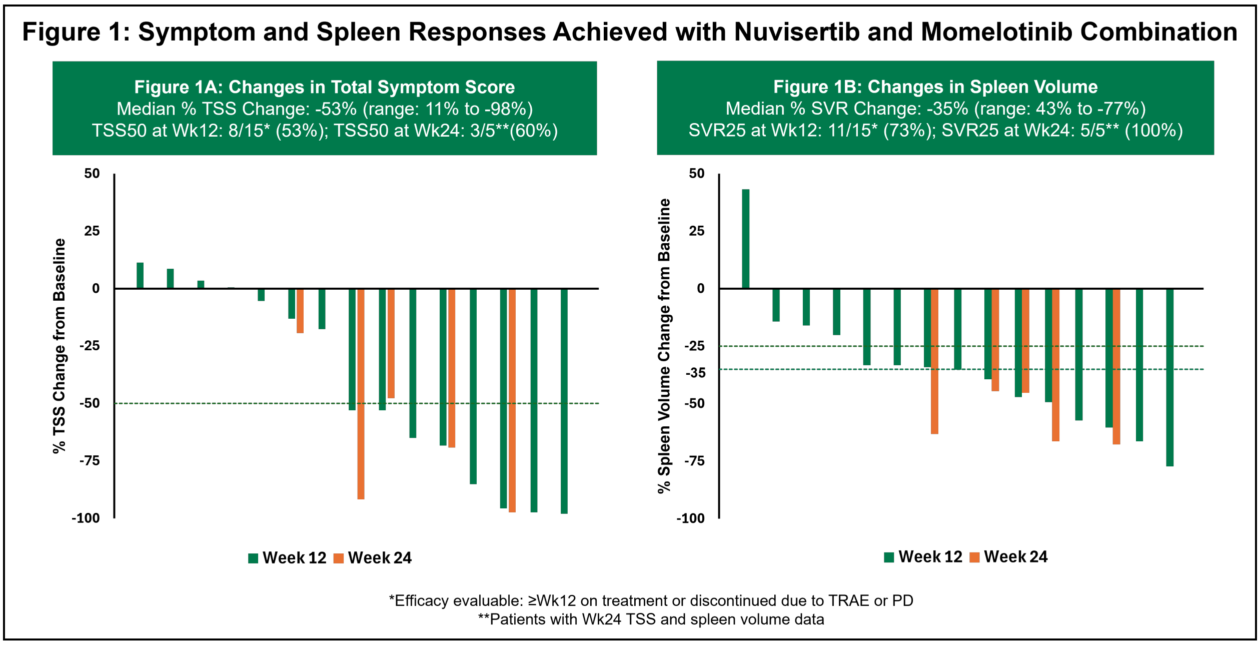

**Copyright Information:** (Online) ISSN: 2572-9241
©2026 The Author(s). HemaSphere is published by John Wiley & Sons Ltd on behalf of European Hematology Association. This is an open access Abstract Book distributed under the Attribution-NonCommercial-NoDerivs (CC BY-NC-ND), which allows third parties to download the articles and share them with others as long as they credit the author and the Abstract Book, but they cannot change the content in any way or use them commercially.
**Abstract Book Citations:** Authors, Title, HemaSphere, 2026;10:(S1):pages. The abstract book DOIs can be found on the “Author Information” page.
**Disclaimer:** Articles published in the journal HemaSphere exclusively reflect the opinions of the authors. The authors are responsible for all content in their abstracts including accuracy of the facts, statements, citing resources, etc.

### (PS1986) ROPEGINTERFERON ALFA-2B DEMONSTRATES MOLECULAR AND HEMATOLOGIC RESPONSES WITH A FAVORABLE SAFETY PROFILE IN ESSENTIAL THROMBOCYTHEMIA

**Topic:** 16. Myeloproliferative neoplasms - Clinical

Harry Gill*[1], Lucia Masarova[2], Brandi Reeves[3], Albert Qin[4], Zhijian Xiao[5], Jie Jin[6], Norio Komatsu[7], Keita Kirito[8], Firas El Chaer[9], Lynda Foltz[10], Tsewang Tashi[11], Ghaith Abu-Zeinah[12], Lee-Yung Shih[13], Dawn Maze[14], Stephen Oh[15], Oleh Zagrijtschuk[16], Toshiaki Sato[17], Abdulraheem Yacoub[18], John Mascarenhas[19], Ruben Mesa[20]

*[1]LKS Faculty of Medicine, the University of Hong Kong, Hong Kong, Hong Kong;[2]MD Anderson Cancer Center, Houston, United States of America; [3]University of North Carolina at Chapel Hill, Chapel Hill, United States of America;[4]PharmaEssentia Corporation, Taipei, Taiwan; [5]National Clinical Research Centre for Blood Diseases, Institute of Haematology and Blood Diseases Hospital, Chinese Academy of Medical Sciences & Peking Union Medical College, State Key Laboratory of Experimental Haematology, Tianjin, China; [6]The First Affiliated Hospital, Zhejiang University School of Medicine, Hangzhou, China; [7]PharmaEssentia Japan KK, Tokyo, Japan; [8]University of Yamanashi, Department of Haematology and Oncology, Yamanashi, Japan; [9]Miami Cancer Institute, Miami, United States of America; [10]St. Paul's Hospital, University of British Columbia, Vancouver, Canada; [11]Huntsman Cancer Institute University of Utah, Salt Lake City, United States of America;[12]Division of Hematology and Medical Oncology, Weill Cornell Medicine, New York, United States of America; [13]15Division of Hematology-Oncology, Chang Gung Memorial Hospital, Chang Gung University, , Taiwan, Taipei City, Taiwan; [14]University of Toronto, Toronto, Canada; [15]Washington University School Of Medicine, St. Louis, United States of America; [16]PharmaEssentia Corporation, Burlington, United States of America; [17]PharmaEssentia Japan K.K, Tokyo, Japan; [18]University of Kansas Cancer Center, Westwood, United States of America; [19]Icahn School of Medicine at Mount Sinai, Tisch Cancer Institute, New York, United States of America; [20]Atrium Health Wake Forest Baptist Comprehensive Cancer Center, Winston-Salem, NC, United States of America;*

### Background

Disease-modifying options for essential thrombocythemia (ET) remain limited. Ropeginterferon alfa-2b (BESREMi, P1101) represents the latest generation interferon-based therapy.

### Aims

SURPASS and EXCEED studies aim to evaluate the safety and efficacy of ropeginterferon alfa-2b in the treatment of essential thrombocythemia (ET) across treatment lines, including treatment-naïve and pretreated populations.

### Methods

Data of 182 patients were analyzed from the phase 3, randomized active-controlled study SURPASS-ET (ropeginterferon alfa-2b vs anagrelide in hydroxyurea-resistant/intolerant high-risk ET) and the phase 2b single-arm EXCEED-ET study, which enrolled both treatment-naïve and previously treated patients in North America. Endpoints included thromboembolic events, durable modified European LeukemiaNet (ELN) response, hematologic control, molecular response (*JAK2*V617F, *CALR*, *MPL*), and safety/tolerability.

**Copyright Information:** (Online) ISSN: 2572-9241
©2026 The Author(s). HemaSphere is published by John Wiley & Sons Ltd on behalf of European Hematology Association. This is an open access Abstract Book distributed under the Attribution-NonCommercial-NoDerivs (CC BY-NC-ND), which allows third parties to download the articles and share them with others as long as they credit the author and the Abstract Book, but they cannot change the content in any way or use them commercially.
**Abstract Book Citations:** Authors, Title, HemaSphere, 2026;10:(S1):pages. The abstract book DOIs can be found on the "Author Information" page.
**Disclaimer:** Articles published in the journal HemaSphere exclusively reflect the opinions of the authors. The authors are responsible for all content in their abstracts including accuracy of the facts, statements, citing resources, etc.

**Results**

In SURPASS-ET, ropeginterferon alfa-2b achieved a significantly higher durable modified ELN response at months 9 and 12 compared with anagrelide (42.9% vs 6.0%; p<0.0001), with superior platelet and white blood cell control, significant reductions in *JAK2*V617F allelic burden, and fewer thromboembolic events (1.2% vs 19.6%). In EXCEED-ET, durable objective modified ELN responses of up to 60.2% were observed, confirming robust hematologic and molecular response in a North American population (Table 1). The rates of molecular response as defined by ELN2009 criteria were 35.0% (7/20) for patients with *JAK2*V617F, 16.0% (4/25) for *CALR*, and 25.0% (2/8) for *MPL* mutations. Importantly, clinically meaningful responses were demonstrated in both treatment-naïve and previously treated patients, supporting the expectation of benefit across lines of therapy. Efficacy was consistent across driver mutation subtypes. Analysis of non-driver mutations supported the use of ropeginterferon alfa-2b.
An integrated safety analysis across studies showed that grade ≥3 treatment emergent adverse events (TEAEs) occurred in 28.6% and 33.8% patients receiving ropeginterferon alfa-2b and anagrelide, respectively. Low rates of TEAE leading to treatment discontinuation (8.8% vs 20.0%) and serious adverse events (12.6% vs 30.0%) were observed in ropeginterferon alfa-2b group. AEs with fatal outcome were observed only in three (3.8%) patients receiving anagrelide but not in ropeginterferon alfa-2b group.

**Summary/Conclusion**

Findings from SURPASS-ET and EXCEED-ET show that ropeginterferon alfa-2b delivers durable hematologic control and molecular activity with acceptable tolerability across lines of therapy, geographic populations, and driver-mutation subtypes. These data support ropeginterferon alfa-2b as a disease-modifying treatment option in ET.

**Copyright Information:** (Online) ISSN: 2572-9241
©2026 The Author(s). HemaSphere is published by John Wiley & Sons Ltd on behalf of European Hematology Association. This is an open access Abstract Book distributed under the Attribution-NonCommercial-NoDerivs (CC BY-NC-ND), which allows third parties to download the articles and share them with others as long as they credit the author and the Abstract Book, but they cannot change the content in any way or use them commercially.
**Abstract Book Citations:** Authors, Title, HemaSphere, 2026;10:(S1):pages. The abstract book DOIs can be found on the “Author Information” page.
**Disclaimer:** Articles published in the journal HemaSphere exclusively reflect the opinions of the authors. The authors are responsible for all content in their abstracts including accuracy of the facts, statements, citing resources, etc.

### (PS1987) PELABRESIB MONOTHERAPY IN MYELOFIBROSIS AFTER JANUS KINASE INHIBITOR FAILURE: RESULTS FROM ARM 1 OF THE OPEN-LABEL, PHASE 2 MANIFEST STUDY

**Topic:** 16. Myeloproliferative neoplasms - Clinical

Moshe Talpaz*[1], Marina Kremyanskaya[2], Raajit K Rampal[3], Claire Harrison[4], Vikas Gupta[5], John Mascarenhas[2], Ruben Mesa[6], Andrew Kuykendall[7], Francesca Palandri[8], Joseph M Scandura[9], Prithviraj Bose[10], Gary Schiller[11], Claudia Gorcea-Carson[12], Monika Wroclawska[13], Grace Cleary[14], Soorya Surendran[15], Alessandro Maria Vannucchi[16]

*[1]University of Michigan, Ann Arbor, MI, United States of America; [2]Tisch Cancer Institute, New York, NY, United States of America; [3]Memorial Sloan Kettering Cancer Center, New York, NY, United States of America;[4]Guy's and St. Thomas' NHS Foundation Trust, London, United Kingdom; [5]Princess Margaret Cancer Centre, Toronto, Canada;[6]Wake Forest University School of Medicine, Winston-Salem, NC, United States of America; [7]Moffitt Cancer Center, Tampa, FL, United States of America; [8]IRCCS Azienda Ospedaliero-Universitaria di Bologna, Istituto di Ematologia "Seràgnoli", Bologna, Italy;[9]Weill Cornell Medicine, New York, NY, United States of America; [10]University of Texas MD Anderson Cancer Center, Houston, TX, United States of America; [11]David Geffen School of Medicine at UCLA (University of California, Los Angeles), Los Angeles, CA, United States of America; [12]Novartis Pharmaceuticals UK Limited, London, United Kingdom; [13]Novartis Pharma AG, Basel, Switzerland; [14]Novartis Ireland Limited, Dublin, Ireland; [15]Novartis Pharmaceuticals Corporation, Hyderabad, India; [16]Azienda Ospedaliero-Universitaria Careggi, University of Florence, Florence, Italy;*

### Background

Myelofibrosis (MF) is a chronic, progressive myeloproliferative neoplasm linked to dysregulated Janus kinase (JAK)-STAT signaling, characterized by splenomegaly, cytopenias, and substantial symptom burden (Mughal. *Int J Gen Med.* 2014). Monotherapy JAK inhibitors (JAKi), as current standard of care, offer inadequate durability of treatment response in a proportion of patients (Thaw. *Curr Hematol Malig Rep.* 2024). JAKi failure is associated with poor outcomes and reduced survival (Gill. *Hematology Am Soc Hematol Educ Program.* 2023), highlighting the unmet need for novel therapeutic approaches offering deep, durable responses, and potential to alter MF disease biology. Pelabresib (PELA), an oral, investigational, small molecule bromodomain and extra-terminal domain inhibitor, epigenetically modulates the dysregulated transcriptional and inflammatory pathways implicated in MF biology (Gomes & Harrison. *Curr Hematol Malig Rep.* 2023). Arm 1 of the 4-arm, open-label, Phase 2 MANIFEST study (NCT02158858) investigated PELA monotherapy in primary or post-essential thrombocythemia/-polycythemia vera MF.

### Aims

To evaluate the safety, tolerability, and clinical activity of PELA monotherapy in JAKi-pretreated or JAKi-ineligible MF patients.

### Methods

Arm 1 patients were relapsed/refractory, intolerant, or ineligible for JAKi therapy and were assigned by transfusion dependence (TD) into cohorts 1A (TD) or 1B (non-TD). Patients received a starting dose of PELA 125 mg once daily for 14 days of every 21-day cycle. Key inclusion criteria included dynamic international prognostic scoring system intermediate 2 (INT-2) or high-risk (HR), platelets $\geq 75\times10^9$/L, blasts <10%, and ≥2 measurable MF symptoms using the Myelofibrosis Symptom Assessment Form v4.0; 1A required ≥6 red blood cell transfusions over the prior 12 wks; 1B required spleen volume ≥450 $cm^3$. Primary endpoints were TD to transfusion independence (TI; no transfusion for ≥12 wks) (1A) and ≥35% spleen volume reduction (SVR35) at wk24 (1B). Secondary and exploratory endpoints included ≥50% symptom improvement (TSS50), bone marrow fibrosis (BMF) improvement, and safety.

**Copyright Information:** (Online) ISSN: 2572-9241
©2026 The Author(s). HemaSphere is published by John Wiley & Sons Ltd on behalf of European Hematology Association. This is an open access Abstract Book distributed under the Attribution-NonCommercial-NoDerivs (CC BY-NC-ND), which allows third parties to download the articles and share them with others as long as they credit the author and the Abstract Book, but they cannot change the content in any way or use them commercially.
**Abstract Book Citations:** Authors, Title, HemaSphere, 2026;10:(S1):pages. The abstract book DOIs can be found on the "Author Information" page.
**Disclaimer:** Articles published in the journal HemaSphere exclusively reflect the opinions of the authors. The authors are responsible for all content in their abstracts including accuracy of the facts, statements, citing resources, etc.

**Results**

A total of 100 patients (1A = 48, 1B = 52) were treated for a median duration of 46 wks (1, 364). Baseline characteristics showed a heavily pretreated, advanced MF population with substantial symptom and spleen burden: median age 71 years, 90% INT-2/HR, median spleen volume 1794 $cm^3$ (281, 7371), median TSS 21 (1, 56), median hemoglobin 8.8 g/dL (6.1, 15.3). PELA demonstrated clinical activity in both cohorts with 26% (95% CI 13.5, 41.2; 11/43) TI in 1A and 19% (95% CI 9.6, 32.5; 10/52) SVR35 in 1B at wk24. Across both cohorts, TSS50 was 26% (24/94) at wk24, reflecting meaningful symptom improvement; mean absolute change was –8.92. Among evaluable patients, BMF improved by ≥1 grade in 18/54 (33.3%) at wk24 and 10/31 (32.3%) at wk48. Hemoglobin response was achieved in 24/52 (47.1%) patients in 1B. The safety profile was manageable; most common grade ≥3 treatment-emergent adverse events were anemia (24.0%) and thrombocytopenia (16.0%).

**Summary/Conclusion**

PELA monotherapy demonstrated clinically meaningful single-agent activity and manageable safety profile in advanced MF patients, with improvements across MF hallmarks of splenomegaly, symptoms, anemia, and BMF. These results support the biological and clinical activity of PELA in this difficult-to-treat patient population and the continued development of PELA in MF.

**Copyright Information:** (Online) ISSN: 2572-9241
©2026 The Author(s). HemaSphere is published by John Wiley & Sons Ltd on behalf of European Hematology Association. This is an open access Abstract Book distributed under the Attribution-NonCommercial-NoDerivs (CC BY-NC-ND), which allows third parties to download the articles and share them with others as long as they credit the author and the Abstract Book, but they cannot change the content in any way or use them commercially.
**Abstract Book Citations:** Authors, Title, HemaSphere, 2026;10:(S1):pages. The abstract book DOIs can be found on the "Author Information" page.
**Disclaimer:** Articles published in the journal HemaSphere exclusively reflect the opinions of the authors. The authors are responsible for all content in their abstracts including accuracy of the facts, statements, citing resources, etc.

### (PS1988) SPLICING MUTATIONS PREDICT ANEMIA PROGRESSION AND INFERIOR SURVIVAL IN RUXOLITINIB-TREATED MYELOFIBROSIS PATIENTS

**Topic:** 16. Myeloproliferative neoplasms - Clinical

Chiara Sartor*[1], Filippo Branzanti[2], Massimiliano Bonifacio[3], Elena Elli[4], Elisabetta Abruzzese[5], Mario Tiribelli[6], Erika Morsia[7], Mirko Farina[8], Giulia Benevolo[9], Eloise Beggiato[10], Novella Pugliese[11], Alessia Tieghi[12], Emilia Scalzulli[13], Francesco Cavazzini[14], Alessandra Iurlo[15], Monica Crugnola[16], Andrea Duminuco[17], Daniele Cattaneo[15], Francesca Duca[4], Costanza Bosi[18], Alessandra Dedola[1, 2], Flavia Salvatore[1, 2], Daniele Cavallina[1, 2], Nicola Severi[1, 2], Daniela Cilloni[19], Monica Bocchia[20], Fabrizio Pane[11], Florian Heidel[21], Giuseppe Palumbo[22], Massimo Breccia[13], Francesca Palandri[1]

*[1]IRCCS Azienda Ospedaliero-Universitaria di Bologna, Istituto di Ematologia "Seràgnoli", Bologna, Italy;[2]Dipartimento di Scienze Mediche e Chirurgiche, Università di Bologna, Bologna, Italy; [3]Department of Engineering for Innovation Medicine, Section of Innovation Biomedicine, Hematology Area, University of Verona, Verona, Italy; [4]Fondazione IRCCS San Gerardo dei Tintori, Divisione di ematologia e unità trapianto di midollo, Monza, Italy; [5]Division of Hematology, Ospedale S. Eugenio, Roma, Italy; [6]Division of Hematology and BMT, Department of Medical Area, University of Udine, Udine, Italy;[7]Hematology Unit, Department of Clinical and Molecular Sciences, DISCLIMO, Università Politecnica delle Marche, Ancona, Italy; [8]Unit of Blood Diseases and Stem Cells Transplantation, Department of Clinical and Experimental Sciences, University of Brescia, ASST Spedali Civili of Brescia, Brescia, Italy; [9]University Hematology Division, Città della Salute e della Scienza Hospital, Torino, Italy; [10]Unit of Hematology, Department of Oncology, University of Torino,, Torino, Italy; [11]Department of Clinical Medicine and Surgery, Federico II University Medical School, Napoli, Italy; [12]Department of Hematology, Azienda USL - IRCCS di Reggio Emilia, Reggio Emilia, Italy; [13]Hematology, Department of Translational and Precision Medicine, Az. Policlinico Umberto I-Sapienza University,, Roma, Italy; [14]Division of Hematology, University of Ferrara, Ferrara, Italy;[15]Hematology Division, Foundation IRCCS Ca' Granda Ospedale Maggiore Policlinico, Milano, Italy; [16]Hematology and BMT Centre, Azienda Ospedaliero-Universitaria di Parma, Parma, Italy; [17]UO Ematologia, AOU Policlinico "G. Rodolico" – San Marco, Catania, Italy; [18]Division of Hematology, AUSL di Piacenza, Piacenza, Italy; [19]Department of Clinical and Biological Sciences, University of Turin, Torino, Italy;[20]Hematology Unit, Azienda Ospedaliera Universitaria Senese, University of Siena, Siena, Italy; [21]Hematology, Hemostasis, Oncology and Stem Cell Transplantation, Hannover Medical School (MHH), Hannover, Germany; [22]Department of Scienze Mediche, Chirurgiche e Tecnologie Avanzate "G.F. Ingrassia", University of Catania, Catania, Italy;*

### Background

Mutations in the splicing machinery—including *U2AF1, SRSF2, SF3B1*, and *ZRSR2*—play a cross-cutting role across myeloid diseases, occurring in >50% of myelodysplastic syndromes (MDS), in 15–40% of myelofibrosis (MF) and defining the secondary ontogeny of acute myeloid leukemia.
Recently, the therapeutic armamentarium of MF has largely overlapped with MDS, as anemia represents a major challenge—especially in the cytopenic MF phenotype. In MF, anemia—baseline, worsening, or ruxolitinib (RUX)–emergent—carries strong prognostic relevance. While *U2AF1* and *SRSF2* mutations have established prognostic value in primary (P)MF and secondary (S)MF, the impact of the broader spectrum of splicing mutations (SpM) on anemia dynamics and survival remains unclear.

### Aims

To assess the impact of SpM on worsening anemia, RUX-emergent anemia and outcome in RUX-treated MF patients (pts).

**Copyright Information:** (Online) ISSN: 2572-9241
©2026 The Author(s). HemaSphere is published by John Wiley & Sons Ltd on behalf of European Hematology Association. This is an open access Abstract Book distributed under the Attribution-NonCommercial-NoDerivs (CC BY-NC-ND), which allows third parties to download the articles and share them with others as long as they credit the author and the Abstract Book, but they cannot change the content in any way or use them commercially.
**Abstract Book Citations:** Authors, Title, HemaSphere, 2026;10:(S1):pages. The abstract book DOIs can be found on the "Author Information" page.
**Disclaimer:** Articles published in the journal HemaSphere exclusively reflect the opinions of the authors. The authors are responsible for all content in their abstracts including accuracy of the facts, statements, citing resources, etc.

### Methods

This sub-analysis of the multicenter real-world RUX-MF study (NCT06516406) included 243 MF pts with NGS data. Baseline anemia was defined as Hb <10 g/dL; worsening anemia as Hb decline ≥1.5 g/dL; RUX-emergent anemia as Hb <10 g/dL and/or transfusion requirement at 6 months; thrombocytopenia as platelets (plt) < 100 x $10^9$/L. High molecular risk mutations (HMRm) were defined according to MIPSS70v2 (PMF) or MYSEC-mPM (SMF).
Survival was estimated using left-truncated Kaplan–Meier analysis accounting for time from diagnosis to RUX start: OS was assessed from RUX start to death, blast-phase free survival (BPFS) to BP, progression free survival (PFS) to BP or death.

### Results

Among 243 MF pts, 55 (22.6%) carried SpM: 23 (9.5%) *SRSF2*, 17 (7.0%) *U2AF1*, 9 (3.7%) *ZRSR2*, and 8 (3.3%) *SF3B1*; 2 pts carried co-mutations (*U2AF1/ZRSR2* and *SF3B1/ZRSR2*). Median age was similar between SpM and SpM-wild-type (SpMwt) groups (64.6 vs 63.6 years).
Overall, 136 (56%) were male, with male sex enriched in SpM carriers (70.8% vs 51.6%; p .01). MF distribution included 121 PMF (49.8%), with SpM more frequent in PMF (63.6% vs 45.7%, p .048). No significant differences emerged in driver mutations, leukocytes, peripheral blasts, marrow fibrosis, symptom burden, spleen size, transfusion requirement, DIPSS/MYSEC distribution or RUX starting dose.
Conversely, baseline Hb and Plt were lower in SpM pts (Hb 10.8 vs 11.5 g/dL, p .037; plt 260 vs 375 ×$10^9$/L, p .005). Among HMRm, *ASXL1* mutation was more common in SpM pts vs SpMwt (52.7% vs 36.7%, p .033), as was*IDH1* (7.3% vs 1.1%, p .009). Anemia rates were similar (SpM 43.6% vs SpMwt 29.3%, p .055), but thrombocytopenia differed (SpM 10.9% vs SpMwt 3.7%, p .037).
Nonetheless, anemia prevalence at 6 months was higher in SpM (60% vs 37.2%, p .003). Indeed, SpM carriers showed a significantly higher incidence of RUX-emergent or worsening anemia at 6 months (36.4% vs 22.9%; OR 1.93, 95%CI 1.01–3.68, p .047), but not of thrombocytopenia (p .47).
Median OS was shorter in SpM pts (5.1 vs 8.1 years; HR 1.68, 95%CI 1.07–2.64, p .023), as was PFS (4.7 vs 8.1 years; HR 1.83, 95%CI 1.19–2.91, p .006) (**Figure**). Five-year BPFS was also inferior (70.0% vs 91.3%, p .003). All associations remained significant after adjusting for concomitant HMRm.

### Summary/Conclusion

MF pts harboring SpM exhibit a cytopenic phenotype and a two-fold higher risk for RUX-emergent and worsening anemia, identifying a vulnerable MDS-like molecularly defined subgroup with inferior outcomes and higher progression risk.  These findings support considering alternative, anemia-focused strategies for this vulnerable patient subset.

**Copyright Information:** (Online) ISSN: 2572-9241
©2026 The Author(s). HemaSphere is published by John Wiley & Sons Ltd on behalf of European Hematology Association. This is an open access Abstract Book distributed under the Attribution-NonCommercial-NoDerivs (CC BY-NC-ND), which allows third parties to download the articles and share them with others as long as they credit the author and the Abstract Book, but they cannot change the content in any way or use them commercially.
**Abstract Book Citations:** Authors, Title, HemaSphere, 2026;10:(S1):pages. The abstract book DOIs can be found on the "Author Information" page.
**Disclaimer:** Articles published in the journal HemaSphere exclusively reflect the opinions of the authors. The authors are responsible for all content in their abstracts including accuracy of the facts, statements, citing resources, etc.

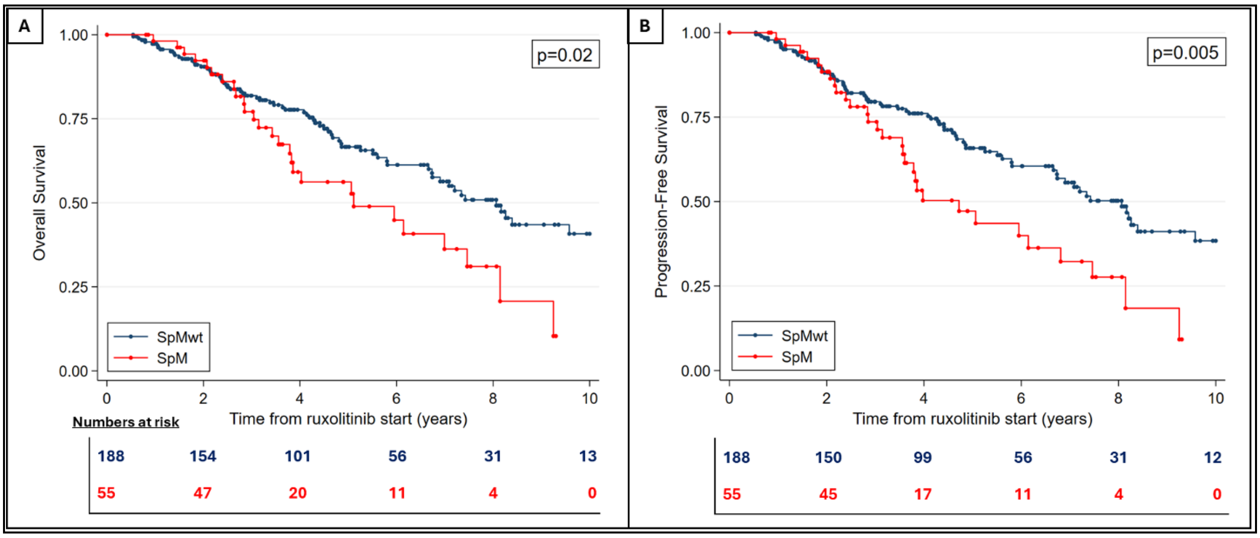

Figure: Kaplan-Meier Curves of Overall Survival (A) and Progression-Free Survival (B) by presence of splicing mutations (SpM)

**Copyright Information:** (Online) ISSN: 2572-9241
©2026 The Author(s). HemaSphere is published by John Wiley & Sons Ltd on behalf of European Hematology Association. This is an open access Abstract Book distributed under the Attribution-NonCommercial-NoDerivs (CC BY-NC-ND), which allows third parties to download the articles and share them with others as long as they credit the author and the Abstract Book, but they cannot change the content in any way or use them commercially.
**Abstract Book Citations:** Authors, Title, HemaSphere, 2026;10:(S1):pages. The abstract book DOIs can be found on the "Author Information" page.
**Disclaimer:** Articles published in the journal HemaSphere exclusively reflect the opinions of the authors. The authors are responsible for all content in their abstracts including accuracy of the facts, statements, citing resources, etc.

### (PS1989) QRISK3 SCORE REINFORCES ITS ROLE IN MPNWITH THE STANDARD 60-YEAR CUT-OFF AND PROVIDES ADDITIVE PROGNOSTIC VALUE TO REVISED-IPSET IN ESSENTIAL THROMBOCYTHEMIA

**Topic:** 16. Myeloproliferative neoplasms - Clinical

Andrea Duminuco*[1], Raj Vaghela[2], Sukhraj Virdee[2], Claire Woodley[2], Susan Asirvatham[2], Natalia Curto-Garcia[2], Benissa Narciso[2], Priya Sriskandarajah[2], Jennifer O'sullivan[2], Hugues De Lavallade[2], Deepti Radia[2], Shahram Kordasti[2], Giuseppe A. Palumbo[1], Claire Harrison[2], Patrick Harrington[2]

*[1]AOU Policlinico "G.Rodolico-San Marco", Catania, Italy; [2]Guy's and St Thomas NHS Foundation Trust, London, United Kingdom;*

**Background**

Thrombotic events (TE) are the leading cause of death in patients with PV and ET. Conventional risk models, based on age and prior thrombosis, incompletely capture cardiovascular (CV) risk. QRISK3 is a prognostic calculator designed to assess an individual's likelihood of experiencing a CV event, incorporating clinical and demographic variables. A QRISK3 threshold of 7.5% can be used to define a high-risk group as per 2021 European Society of Cardiology guidelines. We previously showed that it can refine TE risk stratification in MPNs and that applying cytoreductive therapy to patients with QRISK3 ≥7.5% was associated with a significant reduction in TE.

**Aims**

To evaluate whether QRISK3 evaluation improves TE prediction in ET patients beyond conventional thrombotic risk assessment, and to assess its incremental prognostic value across JAK2 mutational subgroups as well as the revised IPSET-thrombosis score, which incorporates this alongside age and thrombotic history.

**Methods**

We performed additional analysis of our retrospective cohort of ET patients, ,using the inclusion criteria and outcome definitions described previously. Patients were classified as conventional low- or high-risk according to age and prior TE; ET patients were additionally stratified by revised IPSET-T and *JAK2* mutational status. QRISK3 was calculated at diagnosis, and thrombosis-free survival (TFS) was compared across QRISK3 thresholds and combined QRISK3/revised IPSET and QRISK3/*JAK2* strata.

**Copyright Information:** (Online) ISSN: 2572-9241
©2026 The Author(s). HemaSphere is published by John Wiley & Sons Ltd on behalf of European Hematology Association. This is an open access Abstract Book distributed under the Attribution-NonCommercial-NoDerivs (CC BY-NC-ND), which allows third parties to download the articles and share them with others as long as they credit the author and the Abstract Book, but they cannot change the content in any way or use them commercially.
**Abstract Book Citations:** Authors, Title, HemaSphere, 2026;10:(S1):pages. The abstract book DOIs can be found on the "Author Information" page.
**Disclaimer:** Articles published in the journal HemaSphere exclusively reflect the opinions of the authors. The authors are responsible for all content in their abstracts including accuracy of the facts, statements, citing resources, etc.

**Results**

A total of 490 patients with a diagnosis of ET with a median follow up of 85 months (range 3-481) were evaluated. In this group, a total of 46 TEs were recorded (8% in conventional low vs 12.1% in high risk; $p=0.14$); median QRISK3 score at the start of follow-up was higher in patients who experienced TE (10.1% vs 2.8%; $p<0.001$).
Concerning conventional risk score: 50 (15.4%) low risk patients had QRISK3>≥7.5%, and had 18 TEs (36%) versus 8 events (2.9%) among those with QRISK3<7.5%; they also had shorter TFS (277 months vs median not reached; $p<0.001$)
Among 424 ET patients with known driver-mutation status, 253 (59.7%) were *JAK2*-mutated. Combining *JAK2* status with QRISK3 identified four risk classes; TFS differed significantly across groups ($p<0.001$). Patients with QRISK3 ≥7.5% had markedly shorter TFS, in both *JAK2*-mutated and wild-type groups (median 277.1 and 193.1 months, respectively), whereas the median TFS was not reached in either low QRISK3 (<7.5%) group, regardless of *JAK2* status (Figure 1A).
Median TFS according to revised IPSET-T was 385 months for very low-risk (n=132); 397 months for low; not reached for intermediate (n=28), and 301 months for high (n=107, $p=0.04$). Grouping very low/low versus intermediate/high and further stratifying by QRISK3 <7.5% or ≥7.5% generated four distinct prognostic strata ($p<0.001$) where QRISK score was of greater relevance for TFS. Median TFS was 327 months for low IPSET–QRISK3 <7.5%, not reached for high IPSET–QRISK3 <7.5%, 277 for low IPSET–QRISK3 ≥7.5%, and 205 for high IPSET–QRISK3 ≥7.5% (Figure 1B).

**Summary/Conclusion**

QRISK3 provides substantial incremental prognostic information over revised IPSET in ET, identifying a significant subgroup of very low/low IPSET patients who may benefit from earlier cytoreductive intervention. We advise incorporating this assessment into routine clinical practice, particularly for ET patients with a very-low or low revised-IPSET score.

**Copyright Information:** (Online) ISSN: 2572-9241
©2026 The Author(s). HemaSphere is published by John Wiley & Sons Ltd on behalf of European Hematology Association. This is an open access Abstract Book distributed under the Attribution-NonCommercial-NoDerivs (CC BY-NC-ND), which allows third parties to download the articles and share them with others as long as they credit the author and the Abstract Book, but they cannot change the content in any way or use them commercially.
**Abstract Book Citations:** Authors, Title, HemaSphere, 2026;10:(S1):pages. The abstract book DOIs can be found on the "Author Information" page.
**Disclaimer:** Articles published in the journal HemaSphere exclusively reflect the opinions of the authors. The authors are responsible for all content in their abstracts including accuracy of the facts, statements, citing resources, etc.

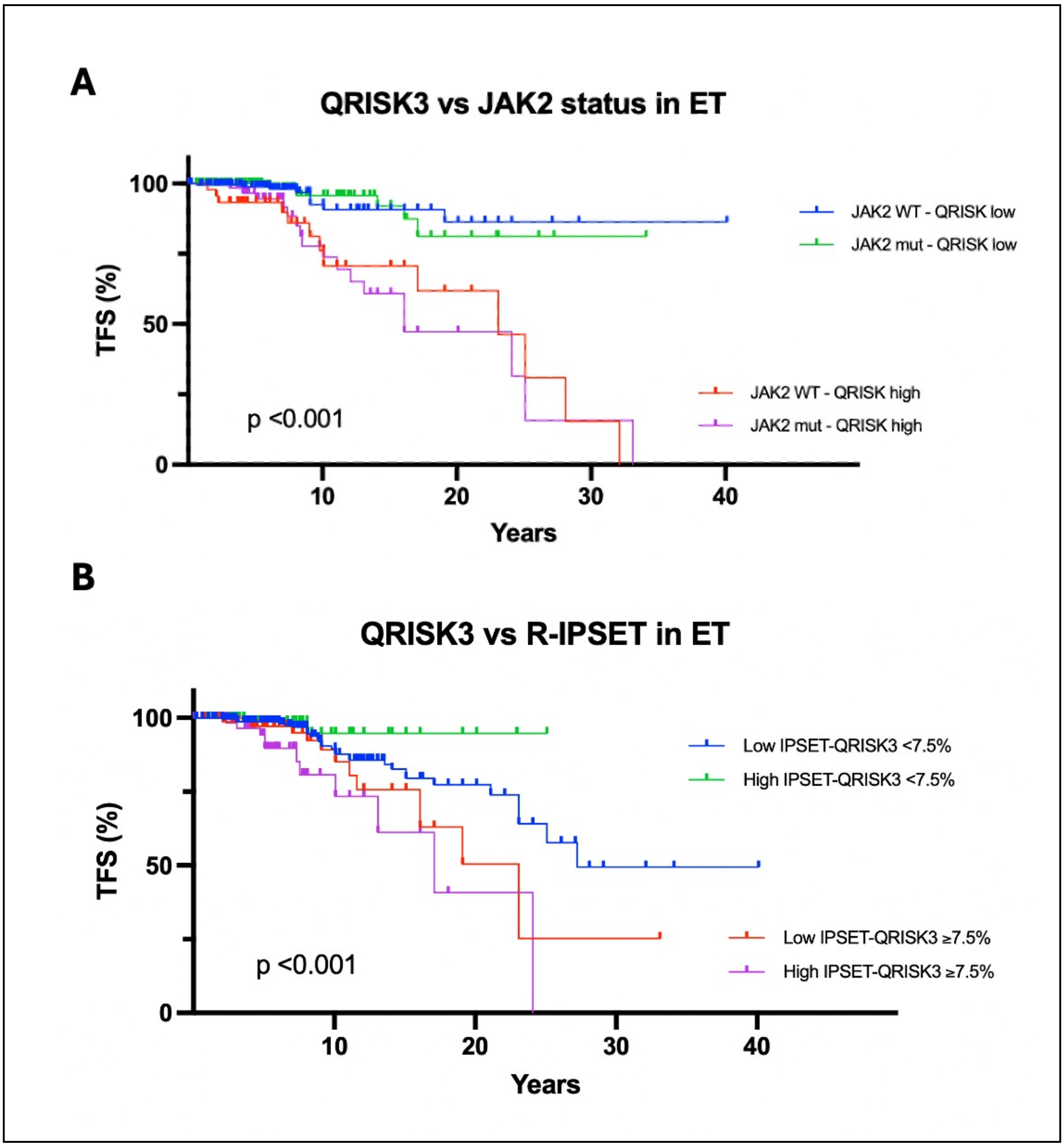

**Copyright Information:** (Online) ISSN: 2572-9241
©2026 The Author(s). HemaSphere is published by John Wiley & Sons Ltd on behalf of European Hematology Association. This is an open access Abstract Book distributed under the Attribution-NonCommercial-NoDerivs (CC BY-NC-ND), which allows third parties to download the articles and share them with others as long as they credit the author and the Abstract Book, but they cannot change the content in any way or use them commercially.
**Abstract Book Citations:** Authors, Title, HemaSphere, 2026;10:(S1):pages. The abstract book DOIs can be found on the "Author Information" page.
**Disclaimer:** Articles published in the journal HemaSphere exclusively reflect the opinions of the authors. The authors are responsible for all content in their abstracts including accuracy of the facts, statements, citing resources, etc.

### (PS1990) A NOVEL JAK2 INHIBITOR OB756 FOR POLYCYTHEMIA VERA WITH SPLENOMEGALY: A PHASE IB/II, MULTICENTER STUDY

**Topic:** 16. Myeloproliferative neoplasms - Clinical

Jian Huang*[1], Bingbing Wen[1, 2], Tiejun Gong[3], Hu Zhou[4], Lihong Shou[5], Qingchi Liu[6], Feng Zhang[7], Cuicui Wu[8], Wei Yang[9], Lidong Zhao[10], Lili Chen[11], Jian Lu[12], Yile Zhou[1], Yi Zhang[1], Jie Jin[1]

*[1]The First Affiliated Hospital, Zhejiang University School of Medicine, Hangzhou, China;[2]Department of Hematology, The Second People's Hospital of Shenzhen, The First Affiliated Hospital of Shenzhen University, Shenzhen, China; [3]Department of Hematology, The First Hospital of Harbin, Harbin, China; [4]Department of Hematology, Henan Provincial Cancer Hospital, Zhengzhou, China; [5]Department of Hematology,Huzhou Central Hospital, Huzhou, China;[6]Department of Hematology, The First Hospital of Hebei Medical University, Shijiazhuang, China; [7]Department of Hematology, The First Affiliated Hospital of Bengbu Medical College, Bengbu, China; [8]Department of Hematology, Yueyang People's Hospital, Yueyang, China;[9]Department of Hematology, Shengjing Hospital affiliated to China Medical University, Shenyang, China; [10]Department of Hematology, The First People's Hospital of Lianyungan, Lianyungan, China; [11]Department of Hematology, Taizhou First People's Hospital, Taizhou, China; [12]Hangzhou Biosun Pharamaceutical Co.,LTD, Hangzhou, China;*

### Background

OB756, is a novel oral, small molecule Janus kinase2JAK2inhibitor, was shown to have a clinical benefit in patients with myeloproliferative neoplasm (MPN) in phase 1 study. Here we conducted a phase Ib/II multicenter study to evaluate the safety and efficacy of OB756 in polycythemia vera (PV) patients with splenomegaly.

### Aims

We conducted a phase Ib/II multicenter study to evaluate the safety and efficacy of OB756 in polycythemia vera (PV) patients with splenomegaly.

### Methods

A multicenter, phase Ib/II trial (CTR20201950) was conducted at 11 sites in China. Eligible patients aged ≥18 years with polycythemia vera (PV) with splenomegaly intolerant or resistant to hydroxyurea or interferon were enrolled in this study. Patients were required to have Eastern Cooperative Oncology Group (ECOG) performance status ≤2. Patients were treated with OB756 16 mg or 20 mg twice daily in continuous 28-day cycles until lack of efficacy, intolerance, or disease progression. The primary endpoint was complete haematologic response (CHR) rate at 24 week. The secondary key endpoints included spleen volume reduction ≥35% (SVR35), haematologic response (HR), molecular response, and safety.

**Copyright Information:** (Online) ISSN: 2572-9241
©2026 The Author(s). HemaSphere is published by John Wiley & Sons Ltd on behalf of European Hematology Association. This is an open access Abstract Book distributed under the Attribution-NonCommercial-NoDerivs (CC BY-NC-ND), which allows third parties to download the articles and share them with others as long as they credit the author and the Abstract Book, but they cannot change the content in any way or use them commercially.
**Abstract Book Citations:** Authors, Title, HemaSphere, 2026;10:(S1):pages. The abstract book DOIs can be found on the "Author Information" page.
**Disclaimer:** Articles published in the journal HemaSphere exclusively reflect the opinions of the authors. The authors are responsible for all content in their abstracts including accuracy of the facts, statements, citing resources, etc.

**Results**

Between November 3, 2020 and January 20, 2026, 51 patients of PV with splenomegaly were enrolled and treated with either OB756 16 mg BID or 20 mg BID. Median treatment duration was 508 (range, 29~1066) days. At baseline, median age was 63 (range, 34-73) years, and 26/51 (52.0%) patients were men. Baseline median spleen volume was 633.8 (rang, 400~4015)cm$^3$. The percentages of intermediate and high risks based on survival risk status in PV patients were 35.3% (18/51) and 52.9% (27/51), respectively **(Figure A)**. A total of 42/51 (82.4%) evaluable patients met the primary endpoint of CHR at week 24. 26.2% (11/42) of patients achieved CHR at week 24, and 26.3% (10/38) of patients achieved CHR at week 48. In addition, 20/49 (40.8%), and 14/32 (43.8%) patients achieved CHR at EOC3, EOC9, respectively **(Figure B)**. High percentage of hematocrit control was achieved with 81.4% (35/43) at week 24. Similarly, HR rates was achieved with 41/50(82.0%), 37/46(80.4%), 28/30(93.3), and 37/39 (94.8%) at EOC3, EOC6, EOC9, and EOC12, respectively **(Figure C)**. Patients with splenomegaly at baseline achieved SVR35 was 65.9% (29/44) at week 24 **(Figure D)**. The median spleen reduction was -40.3% (-83.7% to -0.05%), -39.6% (-83.7% to -0.06%) at EOC6, and EOC12, respectively. MPN-TSS decreased ≥ 50% were recorded with 76.7% (23/30) at week 24 **(Figure F)**. The mean change in the JAK2-V617F allele burden from baseline to 48 week was −9.0% **(Figure E)**. The most common treatment-emergent adverse events (TEAEs) were 1~2 grade, and only 1/51 (2.0%) patients were observed with thrombocytopenia. No death was reported with related to disease progression of PV or OB756. TEAEs were generally manageable.

**Summary/Conclusion**

OB756 was well tolerated and showed meaningful clinical benefits in PV patients with splenomegaly, especially for quick spleen reduction. OB756 may be a new treatment option for PV patients with splenomegaly. Furthermore, a randomized double-blind phase 3 study is ongoing, aiming to assess the efficacy and safety of OB756 compared to hydroxyurea in patients with PV in China (CTR20240873).

Acknowledgement: This research was supported by the National Natural Science Foundation of China (No. 82570191) and the Zhejiang Provincial Health Highlevel Innovative Talent Project (2022-2026).

**Copyright Information:** (Online) ISSN: 2572-9241
©2026 The Author(s). HemaSphere is published by John Wiley & Sons Ltd on behalf of European Hematology Association. This is an open access Abstract Book distributed under the Attribution-NonCommercial-NoDerivs (CC BY-NC-ND), which allows third parties to download the articles and share them with others as long as they credit the author and the Abstract Book, but they cannot change the content in any way or use them commercially.
**Abstract Book Citations:** Authors, Title, HemaSphere, 2026;10:(S1):pages. The abstract book DOIs can be found on the “Author Information” page.
**Disclaimer:** Articles published in the journal HemaSphere exclusively reflect the opinions of the authors. The authors are responsible for all content in their abstracts including accuracy of the facts, statements, citing resources, etc.

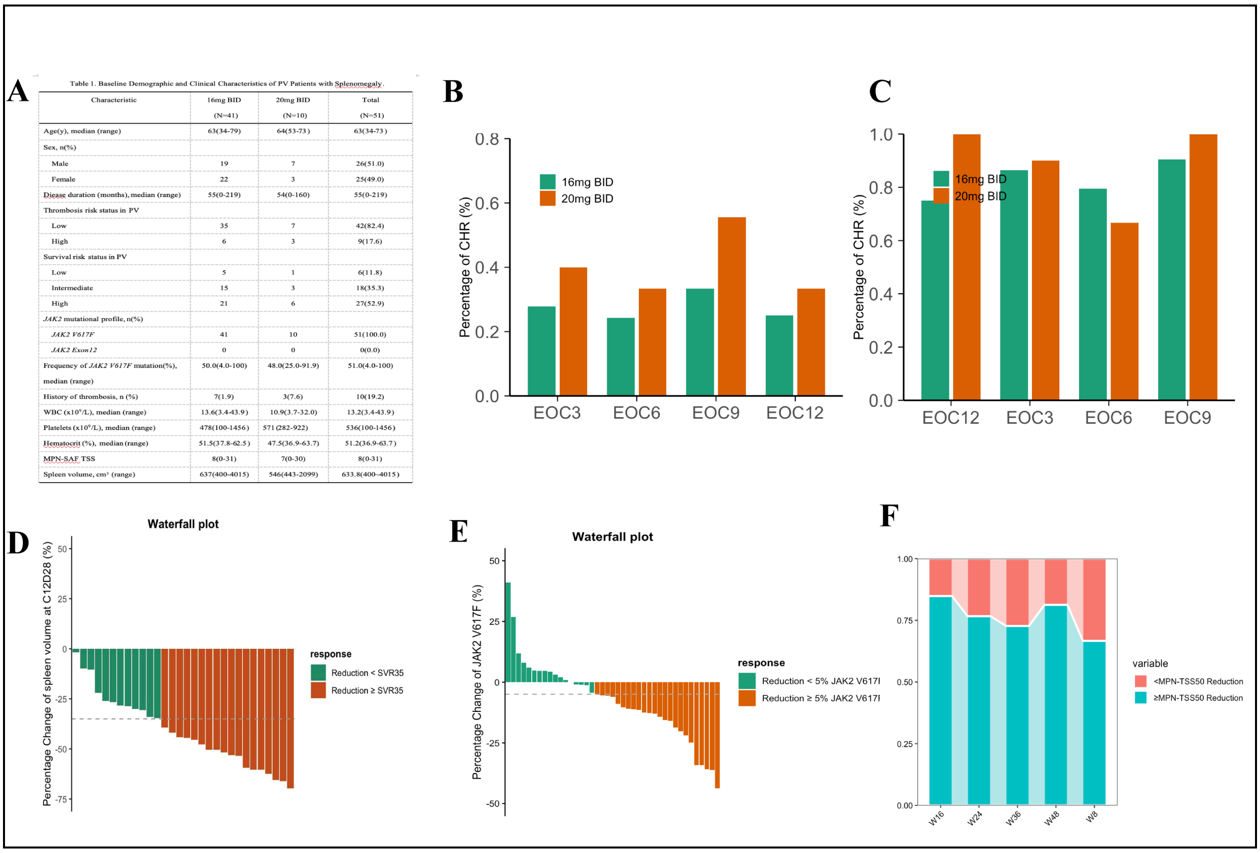

Table 1. Baseline Demographic and Clinical Characteristics of PV Patients with Splenomegaly.

| Characteristic | 16mg BID (N=41) | 20mg BID (N=10) | Total (N=51) |
|---|---|---|---|
| Age(y), median (range) | 63(34-79) | 64(53-73) | 63(34-73) |
| Sex, n(%) | | | |
| Male | 19 | 7 | 26(51.0) |
| Female | 22 | 3 | 25(49.0) |
| Diease duration (months), median (range) | 55(0-219) | 54(0-160) | 55(0-219) |
| Thrombosis risk status in PV | | | |
| Low | 35 | 7 | 42(82.4) |
| High | 6 | 3 | 9(17.6) |
| Survival risk status in PV | | | |
| Low | 5 | 1 | 6(11.8) |
| Intermediate | 15 | 3 | 18(35.3) |
| High | 21 | 6 | 27(52.9) |
| *JAK2* mutational profile, n(%) | | | |
| *JAK2 V617F* | 41 | 10 | 51(100.0) |
| *JAK2 Exon12* | 0 | 0 | 0(0.0) |
| Frequency of *JAK2 V617F* mutation(%), median (range) | 50.0(4.0-100) | 48.0(25.0-91.9) | 51.0(4.0-100) |
| History of thrombosis, n (%) | 7(1.9) | 3(7.6) | 10(19.2) |
| WBC (x$10^9$/L), median (range) | 13.6(3.4-43.9) | 10.9(3.7-32.0) | 13.2(3.4-43.9) |
| Platelets (x$10^9$/L), median (range) | 478(100-1456) | 571 (282-922) | 536(100-1456) |
| Hematocrit (%), median (range) | 51.5(37.8-62.5) | 47.5(36.9-63.7) | 51.2(36.9-63.7) |
| MPN-SAF TSS | 8(0-31) | 7(0-30) | 8(0-31) |
| Spleen volume, $cm^3$ (range) | 637(400-4015) | 546(443-2099) | 633.8(400~4015) |

**Copyright Information:** (Online) ISSN: 2572-9241
©2026 The Author(s). HemaSphere is published by John Wiley & Sons Ltd on behalf of European Hematology Association. This is an open access Abstract Book distributed under the Attribution-NonCommercial-NoDerivs (CC BY-NC-ND), which allows third parties to download the articles and share them with others as long as they credit the author and the Abstract Book, but they cannot change the content in any way or use them commercially.
**Abstract Book Citations:** Authors, Title, HemaSphere, 2026;10:(S1):pages. The abstract book DOIs can be found on the “Author Information” page.
**Disclaimer:** Articles published in the journal HemaSphere exclusively reflect the opinions of the authors. The authors are responsible for all content in their abstracts including accuracy of the facts, statements, citing resources, etc.

### (PS1991) ANTITHROMBOTIC AND CYTOREDUCTION STRATEGIES IN MYELOPROLIFERATIVE NEOPLASM–ASSOCIATED SPLANCHNIC VEIN THROMBOSIS: A RETROSPECTIVE STUDY FROM THE FRENCH INTERGROUP OF MYELOPROLIFERATIVE NEOPLASMS

**Topic:** 16. Myeloproliferative neoplasms - Clinical

Alexandre Guy[1, 2], Camille Hache[3], Jean Christophe Ianotto[4], Eric Lippert[5], Louis Drevon[6], Stephane Giraudier[6], Francoise Boyer Perrard[7], Corentin Orvain[7], Vincent Alcazer[8], Laurence Legros[9], Mathieu Wemeau[10], Dana Ranta[11], Geoffroy Venton[12], Lydia Roy[13], François Girodon[14], Franck Emmanuel Nicolini[15], Damien Luque-Paz[16], Jean-Jacques Kiladjian[17], Juliette Soret-Dulphy*[17], Chloe James[1, 2]

*[1]Univ. Bordeaux, INSERM, BMC, U1034, Pessac, France, Pessac, France;[2]Laboratory of Hematology, Bordeaux University Hospital, Pessac, France; [3]Department of vascular medicine, Bordeaux University Hospital, Bordeaux, France; [4]Department of Hematology and Clinical Hemostasis, Brest University Hospital, Brest, France, Brest, France; [5]Laboratory of Hematology, Brest University Hospital, Brest, France, Brest, France; [6]Université Paris-Cité, Hôpital Saint Louis: Hematological Functional Tests and Cell Biology Laboratory, INSERM U1131, Paris, France; [7]Department of Clinical Hematology and Cellular Therapy, Angers University Hospital, Angers, France, Angers, France; [8]Department of Hematology, Lyon University Hospital, Lyon, France; [9]Department of Clinical Hematology, APHP Paul Brousse Hospital, Villejuif, France; [10]Department of clinical hematology, Roubaix Hospital Center, Roubaix, France; [11]Department of Internal Medicine and Hematology, Nancy University Hospital, Nancy, France; [12]Department of Clinical Hematology, APHM, Marseille, France; [13]Department of Clinical Hematology and Cellular Therapy, APHP Henri Mondor University Hospital, Créteil, France; [14]Laboratory of Hematology, Dijon University Hospital, Dijon, France; [15]Department of Clinical Hematology, Léon Bérard Center, Lyon, France; [16]Univ Angers, Nantes Université, CHU Angers, Inserm, CNRS, CRCI2NA, Angers, France; [17]Clinical Investigations Center, APHP Saint-Louis Hospital, Paris, France;*

### Background

Splanchnic vein thrombosis (SVT) occurs more frequently in patients with myeloproliferative neoplasms (MPNs). Management of MPN-associated SVT remains challenging, as there is no consensus on the optimal diagnostic criteria for underlying MPNs, the appropriate cytoreductive treatment targets, or the choice of anticoagulant therapy. Moreover, data on the efficacy and safety of direct oral anticoagulants (DOACs) in this setting remain limited.

### Aims

Evaluate the impact of cytoreductive therapy and antithrombotic strategies on thrombotic recurrence and bleeding risk in MPN patients with SVT.

### Methods

We conducted a retrospective study including patients with BCR:ABL1-neg MPN and SVT. Patients had given their informed consent. Baseline characteristics at SVT included age, sex, MPN subtype, driver mutation, allelic burden, history of thrombosis, and SVT localization. Outcomes during follow-up were thrombotic recurrence, clinically relevant bleeding, MPN progression, and death. Antithrombotic and cytoreductive treatments were recorded. Multivariable Cox proportional hazards models estimated hazard ratios (HRs) for recurrence, and multivariable logistic regression identified bleeding-associated factors. Time-to-event analysis was performed using the Kaplan-Meier method to estimate rethrombosis-free survival and differences between groups were assessed using log-rank test and pairwise comparisons

**Copyright Information:** (Online) ISSN: 2572-9241
©2026 The Author(s). HemaSphere is published by John Wiley & Sons Ltd on behalf of European Hematology Association. This is an open access Abstract Book distributed under the Attribution-NonCommercial-NoDerivs (CC BY-NC-ND), which allows third parties to download the articles and share them with others as long as they credit the author and the Abstract Book, but they cannot change the content in any way or use them commercially.
**Abstract Book Citations:** Authors, Title, HemaSphere, 2026;10:(S1):pages. The abstract book DOIs can be found on the “Author Information” page.
**Disclaimer:** Articles published in the journal HemaSphere exclusively reflect the opinions of the authors. The authors are responsible for all content in their abstracts including accuracy of the facts, statements, citing resources, etc.

**Results**

358 patients were included (302 portal vein thrombosis, 56 Budd–Chiari syndrome). First-line anticoagulation consisted of vitamin K antagonists (65.6%), DOACs (20.4%), low-molecular-weight heparin (7.3%), while 6.7% received none. First-line CR included hydroxyurea (68.7%), interferon (20.7%), ruxolitinib (1.7%), other agents (2.0%), and none in 6.9%. Thrombotic recurrence occurred in 45 patients (12.6%). The incidence of rethrombosis was 1.7 and 3.4 per 100 patient-years in VKA-treated and DOAC-treated patients, respectively. Multivariate analysis showed that interferon (HR: 0.33, p=0.01) and hydroxyurea (HR: 0.29, p=0.0003) were associated with decreased risk of recurrence versus no CR, whereas DOACs (HR: 2.6, p=0.04) was associated with increased risk of recurrence compared with VKA. Interestingly, in univariate analysis, a platelet count <250 G/L at one year after initiation of cytoreductive therapy was associated with a significantly lower risk of recurrence (HR 0.27; p = 0.005), without an increased risk of bleeding. Survival analysis comparing patients with platelet count < or >250 G/L (with either DOACs or VKAs) revealed that the platelet count was the main determinant of recurrent thrombosis and that a platelet count <250G/L was associated, independently of anticoagulation therapy, with decreased rethrombosis (HR: 0,21, p=0.01) ( **Figure**). Esophageal varices were independently associated with clinically relevant bleeding (OR 4.01, p<0.0001).

**Summary/Conclusion**

This study is the largest to date exploring the efficacy and safety of cytoreduction and antithrombotic strategies in MPN-associated SVT. We showed that cytoreduction reduced thrombotic recurrence, and that a platelet count <250 G/L was associated with lower rethrombosis risk without excess bleeding. Cytoreduction and platelet count appear to be the primary factors associated with the risk of recurrent thrombosis. However, our data suggest that caution is warranted when using DOACs as initial anticoagulation therapy. Prospective studies are thus needed to define optimal anticoagulation strategies in this high-risk population.

**Copyright Information:** (Online) ISSN: 2572-9241
©2026 The Author(s). HemaSphere is published by John Wiley & Sons Ltd on behalf of European Hematology Association. This is an open access Abstract Book distributed under the Attribution-NonCommercial-NoDerivs (CC BY-NC-ND), which allows third parties to download the articles and share them with others as long as they credit the author and the Abstract Book, but they cannot change the content in any way or use them commercially.
**Abstract Book Citations:** Authors, Title, HemaSphere, 2026;10:(S1):pages. The abstract book DOIs can be found on the "Author Information" page.
**Disclaimer:** Articles published in the journal HemaSphere exclusively reflect the opinions of the authors. The authors are responsible for all content in their abstracts including accuracy of the facts, statements, citing resources, etc.

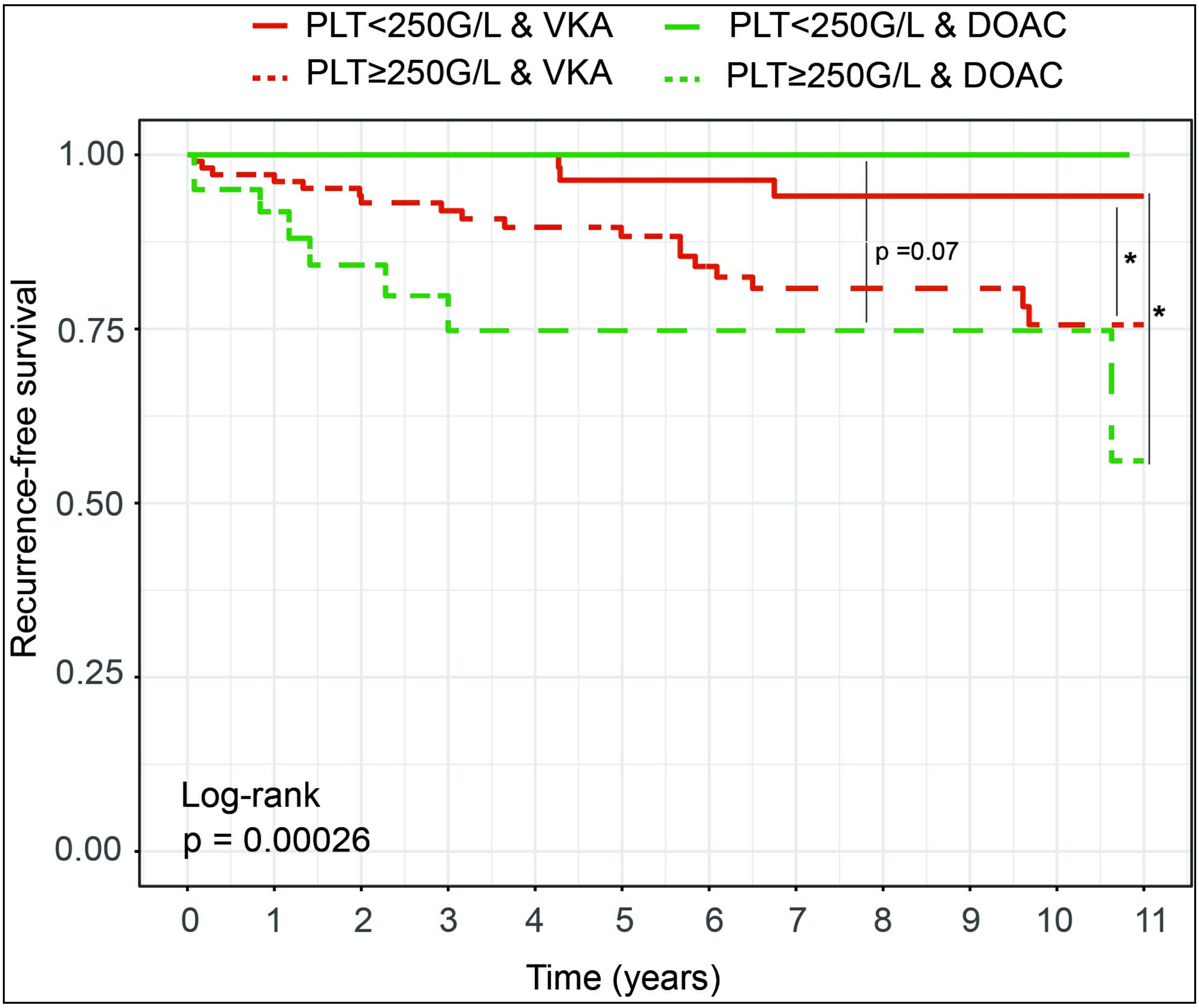

Figure: recurrence free survival analysis comparing MPN patients with SVT according to platelet count (< or >250 G/L) and anticoagulation therapy (DOACs or VKAs)

**Copyright Information:** (Online) ISSN: 2572-9241
©2026 The Author(s). HemaSphere is published by John Wiley & Sons Ltd on behalf of European Hematology Association. This is an open access Abstract Book distributed under the Attribution-NonCommercial-NoDerivs (CC BY-NC-ND), which allows third parties to download the articles and share them with others as long as they credit the author and the Abstract Book, but they cannot change the content in any way or use them commercially.
**Abstract Book Citations:** Authors, Title, HemaSphere, 2026;10:(S1):pages. The abstract book DOIs can be found on the “Author Information” page.
**Disclaimer:** Articles published in the journal HemaSphere exclusively reflect the opinions of the authors. The authors are responsible for all content in their abstracts including accuracy of the facts, statements, citing resources, etc.

### (PS1992) ESTIMATED PULSE WAVE VELOCITY (EPWV) INDEPENDENTLY PREDICTS CARDIOVASCULAR EVENTS IN BCR::ABL1-NEGATIVE MYELOPROLIFERATIVE NEOPLASMS

**Topic:** 16. Myeloproliferative neoplasms - Clinical

Alessandro Costa*[1, 2], Olga Mulas[1, 2], Federica Pilo[2], Martina Pettinau[2], Eleonora Atzeni[1], Daniela Mantovani[2], Giovanni Caocci[1, 2]

*[1]Department of Medical Sciences and Public Health, University of Cagliari, 09121 Cagliari, Italy, Caglari, Italy;[2]Hematology and BMT Unit, "A. Businco" Hospital, ARNAS Brotzu, Cagliari, Cagliari, Italy;*

### Background

Cardiovascular events (CVE) remain a major cause of morbidity in *BCR::ABL1*-negative myeloproliferative neoplasms (MPN). Estimated pulse wave velocity (ePWV), a validated surrogate marker of arterial stiffness, predicts cardiovascular risk in the general population; however, its prognostic relevance in MPN has not been systematically investigated.

### Aims

To evaluate whether baseline ePWV predicts incident CVE in MPN patients, accounting for competing mortality and conventional cardiovascular risk factors.

### Methods

We retrospectively analyzed 568 consecutive ET/PV patients with available baseline ePWV and longitudinal follow-up. ePWV was calculated using the validated equation by Greve et al. Incident post-baseline CVE were analyzed using cause-specific Cox proportional hazards models adjusted for sex and Fine–Gray (FG) competing-risk regression. Sensitivity analyses additionally adjusted for baseline smoking status, BMI, diabetes, and renal function in complete-case models. Subgroup and interaction analyses were performed according to diagnosis and thrombotic risk category. Overall survival (OS) was evaluated using multivariable Cox regression adjusted for age and sex. Statistical significance was defined as $p < 0.05$. Analyses were conducted using R software.

**Copyright Information:** (Online) ISSN: 2572-9241
©2026 The Author(s). HemaSphere is published by John Wiley & Sons Ltd on behalf of European Hematology Association. This is an open access Abstract Book distributed under the Attribution-NonCommercial-NoDerivs (CC BY-NC-ND), which allows third parties to download the articles and share them with others as long as they credit the author and the Abstract Book, but they cannot change the content in any way or use them commercially.
**Abstract Book Citations:** Authors, Title, HemaSphere, 2026;10:(S1):pages. The abstract book DOIs can be found on the "Author Information" page.
**Disclaimer:** Articles published in the journal HemaSphere exclusively reflect the opinions of the authors. The authors are responsible for all content in their abstracts including accuracy of the facts, statements, citing resources, etc.

**Results**

The cohort comprised 568 patients (median age 64.4 years, IQR: 51.6–74.4); 51.7% were female; 77.8% had ET and 22.1% PV. Median ePWV was 11.16 m/s (IQR: 9.12–12.9), with no significant difference between ET and PV (median 11.1 vs 11.4 m/s, $p$=0.44). Over a median follow-up of 63.5 months (IQR: 30.0–125.7), 64 patients developed CVE and 47 died.
In the primary continuous analysis, each 1 m/s increase in ePWV was associated with a 21% higher hazard of CVE (Cox HR 1.21, 95% CI 1.09–1.34, $p$<0.001). The association remained significant after accounting for competing mortality (FG sHR 1.16, 95% CI 1.05–1.28, $p$=0.0033).
Using maximally selected rank statistics, an internal threshold of 11.12 m/s was identified. In the full cohort (sex-adjusted), patients with ePWV ≥11.12 m/s had significantly increased CVE risk (Cox HR 2.51, 95% CI 1.50–4.23, $p$=0.0005; FG sHR 2.11, 95% CI 1.28–3.47, $p$=0.0033), with lower CVE-free survival (log-rank $p$=0.00054, Figure). Results were consistent in 6- and 12-month landmark analyses.
In complete-case models additionally adjusted for cardiovascular risk factors, ePWV remained independently associated with CVE both as a continuous variable (Cox HR 1.17, 95% CI 1.04–1.32, $p$=0.0083; FG sHR 1.13, 95% CI 1.00–1.28, $p$=0.043) and using the internally derived cut-off of 11.12 m/s (Cox HR 2.08, 95% CI 1.19–3.66, $p$=0.0107; FG sHR 1.79, 95% CI 1.04–3.10, $p$=0.037). The association was observed in both ET (Cox HR 2.08, $p$=0.021) and PV (Cox HR 3.74, $p$=0.010); however, no evidence of interaction was observed according to thrombotic risk category or age. Moreover, no significant association was found between ePWV and isolated arterial or venous events. In multivariable OS analysis, ePWV was not independently associated with mortality (HR 0.83, 95% CI 0.58–1.20, $p$=0.33), whereas age and male sex were significant predictors ($p$<0.001).

**Summary/Conclusion**

Baseline ePWV is independently associated with incident cardiovascular events in patients with ET and PV, including after adjustment for conventional cardiovascular risk factors and competing mortality. These findings support prospective evaluation of arterial stiffness assessment to refine cardiovascular risk stratification in MPN.

**Copyright Information:** (Online) ISSN: 2572-9241
©2026 The Author(s). HemaSphere is published by John Wiley & Sons Ltd on behalf of European Hematology Association. This is an open access Abstract Book distributed under the Attribution-NonCommercial-NoDerivs (CC BY-NC-ND), which allows third parties to download the articles and share them with others as long as they credit the author and the Abstract Book, but they cannot change the content in any way or use them commercially.
**Abstract Book Citations:** Authors, Title, HemaSphere, 2026;10:(S1):pages. The abstract book DOIs can be found on the "Author Information" page.
**Disclaimer:** Articles published in the journal HemaSphere exclusively reflect the opinions of the authors. The authors are responsible for all content in their abstracts including accuracy of the facts, statements, citing resources, etc.

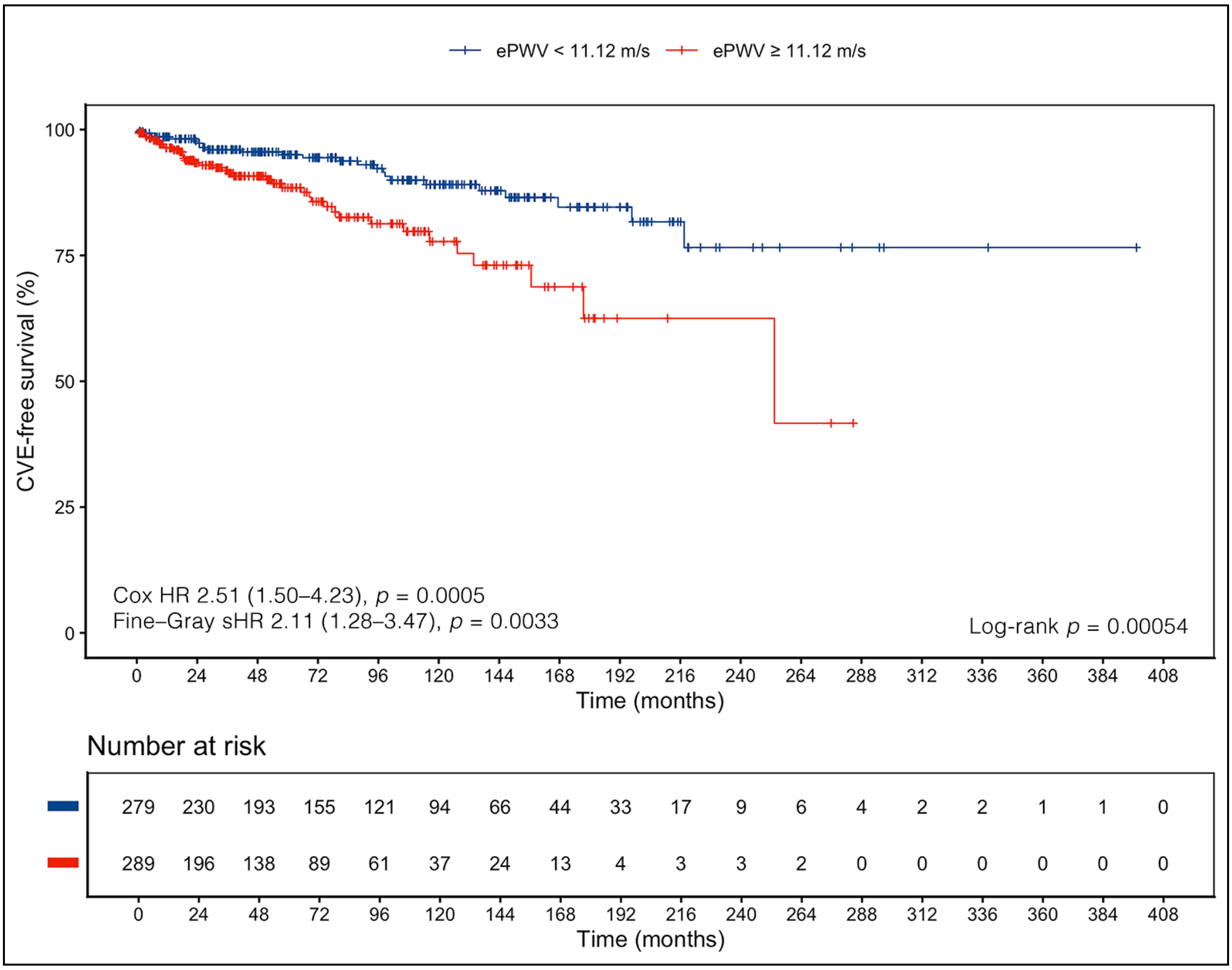

Figure. Kaplan–Meier estimates of CVE-free survival stratified by baseline ePWV (<11.12 vs ≥11.12 m/s). Internal cut-off identified by maximally selected rank statistics. Abbreviations: CVE, cardiovascular events; ePWV, estimated pulse wave velocity; HR, hazard ratio

**Copyright Information:** (Online) ISSN: 2572-9241
©2026 The Author(s). HemaSphere is published by John Wiley & Sons Ltd on behalf of European Hematology Association. This is an open access Abstract Book distributed under the Attribution-NonCommercial-NoDerivs (CC BY-NC-ND), which allows third parties to download the articles and share them with others as long as they credit the author and the Abstract Book, but they cannot change the content in any way or use them commercially.
**Abstract Book Citations:** Authors, Title, HemaSphere, 2026;10:(S1):pages. The abstract book DOIs can be found on the "Author Information" page.
**Disclaimer:** Articles published in the journal HemaSphere exclusively reflect the opinions of the authors. The authors are responsible for all content in their abstracts including accuracy of the facts, statements, citing resources, etc.

### (PS1993) INDIRECT TREATMENT COMPARISON: GECACITINIB VS. PACRITINIB, MOMELOTINIB, AND FEDRATINIB IN FIRST-LINE MYELOFIBROSIS

**Topic:** 16. Myeloproliferative neoplasms - Clinical

Yi Zhang*[1], Hu Zhou[2], Junling Zhuang[3], Zhongxing Jiang[4], Dengshu Wu[5], Linhua Yang[6], Qian Jiang[7], Huanling Zhu[8], Xin Du[9], Wei Li[10], Ai-li He[11], Jinwen Huang[12], Yan Li[13], Xiuli Wang[14], Qingchi Liu[15], Yuqing Chen[16], Sujun Gao[10], Fei Li[17], Yarong Li[14], Wen Wu[18], Feng Zhang[19], Guangsheng He[20], Mei Hong[21], Jianda Hu[22], Chenghao Jin[23], Hongmei Jing[24], Jishi Wang[25], Ruixiang Xia[26], Na Xu[27], Lingling Yue[28], Cuiping Zheng[29], Zeping Zhou[30], Yu Hu[31], Xin Li[32], Jing Liu[33], Lin Liu[34], Kai Sun[16], Jin Zhang[35], Yajing Xu[5], Zhijian Xiao[36], Min Yang[1], Jie Jin[1]

*[1]The First Affiliated Hospital, Zhejiang University School of Medicine, Department of Hematology, Hangzhou, China;[2]The Affiliated Cancer Hospital of Zhengzhou University, Henan Cancer Hospital, Department of Hematology, Zhengzhou, China; [3]Peking Union Medical College Hospital, ChineseAcademy of Medical Sciences, Department of Hematology, Beijing, China;[4]The First Affiliated Hospital of Zhengzhou University, Department of Hematology, Zhengzhou, China; [5]Xiangya hospital, Central South University, Department of Hematology, Changsha, China; [6]The Second Affiliated Hospital of Shanxi Medical University, Department of Hematology, Taiyuan, China; [7]Peking University People's Hospital, Peking University Institute of Hematology, National Clinical Research Center for Hematologic Disease, Beijing Key Laboratory of Hematopoietic Stem Cell Transplantation, Department of Hematology, Beijing, China; [8]West China Hospital, Sichuan University, Department of Hematology, Chengdu, China; [9]Guangdong Provincial People's Hospital, Department of Hematology, Guangzhou, China;[10]The First Hospital of Jilin University, Department of Hematology, Changchun, China; [11]The Second Affiliated Hospital of Xi'an Jiaotong University, Department of Hematology, Xi'an, China; [12]Sir Run Run Shaw Hospital, School of Medicine, Zhejiang University, Department of Hematology, Hangzhou, China; [13]The First Hospital of China Medical University, Department of Hematology, Shenyang, China; [14]The Second Hospital of Jilin University, Department of Hematology, Changchun, China;[15]The First Hospital of Hebei Medical University, Department of Hematology, Shijiazhuang, China; [16]Henan Provincial People's Hospital, Department of Hematology, Zhengzhou, China; [17]The First Affiliated Hopital of Nanchang University, Department of Hematology, nanchang, China; [18]Shanghai Institute of Hematology, State Key Laboratory of Medical Genomics, National Research Center for Translational Medicine at Shanghai, Ruijin Hospital Shanghai Jiao Tong University School of Medicine, Department of Hematology, shanghai, China; [19]The First Affiliated Hospital of Bengbu Medical University, Bengbu, China;[20]The First Affiliated Hospital of Nanjing Medical University, Jiangsu Provincial People's Hospital in Nanjing, Key Laboratory of Hematology of NanjingMedical University, Collaborative Innovation Center for Cancer Personalize, Department of Hematology, Nanjing, China; [21]Union Hospital, Tongji Medical College, Huazhong University of Science and Technology, Wuhan, China; [22]Fujian Institute of Hematology, Fujian Medical University Union Hospital, Department of Hematology, Fuzhou, China; [23]Jiangxi Provincial People's Hospital, Department of Hematology, Nanchang, China; [24]Peking University Third Hospital, Department of Hematology, Beijing, China;[25]Affiliated Hospital of Guizhou Medical University, Department of Hematology, Guizhou, China; [26]the First Affiliated Hospital of Anhui Medical University, Department of Hematology, Anhui, China; [27]Nanfang Hospital, Southern Medical University, Department of Hematology, Guangzhou, China; [28]Lanzhou University Second Hospital, Department of Hematology, Lanzhou, China;[29]Wenzhou Central Hospital, Department of Hematology, Wenzhou, China; [30]The Second Affiliated Hospital of Kunming Medical University, Department of Hematology, Kunming, China; [31]Union Hospital, Tongji Medical College, Huazhong University of Science and Technology, Department of Hematology, Wuhan, China; [32]The Third Xiangya Hospital, Central SouthUniversity, Department of Hematology, Changsha, China; [33]The Third Xiangya Hospital Central South University, Department of Hematology, Changsha, China; [34]The First Affiliated Hospital of Chongqing Medical University, Department of Hematology, Chongqing, China;[35]Sir Run Run Shaw Hospital, Zhejiang University School of Medicine, Department of Hematology, Hangzhou, China; [36]Institute of Hematology and Blood Diseases Hospital, Chinese Academy of Medical Sciences & Peking Union Medical College, Department of Hematology, Tianjin, China;*

**Copyright Information:** (Online) ISSN: 2572-9241
©2026 The Author(s). HemaSphere is published by John Wiley & Sons Ltd on behalf of European Hematology Association. This is an open access Abstract Book distributed under the Attribution-NonCommercial-NoDerivs (CC BY-NC-ND), which allows third parties to download the articles and share them with others as long as they credit the author and the Abstract Book, but they cannot change the content in any way or use them commercially.
**Abstract Book Citations:** Authors, Title, HemaSphere, 2026;10:(S1):pages. The abstract book DOIs can be found on the "Author Information" page.
**Disclaimer:** Articles published in the journal HemaSphere exclusively reflect the opinions of the authors. The authors are responsible for all content in their abstracts including accuracy of the facts, statements, citing resources, etc.

### Background

JAK inhibitors are standard first-line therapy for myelofibrosis, yet no head-to-head trials compare gecacitinib with pacritinib, momelotinib, or fedratinib. Understanding their relative efficacy and safety is critical to inform optimal treatment selection.

### Aims

To indirectly compare the efficacy and safety of gecacitinib versus pacritinib, momelotinib, and fedratinib in treatment-naïve patients with myelofibrosis.

### Methods

We conducted a matching-adjusted indirect comparison (MAIC) using patient-level data from the gecacitinib trials (ZGJAK002/016) and published aggregate data from SIMPLIFY-1, PERSIST-1, and JAKARTA-1. Gecacitinib patients were reweighted to match baseline characteristics of comparator arms (age, MF subtype, IPSS risk, platelet count, hemoglobin, TSS, spleen volume) prioritized by clinical experts. Key endpoints at Week 24 included spleen volume reduction ≥35% (SVR35), total symptom score reduction ≥50% (TSS50), and adverse events (AEs). Odds ratios (ORs) and risk differences (RDs) were calculated for balanced populations. Standard errors were estimated using the robust sandwich estimator method, with 95% confidence intervals (CIs) and P-values derived accordingly. A two-tailed P-value <0.05 was considered statistically significant.

### Results

Main results are presented in Table 1. Effective sample sizes (ESS) were 21.7 for momelotinib, 25.4 for fedratinib, and 72.4 for pacritinib (PERSIST-1).
At Week 24, gecacitinib demonstrated numerically higher odds of achieving SVR35 and TSS50 versus all comparators. Statistically significant advantages for SVR35 were observed against momelotinib (SIMPLIFY-1: OR 3.7, 95% CI 1.8–7.5; P=0.0004) and pacritinib (PERSIST-1: OR 6.2, 95% CI 3.5–11.3; P<0.0001). Similarly, TSS50 favored gecacitinib with significant ORs of 2.8 (95% CI 1.4–5.5; P=0.0031) versus momelotinib and 4.8 (95% CI 2.4–9.7; P<0.0001) versus pacritinib.
Regarding safety, gecacitinib showed significantly lower incidences of any-grade nausea across all comparator cohorts. The risk of any-grade diarrhea was significantly lower with gecacitinib versus pacritinib (RD=-0.3, 95% CI -0.5 to -0.2; P<0.0001) and fedratinib (RD=-0.4, 95% CI -0.6 to -0.2; P=0.0004). However, risks of any-grade or grade 3/4 anemia and grade 3/4 thrombocytopenia were significantly higher with gecacitinib compared to momelotinib and pacritinib. The risk of any-grade or grade 3/4 decreased neutrophil count was significantly lower with gecacitinib versus fedratinib. AEs leading to discontinuation were significantly less frequent with gecacitinib versus momelotinib.

### Summary/Conclusion

Gecacitinib demonstrated superior efficacy versus momelotinib and pacritinib, with significant improvements in SVR35 and TSS50, alongside favorable gastrointestinal tolerability (lower nausea and diarrhea) and reduced discontinuation. These findings support gecacitinib as a promising first-line option for JAK inhibitor-naïve patients with myelofibrosis who prioritize spleen and symptom control, pending head-to-head confirmation.

**Copyright Information:** (Online) ISSN: 2572-9241
©2026 The Author(s). HemaSphere is published by John Wiley & Sons Ltd on behalf of European Hematology Association. This is an open access Abstract Book distributed under the Attribution-NonCommercial-NoDerivs (CC BY-NC-ND), which allows third parties to download the articles and share them with others as long as they credit the author and the Abstract Book, but they cannot change the content in any way or use them commercially.
**Abstract Book Citations:** Authors, Title, HemaSphere, 2026;10:(S1):pages. The abstract book DOIs can be found on the “Author Information” page.
**Disclaimer:** Articles published in the journal HemaSphere exclusively reflect the opinions of the authors. The authors are responsible for all content in their abstracts including accuracy of the facts, statements, citing resources, etc.

**Table 1. Comparative Analysis of Gecacitinib versus Momelotinib, Pacritinib, and Fedratinib on Efficacy and Safety Endpoints**

| Endpoint | Comparator | OR/RD (95% CI) | P-value | Significance |
|---|---|---|---|---|
| **Efficacy Endpoints** | | | | |
| SVR35 at week 24 | SIMPLIFY-I Momelotinib (n=215) | OR: 3.66 (1.79, 7.48 ) | 0.0004 | ** |
| | PERSIST-1 Pacritinib (n=220) | OR: 6.24 (3.45, 11.30) | <0.0001 | ** |
| | JAKARTA-I Fedratinib (n=96) | OR: 1.43 (0.57, 3.55) | 0.4450 | ns |
| TSS50 at week 24 | Momelotinib | OR: 2.79 (1.41, 5.51) | 0.0031 | ** |
| | Pacritinib | OR: 4.84 (2.42, 9.69) | <0.0001 | ** |
| | Fedratinib | OR: 2.09 (0.81, 5.36) | 0.1251 | ns |
| **Safety Endpoints** | | | | |
| Anemia (Grade 3/4 ) | Momelotinib | RD: 0.15 (0.06, 0.25) | 0.0020 | ** |
| | Pacritinib | RD: 0.20 (0.07, 0.32) | 0.0025 | ** |
| | Fedratinib | RD: -0.21 (-0.40, -0.02) | 0.0319 | * |
| Thrombocytopenia (Grade 3/4 ) | Momelotinib | RD: 0.04 (-0.04, 0.11) | 0.3431 | ns |
| | Pacritinib | RD: 0.02 (-0.07, 0.11) | 0.6780 | ns |
| | Fedratinib | RD: -0.00 (-0.18, 0.17) | 0.9622 | ns |
| Decreased Neutrophil Count (Grade 3/4 ) | Momelotinib | RD: 0.01 (-0.04, 0.05) | 0.8023 | ns |
| | Pacritinib | Not available | Not available | Not available |
| | Fedratinib | RD: -0.06 (-0.13, -0.00) | 0.0365 | * |
| Nausea (Any grade) | Momelotinib | RD: -0.12 (-0.18, -0.05) | 0.0004 | ** |
| | Pacritinib | RD: -0.21 (-0.29, -0.13) | <0.0001 | ** |
| | Fedratinib | RD: -0.53 (-0.71, -0.34) | <0.0001 | ** |
| Diarrhea (Any grade) | Momelotinib | RD: -0.00 (-0.13, 0.12) | 0.9645 | ns |
| | Pacritinib | RD: -0.33 (-0.45, -0.20) | <0.0001 | ** |
| | Fedratinib | RD: -0.39 (-0.61, -0.17) | 0.0004 | ** |
| Discontinuation AE | Momelotinib | RD: -0.11 (-0.16, -0.05) | <0.0001 | ** |
| | Pacritinib | RD: -0.04 (-0.12, 0.04) | 0.3359 | ns |
| | Fedratinib | RD: -0.05 (-0.22, 0.12) | 0.5746 | ns |

**Notes**: P-values marked with "*" indicate statistical significance (P < 0.05), "**" indicates highly significant (P < 0.01), and "ns" indicates not significant. "Not available" indicates missing data.

**Copyright Information:** (Online) ISSN: 2572-9241
©2026 The Author(s). HemaSphere is published by John Wiley & Sons Ltd on behalf of European Hematology Association. This is an open access Abstract Book distributed under the Attribution-NonCommercial-NoDerivs (CC BY-NC-ND), which allows third parties to download the articles and share them with others as long as they credit the author and the Abstract Book, but they cannot change the content in any way or use them commercially.
**Abstract Book Citations:** Authors, Title, HemaSphere, 2026;10:(S1):pages. The abstract book DOIs can be found on the "Author Information" page.
**Disclaimer:** Articles published in the journal HemaSphere exclusively reflect the opinions of the authors. The authors are responsible for all content in their abstracts including accuracy of the facts, statements, citing resources, etc.

### (PS1994) MYELOPROLIFERATIVE NEOPLASMS AND MPN-LIKE SYNDROMES WITH NON-CANONICAL JAK2/MPL VARIANTS. PRELIMINARY RESULTS OF THE ELN MPN-OLIVETTI STUDY

**Topic:** 16. Myeloproliferative neoplasms - Clinical

Miguel Navarro Sánchez[1], Gonzalo Carreño Gómez-Tarragona*[1], Gabriel Levy[2], Elisa Rumi[3], Eric Lippert[4], Beatriz Bellosillo Paricio[5], Olivier Mansier[6], Anna Angona Figueras[7], Maria Chiara Finazzi[8], Juan Carlos Hernandez Boluda[9], Monia Marchetti[10], Maria Soledad Noya Pereira[11], Damien Luque-Paz[12], Ruth Stuckey[13], Alessandra Iurlo[14], Kim Kricheldorf[15], Paola Guglielmelli[16], Clémentine Salvado[17], Alberto Hernandez-Sanchez[18], Jennifer O'sullivan[19], Maria Alicia Senin Magan[20], Nikolaos Sousos[21], David Ross[22], Louis Drevon[23], Nicolas Papadopouloos[2], Lurdes Zamora Plana[24], Patricia Velez Tenza[5], Francisca Ferrer Marin[25], Oscar Borsani[3], Rosa María Ayala Díaz[1], Wayne-Corentin Lambert[4], Alessandro Rambaldi[8], Daniela Pietra[3], Juliette Soret-Dulphy[26], Antoine Bonnet[27], Charlotte Lees[21], Kevin James Wattebled[28], Dana Ranta[29], Concepción Fernández-Rodríguez[5], Benjamin Lebecque[30], Lise Willems[31], Steffen Koschmieder[15], Joseph M Scandura[32], Joaquín Martinez-Lopez[1], Adam Mead[21], Claire Harrison[19], Alessandro Maria Vannucchi[16], Bert Van der Reijden[33], Alberto Alvarez Larran[34], Stefan Constantinescu[2], Tiziano Barbui[8]

*[1]Hematology Department. Hospital Universitario 12 de Octubre, imas12, CNIO, Complutense University, CIBERONC, Madrid, Spain., Madrid, Spain; [2]Université Catholique de Louvain and de Duve Institute, Brussels, Belgium, Brussels, Belgium;[3]Unit of Molecular Hematology and Precision Medicine, Fondazione IRCCS Policlinico San Matteo, Pavia, Italy, Pavia, Italy; [4]Service d'hématologie biologique, CHU de Brest, Brest, France, Brest, France; [5]Hematology and Pathology Department. Hospital del Mar, IMIM, Barcelona, Spain, Barcelona, Spain; [6]Laboratory of Hematology, Bordeaux University Hospital, Bordeaux, France, Bardeaux, France; [7]Hematology Department. Hospital Doctor Josep Trueta, Girona, Spain, Girona, Spain;[8]FROM - Research Foundation Bergamo Hospital - ETS, Bergamo, Italy, Bergamo, Italy; [9]Hematology Department. Hospital Clínico, Valencia, Spain, Valencia, Spain; [10]Hematology Unit, University Hospital “SS Antonio e Biagio e Cesare Arrigo”, Alessandria, Italy, Alessandria, Italy; [11]Hematology Department. Complexo Hospitalario Universitario de A Coruña, Spain., A Coruña, Spain;[12]Univ Angers, Nantes Université, CHU Angers, Inserm, CNRS, CRCI2NA, F-49000, Angers, France, Angers, France; [13]Hematology Department. Hospital Universitario de Gran Canaria Dr. Negrín, Instituto de Investigación Sanitaria de Canarias (IISC), Las Palmas de Gran Canaria, Spain., Las Palmas de Gran Canaria, Spain; [14]Hematology Division, Foundation IRCCS Cà Granda Ospedale Maggiore Policlinico., Milan, Italy; [15]Department of Hematology, Oncology, Hemostaseology and Stem Cell Transplantation, Medical Faculty, RWTH Aachen University., Aachen, Germany; [16]Department of Experimental and Clinical Medicine, Center for Research and Innovation of Myeloproliferative Neoplasms, AOU Careggi, University of Florence, Florence, Italy; [17]Médecine Interne-Immuno-Hématologie CH de, Dax, France; [18]Universidad de Salamanca, IBSAL, IBMCC, CSIC, Centro de Investigación del Cáncer, Salamanca, Spain; [19]Guy's and St Thomas' NHS Foundation Trust, London, United Kingdom;[20]Hematology Laboratory Department, Institut Català d'Oncologia (ICO). Josep Carreras Leukaemia Research Institute (IJC)., Barcelona, Spain; [21]Medical Research Council Weatherall Institute of Molecular Medicine and NIHR Biomedical Research Centre, University of Oxford, Oxford, United Kingdom; [22]Department of Haematology Flinders University and Medical Centre Adelaide South Australia Australia, Adelaide, Australia; [23]Service d'Hématologie Clinique Hôpital Saint-Louis, APHP Paris, France, Paris, France;[24]ICO Badalona-Hospital Germans Trias i Pujol, Josep Carreras Leukaemia Research Institute (IJC), Barcelona, Spain; [25]Hematology Department. Hospital Morales Meseguer, CIBERER, UCAM, Murcia, Spain, Murcia, Spain; [26]INSERM CIC 1427, Université Paris Cité, Centre d'Investigations Cliniques, Hôpital Saint-Louis, AP-HP, Paris, France, Paris, France; [27]Hematology Department, CH Bretagne Atlantique, Vannes, France, Vannes, France; [28]Department of Hematology, CH Dunkerque, Dunkerque, France, Dunkerque, France; [29]Department of Internal Medicine and Hematology, Nancy University Hospital, Nancy, France, Nancy, France; [30]Service d'Hématologie Biologique, CHU Clermont-Ferrand, Clermont-Ferrand, France, Clermont-Ferrand, France; [31]Hématologie Clinique, Hôpital Cochin, APHP Paris France, Paris, France;[32]Richard T. Silver Myeloproliferative Neoplasms Center, Weill Cornell Medicine, New York, NY, New York, United States of America; [33]RadboudUMC, Radboud Institute for Medical Innovation, Nijmegen, Netherlands, Nijmegen, The Netherlands; [34]Hematology Department, Hospital Clínic, Institut d'Investigacions Biomèdiques August Pi i Sunyer (IDIBAPS), Barcelona, Spain, Barcelona, Spain;*

**Copyright Information:** (Online) ISSN: 2572-9241
©2026 The Author(s). HemaSphere is published by John Wiley & Sons Ltd on behalf of European Hematology Association. This is an open access Abstract Book distributed under the Attribution-NonCommercial-NoDerivs (CC BY-NC-ND), which allows third parties to download the articles and share them with others as long as they credit the author and the Abstract Book, but they cannot change the content in any way or use them commercially.
**Abstract Book Citations:** Authors, Title, HemaSphere, 2026;10:(S1):pages. The abstract book DOIs can be found on the “Author Information” page.
**Disclaimer:** Articles published in the journal HemaSphere exclusively reflect the opinions of the authors. The authors are responsible for all content in their abstracts including accuracy of the facts, statements, citing resources, etc.

### Background

Up to 20% of MPN are triple-negative, part of them presenting with non-canonical *JAK2/MPL* variants. These cases present a diagnostic and therapeutic challenge as the pathogenicity of these variants in not always clear and some case may be inherited.

### Aims

The aim of the ELN-promoted European Registry of NOn-canonicaL DrIVEr MuTaTIons in MPN (MPN-OLIVETTI) is to provide a comprehensive registry of non-canonical *JAK2*, and *MPL* variants identified in MPN patients and characterize their functionality and clinical consequences.

### Methods

A database including anonymized data from MPN and MPN-like patients with non-canonical JAK2/ /MPL variants was designed. Currently there are 90 and 86 different JAK2 and MPL variants, respectively, from 369 patients from 9 different countries and 33 different centers. This preliminary analysis only includes data from the 261 patients included in the registry with clinical information.

### Results

Median age at diagnosis was 58yo and the median follow-up from diagnosis was 7.6 y. 41.9% (n=103) of patients had a diagnosis of ET, 18.3% (n=45) of PV, 15.9% (n=39) of MF, 10.2% (n=25) of familiar thrombocytosis. 27% (n=71) of patients had a concomitant JAK2 V617F and 7% (n=18) had a MPL canonical variant. 63% (n=172) of patients had additional MPN-associated mutations. 20.6% (n=36) and 8.1% (n=14) of patients had at least one thromboembolic (TE) or major bleeding event, respectively. 13.5% (n=21) and 12.4% (n=20) progressed to MF or AML.

130 different non-canonical *JAK2* and *MPL* variants were identified. 25.4% of them were classified as pathogenic/likely pathogenic (P), 40.7% as VUS and 33.9% as benign/likely benign (B). 75.9% of variants had a VAF of 40-60% and were therefore suspicious of germinality. 33.8% of identified variants were already described in scientific literature, of which 52.3% had some degree of in vitro activity.

28.9% and 14.1% (p= 0.016) of patients with and without a canonical variant suffered a thromboembolic event (23% and 6% of cases at 5 years and 26% and 8% of cases at 10y, respectively). No differences were observed for the rest of events. Patients without canonical variants had a statistically significant better overall survival (OS) than those with canonical variants (p<0.001, Figure 1), and 59% and 83% of OS at 10y. We did not found differences between patients carrying additional mutations.

Excluding patients with *JAK2* V617F or *MPL* canonical variants there were no differences between patients carrying P, VUS or B variants, although patients with P/PP non-canonical variants seemed to have a worse survival at 10y (68.9% vs 84.8% vs 88.5%, p=0.53).
Of patients with essential thrombocythemia 16.4% had TE events, 4.5% had a major bleeding event, 18.8% had a MF progression and 7.8% had an AML progression. There were no differences in outcomes between patients with canonical variants (23.3%) and those without them and no differences in outcomes depending on variant classification.

**Copyright Information:** (Online) ISSN: 2572-9241
©2026 The Author(s). HemaSphere is published by John Wiley & Sons Ltd on behalf of European Hematology Association. This is an open access Abstract Book distributed under the Attribution-NonCommercial-NoDerivs (CC BY-NC-ND), which allows third parties to download the articles and share them with others as long as they credit the author and the Abstract Book, but they cannot change the content in any way or use them commercially.
**Abstract Book Citations:** Authors, Title, HemaSphere, 2026;10:(S1):pages. The abstract book DOIs can be found on the "Author Information" page.
**Disclaimer:** Articles published in the journal HemaSphere exclusively reflect the opinions of the authors. The authors are responsible for all content in their abstracts including accuracy of the facts, statements, citing resources, etc.

### Summary/Conclusion

Around 50% of *JAK2/MPL* variants found in MPN/MPN-like syndromes are of unknown significance and less than 30% have been functionally characterized. Furthermore, 75% of variants seem to be germline. Also, outcomes seem to be better in non-canonical carriers than *JAK2* V617F/canonical *MPL* variants carriers. Functional testing and larger cohorts are warranted to establish recommendations in these patients.

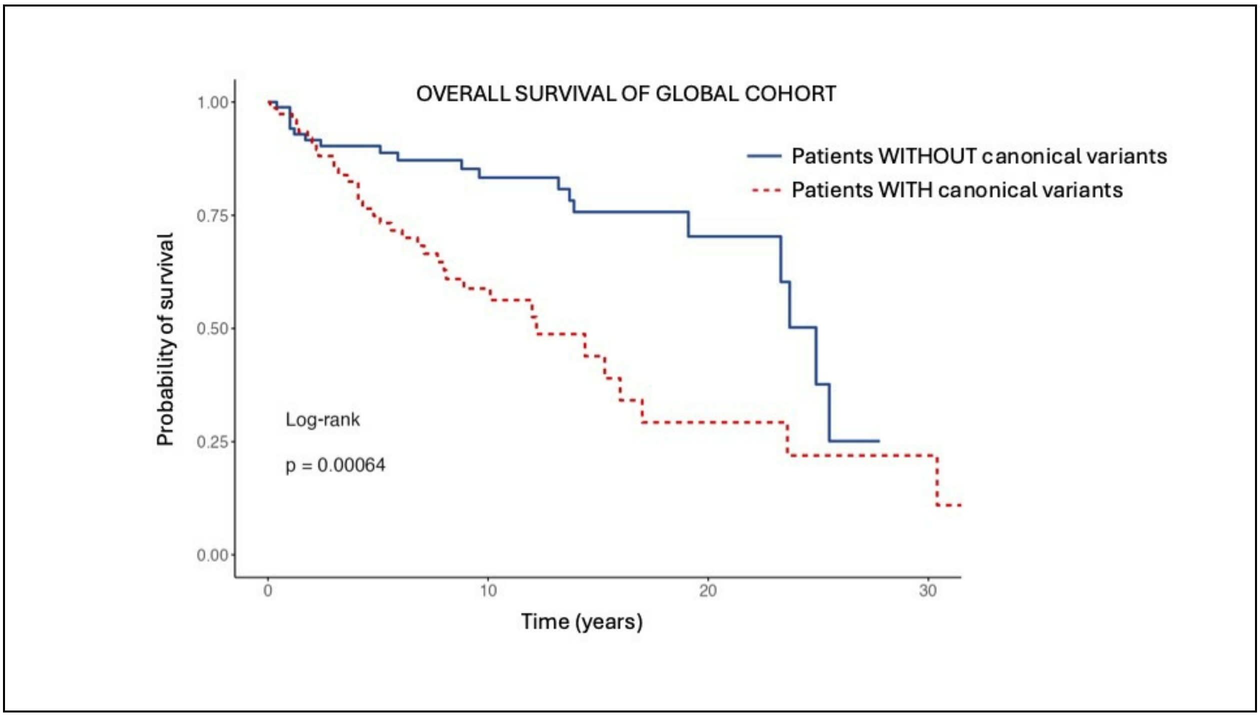

Figure 1: Kaplan-Meier curves of overall survival in the global cohort.

**Copyright Information:** (Online) ISSN: 2572-9241
©2026 The Author(s). HemaSphere is published by John Wiley & Sons Ltd on behalf of European Hematology Association. This is an open access Abstract Book distributed under the Attribution-NonCommercial-NoDerivs (CC BY-NC-ND), which allows third parties to download the articles and share them with others as long as they credit the author and the Abstract Book, but they cannot change the content in any way or use them commercially.
**Abstract Book Citations:** Authors, Title, HemaSphere, 2026;10:(S1):pages. The abstract book DOIs can be found on the "Author Information" page.
**Disclaimer:** Articles published in the journal HemaSphere exclusively reflect the opinions of the authors. The authors are responsible for all content in their abstracts including accuracy of the facts, statements, citing resources, etc.

### (PS1995) OUTCOMES WITH MOMELOTINIB IN PATIENTS WITH INTERMEDIATE-1– VS INTERMEDIATE-2–/HIGH-RISK MYELOFIBROSIS: POST HOC ANALYSES OF THE PHASE 3 SIMPLIFY-1 AND MOMENTUM TRIALS

**Topic:** 16. Myeloproliferative neoplasms - Clinical

Prithviraj Bose*[1], Casey O'connell[2], Donal McLornan[3], Mahshid Azimi[4], Shiyuan Zhang[4], Jessica Lim[4], Dwaipayan Patnaik[5], Francesco Passamonti[6]

*[1]University of Texas MD Anderson Cancer Center, Houston, TX, United States of America;[2]Keck School of Medicine of the University of Southern California, Los Angeles, CA, United States of America; [3]University College London Hospitals NHS Trust, London, United Kingdom; [4]GSK, Collegeville, PA, United States of America;[5]GSK, Baar, Switzerland; [6]Dipartimento di Oncologia ed Onco-Ematologia, Università degli Studi di Milano, Fondazione IRCCS Ca' Granda Ospedale Maggiore Policlinico, Milano, Italy;*

### Background

Patient prognosis in myelofibrosis (MF) may be predicted by a number of risk assessment tools that classify patients as having (in order of increasingly poor prognosis) low-, intermediate-1 (int-1)–, intermediate-2 (int-2)–, or high-risk disease. Janus kinase (JAK) inhibitors are approved for the treatment of patients with intermediate- or high-risk MF; however, data on patients with int-1–risk disease are limited, as the pivotal trials of JAK inhibitors such as ruxolitinib and fedratinib did not enroll patients with this risk category. Momelotinib is a JAK1/JAK2/activin A receptor type 1 inhibitor approved for the treatment of patients with intermediate- or high-risk MF who have anemia based on the phase 3 SIMPLIFY-1 and MOMENTUM trials in JAK inhibitor–naive and –experienced patients, respectively. While SIMPLIFY-1 and MOMENTUM did enroll patients with int-1–risk disease, outcomes specifically in this lowest-risk group for which momelotinib is approved have not been reported.

### Aims

To characterize the spleen, symptom, and anemia-related benefits of momelotinib in patients with int-1– vs int-2–/high-risk MF.

### Methods

SIMPLIFY-1 (NCT01969838) and MOMENTUM (NCT04173494) were randomized, double-blind, phase 3 trials of momelotinib vs ruxolitinib or danazol, respectively; the present post hoc analyses focused on the momelotinib arms of the intent-to-treat (ITT) populations only, given the goal of establishing relative benefit based on risk assessment category. Week 24 endpoints summarized descriptively were spleen volume reduction ≥35% from baseline (SVR35), Total Symptom Score reduction ≥50% from baseline (TSS50), transfusion independence (TI; no transfusions or hemoglobin <8 g/dL in the previous 12 weeks), and achievement of dual SVR35+TI. Risk assignment was based on the assessment tool used in each trial (SIMPLIFY-1, International Prognostic Scoring System [IPSS]; MOMENTUM, Dynamic IPSS [DIPSS]).

### Results

In SIMPLIFY-1, 46/215 (21%) momelotinib-randomized patients were int-1 risk, while in MOMENTUM, only 7/130 (5%) were int-1 risk, reflecting the more advanced disease of this JAK inhibitor–experienced, anemic population; hemoglobin <10 g/dL was an inclusion criterion for MOMENTUM and is assigned 2 points in the DIPSS risk evaluation. Outcomes in the int-1– vs int-2–/high-risk populations of both trials are shown in the **Figure**. Response rates for a majority of the endpoints evaluated were numerically higher in the int-1– vs int-2–/high-risk population, including TSS50 and TI in SIMPLIFY-1 and SVR35, TSS50, and TI in MOMENTUM.

**Copyright Information:** (Online) ISSN: 2572-9241
©2026 The Author(s). HemaSphere is published by John Wiley & Sons Ltd on behalf of European Hematology Association. This is an open access Abstract Book distributed under the Attribution-NonCommercial-NoDerivs (CC BY-NC-ND), which allows third parties to download the articles and share them with others as long as they credit the author and the Abstract Book, but they cannot change the content in any way or use them commercially.
**Abstract Book Citations:** Authors, Title, HemaSphere, 2026;10:(S1):pages. The abstract book DOIs can be found on the "Author Information" page.
**Disclaimer:** Articles published in the journal HemaSphere exclusively reflect the opinions of the authors. The authors are responsible for all content in their abstracts including accuracy of the facts, statements, citing resources, etc.

**Summary/Conclusion**

Spleen, symptom, and anemia-related benefits of momelotinib in both JAK inhibitor–naive and –experienced trial populations were observed across all IPSS/DIPSS risk assessment categories enrolled. TSS50 and TI rates were numerically higher in the int-1–risk population vs the ITT, suggesting that earlier initiation of treatment before progression to int-2–/high-risk disease may be associated with better outcomes. Overall, these findings support use of momelotinib across the full spectrum of int-1–, int-2–, and high-risk patients for which it is approved.

**Funding:** GSK.

**Figure.** Outcomes in Int-1– vs Int-2–/High-Risk Patients With Momelotinib

**SIMPLIFY-1**

**Risk assessment tool:** IPSS
**Population:** JAK inhibitor naive

| Response rate, n/N (%) | SVR35 | TSS50 | TI | SVR35+TI |
|---|---|---|---|---|
| ITT | 57/215 (27) | 60/215 (28) | 143/215 (67) | 53/215 (25) |
| Int-1 | 12/46 (26) | 18/46 (39) | 38/46 (83) | 12/46 (26) |
| Int-2/high | 45/169 (27) | 42/169 (25) | 105/169 (62) | 41/169 (24) |

**MOMENTUM**

**Risk assessment tool:** DIPSS
**Population:** JAK inhibitor experienced, anemic (Hb <10 g/dL), and symptomatic (MFSAF TSS ≥10)

| Response rate, n/N (%) | SVR35 | TSS50 | TI | SVR35+TI |
|---|---|---|---|---|
| ITT | 29/130 (22) | 32/130 (25) | 39/130 (30) | 14/130 (11) |
| Int-1 | 2/7 (29) | 4/7 (57) | 4/7 (57) | 2/7 (29) |
| Int-2/high | 27/122 (22) | 28/122 (23) | 35/122 (29) | 12/122 (10) |

DIPSS, Dynamic International Prognostic Scoring System; Hb, hemoglobin; int, intermediate; IPSS, International Prognostic Scoring System; ITT, intent to treat; JAK, Janus kinase; MFSAF, Myelofibrosis Symptom Assessment Form; SVR35, spleen volume reduction ≥35% from baseline; TI, transfusion independence; TSS, Total Symptom Score; TSS50, Total Symptom Score reduction ≥50% from baseline.

**Copyright Information:** (Online) ISSN: 2572-9241
©2026 The Author(s). HemaSphere is published by John Wiley & Sons Ltd on behalf of European Hematology Association. This is an open access Abstract Book distributed under the Attribution-NonCommercial-NoDerivs (CC BY-NC-ND), which allows third parties to download the articles and share them with others as long as they credit the author and the Abstract Book, but they cannot change the content in any way or use them commercially.
**Abstract Book Citations:** Authors, Title, HemaSphere, 2026;10:(S1):pages. The abstract book DOIs can be found on the “Author Information” page.
**Disclaimer:** Articles published in the journal HemaSphere exclusively reflect the opinions of the authors. The authors are responsible for all content in their abstracts including accuracy of the facts, statements, citing resources, etc.

### (PS1996) BEYOND THROMBOSIS: QRISK3 PREDICTS SURVIVAL IN MPN PATIENTS

**Topic:** 16. Myeloproliferative neoplasms - Clinical

Isidora Arsenovic[1], Andrijana Kojic[1], Andjela Nikolic[1], Nikola Lemajić[1], Mihailo Smiljanic[1], Andrija Bogdanovic[1, 2], Danijela Lekovic*[1, 2]

[1]*Clinic of Hematology, University Clinical Center Serbia, Belgrade,Serbia, Belgrade, Serbia;*[2]*Faculty of Medicine, University of Belgrade, Belgrade, Serbia;*

### Background

Despite advances in disease-specific thrombotic risk scores, cardiovascular comorbidities are not routinely incorporated into MPN risk stratification. Recent data suggest QRISK3 model may improve clinically prognostic potential in these patients.

### Aims

To evaluate QRISK3 score for prediction of thrombosis and overall survival (OS) in consecutive MPN patients, and to compare its performance across MPN subtypes.

### Methods

This retrospective single-center analysis included 273 consecutive MPN patients diagnosed per current WHO criteria. We analyzed demographics, laboratory and cardiovascular comorbidity data and calculated QRISK3 model. Endpoints were any arterial or venous thrombotic event and OS estimated by Kaplan–Meier, predictive performance assessed by ROC (AUC), and subgroup analyses performed for PV, ET and PMF by SPSS 21 statistical program.

### Results

The cohort comprised 123 PV, 35 ET, and 115 PMF patients; 38.8% were male, with a median age of 65 years.During a mean follow-up of 34 months, 18 patients (6.6%) experienced thrombotic events, including CVI (n=6), DVT (n=3), PE (n=2), AMI (n=4), splenic infarction (n=1), and peripheral gangrene (n=2).
Mean QRISK3 score for the entire cohort was 16.9 ± 0.8, without significant differences across MPN subtypes (PV 16.8, ET 14.7 and for PMF 17.9, p=0.442). However, patients who developed thrombosis had significantly higher QRISK3 score compared to those without thrombosis (25.4 vs 16.4, p=0.005).
Subgroup analysis demonstrated that this association was primarily driven by PMF patients, in whom those with thrombosis had markedly higher QRISK3 score than those without (31.1 vs 16.8, p=0.004). No significant association was observed in PV, with only one thrombotic event occurred in ET, precluding meaningful analysis.
ROC analysis showed modest discriminative ability of continuous QRISK3 score for thrombotic events (AUC 0.65). Dichotomization of QRISK3 at ≥20% improved sensitive and specific predictive performance of score. Importantly, 87.5% of thrombotic events in MF occurred in patients with QRISK3score≥20% (p=0.003).
Mean OS was 188 months (m) for the entire cohort (PV 132m, ET 93m, PMF 209m) with death rate of 4.8% (13 pts) caused mostly by AML transformation (n=2), COVID 19 infection, cardiovascular events and heart failure.
Kaplan–Meier analysis demonstrated significantly inferior OS in patients who developed thrombosis compared with those who didn't (p=0.009). Similarly, patients with QRISK3 score ≥20% had significantly worse survival compared to those with QRISK3<20% (p=0.001). This survival difference was particularly pronounced in the PMF subgroup (p<0.001).

**Copyright Information:** (Online) ISSN: 2572-9241
©2026 The Author(s). HemaSphere is published by John Wiley & Sons Ltd on behalf of European Hematology Association. This is an open access Abstract Book distributed under the Attribution-NonCommercial-NoDerivs (CC BY-NC-ND), which allows third parties to download the articles and share them with others as long as they credit the author and the Abstract Book, but they cannot change the content in any way or use them commercially.
**Abstract Book Citations:** Authors, Title, HemaSphere, 2026;10:(S1):pages. The abstract book DOIs can be found on the "Author Information" page.
**Disclaimer:** Articles published in the journal HemaSphere exclusively reflect the opinions of the authors. The authors are responsible for all content in their abstracts including accuracy of the facts, statements, citing resources, etc.

### Summary/Conclusion

Higher QRISK3 was associated with increased risk of thrombosis and reduced overall survival, with the strongest predictive value in myelofibrosis. Cut-off of QRISK3 score ≥20% identified high-risk subgroup of patients with significantly worse survival who required focused treatment of comorbidities and considered early treatment of disease (Figure 1). Although discrimination for incident thrombosis was modest, QRISK3 adds prognostically relevant cardiovascular information to disease-specific MPN scores and may help to redefine thrombotic and survival risk assessment with needed prospective validation.

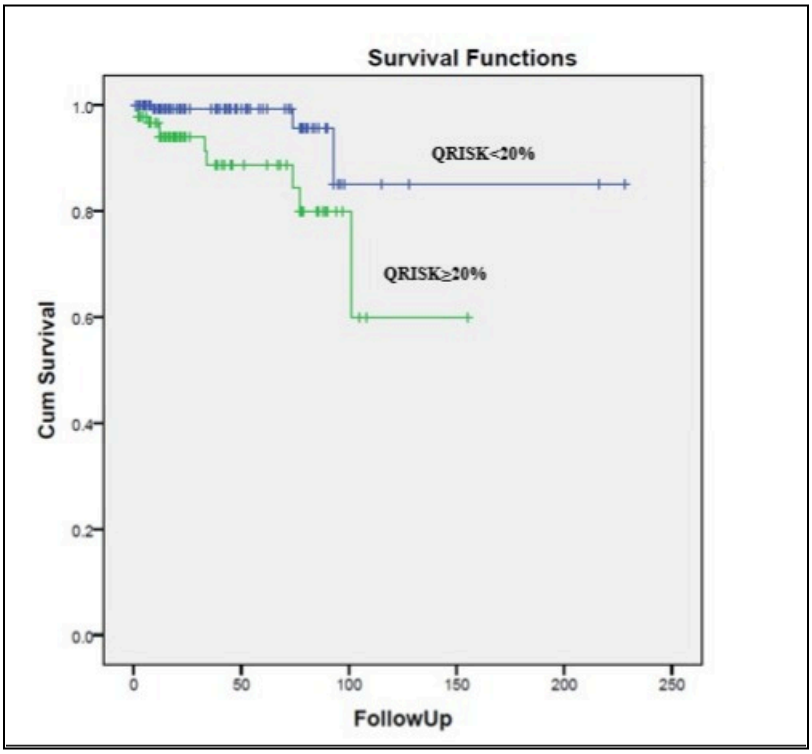

Figure 1. Overall Survival in MPN Patients Stratified by QRISK ≥20%

**Copyright Information:** (Online) ISSN: 2572-9241
©2026 The Author(s). HemaSphere is published by John Wiley & Sons Ltd on behalf of European Hematology Association. This is an open access Abstract Book distributed under the Attribution-NonCommercial-NoDerivs (CC BY-NC-ND), which allows third parties to download the articles and share them with others as long as they credit the author and the Abstract Book, but they cannot change the content in any way or use them commercially.
**Abstract Book Citations:** Authors, Title, HemaSphere, 2026;10:(S1):pages. The abstract book DOIs can be found on the "Author Information" page.
**Disclaimer:** Articles published in the journal HemaSphere exclusively reflect the opinions of the authors. The authors are responsible for all content in their abstracts including accuracy of the facts, statements, citing resources, etc.

### (PS1997) UPDATE FROM THE PHASE 2 RESTORE TRIAL OF ELRITERCEPT IN PATIENTS WITH MYELOFIBROSIS (MF) RECEIVING RUXOLITINIB

**Topic:** 16. Myeloproliferative neoplasms - Clinical

Ciro Rinaldi*[1], David Ross[2], Lynette Chee[3], Alessandra Iurlo[4], Chun Huat Teh[5], Francesca Palandri[6], Maria Laura Fox[7], Jennifer O'sullivan[8], Marco Brociner[9], Xin Tong[10], Varsha Sundaresan[10], Ziji Yu[10], Leopold Sellner[10], Katsu Togami[10], Claire Harrison[8]

*[1]United Lincolnshire Teaching Hospital and University of Lincoln, Haematology, Lincoln, United Kingdom;[2]Flinders Medical Centre and SA Pathology, Adelaide, Australia; [3]Department of Clinical Haematology, The Royal Melbourne Hospital and Peter MacCallum Cancer Centre, Parkville, Australia; [4]Hematology Division, Fondazione IRCCS Ca' Granda Ospedale Maggiore Policlinico, Milan, Italy; [5]St James Hospital, Leeds, United Kingdom;[6]IRCCS Azienda Ospedaliero-Universitaria di Bologna, Policlinico S. Orsola-Malpighi, Istituto di Ematologia "Seràgnoli", Bologna, Italy; [7]Vall d`Hebron Institute of Oncology (VHIO), Vall d'Hebron University Hospital, Barcelona, Spain; [8]Guy's and St Thomas' NHS Foundation Trust, London, United Kingdom; [9]Azienda Socio Sanitaria Territoriale Sette Laghi, Varese, Italy; [10]Takeda Development Center Americas, Inc. (TDCA), Cambridge, United States of America;*

### Background

Anemia is common in patients (pts) with MF, and is associated with fatigue, transfusion dependence, impaired quality of life, and reduced survival. Janus kinase (JAK) inhibitors such as ruxolitinib are current standard of care for MF but can exacerbate anemia, limiting optimal dosing. Elritercept is an investigational, modified activin receptor type IIA ligand trap designed to selectively bind and block downstream signaling of select TGF-β superfamily ligands, including activin A/B and GDF 8/11, with the aim to restore functional hematopoiesis.

### Aims

We report updated findings from the ongoing Phase 2 RESTORE trial in pts with MF (NCT05037760).

### Methods

RESTORE is an open-label study of elritercept as monotherapy or in combination with ruxolitinib in pts with MF and anemia. Eligible patients are transfusion-dependent (TD), defined as having received ≥3 red blood cell (RBC) units/12 weeks (wks); or non-transfusion-dependent (NTD), defined as having received <3 RBC units/12 wks with hemoglobin (Hgb) <10 g/dL. We report data for pts receiving elritercept starting at the recommended phase 2 dose of 3.75 mg/kg every 4 wks in combination with ruxolitinib. The primary endpoint is safety and tolerability of elritercept. Secondary and exploratory endpoints include elritercept treatment effects on hematological parameters, spleen size, Myelofibrosis Symptom Assessment Form Total Symptom Score (MF-SAF TSS), and gene mutations.

**Copyright Information:** (Online) ISSN: 2572-9241
©2026 The Author(s). HemaSphere is published by John Wiley & Sons Ltd on behalf of European Hematology Association. This is an open access Abstract Book distributed under the Attribution-NonCommercial-NoDerivs (CC BY-NC-ND), which allows third parties to download the articles and share them with others as long as they credit the author and the Abstract Book, but they cannot change the content in any way or use them commercially.
**Abstract Book Citations:** Authors, Title, HemaSphere, 2026;10:(S1):pages. The abstract book DOIs can be found on the "Author Information" page.
**Disclaimer:** Articles published in the journal HemaSphere exclusively reflect the opinions of the authors. The authors are responsible for all content in their abstracts including accuracy of the facts, statements, citing resources, etc.

### Results

As of July 2025, 38 pts receiving ruxolitinib were enrolled and treated with elritercept ≥3.75 mg/kg. At baseline, median age was 71 years (range 45–80); median duration of ruxolitinib treatment was 44 wks (range 18–631) at a median daily dose of 20 mg; 27 (71%) pts had spleen volume ≥450 $cm^3$. Median Hgb and platelet counts were 8.5 g/dL and 121 $x10^9$/L. Median RBC transfusion requirement was 4 units/12 wks (range 0–17), with 23 pts (61%) classified as TD. Most patients had a Dynamic International Prognostic Scoring System risk category score of intermediate-2 (n=26, 69%) or high (n=8, 21%). On study, median exposure to elritercept was 10.9 months (range 1.2–20.6); 21 (55%) pts had up-titration to 5 mg/kg. Median ruxolitinib dose intensity was 25 mg/day (range 5–50). The most common adverse events were diarrhea (24%, none grade ≥3; median duration 5.5 days), thrombocytopenia (18%, grade 3 in 10.5%), and fatigue (18%, grade 3 in 5%). By week 24, 8 (35%) TD pts achieved RBC transfusion independence (TI) for ≥12 wks, 13 (57%) had a ≥50% reduction in RBC transfusions over a consecutive 12-week period, and 4 (17%) achieved TI with a concurrent mean Hgb increase of ≥1.5 g/dL. Among 14 NTD pts, 5 (36%) and 7 (50%) achieved mean Hgb increases of ≥1.5 and ≥1 g/dL over any 12-week period. By week 24, 16 of 38 pts (42%) had a mean platelet increase of ≥30 × $10^9$/L over any 12-week period. Among TD and NTD pts, spleen volume reduction of ≥25% was achieved in 5 of 24 (21%) evaluable pts, and ≥50% reduction in MF-SAF TSS was achieved in 13 of 27 (48%) evaluable pts. *JAK2 V617F* variant allele frequency reduction of ≥50% was observed in 3 of 19 evaluable pts at week 24, with sustained reductions observed at week 52.

### Summary/Conclusion

Elritercept as an add-on treatment to ruxolitinib demonstrated clinically meaningful improvements in transfusion burden, spleen volume reduction, thrombocytopenia, and symptoms in pts with MF. These findings support elritercept as a potential novel treatment for pts with MF.

*©American Society of Hematology (2025). Reused with permission.*

**Copyright Information:** (Online) ISSN: 2572-9241
©2026 The Author(s). HemaSphere is published by John Wiley & Sons Ltd on behalf of European Hematology Association. This is an open access Abstract Book distributed under the Attribution-NonCommercial-NoDerivs (CC BY-NC-ND), which allows third parties to download the articles and share them with others as long as they credit the author and the Abstract Book, but they cannot change the content in any way or use them commercially.
**Abstract Book Citations:** Authors, Title, HemaSphere, 2026;10:(S1):pages. The abstract book DOIs can be found on the "Author Information" page.
**Disclaimer:** Articles published in the journal HemaSphere exclusively reflect the opinions of the authors. The authors are responsible for all content in their abstracts including accuracy of the facts, statements, citing resources, etc.

### (PS1998) SUSTAINED CONTROL OF EXCESSIVE ERYTHROPOIESIS BY THE TMPRSS6 ANTISENSE INHIBITOR SAPABLURSEN IN POLYCYTHEMIA VERA: IMPRSSION TRIAL TREATMENT EXTENSION DATA

**Topic:** 16. Myeloproliferative neoplasms - Clinical

Pankit Vachhani*[1], Jeanne Palmer[2], Jonathan How[3], Adam Mead[4], Jonathan Sillar[5], Aaron T. Gerds[6], Angela Fleischman[7], Caitlin O'neill[8], Ross Baker[9], Ronald Hoffman[10], Shiangtung Jung[11], Jon Ruckle[11], Keyvan Yousefi[11], Sanjay Bhanot[11], Terrance Barrett[11], Ruben Mesa[12], Prithviraj Bose[13]

*[1]Hematology Oncology at The Kirklin Clinic of UAB Hospital, Birmingham, United States of America;[2]Mayo Clinic College of Medicine and Science, Division of Hematology/Oncology, Department of Internal Medicine, Mayo Clinic, Phoenix, United States of America; [3]Division of Hematology, Royal Victoria Hospital, McGill University, Montreal, Canada;[4]Medical Research Council Weatherall Institute of Molecular Medicine (MRC WIMM) and NIHR Biomedical Research Centre Hematology Theme, University of Oxford, Oxford, United Kingdom; [5]Calvary Mater Hospital, Department of Haematology, Waratah, Australia;[6]Cleveland Clinic, Department of Hematology and Medical Oncology, Cleveland, United States of America; [7]Department of Medicine, University of California Irvine, Irvine, United States of America; [8]Division of Hematology, Keck School of Medicine, University of Southern California, Los Angeles, United States of America; [9]Perth Blood Institute, Murdoch University, Perth, Australia; [10]Tisch Cancer Institute, Department of Medicine, Mount Sinai School of Medicine, New York, United States of America; [11]Ionis Pharmaceuticals, Carlsbad, United States of America; [12]Atrium Health Levine Cancer Institute and Atrium Health Wake Forest Baptist Comprehensive Cancer Center, Charlotte, United States of America; [13]The University of Texas MD Anderson Cancer Center, Department of Leukemia, Division of Cancer Medicine, Houston, United States of America;*

### Background

Sapablursen (ISIS 702843, ONO-0530) is a liver-directed, ligand-conjugated antisense oligonucleotide inhibitor of transmembrane serine protease 6 (TMPRSS6) that increases hepcidin production and reduces iron availability for erythropoiesis. We recently reported the primary results of the IMPRSSION study (NCT05143957), an ongoing, open-label phase 2 trial of two dose cohorts in participants with phlebotomy dependent polycythemia vera (PV). Treatment with sapablursen met the primary endpoint in both dose cohorts and significantly reduced phlebotomy rate during the primary endpoint window (Weeks 17-37).

### Aims

To report on the long-term safety, and efficacy of sapablursen over 73 weeks, including a 36-week treatment extension period where increases above the originally assigned dose were allowed.

### Methods

Eligible participants met 2016 WHO diagnostic criteria for PV, were required to have≥3 phlebotomies within six months prior to screening, including ≥1 phlebotomy in the last 12 weeks. There were no exclusions for high HCT prior to randomization and phlebotomy was performed for HCT ≥45%, without need for confirmation. Participants received Q4W subcutaneous (SC) doses of sapablursen in Cohort A (120 or 80 mg dose) or Cohort B (40 mg dose), during a 37-week treatment period, followed by an additional 36-week treatment extension period. Dose adjustment based on HCT was implemented during the study so that doses of 20, 40, 80, 120 and 160 mg Q4W were possible. Dose reduction was allowed if HCT≤40% and required if HCT≤38%. Dose increase was allowed after Week 37 if HCT ≥43% and was required if HCT≥45%.

**Copyright Information:** (Online) ISSN: 2572-9241
©2026 The Author(s). HemaSphere is published by John Wiley & Sons Ltd on behalf of European Hematology Association. This is an open access Abstract Book distributed under the Attribution-NonCommercial-NoDerivs (CC BY-NC-ND), which allows third parties to download the articles and share them with others as long as they credit the author and the Abstract Book, but they cannot change the content in any way or use them commercially.
**Abstract Book Citations:** Authors, Title, HemaSphere, 2026;10:(S1):pages. The abstract book DOIs can be found on the "Author Information" page.
**Disclaimer:** Articles published in the journal HemaSphere exclusively reflect the opinions of the authors. The authors are responsible for all content in their abstracts including accuracy of the facts, statements, citing resources, etc.

### Results

Forty-nine (49) participants received at least one dose of sapablursen in either Cohort A (n=32) or Cohort B (n=17). The median age was 61 years, 40 (82%) were male, and 30 (61%) classified as high risk. At baseline, weeks 37 and 73 mean local HCT(±SD) was 46.4±3.6%, 40.5±4.4% and 41.1±4.0%, respectively, in Cohort A and 45.5±3.4%, 43.5±2.2% and 41.8±3.7% in Cohort B. The weekly phlebotomy rate (mean±SD) decreased from baseline of 0.15±0.07 (~7.8/y) to 0.05±0.09 (~2.6/y) during the primary endpoint window (Weeks 17-37) and was maintained at a low level of 0.03±0.05 (~1.6/y) during the 36 treatment extension period (Weeks 37-73) in Cohort A, and from 0.17±0.07 (~8.8/y) to 0.07±0.07 (~3.6/y) and 0.04±0.04 (~2.1/y) in Cohort B at the same time periods. During the treatment extensions period dose was increased based on HCT in 2 and 8 participants in Cohort A and B, respectively, and dose was reduced in 7 and 0 participants in Cohort A and B, respectively. In the 73-week treatment period, anemia was reported in 15 (46.9%) patients in Cohort A, and 7 (41.2%) patients in Cohort B. One participant in Cohort A (3.1%) developed a Grade 3 hemoglobin decrease in the context of an unrelated bleeding gastric ulcer. The incidence of injection site reactions with Q4W administration was 12.2% (6/49). Injection site reactions did not recur following multiple injections, were not progressive and resolved spontaneously.

### Summary/Conclusion

Hematocrit control with sapablursen was durable through 73 weeks of treatment. These findings highlight the sustained control of excessive erythropoiesis by sapablursen and establish 80 mg SC Q4W as an optimal starting dose for future studies.

**Copyright Information:** (Online) ISSN: 2572-9241
©2026 The Author(s). HemaSphere is published by John Wiley & Sons Ltd on behalf of European Hematology Association. This is an open access Abstract Book distributed under the Attribution-NonCommercial-NoDerivs (CC BY-NC-ND), which allows third parties to download the articles and share them with others as long as they credit the author and the Abstract Book, but they cannot change the content in any way or use them commercially.
**Abstract Book Citations:** Authors, Title, HemaSphere, 2026;10:(S1):pages. The abstract book DOIs can be found on the “Author Information” page.
**Disclaimer:** Articles published in the journal HemaSphere exclusively reflect the opinions of the authors. The authors are responsible for all content in their abstracts including accuracy of the facts, statements, citing resources, etc.

### (PS1999) NOVEL MACHINE LEARNING-BASED MODEL PREDICTS PRESENCE OF ASSOCIATED HEMATOLOGIC NEOPLASMS IN ADVANCED SYSTEMIC MASTOCYTOSIS

**Topic:** 16. Myeloproliferative neoplasms - Clinical

Johannes Lübke*[1], Aaron Zakharyan[2], Tracy I. George[3], Jason Gotlib[4], Daniel J. Deangelo[5], Deepti H. Radia[6], Michael Deininger[7], Juliana Schwaab[1], Saša Dimitrijević[8], Andreas Reiter[1]

*[1]University Hospital Mannheim, Department of Hematology and Oncology, Manheim, Germany; [2]Blueprint Medicines Corporation, Cambridge, MA, United States of America; [3]ARUP Laboratories, University of Utah, Salt Lake City, UT, United States of America; [4]Stanford Cancer Institute/Stanford University School of Medicine, Division of Hematology, Stanford, CA, United States of America; [5]Dana-Farber Cancer Institute, Department of Medical Oncology, Boston, MA, United States of America; [6]Guy's and St Thomas' NHS Foundation Trust, London, United Kingdom; [7]Michael Deininger Laboratory, University of Michigan, Division of Hematology/Oncology, Ann Arbor, MI, United States of America; [8]Blueprint Medicines GmbH, Zug, Switzerland;*

### Background

Systemic mastocytosis with associated hematologic neoplasm (SM-AHN) is the most frequent and heterogeneous subtype of advanced SM (AdvSM). Identifying the AHN at diagnosis is challenging as SM can dominate clinico-morphologic findings and obscure the AHN. Accurate diagnosis is critical for adequate clinical management and eventually impacts prognosis.

### Aims

Development and validation of tiered models, adapted to data availability, to identify AdvSM patients (pts) with likelihood of AHN using clinical, morphologic, and genetic variables.

### Methods

Diagnoses of AdvSM (including presence/absence of AHN) were clinically assigned according to 2016 World Health Organization (WHO) criteria. All AHNs were myeloid. Bone marrow (BM) and tissue samples were centrally reviewed by reference pathologists. Baseline variables (n=33) were obtained from 374 pts in the EXPLORER phase 1 and PATHFINDER phase 2 studies of avapritinib in AdvSM (n=167) as well as the German Registry on Disorders of Eosinophils and Mast Cells (GREM, n=207). Data were randomly split (70:30) into training and test cohorts. A composite variable ($AHN^{comp}$), derived from WHO thresholds for neutrophils, monocytes, and eosinophils characterizing AHN subtypes, was used as a binary AHN indicator. Genetic data from BM (n=73) and peripheral blood (PB, n=298) were incorporated as a single weighted composite variable ($MUT^{weighted}$) reflecting the presence/absence of mutations across 11 genes (*SRSF2* and *TET2* modeled separately). *KIT* D816V was quantified by digital PCR in PB (detection limit: 0.17% in EXPLORER, 0.02% in PATHFINDER, 0.1% in GREM). Random forest–guided variable selection, followed by two-step logistic regression, generated models robust to variable data availability. Pts scoring $0.5 \le P < 1.0$ were classified as having AHN.

**Copyright Information:** (Online) ISSN: 2572-9241
©2026 The Author(s). HemaSphere is published by John Wiley & Sons Ltd on behalf of European Hematology Association. This is an open access Abstract Book distributed under the Attribution-NonCommercial-NoDerivs (CC BY-NC-ND), which allows third parties to download the articles and share them with others as long as they credit the author and the Abstract Book, but they cannot change the content in any way or use them commercially.
**Abstract Book Citations:** Authors, Title, HemaSphere, 2026;10:(S1):pages. The abstract book DOIs can be found on the “Author Information” page.
**Disclaimer:** Articles published in the journal HemaSphere exclusively reflect the opinions of the authors. The authors are responsible for all content in their abstracts including accuracy of the facts, statements, citing resources, etc.

### Results

In the full model (A), AHN was predicted by lower basal serum tryptase, higher monocytes, fewer BM mast cell (MC) aggregates, BM hypercellularity, higher $MUT^{weighted}$, $AHN^{comp}$, *SRSF2* mutation, and *TET2* mutation (training sensitivity 0.961; test sensitivity 0.977). Model (B), excluding morphologic but retaining genetic and PB laboratory variables, predicted AHN by lower serum tryptase, higher monocytes, higher $MUT^{weighted}$, higher PB *KIT* D816V allele burden, $AHN^{comp}$, and *SRSF2* and *TET2* mutations (training sensitivity 0.937; test sensitivity 0.954). Model (C), excluding genetic but retaining morphologic and PB laboratory variables, predicted AHN by lower serum tryptase, higher monocytes, fewer BM MC aggregates, lower hemoglobin, $AHN^{comp}$, and BM hypercellularity (training sensitivity 0.942; test sensitivity 0.977). Model (D), excluding morphologic and genetic variables, and retaining PB laboratory variables, predicted AHN by lower serum tryptase, higher monocytes, lower hemoglobin, higher age, and $AHN^{comp}$ (training sensitivity 0.932; test sensitivity 0.966). Model performance was comparable when next-generation sequencing was performed on BM vs PB. Among 28 pts (7.5%) who were clinically classified as AHN-negative but predicted to be AHN-positive by model (A), 21 (75%) had ≥1 parameter suggestive of (obscured) AHN: PB *KIT* D816V allele burden >10% (n=20, 71%), monocytes >0.8 $x10^9$/L (n=8, 29%), eosinophils >0.5 $x10^9$/L (n=5, 18%), and ≥1 additional somatic mutation beyond *KIT* D816V (n=5, 18%).

### Summary/Conclusion

Presence of AHN in AdvSM can be predicted with high accuracy using adaptable models that retain performance even in cases where only a PB sample is available.

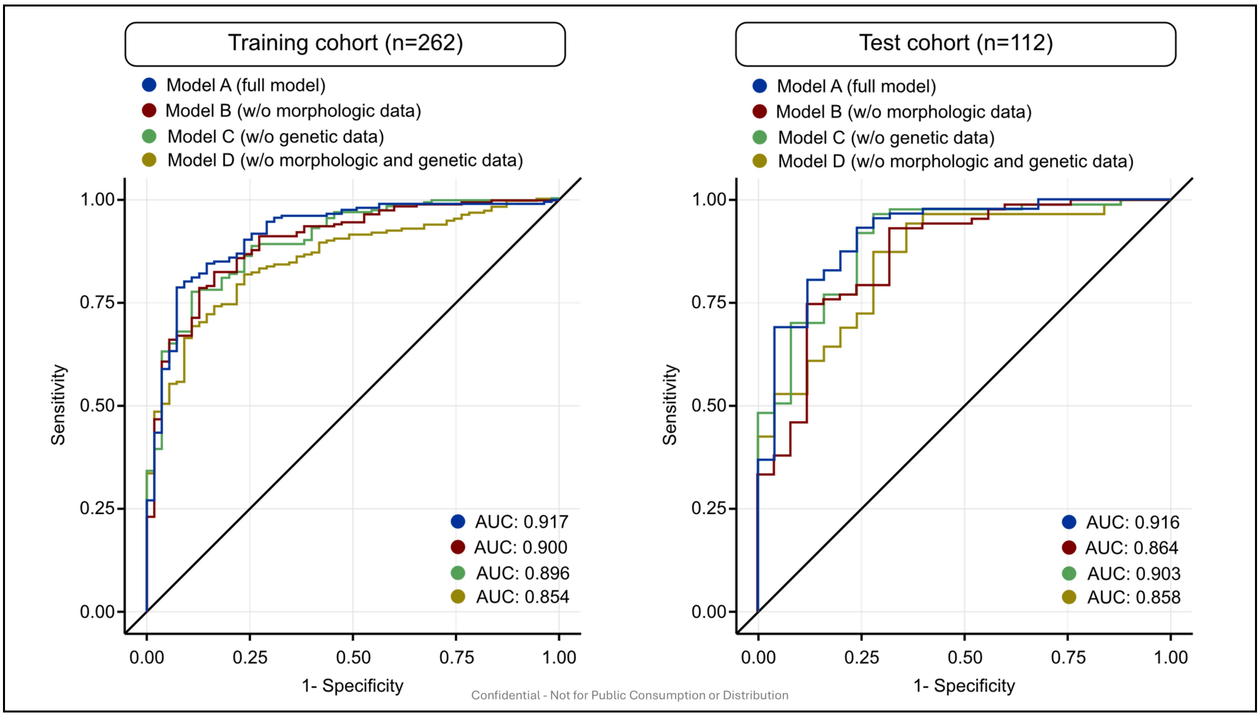

Confidential - Not for Public Consumption or Distribution

Figure. ROC curves for models A–D predicting the presence of an additional hematologic neoplasm in advanced systemic mastocytosis. The left panel shows performance in the training cohort, and the right panel shows performance in the independent test cohort. Sensitivity is plotted against 1–specificity; the gray diagonal line denotes no-discrimination performance (AUC=0.5). AUC denotes area under the ROC curve. ROC, receiver operating characteristic; w/o, without.

**Copyright Information:** (Online) ISSN: 2572-9241
©2026 The Author(s). HemaSphere is published by John Wiley & Sons Ltd on behalf of European Hematology Association. This is an open access Abstract Book distributed under the Attribution-NonCommercial-NoDerivs (CC BY-NC-ND), which allows third parties to download the articles and share them with others as long as they credit the author and the Abstract Book, but they cannot change the content in any way or use them commercially.
**Abstract Book Citations:** Authors, Title, HemaSphere, 2026;10:(S1):pages. The abstract book DOIs can be found on the "Author Information" page.
**Disclaimer:** Articles published in the journal HemaSphere exclusively reflect the opinions of the authors. The authors are responsible for all content in their abstracts including accuracy of the facts, statements, citing resources, etc.

### (PS2000) LONG-TERM HEMATOLOGIC AND MOLECULAR RESPONSE TO ROPEGINTERFERON ALFA-2B AND CLINICAL VALIDATION OF Q-PCR FOR LOW-LEVEL JAK2 MONITORING IN POLYCYTHEMIA VERA

**Topic:** 16. Myeloproliferative neoplasms - Clinical

Sung-Eun Lee*[1], So Yeon Park[1], Seug Yun Yoon[2], Junshik Hong[3], Deok Hwan Yang[4], Gyeong-Won Lee[5], Sang Kyun Sohn[6], Ho-Jin Shin[7], Sunghwa Bae[8], Chul Won Choi[9], Yunsuk Choi[10], June-Won Cheong[11], Soo Mee Bang[12], Joon Seong Park[13], Yong Park[14], Young Hoon Park[15]

*[1]Seoul St. Mary's Hospital, College of Medicine, The Catholic University of Korea, Department of Hematology, Seoul, South Korea; [2]Soonchunhyang University Seoul Hospital, Seoul, South Korea; [3]Seoul National University Hospital, Seoul, South Korea; [4]Chonnam National University Hwasun Hospital, Chonnam National University Medical School, Jeollanam-do, South Korea; [5]Institute of Health Science, Gyeongsang National University Hospital, Gyeongsang National University College of Medicine, Jinju, South Korea; [6]Kyungpook National University Hospital, Daegu, South Korea; [7]Pusan National University Hospital, Busan, South Korea; [8]Daegu Catholic, University Hospital, Daegu Catholic University School of Medicine, Daegu, South Korea; [9]Korea University Medical Center, Seoul, South Korea; [10]Asan Medical Center, University of Ulsan College of Medicine, Seoul, South Korea; [11]Severance Hospital, Yonsei University College of Medicine, Seoul, South Korea; [12]Seoul National University College of Medicine, Seoul National University Bundang Hospital, Seongnam, South Korea; [13]Ajou University School of Medicine, Suwon, South Korea; [14]Korea University College of Medicine, Seoul, South Korea; [15]Ewha Womans University Mokdong Hospital, Seoul, South Korea;*

### Background

Polycythemia vera (PV) is a JAK2-mutated myeloproliferative neoplasm characterized by clonal erythrocytosis, thromboinflammation, and risk of disease progression. Beyond complete hematologic response (CHR), molecular response (MR) is increasingly recognized as a potential indicator of disease modification.

### Aims

We evaluated long-term hematologic and molecular responses to ropeginterferon alfa-2b and compared quantitative PCR (qPCR) with digital PCR (dPCR) for monitoring low JAK2 V617F allele burdens.

### Methods

This multicenter, single-arm extension trial assessed long-term outcomes of ropeginterferon alfa-2b in PV. Among 99 enrolled patients, 79 completed core treatment (Week 48); 77 entered the extension phase, and 76 completed follow-up through Week 168. CHR and MR were assessed longitudinally through Visit 13 (Week 132). JAK2 V617F allele burden was measured by qPCR every 12 weeks. dPCR was performed at Week 132 to validate low-level detection. Correlation between assays was evaluated using Pearson's coefficient.

**Copyright Information:** (Online) ISSN: 2572-9241
©2026 The Author(s). HemaSphere is published by John Wiley & Sons Ltd on behalf of European Hematology Association. This is an open access Abstract Book distributed under the Attribution-NonCommercial-NoDerivs (CC BY-NC-ND), which allows third parties to download the articles and share them with others as long as they credit the author and the Abstract Book, but they cannot change the content in any way or use them commercially.
**Abstract Book Citations:** Authors, Title, HemaSphere, 2026;10:(S1):pages. The abstract book DOIs can be found on the "Author Information" page.
**Disclaimer:** Articles published in the journal HemaSphere exclusively reflect the opinions of the authors. The authors are responsible for all content in their abstracts including accuracy of the facts, statements, citing resources, etc.

**Results**

CHR improved steadily over time, from 26.6% at Week 12 to 62.9% at Week 48, 70.1% at Week 72, 74.0% at Week 96, and 80.0% at Week 132. In parallel, MR rates increased progressively: 31.8% at Week 12, 56.8% at Week 48, 68.5% at Week 72, 75.7% at Week 96, 79.7% at Week 108, and 85.1% at Week 132. Median JAK2 V617F allele burden declined from 69.89% at baseline to 33.82% at Week 48, 16.22% at Week 72, 12.52% at Week 96, and 4.96% at Week 132. By Week 132, 58% of patients achieved <10% allele burden, including 32% with very-low residual disease (VAF <1%). At Week 132, median allele burden measured by dPCR was 4.98%, highly consistent with qPCR. Overall concordance between qPCR and dPCR was near perfect ($r = 0.997$; 95% CI, 0.995–0.998; $p < 0.001$). Agreement remained strong even at VAF <1% ($r = 0.938$; 95% CI, 0.862–0.972; $p < 0.001$), supporting reliable detection of minimal residual alleles.

**Summary/Conclusion**

Ropeginterferon alfa-2b induces deep, durable hematologic and molecular responses over two years of therapy, with convergence of high CHR and MR rates by Week 132. Progressive reduction of JAK2 V617F burden, including achievement of <1% VAF in a subset of patients, supports its potential disease-modifying activity. As qPCR may be limited at very low allele burdens, dPCR was used for confirmation and showed strong concordance. These findings highlight the feasibility of deep molecular monitoring and support further investigation of its clinical implications in PV.

**Copyright Information:** (Online) ISSN: 2572-9241
©2026 The Author(s). HemaSphere is published by John Wiley & Sons Ltd on behalf of European Hematology Association. This is an open access Abstract Book distributed under the Attribution-NonCommercial-NoDerivs (CC BY-NC-ND), which allows third parties to download the articles and share them with others as long as they credit the author and the Abstract Book, but they cannot change the content in any way or use them commercially.
**Abstract Book Citations:** Authors, Title, HemaSphere, 2026;10:(S1):pages. The abstract book DOIs can be found on the “Author Information” page.
**Disclaimer:** Articles published in the journal HemaSphere exclusively reflect the opinions of the authors. The authors are responsible for all content in their abstracts including accuracy of the facts, statements, citing resources, etc.

### (PS2001) CHARACTERIZATION OF SYMPTOMS AFTER IMMEDIATE TRANSITION FROM RUXOLITINIB TO MOMELOTINIB IN PATIENTS WITH MYELOFIBROSIS: POST HOC ANALYSES OF THE PHASE 3 SIMPLIFY-1 AND SIMPLIFY-2 TRIALS

**Topic:** 16. Myeloproliferative neoplasms - Clinical

Pankit Vachhani*[1], Alessandro Lucchesi[2], Andrew Perkins[3], Jasmine Sahni[4], Mahshid Azimi[4], Jessica Lim[4], Shiyuan Zhang[4], Dwaipayan Patnaik[5], Jeanne Palmer[6], Prithviraj Bose[7]

*[1]The University of Alabama at Birmingham, Birmingham, AL, United States of America;[2]IRCCS Istituto Romagnolo per lo Studio dei Tumori "Dino Amadori", Meldola, Italy; [3]The Alfred Hospital and Australian Centre for Blood Diseases, Monash University, Melbourne, VIC, Australia; [4]GSK, Collegeville, PA, United States of America;[5]GSK, Baar, Switzerland; [6]Mayo Clinic, Phoenix, AZ, United States of America; [7]University of Texas MD Anderson Cancer Center, Houston, TX, United States of America;*

### Background

Discontinuation of ruxolitinib (RUX) in myelofibrosis (MF) carries a risk of ruxolitinib discontinuation syndrome (RDS), characterized by acute flare-up of splenomegaly, hematologic abnormalities, hemodynamic decompensation, and, in severe cases, a cytokine storm-like hyperinflammatory response. A previous post hoc analysis of SIMPLIFY-1 demonstrated that patients can be immediately transitioned from RUX to momelotinib without evidence of RDS (Mesa R, et al. *Haematologica*. 2024). Due to limited additional data available on RUX-to-momelotinib sequencing, the present analyses of SIMPLIFY-1 and SIMPLIFY-2 were conducted to more thoroughly characterize adverse events (AEs) resembling MF symptoms during the first 1-2 weeks post transition.

### Aims

To evaluate RUX dosing and symptom patterns in patients who experienced AEs resembling MF symptoms after immediate transition from RUX to momelotinib in SIMPLIFY-1 and SIMPLIFY-2.

### Methods

SIMPLIFY-1 (NCT01969838; Janus kinase [JAK] inhibitor naive) and SIMPLIFY-2 (NCT02101268; JAK inhibitor experienced) were randomized trials of momelotinib vs RUX or best available therapy (88.5% RUX), respectively, with no tapering or washout of prior RUX. These post hoc analyses included SIMPLIFY-1 patients with AEs in the first 2 weeks post crossover from randomized RUX to open-label momelotinib, and momelotinib-randomized SIMPLIFY-2 patients who transitioned directly after ≥12 weeks of prior RUX. In SIMPLIFY-1, RUX dose before crossover and symptoms in 2-week intervals post crossover were assessed. In SIMPLIFY-2, Total Symptom Score (TSS), including individual symptom items, was assessed at momelotinib initiation and at weeks 1-4, 8, 12, and 24. Data are summarized descriptively.

**Copyright Information:** (Online) ISSN: 2572-9241
©2026 The Author(s). HemaSphere is published by John Wiley & Sons Ltd on behalf of European Hematology Association. This is an open access Abstract Book distributed under the Attribution-NonCommercial-NoDerivs (CC BY-NC-ND), which allows third parties to download the articles and share them with others as long as they credit the author and the Abstract Book, but they cannot change the content in any way or use them commercially.
**Abstract Book Citations:** Authors, Title, HemaSphere, 2026;10:(S1):pages. The abstract book DOIs can be found on the “Author Information” page.
**Disclaimer:** Articles published in the journal HemaSphere exclusively reflect the opinions of the authors. The authors are responsible for all content in their abstracts including accuracy of the facts, statements, citing resources, etc.

**Results**

In SIMPLIFY-1, 88/197 patients experienced AEs within 2 weeks post crossover; high doses of prior RUX were observed in this group (median, 34.5 mg/day [range, 10.0-46.2]). Among these 88 patients, AEs that resembled MF symptoms mainly occurred in weeks 1-2 post crossover, including fatigue (11%), dizziness (10%), pruritus (7%), and night sweats (5%); frequency did not increase over the next 24 weeks. The incidence rates (new onset) of these AEs decreased to <5% at weeks 3-4 and then to <3% at weeks 5-6 and thereafter (**Figure**). Additional data on grade, onset, and duration of these AEs will also be evaluated.
As TSS was not assessed after crossover in SIMPLIFY-1, TSS during the randomized part of SIMPLIFY-2 was evaluated in 75 patients who were receiving prior RUX immediately before initiating momelotinib. Mean TSS improved as early as the first assessment time point (week 1) and continued to improve through week 4 and beyond. Over the first 4 weeks of momelotinib treatment, individual TSS items remained stable or improved in most patients (>90%); at week 1, stability or improvement (≥2 points) was reported in tiredness in 96% of patients, night sweats in 99%, and itching in all patients.

**Summary/Conclusion**

Immediate transition from RUX to momelotinib is not associated with severe or acute symptom relapses. Symptom reemergence occurred in few patients after immediate transition from high-dose RUX to momelotinib; events were mostly low grade and transient, and symptoms typically associated with splenomegaly were not reported. Overall, these findings inform clinical expectations for symptom management during treatment switch from RUX to momelotinib in patients with MF, regardless of prior JAK inhibitor experience.
**Funding**: GSK.

**Figure.** Incidence Over Time of AEs Resembling MF Symptoms After Crossover From RUX to Momelotinib in SIMPLIFY-1 (n=88)

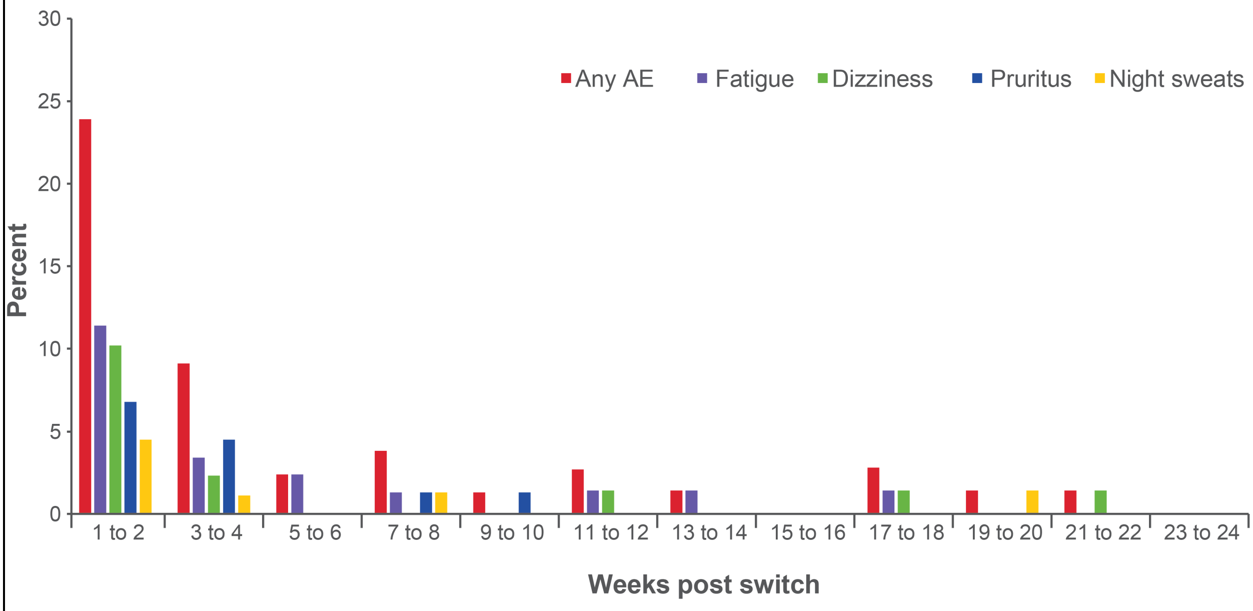

AE, adverse event; MF, myelofibrosis; RUX, ruxolitinib.

Figure. Incidence Over Time of AEs Resembling MF Symptoms After Crossover From RUX to Momelotinib in SIMPLIFY-1 (n=88). AE, adverse event; MF, myelofibrosis; RUX, ruxolitinib.

**Copyright Information:** (Online) ISSN: 2572-9241
©2026 The Author(s). HemaSphere is published by John Wiley & Sons Ltd on behalf of European Hematology Association. This is an open access Abstract Book distributed under the Attribution-NonCommercial-NoDerivs (CC BY-NC-ND), which allows third parties to download the articles and share them with others as long as they credit the author and the Abstract Book, but they cannot change the content in any way or use them commercially.
**Abstract Book Citations:** Authors, Title, HemaSphere, 2026;10:(S1):pages. The abstract book DOIs can be found on the "Author Information" page.
**Disclaimer:** Articles published in the journal HemaSphere exclusively reflect the opinions of the authors. The authors are responsible for all content in their abstracts including accuracy of the facts, statements, citing resources, etc.

### (PS2002) DYNAMIC CYTOPENIA PATTERNS IN MYELOFIBROSIS: A REAL-WORLD ANALYSIS FROM THE ERNEST-3 REGISTRY

**Topic:** 16. Myeloproliferative neoplasms - Clinical

Tiziano Barbui*[1], Arianna Ghirardi[1], Francesca Fenili[1], Danijela Lekovic[2], Novella Pugliese[3], Eduardo Arellano Rodrigo[4], Giuseppe Gaetano Loscocco[5], Valerio De Stefano[6], Annalisa Condorelli[7], Pietro Tumminello[8], Monia Marchetti[9], Iulia Ursuleac[10], Antónia Hatalová[11], Francesca Palandri[12], Marta Sobas[13], Elena Elli[14], Alessandra Iurlo[15], Daniele Sannipoli[16], Andrea Patriarca[17], Giuseppe Carli[18], Massimo Breccia[19], Gonzalo Carreño Gómez-Tarragona[20], Maria Badikyan[21], Barbora Weinbergerová[22], Paola Guglielmelli[5], Alessandro Rambaldi[1], Alberto Alvarez Larran[23], Konstanze Döhner[24], Alessandro Maria Vannucchi[5]

*[1]FROM - Research Foundation Bergamo Hospital - ETS, Bergamo, Italy; [2]Clinic of Hematology, University Clinical Center Serbia; Faculty of Medicine, University of Belgrade, Belgrade, Serbia; [3]Hematology and Stem Cell Trasplant unit, University of Naples, "Federico II", Naples, Italy; [4]Hematology Department, Hospital Clínic Barcelona, Barcelona, Spain;[5]Department of Experimental and Clinical Medicine, Center for Research and Innovation of Myeloproliferative Neoplasms, AOU Careggi, University of Florence, Florence, Italy; [6]Catholic University and Policlinico "A. Gemelli" IRCCS Foundation, Rome, Italy; [7]Hematology Division, ASST Papa Giovanni XXIII, Bergamo, Italy; [8]Department of Molecular Medicine, University of Pavia, Pavia, Italy; [9]Hematology Unit, University Hospital "SS Antonio e Biagio e Cesare Arrigo", Alessandria, Italy; [10]University of Medicine "Carol Davila"; Fundeni Clinical Institute, Bucharest, Romania; [11]Department of Hematology and Transfusiology, Faculty of Medicine, University Hospital Bratislava, Bratislava, Slovakia; [12]Institute of Hematology "L. e A. Seràgnoli" IRCCS S. Orsola-Malpighi Alma Mater Studiorum - University of Bologna, Bologna, Italy; [13]Department of Hematology, Collegium Medicum in Bydgoszcz, Nicolaus Copernicus University in Toruń, Bydgoszcz, Poland; [14]Hematology Division, Fondazione IRCCS "San Gerardo dei Tintori", Monza, Italy; [15]Hematology Division, Foundation IRCCS Cà Granda Ospedale Maggiore Policlinico, Milan, Italy; [16]Hematology and Bone Marrow Trasplantation Unit, IRCCS Ospedale San Raffaele, Milan, Italy; [17]Hematology Unit, AOU maggiore della Carità and Department of Translational Medicine, University of Eastern Piedmont, Novara, Italy; [18]Hematology Unit, San Bortolo Hospital, ULSS8 Berica, Vicenza, Italy; [19]Policlinico Umberto I and Sapienza University, Rome, Italy;[20]Hematology Department, Hospital Universitario 12 de Octubre, Madrid, Spain; [21]Immune Oncology Research Institute; Yeolyan Hematology and Oncology Center, Yerevan, Armenia; [22]Masaryk University and University Hospital Brno, Brno, Czechia; [23]Hematology Department, Hospital Clínic, Institut d'Investigacions Biomèdiques August Pi i Sunyer (IDIBAPS), Barcelona, Spain; [24]University Hospital of Ulm Department of Internal Medicine III, Ulm, Germany;*

### Background

In myelofibrosis (MF), cytopenias identify a disease phenotype (CyP) with poorer prognosis than myeloproliferative presentations (MyP) with anemia and thrombocytopenia predicting mortality. The ELN-promoted ERNEST-3 registry enables real-world characterization of cytopenic patterns, their evolution, and treatment associations in WHO-defined overt MF.

### Aims

To characterize baseline CyP and MyP phenotypes, treatment exposure, their longitudinal dynamics, and clinical outcomes in MF patients enrolled in the ERNEST-3 registry.

**Copyright Information:** (Online) ISSN: 2572-9241
©2026 The Author(s). HemaSphere is published by John Wiley & Sons Ltd on behalf of European Hematology Association. This is an open access Abstract Book distributed under the Attribution-NonCommercial-NoDerivs (CC BY-NC-ND), which allows third parties to download the articles and share them with others as long as they credit the author and the Abstract Book, but they cannot change the content in any way or use them commercially.
**Abstract Book Citations:** Authors, Title, HemaSphere, 2026;10:(S1):pages. The abstract book DOIs can be found on the "Author Information" page.
**Disclaimer:** Articles published in the journal HemaSphere exclusively reflect the opinions of the authors. The authors are responsible for all content in their abstracts including accuracy of the facts, statements, citing resources, etc.

### Methods

Patients diagnosed with MF between January 2018 and January 2026 from 21 European centers were enrolled in this study sponsored by "*FROM Research Foundation Bergamo Hospital*", supported by an unrestricted grant from GSK. CyP at diagnosis was defined by at least one of the following: anemia (Hb <11 g/dL in men and <10 g/dL in women; moderate if Hb 9–10.9 or 8–9.9 g/dL, and severe if Hb <9 or <8 g/dL, respectively), thrombocytopenia (platelets <100 $\times 10^9$/L), or leukopenia (leukocytes <4 $\times 10^9$/L). CyP/MyP transitions and their magnitude were evaluated over a median 3-year follow-up using Sankey diagrams and correlated with major clinical events.

### Results

Baseline data were available for 418/454 patients. At diagnosis, 232 patients (55%) had a MyP and 186 (45%) a CyP, most commonly isolated anemia (27%); isolated leukopenia (3%), thrombocytopenia (4%), and combined cytopenias were rare (≤5% each). CyP patients less frequently had secondary MF than MyP (32% vs 45%). Compared with MyP, CyP was associated with more frequent splenomegaly (73% vs 66%), higher-grade (G3) marrow fibrosis in 41% vs 21%, lower $JAK2^{V617F}$ allele burden (38% vs 60%), fewer *CALR* mutations (17% vs 25%), and a higher prevalence of additional myeloid mutations (69% vs 56%).
Cytoreductive treatments At diagnosis, MyP patients were mainly treated with ruxolitinib (ruxo) (56%) and hydroxyurea (HU) (43%), with limited use of other agents. In CyP patients cytoreductive frequency was: HU 33%, ruxo 55%, fedratinib 11%, momelotinib 11%, interferon 1% and pacritinib 1%. Limited momelotinib use likely reflected recent approval rather than clinical selection.
CyP/MyP dynamics (Sankey analysis) Among MyP patients at diagnosis, 52% remained non-cytopenic while 48% transitioned to CyP: moderate anemia (30%), severe anemia (10%) and combined cytopenias (8%). Patients with moderate anemia at diagnosis (N=115) showed bidirectional dynamics: 21% shifted to MyP, 35% progressed to severe anemia, 3% evolved to other cytopenias, and 42% remained stable. In severe anemia at diagnosis (N=41), 56% remained severely anemic, 17% improved to moderate anemia, and 27% shifted to MyP. Other cytopenias (N=30) showed limited reversibility, with most remaining cytopenic (30%) or evolving to moderate (33%) or severe anemia (10%); only 27% shifting to MyP.
Clinical outcomes Major outcomes in CyP and MyP were recorded in 43% vs 19% (p<0.001), including thrombosis, bleeding, disease progression, second cancers, transplantation, and death. Three-year event-free survival was 52% in CyP versus 83% in MyP patients.

### Summary/Conclusion

In this real-world MF cohort, cytopenias were frequent at diagnosis, influenced treatment selection, and were associated with poorer outcomes, with cytopenic phenotypes often emerging or worsening over time. Expanding ERNEST-3 and newer JAK inhibitors will enable treatment-specific analyses, including real-world momelotinib anemia improvement.

**Copyright Information:** (Online) ISSN: 2572-9241
©2026 The Author(s). HemaSphere is published by John Wiley & Sons Ltd on behalf of European Hematology Association. This is an open access Abstract Book distributed under the Attribution-NonCommercial-NoDerivs (CC BY-NC-ND), which allows third parties to download the articles and share them with others as long as they credit the author and the Abstract Book, but they cannot change the content in any way or use them commercially.
**Abstract Book Citations:** Authors, Title, HemaSphere, 2026;10:(S1):pages. The abstract book DOIs can be found on the "Author Information" page.
**Disclaimer:** Articles published in the journal HemaSphere exclusively reflect the opinions of the authors. The authors are responsible for all content in their abstracts including accuracy of the facts, statements, citing resources, etc.

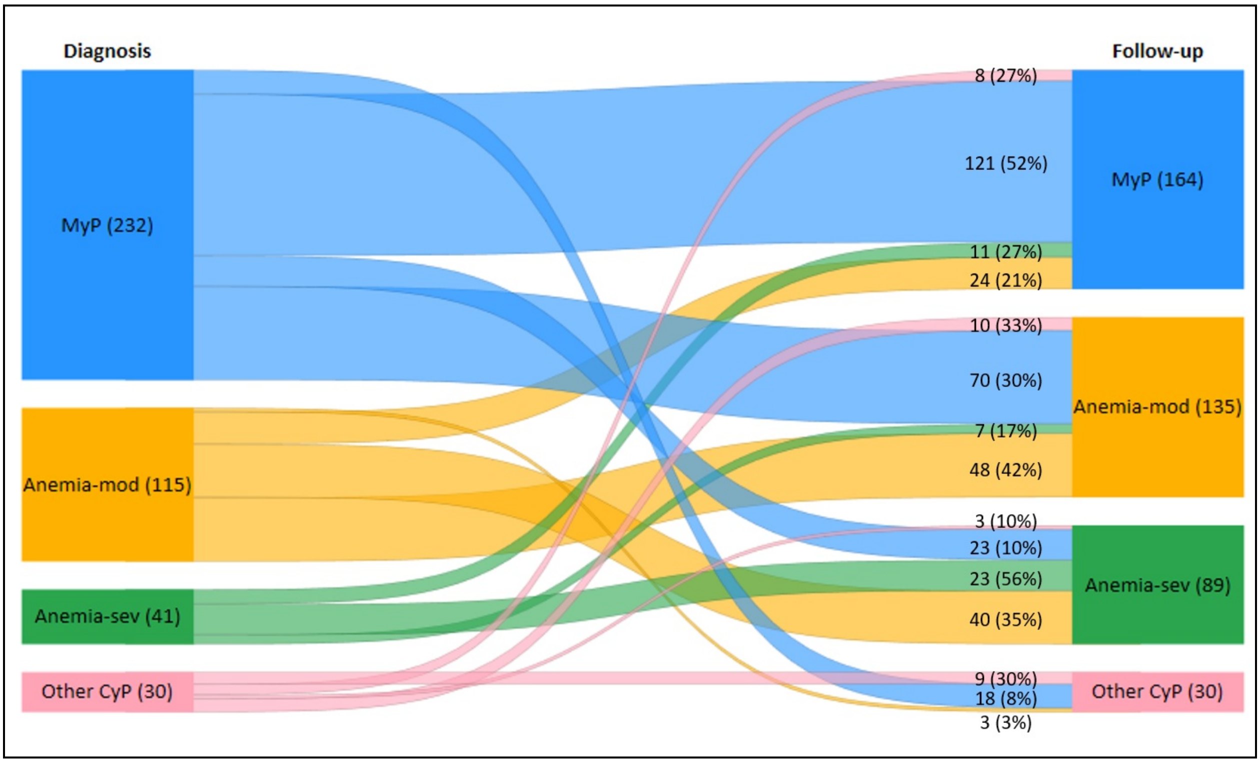

Dynamics of Cytopenia Status Between Diagnosis and Follow-up (Sankey diagram)

**Copyright Information:** (Online) ISSN: 2572-9241
©2026 The Author(s). HemaSphere is published by John Wiley & Sons Ltd on behalf of European Hematology Association. This is an open access Abstract Book distributed under the Attribution-NonCommercial-NoDerivs (CC BY-NC-ND), which allows third parties to download the articles and share them with others as long as they credit the author and the Abstract Book, but they cannot change the content in any way or use them commercially.
**Abstract Book Citations:** Authors, Title, HemaSphere, 2026;10:(S1):pages. The abstract book DOIs can be found on the "Author Information" page.
**Disclaimer:** Articles published in the journal HemaSphere exclusively reflect the opinions of the authors. The authors are responsible for all content in their abstracts including accuracy of the facts, statements, citing resources, etc.

### (PS2003) INTEGRATED CLINICAL AND MOLECULAR REGISTRY WITH LONGITUDINAL BIOREPOSITORY IN SYSTEMIC MASTOCYTOSIS: A SINGLE-INSTITUTION COHORT

**Topic:** 16. Myeloproliferative neoplasms - Clinical

Jennifer Marvin-Peek*[1], Annie Hoai Nguyen[1], Leticia Campoverde[1], Elisabeth Rebboah[1], Wei Xiao[1], Maria Hachem[1], Ivo Veletic[1], Taghi Manshouri[1], Julie Braish[2], Georgina El Hajjar[1], Ghayas Issa[1], Naveen Pemmaraju[1], Lucia Masarova[1], Prithviraj Bose[1], Hussein Abbas[1]

*[1]University of Texas MD Anderson Cancer Center, Leukemia, Houston, United States of America;[2]Moffitt Cancer Center, Tampa, United States of America;*

### Background

Systemic mastocytosis (SM) is a rare myeloid neoplasm marked by clonal mast cell accumulation in the bone marrow and extracutaneous organs. Large, deeply annotated clinical and molecular reference datasets representing the spectrum of SM remain limited.

### Aims

In this single-institution study, we established an integrated SM registry and biorepository to enable cross-institutional comparisons, retrospective analyses, and benchmarking of outcomes as targeted therapies and clinical trials expand.

### Methods

Adults with a confirmed diagnosis of SM according to WHO criteria who presented to our institution between 2001 and 2024 were included. More than 150 clinical and molecular variables were curated (Figure 1). Patients (pts) were stratified by WHO subtype and evaluated according to their SM and/or associated hematologic neoplasm (AHN)-directed therapy. Overall survival (OS) was assessed by Kaplan-Meier method from diagnosis to death or loss of follow up.

**Copyright Information:** (Online) ISSN: 2572-9241
©2026 The Author(s). HemaSphere is published by John Wiley & Sons Ltd on behalf of European Hematology Association. This is an open access Abstract Book distributed under the Attribution-NonCommercial-NoDerivs (CC BY-NC-ND), which allows third parties to download the articles and share them with others as long as they credit the author and the Abstract Book, but they cannot change the content in any way or use them commercially.
**Abstract Book Citations:** Authors, Title, HemaSphere, 2026;10:(S1):pages. The abstract book DOIs can be found on the "Author Information" page.
**Disclaimer:** Articles published in the journal HemaSphere exclusively reflect the opinions of the authors. The authors are responsible for all content in their abstracts including accuracy of the facts, statements, citing resources, etc.

### Results

A diagnosis of SM was confirmed in 340 pts across the >20-year period. Non-advanced SM (non-advSM) accounted for 217 pts (64%), including 199 indolent SM (ISM, 58%) and 18 smoldering SM (SSM, 5%). Advanced SM (advSM) comprised 123 pts (36%), including SM-AHN (24%), aggressive SM (ASM, 11%), and mast cell leukemia (MCL, 1.5%). Most pts with SM-AHN had an associated myeloid neoplasm (AMN, 83%), including CMML (26%), MPN (26%), MDS (25%), AML (16%), and CEL (2%). The remaining 13 pts with SM-AHN (17%) had an associated lymphoid neoplasm. *KITD816V* mutations were detected in 65% of pts. Next-generation sequencing data were available for 151 pts, with recurrent mutations in TET2 (32%), DNMT3A (13%), ASXL1 (12%), and JAK2 (8%). The registry captured expected clinical distinctions across SM subtypes, including higher rates of organ damage in advSM and greater cutaneous involvement and anaphylaxis in non-advSM.
SM-directed therapy was received by 45% of non-advSM and 79% of advSM pts, predominantly with TKI-based therapies. At a median follow-up of 48.4 months, median OS was 135 months for SSM, 73 months for ASM, 80 months for SM-AHN, and 15 months for MCL (p<0.001). Of pts with chronic phase SM-AMN, 11 (18%) progressed to AML. Survival for pts with SM-AHN was greater than historically reported (2-3 years), which may reflect differences in the underlying AHN and treatment approach. All pts with SM-AHN received SM and/or AHN-directed therapy, with a median of one line of SM-directed therapy (range 0-4) and one line of AHN-directed therapy (range 0-4). There were 34 pts (41%) with SM-AHN who received SM-directed therapy alone, 21 (26%) AHN-directed therapy alone, and 27 (33%) both SM and AHN-directed therapy. For pts who received both, the median time between starting SM and AHN-directed therapies was 0.3 months (range 0-108 months). Allogeneic stem cell transplant was performed in 22%.
This clinical dataset is linked to a longitudinal, IRB-approved SM biorepository comprising >3,000 biospecimens from >90% of pts across all SM subtypes, including pre- and post-treatment sampling to support future correlative, translational, and validation studies.

### Summary/Conclusion

Together, this integrated registry and biorepository provide a scalable reference resource spanning the full clinical spectrum of SM. This platform enables cross-institutional and international comparisons, informs clinical trial design and interpretation, and supports efforts to refine risk stratification, biomarker discovery, and therapeutic response assessment in SM.

**Copyright Information:** (Online) ISSN: 2572-9241
©2026 The Author(s). HemaSphere is published by John Wiley & Sons Ltd on behalf of European Hematology Association. This is an open access Abstract Book distributed under the Attribution-NonCommercial-NoDerivs (CC BY-NC-ND), which allows third parties to download the articles and share them with others as long as they credit the author and the Abstract Book, but they cannot change the content in any way or use them commercially.
**Abstract Book Citations:** Authors, Title, HemaSphere, 2026;10:(S1):pages. The abstract book DOIs can be found on the "Author Information" page.
**Disclaimer:** Articles published in the journal HemaSphere exclusively reflect the opinions of the authors. The authors are responsible for all content in their abstracts including accuracy of the facts, statements, citing resources, etc.

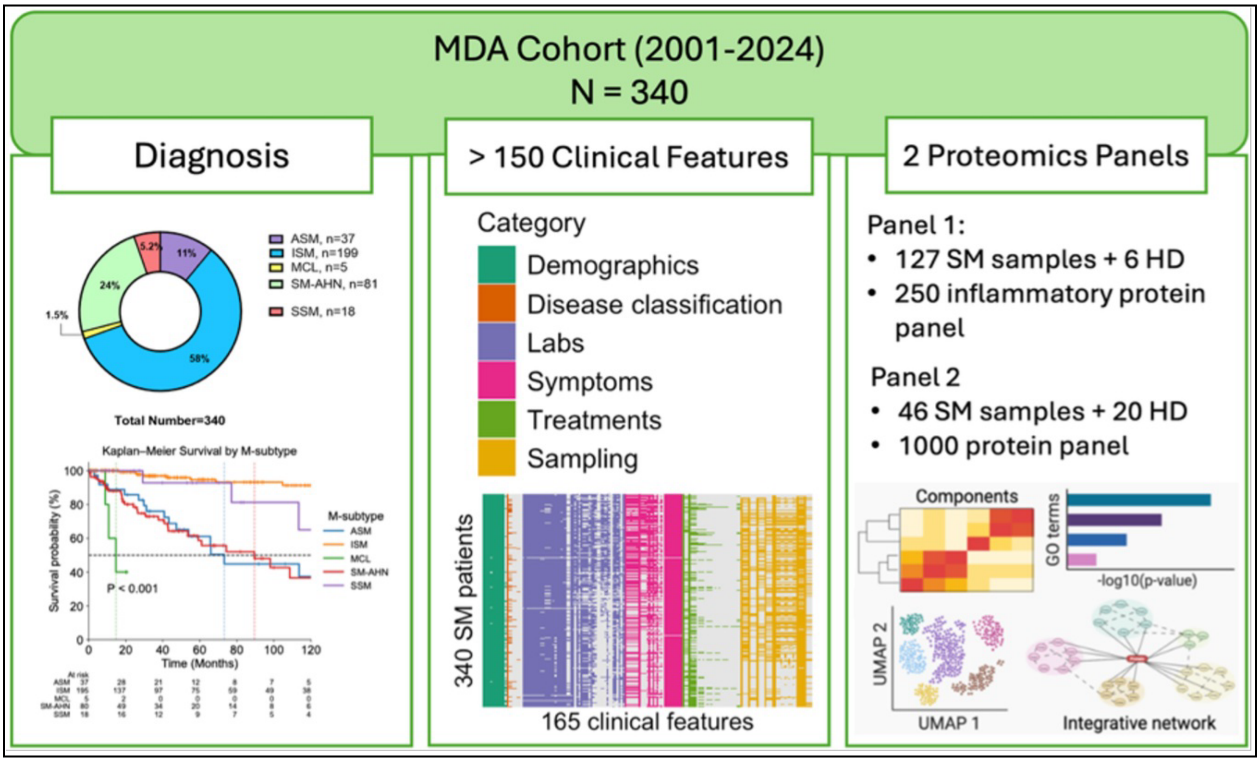

Figure 1. Dataset Overview

**Copyright Information:** (Online) ISSN: 2572-9241
©2026 The Author(s). HemaSphere is published by John Wiley & Sons Ltd on behalf of European Hematology Association. This is an open access Abstract Book distributed under the Attribution-NonCommercial-NoDerivs (CC BY-NC-ND), which allows third parties to download the articles and share them with others as long as they credit the author and the Abstract Book, but they cannot change the content in any way or use them commercially.
**Abstract Book Citations:** Authors, Title, HemaSphere, 2026;10:(S1):pages. The abstract book DOIs can be found on the "Author Information" page.
**Disclaimer:** Articles published in the journal HemaSphere exclusively reflect the opinions of the authors. The authors are responsible for all content in their abstracts including accuracy of the facts, statements, citing resources, etc.

### (PS2004) TP53 MUTATION CHARACTERIZES DIRECT AND ASXL1 AND EZH2 INDIRECT (VIA MYELOFIBROSIS) ET TO AML TRANSITION

**Topic:** 16. Myeloproliferative neoplasms - Clinical

Merry Nakhleh[1], Shivani Udyawar[1], Shraddha Patel[1], Aysel Kaplan Baryraktar[1], Cinthya Zepeda Mendoza[1], Kaaren Reichard[2], David Viswanatha[2], Rong He[2], Animesh Pardanani[1], Giuseppe Loscocco[3], Noushin Farnoud[4], Raajit K Rampal[4], Paola Guglielmelli[3], Alessandro Maria Vannucchi[3], Naseema Gangat*[1], Ayalew Tefferi[1]

*[1]Mayo Clinic, Division of Hematology, Department of Medicine, Rochester, United States of America;[2]Mayo Clinic, Department of Laboratory Medicine and Pathology, Rochester, United States of America; [3]University of Florence, Department of Experimental and Clinical Medicine, Florence, Italy; [4]Memorial Sloan Kettering Cancer Center, Leukemia Service, Department of Medicine, New York, United States of America;*

### Background

Essential thrombocythemia (ET) is the most favorable among myeloproliferative neoplasms, in terms of overall survival (OS; median ~18 years) and risk of disease transformation into acute myeloid leukemia (AML; 10-year rate ~2%) or myelofibrosis (post-ET MF; 10-year rate ~13%) [MCP 2019;94:599]. Previously reported genetic risk factors for post-ET AML include *TP53* and, for post-ET MF, *U2AF1* and *SF3B1* mutations (BJH 2020;189:291).

### Aims

We compared the mutational landscape of ET at different phases of the disease, including post-ET AML stemming directly from ET (direct) or indirectly after an intermediary fibrotic phase (indirect).

### Methods

The current retrospective study was conducted under an institutional review board approved protocol. Pathology diagnoses were rendered by board-certified hematopathologists following the 2022 ICC criteria (Blood 2022. 140:1200). Research-based or CLIA-approved next-generation sequencing (NGS) was used to characterize mutational status. Conventional statistical methods were applied for data analysis (JMP 18 software, Cary, NC, USA).

**Copyright Information:** (Online) ISSN: 2572-9241
©2026 The Author(s). HemaSphere is published by John Wiley & Sons Ltd on behalf of European Hematology Association. This is an open access Abstract Book distributed under the Attribution-NonCommercial-NoDerivs (CC BY-NC-ND), which allows third parties to download the articles and share them with others as long as they credit the author and the Abstract Book, but they cannot change the content in any way or use them commercially.
**Abstract Book Citations:** Authors, Title, HemaSphere, 2026;10:(S1):pages. The abstract book DOIs can be found on the "Author Information" page.
**Disclaimer:** Articles published in the journal HemaSphere exclusively reflect the opinions of the authors. The authors are responsible for all content in their abstracts including accuracy of the facts, statements, citing resources, etc.

**Results**

NGS information was available for 530 Mayo Clinic patients with ET (median age 57 years; females 61%) seen between October 1970 and July 2025. At a median follow-up of 10.3 years, 118 (22.3%) deaths, 138 (26%) fibrotic progressions, and 41 (8%) leukemic transformations, including 18 (3.4%) direct and 23 (4.3%) indirect, were documented. The study population was divided into 5 groups based primarily on timing of NGS testing (figure): Group A: performed at time of diagnosis or in chronic phase disease before fibrotic or leukemic transformation (N=416; 78.5%); Group B: performed during fibrotic phase of the disease without documentation of subsequent leukemic transformation (N=89; 16.8%); Group C: performed during fibrotic phase of the disease with documentation of subsequent leukemic transformation (N=7; 1.3%); Group D: performed at time of AML transition following post-ET MF (N=8; 1.5%); and Group E: performed at time of direct ET to AML transition without intermediary fibrotic phase (N=10; 1.9%). Significant differences in mutational frequencies between the groups were apparent for *TP53* (2.4%, 6%, 29%, 25%, 90%; p<0.01), *ASXL1* (6%, 17%, 43%, 38%, 0%; p<0.01), *IDH2* (1%, 1%, 14%, 25%, 0%; p=0.02), and *CALR* (27%, 37%, 29%, 88%, 10%, 10%; p<0.01), respectively. Significant mutation clustering was apparent for *ASXL1* with *CALR, EZH2, TP53, IDH2,* and *SRSF2*. In univariate analysis limited to group A, *TP53* (p<0.01), *EZH2* (p<0.01), *ASXL1* (p<0.01), *RUNX1* (p<0.01), *SF3B1* (p<0.01), and *SRSF2* (p<0.01) predicted AML transformation; in multivariable analysis, only *EZH2* (p<0.01), and *RUNX1* (p=0.02) remained significant. A similar analysis identified *ASXL1* mutation (p=0.03) to predict fibrotic transformation, *TP53* (p=0.03), *RUNX1* (p<0.01), and *SRSF2* (p=0.02) to predict direct AML transformation, and *EZH2* (p<0.01) to predict indirect AML transformation. The inclusion of cytogenetic information did not alter the above findings.

**Summary/Conclusion**

*TP53* mutation is highly enriched at time of direct ET to AML transformation. Baseline occurrence of *TP53* mutation, along with *RUNX1* and *SRSF2*, also predicts direct AML transition. By contrast, indirect ET to AML transformation appears to be driven by *EZH2* mutation and fibrotic progression by *ASXL1* mutation. It is imperative that these single institution-derived observations be confirmed by larger studies.

**Copyright Information:** (Online) ISSN: 2572-9241
©2026 The Author(s). HemaSphere is published by John Wiley & Sons Ltd on behalf of European Hematology Association. This is an open access Abstract Book distributed under the Attribution-NonCommercial-NoDerivs (CC BY-NC-ND), which allows third parties to download the articles and share them with others as long as they credit the author and the Abstract Book, but they cannot change the content in any way or use them commercially.
**Abstract Book Citations:** Authors, Title, HemaSphere, 2026;10:(S1):pages. The abstract book DOIs can be found on the “Author Information” page.
**Disclaimer:** Articles published in the journal HemaSphere exclusively reflect the opinions of the authors. The authors are responsible for all content in their abstracts including accuracy of the facts, statements, citing resources, etc.

**NGS-derived mutational frequencies in essential thrombocythemia based on timing of NGS testing (total N = 530)**

| | Test performed at time of diagnosis or prior to transformation into MF or AML N=416 | Test performed in fibrotic phase *without* documentation of subsequent AML N=89 | Test performed in fibrotic phase *with* documentation of subsequent AML N=7 | Test performed in AML phase following transition from post-ET MF N=8 | Test performed in AML phase following transition *directly* from ET N=10 | |
|---|---|---|---|---|---|---|
| *CALR* | 27% | 37% | 29% | 88% | 10% | P<0.01 |
| *TP53* | 2.4% | 6% | 29% | 25% | 90% | P<0.01 |
| *ASXL1* | 6% | 17% | 43% | 38% | 0% | P<0.01 |
| *IDH2* | 0.9% | 1% | 14% | 25% | 0% | P=0.02 |
| *EZH2* | 1.4% | 4.5% | 0% | 13% | 10% | P=0.15 |

NGS-derived mutational frequencies in essential thrombocythemia based on timing of NGS testing (total N = 530)

**Copyright Information:** (Online) ISSN: 2572-9241
©2026 The Author(s). HemaSphere is published by John Wiley & Sons Ltd on behalf of European Hematology Association. This is an open access Abstract Book distributed under the Attribution-NonCommercial-NoDerivs (CC BY-NC-ND), which allows third parties to download the articles and share them with others as long as they credit the author and the Abstract Book, but they cannot change the content in any way or use them commercially.
**Abstract Book Citations:** Authors, Title, HemaSphere, 2026;10:(S1):pages. The abstract book DOIs can be found on the "Author Information" page.
**Disclaimer:** Articles published in the journal HemaSphere exclusively reflect the opinions of the authors. The authors are responsible for all content in their abstracts including accuracy of the facts, statements, citing resources, etc.

### (PS2005) SHORESPAN-017: A PHASE 3 EXTENSION STUDY EVALUATING THE SAFETY OF CONTINUED BOMEDEMSTAT IN PARTICIPANTS WITH ESSENTIAL THROMBOCYTHEMIA

**Topic:** 16. Myeloproliferative neoplasms - Clinical

Francesca Palandri*[1], David Ross[2], Andrew Wilson[3], Tara Cochrane[4], William Stevenson[5], James Mao-Chun Liang[6], Marco Brociner[7], Stephen Larsen[8], Merit Hanna[9], Alessandro Maria Vannucchi[10], Kristen Pettit[11], Natalia Curto-Garcia[12], Harry Gill[13], Uche Okoro[14], Jonas Jutzi[14], Patricia Marinello[14], Monia Marchetti[15]

*[1]IRCCS Azienda Ospedaliero-Universitaria di Bologna, Bologna, Italy;[2]Royal Adelaide Hospital and SA Pathology, Adelaide, SA, Australia; [3]University College London Hospitals NHS Foundation Trust, London, United Kingdom;[4]Gold Coast University Hospita, Southport, QLD, Australia; [5]Royal North Shore Hospital, University of Sydney, Sydney, NSW, Australia;[6]Middlemore Clinical Trials, Auckland, New Zealand; [7]ASST Sette Laghi Osp di Circolo e Fond Macchi P O Varese, Varese, Italy;[8]Institute of Haematology, Royal Prince Alfred Hospital; University of Sydney, Sydney, NSW, Australia; [9]North Shore Hospital, Waitemata District Health Board, Auckland, New Zealand; [10]University of Florence, Florence, Italy; [11]University of Michigan, Ann Arbor, MI, United States of America; [12]Guy's and St Thomas' NHS Foundation Trust, London, United Kingdom; [13]School of Clinical Medicine, LKS Faculty of Medicine, the University of Hong Kong, Hong Kong, China; [14]Merck & Co., Inc., Rahway, United States of America; [15]Azienda Ospedaliera Universitaria di Alessandria, Alessandria, Italy;*

### Background

Myeloproliferative neoplasms (MPNs), such as essential thrombocythemia, are characterized by vascular events and systemic symptoms resulting from platelet abnormalities and cytokine imbalance. Despite recent advances in available therapies, novel treatments that can modify the disease trajectory of MPNs are still needed. Bomedemstat is an irreversible inhibitor of lysine-specific demethylase 1 (LSD1) that has shown promising clinical activity and manageable safety in MPNs, including essential thrombocythemia. However, because MPNs are chronic diseases with long-term prognoses, extended follow-up is necessary.

### Aims

Shorespan-017 is an open-label, single-arm, phase 3 extension study (NCT06351631) that was designed to evaluate the long-term safety, tolerability, and efficacy of bomedemstat in participants with MPNs who were continuing treatment after receiving bomedemstat in a prior clinical study. Here, we report safety outcomes for participants with essential thrombocythemia.

### Methods

Eligible participants were aged ≥18 years and had transitioned from a bomedemstat study sponsored by Imago BioSciences, Inc., a subsidiary of Merck & Co., Inc., Rahway, NJ, USA, or MSD (feeder study). Participants from the IMG-7289-CTP-201/MK-3543-003 and IMG-7289-CTP-102/MK-3543-002 studies were required to have completed at least 6 months of treatment with bomedemstat and experienced manageable safety and clinical benefit per the investigator. All participants initiated treatment with bomedemstat at the dose that had been established as safe and effective in their feeder study. The primary end point was the safety and tolerability of bomedemstat.

**Copyright Information:** (Online) ISSN: 2572-9241
©2026 The Author(s). HemaSphere is published by John Wiley & Sons Ltd on behalf of European Hematology Association. This is an open access Abstract Book distributed under the Attribution-NonCommercial-NoDerivs (CC BY-NC-ND), which allows third parties to download the articles and share them with others as long as they credit the author and the Abstract Book, but they cannot change the content in any way or use them commercially.
**Abstract Book Citations:** Authors, Title, HemaSphere, 2026;10:(S1):pages. The abstract book DOIs can be found on the “Author Information” page.
**Disclaimer:** Articles published in the journal HemaSphere exclusively reflect the opinions of the authors. The authors are responsible for all content in their abstracts including accuracy of the facts, statements, citing resources, etc.

**Results**

56 participants received treatment in Shorespan-017, including 40 with essential thrombocythemia. Median follow-up at data cutoff (April 1, 2025) was 8.1 months (range, 3.5-10.3). Median age was 68.5 years (range, 45-88); 23 participants (58%) were female, and 11 (28%) had an Eastern Cooperative Oncology Group performance status score of 1. At the data cutoff, 1 participant (3%) had discontinued treatment due to an adverse event (AE; grade 4 anemia) and 39 participants (98%) remained on treatment. Treatment-emergent AEs of any grade occurred in 35 participants (88%), most frequently (≥10%) diarrhea (23%), contusion (13%), abdominal pain (10%), and upper respiratory tract infection (10%). Grade 3 or 4 treatment-emergent AEs were reported in 8 participants (20%) and included anemia, anaphylactic reaction, atrial flutter, depressed mood, diarrhea, gastroenteritis, hyponatremia, inappropriate antidiuretic hormone secretion, and tachycardia (all n = 1 [3%]). No participants died due to treatment-emergent AEs. Treatment-related AEs of any grade occurred in 7 participants (18%), most frequently (≥5%) contusion (n = 2 [5%]) and anemia (n = 2 [5%]). No grade 3 or 4 treatment-related AEs occurred. No participants discontinued treatment or died due to treatment-related AEs. One serious treatment-related AE of grade 2 constipation was reported.

**Summary/Conclusion**

Results of this analysis show that continued treatment with bomedemstat had manageable safety in participants with essential thrombocythemia who had achieved a clinical benefit with bomedemstat in their feeder study.

**Copyright Information:** (Online) ISSN: 2572-9241
©2026 The Author(s). HemaSphere is published by John Wiley & Sons Ltd on behalf of European Hematology Association. This is an open access Abstract Book distributed under the Attribution-NonCommercial-NoDerivs (CC BY-NC-ND), which allows third parties to download the articles and share them with others as long as they credit the author and the Abstract Book, but they cannot change the content in any way or use them commercially.
**Abstract Book Citations:** Authors, Title, HemaSphere, 2026;10:(S1):pages. The abstract book DOIs can be found on the "Author Information" page.
**Disclaimer:** Articles published in the journal HemaSphere exclusively reflect the opinions of the authors. The authors are responsible for all content in their abstracts including accuracy of the facts, statements, citing resources, etc.

### (PS2006) A PHASE II STUDY OF F182112 IN COMBINATION WITH DARATUMUMAB AND POMALIDOMIDE IN PATIENTS WITH RELAPSED OR REFRACTORY MULTIPLE MYELOMA

**Topic:** 16. Myeloproliferative neoplasms - Clinical

Gang An[1], Guolin Yuan[2], Xiaobing Huang[3], Tangrong Liu*[4], Shaohong Yin[4], Aichun Liu[5], Hui Geng[6], Zhongjun Xia[7], Jun Wang[8], Jun Luo[9], Guitao Jie[10], Meifang Zheng[11], Songfu Jiang[12], Yan Li[13], Hongbin Zhang[14], Jianxiang Zhang[4], Guimin Zhang[4], Lugui Qiu[1]

*[1]Institute of Hematology & Blood Diseases Hospital,Chinese Academy of Medical Sciences, Tianjin, China;[2]Xiangyang Central Hospital, Changsha, China; [3]Sichuan Provincial People's Hospital, Chengdu, China; [4]Lunan Pharmaceutical Group Co. Ltd., Linyi, China; [5]Harbin Medical University Cancer Hospital, Harbin, China; [6]Qinghai University Affiliated Hospital, Xining, China; [7]Sun-Yat-sen University Cancer Center, Guangdong, China; [8]The First People's Hospital of Changde, Changde, China; [9]The First Affiliated Hospital of Guangxi Medical University, Nanning, China; [10]Linyi Central Hospital, Linyi, China; [11]Linyi Cancer Hospital, Linyi, China; [12]The First Affiliated Hospital of Wenzhou Medical University, Wenzhou, China; [13]People's Hospital of Xinjiang Uygur Autonomous Region, Urumqi, China; [14]The First Affiliated Hospital of Chongqing Medical University, Chongqing, China;*

### Background

In a phase 1 trial (NCT04984434), F182112, a bispecific T-cell engager targeting B-cell maturation antigen (BCMA) on myeloma cells and CD3 on T cells, demonstrated durable responses in heavily pretreated patients with relapsed or refractory multiple myeloma (RRMM). Daratumumab and pomalidomide exert immunomodulatory effects that may synergize with F182112 to enhance anti-myeloma activity. Here, we report the safety and efficacy findings from an ongoing phase 2 study (NCT07312188) evaluating the triplet combination of F182112, daratumumab and pomalidomide (F-DP) in patients with RRMM.

### Aims

The objective of this phase 2 study is to evaluate the efficacy, safety and immunogenicity of F182112in combination with daratumumab and pomalidomide in RRMM patients.

### Methods

This open-label, multicenter Phase 2 study including a cohort (Cohort 3) evaluating the F-DP regimen in patients with RRMM. Eligible patients had 1-3 prior lines of therapy (LOT), including a proteasome inhibitor (PI) and lenalidomide. Responses were assessed by investigators assessed per International Myeloma Working Group criteria and adverse events (AEs) were graded per Common Terminology Criteria for Adverse Events (CTCAE) v5.0. Cytokine release syndrome (CRS) and immune effector cell-associated neurotoxicity syndrome (ICANS) were graded per American Society of Transplantation and Cellular Therapy (ASTCT) guidelines.

**Copyright Information:** (Online) ISSN: 2572-9241
©2026 The Author(s). HemaSphere is published by John Wiley & Sons Ltd on behalf of European Hematology Association. This is an open access Abstract Book distributed under the Attribution-NonCommercial-NoDerivs (CC BY-NC-ND), which allows third parties to download the articles and share them with others as long as they credit the author and the Abstract Book, but they cannot change the content in any way or use them commercially.
**Abstract Book Citations:** Authors, Title, HemaSphere, 2026;10:(S1):pages. The abstract book DOIs can be found on the "Author Information" page.
**Disclaimer:** Articles published in the journal HemaSphere exclusively reflect the opinions of the authors. The authors are responsible for all content in their abstracts including accuracy of the facts, statements, citing resources, etc.

### Results

A total of thirty patients had received F-DP. At the data cut-offs (February 20, 2026), the median follow-up period was 98 days (range, 16-273) and the median treatment duration of F-DP was 98 days (range, 1-273). The median age was 62 years (range, 45-77); 40.0% were male. The number of prior LOT was 2, with 33.3% of patients having received >2 prior LOT. According to the Revised International Staging System (R-ISS), 8 of the 30 patients (26.7%) presented with stage III disease. The overall response rate was 96.7% (29/30). A very good partial response or better rate was 75.9%, While a complete response or better rate was 37.9%. The median time to first response was 1.17 months (range, 0.53-1.23). The median duration of response, progression-free survival and overall survival were not reached (NE-NE). The most common treatment-emergent AEs (TEAEs) were leukopenia (70.0%), CRS (66.7%), thrombocytopenia (63.3%), lymphopenia (50.0%), neutropenia (56.7%), and anemia (53.3%). Grade 3/4 AEs occurring in ≥15% of patients included leukopenia (53.3%), neutropenia (50.0%), lymphopenia (46.7%), anemia (33.3%) and thrombocytopenia (33.3%). CRS events were predominantly grade 1/2 (93.3%) and all were resolved. No ICANS was observed. Infections occurred in 13 patients (43.3%; grade 3/4, 33.3%), most commonly upper respiratory infection (20.0%; grade 3/4, 16.7%), pneumonia (16.7%; grade 3/4, 6.7%) and COVID-19 infection (6.7%; grade 3/4, 3.3%).

### Summary/Conclusion

F-DP was feasible and led to promising efficacy in patients with RRMM. Moreover, its safety profile was consistent with F182112 individually. Promising ORR findings support the potential of combined treatment on enhanced early disease control through the addition of F182112.

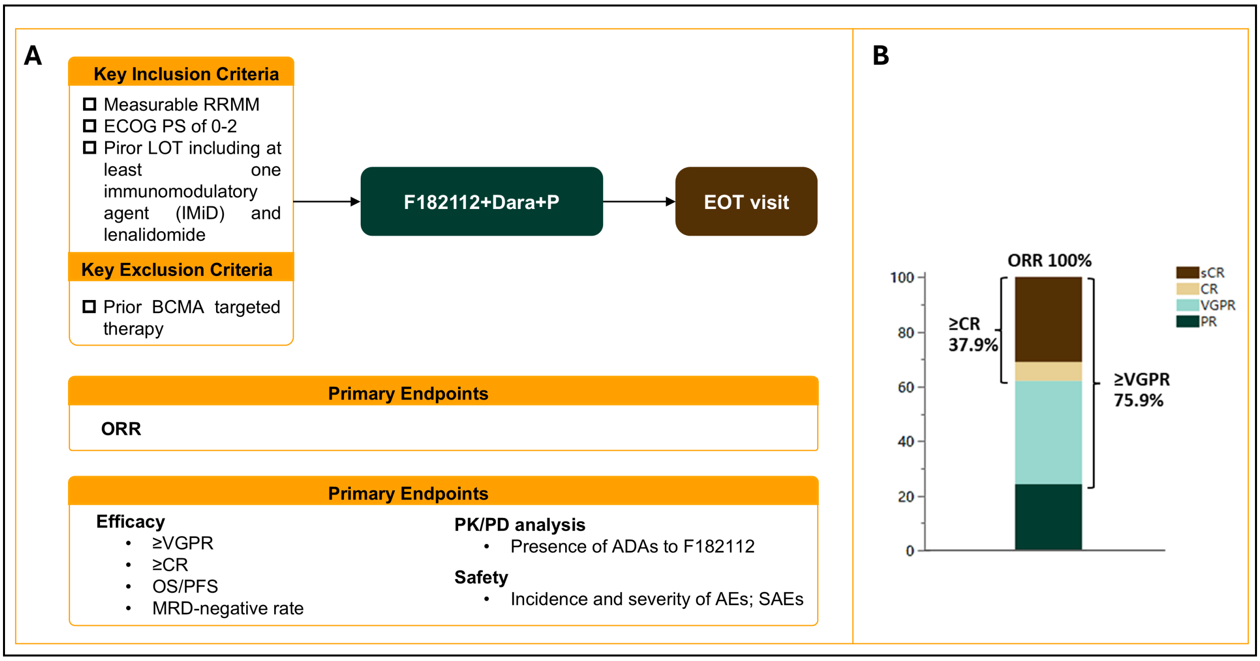

Figure 1. Study design (A) and Response rate (B) in RRMM patients treated with F-DP.

Copyright Information: (Online) ISSN: 2572-9241
©2026 The Author(s). HemaSphere is published by John Wiley & Sons Ltd on behalf of European Hematology Association. This is an open access Abstract Book distributed under the Attribution-NonCommercial-NoDerivs (CC BY-NC-ND), which allows third parties to download the articles and share them with others as long as they credit the author and the Abstract Book, but they cannot change the content in any way or use them commercially.
Abstract Book Citations: Authors, Title, HemaSphere, 2026;10:(S1):pages. The abstract book DOIs can be found on the “Author Information” page.
Disclaimer: Articles published in the journal HemaSphere exclusively reflect the opinions of the authors. The authors are responsible for all content in their abstracts including accuracy of the facts, statements, citing resources, etc.

### (PS2007) PHENOTYPE-SPECIFIC CHOLESTEROL ELEVATION FOLLOWING RUXOLITINIB IN MPN: ENRICHMENT IN POLYCYTHAEMIA VERA

**Topic:** 16. Myeloproliferative neoplasms - Clinical

Elena Torre*[1, 2], Harry Robertson[1], Terrenjit Gill[2], Ipek Baymul[1], Thaw Htin[1], Maria Georgiou[1], Natalia Curto-Garcia[1], Patrick Harrington[1], Priya Sriskandarajah[1], Llywelyn Cadman-Davies[1], Shahram Kordasti[1, 2], Claire Woodley[1], Susan Asirvatham[1], Benissa Narciso[1], Deepti H. Radia[1], Hugues de Lavallade[1], Claire Harrison[1], Jennifer O'sullivan[1]

*[1]Guy's and St Thomas' NHS Foundation Trust, Haematology Department, London, United Kingdom;[2]King's College London, School of Cancer and Pharmaceutical Sciences, London, United Kingdom;*

### Background

Patients with myeloproliferative neoplasms (MPN) have increased cardiovascular risk (CVR) driven by the mutant-clone propagated chronic pro-inflammatory and pro-thrombotic state.
Ruxolitinib, a selective JAK1/JAK2 inhibitor, suppresses JAK-STAT signalling and reduces inflammatory cytokine production. Prior randomised and post-hoc trial analyses have reported metabolic changes with ruxolitinib, including increases in body weight and total cholesterol, consistent with JAK1/JAK2 inhibition affecting lipid homeostasis. JAK2 inhibition impairs macrophage cholesterol efflux, increasing circulating cholesterol levels. According to European Society of Hypertension (ESH) and European Society of Cardiology (ESC) guidelines, total cholesterol >5 mmol/L and low-density lipoprotein (LDL) ≥2 mmol/L represent clinically relevant thresholds depending on CVR category.

### Aims

To evaluate longitudinal changes in lipids across MPN phenotypes in patients treated with ruxolitinib.

### Methods

We retrospectively analysed 400 patients initiating ruxolitinib (2009–2024). Post-treatment lipid profiles were available in 206 patients; 97 had systematically collected paired pre- and post-treatment measurements and formed the primary cohort. Paired analyses assessed longitudinal change. MPN phenotype comparisons and logistic regression evaluated predictors of clinically relevant lipid thresholds.

**Copyright Information:** (Online) ISSN: 2572-9241
©2026 The Author(s). HemaSphere is published by John Wiley & Sons Ltd on behalf of European Hematology Association. This is an open access Abstract Book distributed under the Attribution-NonCommercial-NoDerivs (CC BY-NC-ND), which allows third parties to download the articles and share them with others as long as they credit the author and the Abstract Book, but they cannot change the content in any way or use them commercially.
**Abstract Book Citations:** Authors, Title, HemaSphere, 2026;10:(S1):pages. The abstract book DOIs can be found on the “Author Information” page.
**Disclaimer:** Articles published in the journal HemaSphere exclusively reflect the opinions of the authors. The authors are responsible for all content in their abstracts including accuracy of the facts, statements, citing resources, etc.

**Results**

Of the 97 paired patients (median age 61 years, range 31–85), diagnoses included post-essential thrombocythaemia MF (PET-MF, n=7), post-polycythaemia vera MF (PPV-MF, n=14), primary MF (PMF, n=24), and polycythaemia vera (PV, n=50). Driver mutations consisted of *JAK2*V617F (76.3%), *CALR* (8.2%), and *JAK2* Exon12 (2.1%), with 13.4% triple-negative or undocumented. Median *JAK2* variant allele frequency was 51%.

Over a median interval of 8 months (IQR 3.5–22), mean total cholesterol increased by +0.65 mmol/L (95% CI 0.40–0.90; p<0.001); 70/97 patients (72%) showed an increase. Total cholesterol levels rose in 43% of PET-MF, 100% of PPV-MF, 67% of PMF, and 74% of PV patients (p=0.016).

The proportion with total cholesterol >5 mmol/L increased from 13.4% pre-treatment to 35.9% post-treatment (p=0.0001). In MF, this rose from 6.3% to 29.6% (p=0.004); in PV, from 20% to 45.6% (p=0.016). Post-treatment total cholesterol levels were significantly higher in PV as compared with MF (Figure 1), and the proportion with levels >5 mmol/L was enriched in PV vs MF (46% vs 30%, p=0.028).

Post-treatment LDL ≥2 mmol/L was observed in 60% of MF and 74.7% of PV patients (p=0.059). PV was associated with higher odds of LDL ≥2 mmol/L compared with MF (OR 1.96, 95% CI 1.04–3.82; p=0.042). Cholesterol change was not associated with age (p=0.391) and did not differ by gender (mean difference 0.12 mmol/L, 95% CI −0.42 to 0.66; p=0.663).

Triglycerides ≥1.7 mmol/L were observed more often in MF than PV (52.9% vs 33.8%, p=0.015), without significant paired change (p=0.404). *JAK2*V617F VAF showed a weak positive correlation with cholesterol increase (r=0.27, p=0.040).

**Summary/Conclusion**

In this real-world paired analysis, ruxolitinib was associated with a significant increase in total cholesterol in MPN. While increases occur in all MPNs, PV demonstrated greater cholesterol increases and higher odds of LDL elevation, irrespective of age and gender. Given the intrinsically elevated CVR in MPN, proactive lipid monitoring and cardovascular risk optimisation should be systematically integrated into long-term management.

**Copyright Information:** (Online) ISSN: 2572-9241
©2026 The Author(s). HemaSphere is published by John Wiley & Sons Ltd on behalf of European Hematology Association. This is an open access Abstract Book distributed under the Attribution-NonCommercial-NoDerivs (CC BY-NC-ND), which allows third parties to download the articles and share them with others as long as they credit the author and the Abstract Book, but they cannot change the content in any way or use them commercially.
**Abstract Book Citations:** Authors, Title, HemaSphere, 2026;10:(S1):pages. The abstract book DOIs can be found on the “Author Information” page.
**Disclaimer:** Articles published in the journal HemaSphere exclusively reflect the opinions of the authors. The authors are responsible for all content in their abstracts including accuracy of the facts, statements, citing resources, etc.

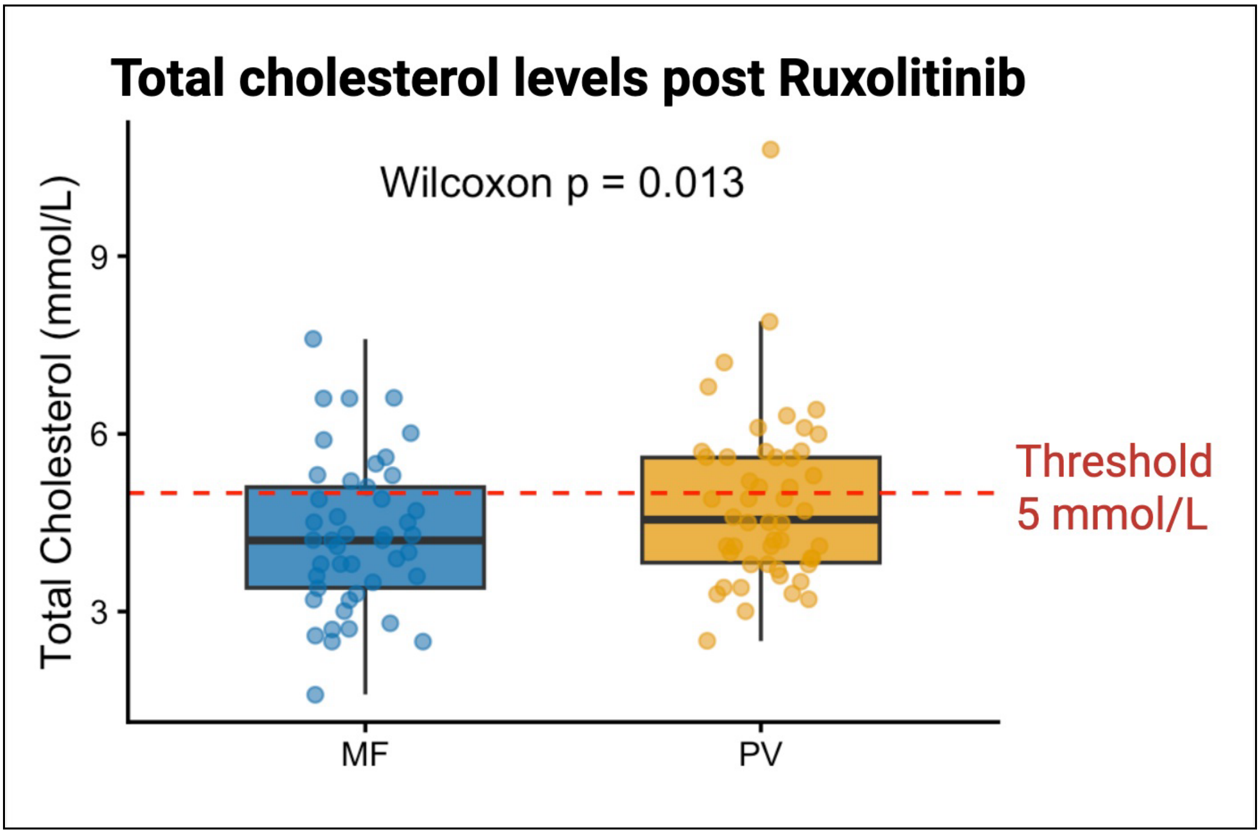

Figure 1. Boxplots show median and interquartile range of post-treatment total cholesterol in MF (median 4.2 mmol/L) and PV (median 4.7 mmol/L) patients. PV exhibited higher post-treatment cholesterol levels (p=0.013), with a greater proportion exceeding the 5 mmol/L threshold (dashed line).

**Copyright Information:** (Online) ISSN: 2572-9241
©2026 The Author(s). HemaSphere is published by John Wiley & Sons Ltd on behalf of European Hematology Association. This is an open access Abstract Book distributed under the Attribution-NonCommercial-NoDerivs (CC BY-NC-ND), which allows third parties to download the articles and share them with others as long as they credit the author and the Abstract Book, but they cannot change the content in any way or use them commercially.
**Abstract Book Citations:** Authors, Title, HemaSphere, 2026;10:(S1):pages. The abstract book DOIs can be found on the "Author Information" page.
**Disclaimer:** Articles published in the journal HemaSphere exclusively reflect the opinions of the authors. The authors are responsible for all content in their abstracts including accuracy of the facts, statements, citing resources, etc.

### (PS2008) ROMACICLIB (RVU120), A SELECTIVE CDK8/19 INHIBITOR, AS MONOTHERAPY OR IN COMBINATION WITH RUXOLITINIB IN PATIENTS WITH MYELOFIBROSIS: UPDATED RESULTS FROM THE PHASE II POTAMI-61 STUDY

**Topic:** 16. Myeloproliferative neoplasms - Clinical

Tomasz Sacha*[1], Sebastian Grosicki[2], Marcin Rymko[3], Alessandro Lucchesi[4], Ewelina Kowalewska[5], Milena Mazan[5], Agnieszka Szymula[5], Nemanja Nikic[5], Malgorzata Garscia[5], Kinga Michalik[5], Agnieszka Sroka-Porada[5], Gary Wilkinson[5], Renata Windak[5], Renata Dudziak[5], Chadi Saba[5], Hendrik Nogai[5], Witold Prejzner[6], Katarzyna Dulik[7], Tomasz Wrobel[8], Aneta Szudy-Szczyrek[9]

*[1]Jagiellonian University Hospital, Kraków, Poland; [2]Department of Cancer Prevention, Medical University of Silesia, Katowice, Poland; [3]Rydygier Provincial Polyclinical Hospital in Toruń, Toruń, Poland; [4]Comprehensive Cancer Care and Research Network (CCCRN) of Romagna, Meldola, Italy; [5]Ryvu Therapeutics, Kraków, Poland; [6]Uniwersyteckie Centrum Kliniczne, Klinika Hematologii i Transplantologii, Gdańsk, Poland; [7]M2M Med, Chorzów, Poland; [8]Wroclaw Medical University, Wrocław, Poland; [9]Lublin Medical University, Lublin, Poland;*

### Background

Myelofibrosis (MF) is driven by dysregulated JAK/STAT signaling. Although ruxolitinib (RUX) improves splenomegaly and symptoms, cytopenias and suboptimal responses remain clinical challenges. Romaciclib (RVU120) is a first-in-class, oral CDK8/19 inhibitor that modulates STAT-dependent transcription. In preclinical MPN models, romaciclib reduced splenomegaly and fibrosis, synergized with RUX, and in combination with RUX decreased mutant clone burden.

### Aims

To evaluate safety and clinical activity of romaciclib as monotherapy or in combination with RUX in patients with intermediate- or high-risk MF.

### Methods

POTAMI-61 (NCT06397313) is an ongoing, open-label Phase II study. In Cohort 1, patients that failed or are ineligible for JAK inhibitor treatment (including thrombocytopenic patients) receive romaciclib as monotherapy. In Cohort 2, patients with suboptimal response to JAK inhibitors receive romaciclib add-on therapy. The primary endpoint is spleen volume reduction (SVR) at Week 24. Secondary endpoints include changes of the total symptom score (TSS), duration of response, reduction of bone marrow (BM) fibrosis, leukemic transformation, hematologic improvement, progression-free survival (PFS), overall survival (OS), and safety. A ramp-up dosing schedule (75 mg QD → 150 mg QD) was introduced to reduce early discontinuations. Data cutoff: 19 February 2026.

**Copyright Information:** (Online) ISSN: 2572-9241
©2026 The Author(s). HemaSphere is published by John Wiley & Sons Ltd on behalf of European Hematology Association. This is an open access Abstract Book distributed under the Attribution-NonCommercial-NoDerivs (CC BY-NC-ND), which allows third parties to download the articles and share them with others as long as they credit the author and the Abstract Book, but they cannot change the content in any way or use them commercially.
**Abstract Book Citations:** Authors, Title, HemaSphere, 2026;10:(S1):pages. The abstract book DOIs can be found on the “Author Information” page.
**Disclaimer:** Articles published in the journal HemaSphere exclusively reflect the opinions of the authors. The authors are responsible for all content in their abstracts including accuracy of the facts, statements, citing resources, etc.

**Results**

Twenty-eight patients were treated across both cohorts (13 in Cohort 1, 15 in Cohort 2). Ten patients remain on treatment, with durations up to 15 months.
Seven patients achieved ≥10% SVR with romaciclib alone or in combination with RUX. All three patients with ASXL1 mutation demonstrated spleen reduction. Clinically meaningful and durable symptom improvements were observed in some patients. One patient (ASXL1mut/CALRmut) achieved BM fibrosis reduction at Week 12 sustained at Week 24; most others had stable fibrosis. No leukemic transformation occurred; peripheral and marrow blast counts remained stable.
Platelet counts remained stable throughout treatment, including in cytopenic patients. Mean hemoglobin levels were maintained. In selected patients, an increase of erythropoietic progenitors were observed with hemoglobin increase (>2 g/dL in one case) and reduction in transfusion burden.
The majority of treatment-emergent adverse events (TEAEs) were Grade 1–2. Nausea and vomiting were the most common TEAEs. No dose-limiting toxicities were observed. No Grade ≥3 thrombocytopenia or neutropenia was attributed to romaciclib alone. No overlapping hematologic toxicity with RUX was observed.

**Summary/Conclusion**

Romaciclib, administered as monotherapy or combined with RUX, demonstrates a manageable safety profile in patients with MF without significant treatment-related cytopenias. Prolonged exposure, spleen volume reductions, including in patients with high molecular risk mutations, and favorable hematologic tolerability support continued clinical development and further evaluation in expansion cohorts.

**Copyright Information:** (Online) ISSN: 2572-9241
©2026 The Author(s). HemaSphere is published by John Wiley & Sons Ltd on behalf of European Hematology Association. This is an open access Abstract Book distributed under the Attribution-NonCommercial-NoDerivs (CC BY-NC-ND), which allows third parties to download the articles and share them with others as long as they credit the author and the Abstract Book, but they cannot change the content in any way or use them commercially.
**Abstract Book Citations:** Authors, Title, HemaSphere, 2026;10:(S1):pages. The abstract book DOIs can be found on the "Author Information" page.
**Disclaimer:** Articles published in the journal HemaSphere exclusively reflect the opinions of the authors. The authors are responsible for all content in their abstracts including accuracy of the facts, statements, citing resources, etc.

### (PS2009) DISEASE MODIFYING EFFECTS OF AVAPRITINIB IN PATIENTS WITH ADVANCED SYSTEMIC MASTOCYTOSIS: IMPACT ON BONE TURNOVER MARKERS (BTMS) AND BONE DISEASE

**Topic:** 16. Myeloproliferative neoplasms - Clinical

Johannes Lübke*[1], Tracy I. George[2], Daniel J. Deangelo[3], Jason Gotlib[4], Deepti H. Radia[5], Michael Deininger[6], Aaron Zakharyan[7], Joana Caetano-Lopes[7], Saša Dimitrijević[8], Rachel L. Erlich[7], Guang Yang[7], Andreas Reiter[1]

*[1]University Hospital Mannheim, Mannheim, Germany; [2]ARUP Laboratories, University of Utah, Salt Lake City, UT, United States of America; [3]Department of Medical Oncology, Dana-Farber Cancer Institute, Boston, MA, United States of America; [4]Division of Hematology, Stanford Cancer Institute/Stanford University School of Medicine, Stanford, CA, United States of America; [5]Guy's and St Thomas' NHS Foundation Trust, London, United Kingdom; [6]Division of Hematology/Oncology, University of Michigan, Ann Arbor, MI, United States of America; [7]Blueprint Medicines Corporation, Cambridge, MA, United States of America; [8]Blueprint Medicines GmbH, Zug, Switzerland;*

### Background

Patients with advanced systemic mastocytosis (AdvSM) can have evidence of bone disease, such as osteosclerosis, osteolytic lesions, osteopenia/osteoporosis, and bone marrow mast cell infiltration. Bone disease can be assessed by several complementary methods, including: 1) bone density (BD), static bone mass measurement via dual-energy X-ray absorptiometry; 2) bone turnover markers (BTMs), dynamic bone-remodeling assessment that includes procollagen I N-propeptide (PINP; bone formation marker) and tartrate-resistant acid phosphatase type 5b (TRAcP-5b; bone resorption marker); and 3) bone histopathology assessment. Improvements in BD were seen in patients with indolent systemic mastocytosis (ISM) and AdvSM receiving avapritinib, a selective *KIT* D816V inhibitor approved for ISM and AdvSM.

### Aims

We aimed to describe the effects of avapritinib on BD, BTMs, bone histopathology, and objective measures of disease burden in patients with AdvSM from PATHFINDER (NCT03580655).

### Methods

In PATHFINDER, 56 patients had serial lumbar spine T-scores (low BD [$BD^{low}$, T-score < –1], high BD [$BD^{high}$, T-score >1], or normal BMD [$BD^{norm}$, T-score ≥ –1 and ≤1]), and 47 of the 56 had paired baseline and on-treatment measurements of plasma BTM levels and bone histopathology assessments. BTMs were compared with healthy volunteer (HV) levels, that match the reference values for post-menopausal females and males over 45 years old, using Welch's t-test. The Wilcoxon signed-rank test was used to compare BTM levels during avapritinib treatment with those at baseline.

**Copyright Information:** (Online) ISSN: 2572-9241
©2026 The Author(s). HemaSphere is published by John Wiley & Sons Ltd on behalf of European Hematology Association. This is an open access Abstract Book distributed under the Attribution-NonCommercial-NoDerivs (CC BY-NC-ND), which allows third parties to download the articles and share them with others as long as they credit the author and the Abstract Book, but they cannot change the content in any way or use them commercially.
**Abstract Book Citations:** Authors, Title, HemaSphere, 2026;10:(S1):pages. The abstract book DOIs can be found on the "Author Information" page.
**Disclaimer:** Articles published in the journal HemaSphere exclusively reflect the opinions of the authors. The authors are responsible for all content in their abstracts including accuracy of the facts, statements, citing resources, etc.

**Results**

Mean lumbar spine T-scores significantly improved in the $BD^{low}$ group (P=0.034; n=12) and remained stable in the $BD^{high}$ (n=21) and $BD^{norm}$ (n=23) groups during avapritinib treatment. Baseline PINP was significantly higher in $BD^{high}$ (P=0.0059; n=15) and $BD^{norm}$ (P=0.0209; n=21) groups than HVs (n=20) and decreased toward the normal range during avapritinib treatment ($BD^{high}$ P<0.0062; $BD^{norm}$ P<0.0081; **Figure 1A**). Baseline TRAcP-5b was significantly lower than HVs in all three BD groups than HV ($BD^{low}$ P=0.0103, n=11; $BD^{high}$ P=0.0275, n=15; $BD^{norm}$ P=0.0028, n=21; HVs n=20) and increased toward normal range in the $BD^{high}$ (P=0.0272) and $BD^{norm}$ (P=0.0036) groups following avapritinib treatment (**Figure 1B**). In addition, *KIT* D816V variant allele fraction in peripheral blood, serum tryptase, bone marrow mast cell burden, and bone histopathology (cellularity, fibrosis, and osteosclerosis score) improved during avapritinib treatment.

**Summary/Conclusion**

Collectively, these encouraging exploratory results provide an impetus for further exploring the potential disease-modifying effects of avapritinib on bone disease in systemic mastocytosis.

**Figure 1. PINP (bone formation marker) and TRAcP-5b (bone resorption marker) in HVs and patients with AdvSM at baseline and during avapritinib treatment**

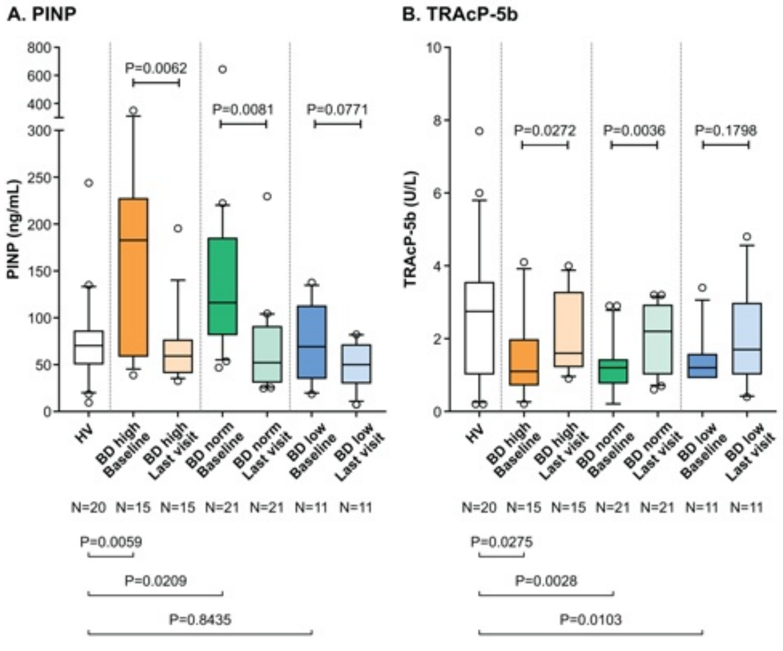

AdvSM, advanced systemic mastocytosis; BD, bone density; HV, healthy volunteer; PINP, procollagen I N-propeptide; TRAcP-5b, tartrate-resistant acid phosphatase type 5b.

**Copyright Information:** (Online) ISSN: 2572-9241
©2026 The Author(s). HemaSphere is published by John Wiley & Sons Ltd on behalf of European Hematology Association. This is an open access Abstract Book distributed under the Attribution-NonCommercial-NoDerivs (CC BY-NC-ND), which allows third parties to download the articles and share them with others as long as they credit the author and the Abstract Book, but they cannot change the content in any way or use them commercially.
**Abstract Book Citations:** Authors, Title, HemaSphere, 2026;10:(S1):pages. The abstract book DOIs can be found on the "Author Information" page.
**Disclaimer:** Articles published in the journal HemaSphere exclusively reflect the opinions of the authors. The authors are responsible for all content in their abstracts including accuracy of the facts, statements, citing resources, etc.

### (PS2010) CONSISTENCY OF RESPONSE BY BASELINE PLATELET COUNT IN PACRITINIB-TREATED PATIENTS IN THE REAL WORLD: MY-PAC ANALYSIS

**Topic:** 16. Myeloproliferative neoplasms - Clinical

James Rossetti*[1], Douglas Tremblay[2], Abiola Oladapo[3], Michael Marrone[3], Caleb Paydar[4], Djibril Liassou[4], Sarah Buckley[3], Purvi Suthar[3], Adina Gepriftí[3], Michael Vredenburg[3], Bruce Feinberg[4], Naveen Pemmaraju[5]

*[1]University of Pittsburgh Medical Center, Pittsburgh, United States of America;[2]Icahn School of Medicine at Mount Sinai, New York, United States of America; [3]Sobi, Inc., Waltham, United States of America;[4]Cardinal Health, Dublin, United States of America; [5]University of Texas MD Anderson Cancer Center, Houston, United States of America;*

### Background

Pacritinib (PAC), a JAK1-sparing inhibitor of JAK2/IRAK1/ACVR1, has demonstrated consistency of spleen and symptom response in patients (pts) with myelofibrosis (MF) across platelet (PLT) strata. There is limited real-world data assessing the consistency of PAC outcomes across PLT counts.

### Aims

To evaluate real-world outcomes in PAC-treated pts with MF stratified by baseline PLT count (<50 x$10^9$/L and ≥50 x$10^9$/L).

### Methods

This was a multisite chart review of pts with MF treated with PAC for ≥1 month between 06/01/2022 and 07/31/2024 with ≥6 months of follow-up through 01/31/2025. Pts were followed from PAC initiation (index) until the end of the study, end of data availability for the pt, or death, whichever came first. Evaluated data included pt characteristics, treatment patterns, palpable spleen length based on ultrasound (total spleen length minus 10 cm), MF-related symptoms, and overall survival through Day 180. Results for pts with PTL <50 x$10^9$/L and ≥50 x$10^9$/L at index are described using counts and percentages, medians and interquartile range (IQR), and Kaplan Meier survival probabilities.

**Copyright Information:** (Online) ISSN: 2572-9241
©2026 The Author(s). HemaSphere is published by John Wiley & Sons Ltd on behalf of European Hematology Association. This is an open access Abstract Book distributed under the Attribution-NonCommercial-NoDerivs (CC BY-NC-ND), which allows third parties to download the articles and share them with others as long as they credit the author and the Abstract Book, but they cannot change the content in any way or use them commercially.
**Abstract Book Citations:** Authors, Title, HemaSphere, 2026;10:(S1):pages. The abstract book DOIs can be found on the "Author Information" page.
**Disclaimer:** Articles published in the journal HemaSphere exclusively reflect the opinions of the authors. The authors are responsible for all content in their abstracts including accuracy of the facts, statements, citing resources, etc.

**Results**

Of 169 PAC-treated pts, 73% (n=123) had PLT <50 x$10^9$/L and 27% (n=45) had PLT ≥50 x$10^9$/L at index. The median (IQR) PLT count at index in each group was 43 (39, 45) and 72 (53, 110), respectively. Compared to pts with higher PLT counts, those with PLT <50 x$10^9$/L were older (median age 73 vs 67 years), had a shorter time from MF diagnosis to index (1.8 vs 6.0 years), and were more likely to receive PAC as first-line therapy (66% vs 51%). Most pts in both groups were anemic at index, with median (IQR) hemoglobin of 9.0 g/dL (8.2, 9.8) and 8.8 g/dL (8.0, 9.7), respectively. Median duration of follow-up from index was 9.8 months (IQR: 7.0-14.0) and 7.6 months (IQR: 6.7-11.0) in PLT <50 and ≥50 x$10^9$/L, respectively. At the end of follow-up, 71% (87/123) and 82% of pts (37/45) were still on PAC in PLT <50 and ≥50 x$10^9$/L, respectively.
Treatment effectiveness outcomes, including spleen response and change in number of MF-related symptoms, were similar across both PLT strata, as shown in the **Table.** The percentage of evaluable pts achieving spleen length reduction ≥50% (SLR-50) was higher in pts with PLT <50 compared to those with PLT ≥ 50 (45% and 30%, respectively). Reduction in the number of MF symptoms was observed in the majority of evaluable pts in both groups. Increases in PLT count and hemoglobin were observed across both PLT strata groups, as shown in the Table. Overall survival by the end of follow-up was 85% (105/123) and 89% (40/45) in pts with PLT <50 x$10^9$/L and PLT ≥50 x$10^9$/L respectively, and 6-month survival probabilities were comparable: 93.5% (95% CI: 87-97) in pts with PLT <50 x$10^9$/L and 93% (95% CI: 81-98) in pts with PLT ≥50 x$10^9$/L.

**Summary/Conclusion**

Real-world outcomes in pts with MF show consistent clinical benefits in pts treated with PAC across baseline PLT counts. These findings may help guide treatment decisions in pts with MF, including those with and without severe thrombocytopenia.

**Copyright Information:** (Online) ISSN: 2572-9241
©2026 The Author(s). HemaSphere is published by John Wiley & Sons Ltd on behalf of European Hematology Association. This is an open access Abstract Book distributed under the Attribution-NonCommercial-NoDerivs (CC BY-NC-ND), which allows third parties to download the articles and share them with others as long as they credit the author and the Abstract Book, but they cannot change the content in any way or use them commercially.
**Abstract Book Citations:** Authors, Title, HemaSphere, 2026;10:(S1):pages. The abstract book DOIs can be found on the “Author Information” page.
**Disclaimer:** Articles published in the journal HemaSphere exclusively reflect the opinions of the authors. The authors are responsible for all content in their abstracts including accuracy of the facts, statements, citing resources, etc.

Table. Spleen, symptom, and hematologic response outcomes in patients treated with pacritinib by baseline platelet count.

| | PLT <50 x10⁹/L (n=123) | PLT ≥50 x10⁹/L (n=45) |
|---|---|---|
| **Spleen and symptom outcomes** | | |
| **Number of pts evaluable for spleen response** | 34 | 12 |
| SLR (%) at Day 180, median (IQR) | 44 (33.3, 66.7) | 39 (30.4, 48.3) |
| Pts achieving SLR ≥50% at Day 180, n (%) | 14 (45%) | 3 (30%) |
| **Number of pts evaluable for symptom response (≥ 1 MF-related symptom at index)** | 117 | 44 |
| Pts with reduction in # of MF symptoms at Day 180, n (%) | 98 (84%) | 35 (80%) |
| Change in # of MF symptoms, median | 67% reduction | 67% reduction |
| **Hematologic response outcome** | | |
| **Number of pts evaluable for platelet measurement** | 102 | 38 |
| Percent change in PLT count (x10⁹/L) from index to day 180, median (IQR) | +62.7% (28.6, 153.6) | +13.9% (0.0, 30.0) |
| Change in PLT count (x10⁹/L) from index to day 180, median (IQR) | 24 (12.0, 62.0) | 10 (0.0, 22.0) |
| **Number of pts evaluable for hemoglobin measurement** | 73 | 29 |
| Change in Hb (g/dL) from index to day 180 at index, median g/dL, (IQR)* | +0.6 (0.0, 1.2) | +0.8 (-0.1, 1.0) |
| Hb response: ≥1.0 g/dL increase from index to Day 180, N (%)* | 26 (35.6%) | 9 (31.0%) |
| Hb response: ≥1.5 g/dL increase from index to Day 180, N (%)* | 16 (21.9%) | 5 (17.2%) |

Hb, hemoglobin; IQR, interquartile range; MF, myelofibrosis; PLT, platelet; pts, patients; SLR, spleen length reduction.
*Restricted to pts with Hb <10 g/dL at index.

Copyright Information: (Online) ISSN: 2572-9241
©2026 The Author(s). HemaSphere is published by John Wiley & Sons Ltd on behalf of European Hematology Association. This is an open access Abstract Book distributed under the Attribution-NonCommercial-NoDerivs (CC BY-NC-ND), which allows third parties to download the articles and share them with others as long as they credit the author and the Abstract Book, but they cannot change the content in any way or use them commercially.
Abstract Book Citations: Authors, Title, HemaSphere, 2026;10:(S1):pages. The abstract book DOIs can be found on the "Author Information" page.
Disclaimer: Articles published in the journal HemaSphere exclusively reflect the opinions of the authors. The authors are responsible for all content in their abstracts including accuracy of the facts, statements, citing resources, etc.

### (PS2011) CLINICAL CHARACTERISTICS, MOLECULAR LANDSCAPE AND OUTCOMES OF NON-CMML MDS/MPN OVERLAP SYNDROMES

**Topic:** 16. Myeloproliferative neoplasms - Clinical

Yael Morgenstern*[1], Dawn Maze[1], Karen Yee[1], Aniket Bankar[1], Guillaume Richard-Carpentier[1], Steven M Chan[1], Aaron Schimmer[1], Andre Schuh[1], Hassan, Sibai[1], Maria Agustina Perusini[1], Vikas Gupta[1], Auro Viswabandya[1], Marta Davidson[1]

[1]*Princess Margaret Cancer Center, Toronto, Canada, Toronto, Canada;*

### Background

MDS/MPN are a rare group of hematological malignancies which consists of chronic myelomonocytic leukemia (CMML), MDS/MPN with neutrophilia (MDS/MPN-N), MDS/MPN with *SF3B1* and thrombocytosis (MDS/MPN-*SF3B1*-T) and MDS/MPN not otherwise specified (MDS/MPN-NOS). While CMML is the most common type of MDS/MPN, data on non-CMML MDS/MPN remains limited.

### Aims

To characterize the clinical features, molecular landscape and clinical outcomes including overall survival (OS) and leukemia free survival (LFS) of MDS/MPN patients (pts) focusing on MDS/MPN-NOS, MDS/MPN-RS-T and MDS/MPN-N at Princess Margaret Cancer Centre.

### Methods

We conducted a retrospective analysis of 131 pts (≥18 years old) diagnosed with MDS/MPN overlap syndrome, treated at Princess Margaret Hospital between March 2003 and June 2024. Pts meeting the criteria of CMML were excluded. Risk stratification was performed using the International Prognostic Scoring System–Revised (IPSS-R), with scores >3.5 indicating a higher-risk disease. OS and LFS were analyzed using Kaplan–Meier estimates and compared by the log-rank test. LFS was defined as time from diagnosis to leukemic transformation or death. Multivariable analysis (MVA) was used to identify independent predictors of OS.

**Copyright Information:** (Online) ISSN: 2572-9241
©2026 The Author(s). HemaSphere is published by John Wiley & Sons Ltd on behalf of European Hematology Association. This is an open access Abstract Book distributed under the Attribution-NonCommercial-NoDerivs (CC BY-NC-ND), which allows third parties to download the articles and share them with others as long as they credit the author and the Abstract Book, but they cannot change the content in any way or use them commercially.
**Abstract Book Citations:** Authors, Title, HemaSphere, 2026;10:(S1):pages. The abstract book DOIs can be found on the "Author Information" page.
**Disclaimer:** Articles published in the journal HemaSphere exclusively reflect the opinions of the authors. The authors are responsible for all content in their abstracts including accuracy of the facts, statements, citing resources, etc.

### Results

Among 131 MDS/MPN pts, 65% (n=85) were classified as lower-risk and 35% (n=46) as higher-risk by IPSS-R. Higher-risk pts were diagnosed more frequently with MDS/MPN-NOS (85% vs. 73%, $p$=0.03), received hypomethylating agents (HMA) more often, and exhibited lower hemoglobin (86 vs. 101 g/L, $p$<0.001) and higher blast percentages in both peripheral blood (PB) (1.5% vs. 0%, $p$<0.001) and marrow (6% vs. 2.5%, $p$<0.001), consistent with more advanced disease. Other features were similar.
Next-generation sequencing was available for 74 pts, revealing frequent mutations in ASXL1 (n=31, 42%), TET2 (n=26, 35%), SF3B1 (n=22, 30%), and SRSF2 (n=19, 26%) mutations. STAG2 mutation was enriched in the higher-risk group (29% vs. 6%, p=0.011). The remaining mutational spectrum was comparable across risk groups.
After a median follow up of 19.3 months (IQR 8.0 – 41.1), higher-risk pts exhibited significantly poorer outcomes compared to lower-risk pts with median OS of 20.0 months (95%CI 14.9-41.2) versus 52.1 months (95%CI 36.1-not reached), $p$=0.003 and median LFS of 14.3 months (95%CI 7.6-30.7) vs 45.4 months (95%CI 31.9-71.6), p<0.001 respectively; Among higher-risk pts, treatment with HMA did not confer a survival advantage compared to patients who did not receive HMA (median OS 19.4 months vs. 31.1 months, p=0.7). MVA showed that advanced age (HR 1.06, 95%CI 1.0-1.1, p<0.001), IPSS-R>3.5 (HR 2.08, 95%CI 1.0-4.1, p=0.038) and increased circulating blasts in PB (HR 1.15, 95%CI 1.1-1.3, p=0.003) predicted inferior OS. Twenty-one pts underwent upfront allogeneic stem cell transplantation (allo-SCT) with a 2-year OS of 72.2% (95%CI 45.6-87.4) and relapse observed in 24% (n=5) of pts post allo-SCT. Nonetheless, even pts categorized as low-risk had suboptimal outcomes with 14% (n=12) of patients progressing to acute myeloid leukemia, underscoring the aggressive nature of these diseases.

### Summary/Conclusion

Non-CMML MDS/MPN subtypes demonstrate poor survival outcomes, including among patients classified as low-risk by conventional scoring systems. These findings underscore the aggressive nature of these diseases and the need to develop refined tools to better identify patients at high risk and guide therapeutic decision-making.

**Copyright Information:** (Online) ISSN: 2572-9241
©2026 The Author(s). HemaSphere is published by John Wiley & Sons Ltd on behalf of European Hematology Association. This is an open access Abstract Book distributed under the Attribution-NonCommercial-NoDerivs (CC BY-NC-ND), which allows third parties to download the articles and share them with others as long as they credit the author and the Abstract Book, but they cannot change the content in any way or use them commercially.
**Abstract Book Citations:** Authors, Title, HemaSphere, 2026;10:(S1):pages. The abstract book DOIs can be found on the "Author Information" page.
**Disclaimer:** Articles published in the journal HemaSphere exclusively reflect the opinions of the authors. The authors are responsible for all content in their abstracts including accuracy of the facts, statements, citing resources, etc.

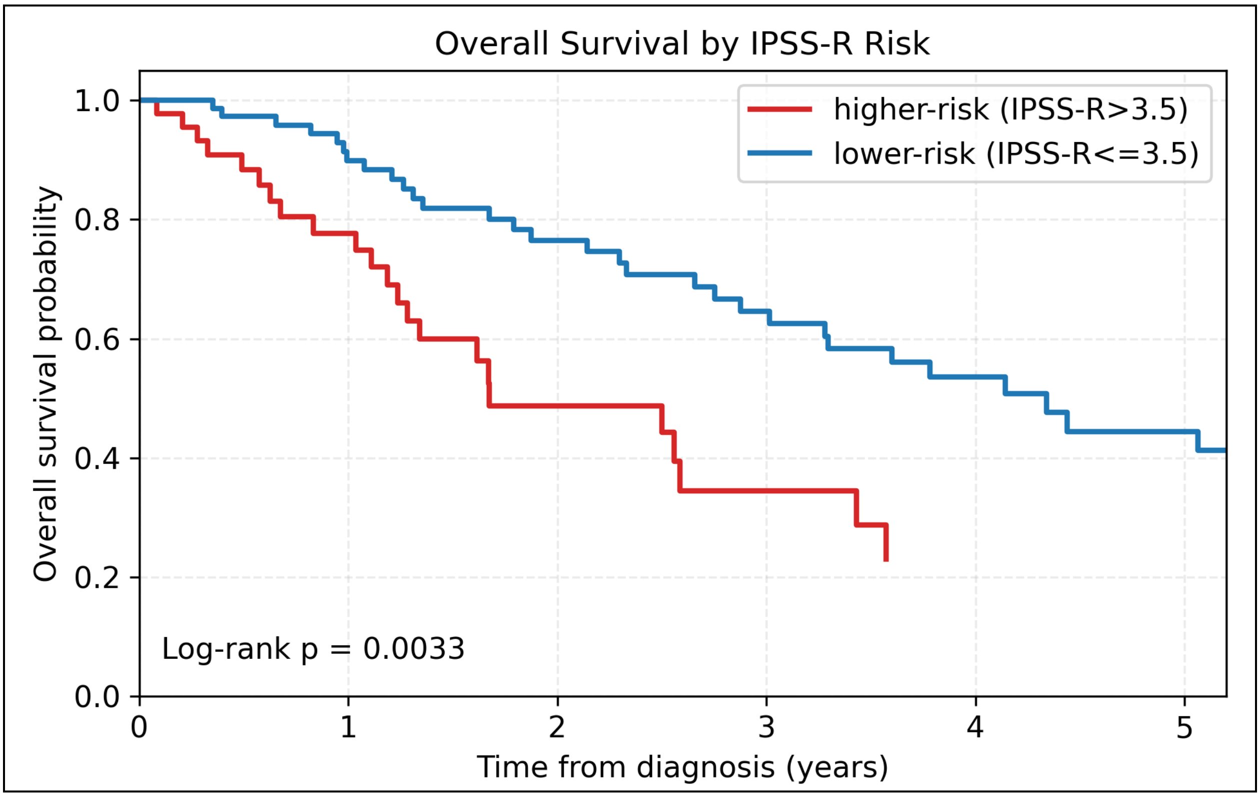

Overall Survival of non-CMML MDS/MPN patients stratified by IPSS-R score

**Copyright Information:** (Online) ISSN: 2572-9241
©2026 The Author(s). HemaSphere is published by John Wiley & Sons Ltd on behalf of European Hematology Association. This is an open access Abstract Book distributed under the Attribution-NonCommercial-NoDerivs (CC BY-NC-ND), which allows third parties to download the articles and share them with others as long as they credit the author and the Abstract Book, but they cannot change the content in any way or use them commercially.
**Abstract Book Citations:** Authors, Title, HemaSphere, 2026;10:(S1):pages. The abstract book DOIs can be found on the "Author Information" page.
**Disclaimer:** Articles published in the journal HemaSphere exclusively reflect the opinions of the authors. The authors are responsible for all content in their abstracts including accuracy of the facts, statements, citing resources, etc.

### (PS2012) ASSOCIATION OF CAR, EASIX, AND SII SCORES WITH JAK2 MUTATION STATUS AND THROMBOTIC EVENTS IN PATIENTS WITH ESSENTIAL THROMBOCYTHEMIA

**Topic:** 16. Myeloproliferative neoplasms - Clinical

Nergiz Usta*[1], Ozlen Balta[2]

*[1]Karadeniz Technical Univercity, Department of Hematology, TRABZON, Türkiye;[2]Karadeniz Technical University, Department of Hematology, TRABZON, Türkiye;*

### Background

Thrombosis is one of the leading causes of morbidity and mortality in patients with essential thrombocythemia (ET).

### Aims

This study aimed to investigate the association between the C-reactive protein–albumin ratio (CAR), endothelial activation and stress index (EASIX), and systemic immune-inflammation index (SII), JAK2 mutation status, and thrombotic events in patients with ET.

### Methods

This retrospective study included patients diagnosed with ET who were followed at Karadeniz Technical University Faculty of Medicine between January 2010 and September 2024. Demographic, clinical, and laboratory data were obtained from medical records. CAR, EASIX, and SII scores were calculated using baseline laboratory parameters at the time of diagnosis. JAK2 mutation status and history of thrombotic events were recorded. The associations between CAR, EASIX, SII scores, JAK2 mutation status, and thrombotic events were analyzed using appropriate statistical methods. A p-value < 0.05 was considered statistically significant.

**Copyright Information:** (Online) ISSN: 2572-9241
©2026 The Author(s). HemaSphere is published by John Wiley & Sons Ltd on behalf of European Hematology Association. This is an open access Abstract Book distributed under the Attribution-NonCommercial-NoDerivs (CC BY-NC-ND), which allows third parties to download the articles and share them with others as long as they credit the author and the Abstract Book, but they cannot change the content in any way or use them commercially.
**Abstract Book Citations:** Authors, Title, HemaSphere, 2026;10:(S1):pages. The abstract book DOIs can be found on the “Author Information” page.
**Disclaimer:** Articles published in the journal HemaSphere exclusively reflect the opinions of the authors. The authors are responsible for all content in their abstracts including accuracy of the facts, statements, citing resources, etc.

### Results

A total of 172 patients, including 92 (54%) females and 80 (46%) males, were included in the study. The median age at diagnosis was 56 (18-87) years. Thrombosis was present in 65 (38%) patients. The presence of thrombosis was associated with advanced age, JAK2 mutation positivity, a high revised IPSET score, and the presence of chronic comorbidities. Lymphocyte count, platelet count, and albumin levels were significantly lower, whereas monocyte count, creatinine, C-reactive protein (CRP) levels, CAR, and EASIX scores were significantly higher in patients with thrombosis compared with those without thrombosis. In ROC analysis evaluating thrombosis prediction, the AUC for CAR was 0.731 (95% CI: 0.655–0.807; p < 0.001), for EASIX was 0.707 (95% CI: 0.630–0.784; p < 0.001), and for SII was 0.533 (95% CI: 0.442–0.623; p = 0.474). Based on cut-off values, high CAR and high EASIX scores were significantly associated with thrombosis. A significant association was observed between thrombosis and the combined JAK–CAR, JAK–EASIX, and JAK–SII groups (p < 0.001, p < 0.001, p = 0.006). In Bonferroni-adjusted pairwise comparisons, within the JAK–CAR classification, the JAK-positive/CAR-low and JAK-positive/CAR-high groups exhibited higher thrombosis rates compared with the JAK-negative/CAR-low group (p = 0.008, p < 0.001,). In the JAK–EASIX classification, the JAK-positive/EASIX-high group demonstrated a higher risk of thrombosis than both the JAK-negative/EASIX-low and JAK-positive/EASIX-low groups (p < 0.001, p = 0.008). Similarly, in the JAK–SII classification, the JAK-positive/SII-high group had a higher thrombosis risk compared with the JAK-negative/SII-low group (p = 0.002).

**Table 1. ROC analysis for CAR, EASIX, and SII in detecting thrombosis in patients with essential thrombocythemia**

| | **CAR** | **EASIX** | **SII** |
|---|---|---|---|
| AUC, (%95 CI) | 0.731 (0.655-0.807) | 0.707 (0.630-0.784) | 0.533 (0.442-0.623) |
| Cut-off value | 0.068 | 0.256 | 2625.861 |
| Sensitivity % | 65 | 63 | 52 |
| Specificity % | 65 | 63 | 52 |
| P value | <0.001* | <0.001* | 0.474 |

CAR: C-reactive protein/albumin ratio, EASIX: Endotelyal activation and stress index, SII Systemic immune-inflammatory index, AUC: Area under the curve.
*p< 0,05 is statistically significant.

### Summary/Conclusion

These findings suggest that the coexistence of JAK2 positivity and elevated inflammatory indices (CAR and EASIX) is associated with an increased risk of thrombosis, highlighting the potential value of combined risk stratification models in identifying high-risk patients.

**Copyright Information:** (Online) ISSN: 2572-9241
©2026 The Author(s). HemaSphere is published by John Wiley & Sons Ltd on behalf of European Hematology Association. This is an open access Abstract Book distributed under the Attribution-NonCommercial-NoDerivs (CC BY-NC-ND), which allows third parties to download the articles and share them with others as long as they credit the author and the Abstract Book, but they cannot change the content in any way or use them commercially.
**Abstract Book Citations:** Authors, Title, HemaSphere, 2026;10:(S1):pages. The abstract book DOIs can be found on the “Author Information” page.
**Disclaimer:** Articles published in the journal HemaSphere exclusively reflect the opinions of the authors. The authors are responsible for all content in their abstracts including accuracy of the facts, statements, citing resources, etc.

### (PS2013) ANALYSIS OF VARIANT ALLELE FREQUENCY OF JAK2V617F IN PATIENTS WITH POLYCYTHEMIA VERA TREATED WITH EITHER RUXOLITINIB OR BEST AVAILABLE THERAPY IN THE RANDOMIZED PHASE 2B RUXOBEAT CLINICAL TRIAL

**Topic:** 16. Myeloproliferative neoplasms - Clinical

Madeline Caduc*[1, 2], Susanne Isfort[1, 2, 3], Lino Lars Teichmann[4], Philippe Schafhausen[5], Florian Heidel[3, 6], Carl Crodel[6], Andreas Hochhaus[6], Martin Griesshammer[7], Katja Sockel[8], Philipp Jost[9, 10], Denise Walther[11], Konstanze Döhner[12], Joachim Goethert[13], Markus Radsak[14], Marinela Augustin[15], Heiko Becker[16], Aristoteles Giagounidis[17], Markus Schaich[18], Martine Klausmann[19], Nikolaschristiancornelius Bubnoff[20], Frank Stegelmann[12], Jennifer Peil[1, 2], Maike Kortmann[2, 21], Michelle Zentis[2, 21], Julia Frank[22], Anna Hagemeier[22], Martin Hellmich[22, 23], Tim Brümmendorf[1, 2], Steffen Koschmieder[1, 2]

*[1]Department of Hematology, Oncology, Hemostaseology, and SCT, Faculty of Medicine, RWTH Aachen University, Aachen, Germany; [2]Center for Integrated Oncology Aachen Bonn Cologne Düsseldorf (CIO ABCD), Aachen, Germany; [3]Hematology, Hemostasis, Oncology and Stem Cell Transplantation, Hannover Medical School, Hannover, Germany; [4]Department of Medicine III, University Hospital Bonn, Bonn, Germany; [5]Department of Oncology, Hematology, and Bone Marrow Transplantation with Section of Pneumology, University Medical Center Hamburg-Eppendorf, Hamburg, Germany; [6]Klinik für Innere Medizin II, Jena University Hospital, Jena, Germany; [7]University Clinic for Haematology, Oncology, Haemostaseology and Palliative Care, Johannes Wesling Medical Centre Minden, University of Bochum, Minden, Germany; [8]Medical Clinic and Policlinic I, University Hospital Dresden, TU Dresden, Dresden, Germany; [9]Medical Department III, Hematology and Oncology, and Center for Translational Cancer Research, Translatum, Technical University of Munich, Munich, Germany; [10]Division of Clinical Oncology, Internal Medicine, Medical University of Graz, Graz, Austria; [11]Department of Hematology/Oncology, Otto-von-Guericke, University Medical Center Magdeburg, Magdeburg, Germany; [12]Department of Internal Medicine III, University Hospital of Ulm, Ulm, Germany; [13]Department of Hematology and Stem Cell Transplantation, West German Cancer Center, University Hospital Essen, Essen, Germany; [14]Department of Hematology and Medical Oncology, University Medical Center; DKTK, UCT Mainz, Johannes Gutenberg University Mainz, Mainz, Germany; [15]Klinikum Nürnberg Nord, Schwerpunkt Onkologie/Hämatologie, Klinik für Innere Medizin 5, Paracelsus Medizinische Privatuniversität, Nürnberg, Germany; [16]Department of Hematology, Oncology and Stem Cell Transplantation, Medical Center, Faculty of Medicine, Freiburg, Freiburg, Germany, and German Cancer Consortium (DKTK), partner site Freiburg, Germany, Freiburg, Germany; [17]Dept. Oncology, Hematology and Palliative Care, Marienhospital Düsseldorf, Düsseldorf, Germany; [18]Department of Hematology, Oncology and Palliative Medicine, Rems-Murr-Klinikum, Winnenden, Germany; [19]Gemeinschaftspraxis, Aschaffenburg, Aschaffenburg, Germany; [20]Department of Hematology and Oncology, University Medical Center Schleswig-Holstein and University Cancer Center Schleswig-Holstein (UCCSH), University of Lübeck, Lübeck, Germany; [21]Center for Translational & Clinical Research (CTC-A), Faculty of Medicine, RWTH Aachen University, Aachen, Germany, Aachen, Germany; [22]Institute of Medical Statistics and Computational Biology, Faculty of Medicine and University Hospital Cologne, University of Cologne, Cologne, Germany; [23]Department of Medical Statistics, University Medical Center Göttingen, Göttingen, Germany;*

### Background

Polycythemia vera (PV) is a BCR::ABL1-negative myeloproliferative neoplasm characterized by clonal trilineage hyperproliferation, risk of thromboembolic events, and progression to myelofibrosis or blast phase. First-line cytoreduction in the EU includes hydroxyurea (HU) or pegylated interferon-α (IFNα), while ruxolitinib (RUX) is approved for HU-resistant or -intolerant PV and has demonstrated molecular activity, but has not been prospectively evaluated in the first-line setting.

### Aims

To assess whether early JAK1/2 inhibition with RUX improves molecular responses compared with best available therapy (BAT) in treatment-naïve, high-risk PV patients.

**Copyright Information:** (Online) ISSN: 2572-9241
©2026 The Author(s). HemaSphere is published by John Wiley & Sons Ltd on behalf of European Hematology Association. This is an open access Abstract Book distributed under the Attribution-NonCommercial-NoDerivs (CC BY-NC-ND), which allows third parties to download the articles and share them with others as long as they credit the author and the Abstract Book, but they cannot change the content in any way or use them commercially.
**Abstract Book Citations:** Authors, Title, HemaSphere, 2026;10:(S1):pages. The abstract book DOIs can be found on the "Author Information" page.
**Disclaimer:** Articles published in the journal HemaSphere exclusively reflect the opinions of the authors. The authors are responsible for all content in their abstracts including accuracy of the facts, statements, citing resources, etc.

**Methods**

High-risk PV patients enrolled in the ongoing multicenter, open-label, randomized phase IIb RuxoBEAT trial (NCT02577926) were assigned 1:1 to RUX or BAT. RUX was initiated at 10 mg BID with dose escalation up to 20 mg BID permitted; BAT was administered as per investigator decision. Up to 6 weeks of prior PV-directed therapy was permitted. Crossover from BAT to RUX (BAT-CO) was allowed after 6 months in eligible patients. Primary (clinico-hematologic complete response) and secondary endpoints were reported previously. As an exploratory endpoint, JAK2V617F variant allele frequency (VAF) was assessed at baseline (BL; month 1 used if BL unavailable in n=5), month 6 (M6; month 9 in n=2), and month 24 or end of treatment (M24). Between-group differences were analyzed using the Mann–Whitney-U test.

**Results**

77 PV patients (RUX n=43; BAT n=34) were included; 17/34 BAT patients crossed over to RUX after 6 months (BAT-CO). BL characteristics were comparable between arms. At BL, JAK2V617F VAF (mean BL VAF was 52% for RUX and 48% for BAT) significantly ($p<0.05$) correlated with WBC ($r=0.56$) and spleen length ($r=0.40$), and inversely with headache ($r=-0.25$), indicating association with disease burden. Clinically relevant co-driver mutations were identified in 4 patients (RUX n=2, BAT-CO n=1, BAT n=1) and were not analyzed further. At M6, VAF remained significantly ($p<0.01$) correlated with WBC ($r=0.55$), HCT ($r=0.32$), and spleen length ($r=0.43$), but not with PROM; similar correlations were observed at M24 for WBC ($r=0.29$), HCT ($r=0.43$), and spleen length ($r=0.46$) (all $p<0.05$). Stratified by treatment, VAF decreased significantly from baseline to M6 in both RUX (mean 45%, mean -15% reduction, $p<0.001$) and BAT (mean 39%, mean -17% reduction, $p=0.013$), with no between-group difference at M6 ($p=0.53$). BAT patients who later crossed over to RUX showed no early VAF reduction at M6 ($p=0.516$), whereas non-cross-over BAT patients showed a trend toward reduction ($p=0.058$). From M6/M9 to M24, RUX induced a sustained VAF reduction (mean -30%, $p<0.001$), whereas VAF remained stable in BAT (mean +6%, $p=0.379$) and BAT-CO (mean –9%, $p=0.625$).

**Summary/Conclusion**

JAK2V617F VAF correlated with objective disease burden (blood counts, spleen length) but not PROM in high-risk PV. Early VAF reductions occurred with both RUX and BAT; however, only first-line ruxolitinib induced a sustained molecular decline, whereas BAT effects were transient and did not deepen after crossover.

**Copyright Information:** (Online) ISSN: 2572-9241
©2026 The Author(s). HemaSphere is published by John Wiley & Sons Ltd on behalf of European Hematology Association. This is an open access Abstract Book distributed under the Attribution-NonCommercial-NoDerivs (CC BY-NC-ND), which allows third parties to download the articles and share them with others as long as they credit the author and the Abstract Book, but they cannot change the content in any way or use them commercially.
**Abstract Book Citations:** Authors, Title, HemaSphere, 2026;10:(S1):pages. The abstract book DOIs can be found on the “Author Information” page.
**Disclaimer:** Articles published in the journal HemaSphere exclusively reflect the opinions of the authors. The authors are responsible for all content in their abstracts including accuracy of the facts, statements, citing resources, etc.

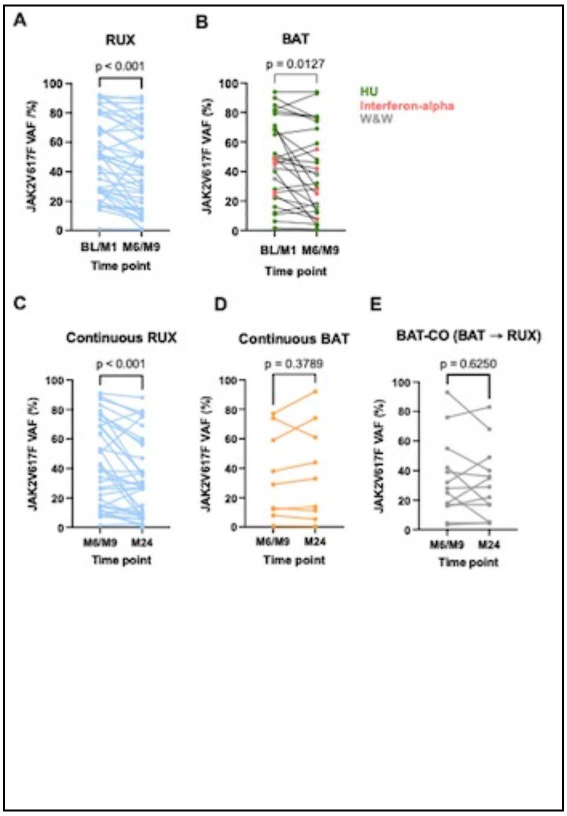

Figure 1. Longitudinal JAK2V617F variant allele frequency (VAF) dynamics in high-risk polycythemia vera patients in the RuxoBEAT trial. (A–B) Paired individual VAF (%) at baseline/month 1 and month 6/9 (M6/M9) in the ruxolitinib (RUX) and best available therapy (BAT) arms. (C–E) VAF (%) from M6/M9 to month 24 (M24) in patients receiving continuous RUX, continuous BAT, or BAT with crossover to RUX after 6 months (BAT-CO).

**Copyright Information:** (Online) ISSN: 2572-9241
©2026 The Author(s). HemaSphere is published by John Wiley & Sons Ltd on behalf of European Hematology Association. This is an open access Abstract Book distributed under the Attribution-NonCommercial-NoDerivs (CC BY-NC-ND), which allows third parties to download the articles and share them with others as long as they credit the author and the Abstract Book, but they cannot change the content in any way or use them commercially.
**Abstract Book Citations:** Authors, Title, HemaSphere, 2026;10:(S1):pages. The abstract book DOIs can be found on the "Author Information" page.
**Disclaimer:** Articles published in the journal HemaSphere exclusively reflect the opinions of the authors. The authors are responsible for all content in their abstracts including accuracy of the facts, statements, citing resources, etc.

### (PS2014) COMPARISON OF JAK2-MUTATED ESSENTIAL THROMBOCYTHAEMIA AND POLYCYTHAEMIA VERA IN ADOLESCENTS AND YOUNG ADULTS: CHARACTERISTICS, VASCULAR EVENTS AND LONG-TERM OUTCOME

**Topic:** 16. Myeloproliferative neoplasms - Clinical

Maria Georgiou*[1], Marta Sobas[2], Jean-Jacques Kiladjian[3], Parvis Sadjadian[4], Lee-Yung Shih[5], Timothy Devos[6], Dorota Krochmalczyk[7], Serena Galli[8], Maria Bieniaszewska[9], Ilona Seferynska[10], Mary Frances McMullin[11], Anna Armatys[12], Adrianna Spalek[13], Joanna Wącław-Koziara[7], Zdrenghea Mihnea Tudor[14], Laurence Legros[15], François Girodon[16], Krzysztof Lewandowski[17], Beatriz Bellosillo Paricio[18], Jan Samuelsson[19], Iman Noumani[1, 20], Thaw Htin[1], Elena Torre[1], Aitor Abuin Blanco[21], Pascale Cony-Makhoul[22], Angela Collins[23], Chloé James[24], Rajko Kušec[25], Marie Lauermannova[26], Maria Soledad Noya Pereira[27], Malgorzata Skowronek[28], Łukasz Szukalski[29], Anna Szmigielska-Kapłon[30], Marielle Wondergem[31], Iryna Dudchenko[32], Joanna Gora-Tybor[30], Kamel Laribi[33], Anna Kulikowska de Nałęcz[34], Jean-Loup Demory[35], Katell Le Du[36], Sonja Zweegman[31], Carlos Besses Raebel[18], Radek Skoda[37], Stephane Giraudier[38], Martin Griesshammer[4], Claire Harrison[1], Jean Christophe Ianotto[39], Yan Beauverd[40]

*[1]Guy's and St Thomas' NHS Foundation Trust, Haematology Department, London, United Kingdom;[2]Collegium Medicum in Bydgoszcz, Nicolaus Copernicus University in Toruń, Department of Hematology, Bydgoszcz, Poland; [3]Hopital Saint-Louis, Paris, France; [4]Johannes Wesling Medical Center, University of Bochum, University Clinic for Hematology, Oncology, Hemostaseology and Palliative Care, Minden, Germany; [5]Chang Gung Memorial Hospital-Linkou and Chang Gung University, Division of Hematology-Oncology, Taoyuan, Taiwan; [6]University Hospitals Leuven and Department of Microbiology and Immunology, Laboratory of Molecular Immunology (Rega Institute), Department of Hematology, Leuven, Belgium; [7]Collegium Medicum, Jagiellonian University, Department of Hematology, Krakow, Poland; [8]University Hospital Zurich, Department of Medical Oncology and Hematology, Zurich, Switzerland; [9]Medical University and Clinical Center, Hematology and Transplantation Department, Gdansk, Poland; [10]Institute of Hematology and Transfusion Medicine, Warsaw, Poland; [11]Belfast City Hospital, Queen's University Belfast, Belfast, United Kingdom; [12]Jagiellonian University Hospital, Krakow, Poland; [13]Jagiellonian University Hospital, Hematology Department, Krakow, Poland; [14]Iuliu Hatieganu University of Medicine and Pharmacy, Department of Hematology, Cluj-Napoca, Romania; [15]University of Paris Saclay, Bicêtre Hospital, Paris, France;[16]University Hospital Dijon, Laboratory of Biological Hematology,, Dijon, France; [17]University of Medical Sciences, Hematology and Bone Marrow Transplantation Department, Poznan, Poland; [18]Hospital del Mar, Hospital del Mar Research Institute, Hematology Department, Barcelona, Spain; [19]University Hospital Linkoping, Linkoping, Sweden;[20]Royal Hospital Oman, Adult Hematology Department, Muscat, Oman; [21]Hospital Universitario Lucus Augusti, Servicio de Hematología, Lugo, Spain;[22]Annecy-Genevois Hospital, Pringy, France; [23]Norfolk and Norwich University Hospitals National Health Service Trust, Norwich, United Kingdom;[24]University of Bordeaux, Biology of Cardiovascular Diseases, Pessac, France; [25]University Hospital Dubrava, School of Medicine,University of Zagreb, Zagreb, Croatia; [26]Institute of Hematology and Blood Transfusion, Prague, Prague, Czechia;[27]University Hospital CHUAC, Hematology Department, A Coruña, Spain; [28]Holy Cross Oncology Center, Kielce, Poland;[29]Collegium Medicum in Bydgoszcz, Nicolaus Copernicus University, Torun, Poland; [30]Medical University, Department of Hematology, Lodz, Poland; [31]Amsterdam University Medical Center, Amsterdam, The Netherlands;[32]Academic and Research Medical Institute, Sumy State University, Department of Internal and Family Medicine, Sumy, Ukraine; [33]Le Mans Hospital, Le Mans, France; [34]State Hospital, Department of Hematology, Opole, Poland; [35]St. Vincent De Paul Hospital, Lille, France;[36]The Confluent, Private Hospital, Nantes, France; [37]University Hospital Basel, Experimental Hematology, Department of Biomedicine, Basel, Switzerland;[38]St Louis Hospital, APHP, Cellular Biology Department, Paris, France; [39]Centre Hospitalier Universitaire de Brest, Brest, France; [40]Geneva University Hospitals, University of Geneva, Hematology Division and Faculty of Medicine, Geneva, Switzerland;*

**Copyright Information:** (Online) ISSN: 2572-9241
©2026 The Author(s). HemaSphere is published by John Wiley & Sons Ltd on behalf of European Hematology Association. This is an open access Abstract Book distributed under the Attribution-NonCommercial-NoDerivs (CC BY-NC-ND), which allows third parties to download the articles and share them with others as long as they credit the author and the Abstract Book, but they cannot change the content in any way or use them commercially.
**Abstract Book Citations:** Authors, Title, HemaSphere, 2026;10:(S1):pages. The abstract book DOIs can be found on the "Author Information" page.
**Disclaimer:** Articles published in the journal HemaSphere exclusively reflect the opinions of the authors. The authors are responsible for all content in their abstracts including accuracy of the facts, statements, citing resources, etc.

### Background

Polycythaemia Vera (PV) and Essential Thrombocythaemia (ET) are rare conditions in young adults. The *JAK2*V617F mutation is present in ~95% of patients with PV, and only in 50%-60% of ET cases. *JAK2* allele burden is typically higher in PV than in ET. Although both conditions carry a comparable thrombotic risk, some studies suggest a higher risk of progression to myelofibrosis in PV.

### Aims

To characterize and compare clinical features, treatment patterns and outcomes of patients diagnosed before the age of 25 years with *JAK2*-mutated PV or ET.

### Methods

We performed a retrospective multicentre study of *JAK2*-mutated PV or ET patients (including *JAK2* exon 14 and exon 12) diagnosed less than 25 years between 1974- 2017. Data were collected from participating centres across Europe and Taiwan. Variables include: baseline characteristics (age, gender, cardiovascular risk factors), thrombotic and haemorrhagic events, treatment strategies (cytoreduction vs observation) and progression to myelofibrosis.

### Results

A total of 217 *JAK2*-mutated PV and ET patients were included (70 PV, 147 ET). Median age at diagnosis was 20 years in PV and 21 years in ET (p=0.32) with median follow-up of 11 and 9 years, respectively (p=0.08). Cardiovascular risk factors were present in 27 patients (16 PV, 11 ET), and 39 patients had history of thrombosis at diagnosis (18 PV, 21 ET). Haemorrhagic events occurred in 9 patients (3 PV, 6 ET). Among ET patients, 89% were classified as low/intermediate risk by IPSET survival risk score. The majority of patients had at least 1 line of cytoreductive treatment.
Ten-year thrombosis-free survival (TFS) was not different: 80.5% in ET and 85.2% in PV (p=0.598). Antiplatelet therapy was not associated with improved TFS in ET (HR 16.2, 95% CI 0.04–17.06; p=0.32) or PV (HR 0.35, 95% CI 0.035–3.5; p=0.37). Elevated WCC >$11\times10^9$/L (p=0.01) and absence of splenomegaly (p=0.03), but neither cardiovascular risk factors (p=0.60), nor platelet count >$1000\times10^9$/L (p=0.80) were associated with thrombotic events.
Ten patients progressed to myelofibrosis (4 PV, 6 ET), with no significant difference between groups (p=0.598). Interferon (IFN) was associated with improved myelofibrosis-free survival (MFS) compared with other treatments or observation in both ET (p=0.045) and PV (p=0.034). Additionally, WCC>$11x10^9$/L was not associated with MFS in either group (p=0.58 in ET, p=0.595 in PV).
Limitations include that as this is a real-world retrospective study both diagnostic and treatment allocations were at the discretion of the local teams and pertinent to the guidance at the time. Furthermore, despite the long follow up event numbers were modest.

### Summary/Conclusion

In this large cohort of young patients with *JAK2*-mutated PV and ET, no significant differences in terms of MFS and TFS were observed. Interferon therapy was associated with improved long-term outcomes, particularly MFS, but did not significantly reduce thrombotic risk. Young *JAK2*-mutated PV and *JAK2*-ET populations appear clinically comparable, raising questions about the relevance of their distinction and supporting a unified management approach

**Copyright Information:** (Online) ISSN: 2572-9241
©2026 The Author(s). HemaSphere is published by John Wiley & Sons Ltd on behalf of European Hematology Association. This is an open access Abstract Book distributed under the Attribution-NonCommercial-NoDerivs (CC BY-NC-ND), which allows third parties to download the articles and share them with others as long as they credit the author and the Abstract Book, but they cannot change the content in any way or use them commercially.
**Abstract Book Citations:** Authors, Title, HemaSphere, 2026;10:(S1):pages. The abstract book DOIs can be found on the “Author Information” page.
**Disclaimer:** Articles published in the journal HemaSphere exclusively reflect the opinions of the authors. The authors are responsible for all content in their abstracts including accuracy of the facts, statements, citing resources, etc.

### (PS2015) PEMBROLIZUMAB IN CHILDREN AND YOUNG ADULTS WITH HIGH-RISK CLASSIC HODGKIN LYMPHOMA WITH SLOW EARLY RESPONSE TO FRONT-LINE CHEMOTHERAPY: UPDATED RESULTS FROM KEYNOTE-667

**Topic:** 17. Hodgkin lymphoma - Clinical

Christine Mauz-Koerholz*[1, 2], Luciana Vinti[3], Stephen Daw[4], Antony Ceraulo[5], Gerard Michel[6], Michaela Cepelova[7], Lorena Valero-Arrese[8], Franca Fagioli[9, 10], Stéphane Ducassou[11], Salvatore Buffardi[12], Marie-Emilie Dourthe[13], Bradford S. Hoppe[14], Kara Kelly[15], Lisa Giulino-Roth[16], Judith Landman-Parker[17], Juan Shen[18], Pallavi Pillai[18], Rushdia Yusuf[18], Auke Beishuizen[19]

*[1]Justus-Liebig University of Giessen, Giessen, Germany; [2]Medical Faculty of the Martin-Luther-University of Halle-Wittenberg, Halle, Germany; [3]IRCCS Ospedale Pediatrico Bambino Gesu, Rome, Italy; [4]University College London Hospitals NHS Foundation Trust, London, United Kingdom; [5]Institut d'Hematologie-Oncologie Pediatrique (IHOPe), Lyon, France; [6]CHU de Marseille Hopital de la Timone Enfants, Marseille, France; [7]University Hospital Motol and Homolka, Prague, Czechia; [8]Vall d'Hebron University Hospital, Barcelona, Spain; [9]Ospedale Infantile Regina Margherita, Turin, Italy; [10]University of Turin, Turin, Italy; [11]CHU de Bordeaux, Hopital Pellegrin, Bordeaux, France; [12]Azienda Ospedaliera di Rilievo Nazionale Santobono Pausilipon, Naples, Italy; [13]Duke University School of Medicine, Durham, United States of America; [14]Mayo Clinic, Jacksonville, United States of America; [15]Roswell Park Comprehensive Cancer Center, Buffalo, United States of America; [16]Weill Cornell Medicine, New York, United States of America; [17]Sorbonne Université APHP Hôpital Armand Trousseau, Paris, France; [18]Merck & Co., Inc., Rahway, United States of America; [19]Princess Máxima Centre, Utrecht, The Netherlands;*

### Background

Children and young adults with classic Hodgkin lymphoma (cHL) who have a slow early response (SER) to front-line chemotherapy are at increased risk of relapse. The open-label, phase 2 KEYNOTE-667 study (NCT03407144) was designed to evaluate pembrolizumab plus chemotherapy consolidation with or without radiotherapy (RT) and pembrolizumab maintenance in participants (pts) with cHL and SER to front-line chemotherapy. Prior analysis of pts with high-risk cHL and SER to front-line OEPA showed that consolidation with pembrolizumab plus COPDAC-28 followed by pembrolizumab maintenance had promising activity and manageable safety.

### Aims

To present updated results with an additional 13 months of follow-up for pts with high-risk cHL.

### Methods

Eligible pts were aged 3-25 years; had newly diagnosed stage IIEB, IIIEA, IIIEB, IIIB, IVA, or IVB cHL; had measurable disease per investigator; and had a performance status score of ≥50. All pts with high-risk cHL received 2 cycles of OEPA induction followed by early response assessment. Pts with rapid early response (Deauville score [DS] 1-3) received nonstudy therapy at investigator discretion. Pts with a SER (DS 4 or 5) received consolidation with 6 doses of pembrolizumab 2 mg/kg up to 200 mg (3-17 years) or 200 mg (18-25 years) IV Q3W plus COPDAC-28 for 4 cycles followed by late-response assessment (LRA; between C4D29 and C4D32 of COPDAC-28). Pts with PET-positive disease at LRA (DS 4 or 5) received RT (28.8 Gy) to late PET-positive residua. Pts with PET-negative disease (DS 1-3) received no RT. All pts with SER continued to receive pembrolizumab for a total of 17 cycles. Primary end point was ORR per Cheson 2007 IWG criteria by blinded independent central review (BICR). Secondary end points were PET negativity after consolidation, 2-year EFS after enrollment, OS, and safety in pts with SER.

**Copyright Information:** (Online) ISSN: 2572-9241
©2026 The Author(s). HemaSphere is published by John Wiley & Sons Ltd on behalf of European Hematology Association. This is an open access Abstract Book distributed under the Attribution-NonCommercial-NoDerivs (CC BY-NC-ND), which allows third parties to download the articles and share them with others as long as they credit the author and the Abstract Book, but they cannot change the content in any way or use them commercially.
**Abstract Book Citations:** Authors, Title, HemaSphere, 2026;10:(S1):pages. The abstract book DOIs can be found on the “Author Information” page.
**Disclaimer:** Articles published in the journal HemaSphere exclusively reflect the opinions of the authors. The authors are responsible for all content in their abstracts including accuracy of the facts, statements, citing resources, etc.

### Results

246 pts with high-risk cHL were enrolled; 84 had SER to front-line OEPA and received consolidation with pembrolizumab + COPDAC-28. At the data cutoff (October 1, 2025), 74 pts with SER had completed consolidation and maintenance, and 10 had discontinued. Pts received a median of 17 cycles of pembrolizumab (range, 2-17). Median time from enrollment to database cutoff was 43.4 months (range, 24.7-67.5). Of 84 pts with SER who received consolidation, 82 (98%) had an LRA and 2 (2%) did not have an LRA. At LRA, 58 of 84 pts (69%) were PET negative by BICR and investigator, of whom 57 were spared RT. The ORR was 98% (95% CI, 92-100; 63 complete response, 19 partial response). Median EFS was not reached (NR; 95% CI, NR-NR); 2-year EFS was 85% and 3-year EFS was 81%. Median OS was NR (95% CI, NR-NR); 3-year OS was 100%. Treatment-related AEs occurred in 64 pts (76%; grade 3 or 4, n = 16 [19%]). Four pts (5%) discontinued treatment due to treatment-related AEs (grade 3 pneumonitis; grade 4 pneumonitis; grade 2 peripheral motor neuropathy [completed 17 cycles of pembrolizumab]; grade 3 AST increased). No grade 5 treatment-related AEs occurred. Immune-mediated AEs and infusion reactions occurred in 12 pts (14%; grade 3 or 4, n = 2 [2%]).

### Summary/Conclusion

With additional follow-up, pembrolizumab plus COPDAC-28 consolidation with or without RT and pembrolizumab maintenance continued to show promising antitumor activity (ORR, 98%: 2-year EFS rate, 85%) and manageable safety in pts with high-risk cHL and SER to front-line OEPA; 58 pts (69%) were PET negative, of whom 57 were spared RT.

**Copyright Information:** (Online) ISSN: 2572-9241
©2026 The Author(s). HemaSphere is published by John Wiley & Sons Ltd on behalf of European Hematology Association. This is an open access Abstract Book distributed under the Attribution-NonCommercial-NoDerivs (CC BY-NC-ND), which allows third parties to download the articles and share them with others as long as they credit the author and the Abstract Book, but they cannot change the content in any way or use them commercially.
**Abstract Book Citations:** Authors, Title, HemaSphere, 2026;10:(S1):pages. The abstract book DOIs can be found on the "Author Information" page.
**Disclaimer:** Articles published in the journal HemaSphere exclusively reflect the opinions of the authors. The authors are responsible for all content in their abstracts including accuracy of the facts, statements, citing resources, etc.

### (PS2016) PROGNOSTIC IMPACT OF METABOLIC TUMOR VOLUME AFTER TWO CYCLES OF ABVD IN EARLY-STAGE FAVORABLE HODGKIN LYMPHOMA: A POST-HOC ANALYSIS OF THE GHSG HD16 TRIAL

**Topic:** 17. Hodgkin lymphoma - Clinical

Justin Ferdinandus*[1, 2], Dominic Ufton[1], Ina Bühnen[1, 2], Janina Selvarajah[1, 2], Anne Robertz[1, 2], Helen Kaul[1, 2], Hans T. Eich[2, 3], Christian Baues[2, 4], Michael Fuchs[1, 2], Peter Borchmann[1, 2], Carsten Kobe[1, 2], Dennis A. Eichenauer[1, 2]

*[1]University Hospital Cologne, Cologne, Germany; [2]German Hodgkin Study Group, Cologne, Germany; [3]University Hospital Muenster, Muenster, Germany; [4]Marienhospital Herne, Ruhr University Bochum, Bochum, Germany;*

### Background

Interim PET after two cycles of ABVD (PET-2) is widely used to stratify treatment in early-stage favorable Hodgkin lymphoma (EF-HL), yet most patients are cured with standard combined-modality treatment. Consequently, PET-guided treatment intensification remains controversial, and better quantitative biomarkers are needed to identify patients who may benefit from escalation in the case of PET-2 positivity.

### Aims

To assess the prognostic impact of metabolic tumor volume after on PET-2 (MTV-2) in the HD16 trial for EF-HL and to test whether MTV-2 refines risk groups, especially among patients with Deauville 4 (DS4).

### Methods

This is a post-hoc analysis of patients from the HD16 trial uniformly treated with combined-modality treatment, with centrally reviewed PET-2, excluding interim PET-negative patients randomized to chemotherapy alone. Residual MTV on PET-2 was quantified using a fixed absolute threshold (SUV 4.0). Residual MTV-2 was defined as MTV-2 >0 mL. Progression-free survival (PFS) and overall survival (OS) were estimated by Kaplan–Meier; hazard ratios (HR) were derived from Cox models. For DS-stratified analysis, patients were grouped as (i) DS1–3 with MTV=0, (ii) DS4 with MTV=0, and (iii) MTV>0.

### Results

The analysis included 677 patients (median observation time 64.5 months). MTV-2 was positive in 86 and negative in 591 patients (including DS1–3: n=550; DS4 with MTV=0 ml: n=41). MTV-2 positivity was associated with an inferior PFS (HR 2.63, 95%CI 1.49–4.64). PFS at 60 months was 80.5% for MTV-2 positive versus 94.0% for MTV-2 negative patients. In the DS-stratified analysis, DS4 patients with MTV=0 ml had a PFS comparable to DS1–3 (60-month PFS 95.7% vs 94.2%; HR 1.79, 95%CI 0.71–4.55), whereas MTV-2 positive patients remained at higher risk (HR 2.76, 95%CI 1.55–4.92). OS was high in all groups with no difference observed (HR 0.93, 95%CI 0.21–4.08; 60-month OS 97.5% [MTV-2 positive] vs 98.6% [MTV-2 negative]).

### Summary/Conclusion

In patients with EF-HL, residual MTV after two cycles of ABVD identifies a small subgroup with clearly inferior PFS. Patients with DS4 but without measurable MTV-2 had outcomes comparable to DS1–3, suggesting a presumably low benefit from treatment intensification in this group. Conversely, residual MTV-2 may serve as an enrichment biomarker to identify the minority in whom escalation may be considered.

**Copyright Information:** (Online) ISSN: 2572-9241
©2026 The Author(s). HemaSphere is published by John Wiley & Sons Ltd on behalf of European Hematology Association. This is an open access Abstract Book distributed under the Attribution-NonCommercial-NoDerivs (CC BY-NC-ND), which allows third parties to download the articles and share them with others as long as they credit the author and the Abstract Book, but they cannot change the content in any way or use them commercially.
**Abstract Book Citations:** Authors, Title, HemaSphere, 2026;10:(S1):pages. The abstract book DOIs can be found on the "Author Information" page.
**Disclaimer:** Articles published in the journal HemaSphere exclusively reflect the opinions of the authors. The authors are responsible for all content in their abstracts including accuracy of the facts, statements, citing resources, etc.

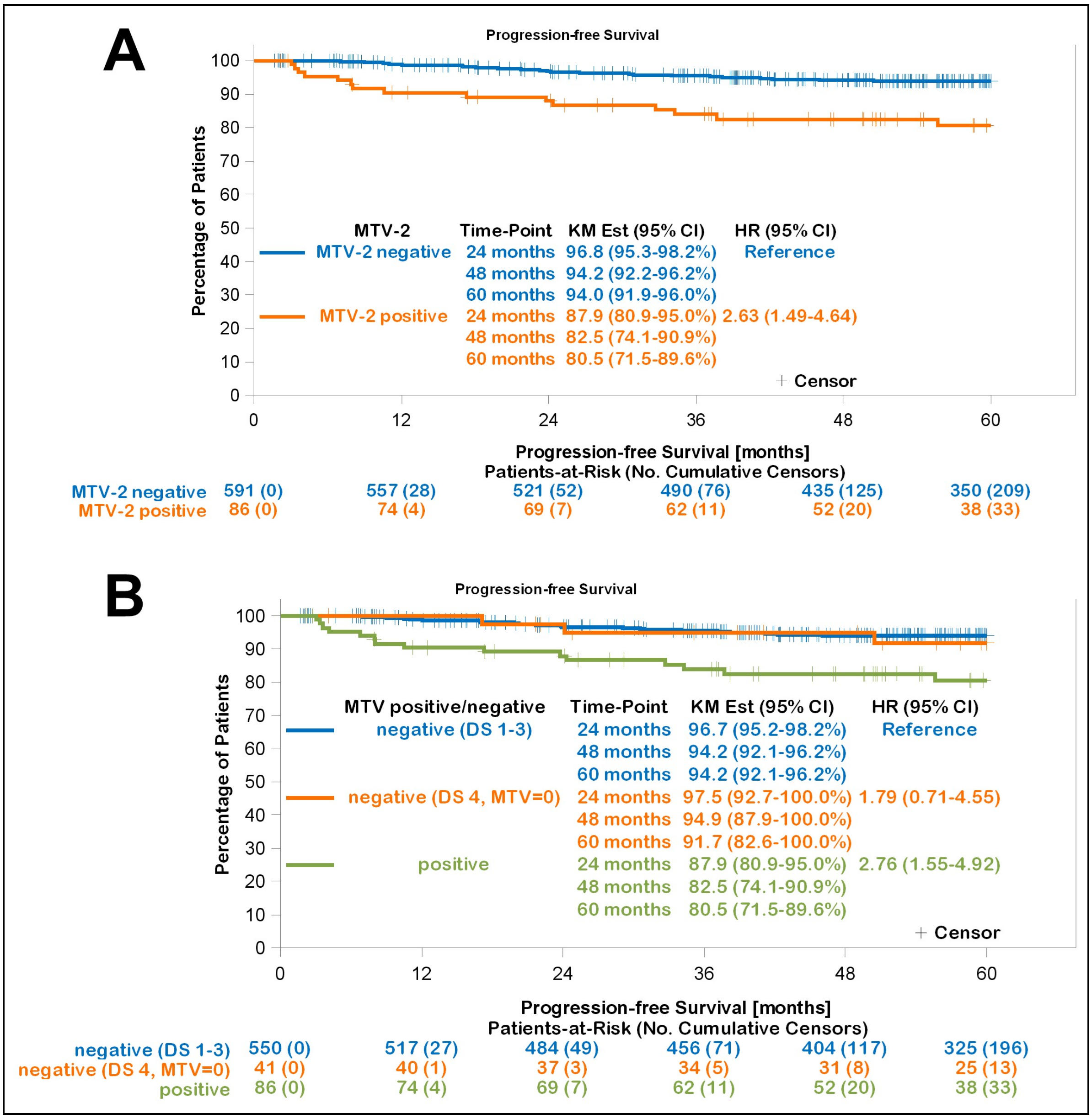

Progression-free survival stratified by MTV-2 (A) and MTV-2 + Deauville Score (B)

**Copyright Information:** (Online) ISSN: 2572-9241
©2026 The Author(s). HemaSphere is published by John Wiley & Sons Ltd on behalf of European Hematology Association. This is an open access Abstract Book distributed under the Attribution-NonCommercial-NoDerivs (CC BY-NC-ND), which allows third parties to download the articles and share them with others as long as they credit the author and the Abstract Book, but they cannot change the content in any way or use them commercially.
**Abstract Book Citations:** Authors, Title, HemaSphere, 2026;10:(S1):pages. The abstract book DOIs can be found on the “Author Information” page.
**Disclaimer:** Articles published in the journal HemaSphere exclusively reflect the opinions of the authors. The authors are responsible for all content in their abstracts including accuracy of the facts, statements, citing resources, etc.

### (PS2017) COMPARISON OF LONG-TERM EFFICACY BETWEEN THE BRECADD AND N+AVD REGIMENS IN ADVANCED-STAGE HODGKIN LYMPHOMA: A MATCHING-ADJUSTED INDIRECT TREATMENT COMPARISON OF THE HD21 AND SWOG S1826 TRIALS

**Topic:** 17. Hodgkin lymphoma - Clinical

Alexa Molinari*[1], Fjoralba Kristo[2], Thomas Melchardt[3], Hishan Tharmaseelan[4], Christian Jaworek[4], Mingxin Liu[5], Hoora Moradian[5], Athanasios Zomas[6], Vanessa Stache[6], Ajibade Ashaye[2], Victoria Federico Paly[1]

*[1]Takeda Pharmaceuticals America, Inc., Lexington, United States of America; [2]Takeda Development Center Americas, Inc. (TDCA), Cambridge, United States of America; [3]Department of Internal Medicine III, Paracelsus Medical University, Salzburg, Austria; [4]Department I of Internal Medicine, Center for Integrated Oncology Aachen Bonn Cologne Duesseldorf (CIO ABCD), German Hodgkin Study Group (GHSG), Faculty of Medicine and University Hospital Cologne, University of Cologne, Cologne, Germany; [5]Cytel Inc., Cambridge, United States of America; [6]Takeda Pharmaceuticals International AG, Zurich, Switzerland;*

### Background

In the phase 3 HD21 trial, positron emission tomography (PET)-guided brentuximab vedotin, etoposide, cyclophosphamide, doxorubicin, dacarbazine, and dexamethasone (BrECADD) demonstrated improved progression-free survival (PFS; median follow-up [FU]: 61.6 months) over PET-guided, escalated bleomycin, etoposide, doxorubicin, cyclophosphamide, vincristine, procarbazine, and prednisone among patients aged 18–60 with advanced-stage Hodgkin lymphoma (aHL). HD21's non-randomized (nR) phase 2 cohort (aged 61–75) only received BrECADD (median FU: 34.3 months). SWOG S1826 (median FU: 37.2 months) evaluated nivolumab (N) + doxorubicin, vinblastine, and dacarbazine (AVD) vs brentuximab vedotin + AVD in stage III/IV disease.

### Aims

This study assessed PFS via indirect comparison to estimate the relative long-term efficacy of BrECADD vs N+AVD in patients with stage III/IV aHL aged ≥18, 18–60, and >60 in HD21 and the SWOG S1826 intent-to-treat (ITT; ≥12) population.

### Methods

Patient-level data pooled for the randomized and nR cohorts (R+nR) from HD21 (5-year datacut) were weighted for comparison with aggregate SWOG S1826 data (3-year datacut; sourced from the literature) using an unanchored matching-adjusted indirect comparison (MAIC) approach. Time-to-event outcome data in SWOG S1826 were recreated via digitization based on available PFS curves for populations aged ≥12 (ITT), 18–60, ≥18, and >60. Analyses were adjusted for baseline age, sex, International Prognostic Score, stage, and B-symptoms and censored at 36 months to inform the base-case comparisons. Weighted Cox regression was used to generate hazard ratios (HR) with 95% confidence intervals (CI). Scenario analyses assessed implications of covariate selection and censoring cutoff timepoints.

### Results

Patient characteristics were well balanced between trials after MAIC reweighting. BrECADD showed significantly improved PFS vs N+AVD in the overall ≥18 aHL population (HD21 R+nR) with an HR of 0.58 (95% CI: 0.36, 0.92; p=0.029) and in the 18–60 group (HD21 R): HR=0.48 (95% CI: 0.29, 0.81; p=0.010). In the >60 group, a non-significant trend favoring N+AVD was observed (HR=1.05; 95% CI: 0.39, 2.80; p=0.925). When compared with the SWOG S1826 ITT population (≥12), BrECADD (HD21 R+nR, ≥18) demonstrated significant benefit in PFS (HR=0.56; 95% CI: 0.35, 0.88; p=0.015). BrECADD wasconsistently favored in scenario analyses regardless of covariate adjustment tested or censoring timepoint cutoff applied in the ≥18 and 18–60 cohorts.

**Copyright Information:** (Online) ISSN: 2572-9241
©2026 The Author(s). HemaSphere is published by John Wiley & Sons Ltd on behalf of European Hematology Association. This is an open access Abstract Book distributed under the Attribution-NonCommercial-NoDerivs (CC BY-NC-ND), which allows third parties to download the articles and share them with others as long as they credit the author and the Abstract Book, but they cannot change the content in any way or use them commercially.
**Abstract Book Citations:** Authors, Title, HemaSphere, 2026;10:(S1):pages. The abstract book DOIs can be found on the “Author Information” page.
**Disclaimer:** Articles published in the journal HemaSphere exclusively reflect the opinions of the authors. The authors are responsible for all content in their abstracts including accuracy of the facts, statements, citing resources, etc.

### Summary/Conclusion

BrECADD is robustly associated with significantly improved PFS vs N+AVD in the≥18 and 18–60 aHL groups in MAIC analyses with mature FU. Interpretation of results for patients aged >60 was challenging given small sample sizes and limited events, even with long-term FU. MAICs based on long-term data can aid decision-makers when no head-to-head data exist. These findings clearly demonstrate that BrECADD delivers statistically improved PFS for target populations.

**Figure 1. Base-case MAIC results for BrECADD vs N+AVD: progression-free survival**

| Comparison | Scenario | Age | Stage | ESS/N | Index arm | Comparator arm | | HR (95% CI) | p-value |
|---|---|---|---|---|---|---|---|---|---|
| Progression-free survival | | | | | | | | | |
| BrECADD vs N+AVD | BC | 18-60 | III,IV | 481.68/631 | HD21 | SWOG S1826 | | 0.48 (0.29, 0.81) | 0.01^ |
| BrECADD vs N+AVD | BC | >60 | III,IV | 53.65/82 | HD21 | SWOG S1826 | | 1.05 (0.39, 2.80) | 0.925 |
| BrECADD vs N+AVD | BC | ≥18 | III,IV | 628.13/713 | HD21 | SWOG S1826 | | 0.58 (0.36, 0.92) | 0.029^ |
| BrECADD vs N+AVD | BC | ITT* | III,IV | 555.21/713 | HD21 | SWOG S1826 | | 0.56 (0.35, 0.88) | 0.015^ |

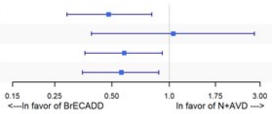

Notes: *The pediatric population (<18 years) was included in the SWOG S1826 ITT population; ^ statistically significant results
Abbreviations: BC, base case; BrECADD, brentuximab vedotin, etoposide, cyclophosphamide, doxorubicin, dacarbazine, and dexamethasone; CI, confidence interval; ESS, effective sample size; HR, hazard ratio; ITT, intent to treat; MAIC, matching-adjusted indirect comparison; N+AVD, nivolumab, doxorubicin, vinblastine, and dacarbazine

Copyright Information: (Online) ISSN: 2572-9241
©2026 The Author(s). HemaSphere is published by John Wiley & Sons Ltd on behalf of European Hematology Association. This is an open access Abstract Book distributed under the Attribution-NonCommercial-NoDerivs (CC BY-NC-ND), which allows third parties to download the articles and share them with others as long as they credit the author and the Abstract Book, but they cannot change the content in any way or use them commercially.
Abstract Book Citations: Authors, Title, HemaSphere, 2026;10:(S1):pages. The abstract book DOIs can be found on the "Author Information" page.
Disclaimer: Articles published in the journal HemaSphere exclusively reflect the opinions of the authors. The authors are responsible for all content in their abstracts including accuracy of the facts, statements, citing resources, etc.

### (PS2018) FIVE-YEAR CONDITIONAL RELATIVE SURVIVAL UP TO 20 YEARS POST-DIAGNOSIS IN ADULT PATIENTS WITH HODGKIN LYMPHOMA: PERSISTENT EXCESS MORTALITY IN OLDER PATIENTS— A CONTEMPORARY, NATIONWIDE DUTCH STUDY

**Topic:** 17. Hodgkin lymphoma - Clinical

Fer de Wit*[1, 2, 3], Otto Visser[4], Eduardus F.M. Posthuma[5, 6], Josée Zijlstra[3, 7], Marie José Kersten[1, 2, 3], Avinash Dinmohamed[1, 8]

*[1]Amsterdam UMC location Universiteit van Amsterdam, Department of Hematology, Amsterdam, The Netherlands;[2]Lymphoma and Myeloma Center Amsterdam (LYMMCARE), Amsterdam, The Netherlands; [3]Cancer Center Amsterdam, Cancer Treatment and Quality of Life, Amsterdam, The Netherlands; [4]Netherlands Comprehensive Cancer Organisation (IKNL), Department of Registration, Utrecht, The Netherlands; [5]Reinier de Graaf Ziekenhuis, Department of Internal Medicine, Delft, The Netherlands; [6]Leiden University Medical Center, Department of Hematology, Leiden, The Netherlands;[7]Amsterdam UMC location Vrije Universiteit, Department of Hematology, Amsterdam, The Netherlands; [8]Netherlands Comprehensive Cancer Organisation (IKNL), Department of Research and Development, Utrecht, The Netherlands;*

### Background

Modern multi-agent chemotherapy regimens (± brentuximab vedotin or nivolumab) cure >90% of young patients with Hodgkin lymphoma (HL). However, late therapy-related cardiovascular disease and second primary malignancies may comprise long-term survival. Conversely, older patients often tolerate intensive therapy poorly and receive attenuated or palliative regimens, yielding inferior outcomes. Clinical trials often exclude older, frail patients and provide limited data on late mortality. Population-based studies are thus crucial to characterize real-world, long-term prognosis.

### Aims

In this nationwide Dutch study, we estimated up-to-date 5-year conditional relative survival (CRS) up to 20 years post-diagnosis in adult patients diagnosed with HL.

### Methods

We included Dutch adults (≥18 years) with HL (excluding nodular lymphocyte-predominant HL) diagnosed between 1989-2023 from the Netherlands Cancer Registry, with follow-up through 2024 (N=12,316; 57% male; median age, 40 years; 57% stage I/II). We estimated 5-year CRS(*t*): relative survival (RS) over the next 5 years among patients surviving *t* years post-diagnosis (*t*=0-20). RS equaled observed survival divided by expected survival from Dutch population life tables matched for age, sex, and calendar year. To generate contemporary long-term survival estimates despite limited follow-up for more recent diagnoses, we used the hybrid approach combining long-term follow-up from earlier diagnostic cohorts with period-specific mortality rates (2013-2024), yielding CRS estimates up to 20 years post-diagnosis (interpretable as 2013-2024 diagnosis-period survival estimates). Analyses were stratified by sex, Ann Arbor stage (I-II vs III-IV), and age at diagnosis (18-39, 40-59, ≥60), with age-specific estimates according to disease stage. Minimal excess mortality was defined as CRS ≥95%. Estimates with standard error >5% were suppressed due to imprecision.

**Copyright Information:** (Online) ISSN: 2572-9241
©2026 The Author(s). HemaSphere is published by John Wiley & Sons Ltd on behalf of European Hematology Association. This is an open access Abstract Book distributed under the Attribution-NonCommercial-NoDerivs (CC BY-NC-ND), which allows third parties to download the articles and share them with others as long as they credit the author and the Abstract Book, but they cannot change the content in any way or use them commercially.
**Abstract Book Citations:** Authors, Title, HemaSphere, 2026;10:(S1):pages. The abstract book DOIs can be found on the "Author Information" page.
**Disclaimer:** Articles published in the journal HemaSphere exclusively reflect the opinions of the authors. The authors are responsible for all content in their abstracts including accuracy of the facts, statements, citing resources, etc.

**Results**

Five-year RS at diagnosis was 87% overall. Minimal excess mortality was reached after 4 years survived and persisted until 13 years survived. Thereafter, it declined slightly. We found no sex specific difference in mortality. Limited-stage (I/II) RS at diagnosis was 94% *v* 81% for advanced-stage (III/IV; Fig A). Minimal excess mortality occurred after 1 year survived in limited-stage patients (5-year CRS 96%) and persisted to 16 years; 5-year CRS then slightly fell to 93% at 20 years survived. Advanced stage patients had persistent slight excess mortality throughout follow-up. Age at diagnosis also had a pronounced effect (Fig B): patients aged 19-39 years showed minimal excess mortality throughout. Those aged 40-59 years maintained this up to 10 years survived, after which 5-year CRS declined to 85% at 20 years post-diagnosis. Patients aged ≥60 years had substantial excess mortality at all time points post-diagnosis. Age-specific patterns by stage were broadly consistent with overall findings, with particularly long-term excess mortality in those aged ≥40 years (Fig C).

**Summary/Conclusion**

Current treatment algorithms lead to near-normal life expectancy for most Dutch patients diagnosed with HL at age 18-39 years. However, long-term excess mortality persists for those diagnosed at age ≥40 years, and is most pronounced for those diagnosed at ≥60 years. These data can inform risk-adapted patient counselling and long-term surveillance and enable benchmarking of the impact of new regimens implemented in routine practice.

**Copyright Information:** (Online) ISSN: 2572-9241
©2026 The Author(s). HemaSphere is published by John Wiley & Sons Ltd on behalf of European Hematology Association. This is an open access Abstract Book distributed under the Attribution-NonCommercial-NoDerivs (CC BY-NC-ND), which allows third parties to download the articles and share them with others as long as they credit the author and the Abstract Book, but they cannot change the content in any way or use them commercially.
**Abstract Book Citations:** Authors, Title, HemaSphere, 2026;10:(S1):pages. The abstract book DOIs can be found on the "Author Information" page.
**Disclaimer:** Articles published in the journal HemaSphere exclusively reflect the opinions of the authors. The authors are responsible for all content in their abstracts including accuracy of the facts, statements, citing resources, etc.

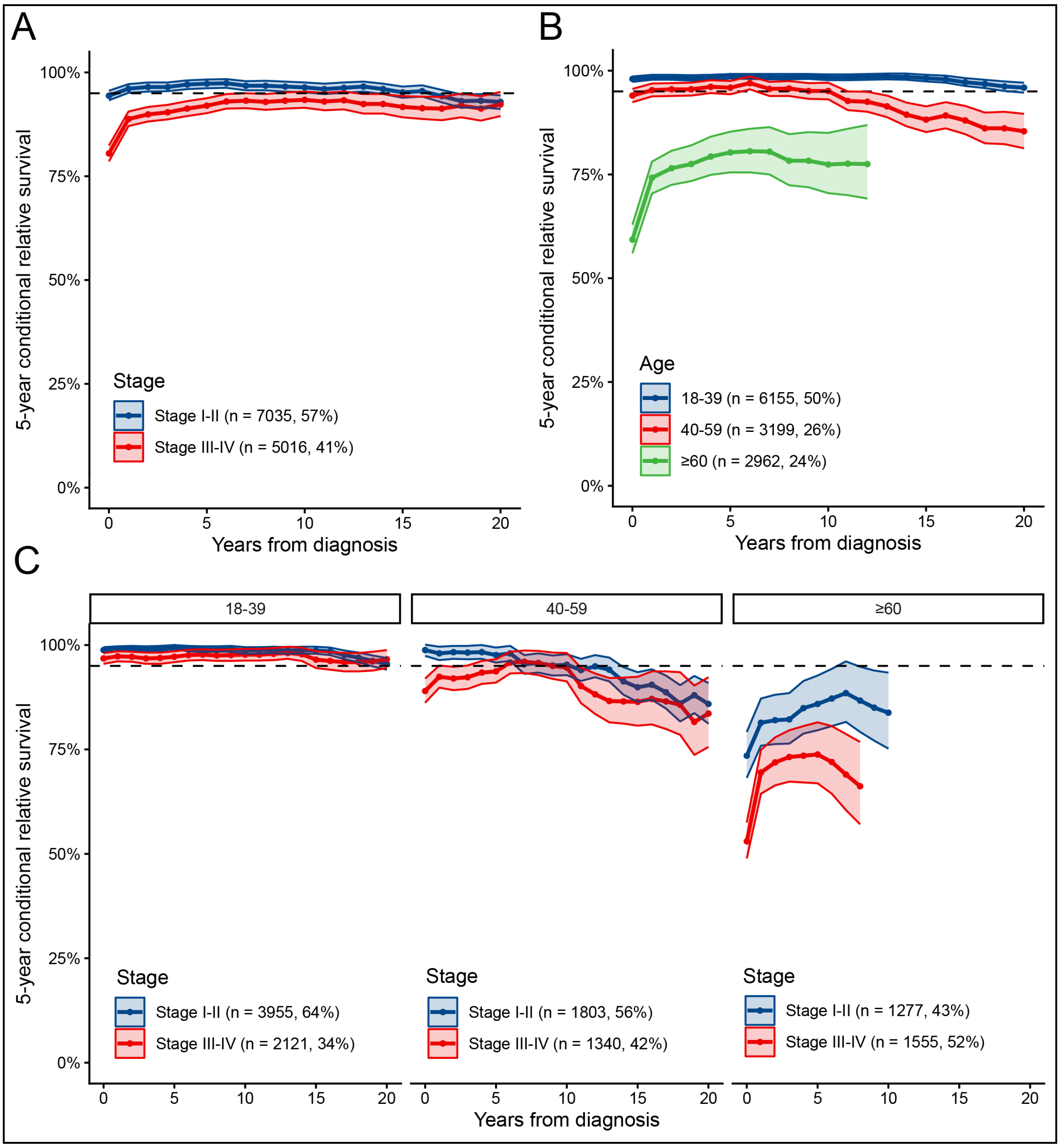

Figure 1: 5-year conditional relative survival in cHL patients stratified according to A) stage, B) age and C) both stage and age

**Copyright Information:** (Online) ISSN: 2572-9241
©2026 The Author(s). HemaSphere is published by John Wiley & Sons Ltd on behalf of European Hematology Association. This is an open access Abstract Book distributed under the Attribution-NonCommercial-NoDerivs (CC BY-NC-ND), which allows third parties to download the articles and share them with others as long as they credit the author and the Abstract Book, but they cannot change the content in any way or use them commercially.
**Abstract Book Citations:** Authors, Title, HemaSphere, 2026;10:(S1):pages. The abstract book DOIs can be found on the "Author Information" page.
**Disclaimer:** Articles published in the journal HemaSphere exclusively reflect the opinions of the authors. The authors are responsible for all content in their abstracts including accuracy of the facts, statements, citing resources, etc.

### (PS2019) PREMENOPAUSAL WOMEN WITH CLASSIC HODGKIN LYMPHOMA HAVE SUPERIOR LYMPHOMA-SPECIFIC SURVIVAL COMPARED TO MEN

**Topic:** 17. Hodgkin lymphoma - Clinical

Johanna Borg Bruchfeld*[1, 2], Caroline Dietrich[1], Karin Ekstrom Smedby[1, 2], Elisavet Syriopoulou[3], Cecilia Radkiewicz[1, 4], Tove Wästerlid[1, 2]

*[1]Karolinska Institutet, Department of Medicine Solna, Division of Clinical Epidemiology, Stockholm, Sweden;[2]Karolinska University Hospital, Department of Hematology, Stockholm, Sweden; [3]Karolinska Institutet, Department of Medical Epidemiology and Biostatistics, Stockholm, Sweden; [4]St Göran Hospital, Department of Oncology, Stockholm, Sweden;*

### Background

Male sex has been associated with inferior survival in classic Hodgkin lymphoma (cHL) in previous studies. The reason for the survival advantage in women remains unclear, but a beneficial effect of female sex hormones has been suggested.

### Aims

- Explore sex differences in lymphoma-specific survival among patients diagnosed with cHL in a population-based setting, stratified by menopausal age (<52 vs ≥52 years).
- Quantify to which degree these can be explained by differences in patient and disease characteristics.

### Methods

Adult patients (18-99 years) with cHL in the Swedish lymphoma register (SLR) diagnosed 2000-2023 were included, with linkage to information from other Swedish health registers. Patients were stratified by age <52 vs ≥52 years, according to mean menopausal age in Sweden. Follow-up ended in June 2024.
Relative survival was used to compare cHL-specific mortality in women and men. Flexible parametric survival models were applied to estimate female-to-male excess mortality ratios (EMRs) with 95% confidence intervals (CIs). Three models were fitted; model 0 (crude), model 1 (including age and calendar year) and model 2 (additionally including potential mediators; cHL stage and subtype, CCI, educational level and performance status). Based on model 2, standardised survival was predicted in men and women, and then applying the empirical covariate distribution of women (age, calendar year, cHL stage and subtype, CCI, educational level and performance status) on men.

**Copyright Information:** (Online) ISSN: 2572-9241
©2026 The Author(s). HemaSphere is published by John Wiley & Sons Ltd on behalf of European Hematology Association. This is an open access Abstract Book distributed under the Attribution-NonCommercial-NoDerivs (CC BY-NC-ND), which allows third parties to download the articles and share them with others as long as they credit the author and the Abstract Book, but they cannot change the content in any way or use them commercially.
**Abstract Book Citations:** Authors, Title, HemaSphere, 2026;10:(S1):pages. The abstract book DOIs can be found on the "Author Information" page.
**Disclaimer:** Articles published in the journal HemaSphere exclusively reflect the opinions of the authors. The authors are responsible for all content in their abstracts including accuracy of the facts, statements, citing resources, etc.

### Results

Among 2,380 patients aged 18-51 years, 1,229 (51.6%) were men and 1,151 (48.4%) were women. Median follow-up was 10.2 (IQR 5.2-16.6) years. Women with cHL had a higher proportion of nodular sclerosis subtype (72.6 vs 59.1%), lower proportion of advanced stage disease (52.8 vs 60.4%) and higher level of education (49 vs 36.7% with ≥13 years of education) compared to men. The cHL-specific mortality was significantly lower in women across models (EMR 0.47 (95% CI: 0.28-0.80) in model 0) although slightly attenuated after adjustments: EMR 0.50 (95% CI: 0.30-0.84) in model 1 and EMR 0.56 (95% CI: 0.34-0.94) in model 2. Women had a superior standardised lymphoma-specific survival compared to men over 10 years of follow-up but when applying the covariate distribution of women to men, the survival difference decreased slightly and no significant survival difference between men and women remained (Figure 1, left panel).
Among 1,669 patients aged 52-99 years, 965 (57.8%) were men and 704 (42.2%) were women. Median follow-up was 4.8 (IQR 1.5-10) years. Baseline characteristics were similar for men and women, apart from a lower proportion of advanced stage disease in women (63.1 vs 67.6%). No sex difference in cHL-specific mortality was seen in model 0 (EMR0 0.97 (95% CI: 0.78-1.21)). When adjusting for age and calendar year women had a lower excess mortality (EMR 0.81, 95% CI: 0.66-0.99), but this was no longer significant after including mediators (EMR 0.85, 95%: 0.70-1.04). The standardised survival curves of men and women overlapped and when applying the covariate distribution of women to men, survival decreased in men (Figure 1, right panel).

### Summary/Conclusion

Premenopausal women (18-51 years) with cHL have a survival benefit compared to men of corresponding age, which is only partly explained by differences in lymphoma and patient characteristics. No sex difference was seen in patients aged 52-99 years. These results indicate underlying differences in tumour biology, potentially due to sex hormones.

Figure 1. Lymphoma-specific survival curve of patients diagnosed with classic Hodgkin lymphoma year 2000-2023*

Standardised to the actual covariate distribution of men (dashed line) and women (full line) vs. men standardised to the covariate distribution of women (red line)

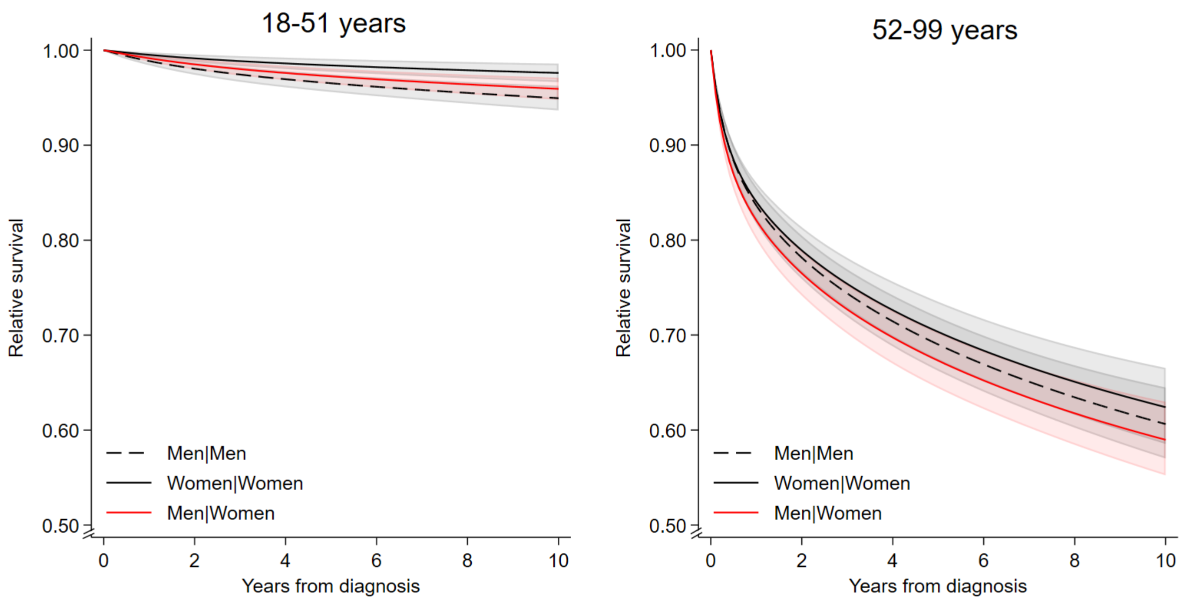

*adjusted for age, calendar period, stage, cHL subtype, education, comorbidity and performance status (model 2)

**Copyright Information:** (Online) ISSN: 2572-9241
©2026 The Author(s). HemaSphere is published by John Wiley & Sons Ltd on behalf of European Hematology Association. This is an open access Abstract Book distributed under the Attribution-NonCommercial-NoDerivs (CC BY-NC-ND), which allows third parties to download the articles and share them with others as long as they credit the author and the Abstract Book, but they cannot change the content in any way or use them commercially.
**Abstract Book Citations:** Authors, Title, HemaSphere, 2026;10:(S1):pages. The abstract book DOIs can be found on the "Author Information" page.
**Disclaimer:** Articles published in the journal HemaSphere exclusively reflect the opinions of the authors. The authors are responsible for all content in their abstracts including accuracy of the facts, statements, citing resources, etc.

### (PS2020) AUTOLOGOUS STEM CELL TRANSPLANTATION OUTCOMES AFTER ANTI-PD-1 TREATMENT IN THIRD AND SUBSEQUENT LINES FOR RELAPSED/REFRACTORY CLASSICAL HODGKIN LYMPHOMA

**Topic:** 17. Hodgkin lymphoma - Clinical

Yulia Protopopova*[1], Nikita Mochkin[1], Vladislav Sarzhevskij[1], Vladimir Melnichenko[1], Elena Demina[1], Nikita Shorokhov[1], Anastasiia Samoylova[1], Vladimir Bogatyrev[1], Aysel Mamedova[1], Anatoliy Rukavitsyn[1], Anna Bannikova[1], Elena Smirnova[1]

*[1]Federal State Budgetary Institution "National Medical and Surgical Center named after N.I. Pirogov" of the Ministry of Health of the Russian Federation, Moscow, Russia;*

### Background

High-dose chemotherapy followed by autologous stem cell transplantation (ASCT) is a standard consolidation therapy for relapsed/refractory (r/r) classical Hodgkin lymphoma (cHL). Most international clinical guidelines recommend ASCT as second-line consolidation. However, real-world practice frequently limits ASCT to third and subsequent lines, reflecting disrepancies between guidelines and clinical implementation.

### Aims

1. To report outcomes of ASCT after anti-PD-1 therapy in third and subsequent lines.
2. To identify predictive factors for ASCT outcomes.
3. To characterize relapses after ASCT.

### Methods

We retrospectively analyzed patients received anti-PD-1 based treatment as third-line or later therapy and subsequently underwent ASCT between 2018 and 2024. Patients receiving salvage therapy between anti-PD-1 and ASCT were excluded. Response was assessed using Lugano criteria and LYRIC. PFS and OS were estimated using the Kaplan-Meier analysis. Prognostic factors analyzed included: number of prior systemic therapies before anti-PD1 and ASCT, time interval from anti-PD-1 to ASCT (<60 vs >60 days) and refractory status to the last line prior to anti-PD-1.

**Copyright Information:** (Online) ISSN: 2572-9241
©2026 The Author(s). HemaSphere is published by John Wiley & Sons Ltd on behalf of European Hematology Association. This is an open access Abstract Book distributed under the Attribution-NonCommercial-NoDerivs (CC BY-NC-ND), which allows third parties to download the articles and share them with others as long as they credit the author and the Abstract Book, but they cannot change the content in any way or use them commercially.
**Abstract Book Citations:** Authors, Title, HemaSphere, 2026;10:(S1):pages. The abstract book DOIs can be found on the "Author Information" page.
**Disclaimer:** Articles published in the journal HemaSphere exclusively reflect the opinions of the authors. The authors are responsible for all content in their abstracts including accuracy of the facts, statements, citing resources, etc.

**Results**

90 patients were analyzed (median age 36 years, range 19–56). The median number of prior systemic therapies before anti-PD-1 followed by ASCT was 2 (range, 2–7). 60% of patients were refractory to one, 42.2% to two and 17.8% to three consecutive lines of therapy immediately before pretransplant anti-PD-1. Prior brentuximab vedotin (BV) exposure was documented in 34.4% of patients, 55.0% of whom were BV-refractory.
Anti-PD-1 was administered as monotherapy (53.3%, n=48) or combined with chemotherapy (46.7%, n=42). The median number of anti-PD-1 cycles was 6 (range, 2–52) with median time from the last anti-PD-1 dose to ASCT of 84 days (range, 14–365). Pre-ASCT response: complete response (82.3%), partial response (13.3%) and indeterminate response (4.4%). Conditioning regimens: BEAM (31.1%) and BeEAC (68.9%). Post-ASCT maintenance: BV (13.3%) and anti-PD-1 (16.7%).
At a median follow-up of 31 months (range, 1–84), 2-year OS and PFS were 90% and 79%, respectively (Figure 1). Complete remission was maintained in 55.6% (50/90) of patients beyond 2 years, indicating a distinct survival plateau in this heavily pretreated cohort.
No significant PFS differences were observed based on the number of prior therapies and time to ASCT. A trend toward shorter PFS emerged in patients who were refractory to the pre-anti-PD-1 line (p=0.098).
9 relapses occurred; 8 within 12 months of ASCT (range, 4–17 months). All relapsed patients achieved complete response pre-ASCT. 2 patients relapsed during maintenance with BV.
Causes of death included transplant-related toxicity (5 patients), late chronic heart failure (2 patients) and progressive cHL (2 patients).

**Summary/Conclusion**

ASCT following anti-PD-1 therapy enables long-term remission in pretreated patients with refractory disease and multiple relapses. Most relapses are early, occurring within the first year after transplant. Currently, specific predictors adversely affecting outcomes remain unidentified. Further studies may identify refractory status prior to anti-PD-1 therapy as an independent prognostic factor for ASCT outcomes

**Copyright Information:** (Online) ISSN: 2572-9241
©2026 The Author(s). HemaSphere is published by John Wiley & Sons Ltd on behalf of European Hematology Association. This is an open access Abstract Book distributed under the Attribution-NonCommercial-NoDerivs (CC BY-NC-ND), which allows third parties to download the articles and share them with others as long as they credit the author and the Abstract Book, but they cannot change the content in any way or use them commercially.
**Abstract Book Citations:** Authors, Title, HemaSphere, 2026;10:(S1):pages. The abstract book DOIs can be found on the “Author Information” page.
**Disclaimer:** Articles published in the journal HemaSphere exclusively reflect the opinions of the authors. The authors are responsible for all content in their abstracts including accuracy of the facts, statements, citing resources, etc.

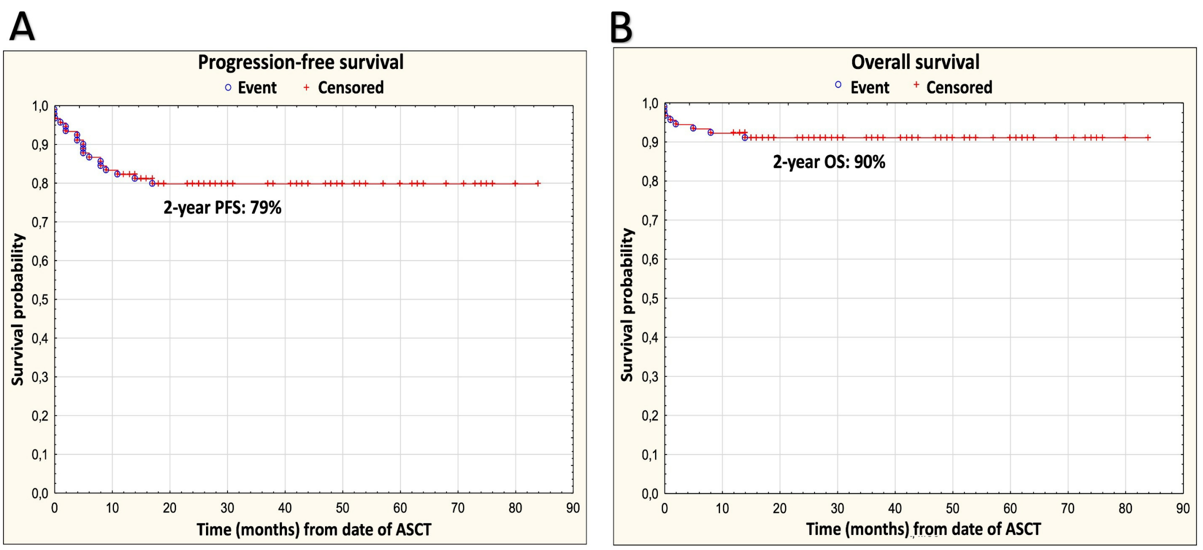

**Figure 1. Survival outcomes (n = 90).**
(A) Progression-free survival in all transplanted patients.
(B) Overall survival in all transplanted patients.

**Copyright Information:** (Online) ISSN: 2572-9241
©2026 The Author(s). HemaSphere is published by John Wiley & Sons Ltd on behalf of European Hematology Association. This is an open access Abstract Book distributed under the Attribution-NonCommercial-NoDerivs (CC BY-NC-ND), which allows third parties to download the articles and share them with others as long as they credit the author and the Abstract Book, but they cannot change the content in any way or use them commercially.
**Abstract Book Citations:** Authors, Title, HemaSphere, 2026;10:(S1):pages. The abstract book DOIs can be found on the "Author Information" page.
**Disclaimer:** Articles published in the journal HemaSphere exclusively reflect the opinions of the authors. The authors are responsible for all content in their abstracts including accuracy of the facts, statements, citing resources, etc.

### (PS2021) PHARMACOKINETICS, EFFICACY, AND SAFETY OF SUBCUTANEOUS PEMBROLIZUMAB IN RELAPSED OR REFRACTORY CLASSIC HODGKIN LYMPHOMA (CHL) OR PRIMARY MEDIASTINAL LARGE B-CELL LYMPHOMA (PMBCL): A PHASE 2 STUDY

**Topic:** 17. Hodgkin lymphoma - Clinical

Alejandro Berkovits*[1], Muhit Ozcan[2], Gabriel Barragan Ibanez[3], Ewa Paszkiewicz-Kozik[4], Wojciech Jurczak[5], Mauricio Chandía[6], Sergio Portino[7], Elifcan Aladag[8], Sebastian Giebel[9], Anna Sureda[10], Tae Min Kim[11], Brenda Garcia[12], Norma Gutierrez Gutierrez[13], Sebastian Hidalgo[14], Kirit Ardeshna[15], Antonia Rodriguez Izquierdo[16], Bastian von Tresckow[17, 18], Gina Song[19], Jessica Flynn[19], Katherine Ryland[19], Rushdia Yusuf[19], Elizabeth H. Phillips[20]

*[1]Immunocel Chile, Providencia, Chile; [2]Ankara University, School of Medicine, Ankara, Türkiye; [3]Centro de Investigación Clínica de Oaxaca (CICLO), Oaxaca, Mexico; [4]Maria-Skłodowska-Curie National Research Institute of Oncology, Warsaw, Poland; [5]Maria-Skłodowska-Curie National Research Institute of Oncology, Krakow, Poland; [6]Biocenter, Concepción, Chile; [7]Bradford Hill Clinical Research Center and Finis Terrae University, Faculty of Medicine, Santiago, Chile; [8]Hacettepe University Faculty of Medicine, Ankara, Türkiye; [9]Maria Skłodowska-Curie National Research Institute of Oncology, Gliwice, Poland; [10]Institut Català d'Oncologia - L'Hospitalet, IDIBELL, Universitat de Barcelona, Barcelona, Spain; [11]Seoul National University Hospital, Seoul, South Korea; [12]Health Pharma Professional Research, Mexico City, Mexico; [13]Hospital Universitario de Salamanca, IBSAL, Centro de Investigación del Cáncer, Salamanca, Spain; [14]Fundación Arturo López Pérez (FALP), Santiago, Chile; [15]University College London Hospitals NHS Foundation Trust, London, United Kingdom; [16]Hospital Universitario 12 de Octubre, Madrid, Spain; [17]German Hodgkin Study Group, University of Cologne, Cologne, Germany; [18]West German Cancer Center and German Cancer Consortium (DKTK partner site Essen), University Hospital Essen, University of Duisburg-Essen, Essen, Germany; [19]Merck & Co., Inc., Rahway, United States of America; [20]Division of Cancer Sciences, University of Manchester and The Christie NHS Foundation Trust, Manchester, United Kingdom;*

### Background

Pembrolizumab is a well-established intravenous PD-1 inhibitor for relapsed or refractory (R/R) cHL and PMBCL, administered Q3W or Q6W. MK-3475A is pembrolizumab with berahyaluronidase alfa (MK-5180) for subcutaneous administration (subcutaneous pembrolizumab). Berahyaluronidase alfa, a human hyaluronidase variant developed and manufactured by Alteogen Inc., is a permeation enhancer that enables subcutaneous administration of pembrolizumab. Subcutaneous pembrolizumab is approved globally (outside of the US) for the treatment of cHL and PMBCL.

### Aims

To present pharmacokinetics (PK), efficacy, and safety from a single-arm, open-label, phase 2 study (NCT06504394) evaluating subcutaneous pembrolizumab in participants with R/R cHL or R/R PMBCL.

**Copyright Information:** (Online) ISSN: 2572-9241
©2026 The Author(s). HemaSphere is published by John Wiley & Sons Ltd on behalf of European Hematology Association. This is an open access Abstract Book distributed under the Attribution-NonCommercial-NoDerivs (CC BY-NC-ND), which allows third parties to download the articles and share them with others as long as they credit the author and the Abstract Book, but they cannot change the content in any way or use them commercially.
**Abstract Book Citations:** Authors, Title, HemaSphere, 2026;10:(S1):pages. The abstract book DOIs can be found on the “Author Information” page.
**Disclaimer:** Articles published in the journal HemaSphere exclusively reflect the opinions of the authors. The authors are responsible for all content in their abstracts including accuracy of the facts, statements, citing resources, etc.

**Methods**

Eligible participants were ≥18 years with histologically confirmed, FDG-avid cHL or PMBCL per WHO criteria, radiographically measurable disease, and an ECOG PS of 0 or 1. Participants with cHL or PMBCL were anti–PD-1 naive and had disease that was refractory to, did not achieve CR with, or had relapsed after prior therapy. Participants with cHL had received ≥1 multiagent regimen, and participants with PMBCL had received ≥2 prior therapies (including ≥1 rituximab-based regimen). All participants were ineligible for or had relapsed after autologous stem cell transplant. Participants received subcutaneous pembrolizumab 790 mg Q6W for ≤18 cycles (~2 years) or until disease progression or other discontinuation criteria were met. Primary end points were PK exposure measures during cycle 1 (C1) and ORR per Lugano criteria by investigator. Intensive PK samples were collected during C1, and noncompartmental analysis methods were used to determine PK end points based on observed data. Secondary end points included safety. Data cutoff date was June 10, 2025.

**Results**

At the time of this analysis, 53 participants had been enrolled and received ≥1 cycle of subcutaneous pembrolizumab (n=51, cHL; n=2, PMBCL); enrollment was ongoing. Median follow-up was 2.6 months (range, 0.0-7.4). Geometric mean (GM) of C1 overall pembrolizumab exposure of the area under the concentration-time curve from time 0 to 6 weeks ($AUC_{0-6}$; n=21) was 1716 mcg*day/mL, and variability was 47%. GM of C1 maximum concentration ($C_{max}$; n=47) was 75.06 mcg/mL with variability of 48%, and GM of C1 trough concentration ($C_{trough}$; n=25) at the end of dosing interval was 25.17 mcg/mL with variability of 62%. All participants who were efficacy evaluable (baseline and postbaseline assessment available) had cHL (n=21); the ORR was 71.4% (95% CI, 47.8-88.7), with 7 participants (33%) achieving a CR and 8 (38%) a PR. In all participants, treatment-related AEs (TRAEs) of any grade occurred in 27 participants (51%), most commonly (incidence ≥5%) pruritus (9%), rash (8%), arthralgia (6%), hypothyroidism (6%), and myalgia (6%). Grade 3 or 4 TRAEs occurred in 5 participants (9%). No participants discontinued or died due to TRAEs. Immune-mediated AEs and infusion reactions occurred in 5 participants (9%), with grade 3 or 4 events reported for 2 participants (4%).

**Summary/Conclusion**

The PK profile of subcutaneous pembrolizumab in participants with hematologic tumors was similar to that in solid tumors and was consistent with the typical PK profile for subcutaneous monoclonal antibodies (Figure). Subcutaneous pembrolizumab at a dose of 790 mg Q6W showed a manageable safety profile and efficacy consistent with intravenous pembrolizumab.

**Copyright Information:** (Online) ISSN: 2572-9241
©2026 The Author(s). HemaSphere is published by John Wiley & Sons Ltd on behalf of European Hematology Association. This is an open access Abstract Book distributed under the Attribution-NonCommercial-NoDerivs (CC BY-NC-ND), which allows third parties to download the articles and share them with others as long as they credit the author and the Abstract Book, but they cannot change the content in any way or use them commercially.
**Abstract Book Citations:** Authors, Title, HemaSphere, 2026;10:(S1):pages. The abstract book DOIs can be found on the “Author Information” page.
**Disclaimer:** Articles published in the journal HemaSphere exclusively reflect the opinions of the authors. The authors are responsible for all content in their abstracts including accuracy of the facts, statements, citing resources, etc.

**Mean Pharmacokinetic Profile for Subcutaneous Pembrolizumab 790 mg Q6W in Cycle 1.**

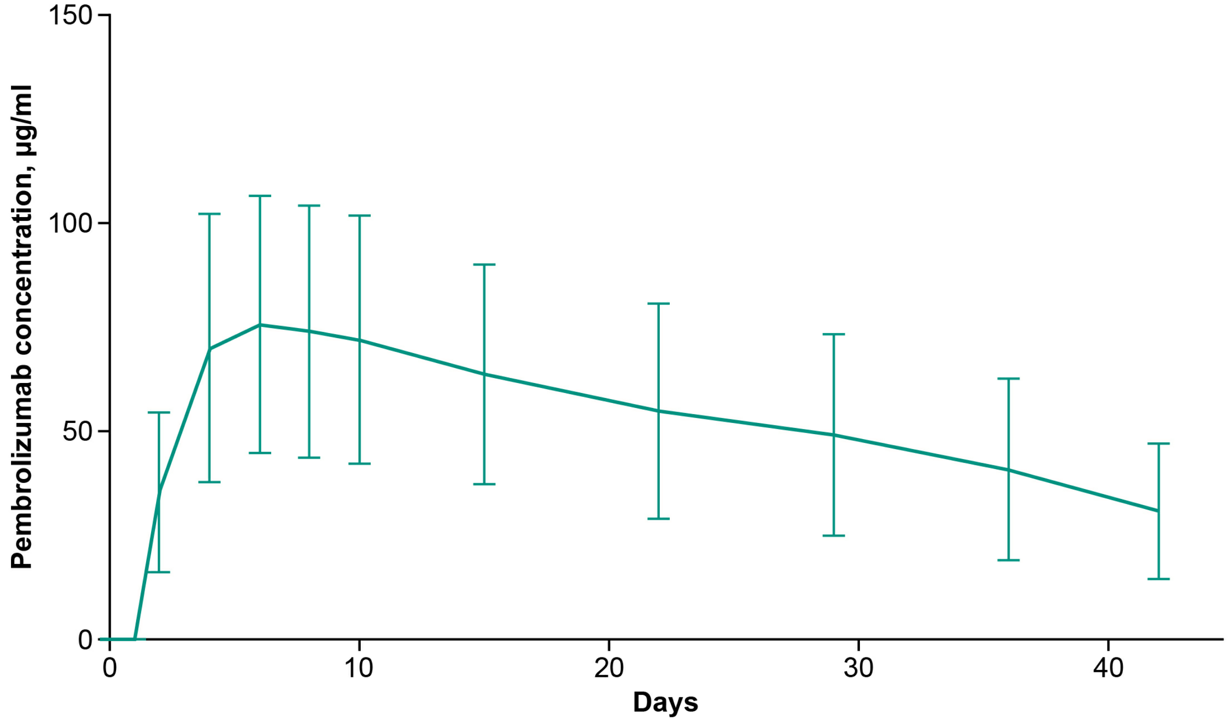

Error bars represent the standard deviation of concentration.

**Copyright Information:** (Online) ISSN: 2572-9241
©2026 The Author(s). HemaSphere is published by John Wiley & Sons Ltd on behalf of European Hematology Association. This is an open access Abstract Book distributed under the Attribution-NonCommercial-NoDerivs (CC BY-NC-ND), which allows third parties to download the articles and share them with others as long as they credit the author and the Abstract Book, but they cannot change the content in any way or use them commercially.
**Abstract Book Citations:** Authors, Title, HemaSphere, 2026;10:(S1):pages. The abstract book DOIs can be found on the "Author Information" page.
**Disclaimer:** Articles published in the journal HemaSphere exclusively reflect the opinions of the authors. The authors are responsible for all content in their abstracts including accuracy of the facts, statements, citing resources, etc.

### (PS2022) LATE CARDIOPULMONARY MORBIDITY IN THE PATIENTS WITH HODGKIN LYMPHOMA: A NATIONWIDE REGISTRY STUDY

**Topic:** 17. Hodgkin lymphoma - Clinical

Noora Hannuksela*[1], Outi Kuittinen[1, 2], Aino Rönkä[1, 2], Anna Anttalainen[3], Liisa Ukkola-Vuoti[3], Iiro Toppila[3], Johanna Vikkula[3], Jan Seppälä[4], Katja Marin[2], Hanne Kuitunen[5], Tatu Miettinen[6], Anu Partanen[7]

*[1]University of Eastern Finland, Oncology, Faculty of Health Medicine, School of Medicine, Institute of Clinical Medicine, Kuopio, Finland; [2]Kuopio University Hospital, Department of Oncology, Kuopio, Finland;[3]Medaffcon Oy, Espoo, Finland; [4]Kuopio University Hospital, Department of Oncology, Department of Radiotherapy, Kuopio, Finland; [5]Oulu University Hospital, Cancer centre, Oulu, Finland; [6]Takeda Oy, Helsinki, Finland; [7]Kuopio University Hospital, Department of Medicine, Kuopio, Finland;*

### Background

Despite good disease control, late morbidity, especially among younger long-term survivors of Hodgkin lymphoma (HL) remains a challenge.

### Aims

This real-world study aimed to clarify the incidence of late-onset cardiopulmonary morbidity and its association with radiotherapy in patients diagnosed with HL in Finland between 2000 and 2019.

### Methods

In this nationwide retrospective registry study, 2912 patients (Table 1) diagnosed with HL between 2000 and 2019 were identified from Finnish four national health care registries and individually matched 1:1 with population controls by age, sex, and municipality. A population of the study was divided into younger (aged <50 years at diagnosis) and older (aged ≥50 years at diagnosis) groups. The occurrence of comorbidities over the follow-up time, differences in comorbidity incidence rates between cases and controls and factors associated with comorbidity occurrence were analyzed according to age and radiation status. Statistical analyses were conducted by using R (version 4.0.3), including multi-state Aalen-Johanssen models, Poisson regression and Cox proportional hazards models.

**Copyright Information:** (Online) ISSN: 2572-9241
©2026 The Author(s). HemaSphere is published by John Wiley & Sons Ltd on behalf of European Hematology Association. This is an open access Abstract Book distributed under the Attribution-NonCommercial-NoDerivs (CC BY-NC-ND), which allows third parties to download the articles and share them with others as long as they credit the author and the Abstract Book, but they cannot change the content in any way or use them commercially.
**Abstract Book Citations:** Authors, Title, HemaSphere, 2026;10:(S1):pages. The abstract book DOIs can be found on the “Author Information” page.
**Disclaimer:** Articles published in the journal HemaSphere exclusively reflect the opinions of the authors. The authors are responsible for all content in their abstracts including accuracy of the facts, statements, citing resources, etc.

Table 1. Characteristics and demographics of HL patients and controls according to two age groups

| Variable | | Younger (<50) cohort Case n=1855 (%) | Younger (<50) cohort Control n=1855 (%) | P-value | Older (≥50) cohort Case n=1057 (%) | Older (≥50) cohort Control n=1057 (%) | P-value |
|---|---|---|---|---|---|---|---|
| Age, years, median [IQR] | | 28.87 [20.65,35.71] | 28.87 [20.65,35.71] | | 66.21 [58.89,75.22] | 66.21 [58.89,75.22] | |
| HL subtype, n (%) | cHL | 1650 (88.9) | - | | 860 (81.4) | - | |
| | NLPHL | 205 (11.1) | - | | 197 (18.6) | - | |
| Sex, n (%) | Male | 994 (53.6) | 994 (53.6) | | 661 (62.5) | 661 (62.5) | |
| | Female | 861 (46.4) | 861 (46.1) | | 396 (37.5) | 396 (37.5) | |
| CCI, n (%) | 0 | 1698 (91.5) | 1792 (96.6) | | 706 (66.8) | 859 (81.3) | |
| | 1-2 | 127 (6.8) | 63 (3.4) | <0.001 | 240 (22.7) | 159 (15.0) | <0.001 |
| | 3+ | 36 (1.6) | 0 | | 111 (10.5) | 39 (3.7) | |
| Baseline comorbidities, n (%) | Diabetes (ICD-10: E10-E11) | 14 (0.8) | 19 (1.0) | 0.484 | 95 (9.0) | 51 (4.8) | <0.001 |
| | Hypertension (ICD-10: I10) | 13 (0.7) | <5 | 0.052 | 171 (16.2) | 101 (9.6) | <0.001 |
| | Dyslipidaemia (ICD-10: E78) | <5 | 0 | 0.479 | 15 (1.4) | 10 (0.9) | 0.421 |
| **Treatment** | | | | | | | |
| Radiotherapy within 1 year | | 539 (29.1) | - | | 253 (23.9) | - | |
| Abbreviations | SD = standard deviation, CCI = Charlson comorbidity index, cHL = classical Hodgkin lymphoma, NLPHL = nodular lymphocyte-predominant Hodgkin lymphoma | | | | | | |

### Results

We observed an increased incidence of coronary artery disease in patients aged <50 years compared with controls (RR 3.80, 95% CI 2.16–6.74; p<0.001). In patients aged ≥50 years, heart failure (RR 1.68, 95% CI 1.28–2.21; p<0.001) and valvular heart disease (RR 1.80, 95% CI 1.17–2.78; p=0.006) were more frequent than in controls. In the younger cohort, arrhythmia occurred more often in patients with HL than in controls (RR 2.25, 95% CI 1.13–4.46; p=0.020). Radiotherapy in younger patients was associated with an over fourfold increased risk of arrhythmia (HR 4.05, 95% CI 1.62–10.14; p=0.0028); however, detailed data regarding radiotherapy dose and treatment fields were not available. In patients aged <50 years, pneumonia (RR 4.89, 95% CI 3.58–6.71; p<0.001) and pulmonary embolism (RR 7.78, 95% CI 2.75–21.95; p<0.001) were markedly more frequent than in controls. In the older cohort, pneumonia incidence remained elevated both at baseline (RR 3.87, 95% CI 2.61–5.71; p<0.001) and during follow-up (RR 2.51, 95% CI 2.02–3.11; p<0.001).

### Summary/Conclusion

In conclusion, late cardiopulmonary morbidity remains a clinically meaningful issue in HL survivors. Increased risks of infections and cardiovascular disease warrant sustained surveillance and targeted preventive strategies.

**Copyright Information:** (Online) ISSN: 2572-9241
©2026 The Author(s). HemaSphere is published by John Wiley & Sons Ltd on behalf of European Hematology Association. This is an open access Abstract Book distributed under the Attribution-NonCommercial-NoDerivs (CC BY-NC-ND), which allows third parties to download the articles and share them with others as long as they credit the author and the Abstract Book, but they cannot change the content in any way or use them commercially.
**Abstract Book Citations:** Authors, Title, HemaSphere, 2026;10:(S1):pages. The abstract book DOIs can be found on the "Author Information" page.
**Disclaimer:** Articles published in the journal HemaSphere exclusively reflect the opinions of the authors. The authors are responsible for all content in their abstracts including accuracy of the facts, statements, citing resources, etc.

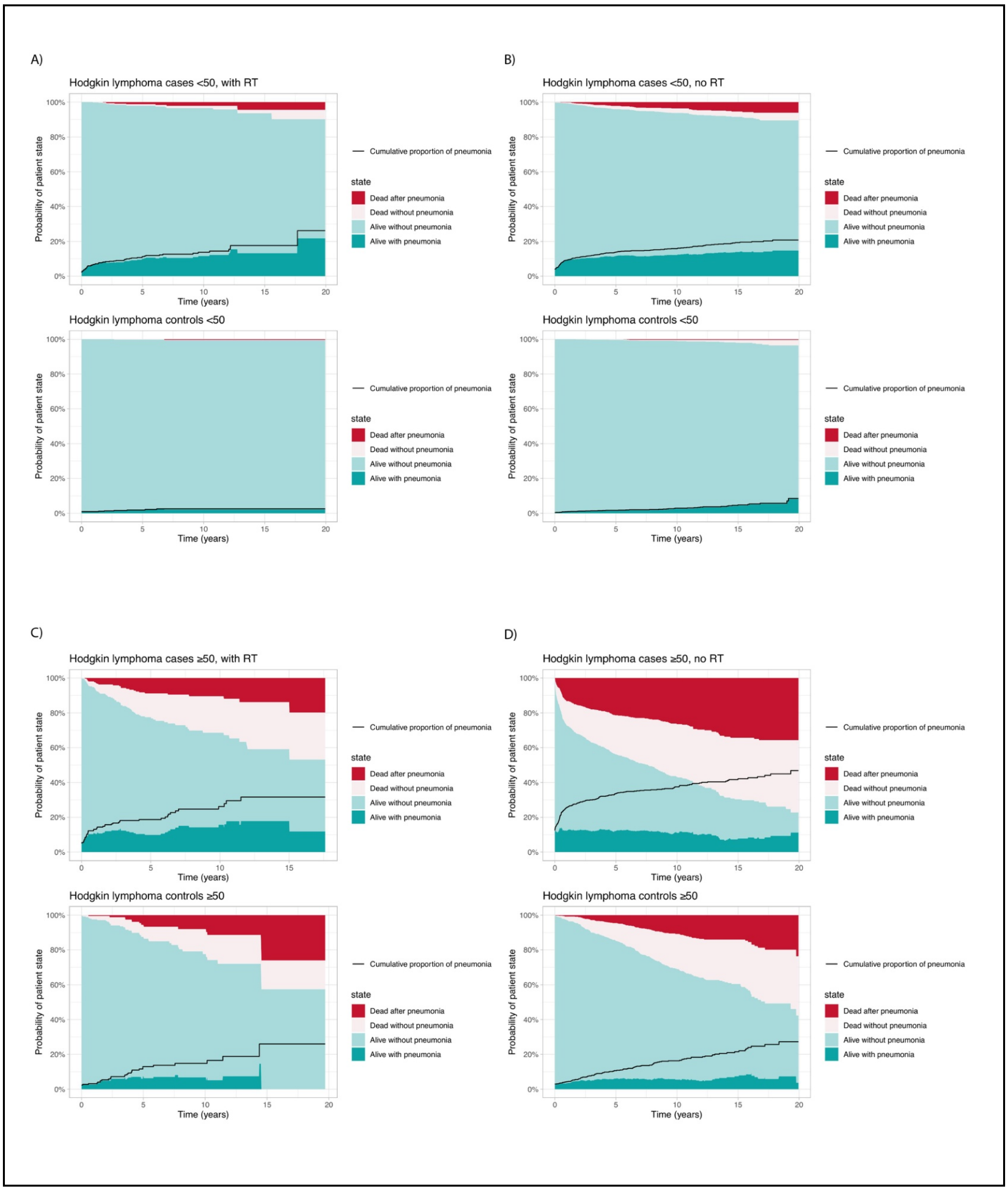

Figure 1. Aalen-Johanssen multi-state model for the occurrence of pneumonia in a) patients <50 years of age with radiotherapy b) patients <50 years without radiotherapy c) patients ≥50 years of age with radiotherapy d) patients ≥50 years without radiotherapy

**Copyright Information:** (Online) ISSN: 2572-9241
©2026 The Author(s). HemaSphere is published by John Wiley & Sons Ltd on behalf of European Hematology Association. This is an open access Abstract Book distributed under the Attribution-NonCommercial-NoDerivs (CC BY-NC-ND), which allows third parties to download the articles and share them with others as long as they credit the author and the Abstract Book, but they cannot change the content in any way or use them commercially.
**Abstract Book Citations:** Authors, Title, HemaSphere, 2026;10:(S1):pages. The abstract book DOIs can be found on the “Author Information” page.
**Disclaimer:** Articles published in the journal HemaSphere exclusively reflect the opinions of the authors. The authors are responsible for all content in their abstracts including accuracy of the facts, statements, citing resources, etc.

### (PS2023) SURFACE TUMOR EXTENSION (STE) EXTRACTED FROM BASELINE PET/CT PREDICTS LONG-TERM ABVD THERAPY OUTCOME IN ADVANCED-STAGE HODGKIN LYMPHOMA WITH A NEGATIVE INTERIM PET.

**Topic:** 17. Hodgkin lymphoma - Clinical

Andrea Gallamini*[1], Andrea Visentin[2], Marco Picardi[3], Caterina Patti[4], Alessandra Romano[5], Anna Guidetti[6], Anna Giulia Zizzari[7], Oppi Sara[8], Silvia Bolis[9], Guido Gini[10], Roberta Battistini[11], Roberto Sorasio[12], Candida Vitale[13], Andrea Bernardelli[13], Schiavotto Corrado[14], Leonardo Flenghi[15], Andrés José María Ferreri[16], Maurizio Miglino[17], Michele Cimminiello[18], Frolli Antonio[19], Carlo Rosanelli[20], Anna Borra[21], Federico Fallanca[22], Luca Guerra[23], Viviani Simonetta[24], Stephane Chauvie[25]

*[1]Centre Antoine Lacassagne, Research and Clinical Innovation, Nice, France; [2]University of Padua, Hematology, Padua, Italy; [3]Policlinico Federico II, Hematology, Naples, Italy; [4]Ospedale Policlinico Vittorio Cervello, Hematology, Palermo, Italy; [5]Azienda Ospedaliera Universitaria Policlinico Vittorio Emanuele, Hematology, Catania, Italy; [6]Istituto Nazionale dei Tumori, Hematology, Milan, Italy; [7]Policlinico Tor Vergata, Hematology, Rome, Italy; [8]Azienda Ospedaliera Universitaria Businco, Hematology, Cagliari, Italy; [9]Ospedale San Gerardo, Hematology Unit, Monza, Italy; [10]Azienda Ospedalera Universitaria delle Marche, Hematology, Ancona, Italy; [11]Azienda Ospedaliera San Camillo Forlanini, Hematology, Rome, Italy; [12]Azienda Ospedaliera Santa Croce e Carle, Hematology, Cuneo, Italy; [13]Azienda Ospedaliera Universitaria Integrata di Verona, Hematology, Verona, Italy; [14]San Bortolo Hospital, Hematology, Vicenza, Italy; [15]Azienda Ospedaliera di Perugia, Hematology, Perugia, Italy; [16]IRCCS San Raffaele, Hematology, Milan, Italy; [17]University Hospital, Hematology, Genoa, Italy; [18]University Hospital S. Carlo, Hematology, Potenza, Italy; [19]Umberto i° Mauriziano Hospital, Hematology, Turin, Italy;[20]Bolzano Hospital, Hematology, Bolzano, Italy; [21]Sanremo Hospital, Onco-Hematology, Sanremo, Italy; [22]IRCCS Ospedale San Raffaele, Nuclear Medicine, Milan, Italy; [23]IRCCS Ospedale S. Gerardo, Nuclear Medicine, Monza, Italy; [24]European Institute of Oncology, Hematology, Milan, Italy;[25]Azienda Ospedaliera S. Croce e Carle, Medical Physics, Cuneo, Italy;*

**Background**

In the HD 0607 Italian GITIL/FIL clinical trial (NCT Id. n° 00795613), 782 advanced-stage (IIB-IVB), classic Hodgkin Lymphoma (cHL) patients (p.) were treated with 2 ABVD cycles. The baseline (bPET) and the interim PET/CT scan after 2 ABVD cycles (PET-2) underwent a blinded independent central review (BICR). PET-2 positive p. with a Deauville Score (DS) 4-5 p. switched to BEACOPP escalated/baseline (4+4), and PET-2 neg. p. with a DS 1-3 p continued with ABVD.

**Aims**

To assess the long-term predictive value on treatment outcome of new bPET-extracted radiomics parameters from bPET in 432/630 PET-2 neg. p. in which PET images underwent a review.

**Methods**

In this p. cohort, treated with six ABVD cycles, all the lymphoma lesions detected in the bPET were contoured using the internationally validated SUV4 workflow for total metabolic tumor volume (TMTV) delineation in lymphoma, upon applying a fixed SUV threshold of 4, and retaining only segmented volumes ≥ 3 ml (Boellard R. JNM 2024). Tumor Surface Extension (STE) was computed from TMTV upon adoption of a three-dimensional surface mesh reconstruction, implemented within an in-house radiomics pipeline following resampling to isotropic voxel spacing. The bPET scans were uploaded in DICOM format into the WIDENÒ website and automatically transferred to two expert nuclear medicine reviewers (LG and FF). Multivariate analysis was applied to clinical and radiomics variables. Radiomics cut-off for PFS were obtained using the maximally selected ranks statistics. PFS were compared with log-rank statistics on Kaplan-Meier curves.

**Copyright Information:** (Online) ISSN: 2572-9241
©2026 The Author(s). HemaSphere is published by John Wiley & Sons Ltd on behalf of European Hematology Association. This is an open access Abstract Book distributed under the Attribution-NonCommercial-NoDerivs (CC BY-NC-ND), which allows third parties to download the articles and share them with others as long as they credit the author and the Abstract Book, but they cannot change the content in any way or use them commercially.
**Abstract Book Citations:** Authors, Title, HemaSphere, 2026;10:(S1):pages. The abstract book DOIs can be found on the "Author Information" page.
**Disclaimer:** Articles published in the journal HemaSphere exclusively reflect the opinions of the authors. The authors are responsible for all content in their abstracts including accuracy of the facts, statements, citing resources, etc.

**Results**

A total of 432 stage IIB-IVB cHL p. were included in the analysis; 42 (9.7%) were aged ≥50 years, 209 (48.4%) were female and 223 (51.6%) male. The Ann Arbor stage was IIB in 154 (35.6%), III in 140 (32.4%), and IV in 138 (31.9%) p. The International Prognostic Score (IPS) breakdown was: 159 p. (36.8%) with a IPS 0-2, 218 (50.5%) with a IPS 3-4, and 55 (12.7%) with a IPS ³ 5. ECOG performance status was 0 in 152 (35.2%) p., 1 in 239 (55.3%), 2 in 37 (8.6%), 3 in 3 (0.7%), and 4 in 1 (0.2%). Bulky was present in 171 (39.6%) p., while B-symptoms were reported in 86 (19.9%) p. The median STE was 426 $cm^2$. Using a STE cut-off of 863.2 $cm^2$, 101/432 (23.3%) p. had a high and 331/432 (76.6%) a low STE. After a median follow-up (f-up) of 11 years (IQR 7-13 years), high STE p. had a significantly inferior 11-Y progression-free survival (PFS) compared with those with a low-STE (log-rank p = 0.00096: Figure 1). The 3-year and 11-year PFS were 67% and 63% in the high-STE group, versus 82% and 79% in the low-STE group, respectively. In multivariate Cox proportional hazards analysis, including age, sex, Ann Arbor stage, ECOG performance status, bulky disease, B-symptoms, IPS, and STE, the overall model turned out statistically significant (likelihood ratio $\chi^2 = 27.85$, p = 0.0010). Both IPS and STE were independently associated with PFS: IPS, with an increased risk of progression (HR = 1.42, 95% CI: 1.00–2.02; p = 0.0488), and STE with a surface area associated with a worse PFS (HR = 1.0003 per $cm^2$; 95% CI: 1.0001–1.0005; p = 0.0051), corresponding to an approximately 3% increase in risk per 100 $cm^2$ and ~19% per interquartile-range (566.7 $cm^2$) increase.

**Summary/Conclusion**

A low STE value in bPET proved able to predict a very good long-term ABVD outcome in three-quarter (76%) of PET-2 negative cHL p. treated with standard ABVD. This information could be relevant when ABVD is the only available treatment for advanced cHL.

**Copyright Information:** (Online) ISSN: 2572-9241
©2026 The Author(s). HemaSphere is published by John Wiley & Sons Ltd on behalf of European Hematology Association. This is an open access Abstract Book distributed under the Attribution-NonCommercial-NoDerivs (CC BY-NC-ND), which allows third parties to download the articles and share them with others as long as they credit the author and the Abstract Book, but they cannot change the content in any way or use them commercially.
**Abstract Book Citations:** Authors, Title, HemaSphere, 2026;10:(S1):pages. The abstract book DOIs can be found on the "Author Information" page.
**Disclaimer:** Articles published in the journal HemaSphere exclusively reflect the opinions of the authors. The authors are responsible for all content in their abstracts including accuracy of the facts, statements, citing resources, etc.

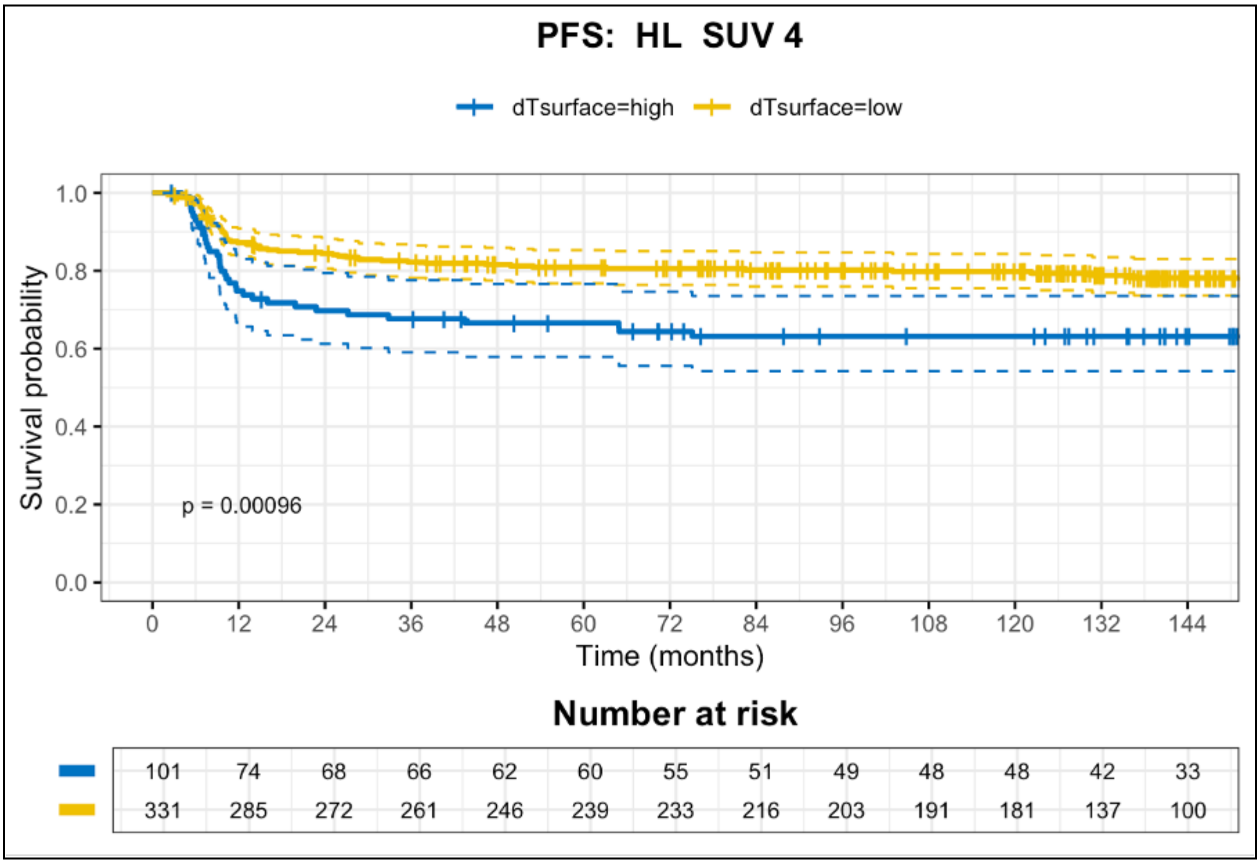

**Copyright Information:** (Online) ISSN: 2572-9241
©2026 The Author(s). HemaSphere is published by John Wiley & Sons Ltd on behalf of European Hematology Association. This is an open access Abstract Book distributed under the Attribution-NonCommercial-NoDerivs (CC BY-NC-ND), which allows third parties to download the articles and share them with others as long as they credit the author and the Abstract Book, but they cannot change the content in any way or use them commercially.
**Abstract Book Citations:** Authors, Title, HemaSphere, 2026;10:(S1):pages. The abstract book DOIs can be found on the "Author Information" page.
**Disclaimer:** Articles published in the journal HemaSphere exclusively reflect the opinions of the authors. The authors are responsible for all content in their abstracts including accuracy of the facts, statements, citing resources, etc.

### (PS2024) LONG-TERM COMPLICATIONS OF THE ABVD PROTOCOL WITH OR WITHOUT RADIOTHERAPY IN PATIENTS WITH CLASSICAL HODGKIN LYMPHOMA

**Topic:** 17. Hodgkin lymphoma - Clinical

Nassima Boulaziz*[1], Malika Allouda[1], Nadia Dali[1], Sabiha Gherras[1], Malek Benakli[2]

*[1]Hospital university Nedir Mohamed, Hematology department, Tizi-Ouzou, Algeria; [2]Pierre and Marie Curie Center, Hematology-Bone marrow transplantation, Algiers, Algeria;*

### Background

The introduction of ABVD chemotherapy, with or without radiotherapy (RT), has significantly improved overall survival in classical Hodgkin lymphoma (cHL). However, this success is tempered by a growing burden of late toxicities. In the era of personalized medicine, accurately identifying these complications is essential to optimizing treatment strategies and survivorship care.

### Aims

This study aimed to evaluate the frequency, cumulative incidence, and severity of late toxicities, as well as their impact on patient survival.

### Methods

We conducted a retrospective study over a 20-year period (2000–2019). Among 620 patients (pts) diagnosed with cHL, 492 had regular follow-up. Of these, 411 pts achieved sustained remission following ABVD (with or without RT) and were included. Late toxicities were graded according to CTCAE v5.0. Toxicity-free survival (TFS) was defined as the interval between treatment completion and the occurrence of the first Grade ≥3 toxic event (G≥3 TE). Cumulative incidence (CI) was estimated, and prognostic factors were analyzed using Cox regression models. Statistical analysis was finalized in January 2026.

### Results

The cohort of 411 evaluable pts had a median age of 27 years (15–78), with 54% males. At diagnosis, 49.1% had localized disease, and 57% received RT. Hypothyroidism was the most frequent complication (70 pts, 17%), strongly associated with cervical irradiation ($p<0.0001$). Secondary Malignancies observed in 23 pts (5.6%), representing 42.6% of G≥3 TE. These included breast cancer (30%), head and neck cancers (13%), and hematologic malignancies (17.4%). Notably, 6 out of 7 breast cancer cases were associated with mediastinal extended-field RT (EFRT) ($p<0.0001$). Eighteen pts developed cardiac complications, primarily cardiomyopathies (55.5%), significantly linked to mediastinal EFRT at doses >30 Gy ($p<0.0001$). Infertility reported in 11 cases (2.67%), significantly associated with abdominopelvic EFRT ($p<0.0001$). After a median follow-up of 134 months (52–293), the 24-year TFS was 77%. The 24-year CI was 23% for G≥3 TE, 11% for secondary cancers, and 8% for cardiac complications. Multivariate analysis identified age > 40 years (HR=3.166; $p<0.0001$) and female sex (HR=2.401; $p=0.011$) as independent predictors of poorer TFS.

### Summary/Conclusion

While ABVD ± RT ensures excellent long-term survival, it carries a significant risk of late toxicities, particularly in pts receiving radiotherapy. These results underscore the necessity for individualized treatment to de-escalate therapy where possible and the importance of lifelong, personalized follow-up.

**Copyright Information:** (Online) ISSN: 2572-9241
©2026 The Author(s). HemaSphere is published by John Wiley & Sons Ltd on behalf of European Hematology Association. This is an open access Abstract Book distributed under the Attribution-NonCommercial-NoDerivs (CC BY-NC-ND), which allows third parties to download the articles and share them with others as long as they credit the author and the Abstract Book, but they cannot change the content in any way or use them commercially.
**Abstract Book Citations:** Authors, Title, HemaSphere, 2026;10:(S1):pages. The abstract book DOIs can be found on the "Author Information" page.
**Disclaimer:** Articles published in the journal HemaSphere exclusively reflect the opinions of the authors. The authors are responsible for all content in their abstracts including accuracy of the facts, statements, citing resources, etc.

### (PS2025) COMPARISON OF NIVO-AVD AND STANDARD PET-ADAPTED APPROACH AS FIRST-LINE THERAPY IN PATIENTS WITH CLASSICAL HODGKIN LYMPHOMA IN REAL-WORLD PRACTICE

**Topic:** 17. Hodgkin lymphoma - Clinical

Sergey Alekseev[1, 2], Evgenii Kunevich*[1], Olga Uspenskaya[1], Ruslan Bekoev[1], George Pavlinov[1], Albina Aidemirova[1], Victoria Basmanova[1], Maria Bashkova[1], Dmitrii Voroshin[1], Maxim Ganzin[1], Victoria Grishko[1], Mark Ivanyugin[1], Olga Ruzhinskaya[1], Natalia Sokolova[1], Victor Solovyov[1], Svetlana Tolstova[1], Tsynzhidma Tsyrenzhabe[1], Sergey Voloshin[1, 3]

*[1]Leningrad Regional Clinical Hospital, Saint-Petersburg, Russia;[2]N.N. Petrov National Medical Research Center of Oncology, Saint-Petersburg, Russia; [3]S.M. Kirov Military Medical Academy, Saint-Petersburg, Russia;*

### Background

The current paradigm in the treatment of lymphoproliferative neoplasms emphasizes the use of innovative therapies at early treatment stages to achieve maximal tumor eradication. The promise of this approach is supported by data reported by A.F. Herrera et al. (2024), demonstrating the efficacy of nivolumab as first-line therapy in classical Hodgkin lymphoma (cHL).

### Aims

To evaluate the efficacy of the Nivo-AVD regimen in patients with cHL compared with standard PET-adapted chemotherapy.

### Methods

This prospective study with a prospective–retrospective control included 158 adult patients (≥18 years) with cHL. Patients were allocated into two groups: the study group (SG) comprised 34 patients (median age 38 years, range 18–84) who received Nivo-AVD as first-line therapy; the control group (CG) included 124 patients (median age 35 years, range 18–76) treated with standard chemotherapy using a PET-adapted approach.
Median follow-up was 18.5 months (mean 20.8 months) in the SG and 48.7 months in the CG. In the SG, patients received four cycles of therapy for stage I–II disease and six cycles for stage III–IV disease. Consolidative radiotherapy was administered to 34.4% of patients in the CG and 22.6% in the SG. Statistical analyses were performed using Jamovi v2.4.8.0 and R v4.4.2.

**Copyright Information:** (Online) ISSN: 2572-9241
©2026 The Author(s). HemaSphere is published by John Wiley & Sons Ltd on behalf of European Hematology Association. This is an open access Abstract Book distributed under the Attribution-NonCommercial-NoDerivs (CC BY-NC-ND), which allows third parties to download the articles and share them with others as long as they credit the author and the Abstract Book, but they cannot change the content in any way or use them commercially.
**Abstract Book Citations:** Authors, Title, HemaSphere, 2026;10:(S1):pages. The abstract book DOIs can be found on the "Author Information" page.
**Disclaimer:** Articles published in the journal HemaSphere exclusively reflect the opinions of the authors. The authors are responsible for all content in their abstracts including accuracy of the facts, statements, citing resources, etc.

### Results

The objective response rate (ORR) in the SG was 97.1%, with a complete response (CR) rate of 85.3%. In the CG, ORR and CR rates were 92.7% (p=0.6913) and 76.9% (p=0.2885), respectively. Disease progression, relapse, or development of refractoriness occurred less frequently in the SG (8.8%) than in the CG (27.4%), p=0.0230.

Despite the longer follow-up in the CG (48.7 vs 18.5 months), most cases of relapse or progression occurred within the first year from treatment initiation (median 9.3 months), partially mitigating the impact of follow-up differences between groups. A trend toward improved 2-year event-free survival (EFS) was observed in the SG: 91.0% (95% CI 81.7–100) versus 74.8% (95% CI 67.1–83.4) in the CG, p=0.12.

Logistic regression analysis was performed with unfavorable outcome as the dependent variable. In the univariable model, treatment with Nivo-AVD was associated with a significantly reduced risk of unfavorable events compared with standard therapy (OR=0.26; 95% CI 0.06–0.81; p=0.038). After adjustment for clinically relevant factors (stage IV disease, B symptoms, radiotherapy), the effect remained statistically significant and even strengthened (OR=0.22; 95% CI 0.05–0.71; p=0.023), corresponding to a 78% reduction in the risk of refractory disease, progression, relapse, or death from any cause with Nivo-AVD.

Nivo-AVD demonstrated good tolerability and a manageable safety profile. Grade 1–3 infectious complications were observed in 32% of patients, predominantly acute respiratory viral infections and episodes of febrile neutropenia. Grade 3–4 hematologic toxicity was mainly represented by neutropenia, occurring in 25% of patients.

### Summary/Conclusion

First-line treatment with Nivo-AVD in cHL is associated with higher complete response rates and a trend toward improved event-free survival compared with the standard PET-adapted approach. The regimen shows good tolerability and a manageable toxicity profile, supporting its potential use in routine clinical practice, including in older patient populations.

**Copyright Information:** (Online) ISSN: 2572-9241
©2026 The Author(s). HemaSphere is published by John Wiley & Sons Ltd on behalf of European Hematology Association. This is an open access Abstract Book distributed under the Attribution-NonCommercial-NoDerivs (CC BY-NC-ND), which allows third parties to download the articles and share them with others as long as they credit the author and the Abstract Book, but they cannot change the content in any way or use them commercially.
**Abstract Book Citations:** Authors, Title, HemaSphere, 2026;10:(S1):pages. The abstract book DOIs can be found on the "Author Information" page.
**Disclaimer:** Articles published in the journal HemaSphere exclusively reflect the opinions of the authors. The authors are responsible for all content in their abstracts including accuracy of the facts, statements, citing resources, etc.

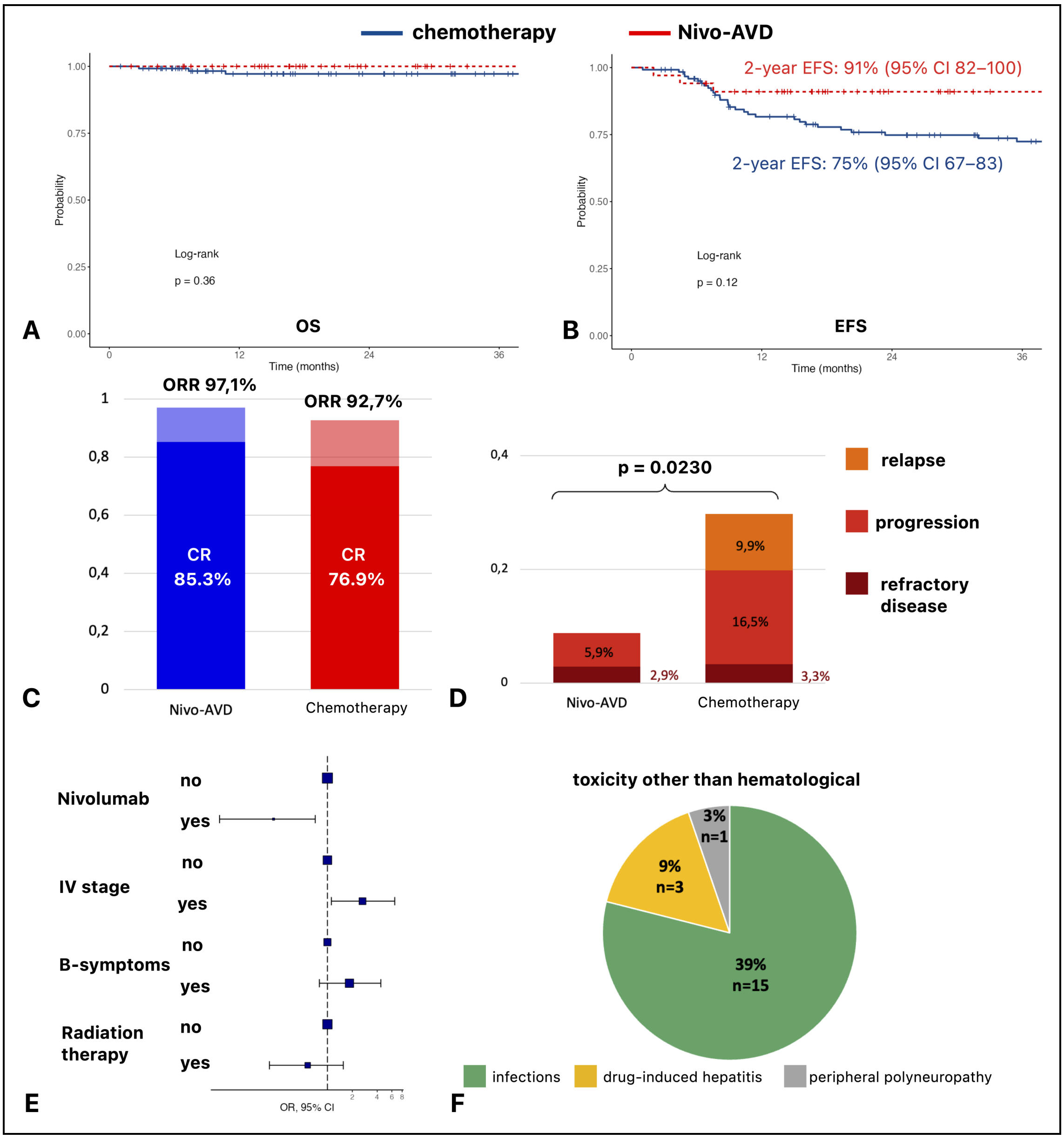

A) Overall survival in the comparison groups. B) Event-free survival in the comparison groups. C) Complete response rate and objective response rate in the comparison groups. D) Rates of refractory disease, progression, and relapse in the comparison groups. E) Forest plot showing results of multivariable logistic regression analysis in the overall patient cohort (n=149) assessing event-free survival. F) Incidence of adverse events (non-hematologic toxicity).

**Copyright Information:** (Online) ISSN: 2572-9241
©2026 The Author(s). HemaSphere is published by John Wiley & Sons Ltd on behalf of European Hematology Association. This is an open access Abstract Book distributed under the Attribution-NonCommercial-NoDerivs (CC BY-NC-ND), which allows third parties to download the articles and share them with others as long as they credit the author and the Abstract Book, but they cannot change the content in any way or use them commercially.
**Abstract Book Citations:** Authors, Title, HemaSphere, 2026;10:(S1):pages. The abstract book DOIs can be found on the "Author Information" page.
**Disclaimer:** Articles published in the journal HemaSphere exclusively reflect the opinions of the authors. The authors are responsible for all content in their abstracts including accuracy of the facts, statements, citing resources, etc.

### (PS2026) HEART FAILURE SURVIVAL IN PATIENTS WITH PRIOR HODGKIN LYMPHOMA: INCREASED ALL-CAUSE BUT NOT CARDIOVASCULAR MORTALITY

**Topic:** 17. Hodgkin lymphoma - Clinical

Sissel Johanne Godtfredsen*[1], Sandra Eloranta[2], Karin Ekstrom Smedby[2, 3], Stefanie Antonilli[2], Joachim Baech[1], Daniel Molin[4], Kristian Hay Kragholm[1, 5], Tarec El-Galaly[2, 6, 7, 8, 9], Caroline Dietrich[2]

*[1]Aalborg University Hospital, Department of Cardiology, Aalborg, Denmark;[2]Karolinska Institutet, Department of Medicine Solna, Clinical Epidemiology Division, Stockholm, Sweden; [3]Karolinska University Hospital, Department of Hematology, Stockholm, Sweden; [4]Uppsala Akademiska Hospital, Department of Immunology, Genetics, and Pathology, Cancer Immunotherapy, Uppsala University, Uppsala, Sweden; [5]Aalborg University, Department of clinical medicine, Aalborg, Denmark;[6]Aarhus University Hospital, Department of Hematology, Aarhus, Denmark; [7]Aarhus University Hospital, Department of Molecular Medicine, Aarhus, Denmark; [8]Aarhus University Hospital, Department of Clinical Epidemiology, Aarhus, Denmark;[9]Odense University Hospital, Department of Hematology, Odense, Denmark;*

### Background

Advances in the treatment of classical Hodgkin lymphoma (cHL) have led to excellent long-term survival. While an increased incidence of heart failure (HF) among cHL survivors is well-documented, the subsequent prognosis once HF has developed remains poorly characterized.

### Aims

To compare all-cause and cardiovascular (CV)-specific mortality following incident HF between patients with a history of cHL and matched lymphoma-free comparators from the general population.

### Methods

Using Swedish nationwide registries, we identified patients diagnosed with cHL between 2000 and 2022 who subsequently developed HF. These patients were matched 1:2 with comparators with incident HF on sex, birth year, year of HF, and contact type (in- vs. outpatient). Follow-up began at date of HF diagnosis and ended upon first of death, emigration, five years of follow-up, or end of study (December 31, 2023). Hazard ratios (HRs) and 95% confidence intervals (CIs) comparing the cHL group to comparators were estimated using flexible parametric models, adjusting for matching variables.

### Results

A total of 257 patients with cHL developed HF and were matched to 514 comparators. Overall, median age at HF diagnosis was 73 years, with a male predominance (64%). Median follow-up was 2.5 years for the cHL group and 3.3 years for the comparators. In the cHL group, 130 deaths occurred; 26% due to CV causes and 40% to lymphoma. For comparators, 192 deaths were recorded (42% from CV causes and 20% from non-lymphoma cancer). This corresponded to a two-fold increase in all-cause mortality among the cHL cases with HF (HR=1.81, 95% CI: 1.44–2.26). A longer duration between cHL and HF was associated with increased all-cause mortality (**Figure 1**). While overall CV-specific mortality did not differ significantly between groups (HR=1.17, 95% CI: 0.78-1.75), patients with ≥10 years between cHL and HF had a markedly elevated mortality due to CV disease (HR=3.13, 95% CI: 1.71-5.73) (**Figure 1**).

**Copyright Information:** (Online) ISSN: 2572-9241
©2026 The Author(s). HemaSphere is published by John Wiley & Sons Ltd on behalf of European Hematology Association. This is an open access Abstract Book distributed under the Attribution-NonCommercial-NoDerivs (CC BY-NC-ND), which allows third parties to download the articles and share them with others as long as they credit the author and the Abstract Book, but they cannot change the content in any way or use them commercially.
**Abstract Book Citations:** Authors, Title, HemaSphere, 2026;10:(S1):pages. The abstract book DOIs can be found on the "Author Information" page.
**Disclaimer:** Articles published in the journal HemaSphere exclusively reflect the opinions of the authors. The authors are responsible for all content in their abstracts including accuracy of the facts, statements, citing resources, etc.

### Summary/Conclusion

Patients with prior cHL experience significantly higher all-cause but not CV-specific mortality following HF than lymphoma-free comparators. The elevated rate of CV-specific death in patients with ≥10 years between cHL and HF may may reflect the delayed emergence of mediastinal radiotherapy-related cardiotoxicity which may involve a potentially more ischemic, valvular, or restrictive HF subtype. These findings suggest that the previously reported high CV-mortality in cHL cohorts may primarily reflect a higher incidence of HF and continued higher all-cause mortality rather than a universally poorer prognosis once HF has developed.

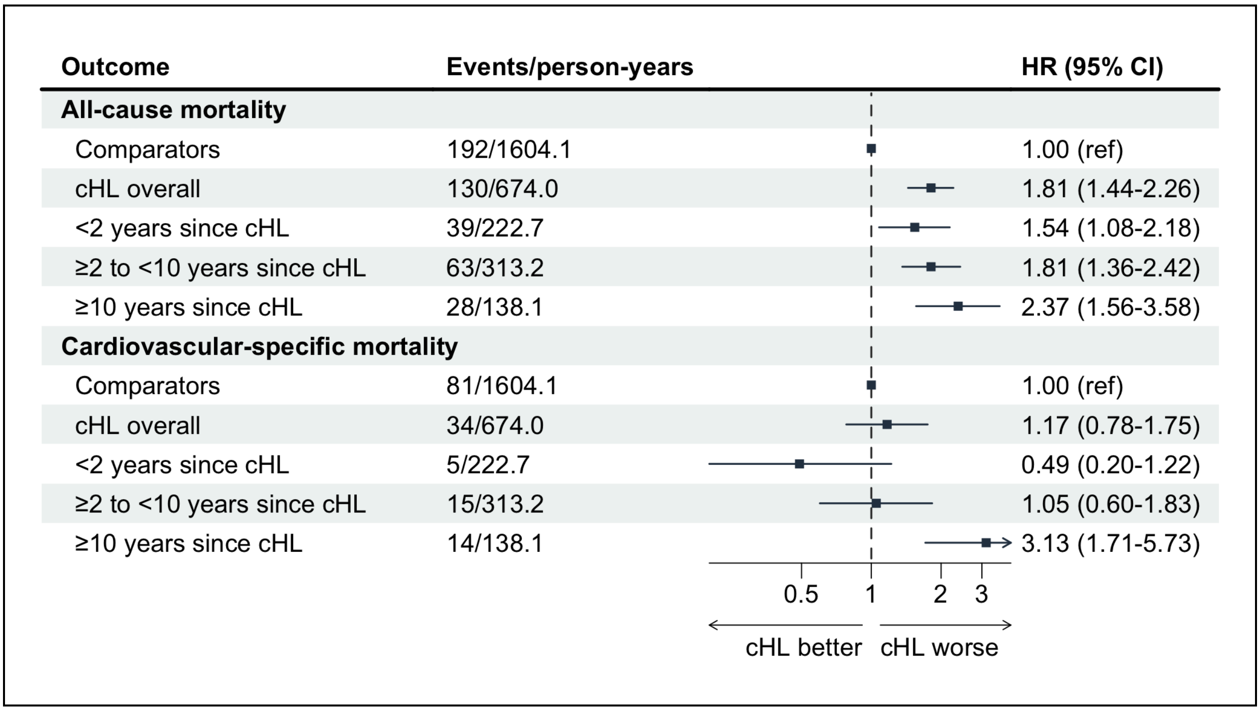

Figure 1: Hazard ratios (HRs) with 95% confidence intervals (CIs) of all-cause and cardiovascular-specific mortality comparing patients with classical Hodgkin lymphoma (cHL) and heart failure to lymphoma-free comparators (reference [ref]) with heart failure.

**Copyright Information:** (Online) ISSN: 2572-9241
©2026 The Author(s). HemaSphere is published by John Wiley & Sons Ltd on behalf of European Hematology Association. This is an open access Abstract Book distributed under the Attribution-NonCommercial-NoDerivs (CC BY-NC-ND), which allows third parties to download the articles and share them with others as long as they credit the author and the Abstract Book, but they cannot change the content in any way or use them commercially.
**Abstract Book Citations:** Authors, Title, HemaSphere, 2026;10:(S1):pages. The abstract book DOIs can be found on the "Author Information" page.
**Disclaimer:** Articles published in the journal HemaSphere exclusively reflect the opinions of the authors. The authors are responsible for all content in their abstracts including accuracy of the facts, statements, citing resources, etc.

### (PS2027) HIGH INCIDENCE AND CLINICAL SIGNIFICANCE OF METABOLIC SYNDROME IN YOUNG HODGKIN LYMPHOMA SURVIVORS: A LARGE MATCHED COHORT STUDY

**Topic:** 17. Hodgkin lymphoma - Clinical

Dor Shpitzer*[1, 2], Dana Barnea[1, 2], Shir Dekel[2], Roy Vitkon[1, 2], Irit Avivi Mazza[1, 2], Chava Perry[1, 2]

*[1]Hematology department., Tel Aviv Sourasky Medical Center, Tel Aviv, Israel;[2]Faculty of Medicine, Tel Aviv University, Tel Aviv, Israel;*

### Background

Although Hodgkin lymphoma (HL) is highly curable with 10-year survival rates exceeding 85%, treatments often cause late toxicities. Notably, the incidence of metabolic syndrome in HL survivors is two- to threefold higher than in the general population. However, comprehensive data on the prevalence and risk factors for these metabolic complications in young survivors remain limited, necessitating focused, long-term studies.

### Aims

We aim to evaluate the long-term incidence and risk factors of metabolic complications, including metabolic syndrome, in young HL survivors and to assess their association with subsequent cardiovascular events.

### Methods

We conducted a retrospective cohort study using electronic medical records from Maccabi Healthcare Services, identifying patients diagnosed with HL between the ages of 18 and 50. Each HL patient was matched with 20 healthy controls based on age and sex. Individuals with prior malignancy, pre-existing metabolic or cardiovascular conditions, or those with incomplete baseline data (BMI or blood pressure) were excluded. Metabolic complications, including type 2 diabetes mellitus (NIDDM), dyslipidemia, hypothyroidism and osteoporosis, were recorded using ICD-9 coding. Metabolic syndrome was defined by the presence of at least three of the following criteria within a 12-month period: Hypertriglyceridemia, low HDL cholesterol, elevated blood pressure and hyperglycemia. Cardiovascular events occurring at least 12 months post HL diagnosis were collected.The study endpoints included incidence and risk factors for each metabolic complication, including metabolic syndrome and its association with cardiovascular complications.

**Copyright Information:** (Online) ISSN: 2572-9241
©2026 The Author(s). HemaSphere is published by John Wiley & Sons Ltd on behalf of European Hematology Association. This is an open access Abstract Book distributed under the Attribution-NonCommercial-NoDerivs (CC BY-NC-ND), which allows third parties to download the articles and share them with others as long as they credit the author and the Abstract Book, but they cannot change the content in any way or use them commercially.
**Abstract Book Citations:** Authors, Title, HemaSphere, 2026;10:(S1):pages. The abstract book DOIs can be found on the “Author Information” page.
**Disclaimer:** Articles published in the journal HemaSphere exclusively reflect the opinions of the authors. The authors are responsible for all content in their abstracts including accuracy of the facts, statements, citing resources, etc.

**Results**

A total of 1,015 HL patients (median age 29 years; IQR 23–36) met the study eligibility criteria and were matched with 20,300 population-based controls. All HL patients were treated with chemotherapy, 12% (n=122) received radiotherapy (RT) and 12% (n=117) underwent autologous hematopoietic cell transplantation.
Over a median follow-up of 13.6 years (IQR 8–20.6), HL survivors exhibited a significantly higher burden of metabolic complications (14.5% vs. 11.5%, p=0.01). The incidences of hypothyroidism (1.9% vs. 0.7%, p<0.001), osteoporosis (4.9% vs. 3.7%, p=0.03), NIDDM (7.4% vs. 6.2%, p=0.05) and metabolic syndrome (21% vs. 18%, p=0.017) were all significantly higher in HL patients compared to their controls.Multivariable Cox analysis identified HL diagnosis as an independent risk factor for the development of metabolic complications (HR 1.33, p=0.004), alongside older age at diagnosis (HR=1.09, p<0.001), higher BMI (HR=1.06, p<0.001), and smoking (HR=1.35, p<0.001). Higher socioeconomic status exerted a protective effect (HR=0.95, p<0.001). HL survivors had a higher incidence of IHD compared with controls (4.2% vs. 2.2%, p<0.001) and underwent more coronary revascularization procedures, including percutaneous coronary intervention (PCI) or coronary artery bypass grafting (CABG) (0.8% vs. 0.3%, p=0.02). Among HL survivors who developed cardiovascular complication, 90% had met the criteria for metabolic syndrome prior to the event.

**Summary/Conclusion**

In this large, matched cohort, young HL survivors demonstrated a significantly higher incidence of metabolic complications, including metabolic syndrome, which is associated with an elevated risk of cardiovascular events. These findings underscore the critical need for lifelong surveillance, early risk-factor modification, and tailored post-treatment care for this population.

**Copyright Information:** (Online) ISSN: 2572-9241
©2026 The Author(s). HemaSphere is published by John Wiley & Sons Ltd on behalf of European Hematology Association. This is an open access Abstract Book distributed under the Attribution-NonCommercial-NoDerivs (CC BY-NC-ND), which allows third parties to download the articles and share them with others as long as they credit the author and the Abstract Book, but they cannot change the content in any way or use them commercially.
**Abstract Book Citations:** Authors, Title, HemaSphere, 2026;10:(S1):pages. The abstract book DOIs can be found on the "Author Information" page.
**Disclaimer:** Articles published in the journal HemaSphere exclusively reflect the opinions of the authors. The authors are responsible for all content in their abstracts including accuracy of the facts, statements, citing resources, etc.

### (PS2028) EVALUATION OF PET-CT METRICS AND PHARMACOKINETICS IN ADULTS RECEIVING TISLELIZUMAB FOR RELAPSED/REFRACTORY CLASSICAL HODGKIN LYMPHOMA: ANCILLARY ANALYSES OF LYSA PHASE 2 TIRHOL STUDY BGB-A317-210

**Topic:** 17. Hodgkin lymphoma - Clinical

Herve Ghesquieres*[1], Kamal Bouabdallah[2], Salim Kanoun[3], Marc André[4], Philippe Quittet[5], Cecile Borel[6], Aspasia Stamatoullas Bastard[7], Michael Gilbertson[8], Fabien Le Bras[9], Catherine Thieblemont[10], Baptiste Delapierre (Moisdon)[11], Mohamed Touati[12], Pierre Feugier[13], Loïc Renaud[14], Nadine Morineau[15], Thomas Gastinne[16], Isabelle Chaillol[17], Rod Ramchandren[18], Harsh Shah[19], Dipenkumar Modi[20], Heather Allewelt[21], Pierre Fustier[22], Xiangyu Liu[21], Jun Zhang[21], Richard Delarue[22], Franck Morschhauser[23], Cedric Rossi[24]

*[1]Lyon Sud Hospital, Pierre Benite, France; [2]Hopital Haut-Leveque, Bordeaux, France; [3]Pixilib SAS, Toulouse, France; [4]CHU UCL Namur, Yvoir, Belgium; [5]CHU Montpellier, Montpellier, France; [6]IUCT Oncopole, Toulouse, France; [7]Centre Henri Becquerel, Rouen, France; [8]Monash Health, Melbourne, Australia; [9]CHU Mondor, Creteil, France; [10]AP-HP, Hopital Saint Louis, Paris, France; [11]Caen University Hospital, Caen, France; [12]CHU Limoges, Limoges, France; [13]CHU Nancy, Nancy, France; [14]APHP, CHU Cochin, Paris, France; [15]CHD de Vendee, La Roche Sur Yon, France; [16]CHU de Nantes, Nantes, France; [17]Lymphoma Academic Research Organisation, Pierre Benite, France; [18]UPMC Hillman Cancer Center, Pittsburgh, United States of America; [19]University of Utah, Salt Lake City, United States of America; [20]Karmanos Cancer Institute, Detroit, United States of America; [21]BeOne Medicines Ltd, San Carlos, United States of America; [22]BeOne Medicines Ltd, Basel, Switzerland; [23]CHU Lille, Lille, France; [24]CHU Dijon, Dijon, France;*

### Background

Conventional methods using PET-CT to assess lymphoma response are suboptimal in patients (pts) treated with PD1 inhibitors; specialized criteria are required to prevent premature treatment discontinuation due to inflammation unrelated to disease progression. In the LYSA phase 2 BGB-A317-210 study (NCT04318080), 45 pts with relapsed/refractory (R/R) classical Hodgkin lymphoma (cHL) were treated with tislelizumab every 3 weeks until progressive disease (PD), unacceptable toxicity, or study withdrawal; disease response was assessed by investigator every 12 weeks according to Lugano 2014 PET-CT classification. At final analysis, the overall response rate (ORR), the primary endpoint of TIRHOL, was 66.7% (95%CI, 51%-80%), and the complete response rate was 31%. PK profiles were comparable between BGB-A317-210 and the prior Chinese study BGB-A317-203.

### Aims

For this correlative analysis, PET-CT imaging in pts in the TIRHOL study was evaluated post-hoc by a central, independent reviewer.

### Methods

Relationships were assessed between baseline PET-CT metrics (TMTV4, TLG4, Dmax4, SUVmean, and SUVmax) and response assessments, progression-free (PFS), and overall survival (OS). The longitudinal evolution of TMTV4 and SUV during tislelizumab therapy was also examined. Tislelizumab PK profiles (cycle 1 post-dose and cycle 2 pre-dose concentrations) were evaluated against baseline clinical and PET-CT characteristics.

**Copyright Information:** (Online) ISSN: 2572-9241
©2026 The Author(s). HemaSphere is published by John Wiley & Sons Ltd on behalf of European Hematology Association. This is an open access Abstract Book distributed under the Attribution-NonCommercial-NoDerivs (CC BY-NC-ND), which allows third parties to download the articles and share them with others as long as they credit the author and the Abstract Book, but they cannot change the content in any way or use them commercially.
**Abstract Book Citations:** Authors, Title, HemaSphere, 2026;10:(S1):pages. The abstract book DOIs can be found on the "Author Information" page.
**Disclaimer:** Articles published in the journal HemaSphere exclusively reflect the opinions of the authors. The authors are responsible for all content in their abstracts including accuracy of the facts, statements, citing resources, etc.

### Results

Baseline and follow-up PET-CT images were available for post-hoc central review for 37 of the 45 pts in TIRHOL. ORR was 70%, with complete metabolic response (CMR) observed in 15 pts (40%) and partial metabolic response (PMR) in 11 (30%); remaining pts had no metabolic response (NMR; stable disease or PD; n=10, [27%]), or were not evaluated (n=1, [3%]). At the first metabolic evaluation at Week 12, 10 pts had CMR (27%), 13 had PMR (35%), 13 had NMR (35%) and 1 (3%) was not evaluated. Pts with best metabolic response of CMR, PMR and NMR had 1-year PFS rates of 60%, 26%, and 8%, and 1-year OS rates of 100%, 100%, and 77%, respectively. Median baseline PET-CT metrics were comparable across Week 12 and best metabolic response categories and were comparable between pts with PFS or OS events and those who remained event-free. Longitudinal evaluations of TMTV4 and SUV showed a rapid decrease of values at first evaluation for pts with CMR and PMR at Week 12. The patterns of TMTV4 and SUV variations during tislelizumab treatment differed between pts with best metabolic response of CMR, PMR, and NMR. No differences in PK results were observed based on key baseline categorical variables (sex, age, B-symptoms, cohort 1/2, Ann Arbor stage, bulk, refractory vs relapse, IPS score, TMTV4, albumin level).

### Summary/Conclusion

Central review of PET-CT imaging confirmed that tislelizumab is an effective therapeutic option for pts with R/R cHL, and efficacy may have been underestimated in the TIRHOL study. Tislelizumab PK and efficacy outcomes did not differ between baseline PET-CT metrics. OS was lower in pts with NMR at Week 12 compared with pts who had CMR or PMR. Analyses of centrally reviewed PET-CT imaging demonstrated a trend for underestimation of local response assessments, and therefore in ORR (70% vs. 66.7%) and CMR rate (40% vs. 31%), likely due to the presence of non-lymphomatous inflammatory lesions. Variations in PET-CT parameters during anti-PD1 therapy should be interpreted with caution to avoid premature treatment discontinuation.

**Copyright Information:** (Online) ISSN: 2572-9241
©2026 The Author(s). HemaSphere is published by John Wiley & Sons Ltd on behalf of European Hematology Association. This is an open access Abstract Book distributed under the Attribution-NonCommercial-NoDerivs (CC BY-NC-ND), which allows third parties to download the articles and share them with others as long as they credit the author and the Abstract Book, but they cannot change the content in any way or use them commercially.
**Abstract Book Citations:** Authors, Title, HemaSphere, 2026;10:(S1):pages. The abstract book DOIs can be found on the “Author Information” page.
**Disclaimer:** Articles published in the journal HemaSphere exclusively reflect the opinions of the authors. The authors are responsible for all content in their abstracts including accuracy of the facts, statements, citing resources, etc.

### (PS2029) INCIDENCE AND SURVIVAL OF CLASSIC HODGKIN LYMPHOMA: AN ENGLAND-WIDE COHORT STUDY FROM THE UNCOVER STUDY GROUP, 2014-2022

**Topic:** 17. Hodgkin lymphoma - Clinical

Sidhant Seth*[1, 2], Indrani Karpha[3, 4], Nurunnahar Akter[4], Aisling Barrett[5], Temitope Erinfolami[4], Toyyib Abdulkareem[4], Brogan Johnston[3], Yeong Jer Lim[3, 4], Marianne Moxon[6], Tess Nowell[6], Catrin Tudur Smith[4], Nagesh Kalakonda[3, 4], Mark Bishton[7, 8], Andrew Pettitt[3, 4], Cathy Burton[9], Mathew Wells[3], Pam McKay[1, 2]

*[1]Beatson West of Scotland Cancer Centre, Glasgow, United Kingdom;[2]School of Cancer Sciences, University of Glasgow, Glasgow, United Kingdom; [3]The Clatterbridge Cancer Centre, Liverpool, United Kingdom; [4]The University of Liverpool, Liverpool, United Kingdom; [5]Nuffield Department of Population Health, University of Oxford, Oxford, United Kingdom;[6]Patient Representative, Oxford, United Kingdom; [7]University of Nottingham, Nottingham, United Kingdom; [8]Nottingham University Hospitals NHS Trust, Nottingham, United Kingdom; [9]The Leeds Teaching Hospitals NHS Trust, Leeds, United Kingdom;*

### Background

Classic Hodgkin lymphoma (cHL) has recognised epidemiological patterns, including age-specific incidence peaks (adolescents/young adults and older adults) and geographical variation. However, contemporary population-based data describing its epidemiology in the UK remain limited. UNCOVER is a blood cancer health data research programme utilising the National Cancer Registration Dataset (NCRD), capturing all cancer diagnoses in England.

### Aims

To characterise epidemiological patterns and survival outcomes of patients with cHL in England using national cancer registry data.

### Methods

Data on all patients in England diagnosed with a blood cancer between January 2014 and December 2022 were obtained from NCRD. Patients with cHL were identified using ICD-O-3 codes (9650-9655/3, 9662-9665/3 and 9667/3). Crude and age-standardised incidence rates (ASIR) were calculated. Incidence rate ratios (IRR) were estimated using Poisson regression. Overall survival (OS) was estimated using Kaplan–Meier methods, with multivariate Cox regression to examine factors associated with OS. Relative survival (RS) was estimated using the Pohar–Perme method with England life tables stratified by age, gender, indices of multiple deprivation (IMD) and government region. Models were adjusted for age, gender, IMD quintile, and government region; Cox models were additionally adjusted for cHL stage, ethnicity and Charlson comorbidity index (CCI). Two-sided p-values $<0.05$ were considered significant. All analyses were conducted in R version 4.4.1.

**Copyright Information:** (Online) ISSN: 2572-9241
©2026 The Author(s). HemaSphere is published by John Wiley & Sons Ltd on behalf of European Hematology Association. This is an open access Abstract Book distributed under the Attribution-NonCommercial-NoDerivs (CC BY-NC-ND), which allows third parties to download the articles and share them with others as long as they credit the author and the Abstract Book, but they cannot change the content in any way or use them commercially.
**Abstract Book Citations:** Authors, Title, HemaSphere, 2026;10:(S1):pages. The abstract book DOIs can be found on the "Author Information" page.
**Disclaimer:** Articles published in the journal HemaSphere exclusively reflect the opinions of the authors. The authors are responsible for all content in their abstracts including accuracy of the facts, statements, citing resources, etc.

**Results**

14,172 patients were registered with cHL between 2014 and 2022 with median follow-up of 5.7 years (IQR 3.6–7.9). The median age at diagnosis was 42 (range 0–101, IQR 26–65) with a male preponderance (55%). Most patients were White (79%) and had a CCI of 0 (81%).
The overall ASIR was 2.72 per 100,000 person-years with a bi-modal age distribution. Compared with White individuals, IRR was lower in Asian (0.78, 95% CI 0.73–0.84), Black (0.67, 0.61–0.74), and mixed/other (0.82, 0.75–0.89) groups. Regional variation was evident (global p<0.001), with ASIR ranging between 2.49 (Yorkshire and The Humber) and 2.88 per 100,000 (East Midlands). Incidence was associated with deprivation (IRR 1.19 for most vs least deprived quintile, 95% CI 1.12–1.26). No significant incidence trends were observed over calendar years, including the COVID-19 pandemic.
Five-year OS and RS were 80% (95% CI 79–81%) and 84% (83–85%) respectively. Advanced stage, male gender, older age, higher deprivation and higher CCI were all independently associated with shorter OS (p<0.001 for all). Age >60 years (HR vs 0–15: 27.6, 95% CI 16.6–46.0) and stage 4 disease (HR vs stage 1: 2.25, 1.94–2.62) had the largest impact. Asian (HR 0.80, 95% CI 0.68–0.95) and mixed/other (0.71, 0.53–0.96) ethnicities had improved OS compared with White (reference) and Black patients (0.98, 0.85–1.12). There was regional variation in survival (global p=0.038) with worse OS in regions outside London, most notably the North East (HR: 1.30, 95% CI: 1.08–1.59).

**Summary/Conclusion**

This population-based study provides contemporary epidemiological and survival data for cHL in England. Incidence varied by age, sex, deprivation, ethnicity, and region. While survival was generally favourable, it was independently influenced by age, stage, gender, deprivation, comorbidity, ethnicity and geographical region. These findings provide an updated national benchmark for incidence and outcomes, informing healthcare planning and future research.

**Copyright Information:** (Online) ISSN: 2572-9241
©2026 The Author(s). HemaSphere is published by John Wiley & Sons Ltd on behalf of European Hematology Association. This is an open access Abstract Book distributed under the Attribution-NonCommercial-NoDerivs (CC BY-NC-ND), which allows third parties to download the articles and share them with others as long as they credit the author and the Abstract Book, but they cannot change the content in any way or use them commercially.
**Abstract Book Citations:** Authors, Title, HemaSphere, 2026;10:(S1):pages. The abstract book DOIs can be found on the "Author Information" page.
**Disclaimer:** Articles published in the journal HemaSphere exclusively reflect the opinions of the authors. The authors are responsible for all content in their abstracts including accuracy of the facts, statements, citing resources, etc.

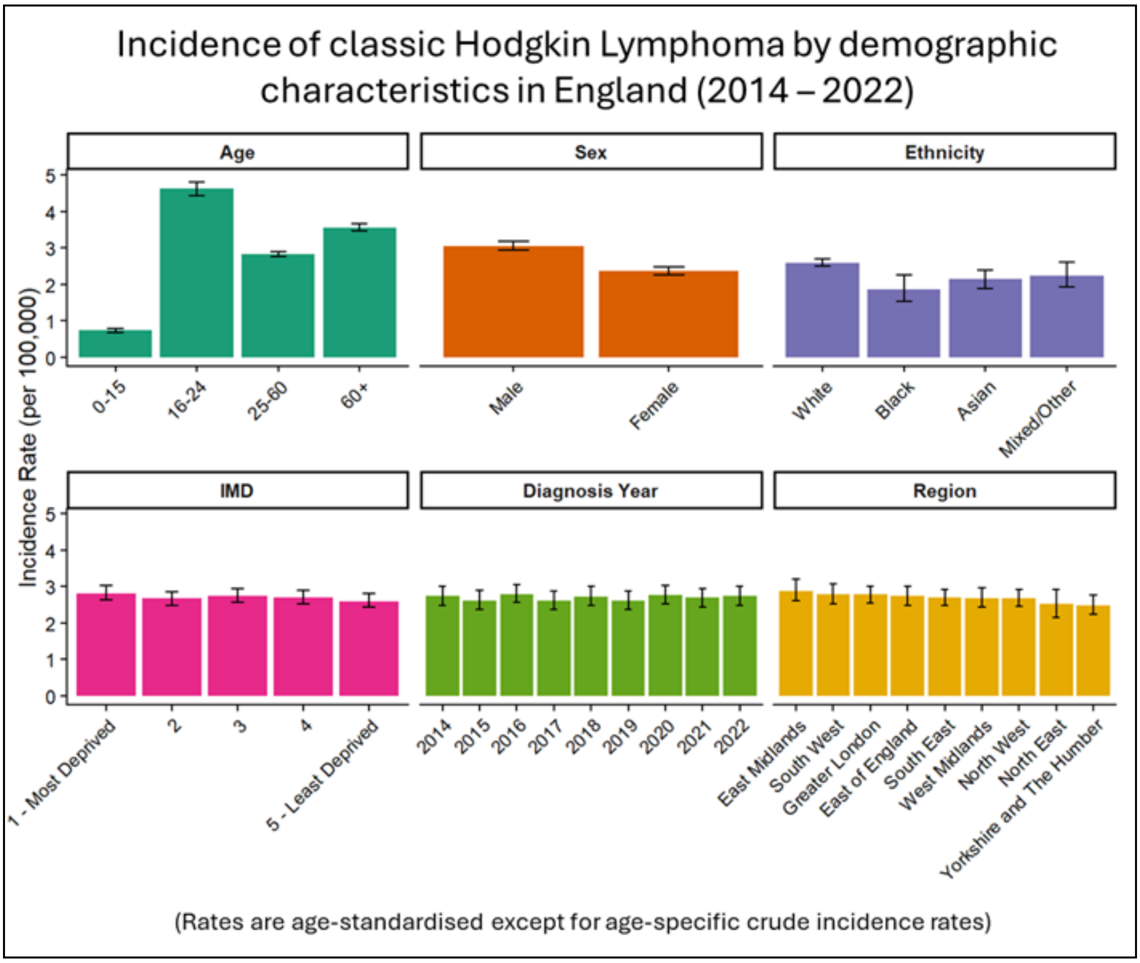

**Copyright Information:** (Online) ISSN: 2572-9241
©2026 The Author(s). HemaSphere is published by John Wiley & Sons Ltd on behalf of European Hematology Association. This is an open access Abstract Book distributed under the Attribution-NonCommercial-NoDerivs (CC BY-NC-ND), which allows third parties to download the articles and share them with others as long as they credit the author and the Abstract Book, but they cannot change the content in any way or use them commercially.
**Abstract Book Citations:** Authors, Title, HemaSphere, 2026;10:(S1):pages. The abstract book DOIs can be found on the “Author Information” page.
**Disclaimer:** Articles published in the journal HemaSphere exclusively reflect the opinions of the authors. The authors are responsible for all content in their abstracts including accuracy of the facts, statements, citing resources, etc.

### (PS2030) MINI-A(B)VD AS FIRST-LINE TREATMENT FOR OLDER PATIENTS WITH CLASSICAL HODGKIN LYMPHOMA: RESULTS FROM A MULTICENTRE RETROSPECTIVE STUDY

**Topic:** 17. Hodgkin lymphoma - Clinical

Silvia Ferrari*[1], Cristina Muzi[1], Silvia Bolis[2], Annalisa Arcari[3], Emanuele Ravano[1], Elisa Bossi[2], Erika Meli[1], Erika Ravelli[1], Giovanni Rindone[2], Lorenzo Del Castello[4], Carlo Gambacorti-Passerini[2, 5], Davide Bernasconi[4, 6], Roberto Cairoli[1, 5], Vittorio Ruggero Zilioli[1]

*[1]Division of Hematology, ASST Grande Ospedale Metropolitano Niguarda, Milano, Italy; [2]Division of Hematology, IRCCS San Gerardo dei Tintori, Monza, Italy; [3]Division of Hematology, Ospedale Guglielmo da Saliceto, Piacenza, Italy;[4]Bicocca Bioinformatics Biostatistics and Bioimaging Centre - B4 School of Medicine and Surgery, University of Milano- Bicocca, Monza, Italy; [5]University of Milano-Bicocca, Milano, Italy; [6]Research Innovation and Technology Transfer, ASST Grande Ospedale Metropolitano Niguarda, Milano, Italy;*

### Background

Management of classical Hodgkin lymphoma (cHL) in older adults is challenging due to frailty, comorbidities, and treatment-related toxicities. While ABVD (Adriamycin, Bleomycin, Vinblastine, Dacarbazine) remains the reference regimen, anthracycline dose reduction and bleomycin omission are often required in this population. However, the real-world clinical impact of these dose modifications remains poorly defined.

### Aims

To assess the frequency of use and clinical outcomes of reduced-dose ABVD/ABVD-like regimens as first-line treatment in older patients with cHL. We further aimed to evaluate the median anthracycline dose administered at cycle 1 in reduced-dose regimens in order to define a “mini-A(B)VD” schedule. Additionally, we explored the prognostic role of geriatric parameters in treatment stratification.

### Methods

We conducted a retrospective, observational, multicentre study including 103 consecutive patients aged ≥ 60 years with newly diagnosed cHL treated with ABVD/ABVD-like regimens between 2010 and 2023 at three haematology centres in Italy. A “mini-A(B)VD” regimen was defined as <80% of the standard anthracycline dose (25 mg/m²) at cycle 1. The primary endpoint was the frequency of reduced-dose use. Secondary endpoints included median anthracycline dose at cycle 1, complete remission rate (CRR), progression-free survival (PFS), overall survival (OS), acute and late toxicities (graded according to CTCAE v5.0), and the prognostic impact of simplified geriatric assessment (sGA) incorporating Activities of Daily Living (ADL), Instrumental Activities of Daily Living (IADL), and the Cumulative Illness Rating Scale for Geriatrics (CIRS-G). Survival was estimated using Kaplan–Meier and Cox models.

**Copyright Information:** (Online) ISSN: 2572-9241
©2026 The Author(s). HemaSphere is published by John Wiley & Sons Ltd on behalf of European Hematology Association. This is an open access Abstract Book distributed under the Attribution-NonCommercial-NoDerivs (CC BY-NC-ND), which allows third parties to download the articles and share them with others as long as they credit the author and the Abstract Book, but they cannot change the content in any way or use them commercially.
**Abstract Book Citations:** Authors, Title, HemaSphere, 2026;10:(S1):pages. The abstract book DOIs can be found on the “Author Information” page.
**Disclaimer:** Articles published in the journal HemaSphere exclusively reflect the opinions of the authors. The authors are responsible for all content in their abstracts including accuracy of the facts, statements, citing resources, etc.

### Results

A total of 103 patients were included (median age 70 years; 45% stage IV), of whom 31 (30%) received reduced-dose therapy. These patients were significantly older and had higher comorbidity burden and greater functional impairment. In the reduced-dose group, the median anthracycline dose administered at cycle 1 was 18.6 mg/m², corresponding to a 25% reduction compared with standard dosing. Overall CRR was 79.6%, with no significant difference between full-dose and reduced-dose groups (80.9% vs 76.7%, p=0.5). Three- and five-year PFS were 69.0% and 53.2%, respectively, and three- and five-year OS were 92.6% and 81.7%, with no significant differences according to treatment intensity. Toxicity rates were comparable between groups. Haematologic toxicity was manageable. Pulmonary toxicity occurred mainly in bleomycin-exposed patients. Cardiac toxicity occurred in 17.5% of patients and was manageable, with no significant differences according to treatment intensity. Treatment-related mortality was 2.9%. In univariate analyses, higher comorbidity burden (CIRS-G) and frail status according to sGA were associated with inferior PFS, while functional impairment (ADL/IADL) and frail status strongly predicted worse OS.

### Summary/Conclusion

In older patients with cHL considered ineligible for full-dose therapy, mini-A(B)VD achieved efficacy outcomes comparable to standard ABVD with an acceptable safety profile, representing an effective and feasible treatment option in this population. Frailty and comorbidities, rather than chemotherapy dose intensity, emerged as the main drivers of outcome in older patients. Therefore, comprehensive geriatric assessment and comorbidity profiling are essential to guide therapeutic decision-making.

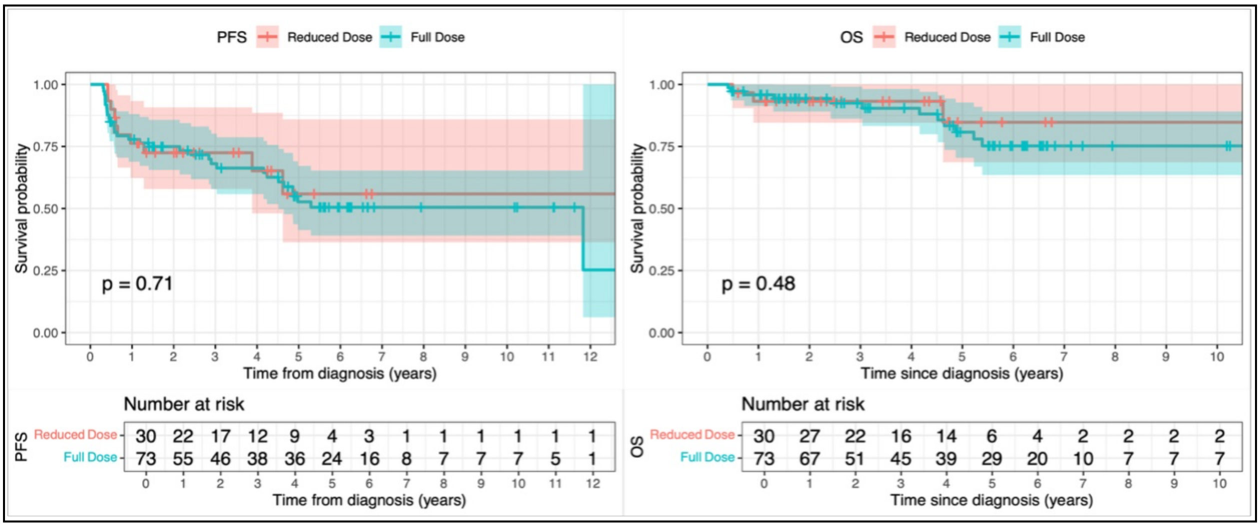

PFS and OS according to treatment intensity in older patients with cHL

**Copyright Information:** (Online) ISSN: 2572-9241
©2026 The Author(s). HemaSphere is published by John Wiley & Sons Ltd on behalf of European Hematology Association. This is an open access Abstract Book distributed under the Attribution-NonCommercial-NoDerivs (CC BY-NC-ND), which allows third parties to download the articles and share them with others as long as they credit the author and the Abstract Book, but they cannot change the content in any way or use them commercially.
**Abstract Book Citations:** Authors, Title, HemaSphere, 2026;10:(S1):pages. The abstract book DOIs can be found on the "Author Information" page.
**Disclaimer:** Articles published in the journal HemaSphere exclusively reflect the opinions of the authors. The authors are responsible for all content in their abstracts including accuracy of the facts, statements, citing resources, etc.

### (PS2031) NON-INVASIVE PROGNOSTICATION AND MINIMAL RESIDUAL DISEASE (MRD) MONITORING OF MYD88L265P IN IGM-GAMMOPATHIES: INSIGHT FROM THE “BIO-WM” PROSPECTIVE TRIAL OF THE FONDAZIONE ITALIANA LINFOMI (FIL)

**Topic:** 18. Indolent and mantle-cell non-Hodgkin lymphoma - Clinical

Daniela Drandi[1], Silvia Zibellini[2], Luigi Marcheselli[3], Enrico Amaducci[1], Chiara Varraso[2], Elisa Genuardi[1], Irene Dogliotti[4], Emilia Cappello[2], Federica Cavallo[1, 4], Camilla Sasso[1], Angela Ferrari[5], Benedetta Bianchi[6], Marina Deodato[7], Luca Laurenti[8], Patrizia Tosi[9], Emanuele Cencini[10], Giacomo Loseto[11], Dario Marino[12], Monia Marchetti[13, 14], Filomena Russo[15], Antonello Sica[16], Jacopo Olivieri[17], Gianmarco Favrin[2], Cristina Jiménez Sánchez[18], Noemi Puig[18], Luca Arcaini[2], Benedetto Bruno[1, 4], Ramón García-Sanz[19], Simone Ferrero*[1, 4], Marzia Varettoni[2]

*[1]Hematology, Department of Molecular Biotechnologies and Health Sciences, University of Torino, Torino, Italy;[2]S.C. Ematologia 1, Fondazione IRCCS Policlinico San Matteo, Pavia, Italy; [3]Fondazione Italiana Linfomi, Clinical Trial Office, Modena, Italy; [4]Division of Hematology, A.O.U. Città della Salute e della Scienza di Torino, Torino, Italy;[5]Ematologia, A.O. Arcispedale Santa Maria Nuova - IRCCS, Reggio Emilia, Italy; [6]U.O.C. Ematologia, Ospedale di Circolo e Fondazione Macchi – ASST Sette Laghi, Varese, Italy; [7]Department of Hematology, ASST Grande Ospedale Metropolitano Niguarda, Niguarda Cancer Center, Milano, Italy; [8]Ematologia - Università Cattolica S. Cuore, Roma, Italy; [9]Ematologia, Ospedale degli Infermi di Rimini, Rimini, Italy; [10]U.O.C. Ematologia, AOU Senese, Siena, Italy; [11]U.O.C. Ematologia, IRCCS Istituto Tumori Giovanni Paolo II, Bari, Italy; [12]U.O.S.D. Trapianto di cellule staminali, Istituto Oncologico Veneto IOV, IRCCS, Padova, Italy;[13]S.O.S. Ematologia, Ospedale Cardinal Massaia, Asti, Italy; [14]Hematology, Unit, University Hospital SS Antonio e Biagio e Cesare Arrigo, Alessandria, Italy; [15]U.O. Ematologia e CTMO, Azienda Ospedaliera Universitaria di Parma, Parma, Italy;[16]Oncologia Medica ed Ematologia, A.O.U. Università degli Studi della Campania Luigi Vanvitelli, Napoli, Italy; [17]Clinica Ematologica, Centro Trapianti e Terapie Cellulari "Carlo Melzi", Azienda Sanitaria Universitaria Friuli Centrale, Udine, Italy; [18]Hospital Universitario de Salamanca (HUSAL), IBSAL, IBMCC (USAL-CSIC), CIBERONC, Salamanca, Spain; [19]Department of Hematology, Hospital General Universitario Gregorio Marañón, CIBERONC, IiSGM, Madrid, Spain;*

### Background

*MYD88*$^{L265P}$ is the hallmark mutation in IgM-gammopathies traditionally assessed through bone marrow (BM) aspirate, an invasive and uncomfortable procedure for patients.

### Aims

The multicenter “BIO-WM” trial (NCT03521596) of the Fondazione Italiana Linfomi (FIL) evaluated droplet digital PCR (ddPCR) for *MYD88*$^{L265P}$ screening and minimal residual disease (MRD) monitoring in minimally invasive specimens (peripheral blood, PB, and plasma-derived cell-free DNA, cfDNA) versus the gold standard BM aspirate.

### Methods

In a retro-prospective series of 300 Waldenstroem Macroglobulinemia (WM) and IgM-monoclonal gammopathy of undetermined significance (MGUS) patients 274 paired BM, PB, and plasma samples were analyzed for *MYD88*$^{L265P}$ by ddPCR at diagnosis and post-treatment. For survival analysis, MRD data were considered as measured in cfDNA or, when not available, in BM.

**Copyright Information:** (Online) ISSN: 2572-9241
©2026 The Author(s). HemaSphere is published by John Wiley & Sons Ltd on behalf of European Hematology Association. This is an open access Abstract Book distributed under the Attribution-NonCommercial-NoDerivs (CC BY-NC-ND), which allows third parties to download the articles and share them with others as long as they credit the author and the Abstract Book, but they cannot change the content in any way or use them commercially.
**Abstract Book Citations:** Authors, Title, HemaSphere, 2026;10:(S1):pages. The abstract book DOIs can be found on the “Author Information” page.
**Disclaimer:** Articles published in the journal HemaSphere exclusively reflect the opinions of the authors. The authors are responsible for all content in their abstracts including accuracy of the facts, statements, citing resources, etc.

### Results

*MYD88*$^{L265P}$ rates and median mutational burden in cfDNA closely mirrored those of BM specimens in WM (91% vs. 96% and 1.4E-02 vs 3E-02, respectively), while genomic DNA from PB cells showed overall lower sensitivity and mutational burden (85% and 1.2E-03, respectively). Similar results were described across tissues in IgM-MGUS, but with significantly lower rates (75% in BM, 58% in PB and 67% in cfDNA) and abundance of *MYD88*$^{L265P}$ mutations (median levels: 1.5E-03 in BM, 8E-04 in PB and 2.6E-03 in cfDNA). Interestingly, the integration of baseline cfDNA *MYD88*$^{L265P}$ levels with serum IgM values by classification & regression trees (CART) models might be able to discriminate WM from IgM-MGUS and to predict the risk of treatment initiation without BM examination. Essentially, patients with a cfDNA *MYD88*$^{L265P}$ > 5.9 displayed a 94% probability of being affected by WM, while patients with a cfDNA *MYD88*$^{L265P}$ < 5.9 and IgM values < 2100 had a 70% probability of being affected by IgM-MGUS. Moreover, patients with basal IgM values < 2100 underwent treatment in 15% of cases if the cfDNA *MYD88*$^{L265P}$ was < 6.9 but in 90% if the value was > 6.9. Finally, MRD was prospectively evaluated tracking *MYD88*$^{L265P}$ in 60 WM patients after chemo-immunotherapy. Progressively increasing MRD negativity rates were observed across different tissues: 33% (17/51) in BM, 55% (29/53) in cfDNA and 91% (52/57) in PB, respectively (P<0.001). Of note, patients in a complete (CR) or very good partial response (VGPR) were far more likely to reach MRD- (69%, 11/16) compared to those achieving a partial (PR) or minimal response (MR) (16%, 16/38). Moreover, MRD negativity was obtained more frequently in patients under bendamustine-rituximab treatment (43%, 12/28) compared to those receiving dexamethasone, rituximab and cyclophosphamide (9%, 2/23). Most importantly, MRD negativity strongly predicted improved progression-free survival (4-year PFS: 93% in MRD- vs. 62% in MRD+, p=0.018), only an initial trend towards overall survival advantage was reported (4-year OS: 94% in MRD- vs. 83% in MRD+, p=0.264). Interestingly, MRD evaluation outperformed conventional clinical response criteria as outcome prognosticator in terms of PFS: HR 3.44 (1.03-11.5, p=0.045) for MRD+ vs 2.59 (0.77-8.71, p=0.123) for <VGPR, see Table 1.

### Summary/Conclusion

These results provide for the first-time prospective, compelling evidence for the utility and clinical impact of *MYD88*$^{L265P}$ assessment by ddPCR as a simple and robust non-invasive tool for diagnosis, outcome prediction and MRD monitoring in IgM gammopathies, supporting its integration into routine clinical practice and future trial design.

**Copyright Information:** (Online) ISSN: 2572-9241
©2026 The Author(s). HemaSphere is published by John Wiley & Sons Ltd on behalf of European Hematology Association. This is an open access Abstract Book distributed under the Attribution-NonCommercial-NoDerivs (CC BY-NC-ND), which allows third parties to download the articles and share them with others as long as they credit the author and the Abstract Book, but they cannot change the content in any way or use them commercially.
**Abstract Book Citations:** Authors, Title, HemaSphere, 2026;10:(S1):pages. The abstract book DOIs can be found on the "Author Information" page.
**Disclaimer:** Articles published in the journal HemaSphere exclusively reflect the opinions of the authors. The authors are responsible for all content in their abstracts including accuracy of the facts, statements, citing resources, etc.

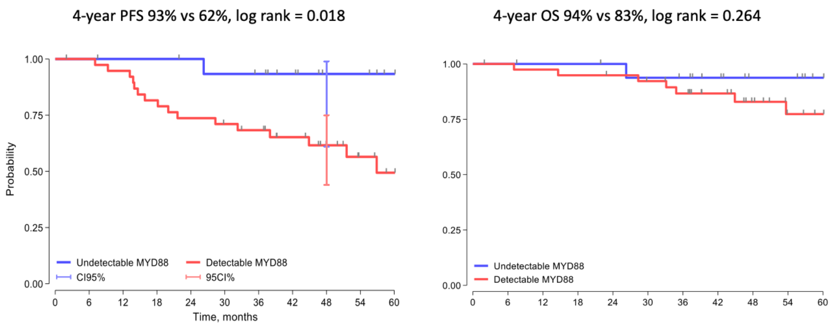

**Figure 1.** Landmark progression-free and overall survival analysis stratified by minimal residual disease at the end of treatment

| n = 55 | n | | 4-yr PFS (95%CI) | HR (95%CI) | P (Wald) | c-Harrell |
|---|---|---|---|---|---|---|
| Response | 16 | CR/VGPR | 87 (57-96) | 1.00 | | |
| | 41 | <VGPR | 60 (43-73) | 2.59 (0.77-8.71) | 0.123 | 56.6 |
| MYD88 | 18 | Negative | 88 (59-97) | 1.00 | | |
| | 39 | Positive | 58(41-72) | 3.44 (1.03-11.5) | 0.045 | 60.6 |

**Table 1**. Progression-free survival according to MRD status and clinical response at the end of treatment

Figure 1. Landmark progression-free and overall survival analysis stratified by minimal residual disease at the end of treatment

Copyright Information: (Online) ISSN: 2572-9241
©2026 The Author(s). HemaSphere is published by John Wiley & Sons Ltd on behalf of European Hematology Association. This is an open access Abstract Book distributed under the Attribution-NonCommercial-NoDerivs (CC BY-NC-ND), which allows third parties to download the articles and share them with others as long as they credit the author and the Abstract Book, but they cannot change the content in any way or use them commercially.
Abstract Book Citations: Authors, Title, HemaSphere, 2026;10:(S1):pages. The abstract book DOIs can be found on the "Author Information" page.
Disclaimer: Articles published in the journal HemaSphere exclusively reflect the opinions of the authors. The authors are responsible for all content in their abstracts including accuracy of the facts, statements, citing resources, etc.

### (PS2033) BGB-16673, A BRUTON TYROSINE KINASE (BTK) DEGRADER, IN PATIENTS WITH RELAPSED/REFRACTORY (R/R) WALDENSTRÖM MACROGLOBULINEMIA (WM): A PHASE 1 CADANCE-101 STUDY UPDATE

**Topic:** 18. Indolent and mantle-cell non-Hodgkin lymphoma - Clinical

Judith Trotman*[1], Chan Y. Cheah[2, 3, 4], John F. Seymour[5], Constantine S. Tam[6], Ricardo D. Parrondo[7], Anna Maria Frustaci[8], Damien Roos-Weil[9], Eugen Tausch[10], Anne-Sophie Michallet[11], Georg Heß[12], Franziska Bach[13], Herbert Eradat[14], Yanan Zhang[15], Linlin Xu[15], Kaiyuan Hua[15], Shannon Fabre[15], Motohisa Takai[15], Shelonitda Rose[15], Mazyar Shadman[16, 17]

*[1]Concord Repatriation General Hospital, University of Sydney, Concord, NSW, Australia;[2]Sir Charles Gairdner Hospital, Nedlands, WA, Australia; [3]Medical School, University of Western Australia, Crawley, WA, Australia;[4]Linear Clinical Research, Nedlands, WA, Australia; [5]Peter MacCallum Cancer Centre, Royal Melbourne Hospital, and University of Melbourne, Melbourne, VIC, Australia; [6]Alfred Hospital and Monash University, Melbourne, VIC, Australia; [7]Mayo Clinic - Jacksonville, Jacksonville, FL, United States of America; [8]ASST Grande Ospedale Metropolitano Niguarda, Milano, Italy; [9]Pitié-Salpêtrière Hospital, Paris, France; [10]Ulm University, Ulm, Germany; [11]Centre Léon Bérard, Lyon, France; [12]Department of Hematology and Medical Oncology, University Medical School Mainz, Mainz, Germany; [13]University of Cologne, Center for Integrated Oncology Aachen Bonn Köln Düsseldorf, Cologne, Germany; [14]David Geffen School of Medicine at UCLA, Los Angeles, CA, United States of America; [15]BeOne Medicines, Ltd, San Carlos, CA, United States of America; [16]Fred Hutchinson Cancer Center, Seattle, WA, United States of America; [17]University of Washington, Seattle, WA, United States of America;*

### Background

BGB-16673 is a BTK degrader that blocks signaling by tagging BTK for degradation through the cell's proteasome pathway, leading to tumor regression. The ongoing open-label, phase 1/2 CaDAnCe-101 (BGB-16673-101; NCT05006716) study is evaluating BGB-16673 monotherapy in patients (pts) with B-cell malignancies.

### Aims

To report updated safety and efficacy results of BGB-16673 in pts with R/R WM in the phase 1 portion of CaDAnCe-101.

### Methods

Eligible pts had confirmed R/R WM (≥2 prior therapies); an ECOG performance status of 0-2 (0/1 in the EU); and previous treatment (tx) with an anti-CD20 antibody and a covalent BTK inhibitor (BTKi). BGB-16673 was administered orally once daily. The primary objectives were to determine the safety/tolerability (NCI-CTCAE v5.0), maximum tolerated dose, and recommended dose for expansion. A key secondary objective was the overall response rate (ORR, defined as ≥minor response; IWWM-11 consensus criteria); the major response rate (MRR) was defined as ≥partial response.

**Copyright Information:** (Online) ISSN: 2572-9241
©2026 The Author(s). HemaSphere is published by John Wiley & Sons Ltd on behalf of European Hematology Association. This is an open access Abstract Book distributed under the Attribution-NonCommercial-NoDerivs (CC BY-NC-ND), which allows third parties to download the articles and share them with others as long as they credit the author and the Abstract Book, but they cannot change the content in any way or use them commercially.
**Abstract Book Citations:** Authors, Title, HemaSphere, 2026;10:(S1):pages. The abstract book DOIs can be found on the “Author Information” page.
**Disclaimer:** Articles published in the journal HemaSphere exclusively reflect the opinions of the authors. The authors are responsible for all content in their abstracts including accuracy of the facts, statements, citing resources, etc.

### Results

As of the Dec 15, 2025, data cutoff, 43 pts with R/R WM received BGB-16673 (100 mg, n=15; 200 mg, n=15; 350 mg, n=13). Median age was 72 (range, 46-81) years, and median number of prior therapies was 3 (range, 2-11). Prior therapies included an anti-CD20 antibody (n=43 [100%]), covalent BTKi (n=43 [100%]), noncovalent BTKi (n=7 [16.3%]), BCL2 inhibitor (n=10 [23.3%]), chemotherapy (n=40 [93.0%]), and proteasome inhibitor (n=14 [32.6%]); 36 pts (83.7%) discontinued prior BTKi due to progressive disease (PD). Baseline mutation status is shown (Table). Median study follow-up was 14.3 (range, 0.2-37.3) months. At data cutoff, 26 pts (60.5%) remained on tx; of the 17 pts who discontinued, the primary reason for discontinuation was PD in 6 pts (35.3%). Overall, 95.3% of pts experienced any-grade tx-emergent adverse events (TEAEs; grade ≥3, 60.5%). The most common any-grade TEAEs in ≥25% of pts were neutropenia/neutrophil count decreased (37.2%), diarrhea (30.2%), and contusion (27.9%). Grade ≥3 TEAEs in ≥10% of pts included neutropenia/neutrophil count decreased (34.9%) and anemia (14.0%). Eight pts (18.6%) had a grade ≥3 infection. No atrial fibrillation occurred. Febrile neutropenia and major hemorrhage (grade 3 hematemesis) occurred in 1 pt (2.3%) each. Five pts (11.6%) had TEAEs that led to tx discontinuation, and 3 (7.0%) pts had TEAEs that led to dose reduction. As previously reported, 3 pts (7.0%) died due to TEAEs.
Among the 42 response-evaluable pts, the ORR was 85.7% (n=36), the MRR was 76.2% (n=32), and the very good partial response rate was 31.0% (n=13). Median time to first overall response was 1.0 (range, 0.9-8.2) months and to best overall response was 2.8 (range, 1.0-12.2) months. Responses were also observed in pts with high-risk features, including *BTK* mutations (Table), and in 29/35 pts (82.9%) who discontinued a prior BTKi due to PD. After a progression-free survival (PFS) median follow-up of 16.6 (95% CI, 13.8-19.5) months, the estimated 15-month PFS rate was 70.4% (95% CI, 52.6%-82.5%).

### Summary/Conclusion

These phase 1 data from the CaDAnCe-101 study demonstrate that the novel BTK degrader BGB-16673 has a manageable safety profile with substantial antitumor activity in heavily pretreated pts with R/R WM, including pts with WM bearing *BTK*, *CXCR4*, and *TP53* mutations. Responses deepened over time, with early evidence of response durability. The phase 2 portion of the study is actively enrolling.

**Table. Response rate by mutation status**

| Mutation status[a] | Patients, n/N (%) | ORR, n/N (%) | MRR, n/N (%) |
|---|---|---|---|
| *BTK* mutated | 13/42 (31.0) | 13/13 (100) | 13/13 (100) |
| *MYD88* mutated | 33/42 (78.6) | 29/33 (87.9) | 25/33 (75.8) |
| *CXCR4* mutated | 19/42 (45.2) | 19/19 (100) | 16/19 (84.2) |
| *TP53* mutated | 23/42 (54.8) | 21/23 (91.3) | 20/23 (87.0) |
| *PLCG2* mutated | 3/42 (7.1) | 3/3 (100) | 2/3 (66.7) |

[a]Mutation status was unknown in 1 patient.
BTK, Bruton tyrosine kinase; ORR, overall response rate (≥minor response); MRR, major response rate (≥partial response).

**Copyright Information:** (Online) ISSN: 2572-9241
©2026 The Author(s). HemaSphere is published by John Wiley & Sons Ltd on behalf of European Hematology Association. This is an open access Abstract Book distributed under the Attribution-NonCommercial-NoDerivs (CC BY-NC-ND), which allows third parties to download the articles and share them with others as long as they credit the author and the Abstract Book, but they cannot change the content in any way or use them commercially.
**Abstract Book Citations:** Authors, Title, HemaSphere, 2026;10:(S1):pages. The abstract book DOIs can be found on the “Author Information” page.
**Disclaimer:** Articles published in the journal HemaSphere exclusively reflect the opinions of the authors. The authors are responsible for all content in their abstracts including accuracy of the facts, statements, citing resources, etc.

### (PS2034) REAL WORLD ANALYSIS OF PIRTOBRUTINIB IN RELAPSED/REFRACTORY MANTLE CELL LYMPHOMA (RRMCL) – AN ANALYSIS FROM THE EUROPEAN MANTLE CELL LYMPHOMA-REGISTRY (EMCL-R)

**Topic:** 18. Indolent and mantle-cell non-Hodgkin lymphoma - Clinical

Linda Simon*[1], Friedrich Schwarz[2], Patrick Marschner[3], Peter Dreger[4], Markus Maulhardt[2], Mareike Tometten[5], Corinna Leng[6], Andrea Kerkhoff[7], Maria Ramos Lopes Gomes Da Silva[8], Martin Dreyling[9], Vladan Vucinic[10], Thomas Melchardt[11], Christiane Pott[12], Ron Jachimowicz[13], Fabian Müller[14], Martin Kaufmann[15], Simon Weisemann[16], Hans C. Reinhardt[17], Marie-Kristin Tilch[1], Stepanie Herold[1], Anke Ohler[1], Georg Heß[1], Enver Aydilek[18]

*[1]University Medical Center, Johannes Gutenberg University, Hematology and medical Oncology, Mainz, Germany;[2]Department for Hematology and Medical Oncology, University Medical Center Göttingen, Göttingen, Germany; [3]Medical Department, iOMEDICO, Freiburg, Germany., Freiburg, Germany; [4]4. Department of Hematology, Oncology and Rheumatology, Heidelberg University Hospital, Heidelberg, Germany; [5]Department of Hematology, Oncology, Hemostaseology and Stem Cell Transplantation, Medical Faculty, RWTH Aachen, Aachen, Germany; [6]Med. Department of Hematology, Oncology and Cancer Immunology, Charité -Universitätsmedizin Berlin, Berlin, Germany; [7]Department for Medicine A, Hematology, Oncology, Hemostaseology and Pneumology, University Hospital Muenster, Muenster, Germany; [8]Instituto Portugues de Oncologia de Lisboa, Lisboa, Portugal; [9]Department of Medicine III, LMU Hospital, Munic, Germany; [10]2. Department of Hematology, Cellular Therapy, Hemostaseology and Infectious Diseases, University of Leipzig, Leipzig, Germany; [11]Third Medical Department with Hematology, Medical Oncology, Rheumatology and Infectiology, Paracelsus Medical University, Salzburg, Austria; [12]Second Medical Department, University Hospital Schleswig-Holstein, Campus Kiel, Kiel, Germany; [13]University Hospital Cologne & Max Planck Institute For Biology Of Ageing, Cologne, Germany; [14]University Hospital of Erlangen, Friedrich-Alexander University of Erlangen-Nuremberg (FAU), Erlangen, Erlangen, Germany; [15]Department for Hematology, Oncology and Palliative Care, Robert-Bosch-Hospital, Stuttgart, Germany; [16]Asclepius Clinic Hamburg, Department for Oncology and Palliative Care, Hamburg, Germany; [17]Department of Hematology and Stem Cell Transplantation, University Hospital Essen, Essen, Germany; [18]Evangelisches Klinikum Bethel, University Hospital Bielefeld, Bielefeld, Germany;*

### Background

MCL is a rare and generally incurable B-cell lymphoma. Relapse and resistance to BTK inhibitors (BTKi) is associated with poor survival. Based on the BRUIN-trial demonstrating a median PFS of 5.6 and median OS of 23.5 months, Pirtobrutinib, a non-covalent BTKi binding reversibly both to wild-type and C481S-mutant BTK has been approved for rrMCL. However, until now there is limited real world-data (RWD) on the efficacy and a comparison to standard options, which we addressed herein.

### Aims

To compare RWD from the compassionate use program of Pirtobrutinib in r/r MCL with a comparator cohort from the EMCL-R (EV Ref. Nr. 2018-13856).

### Methods

A retrospective analysis was conducted with data from the EMCL-R, including all patients having received Pirtobrutinib outside the BRUIN trial, data cut-off was December 2025. We evaluated preliminary tolerability, response, progression and overall survival comparing the different groups using multivariate Cox models. A separate analysis comprised only patients that received Pirtobrutinib as bridging to consolidative CAR-T-therapy. Furthermore, we generated a comparator cohort with alternative treatments after Ibrutinib failure matched for age, sex, risk scores, tumor biology and progression of disease (POD) 6 and 12 after Ibrutinib initiation.

**Copyright Information:** (Online) ISSN: 2572-9241
©2026 The Author(s). HemaSphere is published by John Wiley & Sons Ltd on behalf of European Hematology Association. This is an open access Abstract Book distributed under the Attribution-NonCommercial-NoDerivs (CC BY-NC-ND), which allows third parties to download the articles and share them with others as long as they credit the author and the Abstract Book, but they cannot change the content in any way or use them commercially.
**Abstract Book Citations:** Authors, Title, HemaSphere, 2026;10:(S1):pages. The abstract book DOIs can be found on the “Author Information” page.
**Disclaimer:** Articles published in the journal HemaSphere exclusively reflect the opinions of the authors. The authors are responsible for all content in their abstracts including accuracy of the facts, statements, citing resources, etc.

**Results**

We included 129 Pirtobrutinib patients and 79 in the comparative cohort. For Pirtobrutinib, median prior lines were 3 and most presented with high-risk biology (Ki67 ≥30% in 79%, *TP53* mutation in 55%, high MIPI in 54%) at start of treatment. All patients had previous BTKi exposition and 30% failed within 6 months of initiation. With a median follow-up time of 10.2 months (range 8.0 – 11.5), overall response rate (ORR) was 40%, while median PFS and OS were 4.2 and 12.7 months, respectively. In univariate analysis, Ki67 > 50%, high-risk MIPI and prior CAR-T therapy but not Ibrutinib failure were associated with inferior OS. In the comparative analysis, median PFS was 1.5 months for the current standard of care (SoC) vs. 3.5 for Pirtobrutinib (p <0.001, HR 0.4). Among patients that received subsequent CAR-T therapy (37%) median PFS was 5.5 months and median OS was not reached. The CAR-T infusion rate was 62%. The ORR improved in 74% of documented cases after bridging with Pirtobrutinib (PR – CR: n=12, SD-CR: n=2 and PD-PR/CR: n=6). One third of patients had at least one adverse event (AE) grade ≥ 3. Infections accounted for 41% and hematologic toxicities for 28% of all grade ≥3 AEs. Four AEs of grade 5 occurred, however deaths were dominantly attributable to disease progression.

**Summary/Conclusion**

To our knowledge, this is the largest real-world data analysis of r/r MCL patients to assess efficacy and tolerability of Pirtobrutinib. Survival and ORR were lower as compared to BRUIN likely due to patient selection and higher prevalence of high-risk biology in our cohort. Importantly, our data confirm efficacy in patients refractory to prior BTKi. Whether pre-treatment with Pirtobrutinib improves response to CAR-T-cell therapy, as suggested from our data, needs further exploration. Evaluation of safety suggest better tolerability of Pirtobrutinib as compared to data for other BTKi.
In summary, our data support that Pirtobrutinib imposes as a safe and effective therapy after BTKi failure. Further evidence from either randomized or prospective registry-based analysis may include the direct comparison to CAR-T therapy and the impact on CAR-T infusion rates if systemically used as bridging therapy.

**Copyright Information:** (Online) ISSN: 2572-9241
©2026 The Author(s). HemaSphere is published by John Wiley & Sons Ltd on behalf of European Hematology Association. This is an open access Abstract Book distributed under the Attribution-NonCommercial-NoDerivs (CC BY-NC-ND), which allows third parties to download the articles and share them with others as long as they credit the author and the Abstract Book, but they cannot change the content in any way or use them commercially.
**Abstract Book Citations:** Authors, Title, HemaSphere, 2026;10:(S1):pages. The abstract book DOIs can be found on the "Author Information" page.
**Disclaimer:** Articles published in the journal HemaSphere exclusively reflect the opinions of the authors. The authors are responsible for all content in their abstracts including accuracy of the facts, statements, citing resources, etc.

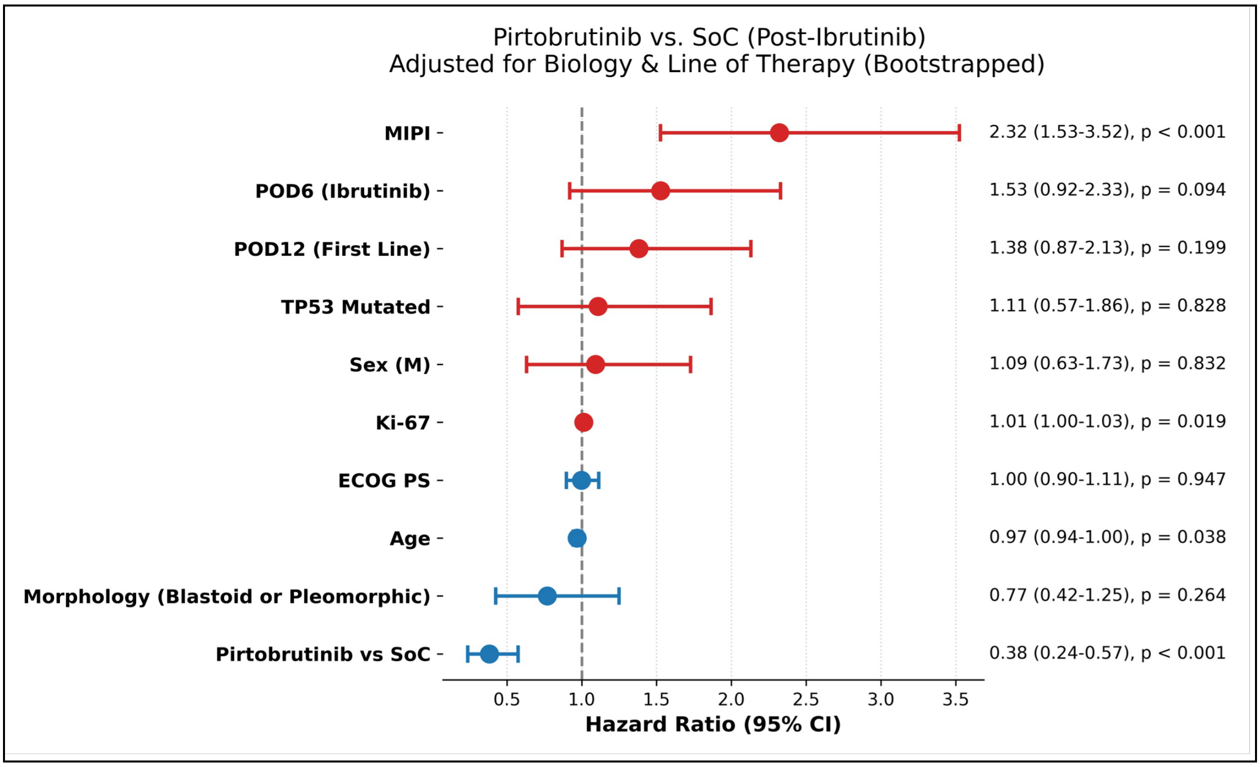

Figure 1: Multivariate Cox analysis for Pirtobrutinib vs. SoC Post-Ibrutinib

**Copyright Information:** (Online) ISSN: 2572-9241
©2026 The Author(s). HemaSphere is published by John Wiley & Sons Ltd on behalf of European Hematology Association. This is an open access Abstract Book distributed under the Attribution-NonCommercial-NoDerivs (CC BY-NC-ND), which allows third parties to download the articles and share them with others as long as they credit the author and the Abstract Book, but they cannot change the content in any way or use them commercially.
**Abstract Book Citations:** Authors, Title, HemaSphere, 2026;10:(S1):pages. The abstract book DOIs can be found on the "Author Information" page.
**Disclaimer:** Articles published in the journal HemaSphere exclusively reflect the opinions of the authors. The authors are responsible for all content in their abstracts including accuracy of the facts, statements, citing resources, etc.

### (PS2035) ANCHORED MATCHING-ADJUSTED INDIRECT COMPARISON OF EPCORITAMAB, LENALIDOMIDE, AND RITUXIMAB VS TAFASITAMAB, LENALIDOMIDE, AND RITUXIMAB IN RELAPSED/REFRACTORY FOLLICULAR LYMPHOMA: EPCORE FL-1 VS INMIND

**Topic:** 18. Indolent and mantle-cell non-Hodgkin lymphoma - Clinical

Kim Linton*[1], Moshe Y. Levy[2], Dai Maruyama[3], Abel Costa Neto[4], Shane Gangatharan[5], Habte A. Yimer[6], Abualbishr Alshreef[7], Wenying Quan[8], Viktor Chirikov[8], Gauri Sunkersett[7], Jian Mei[7], Anthony Wang[7], Savreet Bains Chawla[9], Laura Liao[9], Felipe Marques Goncalves[9], Zhijie Ding[9], Marcel Nijland[10]

*[1]The Christie NHS Foundation Trust, Manchester Cancer Research Centre, and Division of Cancer Sciences, University of Manchester, Manchester, United Kingdom; [2]Texas Oncology–Tyler, US Oncology Research, Tyler, United States of America; [3]Cancer Institute Hospital, Japanese Foundation for Cancer Research, Tokyo, Japan;[4]D'or Institute for Research and Education (IDOR), Department of Hematology, São Paulo, Brazil; [5]Fiona Stanley Hospital, Murdoch, Western Australia, Australia;[6]Texas Oncology-Tyler, US Oncology Research, Tyler, United States of America; [7]AbbVie, Inc., North Chicago, United States of America; [8]OPEN Health, New York, United States of America;[9]Genmab US Inc., Plainsboro, United States of America; [10]University Medical Center Groningen and University of Groningen, Groningen, The Netherlands;*

### Background

The ongoing phase 3 EPCORE FL-1 trial (NCT05409066) compared 12-cycle fixed-duration epcoritamab (epcor) plus rituximab and lenalidomide ($R^2$) vs $R^2$ in relapsed/refractory follicular lymphoma (R/R FL) after ≥1 prior lines of therapy (LOTs). Epcor + $R^2$ resulted in a 79% reduction in risk of progression or death and a higher rate of complete responses (CRs) vs $R^2$ alone along with a manageable safety profile, leading to regulatory approval for this indication. The efficacy and safety of tafasitamab (tafa), a humanized CD19-directed monoclonal antibody, plus $R^2$ after ≥1 LOT in patients with R/R FL have been assessed in the phase 3 inMIND trial (NCT04680052), supporting its regulatory approval.

### Aims

In the absence of head-to-head clinical trials, we conducted an anchored matching-adjusted indirect comparison (MAIC) to estimate the comparative efficacy of epcor + $R^2$ vs tafa + $R^2$ to treat R/R FL.

### Methods

Individual patient data from the intention-to-treat (ITT) populations of EPCORE FL-1 (epcor + $R^2$ and $R^2$); data cutoff: 24 May 2025; median follow-up: 14.8 mo) for epcor + $R^2$ and from inMIND (data cutoff: 23 Feb 2024; median follow-up: 14.1 mo) for tafa + $R^2$ were used for this analysis. An anchored MAIC approach was used with $R^2$ as the common comparator arm. The EPCORE FL-1 trial population was statistically rebalanced using propensity score weights to match the distributions of 5 treatment-effect modifiers that were empirically tested in EPCORE FL-1 and selected in the context of clinical opinion in inMIND: age ≥65 y, ethnicity, Follicular Lymphoma International Prognostic Index score of 3–5, refractory to last treatment, and prior treatment with $R^2$. Anchored odds ratios (ORs) and 95% confidence intervals (CIs) were estimated for overall response rates (ORRs) and CR rates using weighted regression models. Kaplan-Meier plots of progression-free survival (PFS) and time to next treatment (TTNT) were compared, with anchored hazard ratios (HRs) and 95% CIs estimated using weighted stratified Cox proportional hazard models.

**Copyright Information:** (Online) ISSN: 2572-9241
©2026 The Author(s). HemaSphere is published by John Wiley & Sons Ltd on behalf of European Hematology Association. This is an open access Abstract Book distributed under the Attribution-NonCommercial-NoDerivs (CC BY-NC-ND), which allows third parties to download the articles and share them with others as long as they credit the author and the Abstract Book, but they cannot change the content in any way or use them commercially.
**Abstract Book Citations:** Authors, Title, HemaSphere, 2026;10:(S1):pages. The abstract book DOIs can be found on the “Author Information” page.
**Disclaimer:** Articles published in the journal HemaSphere exclusively reflect the opinions of the authors. The authors are responsible for all content in their abstracts including accuracy of the facts, statements, citing resources, etc.

**Results**

After match-adjustment (effective sample size: 208 for epcor + $R^2$; 225 for $R^2$ from the EPCORE FL-1 trial), the ITT populations were well balanced. Epcor + $R^2$ demonstrated a significantly higher likelihood of achieving ORR (94.3% vs 83.5%; OR 2.48; 95% CI 1.12–5.53; *P*=0.03) vs tafa + $R^2$. A significantly higher likelihood of achieving CR was also observed with epcor + $R^2$ vs tafa + $R^2$ (81.2% vs 52.0%; OR 2.94; 95% CI 1.69–5.13; *P*<0.001). Epcor + $R^2$ demonstrated significant PFS benefit over tafa+ $R^2$ (HR 0.54; 95% CI 0.32–0.91; *P*=0.02) (**Figure 1A**). The 24-mo PFS was 72.2% with epcor + $R^2$ and 54.4% with tafa + $R^2$. Epcor + $R^2$ also demonstrated a significant improvement in TTNT (HR 0.50; 95% CI 0.27–0.91; *P*=0.02), with 24-mo TTNT rates of 88.1% and 78.0%, respectively (**Figure 1B**).

**Summary/Conclusion**

Findings from this anchored MAIC demonstrate that epcor + $R^2$ is associated with significantly higher ORR and CR and improved PFS and TTNT compared with tafa + $R^2$ in patients with R/R FL. Safety findings should be interpreted within the context of the respective trials. In the context of evolving phase 3 data beyond $R^2$-based therapy, these findings position epcor + $R^2$ as the new chemotherapy-free standard of care in R/R FL.

**Copyright Information:** (Online) ISSN: 2572-9241
©2026 The Author(s). HemaSphere is published by John Wiley & Sons Ltd on behalf of European Hematology Association. This is an open access Abstract Book distributed under the Attribution-NonCommercial-NoDerivs (CC BY-NC-ND), which allows third parties to download the articles and share them with others as long as they credit the author and the Abstract Book, but they cannot change the content in any way or use them commercially.
**Abstract Book Citations:** Authors, Title, HemaSphere, 2026;10:(S1):pages. The abstract book DOIs can be found on the "Author Information" page.
**Disclaimer:** Articles published in the journal HemaSphere exclusively reflect the opinions of the authors. The authors are responsible for all content in their abstracts including accuracy of the facts, statements, citing resources, etc.

**Figure 1.** Anchored MAIC results. (A) PFS and (B) TTNT for epcor + $R^2$ vs tafa + $R^2$. adj, adjusted; IRC, independent review committee; unadj, unadjusted.

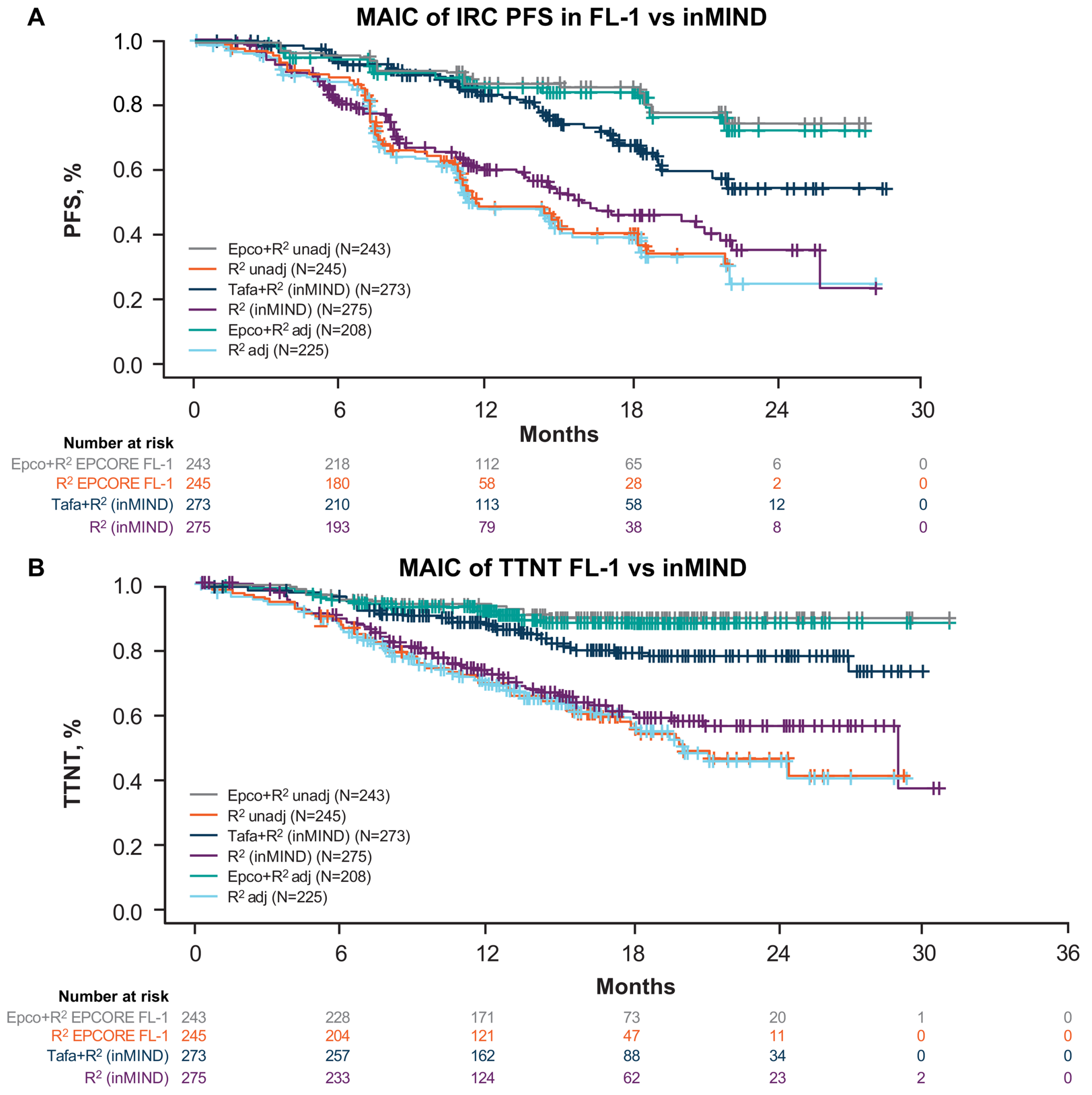

**Copyright Information:** (Online) ISSN: 2572-9241
©2026 The Author(s). HemaSphere is published by John Wiley & Sons Ltd on behalf of European Hematology Association. This is an open access Abstract Book distributed under the Attribution-NonCommercial-NoDerivs (CC BY-NC-ND), which allows third parties to download the articles and share them with others as long as they credit the author and the Abstract Book, but they cannot change the content in any way or use them commercially.
**Abstract Book Citations:** Authors, Title, HemaSphere, 2026;10:(S1):pages. The abstract book DOIs can be found on the "Author Information" page.
**Disclaimer:** Articles published in the journal HemaSphere exclusively reflect the opinions of the authors. The authors are responsible for all content in their abstracts including accuracy of the facts, statements, citing resources, etc.

### (PS2036) CONTEMPORARY TREATMENT AND SURVIVAL OUTCOMES IN 1,101 FOLLICULAR LYMPHOMA PATIENTS INCLUDED IN REALYSA, A PROSPECTIVE REAL-WORLD MULTICENTRIC LYSA COHORT

**Topic:** 18. Indolent and mantle-cell non-Hodgkin lymphoma - Clinical

Anne Loap*[1], Catherine Thieblemont[2], Aurelien Belot[3], Corinne Haioun[4], Fontanet Bijou[5], Cedric Rossi[6], Luc-Matthieu Fornecker[7], Loic Ysebaert[8], Julie Abraham[9], Kamal Bouabdallah[10], Remy Gressin[11], Fanny Cherblanc[3], Alice Pauc[3], Bastien Lesne[3], Nadine Morineau[12], Adrien Chauchet[13], Stephanie Guidez[14], Fabrice Jardin[15], Ludovic Fouillet[16], Gaelle Laboure[17], Clémentine Sarkozy[1], Herve Ghesquieres[18]

*[1]Institut Curie, Saint Cloud, France; [2]Hôpital Saint-Louis, Paris, France; [3]LYSARC, Hôpital Lyon Sud, Pierre Bénite, France; [4]Hopital Henri Mondor, Créteil, France; [5]Institut Bergonié, Bordeaux, France; [6]CHU Dijon Bourgogne, Dijon, France; [7]CHRU de Strasbourg, Strasbourg, France; [8]IUCT Oncopole, Toulouse, France; [9]CHU de Limoges, Limoges, France; [10]CHU de Bordeaux, Bordeaux, France; [11]CHU de Grenoble, Grenoble, France; [12]CHD de Vendée, La Roche sur Yon, France; [13]CHU de Besançon, Besançon, France; [14]CHU de Poitiers, Poitiers, France; [15]Centre Henri Becquerel, Rouen, France; [16]CHU de Saint-Etienne, Saint-Etienne, France; [17]CH de Libourne, Libourne, France; [18]Hôpital Lyon Sud, Pierre Bénite, France;*

### Background

Follicular lymphoma (FL) is the most common indolent lymphoma in adults, with significant heterogeneity in initial presentation, leading to various first-line (L1) strategies. Optimal frontline regimen remains debated, and comparative real-world (RW) evidence is needed.

### Aims

To describe L1 strategies and outcomes of FL patients (pts) in a RW French multicenter prospective REal world dAta in LYmphoma and Survival in Adults (REALYSA) cohort.

### Methods

We analyzed FL grades 1-3A pts included (Nov 2018 -Oct 2023*)* in REALYSA (NCT03869619). L1 strategies and outcomes including PFS, EFS, progression of disease within 24 months (POD24), time to next chemotherapy (TTNC) and OS were analyzed. Pts were grouped as low tumor burden (LTB)-managed (Watch & Wait >3 months [WW] or rituximab monotherapy [RMono]) or high tumor burden (HTB)-managed (anti-CD20 chemotherapy or lenalidomide). Within LTB or HTB groups, outcomes according to L1 (i.e WW *vs* RMono, or CHOP *vs* bendamustine) were compared using propensity score (PS)-based inverse probability of treatment weighting (IPTW) analysis. PS included age, LDH, leukocytes, hemoglobin, Ann Arbor staging, FL grade, and B symptoms. Weighted Cox and logistic regression models were used for outcome analyses.

**Copyright Information:** (Online) ISSN: 2572-9241
©2026 The Author(s). HemaSphere is published by John Wiley & Sons Ltd on behalf of European Hematology Association. This is an open access Abstract Book distributed under the Attribution-NonCommercial-NoDerivs (CC BY-NC-ND), which allows third parties to download the articles and share them with others as long as they credit the author and the Abstract Book, but they cannot change the content in any way or use them commercially.
**Abstract Book Citations:** Authors, Title, HemaSphere, 2026;10:(S1):pages. The abstract book DOIs can be found on the "Author Information" page.
**Disclaimer:** Articles published in the journal HemaSphere exclusively reflect the opinions of the authors. The authors are responsible for all content in their abstracts including accuracy of the facts, statements, citing resources, etc.

**Results**

The cohort included 1,101 pts (median age 63 y, 51% male) in 35 centers with median follow-up of 37 m (Q1-Q3 : 31-48). At diagnosis, 79% (867) had AA stage III-IV, 11% (121) a FL-3A, 58% (635) ≥ 1 GELF criterion, 49% (538) a high FLIPI and 36% (265) a high FLIPI24 score. L1 included radiotherapy alone in 2% (24), a LTB-strategy in 31% (347, WW in 19% [215] and RMono in 12% [132]), and a HTB-strategy in 66% (730, with anti CD20-CHOP [55%, 604], bendamustine [5%, 59], CVP (3%, 34), or lenalidomide [2.5%, 29], followed by maintenance in 87%). Within LTB-treated pts, 20% (68) had ≥ 1 GELF criterion whereas 23% (169) of HTB-treated had no GELF criteria. At 36-m, PFS estimate was 72% [95%CI 69-75%], EFS 71% [95%CI 68-74%] and OS 92% [95%CI 90-94%].

Among LTB-treated pts (347), 36-m PFS was 61% [95%CI 55-67%], EFS 57% [95%CI 52-63%], TTNC 73% [95%CI 68-78%] and OS 95% [95%CI 92-97%]. After IPTW adjustment, TTNC at 36 m was prolonged with RMono (77% [95%CI 69-86%]) *vs* WW (64% [95%CI 56-72%]) (wHR=1.66, 95%CI 1.05-2.62, p=0.03) whereas no significant difference was observed in PFS, EFS and OS.

Among HTB-treated pts (730), 36-m PFS was 77% [95%CI,74-80%], EFS 76% [95%CI 73-80%], OS 90% [95%CI 88-92%]. After IPTW adjustment, a prolonged PFS and EFS were observed after CHOP-based regimens compared to bendamustine-based regimens (wHR=0.58, 95%CI 0.35-0.97, p=0.04; and wHR=0.58, 95%CI 0.34-0.97, p=0.04, respectively).

A POD24 event occurred in 14% (92) and was associated with a shorter 5 y OS in 24m-landmark analysis. In a multivariate regression analysis, high FLIPI24 score (OR=6.20, 95%CI 2.33-21.5, p<0.01) and male sex (OR=1.80, 95%CI 1.02-3.27, p<0.05) were associated with increased risk of POD24.

**Summary/Conclusion**

This large prospective cohort shows that diverse treatment options for FL patients performed in RW setting are not fully consistent with GELF criteria. Prolonged TTNC was observed in RMono-treated vs WW pts with similar EFS/PFS and OS. Within HTB-treated pts, CHOP-based regimens showed a favorable outcome compared to bendamustine. Finally, FLIPI-24 was the strongest prognostic marker for POD24 event. Overall, this high-quality RW data cohort is a source for numerous applications, especially comparisons for innovative treatment strategies.

**Copyright Information:** (Online) ISSN: 2572-9241
©2026 The Author(s). HemaSphere is published by John Wiley & Sons Ltd on behalf of European Hematology Association. This is an open access Abstract Book distributed under the Attribution-NonCommercial-NoDerivs (CC BY-NC-ND), which allows third parties to download the articles and share them with others as long as they credit the author and the Abstract Book, but they cannot change the content in any way or use them commercially.
**Abstract Book Citations:** Authors, Title, HemaSphere, 2026;10:(S1):pages. The abstract book DOIs can be found on the "Author Information" page.
**Disclaimer:** Articles published in the journal HemaSphere exclusively reflect the opinions of the authors. The authors are responsible for all content in their abstracts including accuracy of the facts, statements, citing resources, etc.

### (PS2037) RISK-ADAPTED MANAGEMENT WITH OBINUTUZUMAB AND ORELABRUTINIB WITH OR WITHOUT LENALIDOMIDE IN UNTREATED MARGINAL ZONE LYMPHOMA: FIRST REPORT OF A PROSPECTIVE, PHASE II, MULTI-CENTRE (MAGIC) STUDY

**Topic:** 18. Indolent and mantle-cell non-Hodgkin lymphoma - Clinical

Yujia Huo*[1], Xixi Xiang[2], Lin Shen[3], Yan Huang[4], Lihong Shou[5], Xiaosheng Fang[6], Hongmei Jing[7], Zhao Wang[8], Hongbin Lu[9], Weiying Feng[10], Xiaojing Yan[11], Li Wang[1], Pengpeng Xu[1], Huijuan Zhong[1], Shu Cheng[1], Weili Zhao[1]

*[1]Ruijin Hospital, Shanghai Jiao Tong University School of Medicine, shanghai, China;[2]Medical Center of Hematology, Xinqiao Hospital of Army Medical University, Chongqing, China; [3]Huadong Hospital, Shanghai, China;[4]Jiujiang NO.1 People's Hospital, Jiujiang, China; [5]Huzhou Central Hospital, Hu Zhou, China;[6]Shandong Provincial Hospital, Shandong, China;[7]Peking University Third Hospital, Beijing, China; [8]Beijing Friendship Hospital, Capital Medical University, Beijing, China;[9]Wuxi Branch of Ruijin Hospital, Shanghai Jiao Tong University School of Medicine, Wuxi, China; [10]Shaoxing People's Hospital, Shaoxing, China; [11]The First Affiliated Hospital of China Medical University, Shenyang, China;*

### Background

Marginal zone lymphoma (MZL) comprises a biologically heterogeneous group of indolent B-cell lymphomas, posing substantial challenges to the design and conduct of clinical trials. Although accumulating evidence supports the clinical activity of targeted therapies—particularly anti-CD20 monoclonal antibodies and Bruton's tyrosine kinase inhibitors—the overall evidence base remains limited. The MZL International Prognostic Index (MZL-IPI) provides a validated tool for risk stratification across the spectrum of MZL subtypes; however, clinical trial designs incorporating risk-adapted strategies have yet to be established.

### Aims

To evaluate the efficacy and safety of obinutuzumab plus orelabrutinib with or without lenalidomide followed by orelabrutinib maintenance as an MZL-IPI–guided strategy in untreated MZL.

### Methods

This is a prospective, phase II, multi-centre study (NCT06793189). Eligible patients were aged ≥18 years with previously untreated with systemic therapy, pathologically confirmed MZL who met the indication for systemic therapy. Patients with an MZL-IPI score of 0–2 received obinutuzumab (1000 mg IV on days 1, 8 and 15 of cycle 1 and day 1 of cycles 2–6) plus orelabrutinib (150 mg orally once daily on days 1–21; O2 regimen). Those with a score of 3–5 received obinutuzumab, orelabrutinib and lenalidomide (25 mg orally once daily on days 1–10; RO2 regimen). Both regimens were administered for six 21-day cycles. Patients without disease progression were eligible to receive orelabrutinib maintenance for up to two years. The primary endpoint was 2-year progression-free survival (PFS). Key secondary endpoints included complete response rate (CRR), objective response rate (ORR), overall survival (OS) and safety.

**Copyright Information:** (Online) ISSN: 2572-9241
©2026 The Author(s). HemaSphere is published by John Wiley & Sons Ltd on behalf of European Hematology Association. This is an open access Abstract Book distributed under the Attribution-NonCommercial-NoDerivs (CC BY-NC-ND), which allows third parties to download the articles and share them with others as long as they credit the author and the Abstract Book, but they cannot change the content in any way or use them commercially.
**Abstract Book Citations:** Authors, Title, HemaSphere, 2026;10:(S1):pages. The abstract book DOIs can be found on the "Author Information" page.
**Disclaimer:** Articles published in the journal HemaSphere exclusively reflect the opinions of the authors. The authors are responsible for all content in their abstracts including accuracy of the facts, statements, citing resources, etc.

### Results

As of 31 Dec. 2025, 69 patients had been enrolled (O2: n=55; RO2: n=14). Pathological subtypes comprised extranodal marginal zone lymphoma (ENMZL; 42.0%), disseminated MZL (DissMZL; 33.3%), nodal marginal zone lymphoma (NMZL; 15.9%), and splenic marginal zone lymphoma (SMZL; 8.7%). The majority of patients (84.1%) presented with stage III–IV disease. A Ki-67 proliferation index >20% was observed in 21.7% of patients (Table 1). Twenty-eight patients completed six cycles of induction therapy. The CRR was 85.7% (18/21) in the O2 group and 71.4% (5/7) in the RO2 group, with ORR of 95.3% (20/21) and 85.7% (6/7), respectively. Among 50 patients who received at least one cycle of induction therapy, the best CRR was 78.9% (30/38) in the O2 group and 83.3% (10/12) in the RO2 group, with ORRs of 97.4% (37/38) and 91.7% (11/12), respectively (Table 2). At the data cut-off, the median follow-up was 5.5 months; PFS and OS data were immature. The most frequent grade ≥3 adverse events (AEs) were neutropenia (21.7%) and thrombocytopenia (7.2%). Other grade ≥3 non-haematological AEs included pneumonia (2.9%), febrile neutropenia (1.4%), and elevated alanine aminotransferase (1.4%). Baseline tumour DNA sequencing data were available for 48 patients. The most frequently mutated genes were *IGLL5* (27.1%), *TET2* (22.9%), *MYD88* (20.8%), *CARD11* (14.6%), and *KMT2C*, *KMT2D*, *NOTCH2*, and *TP53* (each 12.5%; Figure 1).

### Summary/Conclusion

The preliminary results of the risk-adapted regimen combining obinutuzumab and orelabrutinib, with or without lenalidomide, demonstrated encouraging efficacy and a manageable safety profile in patients with previously untreated MZL. The study is ongoing, and updated efficacy and safety data will be reported in due course.

**Table 1. Baseline characteristics of enrolled patients (n=69)**

| | Total (n=69) n (%) | O2 (n=55) n (%) | RO2 (n=14) n (%) |
|---|---|---|---|
| **Gender, Male** | 31 (44.9%) | 23 (41.8%) | 8 (57.1%) |
| **Age ≥60 years** | 47 (68.1%) | 36 (65.5%) | 11 (78.6%) |
| **Pathological subtypes** | | | |
| DissMZL | 23 (33.3%) | 13 (23.6%) | 10 (71.4%) |
| ENMZL | 29 (42.0%) | 27 (49.1%) | 2 (14.3%) |
| NMZL | 11 (15.9%) | 11 (20.0%) | 0 (0%) |
| SMZL | 6 (8.7%) | 4 (7.3%) | 2 (14.3%) |
| **BM involvement** | 36 (52.2%) | 25 (45.5%) | 11 (78.6%) |
| **Ann Arbor stage** | | | |
| I-II | 11 (15.9%) | 11 (20.0%) | 0 (0%) |
| III-IV | 58 (84.1%) | 44 (80.0%) | 14 (100%) |
| **MZL-IPI score** | | | |
| 0 | 9 (13.0%) | 9 (16.4%) | 0 (0%) |
| 1-2 | 46 (66.7%) | 46 (83.6%) | 0 (0%) |
| 3-5 | 14 (20.3%) | 0 (0%) | 14 (100%) |
| **Ki67 >20%** | 15 (21.7%) | 12 (21.8%) | 3 (21.4%) |

Abbreviations: BM, bone marrow; DissMZL, disseminated presentation of MZL; ENMZL, extranodal marginal zone lymphoma, MZL-IPI, MZL international prognostic index; NMZL: nodal marginal zone lymphoma; SMZL: splenic marginal zone lymphoma;

**Figure 1. Mutational landscape of enrolled patients (n=48)**

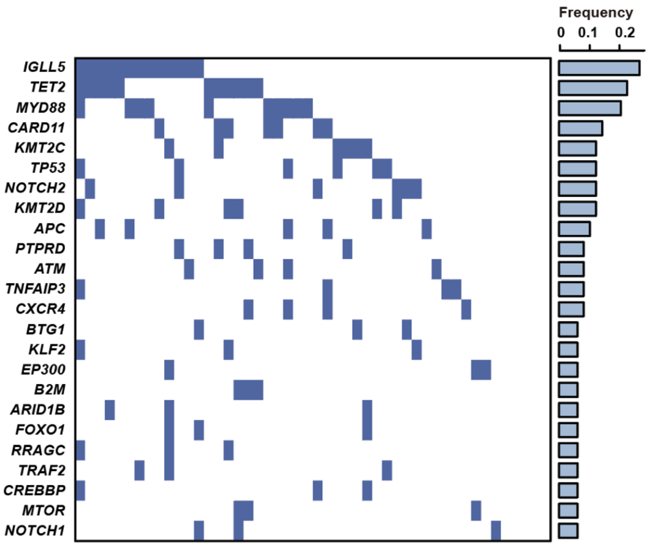

**Table 2. Treatment response among patients who received at least one cycle of induction therapy (n=50)**

| | O2 (n=38) | RO2 (n=12) |
|---|---|---|
| **Complete response rate, n(%[95%CI])** | 30 (78.9 [62.7-90.4]) | 10 (83.3 [51.6-97.9]) |
| **Objective response rate, n(%[95%CI])** | 37 (97.4 [86.2-99.9]) | 11 (91.7 [61.5-99.8]) |
| **Overall response category, n(%)** | | |
| **Complete response** | 30 (78.9) | 10 (83.3) |
| **Partial response** | 7 (18.4) | 1 (8.3) |
| **Stable disease** | 0 (0) | 0 (0) |
| **Progressive disease** | 1 (26.3) | 1 (8.3) |

Abbreviations: CI, confidence interval

**Copyright Information:** (Online) ISSN: 2572-9241
©2026 The Author(s). HemaSphere is published by John Wiley & Sons Ltd on behalf of European Hematology Association. This is an open access Abstract Book distributed under the Attribution-NonCommercial-NoDerivs (CC BY-NC-ND), which allows third parties to download the articles and share them with others as long as they credit the author and the Abstract Book, but they cannot change the content in any way or use them commercially.
**Abstract Book Citations:** Authors, Title, HemaSphere, 2026;10:(S1):pages. The abstract book DOIs can be found on the “Author Information” page.
**Disclaimer:** Articles published in the journal HemaSphere exclusively reflect the opinions of the authors. The authors are responsible for all content in their abstracts including accuracy of the facts, statements, citing resources, etc.

### (PS2038) A PHASE II STUDY OF LENALIDOMIDE, ZANUBRUTINIB PLUS OBINUTUZUMAB (ZGR) IN PATIENTS WITH FLIPI2 HIGH-RISK NEWLY DIAGNOSED FOLLICULAR LYMPHOMA: AN INTERIM ANALYSIS

**Topic:** 18. Indolent and mantle-cell non-Hodgkin lymphoma - Clinical

Li Wang*[1], Zhong Zheng[1], Ruichi Li[1], Pengpeng Xu[1], Shu Cheng[1], Weili Zhao[1]

[1]*Ruijin Hospital Affiliated To Shanghai Jiaotong University School Of Medicine, Shanghai, China;*

### Background

Follicular lymphoma (FL) is the most common indolent non-Hodgkin lymphoma. Chemoimmunotherpy is a standard of care (SOC) for first-line FL with complete response (CR) rates of up to 73% but is associated with significant toxicities. Chemo-free regimens like R2 offer similar efficacy and a more favorable safety profile, highlighting the need for treatments that can induce deeper and more durable responses. The second generation anti-CD20 monoclonal antibody, Obinutuzumab, is approved and demonstrates superior progression-free survival (PFS) compared to first-generation CD20 antibodies in patients with de novo FL. Zanubrutinib is a potent, specific, and irreversible second-generation covalent BTK inhibitor and has demonstrated favorable efficacy in combination with Obinutuzumab in relapse/refractory FL.

### Aims

To improve the prognosis of FL patients with multiple high-risk factors at the time of initial treatment, we therefore launched this single-arm, phase II trial to assess the efficacy and safety of obinutuzumab, lenalidomide, and zanubrutinib (ZGR) in treatment-naïve, high-risk (FLIPI2 score 3-5) FL patients (NCT06549335).

### Methods

Eligible adults (≥18 years) with newly diagnosed grade 1–3a FL and FLIPI-2 scores 3–5 received induction of ZGR (obinutuzumab 1,000 mg intravenously on days 1, 8, and 15 of cycle 1 and day 1 of cycles 2–6; lenalidomide 25 mg orally days 2–11; and zanubrutinib 160 mg orally twice daily continuously). Maintenance consisted of obinutuzumab every 3 months for 2 years and lenalidomide 25 mg days 1–10 monthly  for 6 months. The primary endpoint was overall response rate (ORR) after induction; secondary endpoints included CR rate, duration of response (DOR), PFS, and 2-year overall survival (OS).

### Results

In this study, 60 patients had been enrolled (median age 61 years, range 33–80). All presented with advanced Ann Arbor stage; 78% with Bulky disease; 90% with bone marrow involvement, 44 % had elevated LDH, and 57 % had multiple extranodal sites.  Among 45 response-evaluable patients, ORR was 89% (40/45) and CR rate was 76 % (34/45). Meanwhile, ZGR regimen demonstrated consistent efficacy across all high-risk subgroups. The most common adverse event was neutropenia (57%). No new toxicities were observed with the combination regimen compared to the individual agents. No patients discontinued treatment due to adverse events. Exploratory monitoring of peripheral blood ctDNA dynamics between baseline (C1) and post-cycle 1 (C2) patients achieving CR/PR displayed a marked and rapid reduction in ctDNA concentrations early in treatment relative to those experiencing SD/PD.

**Copyright Information:** (Online) ISSN: 2572-9241
©2026 The Author(s). HemaSphere is published by John Wiley & Sons Ltd on behalf of European Hematology Association. This is an open access Abstract Book distributed under the Attribution-NonCommercial-NoDerivs (CC BY-NC-ND), which allows third parties to download the articles and share them with others as long as they credit the author and the Abstract Book, but they cannot change the content in any way or use them commercially.
**Abstract Book Citations:** Authors, Title, HemaSphere, 2026;10:(S1):pages. The abstract book DOIs can be found on the “Author Information” page.
**Disclaimer:** Articles published in the journal HemaSphere exclusively reflect the opinions of the authors. The authors are responsible for all content in their abstracts including accuracy of the facts, statements, citing resources, etc.

**Summary/Conclusion**

Lenalidomide, Zanubrutinib plus Obinutuzumab achieve rapid and deep responses in patients with high tumor burden in FL. With a median of 7.7 months of follow up, follow-up is ongoing. The safety profile was manageable and remained consistent with the known toxicities of the individual components. Lenalidomide, Zanubrutinib plus Obinutuzumab regimen is a novel chemo-free therapy suitable for high-risk FL patients.

**Copyright Information:** (Online) ISSN: 2572-9241
©2026 The Author(s). HemaSphere is published by John Wiley & Sons Ltd on behalf of European Hematology Association. This is an open access Abstract Book distributed under the Attribution-NonCommercial-NoDerivs (CC BY-NC-ND), which allows third parties to download the articles and share them with others as long as they credit the author and the Abstract Book, but they cannot change the content in any way or use them commercially.
**Abstract Book Citations:** Authors, Title, HemaSphere, 2026;10:(S1):pages. The abstract book DOIs can be found on the “Author Information” page.
**Disclaimer:** Articles published in the journal HemaSphere exclusively reflect the opinions of the authors. The authors are responsible for all content in their abstracts including accuracy of the facts, statements, citing resources, etc.

### (PS2039) TREATMENT AND PROGNOSIS OF PRIMARY OCULAR ADNEXAL MUCOSA-ASSOCIATED LYMPHOID TISSUE LYMPHOMA IN CHINA: A COMPREHENSIVE NATIONAL RETROSPECTIVE STUDY OF 805 PATIENTS

**Topic:** 18. Indolent and mantle-cell non-Hodgkin lymphoma - Clinical

Yuan-Zheng Liang[1], Xin Li[1], Yizhen Liu[2], Jia Wei[3], Shu-Nan Qi[4], Bing-Zong Li[5], Wei Guo[6], Li Wang[7], Yajun Li[8], Xudong Zhang[9], Yong Yang[10], Shu-Kai Qiao[11], Dongfeng Zeng[12], Rong Tao[2], Jian-Min MA[1], Liang Wang*[1]

*[1]Beijing Tongren Hospital, Capital Medical University, Beijing, China;[2]Fudan University Shanghai Cancer Center, Shanghai, China; [3]Tongji Hospital, Tongji Medical College, Huazhong University of Science and Technology, Wuhan, China;[4]National Cancer Center/ National Clinical Research Center for Cancer/Cancer Hospital, Chinese Academy of Medical Sciences (CAMS) and Peking Union Medical College (PUMC), Beijing, China; [5]The Second Affiliated Hospital of Soochow University, Suzhou, China; [6]The First Hospital of Jilin University, Changchun, China;[7]Ruijin Hospital Affiliated to Shanghai Jiao Tong University School of Medicine, Shanghai, China; [8]The Affiliated Cancer Hospital of Xiangya School of Medicine, Central South University/Hunan Cancer Hospital, Changsha, China; [9]The First Affiliated Hospital of Zhengzhou University, Zhengzhou, China; [10]Fujian Medical University Union Hospital, Fuzhou, China; [11]Hebei Medical University Second Hospital, Shijiazhuang, China; [12]Daping Hospital, Army Medical University, Chongqing, China;*

### Background

Primary ocular adnexal mucosa-associated lymphoid tissue lymphoma (POAML) is the most common malignant tumor of the ocular adnexa, characterized by an indolent clinical course and favorable prognosis. However, optimal treatment strategies remain controversial, with significant practice variations across institutions. Large-scale multicenter data from Asian populations are scarce.

### Aims

We conducted the largest retrospective multicenter study in China to evaluate treatment patterns, long-term outcomes, and prognostic factors in POAML patients.

### Methods

We retrospectively analyzed 805 POAML patients from 12 tertiary medical centers across China between May 2001 and June 2024. All cases were pathologically confirmed according to WHO classification. Staging was performed using the Ann Arbor system. Treatment strategies included watch & wait (W&W) after surgical resection, radiotherapy (RT), and chemotherapy-based treatment (chemotherapy alone or immunochemotherapy including R-CHOP, R-CVP, R-lenalidomide, BTK inhibitors, or bendamustine). Treatment response was evaluated using Revised Response Criteria. Progression-free survival (PFS) and overall survival (OS) were analyzed using Kaplan-Meier methods. A nomogram model for PFS prediction was constructed and validated using bootstrap resampling.

**Copyright Information:** (Online) ISSN: 2572-9241
©2026 The Author(s). HemaSphere is published by John Wiley & Sons Ltd on behalf of European Hematology Association. This is an open access Abstract Book distributed under the Attribution-NonCommercial-NoDerivs (CC BY-NC-ND), which allows third parties to download the articles and share them with others as long as they credit the author and the Abstract Book, but they cannot change the content in any way or use them commercially.
**Abstract Book Citations:** Authors, Title, HemaSphere, 2026;10:(S1):pages. The abstract book DOIs can be found on the "Author Information" page.
**Disclaimer:** Articles published in the journal HemaSphere exclusively reflect the opinions of the authors. The authors are responsible for all content in their abstracts including accuracy of the facts, statements, citing resources, etc.

### Results

The cohort comprised 468 males (58.1%) with median age of 51 years (range 18-91). Most patients (88.4%) had Ann Arbor stage I-II disease, and 81.6% presented with unilateral involvement. Following surgical diagnosis, 348 patients (43.2%) underwent W&W, 226 (28.1%) received RT, and 231 (28.7%) received chemotherapy-based treatment. The overall complete response (CR) rate was 51.8%. Notably, the RT group achieved the highest CR rate at 72.1%, compared to 44.2% in the chemotherapy group and 43.7% in the W&W group. After median follow-up of 86 months (range 10-287), 757 patients remained alive. Disease progression/recurrence occurred in 201 patients (172 local, 29 distant). The 10-year PFS and OS rates for the entire cohort were 68.2% (95% CI: 64.2-72.4%) and 96.5% (94.8-98.3%), respectively, with 10-year lymphoma-specific survival of 99.3%. Kaplan-Meier analysis demonstrated significantly superior PFS in the RT group compared to both W&W and chemotherapy groups (all $P<0.05$), with 10-year PFS of 74.1% versus 65.8% and 62.3%, respectively. The 10-year OS rates were excellent across all groups (97.3%, 96.1%, and 96.2%, respectively), with no significant difference ($P=0.97$). Importantly, even among completely resected patients, W&W resulted in significantly inferior PFS compared to RT after incomplete resection, highlighting that local control outweighs surgical completeness. We developed and validated a novel nomogram incorporating age (>60 years), male gender, bilateral disease, and Ann Arbor stage III-IV for PFS prediction. Risk stratification using this model identified low-, intermediate-, and high-risk groups with 10-year PFS rates of 73.6%, 54.4%, and 43.2%, respectively ($P<0.0001$).

### Summary/Conclusion

This largest-to-date POAML cohort confirms excellent long-term survival (96.5% 10-year OS) while revealing significant differences in disease control across treatment modalities. Radiotherapy emerges as the most effective strategy for localized disease, achieving superior CR rates and PFS compared to both watchful waiting and systemic therapy, without compromising quality of life. We recommend radiotherapy as the cornerstone of local control for POAML.

**Copyright Information:** (Online) ISSN: 2572-9241
©2026 The Author(s). HemaSphere is published by John Wiley & Sons Ltd on behalf of European Hematology Association. This is an open access Abstract Book distributed under the Attribution-NonCommercial-NoDerivs (CC BY-NC-ND), which allows third parties to download the articles and share them with others as long as they credit the author and the Abstract Book, but they cannot change the content in any way or use them commercially.
**Abstract Book Citations:** Authors, Title, HemaSphere, 2026;10:(S1):pages. The abstract book DOIs can be found on the "Author Information" page.
**Disclaimer:** Articles published in the journal HemaSphere exclusively reflect the opinions of the authors. The authors are responsible for all content in their abstracts including accuracy of the facts, statements, citing resources, etc.

### (PS2040) A REAL-WORLD COMPARISON OF TREATMENT AND SURVIVAL OUTCOMES WITH ZANUBRUTINIB AND ACALABRUTINIB MONOTHERAPY AMONG PATIENTS WITH RELAPSED OR REFRACTORY MANTLE CELL LYMPHOMA IN THE UNITED STATES

**Topic:** 18. Indolent and mantle-cell non-Hodgkin lymphoma - Clinical

Yucai Wang*[1], Manasi Suryavanshi[2], Gregory Maglinte[2], Rucha Kulkarni[3], Divya Nagpal[4], Melissa Hagan[2], Nishit Jain[4], Erlene Seymour[2], G. Rhys Williams[2], Javier Munoz[5]

*[1]Mayo Clinic, Rochester, MN, United States of America;[2]BeOne Medicines Ltd, San Carlos, CA, United States of America;[3]ZS Associates, San Francisco, CA, United States of America; [4]ZS Associates, Gurugram, Haryana, India;[5]Mayo Clinic, Phoenix, AZ, United States of America;*

### Background

Mantle cell lymphoma (MCL) is a rare and aggressive subtype of B-cell non-Hodgkin lymphoma that remains incurable. Bruton tyrosine kinase (BTK) inhibitors are an established standard of care for relapsed or refractory (R/R) MCL. However, comparative effectiveness data for different BTK inhibitors for R/R MCL are limited.

### Aims

This study evaluated the real-world effectiveness of zanubrutinib (zanu) and acalabrutinib (acala) in United States (US) patients with R/R MCL based on overall survival (OS) and time to next treatment (TTNT).

### Methods

A retrospective cohort study was conducted using Komodo health administrative claims data. Eligible patients were adults (aged ≥18 years) with ≥2 MCL diagnoses and continuous enrollment or activities within 1 year prior to and 3 months following the index date. Patients were required to initiate monotherapy with zanu (index period: November 2019 to August 2025) or acala (index period: October 2017 to August 2025) as a second-line or later (2L+) treatment, with index date defined as the first observed claim for zanu or acala. Patients with evidence of clinical trial participation, end-stage renal disease, or prior stem cell transplant were excluded. Outcomes included OS (time from index date to all-cause mortality) and TTNT (time from index date to subsequent therapy with an allowable gap of 120 days). If an outcome was not observed, patients were censored at the date of last activity or enrollment end date. Survival analyses were conducted using Kaplan-Meier estimates and Cox proportional hazards models. Inverse probability of treatment weighting (IPTW) was adjusted for age, sex, US region, treatment initiation year, and Charlson Comorbidity Index (CCI).

### Results

In total, 2219 patients (zanu, n=931; acala, n=1288) were eligible and included in the study. The mean age was higher in the zanu cohort (72.3 years; SD, 9.4) than the acala cohort (71.4 years; SD, 9.7). Most patients were male (zanu, 71%; acala, 75%), non-Hispanic white (zanu, 73%; acala, 74%), and the mean CCI was 3.5 (SD, 3.2) and 3.6 (SD, 3.1) in the zanu and acala cohorts, respectively. Median follow-up was 14.8 and 18.2 months in zanu and acala cohorts, respectively. Median TTNT was 26.7 months for zanu and 20.8 months for acala. Median OS was not reached for zanu and was 60.6 months for acala. In the unadjusted model, zanu had a longer TTNT (hazard ratio [HR]=0.86; 95% CI, 0.76-0.97; *P*=.012) and OS (HR=0.74; 95% CI, 0.62-0.88; *P*<.001; **Table**). After IPTW adjustment, TTNT and OS favored zanu treatment (TTNT: IPTW-adjusted HR=0.86; 95% CI, 0.76-0.98; *P*=.025; OS: IPTW-adjusted HR=0.81; 95% CI, 0.68-0.98; *P*=.03).

**Copyright Information:** (Online) ISSN: 2572-9241
©2026 The Author(s). HemaSphere is published by John Wiley & Sons Ltd on behalf of European Hematology Association. This is an open access Abstract Book distributed under the Attribution-NonCommercial-NoDerivs (CC BY-NC-ND), which allows third parties to download the articles and share them with others as long as they credit the author and the Abstract Book, but they cannot change the content in any way or use them commercially.
**Abstract Book Citations:** Authors, Title, HemaSphere, 2026;10:(S1):pages. The abstract book DOIs can be found on the "Author Information" page.
**Disclaimer:** Articles published in the journal HemaSphere exclusively reflect the opinions of the authors. The authors are responsible for all content in their abstracts including accuracy of the facts, statements, citing resources, etc.

### Summary/Conclusion

In this real-world analysis of US health claims data, zanu monotherapy demonstrated significantly longer TTNT and improved OS compared with acala in patients receiving 2L+ therapy for MCL. These findings support zanu as an effective BTK inhibitor for R/R MCL.

**Table. TTNT and OS Landmark Rates and HRs**

| | | Zanu | Acala (ref) |
|---|---|---|---|
| **TTNT** | 12 m, % (95% CI) | 66.3 (63.0-69.4) | 60.6 (57.8-63.3) |
| | 18 m, % (95% CI) | 58.3 (54.7-61.8) | 53.8 (50.8-56.7) |
| | 24 m, % (95% CI) | 52.8 (48.9-56.6) | 46.7 (43.7-49.8) |
| | Unadjusted HR (95% CI) | 0.86 (0.76-0.97) | |
| | Adjusted HR (95% CI) | 0.86 (0.76-0.98) | |
| **OS** | 12 m, % (95% CI) | 86.4 (83.9-88.6) | 81.6 (79.3-83.7) |
| | 18 m, % (95% CI) | 81.1 (78.0-83.8) | 74.8 (72.1-77.3) |
| | 24 m, % (95% CI) | 76.3 (72.7-79.6) | 68.6 (65.6-71.4) |
| | Unadjusted HR (95% CI) | 0.74 (0.62-0.88) | |
| | Adjusted HR (95% CI) | 0.81 (0.68-0.98) | |

**Copyright Information:** (Online) ISSN: 2572-9241
©2026 The Author(s). HemaSphere is published by John Wiley & Sons Ltd on behalf of European Hematology Association. This is an open access Abstract Book distributed under the Attribution-NonCommercial-NoDerivs (CC BY-NC-ND), which allows third parties to download the articles and share them with others as long as they credit the author and the Abstract Book, but they cannot change the content in any way or use them commercially.
**Abstract Book Citations:** Authors, Title, HemaSphere, 2026;10:(S1):pages. The abstract book DOIs can be found on the "Author Information" page.
**Disclaimer:** Articles published in the journal HemaSphere exclusively reflect the opinions of the authors. The authors are responsible for all content in their abstracts including accuracy of the facts, statements, citing resources, etc.

### (PS2041) IMPACT OF RECENT BENDAMUSTINE (BENDA) EXPOSURE IN PATIENTS (PTS) WITH RELPASED/REFRACTORY (R/R) FOLLICULAR LYMPHOMA (FL) TREATED WITH ODRONEXTAMAB (ODRO) IN THE PHASE 2 ELM-2 STUDY

**Topic:** 18. Indolent and mantle-cell non-Hodgkin lymphoma - Clinical

Gloria Iacoboni*[1, 2], Giovanni Antico[3], C. Akunna Otele[3], Jon Arnason[4], Cecilia Carpio[1, 2], Jose C. Villasboas[5], Chinjune Lin[3], Alexey Danilov[6]

*[1]Vall d'Hebron University Hospital, Barcelona, Spain;[2]Vall d'Hebron Institute of Oncology (VHIO), Barcelona, Spain;[3]Regeneron Pharmaceuticals, Inc., Tarrytown, NY, United States of America; [4]Beth Israel Deaconess Medical Cente, Boston, MA, United States of America; [5]Mayo Clinic, Rochester, MN, United States of America;[6]City of Hope National Medical Centre, Duarte, CA, United States of America;*

### Background

Prior benda exposure is linked to inferior outcomes in pts with diffuse large B-cell lymphoma receiving CAR T therapy, however, its impact before the use of bispecific antibodies (bsAbs) is unclear. A recent multicenter analysis of pts with R/R FL revealed no significant difference in bsAb efficacy according to time since prior benda (Iacoboni G et al. *Blood Adv* 2026). In the Phase 2 ELM-2 study (NCT03888105), odro showed an objective response rate (ORR) of 80.0%, a complete response (CR) rate of 73.4%, and a generally manageable safety profile in pts with R/R FL (Kim T et al. *Ann Oncol* 2024).

### Aims

To evaluate the efficacy and safety of odro in pts with R/R FL from ELM-2 according to time elapsed since last benda dose.

### Methods

Pts with Grade (Gr) 1–3a FL that was R/R after ≥2 prior lines of therapy (LOTs) were enrolled. Odro was administered intravenously in 21-day cycles until disease progression or unacceptable toxicity, with step-up dosing (0.7/4/20 mg) in Cycle 1. In this retrospective analysis, unadjusted analyses assessed the efficacy of odro in pts treated with benda at various time intervals prior to first odro dose (<3 months [mo] to ≥24 mo). An inverse probability of treatment weighting (IPTW) landmark analysis (≥9 mo vs <9 mo prior to first odro dose) using 9 covariates was applied to balance key baseline characteristics. Data cut-off: Aug 15, 2024.

**Copyright Information:** (Online) ISSN: 2572-9241
©2026 The Author(s). HemaSphere is published by John Wiley & Sons Ltd on behalf of European Hematology Association. This is an open access Abstract Book distributed under the Attribution-NonCommercial-NoDerivs (CC BY-NC-ND), which allows third parties to download the articles and share them with others as long as they credit the author and the Abstract Book, but they cannot change the content in any way or use them commercially.
**Abstract Book Citations:** Authors, Title, HemaSphere, 2026;10:(S1):pages. The abstract book DOIs can be found on the "Author Information" page.
**Disclaimer:** Articles published in the journal HemaSphere exclusively reflect the opinions of the authors. The authors are responsible for all content in their abstracts including accuracy of the facts, statements, citing resources, etc.

**Results**

All 128 efficacy-evaluable pts with FL had received a prior LOT with benda. Unadjusted ORR and CR rates based on time since prior benda showed no clear efficacy trends across groups by time interval (**Figure**). Analyses based on the 9 mo landmark revealed time from last benda to first odro dose was ≥9 mo in 98 pts and <9 mo in 30 pts. Baseline demographic and disease characteristics are shown in the table. Although there were differential response rates between groups (ORR 83.7% in the ≥9 mo group vs 70.0% in the <9 mo group), IPTW analyses showed no significant differences in long term outcomes: median progression free survival (PFS; 25.2 mo in the ≥9 mo group vs 19.4 mo in the <9 mo group; hazard ratio [HR] 1.47 [95% CI 0.79–2.73]) or median overall survival (OS; 54.2 mo in the ≥9 mo group vs not reached in the <9 mo group; HR 1.52 [95% CI 0.71–3.28]). There was no observed effect of time since benda on peripheral T-cell counts and no difference in immune cell marker trends (CD3+CD4+ absolute cell count [ABS] and CD3+CD8+ ABS) among time subgroups and based on ≥9 mo or <9 mo cut-offs. All pts (safety analysis set; N=128) had ≥1 treatment-emergent adverse event (TEAE); the most common were cytokine release syndrome (CRS; Gr ≥3 3.9%) and neutropenia (Gr ≥3 32.8%), with no clear differences in CRS or neutropenia between the <9 mo and ≥9 mo benda groups. Any-Gr infection-related TEAEs occurred in 79.7% of pts (Gr ≥3 43.8%) and were numerically lower in the <9 mo (66.7%) vs ≥9 mo (83.7%) benda group.

**Summary/Conclusion**

In this retrospective analysis from ELM-2 in patients with recent benda exposure, no detriment to odro efficacy was seen, differing from CAR T. Although benda treatment ≥9 mo before odro dosing was linked to higher response rates vs <9 mo, PFS and OS were similar between both groups. Future validation in larger trials is required.

**Table. Baseline characteristics**

| | Time from last benda to first odro dose | |
|---|---|---|
| | <9 mo (n=30) | ≥9 mo (n=98) |
| Median age (range), years | 59.0 (38–84) | 61.5 (22–84) |
| Median prior LOTs (range), n | 2.0 (2–8) | 3.0 (2–13) |
| Ann Arbor Stage III–IV at study entry, n (%) | 24 (80.0) | 85 (86.7) |

**Copyright Information:** (Online) ISSN: 2572-9241
©2026 The Author(s). HemaSphere is published by John Wiley & Sons Ltd on behalf of European Hematology Association. This is an open access Abstract Book distributed under the Attribution-NonCommercial-NoDerivs (CC BY-NC-ND), which allows third parties to download the articles and share them with others as long as they credit the author and the Abstract Book, but they cannot change the content in any way or use them commercially.
**Abstract Book Citations:** Authors, Title, HemaSphere, 2026;10:(S1):pages. The abstract book DOIs can be found on the “Author Information” page.
**Disclaimer:** Articles published in the journal HemaSphere exclusively reflect the opinions of the authors. The authors are responsible for all content in their abstracts including accuracy of the facts, statements, citing resources, etc.

**Figure. Unadjusted ORR and CR rates* (%) as a function of time from last benda dose, and % of prior benda use as 3L+ (full analysis set).**

Included Grade 1–3a FL patients who had the opportunity for response assessment at 12 weeks.

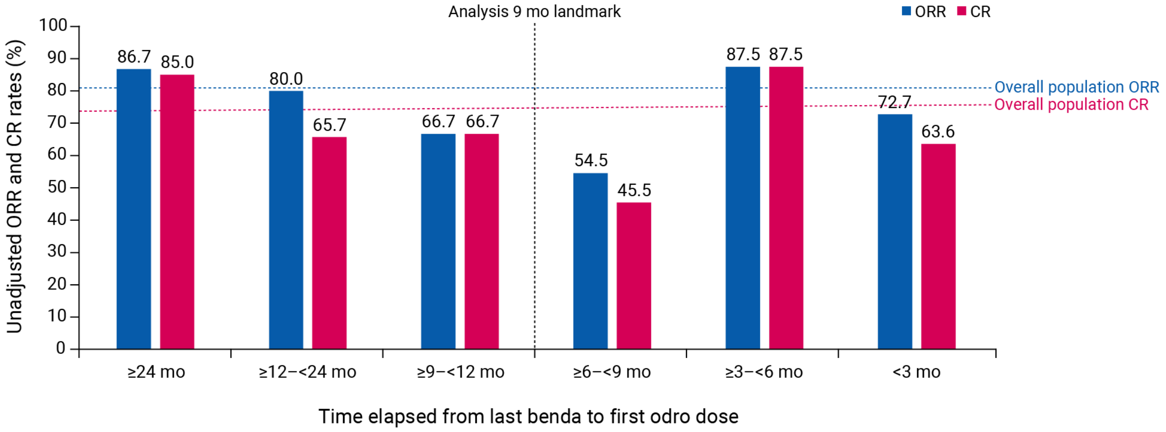

| | ≥24 mo | ≥12–<24 mo | ≥9–<12 mo | ≥6–<9 mo | ≥3–<6 mo | <3 mo |
|---|---|---|---|---|---|---|
| n: | 60 | 35 | 3 | 11 | 8 | 11 |
| % of prior benda use as 3L+: | 21.7 | 45.7 | 100.0 | 63.6 | 25.0 | 45.5 |

3L+, third line and beyond.

*Based on Lugano classification per Independent Central Review.

Copyright Information: (Online) ISSN: 2572-9241
©2026 The Author(s). HemaSphere is published by John Wiley & Sons Ltd on behalf of European Hematology Association. This is an open access Abstract Book distributed under the Attribution-NonCommercial-NoDerivs (CC BY-NC-ND), which allows third parties to download the articles and share them with others as long as they credit the author and the Abstract Book, but they cannot change the content in any way or use them commercially.
Abstract Book Citations: Authors, Title, HemaSphere, 2026;10:(S1):pages. The abstract book DOIs can be found on the “Author Information” page.
Disclaimer: Articles published in the journal HemaSphere exclusively reflect the opinions of the authors. The authors are responsible for all content in their abstracts including accuracy of the facts, statements, citing resources, etc.

### (PS2042) COMPARATIVE EFFECTIVENESS OF EPCORITAMAB, LENALIDOMIDE, AND RITUXIMAB IN EPCORE FL-1 VS REAL-WORLD CHEMOIMMUNOTHERAPY IN RELAPSED/REFRACTORY FOLLICULAR LYMPHOMA

**Topic:** 18. Indolent and mantle-cell non-Hodgkin lymphoma - Clinical

Lorenzo Falchi*[1], Rong Tao[2], Loic Ysebaert[3], Michal Kwiatek[4], Abel Costa Neto[5], Theodoros Vassilakopoulos[6], Marcel Nijland[7], Shane Gangatharan[8], Habte A. Yimer[9], Dai Maruyama[10], Abualbishr Alshreef[11], Anthony Wang[11], Junhua Yu[11], David Tybor[11], Yves Mbous[11], Kavita Sail[11], Zhijie Ding[12], Gauri Sunkersett[11], Jian Mei[11], Kim Linton[13]

*[1]Lymphoma Service, Memorial Sloan Kettering Cancer Center, New York, United States of America;[2]Fudan University Cancer Hospital, Shanghai, China; [3]Hematology Department, Univ Toulouse, CHU Toulouse, IUCT-Oncopole, Toulouse, France;[4]Aidport Clinical Trials Hospital, Skórzewo (Poznan), Poland; [5]Department of Hematology, D'or Institute for Research and Education, São Paulo, Brazil; [6]Laikon General Hospital, National and Kapodistrian University of Athens, Athens, Greece;[7]University Medical Center Groningen, University of Groningen, Groningen, The Netherlands; [8]Fiona Stanley Hospital, Murdoch, Australia; [9]Texas Oncology-Tyler, US Oncology Research, Tyler, United States of America; [10]Department of Hematology Oncology, Cancer Institute Hospital, Japanese Foundation for Cancer Research, Tokyo, Japan; [11]AbbVie Inc., North Chicago, United States of America; [12]Genmab US, Inc, Plainsboro, United States of America; [13]The Christie NHS Foundation Trust, Manchester Cancer Research Centre, and Division of Cancer Sciences, University of Manchester, Manchester, United Kingdom;*

### Background

Chemoimmunotherapy (CIT) is commonly used to treat relapsed/refractory follicular lymphoma (R/R FL). However, cumulative chemotoxicities, treatment burden, and limited response durability with these regimens highlight the need for novel chemotherapy-free immunotherapies that provide deep and durable responses, and longer survival. Epcoritamab (E) is a subcutaneous CD3×CD20 bispecific antibody approved as monotherapy in third-line or later (3L+) FL and recently approved as 12-cycle fixed-duration in combination with lenalidomide and rituximab ($R^2$) in R/R FL based on the randomized Phase 3 EPCORE FL-1 trial (NCT05409066) showing superiority over $R^2$ (progression-free survival hazard ratio [PFS HR] 0.21, 95% CI 0.14–0.31; *P*<.0001) and a manageable safety profile (Falchi et al *Lancet* 2026).

### Aims

To compare efficacy of E+$R^2$ from EPCORE FL-1 with real-world CIT in R/R FL.

### Methods

Individual patient (pt) data from EPCORE FL-1 E+$R^2$ arm (24May2025 cutoff; median follow-up 14.8 mo) and COTA electronic health records for pts with R/R FL treated with CIT at US community and academic centers (Jan 2010–Dec 2024; COTA Healthcare, NY, USA; median follow-up 24.6 mo) were analyzed. Generalized estimation equations were used to match CIT lines of therapy (LOTs). Inverse probability of treatment weighting was used to balance demographic and baseline characteristics (age ≥65 y, sex, ethnicity, ECOG performance status, Ann Arbor stage, prior LOTs, disease progression within 24 mo of initial first-line CIT, double refractoriness, low hemoglobin [<12 g/dL], high lactate dehydrogenase [LDH; >280U/L], refractory to last LOT, refractory to prior anti-CD20). Efficacy outcomes included overall response rate (ORR), complete response (CR), duration of response (DOR), duration of CR (DOCR), PFS, and overall survival (OS). Pts without progression or death (DOR, DOCR, PFS) or without death (OS) were censored at last follow-up. Weighted logistic regression and Cox proportional hazards models estimated relative risks (RRs) and HRs, respectively.

**Copyright Information:** (Online) ISSN: 2572-9241
©2026 The Author(s). HemaSphere is published by John Wiley & Sons Ltd on behalf of European Hematology Association. This is an open access Abstract Book distributed under the Attribution-NonCommercial-NoDerivs (CC BY-NC-ND), which allows third parties to download the articles and share them with others as long as they credit the author and the Abstract Book, but they cannot change the content in any way or use them commercially.
**Abstract Book Citations:** Authors, Title, HemaSphere, 2026;10:(S1):pages. The abstract book DOIs can be found on the "Author Information" page.
**Disclaimer:** Articles published in the journal HemaSphere exclusively reflect the opinions of the authors. The authors are responsible for all content in their abstracts including accuracy of the facts, statements, citing resources, etc.

### Results

The analysis included 243 pts from EPCORE FL-1 who received E+$R^2$ and 274 pts from COTA who received CIT (bendamustine-based 69.3%; CHOP-based 16.4%; CVP-based 6.2%; other chemo 8.0%). Prior to match adjustment, the E+$R^2$ arm included fewer older pts (≥65 y: 36.2% vs 59.1%); fewer Hispanic/Latino pts (11.9% vs 21.2%); more pts with elevated LDH (35.0% vs 18.2%); and more pts with 2+ prior LOTs (≥2: 40.3% vs 28.1%) than the CIT arm. Arms were balanced after weighting. After adjustment, the E+$R^2$ arm showed higher ORR (95.1% vs 82.7%; RR 1.15, 95% CI 1.08–1.23) and CR (84.2% vs 55.8%; RR 1.51, 95% CI 1.32–1.72) vs CIT, with better DOR (16-mo rate: 81.7% vs 53.0%) and DOCR (16-mo rate: 79.3% vs 58.3%). E+$R^2$ significantly improved PFS (HR 0.33, 95% CI 0.21–0.50; $P$<.0001) and OS (HR 0.18, 95% CI 0.09–0.38; $P$<.0001) vs CIT, with E+$R^2$ median PFS and OS not reached (NR; **Table, Figure**).

**Table. Comparative Efficacy**

| **Adjusted outcome (95% CI)** | **E+$R^2$ (n=243, ESS=199)** | **CIT (n=274, ESS=220)** |
|---|---|---|
| **PFS** | | |
| Median, mo | NR | 20.4 (17.4–25.9) |
| 16-mo rate, % | 86.9 (82.1–92.1) | 58.4 (51.5–66.3) |
| **OS** | | |
| Median, mo | NR | 60.7 (44.5–90.2) |
| 16-mo rate, % | 95.6 (92.6–98.7) | 76.4 (70.7–82.5) |

ESS, effective sample size.

### Summary/Conclusion

In this match-adjusted analysis, E+$R^2$ showed significantly improved depth and durability of response and longer PFS and OS compared with CIT regimens. Taken together with superior efficacy vs $R^2$ demonstrated in the trial, these findings position E+$R^2$ as the new chemotherapy-free standard of care for 2L+ FL.

**Copyright Information:** (Online) ISSN: 2572-9241
©2026 The Author(s). HemaSphere is published by John Wiley & Sons Ltd on behalf of European Hematology Association. This is an open access Abstract Book distributed under the Attribution-NonCommercial-NoDerivs (CC BY-NC-ND), which allows third parties to download the articles and share them with others as long as they credit the author and the Abstract Book, but they cannot change the content in any way or use them commercially.
**Abstract Book Citations:** Authors, Title, HemaSphere, 2026;10:(S1):pages. The abstract book DOIs can be found on the "Author Information" page.
**Disclaimer:** Articles published in the journal HemaSphere exclusively reflect the opinions of the authors. The authors are responsible for all content in their abstracts including accuracy of the facts, statements, citing resources, etc.

**Figure. Adjusted PFS (A) and OS (B) for E+R² From EPCORE FL-1 vs CIT From COTA in R/R FL**

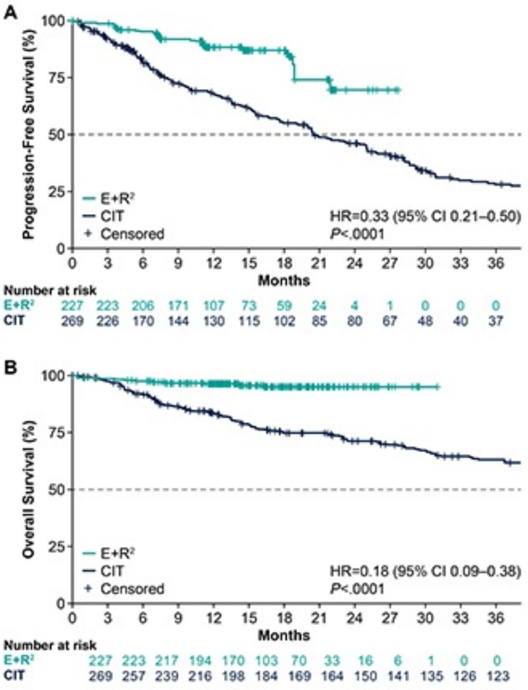

CIT, chemoimmunotherapy; E+R², epcoritamab plus lenalidomide and rituximab; FL, follicular lymphoma; HR, hazard ratio; OS, overall survival; PFS, progression-free survival; R/R, relapsed/refractory.

**Copyright Information:** (Online) ISSN: 2572-9241
©2026 The Author(s). HemaSphere is published by John Wiley & Sons Ltd on behalf of European Hematology Association. This is an open access Abstract Book distributed under the Attribution-NonCommercial-NoDerivs (CC BY-NC-ND), which allows third parties to download the articles and share them with others as long as they credit the author and the Abstract Book, but they cannot change the content in any way or use them commercially.
**Abstract Book Citations:** Authors, Title, HemaSphere, 2026;10:(S1):pages. The abstract book DOIs can be found on the “Author Information” page.
**Disclaimer:** Articles published in the journal HemaSphere exclusively reflect the opinions of the authors. The authors are responsible for all content in their abstracts including accuracy of the facts, statements, citing resources, etc.

### (PS2043) CELESTIMO SINGLE ARM EXTENSION: SAFETY AND EFFICACY OF MOSUNETUZUMAB PLUS LENALIDOMIDE IN PATIENTS WITH R/R FOLLICULAR LYMPHOMA

**Topic:** 18. Indolent and mantle-cell non-Hodgkin lymphoma - Clinical

L. Elizabeth Budde*[1], Dahlio Sano[2], Nancy Bartlett[3], Nina Wagner-Johnston[4], Brett Brinker[5], Rakhee Vaidya[6], Sunil Babu[7], Chijioke Nze[8], Edward Pearson[9], Matthew McKinney[10], Ruemu Birhiray[11], Elicia Penuel[12], Connie Y. MA[12], Andrea Knapp[13], Michelle Doral[12], Vivian Chen[12], Paloma Hauser[12], Michael C. Wei[12], Enkhtsetseg Purev[12], Catherine Diefenbach[14]

*[1]City of Hope National Medical Center, Duarte, CA, United States of America;[2]Division of Hematology/Oncology, University of Michigan, Ann Arbor, MI, United States of America; [3]Siteman Cancer Center, Washington University School of Medicine, St. Louis, MO, United States of America; [4]Johns Hopkins University, Baltimore, MD, United States of America;[5]The Cancer and Hematology Centers of West Michigan, Grand Rapids, MI, United States of America; [6]Wake Forest School of Medicine, 1 Medical Center Blvd, Winston-Salem, NC, United States of America; [7]Fort Wayne Medical Oncology & Hematology, Fort Wayne, IN, United States of America; [8]The University of Texas MD Anderson Cancer Center, Houston, TX, United States of America;[9]Baylor University Medical Center, Dallas, TX, United States of America; [10]Duke University Medical Center, Durham, NC, United States of America; [11]Investigative Clinical Research of Indiana, LLC, Indianapolis, IN, United States of America;[12]Genentech, Inc., South San Francisco, CA, United States of America; [13]F. Hoffmann-La Roche Ltd, Basel, Switzerland; [14]Perlmutter Cancer Center at NYU Langone Health, New York, NY, United States of America;*

### Background

CELESTIMO (NCT04712097) is a randomized, multi-center, Phase 3 study evaluating the efficacy and safety of mosunetuzumab plus lenalidomide (M+Len) vs rituximab plus lenalidomide in patients (pts) with relapsed/refractory (R/R) follicular lymphoma (FL). The non-randomized, single arm extension of CELESTIMO further evaluates the efficacy and safety of M+Len in US patients.

### Aims

Present minimal residual disease (MRD) and updated efficacy, safety data.

### Methods

Pts with R/R FL (≥1 prior therapy) received 12 cycles of M+Len (Cycle [C] 1, 21 days; C2–12, 28 days). In C1, step-up doses of M (IV infusion) are given on C1 Day (D)1 (1mg) and C1D8 (2mg), with the target dose given on C1D15 (30mg) and on D1 of C2–12. Len (20mg orally) is administered on D1–21 of C2–12. No hospitalization is mandated. G-CSF use was optional. Antiviral prophylaxis was per investigator choice. Efficacy was assessed by investigators using Lugano 2014 criteria; safety per CTCAE v5.0 and ASTCT (Lee et al. 2019). MRD was assessed in longitudinal PBMC samples via clonoSEQ next-generation sequencing.

**Copyright Information:** (Online) ISSN: 2572-9241
©2026 The Author(s). HemaSphere is published by John Wiley & Sons Ltd on behalf of European Hematology Association. This is an open access Abstract Book distributed under the Attribution-NonCommercial-NoDerivs (CC BY-NC-ND), which allows third parties to download the articles and share them with others as long as they credit the author and the Abstract Book, but they cannot change the content in any way or use them commercially.
**Abstract Book Citations:** Authors, Title, HemaSphere, 2026;10:(S1):pages. The abstract book DOIs can be found on the “Author Information” page.
**Disclaimer:** Articles published in the journal HemaSphere exclusively reflect the opinions of the authors. The authors are responsible for all content in their abstracts including accuracy of the facts, statements, citing resources, etc.

### Results

At data cut-off (January 12, 2026), 54 pts were enrolled. Median age was 62 years (range: 37-82 years); 32 pts (59.3%) were male. All pts had an ECOG performance status of 0-1 (53 pts, 98.2%) or 2 (1 pts, 1.9%). Median number of prior lines of therapy was 1 (range: 1-5); 83.3% of pts had Ann Arbor Stage III-IV; 40.7% had a FLIPI score of ≥3; 44.4% had POD24 disease; 42.6% were refractory to prior anti-CD20 therapy; and 20.4% of pts had double-refractory disease.
At data cut-off, ORR was 96.3% (n=52/54; 95% CI: 87.25; 99.55) and CRR was 88.9% (n=48/54; 95% CI: 77.37; 95.81). After a median follow-up of 20.0 months (range: 17.5-21.8), median DOR, DOCR, PFS and OS were not reached. The 18-month event-free rates for DOR, DOCR, PFS and OS were 88.2% (95% CI: 79.32; 97.07), 93.6% (95% CI: 86.5; 100), 85% (95% CI: 75.41; 94.59), and 92.3% (95% CI: 85.01; 99.56), respectively. All pts experienced at least one any-grade AE and 63.0% experienced a Grade (Gr) 3–4 AE. Serious AEs occurred in 31.5% of patients, and 13.0% and 24.1% of patients had an AE that led to mosunetuzumab and lenalidomide withdrawal, respectively. One pt had a fatal AE of pneumonia in the setting of prolonged COVID positivity. Cytokine release syndrome (CRS) occurred in 31.5% of pts (Gr 1, 25.9%; Gr 2, 3.7%). One case of Grade 3 CRS occurred after C1D1. The median duration of CRS was 3.5 days (range: 1.0–23.0). All CRS events resolved. No events of Immune Effector Cell-Associated Neurotoxicity Syndrome (ICANS) occurred. Grade 3-4 neutropenia occurred in 35.2% and febrile neutropenia occurred in 5.6% of pts (all Gr3-4). Infections occurred in 63.0% of pts (Gr 3-4, 9.3%). Most common infections: COVID-19, 20.4%; sinusitis, upper respiratory tract infection 18.5% each and were mainly Gr 1-2 in severity. At 1E-5 sensitivity, MRD was evaluable for 38/54 pts (70%); 22 (58%) were detectable and 16 (42%) were undetectable at baseline. For all detectable pts, MRD fell below the detection limit as early as Cycle 3.

### Summary/Conclusion

M+Len resulted in a high and durable response in pts with R/R FL. Adverse events were manageable and consistent with established safety profiles of mosunetuzumab and lenalidomide, with no new safety findings identified. These results demonstrate the fixed-duration, chemotherapy-free, M+Len achieves promising efficacy and a favorable safety profile, supporting the ongoing pivotal randomized cohort of the CELESTIMO trial.

**Copyright Information:** (Online) ISSN: 2572-9241
©2026 The Author(s). HemaSphere is published by John Wiley & Sons Ltd on behalf of European Hematology Association. This is an open access Abstract Book distributed under the Attribution-NonCommercial-NoDerivs (CC BY-NC-ND), which allows third parties to download the articles and share them with others as long as they credit the author and the Abstract Book, but they cannot change the content in any way or use them commercially.
**Abstract Book Citations:** Authors, Title, HemaSphere, 2026;10:(S1):pages. The abstract book DOIs can be found on the “Author Information” page.
**Disclaimer:** Articles published in the journal HemaSphere exclusively reflect the opinions of the authors. The authors are responsible for all content in their abstracts including accuracy of the facts, statements, citing resources, etc.

### (PS2044) HOSPITAL BURDEN OF CAR T INFUSION OF PATIENTS WITH FOLLICULAR LYMPHOMA OR MANTLE CELL LYMPHOMA IN FRANCE: A NATIONWIDE RETROSPECTIVE STUDY

**Topic:** 18. Indolent and mantle-cell non-Hodgkin lymphoma - Clinical

Catherine Thieblemont[1], Florian Colrat*[2], Denis Caillot[3], François-Emery Cotté[2], Alexandre Caron[4], Camille Nevoret[4], Elodie Torreton[4], Sébastien Branchoux[2], Fabien Despas[5]

*[1]APHP, Hopital Saint-Louis, Hemato-oncologie, Paris, France;[2]Bristol Myers Squibb, Rueil Malmaison, France; [3]Institut de Cancérologie de Bourgogne, Dijon, France; [4]CEMKA, Bourg-la-Reine, France; [5]University Hospital Center CHU of Toulouse, Toulouse, France;*

### Background

CAR T therapies have been available in France for the treatment of patients (pts) with mantle cell lymphoma (MCL) since 2021 and for follicular lymphoma (FL) since 2022. Brexu-cel is available for pts with MCL after at least 2 lines of therapy (LOT), tisa-cel for pts with FL after at least 2 LOT, and axi-cel for pts with FL after at least 3 LOT.

### Aims

The objective was to describe infusion stays of all French pts infused with a CAR T for these indications since availability.

### Methods

A retrospective cohort study was conducted using the comprehensive hospital stay database (PMSI). Included pts were hospitalized for a CAR T infusion, had an indication code for MCL or FL, and were discharged between 1/1/2021 and 1/31/2024. Time from hospitalization to the index date (date of CAR T infusion) was the pre-infusion period. Time from the index to the discharge date was the post-infusion period. A descriptive analysis was performed of pts and hospital stay characteristics, including healthcare resource utilization (HCRU), and 3-month rehospitalization rate (at least one hospitalization among pts with at least 3-month follow-up).

### Results

Overall, 495 pts were treated with a CAR T for MCL (n=225) or FL (n=270) and included in the study. All pts with MCL were treated with brexu-cel; mean age 67.2 years (yrs), 79% male. Mean length of stay (LOS) was 27.4 days (d), including 7.2d of pre-infusion period and 20.2d of post-infusion period. During this stay, 25% of patients required intensive care unit (ICU) and 66% received additional high-cost medication (as per French law, e.g., tocilizumab). In the 3 months following discharge, 65% of pts (n=138) were re-hospitalized. Pts with FL treated with CAR T received tisa-cel (n=220) or axi-cel (n=50). Mean age was 62.8 yrs for pts treated with tisa-cel and 62.2 yrs for those treated with axi-cel; 67% and 74% were male. Mean LOS was 18.1d for tisa-cel and 25.0d for axi-cel. Pts treated with tisa-cel had shorter pre-infusion (5.3d) and post-infusion (12.8d) durations than those treated with axi-cel (7.1d and 17.9d). Pts who received tisa-cel required ICU in 5% of cases (n=11) while those treated with axi-cel in 18% of cases (n=9). High-cost medications were administered to 50% pts treated with tisa-cel (n=110) and 66% pts treated with axi-cel (n=33). 3-month rehospitalization rate was 52% (n=102) for pts treated with tisa-cel and 63.0% (n=29) for those treated with axi-cel.

**Copyright Information:** (Online) ISSN: 2572-9241
©2026 The Author(s). HemaSphere is published by John Wiley & Sons Ltd on behalf of European Hematology Association. This is an open access Abstract Book distributed under the Attribution-NonCommercial-NoDerivs (CC BY-NC-ND), which allows third parties to download the articles and share them with others as long as they credit the author and the Abstract Book, but they cannot change the content in any way or use them commercially.
**Abstract Book Citations:** Authors, Title, HemaSphere, 2026;10:(S1):pages. The abstract book DOIs can be found on the “Author Information” page.
**Disclaimer:** Articles published in the journal HemaSphere exclusively reflect the opinions of the authors. The authors are responsible for all content in their abstracts including accuracy of the facts, statements, citing resources, etc.

| | MCL | FL | |
|---|---|---|---|
| | Brexu-cel | Tisa-cel | Axi-cel |
| N | 225 | 220 | 50 |
| **Patient characteristics** | | | |
| Age, years, mean (std) | 67.2 (9.6) | 62.8 (10.1) | 62.2 (10.0) |
| Gender, male, n (%) | 178 (79.1) | 148 (67.3) | 37 (74.0) |
| **Period of duration,** days, mean (std) | | | |
| Pre-infusion | 7.2 (7.0) | 5.3 (2.3) | 7.1 (6.4) |
| Post-infusion | 20.2 (15.0) | 12.8 (6.1) | 17.9 (10.7) |
| From admission to discharge | 27.4 (17.3) | 18.1 (7.0) | 25.0 (16.2) |
| **HCRU,** n (%) | | | |
| Admission to ICU | 56 (24.9) | 11 (5.0) | 9 (18.0) |
| High-cost medication | 149 (66.2) | 110 (50.0) | 33 (66.0) |
| **Rehospitalization** | | | |
| N | 211 | 198 | 46 |
| 3-month rate, n (%) | 138 (65.4) | 102 (51.5) | 29 (63.0) |

### Summary/Conclusion

For MCL and FL pts, this nationwide analysis underscores the burden incurred with currently available CAR Ts. In pts with FL, burden differed according to CAR T, with axi-cel associated with longer hospitalization periods and ICU admissions than tisa-cel, although both resulted in considerable use of high-cost medications and 3-month re-hospitalization rates. These findings underscore the ongoing unmet need for CAR T treatment strategies and care delivery approaches that may reduce hospital based resource utilization in patients with MCL and FL.

**Copyright Information:** (Online) ISSN: 2572-9241
©2026 The Author(s). HemaSphere is published by John Wiley & Sons Ltd on behalf of European Hematology Association. This is an open access Abstract Book distributed under the Attribution-NonCommercial-NoDerivs (CC BY-NC-ND), which allows third parties to download the articles and share them with others as long as they credit the author and the Abstract Book, but they cannot change the content in any way or use them commercially.
**Abstract Book Citations:** Authors, Title, HemaSphere, 2026;10:(S1):pages. The abstract book DOIs can be found on the "Author Information" page.
**Disclaimer:** Articles published in the journal HemaSphere exclusively reflect the opinions of the authors. The authors are responsible for all content in their abstracts including accuracy of the facts, statements, citing resources, etc.

### (PS2045) REAL-WORLD OUTCOMES OF BISPECIFIC ANTIBODIES IN RELAPSED/REFRACTORY FOLLICULAR LYMPHOMA

**Topic:** 18. Indolent and mantle-cell non-Hodgkin lymphoma - Clinical

Rahul Shah*[1], Jessica Allen[2], Lei Feng[3], Lorenzo Falchi[4], Ayushi Chauhan[1], Sonia Godbole[5], Swetha Kambhampati[6], Ajay Major[7], Andrew Jallouk[8], Reid Merryman[9], Amy Ayers[1], Jennifer L. Crombie[9], Shakthi Bhaskar[8], Steven Bair[7], Alex F. Herrera[6], Yazeed Sawalha[5], Gilles Salles[4], Narendranath Epperla[2], Allison Bock[2], Sairah Ahmed[1]

*[1]MD Anderson Cancer Center, Department of Lymphoma and Myeloma, Houston, United States of America;[2]University of Utah/Huntsman Cancer Institute, Salt Lake City, United States of America; [3]MD Anderson Cancer Center, Department of Biostatistics, Houston, United States of America; [4]Memorial Sloan Kettering Cancer Center, New York City, United States of America; [5]Ohio State University, Columbus, United States of America;[6]City of Hope, Duarte, United States of America; [7]University of Colorado, Aurora, United States of America;[8]Vanderbilt University Medical Center, Nashville, United States of America; [9]Dana-Farber Cancer Institute, Boston, United States of America;*

### Background

Mosunetuzumab (mosun) and epcoritamab (epco) are bispecific antibodies (BsAbs) approved for relapsed/refractory (R/R) follicular lymphoma (FL), with the pivotal trials (n=90-128) initially demonstrating 60-63% complete response rate (CRR), 80-82% overall response rate (ORR), and 1-year (yr) progression-free survival (PFS) rate of 58-66%. Real world outcomes remain limited. We report the real-world efficacy and safety of BsAbs for patients (pts) with R/R FL treated in the Collaborative United States Bispecifics Consortium (CUBIC).

### Aims

To characterize real-world efficacy and safety outcomes among patients with R/R FL treated with bispecific antibodies.

### Methods

This is a multicenter retrospective study of adult pts with R/R FL treated with standard of care mosun or epco between 2/2023 and 9/2025 at eight CUBIC centers. Outcomes included ORR, CRR, PFS, and overall survival (OS). PFS and OS were estimated using the Kaplan-Meier method. Safety outcomes included incidence and grade (G) of cytokine release syndrome (CRS) and immune effector cell-associated neurotoxicity syndrome (ICANS).

**Copyright Information:** (Online) ISSN: 2572-9241
©2026 The Author(s). HemaSphere is published by John Wiley & Sons Ltd on behalf of European Hematology Association. This is an open access Abstract Book distributed under the Attribution-NonCommercial-NoDerivs (CC BY-NC-ND), which allows third parties to download the articles and share them with others as long as they credit the author and the Abstract Book, but they cannot change the content in any way or use them commercially.
**Abstract Book Citations:** Authors, Title, HemaSphere, 2026;10:(S1):pages. The abstract book DOIs can be found on the "Author Information" page.
**Disclaimer:** Articles published in the journal HemaSphere exclusively reflect the opinions of the authors. The authors are responsible for all content in their abstracts including accuracy of the facts, statements, citing resources, etc.

**Results**

A total of 99 pts with R/R FL were treated with BsAbs, with 92% receiving mosun (n=91) and 8% (n=8) receiving epco. Median age was 68 years (range 33-101), 61% were ≥65 years (n=60), 63% were male (n=62), 8% were Hispanic (n=8), 92% (n=79) had ECOG performance status (PS) of 0-1, 51% (n=42) had FLIPI ≥3, and 31% (n=30) had elevated LDH. Median prior lines of therapy were 3 (range, 1-14); 15% had received prior CAR-T (n=15), 51% prior bendamustine (n=50), 53% prior lenalidomide (n=52), 63% prior anthracycline (n=62), and all had previously received an anti-CD20 monoclonal antibody. Forty-five (46%) of patients had progression of disease within 24 months (POD24) after frontline therapy.

Among 96 pts with complete response data, the ORR was 87% and CRR was 72%. Median time to best response was 2.9 months (mo) (range 1.2-12.7). Response rates were not significantly associated with BsAb, ECOG PS, FLIPI, elevated LDH, POD24 status, or prior CAR-T. Prior exposure to bendamustine was significantly associated with a lower CRR (58% vs 85%, p=0.0059).

At median follow-up of 16.9 mo, PFS rates were 72% (95%CI 62-83%) at 1 yr and 56% (95%CI 45-70%) at 18 mo. OS rates were 94% (95%CI 90-99%) at 1 yr and 92% (95%CI 86-99%) at 18 mo. PFS was not significantly influenced by BsAb product, elevated LDH, ECOG PS, FLIPI, POD24 status, or prior CAR-T. Patients with ECOG of 2 had inferior OS than pts with ECOG of 0-1 (2-yr OS 43% vs 89%, p=0.02).

Of 96 pts with complete safety data, CRS occurred in 34% of pts (G1 26%; G2 6%), with no ≥G3 events. ICANS occurred in 3% of pts (n=3), all G1 events. The incidence of CRS or ICANS was not significantly associated with age, LDH, ECOG, FLIPI, or prior CAR-T.

**Summary/Conclusion**

In this diverse, heavily pretreated real-world cohort, bispecific antibodies for R/R FL demonstrated high response rates, favorable survival outcomes, lower rates of cytokine release syndrome—primarily due to fewer grade ≥2 events—and ICANS rates comparable to those observed in pivotal clinical trials, supporting their use across heterogeneous populations.

**Copyright Information:** (Online) ISSN: 2572-9241
©2026 The Author(s). HemaSphere is published by John Wiley & Sons Ltd on behalf of European Hematology Association. This is an open access Abstract Book distributed under the Attribution-NonCommercial-NoDerivs (CC BY-NC-ND), which allows third parties to download the articles and share them with others as long as they credit the author and the Abstract Book, but they cannot change the content in any way or use them commercially.
**Abstract Book Citations:** Authors, Title, HemaSphere, 2026;10:(S1):pages. The abstract book DOIs can be found on the "Author Information" page.
**Disclaimer:** Articles published in the journal HemaSphere exclusively reflect the opinions of the authors. The authors are responsible for all content in their abstracts including accuracy of the facts, statements, citing resources, etc.

### (PS2046) A DEEP LEARNING PET/CT BIOMARKER FOR EARLY PROGRESSION (POD24) AND SURVIVAL STRATIFICATION IN FOLLICULAR LYMPHOMA: A MULTICENTER STUDY

**Topic:** 18. Indolent and mantle-cell non-Hodgkin lymphoma - Clinical

Chong Jiang*[1], Zekun Jiang[2], Jing-Yan Xu[3], Ming Jiang[4], Xu Bing[5]

[1]*West China Hospital, Sichuan University, Department of Nuclear Medicine, Chengdu, China;*[2]*West China Hospital, Sichuan University, Chengdu, China;* [3]*Nanjing Drum Tower Hospital, Nanjing University Medical School, Nanjing, China;* [4]*West China Hospital, Sichuan University, Department of Oncology, Chengdu, China;* [5]*the first affiliated hospital of xiamen university, Xiamen, China;*

### Background

Early disease progression within 24 months (POD24) is a critical clinical endpoint in follicular lymphoma (FL), strongly predicting adverse long-term survival. While conventional clinical indices like FLIPI offer prognostic value, they often perform suboptimally for individualized risk assessment. Baseline [$^{18}$F]FDG PET/CT provides valuable metabolic information, but standard metrics often fail to capture the complex spatial heterogeneity associated with treatment resistance.

### Aims

To develop and validate a prognostic imaging biomarker derived from baseline [$^{18}$F]FDG PET/CT using tabular deep learning for the prediction of POD24 and survival risk stratification in patients with FL.

### Methods

This retrospective study included 309 patients with newly diagnosed FL (grades 1-3a) from five medical centers. High-throughput radiomic features (1,106 per modality) were extracted from tumor volumes segmented on baseline PET and CT images. Five conventional machine learning algorithms and four advanced tabular deep learning foundation models (GAMformer, MotherNet, TabPFN, TabFlex) were developed and compared. The predictive output of the best-performing model was defined as the deep learning score (DLS). A multiparametric model was then constructed by integrating the DLS with independent clinical and metabolic predictors identified via multivariate analysis.

### Results

The GAMformer model achieved the best generalization, with an AUC of 0.857 in the training cohort and 0.753 in the validation cohort for POD24 prediction, outperforming other algorithms ($P < 0.05$). Multivariate analysis identified LDH levels and metabolic tumor volume (MTV) as independent prognostic factors. The integrated multiparametric model (DLS + LDH + MTV) further improved performance, achieving AUCs of 0.882 (training) and 0.797 (validation), significantly outperforming FLIPI, FLIPI-2, and PRIMA-PI ($P < 0.01$). Kaplan–Meier analysis showed the DLS significantly stratified overall survival (OS) across all cohorts ($P < 0.0001$) and remained robust within both low-grade (1-2) and high-grade (3a) histological subgroups.

### Summary/Conclusion

The deep learning score (DLS) derived from baseline PET/CT is a robust biomarker for predicting POD24 and stratifying survival risk in FL. The resulting multiparametric model provides superior prognostic accuracy compared to conventional indices, supporting its potential for guiding risk-adapted first-line management and precision oncology.

**Copyright Information:** (Online) ISSN: 2572-9241
©2026 The Author(s). HemaSphere is published by John Wiley & Sons Ltd on behalf of European Hematology Association. This is an open access Abstract Book distributed under the Attribution-NonCommercial-NoDerivs (CC BY-NC-ND), which allows third parties to download the articles and share them with others as long as they credit the author and the Abstract Book, but they cannot change the content in any way or use them commercially.
**Abstract Book Citations:** Authors, Title, HemaSphere, 2026;10:(S1):pages. The abstract book DOIs can be found on the "Author Information" page.
**Disclaimer:** Articles published in the journal HemaSphere exclusively reflect the opinions of the authors. The authors are responsible for all content in their abstracts including accuracy of the facts, statements, citing resources, etc.

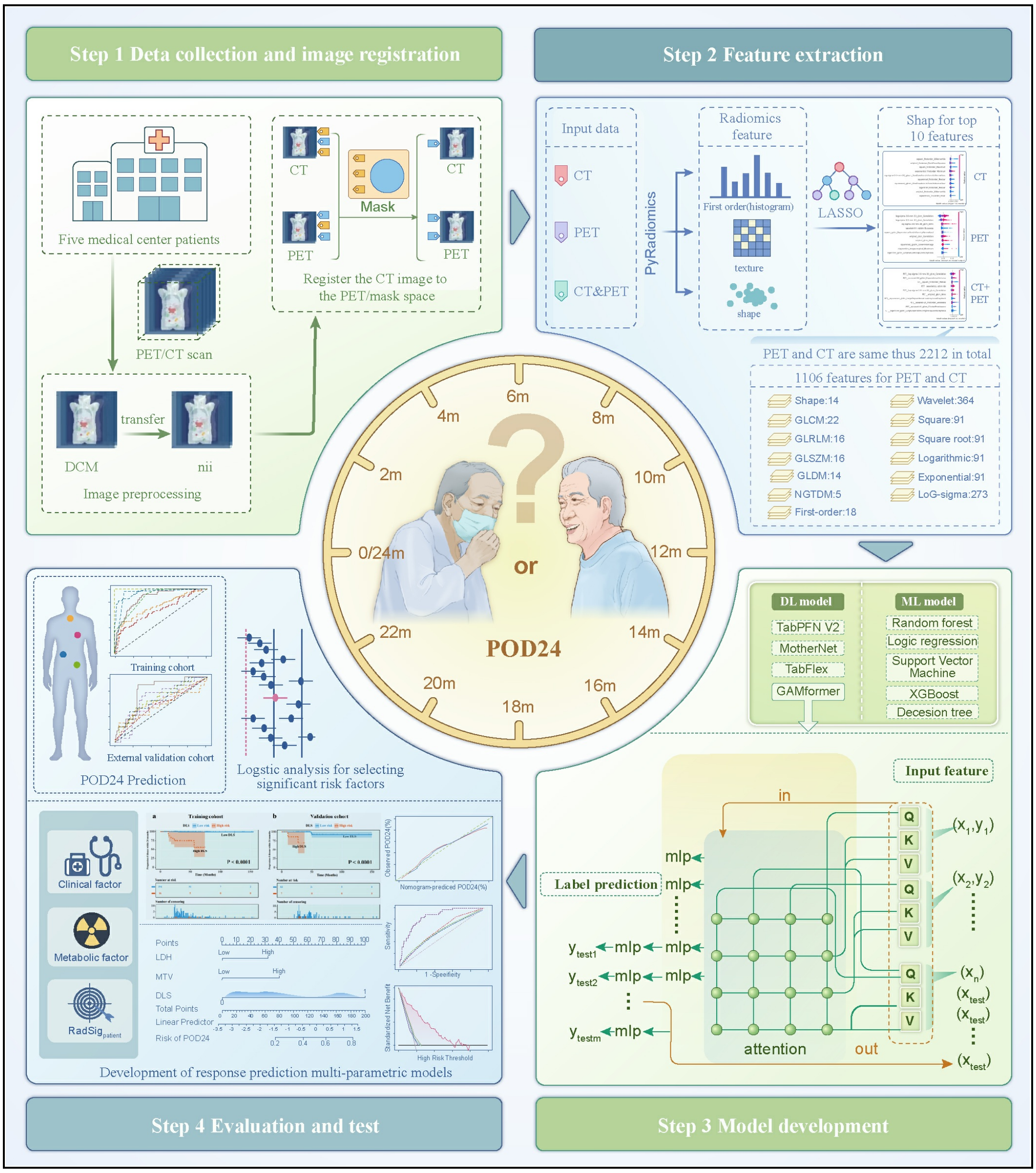

**Copyright Information:** (Online) ISSN: 2572-9241
©2026 The Author(s). HemaSphere is published by John Wiley & Sons Ltd on behalf of European Hematology Association. This is an open access Abstract Book distributed under the Attribution-NonCommercial-NoDerivs (CC BY-NC-ND), which allows third parties to download the articles and share them with others as long as they credit the author and the Abstract Book, but they cannot change the content in any way or use them commercially.
**Abstract Book Citations:** Authors, Title, HemaSphere, 2026;10:(S1):pages. The abstract book DOIs can be found on the “Author Information” page.
**Disclaimer:** Articles published in the journal HemaSphere exclusively reflect the opinions of the authors. The authors are responsible for all content in their abstracts including accuracy of the facts, statements, citing resources, etc.

### (PS2047) REAL-WORLD EPIDEMIOLOGY TRENDS AND SURVIVAL CHARACTERISTICS OF EXTRANODAL MARGINAL ZONE LYMPHOMA BY ANATOMICAL SUBTYPE IN ENGLAND FROM 2014-2022: FINDINGS FROM THE UNCOVER STUDY PROGRAMME.

**Topic:** 18. Indolent and mantle-cell non-Hodgkin lymphoma - Clinical

Li Yuan Chan*[1], Temitope Erinfolami[2], Indrani Karpha[2, 3], Nurunnahar Akter[2], Toyyib Abdulkareem[2], Yeong Jer Lim[2, 3], Brogan Johnston[3], Hanhua Liu[4], Shankara Paneesha[5, 6], Geetha Menon[7], Andrew Wotherspoon[1], Nagesh Kalakonda[2, 3], Mark Bishton[8, 9], Renata Walewska[10], Catrin Tudur Smith[2], Kim Linton[11, 12, 13], Andrew Pettitt[2, 3], Sunil Iyengar[1]

*[1]The Royal Marsden Hospital NHS Foundation Trust, Haemato-oncology, London, United Kingdom;[2]University of Liverpool, Liverpool, United Kingdom; [3]The Clatterbridge Cancer Centre NHS Foundation Trust, Liverpool, United Kingdom;[4]National Disease Registration Service, NHS England, England, United Kingdom; [5]University Hospitals Birmingham NHS Foundation Trust, Birmingham, United Kingdom; [6]University of Birmingham, Birmingham, United Kingdom; [7]Liverpool University Hospitals NHS Foundation Trust, Liverpool, United Kingdom; [8]University of Nottingham, Nottingham, United Kingdom; [9]Nottingham University Hospitals NHS Trust, Nottingham, United Kingdom; [10]Royal Bournemouth Hospital, Bournemouth, United Kingdom; [11]The Christie NHS Foundation Trust, Manchester, United Kingdom; [12]Manchester Cancer Research Centre, Manchester, United Kingdom; [13]University of Manchester, Division of Cancer Sciences, Manchester, United Kingdom;*

### Background

Extranodal marginal zone lymphomas (EMZL) form a group of indolent B-cell neoplasms. subcategorised by their anatomical site. Although survival outcomes are generally favourable, population-based cohort studies have reported site-specific differential outcomes.

### Aims

We aimed to describe the epidemiology and survival of EMZL patients in England within the UNCOVER programme using population-based data from the National Cancer Registration Dataset (NCRD).

### Methods

NCRD data on blood cancer diagnoses made between January 2014 and December 2022 were interrogated using ICD-O-3 code 9699/3. Cases were assigned as EMZL by excluding biopsy sites coded as lymph node, blood, bone marrow or spleen, and subcategorised by anatomical groups. Documentation of associated conditions were extracted using data from linked hospital episode statistics.

Analysis included crude and age-standardised incidence rates (ASIR), incidence rate ratio (IRR) by Poisson regression, overall survival (OS), cumulative incidence of mortality (CIM) and multivariate analysis (MVA) by Cox regression. Models were adjusted for disease subtype, age, gender, ethnicity, region, index of multiple deprivation (IMD), Charlson Comorbidity Index (CCI), and year of diagnosis.

**Copyright Information:** (Online) ISSN: 2572-9241
©2026 The Author(s). HemaSphere is published by John Wiley & Sons Ltd on behalf of European Hematology Association. This is an open access Abstract Book distributed under the Attribution-NonCommercial-NoDerivs (CC BY-NC-ND), which allows third parties to download the articles and share them with others as long as they credit the author and the Abstract Book, but they cannot change the content in any way or use them commercially.
**Abstract Book Citations:** Authors, Title, HemaSphere, 2026;10:(S1):pages. The abstract book DOIs can be found on the "Author Information" page.
**Disclaimer:** Articles published in the journal HemaSphere exclusively reflect the opinions of the authors. The authors are responsible for all content in their abstracts including accuracy of the facts, statements, citing resources, etc.

**Results**

5,605 EMZL cases were identified, with median age 70yrs (IQR:59-78), 55% female and 85% White ethnicity. Median follow up was 70 months (95% CI:64-76). Crude and ASIR per million persons per year were 14.2 (95% CI:13.8-14.5) and 11.8 (95%CI: 11.2-12.5). EMZL cases were subcategorised into 1,078 (19%) gastric, 824 (15%) pulmonary, 794 (14%) ocular, 556 (10%) skin, 371 (7%) salivary gland, 368 (7%) non-gastric gastrointestinal (GI), 262 (5%) bone/connective tissue, 248 (4%) genitourinary, 229 (4%) oropharyngeal, 160 (3%) breast, 93 (2%) thyroid, 146 (3%) others and 476 (8%) unknown.

On MVA, IRR was lower in mixed 0.26 (95% CI:0.19-0.35, p<0.001), Asian 0.56 (95% CI:0.49-0.63, p<0.001), and Black/Caribbean 0.82 (95% CI:0.69-0.96, p<0.001) vs White ethnicity. IRR was also lower in females vs males; for gastric 0.75 (95% CI 0.66-0.85, p<0.001) and other GI sites 0.77 (95% CI:0.62-0.95, p<0.001), and higher for salivary glands 2.41 (95% CI:1.91-3.07, p<0.001), breast 14.2 (95% CI:7.67-30.0, p<0.001) and thyroid 1.88 (95% CI:1.20-2.95, p=0.005).

Helicobacter pylori was documented in 14% of gastric, Sjogren's syndrome in 22% of salivary gland, and rheumatoid arthritis in 8% of salivary gland, 6% of oropharyngeal and 5% of pulmonary EMZL.

5-year OS was highest in skin, salivary gland and ocular EMZL at 86%, 84% and 82% respectively and lowest in non-gastric GI and other EMZL at 68% and 66% respectively (Figure 1). 5-year CIM from EMZL was highest in others and non-gastric GI EMZL at 16% and 14%, and lowest in ocular, skin and gastric EMZL at 4%, 4.1% and 5.9% respectively. On MVA, age≥75 (HR 66.7 vs age 18-44, global p<0.001), CCI≥4 (HR 2.16 vs CCI=0, global p<0.001), higher deprivation IMD=1 (HR 1.75 vs IMD=5, global p<0.001) and the following subtypes: others, non-gastric GI, breast and bone/connective tissue EMZL (HR 2.66, 2.18, 1.84 and 1.81 vs gastric, global p<0.001), were associated with an increased risk of death from EMZL.

**Summary/Conclusion**

To our knowledge, this is the largest consecutive series of EMZL reported in Europe. We observed higher incidence in older age and White ethnicity. Female incidence was higher in salivary gland EMZL likely due to its link with Sjogren syndrome. Skin and ocular EMZL has the best survival outcomes.

**Copyright Information:** (Online) ISSN: 2572-9241
©2026 The Author(s). HemaSphere is published by John Wiley & Sons Ltd on behalf of European Hematology Association. This is an open access Abstract Book distributed under the Attribution-NonCommercial-NoDerivs (CC BY-NC-ND), which allows third parties to download the articles and share them with others as long as they credit the author and the Abstract Book, but they cannot change the content in any way or use them commercially.
**Abstract Book Citations:** Authors, Title, HemaSphere, 2026;10:(S1):pages. The abstract book DOIs can be found on the "Author Information" page.
**Disclaimer:** Articles published in the journal HemaSphere exclusively reflect the opinions of the authors. The authors are responsible for all content in their abstracts including accuracy of the facts, statements, citing resources, etc.

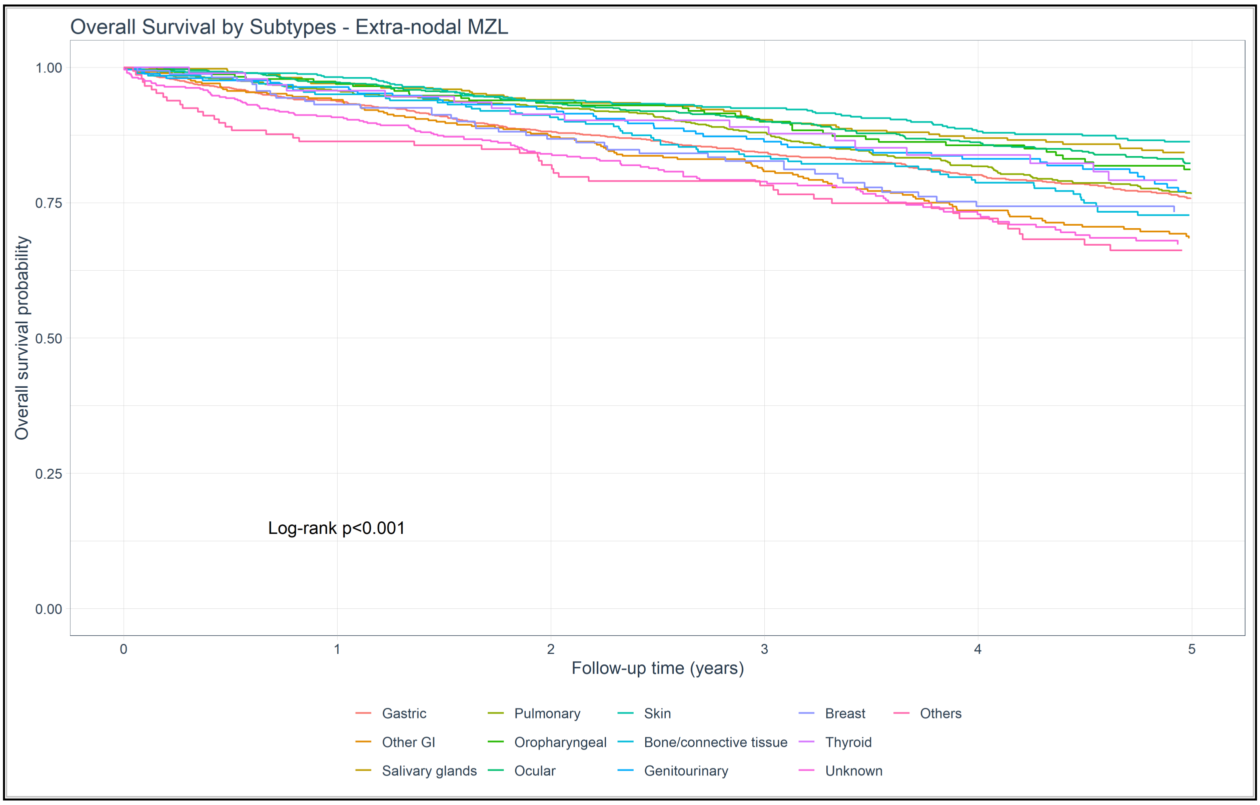

Figure 1. Overall survival of EMZL by subtypes

**Copyright Information:** (Online) ISSN: 2572-9241
©2026 The Author(s). HemaSphere is published by John Wiley & Sons Ltd on behalf of European Hematology Association. This is an open access Abstract Book distributed under the Attribution-NonCommercial-NoDerivs (CC BY-NC-ND), which allows third parties to download the articles and share them with others as long as they credit the author and the Abstract Book, but they cannot change the content in any way or use them commercially.
**Abstract Book Citations:** Authors, Title, HemaSphere, 2026;10:(S1):pages. The abstract book DOIs can be found on the "Author Information" page.
**Disclaimer:** Articles published in the journal HemaSphere exclusively reflect the opinions of the authors. The authors are responsible for all content in their abstracts including accuracy of the facts, statements, citing resources, etc.

### (PS2048) PHASE 3 INMIND STUDY OF TAFASITAMAB (TAFA) PLUS LENALIDOMIDE (LEN) AND RITUXIMAB (R) FOR RELAPSED OR REFRACTORY FOLLICULAR LYMPHOMA (R/R FL): OUTCOMES IN PATIENTS WITH OR WITHOUT HIGH-RISK FACTORS

**Topic:** 18. Indolent and mantle-cell non-Hodgkin lymphoma - Clinical

Christian W. Scholz*[1], Paolo Strati[2], Laurie H. Sehn[3], Julie Wu[4], Philomena Colucci[4], Oscar Bortolami[5], Kai Hübel[6]

*[1]Vivantes Klinikum Am Urban, Berlin, Germany; [2]Department of Lymphoma and Myeloma, Division of Cancer Medicine, The University of Texas MD Anderson Cancer Center, Houston, TX, United States of America; [3]BC Cancer Centre for Lymphoid Cancer and The University of British Columbia, Vancouver, BC, Canada; [4]Incyte Corporation, Wilmington, DE, United States of America; [5]Incyte Biosciences International Sàrl, Morges, Switzerland; [6]University of Cologne and Faculty of Medicine and University Hospital of Cologne, Cologne, Germany;*

### Background

Despite initial response to first-line therapy, most patients with FL relapse and require multiple lines of therapy. Treatments resulting in durable responses are needed for patients with R/R FL, including those with high-risk factors of disease. Tafa, an anti-CD19 mAb, is approved in combination with Len+R for patients with R/R FL based on inMIND (NCT04680052), a phase 3, double-blind, placebo (Pbo)-controlled trial that evaluated addition of Tafa to Len+R in patients with R/R FL or marginal zone lymphoma. Tafa+Len+R resulted in significant improvement in PFS, with a 57% reduction in the risk of progression, relapse or death and a manageable safety profile in patients with R/R FL; benefit was observed regardless of refractoriness to prior anti-CD20 mAb therapy or POD24 status (Sehn L, *Lancet* 2026).

### Aims

Post hoc analyses from the inMIND study to evaluate efficacy outcomes in subgroups of patients with FL based on high-risk disease factors at baseline.

### Methods

Patients aged ≥18 years with R/R CD19$^+$ and CD20$^+$ FL (grade 1-3A), ECOG PS ≤2 and who had received ≥1 prior systemic therapy including an anti-CD20 mAb, were randomized 1:1 to receive Tafa 12 mg/kg IV or Pbo with standard dosing of Len+R up to 12×28-day cycles. Efficacy outcomes (PFS by INV [primary endpoint], PFS by IRC, PET-CR rate, ORR, TTNT) were assessed according to high-risk factors: refractoriness to chemoimmunotherapy (CIT) including SD or PD or achieving a response lasting <6 months in at least 1 prior line; bulky disease (≥7 cm diameter); high risk FLIPI score; progression of disease within 24 months from start of initial treatment (POD24); and elevated LDH level.

**Copyright Information:** (Online) ISSN: 2572-9241
©2026 The Author(s). HemaSphere is published by John Wiley & Sons Ltd on behalf of European Hematology Association. This is an open access Abstract Book distributed under the Attribution-NonCommercial-NoDerivs (CC BY-NC-ND), which allows third parties to download the articles and share them with others as long as they credit the author and the Abstract Book, but they cannot change the content in any way or use them commercially.
**Abstract Book Citations:** Authors, Title, HemaSphere, 2026;10:(S1):pages. The abstract book DOIs can be found on the “Author Information” page.
**Disclaimer:** Articles published in the journal HemaSphere exclusively reflect the opinions of the authors. The authors are responsible for all content in their abstracts including accuracy of the facts, statements, citing resources, etc.

### Results

548 patients were randomized to Tafa (n=273) or Pbo (n=275); baseline characteristics were well balanced between arms. Overall, 454 (83%) met ≥1 GELF criterion, 185 (34%) were CIT refractory, 207 (38%) had bulky disease, 287 (52%) had FLIPI score 3-5, 249 (45%) had POD24, and 110 (20%) had elevated LDH >ULN (250 IU/L); these characteristics were similar in the Tafa and Pbo arms. 170 (31%) patients had ≥3 high-risk factors (CIT refractory, bulky disease, high FLIPI score, POD24 from initial treatment or elevated LDH) at study baseline. Among CIT-refractory patients, median INV-assessed PFS was 14.8 months vs 8.3 months with addition of Tafa vs Pbo (hazard ratio [HR] [95% CI] 0.42 [0.27, 0.65]). Median INV-assessed PFS was also improved in the Tafa arm vs Pbo arm in patients with bulky disease (22.3 months vs 13.0 months; HR [95% CI] 0.46 [0.28, 0.74]), FLIPI score 3-5 (19.2 months vs 11.2 months; HR [95% CI] 0.43 [0.30, 0.62]), POD24-positive disease (19.2 months vs 11.5 months; HR [95% CI] 0.44 [0.30, 0.66]), and in patients with elevated LDH >ULN (19.2 months vs 8.5 months; HR [95% CI] 0.36 [0.20, 0.67]). Improvements in all secondary efficacy outcomes assessed were observed with addition of Tafa vs Pbo for all patients' high-risk subgroups (**Table**). All efficacy outcomes were also improved among patients in lower-risk subgroups (no bulky disease, FLIPI score 0–2, POD24-negative disease, normal LDH ≤ULN; data to be presented). In patients with ≥3 high-risk factors, median INV-assessed PFS was 19.2 months vs 8.5 months with addition of Tafa vs Pbo (HR [95% CI] 0.36 [0.23, 0.58]).

### Summary/Conclusion

This analysis of R/R FL patients from inMIND confirms that addition of Tafa to Len+R improved all efficacy outcomes assessed in patients with or without high-risk disease, including those presenting with multiple risk-factors at baseline.

**Table. Efficacy outcomes in select high-risk patients from inMIND**

| | CIT Refractory | | Bulky Disease | | FLIPI Score 3-5 | | POD24-Positive | | Elevated LDH >ULN | |
|---|---|---|---|---|---|---|---|---|---|---|
| **Study Endpoint** | **Tafa+ Len+R (n=95)** | **Pbo+ Len+R (n=90)** | **Tafa+ Len+R (n=98)** | **Pbo+ Len+R (n=109)** | **Tafa+ Len+R (n=137)** | **Pbo+ Len+R (n=150)** | **Tafa+ Len+R (n=121)** | **Pbo+ Len+R (n=128)** | **Tafa+ Len+R (n=58)** | **Pbo+ Len+R (n=52)** |
| Median PFS by INV | 14.8 | 8.3 | 22.3 | 13.0 | 19.2 | 11.2 | 19.2 | 11.5 | 19.2 | 8.5 |
| (95% CI), months* | (13.8, NE) | (6.9, 11.6) | (17.4, NE) | (10.9, 17.1) | (14.8, 23.6) | (8.4, 15.2) | (14.8, NE) | (8.5, 13.9) | (13.8, NE) | (5.5, 13.6) |
| HR (95% CI) | 0.42 (0.27, 0.65) | | 0.46 (0.28, 0.74) | | 0.43 (0.30, 0.62) | | 0.44 (0.30, 0.66) | | 0.36 (0.20, 0.67) | |
| Median PFS by IRC | 19.1 | 8.4 | NR | 16.4 | 19.3 | 11.2 | 21.5 | 13.6 | 19.2 | 11.1 |
| (95% CI), months* | (14.6, NE) | (6.6, 13.9) | (18.5, NE) | (12.0, 22.2) | (16.1, NE) | (8.4, 15.0) | (17.2, NE) | (8.8, 16.0) | (14.1, NE) | (8.1, 16.0) |
| HR (95% CI) | 0.40 (0.25, 0.66) | | 0.45 (0.26, 0.76) | | 0.41 (0.28, 0.61) | | 0.43 (0.27, 0.66) | | 0.40 (0.20, 0.77) | |
| PET-CR rate | 44.0 | 20.7 | 51.1 | 32.0 | 48.4 | 33.8 | 43.2 | 31.4 | 45.3 | 29.2 |
| (95% CI), %† | (33.2, 55.3) | (12.6, 31.1) | (40.4, 61.7) | (23.0, 42.1) | (39.5, 57.4) | (26.0, 42.3) | (33.9, 53.0) | (23.1, 40.5) | (31.6, 59.6) | (17.0, 44.1) |
| OR (95% CI) | 3.2 (1.6, 6.5) | | 2.2 (1.2, 3.9) | | 1.9 (1.1, 3.1) | | 1.7 (1.0, 2.8) | | 1.9 (0.8, 4.5) | |
| ORR | 72.6 | 60.0 | 83.7 | 66.1 | 81.0 | 70.0 | 79.3 | 64.8 | 74.1 | 55.8 |
| (95% CI), %* | (62.5, 81.3) | (49.1, 70.2) | (74.8, 90.4) | (56.4, 74.9) | (73.4, 87.2) | (62.0, 77.2) | (71.0, 86.2) | (55.9, 73.1) | (61.0, 84.7) | (41.3, 69.5) |
| OR (95% CI) | 1.8 (0.9, 3.4) | | 2.3 (1.2, 4.6) | | 1.9 (1.1, 3.4) | | 2.1 (1.2, 3.7) | | 1.9 (0.9, 4.3) | |
| Median TTNT | 27.8 | 13.4 | NR | NR | NR | 19.5 | NR | 17.9 | NR | 10.7 |
| (95% CI), months* | (17.2, NE) | (9.3, 17.9) | (NE, NE) | (15.0, NE) | (26.8, NE) | (13.6, NE) | (NE, NE) | (13.6, NE) | (14.1, NE) | (7.8, 15.5) |
| HR (95% CI) | 0.53 (0.33, 0.85) | | 0.30 (0.16, 0.56) | | 0.50 (0.32, 0.76) | | 0.51 (0.32, 0.81) | | 0.43 (0.24, 0.78) | |
| 2-year K-M estimate of TTNT (95% CI), %* | 58 (45, 69) | 33 (20, 46) | 79 (66, 88) | 50 (38, 61) | 68 (57, 77) | 49 (38, 59) | 71 (60, 80) | 43 (29, 55) | 61 (43, 74) | 25 (12, 41) |

*ITT population. †FDG-avid population (CIT refractory: Tafa, n=84; Pbo, n=82; bulky disease [≥7 cm diameter]: Tafa, n=92; Pbo, n=100; FLIPI score 3-5: Tafa, n=128; Pbo, n=139; POD24-positive: Tafa, n=111; Pbo, n=118; elevated LDH >ULN [250 IU/L]: Tafa, n=53; Pbo, n=48). PET-CR rate was defined as the proportion of patients who achieved complete metabolic response at any time after start of treatment as per Lugano classification among the patients with a positive PET scan at baseline, by PET-CR results only. CI, confidence interval; CIT, chemoimmunotherapy; FDG, fluorodeoxyglucose; FL, follicular lymphoma; FLIPI, Follicular Lymphoma International Prognostic Index; HR, hazard ratio; INV, investigator; IRC, independent review committee; ITT, intention-to-treat; K-M, Kaplan-Meier; Len, lenalidomide; LDH, lactate dehydrogenase; NE, not estimable; NR, not reached; OR, odds ratio; ORR, overall response rate; Pbo, placebo; PET-CR, positron emission tomography complete response; PFS, progression-free survival; POD24, progression of disease within 24 months of start of initial treatment; R, rituximab; Tafa, Tafasitamab; TTNT, time to next treatment.

**Copyright Information:** (Online) ISSN: 2572-9241
©2026 The Author(s). HemaSphere is published by John Wiley & Sons Ltd on behalf of European Hematology Association. This is an open access Abstract Book distributed under the Attribution-NonCommercial-NoDerivs (CC BY-NC-ND), which allows third parties to download the articles and share them with others as long as they credit the author and the Abstract Book, but they cannot change the content in any way or use them commercially.
**Abstract Book Citations:** Authors, Title, HemaSphere, 2026;10:(S1):pages. The abstract book DOIs can be found on the "Author Information" page.
**Disclaimer:** Articles published in the journal HemaSphere exclusively reflect the opinions of the authors. The authors are responsible for all content in their abstracts including accuracy of the facts, statements, citing resources, etc.

### (PS2049) ZANUBRUTINIB PLUS RITUXIMAB AS FRONT-LINE TREATMENT FOR MUCOSA-ASSOCIATED LYMPHOID TISSUE (MALT) LYMPHOMA: A SINGLE-ARM, PHASE II STUDY (ZAMA)

**Topic:** 18. Indolent and mantle-cell non-Hodgkin lymphoma - Clinical

Qingqing Cai*[1], Yi Xia[1], Huiqiang Huang[1], Nan Chen[2], Junfeng Jiang[3], Zhitao Ying[4], Runhui Zheng[5], Jianbo Liu[5], Yirong Jiang[6], Liang Wang[7], Xiaojie Fang[1], Man Nie[1], Yan Gao[1], Xiaoxiao Wang[1], Yuchen Zhang[1], Jun Cai[1], Yi Cao[1]

*[1]Sun Yat-sen University Cancer Center, Guangzhou, China; [2]The Fifth Affiliated Hospital of Sun Yat-sen University, Zhuhai, China; [3]Sun Yat-sen University Cancer Center Gansu Hospital, Lanzhou, China; [4]Peking University Cancer Hospital & Institute, Beijing, China; [5]The Fifth Affiliated Hospital of Guangzhou Medical University, Guangzhou, China;[6]Dongguan People's Hospital, Dongguan, China; [7]Beijing Tongren Hospital, Beijing, China;*

### Background

Mucosa-associated lymphoid tissue (MALT) lymphoma is the most common subtype of marginal zone lymphoma and follows an indolent clinical course. For patients with advanced-stage disease with an indication for treatment, rituximab-based immunochemotherapy is recommended as frontline therapy; however, toxicity and tolerability remain concerns. Zanubrutinib, a next-generation Bruton tyrosine kinase inhibitor, has shown promising antitumor activity with a manageable safety profile in marginal zone lymphoma.

### Aims

This study aimed to evaluate zanubrutinib plus rituximab as frontline treatment for MALT lymphoma.

### Methods

The ZAMA study (NCT06647732), a multicenter, single-arm, phase II trial, enrolled patients aged ≥18 years with histologically confirmed CD20-positive MALT lymphoma. Eligible patients had newly diagnosed advanced-stage disease or relapsed disease after prior local therapy, had not received prior systemic treatment, and had at least one measurable lesion. Patients received rituximab (375 mg/m$^2$) plus zanubrutinib (160 mg twice daily) in 28-day cycles. Treatment was administered for six cycles, after which patients achieving complete response (CR) entered observation, while those with partial response or stable disease received two additional cycles. The primary endpoint was the CR rate as best response.

**Copyright Information:** (Online) ISSN: 2572-9241
©2026 The Author(s). HemaSphere is published by John Wiley & Sons Ltd on behalf of European Hematology Association. This is an open access Abstract Book distributed under the Attribution-NonCommercial-NoDerivs (CC BY-NC-ND), which allows third parties to download the articles and share them with others as long as they credit the author and the Abstract Book, but they cannot change the content in any way or use them commercially.
**Abstract Book Citations:** Authors, Title, HemaSphere, 2026;10:(S1):pages. The abstract book DOIs can be found on the "Author Information" page.
**Disclaimer:** Articles published in the journal HemaSphere exclusively reflect the opinions of the authors. The authors are responsible for all content in their abstracts including accuracy of the facts, statements, citing resources, etc.

### Results

From October 30, 2024 to December 12, 2025, 39 patients were enrolled. At the data cutoff of January 10, 2026, 23 patients had completed protocol-specified treatment, including 14 patients who received six cycles and 9 patients who received eight cycles. The median age was 58 years (range 24–75); 7 patients were males (30.4%). Most patients (n = 21, 91.3%) had an ECOG performance status of 0–1, and 21 patients (91.3%) had Ann Arbor stage III–IV disease; two patients had relapsed disease after prior local therapy. Eighteen patients (78.2%) achieved CR, and the remaining five patients achieved PR. Treatment-emergent adverse events (TEAEs) occurred in 20 patients (87.0%); hematologic toxicity were most common: neutropenia (n = 2, 8.7%), and thrombocytopenia (n = 2, 8.7%). Grade ≥ 3 TEAEs occurred in 4 patients (17.4%), most frequently neutropenia (n = 2, 8.7%), and thrombocytopenia (n = 2, 8.7%).

**Table 1. Baseline characteristics.**

| **Characteristics** | **Patients** (n=23) |
|---|---|
| **Age, years** (median [range]) | 58 (24-75) |
| **Sex** | |
| Male | 7 (30.4%) |
| Female | 16 (69.6%) |
| **ECOG PS** | |
| 0-1 | 21 (91.3%) |
| 2 | 2 (8.7%) |
| **Ann arbor stage** | |
| III-IV | 21 (91.3%) |
| **Prior local therapy** | 2 (8.7%) |
| **MALT-IPI score** | |
| 0 | 1 (4.3%) |
| 1 | 21 (91.3%) |
| 2 | 1 (4.3%) |
| **Bone marrow involvement** | 4 (17.4%) |

### Summary/Conclusion

Preliminary results from the ZAMA study show that frontline zanubrutinib plus rituximab provides encouraging efficacy with manageable safety in MALT lymphoma. The study is ongoing, and patient enrollment is continuing.

**Copyright Information:** (Online) ISSN: 2572-9241
©2026 The Author(s). HemaSphere is published by John Wiley & Sons Ltd on behalf of European Hematology Association. This is an open access Abstract Book distributed under the Attribution-NonCommercial-NoDerivs (CC BY-NC-ND), which allows third parties to download the articles and share them with others as long as they credit the author and the Abstract Book, but they cannot change the content in any way or use them commercially.
**Abstract Book Citations:** Authors, Title, HemaSphere, 2026;10:(S1):pages. The abstract book DOIs can be found on the “Author Information” page.
**Disclaimer:** Articles published in the journal HemaSphere exclusively reflect the opinions of the authors. The authors are responsible for all content in their abstracts including accuracy of the facts, statements, citing resources, etc.

### (PS2050) PRELIMINARY ANALYSIS OF ORELABRUTINIB COMBINED WITH OBINUTUZUMAB IN THE TREATMENT OF MARGINAL ZONE LYMPHOMA (ORION STUDY)

**Topic:** 18. Indolent and mantle-cell non-Hodgkin lymphoma - Clinical

Rui Lv*[1], Tingyu Wang[1], Yuting Yan[1], Tengteng Yu[1], Wenjie Xiong[1], Ying Yu[1], Qi Wang[1], Shuaishuai Zhang[1], Hesong Zou[1], Shuhui Deng[1], Yan Xu[1], Dehui Zou[1], Liang Huang[1], Gang An[1], Lugui Qiu[1], Shuhua Yi[1]

*[1]State Key Laboratory of Experimental Hematology, National Clinical Research Center for Blood Diseases, Haihe Laboratory of Cell Ecosystem, Institute of Hematology & Blood Diseases Hospital Chinese Academy of Medical Sciences & Peking Union Medical College, Tianjin, China;*

### Background

Marginal Zone Lymphoma (MZL) is a group of B-cell malignancies. Orelabrutinib (ORE), the first approved BTKi in China for patients after at least one treatment. This phase II trial evaluates the efficacy and safety of orelabrutinib combined with obinutuzumab (OG) in treatment-naïve MZL.

### Methods

This prospective single-arm phase II trial (NCT06513234) enrolled systemic treatment-naïve patients with MZL, including those who previously received local therapy and/or antimicrobial therapy, and subsequently progressed or relapsed, or are unsuitable for local therapy. Treatment consisted of: Induction phase (6 cycles, 28 days/cycle): Orelabrutinib (150mg/day orally), Obinutuzumab (1000mg IV, C1: D1/D8/D15; C2-6: D1). Maintenance phase (≤18 cycles): Orelabrutinib monotherapy (150mg/day). Primary endpoint: 12-month complete response rate (CRR); Secondary endpoints: ORR, PFS, OS, and safety.

### Results

As of Feb. 2026, 44 patients were enrolled (MALT 11.4%, NMZL 20.5%, SMZL 68.2%), baselinecharacteristics: median age 60.5 years (range 29–80 years), staging: stage IV 90.9% (40/44); 81.8% (36/44) with bone marrow involvement, 75% (33/44) with splenomegaly. MZL-IPI ≥ 2 points 56.8% (25/44). Among 44 efficacy-evaluable patients:43 patients completed interim assessment (3/4 cycles): ORR 97.7% (95%CI 87.7-99.9), CR rate 69.8% (53.9-82.8); 30 patients completed 6 cyclesassessment: ORR 96.7%, CR rate 80.0%. Best response: CR rate 81.8%, ORR 97.7%. By Subtype (Best Response):30 pts of SMZL: CR rate 83.3% (25/30), ORR 96.7% (29/30). 9 pts of NMZL: CR rate 66.7% (6/9), ORR 100% (9/9); 5 pts of MALT all achieved CR(table1).Among the patients who started maintenance therapy, 15 were evaluated at least once, with 86.7% (13/15) achieving CR and 30.77% (4/13). MRD assessment was performed in 24 patients with SMZL at baseline, the positivity rate of 100%. MRD negative rates were 88% at interim (22/25), 90.9% at end of induction therapy (10/11). With a median follow-up of 6.1 months (range 1.8–21.3), one patient (SMZL) experienced disease progression (PD). Median PFS and OS were not reached. The Grade≥3 hematologic toxicity mainly included neutrophil count decreased (29.5%) and platelet count decreased (27.2%). Common adverse events are grade 1-2 hematological toxicity, and no unexpected adverse events have occurred.

### Summary/Conclusion

The OG regimen demonstrated promising antitumor activity in treatment-naïve MZL with no severetoxicities observed. These early results support OG as a potential chemotherapy-free first-line option for MZL. Longer follow-up is needed to assess durability of response and survival outcomes.

**Copyright Information:** (Online) ISSN: 2572-9241
©2026 The Author(s). HemaSphere is published by John Wiley & Sons Ltd on behalf of European Hematology Association. This is an open access Abstract Book distributed under the Attribution-NonCommercial-NoDerivs (CC BY-NC-ND), which allows third parties to download the articles and share them with others as long as they credit the author and the Abstract Book, but they cannot change the content in any way or use them commercially.
**Abstract Book Citations:** Authors, Title, HemaSphere, 2026;10:(S1):pages. The abstract book DOIs can be found on the "Author Information" page.
**Disclaimer:** Articles published in the journal HemaSphere exclusively reflect the opinions of the authors. The authors are responsible for all content in their abstracts including accuracy of the facts, statements, citing resources, etc.

| n(%) | overall(n=44) | MALT(n=5) | NMZL(n=9) | SMZL(n=30) |
|---|---|---|---|---|
| Overall response rate | 43 (97.7) | 5 (100.0) | 9 (100.0) | 29 (96.7) |
| Complete response | 36 (81.8) | 5 (100.0) | 6 (66.7) | 25 (83.3) |
| Partial response | 7 (15.9) | 0 (0.0) | 3 (33.3) | 4 (13.3) |
| Stable disease | 0 (0.0) | 0 (0.0) | 0 (0.0) | 0 (0.0) |
| Progressive disease | 1 (2.3) | 0 (0.0) | 0 (0.0) | 1 (3.3) |

Table 1. Best response in induction treatment

**Copyright Information:** (Online) ISSN: 2572-9241
©2026 The Author(s). HemaSphere is published by John Wiley & Sons Ltd on behalf of European Hematology Association. This is an open access Abstract Book distributed under the Attribution-NonCommercial-NoDerivs (CC BY-NC-ND), which allows third parties to download the articles and share them with others as long as they credit the author and the Abstract Book, but they cannot change the content in any way or use them commercially.
**Abstract Book Citations:** Authors, Title, HemaSphere, 2026;10:(S1):pages. The abstract book DOIs can be found on the "Author Information" page.
**Disclaimer:** Articles published in the journal HemaSphere exclusively reflect the opinions of the authors. The authors are responsible for all content in their abstracts including accuracy of the facts, statements, citing resources, etc.

### (PS2051) CD19-DIRRECTED CAR T-CELL THERAPY FOR RELAPSED/REFRACTORY FOLLICULAR LYMPHOMA: OFF-TRIAL OUTCOMES AND SUBSEQUENT THERAPY

**Topic:** 18. Indolent and mantle-cell non-Hodgkin lymphoma - Clinical

Alfredo Rivas-Delgado*[1], Hari Raman[2], MacIej Kabat[3], Neta Glaubach[4], David Nemirovsky[5], Uri Greenbaum[6], Magdalena Corona[7], Efrat Luttwak[1], Lorenzo Falchi[1, 8], Jennifer Lue[1, 8], Michael Scordo[7, 8], Andrew Zelenetz[1], Miguel-Angel Perales[7, 8], Gunjan Shah[7, 8], Jae H. Park[8], Shimrit Ringelstein-Harlev[4], Ofrat Beyar-Katz[4], Sarah Samorodnitsky[5], Lori Leslie[3], Andrew Ip[3], Philippe Armand[2], Caron Jacobson[2], M. Lia Palomba[1, 8], Roni Shouval[7, 8], Reid Merryman[2], Gilles Salles[1, 8]

*[1]Memorial Sloan Kettering Cancer Center, Lymphoma Service, New York, NY, United States of America;[2]Dana-Farber Cancer Institute, Division of Lymphoma, Boston, MA, United States of America; [3]Hackensack Meridian Health, Lymphoma Service, New Jersey, NJ, United States of America; [4]Rambam Health Care Campus, Department of Hematology and Bone Marrow Transplantation, Haifa, Israel; [5]Memorial Sloan Kettering Cancer Center, Department of Epidemiology and Biostatistics, New York, NY, United States of America; [6]Soroka University Medical Center, Department of Hematology, Beer Sheva, Israel; [7]Memorial Sloan Kettering Cancer Center, Adult Bone Marrow Transplantation Service, New York, NY, United States of America; [8]Memorial Sloan Kettering Cancer Center, Cellular Therapy Service, New York, NY, United States of America;*

### Background

CD19-directed CAR T-cell therapy (CAR-T) has shown excellent efficacy in patients (pts) with refractory/relapsed (R/R) follicular lymphoma (FL). However, off-trial data on pathological evolution, treatment patterns, and outcomes after disease progression (POD) following CAR-T remain limited.

### Aims

To describe off-trial outcomes of pts with R/R FL treated with commercial CD19 CAR T-cell therapy, focusing on pathological features at POD and outcomes of subsequent treatments.

### Methods

Clinical characteristics of 92 pts treated with CAR-T for FL across five international centers were analyzed. Pts with FL grade 3B or prior histologic transformation were excluded.

**Copyright Information:** (Online) ISSN: 2572-9241
©2026 The Author(s). HemaSphere is published by John Wiley & Sons Ltd on behalf of European Hematology Association. This is an open access Abstract Book distributed under the Attribution-NonCommercial-NoDerivs (CC BY-NC-ND), which allows third parties to download the articles and share them with others as long as they credit the author and the Abstract Book, but they cannot change the content in any way or use them commercially.
**Abstract Book Citations:** Authors, Title, HemaSphere, 2026;10:(S1):pages. The abstract book DOIs can be found on the "Author Information" page.
**Disclaimer:** Articles published in the journal HemaSphere exclusively reflect the opinions of the authors. The authors are responsible for all content in their abstracts including accuracy of the facts, statements, citing resources, etc.

**Results**

Median age at infusion was 63 years (range, 34–81), with a median of 3 prior therapy lines (range, 1–9); 13% had undergone prior autologous transplantation. CAR-T products included axi-cel (79%), tisa-cel (20%), and liso-cel (1%). High-risk features were frequent: 53% had experienced POD24, 49% had disease refractory to last therapy, and 18% with primary refractory disease; 42% received bridging therapy.
CRS occurred in 72% of pts (median onset 3 days; median duration 4 days), with grade 3 CRS in 2%. ICANS occurred in 29% (median onset 7 days; median duration 4 days), with grade ≥3 ICANS in 14%; no CRS- or ICANS-related deaths were observed
After a median follow-up of 24 months (range 21-27), the best overall response rate (ORR) and complete response (CR) rate were 93% and 88%, respectively; bendamustine exposure within 24 months of CAR-T was associated with a lower CR rate at day 100 (p=0.002). Median PFS was not reached, with a 2-year PFS of 65% (95% CI, 55–77) and estimated 24-month OS was 93% (95% CI, 88-99). Bone marrow involvement before apheresis and the use of bridging therapy were associated with inferior PFS and OS, whereas recent bendamustine exposure was not.
Twenty-seven pts (29%) relapsed or progressed. Following POD, 22 received subsequent systemic therapy (median 1 line; range, 1–5), 2 remained under active observation, 1 received palliative care, and 2 were lost to follow-up. Median time from POD to next therapy was 1.1 months. The ORR to first post–CAR-T therapy was 71%, with a CR rate of 50%. Treatments included CD3×CD20 bispecific antibodies (n=9; ORR 88%; CR 63%), chemoimmunotherapy (n=7; ORR 85%; CR 57%), tafasitamab-based combinations (n=2), lenalidomide plus obinutuzumab (n=1), tazemetostat monotherapy (n=1) (no responses), and investigational agents (n=2). Three pts underwent consolidative allogeneic SCT following post POD therapy and remain in CR.
Median PFS for the initial post-CAR treatment was 12 months (95% CI, 6.4–NR), with a 1-year PFS of 49%. Five pts (19%) died, all from disease progression, with an estimated 1-year OS of 89% (95% CI, 75–100).
Among 19 pts with post–CAR-T POD biopsies, transformation to DLBCL occurred in 3, FL grade 3B in 1, and relapsed FL (grade 1–3A) in 15. CD19 expression was preserved in all evaluable cases, while CD20-negative disease was identified in 28%.

**Summary/Conclusion**

CD19 CAR-T therapy provides durable disease control in heavily pretreated FL, with outcomes comparable to pivotal clinical trials and a manageable safety profile. Our data highlight the unmet need after CAR-T failure and suggest that bispecific antibodies may represent an effective subsequent therapy in this setting. The occurrence of histologic transformation after CAR-T therapy underscores the need for improved characterization of pre-CAR and post–CAR-T FL biology.

**Copyright Information:** (Online) ISSN: 2572-9241
©2026 The Author(s). HemaSphere is published by John Wiley & Sons Ltd on behalf of European Hematology Association. This is an open access Abstract Book distributed under the Attribution-NonCommercial-NoDerivs (CC BY-NC-ND), which allows third parties to download the articles and share them with others as long as they credit the author and the Abstract Book, but they cannot change the content in any way or use them commercially.
**Abstract Book Citations:** Authors, Title, HemaSphere, 2026;10:(S1):pages. The abstract book DOIs can be found on the "Author Information" page.
**Disclaimer:** Articles published in the journal HemaSphere exclusively reflect the opinions of the authors. The authors are responsible for all content in their abstracts including accuracy of the facts, statements, citing resources, etc.

### (PS2052) COMPARATIVE ANALYSES OF EPCORITAMAB IN COMBINATION WITH LENALIDOMIDE AND RITUXIMAB VS OBINUTUZUMAB AND BENDAMUSTINE IN RELAPSED/REFRACTORY FOLLICULAR LYMPHOMA

**Topic:** 18. Indolent and mantle-cell non-Hodgkin lymphoma - Clinical

Adam Kittai[1], Marshall Patrick Stagg[2], Habte A. Yimer[3], Yves Mbous[4], Junhua Yu[4], Wenying Quan[5], Anthony Wang[4], David Tybor[4], Abualbishr Alshreef[4], Rajesh Kamalakar[4], Gauri Sunkersett[4], Jian Mei[4], Viktor Chirikov[5], Savreet Bains Chawla[6], Laura Liao[6], Fernando Rivas Navarro[7], Seth Nickerson[6], Felipe Marques Goncalves[6], Alex Mutebi[6], Zhijie Ding[6], Johannes Duell*[8]

*[1]Icahn School of Medicine at Mount Sinai, New York, United States of America;[2]Our Lady of the Lake Regional Medical Center, Baton Rouge, United States of America; [3]Texas Oncology–Tyler, US Oncology Research, Tyler, United States of America; [4]AbbVie, Inc., North Chicago, United States of America;[5]OPEN Health, New York, United States of America;[6]Genmab US Inc., Plainsboro, United States of America; [7]Genmab A/S, Copenhagen, Denmark;[8]Medical Clinic and Polyclinic II, University Hospital Würzburg, Würzburg, Germany;*

### Background

Epcoritamab (E), a subcutaneously administered bispecific antibody (CD3xCD20), received FDA approval in combination with lenalidomide and rituximab ($R^2$) for relapsed/refractory follicular lymphoma (R/R FL) based on the phase 3 EPCORE FL-1 trial (NCT05409066), which demonstrated superiority over $R^2$ in progression-free survival (PFS; hazard ratio [HR] 0.21 [95% confidence interval (CI) 0.14–0.31]; *P*<0.0001). Obinutuzumab (Gazyva) + bendamustine (G+B) is a guideline-recommended option for patients (pts) with R/R FL and a commonly used regimen globally. No clinical study to date has directly compared outcomes of E+$R^2$ vs G+B in R/R FL.

### Aims

To compare the efficacy of E+$R^2$ to G+B in pts with R/R FL.

### Methods

Two separate analyses were performed to assess the efficacy of E+$R^2$ vs G+B: one using inverse probability of treatment weighting (IPTW) and the other using matching-adjusted indirect comparison (MAIC). For E+$R^2$, individual pt data (IPD) from the EPCORE FL-1 study (data cutoff May 2025; median follow-up 14.8 months) were used. In the IPTW analysis, IPD from the COTA real-world electronic health records database (2010–2025, COTA Healthcare, NY, USA; median follow-up 27.2 months) were used for the G+B cohort. For the MAIC analysis, the G+B cohort was represented by aggregate data from the final results of the GADOLIN trial (NCT01059630, data cutoff November 2018; median observation time 57.5 months). E+$R^2$ and G+B cohorts were balanced after weighting (IPTW) and match-adjustment (MAIC) of key baseline covariates, respectively. Efficacy outcomes of overall response rates (ORRs), complete response (CR) rates, and PFS were compared between cohorts. CR was not available for the FL subgroup in the GADOLIN trial and was not included in the MAIC. Weighted regressions were used to estimate odds ratios (ORs) and 95% CIs for ORR and CR; weighted Cox proportional-hazards models were used to estimate HRs and 95% CIs for PFS.

**Copyright Information:** (Online) ISSN: 2572-9241
©2026 The Author(s). HemaSphere is published by John Wiley & Sons Ltd on behalf of European Hematology Association. This is an open access Abstract Book distributed under the Attribution-NonCommercial-NoDerivs (CC BY-NC-ND), which allows third parties to download the articles and share them with others as long as they credit the author and the Abstract Book, but they cannot change the content in any way or use them commercially.
**Abstract Book Citations:** Authors, Title, HemaSphere, 2026;10:(S1):pages. The abstract book DOIs can be found on the "Author Information" page.
**Disclaimer:** Articles published in the journal HemaSphere exclusively reflect the opinions of the authors. The authors are responsible for all content in their abstracts including accuracy of the facts, statements, citing resources, etc.

### Results

Before weighting, compared with G+B pts from COTA (N=77), the E+$R^2$ cohort (N=243) at baseline had higher proportions of younger pts (<65 y: 63.8% vs 37.7%), pts with elevated lactate dehydrogenase levels (35.0% vs 14.3%), and pts with Eastern Cooperative Oncology Group performance status of 0 (68.3% vs 53.2%). Before match-adjustment, compared with G+B pts in GADOLIN (N=164), the E+$R^2$ cohort (N=243) at baseline had a higher proportion of younger pts (≤63 y: 60.9% vs 50.0%) and lower proportions of pts who were refractory to last therapy (34.6% vs 93.9%) and double-refractory (37.4% vs 78.7%). In the IPTW analysis of EPCORE FL-1 vs COTA, E+$R^2$ demonstrated a significantly higher likelihood of achieving response outcomes vs G+B (ORR 95.0% vs 81.0%; OR 4.8 [95% CI 1.9–11.7]; *P*<0.001; CR 81.3% vs 66.3%; OR 2.4 [95% CI 1.2–4.4]; *P*=0.003) and PFS was significantly improved with E+$R^2$ (HR 0.44 [95% CI 0.23–0.85]; *P*=0.015). In the MAIC analysis of EPCORE FL-1 vs GADOLIN, E+$R^2$ showed a significantly higher likelihood of achieving ORR (90.4% vs 67.7% [G+B]; OR 4.5 [95% CI 1.8–11.3]; *P*=0.001) after induction, and E+$R^2$ significantly improved PFS (HR 0.42 [95% CI 0.22–0.78]; *P*=0.006) (**Figure**).

### Summary/Conclusion

E+$R^2$ showed higher likelihood of achieving ORR and CR and improved PFS vs G+B in R/R FL. Similar findings were observed using G+B real-world and trial data, and these findings position E+$R^2$ as a new chemotherapy-free standard of care for R/R FL.

**Copyright Information:** (Online) ISSN: 2572-9241
©2026 The Author(s). HemaSphere is published by John Wiley & Sons Ltd on behalf of European Hematology Association. This is an open access Abstract Book distributed under the Attribution-NonCommercial-NoDerivs (CC BY-NC-ND), which allows third parties to download the articles and share them with others as long as they credit the author and the Abstract Book, but they cannot change the content in any way or use them commercially.
**Abstract Book Citations:** Authors, Title, HemaSphere, 2026;10:(S1):pages. The abstract book DOIs can be found on the “Author Information” page.
**Disclaimer:** Articles published in the journal HemaSphere exclusively reflect the opinions of the authors. The authors are responsible for all content in their abstracts including accuracy of the facts, statements, citing resources, etc.

**Figure.** Adjusted PFS for E+R$^2$ from EPCORE FL-1 vs (A) G+B from COTA in R/R FL and (B) G+B from GADOLIN. adj, adjusted; unadj, unadjusted.

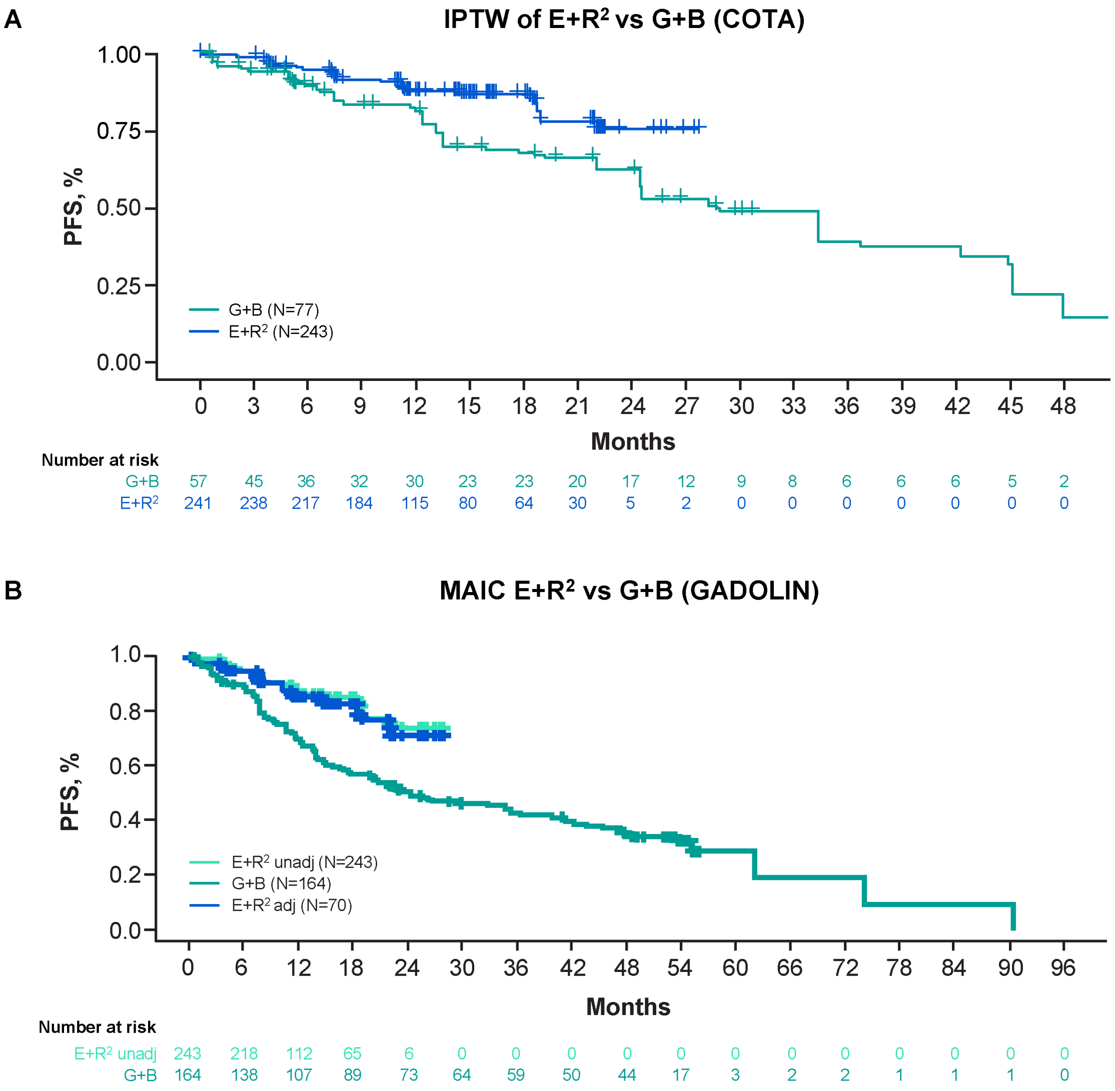

**Copyright Information:** (Online) ISSN: 2572-9241
©2026 The Author(s). HemaSphere is published by John Wiley & Sons Ltd on behalf of European Hematology Association. This is an open access Abstract Book distributed under the Attribution-NonCommercial-NoDerivs (CC BY-NC-ND), which allows third parties to download the articles and share them with others as long as they credit the author and the Abstract Book, but they cannot change the content in any way or use them commercially.
**Abstract Book Citations:** Authors, Title, HemaSphere, 2026;10:(S1):pages. The abstract book DOIs can be found on the "Author Information" page.
**Disclaimer:** Articles published in the journal HemaSphere exclusively reflect the opinions of the authors. The authors are responsible for all content in their abstracts including accuracy of the facts, statements, citing resources, etc.

### (PS2053) PRELIMINARY RESULTS OF ORELABRUTINIB FOLLOWED BY RESPONSE-ADAPTED ULTRA-LOW-DOSE 4GY RADIOTHERAPY AS FIRST-LINE TREATMENT FOR LOCALIZED MALT LYMPHOMA: A PROSPECTIVE, OPEN-LABEL, PHASE II STUDY

**Topic:** 18. Indolent and mantle-cell non-Hodgkin lymphoma - Clinical

Yizhen Liu*[1], Qunling Zhang[1], Chuanxu Liu[1], Wen-Hao Zhang[1], Fangfang Lyu[1], Xuejun MA[2], Rong Tao[1]

*[1]Department of Lymphoma, Fudan University Shanghai Cancer Center; Department of Oncology, Shanghai Medical College, Fudan University, Shanghai 200032, China, Shanghai, China; [2]Department of Radiotherapy, Fudan University Shanghai Cancer Center; Department of Oncology, Shanghai Medical College, Fudan University, Shanghai 200032, China, Shanghai, China;*

### Background

Radiotherapy is the first-line treatment for localized extranodal mucosa-associated lymphoid tissue (MALT) lymphoma. However, standard-dose(24-30Gy) radiotherapy is associated with considerable toxicities. Strategies that maintain treat efficacy while minimizing radiation exposure are therefore needed. Orelabrutinib, a highly selective Bruton's tyrosine kinase (BTK) inhibitor, has demonstrated durable responses with a favorable benefit-risk profile in MALT. This provides a rationale for exploring the potential synergistic effect between targeted therapy and low-dose radiotherapy.

### Aims

To evaluate the efficacy and safety of Orelabrutinib induction followed by response-adapted ultra-low-dose 4 Gy radiotherapy as first-line treatment for patients with stage I/II MALT lymphoma.

### Methods

This is a prospective, open-label, single-arm phase II study (NCT06583837). Patients received Orelabrutinib 150 mg once daily for 12 weeks. Patients without disease progression(PD) proceeded to ultra-low-dose 4 Gy radiotherapy(2 Gy x 2). Patients achieving complete response (CR) or partial response (PR) after radiotherapy were re-evaluated at 3 months. Patients with increased stable disease (SD) or PD at any assessment received additional radiotherapy.The primary endpoint is 1-year event-free survival (EFS). Secondary endpoints included CR rate at 3 months after 4 Gy radiotherapy, 2-year EFS, 2-year progression-free survival (PFS), safety, and quality of life.

**Copyright Information:** (Online) ISSN: 2572-9241
©2026 The Author(s). HemaSphere is published by John Wiley & Sons Ltd on behalf of European Hematology Association. This is an open access Abstract Book distributed under the Attribution-NonCommercial-NoDerivs (CC BY-NC-ND), which allows third parties to download the articles and share them with others as long as they credit the author and the Abstract Book, but they cannot change the content in any way or use them commercially.
**Abstract Book Citations:** Authors, Title, HemaSphere, 2026;10:(S1):pages. The abstract book DOIs can be found on the “Author Information” page.
**Disclaimer:** Articles published in the journal HemaSphere exclusively reflect the opinions of the authors. The authors are responsible for all content in their abstracts including accuracy of the facts, statements, citing resources, etc.

**Results**

Between September 10, 2024, and August 18, 2025, 50 patients were enrolled, including 26 with Helicobacter pylori–negative gastric MALT lymphoma and 24 with non-gastric MALT lymphoma. Median age was 58 years (range, 31–77); 22 patients (44%) were male. Stage distribution was 50% Stage I and 50% Stage II. One patient with gastric MALT lymphoma withdrew due to ulcer bleeding during induction and received standard-dose 36Gy/20Fx radiotherapy. The remaining 49 patients completed 12 weeks of Orelabrutinib. Of these, 31 (63.3%) patients achieved CR, 7(14.3%) patients achieved PR, yielding an ORR of 77.6%. Eight patients (16.3%) had SD. Three patients experienced PD, including one with localized progression who received standard-dose 3060cGy/17Fx radiotherapy and two with distant progression who were managed with watch and wait. Subsequently, 46 patients received ultra-low-dose 4 Gy radiotherapy, with 43 evaluable at 3 months. Post-radiotherapy ORR was 95.3%, with a CR rate of 86.0% (37/43) and PR rate of 9.3% (4/43). One patient (2.3%) with sigmoid colon MALT lymphoma had SD and received additional 2520cGy/14Fx radiotherapy per radiologist decision. One patient with gastric MALT lymphoma who achieved CR after induction experienced progression after 4 Gy and subsequently received additional 20 Gy/10Fx radiotherapy. Treatment-related adverse events related to Orelabrutinib were predominantly grade 1,including subcutaneous bleeding spots (16% [8/50]), elevated transaminase (6% [3/50] ), thrombocytopenia (4%[2/50] ), leukopenia (2%[1/50] ), and hypokalemia (2%[1/50] ). Ultra-low-dose 4 Gy radiation-related adverse events were grade 1 nausea (4.3%[2/46] ) and grade 1 vomiting (4.3%[2/46] ).No grade ≥3 adverse events or treatment-related deaths were observed.

**Summary/Conclusion**

Orelabrutinib induction followed by response-adapted ultra-low-dose 4 Gy radiotherapy represents a promising first-line strategy for early-stage MALT lymphoma, demonstrating a high CR rate with well-tolerated toxicity.

**Copyright Information:** (Online) ISSN: 2572-9241
©2026 The Author(s). HemaSphere is published by John Wiley & Sons Ltd on behalf of European Hematology Association. This is an open access Abstract Book distributed under the Attribution-NonCommercial-NoDerivs (CC BY-NC-ND), which allows third parties to download the articles and share them with others as long as they credit the author and the Abstract Book, but they cannot change the content in any way or use them commercially.
**Abstract Book Citations:** Authors, Title, HemaSphere, 2026;10:(S1):pages. The abstract book DOIs can be found on the “Author Information” page.
**Disclaimer:** Articles published in the journal HemaSphere exclusively reflect the opinions of the authors. The authors are responsible for all content in their abstracts including accuracy of the facts, statements, citing resources, etc.

### (PS2055) MANAGEMENT AND OUTCOMES OF FOLLICULAR LYMPHOMA (FL) WITH GASTROINTESTINAL (GI) INVOLVEMENT: SPANISH REAL WORLD EXPERIENCE FROM GELTAMO GROUP

**Topic:** 18. Indolent and mantle-cell non-Hodgkin lymphoma - Clinical

Paola Villafuerte Gutierrez[1], Monica Molina-Martinez[2], Juan Carlos Caballero[3], Maria Poza[4], Sofía Huerga[5], Ana Garcia Bacelar[6], Raquel Santiago Alonso[7], Alessandra Comai[8], Emilia Pardal[9], Fernando Martin-Moro[10], MA Jimena Cannata-Ortiz*[11], Haridian De La Nuez Melian[12], Eukene Gainza Gonzalez[13], Katia Flores[14], Fatima Salido[15], Alfonso Ortiz Algarra[16], Daniel García-Belmonte[17], Miguel Angel Rodriguez Gil[18], Iñigo Olazabal[19], Samuel Romero[20], Armando Lopez Guillermo[2], Ana Muntañola Prat[21]

*[1]Hospital Universitario Puerta de Hierro, Hematology, Madrid, Spain;[2]Hospital Clinic de Barcelona, Hematology, Barcelona, Spain; [3]Hospital Fundación Jimenez Díaz, Madrid, Spain; [4]Hospital Universitario 12 de Octubre, Madrid, Spain;[5]Clínica Universitaria de Navarra, Pamplona, Spain; [6]Hospital Universitario de Salamanca, Salamanca, Spain;[7]Hospital Sant Joan de Deu de Manresa Fundación Althaia Manresa, Barcelona, Spain; [8]Hospital Moises Broggi, Barcelona, Spain;[9]Hospital Virgen del Puerto, Cáceres, Spain; [10]Hospital Universitario Ramón y Cajal, Madrid, Spain; [11]Hospital Universitario de La Princesa, Madrid, Spain; [12]Hospital Universitario Doctor Negrín, Las Palmas de Gran Canarias, Spain;[13]Hospital Universitario de Galdakao-Usansolo Ospitalea, Vizcaya, Spain; [14]Hospital General de Cataluña, Barcelona, Spain;[15]Complejo Hospitalario Universitario de Ferrol, A Coruña, Spain; [16]Hospital HM Sanchinarro, Madrid, Spain; [17]Hospital Universitario La Zarzuela, Madrid, Spain; [18]Hospital Universitario La Mancha Centro, Alcázar de San Juan, Spain; [19]Hospital Universitario de Donostia, San Sebastián, Spain; [20]Hospital de Manises, Valencia, Spain; [21]Hospital de la Santa Creu i Sant Pau, Barcelona, Spain;*

### Background

Follicular lymphoma (FL) of the gastrointestinal (GI) tract is rare, accounting for less than 7% of all indolent non-Hodgkin's lymphomas. FL primarily or secondarily occurs in the GI tract, with the duodenum being the most frequently affected site of involvement. Since the 2016 World Health Organization (WHO) classification, duodenal-type FL (DFL) has been recognized as a distinct subcategory. Considering the heterogeneity and lack of standard treatment for these lymphomas.

### Aims

We aimed to analyze the clinical features, therapeutic approaches, and outcomes of patients (pts) with FL with primary or secondary GI involvement in routine clinical practice in Spain

### Methods

We retrospectively included adult pts with DFL or systemic grade 1-3A advanced stage FL with secondary GI involvement (SFL), diagnosed between January 1998 and November 2025 across 21 Spanish centers belonging to GELTAMO. DFL was defined according to the WHO classification criteria as involvement of the duodenum and/or other GI sites (jejunum, ileum, colon, or stomach) with pathological characteristics that fit DFL without nodal or extranodal site involvement. All pts underwent enteroscopy to obtain biopsy sample. Computed tomography, positron emission tomography, and bone marrow biopsy were performed at the discretion of the physician. The clinical stage was assigned according to the Lugano Classification. Written informed consent was obtained from all living pts.

**Copyright Information:** (Online) ISSN: 2572-9241
©2026 The Author(s). HemaSphere is published by John Wiley & Sons Ltd on behalf of European Hematology Association. This is an open access Abstract Book distributed under the Attribution-NonCommercial-NoDerivs (CC BY-NC-ND), which allows third parties to download the articles and share them with others as long as they credit the author and the Abstract Book, but they cannot change the content in any way or use them commercially.
**Abstract Book Citations:** Authors, Title, HemaSphere, 2026;10:(S1):pages. The abstract book DOIs can be found on the "Author Information" page.
**Disclaimer:** Articles published in the journal HemaSphere exclusively reflect the opinions of the authors. The authors are responsible for all content in their abstracts including accuracy of the facts, statements, citing resources, etc.

**Results**

One-hundred and eight pts were included. The median age at diagnosis was 61 years (range, 30-93) and 64 pts (59.3%) were male. According to lymphoma subtype, 71% (n=77) were DLF and 29% (n=31) were SFL. Pts with SFL had higher LDH (p=0.03) and β2-microglobulin (p<0.001) levels and lower hemoglobin levels (p=0.04) than those with DFL. At diagnosis, 20 pts (26%) were asymptomatic in the DFL group compared to 3 pts (10%) in the SFL group (*p*=0.061). When present, abdominal discomfort, diarrhea, and gastroesophageal reflux were the most frequent symptoms (40.7%, 10.1%, and 8.3%, respectively), without significant differences between the cohorts. Hemorrhage and intestinal obstruction were rare but significantly higher in pts with SFL than in those with DFL (*p*=0.009 and *p*=0.006, respectively). According to the site of involvement, the duodenum, specifically the 2$^{nd}$ portion, was the most commonly involved site in both subtypes (63.6% in DFL and 48.4% in SFL, *p*=ns). Seven DFL cases presented with more than one affected GI site. Among the DFL cohort, 45/77 (58%) pts were initially managed with an active surveillance approach. With a median follow-up of 69.6 months, only 6 out of 45 pts needed subsequent treatment, with a median time from diagnosis to first treatment of 6.81 months (range, 2.83-28.12). Overall, 36 pts with DFL (44%) received therapy at some point: 24 received rituximab, 10 immunochemotherapy, 1 radiotherapy, and 1 surgical resection. No statistically significant differences were observed in the overall survival (OS) according to the initial management approach (Fig.1A). The median OS was not reached in the overall cohort. Five-year OS was significantly higher in the DFL group than in the SFL group (98% vs. 72%, *p*<0.001) (Fig.1B).

**Summary/Conclusion**

In our experience, this large real-world cohort confirms the indolent course of DFL, with a longer OS than that of SFL. Severe local GI complications (hemorrhage and intestinal obstruction) were rare, supporting the notion that these pts can be safely managed with a conservative approach.

**Copyright Information:** (Online) ISSN: 2572-9241
©2026 The Author(s). HemaSphere is published by John Wiley & Sons Ltd on behalf of European Hematology Association. This is an open access Abstract Book distributed under the Attribution-NonCommercial-NoDerivs (CC BY-NC-ND), which allows third parties to download the articles and share them with others as long as they credit the author and the Abstract Book, but they cannot change the content in any way or use them commercially.
**Abstract Book Citations:** Authors, Title, HemaSphere, 2026;10:(S1):pages. The abstract book DOIs can be found on the "Author Information" page.
**Disclaimer:** Articles published in the journal HemaSphere exclusively reflect the opinions of the authors. The authors are responsible for all content in their abstracts including accuracy of the facts, statements, citing resources, etc.

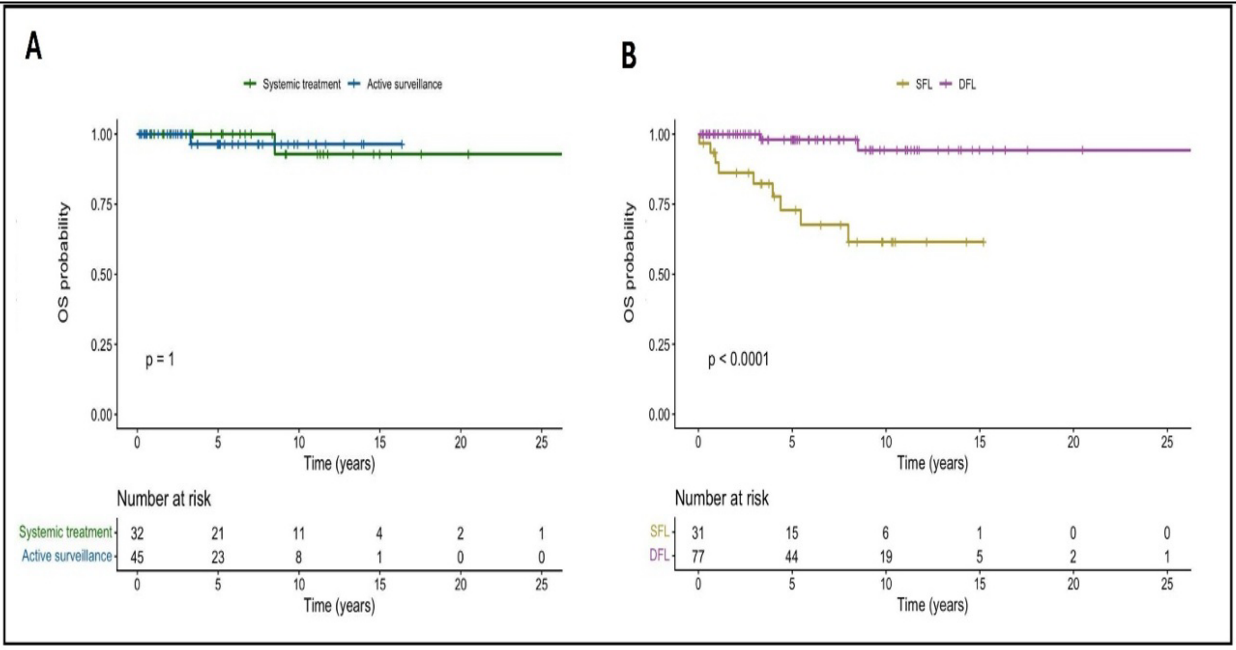

**Figure 1.** Overall survival according to: **A)** initial management in DFL cohort, and **B)** FL subtype in the overall series (DFL: duodenal-type FL and SFL: FL with secondary gastrointestinal involvement).

Figure 1. Overall survival according to:A)initial management in DFL cohort, and B)FL subtype in the overall series(DFL:duodenal-type FL and SFL:FL with secondary gastrointestinal involvement)

**Copyright Information:** (Online) ISSN: 2572-9241
©2026 The Author(s). HemaSphere is published by John Wiley & Sons Ltd on behalf of European Hematology Association. This is an open access Abstract Book distributed under the Attribution-NonCommercial-NoDerivs (CC BY-NC-ND), which allows third parties to download the articles and share them with others as long as they credit the author and the Abstract Book, but they cannot change the content in any way or use them commercially.
**Abstract Book Citations:** Authors, Title, HemaSphere, 2026;10:(S1):pages. The abstract book DOIs can be found on the "Author Information" page.
**Disclaimer:** Articles published in the journal HemaSphere exclusively reflect the opinions of the authors. The authors are responsible for all content in their abstracts including accuracy of the facts, statements, citing resources, etc.

### (PS2056) PHASE 1 FIRST-IN-HUMAN STUDY OF AUR112, A HIGHLY SELECTIVE MALT1 INHIBITOR, IN RELAPSED LYMPHOID MALIGNANCIES

**Topic:** 18. Indolent and mantle-cell non-Hodgkin lymphoma - Clinical

Suchit Kumbhare*[1], Suresh Oduru[1], Manjunath Krishnappa[1], Nirbhary Tiwari[1], Lavanya Krishna[1], Saravanan Thiyagarajan[1], Divya Adiga[1], Sourav Kumar Mishra[2], Manoj Toshniwal[3], Vinod Patil[4], Pradnya Modak[5], Shashikant Janardan Apte[6], Uttam Kumar Nath[7], Lakshmaiah K.C.[8], Akash Kumar[9], Aniket Mohite[10], Murali Ramachandra[1], Akhil Kumar[1]

*[1]Aurigene Oncology Limited, Clinical Development, Bengaluru, India;[2]All India Institute of Medical Sciences, Medical Oncology and Hematology, Bhubaneswar, India; [3]Jeevan Amrut Hematology Centre India Pvt. Ltd., Chhatrapati Sambhaji Nagar, India; [4]Onco-Life Cancer Centre, Satara, India;[5]Siddharth Gupta Memorial Hospital, Wardha, India; [6]Sahyadri Hospital Private Limited, Pune, India; [7]All India Institute of Medical Sciences, Medical Oncology & Hematology, Rishikesh, India;[8]Srinivasam Caner Care Multi-Specialty Hospital India Pvt. Ltd, Bengaluru, India; [9]National Cancer Institute, All India Institute of Medical Sciences, Badsa, India; [10]Novo Solitaire Care, Pune, India;*

### Background

MALT1 (mucosa-associated lymphoid tissue lymphoma translocation protein 1) is a central regulator of B-cell and T-cell receptor signaling and is implicated in the pathogenesis of multiple lymphoid malignancies. Aberrant activation of the CARD11-BCL10-MALT1 (CBM) complex activates canonical NF-κB pathway crucial for cell proliferation and survival, supporting investigation of MALT1-targeted inhibition. AUR112 is an oral selective small-molecule MALT1 inhibitor with acceptable pre-clinical safety, efficacy, and adequate DMPK profile[1].

### Aims

To present early results from a phase 1, first-in-human, dose escalation study (NCT06755450; CTRI/2025/01/078964) of AUR112 in relapsed lymphoid malignancies.

### Methods

Eligible patients aged ≥ 18 years with all different subtypes of lymphoid malignancies, including CLL, Eastern Cooperative Oncology Group performance status (ECOG PS) ≤1 were enrolled in three sequential dose cohorts at doses ranging from 100-400 mg once daily. Safety assessments included adverse events and dose-limiting toxicities (DLTs). PK parameters were evaluated after single and multiple dosing. PD effects were assessed using cytokine inhibition and exploratory biomarkers. Tumor responses were assessed by modified Lugano for lymphoma and International Workshop criteria for CLL (iWCLL).  Based on the efficacy and PK from 100 mg and 200 mg cohorts and following enrollment of a single patient with T-cell lymphoma at 400 mg, further enrollment is being focused on lower dosages.

**Copyright Information:** (Online) ISSN: 2572-9241
©2026 The Author(s). HemaSphere is published by John Wiley & Sons Ltd on behalf of European Hematology Association. This is an open access Abstract Book distributed under the Attribution-NonCommercial-NoDerivs (CC BY-NC-ND), which allows third parties to download the articles and share them with others as long as they credit the author and the Abstract Book, but they cannot change the content in any way or use them commercially.
**Abstract Book Citations:** Authors, Title, HemaSphere, 2026;10:(S1):pages. The abstract book DOIs can be found on the "Author Information" page.
**Disclaimer:** Articles published in the journal HemaSphere exclusively reflect the opinions of the authors. The authors are responsible for all content in their abstracts including accuracy of the facts, statements, citing resources, etc.

**Results**

At a cutoff of 31 Jan 2026, sixteen patients with relapsed lymphoid malignancies were treated: Hodgkin lymphoma (n=7), CLL (n = 2), diffuse large B-cell Lymphoma (n = 3), one patient each with follicular, mantle cell, marginal zone, and T-cell lymphoma. Median age was 36.5 years (range 20-71), and 44% were male. Most patients (81.5%) had received ≥3 prior lines of therapy. Ninety Treatment-emergent AEs were reported in 15/16 (93.75%) patients enrolled. Most AEs (69/90; 76.7%) were lower grades (Grade 1/2). Only one patient developed a DLT of Grade 3 neutropenia (100 mg). Objective responses were observed in 55.6% (5/9) and 75% (3/4) among evaluable patients in 100 mg QD and 200 mg QD cohorts, respectively, with an ORR of 61.5 % (8/13) across the two cohorts. PK analyses demonstrated dose-proportional exposure from 100 mg to 200 mg. Most patients showed IL-2 levels below the lower limit of quantification post-dosing, consistent with PD activity.

**Summary/Conclusion**

AUR112 demonstrated encouraging clinical activity with manageable safety profile in relapsed lymphoid malignancies across histological subtypes. Pharmacokinetics / Pharmacodynamics findings support continued dose expansion at dosages ≤200 mg daily dose. Activity in selected lymphoid malignancies, including Chronic Lymphocytic Leukemia (CLL), Follicular Lymphoma (FL), Marginal Zone Lymphoma (MZL), Mantle Cell Lymphoma (MCL), Waldenstrom's Macroglobulinemia, and Activated B-cell like Diffuse Large B-cell Lymphoma (ABC-DLBCL) is being explored as part of Dose Expansion.

[1] Balasubramanian WR et al. Discovery of AUR-112, a novel MALT1 protease inhibitor for the treatment of B-cell lymphomas. Proc. AACR 2023; Abstract C127

**Copyright Information:** (Online) ISSN: 2572-9241
©2026 The Author(s). HemaSphere is published by John Wiley & Sons Ltd on behalf of European Hematology Association. This is an open access Abstract Book distributed under the Attribution-NonCommercial-NoDerivs (CC BY-NC-ND), which allows third parties to download the articles and share them with others as long as they credit the author and the Abstract Book, but they cannot change the content in any way or use them commercially.
**Abstract Book Citations:** Authors, Title, HemaSphere, 2026;10:(S1):pages. The abstract book DOIs can be found on the "Author Information" page.
**Disclaimer:** Articles published in the journal HemaSphere exclusively reflect the opinions of the authors. The authors are responsible for all content in their abstracts including accuracy of the facts, statements, citing resources, etc.

### (PS2057) CLINICAL CHARACTERISTICS AND TREATMENT PATTERNS IN PATIENTS WITH FOLLICULAR LYMPHOMA MANAGED WITH LONG-TERM INITIAL WATCH-AND-WAIT: A DANISH NATIONWIDE POPULATION-BASED STUDY

**Topic:** 18. Indolent and mantle-cell non-Hodgkin lymphoma - Clinical

Ruth Salim Raaschou*[1, 2], Jennifer Bøgh Jørgensen[3, 4], Anders Maagaard Lyhne[5], Katrin Bamdeg-Hvolbek[6], Per Ishøy Nielsen[7], Uffe Klausen[1], Mette Dahl[1], Tarec El-Galaly[3, 4, 8, 9], Kirsten Grønbæk[1, 2, 10]

*[1]Rigshospitalet, Department of Hematology, Copenhagen, Denmark; [2]University of Copenhagen, Biotech Research and Innovation Centre (BRIC), Copenhagen, Denmark; [3]Aarhus University Hospital, Department of Hematology, Aarhus, Denmark; [4]Aarhus University Hospital, Department of Molecular Medicine, Aarhus, Denmark; [5]Aalborg University Hospital, Department of Hematology, Aalborg, Denmark; [6]Lillebaelt Hospital, Department of Hematology, Vejle, Denmark; [7]Odense University Hospital, Department of Hematology, Odense, Denmark; [8]Aarhus University, Department of Clinical Epidemiology, Aarhus, Denmark; [9]Aarhus University, Faculty of Health Science, Department of Clinical Medicine, Aarhus, Denmark; [10]University of Copenhagen, Faculty of Health Science, Department of Clinical Medicine, Copenhagen, Denmark;*

### Background

Watch-and-wait (WAW) is an accepted management strategy for patients with asymptomatic, advanced-stage, low tumor burden follicular lymphoma (FL). Nonetheless, disease course heterogeneity among patients selected for initial WAW warrants risk-stratified surveillance strategies. Such stratification also requires identification of patients who may never require treatment. While WAW is standard for selected patients, long-term outcomes beyond 5 years and risk factors guiding surveillance intensity remain poorly defined.

### Aims

This study aimed to investigate diagnostic clinical characteristics associated with subsequent treatment patterns in patients with FL managed with long-term WAW (≥5 years; WAW5).

### Methods

Time of WAW was defined from date of the FL diagnosis until lymphoma treatment initiation, clinical decision of terminating WAW in untreated patients or death during WAW. Eligible patients were identified from The Danish National Lymphoma Registry (LYFO), leading to inclusion of 474 patients diagnosed with FL in Denmark from 2000-2020 and followed with WAW for at least 5 years. Clinical variables, treatment data and outcome measures were obtained from LYFO and completed with data from the medical health care records. All survival analyses were performed with a 5-year landmark after diagnosis. Cumulative incidence of treatment initiation accounted for death before treatment as a competing risk.

**Copyright Information:** (Online) ISSN: 2572-9241
©2026 The Author(s). HemaSphere is published by John Wiley & Sons Ltd on behalf of European Hematology Association. This is an open access Abstract Book distributed under the Attribution-NonCommercial-NoDerivs (CC BY-NC-ND), which allows third parties to download the articles and share them with others as long as they credit the author and the Abstract Book, but they cannot change the content in any way or use them commercially.
**Abstract Book Citations:** Authors, Title, HemaSphere, 2026;10:(S1):pages. The abstract book DOIs can be found on the “Author Information” page.
**Disclaimer:** Articles published in the journal HemaSphere exclusively reflect the opinions of the authors. The authors are responsible for all content in their abstracts including accuracy of the facts, statements, citing resources, etc.

### Results

A total of 102 patients (22%) initiated treatment at some point after the WAW5-period. Of these, 78% received chemotherapy (+/- immunotherapy), 14% received immuno-monotherapy and 8% received radiotherapy as first-line therapy. Overall survival did not differ in the treated versus untreated patients (log-rank p-value 0.98, median not reached). In univariable and multivariable cause-specific Cox regression models, several clinical variables at diagnosis were evaluated including age >60 years, male gender, ECOG performance status >1, Ann Arbor stage ≥3, involved nodal regions >4, involved extranodal sites >1, and bone marrow involvement. The cut-off values were defined according to existing prognostic risk scores. In the multiple regression model, Ann Arbor stage ≥3 (HR 3.29, 95% CI 1.54–7.04, p-value 0.002) and involved nodal regions >4 (HR 1.81, 95% CI 1.19–2.76, p-value 0.006) were independently associated with time to treatment. Further investigating the impact of these two risk factors showed that patients without either risk factor (low-risk, 26%) displayed a cumulative incidence of treatment initiation after the WAW5-period of only 4% (95% CI 1%–9%), 8% (95% CI 2%–13%), and 10% (95% CI 3%–18%) at 2, 5, and 10 years after the 5-year landmark, respectively. For the patients with both risk factors (high-risk, 33%) the cumulative incidence of treatment initiation was 24% (95% CI 17%–31%), 35% (95% CI 27%–44%), and 50% (95% CI 38%–61%) at 2, 5, and 10 years respectively after the 5-year landmark (see figure).

### Summary/Conclusion

In this Danish nationwide cohort of patients with FL managed with long-term WAW (≥5 years), treatment initiation beyond the 5-year period was generally low. Ann Arbor stage and number of involved nodal regions at diagnosis were independently associated with subsequent treatment initiation. Patients without either risk factor were at low risk of treatment initiation and may be candidates for earlier surveillance deescalation.

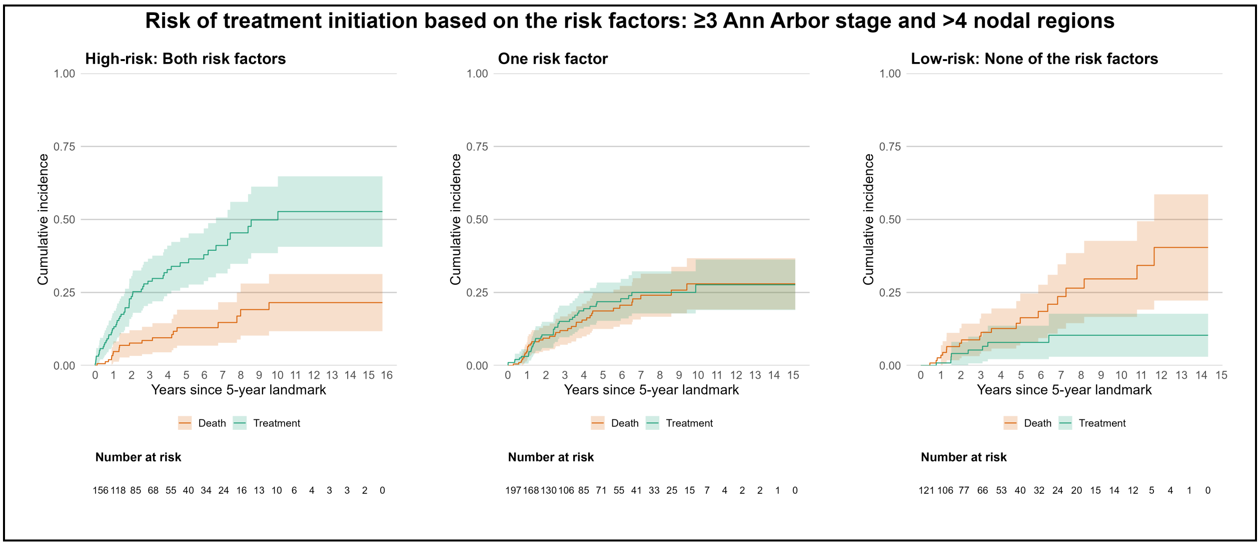

**Copyright Information:** (Online) ISSN: 2572-9241
©2026 The Author(s). HemaSphere is published by John Wiley & Sons Ltd on behalf of European Hematology Association. This is an open access Abstract Book distributed under the Attribution-NonCommercial-NoDerivs (CC BY-NC-ND), which allows third parties to download the articles and share them with others as long as they credit the author and the Abstract Book, but they cannot change the content in any way or use them commercially.
**Abstract Book Citations:** Authors, Title, HemaSphere, 2026;10:(S1):pages. The abstract book DOIs can be found on the “Author Information” page.
**Disclaimer:** Articles published in the journal HemaSphere exclusively reflect the opinions of the authors. The authors are responsible for all content in their abstracts including accuracy of the facts, statements, citing resources, etc.

### (PS2058) MACHINE LEARNING-BASED BODY COMPOSITION ANALYSIS FOR RISK STRATIFICATION IN MANTLE CELL LYMPHOMA (MCL) WITH NEED FOR SYSTEMIC TREATMENT

**Topic:** 18. Indolent and mantle-cell non-Hodgkin lymphoma - Clinical

Marie-Kristin Tilch*[1], Lukas Müller[2], Marietheres Polsak[1], Daniel Pinto Dos Santos[2], Tobias Bäuerle[2], Matthias Theobald[1], Georg Heß[1]

*[1]University Medical Center of the Johannes Gutenberg University Mainz, Hematology and Clinical Oncology, Mainz, Germany;*
*[2]University Medical Center of the Johannes Gutenberg University Mainz, Radiology, Mainz, Germany;*

### Background

Mantle Cell Lymphoma (MCL) is a rare, aggressive, and incurable subtype of non-Hodgkin lymphoma. Most patients (pts) present with advanced-stage disease requiring systemic therapy. Prognosis is
guided by age, performance status, MIPI, and biological disease features. Treatment varies from oral agents to intensive chemoimmunotherapy (CIT), with cellular therapies in relapse.
Despite therapeutic advances, most pts ultimately die from MCL. Imaging-based body composition analysis (BCA) using CT or MRI enables quantitative assessment of metabolic phenotypes, including sarcopenia. Two-dimensional BCA supports risk stratification in several malignancies, but its prognostic relevance in MCL remains undefined.

### Aims

To evaluate the prognostic impact of body composition parameters and sarcopenia in a retrospective MCL cohort.

### Methods

We analyzed 100 MCL pts undergoing CIT at our department. All pts were enrolled within the eMCL registry, giving informed consent for data collection and analysis. Artificial intelligence–based BCA was conducted on routine CT scans at two independent time points. Quantitative metrics for muscle, bone, visceral (VAT), subcutaneous (SAT), total (TAT), and intramuscular adipose tissue (IMAT) were extracted. Associations of BCA parameters with age, sex, and Body Mass Index (BMI) were assessed using general linear models. The prognostic value of individual body composition parameters was evaluated through univariate Cox regression analyses at each time point. To identify body composition phenotypes, principal component analysis (PCA) was performed followed by k-means clustering. Cluster-specific survival was compared using Kaplan–Meier estimation and log-rank testing.

### Results

Pts treated between 2002-2024 with available pre- and post-treatment CT scans were included. Median age was 64.5 years (IQR 45–94); 71% were male, and 86% had stage IV disease. High-risk features comprised 44% high MIPI, 40% Ki-67 >30%, 19% blastoid/pleomorphic morphology, and 11% TP53 alterations. BCA was performed mainly at first-line therapy (88%), with common regimens including alternating CHOP/DHAP (42%), CHOP (27%), or bendamustine (18%) plus rituximab. Body composition metrics significantly correlated with age, sex, and BMI. At both time points, higher VAT, TAT, and IMAT were consistently associated with inferior overall survival, while muscle/bone parameters showed no significant baseline association. Notably, longitudinal decline in muscle volume was significantly linked to shorter overall survival in pts ≥70 years (n=27; p=0.015, see figure). During follow-up, 49% of pts died, predominantly from lymphoma (67%).

**Copyright Information:** (Online) ISSN: 2572-9241
©2026 The Author(s). HemaSphere is published by John Wiley & Sons Ltd on behalf of European Hematology Association. This is an open access Abstract Book distributed under the Attribution-NonCommercial-NoDerivs (CC BY-NC-ND), which allows third parties to download the articles and share them with others as long as they credit the author and the Abstract Book, but they cannot change the content in any way or use them commercially.
**Abstract Book Citations:** Authors, Title, HemaSphere, 2026;10:(S1):pages. The abstract book DOIs can be found on the "Author Information" page.
**Disclaimer:** Articles published in the journal HemaSphere exclusively reflect the opinions of the authors. The authors are responsible for all content in their abstracts including accuracy of the facts, statements, citing resources, etc.

### Summary/Conclusion

CT-based BCA provides clinically relevant prognostic information beyond established risk factors. Adipose tissue distribution was consistently associated with inferior survival, suggesting a role for tumor-host metabolic interactions in disease biology. Longitudinal muscle loss predicted adverse outcomes in pts ≥70 years, highlighting the value of dynamic assessment in the elderly. While baseline muscle/bone metrics were not prognostic, this may reflect its heterogeneity rather than associations seen in aggressive lymphoma. Integration of automated BCA into routine imaging may refine risk stratification and help identify pts who could benefit from metabolism- and frailty-adapted therapeutic strategies. BCA-guided prevention of muscle loss may improve outcomes in vulnerable pts. Prospective validation is ongoing in the phase II MCL Elderly III clinical trial.

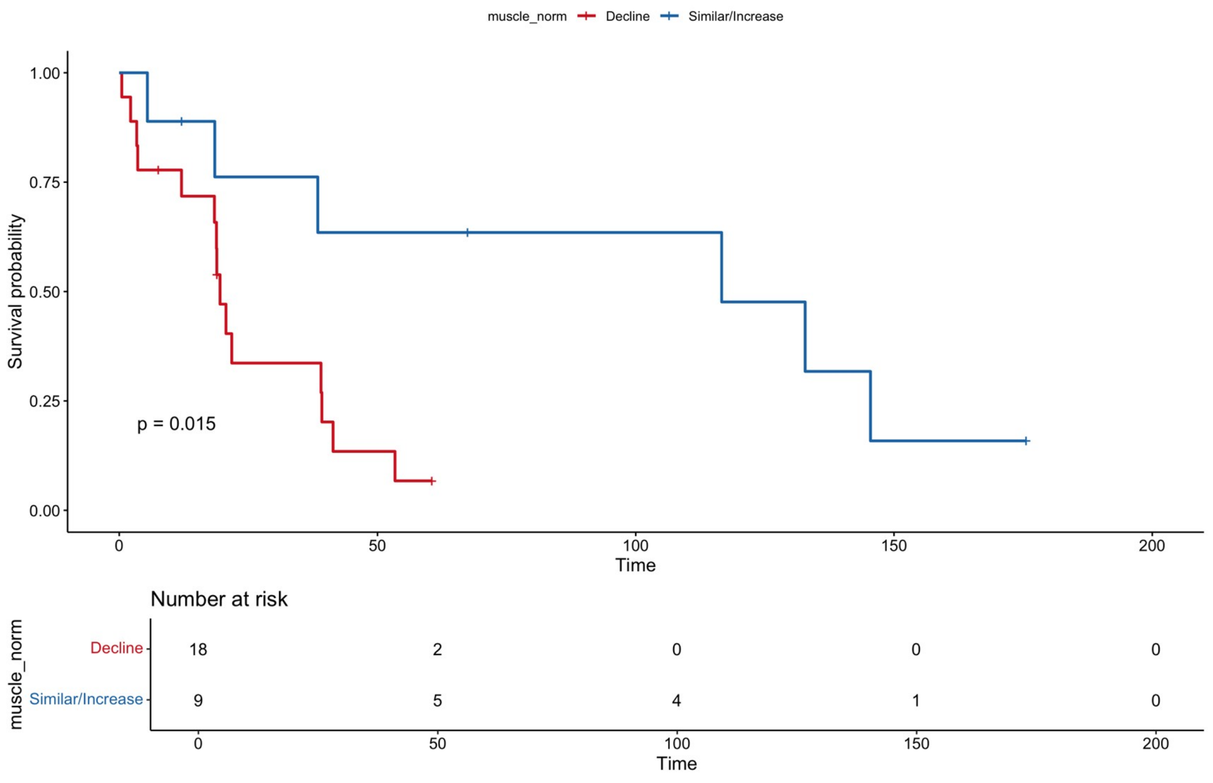

| BCA Parameter (t1) | Hazard Ratio | 95% CI (low) | 95% CI (high) | p-value | Age Group |
|---|---|---|---|---|---|
| bone_norm | 0.93 | 0.57 | 1.52 | 0.7740 | >70 |
| muscle_norm | 0.93 | 0.51 | 1.67 | 0.7980 | >70 |
| tat_norm | 1.70 | 1.08 | 2.66 | 0.0216 | >70 |
| imat_norm | 1.54 | 1.02 | 2.33 | 0.0422 | >70 |
| sat_norm | 1.57 | 1.03 | 2.40 | 0.0378 | >70 |
| vat_norm | 1.70 | 1.05 | 2.73 | 0.0294 | >70 |

| Δ BCA (t2 – t1) | Hazard Ratio | 95% CI (low) | 95% CI (high) | p-value | Age Group |
|---|---|---|---|---|---|
| bone_norm | 0.10 | 0.01 | 1.01 | 0.05120 | >70 |
| muscle_norm | 0.14 | 0.03 | 0.55 | 0.00536 | >70 |
| tat_norm | 0.42 | 0.21 | 0.83 | 0.01350 | >70 |
| imat_norm | 0.45 | 0.20 | 1.03 | 0.06000 | >70 |
| sat_norm | 0.36 | 0.16 | 0.77 | 0.00870 | >70 |
| vat_norm | 0.39 | 0.17 | 0.88 | 0.02330 | >70 |

**Copyright Information:** (Online) ISSN: 2572-9241
©2026 The Author(s). HemaSphere is published by John Wiley & Sons Ltd on behalf of European Hematology Association. This is an open access Abstract Book distributed under the Attribution-NonCommercial-NoDerivs (CC BY-NC-ND), which allows third parties to download the articles and share them with others as long as they credit the author and the Abstract Book, but they cannot change the content in any way or use them commercially.
**Abstract Book Citations:** Authors, Title, HemaSphere, 2026;10:(S1):pages. The abstract book DOIs can be found on the "Author Information" page.
**Disclaimer:** Articles published in the journal HemaSphere exclusively reflect the opinions of the authors. The authors are responsible for all content in their abstracts including accuracy of the facts, statements, citing resources, etc.

### (PS2059) EFFICACY AND SAFETY OF NEMTABRUTINIB IN RELAPSED OR REFRACTORY MARGINAL ZONE LYMPHOMA: COHORT F OF THE PHASE 2 BELLWAVE-003 STUDY

**Topic:** 18. Indolent and mantle-cell non-Hodgkin lymphoma - Clinical

Alessandra Tucci*[1], Yair Herishanu[2], Juan Dupont[3], Sarit Assouline[4], Lori Leslie[5], Thor Høyer[6], Pier Luigi Zinzani[7, 8], Yan Xu[9], Mohammed Farooqui[9], Jing Yang[9], Muhit Ozcan[10]

*[1]UOC Ematologia, Brescia, Italy; [2]Sourasky Medical Center, Tel Aviv, Israel; [3]CEMIC Hospital Universitario, Buenos Aires, Argentina; [4]Jewish General Hospital, Montréal, Canada; [5]John Theurer Cancer Center, Hackensack University Medical Center, Hackensack, United States of America; [6]Klinik Kirurgi og Kreftbehandling, Aalborg, Denmark; [7]IRCCS Azienda Ospedaliero-Universitaria di Bologna Istituto di Ematologia “Seragnoli", Bologna, Italy; [8]Dipartimento di Scienze Mediche e Chirurgiche, Università di Bologna, Bologna, Italy; [9]Merck & Co., Inc., Rahway, United States of America; [10]Ankara University School of Medicine, Ankara, Türkiye;*

### Background

Nemtabrutinib is a potent, noncovalent, reversible Bruton tyrosine inhibitor (BTKi) administered orally (PO) once daily (QD) that has shown antitumor activity across B-cell malignancies, including in models harboring BTK mutations associated with resistance to covalent BTKi's. BELLWAVE-003 (NCT04728893) is a phase 2 study evaluating nemtabrutinib in hematologic malignancies.

### Aims

To report efficacy and safety outcomes from cohort F of the BELLWAVE-003 study, comprising participants with relapsed or refractory (R/R) marginal zone lymphoma (MZL) previously treated with chemoimmunotherapy and a covalent irreversible BTKi and who received nemtabrutinib at the recommended phase 2 dose (RP2D).

### Methods

BELLWAVE-003 is a multicenter, open-label, single-arm phase 2 study enrolling participants with R/R B-cell malignancies. Participants with R/R MZL were required to have failed prior covalent BTKi therapy and chemoimmunotherapy. Eligible participants received nemtabrutinib at the RP2D (65 mg PO QD) until disease progression, unacceptable toxicity, or withdrawal. The primary end point was objective response rate. Secondary end points included duration of response per Lugano 2014 criteria by blinded independent central review (BICR) and safety. Exploratory end points included progression-free survival by BICR and overall survival. The data cut-off date for this analysis was September 8, 2025.

**Copyright Information:** (Online) ISSN: 2572-9241
©2026 The Author(s). HemaSphere is published by John Wiley & Sons Ltd on behalf of European Hematology Association. This is an open access Abstract Book distributed under the Attribution-NonCommercial-NoDerivs (CC BY-NC-ND), which allows third parties to download the articles and share them with others as long as they credit the author and the Abstract Book, but they cannot change the content in any way or use them commercially.
**Abstract Book Citations:** Authors, Title, HemaSphere, 2026;10:(S1):pages. The abstract book DOIs can be found on the “Author Information” page.
**Disclaimer:** Articles published in the journal HemaSphere exclusively reflect the opinions of the authors. The authors are responsible for all content in their abstracts including accuracy of the facts, statements, citing resources, etc.

**Results**

A total of 36 participants with R/R MZL were treated with nemtabrutinib at the RP2D. Median study follow-up was 11.6 months (range, 0.3-31.3). Median age was 69 years (range, 41-86); 14 participants (39%) were male and 23 (64%) had Lugano stage IV disease. Participants had received a median of 3 (range, 1-9) prior lines of therapy. Median duration of treatment was 5.1 months (range, 0.03-27.7), with 15 participants (42%) remaining on treatment at data cutoff. Objective response rate by both BICR and investigator review was 47% (95% CI, 30-65). Median duration of response was 8.5 months (95% CI, 2.8-not reached). Median progression-free survival was 6.5 months by investigator review and 5.6 months by BICR. Median overall survival was not reached, with a 12-month overall survival rate of 86.7% (95% CI, 62.5-95.8). All-cause adverse events (AEs) of any grade were reported in 33 participants (92%). The most common all-cause AEs (≥25%) were pneumonia (31%), pyrexia (31%), and anemia (25%). Grade 3-5 all-cause AEs occurred in 23 participants (64%), most commonly pneumonia (25%) and anemia (17%). Dose reductions due to all-cause AEs were reported in 6 participants (17%), including febrile neutropenia, fatigue, pneumonia, weight decreased, rash, and maculopapular rash (1 participant each). Treatment discontinuation due to all-cause AEs occurred in 6 participants (17%). Two deaths (6%) related to all-cause AEs were reported.

**Summary/Conclusion**

Nemtabrutinib showed clinically meaningful antitumor activity with a manageable safety profile in heavily pretreated participants with R/R MZL who had failed prior covalent BTKi therapy and chemoimmunotherapy. These findings support ongoing clinical evaluation of nemtabrutinib in this population.

**Copyright Information:** (Online) ISSN: 2572-9241
©2026 The Author(s). HemaSphere is published by John Wiley & Sons Ltd on behalf of European Hematology Association. This is an open access Abstract Book distributed under the Attribution-NonCommercial-NoDerivs (CC BY-NC-ND), which allows third parties to download the articles and share them with others as long as they credit the author and the Abstract Book, but they cannot change the content in any way or use them commercially.
**Abstract Book Citations:** Authors, Title, HemaSphere, 2026;10:(S1):pages. The abstract book DOIs can be found on the "Author Information" page.
**Disclaimer:** Articles published in the journal HemaSphere exclusively reflect the opinions of the authors. The authors are responsible for all content in their abstracts including accuracy of the facts, statements, citing resources, etc.

### (PS2060) LONG TERM FOLLOW-UP OF THE FIRST REAL-LIFE SERIES OF OBINUTUZUMAB-CHEMOTHERAPY COMBINATIONS IN FRONT-LINE TREATMENT OF FOLLICULAR LYMPHOMA: A STUDY OF KROHEM

**Topic:** 18. Indolent and mantle-cell non-Hodgkin lymphoma - Clinical

Davor Galusic[1, 2], Sandra Bašić Kinda*[3], Petra Bernes[4], Klara Brcic[5], Bozena Coha[6], Barbara Dreta[3], Neven Franjić[7, 8], Marija Ivić[9], Ivan Krecak[8, 10], Vibor Milunovic[11], Dejan Strahija[12], Ivan Zekanovic[13], Igor Aurer[3, 14]

*[1]University hospital of Split, Hematology, Split, Croatia;[2]University of Split, Split, Croatia;[3]University Hospital Centre Zagreb, Zagreb, Croatia; [4]Pula General Hospital, Pula, Croatia;[5]Sestre Milosrdnice University Hospital Center, Zagreb, Croatia;[6]Dr. "Josip Bencevic" General Hospital, Slavonski Brod, Croatia; [7]University Hospital Centre Rijeka, Rijeka, Croatia; [8]University of Rijeka, Rijeka, Croatia; [9]University Hospital Dubrava, Zagreb, Croatia; [10]General Hospital of Sibenik, Sibenik, Croatia; [11]University Hospital Merkur, Zagreb, Croatia; [12]Varazdin General Hospital, Varazdin, Croatia; [13]General Hospital Zadar, Zadar, Croatia; [14]University of Zagreb, Zagreb, Croatia;*

### Background

Randomized trials have established that obinutuzumab (G) is superior to rituximab (R) and R-bendamustine (B) to R-CHOP infront-line therapy of follicular lymphoma (FL). However, there is a dearth of real-life data on the outcome of G-chemotherapy combinations in this setting. At the EHA 2022 congress we presented and later published the first real-world cohort of patients with FL grade 1-3A treated with G-chemotherapy combinations. After a median follow-up of 17 months OS and PFS of the group treated with G-B were significantly inferior in comparison to the group treated with G-CHOP, mainly due to an increase in COVID19-related deaths, but also deaths from other infections. It remained unclear whether the putative superior lymphoma control achieved with bendamustine will compensate for the difference in susceptibility to infections after longer follow-up. We therefore performed this analysis after a median follow-up of 62 months.

### Aims

Present long-term follow-up data of patients treated with obinutuzumab-chemotherapy combinations outside of clinical trials.

### Methods

We collected and analyzed follow-up data from patients included in our original study who started treatment for FL gr. 1-3A between April 2019 and March 2021 with obinutuzumab combined with CHOP, B or CVP.

**Copyright Information:** (Online) ISSN: 2572-9241
©2026 The Author(s). HemaSphere is published by John Wiley & Sons Ltd on behalf of European Hematology Association. This is an open access Abstract Book distributed under the Attribution-NonCommercial-NoDerivs (CC BY-NC-ND), which allows third parties to download the articles and share them with others as long as they credit the author and the Abstract Book, but they cannot change the content in any way or use them commercially.
**Abstract Book Citations:** Authors, Title, HemaSphere, 2026;10:(S1):pages. The abstract book DOIs can be found on the "Author Information" page.
**Disclaimer:** Articles published in the journal HemaSphere exclusively reflect the opinions of the authors. The authors are responsible for all content in their abstracts including accuracy of the facts, statements, citing resources, etc.

### Results

A total of 114 FL patients were treated with G-chemotherapy. The median age was 63 (range 33 to 86) years. Majority of patients were female (71/114 (62.3%)). 61 (53.5%) patients were treated with G-B, 49 (43%) with G-CHOP and 4 (3.5%) with G-CVP. Overall response rate at 6 months after treatment initiation was 94%; complete remission (CR) in 68% and partial remission (PR) in 25% of patients. After a median follow up of 62 months, median overall survival (OS) and progression free survival (PFS) were not reached, with 5-year rates of 74% and 65%, respectively. Patients treated with G-CHOP retained a statistically significantly superior OS compared to patients treated with G-B (5-year OS rate 84% and 67% respectively, P=0.032, HR=0.44) but failed to retain superiority in PFS (5-year PFS rate 71% and 62% respectively, P=0.34, HR=0.73, Fig 1). A total of 30 (26.3%) patients died during follow up. COVID-19 was the most common cause of death (15 patients), followed by other infections (6 patients), lymphoma (3 patients), secondary malignancy (1 patient) and in 5 patients cause of death was unknown. Only 5 deaths occurred after December 2022. SARS-CoV-2 infection caused a fatal outcome in 16% of patients treated with G-B and 10% of patients treated with G-CHOP. In order to minimize the effect of the COVID19 pandemic, we reanalyzed the results, censoring COVID19-related deaths. This resulted in a 5-year OS and PFS rates of 82% and 77% in the G-B, and 93% and 79% in the G-CHOP group. G-B did not appear to be superior to G-CHOP neither in elderly (>65 years) nor in patients with FL gr. 1-2.

### Summary/Conclusion

After more than 5 years of median follow-up OS of patients with FL gr. 1-3A receiving front-line therapy with G-CHOP remains superior to those treated with G-B. This is largely due to increased COVID19 mortality in the latter group. However, G-B failed to show superiority in anti-lymphoma efficacy compared to G-CHOP, even after COVID-19 deaths were censored, neither in the whole cohort nor in relevant subgroups. Our results suggest that, in a real-life setting, G-CHOP should be considered the treatment of choice for front-line therapy of fit FL patients.

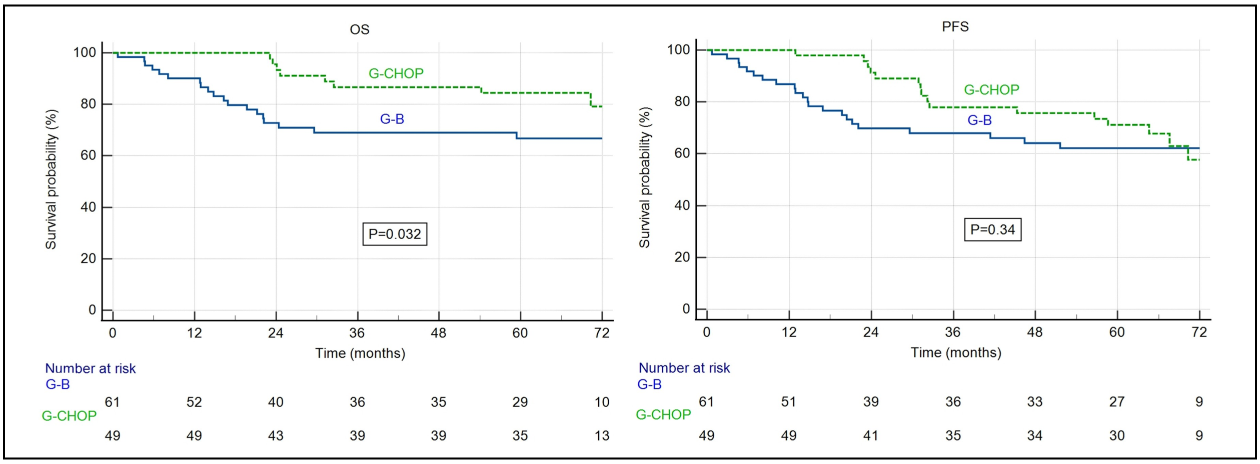

Overall (OS) and progression free survival (PFS) stratified according to the chemotherapy type (G-CHOP and G-Bendamustine).

Copyright Information: (Online) ISSN: 2572-9241
©2026 The Author(s). HemaSphere is published by John Wiley & Sons Ltd on behalf of European Hematology Association. This is an open access Abstract Book distributed under the Attribution-NonCommercial-NoDerivs (CC BY-NC-ND), which allows third parties to download the articles and share them with others as long as they credit the author and the Abstract Book, but they cannot change the content in any way or use them commercially.
Abstract Book Citations: Authors, Title, HemaSphere, 2026;10:(S1):pages. The abstract book DOIs can be found on the "Author Information" page.
Disclaimer: Articles published in the journal HemaSphere exclusively reflect the opinions of the authors. The authors are responsible for all content in their abstracts including accuracy of the facts, statements, citing resources, etc.

### (PS2061) IBRUTINIB IN SECOND-LINE RELAPSED/REFRACTORY MANTLE CELL LYMPHOMA: EFFICACY AND SAFETY RESULTS FROM THE SECOND INTERIM ANALYSIS OF THE OLIMPUS STUDY

**Topic:** 18. Indolent and mantle-cell non-Hodgkin lymphoma - Clinical

Carlo Visco*[1], Alessia Moioli[1], Luca Arcaini[2], Anna Maria Frustaci[3], Cristina Muzi[3], Maura Nicolosi[4], Alessandro Re[5], Gioacchino Catania[6], Emanuele Cencini[7], Annalisa Chiarenza[8], Alice Di Rocco[9], Luca Laurenti[10], Caterina Patti[11], Francesca Gaia Rossi[12], Benedetta Bianchi[13], Andrés José María Ferreri[14], Donato Mannina[15], Federico Meconi[16], Pellegrino Musto[17], Benedetta Puccini[18], Beatrice Casadei[19], Mariapia Pironti[20], Myriam Foglietta[21], Sara Galimberti[22], Maurizio Musso[23], Potito Rosario Scalzulli[24], Michele Spina[25], Piero Maria Stefani[26], Francesco Piazza[27], Ilaria Scortechini[28], Maria Pina Simula[29], Daniele Vallisa[30], Francesco Zaja[31], Giulia Maria Rivolta[32], Maria Palma[32], Danilo Arienti[32], Alessandra Tedeschi[3], Pier Luigi Zinzani[33]

*[1]Hematology Unit, Section of Biomedicine of Innovation, Department of Engineering for Innovative Medicine, Verona University, Verona, Italy; [2]Department of Molecular Medicine, University of Pavia; Division of Hematology, Fondazione IRCCS Policlinico San Matteo, Pavia, Italy; [3]ASST Grande Ospedale Metropolitano Niguarda, Niguarda Cancer Center, Milan, Italy; [4]Hematology Division - A.O.U. Città della Salute e della Scienza di Torino, Torino, Italy; [5]SC Ematologia, Ospedale Carlo Poma, ASST Mantova, Mantova, Italy; [6]AOU SS Antonio e Biagio e C. Arrigo, Alessandria, Italy;[7]Hematology, Azienda Ospedaliera Universitaria Senese & University of Siena, Siena, Italy; [8]Division of Hematology, Azienda Policlinico-S. Marco, University of Catania, Catania, Italy; [9]Hematology, Department of Translational and Precision Medicine, Sapienza University of Rome, Rome, Italy;[10]Ematologia, Fondazione Policlinico Universitario A Gemelli Roma IRCCS, Rome, Italy; [11]Oncohematology Unit, A.O.O.R. Villa Sofia Cervello, Palermo, Italy; [12]Ematologia, IRCCS Ca Granda, Ospedale Maggiore Policlinico, Milan, Italy; [13]Division of Hematology, University Hospital Ospedale di Circolo e Fondazione Macchi, ASST Sette Laghi, University of Insubria, Varese, Italy; [14]Lymphoma Unit, IRCCS San Raffaele Scientific Institute, Milan, Italy; [15]UOC Ematologia, Azienda Ospedaliera Papardo, Messina, Italy; [16]Unità Patologie Linfoproliferative, Dipartimento di Oncoematologia, Fondazione Policlinico di Tor Vergata, Rome, Italy; [17]Unit of Hematology and Stem Cell Transplantation, AOUC Policlinico, University of Bari, Bari, Italy; [18]Hematology Unit, Careggi University Hospital, Florence, Italy; [19]IRCCS Azienda Ospedaliero-Universitaria di Bologna, Istituto di Ematologia "Seràgnoli", Bologna, Italy; [20]Division of Hematology, Department of Molecular Biotechnologies and Health Sciences, University of Torino, Torino, Italy; [21]Hematology Unit, Santa Croce e Carle General Hospital, Cuneo, Italy;[22]Hematology Unit, Department of Clinical and Experimental Medicine, University of Pisa, Pisa, Italy; [23]Onco-Hematology Unit and BMT, La Maddalena Hospital, Palermo, Italy; [24]Department of Hematology and Bone Marrow Transplant, IRCCS Casa Sollievo della Sofferenza Hospital Foundation, San Giovanni Rotondo, Italy; [25]Division of Medical Oncology and Immune-Related Tumors, Centro di Riferimento Oncologico di Aviano IRCCS, Aviano, Italy; [26]Struttura Complessa di Ematologia, Dipartimento Strutturale di Medicina Interna, Presidio Ospedaliero di Treviso, Treviso, Italy; [27]Hematology Unit, Department of Medicine, Azienda Ospedale Università di Padova, Padova, Italy; [28]Clinic of Hematology, Azienda Ospedaliero Universitaria delle Marche, Ancona, Italy;[29]Ematologia e Centro Trapianti Midollo Osseo, Ospedale Oncologico Businco, Cagliari, Italy; [30]Hematology Unit, Department of Oncology and Hematology, Guglielmo da Saliceto Hospital, Piacenza, Italy; [31]Hematology Unit, Azienda Sanitaria Universitaria Giuliano-Isontina, Trieste, Italy; [32]Johnson & Johnson Innovative Medicine, Milan, Italy; [33]IRCCS Azienda Ospedaliero-Universitaria di Bologna Istituto di Ematologia "Seràgnoli" and Dipartimento di Scienze Mediche e Chirurgiche, Bologna, Italy;*

### Background

Mantle cell lymphoma (MCL) is an aggressive and incurable hematological disease, for which ibrutinib is approved in the relapsed/refractory (R/R) setting. While the literature on ibrutinib in R/R MCL clinical trials is broad, published data on prospective series in second line and/or real-world clinical practice is scarce.

**Copyright Information:** (Online) ISSN: 2572-9241
©2026 The Author(s). HemaSphere is published by John Wiley & Sons Ltd on behalf of European Hematology Association. This is an open access Abstract Book distributed under the Attribution-NonCommercial-NoDerivs (CC BY-NC-ND), which allows third parties to download the articles and share them with others as long as they credit the author and the Abstract Book, but they cannot change the content in any way or use them commercially.
**Abstract Book Citations:** Authors, Title, HemaSphere, 2026;10:(S1):pages. The abstract book DOIs can be found on the "Author Information" page.
**Disclaimer:** Articles published in the journal HemaSphere exclusively reflect the opinions of the authors. The authors are responsible for all content in their abstracts including accuracy of the facts, statements, citing resources, etc.

### Aims

The OLIMPUS study is a multicenter prospective collection of patients with first R/R MCL. The objective of the study was to collect the real-world Italian experience and share the efficacy and safety outcome about ibrutinib treatment in the second line. The present interim analysis (IA) updates and expands the previously described population.

### Methods

This study prospectively enrolled 106 patients with R/R MCL from 34 Italian centers. This IA presents data on the total population (106 patients), extrapolated up to the last available visit at the data cut-off, with a median follow-up calculated through Kaplan-Meier analysis of 18.04 months (95%CI: 16.56-21.95). Further, results of the first interim analysis population (IA1 population) of 51 patients are updated to a median follow-up of 24.57 months (95%CI: 22.44-NR). Primary objective was overall response rate (ORR) according to Lugano criteria.

### Results

In both populations, IA1 and IA, median age at enrollment was 71 years (range 35-90) with a predominance of male patients (73.6%). sMIPI score was high in 33%, TP53 mutation was identified in 51% of the tested patients (16/31), and blastoid/pleomorphic histology was observed in 17% (12/71). 58/106 patients (55%) were POD24, defined as R/R within 24 months from start of first line therapy. The median starting dose of ibrutinib was 560 mg. Subjects with significant cardiovascular disease were 22/106 (21%). Overall, the ORR was 73% (95% CI 58.1–85.4), with a complete response rate (CRR) of 50%; results were comparable in IA1 group (CRR 53%). 10% had stable disease, while progressive disease was recorded in 17%. At data cut-off, in the IA and IA1 cohort, progression-free survival (PFS) rate at month 24 was 57.3% (95% CI: 45.5-67.4) and 61.5% (95% CI: 45.5-74.0) respectively; PFS rate at month 24 for POD24 patients was 46.2% (95% CI: 31.1-60.0) in IA and was 48.5% (95% CI: 29.0-65.5) in IA1. Overall survival (OS) rate was 72.6% (95% CI: 59.8-81.9) in IA population and 80.1% (95% CI: 63.9-89.6) in IA1 group. Median duration of response (calculated through Kaplan-Meier analysis) was 28.45 months (95% CI: 23.39-NR) and not reached in IA1. Adverse events (AE) were observed in 68% of patients, 19.8% of grade 3 and 7.5% of grade 4. All grades, infections were the most frequent AE (23.6%) followed by general disorders (20%) such as pyrexia (8.5%) and asthenia (4.7%); and blood disorders (20%), such as neutropenia (9.4%), anemia (7.5%). Cardiac disorders occurred in 16 patients (15%), of which 6.6% of AF and 1.9% of cardiac failure. 39/106 (37%) patients experienced interruption of ibrutinib due to an AE, of these patients only 5/106 (4.7%) had a permanent discontinuation.

### Summary/Conclusion

The second interim analysis of the OLIMPUS prospective study confirmed the efficacy and safety of ibrutinib as a second-line treatment for MCL patients. These findings in the real-world clinical setting are consistent with those emerged from clinical trials and strengthen the role of ibrutinib in second-line R/R MCL.

**Copyright Information:** (Online) ISSN: 2572-9241
©2026 The Author(s). HemaSphere is published by John Wiley & Sons Ltd on behalf of European Hematology Association. This is an open access Abstract Book distributed under the Attribution-NonCommercial-NoDerivs (CC BY-NC-ND), which allows third parties to download the articles and share them with others as long as they credit the author and the Abstract Book, but they cannot change the content in any way or use them commercially.
**Abstract Book Citations:** Authors, Title, HemaSphere, 2026;10:(S1):pages. The abstract book DOIs can be found on the "Author Information" page.
**Disclaimer:** Articles published in the journal HemaSphere exclusively reflect the opinions of the authors. The authors are responsible for all content in their abstracts including accuracy of the facts, statements, citing resources, etc.

### (PS2062) PROFILING THE CSF-CTDNA MUTATIONAL LANDSCAPE IN A LARGE COHORT OF DLBCL: IMPLICATIONS FOR PREDICTING CNS RELAPSE AND CLINICAL OUTCOMES

**Topic:** 19. Large B cell lymphomas - Clinical

Jinhua Liang*[1], Yi-Fan Wu[1], Xin-Yi Zhang[1], Kai-Xin Du[1], Chunyu Shang[1], Li Wang[1], Jianyong Li[1], Wei Xu[1]

*[1]the First Affiliated Hospital of Nanjing Medical University, Jiangsu Province Hospital, Nanjing, China;*

### Background

Central nervous system involvement (CNSi) is a rare but often fatal event in systemic diffuse large B-cell lymphoma (DLBCL) patients. Circulating tumor DNA from cerebrospinal fluid (CSF-ctDNA) has played an important part in the application of liquid biopsy in CNS cancers. Nevertheless, larger-scale studies are needed to further characterize the mutational landscape of CSF-ctDNA and validate its clinical relevance in newly diagnosed (ND) DLBCL (ND-DLBCL).

### Aims

To thoroughly profile the mutational landscape of CSF-ctDNA and to explore its clinical significance in a large ND-DLBCL cohort.

### Methods

Between December, 2021 and January, 2026, 410 ND-DLBCL patients treated in our center were enrolled in the cohort. Baseline CSF, tissue and plasma samples were sequenced using a 475-gene lymphoma-specific panel. All patients were treated with R-CHOP or Pola-R-CHP regimen. Patients were identified as CSF-ctDNA positive (CSF(+)) based on two criteria: (1) at least one mutant variant detected in CSF-ctDNA was shared with tissue; (2) at least one mutant variant detected in CSF-ctDNA was canonical driver mutation in DLBCL (**Figure 1A**).

### Results

Of the 410 ND-DLBCL patients, 98 (23.9%) were identified as CSF(+), among which 63 (64.3%) fulfilled both criteria, while 17 (17.3%) only met criterion 1 and 18 (18.4%) only met criterion 2 (**Figure 1B**). Among the 80 CSF(+) patients with matched tissue, plasma and CSF samples, the top 10 frequently mutated genes were MYD88 (35.8%), PIM1 (34.6%), CD79B (28.8%), KMT2D (24.2%), BTG2 (23.3%), TP53 (23.3%), H1-4 (23.3%), BTG1 (22.1%), BCL6 (20.8%) and ETV6 (19.6%) (**Figure 1C**). The mean variant allele frequency was significantly lower in CSF than in tissue and plasma samples (**Figure 1D**). As to genetic subtypes, CSF(+) patients were mostly distributed in MCD (N=37), Other (N=29), ST2 (N=10), BN2 (N=8) and TP53-disrupted subtypes (N=7), while TP53-disrupted (36.8%) and MCD (34.3%) subtypes showed highest CSF(+) ratio (**Figure 1E**). However, no difference in CSF-ctDNA burden was observed across genetic subtypes (**Figure 1F**). Furthermore, CSF(+) patients exhibited higher plasma ctDNA burden (**Figure 1G**). Patients with MCD subtype, non-GCB orign, specific extranodal sites involvement (reproductive system, kidney/adrenal gland, bone marrow) and higher IPI score were correlated with higher CSF(+) rate, while gastrointestinal tract involvement seemed negatively associated with CSF(+) (**Figure 1H**). In plasma samples, MYD88, CD79B, ETV6, IGLL5, CDKN2A, CARD11, CD274 mutations were associated with higher CSF(+) rate (**Figure 1I**), while in tissue samples, CARD11, PRDM1, MCL1 mutations seemed correlated with higher CSF(+) rate (**Figure 1J**). In RNA level, the dysregulated genes of CSF(+) patients were enriched in cytokine-cytokine receptor interaction, focal adhesion, cytoskeleton, and PI3K-AKT signaling pathways, potentially promoting CNSi in ND-DLBCL (**Figure 1K**). Moreover, CSF(+) significantly correlated with inferior response to initial treatment and worse survival in ND-DLBCL patients (**Figure 1L-1N**). Besides, CSF-ctDNA(+) demonstrated superior prediction capability for CNS relapse compared with high-risk CNS-IPI in ND-DLBCL (**Figure 1O**).

**Copyright Information:** (Online) ISSN: 2572-9241
©2026 The Author(s). HemaSphere is published by John Wiley & Sons Ltd on behalf of European Hematology Association. This is an open access Abstract Book distributed under the Attribution-NonCommercial-NoDerivs (CC BY-NC-ND), which allows third parties to download the articles and share them with others as long as they credit the author and the Abstract Book, but they cannot change the content in any way or use them commercially.
**Abstract Book Citations:** Authors, Title, HemaSphere, 2026;10:(S1):pages. The abstract book DOIs can be found on the "Author Information" page.
**Disclaimer:** Articles published in the journal HemaSphere exclusively reflect the opinions of the authors. The authors are responsible for all content in their abstracts including accuracy of the facts, statements, citing resources, etc.

### Summary/Conclusion

To conclude, in this large-scale cohort, we comprehensively depicted the mutational landscape of CSF-ctDNA, explored the biological characteristics of CSF(+) patients, and highlighted the importance of CSF-ctDNA in predicting clinical outcome and CNS relapse in ND-DLBCL patients.

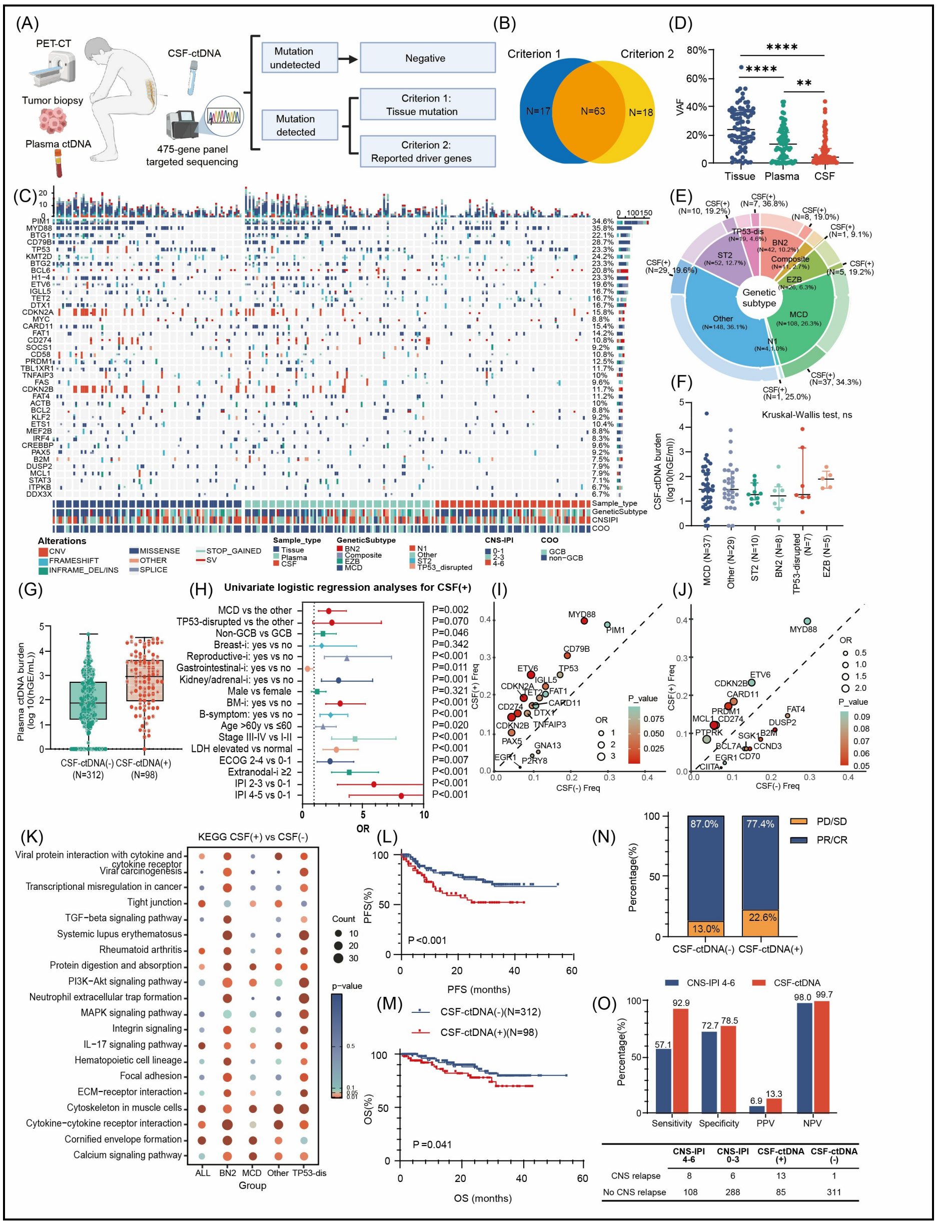

| | CNS-IPI 4-6 | CNS-IPI 0-3 | CSF-ctDNA (+) | CSF-ctDNA (-) |
|---|---|---|---|---|
| CNS relapse | 8 | 6 | 13 | 1 |
| No CNS relapse | 108 | 288 | 85 | 311 |

**Copyright Information:** (Online) ISSN: 2572-9241
©2026 The Author(s). HemaSphere is published by John Wiley & Sons Ltd on behalf of European Hematology Association. This is an open access Abstract Book distributed under the Attribution-NonCommercial-NoDerivs (CC BY-NC-ND), which allows third parties to download the articles and share them with others as long as they credit the author and the Abstract Book, but they cannot change the content in any way or use them commercially.
**Abstract Book Citations:** Authors, Title, HemaSphere, 2026;10:(S1):pages. The abstract book DOIs can be found on the "Author Information" page.
**Disclaimer:** Articles published in the journal HemaSphere exclusively reflect the opinions of the authors. The authors are responsible for all content in their abstracts including accuracy of the facts, statements, citing resources, etc.

### (PS2063) DURABLE REMISSIONS WITH GLOFITAMAB PLUS POLATUZUMAB VEDOTIN IN RELAPSED/REFRACTORY (R/R) LARGE B-CELL LYMPHOMA (LBCL), INCLUDING ELDERLY PATIENTS AND THOSE WITH HIGH-GRADE B-CELL LYMPHOMA (HGBCL)

**Topic:** 19. Large B cell lymphomas - Clinical

Giuseppe Gritti*[1], Anna Sureda[2], Francesc Bosch[3], Thomas Stauffer Larsen[4], Paolo Corradini[5], Abraham Avigdor[6], María José Terol[7], Antonio Rueda Dominguez[8], Antonio Pinto[9], Alan Skarbnik[10], Raul Cordoba[11], Judit Jørgensen[12], Pier Luigi Zinzani[13], Ronit Gurion[14], Neta Goldschmidt[15], Wilfred Leung[16], James Relf[17], Robert Barrier[18], Maneesh Tandon[17], Martine Joanna Kallemeijn[19], Gila Sellam[19], Martin Hutchings[20]

*[1]ASST Ospedale Papa Giovanni XXIII, Bergamo, Italy; [2]Clinical Hematology Department, Institut Català d'Oncologia – L'Hospitalet, IDIBELL, Universitat de Barcelona, Barcelona, Spain; [3]University Hospital Vall d'Hebron, VHIO, Barcelona, Spain; [4]Odense University Hospital, Odense, Denmark; [5]University of Milan and Fondazione IRCCS Istituto Nazionale dei Tumori, Milan, Italy; [6]Sheba Medical Center, Ramat Gan, and Gray Faculty of Medical and Health Sciences, Tel Aviv University, Tel Aviv, Israel; [7]Hospital Clínico Universitario INCLIVA, University of Valencia, Valencia, Spain; [8]Virgen de la Victoria University Hospital, Málaga, Spain; [9]National Cancer Institute Fondazione G. Pascale IRCCS, Naples, Italy; [10]Novant Health Cancer Institute, Charlotte, NC, United States of America; [11]Fundación Jiménez Díaz University Hospital, Madrid, Spain; [12]Aarhus University Hospital, Aarhus, Denmark; [13]University of Bologna, Bologna, Italy; [14]Institute of Hematology, Rabin Medical Center, Petah Tikva, and Gray Faculty of Medical and Health Sciences, Tel Aviv University, Tel Aviv, Israel; [15]Department of Hematology, Hadassah Medical Center, Jerusalem, Israel; [16]Genentech, Inc., South San Francisco, CA, United States of America; [17]Roche Products Ltd, Welwyn Garden City, United Kingdom; [18]Parexel International (on assignment to F. Hoffmann-La Roche AG), Basel, Switzerland; [19]F. Hoffmann-La Roche Ltd, Basel, Switzerland; [20]Rigshospitalet and University of Copenhagen, Copenhagen, Denmark;*

### Background

Despite recent advances, R/R LBCL remains clinically challenging with poor outcomes, particularly in elderly patients, necessitating durable therapeutic options. In an open-label, multicenter, Phase 1b/2 study (NCT03533283), glofitamab plus polatuzumab vedotin (Glofit-Pola; an investigational regimen) demonstrated high response rates with durable clinical benefit, and manageable safety in patients with R/R LBCL after ≥1 prior line of therapy (LOT).

### Aims

Investigate Glofit-Pola efficacy in patients with R/R LBCL, stratified by age, including outcomes for those with HGBCL.

### Methods

Eligible patients were aged ≥18 years (y) with R/R LBCL after ≥1 prior LOT. Methods have been previously described (Hutchings, et al. JCO 2025). All patients provided informed consent. Efficacy endpoints were independent review committee-assessed overall response, durability of response, and survival outcomes.

**Copyright Information:** (Online) ISSN: 2572-9241
©2026 The Author(s). HemaSphere is published by John Wiley & Sons Ltd on behalf of European Hematology Association. This is an open access Abstract Book distributed under the Attribution-NonCommercial-NoDerivs (CC BY-NC-ND), which allows third parties to download the articles and share them with others as long as they credit the author and the Abstract Book, but they cannot change the content in any way or use them commercially.
**Abstract Book Citations:** Authors, Title, HemaSphere, 2026;10:(S1):pages. The abstract book DOIs can be found on the "Author Information" page.
**Disclaimer:** Articles published in the journal HemaSphere exclusively reflect the opinions of the authors. The authors are responsible for all content in their abstracts including accuracy of the facts, statements, citing resources, etc.

**Results**

As of September 1, 2025, 129 patients had received study treatment; 43% were aged <65y, 57% were aged ≥65y, and 27% were aged ≥75y. The proportion of patients with ≥2 prior LOT was 71% for those aged <65y, 50% for those aged ≥65y, and 43% for those aged ≥75y. Median overall survival (mOS) follow-up was 42.2 months (mo; range: 0–67).
The overall response rate (ORR) and complete response (CR) rate were 79.1% (95% confidence interval [CI]: 71.0–85.7) and 60.5% (95% CI: 51.5–69.0), respectively, and were consistent across age groups (<65y: ORR 80.0%, CR 56.4%; ≥65y: ORR 78.4%, CR 63.5%; ≥75y: ORR 85.7%, CR 65.7%). The overall median duration of response (mDOR) was 28.4 mo (95% CI: 14.3–not estimable [NE]) and median duration of CR (mDOCR) was 56.3 mo (95% CI: 28.4–NE). In patients aged <65y, both mDOR and mDOCR were NE (95% CI: 7.4–NE and 37.8–NE, respectively); in patients aged ≥65y, mDOR was 24.1 mo (95% CI: 14.3–NE) and mDOCR was 28.4 mo (95% CI: 19.6–NE); in patients aged ≥75y, mDOR was 26.4 mo (95% CI: 8.3–NE) and mDOCR was NE (95% CI: 16.3–NE).
Median progression-free survival (mPFS) in the overall population was 15.4 mo (95% CI: 8.8–29.7), and was 10.4 mo (95% CI: 5.7–NE) in patients aged <65y, 15.4 mo (95% CI: 8.8–27.7) in patients aged ≥65y, and 17.7 mo (95% CI: 7.7–NE) in patients aged ≥75y. Overall mOS was 35.8 mo (95% CI: 22.5–NE) and was NE (95% CI: 24.1–NE) in patients aged <65y, 24.1 mo (95% CI: 17.1–NE) in patients aged ≥65y, and NE (95% CI: 15.5–NE) in patients aged ≥75y.
Incidence of HGBCL was consistent across age groups (<65y: 35%; ≥65y: 35%; ≥75y: 37%) and among these patients, the response rates were robust (<65y: ORR 94.7%, CR 73.7%; ≥65y: ORR 73.1%, CR 65.4%; ≥75y: ORR 85.7%, CR 65.7%). Both mDOR and mDOCR were NE for all age groups with HGBCL; mPFS and mOS were NE for those aged <65y. In patients with HGBCL, mPFS and mOS, respectively, were 15.5 mo and 22.8 mo for those aged ≥65y, and 15.5 mo and 24.1 mo for those aged ≥75y.
Safety profiles overall and across age groups were comparable, with similar cytokine release syndrome event rates. The serious adverse event (AE) rate was slightly lower in patients aged <65y. No new safety signals or new AE-related deaths were reported.

**Summary/Conclusion**

Fixed-duration Glofit-Pola in patients with R/R LBCL demonstrated high and durable response rates in the elderly population, including those with HGBCL. Results are comparable with the overall population. These findings support the potential of this drug combination as an effective treatment option in elderly patients with limited therapeutic alternatives.

**Copyright Information:** (Online) ISSN: 2572-9241
©2026 The Author(s). HemaSphere is published by John Wiley & Sons Ltd on behalf of European Hematology Association. This is an open access Abstract Book distributed under the Attribution-NonCommercial-NoDerivs (CC BY-NC-ND), which allows third parties to download the articles and share them with others as long as they credit the author and the Abstract Book, but they cannot change the content in any way or use them commercially.
**Abstract Book Citations:** Authors, Title, HemaSphere, 2026;10:(S1):pages. The abstract book DOIs can be found on the "Author Information" page.
**Disclaimer:** Articles published in the journal HemaSphere exclusively reflect the opinions of the authors. The authors are responsible for all content in their abstracts including accuracy of the facts, statements, citing resources, etc.

### (PS2064) FIRST IN HUMAN STUDY OF CT1190B, AN ALLOGENEIC ANTI-CD19/CD20 CAR T-CELL THERAPY, IN PATIENTS WITH RELAPSED OR REFRACTORY B-CELL NON-HODGKIN LYMPHOMA

**Topic:** 19. Large B cell lymphomas - Clinical

Shiguang Ye*[1], Aibin Liang[1], Ping Li[1], Lili Zhou[1], Yan Lu[1], Wenjun Zhang[1], Bing Xiu[1], Yicheng Zhang[2], Jia Wei[2], Xiaoxi Zhou[2], Xinying Zhao[3], Daijing Yuan[3], Yi Luo[3], Zonghai Li[3]

*[1]School of Medicine, Tongji Hospital of Tongji University, Shanghai, China;[2]Tongji Hospital, Tongji Medical College, Huazhong University of Science and Technology, Wuhan, China; [3]CARsgen Therapeutics Co. Ltd., Shanghai, China;*

### Background

CT1190B is an anti-NKG2A CAR armored allogeneic CD19/CD20 dual targeting CAR T-cell product, which has TRAC, B2M and NKG2A knockout to reduce graft-versus-host disease and host immune rejection, and increase anti-tumor activities respectively.

### Aims

This is an exploratory study to evaluate the safety, tolerability, preliminary efficacy and pharmacokinetics (PK) of CT1190B in patients with relapsed/refractory non-Hodgkin lymphoma (R/R NHL).

### Methods

Adult patients with R/R NHL who had received at least two prior lines of therapy were enrolled. An i3+3 design was utilized for dose escalation across four dose-levels: $1.5\times10^8$, $3.0\times10^8$, $4.5\times10^8$, and $6.0\times10^8$ cells. Two lymphodepletion regimens were explored: regimen A (Fludarabine 30 mg/m²/day × 3 days + Cyclophosphamide 1000 mg/m²/day × 2 days) and regimen B (Fludarabine 30 mg/m²/day × 3 days + Cyclophosphamide 500 mg/m²/day × 3 days).

**Copyright Information:** (Online) ISSN: 2572-9241
©2026 The Author(s). HemaSphere is published by John Wiley & Sons Ltd on behalf of European Hematology Association. This is an open access Abstract Book distributed under the Attribution-NonCommercial-NoDerivs (CC BY-NC-ND), which allows third parties to download the articles and share them with others as long as they credit the author and the Abstract Book, but they cannot change the content in any way or use them commercially.
**Abstract Book Citations:** Authors, Title, HemaSphere, 2026;10:(S1):pages. The abstract book DOIs can be found on the “Author Information” page.
**Disclaimer:** Articles published in the journal HemaSphere exclusively reflect the opinions of the authors. The authors are responsible for all content in their abstracts including accuracy of the facts, statements, citing resources, etc.

**Results**

As of Jan 22, 2026, a total of 13 patients (10 large B-cell Lymphoma [LBCL] and 3 Follicular Lymphoma [FL]) received CT1190B infusion, with 1, 2, 4, and 6 patients dosed with $1.5\times10^8$, $3.0\times10^8$, $4.5\times10^8$, and $6.0\times10^8$ cells, respectively. The median age was 55 years (range 35-70). All patients were heavily pretreated with median prior lines of therapy of 3 (range 2-7). One patient received prior CD19 CAR T-cell therapy, 1 received CD20/CD3 bispecific antibody, 2 had prior autologous stem cell transplantation, and 1 had experienced a failure autologous CAR T-cells manufacturing. The median value of sum of the perpendicular diameters at baseline was 1485.13 $mm^2$ (range 237.01-8847.25).
The majority of grade ≥3 adverse events were hematological toxicities, of which most recovered within 28 days. Cytokine release syndrome was observed in 8 patients (7 grade 1-2, 1 grade 3) and all recovered within 11 days. Immune effector cell-associated neurotoxicity syndrome occurred in 2 patients (one grade ≥3 resolving, one grade 1 resolved). No grade ≥3 infections, treatment discontinuations or deaths due to AE were observed.
As of Feb 2, 2026, 11 patients were evaluable for efficacy. The objective response rate was 90.9% (10/11) with complete response (CR) rate of 72.7% (8/11), including: 1 partial response (PR) and 1 CR at $3.0\times10^8$; 3 CR and 1 PR at $4.5\times10^8$; 4 CR at $6.0\times10^8$, as shown in the figure. All 3 FL patients achieved CR. All the 6 LBCL patients at regimen A lymphodepletion achieved response with a CR rate of 83.3%. Notably, responses were observed in patients with prior CAR T-cell or bispecific antibody therapy exposure, and all patients treated at intermediate or higher doses (≥$3.0\times10^8$) achieved response. With a median follow-up of 5.62 months, 7 out of 10 responders maintained ongoing response.
CAR-T cell expansion was observed across all dose levels. The median peak expansion ($C_{max}$) and exposure ($AUC_{0-t}$) were notably higher in patients receiving regimen A lymphodepletion ($C_{max}$: 77903.0 copies/μg gDNA; $AUC_{0-t}$: 575650.3 days*copies/μg gDNA) compared to those receiving regimen B lymphodepletion across all dose levels. In the highest dose cohort ($6.0\times10^8$ cells), the median $C_{max}$ and $AUC_{0-t}$ reached 117863.0 copies/μg gDNA and 643903.5 days*copies/μg gDNA, respectively.

**Summary/Conclusion**

CT1190B showed manageable safety and encouraging efficacy in patients with R/R B-cell NHL. We are excited to see the robust expansion and exposure of the CAR T- cells, especially in patients with intensive dose lymphodepletion. All these data support further investigation on CT1190B.

**Copyright Information:** (Online) ISSN: 2572-9241
©2026 The Author(s). HemaSphere is published by John Wiley & Sons Ltd on behalf of European Hematology Association. This is an open access Abstract Book distributed under the Attribution-NonCommercial-NoDerivs (CC BY-NC-ND), which allows third parties to download the articles and share them with others as long as they credit the author and the Abstract Book, but they cannot change the content in any way or use them commercially.
**Abstract Book Citations:** Authors, Title, HemaSphere, 2026;10:(S1):pages. The abstract book DOIs can be found on the "Author Information" page.
**Disclaimer:** Articles published in the journal HemaSphere exclusively reflect the opinions of the authors. The authors are responsible for all content in their abstracts including accuracy of the facts, statements, citing resources, etc.

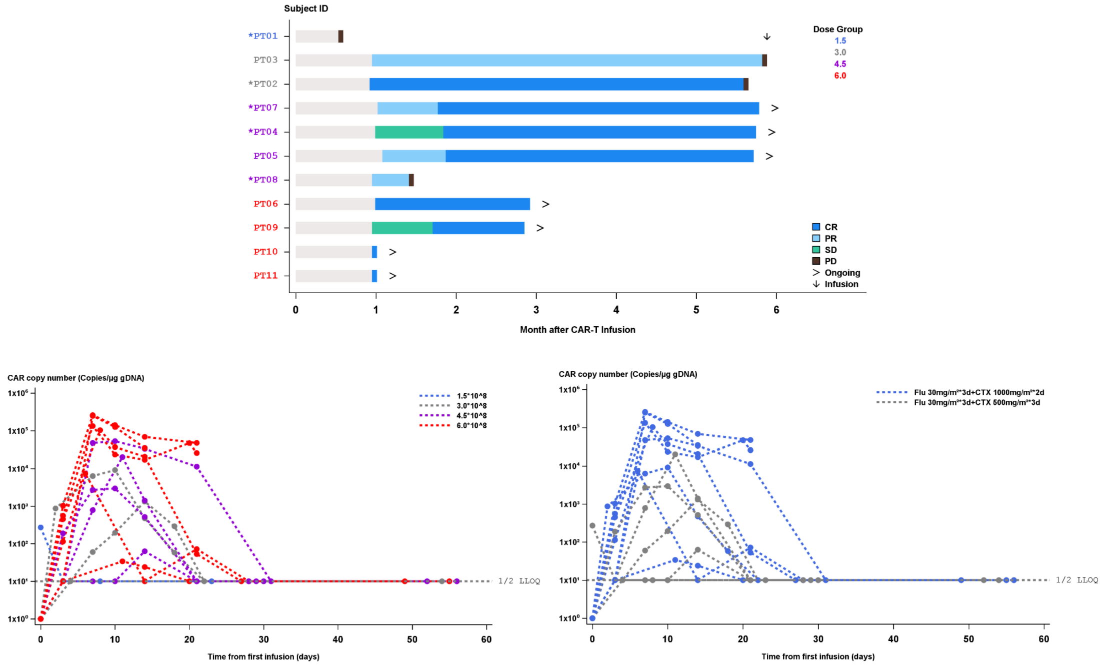

Swimming plot and CAR copy number-time plot

**Copyright Information:** (Online) ISSN: 2572-9241
©2026 The Author(s). HemaSphere is published by John Wiley & Sons Ltd on behalf of European Hematology Association. This is an open access Abstract Book distributed under the Attribution-NonCommercial-NoDerivs (CC BY-NC-ND), which allows third parties to download the articles and share them with others as long as they credit the author and the Abstract Book, but they cannot change the content in any way or use them commercially.
**Abstract Book Citations:** Authors, Title, HemaSphere, 2026;10:(S1):pages. The abstract book DOIs can be found on the “Author Information” page.
**Disclaimer:** Articles published in the journal HemaSphere exclusively reflect the opinions of the authors. The authors are responsible for all content in their abstracts including accuracy of the facts, statements, citing resources, etc.

### (PS2065) SELINEXOR IN COMBINATION WITH THE R-CHOP REGIMEN AS A FIRST-LINE TREATMENT FOR PATIENTS WITH HIGH-RISK GCB-SUBTYPE DIFFUSE LARGE B-CELL LYMPHOMA: A MULTI-CENTER, SINGLE-ARM, PHASE II STUDY

**Topic:** 19. Large B cell lymphomas - Clinical

Hang Yang[1], Peng Sun[1], Panpan Liu[1], Yu Wang[1], Hui Zhou[2], Ying Lu[3], Lu Zhang[4], Keshu Zhou[5], Zhiming Li*[1]

*[1]Sun Yat-Sen University Cancer Center, Department of Medical Oncology, Guangzhou, China;[2]Hunan Cancer Hospital, Changsha, China; [3]The Affiliated People's Hospital of Ningbo University, Department of Hematology, Ningbo, China;[4]Union Hospital, Tongji Medical College, Huazhong University of Science and Technology, Department of Hematology, Wuhan, China; [5]The Affiliated Cancer Hospital of Zhengzhou University, Henan Cancer Hospital, Department of Hematology, Zhengzhou, China;*

### Background

The germinal center B-cell-like (GCB) subtype is the most common molecular classification of diffuse large B-cell lymphoma (DLBCL). Approximately 30% of patients with GCB-DLBCL show resistance to R-CHOP therapy. An International Prognostic Index (IPI) score of 3-5 is recognized as one of the high-risk factors in patients with DLBCL. Current therapeutic strategies for high-risk GCB-DLBCL remain insufficiently defined, underscoring a critical unmet need in first-line treatment approaches. Selinexor, an oral selective inhibitor of nuclear export (SINE), targets exportin 1 (XPO1), leading to nuclear retention and reactivation of tumor suppressor proteins. In a real-world study involving 17 patients, Selinexor in combination with R-CHOP has shown promising results in treating high-risk GCB DLBCL.

### Aims

This phase II trial (NCT05422066) aims to evaluate the efficacy and safety of selinexor in combination with the R-CHOP regimen as a first-line treatment for patients with high-risk (IPI 3-5) GCB-DLBCL.

### Methods

Patients newly diagnosed with untreated GCB-DLBCL (IPI 3-5) were enrolled in this study. Patients will be treated with six cyclesof R-CHOP and selinexor (60 mg qw), with each cycle lasting three weeks. Treatment continue d for six cycles, or until intolerability, inadequate response, disease progression, consent withdrawal or death. Efficacy was evaluated according to the Lugano 2014 criteria. The primary endpoint was the complete response (CR) rate, and the secondary endpoints included the objective response rate (ORR), progression-free survival (PFS), disease-free survival (DFS), overall survival (OS), as well as safety and tolerability.

**Copyright Information:** (Online) ISSN: 2572-9241
©2026 The Author(s). HemaSphere is published by John Wiley & Sons Ltd on behalf of European Hematology Association. This is an open access Abstract Book distributed under the Attribution-NonCommercial-NoDerivs (CC BY-NC-ND), which allows third parties to download the articles and share them with others as long as they credit the author and the Abstract Book, but they cannot change the content in any way or use them commercially.
**Abstract Book Citations:** Authors, Title, HemaSphere, 2026;10:(S1):pages. The abstract book DOIs can be found on the "Author Information" page.
**Disclaimer:** Articles published in the journal HemaSphere exclusively reflect the opinions of the authors. The authors are responsible for all content in their abstracts including accuracy of the facts, statements, citing resources, etc.

### Results

Between August 2022 and February 2026, a total of 45 patients were enrolled, with a median age of 59 years (range, 26-79). The majority of patients (97.8%) presented with stage III-IV disease, and 42.2% were over 60 years. Additionally, 68.9% of patients presented with two or more extranodal involvement, and 51.1% had elevated lactate dehydrogenase levels. The double expression of MYC and BCL2 was detected in 20.0% of patients. The median follow-up period was 21.0 months (95% CI: 16.1 - 25.9), with 42 patients being evaluable for response. The CR rate was 76.2%, and the ORR was 100.0%. The 2-year OS, PFS and DFS rate were 97.6% (95% CI: 93.1%-100%), 66.7% (95% CI: 50.4%-88.1%) and 82.4% (95% CI: 63.9%-100%), respectively. The median PFS, DFS and OS have not yet been reached. The majority of adverse events (AEs) were of grade 1-2. Grade 3-4 AEs included neutropenia (71.1%), leukopenia (60.0%), decreased lymphocyte count (31.1%), and thrombocytopenia (26.7%). All AEs were effectively managed with supportive care and subsequently resolved.

**Table 1: Baseline Characteristics of the Study Population.**

| Baseline characteristics | All patients (n=45) |
|---|---|
| Median age, years (range) | 59 (26-79) |
| Sex, no.(%) | |
| Male | 26 (57.8%) |
| Female | 19 (42.2%) |
| Ann Arbor stage, no.(%) | |
| II | 1 (2.2%) |
| III | 11 (24.4%) |
| IV | 33 (73.3%) |
| IPI risk factors, no. (%) | |
| > 60 years | 19 (42.2%) |
| III-IV stage | 44 (97.8%) |
| ≥ 2 extranodal lesions | 31 (68.9%) |
| ECOG PS ≥ 2 | 19 (42.2%) |
| Elevated lactate dehydrogenase levels | 23 (51.1%) |
| Double-expression of MYC and BCL2, no.(%) | 9 (20.0%) |

### Summary/Conclusion

The combination of selinexor with the R-CHOP as a first-line treatment for high-risk (IPI 3-5) GCB-DLBCL demonstrates encouraging efficacy and and a manageable safety profile.

**Copyright Information:** (Online) ISSN: 2572-9241
©2026 The Author(s). HemaSphere is published by John Wiley & Sons Ltd on behalf of European Hematology Association. This is an open access Abstract Book distributed under the Attribution-NonCommercial-NoDerivs (CC BY-NC-ND), which allows third parties to download the articles and share them with others as long as they credit the author and the Abstract Book, but they cannot change the content in any way or use them commercially.
**Abstract Book Citations:** Authors, Title, HemaSphere, 2026;10:(S1):pages. The abstract book DOIs can be found on the "Author Information" page.
**Disclaimer:** Articles published in the journal HemaSphere exclusively reflect the opinions of the authors. The authors are responsible for all content in their abstracts including accuracy of the facts, statements, citing resources, etc.

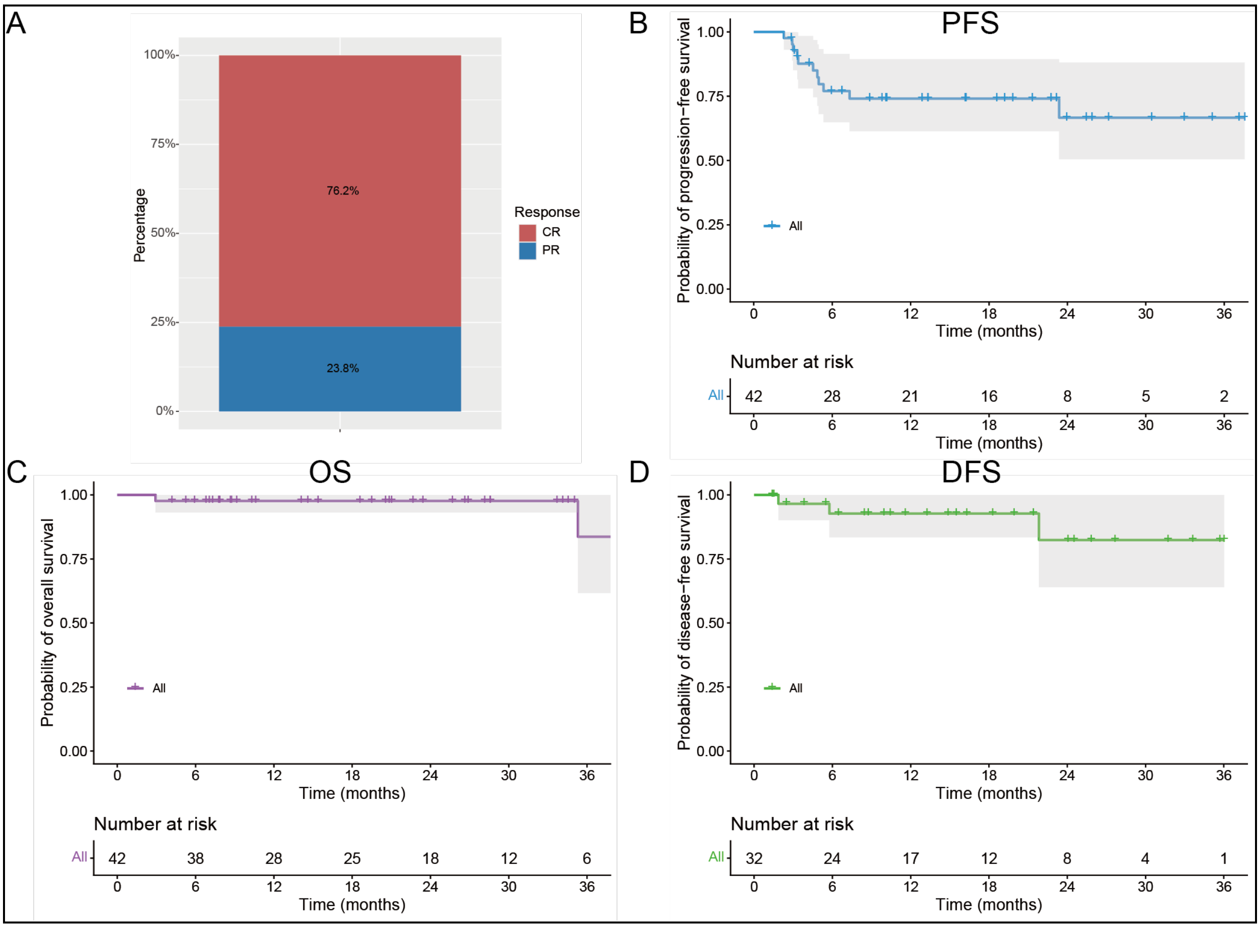

Figure 1 illustrates the best response, survival curves of Progression-Free Survival (PFS), Overall Survival (OS), and Disease-Free Survival (DFS).

**Copyright Information:** (Online) ISSN: 2572-9241
©2026 The Author(s). HemaSphere is published by John Wiley & Sons Ltd on behalf of European Hematology Association. This is an open access Abstract Book distributed under the Attribution-NonCommercial-NoDerivs (CC BY-NC-ND), which allows third parties to download the articles and share them with others as long as they credit the author and the Abstract Book, but they cannot change the content in any way or use them commercially.
**Abstract Book Citations:** Authors, Title, HemaSphere, 2026;10:(S1):pages. The abstract book DOIs can be found on the "Author Information" page.
**Disclaimer:** Articles published in the journal HemaSphere exclusively reflect the opinions of the authors. The authors are responsible for all content in their abstracts including accuracy of the facts, statements, citing resources, etc.

### (PS2066) RADIOTHERAPY AS BRIDGING, SALVAGE OR CONSOLIDATION STRATEGY IN CAR-T–TREATED LYMPHOMA PATIENTS: INSIGHTS FROM THE FRENCH DESCAR-T REGISTRY

**Topic:** 19. Large B cell lymphomas - Clinical

Vincent Camus*[1], Roch Houot[2], Gabriel Brisou[3], Kamal Bouabdallah[4], Franck Morschhauser[5], Cristina Castilla Llorente[6], Pierre Sesques[7], Catherine Thieblemont[8], Laura Herbreteau[9], Thomas Gastinne[10], Francois Lemonnier[11], Loic Ysebaert[12], Charles Herbaux[13], Magalie Joris[14], Cedric Rossi[15], Aline Tanguy-Schmidt[16], Arnaud Campidelli[17], Stephanie Guidez[18], Michael Loschi[19], Gandhi Laurent Damaj[20], Julie Abraham[21], Laure Lebras[22], Olivier Hermine[23], Luc-Matthieu Fornecker[24], Laure Ricard[25], Emmanuel Gyan[26], Sylvain Choquet[27], Adrien Chauchet[28], Sylvain Carras[29], Jean-Valère Malfuson[30], Cécile Pizot[31], Elodie Gat[31], Aurélie Villard[31], Valentine Martin[32], Youssef Ghannam[32], Loïc Renaud[33], Waisse Waissi[34]

*[1]Centre Henri Becquerel, Department of Hematology and INSERM U1245 "Cancer & Brain Genomics", Rouen, France;[2]CHU Rennes – Hôpital Pontchaillou, Department of Hematology, Rennes, France; [3]Institut Paoli Calmettes, Marseille, France; [4]CHU Bordeaux – Centre François Magendie, Bordeaux, France; [5]CHRU de Lille – Hôpital Claude Huriez, Lille, France;[6]Gustave Roussy, Villejuif, France; [7]CHU Lyon Sud - Hospices Civils de Lyon, Pierre-Bénite, France;[8]APHP – Hôpital Saint-Louis, Paris, France; [9]CHU de Brest – Hôpital de la Cavale Blanche, Brest, France;[10]CHU de Nantes – Hôtel-Dieu, Nantes, France; [11]APHP – Hôpital Henri-Mondor, Créteil, France; [12]IUCT Oncopole Toulouse, Toulouse, France; [13]CHU Montpellier, Montpellier, France; [14]CHU d'Amiens – Hôpital Sud, Amiens, France; [15]Université Bourgogne Europe, CHU Dijon Bourgogne, INSERM1231 CTM Equipe Epi2THM, Dijon, France; [16]CHU d'Angers, Angers, France; [17]CHU de Nancy – Hôpital de Brabois, Nancy, France;[18]CHU de Poitiers, Poitiers, France; [19]CHU de Nice – Hôpital de l'Archet, Nice, France;[20]CHU de Caen – Côte de Nacre, Caen, France; [21]CHU de Limoges – Hôpital Universitaire Dupuytren, Limoges, France;[22]Centre Léon Berard; Clinique de la Sauvegarde, Lyon, France; [23]APHP – Hôpital Necker, Paris, France;[24]CHU de Strasbourg, Strasbourg, France;[25]APHP – Hôpital Saint-Antoine, Paris, France; [26]CHU de Tours – Hôpital Bretonneau, Tours, France;[27]APHP – Hôpital Pitié-Salpêtrière, Paris, France; [28]CHU de Besançon – Hôpital Jean Minjoz, Besançon, France; [29]CHU de Grenoble – Hôpital Albert Michallon, Grenoble, France;[30]Hôpital d'Instruction des Armées Percy, Clamart, France; [31]LYSARC, The Lymphoma Academic Research Organisation, Hôpital Lyon Sud - HCL, Pierre-Bénite, France; [32]Gustave Roussy, Department of Radiotherapy, Villejuif, France;[33]APHP – Hôpital Cochin, Paris, France; [34]Centre Léon Berard, Department of Radiotherapy, Lyon, France;*

### Background

Radiotherapy (RT) is frequently used in lymphoma patients (pts) eligible for CAR-T cells, either as holding/bridging therapy before infusion or as salvage/consolidation after infusion. However, large real-world data evaluating its impact on outcomes are limited.

### Aims

We analyzed the role of RT in CAR-T–treated pts from the French national DESCAR-T registry.

### Methods

This study included B-cell lymphoma pts from DESCAR-T (NCT04328298) who received RT either as (i) holding/bridging therapy before first CAR-T infusion (Population 1, pop1) or (ii) salvage (SRT) or consolidation RT (CRT, RT delivered after CAR-T without evidence of progression) within 6 months (mo) after infusion (Population 2, pop2). Survival endpoints (PFS and OS) were assessed from first CAR-T infusion. Patients were censored at their second infusion. Objective response was analyzed through ORR, CRR and DOR. Safety was evaluated through CAR-T specific toxicity, persisting cytopenias (lasting ≥30 days) and death. Irradiated sites were not collected.

**Copyright Information:** (Online) ISSN: 2572-9241
©2026 The Author(s). HemaSphere is published by John Wiley & Sons Ltd on behalf of European Hematology Association. This is an open access Abstract Book distributed under the Attribution-NonCommercial-NoDerivs (CC BY-NC-ND), which allows third parties to download the articles and share them with others as long as they credit the author and the Abstract Book, but they cannot change the content in any way or use them commercially.
**Abstract Book Citations:** Authors, Title, HemaSphere, 2026;10:(S1):pages. The abstract book DOIs can be found on the "Author Information" page.
**Disclaimer:** Articles published in the journal HemaSphere exclusively reflect the opinions of the authors. The authors are responsible for all content in their abstracts including accuracy of the facts, statements, citing resources, etc.

### Results

In pop1 (n=303), 183 pts received RT alone (RTa) and 120 RT combined with systemic therapy (RTc). Median age was 64 years (range 19–81). A majority of LBCL (82%) was observed with small proportions of PMBL (6%), FL (5%), and MCL (4%). Advanced stage was reported in 67.8% of pts (RTa 63% vs RTc 76%), and elevated LDH in 58% (RTa 55% vs RTc 64%). Median prior treatment lines before CAR-T was 2 (range 1-8). Best ORR after CAR-T was 86.6% (95% CI 82.2–90.2), including CRR 72.5% (67–77.5). Responses were higher with bridging RTa vs RTc: ORR 90.7% (85.5–94.5) vs 80.2% (71.7–87); CRR 79.1% (72.5–84.8) vs 62.1% (52.6–70.9). Median follow-up since CAR-T infusion was 24.6 mo. Among responders (n=258), 38.8% had a subsequent event (progression/death). Median DOR was 41.8 mo (29.9–NA), with 12- and 24-month DOR rates of 61.4% (54.7–67.4) and 57.7% (50.6–64.1), respectively. In pop1, 93 deaths (31%) occurred after first infusion; 75% were due to progression. Median PFS and OS were 22.9 mo (8.8–42.5) and 51.2 mo (CI 39.1–NA), respectively (Figure 1). CAR-T toxicities were consistent with expected profiles: CRS occurred in 82.5% vs 83.2% (grade ≥3: 4.4% vs 1.7%) and ICANS in 41% vs 45.4% (grade ≥3: 11.4% vs 12.6%) for RTa vs RTc. Prolonged cytopenias persisted at month 3 (16.5% vs 13.3%) and month 6 (7.1% vs 13%).
In pop2 (n=113), 91 pts received SRT and 22 CRT. Median age was 58 years (range 18–78). A majority of LBCL was observed (86%), with small proportions of PMBL (9%), FL (4%), and MCL (1%). Advanced stage and elevated LDH were reported in 79.6% and 73.9% of pts. Median prior treatment lines before CAR-T was 2 (range 1–6). Median follow-up since CAR-T infusion was 26.8 mo. ORR after RT was not collected. Overall, 49 deaths (43.4%) occurred; 88% were due to progression. Median PFS was 2.1 mo (1.9–2.8) with SRT vs 14.3 mo (4.4–NA) with CRT. Median OS was 15 mo (11.2–37.2) with SRT and was not reached with CRT.

### Summary/Conclusion

In this large national real-world cohort, RT delivered as holding/bridging before CAR-T was associated with high response rates and durable remissions, including in pts with advanced-stage disease. Post-CAR-T RT demonstrated limited efficacy in the salvage setting but encouraging disease control when used as consolidation. No excess CAR-T–related toxicity was observed in pts exposed to RT. These findings support the strategic integration of RT in peri–CAR-T management and warrant prospective evaluation.

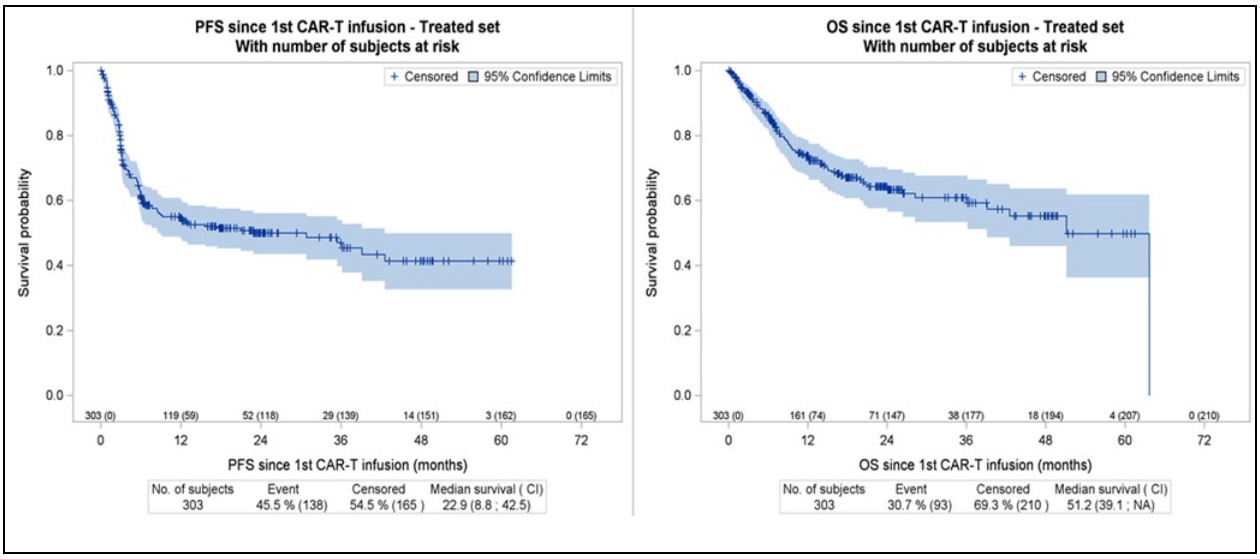

Figure 1: Overall Survival (OS) and Progression-Free Survival (PFS) among patients treated with holding/bridging radiotherapy prior to infusion (Population 1)

Copyright Information: (Online) ISSN: 2572-9241
©2026 The Author(s). HemaSphere is published by John Wiley & Sons Ltd on behalf of European Hematology Association. This is an open access Abstract Book distributed under the Attribution-NonCommercial-NoDerivs (CC BY-NC-ND), which allows third parties to download the articles and share them with others as long as they credit the author and the Abstract Book, but they cannot change the content in any way or use them commercially.
Abstract Book Citations: Authors, Title, HemaSphere, 2026;10:(S1):pages. The abstract book DOIs can be found on the “Author Information” page.
Disclaimer: Articles published in the journal HemaSphere exclusively reflect the opinions of the authors. The authors are responsible for all content in their abstracts including accuracy of the facts, statements, citing resources, etc.

### (PS2067) THE PIVOTAL CLINICAL STUDY OF IM19 CAR-T CELLS IN THE TREATMENT OF RELAPSED OR REFRACTORY AGGRESSIVE B-CELL NON-HODGKIN LYMPHOMAS

**Topic:** 19. Large B cell lymphomas - Clinical

Yan Xie*[1], Yuerong Shuang[2], Zhiming Li[3], Hui Liu[4], Hongmei Jing[5], Liang Wang[6], Jianqiu Wu[7], Yufei Liu[7], Yafei Wang[8], Hui Zhou[9], Yajun Li[10], Wenyu Li[11], Xiuhua Sun[12], Chunkang Chang[13], Xiaojing Yan[14], Jun Luo[15], Hai-Yan Yang[16], Shan Wang[17], Fei Wu[17], Xinan Lu[17], Ting He[17], Yuqin Song[1]

*[1]Beijing Cancer Hospital, Beijing, China; [2]jiangxi Cancer Hospital, Nanchang], China; [3]Sun Yat-sen University Cancer Center, Guangzhou, China; [4]Beijing Hospital, Beijing, China; [5]Peking University Third Hospital, Beijing, China; [6]Beijing Tongren Hospital, Capital Medical University, Beijing, China; [7]Jiangsu Cancer Hospital, Nanjing, China; [8]Tianjin Medical University Cancer institute and Hospital,National Clinical Research Center for Cancer, Tianjin's Clinical Research Center for Cancer, Tianjin, China; [9]Hunan Cancer Hospital, The Affiliated Cancer Hospital of Xiangya School of Medicine, Central South University, changsha, China; [10]the Afliated Cancer Hospital of Xiangya School of Medicine, Central South University, Hunan Cancer Hospital, Changsha, China; [11]Guangdong Provincial People's Hospital, Guangzhou, China; [12]The Second Hospital of Dalian Medical University, Dalian, China; [13]Shanghai Sixth People's Hospital Affiliated to Shanghai Jiao Tong University School of Medicine, Shanghai, China; [14]First Hospital of China Medical University, shenyang, China; [15]The First Affiliated Hospital Of Guangxi Medical University, Nanning, China; [16]Zhejiang Cancer Hospital, Hangzhou, China; [17]IMUNOPHARM Technology Co., Ltd., beij, China;*

### Background

IM19 is a CD19/4-1BB CAR-T cell product enriched in naïve and memory T cells, which is manufactured in China using a commercial process.

### Aims

SD45 is a multicenter, single-arm, open-label phase II pivotal study evaluating the safety and efficacy of IM19 in patients with relapsed/refractory aggressive non-Hodgkin's lymphoma.

### Methods

Eligible patients (≥18 years) had histologically confirmed aggressive large B-cell lymphoma (DLBCL NOS, double/triple-hit high-grade B-cell lymphoma, transformed DLBCL, primary mediastinal B-cell lymphoma, follicular lymphoma grade 3B) with ≥2 prior lines of therapy; those with active central nervous system DLBCL were excluded. Written informed consent was obtained from all patients, who underwent leukapheresis with optional investigator-chosen bridging therapy. Lymphodepletion included fludarabine (25-30 mg/m²/day) + cyclophosphamide (250-300 mg/m²/day) for 3 days, followed by a single IM19 infusion ($3 \times 10^6$/kg). The primary endpoint was 3-month objective response rate (ORR) .

**Copyright Information:** (Online) ISSN: 2572-9241
©2026 The Author(s). HemaSphere is published by John Wiley & Sons Ltd on behalf of European Hematology Association. This is an open access Abstract Book distributed under the Attribution-NonCommercial-NoDerivs (CC BY-NC-ND), which allows third parties to download the articles and share them with others as long as they credit the author and the Abstract Book, but they cannot change the content in any way or use them commercially.
**Abstract Book Citations:** Authors, Title, HemaSphere, 2026;10:(S1):pages. The abstract book DOIs can be found on the “Author Information” page.
**Disclaimer:** Articles published in the journal HemaSphere exclusively reflect the opinions of the authors. The authors are responsible for all content in their abstracts including accuracy of the facts, statements, citing resources, etc.

### Results

As of data cutoff (31 May 2025), 160 patients were screened at 15 Chinese sites, 116 underwent successful leukapheresis, with an IM19 production success rate of 99.1% (115/116).A total of 101 patients received IM19 infusion (100 standard dose, 1 reduced dose due to infusion set error). Of these, 94 (94%) had DLBCL, 1 (1%) FL grade 3B, 1 (1%) transformed DLBCL, 2 (2%) HGBL, and 2 (2%) other lymphoma (DLBCL combined with FL).Median age was 60 years (21–79), median prior lines 2 (2–9), median production time 8 days, and naïve/memory T-cell proportion >80%.Bridging therapy was used in 69% of patients (median vein-to-vein time: 34 vs. 29 days).
In the efficacy-evaluable population (100 patients), 3-month ORR was 57% (95%CI:46.7%-66.9%); best ORR and CR were 71% (95%CI:61.1%-79.6%) and 41% (95%CI:31.3-51.3%), respectively. Median PFS was 5.98 months (95%CI:5.68m-11.89m), median DOR 10.94 months (95%CI:5.03m-NA).The 1-year OS rate was 63.7% (95%CI: 53.0%–72.7%).Of note, among 10 patients (10%) with post-ASCT relapse, best ORR was 90.0% and best CRR was 80.0%. Among 28 patients aged ≥65 years, best ORR was 75.0%.Among 31 patients (31%) without bridging therapy, best ORR was 77.4% and best CRR was 61.3%.
In the safety population (101 patients), 44.6% developed cytokine release syndrome (CRS), with only 1 grade 3 event (0.9%); immune effector cell-associated neurotoxicity syndrome (ICANS) occurred in 5.0% (all grade 1-2). Grade ≥3 adverse events occurring in ≥10% of patients were neutropenia, leukopenia, lymphopenia, thrombocytopenia, and anemia. No IM19-related deaths were reported.
Among 101 patients with evaluable cellular kinetics, median time to CAR-T peak expansion was 13 days, median peak concentration (Cmax) 96.88cells/μL, and median AUClast 725.37 day×cells/μL. Bridging therapy and lymphodepletion dosage had no significant impact on PK parameters; patients with CRS/ICANS had higher Cmax and AUC0-28d. PK parameters did not differ between patients with 90-day remission and those without.

### Summary/Conclusion

IM19 CAR-T demonstrated favorable efficacy and a favorable safety profile (low grade ≥3 CRS/ICANS) in R/R aggressive B-cell NHL patients with diverse histological subtypes and high-risk features. The study population had a high proportion of DLBCL (94%). The slower CAR-T peak expansion (13 days) – potentially due to the high naïve/memory T cell content – may contribute to the excellent safety of IM19, distinguishing it from other CD19 CAR-T products.

**Copyright Information:** (Online) ISSN: 2572-9241
©2026 The Author(s). HemaSphere is published by John Wiley & Sons Ltd on behalf of European Hematology Association. This is an open access Abstract Book distributed under the Attribution-NonCommercial-NoDerivs (CC BY-NC-ND), which allows third parties to download the articles and share them with others as long as they credit the author and the Abstract Book, but they cannot change the content in any way or use them commercially.
**Abstract Book Citations:** Authors, Title, HemaSphere, 2026;10:(S1):pages. The abstract book DOIs can be found on the “Author Information” page.
**Disclaimer:** Articles published in the journal HemaSphere exclusively reflect the opinions of the authors. The authors are responsible for all content in their abstracts including accuracy of the facts, statements, citing resources, etc.

### (PS2068) A PHASE 1 STUDY OF KITE-753 IN PATIENTS (PTS) WITH RELAPSED/REFRACTORY (R/R) B-CELL LYMPHOMA: UPDATED SAFETY AND EFFICACY RESULTS

**Topic:** 19. Large B cell lymphomas - Clinical

Saurabh Dahiya[1], Matthew Ulrickson[2], Jean A. Yared[3], Patrick Reagan[4], Timothy Voorhees[5], Andre Goy[6], Cameron Turtle[7, 8], Lizamarie Bachier-Rodriguez[9], Gary Simmons[10], Ran Reshef[11], Marie José Kersten[12], Max Topp[13], Robin Sanderson[14], Loretta Nastoupil[15], A. Scott Jung[16], Jinghui Dong[16], Joshua Winters[16], Rhine Shen[16], Justyna Kanska[16], Myrna Nahas[16], Sairah Ahmed*[17]

*[1]Stanford University School of Medicine, Stanford, CA, United States of America; [2]Banner MD Anderson Cancer Center, Gilbert, AZ, United States of America; [3]University of Maryland School of Medicine, Greenebaum Comprehensive Cancer Center, Baltimore, MD, United States of America; [4]University of Rochester School of Medicine, Rochester, NY, United States of America; [5]The Ohio State University, James Comprehensive Cancer Center, Columbus, OH, United States of America; [6]John Theurer Cancer Center, Hackensack, NJ, United States of America; [7]Royal North Shore Hospital, St. Leonards, NSW, Australia; [8]University of Sydney, Camperdown, NSW, Australia; [9]The Bone and Marrow Transplant Group of Georgia and Northside Hospital, Altanta, GA, United States of America; [10]Virginia Oncology Associates, Norfolk, VA, United States of America; [11]Columbia University Irving Medical Center, New York, NY, United States of America; [12]Amsterdam UMC, University of Amsterdam, Cancer Center Amsterdam, Amsterdam, The Netherlands; [13]Medizinische Klinik und Poliklinik II, Universitätsklinikum Würzburg, Würzburg, Germany; [14]King's College Hospital, London, United Kingdom; [15]CommonSpirit Oncology Mercy, Durango, CO, United States of America; [16]Kite, a Gilead Company, Santa Monica, CA, United States of America; [17]The University of Texas MD Anderson Cancer Center, Houston, TX, United States of America;*

### Background

KITE-753 is an autologous CD19/CD20 chimeric antigen receptor (CAR) T-cell therapy under investigation for pts with R/R B-cell lymphoma (BCL). KITE-753 uses a unique bicistronic design to optimally engage CD19 and CD20, synergistic CD28/4-1BB co-stimulation to enhance T-cell function, and a rapid manufacturing process that preserves naive and stem cell memory T cells. In a multicenter, Phase 1 study in pts with R/R BCL (4.0 months median follow-up), KITE-753 demonstrated an overall response rate (ORR) of 86% and a complete response (CR) rate of 79% in CAR-naive pts at the highest dose level (DL; n=14), with a safety profile of low-grade cytokine release syndrome (CRS) and 2 cases of low-grade immune effector cell-associated neurotoxicity syndrome (ICANS) at the highest DL (n=19; Dahiya et al. ASH 2025).

### Aims

To report updated safety and efficacy results from the Phase 1 study of KITE-753.

### Methods

Adults with BCL R/R after ≥2 lines of therapy or with second-line primary refractory large BCL (LBCL) were eligible. The trial included dose escalation (1A) and dose expansion (1B; LBCL only) cohorts. After lymphodepletion, pts received KITE-753 at 1 of 3 DLs ($0.3\times10^5$, $1\times10^5$, or $2\times10^5$ CAR T cells/kg in DL 1, 2, or 3, respectively). The primary endpoints were incidence of dose-limiting toxicities (DLTs; 1A) and investigator-assessed ORR (per Lugano; 1B).

**Copyright Information:** (Online) ISSN: 2572-9241
©2026 The Author(s). HemaSphere is published by John Wiley & Sons Ltd on behalf of European Hematology Association. This is an open access Abstract Book distributed under the Attribution-NonCommercial-NoDerivs (CC BY-NC-ND), which allows third parties to download the articles and share them with others as long as they credit the author and the Abstract Book, but they cannot change the content in any way or use them commercially.
**Abstract Book Citations:** Authors, Title, HemaSphere, 2026;10:(S1):pages. The abstract book DOIs can be found on the "Author Information" page.
**Disclaimer:** Articles published in the journal HemaSphere exclusively reflect the opinions of the authors. The authors are responsible for all content in their abstracts including accuracy of the facts, statements, citing resources, etc.

### Results

As of 12/03/25, 33 pts received KITE-753 (3 in DL 1, 8 in DL 2, 22 in DL 3). Median follow-up was 7.4 months (range, 5.8-12.7). Median age at analysis was 59.0 years (range, 25-84); 88% of pts had LBCL and 12% had indolent non-Hodgkin lymphoma, and 76% had Stage III/IV disease. Nine pts (27% of all; 31% of LBCL) were primary refractory and 10 (30%) had received prior CAR T-cell therapy.

No DLTs occurred in dose escalation. Grade ≥3 adverse events occurred in 85% of pts (91% in DL 3), primarily cytopenias. Eight pts (24%) had max Grade 1 CRS, 8 (24%) had max Grade 2 CRS, 1 had max Grade 3 CRS (LBCL, DL 2), and none had Grade 4/5 CRS. Median time to CRS onset was 6.0 days; median duration was 6.0 days. Two pts had Grade 1 ICANS (1 each in DL 2 and DL 3), 4 had Grade 2 ICANS (1 in DL 2, 3 in DL 3), and none had Grade ≥3 ICANS. Median time to ICANS onset was 10.5 days; median duration was 3.5 days. Five pts died: 2 due to infections unrelated to KITE-753 per investigator assessment and 3 due to progression.

ORR in CAR-naive pts at DL 3 (n=17) was 88%; CR rate was 71% (12/17) at the data cutoff and 76% (13/17) post-data cutoff, with 1 pt converting from a partial response (PR) to a CR. In pts with prior CAR exposure (all DLs; n=10), 3 (30%) had a CR and 2 (20%) had a PR.

In a comparative analysis of KITE-753 vs axicabtagene ciloleucel (axi-cel; ZUMA-1), levels of the neurotoxicity-associated cytokine, IL-15, were lower with KITE-753, coinciding with an improved toxicity profile. Peak CAR T-cell expansion was delayed with KITE-753 vs axi-cel; IL-2, INF-γ, and granzyme B kinetics correlated with delayed time to peak. These favorable pharmacokinetic and pharmacodynamic profiles may reflect KITE-753's optimized CAR T-cell dose that mitigates toxicity and its rapid manufacturing process that preserves CAR T-cell fitness.

### Summary/Conclusion

KITE-753, with a unique CAR design and rapid manufacturing process preserving a naive T-cell phenotype, at the highest DL showed high CR rates in CAR-naive pts and a favorable safety profile with only low-grade CRS and ICANS.

**Copyright Information:** (Online) ISSN: 2572-9241
©2026 The Author(s). HemaSphere is published by John Wiley & Sons Ltd on behalf of European Hematology Association. This is an open access Abstract Book distributed under the Attribution-NonCommercial-NoDerivs (CC BY-NC-ND), which allows third parties to download the articles and share them with others as long as they credit the author and the Abstract Book, but they cannot change the content in any way or use them commercially.
**Abstract Book Citations:** Authors, Title, HemaSphere, 2026;10:(S1):pages. The abstract book DOIs can be found on the "Author Information" page.
**Disclaimer:** Articles published in the journal HemaSphere exclusively reflect the opinions of the authors. The authors are responsible for all content in their abstracts including accuracy of the facts, statements, citing resources, etc.

### (PS2069) INTEGRATING VAF AND IMMUNE-COLLAGEN SUBTYPES IDENTIFIES A HIGH-RISK “SOFT” SUBTYPE IN TP53-MUTANT DLBCL WITH PRIMARY DOXORUBICIN RESISTANCE

**Topic:** 19. Large B cell lymphomas - Clinical

Jinhua Liang*[1], Kai-Xin Du[1], Guozhong Xi[1], Yi-Fan Wu[1], Chunyu Shang[1], Li Wang[1], Jianyong Li[1], Wei Xu[1]

[1]*the First Affiliated Hospital of Nanjing Medical University, Jiangsu Province Hospital, Nanjing, China;*

### Background

Diffuse large B-cell lymphoma (DLBCL) exhibits significant genetic and clinical heterogeneity, and while TP53 mutations (TP53mut) occur in approximately 33% of cases, their prognostic impact remains controversial, warranting precise risk stratification.

### Aims

This study aims to refine risk stratification within TP53mut DLBCL by integrating variant allele frequency (VAF) and immune-collagen features.

### Methods

We analyzed data from 2764 DLBCL patients treated with R-CHOP from 7 public cohorts and 54 TP53mut DLBCL patients from our center cohort. This included 192 TP53mut patients with transcriptomic data and an additional 48 TP53mut cases from our center for validation. Six cases from our center were analyzed by scRNA sequencing. Unsupervised clustering of functional modules defined immune-collagen subtypes. Multiplex immunofluorescence was performed. In vivo doxorubicin response was assessed in a SUDHL8 xenograft model **(Fig. 1A)**.

**Copyright Information:** (Online) ISSN: 2572-9241
©2026 The Author(s). HemaSphere is published by John Wiley & Sons Ltd on behalf of European Hematology Association. This is an open access Abstract Book distributed under the Attribution-NonCommercial-NoDerivs (CC BY-NC-ND), which allows third parties to download the articles and share them with others as long as they credit the author and the Abstract Book, but they cannot change the content in any way or use them commercially.
**Abstract Book Citations:** Authors, Title, HemaSphere, 2026;10:(S1):pages. The abstract book DOIs can be found on the “Author Information” page.
**Disclaimer:** Articles published in the journal HemaSphere exclusively reflect the opinions of the authors. The authors are responsible for all content in their abstracts including accuracy of the facts, statements, citing resources, etc.

**Results**

TP53mut was confirmed as an adverse prognostic factor correlated with refractory disease and early progression **(Fig. 1B-C)**, with an associated exhausted TME **(Fig. 1D)**. To refine risk stratification within TP53mut DLBCL, we systematically evaluated prognostic indicators **(Fig. 1E)**. A 40% VAF cutoff was established as optimal using Youden index, median, X-tile, and CRT decision tree analyses, confirming VAF≥40% as a robust prognostic indicator in TP53mut patients **(Fig. 1F)**. Multivariate Cox regression identified VAF and IPI as independent prognostic factors, forming the VAFIPI model **(Fig. 1G)**. In the training set, VAFIPI effectively discriminated high-risk TP53mut patients **(Fig. 1H)**, with robustness confirmed in the independent validation cohort **(Fig. 1I)**. ROC analysis demonstrated superior performance of VAFIPI over IPI alone **(Fig. 1J)**. Sankey plot revealed that refractory disease and early progression predominantly clustered within VAFIPI intermediate/high-risk groups **(Fig. 1K)**.
Mechanistically, while no significant mutational landscape differences emerged across VAFIPI strata, transcriptomic profiling revealed striking disparities: the VAFIPI-high group exhibited marked downregulation of immune and extracellular matrix (ECM) pathways, validated in the our center cohort **(Fig. 1L-M)**. ScRNA sequencing corroborated these findings, demonstrating depletion of T/NK cells and, notably, fibroblasts within the TP53mut TME **(Fig. 1N-O)**. We therefore constructed 10 ECM/collagen-feature and 19 immune-feature modules. Correlation matrices revealed non-reciprocal relationships between immune signatures and collagen modules **(Fig. 1P)**. Unsupervised clustering of these 29 modules identified three distinct collagen-immune subtypes: Hot-armored, Cold-armored, and Soft **(Fig. 1Q)**. The Soft subtype TME conferred significantly inferior survival **(Fig. 1R)** and was significantly enriched in the VAFIPI-high group, findings validated in the our center cohort **(Fig. 1S-T)**.
For clinical translation, we selected CD3, PDL1, and COL1 for immunohistochemical staining in 9 cases, which demonstrated excellent concordance with the transcriptomic subtypes **(Fig. 1U)**. Functional validation using a Soft-subtype, TP53mut, CD20-negative SUDHL8 xenograft model in NOD/SCID mice confirmed primary resistance to doxorubicin, underscoring the therapeutic implications of this novel classification **(Fig. 1V)**.

**Summary/Conclusion**

Integration of VAF, IPI, and collagen-immune subtyping refines risk stratification inTP53mut DLBCL, identifying a high-risk Soft subtype with therapeutic resistance **(Fig. 1W)**.

**Copyright Information:** (Online) ISSN: 2572-9241
©2026 The Author(s). HemaSphere is published by John Wiley & Sons Ltd on behalf of European Hematology Association. This is an open access Abstract Book distributed under the Attribution-NonCommercial-NoDerivs (CC BY-NC-ND), which allows third parties to download the articles and share them with others as long as they credit the author and the Abstract Book, but they cannot change the content in any way or use them commercially.
**Abstract Book Citations:** Authors, Title, HemaSphere, 2026;10:(S1):pages. The abstract book DOIs can be found on the "Author Information" page.
**Disclaimer:** Articles published in the journal HemaSphere exclusively reflect the opinions of the authors. The authors are responsible for all content in their abstracts including accuracy of the facts, statements, citing resources, etc.

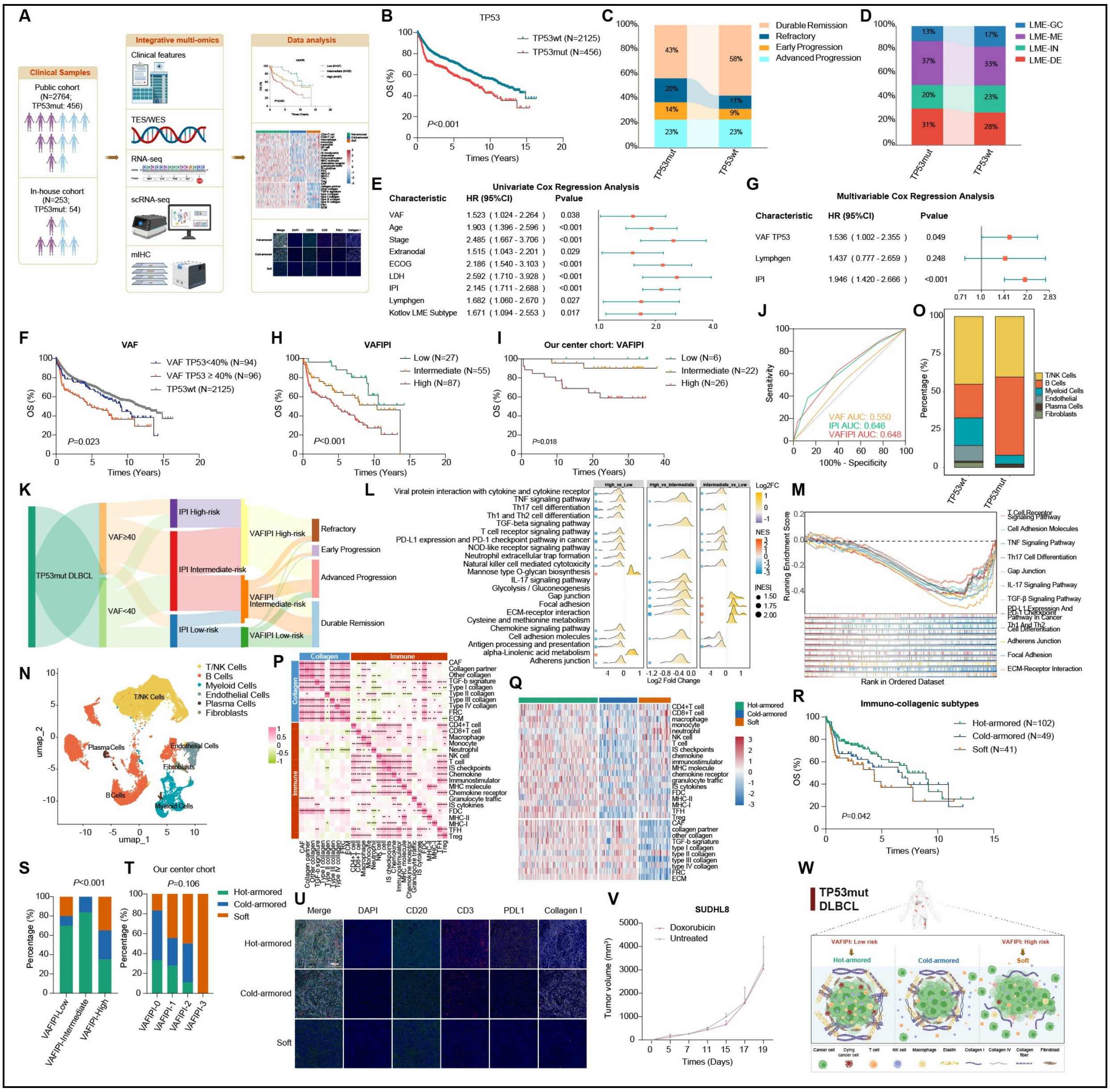

**Copyright Information:** (Online) ISSN: 2572-9241
©2026 The Author(s). HemaSphere is published by John Wiley & Sons Ltd on behalf of European Hematology Association. This is an open access Abstract Book distributed under the Attribution-NonCommercial-NoDerivs (CC BY-NC-ND), which allows third parties to download the articles and share them with others as long as they credit the author and the Abstract Book, but they cannot change the content in any way or use them commercially.
**Abstract Book Citations:** Authors, Title, HemaSphere, 2026;10:(S1):pages. The abstract book DOIs can be found on the "Author Information" page.
**Disclaimer:** Articles published in the journal HemaSphere exclusively reflect the opinions of the authors. The authors are responsible for all content in their abstracts including accuracy of the facts, statements, citing resources, etc.

### (PS2070) EPCORITAMAB + CHEMOIMMUNOTHERAPY IN PATIENTS WITH RELAPSED/REFRACTORY LARGE B-CELL LYMPHOMA ELIGIBLE FOR AUTOLOGOUS STEM CELL TRANSPLANT: POOLED RESULTS FROM ARMS 4 AND 10 OF EPCORE NHL-2

**Topic:** 19. Large B cell lymphomas - Clinical

Raul Cordoba*[1], Pau Abrisqueta[2], Charalambos Andreadis[3], Chan Y. Cheah[4], Justin Darrah[5], Sven de Vos[6], Lorenzo Falchi[7], Gerardo Musuraca[8], Marek Trněný[9], Anna Sureda[10], Charles Kearns[11], Kimberly Archer[12], Milan Geybels[13], Malene Risum[13], Jon Ukropec[12], Mohammad Atiya[12], Yasmin Karimi[14]

*[1]Fundación Jiménez Díaz University Hospital, Madrid, Spain; [2]Hospital Universitario Vall d'Hebron, Barcelona, Spain; [3]Division of Hematology and Oncology, University of California San Francisco, San Francisco, CA, United States of America; [4]Sir Charles Gairdner Hospital and the University of Western Australia, Perth, WA, Australia; [5]Cedars-Sinai Medical Center, Division of Hematology and Cellular Therapy, Department of Medicine, Los Angeles, CA, United States of America; [6]Ronald Reagan University of California Los Angeles Medical Center, Los Angeles, CA, United States of America; [7]Lymphoma Service, Memorial Sloan Kettering Cancer Center, New York, NY, United States of America; [8]IRCCS Istituto Romagnolo per lo Studio dei Tumori (IRST) "Dino Amadori", Hematology Unit, Meldola, Italy; [9]Charles University and General University Hospital, Prague, Czechia; [10]Institut Català d'Oncologia-Hospitalet, IDIBELL, Clinical Hematology Department, Barcelona, Spain; [11]AbbVie, North Chicago, IL, United States of America; [12]Genmab, Plainsboro, NJ, United States of America; [13]Genmab, Copenhagen, Denmark; [14]University of Michigan, Division of Hematology/Oncology, Ann Arbor, MI, United States of America;*

### Background

Patients (pts) with R/R LBCL can have inadequate response to salvage treatment (tx), precluding autologous stem cell transplant (ASCT). While CAR T therapies are standard of care in the 2L setting for pts with early relapse (< 12 months of 1L tx), their use is limited by eligibility, accessibility, or cost. Regimens that enhance salvage tx response rates, enabling pts to proceed to ASCT, are needed. In Arms 4 and 10 of the phase 1b/2 EPCORE® NHL-2 trial (NCT04663347), epcoritamab + chemoimmunotherapy (CIT) showed high response rates in ASCT-eligible R/R LBCL.

### Aims

Here, we report a pooled analysis of EPCORE NHL-2 Arms 4 and 10 data.

### Methods

ASCT-eligible pts received epcoritamab + rituximab; dexamethasone, cytarabine, and oxaliplatin/carboplatin (R-DHAX/C) (Arm 4); or epcoritamab + rituximab, ifosfamide, carboplatin, and etoposide (R-ICE) (Arm 10). After salvage tx, pts could proceed to ASCT or remain on epcoritamab monotherapy. ORR per investigator assessment, DOCR, PFS, OS, and safety are reported.

**Copyright Information:** (Online) ISSN: 2572-9241
©2026 The Author(s). HemaSphere is published by John Wiley & Sons Ltd on behalf of European Hematology Association. This is an open access Abstract Book distributed under the Attribution-NonCommercial-NoDerivs (CC BY-NC-ND), which allows third parties to download the articles and share them with others as long as they credit the author and the Abstract Book, but they cannot change the content in any way or use them commercially.
**Abstract Book Citations:** Authors, Title, HemaSphere, 2026;10:(S1):pages. The abstract book DOIs can be found on the "Author Information" page.
**Disclaimer:** Articles published in the journal HemaSphere exclusively reflect the opinions of the authors. The authors are responsible for all content in their abstracts including accuracy of the facts, statements, citing resources, etc.

**Results**

The analysis included 63 pts (Arm 4: n = 29; Arm 10: n = 34). Baseline characteristics: median age 61 years (range, 28–77), 68% male, 71% stage III/IV, 13% (8/33 assessed) high-grade B-cell lymphoma with *MYC* and *BCL2* and/or *BCL6* translocations (central lab), and 8% prior CAR T therapy. In total, 79% (n = 50) were receiving 2L tx; 57% (n = 36) were receiving 2L tx and were refractory or had early relapse to 1L tx. Median relative CIT dose intensity was ≥ 80%.

At 18.3 months median follow-up, ORR and CR rates were 84% and 67%, respectively (**Table**). Additionally, 57% (n = 36) proceeded to ASCT; 16% (n = 10) remained on epcoritamab monotherapy due to pt/physician decision. Median DOCR, PFS, and OS were not reached in pts who proceeded to ASCT or who remained on epcoritamab. Similar outcomes were seen in pts in 2L therapy and across pts in 2L therapy with early and late relapse.

Overall, the most common any-grade TEAEs were anemia (68%), thrombocytopenia (68%), and neutropenia (62%). Cytokine release syndrome occurred in 48% of pts and ICANS in 3%; all events were grade 1–2. Serious infections occurred in 17%. TEAEs led to tx discontinuation in 11%. There were no fatal TEAEs.

**Copyright Information:** (Online) ISSN: 2572-9241
©2026 The Author(s). HemaSphere is published by John Wiley & Sons Ltd on behalf of European Hematology Association. This is an open access Abstract Book distributed under the Attribution-NonCommercial-NoDerivs (CC BY-NC-ND), which allows third parties to download the articles and share them with others as long as they credit the author and the Abstract Book, but they cannot change the content in any way or use them commercially.
**Abstract Book Citations:** Authors, Title, HemaSphere, 2026;10:(S1):pages. The abstract book DOIs can be found on the “Author Information” page.
**Disclaimer:** Articles published in the journal HemaSphere exclusively reflect the opinions of the authors. The authors are responsible for all content in their abstracts including accuracy of the facts, statements, citing resources, etc.

### Summary/Conclusion

In this pooled analysis of ASCT-eligible pts with R/R LBCL, tx with epcoritamab + salvage CIT resulted in high CR rates, enabling a large proportion of pts to proceed to ASCT. Deep, durable responses were seen in pts who underwent ASCT and those who continued epcoritamab monotherapy. A high CR rate was also observed in 2L pts with early relapse. Safety was consistent with prior reports. Dose intensity of CIT was not impacted. These findings support epcoritamab-based combination salvage strategies to increase response depth and facilitate higher ASCT rates and possible cure in ASCT-eligible pts with DLBCL.

### Table

| | All pts N = 63 | 2L n = 50 | 2L with early relapse[a] n = 36 | Proceeded to ASCT n = 36 | Remained on epcoritamab monotherapy n = 10 |
|---|---|---|---|---|---|
| **ORR/CR, %** | 84/67 | 84/68 | 81/61 | 100/94 | 100/80 |
| **DOCR, 18-month rate, %** | 79 | 80 | 81 | 83 | 64 |
| **PFS, 18-month rate, %** | 62 | 62 | 58 | 80 | 67 |
| **OS, 18-month rate, %** | 82 | 82 | 77 | 93 | 100 |

[a]Pts receiving 2L tx who were refractory to or progressed <- 12 months of 1L tx. ASCT, autologous stem cell transplant; pt, patient; tx, treatment.

© 2026 American Society of Clinical Oncology, Inc. Reused with permission. This abstract was accepted and previously presented at the 2026 ASCO Annual Meeting. All rights reserved.

**Copyright Information:** (Online) ISSN: 2572-9241
©2026 The Author(s). HemaSphere is published by John Wiley & Sons Ltd on behalf of European Hematology Association. This is an open access Abstract Book distributed under the Attribution-NonCommercial-NoDerivs (CC BY-NC-ND), which allows third parties to download the articles and share them with others as long as they credit the author and the Abstract Book, but they cannot change the content in any way or use them commercially.
**Abstract Book Citations:** Authors, Title, HemaSphere, 2026;10:(S1):pages. The abstract book DOIs can be found on the "Author Information" page.
**Disclaimer:** Articles published in the journal HemaSphere exclusively reflect the opinions of the authors. The authors are responsible for all content in their abstracts including accuracy of the facts, statements, citing resources, etc.

### (PS2071) IMPACT OF TP53 MUTATION ON CD19-CHIMERIC ANTIGEN RECEPTOR T-CELL THERAPY IN LARGE B-CELL LYMPHOMA

**Topic:** 19. Large B cell lymphomas - Clinical

Lingshuang Sheng*[1], Yan Yu[1], Rong Shen[1], Zixun Yan[1], Yaohui Huang[1], Wen Wu[1], Weili Zhao[1], Li Wang[1]

[1]*Shanghai Institute of Hematology, State Key Laboratory of Medical Genomics, National Research Center for Translational Medicine at Shanghai, Ruijin Hospital、Shanghai Jiao Tong University School of Medicine, Shanghai, China, Shanghai, China;*

### Background

*TP53* mutation is a well-established adverse prognostic factor in large B-cell lymphoma (LBCL). However, its role in predicting response to CD19-chimeric antigen receptor T-cell (CAR-T) therapy remains controversial. While several studies have suggested that *TP53* mutation confers inferior outcomes after CAR-T treatment, others have reported no significant association. The underlying mechanisms and potential modulating factors, such as CAR costimulatory domains, are poorly understood.

### Aims

This study aimed to: (1) comprehensively characterize the *TP53* mutation spectrum in relapsed/refractory LBCL (R/R LBCL) patients receiving CD19-CAR-T;(2) evaluate the independent prognostic value of *TP53* mutation for progression-free survival (PFS); (3) investigate the impact of *TP53* mutation on CAR-T cell expansion, toxicity, peripheral blood T-cell subsets, and tumor microenvironment T-cell functionality using flow cytometry, CyTOF, and single-cell RNA sequencing.

### Methods

We retrospectively analyzed 139 R/R LBCL patients treated with CD19-CAR-T at Ruijin Hospital (2018–2024). *TP53* mutations were identified via next-generation sequencing (152-gene panel) from tumor tissues. Mutation types and loci were annotated. CAR-T cell expansion (Cmax) was assessed by flow cytometry. Peripheral blood T-cell subsets were analyzed by CyTOF prior to leukapheresis. Single-cell RNA sequencing was performed on tumor tissues to evaluate T-cell functional status. Univariate and multivariate Cox regression were performed to identify predictors of PFS.

### Results

Among 139 patients, 55 (39.6%) harbored *TP53* mutations, comprising 48 unique mutation sites, with missense mutations predominating (51/55, 92.7%; Figure 1A).
In univariate analysis of *TP53* mutated patients, only CAR-T product type (CD28 vs. 41BB) was significantly associated with PFS (HR 0.34, 95% CI 0.15-0.76, p=0.008). Multivariate analysis confirmed product type as an independent predictor (HR 0.35, 95% CI 0.16-0.78, p=0.010)(Figure 1B). Among patients treated with CAR T-cells incorporating a CD28 costimulatory domain, there was no significant difference in PFS between those with TP53 mutations and those with wild-type *TP53.* In contrast, among patients treated with 4-1BB–based CAR T-cell products, *TP53*-mutated patients experienced significantly inferior PFS compared with *TP53* wild-type patients (median 6.3 vs. not reached, p=0.024; Figure 1C).
With respect to CAR T-cell expansion capacity, patients with wild-type *TP53* exhibited a significantly higher Cmax compared with those harboring *TP53* mutations. In addition, analysis of $CD3^+$ T-cell subsets prior to leukapheresis revealed a marked reduction in γδ T cells in patients with *TP53* mutations. Furthermore, single-cell RNA sequencing data of tumor tissues demonstrated that T-cell function was significantly impaired in *TP53*-mutated patients, including reduced activation, effector function, migration, and adhesion capacity.

**Copyright Information:** (Online) ISSN: 2572-9241
©2026 The Author(s). HemaSphere is published by John Wiley & Sons Ltd on behalf of European Hematology Association. This is an open access Abstract Book distributed under the Attribution-NonCommercial-NoDerivs (CC BY-NC-ND), which allows third parties to download the articles and share them with others as long as they credit the author and the Abstract Book, but they cannot change the content in any way or use them commercially.
**Abstract Book Citations:** Authors, Title, HemaSphere, 2026;10:(S1):pages. The abstract book DOIs can be found on the "Author Information" page.
**Disclaimer:** Articles published in the journal HemaSphere exclusively reflect the opinions of the authors. The authors are responsible for all content in their abstracts including accuracy of the facts, statements, citing resources, etc.

### Summary/Conclusion

Our data demonstrate that *TP53* mutation is frequent in R/R LBCL and is associated with reduced CAR-T expansion, altered peripheral T-cell subsets (notably γδ T-cell depletion), and impaired T-cell functionality in the tumor microenvironment. Despite these effects, *TP53* mutation does not independently predict toxicity. Importantly, the negative prognostic impact of *TP53* mutation on PFS was observed only in patients receiving 41BB-costimulated CAR-T, whereas CD28-based products appeared to overcome this effect.

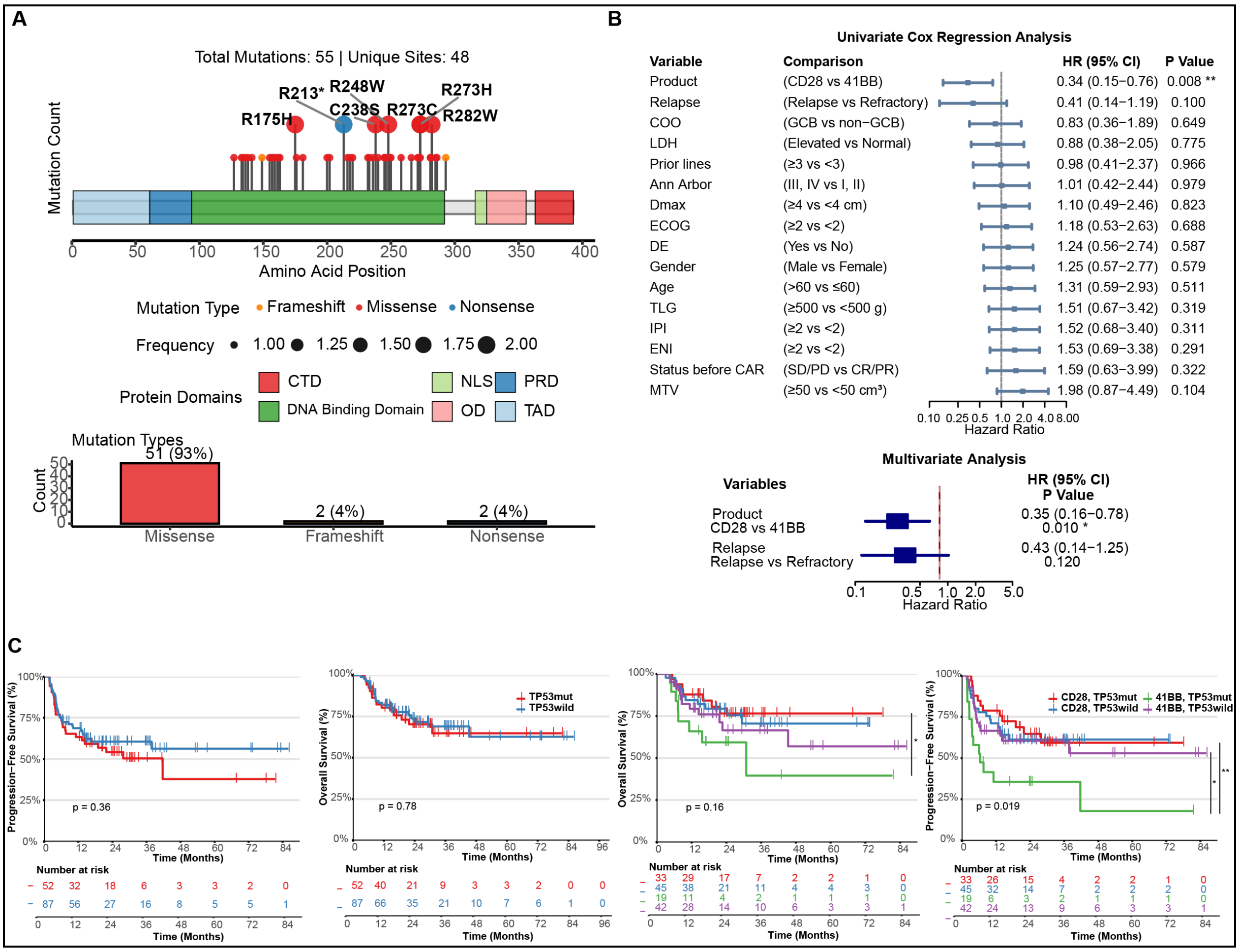

Figure 1. TP53 mutation spectrum, clinical outcomes, CAR-T expansion, and safety profile. (A) Distribution of TP53 mutation types and loci in 55 patients. (B) Forest plot of univariate and multivariate Cox regression for PFS of TP53 mutated patients. (C) PFS stratified by TP53 mutation status and CAR product type.

**Copyright Information:** (Online) ISSN: 2572-9241
©2026 The Author(s). HemaSphere is published by John Wiley & Sons Ltd on behalf of European Hematology Association. This is an open access Abstract Book distributed under the Attribution-NonCommercial-NoDerivs (CC BY-NC-ND), which allows third parties to download the articles and share them with others as long as they credit the author and the Abstract Book, but they cannot change the content in any way or use them commercially.
**Abstract Book Citations:** Authors, Title, HemaSphere, 2026;10:(S1):pages. The abstract book DOIs can be found on the "Author Information" page.
**Disclaimer:** Articles published in the journal HemaSphere exclusively reflect the opinions of the authors. The authors are responsible for all content in their abstracts including accuracy of the facts, statements, citing resources, etc.

### (PS2072) POLATUZUMAB VEDOTIN COMBINED WITH ORELABRUTINIB AND RITUXIMAB (PRO REGIMEN) AS FRONTLINE THERAPY IN VERY ELDERLY AND FRAIL PATIENTS WITH DLBCL: UPDATED RESULTS FROM A PHASE II STUDY

**Topic:** 19. Large B cell lymphomas - Clinical

Wenyu Shi*[1], Huayuan Zhu[2], Xiaoyan Xie[3], Miao Sun[4], Zhuxia Jia[5], Hui Chen[6], Lei Cao[2], Jianyong Li[2], Yi Xia[2]

*[1]Affiliated Hospital of Nantong University, Nantong, China;[2]the First Affiliated Hospital of Nanjing Medical University, Nanjing, China; [3]Northern Jiangsu People's Hospital Affiliated to Yangzhou University, Yangzhou, China;[4]Jingjiang People's Hospital Affiliated to Yangzhou University, Jingjiang, China; [5]The Second people's hospital of Changzhou, Changzhou, China;[6]Yancheng First People's Hospital, Yancheng, China;*

### Background

The management of very elderly patients (aged ≥75-80 years) with diffuse large B-cell lymphoma (DLBCL) remains a significant clinical challenge due to comorbidities, frailty, and poor tolerance to standard immunochemotherapy. There is a critical need for effective, reduced-intensity, or chemotherapy-free regimens.

### Aims

The PRO regimen (Polatuzumab vedotin, Orelabrutinib, and Rituximab) was designed to address this unmet need. We present updated results from an ongoing Phase II study focusing on this vulnerable population.

### Methods

This open-label, multicenter Phase II study enrolled previously untreated elderly and frail patients with DLBCL. Patients received Polatuzumab vedotin (1.8 mg/kg), Orelabrutinib (150 mg daily), and Rituximab (375 mg/m²) every 3 weeks for up to six cycles, followed by two additional Rituximab cycles in responders. Tumor response was evaluated every three cycles by PET/CT or contrast-enhanced CT according to Lugano 2014 criteria. The primary endpoint was end-of-treatment CR rate; secondary endpoints included ORR, PFS, DoR, OS, and safety.

**Copyright Information:** (Online) ISSN: 2572-9241
©2026 The Author(s). HemaSphere is published by John Wiley & Sons Ltd on behalf of European Hematology Association. This is an open access Abstract Book distributed under the Attribution-NonCommercial-NoDerivs (CC BY-NC-ND), which allows third parties to download the articles and share them with others as long as they credit the author and the Abstract Book, but they cannot change the content in any way or use them commercially.
**Abstract Book Citations:** Authors, Title, HemaSphere, 2026;10:(S1):pages. The abstract book DOIs can be found on the "Author Information" page.
**Disclaimer:** Articles published in the journal HemaSphere exclusively reflect the opinions of the authors. The authors are responsible for all content in their abstracts including accuracy of the facts, statements, citing resources, etc.

### Results

By February 2026, 39 patients with a median age of 78.0 years (IQR: 75.0–84.0) were evaluable, including 43.6% (n=17) aged ≥80. High-risk features were prevalent: 69.2% each presented with an IPI score of 3–5, stage III–IV disease, or elevated LDH. Histologically, 97.4% had DLBCL, and one (2.6%) had primary cutaneous DLBCL, leg type (PCDLBCL, LT); 77.0% were of the non-GCB/ABC subtype. Comorbidities affected 74.3% (29/39) of this ultra-elderly cohort, most commonly cardiovascular diseases (41.0%), hypertension (35.9%), diabetes (15.4%), cerebrovascular diseases (15.4%), and prostatic hypertrophy (5.1%).
At the data cutoff, of the 39 enrolled patients, 29 have completed the interim efficacy assessment, the CR rate was 58.6% (17/29), with an ORR of 96.5%. Among these, 37.9% (11/29) achieved a partial response, while 3.4% (1/29) experienced disease progression. Notably, in the 12 patients identified with the MCD subtype, the ORR rate was 100% (12/12), the CR rate was 66.7% (8/12). At the completion of combination therapy (C6-response), the CR rate was 91.7% (22/24), the ORR rate was 95.8 % (23/24). With a median follow-up of 7.0 months (range, 0.4–21.5), neither the median progression-free survival (PFS) nor the median overall survival (OS) has been reached. The estimated 9-month PFS rate was 92.8%. Subgroup analysis of patients aged ≥80 years (n=17) demonstrated survival outcomes comparable to the overall cohort, suggesting the sustained benefit of the chemo-free PRO regimen in the oldest-old.
The PRO regimen demonstrated a manageable safety profile in this ultra-elderly and frail population. Grade 3-4 adverse events (AEs) were primarily pulmonary infections (17.9%) and neutropenia (10.3%). Most other hematological and non-hematological toxicities were confined to Grade 1-2 and were clinically manageable with supportive care.

### Summary/Conclusion

In summary, despite a high comorbidity burden (74.3%), updated data from 39 patients indicate that the chemotherapy-free PRO regimen is an effective and tolerable frontline option for elderly and frail DLBCL patients. It achieved a 91.7% CR rate at treatment completion and an estimated 9-month PFS of 92.8%, with 100% ORR in the MCD subtype. These results support PRO as a feasible strategy for vulnerable patients, including those ≥80 years who are unfit for intensive immunochemotherapy.

**Copyright Information:** (Online) ISSN: 2572-9241
©2026 The Author(s). HemaSphere is published by John Wiley & Sons Ltd on behalf of European Hematology Association. This is an open access Abstract Book distributed under the Attribution-NonCommercial-NoDerivs (CC BY-NC-ND), which allows third parties to download the articles and share them with others as long as they credit the author and the Abstract Book, but they cannot change the content in any way or use them commercially.
**Abstract Book Citations:** Authors, Title, HemaSphere, 2026;10:(S1):pages. The abstract book DOIs can be found on the “Author Information” page.
**Disclaimer:** Articles published in the journal HemaSphere exclusively reflect the opinions of the authors. The authors are responsible for all content in their abstracts including accuracy of the facts, statements, citing resources, etc.

### (PS2073) EXTRANODAL INVOLVEMENT AND SITE-SPECIFIC SURVIVAL IN EARLY-STAGE DIFFUSE LARGE B-CELL LYMPHOMA: A POPULATION-BASED STUDY

**Topic:** 19. Large B cell lymphomas - Clinical

Antonios Balantinakis*[1, 2], Enoch Chen[1], Sara Harrysson[1, 3], Gunilla Enblad[4], Sandra Eloranta[1], Karin Ekstrom Smedby[1, 3]

*[1]Division of Clinical Epidemiology, Department of Medicine Solna, Karolinska Institutet, Stockholm, Sweden;[2]Department of Medicine, University of Athens, Athens, Greece; [3]Department of Hematology, Karolinska University Hospital, Solna, Sweden; [4]Department of Immunology, Genetics and Pathology, Uppsala University, Uppsala, Sweden;*

### Background

Understanding prognostic differences within heterogenous diseases can potentially change the way we approach individual patients. In early-stage Diffuse Large B-cell Lymphoma (DLBCL), extranodal sites such as testis, kidney and lung have been linked to poorer survival, but evidence supporting this association remains unclear.

### Aims

This study aimed to compare survival between primary extranodal and nodal-only disease and evaluate survival by extranodal site in early-stage (Ann Arbor stage I-II) DLBCL.

### Methods

We conducted a population-based cohort study, using data from the Swedish Lymphoma Register. The cohort consisted of adult patients diagnosed with stage I and II DLBCL in Sweden between 2007 and 2023, with follow-up through May 16$^{th}$ 2024. Patients with involvement in the bone marrow, liver, pleura, ocular or central nervous system (CNS), as well as those with multiple extranodal sites, were excluded, as these features are consistent with stage IV DLBCL. Overall survival (OS) was estimated using the Kaplan-Meier method and compared using the log-rank test. Hazard ratios (HRs) with 95% confidence intervals, adjusted for potential confounding factors, were estimated using Cox proportional hazards models.

### Results

Among the 3804 patients included in the study, 31% (n=1170) presented with extranodal disease. Baseline patient characteristics are summarized in Table 1.
No significant difference in OS (5-year OS: 69% vs. 71%, p = 0.072) was observed between patients with extranodal and nodal-only disease (Figure 1a), or when contrasting the groups with adjustment for age at diagnosis, sex, stage at diagnosis, Charlson Comorbidity Index, health region and level (HR=1.08, 95% CI: 0.96–1.2). However, OS varied substantially by site within the extranodal group (Figure 1b). Among the six most frequent sites (intestine, stomach, bone, head and neck, testis and skin), skin demonstrated the poorest prognosis (5-year OS: 55%), while bone involvement was associated with the most favorable outcomes (5-year OS: 77%).

**Copyright Information:** (Online) ISSN: 2572-9241
©2026 The Author(s). HemaSphere is published by John Wiley & Sons Ltd on behalf of European Hematology Association. This is an open access Abstract Book distributed under the Attribution-NonCommercial-NoDerivs (CC BY-NC-ND), which allows third parties to download the articles and share them with others as long as they credit the author and the Abstract Book, but they cannot change the content in any way or use them commercially.
**Abstract Book Citations:** Authors, Title, HemaSphere, 2026;10:(S1):pages. The abstract book DOIs can be found on the “Author Information” page.
**Disclaimer:** Articles published in the journal HemaSphere exclusively reflect the opinions of the authors. The authors are responsible for all content in their abstracts including accuracy of the facts, statements, citing resources, etc.

### Summary/Conclusion

In this nationwide cohort of patients with early-stage DLBCL, the results suggest that extranodal involvement is not associated with poorer survival compared to nodal-limited disease. However, substantial site-specific heterogeneity exists, indicating that early-stage DLBCL is a clinical diverse group rather than a uniform entity. Understanding site-specific survival can be useful in improving prognostic accuracy and guiding management strategies.

| **Variable** | **Total** N = 3804 | **Nodal** N = 2,634 | **Extranodal** N = 1,170 |
|---|---|---|---|
| **Age at diagnosis, years. Median (range)** | 71 (18, 102) | 71 (18, 99) | 72 (20, 102) |
| **Sex** | | | |
| Male | 2,134 (56.1%) | 1,473 (55.9%) | 661 (56.5%) |
| Female | 1,670 (43.9%) | 1,161 (44.1%) | 509 (43.5%) |
| **Ann Arbor Stage** | | | |
| I | 1,839 (48.3%) | 1,139 (43.2%) | 700 (59.8%) |
| II | 1,965 (51.7%) | 1,495 (56.8%) | 470 (40.2%) |

Table 1: Clinical characteristics among all patients diagnosed with DLBCL 2007–2023 in Sweden and in nodal only vs extranodal patients separately.

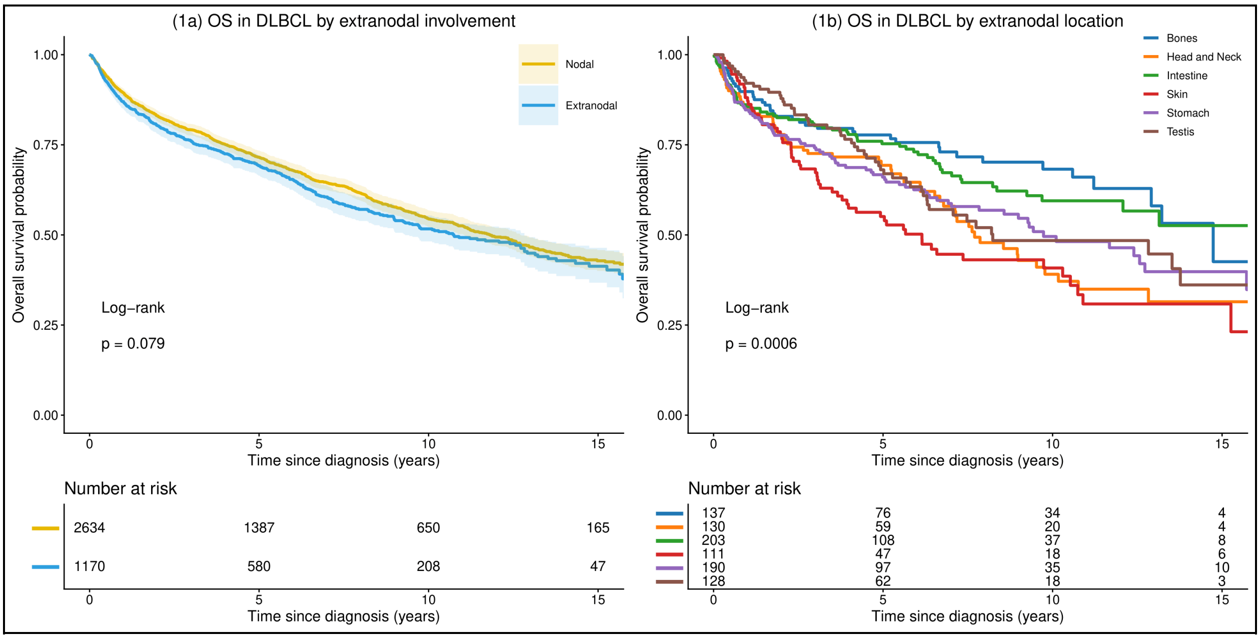

Kaplan–Meier curves for overall survival according to extranodal involvement (1a) and extranodal site (1b). Numbers at risk are shown below.

**Copyright Information:** (Online) ISSN: 2572-9241
©2026 The Author(s). HemaSphere is published by John Wiley & Sons Ltd on behalf of European Hematology Association. This is an open access Abstract Book distributed under the Attribution-NonCommercial-NoDerivs (CC BY-NC-ND), which allows third parties to download the articles and share them with others as long as they credit the author and the Abstract Book, but they cannot change the content in any way or use them commercially.
**Abstract Book Citations:** Authors, Title, HemaSphere, 2026;10:(S1):pages. The abstract book DOIs can be found on the “Author Information” page.
**Disclaimer:** Articles published in the journal HemaSphere exclusively reflect the opinions of the authors. The authors are responsible for all content in their abstracts including accuracy of the facts, statements, citing resources, etc.

### (PS2074) REAL-WORLD OUTCOMES OF GLOFITAMAB PLUS GEMCITABINE AND OXALIPLATIN IN RELAPSED/REFRACTORY LARGE B-CELL LYMPHOMA: A EUROPEAN MULTICENTER COHORT

**Topic:** 19. Large B cell lymphomas - Clinical

Elisa Bozzotto*[1], Rebecca Wurm-Kuczera *[2], Evgenii Shumilov[3], Nina Malburg[3], Joseph Kauer[4], Zoi Saxonis[2], Marcel Teichert[5, 6, 7, 8], Ulf Schnetzke[9], Ellen Obstfelder[9], Theodoros Vassilakopoulos[10], Susanne Ghandili[11], Francis Ayuk[11], Sascha Dietrich[12], Giulia Magno[13], Philipp Nakov[14], Papageorgiou Sotirios[15], Thomas Pabst[16, 17], Normann Steiner[18], Josia Fauser[18], Enrique D ´harcourt Rowold[19], Vladan Vucinic[19], Michael Panny[20], Dominik Kiem[1], Michael Leisch[1], Petra Pichler[21], Thamer Sliwa[22], Henriette Huber[23], Semra Aydin[24], Noëlle Sieg[25, 26, 27], Niklas Gebauer[28], Julia Katharina Scholz[29], Fabian Müller[29], Christoph Kresse[30], Kai Wille[31], Annkristin Heine[32], Katharina Prochzaka[32], Marion Subklewe[13, 33, 34], Christiane Pott[14], Alexander Egle[1, 35], Bastian von Tresckow[5], Georg Lenz[3], Björn Chapuy[2], Thomas Melchardt*[1]

*[1]Paracelsus Medical University, University Clinic, Department of Internal Medicine III with Haematology, Medical Oncology, Haemostaseology, Infectiology and Rheumatology, Salzburg, Austria; [2]Charité-Universitätsmedizin, Department of Hematology, Oncology, and Cancer Immunology, Campus Benjamin Franklin, Berlin, Germany; [3]University Hospital Muenster (UKM), Muenster, Germany., Department of Medicine A, Hematology, Oncology and Pneumology, Münster, Germany; [4]Heidelberg University Hospital, Internal Medicine V, Hematology, Oncology and Rheumatology,, Heidelberg, Germany; [5]University Hospital Essen, Klinik für Hämatologie und Stammzelltransplantation, Essen, Germany; [6]Department of Hematology and Stem Cell Transplantation, Essen, Germany; [7]West German Cancer Center and German Cancer consortium (DKTK partner site Essen), Essen, Germany; [8]University of Duisburg-Essen, Essen, Germany; [9]Universitätsklinikum Jena, Klinik für Innere Medizin II, Hämatologie und Internistische Onkologie, Jena, Germany; [10]National and Kapodistrian University of Athens, Laikon General Hospital, Department of Haematology and Bone Marrow Transplantation, Athens,, Greece; [11]University Hospital Hamburg Eppendorf, Department of Hematology and Oncology, Hamburg, Germany, Hamburg, Germany; [12]Haematology and Clinical Immunology Universitätsklinikum Düsseldorf, Department of Oncology, Düsseldorf, Germany; [13]LMU University Hospital, Department of Medicine III - Hematology/Oncology, Munich, Germany; [14]University Hospital Schleswig-Holstein, Department of Internal Medicine II, Kiel, Germany; [15]Attikon University General Hospital, Second Department of Internal Medicine and Research Institute, Hematology Unit, Athene, Greece; [16]University Hospital, Inselspital, Bern, Switzerland; [17]Swiss Group for Clinical Cancer Research (SAKK), Bern, Switzerland; [18]Medical University of Innsbruck, innsbruck, Austria, Department of Internal Medicine V, Hematology and Oncology, Innsbruck, Austria; [19]Leipzig University, Department for Hematology, Leipzig, Germany; [20]Hanusch Krankenhaus, 3. Medizinische Abteilung für Hämatologie und Onkologie, Wien, Austria;[21]Fachbereich Molekulare Onkologie und HämatologieKlinische Abteilung für Innere Medizin 2 (UK Krems), Klinische Abteilung für Innere Medizin I (UK St. Pölten), St Pölten, Austria; [22]LKH Hochsteiermark, Department of Hemato-Oncology, Leoben, Austria;[23]Städtisches Klinikum Karlsruhe, Medizinische Klinik III, Karlsruhe, Germany; [24]University Hospital of Bonn, Department of Oncology, Hematology, Immuno-Oncology and Rheumatology,, Bonn, Germany; [25]Medical Faculty and University Hospital of Cologne, University of Cologne, Cologne, Germany; [26]Center for Integrated Oncology Aachen Bonn Köln Düsseldorf (CIO ABCD), Germany, Aachen Bonn Köln Düsseldorf (CIO ABCD), Germany; [27]Cologne Lymphoma Working Group, Cologne, Germany; [28]University Hospital of Schleswig-Holstein, Campus Lübeck, Department of Hematology and Oncology,, Lübeck, Germany; [29]University Hospital of Erlangen, Department of Internal Medicine 5 - Hematology and Oncology, Erlangen, Germany; [30]Klinikum Chemnitz gGmbH, Chemnitz, Germany; [31]Johannes Wesling Medical Center Minden, University of Bochum, University Clinic for Hematology, Oncology, Hemostaseology and Palliative Care, Minden, Germany; [32]Medical University of Graz, Clinical Division of Hematology, Department of Internal Medicine, Graz, Austria; [33]Laboratory for Translational Cancer Immunology, LMU Gene Center, Munich, Germany; [34]German Cancer Consortium (DKTK), Munich, Germany;[35]Cancer Cluster Salzburg, Salzburg, Austria;*

**Copyright Information:** (Online) ISSN: 2572-9241
©2026 The Author(s). HemaSphere is published by John Wiley & Sons Ltd on behalf of European Hematology Association. This is an open access Abstract Book distributed under the Attribution-NonCommercial-NoDerivs (CC BY-NC-ND), which allows third parties to download the articles and share them with others as long as they credit the author and the Abstract Book, but they cannot change the content in any way or use them commercially.
**Abstract Book Citations:** Authors, Title, HemaSphere, 2026;10:(S1):pages. The abstract book DOIs can be found on the "Author Information" page.
**Disclaimer:** Articles published in the journal HemaSphere exclusively reflect the opinions of the authors. The authors are responsible for all content in their abstracts including accuracy of the facts, statements, citing resources, etc.

### Background

*Contributed equally

Gemcitabine and oxaliplatin with glofitamab (Glo-Gem-Ox, STARGLO regimen) is now approved in relapsed/refractory (r/r) diffuse large B-cell lymphoma (DLBCL) in Europe.
Nevertheless real-world evidence regarding this regimen is sparse and important sub-groups such as transformed indolent or double hit (DH) - lymphoma were not included in the privotal trial.

### Aims

To evaluate response, progression-free survival (PFS), overall survival (OS), safety/toxicity and bridging intent in a multicenter real-world cohort treated with Glo-Gem-Ox, including transformed low grade and DH - lymphoma.

### Methods

We retrospectively analyzed 149 consecutive patients with r/r LBCL treated with Glo-Gem-Ox across multiple European centers. Transformed low-grade and DH cases were included according to the recorded histopathologic diagnosis. Baseline characteristics included age, Ann Arbor stage, LDH, IPI, number of prior therapy lines, and prior CAR-T therapy. Best overall response (BOR) was categorized as CR (including metabolic CR) and PR. PFS was calculated from treatment initiation to documented PD or death from any cause. Overall survival (OS) was defined as the time from treatment initiation to death from any cause and was estimated using the Kaplan–Meier method. Median follow-up was estimated using the reverse Kaplan–Meier method.

### Results

Median age before treatment start was 67 years (range 22-90); 73.1% had stage III–IV disease, 73.2% elevated LDH, and 65.5% IPI ≥3, respectively.
Patients received a median of 2 prior therapy lines (range 0 to 5); 26.7% had prior CAR-T. Transformed DLBCL accounted for 16.1% and DH - lymphoma for 6% of cases, respectively. Glo-Gem-Ox was administered with bridging intent to CAR-T in 52 patients (34.9%) and to allogeneic transplantation in 15 patients (10.1%).

Given the very recent regulatory approval of this therapy, follow-up is at data cut still immature (median 5 months), and survival outcomes need to be interpreted in the light of this. Therapy was ongoing at data cut-off in 36% of patients. Nevertheless, response to treatment was evaluable in 82% of the patients.
Overall, the BOR was 61.1% (CR: 28.9% and PR 32.2%) in all patients. Notably, Glo-Gem-Ox was also efficient in patients treated with bridging-intent to CAR-T (BOR: 57.7%) or bridging to allogeneic transplantation (alloPBSCT; BOR: 73.4%), respectively.
Patients with transformed or DH - lymphoma achieved a BOR of 65% and 77.8%, respectively. In patients pre-treated with CAR-T a BOR of 76.3% (CR: 47.3%, PR: 29%) was observed.
Despite the limitation of immature follow-up, the median PFS was 13.2 months in the overall population reflecting the results of the pivotal trial.
Safety was consistent with the known profile of CD20xCD3 bispecific therapy. Among evaluable patients, any-grade CRS occurred in 51% (5% grade ≥3), while ICANS was observed in 11.1% (2.8% grade ≥3). ICU admission related to immune-mediated toxicity was infrequent (3.9%). Infections were reported in 43.7% of evaluable patients, reflecting the heavily pretreated population.

**Copyright Information:** (Online) ISSN: 2572-9241
©2026 The Author(s). HemaSphere is published by John Wiley & Sons Ltd on behalf of European Hematology Association. This is an open access Abstract Book distributed under the Attribution-NonCommercial-NoDerivs (CC BY-NC-ND), which allows third parties to download the articles and share them with others as long as they credit the author and the Abstract Book, but they cannot change the content in any way or use them commercially.
**Abstract Book Citations:** Authors, Title, HemaSphere, 2026;10:(S1):pages. The abstract book DOIs can be found on the “Author Information” page.
**Disclaimer:** Articles published in the journal HemaSphere exclusively reflect the opinions of the authors. The authors are responsible for all content in their abstracts including accuracy of the facts, statements, citing resources, etc.

**Summary/Conclusion**

Glo-Gem-Ox demonstrated high response rates and promising outcomes despite short follow-up in this high-risk real-world r/r LBCL cohort including transformed and DH - cases that were excluded from the pivotal trial. The treatment was frequently used as a bridging therapy to CART-T or alloPBSCT. These findings support its relevance in routine practice and warrant further evaluation.

**Copyright Information:** (Online) ISSN: 2572-9241
©2026 The Author(s). HemaSphere is published by John Wiley & Sons Ltd on behalf of European Hematology Association. This is an open access Abstract Book distributed under the Attribution-NonCommercial-NoDerivs (CC BY-NC-ND), which allows third parties to download the articles and share them with others as long as they credit the author and the Abstract Book, but they cannot change the content in any way or use them commercially.
**Abstract Book Citations:** Authors, Title, HemaSphere, 2026;10:(S1):pages. The abstract book DOIs can be found on the "Author Information" page.
**Disclaimer:** Articles published in the journal HemaSphere exclusively reflect the opinions of the authors. The authors are responsible for all content in their abstracts including accuracy of the facts, statements, citing resources, etc.

### (PS2075) ORELABRUTINIB PLUS R-CHOP FOR THE TREATMENT OF NEWLY DIAGNOSED NON-GCB DOUBLE-EXPRESSOR DIFFUSE LARGE B-CELL LYMPHOMA: A MULTICENTER, SINGLE-ARM, PHASE II STUDY

**Topic:** 19. Large B cell lymphomas - Clinical

Yang Yang*[1], Yi Zhao[1], Wenjun Wu[1], Yi Li[1], Jingsong He[1], Zhen Cai[1]

[1] *The First Affiliated Hospital, School of Medicine, Zhejiang University, Bone Marrow Transplantation Center, Department of Hematology, Hangzhou, China;*

### Background

Diffuse large B-cell lymphoma (DLBCL) patients with co-expression of MYC (≥40%) and BCL2 (≥50%), defined as double-expressor DLBCL (DE-DLBCL), typically have poor outcomes with the traditional first-line R-CHOP regimen. Given that the optimal first-line treatment strategy for DE-DLBCL patients remains unclear, there is an urgent need to explore more effective and therapeutic approaches. Orelabrutinib (O), a novel, potent, and highly selective Bruton's tyrosine kinase (BTK) inhibitor, has demonstrated encouraging therapeutic potential in patients with DLBCL when combined with the R-CHOP regimen.

### Aims

We conducted a multicenter, single-arm, phase II study to evaluate the efficacy and safety of the O plus R-CHOP (O-RCHOP) regimen in newly diagnosed patients with non-germinal center B-cell-like (non-GCB) DE-DLBCL.

### Methods

All eligible patients received 6-8 cycles of 21-day treatment, including O 150 mg once daily on days 1-14 combined with R-CHOP (rituximab 375 mg/m$^2$ on day 0; cyclophosphamide 750 mg/m$^2$ on day 1; doxorubicin 50 mg/m$^2$ on day 1; vindesine 4 mg on day 1; prednisone 100 mg on days 1-5). The primary endpoint was event-free survival (EFS). The secondary endpoints included progression-free survival (PFS), complete response rate (CRR), objective response rate (ORR), 3-year overall survival (OS) rate, and safety.

### Results

From November 2, 2021, to February 6, 2025, 49 patients were enrolled, with a median age of 59 years (range: 24-70), with 46.9 % of patients over 60 years old. Advanced-stage (III/IV) disease was present in 71.4 % of patients at diagnosis, and 32.7 % exhibited an International Prognostic Index score≥3. The CRR was 75.5% (37/49) and ORR was 98.0% (48/49) after four cycles of treatment. At the end of eight cycles of treatment, the CRR and ORR were 89.8% (44/49) and 95.9% (47/49), respectively. Median follow-up was 26.6 months (range: 5.6-43.9). The median EFS, PFS, and OS were not reached, and the estimated 2-year EFS, PFS, and OS rates were 82.0% (95% confidence interval [CI]: 71.2-94.3%), 82.0% (95% CI: 71.2-94.3%), and 97.9% (95% CI: 94.0-100.0%), respectively. The central nervous system (CNS) relapse occurred in 2 (4.1 %) patients, the 1-year and 2-year cumulative incidence of CNS relapse was 2.1% and 5.6%. Adverse events (AEs) of 1-2 grade were reported in 85.7% of patients. The grade ≥3 AEs occurred in 51.0%, with the neutropenia (38.8%) being the most common. No BTK inhibitor–associated atrial fibrillation or bradycardia was observed. Genetic alteration profiling performed in 33 patients with available baseline tumor samples showed that the frequently mutated genes were *PIM1* (63.6%), *MYD88* (51.5%), and *CD79B* (48.5%). *CD79B* mutations activate the B-cell receptor (BCR) signaling pathway, whereas *MYD88* mutations drive NF-κB signaling; both mechanisms support the rationale for orelabrutinib-based therapy. All five patients harboring *TP53* mutations achieved CR, indicating the effectiveness of O-RCHOP for patients with poor prognosis. According to the LymphGen algorithm, the MCD-like subtype was the most prevalent (51.5%), followed by BN2-like (27.3%) and EZB-like (6.1%) subtypes; no significant differences in EFS were observed among genetic subgroups.

**Copyright Information:** (Online) ISSN: 2572-9241
©2026 The Author(s). HemaSphere is published by John Wiley & Sons Ltd on behalf of European Hematology Association. This is an open access Abstract Book distributed under the Attribution-NonCommercial-NoDerivs (CC BY-NC-ND), which allows third parties to download the articles and share them with others as long as they credit the author and the Abstract Book, but they cannot change the content in any way or use them commercially.
**Abstract Book Citations:** Authors, Title, HemaSphere, 2026;10:(S1):pages. The abstract book DOIs can be found on the "Author Information" page.
**Disclaimer:** Articles published in the journal HemaSphere exclusively reflect the opinions of the authors. The authors are responsible for all content in their abstracts including accuracy of the facts, statements, citing resources, etc.

### Summary/Conclusion

In previously untreated non-GCB DE-DLBCL patients, O-RCHOP achieved promising efficacy with a manageable safety profile, supporting its promise as a feasible first-line regimen.

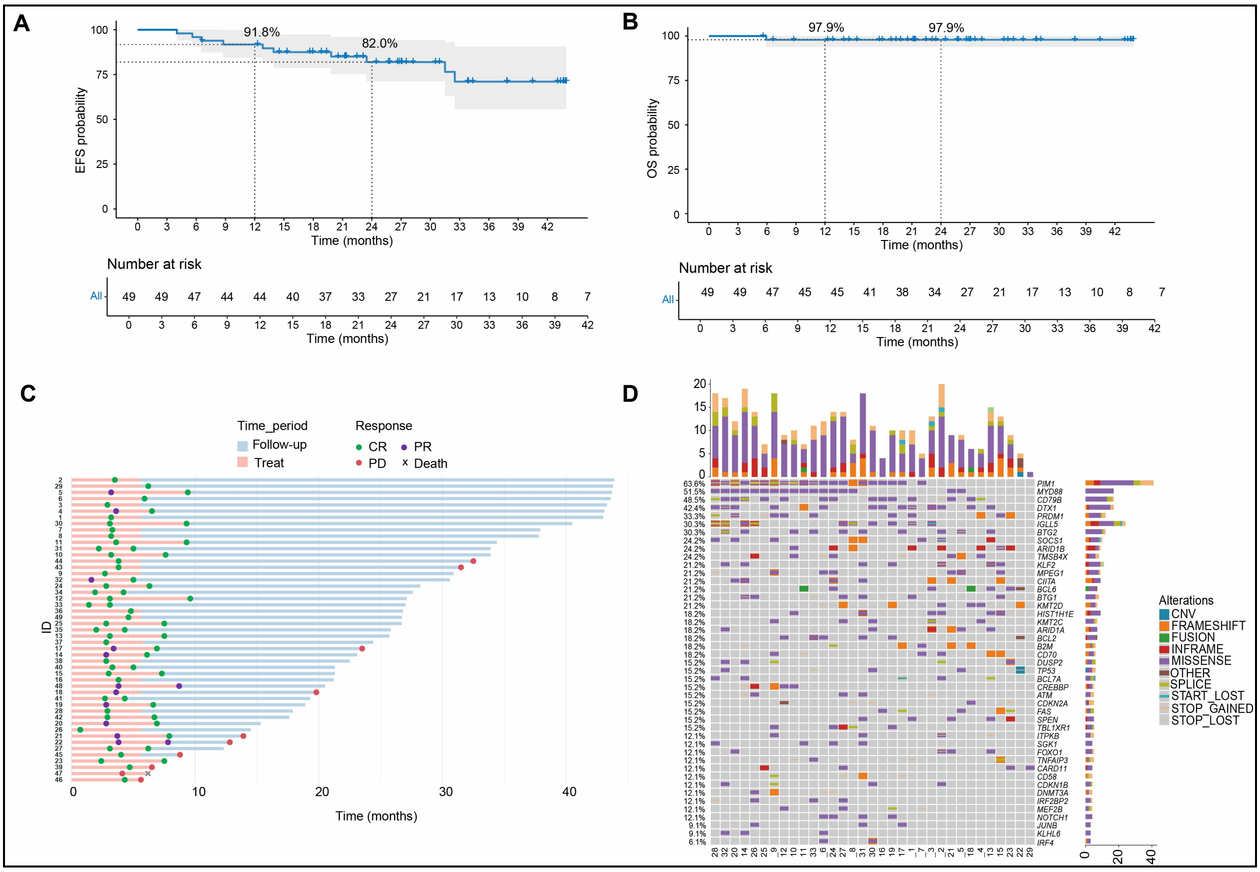

**Copyright Information:** (Online) ISSN: 2572-9241
©2026 The Author(s). HemaSphere is published by John Wiley & Sons Ltd on behalf of European Hematology Association. This is an open access Abstract Book distributed under the Attribution-NonCommercial-NoDerivs (CC BY-NC-ND), which allows third parties to download the articles and share them with others as long as they credit the author and the Abstract Book, but they cannot change the content in any way or use them commercially.
**Abstract Book Citations:** Authors, Title, HemaSphere, 2026;10:(S1):pages. The abstract book DOIs can be found on the "Author Information" page.
**Disclaimer:** Articles published in the journal HemaSphere exclusively reflect the opinions of the authors. The authors are responsible for all content in their abstracts including accuracy of the facts, statements, citing resources, etc.

### (PS2076) FIXED-DURATION SUBCUTANEOUS MOSUNETUZUMAB IN ELDERLY/UNFIT PATIENTS WITH PREVIOUSLY UNTREATED DIFFUSE LARGE B-CELL LYMPHOMA: PHASE 2 MORNINGSUN STUDY INTERIM ANALYSIS AND PATIENT-REPORTED OUTCOMES

**Topic:** 19. Large B cell lymphomas - Clinical

Ian W. Flinn[1], John M. Burke*[2], Aung M. Tun[3], Ayed Ayed[4], Juliana M. L. Biondo[5], Mei Wu[5], Farah Hossain[5], Yong Mun[5], Rona Farighi[5], Avrita Singh[5], Jamal Misleh[6], Shachar Peles[7], L. Elizabeth Budde[8], Jose C. Villasboas[9], Jeff P. Sharman[10]

*[1]Tennessee Oncology and OneOncology, Nashville, TN, United States of America;[2]SCRI at Rocky Mountain Cancer Centers, Aurora, CO, United States of America; [3]University of Kansas Cancer Center, Kansas City, KS, United States of America;[4]Cancer Specialists of North Florida, Jacksonville, FL, United States of America; [5]Genentech, Inc., South San Francisco, CA, United States of America; [6]Delaware/Christiana Care National Cancer Institute Community Oncology Research Program, Helen Graham Cancer Center, Newark, DE, United States of America; [7]Florida Cancer Specialists and Research Institute, Tampa, FL, United States of America; [8]City of Hope National Medical Center, Duarte, CA, United States of America;[9]Mayo Clinic, Rochester, MN, United States of America; [10]Willamette Valley Cancer Institute, Eugene, OR, United States of America;*

### Background

Mosunetuzumab, a CD20xCD3 T-cell engaging bispecific antibody, is approved for relapsed/refractory follicular lymphoma after ≥2 prior lines of therapy. We report an interim analysis and patient-reported outcomes (PROs) with subcutaneous (SC) mosunetuzumab monotherapy in elderly/unfit patients with previously untreated diffuse large B-cell lymphoma (DLBCL).

### Methods

MorningSun (NCT05207670) is a Phase 2, basket study of mosunetuzumab SC in patients with B-cell non-Hodgkin lymphoma. In the DLBCL cohort, eligible patients (aged ≥80 years, or 65–79 years and ineligible for chemoimmunotherapy) received outpatient mosunetuzumab SC as an investigational regimen: Day (D) 1 (5mg), D8 (45mg) and D15 (45mg) of Cycle (C) 1, then 45mg on D1 for up to 17 (21-day cycles). The primary endpoint was progression-free survival (PFS) rate at 24 months. Other endpoints included overall survival (OS), objective response rate (ORR), complete response (CR) rate, and safety. PROs were assessed by the Functional Assessment of Cancer Therapy-Lymphoma subscale (FACT-Lym LYMS; scores range from 0–60 with higher scores indicating improvement, and a ≥3-point increase is considered clinically meaningful) with an endpoint of change from baseline (CfB) in lymphoma symptoms. All patients provided written informed consent.

**Copyright Information:** (Online) ISSN: 2572-9241
©2026 The Author(s). HemaSphere is published by John Wiley & Sons Ltd on behalf of European Hematology Association. This is an open access Abstract Book distributed under the Attribution-NonCommercial-NoDerivs (CC BY-NC-ND), which allows third parties to download the articles and share them with others as long as they credit the author and the Abstract Book, but they cannot change the content in any way or use them commercially.
**Abstract Book Citations:** Authors, Title, HemaSphere, 2026;10:(S1):pages. The abstract book DOIs can be found on the “Author Information” page.
**Disclaimer:** Articles published in the journal HemaSphere exclusively reflect the opinions of the authors. The authors are responsible for all content in their abstracts including accuracy of the facts, statements, citing resources, etc.

### Results

Forty-nine patients were enrolled, primarily from community sites (n=34). Median age was 83 years (range: 71–101). International Prognostic Index was 3–5 in 53.1% patients and 18.4% of patients had bulky disease. As of February 10, 2025, the median duration of follow-up was 12.5 months (range: 1.4–30.8). PFS and OS rates at 12 months were 71.3% (95% confidence interval [CI]: 54.8–82.7) and 74.7% (95% CI: 58.5–85.4), respectively. The ORR was 73.5% and the CR rate was 59.2%. Completion rates for FACT-Lym LYMS were ≥95% at baseline and remained ≥87% at each subsequent timepoint. The FACT-Lym LYMS baseline mean score was 44.5 (standard deviation [SD], 9.54) and was reflective of moderate symptom burden. Clinically meaningful improvements in lymphoma symptoms were observed in 53.1% (95% CI: 38.3–67.5) of patients; improvements were demonstrated during treatment by mean CfB FACT-Lym LYMS scores: C4D1, 5.1 (SD, 9.8); C9D1, 5.6 (SD, 11.8); C17D1, 9.8 (SD, 11.4). The most common any-grade adverse event (AE) was injection site reaction (53.1%; all Grade 1/2). Grade 3/4 AEs were reported in 65.3% of patients and 8.2% experienced a Grade 5 AE (no Grade 5 AEs were mosunetuzumab related, per investigator assessment). Serious AEs occurred in 42.9% of patients. Infection AEs occurred in 49.0% of patients (Grade 1: 4.1%; Grade 2: 28.6%; Grade 3: 16.3%) and resolved in 91.7%. Cytokine release syndrome occurred in 12.2% of patients (Grade 1: 10.2%; Grade 2: 2.0%; all resolved). Updated data with around one additional year of follow-up will be presented.

### Summary/Conclusion

Mosunetuzumab SC showed promising efficacy with manageable safety in elderly/unfit patients with previously untreated DLBCL in an outpatient setting. Clinically meaningful improvements in lymphoma symptoms suggest that mosunetuzumab SC improves aspects of health-related quality of life in this population.

© 2026 American Society of Clinical Oncology, Inc. Reused with permission. This abstract was accepted and previously presented at the 2026 ASCO Annual Meeting. All rights reserved.

**Copyright Information:** (Online) ISSN: 2572-9241
©2026 The Author(s). HemaSphere is published by John Wiley & Sons Ltd on behalf of European Hematology Association. This is an open access Abstract Book distributed under the Attribution-NonCommercial-NoDerivs (CC BY-NC-ND), which allows third parties to download the articles and share them with others as long as they credit the author and the Abstract Book, but they cannot change the content in any way or use them commercially.
**Abstract Book Citations:** Authors, Title, HemaSphere, 2026;10:(S1):pages. The abstract book DOIs can be found on the “Author Information” page.
**Disclaimer:** Articles published in the journal HemaSphere exclusively reflect the opinions of the authors. The authors are responsible for all content in their abstracts including accuracy of the facts, statements, citing resources, etc.

### (PS2077) RISK FOR CYTOKINE RELEASE SYNDROME IN PATIENTS WITH LARGE B-CELL LYMPHOMA TREATED WITH CD3XCD20 BISPECIFIC ANTIBODIES: AN INTERNATIONAL MULTICENTER RETROSPECTIVE STUDY

**Topic:** 19. Large B cell lymphomas - Clinical

Giuseppe Gritti*[1], Monica Rondi[1], Chiara Bancone[2, 3], Simon Husby[2], James Paterson[4], Emil Kyvsgaard[2], Carsten Niemann[2], Rita Tavarozzi[5], Silvia Ferrari[1], Michele Clerico[6], Giulio Cassanello[7], Francesco Piazza[8], Alice Di Rocco[9], Luca Arcaini[10], Alessandro Re[11], Marika Porrazzo[12], Antonio Pinto[13], Wonseog Kim[14], Sang Eun Yoon[14], Chiara Pavoni[1, 15], Alessia Moioli[16], Carlo Visco[16], Beatrice Casadei[17], Pier Luigi Zinzani[17], William Townsend[4], Jean-Marie Michot[18], Jacopo Calabrese De Feo[19], Carmelo Carlo-Stella[19], Michael Dickinson[20], Martin Hutchings[2], Lorenzo Falchi[21]

*[1]ASST Papa Giovanni XXIII, Bergamo, Italy; [2]Rigshospitalet, Copenhagen, Denmark; [3]Università Campus Bio-Medico, Roma, Italy; [4]University College Hospital, London, United Kingdom; [5]AOU SS. Antonio e Biagio e Cesare Arrigo, Alessandria, Italy; [6]Ospedale Molinette, Torino, Italy; [7]Ospedale San Raffaele, Milan, Italy; [8]University of Padova and Azienda Ospedale-Università, Padova, Italy; [9]Policlinico Umberto I, Roma, Italy; [10]Fondazione IRCCS Policlinico San Matteo, Pavia, Italy; [11]ASST Spedali Civili, Brescia, Italy; [12]Ospedali Riuniti Villa Sofia-Cervello, Palermo, Italy; [13]Istituto Nazionale Tumori "G. Pascale", Napoli, Italy; [14]Samsung Medical Center, Seoul, South Korea; [15]Fondazione FROM, Bergamo, Italy; [16]AOUI di Verona – Ospedale di Borgo Roma, Verona, Italy; [17]IRCCS Azienda Ospedaliero-Universitaria, Bologna, Italy; [18]Institute Gustave Roussy, Paris, France; [19]IRCCS Humanitas Research Hospital and Humanitas University, Rozzano, Italy; [20]Peter MacCallum Cancer Centre, Melbourne, Australia; [21]Memorial Sloan Kettering Cancer Center, New York, United States of America;*

### Background

CD3xCD20 bispecific antibodies (BsAbs) have emerged as a highly effective "off-the-shelf" immunotherapy for relapsed/refractory (R/R) large B-cell lymphoma (LBCL). However, cytokine release syndrome (CRS) remains the primary toxicity limiting their clinical use. While a predictive model for CRS risk was recently developed for glofitamab (Gritti et al., Blood Adv. 2024), its utility is limited by the need for specific parameters and a lack of validation across the broader class of CD3xCD20 BsAbs. There is a critical need for an easily applicable, universal clinical tool to identify patients at low risk for clinically significant (grade 2+) CRS to optimize management strategies and potentially facilitate outpatient administration.

### Aims

The primary objective of this multicenter retrospective study is to develop and validate a clinical score to identify patients with R/R LBCL at low risk of developing grade 2+ CRS following the first or full therapeutic dose of a CD3xCD20 BsAb. In this abstract preliminary analysis and characterization of the incidence of CRS will be presented.

### Methods

This is a retrospective, international, multicenter study. Data were collected from adult patients with R/R LBCL, including diffuse large B-cell lymphoma (DLBCL), LBCL transformed from indolent lymphoma (t-LBCL), high-grade B-cell lymphoma (HGBCL), and primary mediastinal B-cell lymphoma (PMBCL) treated with CD3xCD20 BsAbs, including glofitamab and epcoritamab. CRS was graded according to the 2019 ASTCT criteria. Pre-treatment clinical factors, laboratory parameters, and radiologic data were analyzed. Missing data were handled using multiple imputation by chained equations. Candidate predictors were selected using penalized regression (LASSO). Variables identified as stable predictors were subsequently entered into a multivariable logistic regression model to identify independent factors associated with CRS.

**Copyright Information:** (Online) ISSN: 2572-9241
©2026 The Author(s). HemaSphere is published by John Wiley & Sons Ltd on behalf of European Hematology Association. This is an open access Abstract Book distributed under the Attribution-NonCommercial-NoDerivs (CC BY-NC-ND), which allows third parties to download the articles and share them with others as long as they credit the author and the Abstract Book, but they cannot change the content in any way or use them commercially.
**Abstract Book Citations:** Authors, Title, HemaSphere, 2026;10:(S1):pages. The abstract book DOIs can be found on the "Author Information" page.
**Disclaimer:** Articles published in the journal HemaSphere exclusively reflect the opinions of the authors. The authors are responsible for all content in their abstracts including accuracy of the facts, statements, citing resources, etc.

### Results

A total of 440 patients were included in the analysis. The median age was 68 years (range 18-90) with 60% of male patients. Diagnosis was DLBCL (n=266, 60%), t-LBCL (n= 113, 26%), HGBCL (n=52, 12%) and PMBCL (n=9, 2%). The median number of previous lines of therapy was 2 (range 1-11) and most patients (71%) were refractory to the last line of treatment. The most common BsAbs administered were glofitamab (n=353) and epcoritamab (n=87). In the first cycle of treatment, CRS of any grade occurred in 46% (n=204) of patients, while grade >1 CRS was observed in 18% (n=78). Five patients treated with epcoritamab did not receive the full dose. After administration of the first/first full dose of BsAb, CRS of any grade occurred in 43% (n=185/435) of patients, while grade >1 CRS was observed in 16% (n=71/435).
In univariate analysis, stage, IPI, bulky disease (≥7.5 cm), extranodal disease, LDH and WBC count were associated with grade 2+ CRS. Preliminary analysis indicates that among factors independently associated with grade 2+ CRS at multivariable analysis, bulky disease (OR 2.45, 95% CI 1.33–4.52; P=0.004), Ki67 ≥80% (OR 0.52, 95% CI 0.29–0.95; P=0.033), and WBC ≥5 x$10^9$/L (OR 2.27, 95% CI 1.23–4.18; P=0.009) were most strongly predictive of the outcome.

### Summary/Conclusion

In LBCL, CRS risk factors largely converge on disease burden and kinetics. Analysis of this multi-national large cohort of patients is ongoing, full results and the predictive score will be presented at the meeting.

**Copyright Information:** (Online) ISSN: 2572-9241
©2026 The Author(s). HemaSphere is published by John Wiley & Sons Ltd on behalf of European Hematology Association. This is an open access Abstract Book distributed under the Attribution-NonCommercial-NoDerivs (CC BY-NC-ND), which allows third parties to download the articles and share them with others as long as they credit the author and the Abstract Book, but they cannot change the content in any way or use them commercially.
**Abstract Book Citations:** Authors, Title, HemaSphere, 2026;10:(S1):pages. The abstract book DOIs can be found on the "Author Information" page.
**Disclaimer:** Articles published in the journal HemaSphere exclusively reflect the opinions of the authors. The authors are responsible for all content in their abstracts including accuracy of the facts, statements, citing resources, etc.

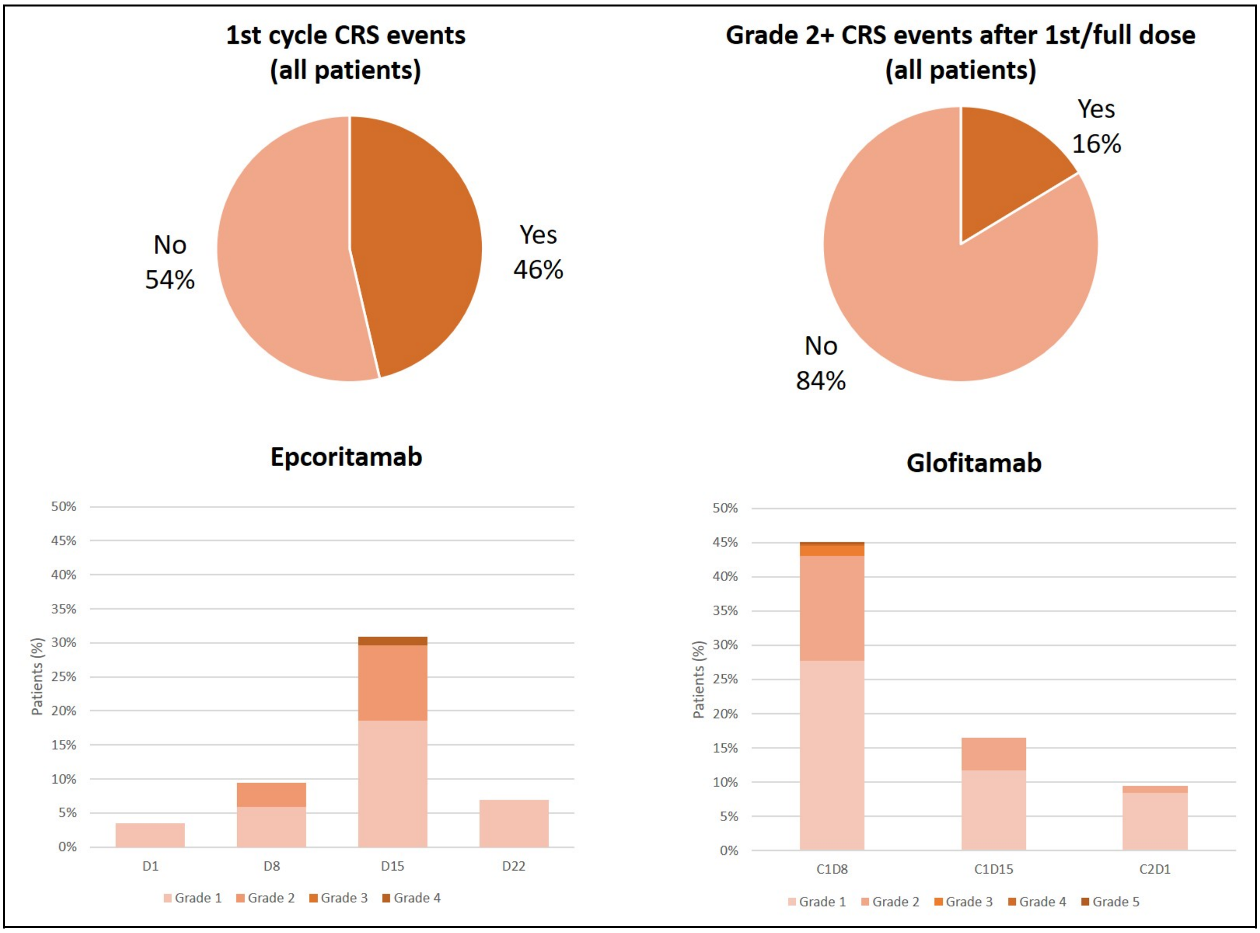

Incidence of any-grade and grade 2+ CRS events in the full cohort and by administration day for epcoritamab and glofitamab

**Copyright Information:** (Online) ISSN: 2572-9241
©2026 The Author(s). HemaSphere is published by John Wiley & Sons Ltd on behalf of European Hematology Association. This is an open access Abstract Book distributed under the Attribution-NonCommercial-NoDerivs (CC BY-NC-ND), which allows third parties to download the articles and share them with others as long as they credit the author and the Abstract Book, but they cannot change the content in any way or use them commercially.
**Abstract Book Citations:** Authors, Title, HemaSphere, 2026;10:(S1):pages. The abstract book DOIs can be found on the "Author Information" page.
**Disclaimer:** Articles published in the journal HemaSphere exclusively reflect the opinions of the authors. The authors are responsible for all content in their abstracts including accuracy of the facts, statements, citing resources, etc.

### (PS2078) NUTRITIONAL PARAMETERS AS PROGNOSTIC MARKERS IN OLDER PATIENTS WITH DIFFUSE LARGE B-CELL LYMPHOMA: A POST-HOC ANALYSIS OF THE ELDERLY PROJECT BY FONDAZIONE ITALIANA LINFOMI

**Topic:** 19. Large B cell lymphomas - Clinical

Salvatrice Mancuso*[1], Annalisa Arcari[2], Luigi Marcheselli[3], Alessandra Tucci[4], Caterina Mammi[5], Francesco Merli[6], Michele Spina[7]

*[1]University of Palermo, Promozione della Salute, Materno-Infantile, di Medicina Interna e Specialistica di Eccellenza “G. D’Alessandro”, Palermo, Italy; [2]Ospedale Guglielmo da Saliceto, Hematology Unit, Piacenza, Italy; [3]Fondazione Italiana Linfomi Onlus, Modena, Italy; [4]ASST Spedali Civili Brescia, Hematology Division, Brescia, Italy; [5]Gruppo Amici dell'Ematologia GRADE-Onlus Foundation, Reggio Emilia, Italy; [6]Hematology Unit, Azienda Unità Sanitaria Locale-IRCCS, Reggio Emilia, Italy; [7]Centro di Riferimento Oncologico di Aviano (CRO) IRCCS Aviano (PN), Italy., Division of Medical Oncology and Immune-related Tumors,, Aviano (PN), Italy;*

### Background

Diffuse Large B Cell Lymphoma (DLBCL) is the most common lymphoma in the elderly, with variable response to immunochemotherapy. Several prognostic systems have been developed to guide treatment. In this setting, comprehensive geriatric assessment (CGA) enables risk stratification and correlates with outcomes, with nutritional status representing a key evaluated component.Albumin, Body Mass index (BMI) and Geriatric Nutritional Risk Index (GNRI) are indices that reflect nutritional status and have been variably employed across different disease settings to explore their prognostic significance.To date, few studies have investigated the prognostic potential of these parameters in the elderly population undergoing treatment for DLBCL.

### Aims

To evaluate the predictive value of albumin, BMI and GNRI on outcomes of patients aged > 65 years undergoing first-line therapy for DLBCL.

### Methods

This is an ancillary analysis of Elderly Project of the Fondazione Italiana Linfomi, a prospective multicenter observational study aimed at validating a simplified geriatric assessment tool and a new prognostic score, the EPI. In this study, we selected patients whose complete anthropometric and laboratory parameters were available at baseline.The primary endpoint was the correlation between serum albumin levels, BMI and GNRI and overall survival

**Copyright Information:** (Online) ISSN: 2572-9241
©2026 The Author(s). HemaSphere is published by John Wiley & Sons Ltd on behalf of European Hematology Association. This is an open access Abstract Book distributed under the Attribution-NonCommercial-NoDerivs (CC BY-NC-ND), which allows third parties to download the articles and share them with others as long as they credit the author and the Abstract Book, but they cannot change the content in any way or use them commercially.
**Abstract Book Citations:** Authors, Title, HemaSphere, 2026;10:(S1):pages. The abstract book DOIs can be found on the “Author Information” page.
**Disclaimer:** Articles published in the journal HemaSphere exclusively reflect the opinions of the authors. The authors are responsible for all content in their abstracts including accuracy of the facts, statements, citing resources, etc.

### Results

Based on the inclusion criteria, 688 out of total of 1207 patients (59%) were deemed eligible, with median age of 75 years.In univariate analysis, albumin showed statistical correlation with 3-yr OS (HR, 0.43; 95% CI, 0.34-0.55; P<0.001). In addition, patients with serum albumin concentration<3.5 g/dl presented 3-yr OS of 56% vs 73% of subjects with values >3,5 gr/dl (P<0.001). In a multivariate Cox proportional hazard regression, we observed a HR of 0.58 (95% CI, 0.44-0.75, P<0.001). As regards BMI, it correlates with 3-yr OS (p-value: 0.003, HR: 0.95, 0.91-0.96 CI). With a cut-off value of 25, patients with BMI < 25 showed worst survival compared with BMI ≥25 (p 0.025). In a multivariate Cox regression analysis, BMI was an independent prognostic factor with an HR of 0.96 (95% CI, 0.92-0.99; P= 0.002). We have identified four groups of patients stratified according to GNRI values in relationship to risk of death: none risk (GNRI >98), low risk (GNRI 92-98), moderate (GNRI 82-92), high risk (GNRI <82). Regarding GNRI as continuous variable, there was a significant correlation with 3-yr OS (P<0.001, HR 0.65, 95% CI 0.58-0.74). A correlation was also observed between different GNRI patients groups and 3-yr OS (Tab.1). Finally, high statistical association of albumin, BMI and GNRI with EPI was observed.

Tab. 1: GNRI in DLBCL

| **GNRI** | **N (fail)** | **3-yr OS % (95%CI)** | **HR (95%CI)** | **p-value** |
|---|---|---|---|---|
| Overall Survival | 688 (184) | 68 (63-72) | - | - |
| Continuous (increase 10 points) | - | - | 0.65 (0.58-0.74) | <0.001 |
| Adjusted by EPI | | | 0,73 (0,64-0,83) | <0.001 |
| **GNRI group** | **N (%) [fail]** | **3-yr OS % (95%CI)** | **HR (95%CI)** | **p-value** |
| None | 408 (59) [79] | 76 (70-80) | 1.00 | |
| Low | 131 (39) [39] | 67 (57-75) | 1.62 (1.10-2.38) | 0.013 |
| Moderate | 109 (16) [47] | 50 (39-75) | 2.70 (1.88-3.87) | <0.001 |
| High | 40 (6) [19] | 35 (13-58) | 3.65 (2.21-6.03) | <0.001 |

### Summary/Conclusion

In the current therapeutic landscape, treatment personalization requires accurate risk stratification. In elderly patients with DLBCL undergoing antineoplastic treatment, albumin, BMI and GNRI represent independent prognostic parameters for survival, which are simple and easily applicable

**Copyright Information:** (Online) ISSN: 2572-9241
©2026 The Author(s). HemaSphere is published by John Wiley & Sons Ltd on behalf of European Hematology Association. This is an open access Abstract Book distributed under the Attribution-NonCommercial-NoDerivs (CC BY-NC-ND), which allows third parties to download the articles and share them with others as long as they credit the author and the Abstract Book, but they cannot change the content in any way or use them commercially.
**Abstract Book Citations:** Authors, Title, HemaSphere, 2026;10:(S1):pages. The abstract book DOIs can be found on the “Author Information” page.
**Disclaimer:** Articles published in the journal HemaSphere exclusively reflect the opinions of the authors. The authors are responsible for all content in their abstracts including accuracy of the facts, statements, citing resources, etc.

### (PS2079) DYNAMIC CHANGES IN MONONUCLEOSOMAL CFDNA PROFILES IDENTIFY HIGH-RISK PATIENTS WITH LARGE B-CELL LYMPHOMA

**Topic:** 19. Large B cell lymphomas - Clinical

Amos Bonsdorff*[1, 2], Maare Arffman[1, 2], Leo Meriranta[1, 2], Judit Jørgensen[3], Kristina Drott[4], Øystein Fluge[5], Sirkku Jyrkkiö[6], Peter Brown[7], Harald Holte[8], Sirpa Leppä[1, 2]

*[1]University of Helsinki, Research Programs Unit, Applied Tumor Genomics, Helsinki, Finland;[2]Helsinki University Hospital Comprehensive Cancer Centre, Department of Oncology, Helsinki, Finland; [3]Aarhus University Hospital, Department of Hematology, Aarhus, Denmark; [4]Skane University Hospital, Department of Oncology, Lund, Sweden;[5]Haukeland University Hospital, Department of Oncology, Bergen, Norway; [6]Turku University Hospital, Department of Oncology, Turku, Finland; [7]Rigshospitalet, Department of Hematology, Copenhagen, Denmark; [8]Oslo University Hospital, Department of Oncology, Oslo, Norway;*

### Background

Large B-cell lymphoma (LBCL) represents a heterogeneous group of aggressive lymphoid malignancies with diverse molecular features and clinical outcomes. Approximately 30% of patients develop relapsed or refractory disease. Current diagnostic approaches rely on invasive tissue biopsy, which captures only a single tumor site and time point, and thus fail to capture the full heterogeneity of the disease. Liquid biopsy approaches based on circulating tumor DNA (ctDNA) allow non-invasive disease monitoring but require ultra-sensitive sequencing to detect tumor-derived fragments among abundant cell-free DNA (cfDNA). Length distribution of cfDNA, reflecting the underlying chromatin architecture and fragmentation biology, is non-random, typically peaking at ~166 base pairs (bp). Tumor-derived DNA fragment distributions tend to be enriched with shorter fragments, suggesting that cfDNA fragment length profiles may provide additional biologically and clinically relevant information for cancer care.

### Aims

The aim of the study was to investigate whether cfDNA fragment length profiles and their temporal changes during therapy are associated with clinical outcomes in high-risk LBCL patients.

### Methods

Plasma samples from 123 high-risk LBCL patients enrolled in the Nordic Lymphoma Group LBC-06 trial were collected before treatment (BL), during therapy, at end-of-treatment (EOT), and during follow-up. cfDNA was sequenced using a targeted ~750 kb panel. Analyses of fragment length distributions were performed in R environment. Fragment counts were Z-standardized to enable profile shape comparisons across timepoints. The short fragment fraction (SFF) was defined as the proportion of fragments <150 bp out of all 100bp-220bp fragments. Temporal changes in fragmentation were quantified as ΔSFF between BL and EOT. Fragmentome shift risk (FSR) groups were defined using ROC-derived optimal cut-offs based on Youden's J statistic, identifying thresholds for large decreases (ΔSFF < −44.2%) and increases (ΔSFF > +12.3%) in SFF.

**Copyright Information:** (Online) ISSN: 2572-9241
©2026 The Author(s). HemaSphere is published by John Wiley & Sons Ltd on behalf of European Hematology Association. This is an open access Abstract Book distributed under the Attribution-NonCommercial-NoDerivs (CC BY-NC-ND), which allows third parties to download the articles and share them with others as long as they credit the author and the Abstract Book, but they cannot change the content in any way or use them commercially.
**Abstract Book Citations:** Authors, Title, HemaSphere, 2026;10:(S1):pages. The abstract book DOIs can be found on the "Author Information" page.
**Disclaimer:** Articles published in the journal HemaSphere exclusively reflect the opinions of the authors. The authors are responsible for all content in their abstracts including accuracy of the facts, statements, citing resources, etc.

### Results

Fragment length profiles showed marked inter-patient heterogeneity and dynamic changes during treatment. Both large decreases and increases in SFF between baseline and EOT were associated with inferior survival. Patients classified as FSR had significantly worse progression-free survival (PFS) (p=0.00023) and overall survival (OS) (p<0.0001) compared with non-FSR patients. In Kaplan–Meier analyses, non-FSR patients (n=53) had only two PFS events and no OS events, whereas FSR patients (n=45) experienced 17 PFS and 10 OS events (Figure 1A). Changes in fragment length profiles were consistent across the targeted sequencing panel (Figure 1B). In the Cox multivariable model with age, age-adjusted International Prognostic Index, and minimal residual disease status, FSR remained independently prognostic for PFS (Figure 1C).

### Summary/Conclusion

The proposed fragmentome shift risk (FSR) metric may represent a novel non-invasive biomarker for outcome prediction. Integration of cfDNA fragmentomics with ctDNA mutation profiling and minimal residual disease assessment could improve disease monitoring and risk stratification in LBCL. Independent validation in additional cohorts is needed. Further insight into differences in fragmentation could be provided by studying the end-motif enrichment across distinct fragment lengths.

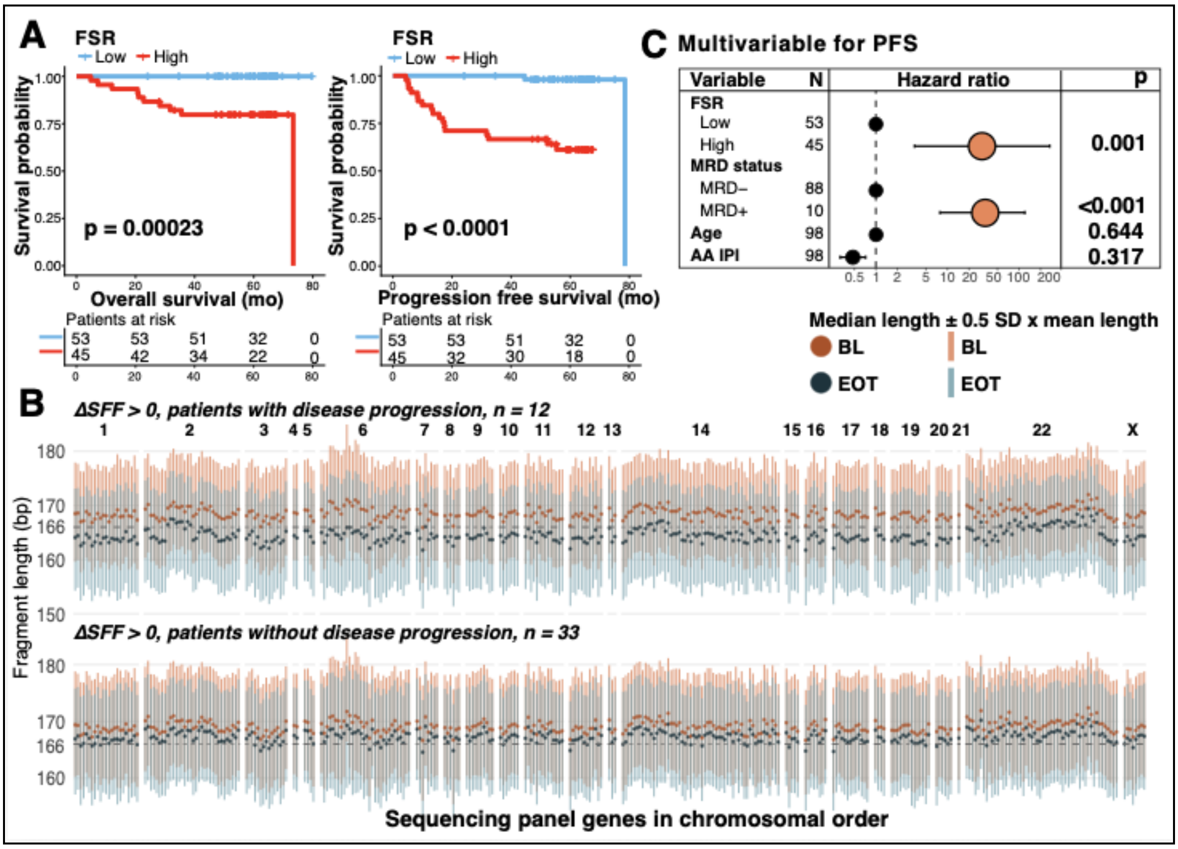

Figure 1. A Kaplan-Meier survival estimates for PFS and OS for FSR stratified patients. B Gene-level dot plot showing the median length and length standard deviation of cfDNA fragments at BL and EOT. C Multivariable model for PFS according to FSR, MRD status at EOT, Age and aaIPI. aaIPI, age-adjusted international prognostic index; BL, baseline; EOT, end-of-treatment; FSR, fragmentome shift risk; MRD, minimal residual disease; SD, standard deviation; ΔSFF, short fragment fraction deviation.

Copyright Information: (Online) ISSN: 2572-9241
©2026 The Author(s). HemaSphere is published by John Wiley & Sons Ltd on behalf of European Hematology Association. This is an open access Abstract Book distributed under the Attribution-NonCommercial-NoDerivs (CC BY-NC-ND), which allows third parties to download the articles and share them with others as long as they credit the author and the Abstract Book, but they cannot change the content in any way or use them commercially.
Abstract Book Citations: Authors, Title, HemaSphere, 2026;10:(S1):pages. The abstract book DOIs can be found on the "Author Information" page.
Disclaimer: Articles published in the journal HemaSphere exclusively reflect the opinions of the authors. The authors are responsible for all content in their abstracts including accuracy of the facts, statements, citing resources, etc.

### (PS2080) ALEMTUZUMAB EXPOSURE AND SUSTAINED IL-2 DRIVE UCART20X22 EXPANSION AND CLINICAL RESPONSE INADULTS WITH RELAPSED OR REFRACTORY B-CELL NON-HODGKIN LYMPHOMA: NATHALI-01 STUDY

**Topic:** 19. Large B cell lymphomas - Clinical

Emmanuel Bachy*[1], Aravind Ramakrishnan[2], Simon Metenou[3], Matthew Matasar[4], Laurent Poirot[5], Guillaume Cartron[6], Xenia Peluffo[5], Catherine Thieblemont[7], Vivian Dai[3], Peter Riedell[8], Narayan Lebaka[3], Thomas Gastinne[9], Caroline Roudet[3], Ana Alfonso Pierola[10], Adrian Kilcoyne[3], Jeremy S. Abramson[11]

*[1]Department of Hematology, Hospices Civils de Lyon and Université Claude Bernard Lyon 1, Lyon, France;[2]South Austin Medical Center, Austin, United States of America; [3]Cellectis Inc, New York, United States of America;[4]Rutgers Cancer Institute of New Jersey, New Brunswick, United States of America; [5]Cellectis, Paris, France; [6]Centre Hospitalier Universitaire de Montpellier, Montpellier, France; [7]Hopital Saint-Louis, Paris, France;[8]David and Etta Jonas Center for Cellular Therapy, University of Chicago, Chicago, United States of America; [9]Centre Hospitalier Universitaire de Nantes, Nantes, France; [10]Universidad de Navarra - Clinica Universidad de Navarra – Pamplona,, Pamplona, Spain; [11]Harvard Medical School, Massachusetts General Hospital, Boston, United States of America;*

### Background

UCART20x22 is a first-in-class allogeneic, CAR T-cell product with dual CAR targeting CD20/CD22. TALEN® gene editing technology is used to inactivate *TRAC* and *CD52* to minimize the risk of GvHD and enable the use of alemtuzumab to optimize lymphodepletion (LD). NatHaLi-01 is an open-label, dose-finding and expansion study of UCART20x22 (etivelcabtagene erigedleucel) in pts with 3L+ R/R B-NHL. Fludarabine, cyclophosphamide, and alemtuzumab (FCA) was investigated as the LD regimen.

### Aims

This preliminary - analysis is to explore alemtuzumab exposure, UCART20x22 cellular kinetics, cytokine responses, and clinical outcomes in patients receiving UCART20x22 in R/R B-NHL.

### Methods

Alemtuzumab pharmacokinetics (Cmax, AUC), UCART20x22 expansion, cytokine dynamics, endogenous T cell recovery, and best objective response (BOR) were analyzed to understand how these parameters influence clinical outcomes.

**Copyright Information:** (Online) ISSN: 2572-9241
©2026 The Author(s). HemaSphere is published by John Wiley & Sons Ltd on behalf of European Hematology Association. This is an open access Abstract Book distributed under the Attribution-NonCommercial-NoDerivs (CC BY-NC-ND), which allows third parties to download the articles and share them with others as long as they credit the author and the Abstract Book, but they cannot change the content in any way or use them commercially.
**Abstract Book Citations:** Authors, Title, HemaSphere, 2026;10:(S1):pages. The abstract book DOIs can be found on the “Author Information” page.
**Disclaimer:** Articles published in the journal HemaSphere exclusively reflect the opinions of the authors. The authors are responsible for all content in their abstracts including accuracy of the facts, statements, citing resources, etc.

**Results**

At the data cutoff on 3rd February 2026, 14 pts (median age 61.5 y) with R/R B-NHL (DLBCL n=12, HGBCL n=1, tMZL n=1) were treated. 12 pts received LD FCA1 with flat dose alemtuzumab (60 mg) and UCART20x22 at an assigned dose (DL1 $50x10^6$ cells n=6, DL2 $150x10^6$ cells n=3, DL3 $450x10^6$ cells n=3). Given that exposure to alemtuzumab is weight dependent, a weight-based alemtuzumab dosing LD regimen, FCA3, was subsequently implemented at DL1 to optimize the LD regimen and mitigate risks of over-exposure and improve efficacy by limiting under-exposure to alemtuzumab. Two pts received FCA3 and UCART20x22 at DL1. Across all patients in the study, median prior lines of tx was 3 (range 2-8), 93.0% had prior CD19 CART, 71.4% had elevated LDH, and all 14-pts had stage IV disease at baseline. ORRs are 87.5%, 66.7% and 66.7% for DL1 (n=8), DL2 (n=3) and, DL3 (n=3), respectively.

There were strong statistical correlations between alemtuzumab dose per kilogram of body weight and the PK parameters **Cmax** and **AUC** (alemtuzumab exposure). Flat-dose administration of alemtuzumab resulted in notable inter-subject variability in exposure. The realized dose per kilogram, Cmax, and $AUC_{D\text{-}5-28}$ had median values (25th–75th percentiles) of **0.83 mg/kg** (0.74–0.89), **4,096 pg/mL** (3,178–5,297), and **84,454 pg·day/mL** (61,186–117,878), respectively. High exposure was associated with delayed host $CD4^+$ T-cell recovery, enhanced UCART20x22 expansion, lower inflammatory markers post LD before UCART20x22 infusion, and higher response rates. Conversely, low exposure was associated with reduced lymphodepletion and a higher inflammatory milieu (IFN-γ, TNF-α, IL-6, IL-8 and IL-12p40) before UCART20x22 infusion, reduced UCART20x22 expansion, and lower response rates. Cytokine kinetics differed between response groups: Non-responders (SD + PD) showed higher baseline concentrations of TNF-α and IFN-γ and earlier IL-2 loss of production, while responders (CR +PR) exhibited more sustained TNF-α, IFN-γ and IL-2 production.

**Summary/Conclusion**

These preliminary findings highlight that alemtuzumab exposure and sustained IL-2 production as well as delayed and sustained production of IFN-γ, TNF-α, might be key correlates of UCART20x22 expansion/persistence and clinical response. This observation supports the need to optimize LD and we are also currently investigating the use of subcutaneous low dose IL-2 to further enhance treatment response [NCT05607420]. Weight-based alemtuzumab might optimize trade-off between adequate exposure and toxicity mitigation.

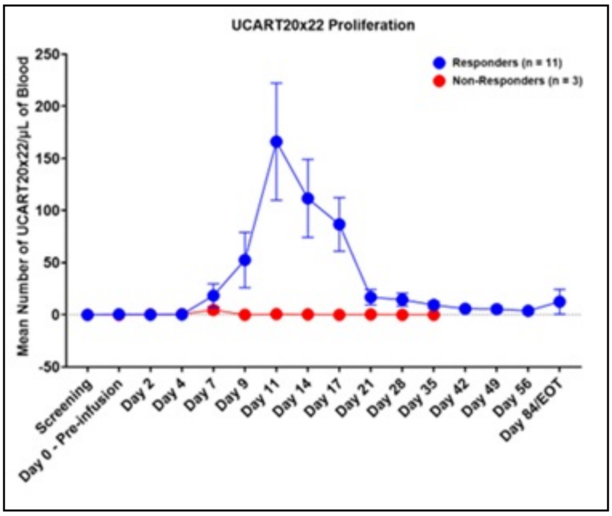

**Copyright Information:** (Online) ISSN: 2572-9241
©2026 The Author(s). HemaSphere is published by John Wiley & Sons Ltd on behalf of European Hematology Association. This is an open access Abstract Book distributed under the Attribution-NonCommercial-NoDerivs (CC BY-NC-ND), which allows third parties to download the articles and share them with others as long as they credit the author and the Abstract Book, but they cannot change the content in any way or use them commercially.
**Abstract Book Citations:** Authors, Title, HemaSphere, 2026;10:(S1):pages. The abstract book DOIs can be found on the "Author Information" page.
**Disclaimer:** Articles published in the journal HemaSphere exclusively reflect the opinions of the authors. The authors are responsible for all content in their abstracts including accuracy of the facts, statements, citing resources, etc.

### (PS2081) LONGITUDINAL SAFETY OF LISOCABTAGENE MARALEUCEL (LISO-CEL) IN PATIENTS WITH SECOND-LINE (2L) OR LATER (2L+) LARGE B-CELL LYMPHOMA (LBCL)

**Topic:** 19. Large B cell lymphomas - Clinical

Stephan Mielke*[1], Manali Kamdar[2], Jeremy S. Abramson[3], Alison Sehgal[4], Scott Solomon[5], Leo I. Gordon[6], Matthew Lunning[7], Nilanjan Ghosh[8], Bertram Glass[9], Pim G. N. J. Mutsaers[10], Franck Morschhauser[11], Koji Izutsu[12], Robert Braun[13], Maria Strocchia[13], Muskan Mittal[14], Giovanna Andreola[13], Jon Arnason[15]

*[1]Karolinska Institutet and University Hospital; Karolinska Comprehensive Cancer Center, Karolinska ATMP Center, Stockholm, Sweden; [2]University of Colorado Cancer Center, Aurora, United States of America;[3]Massachusetts General Hospital Cancer Center, Harvard Medical School, Boston, United States of America; [4]University of Pittsburgh Medical Center: Hillman Cancer Center, Pittsburgh, United States of America; [5]Northside Hospital Cancer Institute, Atlanta, United States of America; [6]Northwestern University, Feinberg School of Medicine, Robert H. Lurie Comprehensive Cancer Center, Chicago, United States of America; [7]University of Nebraska Medical Center, Omaha, United States of America;[8]Atrium Health, Levine Cancer Institute, Wake Forest University School of Medicine, Charlotte, United States of America; [9]Helios Klinikum Berlin-Buch, Berlin, Germany; [10]Erasmus University Medical Center, Rotterdam, The Netherlands; [11]Centre Hospitalier Universitaire de Lille, Groupe de Recherche sur les formes Injectables et les Technologies Associées, Lille, France; [12]National Cancer Center Hospital, Tokyo, Japan; [13]Bristol Myers Squibb, Boudry, Switzerland; [14]Syneos Health, Haryana, India; [15]Beth Israel Deaconess Medical Center, Boston, United States of America;*

### Background

Liso-cel has shown favorable efficacy and a manageable, consistent safety profile across B-cell malignancies, including R/R LBCL.

### Aims

To present pooled longitudinal safety results in patients who received liso-cel as 2L+ treatment for LBCL in 3 pivotal clinical trials.

### Methods

Data from liso-cel–treated patients in the TRANSFORM (2L transplant-eligible LBCL, NCT03575351), PILOT (2L transplant-noneligible LBCL, NCT03483103), and TRANSCEND NHL 001 (third-line or later LBCL, NCT02631044) trials were pooled. Informed consent was obtained. Incidences of infections, laboratory parameters, and concomitant medication/procedure use were assessed from Day 0–15, Day 16–30, Month 2–3, Month 4–6, Month 7–9, Month 10–12, Month 13–18, Month 19–24, and Month 25 to end of study. Incidences were calculated as patients with ≥ 1 event divided by ongoing patients during that period.

**Copyright Information:** (Online) ISSN: 2572-9241
©2026 The Author(s). HemaSphere is published by John Wiley & Sons Ltd on behalf of European Hematology Association. This is an open access Abstract Book distributed under the Attribution-NonCommercial-NoDerivs (CC BY-NC-ND), which allows third parties to download the articles and share them with others as long as they credit the author and the Abstract Book, but they cannot change the content in any way or use them commercially.
**Abstract Book Citations:** Authors, Title, HemaSphere, 2026;10:(S1):pages. The abstract book DOIs can be found on the "Author Information" page.
**Disclaimer:** Articles published in the journal HemaSphere exclusively reflect the opinions of the authors. The authors are responsible for all content in their abstracts including accuracy of the facts, statements, citing resources, etc.

### Results

A total of 420 patients were included. Incidence of grade ≥ 3 neutropenia was highest from Day 0–15 (**Table**), decreased sharply after Month 6, and then remained low. Overall, 62% of patients received growth factors for neutropenia, with the highest use from Day 0–15 and minimal use after Month 3. Rate of grade ≥ 3 anemia was highest from Day 0–15, declined by Month 9, and then remained low. A total of 49% of patients received red blood cell transfusions at any point, with the highest use from Day 0–15 and minimal use after Month 3. Incidence of grade ≥ 3 thrombocytopenia was highest from Day 0–Month 3, declined by Month 9, and then remained low. Platelet transfusions were received by 31% of patients at any point, with the highest use from Day 0–Month 3 and minimal use after Month 3. Use of erythropoiesis- and thrombopoiesis-stimulating agents was rare (1% and 2% at any point, respectively). Incidence of grade ≥ 3 infections was 15% overall and remained low in all time periods (**Table**). Hypogammaglobulinemia (serum immunoglobulin < 500 mg/dL) ranged from 43% to 63% across time periods with intravenous immunoglobulin use in 20% of patients at any point on study. B-cell aplasia declined over time from 99% from Day 0–15 to 73% by Month 12 and < 50% beyond Month 18. The overall incidence of second primary malignancies was 6%.

### Summary/Conclusion

In these longitudinal analyses in liso-cel–treated patients with 2L+ LBCL, grade ≥ 3 cytopenias and concomitant medication use decreased over time with minimal needs for growth factors or transfusions after Month 3. Grade ≥ 3 infections remained low in the short- and long-term periods despite persistent hypogammaglobulinemia and B-cell aplasia in most patients at Month 12. These data underscore the favorable long-term safety of liso-cel in patients with 2L+ LBCL, reinforcing the feasibility of outpatient management and the potential for low health care resource utilization in clinical practice.
©2026 American Society of Clinical Oncology, Inc. Reused with permission. This abstract was submitted to ASCO 2026. All rights reserved.

**Table. Select longitudinal safety results**

| Time period | Grade ≥ 3 neutropenia, % | Growth factor for neutropenia, % | Grade ≥ 3 anemia, % | RBC transfusion, % | Grade ≥ 3 thrombo-cytopenia, % | Platelet transfusion, % | Grade ≥ 3 infection, % |
|---|---|---|---|---|---|---|---|
| Day 0–15 | 87 | 42 | 29 | 35 | 23 | 12 | 6 |
| Day 16–30 | 36 | 18 | 12 | 19 | 35 | 22 | 2 |
| Month 2–3 | 33 | 16 | 17 | 20 | 32 | 18 | 5 |
| Month 4–6 | 26 | 5 | 12 | 5 | 16 | 3 | 2 |
| Month 7–9 | 12 | 1 | 4 | 2 | 9 | 2 | < 1 |
| Month 10–12 | 15 | 2 | 2 | 1 | 1 | < 1 | 1 |
| Month 13–18 | 10 | < 1 | 1 | < 1 | 3 | 0 | < 1 |
| Month 19–24 | 4 | 1 | 3 | < 1 | 3 | 0 | 2 |
| Month 25–EOS | 6 | 1 | 2 | 1 | 1 | 1 | 1 |

EOS, end of study; RBC, red blood cell.

**Copyright Information:** (Online) ISSN: 2572-9241
©2026 The Author(s). HemaSphere is published by John Wiley & Sons Ltd on behalf of European Hematology Association. This is an open access Abstract Book distributed under the Attribution-NonCommercial-NoDerivs (CC BY-NC-ND), which allows third parties to download the articles and share them with others as long as they credit the author and the Abstract Book, but they cannot change the content in any way or use them commercially.
**Abstract Book Citations:** Authors, Title, HemaSphere, 2026;10:(S1):pages. The abstract book DOIs can be found on the "Author Information" page.
**Disclaimer:** Articles published in the journal HemaSphere exclusively reflect the opinions of the authors. The authors are responsible for all content in their abstracts including accuracy of the facts, statements, citing resources, etc.

### (PS2082) FIXED-DURATION EPCORITAMAB MONOTHERAPY INDUCES HIGH RESPONSE AND MRD-NEGATIVITY RATES IN ELDERLY PATIENTS WITH NEWLY DIAGNOSED LARGE B-CELL LYMPHOMA AND COMORBIDITIES: RESULTS FROM EPCORE DLBCL-3

**Topic:** 19. Large B cell lymphomas - Clinical

Umberto Vitolo*[1], Juan Miguel Bergua Burgues[2], Johannes Duell[3], Michal Kwiatek[4], David Belada[5], Wojciech Jurczak[6], Marie Maerevoet[7], David Sibon[8], Richard Greil[9], Takahiro Kumode[10], Javier Lopez Jimenez[11], Caressa Meert[12], Juan-Manuel Sancho Cia[13], Catherine Thieblemont[14], Sergio Ortegon Alcaide[15], Won Seog Kim[16], Raul Cordoba[17], Monica Wielgos-Bonvallet[18], Tony Jiang[19], Yanli Wang[18], Stephanie McGoldrick[18], Evelyn Guo[18], Franck Morschhauser[20], Sherida Woei-A-Jin[21]

*[1]Candiolo Cancer Institute, FPO-IRCCS, Candiolo (Turin), Italy; [2]Servicio De Hematologia Y Hemoterapia, Hospital San Pedro de Alcántara, Cáceres, Spain; [3]Medizinische Klinik und Poliklinik II, Universitätsklinikum Würzburg, Würzburg, Germany; [4]AidPort Clinical Trials Hospital, Skórzewo, Poland; [5]Charles University, Hospital and Faculty of Medicine, 4th Department of Internal Medicine – Haematology, Hradec Králové, Czechia; [6]Maria Skłodowska-Curie National Research Institute of Oncology, Kraków, Poland; [7]Institut Jules Bordet, Brussels, Belgium; [8]Henri Mondor University Hospital, Assistance Publique - Hôpitaux de Paris, Paris-Est Creteil University, Lymphoid Hematology Department, Créteil, France; [9]Uniklinikum Salzburg, Universitätsklinik für Innere Medizin, Salzburg, Austria; [10]Kindai University, Department of Hematology and Rheumatology, Osaka, Japan; [11]Servicio de Hematología y Hemoterapia, Hospital Universitario Ramón y Cajal, IRYCIS, Universidad Alcalá Henares, Madrid, Spain; [12]Algemeen Ziekenhuis Delta, Roeselare, Belgium; [13]Institut Catala d Oncologia, Hospital Germans Trias i Pujol, Badalona, Spain; [14]Assistance Publique Hôpitaux de Paris - Hôpital Saint-Louis, University of Paris, Department of Hematology, Paris, France; [15]Hospital Universitario Virgen Macarena, Sevilla, Spain; [16]Samsung Medical Center, Seoul, South Korea; [17]Fundación Jiménez Díaz University Hospital, Madrid, Spain; [18]Genmab, Plainsboro, NJ, United States of America; [19]AbbVie, North Chicago, IL, United States of America; [20]University of Lille, CHU Lille, Department of Hematology, Lille, France; [21]Department of General Medical Oncology, University Hospitals Leuven, Leuven, Belgium;*

### Background

A subset of newly diagnosed (1L) patients with LBCL are ineligible for curative chemotherapy due to age or comorbidities. EPCORE® DLBCL-3 (NCT05660967) is a phase 2 trial evaluating the efficacy and safety of fixed-duration epcoritamab (epcor), a CD3×CD20 bispecific antibody, in elderly patients with 1L LBCL and comorbidities.

### Aims

To report efficacy and safety of fixed-duration epcor monotherapy after 22 months of follow-up in EPCORE DLBCL-3.

### Methods

Patients with 1L CD20+ LBCL ineligible for anthracycline-based chemotherapy due to age ≥ 80 years or ≥ 75 years with comorbidities received SC epcor monotherapy: step-up dosing (0.16 mg on cycle 1 day 1 [C1D1]; 0.8 mg on C1D8) followed by 48 mg QW in C1–3 and Q4W in C4–12, for up to 1 year. Primary endpoint was CR rate per investigator assessment (Lugano criteria). MRD negativity (MRD-neg) was assessed in responders by circulating tumor DNA (cutoff, < 1 mutant molecules per mL).

**Copyright Information:** (Online) ISSN: 2572-9241
©2026 The Author(s). HemaSphere is published by John Wiley & Sons Ltd on behalf of European Hematology Association. This is an open access Abstract Book distributed under the Attribution-NonCommercial-NoDerivs (CC BY-NC-ND), which allows third parties to download the articles and share them with others as long as they credit the author and the Abstract Book, but they cannot change the content in any way or use them commercially.
**Abstract Book Citations:** Authors, Title, HemaSphere, 2026;10:(S1):pages. The abstract book DOIs can be found on the “Author Information” page.
**Disclaimer:** Articles published in the journal HemaSphere exclusively reflect the opinions of the authors. The authors are responsible for all content in their abstracts including accuracy of the facts, statements, citing resources, etc.

**Results**

As of December 5, 2025, 66 patients had received epcor monotherapy. Median age was 82.5 years (range, 76–95); 36% of patients were ≥ 85 years. 23% had ECOG PS score of 2, 71% had significant renal impairment, 62% had Ann Arbor stage IV, 64% had IPI score ≥ 3, and 35% had bulky disease (≥ 7 cm). All patients had comorbid conditions, with 94% having ≥ 3 comorbidities at baseline. 97% of patients had cardiovascular comorbidities.

Median follow-up was 22 months (95% CI, 11.1–22.5). At cutoff, 30% of patients had completed treatment (tx) per protocol; tx was ongoing in 12% and 58% had discontinued, mainly due to disease progression (29%) or AEs (17%).

ORR was 73% in the response-evaluable patients (n = 60); 63% had a CR. Median time to response was 1.5 months (range, 1.2–5.4), and median time to CR was 2.2 months (range, 1.2–5.6); 11/17 patients with a PR or SD at 1st assessment had a subsequent CR. Median DOR and DOCR were not reached (NR). An estimated 67% of responses and 73% of CRs were ongoing at 12 months. In all patients (N = 66), median PFS was 13.0 months (95% CI, 5.6–NR) and median OS was NR (95% CI, 13.0–NR). An estimated 50% of patients were progression free and 66% were alive at 12 months. 92% (33/36) of MRD-evaluable responders were MRD-neg at any time point. MRD-neg occurred by C3D1 in most complete responders and was sustained through C12D1 in a majority of those with available longitudinal samples.

TEAEs of any grade (G) occurred in 97% of patients, with cytokine release syndrome (CRS; 71%), fatigue (23%), peripheral edema, diarrhea, and constipation (21% each) being most frequent; 67% had a G ≥ 3 TEAE. Most CRS events were low G (G1: 38%; G2: 29%; G3: 5%), with 92% occurring in C1. ICANS occurred in 18% of patients (G1: 8%; G2: 8%; G3: 3%). Neutropenia was reported in 18%. There was no febrile neutropenia or clinical tumor lysis syndrome. 68% of patients had an infection of any G; 24% had a G ≥3 infection. There were 8 G5 TEAEs; 2 were considered tx related by investigator.

**Summary/Conclusion**

Fixed-duration epcor monotherapy showed robust efficacy, with early, deep, and durable responses in elderly patients with 1L LBCL and comorbidities. Safety outcomes, including the occurrence of CRS and ICANS, were consistent with expected rates in this elderly population. These findings suggest that epcor offers a single-agent, chemotherapy-free option for pts with 1L DLBCL who would be excluded from standard chemoimmunotherapy regimens due to age or comorbidities.

**Copyright Information:** (Online) ISSN: 2572-9241
©2026 The Author(s). HemaSphere is published by John Wiley & Sons Ltd on behalf of European Hematology Association. This is an open access Abstract Book distributed under the Attribution-NonCommercial-NoDerivs (CC BY-NC-ND), which allows third parties to download the articles and share them with others as long as they credit the author and the Abstract Book, but they cannot change the content in any way or use them commercially.
**Abstract Book Citations:** Authors, Title, HemaSphere, 2026;10:(S1):pages. The abstract book DOIs can be found on the “Author Information” page.
**Disclaimer:** Articles published in the journal HemaSphere exclusively reflect the opinions of the authors. The authors are responsible for all content in their abstracts including accuracy of the facts, statements, citing resources, etc.

### (PS2083) IMMUNE RECONSTITUTION AFTER CD19-DIRECTED CAR-T THERAPY IN B-CELL NON-HODGKIN LYMPHOMA: PROGNOSTIC IMPACT, PRODUCT-SPECIFIC PATTERNS, AND ASSOCIATIONS WITH CAR-T PERSISTENCE AND SUBSETS

**Topic:** 19. Large B cell lymphomas - Clinical

Yu Yagi*[1], Yusuke Kanemasa[1], Yuki Sasaki[1], Kaho Fujino[1], Takahiro Kuga[1], Nozomi Kanai[1], Yusuke Masuda[1], Kumiko Fujita[1], Kento Ishimine[1], Taro Sawabe[1], Mano Mino[1], Ai Takahara[1], Taichi Tamura[1], Shohei Nakamura[1], Toshihiro Okuya[1], Shota Kusuhara[1], Shinichiro Matsuda[1], Fumihiko Ouchi[2], Masashi Shimabukuro[2], Atsushi Jinguji[2], Shuhei Kurosawa[3], Takuya Shimizuguchi[4], Daichi Sadato[5], Kaoru Morita[2], Tomomi Toubai[2], Hiroaki Shimizu[2], Yuho Najima[2], Yuka Harada[5], Kyoko Haraguchi[6], Noriko Doki[2], Tatsu Shimoyama[1]

*[1]Tokyo Metropolitan Komagome Hospital, Department of Medical Oncology, Tokyo, Japan;[2]Tokyo Metropolitan Komagome Hospital, Hematology Division, Tokyo, Japan; [3]Tokyo Metropolitan Komagome Hospital, Department of Clinical Laboratory, Tokyo, Japan; [4]Tokyo Metropolitan Komagome Hospital, Department of Radiation Oncology, Tokyo, Japan;[5]Tokyo Metropolitan Komagome Hospital, Clinical Research and Trial Center, Tokyo, Japan; [6]Tokyo Metropolitan Komagome Hospital, Division of Transfusion and Cell Therapy, Tokyo, Japan;*

### Background

Prolonged immune dysfunction may persist after CD19-directed chimeric antigen receptor T-cell (CAR-T) therapy, but its mechanisms remain poorly understood. In B-cell non-Hodgkin lymphoma (B-NHL), the prognostic relevance of immune reconstitution (IR), differences across CAR-T products, and the link between CAR-T kinetics/persistence and IR remain incompletely characterized.

### Aims

To characterize IR after CD19-directed CAR-T therapy in B-NHL and evaluate its association with progression-free survival (PFS), differences across CAR-T products, and relationships with CAR-T cell expansion (day 14) and subsequent CAR-T cell loss.

### Methods

We retrospectively analyzed consecutive patients with relapsed/refractory B-NHL treated at our center from April 2020 to December 2025 with tisagenlecleucel (tisa-cel), lisocabtagene maraleucel (liso-cel), or axicabtagene ciloleucel (axi-cel). Peripheral blood lymphocyte subsets and serum immunoglobulins were assessed before infusion and at prespecified post-infusion time points (1 and 3 months, then every 3 months for 2 years, and every 3–6 months thereafter). Peripheral blood CAR-T cells were quantified by flow cytometry at days 3, 14, and 28, at 3 months, and then every 3 months for 2 years (every 3–6 months thereafter). IR was summarized as a 3-point score: CD4+ T-cell recovery (>200/μL), detectable CD19+ B cells, and immunoglobulin G (IgG) recovery (>4 g/L with intravenous immunoglobulin [IVIG] discontinuation or dosing interval ≥3 months); recovery was defined as a score of 2–3. Associations were evaluated using cumulative incidence methods, landmark analyses, time-dependent Cox models, and inverse probability of treatment weighting (IPTW).

**Copyright Information:** (Online) ISSN: 2572-9241
©2026 The Author(s). HemaSphere is published by John Wiley & Sons Ltd on behalf of European Hematology Association. This is an open access Abstract Book distributed under the Attribution-NonCommercial-NoDerivs (CC BY-NC-ND), which allows third parties to download the articles and share them with others as long as they credit the author and the Abstract Book, but they cannot change the content in any way or use them commercially.
**Abstract Book Citations:** Authors, Title, HemaSphere, 2026;10:(S1):pages. The abstract book DOIs can be found on the "Author Information" page.
**Disclaimer:** Articles published in the journal HemaSphere exclusively reflect the opinions of the authors. The authors are responsible for all content in their abstracts including accuracy of the facts, statements, citing resources, etc.

### Results

Among 157 patients (median age, 62 years; median follow-up, 19.9 months), 141 had large B-cell lymphoma and 16 had follicular lymphoma. Products were tisa-cel (n=59), liso-cel (n=57), and axi-cel (n=41). At 6 months, recovery rates for B cells, CD4+ T cells, and IgG were 15.5%, 37.3%, and 32.4%, respectively. Compared with tisa-cel, axi-cel showed slower CD4+ T-cell recovery but faster IgG recovery, while liso-cel showed an intermediate pattern. At 6 months, 22.4% achieved recovery (IR score 2–3). In a time-dependent Cox model, recovery was associated with longer PFS (hazard ratio [HR], 0.14; 95% confidence interval [CI], 0.06–0.33; p<0.001); 3- and 6-month landmark analyses and IPTW results were consistent (Figure A–F). Peripheral blood CAR-T cells were quantified in 128 patients; 17 lost detectable CAR-T cells (12-month cumulative incidence, 15.6%). In a day-14 landmark analysis, CAR-T cell counts above the median were associated with longer PFS (HR, 0.48; 95% CI, 0.24–0.94; p=0.031). CAR-T cell loss was not associated with PFS at 3 or 6 months (HR, 1.29; 95% CI, 0.41–4.05; and HR, 1.08; 95% CI, 0.30–3.92; p=0.91). In multivariable analyses, CAR-T cell loss was independently associated with a higher likelihood of B-cell, CD4+ T-cell, and IgG recovery and recovery (IR score 2–3).

### Summary/Conclusion

IR after CD19-directed CAR-T therapy in B-NHL was delayed and heterogeneous, with distinct patterns across CAR-T products. Recovery (IR score 2–3) was strongly associated with prolonged PFS and remained robust in landmark and IPTW analyses. CAR-T cell loss was associated with subsequent IR but not PFS, whereas higher day-14 CAR-T cell counts were associated with improved PFS, highlighting an interplay between CAR-T kinetics and host immune recovery.

**Copyright Information:** (Online) ISSN: 2572-9241
©2026 The Author(s). HemaSphere is published by John Wiley & Sons Ltd on behalf of European Hematology Association. This is an open access Abstract Book distributed under the Attribution-NonCommercial-NoDerivs (CC BY-NC-ND), which allows third parties to download the articles and share them with others as long as they credit the author and the Abstract Book, but they cannot change the content in any way or use them commercially.
**Abstract Book Citations:** Authors, Title, HemaSphere, 2026;10:(S1):pages. The abstract book DOIs can be found on the "Author Information" page.
**Disclaimer:** Articles published in the journal HemaSphere exclusively reflect the opinions of the authors. The authors are responsible for all content in their abstracts including accuracy of the facts, statements, citing resources, etc.

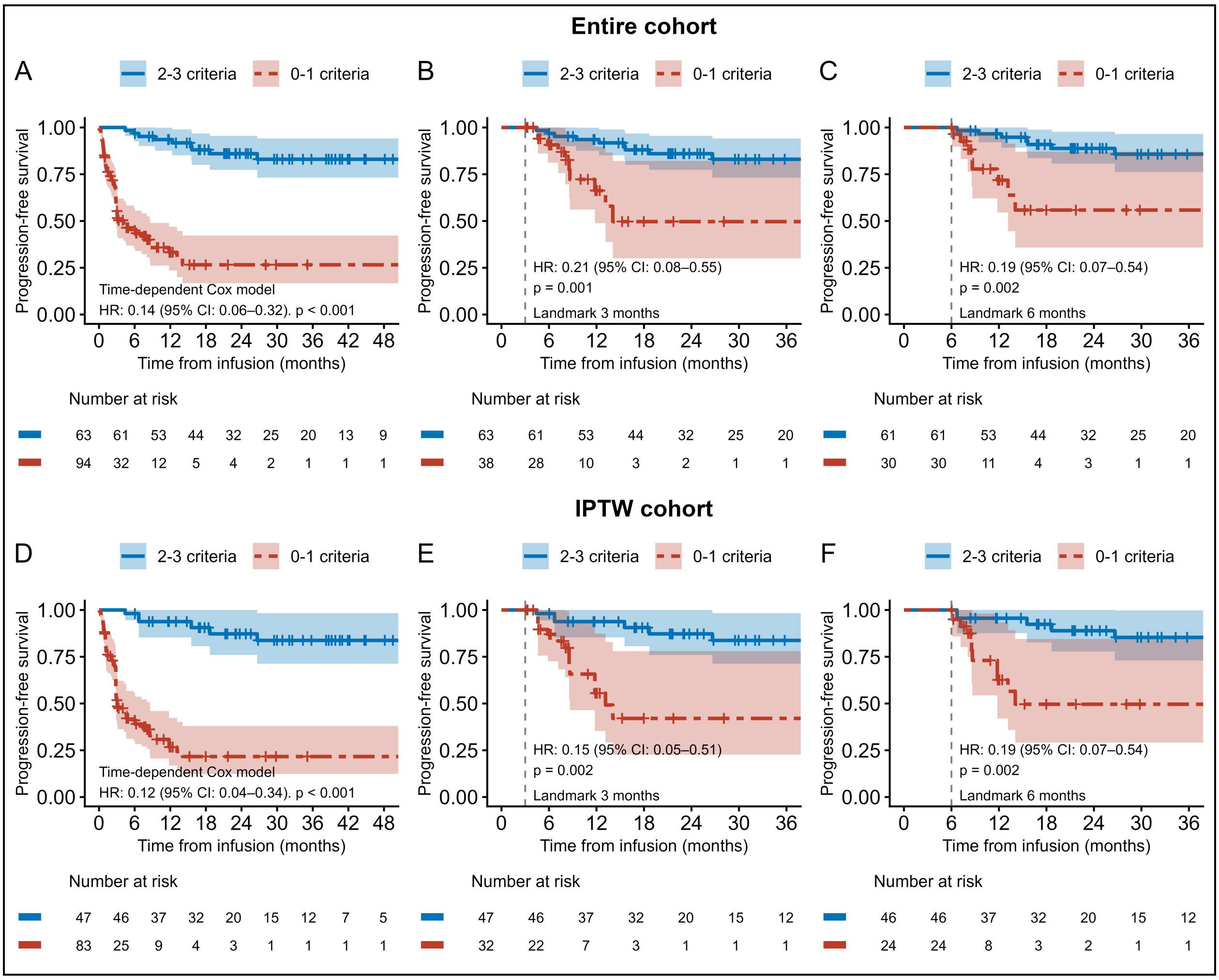

Kaplan-Meier estimates of PFS stratified by immune recovery criteria (recovery of ≥2 versus 0–1 immune reconstitution criteria). (A) PFS in entire cohort . (B) Landmark analysis including only the patients without progression by day30 after CAR T-cell infusion and (C) day 90 after infusion. Patients. (D) Inverse probability of treatment weighting–adjusted Kaplan-Meier curves depicting PFS. (E) Landmark analysis of IPTW cohort including patients without progression by day30 and (F) by day 90 after CAR T-cell infusion. The respective HR and p values are provided in each graph. P values determined by log-rank method; HRs determined using a univariable Cox proportional hazards model. PFS, progression--free survival; CAR T, chimeric antigen receptor T cell; IPTW; Inverse probability of treatment weighting; HR, hazard ratio; CI, confidence interval; Headlines are highlighted in bold.

**Copyright Information:** (Online) ISSN: 2572-9241
©2026 The Author(s). HemaSphere is published by John Wiley & Sons Ltd on behalf of European Hematology Association. This is an open access Abstract Book distributed under the Attribution-NonCommercial-NoDerivs (CC BY-NC-ND), which allows third parties to download the articles and share them with others as long as they credit the author and the Abstract Book, but they cannot change the content in any way or use them commercially.
**Abstract Book Citations:** Authors, Title, HemaSphere, 2026;10:(S1):pages. The abstract book DOIs can be found on the "Author Information" page.
**Disclaimer:** Articles published in the journal HemaSphere exclusively reflect the opinions of the authors. The authors are responsible for all content in their abstracts including accuracy of the facts, statements, citing resources, etc.

### (PS2084) EFFICACY AND SAFETY OF TIRABRUTINIB FOR RELAPSED OR REFRACTORY SECONDARY CENTRAL NERVOUS SYSTEM LYMPHOMA: RESULTS OF THE PHASE II CRYSTAL/NCCH2201 STUDY

**Topic:** 19. Large B cell lymphomas - Clinical

Noriko Fukuhara*[1], Wataru Munakata[2], Shunsuke Hatta[1], Hiroshi Arima[3], Momoko Nishikori[3], Hirokazu Nagai[4], Tatsuya Adachi[4], Hideki Goto[5], Koji Kato[6], Noriko Iwaki[2], Akiko Maeshima[7], Reiko Isomura[8], Yayoi Ando[8], Yoshie Shuda[8], Kazumi Kurishita[8], Riku Kajikawa[8], Ryunosuke MacHida[8], Natsuko Okita[8], Shinji Kohsaka[9], Koji Izutsu[2]

*[1]Tohoku University, Hematology, Sendai, Japan; [2]National Cancer Center Hospital, Hematology, Tokyo, Japan; [3]Kyoto Univerisity, Hematology, Kyoto, Japan; [4]National Hospital Organization Nagoya Medical Center, Hematology, Nagoya, Japan; [5]Hokkaido University Hospital, Laboratory and Transfusion Medicine, Sapporo, Japan; [6]Kyushu University Hospital, Hematology, Oncology and Cardiovascular Medicine, Fukuoka, Japan; [7]National Cancer Center Hospital, Diagnostic Pathology, Tokyo, Japan; [8]National Cancer Center Hospital, Clinical Research Support Office, Tokyo, Japan; [9]National Cancer Center Research Institute, Cellular Signaling, Tokyo, Japan;*

### Background

Secondary central nervous system lymphoma (SCNSL) is a devastating complication of systemic lymphoma with limited treatment options. Recent real-world data suggest a median overall survival (OS) of 3.2 months after SCNSL, highlighting a substantial unmet need (Blood Adv. 2025;9:893–905). Diffuse large B-cell lymphoma (DLBCL) represents the most common histology of SCNSL and is frequently enriched for activating mutations in *MYD88* and *CD79B*, providing a molecular rationale for Bruton's tyrosine kinase (BTK) inhibition. Tirabrutinib, a selective second-generation BTK inhibitor, has demonstrated clinical activity in primary CNS lymphoma and is approved in Japan for the relapsed or refractory (R/R) setting.

### Aims

To evaluate the efficacy and safety of tirabrutinib in patients with R/R SCNSL.

### Methods

This trial was an investigator-initiated phase II CRYSTAL/NCCH2201 study (jRCT2031220529). Eligible patients had histologically confirmed B-cell non-Hodgkin lymphoma with R/R secondary CNS involvement. Patients with active systemic disease outside the CNS were excluded. Patients were required to have received high-dose methotrexate–based therapy or be intolerant to it. Tirabrutinib was administered orally at 480 mg once daily until disease progression or unacceptable toxicity. The primary endpoint was objective response rate (ORR) as determined by the IPCG criteria, assessed by independent central review (ICR). Blood and tumor samples were collected for exploratory biomarker analyses. The expected ORR was set at 40% and the threshold ORR at 10%. Accounting for up to four ineligible or untreated patients, the planned sample size was set at 20 to achieve 80% power under a binomial test with a one-sided alpha of 2.5%.

**Copyright Information:** (Online) ISSN: 2572-9241
©2026 The Author(s). HemaSphere is published by John Wiley & Sons Ltd on behalf of European Hematology Association. This is an open access Abstract Book distributed under the Attribution-NonCommercial-NoDerivs (CC BY-NC-ND), which allows third parties to download the articles and share them with others as long as they credit the author and the Abstract Book, but they cannot change the content in any way or use them commercially.
**Abstract Book Citations:** Authors, Title, HemaSphere, 2026;10:(S1):pages. The abstract book DOIs can be found on the "Author Information" page.
**Disclaimer:** Articles published in the journal HemaSphere exclusively reflect the opinions of the authors. The authors are responsible for all content in their abstracts including accuracy of the facts, statements, citing resources, etc.

### Results

Twenty patients were enrolled between February 2023 and December 2024. The median age was 75 years (range, 48–81), and all patients had DLBCL, not otherwise specified (DLBCL-NOS) with secondary CNS involvement. Central pathological review classified 9 patients as the germinal center B-cell–like (GCB) subtype, 10 as the non-GCB subtype, and 1 patient as unclassifiable. Three patients had cerebrospinal fluid disease, including one without parenchymal lesions. The median number of prior therapies was 3.5 (range, 1–8). Eighteen patients were evaluable for efficacy by ICR. The ORR was 66.7% (95% CI, 41.0–86.7; p<0.0001), including CR/CRu in 4 patients and partial response in 8 patients. Median DOR was 2.0 months (95% CI, 1.7–3.8). At a median follow-up of 6.7 months (range 2.1-28.1 months) for OS, median PFS was 2.7 months (95% CI, 1.7–3.3), and median OS was 6.7 months (95% CI, 4.1–not estimable). All patients experienced at least one adverse event (AE); grade ≥3 events occurred in 75.0%. The most common AEs were thrombocytopenia (70%), lymphocytopenia (40%), leukocytopenia (30%), itching (30%), anemia (30%), rash (25%), and neutropenia (25%). Grade 4 AEs included neutropenia (10%) and leukocytopenia (5%). No grade 5 events were observed. Tirabrutinib was discontinued due to progressive disease in 14 patients and AEs in 2 patients.

### Summary/Conclusion

Tirabrutinib demonstrated clinically meaningful activity with a manageable safety profile in heavily pretreated patients with R/R SCNSL. The phase II CRYSTAL/NCCH2201 study supports tirabrutinib as a promising therapeutic option for this disease with extremely poor prognosis.

**Copyright Information:** (Online) ISSN: 2572-9241
©2026 The Author(s). HemaSphere is published by John Wiley & Sons Ltd on behalf of European Hematology Association. This is an open access Abstract Book distributed under the Attribution-NonCommercial-NoDerivs (CC BY-NC-ND), which allows third parties to download the articles and share them with others as long as they credit the author and the Abstract Book, but they cannot change the content in any way or use them commercially.
**Abstract Book Citations:** Authors, Title, HemaSphere, 2026;10:(S1):pages. The abstract book DOIs can be found on the "Author Information" page.
**Disclaimer:** Articles published in the journal HemaSphere exclusively reflect the opinions of the authors. The authors are responsible for all content in their abstracts including accuracy of the facts, statements, citing resources, etc.

### (PS2085) INFECTION RATES IN PATIENTS TREATED WITH CAR-T VERSUS AUTOLOGOUS STEM CELL TRANSPLANT FOR RELAPSED/REFRACTORY LARGE B-CELL LYMPHOMA AND IN THE GENERAL POPULATION: A SWEDISH POPULATION-BASED STUDY

**Topic:** 19. Large B cell lymphomas - Clinical

Kristina Noring*[1, 2], Sara Ekberg[3], Sara Harrysson[2, 4], Sandra Eloranta[4], Kristina Sonnevi[5, 6], Per-Ola Andersson[7], Mats Jerkeman[8], Gunilla Enblad[9], Karin Ekstrom Smedby[4, 10]

*[1]Karolinska Institute, Department of Medicine, Solna, Stockholm, Sweden;[2]Karolinska Univeristy Hospital, Stockholm, Sweden; [3]Red Door Analytics, Stockholm, Sweden;[4]Karolinska Institute, Department of Medicine Solna, Stockholm, Sweden;[5]Karolinska Institute, Department of Medicine Huddinge, Stockholm, Sweden; [6]Karolinska Univeristy Hospital, Department of Medicine Huddinge, Stockholm, Sweden; [7]Sahlgrenska University Hospital, Department of Hematology and Coagulation, Gothenburg, Sweden; [8]Lund University, Department of Oncology, Lund, Sweden;[9]Uppsala University, Department of Immunology, Genetics and Pathology, Stockholm, Sweden; [10]Karolinska Univeristy Hospital, Department of Medicine Solna, Stockholm, Sweden;*

### Background

Infectious diseases are the leading cause of non-relapse mortality (NRM) in patients with relapsed/refractory large B-cell lymphoma (R/R LBCL) treated with chimeric antigen receptor T-cell (CAR-T) therapy or autologous stem cell transplant (ASCT). Direct population-based comparisons of infection rates among patients treated in clinical routine and the general population are limited.

### Aims

To compare incidence rates of infection in R/R LBCL patients treated with CAR-T versus ASCT, and versus matched comparators from the general population.

### Methods

LBCL patients treated with CAR-T or ASCT for between September 2019 – February 2024 were identified through the Swedish Lymphoma register and the National Patient Register (NPR). Patients were matched 1:10 according to birth year, sex and year of CAR-T/ASCT to comparators from the general population. Infectious disease according to ICD-10 codes from hospital in- and outpatient visits in the NPR or dispensing of an anti-infective medication (excluding standard prophylaxis medications) in the National Prescribed Drug Register was counted as an infectious episode. Follow-up started at CAR-T/ASCT (or matched index date) and ended at death, allogeneic transplantation, treatment switch between CAR-T/ASCT, or administrative censoring (March 1, 2024). Infection incidence rates (IR) and incidence rate ratios (IRR) were estimated using Poisson regression with log person-time offset and robust standard errors. Time since treatment was modelled using restricted cubic splines.

**Copyright Information:** (Online) ISSN: 2572-9241
©2026 The Author(s). HemaSphere is published by John Wiley & Sons Ltd on behalf of European Hematology Association. This is an open access Abstract Book distributed under the Attribution-NonCommercial-NoDerivs (CC BY-NC-ND), which allows third parties to download the articles and share them with others as long as they credit the author and the Abstract Book, but they cannot change the content in any way or use them commercially.
**Abstract Book Citations:** Authors, Title, HemaSphere, 2026;10:(S1):pages. The abstract book DOIs can be found on the "Author Information" page.
**Disclaimer:** Articles published in the journal HemaSphere exclusively reflect the opinions of the authors. The authors are responsible for all content in their abstracts including accuracy of the facts, statements, citing resources, etc.

### Results

We identified 79 R/R LBCL treated with CAR-T, 221 treated with ASCT and 2,964 comparators, see Table 1.Overall IR per person-year was 2.96 (2.63–3.32) for CAR-T patients, 1.05 (0.96–1.15) for ASCT patients and 0.28 (0.26–0.29) for comparators. CAR-T patients had a 2.5 fold higher relative rate of infections compared with ASCT (IRR 2.52 (95% CI 2.15-2.96)) and 9-fold higher than the general population (IRR 9.26 (95% CI 7.96-10.76)). Over follow-up, the rate of infections remained high among CAR-T treated patients (Figure 1). The most common type of infection was bacterial in all groups, while viral and fungal infections were more common in both CAR-T and ASCT patients compared to the general population (Figure 1). Lymphoma relapse was the leading cause of death after CAR-T (96%) and ASCT (76%). Infections were the leading cause of NRM in ASCT patients (14%), no patients died from infections after CAR-T.

**Table 1: Patient and comparator characteristics**

| | **CAR-T** | **ASCT** | **Comparators** |
|---|---|---|---|
| | N = 79 | N =221 | N =2,964 |
| Follow-up years, median (range) | 0.9 (0.0-4.5) | 1.9 (0.1-4.5) | 2.2 (0.0-4.5) |
| Age years, median (range) | 64 (17-75) | 61 (21-74) | 61 (17-76) |
| Sex, male | 63% | 57% | 59% |
| *Lymphoma subtype*: | | | |
| DLBCL | 85% | 89% | N/A |
| Low-grade B-cell lymphoma with transformation | 0% | 4% | |
| Other LBCL | 15% | 7% | |

### Summary/Conclusion

R/R LBCL patients treated with CAR-T had several-fold higher infection rates compared with ASCT and the general population. The higher incidence of infections appeared to persist for at least four years after CAR-T. A hypothesized mechanism for this prolonged risk of infections in CAR-T patients may be the persistence of functional CAR-T cells and off-target B-cell aplasia in relapse-free patients. As expected, the leading cause of NRM in ASCT patients was infections. Contrary to previous studies, there was no mortality from infections after CAR-T. This may be attributable to an increased use of immunoglobulin treatment or improved management of infectious complications.

**Copyright Information:** (Online) ISSN: 2572-9241
©2026 The Author(s). HemaSphere is published by John Wiley & Sons Ltd on behalf of European Hematology Association. This is an open access Abstract Book distributed under the Attribution-NonCommercial-NoDerivs (CC BY-NC-ND), which allows third parties to download the articles and share them with others as long as they credit the author and the Abstract Book, but they cannot change the content in any way or use them commercially.
**Abstract Book Citations:** Authors, Title, HemaSphere, 2026;10:(S1):pages. The abstract book DOIs can be found on the “Author Information” page.
**Disclaimer:** Articles published in the journal HemaSphere exclusively reflect the opinions of the authors. The authors are responsible for all content in their abstracts including accuracy of the facts, statements, citing resources, etc.

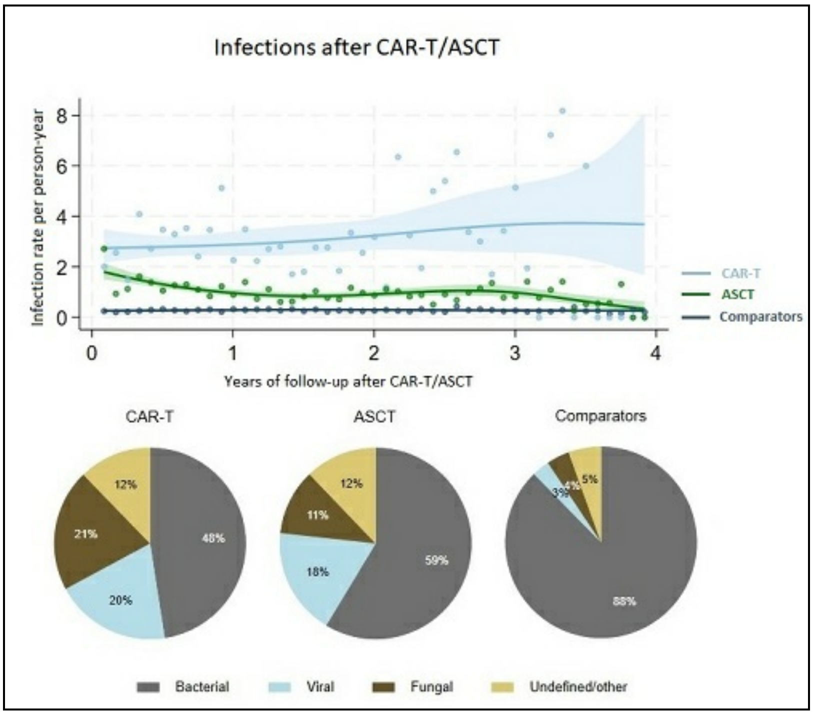

Figure 1: Infection rate per person-year and infectious disease type distribution.

**Copyright Information:** (Online) ISSN: 2572-9241
©2026 The Author(s). HemaSphere is published by John Wiley & Sons Ltd on behalf of European Hematology Association. This is an open access Abstract Book distributed under the Attribution-NonCommercial-NoDerivs (CC BY-NC-ND), which allows third parties to download the articles and share them with others as long as they credit the author and the Abstract Book, but they cannot change the content in any way or use them commercially.
**Abstract Book Citations:** Authors, Title, HemaSphere, 2026;10:(S1):pages. The abstract book DOIs can be found on the "Author Information" page.
**Disclaimer:** Articles published in the journal HemaSphere exclusively reflect the opinions of the authors. The authors are responsible for all content in their abstracts including accuracy of the facts, statements, citing resources, etc.

### (PS2086) EPCORITAMAB IN RELAPSED/REFRACTORY DIFFUSE LARGE B-CELL LYMPHOMA (R/R DLBCL): INSIGHTS FROM THE REAL-WORLD EPCORITAMAB PATIENT CHARACTERISTICS AND OUTCOMES RESEARCH (REAL-EPCOR) STUDY

**Topic:** 19. Large B cell lymphomas - Clinical

Yumeng Zhang*[1], Erin Mulvey[2], Amir Ali[3], Nausheen Ahmed[4], Javier Munoz[5], Muhamad Alhaj Moustafa[6], Monika Jun[7], Alex Mutebi[7], Tychell Branchcomb[7], Abualbishr Alshreef[8], Anthony Wang[8], David Tybor[8], Lynn Huynh[9], Angela Lax[9], Mei Sheng Duh[9], Pallawi Torka[10], Tara Graff[11], Yucai Wang[12]

*[1]Moffitt Cancer Center, Tampa, United States of America;[2]Weill Cornell Medicine, New York, United States of America;[3]Jane Anne Nohl Division of Hematology and Center for the Study of Blood Diseases, Norris Comprehensive Cancer Center, University of Southern California, Los Angeles, United States of America; [4]The University of Kansas Medical Center, Westwood, United States of America; [5]Mayo Clinic, Phoenix, United States of America;[6]Mayo Clinic, Jacksonville, United States of America; [7]Gnemab, Plainsboro, United States of America;[8]AbbVie, Worcester, United States of America;[9]Analysis Group, Inc., Boston, United States of America; [10]Memorial Sloan Kettering Cancer Center, Middletown, United States of America; [11]Mission Cancer and Blood/UIHSMG, Des Moines, United States of America; [12]Mayo Clinic, Rochester, United States of America;*

### Background

Epcoritamab (Epcor) is a subcutaneous CD3×CD20 bispecific antibody approved for the treatment of R/R DLBCL and R/R FL that demonstrated efficacy and manageable safety in the EPCORE NHL-1 trial. R/R DLBCL is an aggressive disease with heterogeneity across patients, and a subset of patients (pts) may present with more rapidly progressive disease and worse prognosis than other DLBCL pts. Characterizing pts receiving Epcor, and evaluating its effectiveness in the real-world, provides insight into its clinical performance among different patient types and informs its use in clinical practice.

### Aims

To characterize pts with R/R DLBCL treated with Epcor in the real-world and evaluate clinical response across clinically relevant subgroups.

### Methods

Real-EPCOR is an ongoing, multicenter US retrospective chart review study of real-world use of Epcor at academic and community oncology centers. Eligible pts are adults (≥18 years) with R/R DLBCL who initiated Epcor in third or later line (3L+) on or after May 19, 2023. Outcomes include overall response rate (ORR) and complete response rate (CRR). Responses are based on the treating physician assessment documented in the chart (imaging and/or clinical assessment). For this analysis, ORR and pt characteristics were evaluated among all pts, the response-evaluable population (pts with a response assessment or death due to disease progression prior to assessment), and pts who received <2 and ≥2 cycles of Epcor, consistent with the analysis by Haynes et al. (2025 British Society for Haematology meeting).

**Copyright Information:** (Online) ISSN: 2572-9241
©2026 The Author(s). HemaSphere is published by John Wiley & Sons Ltd on behalf of European Hematology Association. This is an open access Abstract Book distributed under the Attribution-NonCommercial-NoDerivs (CC BY-NC-ND), which allows third parties to download the articles and share them with others as long as they credit the author and the Abstract Book, but they cannot change the content in any way or use them commercially.
**Abstract Book Citations:** Authors, Title, HemaSphere, 2026;10:(S1):pages. The abstract book DOIs can be found on the “Author Information” page.
**Disclaimer:** Articles published in the journal HemaSphere exclusively reflect the opinions of the authors. The authors are responsible for all content in their abstracts including accuracy of the facts, statements, citing resources, etc.

### Results

This analysis included 132 R/R DLBCL pts from 7 centers. Median age was 68.6 years. Median number of prior lines of therapy (LOT) was 3 (interquartile range: 2-4) and 65.9% received prior CAR T. Median time from end of last LOT to Epcor initiation was 39 days. 32.0% had lactate dehydrogenase (LDH) ≥2x upper limit of normal (ULN), 22.7% had double/triple-hit, 57.9% had primary refractory disease, and 71.8% were refractory to their last LOT. 34.1% received first Epcor dose inpatient.
Pts who received <2 cycles (N=59) had higher risk features compared to pts who received≥2 cycles (N=73) including descriptively shorter time from last prior LOT to Epcor initiation (median 25 vs. 48 days), and higher proportions with ECOG PS ≥2 (40.7% vs 16.4%), primary refractory disease (64.2% vs 52.9%), LDH ≥2x ULN (48.3% vs 18.6%), and double/triple-hit (30.5% vs 16.4%). Median follow-up among all patients was 5.8 months. ORR and CRR for each cohort are presented in**Table 1**.

**Table 1. ORR and CRR among pts treated with Epcor**

| | **ORR, n (%)** | **CRR, n (%)** |
|---|---|---|
| **Real-world pts** | | |
| All pts (n=132) | 50 (37.9) | 30 (22.7) |
| Pts who received ≥2 cycles (n=73) | 49 (67.1) | 30 (41.1) |
| **Response-evaluable pts** | | |
| All pts (n=92) | 50 (54.3) | 30 (32.6) |
| Pts who received ≥2 cycles (n=67) | 49 (73.1) | 30 (44.8) |

### Summary/Conclusion

Real-world pts with R/R DLBCL treated with Epcor in 3L+ were characterized by a high prevalence of disease characteristics associated with poor prognosis. Pts with <2 cycles of Epcor displayed more rapidly progressive disease characteristics than those who received ≥2 cycles of Epcor. These characteristics may influence both treatment course and observed outcomes in routine practice. ORR was highly meaningful in the real-world, particularly in pts who received ≥2 cycles of Epcor and in the response-evaluable population and was consistent with clinical trial results in those subgroups. Further research is needed to better understand factors associated with completion of ≥2 cycles of Epcor.

**Copyright Information:** (Online) ISSN: 2572-9241
©2026 The Author(s). HemaSphere is published by John Wiley & Sons Ltd on behalf of European Hematology Association. This is an open access Abstract Book distributed under the Attribution-NonCommercial-NoDerivs (CC BY-NC-ND), which allows third parties to download the articles and share them with others as long as they credit the author and the Abstract Book, but they cannot change the content in any way or use them commercially.
**Abstract Book Citations:** Authors, Title, HemaSphere, 2026;10:(S1):pages. The abstract book DOIs can be found on the "Author Information" page.
**Disclaimer:** Articles published in the journal HemaSphere exclusively reflect the opinions of the authors. The authors are responsible for all content in their abstracts including accuracy of the facts, statements, citing resources, etc.

### (PS2087) IDENTIFICATION OF AN OPTIMAL ABSOLUTE LYMPHOCYTE COUNT BEFORE BISPECIFIC ANTIBODY THERAPY FOR LARGE B-CELL LYMPHOMAS: A COLLABORATIVE U.S. BISPECIFIC CONSORTIUM (CUBIC) STUDY

**Topic:** 19. Large B cell lymphomas - Clinical

Zachary Hunzeker*[1], Kunhwa Kim[1], Zhouxuan Li[2], Wei Qiao[2], Amy Ayers[3], Allison Bock[4], Ayushi Chauhan[3], Lorenzo Falchi[5], Reid Merryman[6], Swetha Thiruvengadam[7], Andrew Jallouk[8], Omnia Farahat[1], Steven Bair[9], Matthew Lunning[10], Shakthi Bhaskar[8], Yazeed Sawalha[11], Jennifer L. Crombie[6], Narendranath Epperla[4], Geoffrey Shouse[7], Sairah Ahmed[3], Paolo Strati[3], Ajay Major[9]

*[1]The University of Texas MD Anderson Cancer Center, Division of Cancer Medicine, Houston, United States of America;[2]The University of Texas MD Anderson Cancer Center, Department of Biostatistics, Houston, United States of America; [3]The University of Texas MD Anderson Cancer Center, Department of Lymphoma & Myeloma, Houston, United States of America; [4]The University of Utah, Division of Hematology and Hematologic Malignancies, Salt Lake City, United States of America; [5]Memorial Sloan Kettering Cancer Center, Medicine, Lymphoma Service, New York City, United States of America; [6]Dana-Farber Cancer Institute, Department of Medical Oncology, Boston, United States of America; [7]City of Hope Comprehensive Cancer Center, Department of Hematology & Hematopoietic Cell Transplantation, Division of Lymphoma, Duarte, United States of America; [8]Vanderbilt University Medical Center, Department of Hematology and Oncology, Nashville, United States of America; [9]University of Colorado, Department of Medicine, Division of Hematology, Aurora, United States of America; [10]University of Nebraska Medical Center, Division of Hematology and Oncology, Omaha, United States of America; [11]Ohio State University Comprehensive Cancer Center, Division of Hematology, Columbus, United States of America;*

### Background

CD3xCD20 bispecific antibodies (BsAb), approved in the third-line or later setting for relapsed/refractory (R/R) large B-cell lymphomas (LBCL), harness CD3+ T cells to target CD20-expressing tumor cells. Thus, quantitative and functional endogenous T-cell impairments may influence their anti-lymphoma efficacy.

### Aims

We hypothesize that pre-treatment absolute lymphocyte counts (ALC) are associated with clinical outcomes in patients receiving BsAb for R/R LBCL, and aimed to identify an ALC threshold associated with superior progression-free survival (PFS).

### Methods

We conducted a multicenter retrospective study of adult patients with R/R LBCL who received commercial glofitamab (glofit) or epcoritamab (epco) between January 2023 and July 2025 at 10 U.S. cancer centers in the Collaborative US Bispecific Consortium (CUBIC). Baseline characteristics, including ALC, were collected prior to BsAb initiation. Cytokine release syndrome (CRS) and immune effector cell-associated neurotoxicity syndrome (ICANS) were graded per ASTCT consensus grading. Treatment response and survival were assessed per Lugano 2014 criteria. The dataset was randomly divided into training (60%) and validation (40%) datasets. An optimal ALC cutoff value for PFS was identified in the training dataset using maximally selected rank statistics and validated using univariate Cox regression with bootstrap resampling. Patients were then stratified by this threshold, and baseline characteristics, response rates (ORR, CR), and toxicities (CRS, ICANS) were compared between ALC groups. Fisher's exact test or Wilcoxon rank-sum test were used for univariate associations of variables, and logistic regression for multivariate analysis.

**Copyright Information:** (Online) ISSN: 2572-9241
©2026 The Author(s). HemaSphere is published by John Wiley & Sons Ltd on behalf of European Hematology Association. This is an open access Abstract Book distributed under the Attribution-NonCommercial-NoDerivs (CC BY-NC-ND), which allows third parties to download the articles and share them with others as long as they credit the author and the Abstract Book, but they cannot change the content in any way or use them commercially.
**Abstract Book Citations:** Authors, Title, HemaSphere, 2026;10:(S1):pages. The abstract book DOIs can be found on the "Author Information" page.
**Disclaimer:** Articles published in the journal HemaSphere exclusively reflect the opinions of the authors. The authors are responsible for all content in their abstracts including accuracy of the facts, statements, citing resources, etc.

**Results**

A total of 317 patients were included in the analysis: 165 (52%) received glofit and 152 (48%) epco. At BsAb initiation, median age was 70 years (range, 22-93), 13% had double-hit LBCL, 69% had an international prognostic index score of ≥3, median prior lines of therapy (LOT) was 3 (range, 1-9), and median ALC was $0.6\times10^3/\mu L$ (IQR, 0.3-1.0; range, $0\text{-}39.1\times10^3/\mu L$). Any-grade CRS and ICANS occurred in 112 (36%) and 37 (12%) of patients, respectively, with grade ≥3 occurring in 13 (4%) and 6 (2%). Overall response rates (ORR) and complete response rates (CR) were 51% and 34%, respectively. After a median follow-up of 12.1 months, median PFS was 3.9 months. The optimal ALC threshold in the training dataset was $0.25\times10^3/\mu L$. In the overall cohort, 260 patients (82%) had high ALC. Compared to the low pre-treatment ALC (<0.25) group, high ALC (≥0.25) predicted improved PFS in the training (HR 0.42, 95% CI 0.25-0.69) and validation cohorts (HR 0.58, 95% CI 0.33-0.97), confirmed by bootstrap resampling (power = 0.59). There were no significant differences in the rates of any-grade CRS/ICANS or grade ≥3 CRS/ICANS between the low and high ALC cohorts. Patients with low ALC experienced significantly lower ORR (33% vs 55%, p=.004) and CR (21% vs 37%, p=.020). Median PFS was 1.5 vs 4.7 months for low vs high ALC (p<.0001).

**Summary/Conclusion**

An optimal ALC threshold of $0.25\times10^3/\mu L$ could identify patients with R/R LBCL who may experience deeper responses and longer PFS with BsAb. Biological analysis, aimed at investigating clinically relevant T-cell subgroups, and prospective validation of these findings are warranted.

**Copyright Information:** (Online) ISSN: 2572-9241
©2026 The Author(s). HemaSphere is published by John Wiley & Sons Ltd on behalf of European Hematology Association. This is an open access Abstract Book distributed under the Attribution-NonCommercial-NoDerivs (CC BY-NC-ND), which allows third parties to download the articles and share them with others as long as they credit the author and the Abstract Book, but they cannot change the content in any way or use them commercially.
**Abstract Book Citations:** Authors, Title, HemaSphere, 2026;10:(S1):pages. The abstract book DOIs can be found on the "Author Information" page.
**Disclaimer:** Articles published in the journal HemaSphere exclusively reflect the opinions of the authors. The authors are responsible for all content in their abstracts including accuracy of the facts, statements, citing resources, etc.

### (PS2088) ORELABRUTINIB, RITUXIMAB, AND THIOTEPA (ORT) IN COMBINATION WITH OR WITHOUT HIGH-DOSE METHOTREXATE IN UNTREATED PRIMARY CENTRAL NERVOUS SYSTEM LYMPHOMA

**Topic:** 19. Large B cell lymphomas - Clinical

Chunmei Yang*[1], Juying Wei[1], Ying Lu[2], Liping Mao[1], Min Yang[1], Huafeng Wang[1], De Zhou[1], Hongyan Tong[1], Jie Jin[1], Wenjuan Yu[1]

*[1]The First Affiliated Hospital, Zhejiang University School of Medicine, Department of Hematology, Hangzhou, China;[2]Yinzhou People's Hospital, Department of Hematology, Ningbo, China;*

### Background

Primary central nervous system lymphoma (PCNSL) is a rare form of non-Hodgkin's lymphoma, predominantly diffuse large B-cell lymphomas. High-dose methotrexate (HD-MTX) is the cornerstone of chemotherapy for newly diagnosed PCNSL. Orelabrutinib is a novel BTKi with high blood-brain barrier permeability and has demonstrated efficacy on PCNSL with minimal off-target effects and higher safety.

### Aims

This study aimed to evaluate the efficacy and safety of a combination regimen consisting of ORT, with or without HD-MTX, in untreated PCNSL patients (pts).

### Methods

This prospective, multicenter, single-arm, study enrolled untreated PCNSL pts (≥18 years) who received ORT therapy: orelabrutinib 150 mg/day orally, rituximab 375 mg/m² intravenously on day 0, and thiotepa 40mg/m² intravenously on day 1, in a 21-day cycle. HD-MTX (3.5g/m² IV) was administered every 3 weeks to pts ≤60 years with normal renal function. After 4 cycles, pts with a response continued the same dose for up to 4 more cycles, with autologous stem cell transplantation (ASCT) recommended for those ≤60 years. Maintenance therapy with orelabrutinib continued until 2 years of treatment. Adverse events (AEs) were graded according to Standard 5.0 Common Terminology for Adverse Events. The primary endpoint was the objective response rate (ORR) with secondary endpoints including progression-free survival (PFS) and overall survival (OS).

**Copyright Information:** (Online) ISSN: 2572-9241
©2026 The Author(s). HemaSphere is published by John Wiley & Sons Ltd on behalf of European Hematology Association. This is an open access Abstract Book distributed under the Attribution-NonCommercial-NoDerivs (CC BY-NC-ND), which allows third parties to download the articles and share them with others as long as they credit the author and the Abstract Book, but they cannot change the content in any way or use them commercially.
**Abstract Book Citations:** Authors, Title, HemaSphere, 2026;10:(S1):pages. The abstract book DOIs can be found on the "Author Information" page.
**Disclaimer:** Articles published in the journal HemaSphere exclusively reflect the opinions of the authors. The authors are responsible for all content in their abstracts including accuracy of the facts, statements, citing resources, etc.

### Results

From August 2022 to June 2025, 39 untreated PCNSL pts were enrolled, with a median age of 61 years (range, 31-88 years). Among them, 53.8% were older than 60 years, and 56.4% were male. Next-generation sequencing (NGS) was performed on 26 pts, revealing that 69.2% (18/26) had MCD-like subtypes, 15.4% (4/26) had TP53 mutations, and 1 patient had the BN2 subtype. 30.8% (12/39) received ORT and 69.2% (27/39) received ORT+HD-MTX. Among the 37 pts who completed all induction therapies, the ORR was 83.8% (31/37) with a CR rate of 78.4% (29/37). Subgroup analysis showed an 83.3% (15/18) complete response rate in MCD subtypes. The median follow-up was 16.3 months, the median PFS was not reached, with 1- and 2-year PFS rates of 85.8% and 73.1%, respectively. The median OS was not reached, with 1- and 2-year OS rates of 96.4% and 92.7%, respectively. In the patient cohort treated with ORT, the ORR was 72.7% (8/11) with a CR rate of 63.6% (7/11). The median PFS was 18.3 months, with 1- and 2-year PFS rates of 81.8% and 43.8%, respectively. In the group receiving ORT+HD-MTX, the ORR was 88.5% (23/26) with a CR rate of 84.6% (22/26). The median PFS was not reached, and the 1- and 2-year PFS rates were both 87.4%. Statistical analysis revealed significant differences in PFS between the two treatment groups (p=0.016). 34.5% (10/29) of CR pts who finished chemotherapy received ASCT. 58.6% (17/29) of CR pts received subsequent orelabrutinib maintenance therapy, with a median maintenance duration of 17.8 months. Among the 39 pts, 79.5% (31/39) experienced grade 1-2 adverse reactions; grade 3-4 adverse reactions occurred in 28.2% (11/39). The most common grade 3-4 adverse events were leukopenia and neutropenia.

### Summary/Conclusion

This study demonstrates that ORT plus HD-MTX can serve as the preferred regimen for patients ≤60 years of age, whereas ORT monotherapy represents a viable alternative for patients >60 years or with renal insufficiency; nevertheless, the rational use of MTX remains a critical determinant for achieving long-term cure in PCNSL.

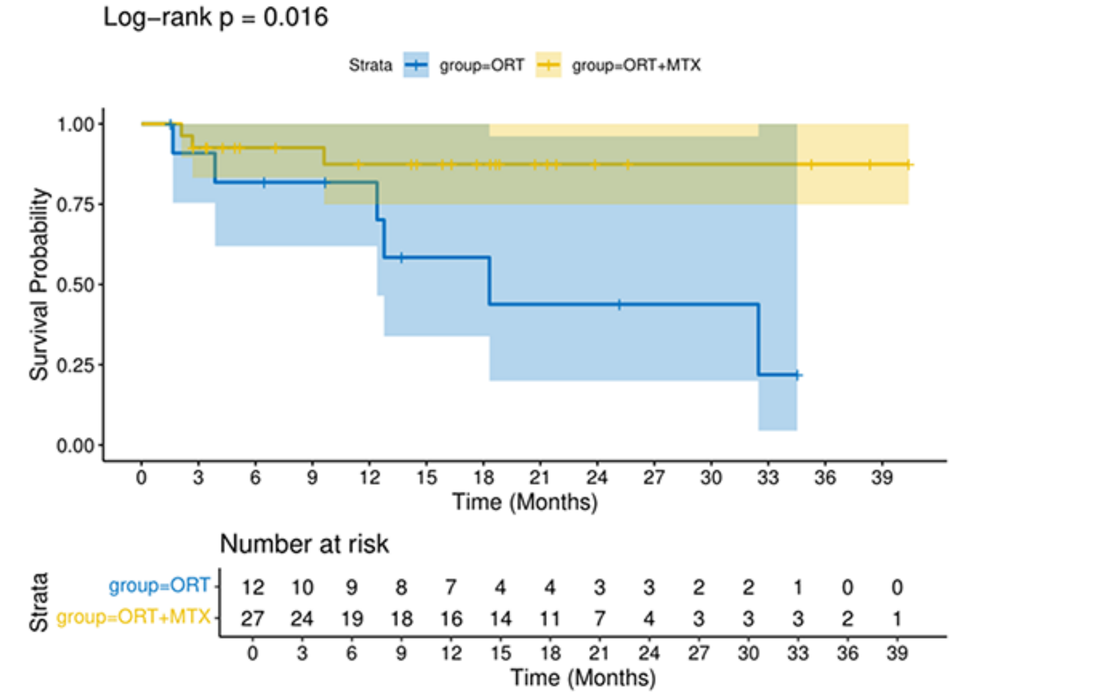

Fig. 1. Progression-free survival (PFS) in patients receiving ORT versus ORT+HD-MTX

Copyright Information: (Online) ISSN: 2572-9241
©2026 The Author(s). HemaSphere is published by John Wiley & Sons Ltd on behalf of European Hematology Association. This is an open access Abstract Book distributed under the Attribution-NonCommercial-NoDerivs (CC BY-NC-ND), which allows third parties to download the articles and share them with others as long as they credit the author and the Abstract Book, but they cannot change the content in any way or use them commercially.
Abstract Book Citations: Authors, Title, HemaSphere, 2026;10:(S1):pages. The abstract book DOIs can be found on the "Author Information" page.
Disclaimer: Articles published in the journal HemaSphere exclusively reflect the opinions of the authors. The authors are responsible for all content in their abstracts including accuracy of the facts, statements, citing resources, etc.

### (PS2089) EFFECTIVENESS AND SAFETY OF CHIMERIC ANTIGEN RECEPTOR T-CELL (CAR-T) THERAPY AFTER TAFASITAMAB (TAFA) IN RELAPSED/REFRACTORY DIFFUSE LARGE B-CELL LYMPHOMA (R/R DLBCL): A REAL-WORLD STUDY

**Topic:** 19. Large B cell lymphomas - Clinical

Narendranath Epperla[1], Kimberly Saverno[2], Irina Pivneva[3], Sakshi Sethi[3], Lucila Sackmann[4], Andrea Zaffalon[4], Julie Wu[2], Mariana Bastos-Oreiro*[5]

*[1]Division of Hematology and Hematologic Malignancies, Huntsman Cancer Institute, University of Utah, Salt Lake City, UT, United States of America; [2]Incyte Corporation, Wilmington, DE, United States of America;[3]Analysis Group, Inc., New York, NY, United States of America; [4]Incyte Biosciences International Sàrl, Morges, Switzerland; [5]Gregorio Marañon Hospital, Health Research Institute of Hospital General Universitario Gregorio Marañón, Hematology Department, Madrid, Spain;*

### Background

The expanding landscape of CD19-directed therapies for DLBCL, including tafa and CAR-T, holds promise for patients (pts). However, there exist theoretical concerns that prior exposure to anti-CD19 therapy may compromise the efficacy of subsequent treatment with CAR-T. Several studies have demonstrated retention of CD19 expression following the use of tafa, with limited evidence on the effectiveness and safety of sequential use of CAR-T post tafa.

### Aims

Assess effectiveness and safety of CAR-T post tafa among adult pts with R/R DLBCL.

### Methods

A retrospective, US and EU panel-based chart review collected data for adults with R/R DLBCL who received tafa (± lenalidomide) and also subsequent CAR-T (index date) in second line (2L)/3L/4L. Pts had to have at least 6 months of follow-up post CAR-T except in the event of death; pts were followed until the earlier of either start of new anticancer therapy after CAR-T, end of data availability, or death. Outcomes were overall response rate to CAR-T (ORR, proportion of pts with physician-assessed complete/partial response [CR/PR]), proportion of pts with cytokine release syndrome (CRS) within 6 weeks, and immune effector cell–associated neurotoxicity syndrome (ICANS) within 3 months after CAR-T. Outcomes were reported overall, by CAR-T line, and by tafa/CAR-T treatment sequence.

**Copyright Information:** (Online) ISSN: 2572-9241
©2026 The Author(s). HemaSphere is published by John Wiley & Sons Ltd on behalf of European Hematology Association. This is an open access Abstract Book distributed under the Attribution-NonCommercial-NoDerivs (CC BY-NC-ND), which allows third parties to download the articles and share them with others as long as they credit the author and the Abstract Book, but they cannot change the content in any way or use them commercially.
**Abstract Book Citations:** Authors, Title, HemaSphere, 2026;10:(S1):pages. The abstract book DOIs can be found on the “Author Information” page.
**Disclaimer:** Articles published in the journal HemaSphere exclusively reflect the opinions of the authors. The authors are responsible for all content in their abstracts including accuracy of the facts, statements, citing resources, etc.

### Results

A total of 338 pt charts (US: 168 [49.7%]; EU: 170 [50.3%]) were abstracted by 145 physicians (US: 73 [50.3%]; EU: 72 [49.7%]). Pts were 66.0% male with median (IQR) age 61.5 (55.1, 67.4) years at CAR-T infusion, 51.2% had double/triple-hit disease, 72.8% were primary refractory, and 53.0% had revised International Prognostic Index score ≥3.

Most pts received axicabtagene ciloleucel (65.1%), followed by lisocabtagene maraleucel (21.9%) and tisagenlecleucel (13.0%) CAR-T. Median (IQR) time from the last tafa dose until CAR-T was 1.2 (0.6-2.3) months; follow-up time post CAR-T was 7.4 (3.3-10.6) months; and 73.1% were still alive at the time of last contact.

Overall, ORR for CAR-T was 81.1%, and grade ≥3 (severe) CRS and ICANS occurred in 7.4% and 3.8% of pts, respectively. ORR by CAR-T line and the observed tafa/CAR-T treatment sequences are reported in the **Table.** CRS and ICANS by CAR-T line are reported in the **Figure**.

**Table. ORR to CAR-T by CAR-T line and treatment sequence**

| | N | ORR, n (%) | CR, n (%) | PR, n (%) |
|---|---|---|---|---|
| **Overall** | **338** | 274 (81.1) | 198 (58.6) | 76 (22.5) |
| **2L CAR-T** (tafa as bridge)* | **37** | **34 (91.9)** | **22 (59.5)** | **12 (32.4)** |
| **3L CAR-T** | **243** | **195 (80.2)** | **146 (60.1)** | **49 (20.2)** |
| 2L tafa | 178 | 142 (79.8) | 112 (62.9) | 30 (16.9) |
| Tafa as bridge | 32 | 27 (84.4) | 16 (50.0) | 11 (34.4) |
| 2L tafa + bridge† | 33 | 26 (78.8) | 18 (54.5) | 8 (24.2) |
| **4L CAR-T** | **58** | **45 (77.6)** | **30 (51.7)** | **15 (25.9)** |
| 2L tafa | 31 | 23 (74.2) | 16 (51.6) | 7 (22.6) |
| 3L tafa | 14 | 13 (92.9) | 8 (57.1) | 5 (35.7) |
| Tafa as bridge | 3 | 1 (33.3) | 0 (0.0) | 1 (33.3) |
| 3L tafa + bridge† | 10 | 8 (80.0) | 6 (60.0) | 2 (20.0) |

*All pts received tafa as bridging therapy to CAR-T.

†Tafa was used both as line of therapy and as bridging therapy to CAR-T.

### Summary/Conclusion

This large, global, real-world study shows that prior exposure to tafa does not appear to adversely affect the effectiveness nor safety of subsequent CAR-T therapy in patients with R/R DLBCL. These results are particularly relevant with recent phase 3 data for tafa in the front-line setting of DLBCL. While these real-world study findings should be interpreted in the context of an observational cohort, they support tafa followed by CAR-T therapy as a feasible and potentially favorable sequencing strategy.

**Copyright Information:** (Online) ISSN: 2572-9241
©2026 The Author(s). HemaSphere is published by John Wiley & Sons Ltd on behalf of European Hematology Association. This is an open access Abstract Book distributed under the Attribution-NonCommercial-NoDerivs (CC BY-NC-ND), which allows third parties to download the articles and share them with others as long as they credit the author and the Abstract Book, but they cannot change the content in any way or use them commercially.
**Abstract Book Citations:** Authors, Title, HemaSphere, 2026;10:(S1):pages. The abstract book DOIs can be found on the "Author Information" page.
**Disclaimer:** Articles published in the journal HemaSphere exclusively reflect the opinions of the authors. The authors are responsible for all content in their abstracts including accuracy of the facts, statements, citing resources, etc.

**Figure. CAR-T safety outcomes among patients who received CAR-T cell therapy post tafasitamab for the treatment of R/R DLBCL, stratified by line of CAR-T cell therapy***

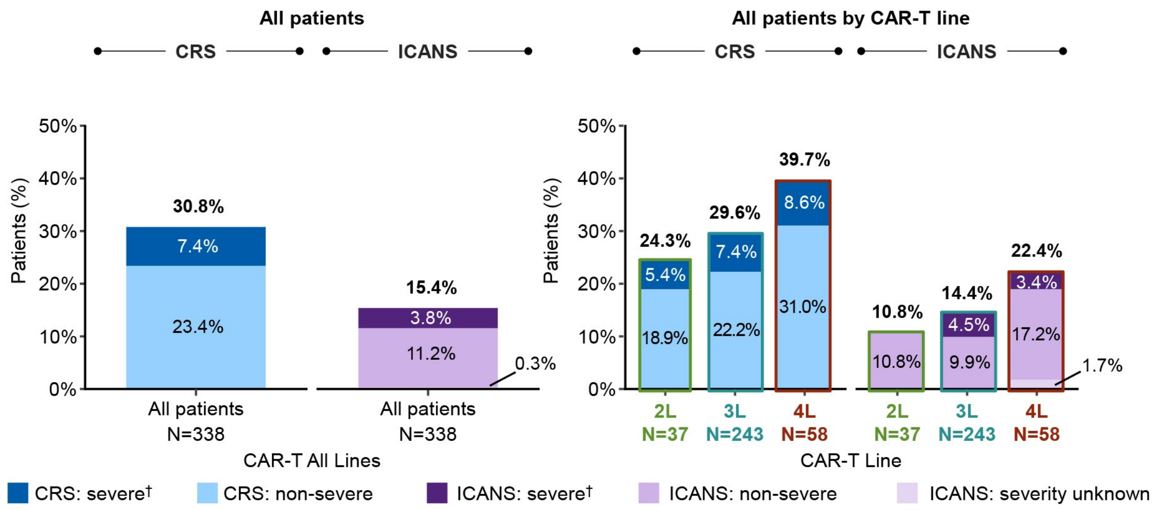

*Safety outcomes stratified by severity may not sum exactly to the reported totals due to rounding.
†Grade ≥3 CRS and ICANS were classified as severe.
2L, second line; 3L, third line; 4L, fourth line; CAR-T, chimeric antigen receptor T-cell; CRS, cytokine release syndrome; DLBCL, diffuse large B-cell lymphoma; ICANS, immune effector cell–associated neurotoxicity syndrome; R/R, relapsed/refractory.

Copyright Information: (Online) ISSN: 2572-9241
©2026 The Author(s). HemaSphere is published by John Wiley & Sons Ltd on behalf of European Hematology Association. This is an open access Abstract Book distributed under the Attribution-NonCommercial-NoDerivs (CC BY-NC-ND), which allows third parties to download the articles and share them with others as long as they credit the author and the Abstract Book, but they cannot change the content in any way or use them commercially.
Abstract Book Citations: Authors, Title, HemaSphere, 2026;10:(S1):pages. The abstract book DOIs can be found on the “Author Information” page.
Disclaimer: Articles published in the journal HemaSphere exclusively reflect the opinions of the authors. The authors are responsible for all content in their abstracts including accuracy of the facts, statements, citing resources, etc.

### (PS2090) ORELABRUTINIB IN PATIENTS WITH RELAPSED OR REFRACTORY PRIMARY OR SECONDARY CENTRAL NERVOUS SYSTEM LYMPHOMA: A MULTICENTER, OPEN-LABEL, PHASE II STUDY

**Topic:** 19. Large B cell lymphomas - Clinical

Lijuan Deng*[1], Song Lin[2], Hui Zhou[3], Wenyu Li[4], Zhiming Li[5], Tong Chen[6], Li Su[7], Kaiyang Ding[8], Yuqin Song[1]

*[1]Peking University Cancer Hospital & Institute, Beijing, China; [2]Beijing Tiantan Hospital, Capital Medical University, Beijing, China; [3]Hunan cancer Hospital, Changsha, Hunan, China; [4]Guangdong Academy of Medical Sciences, Guangdong Provincial People's Hospital, Guangzhou, Guangdong, China; [5]Sun Yat-sen University Cancer Center, Guangzhou, Guangdong, China; [6]Huashan Hospital, Fudan University, Shanghai, China; [7]Xuanwu Hospital Capital Medical University, Beijing, China; [8]the First Affiliated Hospital of USTC, Hefei, China;*

### Background

Patients with relapsed or refractory primary or secondary central nervous system lymphoma (r/r PCNSL/SCNSL) have a poor prognosis and limited treatment options. Orelabrutinib is a highly selective, irreversible Bruton's tyrosine kinase (BTK) inhibitor with promising preclinical blood-brain barrier penetration.

### Aims

Evaluate the antitumor efficacy, safety and pharmacokinetics of orelabrutinib in r/r PCNSL/SCNSL patients.

### Methods

This was a single-arm, multicenter, open-label, phase II trial (Clinical Trial Registration No.: CTR20190854) conducted at 8 sites in China. Employing a Simon's two-stage design, 16 patients were initially enrolled to receive orelabrutinib 150 mg once daily (QD), with ≥4 responders required to proceed to stage two. After meeting the pre-specified efficacy threshold, the study proceeded to stage two, expanding to a total of 39 patients at 150 mg QD and adding 22 patients at an exploratory dose of 200 mg QD. The primary endpoint was objective response rate (ORR) assessed by investigators per International Primary CNS Lymphoma Collaborative Group (IPCG) criteria. Secondary endpoints included disease control rate (DCR), duration of response (DOR), progression-free survival (PFS), overall survival (OS), safety, and pharmacokinetics (PK).

**Copyright Information:** (Online) ISSN: 2572-9241
©2026 The Author(s). HemaSphere is published by John Wiley & Sons Ltd on behalf of European Hematology Association. This is an open access Abstract Book distributed under the Attribution-NonCommercial-NoDerivs (CC BY-NC-ND), which allows third parties to download the articles and share them with others as long as they credit the author and the Abstract Book, but they cannot change the content in any way or use them commercially.
**Abstract Book Citations:** Authors, Title, HemaSphere, 2026;10:(S1):pages. The abstract book DOIs can be found on the “Author Information” page.
**Disclaimer:** Articles published in the journal HemaSphere exclusively reflect the opinions of the authors. The authors are responsible for all content in their abstracts including accuracy of the facts, statements, citing resources, etc.

**Results**

61 patients were enrolled and constituted the full analysis set, representing a heavily pretreated, poor-prognosis population. Notably, 50.8% of the subjects had received two or more lines of therapy. The detailed baseline characteristics are presented in Table 1.
At data cutoff, with a median follow-up of 7.3 months (range, 1.1-21.9 months), the ORR was 28.2% (95% CI: 15.0–44.9) in the 150 mg group and 27.3% (10.7–50.2) in the 200 mg group. The DCR was 53.8% (37.2–69.9) and 54.5% (32.2–75.6), respectively. Median PFS was 2.9 months (95% CI: 1.9–3.6) for the 150 mg group and 2.9 months (1.8–3.7) for the 200 mg group. Importantly, the observed PFS outcomes were comparable to those reported with tirabrutinib (median PFS 2.9 months), a BTK inhibitor approved in Japan for this indication. Median OS was 16.9 months (7.1–not reached) for the 150 mg group and not reached for the 200 mg group.
Treatment-emergent adverse events (TEAEs) occurred in 72.1% of patients, predominantly grades 1-2. Grade≥3 TEAEs and serious TEAEs occurred in 24.6% and 14.8% of patients, respectively. The most common TEAEs (≥10%) were Leukopenia (21.3%), neutropenia (16.4%), and increased alanine aminotransferase (14.8%). PK analysis in a subset of patients (n=7) receiving 150 mg QD showed a mean cerebrospinal fluid (CSF) concentration of 31.3 ng/mL at 2 hours post-dose, with a CSF-to-unbound plasma concentration ratio of approximately 47.7%, indicating favorable blood-brain barrier penetration.
Table 1. Patient demographics and baseline characteristics

| Characteristics | 150mg QD(N=39) | 200mg QD (N=22) | ALL(N=61) |
|---|---|---|---|
| Median age(Q1, Q3),years | 56.9(50.9,66.0) | 60.4(57.6,66.7) | 57.9(52.5,66.0) |
| Male,n(%) | 20(51.3) | 13(59.1) | 33(54.1) |
| ECOG,n(%) | | | |
| 0-1 | 21(53.8) | 12(54.5) | 33(54.1) |
| ≥2 | 18(46.2) | 10(45.5) | 28(45.9) |
| R/R PCNSL,n(%) | 34(87.2) | 22(100.0) | 56(91.8) |
| R/R SCNSL,n(%) | 5(12.8) | 0 | 5(8.2) |
| Prior therapy,n(%) | | | |
| Radiotherapy | 9(23.1) | 6(27.3) | 15(24.6) |
| Surgical treatment for cancer | 38(97.4) | 18(81.8) | 56(91.8) |
| HCT-ASCT | 1(2.6) | 0 | 1(1.6) |

**Copyright Information:** (Online) ISSN: 2572-9241
©2026 The Author(s). HemaSphere is published by John Wiley & Sons Ltd on behalf of European Hematology Association. This is an open access Abstract Book distributed under the Attribution-NonCommercial-NoDerivs (CC BY-NC-ND), which allows third parties to download the articles and share them with others as long as they credit the author and the Abstract Book, but they cannot change the content in any way or use them commercially.
**Abstract Book Citations:** Authors, Title, HemaSphere, 2026;10:(S1):pages. The abstract book DOIs can be found on the “Author Information” page.
**Disclaimer:** Articles published in the journal HemaSphere exclusively reflect the opinions of the authors. The authors are responsible for all content in their abstracts including accuracy of the facts, statements, citing resources, etc.

**Summary/Conclusion**

Orelabrutinib monotherapy demonstrated preliminary antitumor activity and a manageable safety profile in patients with r/r PCNSL/SCNSL, a population with high unmet medical need. Its favorable blood-brain barrier penetration supports further investigation in CNS lymphomas.

**Copyright Information:** (Online) ISSN: 2572-9241
©2026 The Author(s). HemaSphere is published by John Wiley & Sons Ltd on behalf of European Hematology Association. This is an open access Abstract Book distributed under the Attribution-NonCommercial-NoDerivs (CC BY-NC-ND), which allows third parties to download the articles and share them with others as long as they credit the author and the Abstract Book, but they cannot change the content in any way or use them commercially.
**Abstract Book Citations:** Authors, Title, HemaSphere, 2026;10:(S1):pages. The abstract book DOIs can be found on the "Author Information" page.
**Disclaimer:** Articles published in the journal HemaSphere exclusively reflect the opinions of the authors. The authors are responsible for all content in their abstracts including accuracy of the facts, statements, citing resources, etc.

### (PS2091) REAL-WORLD EFFICACY AND SAFETY OF POLATUZUMAB VEDOTIN WITH R-CHP IN PREVIOUSLY UNTREATED DIFFUSE LARGE B-CELL LYMPHOMA: A UK MULTI-CENTRE STUDY

**Topic:** 19. Large B cell lymphomas - Clinical

James Paterson*[1], Amy Kirkwood[2], Girgis Girgis[3], Vicky Calvert[4], Bernard Maybury[5], Victoria Kerr[1], Wendy Osborne[4], Anna Santarsieri[6], Imran Siddiqui[7], Aisling Barrett[8], Toby A. Eyre[9], Samuel Wiltshire[10], David Burns[10], Elizabeth Nelmes[11], Rebecca Auer[11], Oscar Atkins[12], Lucy Cook[13], Tatianna Fernandes[5], Rory McCulloch[14], Mahina Baloch[14], Kevin Joyce[15], Dima El-Sharkawi[3], Jeff Smith[15], Adrian Choy[1], Sajir Mohamedbhai[16], Ramsha Nabeel[17], Aparna Chitharanjan[17], Andrea Kuhnl[17], Alberto Schena[18], Amal Singh[3], Claire Burney[19], Christopher Fox[18], William Townsend[1]

*[1]University College Hospital London, Haematology, London, United Kingdom; [2]CR UK & UCL Cancer Trials Centre, London, United Kingdom; [3]Royal Marsden Hospital, london, United Kingdom; [4]The Newcastle upon Tyne Hospitals NHS Foundation Trust and Newcastle University, Newcastle, United Kingdom; [5]University Hospitals Birmingham NHS Foundation Trust, Haematology, Birmingham, United Kingdom; [6]Addenbrooke's Hospital, Cambridge, United Kingdom; [7]CAMBRIDGE UNIVERSITY HOSPITALS NHS FOUNDATION TRUST, CAMBRIDGE, United Kingdom; [8]University of Oxford, Oxford, United Kingdom; [9]Oxford University Hospitals, Oxford, United Kingdom; [10]UNIVERSITY HOSPITALS PLYMOUTH NHS TRUST, Plymouth, United Kingdom; [11]Barts Health NHS Trust St Bartholomew's Hospital, London, United Kingdom; [12]LONDON NORTH WEST UNIVERSITY HEALTHCARE NHS TRUST, London, United Kingdom; [13]Imperial College London, London, United Kingdom; [14]Gloucestershire Hospitals NHS Foundation Trust, Gloucestershire, United Kingdom; [15]THE CLATTERBRIDGE CANCER CENTRE NHS FOUNDATION TRUST, Liverpool, United Kingdom; [16]North Middlessex University Hospital, london, United Kingdom; [17]Kings College Hospital, London, United Kingdom; [18]NOTTINGHAM UNIVERSITY HOSPITALS NHS TRUST, Nottingham, United Kingdom; [19]University Hospitals Bristol and Weston NHS Foundation Trust, Bristol, United Kingdom;*

### Background

The Phase III POLARIX trial established Polatuzumab Vedotin plus R-CHP (Pola-R-CHP) as a new standard for frontline diffuse large B-cell lymphoma (DLBCL) with IPI 2-5.[1] However, real-world data (RWD) are essential to confirm these findings in broader patient demographics, including those with significant comorbidities and high-risk features often excluded from clinical trials.

### Aims

To assess the real-world efficacy (ORR, CRR) and safety profile of Pola-R-CHP in a large, multicentre UK cohort, including specific sub-group analyses with regards to age, Charlson Comorbidity Index (CCI), cell of origin (COO), transformed disease and HIV status.

### Methods

This retrospective study included 437 adults with previously untreated DLBCL receiving Pola-R-CHP across multiple UK centres (2023–2025). Data were collected on baseline characteristics, treatment compliance, best response (PET-CT), and key toxicities. Survival analysis, including progression-free survival (PFS) and overall survival (OS), is ongoing.

**Copyright Information:** (Online) ISSN: 2572-9241
©2026 The Author(s). HemaSphere is published by John Wiley & Sons Ltd on behalf of European Hematology Association. This is an open access Abstract Book distributed under the Attribution-NonCommercial-NoDerivs (CC BY-NC-ND), which allows third parties to download the articles and share them with others as long as they credit the author and the Abstract Book, but they cannot change the content in any way or use them commercially.
**Abstract Book Citations:** Authors, Title, HemaSphere, 2026;10:(S1):pages. The abstract book DOIs can be found on the "Author Information" page.
**Disclaimer:** Articles published in the journal HemaSphere exclusively reflect the opinions of the authors. The authors are responsible for all content in their abstracts including accuracy of the facts, statements, citing resources, etc.

### Results

The median age was 66 years (range 17–87), with 70.2% of patients aged ≥60 years. The cohort had high-risk features including: 67.2% had stage IV disease, 65.6% had an IPI score ≥3, 11.5% double/triple-hit biology,15.0% transformed disease and 21.3% were ECOG ≥2. The median CCI was 4 (IQR 3-5).

- **Efficacy:** The overall response rate (ORR) was 93.1%, with a complete response rate (CRR) of 79.1%. Efficacy was consistent across high-risk subgroups. ORR remained high regardless of COO (GCB 92.9% vs. non-GCB 92.5%, p>0.99) or patients with transformations of low-grade lymphoma (96.9% vs. 92.4% de novo, p=0.63). There was no significant difference in CRR between patients with CCI<5 vs. ≥5 (79.2% vs. 79.5%, p>0.99) or age <70 vs. ≥70 (77.2% vs. 82.1%, p=0.27).
- **Compliance:** 83.3% of patients completed all 6 cycles. For those that did not complete all 6 cycles reasons for stopping early included: adverse events (35.7%), death (25.0%), and progressive disease (23.2%). Cycle 1 dose reductions were significantly more frequent in patients ≥70 years (13.6% vs 5.9%, p=0.011) and those with CCI ≥5 were significantly less likely to complete 6 cycles (76.2% vs 89.6%, p<0.001).
- **Safety:** Grade ≥3 infections occurred in 35.8% of patients, with significantly higher rates in those patients ≥70 years (42.4% vs 31.9%, p=0.035). Febrile neutropenia (FN) was reported in 29.6%. Diarrhoea occurred in 48.0% (Grade 3-4: 7.7%). Peripheral neuropathy (PN) was reported in 32.1% (grade 3: 3.0%). Opportunistic infections occurred in 10.8%, including PCP (N=8) and shingles (N=5).

### Summary/Conclusion

In this large real-world UK cohort, Pola-R-CHP demonstrated efficacy and deliverability. The CR rate in this series appears comparable to the trial despite being used in a cohort of patients with high-risk features and a broader range of indications than were included in the POLARIX trial. These data support Pola-R-CHP as a first line standard of care in the real-world setting, however the higher rates of grade ≥3 infections and reduced treatment intensity in patients with advanced age and comorbidities demonstrates a need for vigilant supportive care in this demographic. Survival outcomes will be presented.

**References:**

1. Tilly H, et al. Polatuzumab Vedotin in Previously Untreated Diffuse Large B-Cell Lymphoma. N Engl J Med 2022; 386:351-363. DOI: 10.1056/NEJMoa2115304

**Copyright Information:** (Online) ISSN: 2572-9241
©2026 The Author(s). HemaSphere is published by John Wiley & Sons Ltd on behalf of European Hematology Association. This is an open access Abstract Book distributed under the Attribution-NonCommercial-NoDerivs (CC BY-NC-ND), which allows third parties to download the articles and share them with others as long as they credit the author and the Abstract Book, but they cannot change the content in any way or use them commercially.
**Abstract Book Citations:** Authors, Title, HemaSphere, 2026;10:(S1):pages. The abstract book DOIs can be found on the “Author Information” page.
**Disclaimer:** Articles published in the journal HemaSphere exclusively reflect the opinions of the authors. The authors are responsible for all content in their abstracts including accuracy of the facts, statements, citing resources, etc.

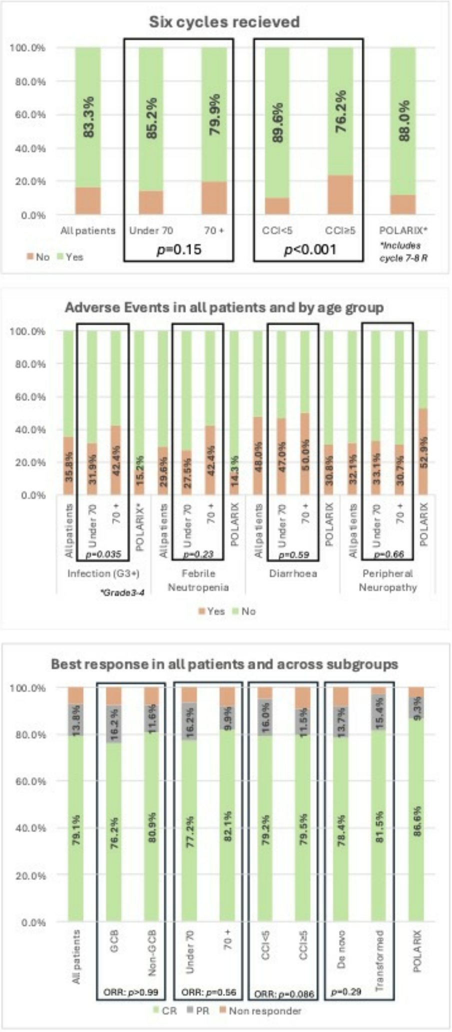

Figure 1 Legend This figure displays the comparative outcomes for previously untreated DLBCL patients (N=437). Panel A shows Overall Response Rate (ORR) consistency across cell of origin ($p > 0.99$) and transformation status ($p = 0.56$). Panel B highlights treatment compliance, noting a significant decrease in 6-cycle completion for patients with a Charlson Comorbidity Index (CCI) ≥5 ($p < 0.001$). Panel C details Grade ≥3 toxicities, showing a significantly higher infection rate in patients aged ≥ 70 years ($p = 0.035$).

Copyright Information: (Online) ISSN: 2572-9241
©2026 The Author(s). HemaSphere is published by John Wiley & Sons Ltd on behalf of European Hematology Association. This is an open access Abstract Book distributed under the Attribution-NonCommercial-NoDerivs (CC BY-NC-ND), which allows third parties to download the articles and share them with others as long as they credit the author and the Abstract Book, but they cannot change the content in any way or use them commercially.
Abstract Book Citations: Authors, Title, HemaSphere, 2026;10:(S1):pages. The abstract book DOIs can be found on the "Author Information" page.
Disclaimer: Articles published in the journal HemaSphere exclusively reflect the opinions of the authors. The authors are responsible for all content in their abstracts including accuracy of the facts, statements, citing resources, etc.

### (PS2092) GLOFITAMAB MONOTHERAPY OR COMBINATION THERAPY IN PATIENTS WITH RELAPSED/REFRACTORY PRIMARY OR SECONDARY CENTRAL NERVOUS SYSTEM LYMPHOMA: A MULTICENTER RETROSPECTIVE STUDY

**Topic:** 19. Large B cell lymphomas - Clinical

Ruixian Xing[1], Apeng Yang[2], Juan Huang[3], Liang Wang[4], Wei Guo[5], Qu Cui[1], Xiaoqiong Tang[6], Yuanyuan MA[7], Kai Hu[8], Tianheng He[9], Danyang Wu[10], Xiaofang Deng[11], Yajing Zhang[8], Kaili Zhong[12], Yang Liu[13], Yixian Guo[14], Ying Wang[15], Chan Long[16], Chongyang Wu[17], Xin Lu[18], Fengmin Li[19], Jie-Fei Bai[20], Jun Qian[1], Xuefei Sun[1], Wenrong Huang[21], Hui Zhou[22], Hui-Lai Zhang[23], Ou Bai[24], Xiaobing Huang[3], Zhiyong Zeng[25], Lin Fu*[1]

*[1]Beijing Tiantan Hospital, Capital Medical University, Department of Hematology, Beijing, China;[2]the First Affiliated Hospital of Fujian Medical University, Fuzhou, China; [3]Sichuan Academy of Medical Sciences and Sichuan Provincial People's Hospital, University of Electronic Science and Technology of China, Chengdu, China; [4]Beijing Tongren Hospital, Capital Medical University, Beijing, China; [5]The First Hospital of Jilin University, Changchun, China;[6]The First Affiliated Hospital of Chongqing Medical University, Chongqing, China; [7]The Fifth Medical Center of People's Liberation Army(PLA), General Hospital, Beijing, China; [8]Beijing GoBroad Boren Hospital, Beijing, China; [9]Peking University International Hospital, Beijing, China; [10]First Affiliated Hospital of China Medical University, Shenyang, China; [11]the Affiliated Cancer Hospital, Guangzhou Medical University, Guangzhou, China; [12]Beijing Shijitan Hospital, Capital Medical University, Beijing, China;[13]the Fifth Medical Center, Chinese PLA General Hospital., Beijing, China; [14]Xuanwu Hospital, Capital Medical University, Beijing, China;[15]The Affiliated Hospital of Xuzhou Medical University, Xuzhou, China; [16]Huizhou First Hospital, Huizhou, China; [17]Lanzhou University Second Hospital, Lanzhou, China; [18]Xingtai People's Hospital, Xingtai, China; [19]the First Hospital of QinHuangdao, QinHuangdao, China;[20]Beijing Hospital, National Center of Gerontology, Institute of Geriatric Medicine, Chinese Academy of Medical Sciences, Beijing, China; [21]The fifth Medical Center,Chinese PLA General Hospital, Beijing, China;[22]Hunan Cancer Hospital, The Affiliated Cancer Hospital of Xiangya School of Medicine, Central South University, Changsha, China; [23]Tianjin Medical University Cancer Institute and Hospital, Tianjin, China; [24]The first hospital of Jilin University, Changchun, China;[25]The First Affiliated Hospital of Fujian Medical University, Fuzhou, China;*

### Background

Patients with relapsed or refractory primary or secondary central nervous system lymphoma (R/R PCNSL/SCNSL) have a dismal prognosis with limited subsequent treatment options. The efficacy and safety profiles of bispecific antibody therapy in this population remain ambiguous.

### Aims

To evaluate the efficacy and safety of glofitamab-based therapy in patients with relapsed/refractory central nervous system lymphoma (R/R CNSL).

**Copyright Information:** (Online) ISSN: 2572-9241
©2026 The Author(s). HemaSphere is published by John Wiley & Sons Ltd on behalf of European Hematology Association. This is an open access Abstract Book distributed under the Attribution-NonCommercial-NoDerivs (CC BY-NC-ND), which allows third parties to download the articles and share them with others as long as they credit the author and the Abstract Book, but they cannot change the content in any way or use them commercially.
**Abstract Book Citations:** Authors, Title, HemaSphere, 2026;10:(S1):pages. The abstract book DOIs can be found on the "Author Information" page.
**Disclaimer:** Articles published in the journal HemaSphere exclusively reflect the opinions of the authors. The authors are responsible for all content in their abstracts including accuracy of the facts, statements, citing resources, etc.

### Methods

R/R CNSL patients who received at least one dose of glofitamab across 24 Chinese medical facilities were investigated. All patients had histologically confirmed diffuse large B-cell lymphoma with documented CNS involvement and were heavily pretreated by chemotherapy, whole brain radiotherapy, or CAR-T therapy. After providing informed consent, patients received obinutuzumab (1000 mg on cycle 1 day 1) as premedication, followed by glofitamab step-up dosing: 2.5 mg on cycle 1 day 8, 10 mg on cycle 1 day 15, and 30 mg on cycle 2 day 1, then 30 mg every 3 weeks thereafter for up to 12 cycles, with or without combination agents at the physician's discretion. CNS response was assessed by IPCG criteria using contrast-enhanced brain/spine MRI and CSF analysis. For SCNSL, systemic disease was evaluated by whole-body PET-CT scan per Lugano 2014. The primary endpoint was the objective response rate (ORR), defined as the proportion of patients who achieved a confirmed best overall response (BOR) of complete response (CR) or partial response (PR). Secondary endpoints included complete response rate (CRR), progression-free survival (PFS), overall survival (OS), and safety profiles (incidences of CRS and ICANS).

### Results

In this multicenter retrospective cohort study, 96 consecutive adult patients treated with glofitamab-based therapy were enrolled; the median age was 59 years (IQR 54-68), with a median of 2.5 prior lines of therapy. Primary CNS lymphoma was present in 60 patients (62.5%) and secondary CNS involvement in 36 patients (37.5%), with elevated serum LDH in 36 patients (37.5%). Glofitamab was administered as monotherapy in 38 patients (39.6%), in combination with other therapies in 58 patients (60.4%). The median number of glofitamab cycles was 6 (IQR 3-7) and an objective response rate (ORR) 91.8% (95% CI, 84.0%-96.8%) was achieved with a complete response rate (CRR) of 66.6% (95% CI, 55.8%-75.8%). At a median follow-up of 8.2 months (95% CI, 7.0-9.6), median progression-free survival was 15.8 months (95% CI, 12.8-not reached). The 6-month and 12-month PFS rates were 78.8% (95% CI, 71.0%-88.7%) and 62.0% (95% CI, 50.2%-77.0%), respectively. Cytokine release syndrome occurred in 40.6% of patients (all grade 1-2), and ICANS in 8%. Further analysis showed elevated baseline serum LDH was independently associated with shorter PFS (HR 2.24, 95% CI 1.04-4.84; p=0.04), while combination therapy was associated with a higher likelihood of achieving CR (OR 0.38, 95% CI 0.11-0.98; p=0.04, monotherapy as reference).

### Summary/Conclusion

These findings demonstrate that glofitamab-based therapy yields substantial antitumor activity with a manageable safety profile in heavily treatment-experienced R/R CNSL patients, indicating a potentially promising treatment paradigm.

**Copyright Information:** (Online) ISSN: 2572-9241
©2026 The Author(s). HemaSphere is published by John Wiley & Sons Ltd on behalf of European Hematology Association. This is an open access Abstract Book distributed under the Attribution-NonCommercial-NoDerivs (CC BY-NC-ND), which allows third parties to download the articles and share them with others as long as they credit the author and the Abstract Book, but they cannot change the content in any way or use them commercially.
**Abstract Book Citations:** Authors, Title, HemaSphere, 2026;10:(S1):pages. The abstract book DOIs can be found on the "Author Information" page.
**Disclaimer:** Articles published in the journal HemaSphere exclusively reflect the opinions of the authors. The authors are responsible for all content in their abstracts including accuracy of the facts, statements, citing resources, etc.

### (PS2093) FIRST-LINE SELINEXOR IN COMBINATION WITH R-CHOP FOR EBV-POSITIVE DIFFUSE LARGE B-CELL LYMPHOMA (XPLORE): A SINGLE-ARM, PHASE IB/II TRIAL

**Topic:** 19. Large B cell lymphomas - Clinical

Qingqing Cai*[1], Yi Xia[1], Huiqiang Huang[1], Rong Tao[2], Chuanxu Liu[2], Wen-Hao Zhang[2], Qike Zhang[3], Yin Qingsong[4], Runhui Zheng[5], Jianbo Liu[5], Qingfang Zeng[6], Xiaoxiao Wang[1], Xiaojie Fang[1], Bing Bai[1], Man Nie[1], Yuchen Zhang[1], Qihua Zou[1], Shenrui Bai[1]

*[1]Sun Yat-sen University Cancer Center, Guangzhou, China; [2]Fudan University Shanghai Cancer Center, Shanghai, China; [3]Gansu Provincial Hospital, Department of Hematology, Lanzhou, China; [4]The Affiliated Cancer Hospital of Zhengzhou University and Henan Cancer Hospital, Zhengzhou, China; [5]The Fifth Affiliated Hospital of Guangzhou Medical University, Guangzhou, China; [6]Ganzhou Cancer Hospital, Ganzhou, China;*

### Background

Diffuse large B-cell lymphoma (DLBCL) is genetically and clinically heterogeneous. Epstein–Barr virus (EBV)–positive DLBCL accounts for ~8% of cases and is associated with inferior outcomes. Selinexor, an oral selective inhibitor of nuclear export (XPO1), has demonstrated activity in non-Hodgkin lymphoma and showed encouraging efficacy when combined with R-CHOP in early-phase clinical studies. This multicenter, single-arm phase Ib/II study (NCT05577364) aimed to assess the efficacy and safety of selinexor plus R-CHOP as frontline therapy in patients with EBV-positive DLBCL.

### Aims

To evaluate the safety and effectiveness of selinexor in combination with R-CHOP as first-line treatment in patients with EBV-positive DLBCL.

### Methods

Eligible patients were aged 18–80 years with pathologically confirmed DLBCL and EBV positivity defined by EBER in situ hybridization. Previously untreated patients with EBV-positive DLBCL received standard-dose R-CHOP every 21 days for up to six cycles, with selinexor initiated from cycle 2. The phase Ib portion followed a standard 3+3 dose-escalation design: selinexor was administered at an initial dose of 40 mg once weekly during cycle 2 and increased to 60 mg once weekly from cycle 3 onward in the absence of dose-limiting toxicities (DLTs). Following induction, patients received two consolidation cycles of rituximab plus selinexor. Patients achieving complete response (CR) after induction therapy proceeded to selinexor maintenance, with the overall treatment duration up to 2 years. Primary endpoints were the maximum tolerated dose (MTD) and recommended phase II dose (RP2D) for phase Ib, and the CR rate for phase II.

**Copyright Information:** (Online) ISSN: 2572-9241
©2026 The Author(s). HemaSphere is published by John Wiley & Sons Ltd on behalf of European Hematology Association. This is an open access Abstract Book distributed under the Attribution-NonCommercial-NoDerivs (CC BY-NC-ND), which allows third parties to download the articles and share them with others as long as they credit the author and the Abstract Book, but they cannot change the content in any way or use them commercially.
**Abstract Book Citations:** Authors, Title, HemaSphere, 2026;10:(S1):pages. The abstract book DOIs can be found on the "Author Information" page.
**Disclaimer:** Articles published in the journal HemaSphere exclusively reflect the opinions of the authors. The authors are responsible for all content in their abstracts including accuracy of the facts, statements, citing resources, etc.

### Results

As of Jan 1, 2026, 27 patients were enrolled. The median age was 58.0 years, and 19 patients (70.4%) had Ann Arbor stage III–IV disease. Plasma EBV-DNA was detectable at baseline in 21 patients (77.8%). With a median follow-up of 8.1 months (range, 7.0–14.8), 13 patients (48.1%) completed the planned 8 cycles of combination treatment (range, 2–8 cycles). In phase Ib, the RP2D of selinexor was determined as 60 mg once weekly. Among 25 response-evaluable patients, the best CR rate was 72.0% (18/25), and the best overall response rate (ORR) was 96.0% (24/25). Treatment-related adverse events (TRAEs) of any grade including leukopenia (92.5%), neutropenia (92.5%), thrombocytopenia (74.0%), anemia (77.8%), and nausea (55.6%). The most frequent grade 3–4 TRAEs were leukopenia (77.8%) and neutropenia (74.0%). TRAE-related treatment interruption occurred in 8 patients (29.6%). No treatment-related deaths were observed.

**Table 1. Baseline characteristics (A)and safety (B)in patients with EBV-positive DLBCL treated with selinexor in combination with R-CHOP**

**(A)**

| **Characteristics** | **n (%)** |
|---|---|
| **Age, years** (median [IQR]) | 58 (52-62) |
| **Sex** | |
| Male | 15 (55.5) |
| Female | 12 (44.4) |
| **IPI score** | |
| 0-2 | 22 (81.5) |
| ≥3 | 5 (18.5) |
| **Ann Arbor Stage** | |
| I-II | 8 (29.6) |
| III-IV | 19 (70.3) |
| **Plasma EBV-DNA** | |
| Undetectable | 6 (22.2) |
| Detectable | 21 (77.8) |

**(B)**

| **TRAEs,** | **Any grade** | **Grade 3-4** |
|---|---|---|
| **Leukopenia** | 25 (92.6) | 21 (77.8) |
| **Neutropenia** | 25 (92.6) | 20 (74.0) |
| **Thrombocytopenia** | 20 (74.0) | 14 (51.9) |
| **Anemia** | 20 (74.0) | 5 (18.5) |
| **Nausea** | 15 (55.6) | 5 (18.5) |
| **Infection** | 8 (29.6) | 5 (18.5) |

Data are shown as number (%).

### Summary/Conclusion

Frontline selinexor plus R-CHOP showed promising antitumor activity with a manageable safety profile in patients with EBV-positive DLBCL. Continued monitoring and optimized management of selinexor-related toxicities are warranted, and patient enrollment is ongoing to further validate these findings.

**Copyright Information:** (Online) ISSN: 2572-9241
©2026 The Author(s). HemaSphere is published by John Wiley & Sons Ltd on behalf of European Hematology Association. This is an open access Abstract Book distributed under the Attribution-NonCommercial-NoDerivs (CC BY-NC-ND), which allows third parties to download the articles and share them with others as long as they credit the author and the Abstract Book, but they cannot change the content in any way or use them commercially.
**Abstract Book Citations:** Authors, Title, HemaSphere, 2026;10:(S1):pages. The abstract book DOIs can be found on the "Author Information" page.
**Disclaimer:** Articles published in the journal HemaSphere exclusively reflect the opinions of the authors. The authors are responsible for all content in their abstracts including accuracy of the facts, statements, citing resources, etc.

### (PS2094) HIGH DOSES OF METHOTREXATE AND CYTARABINE WITH OR WITHOUT INTRAVITREAL METHOTREXATE INJECTIONS IN A COHORT OF 31 HIV-NEGATIVE PATIENTS WITH PRIMARY VITREORETINAL LYMPHOMA

**Topic:** 19. Large B cell lymphomas - Clinical

Teresa Calimeri*[1], Alberto Bavieri[2, 3], Luca Saliani[1, 4], Fabrizio Marino[1], Fabrizio Gozzi[5], Fiorella Ilariucci[2], Federico Rissotto[6], Gilda Magliacane[7], Paolo Fiore[1, 4], Fanny Erika Palumbo[8], Federico Erbella[1], Elena Flospergher[1], Piera Angelillo[1], Giulio Cassanello[1], Tanja Lazic[2, 3], Silvia Gambara[2, 3], Rita Daverio[9], Davide Nicoli[10], Enrico Farnetti[10], Patrizia Ciammella[11], Anna Chiara[12], Sara Mastaglio[13], Giuseppina D'elia[1], Riccardo Valli[10], Maria Giulia Cangi[7], Elisabetta Miserocchi[4, 6], Francesco Merli[2], Maurilio Ponzoni[4, 7], Luca Cimino[5, 14], Stefano Luminari[2, 14], Andrés José María Ferreri[1, 4]

*[1]Strategic Program on Lymphomas, IRCCS San Raffaele Scientific Institute, Milan, Italy; [2]Hematology Unit, Azienda USL-IRCCS Reggio Emilia, Reggio Emilia, Italy; [3]Clinical and Experimental Medicine PhD Program, University of Modena and Reggio Emilia, Modena, Italy; [4]University Vita-Salute San Raffaele, Milan, Italy; [5]Ocular-Immunology Unit, Azienda USL-IRCCS Reggio Emilia, Reggio Emilia, Italy; [6]Ophthalmology Unit, IRCCS San Raffaele Scientific Institute, Milan, Italy; [7]Pathology Unit, IRCCS San Raffaele Scientific Institute, Milan, Italy; [8]Division of Hematology, A.O.U. Policlinico "G.Rodolico-S. Marco", Catania, Italy;[9]Division of Lab Medicine, IRCCS San Raffaele Scientific Institute, Milan, Italy; [10]Pathology Unit, Azienda USL-IRCCS Reggio Emilia, Reggio Emilia, Italy; [11]Radiation Oncology Unit, Azienda USL-IRCCS Reggio Emilia, Reggio Emilia, Italy;[12]Radiotherapy and Tomotherapy Unit, IRCCS San Raffaele Scientific Institute, Milan, Italy; [13]Hematology and Bone Marrow Transplantation Unit, IRCCS San Raffaele Scientific Institute, Milan, Italy; [14]CHIMONO Department, University of Modena and Reggio Emilia, Reggio Emilia, Italy;*

### Background

Primary vitreoretinal lymphoma (PVRL) is a rare large B-cell lymphoma involving the vitreous and/or retina, with a high risk of CNS dissemination. Optimal management remains undefined: most reported series have been managed with local treatments (intravitreal methotrexate [IVT-MTX] and ocular radiotherapy [ORT]), which offer limited CNS protection, while the benefit of intensified systemic regimens adapted from PCNSL is still uncertain.

### Aims

To evaluate efficacy and tolerability of high-dose methotrexate–cytarabine (MA)–based chemotherapy ± IVT-MTX in PVRL patients (pts) treated in two referral Italian centres.

### Methods

HIV-negative pts with ocular symptom-driven referral treated between 2015 and 2025 were included based on: 1) diagnosis of PVRL via ophthalmologic assessment, with confirmation by conventional cytology and/or biomarkers (e.g., MYD88$^{L265P}$, elevated IL-10, clonal IGH) in ocular fluids; 2) MA-based first-line treatment ± IVT-MTX; pts with extra-ocular disease (inside or outside the CNS) were excluded. Responses per IPCG criteria. Biomarker groups were defined as single positive (MYD88$^{L265P}$ or high IL-10) or double-positive.

**Copyright Information:** (Online) ISSN: 2572-9241
©2026 The Author(s). HemaSphere is published by John Wiley & Sons Ltd on behalf of European Hematology Association. This is an open access Abstract Book distributed under the Attribution-NonCommercial-NoDerivs (CC BY-NC-ND), which allows third parties to download the articles and share them with others as long as they credit the author and the Abstract Book, but they cannot change the content in any way or use them commercially.
**Abstract Book Citations:** Authors, Title, HemaSphere, 2026;10:(S1):pages. The abstract book DOIs can be found on the "Author Information" page.
**Disclaimer:** Articles published in the journal HemaSphere exclusively reflect the opinions of the authors. The authors are responsible for all content in their abstracts including accuracy of the facts, statements, citing resources, etc.

**Results**

Thirty-one pts (median age 61; range 39–74; 14 males) were analyzed. Median time from symptoms to diagnosis was 13 months (range 1–48). Vitrectomy was performed in all pts (6 bilateral; 19%). PVRL diagnosis was confirmed by cytology in 11 pts (35%), by biomarkers in 5 (16%), and by both in 15 (48%). Brain MRI was performed in all pts. At least one molecular marker was assessed in CSF of 18 pts and resulted abnormal in 13: MYD88$^{L265P}$ alone in 3, IL-10 alone in 6, and double-positive in 4 **(Table 1).**
MATRix (methotrexate, cytarabine, thiotepa, rituximab) was the most frequent systemic chemo (n=18; 58%). IVT-MTX was given to 26 pts (81%) (median 25 injections, range 3–25). Consolidation was delivered in 10 pts (32%): ORT in 8 and ASCT in 2.
IVT-MTX was well tolerated (no grade ≥3 ocular toxicity; no discontinuations) and no unexpected toxicity occurred with MA-based chemo or consolidation. Best ORR was 97% (30/31; 95% CI 78–99), with 28 CRs (90%).
At a median follow-up of 30 months (range 5–112), relapse occurred in 15 pts (48%): ocular only in 2 (13%), brain only in 6 (40%), and both in 7 (47%). Two-year PFS and OS were 49% (95% CI 28.2–66.6) and 85% (95% CI 64.4–94.0), respectively. Nine pts (28%) died (lymphoma 6; sepsis 1; unknown 2). Seven of the 10 pts treated with consolidative ORT or ASCT remain relapse-free (relapse: brain + ocular 1; ocular only 2). All pts who received consolidation are alive at 5–58 months (median 22+). Acknowledging limited sample size/missingness, neither CSF nor intraocular MYD88 $^{L265P}$/IL-10 status impacted outcome.

**Summary/Conclusion**

MA–based chemotherapy ±IVT-MTX achieved high response rates with acceptable toxicity in PVRL. Relapse occurred in 48% of patients, predominantly involving the brain—alone or combined with ocular disease—whereas isolated ocular relapse was uncommon. The potential benefit of consolidative approaches (ORT or ASCT) and the high detectability of CSF molecular biomarkers warrant prospective evaluation.

**Table 1. Biomarkers tested**

| Assay | N tested | Positive (n) | % positive |
|---|---|---|---|
| Vitreous citology | 31 | 26 | 83.9% |
| MYD88 (vitreous) | 25 | 18 | 72.0% |
| MYD88 (aqueous) | 7 | 5 | 71.4% |
| IL-10 (vitreous) | 20 | 17 | 85.0% |
| IL-10 (aqueous) | 4 | 3 | 75.0% |
| IGH clonality (vitreous) | 17 | 16 | 94.1% |
| MYD88 (CSF) | 17 | 7 | 41.2% |
| IL-10 (CSF) | 12 | 10 | 83.3% |

**Copyright Information:** (Online) ISSN: 2572-9241
©2026 The Author(s). HemaSphere is published by John Wiley & Sons Ltd on behalf of European Hematology Association. This is an open access Abstract Book distributed under the Attribution-NonCommercial-NoDerivs (CC BY-NC-ND), which allows third parties to download the articles and share them with others as long as they credit the author and the Abstract Book, but they cannot change the content in any way or use them commercially.
**Abstract Book Citations:** Authors, Title, HemaSphere, 2026;10:(S1):pages. The abstract book DOIs can be found on the "Author Information" page.
**Disclaimer:** Articles published in the journal HemaSphere exclusively reflect the opinions of the authors. The authors are responsible for all content in their abstracts including accuracy of the facts, statements, citing resources, etc.

### (PS2095) REAL-WORLD TREATMENT PATTERNS AND OUTCOMES OF FIRST-LINE (1L) TREATMENTS IN PATIENTS WITH NEWLY DIAGNOSED DIFFUSE LARGE B-CELL LYMPHOMA (DLBCL) IN THE UNITED STATES (US) COMMUNITY ONCOLOGY SETTING

**Topic:** 19. Large B cell lymphomas - Clinical

David J. Andorsky*[1], Barbara Furtado[2], Helen Latimer[3], Anna Teschemaker[2], Marc Purazo[2], Debbie Adkins[4], Ira Zackon[3], Jinhong Guo[3], Helen Engle[3]

*[1]Rocky Mountain Cancer Centers, Boulder, United States of America; [2]AstraZeneca, Gaithersburg, United States of America; [3]Ontada, Boston, United States of America; [4]AstraZeneca, Cambridge, United Kingdom;*

### Background

The majority of US DLBCL patients are diagnosed and receive cancer care in community settings (Sineshaw et al., 2023), where patient characteristics, care services, and resources can vary; these factors can influence treatment management and outcomes. R-CHOP has been the preferred 1L therapy recommended by clinical guidelines. As of 2023, the introduction of Pola-R-CHP has expanded treatment options. However, there has been limited real-world data published on 1L utilization in the community oncology settings.

### Aims

The goal of this analysis was to characterize treatment patterns and outcomes by 1L treatment in this setting.

### Methods

This study included patients with DLBCL (not otherwise specified) who initiated a 1L regimen within the US Oncology Network from January 1, 2020, to January 31, 2025. Structured electronic health record data was analyzed until death or the last contact by September 2025. Time-to-next-treatment or death (TTNTD) was estimated using Kaplan-Meier methods with corresponding 95% confidence intervals (CIs). Refractory disease was defined as initiation of second-line (2L) therapy ≤ 6 months of the end of 1L. Relapsed disease was defined as initiation of 2L > 6 months after the end of 1L. Line of therapy was based on regimen start and stop dates. Treatment patterns and outcomes were presented overall and by 1L regimen (R-CHOP, Pola-R-CHP, or Other).

### Results

Among the 4,291 eligible patients in this study, 1L regimens included 3,138 (73%) R-CHOP, 411 (10%) Pola-R-CHP, and 742 Other (17% overall; 83% R-mini-CHOP and 17% other chemotherapy). Median age was 70 years overall, 68 for R-CHOP-treated patients, 69 for Pola-R-CHP-treated patients, and 82 for patients treated with Other regimens. Adoption of Pola-R-CHP increased from 2% to (2022) to 28% (2024), while R-CHOP declined but remained most common across years (86%-54%). Median time from diagnosis to 1L was 0.69 months overall (0.69 for R-CHOP, 0.56 for Pola-R-CHP, and 0.66 for Other). A total of 561 patients (13.1%) initiated 2L therapy. Within each treatment subgroup, 402 (12.8%) R-CHOP, 39 (9.5%) Pola-R-CHP, and 120 (16.2%) Other initiated 2L. Among those with 2L, 162 (28.9%) relapsed and 399 (71.1%) were refractory overall; relapse and refractory proportions were 121 (30.1%) and 281 (69.9%) for R-CHOP, 3 (7.7%) and 36 (92.3%) for Pola-R-CHP, and 38 (31.7%) and 82 (68.3%) for Other, respectively. Median follow-up time was 37.6 months overall, 42.0 months for R-CHOP, 17.8 months for Pola-R-CHP, and 36.1 months for Other. Median TTNTD was 63.5 months overall (95% CI: 59.4, not reached [NR]), 68.5 months (95% CI: 63.5, NR) for R-CHOP, not reached (95% CI: 34.2, NR) for Pola-R-CHP, and 17.9 months (95% CI: 13.5, 26.0) for Other. 2-year TTNTD was 66.8% (95% CI: 65.2%, 68.3%) overall, 70.6% (95% CI: 68.8%, 72.3%) for R-CHOP, 73.6% (95% CI: 68.4%, 78.1%) for Pola-R-CHP, and 47.3% (95% CI: 43.2%, 51.3%) for Other.

**Copyright Information:** (Online) ISSN: 2572-9241
©2026 The Author(s). HemaSphere is published by John Wiley & Sons Ltd on behalf of European Hematology Association. This is an open access Abstract Book distributed under the Attribution-NonCommercial-NoDerivs (CC BY-NC-ND), which allows third parties to download the articles and share them with others as long as they credit the author and the Abstract Book, but they cannot change the content in any way or use them commercially.
**Abstract Book Citations:** Authors, Title, HemaSphere, 2026;10:(S1):pages. The abstract book DOIs can be found on the "Author Information" page.
**Disclaimer:** Articles published in the journal HemaSphere exclusively reflect the opinions of the authors. The authors are responsible for all content in their abstracts including accuracy of the facts, statements, citing resources, etc.

### Summary/Conclusion

In the US community setting, Pola-R-CHP utilization is increasing over time, but R-CHOP remains the most common 1L treatment. 2-year TTNTD was similar for R-CHOP and Pola-R-CHP, although additional follow-up is needed to assess the effectiveness of newer regimens, such as Pola-R-CHP. This data highlights an unmet need for optimizing frontline therapy selection and supportive pathways, as well as continued novel therapy development, to improve real-world effectiveness.

**Figure 1. Median TTNTD Overall and by 1L Treatment Subgroup in Months**

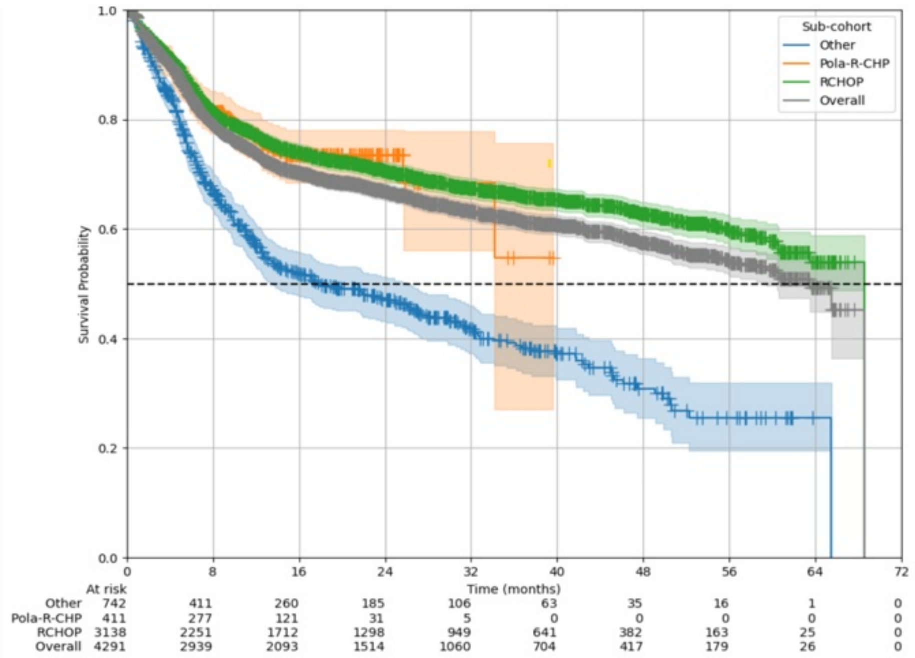

**Copyright Information:** (Online) ISSN: 2572-9241
©2026 The Author(s). HemaSphere is published by John Wiley & Sons Ltd on behalf of European Hematology Association. This is an open access Abstract Book distributed under the Attribution-NonCommercial-NoDerivs (CC BY-NC-ND), which allows third parties to download the articles and share them with others as long as they credit the author and the Abstract Book, but they cannot change the content in any way or use them commercially.
**Abstract Book Citations:** Authors, Title, HemaSphere, 2026;10:(S1):pages. The abstract book DOIs can be found on the "Author Information" page.
**Disclaimer:** Articles published in the journal HemaSphere exclusively reflect the opinions of the authors. The authors are responsible for all content in their abstracts including accuracy of the facts, statements, citing resources, etc.

### (PS2096) TREATMENT FOR MALIGNANT LYMPHOMAS AND RISK OF THERAPY-RELATED MYELOID NEOPLASMS OVERALL AND WITH HIGH-RISK FEATURES

**Topic:** 19. Large B cell lymphomas - Clinical

Joel Joelsson*[1], Stefanie Antonilli[1], Joshua Entrop[1], Elisabeth Ejerblad[2], Christer Nilsson[1], Sören Lehmann[1], Kristina Noring[1], Eva Hellström Lindberg[1], Gunnar Juliusson[3], Tove Wästerlid[1], Richard Rosenquist[1], Sandra Eloranta[1], Karin Ekstrom Smedby[1]

[1]*Karolinska Institutet, Stockholm, Sweden;* [2]*Uppsala University, Uppsala, Sweden;* [3]*Lund university, Lund, Sweden;*

### Background

As lymphoma survival is improving, there is a need to better characterize risk factors for therapy-related myeloid neoplasms (t-MN) to support the development of preventive strategies. While an increased risk of t-MN is established in lymphoma, few studies have examined associations with specific treatments and by t-MN features.

### Aims

To determine risk of t-MN overall and of high risk by treatment/management of lymphoma in a population-based setting.

### Methods

We identified patients with mature B- and T-cell lymphomas in the Swedish Lymphoma register 2007-2022. Population comparators were matched 10:1 to the patients by age, sex and year. Primary lymphoma treatment/management was categorized as watch-and-wait, radiotherapy only, anti-CD20 antibodies only, chemoimmunotherapy (CIT) with alkylating agents only, with anthracyklines/etoposide, intensive regimens (e.g., R-Hyper-CVAD) and other. t-MNs were identified through linkage to the cancer register, the myelodysplastic syndrome (MDS) and acute myeloid leukemia (AML) quality registers with follow-up through 2023. We classified high-risk t-MN as adverse risk AML according to ELN 2022, and high/very high risk MDS according to IPSS-R. We used Cox regression to estimate hazard ratios (HR) with 95% confidence intervals (CI), adjusting for age, sex, year and lymphoma subtype.

**Copyright Information:** (Online) ISSN: 2572-9241
©2026 The Author(s). HemaSphere is published by John Wiley & Sons Ltd on behalf of European Hematology Association. This is an open access Abstract Book distributed under the Attribution-NonCommercial-NoDerivs (CC BY-NC-ND), which allows third parties to download the articles and share them with others as long as they credit the author and the Abstract Book, but they cannot change the content in any way or use them commercially.
**Abstract Book Citations:** Authors, Title, HemaSphere, 2026;10:(S1):pages. The abstract book DOIs can be found on the “Author Information” page.
**Disclaimer:** Articles published in the journal HemaSphere exclusively reflect the opinions of the authors. The authors are responsible for all content in their abstracts including accuracy of the facts, statements, citing resources, etc.

### Results

We identified 27,694 lymphoma patients and 275,473 comparators. Median age at lymphoma diagnosis was 70 years, 57% were male. During a median follow-up of 6.1 years (range 0-16.9), 194 patients had a t-MN (median time to t-MN 3.1 years) and 530 comparators (Figure 1). Risk of t-MN was 4.7-fold increased (HR 4.7, 95% CI 4.0-5.6) in lymphoma patients versus comparators (highest for aggressive T-cell lymphoma: HR 13.1, 95% CI 7.6-22.7). Treatment data was available for 24,075 lymphoma patients in whom 185 t-MN occurred (71, 37%, were high-risk, 15% were unclassifiable). Standard CIT with alkylating agents+anthracycline (i.e., R-CHOP) was associated with a 2.2-fold increased t-MN risk compared to watch-and-wait (Table 1). When adding etoposide, a 4.7-fold increased risk was observed. Intensive regimens conferred an 8-fold risk. HRs were more pronounced for high-risk t-MN, especially following the most intensive regimens (Table 1). Six % of patients were treated with autologous stem-cell transplant (ASCT) as consolidation or at relapse, after which 22% of the t-MNs occurred (N=41, 22 high-risk). Few patients were treated with CAR-T (n=66) with 2 t-MN during follow-up.

**Table 1:** Primary treatment/management of lymphoma and risk of t-MN (MDS/AML) overall and of high-risk.

| Treatment groups | Patients N | All t-MN N | | | HR (95% CI) | | High-risk t-MN N | | | HR (95% CI) | |
|---|---|---|---|---|---|---|---|---|---|---|---|
| | | Total | AML | MDS | Adjusted* | | Total | AML | MDS | Adjusted* | |
| Watch and wait | 2 806 | 11 | 5 | 6 | 1.0 | (ref) | 3 | 2 | 1 | 1.0 | (ref) |
| Radiotherapy only | 1 789 | 3 | 1 | 2 | 0.4 | (0.1-1.6) | 0 | 0 | 0 | 0.0 | - |
| Anti-CD20 only | 1 987 | 7 | 1 | 6 | 1.0 | (0.4-2.5) | 1 | 0 | 1 | 0.5 | (0.0-4.5) |
| Alkylating only | 3 709 | 32 | 12 | 20 | 2.6 | (1.3-5.1) | 12 | 6 | 6 | 3.8 | (1.1-13) |
| Alkylating+anthracyclines | 9 909 | 86 | 47 | 39 | 2.2 | (1.1-4.6) | 33 | 20 | 13 | 3.7 | (1.0-14) |
| Alkylating+anthracyclines+etoposide | 1 939 | 26 | 10 | 16 | 4.7 | (2.0-11) | 9 | 5 | 4 | 6.6 | (1.5-29) |
| Intensive regimens** | 464 | 7 | 2 | 5 | 8.1 | (2.8-23) | 5 | 1 | 4 | 23 | (4.5-118) |
| Other CIT agents/combinations | 1 472 | 13 | 5 | 8 | 2.2 | (1.0-4.9) | 8 | 3 | 5 | 5.0 | (1.3-19) |

** Adjusted for age at diagnosis, sex, lymphoma subtype **R-Hyper-CVAD, BFM, GMALL*

### Summary/Conclusion

Intensity of primary lymphoma treatment was strongly linked to risk of t-MN, especially with high-risk features, although absolute risk of t-MN was low overall.

**Copyright Information:** (Online) ISSN: 2572-9241
©2026 The Author(s). HemaSphere is published by John Wiley & Sons Ltd on behalf of European Hematology Association. This is an open access Abstract Book distributed under the Attribution-NonCommercial-NoDerivs (CC BY-NC-ND), which allows third parties to download the articles and share them with others as long as they credit the author and the Abstract Book, but they cannot change the content in any way or use them commercially.
**Abstract Book Citations:** Authors, Title, HemaSphere, 2026;10:(S1):pages. The abstract book DOIs can be found on the "Author Information" page.
**Disclaimer:** Articles published in the journal HemaSphere exclusively reflect the opinions of the authors. The authors are responsible for all content in their abstracts including accuracy of the facts, statements, citing resources, etc.

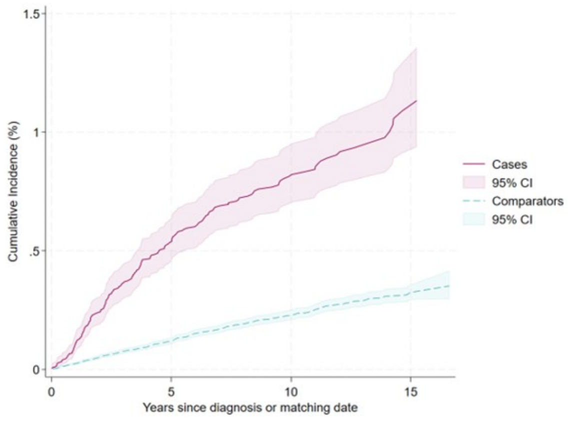

**Figure 1.** Cumulative incidence of myelodysplastic syndromes (MDS) and acute myeloid leukemia (AML) among lymphoma patients (n = 27,694) and matched comparators (n = 275,473), with 95% confidence intervals (CI). The cumulative incidence accounts for death as a competing risk.

**Copyright Information:** (Online) ISSN: 2572-9241
©2026 The Author(s). HemaSphere is published by John Wiley & Sons Ltd on behalf of European Hematology Association. This is an open access Abstract Book distributed under the Attribution-NonCommercial-NoDerivs (CC BY-NC-ND), which allows third parties to download the articles and share them with others as long as they credit the author and the Abstract Book, but they cannot change the content in any way or use them commercially.
**Abstract Book Citations:** Authors, Title, HemaSphere, 2026;10:(S1):pages. The abstract book DOIs can be found on the "Author Information" page.
**Disclaimer:** Articles published in the journal HemaSphere exclusively reflect the opinions of the authors. The authors are responsible for all content in their abstracts including accuracy of the facts, statements, citing resources, etc.

HemaSphere

### (PS2097) REAL-WORLD EFFICACY AND GENOMIC LANDSCAPE OF POLATUZUMA VEDOTIN-BASED FIRST-LINE THERAPY IN DIFFUSE LARGE B-CELL LYMPHOMA: A FOCUS ON TP53 MUTATIONS AND TREATMENT RESPONSE

**Topic:** 19. Large B cell lymphomas - Clinical

Jia Liu*[1], Lan Xu[1], Jun Du[1], Fei Xiao[1], Hua Zhong[1], Xiaofeng Han[1], Jianchen Fang[2], Zebin Liu[2], Honghui Huang[1], Juan Du[1], Ting Wang[1]

*[1]Renji Hospital, School of Medicine, Shanghai Jiao Tong University, Department of Hematology, Shanghai, China;[2]Renji Hospital, School of Medicine, Shanghai Jiao Tong University, Department of Pathology, Shanghai, China;*

### Background

The POLARIX trial established the Pola-R-CHP regimen as the standard first-line treatment for newly diagnosed diffuse large B-cell lymphoma (DLBCL). However, its efficacy in real-world populations, especially in patients with TP53 mutations, remains to be fully elucidated.

### Aims

To compare the efficacy and survival outcomes of Pola-R-CHP and R-CHOP in newly diagnosed DLBCL patients in a real-world cohort using inverse probability treatment weighting (IPTW). The study also explores the molecular characteristics and prognostic impact of TP53 mutations using next-generation sequencing (NGS).

### Methods

We retrospectively included 236 newly diagnosed DLBCL patients who received Pola-R-CHP or R-CHOP as first-line treatment at Renji Hospital from February 2023 to June 2025. IPTW were used to balance baseline confounders. Extreme weights were trimmed at the 99th percentile to ensure model stability. NGS analysis was performed on available tissue samples to assess molecular features.

### Results

The median follow-up time was 14.8 months (IQR, 9.7-22.7 months). After IPTW, Pola-R-CHP showed significantly higher CRR (81.6% *vs.* 66.3%, weighted OR = 2.26, 95% CI 1.16-4.39, $P = 0.017$) and better PFS (weighted HR = 0.41, 95% CI 0.21-0.80, $P = 0.009$) compared to the R-CHOP group, with an estimated 1-year PFS of 91.4% (95% CI 86.8%-96.3%) *vs.* 74.2% (95% CI 64.0%-86.1%) and 2-year PFS of 84.6% (95% CI 76.5%-93.5%) *vs.* 63.6% (95% CI 50.8%-79.5%). Subgroup analysis showed significant benefits of Pola-R-CHP in high-risk groups, including patients with age > 60, ECOG ≥ 2, Ann Arbor stage III-IV, extranodal site ≥ 2, IPI 2-5, and double expresser. Notably, Pola-R-CHP provided marked PFS benefit in the TP53-mutant patients (weighted HR = 0.12; 95% CI: 0.031–0.46, $P = 0.007$). To further delineate the impact of mutation-related characteristics, subgroup analyses were performed among patients harboring TP53 alterations, stratified by mutation multiplicity, variant allele frequency (VAF), and variant type. Pola-R-CHP outperformed R-CHOP with superior PFS across patients with single-hit or multi-hit TP53 mutations, high-VAF or low-VAF, and missense mutations. Conversely, patients with non-missense TP53 alterations derived no significant PFS benefit (weighted Cox $P = 0.270$), identifying this as an ultra-high-risk subgroup that requires further therapeutic optimization.

### Summary/Conclusion

This real-world study confirms that the Pola-R-CHP regimen induces deeper remission and significantly improves PFS compared to R-CHOP in newly diagnosed DLBCL patients, especially those with TP53 mutations.

**Copyright Information:** (Online) ISSN: 2572-9241
©2026 The Author(s). HemaSphere is published by John Wiley & Sons Ltd on behalf of European Hematology Association. This is an open access Abstract Book distributed under the Attribution-NonCommercial-NoDerivs (CC BY-NC-ND), which allows third parties to download the articles and share them with others as long as they credit the author and the Abstract Book, but they cannot change the content in any way or use them commercially.
**Abstract Book Citations:** Authors, Title, HemaSphere, 2026;10:(S1):pages. The abstract book DOIs can be found on the "Author Information" page.
**Disclaimer:** Articles published in the journal HemaSphere exclusively reflect the opinions of the authors. The authors are responsible for all content in their abstracts including accuracy of the facts, statements, citing resources, etc.

### (PS2098) IMPACT OF BASELINE NEUTROPHIL-TO-LYMPHOCYTE RATIO (NLR) ON SURVIVAL OUTCOMES IN PATIENTS WITH LYMPHOMA FOLLOWING BISPECIFIC ANTIBODY TREATMENT

**Topic:** 19. Large B cell lymphomas - Clinical

Allison Bock*[1], Ruitao Liu[2], Xi Qiao[2], Ayushi Chauhan[3], Lorenzo Falchi[4], Ajay Major[5], Swetha Thiruvengadam[6], Reid Merryman[7], Sonia Godbole[8], Andrew Jallouk[9], Christopher D'angelo[10], Amy Ayers[3], Geoffrey Shouse[6], Matthew Lunning[10], Shakthi Bhaskar[9], Alex F. Herrera[6], Yazeed Sawalha[8], Jennifer L. Crombie[7], Steven Bair[5], Gilles Salles[4], Sairah Ahmed[3], Narendranath Epperla[1]

*[1]University of Utah, Salt Lake City, United States of America;[2]University of Utah, Internal Medicine, Division of Epidemiology, Salt Lake City, United States of America; [3]MDACC, Houston, United States of America; [4]Memorial Sloan Kettering Cancer Center, New York, United States of America; [5]University of Colorado, Aurora, United States of America;[6]City of Hope National Medical Center, Duarte, United States of America; [7]Dana-Farber Cancer Institute, Boston, United States of America; [8]The Ohio State University, Columbus, United States of America; [9]Vanderbilt University, Nashville, United States of America; [10]University of Nebraska Medical Center, Omaha, United States of America;*

### Background

Bispecific antibodies (BsAbs) approved for relapsed/refractory (R/R) follicular lymphoma (FL) and diffuse large B-cell lymphomas (DLBCL) harness T-cells to mediate anti-tumor activity. T-cell fitness and the immunosuppressive tumor microenvironment may influence treatment response and outcomes. The NLR is a readily available biomarker that correlates with inflammation and immune suppression by myeloid cells. While elevated NLR at diagnosis is associated with inferior outcomes in DLBCL, its prognostic relevance in patients (pts) treated with BsAbs remains unknown.

### Aims

We sought to evaluate whether the baseline NLR may be prognostic of BsAb efficacy and aimed to identify NLR thresholds associated with response and survival outcomes.

### Methods

We conducted a multicenter retrospective study of adult pts with R/R LBCL who received BsAbs (glofitamab (glofit), epcoritamab (epco) or mosunetuzumab (mosu)) outside of a clinical trial between 2023 and 2026 at 10 centers in the Collaborative US Bispecifics Consortium (CUBIC). Efficacy outcomes included overall response rate (ORR), complete response rate (CRR), progression free survival (PFS) and overall survival (OS). Optimal NLR cutoffs were identified using receiver operating characteristic (ROC) curve analysis for ORR/CRR, and using maximally selected rank statistics for PFS/OS, with performance assessed by 5-fold cross-validation. Logistic regression was used to evaluate NLR (modeled continuously and dichotomized) in relation to ORR /CRR, and Cox proportional hazards models were used to assess its association with PFS/OS. All models were adjusted for baseline clinical and pathological covariates, with statistical significance defined as $\alpha = 0.05$.

**Copyright Information:** (Online) ISSN: 2572-9241
©2026 The Author(s). HemaSphere is published by John Wiley & Sons Ltd on behalf of European Hematology Association. This is an open access Abstract Book distributed under the Attribution-NonCommercial-NoDerivs (CC BY-NC-ND), which allows third parties to download the articles and share them with others as long as they credit the author and the Abstract Book, but they cannot change the content in any way or use them commercially.
**Abstract Book Citations:** Authors, Title, HemaSphere, 2026;10:(S1):pages. The abstract book DOIs can be found on the “Author Information” page.
**Disclaimer:** Articles published in the journal HemaSphere exclusively reflect the opinions of the authors. The authors are responsible for all content in their abstracts including accuracy of the facts, statements, citing resources, etc.

### Results

494 pts were included; 68% (n=338) with LBCL/HGBCL (25% transformed), 19% FL (n=94), 13% other histologies (n=62). Pts received glofit (41%), epco (35%), or mosu (24%). Prior to BsAb, median age was 69 yrs (range 22-93) and 64% had stage 4 disease. The median pre-treatment (tx) WBC was 5.4 (range 0.1-205), ANC was 3.5 (range 0-82), ALC was 0.70 (range 0-197), and NLR was 10 (range 0-274). There was no difference among age, sex, stage, elevated LDH, or prior therapies (CAR-T, auto-SCT, number of prior lines of therapy) among pts with pre-tx high NLR vs low NLR. Use of BsAb was similar other than lower use of mosu among pts with high NLR vs low NLR (13% vs 31%). At a median follow up of 13 months, the median PFS and OS among all pts were 6.2 months and 21.5 months, respectively. The optimal cutoff for a high NLR was determined to be 5.0 for PFS and 5.3 for OS. For high vs low NLR, the median PFS and OS were 3.8 vs 12.1 months and 8.8 vs 21.5 months, respectively. After adjusting for baseline clinico-pathological variables, high NLR was independently associated with an inferior PFS (NLR>5.0, HR 1.45, 95% CI 1.1-1.8, p<0.01) and OS (NLR>5.3, HR 1.4, 95% CI 1.06-2.05, p=0.02) compared to low NLR (Figure 1). NLR as a continuous variable was also significantly associated with PFS (p=0.02) and OS (p<0.01), but not CRR or ORR.

### Summary/Conclusion

A high pre-tx NLR (>5.0) is associated with inferior PFS and OS in pts treated with BsAbs, independent of disease subtype or specific agent. NLR is a readily available prognostic marker of response and survival, unlike more complex assays of T-cell phenotype and function. Further investigation into the immunobiological mechanisms driving BsAb resistance in non-responders is warranted.

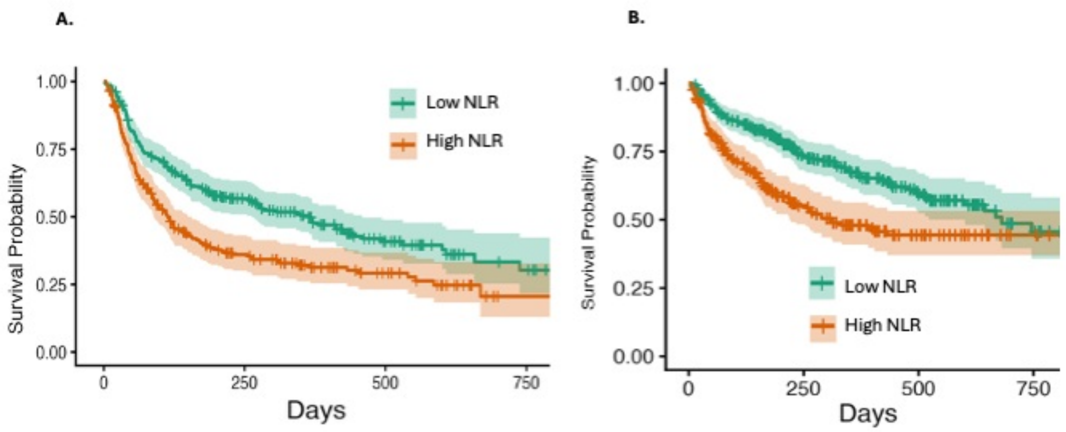

**Figure 1.** A) Adjusted PFS and B) Adjusted OS stratified by baseline NLR

**Copyright Information:** (Online) ISSN: 2572-9241
©2026 The Author(s). HemaSphere is published by John Wiley & Sons Ltd on behalf of European Hematology Association. This is an open access Abstract Book distributed under the Attribution-NonCommercial-NoDerivs (CC BY-NC-ND), which allows third parties to download the articles and share them with others as long as they credit the author and the Abstract Book, but they cannot change the content in any way or use them commercially.
**Abstract Book Citations:** Authors, Title, HemaSphere, 2026;10:(S1):pages. The abstract book DOIs can be found on the "Author Information" page.
**Disclaimer:** Articles published in the journal HemaSphere exclusively reflect the opinions of the authors. The authors are responsible for all content in their abstracts including accuracy of the facts, statements, citing resources, etc.

### (PS2099) LENGTH OF STAY, HEALTHCARE RESOURCE UTILIZATION (HCRU) AND REHOSPITALIZATION OF PATIENTS TREATED WITH AXI-CEL OR LISO-CEL FOR SECOND-LINE LARGE B-CELL LYMPHOMA (LBCL) IN FRANCE

**Topic:** 19. Large B cell lymphomas - Clinical

Catherine Thieblemont[1], Florian Colrat*[2], Denis Caillot[3], Morgane Thevenin[2], François-Emery Cotté[2], Alexandre Caron[4], Camille Nevoret[4], Elodie Torreton[4], Sébastien Branchoux[2], Fabien Despas[5]

*[1]APHP, Hopital Saint-Louis, Hemato-oncologie, Paris, France;[2]Bristol Myers Squibb, Rueil Malmaison, France; [3]Institut de Cancérologie de Bourgogne, Dijon, France; [4]CEMKA, Bourg-la-Reine, France; [5]University Hospital Center CHU of Toulouse, Toulouse, France;*

### Background

Since 2024 the CAR-T cell therapies axicabtagene ciloleucel (axi-cel) and lisocabtagene maraleucel (liso-cel) have been routinely reimbursed in France for the treatment of adult patients (pts) with large B-cell lymphoma (LBCL), who are refractory or relapsed within 12 months of first line immunochemotherapy (LBCL RR≤12).

### Aims

The objectives of this study were to describe the characteristics of pts treated with axi-cel or liso-cel for LBCL RR≤12 and assess the length of stay (LOS) for infusion and associated HCRU.

### Methods

A retrospective cohort study was conducted using the French comprehensive hospital stay databases (PMSI). Included pts were hospitalized for axi-cel or liso-cel infusion, with a specific code for LBCL RR≤12, and discharged from hospital between January 1, and December 31, 2024 (complete stay). The index date was date of CAR-T infusion, the pre-infusion period was the time from hospitalization to index date and time from index date to discharge was post-infusion period. Descriptive analyses were conducted of pts and infusion stay characteristics, HCRU, and 3-month rehospitalization rates.

**Copyright Information:** (Online) ISSN: 2572-9241
©2026 The Author(s). HemaSphere is published by John Wiley & Sons Ltd on behalf of European Hematology Association. This is an open access Abstract Book distributed under the Attribution-NonCommercial-NoDerivs (CC BY-NC-ND), which allows third parties to download the articles and share them with others as long as they credit the author and the Abstract Book, but they cannot change the content in any way or use them commercially.
**Abstract Book Citations:** Authors, Title, HemaSphere, 2026;10:(S1):pages. The abstract book DOIs can be found on the "Author Information" page.
**Disclaimer:** Articles published in the journal HemaSphere exclusively reflect the opinions of the authors. The authors are responsible for all content in their abstracts including accuracy of the facts, statements, citing resources, etc.

**Results**

We included 443 pts, 288 treated with axi-cel (65%) and 155 with liso-cel (35%). Pts treated with axi-cel were younger than those with liso-cel (58 years (±14) 65 years (±13)) (table). The proportion of males was similar between axi-cel (65%, n=186) and liso-cel (60%, n=93). Mean LOS for infusion was 23.1 days (±14.0) for axi-cel and 16.0 days (±5.9) for liso-cel. Post-infusion stay was shorter with liso-cel (10.9 days (±4.2 days) than axi-cel (17.3 days (±12.0)). During this stay, 15% (n=43) of pts treated with axi-cel and 2% (n=3) of pts with liso-cel were admitted to intensive care unit (ICU); overall mean 5.5 days (±5.5; n=46). High-cost drugs (as defined by the French Ministry of Health) were administered to 55% pts treated with axi-cel (n=157) and 12% with liso-cel (n=19). Most were monoclonal antibodies (tocilizumab), for 49% of pts with axi-cel (n=142) and 6% with liso-cel (n=10). Immunoglobulins were administered to 8% (axi-cel, n=24) and 5% (liso-cel, n=7) of pts. 72% of pts treated with axi-cel (n=207) and 81% of pts treated with liso-cel (n=126) were discharged directly home. 3-month rehospitalization rates were 77% for those treated with axi-cel (n=204) and 54% for pts treated with liso-cel (n=58).

| | Overall | Axi-cel | Liso-cel |
|---|---|---|---|
| N | 443 | 288 | 155 |
| **Pt characteristics** | | | |
| Age, years, mean (std) | 60.7 (14.0) | 58.2 (13.8) | 65.3 (13.1) |
| Gender, male, n (%) | 279 (63) | 186 (65) | 93 (60) |
| **Period of duration, days, mean (std)** | | | |
| Pre-infusion | 5.6 (5.4) | 5.9 (6.2) | 5.1 (3.3) |
| Post-infusion | 15.0 (10.5) | 17.3 (12.0) | 10.9 (4.2) |
| From admission to discharge | 20.6 (12.3) | 23.1 (14.0) | 16.0 (5.9) |
| **HCRU, n (%)** | | | |
| Admission to ICU | 46 (10) | 43 (15) | 3 (2) |
| High-cost medication | 176 (40) | 157 (55) | 19 (12) |
| **Rehospitalization** | | | |
| N | 358 | 250 | 108 |
| 3-month rate, n (%) | 262 (73) | 204 (77) | 58 (54) |

**Summary/Conclusion**

With patients experiencing a shorter length of hospital stay and lower HCRU, liso-cel treatment streamlines the patient care pathway, and lowers the intra-hospital burden associated with CAR-T cell therapy.

**Copyright Information:** (Online) ISSN: 2572-9241
©2026 The Author(s). HemaSphere is published by John Wiley & Sons Ltd on behalf of European Hematology Association. This is an open access Abstract Book distributed under the Attribution-NonCommercial-NoDerivs (CC BY-NC-ND), which allows third parties to download the articles and share them with others as long as they credit the author and the Abstract Book, but they cannot change the content in any way or use them commercially.
**Abstract Book Citations:** Authors, Title, HemaSphere, 2026;10:(S1):pages. The abstract book DOIs can be found on the “Author Information” page.
**Disclaimer:** Articles published in the journal HemaSphere exclusively reflect the opinions of the authors. The authors are responsible for all content in their abstracts including accuracy of the facts, statements, citing resources, etc.

### (PS2100) OUTCOMES OF SECOND-LINE AXICABTAGENE CILOLEUCEL AND COMPARISON TO AUTOLOGOUS STEM CELL TRANSPLANTATION IN EARLY RELAPSED/PRIMARY REFRACTORY LARGE B-CELL LYMPHOMA PATIENTS

**Topic:** 19. Large B cell lymphomas - Clinical

Lina Kolloch*[1], Johanna Patt[1], Inna Shaforostova[2], Hristo Boyadzhiev[2, 3], Markus Maulhardt[4], Michael Leisch[5], Enver Aydilek[4, 6], Christian Reicherts[1], Valeria Carobin[1], Julia Marx[1], Matthias Stelljes[1], Norbert Schmitz[1], Simon Call[1], Thomas Melchardt[5], Gerald Wulf[4], Thomas Pabst[2], Urban Novak[2], Evgenii Shumilov[1], Georg Lenz[1]

*[1]University Hospital Muenster, Department of Medicine A, Hematology, Oncology and Pneumology, Muenster, Germany; [2]Inselspital, University Hospital Bern, University of Bern, Department of Medical Oncology, Bern, Switzerland; [3]Habichtswald Hospital, Kassel, Germany; [4]University Hospital Goettingen, Department of Hematology and Medical Oncology, Goettingen, Germany; [5]Paracelsus Medical University, Department of Internal Medicine III, Salzburg, Austria; [6]University Hospital Bielefeld, Evangelisches Klinikum Bethel, Bielefeld, Germany;*

### Background

CD19-directed chimeric antigen receptor T (CAR-T) cells represent the standard of care (SOC) approach in the second-line treatment (2L) for patients with primary refractory/early relapsed large B-cell lymphoma (R/R LBCL). However, real-world data on the safety and efficacy of CAR-T in 2L are limited, particularly in comparison to high-dose chemotherapy and autologous stem cell transplantation (HDCT/ASCT) administered for patients with a chemosensitive disease in 2L.

### Aims

Here we provide real-world data on R/R LBCL patients treated with CAR-T cells in 2L and compare them to the former SOC HDCT/ASCT. CAR-T patients in this analysis were treated with axicabtagene ciloleucel (Axi-Cel) due to its initially better availability in Europe.

### Methods

We conducted a retrospective analysis of 194 R/R LBCL patients of whom 112 were treated with Axi-Cel, and 82 with HDCT/ASCT. The HDCT/ASCT group presented with a chemosensitive disease after bridging therapy in their last staging prior to HDCT/ASCT, while the Axi-Cel group included both, patients with chemosensitive and -refractory disease. Patients with a late relapse (> 12 months) and/or more than one line of prior therapy were excluded. To enable a comparison between the treatment groups, propensity score matching (PSM) was performed via logistic regression with parameters significantly associated with OS in univariate analysis (bulky disease and secondary International Prognostic Index (sIPI)) as covariates.

**Copyright Information:** (Online) ISSN: 2572-9241
©2026 The Author(s). HemaSphere is published by John Wiley & Sons Ltd on behalf of European Hematology Association. This is an open access Abstract Book distributed under the Attribution-NonCommercial-NoDerivs (CC BY-NC-ND), which allows third parties to download the articles and share them with others as long as they credit the author and the Abstract Book, but they cannot change the content in any way or use them commercially.
**Abstract Book Citations:** Authors, Title, HemaSphere, 2026;10:(S1):pages. The abstract book DOIs can be found on the “Author Information” page.
**Disclaimer:** Articles published in the journal HemaSphere exclusively reflect the opinions of the authors. The authors are responsible for all content in their abstracts including accuracy of the facts, statements, citing resources, etc.

### Results

The objective response rate following Axi-Cel was 81%, with a complete remission in 52% of patients. With a median follow up of 9 months, the estimated 2-year progression-free survival (PFS) was 51%, whereas the 2-year overall survival (OS) was 60%. Factors associated with worse survival outcomes following Axi-Cel infusion included higher sIPI and necessity of a bridging therapy (multivariate analysis: HR≥1.39, p≤0.048 for both). Remission status before cellular therapy did not influence survival outcomes in these patients. Grade ≥3 neurotoxicity occurred in 19%, whereas high-grade cytokine release syndrome was documented in 16% of patients.
Significant differences between the CAR-T and the HDCT/ASCT groups included age (≥65 years: 49% of CAR-T vs. 33% of HDCT/ASCT patients, p=0.025), sIPI (≥3: 49% vs. 27%, p=0.01), bulky disease (22% vs. 7%, p=0.005), and response to 1L therapy (primary refractory: 49% vs. 65%, p=0.032). Notably, 24% of the HDCT/ASCT patients received CAR-T cells in a later treatment line.
A comparison of both groups revealed better OS (1-year OS: 63% vs. 77%, p=0.046) for the HDCT/ASCT patients. However, after PSM, resulting in two cohorts with 64 patients each and balanced baseline characteristics, we found comparable OS (1-year OS: 76% vs. 72%, p=0.94) with a trend towards better PFS (1-year PFS: 57% vs. 43%, p=0.093) for patients treated with Axi-Cel (Figure 1).
Infections (during hospitalization: 41% vs. 89%; after day +100: 6% vs. 30%, both p<0.001) and long-term thrombocytopenia (13% vs. 30%, p=0.006) were more frequent in the HDCT/ASCT group.

### Summary/Conclusion

Our real-world data on the safety and efficacy of Axi-Cel align with findings from the ZUMA-7 approval study, demonstrating the efficacy and safety of Axi-Cel in 2L R/R LBCL treatment. Despite the selection of only chemosensitive patients in the HDCT/ASCT group, treatment with Axi-Cel demonstrated comparable OS, with a trend towards superior PFS compared to patients treated with HDCT/ASCT.

**Copyright Information:** (Online) ISSN: 2572-9241
©2026 The Author(s). HemaSphere is published by John Wiley & Sons Ltd on behalf of European Hematology Association. This is an open access Abstract Book distributed under the Attribution-NonCommercial-NoDerivs (CC BY-NC-ND), which allows third parties to download the articles and share them with others as long as they credit the author and the Abstract Book, but they cannot change the content in any way or use them commercially.
**Abstract Book Citations:** Authors, Title, HemaSphere, 2026;10:(S1):pages. The abstract book DOIs can be found on the "Author Information" page.
**Disclaimer:** Articles published in the journal HemaSphere exclusively reflect the opinions of the authors. The authors are responsible for all content in their abstracts including accuracy of the facts, statements, citing resources, etc.

**Figure 1: Survival outcomes following Axi-Cel/ASCT in patients with R/R aggressive lymphomas after propensity score matching.**

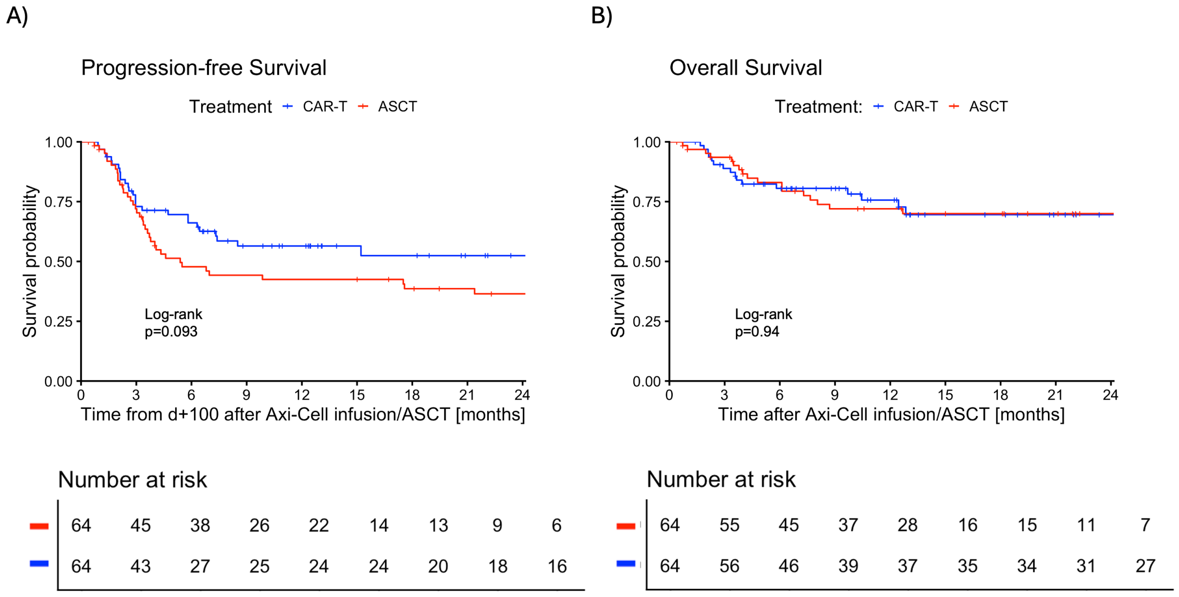

Kaplan-Meier estimates for progression-free survival (A) and overall survival (B) for R/R patients treated with Axi-Cel/ASCT in second line after propensity score matching. Axi-Cel, axicabtagen ciloleucel; ASCT, autologous stem cell transplantation; CAR-T, chimeric antigen receptor T; R/R relapsed/refractory.

**Copyright Information:** (Online) ISSN: 2572-9241
©2026 The Author(s). HemaSphere is published by John Wiley & Sons Ltd on behalf of European Hematology Association. This is an open access Abstract Book distributed under the Attribution-NonCommercial-NoDerivs (CC BY-NC-ND), which allows third parties to download the articles and share them with others as long as they credit the author and the Abstract Book, but they cannot change the content in any way or use them commercially.
**Abstract Book Citations:** Authors, Title, HemaSphere, 2026;10:(S1):pages. The abstract book DOIs can be found on the "Author Information" page.
**Disclaimer:** Articles published in the journal HemaSphere exclusively reflect the opinions of the authors. The authors are responsible for all content in their abstracts including accuracy of the facts, statements, citing resources, etc.

### (PS2101) INCIDENCE AND OUTCOME OF RELAPSED/REFRACTORY LARGE B-CELL LYMPHOMA AND IMPLEMENTATION OF NOVEL T-CELL-ENGAGING THERAPIES IN A POPULATION-BASED SETTING

**Topic:** 19. Large B cell lymphomas - Clinical

Sara Harrysson*[1], Kristina Noring[1], Sandra Eloranta[2], Ann-Sofi Hörstedt[3], Victor Falini[3], Kristina Sonnevi[4], Per-Ola Andersson[5], Gunilla Enblad[6], Mats Jerkeman[7], Karin Ekstrom Smedby[1]

*[1]Division of Clinical Epidemiology, Department of Medicine Solna, Karolinska Institutet and Department of Hematology, Karolinska University Hospital, Stockholm, Sweden; [2]Division of Clinical Epidemiology, Department of Medicine Solna, Karolinska Institutet, Stockholm, Sweden; [3]Regional Cancer Centre South, Lund, Sweden;[4]Department of Medicine Huddinge, Karolinska Institutet and Department of Hematology, Karolinska University Hospital, Stockholm, Sweden; [5]Department of Hematology and Coagulation, Sahlgrenska University Hospital and Sahlgrenska Academy, Gothenburg University, Gothenburg, Sweden; [6]Department of Immunology, Genetics and Pathology, Uppsala University, Uppsala, Sweden; [7]Department of Oncology, Lund University, Lund, Sweden;*

### Background

T-cell-engaging therapies have substantially changed the treatment landscape for patients with large B cell lymphoma (LBCL) in recent years. The logistical challenges and financial burden of these therapies underscore the importance of evaluating their implementation in clinical routine. In Sweden, chimeric antigen receptor (CAR) T-cell therapy was approved for third-line treatment in 2019 and for early second-line in 2024. During the study period, access to bispecific antibodies was limited to clinical trial settings and compassionate use programs.

### Aims

Using Swedish population-based registers, we aimed to report contemporary outcomes for patients with LBCL from primary diagnosis through first and subsequent relapses, and evaluate uptake of novel therapies.

### Methods

We identified all patients with a primary diagnosis of LBCL in 2019-2024 in the Swedish Lymphoma Register (SLR). Patients were followed until death or end of study (16 Dec 2025). Through a development project, the SLR is being updated with complete information regarding R/R disease and treatment from medical records, and here we present data for two thirds of the population. The cumulative incidence of R/R disease was estimated in the presence of the competing risk of death. Overall survival (OS) and progression-free survival (PFS) were estimated from the date of diagnosis using the Kaplan-Meier method. Separate analyses were performed for patients with R/R disease by age group.

**Copyright Information:** (Online) ISSN: 2572-9241
©2026 The Author(s). HemaSphere is published by John Wiley & Sons Ltd on behalf of European Hematology Association. This is an open access Abstract Book distributed under the Attribution-NonCommercial-NoDerivs (CC BY-NC-ND), which allows third parties to download the articles and share them with others as long as they credit the author and the Abstract Book, but they cannot change the content in any way or use them commercially.
**Abstract Book Citations:** Authors, Title, HemaSphere, 2026;10:(S1):pages. The abstract book DOIs can be found on the "Author Information" page.
**Disclaimer:** Articles published in the journal HemaSphere exclusively reflect the opinions of the authors. The authors are responsible for all content in their abstracts including accuracy of the facts, statements, citing resources, etc.

### Results

In this preliminary analysis of 2,259 patients with LBCL, median age at diagnosis was 73 years (range 18-102), 60% had stage III/IV disease and 42% IPI≥3. Most patients (91%, n=1991) received primary treatment with curative intent (i.e., R-CHOP). Overall response rate was 90% (complete response 73%; partial response 17%) and 9% had stable/progressive disease. Two-year OS among curatively treated patients was 78% (95% confidence interval, CI, 76-80) (fig 1A) and 2-year PFS was 69% (95% CI 67-71). The cumulative incidence of R/R disease was 23% (95% CI 21-25) at five years (fig 1B). Most relapses occurred within one year from diagnosis (72%). Among patients with R/R disease (n=450), 67% were older than 70 years (27% >80 years). Overall, 57% of R/R patients received active second-line treatment. Two-year OS was 38% (95% CI 34-43) and 2-year PFS was 26% (95% CI 22-30) for R/R patients. Outcomes differed by age with 2-year OS ranging from 63% (95% CI 53-74) in patients ≤60 years to 17% (95% CI 11-27) in patients >80 years (fig 1C). In total, 15% of all R/R patients received T-cell-engaging therapies at first or later relapses (CAR T: n=53; bispecific antibodies: n=16). Most patients were treated with CAR T in third line (47%). Among patients with a second relapse (n=196), 40% of patients ≤70 years (28/70) received CAR T, and only 6% of patients >70 years (8/126).

### Summary/Conclusion

In this population-based study of LBCL patients treated in 2019-2025 we confirm a 5-year incidence of R/R disease of 23%. This is similar to previous population-based assessments from our group, but lower than what is generally stated in the literature. Two-thirds of R/R patients were older than 70 years and outcomes differed markedly between age groups. Only a minority (15%) of all R/R patients received T-cell-engaging therapies, which may reflect that CAR T was limited to third or later lines during most of the study period, as well as the advanced age distribution in this real-world population.

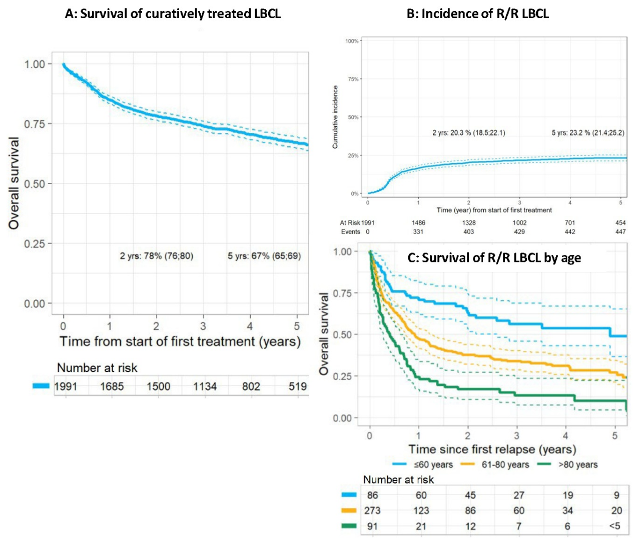

Figure 1. Overall survival among curatively treated patients with Large B-cell lymphoma (LBCL) diagnosed 2019-2024 in Sweden (A), incidence of relapsed/refractory (R/R) disease (accounting for the competing risk of death) (B), and overall survival among patients with R/R disease by age (≤60, 61-80, >80 years) (C).

Copyright Information: (Online) ISSN: 2572-9241
©2026 The Author(s). HemaSphere is published by John Wiley & Sons Ltd on behalf of European Hematology Association. This is an open access Abstract Book distributed under the Attribution-NonCommercial-NoDerivs (CC BY-NC-ND), which allows third parties to download the articles and share them with others as long as they credit the author and the Abstract Book, but they cannot change the content in any way or use them commercially.
Abstract Book Citations: Authors, Title, HemaSphere, 2026;10:(S1):pages. The abstract book DOIs can be found on the “Author Information” page.
Disclaimer: Articles published in the journal HemaSphere exclusively reflect the opinions of the authors. The authors are responsible for all content in their abstracts including accuracy of the facts, statements, citing resources, etc.

### (PS2102) A REAL-WORLD COMPARISON OF CD3XCD20 BISPECIFIC ANTIBODY (BSAB) MONOTHERAPY VS BSAB COMBINATIONS IN RELAPSED/REFRACTORY LARGE B-CELL LYMPHOMA: A COLLABORATIVE US BISPECIFIC CONSORTIUM (CUBIC) STUDY

**Topic:** 19. Large B cell lymphomas - Clinical

Karan Chohan*[1], Sairah Ahmed[1], Allison Bock[2], Narendranath Epperla[2], Sonia Godbole[3], Yazeed Sawalha[3], Andrew Jallouk[4], Shakthi Bhaskar[4], Gilles Salles[5], Matthew Lunning[6], Jennifer L. Crombie[7], Reid Merryman[7], Steven Bair[8], Ajay Major[8], Amy Ayers[1], Geoffrey Shouse[9], Christopher Flowers[1], Alexey Danilov[9], Alex F. Herrera[9], Ayushi Chauhan[1], Swetha Thiruvengadam[9], Lorenzo Falchi[5]

*[1]MD Anderson Cancer Center, Houston, United States of America;[2]University of Utah, Salt Lake City, United States of America; [3]The Ohio State University Comprehensive Cancer Center, Columbus, United States of America;[4]Vanderbilt University, Nashville, United States of America; [5]Memorial Sloan Kettering Cancer Center, New York City, United States of America;[6]University of Nebraska Medical Center, Omaha, United States of America; [7]Dana-Farber Cancer Institute, Boston, United States of America; [8]University of Colorado Anschutz, Colorado, United States of America; [9]City of Hope Comprehensive Cancer Center, Duarte, United States of America;*

### Background

CD3xCD20 bispecific antibodies (BsAb) demonstrate substantial activity in relapsed/refractory (R/R) large B-cell lymphoma (LBCL) and are being combined with chemotherapy, antibody-drug conjugates (ADCs), and targeted agents; however, without prospective comparisons, real-world data are required to inform comparative efficacy, safety, and regimen selection.

### Aims

To compare real-world efficacy and safety outcomes of BsAb monotherapy versus BsAb-based combination regimens in patients (pts) with R/R LBCL.

### Methods

This CUBIC multicenter retrospective consortium study included adult pts with R/R LBCL treated with BsAb alone or in combination across 9 centers. Endpoints included overall response rate (ORR), complete response (CR), progression-free survival (PFS), overall survival (OS), and safety outcomes of cytokine release syndrome (CRS) and immune effector cell-associated neurotoxicity syndrome (ICANS). Multivariable logistic regression and Cox proportional hazards models adjusted for age, sex, ECOG status, stage, extranodal involvement, lactate dehydrogenase (LDH), line of therapy, prior chimeric antigen receptor T-cell (CAR-T), and treatment regimen.

**Copyright Information:** (Online) ISSN: 2572-9241
©2026 The Author(s). HemaSphere is published by John Wiley & Sons Ltd on behalf of European Hematology Association. This is an open access Abstract Book distributed under the Attribution-NonCommercial-NoDerivs (CC BY-NC-ND), which allows third parties to download the articles and share them with others as long as they credit the author and the Abstract Book, but they cannot change the content in any way or use them commercially.
**Abstract Book Citations:** Authors, Title, HemaSphere, 2026;10:(S1):pages. The abstract book DOIs can be found on the “Author Information” page.
**Disclaimer:** Articles published in the journal HemaSphere exclusively reflect the opinions of the authors. The authors are responsible for all content in their abstracts including accuracy of the facts, statements, citing resources, etc.

**Results**

A total of 352 pts received BsAb therapy between 5/2023–11/2025; 90 (25.6%) received BsAb-combination and 262 (74.4%) BsAb alone. Median age at BsAb initiation was 70 years (range, 22–93); 33.2% were female, 70.9% had ECOG 0–1, 87.2% had stage III/IV disease, 84.6% had extranodal involvement, and 64.9% had elevated LDH. Prior CAR-T had been administered in 50.3% of pts. BsAb was administered in the second line in 9.7% of pts. BsAb included glofitamab (53.7%), epcoritamab (44.0%), and mosunetuzumab (2.3%). BsAb-combination regimens comprised BsAb-chemotherapy (11.9%), BsAb-ADC (8.2%), and BsAb-other combinations (5.4%).
Pts receiving BsAb-combination were younger (median 67 vs 71 years; p=0.003), more often treated in second line (24.4% vs 4.6%, p<0.001), and more frequently received glofitamab (66.7% vs 49.2%, p<0.001); other baseline characteristics were comparable.
BsAb-combination produced numerically higher CR rates (44.6% vs 35.0%; p=0.12) and ORR (63.9% vs 54.0%; p=0.12) compared with BsAb alone (**Figure**). On multivariable analysis, BsAb-ADC was the only regimen associated with higher odds of CR compared with BsAb monotherapy (OR 3.21; p=0.026) and showed a trend toward improved ORR (OR 2.48; p=0.096).
Follow-up was shorter with BsAb-combination vs BsAb alone (median 7.5 vs 14.3 months; p<0.001). While not statistically significant, median PFS and OS were longer with BsAb-combination vs BsAb alone (PFS: 5.5 vs 3.5 months, p=0.37; OS: 16.1 vs 9.3 months, p=0.08).
In multivariable models, treatment strategy was not independently associated with OS or PFS. Elevated LDH, poorer ECOG, and advanced stage were independently associated with inferior response and survival.
Safety was comparable between BsAb alone and BsAb-combination, with no differences in CRS (38.8% vs 31.5%), ICANS (13.1% vs 12.6%), post-treatment infections (33.6% vs 30.0%), or ICU admissions (15.2% vs 12.5%) (all p>0.05).

**Summary/Conclusion**

In this large real-world multicenter consortium analysis, BsAb-combination approaches demonstrated numerically higher response rates, with significantly higher CR observed with BsAb-ADC combinations vs monotherapy after adjustment for baseline factors. Safety profiles and early survival were comparable across treatment strategies. Limitations include short follow-up and small sample sizes in the BsAb-combination subgroups.

**Copyright Information:** (Online) ISSN: 2572-9241
©2026 The Author(s). HemaSphere is published by John Wiley & Sons Ltd on behalf of European Hematology Association. This is an open access Abstract Book distributed under the Attribution-NonCommercial-NoDerivs (CC BY-NC-ND), which allows third parties to download the articles and share them with others as long as they credit the author and the Abstract Book, but they cannot change the content in any way or use them commercially.
**Abstract Book Citations:** Authors, Title, HemaSphere, 2026;10:(S1):pages. The abstract book DOIs can be found on the “Author Information” page.
**Disclaimer:** Articles published in the journal HemaSphere exclusively reflect the opinions of the authors. The authors are responsible for all content in their abstracts including accuracy of the facts, statements, citing resources, etc.

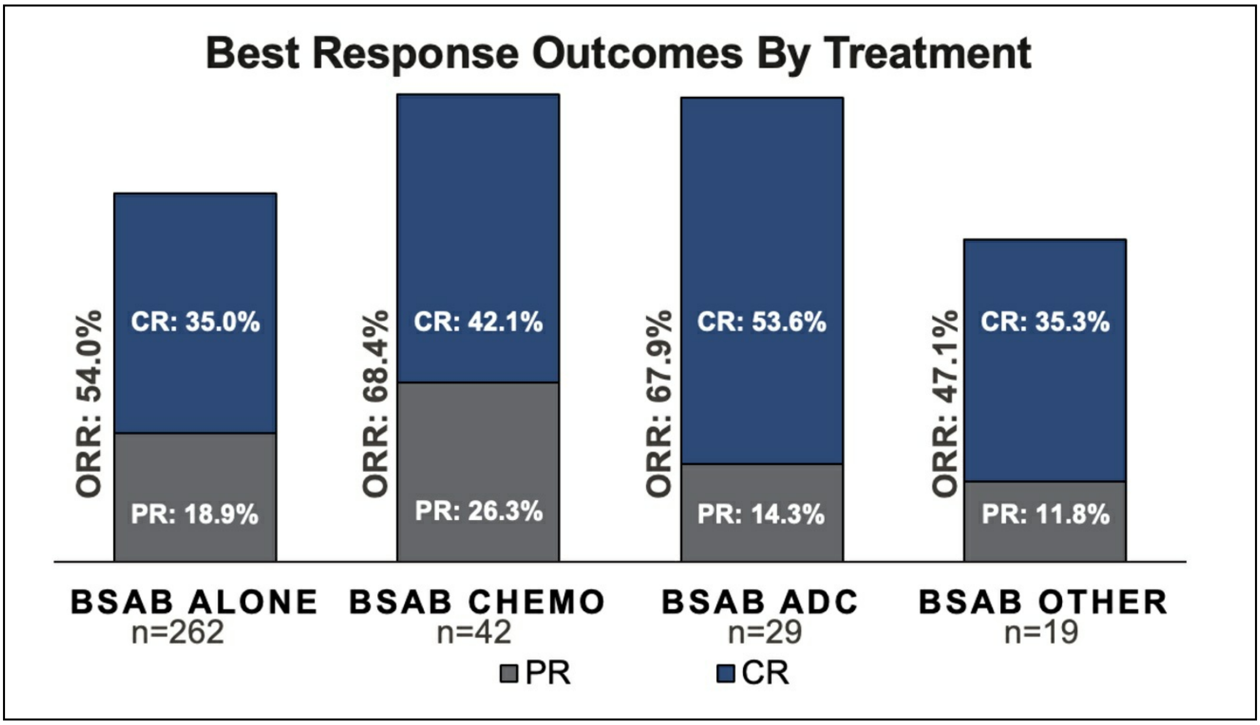

ORR defined as CR + PR. Best overall response assessed per treating investigator. Percentages calculated within each treatment group. Abbreviations: BsAb, bispecific antibody; CR, complete response; PR, partial response; ADC, antibody-drug conjugate.

**Copyright Information:** (Online) ISSN: 2572-9241
©2026 The Author(s). HemaSphere is published by John Wiley & Sons Ltd on behalf of European Hematology Association. This is an open access Abstract Book distributed under the Attribution-NonCommercial-NoDerivs (CC BY-NC-ND), which allows third parties to download the articles and share them with others as long as they credit the author and the Abstract Book, but they cannot change the content in any way or use them commercially.
**Abstract Book Citations:** Authors, Title, HemaSphere, 2026;10:(S1):pages. The abstract book DOIs can be found on the "Author Information" page.
**Disclaimer:** Articles published in the journal HemaSphere exclusively reflect the opinions of the authors. The authors are responsible for all content in their abstracts including accuracy of the facts, statements, citing resources, etc.

### (PS2103) CLINICAL CHARACTERISTICS AND EFFICACY OF MYC/BCL-2 DOUBLE-EXPRESSING DIFFUSE LARGE B-CELL LYMPHOMA: REAL-WORLD DATA FROM THE BELIEVE STUDY

**Topic:** 19. Large B cell lymphomas - Clinical

Pengpeng Xu*[1], Hongmei Jing[2], Keshu Zhou[3], Wenrong Huang[4], Jianzhen Shen[5], Songfu Jiang[6], Xiaoxi Zhou[7], Xiaojing Yan[8], Ting Niu[9], Xiuhua Sun[10], Xiaobo Wang[10], Wuping Li[11], Haisheng Liu[12], Sujiang Zhang[13], Ou Bai[14], Bing Xiu[15], Wei Sang[16], Guohui Cui[17], Jing-Yan Xu[18], Yingyu Nan[19], Yao Liu[19], Xiaolei Wei[20], Hongling Peng[21], Yanli Yang[22], Meiyun Fang[23], Chunling Wang[24], Xudong Zhang[25], Ying Lu[26], Li-Jie Xing[27], Qianqian Cai[28], Jinhai Ren[29], Zhengzi Qian[30], Zhimin Zhai[31], Zhenling Li[32], Weili Zhao[1]

*[1]Shanghai Institute of Hematology, Shanghai, China;[2]Peking University Third Hospital, Beijing, China;[3]Henan Cancer Hospital, Affiliated Cancer Hospital of Zhengzhou University, Zhengzhou, China; [4]The fifth Medical Center,Chinese PLA General Hospital, Beijing, China; [5]Fujian Medical University Union Hospita, Fuzhou, China; [6]The First Affiliated Hospital of Wenzhou Medical University, Wenzhou, China; [7]Tongji Hospital Affiliated to Tongji Medical College, Huazhong University of Science and Technology, Wuhan, China; [8]The First Hospital of China Medial University, Shenyang, China;[9]West China Hospital of Sichuan University, Chengdu, China; [10]The Second Hospital of Dalian Medical University, Dalian, China; [11]Jiangxi Cancer Hospital, Nanchang, China; [12]The Fourth Hospital of Hebei Medical University, Shijiazhuang, China;[13]Ruijin North Hospital, Shanghai Jiao Tong University School of Medicine, Shanghai, China; [14]The First Hospital of Jilin University, Changchun, China;[15]Tongji Hospital, Shanghai, China; [16]The Affiliated Hospital of Xuzhou Medical University, Xuzhou, China;[17]Union Hospital, Tongji Medical College, Huazhong University of Science and Technology, Wuhan, China; [18]Nanjing Drum Tower Hospital, Nanjing, China; [19]Chongqing Cancer Hospital, Chongqing, China;[20]Nanfang Hospital, Southern Medical University, Guangzhou, China; [21]The Second Xiangya Hospital of Central South University, Changsha, China;[22]The First Affiliated Hospital of Bengbu Medical College, Bengbu, China; [23]Affiliated Zhongshan Hospital Of Dalian University, Dalian, China;[24]Huai'an First People's Hospital, Huai'an, China; [25]The First Affliated Hospital of Zhengzhou University, Zhengzhou, China;[26]The Affiliated People's Hospital of Ningbo University, Ningbo, China; [27]Shandong Cancer Hospital and Institute, Shandong University, Jinan, China;[28]Ningbo Medical Treatment Center Lihuili Hospital, Ningbo, China; [29]The Second Hospital of Hebei Medical University, Shijiazhuang, China; [30]Tianjin Medical University Cancer Institute and Hospital, Tianjin, China;[31]The Second Affiliated Hospital of Anhui Medical University, Hefei, China; [32]China-Japan Friendship Hospital, Beijing, China;*

### Background

Diffuse large B-cell lymphoma (DLBCL) displays marked molecular heterogeneity. The phase Ⅱ GUIDANCE-01 trial showed that subtype-guided immunochemotherapy (R-CHOP-X) improves anti-tumor efficacy with manageable safety *(Cancer Cell 2023:41:1705-1716)*. The phase Ⅲ DEB trial confirmed that R-CHOP plus chidamide significantly prolongs survival in MYC/BCL-2 double-expressing DLBCL (DEL-DLBCL) *(ASH 2025 oral 475)*.

### Aims

We present the large-scale Chinese real-world BELIEVE study (NCT06026488) to assess the effectiveness of different therapeutic strategies in DEL-DLBCL and generate real-world evidence to support clinical decision-making.

### Methods

This retrospective study enrolled DLBCL patients who underwent next-generation sequencing (NGS). Baseline characteristics, treatments, efficacy, and survival data were collected. The primary endpoint was progression-free survival (PFS); key secondary endpoints included objective response rate (ORR), complete response rate (CRR), and overall survival (OS).

**Copyright Information:** (Online) ISSN: 2572-9241
©2026 The Author(s). HemaSphere is published by John Wiley & Sons Ltd on behalf of European Hematology Association. This is an open access Abstract Book distributed under the Attribution-NonCommercial-NoDerivs (CC BY-NC-ND), which allows third parties to download the articles and share them with others as long as they credit the author and the Abstract Book, but they cannot change the content in any way or use them commercially.
**Abstract Book Citations:** Authors, Title, HemaSphere, 2026;10:(S1):pages. The abstract book DOIs can be found on the "Author Information" page.
**Disclaimer:** Articles published in the journal HemaSphere exclusively reflect the opinions of the authors. The authors are responsible for all content in their abstracts including accuracy of the facts, statements, citing resources, etc.

**Results**

Among 3773 DLBCL patients, 28.8% (1088/3773) were classified as DEL. Median age was 61 years (IQR 51–69); 51.5% male; 60.5% had Ann Arbor stage Ⅲ–Ⅳ disease; 34.4% had ≥2 extranodal sites; 69.5% had non-GCB phenotype; and 42.2% had IPI score 3–5. R-CHOP-like was the most frequent treatment regimen (52.5%), followed by R-CHOP plus target agents (16.4%).
Among 1051 DEL-DLBCL patients evaluable for efficacy, ORR and CRR were 75.9% and 57.5%, lower than non-DEL-DLBCL patients (ORR 84.1%, CRR 68.4%). R-CHOP plus target agents achieved higher ORR than R-CHOP (84.2% vs. 75.6%). Within R-CHOP plus target agents combinations, BTK inhibitor was commonly used. Among patients prescribed R-CHOP+BTKi regimens (n=69), ORR and CRR were 88.4% and 75.4%. Orelabrutinib was the commonly used BTKi (n=43) with ORR and CRR were 88.3% and 76.7%.

**Summary/Conclusion**

In this large Chinese real-world cohort, DEL-DLBCL accounts for ~30% of DLBCL and is characterized by aggressive clinicopathological features and inferior outcomes compared with non-DEL DLBCL, confirming its high-risk nature. R-CHOP-like therapy shows limited efficacy in DEL-DLBCL, whereas R-CHOP plus BTKi show promising efficacy, supporting individualized, evidence-based treatment options for DEL-DLBCL. This study provides real-world evidence to optimize DEL-DLBCL management. Long-term survival analyses including PFS are ongoing.

**Copyright Information:** (Online) ISSN: 2572-9241
©2026 The Author(s). HemaSphere is published by John Wiley & Sons Ltd on behalf of European Hematology Association. This is an open access Abstract Book distributed under the Attribution-NonCommercial-NoDerivs (CC BY-NC-ND), which allows third parties to download the articles and share them with others as long as they credit the author and the Abstract Book, but they cannot change the content in any way or use them commercially.
**Abstract Book Citations:** Authors, Title, HemaSphere, 2026;10:(S1):pages. The abstract book DOIs can be found on the “Author Information” page.
**Disclaimer:** Articles published in the journal HemaSphere exclusively reflect the opinions of the authors. The authors are responsible for all content in their abstracts including accuracy of the facts, statements, citing resources, etc.

### (PS2104) PHASE II STUDY OF LENALIDOMIDE MAINTENANCE AFTER RITUXIMAB-METHOTREXATE-BASED INDUCTION THERAPY IN PRIMARY CNS LYMPHOMA

**Topic:** 19. Large B cell lymphomas - Clinical

Ji Yun Lee*[1], Sang-A Kim[1], Deok Hwan Yang[2], Hyeon-Seok Eom[3], Jae-Yong Kwak[6], Myung-Won Lee[4], Hyun Jung Lee[5], Young Hoon Park[7], Young Rok Do[8], Jeong-Ok Lee[1]

*[1]Seoul National University Bundang Hospital, Gyeonggi-do Seongnam-si, South Korea;[2]Chonnam National University Hwasun Hospital, Hwasun, South Korea; [3]National Cancer Center, Goyang, South Korea;[4]Chungnam National University Hospital, Daejeon, South Korea; [5]Kyung Hee University Hospital, Seoul, South Korea;[6]Jeonbuk National University Hospital, Jeonju, South Korea; [7]Ewha Womans University Mokdong Hospital, Seoul, South Korea;[8]Dongsan Medical Center, Daegu, South Korea;*

### Background

Elderly patients with primary CNS lymphoma (PCNSL) are frequently unsuitable for consolidative whole-brain radiotherapy (WBRT) or autologous stem-cell transplantation (ASCT) because of neurotoxicity and treatment-related risks. Lenalidomide is active in relapsed PCNSL, but prospective data on its use as maintenance are limited.

### Aims

This study aimed to assess the efficacy and tolerability of lenalidomide maintenance in elderly, consolidation-ineligible patients with primary CNS lymphoma following methotrexate-based chemoimmunotherapy.

### Methods

We conducted a multicenter phase II trial of lenalidomide maintenance in adults with PCNSL who achieved partial response after 2–6 cycles of methotrexate- and rituximab-based chemoimmunotherapy and were not candidates for WBRT/ASCT. Lenalidomide 15 mg was given orally on days 1–21 of 28-day cycles for up to 12 cycles or until progression or toxicity. The primary endpoint was 2-year progression-free survival (PFS) from maintenance initiation.

### Results

Thirty-one patients were enrolled (median age 72 years); 30 received lenalidomide and received a median of 11 cycles, with 50% completing all 12. At a median follow-up of 20.7 months, 2-year PFS and overall survival were 58.8% and 83.9%, respectively. Grade 3–4 neutropenia occurred in 56%, whereas most non-hematologic adverse events were grade 1–2. Treatment was discontinued for toxicity in 13% of patients; one grade 5 pneumonia occurred, and no thromboembolic events were observed.

### Summary/Conclusion

Lenalidomide maintenance after chemoimmunotherapy is feasible in elderly, consolidation-unsuitable PCNSL patients and warrants further evaluation in comparative trials.

**Copyright Information:** (Online) ISSN: 2572-9241
©2026 The Author(s). HemaSphere is published by John Wiley & Sons Ltd on behalf of European Hematology Association. This is an open access Abstract Book distributed under the Attribution-NonCommercial-NoDerivs (CC BY-NC-ND), which allows third parties to download the articles and share them with others as long as they credit the author and the Abstract Book, but they cannot change the content in any way or use them commercially.
**Abstract Book Citations:** Authors, Title, HemaSphere, 2026;10:(S1):pages. The abstract book DOIs can be found on the “Author Information” page.
**Disclaimer:** Articles published in the journal HemaSphere exclusively reflect the opinions of the authors. The authors are responsible for all content in their abstracts including accuracy of the facts, statements, citing resources, etc.

### (PS2105) ESTABLISHMENT AND VALIDATION OF A PROGNOSTIC NOMOGRAM BASED ON THE PROGNOSTIC NUTRITIONAL INDEX FOR CAR-T THERAPY IN RELAPSED/REFRACTORY LYMPHOMA

**Topic:** 19. Large B cell lymphomas - Clinical

Na Zhang[1], Rui Liu[1], Shilin Gan[1], Chen Chen[1], Zhonghua Fu[1], Menglu Pei[1], Jing Liu[1], Fan Yang*[1], Yuelu Guo[1], Miaomiao Cao[1], Lixia MA[1], Biping Deng[2], Alex Chang[3], Xiaoyan Ke[1], Kai Hu[1]

*[1]Beijing GoBroad Hospital, Department of Lymphoma and Myeloma Research Center, Beijing, China; [2]Beijing GoBroad Boren Hospital, Cytology Laboratory, Beijing, China; [3]Shanghai YaKe Biotechnology Ltd., Engineering Research Center of Gene Technology, Ministry of Education, Institute of Genetics, School of Life Sciences, Shanghai, China;*

### Background

Chimeric antigen receptor T-cell (CAR-T) therapy has become a cornerstone treatment for relapsed/refractory (R/R) lymphoma. However, existing prognostic indicators, such as performance status, tumor burden, and routine laboratory parameters, often fail to accurately predict long-term survival. The Prognostic Nutritional Index (PNI), reflecting both nutritional and immune status, has demonstrated prognostic value in multiple solid and hematologic malignancies, yet its role in CAR-T–treated lymphoma remains poorly characterized.

### Aims

This study aimed to develop and validate a risk prediction model integrating PNI with established clinical parameters—including the International Prognostic Index (IPI), Eastern Cooperative Oncology Group (ECOG) performance status, and body mass index (BMI)—to predict survival outcomes in R/R lymphoma patients undergoing CAR-T therapy, thereby facilitating early identification of high-risk patients and personalized intervention.

### Methods

We retrospectively analyzed 503 R/R lymphoma patients treated with CAR-T therapy between December 2018 and January 2024. Patients were randomly divided into a training set (n=353) and a validation set (n=150) at a 7:3 ratio. PNI was calculated as: serum albumin (g/L) + 5 × peripheral blood lymphocyte count (×$10^9$/L). Independent prognostic factors were identified using multivariate Cox regression analysis, and a nomogram was constructed. Model performance was evaluated using the time-dependent area under the receiver operating characteristic curve (AUC) for discrimination, calibration curves for goodness-of-fit, and decision curve analysis (DCA) for clinical net benefit.

**Copyright Information:** (Online) ISSN: 2572-9241
©2026 The Author(s). HemaSphere is published by John Wiley & Sons Ltd on behalf of European Hematology Association. This is an open access Abstract Book distributed under the Attribution-NonCommercial-NoDerivs (CC BY-NC-ND), which allows third parties to download the articles and share them with others as long as they credit the author and the Abstract Book, but they cannot change the content in any way or use them commercially.
**Abstract Book Citations:** Authors, Title, HemaSphere, 2026;10:(S1):pages. The abstract book DOIs can be found on the “Author Information” page.
**Disclaimer:** Articles published in the journal HemaSphere exclusively reflect the opinions of the authors. The authors are responsible for all content in their abstracts including accuracy of the facts, statements, citing resources, etc.

### Results

Among 503 patients (median age 51 years; 46.1% female), 96.8% had B-cell lymphoma and 3.2% T-cell lymphoma. Clinically, 34.4% had ECOG ≥2, 91.1% were stage III–IV, and 40.6% had IPI ≥3. Median follow-up was 32 months, with median OS 37.2 months (95% CI: 26.6–not reached) and 5-year OS 47.1%. Deaths occurred in 46.5% and 43.3% of patients in the training and validation sets, respectively.
Multivariate Cox regression in the training set identified PNI (<0.0001), IPI (0.0014), and BMI (0.0284) as independent predictors of OS; ECOG (0.2814) was not statistically significant but was included in the model for clinical relevance. A nomogram incorporating PNI, IPI, ECOG, and BMI was developed, where total points correspond to predicted OS risk, with higher scores indicating increased mortality risk.
Bootstrap resampling (1,000 iterations) demonstrated AUCs of 0.720 (95% CI: 0.631–0.810), 0.710 (95% CI: 0.619–0.801), and 0.704 (95% CI: 0.600–0.811) for 1-, 2-, and 3-year OS in the validation set, indicating good discriminative ability. Calibration curves showed excellent agreement between predicted and observed survival. DCA revealed net clinical benefit across a threshold probability range of 0.3–0.9, outperforming "treat-all" or "treat-none" strategies, supporting the model's clinical utility (Figure 1).

### Summary/Conclusion

A nomogram integrating PNI with IPI, ECOG, and BMI effectively predicts survival risk in R/R lymphoma patients undergoing CAR-T therapy, demonstrating robust discrimination, calibration, and clinical applicability in internal validation. This tool provides a simple, intuitive approach for pre-treatment risk stratification and individualized clinical decision-making. External validation is warranted to confirm generalizability

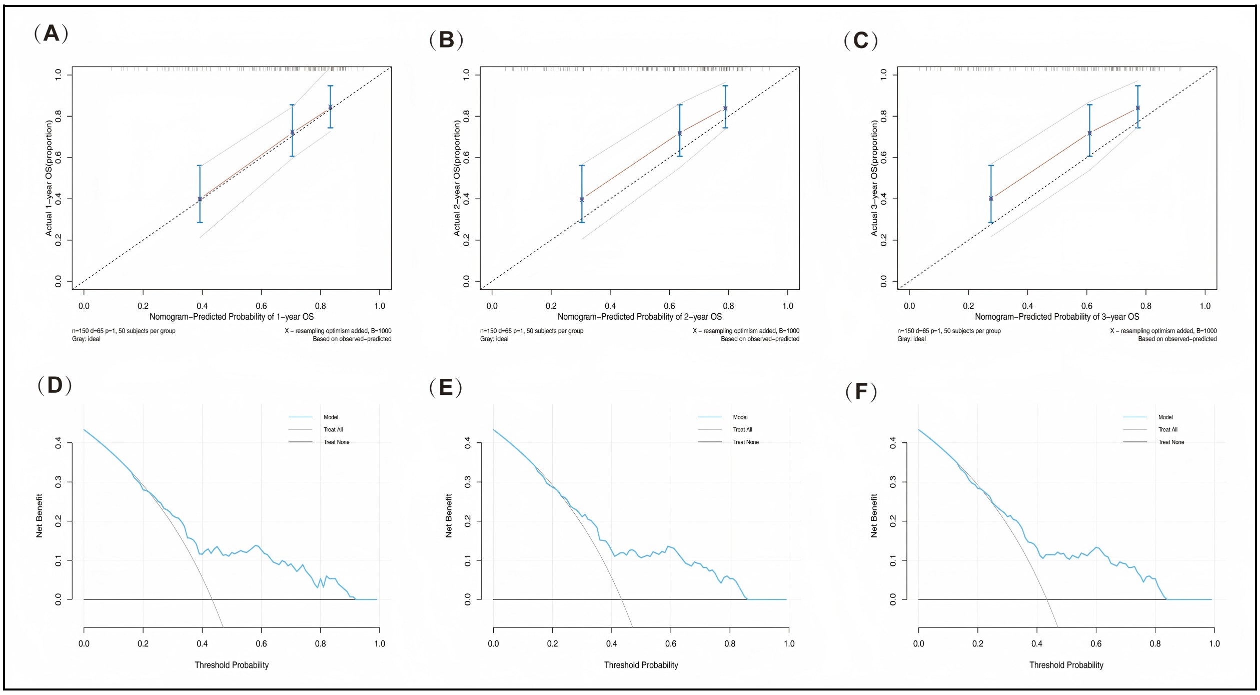

Figure 1. Calibration and decision curves of the nomogram. A–C: Calibration curves for 1-, 2-, and 3-year overall survival predictions. D–F: Corresponding decision curves demonstrating clinical net benefit.

**Copyright Information:** (Online) ISSN: 2572-9241
©2026 The Author(s). HemaSphere is published by John Wiley & Sons Ltd on behalf of European Hematology Association. This is an open access Abstract Book distributed under the Attribution-NonCommercial-NoDerivs (CC BY-NC-ND), which allows third parties to download the articles and share them with others as long as they credit the author and the Abstract Book, but they cannot change the content in any way or use them commercially.
**Abstract Book Citations:** Authors, Title, HemaSphere, 2026;10:(S1):pages. The abstract book DOIs can be found on the "Author Information" page.
**Disclaimer:** Articles published in the journal HemaSphere exclusively reflect the opinions of the authors. The authors are responsible for all content in their abstracts including accuracy of the facts, statements, citing resources, etc.

### (PS2106) THE PHS W-INDEX: A NOVEL PROGNOSTIC FRAMEWORK INTEGRATING NUTRITIONAL AND HEMATOLOGICAL MARKERS TO PREDICT OUTCOMES IN DLBCL PATIENTS TREATED WITH GLOFITAMAB

**Topic:** 19. Large B cell lymphomas - Clinical

Vít Procházka*[1], Prokop Vodicka[2], Kamila Polgarova[2], David Belada[3], David Salek[4], Heidi Mociková[5], Juraj Ďuraš[6], Petra Blahovcova[7], Ales Obr[1], Katerina Steinerova[8], Pavel Klener[2], Andrea Hrušková[1], Tomáš Kozák[5], Barbara Břízová[8], Alice Sykorova[3], Michal Kascak[6], Jan Pirnos[9], Tomas Papajik[1], Frantisek Folber[4], Jozef Michalka[4], Marek Trněný[2]

*[1]University Hospital Olomouc, Dept. of Hemato-Oncology, Olomouc, Czechia;[2]First Faculty of Medicine, Charles University and General Hospital, First Department of Medicine, Prague, Czechia; [3]Faculty of Medicine, Charles University and University Hospital Hradec Kralove, Fourth Department of Internal Medicine – Hematology, Hradec Kralove, Czechia; [4]University Hospital Brno and Faculty of Medicine, Masaryk University, Department of Internal Medicine, Hematology and Oncology, Brno, Czechia; [5]Fakultni nemocnice Kralovske Vinohrady and Third Faculty of Medicine, Charles University, Department of Hematology, Prague, Czechia; [6]Medical Faculty of the Ostrava University and University Hospital, Department of Hematology, Ostrava, Czechia; [7]Czech Lymphoma Study Group, Datacenter, Prague, Czechia;[8]Faculty of Medicine, Charles University and University Hospital Pilsen, Department of Haematology and Oncology, Pilsen, Czechia; [9]Hospital České Budějovice, Dept of Oncology, České Budějovice, Czechia;*

### Background

Diffuse large B-cell lymphoma (DLBCL) management has been transformed by T-cell–engaging bispecific antibodies such as glofitamab, yet heterogeneity in response persists. Although the secondary International Prognostic Index (IPI), revised IPI (R-IPI), and NCCN-IPI are standards for outcome prediction in relapsed/refractory (R/R) disease, they do not fully capture host-related factors—such as nutritional status, anemia, and lymphopenia—that are highly relevant to T-cell–engager therapy. These host factors can be assessed through validated tools like the Prognostic Nutritional Index (PNI  = (10 × serum albumin [g/L] ÷ 10) + (0.005 × total lymphocyte count [$\times 10^9$/L] × $10^6$)).

### Aims

To develop and validate a weighted prognostic score for predicting progression-free survival (PFS) and overall survival (OS) in DLBCL patients treated with glofitamab, and to compare its performance with established indices.

### Methods

Consecutive *de novo* LBCL patients from the Czech NiHiL registry (NCT03199066) were screened. 101 R/R DLBCL patients receiving glofitamab (Apr 2019–Sep 2025) were analyzed: 3 (3.0%) in 2nd, 44 (43.6%) in 3rd, and 54 (53.5%) in ≥4th line. 89 (88.1%) had monotherapy, 12 (11.9%) systemic combinations, mostly R-GemOx (n=7), then Loncastuximab (n=2), Pola-BR, R-ICE, and Gemcitabine (all n=1). Fourteen baseline variables at glofitamab initiation (stage, IPI, Hb, lymphocytes, LDH, age, B-symptoms, ECOG, extranodal sites, WBC/platelets, albumin, sex, and bulk >7.5 cm) were assessed for impact on PFS. Weighted points were assigned based on ROC-defined cut-offs (Hb ≤120 g/L, PNI ≤71.1). The resulting PHS w-Index assigns 2 points for low PNI, 2 for stage III–IV, and 1 for low Hb, stratifying patients into low- (0–1), intermediate- (2–3), and high-risk (4–5) groups. Validation was performed using 1,000 bootstrap iterations for optimism-corrected C-indices.

**Copyright Information:** (Online) ISSN: 2572-9241
©2026 The Author(s). HemaSphere is published by John Wiley & Sons Ltd on behalf of European Hematology Association. This is an open access Abstract Book distributed under the Attribution-NonCommercial-NoDerivs (CC BY-NC-ND), which allows third parties to download the articles and share them with others as long as they credit the author and the Abstract Book, but they cannot change the content in any way or use them commercially.
**Abstract Book Citations:** Authors, Title, HemaSphere, 2026;10:(S1):pages. The abstract book DOIs can be found on the "Author Information" page.
**Disclaimer:** Articles published in the journal HemaSphere exclusively reflect the opinions of the authors. The authors are responsible for all content in their abstracts including accuracy of the facts, statements, citing resources, etc.

### Results

The treatment response was evaluable in 81 patients (80%), overall response rate was 48.1% (n=39) with 35.8% complete responses (n=29). In the whole cohort, 12-month PFS and OS were 41.0% (95% CI 29.3–52.3) and 52.9% (95% CI 40.3–64.0). Of the 43 patients who died during follow-up, the primary cause of death was lymphoma progression in 34 cases (79.1%), treatment-related in 4 (9.3%), unknown in 5 (11.6%). In the internal validation, the PHS w-Index demonstrated superior discriminatory power over all standard clinical indices. The optimism-corrected C-index for the PHS w-Index was 0.659 for PFS and 0.681 for OS. In contrast, standard indices showed significantly lower predictive accuracy in this cohort: original IPI (corrected C-index: PFS 0.544, OS 0.615), R-IPI (PFS 0.557, OS 0.595), and NCCN-IPI (PFS 0.485, OS 0.563). The minimal difference between the apparent and optimism-corrected C-indices (<0.005) across all endpoints suggests high model robustness and minimal risk of overfitting. Twelve-month PFS was 70.9%, 36.3%, and 9.1% in low- (n=27), intermediate- (n=63), and high-risk (n=11) PHS w-Index groups (log-rank p<0.001). Corresponding 12-month OS was 81.5%, 55.6%, and 18.2% (p<0.001).

### Summary/Conclusion

The PHS w-Index is a simple and promising tool that outperforms standard prognostic indices in glofitamab-treated DLBCL. By integrating host fitness (nutrition, anemia, lymphopenia) with tumor burden, it enables improved risk stratification and early identification of high-risk patients for tailored management.
**Acknowledgement:** Supported by the Ministry of Health of the Czech Republic (NU21-03-00411) DRO (FNOL, 00098892).

| Variable | Total (N=101) | Low (n=27) | Intermed. (n=63) | High (n=11) | p-value |
|---|---|---|---|---|---|
| Patients, n (%) | 101 (100%) | 27 (26.7%) | 63 (62.4%) | 11 (10.9%) | — |
| Age, median (IQR) | 70 (56–75) | 63 (56–75) | 71 (58–75) | 63 (51–69) | 0.297 |
| Sex, n (%) | | | | | 0.792 |
| Male | 58 (57.4%) | 17 (63.0%) | 35 (55.6%) | 6 (54.5%) | |
| Female | 43 (42.6%) | 10 (37.0%) | 28 (44.4%) | 5 (45.5%) | |
| Clinical Stage, n (%) | | | | | <0.001 |
| I–II | 27 (26.7%) | 26 (96.3%) | 1 (1.6%) | 0 (0.0%) | |
| III–IV | 74 (73.3%) | 1 (3.7%) | 62 (98.4%) | 11 (100.0%) | |
| ECOG PS, n (%) | | | | | 0.887 |
| 0–1 | 75 (74.3%) | 21 (77.8%) | 46 (73.0%) | 8 (72.7%) | |
| 2+ | 26 (25.7%) | 6 (22.2%) | 17 (27.0%) | 3 (27.3%) | |
| LDH, n (%) | | | | | 0.454 |
| Normal | 28 (28.9%) | 10 (37.0%) | 16 (27.1%) | 2 (18.2%) | |
| Elevated | 69 (71.1%) | 17 (63.0%) | 43 (72.9%) | 9 (81.8%) | |
| Line of therapy, n (%) | | | | | 0.045 |
| L2 | 3 (3.0%) | 2 (7.4%) | 1 (1.6%) | 0 (0.0%) | |
| L3 | 44 (43.6%) | 14 (51.9%) | 29 (46.0%) | 1 (9.1%) | |
| L4+ | 54 (53.5%) | 11 (40.7%) | 33 (52.4%) | 10 (90.9%) | |

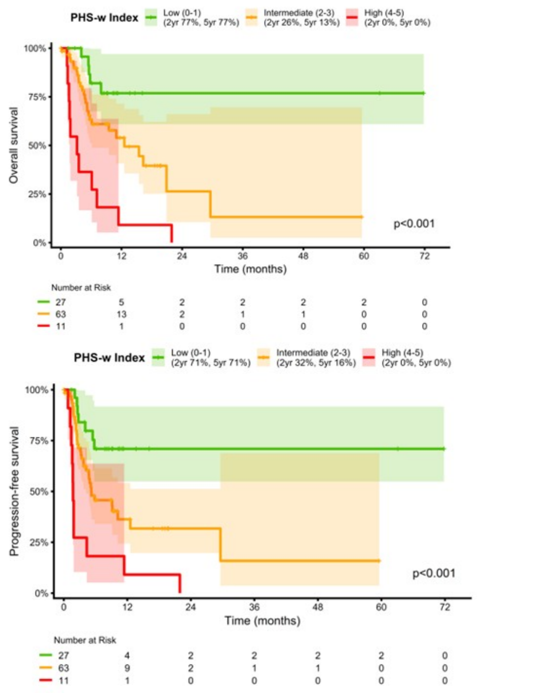

**Copyright Information:** (Online) ISSN: 2572-9241
©2026 The Author(s). HemaSphere is published by John Wiley & Sons Ltd on behalf of European Hematology Association. This is an open access Abstract Book distributed under the Attribution-NonCommercial-NoDerivs (CC BY-NC-ND), which allows third parties to download the articles and share them with others as long as they credit the author and the Abstract Book, but they cannot change the content in any way or use them commercially.
**Abstract Book Citations:** Authors, Title, HemaSphere, 2026;10:(S1):pages. The abstract book DOIs can be found on the "Author Information" page.
**Disclaimer:** Articles published in the journal HemaSphere exclusively reflect the opinions of the authors. The authors are responsible for all content in their abstracts including accuracy of the facts, statements, citing resources, etc.

### (PS2107) PHASE 2 TRIAL OF GLOFITAMAB, POLATUZUMAB VEDOTIN AND ZANUBRUTINIB IN UNTREATED ELDERLY DLBCL PATIENTS: SAFETY RUN-IN RESULTS

**Topic:** 19. Large B cell lymphomas - Clinical

Yuhong Ren*[1], Ling Yuan[1], Yang Ke[1], Yian Zhang[1], Lili Ji[1], Jing Li[1], Peng Liu[1]

*[1]Zhongshan Hospital Affiliated to Fudan University, Department of Hematology, Shanghai, China;*

### Background

R-miniCHOP is the current standard of care for elderly diffuse large B-cell lymphoma (DLBCL), however, treatment-related toxicities remained challenging. There is an unmet need for effective and safe first-line regimens for elderly patients. Glofitamab is now being actively investigated in combination with standard immunochemotherapy for first-line adult DLBCL and exhibited high response rates. Therefore, we designed and initiated a prospective phase 2 clinical trial (NCT07012980) to investigate the efficacy and safety of glofitamab, polatuzumab vedotin and zanubrutinib in untreated elderly DLBCL patients.

### Aims

In this trial, we aim to investigate safety and efficacy of glofitamab, polatuzumab vedotin and zanubrutinib in untreated elderly DLBCL patients. Here, we present the results of safety run-in.

### Methods

Untreated DLBCL patients aged over 70 years old will be enrolled in this study. Central nervous system involvement is excluded. All patients will receive zanubrutinib lead-in 7 days prior to the first cycle. Twenty-one days is a cycle. The regimen consists of 12 cycles, divided into three phases. Zanubrutinib is given 160 mg twice a day orally from day 1 to day 21 throughout all 12 cycles. For cycle 1, the set-up phase, obinutuzumab is administered 1,000 mg intravenously on day 1, polatuzumab vedotin 1.8 mg/KG intravenously on day 2, and glofitamab 2.5mg intravenously on day 8 and 10 mg on day 15. For cycle 2 to 6, target dose phase, polatuzumab vedotin is administered 1.8 mg/KG intravenously on day 1, and glofitamab 30 mg intravenously on day 1. For cycle 7 to 12, consolidation phase, only zanubrutinib remains as maintenance. Whole body 18F-FDG PET/CT scan or contrast-enhanced CT scan is scheduled every 3 cycles. Adverse events are graded by NCI-CTCAE v5.0. The primary end point is 1-year progression-free survival rate. The secondary end points are safety, complete response rate and overall survival. The first 10 patients enrolled in this study will be assigned in the safety run-in phase and reviewed after 28 days of initial treatment.

### Results

Twelve DLBCL patients were screened from June 4, 2025, to January 17, 2026, and 10 patients were enrolled and received at least one cycle of triplet treatment. The median age was 77 years old (range: 70-87). Five (50%) patients had an Eastern Cooperative Oncology Group Performance Status score ≥ 2. Seven (70%) patients had an international prognostic index score of 2 to 5. At the cut-off date February 21, 2026, all 10 patients have completed the 28-day safety run-in observation. Nine patients (90%) had grade 1 to 2 cytokine release syndrome (CRS). One (10%) patient with small intestinal involvement at baseline had both grade 3 lower gastrointestinal hemorrhage and grade 4 tumor lysis syndrome (TLS) and the triplet regimen was discontinued. After best supportive care, this patient was switched to second-line therapy. Two (20%) patients had grade 2 lung infection and 1 (10%) patient had grade 3 lung infection. No patient had tumor flare during safety run-in. Except for the one aforementioned patient, treatment was not discontinued in any of the remaining patients.

**Copyright Information:** (Online) ISSN: 2572-9241
©2026 The Author(s). HemaSphere is published by John Wiley & Sons Ltd on behalf of European Hematology Association. This is an open access Abstract Book distributed under the Attribution-NonCommercial-NoDerivs (CC BY-NC-ND), which allows third parties to download the articles and share them with others as long as they credit the author and the Abstract Book, but they cannot change the content in any way or use them commercially.
**Abstract Book Citations:** Authors, Title, HemaSphere, 2026;10:(S1):pages. The abstract book DOIs can be found on the "Author Information" page.
**Disclaimer:** Articles published in the journal HemaSphere exclusively reflect the opinions of the authors. The authors are responsible for all content in their abstracts including accuracy of the facts, statements, citing resources, etc.

**Summary/Conclusion**

In general, the combination of glofitamab, polatuzumab vedotin and zanubrutinib were considered tolerable in untreated elderly DLBCL patients. The trial is still ongoing, and the follow-up will be further updated.

**Figure 1.** (A) Trial design and (B) safety run-in results.

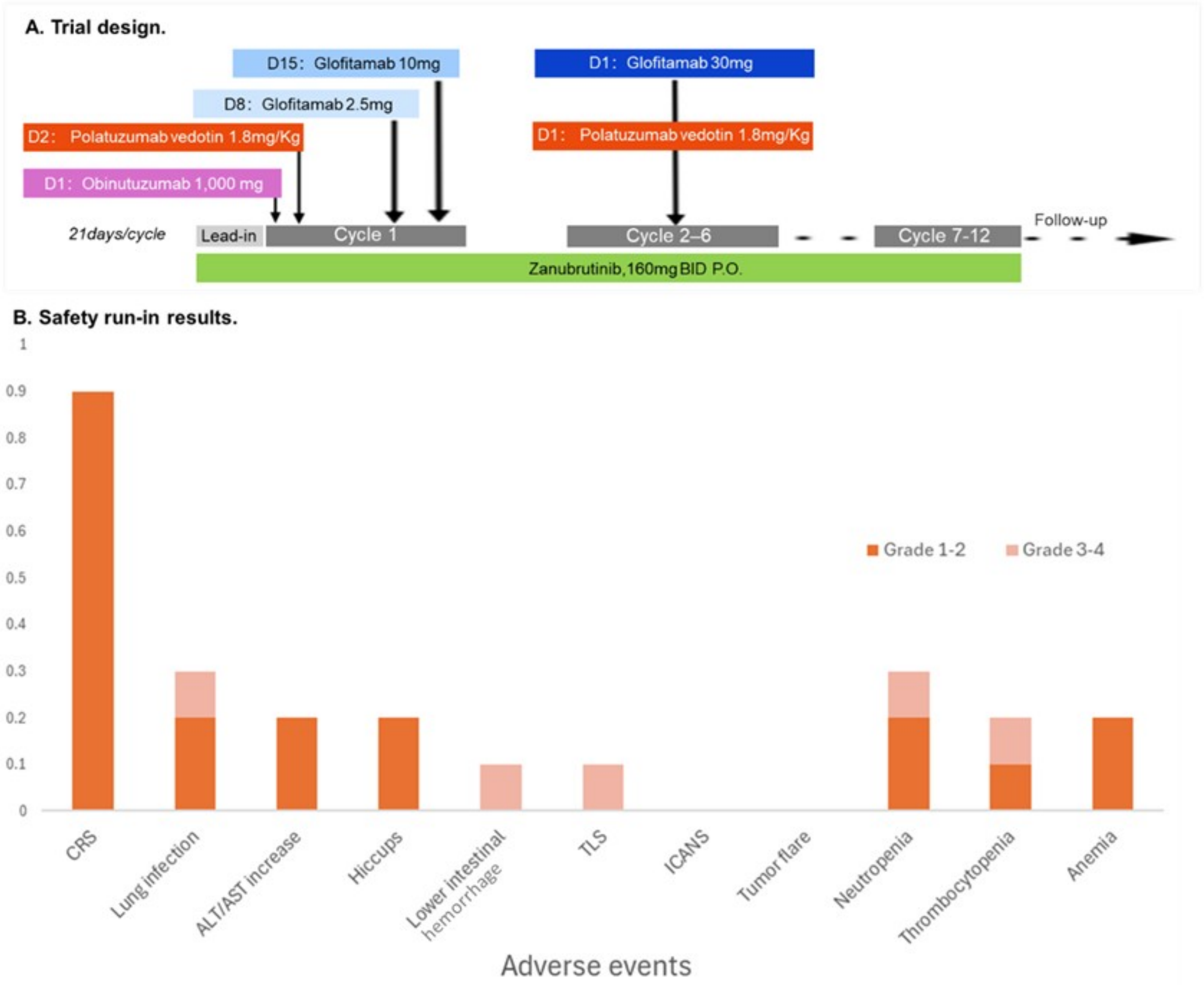

**Copyright Information:** (Online) ISSN: 2572-9241
©2026 The Author(s). HemaSphere is published by John Wiley & Sons Ltd on behalf of European Hematology Association. This is an open access Abstract Book distributed under the Attribution-NonCommercial-NoDerivs (CC BY-NC-ND), which allows third parties to download the articles and share them with others as long as they credit the author and the Abstract Book, but they cannot change the content in any way or use them commercially.
**Abstract Book Citations:** Authors, Title, HemaSphere, 2026;10:(S1):pages. The abstract book DOIs can be found on the "Author Information" page.
**Disclaimer:** Articles published in the journal HemaSphere exclusively reflect the opinions of the authors. The authors are responsible for all content in their abstracts including accuracy of the facts, statements, citing resources, etc.

### (PS2108) EFFICACY AND SAFETY OF GLOFITAMAB IN RELAPSED/REFRACTORY B-CELL NON HODGKIN LYMPHOMA: RESULTS FROM THE RETROSPECTIVE OBSERVATIONAL GIMEMA-FIL IMM0123 STUDY

**Topic:** 19. Large B cell lymphomas - Clinical

Anna Guidetti*[1], Angelica Talone[2], Beatrice Casadei[3], Potito Rosario Scalzulli[4], Luca Nassi[5], Maria Chiara Tisi[6], Vittorio Bolis[7], Emanuele Ravano[8], Francesco D'alò[9], Francesco Piazza[10], Elisa Lucchini[11], Elsa Pennese[12], Filippo Ballerini[13], Alessia Castellino[14], Guido Gini[15], Alice Di Rocco[16], Maurizio Musso[17], Mirko Frascione[18], Angelo Michele Carella[19], Mariangela Iodice[2], Alfonso Piciocchi[2], Pier Luigi Zinzani[20], Paolo Corradini[1]

*[1]Fondazione IRCCS Istituto Nazionale dei Tumori and University of Milano, Milano, Italy;[2]GIMEMA Foundation, Roma, Italy; [3]IRCCS Azienda Ospedaliro-Universitaria di Bologna, Istituto di Ematologia "Seragnoli", Bologna, Italy;[4]IRCCS Fondazione Casa Sollievo della Sofferenza, San Giovanni Rotondo, Italy; [5]Careggi Hospital and University of Firenze, Firenze, Italy;[6]San Bortolo Hospital, Vicenza, Italy; [7]University of Milano, Milano, Italy;[8]ASST Grande Ospedale Metropolitano Niguarda, Milano, Italy; [9]IRCCS Fondazione Policlinico Universitario Agostino Gemelli, Roma, Italy; [10]University of Padova, Padova, Italy; [11]Azienda Sanitaria Universitaria Giuliano Isontina, Trieste, Italy; [12]Presidio Ospedaliero Spirito Santo, Pescara, Italy; [13]IRCCS San Martino Hospital, University of Genova, Genova, Italy; [14]Azienda Ospedaliera Santa Croce e Carle, Cuneo, Italy; [15]AOU delle Marche, Università Politecnica delle Marche, Ancona, Italy; [16]Università degli Studi di Roma "Sapienza" Dipartimento di Medicina Traslazionale e di Precisione, Roma, Italy; [17]Clinica La Maddalena, Palermo, Italy; [18]IRCCS Candiolo Cancer Institute, Candiolo, Italy; [19]IRCCS Casa Sollievo della Sofferenza, San Giovanni Rotondo, Italy; [20]IRCCS Azienda Ospedaliro-Universitaria di Bologna, Istituto di Ematologia "Seragnoli" and University of Bologna, Bologna, Italy;*

### Background

Patients with relapsed and refractory (R/R) aggressive B Cell Non Hodgkin Lymphoma (NHL) have limited survival options beyond second-line treatment. Glofitamab, a T-cell bispecific antibody targeting CD20 and CD3, has demonstrated clinically meaningful activity with a good safety profile. Before commercialization it was available in Italy through a compassionate use program.

### Aims

To evaluate real-world effectiveness and safety of Glofitamab in patients with R/R B-cell lymphoma.

### Methods

IMM0123 is a multicenter, retrospective observational study promoted by GIMEMA-FIL. Adult patients who received at least one dose of Glofitamab in the context of the compassionate use program in Italy between March 2022 and March 2024 were included. Patients were eligible for the study if they had completed at least one year of observation after last Glofitamab administration unless patients died or were lost to follow up. The primary endpoint was complete remission (CR), secondary endpoints included overall response rate (ORR), progression free survival (PFS), overall survival (OS) and safety.

**Copyright Information:** (Online) ISSN: 2572-9241
©2026 The Author(s). HemaSphere is published by John Wiley & Sons Ltd on behalf of European Hematology Association. This is an open access Abstract Book distributed under the Attribution-NonCommercial-NoDerivs (CC BY-NC-ND), which allows third parties to download the articles and share them with others as long as they credit the author and the Abstract Book, but they cannot change the content in any way or use them commercially.
**Abstract Book Citations:** Authors, Title, HemaSphere, 2026;10:(S1):pages. The abstract book DOIs can be found on the "Author Information" page.
**Disclaimer:** Articles published in the journal HemaSphere exclusively reflect the opinions of the authors. The authors are responsible for all content in their abstracts including accuracy of the facts, statements, citing resources, etc.

### Results

Fifty-eight patients treated in 16 Hematological Divisions in Italy were included, median age was 64 years (range, 29-85). Most patients had advanced-stage disease with stage III-IV in 83% (48/58) and high-intermediate/high IPI in 49% of patients (26/58). Histology was diffuse large B-cell lymphoma (DLBCL) in 47 patients (81%), high grade lymphoma in 6 (10%) and other histologies in 5 (8.6%); no patients with primary mediastinal B-cell lymphoma were included. Patients have received a median of 4 previous line of therapy (range, 2 – 9). Forty-five patients (78%) were refractory at last therapy and 32 patients (55%) had failed CAR T-cell therapy. All patients received Obinotuzumab pretreatment. The median number of Glofitamab cycles administered was 4 (range, 1-12). The best ORR was 48% (28/58) including CR in 29% (17/58) and PR in 19% (11/58). With a median follow-up of 24 months (range, 0.29 –33.04), the estimated 2-years OS was 40% (95% CI:28.7-56) and 2-years PFS was 32% (95% CI:21.8-47.6). Patients who achieved CR showed significantly improved 2-years OS (80.1% vs 21.5%, p<0.0001) and PFS (81.4% vs 10.3%, p< 0.0001) compared to those who did not achieve CR (Figure 1). Reasons for discontinuation were treatment completion in 24% of patients (n=14), treatment failure in 17% (n=26), death during treatment in 7 patients (12%), adverse event in 6 patients (10%) and medical decision in 4 (6.9%). Twenty-five patients died due to lymphoma (44%) and 5 (9%) due to infections. Adverse events occurred in 66% of patients (37/56), mainly grade 1-2 cytokine release syndrome (CRS) (26 events) and infections (28 events), 8 patients presented a grade $\geq 3$ infection. Following Glofitamab, 3 patients underwent CAR T-cell therapy and 5 underwent allogeneic stem cell transplantation.

### Summary/Conclusion

In this real-world compassionate-use setting, Glofitamab demonstrated a meaningful clinical activity and a manageable safety profile in heavily pretreated patients with R/R B cell NHL. In this nationwide Italian cohort approximately half of the patients achieved a clinical response, consistent with findings from other real-world studies. Among patients who achieved CR, sustained OS and PFS at 2 years of follow-up further support these promising results, showing Glofitamab as an effective salvage treatment option for R/R NHL patients.

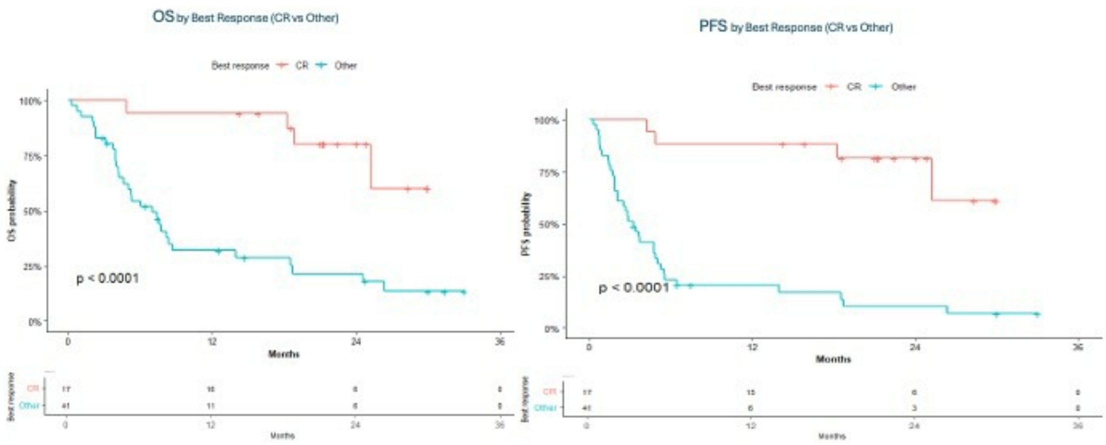

**Copyright Information:** (Online) ISSN: 2572-9241
©2026 The Author(s). HemaSphere is published by John Wiley & Sons Ltd on behalf of European Hematology Association. This is an open access Abstract Book distributed under the Attribution-NonCommercial-NoDerivs (CC BY-NC-ND), which allows third parties to download the articles and share them with others as long as they credit the author and the Abstract Book, but they cannot change the content in any way or use them commercially.
**Abstract Book Citations:** Authors, Title, HemaSphere, 2026;10:(S1):pages. The abstract book DOIs can be found on the "Author Information" page.
**Disclaimer:** Articles published in the journal HemaSphere exclusively reflect the opinions of the authors. The authors are responsible for all content in their abstracts including accuracy of the facts, statements, citing resources, etc.

### (PS2109) SERUM MIR-155-3P, MIR-142-3P AND MIR-22-3P AS DIAGNOSTIC AND PROGNOSTIC BIOMARKERS IN PATIENTS WITH LARGE B-CELL LYMPHOMA (LBCL): A SINGLE CENTER STUDY

**Topic:** 19. Large B cell lymphomas - Clinical

Andreas-Epameinondas Koumpis*[1], Eirini Panteli[1], Vasileios Georgoulis[1], Elisavet Apostolidou[1], Dimitrios Leonardos[1], Konstantina Papathanasiou[1], Konstantina Kelaidoni[1], Lydia Kyriazopoulou[1], Dora Gougopoulou[1], Leonidas Benetatos[1], Panagiotis Kanavaros[2], Evangelos Kolettas[3, 4], Eleni Kapsali[1], Eleftheria Hatzimichael[1]

*[1]Department of Haematology, University General Hospital of Ioannina, Ioannina, Greece;[2]Department of Anatomy-Histology-Embryology, Faculty of Medicine, School of Health Sciences, University of Ioannina, Ioannina, Greece; [3]Laboratory of Biology, Faculty of Medicine, School of Health Sciences, Institute of Biosciences, University Centre for Research and Innovation, University of Ioannina, Ioannina, Greece; [4]Biomedical Research Institute, Foundation for Research and Technology, Ioannina, Greece;*

### Background

Large B-cell lymphoma (LBCL) is a biologically and clinically heterogeneous disease, with many patients relapsing despite standard immunochemotherapy such as R-CHOP. Prognosis relies on the IPI and biological markers (e.g., GCB/ABC subtype and MYC/BCL2 expression), but new non-invasive biomarkers are needed. MicroRNAs (miRNAs) are small (~22-nucleotide) regulatory molecules that modulate gene expression at the post-transcriptional level and circulating miRNAs have been proposed as potential diagnostic and prognostic biomarkers in LBCL.

### Aims

The aim of this study was to evaluate serum expression levels of miR-155-3p, miR-142-3p, and miR-22-3p at diagnosis and to investigate their associations with clinicopathological parameters, progression-free survival (PFS) and overall survival (OS).

### Methods

Serum samples were prospectively collected and analyzed from 45 newly diagnosed LBCL patients (age range 34–84 years; 26 males) treated at our center over a 4.5-year period, along with 26 healthy controls. All patients received immunochemotherapy. RNA isolation was performed using the miRNeasy Serum/Plasma Kit, and cDNA synthesis using the TaqMan Advanced miRNA cDNA Synthesis Kit (Applied Biosystems). Quantitative RT-PCR was conducted on a LightCycler 480 platform using TaqMan™ Advanced miRNA assays for miR-155-3p, miR-142-3p, and miR-22-3p. Exogenous spike-in Ath-miR-159 from Arabidopsis thaliana was used for normalization, and all samples were analyzed in duplicate. The 25th and 75th percentiles of controls were used as cutoffs for low and high expression. ROC curve analysis assessed the diagnostic accuracy of miRNAs. Statistical analyses included Mann–Whitney U and $\chi^2$ tests, Kaplan–Meier survival analysis with log-rank testing, and multivariable Cox proportional hazards modeling for variables with $p<0.05$ in univariable analysis (SPSS v29).

**Copyright Information:** (Online) ISSN: 2572-9241
©2026 The Author(s). HemaSphere is published by John Wiley & Sons Ltd on behalf of European Hematology Association. This is an open access Abstract Book distributed under the Attribution-NonCommercial-NoDerivs (CC BY-NC-ND), which allows third parties to download the articles and share them with others as long as they credit the author and the Abstract Book, but they cannot change the content in any way or use them commercially.
**Abstract Book Citations:** Authors, Title, HemaSphere, 2026;10:(S1):pages. The abstract book DOIs can be found on the "Author Information" page.
**Disclaimer:** Articles published in the journal HemaSphere exclusively reflect the opinions of the authors. The authors are responsible for all content in their abstracts including accuracy of the facts, statements, citing resources, etc.

### Results

The median patient age was 67 years (58% male), with a median follow-up of 26 months; 13 patients died during the study period. Serum miR-155-3p and miR-142-3p levels did not differ significantly between patients and controls, whereas miR-22-3p was significantly elevated in LBCL patients ($p<0.001$). Among the three miRNAs, miR-22-3p demonstrated the best diagnostic performance with an area under the curve (AUC) of 0.784 (95% CI: 0.649-0.899), indicating acceptable discrimination. Low miR-142 expression was associated with increased MYC expression ($p=0.023$) and a lower frequency of the non-GCB phenotype ($p=0.026$), while high miR-22 expression was more common in non-GCB cases ($p=0.046$). Univariate analysis linked hypoalbuminemia, high IPI (3–5), B symptoms, neutropenic fever, and low miR-142-3p with poorer 2-year PFS and multivariable analysis identified hypoalbuminemia (HR 4.53) and low miR-142-3p (HR 3.97) as independent predictors. For OS, previous factors as well as high miR-155-3p and hyponatremia were significant in univariable analysis, while multivariable analysis confirmed high miR-155-3p (HR 6.24) and low miR-142-3p (HR 7.52) as independent adverse prognostic factors.

### Summary/Conclusion

Elevated serum miR-22-3p is characteristic of LBCL but not linked to OS, while high miR-155-3p and low miR-142-3p are independent adverse prognostic factors, supporting their potential as circulating risk-stratification biomarkers. These preliminary findings require validation in larger multicenter studies and further exploration of their integration into existing prognostic models.

**Copyright Information:** (Online) ISSN: 2572-9241
©2026 The Author(s). HemaSphere is published by John Wiley & Sons Ltd on behalf of European Hematology Association. This is an open access Abstract Book distributed under the Attribution-NonCommercial-NoDerivs (CC BY-NC-ND), which allows third parties to download the articles and share them with others as long as they credit the author and the Abstract Book, but they cannot change the content in any way or use them commercially.
**Abstract Book Citations:** Authors, Title, HemaSphere, 2026;10:(S1):pages. The abstract book DOIs can be found on the “Author Information” page.
**Disclaimer:** Articles published in the journal HemaSphere exclusively reflect the opinions of the authors. The authors are responsible for all content in their abstracts including accuracy of the facts, statements, citing resources, etc.

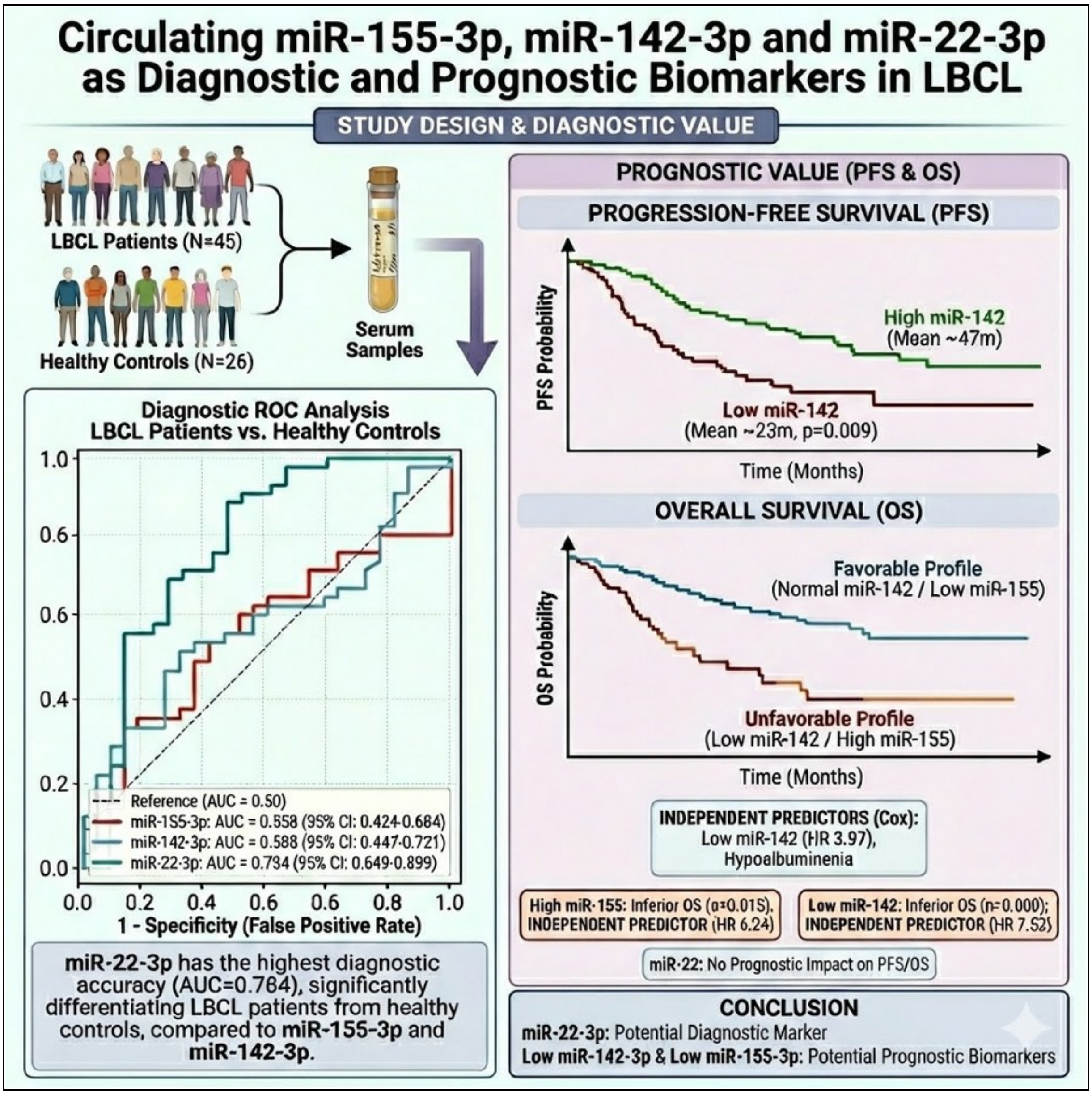

Circulating miR-155-3p, miR-142-3p and miR-22-3p as diagnostic and prognostic biomarkers in LBCL

**Copyright Information:** (Online) ISSN: 2572-9241
©2026 The Author(s). HemaSphere is published by John Wiley & Sons Ltd on behalf of European Hematology Association. This is an open access Abstract Book distributed under the Attribution-NonCommercial-NoDerivs (CC BY-NC-ND), which allows third parties to download the articles and share them with others as long as they credit the author and the Abstract Book, but they cannot change the content in any way or use them commercially.
**Abstract Book Citations:** Authors, Title, HemaSphere, 2026;10:(S1):pages. The abstract book DOIs can be found on the "Author Information" page.
**Disclaimer:** Articles published in the journal HemaSphere exclusively reflect the opinions of the authors. The authors are responsible for all content in their abstracts including accuracy of the facts, statements, citing resources, etc.

### (PS2110) GLOFITAMAB IN RELAPSED/REFRACTORY LARGE B-CELL LYMPHOMA

**Topic:** 19. Large B cell lymphomas - Clinical

Jiayin He*[1], Muchen Zhang[1], Ying Qian[1], Huijuan Zhong[1], Shu Cheng[1], Pengpeng Xu[1], Weili Zhao[1], Li Wang[1]

*[1]Shanghai Institute of Hematology; State Key Laboratory of Medical Genomics; National Research Center for Translational Medicine at Shanghai, Ruijin Hospital Affiliated to Shanghai Jiao Tong University School of Medicine, Shanghai, China;*

### Background

Glofitamab, a CD20×CD3 bispecific antibody, has demonstrated encouraging efficacy in relapsed/refractory (R/R) large B-cell lymphoma (LBCL). We conducted a prospective single-center analysis to evaluate clinical outcomes and exploratory biomarkers in routine practice.

### Aims

To evaluate clinical outcomes of glofitamab in a high-risk R/R LBCL cohort and to explore early circulating tumor DNA (ctDNA) dynamics and immune microenvironment characteristics associated with treatment response.

### Methods

Tumor-informed ctDNA profiling using a 152-gene DLBCL panel was performed at baseline and cycle 2 day 1 (C2D1) in 48 patients. Log-fold change (LFC) between baseline and C2D1 was calculated. Bulk RNA sequencing of tumor biopsies obtained at relapse or progression (n=57) was analyzed using TIMER, CIBERSORT, and xCell to evaluate immune infiltration patterns.

### Results

As of February 28 2026, 114 evaluable patients with R/R LBCL treated with glofitamab at Ruijin Hospital were included. Median prior lines were 3 (range 2–9). 92.1% had International Prognostic Index (IPI) scores of 2–5, and 43.9% had Eastern Cooperative Oncology Group (ECOG) performance status ≥2. Best overall response rate (ORR) was 80%, with a complete response (CR) rate of 42%. Early response (cycle 2 or 3) ORR was 78%, demonstrating clinical activity in this high-risk cohort.
Among patients with paired ctDNA data, responders (CR or partial response [PR]) showed significantly lower ctDNA levels at C2D1 and greater LFC reduction compared with non-responders (stable disease [SD] or progressive disease [PD]). A greater magnitude of ctDNA decline at C2D1 was associated with longer progression-free survival, with no progression events observed in patients achieving deep molecular reduction during follow-up. These findings warrant validation in larger cohorts.
Immune profiling of relapsed/progressive tumor samples revealed higher infiltration of dendritic cells, CD4+ and CD8+ T cells, activated memory CD4+ T cells, and M1 macrophages in responders, suggesting that an inflamed tumor microenvironment may be associated with sensitivity to bispecific antibody therapy.
Safety was favorable. Cytokine release syndrome (CRS) occurred in 43.2% of patients (≥Grade 3: 2.7%), predominantly during cycle 1 (any grade: 35.1%; ≥Grade 3: 1.4%), with only one Grade 1 event observed after cycle 5 and no Grade 5 events. Immune effector cell-associated neurotoxicity syndrome (ICANS) occurred in 4.1% (≥Grade 3: 1.4%), with no Grade 5 events reported.

**Copyright Information:** (Online) ISSN: 2572-9241
©2026 The Author(s). HemaSphere is published by John Wiley & Sons Ltd on behalf of European Hematology Association. This is an open access Abstract Book distributed under the Attribution-NonCommercial-NoDerivs (CC BY-NC-ND), which allows third parties to download the articles and share them with others as long as they credit the author and the Abstract Book, but they cannot change the content in any way or use them commercially.
**Abstract Book Citations:** Authors, Title, HemaSphere, 2026;10:(S1):pages. The abstract book DOIs can be found on the "Author Information" page.
**Disclaimer:** Articles published in the journal HemaSphere exclusively reflect the opinions of the authors. The authors are responsible for all content in their abstracts including accuracy of the facts, statements, citing resources, etc.

**Summary/Conclusion**

In this high-risk R/R LBCL cohort, including patients commonly underrepresented in clinical trials, glofitamab achieved meaningful response rates with manageable toxicity. Early ctDNA reduction after one treatment cycle was associated with improved progression-free survival. Immune profiling further indicates that an inflamed tumor microenvironment may underlie sensitivity to bispecific antibody therapy. Together, these findings expand the clinical applicability of glofitamab and support the integration of early molecular monitoring and biological stratification in future treatment strategies.

**Copyright Information:** (Online) ISSN: 2572-9241
©2026 The Author(s). HemaSphere is published by John Wiley & Sons Ltd on behalf of European Hematology Association. This is an open access Abstract Book distributed under the Attribution-NonCommercial-NoDerivs (CC BY-NC-ND), which allows third parties to download the articles and share them with others as long as they credit the author and the Abstract Book, but they cannot change the content in any way or use them commercially.
**Abstract Book Citations:** Authors, Title, HemaSphere, 2026;10:(S1):pages. The abstract book DOIs can be found on the “Author Information” page.
**Disclaimer:** Articles published in the journal HemaSphere exclusively reflect the opinions of the authors. The authors are responsible for all content in their abstracts including accuracy of the facts, statements, citing resources, etc.

### (PS2111) SINGLE-TARGET (CD19) VERSUS DUAL-TARGET (CD19/CD22 OR CD19/CD20) CAR T-CELL THERAPY IN RELAPSED/REFRACTORY DIFFUSE LARGE B-CELL LYMPHOMA: A SYSTEMATIC REVIEW AND META-ANALYSIS

**Topic:** 19. Large B cell lymphomas - Clinical

Ying He*[1], Yaxing Xiong[2], Dan Chen[1], Mengjiao Li[1, 3], Baitao Dou[1, 4], Yanling Li[1, 3], Jiajia Li[1, 3], Ling Qiu[1], Shihui Ren[1], Xiao Wang[1, 5], Mingqiang Ren[6], Yunming Li[7], Hao Yao[1, 5, 8, 9], Fangyi Fan[1, 3]

*[1]Department of Hematology, The General Hospital of Western Theater Command, Chengdu, China;[2]School of Public Health, North Sichuan Medical College, Nanchong, China; [3]Department of Clinical Medicine, North Sichuan Medical College, Nanchong, China; [4]Department of Hematology, Chongqing University FuLing Hospital, Chongqing, China;[5]College of Medicine, Southwest Jiaotong University, Chengdu, China; [6]Department of Hematology, Affiliated Hospital of Zunyi Medical Univercity, Zunyi, China; [7]Department of Information, Medical Support Center, The General Hospital of Western Theater Command,, Chengdu, China; [8]Institute of Basic Medicine, North Sichuan Medical College, Nanchong, China;[9]Tissue Stress Injury and Functional Repair Key Laboratory of Sichuan Province, Chengdu, China;*

### Background

Chimeric antigen receptor (CAR) T-cell therapy has become a pivotal treatment option for patients with relapsed/refractory diffuse large B-cell lymphoma (R/R DLBCL). While single-target CD19 CAR T-cell therapy is widely used in clinical practice, dual-target strategies (e.g., CD19/CD22 or CD19/CD20) have been developed to reduce antigen escape and potentially improve treatment durability. However, direct comparisons between these approaches remain limited.

### Aims

This meta-analysis aimed to systematically compare the efficacy and safety of single-target (CD19) versus dual-target (CD19/CD22 or CD19/CD20) CAR T-cell therapy in patients with R/R DLBCL.

### Methods

A systematic review and meta-analysis were conducted following predefined inclusion criteria. PubMed, Embase, Cochrane Library, Web of Science, and major conference proceedings (ASH, EHA, ASCO, ICML) were searched from database inception to December 31, 2025. Clinical studies evaluating single-target (CD19) or dual-target (CD19/CD22 or CD19/CD20) CAR T-cell therapies in R/R DLBCL were included. Primary efficacy outcomes included overall response rate (ORR), complete response (CR) rate, and 1-year progression-free survival (PFS). Safety outcomes included grade ≥3 cytokine release syndrome (CRS), immune effector cell-associated neurotoxicity syndrome (ICANS), infections, and hematologic toxicities. Literature screening, data extraction, and risk-of-bias assessment were independently performed by two investigators. Meta-analysis was conducted using R (version 4.3.1) with the *meta* and *metafor* packages under a random-effects model.

**Copyright Information:** (Online) ISSN: 2572-9241
©2026 The Author(s). HemaSphere is published by John Wiley & Sons Ltd on behalf of European Hematology Association. This is an open access Abstract Book distributed under the Attribution-NonCommercial-NoDerivs (CC BY-NC-ND), which allows third parties to download the articles and share them with others as long as they credit the author and the Abstract Book, but they cannot change the content in any way or use them commercially.
**Abstract Book Citations:** Authors, Title, HemaSphere, 2026;10:(S1):pages. The abstract book DOIs can be found on the "Author Information" page.
**Disclaimer:** Articles published in the journal HemaSphere exclusively reflect the opinions of the authors. The authors are responsible for all content in their abstracts including accuracy of the facts, statements, citing resources, etc.

**Results**

A total of 18 studies comprising 1,425 patients were included (11 single-target studies, n=1,286; 7 dual-target studies, n=139). Median follow-up ranged from 6.6 to 28.6 months, and median age ranged from 54 to 74 years. Dual-target CAR T-cell therapy demonstrated numerically higher ORR compared with single-target therapy (86% vs. 75%, P=0.06), although the difference did not reach statistical significance. Similarly, CR rates (62% vs. 53%, P=0.22) and 1-year PFS rates (67% vs. 48%, P=0.23) were higher in the dual-target group, without statistically significant differences. Grade ≥3 hematologic toxicity was the most frequent severe adverse event in both groups and was significantly higher with dual-target CAR T-cell therapy compared with single-target therapy (81% vs. 55%, P=0.02). In contrast, the incidence of grade ≥3 infections was significantly lower in the dual-target group (4% vs. 13%, P<0.001). Severe CRS (10% vs. 8%, P=0.55) and neurological events (7% vs. 13%, P=0.15) were comparable between groups. Overall, most adverse events were grade 1–2 and manageable.

**Summary/Conclusion**

This meta-analysis suggests that dual-target (CD19/CD22 or CD19/CD20) CAR T-cell therapy may provide numerically improved response rates compared with single-target CD19 therapy in R/R DLBCL, although differences in CR and 1-year PFS were not statistically significant. Dual-target therapy was associated with higher rates of grade ≥3 hematologic toxicity but a lower incidence of severe infections, while other major adverse events were comparable between groups. Given the indirect nature of comparisons and potential heterogeneity across studies, these findings should be interpreted cautiously. Prospective randomized trials are warranted to determine the optimal CAR T-cell strategy in R/R DLBCL.

**Copyright Information:** (Online) ISSN: 2572-9241
©2026 The Author(s). HemaSphere is published by John Wiley & Sons Ltd on behalf of European Hematology Association. This is an open access Abstract Book distributed under the Attribution-NonCommercial-NoDerivs (CC BY-NC-ND), which allows third parties to download the articles and share them with others as long as they credit the author and the Abstract Book, but they cannot change the content in any way or use them commercially.
**Abstract Book Citations:** Authors, Title, HemaSphere, 2026;10:(S1):pages. The abstract book DOIs can be found on the "Author Information" page.
**Disclaimer:** Articles published in the journal HemaSphere exclusively reflect the opinions of the authors. The authors are responsible for all content in their abstracts including accuracy of the facts, statements, citing resources, etc.

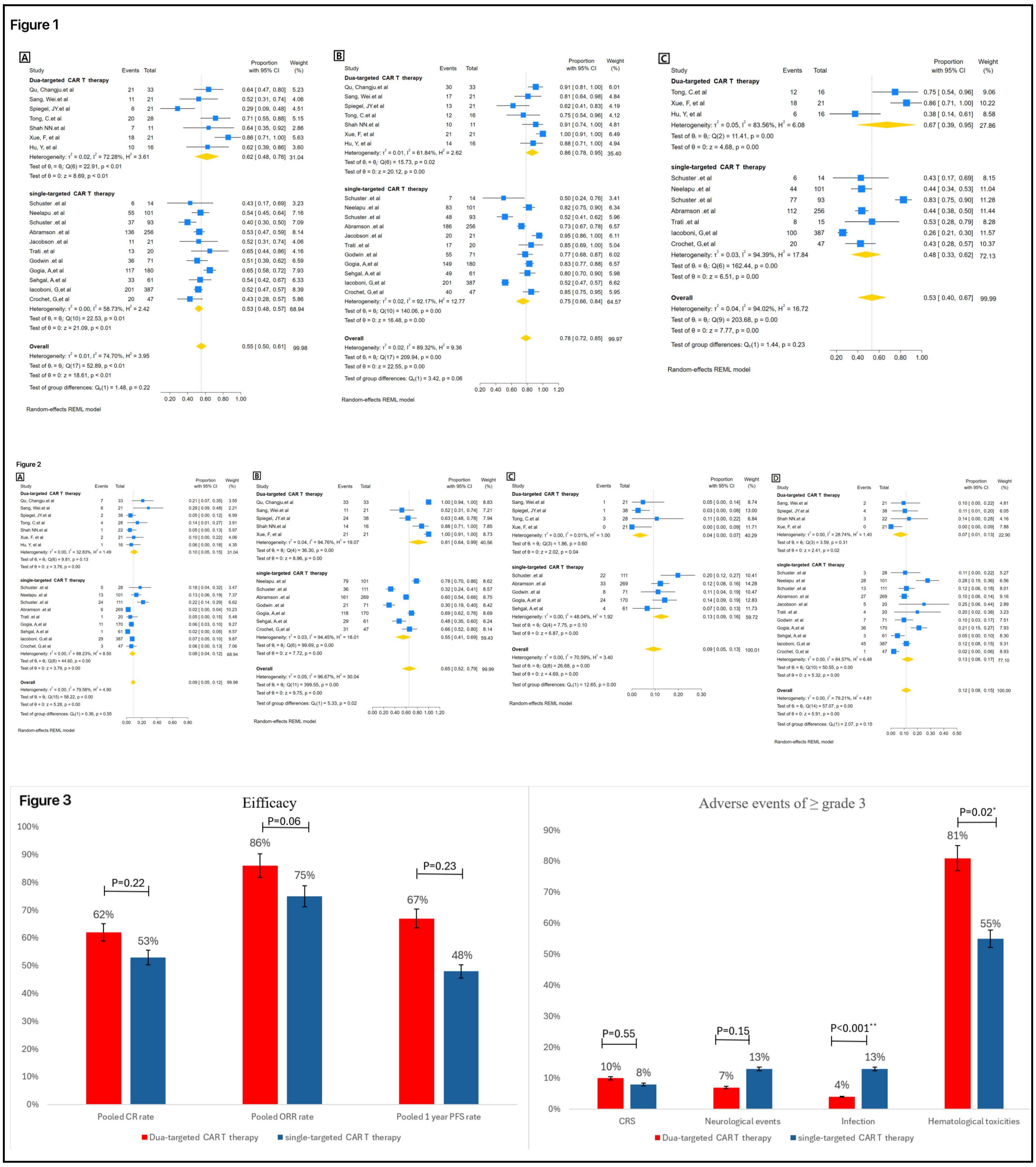

Figure 1. Pooled efficacy outcomes of single-target versus dual-target CAR T-cell therapy in relapsed/refractory diffuse large B-cell lymphoma. (A) Pooled complete response (CR) rate. (B) Pooled overall response rate (ORR). (C) Pooled 1-year progression-free survival (PFS) rate. Dual-target CAR T-cell therapy demonstrated numerically higher CR (62% vs. 53%, P=0.22), ORR (86% vs. 75%, P=0.06), and 1-year PFS (67% vs. 48%, P=0.23) compared with single-target therapy; however, intergroup differences were not statistically significant. Diamonds represent pooled estimates calculated using a random-effects model; horizontal lines indicate 95% confidence intervals. Figure 2. Pooled incidence of grade ≥3 adverse events. (A) Cytokine release syndrome (CRS). (B) Hematologic toxicities. (C) Infections. (D) Neurological events. Grade ≥3 hematologic toxicity was significantly higher in the dual-target group (81% vs. 55%, P=0.02), whereas infection was more frequent in the single-target group (13% vs. 4%, P<0.001). Severe CRS (10% vs. 8%, P=0.55) and neurological events (7% vs. 13%, P=0.15) were comparable between groups. Figure 3. Summary of pooled efficacy and grade ≥3 adverse events. Bar charts illustrate pooled response rates and severe adverse event rates for dual-target and single-target CAR T-cell therapy. Error bars indicate 95% confidence intervals.

Copyright Information: (Online) ISSN: 2572-9241
©2026 The Author(s). HemaSphere is published by John Wiley & Sons Ltd on behalf of European Hematology Association. This is an open access Abstract Book distributed under the Attribution-NonCommercial-NoDerivs (CC BY-NC-ND), which allows third parties to download the articles and share them with others as long as they credit the author and the Abstract Book, but they cannot change the content in any way or use them commercially.
Abstract Book Citations: Authors, Title, HemaSphere, 2026;10:(S1):pages. The abstract book DOIs can be found on the “Author Information” page.
Disclaimer: Articles published in the journal HemaSphere exclusively reflect the opinions of the authors. The authors are responsible for all content in their abstracts including accuracy of the facts, statements, citing resources, etc.

### (PS2112) FOLLOW-UP-TIME–ADJUSTED NON-RELAPSE MORTALITY (NRM) IN PATIENTS WITH NON-HODGKIN LYMPHOMA FOLLOWING CHIMERIC ANTIGEN RECEPTOR (CAR) T-CELL THERAPY WITHIN CLINICAL TRIALS

**Topic:** 19. Large B cell lymphomas - Clinical

Michael Leisch*[1], Thomas Melchardt[1], Guido Ghilardi[2], Caroline Arber[3], Ulrich Jäger[4], Normann Steiner[5], Björn Chapuy[6], Robin Sanderson[7], Bernd Schoepf[8], Graeme Ball[9], Tsveta Hadjivassileva[10], Saurabh Dahiya[11]

*[1]Department of Internal Medicine III, Paracelsus Medical University, Salzburg, Austria; [2]Oncology Institute of Southern Switzerland, Bellinzona, Switzerland; [3]Department of Oncology, Lausanne University Hospital (CHUV), Lausanne, Switzerland; [4]Medical University of Vienna, Vienna, Austria; [5]Universitätsklinik für Innere Medizin V, Medizinnischen Innsbruck, Innsbruck, Austria; [6]Charité - Universitätsmedizin Berlin, Berlin, Germany; [7]King's College Hospital, London, United Kingdom; [8]Kite, a Gilead Company, Vienna, Austria; [9]Kite, a Gilead Company, Santa Monica, CA, United States of America; [10]Kite, a Gilead Company, London, United Kingdom; [11]Stanford University, Stanford, CA, United States of America;*

### Background

CAR T-cell therapy has transformed the treatment landscape and significantly extended survival for patients (pts) with relapsed or refractory non-Hodgkin lymphoma (NHL). Rare toxicities of CAR T-cell therapies, beyond cytokine release syndrome (CRS) and immune effector cell-associated neurotoxicity syndrome, may impact long-term survivorship. As such, it is important for clinical decision-making to consistently capture NRM, defined as any death not primarily caused by disease progression. Due to the substantial impact of follow-up time on NRM, comparative analyses must include adjustments for differences to be considered valid.

### Aims

To characterize follow-up–adjusted, product-specific NRM rates in pts with NHL who received CAR T-cell therapy in a clinical trial. To assess types and proportions of NRM.

### Methods

In this systematic literature review and meta-analysis, selected references/studies met pre-defined eligibility criteria and included adult pts with NHL (large B-cell lymphoma [LBCL], follicular lymphoma [FL], or mantle cell lymphoma [MCL]) treated with an approved anti-CD19 CAR T-cell therapy (axicabtagene ciloleucel [axi-cel], brexucabtagene autoleucel [brexu-cel], lisocabtagene maraleucel [liso-cel], tisagenlecleucel [tisa-cel]) in a prospective clinical trial. Variables were extracted manually; only studies with comparable follow-up were included in the follow-up–adjusted analysis. The primary variable was the number of pts with an NRM event; subgroup analyses were conducted by NHL and product type. NRM rates were determined via a meta-analysis of prevalence (Barendregt, et al. *JECH.* 2013).

**Copyright Information:** (Online) ISSN: 2572-9241
©2026 The Author(s). HemaSphere is published by John Wiley & Sons Ltd on behalf of European Hematology Association. This is an open access Abstract Book distributed under the Attribution-NonCommercial-NoDerivs (CC BY-NC-ND), which allows third parties to download the articles and share them with others as long as they credit the author and the Abstract Book, but they cannot change the content in any way or use them commercially.
**Abstract Book Citations:** Authors, Title, HemaSphere, 2026;10:(S1):pages. The abstract book DOIs can be found on the "Author Information" page.
**Disclaimer:** Articles published in the journal HemaSphere exclusively reflect the opinions of the authors. The authors are responsible for all content in their abstracts including accuracy of the facts, statements, citing resources, etc.

### Results

In total, 21 clinical trials met the inclusion criteria, among which 1810 pts were infused, and 130 NRM events were reported. NRM rates (95% CI) for the shortest and longest follow-up timepoints were 5.4% (3.4-7.8) at 17.3 mo and 12.0% (8.5-16.0) at 43.3 mo. After adjusting for follow-up time, the median follow-up time (SD) was 16.7 ± 6.8 mo. At this timepoint, the NRM rate (95% CI) was 6.4% (4.8-8.3) with 3.8% (1.8-4.4), 6.7% (5.3-8.3), and 9.4% (2.5-19.6) for FL, LBCL, and MCL, respectively. NRM rates for axi-cel, brexu-cel, liso-cel, and tisa-cel at 16.7 mo were 5.4% (3.3-8.0), 5.9% (1.2-13.4), 9.1% (5.9-12.8), and 4.2% (1.0-9.3), respectively. Compared with axi-cel and brexu-cel (same construct), tisa-cel had a lower NRM rate (*P*=.477; 95% CI, -0.261 to 0.122) and liso-cel had a higher NRM rate (*P*=.089; 95% CI, -0.018 to 0.254). When data were normalized for average follow-up time, axi-cel and liso-cel had similar NRM rate increases of 0.30% and 0.32% per mo, respectively.
The main cause of all 130 NRM events were infections (n=59). Further causes of NRM were organ-related toxicity (n=32), secondary primary malignancy (n=11), CRS or hemophagocytic lymphohistiocytosis (n=4), neurotoxicity (NT; n=1), and other (n=12). For 11 events, NRM causes were unspecified. Only 0.85% of pts with FL died due to infections compared with 3.45% and 5.79% of pts with LBCL and MCL, respectively. Rates of Grade 5 infections were largely comparable across all products but were numerically highest for axi-cel/brexu-cel; organ-related NRM events were numerically highest for liso-cel (Figure).

### Summary/Conclusion

Follow-up time significantly impacted NRM rates. NRM rates differed by NHL type (lowest, FL; highest, MCL) and product (lowest, tisa-cel; highest, liso-cel). The most common NRM cause was infection; very few pts died due to CRS or NT.

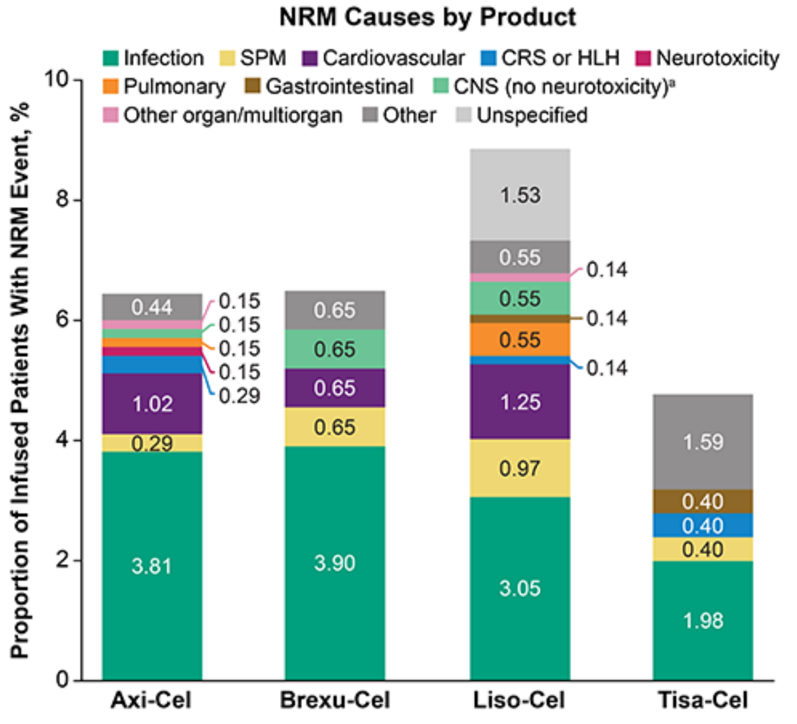

ª Includes fludarabine-associated leukoencephalopathy and progressive multifocal leukoencephalopathy.
Abbreviations: CNS, central nervous system; CRS, cytokine release syndrome; HLH, hemophagocytic lymphohistiocytosis; SPM, secondary primary malignancy.

**Copyright Information:** (Online) ISSN: 2572-9241
©2026 The Author(s). HemaSphere is published by John Wiley & Sons Ltd on behalf of European Hematology Association. This is an open access Abstract Book distributed under the Attribution-NonCommercial-NoDerivs (CC BY-NC-ND), which allows third parties to download the articles and share them with others as long as they credit the author and the Abstract Book, but they cannot change the content in any way or use them commercially.
**Abstract Book Citations:** Authors, Title, HemaSphere, 2026;10:(S1):pages. The abstract book DOIs can be found on the "Author Information" page.
**Disclaimer:** Articles published in the journal HemaSphere exclusively reflect the opinions of the authors. The authors are responsible for all content in their abstracts including accuracy of the facts, statements, citing resources, etc.

### (PS2113) SAFETY AND EFFICACY OF RELMACABTAGENE AUTOLEUCEL (RELMA-CEL) IN ADULT PATIENTS WITH RELAPSED/REFRACTORY LARGE B-CELL LYMPHOMA RESULTS FROM AN OPEN-LABEL, SINGLE-ARM STUDY

**Topic:** 19. Large B cell lymphomas - Clinical

Xiangrui Meng*[1], Lanfang Li[1], Zheng Song[1], Zhengzi Qian[1], Lihua Qiu[1], Yongen Gu[2], Mengmeng Chen[2], Hui-Lai Zhang[1]

*[1]Tianjin Medical University Cancer Institute and Hospital, Tianjin, China;[2]JW Therapeutics (Shanghai) Co. Ltd, Shanghai, China;*

### Background

Relma-cel, a CD19-directed CAR-T therapy, is an approved treatment for relapsed/refractory (R/R) large B-Cell lymphoma (LBCL). To reduce manufacturing costs and enhance supply-chain stability, an in-house lentiviral vector manufacturing process was established. This study was a post-approval bridging study to evaluate the relma-cel manufactured using the domestically produced lentiviral vector (JWLV011).

### Aims

To assess the efficacy, safety and pharmacokinetic (PK)/ pharmcodynamic (PD) profiles of relma-cel manufactured with JWLV011 in adult patients with R/R LBCL.

### Methods

This phase II study enrolled adults (≥18 years) with histologically confirmed R/R LBCL who had relapsed or progressed after ≥2 prior systemic therapies, including an anthracycline-containing regimen and anti-CD20 monoclonal antibody, or following autologous stem cell transplantation. Following by lymphodepleting chemotherapy with cyclophosphamide (250 mg/m$^2$/day × 3 days) and fludarabine (25 mg/m$^2$/day × 3 days), and then received a single infusion of relma-cel at a dose of 100 × 10$^6$ CAR$^+$ T cells. The primary endpoint was investigator-assessed objective response rate (ORR) at 3 months post-infusion.

### Results

As of July 15, 2025, 14 patients underwent leukapheresis and 12 received relma-cel. The median age was 61.5 years (range, 46–76; 41.7% ≥65 years), and 58.3% were male. High-risk disease features were common: 50.0% had an International Prognostic Index ≥3, 83.3% had Ann Arbor stage III/IV disease, 41.7% had elevated baseline lactate dehydrogenase, and 83.3% had the non-germinal center B-cell-like subtype. Prior therapies included ≥3 lines in 41.7% of patients; 25.0% had received polatuzumab vedotin and 25.0% had received CD3/CD20 bispecific antibodies (one patient was primary refractoriness, two patients were relapsed within 12 months of first-line therapy, and three patients were subsequently treated with bispecific antibodies, and all were refractory to bispecific antibody). Overall, 33.3% had relapsed disease, 66.7% were refractory to their most recent therapy. 41.7% of patients received bridging therapy. With a median follow-up was 4.6 months (range, 2.79–5.85), in the modified intent to treatment (mITT) population (n=12), the investigator-assessed ORR at 3 months was 58.33%, with a CRR of 41.67%, meeting the primary endpoint. The best investigator-assessed ORR was 66.67%, with a best CRR of 41.67%. Independent review committee (IRC)-assessed ORR at 3 months was 58.33%, with a CRR of 58.33%. The best ORR was 75.00%, with a best CRR of 58.33% (**Figure 1**). Three patients refractory to bispecific antibodies achieved PR, PD and CR, respectively, at 1 month; excluding the patient with PD, the other two patients had PD and CR respectively at 3 months. Median DOR, PFS, and OS have not been reached. Relma-cel manufactured with JWLV011 demonstrated a manageable safety profile with no treatment-related deaths, or study discontinuations due to adverse events. All patients experienced treatment-related adverse events, 8.3% developed serious treatment-emergent adverse events. Cytokine release syndrome occurred in 83.3% of patients, all grade 1-2. No patient developed neurological toxicities.

**Copyright Information:** (Online) ISSN: 2572-9241
©2026 The Author(s). HemaSphere is published by John Wiley & Sons Ltd on behalf of European Hematology Association. This is an open access Abstract Book distributed under the Attribution-NonCommercial-NoDerivs (CC BY-NC-ND), which allows third parties to download the articles and share them with others as long as they credit the author and the Abstract Book, but they cannot change the content in any way or use them commercially.
**Abstract Book Citations:** Authors, Title, HemaSphere, 2026;10:(S1):pages. The abstract book DOIs can be found on the "Author Information" page.
**Disclaimer:** Articles published in the journal HemaSphere exclusively reflect the opinions of the authors. The authors are responsible for all content in their abstracts including accuracy of the facts, statements, citing resources, etc.

**Summary/Conclusion**

Relma-cel manufactured using the domestically produced lentiviral vector demonstrated favorable efficacy and a manageable safety profile in adult patients with R/R LBCL. These findings support the feasibility of in-house lentiviral vector manufacturing without compromising clinical performance.

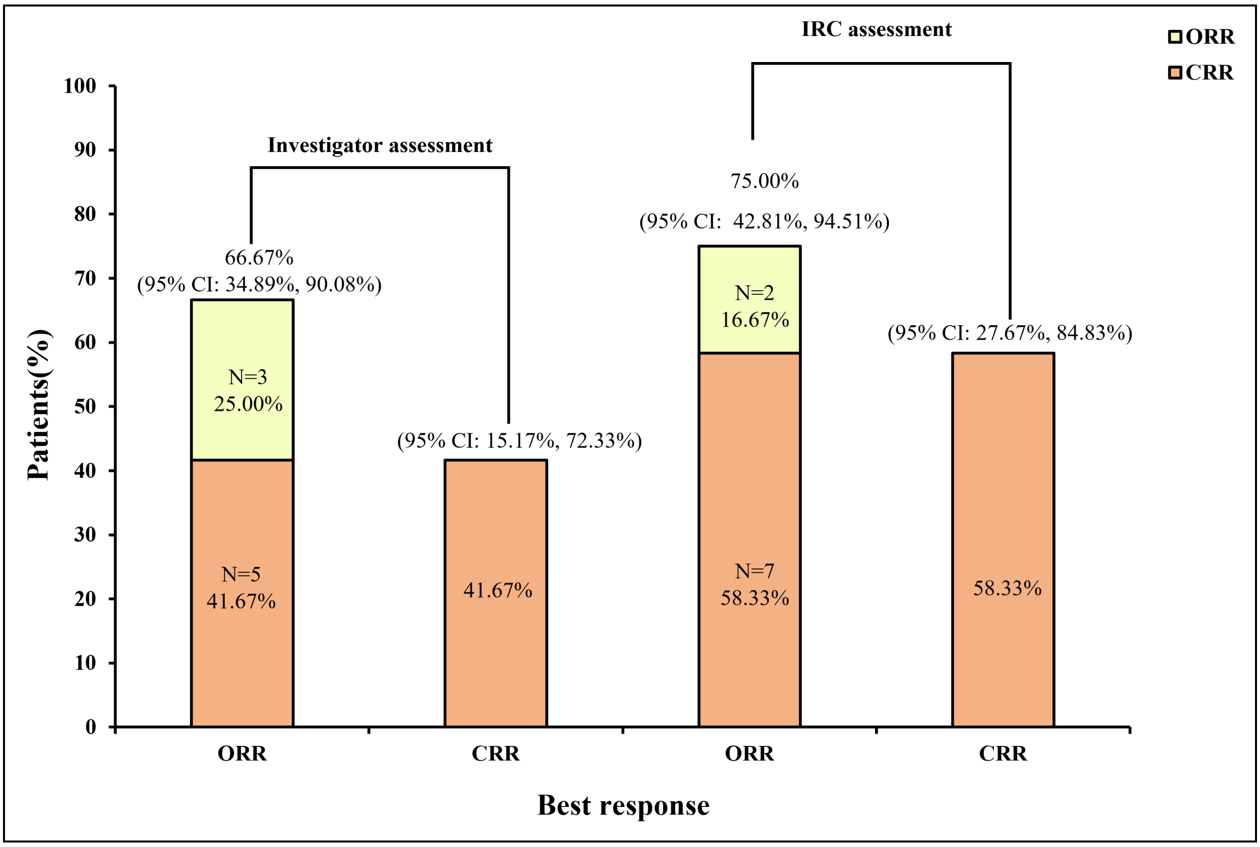

**Copyright Information:** (Online) ISSN: 2572-9241
©2026 The Author(s). HemaSphere is published by John Wiley & Sons Ltd on behalf of European Hematology Association. This is an open access Abstract Book distributed under the Attribution-NonCommercial-NoDerivs (CC BY-NC-ND), which allows third parties to download the articles and share them with others as long as they credit the author and the Abstract Book, but they cannot change the content in any way or use them commercially.
**Abstract Book Citations:** Authors, Title, HemaSphere, 2026;10:(S1):pages. The abstract book DOIs can be found on the "Author Information" page.
**Disclaimer:** Articles published in the journal HemaSphere exclusively reflect the opinions of the authors. The authors are responsible for all content in their abstracts including accuracy of the facts, statements, citing resources, etc.

### (PS2114) EARLY SILTUXIMAB RESCUE FOR PERSISTENT CRS IN PATIENTS WITH DIFFUSE LARGE B CELL LYMPHOMA RECEIVING AXICABTAGENE CILOLEUCEL: A REAL-WORLD EXPERIENCE

**Topic:** 19. Large B cell lymphomas - Clinical

José María Sánchez-Pina[1], Adolfo Jesus Saez Marin*[1], Daniel Gil-Alós[1], Maria Nieves Lopez Muñoz[1], Silvia Chacón Alves[2], Guillermo Ramos Moreno[1], Magdalena Corona[1], Maria Pilar Martinez Sanchez[1], Maria Puy Goyache[3], Susana Cortijo[3], Ana Isabel Jimenez Ubieto[1], Joaquín Martinez-Lopez[1], Maria Calbacho Robles[1]

*[1]Hospital 12 de Octubre, Haematology, Madrid, Spain;[2]Hospital 12 de Octubre, Intensive Care Unit, Madrid, Spain;[3]Hospital 12 de Octubre, Pharmacy, Madrid, Spain;*

### Background

Chimeric Antigen Receptor (CAR) T-cell therapy is associated with significant and potentially life-threatening toxicities, such as CRS and ICANS. 5-17% of patients treated with Axi-cel will experience grade ≥3 CRS and 9%-20% grade ≥3 ICANS, ICU admission rates can reach up to 21%. Studies have explored strategies to mitigate this toxicity, with contradictory outcomes.

### Aims

The primary objective of this study was to evaluate the impact of early siltuximab rescue for persistent CRS in patients with R/R DLBCL undergoing CAR T-cell therapy with Axi-cel.

### Methods

Retrospective, single-center analysis of 87 adult patients with R/R B-cell lymphomas treated with Axi-cel between November 2022 and December 2025 comparing two clinical protocols implemented according to institutional policy. Patients were divided into two cohorts. In both, patients received two doses of tocilizumab (if CRS persisted administered 8 hours apart starting at the onset of grade 1 CRS). In the first cohort (Siltuximab group), patients with persistent CRS received up to two 11mg/kg doses of siltuximab combined with dexamethasone. In the second historical cohort (Control group) patients with persistent CRS continued with up to two subsequent doses of tocilizumab, also in combination with dexamethasone.

**Copyright Information:** (Online) ISSN: 2572-9241
©2026 The Author(s). HemaSphere is published by John Wiley & Sons Ltd on behalf of European Hematology Association. This is an open access Abstract Book distributed under the Attribution-NonCommercial-NoDerivs (CC BY-NC-ND), which allows third parties to download the articles and share them with others as long as they credit the author and the Abstract Book, but they cannot change the content in any way or use them commercially.
**Abstract Book Citations:** Authors, Title, HemaSphere, 2026;10:(S1):pages. The abstract book DOIs can be found on the “Author Information” page.
**Disclaimer:** Articles published in the journal HemaSphere exclusively reflect the opinions of the authors. The authors are responsible for all content in their abstracts including accuracy of the facts, statements, citing resources, etc.

### Results

A total of 87 patients were included in the analysis. The Siltuximab group (n=26) was compared to the Control group (n=61). 84.6% of patients treated with siltuximab did not progress beyond grade 1 CRS, compared to 59.1%. Patients who did not receive siltuximab had a 3.8-fold higher probability of developing grade ≥ 2 CRS (OR = 3.82 [95% CI: 1.17–12.45], p = 0.026). The incidence of ICANS was reduced in the Siltuximab group (26.9% vs. 45.9%). Patients in the Control group had a 9.66-fold higher likelihood of developing severe (grade ≥2) ICANS. (3,8% vs 27,9% OR = 9.66 [95% CI: 1.21–76.98], p = 0.010).Patients treated with siltuximab had a significantly shorter median hospital stay (16 days vs. 22.5 days). The probability of requiring ICU admission was 9.66 times higher for patients in the Control group (OR = 9.66 [95% CI: 1.21–76.98], p = 0.010). No increased risk of CMV reactivation, other infections or prolonged cytopenias was observed with the use of siltuximab. Response rates, PFS and OS were similar between groups, although the follow-up for patients treated with siltuximab remains relatively short, 3.43 months (0.56 - 10.22).

| Variable | Siltuximab (n=26) | No Siltuximab (n=61) | p-value |
|---|---|---|---|
| **CRS Grade, n (%)** | | | |
| Grade 1 | 22 (84.6%) | 36 (59.0%) | **0.026** |
| Grade 2 | 4 (15.4%) | 19 (31.1%) | |
| Grade 3 | 0 (0%) | 5 (8.2%) | |
| Grade 4 | 0 (0%) | 1 (1.6%) | |
| Grade ≥2 | 4 (15.4%) | 25 (41.0%) | **0.026** |
| **ICANS, n (%)** | | | |
| Yes | 7 (26.9%) | 28 (45.9%) | 0.151 |
| No | 19 (73.1%) | 33 (54.1%) | |
| **ICANS Grade (n=35)** | | | |
| Grade 1 | 6 (85.7%) | 11 (39.3%) | **0.041** |
| Grade 2 | 0 (0%) | 11 (39.3%) | |
| Grade 3 | 1 (14.3%) | 4 (14.3%) | |
| Grade 4 | 0 (0%) | 2 (7.1%) | |
| Grade ≥2 | 1 (3.8%) | 17 (27.9%) | **0.010** |
| **Other Complications** | | | |
| CMV Reactivation | 8 (32.0%) | 28 (45.9%) | 0.336 |
| Cytopenias | 15 (57.7%) | 28 (45.9%) | 0.355 |
| Grade 3 Infection | 2 (7.7%) | 11 (18.0%) | 0.328 |
| **Hospital Stay** | | | |
| Days, median (IQR) | 14 (13-15) | 18 (15-25) | **0.0002** |
| Range | 10-38 | 10-180 | |
| **ICU Admission** | | | |
| Yes, n (%) | 1 (3.8%) | 17 (27.9%) | **0.010** |

### Summary/Conclusion

Early switch to siltuximab seems to ameliorate Axi-cel cytokine-related toxicity without increasing adverse events in comparison to continue with tocilizumab. These findings support the development of clinical trials with this promising strategy to mitigate CAR T-cell-related toxicities.

**Copyright Information:** (Online) ISSN: 2572-9241
©2026 The Author(s). HemaSphere is published by John Wiley & Sons Ltd on behalf of European Hematology Association. This is an open access Abstract Book distributed under the Attribution-NonCommercial-NoDerivs (CC BY-NC-ND), which allows third parties to download the articles and share them with others as long as they credit the author and the Abstract Book, but they cannot change the content in any way or use them commercially.
**Abstract Book Citations:** Authors, Title, HemaSphere, 2026;10:(S1):pages. The abstract book DOIs can be found on the “Author Information” page.
**Disclaimer:** Articles published in the journal HemaSphere exclusively reflect the opinions of the authors. The authors are responsible for all content in their abstracts including accuracy of the facts, statements, citing resources, etc.

### (PS2115) EVALUATING THE CLINICAL OUTCOMES OF RIPERTAMAB IN NON-HODGKIN'S LYMPHOMA: AN OPEN-LABLE, MULTI-CENTER, PERSPECTIVE STUDY

**Topic:** 19. Large B cell lymphomas - Clinical

Jun MA*[1], Donglu Zhao[1], Keshu Zhou[2], Shifeng Lou[3], Haisheng Liu[4], Liping Su[5], Liqun Zou[6], Dengke Chen[7], Shuling Hou[8], Xiao-Ling Guo[9], Jing Le[10], Jie Bai[11], Zunmin Zhu[12], Xiuhua Sun[13], Yunhong Huang[14], Chan Long[15], Quanfang Luo[16], Shihua Huang[17], Hongyun Xing[18], Ruifeng Jiang[19], Zhigang Peng[20], Yan Yi[21], Qi Zhong[22], Haiping Yang[23], Ou Bai[24], Mei Sun[25], Dongmei Wang[26], Yini Wang[27]

*[1]Institute of Hematology and Oncology, Harbin The First Hospital, Harbin, China;[2]Henan Cancer Hospital, Affiliated Cancer Hospital of Zhengzhou University, Zhengzhou, China, zhengzhou, China; [3]The Second Affiliated Hospital of Chongqing Medical University, chongqing, China; [4]Hebei Tumor Hospital, shijiazhuang, China;[5]Shanxi Cancer Hospital, taiyuan, China;[6]West China Hospital of Sichuan University, chengdu, China; [7]The First People's Hospital of Zunyi, zunyi, China;[8]Sanxi Bethune Hospital, taiyuan, China; [9]The second hospital of Hebei Medical Univerisity, shijiazhuang, China;[10]Ningbo Medical Center Lihuili Hospital, ningbo, China; [11]The Second Hospital of Tianjin Medical University, tianjin, China;[12]Henan Provincial People's Hospital, zhengzhou, China; [13]The Second Hospital of Dalian Medical University, dalian, China;[14]Affiliated hospital of Guizhou medical university, guiyang, China; [15]Huizhou First Hospital, huizhou, China; [16]Nanxishan Hospital of Guangxi Zhuang Autonomous Region, guilin, China; [17]Yibin Second People's Hospital, Yibin, China;[18]The Affiliated Hospital of Southwest Medical University, luzhou, China; [19]Liuzhou Worker's Hospital, liuzhou, China;[20]The First Affiliated Hospital of Guangxi Medical University, nanning, China; [21]Southern Medical Third Hospital, guangzhou, China;[22]Guangdong Second People's Hospital, guangzhou, China; [23]The First Affiliated Hospital, and College of Clinical Medicine of Henan University of Science and Technology, luoyang, China; [24]The first hospital of Jilin University, changchun, China; [25]Northern Jiangsu People's Hospital, yangzhou, China;[26]Hengshui People's Hospital, hengshui, China; [27]Beijing Anzhen Hospital, beijing, China;*

### Background

Non-Hodgkin's lymphoma accounts for approximately 85%- 90% of lymphoma cases, with treatment regimens varying across different pathological subtypes. CD20-directed antibody therapy in combination with a conventional chemotherapy backbone is still considered standard of care in most B-NHL subtypes, achieving long-lasting responses in most patients. Ripertamab is a novel chimeric monoclonal IgG1 antibody, with an amino acid difference in the constant region sequence compared to rituximab. However, the response resulting from the combination of ripertamab-based induction is still to be determined.

### Aims

To describe clinical characteristics, contemporaneous treatment patterns among adult patients diagnosed with non-Hodgkin's lymphoma in real-world settings, as well as the efficacy and safety of the novel anti-CD20 monoclonal antibody ripertamab.

### Methods

A prospective cohort of adult patients diagnosed with non-Hodgkin's lymphoma were enrolled.Patients were eligible if they were new diagnosed or ≤2 lines treatment. Lymphomas were mainly classified as diffuse large B-cell lymphoma, mantle cell lymphoma, or follicular lymphoma, marginal zone lymphoma. Treatment patterns, response (per Lugano 2014 Criteria), and survival outcomes were analyzed using descriptive statistics, Kaplan-Meier survival analysis.

**Copyright Information:** (Online) ISSN: 2572-9241
©2026 The Author(s). HemaSphere is published by John Wiley & Sons Ltd on behalf of European Hematology Association. This is an open access Abstract Book distributed under the Attribution-NonCommercial-NoDerivs (CC BY-NC-ND), which allows third parties to download the articles and share them with others as long as they credit the author and the Abstract Book, but they cannot change the content in any way or use them commercially.
**Abstract Book Citations:** Authors, Title, HemaSphere, 2026;10:(S1):pages. The abstract book DOIs can be found on the "Author Information" page.
**Disclaimer:** Articles published in the journal HemaSphere exclusively reflect the opinions of the authors. The authors are responsible for all content in their abstracts including accuracy of the facts, statements, citing resources, etc.

**Results**

At the data cutoff (Jan 28, 2026), 410 patients have been treated, receiving a median of 5 (range, 1-12) treatment cycles. The median age was 62 years (IQR 55, 70). Patients were predominantly male (58%), Ann Arbor stage III/IV disease (64%). In overall population, diffuse large B-cell lymphoma has 134(33%), marginal zone lymphoma 116(28%), mantle cell lymphoma 83(20%), the subtypes and baseline characteristics of these patients are presented in Table 1.
As of Jan 28, 2026, 231(56%) patients completed follow-up. Median duration of follow-up was 12.0 months (IQR 7.3, 14.4), the median PFS and OS were not reached. 6-month PFS 89% and 1-year PFS 86%, 6-month OS 96% and 1-year OS 95%. Among 268 efficacy-evaluable patients who completed ≥3 cycles of study treatments, the overall ORR was 84.3%, with a CR rate of 54.9%. At the EOT, ORR was 90.0% with a CR of 63.0%. Median duration of response was 10.1 months (IQR 8.0, 12.6) at the data cutoff. In subtypes analysis, diffuse large B-cell lymphoma classified as IPI 0-2 group and IPI≧3 group. At two groups, the ORR was 72.4% and 80.0%, respectively. In mantle cell lymphoma, the ORR was 89.7%, the CR rate was 63.8%. In marginal zone lymphoma, the ORR was 83.5%, with a CR rate of 55.3%.
Overall, the majority of patients were treated with ripertamab-CHOP or ripertamab-CHOP like regimens (37%), followed by BR regimens (21%), subsequently ripertamab alone regimens (13%). The most hematological frequent AEs were lymphocytopenia (10.0%), thrombocytopenia (9.7%), neutropenia (8.8%).The other most common events included immunoglobulin reduction (5.6%), lung infection (4.0%), allergic reactions (3.7%). In this study, AEs were mostly mild-moderate grade, overall adverse events were manageable.

**Summary/Conclusion**

The ripertamab-based regimen exhibited encouraging efficacy in NHL, while ripertamab demonstrates a favorable safety profile.

**Copyright Information:** (Online) ISSN: 2572-9241
©2026 The Author(s). HemaSphere is published by John Wiley & Sons Ltd on behalf of European Hematology Association. This is an open access Abstract Book distributed under the Attribution-NonCommercial-NoDerivs (CC BY-NC-ND), which allows third parties to download the articles and share them with others as long as they credit the author and the Abstract Book, but they cannot change the content in any way or use them commercially.
**Abstract Book Citations:** Authors, Title, HemaSphere, 2026;10:(S1):pages. The abstract book DOIs can be found on the “Author Information” page.
**Disclaimer:** Articles published in the journal HemaSphere exclusively reflect the opinions of the authors. The authors are responsible for all content in their abstracts including accuracy of the facts, statements, citing resources, etc.

**Table 1： Baseline characteristics of patients**

| Baseline characteristics | Patients （n=410) |
|---|---|
| **Age，median（IQR)** | 62（55-70) |
| **Male, n(%)** | 237(58%) |
| **Ann Arbor staging , n(%)** | |
| Ⅰ | 63(15.4%) |
| Ⅱ | 72(17.6%) |
| Ⅲ | 57(13.9%) |
| Ⅳ | 206( 50.2%) |
| Unknown | 12(2.9%) |
| **Histological subtypes, n(%)** | |
| Diffuse large B-cell lymphoma | 134(32.7%) |
| Marginal zone lymphoma | 116( 28.3%) |
| Mantle cell lymphoma | 83(20.2%) |
| High-grade B-cell lymphoma | 13(3.2%) |
| Waldenstrom macroglobulinemia | 13(3.2%) |
| Follicular Lymphoma | 11(2.7%) |
| Chronic lymphocyte leukemia | 11(2.7%) |
| Other mature B-cell lymphoma | 29(7.1%) |

**Figure 1: Investigator-Assessed Progression free Survival and Overall Survival**

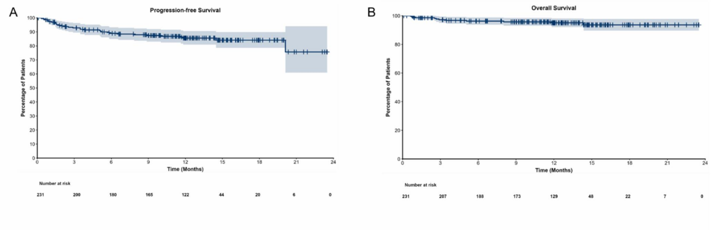

**Copyright Information:** (Online) ISSN: 2572-9241
©2026 The Author(s). HemaSphere is published by John Wiley & Sons Ltd on behalf of European Hematology Association. This is an open access Abstract Book distributed under the Attribution-NonCommercial-NoDerivs (CC BY-NC-ND), which allows third parties to download the articles and share them with others as long as they credit the author and the Abstract Book, but they cannot change the content in any way or use them commercially.
**Abstract Book Citations:** Authors, Title, HemaSphere, 2026;10:(S1):pages. The abstract book DOIs can be found on the "Author Information" page.
**Disclaimer:** Articles published in the journal HemaSphere exclusively reflect the opinions of the authors. The authors are responsible for all content in their abstracts including accuracy of the facts, statements, citing resources, etc.

### (PS2116) PROGNOSTIC VALUE OF DEAUVILLE 5-POINT SCALE ASSESSED ON PET-CT FOLLOWING FIRST-LINE TREATMENT IN DLBCL: A RETROSPECTIVE STUDY

**Topic:** 19. Large B cell lymphomas - Clinical

Henry Kouva*[1], Susanna Tokola[2], Elias Vaattovaara[2], Joonas Kuitunen[3], Henri Kurttila[3], Atte Karhu[1], Eetu Pellonperä[1], Juho Kangas[1], Miko Pietilä[1], Outi Kuittinen[3], Hanne Kuitunen[2], Milla Kuusisto[1]

[1]*University of Oulu, Biomedicine and Internal Medicine Research Unit, Oulu, Finland;* [2]*Oulu University Hospital, Oulu, Finland;* [3]*University of Eastern Finland, Kuopio, Finland;*

### Background

Diffuse large B-cell lymphoma (DLBCL) is treated with immune-chemotherapy with curative intent. Fluorodeoxyglucose positron emission tomography combined with computed tomography (FDG-PET-CT) is the preferred modality to assess treatment response using the Deauville five-point scale (DS). It has a high negative predictive value after first-line treatment. DS score from 1 to 3 are widely considered as PET-negative, and scores of 4 and 5 as PET-positive indicating residual disease, however, false-positive results are relatively common.

### Aims

To assess the prognostic value of DS after first-line treatment in real-world DLBCL patients.

### Methods

This retrospective registry study included DLBCL patients with data collected from the medical records of three university hospitals in Finland. Adult patients who had been diagnosed with DLBCL between years 2000 and 2020, and had PET-CT results at the end of first-line treatment, were included in the study. Patients were excluded if they had primary DLBCL of the central nervous system, received radiotherapy to brain, or had immunosuppressive condition, or their PET-CT was positive, but DS could not be obtained. The survival endpoints were overall survival (OS), disease-specific survival (DSS) and progression-free survival (PFS), and they were calculated using the Kaplan-Meier method, and compared using the log-rank test. The Cox regression model was used for the multivariate analysis.

**Copyright Information:** (Online) ISSN: 2572-9241
©2026 The Author(s). HemaSphere is published by John Wiley & Sons Ltd on behalf of European Hematology Association. This is an open access Abstract Book distributed under the Attribution-NonCommercial-NoDerivs (CC BY-NC-ND), which allows third parties to download the articles and share them with others as long as they credit the author and the Abstract Book, but they cannot change the content in any way or use them commercially.
**Abstract Book Citations:** Authors, Title, HemaSphere, 2026;10:(S1):pages. The abstract book DOIs can be found on the “Author Information” page.
**Disclaimer:** Articles published in the journal HemaSphere exclusively reflect the opinions of the authors. The authors are responsible for all content in their abstracts including accuracy of the facts, statements, citing resources, etc.

### Results

504 patients were included. The median age at diagnosis was 64 years (range 19 – 91), with a slight male predominance (53.4%). A majority of patients had disease stage of III or IV (74.1%), IPI score of 3 to 5 (59.4%), elevated LDH level (72.8%), and WHO performance status score of 0 or 1 (69.3%). More than a quarter of the patients (28.4%) had two or more sites of extranodal involvement. After the first-line therapy, 415 (82.3%) patients had DS score <4, 49 (9.7%) patients DS 4, and 40 (7.9%) patients DS 5.
Overall, 114 (22.6%) patients had at least one relapse, and 82 (16.3%) of the patients died of DLBCL. After a median follow-up of 71 months, the 2-year OS rate was 91.1% for the DS<4 group, 75.4% for DS4, and 49.3% for DS5. The corresponding 5-yr OS rates were 82.1% vs. 69.8% vs. 43.8%. The 2-yr DSS rates 93.9% vs. 77.0% vs. 53.5%; and 5-yr DSS 88.0% vs. 74.2% vs. 53.5%, respectively. The 2-yr PFS 87.4% vs. 69.5% vs. 32.1%; and 5-yr PFS 80.7% vs. 64.2% vs. 29.2%, respectively. All of the estimates were significant (p<0.001).

The multivariate analysis showed that DS4 and DS5 were significant risk factors when compared to DS<4 as baseline, and that DS5 had clearly higher risk, especially in PFS (DS4 vs. DS5: OS HR 2.26 (p=0.015) vs. 5.81 (p<0.001); DSS HR 2.59 (p=0.011) vs. 5.37 (p<0.001); PFS HR 2.18 (p=0.020) vs. 9.21 (p<0.001); when DS<4 as reference). Other significant risk factors were age ≥60 (OS HR 2.21, p=0.010), elevated LDH (OS HR 2.14, p=0.026; DSS HR 2.44, p=0.035), disease stage (DSS HR 3.63, p=0.025; PFS HR 2.56, p=0.025), and extranodal sites ≥ 2 (OS HR 1.88, p=0.007; DSS HR 2.16, p=0.005).

### Summary/Conclusion

DLBCL patients with DS <4 after the first-line therapy had superior outcomes in OS, DSS and PFS, when compared to DS4 and DS5 groups. The DS5 group had clearly inferior prognosis. There appears to be additional prognostic value in further stratifying DLBCL patients according to the Deauville score, rather than treating DS 4 and 5 as a single homogenous PET-CT positive group.

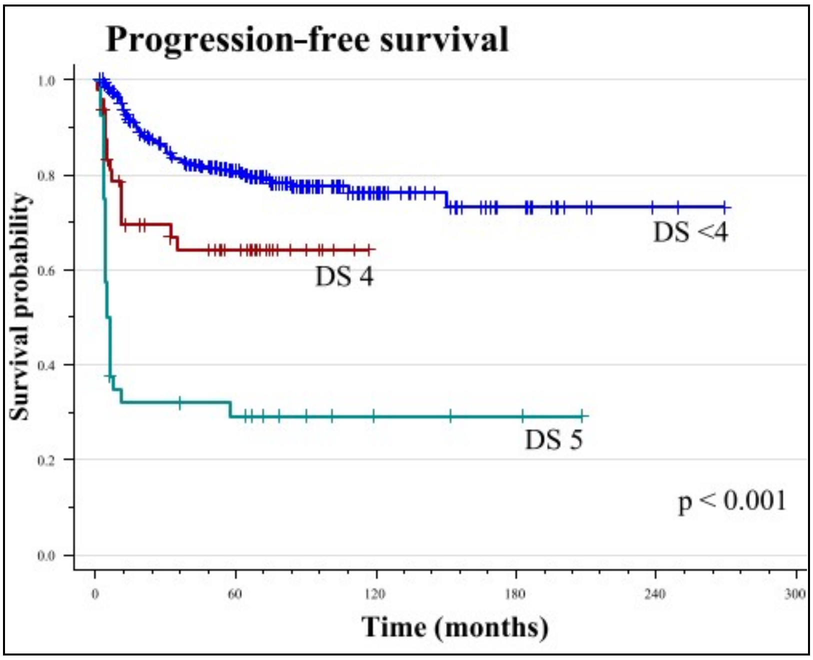

Figure 1. Progression-free survival (PFS) on DLBCL patients divided by their end-of treatment PET-TT results.

Copyright Information: (Online) ISSN: 2572-9241
©2026 The Author(s). HemaSphere is published by John Wiley & Sons Ltd on behalf of European Hematology Association. This is an open access Abstract Book distributed under the Attribution-NonCommercial-NoDerivs (CC BY-NC-ND), which allows third parties to download the articles and share them with others as long as they credit the author and the Abstract Book, but they cannot change the content in any way or use them commercially.
Abstract Book Citations: Authors, Title, HemaSphere, 2026;10:(S1):pages. The abstract book DOIs can be found on the "Author Information" page.
Disclaimer: Articles published in the journal HemaSphere exclusively reflect the opinions of the authors. The authors are responsible for all content in their abstracts including accuracy of the facts, statements, citing resources, etc.

### (PS2117) OUTCOMES OF SECOND-LINE CAR-T THERAPY IN STARGLO-ELIGIBLE PATIENTS: A COMPARATIVE ANALYSIS

**Topic:** 19. Large B cell lymphomas - Clinical

Gil Fridberg*[1, 2], Odelia Amit[1, 2], Muhamad Ganaim[1, 2], Chava Perry[1, 2], Ronit Gold[1], Irit Avivi Mazza[1, 2], Ron Ram[1, 2]

*[1]Tel Aviv Sourasky University Medical Center, BMT Unit, Hematology Division, Tel Aviv, Israel;[2]Gray Faculty of Medicine Tel Aviv University, Tel Aviv, Israel;*

### Background

CAR-T therapy has redefined the treatment paradigm for relapsed or refractory (R/R) DLBCL and is the established second-line (2L) standard of care. However, this is based on pivotal trials comparing CAR-T to chemoimmunotherapy rather than bispecific antibodies (bsAbs). The Phase III STARGLO trial demonstrated the efficacy of glofitamab plus gemcitabine and oxaliplatin (Glofit-GemOx) in the 2L setting for autologous stem cell transplant (ASCT) ineligible patients.

### Aims

In the absence of direct comparative data, we aimed to evaluate whether 2L CAR-T patients meeting STARGLO inclusion criteria achieved comparable outcomes to the Glofit-GemOx arm benchmarks.

### Methods

We analyzed a real-world cohort of patients receiving 2L CAR-T therapy between 12/2022 and 09/2025. Patients were selected based on the specific inclusion and exclusion criteria of the Phase III STARGLO trial. To facilitate a rigorous comparison, individual patient data (IPD) were reconstructed from the published STARGLO 2L sub-analysis Kaplan-Meier curves using the Guyot iterative algorithm. Curves were digitized via WebPlotDigitizer using an automated extraction process with high-density coordinate sampling (ΔX and ΔY of 1 pixel) to capture all granular step changes in survival trajectories. Survival estimates were further refined using the published Number at Risk (NAR) for both progression-free survival (PFS) and overall survival (OS) to account for censoring.

### Results

A total of 38 patients were included, median age 72.3 years (range, 30.3–84.2); 52.6% female; 47.4% ECOG 0. At a median follow-up of 17.8 months, the 2L CAR-T cohort achieved a complete response (CR) rate of 65.8% vs. 63.5% in STARGLO 2L. In the general 2L population, the 24-month PFS was 38.9% (95% CI: 24.9–60.6) for CAR-T versus 49.9% (95% CI: 38.8–61.0) for the reconstructed STARGLO data, while 24-month OS was 50.1% (95% CI: 34.2–73.4) and 57.4% (95% CI: 47.9–66.9), respectively. In a sub-analysis of patients with early relapse, the 24-month PFS was 45.0% (95% CI: 28.4–71.3) for CAR-T versus 40.2% (95% CI: 27.2–53.2) for STARGLO. The 24-month OS in this group was 60.9% (95% CI: 42.1–88.2) for CAR-T compared to 46.5% (95% CI: 35.2–57.8) for the reconstructed STARGLO early-relapse cohort.

### Summary/Conclusion

In this "STARGLO-eligible" real-world cohort, 2L CAR-T achieved high initial response rates. At 24 months, numerical differences in PFS and OS favored STARGLO in the general population, whereas in the high-risk early-relapse sub-population, 24-month PFS and OS were higher in the CAR-T cohort. The overlapping confidence intervals suggest comparable survival outcomes between these regimens in this sample. These signals warrant exploration in larger prospective cohorts to better define the 2L treatment landscape and refine patient selection.

**Copyright Information:** (Online) ISSN: 2572-9241
©2026 The Author(s). HemaSphere is published by John Wiley & Sons Ltd on behalf of European Hematology Association. This is an open access Abstract Book distributed under the Attribution-NonCommercial-NoDerivs (CC BY-NC-ND), which allows third parties to download the articles and share them with others as long as they credit the author and the Abstract Book, but they cannot change the content in any way or use them commercially.
**Abstract Book Citations:** Authors, Title, HemaSphere, 2026;10:(S1):pages. The abstract book DOIs can be found on the “Author Information” page.
**Disclaimer:** Articles published in the journal HemaSphere exclusively reflect the opinions of the authors. The authors are responsible for all content in their abstracts including accuracy of the facts, statements, citing resources, etc.

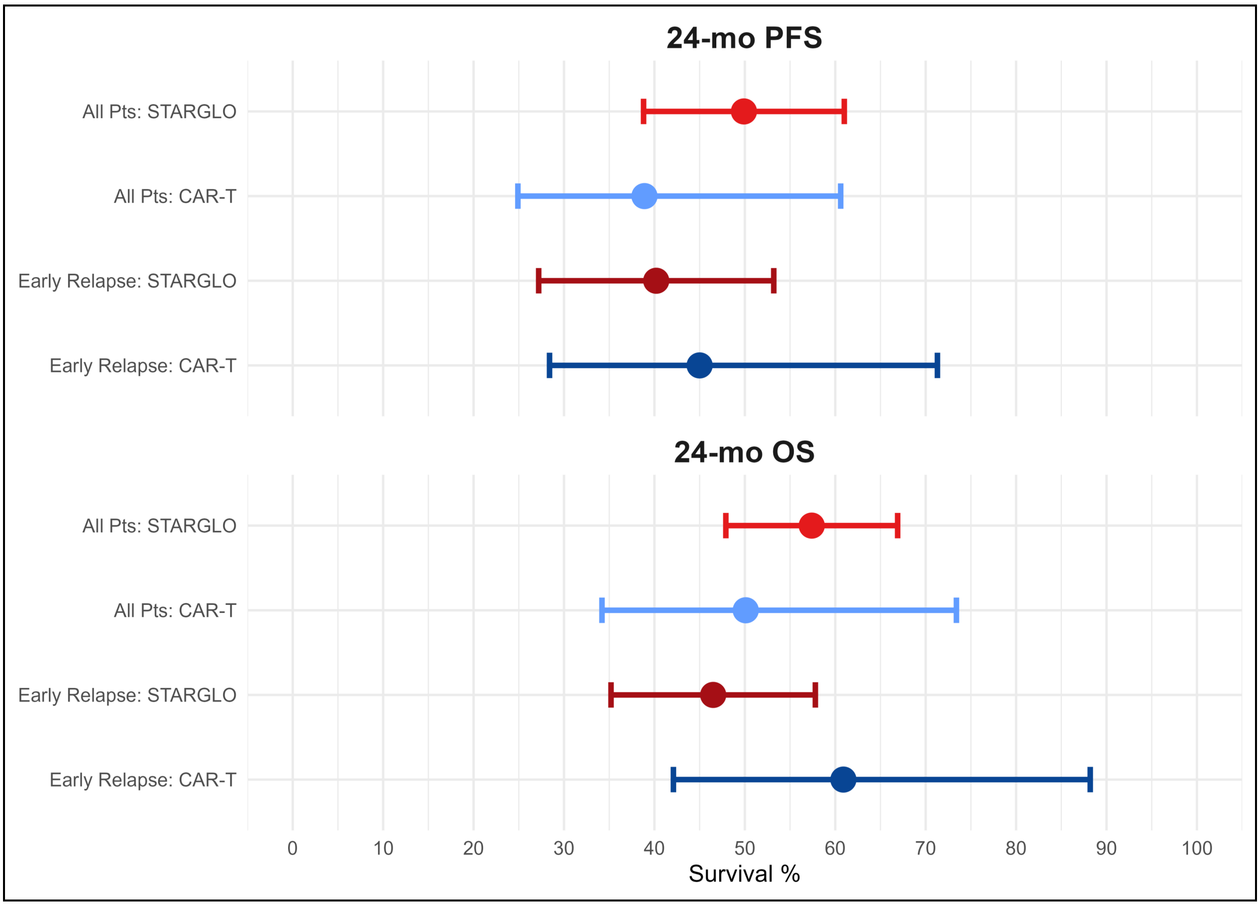

Figure 1. 24 Month Survival Comparison

**Copyright Information:** (Online) ISSN: 2572-9241
©2026 The Author(s). HemaSphere is published by John Wiley & Sons Ltd on behalf of European Hematology Association. This is an open access Abstract Book distributed under the Attribution-NonCommercial-NoDerivs (CC BY-NC-ND), which allows third parties to download the articles and share them with others as long as they credit the author and the Abstract Book, but they cannot change the content in any way or use them commercially.
**Abstract Book Citations:** Authors, Title, HemaSphere, 2026;10:(S1):pages. The abstract book DOIs can be found on the "Author Information" page.
**Disclaimer:** Articles published in the journal HemaSphere exclusively reflect the opinions of the authors. The authors are responsible for all content in their abstracts including accuracy of the facts, statements, citing resources, etc.

### (PS2118) IMPACT OF VINCRISTINE DOSE MODIFICATIONS OR SUBSTITUTION ON CLINICAL OUTCOMES IN R-CHOP TREATED PATIENTS WITH DLBCL

**Topic:** 19. Large B cell lymphomas - Clinical

Tine Bennedsen[1, 2], Jacob Nissen[1], Tobias Eberlein[1], Ida Aagaard[1], Rasmus Rask Kragh Jørgensen[1, 3], Paw Jensen*[1], Tarec El-Galaly[4, 5], Daniel Molin[6], Inger Lise Gade[1, 7], Eva Maksten[1]

*[1]Aalborg University Hospital, Department of Hematology, Clinical Cancer Research Center, Aalborg, Denmark;[2]Aalborg University Hospital, Department of Internal Medicine, Thisted, Denmark; [3]Aalborg University, Center for Clinical Data Science, Aalborg, Denmark; [4]Aarhus University, Department of Clinical Medicine, Aarhus, Denmark;[5]Aarhus University Hospital, Departments of Hematology, Clinical Epidemiology, and Molecular Medicine, Aarhus, Denmark; [6]Uppsala University, Department of Immunology, Genetics, and Pathology; Cancer Immunotherapy, Uppsala, Sweden; [7]Aalborg University, Department of Clinical Medicine, Aalborg, Denmark;*

### Background

Standard 1$^{st}$ line treatment for diffuse large B-cell lymphoma (DLBCL) is the R-CHOP regimen (rituximab, cyclophosphamide, doxorubicin, vincristine, and prednisone). Vincristine is for many patients associated with treatment-limiting neurotoxicity. In clinical practice, management options include dose reduction or omission of vincristine or substitution with vinblastine in case neurotoxicity occurs. Several registry studies have shown that omission of vincristine from R-CHOP does not compromise survival.

### Aims

This study compares clinical outcomes in patients with DLBCL treated with R-CHOP with or without vincristine dose modifications or substitution with vinblastine.

### Methods

Patients aged ≥18 years diagnosed with de novo DLBCL between January 2006 and December 2020 and treated with R-CHOP–based 1$^{st}$ line therapy in the North Denmark Region were identified through the Danish National Lymphoma Registry. Treatment cohorts were defined according to continuation of standard R-CHOP, reduction or omission of vincristine, or substitution with vinblastine. A complete-case landmark analysis was performed with a landmark set at 105 days after diagnosis, corresponding to the time where all eventual treatment modifications to vincristine were observed completed. Overall survival (OS) and disease-free survival (DFS) were assessed using the Kaplan-Meier estimator in patients who continued standard R-CHOP versus those who received modified treatment with vincristine. Both crude and adjusted (age, sex, and stage) OS and DFS estimates were presented and compared using the log-rank tests.

**Copyright Information:** (Online) ISSN: 2572-9241
©2026 The Author(s). HemaSphere is published by John Wiley & Sons Ltd on behalf of European Hematology Association. This is an open access Abstract Book distributed under the Attribution-NonCommercial-NoDerivs (CC BY-NC-ND), which allows third parties to download the articles and share them with others as long as they credit the author and the Abstract Book, but they cannot change the content in any way or use them commercially.
**Abstract Book Citations:** Authors, Title, HemaSphere, 2026;10:(S1):pages. The abstract book DOIs can be found on the “Author Information” page.
**Disclaimer:** Articles published in the journal HemaSphere exclusively reflect the opinions of the authors. The authors are responsible for all content in their abstracts including accuracy of the facts, statements, citing resources, etc.

**Results**

A total of 294 patients were included. Among these, 210 patients (71%) with a median age of 68 years (range 19-84) continued full-dose vincristine throughout the R-CHOP-therapy, while 84 patients (29%) with a median age of 67 years (range 19-84) transitioned to modified treatment. Baseline demographics and disease characteristics were largely comparable between groups. In both groups, the majority were male (58%) and had performance status 0-1 (88%). Extra nodal disease was present in 60% and 62% of cases, respectively, and low-intermediate risk disease (IPI 0-2) in 56% and 54%. In the modified cohort, 14 (17%) underwent vincristine dose reduction, 10 (12%) had vincristine omitted, 38 (45%) received vinblastine substitution, and 22 (26%) experienced both reduction and substitution.
The landmark analysis showed no statistically significant difference in adjusted OS (p=0.90) or DFS (p=0.79). The two years OS estimates were 80% (75%, 86%) and 83% (76%, 92%) between patients remaining on R-CHOP and those who received modified vincristine treatment, respectively (Figure 1). The corresponding two years DFS estimates were 77% (72%, 83%) and 78% (69, 88%). The five years OS estimates were 72% (65%,79%) and 72% (62%,84%) and the five years DFS estimates were 68% (61%,75%) and 67% (56%,79%). Both curves demonstrated a gradual decline over 10 years without clear separation between treatment strategies.

**Summary/Conclusion**

In this population-based cohort of patients with de novo DLBCL, modification of vincristine during R-CHOP therapy either through dose reduction, omission, or substitution with vinblastine, no significant differences in overall survival or disease-free survival compared with continued standard full-dose vincristine throughout the R-CHOP-therapy were found. These findings suggest that adjustments of vincristine doses can be implemented without compromising long-term clinical outcomes.

**Copyright Information:** (Online) ISSN: 2572-9241
©2026 The Author(s). HemaSphere is published by John Wiley & Sons Ltd on behalf of European Hematology Association. This is an open access Abstract Book distributed under the Attribution-NonCommercial-NoDerivs (CC BY-NC-ND), which allows third parties to download the articles and share them with others as long as they credit the author and the Abstract Book, but they cannot change the content in any way or use them commercially.
**Abstract Book Citations:** Authors, Title, HemaSphere, 2026;10:(S1):pages. The abstract book DOIs can be found on the “Author Information” page.
**Disclaimer:** Articles published in the journal HemaSphere exclusively reflect the opinions of the authors. The authors are responsible for all content in their abstracts including accuracy of the facts, statements, citing resources, etc.

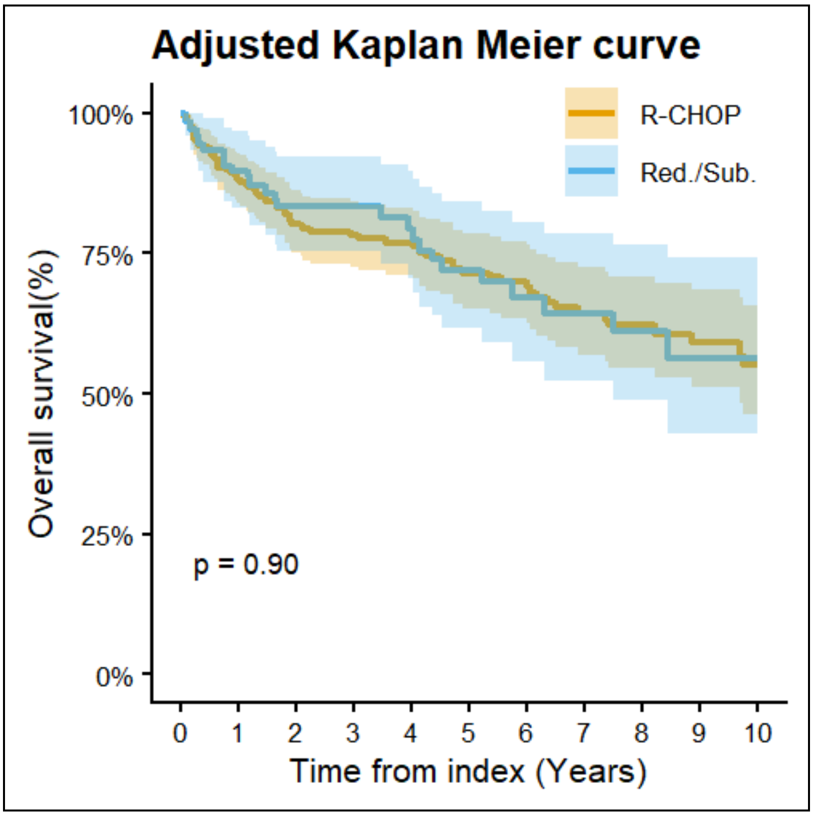

Figure 1: Yellow: Full-dose vincristine throughout all R-CHOP cycles. Blue: Modified vincristine treatment during R-CHOP (dose reduction, omission, or substitution with vinblastine)

**Copyright Information:** (Online) ISSN: 2572-9241
©2026 The Author(s). HemaSphere is published by John Wiley & Sons Ltd on behalf of European Hematology Association. This is an open access Abstract Book distributed under the Attribution-NonCommercial-NoDerivs (CC BY-NC-ND), which allows third parties to download the articles and share them with others as long as they credit the author and the Abstract Book, but they cannot change the content in any way or use them commercially.
**Abstract Book Citations:** Authors, Title, HemaSphere, 2026;10:(S1):pages. The abstract book DOIs can be found on the "Author Information" page.
**Disclaimer:** Articles published in the journal HemaSphere exclusively reflect the opinions of the authors. The authors are responsible for all content in their abstracts including accuracy of the facts, statements, citing resources, etc.

### (PS2119) EFFECTS OF ANTIBIOTIC-EXPOSURE AND LEVOFLOXACIN-PROPHYLAXIS ON OUTCOMES AFTER CART IN LARGE B-CELL LYMPHOMAS

**Topic:** 19. Large B cell lymphomas - Clinical

Giulia Gabrielli*[1, 2], Beatrice Casadei[2], Marianna Gentilini[1, 2], Francesco De Felice[3], Marcello Roberto[3], Giovanni Culpo[1], Cinzia Pellegrini[2], Alessandro Broccoli[1, 2], Vittorio Stefoni[1, 2], Gabriele Gugliotta[2], Matteo Carella[1, 2], Nicole Fabbri[1, 2], Gianmarco Bagnato[1, 2], Camilla Mazzoni[1, 2], Martina Mutti[1, 2], Alessandro Desiderio[1, 2], Francesco Visini[1, 2], Simona Citro[1, 2], Flavia D'aniello[1, 2], Lisa Argnani[1], Francesca Bonifazi[3], Pier Luigi Zinzani[1, 2]

*[1]University of Bologna, Department of Medical and Surgical sciences (DIMEC), Bologna, Italy;[2]IRCCS Azienda Ospedaliero-Universitaria di Bologna, Istituto di Ematologia "Seràgnoli", Bologna, Italy; [3]IRCCS Azienda Ospedaliero-Universitaria di Bologna, Bologna, Italy;*

### Background

Antibiotics (AB) are major drivers of dysbiosis, which has been shown to affect outcomes after immunotherapies, including chimeric antigen receptor T-cell (CART) therapy. The role of prophylactic treatment with levofloxacin (levo) in hematologic patients remains controversial, being effective and well-tolerated strategy to prevent febrile episodes but also associated with detrimental effects after allogeneic transplantation. Moreover, the emergence of multi-resistant pathogens has risen concerns on its widespread use.

### Aims

We investigated the impact of AB-treatment and levo-prophylaxis on outcomes after CART in large B-cell lymphoma (LBCL).

### Methods

We retrospectively analyzed 124 LBCL patients treated with anti-CD19 CART at the Hematology Institute of Bologna. Considering the effect on dysbiosis, we evaluated the AB-exposure within 30 days before CART, whereas levo-prophylaxis (500 mg/day) within 30 days after CART. Analyses were performed using R.

### Results

The cohort included 124 LBCL patients: 94 (75.8%) received axi-cel and 30 (24.2%) tisa-cel, reflecting the most frequent use of axi-cel in Europe. Median age was 59 years. Most of patients had stage III/IV disease (71.0%) at leukapheresis, were primary refractory to last therapy (89.5%) and received bridging therapy during CART manufacturing (83.1%).
After a median follow-up of 24.0 months, median progression-free survival (mPFS) was 12.0 months, and median overall survival (mOS) was not reached. Baseline characteristics were balanced both between AB-exposed/-naïve and levo-exposed/-naïve groups (table 1).
Responses at 3 months and mPFS were similar between AB-exposed and -naïve patients, while mOS was longer in AB-naïve than AB-exposed (not reached vs 35.0 months, respectively, p=0.003). Moreover, exposure to AB pre-CART was significantly associated with neurotoxicity (NT) occurrence (AB-naïve 20.4% vs AB-exposed 50.0%, p=0.010), but not with cytokine-release syndrome. A multivariable analysis confirmed the predictive role of tumor burden on survivals and AB-exposure remained a strong predictor of shorter OS.
Levo-prophylaxis after CART did not affect survivals nor the occurrence of toxicities after infusion. Notably, infection rates within 30 days after CART were similar between the two groups (figure 1).

**Copyright Information:** (Online) ISSN: 2572-9241
©2026 The Author(s). HemaSphere is published by John Wiley & Sons Ltd on behalf of European Hematology Association. This is an open access Abstract Book distributed under the Attribution-NonCommercial-NoDerivs (CC BY-NC-ND), which allows third parties to download the articles and share them with others as long as they credit the author and the Abstract Book, but they cannot change the content in any way or use them commercially.
**Abstract Book Citations:** Authors, Title, HemaSphere, 2026;10:(S1):pages. The abstract book DOIs can be found on the "Author Information" page.
**Disclaimer:** Articles published in the journal HemaSphere exclusively reflect the opinions of the authors. The authors are responsible for all content in their abstracts including accuracy of the facts, statements, citing resources, etc.

**Table 1.** Patients' characteristics

| Variable, n (%) | AB-exposed pre-CART (77) | AB-naïve pre-CART (47) | p-value | LV-exposed after CART (50) | LV-naïve after CART (74) | p-value |
|---|---|---|---|---|---|---|
| **Female** | 30 (39.0) | 16 (34.0) | 0.582 | 19 (38.0) | 27 (36.5) | 1.000 |
| **Age >65y at apheresis** | 23 (29.9) | 12 (26.1) | 0.653 | 15 (30.0) | 20 (27.0) | 0.875 |
| **Axicel**<br>**Tisacel** | 52 (67.5)<br>25 (32.5) | 42 (89.4)<br>5 (10.6) | **0.009** | 43 (86.0)<br>7 (14.0) | 51 (68.9)<br>23 (31.1) | **0.049** |
| **N° of previous therapies, median [range]** | 2 [1-8] | 2 [1-7] | 0.116 | 2 [1-6] | 2 [1-8] | 0.659 |
| **Previous ASCT** | 19 (24.7) | 7 (14.4) | 0.194 | 9 (18.0) | 17 (23.0) | 0.658 |
| **Primary refractory** | 73 (94.8) | 38 (84.4) | 0.097 | 45 (90.0) | 66 (89.2) | 1.000 |
| **Stage III-IV** | 56 (72.7) | 32 (68.1) | 0.581 | 38 (76.0) | 50 (67.6) | 0.416 |
| **Bridging therapy** | 65 (84.4) | 38 (80.9) | 0.608 | 42 (84.0) | 61 (82.4) | 1.000 |
| **CR-PR pre-CART** | 16 (20.8) | 13 (27.7) | 0.380 | 8 (16.0) | 21 (28.4) | 0.167 |

### Summary/Conclusion

Our study confirms the detrimental effects of AB-exposure on survival and NT occurrence after CART, as reported in the literature. To the best of our knowledge, this is the first study to evaluate levofloxacin prophylaxis after CART, showing no significant effect on outcomes nor advantages on infection occurrence after CART. Further investigation is needed to understand the long-term effects of prophylaxis and the interactions with the microbiome.

**Copyright Information:** (Online) ISSN: 2572-9241
©2026 The Author(s). HemaSphere is published by John Wiley & Sons Ltd on behalf of European Hematology Association. This is an open access Abstract Book distributed under the Attribution-NonCommercial-NoDerivs (CC BY-NC-ND), which allows third parties to download the articles and share them with others as long as they credit the author and the Abstract Book, but they cannot change the content in any way or use them commercially.
**Abstract Book Citations:** Authors, Title, HemaSphere, 2026;10:(S1):pages. The abstract book DOIs can be found on the "Author Information" page.
**Disclaimer:** Articles published in the journal HemaSphere exclusively reflect the opinions of the authors. The authors are responsible for all content in their abstracts including accuracy of the facts, statements, citing resources, etc.

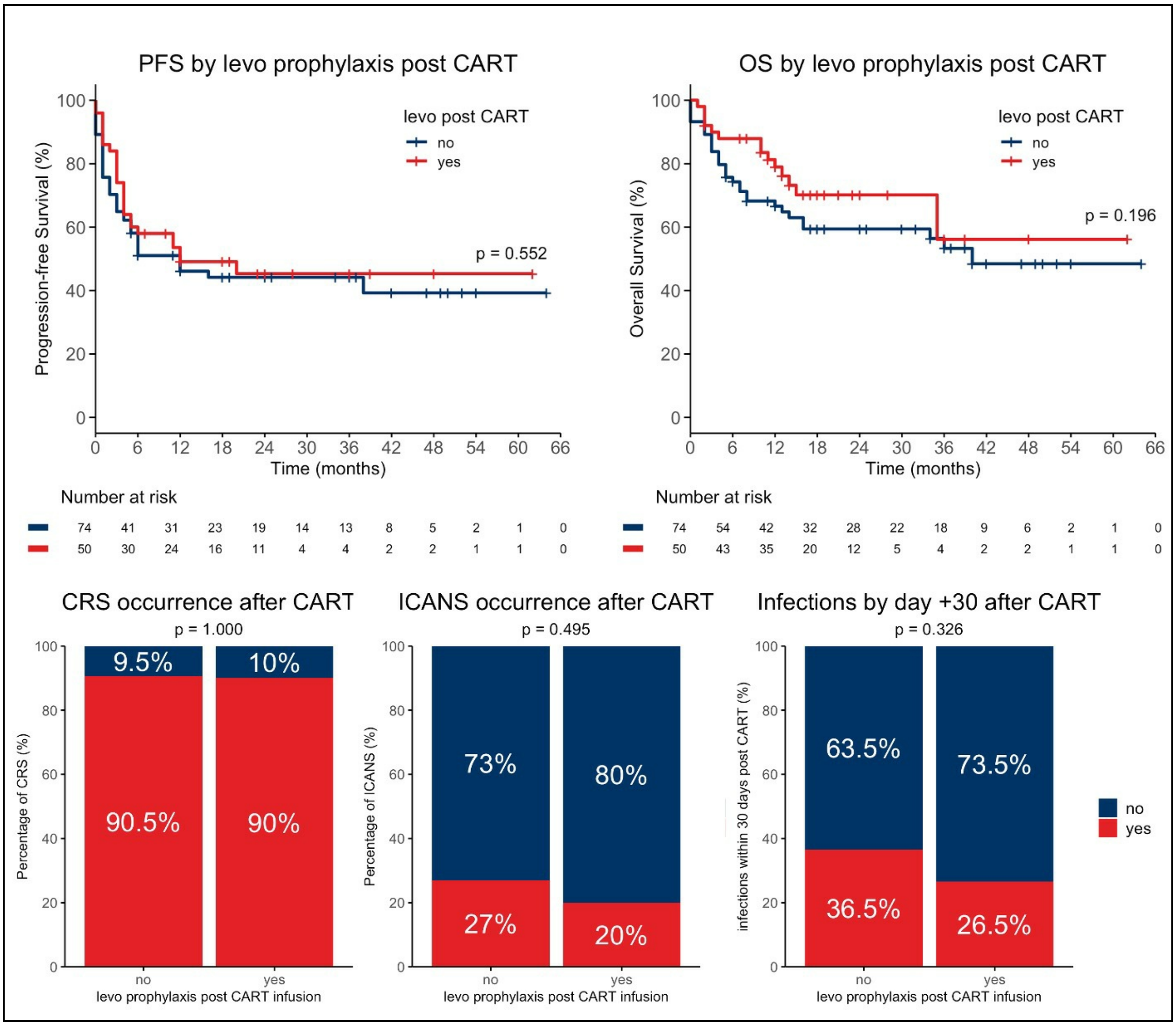

Figure 1. Effects of levofloxacin (levo) exposure on survivals and toxicities after CART.

**Copyright Information:** (Online) ISSN: 2572-9241
©2026 The Author(s). HemaSphere is published by John Wiley & Sons Ltd on behalf of European Hematology Association. This is an open access Abstract Book distributed under the Attribution-NonCommercial-NoDerivs (CC BY-NC-ND), which allows third parties to download the articles and share them with others as long as they credit the author and the Abstract Book, but they cannot change the content in any way or use them commercially.
**Abstract Book Citations:** Authors, Title, HemaSphere, 2026;10:(S1):pages. The abstract book DOIs can be found on the "Author Information" page.
**Disclaimer:** Articles published in the journal HemaSphere exclusively reflect the opinions of the authors. The authors are responsible for all content in their abstracts including accuracy of the facts, statements, citing resources, etc.

### (PS2120) REAL-WORLD EFFICACY, SAFETY, AND MOLECULAR CHARACTERIZATION OF POLATUZUMAB VEDOTIN–BASED FIRST-LINE THERAPY IN DIFFUSE LARGE B-CELL LYMPHOMA: A SINGLE-CENTER STUDY WITH COMPREHENSIVE NGS PROFILING

**Topic:** 19. Large B cell lymphomas - Clinical

Li-Wei Lyu[1], Jia Cong[1], Jin Ye[1], Na Yao[1], Xin Li[1], Liqiang Wei[1], Liang Wang*[1], Lei Yang[1]

[1]*Beijing Tongren Hospital, Capital Medical University, Beijing, China;*

### Background

Optimizing first-line treatment for diffuse large B-cell lymphoma (DLBCL) remains clinically challenging, particularly in older and frail patients who are frequently underrepresented in prospective clinical trials. While the POLARIX trial established polatuzumab vedotin (Pola) plus R-CHP as a new standard for selected patients, real-world evidence regarding its efficacy across diverse patient populations and the predictive value of molecular subtyping remains limited. Integration of next-generation sequencing (NGS) may refine risk stratification and identify genetic determinants of treatment resistance.

### Aims

To evaluate the real-world efficacy, safety, and progression-free survival (PFS) of Pola-based first-line regimens in an unselected DLBCL cohort, and to explore associations between genetic subtypes, somatic variants, and treatment outcomes.

### Methods

We conducted a single-center retrospective analysis of 51 DLBCL patients treated with Pola-based first-line therapy between April 2023 and August 2025. Treatment allocation was stratified by age and geriatric assessment: Pola-R-CHP was administered to patients ≤60 years and fit patients >60 years, while unfit/frail elderly patients received Pola-R-miniCHP or Pola-ZR. Response assessment utilized whole-body 18F-FDG PET/CT after cycles 4 and 6, classified per Lugano 2014 criteria. Adverse events were graded using CTCAE v5.0. Tumor DNA underwent targeted capture NGS using a 169-gene panel to identify coding SNVs/indels and enable LymphGen molecular subtyping. An exploratory case-control analysis compared genomic profiles between progressors (PD) and responders (CR/PR). PFS was estimated using the Kaplan-Meier method.

### Results

The cohort comprised 51 patients (median age 66 years; range, 52-78). After a median follow-up of 13.2 months, the best overall response rate (ORR) was 90.2%, with complete response (CR) in 43 patients (84.3%), partial response (PR) in 3 (5.9%), and progressive disease (PD) in 5 (9.8%). The 12-month PFS was 88.6% (median not reached). No deaths occurred during follow-up. Efficacy was maintained across high-risk subgroups: stage III/IV (ORR 86.2%; CR 75.9%), IPI ≥2 (ORR 86.7%; CR 76.7%), multiple extranodal sites (ORR 84.0%; CR 72.0%), and double-expressor lymphoma (ORR 90.9%; CR 86.4%). Molecular subtyping revealed NOS (n=21), A53 (n=12), ST2 (n=7), MCD (n=7), EZB (n=2), and BN2 (n=2). Notably, the A53 subtype demonstrated a higher PD rate (25.0%) compared with NOS (4.8%), suggesting reduced Pola sensitivity. Exploratory genomic analysis identified CCND3 mutations exclusively in PD cases (0% in responders), implicating cell-cycle dysregulation in primary resistance. SOCS1 and SGK1 alterations were also enriched in progressors, though these findings require validation. Grade 3-4 hematologic toxicity occurred in 51.0% (predominantly neutropenia); pulmonary infection in 9.8%; and peripheral neuropathy in 19.6% (all grade 1-2), consistent with expected safety profiles.

**Copyright Information:** (Online) ISSN: 2572-9241
©2026 The Author(s). HemaSphere is published by John Wiley & Sons Ltd on behalf of European Hematology Association. This is an open access Abstract Book distributed under the Attribution-NonCommercial-NoDerivs (CC BY-NC-ND), which allows third parties to download the articles and share them with others as long as they credit the author and the Abstract Book, but they cannot change the content in any way or use them commercially.
**Abstract Book Citations:** Authors, Title, HemaSphere, 2026;10:(S1):pages. The abstract book DOIs can be found on the “Author Information” page.
**Disclaimer:** Articles published in the journal HemaSphere exclusively reflect the opinions of the authors. The authors are responsible for all content in their abstracts including accuracy of the facts, statements, citing resources, etc.

**Summary/Conclusion**

In this real-world cohort, Pola-based first-line therapy achieved high response rates with durable disease control and manageable toxicity, extending benefit to patients traditionally excluded from clinical trials. The A53 molecular subtype may predict inferior outcomes, while CCND3 mutations emerge as a potential biomarker of primary resistance. These findings support the integration of genomic profiling into treatment algorithms and warrant prospective validation in larger cohorts to personalize Pola-based therapy in DLBCL.

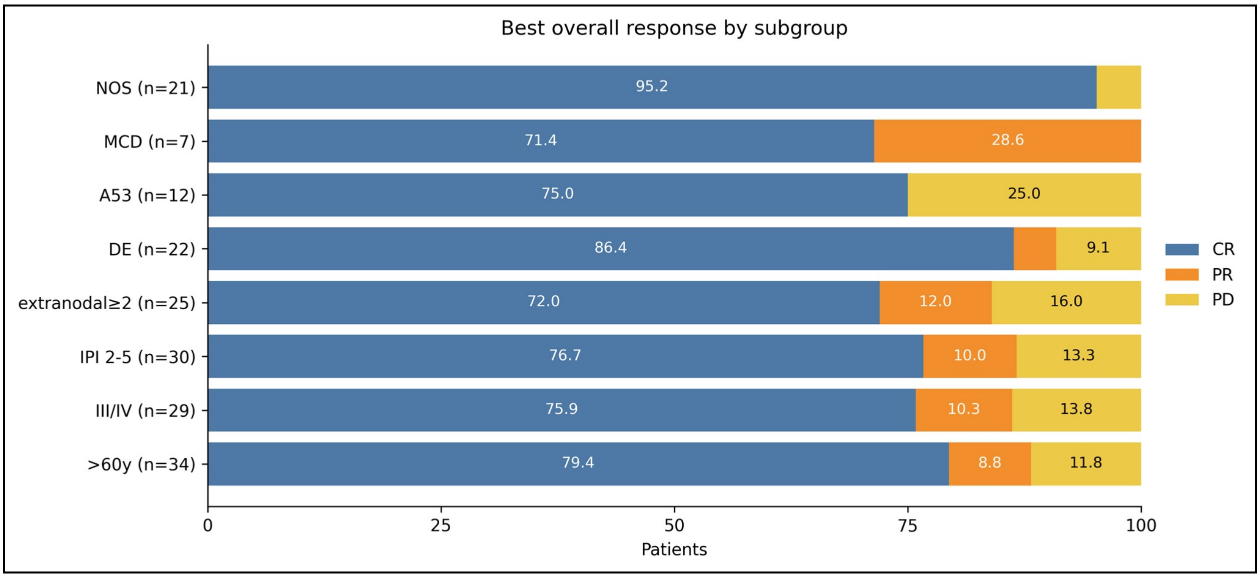

**Copyright Information:** (Online) ISSN: 2572-9241
©2026 The Author(s). HemaSphere is published by John Wiley & Sons Ltd on behalf of European Hematology Association. This is an open access Abstract Book distributed under the Attribution-NonCommercial-NoDerivs (CC BY-NC-ND), which allows third parties to download the articles and share them with others as long as they credit the author and the Abstract Book, but they cannot change the content in any way or use them commercially.
**Abstract Book Citations:** Authors, Title, HemaSphere, 2026;10:(S1):pages. The abstract book DOIs can be found on the "Author Information" page.
**Disclaimer:** Articles published in the journal HemaSphere exclusively reflect the opinions of the authors. The authors are responsible for all content in their abstracts including accuracy of the facts, statements, citing resources, etc.

### (PS2121) AGE-INDEPENDENT OUTCOMES AND COMPETING RISK DYNAMICS IN RELAPSED/REFRACTORY LBCL: A REAL-WORLD ANALYSIS

**Topic:** 19. Large B cell lymphomas - Clinical

Massimiliano Marinoni*[1, 2], Inna Gong[2], Maria Azab[3], Tomohiro Aoki[2], Sita Bhella[2], Christine Chen[2], Katrina Hueniken[4], Robert Kridel[2], Vishal Kukreti[2], Anca Prica[2], Abi Vijenthira[2], Chloe Yang[2], Nauman Malik[5], Danielle Rodin[5], Woodrow Wells[5], David Hodgson[5], Michael Crump[2], John Kuruvilla[2]

*[1]Policlinico Tor Vergata, Hematology, Rome, Italy; [2]Princess Margaret Cancer Centre, Division of Medical Oncology and Hematology, Toronto, Canada; [3]University of Toronto, Faculty of Medicine, Toronto, Canada;[4]Princess Margaret Cancer Centre, Department of Biostatistics, Toronto, Canada; [5]Princess Margaret Cancer Centre, Department of Radiation Medicine, Toronto, Canada;*

### Background

In relapsed/refractory large B-cell lymphoma (R/R LBCL), age is a standard surrogate for "fitness for curative therapy". However, the degree to which age-related survival differences reflect competing risk dynamics—specifically disease relapse/progression (R/PD), lymphoma-related and non-lymphoma related death —versus treatment selection remains unclear.

### Aims

We aimed to characterize this relationship by investigating if age independently influences the trajectory of R/R LBCL outcomes through an analysis of causes of mortality in a large real-world cohort.

### Methods

We performed a retrospective analysis of patients (pts) with R/R LBCL treated at Princess Margaret between 2017-2024. Pts were stratified by age (<70: Younger vs. ≥70: Older). Outcomes from first R/PD included progression-free survival (PFS), competing risk estimates of lymphoma-related vs. non-lymphoma-related death, and non-relapse mortality (NRM) defined as time from first R/PD to death without secondary R/PD, and overall survival (OS) post-autologous stem cell transplantation (ASCT).

**Copyright Information:** (Online) ISSN: 2572-9241
©2026 The Author(s). HemaSphere is published by John Wiley & Sons Ltd on behalf of European Hematology Association. This is an open access Abstract Book distributed under the Attribution-NonCommercial-NoDerivs (CC BY-NC-ND), which allows third parties to download the articles and share them with others as long as they credit the author and the Abstract Book, but they cannot change the content in any way or use them commercially.
**Abstract Book Citations:** Authors, Title, HemaSphere, 2026;10:(S1):pages. The abstract book DOIs can be found on the "Author Information" page.
**Disclaimer:** Articles published in the journal HemaSphere exclusively reflect the opinions of the authors. The authors are responsible for all content in their abstracts including accuracy of the facts, statements, citing resources, etc.

### Results

401 R/R LBCL pts were identified; median age 61 y (range: 19-89); 63% male. 69 pts (17%; median age 73.9 y, range: 70-89) were Older. At first R/PD: 72% Stage III-IV, 31% extensive extranodal involvement (EN≥2), 51% high-risk IPI scores (3-5). Older were more likely to have ECOG ≥2 (37% vs. 18%, *p=0.001*), while Younger experienced R/PD within 12 months ("early relapse") more often (71% vs 55%, *p=0.016*) as well as primary refractory disease (PRD; 46% vs. 26%, *p=0.003*).
The median follow-up time was 32 months (range, 1 – 183). Older patients were markedly less likely to receive curative-intent (CI) therapy (39% vs 97%, *p<0.001*) or proceed to ASCT (25% vs 56%, *p<0.001*). Median OS from relapse differed between age groups (24 vs. 91 m, *p=0.008*). Despite this, median PFS was similar (7 vs 8 m,*p=0.068*) [**Fig 1A**].
At 2 years, lymphoma-related mortality (37% vs. 31%, *p=0.4*) and non-lymphoma mortality (13% vs. 7.3%, *p=0.1*) did not significantly differ [**Fig 1B**]. In multivariable Cox regression—adjusting for curative intent, diagnostic subtype, IPI, LDH, ECOG, stage, extranodal disease, and time-to-relapse—age was not independently associated with lymphoma-related death (HR 0.67, 95% CI 0.31-1.43, *p=0.3*). NRM was comparable between cohorts (*p=0.1*). When restricted to the period prior to second relapse, the 2-year cumulative incidence of lymphoma-death was 7.5% in Older vs. 3.2% in Younger (*p=0.086*), while non-lymphoma-related death was 1.7% and 2.7%, respectively (*p=0.54*) [**Fig 1C**].
Older pts were less likely to be eligible or proceed to ASCT: 39% vs. 96%,*p<0.0001*; and 25% vs. 56%, *p<0.0001*, respectively. Within the subgroup of pts who successfully proceeded to ASCT (n=201, 51%), 2-year OS: 53% Older and 73% Younger (*p = 0.15*) [**Fig 1D**].

### Summary/Conclusion

Factors influencing competing risk dynamics for R/R LBCL pts include disease, treatment toxicity, and comorbidities. In our cohort, lymphoma remains the primary cause of death regardless of age. Results were similar in lymphoma-related or non-lymphoma mortality between age groups; however, results may be influenced by sample size. While non-lymphoma related mortality and NRM appear numerically higher in Older, these results do not appear prohibitive - PFS outcomes also look inferior. Prospective controlled data are needed to better define the benefit of 2L curative approaches in older LBCL.

**Copyright Information:** (Online) ISSN: 2572-9241
©2026 The Author(s). HemaSphere is published by John Wiley & Sons Ltd on behalf of European Hematology Association. This is an open access Abstract Book distributed under the Attribution-NonCommercial-NoDerivs (CC BY-NC-ND), which allows third parties to download the articles and share them with others as long as they credit the author and the Abstract Book, but they cannot change the content in any way or use them commercially.
**Abstract Book Citations:** Authors, Title, HemaSphere, 2026;10:(S1):pages. The abstract book DOIs can be found on the "Author Information" page.
**Disclaimer:** Articles published in the journal HemaSphere exclusively reflect the opinions of the authors. The authors are responsible for all content in their abstracts including accuracy of the facts, statements, citing resources, etc.

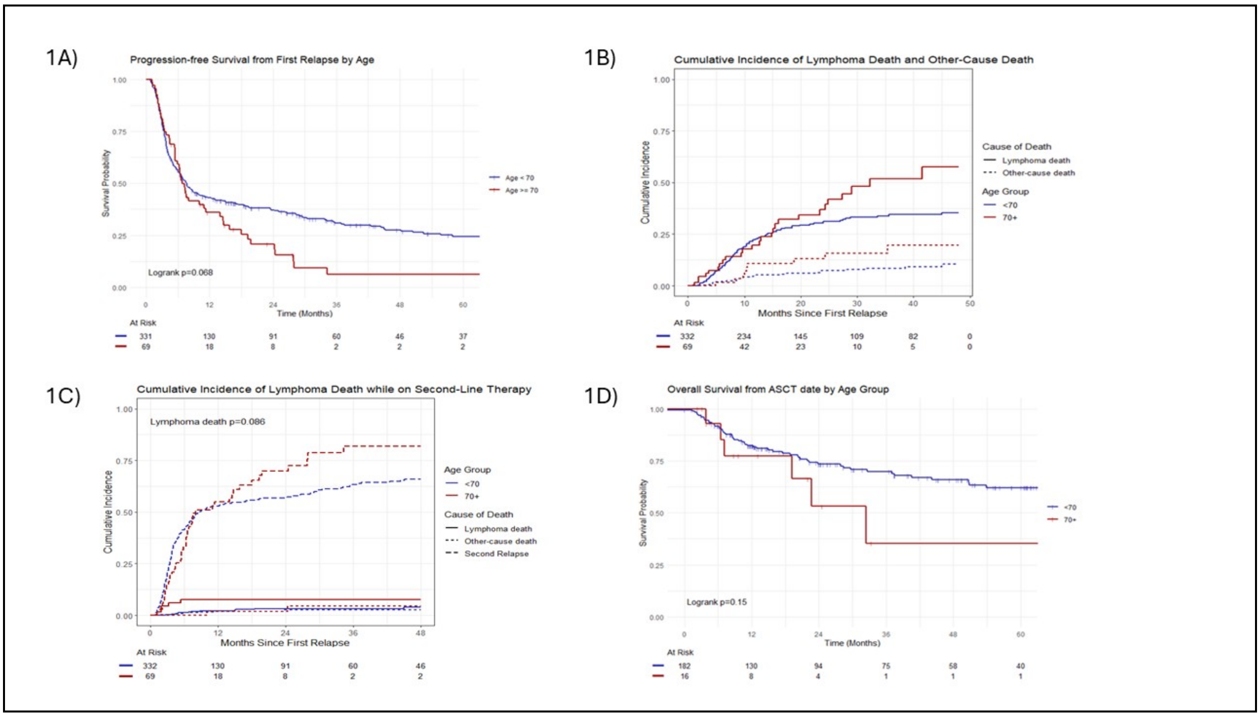

**Copyright Information:** (Online) ISSN: 2572-9241
©2026 The Author(s). HemaSphere is published by John Wiley & Sons Ltd on behalf of European Hematology Association. This is an open access Abstract Book distributed under the Attribution-NonCommercial-NoDerivs (CC BY-NC-ND), which allows third parties to download the articles and share them with others as long as they credit the author and the Abstract Book, but they cannot change the content in any way or use them commercially.
**Abstract Book Citations:** Authors, Title, HemaSphere, 2026;10:(S1):pages. The abstract book DOIs can be found on the "Author Information" page.
**Disclaimer:** Articles published in the journal HemaSphere exclusively reflect the opinions of the authors. The authors are responsible for all content in their abstracts including accuracy of the facts, statements, citing resources, etc.

### (PS2122) PLASMA COPPER AS PROGNOSTIC BIOMARKER IN DIFFUSE LARGE B-CELL LYMPHOMA

**Topic:** 19. Large B cell lymphomas - Clinical

Jinghan Wang*[1], Zhixin MA[2], Qiaoli Li[1], Jiayuan Gu[1], Fang Yu[1], Wenjuan Yu[1], Wanzhuo Xie[1], Jun Tang[2], Hongyan Tong[1], Jie Jin[1]

*[1]The First Affiliated Hospital, Zhejiang University School of Medicine, Hangzhou, China;[2]Clinical Prenatal Diagnosis Center, Women's Hospital, School of Medicine, Zhejiang University, Hangzhou, China;*

### Background

Diffuse large B-cell lymphoma (DLBCL) exhibits significant clinical heterogeneity, necessitating improved prognostic biomarkers beyond conventional risk stratification systems.Recent research highlights cuproptosis, a novel form of regulated cell death triggered by abnormal intracellular copper accumulation affecting mitochondrial function, as a promising target in cancer therapy. Its susceptibility depends on tumor metabolism and microenvironmental factors, including inflammatory signals. In DLBCL, cuproptosis-related genes and copper transporters influence prognosis and treatment response, but the role of systemic plasma copper levels remains unclear. This research aims to establish plasma copper as a prognostic biomarker and inform therapeutic strategies targeting cuproptosis and copper homeostasis to improve patient outcomes.

### Aims

This study aims to evaluate plasma copper levels as a prognostic biomarker in DLBCL. By integrating published genetic datasets and our patiients' cytokine profiling, it seeks to enhance understanding of copper's role in lymphoma progression and guide novel therapeutic strategies.

### Methods

We analyzed 600 newly diagnosed DLBCL patients divided into training (n=360) and validation (n=240) cohorts. plasma copper levels were evaluated alongside comprehensive clinical, molecular, and inflammatory parameters. Copper chelator-related genes were systematically identified through bioinformatic analysis, with meta-analysis performed across six independent cohorts.

### Results

Elevated plasma copper levels independently predicted inferior survival outcomes in both cohorts. High copper patients demonstrated dramatically shorter median PFS (467 vs 3,295 days, P<0.001) and OS (1,114 days vs not reached, P<0.001). Multivariate analysis confirmed copper as an independent prognostic factor beyond established clinical risk indices (IPI, LMR and HP) and molecular subtypes (non-GCB, DEL). High copper patients exhibited distinct inflammatory profiles with elevated IL-6 and IL-10, with propensity score-matched analysis confirming the copper-IL-6 association. Systematic analysis identified SOD1 as a key copper-chelating enzyme, with meta-analysis revealing its prognostic significance (HR=1.847, 95% CI: 1.491-2.288, P<0.001). The protein expression of Superoxide Dismutase1 (SOD1) was assessed using immunohistochemistry (IHC) on representative tumor tissue sections stratified into high and low plasma copper groups. IHC demonstrated patients with high copper levels with higher SOD1 expression.

**Copyright Information:** (Online) ISSN: 2572-9241
©2026 The Author(s). HemaSphere is published by John Wiley & Sons Ltd on behalf of European Hematology Association. This is an open access Abstract Book distributed under the Attribution-NonCommercial-NoDerivs (CC BY-NC-ND), which allows third parties to download the articles and share them with others as long as they credit the author and the Abstract Book, but they cannot change the content in any way or use them commercially.
**Abstract Book Citations:** Authors, Title, HemaSphere, 2026;10:(S1):pages. The abstract book DOIs can be found on the "Author Information" page.
**Disclaimer:** Articles published in the journal HemaSphere exclusively reflect the opinions of the authors. The authors are responsible for all content in their abstracts including accuracy of the facts, statements, citing resources, etc.

**Summary/Conclusion**

Plasma copper represents a novel, independent prognostic biomarker in DLBCL that captures unique disease biology through its association with inflammatory signaling. Integration of copper assessment into clinical practice could guide personalized treatment strategies in DLBCL.

**Copyright Information:** (Online) ISSN: 2572-9241
©2026 The Author(s). HemaSphere is published by John Wiley & Sons Ltd on behalf of European Hematology Association. This is an open access Abstract Book distributed under the Attribution-NonCommercial-NoDerivs (CC BY-NC-ND), which allows third parties to download the articles and share them with others as long as they credit the author and the Abstract Book, but they cannot change the content in any way or use them commercially.
**Abstract Book Citations:** Authors, Title, HemaSphere, 2026;10:(S1):pages. The abstract book DOIs can be found on the “Author Information” page.
**Disclaimer:** Articles published in the journal HemaSphere exclusively reflect the opinions of the authors. The authors are responsible for all content in their abstracts including accuracy of the facts, statements, citing resources, etc.

### (PS2123) HIGH PROPORTION OF INTRATUMORAL TCF1+ T-CELLS PREDICTS FAVORABLE IMMUNOTHERAPY RESPONSE IN RELAPSED/REFRACTORY PRIMARY MEDIASTINAL LARGE B-CELL LYMPHOMA

**Topic:** 19. Large B cell lymphomas - Clinical

Xia Zhao*[1], Zhihe Liu[1]

[1]*the affiliated hospital of Qingdao university, Qingdao, China;*

### Background

Primary Mediastinal Large B-cell Lymphoma (PMBCL) is an aggressive B-cell lymphoma predominantly affecting young adults. While frontline chemoimmunotherapy achieves favorable outcomes, patients with relapsed/refractory (R/R) disease face poor prognosis. Immunotherapy, such as PD-1 inhibitors, has demonstrated clinical activity, but response rates remain heterogeneous. Recent studies have identified TCF1+ progenitor exhausted T-cells within the tumor microenvironment (TME) as a critical determinant of immunotherapy efficacy in various malignancies. These TCF1+ T-cells possess stem-like properties and self-renewal capacity, enabling sustained anti-tumor responses upon immune checkpoint blockade. However, their predictive value in R/R PMBCL remains poorly characterized.

### Aims

This study aimed to investigate the association between the proportion of intratumoral TCF1+ T-lymphocytes and clinical outcomes in patients with R/R PMBCL receiving immunotherapy.

### Methods

We analyzed 27 patients with R/R PMBCL who received immunotherapy as second-line therapy (anti-PD-1 monotherapy or PD1 plus chemotherapy). Patients were stratified based on the proportion of TCF1+ cells among CD8+ tumor-infiltrating lymphocytes (TILs) assessed by immunohistochemistry: high TCF1+ group (n=12, TCF1+ ≥25%) and normal/low TCF1+ group (n=15, TCF1+ <25%). Baseline clinicopathologic characteristics, treatment modalities, and outcomes were recorded. Primary endpoints included Overall Response Rate (ORR), Complete Response (CR) rate and Progression-Free Survival (PFS). Secondary endpoints included 12-month PFS rate and treatment-related toxicity.

### Results

Baseline characteristics were balanced between groups, including median age (30 vs 31 years) and gender distribution (50% vs 47% female). The mean proportion of TCF1+ T-cells was 39.4% ± 5.9% in the high group versus 11.0% ± 4.6% in the normal/low group. CD8+ TIL density was significantly higher in the high TCF1+ group (1177 ± 262 vs 704 ± 193 cells/mm², p<0.001). Patients with high TCF1+ infiltration demonstrated superior response to immunotherapy, with an ORR of 83.3% versus 73.3% in the normal/low group. The CR rate was notably higher in the high TCF1+ cohort (66.7% vs 53.3%). Survival outcomes were markedly improved in the high TCF1+ group, with median PFS of 24.1 months compared to 12.4 months in the normal/low group (hazard ratio 0.42, p=0.018). The 12-month PFS rate was 66.7% in the high TCF1+ group versus 53.3% in the normal/low group. Grade 3-4 treatment-related toxicities were comparable between groups (16.7% vs 13.3%, p=0.78).

**Copyright Information:** (Online) ISSN: 2572-9241
©2026 The Author(s). HemaSphere is published by John Wiley & Sons Ltd on behalf of European Hematology Association. This is an open access Abstract Book distributed under the Attribution-NonCommercial-NoDerivs (CC BY-NC-ND), which allows third parties to download the articles and share them with others as long as they credit the author and the Abstract Book, but they cannot change the content in any way or use them commercially.
**Abstract Book Citations:** Authors, Title, HemaSphere, 2026;10:(S1):pages. The abstract book DOIs can be found on the "Author Information" page.
**Disclaimer:** Articles published in the journal HemaSphere exclusively reflect the opinions of the authors. The authors are responsible for all content in their abstracts including accuracy of the facts, statements, citing resources, etc.

**Summary/Conclusion**

A high proportion of intratumoral TCF1+ T-lymphocytes is strongly associated with superior response rates and significantly prolonged survival in patients with R/R PMBCL treated with immunotherapy. These findings support the integration of TCF1+ T-cell assessment into clinical decision-making and may facilitate the development of personalized treatment strategies in R/R PMBCL.

**Copyright Information:** (Online) ISSN: 2572-9241
©2026 The Author(s). HemaSphere is published by John Wiley & Sons Ltd on behalf of European Hematology Association. This is an open access Abstract Book distributed under the Attribution-NonCommercial-NoDerivs (CC BY-NC-ND), which allows third parties to download the articles and share them with others as long as they credit the author and the Abstract Book, but they cannot change the content in any way or use them commercially.
**Abstract Book Citations:** Authors, Title, HemaSphere, 2026;10:(S1):pages. The abstract book DOIs can be found on the "Author Information" page.
**Disclaimer:** Articles published in the journal HemaSphere exclusively reflect the opinions of the authors. The authors are responsible for all content in their abstracts including accuracy of the facts, statements, citing resources, etc.

### (PS2124) RESISTIN ASSOCIATES WITH POOR PROGNOSIS AND PROMOTES TUMOR GROWTH IN DIFFUSE LARGE B-CELL LYMPHOMA

**Topic:** 19. Large B cell lymphomas - Clinical

Wenyu Shi*[1], Beiqi Gong[1], Yaping Zhang[1]

[1] *Affiliated Hospital of Nantong University, Nantong, China;*

### Background

Diffuse large B-cell lymphoma (DLBCL) is an aggressive and heterogeneous malignancy. Although R-CHOP is curative for many patients, a substantial fraction relapse or are refractory. Tumor microenvironment (TME)-derived soluble factors may contribute to disease aggressiveness. Resistin (encoded by RETN) is a pro-inflammatory adipokine predominantly produced by myeloid cells, but its clinical relevance and functional role in DLBCL remain unclear.

### Aims

This study aims to investigate the expression pattern of Resistin in DLBCL patients, evaluate its prognostic value, and characterize its functional impact on DLBCL cell proliferation both *in vitro* and *in vivo*.

### Methods

Plasma Resistin was quantified in DLBCL patients and healthy donors by proteomic analysis, including subgroup assessment by cell-of-origin. Prognostic value of RETN was evaluated in independent GEO cohorts (GSE10846, GSE32918, GSE31312) using overall survival analyses. Recombinant Resistin was added to GCB and non-GCB DLBCL cell lines and proliferation was assessed by CCK-8 in a dose-dependent manner. RETN mRNA was measured by qPCR in DLBCL cell lines and peripheral blood mononuclear cells (PBMC). For *in vivo* validation, 12 female BALB/c mice (6/group) were subcutaneously inoculated with A20 murine DLBCL cells ($2\times10^6$). Mice received intraperitoneal recombinant Resistin (400 ng/mouse) every other day starting prior to inoculation, or vehicle control. Tumor volume was monitored and tumors were weighed at endpoint.

### Results

Plasma Resistin levels were significantly elevated in DLBCL patients compared to healthy doners, with particularly high expression observed in the non-GCB subtype. Survival analysis of GEO data revealed that high *RETN* expression was significantly associated with shorter overall survival (OS) across multiple cohorts ($p<0.05$). *In vitro,* Resistin significantly enhanced proliferation of both GCB (OCI-Ly19, S8) and non-GCB (RIVA, U2932) DLBCL cell lines in a dose-dependent fashion. Interestingly, qPCR showed low *RETN* expression in DLBCL tumor cells but significantly higher levels in PBMCs, suggesting that non-tumor cells in the TME, such as macrophages, are the primary source of Resistin. *In vivo* experiments confirmed these findings: the Resistin-treated group exhibited a significantly faster tumor growth rate ($p<0.01$), and significantly higher final tumor weights compared to the control group.

**Copyright Information:** (Online) ISSN: 2572-9241
©2026 The Author(s). HemaSphere is published by John Wiley & Sons Ltd on behalf of European Hematology Association. This is an open access Abstract Book distributed under the Attribution-NonCommercial-NoDerivs (CC BY-NC-ND), which allows third parties to download the articles and share them with others as long as they credit the author and the Abstract Book, but they cannot change the content in any way or use them commercially.
**Abstract Book Citations:** Authors, Title, HemaSphere, 2026;10:(S1):pages. The abstract book DOIs can be found on the "Author Information" page.
**Disclaimer:** Articles published in the journal HemaSphere exclusively reflect the opinions of the authors. The authors are responsible for all content in their abstracts including accuracy of the facts, statements, citing resources, etc.

**Summary/Conclusion**

Resistin is a potent pro-proliferative factor in DLBCL and serves as a robust biomarker for poor prognosis. Our findings suggest that Resistin, primarily secreted by non-tumor cells in the microenvironment rather than the lymphoma cells themselves, drives tumor progression. Targeting the Resistin-mediated signaling pathway or its cellular sources in the TME may represent a novel therapeutic strategy for DLBCL, especially for the non-GCB subtype.

**Copyright Information:** (Online) ISSN: 2572-9241
©2026 The Author(s). HemaSphere is published by John Wiley & Sons Ltd on behalf of European Hematology Association. This is an open access Abstract Book distributed under the Attribution-NonCommercial-NoDerivs (CC BY-NC-ND), which allows third parties to download the articles and share them with others as long as they credit the author and the Abstract Book, but they cannot change the content in any way or use them commercially.
**Abstract Book Citations:** Authors, Title, HemaSphere, 2026;10:(S1):pages. The abstract book DOIs can be found on the "Author Information" page.
**Disclaimer:** Articles published in the journal HemaSphere exclusively reflect the opinions of the authors. The authors are responsible for all content in their abstracts including accuracy of the facts, statements, citing resources, etc.

### (PS2125) CONTIGUOUS SPREAD, NOT HEMATOGENOUS DISSEMINATION: RETHINKING CNS RISK AND TREATMENT INTENSITY IN PRIMARY SINONASAL DLBCL—A 94-PATIENT REAL-WORLD EVIDENCE STUDY

**Topic:** 19. Large B cell lymphomas - Clinical

Na Yao*[1], Cheng-Ji Wang[1], Liang Wang[1]

[1]*Beijing Tongren Hospital, Capital Medical University, Beijing, China;*

### Background

Primary sinonasal diffuse large B-cell lymphoma (PSN-DLBCL) is an exceptionally rare extranodal lymphoma (incidence ~1 per million) with undefined optimal management. Critical uncertainties persist regarding treatment intensity and central nervous system (CNS) prophylaxis necessity. Notably, CNS involvement at diagnosis appears more frequent than in nodal DLBCL, yet the mechanism—hematogenous versus direct extension—and appropriate prophylactic strategy remain uncharacterized.

### Aims

We conducted this large single-center retrospective study to delineate clinical features, CNS risk patterns, and real-world treatment outcomes.

### Methods

We enrolled 94 treatment-naïve PSN-DLBCL patients diagnosed at Beijing Tongren Hospital (2009–2024). CNS involvement required positive CSF flow cytometry or parenchymal brain lesions. Extensive local invasion (ELI) was defined as tumor extension beyond sinonasal bony confines into ≥2 adjacent structures. Treatment regimens included R-CHOP (60.6%), da-R-EPOCH (25.5%), and Pola-R-CHP (6.4%). CNS-directed strategies comprised intrathecal chemotherapy (52.1%), high-dose methotrexate (HD-MTX, 21.3%), or both.

### Results

Median age was 68.5 years (range 31–90); 61.7% had limited-stage (I–II) disease. Remarkably, 76.6% demonstrated ELI with frequent orbital (59.6%) and bone (77.7%) invasion. Baseline CNS involvement occurred in 9 patients (9.6%), significantly exceeding nodal DLBCL rates (2–2.5%). However, CNS-IPI failed to stratify this risk ($P$=0.205), and only 3 patients (3.2%) experienced CNS progression during follow-up (median 43.7 months). Estimated 5-year progression-free survival (PFS) and overall survival (OS) were 67.0% and 73.1%, respectively—substantially superior to nodal DLBCL.

Crucially, survival did not differ significantly by treatment regimen (R-CHOP vs. da-R-EPOCH vs. Pola-R-CHP; $P$=0.60 for PFS, $P$=0.47 for OS) or primary anatomical site. High IPI scores independently predicted inferior OS (HR 2.96, 95%CI 1.25–7.00, $P$=0.014) and PFS (HR 2.44, 95%CI 1.05–5.67, $P$=0.038), while complete response achievement strongly correlated with favorable outcomes. Intriguingly, ELI was associated with improved PFS (HR 0.48, $P$=0.046), suggesting immunogenic tumor microenvironment effects. Neither HD-MTX nor intrathecal therapy demonstrated significant survival benefit in multivariate analysis, though these were predominantly administered to high-risk patients. Grade ≥3 neutropenia occurred in 83.0% across all groups, with no significant difference by CNS prophylaxis intensity.

**Copyright Information:** (Online) ISSN: 2572-9241
©2026 The Author(s). HemaSphere is published by John Wiley & Sons Ltd on behalf of European Hematology Association. This is an open access Abstract Book distributed under the Attribution-NonCommercial-NoDerivs (CC BY-NC-ND), which allows third parties to download the articles and share them with others as long as they credit the author and the Abstract Book, but they cannot change the content in any way or use them commercially.
**Abstract Book Citations:** Authors, Title, HemaSphere, 2026;10:(S1):pages. The abstract book DOIs can be found on the “Author Information” page.
**Disclaimer:** Articles published in the journal HemaSphere exclusively reflect the opinions of the authors. The authors are responsible for all content in their abstracts including accuracy of the facts, statements, citing resources, etc.

### Summary/Conclusion

This study provides substantive real-world evidence that PSN-DLBCL achieves excellent outcomes with standard R-CHOP immunochemotherapy, without incremental benefit from intensified regimens. The dissociation between relatively common baseline CNS involvement (9.6%) and rare CNS relapse (3.2%) suggests contiguous anatomical extension—via skull base erosion and cranial foramina—rather than hematogenous dissemination as the predominant mechanism. This pathophysiological insight carries direct therapeutic implications: intrathecal chemotherapy, which achieves limited parenchymal penetration, should not substitute for systemic HD-MTX when CNS-directed therapy is indicated. The favorable prognosis of ELI challenges conventional assumptions about local aggression. These findings support de-escalation strategies for most patients while highlighting the need for prospective studies to refine risk-adapted CNS prophylaxis in this rare extranodal subtype.

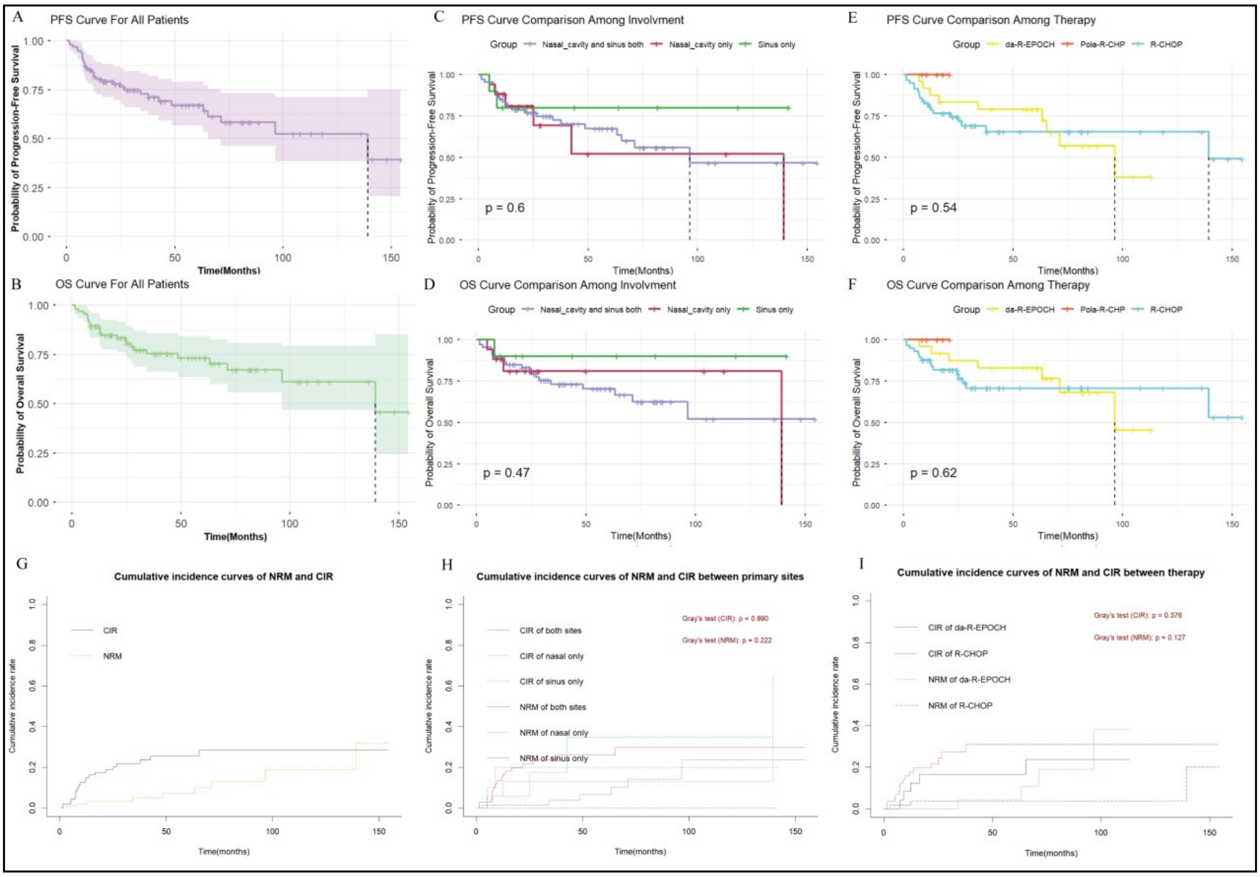

**Copyright Information:** (Online) ISSN: 2572-9241
©2026 The Author(s). HemaSphere is published by John Wiley & Sons Ltd on behalf of European Hematology Association. This is an open access Abstract Book distributed under the Attribution-NonCommercial-NoDerivs (CC BY-NC-ND), which allows third parties to download the articles and share them with others as long as they credit the author and the Abstract Book, but they cannot change the content in any way or use them commercially.
**Abstract Book Citations:** Authors, Title, HemaSphere, 2026;10:(S1):pages. The abstract book DOIs can be found on the "Author Information" page.
**Disclaimer:** Articles published in the journal HemaSphere exclusively reflect the opinions of the authors. The authors are responsible for all content in their abstracts including accuracy of the facts, statements, citing resources, etc.

### (PS2126) EVALUATION AND VALIDATION OF AUTOMATED MULTIPLEXED IMMUNOFLUORESCENCE FOR CLINICAL LYMPHOMA DIAGNOSES

**Topic:** 19. Large B cell lymphomas - Clinical

Antonio Sorrentino*[1], Joseph Lownik[2], Anton Villamejor[2], Akil Merchant[2]

*[1]Bio-Techne Spatial, Tolochenaz, Switzerland; [2]Cedars-Sinai Medical Center, Losa Angeles, United States of America;*

### Background

B-cell lymphomas encompass a wide range of malignancies, with clinical features ranging from indolent to aggressive. The diagnosis of these malignancies can routinely entail the evaluation of up to 20 immunohistochemistry (IHC) markers for complete characterization. An additional challenge is that analysis of these lymphomas requires inference of co-expression patterns from single-plex IHC on serial tissue sections, leading to significant subjectivity and inaccuracy. At the same time, core needle biopsies, rather than full excisional biopsies, have become standard for lymphoma diagnosis, further limiting the material available for analysis (Seviar et al., 2020).

### Aims

Here, we aim to develop, validate, and clinically evaluate a 22-marker immunofluorescence panel for diagnosis and prognostication of B-cell lymphomas from a single tissue section, and to benchmark performance against routine single-plex IHC.

### Methods

Formalin fixed paraffin embedded (FFPE) human lymphoma tissue microarray (TMA) and tissue sections were stained and imaged on the COMET automated platform by sequential immunofluorescence (seqIF) (Rivest et al., 2023) for multiplexed protein profiling.

### Results

Here, we present the development, validation, and evaluation of a 22-marker seqIF panel for the diagnosis and prognosis of B-cell lymphomas that can be performed on a single FFPE section on the Lunaphore COMET automated staining and imaging platform. Each marker was robustly validated through blinded hematopathologist review and compared to clinical IHC results from the same case. Validation was performed by converting each single marker expression from fluorescence to an RGB image with nuclei represented in a color similar to hematoxylin and the marker of interest in a color similar to DAB, which is routinely used in clinical practice. These sets of single-plex pseudo-colored images were then evaluated by a hematopathologist for diagnosis and classification, as well as comparison to the original IHC stains for that case.
We next evaluated the utility of this seqIF panel in the classification and diagnosis of indolent and aggressive B-cell lymphoma subtypes. We constructed a TMA with a broad selection of subtypes and controls (n=85) to recapitulate the limited diagnostic material received from core biopsies. Again, a blinded hematopathology review of pseudo single-plex images was performed, leading to a 100% concordance (marker positive/negative) between the COMET generated data and diagnostic IHC at the single marker level. Furthermore, there was no discordance between classification of a tissue as malignant vs benign, or indolent vs aggressive between the seqIF images and the clinical pathology report.

**Copyright Information:** (Online) ISSN: 2572-9241
©2026 The Author(s). HemaSphere is published by John Wiley & Sons Ltd on behalf of European Hematology Association. This is an open access Abstract Book distributed under the Attribution-NonCommercial-NoDerivs (CC BY-NC-ND), which allows third parties to download the articles and share them with others as long as they credit the author and the Abstract Book, but they cannot change the content in any way or use them commercially.
**Abstract Book Citations:** Authors, Title, HemaSphere, 2026;10:(S1):pages. The abstract book DOIs can be found on the "Author Information" page.
**Disclaimer:** Articles published in the journal HemaSphere exclusively reflect the opinions of the authors. The authors are responsible for all content in their abstracts including accuracy of the facts, statements, citing resources, etc.

**Summary/Conclusion**

Overall, we demonstrate the utility of a seqIF panel in the classification and prognostication of B-cell lymphomas. This panel has equal performance characteristics to single-plex clinical IHC in both qualitative marker assessment, tissue classification, and B-cell lymphoma classification. Additionally, as the system performs the analysis on a single FFPE tissue section that can be reused for downstream applications, it preserves precious material for cytogenetic and molecular prognostic studies. Automated image analysis with the help of machine learning classifiers to assess potential clinical diagnostic use and independent validation on a second set of diagnostic samples is ongoing.

**Copyright Information:** (Online) ISSN: 2572-9241
©2026 The Author(s). HemaSphere is published by John Wiley & Sons Ltd on behalf of European Hematology Association. This is an open access Abstract Book distributed under the Attribution-NonCommercial-NoDerivs (CC BY-NC-ND), which allows third parties to download the articles and share them with others as long as they credit the author and the Abstract Book, but they cannot change the content in any way or use them commercially.
**Abstract Book Citations:** Authors, Title, HemaSphere, 2026;10:(S1):pages. The abstract book DOIs can be found on the “Author Information” page.
**Disclaimer:** Articles published in the journal HemaSphere exclusively reflect the opinions of the authors. The authors are responsible for all content in their abstracts including accuracy of the facts, statements, citing resources, etc.

### (PS2127) BRUTON TYROSINE KINASE INHIBITOR MAINTENANCE THERAPY IN FIRST-LINE DIFFUSE LARGE B-CELL LYMPHOMA: A MULTICENTER REAL-WORLD STUDY CHALLENGING CONVENTIONAL PARADIGMS

**Topic:** 19. Large B cell lymphomas - Clinical

Li-Wei Lyu[1], Na Yao*[1], Zi-Xian Liu[2], Wang Yanying[3], Jie Li[4], Hebing Zhou[4], Lihong Li[3], Zhenling Li[2], Liang Wang[1]

*[1]Beijing Tongren Hospital, Capital Medical University, Beijing, China;[2]China-Japan Friendship Hospital, beijing, China;[3]Beijing Tsinghua Changgung Hospital, School of Clinical Medicine, Tsinghua University, beijing, China; [4]Beijing Luhe Hospital, Capital Medical University, beijing, China;*

### Background

Diffuse large B-cell lymphoma (DLBCL) remains the most common aggressive non-Hodgkin lymphoma, with approximately 30% of patients experiencing relapse following standard R-CHOP induction therapy.The role of maintenance strategies in DLBCL has been controversial, with rituximab and lenalidomide maintenance failing to demonstrate consistent overall survival (OS) benefits. The emergence of novel targeted agents, particularly Bruton tyrosine kinase inhibitors (BTKi), has potentially reshaped first-line treatment paradigms, yet their efficacy as maintenance therapy remains inadequately characterized.

### Aims

This multicenter retrospective study aims to evaluate the prognostic impact of BTKi-based maintenance therapy in the context of modern DLBCL treatment.

### Methods

We conducted a retrospective analysis of 106 DLBCL patients who received first-line maintenance therapy. Eligible patients achieved complete (CR) or partial response (PR) following induction therapy. BTKi maintenance (zanubrutinib or orelabrutinib) was administered to 73.6% of patients. Using propensity score matching (PSM), we selected 44 patients from the publicly available REMoDL-B trial database as a control group. Next-generation sequencing was performed for molecular subtyping according to Wright's "seven-gene" model. Survival outcomes were analyzed using Kaplan-Meier methods and Cox proportional hazards regression.

### Results

With median follow-up of 25.4 months, the maintenance group demonstrated exceptional outcomes: 2-year progression-free survival (PFS) and OS rates were 90% and 98%, respectively. Notably, even in high-risk subgroups—IPI ≥3 (87.0% 2-year PFS) and CNS-IPI ≥4 (86.5% 2-year PFS)—outcomes remained superior to historical benchmarks. Multivariate analysis identified maintenance therapy as an independent favorable prognostic factor for PFS (HR 0.42, 95% CI 0.20-0.88; P=0.019). Compared to the PSM control group, the maintenance cohort showed significantly improved PFS (P=0.024) and OS (P=0.008). Among BTKi-treated patients (n=78), 2-year PFS and OS were 87.6% and 97.2%, respectively. Strikingly, despite 43.6% being high-risk for CNS relapse by CNS-IPI criteria, none CNS recurrences were observed during follow-up. Maintenance duration ≥12 months correlated with superior PFS (P=0.042). Patients achieving only PR at end-of-induction demonstrated comparable PFS to those achieving CR when receiving maintenance therapy—a finding with particular relevance for elderly, chemotherapy-intolerant populations. Molecular subtyping revealed differential BTKi efficacy: MCD subtype patients trended toward better PFS than A53 subtype (P=0.078), suggesting BTKi maintenance may be particularly beneficial for MCD-molecular DLBCL. Safety profiles were favorable, with grade ≥3 thrombocytopenia in only 2.6% and no grade ≥3 non-hematologic toxicities. All adverse events were manageable through dose modification.

**Copyright Information:** (Online) ISSN: 2572-9241
©2026 The Author(s). HemaSphere is published by John Wiley & Sons Ltd on behalf of European Hematology Association. This is an open access Abstract Book distributed under the Attribution-NonCommercial-NoDerivs (CC BY-NC-ND), which allows third parties to download the articles and share them with others as long as they credit the author and the Abstract Book, but they cannot change the content in any way or use them commercially.
**Abstract Book Citations:** Authors, Title, HemaSphere, 2026;10:(S1):pages. The abstract book DOIs can be found on the "Author Information" page.
**Disclaimer:** Articles published in the journal HemaSphere exclusively reflect the opinions of the authors. The authors are responsible for all content in their abstracts including accuracy of the facts, statements, citing resources, etc.

### Summary/Conclusion

This real-world study challenges established dogma by demonstrating that BTKi-based maintenance therapy significantly improves PFS and OS in DLBCL, particularly for high-risk patients and those achieving suboptimal responses to induction therapy. The absence of CNS relapses in high-risk patients and potential enhanced efficacy in MCD molecular subtype support BTKi maintenance as a promising strategy. These findings warrant prospective validation in randomized trials and suggest reconsideration of maintenance therapy's role in modern DLBCL management.

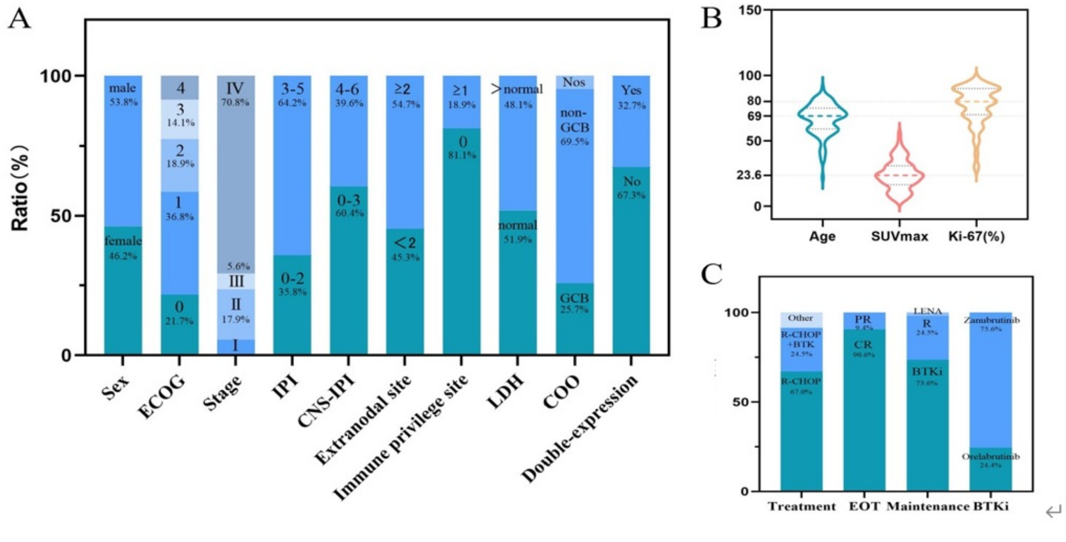

Figure 1 Clinical characteristics of DLBCL patients receiving maintenance therapy.

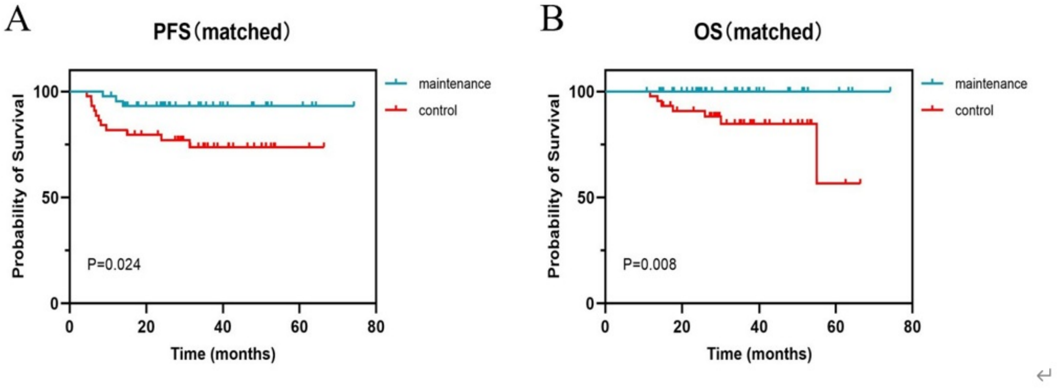

Figure 2 Survival analysis of maintenance therapy group and control group.

**Copyright Information:** (Online) ISSN: 2572-9241
©2026 The Author(s). HemaSphere is published by John Wiley & Sons Ltd on behalf of European Hematology Association. This is an open access Abstract Book distributed under the Attribution-NonCommercial-NoDerivs (CC BY-NC-ND), which allows third parties to download the articles and share them with others as long as they credit the author and the Abstract Book, but they cannot change the content in any way or use them commercially.
**Abstract Book Citations:** Authors, Title, HemaSphere, 2026;10:(S1):pages. The abstract book DOIs can be found on the "Author Information" page.
**Disclaimer:** Articles published in the journal HemaSphere exclusively reflect the opinions of the authors. The authors are responsible for all content in their abstracts including accuracy of the facts, statements, citing resources, etc.

### (PS2128) MAPPING THE METHODOLOGY & ATTRIBUTES OF GLOBAL LYMPHOMA REGISTRIES: A LYMPHOMA REGISTRY ALLIANCE (LYRA) INITIATIVE

**Topic:** 19. Large B cell lymphomas - Clinical

Eliza Chung*[1], Mark Bishton[2, 3], Fanny Cherblanc[4], Theodora Agnes Maria Claushuis[5], Martine Chamuleau[5], Tarec El-Galaly[6], Lise Enerstvedt[7], Massimo Federico[8], Herve Ghesquieres[9], Georg Heß[10], Tai-Chung Huang[11], Tom Johannesen[7], Iryna Kryachok[12], Zoe McQuilten[1], Anke Ohler[10], Alice Pauc[4], Nazarii Shokun[12], Karin Ekstrom Smedby[13, 14], Yana Stepanishyna[15], Alice Sykorova[16], Ciprian Tomuleasa[17], Marek Trněný[18], Yu-Hsuan Tuan[11], Erica Wood[1, 19], Eliza Hawkes[1, 20]

*[1]Monash University, School of Public Health and Preventive Medicine, Melbourne, Australia;[2]University of Nottingham, Translational Medical Sciences, Nottingham, United Kingdom; [3]Nottingham University Hospitals NHS Trust, Nottingham, United Kingdom; [4]Lymphoma Academic Research Organisation, Innovation Hub, Pierre-Benite, France;[5]Amsterdam UMC, Department of Hematology, Cancer Center Amsterdam, Amsterdam, The Netherlands; [6]Aarhus University Hospital and Aarhus University, Departments of Hematology, Clinical Epidemiology, and Molecular Medicine, Aarhus, Denmark; [7]Norwegian Institute of Public Health, Cancer Registry of Norway, Oslo, Norway; [8]University of Modena and Reggio Emilia, CHIMOMO Department, Modena, Italy; [9]Hopital Lyon Sud, Department of Hematology, Claude Bernard Lyon 1 University, Pierre-Benite, France;[10]University of Mainz, Department of Hematology and Medical Oncology, German Lymphoma Alliance and European MCL-network, Mainz, Germany; [11]National Taiwan University Hospital, Division of Hematology, Department of Internal Medicine, Taipei, Taiwan; [12]National Cancer Institute, Kyiv, Ukraine; [13]Karolinska Institutet, Division of clinical epidemiology, Department of Medicine Solna, Stockholm, Sweden; [14]Karolinska University Hospital, Department of hematology, Stockholm, Sweden;[15]Institut Jules Bordet, Hematology Department, Brussels, Belgium; [16]University Hospital and Faculty of Medicine, 4th Department of Internal Medicine – Hematology, Hradec Kralove, Czechia; [17]Medfuture Institute for Biomedical Research, Iuliu Hațieganu University of Medicine and Pharmacy, Department of Hematology/Personalized Medicine and Rare Diseases, Cluj-Napoca, Romania; [18]Charles University, General Hospital, 1st Department of Medicine – Department of Hematology, Prague, Czechia;[19]Monash Health, Department of Clinical Haematology, Melbourne, Australia; [20]Olivia Newton-John Cancer Research Institute at Austin Health, Melbourne, Australia;*

### Background

The Global LyRA unites stakeholders from 48 real-world lymphoma datasets (e.g. population-based cancer registries, disease registries, institutional databases and research consortia). Registries are longitudinal datasets monitoring the course of disease. The methodological diversity and maturity of lymphoma registries worldwide have never been examined but are fundamental to collaborations in benchmarking and real-world research.

### Aims

To identify and report characteristics of the LyRA member datasets.

### Methods

All LyRA member organisations were invited to provide administrative information on their registries or consortia from Jun 2025 to Jan 2026. Member registry name, dataset type, established year, leading country or jurisdiction, mode of patient consent and data entry, patient accrual, availability of clinical quality indicators (CQIs) and capacity for data sharing were collected and descriptively analysed. Interviews were conducted to gain further clarity on responses.

**Copyright Information:** (Online) ISSN: 2572-9241
©2026 The Author(s). HemaSphere is published by John Wiley & Sons Ltd on behalf of European Hematology Association. This is an open access Abstract Book distributed under the Attribution-NonCommercial-NoDerivs (CC BY-NC-ND), which allows third parties to download the articles and share them with others as long as they credit the author and the Abstract Book, but they cannot change the content in any way or use them commercially.
**Abstract Book Citations:** Authors, Title, HemaSphere, 2026;10:(S1):pages. The abstract book DOIs can be found on the "Author Information" page.
**Disclaimer:** Articles published in the journal HemaSphere exclusively reflect the opinions of the authors. The authors are responsible for all content in their abstracts including accuracy of the facts, statements, citing resources, etc.

### Results

Twenty-one LyRA members (44%) from 16 jurisdictions provided data by the cut off, including 7 population-based cancer registries (33%), 7 disease registries (33%), 3 research consortia (14%), 2 institutional databases (10%) and 2 cohort studies (10%) (Table 1). 71% (15/21) also had clinical or operational leads attending an interview. Geographical representation was from Europe, Asia & Oceania. Information about one additional planned registry was also provided. The oldest population-based cancer registry was 73 years old and the largest had accrued 703715 patients.
80% of organisations utilised waived consent for recruitment, 60% captured all lymphomas, 67% collected data manually (Table 1). The 14 members collecting data manually also obtained regular follow-up semi-annually (n=4), or on a rolling basis (n=10) (e.g. upon clinical reviews). Other members routinely collect data manually from hospital medical records in addition to either linkage with administrative datasets held by government agencies (n=5), or dataset extraction (n=2).
33% (7/21) of the member datasets were established as clinical quality registries (CQRs) that report for quality improvement and benchmarking purposes. Six captured CQIs defined by expert consensus or national guidelines, categorised into structure (e.g. reporting of required clinical information such as stage), process (e.g. adherence to guidelines, wait times to treatment decisions, inclusion on clinical trials), and outcome (e.g. survival rate at pre-determined timeframe). The remaining CQR regularly fed back benchmarking reports to the registry-affiliated hospitals on clinical outcomes without defined CQIs.
All participating datasets expressed capacity in data sharing at various sensitivity levels permitted by regulatory requirements. Visualisations (33%) or aggregated data (38%) are publicly available without requiring approvals. De-identified or anonymised datasets with registry governance or ethics approval could be provided (20%), accessed in a secure portal (52%), or by file transfer (95%). Requests of identifiable data were only available in 15% upon similar approval.

### Summary/Conclusion

Lymphoma registries and consortia have significant variation in all aspects, including operations, consent model, data collection and sharing, and capacity for benchmarking. This mapped information will present opportunities for LyRA members to share knowledge and encourage large-scale collaborative research and benchmarking in lymphoma care.

**Table 1: Member characteristics of the Global Lymphoma Registry Alliance.**

| Member type | Number of pts (range) | Established year (range) | Consent (%) | | | | Disease (%) | | | | Mode of data collection (%) | | | | Collection of clinical quality indicators (%) | | | |
|---|---|---|---|---|---|---|---|---|---|---|---|---|---|---|---|---|---|---|
| | | | Waived^ | | Opt-in | | All lymphomas | | Subtypes | | Manual entry | | Multi-modal | | No | | Yes | |
| **N=21** | N=973899 | | **17** | **(80)** | **4** | **(20)** | **13** | **(60)** | **8** | **(40)** | **14** | **(67)** | **7** | **(33)** | **15** | **(72)** | **6** | **(28)** |
| **Population-based cancer registry (n=7)** | 1451-703715 | 1953-2025 | 6 | (28) | 1 | (5) | 7 | (32) | 0 | (0) | 2 | (10) | 5 | (23) | 2 | (10) | 5 | (23) |
| **Disease registry* (n=7)** | 0-9071 | 2015-2024 | 5 | (23) | 2 | (10) | 5 | (23) | 2 | (10) | 7 | (32) | 0 | (0) | 6 | (28) | 1 | (5) |
| **Consortium (n=3)** | 121-3000 | 2016-2025 | 3 | (14) | 0 | (0) | 1 | (5) | 2 | (10) | 2 | (10) | 1 | (5) | 3 | (14) | 0 | (0) |
| **Institutional database (n=2)** | 135-158 | 2023 | 2 | (10) | 0 | (0) | 0 | (0) | 2 | (10) | 1 | (5) | 1 | (5) | 2 | (10) | 0 | (0) |
| **Cohort study (n=2)** | 600-6000 | 2018-2023 | 1 | (5) | 1 | (5) | 0 | (0) | 2 | (10) | 2 | (10) | 0 | (0) | 2 | (10) | 0 | (0) |

*Including one planned registry not yet in operation.
^Waived consent includes opt-out approach and compulsory registration abide by the law.

Copyright Information: (Online) ISSN: 2572-9241
©2026 The Author(s). HemaSphere is published by John Wiley & Sons Ltd on behalf of European Hematology Association. This is an open access Abstract Book distributed under the Attribution-NonCommercial-NoDerivs (CC BY-NC-ND), which allows third parties to download the articles and share them with others as long as they credit the author and the Abstract Book, but they cannot change the content in any way or use them commercially.
Abstract Book Citations: Authors, Title, HemaSphere, 2026;10:(S1):pages. The abstract book DOIs can be found on the "Author Information" page.
Disclaimer: Articles published in the journal HemaSphere exclusively reflect the opinions of the authors. The authors are responsible for all content in their abstracts including accuracy of the facts, statements, citing resources, etc.

### (PS2129) GOLIDOCITINIB COMBINED WITH CHOP IN NEWLY-DIAGNOSED PERIPHERAL T-CELL LYMPHOMA: PRELIMINARY RESULTS FROM A PHASE 1/2 CLINICAL TRIAL(JACKPOT 53)

**Topic:** 20. Other aggressive lymphomas

Chong Wei*[1], Wei Zhang[1], Jian Li[1], Dao-Bin Zhou[1], Yan Zhang[1], Kaini Shen[1], Wei Wang[1], Zi Wang[1], Danqing Zhao[1], Jing Ruan[1], Yueying Mao[2], Huacong Cai[1]

*[1]Department of Hematology, Peking Union Medical College Hospital, Chinese Academy of Medical Sciences & Peking Union Medical College, Beijing, China; [2]Beijing Friendship Hospital, Capital Medical University, Beijing, China;*

**Background**

Peripheral T-cell lymphoma (PTCL) represents a heterogeneous group of aggressive non-Hodgkin lymphomas with poor clinical outcomes. Although CHOP remains the standard first-line therapy, long-term survival remains unsatisfactory. Golidocitinib, a highly selective JAK1 inhibitor, has demonstrated promising anti-tumor activity and a favorable safety profile in relapsed/refractory PTCL.

**Aims**

To evaluate the safety, tolerability, and preliminary efficacy of golidocitinib combined with CHOP (Go-CHOP) as frontline therapy for newly diagnosed PTCL.

**Methods**

This is an ongoing phase 1/2, single-center, single-arm clinical trial investigating Go-CHOP as first-line treatment in adult patients with histologically confirmed previously untreated PTCL. The study comprises two sequential phases. Phase 1 follows a 3+3 dose-escalation design to determine the maximum tolerated dose (MTD) and recommended phase 2 dose (RP2D). Phase 2 is a dose-expansion cohort evaluating efficacy and safety at the RP2D. During Phase 1, patients receive six cycles of Go-CHOP every 21 days, with golidocitinib administered at two prespecified dose levels (150 mg once daily and 150 mg every other day) in combination with standard CHOP. In Phase 2, patients receive Go-CHOP at the RP2D. Patients achieving response after Go-CHOP may undergo consolidative autologous stem cell transplantation (ASCT) if eligible, while transplant-ineligible patients receive golidocitinib maintenance for up to 24 months or until disease progression or unacceptable toxicity. The primary endpoints are MTD and RP2D in Phase 1 and complete response rate (CRR) in Phase 2.

**Copyright Information:** (Online) ISSN: 2572-9241
©2026 The Author(s). HemaSphere is published by John Wiley & Sons Ltd on behalf of European Hematology Association. This is an open access Abstract Book distributed under the Attribution-NonCommercial-NoDerivs (CC BY-NC-ND), which allows third parties to download the articles and share them with others as long as they credit the author and the Abstract Book, but they cannot change the content in any way or use them commercially.
**Abstract Book Citations:** Authors, Title, HemaSphere, 2026;10:(S1):pages. The abstract book DOIs can be found on the “Author Information” page.
**Disclaimer:** Articles published in the journal HemaSphere exclusively reflect the opinions of the authors. The authors are responsible for all content in their abstracts including accuracy of the facts, statements, citing resources, etc.

**Results**

As of the data cutoff on Feb 28th, 2026, 34 patients were enrolled and received at least one cycle of Go-CHOP, including 6 patients in Phase 1 and 28 in Phase 2. The median age was 52 years (range, 18–70), and 26 patients (76.4%) were male. Thirty-three patients (97.1%) had stage III–IV disease, and 19 (55.8%) had an IPI score of 3–5. Diagnoses included TFHL in 15 patients (44.1%), PTCL-NOS in 15 (44.1%), ALCL in 2 (5.9%), and MEITL in 2 (5.9%). No DLTs were observed during dose escalation, establishing golidocitinib 150 mg once daily as the RP2D. Treatment-related adverse events (TRAEs) occurred in 32 patients (94.1%), with neutropenia being the most common (88.2%), followed by CMV reactivation (41.2%), thrombocytopenia (23.5%), and hepatic impairment (20.5%). Grade 3/4 TRAEs were mainly neutropenia (64.7%). CMV reactivation occurred in 14 patients (41.2%), including CMV retinitis (n=2), enterocolitis (n=2), pneumonia (n=2), and viremia (n=8), highlighting the need for close monitoring. Additionally, one case of hepatitis B virus reactivation and one case of pulmonary cryptococcosis were observed, with the latter leading to treatment discontinuation. Among 26 efficacy-evaluable patients, 22 achieved objective responses, including 15 complete responses, yielding an overall response rate (ORR) of 84.6% and a CRR of 62.5%. Four patients underwent consolidative ASCT, and eight patients initiated golidocitinib maintenance therapy. With a median follow-up of 7.5 months, median progression-free survival and overall survival were not reached.

**Summary/Conclusion**

Go-CHOP demonstrated manageable safety and encouraging anti-tumor activity as frontline therapy for PTCL, with a high CRR and a feasible treatment strategy incorporating consolidative ASCT and golidocitinib maintenance. Longer follow-up is warranted to confirm survival benefits.

**Copyright Information:** (Online) ISSN: 2572-9241
©2026 The Author(s). HemaSphere is published by John Wiley & Sons Ltd on behalf of European Hematology Association. This is an open access Abstract Book distributed under the Attribution-NonCommercial-NoDerivs (CC BY-NC-ND), which allows third parties to download the articles and share them with others as long as they credit the author and the Abstract Book, but they cannot change the content in any way or use them commercially.
**Abstract Book Citations:** Authors, Title, HemaSphere, 2026;10:(S1):pages. The abstract book DOIs can be found on the “Author Information” page.
**Disclaimer:** Articles published in the journal HemaSphere exclusively reflect the opinions of the authors. The authors are responsible for all content in their abstracts including accuracy of the facts, statements, citing resources, etc.

### (PS2130) EFFICACY AND SAFETY OF ORELABRUTINIB COMBINED WITH SINTILIMAB IN PATIENTS WITH RELAPSED/REFRACTORY PRIMARY CENTRAL NERVOUS SYSTEM LYMPHOMA (R/R PCNSL): A PROSPECTIVE MULTICENTER PHASE II STUDY

**Topic:** 20. Other aggressive lymphomas

Yan Zhang*[1], Chong Wei[1], Danqing Zhao[1], Jing Ruan[1], Huiying Zhu[1], Zhangyuting He[1], Wei Zhang[1], Dao-Bin Zhou[1]

[1] *Chinese Academy of Medical College & Peking Union Medical College Hospital, Department of Hematology, Beijing, China;*

### Background

PCNSL is an aggressive non-Hodgkin lymphoma. While high-dose methotrexate (HD-MTX)-based regimens are first-line standards for newly diagnosed patients, disease relapse remains a major clinical challenge. R/R PCNSL has poor outcomes with high recurrence and limited therapies, and no standard treatment exists, representing an unmet need. Preclinical studies have shown synergistic efficacy of BTK inhibitors (BTKi) plus anti-PD-1 antibodies in B-cell lymphoma (Vincent-Fabert et al. Cell Commun Signal. 2019). Orelabrutinib, a novel BTKi with high CNS penetrance, exhibits efficacy in PCNSL with minimal off-target effects and acceptable safety; sintilimab is a next-generation anti-PD-1 monoclonal antibody.

### Aims

To evaluate the safety and efficacy of orelabrutinib combined with sintilimab in patients with R/R PCNSL.

### Methods

This prospective, multicenter, single-arm phase II study (NCT04899427) enrolled immunocompetent adults with R/R PCNSL and eligible organ function. Patients received orelabrutinib (150 mg once daily) plus sintilimab (200 mg on day 1 of each cycle) every 3 weeks for up to 2 years, until progression, unacceptable toxicity, or death. The primary endpoint was the overall response rate (ORR), defined as achievement of partial response (PR) or better, following four cycles of treatment. Secondary endpoints included the 1-year progression-free survival (PFS) rate and safety.

### Results

From March 2021 to December 2023, 28 patients were enrolled (median age 61.0 years, 48-75; 51.7% male). All patients had prior exposure to HD-MTX; 65.5% had relapsed disease, and 34.5% were refractory. The cohort was heavily pretreated, with a median of two prior therapy lines (range: 1-5), and 44.8% had received≥3 prior lines. 48% of patients were classified as high-risk (IELSG score 3-4).
Among 28 evaluable patients, the ORR after 4 cycles was 60.7% (17/28), including 28.6% achieving CR, 7.1% unconfirmed CR (CRu), and 25.0% PR. The disease control rate (DCR) was 78.6% (22/28). ORR increased to 77.8% at cycle 6 in ongoing patients. At a median follow-up of 22.9 months, the median PFS was 8.4 months (Figure 1A), with 1-year and 2-year PFS rates of 49.7% (95% CI: 34.2-72.3%) and 40.7% (95% CI: 25.5-64.9%), respectively. Median overall survival (OS) was not reached (Figure 1B), and the 1-year and 2-year OS rates were 77.4% (95% CI: 63.0-95.1%) and 73.3% (95% CI: 58.2-92.5%), respectively. A trend toward longer PFS was observed in patients with the GCB subtype (median PFS of 14.8 months) compared to the non-GCB subtype (median PFS of 3.2 months; log-rank p=0.1) (Figure 1C). Among the 15 responders, the median duration of response (DOR) was 49.7 months, indicating sustained efficacy (Figure 1D).
Treatment-emergent adverse events (TEAEs) occurred in 72.4% (21/29) of patients, with grade 3-4 events in 13.8% (4/29); no grade 5 events were reported. Common any-grade TEAEs included fatigue (39.3%), bleeding events (35.7%), leukopenia (32.1%), and infections (35.7%).

**Copyright Information:** (Online) ISSN: 2572-9241
©2026 The Author(s). HemaSphere is published by John Wiley & Sons Ltd on behalf of European Hematology Association. This is an open access Abstract Book distributed under the Attribution-NonCommercial-NoDerivs (CC BY-NC-ND), which allows third parties to download the articles and share them with others as long as they credit the author and the Abstract Book, but they cannot change the content in any way or use them commercially.
**Abstract Book Citations:** Authors, Title, HemaSphere, 2026;10:(S1):pages. The abstract book DOIs can be found on the “Author Information” page.
**Disclaimer:** Articles published in the journal HemaSphere exclusively reflect the opinions of the authors. The authors are responsible for all content in their abstracts including accuracy of the facts, statements, citing resources, etc.

### Summary/Conclusion

This prospective phase II study demonstrates that combining orelabrutinib with sintilimab provided significant clinical benefits for patients with R/R PCNSL, including strong response rates and notably durable responses. The manageable safety profile further supports this combination as a valuable treatment option for this challenging population.

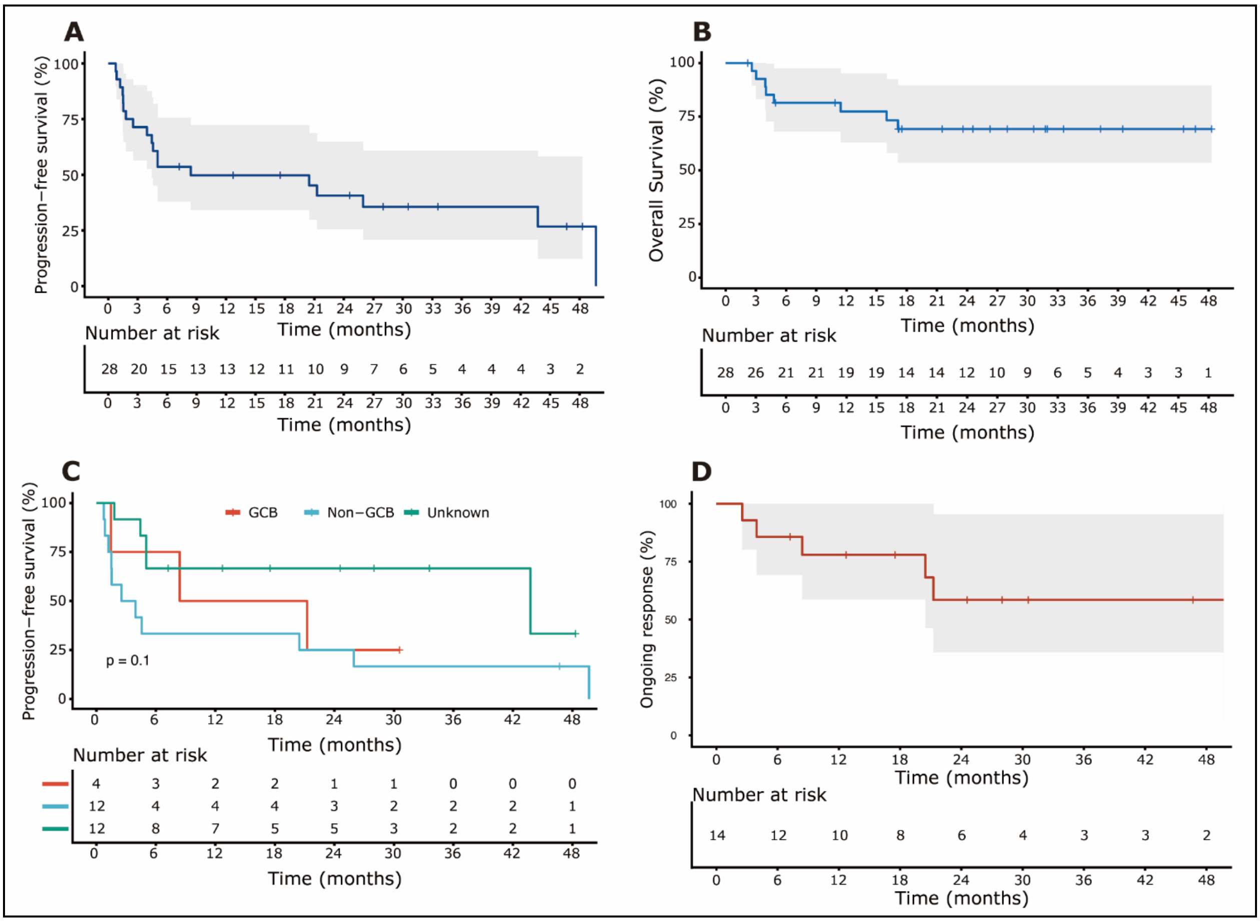

Figure 1. Survival Outcomes: (A) PFS; (B) OS; (C) by molecular subtype; (D) DOR .

**Copyright Information:** (Online) ISSN: 2572-9241
©2026 The Author(s). HemaSphere is published by John Wiley & Sons Ltd on behalf of European Hematology Association. This is an open access Abstract Book distributed under the Attribution-NonCommercial-NoDerivs (CC BY-NC-ND), which allows third parties to download the articles and share them with others as long as they credit the author and the Abstract Book, but they cannot change the content in any way or use them commercially.
**Abstract Book Citations:** Authors, Title, HemaSphere, 2026;10:(S1):pages. The abstract book DOIs can be found on the "Author Information" page.
**Disclaimer:** Articles published in the journal HemaSphere exclusively reflect the opinions of the authors. The authors are responsible for all content in their abstracts including accuracy of the facts, statements, citing resources, etc.

### (PS2131) ANTI-PD-1-ANTIBODY (TISLELIZUMAB) PLUS CHIDAMIDE, LENALIDOMIDE AND ETOPOSIDE FOR REFRACTORY/RELAPSED EXTRANODAL NK/T-CELL LYMPHOMA, NASAL TYPE : A PROSPECTIVE, MULTICENTER, SINGLE-ARM TRIAL

**Topic:** 20. Other aggressive lymphomas

Yu Chang*[1], Ling Li[1], Xin Li[1], Xinhua Wang[1], Xiaorui Fu[1], Zhenchang Sun[1], Xudong Zhang[1], Linan Zhu[1], Songtao Niu[1], Feifei Nan[1], Shuli Guo[2], Qiuli Meng[3], Wei Sang[4], Lei Zhang[1], Mingzhi Zhang[1]

*[1]First Affiliated Hospital of Zhengzhou University, Zhengzhou, China;[2]Luoyang Central Hospital, Luoyang, China;[3]Gongyi City People's Hospital, Gongyi, China; [4]The Affiliated Hospital of Xuzhou Medical University, Xuzhou, China;*

### Background

Extranodal Natural Killer/T-Cell Lymphoma (ENKTL) is a highly aggressive  subtype of non-Hodgkin lymphoma with a markedly higher incidence in Asia. Patients with Refractory/Relapsed-ENKTL(r/r-ENKTL) face a dismal prognosis and have limited therapeutic options.

### Aims

To evaluate the efficacy and safety of a novel regimen comprising Tislelizumab, Chidamide, Lenalidomide and Etoposide in patients with r/r-ENKTL.

### Methods

This was a prospective, single-arm, open-label, multicenter clinical trial enrolling adult patients with r/r-ENKTL who had received at least one prior systemic therapy. Patients received 6 cycles of combination therapy comprising Tislelizumab (200mg, Day 1), Chidamide (20mg, biw), Lenalidomide (25mg, Days 1-10) and Etoposide (100mg/m$^2$, Days 1-3) every 21 days, followed by 10 cycles of Tislelizumab maintenance (200mg, Day1) every 21 days. The primary endpoints were objective response rate (ORR) and progression-free survival (PFS). This trial is registered at ClinicalTrials.gov (NCT04038411).

**Copyright Information:** (Online) ISSN: 2572-9241
©2026 The Author(s). HemaSphere is published by John Wiley & Sons Ltd on behalf of European Hematology Association. This is an open access Abstract Book distributed under the Attribution-NonCommercial-NoDerivs (CC BY-NC-ND), which allows third parties to download the articles and share them with others as long as they credit the author and the Abstract Book, but they cannot change the content in any way or use them commercially.
**Abstract Book Citations:** Authors, Title, HemaSphere, 2026;10:(S1):pages. The abstract book DOIs can be found on the "Author Information" page.
**Disclaimer:** Articles published in the journal HemaSphere exclusively reflect the opinions of the authors. The authors are responsible for all content in their abstracts including accuracy of the facts, statements, citing resources, etc.

### Results

From June 2020 to December 2025, 38 eligible patients with r/r-ENKTL were enrolled from 5 centers. The final follow-up occurred on February 20, 2026, with a median follow-up of 32 months (range: 2-67 months). The median age of the cohort was 39 years (range, 17-63 years). All patients received at least four cycles of induction chemotherapy. Among the 38 efficacy-evaluable patients, the overall response rate (ORR) was 84.2% (32/38), including a complete response (CR) rate of 60.5% (23/38). During Tislelizumab maintenance therapy, an additional one patients achieved CR, resulting in a cumulative CR rate of 65.8% (25/38) at the end of maintenance treatment. The median progression-free survival (PFS) was 49.0 months (95%CI: 24.3-3.7). Estimated PFS rates at 1, 2, and 5 years were 72.7%, 55.5%, and 42.3%, respectively. Median overall survival (OS) was not reached; corresponding 1-year, 2-year, and 5-year OS rates were 81.1%, 69.9%, and 54.9%. At last follow-up, 17 patients remained in continuous CR. The most common treatment-related adverse events (TRAEs) were anemia (83.3%), leukopenia (80.0%), neutropenia (80.8%), and thrombocytopenia (56.7%). The most frequent grade ≥3 TRAE was neutropenia (46.7%). Immune-related adverse events of grade 3 or higher included one case each of hypothyroidism and hyperglycemia.

| Characteristic | Total (%) |
|---|---|
| Sex (male) | 27(71.1) |
| B symptoms (Yes) | 14(36.8) |
| CA Stage(Ⅲ-Ⅳ) | 36(94.7) |
| PINK score<br>0-2<br>3-4 | <br>27(71.1)<br>11(28.9) |
| Prior PD-1 mAb use | 3(7.9) |

### Summary/Conclusion

This regimen demonstrated promising efficacy and a favorable safety profile in patients with r/r-ENKTL.

**Copyright Information:** (Online) ISSN: 2572-9241
©2026 The Author(s). HemaSphere is published by John Wiley & Sons Ltd on behalf of European Hematology Association. This is an open access Abstract Book distributed under the Attribution-NonCommercial-NoDerivs (CC BY-NC-ND), which allows third parties to download the articles and share them with others as long as they credit the author and the Abstract Book, but they cannot change the content in any way or use them commercially.
**Abstract Book Citations:** Authors, Title, HemaSphere, 2026;10:(S1):pages. The abstract book DOIs can be found on the "Author Information" page.
**Disclaimer:** Articles published in the journal HemaSphere exclusively reflect the opinions of the authors. The authors are responsible for all content in their abstracts including accuracy of the facts, statements, citing resources, etc.

### (PS2132) REAL-WORLD INSIGHTS INTO CLINICAL FEATURES AND OUTCOMES IN EXTRANODAL T-CELL LYMPHOMAS: RESULTS FROM THE INTERNATIONAL T-CELL PROJECT 2.0 PROSPECTIVE COHORT STUDY.

**Topic:** 20. Other aggressive lymphomas

Maryna Bushuieva*[1], Luana Conte[2], Monica Civallero[3], Nazarii Shokun[4], Ciprian Tomuleasa[5], Tetiana Skrypets[6], Ranjana Advani[7], Ilias Stagakis[8], Neeraj Arora[9], Julie Vose[10], Wonseog Kim[11], Stefano Luminari[12], Carolina Mahuad[13], Camila Pena[14], Raimundo Gazitua[15], Eliza Chung[16], Thais Fischer[17], Cameron Wellard[16], Anastasiia Maliar[18], Carlos Sergio Chiattone[19], Eliza Hawkes[16], Astrid Pavlovsky[20], Massimo Federico[3], Iryna Kryachok[18]

*[1]National specialized childrens' hospital "Ohmatdyt", Kyiv, Ukraine;[2]University of Palermo, Department of Physics and Chemistry, Palermo, Italy; [3]University of Modena and Reggio Emilia, Modena, Italy; [4]University of Salento, Lecce, Italy; [5]Iuliu Hatieganu University of Medicine and Pharmacy, Department of Hematology, Cluj Napoca, Romania; [6]IRCCS - Istituto Tumori Giovanni Paolo II, Hematology Unit, Bari, Italy; [7]Stanford University, Division of Oncology, Department of Medicine, Stanford, United States of America; [8]Kantonsspital St. Gallen, St. Gallen, Switzerland;[9]Tata Medical Center, Kolkata, India; [10]University of Nebraska Medical Center, Omaha, United States of America; [11]Samsung Medical Center, Seoul, South Korea; [12]Azienda USL-IRCCS di Reggio Emilia, Hematology Unit, Reggio Emilia, Italy; [13]Hospital Alemán, Buenos Aires, Argentina; [14]Hospital del Salvador, Santiago, Chile; [15]Instituto Oncológico Fundación Arturo López Pérez, Santiago, Chile;[16]Monash University, School of Public Health and Preventive Medicine, Melbourne, Australia; [17]Ac Camargo Cancer Center - ACCCC, São Paulo, Brazil; [18]National cancer institute, Kyiv, Ukraine; [19]Universidade Estadual de Campinas-UNICAMP, Sao Paulo, Brazil;[20]Buenos Aires Fundaleu, Departamento de Hematologia, Buenos Aires, Argentina;*

### Background

Extranodal T-cell lymphomas (EN-TCL) comprise a complex and heterogeneous group of malignancies often associated with an aggressive clinical course. Despite improvements largely due to asparaginase-based therapies in Natural killer/T-cell subtypes, real world data suggests that outcomes for advances disease remain poor. Utilizing data from the International T-cell Project 2.0 (TCP2.0), this study aimed to evaluate current treatment modalities and survival outcomes to better characterize this patient population.

### Aims

To analyze the clinical characteristics, frontline treatment patterns, and survival outcomes of patients with extranodal T-cell lymphomas within the prospective TCP2.0 cohort.

### Methods

We conducted a prospective analysis of 323 patients with EN-TCL enrolled in the TCP2.0 registry between 2018 and 2026. All cases were classified according to 2016 WHO criteria. The primary endpoint was 2-year progression-free survival (PFS), estimated using the Kaplan-Meier method with 95% Confidence Intervals (CI).

**Copyright Information:** (Online) ISSN: 2572-9241
©2026 The Author(s). HemaSphere is published by John Wiley & Sons Ltd on behalf of European Hematology Association. This is an open access Abstract Book distributed under the Attribution-NonCommercial-NoDerivs (CC BY-NC-ND), which allows third parties to download the articles and share them with others as long as they credit the author and the Abstract Book, but they cannot change the content in any way or use them commercially.
**Abstract Book Citations:** Authors, Title, HemaSphere, 2026;10:(S1):pages. The abstract book DOIs can be found on the "Author Information" page.
**Disclaimer:** Articles published in the journal HemaSphere exclusively reflect the opinions of the authors. The authors are responsible for all content in their abstracts including accuracy of the facts, statements, citing resources, etc.

**Results**

Among the 323 patients, the most frequent subtypes were Extranodal NK/T-cell lymphoma, nasal type (ENKTL; N=145), Intestinal T-cell lymphoma, NOS (ITCL; N=92), Enteropathy-associated T-cell lymphoma (EATL; N=27), Breast implant-associated anaplastic large cell lymphoma (BIA-ALCL; N=15), Hepatosplenic T-cell lymphoma (HSTCL; N=23). Advanced-stage disease (Stage III-IV) was present in 51% of patients, and 47% exhibited B-symptoms at diagnosis, and 81% of the cohort maintained a favorable ECOG performance status (0-1). The high-risk group comprises 18% (N=38) and 13% (N=33) according to IPI and PIT, respectively. Frontline therapy was predominantly anthracycline-based, with CHOP (23%) and CHOEP (18%) being the most utilized regimens. Asparaginase-containing protocols were administered to 22% of patients. Consolidation with stem cell transplantation was performed in a subset of 35 cases, of which 27 (77%) were autologous (ASCT). The overall response rate (ORR) was 65%, with a complete response (CR) rate of 49%.
The 2-year PFS demonstrated significant variability across histological subtypes: for BIA-ALCL 92% (95% CI: 77–100), for ENKTL 45% (95% CI: 37–53), for EATL 32% (95% CI: 18–58), for ITCL, NOS 21% (95% CI: 13–32), for HSTCL 12% (95% CI: 3–43). Information about relapse was recorded in 122 (37.7%), and the second-line therapy information was presented in 85 (69.6%). The three most frequent regimes were Gemcitabine-based 17(20%), Asparginase-based 14(16%) and Platinum-based 11(13%). Only 2 patients received BV, and 9 received Pralatrexate. The response to second-line therapy was recorded in 34/85 (40%), with an ORR 36%, where 21% reached CR and 15% PR.

**Summary/Conclusion**

Our findings confirm that extranodal T-cell lymphomas, with the exception of BIA-ALCL, continue to carry a poor prognosis. The low utilization of transplant consolidation and a high rate of primary disease progression highlight a critical need for the integration of novel agents and intensified frontline strategies to improve long-term survival in this heterogeneous group.

**Copyright Information:** (Online) ISSN: 2572-9241
©2026 The Author(s). HemaSphere is published by John Wiley & Sons Ltd on behalf of European Hematology Association. This is an open access Abstract Book distributed under the Attribution-NonCommercial-NoDerivs (CC BY-NC-ND), which allows third parties to download the articles and share them with others as long as they credit the author and the Abstract Book, but they cannot change the content in any way or use them commercially.
**Abstract Book Citations:** Authors, Title, HemaSphere, 2026;10:(S1):pages. The abstract book DOIs can be found on the "Author Information" page.
**Disclaimer:** Articles published in the journal HemaSphere exclusively reflect the opinions of the authors. The authors are responsible for all content in their abstracts including accuracy of the facts, statements, citing resources, etc.

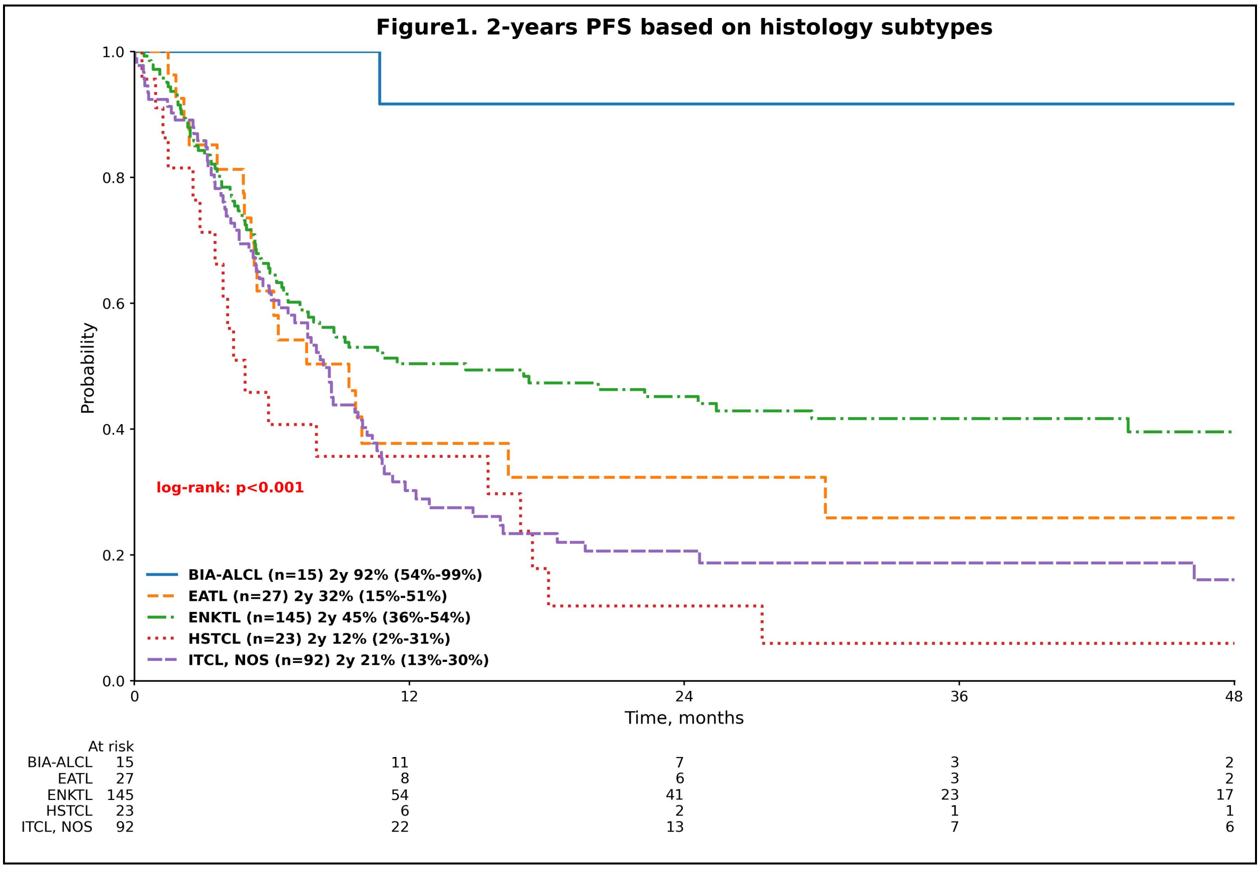

**Copyright Information:** (Online) ISSN: 2572-9241
©2026 The Author(s). HemaSphere is published by John Wiley & Sons Ltd on behalf of European Hematology Association. This is an open access Abstract Book distributed under the Attribution-NonCommercial-NoDerivs (CC BY-NC-ND), which allows third parties to download the articles and share them with others as long as they credit the author and the Abstract Book, but they cannot change the content in any way or use them commercially.
**Abstract Book Citations:** Authors, Title, HemaSphere, 2026;10:(S1):pages. The abstract book DOIs can be found on the "Author Information" page.
**Disclaimer:** Articles published in the journal HemaSphere exclusively reflect the opinions of the authors. The authors are responsible for all content in their abstracts including accuracy of the facts, statements, citing resources, etc.

### (PS2133) TEMPORAL TRENDS AND RISK FACTORS FOR TUBERCULOSIS IN LYMPHOMA PATIENTS: A SWEDISH POPULATION-BASED STUDY (2002–2024)

**Topic:** 20. Other aggressive lymphomas

Torsten Dahlén*[1], Karin Ekstrom Smedby[1]

[1]*Karolinska Institutet, Solna, Sweden;*

### Background

Patients with lymphoma face a heightened risk of Tuberculosis (TB) reactivation due to malignancy-associated immune dysfunction and increasingly intensive immunosuppressive treatments. In low-prevalence regions like Sweden, the cost-effectiveness and necessity of universal screening remain subjects of clinical debate. This study aimed to identify high-risk subgroups to inform targeted screening strategies.

### Aims

This study aimed to (1) quantify the excess risk of TB in lymphoma patients compared to the general population, (2) characterize temporal trends in TB incidence across diagnosis periods, and (3) develop a targeted risk-stratification matrix based on birth cohort and geographic origin to identify high-risk subgroups for pre-therapy screening.

### Methods

We analysed a nationwide cohort of 63,782 lymphoma patients diagnosed between 2002 and 2024 and 594,957 matched general population comparators. Cumulative incidence of TB was estimated starting from the diagnosis/index date, with death treated as a competing risk. Poisson regression was used to calculate incidence rate ratios (IRRs) to compare the risk between the cohorts. Absolute 2-year TB risk was predicted using a Cox proportional hazards model with restricted cubic splines for birth cohort and region of origin.

### Results

During a total follow-up of over 5.4 million person-years, we identified 256 TB events among lymphoma patients and 1,158 events among comparators. The incidence rate was significantly higher in the lymphoma cohort at 62.7 per 100,000 person-years, compared to 22.9 per 100,000 person-years in the general population. Lymphoma patients had a significantly higher risk of TB compared to the population comparators, with an IRR of 2.74 (95% CI 2.39–3.14; $p < 0.0001$). Among treated lymphoma patients, the 5 year risk of TB has declined over time, with the highest incidence observed in the 2002–2004 cohort, 0.3%, and the lowest in the 2020–2024 cohort, 0.1%. TB risk was strongly associated with birth cohort and geographic origin. Specifically, the predicted 2-year absolute risk reached 2.45% for patients born in Africa in the 1940s, compared to a negligible 0.03% among native-born Swedish patients in younger cohorts (born 1990+). A clear risk gradient was observed: Africa > Asia > South America > Europe (non-Nordic) > Sweden.

### Summary/Conclusion

While the overall incidence of TB in lymphoma patients is declining, it remains nearly three times higher than in the general population. Our findings suggest that a universal screening strategy is inefficient in low-prevalence settings. Instead, a targeted approach (e.g., IGRA) possibly based on geographic origin and birth year is warranted, prioritizing patients from high-prevalence regions and older birth cohorts to mitigate reactivation risk before initiating therapy

**Copyright Information:** (Online) ISSN: 2572-9241
©2026 The Author(s). HemaSphere is published by John Wiley & Sons Ltd on behalf of European Hematology Association. This is an open access Abstract Book distributed under the Attribution-NonCommercial-NoDerivs (CC BY-NC-ND), which allows third parties to download the articles and share them with others as long as they credit the author and the Abstract Book, but they cannot change the content in any way or use them commercially.
**Abstract Book Citations:** Authors, Title, HemaSphere, 2026;10:(S1):pages. The abstract book DOIs can be found on the "Author Information" page.
**Disclaimer:** Articles published in the journal HemaSphere exclusively reflect the opinions of the authors. The authors are responsible for all content in their abstracts including accuracy of the facts, statements, citing resources, etc.

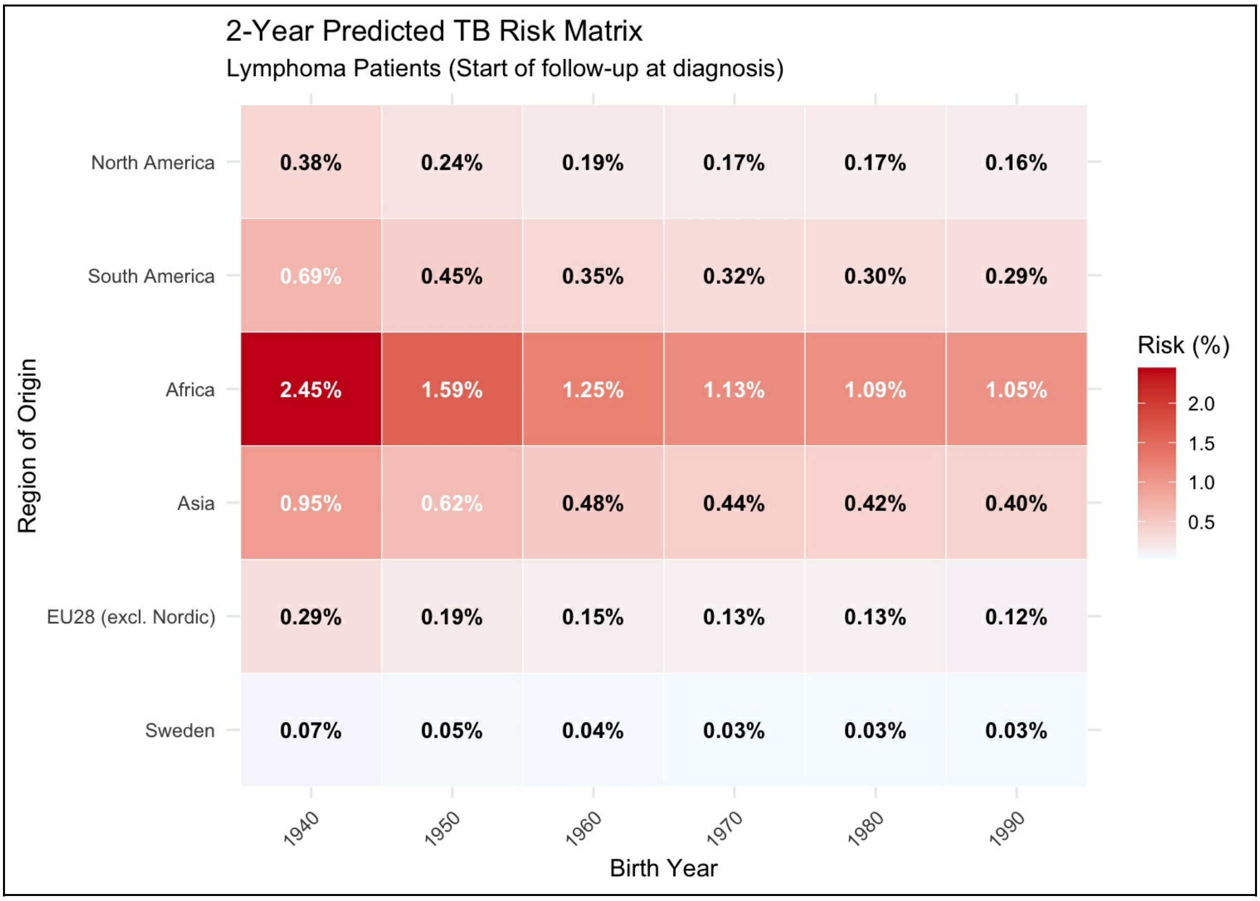

2-Year Predicted TB Risk Matrix by birth-year

**Copyright Information:** (Online) ISSN: 2572-9241
©2026 The Author(s). HemaSphere is published by John Wiley & Sons Ltd on behalf of European Hematology Association. This is an open access Abstract Book distributed under the Attribution-NonCommercial-NoDerivs (CC BY-NC-ND), which allows third parties to download the articles and share them with others as long as they credit the author and the Abstract Book, but they cannot change the content in any way or use them commercially.
**Abstract Book Citations:** Authors, Title, HemaSphere, 2026;10:(S1):pages. The abstract book DOIs can be found on the "Author Information" page.
**Disclaimer:** Articles published in the journal HemaSphere exclusively reflect the opinions of the authors. The authors are responsible for all content in their abstracts including accuracy of the facts, statements, citing resources, etc.

### (PS2134) LYMPHOMA-ASSOCIATED HEMOPHAGOCYTIC LYMPHOHISTIOCYTOSIS: INCIDENCE, CLINICAL PHENOTYPE, AND OUTCOMES IN A GLOBAL DATABASE STUDY

**Topic:** 20. Other aggressive lymphomas

Phyo Thazin Myint*[1], Siming MA[2], Ajay Iyer[1], Aye Thet[2], Zhiting Tang[1], Gerhard Hildebrandt[1]

*[1]University of Missouri, Columbia, Columbia, United States of America;[2]Case Western Reserve University School of Medicine, Cleveland, United States of America;*

### Background

Lymphoma-associated hemophagocytic lymphohistiocytosis (LA-HLH) is a life-threatening hyperinflammatory syndrome associated with high mortality. Despite poor survival outcomes reported in prior studies, clinical recognition of malignancy-associated HLH remains limited. Our study aimed to evaluate the incidence, clinical characteristics, and outcomes of LA-HLH in a global population.

### Methods

A retrospective cohort study was conducted using the TriNetX global electronic health records network. Adult patients (≥18 years) with a lymphoma diagnosis between 01/01 2005 and 12/31/2024 were included. LA-HLH was identified by ICD-10 codes D76.1 or D76.2 occurring within a window of −30 to +180 days relative to the lymphoma diagnosis. The incidence, diagnostic timing of LA-HLH, and clinical phenotypes were evaluated using descriptive analysis. 1:1 propensity matching was performed between patients with LA-HLH and the overall lymphoma population, as well as between the subgroups, to balance confounders. Survival probabilities were estimated using the Kaplan–Meier method, and hazard ratios (HR) were calculated using Cox regression. Risk ratios (RR) were used to compare 30-day and 1-year mortality across lymphoma subtypes, racial groups, and those with or without etoposide exposure within 30 days of HLH diagnosis.

### Results

A total of 1,177 patients with LA-HLH were identified from about 313,000 patients with lymphoma. The incidence of LA-HLH among adult lymphoma patients was approximately 0.37%. Among LA-HLH cases, T/NK-cell non-Hodgkin lymphoma was the predominant phenotype (58.8%), followed by B-cell non-Hodgkin lymphoma (35.3%) and Hodgkin lymphoma (5.9%).
Most LA-HLH cases (67.6%) were diagnosed within 30 days of lymphoma diagnosis, with 20.6% between 31–90 days and 11.8% between 91–180 days. Advanced-stage disease (stage III/IV) was more common in patients with LA-HLH compared with the overall lymphoma cohort (78.2% vs 62.4%). The mean age at diagnosis was similar between groups (52.2 vs 53.5 years). The majority of patients were White (59.5% in the LA-HLH cohort vs 66.3% in the overall lymphoma cohort), with no significant differences in age, sex, or race distribution between groups.
Within the LA-HLH cohort, mortality rates were 26.8% at 30 days, 54.3% at 90 days, and 68.5% at 1 year. In propensity score–matched analyses, one-year survival probability was significantly lower in the LA-HLH group compared with the overall lymphoma cohort (42.3% vs 81.7%), with HR of 5.27 (CI: 4.421-6.283, $p<0.001$).
Upon subgroup analysis, the 30-day mortality rate was higher in patients with LA-HLH associated with B-cell lymphomas compared with T/NK-cell lymphomas (RR 0.69, 95% CI 0.51–0.93; $p < 0.05$). However, there was no significant difference in 1-year mortality between the two groups. Additionally, 30-day mortality did not differ significantly across racial groups or based on exposure to etoposide within 30 days of HLH diagnosis.

**Copyright Information:** (Online) ISSN: 2572-9241
©2026 The Author(s). HemaSphere is published by John Wiley & Sons Ltd on behalf of European Hematology Association. This is an open access Abstract Book distributed under the Attribution-NonCommercial-NoDerivs (CC BY-NC-ND), which allows third parties to download the articles and share them with others as long as they credit the author and the Abstract Book, but they cannot change the content in any way or use them commercially.
**Abstract Book Citations:** Authors, Title, HemaSphere, 2026;10:(S1):pages. The abstract book DOIs can be found on the “Author Information” page.
**Disclaimer:** Articles published in the journal HemaSphere exclusively reflect the opinions of the authors. The authors are responsible for all content in their abstracts including accuracy of the facts, statements, citing resources, etc.

**Summary/Conclusion**

Our study highlights the global burden of LA-HLH, especially in T/NK cell lymphomas and advanced-stage disease. Given that more than two-thirds of cases occurred within 30 days of lymphoma diagnosis with a high early mortality rate, it is crucial to have improved clinical awareness. Further studies investigating factors associated with poor prognosis are also warranted.

**Copyright Information:** (Online) ISSN: 2572-9241
©2026 The Author(s). HemaSphere is published by John Wiley & Sons Ltd on behalf of European Hematology Association. This is an open access Abstract Book distributed under the Attribution-NonCommercial-NoDerivs (CC BY-NC-ND), which allows third parties to download the articles and share them with others as long as they credit the author and the Abstract Book, but they cannot change the content in any way or use them commercially.
**Abstract Book Citations:** Authors, Title, HemaSphere, 2026;10:(S1):pages. The abstract book DOIs can be found on the "Author Information" page.
**Disclaimer:** Articles published in the journal HemaSphere exclusively reflect the opinions of the authors. The authors are responsible for all content in their abstracts including accuracy of the facts, statements, citing resources, etc.

### (PS2135) OUTCOMES AND PROGNOSTIC RISK FACTORS IN NODAL PERIPHERAL T-CELL LYMPHOMA. RESULTS FROM THE INTERNATIONAL T-CELL PROJECT 2.0 PROSPECTIVE COHORT STUDY

**Topic:** 20. Other aggressive lymphomas

Stefano Luminari*[1], Nazarii Shokun[2], Luana Conte[3], Kateryna Filonenko[4], Giorgio De Nunzio[5], Carlos Sergio Chiattone[6], H. Miles Prince[7], Astrid Pavlovsky[8], Ciprian Tomuleasa[9], Yasodha Natkunan[10], Iryna Kryachok[11], Ilias Stagakis[12], Tetiana Skrypets[13], Neeraj Arora[14], Wonseog Kim[15], Eliza Chung[16], Sabina Ioana Doinita Iluta[17], Cameron Wellard[16], Carolina Mahuad[18], Lorena Fiad[19], Fernando Warley[20], Graciela Monica Alfonso[21], Thais Fischer[22], Iryna Tytorenko[11], Danielle de Farias[23], Eliza Hawkes[16], Ranjana Advani[10], Francine Foss[24], Julie Vose[25], Massimo Federico[26]

*[1]Azienda USL-IRCCS di Reggio Emilia, Hematology Unit, Reggio Emila, Italy;[2]University of Salento, Department of Experimental Medicine, Lecce, Italy; [3]University of Palermo, Department of Physics and Chemistry, Palermo, Italy;[4]University Medical Center, Department of Hematology, Transplantology and Cell Therapies, Gdansk, Poland; [5]University of Salento, Department of Mathematics and Physics, Lecce, Italy; [6]Universidade Estadual de Campinas-UNICAMP, Sao Paulo, Brazil;[7]Peter MacCallum Cancer Centre, Department of Pathology, Melbourne, Australia; [8]Buenos Aires Fundaleu, Departamento de Hematologia, Buenos Aires, Argentina; [9]Iuliu Hatieganu University of Medicine and Pharmacy, Department of Hematology, Cluj Napoca, Romania; [10]Stanford University, Division of Oncology, Department of Medicine, Stanford, United States of America;[11]National Cancer Institute, Department of Hematology, Kyiv, Ukraine; [12]Kantonsspital St. Gallen, St. Gallen, Switzerland;[13]IRCCS - Istituto Tumori Giovanni Paolo II, Hematology Unit, Bari, Italy; [14]Tata Medical Center, Kolkata, India; [15]Samsung Medical Center, Seoul, South Korea; [16]Monash University, School of Public Health and Preventive Medicine, Melbourne, Australia;[17]Bistrita County Emergency Hospital, Department of Medical Oncology, Bistrita, Romania; [18]Hospital Alemán, Buenos Aires, Argentina; [19]Hospital Italiano de La Plata, La Plata, Argentina; [20]Hospital Italiano de Buenos Aires, Buenos Aires, Argentina;[21]Hospital Nacional Profesor Alejandro Posadas, El Palomar, Argentina; [22]Ac Camargo Cancer Center - ACCCC, São Paulo, Brazil;[23]A Beneficência Portuguesa de São Paulo, São Paulo, Brazil; [24]Yale University School of Medicine, New Haven, United States of America; [25]University of Nebraska Medical Center, Omaha, United States of America;[26]University of Modena and Reggio Emilia, Modena, Italy;*

### Background

Nodal peripheral T-cell lymphomas (PTCL) are a complex, heterogeneous group of aggressive malignancies that historically present significant therapeutic challenges. While standard anthracycline-based therapies have been the mainstay of treatment, real-world outcomes remain suboptimal for many patients. Investigating the outcome of patients with nodal PTCL in real-world settings is essential to refining clinical management with the goal of improving patient survival

### Aims

The aims of the present study was to investigate the outcome of 875 patients with nodal PTCL diagnosed between 2018 and 2025 and prospectively registered in the T-Cell Project 2 (TCP2) at 135 centers worldwide, to verify whether a prospective collection of real-world data would allow a better understanding of the efficacy of current therapeutic strategies, and to identify the most relevant prognostic factors.

### Methods

Clinical information and pathology reports were centrally reviewed, with cases classified according to the 2008 WHO Classification of Tumours of Haematopoietic and Lymphoid Tissues criteria. The primary endpoint was 2-year progression-free survival (PFS), with overall survival (OS) as a key secondary endpoint. A multivariate Cox regression model was employed to identify independent prognostic factors and to develop a novel risk stratification index

**Copyright Information:** (Online) ISSN: 2572-9241
©2026 The Author(s). HemaSphere is published by John Wiley & Sons Ltd on behalf of European Hematology Association. This is an open access Abstract Book distributed under the Attribution-NonCommercial-NoDerivs (CC BY-NC-ND), which allows third parties to download the articles and share them with others as long as they credit the author and the Abstract Book, but they cannot change the content in any way or use them commercially.
**Abstract Book Citations:** Authors, Title, HemaSphere, 2026;10:(S1):pages. The abstract book DOIs can be found on the “Author Information” page.
**Disclaimer:** Articles published in the journal HemaSphere exclusively reflect the opinions of the authors. The authors are responsible for all content in their abstracts including accuracy of the facts, statements, citing resources, etc.

**Results**

PTCL-NOS was the most frequent subtype (36%), followed by AITL (27%), ALK-negative (24%), and ALK-positive ALCL (10%). Most patients (78%) presented with advanced-stage disease, although 77% had a favorable ECOG performance status (0-1). B symptoms were present in 51% of patients.
First-line therapy was mainly anthracycline-based (710 cases, 81%); CHOEP was the most frequently adopted regimen (319, 36%), followed by CHOP (229, 26%) and BV-CHP (133, 15%). ASCT as consolidation of first-line therapy was reported in 149 cases. Three hundred and seventy-five patients (58%) achieved complete remission (CR) and 70 (11%) partial remission (PR), with an overall response rate (ORR) of 69%. The CR rate was 74%, 57%, and 42% for patients treated with BV-CHP, CHOEP, and CHOP, respectively ($p<0.001$).
After a median follow-up of 26 months, the 2-year PFS and OS were 37% and 55%, respectively.
Two-year PFS was 53%, 41.2%, and 29.9 for BV-CHP, CHOEP, and CHOP, respectively; the differences in PFS were significant for BV-CHP vs CHOEP vs CHOP ($p<0.0001$).
On univariate analysis, several prognostic factors affected outcome. ECOG performance status ≥2, histology other than ALCL, extranodal involvement, bone marrow involvement, and high LDH retained an independent value on multivariate analysis. Using these variables, a prognostic model was devised to identify three groups at different levels of risk. The 2-year PFS rate was 60.2%, 41.4%, and 22.5% for patients at low (0-1), intermediate (2), and high risk (3 or more), respectively ($p<0.001$) (Figure 1). Refractory/relapsed patients had a very dismal outcome, with a 2-year PFS of 27.2%.

**Summary/Conclusion**

Because the outcome of patients with nodal PTCL continues to be quite poor with current therapeutic approaches, a broader use of novel agents as well as new treatment modalities are needed to improve outcome. However, as the prognostic index developed for these patients shows a robust discriminating power, it may represent a promising new tool to identify patients at different risk and to investigate the most adequate treatment strategies for these neoplasms.

**Copyright Information:** (Online) ISSN: 2572-9241
©2026 The Author(s). HemaSphere is published by John Wiley & Sons Ltd on behalf of European Hematology Association. This is an open access Abstract Book distributed under the Attribution-NonCommercial-NoDerivs (CC BY-NC-ND), which allows third parties to download the articles and share them with others as long as they credit the author and the Abstract Book, but they cannot change the content in any way or use them commercially.
**Abstract Book Citations:** Authors, Title, HemaSphere, 2026;10:(S1):pages. The abstract book DOIs can be found on the "Author Information" page.
**Disclaimer:** Articles published in the journal HemaSphere exclusively reflect the opinions of the authors. The authors are responsible for all content in their abstracts including accuracy of the facts, statements, citing resources, etc.

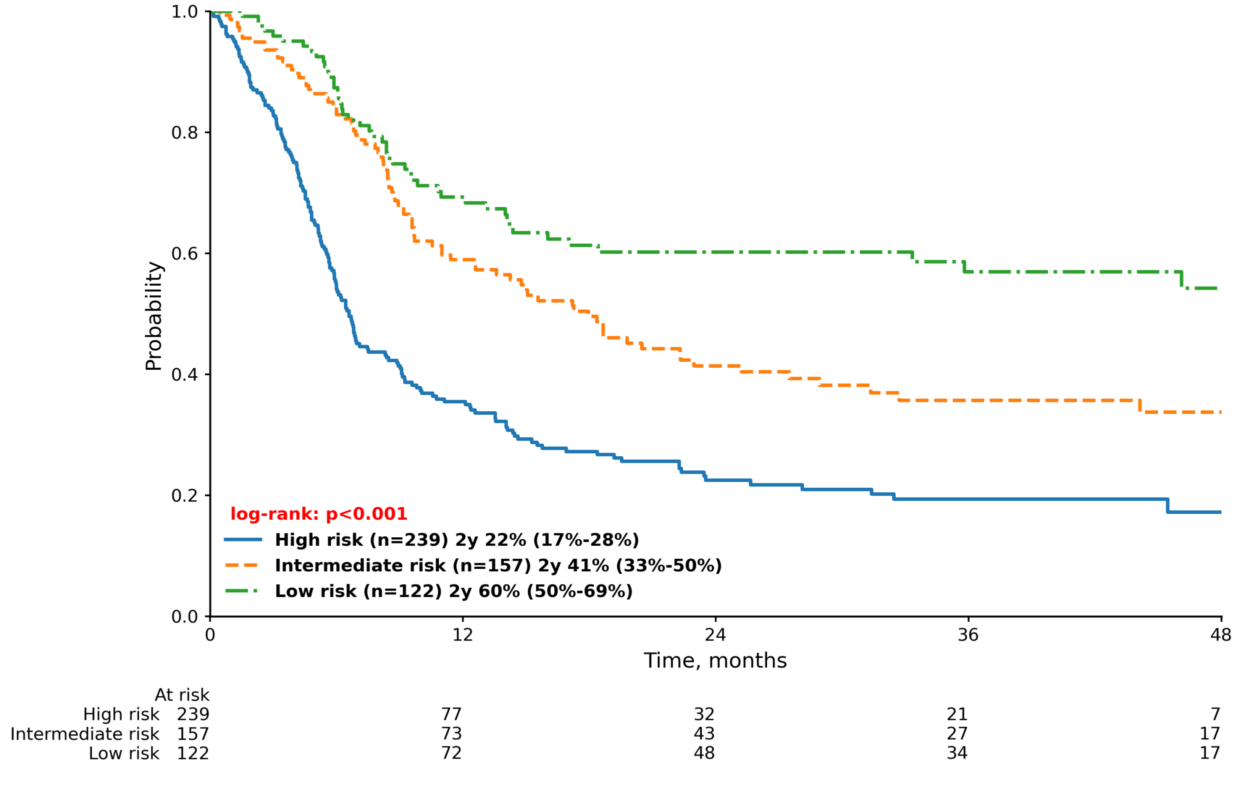

**Figure 1. Two-year PFS according to the new prognostic risk stratification model**

**Copyright Information:** (Online) ISSN: 2572-9241
©2026 The Author(s). HemaSphere is published by John Wiley & Sons Ltd on behalf of European Hematology Association. This is an open access Abstract Book distributed under the Attribution-NonCommercial-NoDerivs (CC BY-NC-ND), which allows third parties to download the articles and share them with others as long as they credit the author and the Abstract Book, but they cannot change the content in any way or use them commercially.
**Abstract Book Citations:** Authors, Title, HemaSphere, 2026;10:(S1):pages. The abstract book DOIs can be found on the "Author Information" page.
**Disclaimer:** Articles published in the journal HemaSphere exclusively reflect the opinions of the authors. The authors are responsible for all content in their abstracts including accuracy of the facts, statements, citing resources, etc.

### (PS2136) AUTOLOGOUS HEMATOPOIETIC CELL TRANSPLANTATION FOR PLASMABLASTIC LYMPHOMA: A STUDY FROM THE EBMT LYMPHOMA WORKING PARTY

**Topic:** 20. Other aggressive lymphomas

Joseph Kauer*[1], Maud Ngoya[2], Irma Khvedelize[2], Hervé Finel[2], Ariane Boumendil[2], Tobias Holderried[3], Bruno Lioure[4], Jiří Mayer[5], Robert Zeiser[6], Jan MacIej Zaucha[7], Lorenz Thurner[8], Eva Wagner-Drouet[9], Anoek Broers[10], Alexander Kulagin[11], Anne Huynh[12], Edgar Jost[13], Anna Ossami Saidy[14], Rémy Duléry[15], Anna Sureda[16], Norbert Schmitz[17], Bertram Glass[14], Ali Bazarbachi[18], Wolfram Klapper[19], Peter Dreger[1]

*[1]Heidelberg University Hospital, Internal Medicine V, Hematology, Oncology and Rheumatology, Heidelberg, Germany; [2]Lymphoma Working Party, EBMT Central Registry Office, Paris, France;[3]Department of Hematology, Oncology, Stem Cell Transplantation, Immune and Cell Therapy, Clinical Immunology and Rheumatology, University Hospital Bonn, Bonn, Germany; [4]Service d'Hématologie, Hôpital Hautepierre, Hôpitaux Universitaire de Strasbourg, Strasbourg, France;[5]University Hospital Brno and Masaryk University, Brno, Czechia; [6]Department of Medicine I, Medical Center—University of Freiburg, Faculty of Medicine, University of Freiburg, Freiburg, Germany; [7]Department of Haematology and Transplantology, Medical University of Gdańsk, Gdańsk, Poland; [8]Department of Oncology, Hematology, Rheumatology and Clinical Immunology, Saarland University Medical Center, Homburg, Germany; [9]Department of Medicine III, University Medical Center, Johannes Gutenberg University Mainz, Mainz, Germany; [10]Erasmus MC Cancer Institute Rotterdam, Rotterdam, The Netherlands; [11]RM Gorbacheva Research Institute, Pavlov University, St Petersburg, Russia; [12]Hematology Department, Institut Universitaire du Cancer Toulouse-Oncopole, Toulouse, France; [13]Department of Hematology, Oncology, Hemostaseology, and Stem Cell Transplantation, Faculty of Medicine, RWTH Aachen University, Aachen, Germany; [14]Department for Hematology, Oncology, Tumor Immunology, and Palliative Care, Helios Klinikum Berlin-Buch, Berlin, Germany; [15]Department of Clinical Hematology and Cellular Therapy, Sorbonne University, Hôpital Saint-Antoine, Assistance Publique-Hôpitaux de Paris, INSERM, UMRs 938, Centre de recherche Saint-Antoine (CRSA), Paris, France; [16]Catalan Institute of Oncology-Hospitalet, Barcelona, Spain; [17]Department of Medicine A, University Hospital Muenster, Münster, Germany; [18]Bone Marrow Transplantation Program, Department of Internal Medicine, American University of Beirut Medical Center, Beirut, Lebanon; [19]Department of Pathology, Hematopathology Section and Lymph Node Registry, University Hospital Schleswig-Holstein, Christian Albrechts Universität Kiel, Kiel, Germany;*

### Background

PBL is a rare form of aggressive B-cell Non-Hodgkin lymphomas. PBL lacks classic B-cell markers and expresses plasmacytic markers. It is commonly associated with HIV infection. The first-line therapy most commonly consists of CHOP chemotherapy, although long-term remission can only be achieved in a minority of patients. Due to its aggressive clinical course, autoHCT is often performed as consolidation therapy after first-line or salvage therapy. However, this practice is currently supported only by small retrospective analyses which showed a 2-year overall survival (OS) rate of 53%.

### Aims

We therefore aimed to study the outcome of PBL after autoHCT in the largest cohort to date.

### Methods

This EBMT registry analysis included adult patients who underwent a first autoHCT for PBL between 2016 and 2022. Pathological reports were collected for all patients and were grouped by an expert pathologist according to diagnostic certainty: high, moderate or low. Criteria for high certainty were (1) PBL diagnosis explicitly indicated in the report, (2) a compatible clinical presentation and (3) an absence of CD20 or the presence of EBV. Statistical analysis was descriptive, employing univariate and multivariate comparisons to assess the impact of baseline characteristics on survival endpoints.

**Copyright Information:** (Online) ISSN: 2572-9241
©2026 The Author(s). HemaSphere is published by John Wiley & Sons Ltd on behalf of European Hematology Association. This is an open access Abstract Book distributed under the Attribution-NonCommercial-NoDerivs (CC BY-NC-ND), which allows third parties to download the articles and share them with others as long as they credit the author and the Abstract Book, but they cannot change the content in any way or use them commercially.
**Abstract Book Citations:** Authors, Title, HemaSphere, 2026;10:(S1):pages. The abstract book DOIs can be found on the "Author Information" page.
**Disclaimer:** Articles published in the journal HemaSphere exclusively reflect the opinions of the authors. The authors are responsible for all content in their abstracts including accuracy of the facts, statements, citing resources, etc.

**Results**

A total of 78 patients with available pathology reports were identified. Eighty-three per cent were male, and 32% were HIV positive. The median age at autoHCT was 50.7 years (range 23 - 74). AutoHCT was performed after first-line therapy in 56.4% of patients and after second-line therapy in 32.4% of patients. The majority of patients were in partial (39.7%) or complete (55.1%) remission prior to AutoHCT. Conditioning was performed according to BEAM or similar protocols in 73.1% of patients. Diagnostic certainty was high in 44.9%of patients and moderate in 23.1% of patients. After a median follow-up period of 4.5 years, the 2-year OS rate was 78.7%. Lymphoma-free survival was 64.1% for the entire cohort (**Figure 1**). The majority of patients who died did so due to relapse with a two-year relapse incidence (RI) of 30.7% and a low two-year non-relapse mortality (NRM) rate of 5.2%.
A univariate analysis incorporating baseline characteristics revealed that high diagnostic certainty was associated with favourable outcomes (20% RI versus 50.1% RI in cases of low certainty), suggesting that PBL with typical features may be highly susceptible to high-dose chemotherapy.
Notably, relapse incidence was lower in HIV-positive patients in a multivariate analysis (hazard ratio (HR) 0.35, 95% confidence interval (CI) 0.1–1.25, p = 0.11). Age, sex, and lines of treatment did not correlate with disease-related outcomes. Neither baseline characteristic correlated with NRM.

**Summary/Conclusion**

AutoHCT is an effective consolidation therapy for PBL after first- or second-line therapy. Treatment resulted in an impressive two-year LFS of 64.1%, suggesting added efficacy compared with historical chemotherapy data without consolidation. This retrospective analysis supports the use of autoHCT in eligible patients with PBL.

**Copyright Information:** (Online) ISSN: 2572-9241
©2026 The Author(s). HemaSphere is published by John Wiley & Sons Ltd on behalf of European Hematology Association. This is an open access Abstract Book distributed under the Attribution-NonCommercial-NoDerivs (CC BY-NC-ND), which allows third parties to download the articles and share them with others as long as they credit the author and the Abstract Book, but they cannot change the content in any way or use them commercially.
**Abstract Book Citations:** Authors, Title, HemaSphere, 2026;10:(S1):pages. The abstract book DOIs can be found on the “Author Information” page.
**Disclaimer:** Articles published in the journal HemaSphere exclusively reflect the opinions of the authors. The authors are responsible for all content in their abstracts including accuracy of the facts, statements, citing resources, etc.

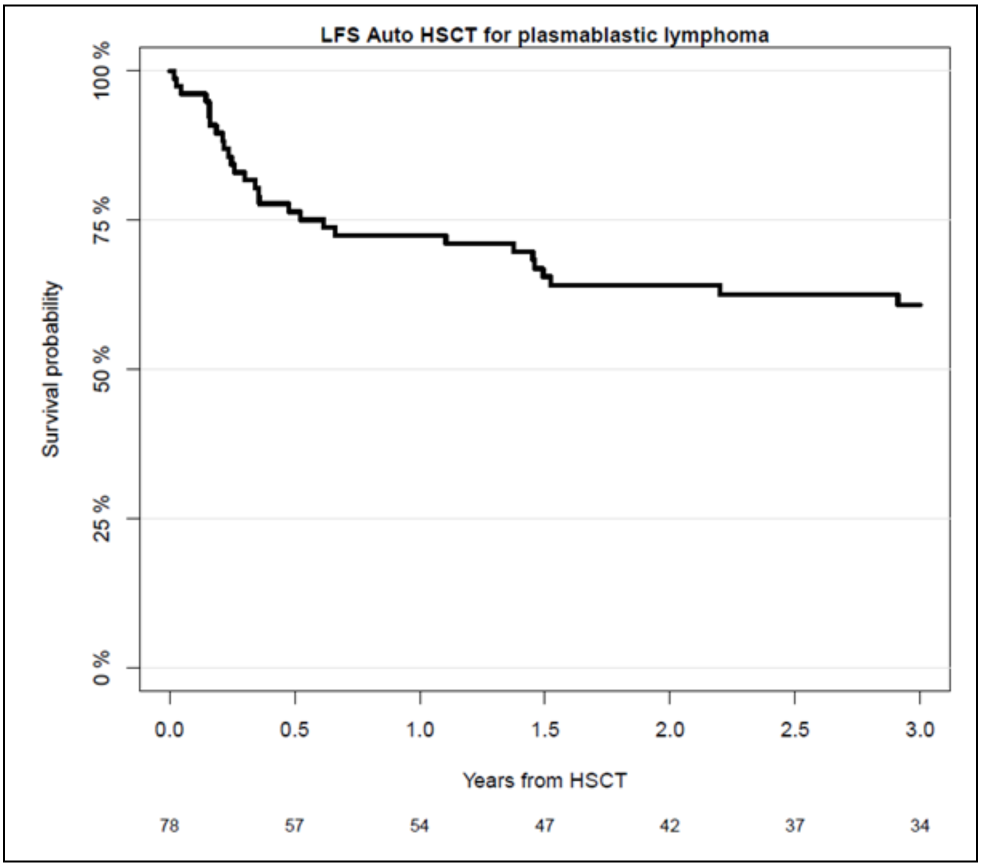

**Copyright Information:** (Online) ISSN: 2572-9241
©2026 The Author(s). HemaSphere is published by John Wiley & Sons Ltd on behalf of European Hematology Association. This is an open access Abstract Book distributed under the Attribution-NonCommercial-NoDerivs (CC BY-NC-ND), which allows third parties to download the articles and share them with others as long as they credit the author and the Abstract Book, but they cannot change the content in any way or use them commercially.
**Abstract Book Citations:** Authors, Title, HemaSphere, 2026;10:(S1):pages. The abstract book DOIs can be found on the "Author Information" page.
**Disclaimer:** Articles published in the journal HemaSphere exclusively reflect the opinions of the authors. The authors are responsible for all content in their abstracts including accuracy of the facts, statements, citing resources, etc.

### (PS2137) AUTOLOGOUS BONE MARROW TRANSPLANTATION. INSIGHTS FROM INTERNATIONAL T-CELL PROJECT 2.0

**Topic:** 20. Other aggressive lymphomas

Nazarii Shokun*[1], Ranjana Advani[2], Iryna Kryachok[3], Luana Conte[4], Ilias Stagakis[5], Neeraj Arora[6], Julie Vose[7], Wonseog Kim[8], Stefano Luminari[9], Astrid Pavlovsky[10], Carolina Mahuad[11], Camila Pena[12], Raimundo Gazitua[13], Eliza Chung[14], Thais Fischer[15], Cameron Wellard[14], Anastasiia Maliar[3], Carlos Sergio Chiattone[16], Eliza Hawkes[14], Monica Civallero[17], Massimo Federico[17], Alejandro Martín García-Sancho[18]

*[1]University of Salento, Lecce, Italy; [2]Stanford University, Division of Oncology, Department of Medicine, Stanford, United States of America; [3]National cancer institute, Kyiv, Ukraine; [4]University of Palermo, Department of Physics and Chemistry, Palermo, Italy; [5]Kantonsspital St. Gallen, St. Gallen, Switzerland;[6]Tata Medical Center, Kolkata, India;[7]University of Nebraska Medical Center, Omaha, United States of America; [8]Samsung Medical Center, Seoul, South Korea;[9]Azienda USL-IRCCS di Reggio Emilia, Hematology Unit, Reggio Emila, Italy; [10]Buenos Aires Fundaleu, Departamento de Hematologia, Buenos Aires, Argentina; [11]Hospital Alemán, Buenos Aires, Argentina; [12]Hospital del Salvador, Santiago, Chile; [13]Instituto Oncológico Fundación Arturo López Pérez, Santiago, Chile; [14]Monash University, School of Public Health and Preventive Medicine, Melbourne, Australia; [15]Ac Camargo Cancer Center - ACCCC, São Paulo, Brazil; [16]Universidade Estadual de Campinas-UNICAMP, Sao Paulo, Brazil; [17]University of Modena and Reggio Emilia, Modena, Italy; [18]Hospital Universitario de Salamanca, Department of Hematology, Salamanca, Spain;*

### Background

The role of autologous stem cell transplantation (ASCT) in patients with PTCL responding to first line therapy is not yet well defined.

### Aims

The aim of this study was to investigate the impact of ASCT on the outcome of patients with chemosensitive disease responding to first-line therapy and enrolled in the international T-cell Project 2.0 (TCP2) study

### Methods

Patients with aggressive, nodal and extranodal subtypes of PTCLs, aged ≤65 years and responding to first-line therapy were considered eligible for this sub-study. ALK-positive ALCL, hepatosplenic T-cell lymphoma, breast-associated T-cell lymphoma, and those who relapsed within five months were excluded. A total of 296 cases fulfilled inclusion/exclusion criteria.
The primary endpoint was 2-year progression-free survival (PFS), with overall survival (OS) being a key secondary endpoint. Survival was compared between patients receiving ASCT consolidation and those who did not. Prognostic factors were evaluated using Cox regression.

**Copyright Information:** (Online) ISSN: 2572-9241
©2026 The Author(s). HemaSphere is published by John Wiley & Sons Ltd on behalf of European Hematology Association. This is an open access Abstract Book distributed under the Attribution-NonCommercial-NoDerivs (CC BY-NC-ND), which allows third parties to download the articles and share them with others as long as they credit the author and the Abstract Book, but they cannot change the content in any way or use them commercially.
**Abstract Book Citations:** Authors, Title, HemaSphere, 2026;10:(S1):pages. The abstract book DOIs can be found on the “Author Information” page.
**Disclaimer:** Articles published in the journal HemaSphere exclusively reflect the opinions of the authors. The authors are responsible for all content in their abstracts including accuracy of the facts, statements, citing resources, etc.

### Results

Of the study population of 296 patients, 127 (42%) underwent ASCT and 169 (58%) did not. PTCL-NOS was the most frequent histologic subtype (37% in non-ASCT vs 35% in ASCT), followed by ALK-negative ALCL (24% in both groups). T follicular helper lymphoma was more common in the ASCT group (25% vs 14%), whereas extranodal NK/T-cell lymphoma was more frequent in the non-ASCT group (20% vs 8.7%).
Most patients (66%) presented with advanced-stage disease, yet 84% had favorable ECOG performance status (0-1). The ASCT group had a higher prevalence of adverse baseline characteristics, including advanced-stage disease (78% vs 57%; $p<0.001$), bone marrow involvement (28% vs 18%; $p=0.05$), and thrombocytopenia (20% vs 10%; $p=0.019$). Low IPI risk was more frequent in the non-ASCT group (39% vs 29%).
First-line therapy was mainly anthracycline-based (82%), most commonly CHOEP (48%), followed by CHOP (18%) and BV-CHP (16%). After first-line therapy, complete remission (CR) was achieved in 266 patients (90%) and partial remission (PR) in 30 (10%).
After a median follow-up of 27.1 months (2–93 months), 2-year PFS was 56.5% and 66.4% ($p=0.025$), and 2-year OS was 73.4% and 83.7% ($p=0.019$) for the non-ASCT and ASCT groups, respectively. In patients with advanced-stage disease, 2-year PFS was 41.5% for the non-ASCT group versus 63.3% for the ASCT group. Among patients with elevated LDH, 2-year PFS was 44.5% for the non-ASCT group versus 66.3% for the ASCT group ($p=0.009$).
No significant difference in 1-year PFS was observed across first-line regimens within either group. On univariate analysis, advanced stage, elevated LDH, IPI risk, response status, and ASCT consolidation were associated with outcome.

### Summary/Conclusion

Our data indicate that outcomes of patients with PTCL remain poor. However, ASCT consolidation in first-line therapy was associated with significantly improved PFS and OS compared to non-transplanted patients. Notably, this benefit was observed despite a higher prevalence of adverse baseline characteristics in the ASCT group, including advanced-stage disease, bone marrow involvement, and thrombocytopenia. This suggests that a subgroup of patients with adverse baseline clinical characteristics may benefit more from this approach. Further prospective studies are warranted to validate these findings.

**Copyright Information:** (Online) ISSN: 2572-9241
©2026 The Author(s). HemaSphere is published by John Wiley & Sons Ltd on behalf of European Hematology Association. This is an open access Abstract Book distributed under the Attribution-NonCommercial-NoDerivs (CC BY-NC-ND), which allows third parties to download the articles and share them with others as long as they credit the author and the Abstract Book, but they cannot change the content in any way or use them commercially.
**Abstract Book Citations:** Authors, Title, HemaSphere, 2026;10:(S1):pages. The abstract book DOIs can be found on the “Author Information” page.
**Disclaimer:** Articles published in the journal HemaSphere exclusively reflect the opinions of the authors. The authors are responsible for all content in their abstracts including accuracy of the facts, statements, citing resources, etc.

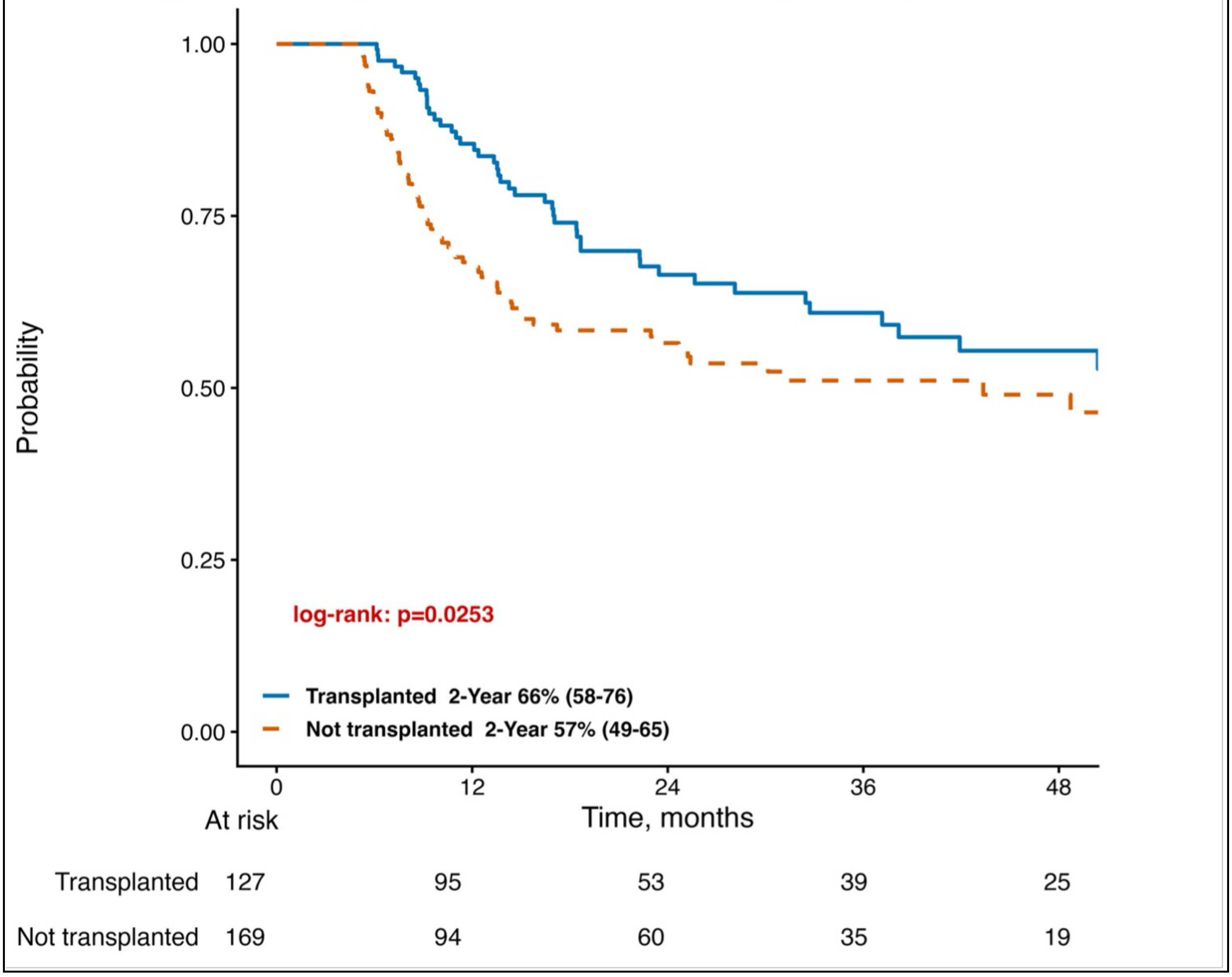

**Figure1. Progression free survival by transplantation status**

**Copyright Information:** (Online) ISSN: 2572-9241
©2026 The Author(s). HemaSphere is published by John Wiley & Sons Ltd on behalf of European Hematology Association. This is an open access Abstract Book distributed under the Attribution-NonCommercial-NoDerivs (CC BY-NC-ND), which allows third parties to download the articles and share them with others as long as they credit the author and the Abstract Book, but they cannot change the content in any way or use them commercially.
**Abstract Book Citations:** Authors, Title, HemaSphere, 2026;10:(S1):pages. The abstract book DOIs can be found on the "Author Information" page.
**Disclaimer:** Articles published in the journal HemaSphere exclusively reflect the opinions of the authors. The authors are responsible for all content in their abstracts including accuracy of the facts, statements, citing resources, etc.

### (PS2138) PHASE I STUDY OF TR115, A NOVEL SELECTIVE EZH2 INHIBITOR, IN RELAPSED/REFRACTORY NON-HODGKIN LYMPHOMA AND ADVANCED SOLID TUMORS

**Topic:** 20. Other aggressive lymphomas

Juying Wei*[1], Keshu Zhou[2], Huan Zhou[3], Yuzhi Li[3], Yan Zhang[4], Xiaochun MA[4], Aoli Wang[4], Li Wang[4], Jie Jin[1]

*[1]Department of Hematology, The First Affiliated Hospital, Zhejiang University School of Medicine, Hangzhou, China;[2]Department of Hematology, Henan Cancer Hospital, Zhengzhou, China; [3]Clinical Trial Research Center, The First Affiliated Hospital of Bengbu Medical University, Bengbu, China; [4]Tarapeutics Pharmaceutical Science Inc, Bengbu, China;*

### Background

Gain-of-function mutations and overexpression of EZH2 lead to aberrant H3K27me3 levels, promoting tumorigenesis and metastasis in lymphoid malignancies and solid tumors. TR115 is a novel, highly selective EZH2 inhibitor with potent preclinical antitumor activity.

### Aims

This Phase I study evaluated the safety, tolerability, and preliminary efficacy of TR115 in patients with relapsed/refractory (R/R) non-Hodgkin lymphoma (NHL) and advanced solid tumors.

### Methods

Six TR115 dose levels (100–1600 mg, oral twice daily, BID) were assessed via a standard 3+3 dose-escalation design with an accelerated titration phase. Based on dose-escalation safety and tolerability data, 1200 mg BID was selected for the dose-expansion cohort, which enrolled patients with peripheral T-cell lymphoma (PTCL).

### Results

By November 17, 2025, 36 patients were enrolled (20 dose-escalation; 16 dose-expansion), comprising 22 PTCL, 6 mature B-cell lymphoma, 5 solid tumors, 1 mycosis fungoides, 1 subcutaneous panniculitis-like T-cell lymphoma (SPTCL), and 1 extranodal NK/T-cell lymphoma (ENKTL). Most patients were heavily pretreated (median 3 prior systemic therapy lines).
One ovarian cancer patient achieved stable disease (SD) at 800 mg, demonstrating TR115's preliminary efficacy in solid tumors. In 20 evaluable R/R PTCL patients treated with TR115 ≥1200 mg BID, the objective response rate (ORR) was 75% (complete response [CR], 40%) and disease control rate (DCR) 90%. Median progression-free survival (PFS), duration of response (DOR), and overall survival (OS) were not reached at data cutoff. Time to response (TTR) ranged from 1.6 to 5.6 months.
Subtype-specific ORRs: PTCL not otherwise specified (PTCL-NOS), 80% (4/5); anaplastic large-cell lymphoma, ALK-negative (ALCL, ALK⁻), 100% (2/2); angioimmunoblastic T-cell lymphoma (AITL), 72.7% (8/11); PTCL with T-follicular helper (TFH) phenotype, 0% (0/1); nodal TFH cell lymphoma, NOS, 100% (1/1).
TR115 exhibited a more favorable tolerability profile than historical data of other EZH2 inhibitors, with significantly lower incidence/severity of hepatotoxicity, pneumonia, and gastrointestinal adverse events, and comparable myelosuppression incidence.
Most common any-grade treatment-emergent adverse events (TEAEs; incidence ≥20%) included cytopenias (thrombocytopenia, leukopenia, neutropenia, anemia, lymphocytopenia), hypertriglyceridemia, and elevated blood creatinine.

**Copyright Information:** (Online) ISSN: 2572-9241
©2026 The Author(s). HemaSphere is published by John Wiley & Sons Ltd on behalf of European Hematology Association. This is an open access Abstract Book distributed under the Attribution-NonCommercial-NoDerivs (CC BY-NC-ND), which allows third parties to download the articles and share them with others as long as they credit the author and the Abstract Book, but they cannot change the content in any way or use them commercially.
**Abstract Book Citations:** Authors, Title, HemaSphere, 2026;10:(S1):pages. The abstract book DOIs can be found on the "Author Information" page.
**Disclaimer:** Articles published in the journal HemaSphere exclusively reflect the opinions of the authors. The authors are responsible for all content in their abstracts including accuracy of the facts, statements, citing resources, etc.

### Summary/Conclusion

TR115 demonstrates a favorable safety profile and promising antitumor activity in R/R NHL, particularly across PTCL subtypes. These data support further evaluation of TR115 as monotherapy and in rational combination regimens. (NCT05650580)

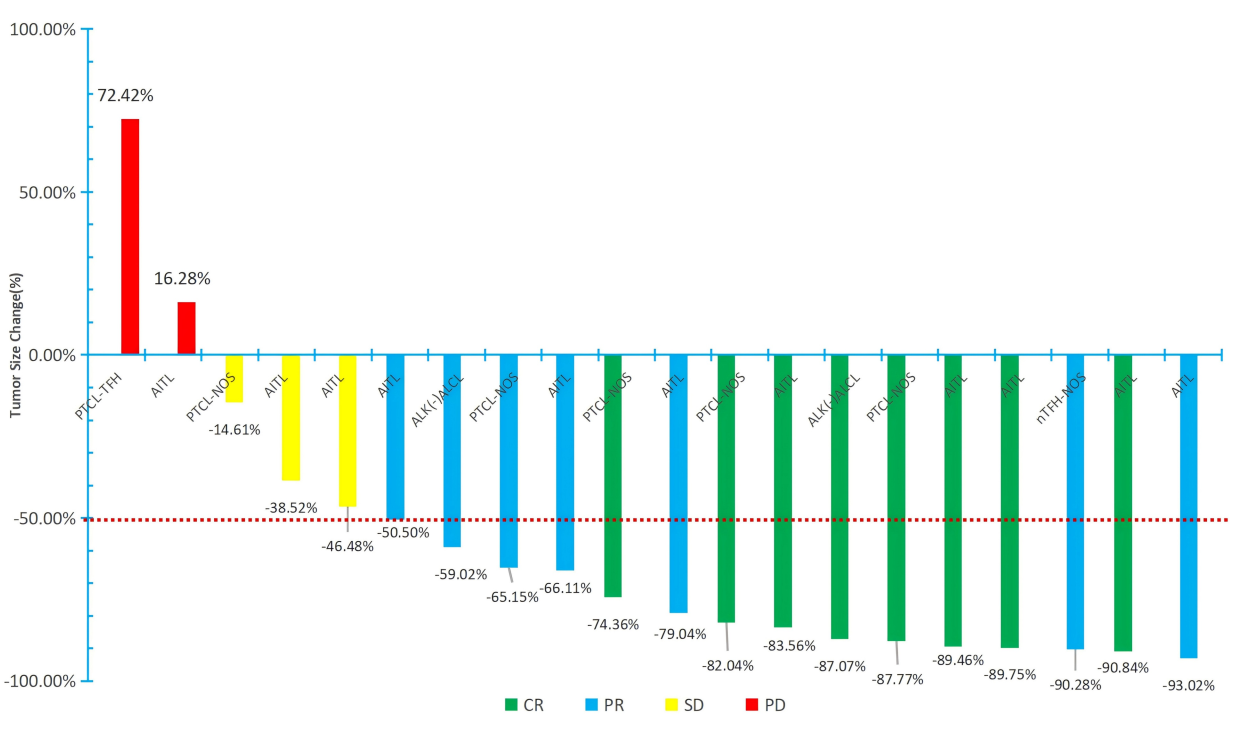

**Figure. Target Lesion Best Percentage Change from Baseline with TR115 in R/R PTCL (N=20)**

**Copyright Information:** (Online) ISSN: 2572-9241
©2026 The Author(s). HemaSphere is published by John Wiley & Sons Ltd on behalf of European Hematology Association. This is an open access Abstract Book distributed under the Attribution-NonCommercial-NoDerivs (CC BY-NC-ND), which allows third parties to download the articles and share them with others as long as they credit the author and the Abstract Book, but they cannot change the content in any way or use them commercially.
**Abstract Book Citations:** Authors, Title, HemaSphere, 2026;10:(S1):pages. The abstract book DOIs can be found on the "Author Information" page.
**Disclaimer:** Articles published in the journal HemaSphere exclusively reflect the opinions of the authors. The authors are responsible for all content in their abstracts including accuracy of the facts, statements, citing resources, etc.

### (PS2139) INCIDENCE AND OUTCOMES OF BURKITT LYMPHOMA: AN ENGLAND-WIDE COHORT STUDY FROM THE UNCOVER STUDY GROUP

**Topic:** 20. Other aggressive lymphomas

Nurunnahar Akter[1], Mark Bishton[2, 3], Christopher P. Fox[2, 3], Indrani Karpha[4, 5], Yeong Lim[4, 5], Brogan Johnston[5], Temitope Erinfolami[1], Toyyib Abdulkareem[1], Nagesh Kalakonda[4, 5], Andrew Pettitt[4, 5], Catrin Tudur Smith[1], Sridhar Chaganti[6], Matthew R. Wilson*[7]

*[1]University of Liverpool, Department of Health Data Science, Institute of Population Health, Liverpool, United Kingdom;[2]School of Medicine, University of Nottingham, Nottingham, United Kingdom; [3]Nottingham University Hospitals NHS Trust, Nottingham, United Kingdom; [4]University of Liverpool, Department of Molecular and Clinical Cancer Medicine, Institute of Systems, Molecular and Integrative Biology, Liverpool, United Kingdom; [5]The Clatterbridge Cancer Centre NHS Foundation Trust, Liverpool, United Kingdom; [6]University Hospitals Birmingham, Birmingham, United Kingdom; [7]Beatson West of Scotland Cancer Centre, Glasgow, United Kingdom;*

### Background

UNCOVER is a health data research programme that utilises the National Cancer Registration Dataset (NCRD). NCRD is mandated to record information on all patients (pts) diagnosed with cancer at all NHS hospitals in England.

### Aims

We identified consecutive cases of Burkitt lymphoma (BL) to analyse large scale, nationwide data on factors influencing incidence and survival.

### Methods

Pts in England with BL diagnosed 2014-2022 were identified using ICD-03 code (9687/3). Crude and age-standardised incidence ratios (ASIR) were calculated and incidence rate ratios (IRR) estimated by Poisson regression using denominator population of England. Overall survival (OS) was estimated using Kaplan–Meier methods and multivariable Cox regression models fitted to examine factors independently associated with survival. An adjusted Fine-Gray model assessed sub-distribution of hazards in the presence of competing risk (cause-specific vs other death). All-cause 30-day fatality was estimated using multivariable logistic regression. The Poisson model was adjusted for age, gender, deprivation, ethnicity and government region while survival models were additionally adjusted for Charlson comorbidity index (CCI).

**Copyright Information:** (Online) ISSN: 2572-9241
©2026 The Author(s). HemaSphere is published by John Wiley & Sons Ltd on behalf of European Hematology Association. This is an open access Abstract Book distributed under the Attribution-NonCommercial-NoDerivs (CC BY-NC-ND), which allows third parties to download the articles and share them with others as long as they credit the author and the Abstract Book, but they cannot change the content in any way or use them commercially.
**Abstract Book Citations:** Authors, Title, HemaSphere, 2026;10:(S1):pages. The abstract book DOIs can be found on the “Author Information” page.
**Disclaimer:** Articles published in the journal HemaSphere exclusively reflect the opinions of the authors. The authors are responsible for all content in their abstracts including accuracy of the facts, statements, citing resources, etc.

**Results**

1578 pts with BL (74% male) were identified. Median age at diagnosis was 47y (range 0-93, IQR:19-68). Data for route to diagnosis were available for all pts until 2018, of whom 838/1061 (79%) presented as emergency. Overall ASIR was 0.30/100,000 persons/year (95%CI: 0.27, 0.36) with no significant change over time including during the COVID-19 pandemic. Gender (p<0.001), age (p<0.001), ethnicity (p=0.001) and government region (p=0.001) were independently associated with incidence whereas deprivation (p=0.392) was not. The adjusted IRR was significantly lower in Asian and Black populations (0.70 (95%CI 0.56-0.85) and 0.73 (95% CI 0.54-0.98) respectively) vs White population. Geographical variation in adjusted IRR was seen with the North East region showing significantly lower IRR (0.62 (95% CI 0.45-0.85)) vs reference Greater London.
Survival data were available until July 2024 with median follow-up 5.7y (IQR 3.6-7.9). 633 (40%) pts died, of whom 201 died within 30 days (12.7% of study population, 31.8% of deaths). Haem cancer was cause of death in 501 (79%). Increasing age (p<0.001), Black ethnicity (0.001) and government region (p<0.001) were independently associated with higher 30-day mortality risk. 30-day mortality was 12 and 52 times higher in pts aged 46-55y and >75y respectively vs pts <18y. One and 5-yr OS for all patients was 67% (95% CI: 65,69) and 60.5% (95% CI: 58,63) respectively. Independent risk factors for OS were increasing age (p<0.001), Black ethnicity (p<0.006), government region (p=0.023) and higher CCI (p=0.005). Pts aged <18y and >75y had 5y OS 92% (95%CI: 90-95) and 17% (95%CI: 13-23) respectively (figure 1).

**Summary/Conclusion**

This is the largest contemporary series of consecutive pts with BL, providing valuable insight into factors influencing incidence and survival. The majority of deaths occurred within 1y of diagnosis with pts surviving beyond this point having excellent prognosis. Increasing age had a significant impact on early mortality and longer term OS; pts <18yo had excellent outcomes. Older pts represent a group with clear need for more effective treatments. Incidence and survival were also significantly influenced by ethnicity and geographical location, raising important questions around the uniformity of diagnostic and therapeutic pathways.

**Copyright Information:** (Online) ISSN: 2572-9241
©2026 The Author(s). HemaSphere is published by John Wiley & Sons Ltd on behalf of European Hematology Association. This is an open access Abstract Book distributed under the Attribution-NonCommercial-NoDerivs (CC BY-NC-ND), which allows third parties to download the articles and share them with others as long as they credit the author and the Abstract Book, but they cannot change the content in any way or use them commercially.
**Abstract Book Citations:** Authors, Title, HemaSphere, 2026;10:(S1):pages. The abstract book DOIs can be found on the “Author Information” page.
**Disclaimer:** Articles published in the journal HemaSphere exclusively reflect the opinions of the authors. The authors are responsible for all content in their abstracts including accuracy of the facts, statements, citing resources, etc.

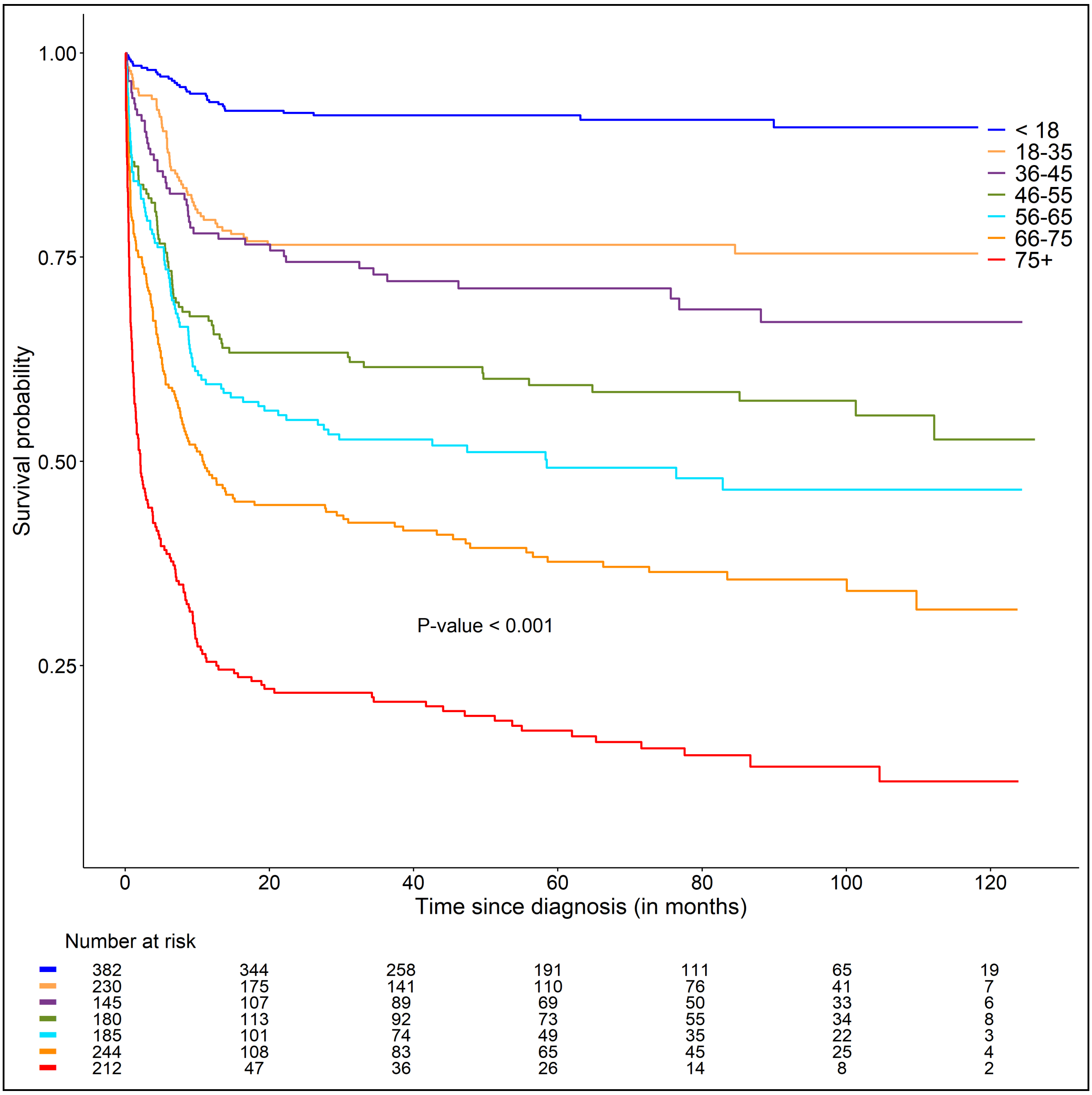

Figure 1: Overall survival by age in patients with newly diagnosed Burkitt lymphoma in England between 2014-2022

**Copyright Information:** (Online) ISSN: 2572-9241
©2026 The Author(s). HemaSphere is published by John Wiley & Sons Ltd on behalf of European Hematology Association. This is an open access Abstract Book distributed under the Attribution-NonCommercial-NoDerivs (CC BY-NC-ND), which allows third parties to download the articles and share them with others as long as they credit the author and the Abstract Book, but they cannot change the content in any way or use them commercially.
**Abstract Book Citations:** Authors, Title, HemaSphere, 2026;10:(S1):pages. The abstract book DOIs can be found on the “Author Information” page.
**Disclaimer:** Articles published in the journal HemaSphere exclusively reflect the opinions of the authors. The authors are responsible for all content in their abstracts including accuracy of the facts, statements, citing resources, etc.

### (PS2140) LINPERLISIB PLUS CHOP FOR NEWLY DIAGNOSED PERIPHERAL T-CELL LYMPHOMA: A SINGLE-ARM, PHASE IB/II STUDY (LINCH TRIAL)

**Topic:** 20. Other aggressive lymphomas

Qingqing Cai*[1], Yi Xia[1], Huiqiang Huang[1], Ying Zhao[2], Hongyan Tong[3], Tao Wu[4], Rong Tao[5], Xiuhua Sun[6], Runhui Zheng[7], Jianbo Liu[7], Fan Yang[2], Wenning Xu[2], Chunmei Yang[3], Xiaojie Fang[1], Man Nie[1], Bing Bai[1], Hang Yang[1], Yu Fang[1], Yuchen Zhang[1], Yi Cao[1]

*[1]Sun Yat-sen University Cancer Center, Guangzhou, China; [2]The First People's Hospital of Foshan, Foshan, China; [3]The First Affiliated Hospital, Zhejiang University School of Medicine, Hangzhou, China; [4]Affiliated Cancer Hospital of Guizhou Medical University, Guiyang, China; [5]Fudan University Shanghai Cancer Center, Shanghai, China; [6]The Second Hospital of Dalian Medical University, Dalian, China; [7]The Fifth Affiliated Hospital of Guangzhou Medical University, Guangzhou, China;*

### Background

Peripheral T-cell lymphoma (PTCL) is a heterogeneous type of aggressive non-Hodgkin lymphoma with poor prognosis. CHOP-based regimens are most widely used for PTCL yet response rates and long-term survival remain unsatisfactory. Linperlisib, a selective PI3Kδ inhibitor, has shown encouraging antitumor activity with manageable safety profile in relapsed or refractory PTCL.

### Aims

This study aimed to evaluate the efficacy and safety of linperlisib plus CHOP (L-CHOP) as first-line treatment for newly diagnosed PTCL.

### Methods

The LINCH trial (NCT05949944), a multi-center, single-arm phase Ib/II study, enrolled patients aged ≥ 18 years with newly diagnosed PTCL. In phase Ib, 6 patients received 6 cycles of L-CHOP to determine the recommended phase II dose (RP2D). In phase II, patients received L-CHOP at the RP2D. Following 6 cycles, patients achieving complete response (CR) or partial response (PR) continued linperlisib maintenance until progression or intolerable toxicity or up to 24 months. The primary endpoints were the DLTs incidence (phase Ib) and the CR rate after 6 cycles of L-CHOP (phase II). Preliminary results were reported.

**Copyright Information:** (Online) ISSN: 2572-9241
©2026 The Author(s). HemaSphere is published by John Wiley & Sons Ltd on behalf of European Hematology Association. This is an open access Abstract Book distributed under the Attribution-NonCommercial-NoDerivs (CC BY-NC-ND), which allows third parties to download the articles and share them with others as long as they credit the author and the Abstract Book, but they cannot change the content in any way or use them commercially.
**Abstract Book Citations:** Authors, Title, HemaSphere, 2026;10:(S1):pages. The abstract book DOIs can be found on the "Author Information" page.
**Disclaimer:** Articles published in the journal HemaSphere exclusively reflect the opinions of the authors. The authors are responsible for all content in their abstracts including accuracy of the facts, statements, citing resources, etc.

### Results

From August 15, 2023 to November 15, 2025, 44 patients were enrolled, including 6 patients in phase Ib. The median age was 57 years (range 18–77); 28 patients were males (63.6%). Most patients (n = 35, 79.5%) had stage III-IV disease, and 17 (38.6%) had an IPI score of 3–5. In phase Ib, one DLT (grade 3 febrile neutropenia) occurred in cycle 1, confirming linperlisib 80 mg once daily as RP2D. At the data cutoff on December 30, 2025, efficacy was evaluable in 34 patients (26 completed six cycles; 8 discontinued early), with 10 still receiving induction therapy. After six cycles of combination therapy, 19 patients (55.9%) achieved CR and four achieved PR, resulting in an objective response rate (ORR) of 67.6%. Treatment-emergent adverse events (TEAEs) occurred in 32 patients (94.1%); hematologic toxicity were common: neutropenia (n = 24, 70.6%), leukopenia (n = 21, 61.8%), anemia (n =14, 41.2%). Grade ≥ 3 TEAEs occurred in 19 patients (55.9%), most frequently neutropenia (n = 13, 38.2%), leukopenia (n = 10, 29.4%) and pneumonia (n = 4, 11.8%).

**Table 1. Baseline characteristics.**

| **Characteristics** | **Patients** (n=44) |
|---|---|
| **Age, years** (median [IQR]) | 57 (18-77) |
| **Sex** | |
| Male | 28 (63.6%) |
| Female | 16 (36.4%) |
| **ECOG PS** | |
| 0-1 | 39 (88.6%) |
| 2 | 5 (11.4%) |
| **Lugano stage** | |
| I-II | 9 (20.5%) |
| III-IV | 35 (79.5%) |
| **Pathological types** | |
| AITL | 23 (52.3%) |
| PTCL-NOS | 14 (31.8%) |
| ALCL | 3 (6.8%) |
| TFHL-NOS | 2 (4.5%) |
| MEITL | 1 (2.3%) |
| SPTCL | 1 (2.3%) |

### Summary/Conclusion

Preliminary results from LINCH study suggest that linperlisib combined with CHOP as first-line treatment for PTCL achieved promising efficacy and manageable safety. A randomized controlled trial evaluating linperlisib plus CHOP versus CHOP in patients with newly diagnosed PTCL has been initiated (NCT06548347).

**Copyright Information:** (Online) ISSN: 2572-9241
©2026 The Author(s). HemaSphere is published by John Wiley & Sons Ltd on behalf of European Hematology Association. This is an open access Abstract Book distributed under the Attribution-NonCommercial-NoDerivs (CC BY-NC-ND), which allows third parties to download the articles and share them with others as long as they credit the author and the Abstract Book, but they cannot change the content in any way or use them commercially.
**Abstract Book Citations:** Authors, Title, HemaSphere, 2026;10:(S1):pages. The abstract book DOIs can be found on the "Author Information" page.
**Disclaimer:** Articles published in the journal HemaSphere exclusively reflect the opinions of the authors. The authors are responsible for all content in their abstracts including accuracy of the facts, statements, citing resources, etc.

### (PS2141) RUXOLITINIB COMBINED WITH P-GEMOX IN ADULT PATIENTS WITH NEWLY DIAGNOSED AGGRESSIVE NATURAL KILLER CELL LEUKEMIA

**Topic:** 20. Other aggressive lymphomas

Xueying Yang*[1], Jiejin Qian[1], Xin Huang[1], Xingnong Ye[1], Wenjuan Yu[1], Gaixiang Xu[1], Mixue Xie[1], Xuelian Hu[1], Wanzhuo Xie[1], Hongyan Tong[1], De Zhou[1]

[1] *The First Affiliated Hospital, Zhejiang University School of Medicine, Hangzhou, China;*

### Background

Aggressive natural killer cell leukemia (ANKL) is a rare and fulminant hematologic malignancy characterized by a median overall survival of less than two months, even with intensive chemotherapy. Patients typically present acutely with fever, constitutional symptoms, hepatosplenomegaly, and hemophagocytic lymphohistiocytosis (HLH). Recent studies have identified recurrent genomic alterations involving genes in the JAK/STAT signaling pathway in ANKL. In addition, our previous work has demonstrated that the JAK inhibitor ruxolitinib is effective and well tolerated in patients with secondary HLH. These findings provide a strong rationale for combining ruxolitinib with P-Gemox (pegaspargase, gemcitabine, and oxaliplatin) to improve outcomes in ANKL. Based on this rationale, we conducted a pilot trial of this combination regimen in adult patients with ANKL (chictr.org.cn identifier: ChiCTR2400086092).

### Aims

To evaluate the efficacy and safety of ruxolitinib combined with P-Gemox in adult patients with newly diagnosed aggressive natural killer cell leukemia.

### Methods

Adult patients (18–80 years) with newly diagnosed ANKL were enrolled. Key exclusion criteria included severe mental illness, HIV infection, severe renal dysfunction, severe cirrhosis, and active malignancies other than ANKL. All enrolled patients received up to six cycles of combination therapy every three weeks, consisting of ruxolitinib 10 mg orally twice daily on days 1–21; pegaspargase 2500 IU/m² on day 2; gemcitabine 1000 mg/m² on days 1 and 8; and oxaliplatin 100 mg/m² on day 1. Patients achieving complete remission (CR) or partial remission (PR) with available donors proceeded to allogeneic hematopoietic stem cell transplantation (allo-HSCT) as soon as feasible.

### Results

From October 18, 2024 to February 23, 2026, ten patients with newly diagnosed ANKL were enrolled in this study. The median age was 45 years (range, 26–71), and six patients were female. At diagnosis, nine patients (90.0%) presented with HLH, and all patients were Epstein-Barr virus (EBV) positive. Therapeutic responses were evaluable in ten patients. After one cycle of therapy, the overall response rate (ORR) was 80.0% (8/10), including 6 patients (60.0%) with CR, 2 patient (20.0%) with PR, and 2 patient (20.0%) with no response (NR). After two cycles, the ORR was 70.0% (7/10), with all seven responders achieving CR. Three non-responders respectively died on day 25, day31, and day 95 due to disease progression (PD). Among those who achieved CR, one patient died on day 86 from H1N1 influenza and disease relapse secondary to treatment delay. Additionally, another patient discontinued further treatment for financial reasons after 2 cycles and currently shows no PD. The remaining five patients (50.0%) underwent allo-HSCT and remained alive at last follow-up, with the longest survival reaching 16.3 months.

**Copyright Information:** (Online) ISSN: 2572-9241
©2026 The Author(s). HemaSphere is published by John Wiley & Sons Ltd on behalf of European Hematology Association. This is an open access Abstract Book distributed under the Attribution-NonCommercial-NoDerivs (CC BY-NC-ND), which allows third parties to download the articles and share them with others as long as they credit the author and the Abstract Book, but they cannot change the content in any way or use them commercially.
**Abstract Book Citations:** Authors, Title, HemaSphere, 2026;10:(S1):pages. The abstract book DOIs can be found on the "Author Information" page.
**Disclaimer:** Articles published in the journal HemaSphere exclusively reflect the opinions of the authors. The authors are responsible for all content in their abstracts including accuracy of the facts, statements, citing resources, etc.

**Summary/Conclusion**

In this study, the combination of ruxolitinib and P-Gemox demonstrated encouraging efficacy in newly diagnosed adult patients with ANKL. This regimen appears to be an effective and well-tolerated induction strategy while bridging to allo-HSCT. These preliminary findings warrant further evaluation in larger, prospective studies.

**Copyright Information:** (Online) ISSN: 2572-9241
©2026 The Author(s). HemaSphere is published by John Wiley & Sons Ltd on behalf of European Hematology Association. This is an open access Abstract Book distributed under the Attribution-NonCommercial-NoDerivs (CC BY-NC-ND), which allows third parties to download the articles and share them with others as long as they credit the author and the Abstract Book, but they cannot change the content in any way or use them commercially.
**Abstract Book Citations:** Authors, Title, HemaSphere, 2026;10:(S1):pages. The abstract book DOIs can be found on the "Author Information" page.
**Disclaimer:** Articles published in the journal HemaSphere exclusively reflect the opinions of the authors. The authors are responsible for all content in their abstracts including accuracy of the facts, statements, citing resources, etc.

### (PS2142) DIFFERENCES IN OUTCOMES FOR ELDERLY PATIENTS WITH DLBCL TREATED AT AMERICAN ACADEMIC VS COMMUNITY PROGRAMS: A 10-YEAR SURVIVAL ANALYSIS

**Topic:** 20. Other aggressive lymphomas

Luis Villela*[1], Carolina Velez-Mejia[2], Joel Michalek[3], Qianqian Liu[3], Perla Colunga[4], Rocio Rodriguez-Arche[1], Kassandra German-Ruiz[1], Juan Rangel Patiño[5], Brady Beltran Garate[6], Rafael Pichardo[7], Henry Idrobo[8], Maria A. Torres Viera[9], Ana Oliver[10], Adolfo Diaz Duque[11]

*[1]Hospital General Dr. Fernando Ocaranza ISSSTE, Hermosillo, Mexico;[2]VCU Health, San Antonio, United States of America;[3]UT HEALTH SAN ANTONIO, San Antonio, United States of America;[4]Hospital Universitario de Monterrey, Monterrey, Mexico;[5]Instituto Nacional de Ciencias Médicas y Nutrición Salvador Zubiran, Ciudad de México, Mexico;[6]HOSPITAL NACIONAL EDGARDO REBAGLIATI MARTINS, LIMA, Peru;[7]Hospital de Apoyo II-2 de Sullana, Sullana, Peru;[8]Universidad Tecnologica de Pereira, Clinica Central del eje, Pereira, Colombia;[9]CLINICA SANTA SOFIA, CARACAS, Venezuela;[10]Hospital Britanico. Casmu, Montevideo, Uruguay;[11]UT HEALTH SAN ANTONIO / MAYS CANCER CENTER, San Antonio, United States of America;*

### Background

Diffuse large B-cell lymphoma (DLBCL) is an aggressive B-cell malignancy that primarily affects older adults. Optimal treatment management in the geriatric population is challenging due to the high-risk disease biology and their age-specific vulnerabilities. Combination chemotherapy is effective, however, due to frailty often a dose-reduced regimen is administered. This large population real-world study compares demographic, clinical and survival characteristics of patients older than 75 years and compares Academic Cancer Programs (ACP) vs Community Cancer Programs (CCP).

### Aims

To determine whether the treatment approach (adjusting for: age, race/ethnicity, insurance status, Charlson-Deyo comorbidity score, and distance from the treating facility) among elderly patients (equal or over 75 years old) with DLBCL in academic versus community hospitals influences overall survival in this lymphoma population.

### Methods

A retrospective analysis using the National Cancer Database was conducted on 103,671 patients (ACP: 51,848; CCP: 51,823) diagnosed with DLBCL between 2004 and 2022 was carried out. ACP included academic and National Cancer Institute-designated research centers; CCP included community, comprehensive community, and integrated network cancer programs. Kaplan-Meier and Cox regression models were used to compare overall survival (OS), adjusting for: age, race/ethnicity, insurance status, Charlson-Deyo comorbidity score, and distance from treating facility.

**Copyright Information:** (Online) ISSN: 2572-9241
©2026 The Author(s). HemaSphere is published by John Wiley & Sons Ltd on behalf of European Hematology Association. This is an open access Abstract Book distributed under the Attribution-NonCommercial-NoDerivs (CC BY-NC-ND), which allows third parties to download the articles and share them with others as long as they credit the author and the Abstract Book, but they cannot change the content in any way or use them commercially.
**Abstract Book Citations:** Authors, Title, HemaSphere, 2026;10:(S1):pages. The abstract book DOIs can be found on the "Author Information" page.
**Disclaimer:** Articles published in the journal HemaSphere exclusively reflect the opinions of the authors. The authors are responsible for all content in their abstracts including accuracy of the facts, statements, citing resources, etc.

**Results**

Patients in ACP and CCP were mostly females with a median age of 81 years. For both cohorts the majority were non-Hispanics and Whites; however, for ACP it was noted a higher rate of Hispanics (6% vs 5%) and Blacks (5% vs 3%) compared to CCP. Most of the patients in both groups were from the Metropolitan area, but the median distance in miles for ACP was 9.0 vs 7.4 in CCP.Treatment rates were higher for ACP vs community settings (63% vs 59%, p<0.001), with more patients presenting at Stage IV (36% vs 33%, p<0.001).
Better OS was noted in elderly patients treated at ACP, p<0.001.The median OS for ACP was of 1.79 years vs1.50 years at CCP, which was consistent with a longer median follow up time of 17.7 months vs 14.1, p<0.001 (Figure 1). This survival advantage remained consistent throughout long-term observation, with 2-year OS rates of 49% at ACP [CI: 0.48,0.49] vs 46% at CCP [CI: 0.459,0.468], 5-year OS of 34% [CI: 0.336,0.346] vs 32% [CI: 0.317,0.327], and 10-year OS of 16% [CI: 0.154,0.164] vs 15% [CI: 0.142,0.151], respectively.

**Summary/Conclusion**

Among patients aged ≥75 years with DLBCL, OS differed by treatment facility type, with a statistically significant but modest survival advantage observed among patients treated at ACP compared with CCP (log-rank p<0.001). This difference occurred in the context of higher treatment rates and greater disease severity at presentation. Given the relatively small absolute survival differences, these findings suggest that care for older adults with DLBCL is largely standardized across practice settings; however, this highlights an opportunity to optimize treatment delivery and intensity, supportive care, and geriatric-focused management in both academic and community programs.

**Copyright Information:** (Online) ISSN: 2572-9241
©2026 The Author(s). HemaSphere is published by John Wiley & Sons Ltd on behalf of European Hematology Association. This is an open access Abstract Book distributed under the Attribution-NonCommercial-NoDerivs (CC BY-NC-ND), which allows third parties to download the articles and share them with others as long as they credit the author and the Abstract Book, but they cannot change the content in any way or use them commercially.
**Abstract Book Citations:** Authors, Title, HemaSphere, 2026;10:(S1):pages. The abstract book DOIs can be found on the "Author Information" page.
**Disclaimer:** Articles published in the journal HemaSphere exclusively reflect the opinions of the authors. The authors are responsible for all content in their abstracts including accuracy of the facts, statements, citing resources, etc.

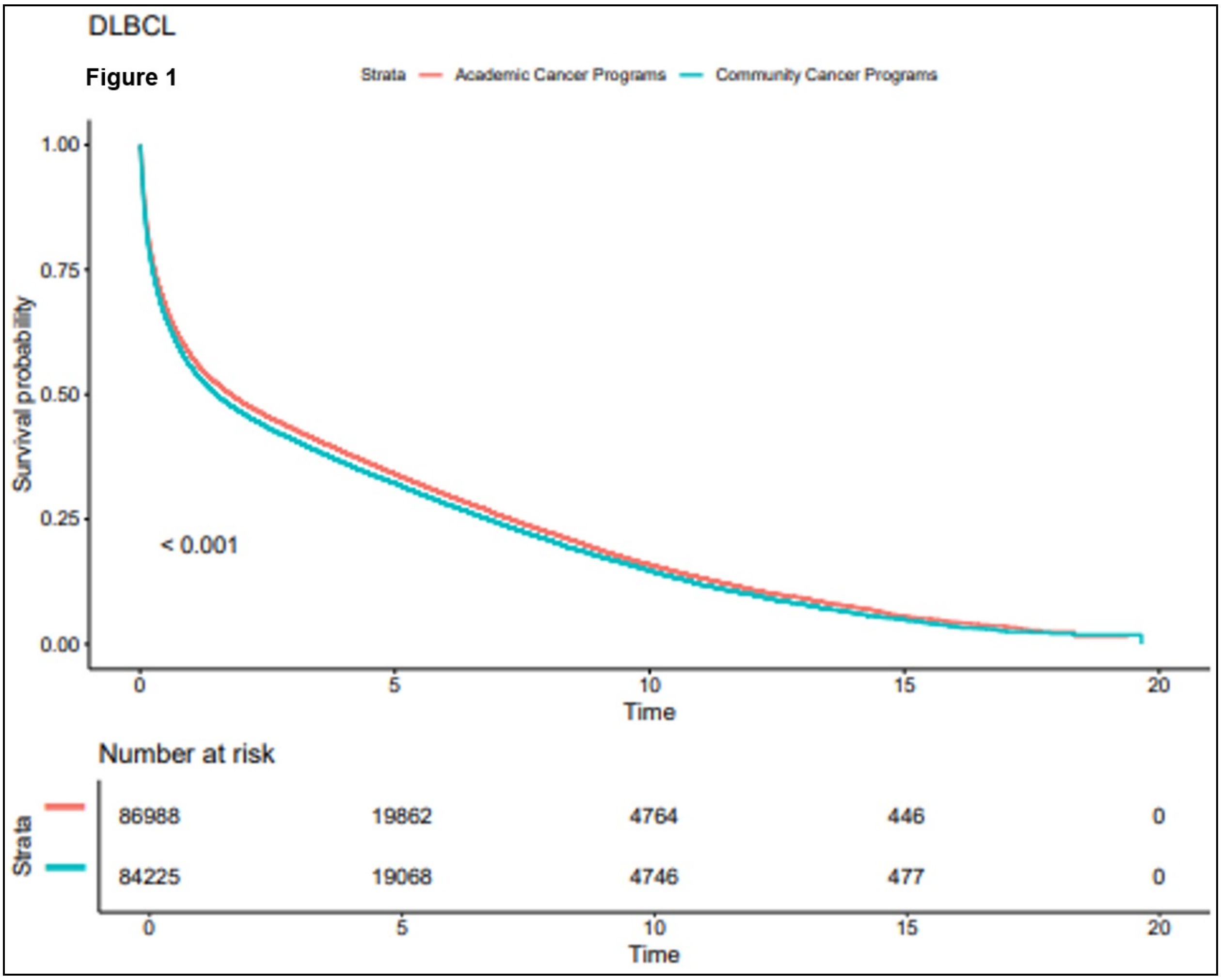

Figure 1. OS in elderly DLBCL patients treated in academic centers vs. community hospitals

**Copyright Information:** (Online) ISSN: 2572-9241
©2026 The Author(s). HemaSphere is published by John Wiley & Sons Ltd on behalf of European Hematology Association. This is an open access Abstract Book distributed under the Attribution-NonCommercial-NoDerivs (CC BY-NC-ND), which allows third parties to download the articles and share them with others as long as they credit the author and the Abstract Book, but they cannot change the content in any way or use them commercially.
**Abstract Book Citations:** Authors, Title, HemaSphere, 2026;10:(S1):pages. The abstract book DOIs can be found on the "Author Information" page.
**Disclaimer:** Articles published in the journal HemaSphere exclusively reflect the opinions of the authors. The authors are responsible for all content in their abstracts including accuracy of the facts, statements, citing resources, etc.

### (PS2143) TUMOR HETEROGENEITY DECIPHERING FOR INTERPRETABLE EARLY WARNING OF ADVERSE THERAPY RESPONSE IN EXTRANODAL NK/T-CELL LYMPHOMA

**Topic:** 20. Other aggressive lymphomas

Chong Jiang*[1], Zekun Jiang[2], Chongyang Ding[3], Rui Guo[4], Ming Jiang[5]

*[1]West China Hospital, Sichuan University, Department of Nuclear Medicine, Chengdu, China;[2]West China Hospital, Sichuan University, Chengdu, China; [3]Jiangsu Province Hospital, The First Affiliated Hospital of Nanjing Medical University, Nanjing, China; [4]Ruijin Hospital, Shanghai Jiao Tong University School of Medicine, Shanghai, China; [5]West China Hospital, Sichuan University, Department of Oncology, Chengdu, China;*

### Background

Extranodal natural killer (NK)/T-cell lymphoma (ENKTCL) is a highly aggressive malignancy with pronounced intratumoral heterogeneity. This heterogeneity, driven by diverse gene expression and signaling pathways, often leads to therapeutic resistance and disease relapse. While traditional imaging focuses on whole-tumor metrics, these approaches often fail to capture the spatial variations that dictate treatment failure. Tumor habitat analysis has emerged as a promising method to unravel this complexity, yet its application in ENKTCL remains unexplored.

### Aims

This multicenter study aimed to develop a PET/CT-based habitat analysis framework to decode patterns of intratumoral heterogeneity and provide an interpretable early warning system for predicting adverse therapy response and long-term prognosis in ENKTCL.

### Methods

Baseline 18F-FDG PET/CT data from 366 ENKTCL patients across four centers were retrospectively analyzed (Training: n=252; Validation: n=114). We applied a super-voxel partitioning and unsupervised K-means clustering approach to identify distinct tumor habitats based on four metrics: PET SUV, CT intensity, and local entropy for both modalities. Radiomics models were developed for each habitat using XGBoost with SHAP-based feature selection. A multiparametric model was further constructed by integrating the key habitat score with cell-free EBV (cfEBV) DNA status. Biological interpretability was assessed via histopathologic correlations and RNA-seq immune/molecular profiling.

### Results

Three distinct tumor habitats were identified: Habitat 1 (metabolically active/complex core), Habitat 2 (high CT intensity/stromal-like), and Habitat 3 (low activity/necrotic).Habitat 1 emerged as the strongest predictor of adverse response, achieving an AUC of 0.853 in the training cohort and 0.826 in the validation cohort, significantly outperforming whole-tumor and other habitat models (P < 0.05) .The Habitat 1 RadScore was an independent predictor of progression-free survival (HR 1.577) and overall survival (HR 1.602).Combining RadScore with cfEBV DNA status yielded a nomogram with robust discrimination (Validation AUC 0.834) and high clinical utility in decision curve analysis.High RadScoreHabitat1 was strongly associated with vascular invasion (Spearman's ρ = 0.587) and enrichment of mutations in DNMT3A, STAT3, and NOTCH1.Functional enrichment linked high-risk habitats to upregulated VEGF signaling and cytokine interactions.

**Copyright Information:** (Online) ISSN: 2572-9241
©2026 The Author(s). HemaSphere is published by John Wiley & Sons Ltd on behalf of European Hematology Association. This is an open access Abstract Book distributed under the Attribution-NonCommercial-NoDerivs (CC BY-NC-ND), which allows third parties to download the articles and share them with others as long as they credit the author and the Abstract Book, but they cannot change the content in any way or use them commercially.
**Abstract Book Citations:** Authors, Title, HemaSphere, 2026;10:(S1):pages. The abstract book DOIs can be found on the "Author Information" page.
**Disclaimer:** Articles published in the journal HemaSphere exclusively reflect the opinions of the authors. The authors are responsible for all content in their abstracts including accuracy of the facts, statements, citing resources, etc.

### Summary/Conclusion

We established the first PET/CT-based habitat framework for ENKTCL, identifying a metabolically active "tumor core" (Habitat 1) as a critical determinant of treatment resistance. By bridging the gap between macroscopic imaging signatures and microscopic vascular/genomic profiles, this interpretable model provides a novel paradigm for early risk stratification and personalized therapeutic intervention in ENKTCL.

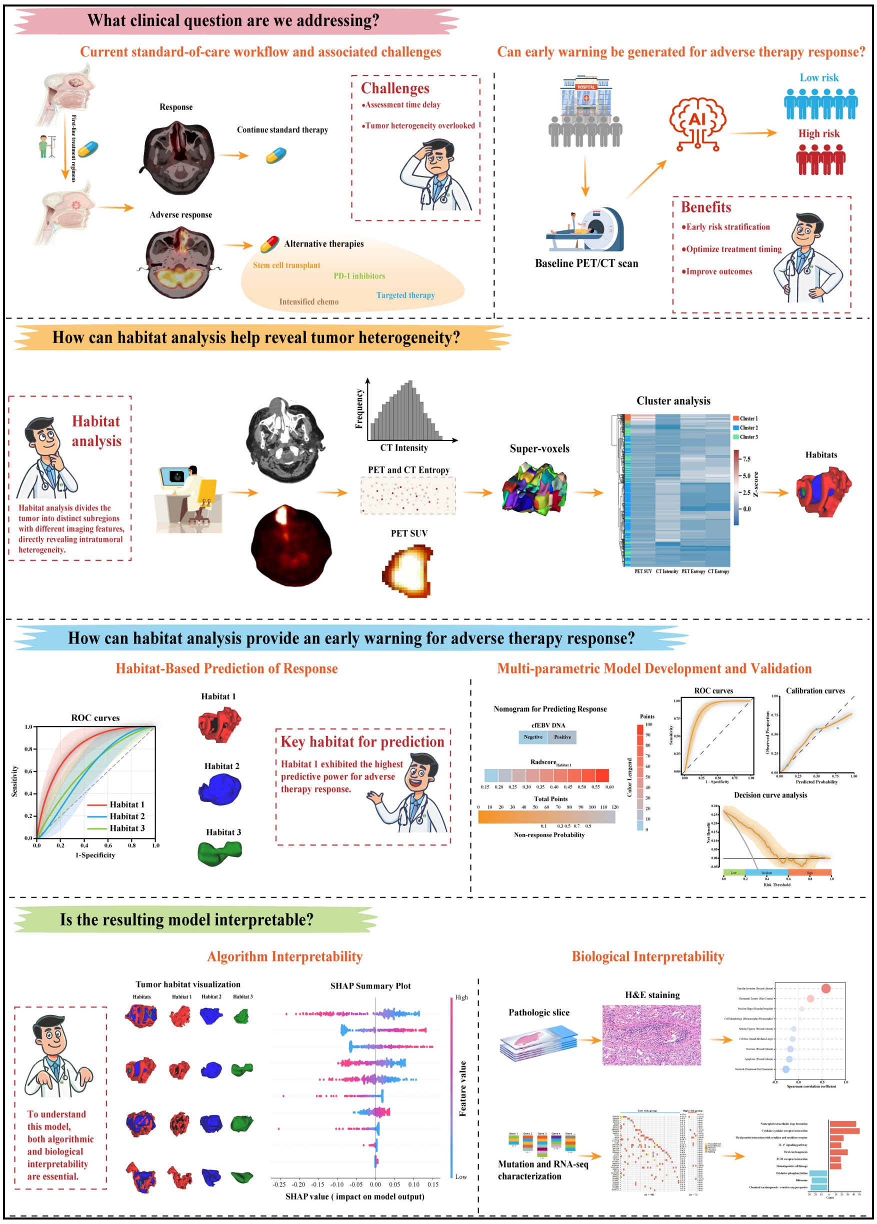

**Copyright Information:** (Online) ISSN: 2572-9241
©2026 The Author(s). HemaSphere is published by John Wiley & Sons Ltd on behalf of European Hematology Association. This is an open access Abstract Book distributed under the Attribution-NonCommercial-NoDerivs (CC BY-NC-ND), which allows third parties to download the articles and share them with others as long as they credit the author and the Abstract Book, but they cannot change the content in any way or use them commercially.
**Abstract Book Citations:** Authors, Title, HemaSphere, 2026;10:(S1):pages. The abstract book DOIs can be found on the "Author Information" page.
**Disclaimer:** Articles published in the journal HemaSphere exclusively reflect the opinions of the authors. The authors are responsible for all content in their abstracts including accuracy of the facts, statements, citing resources, etc.

### (PS2144) RANDOMIZED PHASE 3 TRIAL OF WATCHFUL WAITING VERSUS INTERFERON-ALFA AND ZIDOVUDINE FOR INDOLENT ADULT T-CELL LEUKEMIA-LYMPHOMA: JCOG1111C

**Topic:** 20. Other aggressive lymphomas

Kenji Ishitsuka*[1], Ryunosuke MacHida[2], Wataru Munakata[2], Hirotaka Nakamura[3], Satoshi Yamasaki[4], Kisato Nosaka[5], Yoshikiyo Ito[6], Kazuho Morichika[7], Kana Miyazaki[8], Makoto Yoshimitsu[1], Takuya Fukushima[9], Kunihiro Tsukasaki[10], Yosuke Minami[3], Hirokazu Nagai[11], Dai Maruyama[12]

*[1]Kagoshima University Hospital, Department of Hematology and Rheumatology, Kagoshima, Japan; [2]National Cancer Center Hospital, Tokyo, Japan; [3]National Cancer Center Hospital East, Kashiwa, Japan; [4]NHO Kyushu Medical Center, Fukuoka, Japan; [5]Kumamoto University Hospital, Kumamoto, Japan; [6]Imamura General Hospital, Kagoshima, Japan; [7]Ryukyu University Hospital, Ginowan, Japan; [8]Mie University Hospital, Tsu, Japan; [9]Ryukyu University, Ginowan, Japan; [10]International Medical Center, Saitama Medical University, Hidaka, Japan; [11]NHO Nagoya Medical Center, Nagoya, Japan; [12]Cancer Institute Hospital, Japanese Foundation For Cancer Research, Tokyo, Japan;*

### Background

Adult T-cell leukemia-lymphoma (ATL) is a peripheral T-cell malignancy caused by human T-cell leukemia virus type I (HTLV-1). It is clinically categorized into aggressive and indolent phenotypes. Indolent ATL includes smoldering and chronic types without unfavorable prognostic factors (defined by serum LDH, BUN, and albumin levels). Management of indolent ATL remains controversial: watchful waiting (WW) is the standard in Japan, despite limited retrospective follow-up data, whereas interferon-alfa and zidovudine (IFN/AZT) is recommended elsewhere based on retrospective data reporting a 100% 5-year survival proportion.

### Aims

To compare WW with IFN/AZT in a randomized phase 3 trial conducted by the Japan Clinical Oncology Group, aiming to establish an evidence-based standard of care for indolent ATL (JCOG1111C, Clinical trial identifier: jRCTs031180169).

### Methods

Patients aged 20–75 years with cytotoxic agent-naive indolent ATL, excluding asymptomatic smoldering type, were randomized to the WW or IFN/AZT arm. In the IFN/AZT arm, IFN-alfa (human lymphoblastoid interferon) was administered subcutaneously at $3x10^6$ units (U) on days 1–7, followed by $6x10^6$ U/day until day 84, and $3x10^6$ U/day thereafter. AZT was administered orally at 600 mg/day in three divided doses until day 84 and 400 mg/day in two divided doses thereafter. The primary endpoint was event-free survival (EFS), defined as progression to aggressive ATL or death from any cause. Assuming a 2-year EFS of 60% in the WW arm and an increase to 80% in the IFN/AZT arm (hazard ratio [HR]: 0.44), the initial total sample size was 74 patients with one-sided alpha of 5% and power of 70%. Due to poor accrual, the target total sample size was reduced to 38 patients, and the follow-up period was extended from 2 to 7 years.

**Copyright Information:** (Online) ISSN: 2572-9241
©2026 The Author(s). HemaSphere is published by John Wiley & Sons Ltd on behalf of European Hematology Association. This is an open access Abstract Book distributed under the Attribution-NonCommercial-NoDerivs (CC BY-NC-ND), which allows third parties to download the articles and share them with others as long as they credit the author and the Abstract Book, but they cannot change the content in any way or use them commercially.
**Abstract Book Citations:** Authors, Title, HemaSphere, 2026;10:(S1):pages. The abstract book DOIs can be found on the “Author Information” page.
**Disclaimer:** Articles published in the journal HemaSphere exclusively reflect the opinions of the authors. The authors are responsible for all content in their abstracts including accuracy of the facts, statements, citing resources, etc.

### Results

Between September 2013 and March 2018, 38 patients from 18 hospitals in Japan were randomized (WW, n=18; IFN/AZT, n=20). Baseline characteristics (WW vs. IFN/AZT) were: median age, 55.5 vs. 60.0 years; male, 44.4% vs. 30.0%; PS 0/1, 94.4/5.6% vs. 85.0/15.0%; and favorable chronic type, 50.0% vs. 40.0%. As of the March 18, 2025 data cut-off, with a median follow-up of 3.8 years for all registered patients and 8.5 years for those without events, the median EFS was 2.3 years (95% CI: 1.3–not estimable [NE]) in the WW arm and 7.1 years (95% CI: 2.1–9.7) in the IFN/AZT arm (HR: 0.84, 95% CI: 0.35–1.99; p=0.34). The response rate was 22.2% vs. 55.0%. Median overall survival (OS) was NE in both arms (HR: 0.94, 95% CI: 0.33–2.67). Median acute transformation-free survival (ATFS) was 1.8 years (95% CI: 0.9–NE) vs. 7.0 years (95% CI: 2.1–9.7) (HR: 0.68, 95% CI: 0.31–1.53). Relative dose intensities for IFN and AZT were 36.4% (IQR: 30.3–53.7%) and 83.0% (IQR: 65.3–97.6%), respectively. Adverse events (AEs) were more frequent in the IFN/AZT arm. Grade 3 AEs in the IFN/AZT arm included febrile neutropenia, pyrexia, injection site reaction, infectious enterocolitis, pulmonary infection (n=1 each), and anorexia (n=2). No Grade 4 AEs occurred.

### Summary/Conclusion

This trial demonstrated that EFS, OS, and ATFS favored the IFN/AZT arm; however, the clinical advantage over watchful waiting did not reach statistical significance. Despite the small sample size and limited number of events, these results indicate that watchful waiting remains an appropriate standard management strategy for indolent ATL.

**Copyright Information:** (Online) ISSN: 2572-9241
©2026 The Author(s). HemaSphere is published by John Wiley & Sons Ltd on behalf of European Hematology Association. This is an open access Abstract Book distributed under the Attribution-NonCommercial-NoDerivs (CC BY-NC-ND), which allows third parties to download the articles and share them with others as long as they credit the author and the Abstract Book, but they cannot change the content in any way or use them commercially.
**Abstract Book Citations:** Authors, Title, HemaSphere, 2026;10:(S1):pages. The abstract book DOIs can be found on the "Author Information" page.
**Disclaimer:** Articles published in the journal HemaSphere exclusively reflect the opinions of the authors. The authors are responsible for all content in their abstracts including accuracy of the facts, statements, citing resources, etc.

### (PS2145) DETERMINANTS OF SURVIVAL IN AGGRESSIVE NK-CELL LEUKEMIA (ANKL): ANALYSIS OF AN UPDATED REAL-WORLD POOLED DATABASE

**Topic:** 20. Other aggressive lymphomas

Philip Haddad*[1], Supriya Gupta[2], Christopher Graham[2]

*[1]LSUHSC-S/Overton Brooks VAMC, Hematology-Oncology, Shreveport, United States of America; [2]University of Minnesota, Minneapolis, United States of America;*

### Background

Aggressive NK-cell leukemia (ANKL) is a rare, highly aggressive mature NK-cell neoplasm characterized by systemic symptoms, frequent hemophagocytic syndrome, multiorgan involvement, and rapid clinical decline. Although recognition has increased and ANKL has been incorporated into contemporary lymphoma classifications, prognostic determinants remain inadequately defined due to reliance on small case series and heterogeneous treatment approaches.

### Aims

The aim of this study was to evaluate demographic, immunophenotypic, cytogenetic, and treatment-related determinants of overall survival (OS) in a real-world ANKL cohort.

### Methods

An updated pooled database of 195 ANKL cases meeting established diagnostic criteria was compiled. Overall survival was estimated using Kaplan–Meier methodology. Cox proportional hazards models assessed survival associations. Time-dependent Cox models accounted for stem cell transplantation (SCT) as a time-varying exposure to mitigate immortal-time bias. Immunophenotypic and cytogenetic variables were extracted from source reports and analyzed as binary exposures when explicitly reported.

### Results

The cohort included 195 ANKL patients, with a median overall survival (OS) of 2 months. Older age correlated with poorer OS (p=0.0003). Exposure to asparaginase and stem cell transplantation (analyzed as time-fixed exposure) correlated with improved OS (p<0.0001). When SCT was analyzed as a time-dependent variable, it remained independently associated with reduced mortality (HR=0.43, p=0.03). Immunophenotype significantly influenced survival: CD56 positivity was independently associated with improved OS (HR 0.54 after adjustment for age and time-dependent SCT, p=0.001), whereas cytoplasmic CD3ε positivity correlated with inferior OS (adjusted HR 1.57, p=0.010). Phenotypic stratification revealed distinct outcomes: CD56+/cCD3ε– had the longest median OS (3.0 months), whereas CD56+/cCD3ε+ and CD56–/cCD3ε+ had similarly short OS (~1.6 months) and double-negative cases fared worst (1.0 month). A significant CD56×cCD3ε interaction was observed (interaction HR 2.69, p=0.009), indicating that CD56-associated survival advantage is attenuated in cCD3ε-positive disease. Other markers, including CD2, CD16, CD57, granzyme B, perforin, and EBER status, showed no significant association with OS. No cytogenetic abnormalities, including i(7q), trisomy 8, chromosome 7 abnormalities, del(6q), complex karyotype, or composite abnormal cytogenetics, demonstrated significant survival associations.

**Copyright Information:** (Online) ISSN: 2572-9241
©2026 The Author(s). HemaSphere is published by John Wiley & Sons Ltd on behalf of European Hematology Association. This is an open access Abstract Book distributed under the Attribution-NonCommercial-NoDerivs (CC BY-NC-ND), which allows third parties to download the articles and share them with others as long as they credit the author and the Abstract Book, but they cannot change the content in any way or use them commercially.
**Abstract Book Citations:** Authors, Title, HemaSphere, 2026;10:(S1):pages. The abstract book DOIs can be found on the "Author Information" page.
**Disclaimer:** Articles published in the journal HemaSphere exclusively reflect the opinions of the authors. The authors are responsible for all content in their abstracts including accuracy of the facts, statements, citing resources, etc.

**Summary/Conclusion**

In this updated real-world pooled ANKL cohort, survival is primarily influenced by treatment exposure and immunophenotypic characteristics. Asparaginase-based therapy and SCT are associated with improved survival. CD56 positivity confers a favorable prognosis, whereas cytoplasmic CD3ε expression identifies a higher-risk subgroup. These findings underscore biologically distinct immunophenotypic subsets within ANKL and support the use of asparaginase-based regimens alongside early transplant-integrated treatment strategies.

**Copyright Information:** (Online) ISSN: 2572-9241
©2026 The Author(s). HemaSphere is published by John Wiley & Sons Ltd on behalf of European Hematology Association. This is an open access Abstract Book distributed under the Attribution-NonCommercial-NoDerivs (CC BY-NC-ND), which allows third parties to download the articles and share them with others as long as they credit the author and the Abstract Book, but they cannot change the content in any way or use them commercially.
**Abstract Book Citations:** Authors, Title, HemaSphere, 2026;10:(S1):pages. The abstract book DOIs can be found on the "Author Information" page.
**Disclaimer:** Articles published in the journal HemaSphere exclusively reflect the opinions of the authors. The authors are responsible for all content in their abstracts including accuracy of the facts, statements, citing resources, etc.

### (PS2146) A NOVEL TRIPLET REGIMEN OF TISLELIZUMAB, GOLIDOCITINIB, AND SELINEXOR FOR RELAPSED/REFRACTORY EXTRANODAL NK/T-CELL LYMPHOMA: PRELIMINARY SAFETY AND EFFICACY FROM A PHASE IB/II STUDY

**Topic:** 20. Other aggressive lymphomas

Rong Tao*[1, 2], Chuanxu Liu[1, 2], Da-Hui Li[1, 2], Wen-Hao Zhang[1, 2], Shiyu Jiang[1, 2]

*[1]Department of Lymphoma and Medical Oncology, Fudan University Shanghai Cancer Center, Shanghai, China;[2]Research Center for Lymphoma, Fudan University, Shanghai, China;*

### Background

Relapsed/refractory (R/R) extranodal NK/T-cell lymphoma (ENKTL) has a dismal prognosis, with no established standard salvage therapy. PD-1 blockade has shown efficacy in this setting, but durable responses are rare with monotherapy. Aberrant JAK/STAT signaling and XPO1 overexpression are established oncogenic drivers in ENKTL. Dysregulated JAK/STAT signaling drives malignant cell proliferation, survival and apoptosis resistance. XPO1 upregulation mediates aberrant nuclear export of key tumor suppressor proteins. Concurrent targeting of these complementary pathways has a strong scientific rationale. This study evaluates the antitumor synergy of tislelizumab (PD-1 inhibitor), golidocitinib (JAK1 inhibitor), and selinexor (XPO1 inhibitor) in R/R ENKTL.

### Aims

The primary objectives of the Phase Ib dose-escalation part included the assessment of safety, tolerability, and determination of recommended phase 2 dose (RP2D) of this novel triplet combination. The Phase II expansion part aims to further evaluate the preliminary antitumor activity, focusing on the objective response rate (ORR) and complete response rate (CRR) as the key endpoints.

### Methods

This is an open-label, multicenter, Phase Ib/II study enrolling patients with R/R ENKTL. The Phase Ib stage employed a Bayesian Optimal Interval (BOIN) design to determine the maximum tolerated dose (MTD) and RP2D. Patients received escalating doses of oral golidocitinib and selinexor in combination with fixed-dose intravenous tislelizumab. Safety was assessed per CTCAE v5.0, with dose-limiting toxicities (DLTs) evaluated during the first 21-day cycle. Tumor response was assessed by PET-CT according to Lugano 2014 criteria. The study has completed Phase Ib enrollment and is proceeding to the Phase II expansion at the RP2D.

### Results

As of the data cut-off, 16 patients were enrolled in the Phase Ib cohort. The baseline characteristics indicated a high-risk and heavily pretreated population. The median age was 47 years (range: 25-65), 56.3% (9/16) had Stage III/IV disease, and 81.3% (13/16) presented with a PINK-E score of ≥3. Patients had received a median of 1.5 prior lines of therapy (range: 1–3). Notably, 62.5% (10/16) were refractory to the last line of treatment, and 43.8% (7/16) had prior exposure to PD-1 inhibitors. Among the 14 patients evaluable for DLT assessment, the triplet regimen was generally well-tolerated. Only one DLT event was observed, characterized by Grade 3 fatigue and Grade 3 anorexia, which resolved with supportive care. No Grade 4 or 5 treatment-related adverse events (TRAEs) occurred during the DLT observation period. CMV reactivation was reported in 3 patients. Preliminary efficacy was evaluable in 9 patients who completed at least one post-baseline tumor assessment. The combination demonstrated robust antitumor activity with an ORR of 66.7% (6/9) and a CRR of 33.3% (3/9). Notably, the responses were rapid and durable; the median duration of response (DOR) was not reached, with all 6 responders remaining on treatment and maintaining response at the time of analysis

**Copyright Information:** (Online) ISSN: 2572-9241
©2026 The Author(s). HemaSphere is published by John Wiley & Sons Ltd on behalf of European Hematology Association. This is an open access Abstract Book distributed under the Attribution-NonCommercial-NoDerivs (CC BY-NC-ND), which allows third parties to download the articles and share them with others as long as they credit the author and the Abstract Book, but they cannot change the content in any way or use them commercially.
**Abstract Book Citations:** Authors, Title, HemaSphere, 2026;10:(S1):pages. The abstract book DOIs can be found on the "Author Information" page.
**Disclaimer:** Articles published in the journal HemaSphere exclusively reflect the opinions of the authors. The authors are responsible for all content in their abstracts including accuracy of the facts, statements, citing resources, etc.

### Summary/Conclusion

The triplet regimen of tislelizumab, golidocitinib, and selinexor demonstrates a manageable safety profile and promising preliminary efficacy in heavily pretreated patients with R/R ENKTL. The high response rate and durability observed support the scientific rationale of dual-targeting JAK/STAT and XPO1 pathways alongside PD-1 blockade. The Phase II expansion study is ongoing to further validate these encouraging findings.

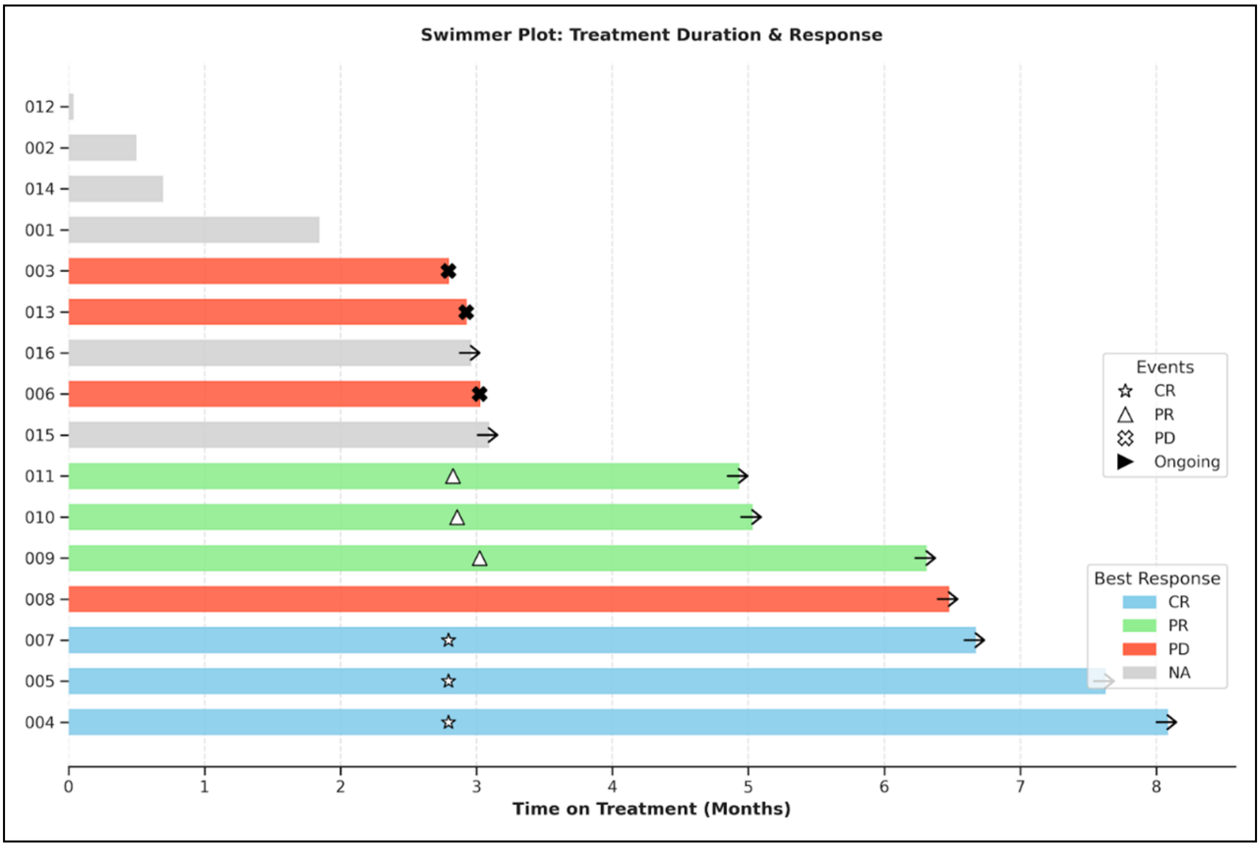

**Copyright Information:** (Online) ISSN: 2572-9241
©2026 The Author(s). HemaSphere is published by John Wiley & Sons Ltd on behalf of European Hematology Association. This is an open access Abstract Book distributed under the Attribution-NonCommercial-NoDerivs (CC BY-NC-ND), which allows third parties to download the articles and share them with others as long as they credit the author and the Abstract Book, but they cannot change the content in any way or use them commercially.
**Abstract Book Citations:** Authors, Title, HemaSphere, 2026;10:(S1):pages. The abstract book DOIs can be found on the "Author Information" page.
**Disclaimer:** Articles published in the journal HemaSphere exclusively reflect the opinions of the authors. The authors are responsible for all content in their abstracts including accuracy of the facts, statements, citing resources, etc.

### (PS2147) CHARACTERIZING CASTLEMAN'S DISEASE IN GERMANY: INTERIM RESULTS FROM THE ABC-CD STUDY

**Topic:** 20. Other aggressive lymphomas

Katharina Cron*[1], Pascal Migaud[2], Markus Mueller[2], Marcus Hentrich[3], Wolfram Klapper[4], Ingram Iaccarino[4], Hauke Walter[5], Tobias Projahn[5], Carsten Marr[6], Christian Hoffmann[1]

*[1]ICH Study Center, Hamburg, Germany; [2]St. Joseph Krankenhaus Berlin-Tempelhof, Klinik für Infektiologie, Berlin, Germany; [3]Rotkreuzklinikum München, Chefarzt Innere Medizin III – Hämatologie und Onkologie Leiter Onkologisches Zentrum Leiter Zentrum für Hämatologische Neoplasien, München, Germany; [4]Universitätsklinikum Schleswig-Holstein, Sektion Hämatopathologie und Lymphknotenregister (LKR-Kiel) im Institut für Pathologie Kiel, Kiel, Germany; [5]ICH Medlab Stendal, Stendal, Germany; [6]Helmholtz Zentrum München, München, Germany;*

### Background

Castleman's disease (CD) encompasses a spectrum of rare inflammatory diseases with heterogeneous pathogenetic mechanisms. Despite characteristic histopathological features, diagnosis remains challenging.

### Aims

The ABC-CD study, the first nationwide German CD cohort, aims to describe clinical symptom frequencies and associations between clinical and laboratory features, comorbidities, biomarkers, and AI-generated histopathological findings.

### Methods

This ongoing, multicentric study investigates all histologically proven cases of unicentric (UCD), idiopathic multicentric (iMCD), and HHV-8 positive MCD (HHV-8-MCD). Following written informed consent, detailed clinical data and biomarkers are collected. In a second phase, histopathological findings will be evaluated centrally and AI-based learning models developed. This analysis compared demographic and clinical features with a recent systematic review (Blood Adv 2024; 8:4924-35)

### Results

Between December 2024 and February 2026, 88 patients were enrolled, including 32 with UCD, 33 with iMCD (including 8 cases of POEMS-associated MCD), and 23 with HHV-8-MCD. UCD predominantly affected younger females (66% female; median age 37 years, range 11–64), while iMCD showed a slight male predominance with higher median age (39% female; median age 53 years, range 20–83; median diagnostic delay 5.1 months). HHV-8-MCD occurred almost exclusively in males (91%) and in people living with HIV (96%), with a median age of 48 years (range 33–81). The time from symptom onset to diagnosis exceeded one year in 19%, 25%, and 5% of UCD, iMCD, and HHV-8-MCD cases, respectively. German nationality was comparable across groups (75%, 79%, 74%). Inflammatory and hematological abnormalities, organ dysfunction, and immune activation were frequent in iMCD and HHV-8-MCD, while systemic features were also observed in a subset of UCD cases (Table). Most diagnostic criteria rates closely mirrored those reported in international cohorts. However, the ABC-CD cohort appears enriched for severe TAFRO-subtype iMCD — characterized by thrombocytopenia, anasarca, fever, renal dysfunction, and organomegaly — with underrepresentation of the milder IPL (idiopathic plasmacytic lymphadenopathy) subtype, which presents predominantly with hypergammaglobulinemia and less severe systemic involvement.

**Copyright Information:** (Online) ISSN: 2572-9241
©2026 The Author(s). HemaSphere is published by John Wiley & Sons Ltd on behalf of European Hematology Association. This is an open access Abstract Book distributed under the Attribution-NonCommercial-NoDerivs (CC BY-NC-ND), which allows third parties to download the articles and share them with others as long as they credit the author and the Abstract Book, but they cannot change the content in any way or use them commercially.
**Abstract Book Citations:** Authors, Title, HemaSphere, 2026;10:(S1):pages. The abstract book DOIs can be found on the "Author Information" page.
**Disclaimer:** Articles published in the journal HemaSphere exclusively reflect the opinions of the authors. The authors are responsible for all content in their abstracts including accuracy of the facts, statements, citing resources, etc.

**Summary/Conclusion**

These preliminary data from the German ABC-CD study confirm clinical features largely consistent with international cohorts across all three CD subtypes. The enrichment of TAFRO-subtype iMCD and underrepresentation of the milder IPL subtype may reflect referral bias, but could also point to genuine ethnic differences in disease expression, given that 92% of iMCD cases in the reference review were of Asian ethnicity. Diagnostic delay remains a relevant challenge, particularly in iMCD.
(This project was funded by Recordati UK Ltd, which provided financial support to the ICH Study Center for the development and implementation of the project. Recordati UK Ltd had no role in the study design, data collection, data analysis, interpretation of results, or the preparation of this material.)

| | UCD | UCD | iMCD | iMCD | HHV-8 MCD | HHV-8 MCD |
|---|---|---|---|---|---|---|
| | ABC-CD | Review | ABC-CD | Review | ABC-CD | Review |
| N | 28 | 53-327 | 32 | 31-647 | 21 | 12-355 |
| ***Clinical Criteria*** | | | | | | |
| Constitutional symptoms | 30% | 5% (2-12) | 100% | 47% (40-53) | 100% | 99% (66-100) |
| Splenomegaly | 10% | 4% (2-7) | 87% | 48% (40-57) | 100% | 89% (60-98) |
| Fluid accumulation | 0% | 2% (1-5) | 77% | 27% (19-37) | 33% | 50% (21-79) |
| Pulmonary symptoms | 10% | 15% (9-27) | 38% | 34% (21-50) | 40% | 52% (35-68) |
| Renal dysfunction | 0% | 1% (0-4) | 29% | 37% (30-45) | 11% | 17% (11-25) |
| Polyneuropathy | 3% | 6% (1-16) | 59% | 31% (17-50) | 22% | 28% (12-52) |
| ***Laboratory Criteria*** | | | | | | |
| CRP elevation | 17% | 19% (11-31) | 85% | 92% (65-99) | 100% | 90% (67-97) |
| Anemia | 21% | 18% (13-25) | 83% | 89% (60-98) | 85% | 75% (56-87) |
| Hypoalbuminemia | 0% | 2% (0-57) | 83% | 61% (40-78) | 71% | 77% (61-88) |
| Thrombocytopenia | 4% | 2% (1-6) | 41% | 17% (7-37) | 55% | 37% (22-56) |
| Hypergammaglobulinemia | 8% | 18% (10-29) | 53% | 97% (97-97) | 82% | 100% (48-100) |

Table. Frequency of diagnostic criteria in the ABC-CD study compared with a systematic review of 1,998 cases from 32 cohort studies, 1995–2021. Note: 92% of iMCD and 85% of UCD cases in the reference cohort were of Asian ethnicity.

**Copyright Information:** (Online) ISSN: 2572-9241
©2026 The Author(s). HemaSphere is published by John Wiley & Sons Ltd on behalf of European Hematology Association. This is an open access Abstract Book distributed under the Attribution-NonCommercial-NoDerivs (CC BY-NC-ND), which allows third parties to download the articles and share them with others as long as they credit the author and the Abstract Book, but they cannot change the content in any way or use them commercially.
**Abstract Book Citations:** Authors, Title, HemaSphere, 2026;10:(S1):pages. The abstract book DOIs can be found on the "Author Information" page.
**Disclaimer:** Articles published in the journal HemaSphere exclusively reflect the opinions of the authors. The authors are responsible for all content in their abstracts including accuracy of the facts, statements, citing resources, etc.

### (PS2148) PROGNOSTIC STRATIFICATION AND CLINICAL CHARACTERIZATION OF EPSTEIN-BARR VIRUS STATUS IN PERIPHERAL T-CELL LYMPHOMA: A RE-EVALUATION OF THE “LOW-RISK” PATIENT

**Topic:** 20. Other aggressive lymphomas

Rong Wei*[1], Shenmiao Yang[1]

[1]*Peking University People's Hospital, Peking University Institute of Hematology, National Clinical Research Center for Treatment of Hematologic Disease, Beijing, China;*

### Background

Epstein-Barr virus (EBV) infection is a known adverse factor in specific lymphoma subtypes, yet its distinct impact on the clinical course and risk stratification of peripheral T-cell lymphoma (PTCL) remains to be fully understood.

### Aims

This study aimed to characterize the clinicopathological features, immunological profiles, and prognostic significance of EBV infection in a cohort of newly diagnosed PTCL patients, with specific emphasis on its role in re-stratifying standard prognostic risk groups.

### Methods

We performed a retrospective analysis of 121 newly diagnosed PTCL patients admitted at Peking University Peoples' Hospital from January 2016 to December 2022. Patients were categorized based on EBV status. EBV-positive was defined as peripheral blood EBV-DNA > 500 copies/ml by PCR or tissue EBER positive by ISH. Clinical characteristics, laboratory index, and survival outcomes were compared.

### Results

Of the 121 patients, 39 (32.2%) were EBV-positive, with the phenotype predominantly observed in angioimmunoblastic T-cell lymphoma (AITL, 21 cases) and PTCL not otherwise specified (PTCL-NOS, 14 cases). Compared with the EBV-negative cohort, EBV-positive patients exhibited significantly more aggressive clinical features, including advanced Ann Arbor stage, higher IPI and PIT scores, and extensive nodal and extranodal involvement. Notably, EBV positivity was associated with profound systemic immunosuppression, evidenced by significantly lower serum IgA levels (p=0.033) and a higher incidence of cytomegalovirus (CMV) co-infection (15.4% vs. 2.4%, p=0.007). In survival analysis, EBV positivity was significantly associated with inferior progression-free survival (mPFS: 5.0 vs. 17.0 months, p=0.04). While overall survival did not differ significantly in the overall cohort (mOS 21.0 vs. 47.0 months, p=0.2464). Subgroup analysis revealed a critical divergence: among patients classified as “low-risk” (PIT score <2), EBV positivity significantly compromised both PFS (mPFS 8.0 vs. 101.0 months, p=0.0026) and OS (mOS 2.0 vs. 30.0 months, p<0.0001).

### Summary/Conclusion

EBV infection in PTCL defines a distinct biological subset characterized by high tumor burden, profound humoral immunodeficiency, and chemo-refractoriness. Crucially, EBV status effectively re-stratifies clinically “low-risk” (PIT score <2) patients into a poor-prognosis category, suggesting an underestimated role of EBV status in current risk assessment and the need for intensified or novel therapeutic strategies for the particular population.

**Copyright Information:** (Online) ISSN: 2572-9241
©2026 The Author(s). HemaSphere is published by John Wiley & Sons Ltd on behalf of European Hematology Association. This is an open access Abstract Book distributed under the Attribution-NonCommercial-NoDerivs (CC BY-NC-ND), which allows third parties to download the articles and share them with others as long as they credit the author and the Abstract Book, but they cannot change the content in any way or use them commercially.
**Abstract Book Citations:** Authors, Title, HemaSphere, 2026;10:(S1):pages. The abstract book DOIs can be found on the “Author Information” page.
**Disclaimer:** Articles published in the journal HemaSphere exclusively reflect the opinions of the authors. The authors are responsible for all content in their abstracts including accuracy of the facts, statements, citing resources, etc.

### (PS2149) HIGH PD-L1 EXPRESSION PREDICTS RELAPSE AND WORSE EVENT-FREE SURVIVAL IN PEDIATRIC PATIENTS DIAGNOSED WITH ANAPLASTIC LARGE CELL LYMPHOMA: A RETROSPECTIVE STUDY OF 117 PATIENTS

**Topic:** 20. Other aggressive lymphomas

Mariam Elsherif*[1], Asmaa Hamoda[1, 2], Hani Abdel Rahman Sayed[1, 2], Sally Talaat[3, 4], Abeer Mokhles[3, 5], Eman Khorshed[6, 7], Iman Zaky[8], Ahmed Mosa[9], Samah Semary[3, 5]

*[1]Children's Cancer Hospital Egypt,57357, Pediatric Oncology, Cairo, Egypt;[2]National Cancer Institute, Cairo University, Pediatric Oncology, Cairo, Egypt; [3]Children's Cancer Hospital Egypt,57357, Cairo, Egypt;[4]National Cancer Institute, Cairo University, Cairo, Egypt; [5]Faculty of Medicine, Beni-Seuf University, Cairo, Egypt;[6]National Cancer Institute, Cairo University, Surgical Pathology, Cairo, Egypt; [7]Children's Cancer Hospital Egypt,57357, Surgical Pathology, Cairo, Egypt;[8]Children's Cancer Hospital Egypt,57357, Radiology, Cairo, Egypt; [9]Children's Cancer Hospital Egypt,57357, Clinical Research and Biostatistics, Cairo, Egypt;*

### Background

Pediatric anaplastic large cell lymphoma (ALCL) is generally chemosensitive, but 25–35% relapse. High PD-L1 expression has been implicated in poorer outcomes and may identify patients who need novel therapies.

### Aims

To identify prognostic factors for relapse in pediatric ALCL, focusing on the incidence and impact of PD-L1 expression (H-score and TPS) on disease progression and survival.

### Methods

This was a retrospective cohort of 117 pediatric patients diagnosed with ALCL patients (median age 10.3 years; 87% ALK-positive). PD-L1 was quantified by immunohistochemistry using H-score and TPS; predefined cutoffs were H-score ≥3.5 and TPS ≥17.5% . Primary endpoints: relapse incidence, event-free survival (EFS) and overall survival (OS). Survival probabilities and cumulative incidence of relapse (CIR) were estimated; group comparisons reported with p-values. ROC analysis evaluated predictive performance of PD-L1 thresholds.

### Results

Of 117 pediatric ALCL patients, high PD-L1 expression (H-score ≥3.5 and/or TPS ≥17.5) was observed in 65–70% of patients, and 26/117 (22%) experienced relapse. The Five-year OS and EFS of the entire cohort was 83% (95% CI 76–90%) and 72.4% (SE 0.042) respectively. High PD-L1 by H-score (≥3.5) was associated with higher relapse risk (28% vs 12%; p=0.05) and significatly worse 5-year EFS (66.6% vs 82.9%, p=0.04). Similarly, TPS ≥17.5% was linked to increase risk of relapse (27% vs 9.1%; p=0.032) and inferior 5-year EFS (66.2% vs 87.9%, p=0.01). Patients with high PDL1 expression had significantly higher CIR at 100 months as compared with those with low H-score (<3.5) and/or TPS (<17.5) (H score; CIR was 29.7% vs 12.2% p=0.04 ; TPS score CIR 29.1% vs 9.1%, p=0.03). ROC thresholds showed reasonable sensitivity and high negative predictive value for relapse (H-score ≥3.5: sensitivity 80.8%, NPV 87.8%; TPS ≥17.5: sensitivity 88.5%, NPV 90.9%). Most patients were ALK-positive and baseline stage distribution, age, and sex did not differ significantly by PD-L1 status. Among relapsed cases, progression-free survival from first relapse was poor (5-year PFS ~31%). Moreover patients that experienced progression and/or a second relapse had high PD-L1 expression.

**Copyright Information:** (Online) ISSN: 2572-9241
©2026 The Author(s). HemaSphere is published by John Wiley & Sons Ltd on behalf of European Hematology Association. This is an open access Abstract Book distributed under the Attribution-NonCommercial-NoDerivs (CC BY-NC-ND), which allows third parties to download the articles and share them with others as long as they credit the author and the Abstract Book, but they cannot change the content in any way or use them commercially.
**Abstract Book Citations:** Authors, Title, HemaSphere, 2026;10:(S1):pages. The abstract book DOIs can be found on the “Author Information” page.
**Disclaimer:** Articles published in the journal HemaSphere exclusively reflect the opinions of the authors. The authors are responsible for all content in their abstracts including accuracy of the facts, statements, citing resources, etc.

**Summary/Conclusion**

Elevated PD-L1 expression level (H-score ≥3.5 or TPS ≥17.5%) was associated with significantly higher cumulative relapse and inferior EFS. PD-L1 IHC showed high sensitivity and NPV for predicting relapse, supporting its potential role as a prognostic biomarker to stratify patients for intensified or immunotherapy-directed approaches. Prospective validation is warranted.

**Copyright Information:** (Online) ISSN: 2572-9241
©2026 The Author(s). HemaSphere is published by John Wiley & Sons Ltd on behalf of European Hematology Association. This is an open access Abstract Book distributed under the Attribution-NonCommercial-NoDerivs (CC BY-NC-ND), which allows third parties to download the articles and share them with others as long as they credit the author and the Abstract Book, but they cannot change the content in any way or use them commercially.
**Abstract Book Citations:** Authors, Title, HemaSphere, 2026;10:(S1):pages. The abstract book DOIs can be found on the "Author Information" page.
**Disclaimer:** Articles published in the journal HemaSphere exclusively reflect the opinions of the authors. The authors are responsible for all content in their abstracts including accuracy of the facts, statements, citing resources, etc.

### (PS2150) PROVISIONAL PROGNOSTIC SCORE FOR HEPATOSPLENIC T-CELL LYMPHOMA

**Topic:** 20. Other aggressive lymphomas

Philip Haddad*[1], Supriya Gupta[2], Christopher Graham[2]

*[1]LSUHSC-S/Overton Brooks VAMC, Hematology-Oncology, Shreveport, United States of America; [2]University of Minnesota, Minneapolis, United States of America;*

### Background

Hepatosplenic T-cell lymphoma (HSTCL) is a rare, highly aggressive peripheral T-cell lymphoma characterized by sinusoidal infiltration of the liver, spleen, and bone marrow, severe cytopenias, and poor survival outcomes. Despite advances in molecular characterization and transplant-based treatments, patient outcomes remain heterogeneous. Conventional prognostic indices for aggressive lymphomas demonstrate limited discriminatory power in HSTCL, highlighting the necessity for a disease-specific risk stratification model.

### Aims

This study aims to identify independent clinicopathologic predictors of overall survival (OS) and to develop a pragmatic provisional prognostic score for risk stratification in HSTCL.

### Methods

A pooled retrospective database comprising 526 HSTCL cases was analyzed. Data collected included demographics, clinical presentation, laboratory parameters, immunophenotype, molecular features, treatment modalities, and survival outcomes. Complete survival data were available for 403 patients. Kaplan–Meier estimates, log-rank tests, and Cox proportional hazards models were employed to assess predictors of OS.

### Results

The median overall survival for the cohort was 12 months. Univariate analysis identified male sex ($p=0.004$), hemoglobin (Hgb) levels below 8 g/dL ($p=0.01$), underlying immunosuppression ($p<0.0001$), and biclonal T-cell receptor (TCR) subtype ($p=0.01$) as factors associated with inferior OS. On multivariable analysis, only Hgb <8 g/dL ($p=0.006$) and immunosuppression ($p=0.02$) remained independently associated with mortality. A 2-point score (1 point each for Hgb <8 g/dL and immunosuppression) stratified patients into low- (0), intermediate- (1), and high-risk (2) groups with median OS of 26 months, 5 months, and 3 months, respectively (log-rank $p<0.0001$). The Cox-based model demonstrated good discrimination (Harrell C-index 0.706; bootstrap 95% CI, 0.671–0.742) with negligible optimism (optimism-corrected C-index 0.706). Time-dependent discrimination was strongest early, with IPCW AUCs of 0.834 at 6 months, 0.799 at 12 months, and 0.753 at 24 months. Calibration was preserved across clinically relevant horizons. Decision curve analysis showed net benefit across a broad range of threshold probabilities relative to treat-all or treat-none strategies.

### Summary/Conclusion

We propose a simple, disease-specific provisional prognostic score based on severe anemia and underlying immunosuppression. This model effectively stratifies patients into clinically meaningful risk groups and may guide therapeutic decision-making. Prospective validation is warranted.

**Copyright Information:** (Online) ISSN: 2572-9241
©2026 The Author(s). HemaSphere is published by John Wiley & Sons Ltd on behalf of European Hematology Association. This is an open access Abstract Book distributed under the Attribution-NonCommercial-NoDerivs (CC BY-NC-ND), which allows third parties to download the articles and share them with others as long as they credit the author and the Abstract Book, but they cannot change the content in any way or use them commercially.
**Abstract Book Citations:** Authors, Title, HemaSphere, 2026;10:(S1):pages. The abstract book DOIs can be found on the "Author Information" page.
**Disclaimer:** Articles published in the journal HemaSphere exclusively reflect the opinions of the authors. The authors are responsible for all content in their abstracts including accuracy of the facts, statements, citing resources, etc.

### (PS2151) REAL-WORLD DURABLE CLINICAL BENEFITS OF TIRABRUTINIB AND ITS SAFETY PROFILE IN RELAPSED OR REFRACTORY PRIMARY CENTRAL NERVOUS SYSTEM LYMPHOMA: INTERIM ANALYSIS FROM THE REVEAL STUDY

**Topic:** 20. Other aggressive lymphomas

Chieh-Lung Cheng[1], Tsai Yun Chen[2], Ming-Chung Kuo[3], Su Peng Yeh[4], Hung-Lin Liu[5], Wan-Hsuan Hsu[6], Liang-Tsai Hsiao*[7]

*[1]National Taiwan University Hospital, Division of Hematology, Department of Internal Medicine, Taipei, Taiwan;[2]National Cheng Kung University Hospital, Division of Hematology, Department of Internal Medicine, Tainan, Taiwan; [3]Chang Gung Memorial Hospital, Linkou Branch, Division of Hematology, Department of Internal Medicine, Taoyuan, Taiwan; [4]China Medical University Hospital, Division of Hematology and Oncology, Department of Internal Medicine, Taichung, Taiwan; [5]Chang Gung Memorial Hospital, Kaohsiung Branch, Division of Hematology and Oncology, Department of Internal Medicine, Kaohsiung, Taiwan; [6]Ono Pharma Taiwan Co., Ltd., Medical Affairs, Taipei, Taiwan; [7]Taipei Veterans General Hospital, Division of Hematology, Department of Internal Medicine, Taipei, Taiwan;*

### Background

Tirabrutinib is a potent, second-generation, highly selective Bruton's tyrosine kinase inhibitor approved in Taiwan for relapsed or refractory (r/r) primary central nervous system lymphoma (PCNSL) based on ONO-4059-02. While clinical trials are promising, real-world evidence regarding the durability of clinical benefit and safety management patterns in routine practice is essential to consolidate its therapeutic role.

### Aims

We conducted the first nationwide study (the REVEAL study; NCT06714370) in Taiwan to evaluate the real-world effectiveness of tirabrutinib, focusing on response characteristics, and its safety profile.

### Methods

This REVEAL study enrolled patients aged ≥18 years with r/r PCNSL receiving NHI (National Health Insurance)-reimbursed tirabrutinib monotherapy across 6 medical centers. Efficacy endpoints included objective response rate (ORR), time-to-response (TTR), and duration of response (DOR). Safety endpoints included incidence of adverse events (AEs).

**Copyright Information:** (Online) ISSN: 2572-9241
©2026 The Author(s). HemaSphere is published by John Wiley & Sons Ltd on behalf of European Hematology Association. This is an open access Abstract Book distributed under the Attribution-NonCommercial-NoDerivs (CC BY-NC-ND), which allows third parties to download the articles and share them with others as long as they credit the author and the Abstract Book, but they cannot change the content in any way or use them commercially.
**Abstract Book Citations:** Authors, Title, HemaSphere, 2026;10:(S1):pages. The abstract book DOIs can be found on the "Author Information" page.
**Disclaimer:** Articles published in the journal HemaSphere exclusively reflect the opinions of the authors. The authors are responsible for all content in their abstracts including accuracy of the facts, statements, citing resources, etc.

**Results**

At the data cutoff (September 2025) for the first interim analysis, 19 patients were enrolled. With median follow-up of 10.9 months, tirabrutinib provided promising effectiveness with an ORR of 63.2% (12/19 [complete response in 3, unconfirmed CR in 1, partial response in 8 patients]). The response was characterized by a rapid onset, with a median TTR of 2.2 months. Notably, treatment responses were resilient: among 12 responders, 10 maintained their response during the follow-up period, resulting in a cumulative durable response rate of 83.3% (95% CI: 51.6–97.9). The median DOR has not yet been reached (lower bound 95%CI: 10.1 months).
Swimmer plot revealed unique real-world patterns where 9 patients (47.4%) derived continued clinical benefit from ongoing treatment despite initial radiographic assessments of stable disease or initial progression, highlighting that symptomatic stability often guides treatment continuation beyond imaging results.
The safety profile was manageable (treatment-emergent AEs 73.7%); common events were urinary tract infection (21.1%), white blood cell count decrease (15.8%), and rash (15.8%). No new safety signals or significant cardiovascular toxicities were observed. Treatment-related AEs (TRAEs) occurred in 57.9% of patients with the majority of onsets occurring within the first 3 months. Treatment-related serious AEs occurred in 2 patients (urinary tract infection in 1 and cerebral haemorrhage in 1 patient). TRAEs were mostly transient; specifically, skin rashes resolved with a median time of 11.5 days. Two Grade 5 events were reported but assessed as unrelated to the treatment.

**Summary/Conclusion**

Tirabrutinib provides rapid and durable treatment responses over a 10.9-month follow-up period in r/r PCNSL patients. These results, along with high cumulative durability and a consistent safety profile, support its role as a key targeted therapy in real-world clinical practice in Taiwan.

**Copyright Information:** (Online) ISSN: 2572-9241
©2026 The Author(s). HemaSphere is published by John Wiley & Sons Ltd on behalf of European Hematology Association. This is an open access Abstract Book distributed under the Attribution-NonCommercial-NoDerivs (CC BY-NC-ND), which allows third parties to download the articles and share them with others as long as they credit the author and the Abstract Book, but they cannot change the content in any way or use them commercially.
**Abstract Book Citations:** Authors, Title, HemaSphere, 2026;10:(S1):pages. The abstract book DOIs can be found on the "Author Information" page.
**Disclaimer:** Articles published in the journal HemaSphere exclusively reflect the opinions of the authors. The authors are responsible for all content in their abstracts including accuracy of the facts, statements, citing resources, etc.

### (PS2152) EXCEPTIONAL EFFICACY AND MANAGEABLE SAFETY OF GOLIDOCITINIB COMBINED WITH CHOP AS FIRST-LINE TREATMENT FOR PERIPHERAL T-CELL LYMPHOMA (JACKPOT52): PRELIMINARY RESULTS FROM A PHASE I/II STUDY

**Topic:** 20. Other aggressive lymphomas

He Li*[1], Jinrong Yang[1], Pan Zhao[2], Ying Yuan[3], Yan Zeng[4], Li Zhang[5], Jing Tan[6], Jie Zhou[7], Tao Jiang[8], Shihua Huang[9], Fang Xu[10], Hongyuan Xing[11], Kaiji Zhang[12], Li Wei[13], Jian Xiao[14], Xiang Bing[1], Jie Wang[1], Ailin Zhao[1], Zhongwang Wang[1], Yang Dai[1], Pu Kuang[1], Jie Huang[1], Ting Niu[1, 15, 16]

*[1]Department of Hematology, Institute of Hematology,and Center for High Altitude Medicine, West China Hospital, Sichuan University, Chengdu, China; [2]Affiliated Hospital of North Sichuan Medical College, Nanchong, China;[3]Guangyuan Central Hospital, Guangyuan, China; [4]Chengdu Second People's Hospital, Chengdu, China;[5]Chengdu Seventh People's Hospital, Chengdu, China; [6]Chengdu Third People's Hospital, Chengdu, China;[7]Deyang People's Hospital, Deyang, China;[8]Sichuan Provincial People's Hospital, Chengdu, China; [9]The Second People's Hospital of Yibin, Yibin, China;[10]Mianyang Central Hospital, Mianyang, China; [11]The Affiliated Hospital of Southwest Medical University, Luzhou, China;[12]Chengdu First People's Hospital, Chengdu, China; [13]Chengdu Fifth People's Hospital, Chengdu, China; [14]The Fourth People's Hospital of Zigong, Zigong, China; [15]State Key Laboratory of Biotherapy, Collaborative Innovation Center of Biotherapy, West China Hospital, Sichuan University., Chengdu, China; [16]National Facility for Translational Medicine (Sichuan), West China Hospital, Sichuan University, Chengdu, China;*

### Background

Peripheral T-cell lymphoma (PTCL) is a heterogeneous group of aggressive non-Hodgkin lymphomas associated with poor prognosis. The current standard first-line regimen, CHOP (cyclophosphamide, doxorubicin, vincristine, and prednisone), often results in suboptimal complete response (CR) rates and limited long-term survival. Golidocitinib, a potent and highly selective JAK1 inhibitor, has demonstrated promising single-agent activity in relapsed/refractory settings. Strategies to improve frontline outcomes by integrating novel agents into the CHOP backbone are urgently needed.

### Aims

The JACKPOT52 study (ChiCTR2500107078) aims to evaluate the safety, tolerability, and recommended phase 2 dose (RP2D) of golidocitinib in combination with CHOP for previously untreated patients with PTCL. Secondary objectives include the assessment of preliminary antitumor activity, specifically Objective Response Rate (ORR) and Complete Response Rate (CRR).

### Methods

This is an ongoing, open-label, multicenter, Phase I/II study enrolling patients with newly diagnosed PTCL. A standard "3+3" dose-escalation design was utilized. Patients received oral golidocitinib (Cohort 1: 150 mg every other day; Cohort 2: 150 mg once daily QD) in combination with standard CHOP for 6 cycles, followed by Golidocitinib monotherapy maintenance for up to 2 years. Safety was assessed continuously, and tumor response was evaluated according to Lugano 2014 criteria. The data cut-off date for this analysis was February 5, 2026. Here we reportthe safety and efficacy data of both cohorts.

**Copyright Information:** (Online) ISSN: 2572-9241
©2026 The Author(s). HemaSphere is published by John Wiley & Sons Ltd on behalf of European Hematology Association. This is an open access Abstract Book distributed under the Attribution-NonCommercial-NoDerivs (CC BY-NC-ND), which allows third parties to download the articles and share them with others as long as they credit the author and the Abstract Book, but they cannot change the content in any way or use them commercially.
**Abstract Book Citations:** Authors, Title, HemaSphere, 2026;10:(S1):pages. The abstract book DOIs can be found on the “Author Information” page.
**Disclaimer:** Articles published in the journal HemaSphere exclusively reflect the opinions of the authors. The authors are responsible for all content in their abstracts including accuracy of the facts, statements, citing resources, etc.

### Results

As of the data cut-off, 15 patients were enrolled. In the dose-escalation phase, the combination of golidocitinib 150 mg once daily and CHOP (Cohort 2) was well-tolerated, with no dose-limiting toxicities (DLTs) observed. Consequently, golidocitinib 150 mg once daily was determined as the RP2D. Among the 15 patients evaluable for safety, the toxicity profile was manageable and comparable to standard CHOP. The most common Grade ≥3 treatment-related adverse events (TRAEs) were neutropenia (33.3%), leukopenia (26.7%), and thrombocytopenia (6.7%). Grade 3 infections included pneumonia (13.3%) and CMV infection (13.3%), which were resolved with standard management. Preliminary efficacy was evaluable in 9 patients at the RP2D level. The regimen demonstrated encouraging antitumor activity with an ORR of 88.9% (8/9) and a high CRR of 66.7% (6/9). Two additional patients with PR only completed the 3-cycle treatment and a deeper response is anticipated at the end of 6-cycle treatment. Furthermore, the response was rapid, all 6 patients who achieved CR reached deep response at the first interim assessment (after 2-4 cycles).

### Summary/Conclusion

The combination of golidocitinib 150 mg QD and CHOP demonstrates a manageable safety profile and promising efficacy in the first-line treatment of PTCL. The high rate of rapid Complete Responses observed in this preliminary analysis highlights the synergistic potential of this regimen to improve the current standard of care. Expansion cohorts are ongoing to further validate these findings.

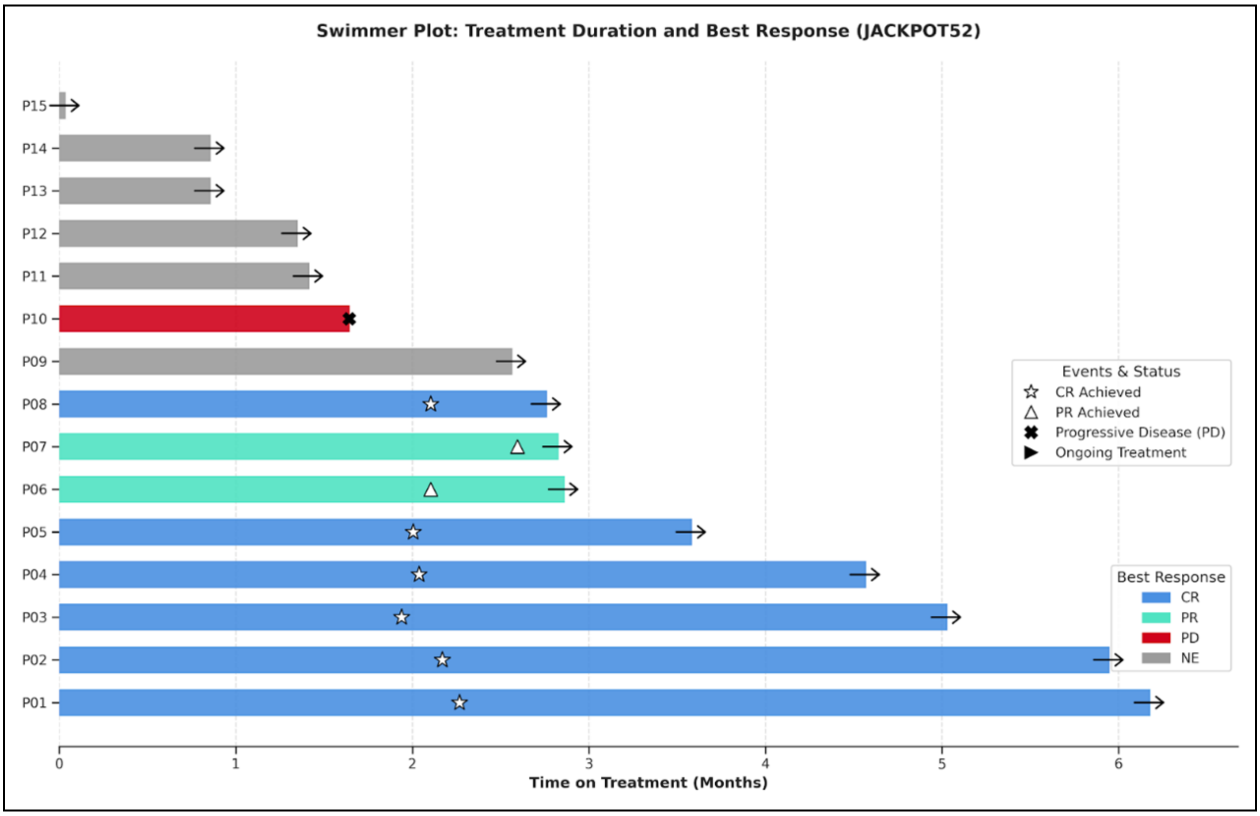

**Copyright Information:** (Online) ISSN: 2572-9241
©2026 The Author(s). HemaSphere is published by John Wiley & Sons Ltd on behalf of European Hematology Association. This is an open access Abstract Book distributed under the Attribution-NonCommercial-NoDerivs (CC BY-NC-ND), which allows third parties to download the articles and share them with others as long as they credit the author and the Abstract Book, but they cannot change the content in any way or use them commercially.
**Abstract Book Citations:** Authors, Title, HemaSphere, 2026;10:(S1):pages. The abstract book DOIs can be found on the "Author Information" page.
**Disclaimer:** Articles published in the journal HemaSphere exclusively reflect the opinions of the authors. The authors are responsible for all content in their abstracts including accuracy of the facts, statements, citing resources, etc.

### (PS2153) BRENTUXIMAB VEDOTIN WITH CHEMOTHERAPY IN FRONTLINE TREATMENT FOR CHINESE PATIENTS WITH CD30-POSITIVE PERIPHERAL T-CELL LYMPHOMA

**Topic:** 20. Other aggressive lymphomas

Yuqin Song*[1], Rong Tao[2], Zengjun Li[3], Fei Li[4], Wenyu Li[5], Xudong Wei[6], Chenlu Yang[7], Ou Bai[8], Jianzhen Shen[9], Zhengming Jin[10], Zhengzi Qian[11], Qiying Li[12], Hongmei Jing[13], Jie Jin[14], Meng Ji[15], Hui Wang[15], Wenhan Jiang[15], Jun Zhu[1]

*[1]Department of Lymphoma, Peking University Cancer Hospital, Beijing, China;[2]Department of Lymphoma, Fudan University Shanghai Cancer Center, Shanghai, China; [3]Department of Lymphoma and Hematology, Cancer Hospital of Shandong First Medical University, Shandong Cancer Institute, Shandong Cancer Hospital, Jinan, China; [4]Department of Hematology, The First Affiliated Hospital of Nanchang University, Nanchang, China; [5]Department of Hematology, Guangdong Provincial People's Hospital, Guangzhou, China; [6]Department of Hematology and Oncology, Henan Cancer Hospital, Affiliated Cancer Hospital of Zhengzhou University, Zhengzhou, China; [7]Department of Haematology, West China School of Medicine, West China Hospital of Sichuan University, Chengdu, China; [8]Department of Hematology, The First Hospital of Jilin University, Changchun, China; [9]Department of Hematology, Fujian Medical University Union Hospital, Fuzhou, China;[10]Department of Hematology, The First Affiliated Hospital of Soochow University, Suzhou, China; [11]Department of Lymphoma, Tianjin Medical University Cancer Institute & Hospital, Tianjin, China; [12]Department of Hematology-Oncology, Chongqing Key Laboratory for the Mechanism and Intervention of Cancer Metastasis, Chongqing University Cancer Hospital, Chongqing, China; [13]Department of Hematology, Peking University Third Hospital, Beijing, China; [14]Department of Haematology, The First Affiliated Hospital, Zhejiang University School of Medicine, Hangzhou, China; [15]Takeda APAC Biopharmaceutical Research and Development Company Limited, Shanghai, China;*

### Background

Peripheral T-cell lymphoma (PTCL) represents a heterogeneous group of aggressive non-Hodgkin lymphoma, with various CD30 expressions across subtypes. Brentuximab vedotin (BV), a CD30-directed ADC, in combination with cyclophosphamide, doxorubicin, and prednisone (BV+CHP), has been approved for adult patients with previously untreated CD30-positive (CD30+) PTCL globally based on the ECHELON-2 trial. However, the efficacy and safety of BV+CHP in the Chinese PTCL patients has not been explored.

### Aims

We aim to evaluate the efficacy and safety of BV+CHP in previously untreated adult Chinese participants with CD30+ PTCL.

### Methods

C25024 (NCT05673785) was a single-arm, open-label, multicenter phase 2 trial conducted in China. Eligible participants were Chinese adults aged ≥18 years with newly diagnosed CD30+ PTCL and ECOG performance status ≤2 and assigned to receive 6-8 cycles of BV+CHP. The primary endpoints were overall response rate (ORR) at EOT as assessed by independent review facility (IRF) and adverse events (AEs). The secondary endpoints were complete response (CR) rate, 1-year PFS and OS rates, time to response, and duration of response.

**Copyright Information:** (Online) ISSN: 2572-9241
©2026 The Author(s). HemaSphere is published by John Wiley & Sons Ltd on behalf of European Hematology Association. This is an open access Abstract Book distributed under the Attribution-NonCommercial-NoDerivs (CC BY-NC-ND), which allows third parties to download the articles and share them with others as long as they credit the author and the Abstract Book, but they cannot change the content in any way or use them commercially.
**Abstract Book Citations:** Authors, Title, HemaSphere, 2026;10:(S1):pages. The abstract book DOIs can be found on the "Author Information" page.
**Disclaimer:** Articles published in the journal HemaSphere exclusively reflect the opinions of the authors. The authors are responsible for all content in their abstracts including accuracy of the facts, statements, citing resources, etc.

**Results**

As data cutoff of 25 October 2025, a total of 52 participants were included in the full analysis set. The median age was 53 years, with 55.8% being male. Nearly half (46.2%) participants had an ECOG PS of 1-2 and 42.3% had an IPI score of 2. The most common subtype of enrolled participants was systemic anaplastic large cell lymphoma (sALCL; ALK-negative [ALK-] 42.3%, ALK-positive [ALK+ 26.9%), followed by angioimmunoblastic T-cell lymphoma (AITL; 19.2%) and PTCL not otherwise specified (PTCL-NOS; 11.5%).
Fifty participants received either 6 (50.0%) or 8 (46.2%) treatment cycles according to plan, with a median of 6 cycles of BV+CHP at a median dose intensity of 1.8 mg/kg/cycle for BV.
By IRF assessment per the Cheson criteria, the ORR and CR rate at EOT were 96.2% and 75%, respectively, with similar results reported in the ALK+ sALCL (100%; 71.4%), ALK- sALCL (95.5%; 72.7%), AITL (90.0%; 90.0%), and PTCL-NOS (100%; 66.7%) subgroups. High response rates were also observed when assessed by IRF per the Lugano criteria.
The median PFS was 26.58 months at a follow-up of 21.03 months and the 1-year PFS rate was 79.69% by IRF assessment per the Cheson criteria. Similar result of 1-year PFS rate of 83.67% was assessed by IRF according to the Lugano criteria. The median OS was not estimable at a follow-up of 18.17 months and the 1-year OS rate was 93.88%.
TEAEs were observed in all participants, with 76.9% experiencing ≥grade 3 TEAEs. Pneumonia was the most frequent SAE (23.1%), followed by febrile neutropenia and neutrophil count decrease (5.8% for each). Peripheral neuropathy was reported in 34.6% of participants; neutropenia, anemia, and thrombocytopenia occurred in 76.9%, 69.2%, and 42.3%, respectively. TEAEs leading to BV discontinuation were reported in 2 patients, grade 4 haemophagocytic lymphohistiocytosis and grade 3 peripheral neuropathy for each. No on-study deaths were reported in the trial.

**Summary/Conclusion**

This trial demonstrates favorable efficacy and well-tolerable safety of A+CHP in Chinese adult participants with newly diagnosed CD30+ PTCL, consistent with the global ECHELON-2 trial. Moreover, consistently high response rates are observed across subtypes, including AITL and PTCL-NOS. These findings underscore a new treatment option of BV+CHP as frontline therapy for patients with CD30+ PTCL in China.

**Copyright Information:** (Online) ISSN: 2572-9241
©2026 The Author(s). HemaSphere is published by John Wiley & Sons Ltd on behalf of European Hematology Association. This is an open access Abstract Book distributed under the Attribution-NonCommercial-NoDerivs (CC BY-NC-ND), which allows third parties to download the articles and share them with others as long as they credit the author and the Abstract Book, but they cannot change the content in any way or use them commercially.
**Abstract Book Citations:** Authors, Title, HemaSphere, 2026;10:(S1):pages. The abstract book DOIs can be found on the “Author Information” page.
**Disclaimer:** Articles published in the journal HemaSphere exclusively reflect the opinions of the authors. The authors are responsible for all content in their abstracts including accuracy of the facts, statements, citing resources, etc.

**Table 1: Efficacy and safety results**

| Endpoints | FAS (N=52) |
|---|---|
| Efficacy according to Lugano 2014 criteria* | |
| ORR at EOT, % (95% CI) | 92.3 (81.5, 97.9) |
| CR at EOT, % (95% CI) | 76.9 (63.2, 87.5) |
| TTR, median (min, max), months | 2.73 (2.56, 6.08) |
| DOR, median (95% CI), months# | 23.95 (15.18, NE) |
| PFS, median (95% CI), months | 26.58 (18.10, NE) |
| PFS follow-up, median (min, max), months | 20.93 (0.03+, 30.06+) |
| 1-year PFS, % (95% CI) | 83.67 (69.95, 91.48) |
| Safety | |
| TEAEs, n (%) | 52 (100) |
| White blood cell count decreased | 38 (73.1) |
| Anemia | 35 (67.3) |
| Neutrophil count decreased | 35 (67.3) |
| Lymphocyte count decreased | 27 (51.9) |
| Alanine aminotransferase increased | 26 (50.0) |
| Aspartate aminotransferase increased | 22 (42.3) |
| Platelet count decreased | 21 (40.4) |
| Grade ≥3 TEAEs, n (%) | 40 (76.9) |
| Neutrophil count decreased | 26 (50.0) |
| White blood cell count decreased | 26 (50.0) |
| Lymphocyte count decreased | 15 (28.8) |
| Pneumonia | 13 (25.0) |
| SAEs, n (%) | 18 (34.6) |
| Pneumonia | 12 (23.1) |
| Febrile neutropenia | 3 (5.8) |
| Neutrophil count decreased | 3 (5.8) |

*Assessed by IRF per the Lugano 2014 criteria; #Assessed by INV per the Lugano 2014 criteria; + A censored observation. Safety is reported using Medical Dictionary for Regulatory Activities (MedDRA) Version 28.0 by Preferred Term.

FAS, full analysis set; CI, confidence interval; NE, not estimable; ORR, overall response rate; CR, complete response; TTR, time to response; DOR, duration of response; PFS, progression-free survival; TEAEs, treatment-emergent adverse events; SAEs, serious adverse events; IRF, independent review facility; INV, investigator assessment.

Copyright Information: (Online) ISSN: 2572-9241
©2026 The Author(s). HemaSphere is published by John Wiley & Sons Ltd on behalf of European Hematology Association. This is an open access Abstract Book distributed under the Attribution-NonCommercial-NoDerivs (CC BY-NC-ND), which allows third parties to download the articles and share them with others as long as they credit the author and the Abstract Book, but they cannot change the content in any way or use them commercially.
Abstract Book Citations: Authors, Title, HemaSphere, 2026;10:(S1):pages. The abstract book DOIs can be found on the "Author Information" page.
Disclaimer: Articles published in the journal HemaSphere exclusively reflect the opinions of the authors. The authors are responsible for all content in their abstracts including accuracy of the facts, statements, citing resources, etc.

### (PS2154) HIGH PRETREATMENT ABSOLUTE LYMPHOCYTE COUNT PREDICTS INFERIOR OUTCOMES IN NK/T-CELL LYMPHOMA PATIENTS TREATED WITH PD-1 INHIBITORS: DEVELOPMENT AND VALIDATION OF A NOVEL PROGNOSTIC MODEL

**Topic:** 20. Other aggressive lymphomas

Xindi Liu*[1], Na Yao[1], Liang Wang[1]

[1]*Beijing Tongren Hospital, Capital Medical University, Beijing, China;*

### Background

Natural killer/T-cell lymphoma (NKTCL) is an aggressive non-Hodgkin lymphoma with poor prognosis, particularly in relapsed/refractory cases. While PD-1 inhibitors have emerged as promising therapeutic options with objective response rates around 70%, significant heterogeneity in treatment response necessitates reliable biomarkers for patient stratification. Absolute lymphocyte count (ALC), a readily accessible marker of systemic immune status, has shown prognostic value in various malignancies. However, its predictive significance in the context of PD-1 inhibitor therapy for NKTCL remains unclear, with potential paradoxical effects given the unique immunobiology of Epstein-Barr virus (EBV)-associated lymphomas.

### Aims

To investigate the prognostic value of pretreatment ALC in NKTCL patients receiving PD-1 inhibitor combination therapy and to develop a clinically applicable prognostic scoring model for progression-free survival (PFS).

### Methods

We conducted a single-center retrospective analysis of 41 NKTCL patients treated with PD-1 inhibitors plus chemotherapy (P-GEMOX, GPED, or other regimens) at Beijing Tongren Hospital between June 2019 and June 2023. The optimal ALC cutoff was determined using maximally selected rank statistics. Cox proportional hazards regression was employed to identify independent prognostic factors. A novel prognostic score was constructed based on multivariate analysis results, with performance evaluated by receiver operating characteristic (ROC) curves, concordance index (C-index), and bootstrap validation (1,000 resamples), and compared against conventional Nomogram-revised Risk Index (NRI) and PINK scores using DeLong test.

### Results

The optimal ALC cutoff was $1.35\times10^9$/L. Contrary to conventional wisdom, high ALC ($\geq1.35\times10^9$/L) was associated with significantly inferior PFS and overall survival (OS) (both $P<0.001$). Median PFS was 216 days in high-ALC group versus not reached in low-ALC group; median OS was 343 days versus not reached, respectively. Multivariate analysis confirmed ALC$\geq1.35\times10^9$/L as an independent adverse prognostic factor for PFS (HR=3.185, 95% CI: 1.146-8.851, $P=0.026$), alongside age >55 years, relapsed/refractory disease, and Ann Arbor stage III-IV.
The novel prognostic model incorporating these four variables (scoring: age >55=1 point; ALC$\geq1.35\times10^9$/L=1 point; stage III-IV=2 points; relapsed/refractory=2 points; range 0-6) demonstrated superior discriminative ability with AUC of 0.924 (95% CI: 0.853-0.995) for PFS prediction, significantly outperforming NRI (AUC=0.545, $P<0.001$) and PINK scores (AUC=0.710, $P=0.005$). The C-index was 0.844 (bootstrap-corrected: 0.826, 95% CI: 0.716-0.885). Patients classified as low-risk (≤3 points) showed higher clinical benefit rate (83.3% vs 43.5%, $P=0.012$) compared to high-risk (>3 points) group.

**Copyright Information:** (Online) ISSN: 2572-9241
©2026 The Author(s). HemaSphere is published by John Wiley & Sons Ltd on behalf of European Hematology Association. This is an open access Abstract Book distributed under the Attribution-NonCommercial-NoDerivs (CC BY-NC-ND), which allows third parties to download the articles and share them with others as long as they credit the author and the Abstract Book, but they cannot change the content in any way or use them commercially.
**Abstract Book Citations:** Authors, Title, HemaSphere, 2026;10:(S1):pages. The abstract book DOIs can be found on the “Author Information” page.
**Disclaimer:** Articles published in the journal HemaSphere exclusively reflect the opinions of the authors. The authors are responsible for all content in their abstracts including accuracy of the facts, statements, citing resources, etc.

### Summary/Conclusion

Pretreatment ALC≥1.35×10⁹/L paradoxically predicts poor outcomes in NKTCL patients receiving PD-1 inhibitor therapy, likely reflecting EBV-driven dysfunctional lymphocyte proliferation rather than effective antitumor immunity. Our novel prognostic model enables accurate risk stratification using readily available clinical parameters, facilitating individualized treatment decisions in this challenging disease. External validation in larger, multicenter cohorts is warranted.

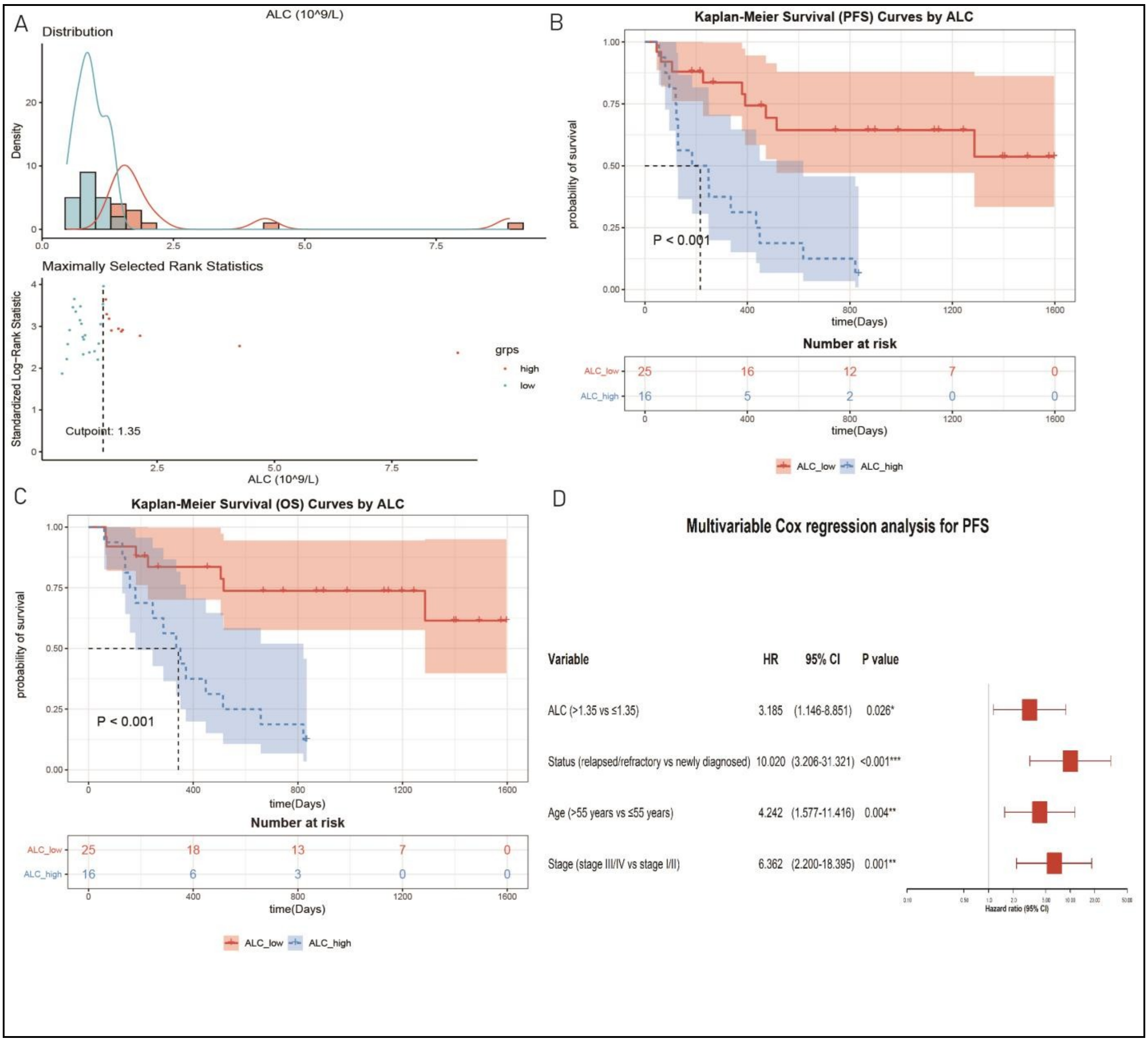

**Copyright Information:** (Online) ISSN: 2572-9241
©2026 The Author(s). HemaSphere is published by John Wiley & Sons Ltd on behalf of European Hematology Association. This is an open access Abstract Book distributed under the Attribution-NonCommercial-NoDerivs (CC BY-NC-ND), which allows third parties to download the articles and share them with others as long as they credit the author and the Abstract Book, but they cannot change the content in any way or use them commercially.
**Abstract Book Citations:** Authors, Title, HemaSphere, 2026;10:(S1):pages. The abstract book DOIs can be found on the "Author Information" page.
**Disclaimer:** Articles published in the journal HemaSphere exclusively reflect the opinions of the authors. The authors are responsible for all content in their abstracts including accuracy of the facts, statements, citing resources, etc.

### (PS2155) PRIMARY THYROID LYMPHOMA: CLINICAL FEATURES AND OUTCOME ANALYSIS IN A MULTICENTRIC RETROSPECTIVE ITALIAN STUDY

**Topic:** 20. Other aggressive lymphomas

Mariella Lo Schirico*[1], Marco Basso[2], Giorgia Marcato[1], Dario Marino[3], Chiara Pagani[4], Giovanni Rindone[5], Emanuele Ravano[6], Valentina Bozzoli[7], Tanja Lazic[8], Maurizio Cavallari[9], Giuseppina Greco[10], Salvatrice Mancuso[11], Samantha Pozzi[12], Michele Gottardi[1]

*[1]Onco Hematology Unit, Veneto Institute of Oncology IOV-IRCCS, Padua, Italy;[2]Pharmacy Unit, Veneto Institute of Oncology IOV-IRCCS, Padua, Italy; [3]Transplant Unit, Veneto Institute of Oncology IOV-IRCCS,, Padua, Italy;[4]Hematology Division ASST Spedali Civili Brescia, Brescia, Italy; [5]Clinica Ematologica, Fondazione IRCCS San Gerardo dei Tintori, Monza, Italy;[6]SC Ematologia, ASST Grande Ospedale Metropolitano Niguarda, Milano, Italy; [7]UO Ematologia e Trapianto di Cellule Staminali, Ospedale Vito Fazzi, ASL Lecce, Lecce, Italy; [8]Hematology Unit, Azienda Unità Sanitaria Locale-IRCCS, Reggio Emilia; Clinical and Experimental Medicine PhD Program, University of Modena and Reggio Emilia, Modena, Italy, Reggio Emilia, Italy; [9]Hematology Section, Ospedale dell'Angelo, Venice Mestre, Italy; [10]Hematology and Transplant Unit, Cardinal Panico Hospital, Tricase, Italy; [11]Division of Hematology, Department PROMISE, University of Palermo, Palermo, Italy;[12]Department of Medical and Surgical Science, University of Modena and Reggio Emilia, Cancer Center - Policlinico di Modena, Modena, Italy;*

### Background

Primary thyroid lymphoma (PTL) accounts for 1–5% of all thyroid malignancies and 1–2.5% of all lymphomas. The majority of PTLs are B-cell non-Hodgkin lymphomas (NHL), specifically diffuse large B-cell lymphoma (DLBCL) and marginal zone lymphoma (MZL). The therapeutic approach was once based solely on surgery, but since the introduction of effective chemotherapy (CT) regimens, the therapeutic paradigm is currently strictly determined by the histopathological subtype. Current literature, however, is often limited by data from the pre-rituximab era or non-Caucasian populations.

### Aims

To comprehensively describe epidemiological, clinical, and diagnostic features of PTL in a contemporary real-world cohort and to estimate overall survival (OS).

### Methods

We conducted a multicentric retrospective study on patients diagnosed with PTL between January 2005 and December 2025 across 11 Italian sites. PTL was defined as a lymphoma originating in the thyroid gland, with or without involvement of locoregional lymph nodes or contiguous extranodal sites. All histological subtypes according to the WHO classification were included.

**Copyright Information:** (Online) ISSN: 2572-9241
©2026 The Author(s). HemaSphere is published by John Wiley & Sons Ltd on behalf of European Hematology Association. This is an open access Abstract Book distributed under the Attribution-NonCommercial-NoDerivs (CC BY-NC-ND), which allows third parties to download the articles and share them with others as long as they credit the author and the Abstract Book, but they cannot change the content in any way or use them commercially.
**Abstract Book Citations:** Authors, Title, HemaSphere, 2026;10:(S1):pages. The abstract book DOIs can be found on the "Author Information" page.
**Disclaimer:** Articles published in the journal HemaSphere exclusively reflect the opinions of the authors. The authors are responsible for all content in their abstracts including accuracy of the facts, statements, citing resources, etc.

### Results

The cohort of 40 patients has a median age of 57 years (range: 25-83) at diagnosis, with a female predominance (66%). The histological subtypes were DLBCL (55%), MZL (13%), Hodgkin Lymphoma (HL, 7.5%), Follicular Lymphoma (FL, 7.5%), Burkitt Lymphoma (BL, 5%), lymphoproliferative disorder NOS (5%), High Grade B-cell Lymphoma (HGBCL, 5%), and Richter's syndrome (RS, 2.5%).

Most patients (n=26) had a clinical history of thyropathy, which in 17 cases was Hashimoto's thyroiditis. The median size of the tumoral lesion at diagnosis was 55 mm (range: 20-140); a quarter of patients presented with bulky disease (>7 cm). FDG-PET scans, performed in 85% of patients, showed an elevated median SUV max (21.7, range: 4.2-56) with no significant difference between histologies. ECOG performance status was 0-2 in all patients, while the majority of them (81%) were at low-risk according to the International Prognostic Index.

Treatment approaches consisted of surgery alone (20%), surgery and radiotherapy (RT) (2.5%), CT alone (38%) and CT combined with surgery and/or RT (40%). Thyroidectomy was performed in half of the patients: total in 14 cases, partial in 6.

Notably, 92.6% of aggressive histotypes received systemic therapy, compared to only 30% of indolent lymphomas ($p<0.001$), which primarily benefited from localized approaches. The preferred CT regimens were CHOP-like (24/31), except for BL and HGBCL that received high-dose CT; in all NHL patients CT was combined with rituximab. HL patients were treated with ABVD-like CT, also in combination.

The overall response rate was 95% with 36 CR and 2 PR, while it is not evaluable in 2 patients due to ongoing treatment at the time of the analysis. At a median follow-up of 36 months (range, 4-197), only one patient relapsed and died of lymphoma. Two other deaths were related to COVID-19 infection. Median OS was not reached (95% CI: 141.1–NA).

### Summary/Conclusion

PTL is a rare and heterogeneous disease. In our contemporary cohort, baseline FDG-PET scans showed elevated metabolic activity regardless of histological subtype. Treatment strategies followed a risk-adapted approach reflecting the specific histotype: systemic therapy for aggressive cases and localized treatment for indolent ones. Both strategies yielded excellent outcomes, with a 95% overall response rate and outstanding survival.

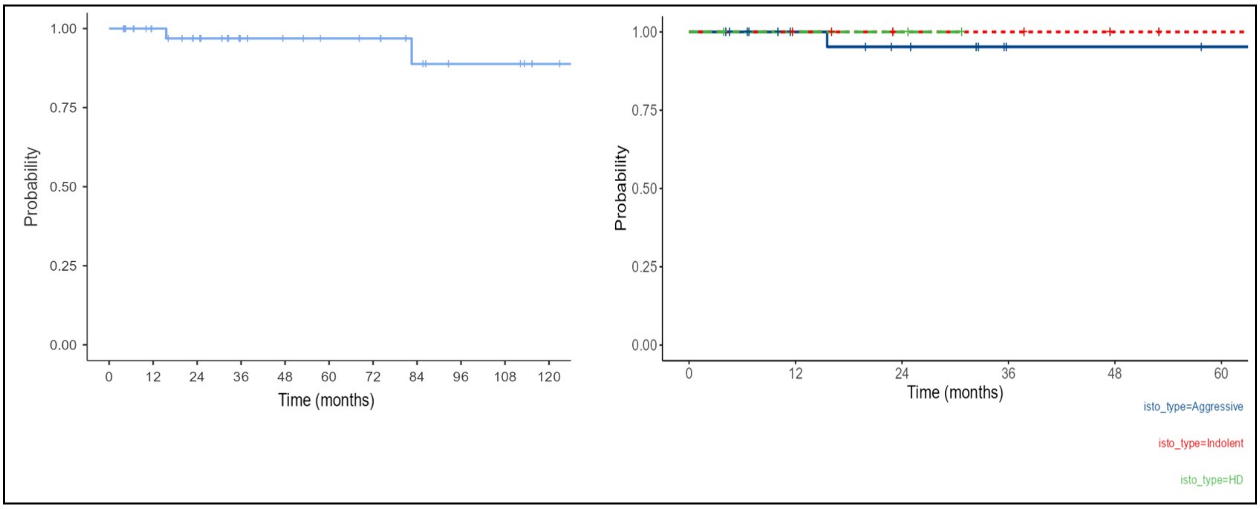

Figure 1A: OS of the whole cohort; Figure 1B: OS according to histotype

**Copyright Information:** (Online) ISSN: 2572-9241
©2026 The Author(s). HemaSphere is published by John Wiley & Sons Ltd on behalf of European Hematology Association. This is an open access Abstract Book distributed under the Attribution-NonCommercial-NoDerivs (CC BY-NC-ND), which allows third parties to download the articles and share them with others as long as they credit the author and the Abstract Book, but they cannot change the content in any way or use them commercially.
**Abstract Book Citations:** Authors, Title, HemaSphere, 2026;10:(S1):pages. The abstract book DOIs can be found on the "Author Information" page.
**Disclaimer:** Articles published in the journal HemaSphere exclusively reflect the opinions of the authors. The authors are responsible for all content in their abstracts including accuracy of the facts, statements, citing resources, etc.

### (PS2156) PREDICTORS OF CHEMORESISTANCE IN ANGIOIMMUNOBLASTIC T-CELL LYMPHOMA: A MULTICENTER RETROSPECTIVE STUDY FROM THREE FRENCH UNIVERSITY HOSPITALS

**Topic:** 20. Other aggressive lymphomas

Aurélia Chacon*[1], Vincent Delwail[1], Cecile Gruchet[1], Kamal Bouabdallah[2], Julie Abraham[3], Cecile Tomowiak[1], Celine Debiais Delpech[1], Marie Parrens[2], Xavier Leleu[1], Stephanie Guidez[1]

*[1]CHU de Poitiers, 86000, Poitiers, France;[2]CHU de Bordeaux, Bordeaux, France;[3]CHU de Limoges, Limoges, France;*

### Background

Angioimmunoblastic T-cell lymphoma (AITL) is a rare disease associated with a poor prognosis. Its clinical and biological presentations are heterogeneous, often leading to diagnostic delays. Standard therapeutic management consists of polychemotherapy, sometimes followed by consolidation with autologous stem cell transplantation (ASCT). However, therapeutic response remains highly variable, and limited data are available to identify the specific prognostic factors underlying the unfavorable outcomes in AITL.

### Aims

This study aimed to identify predictors of chemoresistance based on the clinico-biological profiles of patients with angioimmunoblastic T-cell lymphoma (AITL) and to determine optimal therapeutic strategies.

### Methods

We conducted a retrospective, multicenter observational study across three French university hospitals from January 2015 to August 2023. Patients were eligible if the diagnosis of AITL was histopathologically confirmed.

### Results

We analyzed 184 patients with a median age of 71 years (range: 32–92) and a male predominance (sex ratio 4:1). The median time from first symptoms to diagnosis was 2 months (range: 2–25). Most patients presented with advanced-stage disease, constitutional symptoms, and cytopenias. The PTCL-U prognostic score was 2 in the majority of cases. Over 80% of patients received CHOP-like chemotherapy. After a median follow-up of 52.4 months (95% CI: 44.7–59.8), the median overall survival (OS) was 22.8 months (95% CI: 17.7–40.5) and the median progression-free survival (PFS) was 6.5 months (95% CI: 5.4–8.6). More than 90% of patients met the "POD24" criteria. No significant difference in PFS was observed between CHOP-type first-line therapy and other regimens (p=0.06). Second-line treatments, including immunochemotherapy or non-anthracycline-based polychemotherapy, provided a survival benefit. Consolidation with ASCT in the first line or allogeneic stem cell transplantation at relapse showed an advantage in both OS and PFS. The presence of hemophagocytic lymphohistiocytosis (HLH) (p=0.025) and the concurrent expression of all five TFH markers (p=0.025) significantly impacted PFS.

### Summary/Conclusion

This study highlights that standard CHOP treatment did not improve survival compared to non-anthracycline-based regimens in our cohort. The use of these alternative treatments in the second line improved survival, suggesting they could be considered for first-line therapy. Furthermore, ASCT in first remission and allogeneic transplantation at relapse improved outcomes. Factors such as Performance Status (PS), POD24 status, and the presence of HLH at relapse influenced OS, while the presence of the five TFH markers appeared to improve PFS at relapse.

**Copyright Information:** (Online) ISSN: 2572-9241
©2026 The Author(s). HemaSphere is published by John Wiley & Sons Ltd on behalf of European Hematology Association. This is an open access Abstract Book distributed under the Attribution-NonCommercial-NoDerivs (CC BY-NC-ND), which allows third parties to download the articles and share them with others as long as they credit the author and the Abstract Book, but they cannot change the content in any way or use them commercially.
**Abstract Book Citations:** Authors, Title, HemaSphere, 2026;10:(S1):pages. The abstract book DOIs can be found on the "Author Information" page.
**Disclaimer:** Articles published in the journal HemaSphere exclusively reflect the opinions of the authors. The authors are responsible for all content in their abstracts including accuracy of the facts, statements, citing resources, etc.

### (PS2157) 12 YEARS UPDATE ON OVERALL SURVIVAL IN PATIENTS WITH PRIMARY CENTRAL NERVOUS SYSTEM LYMPHOMA (PCNSL) TREATED WITH HIGH-DOSE CHEMOTHERAPY AND TEMOZOLOMIDE

**Topic:** 20. Other aggressive lymphomas

Maher Salamoon*[1]

[1]*al bairouni university cancer center, hematology/oncology, Damascus, Syrian Arab Republic;*

### Background

Treatment of primary central nervous system lymphoma (PCNSL) is associated with low response rates and poor survival using conventional radio and chemotherapy. Due to its favorable toxicity profile, temozolomide has emerged as a new option for treatment of PCNSL in young patients. In this study, we report a series of PCNSL patients treated with an innovative regimen combining high dose of both cytarabine and methotrexate with temozolomide without radiotherapy or intrathecal chemotherapy.

### Aims

It is to evaluate the combination of Temozolomide, high dose of both Cytarabine and Methotrexate without radiotherapy. This study evaluates the overall survival after 12 years of publication of our first report.

### Methods

The study was performed at that time at Almwassat charity hospital and al Bairouni university cancer center. Forty patients with histologically confirmed PCNSL median age 52 years (range 20-65) years were included. Biopsies were cultured, and a karyotyping was made in 32 patients. An induction chemotherapy was started, and methotrexate 3 $gr/m^2$ over 12 h on day 1, cytarabine 3 $gr/m^2$ every 12 h on day 1 and temozolomide 150 $mg/m^2$ from day 2 through day 6 with a total of 6 cycles were given monthly. Patients were evaluated every 3 months by magnetic resonance (MRI) with contrast.

### Results

At that time in 2013, Among the 40 patients included in the study, a complete response was observed in 34 patients (85%) and a partial response in the remaining 6 patients (15%). Disease progressed in 8 out of 40 patients (20%) while 32 patients are still living at 5 years making the overall survival (OS) reach 77%. Grade II nephrotoxicity was observed in 2 patients while grade III and IV hematotoxicity were observed in 5 patients. After 12 years of follow-up, we documented 5 deaths, one of which was documented as a complication of Covid-19 in 2020 while four deaths were correlated to relapse treated with radiotherapy (whole brain irradiation) then chemotherapy accompanied with anti-CD20. The new statistical studies demonstrate an overall survival of 62.5% after 12 years of follow-up (95% CI: 57.8-66.3). all 25 alive patients are doing well with no cognitive disorders nor neurological symptoms related to the previous treatment before 2013.

### Summary/Conclusion

After the publication of our first report in 2013, treatment of PCNSL with high dose chemotherapy and Temozolomide seems to give a prolonged overall survival rate compared with other studies with minimal long-term toxicity.

**Copyright Information:** (Online) ISSN: 2572-9241
©2026 The Author(s). HemaSphere is published by John Wiley & Sons Ltd on behalf of European Hematology Association. This is an open access Abstract Book distributed under the Attribution-NonCommercial-NoDerivs (CC BY-NC-ND), which allows third parties to download the articles and share them with others as long as they credit the author and the Abstract Book, but they cannot change the content in any way or use them commercially.
**Abstract Book Citations:** Authors, Title, HemaSphere, 2026;10:(S1):pages. The abstract book DOIs can be found on the "Author Information" page.
**Disclaimer:** Articles published in the journal HemaSphere exclusively reflect the opinions of the authors. The authors are responsible for all content in their abstracts including accuracy of the facts, statements, citing resources, etc.

### (PS2158) SAFETY AND PRELIMINARY EFFICACY OF GOLIDOCITINIB COMBINED WITH POMALIDOMIDE IN RELAPSED OR REFRACTORY PERIPHERAL T-CELL LYMPHOMA (JACKPOT25): A MULTICENTER, PHASE I/II STUDY

**Topic:** 20. Other aggressive lymphomas

Peng Sun*[1], Panpan Liu[1], Hang Yang[1], Yu Wang[1], Chuanxu Liu[2, 3], Rong Tao[2, 3], Zhiming Li[1]

*[1]Department of Medical Oncology, State Key Laboratory of Oncology in South China, Collaborative Innovation Center for Cancer Medicine, Sun Yat-sen University Cancer Center, Guangzhou, People's Republic of China, Guangzhou, China; [2]Department of Lymphoma and Medical Oncology, Fudan University Shanghai Cancer Center, Shanghai, China; [3]Research Center for Lymphoma, Fudan University, Shanghai, China;*

### Background

Peripheral T-cell lymphoma (PTCL) is a heterogeneous group of aggressive non-Hodgkin lymphomas with poor prognosis, particularly in the relapsed or refractory (r/r) setting. Golidocitinib, a potent and highly selective JAK1 inhibitor, has demonstrated promising single-agent activity in PTCL. Pomalidomide, an immunomodulatory agent targeting cereblon (CRBN), modulates the tumor microenvironment. The synergistic potential of targeting the JAK/STAT pathway alongside immunomodulation warrants clinical investigation to improve outcomes in this heavily pre-treated population.

### Aims

This study (NCT06855823) aims to assess the safety, tolerability, recommended phase 2 dose (RP2D), and preliminary efficacy of golidocitinib in combination with pomalidomide in patients with r/r PTCL.

### Methods

JACKPOT25 is an ongoing, open-label, multicenter Phase I/II dose-escalation and expansion study. Eligible patients (age ≥18, ECOG PS ≤2) with R/R PTCL received continuous oral golidocitinib 150 mg QD, plus pomalidomide QD on days 1–21 of each 28-day cycle. A standard 3+3 design was used for pomalidomide dose escalation (2 mg, 3 mg, 4 mg). The primary endpoints were dose-limiting toxicities (DLTs) and recommended Phase 2 dose (RP2D) determination for Phase I, and objective response rate (ORR) for Phase II.

**Copyright Information:** (Online) ISSN: 2572-9241
©2026 The Author(s). HemaSphere is published by John Wiley & Sons Ltd on behalf of European Hematology Association. This is an open access Abstract Book distributed under the Attribution-NonCommercial-NoDerivs (CC BY-NC-ND), which allows third parties to download the articles and share them with others as long as they credit the author and the Abstract Book, but they cannot change the content in any way or use them commercially.
**Abstract Book Citations:** Authors, Title, HemaSphere, 2026;10:(S1):pages. The abstract book DOIs can be found on the “Author Information” page.
**Disclaimer:** Articles published in the journal HemaSphere exclusively reflect the opinions of the authors. The authors are responsible for all content in their abstracts including accuracy of the facts, statements, citing resources, etc.

**Results**

As of the data cut-off date on February 2, 2026, 14 patients were enrolled. Baseline characteristics indicated a high-risk population with poor prognosis. The median age was 64 years (range: 35-77), with a significant proportion of elderly patients. The majority of patients presented with advanced-stage disease (Ann Arbor Stage III/IV: 64.3%). Patients were heavily pre-treated, with a median of 2 prior lines of therapy (range: 1-5); notably, 28.6% of patients had received ≥3 prior lines of systemic therapy. In the dose-escalation phase, patients were treated at 2 mg and 3 mg Pomalidomide dose levels. At the 3 mg dose level, one dose-limiting toxicity (DLT) of Grade 4 thrombocytopenia occurred. Consequently, the combination of golidocitinib 150 mg and pomalidomide 3 mg was determined as the RP2D. Among the 13 patients evaluable for safety, treatment-related adverse events (TRAEs) of any grade occurred in 46.2% of patients. The most common TRAEs (≥20%) included thrombocytopenia (38.5%), lymphopenia (30.8%), neutropenia (23.1%), diarrhea (23.1%), and anemia (23.1%). Grade 3/4 TRAEs were reported in 30.8% of patients, primarily cytopenias, including lymphopenia (23.1%) and neutropenia (15.4%). Serious adverse events (SAEs) occurred in 15.4% of patients, including severe pneumonia. The safety profile was generally manageable with dose modifications and supportive care. Preliminary efficacy was evaluable in 9 patients. The Objective Response Rate (ORR) was 44.4% (4/9), including 2 Complete Responses (CR) and 2 Partial Responses (PR). The Disease Control Rate (DCR) was 55.6% (5/9). Notable responses were observed in patients with AITL subtypes (Figure 1). The longest duration of treatment was longer than 9 months.

**Summary/Conclusion**

Despite the poor baseline characteristics and heavy pre-treatment of the study population, the combination of golidocitinib and pomalidomide demonstrates a manageable safety profile and encouraging preliminary antitumor activity. The RP2D was established as golidocitinib 150 mg plus pomalidomide 3 mg. These findings support the ongoing Phase II expansion to further evaluate this chemotherapy-free regimen in this difficult-to-treat population.

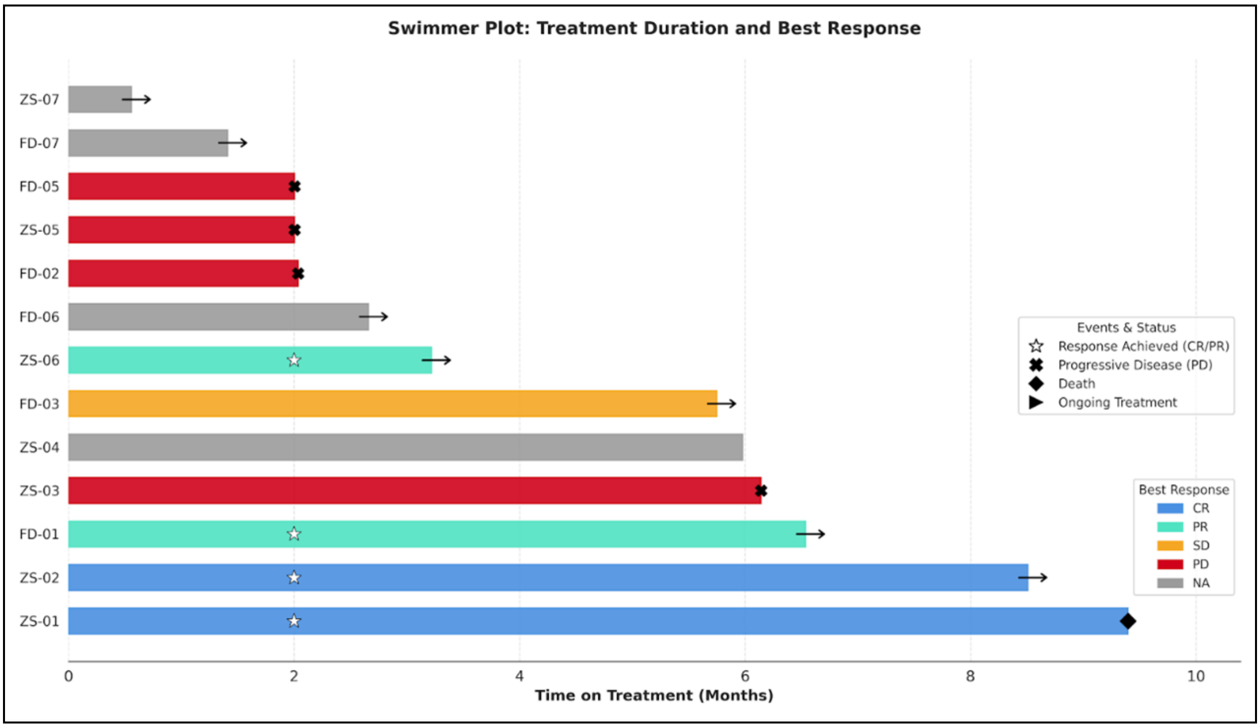

**Copyright Information:** (Online) ISSN: 2572-9241
©2026 The Author(s). HemaSphere is published by John Wiley & Sons Ltd on behalf of European Hematology Association. This is an open access Abstract Book distributed under the Attribution-NonCommercial-NoDerivs (CC BY-NC-ND), which allows third parties to download the articles and share them with others as long as they credit the author and the Abstract Book, but they cannot change the content in any way or use them commercially.
**Abstract Book Citations:** Authors, Title, HemaSphere, 2026;10:(S1):pages. The abstract book DOIs can be found on the "Author Information" page.
**Disclaimer:** Articles published in the journal HemaSphere exclusively reflect the opinions of the authors. The authors are responsible for all content in their abstracts including accuracy of the facts, statements, citing resources, etc.

### (PS2159) CLINICAL FEATURES, TREATMENTS AND OUTCOME OF PLASMABLASTIC LYMPHOMA: RESULTS FROM A PRELIMINARY ANALYSIS OF THE RETROSPECTIVE INTERNATIONAL PLALY STUDY ON BEHALF OF FIL, LEO/MER REGISTRY AND PLRG

**Topic:** 20. Other aggressive lymphomas

Emanuele Ravano*[1], Alessia Castellino[2], Luigi Marcheselli[3], Giovanni Rindone[4], Michele Spina[5], Marcello Riva[6], Angela Ferrari[7], Luca Nassi[8], Chandrakala Toldo[9], Piero Galieni[10], Agostino Tafuri[11], Livia Donzelli[12], Francesca Gaia Rossi[13], Davide Dalu[14], Bruna Riccardo[15], Emanuele Cencini[16], Barbara Botto[17], Maria Vittoria Sacchi[18], Stefano Lazzi[16], Maurilio Ponzoni[19], Anna Dabrowska-Iwanicka[20], Grzegorz Nowakowski[21], Andrés José María Ferreri[22], Alessandro Re[23]

*[1]ASST Grande Ospedale Metropolitano Niguarda, Division of Hematology, Milano, Italy; [2]A.O.S. Croce e Carle, Division of Hematology, Cuneo, Italy; [3]Fondazione Italiana Linfomi, Clinical Trial Office, Modena, Italy;[4]Fondazione IRCCS San Gerardo dei Tintori, Ematologia, Monza, Italy; [5]IRCCS Centro di Riferimento Oncologico di Aviano – S.O.C. di Oncologia Medica e dei Tumori immunocorrelati, Aviano, Italy; [6]ULSS 8 Berica - Ospedale S. Bortolo, Ematologia, Vicenza, Italy;[7]Azienda Unita Sanitaria Locale-IRCCS-Arcispedale Santa Maria Nuova Ematologia, Reggio Emilia, Italy; [8]AOU Careggi and University of Florence., Hematology Department, Firenze, Italy; [9]Ospedale S. Chiara, S.S. Ematologia, Trento, Italy; [10]Ospedale C.G. Mazzoni, U.O.C. di Ematologia, Ascoli Piceno, Italy; [11]Azienda Ospedaliera-Universitaria Sant'Andrea, Ematologia, Roma, Italy; [12]Policlinico Umberto I - Universita "La Sapienza", Istituto Ematologia -Dipartimento di Medicina Traslazionale e di Precisione, Roma, Italy; [13]Fondazione IRCCS Cà Granda Ospedale Maggiore Policlinico di Milano, Ematologia, Milano, Italy; [14]ASST Fatebenefratelli - Polo Luigi Sacco, Oncologia, Milano, Italy; [15]"Amedeo Avogadro" University of Eastern Piedmont, SCDU Ematologia, Novara, Italy; [16]AOU Senese, Division of Anatomo-Pathology, Siena, Italy; [17]AO Città della Salute e della Scienza, S.C.Ematologia, Torino, Italy; [18]Fondazione Italiana Linfomi, Alessandria, Italy; [19]Ateneo Vita Salute San Raffaele University, Pathology Unit, Milano, Italy; [20]Maria Sklodowska-curie National Research Institute Of Oncology, Warzaw, Poland; [21]Mayo Clinic, Rochester, MN, United States of America; [22]IRCCS San Raffaele Scientific Institute, Unit of Clinical Hematology/Lymphoma Research Unit, Milano, Italy; [23]Spedali Civili di Brescia, Unità Operativa di Ematologia, Brescia, Italy;*

### Background

Plasmablastic Lymphoma (PBL) is a rare aggressive B-cell malignancy with unique features, correlated with immunosuppression, typically HIV-related. Optimal first-line therapy is not established and few prognostic factors are known, without clear consensus. On these grounds, we are carrying an international multicenter retrospective study on Plasmablastic Lymphoma patients (PlaLy), treated in
Italy in centers of the Fondazione Italiana Linfomi (FIL), in U.S. centers (LEO/MER registry) and Polish centers of the Polish Lymphoma Research Group (PLRG).

### Aims

This is an observational, multicenter, international, retrospective study, aimed to describe clinical and pathological characteristics, treatments, outcome and prognostic factors in patients affected by PBL.

### Methods

All patients locally diagnosed with PBL between 2000 and 2022, with available histopathological samples are included in the study. Here we present a preliminary analysis on the first 50 patients enrolled. A comprehensive histopathological central review is currently ongoing.

**Copyright Information:** (Online) ISSN: 2572-9241
©2026 The Author(s). HemaSphere is published by John Wiley & Sons Ltd on behalf of European Hematology Association. This is an open access Abstract Book distributed under the Attribution-NonCommercial-NoDerivs (CC BY-NC-ND), which allows third parties to download the articles and share them with others as long as they credit the author and the Abstract Book, but they cannot change the content in any way or use them commercially.
**Abstract Book Citations:** Authors, Title, HemaSphere, 2026;10:(S1):pages. The abstract book DOIs can be found on the "Author Information" page.
**Disclaimer:** Articles published in the journal HemaSphere exclusively reflect the opinions of the authors. The authors are responsible for all content in their abstracts including accuracy of the facts, statements, citing resources, etc.

### Results

At the time of analysis, 50 patients were enrolled: 40% diagnosed in the timeframe from 2000 and 2014 and 60% diagnosed from 2015 to 2022. Median age was 56, with 46% of patients older than 60 years, 82% were male. B symptoms were recorded in 28%, advanced stage in 62%, ECOG PS > 1 in 23% cases. Nineteen (40%) presented with more than 1 extranodal site. In the whole cohort, R-IPI was low (0) in 11%, intermediate (1-2) in 39%, high (3-5) in 50%, while CNS-IPI was low (0-1) in 34%, intermediate (2-3) in 43%, high (4-6) in 23%. HIV serology was positive in 20 (44%) patients, nearly all under antiretroviral treatment at the time of lymphoma onset. In 52% of cases the interval between antiretroviral therapy starting and PBL diagnosis was shorter than 12 months. Median CD4+ count was 99 cells/
uL and 4 patients presented a concomitant opportunistic infection at the time of PBL diagnosis. EBV viremia was positive in 57% of cases, while EBER positivity was detected in 72% of samples. Front line treatment regimens were: CHOP-like in 25, EPOCH in 15, MAGRATH/GMALL in 2, others in 3 cases. 20% patients received bortezomib in association to chemotherapy, 20% radiotherapy (RT) and 14% high dose-chemotherapy (HD-CT) and/or autologous stem cell transplantation (ASCT) consolidation. Response was evaluated in 45 patients, with ORR 71% and CR 60%.
At a median follow-up of 134 months, 10-year PFS and OS were 42% (95% CI, 26–56) and 55% (95% CI, 38–69), respectively. On univariate analysis, ECOG PS >1, >1 extranodal site and diagnosis before 2015 (for subsequent bortezomib incorporation) were associated with worse outcomes. IPI, R-IPI, and CNS-IPI strongly retained their prognostic role (p=0.016). We recorded 10 relapses, one of which in CNS. Patients receiving upfront consolidation therapy (RT, HD-CT or ASCT) showed better outcomes than those treated with CT alone (Figure 1); the trend remained favorable for ASCT alone (p=0.09). Stage I/II patients were more frequently consolidated with RT, while stage distribution did not differ for ASCT. Twenty-one deaths occurred: 71% due to disease progression.

### Summary/Conclusion

In this preliminary analysis new risk factors, such as ECOG PS >1, >1 extranodal site and diagnosis before 2015, have been identified; patients treated with upfront consolidation therapy tend to have better survival, which needs to be confirmed in the whole cohort we expected to enroll in the study.

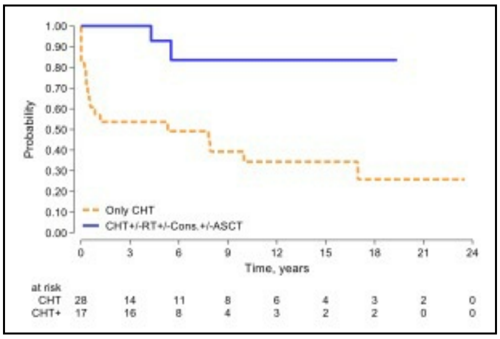

Copyright Information: (Online) ISSN: 2572-9241
©2026 The Author(s). HemaSphere is published by John Wiley & Sons Ltd on behalf of European Hematology Association. This is an open access Abstract Book distributed under the Attribution-NonCommercial-NoDerivs (CC BY-NC-ND), which allows third parties to download the articles and share them with others as long as they credit the author and the Abstract Book, but they cannot change the content in any way or use them commercially.
Abstract Book Citations: Authors, Title, HemaSphere, 2026;10:(S1):pages. The abstract book DOIs can be found on the "Author Information" page.
Disclaimer: Articles published in the journal HemaSphere exclusively reflect the opinions of the authors. The authors are responsible for all content in their abstracts including accuracy of the facts, statements, citing resources, etc.

### (PS2160) AN INTEGRATIVE CLINICAL-IMMUNOGENETIC-GENOMIC INDEX (MCL-CIGI) IMPROVES THE RISK STRATIFICATION OF PATIENTS WITH MANTLE CELL LYMPHOMA

**Topic:** 21. Lymphoma biology & translational research

Ferran Nadeu*[1, 2], Cristina Lopez[1, 2, 3, 4], Amanda Perez-Valencia[3], Anna Montaner[1], Guillem Clot[1, 2, 4], Gerard Frigola[1, 2, 3], Kostiantyn Dreval[5, 6], Krysta Coyle[5, 6], Shuhua Yi[7], Yuting Yan[7], Melika Bashiri[1], Marco Matteo Bühler[3, 8], Aleix Real[1, 2], Luis Luizaga[9], Ana Muntañola Prat[10], Silvia Ruiz-Gaspà[1], Olga Balagué[1, 2, 3], José Ignacio Martín-Subero[1, 2, 4], Fina Climent[11], Patricia López[11], Lili Wang[12], Ryan Morin[5, 6], Jose Manuel Valente Sequeira Cabecadas[13], Maria Ramos Lopes Gomes Da Silva[13], Pedro Jares[1, 2, 3, 4], Armando Lopez Guillermo[1, 3, 4], Eva Gine[1, 3], Elias Campo[1, 2, 3, 4], Silvia Bea[1, 2, 3, 4]

*[1]Institut d'Investigacions Biomèdiques August Pi i Sunyer (IDIBAPS), Barcelona, Spain;[2]Centro de Investigación Biomédica en Red de Cáncer (CIBERONC), Madrid, Spain; [3]Hospital Clínic de Barcelona, Barcelona, Spain;[4]Universitat de Barcelona, Barcelona, Spain; [5]Simon Fraser University, Burnaby, Canada; [6]Genome Sciences Centre - BC Cancer, Vancouver, Canada; [7]Chinese Academy of Medical Sciences and Peking Union Medical College, Tianjin, China;[8]University Hospital Zurich, Zurich, Switzerland; [9]Hospital Universitari Mutua de Terrassa, Terrassa, Spain; [10]Hospital de la Santa Creu i Sant Pau, Barcelona, Spain; [11]Hospital Universitari de Bellvitge - IDIBELL, L'Hospitalet de Llobregat, Spain;[12]Columbia University Irving Medical Center, New York, United States of America; [13]Portuguese Institute of Oncology, Lisbon, Portugal;*

### Background

The clinical course of mantle cell lymphoma (MCL) is heterogeneous and may require distinct management strategies tailored to the underlying pathobiology. Different biological features have been associated with patients' outcomes, including the two molecular subtypes –conventional (cMCL) and non-nodal (nnMCL)–, IGHV status, and *TP53* aberrations. Genomic alterations in other genes such as *MYC* and *CDKN2A/B* could also have prognostic value. Nonetheless, current prognostic indexes, including MIPI and MIPIc –MIPI + Ki-67–, rely mostly on clinical variables. We hypothesized that an integrative prognostic model would improve risk stratification in MCL.

### Aims

To determine the prognostic value of immunogenetic and genomic features in MCL, and to create and validate an integrative index for patient risk stratification.

### Methods

A training cohort of 275 patients treated front-line with chemoimmunotherapy (CIT; 74%) or ibrutinib-based regimens (26%) was analyzed using an NGS panel capturing the *IG::CCND1* translocation, IGHV status, and alterations in ten MCL driver genes from FFPE, frozen, and blood samples. Two independent cohorts of 116 and 130 patients from different geographic regions, each profiled by whole-genome/exome sequencing, were used for validation. The end point was overall survival (OS).

**Copyright Information:** (Online) ISSN: 2572-9241
©2026 The Author(s). HemaSphere is published by John Wiley & Sons Ltd on behalf of European Hematology Association. This is an open access Abstract Book distributed under the Attribution-NonCommercial-NoDerivs (CC BY-NC-ND), which allows third parties to download the articles and share them with others as long as they credit the author and the Abstract Book, but they cannot change the content in any way or use them commercially.
**Abstract Book Citations:** Authors, Title, HemaSphere, 2026;10:(S1):pages. The abstract book DOIs can be found on the "Author Information" page.
**Disclaimer:** Articles published in the journal HemaSphere exclusively reflect the opinions of the authors. The authors are responsible for all content in their abstracts including accuracy of the facts, statements, citing resources, etc.

### Results

The training cohort included 204 (74%) cMCL, 53 (19%) nnMCL, and 18 undetermined MCL. A cutoff of 97% of IGHV identity maximized the difference in OS and had a concordance of 85% with the molecular subtypes. IGHV status –unmutated (U-IGHV) vs mutated– and molecular subtypes showed comparable prognostic value, with U-IGHV/cMCL significantly associated with poorer outcomes.

*TP53* was the most frequently altered gene (31%) followed by *CCND1* 3'UTR alterations (22%), which included translocations, focal copy number alterations, and functional mutations identified using a new algorithm (*cyclinDecodeR*) (Figure).

Alterations in *TP53*, *MYC*, *SP140*, *CDKN2A/B*, and *CCND1* 3'UTR were independently associated with OS in a multivariable analysis (p<0.05). All alterations except *CDKN2A/B* retained a strong association with OS after accounting for clinical (MIPI) and immunogenetic (IGHV status) features (p<0.07, HR>1.6). Therefore, we next derived an integrative clinical-immunogenetic-genomic index (MCL-CIGI) using a weighted grading of the following factors: MIPI high risk, U-IGHV, *TP53*, *MYC*, *SP140*, and *CCND1* 3'UTR. The MCL-CIGI identified four risk groups with significantly different OS (p<0.001; C=0.75; OS at 5 years 100, 87, 59, and 18%). The median OS for the high-risk group (20% of patients) was 2.1 years (Figure). The MCL-CIGI had strong prognostic value within molecular subtypes, MIPI risk groups, patients treated with CIT, and patients treated with ibrutinib (p<0.01). Similarly, the prognostic value of MCL-CIGI was independent of blastoid morphology, Ki-67, MIPIc, and *TP53* alterations, all of which did not retain independent prognostic value once MCL-CIGI was considered.

In the validation cohorts, we confirmed our mutational and clinical observations, including the superior prognostic value of the MCL-CIGI risk groups (Figure).

### Summary/Conclusion

The MCL-CIGI, which relies on a simple NGS panel and is applicable to nodal and blood samples as well as FFPE and frozen material, allows a more precise risk stratification of MCL compared to current prognostic models and *TP53* alterations alone, recognizing both patients with very indolent and very aggressive diseases in the context of CIT and BTK inhibitors.

**Copyright Information:** (Online) ISSN: 2572-9241
©2026 The Author(s). HemaSphere is published by John Wiley & Sons Ltd on behalf of European Hematology Association. This is an open access Abstract Book distributed under the Attribution-NonCommercial-NoDerivs (CC BY-NC-ND), which allows third parties to download the articles and share them with others as long as they credit the author and the Abstract Book, but they cannot change the content in any way or use them commercially.
**Abstract Book Citations:** Authors, Title, HemaSphere, 2026;10:(S1):pages. The abstract book DOIs can be found on the "Author Information" page.
**Disclaimer:** Articles published in the journal HemaSphere exclusively reflect the opinions of the authors. The authors are responsible for all content in their abstracts including accuracy of the facts, statements, citing resources, etc.

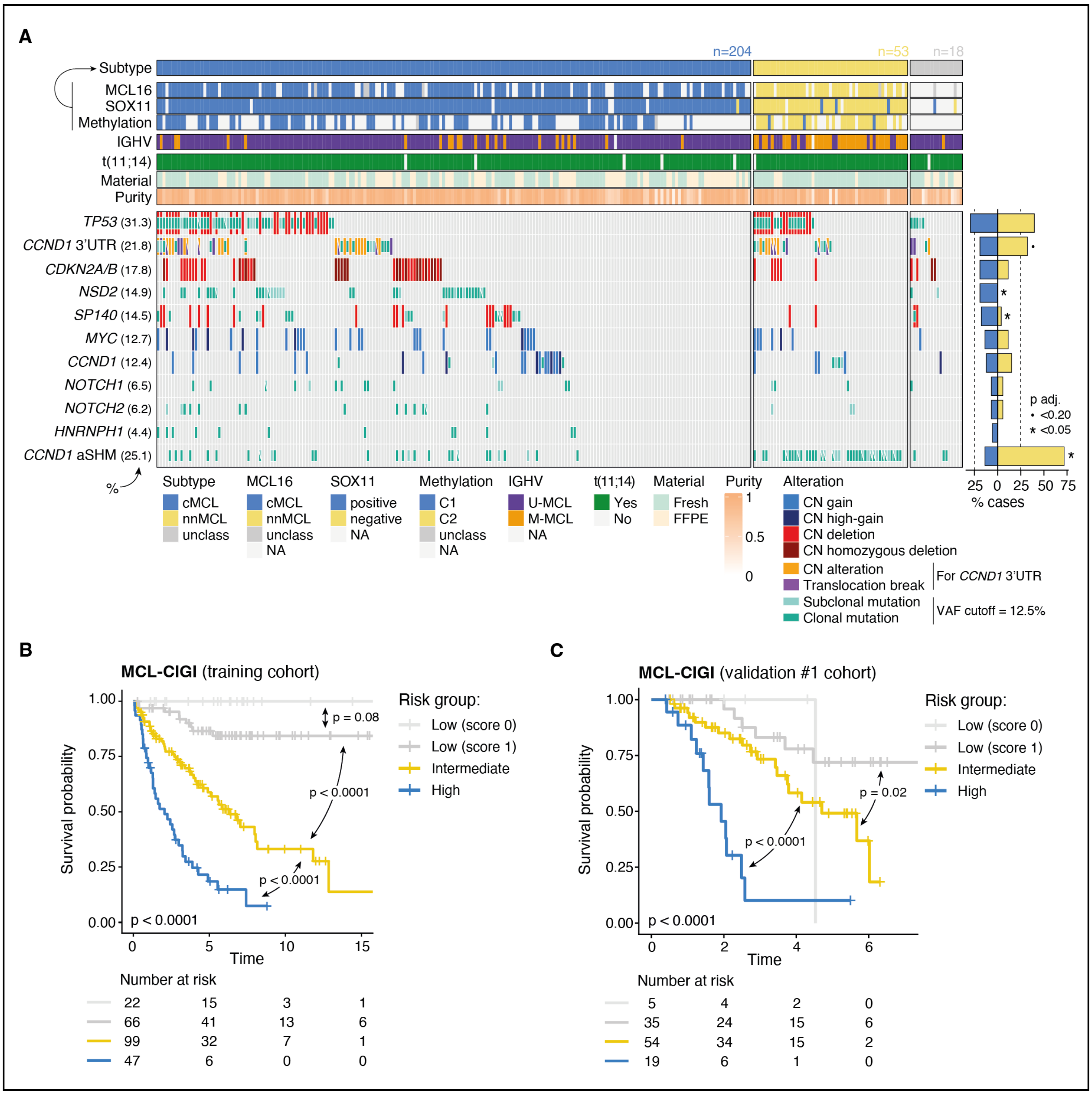

Figure. Genomic characterization of MCL and overall survival according to the MCL-CIGI risk groups. A. Oncoprint showing the (immuno)genomic characterization of the training cohort. B-C. Overall survival according to the MCL-CIGI risk groups in the training (B) and validation #1 (C) cohorts.

**Copyright Information:** (Online) ISSN: 2572-9241
©2026 The Author(s). HemaSphere is published by John Wiley & Sons Ltd on behalf of European Hematology Association. This is an open access Abstract Book distributed under the Attribution-NonCommercial-NoDerivs (CC BY-NC-ND), which allows third parties to download the articles and share them with others as long as they credit the author and the Abstract Book, but they cannot change the content in any way or use them commercially.
**Abstract Book Citations:** Authors, Title, HemaSphere, 2026;10:(S1):pages. The abstract book DOIs can be found on the "Author Information" page.
**Disclaimer:** Articles published in the journal HemaSphere exclusively reflect the opinions of the authors. The authors are responsible for all content in their abstracts including accuracy of the facts, statements, citing resources, etc.

### (PS2161) CTPS1 PROMOTES DIFFUSE LARGE B-CELL LYMPHOMA PROGRESSION BY ENHANCING MITOPHAGY THROUGH CEPT1-MEDIATED PHOSPHOLIPID METABOLIC REMODELING

**Topic:** 21. Lymphoma biology & translational research

Jinhua Liang*[1], Chunyu Shang[1], Kai-Xin Du[1], Zi-Wen Duan[1], Yi-Fan Wu[1], Li Wang[1], Jianyong Li[1], Wei Xu[1]

[1]*the First Affiliated Hospital of Nanjing Medical University, Jiangsu Province Hospital, Nanjing, China;*

### Background

Despite effective first-line regimens, some patients with diffuse large B-cell lymphoma (DLBCL) still experience relapse or resistance, emphasizing the urgent need for innovative treatment approaches. DepMap CRISPR data showed that B-cell lymphoma cell lines were strictly dependent on CTPS1 **(Fig. 1A)**. CTPS1 is a key regulatory and rate-limiting enzyme for de novo nucleotide synthesis pathway. Currently, a highly selective CTPS1 inhibitor (CTPS1i) is undergoing a phase 1/2 clinical trial (NCT05463263) for patients with relapsed/refractory B/T-cell lymphoma, heralding new prospects for targeted therapy and potential clinical translation of CTPS1. The tumor-promoting and drug-resistant roles of CTPS1 in various tumors is widely recognized. However, the role of CTPS1 in DLBCL and its potential therapeutic value remain unknown.

### Aims

To explore the role and underlying mechanism of CTPS1 and the efficacy of CTPS1i in DLBCL.

### Methods

Paraffin-embedded sections from DLBCL patients were collected from the our center with written informed consent. CRISPR/Cas9 editing was used to engineer the loss-of-function models of CTPS1 deficiency in DLBCL cell lines. RNA-seq, untargeted metabolomics, lipidomics and scRNA-seq were performed to investigate how CTPS1 exerts effects on biological processes in DLBCL. Wild-type cells were injected subcutaneously in NOD/SCID male mice. Mice were randomized when tumors reached 50 to 150 $mm^3$. CTPS1i R80 was dosed at 30 or 50mg/kg/day subcutaneously on days 1-4 of a 7-day cycle for three cycles.

### Results

Immunohistochemical analysis of tumor tissues from 111 DLBCL patients demonstrated that elevated CTPS1 expression correlated with poor prognosis **(Fig. 1B-C)**. Functional experiments indicated that CTPS1 depletion inhibited tumor cell proliferation and promoted apoptosis **(Fig. 1D-F)**. KEGG analysis identified significant enrichment in the Mitophagy pathway upon CTPS1 knockout **(Fig. 1G)**. Seahorse stress assays confirmed impaired mitochondrial function **(Fig. 1H)**. Electron micrographs showed the accumulation of damaged swollen mitochondria in CTPS1-deficient cells **(Fig. 1I)**, accompanied by reduced mitochondrial LC3-II expression **(Fig. 1J-K)**. Furthermore, CTPS1 loss disrupted BNIP3-mediated mitophagy **(Fig. 1L)**. Untargeted metabolomics and lipidomics revealed that CTPS1 increased glycerophospholipid levels and disrupted phospholipid metabolism **(Fig. 1M-N)**. The scRNA-seq revealed activation of phospholipid metabolism and mitophagy pathways in CTPS1 high expression DLBCL tumors **(Fig. 1O)**. Integrative analysis of DepMap, KEGG databases and RNA-seq discovered that CTPS1 enhanced mitophagy by CEPT1-mediated phospholipid metabolism remodeling **(Fig. 1P-S)**. CTPS1 knockout downregulated CEPT1 by reducing CTP availability **(Fig. 1T-U)**. Lipidomics of mitochondrial immunoprecipitation revealed that CEPT1 loss specifically depleted mitochondrial membrane phospholipids **(Fig. 1V)**. Supplementation of phospholipids restored mitochondrial homeostasis and BNIP3-mediated mitophagy **(Fig. 1W)**. Moreover, highly selective CTPS1i R80 reduced DLBCL cell viability **(Fig. 1X)** and significantly suppressed tumor growth *in vivo* **(Fig. 1Y)**.

**Copyright Information:** (Online) ISSN: 2572-9241
©2026 The Author(s). HemaSphere is published by John Wiley & Sons Ltd on behalf of European Hematology Association. This is an open access Abstract Book distributed under the Attribution-NonCommercial-NoDerivs (CC BY-NC-ND), which allows third parties to download the articles and share them with others as long as they credit the author and the Abstract Book, but they cannot change the content in any way or use them commercially.
**Abstract Book Citations:** Authors, Title, HemaSphere, 2026;10:(S1):pages. The abstract book DOIs can be found on the "Author Information" page.
**Disclaimer:** Articles published in the journal HemaSphere exclusively reflect the opinions of the authors. The authors are responsible for all content in their abstracts including accuracy of the facts, statements, citing resources, etc.

### Summary/Conclusion

We clarified the specific molecular mechanism by which CTPS1 participates in the occurrence and development of DLBCL by reshaping phospholipid metabolism (**Fig. 1Z**), explored the potential clinical value of CTPS1i in DLBCL.

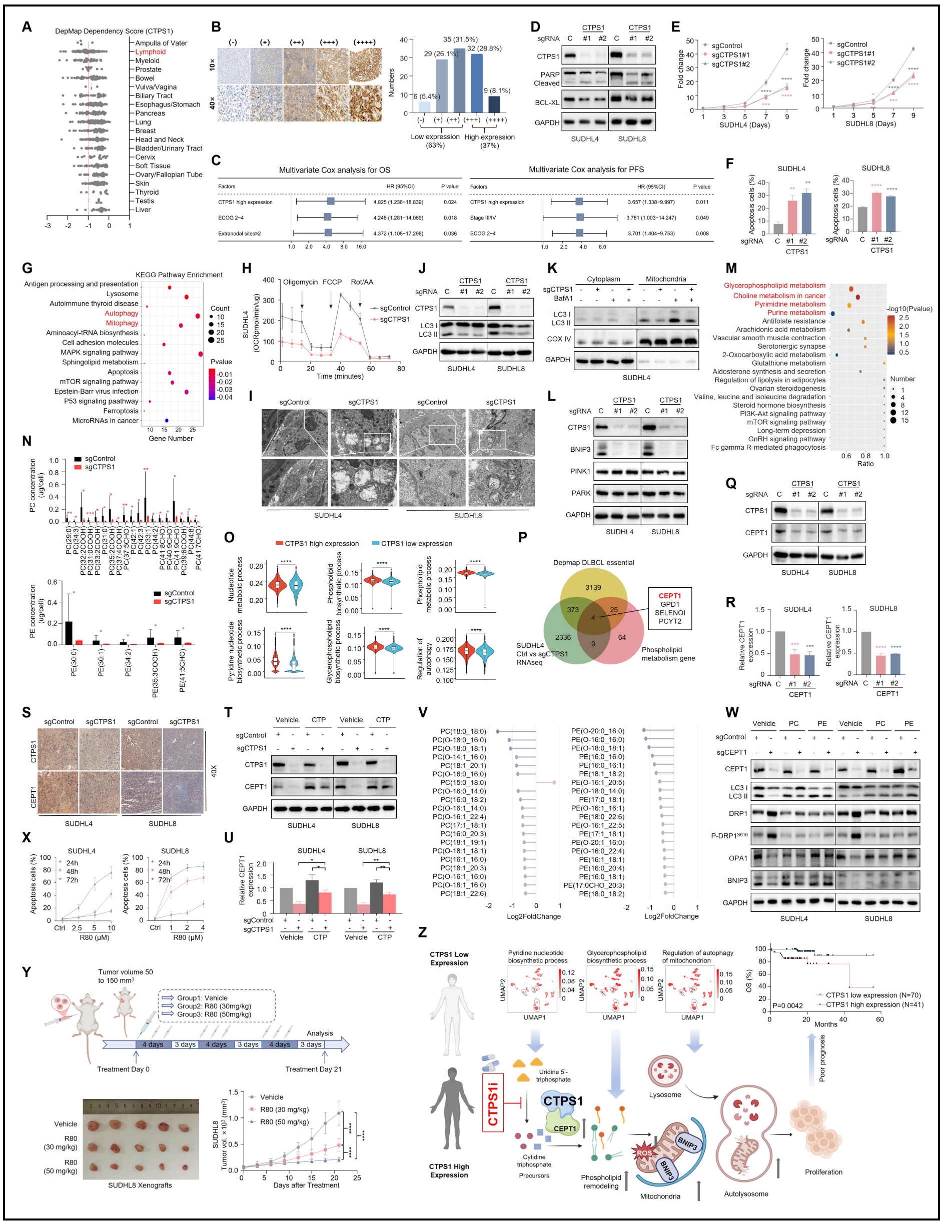

**Copyright Information:** (Online) ISSN: 2572-9241
©2026 The Author(s). HemaSphere is published by John Wiley & Sons Ltd on behalf of European Hematology Association. This is an open access Abstract Book distributed under the Attribution-NonCommercial-NoDerivs (CC BY-NC-ND), which allows third parties to download the articles and share them with others as long as they credit the author and the Abstract Book, but they cannot change the content in any way or use them commercially.
**Abstract Book Citations:** Authors, Title, HemaSphere, 2026;10:(S1):pages. The abstract book DOIs can be found on the "Author Information" page.
**Disclaimer:** Articles published in the journal HemaSphere exclusively reflect the opinions of the authors. The authors are responsible for all content in their abstracts including accuracy of the facts, statements, citing resources, etc.

### (PS2162) MEASURABLE RESIDUAL DISEASE (MRD) BY CIRCULATING TUMOR (CT) DNA IN PATIENTS WITH RELAPSED/REFRACTORY (R/R) FOLLICULAR LYMPHOMA (FL) TREATED WITH LISOCABTAGENE MARALEUCEL (LISO-CEL) IN TRANSCEND FL

**Topic:** 21. Lymphoma biology & translational research

Ariel Avilion[1], Sahar Ansari[1], Abood Okal[2], Ethan Thompson[1], Maria Strocchia[3], Merav Bar[3], Luciana Moro Bueno[3], David Kurtz[4], Alejandro Martín García-Sancho[5], Juan Luis Reguera Ortega[6], Saurabh Dahiya[7, 8], Guillaume Cartron[9], Loretta Nastoupil[10], M. Lia Palomba[11], Kirit Ardeshna[12], Jeremy S. Abramson[13], Koji Izutsu[14], Martin Dreyling[15], Franck Morschhauser*[16], Leanne Peiser[1]

*[1]Bristol Myers Squibb, Seattle, United States of America; [2]Bristol Myers Squibb, Cambridge, United States of America; [3]Bristol Myers Squibb, Boudry, Switzerland; [4]Natera, Inc., Austin, United States of America; [5]Hospital Universitario de Salamanca, IBSAL, CIBERONC, Universidad de Salamanca, Salamanca, Spain; [6]Hospital Virgen del Rocío, Instituto de Biomedicina de Sevilla (IBIS) / CSIC, Universidad de Sevilla, Seville, Spain; [7]Stanford University School of Medicine, Stanford, United States of America; [8]University of Maryland Greenebaum Comprehensive Cancer Center, Baltimore, United States of America; [9]Montpellier University Hospital Center, UMR CNRS 5535, Montpellier, United States of America; [10]Southwest Oncology, CommonSpirit Mercy, Durango, United States of America; [11]Memorial Sloan Kettering Cancer Center, New York, United States of America; [12]University College London Hospitals NHS Foundation Trust, London, United Kingdom; [13]Massachusetts General Hospital Cancer Center, Harvard Medical School, Boston, United States of America; [14]National Cancer Center Hospital, Tokyo, Japan; [15]Medizinische Klinik III Klinikum der Universität LMU München, München, Germany; [16]Centre Hospitalier Universitaire de Lille, Groupe de Recherche sur les formes Injectables et les Technologies Associées, Lille, France;*

### Background ctDNA is a minimally invasive method for detecting MRD in various malignancies. MRD negativity ($MRD^{neg}$) has been linked to improved PFS and durable disease control, including after liso-cel treatment in R/R large B-cell lymphoma. The prognostic value of MRD after CAR T cell therapy in R/R FL has not been established.

### Aims

To report an exploratory MRD analysis from TRANSCEND FL (NCT04245839), in which patients had high rates of deep and durable responses after liso-cel.

### Methods

Of 103 efficacy-evaluable patients with third-line or later (3L+) FL who provided informed consent in TRANSCEND FL, 89 (86%) had ctDNA samples, of whom 90% (80/89) had evaluable ctDNA and efficacy data after liso-cel infusion. Tumor-derived phased variants were primarily identified from screening (baseline) plasma or tumor tissue when plasma was insufficient, using PhasED-Seq. Posttreatment ctDNA-MRD was assessed at Months 1 and 3 after infusion for all patients, and at Month 24 for ongoing responders. MRD positivity ($MRD^{pos}$) was defined as ctDNA above the assay detection threshold (lower limit of detection of 0.7 parts per million, with sample-specific sensitivity dependent on DNA input).

**Copyright Information:** (Online) ISSN: 2572-9241
©2026 The Author(s). HemaSphere is published by John Wiley & Sons Ltd on behalf of European Hematology Association. This is an open access Abstract Book distributed under the Attribution-NonCommercial-NoDerivs (CC BY-NC-ND), which allows third parties to download the articles and share them with others as long as they credit the author and the Abstract Book, but they cannot change the content in any way or use them commercially.
**Abstract Book Citations:** Authors, Title, HemaSphere, 2026;10:(S1):pages. The abstract book DOIs can be found on the “Author Information” page.
**Disclaimer:** Articles published in the journal HemaSphere exclusively reflect the opinions of the authors. The authors are responsible for all content in their abstracts including accuracy of the facts, statements, citing resources, etc.

### Results

MRD$^{neg}$ was achieved by 89% (71/80) of evaluable patients from TRANSCEND FL. Among patients with a best overall response of CR, 93% (71/76) of them achieved MRD$^{neg}$. ctDNA clearance increased over time, with MRD$^{neg}$ rates of 68% (52/76) at Month 1, 83% (63/67) at Month 3, and 97% (57/59) at Month 24. In contrast, patients with PR, SD, or PD (n=4) never achieved MRD$^{neg}$. Baseline ctDNA levels were not associated with PFS after liso-cel treatment. MRD$^{neg}$ after infusion was associated with longer PFS compared with MRD$^{pos}$ ($P$<0.001). In addition, MRD status at Month 3 (HR, 7.0 [95% CI, 3.0–16.2]) showed a stronger correlation with PFS than at Month 1 (HR, 3.0 [95% CI, 1.3–6.8]), with 36-month PFS rates of 80% for MRD$^{neg}$ (n=63) versus 38% for MRD$^{pos}$ (n=13) patients. Multivariate analysis controlling for PET findings confirmed the independent prognostic value of MRD for PFS. Importantly, PFS was longer for patients with both CR by PET and MRD$^{neg}$ at Month 3 (n=62) compared with patients with CR and MRD$^{pos}$ (n=9; HR, 5.0 [95% CI, 1.8–13.4]), with 36-month PFS rates of 81% versus 56%, respectively, suggesting that combining MRD with PET may provide additional prognostic information for long-term outcomes.

### Summary/Conclusion

Liso-cel induced deep molecular responses in patients with 3L+ FL with most patients (89%) achieving MRD$^{neg}$ after infusion. Although baseline ctDNA levels were not associated with PFS, MRD$^{neg}$ after infusion was significantly associated with improved PFS, highlighting the therapeutic benefit of liso-cel regardless of tumor burden before treatment. These findings support the potential of ctDNA-based MRD as a prognostic biomarker in FL and underscore that liso-cel is a key treatment option capable of inducing MRD$^{neg}$ and durable clinical outcomes in patients with 3L+ R/R FL.

©2026 American Society of Clinical Oncology, Inc. Reused with permission. This abstract was submitted to ASCO 2026. All rights reserved.

**Copyright Information:** (Online) ISSN: 2572-9241
©2026 The Author(s). HemaSphere is published by John Wiley & Sons Ltd on behalf of European Hematology Association. This is an open access Abstract Book distributed under the Attribution-NonCommercial-NoDerivs (CC BY-NC-ND), which allows third parties to download the articles and share them with others as long as they credit the author and the Abstract Book, but they cannot change the content in any way or use them commercially.
**Abstract Book Citations:** Authors, Title, HemaSphere, 2026;10:(S1):pages. The abstract book DOIs can be found on the "Author Information" page.
**Disclaimer:** Articles published in the journal HemaSphere exclusively reflect the opinions of the authors. The authors are responsible for all content in their abstracts including accuracy of the facts, statements, citing resources, etc.

### (PS2163) GENOME-WIDE MUTATIONAL SIGNATURES REVEAL STAGE-SPECIFIC MUTAGENESIS ACROSS B-CELL MALIGNANCIES

**Topic:** 21. Lymphoma biology & translational research

Hui Wan*[1], Weicheng Ren[1], Mingyu Yang[1], Birgitta Sander[1], Francesco Maura[2], Ryan Morin[3], Zhiming Li[4], Hui-Lai Zhang[5], Leng-Siew Yeap[6], Qiang Pan-Hammarstrom[1]

*[1]Karolinska Institutet, Department of Medical Biochemistry and Biophysics, Stockholm, Sweden;[2]University of Miami, Division of Myeloma, Sylvester Comprehensive Cancer Center, Miami, United States of America; [3]Simon Fraser University, Department of Molecular Biology and Biochemistry, Burnaby, Canada; [4]Sun Yat-sen University Cancer Center, Department of Medical Oncology, Guangzhou, China; [5]Tianjin Medical University Cancer Institute and Hospital, Tianjin, China;[6]Shanghai Jiao Tong University School of Medicine, Department of Immunology and Microbiology, Shanghai, China;*

### Background

Somatic mutations in cancer genomes arise from distinct mutational processes that leave characteristic genomic signatures reflecting their underlying etiologies. B-cell malignancies arise at distinct stages along B-cell development, yet how developmentally programmed mutagenesis contribute to malignant transformation and shape cancer genomes across B-cell malignancies reamains incomepletely understood. Although mutatinal profiles individual B-cell tumor types have been reported, most studies have focused on specific entities or single mutation classes. Therefore, a unified cross-entity view of the mutational processes in B-cell cancers, particularly across multiple mutation class and evolutionary timing, remains lacking.

### Aims

To systematically investigate mutagenesis in B-cell malignancies by characterizing the landscape, temporal dynamics and clinical relevance of mutational signatures across B-cell malignancies using large-scale whole-genome sequencing (WGS) data. Specifically, we aim to define shared and subtype-specific mutational processes across disease entities and establish a comprehensive reference atlas of mutagenesis in B-cell malignancies.

### Methods

We analyzed somatic mutations from 929 paired tumor–normal WGS samples across six B-cell malignancies: chronic lymphocytic leukemia (CLL), mantle cell lymphoma (MCL), Burkitt's lymphoma (BL), follicular lymphoma (FL), diffuse large B-cell lymphoma (DLBCL), and multiple myeloma (MM). Mutational signatures were extracted *de novo* across single-base substitutions (SBS), doublet-base substitutions (DBS), insertions/deletions (ID), and kataegis events(clustered mutations). We futher characterized their putative etiologies, co-occurrence patterns and temporal activities, as well as the prognosis associations.

**Copyright Information:** (Online) ISSN: 2572-9241
©2026 The Author(s). HemaSphere is published by John Wiley & Sons Ltd on behalf of European Hematology Association. This is an open access Abstract Book distributed under the Attribution-NonCommercial-NoDerivs (CC BY-NC-ND), which allows third parties to download the articles and share them with others as long as they credit the author and the Abstract Book, but they cannot change the content in any way or use them commercially.
**Abstract Book Citations:** Authors, Title, HemaSphere, 2026;10:(S1):pages. The abstract book DOIs can be found on the "Author Information" page.
**Disclaimer:** Articles published in the journal HemaSphere exclusively reflect the opinions of the authors. The authors are responsible for all content in their abstracts including accuracy of the facts, statements, citing resources, etc.

**Results**

We identified 16 SBS, four DBS, three ID, and three kataegis signatures (**Figure**), representing mutational processes associated with aging, defective DNA damage response and repair, and lymphoid-specific processes linked to activation-induced cytidine deaminase (AID) and DNA polymerase eta (POLH). Unsupervised clustering analysis revealed that mutational signatures robustly capture developmental stage-specific mutagenesis along germinal centre reactions and further reveal additional heterogeneity beyond conventional histological classification. Temporal analyses indicate that most mutational processes were acquired early during tumor evolution, with a defective mismatch repair signature particularly enriched in early-onset cases. Notably, elevated activation induced deaminase (AID)-driven mutational process is preferentially enriched in aggressive subgroups amd correlated adverse clinical outcomes. To facilitate broader application, we developed an interactive web-based platform (LymphMutSig, ) that enables exploration of the reference atlas and mutational signature assignment with user-provided datasets.

**Summary/Conclusion**

This large-scale integrative analysis delineates the mutational signature landscape of B-cell malignancies and highlights subtype-specific mutagenic processes shaped by germinal center biology and DNA repair defects, contributing to the understanding of mutagenesis in B-cell malignancies. The accompanying LymphMutSig platform provides a practical resource for interpreting mutational processes in individual tumors and supports future biological and translational investigations.

**Copyright Information:** (Online) ISSN: 2572-9241
©2026 The Author(s). HemaSphere is published by John Wiley & Sons Ltd on behalf of European Hematology Association. This is an open access Abstract Book distributed under the Attribution-NonCommercial-NoDerivs (CC BY-NC-ND), which allows third parties to download the articles and share them with others as long as they credit the author and the Abstract Book, but they cannot change the content in any way or use them commercially.
**Abstract Book Citations:** Authors, Title, HemaSphere, 2026;10:(S1):pages. The abstract book DOIs can be found on the "Author Information" page.
**Disclaimer:** Articles published in the journal HemaSphere exclusively reflect the opinions of the authors. The authors are responsible for all content in their abstracts including accuracy of the facts, statements, citing resources, etc.

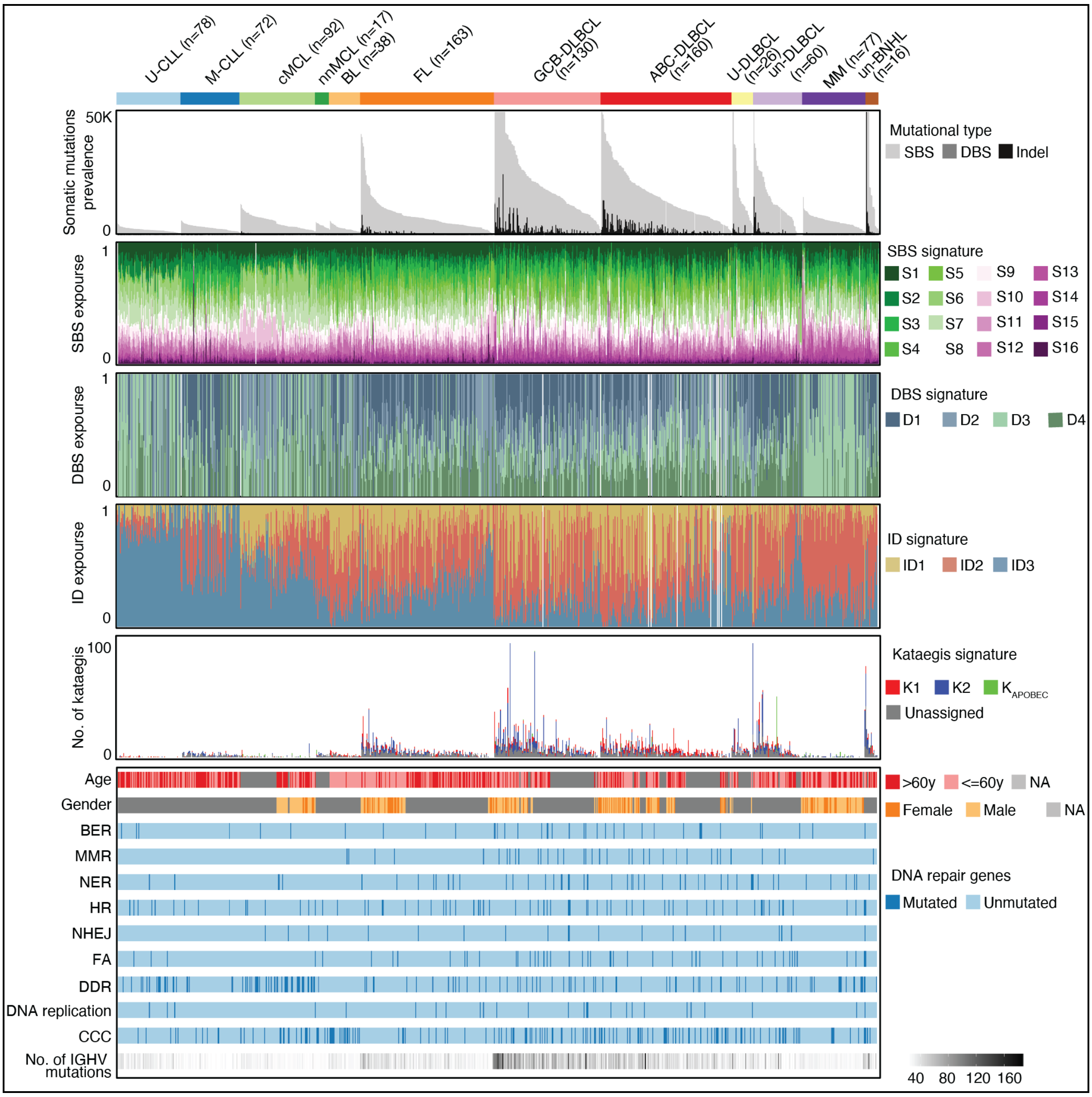

**Copyright Information:** (Online) ISSN: 2572-9241
©2026 The Author(s). HemaSphere is published by John Wiley & Sons Ltd on behalf of European Hematology Association. This is an open access Abstract Book distributed under the Attribution-NonCommercial-NoDerivs (CC BY-NC-ND), which allows third parties to download the articles and share them with others as long as they credit the author and the Abstract Book, but they cannot change the content in any way or use them commercially.
**Abstract Book Citations:** Authors, Title, HemaSphere, 2026;10:(S1):pages. The abstract book DOIs can be found on the "Author Information" page.
**Disclaimer:** Articles published in the journal HemaSphere exclusively reflect the opinions of the authors. The authors are responsible for all content in their abstracts including accuracy of the facts, statements, citing resources, etc.

### (PS2164) SPATIALLY ORGANIZED MACROPHAGE PHENOTYPES DEFINE TREATMENT RESPONSE IN AGGRESSIVE B-NHL

**Topic:** 21. Lymphoma biology & translational research

Christina Schniederjohann*[1, 2, 3, 4], Erin Chung[3, 5], Peter-Martin Bruch[1, 2, 3, 4], Matthias Meyer-Bender[3, 5], Sarah Koziel[1, 2], Harald Vohringer[3, 4, 5], Ekaterina Popova[1, 2], Marc Seifert[1, 2], Hilka Rauert-Wunderlich[6], Marita Ziepert[7], Gerhard Held[8, 9], German Ott[10], Andreas Rosenwald[6], Wolfgang Huber[3, 5], Sascha Dietrich[1, 2, 3, 4, 5]

*[1]University Hospital Düsseldorf, Department of Hematology, Oncology and Clinical Immunology, Düsseldorf, Germany; [2]Center for Integrated Oncology Aachen-Bonn-Cologne-Düsseldorf (CIO ABCD), Düsseldorf, Germany; [3]Molecular Medicine Partnership Unit (MMPU), Heidelberg, Germany; [4]Heidelberg University Hospital, Department of Medicine V, Heidelberg, Germany; [5]European Molecular Biology Laboratory, Heidelberg, Germany; [6]Institute of Pathology, University of Würzburg, Würzburg, Germany; [7]Institute for Medical Informatics, Statistics, and Epidemiology, University of Leipzig, Leipzig, Germany; [8]Westpfalzklinikum Kaiserslautern, Department Internal Medicine I, Kaiserslautern, Germany; [9]Saarland University Medical School, Department of Internal Medicine 1 (Oncology, Hematology, Clinical Immunology, and Rheumatology), Homburg, Germany; [10]Robert-Bosch-Krankenhaus, Stuttgart, Department of Clinical Pathology, Stuttgart, Germany;*

### Background

The tumor microenvironment (TME) is increasingly recognized as an important contributor to therapeutic response across malignancies. While cellular composition has been studied in diffuse large B-cell lymphoma (DLBCL), the spatial organization of tumor and immune cells and its link to clinical outcome remain insufficiently characterized.

### Aims

This study aimed to characterize the cellular composition and spatial architecture of the DLBCL microenvironment and correlate these features with clinical outcomes following frontline chemotherapy.

### Methods

We analyzed diagnostic tumor samples from patients with aggressive B-cell non-Hodgkin lymphoma (B-NHL) treated with standard chemo- or chemoimmunotherapy. Multiplexed immunofluorescence based on CO-Detection by indEXing (CODEX, n=156) and Xenium spatial transcriptomics (n=68) were used to characterize the spatial proteome and transcriptome respectively. Cellular composition and spatial relationships were assessed using radius-based cellular neighborhoods, pairwise cell–cell proximities, and ligand-receptor analysis.

**Copyright Information:** (Online) ISSN: 2572-9241
©2026 The Author(s). HemaSphere is published by John Wiley & Sons Ltd on behalf of European Hematology Association. This is an open access Abstract Book distributed under the Attribution-NonCommercial-NoDerivs (CC BY-NC-ND), which allows third parties to download the articles and share them with others as long as they credit the author and the Abstract Book, but they cannot change the content in any way or use them commercially.
**Abstract Book Citations:** Authors, Title, HemaSphere, 2026;10:(S1):pages. The abstract book DOIs can be found on the “Author Information” page.
**Disclaimer:** Articles published in the journal HemaSphere exclusively reflect the opinions of the authors. The authors are responsible for all content in their abstracts including accuracy of the facts, statements, citing resources, etc.

**Results**

The tumor microenvironment exhibited substantial inter-patient heterogeneity, while technical replicates were highly consistent. Lymphoma-infiltrating T cells ranged from 0.7% to 82% of all cells (mean 24.9%). Higher frequencies of cytotoxic T cells appeared to be associated with longer progression-free survival (PFS), whereas increased proportions of exhausted cytotoxic T cells trended toward shorter PFS. Elevated macrophage levels were linked to longer PFS (p=0.009). Spatial analysis identified five recurrent cellular neighborhoods across patients, including B cell- and immune cell–enriched niches, reflecting distinct tumor–microenvironment patterns. Pairwise proximity analysis revealed that macrophages preferentially clustered together or with dendritic cells. Clustered macrophages formed localized niches characterized by transcriptional programs related to immune recruitment and signaling (CCL3, IL3RA), adhesion and extracellular matrix interactions (SPP1, SDC4, SPN), and checkpoint signaling (CD274, IL1R2). Notably, SPP1-CD44 signaling was enriched in these macrophage clusters. Interestingly, spatially organized macrophage clusters provided additional prognostic information beyond overall macrophage frequency: within the group of patients with higher macrophage frequency, closer spatial proximity between macrophages was associated longer PFS (p=0.04), suggesting that both cellular frequency and spatial organization contribute to the immune control of the tumor. In contrast, proximity between exhausted T cells and proliferating B cells was associated with inferior prognosis (p=0.00006). Exhausted T cells adjacent to proliferating B cells showed elevated CD70-CD27 signaling, potentially reflecting chronic antigen stimulation and local immune suppression.

**Summary/Conclusion**

The study demonstrates that microenvironmental features, including immune cell composition and spatial organization, have prognostic relevance in aggressive B-NHL. Beyond overall cell frequencies, spatial proximities provide additional stratification of outcomes, highlighting the importance of spatially resolved phenotypes for predicting therapy response and guiding biomarker development.

**Copyright Information:** (Online) ISSN: 2572-9241
©2026 The Author(s). HemaSphere is published by John Wiley & Sons Ltd on behalf of European Hematology Association. This is an open access Abstract Book distributed under the Attribution-NonCommercial-NoDerivs (CC BY-NC-ND), which allows third parties to download the articles and share them with others as long as they credit the author and the Abstract Book, but they cannot change the content in any way or use them commercially.
**Abstract Book Citations:** Authors, Title, HemaSphere, 2026;10:(S1):pages. The abstract book DOIs can be found on the "Author Information" page.
**Disclaimer:** Articles published in the journal HemaSphere exclusively reflect the opinions of the authors. The authors are responsible for all content in their abstracts including accuracy of the facts, statements, citing resources, etc.

### (PS2165) PET-GUIDED SINGLE-CELL PROFILING REVEALS MICROENVIRONMENT-DRIVEN METABOLIC HETEROGENEITY IN UNTREATED FOLLICULAR LYMPHOMA

**Topic:** 21. Lymphoma biology & translational research

Danqing Zhao*[1], Ning Lou[2], Jing Ruan[1], Chong Wei[1], Yan Zhang[1], Jian Li[1], Dao-Bin Zhou[1], Xiaohong Han[2], Wei Zhang[1]

*[1]Department of Hematology, Peking Union Medical College Hospital, Chinese Academy of Medical Sciences and Peking Union Medical College (CAMS & PUMC), Hematology, Beijing, China; [2]Clinical Pharmacology Research Center, Peking Union Medical College Hospital, State Key Laboratory of Complex Severe and Rare Diseases, NMPA Key Laboratory for Clinical Research and Evaluation of Drug, Beijing Key Laboratory of Clinical PK & PD Investigation for Innovative Drugs, Chinese Academy of Medical Sciences & Peking Union Medical College, Beijing, China;*

### Background

FDG PET-CT metrics such as SUVmax have been associated with outcome in follicular lymphoma (FL), yet their biological basis and ability to indicate transformation remain controversial.

### Aims

To map the cellular and metabolic correlates of FDG metabolic heterogeneity in untreated FL using PET-guided single-cell transcriptomics.

### Methods

Six treatment-naïve FL patients were analyzed. Lesions with highest FDG uptake were sampled and stratified by metabolic activity (SUVmax>14 vs ≤14) and histology (low-grade: grade 1–2/3a; high-grade: grade 3b/suspected transformation). Single-cell transcriptomic analyses compared cell composition, metabolic pathway activity and inferred cell–cell communication across strata.

**Copyright Information:** (Online) ISSN: 2572-9241
©2026 The Author(s). HemaSphere is published by John Wiley & Sons Ltd on behalf of European Hematology Association. This is an open access Abstract Book distributed under the Attribution-NonCommercial-NoDerivs (CC BY-NC-ND), which allows third parties to download the articles and share them with others as long as they credit the author and the Abstract Book, but they cannot change the content in any way or use them commercially.
**Abstract Book Citations:** Authors, Title, HemaSphere, 2026;10:(S1):pages. The abstract book DOIs can be found on the “Author Information” page.
**Disclaimer:** Articles published in the journal HemaSphere exclusively reflect the opinions of the authors. The authors are responsible for all content in their abstracts including accuracy of the facts, statements, citing resources, etc.

### Results

Single-cell transcriptomic profiling across six untreated FL samples identified major cellular compartments including malignant B cells, cycling B cells, macrophages, and $CD4^+$/$CD8^+$ T cells. High-FDG lesions exhibited a remodeled tumor microenvironment compared with low-FDG lesions, characterized by shifts in immune cell composition and increased representation of cycling B cells, suggesting an ecosystem-level response associated with elevated metabolic activity.
Metabolic pathway scoring indicated that glucose-related metabolic programs were more prominently enriched in immune and stromal compartments than in malignant B cells, implying that FDG uptake is largely driven by microenvironmental metabolic activity rather than tumor cell–intrinsic glycolysis.
When stratified by histologic aggressiveness, high-FDG high-grade cases displayed relatively increased enrichment of glycolysis-related and broader metabolic programs, whereas high-FDG low-grade cases preferentially showed enrichment of lipid-associated metabolic pathways, consistent with grade-dependent metabolic rewiring.
Importantly, even within low-grade FL (grade 1–2/3a), high-FDG lesions demonstrated distinct metabolic pathway enrichment compared with low-FDG lesions. These differences were predominantly observed in immune and stromal cell populations rather than malignant B cells, indicating cell-type–specific metabolic rewiring within the tumor microenvironment independent of histologic grade.
Inferred cell–cell communication analysis revealed extracellular matrix–related signaling (COLLAGEN, FN1, THBS, VTN) distributed across immune and malignant compartments, supporting a matrix-associated interaction network within FL lesions.

**Table 1. Patients characterisitic**

| Gender | Age | Grade | SUVmax |
|---|---|---|---|
| Female | 74 | 1-2 | 5.3 |
| Female | 71 | 3b | 19.1 |
| Female | 43 | tLBCL | 41 |
| Female | 49 | 3a | 26 |
| Female | 41 | tLBCL | 23.7 |
| Female | 39 | 3a | 16.2 |

### Summary/Conclusion

In this pilot, hypothesis-generating PET-guided single-cell study, FDG metabolic heterogeneity in untreated FL appears to be closely associated with microenvironmental remodeling rather than tumor cell–intrinsic glycolysis. High-FDG lesions were characterized by coordinated metabolic programs across specific immune and stromal cell populations, accompanied by enhanced malignant–microenvironment crosstalk.
These findings suggest that FDG uptake represents an ecosystem-level metabolic state involving defined cellular components of the tumor microenvironment, and may provide biological rationale for PET-guided risk stratification. Larger cohorts with longitudinal clinical outcomes are required to validate the clinical relevance of these observations.

**Copyright Information:** (Online) ISSN: 2572-9241
©2026 The Author(s). HemaSphere is published by John Wiley & Sons Ltd on behalf of European Hematology Association. This is an open access Abstract Book distributed under the Attribution-NonCommercial-NoDerivs (CC BY-NC-ND), which allows third parties to download the articles and share them with others as long as they credit the author and the Abstract Book, but they cannot change the content in any way or use them commercially.
**Abstract Book Citations:** Authors, Title, HemaSphere, 2026;10:(S1):pages. The abstract book DOIs can be found on the "Author Information" page.
**Disclaimer:** Articles published in the journal HemaSphere exclusively reflect the opinions of the authors. The authors are responsible for all content in their abstracts including accuracy of the facts, statements, citing resources, etc.

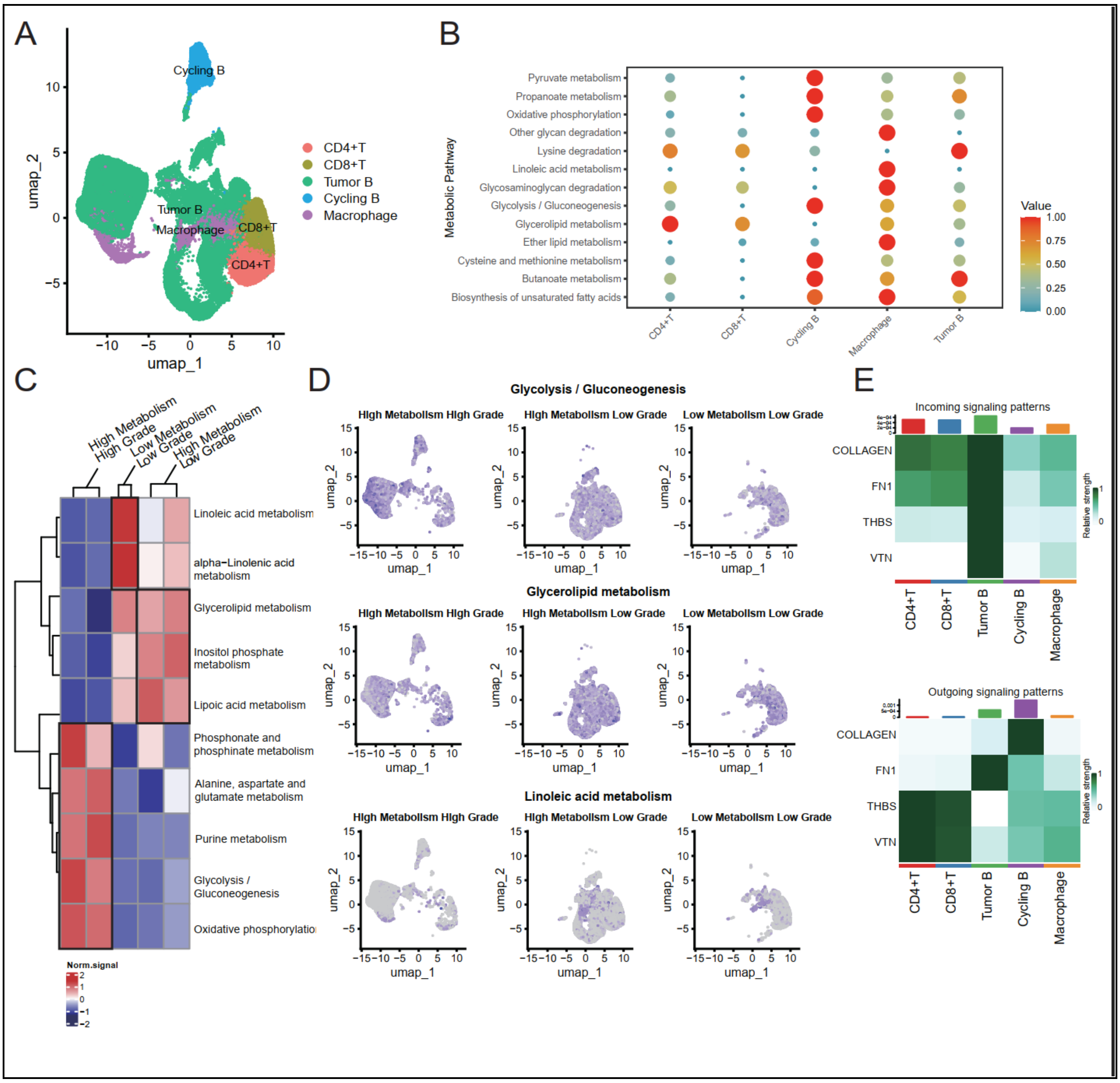

Figure. PET-guided single-cell landscape of metabolic heterogeneity in untreated follicular lymphoma. (A) UMAP embedding showing major cellular compartments identified by single-cell transcriptomics, including malignant B cells, cycling B cells, macrophages, and $CD4^+$/$CD8^+$ T cells. (B) Dot plot of metabolic pathway activity across cell types, indicating cell-type–specific enrichment patterns of glycolytic, lipid, and amino acid metabolism–related pathways. Color indicates relative activity. (C) Heatmap of pathway enrichment across metabolic–histologic strata (high metabolism high grade, high metabolism low grade, low metabolism low grade), highlighting differential metabolic programs associated with metabolic activity and histologic grade. (D) UMAP feature plots illustrating spatial distribution of representative pathways (glycolysis/gluconeogenesis, glycerolipid metabolism, linoleic acid metabolism) across metabolic–grade groups, demonstrating distinct metabolic states within the tumor ecosystem. (E) Inferred cell–cell communication analysis showing incoming and outgoing extracellular matrix–related signaling patterns (COLLAGEN, FN1, THBS, VTN) across cellular compartments, indicating matrix-associated interaction networks within the FL microenvironment.

**Copyright Information:** (Online) ISSN: 2572-9241
©2026 The Author(s). HemaSphere is published by John Wiley & Sons Ltd on behalf of European Hematology Association. This is an open access Abstract Book distributed under the Attribution-NonCommercial-NoDerivs (CC BY-NC-ND), which allows third parties to download the articles and share them with others as long as they credit the author and the Abstract Book, but they cannot change the content in any way or use them commercially.
**Abstract Book Citations:** Authors, Title, HemaSphere, 2026;10:(S1):pages. The abstract book DOIs can be found on the “Author Information” page.
**Disclaimer:** Articles published in the journal HemaSphere exclusively reflect the opinions of the authors. The authors are responsible for all content in their abstracts including accuracy of the facts, statements, citing resources, etc.

### (PS2166) SINGLE-CELL TRANSCRIPTOMIC PROFILING UNCOVERS THE PANORAMIC LANDSCAPE OF HETEROGENEOUS TUMOR CELLS AND IMMUNE MICROENVIRONMENT ACROSS DISEASE STAGES IN EXTRANODAL NATURAL KILLER/T-CELL LYMPHOMA

**Topic:** 21. Lymphoma biology & translational research

Xingcheng Yi[1], Hua Wang[2], Yunpeng Wu[3], Yan Zhang[1], Mengtuan Long[1], Yue Sun[1], Maozhuo Lan[1], Yupeng Li[1], Xinyue Liang[1], Mengru Tian[1], Zhang Yingjie[1], Yurong Yan[1], Weiya Li[1], Xu Chen[1], Yufeng Zhu[1], Shunan Qi[3], Fengyan Jin[1], Yun Dai*[1]

*[1]First Hospital of Jilin University, Hematology, Changchun, China;[2]Sun Yet-Sen University Cancer Center, Guangzhou, China; [3]National Cancer Center/National Clinical Research Center for Cancer/Cancer Hospital, Chinese Academy of Medical Sciences (CAMS) and Peking Union Medical College (PUMC), Department of Radiation Oncology, Beijing, China;*

### Background

Extranodal natural killer/T-cell lymphoma (ENKTCL) is an aggressive malignancy, in which the Ann Arbor staging system provides the cornerstone of clinical management. While this system is critical for prognostication, it is based solely on anatomical spread but fails to explain the biological basis for the clinical dichotomy of ENKTCL (early versus advanced stages). Yet an understanding of comprehensive stage-specific transcriptome remains elusive.

### Aims

We aim to unveil the diversity of tumor cells and the tumor immune microenvironment (TIME) across disease stages of ENKTCL and to explore the cellular/molecular basis for clinical heterogeneity at different stages.

### Methods

We first verified the heterogeneity of clinical outcomes (progression-free survival, PFS, and overall survival, OS) of patients with ENKTCL at early (I and II) and advanced (III and IV) stages. We then analyzed the single-cell RNA sequencing (scRNA-seq) data derived from paired peripheral blood and tumor tissues in the cohort of treatment-naïve ENKTCL patients at stages I (n = 4), II (n = 3), and IV (n = 3). Integrated bioinformatic analyses were employed to delineate the stage-dependent transcriptomic features of malignant NK/T cells and various immune cells within the TIME. The findings were further validated using scRNA-seq data obtained from an independent set of ENKTCL samples (n = 11).

**Copyright Information:** (Online) ISSN: 2572-9241
©2026 The Author(s). HemaSphere is published by John Wiley & Sons Ltd on behalf of European Hematology Association. This is an open access Abstract Book distributed under the Attribution-NonCommercial-NoDerivs (CC BY-NC-ND), which allows third parties to download the articles and share them with others as long as they credit the author and the Abstract Book, but they cannot change the content in any way or use them commercially.
**Abstract Book Citations:** Authors, Title, HemaSphere, 2026;10:(S1):pages. The abstract book DOIs can be found on the "Author Information" page.
**Disclaimer:** Articles published in the journal HemaSphere exclusively reflect the opinions of the authors. The authors are responsible for all content in their abstracts including accuracy of the facts, statements, citing resources, etc.

### Results

In a cohort of 1,208 patients with ENKTCL, median PFS and OS of patients at advanced stage were substantially shorter than those at early stage ($P < 0.0001$ for both PFS and OS). We successfully constructed a high-resolution cellular atlas of ENKTCL, revealing stage-specific phenotypes and functions of tumor cells, with the origin of NK cells for stages I and IV but T cells for stage II. Concurrently, the TIME also displayed a profound diversity in a stage-dependent manner, with the highest proportions of all immune cell types in stage II tumor tissues, substantially more abundant than stages I and IV. Although the overall functions of non-malignant NK cells are impaired, those at advanced stage were more matured/activated than those at early stage; the majority of T cells at stage II were CD4+ memory and Treg cells, while CD8+ T cells were predominant at stage I (memory/activated or exhausted) and IV (resting); NKT cells were activated at stage II, while functionally impaired in the regulation of lymphocyte activation at stages I and IV; TAMs were apt to more pro-inflammatory/angiogenic at stage I or immunosuppressive at stage II, but interferon-responsive, lipid-associated, and proliferative at stage IV; B cell subsets were unique for each stage (I: CD19–/CD20+; II: CD19–/CD20–; IV: CD19+/CD20–); DCs resembled the regulatory phenotype at stage I, and expressed multiple TLRs at stage II but maturation markers at stage IV. All three stages shared the communication between various immune cells and tumor cells via the interaction between B2M and inhibitory NK cells receptors (NKG2A or CD94). Despite the common immune checkpoints (HAVCR2-LGALS9 and B7.2-CTLA4) for all three stages, there were also stage-specific checkpoints (e.g., CD28-CD86 and CD70-CD27 for II; CTLA4-CD86, CD247-BTLA, and ITGB2-CD40LG for IV).

### Summary/Conclusion

This study provides a panoramic landscape with single-cell resolution and molecular insights into distinct biology of tumor cells and TIME in ENKTCL across disease stages, laying a foundation for developing stage-specific therapies.

**Copyright Information:** (Online) ISSN: 2572-9241
©2026 The Author(s). HemaSphere is published by John Wiley & Sons Ltd on behalf of European Hematology Association. This is an open access Abstract Book distributed under the Attribution-NonCommercial-NoDerivs (CC BY-NC-ND), which allows third parties to download the articles and share them with others as long as they credit the author and the Abstract Book, but they cannot change the content in any way or use them commercially.
**Abstract Book Citations:** Authors, Title, HemaSphere, 2026;10:(S1):pages. The abstract book DOIs can be found on the "Author Information" page.
**Disclaimer:** Articles published in the journal HemaSphere exclusively reflect the opinions of the authors. The authors are responsible for all content in their abstracts including accuracy of the facts, statements, citing resources, etc.

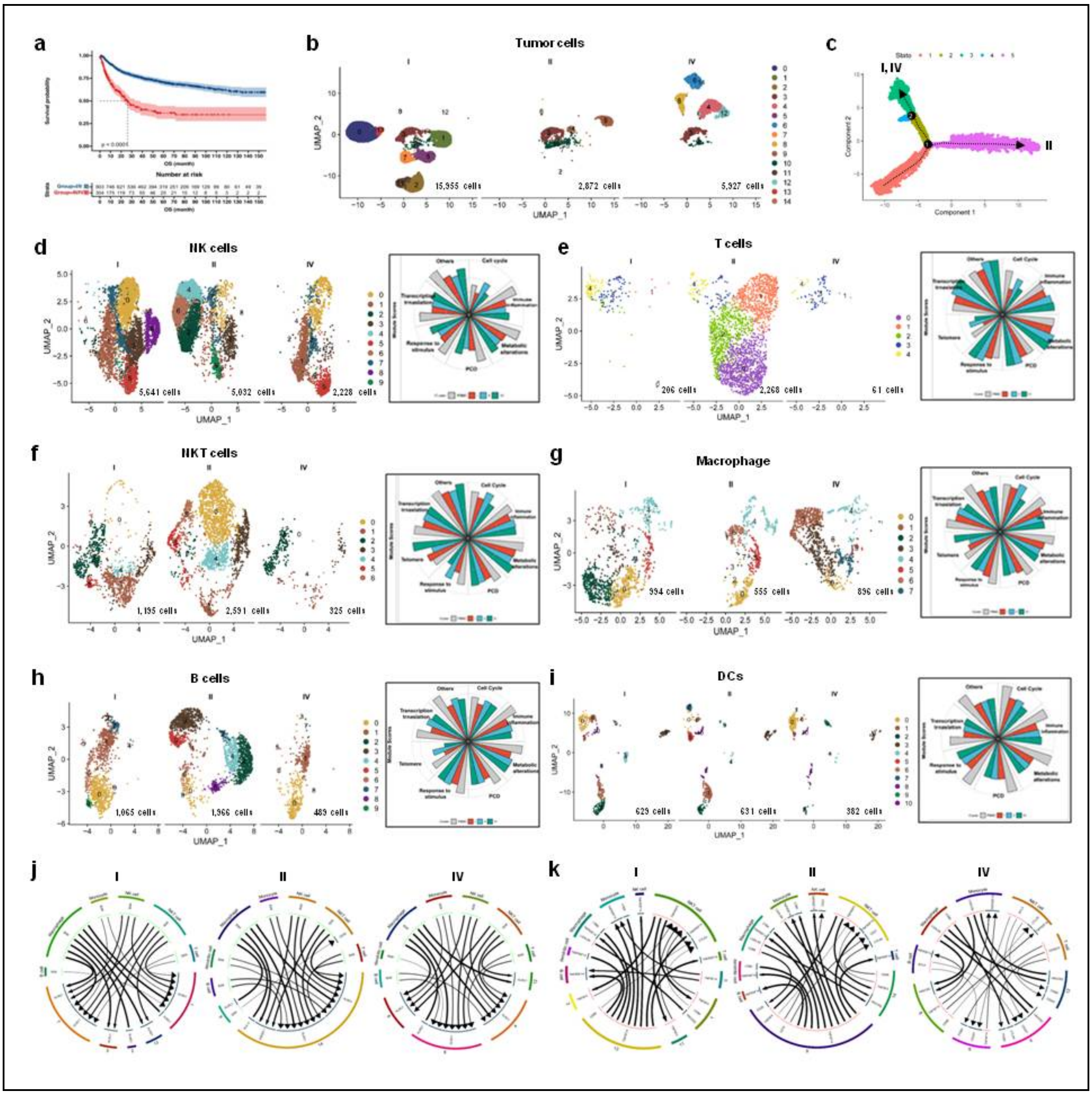

**Copyright Information:** (Online) ISSN: 2572-9241
©2026 The Author(s). HemaSphere is published by John Wiley & Sons Ltd on behalf of European Hematology Association. This is an open access Abstract Book distributed under the Attribution-NonCommercial-NoDerivs (CC BY-NC-ND), which allows third parties to download the articles and share them with others as long as they credit the author and the Abstract Book, but they cannot change the content in any way or use them commercially.
**Abstract Book Citations:** Authors, Title, HemaSphere, 2026;10:(S1):pages. The abstract book DOIs can be found on the "Author Information" page.
**Disclaimer:** Articles published in the journal HemaSphere exclusively reflect the opinions of the authors. The authors are responsible for all content in their abstracts including accuracy of the facts, statements, citing resources, etc.

### (PS2167) EARLY TUMOR-INFORMED CTDNA MONITORING IMPROVES RISK STRATIFICATION AFTER CAR-T THERAPY IN LARGE B-CELL LYMPHOMA

**Topic:** 21. Lymphoma biology & translational research

Ana Isabel Jimenez Ubieto[1], Carolina Martinez Laperche[2], Alejandro Martín Muñoz[3], Ismael De La Iglesia San Sebastián[2], Pablo Silva[2], Margarita Rodriguez[3], Julia Parra[2], Rebeca Bailen[2], Sara Fernández de Córdoba Oñate[2], Francisco Díaz Crespo[2], Beatriz Barranco[2], Alvaro Bienert Garcia[1], Daniel Gil Alós[1], Davide Di-Lernia[4], Angel I. Perez-Santiago[4], Lucía Medina de Alba[1], José María Sánchez-Pina[1], Natalia Toledano[3], Enrique Revilla[1], Laura Astilleros[1], Pilar Sarandeses[1], Antonio Valeri[1], Tycho Stephan Baumann[1], Antonia Rodriguez Izquierdo[1], Mi Kwon[2], Margarita Sanchez-Beato[4], Ismael Buño[2], Rosa María Ayala Díaz[1], Ramón García-Sanz[2], Joaquín Martinez-Lopez[1], Santiago Barrio[4], Mariana Bastos-Oreiro*[2]

*[1]Hospital 12 de Octubre, Hematology, Madrid, Spain; [2]Hospital General Universitario Gregorio Marañón, Hematology, Madrid, Spain; [3]Altum sequencing S.L, Madrid, Spain; [4]Hospital U. Puerta de Hierro-Majadahonda, IDIPHISA, Medical Oncology Department, Majadahonda, Spain;*

### Background

Relapsed or refractory large B-cell lymphoma (LBCL) treated with CAR-T therapy shows heterogeneous outcomes, and early identification of patients at risk of progression remains challenging. Tumor-informed circulating tumor DNA (ctDNA) assays enable highly sensitive minimal residual disease (MRD) detection and offer a noninvasive strategy for dynamic monitoring.

### Aims

We aim to evaluate the prognostic impact of early and longitudinal patient-specific ctDNA assessment in LBCL patients receiving CAR-T therapy and its complementary role alongside PET/CT.

### Methods

Seventy-nine LBCL patients treated with CD19 CAR-T therapy and available liquid biopsy at day +7, +14, or +30 were included. Six lacked suitable molecular markers for MRD analysis, and 10 of the remaining 73 were excluded due to insufficient cfDNA input (<15 ng) or gDNA contamination, resulting in 63 evaluable patients. Somatic mutations were identified at relapse using FFPE tumor tissue and cfDNA analyzed with a custom 134-gene capture panel (Twist Bioscience) sequenced on the Illumina NextSeq platform. Patient-specific mutations were used to develop individualized MRD assays (TrackSeq, Altum Sequencing), as previously reported (Jiménez-Ubieto et al., Leukemia, 2023). The amplicon-based panel also quantified the CAR-T Phi construct relative to albumin cfDNA. A total of 165 follow-up cfDNA samples were analyzed: 59 patients at days +7/+14, 38 at day +30, and 60 additional samples between days 60 and 270. PET/CT was performed at days +30, +90, +180, +365, and every six months thereafter. Paradoxical metabolic response was defined as marked reduction of baseline lesions with concurrent emergence of new FDG-avid lesions.

**Copyright Information:** (Online) ISSN: 2572-9241
©2026 The Author(s). HemaSphere is published by John Wiley & Sons Ltd on behalf of European Hematology Association. This is an open access Abstract Book distributed under the Attribution-NonCommercial-NoDerivs (CC BY-NC-ND), which allows third parties to download the articles and share them with others as long as they credit the author and the Abstract Book, but they cannot change the content in any way or use them commercially.
**Abstract Book Citations:** Authors, Title, HemaSphere, 2026;10:(S1):pages. The abstract book DOIs can be found on the “Author Information” page.
**Disclaimer:** Articles published in the journal HemaSphere exclusively reflect the opinions of the authors. The authors are responsible for all content in their abstracts including accuracy of the facts, statements, citing resources, etc.

### Results

The median age of the final cohort was 57 years; 41% were female, and patients had received a median of two prior lines of therapy. At baseline, 401 trackable somatic mutations were identified for MRD monitoring (mean: 6 per patient). The most frequently mutated genes were *TP53* (44%), *CREBBP* (30%), *KMT2D* (24%), *IGLL5* (22%), and *BCL2* (20%). Early MRD assessment (day +7/+14) identified patients at increased risk of progression, with ctDNA positivity demonstrating significant prognostic impact (one-year PFS: 23% vs 100%; HR, 10; 95% CI, 1.37–72.48; p=0.02), indicating that molecular kinetics precede conventional imaging (Figure A). At day +30, ctDNA MRD positivity retained independent prognostic value (HR 4.5, 95% CI 1.03–19.37; p=0.02), comparable to PET/CT (HR 8.3, 95% CI 4.41–15.6; p<0.005). Among 33 patients with both assessments available, 13 were concordantly positive, 6 concordantly negative, and 14 discordant (Figure B). 10/13 of ctDNA+/PET+ patients progressed within one year (median PFS 77 days vs. not reached for ctDNA–/PET–; p<0.001). Notably, all five patients with paradoxical PET findings were ctDNA-positive at day +30 and subsequently progressed.

### Summary/Conclusion

Tumor-informed ctDNA monitoring enables very early risk stratification after CAR-T therapy in LBCL, identifying high-risk patients within the first two weeks post-infusion, before radiologic assessment. At day +30, ctDNA MRD positivity shows strong prognostic value, and integration of molecular and metabolic response improves outcome discrimination. ctDNA analysis is particularly useful when PET findings generate uncertainty, clarifying disease status. These results support incorporating longitudinal ctDNA monitoring into prospective CAR-T trials to enable earlier, biology-driven, risk-adapted strategies.

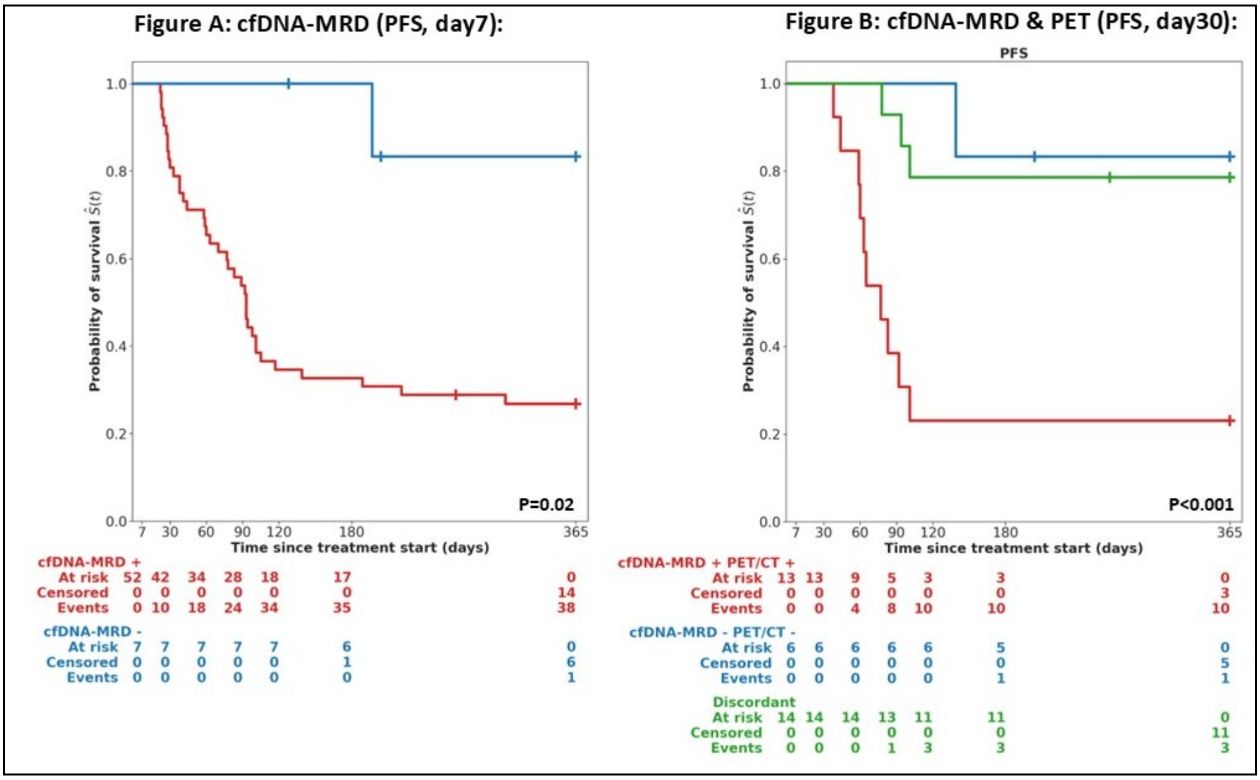

Copyright Information: (Online) ISSN: 2572-9241
©2026 The Author(s). HemaSphere is published by John Wiley & Sons Ltd on behalf of European Hematology Association. This is an open access Abstract Book distributed under the Attribution-NonCommercial-NoDerivs (CC BY-NC-ND), which allows third parties to download the articles and share them with others as long as they credit the author and the Abstract Book, but they cannot change the content in any way or use them commercially.
Abstract Book Citations: Authors, Title, HemaSphere, 2026;10:(S1):pages. The abstract book DOIs can be found on the "Author Information" page.
Disclaimer: Articles published in the journal HemaSphere exclusively reflect the opinions of the authors. The authors are responsible for all content in their abstracts including accuracy of the facts, statements, citing resources, etc.

### (PS2168) LONGITUDINAL CTDNA DYNAMICS AROUND CAR-T CELL THERAPY AND THEIR IMPACT ON TREATMENT EFFICACY IN LARGE B-CELL LYMPHOMA.

**Topic:** 21. Lymphoma biology & translational research

Carolina Martinez Laperche*[1], Sara Fernández de Córdoba Oñate[1], Ismael De La Iglesia San Sebastián[1], Miguel López-Esteban[1], Santiago Barrio[2], Alejandro Martín Muñoz[3], Pablo Silva[1], Julia Parra[1], Mª Jose Llacer[1], Beatriz Barranco[1], Raquel Fernández-González[1], Rebeca Bailen[1], Francisco Díaz Crespo[4], Mi Kwon[1], Ana Isabel Jimenez Ubieto[5], Ismael Buño[1], Ramón García-Sanz[1], Mariana Bastos-Oreiro[1]

*[1]Hospital General Universitario Gregorio Marañón, Instituto de Investigación Sanitaria Gregorio Marañón, Hematology, Madrid, Spain; [2]Lymphoma Research Group, Medical Oncology Department, Hospital U. Puerta de Hierro-Majadahonda, IDIPHISA, Madrid, Madrid, Spain; [3]Altum sequencing Co., Madrid, Spain., Madrid, Spain; [4]Servicio de Anatomía Patológica, Hospital General Universitario Gregorio Marañón, Instituto de investigación Sanitaria Gregorio, Madrid, Spain., Madrid, Spain; [5]Department of Hematology, Hospital Universitario 12 de Octubre, Instituto de Investigación Sanitaria Hospital 12 de Octubre (imas12), CNIO, CIBERONC, Madrid, Spain., Madrid, Spain;*

**Background**

Clonal dynamics and the emergence of resistant subclones are key drivers of relapse after CD19-directed CAR-T therapy. Circulating tumor DNA (ctDNA) enables longitudinal tracking of these molecular changes; however, the relationship between variants detected before CAR-T apheresis, infusion and those present at relapse remains incompletely defined

**Aims**

Our aim was to characterize ctDNA variant dynamics across CAR-T treatment timepoints and to assess clonal overlap between pre-treatment and relapse samples in large B cell lymphoma (LBCL)

**Methods**

We analysed 127 LBCL patients treated with CAR-T. cfDNA was profiled from 224 plasma samples collected at pre-apheresis (PA) (n=103), pre-infusion (PI) (n=94), and relapse (n=24); 70 patients had paired PA–PI samples. Targeted cfDNA sequencing used the CAPP-Seq Lymph panel (135 genes). Somatic variants required PASS, ≥100×, VAF ≥1%, and >10 supporting reads, filtering for artifacts, germline/benign variants, and population frequency ≥1%; analyses were restricted to non-synonymous and splice-site variants. Statistics were performed in RStudio

**Copyright Information:** (Online) ISSN: 2572-9241
©2026 The Author(s). HemaSphere is published by John Wiley & Sons Ltd on behalf of European Hematology Association. This is an open access Abstract Book distributed under the Attribution-NonCommercial-NoDerivs (CC BY-NC-ND), which allows third parties to download the articles and share them with others as long as they credit the author and the Abstract Book, but they cannot change the content in any way or use them commercially.
**Abstract Book Citations:** Authors, Title, HemaSphere, 2026;10:(S1):pages. The abstract book DOIs can be found on the “Author Information” page.
**Disclaimer:** Articles published in the journal HemaSphere exclusively reflect the opinions of the authors. The authors are responsible for all content in their abstracts including accuracy of the facts, statements, citing resources, etc.

### Results

A total of 2,742 unique variants were detected across 132 genes. The most frequently altered genes were *KMT2D* (62% pre–CAR-T; 58% relapse), *TP53* (50%; 54%), *CREBBP* (50%; 42%), with *ARID1A* (37%) whereas IGLL5 (46%) and B2M (29%) predominated at relapse.
Among 127 patients, the median progression-free survival (PFS) was 10.1 months (95% CI, 8.5–NR),, with early relapse (within the first 3 months) occurring in 41.7% (n=53) of patients. Early relapse was associated with elevated LDH, ferritin, and CRP levels, and the presence of variants in *BTG1, IGLL5, HIST1H1C/HIST1H1E, FOXO1, CD58*, and ≥2 in *TP53* (all p<0.001). Relapse was also associated with a higher ctDNA burden, as evidenced by higher mean VAF (PA>10, PI>15), as well as higher mean VAF (PA >8%, PI >2%) (all p<0.001).
Comparison of PA and PI samples revealed marked clonal dynamics: in 42% of patients, at least half of PI variants were not detectable at PA. Variants shared between PA and PI samples showed higher VAF (median 0.084 [0.00512–0.997]) than variants detected exclusively at PI (median 0.011 [0.00508–0.389) Figure A. In addition, 56 of 70 patients developed > 2 newly detectable variants at PI. A composite score integrating PI VAF, new PI variants, and PA-to-PI clonal VAF shifts (capturing clonal expansion and appearance of new variants) was associated with shorter PFS (p<0.001) (Figure 1B).
At relapse, ≥50% overlap between relapse and pre-treatment variants was observed in 60% of patients, while 90% showed previously undetectable variants at relapse. Variants detected exclusively at relapse exhibited lower VAF (median 0.0099 [0.0050–0.141]) compared with variants shared with pre-treatment samples (median 0.116 [0.0056–0.827]). Below, we present two representative cases illustrating genetic variability across different timepoints (Figure 1C, 1D)

### Summary/Conclusion

Longitudinal ctDNA profiling reveals marked clonal dynamics across CAR-T treatment in LBCL, with both clonal expansion and the emergence of previously undetectable variants contributing to treatment failure. Molecular changes occurring between PA and PI, as well as patterns of clonal overlap and evolution at relapse, are strongly associated with CAR-T efficacy. Importantly, these findings demonstrate that PA and PI samples are not interchangeable, underscoring that variant selection for ctDNA monitoring is more complex than commonly assumed and critical for anticipating relapse

**Copyright Information:** (Online) ISSN: 2572-9241
©2026 The Author(s). HemaSphere is published by John Wiley & Sons Ltd on behalf of European Hematology Association. This is an open access Abstract Book distributed under the Attribution-NonCommercial-NoDerivs (CC BY-NC-ND), which allows third parties to download the articles and share them with others as long as they credit the author and the Abstract Book, but they cannot change the content in any way or use them commercially.
**Abstract Book Citations:** Authors, Title, HemaSphere, 2026;10:(S1):pages. The abstract book DOIs can be found on the "Author Information" page.
**Disclaimer:** Articles published in the journal HemaSphere exclusively reflect the opinions of the authors. The authors are responsible for all content in their abstracts including accuracy of the facts, statements, citing resources, etc.

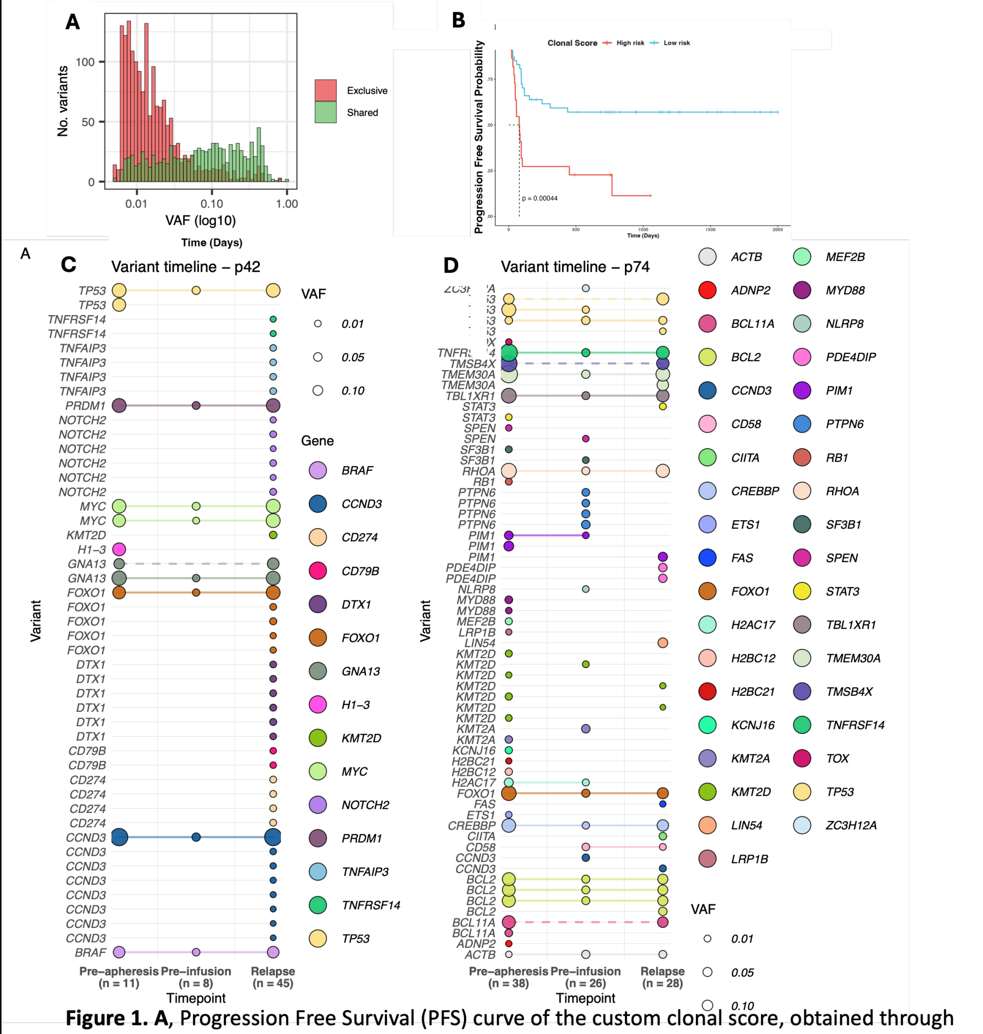

**Figure 1. A**, Progression Free Survival (PFS) curve of the custom clonal score, obtained through generalize lineal models, capturing clonal expansion and the appearance of new variants. **B**, Distribution of variant allele frequencies (VAFs) for shared versus exclusive variants between PA and PI samples. **C**, Longitudinal landscape of detected variants in tow patients (p42 and p74) across pre-apheresis, pre-infusion, and relapse timepoints. Variants are shown as gene-colored dots, with size proportional to VAF.

**Copyright Information:** (Online) ISSN: 2572-9241
©2026 The Author(s). HemaSphere is published by John Wiley & Sons Ltd on behalf of European Hematology Association. This is an open access Abstract Book distributed under the Attribution-NonCommercial-NoDerivs (CC BY-NC-ND), which allows third parties to download the articles and share them with others as long as they credit the author and the Abstract Book, but they cannot change the content in any way or use them commercially.
**Abstract Book Citations:** Authors, Title, HemaSphere, 2026;10:(S1):pages. The abstract book DOIs can be found on the "Author Information" page.
**Disclaimer:** Articles published in the journal HemaSphere exclusively reflect the opinions of the authors. The authors are responsible for all content in their abstracts including accuracy of the facts, statements, citing resources, etc.

### (PS2169) DISSECTING THE REGULATORY MECHANISMS OF CD47 EXPRESSION AND ITS THERAPEUTIC TARGETING IN CENTRAL NERVOUS SYSTEM LYMPHOMA

**Topic:** 21. Lymphoma biology & translational research

Yusuke Kubota*[1, 2], Junji Koya[1, 2], Haroon Kim[1, 2], Sumito Shingaki[1], Hirokuni Honma[3, 4], Yasunori Kogure[1], Ami Inokuchi[1, 2], Yosuke Mizukami[1, 5], Sara Horie[1], Kota Mizuno[1, 2], Mitsuhiro Yuasa[1], Yuta Ito[1, 6], Kentaro Yamaguchi[1, 2], Yuki Saito[1], Koichi Murakami[1, 2], Nobuyoshi Sasaki[7], Akiko Maeshima[8], Daisuke Ennishi[9], Daisuke Kaji[10], Fumihito Tajima[11], Kensuke Tateishi[3, 4], Motoo Nagane[7], Keisuke Kataoka[1, 2]

*[1]National Cancer Center Research Institute, Division of Molecular Oncology, Tokyo, Japan;[2]Keio University School of Medicine, Division of Hematology, Department of Medicine, Tokyo, Japan; [3]Yokohama City University, Department of Neurosurgery, Graduate School of Medicine, Yokohama, Japan; [4]Yokohama City University, Neurosurgical-Oncology Laboratory, Graduate School of Medicine, Yokohama, Japan; [5]Keio University School of Medicine, Division of Gastroenterology and Hepatology, Department of Internal Medicine, Tokyo, Japan; [6]National Cancer Center Hospital, Department of Hematology, Tokyo, Japan; [7]Kyorin University School of Medicine, Department of Neurosurgery, Tokyo, Japan;[8]National Cancer Center Hospital, Department of Diagnostic Pathology, Tokyo, Japan; [9]Okayama University Graduate School of Medicine, Dentistry and Pharmaceutical Sciences, Department of Medical Oncology, Okayama, Japan; [10]Toranomon Hospital, Department of Hematology, Tokyo, Japan; [11]Yonago Medical Center, Department of Hematology and Oncology, Yonago, Japan;*

### Background

CD47 is an anti-phagocytic immune checkpoint that facilitates tumor immune evasion by engaging signal regulatory protein α (SIRPα) on macrophages. In diffuse large B-cell lymphoma (DLBCL), CD47 upregulation is well-documented and CD47-targeted immunotherapies, such as magrolimab, have demonstrated encouraging clinical activity with manageable safety profiles. Although several molecules, such as QPCTL and MYC, have been shown to regulate CD47 expression in different contexts, the regulatory mechanisms of CD47 expression in B-cell lymphomas remain poorly understood. Importantly, given the immune-privileged nature of the central nervous system (CNS), it has not yet been established whether CD47-targeted immunotherapies are effective in CNS lymphoma (CNSL).

### Aims

We aim to define CD47 expression patterns and regulatory mechanisms in B-cell lymphomas and to evaluate the therapeutic potential of CD47-targeted immunotherapy in CNSL.

### Methods

We analyzed our multi-omics single-cell dataset derived from clinical samples, comprising 18 DLBCL tumors, 16 CNSL tumors, 5 reactive lymph nodes, and 4 peripheral blood samples from healthy volunteers. We conducted a genome-wide CRISPR–Cas9 knockout screen targeting 19,050 genes in the DLBCL cell line SU-DHL-4 by comparing guide RNA representation in flow-sorted CD47-high and CD47-low cell populations, followed by validation using genetic and pharmacological perturbation in DLBCL and CNSL cell lines. Finally, we evaluated the efficacy of magrolimab in a patient-derived xenograft (PDX) model established by intracerebral implantation of CNSL tumor cells into immunodeficient mice.

**Copyright Information:** (Online) ISSN: 2572-9241
©2026 The Author(s). HemaSphere is published by John Wiley & Sons Ltd on behalf of European Hematology Association. This is an open access Abstract Book distributed under the Attribution-NonCommercial-NoDerivs (CC BY-NC-ND), which allows third parties to download the articles and share them with others as long as they credit the author and the Abstract Book, but they cannot change the content in any way or use them commercially.
**Abstract Book Citations:** Authors, Title, HemaSphere, 2026;10:(S1):pages. The abstract book DOIs can be found on the "Author Information" page.
**Disclaimer:** Articles published in the journal HemaSphere exclusively reflect the opinions of the authors. The authors are responsible for all content in their abstracts including accuracy of the facts, statements, citing resources, etc.

**Results**

Single-cell CITE-seq analyses revealed that CD47 protein expression was markedly increased in CNSL compared with DLBCL and its upregulation was independent of mRNA expression. This discordance was confined to malignant B cells in CNSL and a subset of DLBCL, whereas mRNA and protein expression levels were well correlated in non-malignant immune cell populations, suggesting a B-cell lymphoma-specific post-transcriptional regulatory mechanism of CD47 expression. The CRISPR screen identified 124 positive regulators of CD47 expression, with significant overrepresentation of genes involved in BCR signaling (e.g., *CD79A* and *CD19*), downstream AKT–mTOR signaling (e.g., *AKT2* and *MTOR*), and actin cytoskeleton regulation (e.g., *ACTB* and *ACTR3*). Individual CRISPR-mediated knockout of representative genes within these pathways reduced CD47 protein expression without affecting CD47 mRNA expression. Consistently, pharmacological inhibition of BCR signaling (ibrutinib) or actin cytoskeleton regulation (latrunculin B) decreased CD47 protein expression across DLBCL and CNSL cell lines. Single-cell transcriptomic analysis showed that BCR pathway signatures were significantly upregulated in CNSL compared with DLBCL and were strongly correlated with CD47 protein expression but not with mRNA expression. Myeloid subcluster analysis revealed increased *SIRPA*-expressing macrophages in CNSL, suggesting a permissive niche for the CD47–SIRPα axis in the tumor microenvironment. In the CNSL PDX model, treatment with magrolimab significantly prolonged survival.

**Summary/Conclusion**

Our study uncovers a novel post-transcriptional regulatory mechanism of CD47 expression in B-cell lymphomas, in which BCR–AKT–mTOR signaling and actin cytoskeleton regulation are its key regulators. Furthermore, our findings provide fundamental evidence supporting CD47 blockade as a promising therapeutic strategy for CNSL.

**Copyright Information:** (Online) ISSN: 2572-9241
©2026 The Author(s). HemaSphere is published by John Wiley & Sons Ltd on behalf of European Hematology Association. This is an open access Abstract Book distributed under the Attribution-NonCommercial-NoDerivs (CC BY-NC-ND), which allows third parties to download the articles and share them with others as long as they credit the author and the Abstract Book, but they cannot change the content in any way or use them commercially.
**Abstract Book Citations:** Authors, Title, HemaSphere, 2026;10:(S1):pages. The abstract book DOIs can be found on the “Author Information” page.
**Disclaimer:** Articles published in the journal HemaSphere exclusively reflect the opinions of the authors. The authors are responsible for all content in their abstracts including accuracy of the facts, statements, citing resources, etc.

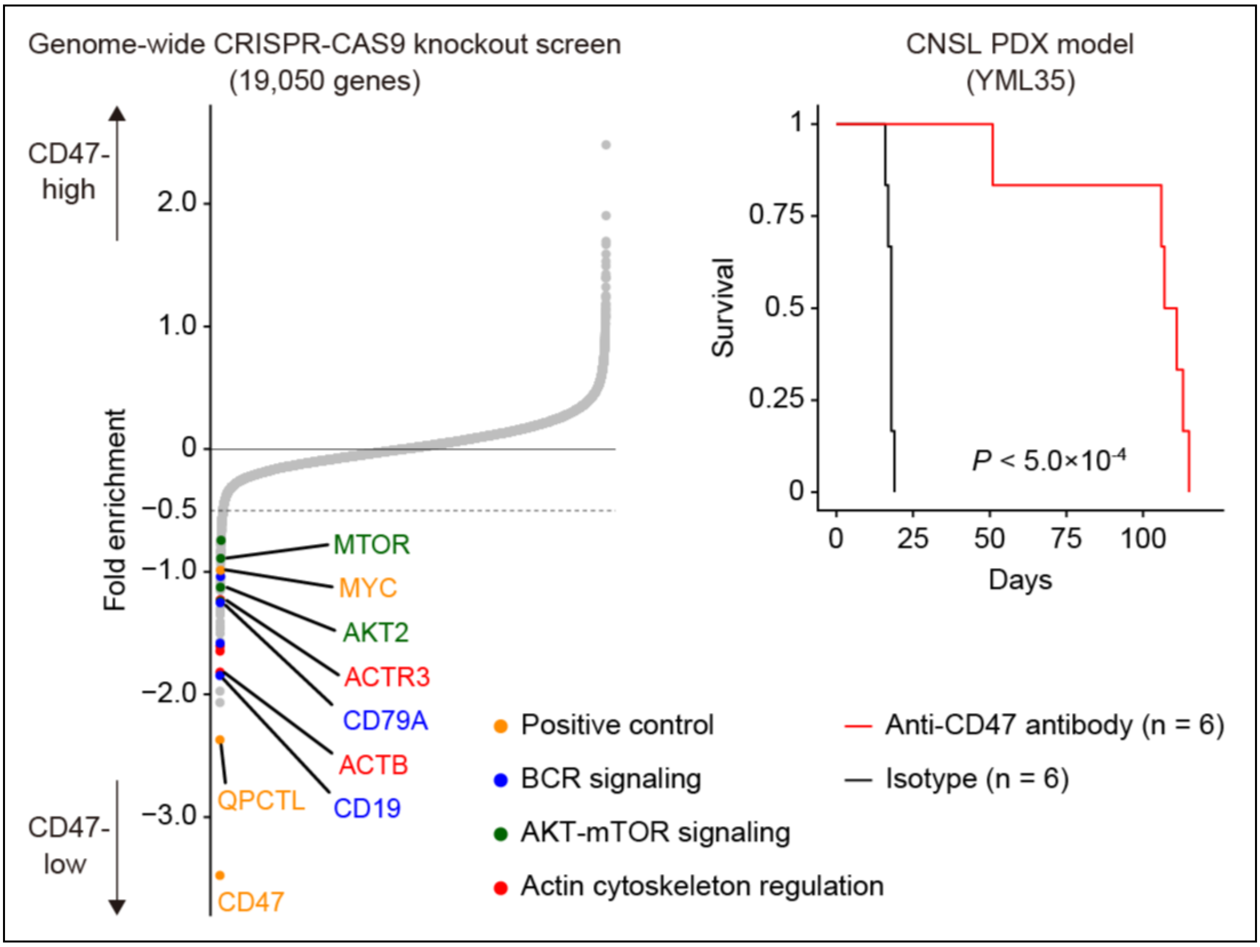

Left panel: Genome-wide CRISPR–Cas9 knockout screen identified positive regulators of CD47 expression, with significant enrichment of genes involved in BCR signaling, AKT–mTOR signaling, and actin cytoskeleton regulation. Right panel: Treatment with anti-CD47 antibody magrolimab significantly prolonged survival in the CNSL PDX model.

**Copyright Information:** (Online) ISSN: 2572-9241
©2026 The Author(s). HemaSphere is published by John Wiley & Sons Ltd on behalf of European Hematology Association. This is an open access Abstract Book distributed under the Attribution-NonCommercial-NoDerivs (CC BY-NC-ND), which allows third parties to download the articles and share them with others as long as they credit the author and the Abstract Book, but they cannot change the content in any way or use them commercially.
**Abstract Book Citations:** Authors, Title, HemaSphere, 2026;10:(S1):pages. The abstract book DOIs can be found on the "Author Information" page.
**Disclaimer:** Articles published in the journal HemaSphere exclusively reflect the opinions of the authors. The authors are responsible for all content in their abstracts including accuracy of the facts, statements, citing resources, etc.

### (PS2170) ATP1B3 REGULATES ALDOC-MEDIATED FRUCTOSE METABOLISM IN ALK-POSITIVE ANAPLASTIC LARGE CELL LYMPHOMA

**Topic:** 21. Lymphoma biology & translational research

Jinhua Liang*[1], Yuanli Gong[1], Tong-Yao Xing[1, 1], Chunyu Shang[1], Kai-Xin Du[1], Yi-Fan Wu[1], Li Wang[1], Jianyong Li[1], Wei Xu[1]

[1]*the First Affiliated Hospital of Nanjing Medical University, Jiangsu Province Hospital, Nanjing, China;*

### Background

ALK-positive anaplastic large cell lymphoma (ALK+ALCL) is a CD30-positive aggressive lymphoma. Although the prognosis is generally favorable, 30-40% of ALK+ALCL patients experience relapses or disease progression. Therefore, elucidating the molecular mechanisms underlying ALK+ ALCL and identifying novel therapeutic targets remain of substantial clinical importance. In this study, we identified sodium/potassium-transporting ATPase subunit beta-3 (ATP1B3) as the essential gene for the growth and survival of ALK+ALCL by CRISPR/Cas9 genome-wide loss-of-function screening **(Fig. 1A)**.

### Aims

To elucidates the role of ATP1B3 in ALK+ALCL progression, providing theoretical foundation for developing targeted strategies.

### Methods

Gene knockout was achieved by CRISPR/Cas9. Animal studies were approved by the Institutional Animal Care and Use Committee. RNA-seq, scRNA-seq, Deep-DIA and CUT-Tag sequencing were performed to investigate how ATP1B3 exerts effects on biological processes in ALK+ALCL.

### Results

In vitro and in vivo functional assays demonstrated that ATP1B3 knockout significantly inhibited the proliferation of Karpas299 and SUDHL1 cells and promoted apoptosis **(Fig. 1B-F)**. RNA-seq analysis of stable ATP1B3 knockout cell lines revealed that fructose metabolism and the HIF-1 hypoxia signaling pathway were significantly activated **(Fig. 1G)**. Differential gene expression analysis identified ALDOC as one of the most significantly differentially expressed genes between the ATP1B3 knockout and control groups **(Fig. 1H)**. In ATP1B3 knockout cells, both HIF-1α and ALDOC expression were downregulated (**Fig. 1I**), while intracellular fructose levels decreased, and fructose consumption in the culture medium was reduced **(Fig. 1J-K)**. Further studies showed that ALDOC knockout significantly decreased fructose utilization and inhibited cell proliferation **(Fig. 1L-N)**, indicating that ATP1B3 regulates fructose metabolism through ALDOC. Hypoxic culture conditions partially restored ALDOC expression in ATP1B3 knockout cells, but this recovery was abolished in HIF-1α knockout cells **(Fig. 1O-P)**, suggesting that HIF-1α mediates the ATP1B3-dependent regulation of ALDOC. Dual-luciferase reporter assays confirmed that ALDOC directly binds to the promoter region of HIF-1α and identified its specific binding sites **(Fig. 1Q-R)**. After fructose treatment, ALDOC expression and its O-GlcNAcylation levels were significantly increased in Karpas299 and SUDHL1 cells. These effects were reversed by ATP1B3 knockout or treatment with an OGT inhibitor **(Fig. 1S-T)**, suggesting that ATP1B3 promotes ALK+ ALCL proliferation via ALDOC-mediated fructose metabolism.Integrative analysis of RNA-seq data from ATP1B3 knockout cells and Deep DIA proteomic data from ALDOC knockout cells identified PGK1 as a key downstream target for ALDOC O-GlcNAc modification **(Fig. 1U)**. Co-immunoprecipitation experiments further confirmed the interaction between PGK1 and ALDOC **(Fig. 1V)**. PGK1 knockout significantly reduced ALDOC O-GlcNAcylation, and exogenous fructose treatment partially restored this modification **(Fig. 1W)**.

**Copyright Information:** (Online) ISSN: 2572-9241
©2026 The Author(s). HemaSphere is published by John Wiley & Sons Ltd on behalf of European Hematology Association. This is an open access Abstract Book distributed under the Attribution-NonCommercial-NoDerivs (CC BY-NC-ND), which allows third parties to download the articles and share them with others as long as they credit the author and the Abstract Book, but they cannot change the content in any way or use them commercially.
**Abstract Book Citations:** Authors, Title, HemaSphere, 2026;10:(S1):pages. The abstract book DOIs can be found on the "Author Information" page.
**Disclaimer:** Articles published in the journal HemaSphere exclusively reflect the opinions of the authors. The authors are responsible for all content in their abstracts including accuracy of the facts, statements, citing resources, etc.

### Summary/Conclusion

This study demonstrates that ATP1B3 promotes the proliferation of ALK+ ALCL cells by regulating the ALDOC-mediated fructolysis pathway **(Fig. 1X)**. From the perspective of metabolic reprogramming, this provides new molecular insights into the pathogenesis of ALK+ALCL and lays a potential theoretical foundation for targeted interventions in its treatment.

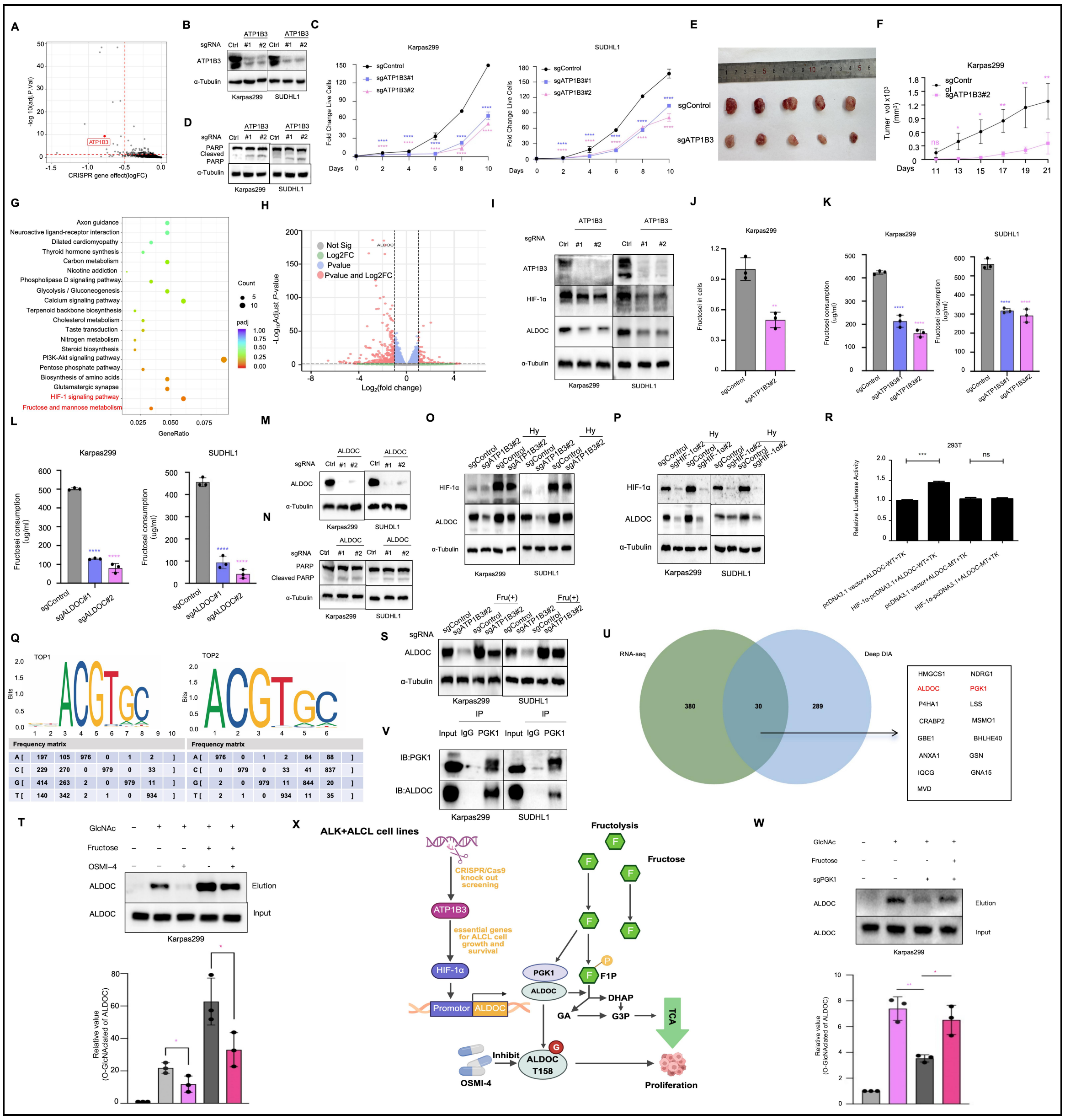

**Copyright Information:** (Online) ISSN: 2572-9241
©2026 The Author(s). HemaSphere is published by John Wiley & Sons Ltd on behalf of European Hematology Association. This is an open access Abstract Book distributed under the Attribution-NonCommercial-NoDerivs (CC BY-NC-ND), which allows third parties to download the articles and share them with others as long as they credit the author and the Abstract Book, but they cannot change the content in any way or use them commercially.
**Abstract Book Citations:** Authors, Title, HemaSphere, 2026;10:(S1):pages. The abstract book DOIs can be found on the "Author Information" page.
**Disclaimer:** Articles published in the journal HemaSphere exclusively reflect the opinions of the authors. The authors are responsible for all content in their abstracts including accuracy of the facts, statements, citing resources, etc.

### (PS2171) SPATIAL PROTEOMICS IDENTIFIES T CELL CENTERED SPATIAL PATTERNS IN HIGH-RISK MANTLE CELL LYMPHOMA BY THE MULTIPLY CONSORTIUM

**Topic:** 21. Lymphoma biology & translational research

Sarah Koziel*[1, 2, 3], Matthias Meyer-Bender[4, 5], Paul J. Bröckelmann[2, 6, 7], Christina Schniederjohann[1, 2, 4, 8], Erin Chung[4, 5], Ekaterina Popova[1, 2], Sarah Reinke[9], Andreas Rosenwald[10], Marc Seifert[1, 2], Wolfram Klapper[9], Silvia Bea[11], Marco Ladetto[12], Eva Hoster[13], Simone Ferrero[14], Martin Dreyling[15], Ron Jachimowicz[2, 6, 7, 16], Wolfgang Huber[4, 5], Sascha Dietrich[1, 2, 4, 5, 8], Peter-Martin Bruch[1, 2, 4, 8]

*[1]Medical Faculty and University Hospital Düsseldorf, Heinrich Heine University Düsseldorf, Department of Hematology, Oncology and Clinical Immunology, Düsseldorf, Germany; [2]Center for Integrated Oncology Aachen-Bonn-Cologne-Düsseldorf (CIO ABCD), Cologne and Düsseldorf, Germany; [3]University Hospital Düsseldorf, Düsseldorf School of Oncology (DSO), Düsseldorf, Germany; [4]Molecular Medicine Partnership Unit (MMPU), Heidelberg, Germany; [5]European Molecular Biology Laboratory, Heidelberg, Germany; [6]Faculty of Medicine and University Hospital Cologne, University of Cologne, Department I of Internal Medicine, Cologne, Germany; [7]Max Planck Institute for Biology of Ageing, Max Planck Research Group Mechanisms of DNA Repair, Cologne, Germany; [8]Heidelberg University Hospital, Department of Medicine V, Heidelberg, Germany; [9]University Hospital Schleswig-Holstein, Campus Kiel, Department of Pathology, Hematopathology Section and Lymph Node Registry, Kiel, Germany; [10]University of Würzburg, Department of Pathology, Würzburg, Germany; [11]Hospital Clínic de Barcelona, Institut d'Investigacions Biomèdiques August Pi i Sunyer, Pathology Department, Barcelona, Spain; [12]Università del Piemonte Orientale, AOU Santi Antonio e Biagio e Cesare Arrigo, Dipartimento di Medicina Traslazionale, SCDU Ematologia, Alessandria, Italy; [13]Ludwig-Maximilians-University, Institute for Medical Information Processing, Biometry, and Epidemiology (IBE), Munich, Germany; [14]University of Torino, Hematology Division, Department of Molecular Biotechnology and Health Sciences, Torino, Italy; [15]Ludwig-Maximilians-University, University Hospital, Department of Medicine III, Munich, Germany; [16]University of Cologne, Cologne Excellence Cluster on Cellular Stress Response in Aging-Associated Diseases (CECAD), Cologne, Germany;*

### Background

Mantle cell lymphoma (MCL) exhibits heterogeneous clinical outcomes, necessitating robust risk stratification. While the transcriptomic MCL35 cell proliferation score assesses lymphoma aggressiveness, it remains unclear how microenvironmental risk factors and the spatial organization of malignant cells and the tumor microenvironment (TME) impact the clinical outcome.

### Aims

Using spatial proteomics on baseline MCL biopsies from patients treated with chemoimmunotherapy, we mapped the MCL TME at single-cell resolution to identify cellular composition, cell states as well as spatial patterns and link these features to MCL35 risk categories and clinical outcome.

### Methods

Diagnostic formalin-fixed, paraffin-embedded primary tissue biopsies from 178 patients enrolled in the MCL Younger (n = 108) and MCL Elderly (n = 70) trials were profiled by CO-detection and indexing (CODEX) with a 59-plex marker panel. Spatial analyses including segmentation, protein quantification, cell phenotyping, and colocalization analysis were performed utilizing the Python toolbox *spatialproteomics*. Associations with MCL35 risk categories were tested using nonparametric Kruskal–Wallis tests followed by Dunn's post-hoc comparisons with FDR correction. Primary analyses focused on comparisons between MCL35 low- and high-risk groups. Associations with overall survival (OS) were evaluated using Cox proportional hazards models.

**Copyright Information:** (Online) ISSN: 2572-9241
©2026 The Author(s). HemaSphere is published by John Wiley & Sons Ltd on behalf of European Hematology Association. This is an open access Abstract Book distributed under the Attribution-NonCommercial-NoDerivs (CC BY-NC-ND), which allows third parties to download the articles and share them with others as long as they credit the author and the Abstract Book, but they cannot change the content in any way or use them commercially.
**Abstract Book Citations:** Authors, Title, HemaSphere, 2026;10:(S1):pages. The abstract book DOIs can be found on the "Author Information" page.
**Disclaimer:** Articles published in the journal HemaSphere exclusively reflect the opinions of the authors. The authors are responsible for all content in their abstracts including accuracy of the facts, statements, citing resources, etc.

### Results

High-risk MCL samples, classified by MCL35 scoring, exhibited changes in TME composition, including increased infiltration of macrophages and exhausted cytotoxic T cells. They were also characterized by spatial reorganization of T cell compartments. Reduced spatial colocalization of T helper cells with antigen-presenting macrophages ($p = 0.025$) and dendritic cells ($p = 0.023$) contrasted with increased tumor-proximal localization of T helper ($p < 0.001$) and regulatory T cells ($p < 0.001$). In multivariable Cox models adjusting for MCL35 risk category, reduced TCF1 expression in progenitor exhausted cytotoxic T cells remained associated with inferior OS (HR = 0.51, 95% CI 0.37 – 0.70; $p < 0.001$). Reduced TCF1 expression indicates compromised stem-like characteristics of progenitor exhausted T cells. In addition, lower spatial proximity between cytotoxic T cells and endothelial cells was associated with inferior OS (HR = 0.84, 95% CI 0.70 – 1.00, $p = 0.044$), suggesting altered immune trafficking.

### Summary/Conclusion

High MCL35 risk score is accompanied by an altered spatial coupling of T cells rather than a classical cold immune microenvironment. In parallel, reduced TCF1 expression in progenitor exhausted cytotoxic T cells represents an independent immune state linked to inferior survival. Together, spatially altered T cell–tumor and T cell–APC colocalization and impaired stem-like features of progenitor exhausted T cells may reflect compromised immune fitness contributing to adverse clinical outcome. Extension of these analyses to BTK inhibitor containing treatment arms within the TRIANGLE trial will assess whether therapeutic intervention can modulate these spatial immune dysfunction signatures and inform immune fitness–based patient stratification.

This study was funded by Blood Cancer United (LLS No. MCL 7005-24).

**Copyright Information:** (Online) ISSN: 2572-9241
©2026 The Author(s). HemaSphere is published by John Wiley & Sons Ltd on behalf of European Hematology Association. This is an open access Abstract Book distributed under the Attribution-NonCommercial-NoDerivs (CC BY-NC-ND), which allows third parties to download the articles and share them with others as long as they credit the author and the Abstract Book, but they cannot change the content in any way or use them commercially.
**Abstract Book Citations:** Authors, Title, HemaSphere, 2026;10:(S1):pages. The abstract book DOIs can be found on the "Author Information" page.
**Disclaimer:** Articles published in the journal HemaSphere exclusively reflect the opinions of the authors. The authors are responsible for all content in their abstracts including accuracy of the facts, statements, citing resources, etc.

### (PS2172) SINGLE-CELL AND SPATIAL TRANSCRIPTOMICS ANALYSIS OF PRIMARY CNS LYMPHOMA

**Topic:** 21. Lymphoma biology & translational research

Cong Wang*[1], Tiange Lu[1], Xiangrong Zheng[2], Yue Li[1], Kang Lu[1], Xinyu Yang[1], Xinting Hu[1], Xiangxiang Zhou[1]

[1]*Shandong Provincial Hospital Affiliated to Shandong First Medical University, Department of Hematology, Jinan, China;*
[2]*Shandong Provincial Hospital Affiliated to Shandong First Medical University, Department of Neurosurgery, Jinan, China;*

### Background

Although a majority of primary central nervous system lymphoma (PCNSL) cases histologically stem from diffuse large B-cell lymphoma (DLBCL), their long-term survival remains markedly inferior to systemic DLBCL. This disparity likely reflects its molecular heterogeneity, immune-privileged niche and immunosuppressive microenvironment, yet the pathophysiological mechanism remains poorly defined.

### Aims

This study aims to systematically define the intratumoral heterogeneity and immune microenvironment of PCNSL, providing novel insights into its pathogenesis and establishing a theoretical framework for identification of potential therapeutic targets.

### Methods

We integrated single cell and spatial transcriptomic profiling of PCNSL samples with bulk RNA sequencing data from multiple tumor cohorts. Spatial transcriptomes were deconvolved using SPOTlight. Trajectory inference, pathway enrichment, and scMetabolism analyses characterized transcriptional programs, spatial features, and metabolic states. BayesPrism based deconvolution of DLBCL bulk RNA sequencing followed by survival analysis assessed clinical relevance. Cell communication networks were reconstructed using CellChat, and spatial expression patterns were used to identify key ligand-receptor axes.

### Results

We identified four transcriptionally and spatially distinct malignant B cell (mBc) subpopulations within PCNSL. Pseudotime trajectory analysis revealed a developmental continuum originating from mBc3, progressing through mBc4 and mBc1, and culminating in a terminal mBc2 state. The mBc2 subset exhibited a pronounced oxidative phosphorylation (OXPHOS) metabolic signature and was significantly associated with adverse clinical outcomes ($p < 0.001$). Spatial transcriptomic analyses confirmed that mBc2 localized to regions characterized by elevated OXPHOS activity. Notably, COX7B, a structural subunit of mitochondrial respiratory chain complex IV, was identified as a specific marker of mBc2, markedly upregulated in this terminally differentiated state. Functional assays demonstrated that stable knockdown or pharmacologic targeting of COX7B significantly impaired tumor cell proliferation and viability via inducing apoptosis, underscoring its therapeutic potential.

Concomitantly, we further clarified the immunosuppressive features of the PCNSL microenvironment and the regulatory mechanisms mediated by malignant B cells. Exhausted T cell subgroup (exhTc) was markedly enriched, accompanied by upregulation of multiple immune checkpoint molecules, including HAVCR2/TIM-3 and CTLA4, consistent with a canonical exhaustion phenotype. Cell-cell communication analysis revealed that mBc subsets engaged in strong interactions with exhTc and regulatory $CD4^+$ T cells (TregCD4) predominantly through the MIF signaling pathway, mediated via the CD74-CXCR4/CD44 axis. Spatial analyses further demonstrated sustained activation of this signaling axis within tumor regions, highlighting its central role in maintaining immune suppression.

**Copyright Information:** (Online) ISSN: 2572-9241
©2026 The Author(s). HemaSphere is published by John Wiley & Sons Ltd on behalf of European Hematology Association. This is an open access Abstract Book distributed under the Attribution-NonCommercial-NoDerivs (CC BY-NC-ND), which allows third parties to download the articles and share them with others as long as they credit the author and the Abstract Book, but they cannot change the content in any way or use them commercially.
**Abstract Book Citations:** Authors, Title, HemaSphere, 2026;10:(S1):pages. The abstract book DOIs can be found on the "Author Information" page.
**Disclaimer:** Articles published in the journal HemaSphere exclusively reflect the opinions of the authors. The authors are responsible for all content in their abstracts including accuracy of the facts, statements, citing resources, etc.

**Summary/Conclusion**

Collectively, our study revealed the intratumoral heterogeneity and immunosuppressive features of PCNSL microenvironment, identified a high-risk COX7B+ mBc2 subset with high OXPHOS activity and verified the therapeutic potential of COX7B. Furthermore, we elucidated the critical role of MIF signaling pathway in shaping the immunosuppressive microenvironment, thereby providing new directions for targeted and immunotherapeutic strategies in PCNSL.

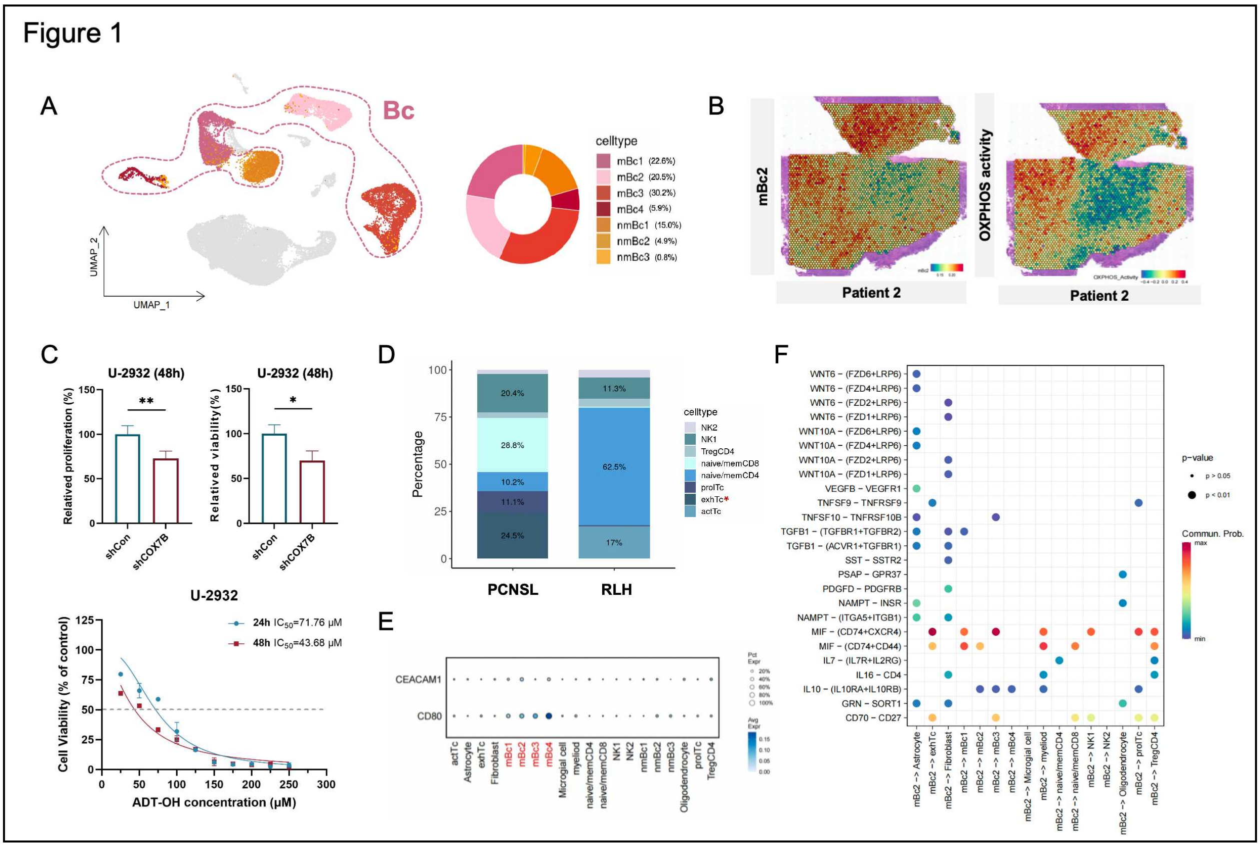

**Copyright Information:** (Online) ISSN: 2572-9241
©2026 The Author(s). HemaSphere is published by John Wiley & Sons Ltd on behalf of European Hematology Association. This is an open access Abstract Book distributed under the Attribution-NonCommercial-NoDerivs (CC BY-NC-ND), which allows third parties to download the articles and share them with others as long as they credit the author and the Abstract Book, but they cannot change the content in any way or use them commercially.
**Abstract Book Citations:** Authors, Title, HemaSphere, 2026;10:(S1):pages. The abstract book DOIs can be found on the "Author Information" page.
**Disclaimer:** Articles published in the journal HemaSphere exclusively reflect the opinions of the authors. The authors are responsible for all content in their abstracts including accuracy of the facts, statements, citing resources, etc.

### (PS2173) INTEGRATIVE MULTI-OMICS ANALYSIS OF MICRORNA REGULATION IN RICHTER TRANSFORMATION

**Topic:** 21. Lymphoma biology & translational research

Romain Piucco[1], Sébastien Hergalant[1], Ghislain Fievet[1], Stéphanie Lacomme[2], Sandra Lomazzi[2], Guillaume Gauchotte[2], Aurélien Desvilles[1], Reiner Siebert[3], Stephan Stilgenbauer[3], Pierre Feugier[2], Julien Broseus*[2]

*[1]INSERM U1256, Nancy, France; [2]Hospital Center Régional And University de Nancy Hospital Central, Nancy, France; [3]University of Ulm, Ulm, Germany;*

### Background

Richter transformation (RT) refers to the emergence of aggressive histology, usually diffuse large B-cell lymphoma (DLBCL), in patients with chronic lymphocytic leukemia (CLL). RT is associated with chemoresistance and poor survival. Most RT cases (~80-90%) arise from a pre-existing CLL clone, whereas a minority are clonally unrelated. No histological, genetic, or molecular features reliably predict transformation, and distinguishing RT from *de novo* DLBCL remains challenging. Characterisation of RT genetic and transcriptomic landscapes revealed recurrent alterations in PI3K/AKT, B-cell receptor, NOTCH, MYC, MAPK, and cell cycle pathways. Yet, these layers alone cannot fully explain aggressiveness, heterogeneity, or therapy resistance. Recent proteomic analyses have shown discrepancies with transcriptomic profiles, indicating implication of post-transcriptional processes.
MicroRNAs (miRNAs) are non-coding RNAs that regulate protein translation and play central roles in B-cell biology and epigenetic regulation. However, their contribution to RT pathogenesis remains poorly understood.

### Aims

This study aimed to characterize miRNA deregulation in RT and its molecular subgroups, define features distinguishing RT from *de novo* DLBCL, identify the genomic and epigenetic mechanisms shaping the RT miRNome, and integrate transcriptomic and proteomic data to assess the functional impact of miRNAs on post-transcriptional regulation.

### Methods

Small RNA sequencing was performed on 64 tumor biopsies (36 RT, 18 *de novo* DLBCL, 3 paired CLL/RT, 7 normal lymph nodes). A dedicated pipeline was developped to reconstruct the miRNome and quantified mature miRNAs and isomiRs. Profiles were integrated with copy-number variation, DNA methylation, and matched transcriptomic and proteomic datasets. Differential analyses combined unsupervised and supervised approaches and were correlated with clinical and molecular variables.

### Results

RT exhibited a distinct miRNA signature compared with *de novo* DLBCL, involving regulators of PI3K/AKT, BCR signaling, proliferation, apoptosis, and metabolism. Clonally related RT showed reduced expression of miRNAs controlling proliferation, metabolism, antigen presentation, and receptor-mediated cell death, whereas clonally unrelated RT displayed increased expression of miRNAs targeting TLR and MYD88 pathways. IsomiR abundance was condition-specific and may reflect the impact of *SF3B1* mutation.
A substantial proportion of deregulated miRNAs were associated with concordant copy-number variations and/or DNA methylation alterations at their precursor loci. Integration with matched transcriptomic and proteomic datasets revealed context-dependent disruption of post-transcriptional control. Selected transcripts (CDK6, BID) remained repressed despite a permissive molecular environment, whereas others (PRKCB, PDCD4) appeared to escape miRNA-mediated regulation and maintained elevated expression, supporting a functional contribution of miRNA deregulation to the transcriptional and proteomic imbalance observed in RT.

**Copyright Information:** (Online) ISSN: 2572-9241
©2026 The Author(s). HemaSphere is published by John Wiley & Sons Ltd on behalf of European Hematology Association. This is an open access Abstract Book distributed under the Attribution-NonCommercial-NoDerivs (CC BY-NC-ND), which allows third parties to download the articles and share them with others as long as they credit the author and the Abstract Book, but they cannot change the content in any way or use them commercially.
**Abstract Book Citations:** Authors, Title, HemaSphere, 2026;10:(S1):pages. The abstract book DOIs can be found on the "Author Information" page.
**Disclaimer:** Articles published in the journal HemaSphere exclusively reflect the opinions of the authors. The authors are responsible for all content in their abstracts including accuracy of the facts, statements, citing resources, etc.

### Summary/Conclusion

Together, these results demonstrate that miRNA deregulation in RT is shaped by coordinated genomic and epigenetic alterations and functionally impacts mRNA and protein expression in a biologically context-dependent manner. Profound differences were observed between CLL-related and unrelated RT, the latter closer to *de novo* DLBCL, highlighting distinct regulatory architectures.

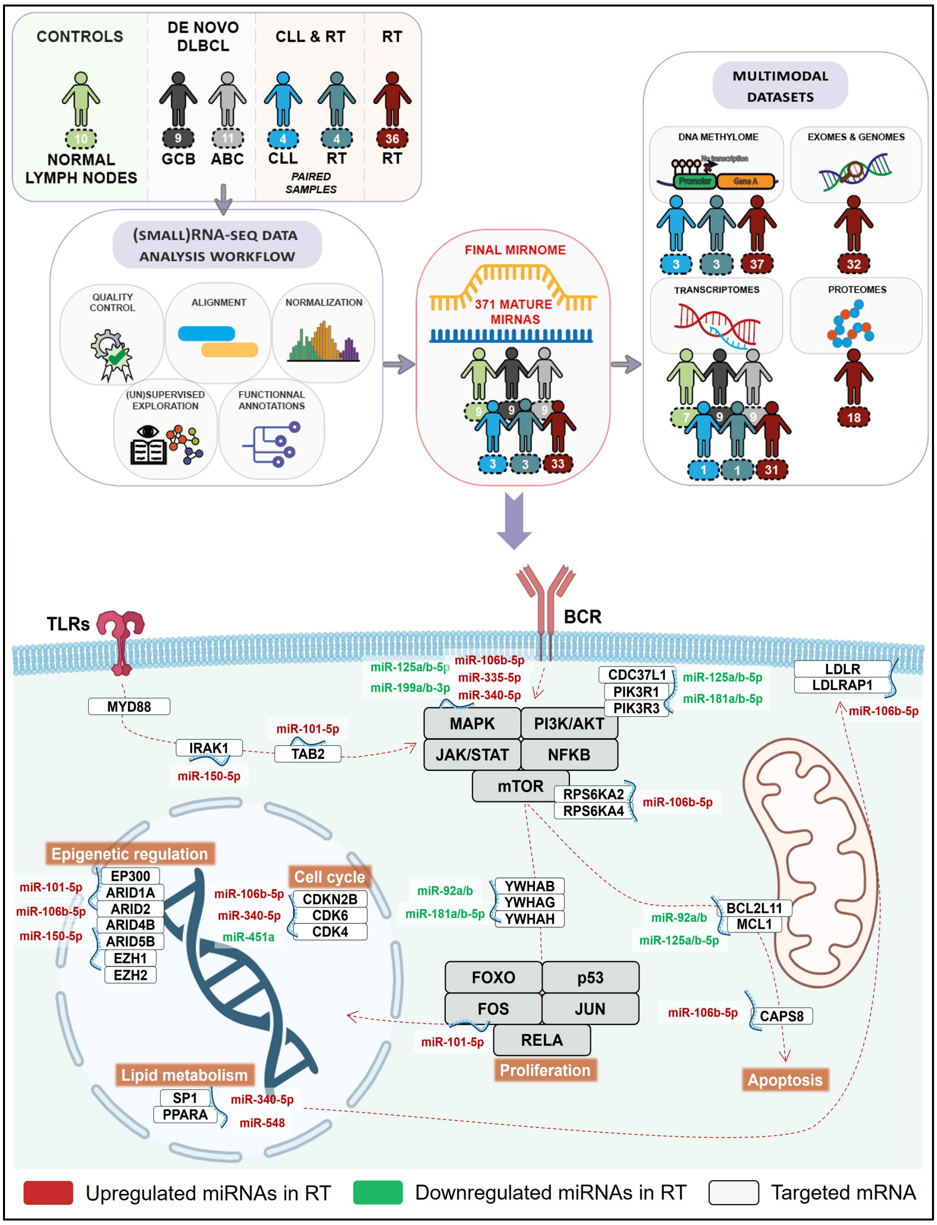

**Copyright Information:** (Online) ISSN: 2572-9241
©2026 The Author(s). HemaSphere is published by John Wiley & Sons Ltd on behalf of European Hematology Association. This is an open access Abstract Book distributed under the Attribution-NonCommercial-NoDerivs (CC BY-NC-ND), which allows third parties to download the articles and share them with others as long as they credit the author and the Abstract Book, but they cannot change the content in any way or use them commercially.
**Abstract Book Citations:** Authors, Title, HemaSphere, 2026;10:(S1):pages. The abstract book DOIs can be found on the "Author Information" page.
**Disclaimer:** Articles published in the journal HemaSphere exclusively reflect the opinions of the authors. The authors are responsible for all content in their abstracts including accuracy of the facts, statements, citing resources, etc.

### (PS2174) TUMOR EVOLUTION AND IMMUNE MICROENVIRONMENT DYNAMICS IN PRIMARY AND RELAPSED MANTLE CELL LYMPHOMA

**Topic:** 21. Lymphoma biology & translational research

Hui Wan*[1], Weicheng Ren[1], Birgitta Sander[2], Qiang Pan-Hammarstrom[1]

*[1]Karolinska Institutet, Department of Medical Biochemistry and Biophysics, Stockholm, Sweden;[2]Laboratory Medicine, Division of Pathology, Laboratory Medicine, Division of Pathology, Stockholm, Sweden;*

### Background

Mantle cell lymphoma (MCL) is an aggressive and largely incurable subtype of non-Hodgkin lymphoma characterized by significant clinical and genetic heterogeneity. Although intensive frontline therapies can extend the median progression-free survival to 7-10 years, most patients eventually experience disease relapse, facing particularly challenging treatment options and a very poor prognosis thereafter. However, the molecular and clonal mechanisms underlying disease relapse and therapeutic resistance remain inadequately understood.

### Aims

To elucidate the clonal architecture and dynamic molecular evolution of tumor B cells from during the disease progression and therapeutic resistance in MCL.

### Methods

We performed single-cell RNA sequencing (scRNA-seq) and single-cell B-cell receptor sequencing (scBCR-seq), and whole-genome sequencing (WGS) on 20 longitudinal samples collected at diagnosis and/or relapse from 11 MCL patients. By integrating somatic mutations, copy-number alterations, BCR clonotypes, and transcriptional states, we inferred clonal composition, evolutionary relationships, and tumor–microenvironment interactions across different disease stages.

### Results

Substantial intra-tumor heterogeneity was observed at diagnosis, with multiple malignant subclones coexisting within individual patients, potentially contributing to limited therapeutic efficacy. Longitudinal analyses demonstrated patient-specific evolutionary trajectories during progression. Residual or dormant tumor cells present at diagnosis may acquire new mutations and copy number variations, such as *TP53* deletion and *CDKN2A* mutations, and/or migrate to favorable microenvironments for expansion, ultimately driving disease relapse. Despite marked interpatient variability, recurrent alterations, transcriptomic changes in tumor cells affecting key signaling pathways and alterations in the tumor microenrivroment were consistently observed during diesase progression. Notably, CD70–CD27 interactions between MCL cells and T-cell subsets emerged as a potential mechanism of tumor–immune crosstalk and a candidate therapeutic target in relapsed MCL.

### Summary/Conclusion

Our integrative multi-omics analysis reveals substantial early clonal complexity in MCL and demonstrates that relapse arises through diverse, patient-specific evolutionary routes involving genetic evolution and microenvironmental adaptation, providing insights to understanding tumor–immune co-evolution in MCL and may inform strategies for precision monitoring and therapeutic intervention.

**Copyright Information:** (Online) ISSN: 2572-9241
©2026 The Author(s). HemaSphere is published by John Wiley & Sons Ltd on behalf of European Hematology Association. This is an open access Abstract Book distributed under the Attribution-NonCommercial-NoDerivs (CC BY-NC-ND), which allows third parties to download the articles and share them with others as long as they credit the author and the Abstract Book, but they cannot change the content in any way or use them commercially.
**Abstract Book Citations:** Authors, Title, HemaSphere, 2026;10:(S1):pages. The abstract book DOIs can be found on the “Author Information” page.
**Disclaimer:** Articles published in the journal HemaSphere exclusively reflect the opinions of the authors. The authors are responsible for all content in their abstracts including accuracy of the facts, statements, citing resources, etc.

### (PS2175) CIRCULATING TUMOR DNA BASED MOLECULAR TUMOR BURDEN DYNAMICS IN A REAL-WORLD COHORT OF PATIENTS WITH FOLLICULAR LYMPHOMA

**Topic:** 21. Lymphoma biology & translational research

Ana Isabel Jimenez Ubieto[1], Alejandro Martín Muñoz[2], Alvaro Bienert Garcia[1], Davide Di-Lernia[3], Angel I. Perez-Santiago[3], Maria Poza[1], Gloria Figaredo Garcia-Mina[4], Paula Sanchez-Llorca[1], Daniel Gil-Alós[1], Enrique Revilla[1], Laura Astilleros[1], Pilar Sarandeses[1], Margarita Rodriguez[2], Tycho Stephan Baumann[1], Antonia Rodriguez Izquierdo[1], Carolina Martinez Laperche[4], Margarita Sanchez-Beato[3], Inmaculada Rapado[1], Mariana Bastos-Oreiro[4], Joaquín Martinez-Lopez[1], Santiago Barrio*[3]

*[1]Hospital 12 de Octubre, Hematology, Madrid, Spain; [2]Altum sequencing S.L, Madrid, Spain; [3]Hospital U. Puerta de Hierro-Majadahonda, IDIPHISA, Medical Oncology Department, Majadahonda, Spain; [4]Hospital General Universitario Gregorio Marañón, Hematology, Madrid, Spain;*

### Background

Circulating tumor DNA (ctDNA) is an emerging prognostic biomarker in follicular lymphoma (FL) enabling minimally invasive disease monitoring. However, the clinical relevance of longitudinal molecular tumor burden (MTB) dynamics remains incompletely defined.

### Aims

To evaluate MTB dynamics in a real-world FL cohort using a personalized ctDNA assay combined with an unsupervised Hidden Markov Model (HMM)–based longitudinal framework, and to compare ctDNA- and PET-based response assessments.

### Methods

Serial plasma samples from 113 FL patients treated between 2018 and 2024 were analyzed in frontline and relapsed/refractory (R/R) settings (432 samples collected at baseline, interim evaluation, end of treatment [EOT], and every 6 months thereafter). Frontline patients received immunochemotherapy (90% R-CHOP). R/R patients were treated with CAR-T (n=16), bispecific antibodies (n=7), or rituximab-lenalidomide (n=12). PET-CT was performed at standard time points.
MRD was quantified using a personalized NGS assay targeting patient-specific variants (sensitivity $10^{-4}$). ctDNA dynamics were assessed using investigator-defined criteria and unsupervised HMM modeling. Models with 2–4 states were evaluated; a three-state model was selected based on AIC/BIC and likelihood metrics. The Viterbi algorithm assigned patient-specific state trajectories. Analyses included cases with ≥2 time points. Validation incorporated case-centric partitioning, cross-validation, and bootstrap out-of-bag testing. ctDNA kinetic profiles were classified according to MRD status and ≥20% slope variation.

**Copyright Information:** (Online) ISSN: 2572-9241
©2026 The Author(s). HemaSphere is published by John Wiley & Sons Ltd on behalf of European Hematology Association. This is an open access Abstract Book distributed under the Attribution-NonCommercial-NoDerivs (CC BY-NC-ND), which allows third parties to download the articles and share them with others as long as they credit the author and the Abstract Book, but they cannot change the content in any way or use them commercially.
**Abstract Book Citations:** Authors, Title, HemaSphere, 2026;10:(S1):pages. The abstract book DOIs can be found on the “Author Information” page.
**Disclaimer:** Articles published in the journal HemaSphere exclusively reflect the opinions of the authors. The authors are responsible for all content in their abstracts including accuracy of the facts, statements, citing resources, etc.

### Results

Median age was 64 years; 46% had high-risk FLIPI at treatment initiation. At EOT, detectable ctDNA predicted significantly inferior 3-year PFS in both frontline (38% vs 100%) and R/R cohorts (15% vs 72%). PET positivity was also associated with inferior outcomes. Combined ctDNA/PET assessment improved risk stratification: ctDNA-/PET- patients had the most favorable outcomes, whereas ctDNA+/PET+ patients had the poorest prognosis ($p<0.0001$).
Unsupervised longitudinal modeling identified three distinct molecular dynamic states with strong concordance to investigator-defined categories, supporting biological validity. Importantly, the HMM framework reclassified 4 of 6 cases initially categorized as low risk into the intermediate-risk group; all subsequently progressed, indicating that trajectory-based molecular patterns capture clinically relevant risk not detected by conventional assessment. Clinical outcomes differed markedly across states. Most patients (n=76) were classified as low risk, with only three progressions. Intermediate-risk patients (n=24) had median PFS of 42 months (HR 13.5, 95% CI 2.9–63.9; $p<0.001$), while high-risk patients (n=13) had median PFS of 9 months (HR 34.3, 95% CI 7.7–163.5; $p<0.001$ vs low risk). Overall, HMM-derived trajectories improved prognostic discrimination beyond static response assessment.

### Summary/Conclusion ctDNA-guided MTB dynamics enable early risk stratification and provide clinically meaningful prognostic information in FL. Incorporation of longitudinal ctDNA modeling into prospective trials may facilitate earlier intervention strategies and biologically informed treatment personalization.

**Copyright Information:** (Online) ISSN: 2572-9241
©2026 The Author(s). HemaSphere is published by John Wiley & Sons Ltd on behalf of European Hematology Association. This is an open access Abstract Book distributed under the Attribution-NonCommercial-NoDerivs (CC BY-NC-ND), which allows third parties to download the articles and share them with others as long as they credit the author and the Abstract Book, but they cannot change the content in any way or use them commercially.
**Abstract Book Citations:** Authors, Title, HemaSphere, 2026;10:(S1):pages. The abstract book DOIs can be found on the “Author Information” page.
**Disclaimer:** Articles published in the journal HemaSphere exclusively reflect the opinions of the authors. The authors are responsible for all content in their abstracts including accuracy of the facts, statements, citing resources, etc.

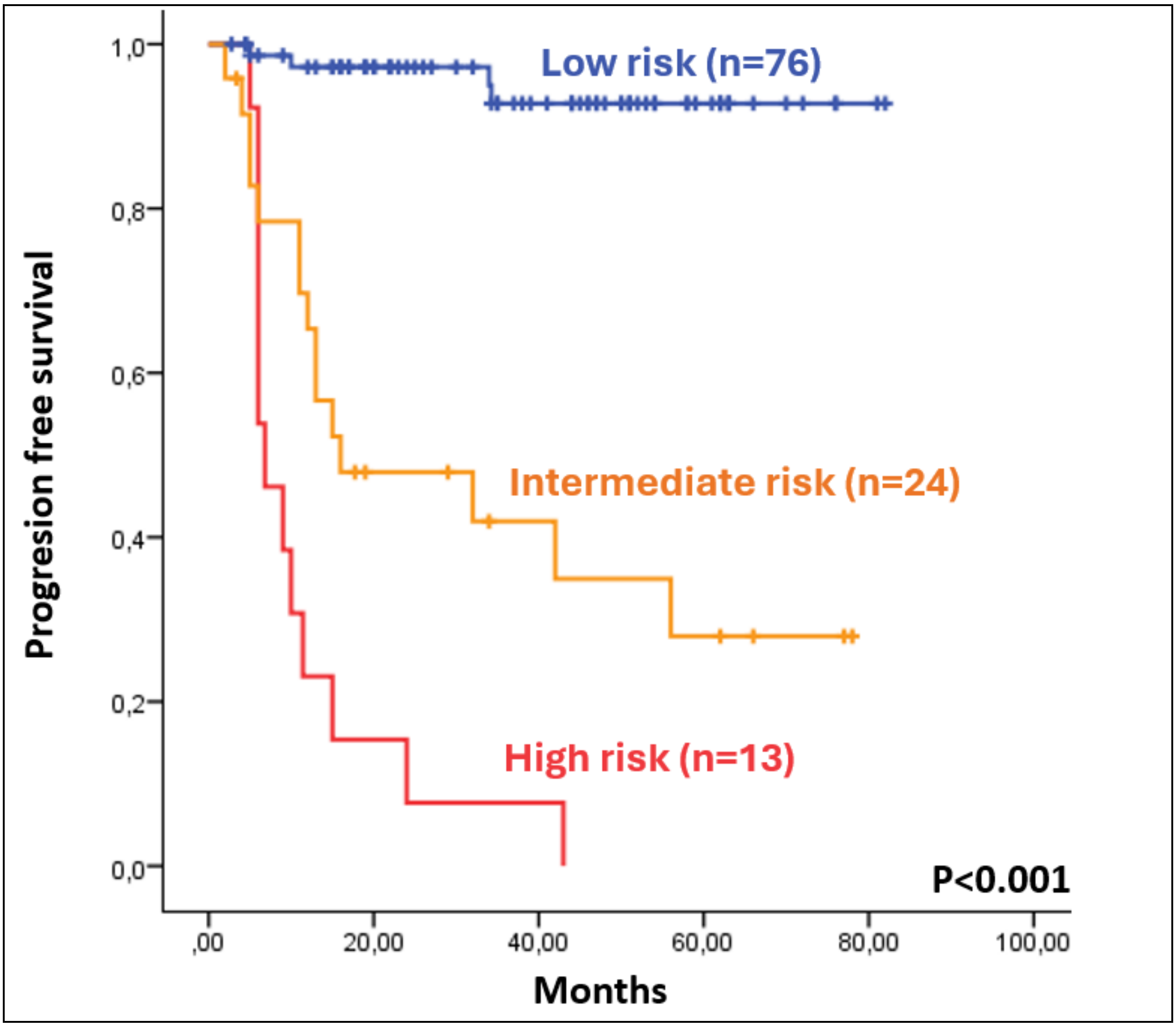

Copyright Information: (Online) ISSN: 2572-9241
©2026 The Author(s). HemaSphere is published by John Wiley & Sons Ltd on behalf of European Hematology Association. This is an open access Abstract Book distributed under the Attribution-NonCommercial-NoDerivs (CC BY-NC-ND), which allows third parties to download the articles and share them with others as long as they credit the author and the Abstract Book, but they cannot change the content in any way or use them commercially.
Abstract Book Citations: Authors, Title, HemaSphere, 2026;10:(S1):pages. The abstract book DOIs can be found on the "Author Information" page.
Disclaimer: Articles published in the journal HemaSphere exclusively reflect the opinions of the authors. The authors are responsible for all content in their abstracts including accuracy of the facts, statements, citing resources, etc.

### (PS2176) SH3GL1 ACTIVATE GLYCOLYTIC GENE PFKFB4 TO PROMOTES H3K18LA MODIFICATION IN DIFFUSE LARGE B-CELL LYMPHOMA PATHOGENESIS

**Topic:** 21. Lymphoma biology & translational research

Jinhua Liang*[1], Zi-Wen Duan[1], Chunyu Shang[1], Kai-Xin Du[1], Tong-Yao Xing[1], Li Wang[1], Jianyong Li[1], Wei Xu[1]

[1]*the First Affiliated Hospital of Nanjing Medical University, Jiangsu Province Hospital, Nanjing, China;*

### Background

Around 40% of diffuse large B-cell lymphoma (DLBCL) patients experiences refractory or relapsed, highlighting the need for new treatments. In our previous study, we identified SH3GL1 as the essential gene for the growth and survival of DLBCL by CRISPR/Cas9 genome-wide loss-of-function screening. With the deepening of study, we have found that SH3GL1 regulate glycolysis, contributing to DLBCL progression.

### Aims

To elucidates the role of SH3GL1 in DLBCL progression, providing theoretical foundation for developing targeted strategies.

### Methods

Gene knockout was achieved by CRISPR/Cas9. Animal studies were approved bythe Institutional AnimalRNA-seq,CareandUse Committee. scRNA-seq, Deep-DIA and CUT-Tag sequencing were performed to investigate how SH3GLl exerts effects on biological processes in DLBCL. Wild-type cells were injected subcutaneously in NOD/SCID male mice.

### Results

In our study, scRNA-seq revealed that SH3GL1 knockout inhibited glycolysis while activating apoptosis in both vivo and in vitro experiments (Fig. A-D). Glycolysis inhibitor (2-DG) greatly suppressed FARAGE growth, highlighting glycolysis's importance in DLBCL (Fig. E). Notably, SH3GL1 knockout reduced Lactate (Lac) production, while exogenous Lac supplementation attenuated SH3GL1 deficiency-induced cell death (Fig. F).
To investigate how SH3GL1 influences glycolysis, SH3GL1 was knocked out in BJAB, FARAGE, and SUDHL8 cells, followed by RNA-seq to find common differentially expressed genes. PFKFB4, a key glycolysis gene, was notably identified (Fig. G). WB showed that SH3GL1 knockout reduced PFKFB4, while overexpression increased them (Fig. H). Treatment with 5-MPN (PFKFB4 inhibitor), inhibited FARAGE growth (Fig. I) and Lac levels (Fig. J). Importantly, supplementation with Lac attenuated 5-MPN-induced apoptosis (Fig. K). To clarify how SH3GL1 regulates PFKFB4, Co-IP confirmed no direct interaction between them (Fig. L). Bioinformatic and WB identified POU2F1 as the transcriptional regulator linking SH3GL1 to PFKFB4 (Fig. M). Literature review uncovered POU2AF1 as POU2F1's canonical binding partner. Subsequent WB showed SH3GL1 knockout reduced POU2AF1 (Fig. N). To clarify how SH3GL1 regulates POU2AF1, Co-IP proved direct SH3GL1-POU2AF1 interaction with predicted domains (Fig. O). Furthermore, POU2AF1 depletion suppressed FARAGE growth and glycolysis, and adding Lac rescued cell death caused by POU2AF1 knockout (Fig. P-Q). These results suggest that SH3GL1 directly binds POU2AF1 to stabilize the POU2F1-PFKFB4 transcriptional complex, driving glycolytic flux and DLBCL tumorigenesis.
Lac, a glycolysis byproduct, is crucial in disease development. Our results showed adding Lac prevent cell death caused by SH3GL1 or POU2AF1 knockout-induced cell death. Mechanistically, ablation of either SH3GL1 or POU2AF1 reduced H3K18la (Fig. R). CUT&Tag sequencing further validated it (Fig. S) while concomitantly activating MAPK pathways (Fig. T-U). Through integrated multi-omics analysis, we identified PRKCB as the key downstream gene (Fig. V).

**Copyright Information:** (Online) ISSN: 2572-9241
©2026 The Author(s). HemaSphere is published by John Wiley & Sons Ltd on behalf of European Hematology Association. This is an open access Abstract Book distributed under the Attribution-NonCommercial-NoDerivs (CC BY-NC-ND), which allows third parties to download the articles and share them with others as long as they credit the author and the Abstract Book, but they cannot change the content in any way or use them commercially.
**Abstract Book Citations:** Authors, Title, HemaSphere, 2026;10:(S1):pages. The abstract book DOIs can be found on the “Author Information” page.
**Disclaimer:** Articles published in the journal HemaSphere exclusively reflect the opinions of the authors. The authors are responsible for all content in their abstracts including accuracy of the facts, statements, citing resources, etc.

### Summary/Conclusion

Our study shows that SH3GL1 interacts with the critical gene POU2AF1, activating POU2F1 levels. This molecular axis leads to increased PFKFB4 levels, enhancing glycolysis. The excess Lac boosts H3K18la, which suppresses downstream gene PRKCB. This pathway drives metabolic changes that promote cell growth, offering a new therapeutic target for DLBCL intervention (Fig. W).

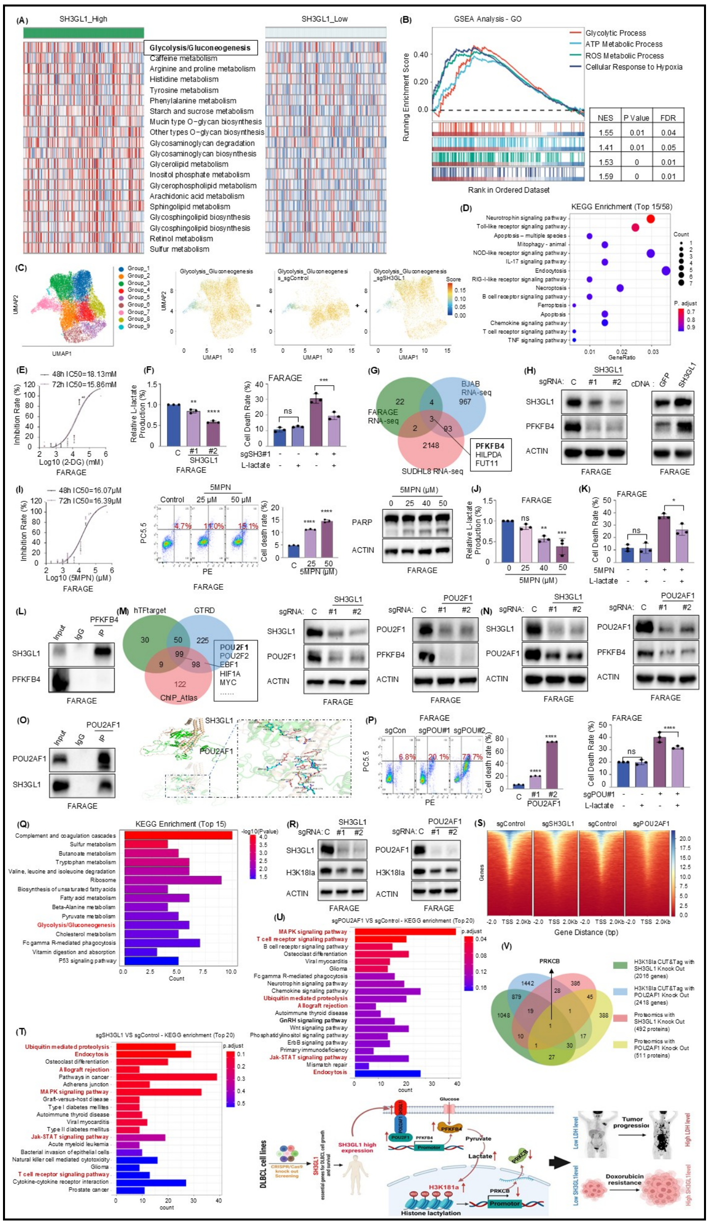

**Copyright Information:** (Online) ISSN: 2572-9241
©2026 The Author(s). HemaSphere is published by John Wiley & Sons Ltd on behalf of European Hematology Association. This is an open access Abstract Book distributed under the Attribution-NonCommercial-NoDerivs (CC BY-NC-ND), which allows third parties to download the articles and share them with others as long as they credit the author and the Abstract Book, but they cannot change the content in any way or use them commercially.
**Abstract Book Citations:** Authors, Title, HemaSphere, 2026;10:(S1):pages. The abstract book DOIs can be found on the "Author Information" page.
**Disclaimer:** Articles published in the journal HemaSphere exclusively reflect the opinions of the authors. The authors are responsible for all content in their abstracts including accuracy of the facts, statements, citing resources, etc.

### (PS2177) INTEGRATED GENOMIC AND TRANSCRIPTOMIC PROFILING REVEALS DISTINCT MECHANISMS IN EARLY- AND LATE-ONSET PT-DLBCL

**Topic:** 21. Lymphoma biology & translational research

Ana-Lucía Rocha*[1, 2, 3], Marlies Vanden Bempt[4, 5], Daan Dierickx[4, 6, 7]

*[1]KU Leuven, Leuven, Belgium;[2]VIB-KU Leuven Center for Cancer Biology, Leuven, Belgium;[3]Leuven Kankerinstituut (LKI), Leuven, Belgium; [4]KU Leuven, Oncology, Leuven, Belgium;[5]Leuven Kanker Instituut, Leuven, Belgium;[6]KU Leuven, Human Genetics, Leuven, Belgium; [7]UZ Leuven Hematology Unit, Leuven, Belgium;*

### Background

Post-transplant lymphoproliferative disorders (PTLDs) are rare hematological cancers occurring in up to 20% of organ or stem cell transplant patients. The oncogenic Epstein Barr Virus (EBV) is involved in ~60% of PTLD cases, while the biology of EBV-negative PTLDs remains elusive. EBV-negative PTLDs typically emerge >1 year post-transplantation (late-onset), while EBV-positive cases may occur early or late. However, the **relation between time-of-onset and PTLD pathogenesis is unknown**. Notably, post-transplant diffuse large B-cell lymphoma (PT-DLBCL) – the most common PTLD subtype – has a five-year overall survival of only 30%. Diagnosis currently relies solely on histopathological examination, offering **limited insights into underlying disease mechanisms**.

### Aims

Our aims were to: (i) define how genomic instability, mutational processes, and viral activity contribute differently to early- versus late-onset PT-DLBCL; and (ii) characterize the cellular and transcriptional states of the tumor microenvironment across subtypes. We hypothesize that early-onset PT-DLBCL is driven mainly by viral oncogenic programs, whereas late-onset cases depend more on genomic instability and loss of tumor suppressor function.

### Methods

We implemented a novel multimodal strategy to characterize the genomic and transcriptomic landscape of**25 PT-DLBCL tumor samples** (12 of these included a matched normal sample). **PacBio long-read Fiber-seq** was used to simultaneously assess genome-wide sequence variation, DNA methylation, and chromatin accessibility. In parallel, we performed **10X Genomics single-nucleus RNA sequencing** to analyze gene expression and tumor microenvironment features from the same samples. Additionally, EBV-negative and EBV-positive non-immunosuppressed DLBCL tumor samples were sequenced for comparison.

### Results

Fiber-seq revealed marked genomic differences between early and late-onset cases. While early-onset cases were predominantly diploid, late-onset cases – regardless of EBV status – showed widespread copy number alterations. Mutational signature analysis further indicated that late-onset cases were enriched for signatures associated with DNA mismatch repair deficiency and defective *POLD1* proofreading, while early-onset cases displayed only clock-like signatures. In addition, *TP53* mutations were more frequently observed in late-onset compared to early-onset cases. Together, these findings support the hypothesis that early-onset EBV-positive PT-DLBCL relies primarily on viral oncogenic activity, while genomic instability and tumor suppressor inactivation predominate in late-onset cases.
We identified diverse cell populations within the tumor microenvironment, including cancer associated fibroblast, endothelial cells, and myeloid cells. Notably, T-cells from early-onset cases clustered separately from those in late-onset cases, indicating distinct underlying transcriptional programs. Ongoing comparison with non-immunosuppressed DLBCL samples will clarify how immunosuppression influences T-cell gene expression differences across subtypes.

**Copyright Information:** (Online) ISSN: 2572-9241
©2026 The Author(s). HemaSphere is published by John Wiley & Sons Ltd on behalf of European Hematology Association. This is an open access Abstract Book distributed under the Attribution-NonCommercial-NoDerivs (CC BY-NC-ND), which allows third parties to download the articles and share them with others as long as they credit the author and the Abstract Book, but they cannot change the content in any way or use them commercially.
**Abstract Book Citations:** Authors, Title, HemaSphere, 2026;10:(S1):pages. The abstract book DOIs can be found on the "Author Information" page.
**Disclaimer:** Articles published in the journal HemaSphere exclusively reflect the opinions of the authors. The authors are responsible for all content in their abstracts including accuracy of the facts, statements, citing resources, etc.

**Summary/Conclusion**

Our findings indicate that PT-DLBCL pathogenesis differs substantially by time of onset. This integrated genomic and transcriptomic framework improves our understanding of PT-DLBCL biology and reveals molecular features with potential diagnostic and therapeutic relevance, supporting future efforts towards more accurate molecular classification and personalized treatments in clinical settings.

**Copyright Information:** (Online) ISSN: 2572-9241
©2026 The Author(s). HemaSphere is published by John Wiley & Sons Ltd on behalf of European Hematology Association. This is an open access Abstract Book distributed under the Attribution-NonCommercial-NoDerivs (CC BY-NC-ND), which allows third parties to download the articles and share them with others as long as they credit the author and the Abstract Book, but they cannot change the content in any way or use them commercially.
**Abstract Book Citations:** Authors, Title, HemaSphere, 2026;10:(S1):pages. The abstract book DOIs can be found on the "Author Information" page.
**Disclaimer:** Articles published in the journal HemaSphere exclusively reflect the opinions of the authors. The authors are responsible for all content in their abstracts including accuracy of the facts, statements, citing resources, etc.

### (PS2178) PROFILING ANTIGEN REACTIVITY OF TUMOR B-CELL RECEPTORS DERIVED FROM HBV-ASSOCIATED DIFFUSE LARGE B CELL LYMPHOMA

**Topic:** 21. Lymphoma biology & translational research

Rui Sun*[1], Anish Behere[2], Minxiang Xie[3], Likun Du[1], Fanglei Zuo[1], Hui Wan[1], Stelios Vlachiotis[1], Mingyu Yang[1], Qiao Wang[3], Nils Landergren[2], Harold Marcotte[1], Weicheng Ren[1], Qiang Pan-Hammarstrom[1]

*[1]Karolinska Institutet, Stockholm, Sweden; [2]Uppsala University, Uppsala, Sweden; [3]Fudan University, Shanghai, China;*

### Background

Since the discovery of the first human oncogenic virus in 1964, approximately 15% of human cancers have been attributed to viral infections. Emerging evidence links hepatitis B virus (HBV) infection to various hematologic malignancies, including non-Hodgkin lymphoma (NHL), one of the most common types of hematological cancers. Among NHL subtypes, diffuse large B-cell lymphoma (DLBCL) is the most prevalent. Epidemiological studies have consistently demonstrated an association between HBV infection and NHL. Our previous studies identified distinct mutational and transcriptomic features in DLBCL patients with concomitant HBV infection ($HBsAg^+$). However, the biological mechanisms underlying this association remain largely unclear.

### Aims

This study aims to provide evidence of how HBV contributes to B-cell lymphomagenesis by characterizing the antigen reactivity of B-cell receptors (BCRs) expressed by dominant malignant B-cell clones in HBV-associated DLBCL.

### Methods

A cohort of 348 DLBCL patients (92 $HBsAg^+$, 26.4%) was analyzed using bulk RNA sequencing. Tumor BCR heavy- and light-chain sequences were extracted and annotated using MiXCR and IMGT to assess immunoglobulin (Ig) gene usage, somatic hypermutation (SHM), and CDR3 length and charge characteristics. To investigate antigen selection pressure during lymphomagenesis, dominant tumor BCRs were reconstructed by gene synthesis followed by recombinant antibody expression in the Expi293F system. Antigen reactivity was assessed using anti-HBV ELISA, polyreactivity assays, and a high-throughput autoantigen array to characterize autoantigen-specific binding profiles.

### Results

$HBsAg^+$ and $HBsAg^-$ DLBCL exhibited distinct IGHV gene usage, with enrichment of IGHV1-69 and IGHV1-18 in $HBsAg^+$ DLBCL. Both groups demonstrated biased usage of IGHV4-34, a gene frequently associated with autoreactivity and autoimmune disorders. Anti-HBV ELISA identified a subset of tumor BCRs reactive to HBV surface and core antigens, suggesting that HBV-derived antigens may contribute to antigen-driven selection during lymphomagenesis. Additionally, these BCRs exhibited high autoreactivity and polyreactivity against a broad range of self-antigens, including DNA, insulin, cytokines, and autoimmune disease-associated antigens. These findings suggest that engagement with multiple antigen sources may confer BCR-mediated growth advantages to dominant tumor clones.

### Summary/Conclusion

Dominant BCRs in DLBCL exhibited broad reactivity against cytokines, autoantigens, and HBV-associated antigens, collectively suggesting that multi-antigen engagement may promote tumor growth and clonal expansion in DLBCL.

**Copyright Information:** (Online) ISSN: 2572-9241
©2026 The Author(s). HemaSphere is published by John Wiley & Sons Ltd on behalf of European Hematology Association. This is an open access Abstract Book distributed under the Attribution-NonCommercial-NoDerivs (CC BY-NC-ND), which allows third parties to download the articles and share them with others as long as they credit the author and the Abstract Book, but they cannot change the content in any way or use them commercially.
**Abstract Book Citations:** Authors, Title, HemaSphere, 2026;10:(S1):pages. The abstract book DOIs can be found on the “Author Information” page.
**Disclaimer:** Articles published in the journal HemaSphere exclusively reflect the opinions of the authors. The authors are responsible for all content in their abstracts including accuracy of the facts, statements, citing resources, etc.

### (PS2179) CTDNA SEQUENCING REVEALS MUTATIONAL SIGNATURES WITH PROGNOSTIC RELEVANCE IN LBCL PATIENTS

**Topic:** 21. Lymphoma biology & translational research

Sophie Heidenreich[1], Jan-Michel Heger[1], Maare Arffman[2, 3], Julia Mattlener[1], Jessica Schneider[1], Julia K. Schleifenbaum[1], Peter Borchmann[1], Bastian von Tresckow[4], Sirpa Leppä[2, 3], Sven Borchmann*[1, 5]

*[1]Department I of Internal Medicine, Center for Integrated Oncology Aachen Bonn Cologne Duesseldorf (CIO ABCD), University of Cologne, Faculty of Medicine and University Hospital of Cologne, Cologne, Germany; [2]Research Programs Unit, Applied Tumor Genomics, University of Helsinki, Helsinki, Finland; [3]Department of Oncology, Helsinki University Hospital Comprehensive Cancer Centre, Helsinki, Finland; [4]Department of Hematology and Stem Cell Transplantation, West German Cancer Center and German Cancer consortium (DKTK partner site Essen), University Hospital Essen, University of Duisburg-Essen, Essen, Germany; [5]Liqomics GmbH, Cologne, Germany;*

### Background

Despite recent advances in treatment strategies, patients with relapsed/refractory (R/R) large B-cell lymphoma (LBCL) are at high risk of further relapses and death. Accordingly, the development of novel biomarkers and the identification of patients who might benefit from alternative treatment strategies remain important unmet medical needs.
Circulating tumor (ct)DNA sequencing has emerged as a reliable tool for genotyping and response assessment in patients with LBCL. Its prognostic value could be further enhanced by integrating mutational signatures that reveal the underlying biological processes.

### Aims

The aim of this study is to extract and characterize single base substitution (SBS) mutational signatures from ctDNA in patients with LBCL, to identify distinct patterns and to assess their impact on progression free survival (PFS).

### Methods

We applied our previously published ctDNA sequencing approach (Sobesky et al., 2021; Heger et al., 2024) to 124 blood samples obtained from 88 patients with R/R LBCL across multiple treatment lines. All samples were obtained prior to treatment initiation and constituted the discovery cohort. Mutational signature analysis was performed with a customized pipeline developed around SigProfiler (Díaz-Gay et al., 2023). The prognostic impact of identified mutational signature subgroups was validated in two independent cohorts of patients with previously untreated LBCL from the Nordic phase II LBC-06 (n = 123; Leppä et al., 2025) and LBC-05 (n = 99; Leppä et al., 2020) trials.

**Copyright Information:** (Online) ISSN: 2572-9241
©2026 The Author(s). HemaSphere is published by John Wiley & Sons Ltd on behalf of European Hematology Association. This is an open access Abstract Book distributed under the Attribution-NonCommercial-NoDerivs (CC BY-NC-ND), which allows third parties to download the articles and share them with others as long as they credit the author and the Abstract Book, but they cannot change the content in any way or use them commercially.
**Abstract Book Citations:** Authors, Title, HemaSphere, 2026;10:(S1):pages. The abstract book DOIs can be found on the "Author Information" page.
**Disclaimer:** Articles published in the journal HemaSphere exclusively reflect the opinions of the authors. The authors are responsible for all content in their abstracts including accuracy of the facts, statements, citing resources, etc.

### Results

We identified four distinct mutational signatures: SBS1 and SBS5 both indicative of age, SBS39 of unknown etiology, and SBS84 induced by cytidine deaminase (AID) activity.
Unsupervised k-means clustering of mutational signature profiles revealed two distinct molecular subgroups. The first subgroup, termed “AID-associated” is characterized by a predominance of the AID-related mutational signature, whereas the second “diverse signature” subgroup exhibited lower proportion of the AID related signature SBS84 (mean 23% vs 68% SBS84 proportion/sample, p<0.001 Mann-Whitney-U-Test) and greater mutational heterogeneity (mean 3 vs 4 signatures/sample, p<0.001, Mann-Whitney-U-Test). Survival analysis demonstrated a trend towards inferior PFS in the AID-associated subgroup (HR 1.78, 95%CI 0.98-3.25, Cox regression). To further investigate the prognostic impact of the two subgroups, validation analyses were conducted using the two independent external cohorts. Across both cohorts the AID-associated subgroup demonstrated significantly inferior PFS (LBC-06: HR 4.87, 95%CI 1.77-13.41; LBC-05: HR 2.61, 95%CI 1.01-6.72, Cox regression). This effect was consistent in a multivariable analysis for each validation cohort adjusting for age and IPI (age-adjusted IPI for LBC-06; LBC-06: HR 4.87, 95%CI 1.77-13.42; LBC-05: HR 2.19, 95%CI 0.82-5.86, multivariable Cox regression).

### Summary/Conclusion ctDNA sequencing enables robust and reproducible identification of distinct mutational signature patterns, independent of technical workflows and targeted gene panel designs. Classification based on these signatures delineates two distinct subgroups that are reproducible and independently associated with PFS. Overall, incorporating the biological context of mutations could further enhance the prognostic power of ctDNA profiling, enabling earlier and more precise identification of patients at high risk for progressive disease.

**Copyright Information:** (Online) ISSN: 2572-9241
©2026 The Author(s). HemaSphere is published by John Wiley & Sons Ltd on behalf of European Hematology Association. This is an open access Abstract Book distributed under the Attribution-NonCommercial-NoDerivs (CC BY-NC-ND), which allows third parties to download the articles and share them with others as long as they credit the author and the Abstract Book, but they cannot change the content in any way or use them commercially.
**Abstract Book Citations:** Authors, Title, HemaSphere, 2026;10:(S1):pages. The abstract book DOIs can be found on the “Author Information” page.
**Disclaimer:** Articles published in the journal HemaSphere exclusively reflect the opinions of the authors. The authors are responsible for all content in their abstracts including accuracy of the facts, statements, citing resources, etc.

### (PS2180) TH22-LIKE SIGNATURE OF CD4+ T CELLS IS ASSOCIATED WITH RESISTANCE TO IMMUNE CHECKPOINT BLOCKADE IN A MOUSE MODEL OF ANAPLASTIC LARGE CELL LYMPHOMA

**Topic:** 21. Lymphoma biology & translational research

Linda Gräßel*[1, 2, 3, 4], Teresa Poggio[2, 3, 5, 6], Geoffroy Andrieux[7], Ana Gómez Martínez[1, 8, 9], Kerstin Stock[2, 3, 5], Magdalena Kršić[8, 9], Irene Gonzalez-Menendez[10, 11], Stefanie Kreutmair[2, 3, 4, 12, 13, 14], Catarina Reis Orcinha[2, 3], Marissa Russ[2, 5], Salome Yacob[1, 9, 9], Cornelius Miething[2, 3], Federico Bonofiglio[15], Ivonne Montes-Mojarro[10], Marie Follo[2], Dietmar Pfeifer[2], Falko Fend[6, 10], Leticia Quintanilla-Fend[6, 10, 11, 16], Roberto Chiarle[17, 18], Claudia Voena[17], Robert Zeiser[2, 3, 4], Suzanne Turner[19, 20], Melanie Boerries[3, 7], Justus Duyster[2, 3, 4], Anna Lena Illert[1, 2, 3, 4, 6, 8, 9]

*[1]Universitätsmedizin Göttingen, Clinic for Hematology and Medical Oncology, Göttingen, Germany; [2]Universitätsklinikum Freiburg, Department of Internal Medicine I, Haematology, Oncology and Stem Cell Transplantation, Freiburg im Breisgau, Germany; [3]German Cancer Consortium (DKTK), Partner site Freiburg – a partnership between German Cancer Research Center (DKFZ) and Medical Center – University of Freiburg, Freiburg, Germany; [4]Faculty of Medicine, Albert Ludwigs University of Freiburg, Freiburg, Germany; [5]Faculty of Biology, Albert Ludwigs University of Freiburg,, Freiburg, Germany; [6]European Research Initiative for ALK related malignancies (www.erialcl.net), Cambridge, United Kingdom; [7]Universitätsklinikum Freiburg, Institute of Medical Bioinformatics and Systems Medicine, Freiburg im Breisgau, Germany; [8]Klinikum rechts der Isar der TU München, Center for Personalized Medicine, München, Germany; [9]Klinikum rechts der Isar der TU München, Clinic and Polyclinic for Haematology and Oncology, Faculty of Medicine, München, Germany; [10]Universitätsklinikum Tübingen, Institute of Pathology and Neuropathology, Tübingen, Germany; [11]Universitätsklinikum Tübingen, Core Facility Histology, Faculty of Medicine Tübingen, Tübingen, Germany; [12]Universitätsspital Zürich, Medical Department of Haematology and Oncology, Zürich, Switzerland; [13]University of Zurich, Institute of Experimental Immunology, Zürich, Switzerland; [14]The LOOP Zurich, University of Zurich, Zürich, Switzerland; [15]National Research Council, Lerici, Italy; [16]Cluster of Excellence iFIT (EXC2180) "Image-guided and Functionally Instructed Tumor Therapies", University of Tübingen, Tübingen, Germany; [17]University of Turin, Department of Molecular Biotechnology and Health Sciences, Torino, Italy; [18]Children's Hospital and Harvard Medical School, Department of Pathology, Boston, United States of America; [19]University of Cambridge, Department of Pathology, Cambridge, United Kingdom; [20]CEITEC, Masaryk University, Faculty of Medicine, Brno, Czechia;*

### Background

T-cell non-Hodgkin lymphomas (T-NHL) are heterogeneous and usually aggressive. Immune checkpoint blockade (ICB) revolutionized solid-tumor therapy, yet case reports and clinical trials in T-NHLs have yielded highly variable outcomes, from hyperprogression to complete remission, leaving its role in T-NHLs incompletely defined.

### Aims

Our aim is to understand the role of ICB in Anaplastic Large Cell Lymphoma (ALCL) as a subtype of T-NHL affecting children and adults, with high medical need in case of relapse to standard therapies.

### Methods

We utilize a syngeneic retroviral bone marrow (BM) transplantation model of ALK+ ALCL. Using LckCre-transgenic murine bone marrow transduced with the MSCV-Stop-NPM-ALK-IRES-EGFP vector, we induce NPM-ALK expression in early T-cells, leading to human-like thymic and systemic ALCL that can be re-transplanted in a serial transplantation and combined with targeted depletion of immune effector cells or therapeutic strategies such as ICB.

**Copyright Information:** (Online) ISSN: 2572-9241
©2026 The Author(s). HemaSphere is published by John Wiley & Sons Ltd on behalf of European Hematology Association. This is an open access Abstract Book distributed under the Attribution-NonCommercial-NoDerivs (CC BY-NC-ND), which allows third parties to download the articles and share them with others as long as they credit the author and the Abstract Book, but they cannot change the content in any way or use them commercially.
**Abstract Book Citations:** Authors, Title, HemaSphere, 2026;10:(S1):pages. The abstract book DOIs can be found on the "Author Information" page.
**Disclaimer:** Articles published in the journal HemaSphere exclusively reflect the opinions of the authors. The authors are responsible for all content in their abstracts including accuracy of the facts, statements, citing resources, etc.

**Results**

The serial transplantation of murine ALK+ ALCL cells in immunocompetent and immunodeficient mice results in significantly prolonged survival in the immunocompetent setting, demonstrating ALCL immunogenicity. By using targeted depletion of distinct immune compartments (CD4+ T cells, CD8+ T cells and NK cells, either individually or in combination), we discover that CD4+ T cells and NK cells are the main effector populations mediating ALCL immune response. Immune checkpoint blockade using aPD-L1 monotherapy leads to complete eradication of the ALCL in 50% of animals. In others, it only delays the ALCL progression and some recipients do not respond to the treatment at all, indicating the presence of resistance mechanisms. Combining ICB with immune-cell depletion revealed that CD4+ T cells are the main effectors driving ICB-induced anti-lymphoma immune response. RNA microarray transcriptome analysis and subsequent Gene Set Variation Analysis of CD4+ and CD8+ T cells, which were extracted and sorted from peripheral blood on day 20 after the start of ICB treatment, showed a significantly lower activity score for “Th1-like” signature in CD4+ T cells from non-responders. In contrast, a significantly higher activity score of a “Th22-like” signature clearly distinguished non-responders from responders. This suggests that dysfunctional CD4+ T cell polarization drives ICB resistance in ALK+ ALCL.

**Summary/Conclusion**

In this preclinical proof-of-concept study, we demonstrate that ICB can be a safe and effective treatment strategy for a subtype of T-NHL. We establish CD4+ T cells as key regulators of spontaneous and checkpoint-induced immune response in ALCL and show that dysfunctional T-helper-cell polarization towards a Th22-like signature is associated with ICI non-response. Notably, we did not observe signs of ALCL hyperprogression following immune checkpoint blockade.

**Copyright Information:** (Online) ISSN: 2572-9241
©2026 The Author(s). HemaSphere is published by John Wiley & Sons Ltd on behalf of European Hematology Association. This is an open access Abstract Book distributed under the Attribution-NonCommercial-NoDerivs (CC BY-NC-ND), which allows third parties to download the articles and share them with others as long as they credit the author and the Abstract Book, but they cannot change the content in any way or use them commercially.
**Abstract Book Citations:** Authors, Title, HemaSphere, 2026;10:(S1):pages. The abstract book DOIs can be found on the “Author Information” page.
**Disclaimer:** Articles published in the journal HemaSphere exclusively reflect the opinions of the authors. The authors are responsible for all content in their abstracts including accuracy of the facts, statements, citing resources, etc.

### (PS2181) SPATIAL NICHE ORGANIZATION IMPLICATES ANTIGEN-PRESENTING–LIKE EPITHELIAL CELLS AS AN ACTIVE COMPONENT OF THE GASTRIC MALT LYMPHOMA MICROENVIRONMENT

**Topic:** 21. Lymphoma biology & translational research

Miki Joyce*[1, 2], Sakurako Suma[2], Kyohei Sano[3], Ayumu Tsubosaka[3], Yuri Tsuboi[1, 2], Kenichi Makishima[2], Keiichiro Hattori[2, 4], Yasuhito Suehara[2, 4], Tatsuhiro Sakamoto[2, 4], Yasunori Ota[5], Miwako Kakiuchi[3], Shumpei Ishikawa[3], Mamiko Sakata-Yanagimoto[2, 4, 6]

*[1]Graduate School of Comprehensive Human Sciences, University of Tsukuba, Department of Hematology, Tsukuba, Japan; [2]University of Tsukuba Hospital, Department of Hematology, Tsukuba, Japan;[3]Graduate School of Medicine, The University of Tokyo, Department of Preventive Medicine, Tokyo, Japan; [4]Institute of Medicine, University of Tsukuba, Department of Hematology, Tsukuba, Japan; [5]The Institute of Medical Science, The University of Tokyo, Department of Pathology, Tokyo, Japan; [6]Division of Advanced Hemato-Oncology, Transborder Medical Research Center, Tsukuba, Japan;*

### Background

Mucosa-associated lymphoid tissue (MALT) lymphoma is an indolent extranodal B-cell lymphoma, most commonly involving the stomach. Its pathogenesis is closely linked to chronic antigenic stimulation in the context of persistent inflammation, including *Helicobacter pylori* infection. Despite well-defined clinicopathologic features, the tumor microenvironment (TME) and intratumoral heterogeneity of MALT lymphomas remain incompletely characterized at single-cell resolution.

### Aims

We hypothesize that inflammation remodels the TME and contributes to the formation of tumor-supportive niches in MALT lymphomas, which may promote tumor initiation and progression. To investigate this, we applied high-plex, single-cell-resolution spatial transcriptomics to gastric MALT lymphoma samples to characterize the TME.

### Methods

We performed CosMx Spatial Molecular Imaging with a 6K RNA panel (NanoString Technologies) on formalin-fixed, paraffin-embedded sections from 10 gastric MALT lymphoma endoscopic biopsy specimens and 3 normal gastric mucosa samples obtained from gastrectomy specimens. Image-based cell segmentation was conducted using *Proseg*. Data processing, quality control (QC), integration, and clustering were performed with *Scanpy* and *Squidpy*. Cell types were annotated based on canonical marker expression and spatial neighborhood context. Putative malignant B-cell populations were identified by B-cell markers and immunoglobulin light-chain gene expression patterns. To characterize spatial tissue organization, we performed niche analysis using *CellCharter* across all samples and assessed inter-niche spatial proximity. Differential gene expression within epithelial-lineage cells was tested using the *Scanpy* rank_genes_groups function (Wilcoxon rank-sum test).

**Copyright Information:** (Online) ISSN: 2572-9241
©2026 The Author(s). HemaSphere is published by John Wiley & Sons Ltd on behalf of European Hematology Association. This is an open access Abstract Book distributed under the Attribution-NonCommercial-NoDerivs (CC BY-NC-ND), which allows third parties to download the articles and share them with others as long as they credit the author and the Abstract Book, but they cannot change the content in any way or use them commercially.
**Abstract Book Citations:** Authors, Title, HemaSphere, 2026;10:(S1):pages. The abstract book DOIs can be found on the "Author Information" page.
**Disclaimer:** Articles published in the journal HemaSphere exclusively reflect the opinions of the authors. The authors are responsible for all content in their abstracts including accuracy of the facts, statements, citing resources, etc.

### Results

After QC, we analyzed 329,592 cells, including 86,587 immune and 106,956 epithelial cells, with the remaining cells corresponding to stromal and endothelial populations. CosMx spatial single-cell profiling revealed substantial inter-patient heterogeneity among tumor cells. Notably, we identified an epithelial cluster present exclusively in MALT lymphoma and absent in normal gastric mucosa. This *KRT18*$^+$ epithelial cluster exhibited upregulation of antigen processing and presentation genes, including HLA class II genes and *CD74*, as well as immune-response and cytokine-signaling-related genes (*CAMK2D*, *ICAM1*, *ITGB2*, *STAT4*, *IRS2*, and *TNFAIP3*). In a subset of cases, this cluster localized spatially adjacent to infiltrating tumor cells, suggesting a potential role in supporting tumor growth. We therefore termed this population "antigen-presenting-like epithelial cells" (APepi). *CellCharter* niche analysis identified 12 distinct niches, among which niches 0, 3, 7, and 10 contained both tumor cells and APepi.

### Summary/Conclusion

Spatial single-cell transcriptomics identified a MALT-specific *KRT18*$^+$ epithelial state enriched for antigen-presentation and proinflammatory programs and spatially associated with tumor-rich niches. These findings indicate that epithelial components are integral to the spatial organization of tumor-supportive niches in the gastric MALT lymphoma microenvironment. Further studies are warranted to clarify the functional interactions between tumor cells and epithelial niche elements.

**Copyright Information:** (Online) ISSN: 2572-9241
©2026 The Author(s). HemaSphere is published by John Wiley & Sons Ltd on behalf of European Hematology Association. This is an open access Abstract Book distributed under the Attribution-NonCommercial-NoDerivs (CC BY-NC-ND), which allows third parties to download the articles and share them with others as long as they credit the author and the Abstract Book, but they cannot change the content in any way or use them commercially.
**Abstract Book Citations:** Authors, Title, HemaSphere, 2026;10:(S1):pages. The abstract book DOIs can be found on the "Author Information" page.
**Disclaimer:** Articles published in the journal HemaSphere exclusively reflect the opinions of the authors. The authors are responsible for all content in their abstracts including accuracy of the facts, statements, citing resources, etc.

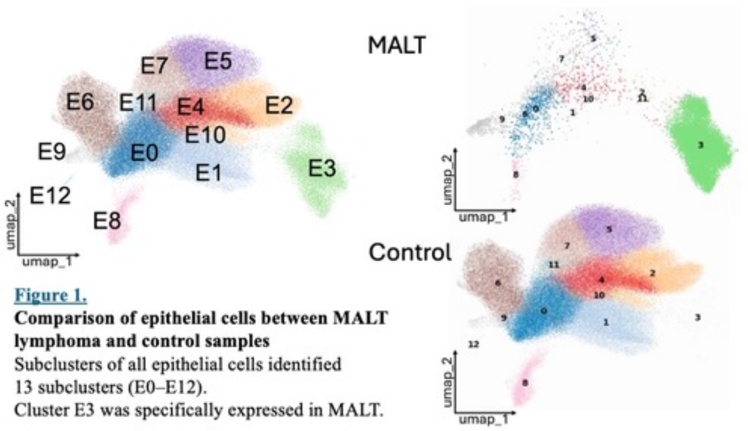

**Figure 1.**
**Comparison of epithelial cells between MALT lymphoma and control samples**
Subclusters of all epithelial cells identified
13 subclusters (E0–E12).
Cluster E3 was specifically expressed in MALT.

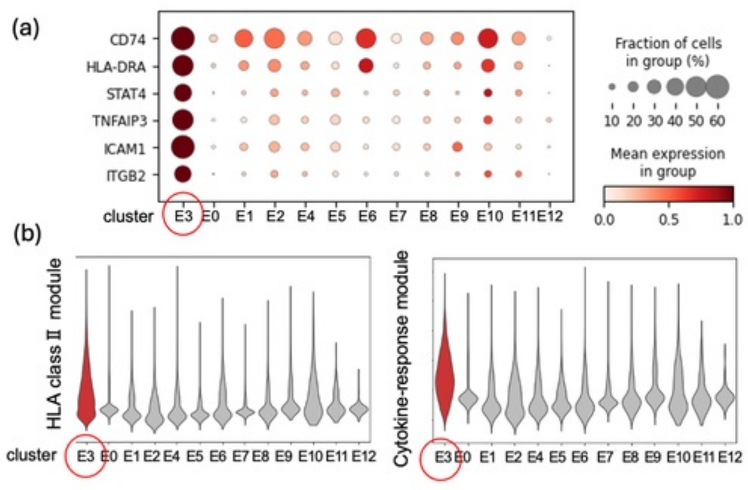

**Figure 2.**
**Antigen-presentation and cytokine-response signatures in epithelial subclusters.**
**(a)** Dot plot showing selected genes (*CD74*, *HLA-DRA*, *STAT4*, *TNFAIP3*, *ICAM1*, and *ITGB2*) across epithelial subclusters.
**(b)** Violin plots of the HLA class II/cytokine-immune response module score per epithelial subcluster.
HLA class II module: *HLA-DRA*, *HLA-DRB1*, *HLA-DPA1*, *HLA-DPB1*, *CD74*.
Cytokine-response module: *CAMK2D*, *ICAM1*, *ITGB2*, *STAT4*, *IRS2*, *TNFAIP3*.

**Copyright Information:** (Online) ISSN: 2572-9241
©2026 The Author(s). HemaSphere is published by John Wiley & Sons Ltd on behalf of European Hematology Association. This is an open access Abstract Book distributed under the Attribution-NonCommercial-NoDerivs (CC BY-NC-ND), which allows third parties to download the articles and share them with others as long as they credit the author and the Abstract Book, but they cannot change the content in any way or use them commercially.
**Abstract Book Citations:** Authors, Title, HemaSphere, 2026;10:(S1):pages. The abstract book DOIs can be found on the "Author Information" page.
**Disclaimer:** Articles published in the journal HemaSphere exclusively reflect the opinions of the authors. The authors are responsible for all content in their abstracts including accuracy of the facts, statements, citing resources, etc.

### (PS2182) THE MSZ REGIMEN (SELINEXOR, HIGH-DOSE METHOTREXATE, AND ZANUBRUTINIB) IN CENTRAL NERVOUS SYSTEM LYMPHOMA: A PHASE II TRIAL WITH NOVEL CNS PHARMACOKINETIC PROFILING

**Topic:** 21. Lymphoma biology & translational research

Li Li*[1], Bing Pan[1], Kaiyi Liu[1], Wenjing Yang[1], Xin Gao[1], Qi Zhang[1], Xiaobo Wang[1]

[1] *The Second Hospital of Dalian Medical University, Dalian, China;*

### Background

Central nervous system lymphoma (CNSL) is an aggressive malignancy with limited options and poor prognosis. High-dose methotrexate (HD-MTX) remains standard, though complete remission (CR) rates are suboptimal. Bruton's tyrosine kinase inhibitors (BTKis) demonstrate efficacy but face resistance. Selinexor, an oral exportin-1 (XPO1) inhibitor, penetrates the blood-brain barrier (BBB); however, its synergy with BTKis and CNS pharmacokinetics remain insufficiently characterized. Preclinically, selinexor promotes FOXO3a nuclear retention via XPO1 inhibition and synergizes with BTKis to suppress PI3K/AKT/NF-κB signaling, potentially overcoming resistance. Nevertheless, the efficacy and safety of this combination in CNSL require further validation.

### Aims

To evaluate the in vitro synergistic effect of selinexor with BTK inhibitors and to analyze the safety, efficacy, and CNS pharmacokinetics of this combination in patients with CNSL.

### Methods

Preclinically, OCI-LY10 cells received selinexor and BTKi; synergy was assessed via Chou-Talalay, and immune modulation was evaluated in A20 tumor-bearing mice. In a phase II trial (ChiCTR2200062154), 25 CNSL patients received six 21-day cycles of HD-MTX (3.5 g/m²), selinexor (40 mg twice weekly), and zanubrutinib (160 mg twice daily). The primary endpoint was objective response rate (ORR). Pharmacokinetic sampling of plasma and cerebrospinal fluid (CSF) quantified selinexor and zanubrutinib concentrations.

### Results

Selinexor and BTKi demonstrated strong synergy in vitro (CI=0.41), suppressing proliferation and inducing apoptosis dose- and time-dependently. The combination inhibited JAK/STAT3 and NFATC1 pathways, with compensatory NOTCH1 and BCL-2 upregulation. In the A20 mouse model, tumor volume reduction exceeded 80% ($P<0.001$), accompanied by 2.5-fold increased $CD3^+$ T-cell infiltration, 60% reduced $CD163^+/CD68^+$ macrophages, PD-1/TIM-3 downregulation, and splenic $CD4^+/CD8^+$ T-cell expansion.

Clinically, 25 patients (median age 64) were enrolled. Currently, 24 out of 25 patients (96.0%) have achieved a complete response, with 19 patients (76.0%) attaining early, deep remissions after either two or four cycles. The 1-year progression-free survival and overall survival rates are 96.0% and 100%, respectively.

Pharmacokinetically, selinexor plasma concentrations peaked at 4h (251500 pg/mL) with rapid clearance, while CSF peaked at 6-8h (813.0 pg/mL), remaining elevated at 25h (639.0 pg/mL). The BBB penetration ratio increased from 0.03% at 2h to 2.75% at 25h, indicating sustained CNS retention. Zanubrutinib showed rapid plasma clearance but stable CSF levels, with CSF-to-plasma ratio rising from 29.9% to 40.9%, confirming sustained CNS exposure.

**Copyright Information:** (Online) ISSN: 2572-9241
©2026 The Author(s). HemaSphere is published by John Wiley & Sons Ltd on behalf of European Hematology Association. This is an open access Abstract Book distributed under the Attribution-NonCommercial-NoDerivs (CC BY-NC-ND), which allows third parties to download the articles and share them with others as long as they credit the author and the Abstract Book, but they cannot change the content in any way or use them commercially.
**Abstract Book Citations:** Authors, Title, HemaSphere, 2026;10:(S1):pages. The abstract book DOIs can be found on the "Author Information" page.
**Disclaimer:** Articles published in the journal HemaSphere exclusively reflect the opinions of the authors. The authors are responsible for all content in their abstracts including accuracy of the facts, statements, citing resources, etc.

### Summary/Conclusion

Our findings confirm that selinexor, alone or with BTKi, exerts potent anti-tumor effects in ABC-DLBCL by inhibiting proliferation, inducing apoptosis, blocking JAK/STAT3 and NFATC1 signaling, and remodeling the immune microenvironment via T-cell activation and macrophage reprogramming. This phase II trial demonstrates that the MSZ regimen yields unprecedented efficacy in CNSL (100% ORR, 96.0% CR) with favorable tolerability. We provide first direct evidence of CNS penetration for both selinexor and zanubrutinib—zanubrutinib exceeding IC50 thresholds in CSF, selinexor demonstrating sustained CSF exposure. Supported by mechanistic synergy and immune modulation, the MSZ regimen represents a highly promising first-line or salvage strategy in CNSL, warranting further validation.

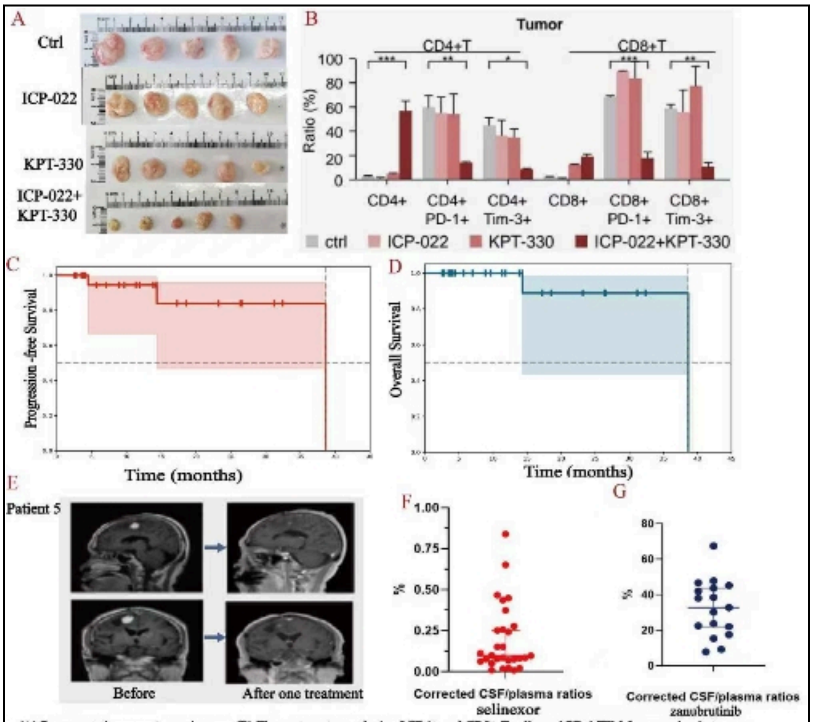

(A) Representative gross tumor images. (B) Flow cytometry analysis of CD4+ and CD8+ T cells, and PD-1/TIM-3 expression in tumors.
(C) Progression-free survival. (D) Overall survival.(E) Early deep radiographic response (≥75% tumor reduction) in one patient.
(F) Zanubrutinib CSF-to-plasma ratio corrected for protein binding.(G) Selinexor CSF-to-plasma ratio corrected for protein binding.

**Copyright Information:** (Online) ISSN: 2572-9241
©2026 The Author(s). HemaSphere is published by John Wiley & Sons Ltd on behalf of European Hematology Association. This is an open access Abstract Book distributed under the Attribution-NonCommercial-NoDerivs (CC BY-NC-ND), which allows third parties to download the articles and share them with others as long as they credit the author and the Abstract Book, but they cannot change the content in any way or use them commercially.
**Abstract Book Citations:** Authors, Title, HemaSphere, 2026;10:(S1):pages. The abstract book DOIs can be found on the "Author Information" page.
**Disclaimer:** Articles published in the journal HemaSphere exclusively reflect the opinions of the authors. The authors are responsible for all content in their abstracts including accuracy of the facts, statements, citing resources, etc.

### (PS2183) SYNERGY BETWEEN REL DEREGULATION AND TNFRSF14/HVEM LOSS IN THE TRANSFORMATION OF GERMINAL CENTER B CELLS

**Topic:** 21. Lymphoma biology & translational research

Karima Ferhat*[1], Jean Feuillard[2], Christelle Vincent-Fabert[1], Nathalie Faumont[1]

*[1]Laboratoire CRIBL UMR CNRS 7276 Inserm 1262 ; Equipe 2MB2C , Centre de Biologie et de Recherche en Santé, Université de Limoges, Limoges, France; [2]Laboratoire CRIBL UMR CNRS 7276 INSERM 1262 ; Equipe 2MB2C , Centre de Biologie et de Recherche en Santé, Université de Limoges et CHU Dupuytren, Limoges, France;*

### Background

In diffuse large B-cell lymphomas (DLBCL), the 2p15-16 amplified region, which includes the *REL* gene, is most frequently associated with the germinal center B-cell-like (GCB)-DLBCL subgroup. The c-Rel protein, encoded by *REL*, is a key subunit of the canonical NF-κB transcription system, which is particularly active in GCB-DLBCL. *In vivo,* c-Rel overexpression is sufficient to generate a persistent pool of GC-derived B cells with lymphoma-initiating potential. However, the oncogenic partners that cooperate with c-Rel in the malignant transformation of GCB-DLBCL remain poorly understood. Loss of *TNFRSF14* gene, as known as herpesvirus entry mediator (HVEM), is associated with alteration in the tumor microenvironment that promote tumor growth in GCB-DLBCL.

### Aims

Because HVEM alterations correlate with a c-Rel-associated transcriptomic signature, our project aims to investigate the cooperation between these two pathways in lymphoma development.

### Methods

We developed a dual-color conditional mouse model allowing *REL* overexpression in a limited pool of GC-derived B cells via the AID.Cre$_{ert2}$/LoxP system. Fluorescent proteins (YFP and tdTomato) enable *in situ* and longitudinal tracking of these B-cells following induction with tamoxifen and immunization with sheep red blood cells.
Using a LoxP/knockout-TNFRSF14 model, we generated a new mouse model that combines *REL* dysregulation with biallelic TNFRSF14/Hvem loss. To understand the role of *REL* in the context of Hvem deficiency during GC transformation, we performed cellular, molecular, and histological analyses.

### Results

REL overexpression conferred a long-term competitive advantage, allowing GC persistence and continuous recirculation of REL-overexpressing B cells. IgHV clonality analysis at the DNA level indicated clonal B-cell expansion in 50% of cases 12 months after induction. At 4 months post-induction, IgHV monoclonality is observed in 75% of REL| TNFRSF14$^{-/-}$ spleens compared with only 25% in TNFRSF14$^{-/-}$ controls.
Immunofluorescence analysis on spleen sections revealed a structural disruption of the GC, with diffuse dispersion of YFP and tdTomato-labeled B cells in the REL| TNFRSF14$^{-/-}$ mouse model.
The co-occurrence of the two oncogenic pathways also caused a significant increase in the proportion of PD-L1 and PD-L2-positive B cells, accompanied by a CD86 overexpression and reduced CD80 expression. Notably, these effects are not restricted to the REL-overexpressing HVEM-deficient B cell population but  affected the entire splenic B cell compartment.
Collectively, these findings suggest that T follicular helper (Tfh) support becomes excessive during GC reaction only when c-Rel is dysregulated in the absence of HVEM, thereby generating a strongly pro-lymphoma microenvironment.

**Copyright Information:** (Online) ISSN: 2572-9241
©2026 The Author(s). HemaSphere is published by John Wiley & Sons Ltd on behalf of European Hematology Association. This is an open access Abstract Book distributed under the Attribution-NonCommercial-NoDerivs (CC BY-NC-ND), which allows third parties to download the articles and share them with others as long as they credit the author and the Abstract Book, but they cannot change the content in any way or use them commercially.
**Abstract Book Citations:** Authors, Title, HemaSphere, 2026;10:(S1):pages. The abstract book DOIs can be found on the "Author Information" page.
**Disclaimer:** Articles published in the journal HemaSphere exclusively reflect the opinions of the authors. The authors are responsible for all content in their abstracts including accuracy of the facts, statements, citing resources, etc.

**Summary/Conclusion**

Our results support the hypothesis that c-Rel overexpression combined with HVEM loss profoundly alters the microenvironment of GC-derived B cells. In this context, the PD-L1/PD-L2 - PD1 and CD80/CD86 - CTLA4 immune-checkpoint axes appear to be heavily involved. Such alterations may amplify and dysregulate the humoral immune response, facilitating the clonal expansion of emerging pre-tumoral B cell clones. Ultimately, this cooperative mechanism may lead to the suppression of anti-tumor immunity.

**Copyright Information:** (Online) ISSN: 2572-9241
©2026 The Author(s). HemaSphere is published by John Wiley & Sons Ltd on behalf of European Hematology Association. This is an open access Abstract Book distributed under the Attribution-NonCommercial-NoDerivs (CC BY-NC-ND), which allows third parties to download the articles and share them with others as long as they credit the author and the Abstract Book, but they cannot change the content in any way or use them commercially.
**Abstract Book Citations:** Authors, Title, HemaSphere, 2026;10:(S1):pages. The abstract book DOIs can be found on the "Author Information" page.
**Disclaimer:** Articles published in the journal HemaSphere exclusively reflect the opinions of the authors. The authors are responsible for all content in their abstracts including accuracy of the facts, statements, citing resources, etc.

### (PS2184) CLONAL HEMATOPOIESIS IS ASSOCIATED WITH PERSISTING ANEMIA IN ELDERLY PATIENTS TREATED FOR DLBCL: ANALYSIS OF THE PHASE 3 POLAR BEAR TRIAL

**Topic:** 21. Lymphoma biology & translational research

Simon Husby*[1], Heidi Naomi Ottesen[1], Jakob Schmidt Jespersen[2], Emilie Skou[3], Julian Hamfjord[4], Matthew Ku[5], Sirpa Leppä[6], Emil Kyvsgaard[1], Ruth Salim Raaschou[1], Elisa Lucchini[7], Alessandro Morotti[8], Alessandro Severino[9], Carlo Visco[10], Karen Juul-Jensen[11], Peter Brown[1], Andrés José María Ferreri[12], Kirsten Grønbæk[1], Mats Jerkeman[13]

*[1]Rigshospitalet, Dept. of Hematology, Copenhagen, Denmark; [2]Biotech Research and Innovation Center (BRIC), Finsen laboratory, Copenhagen, Denmark; [3]Aarhus University Hospital, Dept. of Hematology, Aarhus, Denmark; [4]Radiumhospitalet, Oslo, Norway; [5]St Vincents Hospital, Melbourne, Australia; [6]Helsinki University Hospital, Dept of Oncology, Helsinki, Finland; [7]Azienda Sanitaria Universitaria Giuliano-Isontina, Trieste, Italy; [8]University of Turin, Turino, Italy; [9]Universitaria Federico, Napoli, Italy; [10]Section of Hematology, Department of Medicine,, Verona, Italy; [11]Odense Universitetshospital, Odense, Denmark; [12]IRCCS San Raffaele Scientific Institute, Milano, Italy; [13]Lund University Hospital, Lund, Sweden;*

### Background

Cytopenias are common during immunochemotherapy, particularly among elderly patients (pts), and often result in dose reductions, diminishing treatment efficacy. The factors predicting these toxicities remain to be identified. Clonal hematopoiesis of indeterminate potential (CHIP), defined as somatic mutations in myeloid cells, in pts with lymphoma <65 years has been found to correlate with the need for intensive care, blood transfusions, and the development of therapy-related leukemia in lymphoma pts <65 years (Husby S. *et al*. Leukemia 2020). In particular, mutations in DNA repair pathway genes have been associated with poor survival. To our knowledge, the impact of CHIP on elderly pts with lymphoma has not been extensively studied.

### Aims

To investigate the impact of clonal hematopoiesis on toxicity in elderly patients with aggressive lymphoma undergoing curative-intent chemotherapy.

### Methods

A total of 393 blood samples - peripheral granulocyte pellets – from 228 pts in the phase 3 POLAR BEAR trial (NCT04332822; elderly pts with DLBCL, 1st line, randomized 1:1 to R-miniCHOP or R-miniCHP-polatuzumab vedotin) were collected prospectively and analyzed for CHIP by targeted next-generation sequencing of 31 genes associated with clonal hematopoiesis (min. depth of 1000X). The cut-off for mutation detection was variant allele frequency (VAF) 2.0%. Paired baseline and post-treatment samples were available for 161 pts. The study arm of patients has not yet been unblinded.

**Copyright Information:** (Online) ISSN: 2572-9241
©2026 The Author(s). HemaSphere is published by John Wiley & Sons Ltd on behalf of European Hematology Association. This is an open access Abstract Book distributed under the Attribution-NonCommercial-NoDerivs (CC BY-NC-ND), which allows third parties to download the articles and share them with others as long as they credit the author and the Abstract Book, but they cannot change the content in any way or use them commercially.
**Abstract Book Citations:** Authors, Title, HemaSphere, 2026;10:(S1):pages. The abstract book DOIs can be found on the “Author Information” page.
**Disclaimer:** Articles published in the journal HemaSphere exclusively reflect the opinions of the authors. The authors are responsible for all content in their abstracts including accuracy of the facts, statements, citing resources, etc.

### Results

The median age of the cohort was 82 years. At baseline, 40% (n=92) of the pts had detectable CHIP. The most frequently mutated genes at baseline were *DNMT3A* (n=55), *TET2* (n=45), and *SF3B1* (n=15), which is similar to previous findings in healthy elderly individuals. Baseline characteristics did not differ between pts with and without CHIP. Pts with large CHIP clones at baseline (VAF≥10%, n=33) had persistent anemia, which did not resolve after end of treatment (Figure 1, p<0.0001 and p=0.0002 for on-treatment and post-treatment levels, respectively). Similarly, thrombocytopenia during treatment seemed more pronounced in pts with large CHIP clones; however, it was only borderline significant (p=0.09). Neutropenia was not affected by the presence of CHIP, possibly due to the use of G-CSF. So far, there has been no difference in progression-free or overall survival between pts with and without CHIP.

By analyzing sequential samples, we found two evolutionary patterns. Post-treatment, we identified the appearance/marked growth of mutations associated with therapy-related leukemia (*TP53* and *PPM1D*, n=7) and, interestingly, a rapid decline in the size of *SF3B1* mutations (n=9), all of which decreased by at least 40% by VAF.

### Summary/Conclusion

CHIP is very common in elderly pts with DLBCL treated with immunochemotherapy and large clones are associated with persistent anemia. In sequential samples we identify clonal evolutionary patterns with growth/gain of mutations associated with therapy-related leukemia. We also find that *SF3B1*-mutated CHIP clones shrink during immunochemotherapy, which to our knowledge has not been described previously.

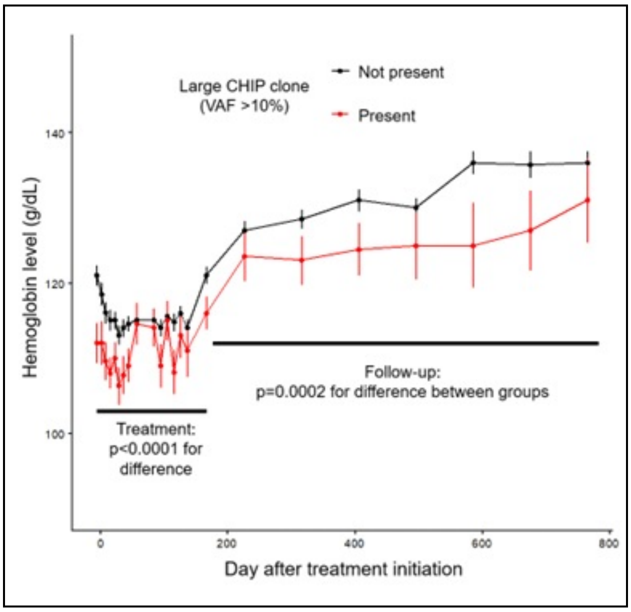

**Copyright Information:** (Online) ISSN: 2572-9241
©2026 The Author(s). HemaSphere is published by John Wiley & Sons Ltd on behalf of European Hematology Association. This is an open access Abstract Book distributed under the Attribution-NonCommercial-NoDerivs (CC BY-NC-ND), which allows third parties to download the articles and share them with others as long as they credit the author and the Abstract Book, but they cannot change the content in any way or use them commercially.
**Abstract Book Citations:** Authors, Title, HemaSphere, 2026;10:(S1):pages. The abstract book DOIs can be found on the "Author Information" page.
**Disclaimer:** Articles published in the journal HemaSphere exclusively reflect the opinions of the authors. The authors are responsible for all content in their abstracts including accuracy of the facts, statements, citing resources, etc.

### (PS2185) ST6GALNAC4 DRIVES DLBCL PROGRESSION BY SIALYLATING LDHA TO ENHANCE GLYCOLYSIS

**Topic:** 21. Lymphoma biology & translational research

Anqi Zhou*[1], Xinyu Yang[1], Guangcai Zhong[1], Ran Kong[1], Yue Li[1], Xiangxiang Zhou[1]

[1]*Shandong Provincial Hospital Affiliated to Shandong First Medical University, Jinan, China;*

### Background

Targeting epigenetic modifications has emerged as a key therapeutic strategy in hematological malignancies. Recent studies have revealed that sialylation plays a central role in tumor progression by regulating protein-protein interactions, subcellular localization, vesicle trafficking, and signal transduction. Our study confirms that sialylation is significantly elevated in diffuse large B-cell lymphoma (DLBCL). The sialyltransferase ST6GALNAC4 is significantly overexpressed in DLBCL and correlates with poor prognosis. Further investigation identified that lactate dehydrogenase A (LDHA) is a critical target of ST6GALNAC4. ST6GALNAC4 sialylates LDHA and regulates its activity, thereby promoting glycolysis and lactate production to drive tumor progression.

### Aims

This study aims to elucidate the molecular mechanism by which ST6GALNAC4 enhances glycolysis and lactate production through sialylating LDHA, and provide new potential therapeutic targets for DLBCL treatment.

### Methods

We analyzed α-2,6-sialylation and ST6GALNAC4 expression in DLBCL cohorts and cell lines, and validated the therapeutic potential of targeting sialylation using the sialyltransferase inhibitor 3FAx-Neu5Ac both in vitro and in vivo. Potential substrates were further screened by cross-analysis of sialylated proteins and ST6GALNAC4-binding proteins in DLBCL cells. Immunofluorescence co-localization, lectin-based co-immunoprecipitation, and molecular docking were used to validate the interaction between ST6GALNAC4 and LDHA and the regulation of LDHA sialylation by ST6GALNAC4. Mitochondrial tracker labeling, immunofluorescence, enzyme activity assays, ATP/lactate/$NAD^+$/NADH measurements, and Seahorse assays were conducted to evaluate LDHA mitochondrial localization, enzyme activity, and glycolytic function.

### Results

We found that ST6GALNAC4 expression was significantly elevated in DLBCL patients and correlated with poor prognosis (Fig1 A-B). In vivo and in vitro experiments confirmed that ST6GALNAC4 promotes DLBCL progression (Fig1 C-D). We observed that LDHA significantly binds to ST6GALNAC4 (Fig1 E), and its activity is regulated by ST6GALNAC4-mediated sialylation. Downregulation of LDHA significantly suppressed the pro-tumor effect of ST6GALNAC4 (Fig1 F). Lectin-based co-immunoprecipitation confirmed that knockdown of ST6GALNAC4 reduced the sialylation level of LDHA. Protein molecular docking revealed high embedding of both proteins in their active pocket regions, further supporting a direct interaction (Fig1 G). Moreover, knockout of ST6GALNAC4 significantly reduced the co-localization of LDHA with mitochondria (Fig1 H), accompanied by decreased ATP and lactate production (Fig1 I-J). Seahorse assays demonstrated that ST6GALNAC4 overexpression significantly increased basal glycolysis, glycolytic capacity, and glycolytic reserve, while knockdown of LDHA significantly reversed this effect (Fig1 K).

**Copyright Information:** (Online) ISSN: 2572-9241
©2026 The Author(s). HemaSphere is published by John Wiley & Sons Ltd on behalf of European Hematology Association. This is an open access Abstract Book distributed under the Attribution-NonCommercial-NoDerivs (CC BY-NC-ND), which allows third parties to download the articles and share them with others as long as they credit the author and the Abstract Book, but they cannot change the content in any way or use them commercially.
**Abstract Book Citations:** Authors, Title, HemaSphere, 2026;10:(S1):pages. The abstract book DOIs can be found on the "Author Information" page.
**Disclaimer:** Articles published in the journal HemaSphere exclusively reflect the opinions of the authors. The authors are responsible for all content in their abstracts including accuracy of the facts, statements, citing resources, etc.

### Summary/Conclusion

This study elucidated that ST6GALNAC4 promotes glycolytic metabolic reprogramming and lactate accumulation by sialylating LDHA, thereby facilitating the progression of DLBCL. Targeting ST6GALNAC4 and its regulation of LDHA sialylation represents a promising therapeutic strategy for DLBCL.

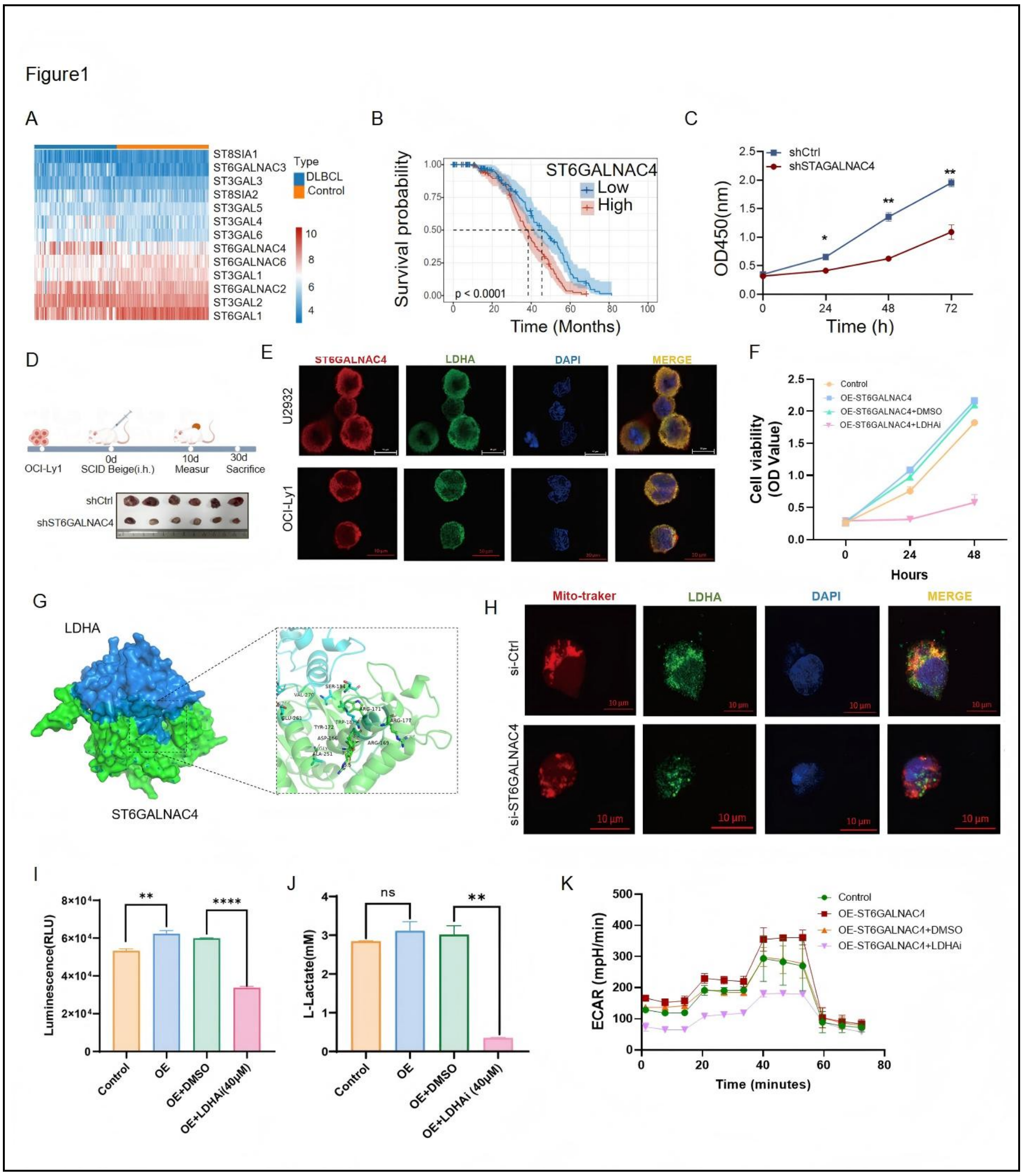

**Copyright Information:** (Online) ISSN: 2572-9241
©2026 The Author(s). HemaSphere is published by John Wiley & Sons Ltd on behalf of European Hematology Association. This is an open access Abstract Book distributed under the Attribution-NonCommercial-NoDerivs (CC BY-NC-ND), which allows third parties to download the articles and share them with others as long as they credit the author and the Abstract Book, but they cannot change the content in any way or use them commercially.
**Abstract Book Citations:** Authors, Title, HemaSphere, 2026;10:(S1):pages. The abstract book DOIs can be found on the "Author Information" page.
**Disclaimer:** Articles published in the journal HemaSphere exclusively reflect the opinions of the authors. The authors are responsible for all content in their abstracts including accuracy of the facts, statements, citing resources, etc.

### (PS2186) SPATIAL TRANSCRIPTOMICS REVEALS GENE EXPRESSION PATTERNS ASSOCIATED WITH BONE MARROW INVOLVEMENT OF FOLLICULAR LYMPHOMA

**Topic:** 21. Lymphoma biology & translational research

Gabriella Szepesi*[1], Bence Bátai[1, 2], Ildikó Krencz[1], Tamás László[1], Richárd Hanza[1], Tamas Schneider[3], Andras Masszi[3], Krenács Tibor[1], Botond Timár[1], Csaba Bödör[1]

*[1]Semmelweis University, MTA SE Lendület Molecular Oncohematology Research Group, Department of Pathology and Experimental Cancer Research, Budapest, Hungary; [2]Semmelweis University, Department of Internal Medicine and Hematology, Budapest, Hungary; [3]National Institute of Oncology, Budapest, Hungary;*

### Background

Follicular lymphoma (FL) comprises approximately 30% of non-Hodgkin lymphoma cases and typically follows an indolent clinical course, yet early relapses remain an unmet clinical need with a dismal prognosis. Bone marrow (BM) involvement occurs in ~40% of patients, which represents an adverse prognostic factor incorporated into the FLIPI-2 score and is associated with inferior outcomes.

### Aims

We aimed to characterize the molecular mechanisms underlying BM involvement in FL by spatially resolving gene expression patterns of tumor B cells and their microenvironment, performing a spatial transcriptomic analysis using the NanoString GeoMx Digital Spatial Profiler (DSP).

### Methods

We analyzed 100 diagnostic lymph node (LN) and bone marrow (BM) samples from 50 patients with follicular lymphoma, including 30 with BM involvement and 20 without at diagnosis. After expert hematopathological review, two 2-µm cores from each LN and BM sample were incorporated into tissue microarrays. Slides were stained with CD20 and SYTO13, followed by hybridization with Whole Transcriptome Atlas barcoded probes. CD20-based segmentation enabled barcode collection from 647 Areas of Interest (AOIs), encompassing follicular and interfollicular regions in LNs and infiltrated and non-infiltrated regions in BM. Transcriptomic profiles were quantified by next-generation sequencing and analyzed using the GeoMxTools v3.12.0 R package.

### Results

The CD20-positive LN cells of patients with confirmed BM infiltration at the time of diagnosis exhibited a significant increase in the expression of anti-apoptotic, pro-survival, and homing genes involving *BCL2* ($\log_2$FC=0,922, FDR<0,001), *IGLL5* ($\log_2$FC=0,799, FDR<0,01) and *KLF2* ($\log_2$FC= 0,664, FDR<0,001). On the other hand, in patients without BM infiltration, the overexpression of genes linked to germinal center phenotype and cell motility control dominated (*MARCKSL1*, $\log_2$FC=0,805, FDR<0,001 and *LMO2*, $\log_2$FC=0,766, FDR<0,001) as seen in Figure 1. Upon separating the CD20-positive lymph node cells into follicular and interfollicular compartments, both the interfollicular and follicular B-cells retained *BCL2* overexpression with the additional overexpression of *KLF2* in interfollicular B-cells in patients with bone marrow infiltration, while *MARCKSL1* was overexpressed in the interfollicular cells in infiltration-negative cases, with the overexpression of *VASP* and *NLRP4* in the follicular region in addition to *LMO2* and *MARCKSL1.*

**Copyright Information:** (Online) ISSN: 2572-9241
©2026 The Author(s). HemaSphere is published by John Wiley & Sons Ltd on behalf of European Hematology Association. This is an open access Abstract Book distributed under the Attribution-NonCommercial-NoDerivs (CC BY-NC-ND), which allows third parties to download the articles and share them with others as long as they credit the author and the Abstract Book, but they cannot change the content in any way or use them commercially.
**Abstract Book Citations:** Authors, Title, HemaSphere, 2026;10:(S1):pages. The abstract book DOIs can be found on the "Author Information" page.
**Disclaimer:** Articles published in the journal HemaSphere exclusively reflect the opinions of the authors. The authors are responsible for all content in their abstracts including accuracy of the facts, statements, citing resources, etc.

**Summary/Conclusion**

Based on our findings, the gene expression pattern of LN B-cells is a critical determinant in the development of BM infiltration. B-cells of patients who presented with BM infiltration at the time of diagnosis overexpressed genes associated with antiapoptosis, B-cell survival, and homing, whereas cases without bone marrow infiltration displayed a gene expression pattern characteristic of the germinal center phenotype and cell motility control. Our findings highlight the tumor cell-intrinsic capacity for BM infiltration and pave the way for functional studies exploring potential targets in the lymphoma dissemination process.

**Funding**
FK20_134253, K21_137948, H2020-739593, BO/00125/22, LP2024-3, TKP2021-EGA-24, TKP2021-NVA-15, ELIXIR Hungary

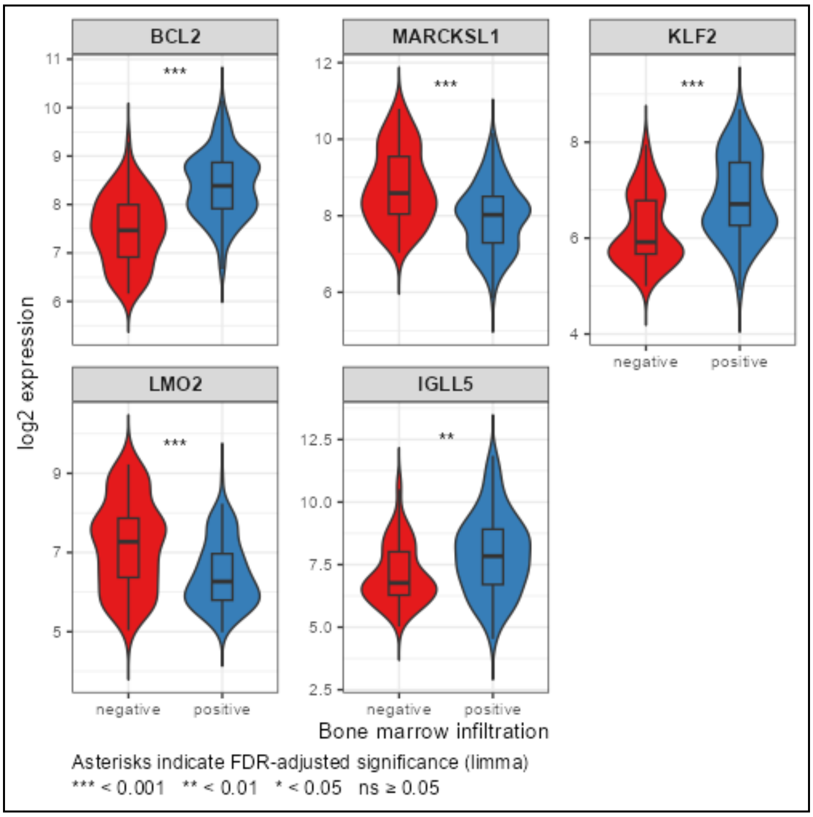

Figure 1. Differentially expressed genes in lymph node B-cells of follicular lymphoma patients with or without bone marrow infiltration at the time of diagnosis

**Copyright Information:** (Online) ISSN: 2572-9241
©2026 The Author(s). HemaSphere is published by John Wiley & Sons Ltd on behalf of European Hematology Association. This is an open access Abstract Book distributed under the Attribution-NonCommercial-NoDerivs (CC BY-NC-ND), which allows third parties to download the articles and share them with others as long as they credit the author and the Abstract Book, but they cannot change the content in any way or use them commercially.
**Abstract Book Citations:** Authors, Title, HemaSphere, 2026;10:(S1):pages. The abstract book DOIs can be found on the "Author Information" page.
**Disclaimer:** Articles published in the journal HemaSphere exclusively reflect the opinions of the authors. The authors are responsible for all content in their abstracts including accuracy of the facts, statements, citing resources, etc.

### (PS2187) GENOMIC DETERMINANTS OF RESPONSE AND RESISTANCE TO ZANUBRUTINIB IN RELAPSED/REFRACTORY PRIMARY CNS LYMPHOMA: BASELINE BIOMARKER ANALYSIS FROM THE PRIZM+ TRIAL

**Topic:** 21. Lymphoma biology & translational research

Donatella Talotta*[1], Findlay Copley[1], Graham McIlroy[2], Jiayi Wang[2], Edward Poynton[1], William Lavelle[1], Francesca Yates[2], Emma Homer[2], Rebecca Boucher[2], Rebecca Collings[2], Jahanzaib Khwaja[3], Thomas Cummin[4], Andrew Davies[5], Graham Collins[6], Pam McKay[7], Shireen Kassam[8], David Lewis[9], Helen Marr[10], Aimee Jackson[2], Nicolas Martinez-Calle[11], Elizabeth H. Phillips[12], Jeff Smith[13], Steffi Thust[14], Dorothee Auer[14], Kate Cwynarski[3], Christopher P. Fox[15], Jessica Okosun[1]

*[1]Barts Cancer Institute, Queen Mary University of London, Centre for Haemato-Oncology, London, United Kingdom;[2]University of Birmingham, Cancer Research UK Clinical Trials Unit, Birmingham, United Kingdom; [3]University College London Hospitals NHS Foundation Trust, London, United Kingdom; [4]Portsmouth Hospitals University NHS Trust, Portsmouth, United Kingdom; [5]University of Southampton, NIHR/Cancer Research UK Southampton Experimental Cancer Medicines Centre, Faculty of Medicine, Southampton, United Kingdom; [6]Oxford University Hospitals NHS Foundation Trust, Churchill Hospital, Oxford Cancer and Haematology Centre, Oxford, United Kingdom; [7]Beatson West Of Scotland Cancer Centre, Glasgow, United Kingdom; [8]King's College Hospital NHS Foundation Trust, London, United Kingdom; [9]University Hospitals Plymouth NHS Trust, Plymouth, United Kingdom; [10]Newcastle upon Tyne Hospitals NHS Foundation Trust, Freeman Hospital, Newcastle, United Kingdom; [11]Nottingham University Hospitals NHS Trust, Clinical Haematology, Nottingham, United Kingdom; [12]University of Manchester, Division of Cancer Sciences, Manchester, United Kingdom; [13]The Clatterbridge Cancer Centre, Liverpool, United Kingdom; [14]University of Nottingham, School of Medicine, Precision Imaging, Nottingham, United Kingdom; [15]University of Nottingham, School of Medicine, Nottingham, United Kingdom;*

### Background

Primary central nervous system lymphoma (PCNSL) is a rare, aggressive B-cell lymphoma confined to the CNS with frequent relapse after initial therapy and poor outcomes in the relapsed/refractory (rr) setting, particularly among older or transplant-ineligible patients. PRiZM+ is a phase II platform trial in rrPCNSL; cohort 1 evaluates Zanubrutinib monotherapy, targeting constitutive B-cell receptor (BCR) signalling that characterizes this disease. Response assessment relies primarily on conventional MRI, with recognised limitations in distinguishing viable tumour from treatment-related effects, and no other prognostic or predictive biomarkers currently guide therapy. Repeat brain biopsies at relapse are rarely feasible due to procedural risk, making archival diagnostic tissue the only feasible source for tissue-based biomarker evaluation.

### Aims

T o determine whether genomic features of diagnostic PCNSL biopsies predict response and durability to Zanubrutinib in rrPCNSL patients enrolled in PRiZM+ cohort 1.

### Methods

Genomic DNA from formalin-fixed diagnostic brain biopsies from 19 of 20 enrolled patients (pts) in PRiZM+ cohort 1 underwent whole-exome sequencing for mutation profiling, copy number analysis and molecular subtyping using LymphGen and DLBclass classifiers. Primary endpoint was overall response rate after two Zanubrutinib cycles. Associations with progression-free survival (PFS) were evaluated using univariable and multivariable analyses.

Copyright Information: (Online) ISSN: 2572-9241
©2026 The Author(s). HemaSphere is published by John Wiley & Sons Ltd on behalf of European Hematology Association. This is an open access Abstract Book distributed under the Attribution-NonCommercial-NoDerivs (CC BY-NC-ND), which allows third parties to download the articles and share them with others as long as they credit the author and the Abstract Book, but they cannot change the content in any way or use them commercially.
Abstract Book Citations: Authors, Title, HemaSphere, 2026;10:(S1):pages. The abstract book DOIs can be found on the "Author Information" page.
Disclaimer: Articles published in the journal HemaSphere exclusively reflect the opinions of the authors. The authors are responsible for all content in their abstracts including accuracy of the facts, statements, citing resources, etc.

### Results

Baseline genomic landscape was consistent with prior PCNSL studies, with recurrent mutations in *MYD88* (53%), *CD79B* (47%) and *CARD11* (47%). Recurrent copy-number alterations included gains at 3p22.3, 3q29, and 7q21.3 and deletions at 1p13.1, 6p21.32, and 6q21, affecting genes such as *BTG2*, *CD58*, *PRDM1*, and HLA class I/II loci. Whole-genome duplication (WGD) occurred in four tumours and was associated with markedly increased genomic instability. Molecular subtyping assigned 52.6% (10/19) of cases to the DLBclass MYD88/CD79B-driven C5 subtype and 10.5% (2/19) being C2 tumours enriched for *TP53* alterations, genomic instability and disruption of MHC class I genes. Concordance between DLBclass and LymphGen classifiers was high.

With median follow up of 23 months, 6 pts experienced durable responses (including one after transplant), while the remainder experienced early progression of disease within 12 months of therapy initiation (ePOD). Although 11 pts had complete or partial response by MRI after two cycles, only 4 out of 11 were durable responders (DR), one of whom with imaging-confirmed complete remission (**Fig. 1A**). DR were enriched for BCR pathway alterations, including *CD79B* mutations (80% vs 38% in ePOD) and C5 subtype (60% vs 23%). By contrast, loss of MHC class I-associated genes and WGD occurred exclusively in pts with ePOD (69% and 31%, respectively) and were absent in DR (**Fig. 1B**). Alterations affecting MHC class I genes were significantly associated with shorter PFS in univariate analyses and was the only independent predictor of inferior PFS in multivariable analysis (HR 4.31, 95% CI 1.12-16.64, $P = 0.03$), including molecular subtype and MRI response.

### Summary/Conclusion

Disruption of MHC class I-associated genes in diagnostic tumour tissue predicts ePOD on BTKi therapy and appears more prognostic than early MRI response. These findings implicate immune evasion as a potential mechanism of resistance to BTK inhibition in rrPCNSL and support the value of genomic profiling of diagnostic tissue to aid response prediction.

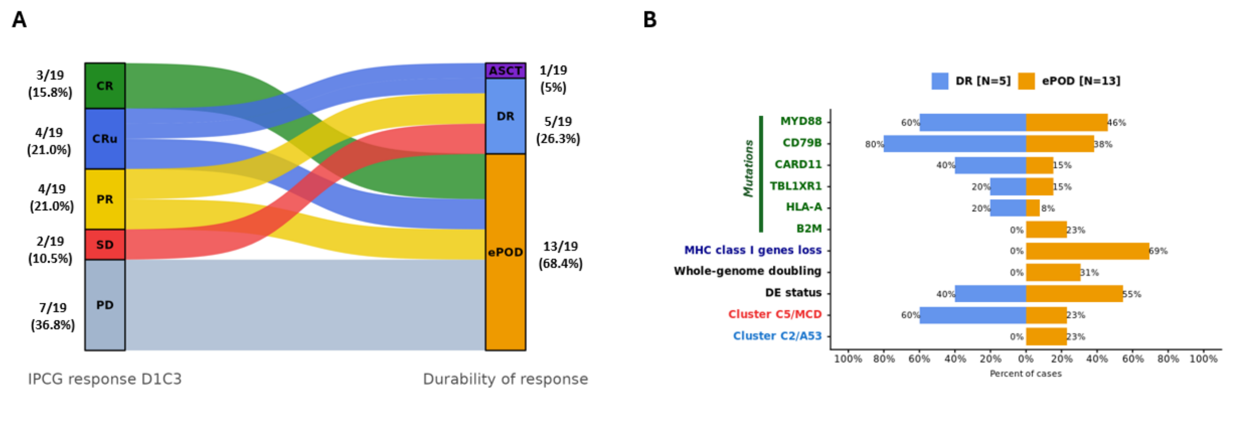

IPCG: International Primary CNS Lymphoma Collaborative Group; CR/CRu: Complete Response; PR: Partial Response; SD: Stable Disease; PD: Progressive Disease; ASCT: Autologous Stem Cell Transplant; DR: Durable Responders; ePOD: Early Progression of Disease; DE: Double Expressor; MHC: Major Histocompatibility Complex.

**Copyright Information:** (Online) ISSN: 2572-9241
©2026 The Author(s). HemaSphere is published by John Wiley & Sons Ltd on behalf of European Hematology Association. This is an open access Abstract Book distributed under the Attribution-NonCommercial-NoDerivs (CC BY-NC-ND), which allows third parties to download the articles and share them with others as long as they credit the author and the Abstract Book, but they cannot change the content in any way or use them commercially.
**Abstract Book Citations:** Authors, Title, HemaSphere, 2026;10:(S1):pages. The abstract book DOIs can be found on the "Author Information" page.
**Disclaimer:** Articles published in the journal HemaSphere exclusively reflect the opinions of the authors. The authors are responsible for all content in their abstracts including accuracy of the facts, statements, citing resources, etc.

### (PS2188) OVERLAYING TP53 LOSS AND ANEUPLOIDY WITH DISTINCT DIFFUSE LARGE B-CELL LYMPHOMA SUBTYPES REFINES TUMOR CLASSIFICATION AND ENHANCES RISK STRATIFICATION

**Topic:** 21. Lymphoma biology & translational research

Andrey Suponin[1], Pavel Zemskiy[1], Dmitrii Snitkin[1], Mark Meerson[1], Alexander Bagaev[1], Alexander Nesmelov[1], Konstantin Chernyshov[1], Nikita Kotlov*[1], Louis Staudt[2]

*[1]BostonGene Corporation, Waltham, United States of America;[2]Center for Cancer Research National Cancer Institute, Bethesda, MD, United States of America;*

### Background

Genetic subtypes are widely used to stratify diffuse large B-cell lymphoma (DLBCL). Yet, patients within the same subtype often exhibit heterogeneous outcomes. While chromosome number alteration (CNA)-driven subtypes such as A53 and C2 capture genomic instability, they encompass tumors with variable degrees of aneuploidy. Loss-of-function (LOF) *TP53* alterations and aneuploidy have each been associated with adverse prognosis, but their independent and combined effects in DLBCL remain insufficiently defined.

### Aims

To refine genomic stratification of DLBCL by disentangling the effects of *TP53* loss and quantitative aneuploidy, we introduced two novel genomic instability statuses into Lymphly, our previously developed DLBCL genetic subtype classification framework. These statuses are TP53+/–, denoting *TP53* LOF alterations, and A+/–, an aneuploidy-based status. This approach explicitly distinguishes *TP53* loss from aneuploidy within Lymphly subtypes.

### Methods

We analyzed 1,123 DLBCL samples with genome-wide CNA profiling. Survival data were available for 579 samples. The aneuploidy score was defined as the number of chromosome arms deviating from average ploidy (excluding acentric arms). The distribution was bimodal (range 0–39), with a nadir at ~15. K-means clustering defined A+ tumors as those with >14 arm-level CNAs. TP53+ status was defined as *TP53* LOF mutation or deletion. We also analyzed previously assigned A53/C2 cases to assess the concordance of these existing CNA-based subtypes with the Lymphly statuses. To evaluate the utility of the Lymphly statuses, we characterized their expression profiles and assessed their ability to stratify patients based on survival outcomes.

### Results

TP53+ tumors had significantly higher aneuploidy scores than TP53– tumors (p<0.0001). However, many individual tumors in the two groups had similar aneuploidy values. A+ cases comprised 20.7% of the analyzed samples and were evenly distributed across TP53 strata: 11.7% TP53+/A+ and 9.1% TP53–/A+. TP53+/A– and TP53–/A– cases made up 23.3% and 55.9%, respectively. Together, TP53+/– and A+/– statuses captured most of the A53/C2 cases analyzed, demonstrating the ability of these two Lymphly statuses to delineate the sample composition and underlying biology of the A53 and C2 subtypes.
Biologically, A+ tumors were enriched for the germinal center B-cell–like cell-of-origin (GCB COO), exhibited reduced B-cell content, and showed altered DNA replication and repair programs. Clinically, TP53+ was associated with primary refractoriness and inferior survival, whereas A+ was associated with improved prognosis, particularly among TP53+ tumors.

**Copyright Information:** (Online) ISSN: 2572-9241
©2026 The Author(s). HemaSphere is published by John Wiley & Sons Ltd on behalf of European Hematology Association. This is an open access Abstract Book distributed under the Attribution-NonCommercial-NoDerivs (CC BY-NC-ND), which allows third parties to download the articles and share them with others as long as they credit the author and the Abstract Book, but they cannot change the content in any way or use them commercially.
**Abstract Book Citations:** Authors, Title, HemaSphere, 2026;10:(S1):pages. The abstract book DOIs can be found on the “Author Information” page.
**Disclaimer:** Articles published in the journal HemaSphere exclusively reflect the opinions of the authors. The authors are responsible for all content in their abstracts including accuracy of the facts, statements, citing resources, etc.

**Summary/Conclusion**

*TP53* loss and high aneuploidy represent independent and clinically meaningful genomic dimensions in DLBCL, in addition to Lymphly subtypes. Their integration into Lymphly refines the existing A53/C2 classification and improves risk stratification based on biologically grounded, actionable insights. Our findings provide a robust framework for patient stratification based on genomic features, offering potential benefits for trial design, patient selection, and treatment decision-making.

**Copyright Information:** (Online) ISSN: 2572-9241
©2026 The Author(s). HemaSphere is published by John Wiley & Sons Ltd on behalf of European Hematology Association. This is an open access Abstract Book distributed under the Attribution-NonCommercial-NoDerivs (CC BY-NC-ND), which allows third parties to download the articles and share them with others as long as they credit the author and the Abstract Book, but they cannot change the content in any way or use them commercially.
**Abstract Book Citations:** Authors, Title, HemaSphere, 2026;10:(S1):pages. The abstract book DOIs can be found on the "Author Information" page.
**Disclaimer:** Articles published in the journal HemaSphere exclusively reflect the opinions of the authors. The authors are responsible for all content in their abstracts including accuracy of the facts, statements, citing resources, etc.

### (PS2189) ESTABLISHING ADDITIONAL PATIENT-DERIVED LYMPHOMA XENOGRAFTS AND CELL LINES OF LARGE B-CELL LYMPHOMA: GENETIC, TRANSCRIPTIONAL, AND FUNCTIONAL RELEVANCE, AND APPLICABILITY FOR PRECLINICAL RESEARCH

**Topic:** 21. Lymphoma biology & translational research

Alexandra Dolnikova*[1], Dmitry Manakov[1], Nazli Serin[2], Shashwat Sahay[2], Lucie Winkowska[3], Eva Pokorna[1], Nicol Renesova[1], Liliana Tuskova[1, 4], Zuzana Zemanova[5], Katerina MacHova Polakova[6], Adam Laznicka[6], Michaela Vatolikova[7], Helena Urbankova[7], Jan Galko[8], Ondrej Havranek[9], Kristina Forsterová[4], Magdalena Klanova[1, 4], Vít Procházka[7], Marek Trněný[4], Björn Chapuy[2, 10], Pavel Klener[1, 4]

*[1]Institute of Pathological Physiology, First Faculty of Medicine, Charles University, Prague Lymphoma Lab - Laboratory of Experimental and Translational Hematology, Prague, Czechia; [2]Department of Hematology, Oncology, and Cancer Immunology, Charité – Universitätsmedizin, Berlin, Germany; [3]CLIP, Department of Pediatric Hematology/Oncology, Second Faculty of Medicine and University Hospital Motol and Homolka, Prague, Czechia; [4]First Department of Medicine-Hematology, University General Hospital Prague and First Faculty of Medicine, Charles University, Prague, Czechia; [5]Center for Oncocytogenetics, Institute of Medical Biochemistry and Laboratory Diagnostics, University General Hospital, Prague, Czechia; [6]Institute of Hematology and Blood Transfusion, Prague, Czechia; [7]Department of Hemato-Oncology, Faculty of Medicine and Dentistry, Palacky University and University Hospital, Olomouc, Czechia; [8]Institute of Pathology, University General Hospital and First Faculty of Medicine, Charles University, Prague, Czechia; [9]BIOCEV LF1 - Biotechnology and Biomedicine Centre, First Faculty of Medicine, Charles University, Vestec, Czechia; [10]German Consortium for Translational Cancer Research (DKTK), partner site Berlin, German Cancer Research Center (DKFZ), Heidelberg, Germany;*

### Background

Most aggressive large B-cell lymphoma (LBCL) cell lines were established decades ago, often after numerous undocumented passages, and exhibit discrepancies in key characteristics, raising concerns about their authenticity. These limitations, along with genomic instability and the accumulation of culture-induced genetic alterations, constrain lymphoma research. To better capture genetic heterogeneity and lineage specificity, systematic efforts have focused on developing patient-derived models that more faithfully recapitulate the properties of the original lymphoma.

### Aims

To establish and characterize additional preclinical LBCL models with verified cellular origin and defined genetic backgrounds, validated against corresponding primary cells to ensure biological fidelity and clinical relevance.

### Methods

Patient-derived xenografts (PDXs) of pathology-confirmed primary or relapsed/refractory LBCL were propagated *in vivo* in NSG mice, and patient-derived cell lines (PDCLs) were established from the same LBCL by *in vitro* culture. Mycoplasma and Epstein-Barr virus (EBV) were assessed by PCR, with EBER ISH performed in PCR-positive cases. Each model was characterized head-to-head with the corresponding primary lymphoma using whole exome sequencing (WES), bulk and single-cell RNA sequencing, targeted sequencing, cytogenetics, flow cytometry, and drug-sensitivity assays. The metabolic assays were performed using the Seahorse Extracellular Flux Analyzer. Classification included the cell-of-origin (COO) transcriptional classification, dark-zone signature (DZsig), genetic subtype (DLB*class*, LymphGen), and consensus clustering.

**Copyright Information:** (Online) ISSN: 2572-9241
©2026 The Author(s). HemaSphere is published by John Wiley & Sons Ltd on behalf of European Hematology Association. This is an open access Abstract Book distributed under the Attribution-NonCommercial-NoDerivs (CC BY-NC-ND), which allows third parties to download the articles and share them with others as long as they credit the author and the Abstract Book, but they cannot change the content in any way or use them commercially.
**Abstract Book Citations:** Authors, Title, HemaSphere, 2026;10:(S1):pages. The abstract book DOIs can be found on the "Author Information" page.
**Disclaimer:** Articles published in the journal HemaSphere exclusively reflect the opinions of the authors. The authors are responsible for all content in their abstracts including accuracy of the facts, statements, citing resources, etc.

**Results**

Nine PDCLs and 20 PDXs were established from primary LBCL samples, including 7 paired models, where both PDCL and PDX were established simultaneously from the same patient. WES confirmed that both PDCLs and PDXs kept the majority of genetic alterations. Two out of nine PDCLs and one out of 20 PDXs were EBER positive. Based on the COO, 6 PDCLs were germinal-center B-cell (GCB)-type, 2 activated B-cell (ABC)-type, and 1 unclassified. In contrast, 12 out of 20 PDXs were ABC-LBCL. At the metabolic level, we found that the glycolysis-to-oxidative phosphorylation (GLYCO/OXPHOS) ratio remained stable throughout cultivation. Bioenergetic profiling of 9 PDCLs uncovered metabolic heterogeneity, with one cell line exhibiting a marked reliance on OXPHOS compared to the others. Pharmacologic inhibition confirmed this dependency, exposing a distinct vulnerability to respiratory chain perturbation. CD20 negativity was detected in 2 PDCLs and 5 PDXs by flow cytometry and subsequently confirmed at both mRNA and protein levels. In addition, 2 cell lines derived from patients with CD20-negative LBCL showed variable CD20 surface expression. In the CD20-negative models, WES revealed no coding *MS4A1* mutations, though one PDCL harbored a 3′-UTR deletion, suggesting regulatory alterations may drive CD20 loss under anti-CD20 therapy. Epigenetic modulators (azacytidine, vorinostat) restored CD20 expression only in a minority of models.

**Summary/Conclusion**

Here, we demonstrate that PDCLs and PDXs are equally genetically relevant models for studying LBCL. Differential adaptation to the *in vivo* and *in vitro* microenvironments, however, leads to distinct transcriptomic and metabolic states that should be considered for specific research objectives. Notably, PDCLs were enriched for DZsig+ GC-LBCL, while PDXs were slightly enriched for ABC-LBCL, thus broadening the repository of LBCL models.

**Copyright Information:** (Online) ISSN: 2572-9241
©2026 The Author(s). HemaSphere is published by John Wiley & Sons Ltd on behalf of European Hematology Association. This is an open access Abstract Book distributed under the Attribution-NonCommercial-NoDerivs (CC BY-NC-ND), which allows third parties to download the articles and share them with others as long as they credit the author and the Abstract Book, but they cannot change the content in any way or use them commercially.
**Abstract Book Citations:** Authors, Title, HemaSphere, 2026;10:(S1):pages. The abstract book DOIs can be found on the “Author Information” page.
**Disclaimer:** Articles published in the journal HemaSphere exclusively reflect the opinions of the authors. The authors are responsible for all content in their abstracts including accuracy of the facts, statements, citing resources, etc.

### (PS2190) CD20-POSITIVE EXTRACELLULAR VESICLES ACT AS ANTIGEN DECOYS AND REDUCE RITUXIMAB ACTIVITY IN DIFFUSE LARGE B-CELL LYMPHOMA

**Topic:** 21. Lymphoma biology & translational research

Seok Jin Kim*[1], Bo Kyoung Seo[2], Bon Park[3], Kyung Ju Ryu[3], Sang Eun Yoon[1], Won Seog Kim[1]

*[1]Samsung Medical Center, Sungkyunkwan University School of Medicine, Deaprtment of Medicine, Division of Hematology-Oncology, Seoul, South Korea; [2]Korea University Ansan Hospital, Ansan city, South Korea;[3]Samsung Advanced Institute of Health Sciences and Technology, Sungkyunkwan University, Department of Health Sciences and Technology, Seoul, South Korea;*

### Background

Loss or downregulation of CD20 has been considered a major mechanism of resistance to rituximab, an anti-CD20 monoclonal antibody in diffuse large B-cell lymphoma (DLBCL). However, tumor-derived extracellular vesicles (EVs) may also contribute to antibody resistance. EVs are operationally categorized by size into small EVs (<200 nm) and large EVs (>200 nm). Because large EVs are shed directly from the plasma membrane, tumor-derived large EVs circulating in the bloodstream may reflect tumor burden and contribute to treatment resistance. Given that CD20 is abundantly expressed on DLBCL cells, tumor-derived CD20-positive large EVs may act as antigen sinks for rituximab and thereby reduce effective antibody exposure to malignant B cells. We therefore hypothesized that circulating CD20-positive large EVs attenuate rituximab activity and predict treatment outcomes. Accordingly, we investigated the association between circulating CD20-positive large EVs and clinical outcomes in newly diagnosed DLBCL patients treated with R-CHOP.

### Aims

The aim of this study was to evaluate the prognostic impact of circulating CD20-positive large EVs in newly diagnosed DLBCL patients treated with standard R-CHOP therapy in routine clinical practice.

### Methods

CD20-positive large and small EVs were isolated from archived serum samples of patients with DLBCL and from supernatants of cultured primary tumor cells. The training cohort consisted of 26 patients selected from our second prospective cohort study (2012–2017). Patients were dichotomized into high and low groups according to the median levels of CD20-positive large EVs and small EVs. The prognostic value of circulating EVs was evaluated in an independent validation cohort with long-term clinical follow-up from our first prospective cohort study (n = 90, 2008–2011).

**Copyright Information:** (Online) ISSN: 2572-9241
©2026 The Author(s). HemaSphere is published by John Wiley & Sons Ltd on behalf of European Hematology Association. This is an open access Abstract Book distributed under the Attribution-NonCommercial-NoDerivs (CC BY-NC-ND), which allows third parties to download the articles and share them with others as long as they credit the author and the Abstract Book, but they cannot change the content in any way or use them commercially.
**Abstract Book Citations:** Authors, Title, HemaSphere, 2026;10:(S1):pages. The abstract book DOIs can be found on the “Author Information” page.
**Disclaimer:** Articles published in the journal HemaSphere exclusively reflect the opinions of the authors. The authors are responsible for all content in their abstracts including accuracy of the facts, statements, citing resources, etc.

### Results

CD20-positive large and small EVs were characterized using B-cell lymphoma cell lines. Raji cells showed the highest CD20 positivity and were used to define gating thresholds. CD20-positive EVs were successfully isolated from patient serum samples and primary tumor culture supernatants. In the training cohort (n=26), the median level of CD20-positive large EV was 3.66%. All patients with progressive disease after R-CHOP belonged to the high large EV group, which showed significantly inferior disease-specific (DSS) and overall survival (OS) despite similar baseline characteristics. CD20-positive small EV levels were not associated with outcomes. Functionally, patient-derived CD20-positive large EV enhanced proliferation of OCI-Ly1 cells and attenuated rituximab-mediated growth inhibition. In T cell co-culture assays using OCI-Ly1 and U2932 cells, large EV significantly reduced rituximab-mediated cytotoxicity and increased tumor cell survival. These findings indicate that tumor-derived CD20-positive large EV promote tumor growth and impair rituximab activity, consistent with an antigen-decoy mechanism. In the validation cohort, high CD20-positive large EV levels were associated with more progressive disease and significantly worse DSS and OS. CD20-positive large EV remained an independent prognostic factor after adjustment for IPI risk.

### Summary/Conclusion

CD20-positive large EVs act as decoys that neutralize rituximab and suppress immune cytotoxicity, promoting resistance and immune evasion in DLBCL. Their serum levels may serve as a novel biomarker for predicting treatment response and prognosis.

**Copyright Information:** (Online) ISSN: 2572-9241
©2026 The Author(s). HemaSphere is published by John Wiley & Sons Ltd on behalf of European Hematology Association. This is an open access Abstract Book distributed under the Attribution-NonCommercial-NoDerivs (CC BY-NC-ND), which allows third parties to download the articles and share them with others as long as they credit the author and the Abstract Book, but they cannot change the content in any way or use them commercially.
**Abstract Book Citations:** Authors, Title, HemaSphere, 2026;10:(S1):pages. The abstract book DOIs can be found on the "Author Information" page.
**Disclaimer:** Articles published in the journal HemaSphere exclusively reflect the opinions of the authors. The authors are responsible for all content in their abstracts including accuracy of the facts, statements, citing resources, etc.

### (PS2191) ALTERED CHROMATIN PATTERNING AS A PREDISPOSING FACTOR FOR ERRONEOUS REARRANGEMENTS IN B-CELL LYMPHOMAS

**Topic:** 21. Lymphoma biology & translational research

Prakruti Uday*[1], Ann Sun[1], Andrew Chen[1], Rui Xu[1], Tianyu Duan[1], Laura Hilton[2], David Scott[2], Andrew Weng[1, 3]

*[1]BC Cancer Research Institute, Vancouver, Canada; [2]BC Cancer Research Institute, Centre for Lymphoid Cancers, Vancouver, Canada; [3]University of British Columbia, Pathology and Laboratory Medicine, Vancouver, Canada;*

### Background

Class-switch recombination (CSR) is a naturally occurring DNA rearrangement event that takes place in activated B-cells entering the germinal centre (GC) to generate antibodies with specific effector function. Successful CSR requires resolution of distant double-stranded breaks (DSBs) introduced by activation-induced deaminase (AID) within the IGH locus. Chromosomal translocations involving the IG loci, the majority of which are introduced in IGH during CSR, are known drivers of lymphomagenesis associated with poor clinical outcomes.
Chromatin modifying genes (CMGs) modulate gene expression by enzymatically altering histones or DNA, or by reshuffling nucleosomes. Loss-of-function (LOF) mutations in CMGs such as *KMT2D* (lysine methyltransferase 2D) and *ARID1A* (AT-rich interacting domain 1A) are recurrent across B-cell lymphomas and their deletion in mouse models show CSR impairment and altered GC B-cell fate dynamics. CMGs may play an as yet uncharacterised but important role in distorting chromatin and altering epigenetic dynamics in a manner that renders GC B-cells prone to chromosomal translocation via erroneous CSR.

### Aims

To elucidate the mechanisms by which LOF in CMGs such as*KMT2D* or *ARID1A* impairs successful CSR and potentiates chromosomal translocations involving the IGH heavy chain locus.

### Methods

*In vitro* models of human B-cell CSR were established using B-cell lymphoma cell lines, Mino and JEKO, and primary naïve B-cells isolated from reactive lymph node samples. CSR induction was confirmed by increased germline transcript (GLT) levels (measured by droplet digital (dd) RT-PCR assay) and successful completion by conversion from surface immunoglobulin (sIg) M+ to sIgG+ phenotype (measured by flow cytometry). LOF *KMT2D* and *ARID1A* mutations were modelled via CRISPR-mediated gene knockout (KO) or lentiviral-delivered shRNA-mediated gene knockdown (KD). Chromatin alterations were interrogated via histone ChIP-seq and ATAC-seq. DSB induction & resolution were assessed by intracellular γH2AX flow cytometry following irradiation, and a custom ddPCR assay to quantify DSB frequency within the IGH locus.

### Results

Loss of *KMT2D* or *ARID1A* impaired CSR in *in vitro* human B-cell lymphoma cell lines Mino and JEKO, and primary B-cells as evidenced by reduced IGHG1 & IGHG3 GLT levels and lower efficiency of sIgM-to-sIgG conversion. While CSR to IgG decreased, switching to IgA increased in *KMT2D* KD but not *ARID1A* KD cells suggesting that they impair CSR by different modes. Expression of AID, which is required for CSR to occur, was minimally perturbed by either KD and reduced sIgM-to-sIgG conversion could not be rescued by AID overexpression. Both *KMT2D* and *ARID1A* KO cells displayed altered DSB repair as indicated by delayed reduction in γH2AX signal after irradiation. ATAC-seq revealed minimal changes over the IGH locus suggesting reduced CSR was not due to impairment of AID's access to DNA. Histone ChIP-seq showed focally increased H3K27ac, an epigenetic feature associated with AID targets, over regions not canonically involved in normal CSR.

**Copyright Information:** (Online) ISSN: 2572-9241
©2026 The Author(s). HemaSphere is published by John Wiley & Sons Ltd on behalf of European Hematology Association. This is an open access Abstract Book distributed under the Attribution-NonCommercial-NoDerivs (CC BY-NC-ND), which allows third parties to download the articles and share them with others as long as they credit the author and the Abstract Book, but they cannot change the content in any way or use them commercially.
**Abstract Book Citations:** Authors, Title, HemaSphere, 2026;10:(S1):pages. The abstract book DOIs can be found on the "Author Information" page.
**Disclaimer:** Articles published in the journal HemaSphere exclusively reflect the opinions of the authors. The authors are responsible for all content in their abstracts including accuracy of the facts, statements, citing resources, etc.

**Summary/Conclusion**

Taken together, the results suggest that altered chromatin structure induced by *KMT2D* and *ARID1A* LOF mutations may reduce normal CSR by redirecting AID to alternate DNA targets in the genome. Additional studies will ascertain AID occupancy, DSB induction, and chromosomal translocation at these alternate loci. Alterations in CSR-related mechanisms in *KMT2D* and *ARID1A* mutated cells may thus contribute to the lymphomagenic process.

**Copyright Information:** (Online) ISSN: 2572-9241
©2026 The Author(s). HemaSphere is published by John Wiley & Sons Ltd on behalf of European Hematology Association. This is an open access Abstract Book distributed under the Attribution-NonCommercial-NoDerivs (CC BY-NC-ND), which allows third parties to download the articles and share them with others as long as they credit the author and the Abstract Book, but they cannot change the content in any way or use them commercially.
**Abstract Book Citations:** Authors, Title, HemaSphere, 2026;10:(S1):pages. The abstract book DOIs can be found on the "Author Information" page.
**Disclaimer:** Articles published in the journal HemaSphere exclusively reflect the opinions of the authors. The authors are responsible for all content in their abstracts including accuracy of the facts, statements, citing resources, etc.

### (PS2192) DECIPHERING THE SPATIAL ARCHITECTURE OF PRIMARY BREAST DLBCL: IDENTIFICATION OF ECTOPIC MICROGLIA AND SPECIALIZED BOUNDARY IMMUNE HUBS

**Topic:** 21. Lymphoma biology & translational research

Chaowei Zhang*[1, 2], Weiyi Yao[1, 2], Yuxuan Han[1, 2], Qing Hong[1, 2], Yanan Zhu[1, 2], Xinyu Tang[1, 2], Na Chen[1]

*[1]Beijing Luhe Hospital, Capital Medical University, Beijing, China;[2]Shandong First Medical University, Jinan, China;*

### Background

Primary breast diffuse large B-cell lymphoma (PB-DLBCL) is a rare and clinically aggressive extranodal lymphoma. While single-cell RNA sequencing (scRNA-seq) has expanded our understanding of lymphoma heterogeneity, the precise spatial architecture and cellular crosstalk within the extranodal tumor microenvironment (TME) remain poorly defined. High-resolution spatial context is crucial for understanding how immune cells organize at the tumor-stroma interface to influence disease progression and immune evasion.

### Aims

This study aims to construct a high-resolution spatial and single-cell atlas of PB-DLBCL to resolve its cellular composition, identify novel niche-specific immune populations, and decode the complex spatial interactions at the tumor boundary.

### Methods

We performed an integrated analysis using scRNA-seq data from 9 DLBCL patients (63,891 high-quality cells) and cutting-edge high-definition spatial transcriptomics (Visium HD) from 3 PB-DLBCL samples. Visium HD utilizes gapless 2μm × 2μm squares, achieving subcellular-level resolution. Robust Cell Type Decomposition (RCTD) was employed to map cell types spatially. Key biological findings were corroborated via immunohistochemistry (IHC) in an independent cohort of PB-DLBCL patients (n=5).

### Results

Integration of scRNA-seq and Visium HD data resolved 12 major cell types and their spatial distributions. Most notably, we identified and validated the presence of ectopic microglia (expressing TMEM119, P2RY12, and CX3CR1) within the breast tumor tissue, challenging the paradigm that these cells are restricted to the central nervous system. Further sub-clustering revealed six microglial functional states, including antigen-presenting (MHC-II+), metabolically active, and a specific subset showing intense physical interaction with cytotoxic T cells. Spatially, we identified a critical "tumor boundary" niche characterized by the enrichment of polarized macrophages and NK cells. We identified three boundary-specific macrophage subsets: C13 (SPP1+/TREM2+), associated with immunosuppression and lipid metabolism; C17 (FRMD4A+), linked to autophagy; and C20 (CCL3+/MHC-II high), active in antigen presentation. NK cells at the boundary exhibited potent cytotoxic potential (high GNLY expression) but also showed significant enrichment in "negative regulation of macrophage cytokine production." This suggests a checkpoint-like feedback mechanism where NK cells modulate local inflammatory responses at the tumor edge.

**Copyright Information:** (Online) ISSN: 2572-9241
©2026 The Author(s). HemaSphere is published by John Wiley & Sons Ltd on behalf of European Hematology Association. This is an open access Abstract Book distributed under the Attribution-NonCommercial-NoDerivs (CC BY-NC-ND), which allows third parties to download the articles and share them with others as long as they credit the author and the Abstract Book, but they cannot change the content in any way or use them commercially.
**Abstract Book Citations:** Authors, Title, HemaSphere, 2026;10:(S1):pages. The abstract book DOIs can be found on the "Author Information" page.
**Disclaimer:** Articles published in the journal HemaSphere exclusively reflect the opinions of the authors. The authors are responsible for all content in their abstracts including accuracy of the facts, statements, citing resources, etc.

### Summary/Conclusion

We present the first high-definition spatial immune map of PB-DLBCL. The discovery of ectopic microglia within breast tissue suggests a unique role for these cells in extranodal lymphoma immunoediting. Furthermore, the characterization of specialized macrophage subpopulations and the NK-macrophage regulatory axis at the tumor boundary provides novel insights into immune evasion mechanisms. These findings identify potential spatial biomarkers and therapeutic targets for precision immunotherapy in PB-DLBCL.

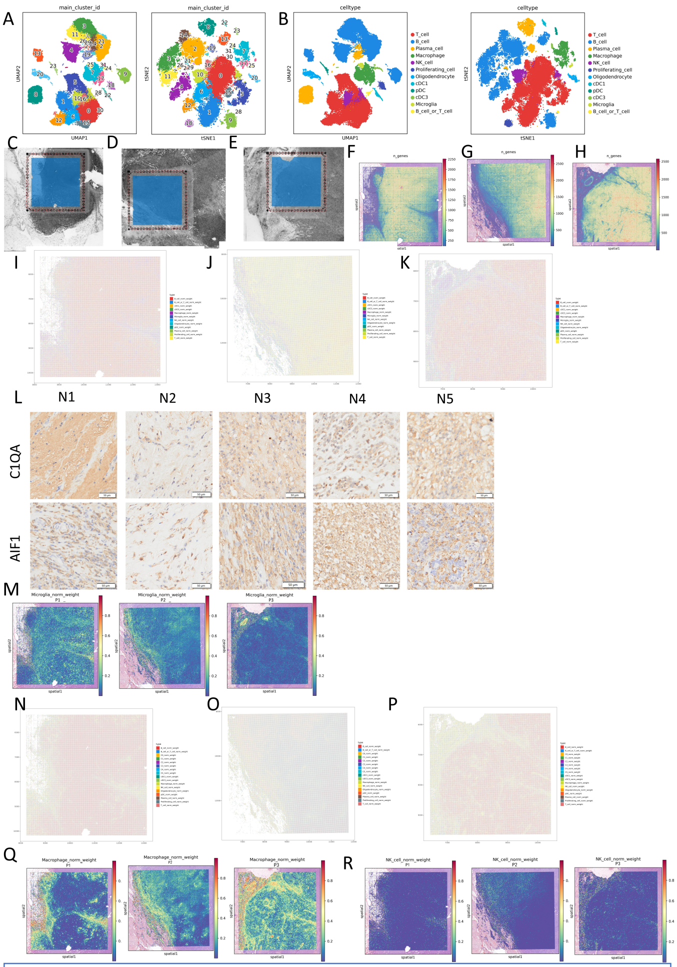

A. Two-dimensional dimensionality reduction and unsupervised clustering of spatial transcriptomic data visualized via UMAP and t-SNE algorithms. B. UMAP visualization of annotated cell types, with distinct colors representing different cell lineages. C–E. Formalin-fixed paraffin-embedded (FFPE) tissue sections with blue shaded regions indicating the specific capture areas for patients P1 (C), P2 (D), and P3 (E). F–H. Spatial distribution maps of gene counts per square after quality control (QC) for P1 (F), P2 (G), and P3 (H). These maps illustrate the variation in the number of detected genes (n_genes) across different spatial coordinates within the capture area. I–K. High-resolution spatial mapping of the major cell type compositions for P1 (I), P2 (J), and P3 (K). L. Representative immunohistochemistry (IHC) images of tumor boundary regions from an independent validation cohort of five PB-DLBCL patients. M. Spatial abundance maps of Microglia across the tissue sections of P1, P2, and P3. N–P. Spatial distribution maps of refined Microglia subpopulations for P1 (N), P2 (O), and P3 (P), illustrating the high-resolution localization of microglial functional states. Q. Spatial abundance maps of Macrophages for P1, P2, and P3. R. Spatial abundance maps of Natural Killer (NK) cells for P1, P2, and P3.

**Copyright Information:** (Online) ISSN: 2572-9241
©2026 The Author(s). HemaSphere is published by John Wiley & Sons Ltd on behalf of European Hematology Association. This is an open access Abstract Book distributed under the Attribution-NonCommercial-NoDerivs (CC BY-NC-ND), which allows third parties to download the articles and share them with others as long as they credit the author and the Abstract Book, but they cannot change the content in any way or use them commercially.
**Abstract Book Citations:** Authors, Title, HemaSphere, 2026;10:(S1):pages. The abstract book DOIs can be found on the "Author Information" page.
**Disclaimer:** Articles published in the journal HemaSphere exclusively reflect the opinions of the authors. The authors are responsible for all content in their abstracts including accuracy of the facts, statements, citing resources, etc.

### (PS2193) TARGETING CD49D SYNERGIZES WITH ACD3/ACD20 BSAB IN A NOVEL AGGRESSIVE TRANSPLANTABLE DLBCL MOUSE MODEL WITH EXTENSIVE BONE MARROW INVOLVEMENT.

**Topic:** 21. Lymphoma biology & translational research

Nicolas Aubert*[1], Hannah Briesch[1], Alessia Floerchinger[1], Dinis Calado[2], Martina Seiffert[1]

*[1]DKFZ, Immune Modulation in Cancer, Heidelberg, Germany;[2]Francis Crick Institute, Immunity & Cancer laboratory, London, United Kingdom;*

### Background

Diffuse large B-cell lymphoma (DLBCL) is the most prevalent aggressive non-Hodgkin lymphoma in adults. Bone marrow (BM) involvement signals poor outcomes, stromal protection and immune exclusion that may limit T-cell-redirecting immunotherapies. CD49d/VLA-4 (ITGA4), via VCAM-1 and extracellular matrix, mediates malignant B-cell adhesion and survival in these immune-restricted niches.

### Aims

This study investigates whether targeting the integrin CD49d can'evicts' lymphoma cells from protective niches and sensitises them to CD3×CD20 bispecific killing.

### Methods

We used a novel transplantable DLBCL mouse model (DCGL), spontaneously arising from T-cell lymphoma passage in C57BL/6 mice. This model reaches end-stage disease within 2 weeks, primarily involving BM, spleen, and liver. The immune landscape was characterized by spectral flow cytometry. We tested CD3×CD20 BsAb in combination with anti-CD49d blockade, elucidating mechanism via in vivo Fc-receptor blocking. Clinical relevance of CD49d was assessed by investigating its expression in DLBCL patient samples and correlating gene expression to survival in TCGA database.

### Results

Disease progression in the DCGL model was marked by rapid tumor expansion, global immune activation, and progressive CD8+ T-cell exhaustion. While CAR-T efficacy was restricted by rapid tumor expansion, BsAb monotherapy significantly reduced tumor load within the BM but had limited peripheral impact. Conversely, aCD49d monotherapy induced dramatic tumor clearance in the spleen and liver through FC-independant mechanism but showed more limited BM efficacy, suggesting a mechanism based on disrupting tumor-microenvironment interactions to promote mobilization. Consequently, the combination of BsAb and aCD49d synergistically reduced tumor load across all compartments and significantly prolonged survival (HR = 0.13 [0.025-0.66], p = 0.014). In patients, high CD49d expression was identified in 5/17 lymph node samples and correlated with poor survival outcomes.

### Summary/Conclusion

CD49d targeting mobilizes DLBCL cells, abrogating niche survival signals. Despite systemic T-cell activation, the BM microenvironment remains T-cell poor with effector exclusion, limiting CD3×CD20 BsAb access. CD49d blockade overcomes this immune restriction, unlocking bispecific potential in this sanctuary site. This combinatorial strategy potently debulks CD49d+ BM-involved DLBCL, overcoming protective niche resistance.

**Copyright Information:** (Online) ISSN: 2572-9241
©2026 The Author(s). HemaSphere is published by John Wiley & Sons Ltd on behalf of European Hematology Association. This is an open access Abstract Book distributed under the Attribution-NonCommercial-NoDerivs (CC BY-NC-ND), which allows third parties to download the articles and share them with others as long as they credit the author and the Abstract Book, but they cannot change the content in any way or use them commercially.
**Abstract Book Citations:** Authors, Title, HemaSphere, 2026;10:(S1):pages. The abstract book DOIs can be found on the “Author Information” page.
**Disclaimer:** Articles published in the journal HemaSphere exclusively reflect the opinions of the authors. The authors are responsible for all content in their abstracts including accuracy of the facts, statements, citing resources, etc.

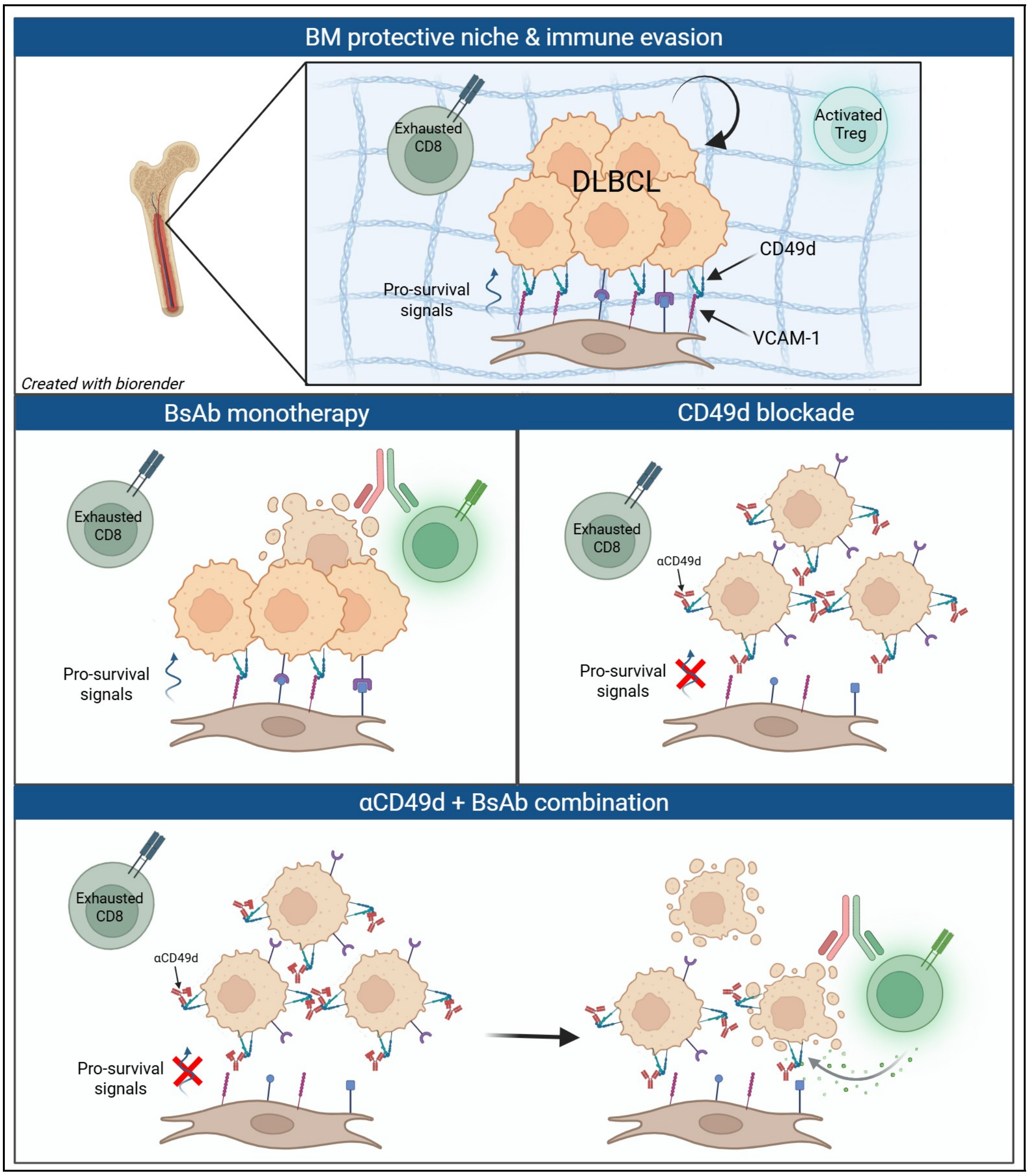

**Copyright Information:** (Online) ISSN: 2572-9241
©2026 The Author(s). HemaSphere is published by John Wiley & Sons Ltd on behalf of European Hematology Association. This is an open access Abstract Book distributed under the Attribution-NonCommercial-NoDerivs (CC BY-NC-ND), which allows third parties to download the articles and share them with others as long as they credit the author and the Abstract Book, but they cannot change the content in any way or use them commercially.
**Abstract Book Citations:** Authors, Title, HemaSphere, 2026;10:(S1):pages. The abstract book DOIs can be found on the "Author Information" page.
**Disclaimer:** Articles published in the journal HemaSphere exclusively reflect the opinions of the authors. The authors are responsible for all content in their abstracts including accuracy of the facts, statements, citing resources, etc.

### (PS2194) FRAGMENTOMICS ANALYSIS TO ENHANCE CTDNA BASED RISK STRATIFICATION IN HODGKIN LYMPHOMA

**Topic:** 21. Lymphoma biology & translational research

Jessica Schneider*[1], Julia Mattlener[1], Sophie Heidenreich[1], Julia K. Schleifenbaum[1], Max Freihammer[1], Daniel Schütte[1], Peter Borchmann[1], Philipp Gödel[1], Hyatt Balke-Want[1], Hans C. Reinhardt[2], Bastian von Tresckow[2], Roland Schwarz[3], Jan-Michel Heger[1], Sven Borchmann[1]

*[1]Department I of Internal Medicine, Center for Integrated Oncology Aachen Bonn Cologne Duesseldorf (CIO ABCD), University of Cologne, Faculty of Medicine and University Hospital of Cologne, Cologne, Germany, Cologne, Germany; [2]Department of Hematology and Stem Cell Transplantation, West German Cancer Center and German Cancer consortium (DKTK partner site Essen), University Hospital Essen, University of Duisburg-Essen, Essen, Germany, Essen, Germany; [3]Institute for Computational Cancer Biology (ICCB), Center for Integrated Oncology, University of Cologne, Faculty of Medicine and University Hospital of Cologne, Germany, Cologne, Germany;*

### Background

Current clinical challenges in Hodgkin lymphoma (HL) include long-term treatment-related toxicities and relapsed/refractory disease. Up to now, risk stratification tools mainly rely on clinical characteristics and lack sensitivity. Recent studies, including ours, demonstrated that circulating tumor (ct)DNA sequencing offers the potential to overcome this limitation.

### Aims

We aim to increase sensitivity to distinguish tumor (ctDNA) from non-tumor derived cell-free (cf)DNA and thus refine detection of minimal residual disease (MRD) (Sobesky, Med, 2021; Heger, J Clin Oncol, 2024; Heger, Blood, 2026) by incorporating fragmentomics analysis.

### Methods

We developed a software tool to extract advanced fragmentomics data at the read level and incorporate mutational information.  The tool was applied to 154 plasma samples from 71 patients with previously untreated HL (sampled from the HD21 trial) and 10 healthy controls.

**Copyright Information:** (Online) ISSN: 2572-9241
©2026 The Author(s). HemaSphere is published by John Wiley & Sons Ltd on behalf of European Hematology Association. This is an open access Abstract Book distributed under the Attribution-NonCommercial-NoDerivs (CC BY-NC-ND), which allows third parties to download the articles and share them with others as long as they credit the author and the Abstract Book, but they cannot change the content in any way or use them commercially.
**Abstract Book Citations:** Authors, Title, HemaSphere, 2026;10:(S1):pages. The abstract book DOIs can be found on the "Author Information" page.
**Disclaimer:** Articles published in the journal HemaSphere exclusively reflect the opinions of the authors. The authors are responsible for all content in their abstracts including accuracy of the facts, statements, citing resources, etc.

### Results

We found significant differences in cell-free DNA fragment length distribution between pre-treatment HL samples and healthy controls (mean 180 vs. 200bp, Mann-Whitney U test, p<0.01). Interestingly, fragment length increased with more treatment applied, ultimately approximating healthy controls (healthy control=200bp; pre-treatment=180bp; mid-treatment=185bp; end-of-treatment=187bp) Not only is this shift noticeable in the mean fragment length but furthermore one can see that it is present for the peak corresponding to mono-nucelosmal length as well as in the second peak (corresponding to di-nucleomal length) when comparing HL samples (Fig 1 (A), pre-treatment (blue; BL), mid-treatment (yellow;2CX), end-of-treatment (grey; EOT)) to healthy controls (red; ctrl) As already gathered from the mean fragment length this effect becomes less pronounced with increasing treatment applied. Facilitating these observations, we found that shorter mean fragment length (<182bp) in pre-treatment HL samples was associated with shorter progression free survival (PFS) (Logrank P-value <0.01).

Compared with healthy controls, we observed significant depletion of "AA", "TA", "TC", "CC", GA", "GC" (3' end) and "TT", "TC", "GA", "GC", "GG" (5' end) end motif frequency (Mann-Whitney U test, multiple-testing corrected, q<0.01) in pre-treatment samples. Additionally, we found enrichment in "AG", "TG", "CG", "GG" (3' end) and "CA", "CC, "CG" (5' end) end motif frequency (Mann-Whitney U test, multiple-testing corrected, q<0.01) in pre-treatment samples.
The analysis of a large B-cell lymphoma cohort (88 patients, 326 samples) is ongoing and will be presented at the meeting

### Summary/Conclusion

Fragmentomics analysis in our HL cohort showed shorter mean pre-treatment fragment length compared to healthy controls, with shorter mean length associated with inferior PFS. These findings suggest a potential use of fragment size and motif patterns for dynamic risk stratification in patients with HL.

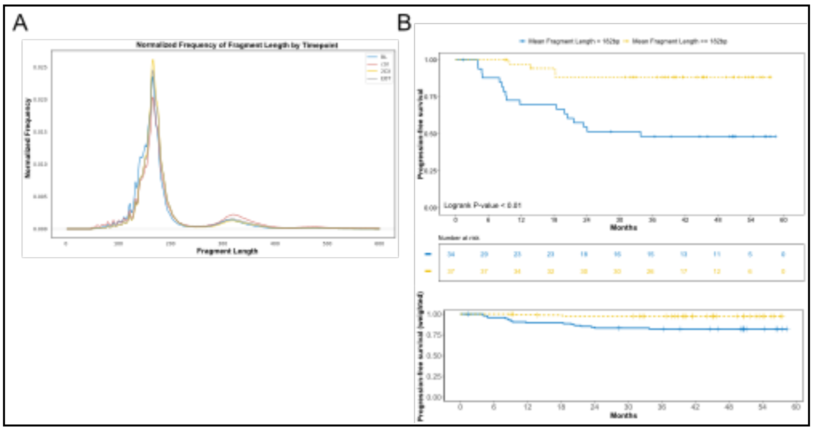

Figure 1: (A) Size profile of samples taken from HL patients before treatment start (blue; BL), after two cycles of therapy (yellow, 2CX) as well as healthy controls (red, ctrl). (B) Kaplan-Meier curve before start of therapy stratified by mean fragment length

**Copyright Information:** (Online) ISSN: 2572-9241
©2026 The Author(s). HemaSphere is published by John Wiley & Sons Ltd on behalf of European Hematology Association. This is an open access Abstract Book distributed under the Attribution-NonCommercial-NoDerivs (CC BY-NC-ND), which allows third parties to download the articles and share them with others as long as they credit the author and the Abstract Book, but they cannot change the content in any way or use them commercially.
**Abstract Book Citations:** Authors, Title, HemaSphere, 2026;10:(S1):pages. The abstract book DOIs can be found on the "Author Information" page.
**Disclaimer:** Articles published in the journal HemaSphere exclusively reflect the opinions of the authors. The authors are responsible for all content in their abstracts including accuracy of the facts, statements, citing resources, etc.

### (PS2195) MECHANISMS OF BCOR MUTATION IN PROMOTING RESISTANCE TO ANTI-PD-1 THERAPY IN NK/T-CELL LYMPHOMA

**Topic:** 21. Lymphoma biology & translational research

Linna Cheng*[1], Liqun Guo[1], Mengni Li[1], Huiyang Zhang[1], Lei Zhang[1], Rick Thorne[2], Zunmin Zhu[1]

*[1]Institution of Hematology⊠Henan Engineering Research Center for CAR-T Cell Therapy and Translational Applications⊠Henan Key Laboratory of Stem Cell Clinical Application and Key Technology⊠Henan Provincial People's Hospital of Zhengzhou University⊠School of Clinical Medicine, Henan University, Zhengzhou, China; [2]Translational Research Institute, Henan Provincial People's Hospital, Academy of Medical Science, Zhengzhou University, Zhengzhou, China;*

### Background

Relapsed/refractory Natural Killer/T-cell lymphoma (NKTCL) poses significant therapeutic challenges. Although PD-1 blockade has shown clinical activity, primary resistance remains a major hurdle. The role of epigenetic regulator mutations, particularly *BCOR* (BCL6 corepressor), in modulating this resistance is not well understood.

### Aims

This study aims to elucidate the mechanistic role of *BCOR* mutation in conferring resistance to anti-PD-1 immunotherapy in NKTCL, specifically investigating its impact on downstream signaling pathways, PD-L1 regulation, and tumor immune evasion.

### Methods

We performed whole-genome sequencing (WGS) and transcriptome sequencing (RNA-seq) on tumor specimens from relapsed/refractory NKTCL patients with divergent responses to PD-1 blockade. The correlation between *BCOR* mutational status and clinical resistance was assessed. *BCOR* knockdown and overexpression models were established in NKTCL cell lines. Co-culture systems with $CD3^+$ T cells were utilized to evaluate tumor cell sensitivity to PD-1 blockade via CCK-8 assays, while immune cytotoxicity and T-cell function were measured by LDH release and ELISA. Tumor proliferation was assessed using EdU and CCK-8 assays. KEGG and GSEA enrichment analyses were conducted to identify differentially regulated pathways, which were subsequently validated by Western blotting. The regulatory effect on PD-L1 was explored using dual-luciferase reporter assays, bioinformatic transcription factor prediction (JASPAR, UCSC), and qPCR/Western blotting.

### Results

WGS analysis revealed a notable enrichment of *BCOR* mutations in the PD-1 resistant cohort, suggesting a potential link between *BCOR* loss and poor immunotherapy response. Functionally, *BCOR* knockdown significantly attenuated T-cell mediated cytotoxicity and diminished the efficacy of PD-1 blockade, while promoting tumor cell proliferation.
Conversely, *BCOR* overexpression suppressed tumor growth. Transcriptomic profiling and subsequent validation demonstrated that *BCOR* loss activates the PI3K-AKT and JAK-STAT3 pathways, evidenced by increased phosphorylation of AKT and STAT3. Interestingly, we observed that *BCOR* upregulates PD-L1 transcription and protein expression. Dual-luciferase assays indicated this regulation occurs at the transcriptional level, potentially through cooperation with transcription factors such as STAT3 at the PD-L1 promoter, suggesting that *BCOR* mutation reduces tumor dependence on the PD-1/PD-L1 axis.

**Copyright Information:** (Online) ISSN: 2572-9241
©2026 The Author(s). HemaSphere is published by John Wiley & Sons Ltd on behalf of European Hematology Association. This is an open access Abstract Book distributed under the Attribution-NonCommercial-NoDerivs (CC BY-NC-ND), which allows third parties to download the articles and share them with others as long as they credit the author and the Abstract Book, but they cannot change the content in any way or use them commercially.
**Abstract Book Citations:** Authors, Title, HemaSphere, 2026;10:(S1):pages. The abstract book DOIs can be found on the "Author Information" page.
**Disclaimer:** Articles published in the journal HemaSphere exclusively reflect the opinions of the authors. The authors are responsible for all content in their abstracts including accuracy of the facts, statements, citing resources, etc.

**Summary/Conclusion**

Our findings identify *BCOR* mutation as a driver of resistance to PD-1 blockade in NKTCL through concurrent activation of oncogenic AKT/STAT3 signaling and downregulation of PD-L1 expression. These results position *BCOR* as a potential predictive biomarker for immunotherapy resistance and highlight it as a promising target for therapeutic intervention in NKTCL.

**Copyright Information:** (Online) ISSN: 2572-9241
©2026 The Author(s). HemaSphere is published by John Wiley & Sons Ltd on behalf of European Hematology Association. This is an open access Abstract Book distributed under the Attribution-NonCommercial-NoDerivs (CC BY-NC-ND), which allows third parties to download the articles and share them with others as long as they credit the author and the Abstract Book, but they cannot change the content in any way or use them commercially.
**Abstract Book Citations:** Authors, Title, HemaSphere, 2026;10:(S1):pages. The abstract book DOIs can be found on the "Author Information" page.
**Disclaimer:** Articles published in the journal HemaSphere exclusively reflect the opinions of the authors. The authors are responsible for all content in their abstracts including accuracy of the facts, statements, citing resources, etc.

### (PS2196) COMPOSITION AND PROGNOSTIC IMPACT OF TP53 ALTERATIONS ACROSS MATURE B-CELL NEOPLASMS: CLONE SIZE OF MUTATION AND DEL(17P) BOTH MATTER

**Topic:** 21. Lymphoma biology & translational research

Veronika Ecker*[1], Wencke Walter[1], Miriam Lenk[1], Manja Jargosch[1], Chrysanthi Tsamadou[1], Lorraine Weidner[1], Constance Baer[1], Wolfgang Kern[1], Anna Stengel[1]

[1]*MLL Munich Leukemia Laboratory, Munich, Germany;*

### Background

*TP53* aberrations (mutation (mut), del(17p), loss of heterozygosity (LOH)) are well-documented in chronic lymphocytic leukaemia (CLL) where single-hit (sh), multi-hit (mh), and even low-level variant allele frequency (VAF) mut are associated with shorter progression-free and overall survival. Notably, targeted therapies have shown superior efficacy compared to chemoimmunotherapy in this context. Currently, individual guidelines for other B-NHL entities do not (yet) recognize the significance of *TP53* aberrations.

### Aims

To study *TP53* aberrations across B-NHL entities with respect to sh vs mh composition, mut/del clone size and impact on overall survival (OS).

### Methods

746 B-NHL samples (307 CLL, 97 MZL, 86 MCL, 76 HGBL, 66 FL, 62 LPL, 30 SBLPN, 22 other) were analyzed for %flow cytometric clone size (FC clone) and *TP53* aberrations: %VAF, presence of del(17p) or LOH (WGS, median coverage 100x), %del(17p) (FISH). Subclones with *TP53* aberrations were defined as %del/mut amounting at least 30% less than %FC clone size. OS was investigated by Kaplan-Meier and log-rank test.

### Results

181/746 (24%) B-NHL cases revealed *TP53* aberrations (Fig. A), 68 (38%) were sh and 113 (62%) mh *TP53* aberrations. Median OS was significantly inferior in the mh group as compared to sh (3.4 vs 8.3 years, p=0.012) and in sh compared to wildtype (wt) group (11.2 years, p=0.002). These observations were not only present in CLL but also in the other indolent B-NHL (MZL, MCL, SBLPN, LPL, FL) cases cross-entity (mh/sh/wt: 3.6/9.2/12.9 and 3.8/5.9/11.0 years, respectively).
In the sh group, mut accounted for 63% (43/68), del for 34% (23/68), and LOH for 3% (2/68) of cases. The mh group primarily consisted of cases with mut+del (79%, 89/113), followed by mut+LOH (13%, 15/113), and ≥2mut (8%, 9/113). Notably, 11% of mut+del and 7% of mut+LOH cases also had ≥2mut. Entity-wise, mh aberrations were most common in MZL (73%) and CLL (67%), predominantly mut+del. Sh aberrations were most frequent in HGBL (51%, mostly mut), followed by MCL (43%), and SBLPN (42%), with these last two showing the highest rates of del only cases (6/10 and 5/5).
Cross-entity analysis of *TP53* VAF vs FC clone size showed that *TP53*mut occurred within the main clone in 80% (114/142) of cases (78% of them mh) with a median OS of 3.1 years. The remaining 20% of cases (61% of them mh) had subclonal *TP53*mut with a superior OS of 8.3 years (p=0.011). A complex karyotype (CK), known to adversely affect CLL prognosis, was detected in 56% and 36% of cases with main or minor *TP53*mut clones, respectively, and did not impact the superior OS observed in the latter group. Despite being limited in number, there was a similar trend toward superior median OS for 14% (8/57) of cases with subclonal del(17p), as compared to FC clone size, without any bias in CK distribution. Subclonal mut and del(17p) were observed across all entities, most frequently in CLL (37%) and MCL (32%). Notably, CLL included 3 cases exhibiting both, subclonal mut and del.

**Copyright Information:** (Online) ISSN: 2572-9241
©2026 The Author(s). HemaSphere is published by John Wiley & Sons Ltd on behalf of European Hematology Association. This is an open access Abstract Book distributed under the Attribution-NonCommercial-NoDerivs (CC BY-NC-ND), which allows third parties to download the articles and share them with others as long as they credit the author and the Abstract Book, but they cannot change the content in any way or use them commercially.
**Abstract Book Citations:** Authors, Title, HemaSphere, 2026;10:(S1):pages. The abstract book DOIs can be found on the “Author Information” page.
**Disclaimer:** Articles published in the journal HemaSphere exclusively reflect the opinions of the authors. The authors are responsible for all content in their abstracts including accuracy of the facts, statements, citing resources, etc.

### Summary/Conclusion

*TP53* aberrations define a high-risk subgroup across different B-NHL entities with inferior outcome of mh compared to sh aberrations and wt *TP53*. *TP53* clone size is prognostic and associates with superior prognosis if del or mut do not comprise the whole lymphoma clone. Combined phenotypic and genetic analysis are essential to capture the clonal hierarchy.

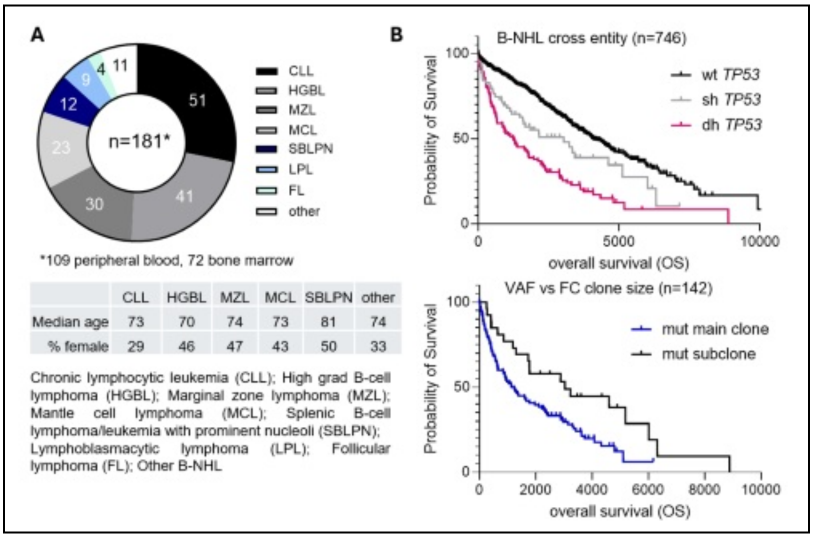

**Copyright Information:** (Online) ISSN: 2572-9241
©2026 The Author(s). HemaSphere is published by John Wiley & Sons Ltd on behalf of European Hematology Association. This is an open access Abstract Book distributed under the Attribution-NonCommercial-NoDerivs (CC BY-NC-ND), which allows third parties to download the articles and share them with others as long as they credit the author and the Abstract Book, but they cannot change the content in any way or use them commercially.
**Abstract Book Citations:** Authors, Title, HemaSphere, 2026;10:(S1):pages. The abstract book DOIs can be found on the "Author Information" page.
**Disclaimer:** Articles published in the journal HemaSphere exclusively reflect the opinions of the authors. The authors are responsible for all content in their abstracts including accuracy of the facts, statements, citing resources, etc.

### (PS2197) DECITABINE RESCUES ANTIGEN PRESENTATION IN EBV-POSITIVE B-CELL LYMPHOMA THROUGH TFAP2A-DEPENDENT MHC-I REACTIVATION

**Topic:** 21. Lymphoma biology & translational research

Chen-Xing Zhao[1], Le Wang*[1], Jingjing Wen[2], Wu-Ting Luan[1]

*[1]Fujian Medical University, The School of Basic Medical Sciences, Fuzhou, Fujian, China;[2]Fujian Provincial Cancer Hospital, Fuzhou, Fujian, China;*

### Background

Epstein-Barr virus (EBV)-associated B-cell lymphomas are characterized by aggressive clinical course, high relapse rates, and poor prognosis. A critical mechanism of immune evasion in these malignancies is the epigenetic silencing of major histocompatibility complex class I (MHC-I) molecules, which impairs antigen presentation and $CD8^+$ T-cell recognition, leading to tumor progression and therapeutic resistance.

### Aims

This study aims to elucidate whether DNA demethylating agent decitabine can restore MHC-I expression and antigen presentation function and identify the key transcriptional regulators mediating decitabine-induced MHC-I reactivation; and develop and optimize combinatorial immunotherapy strategies by integrating epigenetic priming with adoptive cell transfer.

### Methods

We integrated multi-omics data from TCGA, GEO, BioProject, and GTEx databases to characterize the immune microenvironment of EBV-positive B-cell lymphoma. In vitro experiments were performed using EBV-positive B-cell lymphoma cell lines treated with decitabine to assess DNA demethylation, MHC-I expression, and EBV viral replication status. RNA-seq and bisulfite sequencing (BS-seq) were conducted to identify signaling pathways and differentially methylated regions. Key transcription factors were validated by quantitative PCR, methylation-specific PCR (MSP), and chromatin immunoprecipitation (ChIP). The functional impact of decitabine-primed tumor cells on EBV-specific cytotoxic T lymphocytes (EBV-CTLs) was evaluated by co-culture assays.

### Results

Bioinformatics analysis revealed that EBV-positive patients exhibited defective MHC-I antigen presentation associated with disease relapse. Low-dose decitabine (1 μM, 5 days) ,a DNA methyltransferase inhibitor, significantly upregulated MHC-I molecules (HLA-A, HLA-B, HLA-C) and co-stimulatory molecule CD80 through genome-wide CG demethylation, without inducing EBV lytic replication. Mechanistically, decitabine activated the transcription factor TFAP2A via promoter demethylation, and TFAP2A directly bound to HLA-A promoter to drive transcription.  Consequently, decitabine-primed lymphoma cells exhibit significantly improved susceptibility to EBV-specific cytotoxic T lymphocytes (EBV-CTLs), as evidenced by enhanced T-cell activation, cytokine secretion, and cytotoxicity.

### Summary/Conclusion

Our findings establish the decitabine-TFAP2A-MHC-I axis as a critical pathway for restoring antigen presentation in EBV-positive B-cell lymphoma. Epigenetic priming with decitabine represents a promising strategy to enhance the efficacy of adoptive cell therapy and immune checkpoint blockade, providing a mechanistic rationale for clinical translation in EBV-associated B-cell malignancies.

**Copyright Information:** (Online) ISSN: 2572-9241
©2026 The Author(s). HemaSphere is published by John Wiley & Sons Ltd on behalf of European Hematology Association. This is an open access Abstract Book distributed under the Attribution-NonCommercial-NoDerivs (CC BY-NC-ND), which allows third parties to download the articles and share them with others as long as they credit the author and the Abstract Book, but they cannot change the content in any way or use them commercially.
**Abstract Book Citations:** Authors, Title, HemaSphere, 2026;10:(S1):pages. The abstract book DOIs can be found on the "Author Information" page.
**Disclaimer:** Articles published in the journal HemaSphere exclusively reflect the opinions of the authors. The authors are responsible for all content in their abstracts including accuracy of the facts, statements, citing resources, etc.

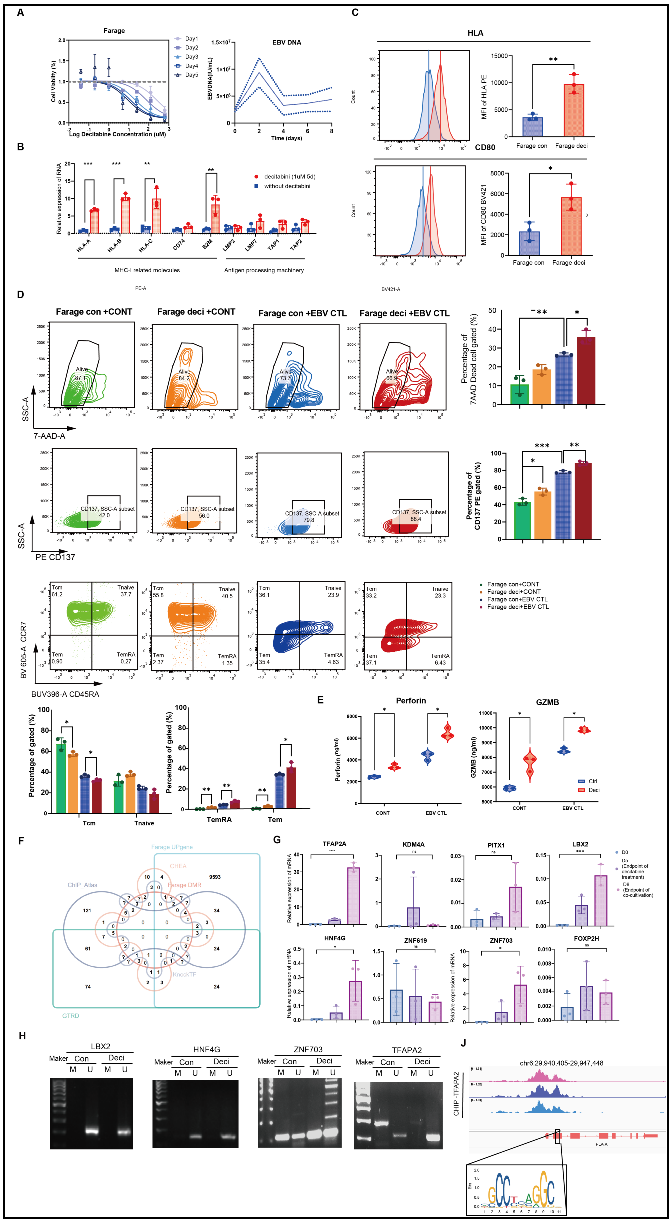

**Copyright Information:** (Online) ISSN: 2572-9241
©2026 The Author(s). HemaSphere is published by John Wiley & Sons Ltd on behalf of European Hematology Association. This is an open access Abstract Book distributed under the Attribution-NonCommercial-NoDerivs (CC BY-NC-ND), which allows third parties to download the articles and share them with others as long as they credit the author and the Abstract Book, but they cannot change the content in any way or use them commercially.
**Abstract Book Citations:** Authors, Title, HemaSphere, 2026;10:(S1):pages. The abstract book DOIs can be found on the "Author Information" page.
**Disclaimer:** Articles published in the journal HemaSphere exclusively reflect the opinions of the authors. The authors are responsible for all content in their abstracts including accuracy of the facts, statements, citing resources, etc.

### (PS2198) PLASMA PROTEOMIC PROFILING OF CLASSICAL HODGKIN LYMPHOMA IDENTIFIES DISTINCT INFLAMMATORY PROTEIN PROFILES

**Topic:** 21. Lymphoma biology & translational research

Ragnhild Risebro*[1], Jeremia Collin[1], Johan Ulfstedt[1], Emma Pettersson[1], Mats Hellstrom[1], Ingrid Glimelius[1], Mattias Berglund[1], Gunilla Enblad[1], Eva Freyhult[2], Daniel Molin[1]

*[1]Uppsala University, Department of Immunology, Genetics, and Pathology, Uppsala, Sweden;[2]National Bioinformatics Infrastructure Sweden, Department of cell and molecular biology, Uppsala, Sweden;*

### Background

Classical Hodgkin lymphoma (cHL) is often associated with signs of inflammation, such as elevated erythrocyte sedimentation rate (ESR) and B-symptoms, although not all patients exhibit these features. While inflammatory response to some extent correlates with tumor stage, it can occur in both limited and advanced stages. We sought to investigate this and the general protein profile of cHL through OlinkÒ plasma proteomics, which is an emerging field that offers insight into disease biology through large-scale data analysis.

### Aims

This study aims to characterize the plasma proteomic profile of cHL in comparison with healthy controls and to identify protein patterns associated with the presence or absence of an inflammatory response.

### Methods

The plasma proteome including 1443 proteins of 115 cHL patients and 122 healthy controls (HC) were compared using linear regression adjusted for age and sex, with correction for multiple hypothesis testing. The cHL inflammatory and non-inflammatory subgroups were defined by elevated versus normal ESR. Protein levels across the inflammatory group, the non-inflammatory group and HC were compared using estimated marginal means based on linear regression with similar adjustments. The supervised multivariate method partial least squares discriminant analysis was applied to identify proteins differing across the three groups. All analyses were done in RStudio version 4.4.2.

### Results

As shown in the figure, the results revealed clear alterations in the plasma proteome of cHL and its inflammatory states with 675 significantly differentially expression proteins. Given the abundance of significant results, we focused on the proteins with the highest statistical significance. In addition to established cHL markers such as TARC, PD1, PDL1, and CD30, we identified von Willebrand factor (VWF) and ATP synthase inhibitor ATP5IF1 as markedly elevated across the entire cHL cohort, while CRTAC1 levels were reduced compared to HC. Interleukin 6 (IL6) was elevated in plasma in both subtypes of cHL, but higher in the inflammatory subtype. The inflammatory cHL subtype was characterized by increased expression of many immune mediators, among them proteins associated with an antibacterial response, including LBP, CD14, DEFA1_DEFA1B, and PLA2G2A. In contrast, the non-inflammatory cHL subtype showed no or minimal elevation of antibacterial proteins but exhibited higher expression of immune suppressive molecules VTCN1, CD83, FASLG and selective upregulation of PLA2G7

**Copyright Information:** (Online) ISSN: 2572-9241
©2026 The Author(s). HemaSphere is published by John Wiley & Sons Ltd on behalf of European Hematology Association. This is an open access Abstract Book distributed under the Attribution-NonCommercial-NoDerivs (CC BY-NC-ND), which allows third parties to download the articles and share them with others as long as they credit the author and the Abstract Book, but they cannot change the content in any way or use them commercially.
**Abstract Book Citations:** Authors, Title, HemaSphere, 2026;10:(S1):pages. The abstract book DOIs can be found on the “Author Information” page.
**Disclaimer:** Articles published in the journal HemaSphere exclusively reflect the opinions of the authors. The authors are responsible for all content in their abstracts including accuracy of the facts, statements, citing resources, etc.

**Summary/Conclusion**

Large-scale data analysis identified plasma proteins altered in cHL, showing novel associations with the disease. Additionally, we found systemic differences in plasma proteome of the inflammatory subtypes of cHL, indicating distinct biological mechanisms.

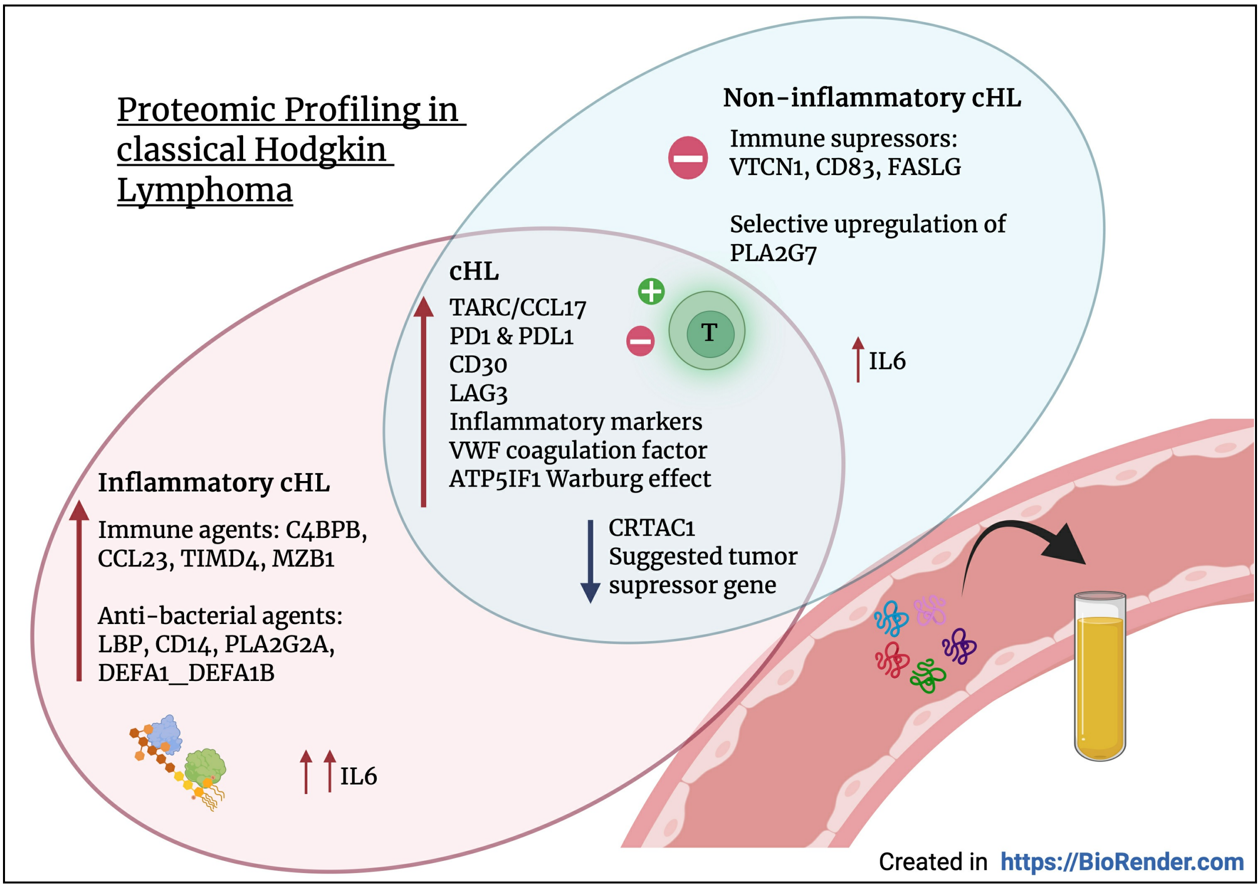

Figure 1: The plasma proteome characterizing classical Hodgkin lymphoma (cHL) and its inflammatory states. Compared to healthy controls, cHL plasma has elevated concentrations of TARC, CD30, immune checkpoint molecules PD1, PDL1, and LAG3, as well as many immune-related proteins. The inflammatory subtype is characterized by abundant immune mediators, including the antibacterial proteins LBP, CD14, PLA2G2A, DEFA1_DEFA1B. IL6 concentrations are elevated in both inflammatory states of cHL, but are higher in the inflammatory subtype. The non-inflammatory subtype has higher expression of immune suppressors VTCN1, CD83, FASL, and a selective upregulation of PLA2G7.

Copyright Information: (Online) ISSN: 2572-9241
©2026 The Author(s). HemaSphere is published by John Wiley & Sons Ltd on behalf of European Hematology Association. This is an open access Abstract Book distributed under the Attribution-NonCommercial-NoDerivs (CC BY-NC-ND), which allows third parties to download the articles and share them with others as long as they credit the author and the Abstract Book, but they cannot change the content in any way or use them commercially.
Abstract Book Citations: Authors, Title, HemaSphere, 2026;10:(S1):pages. The abstract book DOIs can be found on the “Author Information” page.
Disclaimer: Articles published in the journal HemaSphere exclusively reflect the opinions of the authors. The authors are responsible for all content in their abstracts including accuracy of the facts, statements, citing resources, etc.

### (PS2199) EPIGENETIC SILENCING OF MIR-146A-5P DRIVES RESISTANCE TO BTK INHIBITORS IN MANTLE CELL LYMPHOMA

**Topic:** 21. Lymphoma biology & translational research

Nir Cohen*[1, 2], Mali Salmon-Divon1[1, 3], Pavel Klener[4, 5], Tal Zvida-Bloch[1, 2], Maria Ramos Lopes Gomes Da Silva[6], Ana Filipa Moita[6], Jose Manuel Valente Sequeira Cabecadas[7], Marek Trněný[4], May Basood[8], Benjamin Durham[9], Omar Abdel-Wahab[10], Martin Dreyling[11], Ofer Shpilberg[2, 3, 8], Oshrat Hershkovitz-Rokah[1, 2]

*[1]Ariel University, Department of Molecular Biology, Faculty of Natural Sciences, Ariel, Israel;[2]Assuta Medical Center, Translational Research Lab, Tel-Aviv, Israel; [3]Ariel University, Adelson School of Medicine, Ariel, Israel; [4]General University Hospital and First Faculty of Medicine, Charles University, First Department of Internal Medicine-Hematology, Prague, Czechia; [5]Charles University, First Faculty of Medicine, Institute of Pathological Physiology, Prague, Czechia;[6]Instituto Português de Oncologia de Lisboa, Department of Hematology, Lisbon, Portugal; [7]Portuguese Institute of Oncology Lisbon, Department of Pathology, Lisbon, Portugal; [8]Assuta Medical Center, Institute of Hematology, Tel-Aviv, Israel;[9]Rutgers Cancer Institute, Departments of Pediatrics and Pathology and Laboratory Medicine, New Brunswick, NJ, United States of America; [10]Memorial Sloan Kettering Cancer Center, Molecular Pharmacology Program, Sloan Kettering Institute, New York, NY, United States of America; [11]Ludwig Maximilian University, Department of Medicine III, University Hospital, Munich, Germany;*

### Background

Bruton's tyrosine kinase inhibitors (BTKi) have substantially improved clinical outcomes in mantle cell lymphoma (MCL). However, acquired resistance to both covalent and non-covalent BTKi remains a therapeutic challenge. While genetic mechanisms of resistance have been characterized, non-genetic regulatory pathways are less well defined. In our previous work, we demonstrated that microRNAs contribute to ibrutinib resistance in MCL through activation of MAPK and PI3K signaling pathways that promote cell survival. However, the contribution of individual microRNAs and the regulatory mechanisms sustaining their dysregulation in resistant disease remain to be elucidated.

### Aims

To determine whether miR-146a-5p functions as a mechanistic driver of BTKi resistance in MCL and to investigate the epigenetic mechanisms regulating its expression.

### Methods

Ibrutinib- and pirtobrutinib-resistant Jeko-1 MCL cell models were generated and validated by proliferation and apoptosis assays. Patient-derived material included biopsy specimens from ibrutinib-sensitive and -resistant cases, as well as patient-derived xenograft (PDX) mouse models. miR-146a-5p expression was quantified across models. Functional inhibition experiments were performed to assess the phenotypic impact of miR-146a loss. Epigenetic regulation was evaluated by bisulfite sequencing of the miR-146a promoter. Pharmacologic demethylation was induced using 5-azacytidine.

**Copyright Information:** (Online) ISSN: 2572-9241
©2026 The Author(s). HemaSphere is published by John Wiley & Sons Ltd on behalf of European Hematology Association. This is an open access Abstract Book distributed under the Attribution-NonCommercial-NoDerivs (CC BY-NC-ND), which allows third parties to download the articles and share them with others as long as they credit the author and the Abstract Book, but they cannot change the content in any way or use them commercially.
**Abstract Book Citations:** Authors, Title, HemaSphere, 2026;10:(S1):pages. The abstract book DOIs can be found on the "Author Information" page.
**Disclaimer:** Articles published in the journal HemaSphere exclusively reflect the opinions of the authors. The authors are responsible for all content in their abstracts including accuracy of the facts, statements, citing resources, etc.

**Results**

Both ibrutinib- and pirtobrutinib-resistant MCL cell lines exhibited significant downregulation of miR-146a-5p, accompanied by impaired BTKi-induced apoptosis. Consistently, miR-146a-5p expression was significantly reduced in biopsy specimens from ibrutinib-resistant compared to ibrutinib-sensitive cases and PDX models. Functional inhibition of miR-146a in sensitive cells attenuated apoptosis and sustained proliferation during BTKi treatment, partially recapitulating the resistant phenotype. Bisulfite sequencing revealed site-specific CpG hypermethylation within the miR-146a promoter in resistant cells. Treatment with 5-azacytidine reversed promoter hypermethylation, restored miR-146a-5p expression and induced apoptosis.

**Summary/Conclusion**

These findings identify epigenetic silencing of miR-146a-5p contributing to BTKi resistance in MCL. Epigenetic modulation of miR-146a expression may represent a potential strategy to overcome BTKi resistance.

**Copyright Information:** (Online) ISSN: 2572-9241
©2026 The Author(s). HemaSphere is published by John Wiley & Sons Ltd on behalf of European Hematology Association. This is an open access Abstract Book distributed under the Attribution-NonCommercial-NoDerivs (CC BY-NC-ND), which allows third parties to download the articles and share them with others as long as they credit the author and the Abstract Book, but they cannot change the content in any way or use them commercially.
**Abstract Book Citations:** Authors, Title, HemaSphere, 2026;10:(S1):pages. The abstract book DOIs can be found on the "Author Information" page.
**Disclaimer:** Articles published in the journal HemaSphere exclusively reflect the opinions of the authors. The authors are responsible for all content in their abstracts including accuracy of the facts, statements, citing resources, etc.

### (PS2200) CLINICAL AND BIOLOGICAL CHARACTERISTICS OF MATURE AGGRESSIVE B-CELL LYMPHOMA IN ADOLESCENTS AND YOUNG ADULTS USING MULTICENTER REGISTRY DATA IN JAPAN

**Topic:** 21. Lymphoma biology & translational research

Yasuhiro Suzuki*[1], Takahiko Yasuda[2], Akira Satou[3], Akiko Kada[2], Masashi Sanada[2], Katsuya Fujimoto[4], Tohru Izumi[5], Ikuyo Tsutsumi[6], Akio Saito[7], Toshiya Kagoo[8], Naohiro Sekiguchi[9], Terutoshi Hishita[10], Koji Adachi[11], Kazutaka Sunami[12], Takuo Ito[13], Takeshi Shimomura[14], Isao Yoshida[15], Youko Suehiro[16], Ken Takase[17], Mika Kuroiwa[18], Shinichiro Yoshida[19], Toshiro Kawakita[20], Maki Otsuka[21], Kennosuke Karube[3], Masahiro Sekimizu[2], Toshiki I. Saito[2, 22], Akiko M. Saito[2, 22], Hiroatsu Iida[1], Hirokazu Nagai[1]

*[1]Department of Hematology, NHO Nagoya Medical Center, Nagoya, Japan;[2]Clinical Research Center, NHO Nagoya Medical Center, Nagoya, Japan; [3]Department of Pathology and Laboratory Medicine, Nagoya University Hospital, Nagoya, Japan; [4]Department of Hematology, NHO Hokkaido Cancer Center, Sapporo, Japan;[5]Department of Hematology, NHO Sendai Medical Center, Sendai, Japan; [6]Department of Hematology, NHO Mito Medical Center, Ibaraki, Japan;[7]Department of Hematology, NHO Shibukawa Medical Center, Gunma, Japan; [8]Department of Hematology, NHO Tokyo Medical Center, Tokyo, Japan;[9]Department of Hematology, NHO Disaster Medical Center, Tokyo, Japan; [10]Department of Hematology, NHO Himeji Medical Center, Himeji, Japan; [11]Department of Hematology and Oncology, NHO Yonago Medical Center, Yonago, Japan;[12]Department of Hematology, NHO Okayama Medical Center, Okayama, Japan; [13]Department of Hematology, NHO Kure Medical Center, Kure, Japan; [14]Department of Hematology, NHO Hiroshimanishi Medical Center, Hiroshima, Japan;[15]Department of Hematologic Oncology, NHO Shikoku Cancer Center, Matsuyama, Japan; [16]Department of Hematology and Cell Therapy, NHO Kyushu Cancer Center, Fukuoka, Japan; [17]Department of Hematology, NHO Kyushu Medical Center, Fukuoka, Japan;[18]Department of Hematology and Oncology, NHO Fukuokahigashi Medical Center, Fukuoka, Japan; [19]Department of Hematology, NHO Nagasaki Medical Center, Nagasaki, Japan; [20]Department of Hematology, NHO Kumamoto Medical Center, Kumamoto, Japan;[21]Department of Hematology, NHO Kagoshima Medical Center, Kagoshima, Japan; [22]Clinical Research Center, NHO Headquarters, Tokyo, Japan;*

### Background

Adolescents and young adults (AYA) (ages 16–39) with lymphoma require tailored treatment strategies that address unique molecular pathologies, long-term complications, and psychosocial factors. However, due to the low incidence in this age group, comprehensive data regarding the clinical and biological characteristics of AYA aggressive B-cell lymphomas (BCLs) remain limited.

### Aims

To elucidate the clinical, pathological, and molecular landscape of mature aggressive BCLs in the AYA population.

### Methods

We studied 119 cases diagnosed with mature aggressive BCL between 2012 and 2020. Clinical and pathological data were collected from the Japan National Hospital Organization (NHO) registry for AYA patients. Centralized pathological review was performed. Formalin-fixed paraffin-embedded (FFPE) specimens were analyzed using immunohistochemistry (IHC), FISH, targeted sequencing (TS), and RNA sequencing (RNA-seq). This study was approved by the NHO Central Research Ethics Committee and conducted in accordance with the Ethical Guidelines for Life Science and Medical Research in Japan (UMIN000046477).

**Copyright Information:** (Online) ISSN: 2572-9241
©2026 The Author(s). HemaSphere is published by John Wiley & Sons Ltd on behalf of European Hematology Association. This is an open access Abstract Book distributed under the Attribution-NonCommercial-NoDerivs (CC BY-NC-ND), which allows third parties to download the articles and share them with others as long as they credit the author and the Abstract Book, but they cannot change the content in any way or use them commercially.
**Abstract Book Citations:** Authors, Title, HemaSphere, 2026;10:(S1):pages. The abstract book DOIs can be found on the “Author Information” page.
**Disclaimer:** Articles published in the journal HemaSphere exclusively reflect the opinions of the authors. The authors are responsible for all content in their abstracts including accuracy of the facts, statements, citing resources, etc.

**Results**

Central pathological review revealed that 5 of the 119 cases were not aggressive BCL and were therefore excluded from the study. Among the remaining 114 cases, 75 (66%) were classified as diffuse large BCL (DLBCL), including 2 cases of EBV-positive DLBCL. In addition, 22 (19%) cases were diagnosed as primary mediastinal BCL (PMBCL), 11 (9.6%) as Burkitt lymphoma (BL), 4 as LBCL with *IRF4* rearrangement (*IRF4*-R) and 2 (1.8%) as high-grade B-cell lymphoma (HGBL), NOS. IHC analysis of 58 evaluable DLBCL cases revealed 24 (41%) GCB and 34 (59%) non-GCB subtypes by Hans' criteria, showing high concordance with RNA-seq findings. Furthermore, 21 cases (36%) were MYC/BCL2 double-expressors, and an aberrant CD10+/BCL6+/MUM1+ phenotype was observed in 21%. BL cases showed MUM1 positivity of 78% (7/9) and an EBER positivity rate of 22% (2/9).
Using RNA-seq data (n = 34), hierarchical clustering analysis based on 20 GCB/ABC-specific genes categorized cases into four distinct biological groups. Group 1 (n=6) predominantly consisted of LBCL with *IRF4*-R, Group 2 (n=7) comprised GCB-DLBCL, Group 3 (n=6) included BL and *MYC*-R DLBCL, and Group 4 (n=15) contained ABC-DLBCL.
Specifically, Group 1 cases showed a significantly higher burden of *IRF4* mutations, including aberrant somatic hypermutation, compared to other subtypes (mean 10.5 vs. 1.09). In Group 3, *MYC*-R DLBCL cases exhibited mutational profiles (e.g., *ID3, TP53, MYC*) and expression signatures analogous to BL, designating them as "molecular BL." These patients received intensive BL-style chemotherapy and achieved a favorable 5-year OS of 100%.
With a median follow-up of 5.2 years (IQR: 2.7–7.3), the 5-year OS was 87% for DLBCL, 90% for PMBCL, 55% for BL, and 100% for HGBL, indicating a significantly poorer prognosis for BL (p=0.03). These clinical outcomes were consistent with the RNA-seq classification, where the 5-year OS was 100% in Groups 1, 2, and 4, but only 50% in Group 3.

**Summary/Conclusion**

AYA mature aggressive BCLs exhibit significant molecular and histological heterogeneity that may be unique to this age group. Although the overall prognosis for aggressive BCL in the AYA population is relatively favorable, the identification of high-risk or biologically distinct subsets through integrated TS, RNA-seq, and histological analysis underscores the potential for more precise treatment stratification in this population.

**Copyright Information:** (Online) ISSN: 2572-9241
©2026 The Author(s). HemaSphere is published by John Wiley & Sons Ltd on behalf of European Hematology Association. This is an open access Abstract Book distributed under the Attribution-NonCommercial-NoDerivs (CC BY-NC-ND), which allows third parties to download the articles and share them with others as long as they credit the author and the Abstract Book, but they cannot change the content in any way or use them commercially.
**Abstract Book Citations:** Authors, Title, HemaSphere, 2026;10:(S1):pages. The abstract book DOIs can be found on the "Author Information" page.
**Disclaimer:** Articles published in the journal HemaSphere exclusively reflect the opinions of the authors. The authors are responsible for all content in their abstracts including accuracy of the facts, statements, citing resources, etc.

### (PS2201) BIOLOGIC CORRELATES OF LONG-TERM RESPONSE AFTER BREXUCABTAGENE AUTOLEUCEL THERAPY IN MANTLE CELL LYMPHOMA: PRODUCT PHENOTYPE, PK, AND BASELINE FEATURES

**Topic:** 21. Lymphoma biology & translational research

Sabina Adhikary[1], Kelly Speth[1], Colleen Schweitzer[1], Rhine Shen[1], Ioana Kloos[1], Marie José Kersten*[2]

*[1]Kite, A Gilead Company, Santa Monica, United States of America;[2]Amsterdam University Medical Centers, Amsterdam, The Netherlands;*

### Background

Brexucabtagene autoleucel (brexu-cel), a CD19 directed CAR T cell therapy, has demonstrated substantial efficacy in relapsed/refractory mantle cell lymphoma (MCL). In ZUMA 2 cohort 3— enrolling subjects not yet exposed to Bruton Tyrosine Kinase inhibitors (BTKi) the independently assessed overall response rate (ORR) was 91%, with 75% of patients maintaining response at the last data cut-off, which occurred at 18 months. Despite these promising outcomes, the biological and product-related factors associated with durable remission in this population remain poorly defined.

### Aims

These analyses evaluated relationships between CAR T cell product phenotypes, pharmacokinetics (PK), baseline biological features, and duration of response (DOR) in BTKi-naïve cohort of ZUMA 2.

### Methods

The patient population consisted of individuals treated in ZUMA-2 cohort 3 (BTKi-naïve; n=86) who received brexu-cel at the pivotal dose of $2{\times}10^6$ CAR T cells/kg. Analyses were based on investigator assessment. Patients were stratified by disease stage (I–III, n=30; IV, n=56) and by DOR among objective responders: <18 months (n=18) vs ≥18 months (n=55). Patients who died without progression or had ongoing responses but had not reached 18 months were excluded from DOR comparisons. Product immunophenotype, in vivo PK, and baseline clinical and laboratory features were descriptively compared.

### Results

Investigator assessed ORR was 97% across stages. Among stage I–III patients, 3 had DOR <18 months and 22 had DOR ≥18 months; 4 were non evaluable (1 death without progression; 3 ongoing responses at 13.0–17.4 months). Among stage IV patients, 15 had DOR <18 months and 33 had DOR ≥18 months; 6 were non evaluable (5 deaths without progression; 1 ongoing response with follow-up of 17.1 months).
Patients with ≥18 month DOR showed a trend toward a less differentiated product phenotype, including higher proportions of $CCR7^+CD45RA^+$ naïve like subsets (median 40% vs 27%). Earlier stage disease similarly demonstrated higher $CD4^+$ T cell proportions and numerically greater representation of less differentiated subsets ($CCR7^+$ 58% vs 52%), though not statistically significant.
PK profiles differed by DOR. While stage I–III and IV patients had similar overall expansion kinetics, patients with ≥18 month DOR exhibited markedly higher early expansion, as observed at day 7 (median 46.6 vs 0.6 CAR T cells/µL). Additionally, such durable responders showed numerically lower levels of median tumor -burden as measured by sum of products of diameters (or SPD) (1625 vs 2979 $mm^2$), yet higher median LDH (197 vs 178 U/L) at baseline relative to <18-month DOR patients. Interestingly, no meaningful differences in these baseline features were observed when comparing stage I–III vs stage IV, despite visible stage-associated differences in product phenotype.

**Copyright Information:** (Online) ISSN: 2572-9241
©2026 The Author(s). HemaSphere is published by John Wiley & Sons Ltd on behalf of European Hematology Association. This is an open access Abstract Book distributed under the Attribution-NonCommercial-NoDerivs (CC BY-NC-ND), which allows third parties to download the articles and share them with others as long as they credit the author and the Abstract Book, but they cannot change the content in any way or use them commercially.
**Abstract Book Citations:** Authors, Title, HemaSphere, 2026;10:(S1):pages. The abstract book DOIs can be found on the "Author Information" page.
**Disclaimer:** Articles published in the journal HemaSphere exclusively reflect the opinions of the authors. The authors are responsible for all content in their abstracts including accuracy of the facts, statements, citing resources, etc.

**Summary/Conclusion**

In earlier line, BTKi naïve MCL patients treated with brexu-cel, durable responses (≥18 months) occurred across all disease stages. Convergent features—a less differentiated product phenotype, more robust early CAR T cell expansion, and favorable disease characteristics—were associated with long term DOR. The trend towards a less differentiated manufactured product was also evident in earlier-stage disease. These findings support further integrated biomarker analyses to refine predictors of long-term remission.

**Copyright Information:** (Online) ISSN: 2572-9241
©2026 The Author(s). HemaSphere is published by John Wiley & Sons Ltd on behalf of European Hematology Association. This is an open access Abstract Book distributed under the Attribution-NonCommercial-NoDerivs (CC BY-NC-ND), which allows third parties to download the articles and share them with others as long as they credit the author and the Abstract Book, but they cannot change the content in any way or use them commercially.
**Abstract Book Citations:** Authors, Title, HemaSphere, 2026;10:(S1):pages. The abstract book DOIs can be found on the "Author Information" page.
**Disclaimer:** Articles published in the journal HemaSphere exclusively reflect the opinions of the authors. The authors are responsible for all content in their abstracts including accuracy of the facts, statements, citing resources, etc.

### (PS2202) INTEGRATIVE MOLECULAR CHARACTERIZATION OF HIGH-GRADE/LARGE B-CELL LYMPHOMA WITH 11Q ABERRATION

**Topic:** 21. Lymphoma biology & translational research

Vicente Arredondo Prats*[1, 2], Julia Salmerón[1], Ariadna Colmenero[1, 2], Leonie Frauenfeld[1, 2, 3], Sara Mato[1, 2], Noelia García[1], Blanca González Farré[2, 4, 5], Jaume Verdu[4, 6], Mara Andrés[7], Itziar Astigarraga[8], Armando Lopez Guillermo[5], Margarita Llavador[9], Leticia Quintanilla-Fend[3], Elias Campo[1, 2, 4, 5], David Scott[10, 11], Olga Balagué[1, 4, 5], Itziar Salaverria[1, 4]

*[1]Institut d'Investigacions Biomèdiques August Pi i Sunyer (IDIBAPS), Barcelona, Spain;[2]University of Barcelona (UB), Barcelona, Spain; [3]Institute of Pathology and Neuropathology, University Hospital and Comprehensive Cancer Center Tübingen, Tübingen, Germany; [4]Centro de Investigación Biomédica en Red de Cáncer (CIBERONC), Madrid, Spain;[5]Hospital Clínic de Barcelona, Hematopathology Section, Barcelona, Spain; [6]Hospital Clínico Universitario, Pediatric Oncology Department, Valencia, Spain; [7]Hospital La Fe, Pediatric Oncology Department, Valencia, Spain;[8]Biocruces Bizkaia Health Research Institute, Pediatric Oncology Unit, Hospital Universitario Cruces Osakidetza, Barakaldo, Spain; [9]Hospital La Fe, Pathology Department, Valencia, Spain; [10]BC Cancer's Centre for Lymphoid, Vancouver, BC, Canada; [11]University of British Columbia, Division of Medical Oncology, Department of Medicine, Vancouver, BC, Canada;*

### Background

High-grade/Large B-cell lymphoma with 11q aberration (HG/LBCL-11q) is a rare aggressive B-cell non-Hodgkin lymphoma (B-NHL) that predominantly affects children, adolescents and young adults (CAYA) and shows excellent outcomes after standard treatment. It is defined by a characteristic pattern of proximal 11q gains and telomeric losses in the absence of *MYC* rearrangements (*MYC*-R). Despite lacking *MYC*-R, HG/LBCL-11q resembles morphologically and immunophenotypically Burkitt lymphoma (BL) and other germinal center (GC) lymphomas as diffuse large B-cell lymphoma (DLBCL). However, its pathogenesis remains unclear, challenging differential diagnosis and biological classification.

### Aims

To define the genomic landscape of HG/LBCL-11q and refine its diagnostic definition.

### Methods

We evaluated 27 tumors, including 23 HG/LBCL-11q diagnosed in CAYA (median 12, 3-37 years) and 4 adult DLBCL with 11q aberration (median 59.5, 50-76 years). Fluorescence *in situ* hybridization (FISH) was used to detect 11q aberrations and *MYC*-R. All cases were analyzed by OncoScan/CytoScan copy number (CN)-array. Cell-of-origin (COO) and dark zone signature (DZsig) status were determined using the DLBCL90 assay (n=21). The mutational landscape was assessed (n=25) by whole-exome sequencing and/or targeted NGS.

**Copyright Information:** (Online) ISSN: 2572-9241
©2026 The Author(s). HemaSphere is published by John Wiley & Sons Ltd on behalf of European Hematology Association. This is an open access Abstract Book distributed under the Attribution-NonCommercial-NoDerivs (CC BY-NC-ND), which allows third parties to download the articles and share them with others as long as they credit the author and the Abstract Book, but they cannot change the content in any way or use them commercially.
**Abstract Book Citations:** Authors, Title, HemaSphere, 2026;10:(S1):pages. The abstract book DOIs can be found on the "Author Information" page.
**Disclaimer:** Articles published in the journal HemaSphere exclusively reflect the opinions of the authors. The authors are responsible for all content in their abstracts including accuracy of the facts, statements, citing resources, etc.

**Results**

Morphologically, 36% of cases were DLBCL and 64% showed a HG morphology with intermediate features between DLBCL and BL. The cohort showed a male predominance (20/27 cases).
Adults had poorer outcomes than CAYA (5-year EFS 30% vs 100%;$p<0.01$). Four CAYA patients had inborn errors of immunity and one was diagnosed in the context of HIV.
Three subgroups of cases were identified according to the 11q aberration by CN/FISH: prototypical 11q gain-loss pattern (n=19), only telomeric loss (n=6) and proximal gains with telomeric CN-neutral loss of heterozygosity (n=2). Recurrent gains of 13q31.1/*MIR17HG* (33%) and 6q14.1-q22.1/*TMEM30A* (44%) losses were identified. CN profile did not differ across 11q subgroups, but adults showed higher complexity than CAYA (median CN alterations 20 vs 7; $p<0.01$).
All cases displayed a GCB transcriptomic profile, with 48% also showing DZsig expression. MYC expression levels did not differ according to DZsig status or 11q subgroup, and no association between these features was observed.
The mutational landscape was heterogeneous, with recurrent *DDX3X* (30%), *GNA13* and *TP53* mutations (28% each). Interestingly, mutations of *ETS1* and *NFRKB* genes (4/25 and 3/16 cases each), located in the 11q24.3 minimal region of loss, were only present in cases with prototypical 11q with a trend to mutual exclusivity. Conversely, *P2RY8* and *BTG2* mutations were significantly associated with tumors harboring only telomeric 11q loss (OR=21.8 and 19.8, respectively; $p<0.05$). In CAYA, *TP53* mutations in the DNA-binding domain (4/21) did not confer a worse prognosis (5-year EFS 100%). Mutations of BL-related drivers were virtually absent in our cohort.

**Summary/Conclusion**

HG/LBCL-11q, although initially described as sharing BL features, has a mutational profile similar to GCB-DLBCL. Mutations in *ETS1* and *NFRKB* may serve as unique molecular features, particularly in tumors with prototypical 11q aberrations, and could refine their diagnosis. The link between *P2RY8* and *BTG2* mutations with tumors with only telomeric 11q loss and cases exhibiting a DZsig expression reflects subgroup molecular diversity. Outcomes and higher genomic complexity in adult patients suggest age-related differences among these tumors.

**Copyright Information:** (Online) ISSN: 2572-9241
©2026 The Author(s). HemaSphere is published by John Wiley & Sons Ltd on behalf of European Hematology Association. This is an open access Abstract Book distributed under the Attribution-NonCommercial-NoDerivs (CC BY-NC-ND), which allows third parties to download the articles and share them with others as long as they credit the author and the Abstract Book, but they cannot change the content in any way or use them commercially.
**Abstract Book Citations:** Authors, Title, HemaSphere, 2026;10:(S1):pages. The abstract book DOIs can be found on the “Author Information” page.
**Disclaimer:** Articles published in the journal HemaSphere exclusively reflect the opinions of the authors. The authors are responsible for all content in their abstracts including accuracy of the facts, statements, citing resources, etc.

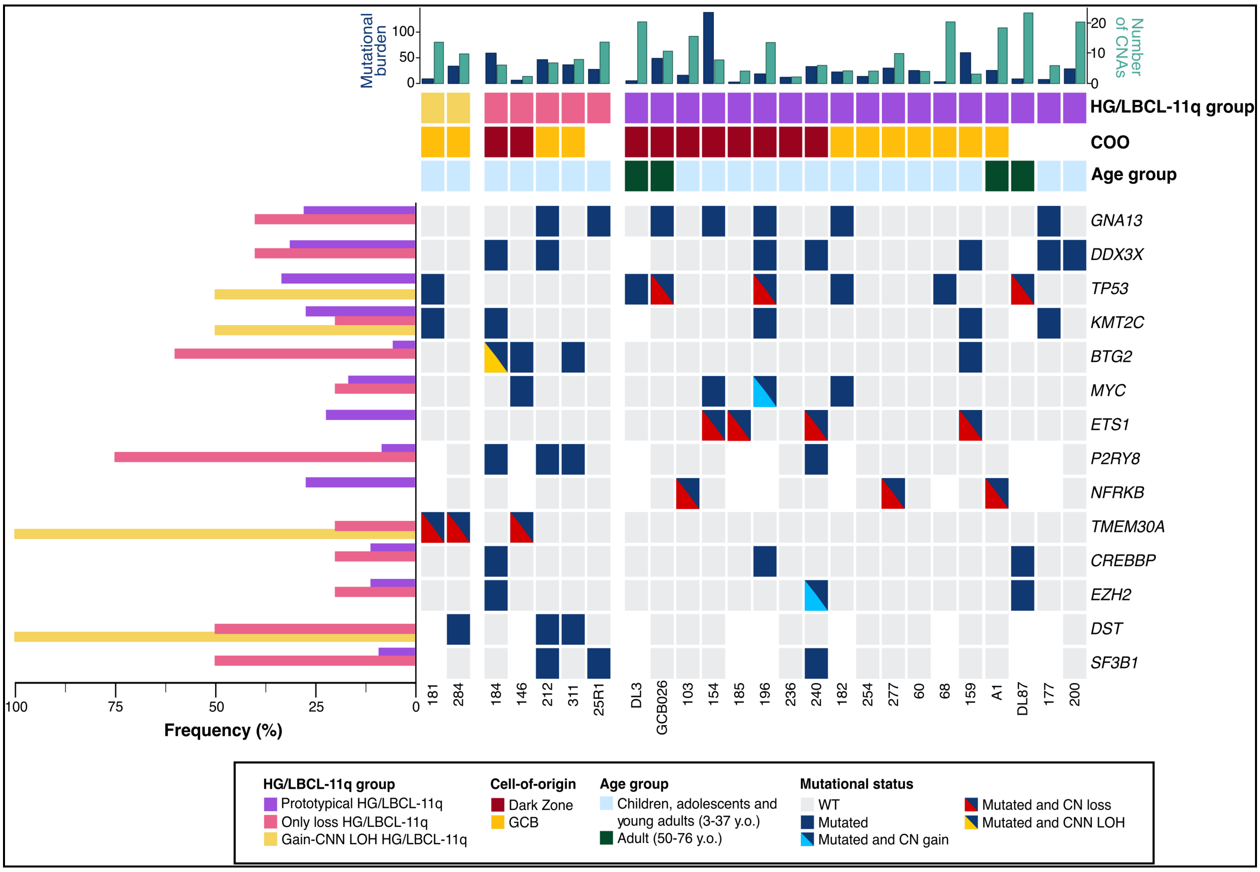

Figure. Oncoprint showing the mutational landscape of 25 HG/LBCL-11q cases. Each column represents an individual case, and each row depicts a recurrently mutated gene. Cases are grouped by 11q aberration pattern, cell-of-origin (COO), and age group (top three rows). The top bar plot indicates the total number of mutations per case (dark blue) and the number of copy number alterations (CNAs; green). The left-hand histogram shows the frequency of each mutation stratified by HG/LBCL-11q group. Only genes with driver mutations in more than three cases are shown.

**Copyright Information:** (Online) ISSN: 2572-9241
©2026 The Author(s). HemaSphere is published by John Wiley & Sons Ltd on behalf of European Hematology Association. This is an open access Abstract Book distributed under the Attribution-NonCommercial-NoDerivs (CC BY-NC-ND), which allows third parties to download the articles and share them with others as long as they credit the author and the Abstract Book, but they cannot change the content in any way or use them commercially.
**Abstract Book Citations:** Authors, Title, HemaSphere, 2026;10:(S1):pages. The abstract book DOIs can be found on the "Author Information" page.
**Disclaimer:** Articles published in the journal HemaSphere exclusively reflect the opinions of the authors. The authors are responsible for all content in their abstracts including accuracy of the facts, statements, citing resources, etc.

### (PS2203) SINGLE-CELL ATLAS OF PCNSL REVEALS A BCR-DOMINANT SIGNALING ARCHITECTURE AND UPREGULATION OF CD79B: A BIOLOGICAL RATIONALE FOR CD79B-DIRECTED THERAPIES

**Topic:** 21. Lymphoma biology & translational research

Byung Woo Yoon*[1], In-Hwan Hwang[1], Je Hyun Seo[2], Young Lee[2], Kwai Han Yoo[3], Jae Hoon Lee[4]

*[1]Gachon University Gil Hospital, Internal Medicine, Division of Hematology, Incheon, South Korea;[2]Veterans Medical Research Institute, Veterans Health Service Medical Center, Department of Ophthalmology, Seoul, South Korea; [3]Seoul National University Bundang Hospital, Internal Medicine, Seongnam, South Korea; [4]EWHA Womans University Seoul Hospital, Internal Medicine, Seoul, South Korea;*

### Background

Primary Central Nervous System Lymphoma (PCNSL) is an aggressive extranodal DLBCL subtype confined to the CNS. While genetic mutations in the *MYD88* pathway are hallmark features, the transcriptional rewiring imposed by the specialized CNS microenvironment—and its specific divergence from systemic DLBCL—remains poorly defined at single-cell resolution.

### Aims

To characterize the high-resolution single-cell landscape of the PCNSL tumor microenvironment (TME).
To delineate transcriptional divergence in malignant B-cell signaling pathways.
To evaluate the biological rationale for targeting CD79B in PCNSL.

### Methods

We analyzed 63,915 cells (including 43,279 malignant B-cells) from integrated PCNSL (*n*=4) and systemic DLBCL (*n*=4) cohorts utilizing public scRNA-seq datasets (GEO: GSE203552, GSE182434). Data were integrated using SCTransform v2 in Seurat v5. We employed a dual-stage annotation strategy to distinguish resident microglia from recruited macrophages. Malignant cells were subtyped using gene module scoring for ABC/GCB programs and expression-based classifiers approximating Chapuy (C1–C5) and LymphGen genetic subtypes. Differential expression (DE) and pathway activity scoring were performed via a malignant pseudobulk approach.

**Copyright Information:** (Online) ISSN: 2572-9241
©2026 The Author(s). HemaSphere is published by John Wiley & Sons Ltd on behalf of European Hematology Association. This is an open access Abstract Book distributed under the Attribution-NonCommercial-NoDerivs (CC BY-NC-ND), which allows third parties to download the articles and share them with others as long as they credit the author and the Abstract Book, but they cannot change the content in any way or use them commercially.
**Abstract Book Citations:** Authors, Title, HemaSphere, 2026;10:(S1):pages. The abstract book DOIs can be found on the "Author Information" page.
**Disclaimer:** Articles published in the journal HemaSphere exclusively reflect the opinions of the authors. The authors are responsible for all content in their abstracts including accuracy of the facts, statements, citing resources, etc.

### Results

The malignant fraction was markedly higher in PCNSL than systemic DLBCL (76.8% vs 36.1%), reflecting a condensed non-malignant microenvironment in CNS tumors. Transcriptional subtyping revealed enrichment of ABC-like and plasmablastic programs in PCNSL. Single-cell genetic classification recapitulated established genomic landscapes (MCD/BN2-like enrichment), validating the cohort's biological fidelity.

Crucially, integrated signaling analysis uncovered a functional **"signaling bottleneck"** unique to the CNS niche. Unlike systemic DLBCL, which maintains multi-pathway survival signaling, PCNSL malignant cells exhibited near-complete transcriptional **attenuation of the TLR/NF-κB axis** ($logFC \approx$ -4.5 for *TNFAIP3*). Instead, these cells demonstrated an obligate reliance on **BCR signaling**, defined by significant upregulation of **CD79B** ($logFC \approx$- +1.2) and *CARD11*. Most importantly, target density profiling confirmed that **CD79B expression was ubiquitous (100% penetrance) and uniform** across PCNSL malignant clusters, whereas *CD19* and *CD20* showed significant heterogeneity and lower expression intensity. Parallel TME analysis placed this malignant program within a specialized niche dominated by $P2RY12^{+}$/TMEM119$^{+}$ **Microglia**.

### Summary/Conclusion

Our single-cell atlas challenges the current reliance on **standard B-cell targets (e.g., CD19, CD20)** in PCNSL by identifying **CD79B as the superior and more uniform therapeutic vulnerability**. The observed transcriptional "addiction" to BCR signaling—driven by the collapse of alternative TLR pathways—provides a definitive biological rationale for prioritizing **CD79B-directed strategies**. While the Blood-Brain Barrier (BBB) poses a delivery challenge for full-sized ADCs like Polatuzumab Vedotin, these data mandate the development of **CNS-penetrant CD79B inhibitors** (e.g., brain-shuttle ADCs, bispecific T-cell engagers) to exploit this critical target.

**Copyright Information:** (Online) ISSN: 2572-9241
©2026 The Author(s). HemaSphere is published by John Wiley & Sons Ltd on behalf of European Hematology Association. This is an open access Abstract Book distributed under the Attribution-NonCommercial-NoDerivs (CC BY-NC-ND), which allows third parties to download the articles and share them with others as long as they credit the author and the Abstract Book, but they cannot change the content in any way or use them commercially.
**Abstract Book Citations:** Authors, Title, HemaSphere, 2026;10:(S1):pages. The abstract book DOIs can be found on the "Author Information" page.
**Disclaimer:** Articles published in the journal HemaSphere exclusively reflect the opinions of the authors. The authors are responsible for all content in their abstracts including accuracy of the facts, statements, citing resources, etc.

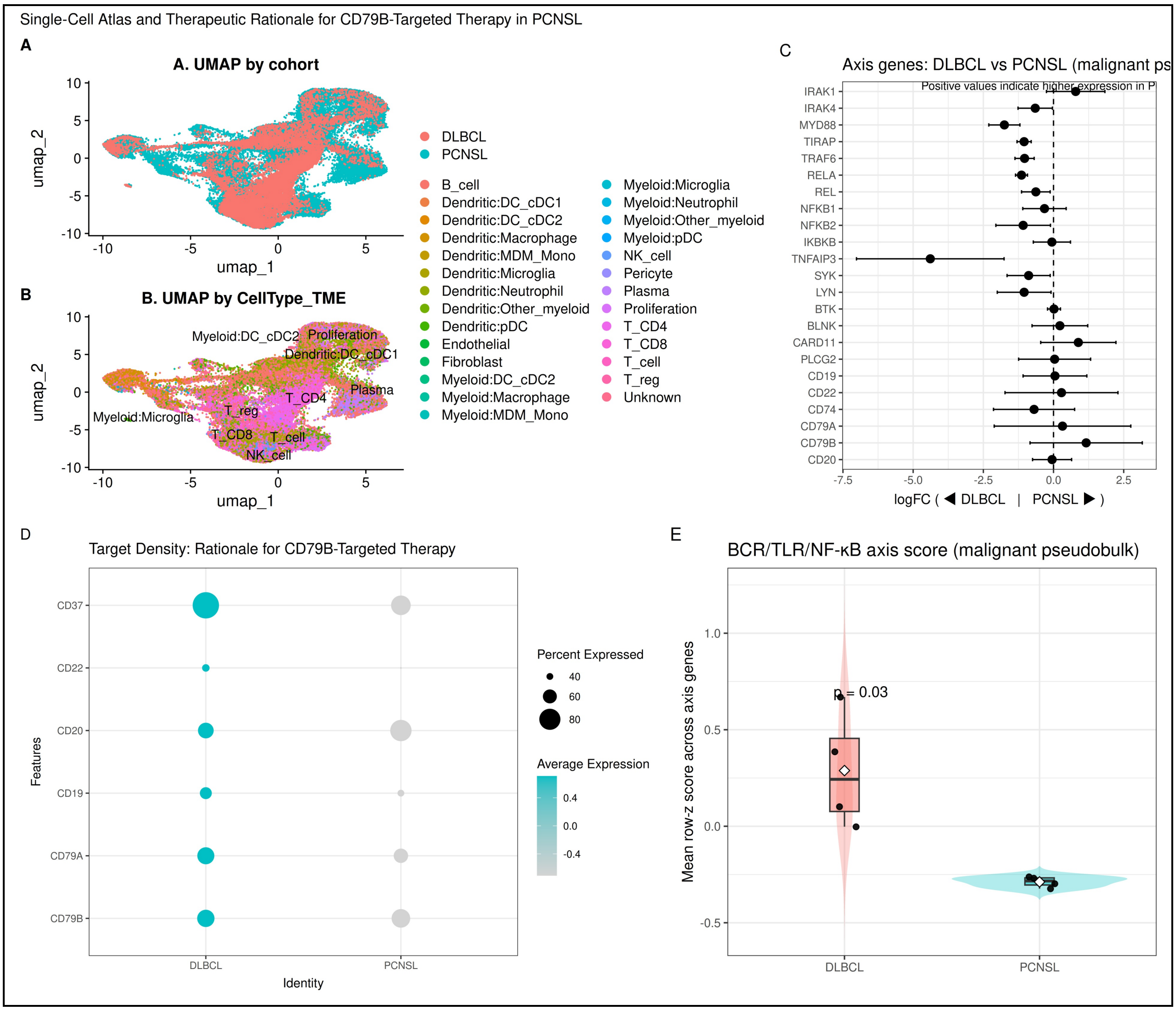

Figure 1: Integrated Single-Cell Landscape and Therapeutic Vulnerabilities in PCNSL. (A-B) UMAP visualizations of 63,915 integrated cells confirm robust cohort alignment and identify specialized brain-resident Microglia unique to the PCNSL niche. (C) Malignant pseudobulk differential expression reveals significant transcriptional attenuation of the TLR axis (TNFAIP3, MYD88) and upregulation of the BCR axis (CD79B) in PCNSL. (D) Target density profiling identifies CD79B as a superior and uniform therapeutic target compared to CD20 and CD19 in PCNSL. (E) Sample-level quantification confirms significant suppression of the global BCR/TLR signaling axis in the CNS niche (p=0.03).

**Copyright Information:** (Online) ISSN: 2572-9241
©2026 The Author(s). HemaSphere is published by John Wiley & Sons Ltd on behalf of European Hematology Association. This is an open access Abstract Book distributed under the Attribution-NonCommercial-NoDerivs (CC BY-NC-ND), which allows third parties to download the articles and share them with others as long as they credit the author and the Abstract Book, but they cannot change the content in any way or use them commercially.
**Abstract Book Citations:** Authors, Title, HemaSphere, 2026;10:(S1):pages. The abstract book DOIs can be found on the "Author Information" page.
**Disclaimer:** Articles published in the journal HemaSphere exclusively reflect the opinions of the authors. The authors are responsible for all content in their abstracts including accuracy of the facts, statements, citing resources, etc.

### (PS2204) SINGLE-CELL ANALYSIS AND FUNCTIONAL PROFILING REVEAL GALECTIN-3–DRIVEN REPROGRAMMING OF THE TUMOR IMMUNE MICROENVIRONMENT IN TRANSFORMED FOLLICULAR LYMPHOMA

**Topic:** 21. Lymphoma biology & translational research

Xiaohui Wang*[1], Huilai Zhang[1]

[1] *Tianjin Medical University Cancer Institute and Hospital,, Tianjin, China;*

### Background

Histological transformation of follicular lymphoma (tFL) represents an aggressive subtype characterized by a complex and immunosuppressive tumor immune microenvironment (TIME). Therefore, it is urgent to investigate the characteristics of TIME and develop more effective immunotherapy strategies for tFL patients.

### Aims

The objective of this study was to comprehensively characterize the tumor immune microenvironment associated with histological transformation of follicular lymphoma and to elucidate the mechanisms underlying immune evasion in tFL. Specifically, we aimed to define T cell exhaustion states and myeloid compartment remodeling during transformation, investigate the role of tumor cell–derived Galectin-3 and its interaction with LAG3 in driving $CD8^+$ T cell dysfunction, and evaluate the therapeutic potential of Galectin-3 inhibition as an immunomodulatory strategy in tFL.

### Methods

Using single-cell RNA sequencing, a high-resolution landscape of the entire TIME ecosystem of follicular lymphoma (FL) and tFL patients is depicted. Findings were validated by multiplex immunofluorescence (mIF) staining, in vitro assays, and a mouse model.

### Results

Compared to FL patients, tFL patients exhibited a higher proportion of exhausted CD8+T cells expressing checkpoint receptors such as LAG3, CTLA4, PD-1, and HAVCR2. Notably, Galectin-3 expression was acquired by tumor cells after transformation, leading to enhanced Galectin-3–LAG3 interactions with $CD8^+$ T cells, as confirmed by mIF staining. In co-culture experiments, Galectin-3-overexpressing tumor cells impaired $CD8^+$ T cell proliferation and cytotoxicity, inducing an exhausted phenotype. Treatment with the Galectin-3 inhibitor GB1107 rescued T cell function. Similar T cell dysfunction and GB1107 response were observed in Galectin-3-overexpressing mouse xenograft model. Additionally, we identified an accumulation of MDSC-like monocytes and M2-like macrophages in tFL patients, further contributing to immune suppression.

### Summary/Conclusion

Galectin-3 drives T cell exhaustion and reshapes the immune microenvironment in tFL patients, promoting immune evasion and disease progression. Targeting Galectin-3 may represent a promising immunotherapeutic strategy for tFL patients.

**Copyright Information:** (Online) ISSN: 2572-9241
©2026 The Author(s). HemaSphere is published by John Wiley & Sons Ltd on behalf of European Hematology Association. This is an open access Abstract Book distributed under the Attribution-NonCommercial-NoDerivs (CC BY-NC-ND), which allows third parties to download the articles and share them with others as long as they credit the author and the Abstract Book, but they cannot change the content in any way or use them commercially.
**Abstract Book Citations:** Authors, Title, HemaSphere, 2026;10:(S1):pages. The abstract book DOIs can be found on the “Author Information” page.
**Disclaimer:** Articles published in the journal HemaSphere exclusively reflect the opinions of the authors. The authors are responsible for all content in their abstracts including accuracy of the facts, statements, citing resources, etc.

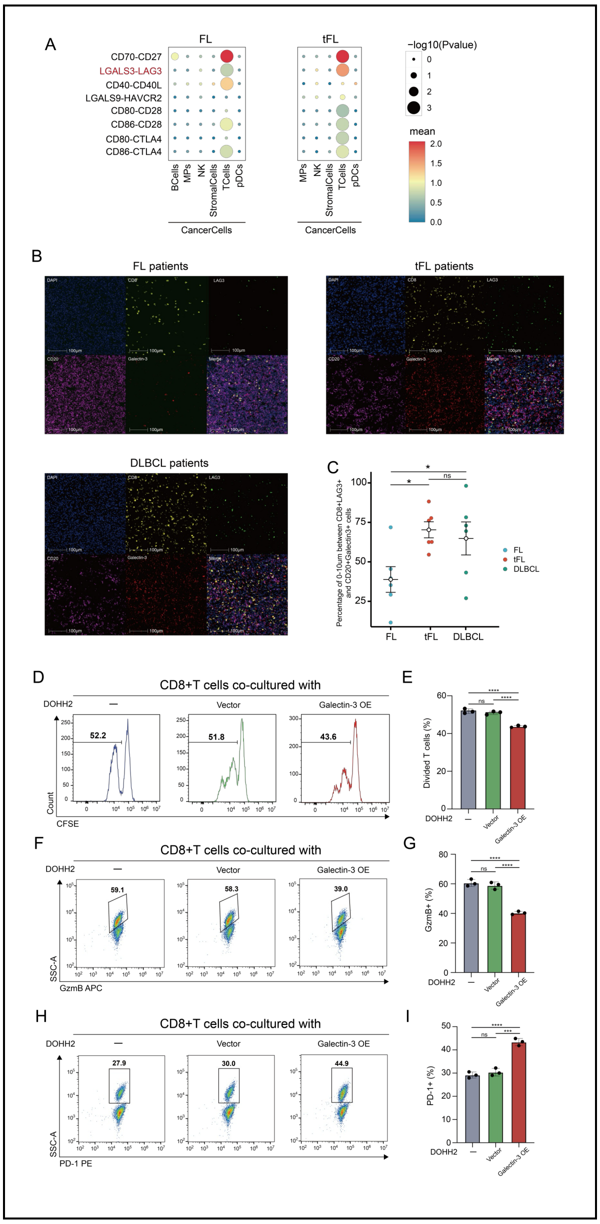

**Copyright Information:** (Online) ISSN: 2572-9241
©2026 The Author(s). HemaSphere is published by John Wiley & Sons Ltd on behalf of European Hematology Association. This is an open access Abstract Book distributed under the Attribution-NonCommercial-NoDerivs (CC BY-NC-ND), which allows third parties to download the articles and share them with others as long as they credit the author and the Abstract Book, but they cannot change the content in any way or use them commercially.
**Abstract Book Citations:** Authors, Title, HemaSphere, 2026;10:(S1):pages. The abstract book DOIs can be found on the "Author Information" page.
**Disclaimer:** Articles published in the journal HemaSphere exclusively reflect the opinions of the authors. The authors are responsible for all content in their abstracts including accuracy of the facts, statements, citing resources, etc.

### (PS2205) GERM LAYER SPECIFICATION AND ORGANOTROPISM IN LYMPHOMA INVASION

**Topic:** 21. Lymphoma biology & translational research

Yan Dong[1], Pengpeng Xu[1], Yuqing Wang[1], Yi Chen[1], Ying Fang[1], Yue Zhu[1], Qing Shi[1], Rong Shen[1], Ziyang Shi[1], Niu Qiao[1], Shu Cheng[1], Li Wang[1], Muchen Zhang*[1], Weili Zhao[1]

[1]*Shanghai Ruijin Hospital, Shanghai, China;*

### Background

Certain relevance exists between tumorigenesis and embryonic development. Representing distinct clinical and molecular features, whether the pattern of lymphoma invasion is related to germ layer development remains unknown.

### Aims

The aim of this study was to determine whether extranodal invasion (ENI) in diffuse large B-cell lymphoma (DLBCL) is specified by germ layer origin and to define its clinical relevance, genomic features, organotropic migratory behavior, and single-cell developmental and immune landscapes.

### Methods

We applied clinical statistics of 2280 newly diagnosed DLBCL patients to identify clinical characteristics among different germ layer-dependent ENI, and further analyzed the association of germ layer-based ENI categories with progression-free survival (PFS) and overall survival (OS) in 1696 patients receiving R-CHOP immunochemotherapy. Oncogenic alterations among different germ layer-based ENI were analyzed in 1190 patients. Zebrafish xenograft model further identified oncogenic characteristics and migration ordering. Single-cell RNA sequencing was applied to determine the temporal disparities and ligand-receptor crosstalk of germ layer-dependent ENI. In vitro assays verified the effect of immune regulatory genes and anti-tumor activity of rituximab of different germ layer-dependent ENI.

### Results

In this study, we identified a germ layer-dependent specification of ENI in diffuse large B-cell lymphoma. Upon R-CHOP treatment, ENI-mesoderm was significantly associated with inferior PFS and OS, as compared to ENI-ectoderm and ENI-endoderm. Representative oncogenic mutations were *MYD88*, *PIM1*, and *TBL1XR1* in ectoderm-originating ENI (ENI-ectoderm), *TP53* and *TET2* in endoderm-originating ENI (ENI-endoderm), and *MYD88*, *PIM1*, *TBL1XR1*, and *CD79B* in mesoderm-originating ENI (ENI-mesoderm). Notably, organotrophic migration exhibited temporal disparities corresponding to germ layer development, occurring from ectoderm- to endoderm- and mesoderm-originating organs, with a similar trajectory to the germ layer process. Single-cell RNA sequencing revealed that malignant B cells follow a developmental trajectory mirroring the germ layer process, differentiating from a progenitor state enriched with NF-κB and T-cell activation signaling into two distinct branches by either upregulated B-cell receptor signaling (Path I) or sustained T-cell activation (Path II). Regarding immune checkpoints, ENI-ectoderm, ENI-endoderm, and ENI-mesoderm exhibited significant upregulation of LGALS9, PD-L1, and B7-H3, respectively.

### Summary/Conclusion

Our findings contributed to a better understanding of crosstalk between lymphoma and embryonic development, providing new insights into targeted approaches against germ layer-specific invasion in cancer treatment.

**Copyright Information:** (Online) ISSN: 2572-9241
©2026 The Author(s). HemaSphere is published by John Wiley & Sons Ltd on behalf of European Hematology Association. This is an open access Abstract Book distributed under the Attribution-NonCommercial-NoDerivs (CC BY-NC-ND), which allows third parties to download the articles and share them with others as long as they credit the author and the Abstract Book, but they cannot change the content in any way or use them commercially.
**Abstract Book Citations:** Authors, Title, HemaSphere, 2026;10:(S1):pages. The abstract book DOIs can be found on the "Author Information" page.
**Disclaimer:** Articles published in the journal HemaSphere exclusively reflect the opinions of the authors. The authors are responsible for all content in their abstracts including accuracy of the facts, statements, citing resources, etc.

### (PS2206) THE MICROBIOME-LYMPHOMA AXIS: A SYSTEMATIC REVIEW AND META-ANALYSIS OF GUT DYSBIOSIS PATTERN IN DIFFUSE LARGE B-CELL LYMPHOMA

**Topic:** 21. Lymphoma biology & translational research

Sarah Elkorashy*[1], Rasha Abu-El-Ruz[2], Medhat Askar[3], Ahmad Hamdan[4], Susu Zughaier[5]

*[1]National Center for Cancer Care & Research (NCCCR), Hamad Medical Corporation (HMC), Hematology and Bone Marrow Transplant, Doha, Qatar; [2]College of Health Science, Qatar University, Biomedical Sciences Department, Doha, Qatar; [3]College of Medicine, Qatar University, Immunology Department, Doha, Qatar; [4]College of Medicine, Qatar University, Doha, Qatar; [5]College of Medicine, Qatar University, Microbiology Department, Doha, Qatar;*

### Background

The gut microbiota plays a pivotal role in immune regulation and cancer biology. Emerging evidence indicates that gut dysbiosis may contribute to the development of diffuse large B-cell lymphoma (DLBCL), the most prevalent and aggressive subtype of non-Hodgkin lymphoma. However, the specific microbial alterations associated with DLBCL have not been comprehensively synthesized.

### Aims

To systematically evaluate and synthesize evidence on gut microbiota (GMB) alterations associated with DLBCL, with a focus on species-level microbial signatures.

### Methods

A systematic review and meta-analysis of thirteen studies involving 4,087 cases was conducted. Both observational and Mendelian randomization (MR) studies were included. Random-effects models were used to calculate pooled odds ratios (ORs) for MR studies, while observational studies contributed to qualitative assessment. Microbial changes were analyzed at the species level to enhance biological interpretation.

### Results

Meta-analysis of MR studies (n = 3,737) yielded a pooled OR of 0.96 (95% CI: 0.93–1.00), indicating a modest trend toward microbial depletion with substantial heterogeneity ($I^2$ = 78.7%, $p < 0.000$). Species-level analyses demonstrated enrichment of pro-inflammatory or potentially oncogenic taxa ($p < 0.05$), including *genus Bilophila* (OR=1.78; 95% CI: 1.05–3.00), *family Desulfovibrionaceae* (OR=1.58; 95% CI: 1.00–2.49), and *genus Coprobacter* (OR=1.37; 95% CI: 1.00–1.86). In contrast, protective commensals with anti-inflammatory and metabolic regulatory roles such as *Eubacterium coprostanoligenes group* (OR=0.19; 95% CI: 0.08–0.45), *genus Alistipes* (OR=0.57; 95% CI: 0.33–0.98), and *genus Ruminococcaceae UCG011* (OR = 0.75; 95% CI: 0.62–0.90) were significantly depleted ($p < 0.05$). Qualitative analysis was conducted to understand the pattern of abundance and depletion of GMB in DLBCL across the included studies.

**Copyright Information:** (Online) ISSN: 2572-9241
©2026 The Author(s). HemaSphere is published by John Wiley & Sons Ltd on behalf of European Hematology Association. This is an open access Abstract Book distributed under the Attribution-NonCommercial-NoDerivs (CC BY-NC-ND), which allows third parties to download the articles and share them with others as long as they credit the author and the Abstract Book, but they cannot change the content in any way or use them commercially.
**Abstract Book Citations:** Authors, Title, HemaSphere, 2026;10:(S1):pages. The abstract book DOIs can be found on the “Author Information” page.
**Disclaimer:** Articles published in the journal HemaSphere exclusively reflect the opinions of the authors. The authors are responsible for all content in their abstracts including accuracy of the facts, statements, citing resources, etc.

### Summary/Conclusion

This first comprehensive synthesis identifies a reproducible species-level microbial signature in DLBCL characterized by loss of beneficial taxa and enrichment of pro-tumorigenic microbes. These alterations may promote lymphomagenesis through inflammatory, metabolic, and immune-modulatory mechanisms. Our findings highlight the gut microbiota as a latent biomarker source and therapeutic target, supporting microbiota-modulating strategies in precision lymphoma care.
Mendelian randomization studies detected a borderline overall association of gut microbiota and DLBCL. However, stratified analyses per taxa showed significant pooled OR shifting towards depletion for; *Alistipes, Ruminococcaceae UCG011, Eubacterium coprostanoligenes* group. Also, shifting towards abundance for; *Bilophila Desulfovibrionaceae, Coprobacter*
Our qualitative analysis revealed abundance of pathogenic and pro-inflammatory taxa in treatment-naïve DLBCL patients, comprising *Bacteroidetes_p, Bacteroides spp., Clostridium difficile, Enterobacteriaceae_f, Enterococcaceae_f, Fusobacteriia_c, Escherichia coli spp., Proteobacteria_p*, and others. Concurrently, there was a marked depletion of beneficial commensals, including *Bifidobacterium_g., Faecalibacterium_g, Roseburia_g., Eubacterium_g, Lachnospira_g and Lactobacillus_g.,* which are critical for maintaining gut barrier integrity and regulating immune homeostasis. The shift toward a microbiota dominated by inflammatory and opportunistic taxa, alongside the loss of beneficial species, may contribute to systemic immune dysregulation and disease progression in DLBCL.

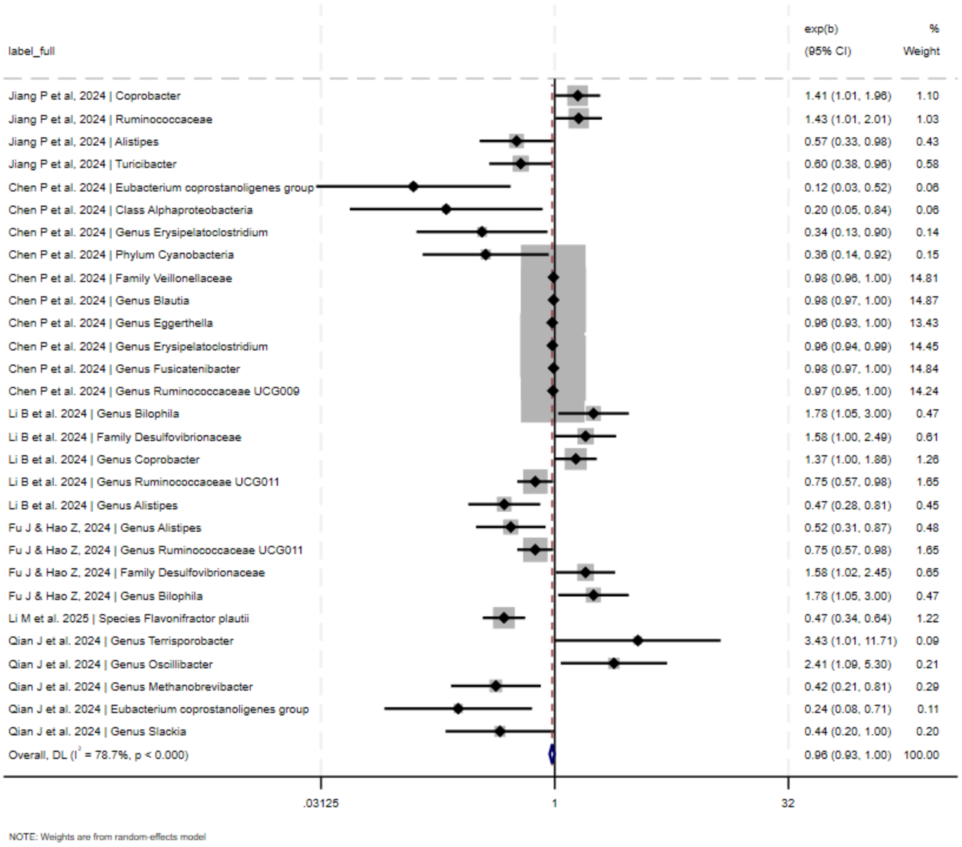

Forest plot of pooled Odds Ratio on MR studies

**Copyright Information:** (Online) ISSN: 2572-9241
©2026 The Author(s). HemaSphere is published by John Wiley & Sons Ltd on behalf of European Hematology Association. This is an open access Abstract Book distributed under the Attribution-NonCommercial-NoDerivs (CC BY-NC-ND), which allows third parties to download the articles and share them with others as long as they credit the author and the Abstract Book, but they cannot change the content in any way or use them commercially.
**Abstract Book Citations:** Authors, Title, HemaSphere, 2026;10:(S1):pages. The abstract book DOIs can be found on the "Author Information" page.
**Disclaimer:** Articles published in the journal HemaSphere exclusively reflect the opinions of the authors. The authors are responsible for all content in their abstracts including accuracy of the facts, statements, citing resources, etc.

### (PS2207) PERIPHERAL CIRCULATING IMMUNE CELL SUBSETS: PROGNOSTIC VALUE AND CORRELATION WITH CLINICAL FEATURES IN TREATMENT-NAIVE DIFFUSE LARGE B-CELL LYMPHOMA

**Topic:** 21. Lymphoma biology & translational research

Ruixin Zheng[1], Kaiyi Liu[1], Bing Pan[1], Yuanyuan Pan[1], Li Li*[1]

[1] *The Second Hospital of Dalian Medical University, Dalian, China;*

### Background

Diffuse large B-cell lymphoma (DLBCL) is the most common aggressive non-Hodgkin lymphoma with marked clinical heterogeneity. Despite R-CHOP-improved survival, 30%-40% of patients have primary resistance or early relapse. TME imbalance drives progression; refined T/B subset correlations with DLBCL clinical features remain unclear, and IPI lacks immune parameters, making exploring immune subsets and developing tools for screening immune-related prognostic markers critical.

### Aims

This study aimed to characterize circulating T/B subsets in treatment-naive DLBCL, clarify their correlations with clinical features, and establish a "clinical-immune" prognostic model.

### Methods

One hundred and eleven treatment-naive DLBCL patients were enrolled and stratified by IPI risk, post-chemotherapy infection, and treatment response (CR/PD). Over 30 T/B lymphocyte subsets in peripheral blood were detected by multicolor flow cytometry. Group comparisons, univariate Logistic and multivariate COX analyses were performed to screen prognostic immune indicators. A prognostic nomogram integrating core immune markers and IPI score was established and validated by ROC, calibration curves and K-M analysis. $P<0.05$ was considered statistically significant.

### Results

This study explored the association between immune subsets and clinical outcomes in diffuse large B-cell lymphoma. High IPI risk patients showed higher DNB I but lower CD4+/CD8+ ratio, helper-inducer T cells, naive CD8+ T cells, DNB II and transitional B cells, with higher relapse rate (all $P<0.05$). These immune indicators were negatively associated with high IPI risk. Patients with post-chemotherapy infection exhibited elevated effector memory CD4+/CD8+ T cells and reduced naive and central memory CD4+ T cells; infection was an independent poor prognostic factor. CR patients had higher naive CD8+ and cytotoxic T cells, while PD patients had higher DNB and Tph levels. A prognostic nomogram combining five immune markers and IPI score was built, with favorable AUCs, good calibration, well-stratified PFS and C-index 0.805.

### Summary/Conclusion

This study examined the relationship between immune subsets and clinical outcomes in diffuse large B-cell lymphoma. High IPI risk was associated with higher DNB I but lower CD4+/CD8+ ratio, helper-inducer T cells, naive CD8+ T cells, DNB II and transitional B cells, accompanied by a higher relapse rate. These immune markers were negatively correlated with high IPI risk. Patients with post-chemotherapy infection showed increased effector memory CD4+/CD8+ T cells and decreased naive and central memory CD4+ T cells; infection was an independent poor prognostic factor. CR patients had higher naive CD8+ and cytotoxic T cells, while PD patients had higher DNB and Tph levels. We established a prognostic nomogram combining five immune indicators and IPI score, which showed satisfactory predictive efficacy with good calibration, well-stratified PFS and a C-index of 0.805.

**Copyright Information:** (Online) ISSN: 2572-9241
©2026 The Author(s). HemaSphere is published by John Wiley & Sons Ltd on behalf of European Hematology Association. This is an open access Abstract Book distributed under the Attribution-NonCommercial-NoDerivs (CC BY-NC-ND), which allows third parties to download the articles and share them with others as long as they credit the author and the Abstract Book, but they cannot change the content in any way or use them commercially.
**Abstract Book Citations:** Authors, Title, HemaSphere, 2026;10:(S1):pages. The abstract book DOIs can be found on the "Author Information" page.
**Disclaimer:** Articles published in the journal HemaSphere exclusively reflect the opinions of the authors. The authors are responsible for all content in their abstracts including accuracy of the facts, statements, citing resources, etc.

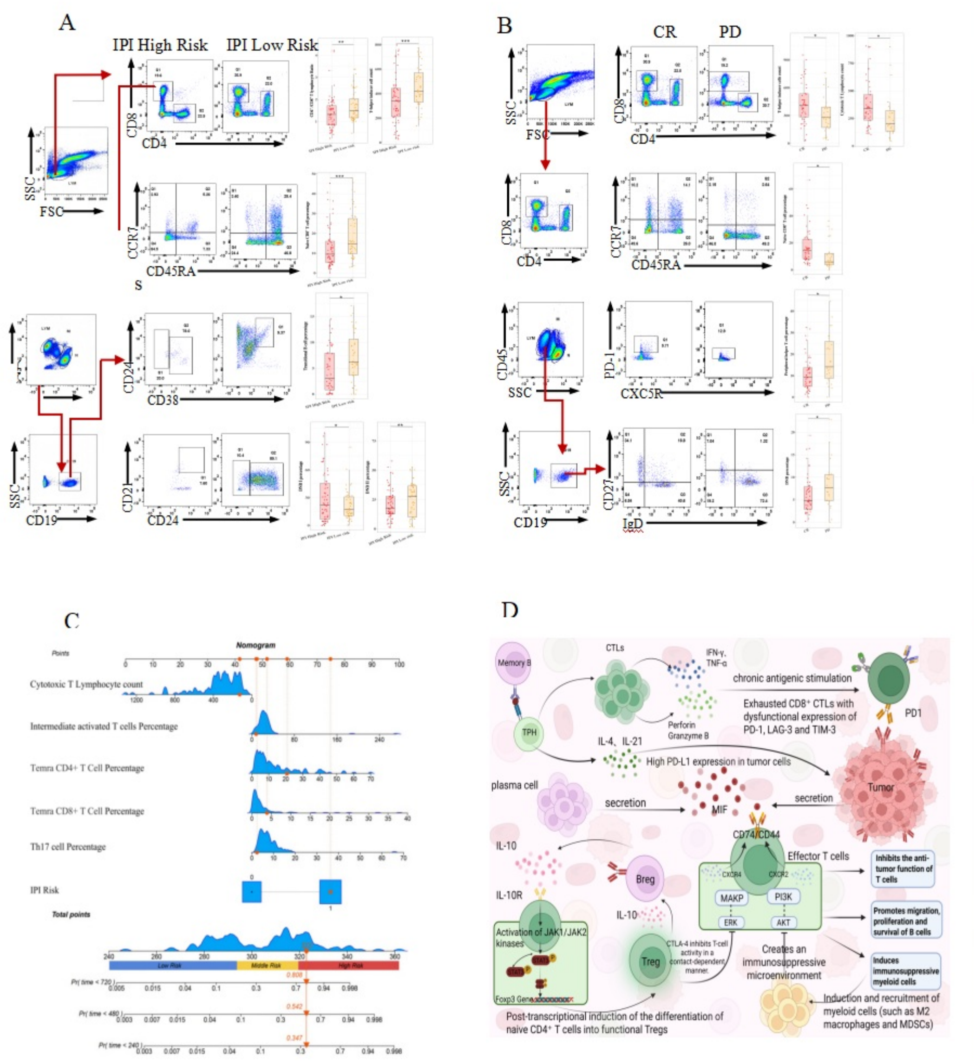

Figure 1. A:Flow cytometry and differential analysis plot of IPI risk stratification; B: Flow cytometry and differential analysis plot of CR vs. PD; C: Prognostic stratification nomogram; D: Schematic diagram of the immune microenvironment in diffuse large B-cell lymphoma (DLBCL)

**Copyright Information:** (Online) ISSN: 2572-9241
©2026 The Author(s). HemaSphere is published by John Wiley & Sons Ltd on behalf of European Hematology Association. This is an open access Abstract Book distributed under the Attribution-NonCommercial-NoDerivs (CC BY-NC-ND), which allows third parties to download the articles and share them with others as long as they credit the author and the Abstract Book, but they cannot change the content in any way or use them commercially.
**Abstract Book Citations:** Authors, Title, HemaSphere, 2026;10:(S1):pages. The abstract book DOIs can be found on the “Author Information” page.
**Disclaimer:** Articles published in the journal HemaSphere exclusively reflect the opinions of the authors. The authors are responsible for all content in their abstracts including accuracy of the facts, statements, citing resources, etc.

### (PS2209) RECIPIENT-ORIGINATED MACROPHAGES REGULATE ACUTE GRAFT-VERSUS-HOST-DISEASE AFTER ALLOGENEIC STEM CELL TRANSPLANTATION

**Topic:** 22. Stem cell transplantation - Experimental

Sisi Zhen*[1], Yuyan Shen[1], Xudong Liao[2, 2], Sizhou Feng[1]

*[1]Institute of Hematology & Blood Diseases Hospital, Chinese Academy of Medical Sciences & Peking Union Medical College, Tianjin, China; [2]Nankai University, Tianjin, China;*

### Background

Acute graft-versus-host disease (aGVHD) remains a major cause of mortality following allogeneic stem cell transplantation (alloHSCT). Current therapeutic strategies predominantly target T cells, but this approach often compromises the graft-versus-tumor (GVT) effect, thereby increasing the risk of malignant relapse.

### Aims

Research on macrophages in the context of GVHD remains relatively underexplored. Therefore, this study focuses on the role of recipient-derived macrophages in the pathogenesis of GVHD. By targeting these cells, it may be possible to develop novel strategies that mitigate GVHD while preserving the graft-versus-leukemia (GVL) effect.

### Methods

We established a fully MHC-mismatched allogeneic hematopoietic stem cell transplantation (allo-HSCT) model using C57BL/6 mice as recipients and BALB/c mice as donors. Both groups received bone marrow (BM) cell infusion and were divided into the GVHD group (BM cells + splenocytes) and the BM-only group (BM cells only) based on whether splenocytes were co-infused. On day 7 post-transplantation, flow cytometry was performed to evaluate changes in the number and phenotype of recipient-derived macrophages. Next, we utilized MerTK knockout mice as recipients to establish the GVHD model again, with wild-type (WT) mice serving as controls. The severity of GVHD was assessed by comparing survival rates, GVHD clinical scores, diarrhea, liver function, and histopathological damage in the liver and intestine between the MerTK knockout and WT groups.
To explore the underlying mechanisms, we measured levels of damage-associated molecular patterns (DAMPs)—specifically cell-free DNA (cfDNA)—in both groups to determine whether differences in cfDNA contributed to the disparity in GVHD severity. Additionally, clinical samples were collected to further validate cfDNA levels in GVHD patients compared with controls. Finally, DNase I was administered to clear cfDNA and evaluate its therapeutic efficacy.

### Results

In this study, we observed significant alterations in the quantity and phenotype (including MerTK, Ly6C, and CD206 expression) of recipient-derived macrophages in GVHD mice compared to those receiving bone marrow alone, suggesting that these cells serve as critical drivers of aGVHD and represent a GVT-sparing therapeutic target (Fig 1A, B). Using a MHC-mismatched alloHSCT model, we demonstrated that recipient mice lacking MerTK—a key phagocytic receptor—developed exacerbated aGVHD (Fig 1C, D, E), likely due to defective efferocytosis by recipient-derived macrophages (Fig 1F). Mechanistically, impaired efferocytosis led to the accumulation of damage-associated molecular patterns (DAMPs), particularly cell-free DNA (cfDNA), which in turn activated antigen-presenting cells and downstream immune effectors to promote aGVHD (Fig 1 G). Notably, administration of DNase I effectively attenuated disease severity, identifying cfDNA as a key DAMP species driving aGVHD pathogenesis and a potential interventional target. Clinically, circulating cfDNA levels were markedly elevated in alloHSCT patients who developed aGVHD compared to those without aGVHD or healthy controls (Fig 1 H, I).

**Copyright Information:** (Online) ISSN: 2572-9241
©2026 The Author(s). HemaSphere is published by John Wiley & Sons Ltd on behalf of European Hematology Association. This is an open access Abstract Book distributed under the Attribution-NonCommercial-NoDerivs (CC BY-NC-ND), which allows third parties to download the articles and share them with others as long as they credit the author and the Abstract Book, but they cannot change the content in any way or use them commercially.
**Abstract Book Citations:** Authors, Title, HemaSphere, 2026;10:(S1):pages. The abstract book DOIs can be found on the “Author Information” page.
**Disclaimer:** Articles published in the journal HemaSphere exclusively reflect the opinions of the authors. The authors are responsible for all content in their abstracts including accuracy of the facts, statements, citing resources, etc.

### Summary/Conclusion

Collectively, our findings highlight recipient-derived macrophages as central pathogenic mediators in aGVHD and propose novel therapeutic strategies targeting these cells or their products, such as cfDNA, which may alleviate aGVHD while preserving T-cell-mediated GVT activity.

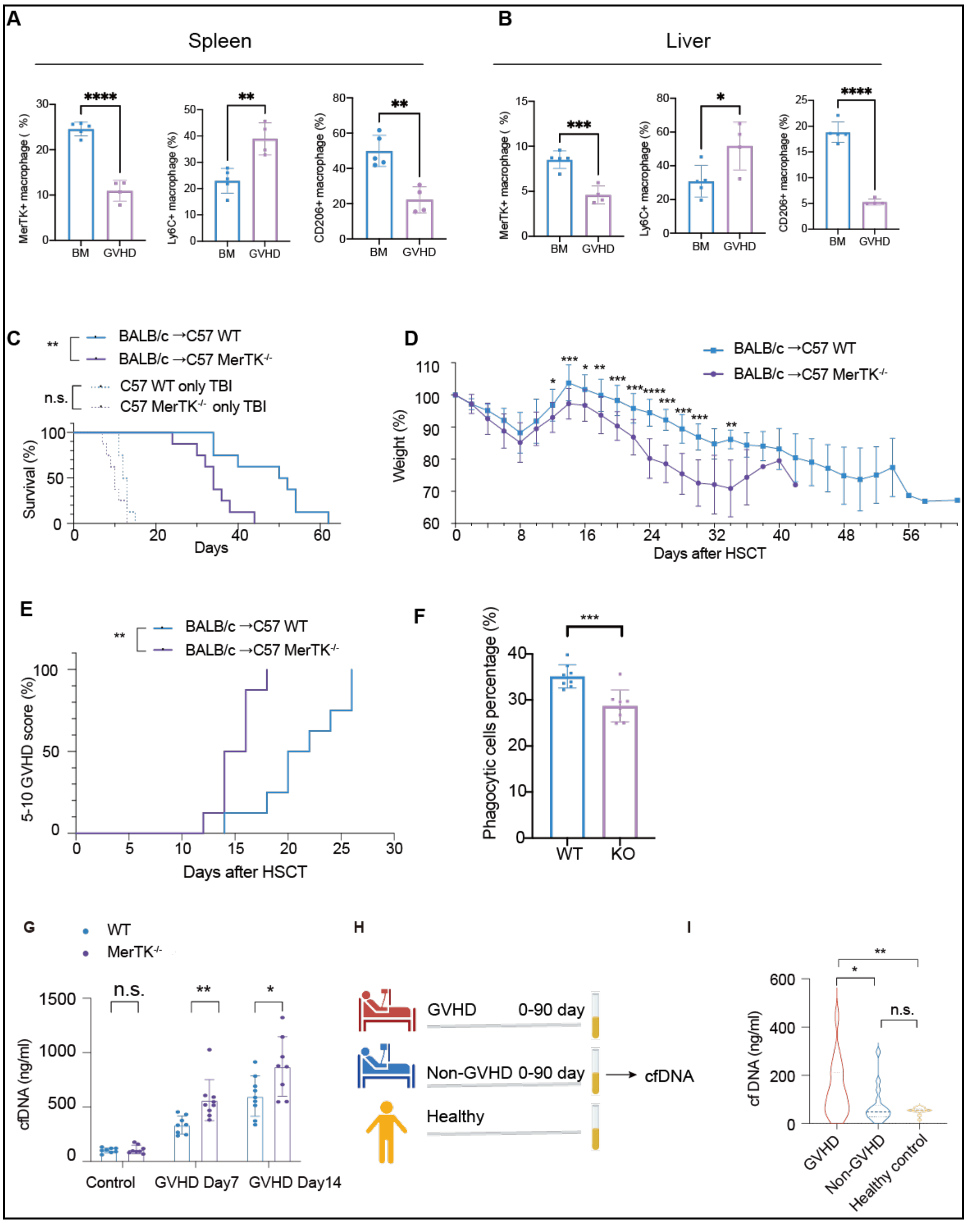

(A, B) Phenotypic changes in recipient-derived macrophages in the spleen (A) and liver (B) of GVHD mice compared with BM-only controls on day 7 post-transplantation. (C–E) MerTK knockout (KO) recipient mice exhibited shorter survival (C), more rapid weight loss (D), and higher GVHD clinical scores (E) compared with wild-type (WT) controls following allo-HSCT. (F) Impaired phagocytosis of apoptotic cells by MerTK-deficient macrophages. (G) Kinetics of cell-free DNA (cfDNA) levels in WT and MerTK KO mice at baseline (untreated) and on days 7 and 14 post-transplantation. (H, I) Circulating cfDNA levels in allo-HSCT recipients with or without GVHD and in healthy controls.

**Copyright Information:** (Online) ISSN: 2572-9241
©2026 The Author(s). HemaSphere is published by John Wiley & Sons Ltd on behalf of European Hematology Association. This is an open access Abstract Book distributed under the Attribution-NonCommercial-NoDerivs (CC BY-NC-ND), which allows third parties to download the articles and share them with others as long as they credit the author and the Abstract Book, but they cannot change the content in any way or use them commercially.
**Abstract Book Citations:** Authors, Title, HemaSphere, 2026;10:(S1):pages. The abstract book DOIs can be found on the "Author Information" page.
**Disclaimer:** Articles published in the journal HemaSphere exclusively reflect the opinions of the authors. The authors are responsible for all content in their abstracts including accuracy of the facts, statements, citing resources, etc.

### (PS2210) BROAD MULTI-TARGET INFLAMMATORY MARKER SILENCING IN A TRANSLATIONAL MODELLING OF OCULAR CHRONIC GRAFT-VERSUS-HOST DISEASE AFTER ALLOGENEIC STEM CELL TRANSPLANTATION

**Topic:** 22. Stem cell transplantation - Experimental

Alessia Cavalleri*[1], Federica Trenta[1], Silvia Mutti[1], Hugo Moreiras[2], Hannah Levis[2], Sara Lancellotti[3], Alessandro Leoni[1], Besjana Xhahysa[1], Luca Garuffo[1], Enrico Morello[1], Vito Romano[4], Domenico Russo[1], Michele Malagola[1], Mirko Farina[1], Simona Bernardi[1]

*[1]University of Brescia, Department of Clinical and Experimental Sciences, Brescia, Italy;[2]University of Liverpool, Institute of Life Course and Medical Sciences, Liverpool, United Kingdom; [3]ASST Spedali Civili di Brescia, Brescia, Italy; [4]University of Brescia, Department of Medical and Surgical Specialties, Radiological Sciences and Public Health, Brescia, Italy;*

### Background

Chronic graft-versus-host disease (cGVHD) is a major cause of long-term morbidity after allogenic stem cell transplantation (allo-HSCT). Ocular involvement (oGVHD) is driven by inflammatory networks causing epithelial damage and barrier dysfunction. Selective modulation of key inflammatory markers may be a precision therapeutic strategy. A robust experimental model is lacking, limiting mechanistic understanding and therapy development.

### Aims

To develop a reproducible inflammatory epithelial model of oGVHD and evaluate silencing of 11 clinically relevant inflammatory markers using two siRNA sequences per target (22 sequences), with translational validation in patient samples.

### Methods

Inflammation was induced in HCET corneal epithelial cells using hyperosmolar media (two protocols) and lipopolysaccharide (LPS, 500 vs1000 ng/mL). IL8, IL6, TNFα, and IL1β were quantified by digital PCR. LPS 1000 ng/mL was used for further experiments. Eleven inflammatory markers were targeted with two siRNA sequences each. Silencing was optimized for IL8 and IL6 and then extended to all targets, individually and combined. Gene expression was measured intracellularly, and delivery validated by fluorescence microscopy (DAPI: nucleus, calcein: cytoskeleton, Alexa: siRNA). Cytotoxicity was assessed via LDH assay.
To achieve a more physiologically model, a multilayer air–liquid interface (ALI) corneal model was established on 1 µm PET inserts and inflamed with LPS, with histological validation. To explore liquid biopsy potential, the 11 targets were quantified in culture supernatants and in tears from 29 subjects (8 healthy, 22 inflammatory dry eye), too.

**Copyright Information:** (Online) ISSN: 2572-9241
©2026 The Author(s). HemaSphere is published by John Wiley & Sons Ltd on behalf of European Hematology Association. This is an open access Abstract Book distributed under the Attribution-NonCommercial-NoDerivs (CC BY-NC-ND), which allows third parties to download the articles and share them with others as long as they credit the author and the Abstract Book, but they cannot change the content in any way or use them commercially.
**Abstract Book Citations:** Authors, Title, HemaSphere, 2026;10:(S1):pages. The abstract book DOIs can be found on the “Author Information” page.
**Disclaimer:** Articles published in the journal HemaSphere exclusively reflect the opinions of the authors. The authors are responsible for all content in their abstracts including accuracy of the facts, statements, citing resources, etc.

### Results

LPS induced robust upregulation of IL8, IL6, TNFα, and IL1β in monolayer cultures. Knockdown of IL8 and IL6 was significant at 24 and 48 h using two siRNA sequences per target, individually and combined.
Extension to all 11 targets resulted in consistent downregulation at 48 h, confirmed by sequence concordance with statistical significance. Marker reduction was detectable intracellularly; immune-cell–restricted markers were not inducible in epithelial-only cultures, supporting specificity.
Fluorescence imaging confirmed efficient intracellular delivery and correct cytoplasmic/perinuclear localization at 24h and 48h (Fig 1A-B). LDH assay showed low cytotoxicity (~7%) without morphological alterations.
The ALI model reproduced epithelial stratification. LPS induced structural alterations consistent with barrier dysfunction and upregulation of IL8, IL6, TNFα, and IL1β, validating inflammatory responsiveness in a complex architecture.
Reduction of targets was observed also in supernatants, highlighting functional modulation of secreted markers and feasibility of liquid biopsy detection, with statistical significance. Consistently, in tear samples, all 11 targets were differentially expressed between healthy controls and patients, including immune-associated markers not detectable in monolayers, supporting translational potential.

### Summary/Conclusion

For the first time, we developed a reproducible oGVHD model in both monolayer and multilayer. Multi-target silencing was robust and reproducible in cultures, with low cytotoxicity and concordant inflammatory signatures in patient samples. This platform provides a foundation for the development of modular, locally delivered RNA interference strategies aimed at selective immunomodulation in ocular cGVHD following allo-HSCT, monitorable by liquid biopsy.

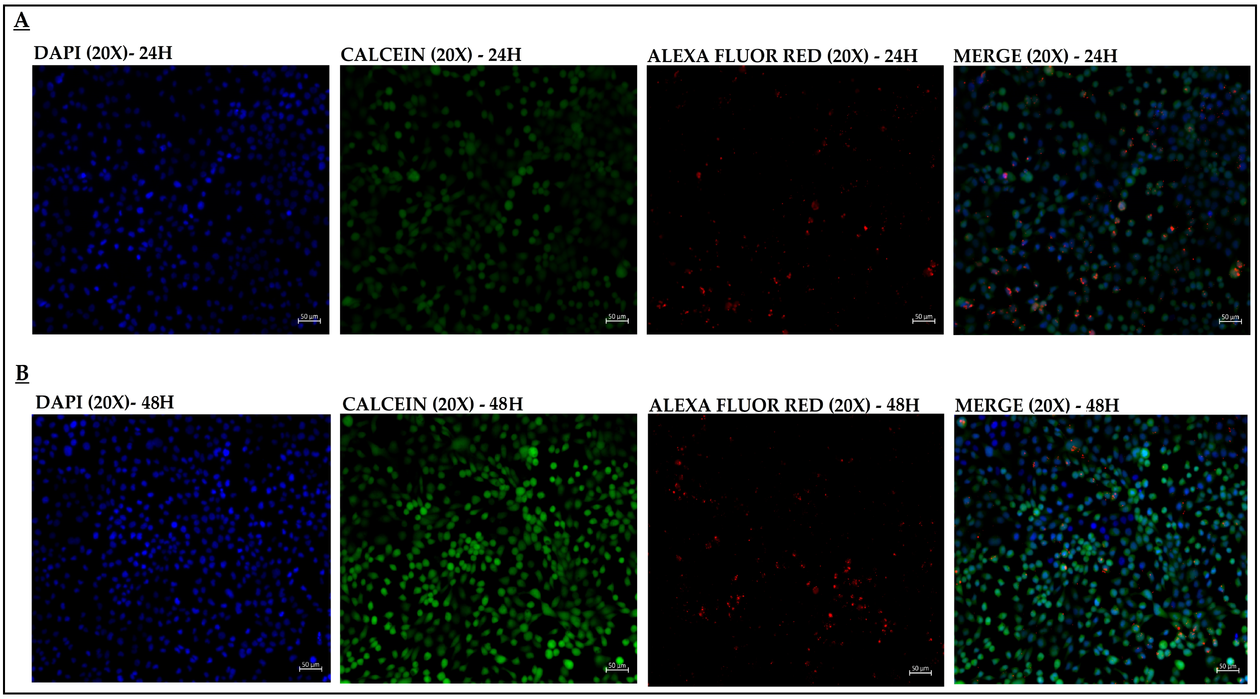

**Copyright Information:** (Online) ISSN: 2572-9241
©2026 The Author(s). HemaSphere is published by John Wiley & Sons Ltd on behalf of European Hematology Association. This is an open access Abstract Book distributed under the Attribution-NonCommercial-NoDerivs (CC BY-NC-ND), which allows third parties to download the articles and share them with others as long as they credit the author and the Abstract Book, but they cannot change the content in any way or use them commercially.
**Abstract Book Citations:** Authors, Title, HemaSphere, 2026;10:(S1):pages. The abstract book DOIs can be found on the "Author Information" page.
**Disclaimer:** Articles published in the journal HemaSphere exclusively reflect the opinions of the authors. The authors are responsible for all content in their abstracts including accuracy of the facts, statements, citing resources, etc.

### (PS2211) MICROBIOTA METABOLITE PHENLYACETYLGLYCINE ALLEVIATES ACUTE GRAFT-VERSUS-HOST DISEASES

**Topic:** 22. Stem cell transplantation - Experimental

Wang Yuqing*[1], Liu Lei[1], Xi Zhang[1]

[1] *The Second Affiliated Hospital, Army Medical University, Chongqing, China;*

### Background

Acute graft-versus-host diseases (aGVHDs) is one of the most important complications after allogenic hematopoietic stem cell transplantation (allo-HSCT), which significantly reduces the overall survival and lower the quality of life of patients. Gut microbiota and their metabolites have been reported to deeply influence aGVHD occurrence and progression.

### Aims

Our research focuses on the mechanism how gut microbiota-derived phenlyacetylglycine (PAGly) plays a protective role against aGVHD, aiming to explore the novel target for aGVHD treatment.

### Methods

We identified key gut microbiota and its metabolites respectively via metagenomic sequencing and liquid chromatography-mass spectrometry based on haploidentical (haplo)-HSCT aGVHD models. Subsequently, flow cytometry, immunofluorescence and gene knockout mice were used to explore the mechanisms how microbiota-derived metabolite PAGly impacts on aGVHD.

### Results

Haplo-HSCT aGVHD mice exhibited a distinguished gut microbiota profiling with non-aGVHD mice. Especially, *Ligilactobacilus* and *Bacteroides* remarkably increased in aGVHD mice with the decline of *Parabacteroides* and *Akkermansia*. Moreover, PAGly, an important metabolite involving phenylalanine metabolism, was significantly decreased. Consistently, the decrease of PAGly was also observed in aGVHD patients from our center. After the supplementary of PAGly, the survival of aGVHD mice was strikingly improved with the mitigation of aGVHD-induced ileum and colon injuries. Further, PAGly treated aGVHD mice exhibited the decreased proportion of Th1 cells and $CD8^+$ T cells in intestinal lamina propria. Simultaneously, PAGly acts as a vital role in maintaining the intestinal permeability via the upregulation of claudin1 and occludin. PAGly also downregulated the expression of MHC-II molecules in intestinal epithelium, which helped epithelial cells to avoid the attack of donor T cells. The knockout of β2-adrenergic receptors (ADRb2) both in donor T cells and recipient intestinal epithelium caused the loss of PAGly protective roles.

### Summary/Conclusion

Gut microbiota-derived metabolite PAGly protects intestine from aGVHD-induced damage via attenuating intestinal inflammation and maintaining intestinal barrier integrity through the ADRb2 signaling.

**Copyright Information:** (Online) ISSN: 2572-9241
©2026 The Author(s). HemaSphere is published by John Wiley & Sons Ltd on behalf of European Hematology Association. This is an open access Abstract Book distributed under the Attribution-NonCommercial-NoDerivs (CC BY-NC-ND), which allows third parties to download the articles and share them with others as long as they credit the author and the Abstract Book, but they cannot change the content in any way or use them commercially.
**Abstract Book Citations:** Authors, Title, HemaSphere, 2026;10:(S1):pages. The abstract book DOIs can be found on the "Author Information" page.
**Disclaimer:** Articles published in the journal HemaSphere exclusively reflect the opinions of the authors. The authors are responsible for all content in their abstracts including accuracy of the facts, statements, citing resources, etc.

### (PS2212) DECODING THE GVL/GVHD PARADOX IN HSCT : A MULTI-OMICS APPROACH FOR SELECTIVE IMMUNOTHERAPY

**Topic:** 22. Stem cell transplantation - Experimental

Romane Bariseel*[1], Caroline Pilon[1], Allan Thiolat[1], Thomas Lajeunesse[1], Sebastien Maury[2], Jimena Tosello Boari[1], José Cohen[1], Mathieu Leclerc[2]

*[1]Inserm U955 - IMRB, Créteil, France; [2]Henri Mondor Hospital AP-HP, Clinical Hematology, Créteil, France;*

**Background**

Allogeneic hematopoietic stem cell transplantation (allo-HSCT) remains the only curative treatment for many high-risk hematologic malignancies, particularly acute myeloid leukemia (AML). Its therapeutic efficacy relies on the graft-versus-leukemia (GVL) effect mediated by donor T cells, but is hindered by two major life-threatening complications: relapse and graft-versus-host disease (GVHD), in which donor T cells attack healthy host tissues. Deciphering the mechanisms governing the balance between GVL and GVHD is therefore essential to identify selective therapeutic targets capable of controlling immune-mediated toxicity without impairing anti-tumor immunity.

**Aims**

This study aimed to characterize the transcriptional and clonal landscape of donor T cells across distinct post-transplant outcomes in a unique murine model of allo-HSCT, in order to identify and prioritize novel selective therapeutic targets.

**Methods**

We developed a semi-allogeneic murine model reproducing the major clinical outcomes of allo-HSCT. Four experimental conditions were generated by modulating the donor T cell dose and introducing AML cells into lethally irradiated recipients: Low GVHD, Severe GVHD, Strong GVL, and Low GVL with AML progression. Single-cell RNA and TCR sequencing using 10x Genomics technology were performed on sorted $CD3^{+}$splenocytes collected 26 days post-transplantation (n = 3 mice per condition), yielding approximately 60,000 T cells. Data were analyzed using unsupervised clustering, differential expression, and pathway enrichment. Candidate therapeutic targets were then prioritized using a scoring pipeline integrating our transcriptomic data with publicly available annotations including subcellular localization, tissue and immune cell expression profiles in human, druggability, and disease associations.

**Results**

Clustering revealed subtle but consistent shifts across experimental groups in distinct $CD4^{+}$ and $CD8^{+}$ T cell subsets including naïve, effector, memory, and exhausted populations. TCR analysis reinforced these findings by showing that the most expanded clones, likely reflecting the dominant alloreactive response, were differentially distributed across conditions. Progenitor exhausted $CD8^{+}$ T cells were enriched in Strong GVL mice, whereas effector and cytotoxic $CD8^{+}$ T cells predominated in Severe GVHD. Differential expression analysis in highly expanded clones identified 279 genes upregulated in GVL and 277 in Severe GVHD. Pathway enrichment implicated integrin and chemokine signaling in Severe GVHD mice, highlighting the role of T cell trafficking in disease pathogenesis. Finally, our scoring pipeline ranked candidate genes based on their potential to selectively target GVHD effector T cells while preserving GVL activity and minimizing off-target toxicity. This provided a prioritized list comprising both known mediators of alloreactive T cell responses, such as S1pr1 and Cxcr3, and novel candidates for functional validation in our murine model.

**Copyright Information:** (Online) ISSN: 2572-9241
©2026 The Author(s). HemaSphere is published by John Wiley & Sons Ltd on behalf of European Hematology Association. This is an open access Abstract Book distributed under the Attribution-NonCommercial-NoDerivs (CC BY-NC-ND), which allows third parties to download the articles and share them with others as long as they credit the author and the Abstract Book, but they cannot change the content in any way or use them commercially.
**Abstract Book Citations:** Authors, Title, HemaSphere, 2026;10:(S1):pages. The abstract book DOIs can be found on the "Author Information" page.
**Disclaimer:** Articles published in the journal HemaSphere exclusively reflect the opinions of the authors. The authors are responsible for all content in their abstracts including accuracy of the facts, statements, citing resources, etc.

**Summary/Conclusion**

This integrative single-cell analysis identified T cell subsets, transcriptional signatures, and signaling pathways distinguishing GVHD from GVL effect. Combined with a multi-dimensional target prioritization framework, this approach revealed candidate genes that could be inhibited to limit GVHD or enhanced to reinforce GVL. Prioritized targets will be further validated in human samples before consideration for clinical translation, offering potential new strategies to improve the efficacy and safety of allo-HSCT.

**Copyright Information:** (Online) ISSN: 2572-9241
©2026 The Author(s). HemaSphere is published by John Wiley & Sons Ltd on behalf of European Hematology Association. This is an open access Abstract Book distributed under the Attribution-NonCommercial-NoDerivs (CC BY-NC-ND), which allows third parties to download the articles and share them with others as long as they credit the author and the Abstract Book, but they cannot change the content in any way or use them commercially.
**Abstract Book Citations:** Authors, Title, HemaSphere, 2026;10:(S1):pages. The abstract book DOIs can be found on the "Author Information" page.
**Disclaimer:** Articles published in the journal HemaSphere exclusively reflect the opinions of the authors. The authors are responsible for all content in their abstracts including accuracy of the facts, statements, citing resources, etc.

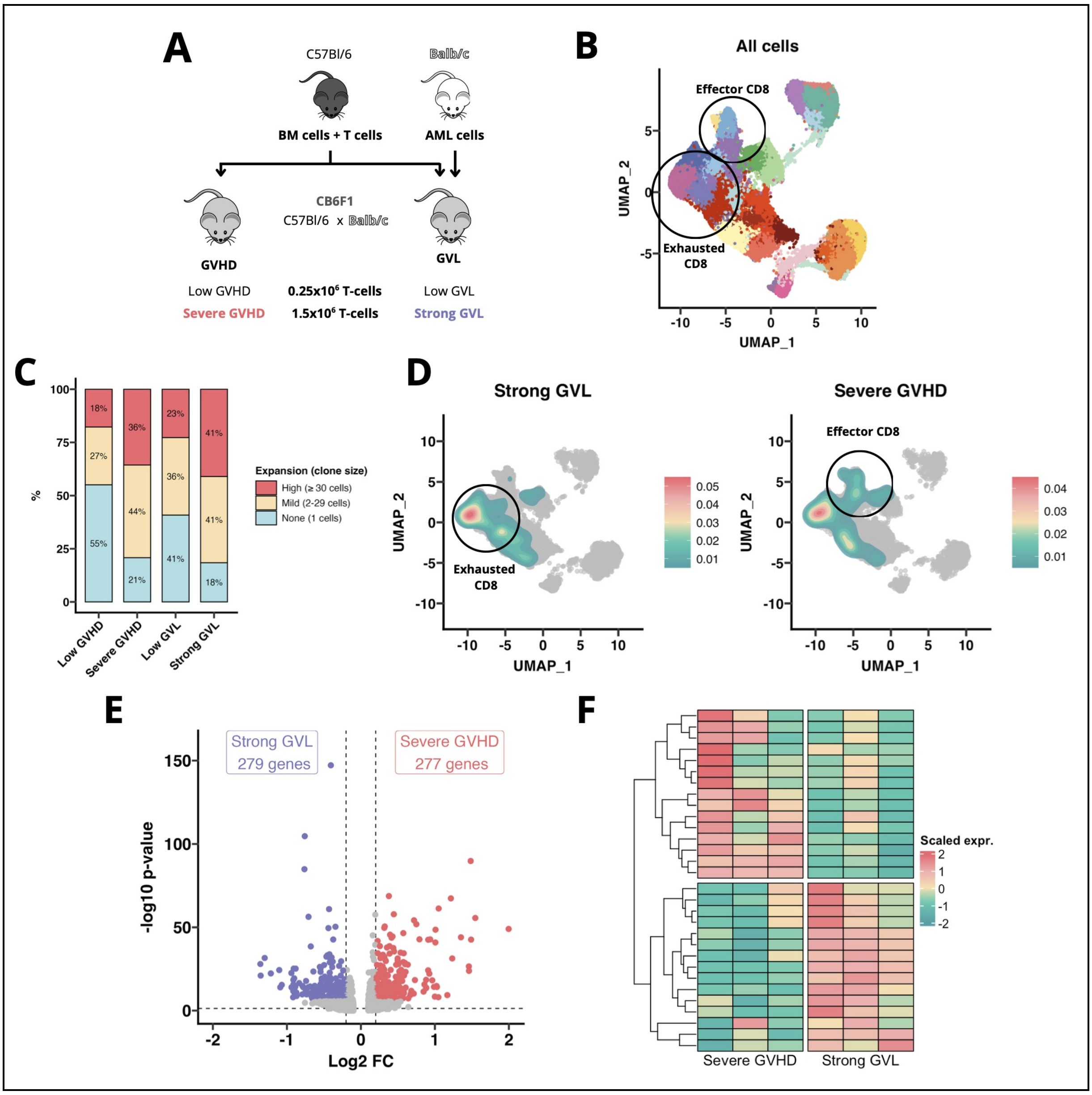

A. Lethally irradiated CB6F1 recipient mice were transplanted with bone marrow (BM) and T cells from C57Bl/6 donors, with or without AML cells of BALB/c origin. Varying the donor T cell dose allowed modulation of the alloimmune response intensity, generating four experimental conditions: Low GVHD, Severe GVHD, Strong GVL, and Low GVL with AML progression. $CD3^{+}$ splenocytes were collected, sorted, and processed for single-cell RNA and TCR sequencing using 10x Genomics technology at day 26 post-transplantation (3 mice per condition). B. UMAP representation of 60221 $CD3^{+}$ T cells colored by cluster identity. Clusters were annotated based on their transcriptional signature. C. Proportion of cells belonging to clonotypes of different expansion levels (highly expanded, mildly expanded, or non-expanded) across the four experimental conditions. T cells were classified based on the size of the clone they belong to: highly expanded (≥30 cells), mildly expanded (2–29 cells), or non-expanded (1 cell). D. UMAP density plots showing the distribution of highly expanded $CD3^{+}$ T cells in Strong GVL (left) and Severe GVHD (right) mice. Color intensity reflects local cell density. E. Volcano plot showing differentially expressed genes in highly expanded T cells (belonging to clones of ≥ 30 cells) between Severe GVHD and Strong GVL conditions. Genes significantly upregulated in Severe GVHD are highlighted in red and genes significantly upregulated in Strong GVL in violet (adjusted p-value < 0.05). F. Heatmap showing the scaled expression of the top 30 genes with the highest log fold change upregulated in Severe GVHD and Strong GVL conditions.

Copyright Information: (Online) ISSN: 2572-9241
©2026 The Author(s). HemaSphere is published by John Wiley & Sons Ltd on behalf of European Hematology Association. This is an open access Abstract Book distributed under the Attribution-NonCommercial-NoDerivs (CC BY-NC-ND), which allows third parties to download the articles and share them with others as long as they credit the author and the Abstract Book, but they cannot change the content in any way or use them commercially.
Abstract Book Citations: Authors, Title, HemaSphere, 2026;10:(S1):pages. The abstract book DOIs can be found on the "Author Information" page.
Disclaimer: Articles published in the journal HemaSphere exclusively reflect the opinions of the authors. The authors are responsible for all content in their abstracts including accuracy of the facts, statements, citing resources, etc.

### (PS2213) CDCP1+ MESENCHYMAL STEM CELLS PREFERENTIALLY INHIBIT ACUTE GRAFT-VERSUS-HOST DISEASE (AGVHD)

**Topic:** 22. Stem cell transplantation - Experimental

Lun Yan*[1], Jing Li[1], Cheng Zhang[1]

[1]*Medical Center of Hematology, Xinqiao Hospital of Army Medical University, Chongqing 400037 China, Chongqing, China;*

### Background

The main complication after hematopoietic stem cells is acute graft-versus-host disease(aGVHD), which affects the success of transplant and survival of patients. It is effective and low-toxicity for mesenchymal stem cells (MSCs) in preventing and treating aGVHD. However, the high heterogeneity of MSCs is unsolved, which leads to instability of clinical outcome and limits the extensive use. Therefore, it is urgent to find a subset of MSCs with advantages in the prevention and treatment of aGVHD while clarifying their roles and mechanisms in regulating aGVHD to solve the bottleneck problem of clinical treatment.

### Aims

This study aims to explore the dominant role and molecular mechanism of CDCP1$^{+}$MSCs in inducing the differentiation of T cells into immunosuppressive cells to control aGVHD, laying a theoretical and experimental foundation for the precise prevention and treatment of aGVHD and promoting the cell therapy of MSCs in clinical practice.

### Methods

Single-cell sequencing and analysis identified the CDCP1$^{+}$MSCs ; Isolate and culture MSCs from umbilical cord tissues; CDCP1$^{+}$MSCs were sorted out by flow cytometry. The migration ability of MSCs was detected by Transwell. The proliferation of T cells was detected by CCK-8 and CSFE proliferation experiments; The changes in the proportion of T cell subsets were detected by flow cytometry; The release of cytokines was detected by ELISA. The expression levels of chemokines were detected by qPCR and Western blot. shCDCP1-MSCs were constructed using lentiviruses that knocked down CDCP1; The gene changes after knockdown of CDCP1 were detected by transcriptome sequencing. Data were analyzed using t test or one-way analysis of variance.

### Results

Through single-cell sequencing and bioinformatics analysis, we found that the CDCP1$^{+}$MSCs derived from the umbilical cord has a stronger potential for immune negative regulation. Through co-culture of CDCP1$^{+}$MSCs and T cells, we found that the CDCP1$^{+}$MSCs group inhibited T cell proliferation compared with the CDCP1 knockdown group. At the same time, it was found that the number of Treg cells increased in the CDCP1$^{+}$MSCs group, while the number of Th2, Th17, Tc1, Tc2, and Tc17 cells decreased. We also found that in the CDCP1$^{+}$MSCs group, the release of cytokines TNF-α, IL-4, IL-6, and IL-21 decreased, while the expression of chemokine CCR2 increased. We found through Transwell detection that CDCP1$^{+}$MSCs have a stronger migration ability. However, after knocking down CDCP1, the migration ability of MSCs decreased significantly. Through RNA-Seq detection, we found that the down-regulation of the CDKN2B gene was the most significant after knockdown of CDCP1, and it might be the key gene affecting the differentiation of T cells into immunosuppressive cells.. Furthermore, through the allo-HSCT aGVHD model mice, we found that the clinical score of aGVHD in mice infused with CDCP1$^{+}$MSCs was lower.

**Copyright Information:** (Online) ISSN: 2572-9241
©2026 The Author(s). HemaSphere is published by John Wiley & Sons Ltd on behalf of European Hematology Association. This is an open access Abstract Book distributed under the Attribution-NonCommercial-NoDerivs (CC BY-NC-ND), which allows third parties to download the articles and share them with others as long as they credit the author and the Abstract Book, but they cannot change the content in any way or use them commercially.
**Abstract Book Citations:** Authors, Title, HemaSphere, 2026;10:(S1):pages. The abstract book DOIs can be found on the "Author Information" page.
**Disclaimer:** Articles published in the journal HemaSphere exclusively reflect the opinions of the authors. The authors are responsible for all content in their abstracts including accuracy of the facts, statements, citing resources, etc.

**Summary/Conclusion**

This study indicates that CDCP1$^+$MSCs derived from the umbilical cord have a stronger immune negative regulatory ability, and they may control aGVHD by inducing T cells to differentiate into immunosuppressive cells through the CDKN2B signaling pathway. The CDCP1$^+$MSCs derived from umbilical cord may be a more effective and stable cell for the prevention and treatment of aGVHD.

**Copyright Information:** (Online) ISSN: 2572-9241
©2026 The Author(s). HemaSphere is published by John Wiley & Sons Ltd on behalf of European Hematology Association. This is an open access Abstract Book distributed under the Attribution-NonCommercial-NoDerivs (CC BY-NC-ND), which allows third parties to download the articles and share them with others as long as they credit the author and the Abstract Book, but they cannot change the content in any way or use them commercially.
**Abstract Book Citations:** Authors, Title, HemaSphere, 2026;10:(S1):pages. The abstract book DOIs can be found on the "Author Information" page.
**Disclaimer:** Articles published in the journal HemaSphere exclusively reflect the opinions of the authors. The authors are responsible for all content in their abstracts including accuracy of the facts, statements, citing resources, etc.

### (PS2214) 6-GINGEROL AMELIORATES CHRONIC GRAFT-VERSUS-HOST DISEASE BY REGULATING TREG/TH17 TRANSDIFFERENCIATION

**Topic:** 22. Stem cell transplantation - Experimental

Yuxuan He*[1], Rui Wang[1], Qi Wang[1], Xu Qing[1], Qingxiao Song[1], Xi Zhang[1]

[1]*Medical Center of Hematology, Xinqiao Hospital of Army Medical University, Chongqing Key Laboratory of Hematology and Microenvironment, Chongqing, China;*

### Background

Allogeneic hematopoietic stem cell transplantation (allo-HSCT) is a potential curative intervention for hematological malignancies.However,its therapeutic efficacy is significantly impaired by the occurrence of graft-versus-host disease (GVHD).Acute GVHD (aGVHD) occurs rapidly following allo-HSCT and predominantly impacts the skin, liver, and gastrointestinal (GI) tract, associated with transplant-related morbidity and mortality.Donor T cells play a central role in the pathogenesis of aGVHD.Among $CD4^+$ T-cell subsets, Th1 and Th17 cells have been reported to drive inflammatory responses and exacerbate aGVHD, whereas regulatory T cells (Tregs) exert protective effects by suppressing excessive immune activation.In herbal medicine, 6-Gingerol has been extensively utilized for the treatment of inflammatory disorders, rheumatic diseases, and gastrointestinal conditions.Purinergic signaling has emerged as a significant modulator of immune cell function. The P2Y family of G protein–coupled receptors, which detect nucleotides such as ATP and ADP, is crucial in influencing T-cell activation, proliferation, and differentiation.Activation of P2Y receptors has been demonstrated to regulate intracellular $Ca^{2+}$ signaling, a pivotal second messenger necessary for T-cell receptor (TCR)–mediated activation and subsequent transcriptional processes.

### Aims

To investigate the immunomodulatory effects and underlying mechanisms of 6-gingerol, a natural monoterpenoid compound, in acute graft-versus-host disease.

### Methods

Two distinct cGVHD mouse models (B6→BALB/c and BALB/c→B6) were established. 6-Gingerol (100 mg/ml, 200 μl/day) was administered via oral gavage from day 0 to day 35 post-HSCT. The body weight change, diarrhea, hair loss and survival of recipients were monitored. cGVHD target organ injuries (Liver, Lung, and gastrointestinal (GI) tract) were examined by H&E staining and quantified using histopathological scoring system. Helper T cell (Th) subsets and regulatory T cell (Treg) in the spleen, liver and GI tract were analyzed by flow cytometry. Serum pro-inflammatory cytokines (TNF-α, IFN-γ, IL-17 etc.) were analyzed by ELISA. The transcriptional profile of in vitro activated T cells with or without gingerol treatment was detected by bulk RNA sequencing.Intracellular $Ca^{2+}$ flux was measured using Fluo-4 AM. A selective P2RY13 agonist was used to assess whether activation of P2RY13 reverses the effects of gingerol.

**Copyright Information:** (Online) ISSN: 2572-9241
©2026 The Author(s). HemaSphere is published by John Wiley & Sons Ltd on behalf of European Hematology Association. This is an open access Abstract Book distributed under the Attribution-NonCommercial-NoDerivs (CC BY-NC-ND), which allows third parties to download the articles and share them with others as long as they credit the author and the Abstract Book, but they cannot change the content in any way or use them commercially.
**Abstract Book Citations:** Authors, Title, HemaSphere, 2026;10:(S1):pages. The abstract book DOIs can be found on the "Author Information" page.
**Disclaimer:** Articles published in the journal HemaSphere exclusively reflect the opinions of the authors. The authors are responsible for all content in their abstracts including accuracy of the facts, statements, citing resources, etc.

**Results**

Gingerol treatment significantly enhanced overall survival, attenuated body weight loss, and reduced clinical aGVHD scores in recipients. Histopathology examination suggested that Gingerol treatment markedly decreased cell infiltration and tissue damage in liver, lung, and GI tract. In addition, serum cytokines IFN-g[illegible] TNF-a and IL-17 were significantly reduced after Gingerol treatment. Mechanistically, Gingerol treatment inhibited Th17 while expanded Tregs in the spleen and aGVHD target organs. RNA sequencing indicated significant changes of the Purinergic signaling and downregulation of P2Y13 that contributing to the Th17 and Treg transdifferenciation.Activation of P2RY13 using a selective agonist restored $Ca^{2+}$ signaling and reversed gingerol-induced changes in Th17 and Treg differentiation.

**Summary/Conclusion**

Gingerol regulates Th17/Treg transdifferentiation via activating butyrate-metabolism-related pathways, therefore, effectively alleviating cGVHD in pre-clinical murine models.

**Copyright Information:** (Online) ISSN: 2572-9241
©2026 The Author(s). HemaSphere is published by John Wiley & Sons Ltd on behalf of European Hematology Association. This is an open access Abstract Book distributed under the Attribution-NonCommercial-NoDerivs (CC BY-NC-ND), which allows third parties to download the articles and share them with others as long as they credit the author and the Abstract Book, but they cannot change the content in any way or use them commercially.
**Abstract Book Citations:** Authors, Title, HemaSphere, 2026;10:(S1):pages. The abstract book DOIs can be found on the “Author Information” page.
**Disclaimer:** Articles published in the journal HemaSphere exclusively reflect the opinions of the authors. The authors are responsible for all content in their abstracts including accuracy of the facts, statements, citing resources, etc.

### (PS2216) LETERMOVIR FOR CYTOMEGALOVIRUS PROPHYLAXIS AFTER ALLOGENEIC HEMATOPOIETIC STEM CELL TRANSPLANTATION: THE RESULTS OF LETREAL STUDY.

**Topic:** 23. Stem cell transplantation - Clinical

Yi Xia*[1], Qi Wen[1], Jinfang Zhao[1], Xiaohui Zhang[1], Lanping Xu[1], Yu Wang[1], Chen-Hua Yan[1], Yu-Hong Chen[1], Wei Han[1], Feng-Rong Wang[1], Jing-Zhi Wang[1], Jun Kong[1], Tingting Han[1], Leqing Cao[1], Dao-Xing Deng[1], Xiao-Dong Mo[1]

[1]*Peking University People's Hospital, Beijing, China;*

### Background

Letermovir is recommended for cytomegalovirus (CMV) prophylaxis after allogeneic hematopoietic stem cell transplantation (allo-HSCT), but its real-world effectiveness in patients receiving haploidentical related donor transplantation with antithymocyte globulin-based graft-versus-host disease (GVHD) prophylaxis remains inconclusive.

### Aims

Thus, in the present real-world study, we aimed to further confirm the efficacy of letermovir prophylaxis in a large allo-HSCT cohort (letermovir prophylaxis in the real-world study, LETreal study) in which many of them received HIDs HSCT with ATG-based protocol for GVHD prophylaxis. We also aimed to further confirmed the association between letermovir prophylaxis and EBV infection after allo-HSCT.

### Methods

We enrolled 1331 acute leukemia patients receiving allo-HSCT in Institute of Hematology, Peking University from January 2023 to March 2025, including 978 who received letermovir prophylaxis. Among them, 254 patients continued letermovir prophylaxis beyond day 100 post-transplant (median time to discontinuation: 119 days). The primary outcome was cs-CMV infection at 100 days and cs-CMV infection between 100 to 200 days after transplantation. Propensity score matching (PSM) was applied to balance baseline characteristics, including age at allo-HSCT (<16 years vs. 16-60 years vs. > 60 years), gender (male vs. female), donor-recipient relationship (matched sibling donor vs. haploidentical related donor vs. matched unrelated donor), and GVHD prophylaxis (without ATG vs. ATG).

**Copyright Information:** (Online) ISSN: 2572-9241
©2026 The Author(s). HemaSphere is published by John Wiley & Sons Ltd on behalf of European Hematology Association. This is an open access Abstract Book distributed under the Attribution-NonCommercial-NoDerivs (CC BY-NC-ND), which allows third parties to download the articles and share them with others as long as they credit the author and the Abstract Book, but they cannot change the content in any way or use them commercially.
**Abstract Book Citations:** Authors, Title, HemaSphere, 2026;10:(S1):pages. The abstract book DOIs can be found on the "Author Information" page.
**Disclaimer:** Articles published in the journal HemaSphere exclusively reflect the opinions of the authors. The authors are responsible for all content in their abstracts including accuracy of the facts, statements, citing resources, etc.

**Results**

Among patients receiving letermovir, the 100-day cumulative incidences of cs-CMV infection (Figure), CMV disease, and refractory CMV infection were 7.1%, 0.1%, and 2.0%, respectively. After propensity score matching, letermovir prophylaxis significantly reduced the 100-day cumulative incidence of cs-CMV infection (7.0% vs. 18.6%, P<0.001) and refractory CMV infection (1.4% vs. 7.0%, P=0.004) compared with no prophylaxis. Letermovir also decreased 100-day cumulative incidence of cs-CMV infection in patients with grade II-IV acute GVHD and those with grade III-IV aGVHD.
Donor-/recipient+ CMV serostatus and age >60 years were associated with a higher risk of cs-CMV infection between days 100 and 200 after allo-HSCT in patients receiving letermovir prophylaxis. After PSM adjustment, patients receiving extended letermovir prophylaxis beyond day 100 experienced comparable late-onset cs-CMV infection, CMV disease and refractory cs-CMV infection between days 100 and 200 after allo-HSCT compared with those without letermovir. For patients experienced chronic GVHD or those experienced moderate-to-severe chronic GVHD, the cumulative incidence of cs-CMV infection between days 100 and 200 was also comparable.
In addition, patients receiving letermovir prophylaxis had significantly higher 2-year cumulative incidences of cs-EBV infection (*P*<0.001) and EBV-post-transplant lymphoproliferative disorder (*P*=0.002) compared with no prophylaxis.
After PSM adjustment, the 2-year clinical outcomes (relapse, non-relapse mortality, leukemia-free survival and overall survival) were comparable among patients among without letermovir and those receiving letermovir prophylaxis.

**Summary/Conclusion**

In summary, we comprehensive present the effectiveness of letermovir prophylaxis through a large allo-HSCT cohort in the real world, and we also confirmed that letermovir prophylaxis increased the risk of cs-EBV infection and PTLD after allo-HSCT.

**Copyright Information:** (Online) ISSN: 2572-9241
©2026 The Author(s). HemaSphere is published by John Wiley & Sons Ltd on behalf of European Hematology Association. This is an open access Abstract Book distributed under the Attribution-NonCommercial-NoDerivs (CC BY-NC-ND), which allows third parties to download the articles and share them with others as long as they credit the author and the Abstract Book, but they cannot change the content in any way or use them commercially.
**Abstract Book Citations:** Authors, Title, HemaSphere, 2026;10:(S1):pages. The abstract book DOIs can be found on the “Author Information” page.
**Disclaimer:** Articles published in the journal HemaSphere exclusively reflect the opinions of the authors. The authors are responsible for all content in their abstracts including accuracy of the facts, statements, citing resources, etc.

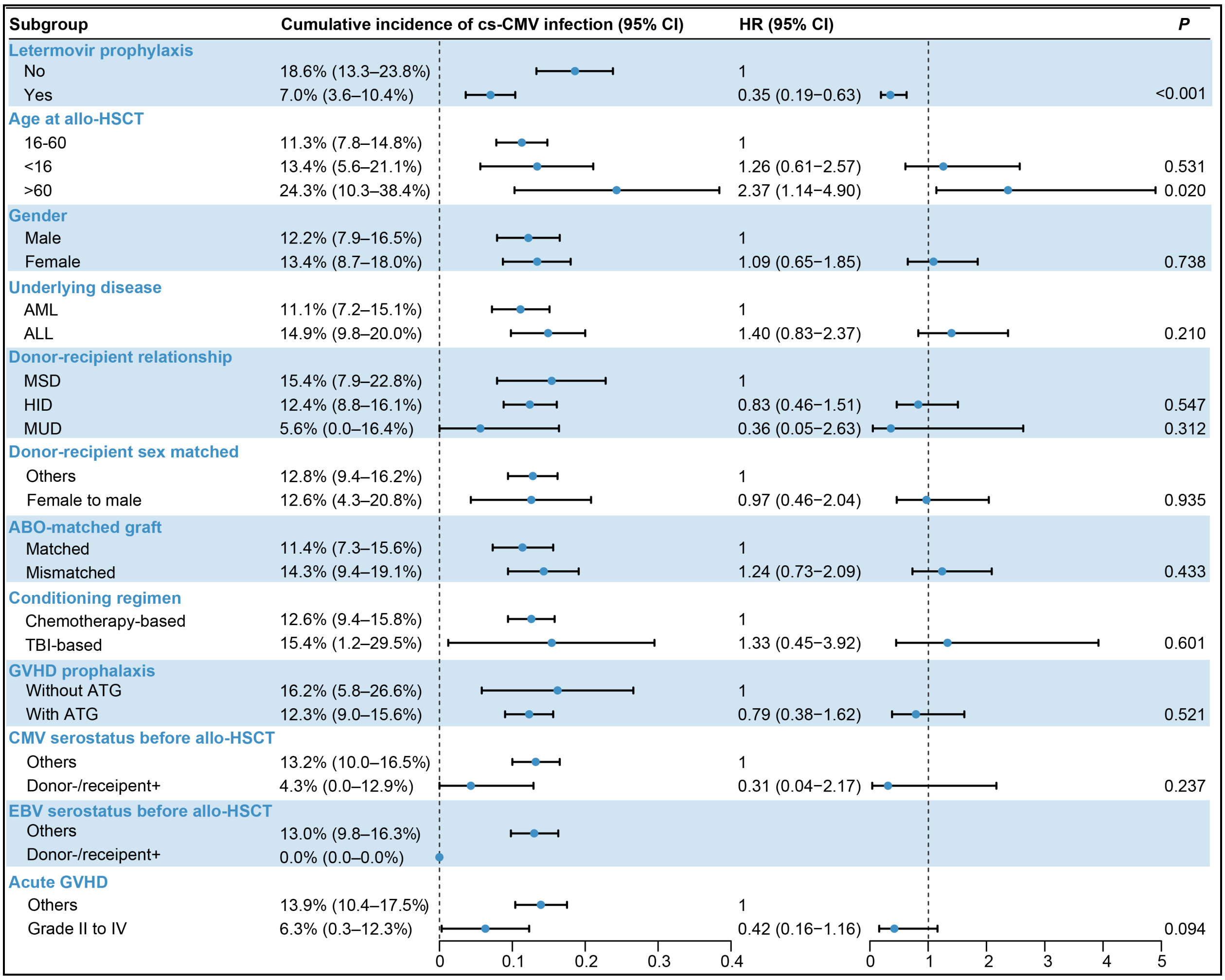

The comparison of cs-CMV infection at 100 days after allo-HSCT after PSM adjusted. Allo-HSCT, allogeneic hematopoietic stem cell transplantation; TBI, total body irradiation; MSD, matched sibling donor; HID, haploidentical related donor; MUD, matched unrelated donor; GVHD, graft-versus-host disease; ATG, antithymocyte globulin; CMV, cytomegalovirus; EBV, Epstein-Barr virus; HR, hazard ratio; 95% CI, 95% confidence interval.

Copyright Information: (Online) ISSN: 2572-9241
©2026 The Author(s). HemaSphere is published by John Wiley & Sons Ltd on behalf of European Hematology Association. This is an open access Abstract Book distributed under the Attribution-NonCommercial-NoDerivs (CC BY-NC-ND), which allows third parties to download the articles and share them with others as long as they credit the author and the Abstract Book, but they cannot change the content in any way or use them commercially.
Abstract Book Citations: Authors, Title, HemaSphere, 2026;10:(S1):pages. The abstract book DOIs can be found on the "Author Information" page.
Disclaimer: Articles published in the journal HemaSphere exclusively reflect the opinions of the authors. The authors are responsible for all content in their abstracts including accuracy of the facts, statements, citing resources, etc.

### (PS2217) PRE-TRANSPLANT HIGH-DOSE METHOTREXATE AND INTENSIFIED INTRATHECAL PROPHYLAXIS DO NOT REDUCE CNS RELAPSE IN B-CELL ACUTE LYMPHOBLASTIC LEUKEMIA

**Topic:** 23. Stem cell transplantation - Clinical

Ni Lu*[1, 2], Mengnan Lv[1, 2], Erlie Jiang[1, 2]

*[1]Institute of Hematology & Blood Diseases Hospital, Chinese Academy of Medical Sciences & Peking Union Medical College, Hematopoietic Stem Cell Transplantation Center, Tianjin, China; [2]State Key Laboratory of Experimental Hematology, National Clinical Research Center for Blood Diseases, Haihe Laboratory of Cell Ecosystem, Tianjin, China;*

### Background

Post-transplant central nervous system (CNS) relapse in B-cell acute lymphoblastic leukemia (B-ALL) carries a dismal prognosis. While pre-transplant consolidation with high-dose methotrexate (HD-MTX) and frequent intrathecal (IT) chemotherapy are standard practices, their efficacy in the context of modern conditioning regimens—particularly those including total body irradiation (TBI)—remains controversial.

### Aims

We aimed to evaluate the impact of pre-transplant CNS-directed consolidation strategies on post-transplant CNS relapse.

### Methods

This single-center retrospective study analyzed 552 B-ALL patients in first complete remission (CR1) who underwent autologous (auto-HSCT, n=154, 27.9%) or allogeneic (allo-HSCT, n=398, 72.1%) transplantation between January 1, 2014, and May 30, 2024. Pre-transplant consolidation regimens were determined by physician discretion: 374 patients (67.8%) received intravenous HD-MTX (median 2 g/m$^2$; range 1–5 g/m$^2$), while the remainder received non-MTX regimens. Standard triple IT prophylaxis (Dexamethasone/Methotrexate/Ara-C) was utilized. Baseline characteristics were balanced between MTX and Non-MTX groups. The primary endpoint was the cumulative incidence of CNS relapse.

**Copyright Information:** (Online) ISSN: 2572-9241
©2026 The Author(s). HemaSphere is published by John Wiley & Sons Ltd on behalf of European Hematology Association. This is an open access Abstract Book distributed under the Attribution-NonCommercial-NoDerivs (CC BY-NC-ND), which allows third parties to download the articles and share them with others as long as they credit the author and the Abstract Book, but they cannot change the content in any way or use them commercially.
**Abstract Book Citations:** Authors, Title, HemaSphere, 2026;10:(S1):pages. The abstract book DOIs can be found on the “Author Information” page.
**Disclaimer:** Articles published in the journal HemaSphere exclusively reflect the opinions of the authors. The authors are responsible for all content in their abstracts including accuracy of the facts, statements, citing resources, etc.

### Results

With a median follow-up of 3.94 years, the 5-year cumulative incidence of CNS relapse was 2.66% (95% CI, 1.36%–4.70%) after allo-HSCT and 3.96% (95% CI, 1.62%–7.97%) after auto-HSCT (P=0.381; Figure a). Median time to relapse was 12 months (range, 3–28). CNS relapse was associated with poor prognosis: 6 patients died (5 from CNS progression, 1 from infection), with numerically inferior 5-year OS compared with those without CNS relapse (Figure e, 58.44% vs. 76.44%; P=0.155).
In univariate analysis, male (P=0.013), prior CNSL (P=0.013), and high-risk disease (P<0.001) predicted CNS relapse, while elevated LDH showed a borderline association (P=0.059). TBI-based conditioning was protective factor (HR 0.27; P=0.029; Figure b). Neither pre-transplant HD-MTX (HR 0.965; P=0.960) nor intensified IT prophylaxis (≥6 courses; HR 1.004; P=1.000) reduced CNS relapse (Figure c–d). To address baseline imbalances—particularly more frequent IT in patients with prior CNSL—we performed IPTW adjustment. After balancing key covariates (prior CNSL, MRD status, risk group, conditioning, and HD-MTX), intensified IT (≥6 courses) remained non-significant (5-year SHR 0.86; 95% CI, 0.29–2.51; P=0.781). Five patients relapsed after 4–6 IT courses, with no difference versus ≥6 courses (P=0.938); no CNS relapse was observed in patients receiving <4 IT courses. There were no significant interactions between TBI and intensified IT (P_interaction=0.669) or HD-MTX (P_interaction=0.279), and the lack of benefit persisted regardless of TBI use(Figure f). HD-MTX did not improve OS (P=0.281) and was associated with substantial non-hematologic toxicity, including mucositis (44.0%) and renal impairment (35.0%). Auto-HSCT and MRD positivity after three chemotherapy cycles predicted overall relapse. Five-year OS was comparable between auto- and allo-HSCT (73.01% vs. 77.15%; P=0.943).

### Summary/Conclusion

Post-transplant CNS relapse is an uncommon event but associated with poor outcomes. Intensified pre-transplant CNS-directed consolidation using HD-MTX or extended IT prophylaxis does not reduce the risk of CNS relapse or improve survival in patients with B-ALL undergoing HSCT. However, TBI-based conditioning provides significant protection against CNS recurrence.

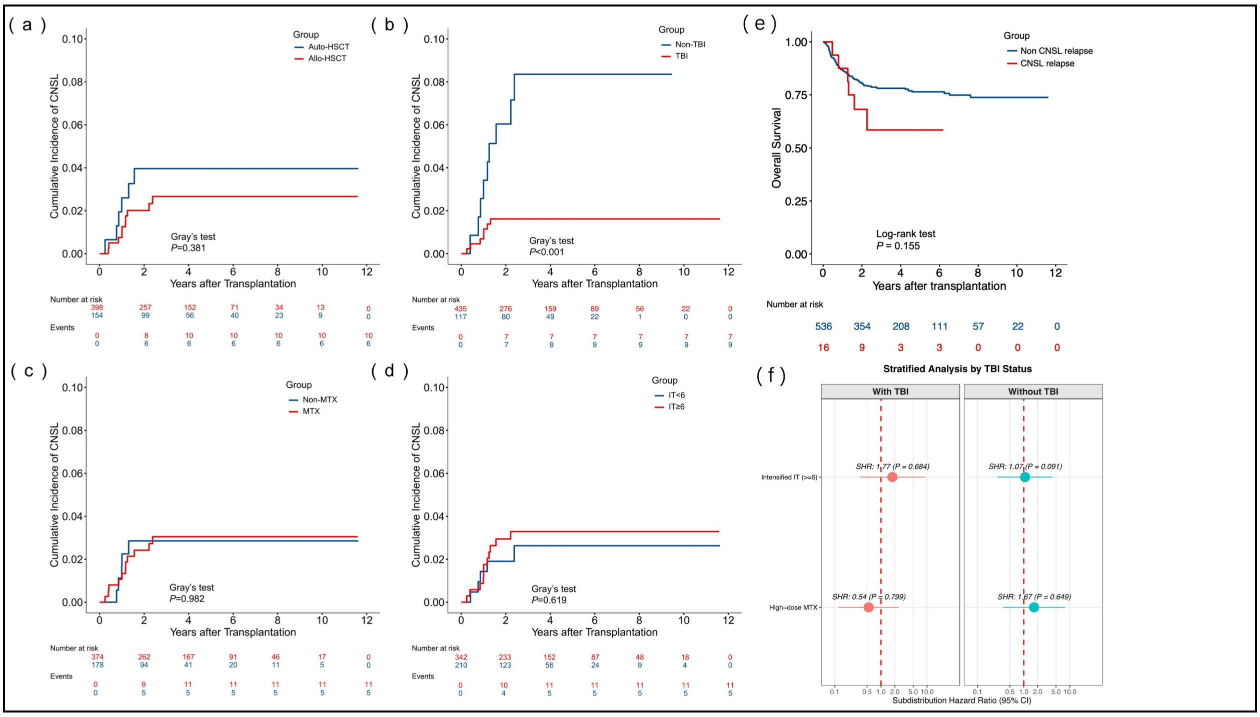

**Copyright Information:** (Online) ISSN: 2572-9241
©2026 The Author(s). HemaSphere is published by John Wiley & Sons Ltd on behalf of European Hematology Association. This is an open access Abstract Book distributed under the Attribution-NonCommercial-NoDerivs (CC BY-NC-ND), which allows third parties to download the articles and share them with others as long as they credit the author and the Abstract Book, but they cannot change the content in any way or use them commercially.
**Abstract Book Citations:** Authors, Title, HemaSphere, 2026;10:(S1):pages. The abstract book DOIs can be found on the "Author Information" page.
**Disclaimer:** Articles published in the journal HemaSphere exclusively reflect the opinions of the authors. The authors are responsible for all content in their abstracts including accuracy of the facts, statements, citing resources, etc.

### (PS2218) EFFICACY OF CAR-T BRIDGING TO ALLO-HSCT IN ADULT TCF3::PBX1+ ALL: IMPACT OF INFUSION FREQUENCY

**Topic:** 23. Stem cell transplantation - Clinical

Jingyu Wang*[1], Jiarui Zhou[1], Lu Huang[1], Chunhui Nie[1], Rui-Juan Sun[1], Xingyu Cao[1], Yan-Li Zhao[1], Yue Lu[1], Jianping Zhang[1], Deyan Liu[1], Zhijie Wei[1], Min Xiong[1], Xian Zhang[2], Junfang Yang[2]

*[1]Hebei Yanda Lu Daopei Hospital, Department of Hematology, langfang, China;[2]Hebei Yanda Lu Daopei Hospital, Hematology and immunotherapy Department, langfang, China;*

### Background

Patients with relapsed/refractory (R/R) TCF3::PBX1-positive acute lymphoblastic leukemia (ALL) face a poor prognosis. In our center, this subtype accounts for 3.26% of all ALL cases. While CAR-T therapy bridging to allogeneic hematopoietic stem cell transplantation (allo-HSCT) is a promising salvage strategy, the impact of repeated CAR-T infusions on transplant outcomes remains undefined.

### Aims

To evaluate the efficacy of CAR-T bridging to allo-HSCT in adult TCF3::PBX1-positive ALL, specifically analyzing the effect of CAR-T infusion frequency (single vs. double) on survival and safety.

### Methods

We retrospectively analyzed 49 adult patients with TCF3::PBX1-positive ALL treated at two centers (2013–2025). Patients were categorized into CAR-T (n=29) and non-CAR-T (n=20) groups. The CAR-T group was subdivided into single CAR-T (n=23) and double CAR-T (n=6, ≥2 infusions). Given the small sample size of the double CAR-T subgroup, Fisher's exact test was used for categorical variables. Multivariate Cox regression calculated hazard ratios (HR).

### Results

The CAR-T group predominantly comprised R/R patients (86.2% in CR2/CR3 vs. 10.0% in non-CAR-T, P<0.001). The CAR-T group showed a trend toward lower chronic GVHD incidence (24.1% vs. 55.0%, P=0.057). Subgroup analysis revealed striking differences:

1. Survival: Single CAR-T bridging achieved a favorable 1-year overall survival (OS) of 68.5%. In contrast, the double CAR-T subgroup had a 1-year OS of 0% (median OS: 4.2 months; Log-rank P<0.001).
2. Risk Analysis: Multivariate Cox regression analysis identified treatment group as a significant independent predictor of mortality. Compared to the non-CAR-T group (reference, HR=1.00), single CAR-T bridging showed no significant difference in mortality risk (HR=0.85, 95% CI: 0.32-2.28, P=0.742), with a 1-year overall survival of 68.5% versus approximately 60% in the non-CAR-T group. However, double CAR-T therapy was associated with a significantly increased mortality risk (HR=7.45, 95% CI: 2.17-25.55, P=0.001), representing a 7.5-fold higher risk of death compared to the non-CAR-T group. When directly comparing the two CAR-T subgroups, double CAR-T remained significantly associated with higher mortality compared to single CAR-T (HR=3.37, 95% CI: 1.16-9.82, P=0.026).
3. Safety: The double CAR-T subgroup exhibited significantly higher rates of platelet engraftment failure (33.3% vs. 4.3%, P=0.048) and severe infections (83.3% vs. 34.8%, P=0.032). Five of six deaths in this group were due to infection combined with poor hematopoietic recovery, suggesting immune exhaustion and microenvironment damage.

**Copyright Information:** (Online) ISSN: 2572-9241
©2026 The Author(s). HemaSphere is published by John Wiley & Sons Ltd on behalf of European Hematology Association. This is an open access Abstract Book distributed under the Attribution-NonCommercial-NoDerivs (CC BY-NC-ND), which allows third parties to download the articles and share them with others as long as they credit the author and the Abstract Book, but they cannot change the content in any way or use them commercially.
**Abstract Book Citations:** Authors, Title, HemaSphere, 2026;10:(S1):pages. The abstract book DOIs can be found on the "Author Information" page.
**Disclaimer:** Articles published in the journal HemaSphere exclusively reflect the opinions of the authors. The authors are responsible for all content in their abstracts including accuracy of the facts, statements, citing resources, etc.

### Summary/Conclusion

Single CAR-T sequential allogeneic hematopoietic stem cell transplantation (allo-HSCT) is an effective strategy for relapsed/refractory (R/R) TCF3::PBX1-positive acute lymphoblastic leukemia (ALL), achieving durable remissions and potentially reducing the risk of chronic graft-versus-host disease (cGVHD). However, in our cohort, two cycles of CAR-T therapy were associated with catastrophic outcomes (100% mortality was observed; while this finding is limited by the small sample size, it necessitates urgent clinical caution). This poor prognosis is most likely attributable to irreversible immune exhaustion and bone marrow microenvironment injury. Clinically, regimens involving multiple CAR-T infusions should be optimized, and alternative therapeutic strategies (e.g., modified dual-target CAR-T regimens, immune checkpoint inhibitors) are urgently needed for refractory patients.

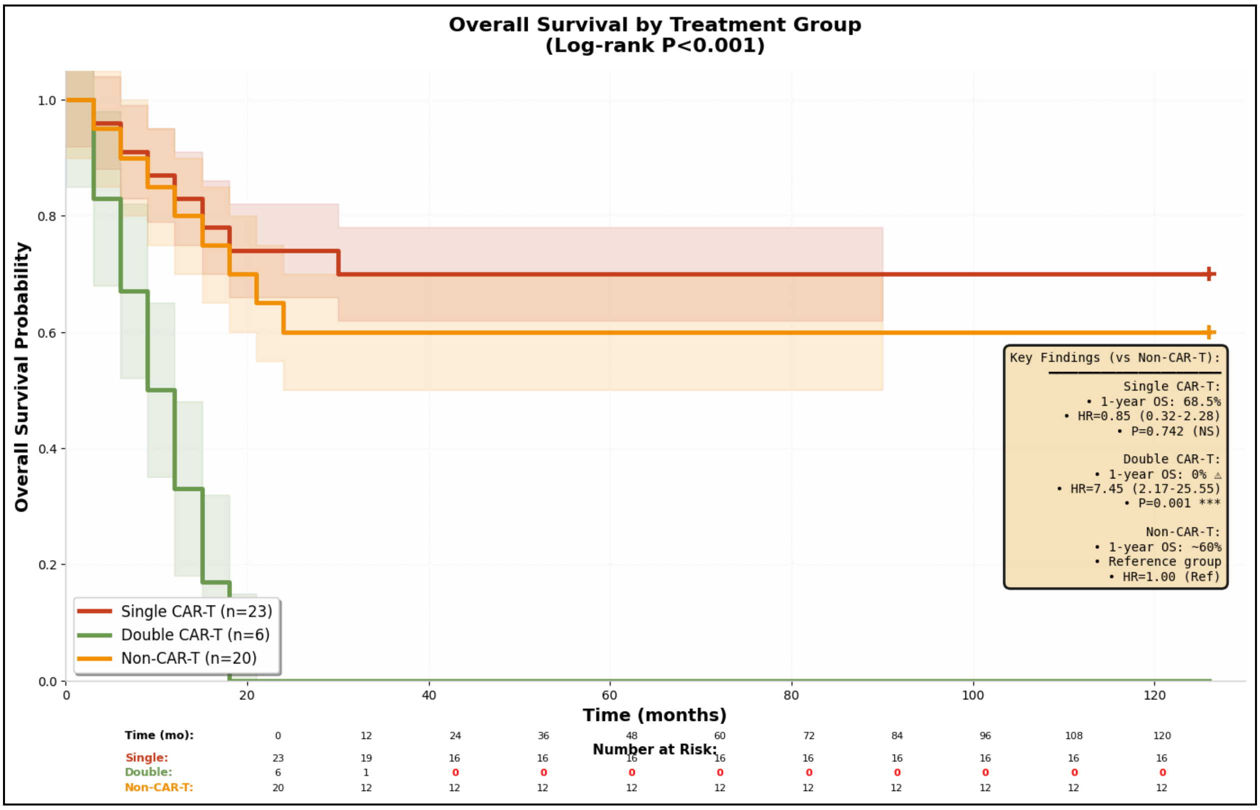

Figure 1: Overall Survival Curve comparing Single CAR-T, Double CAR-T, and Non-CAR-T groups.

**Copyright Information:** (Online) ISSN: 2572-9241
©2026 The Author(s). HemaSphere is published by John Wiley & Sons Ltd on behalf of European Hematology Association. This is an open access Abstract Book distributed under the Attribution-NonCommercial-NoDerivs (CC BY-NC-ND), which allows third parties to download the articles and share them with others as long as they credit the author and the Abstract Book, but they cannot change the content in any way or use them commercially.
**Abstract Book Citations:** Authors, Title, HemaSphere, 2026;10:(S1):pages. The abstract book DOIs can be found on the "Author Information" page.
**Disclaimer:** Articles published in the journal HemaSphere exclusively reflect the opinions of the authors. The authors are responsible for all content in their abstracts including accuracy of the facts, statements, citing resources, etc.

### (PS2219) CLINICAL APPLICATION OF DUAL-TARGET STRATEGY OF NUTRITION AND MICROBIOTA IN HIGH-RISK PATIENTS UNDERGOING TRANSPLANTATION

**Topic:** 23. Stem cell transplantation - Clinical

Shashali Shasha*[1]

[1]*Second Affiliated Hospital of Army Medical University, Blood Disease Medicine Center, Second Affiliated Hospital of Army Medical University, Chongqing, China;*

### Background aiming to validate whether this comprehensive intervention model can improve nutritional status, regulate intestinal microecology, thereby reducing transplantation-related complications and enhancing clinical outcomes.

### Aims

Objective To investigate the clinical feasibility and efficacy of precision nutritional support combined with targeted intestinal microbiota modulation in patients at high nutritional risk following hematopoietic stem cell transplantation (HSCT).

### Methods

This study adopted a prospective cohort design. A total of 80 patients who underwent allogeneic hematopoietic stem cell transplantation and were assessed as having high nutritional risk according to the NRS-2002 score were selected and randomly divided into an intervention group and a control group, with 40 cases in each group. The control group received standard enteral and parenteral nutritional support. The intervention group implemented a comprehensive intervention protocol: 1) Precision nutritional support: Based on indirect energy measurement, body composition analysis, and dynamic nutritional assessment, individualized caloric, protein, and micronutrient targets were formulated and adjusted, with priority given to the use of immunonutritional preparations (rich in glutamine, arginine, and ω-3 fatty acids) administered via nasogastric tube or orally; 2) Targeted modulation of gut microbiota: From the pretreatment period until 30 days post-transplantation, probiotics containing specific strains (e.g., Bifidobacterium, Lactobacillus) and prebiotics (fructooligosaccharides) were administered concurrently. The primary endpoint was the incidence of acute graft-versus-host disease (GVHD) grades III-IV, while secondary endpoints included the incidence of severe oral mucositis, blood infection rate, duration of antibiotic use, changes in nutritional parameters (serum albumin, prealbumin), and dynamic evolution of gut microbiota diversity.

### Results

Compared with the control group, the clinical outcomes of patients in the intervention group showed significant improvement. The incidence of grade III-IV aGVHD, the primary endpoint, was significantly reduced in the intervention group (15.0% vs. 32.5%, $P<0.05$). Regarding secondary endpoints: 1) The incidence of severe oral mucositis (27.5% vs. 47.5%) and bloodstream infection (22.5% vs. 42.5%) was significantly decreased ($P<0.05$); 2) Nutritional indicators recovered faster, with serum prealbumin levels at 30 days post-transplantation significantly higher than those in the control group ($P<0.01$); 3) Gut microbiota analysis revealed that the intervention group maintained better α-diversity and a higher relative abundance of beneficial bacteria such as Bifidobacterium in the early post-transplantation period. Although there was no statistically significant difference in non-relapse mortality and overall survival between the two groups, the intervention group showed an improving trend.

**Copyright Information:** (Online) ISSN: 2572-9241
©2026 The Author(s). HemaSphere is published by John Wiley & Sons Ltd on behalf of European Hematology Association. This is an open access Abstract Book distributed under the Attribution-NonCommercial-NoDerivs (CC BY-NC-ND), which allows third parties to download the articles and share them with others as long as they credit the author and the Abstract Book, but they cannot change the content in any way or use them commercially.
**Abstract Book Citations:** Authors, Title, HemaSphere, 2026;10:(S1):pages. The abstract book DOIs can be found on the "Author Information" page.
**Disclaimer:** Articles published in the journal HemaSphere exclusively reflect the opinions of the authors. The authors are responsible for all content in their abstracts including accuracy of the facts, statements, citing resources, etc.

**Summary/Conclusion**

In high-risk hematopoietic stem cell transplantation patients, an integrated intervention combining precision nutrition with gut microbiota modulation is safe and feasible. This approach improves nutritional status, maintains intestinal homeostasis, and reduces critical complications such as severe aGVHD and infections. These findings support targeting the "nutrition-microbiota" axis to enhance transplant outcomes, though larger multicenter studies are needed to confirm long-term benefits.

**Copyright Information:** (Online) ISSN: 2572-9241
©2026 The Author(s). HemaSphere is published by John Wiley & Sons Ltd on behalf of European Hematology Association. This is an open access Abstract Book distributed under the Attribution-NonCommercial-NoDerivs (CC BY-NC-ND), which allows third parties to download the articles and share them with others as long as they credit the author and the Abstract Book, but they cannot change the content in any way or use them commercially.
**Abstract Book Citations:** Authors, Title, HemaSphere, 2026;10:(S1):pages. The abstract book DOIs can be found on the "Author Information" page.
**Disclaimer:** Articles published in the journal HemaSphere exclusively reflect the opinions of the authors. The authors are responsible for all content in their abstracts including accuracy of the facts, statements, citing resources, etc.

### (PS2220) STEM CELL MOBILIZATION IMPAIRMENT AFTER DARATUMUMAB-BASED QUADRUPLET INDUCTION IN TRANSPLANT-ELIGIBLE NEWLY DIAGNOSED MULTIPLE MYELOMA: A MULTICENTER REAL-WORLD STUDY

**Topic:** 23. Stem cell transplantation - Clinical

Claudia Biosca*[1], Mireia Morgades[1], Carlos Quintero[2], Karla Guillermina Abad[3], Ulises Ferrandiz[1], Marc Bitlloch[1], Maria Alcalde[1], María José Jiménez[1], Merce Aren-Jubany[1], Gladys Virginia Ibarra Fernandez[1], Laura Abril Sabater[1], Itziar Carro Arostegui[2], Nadia García[2], Albert Oriol[1], Juan-Manuel Sancho Cia[1], Rosa Coll Jorda[3], Alberto Mussetti[2], Anna Torrent[1], María Huguet[1]

*[1]ICO - Hospital Universitari Germans Trias i Pujol, Hemathology, Badalona, Spain; [2]ICO-Hospital Universitari Duran i Reynals, Hospitalet de Llobregat, Spain; [3]ICO-Hospital Dr. Josep Trueta, Girona, Spain;*

### Background

While daratumumab-based quadruplet induction improves response depth in transplant-eligible newly diagnosed multiple myeloma (NDMM), its impact on stem cell mobilization in routine clinical practice remains uncertain.

### Aims

To evaluate the real-world effect of daratumumab-based induction on stem cell mobilization outcomes in transplant-eligible NDMM.

### Methods

We conducted a retrospective multicentre study including transplant-eligible NDMM patients undergoing first mobilization between April 2020 and January 2026. Patients were grouped by induction strategy: daratumumab-based quadruplets versus daratumumab-free triplets. Regimens included VRd (bortezomib, lenalidomide, dexamethasone) or VTd (bortezomib, thalidomide, dexamethasone) in the triplet group and daratumumab-VRd or daratumumab-VTd in the quadruplet group. Mobilization parameters and factors associated with plerixafor requirement were analysed.

### Results

A total of 229 patients were included (triplet=126; quadruplet=103). Daratumumab-based quadruplet induction was associated with significantly impaired mobilization, with lower circulating CD34+ counts (12 vs 22 cells/μL, $p<0.001$) and markedly higher plerixafor use (60% vs 23%, $p<0.001$). Platelet count and G-CSF dose distribution were comparable between groups. Among 91 patients requiring plerixafor, shorter lenalidomide washout ($p=0.015$) and lower platelet count ($p=0.001$) were associated with rescue mobilization.

In multivariable analysis (Table 2) including all 229 patients and adjusting for platelet count and G-CSF dose, daratumumab-based quadruplet induction independently predicted plerixafor requirement (OR 5.03, 95% CI 2.79–9.08; $p<0.001$). Platelet count also remained independently associated, whereas G-CSF dose did not. Lenalidomide washout was not included in the primary model as it was not applicable to thalidomide-based regimens, although shorter washout was associated with plerixafor use in exploratory analyses.

**Table 1.** Baseline characteristics and stem cell mobilization parameters according to daratumumab exposure.

**Copyright Information:** (Online) ISSN: 2572-9241
©2026 The Author(s). HemaSphere is published by John Wiley & Sons Ltd on behalf of European Hematology Association. This is an open access Abstract Book distributed under the Attribution-NonCommercial-NoDerivs (CC BY-NC-ND), which allows third parties to download the articles and share them with others as long as they credit the author and the Abstract Book, but they cannot change the content in any way or use them commercially.
**Abstract Book Citations:** Authors, Title, HemaSphere, 2026;10:(S1):pages. The abstract book DOIs can be found on the "Author Information" page.
**Disclaimer:** Articles published in the journal HemaSphere exclusively reflect the opinions of the authors. The authors are responsible for all content in their abstracts including accuracy of the facts, statements, citing resources, etc.

| | | Free-Dara Triplet (n=126) | Dara-Based Quadruplet (n=103) | P |
|---|---|---|---|---|
| **G-CSF dose** | 10µg/kg/24h | 20 (15.9%) | 31 (30.1%) | **0.010** |
| | 20µg/kg/24h | 106 (84.1%) | 72 (69.9%) | |
| **Platelet count, $\times 10^9$/L** | Median [range] | 256 [32 - 608] | 245 [73 - 610] | 0.624 |
| **Lenalidomide washout (days)** | Median [range] | 22 [2 - 194] | 17 [0 - 85] | 0.065 |
| **CD34+ cells pre-apheresis (cells/µL)** | Median [range] | 22 [2 -166] | 12 [0.6 - 68] | **<0.001** |
| **Plerixafor use** | No | 97 (77%) | 41 (39.8%) | **<0.001** |
| | Yes | 29 (23%) | 62 (60.2%) | |

**Table 2.** Multivariate logistic regression for Plerixafor requirement.

| | | N | OR (95% CI) | P |
|---|---|---|---|---|
| **1rst line induction** | **Daratumumab-based quadruplet** | 103 | 5.03 (2.79 – 9.08) | **<0.001** |
| | **Daratumumab-free triplet** | 126 | Reference | |
| **Platelet count (x109/L)** | | 229 | 0.96 (0.93 –0.99) | **0.018** |
| **G-CSF dose** | 10µg/kg/24h | 51 | Reference | 0.41 |
| | 20µg/kg/24h | 178 | 0.75 (0.37 - 1.50) | |

### Summary/Conclusion

In this large real-world multicentre cohort, daratumumab-based quadruplet induction was strongly associated with impaired stem cell mobilization, conferring a fivefold increased likelihood of plerixafor requirement independent of platelet count and G-CSF dose. Despite deeper responses, quadruplet-treated patients exhibited substantially higher rescue mobilization rates, underscoring the need for proactive mobilization planning and early consideration of pre-emptive strategies in the era of quadruplet induction.

**Copyright Information:** (Online) ISSN: 2572-9241
©2026 The Author(s). HemaSphere is published by John Wiley & Sons Ltd on behalf of European Hematology Association. This is an open access Abstract Book distributed under the Attribution-NonCommercial-NoDerivs (CC BY-NC-ND), which allows third parties to download the articles and share them with others as long as they credit the author and the Abstract Book, but they cannot change the content in any way or use them commercially.
**Abstract Book Citations:** Authors, Title, HemaSphere, 2026;10:(S1):pages. The abstract book DOIs can be found on the "Author Information" page.
**Disclaimer:** Articles published in the journal HemaSphere exclusively reflect the opinions of the authors. The authors are responsible for all content in their abstracts including accuracy of the facts, statements, citing resources, etc.

### (PS2221) DONOR-DERIVED DNMT3A MUTATIONS: SUBTYPE, CLONAL BURDEN AND TRANSPLANTATION OUTCOMES AFTER ALLOGENEIC HSCT

**Topic:** 23. Stem cell transplantation - Clinical

Jiawei Zhao*[1], Biao Shen[1], Yujiao Jia[1], Haoxu Wang[1], Bingqing Huang[1], Li Qin[1], Ruixue Zhang[1], Jiao Chang[1], Lijuan Zhang[1], Donglin Yang[1], Qi Sun[1], Erlie Jiang[1, 2], Lihong Shi[1, 2, 3, 4]

*[1]Institute of Hematology & Blood Diseases Hospital, Tian Jin, China;[2]National Clinical Research Center for Blood Diseases, Tian Jin, China; [3]State Key Laboratory of Experimental Hematology,, Tian Jin, China;[4]Haihe Laboratory of Cell Ecosystem, Tian Jin, China;*

### Background

DNMT3A mutations are among the most common drivers of clonal hematopoiesis and are increasingly detected in otherwise healthy stem cell donors. Whether different mutation subtypes or clonal burden influence post-transplant outcomes remains uncertain. We evaluated the clinical relevance of donor-derived DNMT3A mutations in a single-center cohort of allogeneic hematopoietic stem cell transplantation (HSCT).

### Aims

To evaluate the clinical impact of donor-derived DNMT3A mutations on outcomes after allogeneic hematopoietic stem cell transplantation, with particular attention to mutation subtype and clonal burden.

### Methods

Donor–recipient pairs undergoing allogeneic HSCT between 2020 and 2024 were retrospectively analyzed. Donor samples were screened for DNMT3A mutations using next-generation sequencing. Mutations were categorized by pathogenicity into class 1 (pathogenic) and class 2 (likely pathogenic) and stratified according to VAF. Transplant outcomes including engraftment, acute and chronic GVHD, relapse, non-relapse mortality, and survival were assessed and compared according to mutation status, subtype, and clonal burden.

### Results

DNMT3A mutations were detected in 108 of 2,315 screened donors, and 77 mutation-positive individuals proceeded to donation. Most variants showed low clonal burden, with a median VAF of 2.3%. Single-mutation carriers accounted for the majority of donors, and class 2 variants were more frequent than class 1. The distribution of mutation sites demonstrated typical clustering in exon 23 and exon 19, including 15 R882 substitutions.Baseline characteristics of recipients were comparable between DNMT3A-mutated and wild-type donor groups. Neutrophil and platelet engraftment occurred within similar timeframes, and no delays in hematopoietic recovery were observed in recipients of mutation-positive grafts. The incidence of aGVHD and cGVHD did not differ by donor mutation status. When analyzed by mutation subtype, class 1 carriers showed a lower rate of cGVHD compared with both wild-type and class 2 donors, whereas aGVHD rates remained similar across groups.Relapse and non-relapse mortality were comparable between DNMT3A-mutated and wild-type donors in competing-risk analyses. Overall survival and relapse-free survival did not differ significantly by mutation status. When stratified by pathogenicity class, class 2 mutations were associated with longer OS and RFS compared with both wild-type and class 1 donors, while class 1 mutations showed no clear survival advantage. VAF did not influence outcomes within class 1 mutations, whereas class 2 mutations demonstrated a trend toward worse survival with increasing clonal burden. Comparisons between class 1 and class 2 variants did not reveal a uniform pattern of adverse clinical outcomes.

**Copyright Information:** (Online) ISSN: 2572-9241
©2026 The Author(s). HemaSphere is published by John Wiley & Sons Ltd on behalf of European Hematology Association. This is an open access Abstract Book distributed under the Attribution-NonCommercial-NoDerivs (CC BY-NC-ND), which allows third parties to download the articles and share them with others as long as they credit the author and the Abstract Book, but they cannot change the content in any way or use them commercially.
**Abstract Book Citations:** Authors, Title, HemaSphere, 2026;10:(S1):pages. The abstract book DOIs can be found on the "Author Information" page.
**Disclaimer:** Articles published in the journal HemaSphere exclusively reflect the opinions of the authors. The authors are responsible for all content in their abstracts including accuracy of the facts, statements, citing resources, etc.

**Summary/Conclusion**

In this real-world cohort, donor DNMT3A-CH did not increase relapse, non-relapse mortality, or GVHD after allo-HSCT. Survival differed by mutation subtype and clonal burden: class 2 variants were associated with better outcomes than both wild-type donors and class 1 mutations. VAF had little effect within class 1 mutations, whereas class 2 mutations showed a modest tendency toward worse survival with larger clone size. These findings suggest biologically distinct behaviors across DNMT3A mutation classes and a subtype-specific influence of clonal burden. Incorporating both mutation subtype and VAF into donor evaluation may provide a more accurate framework than excluding donors based solely on DNMT3A-CH.

**Copyright Information:** (Online) ISSN: 2572-9241
©2026 The Author(s). HemaSphere is published by John Wiley & Sons Ltd on behalf of European Hematology Association. This is an open access Abstract Book distributed under the Attribution-NonCommercial-NoDerivs (CC BY-NC-ND), which allows third parties to download the articles and share them with others as long as they credit the author and the Abstract Book, but they cannot change the content in any way or use them commercially.
**Abstract Book Citations:** Authors, Title, HemaSphere, 2026;10:(S1):pages. The abstract book DOIs can be found on the “Author Information” page.
**Disclaimer:** Articles published in the journal HemaSphere exclusively reflect the opinions of the authors. The authors are responsible for all content in their abstracts including accuracy of the facts, statements, citing resources, etc.

### (PS2222) DOSE OPTIMIZATION OF ANTI-T LYMPHOCYTE GLOBULIN IN HAPLOIDENTICAL HSCT: A PROSPECTIVE RANDOMIZED NON-INFERIORITY TRIAL

**Topic:** 23. Stem cell transplantation - Clinical

Yigeng Cao*[1], Rongli Zhang[1], Jialin Wei[1], Liu Xiaoliang[2], Li Huang[3], Jianmin Zhang[4], Qiaoling MA[1], Weihua Zhai[1], Wenbin Cao[1], Xin Chen[1], Chen Liang[1], Donglin Yang[1], Aiming Pang[1], Shuangnian Xu[4], Yi He[1], Xiangjun Fu[3], Hai Yi[5], Sujun Gao[2], Erlie Jiang[1]

*[1]Institute of Hematology & Blood Diseases Hospital, National Clinical Research Center for Blood Diseases, Chinese Academy of Medical Sciences & Peking Union Medical College, Hematopoietic Stem Cell Transplantation Center, Tianjin, China; [2]The First Hospital of Jilin University, Changchun, Jilin, China; [3]Hainan Provincial People's Hospital, Hainan, China;[4]Southwest Hospital, Third Military Medical University (Army Medical University), Chongqing, China; [5]People's Liberation Army The General Hospital of Western Theater Command, Chengdu, Sichuan, China;*

### Background

Anti-T lymphocyte globulin (ATLG) is one key component of GVHD prophylaxis in haploidentical hematopoietic stem cell transplantation (haplo-HSCT). While 30 mg/kg is widely used in Europe, 20 mg/kg is frequently adopted in Asia. However, no prospective randomized trial has directly compared these two dosing strategies. This study aimed to determine whether reduced-dose ATLG is non-inferior in preventing clinically significant acute GVHD while preserving immune recovery.

### Aims

To determine whether 20 mg/kg ATLG is non-inferior to 30 mg/kg for preventing grade II–IV acute GVHD after haploidentical HSCT, and to compare immune reconstitution and safety between doses.

### Methods

This multicenter, prospective, randomized, parallel-group non-inferiority trial (NCT06525519) enrolled patients aged 16–65 years with AML, ALL, or MDS undergoing haplo-HSCT. Patients were randomized 1:1 to receive 20 mg/kg or 30 mg/kg ATLG in combination with uniform myeloablative conditioning and standard GVHD prophylaxis consisting of a calcineurin inhibitor (CNI) plus short-course methotrexate (MTX).
The primary endpoint was the cumulative incidence of grade II–IV acute GVHD (aGVHD) within 100 days. A pre-specified absolute non-inferiority margin of 5% was defined. Competing-risk methodology was applied.

**Copyright Information:** (Online) ISSN: 2572-9241
©2026 The Author(s). HemaSphere is published by John Wiley & Sons Ltd on behalf of European Hematology Association. This is an open access Abstract Book distributed under the Attribution-NonCommercial-NoDerivs (CC BY-NC-ND), which allows third parties to download the articles and share them with others as long as they credit the author and the Abstract Book, but they cannot change the content in any way or use them commercially.
**Abstract Book Citations:** Authors, Title, HemaSphere, 2026;10:(S1):pages. The abstract book DOIs can be found on the "Author Information" page.
**Disclaimer:** Articles published in the journal HemaSphere exclusively reflect the opinions of the authors. The authors are responsible for all content in their abstracts including accuracy of the facts, statements, citing resources, etc.

### Results

A total of 105 patients were randomized (20 mg/kg: n=53; 30 mg/kg: n=52). Baseline characteristics were well balanced between groups.
By day 100 post-transplant, grade II–IV acute GVHD occurred in 10% of patients receiving 20 mg/kg ATLG and 8.2% of those receiving 30 mg/kg (P>0.9). Grade III–IV acute GVHD was observed in 10% versus 2.0% of patients, respectively (P=0.2). No statistically significant difference in cumulative incidence of acute GVHD was detected between the two dose groups.
Neutrophil engraftment occurred at a median of 18.0 days in the 20 mg/kg group and 19.0 days in the 30 mg/kg group (P=0.8), while platelet engraftment was achieved at 18.0 and 20.0 days, respectively (P=0.13).
Within the first 100 days, EBV reactivation occurred in 9.4% of patients receiving 20 mg/kg and 9.6% of those receiving 30 mg/kg (P>0.9). CMV reactivation was observed in 1.9% versus 5.8% of patients, respectively (P=0.4).
Adverse events were comparable (77.4% vs 80.8%). Grade ≥3 events occurred in 17.0% vs 25.0%. Serious adverse events were rare (3.8% vs 1.9%), and no ATLG-related treatment discontinuations occurred .
Seven deaths were reported (2 vs 5), primarily infection-related.
Early immune reconstitution differed significantly between dose groups. At day 21 post-transplant, the 20 mg/kg group demonstrated higher proportions of CD3+ (P<0.0001), CD4+ (P<0.001), and CD8+ T cells (P<0.001) compared with the 30 mg/kg group. Activated T cells were also significantly increased at days 14, 21, and 30 (P<0.01 to P<0.05), accompanied by higher regulatory T-cell proportions at early time points.
These differences diminished by day 60–100, indicating accelerated early immune recovery without persistent immune imbalance.

### Summary/Conclusion

In this first prospective randomized comparison of ATLG dosing in haploidentical HSCT, 20 mg/kg demonstrated comparable efficacy to 30 mg/kg for prevention of clinically significant aGVHD, without compromise in engraftment or safety.
These findings support a dose-de-escalation strategy for ATLG in haploidentical transplantation and warrant further validation in larger cohorts.

**Copyright Information:** (Online) ISSN: 2572-9241
©2026 The Author(s). HemaSphere is published by John Wiley & Sons Ltd on behalf of European Hematology Association. This is an open access Abstract Book distributed under the Attribution-NonCommercial-NoDerivs (CC BY-NC-ND), which allows third parties to download the articles and share them with others as long as they credit the author and the Abstract Book, but they cannot change the content in any way or use them commercially.
**Abstract Book Citations:** Authors, Title, HemaSphere, 2026;10:(S1):pages. The abstract book DOIs can be found on the "Author Information" page.
**Disclaimer:** Articles published in the journal HemaSphere exclusively reflect the opinions of the authors. The authors are responsible for all content in their abstracts including accuracy of the facts, statements, citing resources, etc.

### (PS2223) CD7 CAR-T THERAPY AS A BRIDGE TO ALLO-HSCT IN R/R T-ALL/LBL: POST-TRANSPLANT PERSISTENCE OF DONOR-DERIVED CAR-T CELLS PREDICTS POOR OUTCOME

**Topic:** 23. Stem cell transplantation - Clinical

Zhihui Li*[1], Qinlong Zheng[2], Keyan Yang[2], Xianxuan Wang[1], Jing Li[1], Qian Fei[1], Jiahong Zhai[1], Zongyang Ming[1], Xiaopei Wen[1], Lei Wang[1], Tong Wu[1]

*[1]Department of Bone Marrow Transplantation，Beijing Gobroad Boren Hospital, China, Beijing, China;[2]Department of Molecular Diagnostic Laboratory，Beijing Gobroad Boren Hospital, China, Beijing, China;*

### Background

Refractory/relapsed T-cell acute lymphoblastic leukemia/lymphoma (r/r T-ALL/LBL) has a dismal prognosis, with salvage allogeneic hematopoietic stem cell transplantation (allo-HSCT) achieving only 20%-30% disease-free survival (DFS). Although CD7 chimeric antigen receptor T-cell (CAR-T) therapy shows initial efficacy, responses are often transient. Only patients subsequently bridged to allo-HSCT appear to attain superior long-term outcomes.

### Aims

To evaluate the long-term survival and immune recovery after CD7 CAR-T bridging to allo-HSCT in patients with r/r T-ALL/LBL, compared to transplantation in chemotherapy-sensitive remission (CR) or chemotherapy-refractory non-remission (NR) states.

### Methods

We retrospectively analyzed 138 consecutive patients with r/r T-ALL/LBL who underwent allo-HSCT at our center between February 2018 and August 2024. Median age was 13.5 years (range, 2-66). Patients were categorized into three groups based on pre-transplant disease status and treatment: 45 (32.6%) achieved CR with chemotherapy (CR group); 93 (67.4%) were chemotherapy-refractory and in NR pre-HSCT. Among these 93 NR patients, 73 received CD7 CAR-T therapy as a bridge to transplant (CART group), while the remaining 20 proceeded directly to salvage transplant (NR group). Donors were haploidentical (63.8%), unrelated (27.5%), or identical siblings (8.7%). Conditioning was myeloablative, primarily TBI/fludarabine (58.7%) or busulfan/fludarabine (31.9%). Anti-thymocyte globulin was used for haploidentical and unrelated transplants.

**Copyright Information:** (Online) ISSN: 2572-9241
©2026 The Author(s). HemaSphere is published by John Wiley & Sons Ltd on behalf of European Hematology Association. This is an open access Abstract Book distributed under the Attribution-NonCommercial-NoDerivs (CC BY-NC-ND), which allows third parties to download the articles and share them with others as long as they credit the author and the Abstract Book, but they cannot change the content in any way or use them commercially.
**Abstract Book Citations:** Authors, Title, HemaSphere, 2026;10:(S1):pages. The abstract book DOIs can be found on the “Author Information” page.
**Disclaimer:** Articles published in the journal HemaSphere exclusively reflect the opinions of the authors. The authors are responsible for all content in their abstracts including accuracy of the facts, statements, citing resources, etc.

### Results

With a median follow-up of 23.9 months (95%CI:19.6-29.9), the CART group demonstrated a 2-year cumulative incidence of relapse (CIR) of 20.2% (95%CI:12.5-31.8%), significantly lower than the NR group (64.0%; 95%CI:42.5-84.8%; p=0.0011) and comparable to the CR group (25.8%; 95%CI:15.1-41.8%). Non-relapse mortality (NRM) was 19.8% (95%CI:12.0-31.8%) in the CART group, with no significant difference across groups (p=0.176). The CART group achieved superior 2-year DFS (60.0%; 95%CI:49.2-73.1%) and OS (60.4%; 95%CI:49.3-74.1%) compared to the NR group (DFS:21.0%, p=0.00053; OS:37.5%, p=0.013), and outcomes comparable to the CR group (DFS:67.1%; OS:75.0%).
Incidence of II-IV acute GVHD (p=0.75) and chronic GVHD (p=0.58) was similar across groups. However, bacterial infections were significantly higher in the CART group (69.0%) vs. NR (35.0%) and CR (53.3%) groups (p=0.004). No differences in other infections or early immune recovery parameters (WBC, lymphocyte counts, IgG) were observed at +1 month post-HSCT.
Among CART patients, detectable CAR-T cells post-transplant occurred exclusively in recipients of donor-derived CAR-T (100% vs. 0% in Auto-CART, p=0.006) and was associated with a lower fungal infection rate (0% vs. 33.3%, p=0.038). Notably, post-transplant CAR-T positivity was linked to inferior DFS (median 7.3 vs. not reached, p=0.037) and a trend toward inferior OS (p=0.089). Patients with dual positivity for both CAR-T and MRD post-transplant had a significantly increased risk of relapse (DFS: HR=8.0, p=0.004).

### Summary/Conclusion

CD7 CAR-T therapy is an effective bridge to allo-HSCT for chemotherapy-resistant T-ALL/LBL, yielding durable survival outcomes comparable to chemosensitive patients, despite a higher risk of infections. Post-transplant persistence of donor-derived CD7 CAR-T cells, however, identifies a subset with a significantly worse prognosis and may serve as a novel biomarker for impending relapse, complementing MRD monitoring.

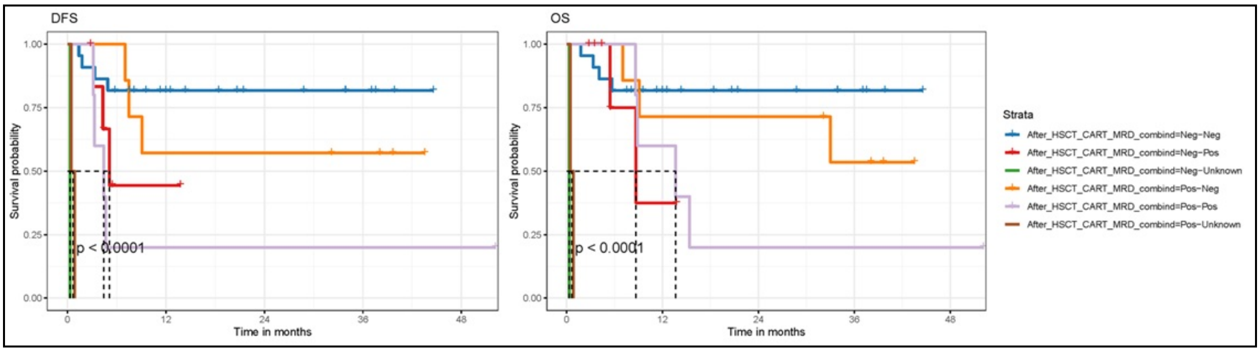

Post-transplantMRDandCAR-TmonitoringdoublepositivesignificantlyincreasetheriskofOSandDFSinpatients

**Copyright Information:** (Online) ISSN: 2572-9241
©2026 The Author(s). HemaSphere is published by John Wiley & Sons Ltd on behalf of European Hematology Association. This is an open access Abstract Book distributed under the Attribution-NonCommercial-NoDerivs (CC BY-NC-ND), which allows third parties to download the articles and share them with others as long as they credit the author and the Abstract Book, but they cannot change the content in any way or use them commercially.
**Abstract Book Citations:** Authors, Title, HemaSphere, 2026;10:(S1):pages. The abstract book DOIs can be found on the “Author Information” page.
**Disclaimer:** Articles published in the journal HemaSphere exclusively reflect the opinions of the authors. The authors are responsible for all content in their abstracts including accuracy of the facts, statements, citing resources, etc.

### (PS2224) REDUCED-DOSE ANTI-THYMOCYTE GLOBULIN PLUS MINI-DOSE POST-TRANSPLANT CYCLOPHOSPHAMIDE FOR GVHD PREVENTION IN HAPLOIDENTICAL DONOR HCT FOR HEMATOLOGIC MALIGNANCY

**Topic:** 23. Stem cell transplantation - Clinical

Yuan-Yuan Zhang*[1, 1]

[1] *Peking University People's Hospital, Peking University Institute of Hematology, National Clinical Research Center for Hematologic Disease, Beijing Key Laboratory of Hematopoietic Stem Cell Transplantation, Collaborative Innovation Center of Hematology, Peking University, BEIJING, China;*

### Background

Using anti-thymocyte globulin (ATG) together with low-dose post-transplant cyclophosphamide (PTCy) is emerging as a potentially valuable approach for graft-versus-host disease (GVHD) prophylaxis in haploidentical stem cell transplantation.

### Aims

The aim of our study was to determine whether the low-dose PTCy (14.5 mg/kg on days 3 and 4) in ATG7.5mg/kg/granulocyte colony-stimulating factor (G-CSF)-based regimen  regimen can reduce the toxicity and infection without increasing the incidence of GVHD.

### Methods

We extended our prospective study in patients with AML or MDS  treated with low-dose PTCy (14.5 mg/kg on days 3 and 4) and ATG 7.5mg/kg/granulocyte colony-stimulating factor (G-CSF)-based regimen and compared the results to the contemporary cohort of patients with ATG 10mg/kg  (ATG cohort).

### Results

We identified 128 consecutive patients (ATG7.5mg-PTCy cohort = 47; ATG 10mg cohort = 81). All patients but one in ATG7.5mg-PTCy cohort achieved myeloid engraftment by day 30 post-HCT.The 100-day cumulative incidence of grade 2-4 aGVHD in ATG7.5mg-PTCy cohort was higher than ATG 10mg cohort (44%95% CI29.49%-58.50% *vs*.25.50%95% CI14.91%-36.08*P*=0.015and  3-4 acute GVHD (6.5%95% CI0.00%-15.32% vs.5.2%95% CI0.00%-10.88%) were comparable.The 1-year incidence of chronic GVHD was 41.7% (27.7-5564) and 46.3% (35.3-57.3) in the 7.5 mg and 10.0 mg groups (P=0.685). The 1-year incidence of CMV and EBV infection  was comparable between the two groups.

### Summary/Conclusion

These results suggested that  ATG7.5mg/kg plus low-dose PTCy regimen demonstrated no advantage over the standard ATG10mg/kg regimen in terms of GVHD prophylaxis. The incidence of acute GVHD was not reduced, and there was no decrease in CMVand EBV infection . These findings suggest that the ATG10mg/kg regimen remains the preferable option.

**Copyright Information:** (Online) ISSN: 2572-9241
©2026 The Author(s). HemaSphere is published by John Wiley & Sons Ltd on behalf of European Hematology Association. This is an open access Abstract Book distributed under the Attribution-NonCommercial-NoDerivs (CC BY-NC-ND), which allows third parties to download the articles and share them with others as long as they credit the author and the Abstract Book, but they cannot change the content in any way or use them commercially.
**Abstract Book Citations:** Authors, Title, HemaSphere, 2026;10:(S1):pages. The abstract book DOIs can be found on the "Author Information" page.
**Disclaimer:** Articles published in the journal HemaSphere exclusively reflect the opinions of the authors. The authors are responsible for all content in their abstracts including accuracy of the facts, statements, citing resources, etc.

### (PS2225) MATCHED SIBLING DONOR TRANSPLANTATION FOR THALASSEMIA MAJOR: A LONG-TERM STUDY EVALUATING ATG FORMULATIONS AND THIOTEPA IN THE CONDITIONING REGIMEN

**Topic:** 23. Stem cell transplantation - Clinical

Qihong Lin*[1], Sixi Liu[1]

[1]*Shenzhen Children's Hospital, SHEN ZHEN, China;*

### Background

Matched sibling donor hematopoietic stem cell transplantation (MSD-HSCT) is curative for thalassemia major (TM). While conditioning regimens have evolved beyond traditional busulfan-cyclophosphamide (BuCy), the impact of specific immunomodulatory components requires further evaluation.

### Aims

This study aimed to evaluate the long-term outcomes of MSD-HSCT using a busulfan-cyclophosphamide-fludarabine (BuCyFlu) based conditioning regimen in children with TM, and to analyze the effects of different anti-thymocyte globulin (ATG) formulations (Fresenius [ATG-F] vs. Thymoglobulin [ATG-T]) and the addition of thiotepa (TT).

### Methods

We retrospectively analyzed 137 consecutive TM patients who underwent MSD-HSCT between January 2012 and December 2021. All patients received a BuCyFlu-based conditioning regimen. GVHD prophylaxis consisted of ATG (ATG-F or ATG-T), cyclosporine A, and short-course methotrexate. Primary endpoints were overall survival (OS) and event-free survival (EFS). Secondary endpoints included engraftment, GVHD, and transplant-related complications.

### Results

With a median follow-up of 7.3 years (range: 4.0–13.9), the estimated 15-year OS and EFS were 99.2% and 98.5%, respectively. Engraftment was rapid, with median times to neutrophil, platelet, and hemoglobin recovery of 16, 13, and 10 days, respectively. SOS occurred in only one patient (0.7%). No cases of obliterative bronchiolitis or heart failure were observed. The cumulative incidences of acute GVHD (aGVHD) and chronic GVHD (cGVHD) were 5.8% and 8.0%, respectively, with severe (grade III-IV) aGVHD in only 1.4% and no extensive cGVHD. Female donor to male recipient was associated with higher aGVHD risk (P=0.044). Common complications included viral reactivations (CMV: 21.9%; EBV: 19.0%), BK virus-associated hemorrhagic cystitis (13.9%), and post-transplant lymphoproliferative disorder (6.0%). Comparative analyses revealed no significant differences between ATG-F (n=35) and ATG-T (n=102) in GVHD or viral reactivation rates. However, the addition of thiotepa (TT group, n=98) was associated with a lower incidence of grade III-IV aGVHD (0% vs. 5.1% in non-TT group [n=39], P=0.02) but a higher incidence of cGVHD (11.2% vs. 0%, P=0.034).

### Summary/Conclusion

MSD-HSCT with a BuCyFlu-based regimen provides outstanding long-term survival for children with TM, with a low incidence of severe complications. Within this highly effective protocol, the choice of ATG formulation did not significantly affect outcomes. The addition of thiotepa modulated GVHD risk, reducing severe acute forms but increasing chronic GVHD, highlighting the need for nuanced conditioning strategy design based on specific risk profiles.

**Copyright Information:** (Online) ISSN: 2572-9241
©2026 The Author(s). HemaSphere is published by John Wiley & Sons Ltd on behalf of European Hematology Association. This is an open access Abstract Book distributed under the Attribution-NonCommercial-NoDerivs (CC BY-NC-ND), which allows third parties to download the articles and share them with others as long as they credit the author and the Abstract Book, but they cannot change the content in any way or use them commercially.
**Abstract Book Citations:** Authors, Title, HemaSphere, 2026;10:(S1):pages. The abstract book DOIs can be found on the "Author Information" page.
**Disclaimer:** Articles published in the journal HemaSphere exclusively reflect the opinions of the authors. The authors are responsible for all content in their abstracts including accuracy of the facts, statements, citing resources, etc.

### (PS2226) REAL-WORLD USE OF POST-TRANSPLANT CYCLOPHOSPHAMIDE FOR GRAFT-VERSUS-HOST DISEASE PROPHYLAXIS AFTER ALLOGENEIC HEMATOPOIETIC STEM CELL TRANSPLANTATION: A SURVEY FROM THE CTIWP OF EBMT

**Topic:** 23. Stem cell transplantation - Clinical

Shlomo Elias*[1], Isabel Sánchez-Ortega[2], Juana Schwartz[3], Mohsen Alzahrani[4], Tobias Gedde-Dahl[5], Grzegorz Helbig[6], Jakob Passweg[7], Raynier Devillier[8], Peter von Dem Borne[9], Igor Wolfgang Blau[10], Henrik Sengeløv[11], Kavita Raj[12], Catherine Flynn[13], Robert Zeiser[14], Edouard Forcade[15], Anoek Broers[16], Stephan Mielke[17], Emma Nicholson[18], Hélène Labussiere Wallet[19], Fabio Ciceri[20], Anne Sirvent[21], Lucía López Corral[22], Linde Morsink[23], Mohamad Mohty[24], Giorgia Battipaglia[25], Jeroen Knippenberg[3], Jorinde Hoogenboom[3], Jurgen Kuball[26], Florent Malard[27], Arnon Nagler[28], Annalisa Ruggeri[29]

*[1]Hebrew Univeristy Hadassah Medical Center, Department of Bone Marrow Transplantation and Cancer Immunotherapy, Jerusalem, Israel; [2]EBMT Executive Office, Barcelona, Spain; [3]EBMT Leiden Study Unit, Leiden, The Netherlands; [4]King Abdul-Aziz Medical City, Riyadh, Saudi Arabia; [5]Oslo University Hospital, Oslo, Norway; [6]Medical University of Silesia, Katowice, Poland; [7]University Hospital Basel, Basel, Switzerland; [8]Programme de Transplantation et Thérapie Cellulaire, Marseille, France; [9]Leiden University Medical Center, Leiden, The Netherlands; [10]Charité – Universitätsmedizin, Berlin, Germany; [11]Rigshospitalet, Copenhagen, Denmark; [12]University College London Hospital, London, United Kingdom; [13]St. James's Hospital, Dublin, Ireland; [14]University of Freiburg, Freiburg, Germany; [15]CHU Bordeaux, Hôpital Haut-Lévêque, Pessac, France; [16]Erasmus MC Cancer Institute, Rotterdam, The Netherlands; [17]Karolinska University Hospital, Stockholm, Sweden; [18]Royal Marsden Hospital, London, United Kingdom; [19]Centre Hospitalier Lyon Sud, Pierre-Bénite, France; [20]IRCCS San Raffaele Hospital Scientific Institute, Milan, Italy; [21]CHU Lapeyronie, Montpellier, France; [22]Hospital Clínico, Salamanca, Spain; [23]University Medical Center Groningen (UMCG), Groningen, The Netherlands; [24]Hôpital Saint-Antoine, Paris, France; [25]Federico II University of Naples, Naples, Italy; [26]University Medical Centre Utrecht, Utrecht, The Netherlands; [27]Sorbonne Université, Hôpital Saint-Antoine, Paris, France; [28]Chaim Sheba Medical Center, Ramat Gan, Israel; [29]San Raffaele Scientific Institute, Milan, Italy;*

### Background

Post-transplant cyclophosphamide (PTCy) is a key platform for graft-versus-host disease (GVHD) prophylaxis in allogeneic hematopoietic stem cell transplantation (allo-HSCT). Initially developed for haploidentical transplantation, its use has expanded to matched sibling and unrelated donor settings. However, important aspects of PTCy implementation, including dosing, dose modifications, supportive care, and combination strategies, remain insufficiently standardized.

### Aims

To characterize real-world practice patterns regarding PTCy use for GVHD prophylaxis across European Society for Blood and Marrow Transplantation (EBMT) centers and to identify areas of consensus and heterogeneity.

### Methods

We conducted a cross-sectional, web-based survey among CTIWP-EBMT- centers. The questionnaire addressed institutional guidance, donor-type-specific use, timing and dosing of PTCy, dose reduction and omission criteria, dose adjustment in overweight or obese patients, cardiac monitoring practices, combination with anti-thymocyte globulin (ATG), adjunct GVHD prophylaxis regimens, calcineurin inhibitor (CNI) target levels, and timing of additional immunosuppression.

**Copyright Information:** (Online) ISSN: 2572-9241
©2026 The Author(s). HemaSphere is published by John Wiley & Sons Ltd on behalf of European Hematology Association. This is an open access Abstract Book distributed under the Attribution-NonCommercial-NoDerivs (CC BY-NC-ND), which allows third parties to download the articles and share them with others as long as they credit the author and the Abstract Book, but they cannot change the content in any way or use them commercially.
**Abstract Book Citations:** Authors, Title, HemaSphere, 2026;10:(S1):pages. The abstract book DOIs can be found on the "Author Information" page.
**Disclaimer:** Articles published in the journal HemaSphere exclusively reflect the opinions of the authors. The authors are responsible for all content in their abstracts including accuracy of the facts, statements, citing resources, etc.

### Results

All EBMT centers were contacted, and 193 responded (**Figure 1A).** Institutional guidance for PTCy use was reported by 84% of centers, while 16% relied on individual physician discretion. PTCy was most commonly used in haploidentical (97%) and mismatched unrelated donor transplantation (71%), although 22% of centers reported use across all major donor types (**Figure 1B**). The predominant PTCY administration schedule was on Days +3/+4 (84%), followed by Days +3/+5 (12%) (**Figure 1C**). The most common standard dose was 50 mg/kg administered on two consecutive days (74%), followed by 40 mg/kg ×2 (12%) (**Figure 1D**). PTCy dose reduction was reported by 78% of centers, primarily driven by cardiac comorbidity and/or older age; the median age threshold for dose reduction was 65 years. Among centers implementing dose reduction (n=150), the most frequently used reduced dose was 40 mg/kg ×2 (57%), followed by 30 mg/kg ×2 (25%), 35 mg/kg ×2 (21%), and 25 mg/kg ×2 (17%), with several centers reporting more than one reduced-dose strategy (**Figure 1E**). Complete omission of PTCy was uncommon and occurred mainly in the context of cardiac comorbidity (41%).

Approaches to dose adjustment in overweight or obese patients and cardiac monitoring practices were highly heterogeneous. Only 18% of centers reported that disease status influenced PTCy use. One-third of centers (33%) combined PTCy with ATG, using Thymoglobulin and Grafalon at similar frequencies. Cyclosporine plus mycophenolate mofetil (58%) and tacrolimus plus mycophenolate mofetil (43%) were the predominant adjunct prophylaxis strategies. Most centers (85%) targeted standard CNI levels, and additional immunosuppression was most frequently initiated on Day +5 (78%) (**Figure 1F**).

### Summary/Conclusion

This large international EBMT survey demonstrates widespread adoption of PTCy for GVHD prophylaxis across different donor types, but reveals substantial heterogeneity in dosing strategies, dose modification, supportive care, and combination approaches. These findings highlight the need for harmonized guidance and prospective studies to optimize and standardize PTCy-based GVHD prophylaxis across donor settings.

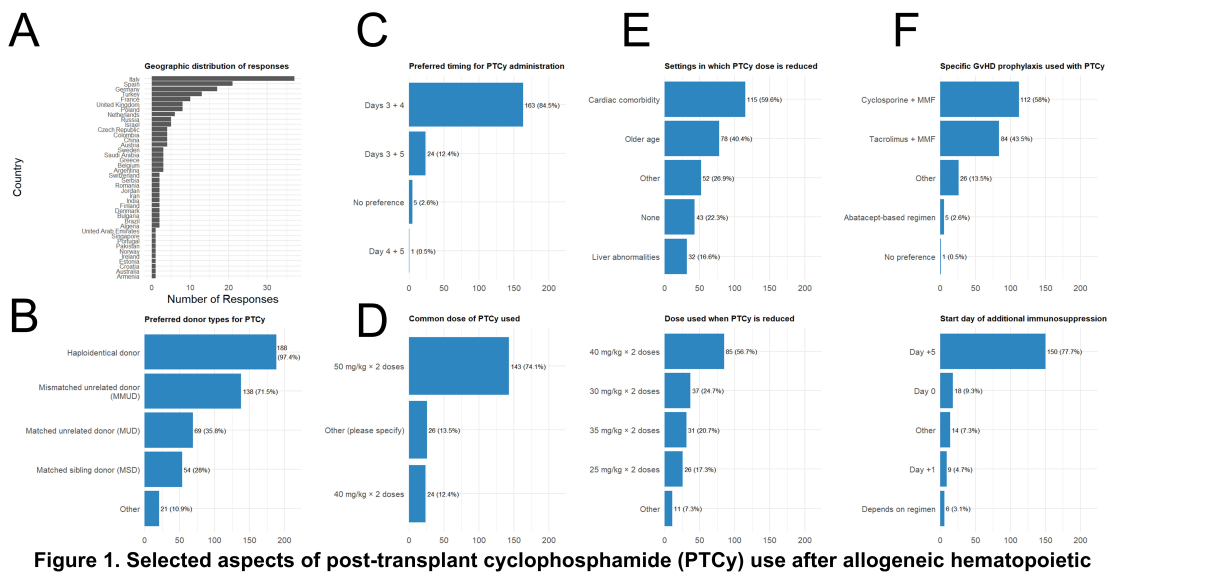

**Figure 1. Selected aspects of post-transplant cyclophosphamide (PTCy) use after allogeneic hematopoietic stem cell transplantation.** (A) Geographic distribution of responding centers; (B) donor-type–specific use of PTCy; (C) preferred timing of PTCy administration; (D) standard dosing strategies for PTCy; (E) clinical settings prompting PTCy dose reduction and corresponding reduced-dose levels used; and (F) adjunct immunosuppressive therapies used in combination with PTCy, including type of immunosuppression and timing of initiation.

**Copyright Information:** (Online) ISSN: 2572-9241
©2026 The Author(s). HemaSphere is published by John Wiley & Sons Ltd on behalf of European Hematology Association. This is an open access Abstract Book distributed under the Attribution-NonCommercial-NoDerivs (CC BY-NC-ND), which allows third parties to download the articles and share them with others as long as they credit the author and the Abstract Book, but they cannot change the content in any way or use them commercially.
**Abstract Book Citations:** Authors, Title, HemaSphere, 2026;10:(S1):pages. The abstract book DOIs can be found on the "Author Information" page.
**Disclaimer:** Articles published in the journal HemaSphere exclusively reflect the opinions of the authors. The authors are responsible for all content in their abstracts including accuracy of the facts, statements, citing resources, etc.

### (PS2228) EARLY CHEMO INDUCED-CARDIOTOXICITY DETECTION AFTER HEMATOPOIETIC STEM CELL TRANSPLANTATION

**Topic:** 23. Stem cell transplantation - Clinical

Jiexian MA*[1], Xinyu Zuo[1]

[1]*Huadong Hospital, Fudan University, Shanghai, China;*

### Background

High-dose chemotherapy followed by autologous hematopoietic stem cell transplantation (auto-HSCT) is a critical curative modality for lymphoma. While mitoxantrone-based conditioning regimens have demonstrated tolerability and survival benefits over traditional BEAM regimens, the dose-dependent cardiotoxicity remains a significant clinical concern. Liposomal mitoxantrone is hypothesized to offer a superior cardiac safety profile; however, comparative studies evaluating its cardiotoxicity and efficacy in the auto-HSCT setting are limited.

### Aims

This study aimed to evaluate and compare the cardiotoxic profiles and therapeutic efficacy of conventional mitoxantrone versus liposomal mitoxantrone as conditioning regimens for lymphoma patients undergoing auto-HSCT, utilizing a translational approach from a pre-clinical murine model to a prospective clinical trial.

### Methods

The study was conducted in two phases. In the pre-clinical phase, mice were administered human HSCT-equivalent doses of either conventional or liposomal mitoxantrone to compare serological cardiotoxicity biomarkers (CK, LDH) and perform cardiomyocyte pathological examinations. Subsequently, a prospective clinical trial (ChiCTR2500099269) was conducted from January 2024 to February 2026. A total of 65 lymphoma patients undergoing auto-HSCT were enrolled and assigned to receive either conventional mitoxantrone (60 mg/m², n=32) or liposomal mitoxantrone (60 mg/m², n=33) as part of their conditioning regimen. Comprehensive cardiac assessments, including 3D echocardiography-derived myocardial work indices (such as Global Longitudinal Strain, GLS) and serum biomarkers (Troponin T, CK-MB, BNP, LDH), were performed at baseline and 4 weeks post-chemotherapy. Multivariable logistic regression analysis was utilized to identify independent predictors of chemotherapy-induced cardiotoxicity.

### Results

In the pre-clinical model, liposomal mitoxantrone demonstrated significantly reduced myocardial structural damage and lower biomarker elevation compared to conventional mitoxantrone. In the clinical cohort, the liposomal mitoxantrone group exhibited significantly lower post-HSCT fluctuations in cardiotoxicity biomarkers. Clinically, 3 patients (9.4%) in the conventional mitoxantrone group developed severe cardiovascular events (heart failure or severe atrial fibrillation) post-HSCT, whereas no such events (0%) occurred in the liposomal group. Both regimens demonstrated comparable antineoplastic efficacy and engraftment success in auto-HSCT. Logistic regression analysis identified a post-HSCT GLS decrease of >10% and elevated serum levels of LDH and TnT as the strongest independent predictors for the onset of chemotherapy-induced cardiotoxicity.

**Copyright Information:** (Online) ISSN: 2572-9241
©2026 The Author(s). HemaSphere is published by John Wiley & Sons Ltd on behalf of European Hematology Association. This is an open access Abstract Book distributed under the Attribution-NonCommercial-NoDerivs (CC BY-NC-ND), which allows third parties to download the articles and share them with others as long as they credit the author and the Abstract Book, but they cannot change the content in any way or use them commercially.
**Abstract Book Citations:** Authors, Title, HemaSphere, 2026;10:(S1):pages. The abstract book DOIs can be found on the "Author Information" page.
**Disclaimer:** Articles published in the journal HemaSphere exclusively reflect the opinions of the authors. The authors are responsible for all content in their abstracts including accuracy of the facts, statements, citing resources, etc.

**Summary/Conclusion**

Liposomal mitoxantrone demonstrates a significantly superior cardiac safety profile with comparable clinical efficacy to conventional mitoxantrone when used as a conditioning regimen for auto-HSCT in lymphoma patients. The integration of 3D echocardiography-derived GLS and specific serological biomarkers into routine clinical protocols is highly recommended for the early detection, dynamic monitoring, and proactive intervention of chemotherapy-related cardiovascular complications.

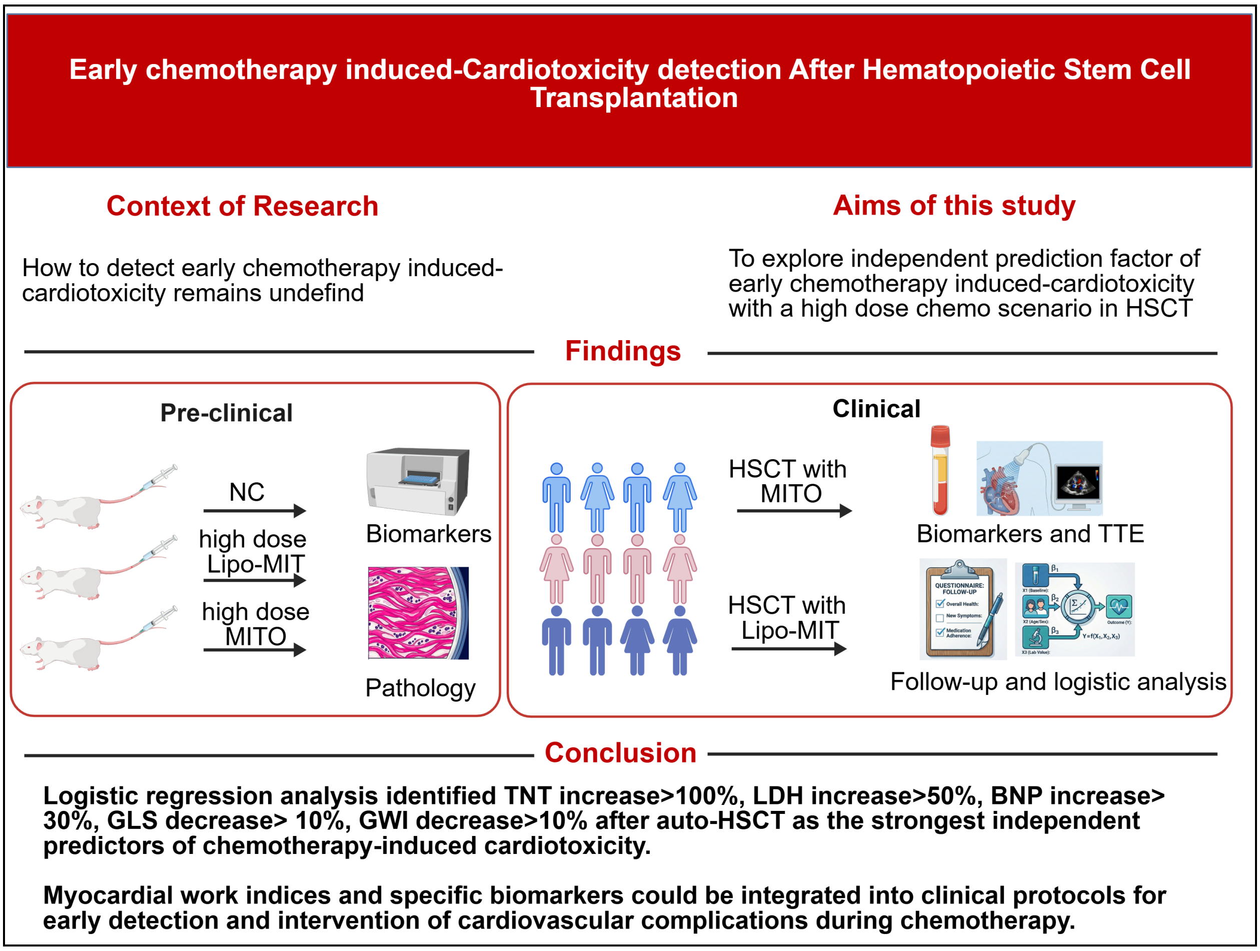

figure abstract

**Copyright Information:** (Online) ISSN: 2572-9241
©2026 The Author(s). HemaSphere is published by John Wiley & Sons Ltd on behalf of European Hematology Association. This is an open access Abstract Book distributed under the Attribution-NonCommercial-NoDerivs (CC BY-NC-ND), which allows third parties to download the articles and share them with others as long as they credit the author and the Abstract Book, but they cannot change the content in any way or use them commercially.
**Abstract Book Citations:** Authors, Title, HemaSphere, 2026;10:(S1):pages. The abstract book DOIs can be found on the "Author Information" page.
**Disclaimer:** Articles published in the journal HemaSphere exclusively reflect the opinions of the authors. The authors are responsible for all content in their abstracts including accuracy of the facts, statements, citing resources, etc.

### (PS2229) POST-TRANSPLANT CYCLOPHOSPHAMIDE DOSE MODULATION BASED ON AGE, COMORBIDITIES AND CARDIOVASCULAR RISK FACTORS: RESULTS FROM A PROSPECTIVE PILOT STUDY.

**Topic:** 23. Stem cell transplantation - Clinical

Francesca Carobolante[1], Giulia Perali[1], Anita Betulla[1], Costanza Fraenza[1], Samanta Bonato[1], Desiree Mencato[2], Silvia Imbergamo[3], Fabio D'amore[3], Stefano Pravato[3], Chiara Adele Cavarretta[3], Michela Mognato[1], Livio Trentin[3], Cristina Skert*[1]

*[1]Ospedale dell'Angelo AULSS3 Serenissima, Venezia, Italy; [2]University of Ferrara, Ferrara, Italy; [3]University of Padova, Padova, Italy;*

### Background

Post-transplant Cyclophosphamide (pTCy) prophylaxis of GVHD has become current clinical practice in haploidentical and mismatched unrelated donor (MMUD) stem cell transplantation. However, age, pluritreated patients, and comorbidities (cardiovascular diseases, especially), may limit the use of pTCy standard dose (100 mg/kg total dose). Previous studies demonstrated the efficacy of reduced pTCy dose mainly in the haploidentical setting.

### Aims

To evaluate the efficacy of pTCy dose modulation in haploidentical and MMUD transplantation, we performed a prospective pilot study on 45 patients, in which pTCy dose was reduced on the basis of patients and disease characteristics.

### Methods

From September 2024 to February 2026, pTCy total dose was reduced (70-80 mg/kg) on the basis of the following criteria: age>60 years, HCT-CI ≥3, cardiovascular disease, pre-transplant ferritin >1000 ng/ml, lines of therapy ≥3, and previous severe infectious complications. Standard total dose of 100 mg/kg was used in patients without the above criteria. Median age of patients was 55 years (range, 19-75) and 42% had HCT-CI ≥3, 14% with cardiovascular comorbidities. Acute Leukemia was the prevailing diagnosis (82%) and 24% of patients were treated with ≥ 3 lines of therapy. Primary endpoint was OS; secondary endpoints were rate of cardio-toxicity, cumulative incidence of TRM, relapse, acute, and chronic GVHD. Analysis included not only patient and transplant variables, but also cardiovascular risk factors, history of inflammatory chronic disease, and indexes of cardiac and pulmonary function.

### Results

Twenty-nine out of 45 patients received a reduced dose of pTCy according to the defined criteria. Haploidentical donor prevailed in the group of reduced-pTCy, whereas other transplant characteristics, such as conditioning regimen and type of calcineurin inhibitor did not differ in comparison with the group of standard-pTCy. Median age, diagnosis and status of disease at transplantation were also not different between the 2 groups. A trend to better OS was observed in the group of reduced pTCy (1y 73 vs 55%; p=0.09) likely due to lower TRM in this group (1y 11 vs 38%; p=0.04), whereas the 1y-cumulative incidence (CI) of relapse was not significantly different (31 vs 20%; p=0.52). CI of acute GVHD did not differ between the 2 groups (28 vs 44%; p=0.24) and chronic GVHD was lower in the pTCy-reduced groups (0 vs 17%; p=0.05). Early bacterial infections were reduced in this group as well (12 vs 46%; p=0.02). The rate of cardiotoxicity was comparable between the two groups (4% pTCy-reduced vs 7% pTCy-standard; p=0.9).

**Copyright Information:** (Online) ISSN: 2572-9241
©2026 The Author(s). HemaSphere is published by John Wiley & Sons Ltd on behalf of European Hematology Association. This is an open access Abstract Book distributed under the Attribution-NonCommercial-NoDerivs (CC BY-NC-ND), which allows third parties to download the articles and share them with others as long as they credit the author and the Abstract Book, but they cannot change the content in any way or use them commercially.
**Abstract Book Citations:** Authors, Title, HemaSphere, 2026;10:(S1):pages. The abstract book DOIs can be found on the “Author Information” page.
**Disclaimer:** Articles published in the journal HemaSphere exclusively reflect the opinions of the authors. The authors are responsible for all content in their abstracts including accuracy of the facts, statements, citing resources, etc.

**Summary/Conclusion**

The dose reduction of pTCy in patients "unfit" for standard dose did not increase the incidence of acute GVHD. On the contrary, it reduced bacterial infections, chronic GVHD, and TRM. Furthermore, the rate of cardiotoxicity was lower than that reported in literature in the setting of the pTCy prophylaxis (up to 19%), contributing to decrease TRM. Reduced pTCy dose seems not only to offset the unfavorable characteristics of "unfit" patients but also to give a certain advantage in OS. The decreased toxicity could not be the single mechanism involved. Distinct effects on PD-1+T cell subsets of reduced versus standard dose of pTCy, such as increased PD1+ CD4+ and CD8+ cells, could explain the efficient immunoregulatory activity and GVHD-prophylaxis efficacy. Further trials with more patients and immuno-reconstitution analysis could confirm our findings.

**Copyright Information:** (Online) ISSN: 2572-9241
©2026 The Author(s). HemaSphere is published by John Wiley & Sons Ltd on behalf of European Hematology Association. This is an open access Abstract Book distributed under the Attribution-NonCommercial-NoDerivs (CC BY-NC-ND), which allows third parties to download the articles and share them with others as long as they credit the author and the Abstract Book, but they cannot change the content in any way or use them commercially.
**Abstract Book Citations:** Authors, Title, HemaSphere, 2026;10:(S1):pages. The abstract book DOIs can be found on the "Author Information" page.
**Disclaimer:** Articles published in the journal HemaSphere exclusively reflect the opinions of the authors. The authors are responsible for all content in their abstracts including accuracy of the facts, statements, citing resources, etc.

### (PS2230) A RETROSPECTIVE STUDY EVALUATING THE ROLE OF ALLOGENEIC STEM CELL TRANSPLANTATION IN PATIENTS WITH MYELODYSPLASTIC SYNDROME INITIALLY DIAGNOSED AS LOWER RISK

**Topic:** 23. Stem cell transplantation - Clinical

Alexandre Bazinet*[1], Uday R. Popat[1], Alex Bataller[1], Kelly Chien[1], Guillermo Montalban-Bravo[1], Ian Bouligny[1], Danielle Hammond[1], Koji Sasaki[1], Mahesh Swaminathan[1], Wei-Ying Jen[1], Eitan Kugler[1], Tapan Kadia[1], Julianne Chen[1], Sherry Pierce[1], Portia Smallbone[1], Richard E. Champlin[1], Elizabeth J. Shpall[1], Chitra Hosing[1], Jeremy L. Ramdial[1], Betul Oran[1], Hagop Kantarjian[1], Guillermo Garcia-Manero[1]

*[1]MD Anderson Cancer Center, Houston, United States of America;*

### Background

Allogeneic stem cell transplantation (SCT) is the only known curative therapy in myelodysplastic syndromes (MDS). Due to the associated morbidity/mortality, SCT is generally reserved for patients (pts) with higher risk MDS or pts with lower risk MDS (LR MDS) who have progressed. The role of SCT in LR MDS is incompletely understood.

### Aims

To evaluate the outcomes of pts with LR MDS who undergo SCT, either upfront or at the time of progression.

### Methods

This was a single center retrospective study. We identified all pts between 2000-2023 with an initial diagnosis of LR MDS (IPSS-R ≤ 3.5) who underwent SCT. Pts were stratified by reason for SCT hierarchically into the following groups: documented physician intent for immediate frontline SCT (FL intent), progression to acute myeloid leukemia (AML), progression to MDS with increased blasts (EB), refractory cytopenias, or clonal evolution (emergence of new mutations or cytogenetic abnormalities). Overall survival (OS) and progression-free survival (PFS) were calculated as time from SCT day (D) 0. Cumulative incidence (CI) analyses were performed using death in remission and disease relapse as competing risks.

**Copyright Information:** (Online) ISSN: 2572-9241
©2026 The Author(s). HemaSphere is published by John Wiley & Sons Ltd on behalf of European Hematology Association. This is an open access Abstract Book distributed under the Attribution-NonCommercial-NoDerivs (CC BY-NC-ND), which allows third parties to download the articles and share them with others as long as they credit the author and the Abstract Book, but they cannot change the content in any way or use them commercially.
**Abstract Book Citations:** Authors, Title, HemaSphere, 2026;10:(S1):pages. The abstract book DOIs can be found on the “Author Information” page.
**Disclaimer:** Articles published in the journal HemaSphere exclusively reflect the opinions of the authors. The authors are responsible for all content in their abstracts including accuracy of the facts, statements, citing resources, etc.

**Results**

1480 pts with LR MDS were identified, of which 126 (9%) underwent SCT. Reasons for SCT were FL intent in 34/126 (27%) pts, AML in 19 (15%), MDS with EB in 33 (26%), refractory cytopenia in 34 (27%), and clonal evolution in 6 (5%). Among pts who underwent SCT, the median age was 58 yrs. Initial IPSS-R risk was very low in 22 (17%), low in 70 (56%), and intermediate in 34 (27%) pts. Complex cytogenetics were present in 5 (4%) patients and *TP53* mutations in 11/62 (18%) pts with testing. By reason for SCT, median time from diagnosis to SCT was 6.2 months (m) in FL intent, 24.8 m in AML, 32 m in MDS with EB, 25.4 m in refractory cytopenia, and 37.4 m in clonal evolution. Patients who underwent SCT as FL intent had significantly higher incidence of therapy-related MDS (56% vs 17% in those transplanted at progression, p<0.001). SCT conditioning was myeloablative (MAC) in 73 (58%) pts and reduced-intensity (RIC) in 53 (42%) pts.
The median OS from SCT D0 was 36.3 m for the full cohort (2-yr 56%, 5-yr 44%). The median PFS was 30.4 m (2-yr 54%, 5-yr 45%). The CI of relapse was 23% at 2-yr and 25% at 5-yr. The cumulative incidence of death was 23% at 2-yr and 30% at 5-yr. No significant differences in OS, PFS, CI of relapse, or CI of death were noted by age strata (<60 yrs, 60-69 yrs, ≥70 yrs). The cumulative incidence of relapse was significantly higher in patients with baseline IPSS-R intermediate risk (2-yr 47%) compared to low risk (9%) or very low risk (30%; p=0.001).
When stratified by reason for SCT, median OS was not reached (NR) for FL intent, 12.3 m for AML, 24.6 m for MDS-EB, 127.2 m for refractory cytopenia, and NR for clonal evolution (p=0.037; Fig 1). Median PFS was not reached (NR) for FL intent, 4.6 m for AML, 19 m for MDS-EB, 127.2 m for refractory cytopenia, and 23.1 m for clonal evolution (p=0.03). CI of relapse at 2-yr was 24% for FL intent, 32% for AML, 33% for MDS-EB, 9% for refractory cytopenia, and 17% for clonal evolution (p=0.17).

**Summary/Conclusion**

SCT is effective in select patients with LR MDS at progression or with high-risk baseline features such as therapy related disease. Outcomes are better in patients transplanted without blast progression. Further studies into the optimal selection of SCT candidates and timing of SCT in LR MDS are required.

**Copyright Information:** (Online) ISSN: 2572-9241
©2026 The Author(s). HemaSphere is published by John Wiley & Sons Ltd on behalf of European Hematology Association. This is an open access Abstract Book distributed under the Attribution-NonCommercial-NoDerivs (CC BY-NC-ND), which allows third parties to download the articles and share them with others as long as they credit the author and the Abstract Book, but they cannot change the content in any way or use them commercially.
**Abstract Book Citations:** Authors, Title, HemaSphere, 2026;10:(S1):pages. The abstract book DOIs can be found on the "Author Information" page.
**Disclaimer:** Articles published in the journal HemaSphere exclusively reflect the opinions of the authors. The authors are responsible for all content in their abstracts including accuracy of the facts, statements, citing resources, etc.

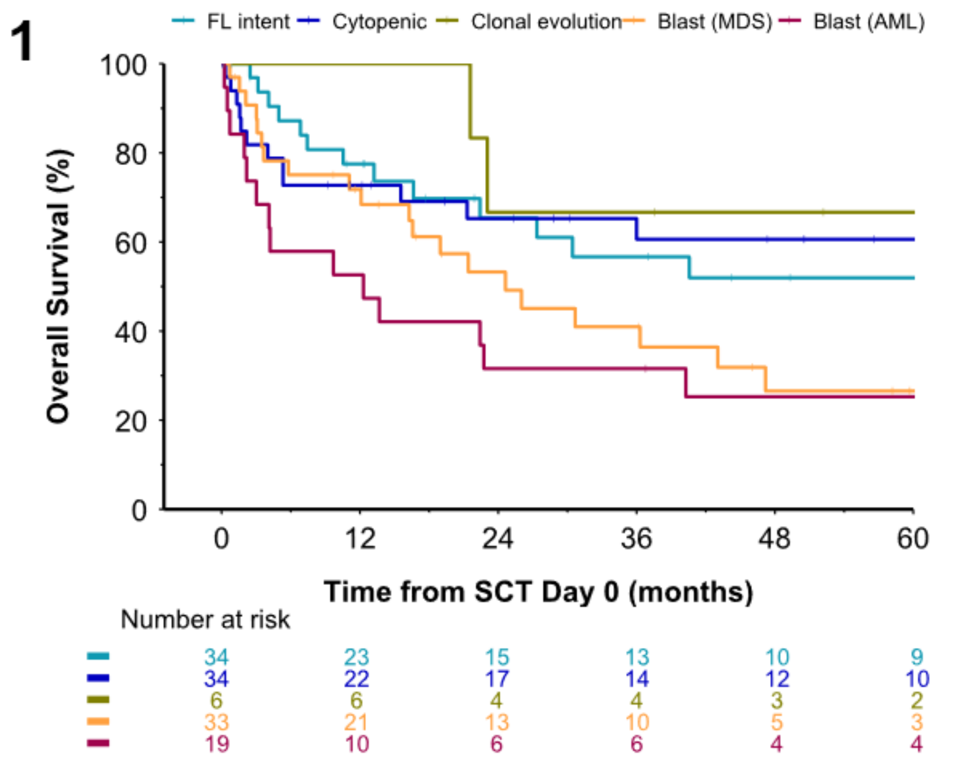

| Reason | median | 2-yr | 5-yr | p |
|---|---|---|---|---|
| Frontline intent | NR | 65% | 52% | |
| Cytopenic | 127.2 | 65% | 61% | |
| Clonal evolution | NR | 67% | 67% | 0.037 |
| Blast prog (MDS) | 24.6 | 53% | 27% | |
| Blast prog (AML) | 12.3 | 32% | 25% | |

**Copyright Information:** (Online) ISSN: 2572-9241
©2026 The Author(s). HemaSphere is published by John Wiley & Sons Ltd on behalf of European Hematology Association. This is an open access Abstract Book distributed under the Attribution-NonCommercial-NoDerivs (CC BY-NC-ND), which allows third parties to download the articles and share them with others as long as they credit the author and the Abstract Book, but they cannot change the content in any way or use them commercially.
**Abstract Book Citations:** Authors, Title, HemaSphere, 2026;10:(S1):pages. The abstract book DOIs can be found on the "Author Information" page.
**Disclaimer:** Articles published in the journal HemaSphere exclusively reflect the opinions of the authors. The authors are responsible for all content in their abstracts including accuracy of the facts, statements, citing resources, etc.

### (PS2231) BELUMOSUDIL IN PEDIATRIC PATIENTS WITH STEROID-REFRACTORY CHRONIC GRAFT-VERSUS-HOST DISEASE

**Topic:** 23. Stem cell transplantation - Clinical

Lipeng Liu*[1], Ning Wang[1], Chen Xia[1], Yang Wan[1], Aoli Zhang[1], Xiaolan Li[1], Xiaoyan Zhang[1], Xiaofan Zhu[1], Wenyu Yang[1], Fang Liu[1]

*[1]Institute of Hematology & Blood Diseases Hospital, Chinese Academy of Medical Sciences & Peking Union Medical College, Division of Pediatric Blood Diseases Center, Tianjin, China;*

### Background

Belumosudil has been introduced to treat steroid-refractory chronic graft-versus-host disease (SR-cGVHD) in adult patients. Similarly, cGVHD occurred in nearly 25% of pediatric allogeneic hematopoietic cell transplant (HCT) recipients. But the lack of data in pediatric SR-cGVHD patients contributes to poor prognosis, especially in young children aged<12.

### Aims

We aim to investigate the efficacy and safety of belumosudil in pediatric patients.

### Methods

This study included 49 HCT pediatric patients with SR-cGVHD failing multiple lines of treatment (LOTs), and all patients received belumosudil. The primary endpoint was the overall response rate (ORR), failure-free survival (FFS), and overall survival (OS), and adverse effects (AEs) during belumosudil administration were documented.

### Results

The median time to response was 6 weeks (range, 3-18), and the ORR was 81.6%, and 38.7% of patients achieved CR. The best ORR was also assessed in other affected organs, and organ-specific analyses demonstrated an ORR of 93.1% in the skin, 90.0% in the mouth, 90.9% in the liver, 87.5% in the eyes, and 94.9% in the gastrointestinal (GI) tract. The overall FFS and OS were 77.0% (95% CI, 70.1-83.9) and 100% in patients who received belumosudil. Belumosudil was well tolerated, and the AE rates were low in patients with belumosudil.

### Summary/Conclusion

Belumosudil was effective and well-tolerated in pediatric patients with SR-cGVHD after failing multiple LOTs as salvage therapy, and most included patients reduced the dose or discontinued using corticosteroids and other immunosuppressive regimens to minimize their side effects and cost burden.

**Copyright Information:** (Online) ISSN: 2572-9241
©2026 The Author(s). HemaSphere is published by John Wiley & Sons Ltd on behalf of European Hematology Association. This is an open access Abstract Book distributed under the Attribution-NonCommercial-NoDerivs (CC BY-NC-ND), which allows third parties to download the articles and share them with others as long as they credit the author and the Abstract Book, but they cannot change the content in any way or use them commercially.
**Abstract Book Citations:** Authors, Title, HemaSphere, 2026;10:(S1):pages. The abstract book DOIs can be found on the "Author Information" page.
**Disclaimer:** Articles published in the journal HemaSphere exclusively reflect the opinions of the authors. The authors are responsible for all content in their abstracts including accuracy of the facts, statements, citing resources, etc.

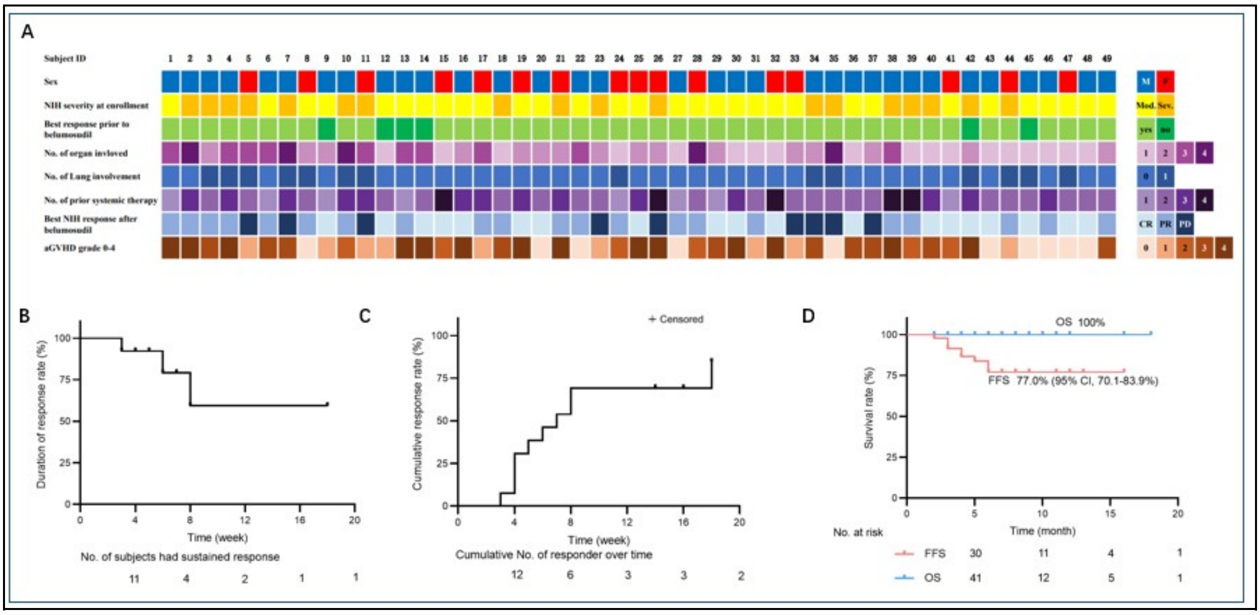

Figure A. Heatmap of disease status and best changes in response metrics in all patients included in the belumosudil group. Baseline characteristics for all 13 subjects and the best improvement of overall response were illustrated. aGVHD, acute graft-versus-host disease; M, male; F, female; Mod, moderate; Sev, severe; PR, partial response; SD, stable disease; PD, progression disease. B. Kaplan-Meier plot of duration of response rate of the belumosudil responder population; C. The time to respond to the belumosudil among responders. DOR, duration of response. D. Kaplan-Meier curves of estimated FFS and OS in patients received belumosudil.

**Copyright Information:** (Online) ISSN: 2572-9241
©2026 The Author(s). HemaSphere is published by John Wiley & Sons Ltd on behalf of European Hematology Association. This is an open access Abstract Book distributed under the Attribution-NonCommercial-NoDerivs (CC BY-NC-ND), which allows third parties to download the articles and share them with others as long as they credit the author and the Abstract Book, but they cannot change the content in any way or use them commercially.
**Abstract Book Citations:** Authors, Title, HemaSphere, 2026;10:(S1):pages. The abstract book DOIs can be found on the "Author Information" page.
**Disclaimer:** Articles published in the journal HemaSphere exclusively reflect the opinions of the authors. The authors are responsible for all content in their abstracts including accuracy of the facts, statements, citing resources, etc.

### (PS2232) A PHASE 2 TRIAL OF MYELOABLATIVE FRACTIONATED BUSULFAN, FLUDARABINE, CLADRIBINE, THIOTEPA, AND VENETOCLAX (CLADILLAC) CONDITIONING FOR HIGH-RISK MDS

**Topic:** 23. Stem cell transplantation - Clinical

Uday R. Popat*[1], Chitra Hosing[1], Roland Bassett[1], Amin Majid Alousi[1], Gheath Al-Atrash[1], Yosra Aljawai[1], Qaiser Bashir[1], George Chen[1], Warren Fingrut[1], Jin Im[1], Partow Kebriaei[1], David Marin[1], Yago Nieto[1], Amanda Olson[1], Betul Oran[1], Muzaffar H. Qazilbash[1], Jeremy L. Ramdial[1], Neeraj Saini[1], Portia Smallbone[1], Samer A. Srour[1], Alexandre Bazinet[1], Guillermo Garcia-Manero[1], Richard E. Champlin[1], Elizabeth J. Shpall[1]

[1] *The University of Texas MD Anderson Cancer Center, Houston, United States of America;*

### Background

Patients with high-risk myelodysplastic syndrome (MDS) have poor outcomes despite the use of stem cell transplantation (SCT). Non-relapse mortality (NRM) post SCT is often driven by conditioning regimen toxicity and graft-versus-host disease (GVHD). To reduce conditioning toxicity, we extended the schedule of myeloablative busulfan (Bu) to 3 weeks. To address GVHD, we used post-transplant cyclophosphamide (PTCy). To reduce relapse, we added cladribine, thiotepa, and venetoclax. This regimen was evaluated in a prospective phase 2 trial in high-risk MDS.

### Aims

To evaluate a myeloablative busulfan conditioning regimen containing cladribine, thiotepa, and venetoclax in patients with high-risk MDS undergoing allogeneic SCT.

### Methods

High-risk MDS was defined as IPSS-R >3.5, poor or very poor risk cytogenetics, bone marrow blasts ≥5%, or mutated TP53 or RAS pathway genes. Eligible patients were 18–70 years old and had a matched or haploidentical related donor, or a 7/8 or 8/8 HLA-matched unrelated donor. The conditioning regimen included outpatient Bu 100 mg/m² on days -20 and -13, fludarabine 10 mg/m², cladribine 10 mg/m², and Bu pharmacokinetically dosed to reach a total systemic exposure of 20,000 ±12% µmol/min on days -6 to -3. Venetoclax 400 mg was given daily from days -22 to -3. GVHD prophylaxis was PTCy 50 mg/kg on days 3 and 4, tacrolimus ± MMF. The primary endpoint was progression-free survival (PFS). A sample size of 50 provided >80% power to detect an increase in 1-year PFS from 30% (historical) to 47%, with a 5% Type I error rate (ClinicalTrials.gov: NCT04708054).

### Results

Fifty patients (23 female, 27 male) with a median age of 63 years (range: 33–69) were enrolled from December 2021 to August 2023. Disease characteristics included IPSS-R high/very high (58%), IPSS-M moderate high/high/very high (72%). TP53 mutations were present in 28% of patients. Donors were matched unrelated (50%), matched sibling (34%), haploidentical (4%), and mismatched unrelated (12%). Median HCT-CI score was 2 (range: 0–8); 38% had a Karnofsky performance score ≤80%.
At a median follow up of 24 months, median PFS was not reached (95% CI: 11 months–NR). One-year and 3-year PFS were 62% (50–77) and 60% (48–75), respectively. Three-year overall survival (OS), NRM, and relapse rates were 60% (48-75), 30% (17-43), and 10% (2-18), respectively. Outcomes in TP53 wildtype patients vs mutated patients at 3 years were: OS of 64% vs 50% (P=0.51), PFS of 64% vs 50% (P=0.34), and relapse rate of 6% vs 21% (P=0.031).
Median time to neutrophil and platelet engraftment were 16 (range: 13–32) and 23 days (range: 10–221), respectively. At day 30, median T cell and myeloid donor chimerism were 100%. Grade 2–4 acute GVHD occurred in 36% (23–50), grade 3–4 in 10% (2–18), chronic GVHD in 56% (42–70), and moderate-to-severe chronic GVHD in 36% (23–50).

**Copyright Information:** (Online) ISSN: 2572-9241
©2026 The Author(s). HemaSphere is published by John Wiley & Sons Ltd on behalf of European Hematology Association. This is an open access Abstract Book distributed under the Attribution-NonCommercial-NoDerivs (CC BY-NC-ND), which allows third parties to download the articles and share them with others as long as they credit the author and the Abstract Book, but they cannot change the content in any way or use them commercially.
**Abstract Book Citations:** Authors, Title, HemaSphere, 2026;10:(S1):pages. The abstract book DOIs can be found on the "Author Information" page.
**Disclaimer:** Articles published in the journal HemaSphere exclusively reflect the opinions of the authors. The authors are responsible for all content in their abstracts including accuracy of the facts, statements, citing resources, etc.

**Summary/Conclusion**

This study met its primary endpoint, demonstrating promising 3-year PFS in patients with high-risk MDS. These findings support further investigation of this regimen.

**Table 1. Summary of outcomes (N=50).**

| | At 1 year | At 2 years | At 3 years |
|---|---|---|---|
| **Overall survival** | 66% | 60% | 60% |
| **Progression-free survival** | 62% | 60% | 60% |
| **Relapse** | 8% | 10% | 10% |
| **Non-relapse mortality** | 30% | 30% | 30% |
| **Median follow-up: 24 months** | | | |
| **Acute GVHD grades 2-4, day 100** | 36% | | |
| **Acute GVHD grades 3-4, day 100** | 10% | | |
| **Moderate/severe chronic GVHD, 3 years** | 36% | | |
| | | | |
| **Neutrophil engraftment: Median (range) days** | 16 (13-32) | | |
| **Platelet engraftment: Median (range) days** | 23 (10-221) | | |
| **T cell chimerism at day 30: Median (range) days** | 100 (79-100) | | |
| **Myeloid chimerism at day 30: Median (range) days** | 100 (100-100) | | |
| | | | |
| **Grade 3-5 toxicity at day 100 (≥10%)** | **N (%)** | | |
| Febrile neutropenia | 28 (56) | | |
| Bacterial infection | 16 (32) | | |
| Diarrhea | 7 (14) | | |
| Creatinine increased | 6 (12) | | |
| Pneumonitis | 5 (10) | | |

**Copyright Information:** (Online) ISSN: 2572-9241
©2026 The Author(s). HemaSphere is published by John Wiley & Sons Ltd on behalf of European Hematology Association. This is an open access Abstract Book distributed under the Attribution-NonCommercial-NoDerivs (CC BY-NC-ND), which allows third parties to download the articles and share them with others as long as they credit the author and the Abstract Book, but they cannot change the content in any way or use them commercially.
**Abstract Book Citations:** Authors, Title, HemaSphere, 2026;10:(S1):pages. The abstract book DOIs can be found on the "Author Information" page.
**Disclaimer:** Articles published in the journal HemaSphere exclusively reflect the opinions of the authors. The authors are responsible for all content in their abstracts including accuracy of the facts, statements, citing resources, etc.

### (PS2233) COMPARISON BETWEEN CHIDAMIDE, CLADRIBINE, GEMCITABINE AND BUSULFAN AS CONDITIONING REGIMEN AND BEAM FOR HIGH RISK NON-HODGKIN LYMPHOMA: A PROPENSITY SCORE MATCH ANALYSIS

**Topic:** 23. Stem cell transplantation - Clinical

Pu Kuang*[1], Chang-Ju Qu[2], Juan Xu[1, 2], Tian Dong[1], Xinchuan Chen[1], Zhigang Liu[1], Ting Liu[1], Depei Wu[2], Zhengming Jin[2], Ting Niu[1], Jie Ji[1]

*[1]West China Hospital, Sichuan University, Chengdu, China;[2]First Affiliated Hospital of Soochow University, Department of Hematology, Suzhou, China;*

### Background

BEAM has been the most widely used conditioning regimen for Hodgkin and non-Hodgkin lymphoma for decades. However, its overall effect remained unsatisfying for high risk lymphomas, with the long-term lymphoma-free survival with the long-term lymphoma-free survival rate of less than 50% in refractory cases.

### Aims

To verify the effectiveness and safety profile of the chi-CGB (chidamide, cladribin, gemcitabine, and busulfan), a novel conditioning regimen for lymphoma. To explore its different anti-lymphoma efficacy across different lymphoma histopathological subtypes.

### Methods

This retrospective study was conducted using electronic medical records from two China university hospitals with propensity score matching method. The **chi-CGB regimen** consists of chidamide 30mg on days -14, -10, -7 and -4, gemcitabine 2000mg/m$^2$ on days -6 and -2, cladribine 5mg/m$^2$ through days -6 to -2, busulfan 3.2mg/kg through days -6 to -3. The**BEAM regimen** is as reported previously. Kaplan-meier method was used to estimate the overall survival (OS), progression free survival (PFS). The cumulative incidences of relapse (CIR) were estimated using competing risk model with death without lymphoma progression considered as the competing event. Log-rank method was used to compare OS and PFS between groups and Gray's test was used to compare the CIRs in the competing risk modelAll statistical analysis were performed using R 4.3.3.

### Results

A total of 831 patients' medical records from two hospitals were screened. Thirty-one patients record were dropped according to the I/E criteria. Patient records were matched using the following parameters: *gender*, *age*, *lymphoma subtype*, *Ann-arbor stage*, *IPI score* at diagnosis, *response* to prior treatment before transplantation. After matching, 309 patients were included in chi-CGB (treatment) and BEAM (control) group respectively. The baseline characteristics of the patients before and after matching are presented in Table 1. All patients achieved neutrophil engraftment. The median time to neutrophil engraftment were 10 days and 9 days in the treatment and the control group respectively. Platelet engraftment was achieved to 99.7% and 99.3% patients in the treatment and the control groups within one month after transplant. The median time to platelet engraftment were 13 days and 11 days respectively. The 4-year PFS (79% vs. 71%, $p < 0.001$ ) and 4-year OS (89% vs. 83%, $p = 0.014$) for the treatment group were superior to the control group. For the subgroup analysis, patients with T cell lymphoma (including peripheral T cell lymphoma NOS, anaplastic large cell lymphoma ,intestinal T-cell lymphoma and angioimmunoblastic T-cell lymphoma) or aggressive B cell lymphoma (mostly diffused large B cell lymphoma) but not natural killer/T-cell lymphoma (NK/T) benefitted from chi-CGB regimen (see Figure 1.). Grade ≤2 hepatic and skin adverse events were more common in the treatment group. Grade III/IV adverse events were similar for both groups.

**Copyright Information:** (Online) ISSN: 2572-9241
©2026 The Author(s). HemaSphere is published by John Wiley & Sons Ltd on behalf of European Hematology Association. This is an open access Abstract Book distributed under the Attribution-NonCommercial-NoDerivs (CC BY-NC-ND), which allows third parties to download the articles and share them with others as long as they credit the author and the Abstract Book, but they cannot change the content in any way or use them commercially.
**Abstract Book Citations:** Authors, Title, HemaSphere, 2026;10:(S1):pages. The abstract book DOIs can be found on the "Author Information" page.
**Disclaimer:** Articles published in the journal HemaSphere exclusively reflect the opinions of the authors. The authors are responsible for all content in their abstracts including accuracy of the facts, statements, citing resources, etc.

### Summary/Conclusion

Based on the results of this study, the chi-CGB regimen can effectively reduce the risk of lymphoma relapse after ASCT with limited adverse effects compared to traditional BEAM regimen. Especially, this regimen exerts pronounced anti-lymphoma effect in two histopathological subtypes: aggressive B NHL and T-NHL.

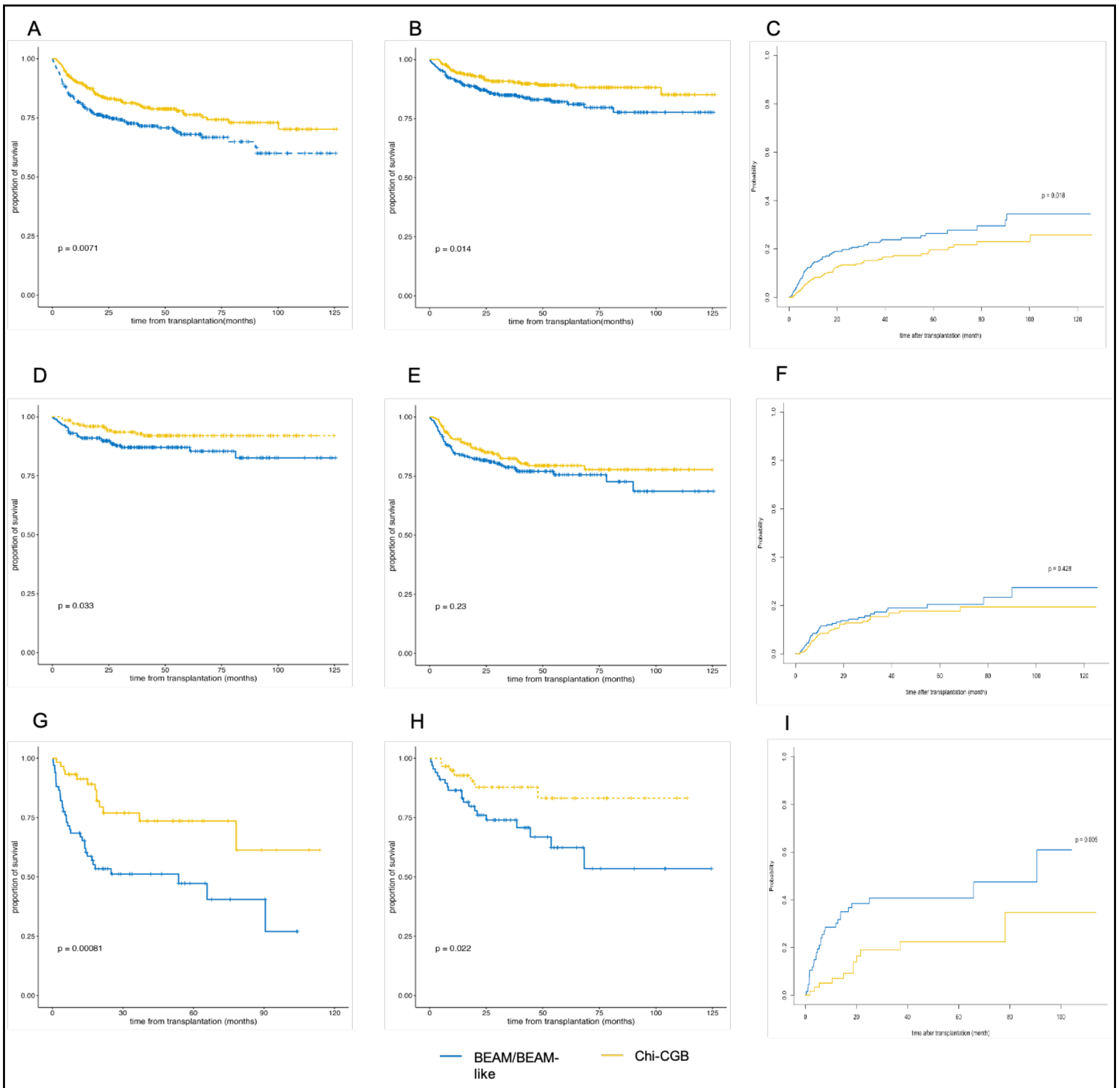

Figure 1. Long-term outcomes of all population and subgroups after propensity score matching.
A. Progression-free survival for all patients; B. Overall survival; C. Cumulative incidence of lymphoma relapse; D. Progression-free survival in the aggressive B-cell lymphoma subgroup; E. Overall survival in the aggressive B-cell lymphoma subgroup; F. Cumulative incidence of lymphoma relapse in the aggressive B-cell lymphoma subgroup; G. Progression-free survival in PTCL-NOS subgroup; H. Overall survival in the PTCL-NOS subgroup; I. Cumulative incidence of lymphoma relapse in the PTCL-NOS subgroup.

**Copyright Information:** (Online) ISSN: 2572-9241
©2026 The Author(s). HemaSphere is published by John Wiley & Sons Ltd on behalf of European Hematology Association. This is an open access Abstract Book distributed under the Attribution-NonCommercial-NoDerivs (CC BY-NC-ND), which allows third parties to download the articles and share them with others as long as they credit the author and the Abstract Book, but they cannot change the content in any way or use them commercially.
**Abstract Book Citations:** Authors, Title, HemaSphere, 2026;10:(S1):pages. The abstract book DOIs can be found on the “Author Information” page.
**Disclaimer:** Articles published in the journal HemaSphere exclusively reflect the opinions of the authors. The authors are responsible for all content in their abstracts including accuracy of the facts, statements, citing resources, etc.

### (PS2234) OUTCOMES AFTER STANDARD- VS. REDUCED-DOSE TREOSULFAN IN ALLOGENEIC HEMATOPOIETIC CELL TRANSPLANTATION

**Topic:** 23. Stem cell transplantation - Clinical

Johanne Schovsbo*[1], Eileen Wedge[1, 2], Henrik Sengeløv[1], Brian Kornblit[1], Bo Mortensen[1], Niels Smedegaard Andersen[1], Søren Lykke Petersen[1], Marietta Nygaard[1], Ida Schjødt[1], Morten Holmström[3], Kirsten Grønbæk[1, 4], Lone Friis[1, 4]

*[1]Rigshospitalet, Copenhagen University Hospital, Department of Hematology, København Ø, Denmark;[2]Rigshospitalet, Copenhagen University Hospital, Department of Genetics, København Ø, Denmark; [3]Herlev Hospital, Copenhagen University Hospital, National Center for Cancer Immune Therapy, Herlev, Denmark; [4]Institute of Clinical Medicine, University of Copenhagen, København N, Denmark;*

### Background

Treosulfan-based conditioning is increasingly used in allogeneic hematopoietic cell transplantation (allo-HCT) due to its myeloablative efficacy and reduced toxicity. However, evidence guiding optimal dosing remains limited and its clinical impact remains insufficiently defined. Conditioning with Fludarabine and Treosulfan (FluTreo) was implemented at Copenhagen University Hospital, Rigshospitalet, in 2014 with a standard dose of 42 g/m². In 2020, a dose-adjusted algorithm introduced 30 g/m² treosulfan for older patients or those with high comorbidity (HCT-CI).

### Aims

This study examines the impact of treosulfan dose on clinical outcomes, aiming to improve understanding of dose optimization in FluTreo conditioning.

### Methods

We performed a retrospective cohort study of all adults receiving FluTreo conditioning prior to allo-HCT for malignant disease at Rigshospitalet 2014–2024. Diagnoses included myelodysplastic syndrome (MDS), high-risk acute myeloid leukemia (AML) and other myeloid malignancies. Patients received either standard- or reduced-dose treosulfan based on time of transplantation, age and HCT-CI. Clinical data were extracted from electronic medical records. Statistical analyses included descriptive statistics, Kaplan–Meier survival analysis, and competing risks models.

**Copyright Information:** (Online) ISSN: 2572-9241
©2026 The Author(s). HemaSphere is published by John Wiley & Sons Ltd on behalf of European Hematology Association. This is an open access Abstract Book distributed under the Attribution-NonCommercial-NoDerivs (CC BY-NC-ND), which allows third parties to download the articles and share them with others as long as they credit the author and the Abstract Book, but they cannot change the content in any way or use them commercially.
**Abstract Book Citations:** Authors, Title, HemaSphere, 2026;10:(S1):pages. The abstract book DOIs can be found on the "Author Information" page.
**Disclaimer:** Articles published in the journal HemaSphere exclusively reflect the opinions of the authors. The authors are responsible for all content in their abstracts including accuracy of the facts, statements, citing resources, etc.

### Results

A total of 252 patients were included (173 standard dose, 79 reduced dose). Reduced-dose recipients were older (median 64.5 years [IQR 52.6–67.8] vs. 57.7 [IQR 52.1–62.2], p=0.00016), while HCT-CI was comparable (median 2 [IQR 0–3] vs. 2 [IQR 0.5–3.5], p=0.27). Diagnostic distribution was similar (~70% MDS, 15% AML, 15% other; p=0.50), with median IPSS-R 4.5 in both groups (p=0.59). Median follow-up was 5.9 and 2.2 years. Overall survival (OS) for the cohort was 87.2% [83.0–91.6%] at 1 year and 77.0% [71.4–83.0%] at 3 years. OS at 1 and 3 years was 86.7% [81.7–92.1%] and 77.0% [70.6–84.0%] with standard dose, and 88.4% [81.1–96.3%] and 76.8% [65.8–89.7%] with reduced dose (p=0.86). Cumulative incidence of relapse (CIR) was 15.8% [11.1–20.5%] at 1 year and 21.4% [16.0–26.9%] at 3 years. CIR at 1 and 3 years was 14.0% [8.7–19.2%] and 18.5% [12.5–24.4%] with standard dose, and 19.3% [10.0–28.8%] and 29.3% [16.8–41.8%] with reduced dose (p=0.12). Non-relapse mortality (NRM) was 6.8% [3.6–10.0%] at 1 year and 8.4% [4.8–12.1%] at 3 years. NRM at 1 and 3 years was 7.3% [3.4–11.4%] and 9.5% [4.9–14.0%] with standard dose, and 4.6% [0.0–9.4%] at both time points with reduced dose (p=0.35). Chronic graft-versus-host disease (cGVHD) was 52.1% [45.1–59.0%] at 1 year and 56.4% [49.5–63.3%] at 3 years. One-year incidences were 53.5% [45.4–61.6%] and 47.6% [33.9–61.3%], and 3-year incidences 58.3% [50.3–66.3%] and 50.0% [36.0–63.7%] for standard and reduced dose, respectively (p=0.35). Acute GVHD (aGVHD) occurred in 31.9% [26.0–37.8%] at day 100 and 33.6% [27.6–39.5%] at 1 year; day-100 incidences were 35.2% [28.0–42.3%] and 23.2% [13.3–33.1%], and 1-year incidences 35.2% [28.0–42.3%] and 29.0% [18.3–39.7%] for standard and reduced dose, respectively.

### Summary/Conclusion

Treosulfan dose adjustment was not associated with differences in OS, CIR, NRM or GVHD. Despite significantly older age in the reduced-dose group, outcomes remained comparable, indicating that reduced-dose treosulfan may be a viable option for selected patients. Ongoing analyses of chimerism and toxicity will help further define the clinical implications of treosulfan dosing and guide individualized conditioning strategies in allo-HCT.

**Copyright Information:** (Online) ISSN: 2572-9241
©2026 The Author(s). HemaSphere is published by John Wiley & Sons Ltd on behalf of European Hematology Association. This is an open access Abstract Book distributed under the Attribution-NonCommercial-NoDerivs (CC BY-NC-ND), which allows third parties to download the articles and share them with others as long as they credit the author and the Abstract Book, but they cannot change the content in any way or use them commercially.
**Abstract Book Citations:** Authors, Title, HemaSphere, 2026;10:(S1):pages. The abstract book DOIs can be found on the "Author Information" page.
**Disclaimer:** Articles published in the journal HemaSphere exclusively reflect the opinions of the authors. The authors are responsible for all content in their abstracts including accuracy of the facts, statements, citing resources, etc.

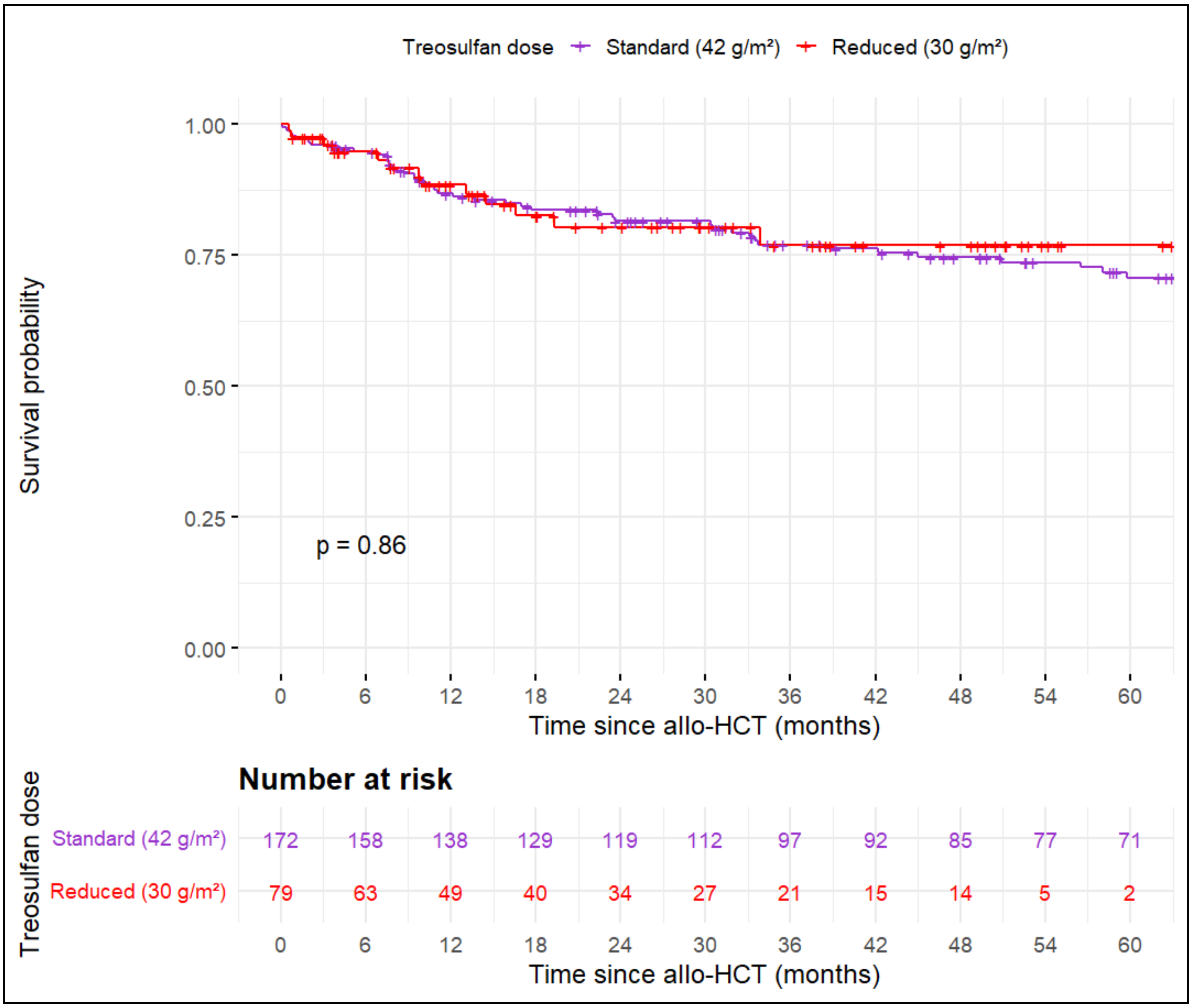

Kaplan–Meier estimates of overall survival by treosulfan dose. Tick marks indicate censored observations. P value calculated using the log-rank test.

**Copyright Information:** (Online) ISSN: 2572-9241
©2026 The Author(s). HemaSphere is published by John Wiley & Sons Ltd on behalf of European Hematology Association. This is an open access Abstract Book distributed under the Attribution-NonCommercial-NoDerivs (CC BY-NC-ND), which allows third parties to download the articles and share them with others as long as they credit the author and the Abstract Book, but they cannot change the content in any way or use them commercially.
**Abstract Book Citations:** Authors, Title, HemaSphere, 2026;10:(S1):pages. The abstract book DOIs can be found on the “Author Information” page.
**Disclaimer:** Articles published in the journal HemaSphere exclusively reflect the opinions of the authors. The authors are responsible for all content in their abstracts including accuracy of the facts, statements, citing resources, etc.

### (PS2235) AUTOLOGOUS HEMATOPOIETIC STEM CELL TRANSPLANTATION COMBINED WITH CD19 CAR-T CELL THERAPY FOR CENTRAL NERVOUS SYSTEM LYMPHOMA: A CLINICAL EFFICACY AND SAFETY ANALYSIS

**Topic:** 23. Stem cell transplantation - Clinical

Xudong Zhang*[1], Jieming Zhang[1], Siyu Qian[1], Wanyue Zhao[1], Zong Feiyang[1], Mingzhi Zhang[1]

*[1]The First affiliated Hospital of Zhengzhou University, zhengzhou, China;*

### Background

Central nervous system lymphoma (CNSL) has an extremely poor prognosis with limited efficacy from conventional therapies. Although CD19-targeted chimeric antigen receptor T-cell (CD19 CAR-T) therapy has achieved breakthroughs in systemic B-cell lymphomas, its application in CNSL faces challenges including the blood-brain barrier, the unique CNS immune microenvironment, and potential neurotoxicity. Autologous hematopoietic stem cell transplantation (ASCT) can reduce tumor burden through myeloablative conditioning and create a favorable environment for CAR-T cell expansion, suggesting synergistic potential with sequential combination. Currently, clinical evidence for ASCT combined with CD19 CAR-T therapy in CNSL remains extremely limited.

### Aims

To evaluate the clinical efficacy and safety of ASCT combined with CD19 CAR-T cell therapy in patients with CNSL.

### Methods

Clinical data were retrospectively collected from CNSL patients who received ASCT combined with CAR-T therapy at the First Affiliated Hospital of Zhengzhou University between July 2023 and August 2025. All patients received myeloablative conditioning chemotherapy followed by autologous stem cell infusion and subsequent sequential infusion of CD19 CAR-T cells. The primary endpoints were objective response rate (ORR) and complete response rate (CRR). Secondary endpoints included overall survival (OS), progression-free survival (PFS), CAR-T cell peak expansion, hematopoietic reconstitution time, and treatment-related adverse events.

### Results

A total of 7 patients were enrolled, with a median age of 49 years (range: 22–56), including 3 males and 4 females, comprising 4 patients with primary CNS lymphoma (PCNSL) and 3 patients with secondary CNS lymphoma (SCNSL). As of February 2026, the median follow-up time was 13.9 months. All patients achieved sustained complete remission after ASCT combined with CAR-T therapy, with ORR and CRR of 100% (7/7). Among them, 4 patients with non-complete response (CR) status (partial response/progressive disease) before treatment all achieved CR after treatment. Three PCNSL patients who had achieved CR before treatment maintained CR without relapse throughout follow-up. Survival analysis showed that median OS and PFS were not reached, with OS and PFS rates of 100% during follow-up. All patients successfully received bridging therapy consisting of fotemustine, pemetrexed, orelabrutinib, and dexamethasone, followed by conditioning regimens, which were BEAM (fotemustine, etoposide, cytarabine, and melphalan) in 2 patients and fotemustine, thiotepa, and teniposide in 5 patients. All patients achieved successful hematopoietic reconstitution after transplantation. The median time to neutrophil and platelet engraftment was 15 days (range: 12–23) and 22 days (range: 16–29), respectively. Among 4 evaluable patients, the median peak absolute CAR-T cell count was 74.3/μL (range: 25.31–137.66/μL), with a median time to peak of 8 days (range: 8–12 days). Regarding safety, cytokine release syndrome (CRS) occurred in 71.4% (5/7) of patients, with no grade ≥3 CRS; one patient developed grade 1 immune effector cell-associated neurotoxicity syndrome (ICANS), with no grade ≥2 ICANS. Two patients developed infections, both of which were controllable.

**Copyright Information:** (Online) ISSN: 2572-9241
©2026 The Author(s). HemaSphere is published by John Wiley & Sons Ltd on behalf of European Hematology Association. This is an open access Abstract Book distributed under the Attribution-NonCommercial-NoDerivs (CC BY-NC-ND), which allows third parties to download the articles and share them with others as long as they credit the author and the Abstract Book, but they cannot change the content in any way or use them commercially.
**Abstract Book Citations:** Authors, Title, HemaSphere, 2026;10:(S1):pages. The abstract book DOIs can be found on the "Author Information" page.
**Disclaimer:** Articles published in the journal HemaSphere exclusively reflect the opinions of the authors. The authors are responsible for all content in their abstracts including accuracy of the facts, statements, citing resources, etc.

**Summary/Conclusion**

ASCT combined with CD19 CAR-T therapy demonstrates excellent efficacy and a favorable safety profile with newly diagnosed and relapsed/refractory CNSL, warranting further validation in larger cohorts with extended follow-up.

**Copyright Information:** (Online) ISSN: 2572-9241
©2026 The Author(s). HemaSphere is published by John Wiley & Sons Ltd on behalf of European Hematology Association. This is an open access Abstract Book distributed under the Attribution-NonCommercial-NoDerivs (CC BY-NC-ND), which allows third parties to download the articles and share them with others as long as they credit the author and the Abstract Book, but they cannot change the content in any way or use them commercially.
**Abstract Book Citations:** Authors, Title, HemaSphere, 2026;10:(S1):pages. The abstract book DOIs can be found on the "Author Information" page.
**Disclaimer:** Articles published in the journal HemaSphere exclusively reflect the opinions of the authors. The authors are responsible for all content in their abstracts including accuracy of the facts, statements, citing resources, etc.

### (PS2236) PROGNOSTIC ANALYSIS OF ALLOGENEIC HEMATOPOIETIC STEM CELL TRANSPLANTATION IN ZNF384-REARRANGED ACUTE LEUKEMIA: A SINGLE-CENTER STUDY

**Topic:** 23. Stem cell transplantation - Clinical

Xueshuang Zhang[1], Yan-Li Zhao[1], Jian-Ping Zhang[1, 1], Deyan Liu[1], Min Xiong[1], Xingyu Cao[1], Zhijie Wei[1], Ruijuan Sun[1], Jiarui Zhou[1, 1], Yue Lu*[1]

[1]*Hebei Yanda Lu Daopei Hospital, langfang, China;*

### Background

ZNF384 rearranged (ZNF384-r) acute leukemia was recognized as a distinct entity in the 2022 WHO and ICC classifications. While allogeneic hematopoietic stem cell transplantation (allo-HSCT) represents a curative approach, prognostic factors specifically within the context of transplantation for this rare subtype remain underexplored.

### Aims

To analyze the clinical characteristics and identify prognostic factors influencing outcomes of allo-HSCT in patients with ZNF384-r acute leukemia.

### Methods

We retrospectively analyzed 100 patients diagnosed with ZNF384-r acute leukemia at our center between April 2020 and December 2025. Of these, 73 patients underwent allo-HSCT and were included in this analysis. Clinical features, transplant outcomes, and prognostic factors were evaluated. The cutoff date for follow-up was December 31, 2025.

### Results

A total of 73 transplanted patients were analyzed (26 children, 47 adults; median age 22 years [range, 3-55]; 52% male). Diagnoses included B-ALL (84.9%), MPAL (B/myeloid, 8.2%), and AML (6.8%). The most common fusion partners were EP300-ZNF384 (50%), TCF3-ZNF384 (27%), and TAF15-ZNF384 (9%). Frequent co-mutations included WT1 (28%) and FLT3 (24%). All patients were classified as high-risk by molecular genetics. At transplantation, 69 patients (94.5%) were in complete remission (CR), 3 (4.1%) were non-remission (NR), 8 (11.6%) were MFC-MRD positive, and 14 (20.2%) were ZNF384-r positive by PCR. Donors were haploidentical in 77.8% of cases. The 3-year overall survival (OS), leukemia-free survival (LFS), cumulative incidence of relapse (CIR), and non-relapse mortality (NRM) for the entire cohort were 78.6%, 76.3%, 14.3%, and 11.9%, respectively (median follow-up 42.4 months).
**Univariate analysis** showed that NR status at transplant was associated with significantly inferior OS (HR 16.41, P<0.001) and LFS (HR 30.22, P<0.01). Pre-transplant MFC-MRD positivity predicted inferior OS (HR 3.30, P=0.047). Pre-transplant ZNF384-r positivity predicted inferior LFS (HR 3.08, P=0.025). The presence of WT1 co-mutation was associated with higher CIR (HR 13.85, P=0.035). Importantly, the detection of ZNF384-r post-transplant was a powerful predictor of poor outcomes, with risk increasing over time: ZNF384-r positivity at 3 months post-transplant was strongly associated with higher CIR (HR 69.38, P=0.0001), inferior LFS (HR 19.14, P=0.0002), and inferior OS (HR 17.31, P=0.0006). In the CR subgroup, patients with both MFC-MRD and ZNF384-r positivity had particularly poor OS (HR 16.8, P=0.001) and LFS (HR 11.84, P=0.0028).
**Multivariable analysis** confirmed NR status as an independent adverse factor for OS, LFS, and CIR. Achieving CR after 1 cycle (CR1) was associated with better LFS compared to ≥CR2 (HR 0.33, P<0.048). Age, sex, fusion partner, and prior immunotherapy were not significant predictors.

**Copyright Information:** (Online) ISSN: 2572-9241
©2026 The Author(s). HemaSphere is published by John Wiley & Sons Ltd on behalf of European Hematology Association. This is an open access Abstract Book distributed under the Attribution-NonCommercial-NoDerivs (CC BY-NC-ND), which allows third parties to download the articles and share them with others as long as they credit the author and the Abstract Book, but they cannot change the content in any way or use them commercially.
**Abstract Book Citations:** Authors, Title, HemaSphere, 2026;10:(S1):pages. The abstract book DOIs can be found on the "Author Information" page.
**Disclaimer:** Articles published in the journal HemaSphere exclusively reflect the opinions of the authors. The authors are responsible for all content in their abstracts including accuracy of the facts, statements, citing resources, etc.

### Summary/Conclusion

Allo-HSCT is a safe and effective curative modality for ZNF384-r acute leukemia. Pre-transplant NR status and combined MRD positivity (MFC and ZNF384-r) in CR patients are associated with dismal outcomes. Early post-transplant detection of ZNF384-r, particularly by 3 months, identifies patients at ultra-high risk of relapse and poor survival, underscoring its value as a dynamic prognostic marker and a potential therapeutic target.

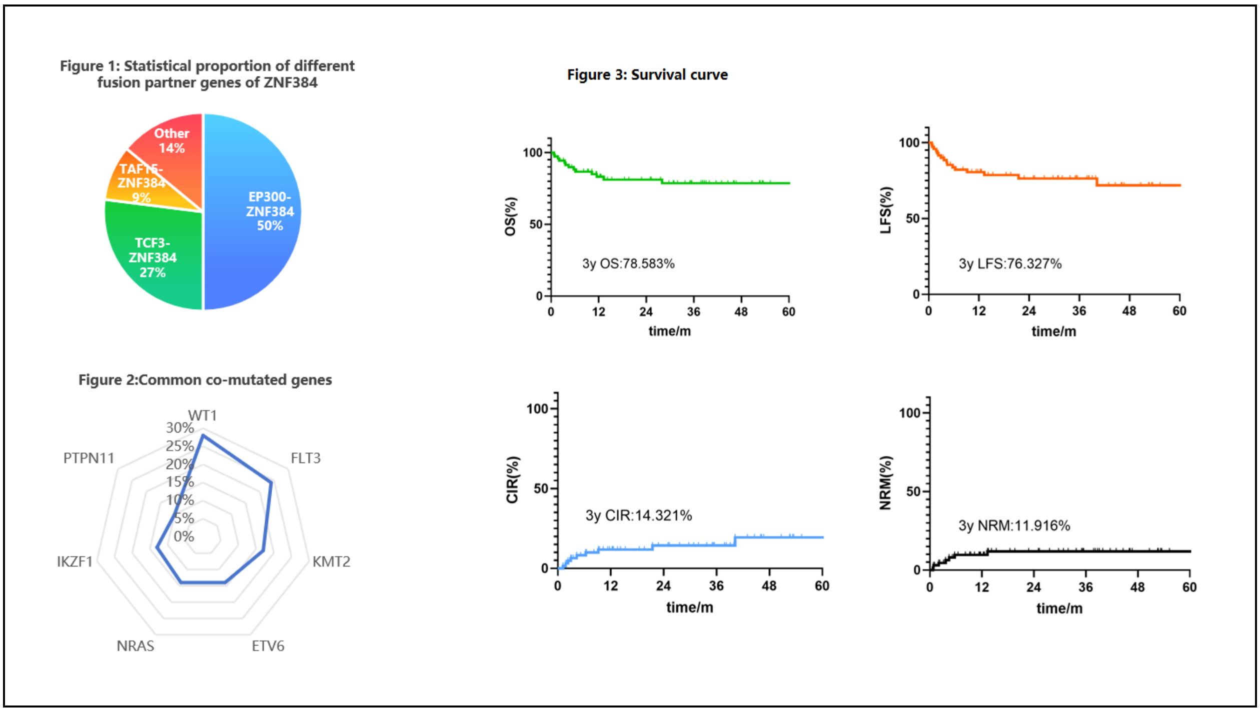

**Copyright Information:** (Online) ISSN: 2572-9241
©2026 The Author(s). HemaSphere is published by John Wiley & Sons Ltd on behalf of European Hematology Association. This is an open access Abstract Book distributed under the Attribution-NonCommercial-NoDerivs (CC BY-NC-ND), which allows third parties to download the articles and share them with others as long as they credit the author and the Abstract Book, but they cannot change the content in any way or use them commercially.
**Abstract Book Citations:** Authors, Title, HemaSphere, 2026;10:(S1):pages. The abstract book DOIs can be found on the "Author Information" page.
**Disclaimer:** Articles published in the journal HemaSphere exclusively reflect the opinions of the authors. The authors are responsible for all content in their abstracts including accuracy of the facts, statements, citing resources, etc.

### (PS2237) LATE RELAPSE RISK AFTER ALLOGENEIC HEMATOPOIETIC STEM CELL TRANSPLANTATION IN KMT2A - REARRANGED ACUTE MYELOID LEUKEMIA- A 20% INCIDENCE BEYOND TWO YEARS.

**Topic:** 23. Stem cell transplantation - Clinical

Yasir Jeelani*[1], Nisham Pandikasala Narikuth[2], Carol Chen[1], Eshrak Al-Shaibani[1], Tommy Alfaro-Moya[1], Igor Novitzky-Basso[1], Ivan Pasic[1], Fotios Michelis[1], Auro Viswabandya[1], Rajat Kumar[1], Jonas Mattsson[1], Dennis Kim[1]

*[1]Princess Margaret Cancer Centre, Medical Oncology and Haematology, Toronto, Canada;[2]Princess Margaret Cancer Centre, Toronto, Canada;*

### Background

**Background:**
KMT2A-rearranged (KMT2Ar) acute myeloid leukemia (AML) features a high-risk molecular subtype defined by 11q23 translocations involving the epigenetic regulator KMT2A. It demonstrates aggressive biology, chemoresistance, high relapse rates, and poor prognosis, supporting allogeneic hematopoietic stem cell transplantation (HCT) in first complete remission (CR1). Despite 50–60% overall survival post-HCT, relapse remains a major barrier to durable remission.
Menin inhibitors were approved by the U.S. FDA in 2024 for relapsed/refractory KMT2Ar AML. By disrupting the Menin–KMT2A interaction, these agents achieved a 63% overall response rate and 23% complete remission in phase II studies. This study shows single-center transplant outcomes in KMT2Ar AML without post-HCT Menin inhibitor maintenance, providing a historical benchmark for this high-risk population.

### Aims

To analyze the incidence of relapse post-HCT in KMT2r AML patients without Menin inhibitor as post-HCT maintenance.

### Methods

**Patients and Methods:**
This is a multicenter retrospective study of patients who received allogeneic HCT at Princess Margaret Cancer Centre between 2010 and 2025. Data were extracted from the institutional BMT database. Parameters analyzed included overall survival (OS), relapse-free survival (RFS), cumulative incidence of relapse (CIR), non-relapse mortality (NRM), and graft-versus-host disease (GvHD).

**Copyright Information:** (Online) ISSN: 2572-9241
©2026 The Author(s). HemaSphere is published by John Wiley & Sons Ltd on behalf of European Hematology Association. This is an open access Abstract Book distributed under the Attribution-NonCommercial-NoDerivs (CC BY-NC-ND), which allows third parties to download the articles and share them with others as long as they credit the author and the Abstract Book, but they cannot change the content in any way or use them commercially.
**Abstract Book Citations:** Authors, Title, HemaSphere, 2026;10:(S1):pages. The abstract book DOIs can be found on the “Author Information” page.
**Disclaimer:** Articles published in the journal HemaSphere exclusively reflect the opinions of the authors. The authors are responsible for all content in their abstracts including accuracy of the facts, statements, citing resources, etc.

### Results

The cohort consisted of 60 patients with a median age of 49.5 years (19–69 years), 32 (53%) were female. Conditioning regimen was myeloablative (MAC) in 33 patients (55%), while donor sources included matched related donors (MRD) in 17 (28%), unrelated donors (URD) in 36 (60%) comprising 10/10 HLA-matched (n=29) and 9/10 mismatched cases (n=7) and haploidentical donors (n=7, 12%).
Among the 26 patients with available cytogenetic information on partner chromosome, the most frequent was chromosome 9 (n=11, 42%), followed by 19 in (n=6, 23%), 6 and 10 each (n=3, 11% each), 17 (n=2, 8%), and 7 (n=1, 4%).

Median follow-up duration was 5.1 years among survivors; a total of 29 deaths (48%) and 22 relapses (37%) were observed. The 5-year OS, RFS, CIR and NRM rate was 45.5% [31.3–58.7%], 44.9% [30.9–57.9%], 37.6% [24.8–50.3%] and 17.5% [8.4–29.5%). Late relapses beyond 2 years post-HCT occurred in 4 of the 22 relapse cases (18%), thus reaching 10-year CIR of 17.5% [8.4–29.5%]. The late relapse was observed at 2.1, 2.2, 3.8, and 9.7 years after HCT, respectively. The incidence of acute and chronic GvHD was 54.5% [40.7–66.4%] by day +100, and 28.3% [17.1–40.6%] at 2-years.
Univariable analysis showed no significant effect of 11q23 partner chromosome, conditioning intensity (myeloablative versus reduced-intensity, or age on OS, RFS, or CIR (all p>0.5). In contrast, chronic GvHD was associated with improved outcomes, with significantly higher 5-year OS (60.4% vs. 39.5%; p=0.05) and favorable trends for RFS and CIR, albeit with borderline significance

### Summary/Conclusion

Allogeneic HCT in KMT2Ar AML achieves meaningful long-term survival but carries substantial relapse risk, particularly late relapse beyond two years, far exceeding the <5% late relapse rate observed in most AML subtypes.
This sustained risk of late relapse warrants incorporation into the design of prospective trials assessing post-HCT, Menin inhibitor maintenance to optimize long-term disease control.

**Copyright Information:** (Online) ISSN: 2572-9241
©2026 The Author(s). HemaSphere is published by John Wiley & Sons Ltd on behalf of European Hematology Association. This is an open access Abstract Book distributed under the Attribution-NonCommercial-NoDerivs (CC BY-NC-ND), which allows third parties to download the articles and share them with others as long as they credit the author and the Abstract Book, but they cannot change the content in any way or use them commercially.
**Abstract Book Citations:** Authors, Title, HemaSphere, 2026;10:(S1):pages. The abstract book DOIs can be found on the "Author Information" page.
**Disclaimer:** Articles published in the journal HemaSphere exclusively reflect the opinions of the authors. The authors are responsible for all content in their abstracts including accuracy of the facts, statements, citing resources, etc.

### (PS2238) UPFRONT ALLOGENEIC STEM CELL TRANSPLANTATION USING ARCHIVE CONDITIONING IN ACUTE MYELOID LEUKEMIA

**Topic:** 23. Stem cell transplantation - Clinical

Andrius Žučenka*[1, 2], Tautvilė Smalinskaitė[1, 2], Guoda Daukėlaitė[1, 2], Milda Mikalonytė[1, 2], Milvydė Tamutytė-Jankauskė[1, 2], Lukas Kevlicius[1], Artūras Slobinas[1], Renata Jucaitienė[1], Inga Šlepikienė[1], Linas Davainis[1], Rita Cekauskiene[1, 2], Vaida Jaraminė[1], Mingaudas Busevičius[1], Justė Strumskytė[1], Gabrielė Buikauskienė[1], Antanas Griškevičius[1], Artūras Jakubauskas[1], Adomas Bukauskas[1], Valdas Pečeliūnas[1, 2], Laimonas Griškevičius[1, 2]

*[1]Vilnius University Hospital Santaros Klinikos, National Cancer Center, Hematology, Oncology, and Transfusion Medicine Center, Vilnius, Lithuania; [2]Vilnius University, Faculty of Medicine, Hematology and Oncology Department, Vilnius, Lithuania;*

### Background

Inefficacy and toxicity of induction regimens result in a proportion of adverse-risk acute myeloid leukemia (AML) patients not being allotransplanted and facing a very poor prognosis. We report a novel conditioning regimen, ARCHIVE (alkylating agents, cytarabine, cladribine, hypomethylating agents, and venetoclax), for upfront allogeneic stem cell transplantation (alloSCT) in patients with newly diagnosed adverse-risk or R/R AML without conventional remission induction.

### Aims

The aim of the study was to evaluate the efficacy and toxicity of upfront alloSCT using ARCHIVE conditioning.

### Methods

This was an observational single-center study. Treatment and data collection were approved by the Vilnius University Hospital Santaros Klinikos Hematology Board and the Vilnius Regional Biomedical Research Ethics Committee. Patients were ≥18 years, had active AML or MDS/AML (>5% blasts and/or biopsy-confirmed extramedullary disease), and were considered alloSCT-eligible. ARCHIVE conditioning regimen consisted of cytarabine 500 or 1000 mg/m$^2$, cladribine 5 mg/m$^2$ (days -8, -7, -6, -5, -4), venetoclax 600 mg/d and HMA (decitabine 20 mg/m$^2$ / azacitidine 75 mg/m$^2$) on days -8, -7, -6, -5, -4, -3, -2. The backbone alkylating agent was either busulfan (3.2 mg/kg on days -3 and/or -2) or melphalan (100 mg/m2 on day -2). Thiotepa 5-10 mg/kg was added in selected cases. Gilteritinib 120 mg/d was administered with ARCHIVE until day -2 in *FLT3*-mutated AML. Idarubicin or etoposide was added on days -8 and -7 in selected cases. GVHD prophylaxis consisted of cyclophosphamide 40 mg/kg (days +3, +4), calcineurin inhibitor, and mycophenolate mofetil.
We collected patient and disease characteristics, CR+CRp rate, MRD negativity rate (by MFC and PCR), time-to-engraftment, grade 3-5 non-hematological adverse events, OS, and RFS.

**Copyright Information:** (Online) ISSN: 2572-9241
©2026 The Author(s). HemaSphere is published by John Wiley & Sons Ltd on behalf of European Hematology Association. This is an open access Abstract Book distributed under the Attribution-NonCommercial-NoDerivs (CC BY-NC-ND), which allows third parties to download the articles and share them with others as long as they credit the author and the Abstract Book, but they cannot change the content in any way or use them commercially.
**Abstract Book Citations:** Authors, Title, HemaSphere, 2026;10:(S1):pages. The abstract book DOIs can be found on the "Author Information" page.
**Disclaimer:** Articles published in the journal HemaSphere exclusively reflect the opinions of the authors. The authors are responsible for all content in their abstracts including accuracy of the facts, statements, citing resources, etc.

### Results

40 patients were enrolled, with their baseline characteristics summarized in Table 1.
The CR+CRp rate at day +30 post-alloSCT was 97% (38/39). MRD negativity was achieved in 95% (35/37) of MRD-evaluable responders. The median time to neutrophil recovery was 17 (13-30) days after the stem cell infusion.
The most common grade 3-5 toxicities were infectious complications (88%, 35/40) and mucositis (40%, 16/40). Adverse events of special interest were VOD (5%, 2/40), secondary graft failure (2%, 1/40), and grade 3-4 acute GVHD (23%, 9/40). Day 30 mortality rate was 5% (2/40).
After the median follow-up of 8.3 months, 2 relapses and 9 deaths were confirmed. The median OS and RFS were not reached (Figure 1).

**Table 1**

| **Characteristics** | **Patients (N40)** |
|---|---|
| Female | 60% (24/40) |
| Age (years), median (min-max) | 57 (23-70) |
| ECOG 0-1 | 72% (29/40) |
| ECOG 2 | 28% (11/40) |
| AML | 83% (33/40) |
| MDS/AML | 17% (7/40) |
| ELN 2022 adverse risk group | 80% (32/40) |
| *MECOM*r | 20% (8/40) |
| *TP53*m | 15% (6/40) |
| ELN 2022 Adverse karyotype | 25% (10/40) |
| Newly diagnosed disease | 18% (7/40) |
| Relapsed/refractory disease | 82% (33/40) |
| 1 prior treatment line | 64% (21/33) |
| >1 prior treatment line | 36% (12/33) |
| Prior Venetoclax | 55% (18/33) |
| Prior *FLT3* inhibitors | 9% (3/33) |
| Prior Revumenib | 6% (2/33) |
| Prior alloSCT | 21% (7/33) |
| Blast count % prior ARCHIVE, median (min-max) | 14 (0-85) |
| Matched sibling donor | 7% (3/40) |
| Matched unrelated donor | 35% (14/40) |
| Mismatched unrelated donor | 13% (5/40) |
| Haploidentical donor | 45% (18/40) |

### Summary/Conclusion

Upfront alloSCT with ARCHIVE conditioning demonstrates high antileukemic efficacy without conventional remission induction in adverse-risk AML. However, high rates of infectious complications and GVHD are notable.

**Copyright Information:** (Online) ISSN: 2572-9241
©2026 The Author(s). HemaSphere is published by John Wiley & Sons Ltd on behalf of European Hematology Association. This is an open access Abstract Book distributed under the Attribution-NonCommercial-NoDerivs (CC BY-NC-ND), which allows third parties to download the articles and share them with others as long as they credit the author and the Abstract Book, but they cannot change the content in any way or use them commercially.
**Abstract Book Citations:** Authors, Title, HemaSphere, 2026;10:(S1):pages. The abstract book DOIs can be found on the "Author Information" page.
**Disclaimer:** Articles published in the journal HemaSphere exclusively reflect the opinions of the authors. The authors are responsible for all content in their abstracts including accuracy of the facts, statements, citing resources, etc.

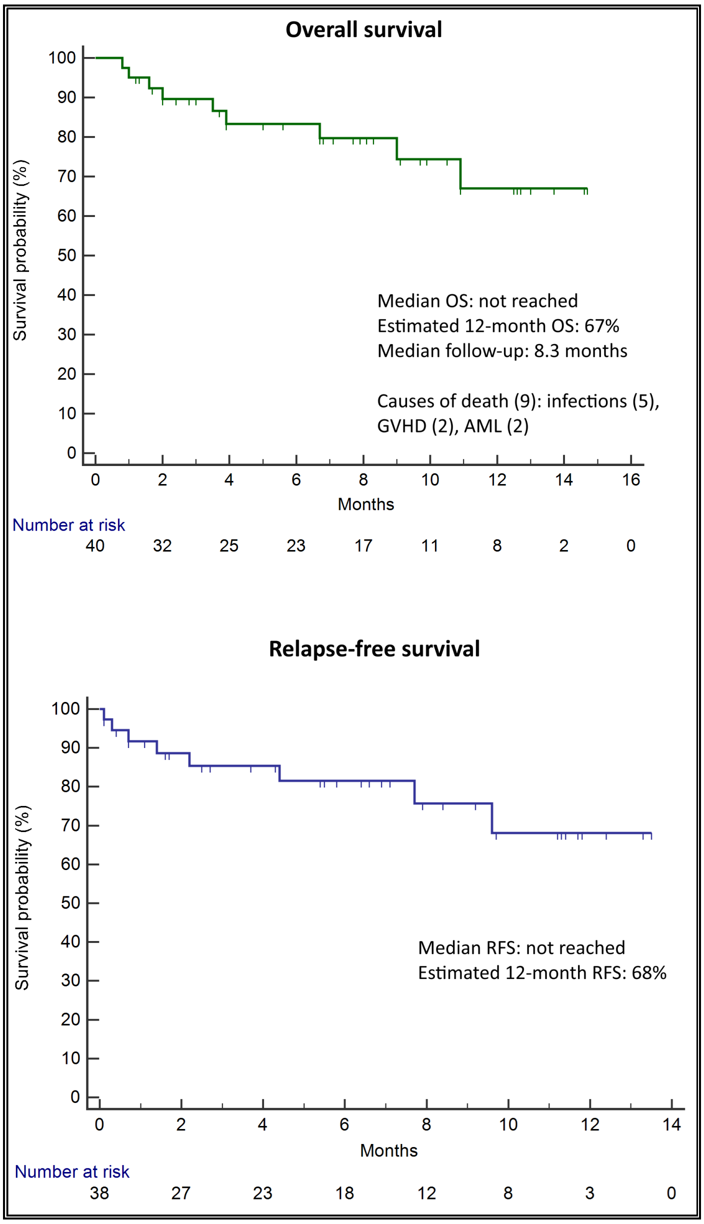

**Copyright Information:** (Online) ISSN: 2572-9241
©2026 The Author(s). HemaSphere is published by John Wiley & Sons Ltd on behalf of European Hematology Association. This is an open access Abstract Book distributed under the Attribution-NonCommercial-NoDerivs (CC BY-NC-ND), which allows third parties to download the articles and share them with others as long as they credit the author and the Abstract Book, but they cannot change the content in any way or use them commercially.
**Abstract Book Citations:** Authors, Title, HemaSphere, 2026;10:(S1):pages. The abstract book DOIs can be found on the "Author Information" page.
**Disclaimer:** Articles published in the journal HemaSphere exclusively reflect the opinions of the authors. The authors are responsible for all content in their abstracts including accuracy of the facts, statements, citing resources, etc.

### (PS2239) LINEAGE-SPECIFIC CHIMERISM ANALYSIS AFTER ALLOGENEIC HEMATOPOIETIC STEM CELL TRANSPLANTATION (ALLO-HSCT) IN LYMPHOPROLIFERATIVE DISORDERS: EVOLUTION, OUTCOMES AND IMPACT OF DONOR LYMPHOCYTE INFUSIONS

**Topic:** 23. Stem cell transplantation - Clinical

Jesus Feliu*[1], Hugues de Lavallade[2], Francesco Grimaldi[3], Victoria Potter[4], Tony Pagliuca[5], Michelle Kenyon[6], Ricardo Francisco Garcia Muñoz[1], Javier Larreina-Perez[1], Montserrat Hernandez-Perez[1], Ada Esteban-Figuerola[1], Andrea Campeny-Najara[1], Sofia Rincon-Lopez[1], Johelys Atencio-Matos[1], Olalla Pereda-Sainz[1], Jone Alberdi Ballina[7,7], Raisa Soraya Peralta Benitez[8]

*[1]Hospital Universitario San Pedro, Hematology Department, Logroño, Spain;[2]Guy's and St Thomas' Foundation Trust, Hematology Department, London, United Kingdom; [3]Divisione di Ematologia, Università Degli Studi Federico II, Hematology Department. U.O.C di Ematologia e Trapianto di midollo, Napoli, Italy;[4]King's College Hospital, Hematology Department, London, United Kingdom; [5]Anthony Nolan, King's College School of Medicine and Dentistry, External Medical Adviser, London, United Kingdom; [6]King's College Hospital, Hematology Department, London, Italy; [7]Hospital Reina Sofía, Hematology Department, Tudela, Spain;[8]Clínica San Miguel, Hematology Department, Pamplona, Spain;*

### Background

Mixed chimerism (MC) is frequently observed after allo-HSCT, particularly in lymphoproliferative disorders (LPD) treated with reduced-intensity conditioning and T-cell depletion. Its prognostic significance remains controversial.

### Aims

Describe the evolution of T-cell chimerism after allo-HSCT for LPD and assess its association with clinical outcomes and the impact of donor lymphocyte infusions (DLI).

### Methods

We retrospectively analyzed 109 LPD patients who underwent allo-HSCT at King's College Hospital between 2002 and 2013 and were alive in CR at 3 months (m) post-transplant. Full Donor Chimerism (FDC) was defined as peripheral blood CD3+ ≥95%; values <95% were considered MC (MC1 50-94%, MC2 <50%). Persistent MC (PMC) was defined as MC beyond 24m. Landmark and survival analyses were performed to evaluate overall survival (OS), event-free survival (EFS) and relapse risk.

**Copyright Information:** (Online) ISSN: 2572-9241
©2026 The Author(s). HemaSphere is published by John Wiley & Sons Ltd on behalf of European Hematology Association. This is an open access Abstract Book distributed under the Attribution-NonCommercial-NoDerivs (CC BY-NC-ND), which allows third parties to download the articles and share them with others as long as they credit the author and the Abstract Book, but they cannot change the content in any way or use them commercially.
**Abstract Book Citations:** Authors, Title, HemaSphere, 2026;10:(S1):pages. The abstract book DOIs can be found on the "Author Information" page.
**Disclaimer:** Articles published in the journal HemaSphere exclusively reflect the opinions of the authors. The authors are responsible for all content in their abstracts including accuracy of the facts, statements, citing resources, etc.

### Results

MC was present in 47%, 42% and 31% of patients at 3, 6 and 12m respectively; 19% had PMC at 24m. Among patients with MC at 3m ($MC_{3m}$) 57% subsequently converted to FDC. **Table 1**

**Table 1:** Chimerism evolution based on 3 month engraftment status.

| | 3m | 6m | | | 12m | | | 24m | | |
|---|---|---|---|---|---|---|---|---|---|---|
| Graft status | | FDC | MC1 | MC2 | FDC | MC1 | MC2 | FDC | MC1 | MC2 |
| FDC | 53 (51%) | 51 (49%) | 1 (1%) | 1 (1%) | 41 (45%) | 7 (8%) | 0 (0%) | 34 (47%) | 2 (3%) | 0 (0%) |
| MC1 | 32 (31%) | 8 (8%) | 20 (19%) | 4 (4%) | 12 (13%) | 10 (11%) | 4 (4%) | 13 (18%) | 8 (11%) | 1 (1%) |
| MC2 | 19 (18%) | 1 (1%) | 6 (6%) | 12 (11%) | 6 (7%) | 7 (8%) | 4 (4%) | 8 (11%) | 6 (8%) | 0 (0%) |

***Impact of early and late chimerism status on outcome***

Early (3m) T-cell chimerism status ($FDC_{3m}$, $MC1_{3m}$ or $MC2_{3m}$) showed no significant impact on OS ($p$=0.396) or EFS (p=0.746) **Figure 1**, and neither conversion from $MC_{3m}$ to FDC nor return from $FDC_{3m}$ to MC influenced relapse risk within the first 24m. Pre-emptive DLI in patients with MC did not reduce relapse risk ($p$=1.000) or improve survival ($p$=0.293).

At 24m post-HSCT 81% patients were FDC and 19% PMC. Relapse risk was not associated with chimerism status ($p$=0.270). Landmark analyses incorporating both chimerism and prior DLI exposure confirmed no differences between the 4 groups (FDC+pDLI, FDC+no pDLI, MC+pDLI, MC+no pDLI) in either OS ($p$=0.222) or EFS (p=0.282).

***Impact of chimerism patterns on outcome***

Among patients alive and in CR at 12m, 4 different chimerism patterns were identified: FDC at all post-HSCT time points; MC at all post-HSCT time points; $FDC_{3m}$ to MC; and $MC_{3m}$ to FDC; while EFS was similar, patients converting from $MC_{3m}$ to FDC had superior OS compared with those with sustained FDC ($p$=0.002) **Figure 2**

***DLI***

29% Patients received either pre-emptive (p) or therapeutic (t) DLI. 79% pDLI patients achieved FDC and never went back to MC; OS and EFS in a pDLI landmark analyses at 24m showed no statistical difference between the 2 groups (pDLI vs no pDLI) in either EFS ($p$=0.912) or OS ($p$=0.233). 62% tDLI patients achieved CR. 4 years after the first tDLI dose >90% patients were still alive

***Chronic graft-versus-host disease (cGvHD)***

cGvHD occurred in 64% patients, was more frequent in those with sustained FDC and moderate/severe cGvHD was associated with worse OS ($p$<0.0001) and EFS ($p$<0.0001).

### Summary/Conclusion

MC is common after allo-HSCT for LPD, and does not adversely affect survival. Conversion from $MC_{3m}$ to FDC is associated with improved OS, whereas sustained FDC correlates with higher incidence of cGvHD and poorer outcomes. DLI promotes FDC without clear survival benefit. Prospective studies are needed to better define the biological and clinical implications of chimerism dynamics.

**Copyright Information:** (Online) ISSN: 2572-9241
©2026 The Author(s). HemaSphere is published by John Wiley & Sons Ltd on behalf of European Hematology Association. This is an open access Abstract Book distributed under the Attribution-NonCommercial-NoDerivs (CC BY-NC-ND), which allows third parties to download the articles and share them with others as long as they credit the author and the Abstract Book, but they cannot change the content in any way or use them commercially.
**Abstract Book Citations:** Authors, Title, HemaSphere, 2026;10:(S1):pages. The abstract book DOIs can be found on the "Author Information" page.
**Disclaimer:** Articles published in the journal HemaSphere exclusively reflect the opinions of the authors. The authors are responsible for all content in their abstracts including accuracy of the facts, statements, citing resources, etc.

**Figure 1:** OS (A) and EFS (B) based on the T-cell engraftment status (FDC / MC1 / MC2) at 3 months

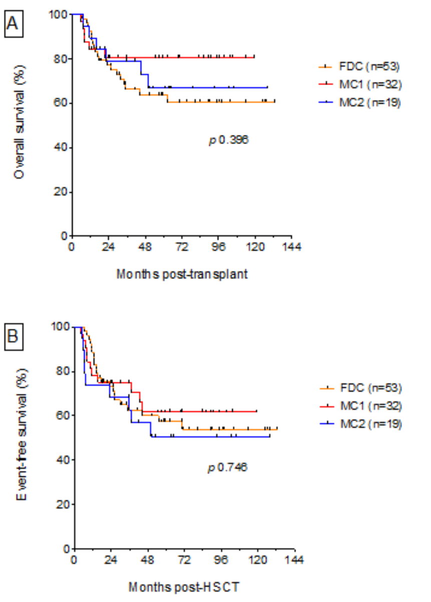

FDC: full donor chimerism (CD3+ = or > 95%)
MC1: mixed chimerism type 1 (CD3+ fraction 50-94%)
MC2: mixed chimerism type 2 (CD3+ fraction <50%).

**Figure 2:** OS (A) and EFS (B) based on the T-cell chimerism patterns MC to FDC / continued FDC / continued MC. Landamark analysis 12m post-Allo

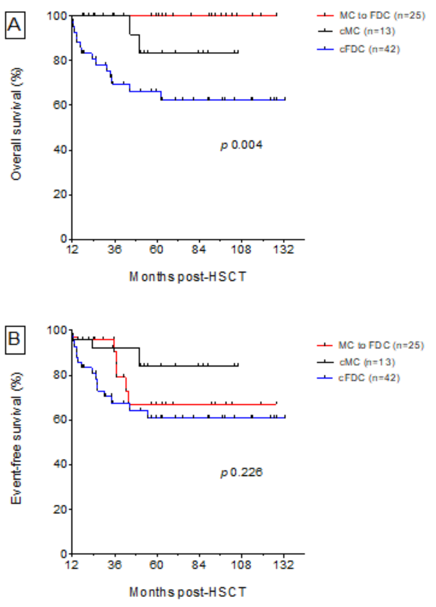

FDC: full donor chimerism (CD3+ = or > 95%)
MC: mixed chimerism (CD3+ fraction <95%)
cFDC: continued FDC; cMC: continued MC.

**Copyright Information:** (Online) ISSN: 2572-9241
©2026 The Author(s). HemaSphere is published by John Wiley & Sons Ltd on behalf of European Hematology Association. This is an open access Abstract Book distributed under the Attribution-NonCommercial-NoDerivs (CC BY-NC-ND), which allows third parties to download the articles and share them with others as long as they credit the author and the Abstract Book, but they cannot change the content in any way or use them commercially.
**Abstract Book Citations:** Authors, Title, HemaSphere, 2026;10:(S1):pages. The abstract book DOIs can be found on the "Author Information" page.
**Disclaimer:** Articles published in the journal HemaSphere exclusively reflect the opinions of the authors. The authors are responsible for all content in their abstracts including accuracy of the facts, statements, citing resources, etc.

### (PS2240) ALLO-HSCT OVERCOME POOR PROGNOSIS IN PATIENTS WITH NUP98::NSD1-POSITIVE AML

**Topic:** 23. Stem cell transplantation - Clinical

Hai He*[1], Yue Lu[1], Jianping Zhang[1], Xingyu Cao[1], Deyan Liu[1], Zhijie Wei[1], Yan-Li Zhao[1], Min Xiong[1], Jiarui Zhou[1], Rui-Juan Sun[1]

[1]*Hebei Yanda Lu Daopei Hospital, Bone Marrow Transplantation, Langfang, China;*

### Background

NUP98::NSD1 is an adverse prognostic factor in acute myeloid leukemia (AML), characterized by a low incidence and poor response to chemotherapy. Its co-expression with FLT3-ITD synergistically worsens survival outcomes. Allogeneic stem cell transplantation (HSCT) may overcome this unfavorable molecular prognosis; however, current investigations in this regard remain limited.

### Aims

To evaluate the safety and efficacy of Allo-HSCT for NUP98::NSD1-positive AML.

### Methods

This study enrolled patients with NUP98::NSD1-positive AML who underwent HSCT between June 2019 and November 2025. The observational endpoints included overall survival (OS), disease-free survival (DFS), cumulative incidence of relapse (CIR), non-relapse mortality (NRM), and the cumulative incidence of acute and chronic graft-versus-host disease (GVHD). All outcome measures were calculated from the time of stem cell infusion.

### Results

A total of 56 patients with NUP98::NSD1-positive AML were enrolled, comprising 31 males and 25 females, with a median age of 30 (14-70 )years. At diagnosis, 21 patients (37.5%) had peripheral blood leukocyte counts exceeding $100\times10^9$/L. The most frequent co-occurring genetic mutation was FLT3-ITD, (n=50, 89.3%), followed by WT1 (n=27, 48.2%), RAS(n=8, 14.3%), CEBPA (n=8, 14.3%), RUNX1 (n=5,8.9%), and TET2 (n=5, 8.9%). The remission rate after the first induction course was 26.8% (15/56), and after the second induction course, it was 28.2% (11/39). 67.9% patients underwent transplantation from a haploidentical donor. Regarding status before HSCT: 29 patients (51.8%) in CR1, 6 (10.7%) in CR2, and 21 (37.5%) in NR status. Myeloablative conditioning regimens administered included BU/Cy in 30 patients (53.6%), BU/Flu in 24 patients (42.9%), and TBI/Cy in 2 patients (3.6%). All patients achieved successful hematopoietic reconstitution post-transplant. 100% leukemia complete remission rate at 30 days post-transplant and 82% (46/56) obtained molecular MRD-. With a median follow-up of 32 (2-79)months for the entire cohort, the 3-year OS and DFS rates were 78.43% (95% CI: 63.5-87.8) and 77.64% (95% CI: 63.3-86.9), respectively. The 3-year CIR and NRM were 10.74% (95% CI: 4.5-25.6) and 11.71% (95% CI: 5.5-24.9), respectively. The 100-day cumulative incidence of grade Ⅱ-Ⅳ aGVHD was25% (95% CI: 15.9-39.3) and 12.5% (95% CI: 6.25-25) for grade Ⅲ-Ⅳ aGVHD. The 3-year cumulative incidence of cGVHD was 44.8% (95% CI: 32.1-62.5), and the cumulative incidence of extensive cGVHD was 9.9% (95% CI: 3.8-25.4). No statistically significant difference was observed in 3-year OS and DFS between patients transplanted in CR versus those in NR (OS: 84.17% vs. 70.59%, *P*=0.40; DFS: 84.27% vs. 67.76%, *P*=0.19). Similarly, outcomes did not differ significantly between patients without and with FLT3-ITD at diagnosis (OS: 83.3% vs. 78.0%, *P*=0.89; DFS: 83.33% vs. 77.2%, *P*=0.79). A total of 9 patients died, with causes of death including infection (n=2), aGVHD (n=1), cGVHD (n=1), relapse (n=4), and unknown causes (n=1).

**Copyright Information:** (Online) ISSN: 2572-9241
©2026 The Author(s). HemaSphere is published by John Wiley & Sons Ltd on behalf of European Hematology Association. This is an open access Abstract Book distributed under the Attribution-NonCommercial-NoDerivs (CC BY-NC-ND), which allows third parties to download the articles and share them with others as long as they credit the author and the Abstract Book, but they cannot change the content in any way or use them commercially.
**Abstract Book Citations:** Authors, Title, HemaSphere, 2026;10:(S1):pages. The abstract book DOIs can be found on the "Author Information" page.
**Disclaimer:** Articles published in the journal HemaSphere exclusively reflect the opinions of the authors. The authors are responsible for all content in their abstracts including accuracy of the facts, statements, citing resources, etc.

### Summary/Conclusion

Allo-HSCT overcome the adverse prognosis NUP98::NSD1-positive AML. Survival outcomes following transplantation were not significantly influenced by pre-transplant disease status or the presence of a concomitant FLT3-ITD mutation.

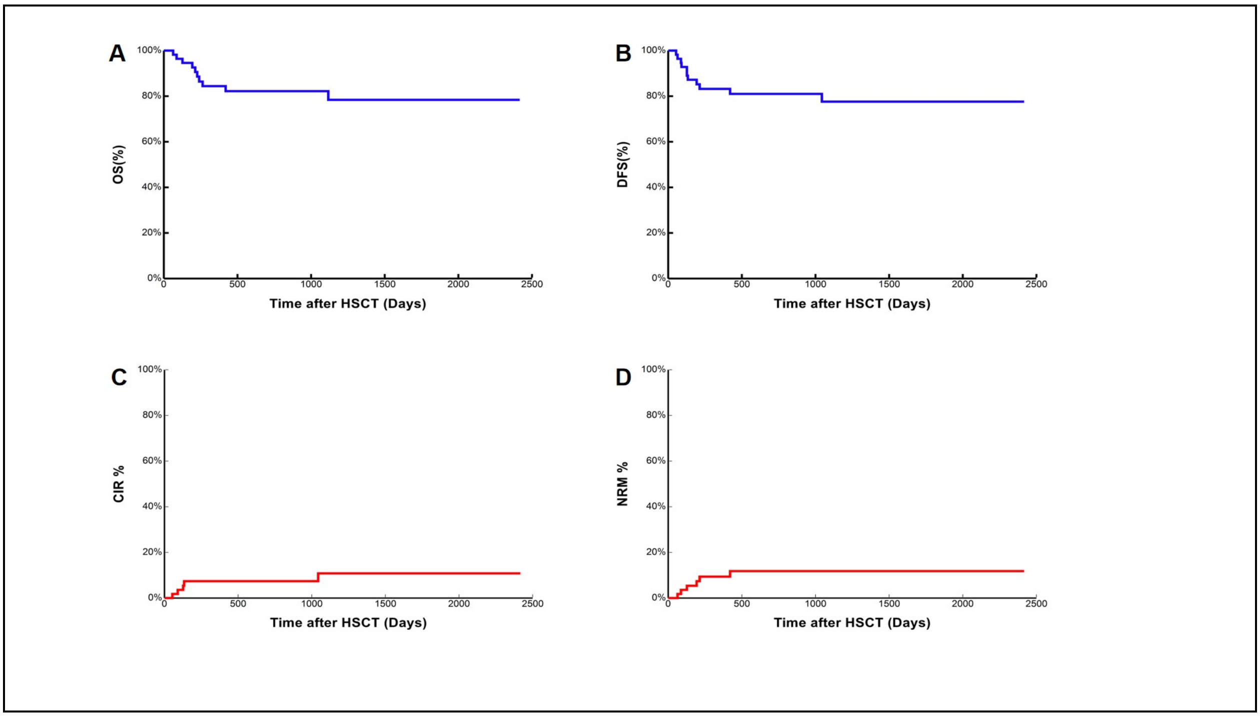

Fig. (A) Overall survival. (B) Disease-free survival. (C) Cumulative incidence of relapse. (D) Non-relapse mortality.

**Copyright Information:** (Online) ISSN: 2572-9241
©2026 The Author(s). HemaSphere is published by John Wiley & Sons Ltd on behalf of European Hematology Association. This is an open access Abstract Book distributed under the Attribution-NonCommercial-NoDerivs (CC BY-NC-ND), which allows third parties to download the articles and share them with others as long as they credit the author and the Abstract Book, but they cannot change the content in any way or use them commercially.
**Abstract Book Citations:** Authors, Title, HemaSphere, 2026;10:(S1):pages. The abstract book DOIs can be found on the "Author Information" page.
**Disclaimer:** Articles published in the journal HemaSphere exclusively reflect the opinions of the authors. The authors are responsible for all content in their abstracts including accuracy of the facts, statements, citing resources, etc.

### (PS2241) MEASURABLE RESIDUAL KMT2A PARTIAL TANDEM DUPLICATION BEFORE ALLOGENEIC STEM CELL TRANSPLANTATION IN ACUTE MYELOID LEUKEMIA

**Topic:** 23. Stem cell transplantation - Clinical

Lulu Wang*[1, 2, 3], Haixia Fu[1, 2, 3], Peng Zhao[1, 2, 3], Zhuo-Yu An[1, 2, 3], Yun He[1, 2, 3], Qiu-Sha Huang[1, 2, 3], Yu-Hong Chen[1, 2, 3], Meng Lv[1, 2, 3], Jun Kong[1, 2, 3], Zhidong Wang[1, 2, 3], Wei Han[1, 2, 3], Yuan-Yuan Zhang[1, 2, 3], Xiao-Dong Mo[1, 2, 3], Tingting Han[1, 2, 3], Chen-Hua Yan[1, 2, 3], Ying-Jun Chang[1, 2, 3], Fengrong Wang[1, 2, 3], Xiangyu Zhao[1, 2, 3], Yu Wang[1, 2, 3], Lan-Ping Xu[1, 2, 3], Xiao-Jun Huang[1, 2, 3], Xiaohui Zhang[1, 2, 3]

*[1]Peking University People's Hospital, Peking University Institute of Hematology, Beijing, China;[2]National Clinical Research Center for Hematologic Disease, Beijing, China; [3]Beijing Key Laboratory of Cell and Gene Therapy for Hematologic Malignancies, Peking University, Beijing, China;*

### Background

The KMT2A partial tandem duplication (*KMT2A-PTD*) results in an in-frame elongated protein and occurs in approximately 5–10% of acute myeloid leukemia (AML) cases. PCR-based *KMT2A-PTD* detection is a reliable measurable residual disease (MRD) marker associated with poor prognosis, but evidence from larger cohorts remains limited.

### Aims

This study evaluated the prognostic impact of pre-transplant MRD assessed by real-time quantitative PCR in*KMT2A-PTD* AML patients undergoing allogeneic hematopoietic stem cell transplantation (HSCT).

### Methods

This retrospective study included 165 adults with *KMT2A-PTD* AML who underwent allogeneic HSCT at Peking University People's Hospital between January 2015 and January 2025. Pre-transplant *KMT2A-PTD* MRD was assessed in bone marrow by real-time quantitative PCR within two months prior to HSCT. Overall survival (OS) and event-free survival (EFS) were estimated by Kaplan-Meier; cumulative incidence of relapse (CIR) and non-relapse mortality (NRM) were analyzed using competing risk models. Multivariable Cox regression was performed to identify independent prognostic factors.

**Copyright Information:** (Online) ISSN: 2572-9241
©2026 The Author(s). HemaSphere is published by John Wiley & Sons Ltd on behalf of European Hematology Association. This is an open access Abstract Book distributed under the Attribution-NonCommercial-NoDerivs (CC BY-NC-ND), which allows third parties to download the articles and share them with others as long as they credit the author and the Abstract Book, but they cannot change the content in any way or use them commercially.
**Abstract Book Citations:** Authors, Title, HemaSphere, 2026;10:(S1):pages. The abstract book DOIs can be found on the "Author Information" page.
**Disclaimer:** Articles published in the journal HemaSphere exclusively reflect the opinions of the authors. The authors are responsible for all content in their abstracts including accuracy of the facts, statements, citing resources, etc.

### Results

Median age at transplantation was 45 years, and median follow-up was 43.8 months. *FLT3-ITD* mutations were present in 51 patients (31%). At HSCT, 135 patients were in first complete remission (CR1), 10 in second remission (CR2), and 20 had refractory disease (RD). For CR patients, 3-year OS/EFS were 73%/64% (CR1) and 70%/60% (CR2). RD patients had 3-year OS/EFS of 53%. Three-year CIR was 30% (CR1), 33% (CR2), and 22% (RD); NRM was 8.5%, 10%, and 32%, respectively. CR patients had significantly better OS and lower NRM than RD patients (P=0.025 and P=0.009), while CIR and EFS did not differ significantly between groups.
Of 145 CR patients, pre-transplant MRD was assessed in 131. A threshold of 1000 copies provided optimal discrimination: 3-year OS 79% (MRD–, n=95) vs. 58% (MRD+, n=36, P=0.01); 3-year EFS 71% vs. 52% (P=0.02). MRD+ patients had higher CIR (37% vs. 25%, P=0.12) and NRM (17% vs. 5.7%, P=0.07).
*FLT3-ITD* positivity was associated with higher 3-year CIR (40% vs. 15%, P=0.006), but not with OS and EFS. In MRD-negative patients, *FLT3-ITD* increased CIR (34% *FLT3*-positive [n=28] vs. 20% *FLT3*-negative [n=67], P=0.044) without affecting OS or EFS. In MRD-positive patients, *FLT3-ITD* showed markedly worse outcomes (3-year OS: 39% vs. 71%, P=0.053; EFS: 39% vs. 61%, P=0.12; CIR: 52% vs. 28%, P=0.1), though did not reach statistical significance, likely due to small sample size (n=14).
Multivariable analysis identified pre-transplant MRD as an independent predictor of inferior OS (HR=3.18, P=0.003) and EFS (HR=2.40, P=0.003). Age >50 years was also independently associated with worse OS (HR=4.24, P<0.001) and EFS (HR=2.95, P=0.001). For CIR, age >50 years was the only independent risk factor (HR=2.14, P=0.037). For NRM, both age >50 years (HR=5.24, P=0.014) and poor platelet engraftment (HR=4.03, P=0.028) retained independent prognostic significance.
The 3-year OS rate of patients transplanted in refractory disease was comparable to that observed in pre-transplant MRD-positive patients (P=0.56).

### Summary/Conclusion

Pre-transplant *KMT2A-PTD* MRD independently predicts post-HSCT outcomes in AML. MRD positivity with *FLT3-ITD* identifies an extremely high-risk population. Refractory disease patients had outcomes similar to MRD-positive patients in remission. These findings support routine MRD assessment and warrant prospective validation.

**Copyright Information:** (Online) ISSN: 2572-9241
©2026 The Author(s). HemaSphere is published by John Wiley & Sons Ltd on behalf of European Hematology Association. This is an open access Abstract Book distributed under the Attribution-NonCommercial-NoDerivs (CC BY-NC-ND), which allows third parties to download the articles and share them with others as long as they credit the author and the Abstract Book, but they cannot change the content in any way or use them commercially.
**Abstract Book Citations:** Authors, Title, HemaSphere, 2026;10:(S1):pages. The abstract book DOIs can be found on the "Author Information" page.
**Disclaimer:** Articles published in the journal HemaSphere exclusively reflect the opinions of the authors. The authors are responsible for all content in their abstracts including accuracy of the facts, statements, citing resources, etc.

### (PS2242) CAN QUANTIFERON CMV® PREDICT THE NEED FOR EXTENDED LETERMOVIR PROPHYLAXIS?

**Topic:** 23. Stem cell transplantation - Clinical

Andrej Pešić[1], Vladimir Perović[2], Nevena Bešević[1], Milena Todorovic Balint*[1, 3]

*[1]Clinic for Hematology, University Clinical Center of Serbia, Bone Marrow Transplant Unit, Belgrade, Serbia;[2]Institute for Microbiology and Immunology, Faculty of Medicine, University of Belgrade, Belgrade, Serbia; [3]Faculty of Medicine, University of Belgrade, Belgrade, Serbia;*

### Background

QuantiFERON CMV®, an assay evaluating CMV-specific cell-mediated immunity, has been demonstrated to predict the risk of CMV infection following allogeneic stem cell transplantation in various clinical settings. Letermovir prophylaxis is routinely employed to prevent early post-transplant CMV infection and has recently received approval for extended use for up to 200 days after transplant in patients at high risk for CMV infection.

### Aims

To assess the clinical utility of QuantiFERON CMV® in identifying patients at high risk for CMV infection who may benefit from extended letermovir prophylaxis.

### Methods

We conducted a retrospective analysis of CMV IgG-positive patients undergoing allogeneic stem cell transplantation who received in vivo T-cell depletion with anti-T-lymphocyte globulin (ATLG) or post-transplant cyclophosphamide (PTCy), along with letermovir prophylaxis for 100 days, prior to the approval of extended prophylaxis. The diagnostic utility of QuantiFERON®-CMV, measured on day 90 post-transplant, was evaluated for predicting subsequent clinically significant CMV infections (csCMVi), defined as CMV reactivations detected by quantitative PCR requiring initiation of anti-CMV therapy. Sensitivity, specificity, positive predictive value and negative predictive value of the test were calculated.

### Results

A total of 43 patients were eligible for the analysis, including three patients previously experiencing breakthrough CMV reactivations during letermovir prophylaxis. The majority of patients received ATLG compared to PTCy (37 and 6 patients, respectively), alongside graft-versus-host disease prophylaxis with cyclosporine A and antimetabolite (methotrexate with ATG or mycophenolate mofetil with PTCy). Most patients were transplanted for acute myeloid leukemia (20/43, 47%), were male (23/43, 53%), received myeloablative conditioning (29/43, 67%), had a matched-unrelated donor (24/43, 56%), and had a CMV seropositive donor (29/43, 67%). The mean patient and donor ages were 43 (range 18-61) and 36 (range 20-63), respectively. A total of 14 patients experienced csCMVi (33%), with only two having a prior positive QuantiFERON CMV® result. Therefore, the positive predictive value of QuantiFERON CMV® in protecting from subsequent CMV reactivation was 90% (95% CI 67-98%), while the negative predictive value was 50% (95% CI 29-70%). The assay demonstrated a specificity of 85% (95% CI 57%-98%) and a sensitivity of 58% (95% CI 39-76%). Among the two patients with positive test who developed CMV reactivation, both were receiving corticosteroids in addition to cyclosporine A, with one of them having a prior CMV infection during letermovir prophylaxis (when preemtive treatment was initiated and letermovir interrupted). Among twelve patients with a negative/indeterminate test and no subsequent CMV reactivation, eight had available CMV serological results prior to any transfusion. Of these, six were initially seronegative, highlighting the importance of serological testing at diagnosis for predicting future CMV reactivations.

**Copyright Information:** (Online) ISSN: 2572-9241
©2026 The Author(s). HemaSphere is published by John Wiley & Sons Ltd on behalf of European Hematology Association. This is an open access Abstract Book distributed under the Attribution-NonCommercial-NoDerivs (CC BY-NC-ND), which allows third parties to download the articles and share them with others as long as they credit the author and the Abstract Book, but they cannot change the content in any way or use them commercially.
**Abstract Book Citations:** Authors, Title, HemaSphere, 2026;10:(S1):pages. The abstract book DOIs can be found on the "Author Information" page.
**Disclaimer:** Articles published in the journal HemaSphere exclusively reflect the opinions of the authors. The authors are responsible for all content in their abstracts including accuracy of the facts, statements, citing resources, etc.

**Summary/Conclusion**

QuantiFERON CMV® has a high positive predictive value for identifying patients with a protective immunity against CMV and can be used as an effective and useful tool in delineating high-risk patients in need of extended letermovir prophylaxis. Accurate determination of true CMV serostatus at diagnosis is essential to define patients at genuine risk of CMV reactivation.

**Copyright Information:** (Online) ISSN: 2572-9241
©2026 The Author(s). HemaSphere is published by John Wiley & Sons Ltd on behalf of European Hematology Association. This is an open access Abstract Book distributed under the Attribution-NonCommercial-NoDerivs (CC BY-NC-ND), which allows third parties to download the articles and share them with others as long as they credit the author and the Abstract Book, but they cannot change the content in any way or use them commercially.
**Abstract Book Citations:** Authors, Title, HemaSphere, 2026;10:(S1):pages. The abstract book DOIs can be found on the "Author Information" page.
**Disclaimer:** Articles published in the journal HemaSphere exclusively reflect the opinions of the authors. The authors are responsible for all content in their abstracts including accuracy of the facts, statements, citing resources, etc.

### (PS2243) AXATILIMAB IN COMBINATION WITH RUXOLITINIB IN PATIENTS WITH NEWLY DIAGNOSED CHRONIC GRAFT-VERSUS-HOST DISEASE: UPDATED SAFETY ANALYSIS OF A RANDOMIZED PHASE 2 STUDY

**Topic:** 23. Stem cell transplantation - Clinical

Robert Zeiser*[1], Amandeep Salhotra[2], Francesca Bonifazi[3], Francesca Kinsella[4], José Luis Piñana[5], Simon Bulley[6], Amin Majid Alousi[7], Mi Kwon[8, 9], Lucía López Corral[10], Alexander Schauwvlieghe[11, 12], Albert Assad[13], Maureen Bleam[13], Peixin Zhang[13], Annie Im[14]

*[1]University of Freiburg, Freiburg, Germany; [2]City of Hope National Medical Center, Hematology, Duarte, United States of America; [3]Università di Bologna, Bologna, Italy; [4]BCCTT, Birmingham, United Kingdom; [5]Hospital Clínico Universitario de Valencia, INCLIVA Research Institute, Hematology, Valencia, Spain; [6]Leeds Cancer Centre and Leeds University, Leeds, United Kingdom; [7]The University of Texas MD Anderson Cancer Center, Stem Cell Transplantation, Houston, United States of America; [8]Hospital Gregorio Marañón, Madrid, Spain; [9]Universidad Complutense de Madrid, Madrid, Spain; [10]Hospital Universitario de Salamanca, Salamanca, Spain; [11]AZ Sint-Jan, Brugge, Belgium; [12]Ghent University, Ghent, Belgium; [13]Incyte Corporation, Wilmington, United States of America; [14]University of Pittsburgh, Pittsburgh, United States of America;*

### Background

Chronic graft-versus-host disease (cGVHD) is a leading cause of morbidity and nonrelapse mortality following allogeneic hematopoietic stem cell transplantation. Axatilimab (AXA), a monoclonal antibody targeting colony-stimulating factor 1 receptor (CSF-1R), depletes the monocytes and macrophages that mediate fibrosis and end-organ damage in cGVHD. Ruxolitinib (RUX) is a selective inhibitor of Janus kinase (JAK)1/JAK2, which mediate cytokine signaling, T-cell activation, and inflammation in cGVHD. Both AXA and RUX have demonstrated clinically meaningful efficacy and were generally well tolerated as single agents in previously treated cGVHD. Owing to their distinct mechanisms of action, combining AXA and RUX may improve efficacy without overlapping safety concerns.

### Aims

To report on the safety of AXA and RUX combined and of RUX alone among patients with newly diagnosed cGVHD.

### Methods

In this ongoing, randomized, open-label, phase 2 study (NCT06388564), eligible patients were aged ≥12 years with new onset, moderate or severe cGVHD with no prior systemic cGVHD treatment. Patients were randomized to AXA 0.3 mg/kg every 2 weeks in combination with RUX 10 mg twice daily (BID; AXA+RUX), RUX 10 mg BID, or corticosteroids for up to 24 months; randomization was stratified by cGVHD severity and use of concomitant systemic immunosuppressants. The primary endpoint is overall response at 6 months in the absence of a new systemic cGVHD therapy. Safety assessments include treatment-emergent adverse events (TEAEs). A prespecified interim safety analysis occurred after ~30 patients received ≥1 cycle of treatment.

**Copyright Information:** (Online) ISSN: 2572-9241
©2026 The Author(s). HemaSphere is published by John Wiley & Sons Ltd on behalf of European Hematology Association. This is an open access Abstract Book distributed under the Attribution-NonCommercial-NoDerivs (CC BY-NC-ND), which allows third parties to download the articles and share them with others as long as they credit the author and the Abstract Book, but they cannot change the content in any way or use them commercially.
**Abstract Book Citations:** Authors, Title, HemaSphere, 2026;10:(S1):pages. The abstract book DOIs can be found on the "Author Information" page.
**Disclaimer:** Articles published in the journal HemaSphere exclusively reflect the opinions of the authors. The authors are responsible for all content in their abstracts including accuracy of the facts, statements, citing resources, etc.

### Results

As of the data cutoff (October 3, 2025), 66 patients were enrolled (AXA+RUX, n=23; RUX, n=24; corticosteroids, n=19), and 56 patients received treatment (AXA+RUX, n=21; RUX, n=19; corticosteroids, n=16; **Figure**). The median age of enrolled patients was 65.0 years (range, 16–77), and 35 patients (53.0%) were male. The median time from transplant to diagnosis of cGVHD was 7.6 months (range, 1.7–72.3), and 31 patients (47.0%) had severe cGVHD at enrollment per the National Institutes of Health consensus criteria. Commonly involved organs included the mouth (71.2%), eyes (65.2%), and skin (62.1%). Nineteen patients discontinued treatment (AXA+RUX, n=4; RUX, n=6; corticosteroids, n=9); 1 patient in the AXA+RUX group discontinued owing to concurrent TEAEs of fatigue and anxiety. Among all treated patients, 44 experienced TEAEs (AXA+RUX, n=15 [75.0%]; RUX, n=16 [84.2%]; corticosteroids, n=13 [81.3%]), and 11 experienced grade ≥3 TEAEs (AXA+RUX, n=5 [25.0%]; RUX, n=3 [15.8%]; corticosteroids, n=3 [18.8%]). TEAEs of any grade occurring in >2 patients in a group were anemia (n=4, 20.0%) in the AXA+RUX group; anemia, increased aspartate aminotransferase (both n=4, 21.1%), decreased platelet count, diarrhea, fatigue, increased amylase, increased alanine aminotransferase, peripheral edema, pyrexia, and upper respiratory tract infection (each n=3, 15.8%) in the RUX group; and insomnia (n=4, 25.0%) and constipation (n=3, 18.8%) in the corticosteroid group. Anemia was the only grade ≥3 TEAE that occurred in 2 patients (n=1 each in AXA+RUX and RUX).

### Summary/Conclusion

In the updated safety analysis of this phase 2 trial, the combination of AXA+RUX was well tolerated and had a similar safety profile to RUX alone, with no evidence of additive toxicity.

**Figure. Patient Disposition**

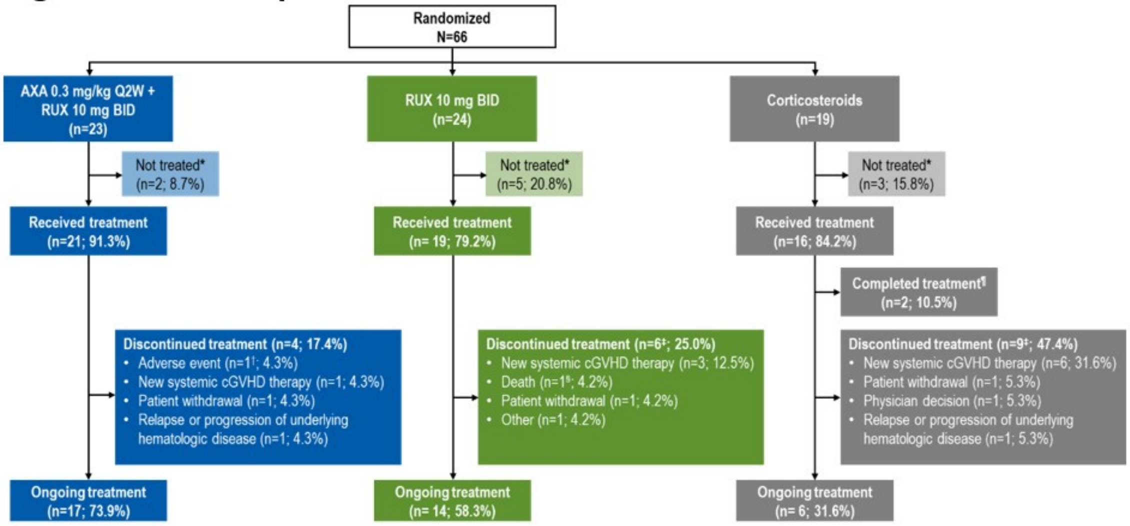

AXA, axatilimab; BID, twice daily; cGVHD, chronic graft-versus-host disease; Q2W, every 2 weeks; RUX, ruxolitinib.
* The majority of the 10 untreated patients were randomized before the data cutoff date and therefore have no dosing information reported yet.
† Treatment-emergent adverse events leading to treatment discontinuation in 1 patient were concurrent fatigue and anxiety.
‡ For ruxolitinib 10 mg BID and corticosteroids, 1 patient in each group discontinued treatment prior to receiving the first dose.
§ Death due to bronchospastic crisis and considered unrelated to treatment.
¶ Treatment is considered complete if a patient achieves a complete or partial response and completes the study treatment taper.

**Copyright Information:** (Online) ISSN: 2572-9241
©2026 The Author(s). HemaSphere is published by John Wiley & Sons Ltd on behalf of European Hematology Association. This is an open access Abstract Book distributed under the Attribution-NonCommercial-NoDerivs (CC BY-NC-ND), which allows third parties to download the articles and share them with others as long as they credit the author and the Abstract Book, but they cannot change the content in any way or use them commercially.
**Abstract Book Citations:** Authors, Title, HemaSphere, 2026;10:(S1):pages. The abstract book DOIs can be found on the “Author Information” page.
**Disclaimer:** Articles published in the journal HemaSphere exclusively reflect the opinions of the authors. The authors are responsible for all content in their abstracts including accuracy of the facts, statements, citing resources, etc.

### (PS2244) SYSTEMIC TREATMENT WITH THE ROCK2 INHIBITOR BELUMOSUDIL IN OCULAR CHRONIC GRAFT VERSUS HOST DISEASE

**Topic:** 23. Stem cell transplantation - Clinical

Yibo Wu*[1, 2, 3, 4], Bei Ye[5], Xiaoyu Lai[1, 2, 3, 4], Lizhen Liu[1, 2, 3, 4], Luxin Yang[1, 2, 3, 4], Xiaolin Yuan[6], Lin Li[1, 2, 3, 4], Ji-Min Shi[1, 2, 3, 4], Yanmin Zhao[1, 2, 3, 4], Panpan Zhu[1, 2, 3, 4], Jian Yu[1, 2, 3, 4], Chixin Du[5], He Huang[1, 2, 3, 4], Jianyong Wang[5], Yi Luo[1, 2, 3, 4]

*[1]Bone Marrow Transplantation Center, the First Affiliated Hospital, Zhejiang University School of Medicine, Hangzhou, China; [2]Liangzhu Laboratory, Hangzhou, China; [3]Institute of Hematology, Zhejiang University, Hangzhou, China;[4]Zhejiang Province Engineering Research Center for Stem Cell and Immunity Therapy,, Hangzhou, China; [5]the First Affiliated Hospital, Zhejiang University School of Medicine, Hangzhou, China, Department of Ophthalmology, Hangzhou, China; [6]Zhejiang Cancer Hospital, Hangzhou Institute of Medicine (HIM), Chinese Academy of Sciences, Department of Hematology,, Hangzhou, China;*

### Background

Chronic graft-versus-host disease (cGVHD) is the most common complication following allogeneic hematopoietic stem cell transplantation (allo-HSCT). Ocular involvement (oGVHD) occurs in 30%–85% of cGVHD patients and significantly diminishes vision and quality of life, and is associated with increased non-relapse mortality (NRM). Despite advances in understanding its pathogenesis, therapeutic progress for oGVHD has remained limited in recent years, underscoring a significant unmet clinical need.

### Aims

To evaluate the efficacy of oral belumosudil on ocular surface disease in patients with steroid-refractory chronic graft-versus-host disease (SR-cGVHD).

### Methods

In this retrospective study, we analyzed patients with SR-cGVHD and ocular involvement (oGVHD) treated with belumosudil (200 mg daily). Ocular assessments at baseline and 6 months included the International Chronic Ocular GVHD Consensus Group severity score, NIH eye score (0-3), corneal fluorescein staining (CFS, 0-4), tear break-up time (TBUT), Schirmer's I test, and the Ocular Surface Disease Index (OSDI). Treatment response rates were assessed per NIH consensus criteria.

**Copyright Information:** (Online) ISSN: 2572-9241
©2026 The Author(s). HemaSphere is published by John Wiley & Sons Ltd on behalf of European Hematology Association. This is an open access Abstract Book distributed under the Attribution-NonCommercial-NoDerivs (CC BY-NC-ND), which allows third parties to download the articles and share them with others as long as they credit the author and the Abstract Book, but they cannot change the content in any way or use them commercially.
**Abstract Book Citations:** Authors, Title, HemaSphere, 2026;10:(S1):pages. The abstract book DOIs can be found on the "Author Information" page.
**Disclaimer:** Articles published in the journal HemaSphere exclusively reflect the opinions of the authors. The authors are responsible for all content in their abstracts including accuracy of the facts, statements, citing resources, etc.

**Results**

Among 78 patients with SR-cGVHD treated with belumosudil, 40 had oGVHD, with a baseline NIH eye score of 2 (1–3). Of these, 28 patients completed both baseline and 6-month assessments. The cohort had high-risk features: 50.0% (14/28) had severe (NIH grade) systemic disease and 82.1% (23/28) had conjunctival fibrosis.
At 6 months, the oGVHD severity score improved in 53.6% (15/28), was stable in 35.7% (10/28), and progressed in 10.7% (3/28). The OSDI improved in 64.3% (18/28), was stable in 25.0% (7/28), and worsened in 10.7% (3/28).
Objective signs also improved from baseline: median CFS decreased from 4 (1–4) to 3 (1–4); median TBUT increased from 1.00 s (0.8–4.8) to 2.00 s (0.0–4.0); and median ST improved from 1.0 mm (0.0–5.0) to 2.0 mm (0.0–22.0).
A greater ocular benefit was observed in the 53.6%(15/28) of patients who achieved systemic overall response, with 66.7%(10/15), showing improved oGVHD severity scores and 80.0%(12/15) reporting better OSDI. Objective signs also improved in this subgroup: median CFS decreased from 4 (1–4) to 3 (1–4); median TBUT increased from 1.00 s (0.0–4.0) to 2.00 s (0.0–4.0); and median ST improved from 1.0 mm (0.0–2.0) to 2.0 mm (0.0–6.0).
Visual acuity remained stable, and 42.9% (12/28) of patients achieved corticosteroid dose reduction. Treatment was well-tolerated.

| Variables | N= 28 |
|---|---|
| Age, median(range),y | 39 (19-64) |
| male | 11 (39.3%) |
| Disease, n(%) | |
| AML | 15 (53.6%) |
| ALL | 6 (21.4%) |
| MDS | 5 (17.9%) |
| Others | 2 (7.1%) |
| Donor type, n(%) | |
| MSD | 7 (25.0%) |
| HRD | 16 (57.1%) |
| URD | 5 (17.9%) |
| Conditioning intensity, n(%) | |
| MAC | 24 (85.7%) |
| RIC | 4 (14.3%) |
| Median time of cGVHD diagnosis from transplantation | 251 (93-898) days |
| NIH cGVHD severity at the strat of belumosudil, n(%) | |
| Severe | n=14 (50.0%) |
| Moderate | n=14 (50.0%) |
| Median NIH oGVHD eye score before belumosudil treatment | 2 (1-3) |
| Median oGVHD severity score before belumosudil treatment | 9.5 (6-10) |
| Median CFS grade score before belumosudil treatment | 4 (1-4) |
| Median modified eye Lee symptom scale before belumosudil treatment | 6.5 (2-12) |
| Median corticosteroid dose (mg) before belumosudil treatment | 0 (0-40) |

Table1 Baseline patient demographics and clinical characteristics

Copyright Information: (Online) ISSN: 2572-9241
©2026 The Author(s). HemaSphere is published by John Wiley & Sons Ltd on behalf of European Hematology Association. This is an open access Abstract Book distributed under the Attribution-NonCommercial-NoDerivs (CC BY-NC-ND), which allows third parties to download the articles and share them with others as long as they credit the author and the Abstract Book, but they cannot change the content in any way or use them commercially.
**Abstract Book Citations:** Authors, Title, HemaSphere, 2026;10:(S1):pages. The abstract book DOIs can be found on the "Author Information" page.
**Disclaimer:** Articles published in the journal HemaSphere exclusively reflect the opinions of the authors. The authors are responsible for all content in their abstracts including accuracy of the facts, statements, citing resources, etc.

### Summary/Conclusion

Systemic belumosudil was associated with improvements in ocular surface parameters and symptoms in steroid-refractory oGVHD, with favorable tolerability. Ocular improvement was closely linked to systemic response, warranting prospective validation.

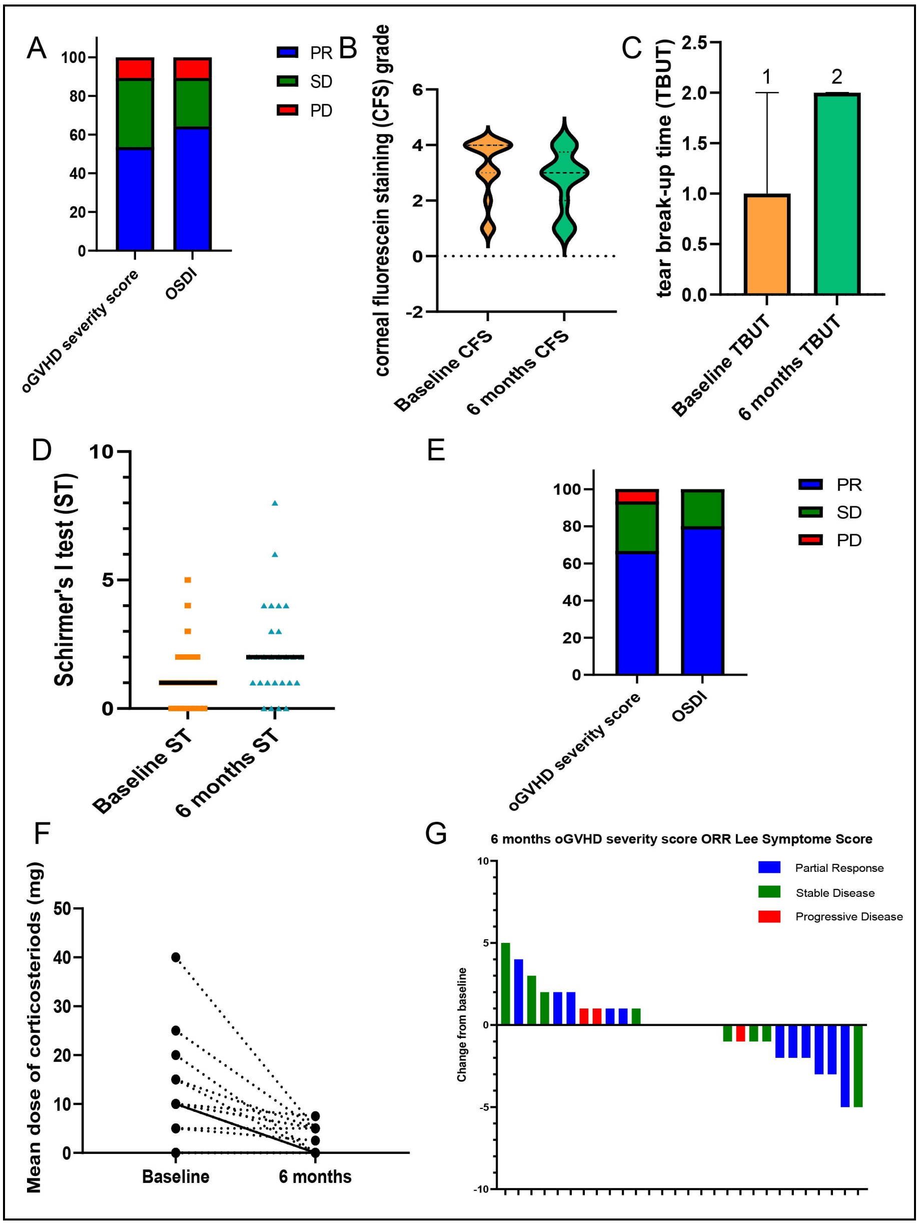

Figure 1. The efficacy of Belumosudil for oGVHD (A) The treatment response beased on oGVHD severity score and OSDI. (B) The baseline and 6-months after belumosudil therapy of CFS grade. (C) The baseline and 6-months after belumosudil therapy of TBUT. (D) The baseline and 6-months after belumosudil therapy of Schirmer's Test. (E) The treatment response based on oGVHD severity score and OSDI in systemic ORR patients. (F) The baseline and 6-months after belumosudil therapy of Corticosteroid reduction. (G) The change of Lee Symptoms Score change at 6-months belumosudil therapy from baseline.

**Copyright Information:** (Online) ISSN: 2572-9241
©2026 The Author(s). HemaSphere is published by John Wiley & Sons Ltd on behalf of European Hematology Association. This is an open access Abstract Book distributed under the Attribution-NonCommercial-NoDerivs (CC BY-NC-ND), which allows third parties to download the articles and share them with others as long as they credit the author and the Abstract Book, but they cannot change the content in any way or use them commercially.
**Abstract Book Citations:** Authors, Title, HemaSphere, 2026;10:(S1):pages. The abstract book DOIs can be found on the "Author Information" page.
**Disclaimer:** Articles published in the journal HemaSphere exclusively reflect the opinions of the authors. The authors are responsible for all content in their abstracts including accuracy of the facts, statements, citing resources, etc.

### (PS2245) STUDY ON THE DIFFERENCE OF FINE IMMUNE INDEXES OF DIFFERENT CONDITIONING REGIMENS IN MICROTRANSPLANTATION FOR ACUTE MYELOID LEUKEMIA

**Topic:** 23. Stem cell transplantation - Clinical

Hanxue Zheng[1], Lijuan Li*[1]

[1]*Lanzhou University Second Hospital, Lanzhou, China;*

### Background

Microtransplantation (MST) represents a unique transplant modality that preserves graft-versus-leukemia (GVL) activity without inducing overt graft-versus-host disease (GVHD). While clinical efficacy has been reported, the immune mechanisms underlying optimized conditioning strategies remain poorly defined. In particular, the interplay between natural killer (NK) cells and regulatory T cells (Tregs) after MST requires clarification.

### Aims

To investigate how different conditioning regimens modulate NK–Treg immune crosstalk and cytokine networks following MST in AML patients.

### Methods

We analyzed 61 AML patients treated with MST and 14 non-MST controls. MST patients received either venetoclax-based conditioning (Group A, n=20) or cytarabine-based conditioning (Group B, n=41). Nineteen immune parameters, including lymphocyte subsets and 12 cytokines, were assessed using flow cytometry and cytometric bead array. Correlation analyses were performed to define immune interaction networks.

### Results

Venetoclax-based conditioning induced a distinct immune remodeling pattern characterized by: (a) Sustained expansion of NK cells after repeated stem cell infusions, (b) Significant elevation of Treg proportions compared with cytarabine conditioning,(c) Increased total T-cell and B-cell recovery, (d) Downregulation of pro-inflammatory cytokines including IL-12p70 and IFN-γ.
Strong immune network correlations were observed. Treg cells showed positive correlations with IL-2, IL-12p70, and TNF-α, and negative correlation with IL-6 ($r>0.7$, $P<0.05$), suggesting coordinated immune tolerance modulation. NK cells demonstrated significant association with IL-6 dynamics, indicating cytokine-dependent cytotoxic regulation. $CD4^+$ and $CD8^+$ T cells exhibited synchronized interactions with TNF-α and IL-5, reflecting adaptive–innate immune integration.

Compared with cytarabine-based conditioning, venetoclax conditioning generated a more balanced immune microenvironment characterized by simultaneous enhancement of NK-mediated cytotoxicity and Treg-mediated immune regulation, potentially preserving GVL effects while limiting inflammatory overactivation.

### Summary/Conclusion

Venetoclax-based conditioning reshapes NK–Treg immune crosstalk after MST, promoting coordinated immune activation and tolerance. This balanced immune remodeling may represent the mechanistic basis for enhanced anti-leukemic efficacy without GVHD. Fine immune monitoring could serve as a mechanistic biomarker platform to optimize MST strategies in AML.

**Copyright Information:** (Online) ISSN: 2572-9241
©2026 The Author(s). HemaSphere is published by John Wiley & Sons Ltd on behalf of European Hematology Association. This is an open access Abstract Book distributed under the Attribution-NonCommercial-NoDerivs (CC BY-NC-ND), which allows third parties to download the articles and share them with others as long as they credit the author and the Abstract Book, but they cannot change the content in any way or use them commercially.
**Abstract Book Citations:** Authors, Title, HemaSphere, 2026;10:(S1):pages. The abstract book DOIs can be found on the "Author Information" page.
**Disclaimer:** Articles published in the journal HemaSphere exclusively reflect the opinions of the authors. The authors are responsible for all content in their abstracts including accuracy of the facts, statements, citing resources, etc.

### (PS2246) DIFFERENTIAL TOXICITY AND IMMUNE RECONSTITUTION PROFILES OF BEAM-ATG VS. CYCLOPHOSPHAMIDE-ATG IN MULTIPLE SCLEROSIS

**Topic:** 23. Stem cell transplantation - Clinical

Christian Koch*[1], Emanuel Rom[1], Tobias Benoit[1], Philipp Hockl[1], Regina Reimann[2], Bettina Sobottka-Brillout[3], Ronny Nienhold[3], Jakob Becker[3], Katja-Daniela Jordan[4], Paulina Will[5], Patrick Roth[5], Ilijas Jelcic[5, 6], Roland Martin[5, 7], Markus G. Manz[1], Veronika Kana[5], Dominik Schneidawind[1, 8, 9]

*[1]University Hospital Zurich and University of Zurich, Department of Medical Oncology and Hematology, Zurich, Switzerland; [2]University Hospital Zurich, Institute of Neuropathology, Zurich, Switzerland; [3]University Hospital Zurich, Department of Pathology and Molecular Pathology, Zurich, Switzerland; [4]University Hospital Zurich, Department of Consultation-Liaison Psychiatry and Psychosomatic Medicine, Zurich, Switzerland; [5]University Hospital Zurich, Department of Neurology, Zurich, Switzerland; [6]Hirslanden Klinik Zurich, Department of Neurology, Zurich, Switzerland; [7]Neurocenter Bellevue, Neuroimmunology Outpatient Clinic, Center for Multiple Sclerosis, Zurich, Switzerland; [8]Comprehensive Cancer Center Zurich, Zurich, Switzerland; [9]University of Tübingen, Faculty of Medicine, Tübingen, Germany;*

### Background

High-dose chemotherapy with ATG followed by autologous hematopoietic stem cell transplantation (ASCT) is an effective treatment option for therapy-refractory multiple sclerosis (MS). In MS, the goal is durable immunomodulation with minimal toxicity. BEAM-ATG and cyclophosphamide-ATG (Cy-ATG) are both being used, but comparative real-world data on detailed toxicity patterns, clinical outcomes, hematologic recovery, and immune reconstitution remain limited.

### Aims

To compare BEAM-ATG versus Cy-ATG with ASCT in persons with MS with respect to early and late serious adverse events (sAEs), infectious complications, engraftment kinetics, transfusion burden, hospitalization duration, weight loss, immune reconstitution and neurological outcomes.

### Methods

We performed a single-center analysis of prospectively collected registry data from persons with MS undergoing treatment with BEAM-ATG (n=43) or Cy-ATG (n=10) in combination with ASCT at the University Hospital Zurich between 2016 and 2025. Adverse events were graded according to CTCAE v5.0 and classified as early (≤ day +100) or late (> day +100). Infectious complications, engraftment kinetics, transfusion requirements, hospitalization duration, and peri-transplant weight loss were analyzed. Immune reconstitution was assessed by longitudinal lymphocyte subset and immunoglobulin measurements, and neurological outcome by Expanded Disability Status Scale (EDSS) at 12 months.

**Copyright Information:** (Online) ISSN: 2572-9241
©2026 The Author(s). HemaSphere is published by John Wiley & Sons Ltd on behalf of European Hematology Association. This is an open access Abstract Book distributed under the Attribution-NonCommercial-NoDerivs (CC BY-NC-ND), which allows third parties to download the articles and share them with others as long as they credit the author and the Abstract Book, but they cannot change the content in any way or use them commercially.
**Abstract Book Citations:** Authors, Title, HemaSphere, 2026;10:(S1):pages. The abstract book DOIs can be found on the "Author Information" page.
**Disclaimer:** Articles published in the journal HemaSphere exclusively reflect the opinions of the authors. The authors are responsible for all content in their abstracts including accuracy of the facts, statements, citing resources, etc.

### Results

Early sAEs were more frequent with BEAM-ATG (median 4 vs. 2 per patient; p=0.015), with significantly higher rates of grade 3/4 febrile neutropenia (95% vs. 40%; p=0.0002), mucositis (51% vs. 0%; p=0.0031) and diarrhea (37% vs. 0%; p=0.0229). Proven bacterial infections within 100 days occurred more often after BEAM-ATG (33% vs 0%; p=0.047), whereas viral infections were comparable between groups. In contrast, Cy-ATG was associated with more frequent clinically relevant fluid overload (30% vs 0%; p=0.005). One early death occurred after Cy-ATG, consistent with presumed cyclophosphamide-associated cardiotoxicity. Hospitalization duration was significantly shorter with Cy-ATG (mean 19 vs 24 days; p=0.0002), and peri-transplant weight loss was lower (1.7% vs 6.2%; p=0.0005). Hematologic recovery was faster after Cy-ATG, with neutrophil (9.7 vs 11.6 days; p=0.008), platelet (11.7 vs 14.5 days; p<0.0001), and lymphocyte recovery (19 vs 29 days; p=0.001). Furthermore, immune reconstitution was accelerated after Cy-ATG, with earlier recovery of $CD3^+$, $CD4^+$, and $CD8^+$ T cells, particularly $CD45RO^+$ memory subsets, whereas $CD45RA^+$ naïve T-cell recovery remained delayed in both regimens. Furthermore, $CD19^+CD27^-CD10^-$ transitional B cells were significantly higher at 12 months after Cy-ATG, while early IgG levels were lower after BEAM-ATG. Neurological disability remained stable at 12 months in both cohorts.

### Summary/Conclusion

Cy-ATG showed a more favorable safety and immune recovery profile than BEAM-ATG, while maintaining short-term neurological efficacy indicating that Cy-ATG may achieve a more favorable balance between efficacy and tolerability. Longer follow-up is needed to confirm comparable durability of clinical benefit and the long-term efficacy profile of Cy-ATG.

**Copyright Information:** (Online) ISSN: 2572-9241
©2026 The Author(s). HemaSphere is published by John Wiley & Sons Ltd on behalf of European Hematology Association. This is an open access Abstract Book distributed under the Attribution-NonCommercial-NoDerivs (CC BY-NC-ND), which allows third parties to download the articles and share them with others as long as they credit the author and the Abstract Book, but they cannot change the content in any way or use them commercially.
**Abstract Book Citations:** Authors, Title, HemaSphere, 2026;10:(S1):pages. The abstract book DOIs can be found on the "Author Information" page.
**Disclaimer:** Articles published in the journal HemaSphere exclusively reflect the opinions of the authors. The authors are responsible for all content in their abstracts including accuracy of the facts, statements, citing resources, etc.

### (PS2247) FACTORS PREDICTING RELAPSE AFTER AUTOLOGOUS HEMATOPOIETIC CELL TRANSPLANTATION IN SYSTEMIC DIFFUSE LARGE B-CELL LYMPHOMA AND PRIMARY CENTRAL NERVOUS SYSTEM LYMPHOMA: A FINNISH MULTICENTER STUDY

**Topic:** 23. Stem cell transplantation - Clinical

Lotta Ravattinen*[1], Anu Partanen[1, 2], Hanne Kuitunen[3, 4], Aino Rönkä[1, 5], Outi Kuittinen[1, 5], Manna Miilunpohja[2], Milla Kuusisto[6, 7], Minna Suominen[8], Alina Kinnunen[9], Juho Kangas[10], Atte Karhu[4, 10], Joonas Kuitunen[10], Henri Kurttila[4, 10], Eetu Pellonperä[10], Miko Pietilä[10], Kaisa Sunela[11]

*[1]University of Eastern Finland, Institute of Clinical Medicine, Kuopio, Finland;[2]Kuopio University Hospital, Department of Medicine, Kuopio, Finland; [3]Oulu University Hospital, Cancer Center, Oulu, Finland;[4]Oulu University Hospital, Medical Research Center, Oulu, Finland; [5]Kuopio University Hospital, Cancer Center, Kuopio, Finland;[6]Länsi-Pohja Central Hospital, Department of Internal Medicine, Kemi, Finland; [7]University of Oulu, Cancer and Translational Research Unit, Oulu, Finland;[8]North Karelia Central Hospital, Cancer Center, Joensuu, Finland; [9]University of Tampere, Faculty of Medicine, Tampere, Finland; [10]University of Oulu, Faculty of Medicine, Oulu, Finland; [11]Finnish Medicines Agency Fimea, Helsinki, Finland;*

### Background

Patients with diffuse large B-cell lymphoma (DLBCL) and primary central nervous system lymphoma (PCNSL) experiencing relapse after autologous hematopoietic cell transplantation (AHCT) have poor outcomes. Factors predicting the risk of post-transplant relapse risk are unknown.

### Aims

The aim of this multicenter real-world study was to identify factors impacting post-transplant outcomes in systemic DLBCL or PCNSL.

### Methods

The study population consisted of 119 patients with systemic DLBCL and 40 patients with PCNSL who underwent AHCT between 2000 and 2019 in three University Hospitals in Finland (Kuopio, Tampere, Oulu). Data were retrospectively collected from patients' medical records including e.g. patient and disease characteristics and outcome. Early relapse was defined as occurring <18 months from diagnosis. SPSS software package was used for data analyses. The research permit was obtained from Findata (THL/1755/14.02.00/2025).

### Results

Patients with PCNSL who underwent AHCT as second-line consolidation with a partial response (PR) prior to mobilization had a 15-fold risk for post-transplant relapse compared to those achieving complete response (CR) (HR = 15.06, 95% CI: 1.08-210.62, p=0.044). Patients with systemic DLBCL had a four-fold risk of post-transplant relapse if they had elevated LDH at the time of primary diagnosis (HR = 3.75, 95% CI: 1.46–9.59, p=0.006) and a three-fold risk if they had ≥2 extranodal lesions (HR = 2.890, 95% CI: 1.21–6.91, p=0.017). Additionally, patients had a two-fold increased risk if they had PR prior to AHCT (HR = 1.97, 95% CI: 1.02–3.80, p=0.042) (**Table 1**).

**Table 1.** Patient characteristics.

**Copyright Information:** (Online) ISSN: 2572-9241
©2026 The Author(s). HemaSphere is published by John Wiley & Sons Ltd on behalf of European Hematology Association. This is an open access Abstract Book distributed under the Attribution-NonCommercial-NoDerivs (CC BY-NC-ND), which allows third parties to download the articles and share them with others as long as they credit the author and the Abstract Book, but they cannot change the content in any way or use them commercially.
**Abstract Book Citations:** Authors, Title, HemaSphere, 2026;10:(S1):pages. The abstract book DOIs can be found on the "Author Information" page.
**Disclaimer:** Articles published in the journal HemaSphere exclusively reflect the opinions of the authors. The authors are responsible for all content in their abstracts including accuracy of the facts, statements, citing resources, etc.

| Characteristic | Systemic DLBCL (n=119), n (%) | PCNSL (n=40), n (%) | |
|---|---|---|---|
| | | AHCT at 1st line (n=28) | AHCT at 2nd line (n=12) |
| **Age, median (range)** | 59 (23–73) | 65.5 (37–75) | 59.5 (36–67) |
| **Sex** | | | |
| Female | 60 (50.4) | 12 (42.9) | 2 (16.7) |
| Male | 59 (49.6) | 16 (57.1) | 10 (83.3) |
| **Extranodal lesions at diagnosis** | | | |
| 1 lesion | 50 (42.0) | 15 (53.6) | 11 (91.7) |
| ≥2 lesions | 30 (25.2) | 3 (10.7) | |
| No lesions | 39 (32.8) | 8 (28.6) | 1 (8.3) |
| | | Unavailable: 2 (7.1) | |
| **LDH levels** | | | |
| Elevated | 81 (68.1) | 9 (32.1) | 5 (41.7) |
| Normal | 20 (16.8) | 13 (46.4) | 5 (41.7) |

**Copyright Information:** (Online) ISSN: 2572-9241
©2026 The Author(s). HemaSphere is published by John Wiley & Sons Ltd on behalf of European Hematology Association. This is an open access Abstract Book distributed under the Attribution-NonCommercial-NoDerivs (CC BY-NC-ND), which allows third parties to download the articles and share them with others as long as they credit the author and the Abstract Book, but they cannot change the content in any way or use them commercially.
**Abstract Book Citations:** Authors, Title, HemaSphere, 2026;10:(S1):pages. The abstract book DOIs can be found on the “Author Information” page.
**Disclaimer:** Articles published in the journal HemaSphere exclusively reflect the opinions of the authors. The authors are responsible for all content in their abstracts including accuracy of the facts, statements, citing resources, etc.

| | | | |
|---|---|---|---|
| | Unavailable: 18 (15.1) | Unavailable: 6 (21.4) | Unavailable: 2 (16.7) |
| **Abbreviations and footnotes**<br>AHCT = autologous hematopoietic cell transplant; DLBCL = diffuse large B-cell lymphoma; LDH = lactatedehydrogenase; PCNSL = primary central nervous system lymphoma | | | |

Based on regression coefficients from multivariate analysis of these three factors, we developed a new risk scoring system for patients with systemic DLBCL. We found that 5-year relapse-free survival (RFS) rates for patients with low-, intermediate- vs. high-risk were 84.9%, 64.4% vs. 47.5%, respectively (p=0.006) (**Figure 1**). When isolating systemic DLBCL patients with early relapse (n=72), the scoring system remained a predictor for post-transplant relapse analyzed by RFS between patients with and without early relapse. The 5-year RFS rates for those low- (n=20), intermediate- (n=17) and high-risk (n=34) groups were 89.7%, 71.4% and 48.5%, respectively (p=0.007).

### Summary/Conclusion

PCNSL patients receiving AHCT after their first relapse were significantly more likely to experience post-transplant relapse if their pre-mobilization response was worse than CR. Our new risk classification for post-transplant relapse divides systemic DLBCL patients into three groups with significantly different RFS rates. Larger studies are needed to validate this classification.

**Copyright Information:** (Online) ISSN: 2572-9241
©2026 The Author(s). HemaSphere is published by John Wiley & Sons Ltd on behalf of European Hematology Association. This is an open access Abstract Book distributed under the Attribution-NonCommercial-NoDerivs (CC BY-NC-ND), which allows third parties to download the articles and share them with others as long as they credit the author and the Abstract Book, but they cannot change the content in any way or use them commercially.
**Abstract Book Citations:** Authors, Title, HemaSphere, 2026;10:(S1):pages. The abstract book DOIs can be found on the "Author Information" page.
**Disclaimer:** Articles published in the journal HemaSphere exclusively reflect the opinions of the authors. The authors are responsible for all content in their abstracts including accuracy of the facts, statements, citing resources, etc.

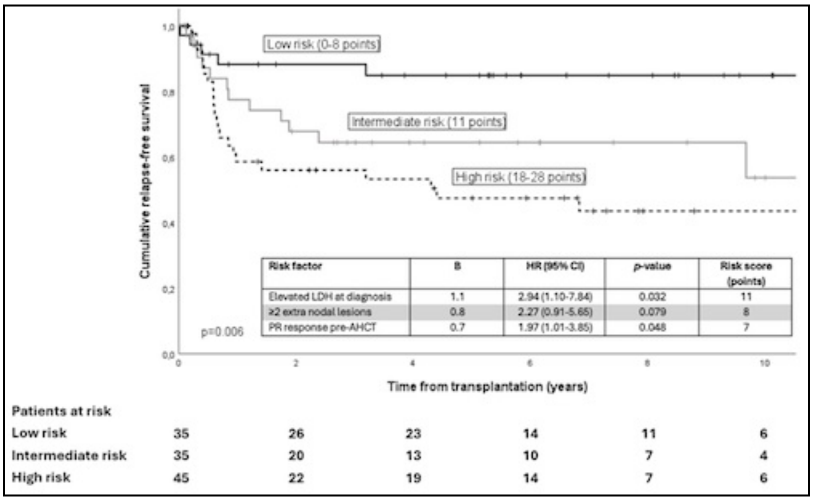

Figure 1. RFS post-AHCT comparing systemic DLBCL patients with 0-3 relapse risk factors based on a new risk score formed by results of multivariate Cox analyses. Risk scores were derived by multiplying the regression coefficients of each risk factor by ten and rounding them to the nearest integer. Patients were then stratified into three approximately equal-sized groups: low risk (n=35), intermediate risk (n=35) and high risk (n=45). Post-AHCT RFS of all systemic DLBCL patients according to new risk-classification. Abbreviations: AHCT = autologous hematopoietic cell transplantation; LDH = lactatedehydrogenase; PR = partial response, RFS = relapse-free survival.

**Copyright Information:** (Online) ISSN: 2572-9241
©2026 The Author(s). HemaSphere is published by John Wiley & Sons Ltd on behalf of European Hematology Association. This is an open access Abstract Book distributed under the Attribution-NonCommercial-NoDerivs (CC BY-NC-ND), which allows third parties to download the articles and share them with others as long as they credit the author and the Abstract Book, but they cannot change the content in any way or use them commercially.
**Abstract Book Citations:** Authors, Title, HemaSphere, 2026;10:(S1):pages. The abstract book DOIs can be found on the “Author Information” page.
**Disclaimer:** Articles published in the journal HemaSphere exclusively reflect the opinions of the authors. The authors are responsible for all content in their abstracts including accuracy of the facts, statements, citing resources, etc.

### (PS2248) VENETOCLAX AND AZACITIDINE TREATMENT FOR HIGH RISK MYELODYSPLASTIC SYNDROME AND CHRONIC MYELOMONOCYTIC LEUKEMIA AS A BRIDGE THERAPY TO TRANSPLANT. REAL-LIFE EXPANDED GESMD STUDY.

**Topic:** 23. Stem cell transplantation - Clinical

Inés Zugasti*[1], Sandra Castaño-Díez[1], Alejandro Avendaño[2], Helena Pomares[3], Ana Perez Gonzalez[4], Felix Lopez Cadenas[4], Sara García Ávila[5], Irene Padilla Conejo[6], Georgina Gener-Ricos[7, 8], Cristina de la Fuente Montes[7], Neus Amer-Salas[9], Lucía García[10], Antonio Garcia Guiñon[11], Alexandra Martínez Roca[1], Ana Triguero[1], Beatriz Merchan[1], Albert Cortés[1], Francesca Guijarro Tomas[1], Carlos Jimenez Vicente[1], Juan José Rodríguez-Sevilla[1], Marina Cornejo[1], Adriana Cuartas[1], Eduardo Francisco Aguilar[1], Marta Moris[1], Paula Sastre[1], Aina Cardús[1], Angela Llop[1], Ulises Ferrandíz[7], Esther Carcelero[1], María Suárez-Lledó[1], Maria Queralt Salas[1], Juan José Bargay Lleonart[9], Fernando Ramos[6], Blanca Xicoy Cirici[7], David Valcárcel[4], Montserrat Arnan Sangerman[3], María Díez Campelo[2], Montserrat Rovira Tarrats[1], Francesc Fernandez Aviles[1], Jordi Esteve Reyner[1], Marina Diaz Beya[1]

*[1]Hospital Clinic de Barcelona, Hematology, Barcelona, Spain;[2]Hospital Universitario de Salamanca, Hematology, Salamanca, Spain; [3]Institut Català d'Oncologia, Hospital Duran i Reynals., Hematology, L´Hospitalet de Llobregat, Spain;[4]Hospital Universitario Vall d´Hebrón, Hematology, Barcelona, Spain; [5]Hospital del Mar, Hematology, Barcelona, Spain; [6]Hospital Universitario de Leon, Hematology, Leon, Spain; [7]Institut Català d'Oncologia (ICO), Hospital Germans Trias i Pujol, Hematology, Badalona, Spain; [8]Institut Català d'Oncologia, Hospital Duran i Reynals, Hematology, Barcelona, Spain;[9]Hospital Son Llatzer, Palma de Mallorca, Spain; [10]Hospital Son Espases, Palma de Mallorca, Spain; [11]Hospital Arnau de Vilanova, Hematology, Lleida, Spain;*

### Background

Venetoclax combined with hypomethylating agents (HMA/VEN), despite improving response rates, has failed to demonstrate survival benefit in high-risk myelodysplastic syndrome (HR-MDS). However, its potential benefit in transplant-eligible patients, particularly in terms of access to transplantation and post-transplant outcomes, remains unclear.

### Aims

To evaluate the impact of HMA/VEN on the likelihood of proceeding to alloHCT among eligible patients. To analyze feasibility, toxicity, efficacy and outcome of HR-MDS treated with HMA/VEN as a bridge to transplant

### Methods

Multicenter retrospective study of HR-MDS/CMML patients treated with HMA/VEN as a bridge to alloHCT. Transplant probability and post-transplant OS were compared with matched cohorts receiving HMA monotherapy (n=32) or other standard bridging therapies (n=57) between 2016–2025, using inverse probability of treatment weighting (IPTW) analysis.

**Copyright Information:** (Online) ISSN: 2572-9241
©2026 The Author(s). HemaSphere is published by John Wiley & Sons Ltd on behalf of European Hematology Association. This is an open access Abstract Book distributed under the Attribution-NonCommercial-NoDerivs (CC BY-NC-ND), which allows third parties to download the articles and share them with others as long as they credit the author and the Abstract Book, but they cannot change the content in any way or use them commercially.
**Abstract Book Citations:** Authors, Title, HemaSphere, 2026;10:(S1):pages. The abstract book DOIs can be found on the "Author Information" page.
**Disclaimer:** Articles published in the journal HemaSphere exclusively reflect the opinions of the authors. The authors are responsible for all content in their abstracts including accuracy of the facts, statements, citing resources, etc.

**Results**

Seventy-two patients were included, 54 MDS and 18 MDS/MPN. Twenty-three patients (32%) received HMA/VEN in a relapsed/refractory (R/R) setting **(Fig1A)**. Grade 3/4 cytopenias were observed in 92%, and 16 patients (22%) developed febrile neutropenia. The 60-day mortality was 1%.

The response rate was: 41 CR (57%), 13 $CR_L$ (18%), 4 $CR_h$ (6%), 9 PR (13%), 1 HI (1%), and 4 NR (6%) (IWG23). The ORR was 94%, and the CRc was 81%. The median time to best response was 1 cycle (range 1-3). In R/R patients, the ORR was 83% and the CRc was 64% (p=NS).

Currently, there are 5 patients awaiting alloHCT. Among the 67 evaluable patients, 57 underwent alloHCT, resulting in an alloHCT rate of 85%. No differences in alloHCT rate were observed between those receiving HMA/VEN as 1L vs. R/R (86% vs. 82.6%, p=0.74). The 10 patients who were not transplanted included 2 MDS-EB, 3 CMML, and 5 MDS/AML. Of these, 7 did not reach alloHCT due to disease progression, two because of loss of eligibility while in CR and MCR, and one died due to infectious complications (response not evaluable). Two patients who did not respond to VEN/AZA were treated with chemotherapy prior to alloHCT achieving CR.

After IPTW adjustment for age and IPSS-M, HMA/VEN was independently associated with an increased probability of proceeding to alloHCT compared with HMA alone (OR 3.6, 95%CI 1.3-10.2; p=0.013).

With a median follow-up of 24.8 months, 2-year post alloHCT OS was 57% (95%CI 43-75)(**Fig1B)**. Eleven patients relapsed after alloHCT, leading to a 2-year CIR of 24.5% (95%CI 11.4-37.6)(**Fig1E**).

Regarding post-transplant OS, in univariable analysis, BM blasts (≥10%; HR 8.3; p=0.04) and conditioning (RIC vs MAC; HR 11.1, p=0.02) were associated with inferior post-transplant OS, whereas *TP53*mut (HR 2.56; p=0.107) and R/R status (HR 2.12; p=0.134) showed a trend. Patients with *TP53mut* had a significantly higher CIR (p=0.028) and those R/R trended toward increased CIR (p=0.052). In multivariable analysis, *TP53*mut (HR 6.52, p=0.010), reduced intensity conditioning (HR 14.9, p=0.014), and blasts ≥10% (HR 12.53, p=0.021) were independently associated with worse post-transplant survival (**Fig1D**). Post-transplant survival was not compromised when compared with other standard bridging therapies (HR 0.84; p=0.57; IPTW age and IPSS-M adjusted).

**Summary/Conclusion**

HMA/VEN represents an effective and safe bridge-to-transplant strategy in HR-MDS/CMML, leading to a increased access to alloHCT even in R/R patients. Prospective randomized studies focused on the transplant setting and longer post alloSCT follow-up are warranted to confirm these findings.

**Copyright Information:** (Online) ISSN: 2572-9241
©2026 The Author(s). HemaSphere is published by John Wiley & Sons Ltd on behalf of European Hematology Association. This is an open access Abstract Book distributed under the Attribution-NonCommercial-NoDerivs (CC BY-NC-ND), which allows third parties to download the articles and share them with others as long as they credit the author and the Abstract Book, but they cannot change the content in any way or use them commercially.
**Abstract Book Citations:** Authors, Title, HemaSphere, 2026;10:(S1):pages. The abstract book DOIs can be found on the “Author Information” page.
**Disclaimer:** Articles published in the journal HemaSphere exclusively reflect the opinions of the authors. The authors are responsible for all content in their abstracts including accuracy of the facts, statements, citing resources, etc.

**Figure 1.A.** Characteristics of the cohort (previous to HMA/VEN). **B.** Post alloHCT Survival of the 57 patients that underwent alloHCT. **C.** Cumulative Incidence of Relapse. **D.** Forest Plot of the Multivariate Cox Model.

A

| **Characteristics** | **n=72** |
|---|---|
| Age, years, median (range) | 63 (39-75) |
| Sex (male), n (%) | 57 (79) |
| Refractory/relapse, n (%) | 23 (32) |
| % Blasts , median (range) | 10 (1–19) |
| ***Cytogenetic risk in MDS, n (%)*** | **n=53** |
| Good | 24 (45) |
| Intermediate | 5 (9) |
| Poor | 8 (15) |
| Very poor | 16 (30) |
| ***Cytogenetic risk in CMML, n (%)*** | **n=15** |
| Low | 11 (73) |
| High | 4 (27) |
| ***2022 ICC Classification, n (%)*** | **n=72** |
| MDS-EB | 12 (17) |
| MDS-NOS with ml. dysplasia | 1 (1) |
| MDS-*TP53*mut | 2 (3) |
| MDS/AML -*TP53*mut | 12 (17) |
| MDS-*SF3B1* | 1 (1) |
| MDS/AML myelodysplasia mut. | 19 (26) |
| MDS/AML myelodysplasia cyt. | 1 (1) |
| MDS/AML NOS | 6 (8) |
| CMML-1 | 4 (6) |
| CMML-2 | 11 (15) |
| aCML | 2 (3) |
| MDS/MPN, NOS | 1 (1) |
| ***IPSS-Mol, n (%)*** | **n=57** |
| Moderate-low | 1(2) |
| Moderate-high | 9 (16) |
| High | 24 (42) |
| Very High | 23 (39) |
| ***CPSS-Mol*, n (%)** | **n=15** |
| Intermediate-1 | 1 (2) |
| Intermediate-2 | 6 (10) |
| High | 8 (53) |

B

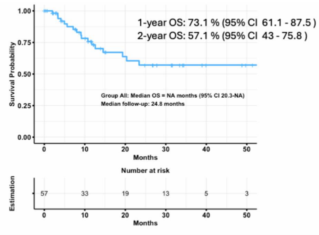

C

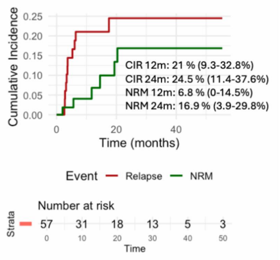

D

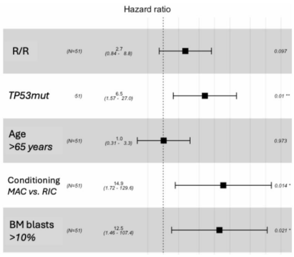

Figure 1.A. Characteristics of the cohort (previous to HMA/VEN). B. Post alloHCT Survival of the 57 patients that underwent alloHCT. C. Cumulative Incidence of Relapse. D. Forest Plot of the Multivariate Cox Model.

**Copyright Information:** (Online) ISSN: 2572-9241
©2026 The Author(s). HemaSphere is published by John Wiley & Sons Ltd on behalf of European Hematology Association. This is an open access Abstract Book distributed under the Attribution-NonCommercial-NoDerivs (CC BY-NC-ND), which allows third parties to download the articles and share them with others as long as they credit the author and the Abstract Book, but they cannot change the content in any way or use them commercially.
**Abstract Book Citations:** Authors, Title, HemaSphere, 2026;10:(S1):pages. The abstract book DOIs can be found on the "Author Information" page.
**Disclaimer:** Articles published in the journal HemaSphere exclusively reflect the opinions of the authors. The authors are responsible for all content in their abstracts including accuracy of the facts, statements, citing resources, etc.

### (PS2249) ALLOGENEIC HEMATOPOIETIC STEM CELL TRANSPLANTATION IN PRIMARY HEMOPHAGOCYTIC LYMPHOHISTIOCYTOSIS WITH CENTRAL NERVOUS SYSTEM INVOLVEMENT FOR CHILDREN

**Topic:** 23. Stem cell transplantation - Clinical

Juan Xiao*[1], Zhouyang Liu[1], Yuan Sun[1]

[1]*Beijing Jingdu Children's Hospital, Department of Hematology, Beijing, China;*

### Background

Pediatric primary hemophagocytic syndrome (PHLH) complicated with central nervous system (CNS) involvement is a critical condition, allogeneic HSCT(allo-HSCT) is an effective treatment for PHLH-CNS.

### Aims

To investigate the efficacy of allo-HSCT in PHLH with central nervous system (CNS) involvement.

### Methods

A retrospective analysis was performed on 74 pediatric patients with PHLH undergoing allo-HSCT at Beijing JingDu Children's Hospital between January 2018 and June 2025. Of these, 18 were CNS-HLH.

### Results

Of the 18 patients, 10 were male and 8 were female, median age was 3.6(1.2-11.2) years. 6 were FHL-2 (PRF1), 6 were FHL-3 (UNC13D), 3 were XLP1 (SH2D1A )2 was FHL-4 (STX11) and 1 was CHS (LYST). 6 patients had CNS symptoms as the first manifestation, and 12 showed central involvement during the course of disease progression. 10 were haplo-HSCT, 6 were unrelated matched HSCT and 2 were sibling compatible HSCT. All patients received induce chemotherapy with HLH-94, HLH-2004 or DEP regimen and intrathecal injection treatment before transplantation, 8 patients achieved complete remission (CR) and 6 patients achieved partial remission (PR), but 4 patients still in active disease (AD).The Conditioning regimen was VP-16 / Bu / Flud / CTX /ATG . The GVHD prophylaxis was either CSA or FK-506+MMF±Basiliximab. All patients achieved Stable hematopoietic reconstruction. With a median follow-up of 28.6 (12 – 72) months, 13 patients are alive and 5 were dead. The 5-year OS was 73.5% (95%CI, 54.9% to 85.4%)and the 5-year FFS was 67.2% (95% CI, 48.4% to 79.5%).The main causes of death were severe infection and TMA. The 13 patients who survived, 4 developed epilepsy requiring drug control, and the remaining 9 had no neurological symptoms with normal intelligence and development of the same age.

### Summary/Conclusion

Allo-HSCT is an effective treatment for primary HLH with CNS involvement. Disease remission before transplantation achieves better survival. Once FHLH diagnosed, allo-HSCT as soon as possible.

**Copyright Information:** (Online) ISSN: 2572-9241
©2026 The Author(s). HemaSphere is published by John Wiley & Sons Ltd on behalf of European Hematology Association. This is an open access Abstract Book distributed under the Attribution-NonCommercial-NoDerivs (CC BY-NC-ND), which allows third parties to download the articles and share them with others as long as they credit the author and the Abstract Book, but they cannot change the content in any way or use them commercially.
**Abstract Book Citations:** Authors, Title, HemaSphere, 2026;10:(S1):pages. The abstract book DOIs can be found on the "Author Information" page.
**Disclaimer:** Articles published in the journal HemaSphere exclusively reflect the opinions of the authors. The authors are responsible for all content in their abstracts including accuracy of the facts, statements, citing resources, etc.

### (PS2250) OPTIMAL DONOR DIRECTION IN HAPLOIDENTICAL HEMATOPOIETIC STEM CELL TRANSPLANTATION: OFFSPRING-TO-PARENT VERSUS PARENT-TO-OFFSPRING

**Topic:** 23. Stem cell transplantation - Clinical

Yunrui Zhang[1], Yu Wang*[1]

[1]*Peking University People's Hospital, Beijing, China;*

### Background

The optimal donor direction for grafts in haploidentical hematopoietic stem cell transplantation (haplo-HSCT), specifically between parent-to-offspring and offspring-to-parent pairings, remains incompletely elucidated in clinical practice. Clarifying the prognostic value of donor-recipient direction is critical for optimizing donor selection strategies and improving transplant outcomes for patients undergoing haplo-HSCT.

### Aims

This study aimed to compare the long-term clinical outcomes of offspring-to-parent versus parent-to-offspring donor direction in haplo-HSCT for acute myeloid leukemia (AML) patients in first complete remission (CR1), and to explore the value of donor-recipient pairing pattern as a prognostic factor for transplant efficacy and safety.

### Methods

A retrospective analysis was conducted on 284 consecutive AML patients who underwent haplo-HSCT in CR1 on a uniform transplantation platform. Patients were stratified into two groups according to donor-recipient relationship: the offspring-to-parent group (n=126) and the parent-to-offspring group (n=158). The primary study endpoints included overall survival (OS), disease-free survival (DFS), GVHD-free and relapse-free survival (GRFS), non-relapse mortality (NRM), and relapse incidence (RI). Secondary endpoints were the cumulative incidence of grade ≥2 acute graft-versus-host disease (aGVHD) and chronic graft-versus-host disease (cGVHD). Single-factor and multivariate Cox regression analyses were used to evaluate the association between donor direction and transplant outcomes, with patient age incorporated as a covariate in multivariate analysis.

### Results

At 5-year follow-up, single-factor analysis showed no statistically significant differences between the offspring-to-parent and parent-to-offspring groups in 5-year OS (86% vs 86%, P=0.7276), DFS (78% vs 73%, P=0.2809), NRM (9% vs 7%, P=0.4241), RI (13% vs 20%, P=0.0577), grade ≥2 aGVHD (21% vs 23%, P=0.6351), and cGVHD (44% vs 46%, P=0.9588) (all P>0.05). Notably, the offspring-to-parent group had a significantly higher 5-year GRFS than the parent-to-offspring group (62% vs 48%, P=0.0275). In multivariate analysis, donor-recipient pairing pattern combined with patient age was associated with transplant outcomes. Offspring-to-parent donation was associated with superior OS (HR=3.73, 95%CI=1.263–11.01, P=0.0172), DFS (HR=2.87, 95%CI=1.258–6.53, P=0.0122), GRFS (HR=2.06, 95%CI=1.109–3.82, P=0.0222), and lower NRM (sdHR=8.32, 95%CI=2.112–32.77, P=0.0025). Donor direction showed no impact on RI, acute GVHD, or chronic GVHD (all P>0.05).

**Copyright Information:** (Online) ISSN: 2572-9241
©2026 The Author(s). HemaSphere is published by John Wiley & Sons Ltd on behalf of European Hematology Association. This is an open access Abstract Book distributed under the Attribution-NonCommercial-NoDerivs (CC BY-NC-ND), which allows third parties to download the articles and share them with others as long as they credit the author and the Abstract Book, but they cannot change the content in any way or use them commercially.
**Abstract Book Citations:** Authors, Title, HemaSphere, 2026;10:(S1):pages. The abstract book DOIs can be found on the "Author Information" page.
**Disclaimer:** Articles published in the journal HemaSphere exclusively reflect the opinions of the authors. The authors are responsible for all content in their abstracts including accuracy of the facts, statements, citing resources, etc.

### Summary/Conclusion

Offspring-to-parent donor direction confers more favorable long-term clinical outcomes than parent-to-offspring donation in haplo-HSCT for AML patients in CR1, with significantly higher GRFS and improved OS, DFS, and lower NRM confirmed in multivariate analysis. The donor-recipient pairing pattern combined with patient age serves as a key prognostic factor for haplo-HSCT outcomes. These findings highlight the clinical importance of incorporating donor direction into individualized donor selection strategies, providing evidence-based guidance for optimizing clinical decision-making in haploidentical transplantation practice.

Baseline Characteristics of Patients and Donors by Haploidentical Donor-Recipient Direction

| Characteristics | levels | parent-to-offspring (N=126) | offspring-to-parent (N=158) | p |
|---|---|---|---|---|
| Patient age | Mean ± SD | 48.5 ± 8.2 | 20.8 ± 10.9 | <.001 |
| Donor age | Mean ± SD | 23.5 ± 8.9 | 47.6 ± 9.6 | <.001 |
| Patient gender | Female | 59 (46.8%) | 72 (45.6%) | .927 |
| | Male | 67 (53.2%) | 86 (54.4%) | |
| Donor gender | Female | 50 (39.7%) | 27 (17.1%) | <.001 |
| | Male | 76 (60.3%) | 131 (82.9%) | |
| Gender match | Female -to- Female | 24 (19%) | 13 (8.2%) | <.001 |
| | Female -to- Male | 26 (20.6%) | 14 (8.9%) | |
| | Male -to- Female | 34 (27%) | 60 (38%) | |
| | Male -to- Male | 42 (33.3%) | 71 (44.9%) | |
| ABO match | Major mismatch | 24 (21.6%) | 32 (21.3%) | .164 |
| | Matched | 60 (54.1%) | 95 (63.3%) | |
| | Minor mismatch | 27 (24.3%) | 23 (15.3%) | |
| Follow up days | Median(range) | 1440(66 - 2095) | 1450(70 - 2062) | |

Baseline Characteristics of Patients and Donors by Haploidentical Donor-Recipient Direction

**Copyright Information:** (Online) ISSN: 2572-9241
©2026 The Author(s). HemaSphere is published by John Wiley & Sons Ltd on behalf of European Hematology Association. This is an open access Abstract Book distributed under the Attribution-NonCommercial-NoDerivs (CC BY-NC-ND), which allows third parties to download the articles and share them with others as long as they credit the author and the Abstract Book, but they cannot change the content in any way or use them commercially.
**Abstract Book Citations:** Authors, Title, HemaSphere, 2026;10:(S1):pages. The abstract book DOIs can be found on the "Author Information" page.
**Disclaimer:** Articles published in the journal HemaSphere exclusively reflect the opinions of the authors. The authors are responsible for all content in their abstracts including accuracy of the facts, statements, citing resources, etc.

### (PS2251) SALVAGE ALLOGENEIC HEMATOPOIETIC STEM CELL TRANSPLANTATION FOR NONHODGKIN LYMPHOMA: A SINGLE-CENTER RETROSPECTIVE ANALYSIS

**Topic:** 23. Stem cell transplantation - Clinical

Linwei Shi*[1], Aiming Pang[1]

[1]*Institute of Hematology & Blood Diseases Hospital, Chinese Academy of Medical Sciences & Peking Union Medical College, Institute of Hematology & Blood Diseases Hospital, Tianjin, China;*

### Background

This study aimed to evaluate the clinical efficacy and prognostic factors of allogeneic hematopoietic stem cell transplantation (allo-HSCT) in patients with non-Hodgkin lymphoma (NHL) through a single-center retrospective analysis.

### Methods

We retrospectively analyzed the clinical data of patients with NHL who underwent allo-HSCT at the Hematopoietic Stem Cell Transplantation Center of the Chinese Academy of Medical Sciences Blood Disease Hospital between July 2021 and June 2025.

### Results

A total of 76 patients were included, with a male-to-female ratio of 3.22:1. The cohort predominantly consisted of young patients with high-risk disease (median age 34 years; 86.8% had Ann Arbor stage III-IV). Among them, 64 patients had T/NK-cell lymphoma and 12 had B-cell lymphoma. The median time from diagnosis to transplantation was 9.6 months (range: 3.6–58.0 months). Nine patients (11.9%) had undergone prior autologous HSCT and experienced early relapse, 7 (9.2%) had received CAR-T therapy, and 35 (46.1%) had received ≥3 lines of prior therapy before allo-HSCT. At the time of transplant, 50 patients (65.8%) had achieved complete remission (CR). Thirty-eight patients (50%) received a total body irradiation (TBI)-based conditioning regimen, while the other half received a chemotherapy-based regimen. Haploidentical donors were used in 61 patients (80.3%), and HLA-matched sibling donors were used in 13 (17.1%). Graft-versus-host disease (GVHD) prophylaxis based on calcineurin inhibitors was administered to 71 patients (93.4%), and anti-thymocyte globulin (ATG) was used for T-cell depletion in 70 patients (92.1%).
With a median follow-up of 17.9 months (cutoff date: November 15, 2025), the median infused mononuclear cell count was $11.41\times10^8$/kg ($5.89–29.45\times10^8$/kg) and the median $CD34^+$ cell count was $4.34\times10^6$/kg ($1.13–14.63\times10^6$/kg). The rates of neutrophil and platelet engraftment were 98.7% and 93.4%, respectively. Grade III-IV acute GVHD occurred in 12 patients (15.8%), and chronic GVHD developed in 19 patients (25.0%), with only one case (1.3%) being severe.
The 1-year overall survival (OS) rate for the entire cohort was 71.09% (95% CI: 59.07–80.15%), and the progression-free survival (PFS) rate was 72.49% (95% CI: 60.57–81.35%). The relapse rate was 5.89% (95% CI: 2.52–14.96%), and the non-relapse mortality (NRM) rate was 24.22% (95% CI: 15.73–36.20%). For patients undergoing salvage transplantation, the 1-year OS was 64.47% (95% CI: 44.53–78.83%) and PFS was 92.94% (95% CI: 74.47–98.20%), with a relapse rate of 7.05% (95% CI: 1.81–25.43) and an NRM rate of 30.75% (95% CI: 17.00–51.55).Multivariable Cox regression analysis identified pre-transplant CR status as an independent risk factor for OS (HR = 3.094, 95% CI 1.305–7.333, $P$= 0.010). A trend towards improved OS was observed with HLA-matched sibling donors compared to haploidentical donors (HR = 0.136, $P$= 0.052).

**Copyright Information:** (Online) ISSN: 2572-9241
©2026 The Author(s). HemaSphere is published by John Wiley & Sons Ltd on behalf of European Hematology Association. This is an open access Abstract Book distributed under the Attribution-NonCommercial-NoDerivs (CC BY-NC-ND), which allows third parties to download the articles and share them with others as long as they credit the author and the Abstract Book, but they cannot change the content in any way or use them commercially.
**Abstract Book Citations:** Authors, Title, HemaSphere, 2026;10:(S1):pages. The abstract book DOIs can be found on the “Author Information” page.
**Disclaimer:** Articles published in the journal HemaSphere exclusively reflect the opinions of the authors. The authors are responsible for all content in their abstracts including accuracy of the facts, statements, citing resources, etc.

**Summary/Conclusion**

Allo-HSCT represents an effective treatment modality for high-risk or refractory NHL, with significantly better outcomes for patients who achieve CR prior to transplantation. In the context of haploidentical transplantation being the predominant approach, donor type and disease remission status emerge as critical factors influencing survival. Longer follow-up is warranted to further validate long-term efficacy.

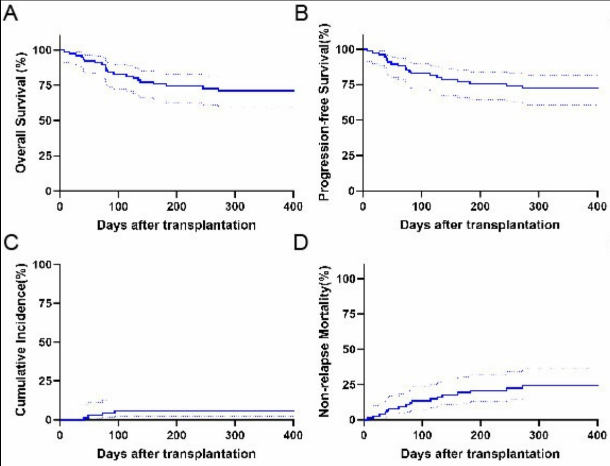

Figure 1 Outcomes of all patients after allo-HSCT

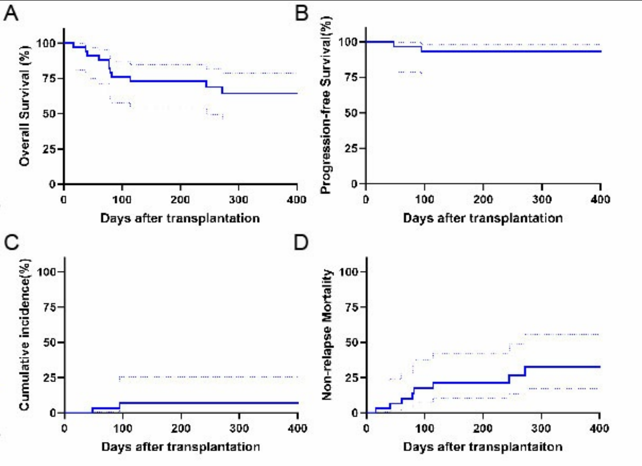

Figure 2 Outcomes of patients undergoing salvage transplantation

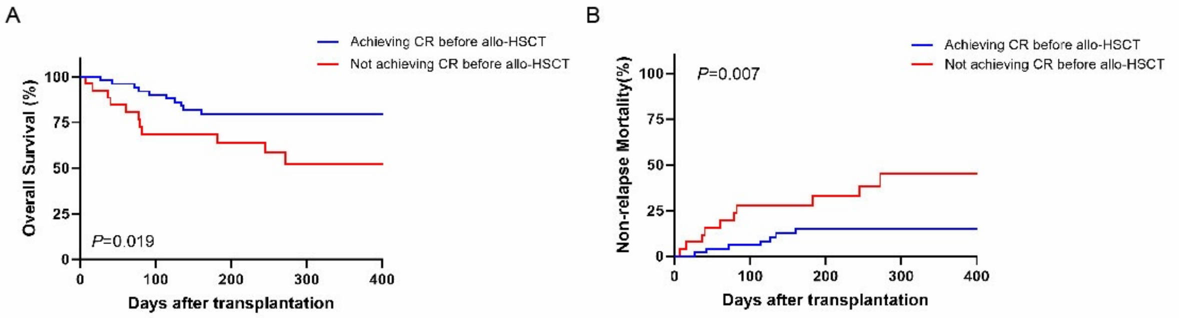

Figure 3 Overall survival and non-relapse mortality for patients according to achieving and not achieving CR before allo-HSCT

**Copyright Information:** (Online) ISSN: 2572-9241
©2026 The Author(s). HemaSphere is published by John Wiley & Sons Ltd on behalf of European Hematology Association. This is an open access Abstract Book distributed under the Attribution-NonCommercial-NoDerivs (CC BY-NC-ND), which allows third parties to download the articles and share them with others as long as they credit the author and the Abstract Book, but they cannot change the content in any way or use them commercially.
**Abstract Book Citations:** Authors, Title, HemaSphere, 2026;10:(S1):pages. The abstract book DOIs can be found on the "Author Information" page.
**Disclaimer:** Articles published in the journal HemaSphere exclusively reflect the opinions of the authors. The authors are responsible for all content in their abstracts including accuracy of the facts, statements, citing resources, etc.

### (PS2252) DONOR SELECTION IN HAPLOIDENTICAL ALLOGENEIC TRANSPLANTATION: THE ROLE OF RECIPIENT AGE AND KINSHIP.

**Topic:** 23. Stem cell transplantation - Clinical

Nisham Pandikasala Narikuth*[1], Nihar Desai[1], Yasir Jeelani[1], Carol Chen[1], Eshrak Al-Shaibani[1], Tommy Alfaro-Moya[1], Igor Novitzky-Basso[1], Ivan Pasic[1], Fotios Michelis[1], Dennis Kim[1], Rajat Kumar[1], Jonas Mattsson[1], Auro Viswabandya[1]

[1]*Princess Margaret Cancer Centre, Medical Oncology and Haematology, Toronto, Canada;*

### Background

Donor selection in haploidentical hematopoietic cell transplantation (haplo-HCT) is increasingly relevant as multiple related donors are often available. There has been guidance from the literature as to which is the ideal Haploidentical donor in terms of multiple non-HLA factors like age, CMV status, and DSA levels. The choice of haploidentical donor is often between parents, siblings or offsprings. Most guidelines prefer young male donors as the preferred choice. EBMT recommends children or offspring over parent donors.

### Aims

We sought to evaluate the impact of donor kinship and donor age on outcomes after haplo-HCT.

### Methods

We performed a retrospective single-center analysis of consecutive adults with AML/MDS undergoing first haplo-HCT using anti-thymocyte globulin (ATG) plus post-transplant cyclophosphamide (PTCy)-based GVHD prophylaxis. Donor kinship included parents, siblings, and offspring. Due to correlation between recipient age, donor age, and kinship, the cohort was stratified at 45 years, corresponding to a marked shift in donor distribution.
The primary endpoint was overall survival (OS). Secondary endpoints included relapse and non-relapse mortality (NRM). Multivariable models included donor kinship, donor age, and conditioning regimen intensity.

### Results

Among the 164 patients, median age was 60 years (IQR: 48-67), of whom 99 (60%) were males. All patients received mobilized peripheral blood grafts. Conditioning regimen was reduced intensity in 138 (84%). Donor selection varied by recipient age: for patients ≤45 years, donors were parents in 48.5%, siblings in 45.5%, and offspring in 6.1%; for those >45 years, donors were primarily offspring (87.5%) and siblings (12.5%), with no parental donors utilized.
With a median follow up of 60.1 months (95% CI: 49.4-69.5), the 2-year OS, NRM, and relapse was 49.5% (95% CI: 41.4-57), 33.2% (95% CI: 26-40.6), and 22.3% (95% CI: 16.1-29.2) respectively.
In recipients aged ≤45 years, parental donors were independently associated with inferior OS (HR: 4.67, 95% CI: 1.1-18.7, p=0.03) and increased NRM (HR: 9.23, 95% CI: 3-27.8, p<0.001) compared with sibling donors.
In recipients aged >45 years, offspring donors were associated with inferior OS (HR: 3.61, 95% CI: 1.3-9.9, p=0.01) and increased NRM (HR: 4.49, 95% CI: 1.1-19.9, p=0.04) compared to sibling haploidentical donors. Increasing donor age was independently associated with higher NRM in this group (HR 1.45 per 10-year increase, 95% CI: 1.1-2.0, p=0.023). Donor kinship did not impact relapse in either group.

**Copyright Information:** (Online) ISSN: 2572-9241
©2026 The Author(s). HemaSphere is published by John Wiley & Sons Ltd on behalf of European Hematology Association. This is an open access Abstract Book distributed under the Attribution-NonCommercial-NoDerivs (CC BY-NC-ND), which allows third parties to download the articles and share them with others as long as they credit the author and the Abstract Book, but they cannot change the content in any way or use them commercially.
**Abstract Book Citations:** Authors, Title, HemaSphere, 2026;10:(S1):pages. The abstract book DOIs can be found on the “Author Information” page.
**Disclaimer:** Articles published in the journal HemaSphere exclusively reflect the opinions of the authors. The authors are responsible for all content in their abstracts including accuracy of the facts, statements, citing resources, etc.

**Summary/Conclusion**

Parental donors in recipients aged ≤45 years and offspring donors in recipients aged >45 years were associated with inferior OS and higher NRM compared with sibling donors, while relapse incidence was not significantly affected by donor kinship. These findings warrant validation in larger, multi-center studies.

**Copyright Information:** (Online) ISSN: 2572-9241
©2026 The Author(s). HemaSphere is published by John Wiley & Sons Ltd on behalf of European Hematology Association. This is an open access Abstract Book distributed under the Attribution-NonCommercial-NoDerivs (CC BY-NC-ND), which allows third parties to download the articles and share them with others as long as they credit the author and the Abstract Book, but they cannot change the content in any way or use them commercially.
**Abstract Book Citations:** Authors, Title, HemaSphere, 2026;10:(S1):pages. The abstract book DOIs can be found on the "Author Information" page.
**Disclaimer:** Articles published in the journal HemaSphere exclusively reflect the opinions of the authors. The authors are responsible for all content in their abstracts including accuracy of the facts, statements, citing resources, etc.

### (PS2253) EFFICACY AND SAFETY OF TOTAL BODY IRRADIATION COMBINED WITH THIOTEPA VERSUS TOTAL BODY IRRADIATION IN ALLO-HSCT FOR PATIENTS WITH RELAPSED/REFRACTORY ACUTE LEUKEMIA

**Topic:** 23. Stem cell transplantation - Clinical

Minglu Li*[1], Jingbo Wang[1], Shuqin Zhang[1], Weijie Zhang[1]

[1]*Aerospace Center Hospital, Hematology, Beijing, China;*

### Background

The prognosis for relapsed/refractory acute leukemia (R/R AL) is extremely poor, and allogeneic hematopoietic stem cell transplantation (allo-HSCT) represents the only treatment with curative potential. However, post-transplant relapse remains the primary cause of treatment failure in this high-risk population. Total body irradiation (TBI) is a cornerstone of myeloablative conditioning, and the addition of thiotepa (TT), an alkylating agent with potent central nervous system and systemic anti-leukemia activity, may synergistically enhance disease control.

### Aims

This study aims to evaluate the efficacy and safety of a conditioning regimen comprising TBI plus thiotepa (TBI+TT) compared to TBI alone in patients with R/R AL undergoing allo-HSCT.

### Methods

In this single-center, retrospective cohort study, 415 patients with R/R AL (TBI+TT group, n=78; TBI group, n=337) were analyzed. To minimize baseline selection bias, propensity score matching using the nearest neighbor matching method was performed. The primary endpoints were overall survival (OS) and disease-free survival (DFS). Secondary endpoints included time to engraftment, mucositis, and other related adverse events. Survival outcomes were assessed using the Kaplan-Meier method and log-rank test, while cumulative incidence functions were used to evaluate competing risks.

### Results

After propensity score matching, 51 matched pairs (n=102) were analyzed, demonstrating good balance in baseline characteristics (all P > 0.05). The median time to neutrophil engraftment was 13 days in the TBI+TT group and 14 days in the TBI group (P = 0.20); median time to platelet engraftment was 15 days and 17 days, respectively (P = 0.93), indicating comparable hematopoietic recovery. Regarding safety, the TBI+TT group had a higher incidence of mucositis-related infections (64.7% vs. 39.2%, P = 0.01), but mucositis-related mortality (17.6% vs. 19.6%) and non-relapse mortality were similar between the two groups. For survival, the TBI+TT group showed numerically higher 1-year OS (59.5% [95% CI: 46.6–76.1] vs. 40.1% [95% CI: 27.5–58.4]) and 1-year DFS (48.2% vs. 44.1%). Although overall OS (P = 0.25) and DFS (P = 0.70) did not reach statistical significance, the TBI+TT cohort achieved an 11.3% 5-year OS, whereas no patients in the TBI group survived beyond 5 years. Notably, the TBI+TT group demonstrated a strong trend towards improved disease control with a lower 3-year cumulative incidence of relapse (6.4% vs. 25.4%, P = 0.09). No significant differences were observed in the incidence of acute/chronic graft-versus-host disease (all P > 0.05).

**Copyright Information:** (Online) ISSN: 2572-9241
©2026 The Author(s). HemaSphere is published by John Wiley & Sons Ltd on behalf of European Hematology Association. This is an open access Abstract Book distributed under the Attribution-NonCommercial-NoDerivs (CC BY-NC-ND), which allows third parties to download the articles and share them with others as long as they credit the author and the Abstract Book, but they cannot change the content in any way or use them commercially.
**Abstract Book Citations:** Authors, Title, HemaSphere, 2026;10:(S1):pages. The abstract book DOIs can be found on the "Author Information" page.
**Disclaimer:** Articles published in the journal HemaSphere exclusively reflect the opinions of the authors. The authors are responsible for all content in their abstracts including accuracy of the facts, statements, citing resources, etc.

**Summary/Conclusion**

In patients with R/R AL undergoing allo-HSCT, the addition of thiotepa to the TBI conditioning regimen is feasible, provides favorable engraftment outcomes, and shows trends towards reduced 3-year relapse rates and improved long-term survival. These findings suggest that TBI+TT represents an effective alternative conditioning strategy for high-risk patients, but large-scale prospective randomized trials are warranted to further validate these survival benefits.

**Copyright Information:** (Online) ISSN: 2572-9241
©2026 The Author(s). HemaSphere is published by John Wiley & Sons Ltd on behalf of European Hematology Association. This is an open access Abstract Book distributed under the Attribution-NonCommercial-NoDerivs (CC BY-NC-ND), which allows third parties to download the articles and share them with others as long as they credit the author and the Abstract Book, but they cannot change the content in any way or use them commercially.
**Abstract Book Citations:** Authors, Title, HemaSphere, 2026;10:(S1):pages. The abstract book DOIs can be found on the "Author Information" page.
**Disclaimer:** Articles published in the journal HemaSphere exclusively reflect the opinions of the authors. The authors are responsible for all content in their abstracts including accuracy of the facts, statements, citing resources, etc.

### (PS2254) A NOVEL CLASSIFICATION OF ENGRAFTMENT SYNDROME AND ITS PROGNOSTIC IMPACT FOLLOWING HAPLOIDENTICAL ALLOGENEIC HAEMATOPOIETIC CELL TRANSPLANTATION

**Topic:** 23. Stem cell transplantation - Clinical

Haixia Fu*[1], Zikang Long[1], Yuan-Yuan Zhang[1], Yu-Qian Sun[1], Xiao-Dong Mo[1], Tingting Han[1], Fengrong Wang[1], Chenhua Yan[1], Yu-Hong Chen[1], Jun Kong[1], Wei Han[1], Huan Chen[1], Yao Chen[1], Yu Wang[1], Lan-Ping Xu[1], Xiao-Jun Huang[1], Xiaohui Zhang[1]

*[1] 1 Peking University People's Hospital, Peking University Institute of Hematology, Beijing, China 2 National Clinical Research Center for Hematologic Disease, Beijing, China 3 Beijing Key Laboratory of Cell and Gene Therapy for Hematologic Malignancies, Peking University, Beijing, China; 4 Collaborative Innovation Center of Hematology，Peking University Institute of Hematology, Beijing, China, beijing, China;*

### Background

Engraftment syndrome (ES) is a common complication following haematopoietic stem cell transplantation (HSCT), and numerous diagnostic criteria are available for ES. Haploidentical HSCT (haplo-HSCT) is generally considered to be associated with a higher incidence of ES. However, it remains unclear which diagnostic criteria for ES are most appropriate for patients undergoing haploidentical transplantation.

### Aims

To investigate the clinical manifestations of ES and its impact on the outcomes of haploidentical haematopoietic stem cell transplantation.

### Methods

We retrospectively studied patients with malignant haematologic diseases who were diagnosed with ES after haplo-HSCT at Peking University People's Hospital from 2020 to 2025. To explore the relationship between the symptoms of ES and its prognosis, we proposed the following classification for ES: definite ES (meeting the Spitzer, Maiolino, or Grant criteria) and probable ES (only fulfilling the criteria used by Dhakal).

### Results

Of the 305 patients, 219 (71.8%) were classified as having definite ES and 86 (28.2%) were classified as having probable ES. The most common ES-related symptom was fever (87%), with other symptoms including skin rash (67%), hepatic dysfunction (33%), diarrhoea (32%), hypoxia (12%) and renal dysfunction (8%). Compared with the probable ES group, the definite ES group had a significantly higher maximum temperature (p=0.037). From conditioning initiation to stem cell infusion, the minimum haemoglobin level was significantly greater (p=0.042), whereas the minimum fibrinogen (FIB) level was significantly lower (p=0.011) in the definite ES group. From 3 days before to 4 days after neutrophil engraftment, the definite ES group also had significantly higher maximum CRP (p=0.025) and minimum FIB (p<0.001) levels but significantly lower maximum LDH (p<0.01) and serum creatinine (p=0.003) levels. Patients with definite ES had a higher incidence of acute graft-versus-host disease (GVHD) (p=0.028). Furthermore, patients with definite ES had significantly poor GVHD-free survival (GRFS) (HR, 0.683; 95% CI, 0.492–0.948; p=0.022).

**Copyright Information:** (Online) ISSN: 2572-9241
©2026 The Author(s). HemaSphere is published by John Wiley & Sons Ltd on behalf of European Hematology Association. This is an open access Abstract Book distributed under the Attribution-NonCommercial-NoDerivs (CC BY-NC-ND), which allows third parties to download the articles and share them with others as long as they credit the author and the Abstract Book, but they cannot change the content in any way or use them commercially.
**Abstract Book Citations:** Authors, Title, HemaSphere, 2026;10:(S1):pages. The abstract book DOIs can be found on the "Author Information" page.
**Disclaimer:** Articles published in the journal HemaSphere exclusively reflect the opinions of the authors. The authors are responsible for all content in their abstracts including accuracy of the facts, statements, citing resources, etc.

**Summary/Conclusion**

Patients with malignant haematologic diseases who meet the definite diagnostic criteria for ES after haplo-HSCT have an increased incidence of acute GVHD and poorer survival outcomes, highlighting the need for more aggressive treatment and close monitoring in this patient population.

**Copyright Information:** (Online) ISSN: 2572-9241
©2026 The Author(s). HemaSphere is published by John Wiley & Sons Ltd on behalf of European Hematology Association. This is an open access Abstract Book distributed under the Attribution-NonCommercial-NoDerivs (CC BY-NC-ND), which allows third parties to download the articles and share them with others as long as they credit the author and the Abstract Book, but they cannot change the content in any way or use them commercially.
**Abstract Book Citations:** Authors, Title, HemaSphere, 2026;10:(S1):pages. The abstract book DOIs can be found on the “Author Information” page.
**Disclaimer:** Articles published in the journal HemaSphere exclusively reflect the opinions of the authors. The authors are responsible for all content in their abstracts including accuracy of the facts, statements, citing resources, etc.

### (PS2255) AZACITIDINE PLUS VENETOCLAX AS POST-TRANSPLANT MAINTENANCE THERAPY IN HIGH-RISK MYELOID MALIGNANCIES: A PROPENSITY SCORE-MATCHED ANALYSIS

**Topic:** 23. Stem cell transplantation - Clinical

Changmeng Zhang[1], Xinxin Xia*[1], Yu Cai[1], Jun Yang[1], Huiying Qiu[1], Kun Zhou[1], Jiahua Niu[1], Chongmei Huang[1], Ying Zhang[1], Yu Wei[1], Baoxia Dong[1], Xiaowei Xu[1], Jie Shao[1], Jingjing Cao[1], Lu Gao[1], Min Yang[1], Yin Tong[1], Xianmin Song[1], Liping Wan[1]

*[1]Shanghai General Hospital, Shanghai Jiao Tong University School of Medicine, Shanghai, China;*

### Background

Allogeneic stem cell transplantation is an effective modality for hematological malignancies. However, disease relapse is still the major barrier for high-risk patients. Hypomethylating Agents azacytidine and venetoclax were effective in myeloid malignancies.

### Aims

To evaluate the efficacy and safety of the Hypomethylating Agents azacytidine and venetoclax in relapse prevention of high-risk myeloid malignancies after allogeneic hematopoietic stem cell transplantation.

### Methods

This is a retrospective study. Patients with high-risk AML or MDS aged between 18-70 years old and underwent allogeneic stem cell transplantation will be included if they fulfill the following criteria: adverse risk group of AML by European LeukemiaNet�MDS with high-risk or very high-risk IPSS-R scores or Presence of TP53 mutation�Presence of measurable residual disease at the time of HSCT, requirement of more than 2 courses of induction chemotherapy to reach complete remission (CR), Extramedullary myeloid malignancy, ≥ CR2. Post-transplant maintenance therapy consisted of azacitidine (32 mg/m²/day, day 1-5) plus venetoclax (400 mg/day, day 1-7), starting from the 60th day posttransplant and repeated every 28 days until up to 1-year posttransplant. Outcomes were compared with a contemporaneous control group constructed via propensity score matching and win ratio analysis. The 1-year disease-free survival rate�1-year cumulative recurrence rate and 1-year overall survival will be analyzed. The study was approved by the institutional review committee of the ethics committee of Shanghai General Hospital.

### Results

This retrospective study enrolled patients with high-risk AML or MDS aged 18 to 70 years who received allogeneic HSCT from January 1, 2022 to December 31, 2024. Outcomes were compared with a contemporaneous control group constructed via propensity score matching (azacitidine-venetoclax, n = 35; control, n = 59). In the matched cohort, eighty-two patients were diagnosed with AML; the other 12 patients were diagnosed with MDS. After a median follow-up of 22.5 months, the 1-year disease-free survival was 88.6% (95% CI, 78.6%-99.8%), the 1-year cumulative incidence of relapse was 5.7% (95% CI, 0%-13.5%). The azacitidine-venetoclax group demonstrated significantly superior 1-year overall survival compared to control (P = 0.037). Win ratio analysis further confirmed a significant overall clinical benefit (P < 0.001). Subgroup analysis revealed a pronounced overall survival benefit for MRD-positive patients (HR, 0.288; P = 0.037). The safety profile was manageable, no significant increase in the incidences of EBV and CMV infection or graft-versus-host disease. The most common side effects were cytopenia. Neutropenia developed in 21 (52.5%) patients, 9 of them had grade 3 neutropenia, 3 patients had grade 4 neutropenia. Thrombocytopenia developed in 14 (35.0%) patients, 3 patients had grad 3 thrombocytopenia, one with grade 4 thrombocytopenia. Immune reconstitution analysis showed delayed B-cell recovery but preserved T-cell and NK-cell recovery.

**Copyright Information:** (Online) ISSN: 2572-9241
©2026 The Author(s). HemaSphere is published by John Wiley & Sons Ltd on behalf of European Hematology Association. This is an open access Abstract Book distributed under the Attribution-NonCommercial-NoDerivs (CC BY-NC-ND), which allows third parties to download the articles and share them with others as long as they credit the author and the Abstract Book, but they cannot change the content in any way or use them commercially.
**Abstract Book Citations:** Authors, Title, HemaSphere, 2026;10:(S1):pages. The abstract book DOIs can be found on the "Author Information" page.
**Disclaimer:** Articles published in the journal HemaSphere exclusively reflect the opinions of the authors. The authors are responsible for all content in their abstracts including accuracy of the facts, statements, citing resources, etc.

**Summary/Conclusion**

Maintenance therapy with low dose azacytidine and venetoclax was effective and well-tolerated in relapse prevention for high-risk myeloid malignancies after allogeneic hematopoietic stem cell transplantation. The majority of adverse events were reversible and manageable.

**Copyright Information:** (Online) ISSN: 2572-9241
©2026 The Author(s). HemaSphere is published by John Wiley & Sons Ltd on behalf of European Hematology Association. This is an open access Abstract Book distributed under the Attribution-NonCommercial-NoDerivs (CC BY-NC-ND), which allows third parties to download the articles and share them with others as long as they credit the author and the Abstract Book, but they cannot change the content in any way or use them commercially.
**Abstract Book Citations:** Authors, Title, HemaSphere, 2026;10:(S1):pages. The abstract book DOIs can be found on the "Author Information" page.
**Disclaimer:** Articles published in the journal HemaSphere exclusively reflect the opinions of the authors. The authors are responsible for all content in their abstracts including accuracy of the facts, statements, citing resources, etc.

### (PS2256) CLINICAL IMPLICATIONS OF SUSTAINED HYPOBARIC HYPOXIA ON NEUTROPHIL ENGRAFTMENT AFTER ALLOGENEIC HEMATOPOIETIC STEM CELL TRANSPLANTATION

**Topic:** 23. Stem cell transplantation - Clinical

Yigeng Cao*[1], Xiaojing MA[2], Yajing Chu[1], Naya MA[3], Mingyue Xu[1], Wei Luo[2], MA Jie[2], Qichao Yin[2], Yujuan Yin[2], Mingyue Zhang[1], Jingni Fan[3], Tingjun Wang[3], Yifei He[1], Mingyue Hao[1], Yansong Ren[1], Dong Su[1], Yueshen MA[1], Qian Ren[1], Mingyang Wang[1], Rongli Zhang[1], Weihua Zhai[1], Qiaoling MA[1], Xin Chen[1], Wenbin Cao[1], Chen Liang[1], Donglin Yang[1], Yi He[1], Aiming Pang[1], Sizhou Feng[1], Mingzhe Han[1], Haizhao MA[4], Yanxia Xing[4], Guoquan Li[5], Haining Fan[2], Sen Cui[2], Zhengping Zhuang[1], Erlie Jiang[1], Hui Geng[2]

*[1]Institute of Hematology & Blood Diseases Hospital, National Clinical Research Center for Blood Diseases, Chinese Academy of Medical Sciences & Peking Union Medical College, Hematopoietic Stem Cell Transplantation Center, Tianjin, China; [2]Affiliated Hospital of Qinghai University, Xining, Qinghai, China; [3]Qinghai University Medical College, Xining, Qinghai, China; [4]The Fifth People's Hospital of Qinghai Province and Qinghai Province Cancer Hospital, Xining, Qinghai, China; [5]The People's Hospital of Qinghai Province, Xining, Qinghai, China;*

**Background**

Allogeneic hematopoietic stem cell transplantation (allo-HSCT) is curative for high-risk hematologic diseases and neutrophil engraftment is a clinically consequential marker of transplant success. Although hypoxia is a fundamental regulator of haematopoietic stem-cell biology, its impact on engraftment kinetics in human remains incompletely defined.

**Aims**

This study aimed to investigate the impact of sustained hypobaric hypoxia on neutrophil engraftment kinetics following allo-HSCT. We sought to compare engraftment outcomes in patients undergoing transplantation at a high-altitude center versus a propensity-matched sea-level cohort, and to validate the biological effect of hypobaric hypoxic exposure on early myeloid recovery using a parallel murine model.

**Methods**

In this prospective registry HALO-SCT study (ClinicalTrials.gov, NCT07205523), patients undergoing allo-HSCT under sustained hypobaric hypoxia at a high-altitude transplant center were compared with a 1:3 propensity-matched cohort transplanted at sea level, selected from the NICHE-BMT registry (ClinicalTrials.gov number NCT04645199) using identical transplantation protocols. The primary end point was the time to neutrophil engraftment. Key secondary endpoints included incidence of graft-versus-host disease (GVHD), infections, relapse and overall survival (OS). Parallel murine transplantation experiments assessed the contribution of hypobaric hypoxic exposure to early neutrophil engraftment.

**Results**

Twelve high-altitude recipients enrolled between September 2023 and September 2025 were matched with 36 low-altitude controls. Neutrophil engraftment occurred in all high-altitude recipients at a median of 11 days, earlier than in the low-altitude cohort (13 days, P=0.03), with comparable rates of acute and chronic GVHD, infectious complications, and 2-year OS. No cases of grade III–IV acute GVHD or relapse were observed in the high-altitude cohort during follow-up. From day 16 to day 21 after transplantation, neutrophil and white blood cells count were consistently higher in the high-altitude cohort. In murine transplantation models, high-altitude–conditioned bone marrow for 4 weeks produced earlier donor chimerism and augmented early myeloid output.

**Copyright Information:** (Online) ISSN: 2572-9241
©2026 The Author(s). HemaSphere is published by John Wiley & Sons Ltd on behalf of European Hematology Association. This is an open access Abstract Book distributed under the Attribution-NonCommercial-NoDerivs (CC BY-NC-ND), which allows third parties to download the articles and share them with others as long as they credit the author and the Abstract Book, but they cannot change the content in any way or use them commercially.
**Abstract Book Citations:** Authors, Title, HemaSphere, 2026;10:(S1):pages. The abstract book DOIs can be found on the "Author Information" page.
**Disclaimer:** Articles published in the journal HemaSphere exclusively reflect the opinions of the authors. The authors are responsible for all content in their abstracts including accuracy of the facts, statements, citing resources, etc.

**Summary/Conclusion**

Sustained hypobaric hypoxia was associated with accelerated neutrophil engraftment after allo-HSCT in humans, representing a consistent early recovery signal. Concordantly, donor hypobaric hypoxic exposure alone accelerated early myeloid recovery in experimental models, supporting further investigation of hypoxia-based strategies to enhance early engraftment.

**Copyright Information:** (Online) ISSN: 2572-9241
©2026 The Author(s). HemaSphere is published by John Wiley & Sons Ltd on behalf of European Hematology Association. This is an open access Abstract Book distributed under the Attribution-NonCommercial-NoDerivs (CC BY-NC-ND), which allows third parties to download the articles and share them with others as long as they credit the author and the Abstract Book, but they cannot change the content in any way or use them commercially.
**Abstract Book Citations:** Authors, Title, HemaSphere, 2026;10:(S1):pages. The abstract book DOIs can be found on the “Author Information” page.
**Disclaimer:** Articles published in the journal HemaSphere exclusively reflect the opinions of the authors. The authors are responsible for all content in their abstracts including accuracy of the facts, statements, citing resources, etc.

### (PS2257) SEQUENTIAL HAPLOIDENTICAL-CORD BLOOD HEMATOPOIETIC STEM-CELL TRANSPLANTATION WITH PT-CY/FLU IN PEDIATRIC ACUTE LEUKEMIA

**Topic:** 23. Stem cell transplantation - Clinical

Zhilin Hu*[1], Yue Li[2], Qian Zhang[2], Xiaohui Zhou[2], Chunlan Yang[2], Chunjing Wang[2], Lulu Wang[2, 3], Sixi Liu[2]

*[1]Affiliated Shenzhen Children's Hospital of Shantou University Medical College, Hematology and Oncology, Shenzhen, China; [2]Shenzhen Children's Hospital, Hematology and Oncology, Shenzhen, China; [3]Dana-Farber Cancer Institute, Department of Cancer Immunology and Virology, Boston, United States of America;*

### Background

Alternative donor Hematopoietic Stem-Cell Transplantation (HSCT) for pediatric acute leukemia (AL) is hampered by delayed engraftment in umbilical cord blood (UCB) transplantation and excessive alloreactivity in haploidentical transplantation. A sequential haplo-UCB transplantation strategy may overcome these limitations by bridging early hematopoiesis, promoting durable UCB predominance, and reducing severe graft-versus-host disease (GVHD).

### Aims

To assess engraftment efficacy, safety, and survival of sequential haplo-UCB HSCT with post-transplant cyclophosphamide and fludarabine (PT-Cy/Flu) in pediatric AL.

### Methods

We retrospectively analyzed 73 consecutive pediatric AL patients (median age 99 months, IQR 44.5–151 months) who underwent myeloablative sequential haplo-UCB HSCT at Shenzhen Children’s Hospital from April 2020 to December 2024. Myeloablative conditioning included busulfan, cyclophosphamide, fludarabine and thiotepa. Haploidentical peripheral blood stem cells were infused on Day 0, with PT-Cy/Flu administered on Days +3 and +4; unrelated UCB was infused on Day +6. GVHD prophylaxis comprised cyclosporine A or tacrolimus combined with methotrexate and mycophenolate mofetil. Cumulative incidence (CI) was calculated for GVHD, viral reactivation and relapse. Survival was analyzed using the Kaplan–Meier estimate.

### Results

No primary graft failure was observed, and 79.5% of patients achieved predominant UCB donor chimerism. For UCB-predominant patients, median infused $CD34^{+}$ cell dose was $1.8\times10^{5}$/kg (range $0.5–7.0\times10^{5}$/kg) and median total nucleated cell (TNC) dose $4.3\times10^{7}$/kg (range $0.5–15.3\times10^{7}$/kg). Median neutrophil and platelet engraftment occurred at Days 25 and 20, respectively. CI of grade II–IV acute GVHD was 32.9% (grade III–IV: 2.7%). Chronic GVHD developed in 20.5% of all patients (17.2%, 10/58 in UCB-predominant chimerism), with no severe cases recorded. CMV viremia and EBV viremia were documented in 50.7% and 2.7% of patients, respectively; no CMV end-organ disease or post-transplant lymphoproliferative disorder was identified. CI of relapse was 13.7%. Over a median follow-up of 24 months, 2-year event-free survival (EFS) and overall survival (OS) were 84.7% and 87.9%, respectively.

### Summary/Conclusion

Sequential haplo-UCB HSCT with PT-Cy/Flu yields high UCB-predominant chimerism, reliable early hematopoietic recovery and low incidence of severe GVHD in pediatric AL patients, with favorable 2-year EFS and OS. This strategy represents an effective alternative donor HSCT option for pediatric AL.

**Copyright Information:** (Online) ISSN: 2572-9241
©2026 The Author(s). HemaSphere is published by John Wiley & Sons Ltd on behalf of European Hematology Association. This is an open access Abstract Book distributed under the Attribution-NonCommercial-NoDerivs (CC BY-NC-ND), which allows third parties to download the articles and share them with others as long as they credit the author and the Abstract Book, but they cannot change the content in any way or use them commercially.
**Abstract Book Citations:** Authors, Title, HemaSphere, 2026;10:(S1):pages. The abstract book DOIs can be found on the “Author Information” page.
**Disclaimer:** Articles published in the journal HemaSphere exclusively reflect the opinions of the authors. The authors are responsible for all content in their abstracts including accuracy of the facts, statements, citing resources, etc.

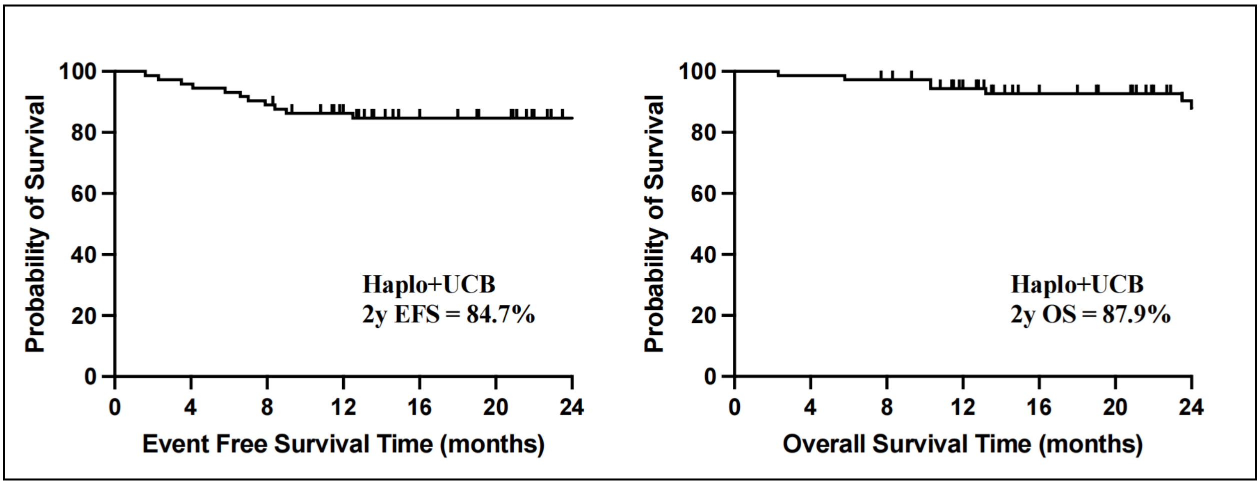

**Copyright Information:** (Online) ISSN: 2572-9241
©2026 The Author(s). HemaSphere is published by John Wiley & Sons Ltd on behalf of European Hematology Association. This is an open access Abstract Book distributed under the Attribution-NonCommercial-NoDerivs (CC BY-NC-ND), which allows third parties to download the articles and share them with others as long as they credit the author and the Abstract Book, but they cannot change the content in any way or use them commercially.
**Abstract Book Citations:** Authors, Title, HemaSphere, 2026;10:(S1):pages. The abstract book DOIs can be found on the "Author Information" page.
**Disclaimer:** Articles published in the journal HemaSphere exclusively reflect the opinions of the authors. The authors are responsible for all content in their abstracts including accuracy of the facts, statements, citing resources, etc.

### (PS2258) COMPARATIVE OUTCOMES OF PATIENTS UNDERGOING ALLOGENEIC HEMATOPOIETIC CELL TRANSPLANTATION FOR MYELODYSPLASTIC NEOPLASM (MDS)/MYELOPROLIFERATIVE NEOPLASM (MPN) OVERLAP VERSUS DE NOVO MDS

**Topic:** 23. Stem cell transplantation - Clinical

Alastair Williams*[1], Amjad Althobaiti[1], Yasser Abou Mourad[1], Reyna Altook[1], Hannah Cherniawsky[1], Shanee Chung[1], Donna Forrest[1], Florian Kuchenbauer[1], Deepesh Lad[1], Stephen Nantel[1], Sujaatha Narayanan[1], Thomas Nevill[1], Judith Rodrigo[1], Claudie Roy[1], Kevin Song[1], Cynthia Toze[1], Jennifer White[1], Eric Zhao[1], Ryan Stubbins[1]

*[1]BC Cancer, Vancouver, Canada;*

### Background

Myelodysplastic neoplasm/myeloproliferative neoplasm (MDS/MPN) overlap syndromes are a rare indication for allogeneic hematopoietic cell transplantation (allo-HCT). Previous studies have suggested relatively poor allo-HCT outcomes for MDS/MPN patients, although currently published data has been non-comparative in nature. Whether MDS/MPN patients have comparatively worse allo-HCT outcomes versus *de novo* MDS in the allo-HCT context remains unknown.

### Aims

We assessed the clinical outcomes and predictors of survival following allo-HCT for patients with MDS/MPN or *de novo* MDS.

### Methods

We retrospectively identified patients receiving a first allo-HCT for MDS or MDS/MPN at a single institution (2010–2023). Patient- and transplant-specific variables were collected by chart review. Diagnoses were classified per the 5th edition WHO classification of haematolymphoid tumors with response and outcome definitions per IWG-2023. Overall survival (OS) was calculated from allo-HCT to death and relapse-free survival (RFS) from allo-HCT to relapse or death. Between-group comparisons used Fisher's exact or Wilcoxon rank-sum tests; survival analyses used Kaplan–Meier (log-rank/absolute risk differences) and multivariable Cox regression. REB approval under H24-01217.

**Copyright Information:** (Online) ISSN: 2572-9241
©2026 The Author(s). HemaSphere is published by John Wiley & Sons Ltd on behalf of European Hematology Association. This is an open access Abstract Book distributed under the Attribution-NonCommercial-NoDerivs (CC BY-NC-ND), which allows third parties to download the articles and share them with others as long as they credit the author and the Abstract Book, but they cannot change the content in any way or use them commercially.
**Abstract Book Citations:** Authors, Title, HemaSphere, 2026;10:(S1):pages. The abstract book DOIs can be found on the "Author Information" page.
**Disclaimer:** Articles published in the journal HemaSphere exclusively reflect the opinions of the authors. The authors are responsible for all content in their abstracts including accuracy of the facts, statements, citing resources, etc.

### Results

We identified 151 allo-HCT recipients: 81% (122/151) with MDS and 19% (29/151) with MDS/MPN, including CMML-1 (24%, 7/29), CMML-2 (28%, 8/29), MDS/MPN-NOS (41%, 12/29), and MDS/MPN with neutrophilia (7%, 2/29). Therapy-related disease rates were comparable (MDS 4.9% vs MDS/MPN 3.4%, $p$=1). Spleen size at diagnosis was smaller in MDS versus MDS/MPN patients (median 8 cm vs 12.6 cm, $p$<0.001). IPSS-R score at diagnosis was higher in MDS versus MDS/MPN patients (median 4.5 vs 3.0, $p$=0.002). NGS was available in 58.2% (71/122) of MDS and 55.2% (16/29) of MDS/MPN patients. Median IPSS-M scores were comparable (0.3 vs 0.3, $p$=0.948). Fully matched sibling or unrelated donors were used in 83% (101/122) of MDS and 86% (25/29) of MDS/MPN ($p$=1) patients. Reduced-intensity conditioning (RIC) was used in 20% (25/122) for MDS versus 24% (7/29) for MDS/MPN patients ($p$=0.623). PTCy GVHD prophylaxis was used in 11% (13/122) for MDS versus 10% (3/29) for MDS/MPN patients ($p$=1).

Estimated 24-month RFS was better in MDS (65%) versus MDS/MPN (41%) ($p$=0.021) while estimated 24-month OS was not significantly different (67% vs 52%, $p$=0.129) (Figure 1A-B). Multivariable Cox for RFS showed significance for IPSS-R (HR 1.20, 95% CI 1.04–1.39, $p$=0.015) and MDS/MPN status (HR 1.82, 95% CI 1.06–3.12, $p$=0.031) with a trend for age (HR 1.02, 95% CI 0.99–1.05, $p$=0.070). For OS, IPSS-R (HR 1.18, 95% CI 1.02–1.36, $p$=0.030) and MDS/MPN (HR 1.84, 95% CI 1.07–3.16, $p$=0.029) were significant. RIC and PTCy were not significant for RFS or OS. In the NGS cohort, only IPSS-M was significant for RFS (HR 1.37, 95% CI 1.05–1.79, $p$=0.022). Graft failure was less common in MDS (3.3%, 4/122) than in MDS/MPN (13.8%, 4/29) patients ($p$=0.045). Relapse was numerically lower in MDS versus MDS/MPN (26% vs 41%, $p$=0.117), with comparable non-relapse mortality (27% vs 24%, $p$=0.8190).

### Summary/Conclusion

Patients receiving allo-HCT for MDS/MPN have worse post-HCT outcomes compared with *de novo* MDS, even after adjustment for baseline features. This may be driven by higher rates of post-HCT relapse. Better approaches are needed for allo-HCT in MDS/MPN patients, and future studies should examine interactions with molecular markers in detail.

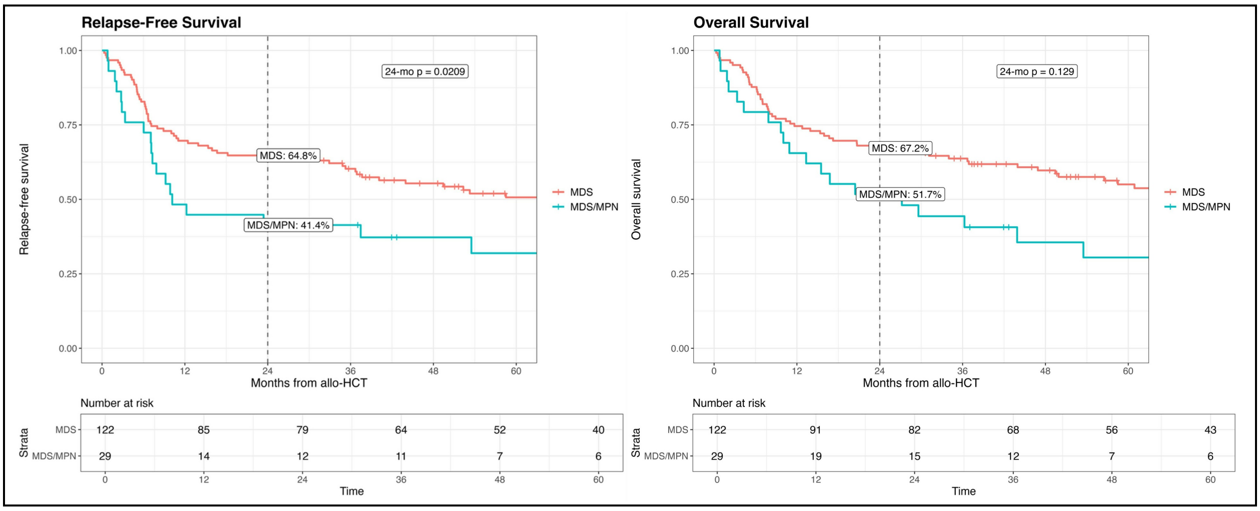

Figure 1. Survival outcomes for patients with MDS/MPN or de novo MDS receiving allogeneic HCT with 24-month outcomes (A) Relapse-free survival (B) Overall survival

**Copyright Information:** (Online) ISSN: 2572-9241
©2026 The Author(s). HemaSphere is published by John Wiley & Sons Ltd on behalf of European Hematology Association. This is an open access Abstract Book distributed under the Attribution-NonCommercial-NoDerivs (CC BY-NC-ND), which allows third parties to download the articles and share them with others as long as they credit the author and the Abstract Book, but they cannot change the content in any way or use them commercially.
**Abstract Book Citations:** Authors, Title, HemaSphere, 2026;10:(S1):pages. The abstract book DOIs can be found on the "Author Information" page.
**Disclaimer:** Articles published in the journal HemaSphere exclusively reflect the opinions of the authors. The authors are responsible for all content in their abstracts including accuracy of the facts, statements, citing resources, etc.

### (PS2259) SURVIVAL IMPACT AND PREDICTORS OF GRAFT DYSFUNCTION AFTER HAPLOIDENTICAL ALLOGENEIC HEMATOPOIETIC STEM CELL TRANSPLANTATION WITH PTCY: A MEXICAN TERTIARY CENTER EXPERIENCE

**Topic:** 23. Stem cell transplantation - Clinical

Mistral Castellanos-Mares*[1], Omar Eduardo Fernandez Vargas[1], Rosa Adriana Alvidrez González[1], Andres Yesid Bonilla Salcedo[1], V. Iván Salinas Herrera[1], Vicky Margarita Moncada Gadea[1], Grecia Pamela Martínez-Vázquez[1], Irma Karen Pellon Tellez[1], Luis M. Valero-Saldaña[1]

*[1]Instituto Nacional de Cancerología, Hematology Department, Ciudad de México, Mexico;*

### Background

Graft dysfunction, including primary/secondary graft failure (GF) and poor graft function (PGF), remains a major cause of early morbidity and mortality after allogeneic hematopoietic cell transplantation (alloHCT), associated with infections and transfusion dependence. Reported incidence ranges from 5–27%. Although haploidentical alloHCT with post-transplant cyclophosphamide (PTCy) has improved engraftment, data on incidence, predictors, and outcomes in real-world cohorts remain limited despite harmonized definitions.

### Aims

To evaluate incidence, predictors, management, and survival impact of graft dysfunction after haploidentical alloHCT with PTCy.

### Methods

Single-center retrospective cohort of consecutive patients undergoing haploidentical alloHCT between August 2017 and December 2025 at our institution in Mexico. All received peripheral blood grafts and uniform GVHD prophylaxis with PTCy (+3/+4), mycophenolate mofetil, and cyclosporine. Variables included recipient/donor characteristics, diagnosis, prior therapy lines, disease status, ferritin ≥1000 µg/L, anti-HLA antibodies and DSA (MFI), donor age, donor–recipient age difference, and sex mismatch. GF and PGF were defined per consensus criteria. Overall survival (OS) was estimated by Kaplan–Meier; predictors were assessed using univariable cause-specific Cox proportional hazards models. Cumulative incidence was analyzed using competing risks with death/relapse as competing events.

**Copyright Information:** (Online) ISSN: 2572-9241
©2026 The Author(s). HemaSphere is published by John Wiley & Sons Ltd on behalf of European Hematology Association. This is an open access Abstract Book distributed under the Attribution-NonCommercial-NoDerivs (CC BY-NC-ND), which allows third parties to download the articles and share them with others as long as they credit the author and the Abstract Book, but they cannot change the content in any way or use them commercially.
**Abstract Book Citations:** Authors, Title, HemaSphere, 2026;10:(S1):pages. The abstract book DOIs can be found on the “Author Information” page.
**Disclaimer:** Articles published in the journal HemaSphere exclusively reflect the opinions of the authors. The authors are responsible for all content in their abstracts including accuracy of the facts, statements, citing resources, etc.

**Results**

Eighty-two patients underwent haploidentical alloHCT. Median age was 27.5 years (IQR 18–43); 36 were female. ALL was the most frequent diagnosis (58.5%), followed by AML (25.6%). Most received ≥1 prior therapy line and were transplanted in complete remission, frequently MRD-positive. Anti-HLA antibodies were detected in 11 patients (4 class I, 1 class II, 6 both); five had DSA ≥2000 MFI. Overall, 24/82 patients (29.2%) developed graft dysfunction. PGF occurred in 15 patients, primary GF in 7, and secondary GF in 2. One patient with major ABO incompatibility developed pure red cell aplasia. Management included transfusion support (10/15 PGF; 6/9 GF), CD34+ boost (n=9; median $1.53\times10^6$/kg, IQR 1.0–2.5), and eltrombopag (n=17; median 100 mg, IQR 50–150).

PGF cumulative incidence was 11.0% (95% CI 5.4–18.9), 14.7% (8.0–23.3), and 17.6% (10.1–26.8) at 3, 6, and 12 months.No significant associations were observed for baseline disease, donor, or transplant-related variables (all p>0.05). However, older recipient age (p<0.001), CMV reactivation (p=0.008), and lower CD34+ dose (p=0.003) predicted PGF.

GF cumulative incidence was 8.5% and 11.1% at 3 and 6–12 months. No significant associations were observed for baseline disease, donor, or transplant-related variables. Older age at HCT (p=0.0008), splenomegaly (p=0.0011), and lower CD34+ dose (p=0.040) predicted GF.

After median follow-up of 31.3 months (95% CI 24.8–36.8), 22 deaths occurred (infection n=10; relapse n=8). Six- and 12-month OS were significantly lower in PGF (73.3% and 59.3%) and particularly in GF (33.3% and 22.2%) (p<0.001).

**Summary/Conclusion**

Graft dysfunction occurred in nearly one-third of haploidentical alloHCT recipients and was associated with inferior survival, particularly in GF. Older age and lower CD34+ dose were consistent predictors. This is the first report from Mexico and Latin America in the haploidentical PTCy setting. Incidence and risk factors were consistent with contemporary literature, confirming its clinical relevance and need for improved risk stratification.

**Copyright Information:** (Online) ISSN: 2572-9241
©2026 The Author(s). HemaSphere is published by John Wiley & Sons Ltd on behalf of European Hematology Association. This is an open access Abstract Book distributed under the Attribution-NonCommercial-NoDerivs (CC BY-NC-ND), which allows third parties to download the articles and share them with others as long as they credit the author and the Abstract Book, but they cannot change the content in any way or use them commercially.
**Abstract Book Citations:** Authors, Title, HemaSphere, 2026;10:(S1):pages. The abstract book DOIs can be found on the "Author Information" page.
**Disclaimer:** Articles published in the journal HemaSphere exclusively reflect the opinions of the authors. The authors are responsible for all content in their abstracts including accuracy of the facts, statements, citing resources, etc.

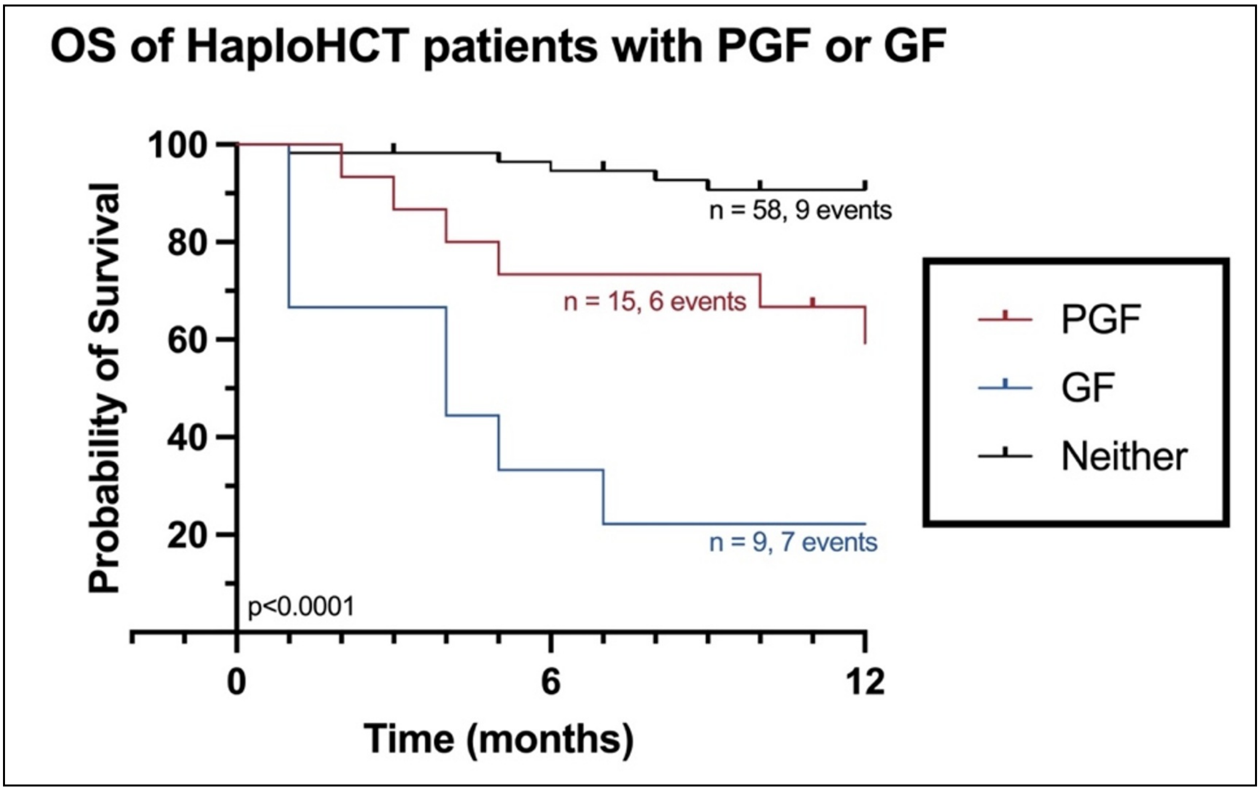

Figure 1. Overall survival (OS) after haploHCT according to graft dysfunction status. GF: Graft Failure, PGF: Poor Graft Function.

**Copyright Information:** (Online) ISSN: 2572-9241
©2026 The Author(s). HemaSphere is published by John Wiley & Sons Ltd on behalf of European Hematology Association. This is an open access Abstract Book distributed under the Attribution-NonCommercial-NoDerivs (CC BY-NC-ND), which allows third parties to download the articles and share them with others as long as they credit the author and the Abstract Book, but they cannot change the content in any way or use them commercially.
**Abstract Book Citations:** Authors, Title, HemaSphere, 2026;10:(S1):pages. The abstract book DOIs can be found on the "Author Information" page.
**Disclaimer:** Articles published in the journal HemaSphere exclusively reflect the opinions of the authors. The authors are responsible for all content in their abstracts including accuracy of the facts, statements, citing resources, etc.

### (PS2260) THE POWER OF DONOR AGE: A SINGLE-CENTER ANALYSIS OF SURVIVAL FOLLOWING ALLOGENEIC HEMATOPOIETIC STEM CELL TRANSPLANTATION

**Topic:** 23. Stem cell transplantation - Clinical

Taner Tan*[1], Umur Topçu[2], Efe Semercioğlu[2], Khayala Mammadova[1], Ümit Üre[1], Sinem Civriz Bozdağ[1]

[1]*Koç University Hospital, Department of Hematology, Istanbul, Türkiye;*[2]*Koç University, School of Medicine, Istanbul, Türkiye;*

### Background

In allogeneic hematopoietic stem cell transplantation (allo-HSCT), donor-related factors, in addition to patient- and disease-specific characteristics, significantly influence transplant outcomes. Transplants from younger donors have been associated with lower rates of transplant-related mortality and graft-versus-host disease (GVHD), with possible implications for overall survival. Nevertheless, the influence of donor age on transplant outcomes remains uncertain and may vary depending on patient and disease characteristics.

### Aims

In this study, we aimed to evaluate the impact of donor age on survival outcomes in patients who underwent allo-HSCT at our center.

### Methods

A total of 156 patients who underwent allo-HSCT at Koç University Hospital between December 2017 and July 2025 were retrospectively reviewed. Eight patients who underwent a second transplantation were excluded, and 148 patients were included in the final analysis. Demographic and clinical data were obtained from patient records. Survival analyses were performed using the Kaplan–Meier method, and groups were compared using the log-rank test.

### Results

The median recipient age of 148 included patients was 48 years (range 19–75), and 60.8% were male. The most common diagnoses were acute myeloid leukemia (36.5%), acute lymphoblastic leukemia (20.3%), and non-Hodgkin lymphoma (18.9%). Median follow-up was 305.5 days. The median donor age was 39 years (range 18–65); 78.4% were fully HLA-matched. During follow-up, 56.8% of patients died. In univariate analysis, donor age was significantly associated with mortality (HR=1.024, 95% CI 1.004–1.044; p=0.016), corresponding to a 2.4% increase in risk per year. Kaplan–Meier analysis showed a median overall survival of 50 months in recipients of donors <40 years versus 10 months in those with donors ≥40 years (log-rank p=0.037).
In diagnosis-specific subgroup analyses, donor age ≥40 years did not significantly affect survival in AML patients ($\chi^2 = 0.164$; p = 0.685). In ALL patients, although not statistically significant, a trend toward inferior survival was observed in the older donor group ($\chi^2 = 2.287$; p = 0.130). In multivariate analysis, donor age (HR=1.027, 95% CI 1.003–1.052; p=0.027) and full HLA matching (HR=0.435, 95% CI 0.233–0.815; p=0.009) remained independently associated with survival. Recipient age and donor relatedness were not significant predictors.

**Copyright Information:** (Online) ISSN: 2572-9241
©2026 The Author(s). HemaSphere is published by John Wiley & Sons Ltd on behalf of European Hematology Association. This is an open access Abstract Book distributed under the Attribution-NonCommercial-NoDerivs (CC BY-NC-ND), which allows third parties to download the articles and share them with others as long as they credit the author and the Abstract Book, but they cannot change the content in any way or use them commercially.
**Abstract Book Citations:** Authors, Title, HemaSphere, 2026;10:(S1):pages. The abstract book DOIs can be found on the “Author Information” page.
**Disclaimer:** Articles published in the journal HemaSphere exclusively reflect the opinions of the authors. The authors are responsible for all content in their abstracts including accuracy of the facts, statements, citing resources, etc.

### Summary/Conclusion

Our study demonstrates that donor age has a significant and independent impact on survival in patients undergoing allogeneic stem cell transplantation, beyond other clinical and disease-related factors. The superior survival outcomes observed with younger donors are consistent with data from the Center for International Blood and Marrow Transplant Research (CIBMTR) and the European Society for Blood and Marrow Transplantation (EBMT) (Abid et al., 2023 ; Canaani et al., 2018). These findings suggest that donor age represents a critical variable in donor selection and should be carefully considered during clinical decision-making. If supported by larger, multicenter datasets within our country, our results may contribute to the development and refinement of donor selection algorithms in the future.

**Table 1.** Multivariate Cox regression analysis of factors affecting survival

| Variable | B | p value | HR (Exp(B)) | 95% CI |
|---|---|---|---|---|
| Donor Age | 0.027 | 0.027 | 1.027 | 1.003 – 1.052 |
| Recipient Age | 0.007 | 0.416 | 1.007 | 0.990 – 1.025 |
| HLA Full Match | -0.832 | 0.009 | 0.435 | 0.233 – 0.815 |
| Related Donor | 0.208 | 0.480 | 1.231 | 0.692 – 2.188 |

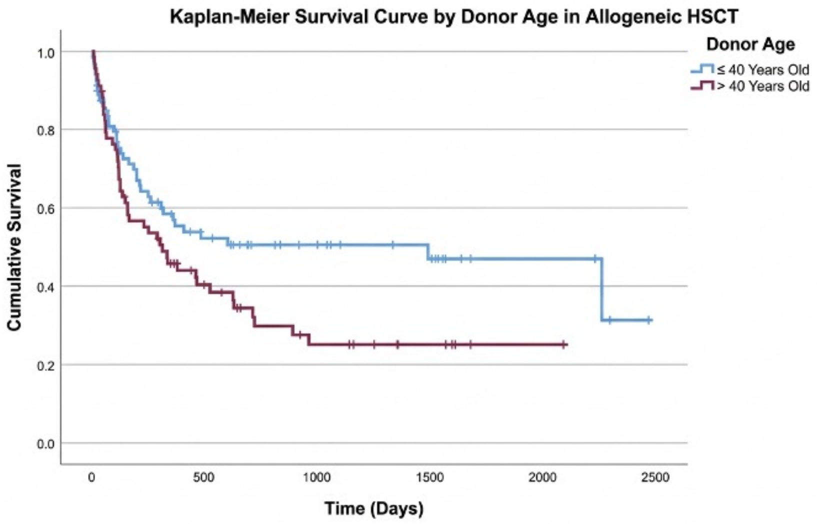

Figure 1

Figure 1. Kaplan-Meier survival curves for allogeneic HSCT patients stratified by donor age.

**Copyright Information:** (Online) ISSN: 2572-9241
©2026 The Author(s). HemaSphere is published by John Wiley & Sons Ltd on behalf of European Hematology Association. This is an open access Abstract Book distributed under the Attribution-NonCommercial-NoDerivs (CC BY-NC-ND), which allows third parties to download the articles and share them with others as long as they credit the author and the Abstract Book, but they cannot change the content in any way or use them commercially.
**Abstract Book Citations:** Authors, Title, HemaSphere, 2026;10:(S1):pages. The abstract book DOIs can be found on the "Author Information" page.
**Disclaimer:** Articles published in the journal HemaSphere exclusively reflect the opinions of the authors. The authors are responsible for all content in their abstracts including accuracy of the facts, statements, citing resources, etc.

### (PS2261) IMPACT OF CPE/ESBL ENTEROBACTERALES COLONIZATION AND ANTIBIOTIC DE-ESCALATION ON OUTCOMES AFTER ALLOGENEIC HEMATOPOIETIC STEM CELL TRANSPLANTATION: A SINGLE-CENTER STUDY

**Topic:** 23. Stem cell transplantation - Clinical

Roberto Bono*[1], Cristina Rotolo[1], Giuseppe Sapienza[1], Corinne Spoto[1], Carmelo Costanza[1], Luca Castagna[1]

[1]*UOSD UTMO, AOR VILLA SOFIA -CERVELLO, PALERMO, Italy;*

### Background

Colonization by carbapenemase-producing Enterobacterales (CPE) or extended-spectrum β-lactamase (ESBL)–producing Enterobacterales has been associated with increased mortality after allogeneic hematopoietic stem cell transplantation (allo-HSCT). In colonized patients  multidrug-resistant (MDR)–directed empirical therapy is standard but its optimal duration is undefined. A strategy based on initial MDR-directed therapy followed by early de-escalation to standard broad-spectrum antibiotics may reduce selective pressure, but its safety in this high-risk population remains uncertain.

### Aims

To evaluate the feasibility and safety of early antibiotic de-escalation in allo-HSCT recipients colonized with CPE or ESBL-producing organisms and to assess its impact on overall survival (OS), non-relapse mortality (NRM).

### Methods

We conducted a single-center retrospective study including consecutive allo-HSCT recipients between January 2021 and October 2025. Colonized patients received MDR-directed empirical therapy at first febrile neutropenia, followed by early de-escalation in the absence of microbiological documentation or clinical instability. OS was estimated by Kaplan–Meier and compared using the log-rank test. NRM was analyzed with competing-risk methodology. Cumulative incidence of CPE and ESBL infections during peri- and post-transplant periods was calculated.

**Copyright Information:** (Online) ISSN: 2572-9241
©2026 The Author(s). HemaSphere is published by John Wiley & Sons Ltd on behalf of European Hematology Association. This is an open access Abstract Book distributed under the Attribution-NonCommercial-NoDerivs (CC BY-NC-ND), which allows third parties to download the articles and share them with others as long as they credit the author and the Abstract Book, but they cannot change the content in any way or use them commercially.
**Abstract Book Citations:** Authors, Title, HemaSphere, 2026;10:(S1):pages. The abstract book DOIs can be found on the "Author Information" page.
**Disclaimer:** Articles published in the journal HemaSphere exclusively reflect the opinions of the authors. The authors are responsible for all content in their abstracts including accuracy of the facts, statements, citing resources, etc.

**Results**

A total of 204 patients were included: 130 (63.7%) were not colonized and 74 (36.3%) were colonized with CPE (mainly KPC-producing Klebsiella pneumoniae) or ESBL-producing organisms (mostly Escherichia coli). Among colonized patients, 88% (65/74) developed fever: 55% (36/65) had fever of unknown origin (FUO), 38% (25/65) developed bloodstream infections (BSI), of which 68% (17/25) were due to MDR bacteriaAmong the 65 colonized patients who developed fever, 61 were evaluable (4 excluded due to alternative infectious etiologies: 2 pneumonia, 1 viral reactivation, 1 Clostridioides difficile infection). Of these 61 patients:74% (45/61) received MDR-directed empirical therapy, 89% (40/45) were successfully de-escalated to piperacillin–tazobactam after a median of 4 days, 11% (5/45) continued MDR-directed therapy for more than 10 days.The remaining 26% (16/61) received empirical piperacillin–tazobactam as first-line therapy
All de-escalated patients were clinically stable and had negative blood cultures 48–72 hours after fever onset.
In the non-colonized group (n=130), 107 patients (82%) developed fever: 42% (54/107) were classified as FUO, 41% (53/107) developed BSI. Among BSIs: 12% (7/53) were caused by CPE pathogens (mainly KPC-producing Klebsiella pneumoniae), 9% (5/53) were caused by ESBL-producing pathogens (mainly Escherichia coli). Among the remaining 23 non-colonized patients:19 had no infectious events, 3 developed pneumonia ,1 developed CMV viremia
The median duration of MDR-directed therapy was 4 days.
Early de-escalation was feasible and not associated with increased short-term mortality.
One-year OS was comparable between colonized and non-colonized patients (68.9% vs 68.1%).100-day NRM: 17% vs 21%. 1-year NRM: 22% vs 30%
No statistically significant differences were observed.

**Summary/Conclusion**

In this cohort, MDR colonization did not adversely impact NRM, or 1-year OS after allo-HSCT.
An early antibiotic de-escalation strategy appears feasible and safe in colonized patients.
Prospective multicenter studies are warranted to define the optimal antimicrobial  strategies in this high-risk population.

**Copyright Information:** (Online) ISSN: 2572-9241
©2026 The Author(s). HemaSphere is published by John Wiley & Sons Ltd on behalf of European Hematology Association. This is an open access Abstract Book distributed under the Attribution-NonCommercial-NoDerivs (CC BY-NC-ND), which allows third parties to download the articles and share them with others as long as they credit the author and the Abstract Book, but they cannot change the content in any way or use them commercially.
**Abstract Book Citations:** Authors, Title, HemaSphere, 2026;10:(S1):pages. The abstract book DOIs can be found on the "Author Information" page.
**Disclaimer:** Articles published in the journal HemaSphere exclusively reflect the opinions of the authors. The authors are responsible for all content in their abstracts including accuracy of the facts, statements, citing resources, etc.

### (PS2262) INITIAL RESULTS OF POSTTRANSPLANTATION LOW-DOSE INOTUZUMAB OZOGAMICIN TO PREVENT RELAPSE OF ACUTE LYMPHOBLASTIC LEUKEMIA

**Topic:** 23. Stem cell transplantation - Clinical

Aiming Pang*[1], Pan Pan[1], Donglin Yang[1], Yi He[1], Yigeng Cao[1], Rongli Zhang[1], Jialin Wei[1], Xin Chen[1], Wenbin Cao[1], Weihua Zhai[1], Qiaoling MA[1], Erlie Jiang[1]

*[1]Institute of Hematology & Blood Diseases Hospital, Chinese Academy of Medical Sciences & Peking Union Medical College, Tianjin, China;*

### Background

The curative potential of allogeneic hematopoietic transplantation (allo-HCT) in patients with acute lymphoblastic leukemia (ALL) is hampered by relapse. Metheny LL, et al had reported Inotuzumab ozogamicin (INO), an anti-CD22 monoclonal antibody bound to calicheamicin, was safe and effective to prolong 1 year PFS after allo-HCT.

### Aims

Here we report lower dose of posttransplantation INO to prevent relapse of high risk B-ALL patients.

### Methods

Adult patients were eligible if they had undergone allo-HCT or auto-HSCT for CD22+ ALL, were in complete remission (CR) after HCT, had high risk of recurrence, were above day 60 after HCT with adequate graft function, and did not have a history of sinusoidal obstruction syndrome (SOS). The treatment consisted of INO given 0.3 mg/m$^2$ on day 1 of Cycle 1 and 0.6 mg/m$^2$ on day 1 of Cycle 2 (Q28D).The objectives of this trial were to determine safety and efficacy of post–HCT INO safety and efficacy. The primary endpoint was 1-year disease-free survival (DFS).

**Copyright Information:** (Online) ISSN: 2572-9241
©2026 The Author(s). HemaSphere is published by John Wiley & Sons Ltd on behalf of European Hematology Association. This is an open access Abstract Book distributed under the Attribution-NonCommercial-NoDerivs (CC BY-NC-ND), which allows third parties to download the articles and share them with others as long as they credit the author and the Abstract Book, but they cannot change the content in any way or use them commercially.
**Abstract Book Citations:** Authors, Title, HemaSphere, 2026;10:(S1):pages. The abstract book DOIs can be found on the "Author Information" page.
**Disclaimer:** Articles published in the journal HemaSphere exclusively reflect the opinions of the authors. The authors are responsible for all content in their abstracts including accuracy of the facts, statements, citing resources, etc.

### Results

| No | Age, years | Sex | Cytogenic character | LOT | Blina | CAR-T | HCT | Donor | Source of stem cell | Preparative regimen |
|---|---|---|---|---|---|---|---|---|---|---|
| 1 | 30 | F | IKZF1, BCR-ABL P190+ | 2 | N | N | Allo | MUD | PB | TBI+CY+Ara-C |
| 2 | 26 | M | TRAF3, FOXC1, CSMD1, PDS5B | 3 | N | N | Allo | MSD | PB | TBI+CY+Ara-C+Flu |
| 3 | 21 | M | p.D91E⊠p.L60I | 1 | N | N | Allo | MSD | PB | TBI+Mel+CTX+ATG |
| 4 | 55 | M | WT1, p.G12D⊠p.V654Sfs*14⊠c.940-2A | 1 | N | N | Auto | Auto | PB | Mel+CY+CLA+Ara-C |
| 5 | 18 | M | CRLF2, ITD, JAK2, XBP1, MTOR | 1 | Y | N | Auto | Auto | PB | TBI+CY+CLA+Ara-C |
| 6 | 55 | M | IDH1, HIST2H2AB | 1 | N | N | Auto | Auto | PB | TBI+CY+CLA+Ara-C |
| 7 | 44 | F | BCR-ABL 190+ | 1 | Y | Y | Auto | Auto | PB | TBI+CTX+Glag+Ara-C |

Median age was 30 years (range, 18-55 years; n = 7). Disease status at transplantation was all CR and MRD negative, with first CR (n = 5) or second CR or beyond (n = 2). 4/5 CR1 patients accepted auto-HCT; the others were allo-HCT. ALL were TBI-based preparative regimen. Median time between HCT and INO was 114 days (range,72-190 days). Most common toxicity was thrombocytopenia and neutropenia. There were no instances of SOS and acute graft versus host disease. With a median follow-up of 12.5 months (range, 7.0-25.5 months), no one relapsed or died.

### Summary/Conclusion

Low-dose INO has a favorable safety profile and was associated with high rates of 1-year DFS.

**Copyright Information:** (Online) ISSN: 2572-9241
©2026 The Author(s). HemaSphere is published by John Wiley & Sons Ltd on behalf of European Hematology Association. This is an open access Abstract Book distributed under the Attribution-NonCommercial-NoDerivs (CC BY-NC-ND), which allows third parties to download the articles and share them with others as long as they credit the author and the Abstract Book, but they cannot change the content in any way or use them commercially.
**Abstract Book Citations:** Authors, Title, HemaSphere, 2026;10:(S1):pages. The abstract book DOIs can be found on the "Author Information" page.
**Disclaimer:** Articles published in the journal HemaSphere exclusively reflect the opinions of the authors. The authors are responsible for all content in their abstracts including accuracy of the facts, statements, citing resources, etc.

### (PS2264) CLINICAL OUTCOMES AND SAFETY OF EXTRACORPOREAL PHOTOPHERESIS ALONE OR COMBINED WITH RUXOLITINIB IN CHRONIC GRAFT-VERSUS-HOST DISEASE

**Topic:** 23. Stem cell transplantation - Clinical

Christian Koch*[1], Markus G. Manz[1], Mirjam Naegeli[2], Dominik Schneidawind[1, 3, 4]

*[1]University Hospital Zurich and University of Zurich, Department of Medical Oncology and Hematology, Zurich, Switzerland; [2]University Hospital Zurich, Department of Dermatology, Zurich, Switzerland; [3]Comprehensive Cancer Center Zurich, Zurich, Switzerland; [4]University of Tübingen, Faculty of Medicine, Tübingen, Germany;*

### Background

Chronic graft-versus-host disease (cGvHD) is a major cause of morbidity after allogeneic hematopoietic stem cell transplantation. Ruxolitinib (RUX) is a standard second-line treatment for steroid-refractory or -dependent cGvHD, while extracorporeal photopheresis (ECP), an autologous cell-based immunomodulatory procedure, is also widely used. However, comparative real-world data on combined immunomodulation with the RUX-ECP combination are scarce.

### Aims

To systematically evaluate the clinical impact and safety of combined immunomodulation with RUX-ECP compared with ECP alone in patients with steroid-refractory or -dependent cGvHD. Specifically, we aimed to assess overall and organ-specific response rates, response kinetics, durability of response, and the ability to reduce or discontinue concomitant immunosuppression, particularly systemic corticosteroids, while also examining survival outcomes and treatment-related toxicities in a real-world setting.

### Methods

We performed a retrospective single-center analysis at the University Hospital Zurich of patients treated between 2012-2025 with RUX-ECP (n=30) or ECP alone (n=21) for steroid-refractory or -dependent cGvHD. cGvHD diagnosis, severity, organ involvement, and response were assessed per NIH consensus criteria. Overall response, organ-specific responses, time to first response, immunosuppression reduction, survival outcomes, relapse incidence, and adverse events were evaluated.

### Results

Overall response rates were 77% with RUX-ECP versus 52% with ECP (p=0.13); CR rates were 17% with RUX-ECP versus 10% with ECP (p=0.69). Median time to first response was significantly shorter with RUX-ECP (2.6 vs 12.3 months; p=0.0249). Organ-specific trends favored RUX-ECP in gastrointestinal, ocular, and cutaneous cGvHD. Pulmonary responses were rare in both cohorts. Overall survival, relapse incidence, non-relapse mortality, and event-free survival were comparable between both groups. Steroid-sparing differed markedly: at 12 months, steroid discontinuation was achieved in 69% with RUX-ECP vs 10% with ECP (p=0.005), and relative corticosteroid reduction was greater for RUX-ECP (88% vs 30%; p=0.0026). Toxicities of RUX-ECP were manageable and consistent with the known ruxolitinib profile, and a substantial proportion of patients discontinued therapy while maintaining stable responses.

**Copyright Information:** (Online) ISSN: 2572-9241
©2026 The Author(s). HemaSphere is published by John Wiley & Sons Ltd on behalf of European Hematology Association. This is an open access Abstract Book distributed under the Attribution-NonCommercial-NoDerivs (CC BY-NC-ND), which allows third parties to download the articles and share them with others as long as they credit the author and the Abstract Book, but they cannot change the content in any way or use them commercially.
**Abstract Book Citations:** Authors, Title, HemaSphere, 2026;10:(S1):pages. The abstract book DOIs can be found on the "Author Information" page.
**Disclaimer:** Articles published in the journal HemaSphere exclusively reflect the opinions of the authors. The authors are responsible for all content in their abstracts including accuracy of the facts, statements, citing resources, etc.

**Summary/Conclusion**

In this real-world steroid-refractory/-dependent cGvHD cohort, combined RUX-ECP was associated with fast responses and high rates of steroid discontinuation, with comparable survival outcomes and manageable toxicity when compared to ECP monotherapy. Responses were durable in the majority of patients. These data support prospective evaluation and systematic integration of RUX-ECP as a combination immunomodulatory strategy in steroid-refractory/-dependent cGvHD.

**Copyright Information:** (Online) ISSN: 2572-9241
©2026 The Author(s). HemaSphere is published by John Wiley & Sons Ltd on behalf of European Hematology Association. This is an open access Abstract Book distributed under the Attribution-NonCommercial-NoDerivs (CC BY-NC-ND), which allows third parties to download the articles and share them with others as long as they credit the author and the Abstract Book, but they cannot change the content in any way or use them commercially.
**Abstract Book Citations:** Authors, Title, HemaSphere, 2026;10:(S1):pages. The abstract book DOIs can be found on the "Author Information" page.
**Disclaimer:** Articles published in the journal HemaSphere exclusively reflect the opinions of the authors. The authors are responsible for all content in their abstracts including accuracy of the facts, statements, citing resources, etc.

### (PS2265) ALLOGENEIC HEMATOPOIETIC STEM CELL TRANSPLANTATION FOR CHILDHOOD B-ALL WITH TCF3::PBX1: A RETROSPECTIVE ANALYSIS OF 70 PATIENTS FROM A SINGLE CENTER

**Topic:** 23. Stem cell transplantation - Clinical

Yun Wang*[1], Yan-Li Zhao[1], Xingyu Cao[1], Jian-Ping Zhang[1], Jiarui Zhou[1], Min Xiong[1], Deyan Liu[1], Zhijie Wei[1], Ruijuan Sun[1], Fang Xu[1], Zhicong Du[1], Baiqing Sun[1], Yue Lu[1]

[1]*Hebei Yanda Ludaopei Hospital, Langfang, China;*

**Background**

The TCF3::PBX1 fusion gene defines a key molecular subtype in pediatric acute lymphoblastic leukemia. Further investigation is required to elucidate its prognostic implications and optimal therapeutic strategies, particularly regarding outcomes in the context of allogeneic hematopoietic stem cell transplantation.

**Aims**

This study aims to evaluate the long-term survival status of children with B-ALL who received allo-HSCT and are TCF3::PBX1 positive, and systematically analyze the key factors influencing their prognosis.

**Methods**

A retrospective analysis included 70 children with TCF3::PBX1-positive B-ALL who underwent allo-HSCT. The clinical characteristics of these patients were analyzed. The primary endpoints were overall survival (OS) and leukemia-free survival (LFS). The cumulative recurrence rate (CIR) and non-relapse mortality rate (NRM) were also evaluated. Survival analysis was conducted using the Kaplan-Meier method, and the differences between groups were assessed using the Log-rank test.

**Copyright Information:** (Online) ISSN: 2572-9241
©2026 The Author(s). HemaSphere is published by John Wiley & Sons Ltd on behalf of European Hematology Association. This is an open access Abstract Book distributed under the Attribution-NonCommercial-NoDerivs (CC BY-NC-ND), which allows third parties to download the articles and share them with others as long as they credit the author and the Abstract Book, but they cannot change the content in any way or use them commercially.
**Abstract Book Citations:** Authors, Title, HemaSphere, 2026;10:(S1):pages. The abstract book DOIs can be found on the "Author Information" page.
**Disclaimer:** Articles published in the journal HemaSphere exclusively reflect the opinions of the authors. The authors are responsible for all content in their abstracts including accuracy of the facts, statements, citing resources, etc.

### Results

The median age at diagnosis was 9.5 (range: 1 - 17 years), with 45 males (64.3%) and 67 cases achieving morphological remission by the end of induction (95.7%), 9 cases being MFC-MRD positive (12.9%); 66 patients had morphological or extramedullary relapses before transplantation (92.3%). The transplantation indications were for patients with morphological or extramedullary relapses or initial induction failure.
Pre-transplant status: 16 CR1 patients (22.9%), 48 CR2 patients (68.6%), and 6 CR3 patients (8.6%); 50 patients had received CART treatment (71.4%), 48 patients had a pre-transplant HCT-CI score of 0 (68.6%), 10 patients had pre-transplant FCM-MRD positive (14.3%), 18 patients had positive gene quantification (25.7%), and 19 patients had a history of extramedullary disease (27.1%); donor type: 60 cases were half-matched (85.6%), 5 cases were full-matched (7.2%), and 5 cases were unrelated (7.2%); stem cell source: 61 cases were a combination of bone marrow and peripheral stem cells (87.1%), 9 cases were solely peripheral stem cells (12.9%); pre-treatment regimen: 61 cases were based on TBI (87.1%), 9 cases were based on BU (12.9%).
The median follow-up time after transplantation was 20.4 months (range: 1.0 - 124.1 months). The 3-year OS was 54.3%, and the 3-year LFS was 53.6%. The 3-year CIR was 29.1%, and the 3-year NRM was 17.2%.
The results of univariate analysis showed that compared with patients with negative gene quantification before transplantation, those with positive gene quantification had lower 3-year OS (35.6% vs 61.2%, P = 0.008) and 3-year LFS (36.4% vs 59.8%, P = 0.015), as well as higher 3-year CIR (46.0% vs 23.2% (P = 0.028)), while the 3-year NRM (17.4% vs 16.9%, P = 0.968) did not show statistical significance.
Patients with a history of extramedullary disease had lower 3-year OS (33.4% vs 52.4%, P = 0.014) and 3-year LFS (28.1% vs 63.5%, P = 0.005), as well as higher 3-year NRM (33.0% vs 11.1% (P = 0.009)), compared with those without a history of extramedullary disease; multivariate analysis results also supported this conclusion.

### Summary/Conclusion

For children with B-ALL carrying the TCF3::PBX1 fusion gene, allo-HSCT can be used as a safe and effective consolidation treatment method. The quantitative positivity of the fusion gene before transplantation and the presence of extramedullary disease history are two independent risk factors affecting prognosis.

**Copyright Information:** (Online) ISSN: 2572-9241
©2026 The Author(s). HemaSphere is published by John Wiley & Sons Ltd on behalf of European Hematology Association. This is an open access Abstract Book distributed under the Attribution-NonCommercial-NoDerivs (CC BY-NC-ND), which allows third parties to download the articles and share them with others as long as they credit the author and the Abstract Book, but they cannot change the content in any way or use them commercially.
**Abstract Book Citations:** Authors, Title, HemaSphere, 2026;10:(S1):pages. The abstract book DOIs can be found on the "Author Information" page.
**Disclaimer:** Articles published in the journal HemaSphere exclusively reflect the opinions of the authors. The authors are responsible for all content in their abstracts including accuracy of the facts, statements, citing resources, etc.

### (PS2266) OUTCOMES OF MYELOFIBROSIS PATIENTS UNDERGOING ALLOGENEIC STEM CELL TRANSPLANTATION WITH TACROLIMUS/METHOTREXATE/RUXOLITINIB AS GRAFT-VERSUS-HOST DISEASE PROPHYLAXIS

**Topic:** 23. Stem cell transplantation - Clinical

Sandro Pino-Ascencio*[1], Shruti Gajjar[1], Julia Keating[2], Richard Hao[3], Steven McAfee[3], Areej El-Jawahri[3], Richard A. Newcomb[3], Jooho Chung[3], Paul O'donnell[3], Thomas Spitzer[3], Peter Westervelt[3], Yi-Bin Chen[3], Zachariah Defilipp[3], Gabriela Hobbs[1]

*[1]Massachusetts General Hospital, Leukemia Department, Boston, United States of America;[2]Dana-Farber Cancer Institute, Department of Data Science, Boston, United States of America; [3]Massachusetts General Hospital, Bone Marrow Transplant Department, Boston, United States of America;*

**Background**

Janus kinase inhibitors (JAKi) have significantly improved care for myelofibrosis (MF) patients. However, allogeneic hematopoietic cell transplantation (HCT) remains the only curative therapy. There is no consensus on how to utilize JAKi at the time of HCT. We recently demonstrated that ruxolitinib (rux) during and following HCT is safe, with low rates of graft-versus-host disease (GVHD) and favorable overall survival (OS) (Hobbs *et al* JCO Onc Adv 2025). Here, we present real-world data from our institution on patients with MF undergoing HCT who received rux with calcineurin inhibitor-based GVHD prophylaxis.

**Aims**

To characterize the outcomes of MF patients who underwent HCT with tacrolimus/methotrexate/rux (Tac/MTX/Rux) GVHD prophylaxis.

**Methods**

We conducted a retrospective study of MF patients that underwent HCT at Massachusetts General Hospital from 2016-2025. Patients with MF undergoing first HCT receiving Tac/MTX/Rux for GVHD prophylaxis were included. MF characteristics, treatment pre-HCT, HCT course and follow-up were collected from the electronic medical record up to January 2026.

**Copyright Information:** (Online) ISSN: 2572-9241
©2026 The Author(s). HemaSphere is published by John Wiley & Sons Ltd on behalf of European Hematology Association. This is an open access Abstract Book distributed under the Attribution-NonCommercial-NoDerivs (CC BY-NC-ND), which allows third parties to download the articles and share them with others as long as they credit the author and the Abstract Book, but they cannot change the content in any way or use them commercially.
**Abstract Book Citations:** Authors, Title, HemaSphere, 2026;10:(S1):pages. The abstract book DOIs can be found on the "Author Information" page.
**Disclaimer:** Articles published in the journal HemaSphere exclusively reflect the opinions of the authors. The authors are responsible for all content in their abstracts including accuracy of the facts, statements, citing resources, etc.

### Results

46 patients were identified; median age at HCT was 69 years (range, 48-79). Most (59%) were male and White (93%). Half (n=23, 50%) had secondary MF. Driver mutations included JAK2 (n=33, 72%), CALR (n=8, 17%), and MPL (n=5, 11%); 29 (63%) had ≥ 1 high molecular risk (HMR) mutation, with ASXL1 in 14 (30%). 27 (59%) patients were high-risk by MIPSS70+ version 2.0. 40 (87%) patients received at least one JAKi prior to HCT, rux given as first-line in 35 (88%). Median time on JAKi pre-HCT was 12 months (range, 2-85). 19 (49%) patients had significant splenomegaly (>18 cm) at HCT, with an average spleen length of 17.9 cm (range, 11.0-28.4).

Most patients had HLA-matched donors (n=39, 85%) or mismatched unrelated donors (n=7, 15%). All but one received reduced-intensity fludarabine/melphalan conditioning, and all received peripheral blood stem cell grafts.

87% of patients received rux at 5 mg twice daily at the start of conditioning. Patients reduced rux by 50% when receiving fluconazole prophylaxis. Median follow-up for survivors was 28.8 months (range, 16.3-44.4). Median time on rux from day 0 was 13 months (range, 1-71), with 24 (52%) patients still on treatment at last follow-up. 11 (24%) patients restarted rux after discontinuation (defined as rux interruption for >1 month), 9 due to new onset GVHD and 2 due to relapse.

All patients successfully engrafted; one developed secondary graft failure. Median time to neutrophil engraftment (ANC ≥0.5x109) was 13 days (range, 11-20) and platelet engraftment (≥20x109) was 16 days (range, 10-366). By Day +180, the cumulative incidence (CI) of grade II-IV acute GVHD was 11% (95%CI, 4-22%) and grade III-IV acute GVHD was 2% (95%CI, 0-10%). The CI of moderate-severe chronic GVHD at 1-year was 17% (95%CI, 7-31%). The CI of NRM at 1 year was 4% (95%CI, 1-13%) and relapse at 1 year was 6% (95%CI, 1-16%). At 1-year OS, PFS, and GRFS were 96% (95%CI, 90-100%), 90% (95%CI, 81-99%), and 76% (95%CI, 62-89%), respectively.

### Summary/Conclusion

The administration of rux during HCT, when used in combination with Tac/MTX, is safe and has no detrimental impact on engraftment. When rux is continued over the first-year post-HCT, the incidence of acute and chronic GVHD is compellingly low, and survival outcomes are high. We believe that the Tac/MTX/Rux platform should be considered a standard of care for patients with MF undergoing HCT from well-matched donors.

**Copyright Information:** (Online) ISSN: 2572-9241
©2026 The Author(s). HemaSphere is published by John Wiley & Sons Ltd on behalf of European Hematology Association. This is an open access Abstract Book distributed under the Attribution-NonCommercial-NoDerivs (CC BY-NC-ND), which allows third parties to download the articles and share them with others as long as they credit the author and the Abstract Book, but they cannot change the content in any way or use them commercially.
**Abstract Book Citations:** Authors, Title, HemaSphere, 2026;10:(S1):pages. The abstract book DOIs can be found on the "Author Information" page.
**Disclaimer:** Articles published in the journal HemaSphere exclusively reflect the opinions of the authors. The authors are responsible for all content in their abstracts including accuracy of the facts, statements, citing resources, etc.

### (PS2267) ALLOGENEIC STEM CELL TRANSPLANTATION FOR ADVANCED CUTANEOUS T-CELL LYMPHOMA: EXPERIENCE WITH POST-TRANSPLANT CYCLOPHOSPHAMIDE-BASED GVHD PROPHYLAXIS

**Topic:** 23. Stem cell transplantation - Clinical

Seda Cakmak*[1], Nihar Desai[1], Sergio Rodriguez[1], Eshrak Al-Shaibani[1], Tommy Alfaro-Moya[1], Igor Novitzky-Basso[1], Ivan Pasic[1], Fotios Michelis[1], Rajat Kumar[1], Jonas Mattsson[1], Dennis Kim[1], Auro Viswabandya[1]

[1]*Princess Margaret Cancer Centre, Toronto, Canada;*

### Background

Allogeneic haematopoietic stem cell transplantation (alloHSCT) remains the only potentially curative therapy for advanced cutaneous T-cell lymphoma (CTCL). Published transplant series are limited, and data describing post-transplant cyclophosphamide (PTCy)-based graft-versus-host disease (GVHD) prophylaxis in CTCL are sparse.

### Aims

To evaluate clinical outcomes of alloHSCT in advanced CTCL in a real-world single-centre cohort, with particular focus on relapse patterns, and the feasibility and outcomes associated with PTCy-based GVHD prophylaxis.

### Methods

We conducted a retrospective single-centre study of consecutive CTCL patients undergoing alloHSCT between January 2010 and October 2025. Data were extracted from our institutional transplant database with detailed clinical variables obtained from electronic patient records. Analyses were performed using EZR.

### Results

Twenty-five patients underwent alloHSCT. Diagnoses included mycosis fungoides (n=8; 4 transformed), Sézary syndrome (n=16), and primary cutaneous ALK-negative anaplastic large cell lymphoma (n=1). Median age at transplant was 58 years (range 20–69), and 72% were male. The majority had advanced stage disease (IVA/IVB 84%). Patients were heavily pre-treated (median 6 prior therapies), with a median interval from diagnosis to transplant of 25.7 months. Pre-transplant disease status was CR 20%, PR 76%, and SD 4%.
All grafts were peripheral blood stem cells. Donors included matched sibling (24%), matched unrelated (36%), mismatched unrelated (24%), and haploidentical (16%). Reduced-intensity conditioning was used in 68%. PTCy-based GVHD prophylaxis was administered in 72% (18/25), most commonly anti-thymocyte globulin (ATG)–PTCy–cyclosporin (60%).
Grade II–IV acute GVHD occurred in 52% of patients (grade III–IV 8%), and moderate–severe chronic GVHD in 20%. Primary graft failure occurred in 12%. Twelve-month non-relapse mortality was 20.6%. Overall, 60% of patients died; principal causes were relapse (24%), infection (20%), and GVHD (16%).
With a median follow-up of 70.4 months, median OS was 22.5 months (12- and 24-month OS 61.8% and 48.1%), and median RFS was 4.86 months (12- and 24-month RFS 29.5% and 21.1%). Relapse was the predominant cause of treatment failure, with a cumulative incidence of 50.1% at 12 months and 58.5% at 24 months. Events were heavily front-loaded, with most relapses occurring within six months post-transplant. Four patients received donor lymphocyte infusion for relapse, with heterogeneous outcomes including one durable long-term remission.

**Copyright Information:** (Online) ISSN: 2572-9241
©2026 The Author(s). HemaSphere is published by John Wiley & Sons Ltd on behalf of European Hematology Association. This is an open access Abstract Book distributed under the Attribution-NonCommercial-NoDerivs (CC BY-NC-ND), which allows third parties to download the articles and share them with others as long as they credit the author and the Abstract Book, but they cannot change the content in any way or use them commercially.
**Abstract Book Citations:** Authors, Title, HemaSphere, 2026;10:(S1):pages. The abstract book DOIs can be found on the "Author Information" page.
**Disclaimer:** Articles published in the journal HemaSphere exclusively reflect the opinions of the authors. The authors are responsible for all content in their abstracts including accuracy of the facts, statements, citing resources, etc.

**Summary/Conclusion**

In this real-world CTCL cohort, alloHSCT provided durable disease control in a minority but was primarily limited by early post-transplant relapse rather than transplant-related mortality. PTCy-based GVHD prophylaxis, frequently delivered with ATG was feasible with low rates of severe acute GVHD. Strategies to improve pre-transplant disease control and mitigate early relapse warrant further evaluation.

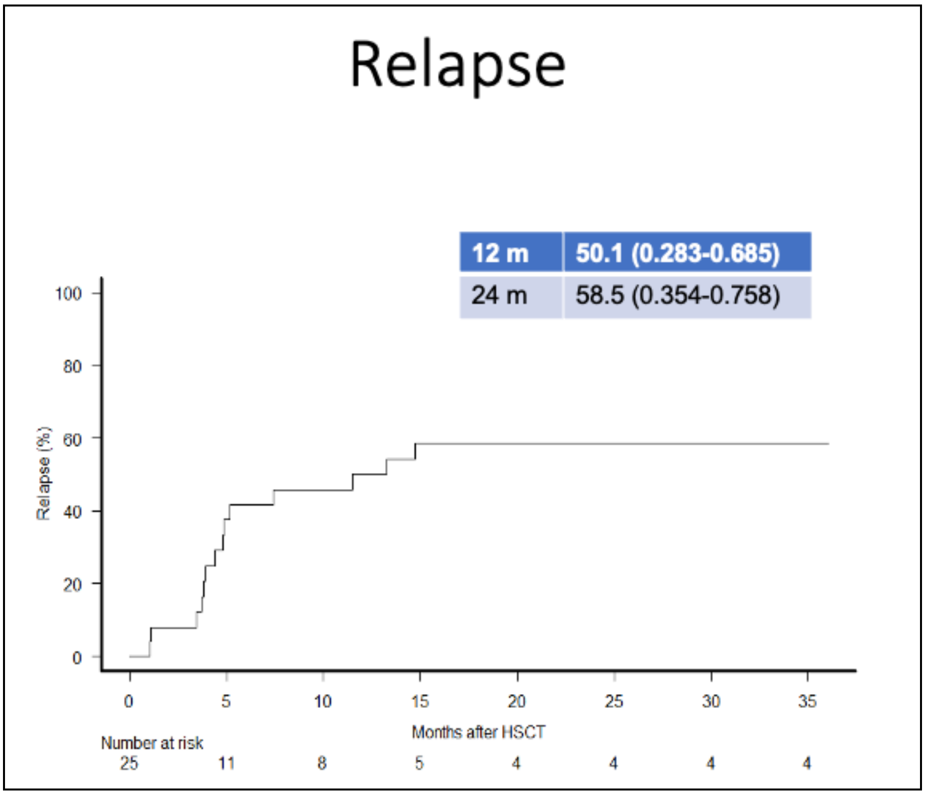

Cumulative incidence of relapse after alloHSCT, with non-relapse mortality as a competing risk. Estimates at 12 and 24 months are shown with 95% confidence intervals

**Copyright Information:** (Online) ISSN: 2572-9241
©2026 The Author(s). HemaSphere is published by John Wiley & Sons Ltd on behalf of European Hematology Association. This is an open access Abstract Book distributed under the Attribution-NonCommercial-NoDerivs (CC BY-NC-ND), which allows third parties to download the articles and share them with others as long as they credit the author and the Abstract Book, but they cannot change the content in any way or use them commercially.
**Abstract Book Citations:** Authors, Title, HemaSphere, 2026;10:(S1):pages. The abstract book DOIs can be found on the "Author Information" page.
**Disclaimer:** Articles published in the journal HemaSphere exclusively reflect the opinions of the authors. The authors are responsible for all content in their abstracts including accuracy of the facts, statements, citing resources, etc.

### (PS2268) YOUNG PATIENTS WITH STANDARD-RISK MULTIPLE MYELOMA UNDERGOING AUTOLOGOUS STEM CELL TRANSPLANTATION ARE EXPECTED TO ACHIEVE FUNCTIONAL CURE A REAL-WORLD MULTICENTER ANALYSIS

**Topic:** 23. Stem cell transplantation - Clinical

Shan Gao[1], Guangzhong Yang[2], Yi MA[3], Yan Li[4], Shuang Gao[4], Zhenling Li[5], Li Bao*[1]

*[1]Beijing Jishuitan Hospital, Capital Medical University, Beijing, China;[2]Beijing Chao-Yang Hospital, Capital Medical University, Beijing, China; [3]The Fifth Medical Center of PLA General Hospital, Beijing, China;[4]Peking University Third Hospital, Beijing, China; [5]China-Japan Friendship Hospital, Beijing, China;*

### Background

Autologous stem cell transplantation (ASCT) was the standard care for young patients with newly diagnosed multiple myeloma (NDMM).In the era of novel agents, patient survival has been prolonged compared to the past. Is it still necessary for standard-risk patients to undergo first-line autologous stem cell transplantation?

### Aims

In the real world of the novel drug era for multiple myeloma,to investigate whether younger standard-risk patients with newly diagnosed multiple myeloma can benefit from undergoing autologous stem cell transplantation.

### Methods

We retrospectively analyzed data from 358 patients with standard-risk NDMM diagnosed between January 1, 2015 and January 1, 2024, across five major medical centers in China. All the patients aged ≤65 years with standard-risk cytogenetics (FISH defined by the absence of t(4;14), t(14;16), del(17p), and gain/amp 1q21) .

### Results

Patients were categorized into two groups: those receiving upfront ASCT following induction therapy (ASCT group, n=214) and those receiving continuous novel agent-based therapy without ASCT (non-ASCT group, n=144). No statistically significant differences were observed in the baseline characteristics between the two groups at the time of diagnosis as shown in Table 1. With a median follow-up of 56 months for PFS,the ASCT group median PFS was not reached versus 29 months in the non-ASCT group (95% CI: 19–40; p<0.001).With a median follow-up of 59.5 months for OS,the ASCT group median OS was not reached versus 81 months in the non-ASCT group (95% CI: 68–NA; p<0.001). Post-ASCT maintenance therapy further extended PFS, the maintenance group median PFS was not reached vs. versus 56 months in the non-maintenance group (p<0.001).The maintenance group median OS was not reached versus 93 months in the non-maintenance group (95%CI:19- NA,p<0.001). Additionally, MRD negativity after transplantation was associated with significantly longer PFS (p=0.043).

**Table 1. Baseline disease characteristics**

**Copyright Information:** (Online) ISSN: 2572-9241
©2026 The Author(s). HemaSphere is published by John Wiley & Sons Ltd on behalf of European Hematology Association. This is an open access Abstract Book distributed under the Attribution-NonCommercial-NoDerivs (CC BY-NC-ND), which allows third parties to download the articles and share them with others as long as they credit the author and the Abstract Book, but they cannot change the content in any way or use them commercially.
**Abstract Book Citations:** Authors, Title, HemaSphere, 2026;10:(S1):pages. The abstract book DOIs can be found on the "Author Information" page.
**Disclaimer:** Articles published in the journal HemaSphere exclusively reflect the opinions of the authors. The authors are responsible for all content in their abstracts including accuracy of the facts, statements, citing resources, etc.

| | non-ASCT N = 144 | ASCT N = 214 | *P* |
|---|---|---|---|
| **Age** | | | |
| *Median (Min,Max)* | 58 (30,65 ) | 57 (27, 65) | |
| **Sex** | | | 0.435 |
| *Female* | 64 (44.4%) | 85 (39.7%) | |
| *Male* | 80 (55.6%) | 129 (60.3%) | |
| **EME** | | | 0.024 |
| | 9 (6.3%) | 31 (14.5%) | |
| **ISS Stage** | | | 0.807 |
| I | 57 (40.1%) | 93 (43.5%) | |
| II | 36 (25.4%) | 53 (24.8%) | |
| III | 49 (34.5%) | 68 (31.8%) | |
| NA | 2 (13.9%) | 0 | |
| **RISS Stage** | | | 0.219 |
| I | 55 (41.0%) | 83 (40.3%) | |
| II | 76 (56.7%) | 110 (53.4%) | |
| III | 3 (2.2%) | 13 (6.3%) | |
| NA | 10 | 8 | |
| **β2-MG** | | | 0.347 |
| *Median (Min,Max)* | 3.95 (0.69, 115.03) | 6.28 (1.21, 52.01) | |
| **Hb** | | | 0.074 |
| *Median (Min,Max)* | 112.00 (49.00, 174.00) | 111.00 (55.00, 167.00) | |
| **Alb** | | | 0.473 |
| *Median (Min,Max)* | 38.20 (22.00, 51.00) | 38.27 (21.40, 50.70 | |
| **Crea** | | | 0.255 |
| *Median (Min,Max)* | 78.50 (36.00, 818.00) | 70.45 (36.00, 890.00) | |
| **LDH** | | | 0.709 |
| *Median (Min,Max)* | 172.00 (137.75, 205.25) | 180.50 (143.00, 197.75) | |

### Summary/Conclusion

In the current era of novel therapeutic agents, as evidenced by significantly prolonged PFS and OS in a real-world multicenter cohort,upfront ASCT continues to provide substantial and clinically meaningful survival benefits for younger standard-risk NDMM patients who are expected to achieve functional cure.

**Copyright Information:** (Online) ISSN: 2572-9241
©2026 The Author(s). HemaSphere is published by John Wiley & Sons Ltd on behalf of European Hematology Association. This is an open access Abstract Book distributed under the Attribution-NonCommercial-NoDerivs (CC BY-NC-ND), which allows third parties to download the articles and share them with others as long as they credit the author and the Abstract Book, but they cannot change the content in any way or use them commercially.
**Abstract Book Citations:** Authors, Title, HemaSphere, 2026;10:(S1):pages. The abstract book DOIs can be found on the "Author Information" page.
**Disclaimer:** Articles published in the journal HemaSphere exclusively reflect the opinions of the authors. The authors are responsible for all content in their abstracts including accuracy of the facts, statements, citing resources, etc.

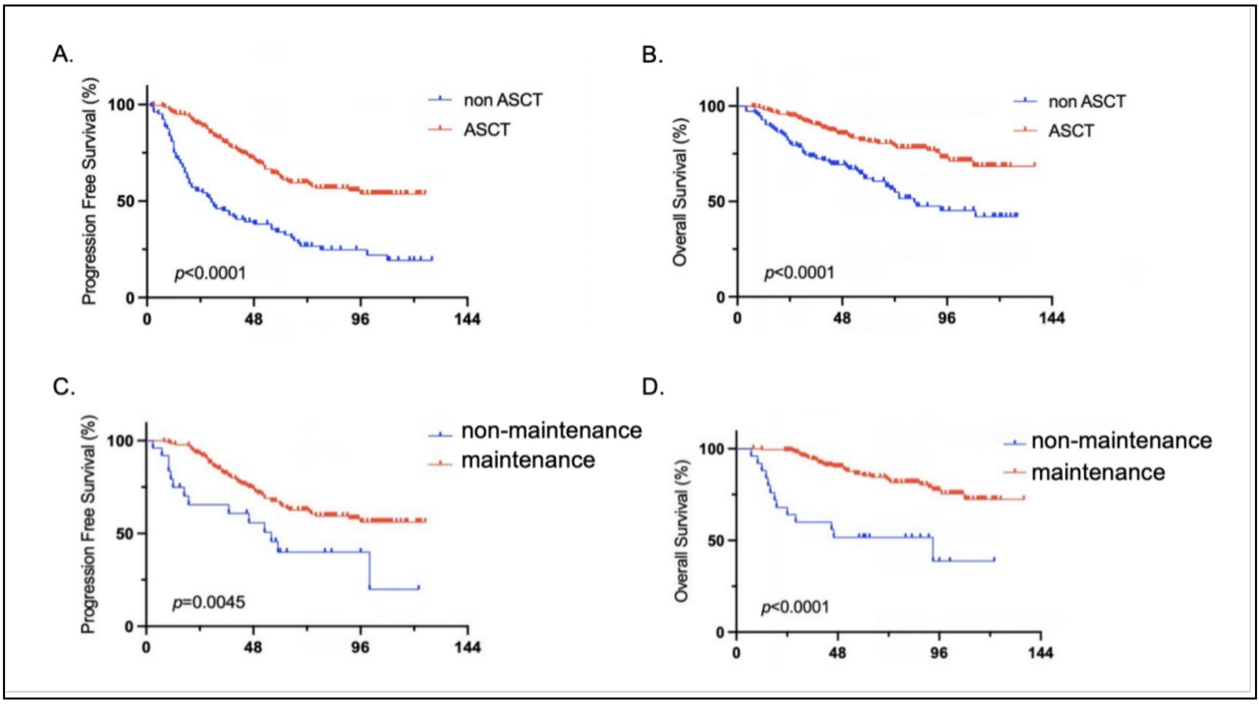

PFS and OS of patients in the non-ASCT and ASCT groups(A and B).PFS and OS of patients in the maintenance and non-maintenance after ASCT groups(C and D).

**Copyright Information:** (Online) ISSN: 2572-9241
©2026 The Author(s). HemaSphere is published by John Wiley & Sons Ltd on behalf of European Hematology Association. This is an open access Abstract Book distributed under the Attribution-NonCommercial-NoDerivs (CC BY-NC-ND), which allows third parties to download the articles and share them with others as long as they credit the author and the Abstract Book, but they cannot change the content in any way or use them commercially.
**Abstract Book Citations:** Authors, Title, HemaSphere, 2026;10:(S1):pages. The abstract book DOIs can be found on the “Author Information” page.
**Disclaimer:** Articles published in the journal HemaSphere exclusively reflect the opinions of the authors. The authors are responsible for all content in their abstracts including accuracy of the facts, statements, citing resources, etc.

### (PS2269) A MULTICENTER CLINICAL STUDY COMPARING HETROMBOPAG AND ELTROMBOPAG FOR PLATELET ENGRAFTMENT AFTER AUTOLOGOUS HEMATOPOIETIC STEM CELL TRANSPLANTATION

**Topic:** 23. Stem cell transplantation - Clinical

Aoxue Tan*[1], Yimei Feng[1], Pan Zhao[2], Mingqiang Ren[3], Peng Zhao[4], Han-Qing Zeng[5], Hongyun Xing[6], Liangliang MA[7], Mingxia Shi[8], Xi Zhang[1]

*[1]Xinqiao Hospital, Army Medical University, Chongqing, China;[2]Affiliated Hospital of North Sichuan Medical College, Nanchong, China; [3]Affiliated Hospital of Zunyi Medical University, Zunyi, China;[4]The Affiliated Hospital of Guizhou Medical University, Guiyang, China; [5]The Second Affiliated Hospital of Chongqing Medical University, Chongqing, China;[6]Department of Hematology, Affiliated Southwest Medical University, Luzhou, China; [7]Department of Hematology, the First Affiliated Hospital of Dalian Medical University, Dalian, China; [8]The First Affiliated Hospital of Kunming Medical University, Kunming, China;*

### Background

Autologous hematopoietic stem cell transplantation (auto-HSCT) is a key therapeutic approach for patients with lymphoma, multiple myeloma, and other hematologic malignancies. However, some patients experience delayed platelet engraftment or poor platelet recovery after auto-HSCT. Persistent thrombocytopenia increases bleeding risk and may compromise subsequent consolidation chemotherapy. Thrombopoietin receptor agonists (TPO-RAs), widely used in immune thrombocytopenia (ITP) and aplastic anemia (AA), have increasingly been applied to manage post-transplant thrombocytopenia after autologous and allogeneic HSCT.

### Aims

This multicenter study aimed to compare the clinical efficacy and safety of two TPO-RAs, hetrombopag and eltrombopag, in promoting platelet reconstitution following auto-HSCT.

### Methods

Clinical data from eight transplantation centers were retrospectively analyzed. Patients who received hetrombopag or eltrombopag within 3–7 days after autologous stem cell infusion to promote platelet recovery were included. The initial dose of hetrombopag ranged from 2.5 to 5.0 mg/day, while eltrombopag was administered at an initial dose of 25–50 mg/day. Treatment was continued until platelet engraftment was achieved. Hematopoietic engraftment kinetics, clinical outcomes, and adverse events were evaluated.

**Copyright Information:** (Online) ISSN: 2572-9241
©2026 The Author(s). HemaSphere is published by John Wiley & Sons Ltd on behalf of European Hematology Association. This is an open access Abstract Book distributed under the Attribution-NonCommercial-NoDerivs (CC BY-NC-ND), which allows third parties to download the articles and share them with others as long as they credit the author and the Abstract Book, but they cannot change the content in any way or use them commercially.
**Abstract Book Citations:** Authors, Title, HemaSphere, 2026;10:(S1):pages. The abstract book DOIs can be found on the "Author Information" page.
**Disclaimer:** Articles published in the journal HemaSphere exclusively reflect the opinions of the authors. The authors are responsible for all content in their abstracts including accuracy of the facts, statements, citing resources, etc.

**Results**

A total of 55 patients were analyzed: 38 received hetrombopag (2.5 mg/day, n=5; 5.0 mg/day, n=33) and 17 received eltrombopag (25 mg/day, n=3; 50 mg/day, n=14). There were 28 males and 27 females, with a median age of 54 years (range, 18–70). Underlying diseases included multiple myeloma (n=32), acute myeloid leukemia (n=3), T-cell lymphoma (n=6), diffuse large B-cell lymphoma (n=8), mantle cell lymphoma (n=4), Hodgkin lymphoma (n=1), and angioimmunoblastic T-cell lymphoma (n=1). Before transplantation, 44 patients achieved complete remission and 11 achieved partial or very good partial remission. The median number of prior chemotherapy cycles was 6 (range, 2–20). The infused $CD34^+$ cell dose was $5.8 \pm 4.2 \times 10^6$/kg in the hetrombopag group and $4.5 \pm 2.1 \times 10^6$/kg in the eltrombopag group, with no significant difference (p=0.26).

The overall mean time to neutrophil engraftment was 11.3±2.3 days, occurring earlier in the hetrombopag group than in the eltrombopag group (11.0±2.1 vs. 12.4±1.9 days, p=0.019). This difference remained significant when comparing hetrombopag 5.0 mg/day with eltrombopag 50 mg/day (p=0.048). The mean time to platelet engraftment was 14.2±4.7 days and was significantly shorter with hetrombopag than with eltrombopag (12.8±2.3 vs. 17.4±6.7 days, p=0.014), with consistent results in dose-matched comparisons (p=0.034).

All adverse events were grade 1–2 and resolved with symptomatic management. Common events included fever, headache, dizziness, diarrhea, fatigue, rash, elevated transaminases, and renal dysfunction. No grade ≥3 adverse events were observed.

**Summary/Conclusion**

This multicenter study demonstrates for the first time that, in patients undergoing autologous HSCT, hetrombopag is associated with significantly faster neutrophil and platelet engraftment compared with eltrombopag, with a favorable safety profile. Hetrombopag may represent a more effective option for promoting hematopoietic recovery following autologous stem cell transplantation.

**Copyright Information:** (Online) ISSN: 2572-9241
©2026 The Author(s). HemaSphere is published by John Wiley & Sons Ltd on behalf of European Hematology Association. This is an open access Abstract Book distributed under the Attribution-NonCommercial-NoDerivs (CC BY-NC-ND), which allows third parties to download the articles and share them with others as long as they credit the author and the Abstract Book, but they cannot change the content in any way or use them commercially.
**Abstract Book Citations:** Authors, Title, HemaSphere, 2026;10:(S1):pages. The abstract book DOIs can be found on the “Author Information” page.
**Disclaimer:** Articles published in the journal HemaSphere exclusively reflect the opinions of the authors. The authors are responsible for all content in their abstracts including accuracy of the facts, statements, citing resources, etc.

### (PS2270) IMPROVED OUTCOMES WITH YOUNGER RELATED HAPLOIDENTICAL DONORS IN PEDIATRIC AML UNDERGOING HAPLO-HSCT

**Topic:** 23. Stem cell transplantation - Clinical

Qingwei Wang*[1], Li Gao[1], Shaoyan Hu[1]

[1]*Children's Hospital of Soochow University, Suzhou, China;*

### Background

Haploidentical hematopoietic stem cell transplantation (haplo-HSCT) has become an important curative strategy for pediatric acute myeloid leukemia (AML), particularly when matched donors are unavailable. Donor-related factors may affect post-transplant immune reconstitution and graft-versus-leukemia effect, thereby influencing survival and GVHD/relapse-free survival. Donor age has been associated with outcomes in adult transplantation; however, evidence in pediatric AML receiving related haploidentical transplantation remains limited.

### Aims

To evaluate the impact of related haploidentical donor age on engraftment, acute graft-versus-host disease (aGVHD), relapse, non-relapse mortality (NRM), and survival outcomes in children with AML undergoing haplo-HSCT.

### Methods

We retrospectively analyzed consecutive pediatric AML patients who underwent first related haplo-HSCT at our center. Patients were stratified by donor age (≤35 years vs >35 years). Baseline recipient and transplant characteristics were compared between groups. Outcomes included neutrophil and platelet engraftment, grade II–IV aGVHD, cumulative incidence of relapse (CIR), NRM, overall survival (OS), relapse-free survival (RFS), and GVHD/relapse-free survival (GRFS). Multivariable Cox regression models were applied to identify independent prognostic factors for OS and GRFS.

### Results

A total of 241 patients were included (donor age ≤35: n=165; donor age >35: n=76). Recipients in the ≤35 group were younger (median 75 months [9.47–185] vs 130 months [20–205]); other baseline characteristics, including sex, ABO compatibility, pre-transplant remission status and MRD, conditioning regimen, and GVHD prophylaxis, were comparable without statistically significant differences. With a median follow-up of 24 months (range 1–90), neutrophil and platelet engraftment were similar between groups, indicating no delay in hematopoietic recovery with younger donors. The ≤35 donor group showed significantly superior survival: OS 87.54% (95% CI 0.824–0.930) vs 71.84% (95% CI 0.607–0.850), P=0.018; RFS 83.9% (95% CI 0.782–0.899) vs 70.5% (95% CI 0.596–0.833), P=0.047; and GRFS 79.1% (95% CI 0.729–0.857) vs 61.6% (95% CI 0.505–0.752), P=0.010. Rates of grade II–IV aGVHD were comparable (27.9% vs 28.3%, P=0.948). No statistically significant differences were observed in CIR (11.9% vs 17.4%, P=0.357) or NRM (8.7% vs 16.7%, P=0.077), although NRM showed a numerical trend toward being lower in the younger-donor group. In multivariable Cox analysis, donor age >35 years independently predicted inferior OS (HR 1.041, 95% CI 1.004–1.079; P=0.030) and inferior GRFS (HR 1.038, 95% CI 1.006–1.064; P=0.023).

**Copyright Information:** (Online) ISSN: 2572-9241
©2026 The Author(s). HemaSphere is published by John Wiley & Sons Ltd on behalf of European Hematology Association. This is an open access Abstract Book distributed under the Attribution-NonCommercial-NoDerivs (CC BY-NC-ND), which allows third parties to download the articles and share them with others as long as they credit the author and the Abstract Book, but they cannot change the content in any way or use them commercially.
**Abstract Book Citations:** Authors, Title, HemaSphere, 2026;10:(S1):pages. The abstract book DOIs can be found on the “Author Information” page.
**Disclaimer:** Articles published in the journal HemaSphere exclusively reflect the opinions of the authors. The authors are responsible for all content in their abstracts including accuracy of the facts, statements, citing resources, etc.

### Summary/Conclusion

In this pediatric AML haplo-HSCT cohort, younger related haploidentical donor age (≤35 years) was associated with improved OS, RFS, and GRFS, without increasing grade II–IV aGVHD or delaying engraftment. These findings support prioritizing younger related haploidentical donors, when multiple donors are available, to optimize long-term transplant outcomes in children with AML.

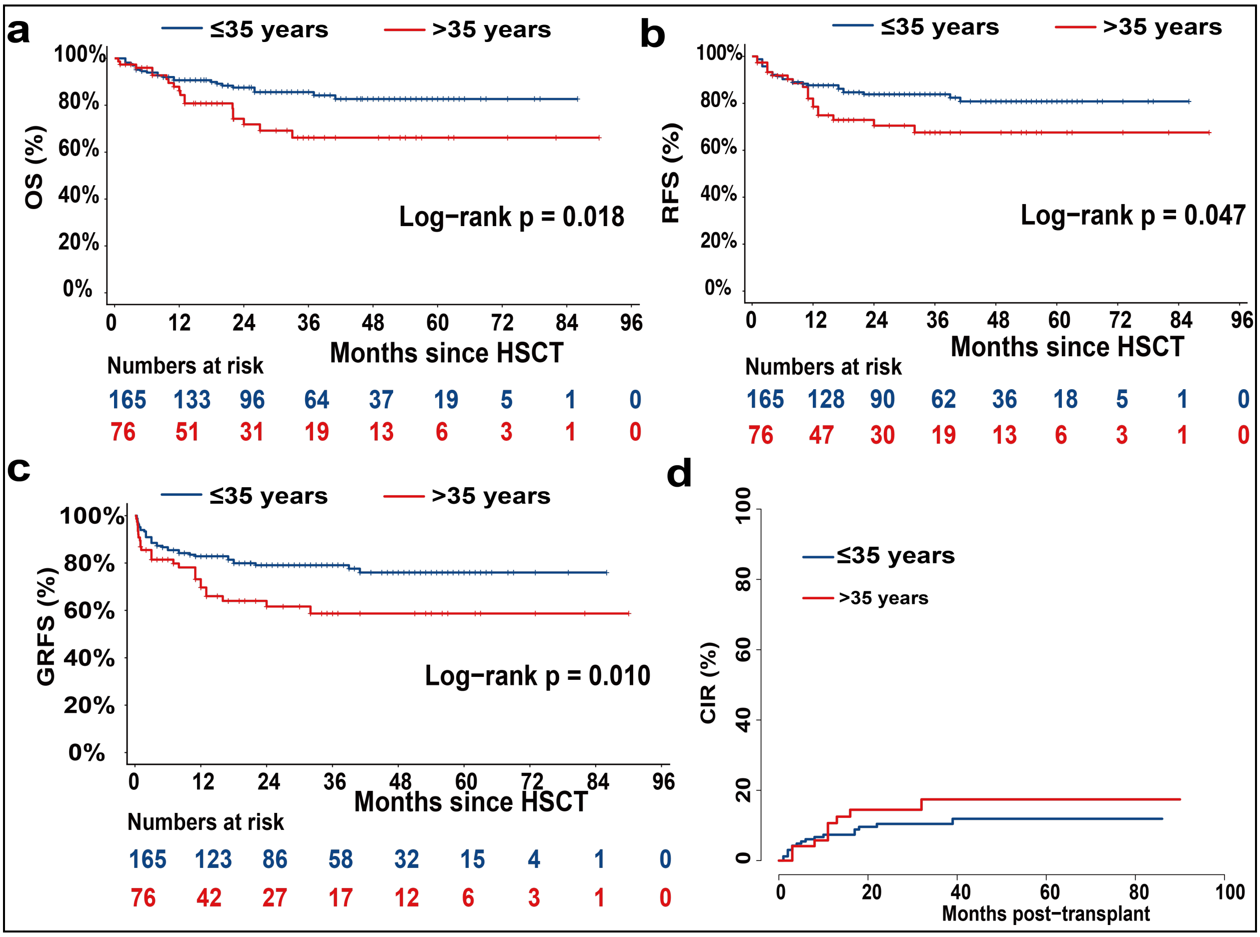

Figure. Outcomes after haplo-HSCT by donor age in pediatric AML. Patients were grouped by related haploidentical donor age (≤35 years, blue; >35 years, red). (a) Overall survival (OS). (b) Relapse-free survival (RFS). (c) GVHD/relapse-free survival (GRFS). (d) Cumulative incidence of relapse (CIR). Numbers at risk are shown below the curves.

**Copyright Information:** (Online) ISSN: 2572-9241
©2026 The Author(s). HemaSphere is published by John Wiley & Sons Ltd on behalf of European Hematology Association. This is an open access Abstract Book distributed under the Attribution-NonCommercial-NoDerivs (CC BY-NC-ND), which allows third parties to download the articles and share them with others as long as they credit the author and the Abstract Book, but they cannot change the content in any way or use them commercially.
**Abstract Book Citations:** Authors, Title, HemaSphere, 2026;10:(S1):pages. The abstract book DOIs can be found on the "Author Information" page.
**Disclaimer:** Articles published in the journal HemaSphere exclusively reflect the opinions of the authors. The authors are responsible for all content in their abstracts including accuracy of the facts, statements, citing resources, etc.

### (PS2271) HAPLOIDENTICAL TRANSPLANTATION WITH UNRELATED CORD BLOOD SUPPORT (SZ-19-TM PROTOCOL) IMPROVES OUTCOMES IN PEDIATRIC TRANSFUSION-DEPENDENT THALASSEMIA: A LARGE-SCALE RETROSPECTIVE COHORT STUDY

**Topic:** 23. Stem cell transplantation - Clinical

Ruihao Zheng*[1], Qihong Lin[1], Chunjing Wang[1], Xiaohui Zhou[1], Xiaoling Zhang[1], Yu Zhang[1], Yue Li[1], Chunlan Yang[1], Qian Zhang[1], Mingjing Luo[1], Zhiheng Ding[1], Jiaming Yu[1], Feiqiu Wen[1], Sixi Liu[1]

*[1]Shenzhen Children's Hospital, Department of Hematology and Oncology, Shenzhen, China;*

### Background

Allogeneic hematopoietic stem cell transplantation (HSCT) is the sole curative modality for pediatric transfusion-dependent thalassemia (TDT), yet its clinical application is severely constrained by the scarcity of HLA-matched donors. Haploidentical HSCT has expanded donor access for TDT patients but is associated with heterogeneous clinical outcomes. We therefore developed the SZ-19-TM protocol: a modified haploidentical HSCT regimen supported by unrelated cord blood (CB), with graft-versus-host disease (GVHD) prophylaxis using post-transplant cyclophosphamide (PTCy) combined with low-dose anti-thymocyte globulin (ATG), which has exhibited promising preliminary clinical efficacy.

### Aims

To evaluate the efficacy of haploidentical HSCT plus unrelated CB infusion in pediatric TDT patients, and to compare clinical outcomes across three distinct graft infusion strategies: haploidentical HSCT alone, competitive haplo-cord transplantation, and supportive haplo-cord transplantation (SZ-19-TM).

### Methods

We performed a retrospective cohort study of pediatric TDT patients who underwent haploidentical HSCT at our center from September 2016 to December 2024. Patients were stratified into three groups: Group A (haploidentical HSCT alone, n=26), Group B (competitive haplo-cord transplantation, CB infusion on day +6, n=81), and Group C (SZ-19-TM, CB infusion on day+1, n=724). All patients received a standardized conditioning regimen of cyclophosphamide, fludarabine, busulfan, thiotepa and rabbit ATG, alongside uniform GVHD prophylaxis with PTCy, tacrolimus and mycophenolate mofetil (MMF). CB units were selected based on HLA matching and a minimum total nucleated cell (TNC) count of $\geq 1\times10^7$/kg. Primary endpoints included overall survival (OS), transfusion-free survival (TFS), neutrophil and platelet engraftment time, and cumulative incidence of acute and chronic GVHD.

### Results

Median follow-up was 1391, 1288 and 883 days for Groups A, B and C, respectively. No statistically significant difference in OS was detected among the three groups (P=0.079), with TFS exceeding 96% across all cohorts. Nine patients died of refractory post-transplant complications (Group B: 3.7%; Group C: 0.8%; no mortality in Group A). Graft failure was observed in 3.8% of Group A and 0.4% of Group C patients, with no cases in Group B. Group B had significantly delayed neutrophil and platelet engraftment compared with Groups A and C (P<0.0001), with no significant difference in engraftment time between Groups A and C. Group C had the lowest cumulative incidence of grade Ⅲ-Ⅳ acute GVHD (2.2% vs. 3.8% in Group A and 22.2% in Group B), and a markedly reduced incidence of chronic GVHD relative to the other two groups(15.7% vs. 33.3% in Group A and 23.0% in Group B). OS rates were 96.15% (A), 96.30% (B) and 99.17% (C); corresponding TFS rates were 96.15%, 96.30% and 98.76%.

**Copyright Information:** (Online) ISSN: 2572-9241
©2026 The Author(s). HemaSphere is published by John Wiley & Sons Ltd on behalf of European Hematology Association. This is an open access Abstract Book distributed under the Attribution-NonCommercial-NoDerivs (CC BY-NC-ND), which allows third parties to download the articles and share them with others as long as they credit the author and the Abstract Book, but they cannot change the content in any way or use them commercially.
**Abstract Book Citations:** Authors, Title, HemaSphere, 2026;10:(S1):pages. The abstract book DOIs can be found on the "Author Information" page.
**Disclaimer:** Articles published in the journal HemaSphere exclusively reflect the opinions of the authors. The authors are responsible for all content in their abstracts including accuracy of the facts, statements, citing resources, etc.

**Summary/Conclusion**

The SZ-19-TM protocol (supportive haplo-cord transplantation with CB infusion on day +1) is a safe and highly effective therapeutic strategy for pediatric TDT patients without available HLA-matched donors. This regimen accelerates hematopoietic engraftment, significantly reduces the incidence of acute and chronic GVHD, and achieves excellent OS and TFS outcomes, thereby representing a promising therapeutic alternative for pediatric TDT patients with limited donor options.

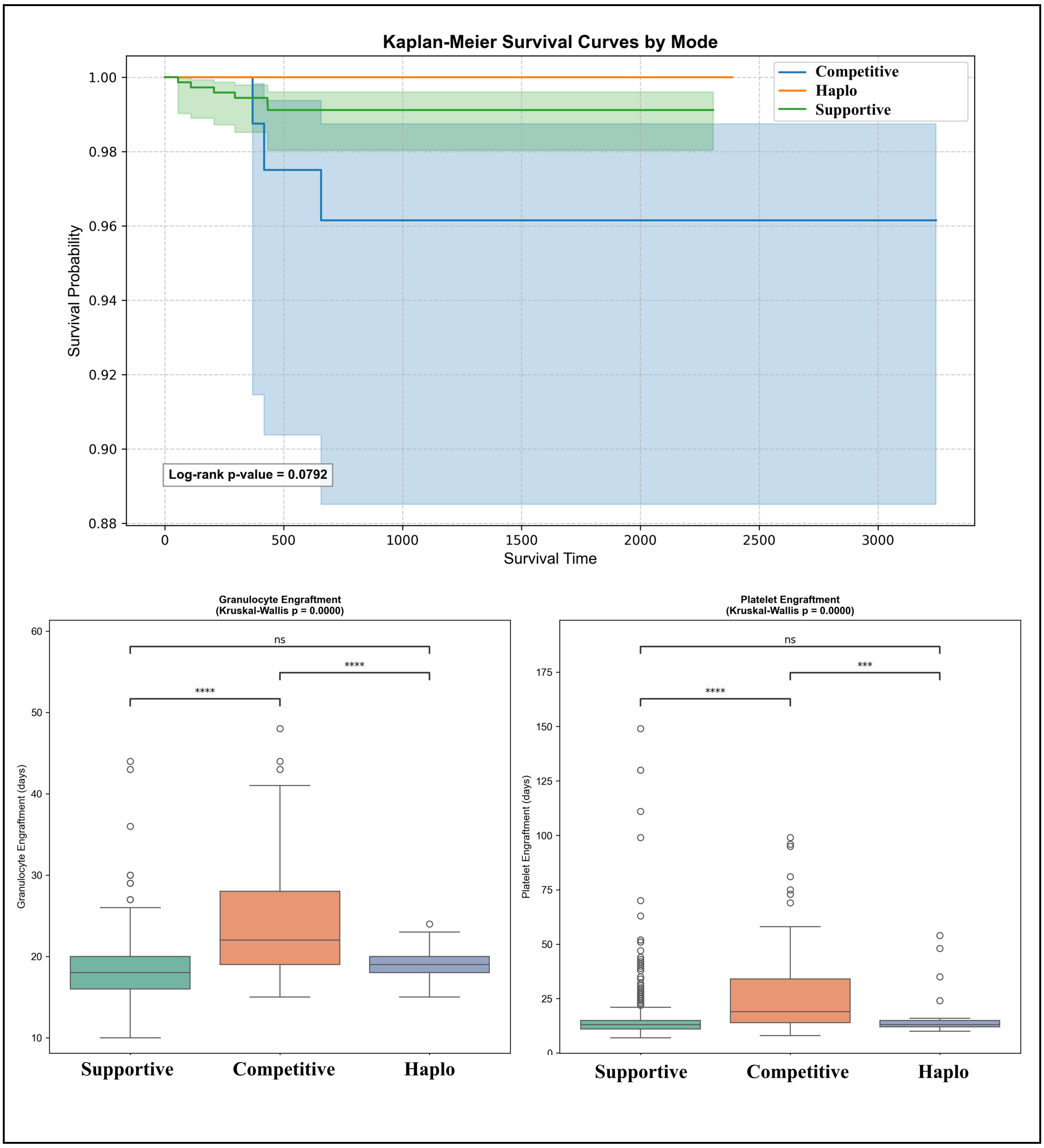

**Copyright Information:** (Online) ISSN: 2572-9241
©2026 The Author(s). HemaSphere is published by John Wiley & Sons Ltd on behalf of European Hematology Association. This is an open access Abstract Book distributed under the Attribution-NonCommercial-NoDerivs (CC BY-NC-ND), which allows third parties to download the articles and share them with others as long as they credit the author and the Abstract Book, but they cannot change the content in any way or use them commercially.
**Abstract Book Citations:** Authors, Title, HemaSphere, 2026;10:(S1):pages. The abstract book DOIs can be found on the "Author Information" page.
**Disclaimer:** Articles published in the journal HemaSphere exclusively reflect the opinions of the authors. The authors are responsible for all content in their abstracts including accuracy of the facts, statements, citing resources, etc.

### (PS2272) IMPACT OF AGE ON OUTCOME OF VENETOCLAX-BASED CONDITIONING REGIMEN FOR OLDER PATIENTS WITH AML/MDS FOLLOWING ALLOGENEIC HEMATOPOIETIC STEM CELL TRANSPLANTATION

**Topic:** 23. Stem cell transplantation - Clinical

Panpan Zhu*[1, 2, 3], Xiaoyu Lai[1, 2, 3], Lizhen Liu[1, 2, 3], Luxin Yang[1, 2, 3], Yibo Wu[1, 2, 3], Ji-Min Shi[1, 2, 3], Jian Yu[1, 2, 3], Yanmin Zhao[1, 2, 3], Guifang Ouyang[4], Yi Chen[5], Ping Zhang[4], Lan Sun[5], Weiyan Zheng[1, 2, 3], Jie Sun[1, 2, 3], He Huang[1, 2, 3], Yi Luo[1, 2, 3]

*[1]Bone Marrow Transplantation Center of The First Affiliated Hospital & Liangzhu Laboratory, Zhejiang University School of Medicine, Hangzhou, China; [2]Institute of Hematology, Zhejiang University, Hangzhou, China;[3]Zhejiang Province Engineering Research Center for Stem Cell and Immunity Therapy, Hangzhou, China; [4]The First Affiliated Hospital of Ningbo University, Ningbo, China; [5]The First Affiliated Hospital of Wenzhou Medical University, Wenzhou, China;*

### Background

Venetoclax is an effective option for patients with acute myeloid leukemia (AML). Recently, it has been incorporated into conditioning regimens for allogeneic hematopoietic stem cell transplantation (allo-HSCT) in myeloid malignancies. Our phase 2 clinical trial (NCT05084027) demonstrated that the combination of venetoclax with fludarabine and melphalan (VFM) as a conditioning regimen yielded promising outcomes with a low incidence of graft-versus-host disease (GVHD), as previously reported (Yi Luo: 50$^{th}$ EBMT and 49$^{th}$ EBMT).

### Aims

This post hoc analysis of the phase 2 trial specifically aims to evaluate the impact of patient age on transplantation outcomes following the VFM conditioning regimen in older patients with acute myeloid leukemia (AML) or myelodysplastic syndrome (MDS).

### Methods

The conditioning regimen consisted of venetoclax (400mg/d on days -8 to -2), fludarabine (30mg/m2/d on days -7 to -3) and melphalan (140mg/m2/d on day-2 for patients < 60 years; 120 mg/m2/d on day-2 for patients ≥ 60 years). Patients aged over 50 years with AML or MDS who received the VFM conditioning regimen were included and stratified into three age cohorts: cohort A (50–54 years), cohort B (55–59 years), and cohort C (≥60 years). Outcomes evaluated included overall survival (OS), disease-free survival (DFS), GVHD-free and relapse-free survival (GRFS), non-relapse mortality (NRM), and cumulative incidence of relapse (CIR).

**Copyright Information:** (Online) ISSN: 2572-9241
©2026 The Author(s). HemaSphere is published by John Wiley & Sons Ltd on behalf of European Hematology Association. This is an open access Abstract Book distributed under the Attribution-NonCommercial-NoDerivs (CC BY-NC-ND), which allows third parties to download the articles and share them with others as long as they credit the author and the Abstract Book, but they cannot change the content in any way or use them commercially.
**Abstract Book Citations:** Authors, Title, HemaSphere, 2026;10:(S1):pages. The abstract book DOIs can be found on the “Author Information” page.
**Disclaimer:** Articles published in the journal HemaSphere exclusively reflect the opinions of the authors. The authors are responsible for all content in their abstracts including accuracy of the facts, statements, citing resources, etc.

### Results

A total of 145 patients were included, with a median follow-up of 539 days (range, 30–1533 days). The median age at transplantation was 58 years (range, 51–68), and 65 patients (44.8%) were female. Sixty-nine patients had an HCT-CI score ≥3, and 75 patients (51.7%) were classified as high or very high risk according to the refined disease risk index (rDRI). Donor types included matched sibling donor (MSD) in 1 patient, unrelated donor (URD) in 19 (13.1%), and haploidentical donor (HID) in 125 (86.2%). The 2-year rates of OS, DFS, GRFS, NRM, and CIR for the entire cohort were 69.90% (95% CI, 62.20–78.50%), 65.80% (95% CI, 57.90–74.80%), 57.10% (95% CI, 48.90-66.70), 16.88% (95% CI, 10.90–23.99%), and 17.27% (95% CI, 11.23–24.42%), respectively. The cumulative incidence of grade II–IV acute GVHD at 100 days and moderate-to-severe chronic GVHD at 2 years were 6.90% (95% CI, 3.52–11.81%) and 9.52% (95% CI, 5.29–35.16%), respectively.
When analyzed by age cohort, the 2-year rates of OS, DFS, GRFS, NRM, and CIR were as follows (Figure 1): cohort A (N=36): 71.48%, 69.36%, 55.57%, 16.67%, and 13.99%; cohort B (N=57): 72.66%, 67.42%, 59.82%, 14.28%, and 18.30%; cohort C (N=52): 68.84%, 61.00%, 54.42%, 21.02%, and 17.99%. No significant differences in transplant outcomes were observed among the three age groups. Notably, patients with a low HCT-CI (≤2) exhibited a trend toward superior OS (77.50% vs. 61.67%, $P$ = 0.065) and a significantly lower incidence of NRM (8.99% vs. 25.69%, $P$ = 0.008) compared to those with a high HCT-CI (≥3). However, no significant differences were observed between these groups in terms of DFS (70.54% vs. 60.94%, $P$ = 0.161), GRFS (58.30% vs. 56.20%, $P$ = 0.710) or CIR (20.47% vs. 13.37%, $P$ = 0.428).

### Summary/Conclusion

In conclusion, the VFM conditioning regimen is feasible and effective for allo-HSCT in patients aged over 50 years with AML or MDS, with patient age demonstrating no significant impact on transplant outcomes. Furthermore, patients with a low HCT-CI experienced superior OS and a reduced incidence of NRM.

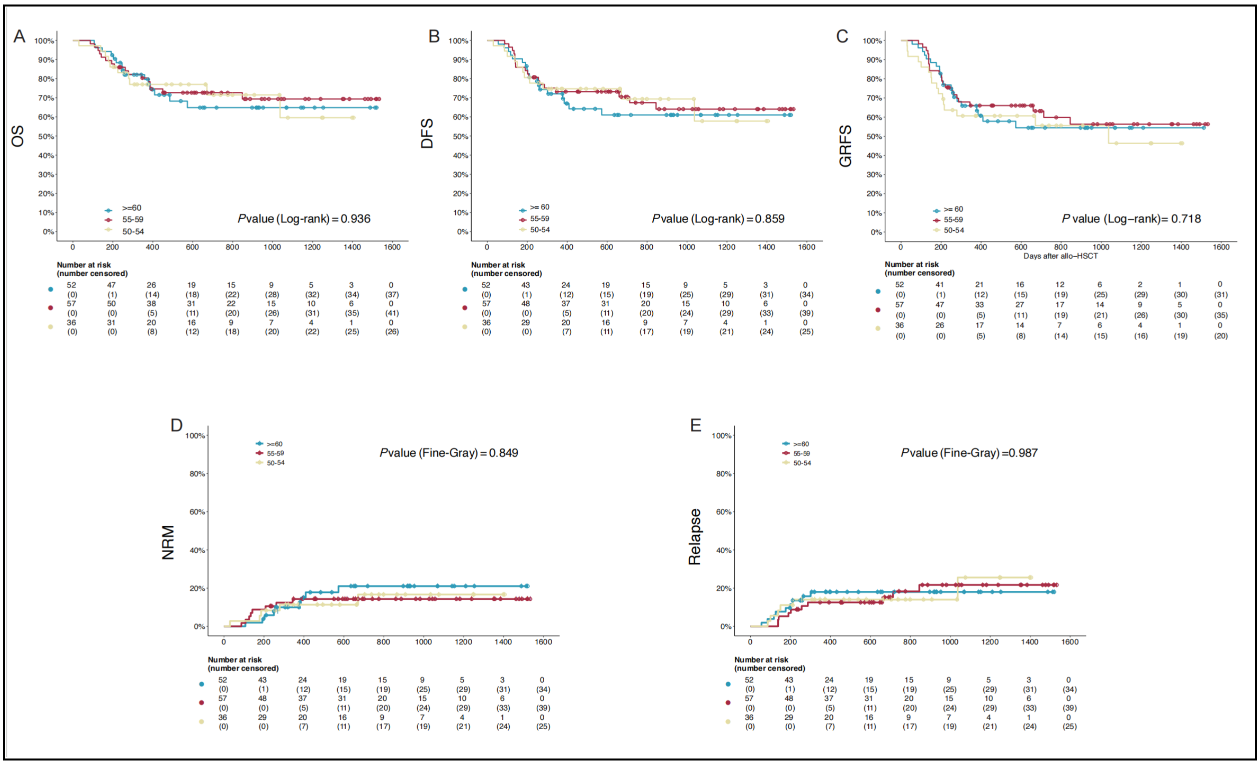

**Copyright Information:** (Online) ISSN: 2572-9241
©2026 The Author(s). HemaSphere is published by John Wiley & Sons Ltd on behalf of European Hematology Association. This is an open access Abstract Book distributed under the Attribution-NonCommercial-NoDerivs (CC BY-NC-ND), which allows third parties to download the articles and share them with others as long as they credit the author and the Abstract Book, but they cannot change the content in any way or use them commercially.
**Abstract Book Citations:** Authors, Title, HemaSphere, 2026;10:(S1):pages. The abstract book DOIs can be found on the "Author Information" page.
**Disclaimer:** Articles published in the journal HemaSphere exclusively reflect the opinions of the authors. The authors are responsible for all content in their abstracts including accuracy of the facts, statements, citing resources, etc.

### (PS2273) MONOCYTE SUBSETS AND MONOCYTE-RELATED CYTOKINES AS POTENTIAL BIOMARKERS IN CHRONIC GRAFT-VERSUS-HOST DISEASE

**Topic:** 23. Stem cell transplantation - Clinical

Antonija Babić*[1], Ana Zelic Kerep[2], Antonela Lelas[2], Josip Knežević[1], Ivan Sabol[3], Lana Desnica[2], Nadira Duraković[2, 4], Radovan Vrhovac[2, 4], Drazen Pulanic[2, 4], Drago Batinić[5], Hrvoje Lalić[1, 5, 6]

*[1]University Hospital Centre Zagreb, Department of Laboratory Immunology, Zagreb, Croatia;[2]University Hospital Centre Zagreb, Division of Hematology, Department of Internal Medicine, Zagreb, Croatia; [3]Rudjer Boskovic Institute, Division of Molecular Medicine, Zagreb, Croatia; [4]University of Zagreb School of Medicine, Department of Internal Medicine, Zagreb, Croatia; [5]University of Zagreb School of Medicine, Department of Physiology, Zagreb, Croatia;[6]Croatian Institute for Brain Research, Laboratory for Cell Biology, Zagreb, Croatia;*

### Background

Chronic graft-versus-host disease (cGVHD) is a major cause of late morbidity after allogeneic hematopoietic stem cell transplantation (allo-HSCT). Reliable biomarkers of disease activity and severity are still lacking. Although monocytes contribute to cGVHD pathogenesis, data on specific subsets and related cytokines remain inconsistent.

### Aims

To evaluate peripheral blood monocyte subsets and monocyte-related cytokines in newly diagnosed and previously diagnosed cGVHD patients compared with allo-HSCT controls, and to assess their associations with clinical characteristics.

### Methods

Peripheral blood samples were obtained from 31 patients with newly diagnosed cGVHD (<1 month from the diagnosis), 38 with previously diagnosed cGVHD, and 32 allo-HSCT recipients without cGVHD. Patients with active infection and overlap cGVHD were excluded. Classical (CD14++CD16−), intermediate (CD14++CD16+), and non-classical (CD14+CD16++) monocytes were quantified by flow cytometry. Plasma levels of 12 cytokines were measured using a microsphere bead array. Clinical assessment included NIH global and organ-specific scores, total NIH score, overall cGVHD severity (0–10 scale), performance status (Karnofsky), and immunosuppressive therapy exposure.

**Copyright Information:** (Online) ISSN: 2572-9241
©2026 The Author(s). HemaSphere is published by John Wiley & Sons Ltd on behalf of European Hematology Association. This is an open access Abstract Book distributed under the Attribution-NonCommercial-NoDerivs (CC BY-NC-ND), which allows third parties to download the articles and share them with others as long as they credit the author and the Abstract Book, but they cannot change the content in any way or use them commercially.
**Abstract Book Citations:** Authors, Title, HemaSphere, 2026;10:(S1):pages. The abstract book DOIs can be found on the “Author Information” page.
**Disclaimer:** Articles published in the journal HemaSphere exclusively reflect the opinions of the authors. The authors are responsible for all content in their abstracts including accuracy of the facts, statements, citing resources, etc.

**Results**

The groups did not differ in age or sex distribution, whereas time from transplantation differed significantly (controls 19.2; newly diagnosed 8.8; and previously diagnosed 58.0 months, $p<0.001$). Immunosuppression intensity was similar between cGVHD groups, but they differed in number of prior therapy lines ($p<0.001$).
In patients with newly diagnosed cGVHD, proinflammatory intermediate monocytes correlated positively with lung involvement (percentage: rs=0.60, p<0.001, and count: rs=0.50, p=0.006) and GI tract involvement (percentage: rs=0.40, p=0.033). Intermediate monocyte count also correlated positively with total NIH score (rs=0.37, p=0.049), and negatively with Karnofsky score (rs=-0.37, p=0.047). In this group, patients had significantly higher CXCL10 levels than both previously diagnosed cGVHD patients and controls (p<0.001), consistent with an early inflammatory milieu.
Patients with previously diagnosed cGVHD had significantly higher classical monocyte counts than controls (p=0.001), which correlated with global NIH score (rs=0.44, p=0.008), overall severity (rs=0.37, p=0.035), total NIH score (rs=0.46, p=0.005), and number of involved organs (rs=0.34, p=0.046), indicating association with cumulative disease burden.  In this group, lower non-classical monocyte proportions were linked to poorer functional status (Karnofsky score rs=0.39, p=0.022) and greater immunosuppressive exposure (number of prior therapy lines (rs=-0.46, p=0.005), and immunosuppression intensity (rs=-0.41, p=0.014)). Proinflammatory cytokines IL-8 and IL-6 strongly correlated with disease severity (rs=0.78, p<0.001; rs=0.59, p=0.004), and inversely with Karnofsky score (rs=-0.51, p=0.010; rs=-0.50, p=0.012). IL-8 also correlated with global (rs=0.66, p<0.001) and total NIH scores (rs=0.60, p=0.002), and number of affected organs (rs=0.54, p=0.007).

**Summary/Conclusion**

Distinct patterns of monocyte subset distribution and cytokine expression are associated with the onset and specific organ involvement of cGVHD (CXCL10, intermediate monocytes) and disease burden and severity (classical monocytes, IL-6, IL-8). These findings support the potential role of monocyte subsets and selected cytokines as biomarkers of disease activity and progression in cGVHD.

**Copyright Information:** (Online) ISSN: 2572-9241
©2026 The Author(s). HemaSphere is published by John Wiley & Sons Ltd on behalf of European Hematology Association. This is an open access Abstract Book distributed under the Attribution-NonCommercial-NoDerivs (CC BY-NC-ND), which allows third parties to download the articles and share them with others as long as they credit the author and the Abstract Book, but they cannot change the content in any way or use them commercially.
**Abstract Book Citations:** Authors, Title, HemaSphere, 2026;10:(S1):pages. The abstract book DOIs can be found on the “Author Information” page.
**Disclaimer:** Articles published in the journal HemaSphere exclusively reflect the opinions of the authors. The authors are responsible for all content in their abstracts including accuracy of the facts, statements, citing resources, etc.

### (PS2274) DOES THE AGE-ADJUSTED HCT-CI RETAIN PROGNOSTIC SIGNIFICANCE IN HAPLOIDENTICAL TRANSPLANTATION WITH PTCY?

**Topic:** 23. Stem cell transplantation - Clinical

Hakan Goker*[1], Elifcan Aladag[1], Haluk Demiroglu[1], Yahya Buyukasik[1]

[1]*Hacettepe, Department of Hematology, Altındağ, Türkiye;*

### Background

The Age-adjusted Hematopoietic Cell Transplantation Comorbidity Index (HCT-CI-age) is widely used to estimate pre-transplant risk in conventional allogeneic hematopoietic stem cell transplantation (allo-HCT). However, its prognostic relevance in the setting of HLA-haploidentical transplantation (haplo-HCT) with post-transplant cyclophosphamide (PTCY) remains uncertain.

### Aims

To assess the prognostic performance and independent predictive value of the HCT-CI-age for overall survival (OS) and non-relapse mortality (NRM) in patients undergoing haplo-HCT with PTCY.

### Methods

We retrospectively analyzed 73 consecutive patients who underwent haplo-HCT with PTCY at a single tertiary transplantation center. Patients were stratified according to HCT-CI-age risk groups as follows: low risk (score 0), intermediate risk (score 1–2), and high risk (score ≥3). The association between HCT-CI-age risk categories and 2-year OS and NRM was evaluated using univariate and multivariate Cox proportional hazards models adjusting for relevant clinical covariates

### Results

The majority of patients had acute myeloid leukemia (68.5%), followed by acute lymphoblastic leukemia (17.8%) and myelodysplastic syndrome (13.7%). Median follow-up was 21.4 months (range 2.3–94).

In univariate analysis, HCT-CI-age risk groups were significantly associated with transplant outcomes. Two-year OS rates were 97.8%, 72.1%, and 50.5% for low-, intermediate-, and high-risk groups, respectively (p=0.006). Corresponding 2-year NRM rates were 0%, 14.8%, and 41.2% (p<0.001).

However, in multivariate analysis adjusting for clinical covariates, HCT-CI-age did not retain independent prognostic significance for either 2-year OS or NRM.

### Summary/Conclusion

Although HCT-CI-age demonstrated discriminatory capacity in univariate analysis, it was not an independent predictor of survival outcomes in the haplo-HCT setting with PTCY. These findings suggest that the predictive utility of conventional comorbidity-based risk models may be attenuated in the contemporary haploidentical transplant platform and warrants validation in larger cohorts.

**Copyright Information:** (Online) ISSN: 2572-9241
©2026 The Author(s). HemaSphere is published by John Wiley & Sons Ltd on behalf of European Hematology Association. This is an open access Abstract Book distributed under the Attribution-NonCommercial-NoDerivs (CC BY-NC-ND), which allows third parties to download the articles and share them with others as long as they credit the author and the Abstract Book, but they cannot change the content in any way or use them commercially.
**Abstract Book Citations:** Authors, Title, HemaSphere, 2026;10:(S1):pages. The abstract book DOIs can be found on the "Author Information" page.
**Disclaimer:** Articles published in the journal HemaSphere exclusively reflect the opinions of the authors. The authors are responsible for all content in their abstracts including accuracy of the facts, statements, citing resources, etc.

### (PS2275) UMBILICAL CORD-DERIVED MESENCHYMAL STEM CELLS: ELEVATING EARLY EFFICACY IN GASTROINTESTINAL ACUTE GRAFT-VERSUS-HOST DISEASE

**Topic:** 23. Stem cell transplantation - Clinical

Fang Zhou*[1], Zhipeng Li[1], Xiaolin Yu[1], Xiaochen Song[1], Lei Deng[1], Bo Xin[1], Fanjun Kong[1], Yuerong Zhao[1]

[1]*The 960th Hospital of The People's Liberation Army (PLA) Joint Logistics Support Force, Jinan, China;*

### Background

Gastrointestinal acute graft-versus-host disease is a potentially life-threatening complication of allogeneic hematopoietic stem cell transplantation (allo-HSCT). Umbilical cord-derived mesenchymal stem cells (UC-MSCs) have potential efficacy in treating gastrointestinal acute graft-versus-host disease.

### Aims

This retrospective study evaluated the 30-day efficacy of UC-MSCs in patients with gastrointestinal acute graft-versus-host disease.

### Methods

In this retrospective cohort study, we analyzed 98 patients with gastrointestinal acute graft-versus-host disease, of whom 57 received UC-MSCs ($1\times10^6$ cells/kg) in addition to standard immunosuppressive therapy, and 41 received standard therapy alone. The primary outcome was the 30-day overall response rate (ORR). Secondary outcomes included overall survival (OS), relapse-free survival (RFS), cumulative incidence of relapse, and chronic GvHD (cGVHD).

### Results

The UC-MSCs group exhibited a significantly higher 30-day ORR compared to the control group (86.0% vs. 68.3%; odds ratio, 2.8; 95% CI, 1.05-7.7; P=0.035). Within the UC-MSCs group, patients with grade I-II gastrointestinal acute graft-versus-host disease achieved a superior ORR of 100% compared to 70.4% in those with grade III-IV disease (P=0.005). However, no statistically significant differences were observed between the two groups in long-term outcomes, including 5-year OS (66.4% vs. 34.4%; HR, 0.75; P=0.42), 5-year RFS (53.8% vs. 33.9%; HR, 0.68; P=0.23), cumulative incidence of relapse (HR, 0.40; P=0.052), or cGVHD (HR, 0.95; P=0.93). UC-MSC infusions were well-tolerated with a favorable safety profile.

### Summary/Conclusion

UC-MSC therapy significantly improves the early clinical response in patients with gastrointestinal acute graft-versus-host disease, particularly in those with mild to moderate disease. While no long-term survival benefits were demonstrated, the enhanced symptom control and favorable safety profile support its potential as a valuable adjunctive treatment. Larger prospective studies are warranted to confirm these findings.

**Copyright Information:** (Online) ISSN: 2572-9241
©2026 The Author(s). HemaSphere is published by John Wiley & Sons Ltd on behalf of European Hematology Association. This is an open access Abstract Book distributed under the Attribution-NonCommercial-NoDerivs (CC BY-NC-ND), which allows third parties to download the articles and share them with others as long as they credit the author and the Abstract Book, but they cannot change the content in any way or use them commercially.
**Abstract Book Citations:** Authors, Title, HemaSphere, 2026;10:(S1):pages. The abstract book DOIs can be found on the "Author Information" page.
**Disclaimer:** Articles published in the journal HemaSphere exclusively reflect the opinions of the authors. The authors are responsible for all content in their abstracts including accuracy of the facts, statements, citing resources, etc.

### (PS2276) PSYCHIATRIC STATUS IN CHRONIC GRAFT-VERSUS-HOST DISEASE: A REAL-WORLD DATA.

**Topic:** 23. Stem cell transplantation - Clinical

Jan Puchowski*[1, 2], Karol Grabowski[2], Jan MacIej Zaucha[1], Agnieszka Piekarska[1]

*[1]University Clinical Centre, Medical University of Gdansk, Poland, Department of Hematology, Transplantology and Cellular Therapies, Gdansk, Poland; [2]University Clinical Centre, Medical University of Gdansk, Poland, Department of Psychiatry, Gdansk, Poland;*

### Background

Chronic graft-versus-host disease (cGvHD) is a potentially life-threatening complication of allogeneic hematopoietic stem cell transplantation. It is associated with a substantial physical symptom burden and reduced quality of life (QoL). Treatment may further impair patients’ well-being and lead to cognitive impairment. The cumulative psychological burden of hospitalization, intensive treatment, and chronic disease may increase vulnerability to depressive symptoms in patients with cGvHD. However, prospective data on the association between psychiatric status and disease symptom severity are limited.

### Aims

This study prospectively examines the bidirectional association between psychiatric status, including depressive symptoms, and patients' perceived burden of cGVHD.

### Methods

Patients with cGvHD underwent psychiatric assessment using standardized instruments for anxiety (GAD-7), depression (IDS-SR, MADRS), quality of life (WHOQOL) and cognitive function (MOCA). Symptom burdens in cGvHD were self-measured by the Lee Symptom Scale (LSS). Assessments were conducted at three-month intervals over a year as part of an ongoing prospective study. When a psychiatric disorder was identified, pharmacological treatment was initiated. Patients were categorized into two groups based on the presence or absence of a psychiatric diagnosis. The Pearson correlation coefficients were calculated to compare patient-reported cGVHD burden with objective psychological implications.

### Results

To date, 47 patients have been enrolled. Among them, 27 (57.4%) met the criteria for a depressive episode on the IDS-SR scale. The diagnosis was confirmed in 23 (48.9%) patients with 16 cases of mild depression, 7 moderate and 1 severe. Approximately 1 in 4 patients (27,7%) met the criteria for general anxiety disorder. Mild and moderate cognitive impairment was observed in 17 (36.2%) and 10 (21%) cases, respectively. However, QoL of the majority (89.3%) of patients was reported as good or very good.
Most of the patients (30 out of 47; 63.83%) were diagnosed with moderate cGvHD; therefore, statistical analysis was limited to this subgroup. Respiratory cGVHD manifestation scored with LSS showed moderate positive correlations with both depressive symptoms (MADRS, r=0.52) and overall well-being (r=0.52). Gastrointestinal symptoms showed a moderate positive correlation with overall well-being (r = 0.50). Muscle symptoms correlated strongly with depressive symptoms (IDS-SR, r=0.65) and moderately with well-being (r = 0.57). Higher scores in the psyche part were positively correlated with the severity of depressive symptoms (MADRS, r=0.62; IDS-SR, r=0.70).

**Copyright Information:** (Online) ISSN: 2572-9241
©2026 The Author(s). HemaSphere is published by John Wiley & Sons Ltd on behalf of European Hematology Association. This is an open access Abstract Book distributed under the Attribution-NonCommercial-NoDerivs (CC BY-NC-ND), which allows third parties to download the articles and share them with others as long as they credit the author and the Abstract Book, but they cannot change the content in any way or use them commercially.
**Abstract Book Citations:** Authors, Title, HemaSphere, 2026;10:(S1):pages. The abstract book DOIs can be found on the “Author Information” page.
**Disclaimer:** Articles published in the journal HemaSphere exclusively reflect the opinions of the authors. The authors are responsible for all content in their abstracts including accuracy of the facts, statements, citing resources, etc.

### Summary/Conclusion

Data suggest that mental health disorders are common among patients with chronic GvHD. Results obtained from our group imply that LSS results correlate to some extent with depressive symptoms. Therefore, LSS may be considered as useful in everyday practice in screening cGVHD patients for depression.

Table 1. Patients' demography and clinical characteristics

| | |
|---|---|
| Study group | 47 |
| Median age (range) | 47 (19-68) years |
| Sex: Females/ Males | 29 /18 |
| Type of hematologic malignancies: | |
| Acute leukemias (AML, ALL) | 25 |
| Myelodysplastic/myeloproliferative neoplasms (MDS, CMML) | 6 |
| Chronic lymphoid malignancies (CLL, NHL, HL, FL, MCL, MM) | 9 |
| Chronic myeloproliferative neoplasms (MF, CML) | 6 |
| SAA/PNH | 1 |
| Conditioning regimen: | |
| MAC/RTC | 28 |
| RIC | 1 |
| NMA | 2 |
| A type of donor: | |
| MSD | 21 |
| MUD | 15 |
| mMUD | 6 |
| Haplo | 5 |
| Chronic GvHD (at study recruitment): | |
| Mild | 8 |
| Moderate | 30 |
| Severe | 9 |
| Number of systemic immunosuppressive agents (at study recruitment): | |
| 0 | 9 |
| 1 | 9 |
| 2 | 19 |
| 3 | 10 |
| ECP: Yes / No | 18 / 29 |
| Lee Symptom Scale (at study recruitment); mean (range): | |
| Skin | 2,69 (0-15) |
| Eye and mouth | 5,55 (0-16) |
| Lung | 2,36 (0-13) |
| Nutrition | 0,81 (0-6) |
| Muscles and joints | 4,08 (0-13) |
| Energy | 2,12 (0-8) |
| Psychic/mental | 2,26 (0-4) |

AML – acute myeloid leukemia; ALL – acute lymphoblastic leukemia; CLL – chronic lymphocytic leukemia; CML – chronic myeloid leukemia; CMML – chronic myelomonocytic leukemia; ECP – extracorporeal photopheresis; FL – follicular lymphoma; GVHD – graft-versus-host disease; Haplo – Haploidentical donor; HL – Hodgkin lymphoma; MAC – Myeloablative Conditioning; MCL – mantle cell lymphoma; MDS – myelodysplastic neoplasm; MF- myelofibrosis; MM – multiple myeloma; MSD – Matched Sibling Donor; MUD – matched unrelated donor; mMUD – mismatched unrelated donor; NHL – non-Hodgkin lymphoma; NMA – non-myeloablative conditioning; RIC – reduced intensity conditioning; RTC – reduced toxicity conditioning; SAA/PNH– severe aplastic anemia/ paroxysmal nocturnal hemoglobinuria

Patients' demography and clinical characteristics - TABLE

**Copyright Information:** (Online) ISSN: 2572-9241
©2026 The Author(s). HemaSphere is published by John Wiley & Sons Ltd on behalf of European Hematology Association. This is an open access Abstract Book distributed under the Attribution-NonCommercial-NoDerivs (CC BY-NC-ND), which allows third parties to download the articles and share them with others as long as they credit the author and the Abstract Book, but they cannot change the content in any way or use them commercially.
**Abstract Book Citations:** Authors, Title, HemaSphere, 2026;10:(S1):pages. The abstract book DOIs can be found on the "Author Information" page.
**Disclaimer:** Articles published in the journal HemaSphere exclusively reflect the opinions of the authors. The authors are responsible for all content in their abstracts including accuracy of the facts, statements, citing resources, etc.

### (PS2277) ABSOLUTE AND RELATIVE CD34+ COUNTS AS PREDICTORS OF MOBILIZATION SUCCESS: EXPERIENCE FROM A BRAZILIAN ONCOLOGY CENTER

**Topic:** 23. Stem cell transplantation - Clinical

Marcos Henrique Fogale Piccoli Silva*[1], Anita Cortez[1], Alfredo Mendrone Junior[1], Ana Paula de Jesus Geraldo[1], Mariana Serpa[1], Erick Xavier[1], Luciana Tucunduva[1], Aliana Meneses[1], Ana Rita Fonseca[1], Maria Cristina Seiwald[1], Yana Novis[1]

*[1]Sírio-Libanês Hospital, São Paulo, Brazil;*

### Background

Autologous stem cell transplantation (ASCT) is a standard therapeutic approach for patients with multiple myeloma, AL amyloidosis, and relapsed or refractory lymphomas. Successful transplantation requires adequate mobilization and collection of CD34+ hematopoietic progenitor cells (HPCs). However, up to 30% of patients experience poor mobilization due to prior therapies and reduced marrow reserve. Peripheral blood CD34+ enumeration is widely used to guide collection timing, but additional predictive markers are needed to optimize mobilization strategies and the cost-effective use of plerixafor.

### Aims

To evaluate absolute and relative peripheral blood CD34+ counts, as well as clinical and laboratory variables, as predictors of successful HPC collection in patients undergoing ASCT mobilization.

### Methods

We conducted a retrospective single-center study including adult patients who underwent peripheral blood HPC collection between 2017 and 2024 at a Brazilian transplant center. Patients received G-CSF mobilization with or without preemptive plerixafor. Clinical, laboratory, and procedural data were analyzed. Successful collection was defined as achieving $\geq 3\times10^6$ CD34+ cells/kg. Logistic regression and ROC curve analyses were performed to identify predictive factors.

### Results

A total of 128 collections were analyzed (median age 59.5 years; 68% male). Diagnoses included multiple myeloma (42%), lymphomas (54%), and AL amyloidosis (4%). Single-day collection success occurred in 72% of procedures and increased to 85% when multiple collection days were allowed. Plerixafor was required in 45% of patients and produced a significantly greater increase in circulating CD34+ cells compared with G-CSF alone ($p<0.001$). Absolute CD34+ count on the day of collection strongly predicted success, with a cutoff of 34 cells/mm³ (AUC 0.94; sensitivity 80%, specificity 99%). The relative CD34+/leukocyte ratio also showed high predictive value, with a cutoff ≥0.065% (AUC 0.919). Day-4 measurements were less discriminative. Patients with lymphoma not in complete remission and those requiring preemptive plerixafor had higher mobilization failure risk. Prior daratumumab exposure did not reduce final yield but increased the need for plerixafor.

### Summary/Conclusion

Peripheral blood CD34+ quantification remains the main predictor of HPC collection success. Our study demonstrates that the relative CD34+/leukocyte ratio is an additional practical biomarker with strong predictive performance, helping refine collection timing and guide rational plerixafor use in real-world transplant settings.

**Copyright Information:** (Online) ISSN: 2572-9241
©2026 The Author(s). HemaSphere is published by John Wiley & Sons Ltd on behalf of European Hematology Association. This is an open access Abstract Book distributed under the Attribution-NonCommercial-NoDerivs (CC BY-NC-ND), which allows third parties to download the articles and share them with others as long as they credit the author and the Abstract Book, but they cannot change the content in any way or use them commercially.
**Abstract Book Citations:** Authors, Title, HemaSphere, 2026;10:(S1):pages. The abstract book DOIs can be found on the "Author Information" page.
**Disclaimer:** Articles published in the journal HemaSphere exclusively reflect the opinions of the authors. The authors are responsible for all content in their abstracts including accuracy of the facts, statements, citing resources, etc.

### (PS2278) EFFECTS OF TWO ANTITHYMOCYTE GLOBULIN CONDITIONING REGIMENS ON IMMUNE RECONSTITUTION AFTER ALLOGENEIC HEMATOPOIETIC STEM CELL TRANSPLANTATION

**Topic:** 23. Stem cell transplantation - Clinical

Man Chen[1], Wei Zhao[1], Jing Long[1], Hui Wang*[2]

[1]*Beijing Lu Daopei Hospital, Beijing, China;* [2]*Hebei Yanda Lu Daopei Hospital, Langfang, China;*

### Background

Antithymocyte globulin (ATG) is commonly used in allogeneic hematopoietic stem cell transplantation (allo-HSCT) to prevent graft-versus-host disease (GVHD) via in vivo T-cell depletion. Two main preparations—rabbit ATG (rATG, Thymoglobulin) and anti-T-lymphocyte globulin (ATLG, Fresenius)—are available, but their differential effects on immune reconstitution, particularly on T-cell subsets and regulatory T cells (Tregs), remain incompletely understood. This study compares the impact of rATG (7.5 mg/kg) and ATLG (20 mg/kg) on immune recovery over 12 months post-transplant.

### Aims

To compare the impact of two ATG regimens—rATG (7.5 mg/kg) and ATLG (20 mg/kg)—on immune reconstitution within 12 months post-transplant.

### Methods

We analyzed 120 patients who underwent allo-HSCT between 2021 and 2025 (ALL=21, AML=68, MDS=31). Conditioning regimens were TBI/CY (n=23) or BU/CY (n=97). Patients received either rATG (Group G, n=77) or ATLG (Group F, n=43). Lymphocyte subsets, including $CD4^+$ and $CD8^+$ T-cell subpopulations (naïve, central memory [TCM], effector memory [TEM], terminally differentiated [TEMRA]) and Tregs, were monitored pre-transplant and at months 1–12 post-transplant.

### Results

**1.Baseline and Clinical Outcomes:** No significant differences were found between groups in baseline characteristics, engraftment, viral infections, or grade III-IV aGVHD (all P>0.05). Group G had a higher incidence of moderate-to-severe cGVHD (15.58% vs. 6.98%, P=0.172). Conditioning regimens differed significantly (P<0.001), with BU/CY more common in Group G (92.2%) and TBI/CY more frequent in Group F (39.5%).**2.Immune Reconstitution Patterns:** Two distinct patterns emerged. Group G exhibited a "quantitative expansion" pattern, with significantly higher $CD4^+$ and $CD8^+$ T-cell counts from month 4 onward ($CD4^+$: 5M P=0.003, 6M P=0.027, 7M P=0.002; $CD8^+$: 4M P=0.040, 5M P=0.003). In contrast, Group F maintained a T-cell pool with greater reserve and diversity. Group F had significantly higher proportions of naïve $CD4^+$ and $CD8^+$ T cells at multiple time points ($CD4^+$ naïve: 1–5M, 7M, 9M P<0.05; $CD8^+$ naïve: 1–7M, 9–10M P<0.01), while in Group G these proportions were mostly <1%. Central memory T cells (TCM) were also significantly higher in Group F ($CD4^+$ TCM: 1–6M, 9M P<0.01; $CD8^+$ TCM: 0–6M P<0.05). In contrast, Group G was dominated by effector memory T cells (TEM), with $CD4^+$ TEM often exceeding 90% and significantly higher than in Group F ($CD4^+$ TEM: 2–7M, 9M P<0.01; $CD8^+$ TEM: 2–6M P<0.05). Regarding regulatory T cells, the proportion of $CD4^+$ Tregs was significantly higher in Group F at multiple time points (1M P=0.000, 3M P=0.023, 4M P=0.021, 8M P=0.005, 10M P=0.002, 12M P=0.017), with an earlier onset and longer persistence, which may correlate with the lower incidence of cGVHD in this group.□fig.1□

**Copyright Information:** (Online) ISSN: 2572-9241
©2026 The Author(s). HemaSphere is published by John Wiley & Sons Ltd on behalf of European Hematology Association. This is an open access Abstract Book distributed under the Attribution-NonCommercial-NoDerivs (CC BY-NC-ND), which allows third parties to download the articles and share them with others as long as they credit the author and the Abstract Book, but they cannot change the content in any way or use them commercially.
**Abstract Book Citations:** Authors, Title, HemaSphere, 2026;10:(S1):pages. The abstract book DOIs can be found on the "Author Information" page.
**Disclaimer:** Articles published in the journal HemaSphere exclusively reflect the opinions of the authors. The authors are responsible for all content in their abstracts including accuracy of the facts, statements, citing resources, etc.

**Summary/Conclusion**

Group G achieved faster quantitative T-cell reconstitution, whereas Group F exhibited superior immune reserve and diversity, coupled with an early and sustained Treg advantage. This "quality-focused" reconstitution in Group F may underlie its lower cGVHD incidence. However, the potential impact of TBI on these immune differences warrants further investigation.

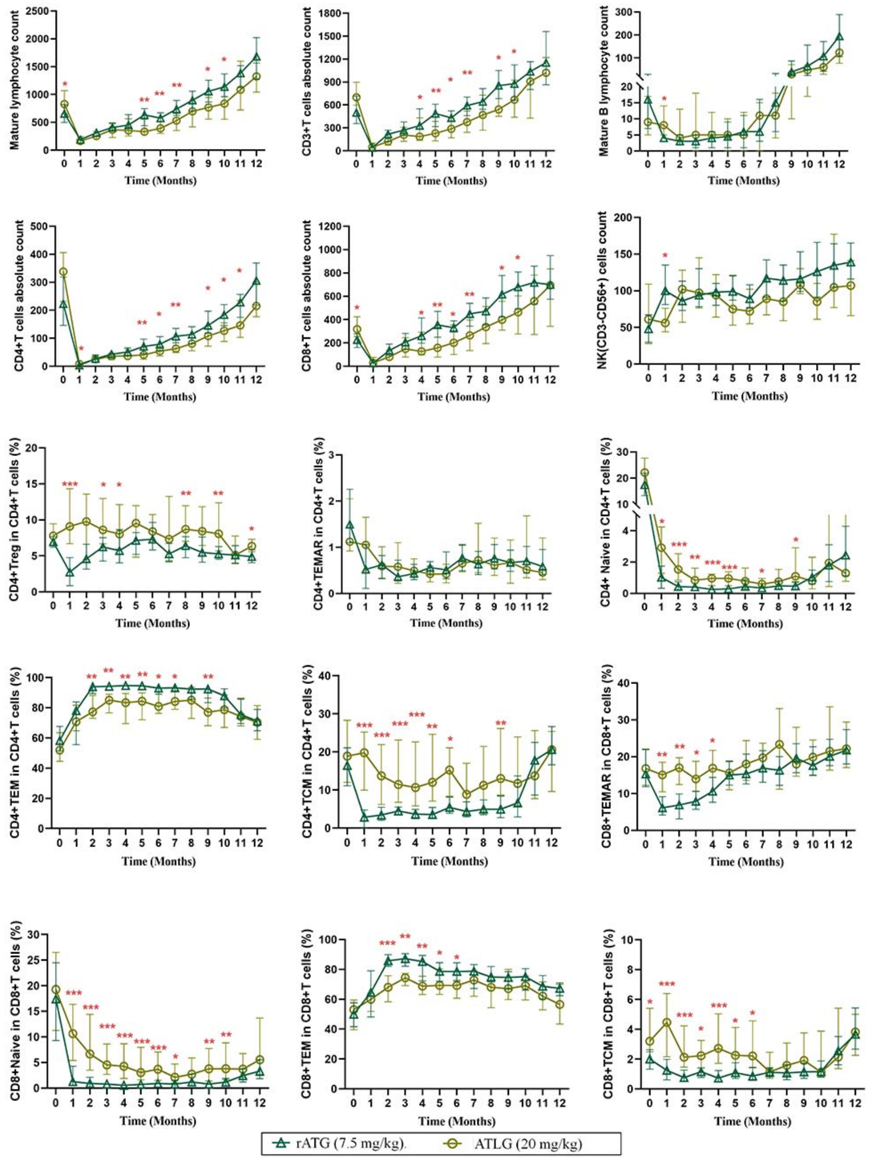

**Fig:** Immune reconstitution kinetics within 12 months post-transplant after ATLG (20 mg/kg) or rATG (7.5 mg/kg). Only lymphocyte subsets with significant intergroup differences are shown.

**Copyright Information:** (Online) ISSN: 2572-9241
©2026 The Author(s). HemaSphere is published by John Wiley & Sons Ltd on behalf of European Hematology Association. This is an open access Abstract Book distributed under the Attribution-NonCommercial-NoDerivs (CC BY-NC-ND), which allows third parties to download the articles and share them with others as long as they credit the author and the Abstract Book, but they cannot change the content in any way or use them commercially.
**Abstract Book Citations:** Authors, Title, HemaSphere, 2026;10:(S1):pages. The abstract book DOIs can be found on the "Author Information" page.
**Disclaimer:** Articles published in the journal HemaSphere exclusively reflect the opinions of the authors. The authors are responsible for all content in their abstracts including accuracy of the facts, statements, citing resources, etc.

### (PS2279) AI BASED FLT3-ITD SENSITIVITY PREDICTION AND ITS CORRELATION WITH CLINICAL OUTCOMES IN FLT3-ITD AML PATIENTS RECEIVED ALLOGENEIC HCT +/- FLT3 INHIBITOR MAINTENANCE

**Topic:** 23. Stem cell transplantation - Clinical

Seda Cakmak*[1], Azin Keshavarz[1], Sergio Rodriguez[1], Yomna Eissa[1, 2], Amirthahowri Ambalavanan[1], Oyeronke Ayansola[1], Dongkyu Kim[1], Eshrak Al-Shaibani[1], Tommy Alfaro-Moya[1], Igor Novitzky-Basso[1], Ivan Pasic[1], Fotios Michelis[1], Auro Viswabandya[1], Rajat Kumar[1], Jonas Mattsson[1], Jose-Mario Capochichi[1], Dennis Kim[1]

*[1]Princess Margaret Cancer Centre, Toronto, Canada;[2]London Health Sciences Centre, London, Canada;*

### Background

FLT3-internal tandem duplication (FLT3-ITD) AML carries a high relapse risk after allogeneic hematopoietic stem cell transplantation (alloHCT). FLT3 inhibitor (FLT3i) post-transplant maintenance (PTM) reduces relapse, yet 20–25% of patients relapse despite PTM. Preclinical data (Yi et al, ASH 2025) identified a pan-resistant subset of juxtamembrane segment (JMS)-spanning FLT3-ITD insertions conferring resistance to both FLT3i and chemotherapy; clinical correlations remain limited.

### Aims

To develop an FLT3-ITD molecular profile–based sensitivity prediction model and evaluate post-HCT outcomes according to variant pattern and FLT3i PTM use.

### Methods

We retrospectively analyzed 73 FLT3-ITD AML patients who underwent alloHCT (2019–2025) with available next-generation sequencing data. An AI-assisted Python algorithm classified FLT3-ITD variants based on insertion start/end codons and length into JMS-spanning pan-resistant variants (Pattern 1) and non-JMS variants (Pattern 2). JMS core involvement was defined as inclusion of residues aa579–592; TKD1 extension as ≥aa610. ITD length was available for 71 patients. Outcomes included cumulative incidence of relapse (CIR), relapse-free survival (RFS), and overall survival (OS), analyzed by variant pattern and PTM use.

### Results

Among 73 patients, 27 received FLT3i PTM (sorafenib n=23; gilteritinib n=4) and 46 did not. Median follow-up among survivors was 36 months. Three-year CIR, RFS, and OS were 29.8%, 54.9%, and 67.7%, respectively.
AI classification identified 21 patients with JMS-spanning variants (Pattern 1) and 52 with non-JMS variants (Pattern 2). Median FLT3-ITD allele frequency was 17% (range 1.1–86.6%). ITD microclones were detected in 7 cases; in all, the dominant clone was JMS-spanning.
Overall CIR did not differ significantly between Pattern 1 and Pattern 2 (3-year CIR 33.7% vs 25.9%, p=0.32). Within Pattern 2, PTM significantly reduced CIR compared with no PTM (12.9% vs 41.9%, p=0.0083). In contrast, among Pattern 1 patients, PTM did not significantly reduce CIR (25% vs 30%, p=0.5), although time to relapse appeared prolonged. No significant differences in OS or RFS were observed between patterns.

**Copyright Information:** (Online) ISSN: 2572-9241
©2026 The Author(s). HemaSphere is published by John Wiley & Sons Ltd on behalf of European Hematology Association. This is an open access Abstract Book distributed under the Attribution-NonCommercial-NoDerivs (CC BY-NC-ND), which allows third parties to download the articles and share them with others as long as they credit the author and the Abstract Book, but they cannot change the content in any way or use them commercially.
**Abstract Book Citations:** Authors, Title, HemaSphere, 2026;10:(S1):pages. The abstract book DOIs can be found on the “Author Information” page.
**Disclaimer:** Articles published in the journal HemaSphere exclusively reflect the opinions of the authors. The authors are responsible for all content in their abstracts including accuracy of the facts, statements, citing resources, etc.

### Summary/Conclusion

Despite high relapse risk nature of FLT3-ITD after HCT, FLT3i PTM could reduce CIR inPattern 2 (non-pan-resistant) variants (12.9% vs. 41.9%, p = 0.0083), but had no impact in Pattern 2 (pan-resistant) cases (25% vs. 30%, p = 0.5).
These preliminary findings support FLT3-ITD pattern-based risk stratification should be further investigated with larger number of patients' NGS data. It could optimize PTM use and highlight the need for novel strategies targeting JMS-pan-resistant variants to further mitigate relapse. Updated data with larger number of expanded cases will be presented in the EHA meeting.

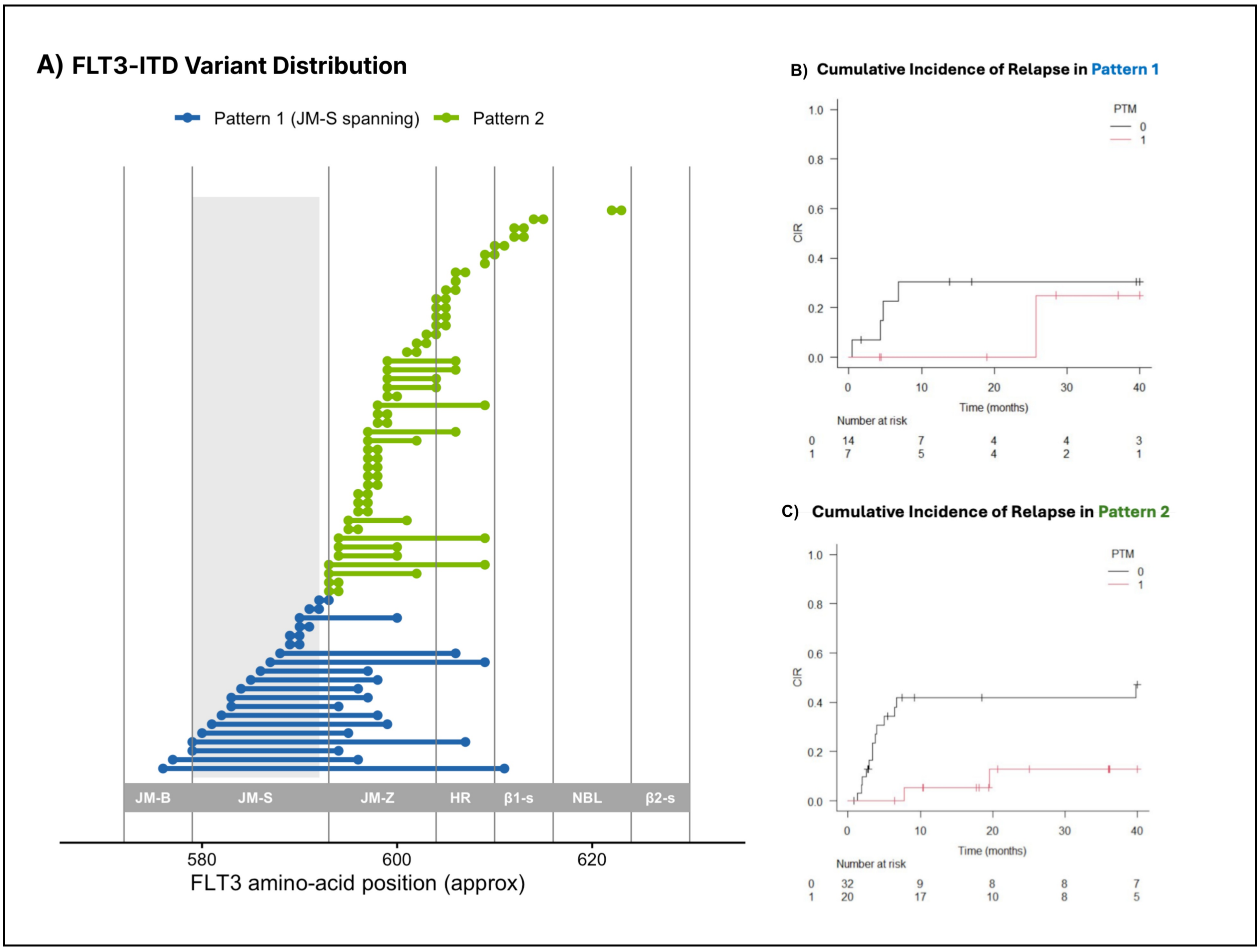

Figure A) Distribution of FLT3-ITD variants mapped along the juxtamembrane and adjacent domains, illustrating Pattern 1 (JM-S–spanning) and Pattern 2 variants. B) Cumulative incidence of relapse (CIR) among patients with Pattern 1 FLT3-ITD following allogeneic HSCT, stratified by receipt of post-transplant FLT3 inhibitor maintenance (PTM). Relapse rates were similar between groups (25% with PTM vs 30% without PTM; p = 0.5). C) CIR among patients with Pattern 2 FLT3-ITD stratified by PTM. PTM was associated with a significantly lower relapse incidence compared with no maintenance (12.9% vs 41.9%; p = 0.0083).

**Copyright Information:** (Online) ISSN: 2572-9241
©2026 The Author(s). HemaSphere is published by John Wiley & Sons Ltd on behalf of European Hematology Association. This is an open access Abstract Book distributed under the Attribution-NonCommercial-NoDerivs (CC BY-NC-ND), which allows third parties to download the articles and share them with others as long as they credit the author and the Abstract Book, but they cannot change the content in any way or use them commercially.
**Abstract Book Citations:** Authors, Title, HemaSphere, 2026;10:(S1):pages. The abstract book DOIs can be found on the "Author Information" page.
**Disclaimer:** Articles published in the journal HemaSphere exclusively reflect the opinions of the authors. The authors are responsible for all content in their abstracts including accuracy of the facts, statements, citing resources, etc.

### (PS2280) THE CRL4-DCAF13 UBIQUITIN E3 LIGASE MAINTAINS RIBOSOME BIOGENESIS AND PROTEIN TRANSLATION IN HEMATOPOIETIC STEM CELLS BY STABILIZING RRS1 THROUGH K27-LINKED NON-DEGRADATIVE UBIQUITINATION.

**Topic:** 24. Hematopoiesis, stem cells and microenvironment

Mengke Li*[1], Yuxin Wu[2], Shuai Zhou[2], Zhenyi Wang[3], Deyang Shi[1], Shengnan Yuan[4], Xiaojing Shi[5], Guangzhi Liu[1], Xiangli Chen[1], Rick Thorne[2], Dongping Wei[3], Zunmin Zhu[1], Song Chen[1]

*[1]Institute of Hematology, Henan Key Laboratory of Stem Cell Differentiation and Modification, Henan Provincial People's Hospital, Zhengzhou University People's Hospital, Zhengzhou, China; [2]Translational Research Institute, Henan International Join Laboratory of Non-coding RNA and Metabolism in Cancer, Henan Provincial People's Hospital, Academy of Medical Sciences, Zhengzhou University, Zhengzhou, China; [3]Medical Research Center, The First Affiliated Hospital of Wenzhou Medical University, Wenzhou, China; [4]School of Medical Technology, Xuzhou Medical University, Xuzhou, China;[5]Laboratory Animal Center, Academy of Medical Sciences, Zhengzhou University, Zhengzhou, China;*

### Background

Hematopoietic stem cells (HSCs) balance quiescence, proliferation, and differentiation to sustain lifelong blood production. Ribosome biogenesis is increasingly recognized as critical for stem cell fate, as mutations in ribosomal components cause bone marrow failure syndromes like Diamond-Blackfan anemia and Shwachman-Diamond syndrome, underscoring HSC sensitivity to ribosomal perturbations. DCAF13, a nucleolar substrate receptor for the CUL4-DDB1 E3 ubiquitin ligase, may participate in ribosome biogenesis, yet its specific role in HSC regulation remains poorly understood.

### Aims

This study aims to systematically delineate the role of Dcaf13 in hematopoiesis and elucidate how DCAF13 couples ribosome biogenesis to HSC homeostasis.

### Methods

To investigate the role of DCAF13 in both embryonic and adult hematopoiesis, we generated conditional knockout mice by crossing *Dcaf13*-floxed mice with Vav-iCre and Mx1-Cre transgenic lines. Following *Dcaf13* deletion, we integrated single-cell RNA sequencing with polysome profiling to assess changes in transcriptomic dynamics, ribosome biogenesis, and translational activity in HSCs. To further dissect the molecular mechanism, quantitative proteomics was employed to screen for potential substrates of DCAF13, and ubiquitination assays were performed to map specific modification sites. Together, these approaches aim to elucidate the DCAF13–substrate–ribosome regulatory axis governing HSC homeostasis.

**Copyright Information:** (Online) ISSN: 2572-9241
©2026 The Author(s). HemaSphere is published by John Wiley & Sons Ltd on behalf of European Hematology Association. This is an open access Abstract Book distributed under the Attribution-NonCommercial-NoDerivs (CC BY-NC-ND), which allows third parties to download the articles and share them with others as long as they credit the author and the Abstract Book, but they cannot change the content in any way or use them commercially.
**Abstract Book Citations:** Authors, Title, HemaSphere, 2026;10:(S1):pages. The abstract book DOIs can be found on the "Author Information" page.
**Disclaimer:** Articles published in the journal HemaSphere exclusively reflect the opinions of the authors. The authors are responsible for all content in their abstracts including accuracy of the facts, statements, citing resources, etc.

**Results**

Using the Vav-iCre system, we found that Dcaf13 deficiency resulted in perinatal lethality, accompanied by pale and hypoplastic fetal livers, indicating a profound impairment of fetal liver hematopoiesis. In adult mice, acute deletion of Dcaf13 via the Mx1-Cre system induced rapid bone marrow failure, with severe pancytopenia and depletion of HSPCs. Dcaf13-konckout HSCs also exhibited cell cycle arrest, increased apoptosis, and impaired BM reconstitution capacity.
Mechanistically, Dcaf13 deletion activated the P53 signaling pathway. Based on reports that DCAF13 can ubiquitinate and degrade P53, we generated *Mx1-Cre; Dcaf13*$^{fl/fl}$ *Trp53*$^{fl/fl}$ mice to determine whether hematopoietic failure was mediated by P53 activation. Trp53 ablation partially restored HSC numbers and improved cell cycle progression in Dcaf13-deficient HSC; however, it failed to prevent lethal hematopoietic failure, indicating the involvement of both p53-dependent and p53-independent mechanisms.
Notably, Dcaf13-deficient HSCs exhibited profound defects in ribosome biogenesis and global protein synthesis. To define the mechanism whereby DCAF13 controls ribosome homeostasis, proteomic analyses identified that Dcaf13 deletion significantly reduced protein levels of RRS1, a key ribosome biogenesis factor, without affecting its mRNA expression. GST pull-down and Co-IP confirmed a direct physical interaction between DCAF13 and RRS1. CHX assays showed that DCAF13 knockout reduced the half-life of RRS1, and ubiquitination assays revealed that DCAF13 mediates non-degradative K27-linked ubiquitination of RRS1. This modification stabilizes RRS1 to support proper ribosome assembly and protein translation in HSC. Consistently, knockdown of RRS1 significantly reduced the rate of protein synthesis.

**Summary/Conclusion**

In summary, our study identifies DCAF13 as an essential regulator of HSC maintenance through the DCAF13-RRS1 axis. These findings provide novel insights into the molecular mechanisms underlying HSC homeostasis and hematopoietic failure.

**Copyright Information:** (Online) ISSN: 2572-9241
©2026 The Author(s). HemaSphere is published by John Wiley & Sons Ltd on behalf of European Hematology Association. This is an open access Abstract Book distributed under the Attribution-NonCommercial-NoDerivs (CC BY-NC-ND), which allows third parties to download the articles and share them with others as long as they credit the author and the Abstract Book, but they cannot change the content in any way or use them commercially.
**Abstract Book Citations:** Authors, Title, HemaSphere, 2026;10:(S1):pages. The abstract book DOIs can be found on the "Author Information" page.
**Disclaimer:** Articles published in the journal HemaSphere exclusively reflect the opinions of the authors. The authors are responsible for all content in their abstracts including accuracy of the facts, statements, citing resources, etc.

### (PS2281) LEUKEMIC STEM CELLS ORCHESTRATE TGFB-DEPENDENT BONE MARROW NICHE REMODELING IN AML XENOGRAFT MODELS

**Topic:** 24. Hematopoiesis, stem cells and microenvironment

Karin Prummel*[1], Anna Mathioudaki[1, 2], Ivan Berest[1, 3], Shubhankar Sood[4], Lixiazi He[2], Thorsten Richter[5], Yagmur Baskan[1], Jonas Rauchhaus[1], Clarissa Holitsch[2], Rim Moussa[1], Robert Reinhardt[6], Simon Renders[2], Sinem Saka[6], Konstantinos Kokkaliaris[5], Marieke Essers[4], Caroline Pabst[2], Judith Zaugg[1, 3]

*[1]European Molecular Biology Laboratory, Molecular Systems Biology, Heidelberg, Germany; [2]University Hospital Heidelberg, Department of Medicine V, Hematology, Oncology and Rheumatology, Heidelberg, Germany; [3]Basel University, Department of Biomedicine, Basel, Switzerland; [4]Heidelberg Institute for Stem Cell Technology and Experimental Medicine (HI-STEM GmbH), Heidelberg, Germany; [5]Goethe University, Institute of Pathology, Frankfurt, Germany;[6]European Molecular Biology Laboratory, Genome Biology, Heidelberg, Germany;*

### Background

The bone marrow (BM) niche, comprising stromal and endothelial cells, regulates hematopoietic stem cell (HSC) fate and is a major determinant of therapy resistance and relapse in acute myeloid leukemia (AML). Leukemic stem cells (LSCs) are thought to remodel their microenvironment to support disease persistence; however, which cell populations and signaling pathways contribute to LSC-mediated niche remodeling remain incompletely defined.

### Aims

To determine how distinct LSC subsets drive BM stromal and vascular remodeling in AML and to identify key signaling pathways mediating leukemic niche reprogramming.

### Methods

We previously identified the transcription factor HLF as a key regulator of LSCs in NPM1/DNMT3A/FLT3-ITD AML; HLF knockout (KO) reduces CD34+ slowly cycling, therapy-resistant LSCs. To model high vs low LSC frequency ($LSC^{high}/LSC^{low}$), we compared isogenic xenografts with HLF KO vs WT in NSGW41 mice. Healthy CD34+ HSCs served as controls. Mouse hematopoietic, human leukemic, and mouse BM niche populations were enriched by flow cytometry followed by scRNA-seq. Cell-cell communication (*MultiNicheNet*) and gene regulatory network analyses (*SCENIC, GRaNPA*) were performed. Histology and multiplexed imaging of femurs assessed LSC localization and overall BM architecture. We validated findings with primary AML-multipotent stromal cell (MSC) co-cultures, subjected to scRNA-seq and pharmacological perturbations.

**Copyright Information:** (Online) ISSN: 2572-9241
©2026 The Author(s). HemaSphere is published by John Wiley & Sons Ltd on behalf of European Hematology Association. This is an open access Abstract Book distributed under the Attribution-NonCommercial-NoDerivs (CC BY-NC-ND), which allows third parties to download the articles and share them with others as long as they credit the author and the Abstract Book, but they cannot change the content in any way or use them commercially.
**Abstract Book Citations:** Authors, Title, HemaSphere, 2026;10:(S1):pages. The abstract book DOIs can be found on the "Author Information" page.
**Disclaimer:** Articles published in the journal HemaSphere exclusively reflect the opinions of the authors. The authors are responsible for all content in their abstracts including accuracy of the facts, statements, citing resources, etc.

### Results

AML engraftment markedly altered BM niche composition, characterized by expansion of *Cxcl12*-abundant reticular adipogenic stromal cells (Adipo-CAR) and reduction in osteo-lineage populations. These changes were significantly more pronounced in LSC$^{high}$ vs LSC$^{low}$ AML and were accompanied by expansion of *Fmod+* and *Cd34+* fibroblasts in LSC$^{high}$ AML. The *Fmod+* fibroblast signature was also elevated in MSC single-cell data from AML patients with NPM1/FLT3-ITD mutations.
Cell-cell communication analysis identified TGFβ as the strongest LSC-associated ligand. We identified particular LSC populations, including slow-cycling LSCs, with high *TGFB1* expression. Stromal populations enriched in LSC$^{high}$ BM, in particular *Fmod+* fibroblasts, displayed high TGFβ signaling activity (KEGG) and high expression of *Tgfbr1/2/3* and downstream *Smad2/3* signaling signatures. We further identified the TGFβ-responsive transcriptional regulator Creb3l1 as a key regulator of the transitions towards the pro-fibrotic *Fmod+* fibroblast state.
In MSC–AML co-cultures, LSC$^{high}$ AML suppressed osteogenic programs and induced extracellular matrix genes in MSCs, recapitulating the *in vivo* findings. Moreover, pharmacologic inhibition of TGFβ signaling (SB431542, SD-208) or CREB3L1 activation in MSCs significantly increased AML cell abundance in the culture supernatant compared to controls, indicating that TGFβ-CREB3L1 activity in MSCs modulates leukemic cell adhesion to their microenvironment.

### Summary/Conclusion

Our study identifies LSCs as key orchestrators of BM niche remodeling via a TGFβ–CREB3L1 signaling axis that promotes fibrotic stromal states. Functional perturbation demonstrates that this pathway affects leukemic cell adhesion to the stromal niche. Integration of single-cell, gene regulatory network, and spatial analyses reveals how distinct AML subpopulations actively reprogram the BM niche, highlighting the LSC–TGFβ-niche axis as a potential therapeutic target in AML.

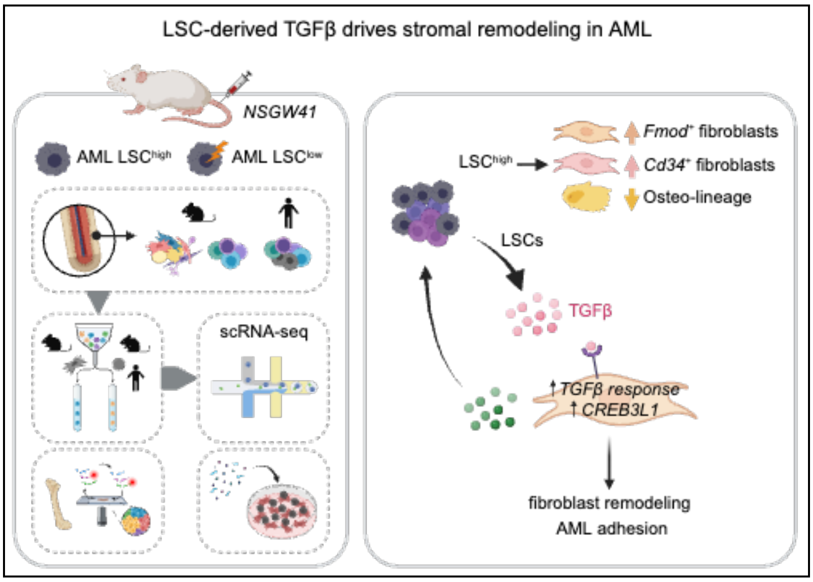

NSGW41 AML xenograft models reveal an LSC-driven TGFβ–CREB3L1 signaling axis that promotes bone marrow niche remodeling.

**Copyright Information:** (Online) ISSN: 2572-9241
©2026 The Author(s). HemaSphere is published by John Wiley & Sons Ltd on behalf of European Hematology Association. This is an open access Abstract Book distributed under the Attribution-NonCommercial-NoDerivs (CC BY-NC-ND), which allows third parties to download the articles and share them with others as long as they credit the author and the Abstract Book, but they cannot change the content in any way or use them commercially.
**Abstract Book Citations:** Authors, Title, HemaSphere, 2026;10:(S1):pages. The abstract book DOIs can be found on the "Author Information" page.
**Disclaimer:** Articles published in the journal HemaSphere exclusively reflect the opinions of the authors. The authors are responsible for all content in their abstracts including accuracy of the facts, statements, citing resources, etc.

### (PS2282) MONOCYTE AND LYMPHOID LINEAGES BIFURCATE FROM COMMON PRECURSORS DISTINCT FROM NEUTROPHIL LINEAGE DURING HEMATOPOIESIS

**Topic:** 24. Hematopoiesis, stem cells and microenvironment

Sujin Chen[1], Yingyun Fu[1], Guoqiang Li[2], Yongjian Yue*[1]

*[1]Shenzhen Institute of Respiratory Disease, Shenzhen People's Hospital (The First Affliated Hospital, Southern University of Science and Technology, Shenzhen, China; [2]Department of Hematology, Shenzhen People's Hospital, Shenzhen, China;*

### Background

Classical hierarchy models proposed that neutrophils and monocytes originate from common precursors of granulocyte-macrophage progenitors (GMPs) or common myeloid progenitors (CMPs) during hematopoiesis. However, early differentiation trajectories proposed by more recent updated hierarchy models remain ambiguous and inconsistent, leaving the ontogenic relationship between neutrophils and monocytes unclear.

### Aims

This study aimed to clarify the developmental origin and differentiation trajectories of neutrophil and monocyte lineages during hematopoiesis.

### Methods

Leveraging a previously refined trilineage hierarchy model of hematopoietic progenitor cells, we revealed the direct commitment of neutrophil progenitors (NMPs) from early multipotent progenitors (MPCs), distinct from lymphoid lineages (Figure 1). HOPX and GATA2 were identified as key regulators during the first bifurcation of lineage commitment. Here, using HOPX-based lineage tracing, we revealed the developmental origin and differentiation trajectory of neutrophils and monocytes.

### Results

Lineage-tracing by conditional Hopx-CreERT2; Rosa26-LSL-tdTomato mice results demonstrated that no detectable tdTomato signal was observed in neutrophils and erythroid cells, which aligns with the established view that CLP-derived lineages are independent of neutrophil and erythroid commitment during hematopoiesis. Interestingly, HOPX-derived tdTomato positive cells are largely restricted to lymphoid and monocyte lineages. Ex vivo primary macrophage cultures derived from bone marrow cells yielded consistent findings with *in vivo* observations. Moreover, we proposed that the classical defined CLPs, redefined here as lymphoid-monocyte progenitors (LMPs), likely represented the common ancestor for both monocyte and lymphoid lineages (Figure 1).

### Summary/Conclusion

Collectively, monocyte and lymphoid lineages bifurcate from a common precursor distinct from the neutrophil lineage during hematopoiesis. Our findings challenge the prevailing hematopoietic dogma and open new avenues for understanding lineage specification. This study provides new perspectives for the precise control of progenitor cell differentiation and offers insights for advances in immunology modulation.

**Copyright Information:** (Online) ISSN: 2572-9241
©2026 The Author(s). HemaSphere is published by John Wiley & Sons Ltd on behalf of European Hematology Association. This is an open access Abstract Book distributed under the Attribution-NonCommercial-NoDerivs (CC BY-NC-ND), which allows third parties to download the articles and share them with others as long as they credit the author and the Abstract Book, but they cannot change the content in any way or use them commercially.
**Abstract Book Citations:** Authors, Title, HemaSphere, 2026;10:(S1):pages. The abstract book DOIs can be found on the "Author Information" page.
**Disclaimer:** Articles published in the journal HemaSphere exclusively reflect the opinions of the authors. The authors are responsible for all content in their abstracts including accuracy of the facts, statements, citing resources, etc.

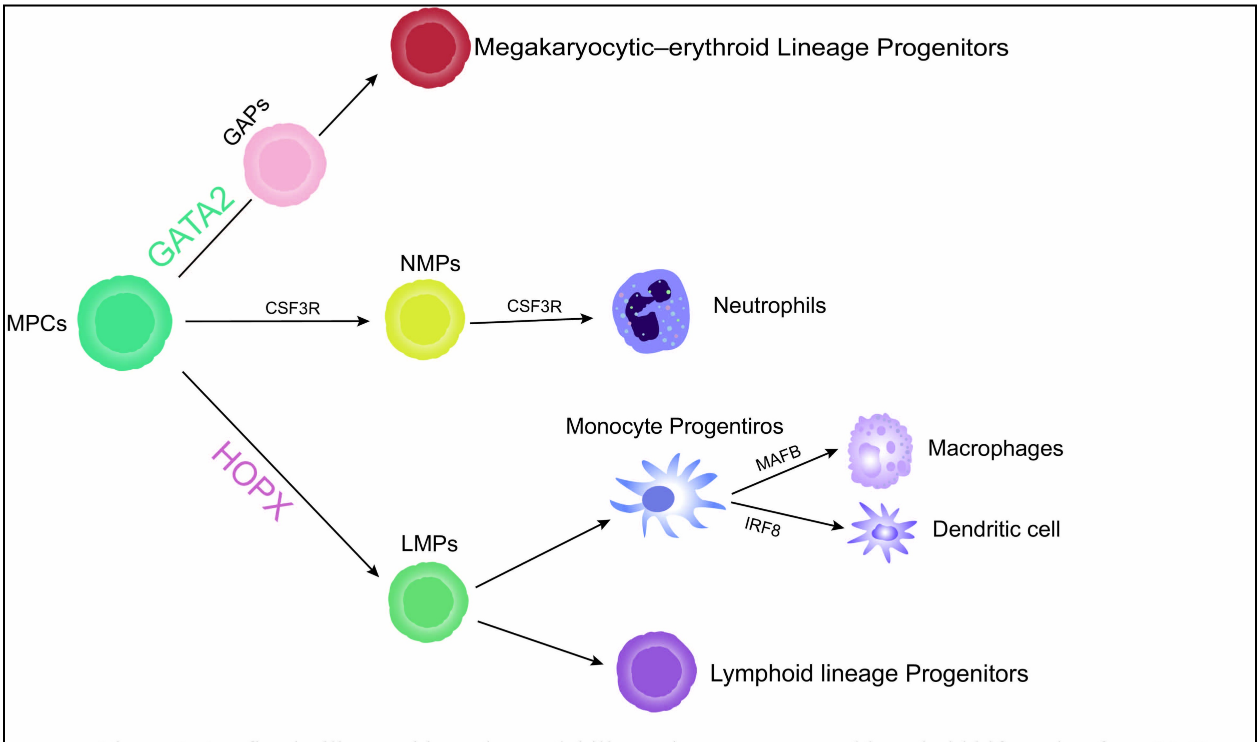

Figure 1, A refined trilineage hierarchy model illustrating monocyte and lymphoid bifurcation from LMPs

Figure 1, A refined trilineage hierarchy model illustrating monocyte and lymphoid bifurcation from LMPs

**Copyright Information:** (Online) ISSN: 2572-9241
©2026 The Author(s). HemaSphere is published by John Wiley & Sons Ltd on behalf of European Hematology Association. This is an open access Abstract Book distributed under the Attribution-NonCommercial-NoDerivs (CC BY-NC-ND), which allows third parties to download the articles and share them with others as long as they credit the author and the Abstract Book, but they cannot change the content in any way or use them commercially.
**Abstract Book Citations:** Authors, Title, HemaSphere, 2026;10:(S1):pages. The abstract book DOIs can be found on the "Author Information" page.
**Disclaimer:** Articles published in the journal HemaSphere exclusively reflect the opinions of the authors. The authors are responsible for all content in their abstracts including accuracy of the facts, statements, citing resources, etc.

### (PS2283) INFLAMED BONE MARROW STROMAL NICHES DRIVE A HIGH-ENTROPY INFLAMMATORY-STEMNESS STATE AND HEMATOPOIETIC STEM CELL DYSFUNCTION IN SICKLE CELL DISEASE

**Topic:** 24. Hematopoiesis, stem cells and microenvironment

Greta Zara[1], Konstancja Urbaniak[2], Charmaine Soco[1], Denis O'meally[3], Karl Gaal[4], Vikram Adhikarla[2], Diana Browning[5], Anna Pawlowska[6], Joseph Rosenthal[6], Angelo Cardoso[5], Sergio Branciamore[2], Nadia Carlesso*[1]

*[1]Department of Stem Cell Biology and Regenerative Medicine, Beckman Research Institute of City of Hope, Duarte, United States of America; [2]Department of Computational and Quantitative Medicine, Beckman Research Institute of City of Hope, Duarte, United States of America; [3]Department of Diabetes and Cancer Discovery, Beckman Research Institute, City of Hope, Duarte, United States of America; [4]Department of Pathology, Beckman Research Institute, City of Hope, Duarte, United States of America; [5]Center for Gene Therapy, Beckman Research Institute, City of Hope, Duarte, United States of America; [6]Department of Pediatrics, City of Hope Medical Center, Duarte, United States of America;*

### Background

Sickle cell disease (SCD) is marked by chronic inflammation and impaired bone marrow (BM) homeostasis, but how these pressures remodel human hematopoietic stem cells (HSCs) and the stromal niche remains unresolved. Through integrated multimodal profiling of 12 SCD patients undergoing hematopoietic cell transplantation (HCT), we identify that SCD HSCs converge into a high-entropy inflammatory-stemness state driven by an inflamed stromal microenvironment.

### Aims

To define cellular and molecular alterations in the SCD BM microenvironment; characterize transcriptional and regulatory rewiring of SCD HSCs; determine how mesenchymal stromal cells (MSCs) and inflammatory plasma contribute to HSC stress programs; and assess BM and HSC recovery following HCT.

### Methods

We analyzed BM biopsies, BM plasma cytokines (34-plex Luminex), immunophenotypes of HSPCs and stromal subsets, and MSC transcriptomes in 9–12 SCD patients. Single-cell RNA sequencing of $CD34^{+}$ cells from SCD (n=3) and age-matched healthy donors (HD; n=3) yielded 11,129 SCD and 19,825 HD transcriptomes, validated using a public dataset (HD n=4; SCD n=4). Regulatory rewiring was assessed by Bayesian network modeling. Functional assays included exposure of HD $CD34^{+}$ cells to SCD plasma or co-culture with SCD MSCs.

**Copyright Information:** (Online) ISSN: 2572-9241
©2026 The Author(s). HemaSphere is published by John Wiley & Sons Ltd on behalf of European Hematology Association. This is an open access Abstract Book distributed under the Attribution-NonCommercial-NoDerivs (CC BY-NC-ND), which allows third parties to download the articles and share them with others as long as they credit the author and the Abstract Book, but they cannot change the content in any way or use them commercially.
**Abstract Book Citations:** Authors, Title, HemaSphere, 2026;10:(S1):pages. The abstract book DOIs can be found on the “Author Information” page.
**Disclaimer:** Articles published in the journal HemaSphere exclusively reflect the opinions of the authors. The authors are responsible for all content in their abstracts including accuracy of the facts, statements, citing resources, etc.

**Results**

SCD BM showed reduced cellularity (0.9–12 × $10^6$ vs. 12–17 × $10^6$ cells/mL in HD) and expansion of $CD45^-CD31^+$ endothelium and $CD45^-CD105^+$ stroma. PCA of 32 cytokines separated all SCD from HD, with elevations in Angiopoietin-2, IL-27, IL-18, TNF-α, and S100A8 ($p<0.01$). This inflammatory milieu corresponded to reduced $CD34^+$ progenitors ($3\times10^4$ vs. $1\times10^5$), HSCs ($CD90^+$; $1.1\times10^3$ vs. $8.4\times10^3$), and overall progenitors ($p<0.01$–0.001).
scRNA-seq identified 1,384 up- and 900 down-regulated genes in SCD HSCs, with activation of AP-1/MAPK, TLR (TLR2/4, MYD88), and DDR pathways (ATM, TP53, BRCA1/2). Stemness regulators (MEIS1, MSI2, PRDM16, IGF1R, NOTCH2) were paradoxically increased, while FTH1 and JUNB/JUND were reduced. PCA of an 88-gene supersignature resolved two HD HSC states but only one in SCD, indicating collapse of quiescence and convergence into a single high-entropy inflammatory-stemness state. Bayesian modeling showed increased regulatory entropy with MSI2 and FTH1 as rewired hubs. SCD MSCs displayed a pro-inflammatory phenotype with elevated VEGFA, miR-155, and IL6/IL8/IL1B/IL10 (4–30-fold) and reduced SCF, JAG1, and CXCL12 (~50%). HD MSCs exposed to SCD plasma adopted similar signatures. Co-culture experiments showed that SCD MSCs induced hybrid inflammatory-stress programs in HD $CD34^+$ cells, partially recapitulating SCD HSC features.
At one year post-HCT, MSC profiles partially normalized (IL7, IL10, JUNB, BCL2 restored), cytokines shifted toward HD patterns, and most HSC supersignature genes recovered, though SCF, JAG1, and some donor HSC stress features persisted.

**Summary/Conclusion**

SCD generates a chronically inflamed, cytokine-rich niche that drives HSCs into a high-entropy inflammatory-stemness state marked by AP-1/TLR activation, DDR induction, loss of quiescence, and stressed self-renewal. Stromal dysfunction reinforces these programs, weakening HSC stability. This inflammatory–DDR–stemness convergence provides mechanistic insight into elevated MDS/AML risk in SCD and reveals intrinsic deficits in HSC fitness relevant to gene therapy. HCT improves—but does not fully restore—niche signaling or HSC states, identifying residual biomarkers important for therapeutic responsiveness and malignant risk assessment.

**Copyright Information:** (Online) ISSN: 2572-9241
©2026 The Author(s). HemaSphere is published by John Wiley & Sons Ltd on behalf of European Hematology Association. This is an open access Abstract Book distributed under the Attribution-NonCommercial-NoDerivs (CC BY-NC-ND), which allows third parties to download the articles and share them with others as long as they credit the author and the Abstract Book, but they cannot change the content in any way or use them commercially.
**Abstract Book Citations:** Authors, Title, HemaSphere, 2026;10:(S1):pages. The abstract book DOIs can be found on the “Author Information” page.
**Disclaimer:** Articles published in the journal HemaSphere exclusively reflect the opinions of the authors. The authors are responsible for all content in their abstracts including accuracy of the facts, statements, citing resources, etc.

### (PS2284) IMPAIRED INTERFERON RESPONSE IN BRAFV600E MYELOID PROGENITORS OF LANGERHANS CELL HISTIOCYTOSIS

**Topic:** 24. Hematopoiesis, stem cells and microenvironment

Maki Sakuma*[1], Wouter van Midden[1], Raphaela Schwentner[1], Giulio Abagnale[1], Philipp Ben Soussia-Weiss[1], Patrick Say[1], Caroline Hutter[1]

[1]*St. Anna Children's Cancer Research Institute, Vienna, Austria;*

### Background

Langerhans cell histiocytosis (LCH) is a myeloid neoplasm primarily driven by the *BRAF*$^{V600E}$ mutation in hematopoietic stem and progenitor cells (HSPCs), frequently associated with regulatory T cell infiltration in lesions. Emerging evidence suggests that inflammatory signals at the HSPC stage create durable transcriptional imprints that shape downstream myeloid function (LaMarche, *Nature* 2024). While *BRAF*$^{V600E}$-mutated HSPCs are inflammatory, it remains unknown whether this progenitor-stage inflammation affects effector myeloid cells and their capacity to shape the tumor microenvironment (TME).

### Aims

To characterize the transcriptional and functional consequences of *BRAF*$^{V600E}$ on dendritic cell (DC) maturation using an isogenic iPSC-based LCH model (Abagnale, *Blood* 2025).

### Methods

*BRAF*$^{V600E}$ and wildtype (WT) iPSCs were differentiated into CD34+ progenitors and myeloid cells. Myeloid cells were stimulated with LPS (6h) or left unstimulated, followed by sorting of CD14, CD1c, and CD1a (mutant only) populations for bulk RNA sequencing. To compare *in vitro* and *in vivo* data, activated myeloid cells were computationally extracted from patient scRNA-seq datasets for comparison, using CellTypist immune models and CD83 expression.

**Copyright Information:** (Online) ISSN: 2572-9241
©2026 The Author(s). HemaSphere is published by John Wiley & Sons Ltd on behalf of European Hematology Association. This is an open access Abstract Book distributed under the Attribution-NonCommercial-NoDerivs (CC BY-NC-ND), which allows third parties to download the articles and share them with others as long as they credit the author and the Abstract Book, but they cannot change the content in any way or use them commercially.
**Abstract Book Citations:** Authors, Title, HemaSphere, 2026;10:(S1):pages. The abstract book DOIs can be found on the "Author Information" page.
**Disclaimer:** Articles published in the journal HemaSphere exclusively reflect the opinions of the authors. The authors are responsible for all content in their abstracts including accuracy of the facts, statements, citing resources, etc.

### Results

We identified 371 LPS-responsive differentially expressed genes across all conditions. PCA on these genes revealed that *BRAF*$^{V600E}$ cells exhibited a consistently attenuated response along PC1, dominated by interferon-stimulated genes (ISGs), regardless of cell type (**Figure 1A**). While mutant cells showed a non-significant but consistently higher baseline expression of the IFN pathway genes, the LPS-induced upregulation of ISGs (*ISG15, IFITs, MXs, OASL*) and antigen-processing genes (*TAP1, PSMB9*) was significantly impaired, particularly at the CD14 stage (**Figure 1BC**). Notably, the induction of *IRF1* upon LPS stimulation was abolished in mutant cells (**Figure 1C**). This phenotype resembles immune paralysis or endotoxin tolerance, where a second LPS challenge results in an attenuated IFN response (Wolk, *Blood* 2000; Keramati, *Nat. Immunol.* 2025). We further investigated this tolerogenic behavior regarding mature regulatory DCs (mregDCs), a conserved mature stage of DC subtypes marked by *CCR7, LAMP3, CD274, CCL19* and *CCL22*, which are known to migrate to the draining lymph nodes to attract T cells but can also be immunosuppressive. In our bulk data, *BRAF*$^{V600E}$ cells upregulated *CCL22,* a mediator of DC-Treg interaction representing the immunosuppressive side of mregDC, prior to LPS stimulation, while other mregDC markers showed attenuated upregulation at the CD14 stage (**Figure 1BC**). Characterization of mregDCs in LCH scRNA-seq (Halbritter, *Cancer Discovery* 2019 and new data for this study) confirmed that *CCL22* expression was not confined to the mregDC population but was also expressed by non-mregDC cells. This broad *CCL22* expression was absent in melanoma (Yang, *Nat. Comm.* 2025) and lung cancer datasets (Liu, *Cell* 2025), where *CCL22* remained comparatively restricted to the mregDC cluster. Thus, tolerogenic DC fate may be imprinted at the DC progenitor stage in LCH.

### Summary/Conclusion

*BRAF*$^{V600E}$ induces chronic inflammatory changes in LCH progenitors, imprinting a cell-intrinsic, differentiation-independent defect in interferon signaling and altered mregDC maturation trajectory. These findings suggest that the LCH TME is shaped by the inflammatory history of clonal progenitors. More broadly, we demonstrate how inflammatory clonal myelopoiesis may impair anti-tumor immunity.

**Copyright Information:** (Online) ISSN: 2572-9241
©2026 The Author(s). HemaSphere is published by John Wiley & Sons Ltd on behalf of European Hematology Association. This is an open access Abstract Book distributed under the Attribution-NonCommercial-NoDerivs (CC BY-NC-ND), which allows third parties to download the articles and share them with others as long as they credit the author and the Abstract Book, but they cannot change the content in any way or use them commercially.
**Abstract Book Citations:** Authors, Title, HemaSphere, 2026;10:(S1):pages. The abstract book DOIs can be found on the "Author Information" page.
**Disclaimer:** Articles published in the journal HemaSphere exclusively reflect the opinions of the authors. The authors are responsible for all content in their abstracts including accuracy of the facts, statements, citing resources, etc.

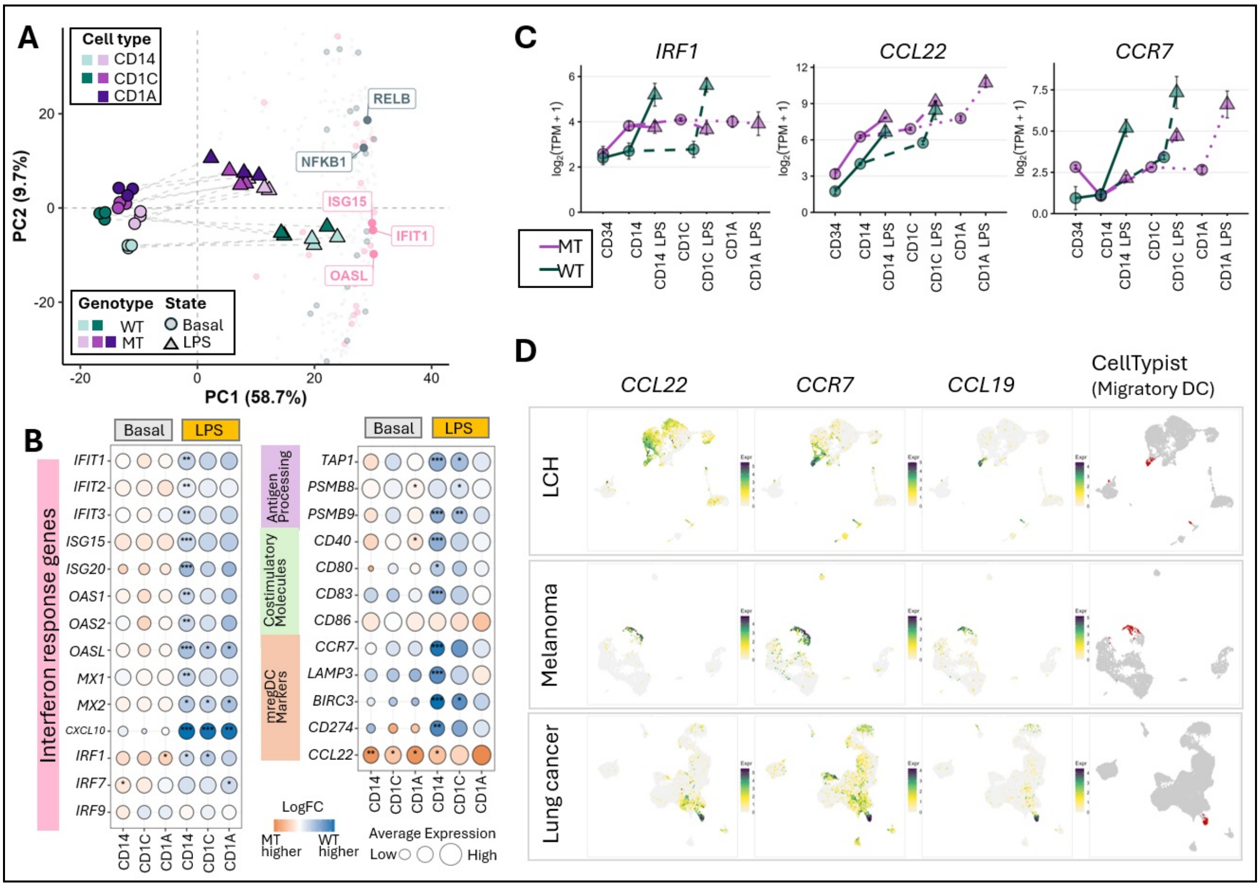

(A) Supervised PCA across all cell types and conditions. Each point represents a sample; PC loadings (gene contributions) are shown as smaller dots. (B) LogFC of DEG comparing wild type and mutant across cell type and conditions. (C) Log2-TPM of representative gene expression across the differentiation and stimulation trajectory. (D) Gene expression level of genes on UMAP and mregDC annotated cells.

**Copyright Information:** (Online) ISSN: 2572-9241
©2026 The Author(s). HemaSphere is published by John Wiley & Sons Ltd on behalf of European Hematology Association. This is an open access Abstract Book distributed under the Attribution-NonCommercial-NoDerivs (CC BY-NC-ND), which allows third parties to download the articles and share them with others as long as they credit the author and the Abstract Book, but they cannot change the content in any way or use them commercially.
**Abstract Book Citations:** Authors, Title, HemaSphere, 2026;10:(S1):pages. The abstract book DOIs can be found on the "Author Information" page.
**Disclaimer:** Articles published in the journal HemaSphere exclusively reflect the opinions of the authors. The authors are responsible for all content in their abstracts including accuracy of the facts, statements, citing resources, etc.

### (PS2285) CHROMATIN ACCESSIBILITY ENCODES LINEAGE PLASTICITY IN FIRST-TRIMESTER FETAL HEMATOPOIETIC STEM AND PROGENITOR CELLS

**Topic:** 24. Hematopoiesis, stem cells and microenvironment

Sara Palo*[1], Keiki Nagaharu[1], Mikael Sommarin[1], Rasmus Olofzon[1], Virginia Turati[2], Shamit Soneji[1], Göran Karlsson[1], Charlotta Böiers[1]

*[1]Lund University, Division of Molecular Hematology, Lund Stem Cell Center, Lund, Sweden;[2]Lund University, Division of Molecular Medicine and Gene Therapy, Lund Stem Cell Center, Lund, Sweden;*

### Background

During ontogeny, the developing hematopoietic system exhibits broad differentiation potential, with certain immune cells arising exclusively during this period. Hematopoietic lineage bias is known to change with age, but the molecular drivers of differences in lineage preference between fetal and adult hematopoietic stem and progenitor cells (HSPCs) remain unclear.
Assessing lineage priming at the single-cell transcriptional level is challenging, as lineage-specific transcription factors (TFs) are often lost to dropout events. Chromatin-level studies of TF motif accessibility can overcome this limitation, but so far no study has directly compared the chromatin landscapes of fetal and adult HSPCs. Ultimately, insights into how lineage bias is driven by epigenetic mechanisms are essential to understand how lineage cues are disrupted in diseases such as leukemia, and why some leukemic driver mutations preferentially arise *in utero*.

### Aims

To investigate how lineage priming and differentiation pathways during human ontogeny differ from those observed in adulthood and to what extent such differences are evident at the chromatin level.

### Methods

Single cell cultures of human fetal liver (FL), cord blood (CB) and adult bone marrow (BM) hematopoietic stem cells (HSCs) were performed in conditions promoting multilineage differentiation to assess lineage output. The chromatin accessibility landscape of first-trimester FL Lin-CD45+CD34+ HSPCs was profiled using single-cell ATAC-sequencing. The data were compared to a corresponding adult dataset and publicly available second-trimester data to evaluate differences in chromatin-level lineage priming and plasticity.

### Results

We found that FL HSCs frequently generate mixed-lineage colonies, while adult HSCs primarily exhibit uni-lineage myeloid output. Contrary to previous report, we also found that first trimester HSCs readily produce B cells. On the chromatin level, adult HSCs showed enrichment of myeloid and megakaryocyte/erythroid-associated TF motifs, while fetal cells lacked such enrichment, in agreement with their broader differentiation potential. Several motifs enriched in adult HSCs also exhibited higher TF occupancy in these cells based on footprinting analysis.
Investigating lymphoid-upregulated TF motifs, we found a developmental-specific plasticity in fetal lymphoid progenitor cells in the form of a hybrid lympho-myeloid chromatin program not present in adult counterparts. Additionally, the motif and putative enhancer elements for PAX5, a master regulator of B cell development, showed reduced accessibility in fetal lymphoid progenitors, indicating a more plastic and less committed lymphoid state.

**Copyright Information:** (Online) ISSN: 2572-9241
©2026 The Author(s). HemaSphere is published by John Wiley & Sons Ltd on behalf of European Hematology Association. This is an open access Abstract Book distributed under the Attribution-NonCommercial-NoDerivs (CC BY-NC-ND), which allows third parties to download the articles and share them with others as long as they credit the author and the Abstract Book, but they cannot change the content in any way or use them commercially.
**Abstract Book Citations:** Authors, Title, HemaSphere, 2026;10:(S1):pages. The abstract book DOIs can be found on the “Author Information” page.
**Disclaimer:** Articles published in the journal HemaSphere exclusively reflect the opinions of the authors. The authors are responsible for all content in their abstracts including accuracy of the facts, statements, citing resources, etc.

**Summary/Conclusion**

Taken together, our results indicate limited lineage priming at the chromatin level in first-trimester HSCs, enabling diverse output of mature blood cells, whereas the myeloid priming of adult HSCs is reflected by the myeloid lineage bias observed *in vitro.* Additionally, our findings suggest that lymphoid progenitor cells exhibit higher chromatin-level lineage flexibility, with reduced B cell priming, during the first trimester. While likely supporting the unique developmental potential of fetal hematopoiesis, this increased flexibility may also contribute to its susceptibility to leukemogenic driver events.

**Copyright Information:** (Online) ISSN: 2572-9241
©2026 The Author(s). HemaSphere is published by John Wiley & Sons Ltd on behalf of European Hematology Association. This is an open access Abstract Book distributed under the Attribution-NonCommercial-NoDerivs (CC BY-NC-ND), which allows third parties to download the articles and share them with others as long as they credit the author and the Abstract Book, but they cannot change the content in any way or use them commercially.
**Abstract Book Citations:** Authors, Title, HemaSphere, 2026;10:(S1):pages. The abstract book DOIs can be found on the “Author Information” page.
**Disclaimer:** Articles published in the journal HemaSphere exclusively reflect the opinions of the authors. The authors are responsible for all content in their abstracts including accuracy of the facts, statements, citing resources, etc.

### (PS2286) QUIESCENT FETAL, NEONATAL, AND ADULT HUMAN HEMATOPOIETIC STEM CELLS ARE SELECTIVELY MAINTAINED IN FLT3-LIGAND CULTURES

**Topic:** 24. Hematopoiesis, stem cells and microenvironment

Margarita MacAldaz*[1], Fangwu Wang[2], Yasmine Yuen Man Lau[2], Kehang Wang[3], Jeremy Shu[2], Connie Eaves[2]

*[1]Institute of Regeneration and Repair, Edinburgh, United Kingdom;[2]University of British Columbia, Vancouver, Canada; [3]University of Toronto, Toronto, Canada;*

### Background

Hematopoietic stem cells (HSCs) generate all blood cell types and reconstitute lifelong hematopoiesis after transplantation, making them indispensable for clinical applications. However, the mechanisms governing their expansion and preservation of stem cell properties remain poorly defined. The earliest detectable HSCs, arising during the first month of human development, display intrinsically high regenerative potential that declines after birth. Studies in mice and humans demonstrate that long-term (>6 months) regenerative HSCs from the fetal liver (FL) possess markedly greater *in vivo* regenerative capacity than those from adult bone marrow (ABM) or cord blood (CB), which are predominantly in a quiescent state. Yet, quiescence in human fetal-derived HSCs remains incompletely characterized. A better understanding of how this impacts the *ex vivo* maintenance of their high regenerative capacity will have important therapeutic implications.

### Aims

Our objective was to determine both functionally and molecularly that human FL HSCs are quiescent and whether their self-renewal can be maintained *in vitro* and if similar preservation strategies can be applied to CB and ABM HSCs.

### Methods

Hematopoietic capability of HSC enriched subsets from first trimester human FL, CB and ABM were assessed in various *in vitro* and *in vivo* assays before and after short exposure in various culture conditions. Long-term hematopoietic output was evaluated in transplanted sublethally irradiated NOD-Rag1-/-IL2Rγc-/- W41/41 mice and in stromal co-culture systems. Single cell initiated cultures were used to assess survival and division kinetics. The culture effects on FL HSC subsets were then analyzed using single-cell CITE-seq RNA profiling and confocal microscopy of cell cycle factors (KI67, CDK6).

### Results

By testing the effects of various growth factors (GFs) and small molecules on the *in vivo* regenerative capability of input FL HSCs, we found that FLT3-L alone in serum free media cultures maintained the transplantable activity and long term hematopoietic output in cocultures as fully equivalent to the unmanipulated input cells and significantly superior ($P<0.05$) to FLT3-L+IL3+SF (3GF) with or without addition of UM171, stemregenin, or eltrombopag. In addition, cell cycle and division analysis demonstrated that surviving cells in the FLT3-L only conditions were better maintained initially in a G0 state and showed delayed and reduced divisions compared to 3GF-based conditions. Single-cell RNA-seq analysis further confirmed the enrichment of G0 features within the HSC-enriched subset of FL cells that was best maintained in FLT3-L alone and uncovered novel factors and pathways that may be important in their maintained self-renewal potential. Furthermore, this single GF culture could similarly maintain long-term hematopoietic output *in vitro* and *in vivo* from HSC enriched populations from CB and ABM.

**Copyright Information:** (Online) ISSN: 2572-9241
©2026 The Author(s). HemaSphere is published by John Wiley & Sons Ltd on behalf of European Hematology Association. This is an open access Abstract Book distributed under the Attribution-NonCommercial-NoDerivs (CC BY-NC-ND), which allows third parties to download the articles and share them with others as long as they credit the author and the Abstract Book, but they cannot change the content in any way or use them commercially.
**Abstract Book Citations:** Authors, Title, HemaSphere, 2026;10:(S1):pages. The abstract book DOIs can be found on the "Author Information" page.
**Disclaimer:** Articles published in the journal HemaSphere exclusively reflect the opinions of the authors. The authors are responsible for all content in their abstracts including accuracy of the facts, statements, citing resources, etc.

**Summary/Conclusion**

Here we identify a quiescent subset of human FL HSCs with high regenerative activity that can be functionally sustained *in vitro* by FLT3-Ligand. Comparable findings in CB and ABM-derived HSCs reinforce quiescence as a defining stem cell trait across development. Furthermore, single-cell transcriptomics of these cultured HSC-enriched subsets enable a strategy for uncovering key molecular regulators required to maintain regenerative potential *ex vivo*. Thus, these findings lay the groundwork for refining HSC expansion approaches while preserving their functional integrity for therapeutic applications

**Copyright Information:** (Online) ISSN: 2572-9241
©2026 The Author(s). HemaSphere is published by John Wiley & Sons Ltd on behalf of European Hematology Association. This is an open access Abstract Book distributed under the Attribution-NonCommercial-NoDerivs (CC BY-NC-ND), which allows third parties to download the articles and share them with others as long as they credit the author and the Abstract Book, but they cannot change the content in any way or use them commercially.
**Abstract Book Citations:** Authors, Title, HemaSphere, 2026;10:(S1):pages. The abstract book DOIs can be found on the “Author Information” page.
**Disclaimer:** Articles published in the journal HemaSphere exclusively reflect the opinions of the authors. The authors are responsible for all content in their abstracts including accuracy of the facts, statements, citing resources, etc.

### (PS2287) ALTERED GLUTAMINE METABOLISM IMPAIRS THE HAEMATOPOIETIC STEM CELL RESPONSE TO INFECTION

**Topic:** 24. Hematopoiesis, stem cells and microenvironment

Charlotte Hellmich*[1, 2], Alyssa Polski-Delve[1], Catherine Chinnery[1, 2], Edyta Wojtowicz[3], Maddie Bristow[1], Dominic Fowler-Shorten[1], Rebecca Maynard[1], Katherine Hampton[1], Charlotte Utting[3], Iain MacAulay[3], Kristian Bowles[1, 2], Stuart Rushworth[1]

*[1]University of East Anglia, Norwich, United Kingdom;[2]Norfork and Norwich University Hospital, Norwich, United Kingdom; [3]Earlham Institute, Norwich, United Kingdom;*

### Background

Haematopoietic stem cell (HSC) function is known to deteriorate with age. This has been associated with impaired HSC expansion in response to infection and subsequent inadequate immune responses. Understanding the metabolic changes that drive HSC ageing will help us to determine if these age-related changes can be targeted and therefore rescued. Glutamine is a conditionally essential amino acid, as the reliance on glutamine rapidly increases during acute illness and infection. Changes in glutamine metabolism have also been implicated in promoting HSC self-renewal and stress response. Glutamine enters cells via the amino acid transporter ASCT2 and can be synthetised from glutamate by glutamine synthetase (GLUL).

### Aims

The aim of this project is to investigate the how changes in glutamine metabolism and availability contribute to the aged HSC phenotype and as a result the impaired response to infection of aged HSCs.

### Methods

Single cell sequencing was performed on HSCs from young (8-12 weeks old) and aged (18-24 months old) mice using the SmartSeq2 protocol. The impact of changes in glutamine availability and metabolism was studied using the GLUL inhibitor Methionine sulfoximine (MSO) and the ACST2 inhibitor V-9302. These drugs were used both *in vitro* in colony forming assays and in an *in vivo* model of infection using LPS in both young and aged mice. An HSC-specific GLUL knock out mouse model was developed by crossing Glultm3Whla/J mice (floxed mice with loxP sites flanking exons 1-7 of the Glul gene) with the Tie2-Cre strain. These mice were then treated with LPS.

### Results

In young mice inhibition of glutamine uptake (V-9302) or its production (MSO) results in reduced colony formation in vitro and impaired HSC expansion in response to LPS in vivo. Furthermore, in the *glul* KO mouse model HSC colony formation capacity is reduced and the HSC response to LPS is impaired. Glutamine is therefore a vital metabolite to support the function of young HSCs.

Single cell sequencing of HSCs revealed that aged mice have two distinct HSC populations, those that are transcriptionally similar to HSCs from young mice and those that are clearly distinct from young HSCs. The most significantly differentially expressed gene between these two populations was glutamine synthetase (*glul*). Despite this increase in *glul*, intracellular glutamine levels in aged HSCs are significantly depleted. This suggests that glutamine is either used rapidly within the HSC or exported from the HSC.

The colony forming capacity of aged HSCs is improved by the presence of exogenous glutamine but inhibition of glutamine uptake (V-9302) almost completely exhausts this capacity. However, targeting of the upregulated GLUL (MSO) in the presence of excess glutamine resulted in improved colony formation in aged HSCs and an improved response to LPS in vivo. This suggests that *glul* upregulation contributes to the impaired function of aged HSCs.

**Copyright Information:** (Online) ISSN: 2572-9241
©2026 The Author(s). HemaSphere is published by John Wiley & Sons Ltd on behalf of European Hematology Association. This is an open access Abstract Book distributed under the Attribution-NonCommercial-NoDerivs (CC BY-NC-ND), which allows third parties to download the articles and share them with others as long as they credit the author and the Abstract Book, but they cannot change the content in any way or use them commercially.
**Abstract Book Citations:** Authors, Title, HemaSphere, 2026;10:(S1):pages. The abstract book DOIs can be found on the “Author Information” page.
**Disclaimer:** Articles published in the journal HemaSphere exclusively reflect the opinions of the authors. The authors are responsible for all content in their abstracts including accuracy of the facts, statements, citing resources, etc.

**Summary/Conclusion**

We demonstrate that glutamine is a key metabolite that contributes to healthy HSC function and that inhibition of its uptake or its synthesis impairs the function of young HSCs both in vitro and in vivo. In aged HSCs changes in *glul* expression and glutamine levels correlate with impaired HSC function. Our work demonstrates that increased *glul* expression negatively impacts on HSC function and that inhibition of GLUL both in vivo and in vitro can improve the function of aged HSCs.

**Copyright Information:** (Online) ISSN: 2572-9241
©2026 The Author(s). HemaSphere is published by John Wiley & Sons Ltd on behalf of European Hematology Association. This is an open access Abstract Book distributed under the Attribution-NonCommercial-NoDerivs (CC BY-NC-ND), which allows third parties to download the articles and share them with others as long as they credit the author and the Abstract Book, but they cannot change the content in any way or use them commercially.
**Abstract Book Citations:** Authors, Title, HemaSphere, 2026;10:(S1):pages. The abstract book DOIs can be found on the "Author Information" page.
**Disclaimer:** Articles published in the journal HemaSphere exclusively reflect the opinions of the authors. The authors are responsible for all content in their abstracts including accuracy of the facts, statements, citing resources, etc.

### (PS2288) MULTIMODAL OMICS IDENTIFY AN AGE RESILIENT MULTIPOTENT PROGENITOR POPULATION IN THE HEMATOPOIETIC SYSTEM

**Topic:** 24. Hematopoiesis, stem cells and microenvironment

Mari Carmen Romero-Mulero*[1], Julian Wolter-Mess[1], Mirijam Egg[1], Nina Cabezas-Wallscheid[1]

[1]*ETH Zürich, Zurich, Switzerland;*

**Background**

Hematopoietic stem cells (HSCs) and multipotent progenitors (MPPs) play critical roles in maintaining hematopoietic homeostasis. Although HSC aging has been a field of major interest, the molecular and functional changes across early progenitor subsets spanning lifetime remain poorly understood.

**Aims**

We aim to provide a comprehensive characterization of the molecular and functional features of HSPCs (HSCs, MPP1, MPP2, MPP3, MPP4, MPP5 and MPP6) in both young (8-12 weeks old) and aged (18-28 months old) mice. Performing the combinatorial integration of multiple omics approaches together with functional characterization assays in this study, we aim to generate a molecular and functional aging framework of HSPCs.

**Methods**

We generated bulk RNA-seq, as well as the first 10x Multiome (single-cell RNA-seq + ATAC-seq), metabolomics and lipidomics of HSCs, MPP1, MPP2, MPP3, MPP4, MPP5 and MPP6 of young and aged mice. We further integrated functional analysis to assess their proliferation and long-term *in vivo* reconstitution potential.

**Results**

Collectively, our multiomic approach deciphers transcriptional, epigenetic and metabolic regulatory hubs of HSPC aging and identifies MPP6 as a uniquely preserved population during this physiological process that retains a youthful transcriptional, epigenetic, metabolic and functional identity.

**Summary/Conclusion**

The main highlights of our study are:

- Multiomic and functional profiling shows that HSC, MPP1, MPP2, MPP3 MPP4 and MPP5 populations undergo molecular and functional decline with age.
- MPP6 retains a youthful transcriptome, chromatin remodeling and lipidome during aging.
- MPP6 maintains reconstitution potential and lymphoid bias in aged hematopoiesis.

These findings highlight the potential of leveraging MPP6 or its regulatory mechanisms to offer therapeutic targets in order to restore hematopoietic balance during aging.

**Copyright Information:** (Online) ISSN: 2572-9241
©2026 The Author(s). HemaSphere is published by John Wiley & Sons Ltd on behalf of European Hematology Association. This is an open access Abstract Book distributed under the Attribution-NonCommercial-NoDerivs (CC BY-NC-ND), which allows third parties to download the articles and share them with others as long as they credit the author and the Abstract Book, but they cannot change the content in any way or use them commercially.
**Abstract Book Citations:** Authors, Title, HemaSphere, 2026;10:(S1):pages. The abstract book DOIs can be found on the “Author Information” page.
**Disclaimer:** Articles published in the journal HemaSphere exclusively reflect the opinions of the authors. The authors are responsible for all content in their abstracts including accuracy of the facts, statements, citing resources, etc.

### (PS2289) CHARACTERIZATION OF THE BONE MARROW NICHE INTERACTOME OF CHEMORESISTANT ACUTE MYELOID LEUKEMIA

**Topic:** 24. Hematopoiesis, stem cells and microenvironment

Maria Pereira*[1, 2], Laura Mosteo[1], Ema Gonçalves[1, 3], George Vassiliou[4], Bruno Cavadas[1], Mireia Lopez[5], Vladimir Benes[5], Delfim Duarte[1, 6]

*[1]Institute for Research and Innovation in Health (i3S), Porto, Portugal;[2]ICBAS- Instituto de Ciências Biomédicas Abel Salazar da Universidade do Porto, Portugal, Porto, Portugal; [3]Université de Paris Cité, Paris, France;[4]Cambridge Stem Cell Institute, University of Cambridge, Cambrige, United Kingdom; [5]European Molecular Biology Laboratory (EMBL), Genomics Core Facility, Heidelberg, Germany; [6]Instituto Português de Oncologia (IPO), Departamento de Hemato-oncologia, Porto, Portugal;*

### Background

Acute myeloid leukemia (AML) is an heterogenous and aggressive disease that develops primarily in the bone marrow (BM). It accounts for about 60% of deaths due to leukemia. Despite the introduction of new drugs, the mainstay of therapy remains intensive chemotherapy for fit patients, who often remain with persistent measurable residual disease (MRD). We previously showed that AML remodels BM vascular microenvironments. However, the regulation of chemoresistant AML cells by BM niches remains elusive.

### Aims

In this project, we hypothesized that specific BM niches and cell types regulate AML chemoresistance. We aim to comprehensively map the niche interactome of AML in the BM, and manipulate key niche-dependent factors that regulate AML expansion and relapse.

### Methods

We used two mouse models of AML that represent a large portion of patients, the aggressive model MLL-AF9 AML and the intermediate prognosis model Npm1/Flt3-ITD AML. We modified these models to express a novel genetic labelling technique, the “Cherry-niche system”, in which AML cells secrete liposoluble mCherry+ particles to interacting niche cells. We injected these leukemias into immunocompetent recipient mice, and analyzed the composition and mCherry labelling of the BM non-malignant immune compartment, hematopoietic stem/progenitor cells and stromal cells during progression and after intensive chemotherapy.

### Results

We observed disease subtype-specific interactions with the immune compartment during progression and a biased labelling of LepR+ mesenchymal stem cells (MSCs) after chemotherapy in both AML models, suggesting the role of a mesenchymal niche that interacts with chemoresistant AML. Plate-based scRNA-sequencing of MSCs revealed the differential expression of genes that are currently being mechanistically explored. Using 2-photon intravital microscopy of the BM, we are also evaluating the *in vivo* dynamics of chemoresistant AML cells interacting with LepR+ MSC niches.

### Summary/Conclusion

In summary, we observed specific AML-immune niche interactions during disease progression, that are dependent on the driver mutation of AML. In contrast, we find MSCs as preferential interacting niches for chemoresistant AML cells. Our study provides a dynamic map of the niche interactome of AML.

**Copyright Information:** (Online) ISSN: 2572-9241
©2026 The Author(s). HemaSphere is published by John Wiley & Sons Ltd on behalf of European Hematology Association. This is an open access Abstract Book distributed under the Attribution-NonCommercial-NoDerivs (CC BY-NC-ND), which allows third parties to download the articles and share them with others as long as they credit the author and the Abstract Book, but they cannot change the content in any way or use them commercially.
**Abstract Book Citations:** Authors, Title, HemaSphere, 2026;10:(S1):pages. The abstract book DOIs can be found on the “Author Information” page.
**Disclaimer:** Articles published in the journal HemaSphere exclusively reflect the opinions of the authors. The authors are responsible for all content in their abstracts including accuracy of the facts, statements, citing resources, etc.

### (PS2290) ABNORMALITIES IN THE BONE MARROW NICHE ARE SUSTAINED AFTER ALLOGENEIC STEM CELL TRANSPLANTATION, SUPPORTING CONTINUED LEUKEMIC DYNAMICS AND RELAPSE OF ACUTE MYELOID LEUKEMIA

**Topic:** 24. Hematopoiesis, stem cells and microenvironment

Shlomit Yehudai-Reshef*[1], Irina Tikhonova[2], Nitzan Davidov[3], Natalia Evtiugina[3], Nabil Ghrayeb[4], Tsila Zuckerman[5]

*[1]Rambam Health Care Campus, Hematology, Haifa, Israel;[2]Rambam Health Care Campus, CRIR, Haifa, Israel;[3]Rambam Health Care Campus, Haifa, Israel; [4]Rambam Health Care Campus, hematology, Haifa, Israel;[5]Rambam Health Care Campus, Hematology and Bone Marrow Transplantation, Haifa, Israel;*

### Background

Disease relapse following allogeneic hematopoietic stem cell transplantation (allo-HSCT) remains a major challenge in the management of acute myeloid leukemia (AML). Increasing evidence suggests that, beyond leukemic stem cells (LSCs), dysfunctional bone marrow microenvironment (BM-ME), particularly mesenchymal stromal cells (MSCs), plays a key role in leukemogenesis. It remains unclear whether BM-ME abnormalities resolve post-allo-HSCT.

### Aims

The current study aimed to determine if morphological, molecular, and functional abnormalities of BM-MSCs in AML persist after allo-HSCT and to define their potential contribution to leukemia support and relapse.

### Methods

BM-MSCs were obtained from AML patients at diagnosis and at least 5 months after allo-HSCT and compared with those collected from healthy donors (HD). Analyses included machine-learning-based morphological profiling (13 parameters), qRT-PCR of key mediators (SDF-1, TGF-β, VCAM-1, FABP4, SPP1), ELISA of BM-derived plasma, wound-healing migration/proliferation assays, transwell migration/invasion assays using OCI-AML-3 and Kasumi-1 cell lines, and ex-vivo co-culture with HD CD34$^+$ hematopoietic stem and progenitor cells (HSPCs).

### Results

The classification using machine-learning modeling demonstrated substantial overlap between the parameters of AML MSCs at diagnosis and post-allo-HSCT, indicating persistent morphological abnormalities months after transplant. Notably, a clear distinction between HD MSCs and patient-derived MSCs at both evaluation time points was observed. Key regulators of the LSC-BM-MSC crosstalk (SDF-1, TGF-β, and VCAM-1) were significantly downregulated in AML MSCs at both timepoints relative to HD cells. Levels of adipogenic (FABP4) and osteogenic (SPP1) markers were also consistently reduced, with decreased FABP4 protein values found in AML patient plasma, suggesting impaired lineage differentiation. MSC migration and proliferation remained significantly impaired after allo-HSCT. Furthermore, leukemia cell lines showed altered migration/invasion towards AML patient-derived serum, whereas HD HSPCs co-cultured with AML MSCs exhibited increased proliferation/viability, indicating niche-induced disruption of normal HSC quiescence. Overall, these findings demonstrate that the post-HSCT BM-ME remains abnormal and leukemia-supportive.

**Copyright Information:** (Online) ISSN: 2572-9241
©2026 The Author(s). HemaSphere is published by John Wiley & Sons Ltd on behalf of European Hematology Association. This is an open access Abstract Book distributed under the Attribution-NonCommercial-NoDerivs (CC BY-NC-ND), which allows third parties to download the articles and share them with others as long as they credit the author and the Abstract Book, but they cannot change the content in any way or use them commercially.
**Abstract Book Citations:** Authors, Title, HemaSphere, 2026;10:(S1):pages. The abstract book DOIs can be found on the "Author Information" page.
**Disclaimer:** Articles published in the journal HemaSphere exclusively reflect the opinions of the authors. The authors are responsible for all content in their abstracts including accuracy of the facts, statements, citing resources, etc.

**Summary/Conclusion**

AML patients retain profound morphological, molecular, and functional BM-MSC abnormalities long after allo-HSCT, preserving a pathological niche that promotes leukemia support and may contribute to disease relapse. Targeting the persistent BM-ME dysfunction represents a promising therapeutic strategy for improving post-transplant outcomes.

**Copyright Information:** (Online) ISSN: 2572-9241
©2026 The Author(s). HemaSphere is published by John Wiley & Sons Ltd on behalf of European Hematology Association. This is an open access Abstract Book distributed under the Attribution-NonCommercial-NoDerivs (CC BY-NC-ND), which allows third parties to download the articles and share them with others as long as they credit the author and the Abstract Book, but they cannot change the content in any way or use them commercially.
**Abstract Book Citations:** Authors, Title, HemaSphere, 2026;10:(S1):pages. The abstract book DOIs can be found on the "Author Information" page.
**Disclaimer:** Articles published in the journal HemaSphere exclusively reflect the opinions of the authors. The authors are responsible for all content in their abstracts including accuracy of the facts, statements, citing resources, etc.

### (PS2291) LEVERAGING THE INTESTINAL MICROBIOTA TO IMPROVE HSC ENGRAFTMENT

**Topic:** 24. Hematopoiesis, stem cells and microenvironment

Christopher Peek*[1], Arushana Maknojia[1], Belinda Candra[1], Leran Wang[2], Megan Baldridge[2], Katherine King[1]

*[1]Baylor College of Medicine, Houston, United States of America;[2]Washington University In Saint Louis, Saint Louis, United States of America;*

### Background

Rapid immune cell reconstitution following hematopoietic stem cell transplantation (HSCT) is vital to improve outcomes and reduce comorbid invasive infections. As a result, time to engraftment (defined as recovery of absolute neutrophil count) is a critical measure of transplant success. The pre-engraftment window, where patients are severely neutropenic, poses a profound risk of life-threatening infections and infection related mortality. Seminal studies reveal the intestinal microbiota as a crucial determinant of HSCT outcomes. Patients with increased intestinal microbial diversity and reduced exposure to broad-spectrum antimicrobial regiments experience improved overall survival post-HSCT. Similarly, patients exposed to long-term antibiotic therapy are known to experience defects in steady state hematopoiesis. Prior work in our lab has revealed a central role for microbially-induced type 1 interferon (IFN-1) in regulating steady state hematopoiesis. Yet, the precise mechanisms that explain how the intestinal microbiota impacts the distant bone marrow through IFN-1 are unknown and limit the development of targeted therapies to improve HSCT outcomes.

### Aims

To decipher the microbes and metabolites contributing to HSC function and engraftment during HSCT.

### Methods

We established a murine model of antibiotic-induced peripheral cytopenia and central bone marrow suppression. We extended this model to murine HSCT. Utilizing untargeted metabolomics, we previously identified serum and fecal metabolites depleted in antibiotic treated mice. Clinical extension of this work revealed depletion of similar fecal metabolites in children with antibiotic-induced neutropenia. We tested candidate metabolites for their ability to induce IFN-1 signaling *in vitro* and *in vivo* using IFN-1 reporter cells and mice and took advantage of small molecules to mechanistically test candidate signaling pathways. We concomitantly utilized 16S and whole genome sequencing to identify bacterial species depleted in patients with antibiotic-associated neutropenia.

### Results

Antibiotic administration led to significant peripheral cytopenias and central bone marrow suppression in mice. Furthermore, antibiotic therapy led to delayed engraftment following HSCT in mice. We identified a candidate metabolite, UDP-glucose, a purinergic ligand, that significantly enhanced IFN-1 production *in vitro* and *in vivo*. UDP-glucose mediated IFN-1 activation occurred independently of $P2Y_{14}R$, a purinergic receptor known to bind UDP-glucose. Administration of UDP-glucose protected mice from antibiotic-associated cytopenia. Our whole genome sequencing data concomitantly identified specific microbial species associated with antibiotic-induced neutropenia in pediatric patients with antibiotic-induced neutropenia.

### Summary/Conclusion

Taken together, these data identify a candidate metabolite, UDP-glucose, and microbes that may support HSC engraftment following HSCT.

**Copyright Information:** (Online) ISSN: 2572-9241
©2026 The Author(s). HemaSphere is published by John Wiley & Sons Ltd on behalf of European Hematology Association. This is an open access Abstract Book distributed under the Attribution-NonCommercial-NoDerivs (CC BY-NC-ND), which allows third parties to download the articles and share them with others as long as they credit the author and the Abstract Book, but they cannot change the content in any way or use them commercially.
**Abstract Book Citations:** Authors, Title, HemaSphere, 2026;10:(S1):pages. The abstract book DOIs can be found on the “Author Information” page.
**Disclaimer:** Articles published in the journal HemaSphere exclusively reflect the opinions of the authors. The authors are responsible for all content in their abstracts including accuracy of the facts, statements, citing resources, etc.

### (PS2292) CLONAL EVOLUTION OF RAS-MAPK MUTATIONS IN HISTIOCYTIC NEOPLASMS REVEALS DISTINCT TRAJECTORIES OF ABERRANT MYELOPOIESIS IN CHILDREN VERSUS ADULTS

**Topic:** 24. Hematopoiesis, stem cells and microenvironment

Antonia Cuff*[1], Kudzai Nyamondo[1], Paul Milne[2], Wing Leung[2], Toni Spence[3], Matthew Mayho[1], Sarah Pagan[2], Nicholas Williams[1], Daniel Leongamornlert[1], Matthew Collin[2], Jyoti Nangalia[1, 4]

*[1]Wellcome Sanger Institute, Somatic Genomics Programme, Hinxton, United Kingdom;[2]Newcastle University, Haematopoiesis and Immunogenomics Lab, Newcastle upon Tyne, United Kingdom; [3]Astrazeneca, Cambridge, United Kingdom; [4]Cambridge Stem Cell Institute, Haematology, Cambridge, United Kingdom;*

### Background

Activating mutations in the RAS-MAPK pathway are recurrent oncogenic drivers of clonal haematopoiesis (CH) and myeloid malignancies. In histiocytic neoplasms (HN) – characterised by lesional accumulation of clonal dendritic cells and macrophages – mutations like *BRAF*$^{\text{V600E}}$ causally drive disease. HN exhibit peculiar spatio-temporal patterns: histiocytic lesions separate widely in space, affecting different organs, and recur over many years, from single-organ to aggressive multisystem disease. Spatial and temporal patterns differ markedly between children and adults, with paediatric HN commonly driven by isolated *BRAF*$^{\text{V600E}}$ or MAPK-pathway mutations, while adult HN frequently co-exists with CH. This raises questions about how identical driver mutations within haematopoietic stem/progenitor cells (HSPCs) produce divergent disease phenotypes.

### Aims

To interrogate the timing and landscape of these mutations across the haematopoietic hierarchy, their clonal fitness within different tissues, and how such factors shape disease patterns.

### Methods

We performed targeted myeloid panel sequencing in 60 paediatric and adult HN patients to characterise the clonal landscape across haematopoietic compartments (lesions, peripheral blood, bone marrow). Next, we reconstructed detailed phylogenies in 5 paediatric (3 months–2 years) and 3 adult patients (58–72 years) with multisystem HN using whole-genome sequencing of HSPC-derived colonies (n=273), and single haematopoietic cells (n>500) following whole-genome DNA amplification.

### Results

Myeloid sequencing revealed frequent co-existing CH in adult HN, with *DNMT3A*, *TET2*, and *ASXL1* mutations in blood and marrow. In contrast, MAPK-pathway mutations were restricted to lesions in children and absent from circulation. *BRAF*$^{\text{V600E}}$, *NRAS* and *KRAS*-mutant clones showed distinct fitness patterns: high competitive advantage in tissue niches but limited marrow expansion, suggesting tissue-specific and gene-specific selection on myeloid progenitors.
Phylogenetic analysis revealed striking age-dependent differences in clonal architecture and mutation timing. In all 3 adults, haematopoiesis was dominated by a complete clonal sweep of biallelic *TET2*-mutant HSPCs occurring decades before disease. Subsequent parallel and independent acquisition of *NRAS*, *KRAS* and *BRAF* mutations in distinct subclones drove rapid expansion prior to clinical onset – a pattern reminiscent of CH-associated secondary malignancies. In contrast, paediatric phylogenies revealed early in utero expansion (~30-40 somatic mutations) of a single *BRAF*$^{\text{V600E}}$ clone without additional cooperating drivers, consistent with fundamentally different clonal selection during foetal and early postnatal haematopoiesis.

**Copyright Information:** (Online) ISSN: 2572-9241
©2026 The Author(s). HemaSphere is published by John Wiley & Sons Ltd on behalf of European Hematology Association. This is an open access Abstract Book distributed under the Attribution-NonCommercial-NoDerivs (CC BY-NC-ND), which allows third parties to download the articles and share them with others as long as they credit the author and the Abstract Book, but they cannot change the content in any way or use them commercially.
**Abstract Book Citations:** Authors, Title, HemaSphere, 2026;10:(S1):pages. The abstract book DOIs can be found on the “Author Information” page.
**Disclaimer:** Articles published in the journal HemaSphere exclusively reflect the opinions of the authors. The authors are responsible for all content in their abstracts including accuracy of the facts, statements, citing resources, etc.

**Summary/Conclusion**

These data are the largest WGS dataset in HN. We show how identical RAS-MAPK oncogenes produce profoundly different trajectories of aberrant myelopoiesis depending on developmental timing, clonal context (CH versus isolated driver), microenvironment, and haematological system age. These findings establish HN as a tractable model for understanding: (1) how developmental mutations shape lifelong clonal dynamics; (2) the interplay between CH and secondary driver acquisition in myeloid disorders; and (3) tissue-specific versus haematopoietic clonal expansion in RAS-MAPK-driven diseases. Understanding these principles has broad implications for predicting CH progression risk, stratifying RAS-pathway inhibitor therapies, and defining age-specific vulnerabilities in myeloid malignancies.

**Copyright Information:** (Online) ISSN: 2572-9241
©2026 The Author(s). HemaSphere is published by John Wiley & Sons Ltd on behalf of European Hematology Association. This is an open access Abstract Book distributed under the Attribution-NonCommercial-NoDerivs (CC BY-NC-ND), which allows third parties to download the articles and share them with others as long as they credit the author and the Abstract Book, but they cannot change the content in any way or use them commercially.
**Abstract Book Citations:** Authors, Title, HemaSphere, 2026;10:(S1):pages. The abstract book DOIs can be found on the "Author Information" page.
**Disclaimer:** Articles published in the journal HemaSphere exclusively reflect the opinions of the authors. The authors are responsible for all content in their abstracts including accuracy of the facts, statements, citing resources, etc.

### (PS2293) WNT10B REGULATES THE HEMATOPOIETIC RESPONSE TO ACUTE AND CHRONIC STRESS

**Topic:** 24. Hematopoiesis, stem cells and microenvironment

Magdalena Havlova*[1, 2], Karolina Vanickova[1, 2], Irina Ribeiro Bas[1, 2], Safaa Andarawi[1, 3], Francesca Matteini[4, 5], Lucie Janeckova[6], Vladimir Korinek[6], Tomas Valenta[6, 7], Maria Carolina Florian[4, 5, 8], Merixtell Alberich Jorda[1, 9]

*[1]Institute of Molecular Genetics of the Czech Academy of Sciences, Laboratory of Hemato-oncology, Prague, Czechia;[2]Charles University, Faculty of Science, Prague, Czechia; [3]Charles University, Biomedical Center, Faculty of Medicine in Pilsen, Pilsen, Czechia; [4]The Bellvitge Institute for Biomedical Research (IDIBELL), Stem Cell Aging Group, Regenerative Medicine Program, L'Hospitalet de Llobregat, Barcelona, Spain; [5]Program for Advancing the Clinical Translation of Regenerative Medicine of Catalonia, P-CMR[C], L'Hospitalet de Llobregat, Barcelona, Spain; [6]Institute of Molecular Genetics of the Czech Academy of Sciences, Laboratory of Cell and Developmental Biology, Prague, Czechia; [7]ETH Zürich, Department of Biosystems Science and Engineering, Basel, Switzerland; [8]The Catalan Institution for Research and Advanced Studies (ICREA), Barcelona, Spain;[9]2nd Faculty of Medicine, Charles University in Prague, Childhood Leukaemia Investigation Prague, Department of Pediatric Haematology and Oncology, Prague, Czechia;*

### Background

All blood cells rise from hematopoietic stem and progenitor cells (HSPCs), which maintain hematopoiesis by tightly regulating the balance between self-renewal, proliferation, and differentiation. This balance becomes especially critical under stress conditions such as infection or injury, when the demand for mature blood cells rises. The Wnt/β catenin signaling pathway is essential for development and hematopoietic regeneration, and its deregulation is linked to human cancers, including leukemia. Our previous work showed that β-catenin-mediated signaling is essential for emergency granulopoiesis, a stress-induced process that rapidly increases granulocyte production to support host defense. However, the identity of the Wnt ligands that mediate this response under acute and chronic stress conditions remains elusive.

### Aims

The aims of this project are (1) to identify which Wnt ligand(s) activate Wnt/β-catenin signaling in HSPCs, and (2) to determine the role of Wnt10b under steady-state, emergency and chronic stress conditions.

### Methods

Wild-type and whole-body Wnt10b knockout (KO) mice were injected intraperitoneally with lipopolysaccharide (LPS) to induce an acute inflammatory response. To investigate the effects of chronic stress, mice were exposed to low repetitive genotoxic irradiation. Bone marrow transplantations were performed, along with cell-culture assays using primary murine and human cells. We used flow cytometry, cell-cycle and proliferation analyses, differentiation assays, immunoassays, single-cell RNA sequencing (scRNA-seq), and bioinformatic analyses.

**Copyright Information:** (Online) ISSN: 2572-9241
©2026 The Author(s). HemaSphere is published by John Wiley & Sons Ltd on behalf of European Hematology Association. This is an open access Abstract Book distributed under the Attribution-NonCommercial-NoDerivs (CC BY-NC-ND), which allows third parties to download the articles and share them with others as long as they credit the author and the Abstract Book, but they cannot change the content in any way or use them commercially.
**Abstract Book Citations:** Authors, Title, HemaSphere, 2026;10:(S1):pages. The abstract book DOIs can be found on the "Author Information" page.
**Disclaimer:** Articles published in the journal HemaSphere exclusively reflect the opinions of the authors. The authors are responsible for all content in their abstracts including accuracy of the facts, statements, citing resources, etc.

**Results**

Our scRNAseq data analysis identified Wnt10b as a Wnt ligand specifically upregulated in a subpopulation of HSCs and multipotent progenitors isolated from LPS-challenged mice and humans. We showed that Wnt10b activates Wnt/β catenin signaling, and that its expression is directly induced through the TLR4-MyD88-NF-kB signaling pathway. To investigate its functional role, we generated a whole-body Wnt10b KO mouse model. Wnt10b deficiency resulted in reduced HSPCs proliferation and impaired myeloid differentiation under both steady-state and LPS-induced emergency conditions. Given that the loss of Wnt10b compromises HSPC maintenance and fate under acute stress, we next investigated its role under chronic stress by inducing sustained genotoxic stress in Wnt10b KO animals. KO animals were more susceptible to chronic stress exposure and exhibited impaired regeneration of the hematopoietic compartment. Remarkably, Wnt10b KO mice succumbed to multiple pathologies, including leukemic malignancies.

**Summary/Conclusion**

We identify Wnt10b as a key regulator of both steady-state and stress-induced hematopoiesis, promoting proliferation and myeloid differentiation. Furthermore, our findings indicate that Wnt10b supports hematopoietic regeneration under chronic genotoxic stress. Given that radiotherapy relies on continuous genotoxic stress, these findings suggest potential clinical relevance for Wnt10b signaling in therapy-associated hematopoietic dysfunction. Altogether, our study establishes Wnt10b as an important upstream activator of Wnt/β-catenin signaling required to maintain hematopoietic function under stress conditions.
This work was partially supported by the Czech Science Foundation (GACR#25-15913S).

**Copyright Information:** (Online) ISSN: 2572-9241
©2026 The Author(s). HemaSphere is published by John Wiley & Sons Ltd on behalf of European Hematology Association. This is an open access Abstract Book distributed under the Attribution-NonCommercial-NoDerivs (CC BY-NC-ND), which allows third parties to download the articles and share them with others as long as they credit the author and the Abstract Book, but they cannot change the content in any way or use them commercially.
**Abstract Book Citations:** Authors, Title, HemaSphere, 2026;10:(S1):pages. The abstract book DOIs can be found on the “Author Information” page.
**Disclaimer:** Articles published in the journal HemaSphere exclusively reflect the opinions of the authors. The authors are responsible for all content in their abstracts including accuracy of the facts, statements, citing resources, etc.

### (PS2294) DEVELOPMENT OF A TRACTABLE EX VIVO MODEL OF HAEMATOPOIESIS ENABLES CRISPR SCREENING OF LINEAGE REGULATORS

**Topic:** 24. Hematopoiesis, stem cells and microenvironment

Elizabeth Brown*[1], Yavor Bozhilov[2], Ian Hsu[1], James Davies[1], Adam Wilkinson[2]

*[1]University of Oxford, Weatherall Institute of Molecular Medicine, Oxford, United Kingdom;[2]University of Cambridge, Stem Cell Institute, Cambridge, United Kingdom;*

### Background

Self-renewing, multipotent haematopoietic stem cells (HSCs) are a rare but indispensable cell population for the life-long maintenance of the haematopoietic system. The loss or dysfunction of haematopoiesis underlies a wide range of disorders which collectively contribute to an estimated 70,000 deaths per year in the United States. HSCs are also central to stem cell transplantation and emerging gene therapies for diseases of the haematopoietic system. However, progress in dissecting the molecular regulation of human haematopoiesis has been limited by the lack of culture protocols that support robust expansion of primary human HSCs that are compatible with next-generation functional genomics approaches.

### Aims

We aim to develop a reproducible, serum-free and feeder-free ex vivo platform for the expansion and directed differentiation of primary human HSCs that is compatible with pooled CRISPR screening and single-cell analyses.

### Methods

Building on recent advances in polymer-based expansion of functional human HSCs, we optimised ex vivo culture conditions for human HSC expansion and the directed differentiation of expanded HSCs into 6 major blood lineages: erythroid, megakaryocytic, monocytic, granulocytic, and B and T lymphoid lineages. We validated ex vivo differentiated populations using flow cytometry, imaging, RNA-sequencing, and lineage specific functional assays.

### Results

Using this ex vivo model of myelopoiesis we have performed CRISPR knockout screens of >4,000 genes to identify genes controlling differentiation in to specific myeloid progenitor populations. The screen revealed candidate regulators of granulocyte-monocyte progenitors (CD34+/CD45RA+/CD123+) and mature neutrophils (CD66b+/CD15+/CD14-). We are now evaluating the roles of these genes in lineage commitment decisions between myeloid and alternative progenitor fates.

### Summary/Conclusion

This tractable human ex vivo haematopoiesis platform enables scalable functional genomics in primary HSCs. It provides a powerful system for dissecting the molecular regulation of normal and diseased haematopoiesis and may facilitate the development of new HSC-based therapies.

**Copyright Information:** (Online) ISSN: 2572-9241
©2026 The Author(s). HemaSphere is published by John Wiley & Sons Ltd on behalf of European Hematology Association. This is an open access Abstract Book distributed under the Attribution-NonCommercial-NoDerivs (CC BY-NC-ND), which allows third parties to download the articles and share them with others as long as they credit the author and the Abstract Book, but they cannot change the content in any way or use them commercially.
**Abstract Book Citations:** Authors, Title, HemaSphere, 2026;10:(S1):pages. The abstract book DOIs can be found on the "Author Information" page.
**Disclaimer:** Articles published in the journal HemaSphere exclusively reflect the opinions of the authors. The authors are responsible for all content in their abstracts including accuracy of the facts, statements, citing resources, etc.

### (PS2295) SKP2-INHIBITION IN HUMAN HSPCS: A NEW PROMISING STRATEGY TO ENHANCE HSC MAINTENANCE AND STRESS TOLERANCE DURING IN-VITRO MANIPULATION

**Topic:** 24. Hematopoiesis, stem cells and microenvironment

Federica Di Biase*[1, 2], Xiaoting Ren[2], Greta Zara[2], Alessia Bortoluzzi[2], Wen Wei Chen[2], Dario Ferrero[1], Benedetto Bruno[1], Angelo Cardoso[3], Nadia Carlesso[2]

*[1]Division of Hematology and Cell Therapy Unit, Department of Molecular Biotechnology and Health Sciences, University of Torino,, Torino, Italy; [2]Department of Stem Cell Biology and Regenerative Medicine – Beckman Research Institute of City of Hope, Duarte CA, United States of America; [3]Center for Gene Therapy - Beckman Research Institute of City of Hope,, Duarte, United States of America;*

### Background

Aging- and therapy-induced inflammation and cellular stress impair hematopoietic stem cell (HSC) quiescence and long-term regenerative capacity. Ex vivo gene therapy protocols similarly activate HSC stress responses, compromising stemness. SKP2, a component of the SCF E3 ubiquitin ligase, promotes cell cycle entry and glycolysis and is therefore a candidate target to preserve HSC integrity under stress. Using SKP2-deficient mouse models, our group previously observed that SKP2 loss enhances HSC quiescence, expands stem cell pools, and improves long-term repopulation in serial transplant assays.

### Aims

To determine whether pharmacological SKP2 inhibition preserves human HSC function during ex vivo culture and protects hematopoietic stem and progenitor cells (HSPCs) from genotoxic stress.

### Methods

Human $CD34^+$ cells were cultured up to 14 days with SCF, IL-3, TPO, and FLT3L ± SKP2 inhibitors DT204 or SCFSkp2 IN-2 (IN2). At days 0, 7, and 14 we assessed differentiation (flow cytometry), colony-forming capacity, cell cycle (BrdU incorporation, and metabolic activity (MitoTracker, Seahorse). To test stress responses, day-7 cultures were irradiated (0–1000 cGy) and analyzed for ROS, γH2AX, and apoptosis. Select assays were repeated in murine HSPCs and leukemia cell lines (HL-60, DND-41). Statistics used unpaired t-tests with Welch's correction.

### Results

SKP2 inhibition by treatment with DT204 or IN-2 preserved significantly higher frequencies of early HSPC subsets at day 7, including $CD34^+/CD38^-/CD90^+$ HSCs (13.2% vs 6.0%) and $CD34^+/CD38^-/CD90^-$ multipotent progenitors (46.4% vs 6.8%). Enrichment decreased by day 14. Colony-forming assays confirmed improved stemness, with higher colony numbers at day 14 (3.78 vs 0.56 colonies/well, $p=0.0044$) and successful secondary replating. Despite these effects, cell cycle status remained largely unchanged in primary HSPCs (S-phase 29.7% vs 29.9%), while malignant cell lines showed strong S-phase reduction (0.7% vs 28.2% control), suggesting a differential sensitivity between normal and transformed cells. Metabolic profiling showed decreased oxidative phosphorylation during SKP2 blockade (basal OCR 30.5 vs 19.2) and reduced glycolysis (basal ECAR 9.5 vs 5.8), accompanied by an increased OCR/ECAR ratio (3.4 vs 3.7, $p<0.0057$), consistent with a shift toward oxidative metabolism. Interestingly, LT-HSCs exhibited substantially higher mitochondrial activation than ST-HSCs at baseline (MitoTracker DR gMFI 14403 vs 40470, $p=0.0058$). SKP2 inhibition improved cellular stress resistance. In murine HSPCs, SKP2 knockdown reduced irradiation-induced DNA damage compared to control ($γH2AX^+$ 28.1% vs 42.7%) and improved viability (62.2% vs 52.8%). Human $CD34^+$ cells treated with SKP2 inhibitors similarly displayed reduced apoptosis after 1000 cGy exposure.

**Copyright Information:** (Online) ISSN: 2572-9241
©2026 The Author(s). HemaSphere is published by John Wiley & Sons Ltd on behalf of European Hematology Association. This is an open access Abstract Book distributed under the Attribution-NonCommercial-NoDerivs (CC BY-NC-ND), which allows third parties to download the articles and share them with others as long as they credit the author and the Abstract Book, but they cannot change the content in any way or use them commercially.
**Abstract Book Citations:** Authors, Title, HemaSphere, 2026;10:(S1):pages. The abstract book DOIs can be found on the "Author Information" page.
**Disclaimer:** Articles published in the journal HemaSphere exclusively reflect the opinions of the authors. The authors are responsible for all content in their abstracts including accuracy of the facts, statements, citing resources, etc.

**Summary/Conclusion**

Pharmacological SKP2 inhibition supported a better preservation of human HSC phenotype and function during ex vivo culture, balanced metabolism, and protected HSPCs from irradiation-induced DNA damage and apoptosis. Although further mechanistic and in-vivo validation are required, these findings establish a rationale for exploring SKP2 inhibition as a strategy to protect and maintain HSCs in both experimental and clinical settings. Indeed, SKP2 inhibition may represent a novel strategy to protect HSCs during ex-vivo manipulation, such as in transplantation or gene therapy.

**Copyright Information:** (Online) ISSN: 2572-9241
©2026 The Author(s). HemaSphere is published by John Wiley & Sons Ltd on behalf of European Hematology Association. This is an open access Abstract Book distributed under the Attribution-NonCommercial-NoDerivs (CC BY-NC-ND), which allows third parties to download the articles and share them with others as long as they credit the author and the Abstract Book, but they cannot change the content in any way or use them commercially.
**Abstract Book Citations:** Authors, Title, HemaSphere, 2026;10:(S1):pages. The abstract book DOIs can be found on the “Author Information” page.
**Disclaimer:** Articles published in the journal HemaSphere exclusively reflect the opinions of the authors. The authors are responsible for all content in their abstracts including accuracy of the facts, statements, citing resources, etc.

### (PS2296) DIVERGENT EFFECTS OF TET2- AND DNMT3A-MUTANT CLONAL HEMATOPOIESIS ON BREAST CANCER PROGRESSION UPON AGING

**Topic:** 24. Hematopoiesis, stem cells and microenvironment

Nagihan Gizay Gönüllü*[1, 2], Jonas Fullin[1, 2], Karolina Zielinska[1, 2], Franceso Prisco[3], Steffen Boettcher[1, 2], Francisco Caiado[1, 2], Markus G. Manz[1, 2]

[1]*University Hospital Zurich, Medical Oncology and Hematology, Zurich, Switzerland;* [2]*University of Zurich, Zurich, Switzerland;* [3]*Institute for Veterinary Pathology, University of Zurich, Zurich, Switzerland;*

### Background

Clonal hematopoiesis of indeterminate potential (CHIP) is defined by the expansion of a somatic blood cell clone carrying mutations in leukemia driver genes, commonly in *DNMT3A* or *TET2*, at a variant allele frequency >2% without other hematological abnormalities. CHIP incidence increases with age and is associated with elevated risks of hematologic malignancies, all-cause mortality which associates with pro-inflammatory immune response. Recent studies report higher CHIP prevalence in solid tumor patients, including elevated breast cancer risk in CHIP carriers. However, whether CHIP-mutant hematopoietic cells influence solid tumor progression, and how, remains poorly understood. We hypothesize that *Tet2*- or *Dnmt3a*-driven CHIP contributes to breast cancer incidence, growth, and/or metastasis by modulating the tumor immune microenvironment.

### Aims

In this project we aimed to detect the influence of *Tet2*- and *Dnmt3a*-driven CHIP on breast cancer progression, to define tumor infiltrating immune populations and underlying molecular pathways mediating the interaction between *Tet2/Dnmt3a* mutant hematopoietic clones and breast cancer cells, to target the molecular pathways involved in the interplay between CHIP carrying immune populations and cancer cells and finally to validate the previous findings on human samples.

### Methods

To test this, we developed transgenic mouse models of CHIP using hematopoietic-specific, where floxed *Tet2* or $Dnmt3a^{fl\text{-}R878H}$ alleles alongside a reporter gene are controlled by inducible *CreER*. These were combined with a syngeneic, orthotopic, and traceable breast cancer model using E0771 cells, in both young (3–4 months) and middle-aged (12–14 months) cohorts, achieving ~70% mutant leukocyte chimerism. Tumor growth and metastasis were monitored longitudinally, supported by histology, flow cytometry, and scRNA-seq to elucidate molecular mechanisms.

### Results

Young *Tet2+/-* CHIP mice exhibited similar primary tumor growth, but increased lung metastasis compared to WT animals. In middle-aged mice, *Tet2*+/- CHIP led to significantly enhanced tumor growth and metastasis, accompanied by reduced tumor-infiltrating *Tet2*+/- CD8+ and NKT cells. In contrast, both young and middle-aged $Dnmt3a^{fl\text{-}R878H}$ CHIP mice showed slower tumor growth with no change in metastasis. Preliminary scRNA-seq revealed suppressed T-cell responses in middle-aged *Tet2*+/- CHIP mice, while $Dnmt3a^{fl\text{-}R878H}$ CHIP animals showed enriched type I interferon signatures.

**Copyright Information:** (Online) ISSN: 2572-9241
©2026 The Author(s). HemaSphere is published by John Wiley & Sons Ltd on behalf of European Hematology Association. This is an open access Abstract Book distributed under the Attribution-NonCommercial-NoDerivs (CC BY-NC-ND), which allows third parties to download the articles and share them with others as long as they credit the author and the Abstract Book, but they cannot change the content in any way or use them commercially.
**Abstract Book Citations:** Authors, Title, HemaSphere, 2026;10:(S1):pages. The abstract book DOIs can be found on the "Author Information" page.
**Disclaimer:** Articles published in the journal HemaSphere exclusively reflect the opinions of the authors. The authors are responsible for all content in their abstracts including accuracy of the facts, statements, citing resources, etc.

**Summary/Conclusion**

These findings suggest age and CHIP mutations synergistically influence breast cancer progression by reshaping tumor-infiltrating immune populations. Further investigation is needed to define the molecular mechanisms and therapeutic implications of CHIP in solid tumor contexts.

**Copyright Information:** (Online) ISSN: 2572-9241
©2026 The Author(s). HemaSphere is published by John Wiley & Sons Ltd on behalf of European Hematology Association. This is an open access Abstract Book distributed under the Attribution-NonCommercial-NoDerivs (CC BY-NC-ND), which allows third parties to download the articles and share them with others as long as they credit the author and the Abstract Book, but they cannot change the content in any way or use them commercially.
**Abstract Book Citations:** Authors, Title, HemaSphere, 2026;10:(S1):pages. The abstract book DOIs can be found on the "Author Information" page.
**Disclaimer:** Articles published in the journal HemaSphere exclusively reflect the opinions of the authors. The authors are responsible for all content in their abstracts including accuracy of the facts, statements, citing resources, etc.

### (PS2297) BONE MARROW MESENCHYMAL CELLS UNDERGO INFLAMMATORY MYOFIBROBLAST DIFFERENTIATION UPON ALLOGENEIC HSCT CORRELATING WITH AN ALTERED SUPPORT OF HEMATOPOIESIS

**Topic:** 24. Hematopoiesis, stem cells and microenvironment

Sara Garaali*[1], Jana Lützkendorf[1], Edith Willscher[1], Katrin Nerger[1], Lisa MacHner[1], Michael Böttcher[2], Cornelia Baum[1], Klara Schwerd[1], Judith Schaffrath[1], Lutz Peter Müller[1]

*[1]Martin-Luther-University Halle-Wittenberg, Dept. of Internal Medicine IV, Halle (Saale), Germany;[2]Martin-Luther-University Halle-Wittenberg, Dept. of Molecular Medicine, Halle (Saale), Germany;*

### Background

The success of allogeneic hematopoietic stem cell transplantation (alloHSCT) relies on engraftment of donor hematopoietic stem and progenitor cells (HSPC) within the recipient bone marrow (BM) niche and subsequent graft-versus-leukemia (GvL) effect. Mesenchymal stromal cells (MSC) are a key component of the BM niche. Our previous data demonstrated alloHSCT-related damage to MSC, which was associated with reduced patient survival. We hypothesize that alloHSCT-related stress promotes a shift toward cancer-associated fibroblast (CAF)-like states in BM-MSC, impairing their niche function.

### Aims

To determine how alloHSCT-related toxicity alters the capacity of BM-MSC to support hematopoietic progenitors and to define MSC subtypes and transcriptional programs associated with alloHSCT.

### Methods

From paired pre-/post-alloHSCT BM aspirates of patients with myeloid neoplasia, CD45-/HLADR-/CD235a- cell fraction was sorted and subjected to single-cell RNA sequencing (scRNA-seq) by 10x Genomics protocol and gene set enrichment analysis. In parallel *in vitro* experiments, we simulated alloHSCT-related stress by irradiation of MSC (irrMSC) and analyzed their interaction with HSPC in 2D cocultures.

### Results

Data of scRNA-seq confirmed the presence of recipient-derived MSC within the sorted cell fraction. Post-alloHSCT samples showed a reduced mesenchymal cell fraction with a shift toward adipogenic and APOD+ MSC, while fibroblastic MSC and osteoblasts were decreased. Pre-alloHSCT stromal cells clustered closer to endothelial cells, whereas a distinct fraction of post-alloHSCT stromal cells exhibited cancer associated fibroblast (CAF) signatures and specifically inflammatory CAF (iCAF) features characterized in particular by upregulation of CFD, CXCL12, IL32 and CCL2.
As expected irrMSC showed reduced proliferation. However, flow cytometry and colony-forming unit assays revealed an enhanced support of HSPC by irrMSC. Specifically, increased IL6 secretion in irrMSC, correlated positively with HSPC counts and negatively with fully differentiated $CD34^{-}/CD45RA^{-}$ cells. Bulk RNA sequencing confirmed that irrMSC can adopt an iCAF and myCAF profile, however this was strinkingly variable between different MSC samples.

**Copyright Information:** (Online) ISSN: 2572-9241
©2026 The Author(s). HemaSphere is published by John Wiley & Sons Ltd on behalf of European Hematology Association. This is an open access Abstract Book distributed under the Attribution-NonCommercial-NoDerivs (CC BY-NC-ND), which allows third parties to download the articles and share them with others as long as they credit the author and the Abstract Book, but they cannot change the content in any way or use them commercially.
**Abstract Book Citations:** Authors, Title, HemaSphere, 2026;10:(S1):pages. The abstract book DOIs can be found on the “Author Information” page.
**Disclaimer:** Articles published in the journal HemaSphere exclusively reflect the opinions of the authors. The authors are responsible for all content in their abstracts including accuracy of the facts, statements, citing resources, etc.

**Summary/Conclusion**

AlloHSCT-related toxicity induces an iCAF phenotype in subsets of BM stromal cells and specifically of BM-MSC. This corresponds to an altered capacity to support hematopoiesis probably relating to the previously observed association of the alloHSCT-induced MSC-damage and patients survival. Future studies should focus on understanding the balance between inflammation-driven progenitor support and relapse-permissive niche remodeling.

**Copyright Information:** (Online) ISSN: 2572-9241
©2026 The Author(s). HemaSphere is published by John Wiley & Sons Ltd on behalf of European Hematology Association. This is an open access Abstract Book distributed under the Attribution-NonCommercial-NoDerivs (CC BY-NC-ND), which allows third parties to download the articles and share them with others as long as they credit the author and the Abstract Book, but they cannot change the content in any way or use them commercially.
**Abstract Book Citations:** Authors, Title, HemaSphere, 2026;10:(S1):pages. The abstract book DOIs can be found on the "Author Information" page.
**Disclaimer:** Articles published in the journal HemaSphere exclusively reflect the opinions of the authors. The authors are responsible for all content in their abstracts including accuracy of the facts, statements, citing resources, etc.

### (PS2298) DECODING BLOOD FORMATION: LINKING CODON USAGE, TRANSFER RNA EXPRESSION AND CELL IDENTITY IN HEMATOPOIESIS

**Topic:** 24. Hematopoiesis, stem cells and microenvironment

Marius Külp*[1, 2, 3], Anna Wiegand[1], Tessa Schmachtel[1], Björn Häupl[1, 3, 4], Thomas Oellerich[1, 3, 4], Halvard Bonig[4, 5, 6], Michael Rieger[1, 2, 3, 4]

*[1]Goethe University Frankfurt, University Hospital, Department of Hematology and Oncology, Frankfurt am Main, Germany; [2]Cardio-Pulmonary Institute, Frankfurt am Main, Germany; [3]German Cancer Consortium (DKTK), Partner site Frankfurt/Mainz, Frankfurt am Main, Germany; [4]Frankfurt Cancer Institute (FCI), Frankfurt am Main, Germany; [5]Goethe University Frankfurt, Institute for Transfusion Medicine and Immunohematology, Frankfurt am Main, Germany; [6]German Red Cross Blood Service Baden-Württemberg-Hessen, Frankfurt am Main, Germany;*

### Background

The genetic code determines the composition and function of RNA and proteins. While all cells rely on the same set of codons, codon usage and transfer RNA (tRNA) expression vary among tissues.

### Aims

We developed a precision method to trace the genetic code from genome to proteome, integrating mRNA and tRNA transcriptomes with the translatome and proteome of cell populations at various stages of hematopoiesis. Thereby, we dissect hematopoietic differentiation at single-codon resolution.

### Methods

Sequencing of tRNAs is challenging due to sequence redundancy, base modifications and rigid secondary structures. We developed a low-input tRNA-sequencing approach including a computational workflow that efficiently captures and aligns full-length tRNAs from a few thousand primary human cells. This allowed us to characterize the tRNA expression profiles of sorted hematopoietic stem cells (HSCs), progenitors and T cells derived from healthy human donors.

### Results tRNA expression profiles alone distinguished hematopoietic stem and progenitor cells (HSPCs) from T cells in unsupervised clustering analyses, underscoring the role of tRNA abundance in defining cellular identity. We detected more than 25 differentially expressed tRNAs between these cell populations.
We integrated tRNA demand, derived from transcriptomic codon usage, with tRNA supply, reflected by tRNA expression, and identified lowly and highly supplied codons. For specific codons, relative tRNA availability varied across the hematopoietic hierarchy. We are currently assessing how these changes influence translational fidelity by aligning translatomic and proteomic data and applying machine learning and perturbation-based analyses.
We validated our approach using peripheral blood samples from ten healthy donors and confirmed codon-specific supply levels. A meta-ORF analysis revealed a depletion of lowly supplied codons at translation start sites, suggesting that codon positioning contributes to the regulation of protein abundance in human blood cells.

**Copyright Information:** (Online) ISSN: 2572-9241
©2026 The Author(s). HemaSphere is published by John Wiley & Sons Ltd on behalf of European Hematology Association. This is an open access Abstract Book distributed under the Attribution-NonCommercial-NoDerivs (CC BY-NC-ND), which allows third parties to download the articles and share them with others as long as they credit the author and the Abstract Book, but they cannot change the content in any way or use them commercially.
**Abstract Book Citations:** Authors, Title, HemaSphere, 2026;10:(S1):pages. The abstract book DOIs can be found on the "Author Information" page.
**Disclaimer:** Articles published in the journal HemaSphere exclusively reflect the opinions of the authors. The authors are responsible for all content in their abstracts including accuracy of the facts, statements, citing resources, etc.

**Summary/Conclusion**

In conclusion, we reveal differential tRNA expression across the hematopoietic system, trace codon usage from genome to proteome, and uncover mechanisms of translational regulation that may shape distinct cellular states.

**Copyright Information:** (Online) ISSN: 2572-9241
©2026 The Author(s). HemaSphere is published by John Wiley & Sons Ltd on behalf of European Hematology Association. This is an open access Abstract Book distributed under the Attribution-NonCommercial-NoDerivs (CC BY-NC-ND), which allows third parties to download the articles and share them with others as long as they credit the author and the Abstract Book, but they cannot change the content in any way or use them commercially.
**Abstract Book Citations:** Authors, Title, HemaSphere, 2026;10:(S1):pages. The abstract book DOIs can be found on the "Author Information" page.
**Disclaimer:** Articles published in the journal HemaSphere exclusively reflect the opinions of the authors. The authors are responsible for all content in their abstracts including accuracy of the facts, statements, citing resources, etc.

### (PS2299) UNCOVERING NEW ROLES FOR H/ACA SNORNPS IN MYC-DRIVEN ONCOGENIC STRESS AND HEMATOPOIESIS

**Topic:** 24. Hematopoiesis, stem cells and microenvironment

Silvia Tucciarone*[1], Gaia Menichincheri[1], Sowndarya Muthukumar[1], Cristian Bellodi[1], Mariel Kleer[1], Phuong Cao Thi Ngoc[2], Joan Yuan[2]

[1]*University of Copenhagen, København N, Denmark;* [2]*Lund University, Lund, Sweden;*

### Background

RNA chemical modifications are conserved RNA hallmarks providing dynamic regulation of gene expression in response to pathophysiological cues. A striking example is pseudouridine (Ψ), the most widespread and evolutionarily conserved RNA modification. A multitude of small ribonucleoproteins (snoRNPs), consisting of a guide H/ACA box small nucleolar RNA bound to four protein components, Dyskerin (DKC1), NOP10, NHP2 and GAR1, are responsible for Ψ catalysis on different types of coding and noncoding RNAs, with important roles in telomere, ribosome, and splicing regulation.

### Aims

Although dysregulation of Ψ is associated with bone marrow failure and cancer in humans, how acute loss of H/ACA snoRNPs impacts HSC function and transformation remains incompletely understood.

### Methods

To dissect the function of Ψ in the hematopoietic system, our team generated a Ψ conditional knock-out (KO) mouse by targeting Gar1, an essential H/ACA snoRNP core component dispensable for telomere maintenance. Strikingly, acute depletion of Gar1 leads to rapid hematopoietic stem cell (HSC) loss, likely independent of defects in major canonical H/ACA snoRNP targets.

### Results

Furthermore, we unveiled a functional link between the H/ACA snoRNP and MYC, a pleiotropic transcription factor and critical regulator of HSC function, homeostasis and survival. Gar1 depletion leads to a rapid reduction of protein synthesis upon MYC activation, independent of ribosome function, which triggers integrated stress response (ISR), an adaptive response that curbs translation upon MYC oncogenic stress. Given the pivotal role of protein synthesis in MYC-driven tumorigenesis, we are currently investigating the molecular mechanism linking Gar1 loss to ISR-mediated translation rewiring in normal and malignant hematopoiesis.

### Summary/Conclusion

Collectively, our work has the potential to uncover novel epitranscriptome-based programs shaping translation and hematopoietic cell-fate commitment, with broad clinical implications for treating aggressive hematological disorders characterized by aberrant protein synthesis and MYC dysregulation.

**Copyright Information:** (Online) ISSN: 2572-9241
©2026 The Author(s). HemaSphere is published by John Wiley & Sons Ltd on behalf of European Hematology Association. This is an open access Abstract Book distributed under the Attribution-NonCommercial-NoDerivs (CC BY-NC-ND), which allows third parties to download the articles and share them with others as long as they credit the author and the Abstract Book, but they cannot change the content in any way or use them commercially.
**Abstract Book Citations:** Authors, Title, HemaSphere, 2026;10:(S1):pages. The abstract book DOIs can be found on the "Author Information" page.
**Disclaimer:** Articles published in the journal HemaSphere exclusively reflect the opinions of the authors. The authors are responsible for all content in their abstracts including accuracy of the facts, statements, citing resources, etc.

### (PS2300) IRON DEFICIENCY IS ASSOCIATED WITH REDUCED TRANSFERRIN RECEPTOR 2 EXPRESSION IN EARLY HEMATOPOIETIC PROGENITORS

**Topic:** 24. Hematopoiesis, stem cells and microenvironment

João Vitor Facco*[1, 2], Karla Vieira Ferro[1, 2], Irene Pereira Santos[1, 2], Nádia Ghinelli Amôr[1, 2], Sara Olalla Saad[1, 2]

*[1]University of Campinas (UNICAMP), Hematology and Transfusion Medicine Center (Hemocentro UNICAMP), Campinas, Brazil; [2]Blood National Institute of Science and Technology (INCT Sangue), Campinas, Brazil;*

### Background

Iron deficiency (ID) is a leading cause of anemia worldwide and is frequently associated with reactive thrombocytosis. While impaired erythropoiesis under ID is well characterized, the biological mechanisms linking systemic iron availability to altered megakaryocytic–erythroid output remain incompletely understood. Recent evidence suggests that iron availability can influence lineage commitment at the level of megakaryocytic–erythroid progenitors, but how early hematopoietic progenitors sense systemic iron status *in vivo* is unclear. Transferrin receptor 2 (TfR2), a key component of systemic iron sensing and erythropoietic regulation, represents a plausible molecular interface through which iron availability could be integrated at the progenitor level.

### Aims

To investigate whether systemic iron deficiency modulates TfR2 expression in early hematopoietic progenitors *in vivo*, with particular focus on the megakaryocytic–erythroid progenitor compartment.

### Methods

C57BL/6 mice received either a standard (Std; Fe >50 ppm) or an iron-deficient (ID; Fe <5 ppm) diet. We assessed hematological parameters by complete blood count and analyzed bone marrow hematopoietic progenitors by flow cytometry. The analyzed populations included hematopoietic stem cells (HSC), megakaryocytic–erythroid progenitors (MEP), primitive megakaryocytic–erythroid progenitors (pMeg-E), megakaryocytic progenitors (MkP), and committed erythroid progenitors (CFU-E). We quantified TfR2 surface expression as both the frequency of $TfR2^+$ cells and mean fluorescence intensity (MFI). Statistical comparisons between groups were performed using Mann–Whitney tests with Holm–Šidák correction for multiple comparisons.

### Results

Mice subjected to dietary iron deficiency developed a microcytic anemia, characterized by reduced hemoglobin levels and mean corpuscular volume, increased red cell distribution width, and elevated platelet counts, consistent with reactive thrombocytosis. Flow cytometric analysis revealed a significant reduction in the frequency of $TfR2^+$ cells across early hematopoietic progenitor compartments in ID mice compared with controls. This effect was most pronounced at the MEP stage. Consistently, TfR2 surface expression per cell was significantly reduced in MEP from iron-deficient mice, as reflected by decreased MFI, whereas changes in other progenitor populations were less marked. These findings indicate that systemic iron deficiency is associated with stage-specific modulation of TfR2 expression in early progenitors.

**Copyright Information:** (Online) ISSN: 2572-9241
©2026 The Author(s). HemaSphere is published by John Wiley & Sons Ltd on behalf of European Hematology Association. This is an open access Abstract Book distributed under the Attribution-NonCommercial-NoDerivs (CC BY-NC-ND), which allows third parties to download the articles and share them with others as long as they credit the author and the Abstract Book, but they cannot change the content in any way or use them commercially.
**Abstract Book Citations:** Authors, Title, HemaSphere, 2026;10:(S1):pages. The abstract book DOIs can be found on the "Author Information" page.
**Disclaimer:** Articles published in the journal HemaSphere exclusively reflect the opinions of the authors. The authors are responsible for all content in their abstracts including accuracy of the facts, statements, citing resources, etc.

**Summary/Conclusion**

Systemic iron deficiency is associated with reduced TfR2 expression in early hematopoietic progenitors *in vivo*, with a preferential effect at the megakaryocytic–erythroid progenitor stage. These data identify TfR2 as a candidate molecular interface linking systemic iron availability to progenitor-level hematopoietic adaptation under iron-deficient conditions. While not establishing causality, our findings provide *in vivo* evidence that iron-dependent cues are integrated at early stages of hematopoiesis and may contribute to the altered megakaryocytic–erythroid balance observed in iron deficiency.

**Copyright Information:** (Online) ISSN: 2572-9241
©2026 The Author(s). HemaSphere is published by John Wiley & Sons Ltd on behalf of European Hematology Association. This is an open access Abstract Book distributed under the Attribution-NonCommercial-NoDerivs (CC BY-NC-ND), which allows third parties to download the articles and share them with others as long as they credit the author and the Abstract Book, but they cannot change the content in any way or use them commercially.
**Abstract Book Citations:** Authors, Title, HemaSphere, 2026;10:(S1):pages. The abstract book DOIs can be found on the “Author Information” page.
**Disclaimer:** Articles published in the journal HemaSphere exclusively reflect the opinions of the authors. The authors are responsible for all content in their abstracts including accuracy of the facts, statements, citing resources, etc.

### (PS2301) SCRNA-SEQ ANALYSIS WORKFLOW TO CHARACTERIZE WNT-MODULATED MESODERM AND HEMATOPOIETIC TRAJECTORIES IN HUMAN GASTRULOIDS

**Topic:** 24. Hematopoiesis, stem cells and microenvironment

Elin Hilpold-Berntsson*[1], Anuntxi Monsalve-Iguiniz[1], Pavan Kumar Mysuru Shivalingappa[1], Anna Hogmalm[1], Robel Tesfaye[1], Carolina Guibentif[1]

[1]*University of Gothenburg, Sahlgrenska Center for Cancer Research / Sahlgrenska Academy, Institute of Biomedicine, Department of Microbiology and Immunology, Gothenburg, Sweden;*

### Background

Human pluripotent stem cells (hPSCs) provide a powerful platform to model early human hematopoietic development *in vitro*, yet the hematopoietic system remains difficult to recapitulate in the dish. This challenge arises from the complex unfolding of the embryonic hematopoiesis, occurring in distinct embryonic anatomical sites via three overlapping sequential waves: primitive, pro-definitive, and definitive. Accurate modelling of human embryonic hematopoiesis will provide a new source of clinically relevant cell types, such as hematopoietic stem cells, and a platform to study molecular mechanisms of congenital diseases and prenatal transformation events leading to childhood leukemia. We explore the hematopoietic potential of a gastruloid-based hPSC differentiation system by examining morphogen-mediated mesoderm patterning.

### Aims

Utilizing defined concentrations of CHIR99021 (CHIR) during gastruloid formation, our aim is to investigate how WNT signaling regulates emergence of distinct mesodermal subtypes driving the hematopoietic waves.

### Methods

At gastruloid formation (day 0), CHIR is pulsed at 1.8, 3, or 5 µM, and at day 3 and day 8 gastruloids are harvested for single-cell RNA sequencing (scRNA-seq). scRNA-seq of early-stage gastruloids is used to benchmark computational workflows such as quality control, normalization, and cell type annotation; this enables identification of reproducible transcriptional trajectories and defines how WNT modulation shapes cell-fate specification in blood development.

### Results

We characterized a prominent *PDGFRA*$^+$/*PRRX1*$^+$ stromal population and a WNT dose-dependent shift in mesodermal lineage composition. At day 3, low CHIR favored extra-embryonic mesoderm and NMPs/PSM, whereas higher CHIR promoted lateral plate mesoderm (LPM) and paraxial mesoderm lineages. By day 8, diverse *VCAN*$^+$/*FN1*$^+$/*COL3A1*$^+$/*ADAM12*$^+$ mesenchymal populations emerged, suggestive of a heterogeneous stromal compartment potentially supportive of hematopoietic specification. Importantly, we identify emergence of endothelial cells, putative developmental precursors to embryonic hematopoietic cells.

### Summary/Conclusion

Together, these findings show that WNT modulation during gastruloid formation reshapes mesodermal patterning and the co-developing niche enabling hematopoietic cell emergence *in vitro*.

**Copyright Information:** (Online) ISSN: 2572-9241
©2026 The Author(s). HemaSphere is published by John Wiley & Sons Ltd on behalf of European Hematology Association. This is an open access Abstract Book distributed under the Attribution-NonCommercial-NoDerivs (CC BY-NC-ND), which allows third parties to download the articles and share them with others as long as they credit the author and the Abstract Book, but they cannot change the content in any way or use them commercially.
**Abstract Book Citations:** Authors, Title, HemaSphere, 2026;10:(S1):pages. The abstract book DOIs can be found on the “Author Information” page.
**Disclaimer:** Articles published in the journal HemaSphere exclusively reflect the opinions of the authors. The authors are responsible for all content in their abstracts including accuracy of the facts, statements, citing resources, etc.

### (PS2302) THE EFFECT OF STATIN THERAPY AND SMALL DENSE LIPOPROTEINS ON CLONAL HAEMATOPOIESIS OF INDETERMINATE POTENTIAL: A TWO SAMPLE MENDELIAN RANDOMISATION STUDY.

**Topic:** 24. Hematopoiesis, stem cells and microenvironment

Ellen Nuttall Musson*[1], Elspeth Payne[1, 2]

[1]*UCL Cancer Institute, Research Department of Haematology, London, United Kingdom;*[2]*University College London Hospitals, London, United Kingdom;*

### Background

There is epidemiological evidence suggesting that lipid homeostasis dysregulation is associated with *TET2* mutated clonal haematopoiesis of indeterminate potential (CHIP). We have previously described that lipid-lowering therapy is associated with reduced *TET2* CHIP clone size and clonal expansion (Nuttall Musson et al). Other longitudinal CHIP cohorts have also described that dyslipidaemia is associated with incident *TET2* CHIP, with a trend towards higher triglyceride/high-density lipoprotein cholesterol (often used to proxy hyper-inflammatory and pro-atherogenic small dense LDL cholesterol (sLDL)) being associated with *TET2* CHIP. However, the degree to which these epidemiological associations correspond to causative relationships is unclear.

### Aims

To leverage a two sample mendelian randomisation approach to assess for causative roles of 1) genetically proxied HMGCR (the primary site of action of statin therapy) inhibition and 2) sLDL lipid parameters in *TET2* and *DNMT3A* CHIP.

### Methods

We used CHIP genome wide association study (GWAS) summary data from 200,453 individuals in the UKBiobank (Kar et al) as outcome data. To assess the effect of statin use, proxied HMGCR inhibition using 5 single nucleotide polymorphisms (SNPs) from a well-established instrumental variable (Ference et al). To assess the effect of sLDL, we used GWAS summary data from a majority Northern European meta-analysis of nuclear magnetic resonance lipid profiles from 136,016 participants (Karjalainen et al) as exposure data for sLDL particle concentration (sLDL-pc, 74 SNPs), sLDL free cholesterol (sLDL-fc, 78 SNPs), sLDL phospholipids (sLDL-pl, 74 SNPs) and sLDL triglycerides (sLDL-tg, 64 SNPs). Inverse variance weighted (IVW) estimates were used as the primary outcome, with analyses undertaken using weighted median and MR-Egger estimates. sLDL analyses were adjusted for multiple comparisons using the Benjamini-Hochberg (BH) procedure.

### Results

HMGCR inhibition was associated with a reduced risk of *DNMT3A* CHIP (IVW (95% confidence interval (CI)): 0.029 (0.013-0.045), $p < 0.001$), with no relationship observed for *TET2* CHIP ((IVW (95%CI): -0.006 (-0.033 - 0.021), p = 0.66). Predicted sLDL-pc, sLDL-fc, sLDL-pl and sLDL-tg showed no relationship with *DNMT3A* CHIP with IVWs (95% CIs) of 0.04 (-0.06 - 0.13, p = 0.49), 0.04 (-0.06 - 0.13, p = 0.44), 0.05 (-0.05 - 0.15, p = 0.32) and 0.10 (-0.03 - 0.22, p = 0.14), respectively. Conversely, predicted sLDL-pc, sLDL-fc and sLDL-pl showed significant putative causal relationships for *TET2* CHIP with IVWs (95% CIs) of 0.21 (0.05 - 0.37, BH q = 0.024), 0.24 (0.09 - 0.40, BH q = 0.01) and 0.19 (0.03 - 0.35, BH q = 0.035), respectively. No relationship was observed between *TET2* CHIP and sLDL-tg (IVW (95% CI): 0.04 (-0.16 - 0.23), p = 0.72).

**Copyright Information:** (Online) ISSN: 2572-9241
©2026 The Author(s). HemaSphere is published by John Wiley & Sons Ltd on behalf of European Hematology Association. This is an open access Abstract Book distributed under the Attribution-NonCommercial-NoDerivs (CC BY-NC-ND), which allows third parties to download the articles and share them with others as long as they credit the author and the Abstract Book, but they cannot change the content in any way or use them commercially.
**Abstract Book Citations:** Authors, Title, HemaSphere, 2026;10:(S1):pages. The abstract book DOIs can be found on the "Author Information" page.
**Disclaimer:** Articles published in the journal HemaSphere exclusively reflect the opinions of the authors. The authors are responsible for all content in their abstracts including accuracy of the facts, statements, citing resources, etc.

### Summary/Conclusion

These results suggest that statin therapy could reduce the risk of *DNMT3A* CHIP. The lack of relationship between HMGCR inhibition and *TET2* CHIP could reflect a limitation of how closely this analysis models high intensity statin treatment later in life, or that the previously described epidemiological effects are HMGCR independent. A number of sLDL lipid parameters, conceivably modified by lipid lowering therapy, showed putative causal relationships with *TET2* CHIP. These findings provide further evidence to support a role for lipid dysregulation in the pathogenesis of CHIP, and support further research into the efficacy of lipid lowering therapy in disease modification in CHIP.

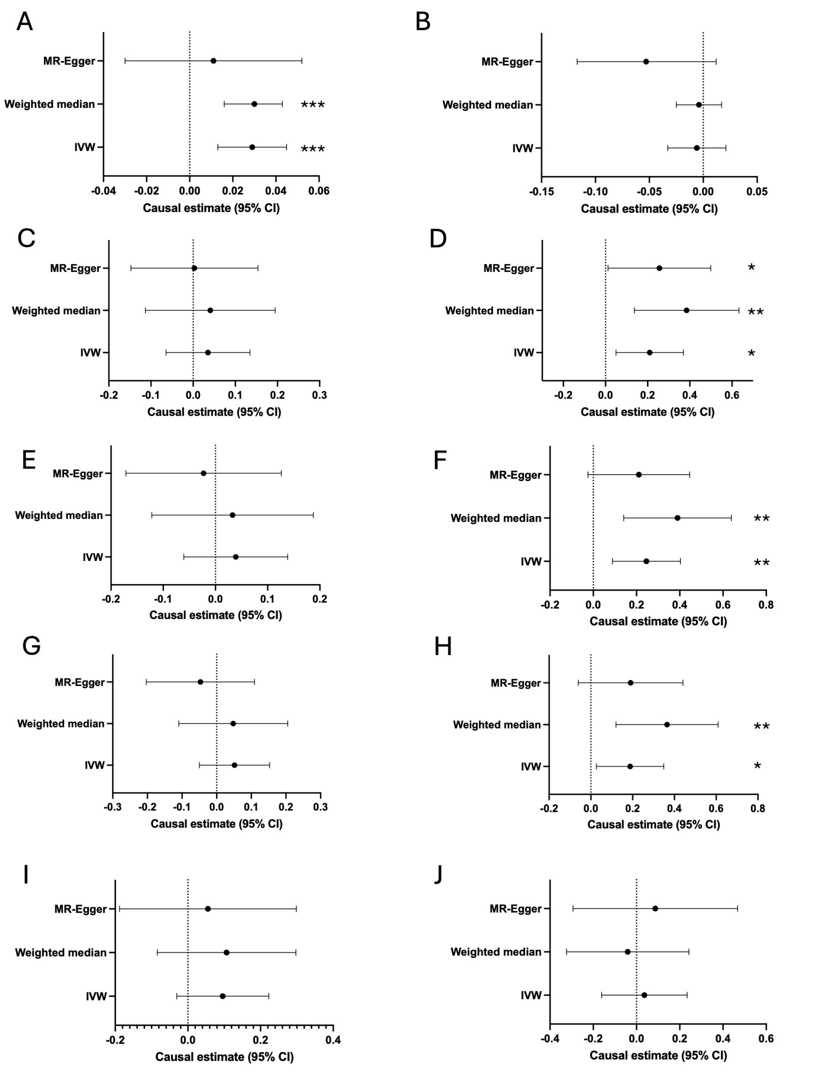

Figure 1: Two sample mendelian randomisation analyses. A) The effect of HMGCR inhibition on *DNMT3A* CHIP. B) The effect of HMGCR inhibition on *TET2* CHIP. C) The effect of sLDL-pc on *DNMT3A* CHIP. D) The effect of sLDL-pc on *TET2* CHIP. E) The effect of sLDL-fc on *DNMT3A* CHIP. F) The effect of sLDL-fc on *TET2* CHIP. G) The effect of sLDL-pl on *DNMT3A* CHIP. H) The effect of sLDL-pl on *TET2* CHIP. I) The effect of sLDL-tg on *DNMT3A* CHIP. J) The effect of sLDL-tg on *TET2* CHIP. ***: p<0.001, **: p<0.01, *: p<0.05.

**Copyright Information:** (Online) ISSN: 2572-9241
©2026 The Author(s). HemaSphere is published by John Wiley & Sons Ltd on behalf of European Hematology Association. This is an open access Abstract Book distributed under the Attribution-NonCommercial-NoDerivs (CC BY-NC-ND), which allows third parties to download the articles and share them with others as long as they credit the author and the Abstract Book, but they cannot change the content in any way or use them commercially.
**Abstract Book Citations:** Authors, Title, HemaSphere, 2026;10:(S1):pages. The abstract book DOIs can be found on the "Author Information" page.
**Disclaimer:** Articles published in the journal HemaSphere exclusively reflect the opinions of the authors. The authors are responsible for all content in their abstracts including accuracy of the facts, statements, citing resources, etc.

### (PS2303) A STANDARDIZED IN VITRO BONE NICHE MODEL FOR QUANTITATIVE ANALYSIS OF OSTEOCLAST-MEDIATED MICROENVIRONMENT REMODELING RELEVANT TO HEMATOLOGIC MALIGNANCIES

**Topic:** 24. Hematopoiesis, stem cells and microenvironment

Min Wu*[1], Yan MA[2], Renren Chen[3], Dandan Cao[3], Yan Shen[3], Shunyuan Lu[3], Chunling Shen[3], Hongxin Zhang[3], Jianqing Mi[3], Zhugang Wang[3], Lingyun Tang[3]

*[1]Shanghai Institute of Hematology, State Key Laboratory of Medical Genomics, National Research Center for Translational Medicine at Shanghai, Ruijin Hospital Affiliated to Shanghai Jiao Tong University School of Medicine, Shanghai, China; [2]Ruijin Hospital Lu Wan Branch, Shanghai Jiaotong University School of Medicine, Shanghai, China; [3]State Key Laboratory of Medical Genomics, Research Center for Experimental Medicine, Ruijin Hospital Affiliated to Shanghai Jiao Tong University School of Medicine, Shanghai, China;*

### Background

Osteoclast-driven bone marrow niche remodeling is an important component of the bone microenvironment and has been implicated in the regulation of hematopoiesis as well as in the progression of hematologic malignancies, including acute myeloid leukemia (AML) and multiple myeloma. Alterations in the endosteal niche may influence leukemic cell survival, proliferation, and response to therapy. However, mechanistic studies of osteoclast-mediated microenvironment remodeling remain limited by the lack of standardized, reproducible, and scalable in vitro models. Current resorption assays relying on natural bone or synthetic substrates are affected by substantial heterogeneity, restricting their application in quantitative studies and high-throughput drug screening.

### Aims

To establish and validate a reproducible, rapidly mineralizing in vitro bone microenvironment platform suitable for quantitative assessment of osteoclast activity and for studying bone marrow niche remodeling relevant to hematopoiesis and leukemia–microenvironment interactions.

### Methods

We developed a uniform mineralized extracellular matrix (ECM) platform using primary and SV40T-immortalized murine dental pulp stem cells (DPSCs). Following rapid osteogenic induction and decellularization, the ECM served as a standardized mineralized substrate for osteoclast differentiation. Osteoclast precursors derived from murine and rat bone marrow mononuclear cells as well as human THP-1 monocytes were cultured on the platform. Osteoclast maturation and resorption activity were evaluated by tartrate-resistant acid phosphatase (TRAP) staining, F-actin ring visualization, scanning electron microscopy, and Von Kossa–based whole-well quantitative analysis. Responsiveness of the system to microenvironmental regulators, including RANKL and IFN-γ, was assessed.

### Results

The engineered ECM formed within 7 days and displayed dentin-like ultrastructural characteristics, providing a highly uniform mineralized substrate compared with conventional bone slices. The platform supported efficient differentiation of hematopoietic precursors from multiple species into TRAP-positive multinucleated osteoclasts with characteristic actin rings and measurable resorption pits. Whole-well quantitative analysis enabled high-throughput evaluation of osteoclast function. Osteoclast activity demonstrated clear dose-dependent modulation in response to RANKL and IFN-γ, confirming the functional sensitivity of the system. Compared with existing assays, the DPSC-derived ECM showed improved reproducibility, accessibility, and suitability for large-scale experiments.

**Copyright Information:** (Online) ISSN: 2572-9241
©2026 The Author(s). HemaSphere is published by John Wiley & Sons Ltd on behalf of European Hematology Association. This is an open access Abstract Book distributed under the Attribution-NonCommercial-NoDerivs (CC BY-NC-ND), which allows third parties to download the articles and share them with others as long as they credit the author and the Abstract Book, but they cannot change the content in any way or use them commercially.
**Abstract Book Citations:** Authors, Title, HemaSphere, 2026;10:(S1):pages. The abstract book DOIs can be found on the "Author Information" page.
**Disclaimer:** Articles published in the journal HemaSphere exclusively reflect the opinions of the authors. The authors are responsible for all content in their abstracts including accuracy of the facts, statements, citing resources, etc.

**Summary/Conclusion**

We established a standardized and scalable in vitro bone microenvironment platform that enables reliable quantitative analysis of osteoclast-mediated matrix resorption. This model provides a practical tool for investigating bone marrow niche remodeling and may facilitate studies of osteoclast–hematopoietic and osteoclast–leukemia interactions, as well as preclinical evaluation of microenvironment-targeting therapies in hematologic diseases.

**Copyright Information:** (Online) ISSN: 2572-9241
©2026 The Author(s). HemaSphere is published by John Wiley & Sons Ltd on behalf of European Hematology Association. This is an open access Abstract Book distributed under the Attribution-NonCommercial-NoDerivs (CC BY-NC-ND), which allows third parties to download the articles and share them with others as long as they credit the author and the Abstract Book, but they cannot change the content in any way or use them commercially.
**Abstract Book Citations:** Authors, Title, HemaSphere, 2026;10:(S1):pages. The abstract book DOIs can be found on the "Author Information" page.
**Disclaimer:** Articles published in the journal HemaSphere exclusively reflect the opinions of the authors. The authors are responsible for all content in their abstracts including accuracy of the facts, statements, citing resources, etc.

### (PS2304) FLT3 INHIBITION SYNERGIZES WITH DNA DAMAGING AGENTS TO ELIMINATE HEMATOPOIETIC STEM AND PROGENITOR CELLS BY SUPPRESSING DNA REPAIR PATHWAYS

**Topic:** 24. Hematopoiesis, stem cells and microenvironment

Samuel Taylor*[1, 2, 3], Catherine Jankovic[3], Catherine Rinaldi[2], Swathi-Rao Narayanagari[3], Johanna Duyvestyn[2], Ulrich Steidl[3], Carolyn Grove[4], Wallace Langdon[2]

*[1]Washington University in St Louis, Pediatrics, St Louis, United States of America;[2]University of Western Australia, School of Biomedical Sciences, Perth, Australia; [3]Albert Einstein College of Medicine, Cell Biology, Bronx, United States of America; [4]Queen Elizabeth II Medical Centre, PathWest Department of Haematology, Perth, Australia;*

### Background

Quizartinib, a FLT3 inhibitor, has been shown to induce quiescence in hematopoietic stem and progenitor cells (HSPCs), affording protection from S-phase-specific chemotherapies. In this study we investigated whether quizartinib can be combined with other chemotherapies and radiotherapy for therapeutic benefit.

### Aims

To categorize the synergism between FLT3 inhibition and DNA damaging therapies upon healthy HSPCs.

### Methods

C57BL/6 mice were treated with quizartinib (30 mg/kg) or vehicle by oral gavage and subsequently exposed to DNA-damaging chemotherapies or total body irradiation (TBI) at 2–6 hours later. Bone marrow (BM) was analyzed via histological examination, flow cytometry (including phospho-flow) and RNA-sequencing at defined timepoints post-treatment. To assess HSC transplantation efficacy, mice were conditioned with sublethal TBI (700 Rads), transplanted with 2 million CD45.1 BM cells, and monitored for donor cell engraftment for 4 months.

**Copyright Information:** (Online) ISSN: 2572-9241
©2026 The Author(s). HemaSphere is published by John Wiley & Sons Ltd on behalf of European Hematology Association. This is an open access Abstract Book distributed under the Attribution-NonCommercial-NoDerivs (CC BY-NC-ND), which allows third parties to download the articles and share them with others as long as they credit the author and the Abstract Book, but they cannot change the content in any way or use them commercially.
**Abstract Book Citations:** Authors, Title, HemaSphere, 2026;10:(S1):pages. The abstract book DOIs can be found on the “Author Information” page.
**Disclaimer:** Articles published in the journal HemaSphere exclusively reflect the opinions of the authors. The authors are responsible for all content in their abstracts including accuracy of the facts, statements, citing resources, etc.

### Results

In contrast to quizartinib's protection of bone marrow cells to S-phase specific chemotherapies quizartinib drastically synergized with DNA damaging chemotherapies cyclophosphamide, carboplatin, cisplatin, temozolomide, and mitoxantrone to deplete HSPC and BM populations. To examine whether quizartinib similarly sensitizes bone marrow cells to the effects of ionizing radiation we dosed mice with vehicle or quizartinib at various times before exposure to TBI. Flow cytometry and histological analysis revealed significant reductions in BM cells, myeloid and multipotent progenitors, and short- and long-term HSCs in quizartinib-primed mice compared to controls (Figure 1). When combined with 5.5 Gy TBI (half a standard conditioning dose), quizartinib resulted in profound myeloablation. Even at 3 Gy, quizartinib maintained potent depletion of total BM, progenitor, and stem cells, with sensitization persisting when dosed 24 hours prior to TBI. Importantly, H&E sections of small intestines revealed no differences in the extent of crypt epithelial injury between vehicle-treated and quizartinib-treated irradiated mice.
To explore underlying mechanisms, we performed RNA-sequencing of sorted LSK and LK cells 20 hours post-treatment. Quizartinib downregulated genes critical for DNA damage repair, including *Brca1*, *Brca2*, *Rad51*, and *Nbn*, in both murine (LSK cells) and human (CD34+) HSPCs. Ionizing radiation exposure in quizartinib-primed HSPCs caused elevated and prolonged γ-H2AX and phospho-ATM signaling, indicating impaired DNA repair and persistence of DNA damage.
We next evaluated the effect of quizartinib upon hematopoeitic stem cell transplant (HSCT) by treating mice with quizartinib, or vehicle, followed by sublethal TBI and BM transplantation. Quizartinib treated mice exhibited enhanced donor-derived multilineage reconstitution compared to vehicle treated mice suggesting that quizartinib enhances myeloablative conditioning by augmenting host HSPC clearance.

### Summary/Conclusion

Quizartinib sensitizes HSPCs to DNA-damaging therapies via downregulation of DNA repair genes and impaired resolution of DNA damage, resulting in enhanced HSPC depletion. This quizartinib-driven sensitization of the host BM leads to superior engraftment after HSCT in murine models. These results provide a rationale for incorporating quizartinib into reduced-intensity, myeloablative conditioning regimens and highlight an unappreciated FLT3-DNA damage repair axis which appears crucial to healthy hematopoiesis.

**Copyright Information:** (Online) ISSN: 2572-9241
©2026 The Author(s). HemaSphere is published by John Wiley & Sons Ltd on behalf of European Hematology Association. This is an open access Abstract Book distributed under the Attribution-NonCommercial-NoDerivs (CC BY-NC-ND), which allows third parties to download the articles and share them with others as long as they credit the author and the Abstract Book, but they cannot change the content in any way or use them commercially.
**Abstract Book Citations:** Authors, Title, HemaSphere, 2026;10:(S1):pages. The abstract book DOIs can be found on the "Author Information" page.
**Disclaimer:** Articles published in the journal HemaSphere exclusively reflect the opinions of the authors. The authors are responsible for all content in their abstracts including accuracy of the facts, statements, citing resources, etc.

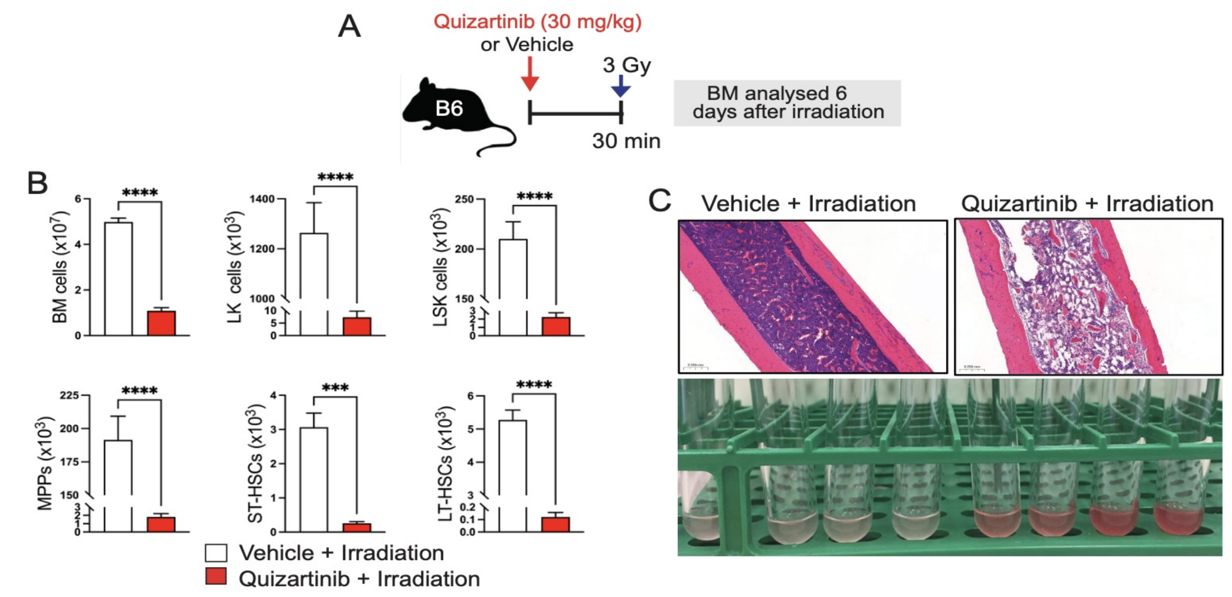

**Figure 1. Quizartinib enhances radiation-induced myeloablation. A**. Mice were dosed with vehicle or quizartinib (30 mg/kg) and exposed to 3 Gy 30 min later. **B**. BM cells were analysed by hemavet counting and flow cytometry after 6 days, and cell numbers are from 2 tibias and 2 femurs. The data are from 4 mice in each group, and the graphs are expressed as mean ± SEM. ***$p<0.001$ and ****$p<0.0001$. **C.** Representative H&E sections of bones and cell suspensions showing the markedly different appearances between the two treatments.

**Copyright Information:** (Online) ISSN: 2572-9241
©2026 The Author(s). HemaSphere is published by John Wiley & Sons Ltd on behalf of European Hematology Association. This is an open access Abstract Book distributed under the Attribution-NonCommercial-NoDerivs (CC BY-NC-ND), which allows third parties to download the articles and share them with others as long as they credit the author and the Abstract Book, but they cannot change the content in any way or use them commercially.
**Abstract Book Citations:** Authors, Title, HemaSphere, 2026;10:(S1):pages. The abstract book DOIs can be found on the "Author Information" page.
**Disclaimer:** Articles published in the journal HemaSphere exclusively reflect the opinions of the authors. The authors are responsible for all content in their abstracts including accuracy of the facts, statements, citing resources, etc.

### (PS2305) P-TERT RELATED ADAPTIVE SOMATIC HEMATOPOIETIC MOSAICISM IN PATIENTS WITH TELOMERE BIOLOGY DISORDERS

**Topic:** 24. Hematopoiesis, stem cells and microenvironment

Madeline Franke*[1], Rachel Kirchner[2], Bianca Barredo[2], Amar Zaidan[3], Jane Churpek[2], Daria Barbushok[3], Terra Lasho[4], Tariq Kewan[4], Olisaemeka Ogbue[4], Abhishek Mangaonkar[4], Alejandro Ferrer[4, 5], Mrinal Patnaik[4]

*[1]Mayo Clinic, Internal Medicine, Rochester, Minnesota, United States of America;[2]University of Wisconsin School of Medicine and Public Health and University of Wisconsin Carbone Cancer Center, Division of Hematology, Medical Oncology, and Palliative Care, Department of Medicine, Madison, Wisconsin, United States of America; [3]Hospital of the University of Pennsylvania, Division of Hematology-Oncology, Department of Medicine, Philadelphia, Pennsylvania, United States of America; [4]Mayo Clinic, Division of Hematology, Department of Internal Medicine, Rochester, Minnesota, United States of America; [5]Mayo Clinic, Pulmonary and Critical Care Medicine, Rochester, Minnesota, United States of America;*

### Background

Telomere biology disorders (TBDs) are hereditary disorders defined by critically short, dysfunctional telomeres. TBDs demonstrate clinical heterogeneity, commonly presenting with bone marrow failure and increased risk of myeloid neoplasms (AML and MDS observed-to-expected ratios of 49.50 and 529.70, respectively [PMID: 39661387]). Emerging literature highlights fitness constraints shortened telomeres impose on hematopoietic stem and progenitor cells (HSPC), creating strong selective pressure favoring clonal lineages that escape this constraint. This phenomenon, in which somatic events in HSPCs partially or completely rescue the phenotype, is termed adaptive somatic mosaicism.

### Aims

Gain-of-function *TERT* promoter (*pTERT*) variants, which increase expression of telomerase, have been described in this setting, and our aim was to investigate this further in a multicenter, natural history study of adult TBD patients.

### Methods

After IRB approval, we identified TBD patients with somatic *pTERT* mutations from Mayo Clinic, University of Pennsylvania, and University of Wisconsin–Madison using error-corrected, high-depth CLIA-approved or research-based NGS with manual review (Mayo, UPenn), or long-read sequencing (UWMadison). Clinical data were abstracted from the medical record.

**Copyright Information:** (Online) ISSN: 2572-9241
©2026 The Author(s). HemaSphere is published by John Wiley & Sons Ltd on behalf of European Hematology Association. This is an open access Abstract Book distributed under the Attribution-NonCommercial-NoDerivs (CC BY-NC-ND), which allows third parties to download the articles and share them with others as long as they credit the author and the Abstract Book, but they cannot change the content in any way or use them commercially.
**Abstract Book Citations:** Authors, Title, HemaSphere, 2026;10:(S1):pages. The abstract book DOIs can be found on the “Author Information” page.
**Disclaimer:** Articles published in the journal HemaSphere exclusively reflect the opinions of the authors. The authors are responsible for all content in their abstracts including accuracy of the facts, statements, citing resources, etc.

**Results**

There were 18 adults with TBD included out of 150 tested (13 male, median age of 62.5 years [range 35-74 years]). All 18 individuals had short telomere lengths (TLs) (lymphocyte TL </= 10th percentile), and 9 had very short TLs (lymphocyte TL </=1st percentile) compared to age matched controls. Seventeen patients (94%) had identifiable germline variants in TBD-associated genes (8 pathogenic or likely pathogenic, 9 VUS - ACMG criteria): *TERT* (n=10), *RTEL1* (n=4), *TERC* (n=2), *PARN* (n=1).
The *pTERT* variants clustered at three hotspots: c.-124C>T (n=15, median VAF 6.1%), c.-57A>C (n=4, median VAF 17.1%), and c.-146C>T (n=2, median VAF 12.45%). Three individuals had >1 *pTERT* variant: two with -124C>T and -146C>T, and one with -124C>T and -57A>C. The remaining somatic landscape included variants in *PPM1D* (n=6, median VAF 6.0%), *DNMT3A* (n=2, median VAF 13%), *U2AF1* S34F (n=1, VAF 3.1%), *TP53* (n=1, VAF 7.0%), and *ASXL1* (n=1, VAF 1.0%).
Median follow up was 24 months [range 12-127 months]. At last follow up, one 59-year-old patient with *RTEL1*-TBD, a small *pTERT* clone (VAF 1.2%), and somatic *TP53* and *PPM1D* variants, had progressed to MDS and passed away. No patients progressed to AML and there were no other deaths. Sankey diagrams outline longitudinal changes in blood counts. Out of 13 baseline bone marrow biopsies, 8 were normocellular, 4 hypocellular/acellular, and 1 with cellularity not reported. At follow up, there were 10 biopsies with 5 reported as normocellular, 4 hypocellular/acellular, and 1 with variable cellularity.
Eleven (61.1%) patients had stable hemoglobin levels, thirteen (72.2%) had stable MCV, sixteen (88.9%) had stable leukocytes, and twelve (66.7%) had stable platelet counts. Four individuals never developed cytopenias or macrocytosis at follow up.

**Summary/Conclusion**

Our findings refine the current understanding of clonal mechanisms shaping the clinical trajectory of TBD patients with bone marrow involvement. The absence of acute leukemia and single MDS progression in this cohort supports adaptive somatic mosaicism driven by gain-of-function *pTERT* mutations. This lower-than-expected risk of myeloid transformation has important implications for risk stratification and clinical management.

**Copyright Information:** (Online) ISSN: 2572-9241
©2026 The Author(s). HemaSphere is published by John Wiley & Sons Ltd on behalf of European Hematology Association. This is an open access Abstract Book distributed under the Attribution-NonCommercial-NoDerivs (CC BY-NC-ND), which allows third parties to download the articles and share them with others as long as they credit the author and the Abstract Book, but they cannot change the content in any way or use them commercially.
**Abstract Book Citations:** Authors, Title, HemaSphere, 2026;10:(S1):pages. The abstract book DOIs can be found on the "Author Information" page.
**Disclaimer:** Articles published in the journal HemaSphere exclusively reflect the opinions of the authors. The authors are responsible for all content in their abstracts including accuracy of the facts, statements, citing resources, etc.

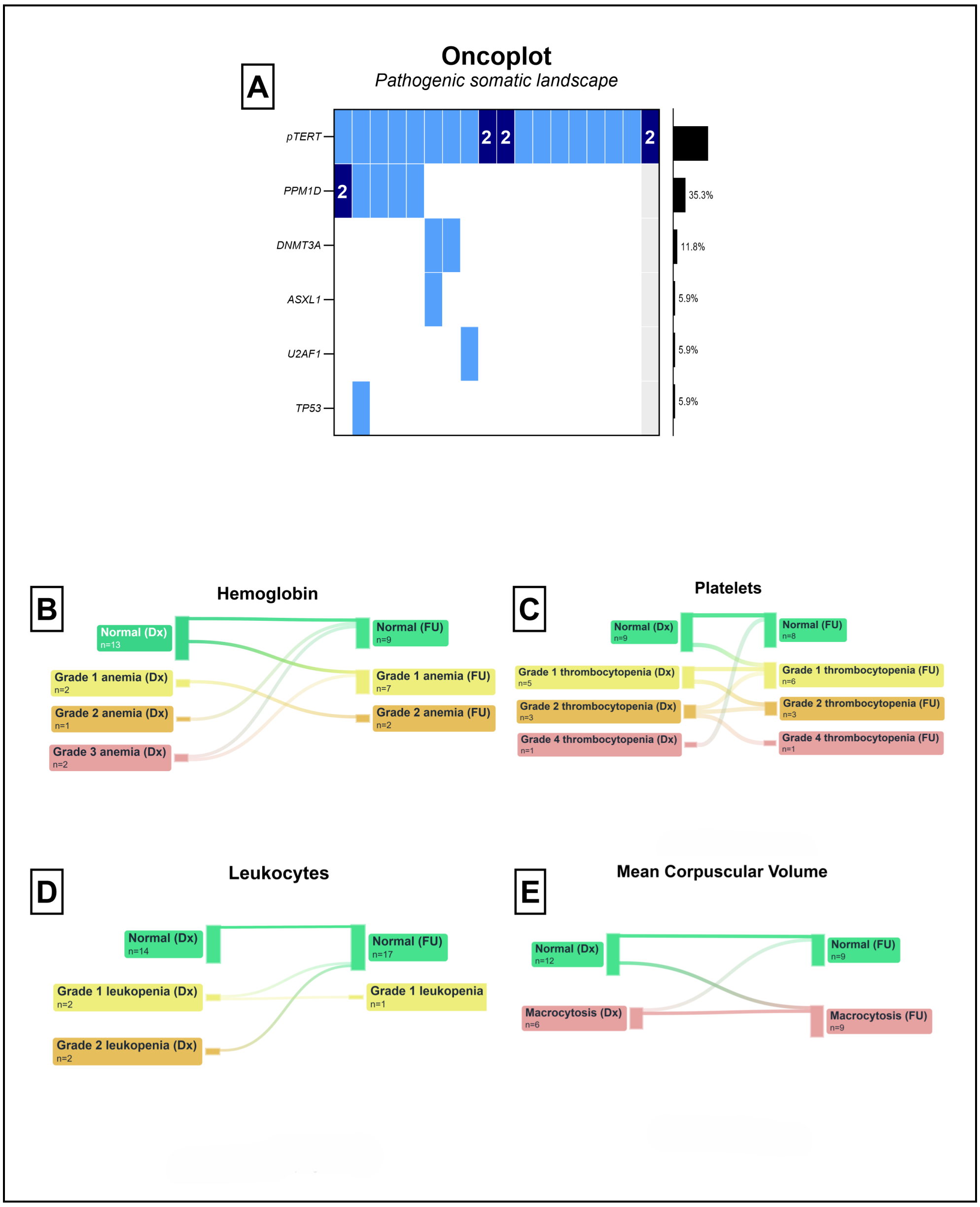

Figure 1A: Oncoplot describing somatic landscape of cohort. Figure 1B-E: Sankey diagrams outline longitudinal changes in blood counts (Time points: Dx = diagnosis; FU = follow up)

**Copyright Information:** (Online) ISSN: 2572-9241
©2026 The Author(s). HemaSphere is published by John Wiley & Sons Ltd on behalf of European Hematology Association. This is an open access Abstract Book distributed under the Attribution-NonCommercial-NoDerivs (CC BY-NC-ND), which allows third parties to download the articles and share them with others as long as they credit the author and the Abstract Book, but they cannot change the content in any way or use them commercially.
**Abstract Book Citations:** Authors, Title, HemaSphere, 2026;10:(S1):pages. The abstract book DOIs can be found on the “Author Information” page.
**Disclaimer:** Articles published in the journal HemaSphere exclusively reflect the opinions of the authors. The authors are responsible for all content in their abstracts including accuracy of the facts, statements, citing resources, etc.

### (PS2306) PRECLINICAL CHARACTERIZATION OF EVX-04, AN AI-DESIGNED OFF-THE-SHELF CANCER VACCINE TARGETING ENDOGENOUS RETROVIRAL ANTIGENS IN ACUTE MYELOID LEUKEMIA

**Topic:** 25. Gene therapy, cellular immunotherapy and vaccination - Biology & translational research

Søren Vester Kofoed*[1], Marina Barrio Calvo[1], Rasmus Ohrt Andersen[1], Christian Garde[1], Thomas Strandet Jepsen[1], Michael S Klausen[1], Jens Kringelum[1], Mads Lausen[1], Birgitte Rønø[1], Kim Theilgaard-Mönch[2], Nadia Viborg[1], Nikolas Thuesen[1], Rasmus Villebro[1], Benjamin Wolthers[1], Daniela Kleine-Kohlbrecher[1]

*[1]Evaxion A/S, Hørsholm, Denmark;[2]Odense Univ. Hospital Clinical Institute, Univ. of Southern Denmark, Dept. of Hematology-X, Odense, Denmark;*

### Background

Outcomes remain poor in patients with acute myeloid leukemia (AML) who are ineligible for intensive chemotherapy and stem cell transplantation, underscoring the need for more effective and less toxic treatment strategies. In this context, immunotherapy for the treatment of AML is currently undergoing a significant transformation. Historically, immune targeting of AML has been challenging due to aggressive disease course and lacking unique leukemia-specific antigens. Most AML-associated surface antigens are also expressed on healthy hematopoietic stem and progenitor cells, creating increasing risks of off-tumor toxicity and prolonged myeloablation. Novel therapies directed against endogenous retroviruses (ERVs) - viral remnants that remain dormant in healthy tissue but become aberrantly expressed in cancer cells - offer tumor-specific therapeutic opportunities. This strategy is particularly relevant for AML, as these patients often exhibit the high expression levels of ERV sequences necessary for effective immune targeting.

### Aims

Here, we present the preclinical characterization of EVX-04, an AI-designed off-the-shelf DNA cancer vaccine targeting ERV antigens in AML. We aimed to validate the vaccine's functional expression through assessment of cellular transcription, translation, and secretion of encoded ERV antigens, and to demonstrate induction of specific T-cell immune responses both *in vitro* and *in vivo*.

### Methods

Genomic and transcriptomic data from 136 AML patients was analyzed by Evaxion's AI-Immunology™ platform to identify tumor-specific ERVs and design immunogenic fragments. The platform integrates ERV sequences with MHC allele frequencies through three steps: identifying population-wide ERVs, detecting high-density MHC-presented antigen regions, and filtering for tumor specificity. The AI Immunology platform analysis resulted in the selection of 16 ERV fragments that were included in the EVX-04 lead design, a DNA plasmid containing a molecular adjuvant and an antigenic cassette encoding the 16 ERV fragments. EVX-04 vaccine functionality was evaluated by characterizing the transcriptional, translational, and secretory expression of the ERV antigen cassette in EVX-04 transfected HEK293 cells using qPCR, immunoblotting and ELISA. The immunogenic potential of the ERV fragments was examined by measuring ERV-specific T-cell activation in healthy donor PBMCs using IFNγ ELISpot following *in vitro* priming and in mouse immunization studies. T-cell mediated cytotoxicity was assessed by co-culturing *in vitro*-primed donor PBMCs with HLA-matched target cells loaded with ERV antigens.

**Copyright Information:** (Online) ISSN: 2572-9241
©2026 The Author(s). HemaSphere is published by John Wiley & Sons Ltd on behalf of European Hematology Association. This is an open access Abstract Book distributed under the Attribution-NonCommercial-NoDerivs (CC BY-NC-ND), which allows third parties to download the articles and share them with others as long as they credit the author and the Abstract Book, but they cannot change the content in any way or use them commercially.
**Abstract Book Citations:** Authors, Title, HemaSphere, 2026;10:(S1):pages. The abstract book DOIs can be found on the "Author Information" page.
**Disclaimer:** Articles published in the journal HemaSphere exclusively reflect the opinions of the authors. The authors are responsible for all content in their abstracts including accuracy of the facts, statements, citing resources, etc.

**Results**

EVX-04´s antigenic cassette containing the 16 ERV fragments, represent at least 15 MHC-presented antigens in 95% of AML patients. The DNA vaccine demonstrated correct transcription and translation in human cells with successful secretion of the EVX-04 protein product. *In vitro* studies showed significant antigen-specific T-cell responses in human PBMCs with induced T cells exhibiting cytotoxic potential against HLA-matched targets. Murine studies confirmed that immunization with the EVX-04 construct induced ERV antigen-specific T-cell responses *in vivo*.

**Summary/Conclusion**

EVX-04 represents a promising therapeutic approach targeting ERV antigens in AML acting by inducing functional antigen-specific T cells with cytotoxic potential. Based on encouraging preclinical results, a first-in-human study combining EVX-04 with azacytidine and venetoclax is in planning.

**Copyright Information:** (Online) ISSN: 2572-9241
©2026 The Author(s). HemaSphere is published by John Wiley & Sons Ltd on behalf of European Hematology Association. This is an open access Abstract Book distributed under the Attribution-NonCommercial-NoDerivs (CC BY-NC-ND), which allows third parties to download the articles and share them with others as long as they credit the author and the Abstract Book, but they cannot change the content in any way or use them commercially.
**Abstract Book Citations:** Authors, Title, HemaSphere, 2026;10:(S1):pages. The abstract book DOIs can be found on the “Author Information” page.
**Disclaimer:** Articles published in the journal HemaSphere exclusively reflect the opinions of the authors. The authors are responsible for all content in their abstracts including accuracy of the facts, statements, citing resources, etc.

### (PS2307) SELECTIVE PRESERVATION OF FYN SIGNALING BY PONATINIB ENHANCES CAR-T CELL PERSISTENCE AND FUNCTIONAL DURABILITY IN B-ALL

**Topic:** 25. Gene therapy, cellular immunotherapy and vaccination - Biology & translational research

Yi Zhou[1], Liwen Wang[1], Yuhan Yan[1], Linzhi Xie[1], Xin Li*[1]

[1] *The Third Xiangya Hospital of Central South University, Department of Hematology, Changsha, China;*

### Background

Although CD19-directed CAR-T therapy induces high remission rates in B-cell acute lymphoblastic leukemia (B-ALL), durable responses remain limited by insufficient persistence and progressive T-cell exhaustion. Second-generation tyrosine kinase inhibitors (TKIs), such as dasatinib, can transiently restrain CAR-T overactivation. However, the immunomodulatory effects of third-generation TKIs, particularly ponatinib, in the context of CAR-T therapy are not well defined.

### Aims

To investigate the clinical efficacy of ponatinib maintenance following CAR-T therapy in patients with Philadelphia chromosome-positive ($Ph^+$) relapsed/refractory (R/R) B-ALL, and compare its effects on CAR-T cells with other TKIs.

### Methods

Clinical outcomes and CAR-T persistence were evaluated in $Ph^+$ R/R B-ALL patients receiving ponatinib or olverembatinib maintenance after CAR-T therapy. *In vitro*, the effects of ponatinib, dasatinib, and olverembatinib on CAR-T cells were assessed using CCK-8, LDH, ELISA, flow cytometry, and Western blotting to evaluate effector function, exhaustion, apoptosis and T cell receptor (TCR)-related signaling.

### Results

All patients achieved complete remission beginning from the second month after CAR-T infusion, and maintained durable remission throughout follow-up during TKI maintenance therapy. Importantly, peripheral blood derived CAR-T cells remained consistently detectable up to day 150 after infusion in all four patients receiving ponatinib maintenance therapy, while no CAR-T copies were detected beyond day 50 in the patient receiving olverembatinib maintenance.
*In vitro*, ponatinib, dasatinib and olverembatinib induced a shared state of CAR-T cells defined by transient restraint of TCR-driven activation, reduced activation-induced apoptosis and decreased intracellular ROS accumulation. Under TCR stimulation, TKI-treated CAR-T cells maintained a higher proportion of functionally competent mitochondria compared with untreated controls, indicating protection against activation-induced metabolic stress. Importantly, ponatinib exerted distinct and durable immunomodulatory effects. Transcriptomic and functional analyses revealed selective enhancement of interferon-γ signaling pathway and sustained resistance to cellular stress during TCR stimulation. Notably, upon re-stimulation following drug washout, ponatinib-treated CAR-T cells exhibited superior recovery of effector function and enhanced resilience to activation-induced stress.
Mechanistically, all three TKIs effectively suppressed proximal TCR signal LCK and downstream signaling pathways activation. However, ponatinib uniquely preserved FYN kinase activity, this selective preservation was accompanied by sustained expression of anti-apoptotic proteins Bcl-2 and Bcl-xL, consistent with reduced apoptotic priming. Importantly, pharmacologic inhibition of FYN significantly attenuated the sustained cytoprotective effects and functional recovery observed following ponatinib treatment, supporting a FYN-dependent mechanism underlying its prolonged cellular protection.

**Copyright Information:** (Online) ISSN: 2572-9241
©2026 The Author(s). HemaSphere is published by John Wiley & Sons Ltd on behalf of European Hematology Association. This is an open access Abstract Book distributed under the Attribution-NonCommercial-NoDerivs (CC BY-NC-ND), which allows third parties to download the articles and share them with others as long as they credit the author and the Abstract Book, but they cannot change the content in any way or use them commercially.
**Abstract Book Citations:** Authors, Title, HemaSphere, 2026;10:(S1):pages. The abstract book DOIs can be found on the "Author Information" page.
**Disclaimer:** Articles published in the journal HemaSphere exclusively reflect the opinions of the authors. The authors are responsible for all content in their abstracts including accuracy of the facts, statements, citing resources, etc.

### Summary/Conclusion

Our study elucidates key mechanisms of TKIs in the context of CAR-T therapy and identifies ponatinib as a promising candidate for post-CAR-T maintenance in R/R Ph⁺ B-ALL. By modulating TCR signaling and enhancing mitochondrial metabolic adaptation, ponatinib enhances CAR-T persistence and therapeutic durability, thereby providing a translational framework for optimizing CAR-T-based treatment strategies.

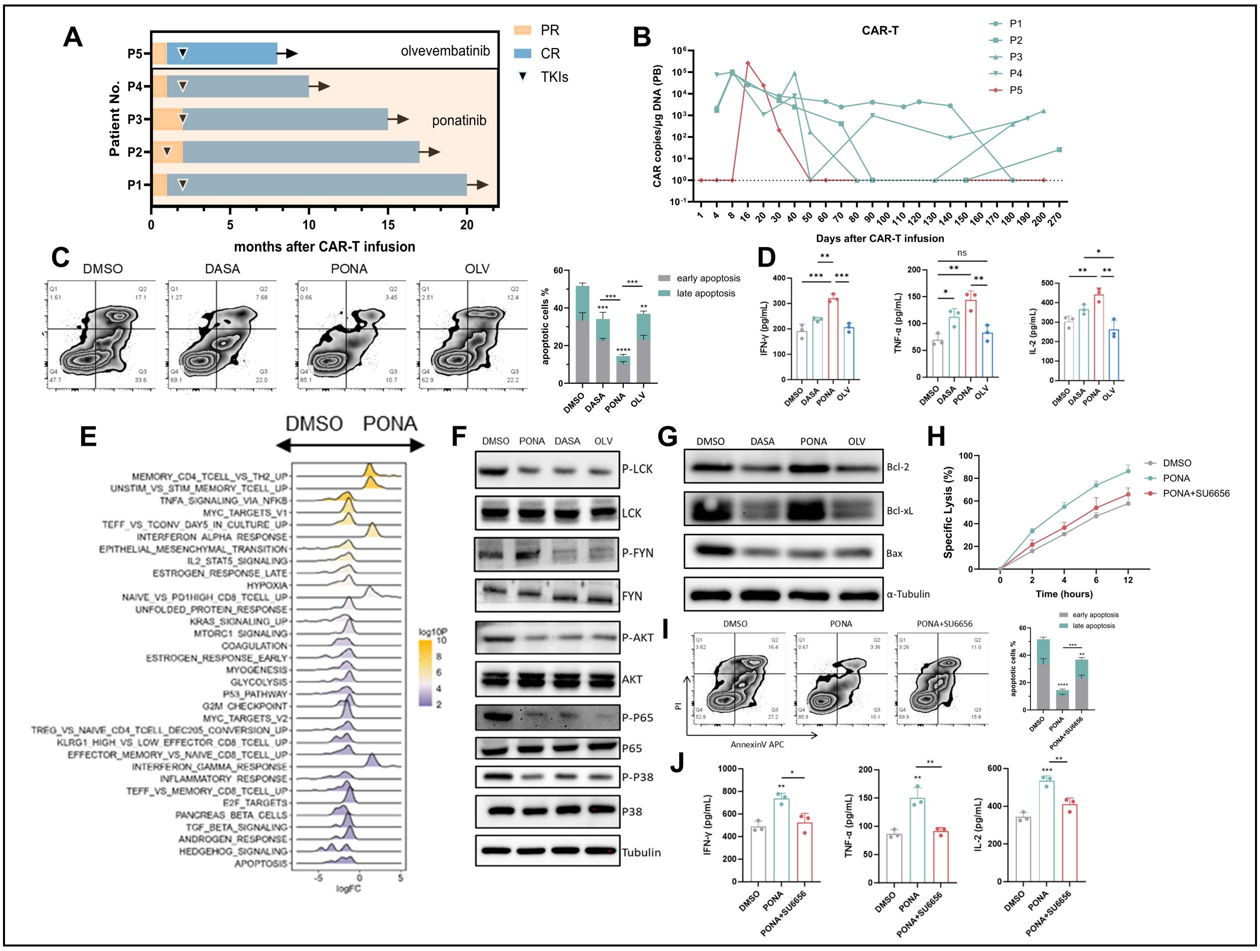

**Copyright Information:** (Online) ISSN: 2572-9241
©2026 The Author(s). HemaSphere is published by John Wiley & Sons Ltd on behalf of European Hematology Association. This is an open access Abstract Book distributed under the Attribution-NonCommercial-NoDerivs (CC BY-NC-ND), which allows third parties to download the articles and share them with others as long as they credit the author and the Abstract Book, but they cannot change the content in any way or use them commercially.
**Abstract Book Citations:** Authors, Title, HemaSphere, 2026;10:(S1):pages. The abstract book DOIs can be found on the "Author Information" page.
**Disclaimer:** Articles published in the journal HemaSphere exclusively reflect the opinions of the authors. The authors are responsible for all content in their abstracts including accuracy of the facts, statements, citing resources, etc.

### (PS2308) CO-RECEPTOR ATTENUATION DEFINES A CONVERGENT TRANSCRIPTIONAL STATE OF LONG-TERM PERSISTING CAR-T CELLS DRIVEN BY INTERMITTENT ANTIGEN STIMULATION

**Topic:** 25. Gene therapy, cellular immunotherapy and vaccination - Biology & translational research

Neel Nabar*[1], Xiaoli Mi[2], Nicholas Midler[3, 4], Jani Huuhtanen[3, 4], Brandon Simone[5], Alexander Lewis[2], Dennis Yuan[3, 4], Rebecca Murray[3, 4], Alyssa Indart[3, 4], Olga Shestova[5], Leonel Torres[5], Elliot Goepfert-Waterman[5], Susan Dewolf[2], Jae H. Park[2], Omar Abdel-Wahab[2], Saar Gill[1, 5], Dan Landau[3, 4]

*[1]Hospital of the University of Pennsylvania, Department of Hematology/Oncology, Philadelphia, United States of America; [2]Memorial Sloan Kettering Cancer Center, New York, United States of America;[3]Weill Cornell Medicine, Division of Hematology and Medical Oncology, New York, United States of America; [4]New York Genome Center, New York, United States of America; [5]University of Pennsylvania, Center for Cellular Immunotherapies, Philadelphia, United States of America;*

### Background

Long-term CAR-T persistence after CD19-directed therapy is associated with durable clinical responses, however the cellular states that sustain persistence and the stimuli that drive them remain undefined. Post-infusion CAR19 dynamics have been characterized within single diseases or constructs, but whether long-term persisting CAR-T cells converge on shared adaptive programs across clinical contexts is unknown. While chronic antigen stimulation induces exhaustion, CAR19 cells likely encounter intermittent antigen exposure following disease eradication as evidenced by sustained deep B-cell aplasia reflecting ongoing deletion of regenerating B-cell precursors. The adaptive states induced by such stimulation are unknown.

### Aims

To define the characteristics of long-term persisting CAR-T cells and determine the mechanisms driving their adaptive states.

### Methods

We analyzed 10 patients from two cohorts (7 LBCL, 3 CLL) treated with distinct CD19 CAR-T products (CD28 vs 4-1BB costimulatory domain), who remained in complete remission with persistent CAR-T cells (19–55 months, ongoing B-cell aplasia). Rare (<1%) long-lived CAR-T cells were sorted 10–40 months post-infusion and profiled using multimodal single-cell sequencing (RNA, surface proteins, TCR). Transcriptional programs were compared across diseases and constructs, and lineage relationships inferred by TCR tracking. Repetitive stimulation assays modeled intermittent antigen exposure in vitro, followed by high-dimensional flow cytometry and functional assays.

**Copyright Information:** (Online) ISSN: 2572-9241
©2026 The Author(s). HemaSphere is published by John Wiley & Sons Ltd on behalf of European Hematology Association. This is an open access Abstract Book distributed under the Attribution-NonCommercial-NoDerivs (CC BY-NC-ND), which allows third parties to download the articles and share them with others as long as they credit the author and the Abstract Book, but they cannot change the content in any way or use them commercially.
**Abstract Book Citations:** Authors, Title, HemaSphere, 2026;10:(S1):pages. The abstract book DOIs can be found on the “Author Information” page.
**Disclaimer:** Articles published in the journal HemaSphere exclusively reflect the opinions of the authors. The authors are responsible for all content in their abstracts including accuracy of the facts, statements, citing resources, etc.

### Results

Long-lived CAR-T cells from patients with LBCL and CLL clustered distinctly from endogenous immune cells and converged on a shared transcriptional program despite differing constructs and disease contexts. This state was characterized by a T-effector memory (TEM) phenotype, differential expression of GZMK, FXYD2, CD95, HMOX1, and GYPC, among others, and enrichment of interferon-γ response, oxidative phosphorylation, and cell cycle pathways. Comparison with independent single-cell datasets from children with ALL treated with obe-cel confirmed reproducibility of this signature across clinical settings.
Notably, a hallmark of this convergent program was co-receptor attenuation. Long-lived CAR-T cells in CLL were predominantly $CD4^-CD8^-$ (double negative, DN), whereas LBCL samples showed heterogeneity ranging from DN to CD4-low states. The shared transcriptional profile resembled naturally occurring $TCR\alpha\beta^+$ DN T cells in healthy blood. TCR lineage tracing demonstrated minimal overlap between peak expansion and long-term persisting clones. However, DN CAR-T cells shared TCR sequences with endogenous $CD4^+$ or $CD8^+$ populations, consistent with co-receptor downregulation rather than a distinct developmental lineage. Gene regulatory network analysis identified strong MYB regulon activity across cohorts.
In vitro modeling of intermittent antigen exposure using huCAR19 led to progressive enrichment of DN CAR-T cells after day 30. These cells maintained a TEM phenotype, upregulated MYB and TCF7, displayed reduced exhaustion features compared with $CD8^+$ counterparts, and retained cytotoxicity with enhanced cytokine production at late timepoints.

### Summary/Conclusion

Long-term persisting CAR-T cells across diseases and constructs converge on a shared co-receptor attenuated state arising from $CD4^+$ and $CD8^+$ T cells through adaptive downregulation. Intermittent antigen stimulation drives this adaptive program, generating less terminally differentiated, functionally preserved DN CAR-T cells.

**Copyright Information:** (Online) ISSN: 2572-9241
©2026 The Author(s). HemaSphere is published by John Wiley & Sons Ltd on behalf of European Hematology Association. This is an open access Abstract Book distributed under the Attribution-NonCommercial-NoDerivs (CC BY-NC-ND), which allows third parties to download the articles and share them with others as long as they credit the author and the Abstract Book, but they cannot change the content in any way or use them commercially.
**Abstract Book Citations:** Authors, Title, HemaSphere, 2026;10:(S1):pages. The abstract book DOIs can be found on the "Author Information" page.
**Disclaimer:** Articles published in the journal HemaSphere exclusively reflect the opinions of the authors. The authors are responsible for all content in their abstracts including accuracy of the facts, statements, citing resources, etc.

### (PS2309) LONG-TERM SAFETY OF CAR T-CELL THERAPY WITH LISOCABTAGENE MARALEUCEL AND IDECABTAGENE VICLEUCEL: ANALYSIS OF SECOND PRIMARY MALIGNANCY (SPM) AND VECTOR-ASSOCIATED OUTCOMES

**Topic:** 25. Gene therapy, cellular immunotherapy and vaccination - Biology & translational research

Matthew Lunning*[1], Larry Anderson[2], Jesus Berdeja[3], Nikhil Munshi[4], Kai Rejeski[5], Jason Westin[6], David Porter[7], Ken Cornetta[8], Alison Yu[9], Adam Omidpanah[9], Jing Wang[9], Amanda Anderson[9], Kenneth Ogasawara[9], Giovanna Andreola[9], Carolyn Tarpey[9], Kristina Jordahl[9], Justine Dell'aringa[9], Amanda Przespolewski[9], Miguel-Angel Perales[10]

*[1]University of Nebraska Medical Center, Omaha, Nebraska, United States of America;[2]UT Southwestern Medical Center, Myeloma, Waldenstrom's, and Amyloidosis Program, Section of Hematologic Malignancies and Cellular Therapy, Simmons Comprehensive Cancer Center, Dallas, Texas, United States of America; [3]Greco-Hainsworth Tennessee Oncology Centers for Research, Nashville, Tennessee, United States of America; [4]Dana-Farber Cancer Institute, Boston, Massachusetts, United States of America; [5]LMU University Hospital, Munich, Germany; [6]The University of Texas MD Anderson Cancer Center, Houston, Texas, United States of America; [7]University of Pennsylvania, Philadelphia, Pennsylvania, United States of America;[8]Indiana University School of Medicine, Indianapolis, Indiana, United States of America; [9]Bristol Myers Squibb, Princeton, New Jersey, United States of America; [10]Memorial Sloan Kettering Cancer Center, Cellular Therapy and Myeloma Services, New York, New York, United States of America;*

### Background

Chimeric antigen receptor (CAR) T cell therapies require long-term safety monitoring due to concerns regarding SPMs and potential lentiviral vector (LVV)-associated genotoxicity. Despite increasing clinical and commercial use, integrated analyses evaluating SPM incidence/timing and vector safety across approved CAR T cell products remain limited.

### Aims

We report SPM incidence and vector safety assessments from clinical trials in patients (pts) treated with lisocabtagene maraleucel (liso-cel) or idecabtagene vicleucel (ide-cel). These analyses are supplemented by post-marketing data in pts treated with liso-cel or ide-cel.

### Methods

Cumulative incidence of SPM, time to first SPM, and follow-up duration were summarized for pts who received liso-cel or ide-cel in registrational clinical trials. Vector safety assessments included replication-competent lentivirus (RCL) testing, quantitative assessment of CAR transgene persistence in peripheral blood, and insertion site analysis (ISA) performed in pts with CAR transgene levels ≥1% at ≥12 mo post-infusion with S-EPTS/LM-PCR–based next-generation sequencing to evaluate clonal dynamics. T-cell SPMs reported in clinical and post-marketing settings were descriptively reviewed, including cases where tumor tissue was available for CAR transgene evaluation. Follow up for T-cell SPMs included data collected from pts enrolled in long-term follow-up trials.

**Copyright Information:** (Online) ISSN: 2572-9241
©2026 The Author(s). HemaSphere is published by John Wiley & Sons Ltd on behalf of European Hematology Association. This is an open access Abstract Book distributed under the Attribution-NonCommercial-NoDerivs (CC BY-NC-ND), which allows third parties to download the articles and share them with others as long as they credit the author and the Abstract Book, but they cannot change the content in any way or use them commercially.
**Abstract Book Citations:** Authors, Title, HemaSphere, 2026;10:(S1):pages. The abstract book DOIs can be found on the "Author Information" page.
**Disclaimer:** Articles published in the journal HemaSphere exclusively reflect the opinions of the authors. The authors are responsible for all content in their abstracts including accuracy of the facts, statements, citing resources, etc.

**Results**

A total of 920 pts treated with liso-cel and 419 pts with ide-cel were included, with a median (range) follow-up of 23.8 (0.2-57.9) and 21.9 (0.2-67.2) mo, respectively. For the liso-cel cohort, SPMs occurred in 74 (8.0%) pts (25 [2.7%] hematologic; 52 [5.7%] solid tumor); overall incidence rate (IR) was 4.9/100 person-years (P-Y; 1.6 hematologic; 3.4 solid). Two cases of T-cell SPMs (PTCL and CTCL) were reported with a time to first onset of 30 and 435 days (median [range] follow-up of 24.4 [0.2-101.0] mo); neither case was considered related to liso-cel vectors following transgene testing. For the ide-cel cohort, SPMs occurred in 37 (8.8%) pts (11 [2.6%] hematologic; 26 [6.2%] solid); overall IR was 4.9/100 P-Y (1.4 hematologic; 3.4 solid). No T-cell SPMs were observed in the ide-cel cohort (median [range] follow-up of 23.1 [0.2-101.2] mo). In the post-marketing setting, 9 T-cell SPMs following liso-cel and 3 following ide-cel were reported. Tumor tissue from 3 of these cases were available for evaluation and no CAR transgene was detected in any sample. Vector safety assessment in clinical trial pts treated with liso-cel or ide-cel confirmed that no RCL cases were identified. Longitudinal monitoring of CAR transgene persistence showed no evidence of abnormal *in vivo* expansion over time. ISA of patient samples identified polyclonal integration patterns, with no sustained clonal dominance observed and no enrichment of vector integration sites proximal to oncogenic loci.

**Summary/Conclusion**

These findings further support the long-term safety of liso-cel and ide-cel. The risk of SPMs, including T-cell SPMs after liso-cel or ide-cel treatment, is consistent with literature rates for similar populations, with no evidence of LVV-mediated insertional oncogenesis observed. Assessment of T-cell malignancies involves over 8 years of clinical trial follow-up across >1,300 pts, and is complemented by post-marketing safety data for commercially treated pts.

**Copyright Information:** (Online) ISSN: 2572-9241
©2026 The Author(s). HemaSphere is published by John Wiley & Sons Ltd on behalf of European Hematology Association. This is an open access Abstract Book distributed under the Attribution-NonCommercial-NoDerivs (CC BY-NC-ND), which allows third parties to download the articles and share them with others as long as they credit the author and the Abstract Book, but they cannot change the content in any way or use them commercially.
**Abstract Book Citations:** Authors, Title, HemaSphere, 2026;10:(S1):pages. The abstract book DOIs can be found on the “Author Information” page.
**Disclaimer:** Articles published in the journal HemaSphere exclusively reflect the opinions of the authors. The authors are responsible for all content in their abstracts including accuracy of the facts, statements, citing resources, etc.

### (PS2310) CD1C-RESTRICTED TCR COMBINED WITH CD38-CCR ENABLES POTENT DUAL-TARGETING IMMUNOTHERAPY IN ACUTE LEUKEMIA

**Topic:** 25. Gene therapy, cellular immunotherapy and vaccination - Biology & translational research

Ivan Civettini*[1, 2], Michela Consonni[2], Claudio Garavaglia[2], Giulia Mondardini[2], Mancino Alessandra[2], Leonie Bourgeois[3], Daniel Häussinger[3], Marta Serafini[4], Sara Mastaglio[4], Fabio Ciceri[4], Maria Themeli[5], Paolo Dellabona[2], Giulia Casorati[2]

*[1]Università Vita-Salute San Raffaele, Experimental Immunology Unit, Milan, Italy; [2]IRCCS San Raffaele Scientific Institute, Experimental Immunology Unit, Milan, Italy; [3]University of Basel, NMR Laboratory, Department of Chemistry, Basel, Switzerland; [4]IRCCS San Raffaele Scientific Institute, Hematology and Bone Marrow Transplantation Unit, Milan, Italy; [5]Amsterdam UMC, Amsterdam, The Netherlands;*

### Background

Adoptive immunotherapy with MHC-restricted TCR-engineered T cells has shown promise in hematologic malignancies but is limited by HLA polymorphism. Targeting CD1c, a monomorphic MHC-like molecule frequently expressed in both myeloid and lymphoblastic acute leukemia, provides an MHC-independent approach. We previously identified DN4.99, a CD1c-restricted TCR recognizing a leukemia associated lipid antigen, which mediates broad anti-leukemic activity in vitro and in xenograft models.

### Aims

Enhance functional avidity and durability, particularly against CD1c-low leukemia via two complementary strategies aimed at: (i) increasing functional avidity of the DN4.99 TCR by selective constant (C) α-chain deglycosylation (DN4.99gly- TCR) (ii) sustaining T-cell reactivity by incorporating a CD38 Chimeric Costimulatory Receptor (CCR)

### Methods

Parental DN4.99 TCR, DN4.99 gly– TCR and CD38-CCR lentiviral constructs were generated. Primary human T cells were transduced with TCR ± CD38-CCR and expanded in IL-7/IL-15. Functional avidity of DN4.99 gly– TCR was assessed using the LUMICKS z-Movi acoustic force assay. Cytotoxicity against $CD1c^+/CD38^+$ leukemia cell lines was evaluated in vitro at multiple E:T ratios using real-time imaging (Incucyte®). In vivo efficacy was tested in NSG xenograft models with luciferase-secreting $CD1c^+$ leukemia, monitoring tumor progression by plasma bioluminescence and immune profiling by flow cytometry.

### Results

The expression of the DN4.99 gly– TCR significantly increased functional avidity of T cells against $CD1c^+$ leukemic targets compared to the parental one, resulting in a more efficient in vitro elimination. In vivo, DN4.99 gly– TCR T cells demonstrated superior anti-leukemic activity compared to parental DN4.99 and the clinically validated WT1C4 TCR, resulting in significantly improved overall survival of treated mice. Leukemic cells recovered from peripheral blood, bone marrow, and spleen of mice at sacrifice showed reduced CD1c expression, while post-infusion T cells exhibited increased exhaustion marker expression, suggesting a potential immune escape mechanism. To improve elimination of CD1c-low leukemia and mitigate exhaustion, DN4.99 gly– TCR T cells were co-transduced with CD38-CCR (98% double-positive purity). CD38-CCR significantly accelerated and enhanced cytotoxicity, with increased IL-2 production, and maintained full tumor control in vitro after six sequential re-stimulations, unlike DN4.99 gly– TCR T cells. CD1c blockade abolished killing and no enhancement was observed against CD38-knockout targets, suggesting that CD38-CCR costimulation (signal 2) required DN4.99-mediated CD1c recognition (signal 1). In vivo, dual-transduced T cells outperformed DN4.99 gly– TCR alone.

**Copyright Information:** (Online) ISSN: 2572-9241
©2026 The Author(s). HemaSphere is published by John Wiley & Sons Ltd on behalf of European Hematology Association. This is an open access Abstract Book distributed under the Attribution-NonCommercial-NoDerivs (CC BY-NC-ND), which allows third parties to download the articles and share them with others as long as they credit the author and the Abstract Book, but they cannot change the content in any way or use them commercially.
**Abstract Book Citations:** Authors, Title, HemaSphere, 2026;10:(S1):pages. The abstract book DOIs can be found on the “Author Information” page.
**Disclaimer:** Articles published in the journal HemaSphere exclusively reflect the opinions of the authors. The authors are responsible for all content in their abstracts including accuracy of the facts, statements, citing resources, etc.

### Summary/Conclusion

Removal of the N-glycosylation site enhances DN4.99 TCR avidity and both in vitro and in vivo efficacy. CD38-CCR co-expression further increases cytotoxicity against CD1c⁺ leukemia while preserving strict signal-1 (CD1c) dependency. Together, these findings support a combinatorial CD1c/CD38-targeted therapeutic platform.

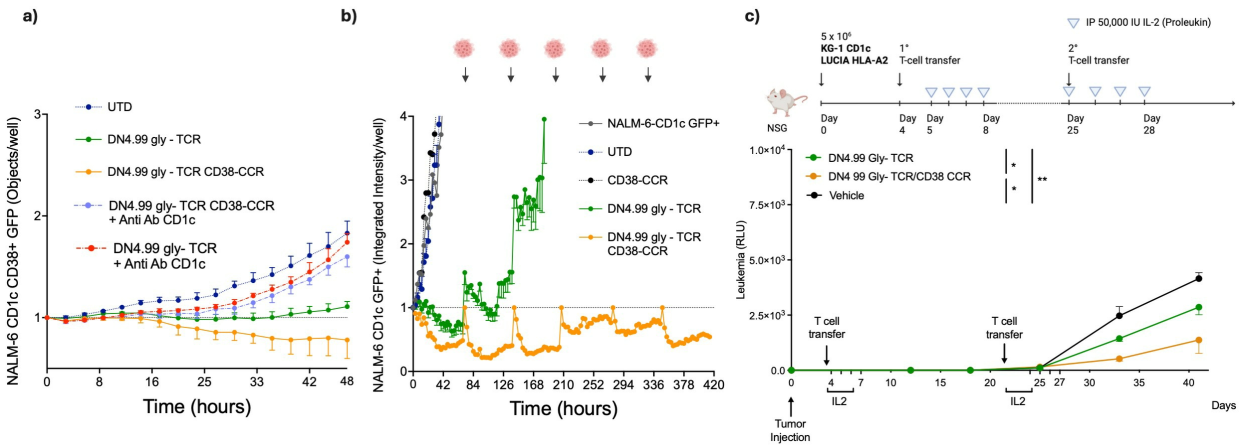

*(a) Cytotoxicity assay against NALM-6 CD1c GFP ⁺CD38 ⁺target cells (E:T 1:3). DN4.99 gly-TCR/CD38-CCR T cells mediated faster and deeper target-cell clearance compared with DN4.99 gly-TCR alone. Blocking CD1c with an anti-CD1c monoclonal antibody abrogated killing by DN4.99 gly-TCR/CD38-CCR. (b) Repeated stimulation assay using NALM-6 CD1c GFP ⁺targets; DN4.99 gly-TCR T cells progressively lost control, with failure by the third restimulation, whereas co-expression of CD38-CCR prolonged target control. (c) In vivo efficacy in NSG mice intravenously injected with KG-1 CD1c ⁺luciferase⁺ leukemia cells: reduced leukemia burden in animals treated with DN4.99 gly-TCR/CD38-CCR compared with DN4.99 gly-TCR alone*

**Copyright Information:** (Online) ISSN: 2572-9241
©2026 The Author(s). HemaSphere is published by John Wiley & Sons Ltd on behalf of European Hematology Association. This is an open access Abstract Book distributed under the Attribution-NonCommercial-NoDerivs (CC BY-NC-ND), which allows third parties to download the articles and share them with others as long as they credit the author and the Abstract Book, but they cannot change the content in any way or use them commercially.
**Abstract Book Citations:** Authors, Title, HemaSphere, 2026;10:(S1):pages. The abstract book DOIs can be found on the "Author Information" page.
**Disclaimer:** Articles published in the journal HemaSphere exclusively reflect the opinions of the authors. The authors are responsible for all content in their abstracts including accuracy of the facts, statements, citing resources, etc.

### (PS2311) AN IN VIVO EPIGENOME EDITING PLATFORM FOR β-HEMOGLOBINOPATHIES USING ADENOVIRAL VECTORS

**Topic:** 25. Gene therapy, cellular immunotherapy and vaccination - Biology & translational research

Kiriaki Paschoudi*[1, 2], Karthik Karupussamy[3], Anna Anderson[2, 2], Chrysanthi Theodorakoglou[1, 2], Efthymia Arizani[1, 2], Aksara Velmurugan[3], Ioanna Sakellari[4], Andre Lieber[3], Evangelia Yannaki[2, 3], Nikoleta Psatha[1]

*[1]School of Biology, Department of Genetics, Development and Molecular Biology, Thessaloniki, Greece;[2]"G. Papanikolaou" General Hospital, Gene and Cell Therapy Center, Hematology Department, Thessaloniki, Greece; [3]University of Washington, Seattle, United States of America; [4]"G. Papanikolaou" Hospital, Hematology Department and BMT Unit, Thessaloniki, Greece;*

### Background

Over the past decade, genome editing approaches have been extensively used in gene therapies. In β-hemoglobinopathies, genome-editing technologies, particularly CRISPR–Cas9 and its derivatives, have successfully reactivated γ-globin expression, highlighting their therapeutic potential. However, double-strand breaks (DSBs) can compromise genomic stability and increase the risk of chromosomal rearrangements and long-term genotoxicity. Additionally, current approaches rely on ex vivo manipulation of hematopoietic stem cells (HSCs), increasing complexity, cost, and limiting clinical accessibility.

### Aims

We have previously established that in vivo HSC gene modification can be succeeded with the use of helper-dependent adenoviral 5/35++ (HDAd5/35++) vectors across multiple gene therapy modalities. Here, we utilized the HDAd5/35++ vector platform to enable in vivo epigenome editing for γ-globin activation, as an alternative gene therapy approach.

### Methods

To establish an in vivo–applicable epigenome editing platform, HDAd5/35++ vectors were used to deliver the epigenome editor dCas9-KRAB-dDNMT3A/L (dCas9-KAL), targeting regulatory regions of the HbF repressors ZNF410 and BCL11A. As a control vector was used a HDAd5/35++ GFP vector . HDAd5/35++ dCas9-KAL-ZNF410 and dCas9-KAL-BCL11A vectors were evaluated in HUDEP-2 cells and in primary cells from healthy donors and β-thalassemia patients. CD34+ HSCs were transduced (MOI 4000) and cultured in erythroid differentiation and semi-liquid culture. Clonogenic potential, expansion, and erythroid maturation were assessed. γ-Globin expression (% HbF+ cells) and reactive oxygen species (ROS) levels were measured at late differentiation stages.

### Results

Transduction of HUDEP-2 cells with either HDAd5/35++ dCas9-KAL-ZNF410 or HDAd5/35++ dCas9-KAL-BCL11A did not affect cell expansion and differentiation capacity. Two weeks post-transduction, γ-globin production was significantly increased with both vectors ($p<0.0001$). Transduction had no impact on the expansion, clonogenicity, or erythroid maturation of healthy donor-derived HSCs. At the end of erythroid differentiation, significantly increased levels of HbF production were observed (HDAd-dCas9-KAL-ZNF410: 31.73 ±2.87% and HDAd5/35++ dCas9-KAL -BCL11A: 24.4 ±2.63% vs control :8.98±2.63% p= 0.008 and p= 0.009, respectively). In a similar manner, epigenetic suppression in thalassemic HSCs significantly restored γ-globin expression (HDAd5/35++ dCas9-KAL-ZNF410: 63.23 ±3.68% and HDAd5/35++ dCas9-KAL -BCL11A: 64.59 ±1.83% vs control: 20.75 ±1.02% p= 0.00001 and p= 0.000001, respectively). Elevated γ-globin levels correlated with improved disease-related cellular features, including higher clonogenic capacity ($p<0.05$), enhanced expansion ($p<0.001$), and reduced ROS accumulation ($p<0.001$).

**Copyright Information:** (Online) ISSN: 2572-9241
©2026 The Author(s). HemaSphere is published by John Wiley & Sons Ltd on behalf of European Hematology Association. This is an open access Abstract Book distributed under the Attribution-NonCommercial-NoDerivs (CC BY-NC-ND), which allows third parties to download the articles and share them with others as long as they credit the author and the Abstract Book, but they cannot change the content in any way or use them commercially.
**Abstract Book Citations:** Authors, Title, HemaSphere, 2026;10:(S1):pages. The abstract book DOIs can be found on the "Author Information" page.
**Disclaimer:** Articles published in the journal HemaSphere exclusively reflect the opinions of the authors. The authors are responsible for all content in their abstracts including accuracy of the facts, statements, citing resources, etc.

**Summary/Conclusion**

Overall, our ex vivo data indicate that this non-integrating epigenome editing platform allows efficient and inheritable epigenetic suppression of HbF repressors in human HSCs, leading to strong γ-globin reactivation and correction of disease-associated cellular defects. Importantly, this approach can be applied *in vivo*, offering a simplified and potentially translatable strategy for β-hemoglobinopathies and HSCs related disorders. In vivo epigenome editing studies in a humanized thalassemia mouse model are ongoing, evaluating long-term efficacy and safety, further supporting the translational potential of this approach.

**Copyright Information:** (Online) ISSN: 2572-9241
©2026 The Author(s). HemaSphere is published by John Wiley & Sons Ltd on behalf of European Hematology Association. This is an open access Abstract Book distributed under the Attribution-NonCommercial-NoDerivs (CC BY-NC-ND), which allows third parties to download the articles and share them with others as long as they credit the author and the Abstract Book, but they cannot change the content in any way or use them commercially.
**Abstract Book Citations:** Authors, Title, HemaSphere, 2026;10:(S1):pages. The abstract book DOIs can be found on the "Author Information" page.
**Disclaimer:** Articles published in the journal HemaSphere exclusively reflect the opinions of the authors. The authors are responsible for all content in their abstracts including accuracy of the facts, statements, citing resources, etc.

### (PS2312) CORD BLOOD-DERIVED NK CELLS ENGINEERED WITH A NOVEL HIGH-AFFINITY CAR RECOGNIZING CLL-1 EXHIBIT SIGNIFICANT ANTI-LEUKEMIA EFFECTS IN VIVO

**Topic:** 25. Gene therapy, cellular immunotherapy and vaccination - Biology & translational research

Makiko Suga*[1], Shuhei Kida[1, 2], Yo Mizutani[1], Yuta Yamaguchi[1], Naoki Hosen[1, 3, 4, 5]

*[1]The University of Osaka Graduate School of Medicine, Hematology and Oncology, Suita, Japan;[2]Osaka General Medical Center, Hematology, Osaka, Japan; [3]The University of Osaka, World Premier International Immunology Frontier Research Center, Suita, Japan; [4]Institute for Open and Transdisciplinary Research Initiatives, The University of Osaka, Integrated Frontier Research for Medical Science Division, Suita, Japan; [5]The University of Osaka, Center for Infectious Disease Education and Research, Suita, Japan;*

### Background

One major limitation of chimeric antigen receptor (CAR) T-cell therapy is the lengthy manufacturing process and high cost. Cord blood (CB)–derived CAR NK cells can be generated as off-the-shelf products for immediate clinical use, potentially reducing manufacturing time and overall cost compared with CAR T-cell therapy. Acute myeloid leukemia (AML) is a hematologic malignancy with a poor prognosis. Although allogeneic hematopoietic stem cell transplantation (allo-HSCT) remains a potentially curative option, relapse rates remain high. These challenges support the development of CB-derived CAR NK cell therapy as a novel immunotherapeutic strategy for AML.

### Aims

We developed and assessed CB-derived NK cells transduced with CARs generated from novel monoclonal antibodies (mAbs) against C-type lectin-like molecule 1 (CLL-1), a candidate target antigen for AML.

### Methods

CAR constructs were generated from thirteen newly established anti–CLL-1 monoclonal antibodies and incorporated CD28–CD3ζ signaling domains with T2A-linked IL-15. To evaluate affinity-dependent differences in CAR-mediated cytotoxicity, CLL-1 knockdown U937 cells were generated using shRNA-mediated silencing to model low–antigen-density targets. CAR NK cells incorporating the scFv derived from the previously reported CLL-1 monoclonal antibody M26 were generated for direct head-to-head evaluation.

### Results

Among the generated constructs, CAR NK cells derived from the 2-23 mAb (2-23 CAR NK cells) demonstrated potent and reproducible anti-leukemic activity. Across independent cord blood donor lots, they showed stable CAR transduction efficiency and sustained anti-leukemic effects, and this activity was consistently observed across multiple AML cell lines, including U937 and HL-60. The 2-23 mAb exhibited higher affinity for CLL-1 than the previously reported anti–CLL-1 mAb M26. The 2-23 CAR NK cells efficiently eliminated CLL-1$^{low}$ U937 cells, whereas M26 CAR NK cells showed minimal cytotoxicity under identical conditions.In AML xenograft models, 2-23 CAR NK cells significantly reduced tumor burden and prolonged survival. Additionally, in AML patient-derived xenograft models generated from primary patient samples, the 2-23 CAR NK cells expanded robustly, persisted for over one month, and eradicated AML cells.

**Copyright Information:** (Online) ISSN: 2572-9241
©2026 The Author(s). HemaSphere is published by John Wiley & Sons Ltd on behalf of European Hematology Association. This is an open access Abstract Book distributed under the Attribution-NonCommercial-NoDerivs (CC BY-NC-ND), which allows third parties to download the articles and share them with others as long as they credit the author and the Abstract Book, but they cannot change the content in any way or use them commercially.
**Abstract Book Citations:** Authors, Title, HemaSphere, 2026;10:(S1):pages. The abstract book DOIs can be found on the “Author Information” page.
**Disclaimer:** Articles published in the journal HemaSphere exclusively reflect the opinions of the authors. The authors are responsible for all content in their abstracts including accuracy of the facts, statements, citing resources, etc.

### Summary/Conclusion

We demonstrate the high therapeutic potential of CB-derived NK cells transduced with a CAR derived from the novel high-affinity monoclonal antibody 2-23. In particular, the 2-23 CAR NK cells exhibited high antigen sensitivity and were effective against AML cells with low CLL-1 expression. These findings support the clinical development of CB-derived 2-23 CAR NK cells as an effective strategy for AML, particularly prior to allogeneic hematopoietic stem cell transplantation.

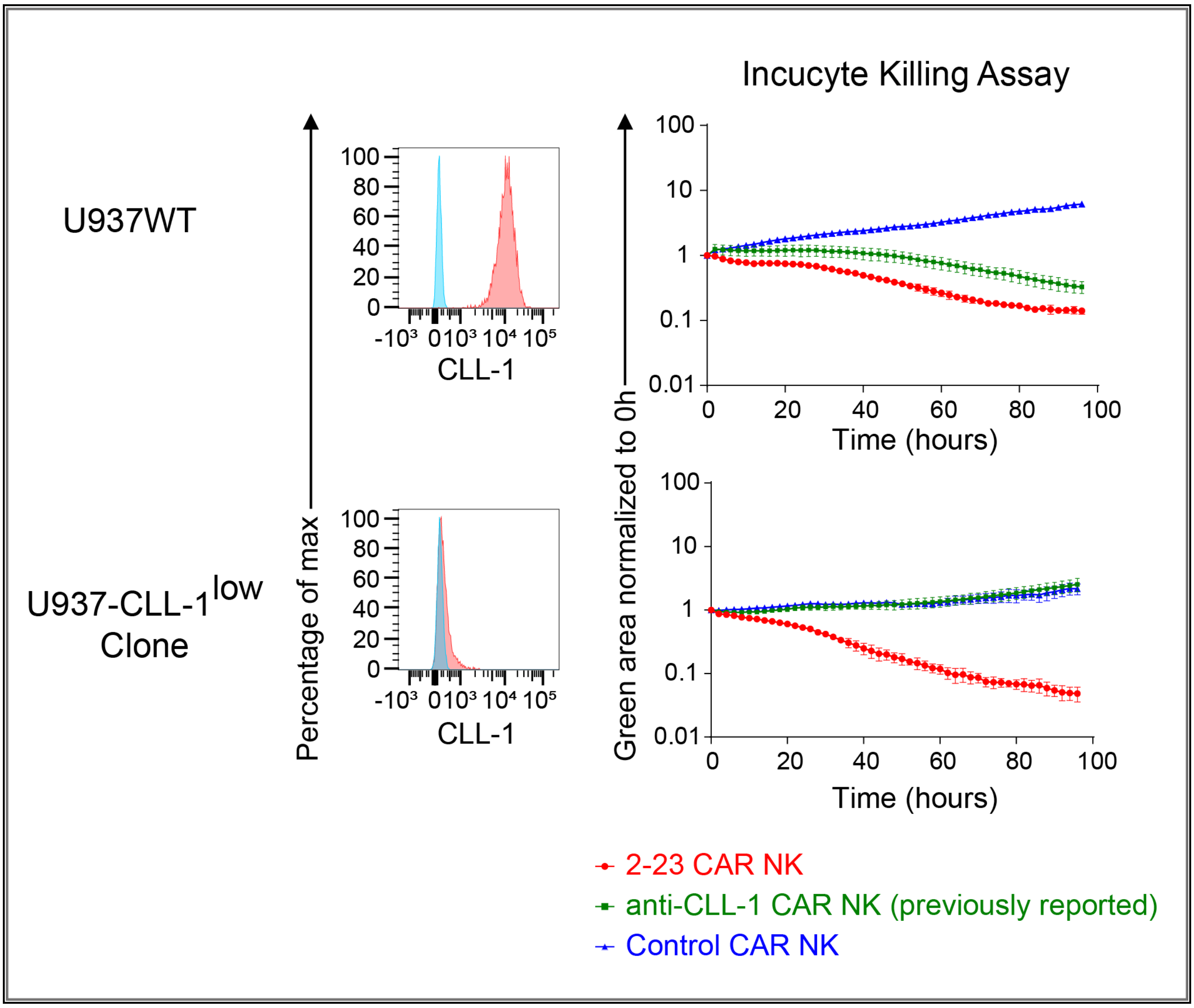

Copyright Information: (Online) ISSN: 2572-9241
©2026 The Author(s). HemaSphere is published by John Wiley & Sons Ltd on behalf of European Hematology Association. This is an open access Abstract Book distributed under the Attribution-NonCommercial-NoDerivs (CC BY-NC-ND), which allows third parties to download the articles and share them with others as long as they credit the author and the Abstract Book, but they cannot change the content in any way or use them commercially.
Abstract Book Citations: Authors, Title, HemaSphere, 2026;10:(S1):pages. The abstract book DOIs can be found on the “Author Information” page.
Disclaimer: Articles published in the journal HemaSphere exclusively reflect the opinions of the authors. The authors are responsible for all content in their abstracts including accuracy of the facts, statements, citing resources, etc.

### (PS2313) PRE-INFUSION IMMUNE SIGNATURES PREDICT SEVERE CYTOPENIA AFTER CAR T-CELL THERAPY: HIGH-DIMENSIONAL LONGITUDINAL PROFILING IN B-CELL MALIGNANCIES

**Topic:** 25. Gene therapy, cellular immunotherapy and vaccination - Biology & translational research

Julian Liebaert*[1], Marie Wesener[1], Schayan Yousefian[1, 2, 3], Maximilian Sprechert[1, 2], Simon Haas[1, 2, 3, 4, 5, 6, 7, 8], Ulrich Keller[1, 4, 6, 7, 8], Uta Demel[1, 4, 9], Antonia Busse[1, 4, 6, 8]

*[1]Charité Universitätsmedizin Berlin, Corporate member of Freie Universität and Humboldt-Universität zu Berlin, Berlin, Germany; [2]Berlin Institute of Health (BIH) at Charité Universitätsmedizin Berlin, Berlin, Germany;[3]Berlin Institute for Medical Systems Biology, Max Delbrück Center for Molecular Medicine in the Helmholtz Association, Berlin, Germany; [4]Max-Delbrück-Center for Molecular Medicine, Berlin, Germany; [5]Precision Healthcare University Research Institute, Queen Mary University of London, London, United Kingdom; [6]German Cancer Consortium (DKTK), partner site Berlin, a partnership between DKFZ and Charité-Universitätsmedizin Berlin, Berlin, Germany; [7]Cluster of Excellence ImmunoPreCept, Charité - Universitätsmedizin Berlin, Berlin, Germany; [8]National Center for Tumor diseases (NCT) Berlin, a partnership between DKFZ and Charité-University Hospital Berlin, Berlin, Germany; [9]Clinician Scientist Program, Berlin Institute of Health (BIH), Berlin, Germany;*

### Background

CAR T-cell therapy has markedly improved outcomes in relapsed/refractory B-cell lymphoma (BCL) and multiple myeloma (MM). However, immune effector cell-associated hematotoxicity (ICAHT) remains frequent and clinically relevant. The immune determinants of prolonged cytopenia and impaired immune reconstitution are insufficiently understood.

### Aims

To identify immune cell signatures and mechanisms associated with impaired immune reconstitution after CAR T-cell therapy.

### Methods

Peripheral blood was prospectively collected from R/R BCL and MM patients treated with axicabtagene ciloleucel, tisagenlecleucel, lisocabtagene maraleucel, or ciltacabtagene autoleucel. Samples were obtained at leukapheresis, pre-infusion, days 1–10, and days 30, 90, and 180 post-infusion. Cytopenia was graded according to CTCAE and ICAHT criteria. High-dimensional immune profiling was performed using 46-plex spectral flow cytometry analyzing ~55 million live cells. Impaired immune reconstitution was defined as CTCAE grade ≥3 anemia, thrombocytopenia, and neutropenia. Early timepoint was defined as 30 days post-CAR T-cell infusion.

**Copyright Information:** (Online) ISSN: 2572-9241
©2026 The Author(s). HemaSphere is published by John Wiley & Sons Ltd on behalf of European Hematology Association. This is an open access Abstract Book distributed under the Attribution-NonCommercial-NoDerivs (CC BY-NC-ND), which allows third parties to download the articles and share them with others as long as they credit the author and the Abstract Book, but they cannot change the content in any way or use them commercially.
**Abstract Book Citations:** Authors, Title, HemaSphere, 2026;10:(S1):pages. The abstract book DOIs can be found on the “Author Information” page.
**Disclaimer:** Articles published in the journal HemaSphere exclusively reflect the opinions of the authors. The authors are responsible for all content in their abstracts including accuracy of the facts, statements, citing resources, etc.

**Results**

We analyzed 107 PB samples from 25 BCL patients (32% axi-cel, 16% tisa-cel, 52% liso-cel) and 83 samples from 17 MM patients (100% cilta-cel). Overall, 29% of patients had a CAR-Hematotox score ≥2. Neutropenia grade ≥3 occurred in 93% of patients, including 14% with early and 34% with late ICAHT grade ≥2. Severe anemia and thrombocytopenia were each observed in 43%.

Reduced γδ T-cell frequencies at leukapheresis were strongly associated with impaired immune reconstitution (p = 0.0095), indicating a pre-infusion immune vulnerability in patients who subsequently developed prolonged cytopenia. γδ T-cells of patients with impaired immune reconstitution had higher mean fluorescence intensities of CD27 (p = 0.012), indicating a shift in the γδ T-cell populations.

In addition, patients with impaired early immune reconstitution (before day 30; CTCAE grade ≥3 for hemoglobin, thrombocytes, and neutrophils) showed reduced relative and absolute classical monocytes pre-infusion (p = 0.0017, 0.012) and during days 1–10 post-infusion (p = 0.031, 0.0071). Late ICAHT and anemia correlated with lower frequencies of intermediate monocytes (p = 0.052, 0.0092).

At day 0, higher frequencies of CD56bright NK cells (p = 0.039) and regulatory T cells (p = 0.032, 0.038) were observed in both early and late impaired immune reconstitution. Furthermore, impaired immune reconstitution was associated with increased ATP5A (p = 0.047) and GLUT1 (p = 0.006) mean fluorescence intensities in regulatory T cells, suggesting enhanced metabolic activation.

**Summary/Conclusion**

Reduced γδ T-cell frequencies at leukapheresis identify patients at risk for prolonged cytopenia following CAR T-cell therapy, suggesting a role for innate-like T-cell immunity in hematologic recovery. In combination with monocyte depletion and metabolically activated Tregs pre-infusion, these findings define an immune vulnerability signature that may enable improved risk stratification and targeted supportive strategies. Further characterization of the pre-infusion γδ T-cells and bone marrow immune landscape are currently underway.

**Copyright Information:** (Online) ISSN: 2572-9241
©2026 The Author(s). HemaSphere is published by John Wiley & Sons Ltd on behalf of European Hematology Association. This is an open access Abstract Book distributed under the Attribution-NonCommercial-NoDerivs (CC BY-NC-ND), which allows third parties to download the articles and share them with others as long as they credit the author and the Abstract Book, but they cannot change the content in any way or use them commercially.
**Abstract Book Citations:** Authors, Title, HemaSphere, 2026;10:(S1):pages. The abstract book DOIs can be found on the “Author Information” page.
**Disclaimer:** Articles published in the journal HemaSphere exclusively reflect the opinions of the authors. The authors are responsible for all content in their abstracts including accuracy of the facts, statements, citing resources, etc.

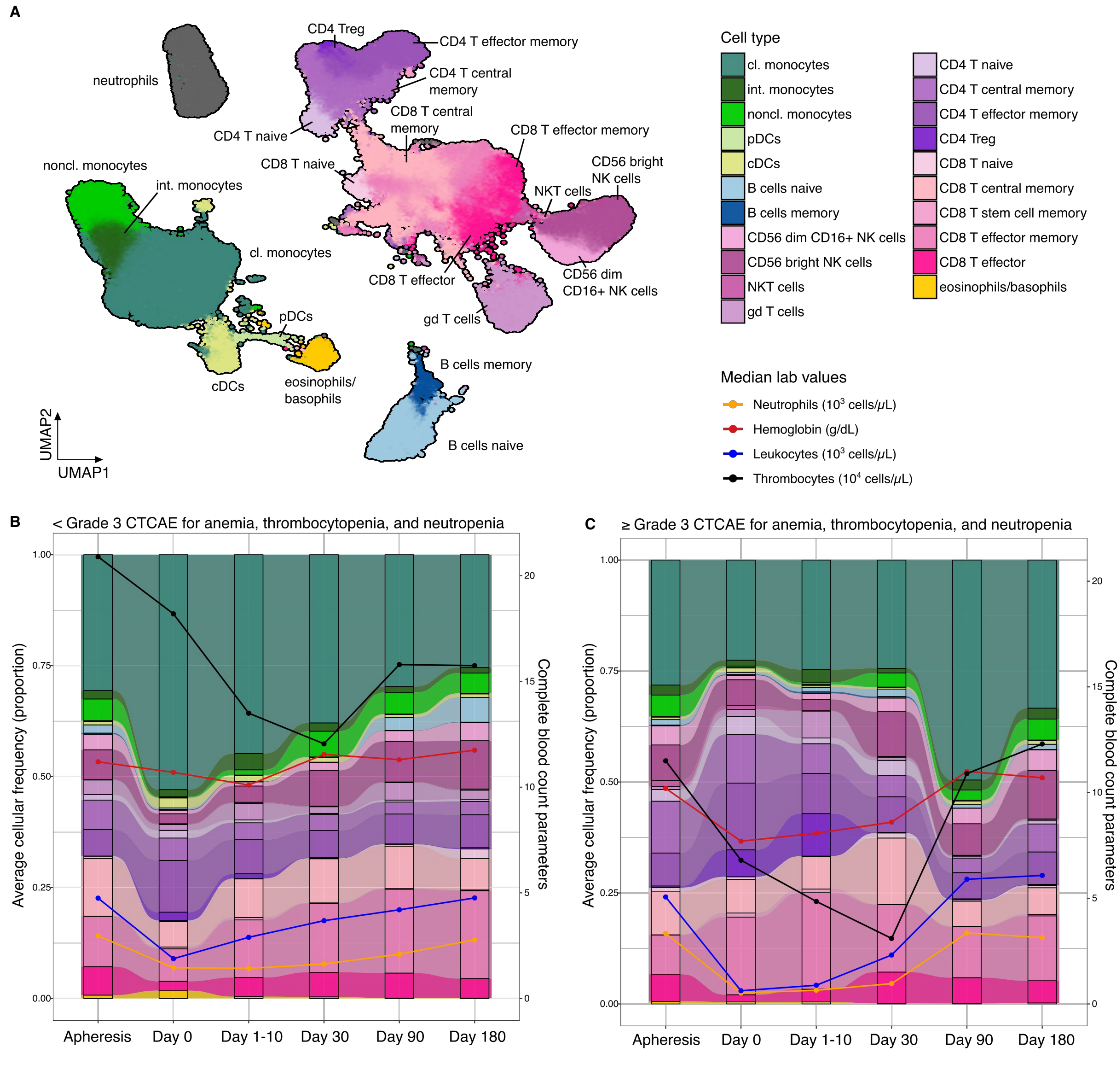

**A:** UMAP representing the immune cell landscape of ~55 million PBMCs of 42 patients at 6 timepoints using a sketch-based approach.
**B & C**: Alluvial and line plots (excluding neutrophils and CAR-positive cells) of patients with CTCAE Grade < 3 for anemia, thrombocytopenia and neutropenia (**B**) and Grade ≥ 3 (**C**). Left y-axis and alluvial plots show average frequency of cell types for each timepoint. The right y-axis shows the median laboratory values of neutrophils (as $10^3$ cells/$\mu$L), hemoglobin (as g/dL), leukocytes (as $10^3$ cells/$\mu$L), and thrombocytes (as $10^4$ cells/$\mu$L)

**Copyright Information:** (Online) ISSN: 2572-9241
©2026 The Author(s). HemaSphere is published by John Wiley & Sons Ltd on behalf of European Hematology Association. This is an open access Abstract Book distributed under the Attribution-NonCommercial-NoDerivs (CC BY-NC-ND), which allows third parties to download the articles and share them with others as long as they credit the author and the Abstract Book, but they cannot change the content in any way or use them commercially.
**Abstract Book Citations:** Authors, Title, HemaSphere, 2026;10:(S1):pages. The abstract book DOIs can be found on the “Author Information” page.
**Disclaimer:** Articles published in the journal HemaSphere exclusively reflect the opinions of the authors. The authors are responsible for all content in their abstracts including accuracy of the facts, statements, citing resources, etc.

### (PS2314) TARGETING TNFA AND IFNG REVERSES CAR-T SECRETOME–INDUCED HUMAN HEMATOPOIETIC STEM CELL IMPAIRMENT

**Topic:** 25. Gene therapy, cellular immunotherapy and vaccination - Biology & translational research

Siva Sai Naga Anurag Muddineni[1], Elad Jacoby[2, 2], Michael Milyavsky*[1, 1]

*[1]Tel Aviv University, Gray Faculty of Medical and Health Sciences, TEL AVIV, Israel;[2]Sheba Medical Center, Department of Pediatric Hematology, Ramat-Gan, Israel;*

### Background

CAR-T cell therapies have transformed outcomes for hematological malignancies, but their broader use is limited by clinically significant toxicities, including prolonged cytopenias that persist for weeks to months. Increasing evidence suggests these persistent cytopenias are linked to impaired hematopoietic stem and progenitor cell (HSPC) function, yet the inflammatory mechanisms driving CAR-T induced HSPC dysfunction remains poorly understood, limiting targeted interventions.

### Aims

To elucidate the molecular mechanisms driving CAR-T–induced hematopoietic injury and to test whether pharmacological blockade of key pro-inflammatory cytokines preserves human HSPC function.

### Methods

We developed complementary *in vitro* and *in vivo* human CAR-T hematotoxicity models. Human HSPC function was assessed through multicolor flow cytometry, apoptosis and differentiation assays, transcriptome analysis and serial NSG mouse repopulation. *In vivo*, we infused CD19-directed CAR-T cells into fully humanized NSGW41 mice and assessed hematotoxicity, HSPC function, and cytokine milieu. Recombinant TNFα and IFNγ, their respective neutralizing antibodies were used to probe mechanistic relationships.

### Results

Short-term (24h) exposure of HSPCs to activated CAR-T enhanced clonogenicity, engraftment, and self-renewal. In contrast, prolonged exposure (7 days) induced functional exhaustion, myeloid-skewed differentiation, and diminished multilineage engraftment. TNFα and IFNγ were identified as key mediators of this deleterious phenotype, and combined neutralization of these cytokines restored HSPC function. CAR-T exposure aberrantly upregulated inflammatory signaling and chronic inflammation program in human HSPCs. In humanized mice, CD19 CAR-T infusion recapitulated clinical hematotoxicity, including human B-cell aplasia, elevated IFNγ, and depletion of HSPCs.

### Summary/Conclusion

Chronic exposure to the CAR-T secretome derived factors drives HSPC dysfunction through a TNFα/IFNγ–inflammatory axis, resulting in myeloid bias and stem cell exhaustion. TNFα and IFNγ are key mediators of CAR-T secretome–induced hematotoxicity; their blockade preserves HSPC phenotype and in vivo repopulating capacity without impairing CAR-T cytotoxicity. These findings support evaluation of clinically approved cytokine-neutralizing biologics, including etanercept (TNFα inhibitor) and emapalumab (anti-IFNγ monoclonal antibody), as potential strategies to prevent or mitigate CAR-T–associated prolonged cytopenias.

**Copyright Information:** (Online) ISSN: 2572-9241
©2026 The Author(s). HemaSphere is published by John Wiley & Sons Ltd on behalf of European Hematology Association. This is an open access Abstract Book distributed under the Attribution-NonCommercial-NoDerivs (CC BY-NC-ND), which allows third parties to download the articles and share them with others as long as they credit the author and the Abstract Book, but they cannot change the content in any way or use them commercially.
**Abstract Book Citations:** Authors, Title, HemaSphere, 2026;10:(S1):pages. The abstract book DOIs can be found on the “Author Information” page.
**Disclaimer:** Articles published in the journal HemaSphere exclusively reflect the opinions of the authors. The authors are responsible for all content in their abstracts including accuracy of the facts, statements, citing resources, etc.

### (PS2315) GAMMA DELTA T-CELL THERAPY NAKU OUTPERFORMS AND ENHANCES AZA/VEN TREATMENT IN PATIENT-DERIVED EX VIVO AML MODELS

**Topic:** 25. Gene therapy, cellular immunotherapy and vaccination - Biology & translational research

Piia Käyhty[1], Risto-Pekka Pölönen[1], Satu Kaipainen[1], Toveann Ahlnäs[2], Tomáš Zárybnický[1], Sara Wojciechowski[1], Kadri Õunap[1], Taina Koponen[1], Katja Sirviö[1], Tanja Ruokoranta[2, 3], Olga Gurvich[1], Tuija Kekarainen[1], Mika Kontro*[2, 3, 4]

*[1]Ferring Ventures Oy, Kuopio, Finland;[2]Institute for Molecular Medicine Finland, University of Helsinki, Helsinki, Finland;[3]Helsinki University Hospital Comprehensive Cancer Center, Department of Hematology, Helsinki, Finland; [4]Foundation for the Finnish Cancer Institute, Helsinki, Finland;*

### Background

Acute myeloid leukemia (AML) patients who are unfit for intensive chemotherapy are typically treated with a combination of venetoclax (VEN) and 5-azacytidine (AZA). Although this regimen has significantly improved outcomes, with approximately 70% of patients achieving remission, the duration of response remains limited, highlighting the need for novel therapeutic strategies. Importantly, previous studies suggest that AZA/VEN does not impair immune cell viability and may even support immune cell proliferation and function, providing a rationale for combining these agents with cellular immunotherapies. Among potential candidates, γδ T cells represent an attractive approach. γδ T cells are an unconventional population of T cells characterized by γ and δ T-cell receptor chains rather than the classical αβ configuration. Vγ9Vδ2 T cells function in a major histocompatibility complex-independent manner via phosphoantigen recognition and have demonstrated potent cytotoxicity against cancer cells, making them ideal candidates for off-the-shelf cell therapy.

### Aims

To evaluate the *ex vivo* efficacy of the unmodified Vγ9Vδ2 T-cell therapy product NAKU (Ferring Ventures Oy, Finland) against patient-derived AML cells, with and without VEN and AZA.

### Methods

AML samples (n=22) were sourced through The Finnish Hematology Registry and Clinical Biobank. The cohort included samples from diagnostic (n = 17) and relapsed (n = 5) AML patients, including those with complex karyotypes (n = 10) and *TP53* mutations (n = 8). Samples were pre-treated with AZA/VEN (n = 22) or AZA only (n = 18), followed by the addition of NAKU and cytotoxic readout after 24h co-culture. Single-treatment controls, i.e. NAKU only and AZA/VEN and AZA only, were included. AML blast cell viability was determined by flow cytometry.

**Copyright Information:** (Online) ISSN: 2572-9241
©2026 The Author(s). HemaSphere is published by John Wiley & Sons Ltd on behalf of European Hematology Association. This is an open access Abstract Book distributed under the Attribution-NonCommercial-NoDerivs (CC BY-NC-ND), which allows third parties to download the articles and share them with others as long as they credit the author and the Abstract Book, but they cannot change the content in any way or use them commercially.
**Abstract Book Citations:** Authors, Title, HemaSphere, 2026;10:(S1):pages. The abstract book DOIs can be found on the “Author Information” page.
**Disclaimer:** Articles published in the journal HemaSphere exclusively reflect the opinions of the authors. The authors are responsible for all content in their abstracts including accuracy of the facts, statements, citing resources, etc.

### Results

AML samples revealed varying sensitivity to AZA/VEN and AZA-only treatments, with a subset of samples being completely resistant to these agents. All samples were sensitive to NAKU-mediated cytotoxicity, regardless of mutation profile, karyotypic complexity, or disease stage. Notably, even drug-resistant blasts exhibited marked sensitivity to NAKU-mediated killing.
Furthermore, combination treatments with NAKU and AZA/VEN or AZA showed a general trend toward enhanced cytotoxicity compared with single treatments, without toxicity to NAKU cells. In 9/22 blasts, NAKU combined with AZA/VEN showed enhanced cytotoxicity, defined as an absolute increase of ≥10 percentage points relative to standalone treatments. Overall, AML blasts from 15/17 diagnostic samples and 3/5 relapse samples demonstrated sensitivity to the NAKU/AZA/VEN combination, exhibiting higher cytotoxicity levels than those achieved with any of the single treatments. NAKU/AZA demonstrated a modest improvement in cytotoxicity against 10/18 blasts (8 diagnostic and 2 relapse) relative to either monotherapy. Furthermore, improved cytotoxicity with NAKU/AZA was observed with AML blasts from 4/6 tested samples with *TP53* mutation.

### Summary/Conclusion

In summary, novel γδ T cell therapy NAKU demonstrates consistent cytotoxicity against all tested AML blasts, including those resistant to AZA/VEN. The synergy of NAKU cells and AZA/VEN provides a solid rationale for advancing this approach into a clinical trial. These robust and uniform responses across diverse AML samples underscore the feasibility and translational readiness of the NAKU product for clinical trials.

**Copyright Information:** (Online) ISSN: 2572-9241
©2026 The Author(s). HemaSphere is published by John Wiley & Sons Ltd on behalf of European Hematology Association. This is an open access Abstract Book distributed under the Attribution-NonCommercial-NoDerivs (CC BY-NC-ND), which allows third parties to download the articles and share them with others as long as they credit the author and the Abstract Book, but they cannot change the content in any way or use them commercially.
**Abstract Book Citations:** Authors, Title, HemaSphere, 2026;10:(S1):pages. The abstract book DOIs can be found on the "Author Information" page.
**Disclaimer:** Articles published in the journal HemaSphere exclusively reflect the opinions of the authors. The authors are responsible for all content in their abstracts including accuracy of the facts, statements, citing resources, etc.

### (PS2316) ADJUSTABLE TUNING OF CHIMERIC PROTEIN-EXPRESSING T CELLS WITH BSABS TO AUGMENT T-CELL REDIRECTION THERAPY FOR HEMATOLOGICAL MALIGNANCIES.

**Topic:** 25. Gene therapy, cellular immunotherapy and vaccination - Biology & translational research

Junichi Kato*[1], Toshiki Ochi[1, 2], Tatsuya Konishi[3], Masaki Maruta[1], Shogo Nabe[1], Yuya Masuda[1], Koji Oka[1], Kento Mori[1], Yukihiro Miyazaki[1], Jun Yamanouchi[3], Katsuto Takenaka[1]

*[1]Ehime University Graduate School of Medicine, Department of Hematology, Clinical Immunology and Infectious Diseases, Toon, Japan; [2]Ehime University, Division of Immune Regulation, Proteo - Science Center, Toon, Japan;[3]Ehime University, Division of Blood Transfusion and Cell Therapy, Toon, Japan;*

### Background

T-cell redirection therapy using chimeric antigen receptor (CAR)-T cells and bispecific antibodies (BsAbs) has shown promise for the treatment of refractory hematological malignancies. Since these two modalities can complementarily work with each other, their sequencing and combination therapy have a potential to further enhance anti-tumor effects. Therefore, their precise assessment will allow us not only to develop new modalities, but also to understand mode of action in more details.

### Aims

In this study, we have generated new chimeric antigen protein (CAP)-expressing T cells, and investigated their functions in combination with a panel of BsAbs specific for CAP and a protein expressed by tumor cells.

### Methods

Deletion mutants of CD19 extracellular domain were individually fused with the CD28/CD3ζ intracellular domain, to generate chimeric antigen proteins (CAP). Each CAP was reconstituted into Jurkat 76 cells which lack the expression of endogenous TCR. Then, selected CAP was transduced into human peripheral blood T cells to assess their functions. Intracellular cytokine assays, memory T-cell phenotypes, CFSE proliferation and killing assays were performed using flow cytometry. On the other hand, CD19-specific mAb, FMC63-based scFv was linked with a target-specific scFv (CD70, CD22, BCMA), respectively. BiTE-based CD19 x CD70, CD19 x CD22, CD19 x BCMA BsAbs as well as CD19 x CD3, CD20 x CD3 BiTE were generated and purified from culture supernatant of 293 cells. In addition, CD70, CD22, BCMA, and CD20-positive K562 cells were prepared as target cells.

**Copyright Information:** (Online) ISSN: 2572-9241
©2026 The Author(s). HemaSphere is published by John Wiley & Sons Ltd on behalf of European Hematology Association. This is an open access Abstract Book distributed under the Attribution-NonCommercial-NoDerivs (CC BY-NC-ND), which allows third parties to download the articles and share them with others as long as they credit the author and the Abstract Book, but they cannot change the content in any way or use them commercially.
**Abstract Book Citations:** Authors, Title, HemaSphere, 2026;10:(S1):pages. The abstract book DOIs can be found on the “Author Information” page.
**Disclaimer:** Articles published in the journal HemaSphere exclusively reflect the opinions of the authors. The authors are responsible for all content in their abstracts including accuracy of the facts, statements, citing resources, etc.

**Results**

Among deletion mutants, $CAP_{del4}$/CD28/CD3ζ was well expressed on T cells and easily detected by anti-CD19 mAb. Then, we investigated differences of two intracellular signals, which are derived from chimeric CD28/CD3ζ (CAP) and endogenous CD3 complexes. By direct comparison, CAP-transduced CD3-negative Jurkat 76 cells recognized CD3-positive Jurkat cells in the presence of CD19 x CD3 BiTE. At the same time, Jurkat cells also activated for Jurkat 76/CAP-T cells, but they responded slowly and weakly than Jurkat 76/CAP-T cells. Next, using CD70 as a model antigen which is expressed by lymphoma cells as well as myeloma cells, $CAP_{del4}$-T cells successfully recognized and activated against $CD70^{+}$ target cells in the presence of CD19 x CD70 BsAb. Reactivity of $CAP_{del4}$-T cells were dependent on the concentration of CD19 x CD70 BsAb. Primary $CAP_{del4}$-T cells dominantly showed naïve T-cell phenotypes, and they produced a panel of cytokines, proliferated, and differentiated after stimulation with CD19 x CD70 BsAb, resulting in sufficient killing of $CD70^{+}$ target cells. Similar results were obtained using CD19 x CD22 and CD19 x BCMA BsAbs. When considered the combination with BiTE, $CAP_{del4}$-T cells were not well activated with CD20 x CD3 BiTE, but once stimulated with CD19 x CD70 BsAb, they adequately responded for $CD20^{+}$ target cells with CD20 x CD3 BiTE.

**Summary/Conclusion**

CAP-T cells were easily detected with anti-CD3 and CD19 antibodies, then rapidly and adjustably responded to, and effectively killed various target cells in combination with CD19-based different BsAbs. This combination strategy will allow us to regulate *in vivo* antitumor CAP-T cell responses and to overcome tumor escapes, resulting in effective and safe T-cell redirection therapy. Moreover, sequencing therapy using CD3-based BiTE following CAP/CD19-BsAb might have a potential to induce further therapeutic effects.

**Copyright Information:** (Online) ISSN: 2572-9241
©2026 The Author(s). HemaSphere is published by John Wiley & Sons Ltd on behalf of European Hematology Association. This is an open access Abstract Book distributed under the Attribution-NonCommercial-NoDerivs (CC BY-NC-ND), which allows third parties to download the articles and share them with others as long as they credit the author and the Abstract Book, but they cannot change the content in any way or use them commercially.
**Abstract Book Citations:** Authors, Title, HemaSphere, 2026;10:(S1):pages. The abstract book DOIs can be found on the “Author Information” page.
**Disclaimer:** Articles published in the journal HemaSphere exclusively reflect the opinions of the authors. The authors are responsible for all content in their abstracts including accuracy of the facts, statements, citing resources, etc.

### (PS2317) DUAL-INPUT LOGIC-GATED CAR NK CIRCUITS HARNESSING IMMUNE SYNAPSE PROTEASE ACTIVITY FOR TUMOR CONFINED MULTI ANTIGEN ENGAGER CASCADES

**Topic:** 25. Gene therapy, cellular immunotherapy and vaccination - Biology & translational research

Didem Cakirsoy Akyoney*[1], Alamdar Hussain[1], Dara Mohammad[1], Safa Bazaz[1], Hans-Gustaf Ljunggren[1], Evren Alici[1]

[1]*Karolinska Institutet, Department of Medicine Huddinge, Stockholm, Sweden;*

### Background

A central challenge in NK cell therapy is achieving tumor-restricted effector activity while preventing systemic toxicity and overcoming antigen heterogeneity. Conventional CAR-NK approaches provide target recognition but lack mechanisms for conditional and spatially confined release of payloads.

### Aims

We developed GAGE, a programmable platform integrating dual-input CAR logic with immune-synapse-coupled protease-gated payload release, to enable multi-antigen therapeutic cascades.

### Methods

NK cells were engineered with logic-gated CAR circuit comprising an NKG2A-based inhibitory CAR and a second-generation CAR recognizing TAA-1. Membrane-tethered payload modules were linked through engineered cleavage substrates responsive to granzyme-B or caspase-3 with limited or no promiscuity, leveraging protease activity enrichment restricted at the immunologic synapse. Target-dependent release was quantified by nanoluciferase kinetics and supported by protease inhibitor sensitivity. We validated function in NK92 cells. To test therapeutic relevance, we utilized bispecific T and NK cell engagers against TAA-2 and additional payloads, including cytokines and checkpoint modulators.

### Results

Dual-input logic-gating coupled TAA-1 recognition to rapid, protease-dependent payload release confined to antigen-positive synapses with minimal background in antigen-negative conditions. Protease gated circuits demonstrated a stringent engagement dependence and synthetic cleavage motifs increased range and signal discrimination. Importantly, released bi-specific engagers mediated secondary activation observed by time-lapse cytotoxicity (Incucyte), increased target killing and demonstrated a programmable cascade beyond CAR engagement.

### Summary/Conclusion

GAGE provides a mechanistic framework for logic-gated, tumor confined release. This strategy demonstrates promise as a controllable and adaptable system for potential targeted therapeutic applications.

**Copyright Information:** (Online) ISSN: 2572-9241
©2026 The Author(s). HemaSphere is published by John Wiley & Sons Ltd on behalf of European Hematology Association. This is an open access Abstract Book distributed under the Attribution-NonCommercial-NoDerivs (CC BY-NC-ND), which allows third parties to download the articles and share them with others as long as they credit the author and the Abstract Book, but they cannot change the content in any way or use them commercially.
**Abstract Book Citations:** Authors, Title, HemaSphere, 2026;10:(S1):pages. The abstract book DOIs can be found on the “Author Information” page.
**Disclaimer:** Articles published in the journal HemaSphere exclusively reflect the opinions of the authors. The authors are responsible for all content in their abstracts including accuracy of the facts, statements, citing resources, etc.

### (PS2318) BIOLOGICAL PROPERTIES AND CLONALITY OF ENGINEERED HEMATOPOIETIC STEM/PROGENITOR CELLS PERSISTING LONG-TERM AFTER GENE THERAPY

**Topic:** 25. Gene therapy, cellular immunotherapy and vaccination - Biology & translational research

Pamela Quaranta[1], Guido Pacini[1], Luca Seffin[1, 2], Luca Basso-Ricci[1], Fabrizio Benedicenti[1], Stefania Giannelli[1], Ilaria Monti[1], Clelia Perini[1], Sara Mazza[1], Silvia Darin[3], Francesca Tucci[3], Francesca Ferrua[1, 3], Valeria Calbi[3], Andrea Calabria[1], Eugenio Montini[1], Maria Ester Bernardo[1, 3], Maria Pia Cicalese[2, 3], Alessandro Aiuti[1, 2, 3], Serena Scala*[1]

*[1]San Raffaele Telethon Institute for Gene Therapy (SR-TIGET), Milan, Italy;[2]Università Vita-Salute San Raffaele, Milan, Italy; [3]IRCCS San Raffaele Scientific Institute, Milan, Italy;*

### Background

Hematopoietic stem/progenitor Cells (HSC) are used in *ex vivo* HSC-based gene therapy (HSC-GT) for treating genetic or acquired disorders, showing a favorable benefit-risk profile in clinical trials, especially when lentiviral vectors (LV) are used. However, with LV HSC-GT patients reaching now >10 years of follow-up, there is still limited information on the biological properties of long-term engrafted engineered HSC.

### Aims

We are studying the functional features, clonality and maintenance of stemness properties of LV-transduced HSC in long-term GT treated (LT-GT) patients with Wiskott-Aldrich Syndrome (WAS, n=6 treated with etuvetidigene autotemcel) and Metachromatic Leukodystrophy (MLD, n=10 treated with atidarsagene autotemcel) reaching a follow-up of >8 years post-GT.

### Methods

To study the functional properties of engineered HSC, we are currently performing phenotypic and molecular characterizations, including integration site (IS) analyses, of bone marrow (BM) and peripheral blood samples from LT-GT patients (follow-up range: 2–12 years). These analyses are complemented with *in vitro* multi-lineage assays and *in vivo* studies. Finally, the application of single-cell multi-ome protocol will allow to retrieve transcriptome (scRNAseq), epigenome (scATACseq), mitochondrial (mtDNA) variants and IS of single HSC.

### Results

Longitudinal analyses of BM HSC content in LT-GT patients show stable HSC count starting from 2-year post-GT, at levels comparable to pre-GT state in all the patients analyzed. Moreover, the composition of the most primitive HSC remains stable over time, implying maintenance of the self-renewal/differentiation rate of primitive HSC. We also measured steady-state transduced cell chimerism and vector copy number in the BM of LT-GT patients. Collection of ~45,000 IS from sorted HSPC and mature lineages in LT-GT patients showed a highly polyclonal pool of engineered HSC. Moreover, we measured a substantial fraction of engrafted HSC, maintaining their number up to the latest follow up post-GT.

To gain insight on the molecular features of LT-GT HSPC we have generated a scRNAseq dataset of almost 65,000 BM HSPC from WAS and MLD patients, and healthy donor(HD) pediatric subjects. Our analyses show that HSC from LT-GT patients express gene signatures associated with stemness properties at levels comparable to HD and no sign of senescence. Moreover, we detected fewer differentially expressed genes in transduced vs. HD HSC than untransduced vs. HD HSC, suggesting the restoration of HSC physiological features upon gene-correction. Finally, HSC isolated from LT-GT patients display consistent lymphoid, myeloid and erythroid/megakaryocytic output *in vitro*, implying preservation of their functionality and multi-lineage production. Moreover, the number of produced cells for each lineage is in line with HSC derived from HD.

**Copyright Information:** (Online) ISSN: 2572-9241
©2026 The Author(s). HemaSphere is published by John Wiley & Sons Ltd on behalf of European Hematology Association. This is an open access Abstract Book distributed under the Attribution-NonCommercial-NoDerivs (CC BY-NC-ND), which allows third parties to download the articles and share them with others as long as they credit the author and the Abstract Book, but they cannot change the content in any way or use them commercially.
**Abstract Book Citations:** Authors, Title, HemaSphere, 2026;10:(S1):pages. The abstract book DOIs can be found on the "Author Information" page.
**Disclaimer:** Articles published in the journal HemaSphere exclusively reflect the opinions of the authors. The authors are responsible for all content in their abstracts including accuracy of the facts, statements, citing resources, etc.

**Summary/Conclusion**

Our preliminary data have shown maintenance of a stable gene-corrected HSC compartment displaying in vitro multilineage properties. Moreover, we provided evidence that the engrafted pool of engineered HSC retain self-renewal properties preserving their number and clonality up to 10 years post-GT. Finally, scRNAseq data suggest that primitive HSC from LT-GT patients express molecular signatures associated with stemnessand biological features in line with HD HSPC.

Overall, this study will shed light on human engineered HSC that enable maintenance a functional and safe long-term graft.

**Copyright Information:** (Online) ISSN: 2572-9241
©2026 The Author(s). HemaSphere is published by John Wiley & Sons Ltd on behalf of European Hematology Association. This is an open access Abstract Book distributed under the Attribution-NonCommercial-NoDerivs (CC BY-NC-ND), which allows third parties to download the articles and share them with others as long as they credit the author and the Abstract Book, but they cannot change the content in any way or use them commercially.
**Abstract Book Citations:** Authors, Title, HemaSphere, 2026;10:(S1):pages. The abstract book DOIs can be found on the "Author Information" page.
**Disclaimer:** Articles published in the journal HemaSphere exclusively reflect the opinions of the authors. The authors are responsible for all content in their abstracts including accuracy of the facts, statements, citing resources, etc.

### (PS2319) INCORPORATING IN VITRO TRANSCRIPTION MRNA TO PROLONG THE HALF-LIFE OF BISPECIFIC ANTIBODY

**Topic:** 25. Gene therapy, cellular immunotherapy and vaccination - Biology & translational research

Yu Yang*[1]

[1]*Center for Integrated Oncology Aachen Bonn Cologne Duesseldorf, University of Cologne, Department I of Internal Medicine, Cologne, Germany;*

### Background

Bispecific T cell engagers (BiTEs) are engineered antibodies with two binding domains: one targeting tumor antigens and the other engaging T cells to redirect cytotoxicity. Despite strong antitumor activity, their clinical use is limited by a short serum half-life due to small size and lack of an Fc domain, leading to rapid renal clearance. For example, the CD3/CD19 BiTE blinatumomab has a half-life of ~2 hours and requires continuous infusion. Fc fusion and PEGylation can extend half-life but increase manufacturing complexity and often show suboptimal in vivo performance. To address this, we developed an mRNA-based strategy for in vivo expression of bispecific antibodies.

### Aims

1.Design DNA templates for in vitro transcripting stabilized mRNA constructs.
2.Verify protein production by transfecting mRNA into various cell lines.
3.Evaluate target-dependent cytotoxicity mediated by in vitro–transcribed mRNA encoding bispecific antibodies.
4.Monitor plasma levels of functional (bi-scFv) $_2$ proteins and assess tumor regression in xenograft mouse models treated with bsAb-encoding mRNA.

### Results

Stabilized mRNA constructs included a 5′ cap, optimized 5′/3′ UTRs, an ORF, and a poly(A) tail. GFP was first cloned and transcribed in vitro; RNA integrity was confirmed, and GFP mRNA was electroporated into PBMCs and tumor cells. GFP was then replaced with anti-CD3/CD30 or anti-CD3/CD19 sequences. mRNA was transfected into CHO cells, and secreted antibodies were detected by Western blot. Flow cytometry confirmed specific binding to $CD3^+$, $CD30^+$, and $CD19^+$ cells. Cytotoxicity was evaluated in PBMC–tumor co-cultures, together with T cell activation (CD25, CD69) and cytokine production (IL-2, IFN-γ). GFP mRNA produced strong fluorescence, validating delivery and translation. Bispecific antibodies were efficiently expressed, specifically bound target cells, and induced antigen-dependent tumor lysis with CD25/CD69 upregulation and increased IL-2 and IFN-γ only in antigen-positive conditions. Prior NSG xenograft studies showed that anti-CD3/CD30 plasmid delivery, but not purified protein, significantly inhibited tumor growth, providing a baseline for future evaluation of mRNA-mediated therapy.

### Summary/Conclusion

Our results show that the mRNA is efficiently translated into functional bispecific antibodies across multiple cell types. In vitro cytotoxicity assays confirmed preserved tumor targeting and T cell–mediated killing activity, accompanied by increased T cell activation and proliferation. Ongoing studies are evaluating the in vivo efficacy and safety of this mRNA-based approach in mouse tumor models.

**Copyright Information:** (Online) ISSN: 2572-9241
©2026 The Author(s). HemaSphere is published by John Wiley & Sons Ltd on behalf of European Hematology Association. This is an open access Abstract Book distributed under the Attribution-NonCommercial-NoDerivs (CC BY-NC-ND), which allows third parties to download the articles and share them with others as long as they credit the author and the Abstract Book, but they cannot change the content in any way or use them commercially.
**Abstract Book Citations:** Authors, Title, HemaSphere, 2026;10:(S1):pages. The abstract book DOIs can be found on the “Author Information” page.
**Disclaimer:** Articles published in the journal HemaSphere exclusively reflect the opinions of the authors. The authors are responsible for all content in their abstracts including accuracy of the facts, statements, citing resources, etc.

### (PS2320) SCREENING AND VALIDATION OF TCR–PEPTIDE INTERACTIONS USING INTEGRATED BULK AND SINGLE-CELL IMMUNE RECEPTOR SEQUENCING

**Topic:** 25. Gene therapy, cellular immunotherapy and vaccination - Biology & translational research

Alex Chenchik*[1, 2], Debbie Deng[1], Kitt Paraiso[1], Guido Stadler[2], Ben Yellen[2]

*[1]Cellecta, Inc., Mountain View, United States of America;[2]CellDom, Durham, United States of America;*

### Background

Identification of cancer-associated antigens and their cognate T cell receptors (TCRs) is essential for advancing immunotherapies, cancer vaccines, and adoptive T-cell treatments. Although antigen candidates can be efficiently predicted using genomic, transcriptomic, and mass-spectrometry–based HLA ligandome profiling, systematic validation of antigen-specific TCRs from blood samples remains challenging. Scalable platforms that integrate quantitative repertoire profiling with functional confirmation of TCR–peptide interactions are needed to accelerate the development of targeted immunotherapies.

### Aims

We aim to establish and validate a high-throughput workflow for discovering antigen-specific TCR clonotypes stimulated by defined peptides in PBMCs, integrating bulk adaptive immune receptor (AIR) RNA sequencing, single-cell analysis of stimulated T cells, bioinformatic prioritization, and functional validation in Jurkat–HLA reporter systems.

### Methods

PBMCs from healthy donors were stimulated for 14 days with synthetic cancer-associated peptides (e.g., NY-ESO-1, MART-1) or DMSO controls. Bulk AIR RNA TCR sequencing quantified clonotype expansion relative to Day 0 and across replicates. Antigen-specific candidates were identified based on ≥100–1,000-fold expansion, enrichment in peptide-treated vs. control samples, and high abundance ranking. Peptide-specific $CD8^+$ T cells were labelled by tetramer staining and expression of activation markers (CD137, IFNG) following re-stimulation. Sorted $tetramer^+$ or $CD137^+/IFNG^+$ T cells underwent single-cell AIR sequencing to obtain full-length paired α/β TCR sequences. Selected TCRs were cloned into Jurkat NFAT-GFP reporter cells and tested against peptide-presenting K562 antigen presenting cells (APCs) expressing matched HLA–β2M–peptide constructs. Clonotype alignment and prioritization were supported by MiXCR and DETECT ImmuneWatch pipelines.

### Results

Bulk AIR-RNA TCR analysis identified 100–300 peptide-responsive clonotypes per peptide treatment, each exhibiting 100–5,000-fold expansion. Peptide-stimulated samples displayed unique CDR3 repertoires in each replicate, reflecting the low baseline abundance of naive antigen-specific T cells. In contrast, nonspecific expansions in DMSO triplicate controls were highly reproducible. Single-cell AIR sequencing of peptide-$tetramer^+$ and activation $marker^+$ $CD8^+$ cells confirmed that most expanded clonotypes were peptide-specific. Seventeen MART-1–responsive TCRs were cloned into Jurkat NFAT-GFP cells; of these, 14 demonstrated strong functional avidity when tested against MART-1 peptide presented by matched HLA APCs.

### Summary/Conclusion

The combination of bulk immune receptor profiling, single-cell immune receptor profiling, and functional validation workflow described can be highly useful for identifying antigen-specific TCR clonotypes stimulated by defined peptides in PBMCs.

**Copyright Information:** (Online) ISSN: 2572-9241
©2026 The Author(s). HemaSphere is published by John Wiley & Sons Ltd on behalf of European Hematology Association. This is an open access Abstract Book distributed under the Attribution-NonCommercial-NoDerivs (CC BY-NC-ND), which allows third parties to download the articles and share them with others as long as they credit the author and the Abstract Book, but they cannot change the content in any way or use them commercially.
**Abstract Book Citations:** Authors, Title, HemaSphere, 2026;10:(S1):pages. The abstract book DOIs can be found on the "Author Information" page.
**Disclaimer:** Articles published in the journal HemaSphere exclusively reflect the opinions of the authors. The authors are responsible for all content in their abstracts including accuracy of the facts, statements, citing resources, etc.

| CDR3 Sequence | DMSO | MART | CD137 | Mart1 | IFNG | Selected for validation | Validated in K562-Jurkat |
|---|---|---|---|---|---|---|---|
| | Day 14 | Day 14 | Day 15 | Day 14 | Day 15 | | |
| CASSQDAIGQPQHF | 71 | 64133 | +++ | +++ | +++ | Cloned | Yes |
| CASSFGNEQFF | 43 | 45125 | +++ | +++ | +++ | Cloned | Yes |
| CAIRRLAGESNGELFF | 31 | 28223 | +++ | +++ | +++ | Cloned | Yes |
| CASSERGVNTEAFF | 35 | 22796 | + | + | - | Cloned | No |
| CASSFQGSGTEAFF | 7 | 12821 | ++ | +++ | +++ | Cloned | Yes |
| CASRPPGLAGDLSYNEQFF | 12 | 15855 | ++ | +++ | +++ | Cloned | Yes |
| CATSPTGLGQPQHF | 2 | 4423 | + | +++ | - | Cloned | No |
| CASSLAGVGQPQHF | 8 | 7881 | ++ | +++ | - | Cloned | Yes |
| CASSLGKAADTQYF | 3 | 4950 | + | +++ | +++ | Cloned | Yes |
| CASSFMGGTYNEQFF | 180 | 4130 | - | - | - | | |
| CASSPLGTGDQETQYF | 4 | 4874 | - | - | - | | |
| CAISEIPSSLTYNEQFF | 2769 | 8064 | - | + | - | | |
| CAISETPEGVRVSEQYF | 2 | 3640 | ++ | +++ | - | Cloned | Yes |
| CASSLSFETQYF | 1886 | 5111 | - | - | - | | |
| CASSGPGTLADTQYF | 3951 | 2016 | - | - | - | | |
| CASSLALGNEQFF | 4 | 4138 | + | +++ | - | Cloned | Yes |
| CASRSGIGQPQHF | 0 | 1937 | - | + | - | | |
| CASGTVYTDTQYF | 905 | 1672 | - | - | - | | |
| CASSEAGTLSWERF | 930 | 1612 | - | - | - | | |
| CASSLTPGQGETQYF | 11 | 5281 | - | - | - | | |
| CASSLGLLSGEQYF | 3 | 2699 | + | ++ | +++ | Cloned | Yes |
| CSARTGYEQYF | 9 | 2716 | - | - | - | | |
| CASSPTPGTAAGEQYF | 7 | 5230 | - | ++ | - | Cloned | No |
| CATSRSTTGELFF | 1 | 1273 | + | + | - | | |
| CASSLDVLAGKEQFF | 7 | 2185 | - | + | - | | |
| CASRTGTGAGEQYF | 2 | 2789 | + | ++ | +++ | Cloned | Yes |
| CASKPLAAGTGELFF | 13583 | 3665 | - | - | - | | |
| CASSLGQLAGNTIYF | 0 | 1843 | + | + | - | | |
| CASSPRGRDFRAPQHF | 8 | 1516 | - | - | - | | |
| CASSTSDLSGNTIYF | 2 | 2034 | + | ++ | - | Clone | No |

Table 1: Columns 1 and 2 (blue) show quantitative analysis of expanded top 30 TCR clonotypes identified by bulk AIR-RNA assays in PBMC samples treated with MART-1 peptide (Day 14). Numbers in DMSO day 14 and MART1 day 14 are fold expansion of each CDR3-specific T cell vs Control at day 0. Non-specifically expanded TCR clonotypes are highlighted in DMSO on day 14 (orange). Columns 3,4 and 5 (pink) show the number of CD8+ cells sorted in a 96-well plate after staining with CD137 or MART1 tetramer or IFNG. These cells were used for scAIR-TCR-RNA assay to generate full-length chain pairing information. Columns 6 and 7 (green) show 14 sequences with the highest score of CD137/MART1 tetramer/INFG markers that were cloned, expressed in Jurkat-NFAT-GFP reporter cells, and validated for functional interaction with K562 cells expressing MART1 peptide-beta-2-microglobin-HLA-A:02:01 constructs.

Copyright Information: (Online) ISSN: 2572-9241
©2026 The Author(s). HemaSphere is published by John Wiley & Sons Ltd on behalf of European Hematology Association. This is an open access Abstract Book distributed under the Attribution-NonCommercial-NoDerivs (CC BY-NC-ND), which allows third parties to download the articles and share them with others as long as they credit the author and the Abstract Book, but they cannot change the content in any way or use them commercially.
Abstract Book Citations: Authors, Title, HemaSphere, 2026;10:(S1):pages. The abstract book DOIs can be found on the "Author Information" page.
Disclaimer: Articles published in the journal HemaSphere exclusively reflect the opinions of the authors. The authors are responsible for all content in their abstracts including accuracy of the facts, statements, citing resources, etc.

### (PS2321) FROM BENCH TO BODY: STABLE IN VIVO CAR T-CELL ENGINEERING WITH A T CELL–TROPIC AAV–TRANSPOSON PLATFORM

**Topic:** 25. Gene therapy, cellular immunotherapy and vaccination - Biology & translational research

Mobina Ostadiasrami*[1], Angela Enrica Araujo[2], Michael Hudecek[3, 4], David Krones[3, 4], Ulrich Hacker[5], Sabrina Friedel[3], Hildegard Buening[6]

[1]*University Hospital Würzburg (Universitätsklinikum Würzburg), Department of Internal Medicine II, Chair of Cellular Immunotherapy, Würzburg, Germany;* [2]*Hannover Medical School (MHH), Institute of Experimental Hematology, Hannover, Germany;* [3]*University Hospital Würzburg, Chair in Cellular Immunotherapy, Department of Internal Medicine II, Würzburg, Germany;* [4]*Fraunhofer Institute for Cell Therapy and Immunology (IZI), Branch for Cellular Immunotherapy, Würzburg, Germany;* [5]*University of Leipzig, Medical Department II, University Cancer Center Leipzig, Leipzig, Germany;*[6]*Hannover Medical School, Institute of Experimental Hematology, Hannover, Germany;*

### Background

Direct in vivo generation of chimeric antigen receptor (CAR) T cells represents a paradigm shift to overcome the logistical complexity, cost, and manufacturing constraints of conventional ex vivo CAR T-cell production. However, current in vivo gene delivery strategies based on adeno-associated virus (AAV) vectors or lipid nanoparticles predominantly result in transient CAR expression, limiting persistence and therapeutic durability in proliferating T cells.

### Aims

We aimed to establish an in vivo–compatible gene-delivery platform combining T cell–tropic AAV vectors with Sleeping Beauty (SB) transposon–mediated genomic integration to enable stable CAR expression and generate functional CAR T cells directly under physiologic conditions within a scalable framework for in-body CAR T-cell engineering.

### Methods

A high-diversity AAV peptide display library (>$10^6$ variants) was screened in non-human primates following intravenous administration to identify capsids with intrinsic T-cell tropism. Lead naturally occurring mutant capsids were used to deliver GFP reporters or CAR constructs targeting hematologic and solid tumor antigens. The AAV-transposon/AAV-transposase system was subsequently evaluated in ex vivo and in vivo models. Genomic integration, CAR expression, lineage selectivity, activation state, functional fitness, and anti-tumor activity were quantified using next-generation sequencing, high-resolution flow cytometry, and a comprehensive suite of functional assays.

### Results

Application of the AAV–SB system directly to human whole blood from multiple donors enabled efficient genetic modification of T cells and allowed optimization of the SB transposase-to-transposon stoichiometry supporting stable, long-term CAR expression. Pronounced lineage restriction and reproducible T-cell–preferential gene transfer were confirmed by high-sensitivity flow cytometry at single-event resolution together with barcode-based genomic sequencing.
Functionally, AAV-engineered CAR T cells displayed robust antigen-specific activation without prior enrichment and were subsequently challenged with multiple antigen-positive tumor cell lines. Strong effector activation was reflected by increased CD25 and CD69 expression, whereas low levels of PD-1, LAG-3, and TIM-3 indicated preservation of a non-exhausted phenotype. In direct comparison with electroporation-based SB delivery, AAV-engineered CAR T cells maintained superior viability and expansion capacity, consistent with reduced cellular stress during gene transfer.

**Copyright Information:** (Online) ISSN: 2572-9241
©2026 The Author(s). HemaSphere is published by John Wiley & Sons Ltd on behalf of European Hematology Association. This is an open access Abstract Book distributed under the Attribution-NonCommercial-NoDerivs (CC BY-NC-ND), which allows third parties to download the articles and share them with others as long as they credit the author and the Abstract Book, but they cannot change the content in any way or use them commercially.
**Abstract Book Citations:** Authors, Title, HemaSphere, 2026;10:(S1):pages. The abstract book DOIs can be found on the "Author Information" page.
**Disclaimer:** Articles published in the journal HemaSphere exclusively reflect the opinions of the authors. The authors are responsible for all content in their abstracts including accuracy of the facts, statements, citing resources, etc.

**Summary/Conclusion**

Using a T-cell–tropic AAV capsid coupled with SB transposon–mediated genomic integration, we established a next-generation AAV–SB platform for durable CAR T-cell engineering. This approach enables stable CAR expression and functional anti-tumor activity under both extracorporeal and intracorporeal conditions while supporting scalable, high-titer manufacturing. Together, these findings define a direct translational route toward in vivo CAR T-cell generation for hematologic and solid malignancies.

**Copyright Information:** (Online) ISSN: 2572-9241
©2026 The Author(s). HemaSphere is published by John Wiley & Sons Ltd on behalf of European Hematology Association. This is an open access Abstract Book distributed under the Attribution-NonCommercial-NoDerivs (CC BY-NC-ND), which allows third parties to download the articles and share them with others as long as they credit the author and the Abstract Book, but they cannot change the content in any way or use them commercially.
**Abstract Book Citations:** Authors, Title, HemaSphere, 2026;10:(S1):pages. The abstract book DOIs can be found on the “Author Information” page.
**Disclaimer:** Articles published in the journal HemaSphere exclusively reflect the opinions of the authors. The authors are responsible for all content in their abstracts including accuracy of the facts, statements, citing resources, etc.

### (PS2322) EARLY IGFBP6 DOWNREGULATION AND ROCK2 ACTIVATION IDENTIFY PATIENTS AT RISK OF ICANS AFTER CD19 CAR-T THERAPY: A REAL-WORLD MOLECULAR MONITORING STUDY

**Topic:** 25. Gene therapy, cellular immunotherapy and vaccination - Biology & translational research

Clara Bono[1], Francesca Guerrini[1], Enrico Orciuolo[2], Gaspare Tancredi[1], La Russa Mattia[1], Ignazio Santo[3], Leoni Giulia[1], Arcangelo Liso[4], Sara Galimberti*[1, 2]

*[1]University of Pisa, Department of Clinical and Experimental Medicine, Hematology., Pisa, Italy;[2]Azienda Ospedaliero Universitaria Pisana, Pisa, Italy; [3]Azienda USL Toscana Nord Ovest - Ospedale Versilia, Lido di Camaiore, Italy;[4]University of Perugia, Perugia, Italy;*

### Background

Immune effector cell–associated neurotoxicity syndrome (ICANS) remains one of the most clinically relevant complications of CD19-directed CAR-T cell therapy. Increasing evidence suggests that endothelial dysfunction represents a central pathogenic mechanism linking systemic inflammation to blood–brain barrier disruption. However, early molecular predictors of neurotoxicity remain poorly defined. IGFBP6 has been described as an endothelial homeostasis–associated mediator with anti-inflammatory properties, whereas ROCK2 is implicated in endothelial-driven inflammatory responses.

### Aims

To investigate CAR-T expansion kinetics and early modulation of ROCK2 and IGFBP6 gene expression in relation to cytokine release syndrome (CRS) and ICANS in a real-world cohort.

### Methods

Fifty patients with B-cell lymphoma treated with commercially available CD19 CAR-T products were prospectively monitored. Peripheral blood samples were collected at +24 h, +72 h, +7 d, +10 d and +14 d post-infusion. CAR transgene copy numbers were quantified by droplet digital PCR (ddPCR). ROCK2 and IGFBP6 expression levels were assessed by multiplex ddPCR and normalized to GAPDH expression. CRS and ICANS were graded according to ASTCT criteria. Inflammatory markers were analyzed at the corresponding time points. Survival outcomes were estimated using the Kaplan–Meier method.

### Results

CRS occurred in 96% of patients, predominantly grade 0–1, whereas ICANS developed in 22%. Median progression-free survival was 18 months, while median overall survival was not reached. No significant associations were observed between toxicity and survival outcomes.CAR-T expansion followed a typical kinetic profile, with a progressive increase from 72 h, peaking at day +7, followed by subsequent contraction. Expansion at 72 h was not associated with CRS severity ($p = 0.963$) or ICANS occurrence ($p = 0.629$). In contrast, day +7 CAR-T copy numbers were significantly higher in patients who developed ICANS ($p = 0.027$). ROC analysis identified a cut-off value of 14,860 CAR copies at day +7 for predicting ICANS (sensitivity 80%, specificity 70%). In patients with high-grade CRS (grade 2–3), ROCK2 expression at 72 h was increased concomitantly with elevated IL-6 levels, supporting the involvement of inflammation-mediated endothelial activation. A distinct temporal pattern was observed for ICANS. At 24 h post-infusion, patients who subsequently developed ICANS exhibited significantly lower IGFBP6 expression together with increased C-reactive protein levels ($p = 0.047$), suggesting an early disruption of endothelial anti-inflammatory homeostasis. At 72 h, ICANS was associated with increased ROCK2 expression concomitant with elevated IL-6 levels ($p = 0.027$), consistent with progressive endothelial activation. No significant associations were observed between IGFBP6 expression and CRS severity.

**Copyright Information:** (Online) ISSN: 2572-9241
©2026 The Author(s). HemaSphere is published by John Wiley & Sons Ltd on behalf of European Hematology Association. This is an open access Abstract Book distributed under the Attribution-NonCommercial-NoDerivs (CC BY-NC-ND), which allows third parties to download the articles and share them with others as long as they credit the author and the Abstract Book, but they cannot change the content in any way or use them commercially.
**Abstract Book Citations:** Authors, Title, HemaSphere, 2026;10:(S1):pages. The abstract book DOIs can be found on the "Author Information" page.
**Disclaimer:** Articles published in the journal HemaSphere exclusively reflect the opinions of the authors. The authors are responsible for all content in their abstracts including accuracy of the facts, statements, citing resources, etc.

### Summary/Conclusion

ICANS appears to arise from a progressive biological continuum characterized by early IGFBP6 downregulation, followed by ROCK2-mediated endothelial activation and a subsequent peak in CAR-T expansion. Integration of CAR-T kinetics with early gene expression profiling may enable preclinical identification of patients at risk of neurotoxicity, thereby supporting a personalized, risk-adapted management strategy.

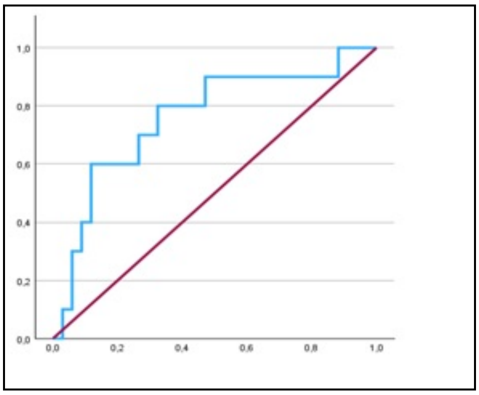

Receiver operating characteristic (ROC) curve of CAR-T cell copy numbers at day 7 for the prediction of ICANS.

**Copyright Information:** (Online) ISSN: 2572-9241
©2026 The Author(s). HemaSphere is published by John Wiley & Sons Ltd on behalf of European Hematology Association. This is an open access Abstract Book distributed under the Attribution-NonCommercial-NoDerivs (CC BY-NC-ND), which allows third parties to download the articles and share them with others as long as they credit the author and the Abstract Book, but they cannot change the content in any way or use them commercially.
**Abstract Book Citations:** Authors, Title, HemaSphere, 2026;10:(S1):pages. The abstract book DOIs can be found on the "Author Information" page.
**Disclaimer:** Articles published in the journal HemaSphere exclusively reflect the opinions of the authors. The authors are responsible for all content in their abstracts including accuracy of the facts, statements, citing resources, etc.

### (PS2323) CD4/CD8 BALANCE IN CIRCULATING CAR T-CELLS SHOWS BIPHASIC PROGNOSTIC IMPACT ON OVERALL SURVIVAL IN LARGE B-CELL LYMPHOMAS

**Topic:** 25. Gene therapy, cellular immunotherapy and vaccination - Biology & translational research

Ludovic Gabellier*[1], Florence Cuschera[2], Caroline Bret[3], Sylvain Lamure[4], Jean-Jacques Tudesq[1], Alexandre Jentzer[5], Houria Hanane Guedon[1], Tarik Kanouni[1], Emmanuelle Tchernonog[1], Franciane Paul[1], Patrice Ceballos[1], Sarah Bonnet[1], Julie Bardol[1], Léa Fornero[1], Laure Vincent[1], Eric Van Den Neste[2], Charles Herbaux[1], Guillaume Cartron[1]

*[1]University Hospital of Montpellier, Clinical Hematolgy, Montpellier, France; [2]Cliniques universitaires Saint-Luc (UC Louvain), Clinical Hematolgy, Bruxelles, Belgium; [3]University Hospital of Montpellier, Hematology laboratory, Montpellier, France; [4]Centre Léon Bérard, Clinical Hematolgy, Lyon, France; [5]University Hospital of Montpellier, Immunology laboratory, Montpellier, France;*

### Background

Anti-CD19 CAR-T expansion/persistence and the balance of CD4+ vs CD8+ CAR T-cells may influence outcomes in large B-cell lymphomas (LBCL), but their temporal dynamics and phase-dependent prognostic value remain unclear.

### Aims

To assess (i) CAR T-cell persistence over 24 months and (ii) the association of CAR-T levels and DiffNorm with response kinetics and survival.

### Methods

In a single-center cohort of 139 consecutive patients treated with anti-CD19 CAR T-cells for R/R LBCL, CAR T-cell levels (total, CAR-CD4, CAR-CD8) were quantified by flow cytometry in peripheral blood from M1 to M24 (LOD 0.1% of lymphocytes). Time-to-complete metabolic response (CMR), PFS and OS were analyzed using a time-varying Cox model. DiffNorm ((CAR-CD4 - CAR-CD8)/(CAR-CD4 + CAR-CD8)) were analyzed using a piecewise time-varying Cox approach (day 21 cutpoint) to account for the sign inversion observed between peak and M3 in timepoint analyses, reflecting early expansion vs post-expansion phases. For time-to-CMR and PFS analyses, CAR T-cell measurements obtained after disease progression were excluded. Holm correction was applied for multiple comparisons.

**Copyright Information:** (Online) ISSN: 2572-9241
©2026 The Author(s). HemaSphere is published by John Wiley & Sons Ltd on behalf of European Hematology Association. This is an open access Abstract Book distributed under the Attribution-NonCommercial-NoDerivs (CC BY-NC-ND), which allows third parties to download the articles and share them with others as long as they credit the author and the Abstract Book, but they cannot change the content in any way or use them commercially.
**Abstract Book Citations:** Authors, Title, HemaSphere, 2026;10:(S1):pages. The abstract book DOIs can be found on the "Author Information" page.
**Disclaimer:** Articles published in the journal HemaSphere exclusively reflect the opinions of the authors. The authors are responsible for all content in their abstracts including accuracy of the facts, statements, citing resources, etc.

### Results

Median age was 66 years; 62.6% were male. Axicabtagene ciloleucel accounted for 86.3% of infusions. CMR was achieved in 103/139 patients (74.1%), ORR was 86.3%. With a median follow-up of 24.5 months, median PFS was 42.5 months and median OS 58.1 months. Among patients with available measurement, CAR T-cells remained detectable in 98.5% at M1, 97.6% at M6, 93.0% at M12, and 94.7% at M24. DiffNorm showed a progressive shift from CAR-CD8 dominance at peak (median -0.26) to a nadir at M1 (median -0.50), followed by a continuous increase through M24 (median +0.02); all paired comparisons vs M1 were significant from M6 onward (Wilcoxon, Holm-adjusted p≤0.02), confirming a population-level inversion of the CAR-CD4/CD8 ratio. In time-varying Cox models for time-to-CMR, higher total CAR T-cell levels were associated with faster CMR achievement (HR 1.12 per doubling, 95%CI 1.04-1.21; Holm-adjusted p=0.022); CAR-CD4 and CAR-CD8 results were consistent but collinear. DiffNorm showed no association with time-to-CMR. Neither CAR T-cell levels nor DiffNorm were associated with PFS. For OS, piecewise DiffNorm revealed opposite phase-dependent effects: early HR 1.31 per +0.1 DiffNorm increase (95%CI 1.10-1.56; Holm-adjusted p=0.006) versus late HR 0.91 (0.85-0.97; p=0.006). In multivariate analysis (**Fig. 1**), covariates were retained based on univariate significance. After adjustment for age, Ann Arbor stage, and pre-lymphodepletion CD4-ALC, DiffNorm (early HR 1.27, p=0.010; late HR 0.93, p=0.019) and pre-lymphodepletion CD4-ALC (HR 0.76 per +0.1 G/L, 95%CI 0.61-0.95; p=0.015) remained independently associated with OS.

### Summary/Conclusion

CAR T-cells remain detectable in over 90% of patients throughout 2 years of follow-up. The CAR-CD4/CD8 balance undergoes a biologically meaningful inversion, from early CD8-dominant expansion to progressive CD4 predominance. Absolute CAR T-cell levels accelerate CMR achievement, while DiffNorm shows opposite phase-dependent associations with OS - early CAR-CD8 dominance favoring cytotoxic expansion, late CAR-CD4 predominance favoring immune memory - independent of baseline immune status. Pre-lymphodepletion CD4 lymphocyte count emerges as an independent prognostic factor. These findings warrant external validation and may inform risk stratification and product optimization.

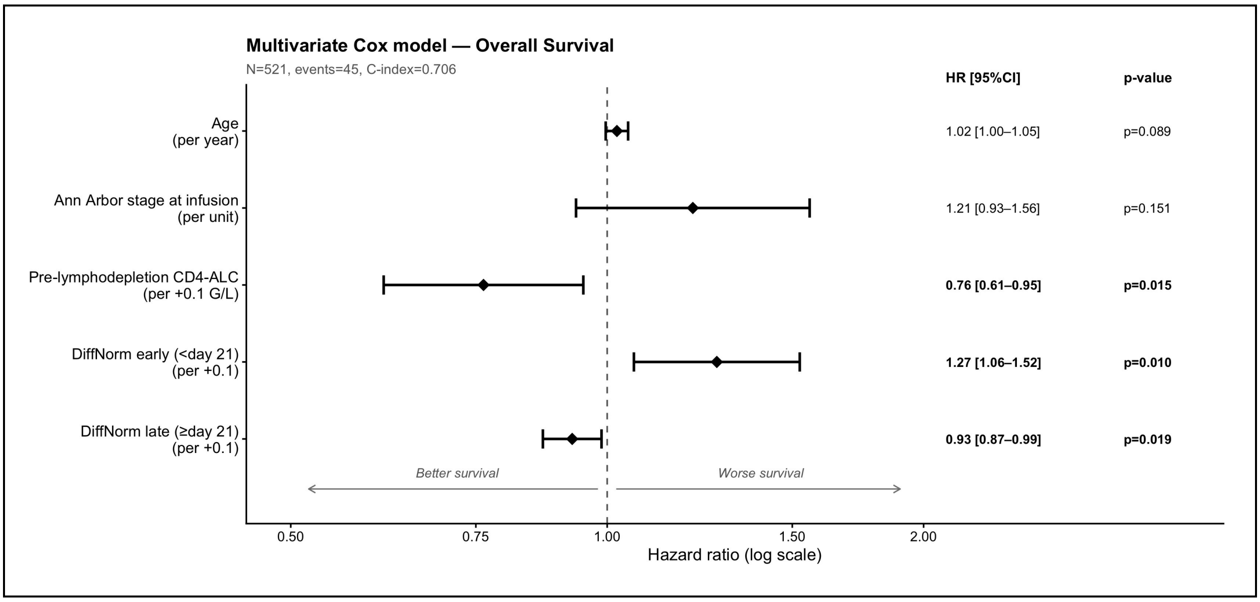

Multivariate Cox Model - Overall Survival

**Copyright Information:** (Online) ISSN: 2572-9241
©2026 The Author(s). HemaSphere is published by John Wiley & Sons Ltd on behalf of European Hematology Association. This is an open access Abstract Book distributed under the Attribution-NonCommercial-NoDerivs (CC BY-NC-ND), which allows third parties to download the articles and share them with others as long as they credit the author and the Abstract Book, but they cannot change the content in any way or use them commercially.
**Abstract Book Citations:** Authors, Title, HemaSphere, 2026;10:(S1):pages. The abstract book DOIs can be found on the "Author Information" page.
**Disclaimer:** Articles published in the journal HemaSphere exclusively reflect the opinions of the authors. The authors are responsible for all content in their abstracts including accuracy of the facts, statements, citing resources, etc.

### (PF2324) A TOOLBOX TO VISUALIZE AND QUANTIFY CHIMERIC ANTIGEN RECEPTORS USING SUPERRESOLUTION MICROSCOPY

**Topic:** 25. Gene therapy, cellular immunotherapy and vaccination - Biology & translational research

Josefine Michael[1], Peter Spieler[1], Fabio Toppeta[1], Leon Gehrke[1], Markus Sauer[2], Hermann Einsele[1], Michael Hudecek[1], Thomas Nerreter*[1]

*[1]University Hospital of Wuerzburg, Internal Medicine II, Wuerzburg, Germany;[2]University of Wuerzburg, Biozentrum, Würzburg, Germany;*

### Background

Chimeric antigen receptor (CAR)-T cells have proven to be a transformative treatment for hematologic malignancies, and concerted efforts in the field aim to translate this success to solid tumors and autoimmune diseases. There is a strong desire to accurately assess CAR organization, spatiotemporal expression, and sensitivity thresholds in order to elucidate the mechanistic details of CAR-T cell-mediated antitumor activity and to evaluate the efficacy and safety of CAR-T cell products.

### Aims

The main objective of this study is to develop a novel, tag-free approach that enables the detection and precise quantification of CAR surface expression on T cells without relying on artificial tags or transfection markers for the visualization of CARs and CAR-T cells. The approach should not be limited to single, specific CAR designs, but should be generally applicable to all CARs based on single-chain variable fragments (scFv).

### Methods

Here, we used super-resolution microscopy by means of *direct* stochastic optical reconstruction microscopy (*d*STORM) to directly visualize CAR and antigens using a toolbox of specific monoclonal antibodies directed against extracellular components of the CAR. We did not only target the spacer regions, but also the linkers present in the CAR binding domain. This allowed us to determine CAR surface expression on primary human T cells with unprecedented detail, independent of CAR specificity and receptor design. In addition, we performed *d*STORM analyses of target antigen expression on tumor cells. We complemented these approaches for CAR and antigen detection with the evaluation of CAR-T cell phenotype and CAR-T cell-mediated effector functions (cytotoxicity, cytokine secretion, and proliferation), thereby demonstrating potential applications for detailed mechanistic insights.

### Results

Using *d*STORM, we were able to visualize and reliably quantify the surface expression of CARs targeting multiple antigens and featuring different backbone designs, even when CAR expression levels were below the detection limit of flow cytometry. Thereby, we could demonstrate that e.g. CARs resembling the globally approved FMC63-based CD19 CAR-T cells products Kymriah (tisagenlecleucel), Yescarta (axicabtagene ciloleucel), Tecartus (brexucabtagene autoleucel), and Breyanzi (lisocabtagene maraleucel) differ in cell surface expression, organization and CAR clustering, and also lead to differences in CAR-T cell phenotype and function. In addition, we were able to visualize extremely low antigen expression on tumor cells with minimal background and high precision, down to single-digit receptors on the cell surface. We identified several factors that influence CAR surface expression and correlate with the functional sensitivity of CAR-T cells.

**Copyright Information:** (Online) ISSN: 2572-9241
©2026 The Author(s). HemaSphere is published by John Wiley & Sons Ltd on behalf of European Hematology Association. This is an open access Abstract Book distributed under the Attribution-NonCommercial-NoDerivs (CC BY-NC-ND), which allows third parties to download the articles and share them with others as long as they credit the author and the Abstract Book, but they cannot change the content in any way or use them commercially.
**Abstract Book Citations:** Authors, Title, HemaSphere, 2026;10:(S1):pages. The abstract book DOIs can be found on the “Author Information” page.
**Disclaimer:** Articles published in the journal HemaSphere exclusively reflect the opinions of the authors. The authors are responsible for all content in their abstracts including accuracy of the facts, statements, citing resources, etc.

**Summary/Conclusion**

Here, we present a novel, tag-free approach to gain insights into the surface expression of CARs and target antigens, and illustrate the potential of super-resolution microscopy for optimizing synthetic immune receptors in CAR-T cell therapy. This lays the foundation for more sophisticated and combinatorial research approaches to further improve the efficacy of CAR-T cell immunotherapy, predict its therapeutic outcome, and ensure optimal patient care.

**Copyright Information:** (Online) ISSN: 2572-9241
©2026 The Author(s). HemaSphere is published by John Wiley & Sons Ltd on behalf of European Hematology Association. This is an open access Abstract Book distributed under the Attribution-NonCommercial-NoDerivs (CC BY-NC-ND), which allows third parties to download the articles and share them with others as long as they credit the author and the Abstract Book, but they cannot change the content in any way or use them commercially.
**Abstract Book Citations:** Authors, Title, HemaSphere, 2026;10:(S1):pages. The abstract book DOIs can be found on the "Author Information" page.
**Disclaimer:** Articles published in the journal HemaSphere exclusively reflect the opinions of the authors. The authors are responsible for all content in their abstracts including accuracy of the facts, statements, citing resources, etc.

### (PS2325) SINGLE-CELL PROFILING REVEALS DIFFERENTIAL ACTIVATION OF EX VIVO EXPANDED NATURAL KILLER CELLS ACROSS PEDIATRIC LEUKEMIA SUBTYPES

**Topic:** 25. Gene therapy, cellular immunotherapy and vaccination - Biology & translational research

Tiina Tuononen*[1], Mouhamad Al Moussawy[1], Charlotte Rinne[2], Anna-Mari Schroderus[3], Jonas Bouhlal[4, 5, 6], Hanna Duàn[4, 5, 6], Sara Gandolfi[4, 5, 6], Andrea Hanel[1], Konstantin Ivanov[1], Tuure Kinnunen[3], Olli Lohi[2], Satu Mustjoki[4, 5, 6], Merja Heinäniemi[1]

*[1]Institute of Biomedicine, School of Medicine, Faculty of Health Sciences, University of Eastern Finland, Kuopio, Finland; [2]Tampere Center for Child, Adolescent, Maternal Health Research, Faculty of Medicine and Health Technology, Tampere University,, Tampere, Finland; [3]Department of Clinical Microbiology, Institute of Clinical Medicine, Faculty of Health Sciences, University of Eastern Finland, Kuopio, Finland; [4]Hematology Research Unit Helsinki, University of Helsinki and Helsinki University Hospital,, Helsinki, Finland; [5]Translational Immunology Research Program, University of Helsinki, Helsinki, Finland;[6]iCAN Digital Precision Cancer Medicine Flagship, Helsinki, Finland;*

**Background**

Pediatric T-lineage acute lymphoblastic leukemia (T-ALL) is associated with worse outcomes compared with B-lineage ALL (B-ALL), including frequent central nervous system involvement and a threefold higher risk of relapse or refractory disease. Relapsed T-ALL has an estimated survival rate of only ~35%, whereas modern immunotherapies have transformed B-ALL treatment, achieving survival rates of >70% even in relapse. Despite these advances, progress in T-ALL remains limited, highlighting the need for novel therapeutic strategies. Allogeneic natural killer (NK) cells are a promising off-the-shelf immunotherapy with demonstrated safety and efficacy in hematological malignancies.

**Aims**

In this study, we aim to evaluate the activity of*ex vivo* expanded NK cells against pediatric acute leukemia and to identify factors underlying patient- and leukemia subtype- specific NK cell responsiveness.

**Methods**

*Ex vivo* expanded NK cells were cultured either alone or in co-culture with patient-derived leukemic cells (T-ALL, n = 18; B-ALL, n = 6; acute myeloid leukemia (AML), n = 4) and leukemia cell lines (K562, Jurkat). NK cell cytotoxicity was assessed by flow cytometry, and transcriptional responses were profiled using multiplexed single-cell RNA sequencing. In addition, existing multi-omics datasets (methylome, bulk and single-cell transcriptome, proteome) will be integrated to identify determinants of leukemic cell sensitivity or resistance to NK-mediated killing.

**Results**

Unsupervised clustering of *9,800* NK cell transcriptomes revealed NK cell phenotypes similar to those previously reported by Dufva et al. (*Immunity*, 2023) in co-cultures with cell lines, including a co-culture–induced activated phenotype associated with NK cell–mediated cytotoxicity and characterized by expression of *TNFRSF9*, *CRTAM*, *HAVCR2*, and *BCL2L11*. This activated state was predominantly induced in T-ALL co-cultures and displayed marked inter-sample heterogeneity, consistent with flow cytometry findings. Importantly, NK cytotoxicity correlated with the overall compositional state of NK cells, particularly the abundance of the activated cluster (Spearman's $\rho$=0.69, p-val= 0.039 in T-ALL). In contrast, B-ALL and AML co-cultures elicited NK activation less frequently, indicating leukemia subtype–specific differences in NK responsiveness.

**Copyright Information:** (Online) ISSN: 2572-9241
©2026 The Author(s). HemaSphere is published by John Wiley & Sons Ltd on behalf of European Hematology Association. This is an open access Abstract Book distributed under the Attribution-NonCommercial-NoDerivs (CC BY-NC-ND), which allows third parties to download the articles and share them with others as long as they credit the author and the Abstract Book, but they cannot change the content in any way or use them commercially.
**Abstract Book Citations:** Authors, Title, HemaSphere, 2026;10:(S1):pages. The abstract book DOIs can be found on the "Author Information" page.
**Disclaimer:** Articles published in the journal HemaSphere exclusively reflect the opinions of the authors. The authors are responsible for all content in their abstracts including accuracy of the facts, statements, citing resources, etc.

**Summary/Conclusion**

Our results demonstrate that *ex vivo* expanded NK cells exhibit enhanced activation and cytotoxic potential against T-ALL compared to other pediatric leukemia subtypes, albeit with considerable patient-to-patient variability. These findings support further investigation of NK cell-based approaches in T-ALL while underscoring notable patient heterogeneity.

**Copyright Information:** (Online) ISSN: 2572-9241
©2026 The Author(s). HemaSphere is published by John Wiley & Sons Ltd on behalf of European Hematology Association. This is an open access Abstract Book distributed under the Attribution-NonCommercial-NoDerivs (CC BY-NC-ND), which allows third parties to download the articles and share them with others as long as they credit the author and the Abstract Book, but they cannot change the content in any way or use them commercially.
**Abstract Book Citations:** Authors, Title, HemaSphere, 2026;10:(S1):pages. The abstract book DOIs can be found on the "Author Information" page.
**Disclaimer:** Articles published in the journal HemaSphere exclusively reflect the opinions of the authors. The authors are responsible for all content in their abstracts including accuracy of the facts, statements, citing resources, etc.

### (PS2326) CAR-T CELL THERAPY FOR THE TREATMENT OF A HIGH-RISK PEDIATRIC AMKL: PRECLINICAL RESULTS

**Topic:** 25. Gene therapy, cellular immunotherapy and vaccination - Biology & translational research

Marta Tassoni*[1], Salvatore Nicola Bertuccio[2], Daria Messelodi[2], Renée Concetta Duardo[2], Francesca Vaglio[1, 2], Serena De Matteis[2], Gianluca Storci[2], Federica Panaccione[2], Cristiano Mauro[2], Giordana Scirè Calabrisotto[2], Giordana Orsino[2], Massimiliano Bonafé[1, 2], Francesca Bonifazi[2]

*[1]University of Bologna, Department of Medical and Surgical Sciences (DIMEC), Bologna, Italy;[2]IRCCS Azienda Ospedaliero-Universitaria di Bologna, Bologna, Italy;*

### Background

Non-Down Syndrome Acute Megakaryoblastic Leukemia (non-DS-AMKL) is a rare pediatric disease associated with poor prognosis. Approximately 20% of cases harbor the *ETO2-GLIS2* fusion gene, which drives the overexpression of CD56 and CD117 (*Masetti R. et al., 2017; Thiollier C. et al., 2012*), thereby providing a strong rationale for CAR-T–based therapy. However, these antigens are also expressed on normal cells, with CD117 present on hematopoietic stem cells (HSCs) and CD56 expressed on multiple normal tissues, including neurons. Depending on clinical timing, an anti-CD117 CAR-T could serve as a potent myeloablative conditioning tool to achieve the MRD-negative remission essential for transplant success, especially in primary refractory cases (*Rettinger E. et al., 2025*). Post-transplant, dual-antigen LINK CAR-T (CD56 AND CD117 logic gating) would then provide a high-precision safeguard to eliminate the leukemic clone while protecting donor-derived marrow and healthy tissues.

### Aims

To develop a novel CAR-T cell therapy for a subgroup for AMKL with poor prognosis to minimize on-target off-tumor toxicities and enhance therapeutic safety.

### Methods

We designed individual second-generation CARs (CD28-CD3ζ) targeting CD56 (m906 scFv;*Feng Y. et al., 2016*) (CAR-CD56) and CD117 (ligand-based; *Branella G.M. et al., 2023*) (CAR-CD117). Then, to implement AND-gate logic, we used the "LINK" system by Tousley A.M. et al. in 2023 and replaced CD3ζ with proximal signalling molecules: the CD56 CAR was fused to GADS-binding domain-deleted LAT (CARCD56 LAT-del) while the CD117 CAR was fused to GADS-binding domain-deleted SLP-76 (CARCD117 SLP76-del). All constructs were delivered via lentiviral transduction into healthy donor CD3+ T cells. A 100% dual-positive LINK population was obtained via sorting. Cytotoxicity against target cells lines was assessed at 24h, 48h, and 6 days. CARCD56 was tested against M07e, WSU-AML, and CRISPR-generated CD56-knockout (KO) M07e cells. Other constructs were evaluated against M07e cells. Untransduced T cells (UT) served as control. Cytokines released in coculture media were measured by flow cytometry at 24h.

### Results

Single-target CAR-CD56 and CAR-CD117 demonstrated robust, antigen-specific cytotoxicity against AMKL cell lines, with specificity further validated by the total sparing of CD56-KO targets. This anti-tumor activity was driven by significant antigen-dependent CAR-T cells expansion and a potent inflammatory response, characterized by marked TNF-α and IFN-γ secretion. Notably, CAR-CD56 maintained high efficacy across multiple AML models, outperforming UT controls which failed to activate or persist. With the final aim to reduce on target off tumor toxicity, the logic-gated preliminary results with the LINK CAR-T system show promising killing data, suggesting effective target discrimination.

**Copyright Information:** (Online) ISSN: 2572-9241
©2026 The Author(s). HemaSphere is published by John Wiley & Sons Ltd on behalf of European Hematology Association. This is an open access Abstract Book distributed under the Attribution-NonCommercial-NoDerivs (CC BY-NC-ND), which allows third parties to download the articles and share them with others as long as they credit the author and the Abstract Book, but they cannot change the content in any way or use them commercially.
**Abstract Book Citations:** Authors, Title, HemaSphere, 2026;10:(S1):pages. The abstract book DOIs can be found on the “Author Information” page.
**Disclaimer:** Articles published in the journal HemaSphere exclusively reflect the opinions of the authors. The authors are responsible for all content in their abstracts including accuracy of the facts, statements, citing resources, etc.

### Summary/Conclusion

Our results demonstrate potent anti-AMKL activity for CAR-CD56 and CD117; the ongoing implementation of the LINK system aims to provide a high-precision strategy to ensure therapeutic safety and minimize off-tumor toxicities.

**Copyright Information:** (Online) ISSN: 2572-9241
©2026 The Author(s). HemaSphere is published by John Wiley & Sons Ltd on behalf of European Hematology Association. This is an open access Abstract Book distributed under the Attribution-NonCommercial-NoDerivs (CC BY-NC-ND), which allows third parties to download the articles and share them with others as long as they credit the author and the Abstract Book, but they cannot change the content in any way or use them commercially.
**Abstract Book Citations:** Authors, Title, HemaSphere, 2026;10:(S1):pages. The abstract book DOIs can be found on the "Author Information" page.
**Disclaimer:** Articles published in the journal HemaSphere exclusively reflect the opinions of the authors. The authors are responsible for all content in their abstracts including accuracy of the facts, statements, citing resources, etc.

### (PS2327) EXPLORING THE THERAPEUTIC POTENTIAL OF TCR GENE THERAPY WITH ARGINASE 1-TARGETED METABOLIC INTERVENTION IN PRECLINICAL TUMOR MODEL

**Topic:** 25. Gene therapy, cellular immunotherapy and vaccination - Biology & translational research

Anita Kansy*[1], Edite Antunes[1], Matthias Theobald[1, 2, 3], Hakim Echchannaoui[1, 3]

*[1]Internal Medicine III, University Cancer Center (UCT), Mainz, Germany; [2]Research Center for Immunotherapy (FZI), University Medical Center (UMC) of the Johannes Gutenberg University Mainz, Mainz, Germany; [3]German Cancer Consortium (DKTK), Partner Site Frankfurt/Mainz, Mainz, Germany;*

### Background

Cancer often exhibits diverse evasion mechanism to subvert immune surveillance. Metabolic remodeling ofthe tumor microenvironment (TME) is a hallmar of tumor evasion. Upregulated secretion of arginase 1 (ARG1) by tumor-infiltrating myeloid-derived cells (MDSCs) and type 2 macrophages (M2) leads to a rapid catabolism of arginine, the resulting depletion suppresses T cell activity by promoting exhaustion and dysfunction. Inhibition of ARG1 can restore the metabolic homeostasis within the TME enhancing functionality and cytotoxicity of tumor antigen-specific T cells.

### Aims

Here, we address the synergistic combinatorial effect of adoptive TCR-gene transfer and ARG1 inhibition in a syngeneic murine tumor model.

### Methods

Spleen derived OT-1 T cells were used as effector cells targeting the H-2Kb-restricted Ovalbumin (OVA) peptide SIINFEKL$_{(257-264)}$, using two different tumor models: the OVA/Luciferase-engineered murine adenocarcinoma cell line MC-38 (MC-38-OVA-Luc) and the murine melanoma cell line B16-F10 (B16-F10-OVA-Luc). Pharmacological inhibition of ARG1 activity was targeted using nor-NOHA, a selective arginase inhibitor. Fluc activity enabled real-time monitoring of tumor growth via bioluminescence imaging (BLI) using Caliper/Xenogen IVIS spectrum device.

### Results

Treamtent with nor-NOHA resulted in inhibited tumor progression in MC-38-OVA engrafted mice. Adoptive TCR-specific cell transfer demonstrates complete tumor control. To characterise changes in the TME upon ARG1 inhibition, we analysed the frequency and ARG1 expression of MDSCs and TAMs, phenotype differences of tumor-infiltrating lymphocytes (TILs), spleen-infiltrating T cells, and frequency of regulatory T cells (Treg) in *ex-vivo* material using flow cytometry. Our data reveal, that mainly monocytic MDSCs ($CD11b^+$/$Ly6C^+Ly6G^-$) and TAMs ($CD11b^+$/$F4|80^+$) infiltrated the TME. While MDSCs also exhibit high ARG1 content, TAMs show the most robust expression, suggesting these infiltrating cells are the primary drivers of arginine-related metabolic remodelling in our model´s TME. Treatment with nor-NOHA successfully reduced both, the frequency of ARG1 positive cells as well as their overall expression levels. Furthermore, despite a lower overall frequency of TILs, the $CD8^+$ T cell population in ex-vivo tumors exhibited reduced expression of different exhaustion markers, such as LAG-3. Regulatory T cells ($CD4^+$/$FoxP3^+$) showed lowered infiltrating upon treatment.

**Copyright Information:** (Online) ISSN: 2572-9241
©2026 The Author(s). HemaSphere is published by John Wiley & Sons Ltd on behalf of European Hematology Association. This is an open access Abstract Book distributed under the Attribution-NonCommercial-NoDerivs (CC BY-NC-ND), which allows third parties to download the articles and share them with others as long as they credit the author and the Abstract Book, but they cannot change the content in any way or use them commercially.
**Abstract Book Citations:** Authors, Title, HemaSphere, 2026;10:(S1):pages. The abstract book DOIs can be found on the "Author Information" page.
**Disclaimer:** Articles published in the journal HemaSphere exclusively reflect the opinions of the authors. The authors are responsible for all content in their abstracts including accuracy of the facts, statements, citing resources, etc.

**Summary/Conclusion**

In our model, we could demonstrate that targeted inhibition of ARG1 within the TME, results in an additive effect on the tumor control. However, since the transfer of antigen-specific OT-1 T cells alone achieved complete tumor regression, the potential for synergistic effects remains to be further evaluated in follow up studies.

**Copyright Information:** (Online) ISSN: 2572-9241
©2026 The Author(s). HemaSphere is published by John Wiley & Sons Ltd on behalf of European Hematology Association. This is an open access Abstract Book distributed under the Attribution-NonCommercial-NoDerivs (CC BY-NC-ND), which allows third parties to download the articles and share them with others as long as they credit the author and the Abstract Book, but they cannot change the content in any way or use them commercially.
**Abstract Book Citations:** Authors, Title, HemaSphere, 2026;10:(S1):pages. The abstract book DOIs can be found on the "Author Information" page.
**Disclaimer:** Articles published in the journal HemaSphere exclusively reflect the opinions of the authors. The authors are responsible for all content in their abstracts including accuracy of the facts, statements, citing resources, etc.

### (PS2328) ALLOGENEIC BCMA-TARGETED CAR-INKT CELLS WITH PD-1 DISRUPTION EXHIBIT ENHANCED ANTITUMOR POTENCY IN A MULTIPLE MYELOMA PRECLINICAL MODEL

**Topic:** 25. Gene therapy, cellular immunotherapy and vaccination - Biology & translational research

Yuhua Wan[1], Dexian Quan[1], Shulin Yang[1], Mo Deng[1], Feng Xu[2], Biao Zhou[2], Yuzhu Cui[2], Linyan Deng[2], Jian Hua[1], Jingyu Li[2], Hua Zhang*[2]

*[1]SPH Biotherapeutics (Shanghai) Limited, Shanghai, China;[2]SPH Biotherapeutics (HK) Limited, Shatin, Hong Kong;*

### Background

Two BCMA-directed CAR-T cell therapies, idecabtagene vicleucel (ide-cel) and ciltacabtagene autoleucel (cilta-cel), are now FDA-approved for relapsed/refractory multiple myeloma (R/R MM) and have shown remarkable clinical benefit, yet autologous manufacturing remains constrained by long production timelines, high costs, delayed treatment initiation, and dependence on patient-derived T-cell quality. To overcome these limitations, we developed an allogeneic "off-the-shelf" platform based on invariant natural killer T (iNKT) cells, which uniquely combine T-cell and NK-cell features, express an invariant Vα24Jα18 TCR and canonical NK receptors, respond to lipid antigens via CD1d and to stress ligands via NKRs, and critically do not induce graft-versus-host disease. Here, we describe a novel PD-1–deleted BCMA-targeted CAR-iNKT product engineered to enhance antitumor potency and improve the therapeutic potential of adoptive cell therapy in multiple myeloma.

### Aims

Developing an allogeneic platform for cell-based therapy to lower overall costs for patients

### Methods

We sought to identify the improvement of CAR designs that would enhance iNKT cells. The VHH CAR construct targeting BCMA and its modified variant incorporating additional PD1 intracellular retention (a non-gene editing technique to remove membrane proteins) were developed to assess and compare their *in vivo* therapeutic efficacy of eliminating malignant myeloma cells. In the study, $3\times10^6$ of H929-luc cells per mouse were inoculated subcutaneously into NOG-hIL15Tg mice, allowed to engraft for 5 days. Subsequently, the mice were treated with one or three injections of $4.5\times10^6$ CAR+ iNKT cells per mouse on days 5, 10, and 15, respectively. This experimental setup enabled comparison of anti-tumor activity between the two CAR designs and assessment of dosing frequency impact.

### Results

The CAR-iNKT with PD1 deletion demonstrated a much better long-term cytotoxicity against H929 cells than the unmodified CAR-iNKT *in vitro* in a 50-hour real-time killing assay by Incucyte at an E:T ratio of 3:1. To further evaluate the activities of both BCMA CAR-iNKT cells *in vivo*, we deployed a xenograft myeloma model using H929 luciferase-expressing cells. Both CAR-iNKT cells along with their controls were i.v transferred to myeloma-bearing mice at one or three injections and disease burden was monitored by bioluminescence. As a result, the CAR-iNKT with PD1 deletion in three doses showed much better tumor control in the animal model study until D35.

**Copyright Information:** (Online) ISSN: 2572-9241
©2026 The Author(s). HemaSphere is published by John Wiley & Sons Ltd on behalf of European Hematology Association. This is an open access Abstract Book distributed under the Attribution-NonCommercial-NoDerivs (CC BY-NC-ND), which allows third parties to download the articles and share them with others as long as they credit the author and the Abstract Book, but they cannot change the content in any way or use them commercially.
**Abstract Book Citations:** Authors, Title, HemaSphere, 2026;10:(S1):pages. The abstract book DOIs can be found on the "Author Information" page.
**Disclaimer:** Articles published in the journal HemaSphere exclusively reflect the opinions of the authors. The authors are responsible for all content in their abstracts including accuracy of the facts, statements, citing resources, etc.

**Summary/Conclusion**

We are developing a novel allogeneic CAR-iNKT cell therapy that incorporates our proprietary PD-1 intracellular retention technology to enhance the potency of adoptive cell treatment. By leveraging an iNKT platform that is naturally devoid of alloreactivity, we enable rapid engineering and large-scale expansion of an off-the-shelf CAR product. iNKT cells also offer intrinsic advantages, including bone marrow homing and BCMA-directed cytotoxicity, further strengthened by CD1d-mediated activation and NK receptor/ligand interactions. Together, these features position our approach to deliver meaningful benefit for patients with relapsed or refractory multiple myeloma, with the potential to surpass the efficacy of current therapeutic options.

**Copyright Information:** (Online) ISSN: 2572-9241
©2026 The Author(s). HemaSphere is published by John Wiley & Sons Ltd on behalf of European Hematology Association. This is an open access Abstract Book distributed under the Attribution-NonCommercial-NoDerivs (CC BY-NC-ND), which allows third parties to download the articles and share them with others as long as they credit the author and the Abstract Book, but they cannot change the content in any way or use them commercially.
**Abstract Book Citations:** Authors, Title, HemaSphere, 2026;10:(S1):pages. The abstract book DOIs can be found on the "Author Information" page.
**Disclaimer:** Articles published in the journal HemaSphere exclusively reflect the opinions of the authors. The authors are responsible for all content in their abstracts including accuracy of the facts, statements, citing resources, etc.

### (PS2330) EFFICACY AND SAFETY OF COMMERCIAL CD19 CAR-T (INATICABTAGENE AUTOLEUCEL) IN RELAPSED/REFRACTORY EXTRAMEDULLARY B-ALL: A LARGE MULTICENTER REAL-WORLD STUDY

**Topic:** 26. Gene therapy, cellular immunotherapy and vaccination - Clinical

Suning Chen*[1], Jin Wang[2], Heng Mei[3], Xianmin Song[4], Yuhua Li[5], Jie Jin[6], Erlie Jiang[7], Jian Li[8], Lei Fan[9], Xudong Wei[10], Bao Hong Ping[11], Liye Zhong[12], Zhenyang Gu[13], Haixia Zhou[1], Shuhua Yi[7], Rong Li[14], Ping Li[15], Zhenshu Xu[16], Lei Gao[17], Ying Wang[7]

*[1]The affiliated first hospital of Soochow University, Suzhou, China;[2]Ruijin Hospital Affiliated to Shanghai Jiao Tong University School of Medicine, Shanghai, China; [3]Institute of Hematology, Union Hospital, Tongji Medical College, Huazhong University of Science and Technology, Wuhan, China; [4]Shanghai General Hospital, Shanghai, China;[5]Zhujiang Hospital of Southern Medical University, Guangzhou, China; [6]The First Affiliated Hospital of Zhejiang University, Hangzhou, China;[7]Institute of Hematology and Blood Diseases Hospital, Chinese Academy of Medical Sciences, Tianjin, China; [8]Peking Union Medical College Hospital, Beijing, China; [9]Jiangsu Province Hospital, Suzhou, China; [10]Henan Cancer Hospital, Zhengzhou, China; [11]Southern Medical University Southern Hospital, Guangzhou, China; [12]The First Affiliated Hospital of Jinan University, Guangzhou, China; [13]Department of Hematology, The Fifth Medical Center of Chinese PLA General Hospital, Beijing, Beijing, China;[14]Naval Medical University of the People's Liberation Army, Shanghai, China; [15]Tongji Hospital of Tongji University, Shanghai, China; [16]Concord Hospital of the Fujian Medical University, Fujian, China; [17]The First Affiliated Hospital of Naval Medical University, Shanghai, China;*

### Background

Adult patients with relapsed/refractory (R/R) extramedullary B-ALL have a dismal prognosis with conventional therapies. Despite the success of CD19 CAR-T in hematologic relapse, its efficacy in extramedullary disease remains underexplored, as pivotal trials often excluded these high-risk pts. This large, multicenter, real-world study aims to define the outcomes of Inaticabtagene autoleucel (Inati-cel) in this challenging population.

### Aims

To evaluate the real-world efficacy and safety of Inati-cel among Chinese adultpatients with relapsed/refractory (R/R) extramedullary B-ALL.

### Methods

Eligible patients had R/R extramedullary B-ALL, underwent apheresis for Inati-cel following its approval (November 7, 2023), and completed at least 30 days of follow-up. The primary endpoints were overall survival (OS) and relapse-free survival (RFS). Secondary outcomes included the complete remission (CR) rate, minimal residual disease (MRD) negativity rate, and toxicity profiles. CR was defined as ≤5% bone marrow blasts morphologically, no circulating lymphoblasts, and resolution of extramedullary disease. MRD was evaluated via flow cytometry, RQ-PCR, and/or NGS. CRS and ICANS were graded according to ASTCT criteria.

**Copyright Information:** (Online) ISSN: 2572-9241
©2026 The Author(s). HemaSphere is published by John Wiley & Sons Ltd on behalf of European Hematology Association. This is an open access Abstract Book distributed under the Attribution-NonCommercial-NoDerivs (CC BY-NC-ND), which allows third parties to download the articles and share them with others as long as they credit the author and the Abstract Book, but they cannot change the content in any way or use them commercially.
**Abstract Book Citations:** Authors, Title, HemaSphere, 2026;10:(S1):pages. The abstract book DOIs can be found on the "Author Information" page.
**Disclaimer:** Articles published in the journal HemaSphere exclusively reflect the opinions of the authors. The authors are responsible for all content in their abstracts including accuracy of the facts, statements, citing resources, etc.

**Results**

As of the data cutoff (December 31, 2025), a total of 50 patients with R/R extramedullary B-ALL from over 20 centers across China received Inati-cel therapy.The median age was 39.5 years (range, 14–79), and 60% were male.Extramedullary disease involved the central nervous system (CNS) in 64%(32/50) of pts, non-CNS disease in 28%(14/50), and both in 8%(4/50). Following infusion, 41 of 48 evaluable pts (85.4%) achieved CR in both bone marrow and EM disease, with 100% of responders achieving bone marrow MRD negativity.Among pts with CNS involvement, the CR rate was 93.3% (28/30), compared with 78.6% (11/14) in those with non-CNS EM disease and 50% (2/4) in pts with both CNS and non-CNS EM disease.
After a median follow-up of 7.2 months, the median OS and RFS had not been reached;Notably, the 1-year OS and RFS rates were 86.8% and 61.9%, respectively. Compared to their Ph-positive counterparts, patients with Ph-negative disease had significantly worse OS (100% vs 76.4%; $P = 0.026$) and RFS (81.2% vs 45.6%; $P = 0.039$). Similarly, IKZF1 deletion was associated with significantly poorer OS (89.4% vs 66.7%; $P = 0.044$) and RFS (67.1% vs 33.3%; $P = 0.004$).
Regarding safety, any-grade CRS occurred in 66% of pts (4% Grade 3–4), and ICANS occurred in 14% (4% Grade 3). All toxicities were manageable and reversible, with no treatment-related deaths.

**Summary/Conclusion**

This largest real-world study demonstrates that commercial CD19 CAR-T therapy with Inati-cel induces high response rates and durable survival in adult patients with high-risk R/R extramedullary B-ALL, exhibiting a manageable safety profile. These findings establish Inati-cel as an effective standard-of-care option for this population and support its use even in settings typically excluded from clinical trials.

**Copyright Information:** (Online) ISSN: 2572-9241
©2026 The Author(s). HemaSphere is published by John Wiley & Sons Ltd on behalf of European Hematology Association. This is an open access Abstract Book distributed under the Attribution-NonCommercial-NoDerivs (CC BY-NC-ND), which allows third parties to download the articles and share them with others as long as they credit the author and the Abstract Book, but they cannot change the content in any way or use them commercially.
**Abstract Book Citations:** Authors, Title, HemaSphere, 2026;10:(S1):pages. The abstract book DOIs can be found on the “Author Information” page.
**Disclaimer:** Articles published in the journal HemaSphere exclusively reflect the opinions of the authors. The authors are responsible for all content in their abstracts including accuracy of the facts, statements, citing resources, etc.

### (PS2331) CTD402 ALLOGENEIC ANTI-CD7 CAR T-CELL THERAPY IS SAFE AND EFFECTIVE IN ADOLESCENT/PEDIATRIC PATIENTS (PTS) WITH RELAPSED/REFRACTORY (R/R) T-ALL/LBL

**Topic:** 26. Gene therapy, cellular immunotherapy and vaccination - Clinical

Xian Zhang*[1], Junfang Yang[1], Xiaofan Zhu[2], Ying Wang[2], Jiang Cao[3], Xiaoyu Zhu[4], Keshu Zhou[5], Sanbin Wang[6], Ming Gao[7], Peng Yu[7], Chengcheng Cai[7], Wengang Ge[7], Jiangtao Ren[7], Maria del Rosario[7], Jan Davidson-Moncada[7], Peihua Lu[1]

*[1]Hebei Yanda Lu Daopei Hospital, Langfang, Hebei, China;[2]Chinese Academy of Medical Sciences Institute of Hematology, Tianjin, China; [3]Xuzhou Medical University Affiliated Hospital, Xuzhou, China;[4]Anhui Provincial Hospital, Hefei, China;[5]The Affiliated Cancer Hospital of Zhengzhou University & Henan Cancer Hospital, Zhengzhou, China; [6]920th Hospital of Joint Logistics Support Force of People's Liberation Army of China, Kunming, China; [7]Imviva Biotech, Wilmington, United States of America;*

### Background

R/R T-ALL/LBL poses significant therapeutic challenges due to paucity, and limited efficacy, of available therapies. Although substantial improvements in the treatment of pediatric T-ALL/LBL have led to cure rates in ~80%, for those with R/R disease prognosis is dismal. CTD402 is a novel universal anti-CD7 CAR-T therapy, with TCR, CD7, and HLA class II knockout to prevent GvHD, fratricide and immune rejection, respectively. Additionally, inhibitory molecules are included to resist T/NK cell rejection. The CAR construct consists of a CD7-binding scFv, a 4-1BB costimulatory domain, a CD3ζ signaling domain, and a γc intracellular domain.

### Aims

Herein we report preliminary outcomes in pediatric R/R T-ALL/LBL pts treated with CTD402.

### Methods

Pooled safety and efficacy data from Phase I/II studies conducted across 5 academic centers in China (NCT04620655; NCT05454241; NCT05907603; NCT05902845; NCT05923541); while the studies were conducted under different protocols, there was uniformity in inclusion/exclusion criteria, clinical and safety data collection and assessments, and product consistency. CTD402, at doses of $2 \times 10^8$ (n=3), $4 \times 10^8$ (n=10), $6 \times 10^8$ (n=1), and $8 \times 10^8$ (n=1) cells, was administered on day (D) 0 following standard lymphodepleting chemotherapy (fludarabine 30 mg/m²/day and cyclophosphamide 500 mg/m²/day from D -5 to -3).

**Copyright Information:** (Online) ISSN: 2572-9241
©2026 The Author(s). HemaSphere is published by John Wiley & Sons Ltd on behalf of European Hematology Association. This is an open access Abstract Book distributed under the Attribution-NonCommercial-NoDerivs (CC BY-NC-ND), which allows third parties to download the articles and share them with others as long as they credit the author and the Abstract Book, but they cannot change the content in any way or use them commercially.
**Abstract Book Citations:** Authors, Title, HemaSphere, 2026;10:(S1):pages. The abstract book DOIs can be found on the “Author Information” page.
**Disclaimer:** Articles published in the journal HemaSphere exclusively reflect the opinions of the authors. The authors are responsible for all content in their abstracts including accuracy of the facts, statements, citing resources, etc.

**Results**

As of data cut of February 6th, 2026, 15 pediatric R/R T-ALL/LBL (11 ALL, 4 LBL) pts were treated. Median (m) age was 15 years (range 10-17), pretreated with m 2 lines of prior therapy (range 1-5), 26.7% were primary refractory, and 73.3% had relapsed disease; 100% of patients were non-ETP, and 60% had high-risk molecular features (*RAS/PTEN* alterations or *NOTCH1/FBXW7*$^{WT}$). Baseline disease burden consisted of m 30% BM blasts (range 6.5-93%), EMD in 60% pts, and 1 CNS leukemia. Post CTD402 66.7% (n=10) pts experienced cytokine release syndrome, mostly (90%) mild Grade [G] 1-2. No pt experienced immune effector cell-associate neurotoxicity, or severe (G ≥3) treatment-related infections. One patient experienced gastric hemorrhage associated with GvHD (G5) on D146 following a stem cell boost. PK analysis revealed mCmax of $1.0 \times 10^6$ copies/µg DNA (range $4.1 \times 10^3$-$3.3 \times 10^6$) at a mTmax at day 10, persistence was ≥ 28 days in 66.7% of pts with CAR-T detected up to 90 or 180 days in some. Among the 15 pts, complete remission rate (CRR) was 80% (n=12, 9 CR, 2 CRi, 1 CRh) with 83.3% (10/12) MRD$^{neg}$ rate. At a m follow-up of 21.94 months (M) (range 0.56-30.88), the mDOR was 7.19 M (95% CI: 1.77 - not estimable). Among pts with high-risk (n=9) or EMD (n=9) disease, the CRR was 66.7% (3 CR, 1 CRh, 2 CRi; MRD$^{neg}$ 66.7%) and 88.9% (5 CR, 1 CRh, 2 CRi; MRD$^{neg}$ 87.5%), respectively. Five (41.7%) pts received consolidative allo-HSCT post-CTD402 in remission (CR/CRh/CRi). Median overall survival was not reached in patients who received consolidative HSCT, with no observed deaths at last follow-up, compared with 4.8 months (95% CI 2.7–NR) in those who did not undergo HSCT (log-rank p=*0.026*).

**Summary/Conclusion**

CTD402 demonstrated an acceptable safety profile and evidence of anti-leukemic activityhigh MRD negative remission rates with lasting benefits. This program advances an "off-the-shelf" ready at point of care CAR-T cell therapy in heavily pre-treated pediatric patients with R/R T-ALL/LBL. A global Phase 1b/2 study is ongoing and will extend to enrolling pediatric patients (NCT# 07070219).

**Copyright Information:** (Online) ISSN: 2572-9241
©2026 The Author(s). HemaSphere is published by John Wiley & Sons Ltd on behalf of European Hematology Association. This is an open access Abstract Book distributed under the Attribution-NonCommercial-NoDerivs (CC BY-NC-ND), which allows third parties to download the articles and share them with others as long as they credit the author and the Abstract Book, but they cannot change the content in any way or use them commercially.
**Abstract Book Citations:** Authors, Title, HemaSphere, 2026;10:(S1):pages. The abstract book DOIs can be found on the "Author Information" page.
**Disclaimer:** Articles published in the journal HemaSphere exclusively reflect the opinions of the authors. The authors are responsible for all content in their abstracts including accuracy of the facts, statements, citing resources, etc.

### (PS2332) RISTOGLOGENE AUTOGETEMCEL TREATMENT RESTORED RED BLOOD CELL HEALTH AND FUNCTION IN PATIENTS WITH SICKLE CELL DISEASE, WITH SICKLING AND RHEOLOGY PARAMETERS COMPARABLE TO SICKLE CELL TRAIT

**Topic:** 26. Gene therapy, cellular immunotherapy and vaccination - Clinical

Priya S. Chockalingam*[1], Ling Lin[1], Yinzhong Chen[1], Bahru Habtemariam[1], Jackson Rutecki[1], Katherine L. Ender[1], Srikanth Ambati[1], Vivien Sheehan[2], Nan Zhang[3], Hélène Vinçon[4], Myriam Armant[4], Aliya Zaidi[5], Robert Goodrich[5], Patrick Hines[5], Sunita Goyal[1], Amy Simon[1]

*[1]Beam Therapeutics Inc., Cambridge, MA, United States of America;[2]Emory University School of Medicine, Atlanta, GA, United States of America; [3]Frontage Laboratories Inc., Exton, PA, United States of America;[4]Boston Children's Hospital, Boston, MA, United States of America; [5]Functional Fluidics, Detroit, MI, United States of America;*

### Background

Ristoglogene autogetemcel (risto-cel, BEAM-101), an investigational cell therapy, consists of autologous CD34+ hematopoietic and progenitor stem cells base edited at the *HBG1/2* gene promotors to disrupt BCL11A repressor binding, enabling re-expression of γ-globin and production of anti-sickling fetal hemoglobin (HbF). Red blood cell (RBC) health and function biomarkers were measured pre- and post- treatment to ascertain the impact of risto-cel on sickle cell disease (SCD) pathophysiology. Biomarkers included cellular HbF, HbS; whole-blood (WB) cell adhesion to vascular cell adhesion molecule 1 (VCAM-1); P-selectin or laminin; real-time sickling kinetics; hemorheology; and RBC morphology.

### Aims

We present exploratory RBC biomarker data from BEACON (NCT05456880), an ongoing, Phase 1/2, single-arm, open-label, multicenter study evaluating the safety and efficacy of risto-cel in patients (pts) with SCD and frequent severe vaso-occlusive crises (VOC's).

### Methods

WB samples were collected and stained for HbF (%F-cells) at screen, and post-risto-cel at months (M)1–6, 9, 12, 15, 18, 21, and 24, and $HbF^+$ vs $HbS^+$ cells at screen, M1, 2, 6, 12, 18, and 24 using singleplex and duplex flow cytometry, respectively. Adhesion and sickling assessment samples were collected at screen, M3, 6, 12, 18, and 24. For adhesion assays, samples were perfused through microfluidic channels coated with VCAM-1, P-selectin or laminin under shear stress and adhered cells were measured. Real-time sickling kinetics were analyzed using a dynamic sickling assay™ to compare pre- and post-treatment study samples along with sickle (HbSS), normal (HbAA) and sickle cell trait (SCT; HbAS) blood reference samples. Oxygen affinity, deformability, RBC density, and cellular morphology were assessed.

**Copyright Information:** (Online) ISSN: 2572-9241
©2026 The Author(s). HemaSphere is published by John Wiley & Sons Ltd on behalf of European Hematology Association. This is an open access Abstract Book distributed under the Attribution-NonCommercial-NoDerivs (CC BY-NC-ND), which allows third parties to download the articles and share them with others as long as they credit the author and the Abstract Book, but they cannot change the content in any way or use them commercially.
**Abstract Book Citations:** Authors, Title, HemaSphere, 2026;10:(S1):pages. The abstract book DOIs can be found on the "Author Information" page.
**Disclaimer:** Articles published in the journal HemaSphere exclusively reflect the opinions of the authors. The authors are responsible for all content in their abstracts including accuracy of the facts, statements, citing resources, etc.

### Results

As of Jan 6, 2026, biomarker data for 43 pts with follow up ranging from M1–24 were available. Post-risto-cel, at M6 (n=26), the mean (standard deviation [SD]) endogenous HbF (HbF/[HbF+HbS]) was 63.7% (5.29) and HbS was 36.3% (5.29). In peripheral blood at M6 (n=15), mean (SD) %F-cells was 99.3% (0.71) with a mean (SD) of 20.57 (1.78) pg HbF per F-cell (n=14), above the 10 pg/cell anti-sickling threshold; these parameters stabilized thereafter. Duplex assay for HbF vs HbS expression showed >98% of $HbF^{+}$ cells, with near elimination of cells that were only $HbS^{+}$, as early as M1, which sustained during follow up to M24. Rate of sickling (Figure 1A) and max induced sickling (Figure 1B) were reduced post-risto-cel and remained at levels comparable to SCT through follow up. Post-risto-cel, adhesion indices for VCAM-1, P-selectin, and laminin were comparable to SCT. RBC density, oxygen affinity, and RBC deformability improved post-risto-cel. RBC morphology normalized post-treatment.

### Summary/Conclusion

Updated RBC biomarker data from the BEACON study demonstrated that risto-cel treatment restored RBC health, function, and hemorheological parameters comparable to SCT levels, with significantly decreased sickling and RBC adhesion. Sickling parameter reductions were less variable than in SCT and even approached HbAA levels, indicating strong correction of SCD pathophysiology. Concurrent improvements were also found in RBC density, oxygen affinity, RBC deformability, and morphology, comparable to established SCT thresholds. Long-term follow-up biomarker data in additional pts continue to support base editing with risto-cel as a potentially transformative therapy for pts with SCD. Further data will be included in the final presentation.

**Copyright Information:** (Online) ISSN: 2572-9241
©2026 The Author(s). HemaSphere is published by John Wiley & Sons Ltd on behalf of European Hematology Association. This is an open access Abstract Book distributed under the Attribution-NonCommercial-NoDerivs (CC BY-NC-ND), which allows third parties to download the articles and share them with others as long as they credit the author and the Abstract Book, but they cannot change the content in any way or use them commercially.
**Abstract Book Citations:** Authors, Title, HemaSphere, 2026;10:(S1):pages. The abstract book DOIs can be found on the "Author Information" page.
**Disclaimer:** Articles published in the journal HemaSphere exclusively reflect the opinions of the authors. The authors are responsible for all content in their abstracts including accuracy of the facts, statements, citing resources, etc.

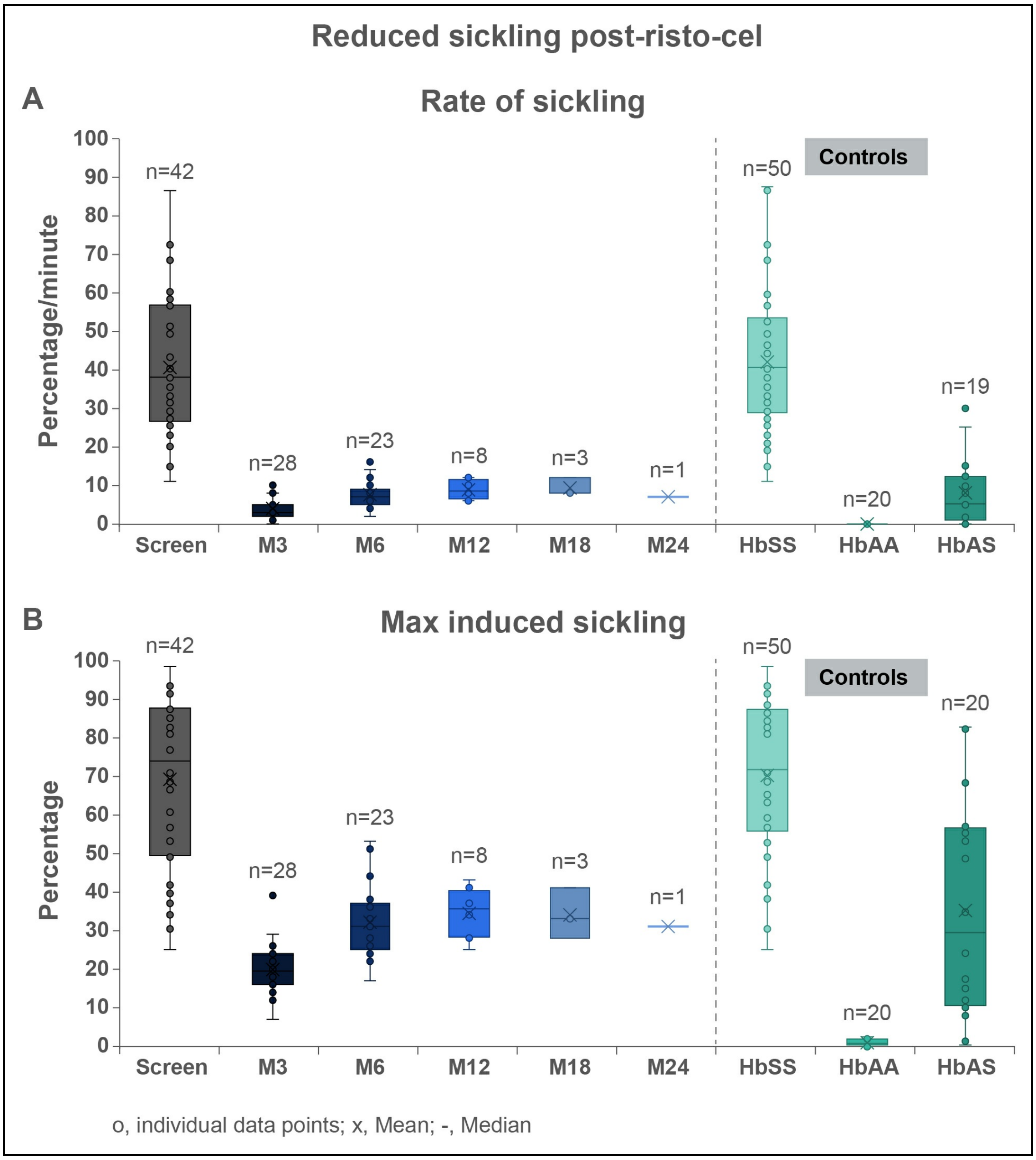

**Copyright Information:** (Online) ISSN: 2572-9241
©2026 The Author(s). HemaSphere is published by John Wiley & Sons Ltd on behalf of European Hematology Association. This is an open access Abstract Book distributed under the Attribution-NonCommercial-NoDerivs (CC BY-NC-ND), which allows third parties to download the articles and share them with others as long as they credit the author and the Abstract Book, but they cannot change the content in any way or use them commercially.
**Abstract Book Citations:** Authors, Title, HemaSphere, 2026;10:(S1):pages. The abstract book DOIs can be found on the "Author Information" page.
**Disclaimer:** Articles published in the journal HemaSphere exclusively reflect the opinions of the authors. The authors are responsible for all content in their abstracts including accuracy of the facts, statements, citing resources, etc.

### (PS2333) LONG-TERM FOLLOW-UP CONFIRMS DURABLE CLINICAL BENEFITS OF EXAGAMGLOGENE AUTOTEMCEL IN TRANSFUSION-DEPENDENT Β-THALASSEMIA: FINAL RESULTS OF THE CLIMB THAL-111 STUDY

**Topic:** 26. Gene therapy, cellular immunotherapy and vaccination - Clinical

Roland Meisel*[1], Haydar Frangoul[2], Selim Corbacioglu[3], Josu De La Fuente[4], Peter Lang[5], Mattia Algeri[6], Joachim Rupprecht[5], Kevin H.M. Kuo[7], Melanie Kirby-Allen[8], Amanda M. Li[9], Ami J. Shah[10], Markus Mapara[11], Ben Carpenter[12], Robert Liem[13], Janet L. Kwiatkowski[14], Hayley Merkeley[15], Stephan Grupp[14], Maria Domenica Cappellini[16], Antonis Kattamis[17], Sujit Sheth[18], Kathleen Lee-Sarwar[19], Bo Tong[19], Sakellarios Zairis[19], William Hobbs[19], Franco Locatelli[20]

*[1]Heinrich-Heine-University, Division of Pediatric Stem Cell Therapy, Department of Pediatric Oncology, Hematology and Clinical Immunology, Medical Faculty, Düsseldorf, Germany; [2]Sarah Cannon Research Institute at The Children's Hospital at TriStar Centennial, Nashville, United States of America; [3]University of Regensburg, Regensburg, Germany; [4]Imperial College Healthcare NHS Trust, St Mary's Hospital, London, United Kingdom; [5]University of Tübingen, Tübingen, Germany; [6]IRCCS, Ospedale Pediatrico Bambino Gesù, Rome, Italy; [7]University of Toronto, Division of Hematology, Toronto, Canada; [8]Hospital for Sick Children, Toronto, Canada; [9]BC Children's Hospital, University of British Columbia, Vancouver, Canada; [10]Stanford University, Palo Alto, United States of America; [11]Columbia University, Division of Hematology and Oncology, New York, United States of America; [12]University College London Hospitals NHS Foundation Trust, London, United Kingdom; [13]Ann & Robert H. Lurie Children's Hospital of Chicago, Chicago, United States of America; [14]Children's Hospital of Philadelphia, Perelman School of Medicine at the University of Pennsylvania, Philadelphia, United States of America; [15]The University of British Columbia/Adult Red Cell Disorder Program of British Columbia, Department of Medicine, Vancouver, Canada; [16]University of Milan, Milan, Italy; [17]National and Kapodistrian University of Athens, Athens, Greece; [18]Joan and Sanford I Weill Medical College of Cornell University, New York, United States of America; [19]Vertex Pharmaceuticals Incorporated, Boston, United States of America; [20]IRCCS, Ospedale Pediatrico Bambino Gesù Rome, Catholic University of the Sacred Heart, Rome, Italy;*

### Background

Exagamglogene autotemcel (exa-cel) is a one-time, non-viral, *ex vivo* CRISPR/Cas9 gene-edited, autologous cell therapy approved for patients ≥12 years old with transfusion-dependent β-thalassemia (TDT).

### Aims

Evaluate longer-term efficacy and safety of exa-cel in TDT participants aged 12 to 35 years and provide final study results of the CLIMB THAL-111 trial.

### Methods

CLIMB THAL-111 is a 2-year, Phase 3 study of exa-cel in TDT participants aged 12 to 35 years. The primary endpoint is transfusion independence defined as proportion of participants maintaining a weighted average hemoglobin (Hb) ≥9 g/dL without RBC transfusion for ≥12 consecutive months (TI12). CLIMB-111 participants then enroll in long-term follow-up study CLIMB-131 for up to 15 years of follow-up after exa-cel infusion. Measures of tissue iron overload (serum ferritin, liver iron concentration [LIC], and T2* cardiac iron content [CIC]) and measures of iron homeostasis (e.g., erythroferrone and hepcidin) were assessed after exa-cel, including data collected before and after discontinuation of iron removal therapy (IRT; namely chelation and/or phlebotomy). Patient-reported outcomes (PROs) were also assessed. Data are from 20 July 2025; data will be updated to reflect the final results from the completed CLIMB THAL-111 trial.

**Copyright Information:** (Online) ISSN: 2572-9241
©2026 The Author(s). HemaSphere is published by John Wiley & Sons Ltd on behalf of European Hematology Association. This is an open access Abstract Book distributed under the Attribution-NonCommercial-NoDerivs (CC BY-NC-ND), which allows third parties to download the articles and share them with others as long as they credit the author and the Abstract Book, but they cannot change the content in any way or use them commercially.
**Abstract Book Citations:** Authors, Title, HemaSphere, 2026;10:(S1):pages. The abstract book DOIs can be found on the "Author Information" page.
**Disclaimer:** Articles published in the journal HemaSphere exclusively reflect the opinions of the authors. The authors are responsible for all content in their abstracts including accuracy of the facts, statements, citing resources, etc.

### Results

56 participants (mean age: 21.2 years [range: 12, 35]) received exa-cel in CLIMB THAL-111 and had median follow-up of 4.1 years (range: 1.6, 6.6). 35/56 participants (62.5%) had severe genotypes ($\beta^0/\beta^0$ or $\beta^0/\beta^0$-like). Median number of mobilization cycles was 1 (range: 1, 4); the mean number of cells infused was 8.4 (range: 3.0, 19.7) × $10^6$ cells/kg. After exa-cel infusion, 98.2% (55/56) achieved TI12 in CLIMB-THAL-111 and CLIMB-131 combined, with a median duration of transfusion independence of 3.9 years (range: 1.4, 6.3). Mean total Hb was maintained at normal/near normal levels of ≥12 g/dL and mean HbF, with pancellular distribution, was ≥11 g/dL from Month 6 onward. Allelic editing in both bone marrow and peripheral blood was stable over time.
In CLIMB THAL-111 and CLIMB-131 combined, all participants received IRT after exa-cel with 69.6% (39/56) discontinuing IRT for ≥6 months. After IRT discontinuation, levels of serum ferritin, LIC, and CIC were generally stable without progressive increase for over 5 years. Erythroferrone and hepcidin levels also remained stable after IRT discontinuation at levels consistent with normalization of iron homeostasis and correction of ineffective erythropoiesis after exa-cel. In addition, durable improvements in PROs after exa-cel were observed in both adults and adolescents.
Exa-cel safety was consistent with myeloablative conditioning and autologous transplant. There were no deaths or malignancies through follow-up in both CLIMB THAL-111 and CLIMB-131.

### Summary/Conclusion

Exa-cel demonstrated durable clinical benefits in adults and adolescents with TDT for up to 6.5 years of follow-up. In addition to durable transfusion independence in 98.2% of participants, there was no evidence of iron reaccumulation after IRT discontinuation. This suggests that, in addition to transfusion independence, exa-cel also has the potential to prevent tissue iron accumulation via correction of underlying ineffective erythropoiesis. The safety profile is consistent with busulfan myeloablative conditioning and autologous transplant. These data continue to support exa-cel as a potential one-time functional cure for TDT.

**Copyright Information:** (Online) ISSN: 2572-9241
©2026 The Author(s). HemaSphere is published by John Wiley & Sons Ltd on behalf of European Hematology Association. This is an open access Abstract Book distributed under the Attribution-NonCommercial-NoDerivs (CC BY-NC-ND), which allows third parties to download the articles and share them with others as long as they credit the author and the Abstract Book, but they cannot change the content in any way or use them commercially.
**Abstract Book Citations:** Authors, Title, HemaSphere, 2026;10:(S1):pages. The abstract book DOIs can be found on the "Author Information" page.
**Disclaimer:** Articles published in the journal HemaSphere exclusively reflect the opinions of the authors. The authors are responsible for all content in their abstracts including accuracy of the facts, statements, citing resources, etc.

### (PS2334) SAFETY AND EFFICACY OF HCD1A-CAR T (OC-1) THERAPY, IN PATIENTS WITH RELAPSED/REFRACTORY (R/R) T-CELL ACUTE LYMPHOBLASTIC LEUKAEMIA/LYMPHOMA (T-ALL/LL): A PRE-SPECIFIED INTERIM ANALYSIS.

**Topic:** 26. Gene therapy, cellular immunotherapy and vaccination - Clinical

Nuria Martinez-Cibrian*[1], Anna Alonso-Saladrigues[2], Nazaret Sánchez-Sierra[2], Valentín Ortiz-Maldonado[1], Ana Triguero[1], Nil Albiol[1], Elena Medina[1], Laura Arqués[2], Verónica Celis[2], Sergio Navarro[3], Leticia Alserawan[3], Marta Español-Rego[3], Daniel Benitez-Ribas[1], Maria Sanchez[1], Diego Sanchez[4], Juan Gonzalo Correa Saldarriaga[1], Mercedes Montoro[1], Pilar Ayora[1], Carla Ramos[1], Paola Charry Espana[1], Miquel Lozano[1], Europa Azucena Gonzalez[1], Wilmar Castillo[5], Avaro Urbano Ispizua[1], Pablo Menendez[4], Manel Juan[1], Julio Delgado[1], Jordi Esteve Reyner[1], Susana Rives[2]

*[1]Hospital Clinic Barcelona, Haematology Department, Barcelona, Spain;[2]Hospital Sant Joan de Déu de Barcelona, Haematology Department, Barcelona, Spain; [3]Hospital Clinic Barcelona, Immunology Department, Barcelona, Spain;[4]Josep Carreras Leukemia Research Institute, Barcelona, Spain; [5]OneChain Immunotherapeutics, Barcelona, Spain;*

### Background

CAR T cell therapy has shown remarkable results in relapsed/refractory (R/R) B-cell acute lymphoblastic leukaemia/lymphoma (B-ALL/LL). However, equivalent progress has not yet been achieved in T-cell ALL/LL, mainly due to fratricide, blast contamination and T-cell aplasia resulting in life-threatening opportunistic infections. CD1a is a selective antigen expressed in cortical T-ALL/LL blasts, cortical thymocytes, skin Langerhans cells and in some circulating myeloid dendritic cells during embryonic development. OC-1 is a humanised second-generation 4-1BB-based, CD1a-directed CAR T cell developed by Josep Carreras Institute and OneChain Immnunotherapeutics. A first in human phase 1 clinical trial is ongoing (EU CT: 2024-514591-40-00). Here we report the first pre-specified interim results.

### Aims

To report interim safety and efficacy data for all patients treated on dose level (DL) 1 and 2.

### Methods

A dose escalation study is ongoing at Hospital Sant Joan de Déu and Hospital Clinic Barcelona to assess safety and efficacy of OC-1. Paediatric and adult patients with R/R T-ALL/LL are being enrolled. After leukapheresis, bridging chemotherapy is administered at physician's discretion. Lymphodepletion consists of fludarabine 30mg/m$^2$/day and cyclophosphamide 300mg/m$^2$/day on days -6 to -4. Four DL are under assessment in a 3+3 design: 0.5, 1.0, 3.0 and 5.0 x10$^6$ CAR T-cells/kg. Target dose is split in 3 fractions (10-30-60%), the first fraction is given on day 0, the following doses are administered on consecutive days provided that no significant toxicity occurs.

**Copyright Information:** (Online) ISSN: 2572-9241
©2026 The Author(s). HemaSphere is published by John Wiley & Sons Ltd on behalf of European Hematology Association. This is an open access Abstract Book distributed under the Attribution-NonCommercial-NoDerivs (CC BY-NC-ND), which allows third parties to download the articles and share them with others as long as they credit the author and the Abstract Book, but they cannot change the content in any way or use them commercially.
**Abstract Book Citations:** Authors, Title, HemaSphere, 2026;10:(S1):pages. The abstract book DOIs can be found on the "Author Information" page.
**Disclaimer:** Articles published in the journal HemaSphere exclusively reflect the opinions of the authors. The authors are responsible for all content in their abstracts including accuracy of the facts, statements, citing resources, etc.

### Results

Twenty-three patients have been screened, 5 were CD1a-negative screening failures, 2 patients did not undergo apheresis due to disease progression. Apheresis was performed in 16 patients. Manufacturing failure occurred in 2 patients due to blast contamination; 5 patients progressed before the infusion. Nine patients received OC-1, 4 children and 5 adults. Among infused patients median age was 24 years (range 11-53), 78% were male, median prior lines were 3 (range 2-6), 67% were exposed to nelarabine and 44% had a previous alloHSCT. Four patients received DL1 and 5 patients DL2. Median follow-up from infusion was 5.5 months (range 5 days – 16.6 months).

CRS occurred in 4 patients (grade ≥3, n=1) and 1 patient grade 1 ICANS. Three patients had grade 1-2 skin rash treated with topical steroids. Grade ≥3 neutropenia was seen in all patients and grade ≥3 thrombocytopenia in all but one. No grade ≥3 infections occurred.

Two patients died due to disease progression on day +5 and +9, both had >50% bone marrow blast before lymphodepletion and developed irreversible lactic acidosis.

Among evaluable patients (n=7) overall responses were seen in 6 patients (3 complete response and 3 partial response), one patient was refractory. Two responders underwent a consolidative alloHSCT and remain in remission. Four patients relapsed, all of them with extramedullary disease despite detectable CAR T cells.

Median duration of response was 2.7 months and median overall survival 6.7 months.

### Summary/Conclusion

Interim data suggests that OC-1 is safe and tolerable in patients with R/R T-ALL/LL.

A high response rate has been seen with OC-1 in a heavily treated population; however, relapses were the main cause of treatment failure.

The trial continues to recruit patients for DL3 and DL4 as there have been no safety concerns and responses have been observed.

**Copyright Information:** (Online) ISSN: 2572-9241
©2026 The Author(s). HemaSphere is published by John Wiley & Sons Ltd on behalf of European Hematology Association. This is an open access Abstract Book distributed under the Attribution-NonCommercial-NoDerivs (CC BY-NC-ND), which allows third parties to download the articles and share them with others as long as they credit the author and the Abstract Book, but they cannot change the content in any way or use them commercially.
**Abstract Book Citations:** Authors, Title, HemaSphere, 2026;10:(S1):pages. The abstract book DOIs can be found on the "Author Information" page.
**Disclaimer:** Articles published in the journal HemaSphere exclusively reflect the opinions of the authors. The authors are responsible for all content in their abstracts including accuracy of the facts, statements, citing resources, etc.

### (PS2335) PD-1AB21-EXPRESSING, ANTI-CD19 CAR T CELLS FOR PATIENTS WITH RELAPSED/REFRACTORY DIFFUSE LARGE B-CELL LYMPHOMA: AN OPEN-LABEL, SINGLE-ARM, PHASE 1 STUDY

**Topic:** 26. Gene therapy, cellular immunotherapy and vaccination - Clinical

Bo Peng*[1], Liping Dou[2], Dai-Hong Liu[2]

*[1]Senior Department of Hematology, Chinese PLA General Hospital, Beijing, China;[2]The Fifth Medical Center of Chinese PLA General Hospital, Chief Department of Hematology, Beijing, China;*

### Background

While CD19-directed chimeric antigen receptor (CAR) T-cell therapy has improved outcomes for relapsed/refractory diffuse large B-cell lymphoma (R/R DLBCL), primary resistance, relapse, and limited response durability remain significant challenges. Strategies to overcome these limitations, including combination with PD-1 blockade, often face issues with systemic toxicity and variable efficacy.

### Aims

We aimed to assess the safety, feasibility, and preliminary efficacy of a novel CD19-directed CAR T-cell product engineered to express a bifunctional fusion protein targeting PD-1 and interleukin-21 (PD-1Ab21-CD19 CAR-T) in patients with R/R DLBCL.

### Methods

In this open-label, single-arm, phase I trial, patients (aged 14–75 years) with CD19-positive R/R DLBCL received lymphodepletion with fludarabine/cyclophosphamide, followed by a single infusion of PD-1Ab21-CD19 CAR-T cells at doses of 0.3, 1, 3, or $9 \times 10^6$ cells/kg. The primary endpoint was the safety and feasibility of PD-1Ab21-CD19 CAR-T cell. Secondary endpoints included efficacy, CAR-T persistence, progression-free survival (PFS), and overall survival (OS).

### Results

Between November 2022 and October 2024, 14 patients received PD-1Ab21-CD19 CAR-T cell infusion. Cytokine release syndrome (CRS) occurred in 11 patients (78.6%; grade 3: 7.1%), with a median onset of 10 days. Immune effector cell-associated neurotoxicity syndrome (ICANS) occurred in 1 patient (grade 3, 7.1%). No treatment-related mortality or unexpected immune-related adverse events were observed. The overall response rate was 71.4%, with 57.1% achieving complete metabolic remission. At a median follow-up of 22.2 months, the 6-month PFS and OS rates were 64.3% and 71.4%, respectively(Figure 1).
Figure 1. Response of R/R DLBCL patients treated with PD-1Ab21-CD19CAR-T. a. Individual patient swimmer plots and their response to PD-1Ab21-CD19CAR-T over time. b. PET/CT scans of patient 3 before and 1 month after PD-1Ab21-CD19CAR-T infusion, demonstrating CMR. c. Overall survival in patients treated with PD-1Ab21-CD19CAR-T. d. Progression-free survival in patients treated with PD-1Ab21-CD19CAR-T.

### Summary/Conclusion

PD-1Ab21-CD19 CAR-T therapy demonstrated a manageable safety profile and promising antitumor activity in patients with heavily pretreated R/R DLBCL. The localized delivery of PD-1 blockade and IL-21 via engineered CAR-T cells may mitigate systemic toxicity while enhancing efficacy. These findings warrant further in

**Copyright Information:** (Online) ISSN: 2572-9241
©2026 The Author(s). HemaSphere is published by John Wiley & Sons Ltd on behalf of European Hematology Association. This is an open access Abstract Book distributed under the Attribution-NonCommercial-NoDerivs (CC BY-NC-ND), which allows third parties to download the articles and share them with others as long as they credit the author and the Abstract Book, but they cannot change the content in any way or use them commercially.
**Abstract Book Citations:** Authors, Title, HemaSphere, 2026;10:(S1):pages. The abstract book DOIs can be found on the "Author Information" page.
**Disclaimer:** Articles published in the journal HemaSphere exclusively reflect the opinions of the authors. The authors are responsible for all content in their abstracts including accuracy of the facts, statements, citing resources, etc.

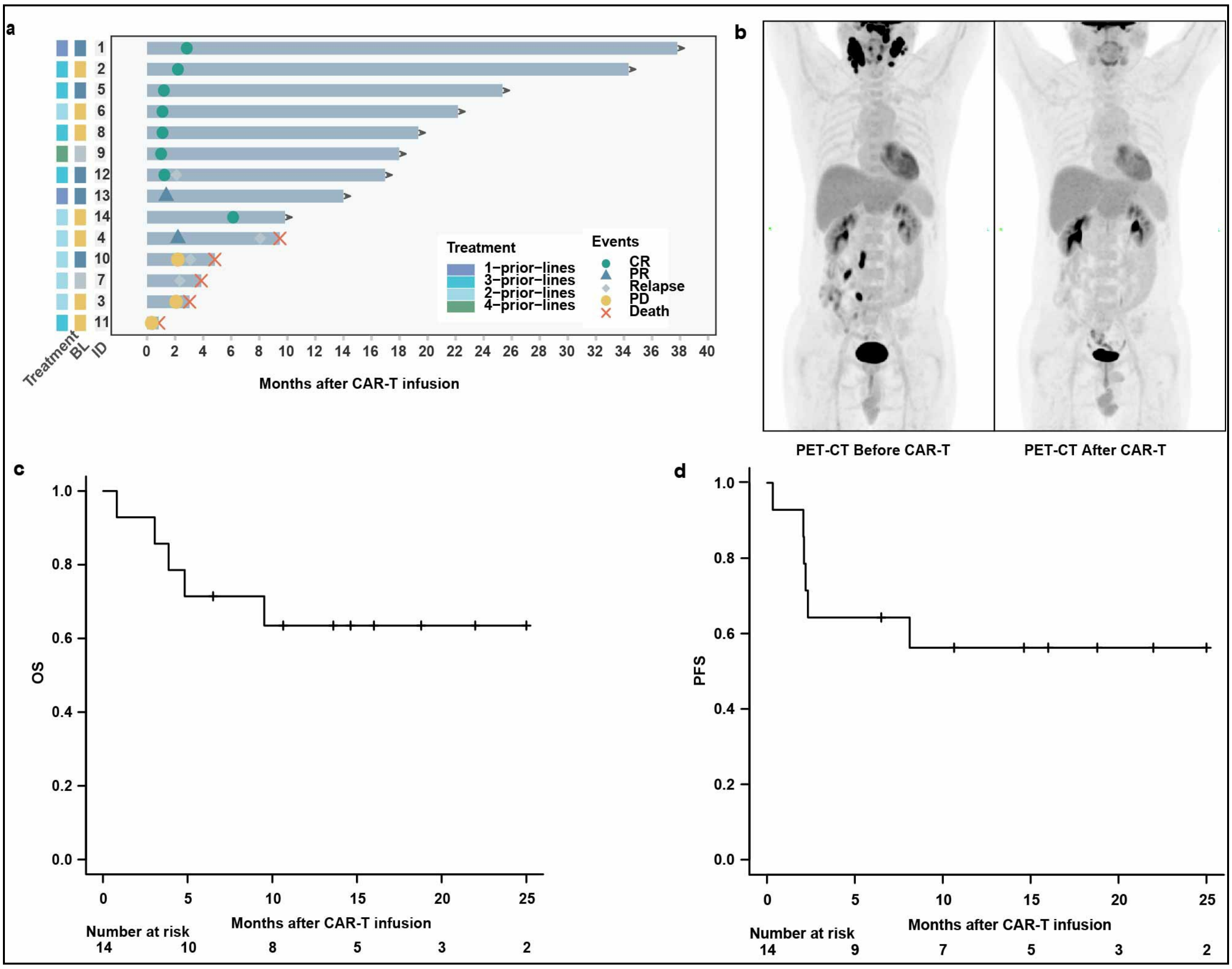

**Copyright Information:** (Online) ISSN: 2572-9241
©2026 The Author(s). HemaSphere is published by John Wiley & Sons Ltd on behalf of European Hematology Association. This is an open access Abstract Book distributed under the Attribution-NonCommercial-NoDerivs (CC BY-NC-ND), which allows third parties to download the articles and share them with others as long as they credit the author and the Abstract Book, but they cannot change the content in any way or use them commercially.
**Abstract Book Citations:** Authors, Title, HemaSphere, 2026;10:(S1):pages. The abstract book DOIs can be found on the "Author Information" page.
**Disclaimer:** Articles published in the journal HemaSphere exclusively reflect the opinions of the authors. The authors are responsible for all content in their abstracts including accuracy of the facts, statements, citing resources, etc.

### (PS2337) SECONDARY MALIGNANCIES AFTER CHIMERIC ANTIGEN RECEPTOR (CAR) T-CELL THERAPY: A REPORT FROM THE EBMT REGISTRY ON 509 CASES.

**Topic:** 26. Gene therapy, cellular immunotherapy and vaccination - Clinical

Annalisa Ruggeri*[1], Jacques-Emmanuel. Galimard[2], Eva Michel[3], Isabel Sánchez-Ortega[4], Neil Rabin[5], Michael Daskalakis[6], Christof Scheid[7], Malte von Bonin[8], Matthew Collin[9], Rajesh Alajangi[10], Helene Schoemans[11], Peter Dreger[12], Robert Zeiser[13], Raynier Devillier[14], Simona Sica[15], Dominik Schneidawind[16], Edouard Forcade[17], Ron Ram[18], Wolfgang Bethge[19], Armin Gerbitz[20], Sylvain Choquet[21], Stéphanie Nguyen-Quoc[22], Donal McLornan[23], Jurgen Kuball[24], Ibrahim Yakoub-Agha[25], Anna Sureda[26], Fabio Ciceri[1]

*[1]IRCCS San Raffaele Hospital, Milano, Italy; [2]EBMT Paris Office, Paris, France; [3]Sorbonne Univ, Paris, France; [4]EBMT, Barcelona, Spain; [5]UCL, London, United Kingdom; [6]University Hospital of Bern, Bern, Switzerland; [7]University of Cologne, Cologne, Germany; [8]Uniklinikum Dresden, Dresden, Germany; [9]Newcastle Upon Tyne Hospitals NHS Foundation Trust, Newcastle, United Kingdom; [10]University Hospitals Bristol and Weston NHSFT, United Kingdom, Bristol, United Kingdom; [11]University Hospital Gasthuisberg, Leuven, Belgium, Leuven, Belgium; [12]University of Heidelberg, Heidelberg, Germany; [13]Freiburg University Medical Center, Freiburg, Germany; [14]Institut Paoli-Calmettes, Marseille, France; [15]Fondazione Policlinico Universitario A. Gemelli IRCCS, Roma, Italy; [16]University Hospital Zürich, Zurich, Switzerland; [17]CHU Bordeaux, Bordeaux, France; [18]Tel Aviv Sourasky Medical Center, Tel Aviv, Israel; [19]University Hospital Tübingen, Tubingen, Germany; [20]uk-erlangen, erlangen, Germany; [21]Sorbonne Université, Hôpital de la Pitié-Salpêtrière, APHP, Paris, France; [22]Pitié-Salpêtrière Hospital – APHP – Sorbonne Université, Paris, France; [23]University College Hospital London, London, United Kingdom; [24]UL, Leyden, The Netherlands; [25]Univ Lille, Inserm UMR1286, CHU de Lille, Lille, France; [26]Institut Català d'Oncologia - Hospital Duran i Reynals, Barcelona, Spain;*

### Background

Secondary malignancies (SM) after chimeric antigen receptor (CAR) T-cell therapy are defined as new, distinct neoplasms arising after CAR T-cell treatment, and are increasingly recognized as a clinically relevant long-term adverse event following this curative treatment. The most common types are hematologic malignancies including myelodysplastic syndrome (MDS) and acute myeloid leukemia (AML), T-cell malignancies, solid tumors (ST), and non-melanoma skin cancers.

### Aims

We report in this analysis all the identified cases of SM diagnosed after CART treatment among the more than 16000 CART infusions registered in the EBMT from 2016 to 2025.

### Methods

This analysis does not allow calculation of the cumulative incidence of secondary malignancy post CART. The OS and incidence of alloHCT after SM were calculated from the date of SM.

### Results

We have identified 509 patients who developed SM after CAR-T (Table-1).
Main diagnosis at CAR-T was DLBCL (n= 318, 61%), MCL (n= 46,9%), FL (n= 27,6%), MM (n= 66,13%), B-ALL (n= 22, 4%).
The type of CAR-T products used was related to the main disease indications: being Yescarta 50%, Kymriah 22%, Tecartus 11%, Abecma 8.3%, and Carvykti 3.6%.
Forty-three % of patients had a previous history of autologous/allogeneic HCT before CAR-T. Twenty patients (4%) received a HCT

**Copyright Information:** (Online) ISSN: 2572-9241
©2026 The Author(s). HemaSphere is published by John Wiley & Sons Ltd on behalf of European Hematology Association. This is an open access Abstract Book distributed under the Attribution-NonCommercial-NoDerivs (CC BY-NC-ND), which allows third parties to download the articles and share them with others as long as they credit the author and the Abstract Book, but they cannot change the content in any way or use them commercially.
**Abstract Book Citations:** Authors, Title, HemaSphere, 2026;10:(S1):pages. The abstract book DOIs can be found on the "Author Information" page.
**Disclaimer:** Articles published in the journal HemaSphere exclusively reflect the opinions of the authors. The authors are responsible for all content in their abstracts including accuracy of the facts, statements, citing resources, etc.

or second CART between the first CART and the SM, with 12 with one alloHCT, 3 with one autoHCT, 4 with a second CART, 1 with a second CART and 2 alloHCT.
Of the 509 reported SM: 98 with AL, 172 MDS/MPN/MDS-MPN overlap/CML, 221 ST (mainly adenocarcinoma and basal cell carcinoma) and 18 Lymphomas/MM/CLL (7 Hodgkin lymphoma, 3 PTCL (2 with documented insertional mutagenesis), 2 ALCL, 2 NKTCL (1 with insertional mutagenesis), 1 Cutaneous T-NHL, 1 NK cell leukemia, 1 CLL, 1 MM).
Of the 172 patients experiencing MDS, few cases reported the occurrence of CCUS and AA as ICHAT, however no data on NGS were available at time of analysis
Probability of 1 year Overall Survival after SM was 54.5% (49.3 - 59.3%) being 16% (8.6 - 25.4%), 51.6% (24.2 - 73.4%) 52.3% ( 43.1 - 60.7%) and 74.6%(67.4 - 80.4) according to the diagnosis of AL, Lymphoma, MDS or ST after CART (Figure 1).
Table 1. Patients and disease characteristics

| **Patients variables** | | **N=509** | **AL** | **MDS/ MPN** | **Lymph/ MM/CLL** | **ST** |
|---|---|---|---|---|---|---|
| | | | **98** | **172** | **18** | **221** |
| Patient sex | female | 159 | 24 | 61 (35.5) | 5 (27.8) | 69 (31.2) |
| | male | 350 | 74 | 111 (64.5) | 13 (72.2) | 152 (68.8) |
| Year at CART | median | 2022 | 2022 | 2022 | 2022 | 2022 |
| | (range) | (2016-2025) | (2019-2025) | (2019-2025) | (2019-2025) | (2016-2025) |
| Age at CART | median | 65.6 | 62.7 | 64.2 | 64.8 | 67.9 |
| | (range) | (1.2-84.4) | (1.2-77.5) | (6.9-80.1) | (20.1-78.5) | (22.3-84.4) |
| Months between diagnosis and CART | median | 2.5 | 1.6 | 3.5 | 2.3 | 2.2 |
| | (range) | (0.2-32.8) | (0.2-19.8) | (0.2-26.5) | (0.5-15.7) | (0.2-32.8) |

**Copyright Information:** (Online) ISSN: 2572-9241
©2026 The Author(s). HemaSphere is published by John Wiley & Sons Ltd on behalf of European Hematology Association. This is an open access Abstract Book distributed under the Attribution-NonCommercial-NoDerivs (CC BY-NC-ND), which allows third parties to download the articles and share them with others as long as they credit the author and the Abstract Book, but they cannot change the content in any way or use them commercially.
**Abstract Book Citations:** Authors, Title, HemaSphere, 2026;10:(S1):pages. The abstract book DOIs can be found on the “Author Information” page.
**Disclaimer:** Articles published in the journal HemaSphere exclusively reflect the opinions of the authors. The authors are responsible for all content in their abstracts including accuracy of the facts, statements, citing resources, etc.

| | | | | | | |
|---|---|---|---|---|---|---|
| Age at SM | median | 67.3 | 64.4 | 66.5 | 65.9 | 68.6 |
| | (range) | (1.5-85.2) | (1.5-81.3) | (7.2-81.1) | (20.4-79) | (25.1-85.2) |
| Months between CART and SM | median | 12.9 | 16.7 | 13.4 | 9.5 | 11.2 |
| | (range) | (0.1-99.4) | (0.7-61.5) | (0.9-52) | (1.9-48.1) | (0.1-99.4) |

### Summary/Conclusion

We report a series of 509 patients experiencing SM after CAR T infusion reported to the EBMT. Information on molecular feature before CAR T and at SM mainly in patients with high burden of pretreatment and disease are needed to understand the main characteristics and kinetics of SM. Active registry collection of adverse events after CAR-T is important to define the long term safety.

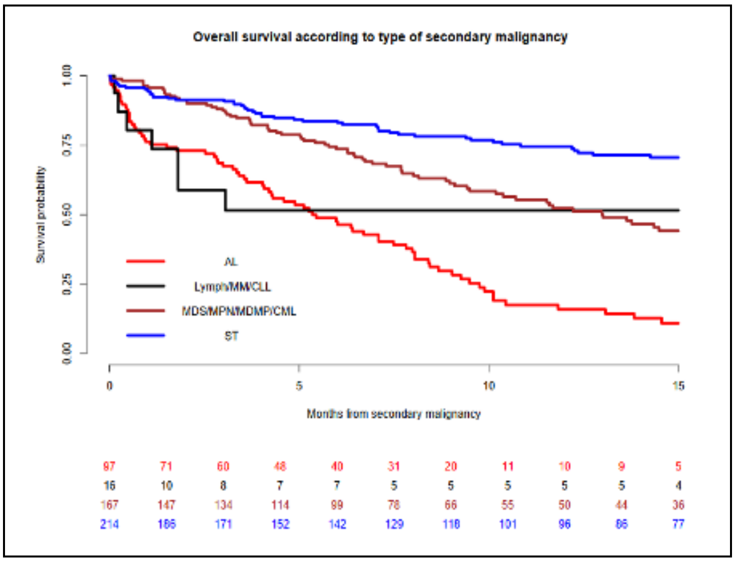

**Copyright Information:** (Online) ISSN: 2572-9241
©2026 The Author(s). HemaSphere is published by John Wiley & Sons Ltd on behalf of European Hematology Association. This is an open access Abstract Book distributed under the Attribution-NonCommercial-NoDerivs (CC BY-NC-ND), which allows third parties to download the articles and share them with others as long as they credit the author and the Abstract Book, but they cannot change the content in any way or use them commercially.
**Abstract Book Citations:** Authors, Title, HemaSphere, 2026;10:(S1):pages. The abstract book DOIs can be found on the “Author Information” page.
**Disclaimer:** Articles published in the journal HemaSphere exclusively reflect the opinions of the authors. The authors are responsible for all content in their abstracts including accuracy of the facts, statements, citing resources, etc.

### (PS2338) PAIRED SINGLE-CELL ANALYSIS OF STARTING MATERIAL AND CAR T PRODUCT IDENTIFIES CD8+ TEMRA SENESCENCE PROGRAMS LINKED TO CLINICAL RESPONSE IN DLBCL

**Topic:** 26. Gene therapy, cellular immunotherapy and vaccination - Clinical

Yue Chun*[1], Wenkang Shen[2], Li Guangmei[1], Lingzhi Wang[1], Qing Li[3], Xiaojian Zhu[1], Anyuan Guo[4]

*[1]Tongji Hospital, Tongji Medical College, Huazhong University of Science and Technology, Department of Hematology, Wuhan, China; [2]Hubei Key Laboratory of Bioinformatics and Molecular-imaging, College of Life Science and Technology, Huazhong University of Science and Technology,, Wuhan, China; [3]Wuhan No. 1 Hospital, Department of Hematology, Wuhan, China; [4]Department of Laboratory Medicine, West China Biomedical Big Data Center, West China Hospital, Sichuan University, Chengdu, China;*

### Background

Responses to CAR T therapy in relapsed/refractory diffuse large B-cell lymphoma (DLBCL) remain heterogeneous. T-cell states in leukapheresis starting material (SMAT) may imprint the manufactured drug product (DP), yet programs propagated from SMAT to DP that govern in vivo expansion and outcome are undefined. We therefore profiled paired SMAT and DP by single-cell RNA sequencing (scRNA-seq) to delineate programs linked to clinical response and nominate actionable targets.

### Aims

Define SMAT T-cell features predicting expansion/response after CD19/22 CAR T in DLBCL, and trace them in DP to guide optimization.

### Methods

We retrospectively reviewed r/r DLBCL patients treated with CD19/22 CAR T at Tongji Hospital (01/2024–06/2025). Response was assessed by PET/CT at month 6 (Lugano 2014). For extreme-phenotype sampling, CR and PD cases were ranked by in vivo expansion AUC0–28 (serial peripheral-blood qPCR of CAR transgene copies): top five CR were GEP (Good Expansion & Prognosis) and bottom five PD were PEP (Poor Expansion & Prognosis). Paired SMAT and DP samples (20 total) underwent 10x Genomics 3′ scRNA-seq. After QC, we obtained 39,927 SMAT T cells and 78,694 DP T cells. UMAP-based clustering, differential abundance and expression, pseudotime trajectory, pathway enrichment, and TCellSI functional state scoring were used to compare T-cell programs between GEP and PEP and across SMAT-to-DP transition, including CD4:CD8 ratio dynamics.

**Copyright Information:** (Online) ISSN: 2572-9241
©2026 The Author(s). HemaSphere is published by John Wiley & Sons Ltd on behalf of European Hematology Association. This is an open access Abstract Book distributed under the Attribution-NonCommercial-NoDerivs (CC BY-NC-ND), which allows third parties to download the articles and share them with others as long as they credit the author and the Abstract Book, but they cannot change the content in any way or use them commercially.
**Abstract Book Citations:** Authors, Title, HemaSphere, 2026;10:(S1):pages. The abstract book DOIs can be found on the "Author Information" page.
**Disclaimer:** Articles published in the journal HemaSphere exclusively reflect the opinions of the authors. The authors are responsible for all content in their abstracts including accuracy of the facts, statements, citing resources, etc.

**Results**

Among 47 r/r DLBCL patients evaluable at 6 months post-CD19/22 CAR T infusion, 23 achieved CR, 8 PR and 16 PD. Paired scRNA-seq of SMAT and DP was performed in outcome extremes defined by expansion and efficacy: GEP (CR, top 5 AUC0–28) vs PEP (PD, bottom 5 AUC0–28) (n=5 each; 10 patients; 20 samples).

SMAT ( $CD4^+$ 15,719; $CD8^+$ 24,208): outcome divergence was CD8-driven, centered on Temra with cluster0 enriched in PEP and cluster1 in GEP. c0-Temra showed senescence programs with preferential MAPK/p38 and AKT-mTOR enrichment, higher senescence/exhaustion scores and senescence gene upregulation. GEP $CD8^+$ T cells enriched cell-cycle/proliferation pathways, whereas PEP $CD8^+$ T cells enriched TCR signaling and CCL4. $CD4^+$ SMAT differences were secondary: GEP showed naïve, memory and cycling programs ($CAPG^+$ memory-like/TCM), while PEP skowed TEMRA-like with higher cytotoxicity, senescence and terminal-exhaustion scores.

DP ($CD4^+$ 49,521; $CD8^+$ 29,173): CD8 drove separation. GEP enriched naïve, cytotoxic and proliferative states; PEP enriched Tisg/Tex exhaustion (LAG3/TOX/TIGIT) with GTPase/actin signatures. CD4 subsets exhibit significant overlap but PEP cells predominantly express $MKI67^+$ proliferative and Th17-like programs with higher senescence/terminal-exhaustion/quiescence scores and lipid-mediator metabolism, whereas GEP cells retained more naïve/effector programs (variable cytotoxic-like CD4).
Lineage balance: SMAT CD4:CD8 ratio was approximately 1 in both groups. In DP, the ratio remained around 1 in GEP but ranged from 1 to 14 in PEP due to CD8 depletion. This consistent with manufacturing-stage lineage imbalance accompanying Temra/exhaustion and poor expansion and outcome.

**Summary/Conclusion**

Paired SMAT/DP scRNA-seq in r/r DLBCL extremes showed CD8 TEMRA drives outcome. PEP had senescent c0-TEMRA (MAPK/p38, AKT–mTOR) and DP CD8 exhaustion (LAG3/TOX/TIGIT) with CD8 loss and CD4:CD8 ratio imbalance. GEP retained proliferative SMAT and naïve/cytotoxic DP CD8 with balanced CD4:CD8 ratio. These features inform biomarkers and targets.

**Copyright Information:** (Online) ISSN: 2572-9241
©2026 The Author(s). HemaSphere is published by John Wiley & Sons Ltd on behalf of European Hematology Association. This is an open access Abstract Book distributed under the Attribution-NonCommercial-NoDerivs (CC BY-NC-ND), which allows third parties to download the articles and share them with others as long as they credit the author and the Abstract Book, but they cannot change the content in any way or use them commercially.
**Abstract Book Citations:** Authors, Title, HemaSphere, 2026;10:(S1):pages. The abstract book DOIs can be found on the "Author Information" page.
**Disclaimer:** Articles published in the journal HemaSphere exclusively reflect the opinions of the authors. The authors are responsible for all content in their abstracts including accuracy of the facts, statements, citing resources, etc.

### (PS2340) SEVERE DELAYED NON-ICANS NEUROTOXICITY AFTER BCMA-DIRECTED CAR-T CELLS IN MULTIPLE MYELOMA: REAL-WORLD OUTCOMES AND IMPLICATIONS FOR STRUCTURED NEUROLOGY–ONCOLOGY CARE

**Topic:** 26. Gene therapy, cellular immunotherapy and vaccination - Clinical

Tim Richardson*[1], Philipp Gödel[1], Elisa Carpinteiro[1], Christoph Scheid[1], Udo Holtick[1], Jan Werner[1], Michael Schroeter[1], Oezguer Onur[1], Norbert Galldiks[1], Gereon Fink[1]

[1]*University hospital of cologne, Cologne, Germany;*

### Background

Beyond cytokine release syndrome (CRS) and immune effector cell-associated neurotoxicity syndrome (ICANS), delayed non-ICANS neurologic toxicities after BCMA-directed CAR-T cells are increasingly recognized but remain poorly characterized in clinical practice.

### Aims

To describe incidence, phenotypes, management, and outcomes of severe non-ICANS neurotoxicity after BCMA-directed CAR-T cells and to derive practical implications for interdisciplinary care pathways.

### Methods

We retrospectively analyzed 117 consecutive patients with multiple myeloma treated with BCMA-directed CAR-T cells at a single center between January 2024 and December 2025. Non-ICANS neurotoxicity was defined per EBMT guidance (onset after CRS resolution) and adjudicated by joint hematology-neurology review after the exclusion of alternative etiologies. Syndromes included cranial nerve palsy, parkinsonism-like movement/neurocognitive toxicity, encephalitis, and Guillain–Barré–Syndrome-like presentations. We analyzed CAR-T expansion and absolute lymphocyte count (ALC) descriptively. All patients provided written informed consent, and the use of BCMA-directed CAR-T cells was per the approved label.

### Results

Severe non-ICANS neurotoxicity occurred in 10/117 patients (8.5%). Median onset was 45 days post-infusion (range, 11-160 days). Neurotoxicity included cranial nerve palsy (n=5), Parkinsonism-like syndromes (n=3), encephalitis (n=2), and Guillain-Barré-Syndrome-like presentations (n=3); overlap and sequential evolution were common. CRS occurred in 90% (grade ≥3 in 10%), and ICANS occurred in 20%, with no grade ≥3. All affected patients achieved a complete response after CAR-T; however, non-relapse mortality among patients with non-ICANS neurotoxicity was 50%, compared with 8.4% among controls. Expansion-kinetics signals were prominent: 9 of 10 patients (90%) had peak ALC $>3\times10^9$/L; median CAR-T copies at neurotoxicity onset were $2.23\times10^5/10^6$ leukocytes (range, $1.24\times10^2$-$1.09\times10^6$). Immune-directed escalation (corticosteroids ± IVIG, cytokine blockade, cytotoxic or intrathecal therapies) was frequently required, with inconsistent neurologic recovery and substantial infectious morbidity.

**Copyright Information:** (Online) ISSN: 2572-9241
©2026 The Author(s). HemaSphere is published by John Wiley & Sons Ltd on behalf of European Hematology Association. This is an open access Abstract Book distributed under the Attribution-NonCommercial-NoDerivs (CC BY-NC-ND), which allows third parties to download the articles and share them with others as long as they credit the author and the Abstract Book, but they cannot change the content in any way or use them commercially.
**Abstract Book Citations:** Authors, Title, HemaSphere, 2026;10:(S1):pages. The abstract book DOIs can be found on the "Author Information" page.
**Disclaimer:** Articles published in the journal HemaSphere exclusively reflect the opinions of the authors. The authors are responsible for all content in their abstracts including accuracy of the facts, statements, citing resources, etc.

### Summary/Conclusion

Delayed non-ICANS neurotoxicity after BCMA-directed CAR-T cells is clinically heterogeneous, often severe, and associated with high non-relapse mortality despite deep myeloma responses. Prominent expansion-kinetics signals support risk-oriented monitoring, but post hoc CAR-T reduction did not reliably result in neurological recovery. These findings argue for standardized, interdisciplinary neurology-oncology pathways with predefined triage, diagnostics, and escalation algorithms to improve recognition and management as CAR-T use broadens beyond specialized centers.

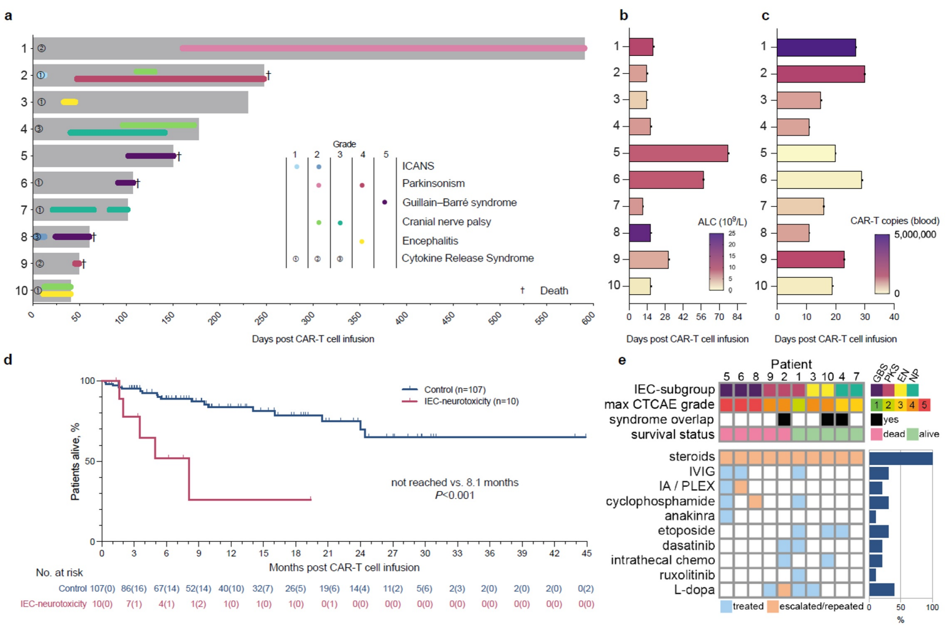

(a) Swimmer plot of the 10 patients with neurotoxicity following CAR-T cell treatment for multiple myeloma. (b) Time to peak of the absolute lymphocyte count (ALC) with visualization of the individual peak ALC-values (range, 1.0-22.2 109/L). (c) Time to peak CAR-T copies in peripheral blood samples after CAR-T cell infusion, color scale visualizing the individual peak CAR-T copy-count (range, 39,553-4,970,000). (d) Kaplan-Meier curves for the overall survival in patients with neurotoxicity and in those without (control). (e) Treatment and outcome oncoprint of neurotoxicity.

**Copyright Information:** (Online) ISSN: 2572-9241
©2026 The Author(s). HemaSphere is published by John Wiley & Sons Ltd on behalf of European Hematology Association. This is an open access Abstract Book distributed under the Attribution-NonCommercial-NoDerivs (CC BY-NC-ND), which allows third parties to download the articles and share them with others as long as they credit the author and the Abstract Book, but they cannot change the content in any way or use them commercially.
**Abstract Book Citations:** Authors, Title, HemaSphere, 2026;10:(S1):pages. The abstract book DOIs can be found on the "Author Information" page.
**Disclaimer:** Articles published in the journal HemaSphere exclusively reflect the opinions of the authors. The authors are responsible for all content in their abstracts including accuracy of the facts, statements, citing resources, etc.

### (PS2341) EFFICACY AND SAFETY OF CD19 CAR-T INATICABTAGENE AUTOLEUCEL IN OLDER ADULTS WITH B-ALL IN FIRST COMPLETE REMISSION: A MULTICENTER REAL-WORLD STUDY FROM CHINA

**Topic:** 26. Gene therapy, cellular immunotherapy and vaccination - Clinical

Jin Wang*[1], Yuhua Li[2], Hongsheng Zhou[3], Ying Wang[4], Yang Liang[5], Juan Du[1], Lei Fan[6], Yi Ding[7], Fei Li[8], Jian Li[9], Bao Hong Ping[3], Xiaobing Huang[10], Xin Du[11], Jing Huang[12], Jingli Zhuang[13], Jianqing Mi[1]

*[1]Ruijin Hospital Affiliated to Shanghai Jiao Tong University School of Medicine, Shanghai, China;[2]Zhujiang Hospital of Southern Medical University, Guangzhou, China; [3]Southern Medical University Southern Hospital, Guangzhou, China;[4]Institute of Hematology and Blood Diseases Hospital, Chinese Academy of Medical Sciences, Tianjin, China; [5]Sun-Yat-Sen-University-Cancer-Center, Guangzhou, China; [6]Jiangsu Province Hospital, Nanjing, China;[7]Tongji Hospital, Affiliated to Tongji University, Shanghai, China; [8]The First Affiliated Hospital of Nanchang University, Nanchang, China;[9]Peking Union Medical College Hospital, Beijing, China; [10]Sichuan academy of medical sciences &Sichuan provincial people's hospital, Sichuan, China; [11]Department of Hematology and Shenzhen Bone Marrow Transplantation Public Service Platform , Shenzhen Second People's Hospital, The First Affiliated Hospital of Shenzhen University, Shenzhen University School of Medicine, Shenzhen, China; [12]Department of Hematology, The Affiliated Guangdong Second Provincial, Guangzhou, China; [13]Zhongshan Hospital Affiliated to Fudan University, Shanghai, China;*

### Background

Older adults with B-cell acute lymphoblastic leukemia (B-ALL) have poor outcomes due to adverse disease biology, treatment-related toxicity, and intolerance to intensive chemotherapy and allo-SCT. While chimeric antigen receptor T-cell (CAR-T) therapy has revolutionized the treatment landscape for relapsed/refractory B-ALL. However, CAR-T consolidation in older B-ALL patients in first complete remission (CR1) remains unexplored. Inaticabtagene Autoleucel (Inati-cel) is the first approved commercial CD19 CAR-T for adult relapsed or refractory B-ALL (r/r B-ALL) in China. This study provides the first real-world evidence of Inati-cel efficacy and safety in this population (NCT07269587).

### Aims

To evaluate the real-world efficacy and safety of Inati-cel among Chinese patients ≥55 years with B-ALL in CR1.

### Methods

As of November 7, 2025, eligible patients were ≥55 years with B-ALL in CR1, underwent apheresis for Inati-cel following commercial approval (November 7, 2023), and at least 30 days of follow-up. Primary endpoints were overall survival (OS) and minimal residual disease (MRD) negativity. Secondary endpoints included leukemia free survival (LFS) and toxicity. CR was defined as ≤5% bone marrow blasts with no circulating blasts or extramedullary disease. MRD was assessed by flow cytometry, RQ-PCR, and/or NGS. CRS and ICANS were graded according to ASTCT criteria.

**Copyright Information:** (Online) ISSN: 2572-9241
©2026 The Author(s). HemaSphere is published by John Wiley & Sons Ltd on behalf of European Hematology Association. This is an open access Abstract Book distributed under the Attribution-NonCommercial-NoDerivs (CC BY-NC-ND), which allows third parties to download the articles and share them with others as long as they credit the author and the Abstract Book, but they cannot change the content in any way or use them commercially.
**Abstract Book Citations:** Authors, Title, HemaSphere, 2026;10:(S1):pages. The abstract book DOIs can be found on the "Author Information" page.
**Disclaimer:** Articles published in the journal HemaSphere exclusively reflect the opinions of the authors. The authors are responsible for all content in their abstracts including accuracy of the facts, statements, citing resources, etc.

### Results

A total of 34 patients aged ≥55 years with B-ALL in CR1 from 15 Chinese centers had received Inati-cel. Median age was 61.5 years (range, 55–76), and 67.6% was female. Before leukapheresis, 38.2% had MRD-positive disease (20.6% flow cytometry-positive; 26.5% molecular-positive), and 55.9% were MRD-negative. Cytogenetic abnormalities included Philadelphia chromosome positivity in 61.8% and IKZF1 aberrations in 26.5%. 88.2% received bridging therapy, and 61.7% remained MRD negativity CR prior to CAR-T infusion. Median time from leukapheresis to infusion was 40 days, with a median dose of 0.60×$10^8$ CAR-T cells (range, 0.5-0.8).

All 34 patients (100%) achieved MRD negativity by flow cytometry within 3 months, 83.3% (20/24) patients achieved MRD negativity by PCR or NGS. After median follow-up of 8.85 months (95%CI, 7.07-11.2), estimated 1-year LFS rate was 92.6%, and 1-year OS rate was 100%. Two patients experienced morphologic relapse at 4.5 and 6.1 months, and two had molecular relapse at 6 and 9 months after CAR-T infusion. Four patients with Ph+ ALL had persistent BCR-ABL positive and received second-/third-generation TKIs maintenance. Any grade CRS occurred in 51.5% (all grade 1–2), no grade ≥3 CRS or ICANS was observed. All toxicities were manageable and reversible with no treatment-related deaths.

After Inati-cel infusion, median peak CAR-T expansion was 29.3 cells/μL (range, 2.21–353), CAR-T expansion peaked on day 14. No significant difference in peak expansion was observed between MRD-positive and MRD-negative patients (P=0.621). The median duration of CAR-T persistence was 3 months (range, 0.5–12), with the longest detectability observed at 12 months post-infusion.

### Summary/Conclusion

Real-world data from the first commercial CD19 CAR-T study in older adults with B-ALL in CR1 in China demonstrate that Inati-cel achieves deep molecular remission, favorable survival outcomes, and an exceptional safety profile. These preliminary results support further exploration of CAR-T consolidation strategy in this population.

**Copyright Information:** (Online) ISSN: 2572-9241
©2026 The Author(s). HemaSphere is published by John Wiley & Sons Ltd on behalf of European Hematology Association. This is an open access Abstract Book distributed under the Attribution-NonCommercial-NoDerivs (CC BY-NC-ND), which allows third parties to download the articles and share them with others as long as they credit the author and the Abstract Book, but they cannot change the content in any way or use them commercially.
**Abstract Book Citations:** Authors, Title, HemaSphere, 2026;10:(S1):pages. The abstract book DOIs can be found on the "Author Information" page.
**Disclaimer:** Articles published in the journal HemaSphere exclusively reflect the opinions of the authors. The authors are responsible for all content in their abstracts including accuracy of the facts, statements, citing resources, etc.

### (PS2342) MAINTENANCE THERAPY WITH PD-1 INHIBITOR AND SELINEXOR FOLLOWING CD19/CD22 BISPECIFIC CAR-T CELL THERAPY IN RELAPSED/REFRACTORY DIFFUSE LARGE B-CELL LYMPHOMA: A RETROSPECTIVE ANALYSIS

**Topic:** 26. Gene therapy, cellular immunotherapy and vaccination - Clinical

Na Yao[1], Lijie Zeng[1], Xindi Liu[1], Liang Wang*[1], Jia Cong[1]

[1]*Beijing Tongren Hospital, Capital Medical University, Beijing, China;*

### Background

Chimeric antigen receptor T-cell (CAR-T) therapy targeting CD19 has revolutionized the treatment landscape for relapsed/refractory diffuse large B-cell lymphoma (R/R DLBCL). Despite impressive initial response rates, disease relapse remains a significant challenge, often attributed to CAR-T cell exhaustion and immune evasion mechanisms. In our prospective phase 2 trial, CD19/CD22 bispecific CAR-T (CAR2219) demonstrated a remarkable 100% best overall response rate; however, strategies to prolong remission duration and prevent relapse are urgently needed. Maintenance therapy represents a promising approach to enhance response persistence by targeting underlying mechanisms of treatment failure. Programmed cell death protein 1 (PD-1) inhibitors have shown potential to reverse CAR-T cell exhaustion and restore anti-tumor immunity, while the exportin 1 (XPO1) inhibitor selinexor exhibits direct anti-lymphoma activity and synergistic effects when combined with immunomodulatory agents.

### Aims

To evaluate the efficacy, safety, and immunomodulatory effects of maintenance therapy combining PD-1 inhibitor with selinexor following CAR2219 therapy in patients with R/R DLBCL.

### Methods

This retrospective analysis included 32 patients enrolled in our prospective phase 2 clinical trial (NCT06081478) who achieved disease control following CAR2219 infusion and subsequently received planned maintenance therapy. The maintenance regimen consisted of PD-1 inhibitor (tislelizumab 200 mg or serplulimab 240 mg intravenously every 3 weeks) plus selinexor (40-60 mg orally once weekly) for 1-2 years duration. The primary endpoint was progression-free survival (PFS). Secondary endpoints included duration of response (DOR), overall survival (OS), safety profiles, and correlative biomarker analyses. Treatment responses were assessed according to Lugano 2014 criteria, and adverse events were graded per CTCAE version 5.0. Longitudinal monitoring of circulating T-cell subsets, including CD3+PD-1+ T-cells, was performed by flow cytometry in a subset of patients.

### Results

A total of 32 patients were included, with a median follow-up of 260 days (range 59-529). Baseline characteristics revealed high-risk disease features. Notably, only 12 patients (38%) had achieved complete response (CR) prior to maintenance initiation. Response rates improved progressively over time during maintenance therapy: overall response rate (ORR) increased from 56% (CR rate 38%) at month 1 to 83% (CR rate 57%) at month 3, and further to 81% (CR rate 71%) at month 6, indicating deepening of responses with continued treatment. With a median PFS of 407 days, the estimated 1-year PFS and OS rates were 56.8% (95%CI: 0.40-0.80) and 71.2% (95%CI: 0.53-0.95), respectively. Median OS was not reached at the time of analysis. Immunophenotypic analysis demonstrated a substantial reduction in circulating CD3+PD-1+ T-cells in all 15 evaluated patients, suggesting effective reversal of T-cell exhaustion. The combination regimen demonstrated manageable toxicity overall.

**Copyright Information:** (Online) ISSN: 2572-9241
©2026 The Author(s). HemaSphere is published by John Wiley & Sons Ltd on behalf of European Hematology Association. This is an open access Abstract Book distributed under the Attribution-NonCommercial-NoDerivs (CC BY-NC-ND), which allows third parties to download the articles and share them with others as long as they credit the author and the Abstract Book, but they cannot change the content in any way or use them commercially.
**Abstract Book Citations:** Authors, Title, HemaSphere, 2026;10:(S1):pages. The abstract book DOIs can be found on the "Author Information" page.
**Disclaimer:** Articles published in the journal HemaSphere exclusively reflect the opinions of the authors. The authors are responsible for all content in their abstracts including accuracy of the facts, statements, citing resources, etc.

### Summary/Conclusion

Maintenance therapy combining PD-1 inhibitor and selinexor following CD19/CD22 bispecific CAR-T cell therapy demonstrates promising clinical activity in R/R DLBCL, characterized by deepening responses over time and durable disease control. The observed reduction in PD-1+ T-cells supports an immunomodulatory mechanism of action. These findings warrant further investigation in prospective randomized trials to establish the optimal maintenance strategy post-CAR-T therapy.

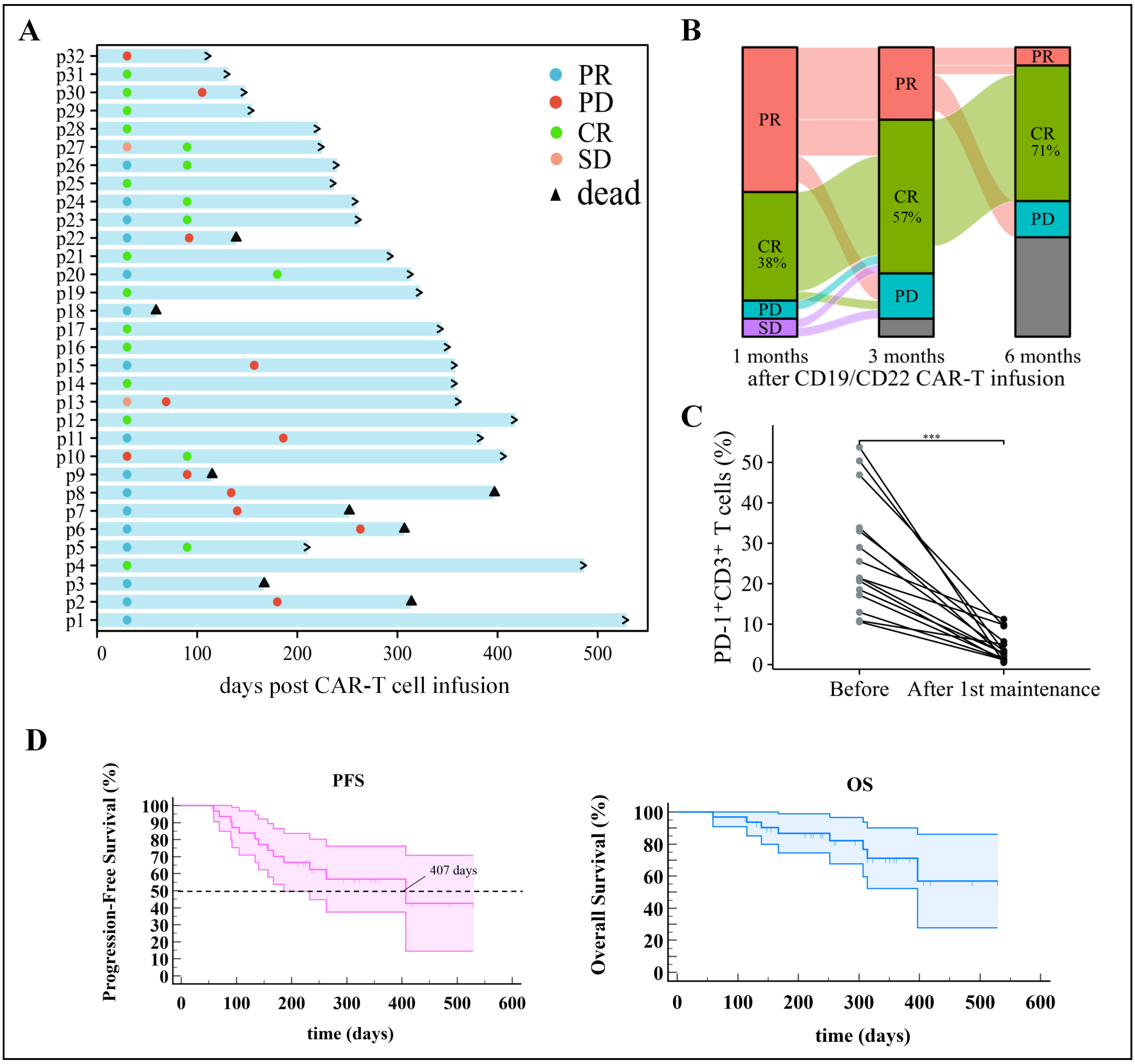

**Copyright Information:** (Online) ISSN: 2572-9241
©2026 The Author(s). HemaSphere is published by John Wiley & Sons Ltd on behalf of European Hematology Association. This is an open access Abstract Book distributed under the Attribution-NonCommercial-NoDerivs (CC BY-NC-ND), which allows third parties to download the articles and share them with others as long as they credit the author and the Abstract Book, but they cannot change the content in any way or use them commercially.
**Abstract Book Citations:** Authors, Title, HemaSphere, 2026;10:(S1):pages. The abstract book DOIs can be found on the "Author Information" page.
**Disclaimer:** Articles published in the journal HemaSphere exclusively reflect the opinions of the authors. The authors are responsible for all content in their abstracts including accuracy of the facts, statements, citing resources, etc.

### (PS2343) THE SUCCESS OF CAR T-CELL THERAPY IS DETERMINED BY PRE-INFUSION DISEASE CONTROL AND POST-INFUSION MRD CLEARANCE MEASURED BY CTDNA IN PATIENTS WITH B-CELL LYMPHOMA

**Topic:** 26. Gene therapy, cellular immunotherapy and vaccination - Clinical

Tim Versteegen*[1], Philipp Nakov[1], Leo Meriranta[1], Guranda Chitadze[1], Britta Kehden[1], Monika Brüggemann[1], Claudia Baldus[1], Friedrich Stölzel[2], Christiane Pott[1], Mouhamad Khouja[1]

*[1]University Cancer Center Schleswig-Holstein (UCCSH), University Hospital Schleswig-Holstein (UKSH) – Campus Kiel, Department of Hematology and Oncology, Kiel, Germany; [2]University Cancer Center Schleswig-Holstein (UCCSH), University Hospital Schleswig-Holstein (UKSH) – Campus Kiel, Division of Stem Cell Transplantation and Cellular Immunotherapies, Kiel, Germany;*

### Background

CD19-directed chimeric antigen-receptor (CAR) T-cell therapy is the new standard of care in refractory/relapsed (r/r) B-cell non-Hodgkin lymphoma (B-NHL). However, treatment failure and therapy-associated toxicity are common and require individualized approaches. High tumor load is a critical negative predictor for CAR T-cell therapy efficacy, leading to lower complete remission rates and poor outcome. Circulating tumor (ct)DNA is a non-invasive and dynamic biomarker to measure this load, providing real-time insights into treatment efficacy, minimal residual disease (MRD) and early relapse detection. We studied ctDNA kinetics in an r/r B-NHL real-world cohort comprising several B-NHL entities to determine the impact of bridging therapy and molecular disease eradication on outcome.

### Aims

To determine the value and prognostic impact of ctDNA kinetics during CAR T-cell therapy to tailor bridging or consolidation strategies prior to and after CAR T-cell administration.

### Methods

In this cohort comprising patients (pts) with large B-cell lymphoma (LBCL, n=59), mantle cell lymphoma (n=10) and follicular lymphoma (n=6), genomic DNA from tumor biopsies (FFPE) and/or ctDNA from plasma samples were sequenced using the EuroClonality NDC assay (Univ8® Genomics). Haplotype variants in hypermutated loci (Kurtz et al., 2021) were analyzed by an in-house pipeline. ctDNA concentration was calculated as genome equivalents (hGE) from mean VAF. ctDNA was assessed at relapse and in follow-up samples on d0, d14 and d28 of CAR T-cell therapy. ctDNA detection was correlated with PET-CT response at month 3 as well as progression-free (PFS) and overall survival (OS). Hazard ratios were derived from Cox proportional hazards regression.

**Copyright Information:** (Online) ISSN: 2572-9241
©2026 The Author(s). HemaSphere is published by John Wiley & Sons Ltd on behalf of European Hematology Association. This is an open access Abstract Book distributed under the Attribution-NonCommercial-NoDerivs (CC BY-NC-ND), which allows third parties to download the articles and share them with others as long as they credit the author and the Abstract Book, but they cannot change the content in any way or use them commercially.
**Abstract Book Citations:** Authors, Title, HemaSphere, 2026;10:(S1):pages. The abstract book DOIs can be found on the “Author Information” page.
**Disclaimer:** Articles published in the journal HemaSphere exclusively reflect the opinions of the authors. The authors are responsible for all content in their abstracts including accuracy of the facts, statements, citing resources, etc.

**Results**

We investigated 75 B-NHL pts treated with CAR T-cell therapy between 09/2019 and 05/2024. Tumor genotypes were obtained from FFPE in 38 pts and from pre-bridging plasma samples (PreBr) in 55 pts with overlapping material in 19 pts. Identification of MRD reporters was successful in 60 (80%) pts with a median of 16 reporters [1 – 143] and a lower number of reporters in MCL pts. In the 50 evaluable LBCL pts, ctDNA negativity at d0 reflecting very low tumor load in 10/48 pts showed a significantly reduced risk of progression (p=0.01, HR 0.21 [0.06-0.68], median PFS not reached (NR) vs. 3.4 months, figure 1). ctDNA-negative pts on d0 remained negative in following timepoints and neither showed higher grades of CRS or ICANS nor early (< 1 year) disease recurrence. We observed a significant difference in ctDNA level reduction from PreBr to d0 between PET-responders and non-responders (p=0.0006, median reduction of 2.75 $\log_{10}$hGE in responders vs. 0.44 in non-responders, Wilcoxon test), whereas the kinetics between later timepoints (i.e. d0 vs. d28 or d0 vs. d14) did not differ. ctDNA positive pts on d0 with low ctDNA reduction from PreBr (< median) had the highest risk of progression (n=12/34, p=0.0002, HR 20.7[4.7-91.2]). ctDNA persistence on d28 (22/39 pts; 56%) was significantly associated with a high risk of progression (p=0.0027, HR 5.3 [2.2–12.9], median PFS 3.3 months vs. NR).

**Summary/Conclusion**

ctDNA monitoring identifies pts at high-risk of CAR T-cell therapy failure. Our data underlines the importance of disease control and tumor reduction by effective bridging therapy prior to CAR T-cell infusion for long-term outcome. ctDNA assessment is a valuable tool to guide the timing of CAR T-cell therapy and the extent of required bridging therapy in a personalized manner.

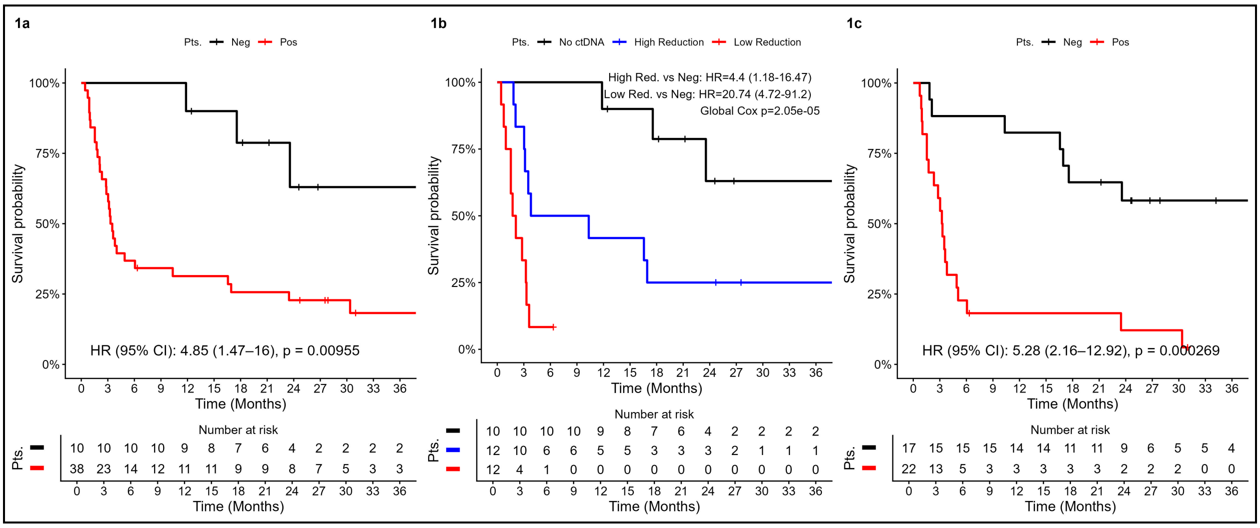

Figure 1: Progression-free survival outcomes stratified by (1a) ctDNA detection on the day of CAR T infusion, (1b) levels of ctDNA reduction from pre-bridging to the day of CAR T infusion, and (1c) ctDNA detection on day 28 after CAR T infusion. Survival is calculated from day of CAR T-cell infusion.

**Copyright Information:** (Online) ISSN: 2572-9241
©2026 The Author(s). HemaSphere is published by John Wiley & Sons Ltd on behalf of European Hematology Association. This is an open access Abstract Book distributed under the Attribution-NonCommercial-NoDerivs (CC BY-NC-ND), which allows third parties to download the articles and share them with others as long as they credit the author and the Abstract Book, but they cannot change the content in any way or use them commercially.
**Abstract Book Citations:** Authors, Title, HemaSphere, 2026;10:(S1):pages. The abstract book DOIs can be found on the "Author Information" page.
**Disclaimer:** Articles published in the journal HemaSphere exclusively reflect the opinions of the authors. The authors are responsible for all content in their abstracts including accuracy of the facts, statements, citing resources, etc.

### (PS2344) TAZEMETOSTAT-MEDIATED IMMUNE REMODELING IN B-CELL LYMPHOMAS RECEIVING CART

**Topic:** 26. Gene therapy, cellular immunotherapy and vaccination - Clinical

Samuel Yamshon*[1], Caitlin Gribbin[1], Yusuke Isshiki[1], Ioannis Karagiannidis[1], Sunjae Lee[1], Jizhi Yan[1], Gwynne Ozkan[1], Anna Alperovich[1], Barbara MA[1], Christopher Hackett[1], Mateo Mejia Saldarriaga[1], Daniel Choi[1], Selina Chen-Kiang[1], John Leonard[1], Ari Melnick[1], Anastasiia Bolshakova[2], Alina Mulyukina[2], Ivan Zubarev[2], Aleksei Tishchenko[2], Nathan Fowler[2], Juliet Barker[1], Giorgio Inghirami[1], Wendy Béguelin[3], Peter Martin[1]

*[1]Weill Cornell Medicine, New York, NY, United States of America;[2]BostonGene Corporation, Waltham, MA, United States of America; [3]NYU Langone Health, New York, NY, United States of America;*

### Background

Chimeric antigen receptor (CAR) T cell therapy has transformed outcomes in B cell lymphoma; however, relapse relapse remains prevalent. Preclinical data suggest EZH2 inhibition may mitigate T-cell exhaustion, promote a T-cell memory phenotype, and enhance response to immune-based approaches.

### Aims

Here, we evaluated whether priming with the oral EZH2 inhibitor tazemetostat could favorably modulate host immunity and improve outcomes in patients receiving standard-of-care CART therapy.

### Methods

Thirteen pts with diffuse large B cell (DLBCL, n = 7), follicular (FL, n = 5), or mantle cell lymphoma (MCL, n = 1) received the EZH2 inhibitor tazemetostat for ≥7 days prior to apheresis and continued to lymphodepletion. Following CART infusion, tazemetostat was resumed at hematologic count recovery and continued for up to 12 mo (6 mo in pts with complete remission [CR]). Peripheral blood mononuclear cells were collected Day -24 and Day -5. Immune modulation was evaluated with multiparametric flow cytometry, transcriptomics, cell deconvolution, differential gene expression patterning, and ssGSEA.

### Results

The median number of prior lines of therapy was 2 (range: 1-4), and 85% were refractory to the most recent therapy.*EZH2* mutations were present in 2 pts. Features included *TP53* mutation or deletion (n = 4), *MYC* translocation (4 of 7 DLBCL pts), and transformed disease (n = 3). Patients received axicabtagene ciloleucel (n = 5), lisocabtagene maraleucel (n = 5), tisagenlecleucel (n = 2), or brexucabtagene autoleucel (n = 1).
The overall response rate was 100%, with 77% achieving CR (DLBCL 71%, FL 100%). At a median follow up of 18 months, 54% remain progression-free, and 77% remain alive. Cytokine release syndrome (CRS) occurred in 10 pts (77%), with 1 grade 3 CRS. Six pts developed transient grade 1-2 ICANS. Grade 3+ neutropenia was seen in 77%, and 3 pts experienced grade 3 infections. Gastrointestinal adverse events were the most common, predominantly grade 1-2.
Immunoprofiling demonstrated an increase in NK cells (14%) and CD16+ monocytes (20%, $p < 0.03$) with a concurrent decrease in Tregs (14%) and CD14+ monocytes (53%,$p < 0.02$) when comparing baseline and pre-CART samples. Gene signature analysis uncovered upregulation of signatures associated with MHC-I antigen presentation ($p = 0.010$), antigen-specific T cell activation ($p = 0.019$), and cellular cytotoxicity ($p = 0.043$), while signatures associated with tolerogenic dendritic cells ($p = 0.011$), myeloid suppression ($p = 0.043$), and coagulation ($p = 0.030$) were downregulated. No detrimental effects on CART transduction efficiency, activation, or expansion were observed.

**Copyright Information:** (Online) ISSN: 2572-9241
©2026 The Author(s). HemaSphere is published by John Wiley & Sons Ltd on behalf of European Hematology Association. This is an open access Abstract Book distributed under the Attribution-NonCommercial-NoDerivs (CC BY-NC-ND), which allows third parties to download the articles and share them with others as long as they credit the author and the Abstract Book, but they cannot change the content in any way or use them commercially.
**Abstract Book Citations:** Authors, Title, HemaSphere, 2026;10:(S1):pages. The abstract book DOIs can be found on the "Author Information" page.
**Disclaimer:** Articles published in the journal HemaSphere exclusively reflect the opinions of the authors. The authors are responsible for all content in their abstracts including accuracy of the facts, statements, citing resources, etc.

**Summary/Conclusion**

EZH2 inhibition with tazemetostat for immune priming ahead of CART is associated with high clinical efficacy and peripheral immune remodeling. Enhanced lymphocyte activation and cytotoxicity paired with decreased myeloid tolerogenicity and immunosuppression may facilitate CART expansion and persistence. Ongoing immune correlates and planned randomized studies will further define the clinical benefits of this approach.

**Copyright Information:** (Online) ISSN: 2572-9241
©2026 The Author(s). HemaSphere is published by John Wiley & Sons Ltd on behalf of European Hematology Association. This is an open access Abstract Book distributed under the Attribution-NonCommercial-NoDerivs (CC BY-NC-ND), which allows third parties to download the articles and share them with others as long as they credit the author and the Abstract Book, but they cannot change the content in any way or use them commercially.
**Abstract Book Citations:** Authors, Title, HemaSphere, 2026;10:(S1):pages. The abstract book DOIs can be found on the "Author Information" page.
**Disclaimer:** Articles published in the journal HemaSphere exclusively reflect the opinions of the authors. The authors are responsible for all content in their abstracts including accuracy of the facts, statements, citing resources, etc.

### (PS2345) DURABLE CLINICAL BENEFITS WITH EXAGAMGLOGENE AUTOTEMCEL FOR PEDIATRIC PATIENTS (5-11 YEARS) WITH TRANSFUSION-DEPENDENT Β-THALASSEMIA AND SICKLE CELL DISEASE WITH RECURRENT VASO-OCCLUSIVE CRISES

**Topic:** 26. Gene therapy, cellular immunotherapy and vaccination - Clinical

Franco Locatelli*[1], Stephan Grupp[2], Josu De La Fuente[3], Yogi Chopra[4], Mattia Algeri[5], Persis Amrolia[6], Akshay Sharma[7], Roland Meisel[8], Maria Domenica Cappellini[9], Selim Corbacioglu[10], Antonis Kattamis[11], Stephan Lobitz[12], Mariane de Montalembert[13], Damiano Rondelli[14], Sujit Sheth[15], Martin Steinberg[16], Mark Walters[17], Kevin Boerner[18], Pengyu Liu[18], Alicia Chang[18], William Hobbs[18], Haydar Frangoul[19]

*[1]IRCCS, Ospedale Pediatrico Bambino Gesù Rome, Catholic University of the Sacred Heart, Rome, Italy;[2]Children's Hospital of Philadelphia, Perelman School of Medicine at the University of Pennsylvania, Philadelphia, United States of America; [3]Imperial College Healthcare NHS Trust, St Mary's Hospital, London, United Kingdom; [4]Hospital for Sick Children, Toronto, Canada; [5]IRCCS, Ospedale Pediatrico Bambino Gesù, Rome, Italy; [6]Great Ormond Street Hospital for Children, London, United Kingdom; [7]St. Jude Children's Research Hospital, Department of Bone Marrow Transplantation and Cellular Therapy, Memphis, United States of America; [8]Heinrich-Heine-University, Division of Pediatric Stem Cell Therapy, Department of Pediatric Oncology, Hematology and Clinical Immunology, Medical Faculty, Düsseldorf, Germany; [9]University of Milan, Milan, Italy; [10]University of Regensburg, Regensburg, Germany; [11]National and Kapodistrian University of Athens, Athens, Greece; [12]Gemeinschaftsklinikum Mittelrhein, Koblenz, Germany; [13]University of Paris-Cité, Necker-Enfants Malades Hospital, Assistance Publique-Hôpitaux de Paris (AP-HP), Paris, France; [14]University of Illinois at Chicago, Chicago, United States of America; [15]Joan and Sanford I Weill Medical College of Cornell University, New York, United States of America; [16]Boston University Chobanian & Avedisian School of Medicine, Boston, United States of America; [17]UCSF Benioff Children's Hospital, Oakland, United States of America; [18]Vertex Pharmaceuticals Incorporated, Boston, United States of America; [19]Sarah Cannon Research Institute at The Children's Hospital at TriStar Centennial, Nashville, United States of America;*

### Background

Exagamglogene autotemcel (exa-cel) is a one-time, autologous cell therapy approved for individuals ≥12 years (y) old with transfusion-dependent β-thalassemia (TDT) or sickle cell disease (SCD) with recurrent vaso-occlusive crises (VOCs).

### Aims

Evaluate safety and efficacy from CLIMB THAL-141 and CLIMB SCD-151 in participants (pts) aged 5-11y.

### Methods

CLIMB THAL-141 (TDT) and CLIMB SCD-151 (SCD) are ongoing 2y, Phase 3 exa-cel trials in pts aged 2-11y with history of ≥100 mL/kg/y RBC transfusions in the 2y before screening (TDT) or ≥2 severe VOCs per y in the 2y before screening (SCD). Data for pts aged 5-11y are from July 20, 2025; updated data will be presented.

Exa-cel was infused following PK-adjusted busulfan myeloablation. Pts were monitored for engraftment, total hemoglobin (Hb), fetal Hb (HbF), allelic editing, transfusions, VOCs (SCD only), and adverse events. CLIMB THAL-141 primary endpoint is TI12, namely proportion of pts maintaining a weighted average Hb ≥9 g/dL without RBC transfusion for ≥12 consecutive months (M). CLIMB SCD-151 primary endpoint is proportion of pts free of severe VOCs for ≥12 consecutive M (VF12). After CLIMB THAL-141 or SCD-151, pts enroll in CLIMB-131 for up to 15y follow-up after exa-cel infusion.

**Copyright Information:** (Online) ISSN: 2572-9241
©2026 The Author(s). HemaSphere is published by John Wiley & Sons Ltd on behalf of European Hematology Association. This is an open access Abstract Book distributed under the Attribution-NonCommercial-NoDerivs (CC BY-NC-ND), which allows third parties to download the articles and share them with others as long as they credit the author and the Abstract Book, but they cannot change the content in any way or use them commercially.
**Abstract Book Citations:** Authors, Title, HemaSphere, 2026;10:(S1):pages. The abstract book DOIs can be found on the "Author Information" page.
**Disclaimer:** Articles published in the journal HemaSphere exclusively reflect the opinions of the authors. The authors are responsible for all content in their abstracts including accuracy of the facts, statements, citing resources, etc.

### Results

13 TDT pts (mean age 7.4y [range 5-11]) and 11 SCD pts (mean age 8.5y [range 5-11]) received exa-cel. 5/13 (38.5%) TDT pts had $\beta^0/\beta^0$ or $\beta^0/\beta^0$-like genotypes; all SCD pts had $\beta^S/\beta^S$ genotype. All 13 TDT pts required ≤2 mobilization cycles (median 1.0 [range 1-2]). 72.7% of SCD pts (8/11) required ≤2 mobilization cycles (median 2.0 [range 1-3]). For SCD pts, higher pre-apheresis blood $CD34^+$ count correlated with higher $CD34^+$ cell collection.
In CLIMB THAL-141, median follow-up after exa-cel was 15.9M (range 4.4-24.3). Pts achieved neutrophil and platelet engraftment at a median of 30 (range 19-38) and 52 (range 22- 82) days, respectively. All 6 evaluable pts achieved TI12; longest transfusion-free duration is 21.4M. Mean HbF increased to >10 g/dL by M6 with pancellular distribution, resulting in normal age adjusted mean Hb that remained stable. In CLIMB SCD-151, median follow-up after exa-cel was 10.9M (range 1.6-23.9). Pts achieved neutrophil and platelet engraftment at median of 28 (range 20-37) and 45.5 (range 24-67) days, respectively. No pts had a VOC post exa-cel infusion and all 4 evaluable pts achieved VF12; longest VOC-free duration was 20.9M. Mean HbF% >40% with pancellular distribution and normal age adjusted mean total Hb was achieved by M6 and remained stable.

TDT and SCD pts had stable allelic editing in bone marrow and blood. Safety profile of exa-cel was consistent with myeloablative conditioning and autologous transplant in TDT and SCD. One TDT pt developed severe veno-occlusive disease (VOD, related to busulfan, not related to exa-cel) with multi-organ failure that was fatal. VOD, including fatal VOD, is a known risk of busulfan therapy and occurs at higher frequency in children compared to adults.

### Summary/Conclusion

Efficacy and safety for pts 5-11y from CLIMB THAL-141 and CLIMB SCD-151 are consistent with pts aged ≥12y. Exa-cel demonstrated benefit in children, with a safety profile consistent with busulfan myeloablative conditioning and autologous transplant. Data support exa-cel as a potential one-time functional cure for children 5-11y with TDT and SCD, with potential for additional benefit with early treatment, before chronic disease complications develop.

**Copyright Information:** (Online) ISSN: 2572-9241
©2026 The Author(s). HemaSphere is published by John Wiley & Sons Ltd on behalf of European Hematology Association. This is an open access Abstract Book distributed under the Attribution-NonCommercial-NoDerivs (CC BY-NC-ND), which allows third parties to download the articles and share them with others as long as they credit the author and the Abstract Book, but they cannot change the content in any way or use them commercially.
**Abstract Book Citations:** Authors, Title, HemaSphere, 2026;10:(S1):pages. The abstract book DOIs can be found on the "Author Information" page.
**Disclaimer:** Articles published in the journal HemaSphere exclusively reflect the opinions of the authors. The authors are responsible for all content in their abstracts including accuracy of the facts, statements, citing resources, etc.

### (PS2346) CAR T-CELL THERAPY FOR POST-TRANSPLANT LYMPHOPROLIFERATIVE DISEASE, A REAL-WORLD ANALYSIS ON BEHALF OF THE EBMT LYMPHOMA WORKING PARTY

**Topic:** 26. Gene therapy, cellular immunotherapy and vaccination - Clinical

Fabio Andreozzi*[1, 2], Julie Castiaux[1], Maud Ngoya[3], Caroline Arber[4], Victoria Potter[5], Jakob Passweg[6], Dominik Schneidawind[7], Rajesh Alajangi[8], Christof Scheid[9], Federico Simonetta[10], Anoek Broers[11], Martin Fehr[12], Andy Peniket[13], Marianne Brodtkorb[14], Urban Novak[15], Henrik Sengeløv[16], Matthew Collin[17], Ron Ram[18], Victoria Campbell[19], Ali Bazarbachi[20]

*[1]Institut Jules Bordet, Brussels, Belgium; [2]Laboratory of Molecular Biology of Leukemia - KULeuven, Leuven, Belgium;[3]European Society for Blood and Marrow Transplantation, Paris Study Unit, Hôpital Saint-Antoine, Paris, France., Paris, France; [4]Department of Oncology, Lausanne University Hospital (CHUV), Lausanne, Switzerland; [5]King's College Hospital, London, United Kingdom; [6]University Hospital, Basel, Switzerland, Basel, Switzerland; [7]University Hospital Zürich, Zürich, Switzerland; [8]University Hospitals Bristol and Weston NHSFT, United Kingdom, Bristol, United Kingdom; [9]University of Cologne, Cologne, Germany; [10]HUG - Hôpitaux universitaires de Genève, Geneve, Switzerland; [11]Erasmus MC Cancer Institute, Rotterdam, The Netherlands; [12]Kantonsspital St.Gallen, St. Gallen, Switzerland; [13]Oxford Radcliffe, Oxford, United Kingdom; [14]Oslo University Hospital, Rikshospitalet, Oslo, Norway; [15]Medical Oncology, Inselspital, Bern University Hospital and University of Bern, Bern, Switzerland; [16]Rigshospitalet, Copenhagen University Hospital, Copenhagen, Denmark; [17]Newcastle Upon Tyne Hospitals NHS Foundation Trust, Newcastle Upon Tyne, United Kingdom; [18]Tel Aviv Sourasky Medical Center, Tel Aviv, Israel; [19]Western General Hospital Edinburgh, Edinburgh, United Kingdom; [20]American University of Beirut, Beirut, Lebanon;*

### Background

Post-transplant lymphoproliferative disease (PTLD) is a rare and difficult to manage condition occurring in immunosuppressed patients after solid organ transplantation or allogeneic stem cell transplantation. PTLD management relies on reduction of immunosuppressive therapy when feasible, administration of rituximab with or without chemotherapy and, more recently, treatment with tabelecleucel in EBV positive cases. In CD19-positive PTLD, anti-CD19 CAR T-cell treatment is emerging as a therapeutic option. However, due to exclusion from clinical trials and rarity of condition, data on CAR T-cells treatment in PTLD are scarce. With this work, we aimed at assessing outcomes in PTLD patients treated with anti-CD19 CAR T-cells.

### Methods

We retrospectively collected all adults patients with a diagnosis of PTLD and who underwent CAR T-cell therapy, reported to the EBMT registry between 2019 and 2025.

### Results

A total of 23 patients were identified. Median age was 41.85 years and most of patients were females (14 out of 23). Fifteen patients had a prior history of solid organ transplantation, including 7 kidney, 2 lung and 2 heart transplantation. CAR T-cell product was autologous in 19/23 cases and allogeneic in remaining 4. At CAR-T infusion, 1 patient was in complete remission, 4 in partial response, 3 had a stable disease, while 13 had refractory disease.
After a median follow up of 1 year, 1year overall survival (OS), progression free survival (PFS), relapse incidence (RI), non-relapse mortality (NRM) were 74.7% (48 - 89), 62.4% (35.7 - 80.5), 32.2% (11.9 - 54.8), 5.4%(0.3 - 22.7), respectively.

**Copyright Information:** (Online) ISSN: 2572-9241
©2026 The Author(s). HemaSphere is published by John Wiley & Sons Ltd on behalf of European Hematology Association. This is an open access Abstract Book distributed under the Attribution-NonCommercial-NoDerivs (CC BY-NC-ND), which allows third parties to download the articles and share them with others as long as they credit the author and the Abstract Book, but they cannot change the content in any way or use them commercially.
**Abstract Book Citations:** Authors, Title, HemaSphere, 2026;10:(S1):pages. The abstract book DOIs can be found on the "Author Information" page.
**Disclaimer:** Articles published in the journal HemaSphere exclusively reflect the opinions of the authors. The authors are responsible for all content in their abstracts including accuracy of the facts, statements, citing resources, etc.

### Summary/Conclusion

In this analysis, patients treated with anti-CD19 CAR T-cells showed encouraging 1-year survival rates, with relatively low relapse/progression incidence and non-relapse mortality. These findings suggest that CAR T-cell therapy represents a feasible and potentially effective option for PTLD patients failing standard therapies.

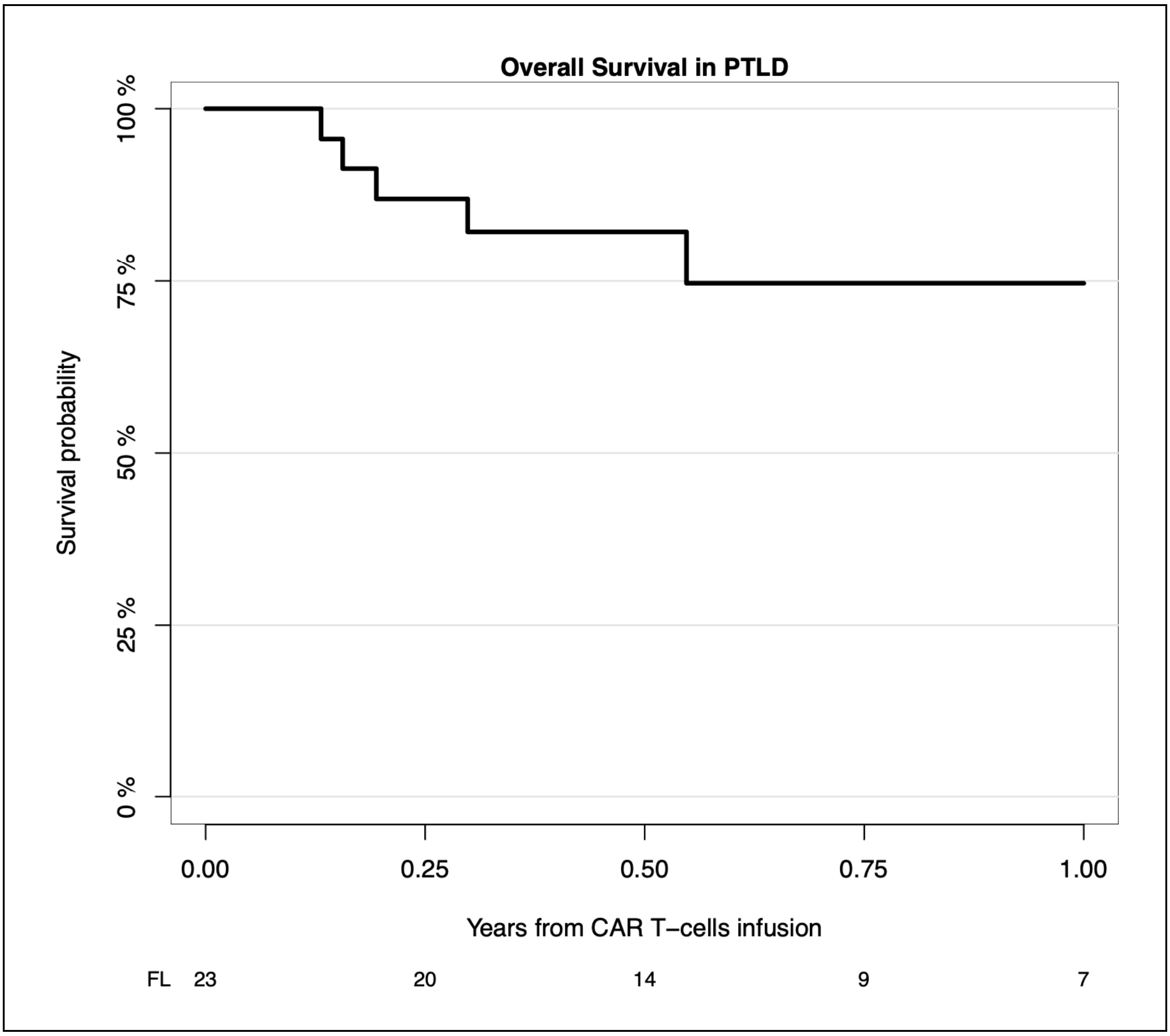

Figure 1 - Overall Survival courbe for patients with post-transplant lymphoproliferative disease after CAR T-cells administration. PTLD- Post-transplant lymphoproliferative disease, FL - follow-up

**Copyright Information:** (Online) ISSN: 2572-9241
©2026 The Author(s). HemaSphere is published by John Wiley & Sons Ltd on behalf of European Hematology Association. This is an open access Abstract Book distributed under the Attribution-NonCommercial-NoDerivs (CC BY-NC-ND), which allows third parties to download the articles and share them with others as long as they credit the author and the Abstract Book, but they cannot change the content in any way or use them commercially.
**Abstract Book Citations:** Authors, Title, HemaSphere, 2026;10:(S1):pages. The abstract book DOIs can be found on the "Author Information" page.
**Disclaimer:** Articles published in the journal HemaSphere exclusively reflect the opinions of the authors. The authors are responsible for all content in their abstracts including accuracy of the facts, statements, citing resources, etc.

### (PS2347) REAL-WORLD USE OF RECOMBINANT HUMAN THROMBOPOIETIN (RHTPO) FOR SEVERE THROMBOCYTOPENIA AFTER CAR T-CELL THERAPY: PLATELET RECOVERY AND TRANSFUSION BURDEN STRATIFIED BY CAR-HEMATOTOX

**Topic:** 26. Gene therapy, cellular immunotherapy and vaccination - Clinical

Xin Zheng*[1], Yin Zhang[1], Wen Wu[1], Zixun Yan[1], Yang He[1]

[1]*Ruijin Hospital Affiliated to Shanghai Jiao Tong University School of Medicine, Shanghai, China;*

### Background

Thrombocytopenia is a major complication after CAR T-cell therapy, occurring early after lymphodepletion or persisting after infusion, with increased bleeding risk and platelet transfusion needs. Current management is mainly supportive,and,when indicated, thrombopoietic agents such as rhTPO and TPO-RAs.However,the optimal timing of intervention remains unclear,and the role of the CAR-HEMATOTOX score in platelet management is unknown.

### Aims

To evaluate the outcomes of rhTPO in severe thrombocytopenia during the peri–CAR T-cell period and to assess the value of the CAR-HEMATOTOX score and baseline platelet count in guiding management.

### Methods

88 lymphoma patients who developed grade≥3 thrombocytopenia after CAR T-cell therapy and received rhTPO were included. The primary endpoint was time to platelet recovery, defined as the interval from the first platelet count $<50\times10^{9}$/L to the first of two consecutive platelet counts ≥50×10^9/L.Platelet transfusion outcomes were also evaluated.Patients were stratified by CAR-HEMATOTOX score, and prespecified subgroup analyses were performed to compare outcomes.

**Copyright Information:** (Online) ISSN: 2572-9241
©2026 The Author(s). HemaSphere is published by John Wiley & Sons Ltd on behalf of European Hematology Association. This is an open access Abstract Book distributed under the Attribution-NonCommercial-NoDerivs (CC BY-NC-ND), which allows third parties to download the articles and share them with others as long as they credit the author and the Abstract Book, but they cannot change the content in any way or use them commercially.
**Abstract Book Citations:** Authors, Title, HemaSphere, 2026;10:(S1):pages. The abstract book DOIs can be found on the "Author Information" page.
**Disclaimer:** Articles published in the journal HemaSphere exclusively reflect the opinions of the authors. The authors are responsible for all content in their abstracts including accuracy of the facts, statements, citing resources, etc.

**Results**

From June 2021 to August 2024,88 patients who developed grade ≥3 thrombocytopenia during the peri–CAR T-cell period and received rhTPO were enrolled.Among them,65.9% received bridging therapy before infusion.

rhTPO was initiated before infusion in 7/88 patients and after infusion in 81/88,while most patients (79.4%) started treatment within 0–10 days post-infusion.The median duration of rhTPO treatment was 20 days.By day 28 after infusion,44/88(50.0%) achieved platelet recovery.Median time to recovery was 14 days from infusion.During rhTPO treatment,13.6% of patients required no platelet transfusions,while among the remaining,the median number of platelet units transfused was 4.

Patients were stratified by CAR-HEMATOTOX score into low-risk(0–1) and high-risk(≥2) groups.Compared with the low-risk group,high-risk patients had a longer time to platelet recovery,longer duration of platelet count<$50\times10^9$/L,lower platelet nadir,more platelet transfusion units,and longer rhTPO treatment duration(all P<0.01).These findings suggest that the CAR-HEMATOTOX score may help predict the severity of thrombocytopenia during CAR T-cell therapy,and that high-risk patients may require earlier and more intensive platelet-directed intervention.

Transfusion burden was further evaluated by baseline platelet strata.Patients with a pre-lymphodepletion platelet count <$100\times10^9$/L required more platelet transfusions than those with counts ≥$100\times10^9$/L (P=0.0008). Similarly, patients with a platelet count <$30\times10^9$/L at rhTPO initiation had a higher transfusion burden than those with counts ≥$30\times10^9$/L(P=0.0005).Overall,lower baseline platelet counts before lymphodepletion and at rhTPO initiation were associated with greater platelet transfusion requirements during treatment, supporting earlier platelet-directed intervention in patients with very low baseline platelet counts.

**Summary/Conclusion**

In this retrospective cohort, rhTPO was a feasible option for grade ≥3 thrombocytopenia during the peri–CAR T-cell period,with half achieved platelet recovery by day 28,and 13.6% remained free of platelet transfusion,indicating a potential clinical benefit through reduced exposure to transfusion-related risks.Higher CAR-HEMATOTOX risk and lower baseline platelet counts were associated with delayed recovery and greater transfusion burden, supporting earlier and more intensive platelet-directed management in high-risk patients.

**Copyright Information:** (Online) ISSN: 2572-9241
©2026 The Author(s). HemaSphere is published by John Wiley & Sons Ltd on behalf of European Hematology Association. This is an open access Abstract Book distributed under the Attribution-NonCommercial-NoDerivs (CC BY-NC-ND), which allows third parties to download the articles and share them with others as long as they credit the author and the Abstract Book, but they cannot change the content in any way or use them commercially.
**Abstract Book Citations:** Authors, Title, HemaSphere, 2026;10:(S1):pages. The abstract book DOIs can be found on the "Author Information" page.
**Disclaimer:** Articles published in the journal HemaSphere exclusively reflect the opinions of the authors. The authors are responsible for all content in their abstracts including accuracy of the facts, statements, citing resources, etc.

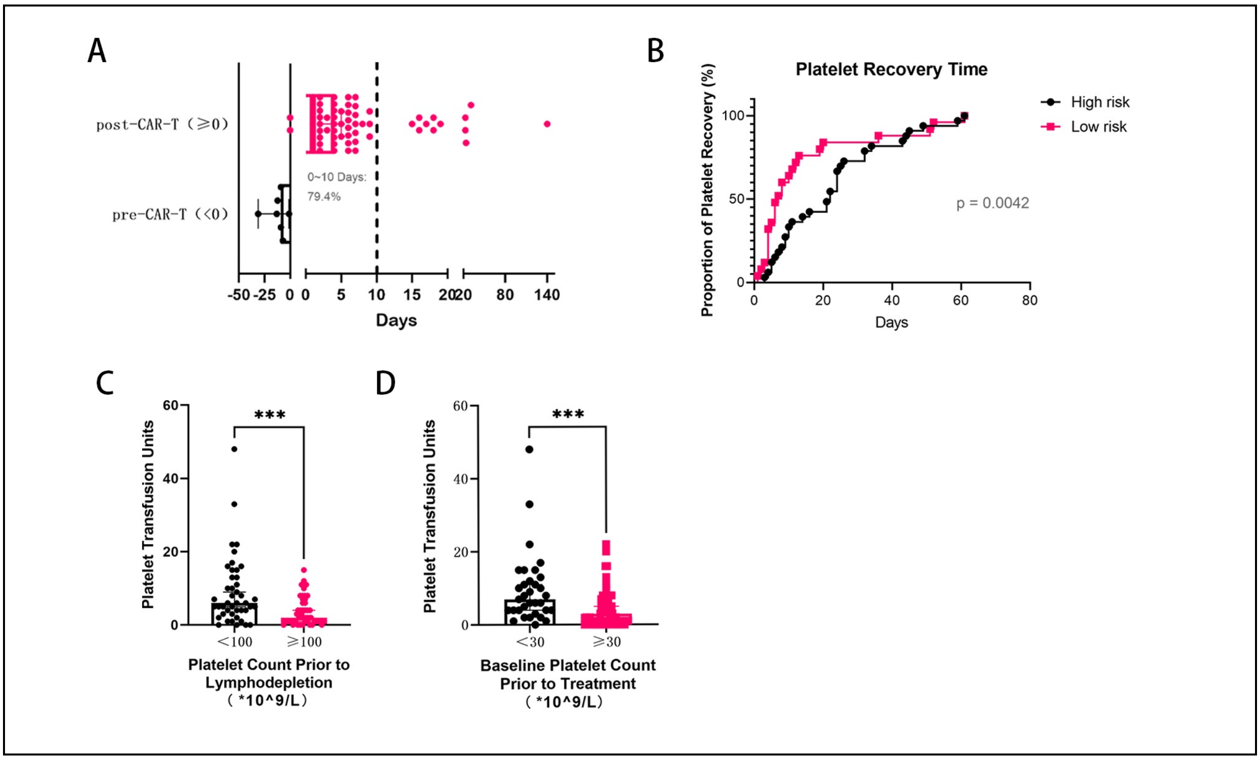

Figure 1 a)Timing of rhTPO initiation relative to CAR T-cell infusion. b)Time to platelet recovery by CAR-HEMATOTOX risk group. c)Platelet transfusion units stratified by pre-lymphodepletion platelet count. d)Platelet transfusion units stratified by platelet count at rhTPO initiation.

**Copyright Information:** (Online) ISSN: 2572-9241
©2026 The Author(s). HemaSphere is published by John Wiley & Sons Ltd on behalf of European Hematology Association. This is an open access Abstract Book distributed under the Attribution-NonCommercial-NoDerivs (CC BY-NC-ND), which allows third parties to download the articles and share them with others as long as they credit the author and the Abstract Book, but they cannot change the content in any way or use them commercially.
**Abstract Book Citations:** Authors, Title, HemaSphere, 2026;10:(S1):pages. The abstract book DOIs can be found on the "Author Information" page.
**Disclaimer:** Articles published in the journal HemaSphere exclusively reflect the opinions of the authors. The authors are responsible for all content in their abstracts including accuracy of the facts, statements, citing resources, etc.

### (PS2348) RELEVANT STUDY BETWEEN CYTOKINE LEVEL OF PATIENTS TREATED WITH CD7 CAR-T THERAPY AND GVHD AFTER ALLOGENEIC HEMATOPOIETIC STEM CELL TRANSPLANTATION

**Topic:** 26. Gene therapy, cellular immunotherapy and vaccination - Clinical

Dongchu Wang*[1], Liyuan Qiu[1], Hui Wang[1]

[1]*Hebei Yanda Lu Daopei Hospital, Langfang, China;*

### Background

CD7 CAR-T therapy has achieved remarkable success in the treatment of T-ALL and T-LBL, with high cure rates. To guarantee treatment outcomes, patients who achieve complete remission (CR) after CD7 CAR-T therapy typically undergo bridging allogeneic hematopoietic stem cell transplantation(allo-HSCT). As the most common complication after allo-HSCT, GVHD is a major cause of mortality. Therefore, predicting post-transplant prognosis is of critical importance.

### Aims

To investigate the correlation between cytokine expression levels during CAR-T therapy and prognosis after bridging allo-HSCT.

### Methods

This study enrolled 54 patients from Hebei Yanda Lu Daopei Hospital who underwent CD7 CAR-T therapy and bridging transplantation between December 2020 and November 2022 . A total of 24 cytokines were measured using CANTO PLUS flow cytometer and microbeads. Statistical analysis was performed using GraphPad Prism 8, and two-way ANOVA was used to analyze the correlation between cytokine levels at 7 different time points and patient prognosis.

### Results

Firstly, this study conducted a horizontal comparison among patients who did not develop GVHD after transplantation(group none), those who developed aGVHD(group aGVHD), and those who developed only cGVHD(group cGVHD). Comparing group none to group aGVHD, statistically significant differences were observed in IL-10(p=0.0282), sCD25(p=0.0079), and TNFRI(p=0.0261) on day 4 post-CAR-T infusion, and in IL-6(p=0.0485) on day 15,see in fig.1. Due to the clinical relevance of aGVHD, ROC curve analysis was performed. On day 15, IL-6 demonstrated an optimal cutoff of 530.2 pg/ml, AUC of 0.9959 (95% CI: 0.9860-1, p<0.0001), with sensitivity of 1.000 and specificity of 0.9565. For IL-10 on day 4, the cutoff was determined as 2.735 pg/ml, AUC of 0.7032 (95% CI: 0.5504-0.8559, p=0.0222), sensitivity of 0.48, and specificity of 0.8947. The cutoff for sCD25 was identified as 359.9 IU/ml, AUC of 0.7432 (95% CI: 0.5924-0.8939, p=0.0062), with sensitivity of 0.84 and specificity of 0.6842. The cutoff for TNFRI was established at 1079 pg/ml, AUC of 0.7347 (95% CI: 0.5874-0.8821, p=0.0082), with sensitivity of 0.60 and specificity of 0.7895(fig.1a,3a). In the comparison between aGVHD and cGVHD patients, statistical significant differences of ST-2, sCD25, and IL-8 in various time points was shown in fig. 2a/b/c. In general, those cytokines were at higher level in group aGVHD. This study further analyzed patients with different grades of aGVHD. Elafin, IL-10, sCD25, IL-8, TNFRI showed significant differences in various grades of aGVHD which need further work(fig.3b). As gastrointestinal GVHD is a common type that often leads to severe outcomes, this study also analyzed varying grades of gastrointestinal GVHD(fig.3c). Finally, the timing of the first onset of aGVHD was concluded as 30(3d.)days after transplantation.

**Copyright Information:** (Online) ISSN: 2572-9241
©2026 The Author(s). HemaSphere is published by John Wiley & Sons Ltd on behalf of European Hematology Association. This is an open access Abstract Book distributed under the Attribution-NonCommercial-NoDerivs (CC BY-NC-ND), which allows third parties to download the articles and share them with others as long as they credit the author and the Abstract Book, but they cannot change the content in any way or use them commercially.
**Abstract Book Citations:** Authors, Title, HemaSphere, 2026;10:(S1):pages. The abstract book DOIs can be found on the "Author Information" page.
**Disclaimer:** Articles published in the journal HemaSphere exclusively reflect the opinions of the authors. The authors are responsible for all content in their abstracts including accuracy of the facts, statements, citing resources, etc.

### Summary/Conclusion

GVHD is a serious complication that typically occurs after transplantation. Therefore, the findings of this study may assist transplant clinicians in predicting potential GVHD post transplantation based on patients' cytokine levels after CAR-T therapy, enabling early preventive measures.

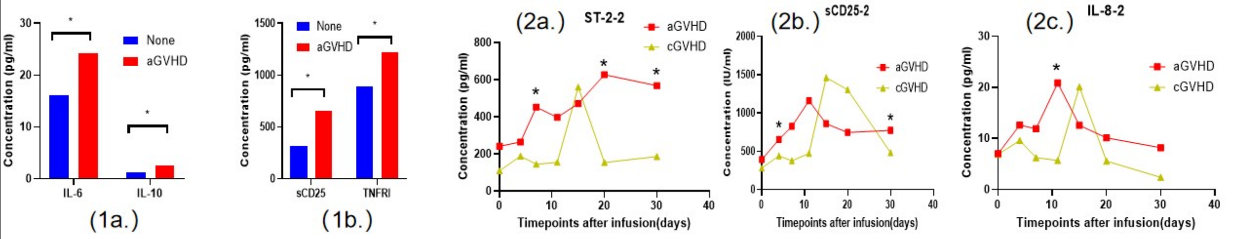

(3a.) Occurrence of GVHD

None vs. aGVHD

| Cytokine | Timepoint | P value | Cutoff value(pg/ml) | AUC | Sensitivity | specificity | 95% confidence interval | P value of ROC |
|---|---|---|---|---|---|---|---|---|
| IL-6 | D15 | 0.0485 | > 530.2 | 0.9959 | 1 | 0.9565 | 0.9860 to 1.000 | <0.0001 |
| IL-10 | D4 | 0.0282 | > 2.735 | 0.7032 | 0.48 | 0.8947 | 0.5504 to 0.8559 | 0.0222 |
| sCD25 | D4 | 0.0079 | > 359.9 | 0.7432 | 0.84 | 0.6842 | 0.5924 to 0.8939 | 0.0062 |
| TNFRI | D4 | 0.0261 | > 1079 | 0.7347 | 0.6 | 0.7895 | 0.5874 to 0.8821 | 0.0082 |

aGVHD vs. cGVHD

| Cytokine | Timepoint | P value |
|---|---|---|
| ST-2 | D7 | 0.0261 |
| | D20 | 0.0157 |
| | D30 | 0.0041 |
| sCD25 | D4 | 0.0139 |
| | D30 | 0.0074 |
| IL-8 | D11 | 0.0027 |

(3b.) Grade of aGVHD

| Cytokine | TIME P value | FACTOR P value |
|---|---|---|
| Elafin | | 0.0489 |
| IL-10 | 0.0248 | |
| sCD25 | 0.0074 | 0.0182 |
| IL-8 | | |
| TNFRI | | 0.0431 |

(3c.) Grade of GI tract GVHD

| | Timepoint | Grade2 vs. Grade3 | Grade2 vs. Grade4 | Grade3 vs. Grade4 |
|---|---|---|---|---|
| IL-10 | D7 | | | 0.0281 |
| IL-8 | D15 | | 0.0162 | |
| MCP-1 | D20 | | | 0.0337 |
| Granzyme B | D4 | 0.0352 | 0.0361 | |
| | D7 | 0.0004 | 0.0013 | |
| TNFRI | D20 | | 0.0462 | |

(3d.) Occurence time of aGVHD

| Grade | Timepoint |
|---|---|
| Grade 1 | 34.5 |
| Grade 2 | 12.5 |
| Grade 3 | 46 |
| Grade 4 | 25.5 |

**Copyright Information:** (Online) ISSN: 2572-9241
©2026 The Author(s). HemaSphere is published by John Wiley & Sons Ltd on behalf of European Hematology Association. This is an open access Abstract Book distributed under the Attribution-NonCommercial-NoDerivs (CC BY-NC-ND), which allows third parties to download the articles and share them with others as long as they credit the author and the Abstract Book, but they cannot change the content in any way or use them commercially.
**Abstract Book Citations:** Authors, Title, HemaSphere, 2026;10:(S1):pages. The abstract book DOIs can be found on the “Author Information” page.
**Disclaimer:** Articles published in the journal HemaSphere exclusively reflect the opinions of the authors. The authors are responsible for all content in their abstracts including accuracy of the facts, statements, citing resources, etc.

### (PS2349) TIME FROM CAR-T EVALUATION TO LEUKAPHERESIS IN B-CELL MALIGNANCIES: ASSOCIATION WITH INFUSION RATES AND OUTCOMES

**Topic:** 26. Gene therapy, cellular immunotherapy and vaccination - Clinical

Justin Desroches*[1], Patrick Johnston[1], Paul Hampel[1], Melinda Tan[1], Arushi Khurana[1], Urshila Durani[1], Jonas Paludo[1], Jose C. Villasboas[1], Andre Demenezes Silva Corraes[1], Saad Kenderian[1], Claire Tiger[1], Jithma Abeykoon[1], N.Nora Bennani[1], Stephen Ansell[1], Hassan Alkhateeb[1], Yucai Wang[1], Yi Lin[1]

[1]*Mayo Clinic, Division of Hematology, Rochester, United States of America;*

### Background

Chimeric antigen receptor T-cell (CAR-T) therapy has improved outcomes in relapsed/refractory B-cell malignancies. The pathway from referral to infusion is complex and resource-intensive, encompassing evaluation, leukapheresis, manufacturing, and infusion. With expanding CAR-T use, many payers now require formal review of evaluation data prior to approval, a process that can take up to two weeks and delay leukapheresis. The effect of time from evaluation to leukapheresis on infusion rates and outcomes remains unclear.

### Aims

To evaluate: (1) the proportion of patients proceeding from evaluation to leukapheresis across commercial CAR-T products; (2) the likelihood of infusion based on time from evaluation to leukapheresis (<=2 vs >2 weeks; <=3 vs >3 weeks); and (3) complete response (CR), progression-free survival (PFS), and overall survival (OS) in large B-cell lymphoma (LBCL) patients according to leukapheresis timing.

### Methods

We retrospectively analyzed all patients with B-cell lymphoma evaluated for commercial CAR-T at Mayo Clinic Rochester since 2018. We calculated rates of leukapheresis and infusion. Patients were stratified by time from evaluation to leukapheresis using 2- and 3-week cutoffs. Among patients with LBCL, CR rates, median PFS, and median OS were compared by leukapheresis timing.

### Results

A total of 409 patients underwent evaluation. Leukapheresis rates were high across products: axi-cel 256/284 (90.1%), brexu-cel 46/50 (92%), liso-cel 55/61 (90.2%), and tisa-cel 14/14 (100%). Overall, approximately 10% did not proceed to leukapheresis. The median time from evaluation to leukapheresis was 10 days (IQR 8); 29% of patients underwent leukapheresis beyond 2 weeks and 12% beyond 3 weeks. Among patients with gaps exceeding 2 weeks, insurance-related barriers were the predominant cause, accounting for approximately 70% of cases.
Earlier leukapheresis was associated with higher infusion rates: 91.5% for <=2 weeks vs 83% for >2 weeks (p=0.02), and 90.7% for <=3 weeks vs 77.8% for >3 weeks (p=0.01).
Among patients with LBCL (n=211) who underwent leukapheresis but did not ultimately receive infusion (n=25), median OS was only 2.2 months (95% CI, 1.8–2.5), underscoring the poor prognosis associated with pre-infusion attrition. At a median follow-up of 23 months from infusion, outcomes among infused patients with LBCL were similar regardless of leukapheresis timing. Using a 2-week cutoff, CR rates were 66.7% vs 68.8% (p=0.53), median PFS 13.0 vs 13.6 months (p=0.94), and median OS 29.4 vs 25.5 months (p=0.69). Using a 3-week cutoff, CR rates were 68.5% vs 63.6% (p=0.31), median PFS 15.1 vs 13.6 months (p=0.78), and median OS 29.0 vs 16.0 months (p=0.47). Thus, leukapheresis timing was not significantly associated with CR, PFS, or OS in infused patients.

**Copyright Information:** (Online) ISSN: 2572-9241
©2026 The Author(s). HemaSphere is published by John Wiley & Sons Ltd on behalf of European Hematology Association. This is an open access Abstract Book distributed under the Attribution-NonCommercial-NoDerivs (CC BY-NC-ND), which allows third parties to download the articles and share them with others as long as they credit the author and the Abstract Book, but they cannot change the content in any way or use them commercially.
**Abstract Book Citations:** Authors, Title, HemaSphere, 2026;10:(S1):pages. The abstract book DOIs can be found on the "Author Information" page.
**Disclaimer:** Articles published in the journal HemaSphere exclusively reflect the opinions of the authors. The authors are responsible for all content in their abstracts including accuracy of the facts, statements, citing resources, etc.

**Summary/Conclusion**

In this large single-center cohort, nearly 90% of evaluated patients proceeded to leukapheresis, with a median time to leukapheresis of 10 days. Earlier leukapheresis was associated with a higher likelihood of infusion, and delayed leukapheresis was significantly associated with attrition to treatment. Although patients with delayed leukapheresis who ultimately received CAR-T had comparable CR, PFS, and OS, prognosis was extremely poor among patients with LBCL who did not proceed to infusion, underscoring the importance of minimizing leukapheresis delays.

**Copyright Information:** (Online) ISSN: 2572-9241
©2026 The Author(s). HemaSphere is published by John Wiley & Sons Ltd on behalf of European Hematology Association. This is an open access Abstract Book distributed under the Attribution-NonCommercial-NoDerivs (CC BY-NC-ND), which allows third parties to download the articles and share them with others as long as they credit the author and the Abstract Book, but they cannot change the content in any way or use them commercially.
**Abstract Book Citations:** Authors, Title, HemaSphere, 2026;10:(S1):pages. The abstract book DOIs can be found on the "Author Information" page.
**Disclaimer:** Articles published in the journal HemaSphere exclusively reflect the opinions of the authors. The authors are responsible for all content in their abstracts including accuracy of the facts, statements, citing resources, etc.

### (PS2350) TENACITY-01 A GLOBAL STUDY OF CTD402, ALLOGENEIC ANTI-CD7 CAR T-CELL, IN RELAPSED OR REFRACTORY (R/R) T-CELL ACUTE LYMPHOBLASTIC LEUKEMIA/LYMPHOBLASTIC LYMPHOMA (T-ALL/LBL)

**Topic:** 26. Gene therapy, cellular immunotherapy and vaccination - Clinical

Lori Muffly*[1], Mark Blaine Geyer[2], Alejandro Sica[3], Nitin Jain[4], Haydar Frangoul[5], Maria del Rosario[6], Eileen McNulty[6], Jan Davidson-Moncada[6], Ming Gao[6], Wengang Ge[6], Yajin Ni[6], Jiangtao Ren[6], Haley Irons[6]

*[1]Stanford Medicine, Palo Alto, United States of America; [2]Memorial Sloan Kettering Cancer Center, New York, NY, United States of America; [3]Montefiore Medical Center, New York, United States of America; [4]MD Anderson Cancer Center, Houston, United States of America; [5]Sarah Cannon Research Institute and The Children's Hospital at TriStar Centennial, Nashville, United States of America; [6]Imviva Biotech, Wilmington, United States of America;*

### Background

Few therapeutic options exist for patients (pts) with relapsed refractory (R/R) T-cell acute lymphoblastic leukemia/lymphoblastic lymphoma (T-ALL/LBL). CTD402 is a CD7 targeted “off-the-shelf” universal CAR-T product with T cell receptor and human leukocyte antigen class II knockout and proprietary ANSWERTM inhibitory ligands designed to resist host immune rejection. CTD402 has demonstrated a favorable safety and efficacy profile in R/R T-ALL/LBL, with an identified recommended phase 2 dose (RP2D) of $400\times10^6$ CAR-T+ cells (Lu et al ASH 2025). These early phase data supported the launch of a global Phase 1b/2 study of CTD402 in R/R T-ALL/LBL-TENACITY-01 (NCT#07070219). Herein we present preliminary data from TENACITY-01 global study.

### Methods

Pts 12 years and older with R/R T-ALL/LBL meeting eligibility criteria receive standard lymphodepletion with fludarabine 30mg/m2/day and cyclophosphamide 500mg/m2/day on Days -5 to –3 followed by a single infusion of CTD402 at the RP2D on Day 0. The primary objectives are safety and efficacy as defined by the rate of overall complete remission (OCR) defined as achieving complete response (CR), CR with partial hematologic recovery (CRh) or CR with incomplete hematologic recovery (CRi).

**Copyright Information:** (Online) ISSN: 2572-9241
©2026 The Author(s). HemaSphere is published by John Wiley & Sons Ltd on behalf of European Hematology Association. This is an open access Abstract Book distributed under the Attribution-NonCommercial-NoDerivs (CC BY-NC-ND), which allows third parties to download the articles and share them with others as long as they credit the author and the Abstract Book, but they cannot change the content in any way or use them commercially.
**Abstract Book Citations:** Authors, Title, HemaSphere, 2026;10:(S1):pages. The abstract book DOIs can be found on the “Author Information” page.
**Disclaimer:** Articles published in the journal HemaSphere exclusively reflect the opinions of the authors. The authors are responsible for all content in their abstracts including accuracy of the facts, statements, citing resources, etc.

**Results**

As of February 28th, 2026, two pts with R/R T-ALL/LBL have been treated with CTD402 in the global TENACITY-01 study. The first pt was a 49-year-old female with a history of R/R T-ALL/LBL who had received 8 prior lines of therapy, including hyper CVAD/nelarabine, FLAG-Ida/venetoclax/asparaginase, decitabine/venetoclax/daratumumab, experimental BCL2/BCLXL inhibitor, POMP, nelarabine/etoposide/cyclophosphamide, and an experimental DNA alkylating agent. At screening, she had involvement of T-ALL/LBL in the bone marrow (62% blasts), peripheral blood, lymph nodes both above and below the diaphragm, skin, and breasts. Following treatment at the RP2D, she experienced grade 1 cytokine release syndrome (CRS) and grade 1 immune effector cell associated hemophagocytic lymphohistiocytosis-like syndrome (IEC-HS); both resolved with therapy. Day 28 response assessment demonstrated CRh with undetectable measurable residual disease (MRD) by flow cytometry and resolution of PET avid uptake. She received consolidative allogeneic hematopoietic cell transplant (allo-HCT) from an unrelated donor and Day 90 post-CAR-T assessment demonstrated ongoing MRD-negative CR.

The second pt was a 30-year-old male with a history of R/R T-ALL/LBL with mutated *TP53* who had received 5 prior lines of therapy including CALGB10403, nelarabine/etoposide/cyclophosphamide, FLAG-Ida, allo-HCT, and mini hyperCVD/venetoclax. At screening, he had involvement of T-ALL/LBL in the bone marrow (52% blasts), peripheral blood, and lymph nodes both above and below the diaphragm, with malignant pericardial and pleural effusions. Following treatment at the RP2D, he experienced grade 2 CRS and grade 2 IEC-HS; both resolved with therapy. Day 28 response assessment demonstrated CRi with undetectable MRD by flow cytometry. He is preparing to receive allo-HCT from an unrelated donor.

**Summary/Conclusion**

CTD402 is a universal CD7 targeted CAR-T with a cumulative experience demonstrating an acceptable safety profile and preliminary evidence of robust anti-leukemic activity in the first two pts treated on the TENACITY-01 trial. Enrollment in the global program is ongoing and supports the development of CTD402 as an “off-the-shelf”, point-of-care-ready CAR-T cell therapy for heavily pre-treated pts with R/R T-ALL/LBL.

**Copyright Information:** (Online) ISSN: 2572-9241
©2026 The Author(s). HemaSphere is published by John Wiley & Sons Ltd on behalf of European Hematology Association. This is an open access Abstract Book distributed under the Attribution-NonCommercial-NoDerivs (CC BY-NC-ND), which allows third parties to download the articles and share them with others as long as they credit the author and the Abstract Book, but they cannot change the content in any way or use them commercially.
**Abstract Book Citations:** Authors, Title, HemaSphere, 2026;10:(S1):pages. The abstract book DOIs can be found on the “Author Information” page.
**Disclaimer:** Articles published in the journal HemaSphere exclusively reflect the opinions of the authors. The authors are responsible for all content in their abstracts including accuracy of the facts, statements, citing resources, etc.

### (PS2351) COMPARATIVE EFFICACY AND SAFETY OF BCMA-TARGETED VERSUS GPRC5D-TARGETED CAR-T CELL THERAPY IN RELAPSED/REFRACTORY MULTIPLE MYELOMA: A META-ANALYSIS

**Topic:** 26. Gene therapy, cellular immunotherapy and vaccination - Clinical

Yanling Li*[1, 2], Ling Qiu[1], Mengjiao Li[1, 2], Jiajia Li[1, 2], Shihui Ren[1], Dan Chen[1], Xiao Wang[1, 3], Mingqiang Ren[4], Hao Yao[1, 3, 5, 6], Fangyi Fan[1, 2]

*[1]Department of Hematology, The General Hospital of Western Theater Command, Chengdu, China;[2]Department of Clinical Medicine, North Sichuan Medical College, Nanchong, China; [3]College of Medicine, Southwest Jiaotong University, Chengdu, China; [4]Department of Hematology, Affiliated Hospital of Zunyi Medical Univercity, Zunyi, China;[5]Institute of Basic Medicine, North Sichuan Medical College, Nanchong, China; [6]Tissue Stress Injury and Functional Repair Key Laboratory of Sichuan Province, Chengdu, China;*

### Background

B-cell maturation antigen (BCMA)- and G protein–coupled receptor class C group 5 member D (GPRC5D)-targeted chimeric antigen receptor (CAR) T-cell therapies have demonstrated promising efficacy in relapsed/refractory multiple myeloma (R/RMM). However, comparative evidence regarding their relative efficacy and safety remains limited.

### Aims

This meta-analysis aimed to systematically evaluate and indirectly compare the efficacy and safety of BCMA-targeted versus GPRC5D-targeted CAR-T therapy in patients with R/RMM.

### Methods

A systematic search of PubMed, Embase, Web of Science, and the Cochrane Library was conducted for English-language studies published up to January 13, 2026. Eligible studies were selected based on predefined inclusion and exclusion criteria. Study quality was assessed using the Newcastle–Ottawa Scale (NOS) and the Methodological Index for Non-Randomized Studies (MINORS). Patients were categorized into BCMA-targeted and GPRC5D-targeted CAR-T groups. Pooled response rates and toxicity outcomes were calculated using a random-effects model in RStudio.

**Copyright Information:** (Online) ISSN: 2572-9241
©2026 The Author(s). HemaSphere is published by John Wiley & Sons Ltd on behalf of European Hematology Association. This is an open access Abstract Book distributed under the Attribution-NonCommercial-NoDerivs (CC BY-NC-ND), which allows third parties to download the articles and share them with others as long as they credit the author and the Abstract Book, but they cannot change the content in any way or use them commercially.
**Abstract Book Citations:** Authors, Title, HemaSphere, 2026;10:(S1):pages. The abstract book DOIs can be found on the “Author Information” page.
**Disclaimer:** Articles published in the journal HemaSphere exclusively reflect the opinions of the authors. The authors are responsible for all content in their abstracts including accuracy of the facts, statements, citing resources, etc.

**Results**

A total of 62 studies comprising 4,645 patients were included. The pooled complete response (CR) and overall response rate (ORR) across all studies were 0.49 (95% CI 0.44–0.53) and 0.86 (95% CI 0.84–0.89), respectively. In subgroup analyses, pooled CR and ORR were 0.40 (95% CI 0.29–0.52) and 0.86 (95% CI 0.71–1.00) in the GPRC5D CAR-T group, and 0.49 (95% CI 0.45–0.54) and 0.86 (95% CI 0.84–0.89) in the BCMA CAR-T group. The pooled incidence of grade ≥3 cytokine release syndrome (CRS) and immune effector cell-associated neurotoxicity syndrome (ICANS) across all studies was 0.09 (95% CI 0.06–0.12) and 0.04 (95% CI 0.03–0.05), respectively.In subgroup analyses, grade ≥3 CRS occurred in 0.06 (95% CI 0.00–0.12) of patients receiving GPRC5D-targeted CAR-T therapy and 0.09 (95% CI 0.06–0.12) in the BCMA-targeted CAR-T group. Grade ≥3 ICANS occurred in 0.06 (95% CI 0.00–0.29) in the GPRC5D CAR-T group and 0.04 (95% CI 0.03–0.05) in the BCMA CAR-T group. For grade <3 toxicities, pooled CRS and ICANS rates were 0.83 (95% CI 0.62–1.00) and 0.03 (95% CI 0.00–0.14) in the GPRC5D group, and 0.75 (95% CI 0.71–0.80) and 0.15 (95% CI 0.09–0.20) in the BCMA group. Because only a single study reported grade <3 CRS and ICANS in the GPRC5D subgroup, these estimates should be interpreted with caution and formal statistical comparison between subgroups was not feasible. Overall, ORR appeared comparable between targets, whereas a higher pooled CR rate was observed in the BCMA subgroup. Differences in CRS and ICANS incidence were noted across subgroups; however, these findings are based on indirect cross-study comparisons.

**Summary/Conclusion**

This meta-analysis suggests that BCMA- and GPRC5D-targeted CAR-T therapies achieve comparable overall response rates in R/RMM, while BCMA-targeted CAR-T appears to be associated with higher pooled complete response rates. Distinct toxicity patterns were observed between targets, with differences in the incidence of CRS and ICANS across severity grades. Given the indirect nature of cross-study comparisons and limited data in certain subgroups, these findings should be interpreted with caution. Prospective head-to-head studies are warranted to better define the optimal CAR-T target in R/RMM.

**Copyright Information:** (Online) ISSN: 2572-9241
©2026 The Author(s). HemaSphere is published by John Wiley & Sons Ltd on behalf of European Hematology Association. This is an open access Abstract Book distributed under the Attribution-NonCommercial-NoDerivs (CC BY-NC-ND), which allows third parties to download the articles and share them with others as long as they credit the author and the Abstract Book, but they cannot change the content in any way or use them commercially.
**Abstract Book Citations:** Authors, Title, HemaSphere, 2026;10:(S1):pages. The abstract book DOIs can be found on the “Author Information” page.
**Disclaimer:** Articles published in the journal HemaSphere exclusively reflect the opinions of the authors. The authors are responsible for all content in their abstracts including accuracy of the facts, statements, citing resources, etc.

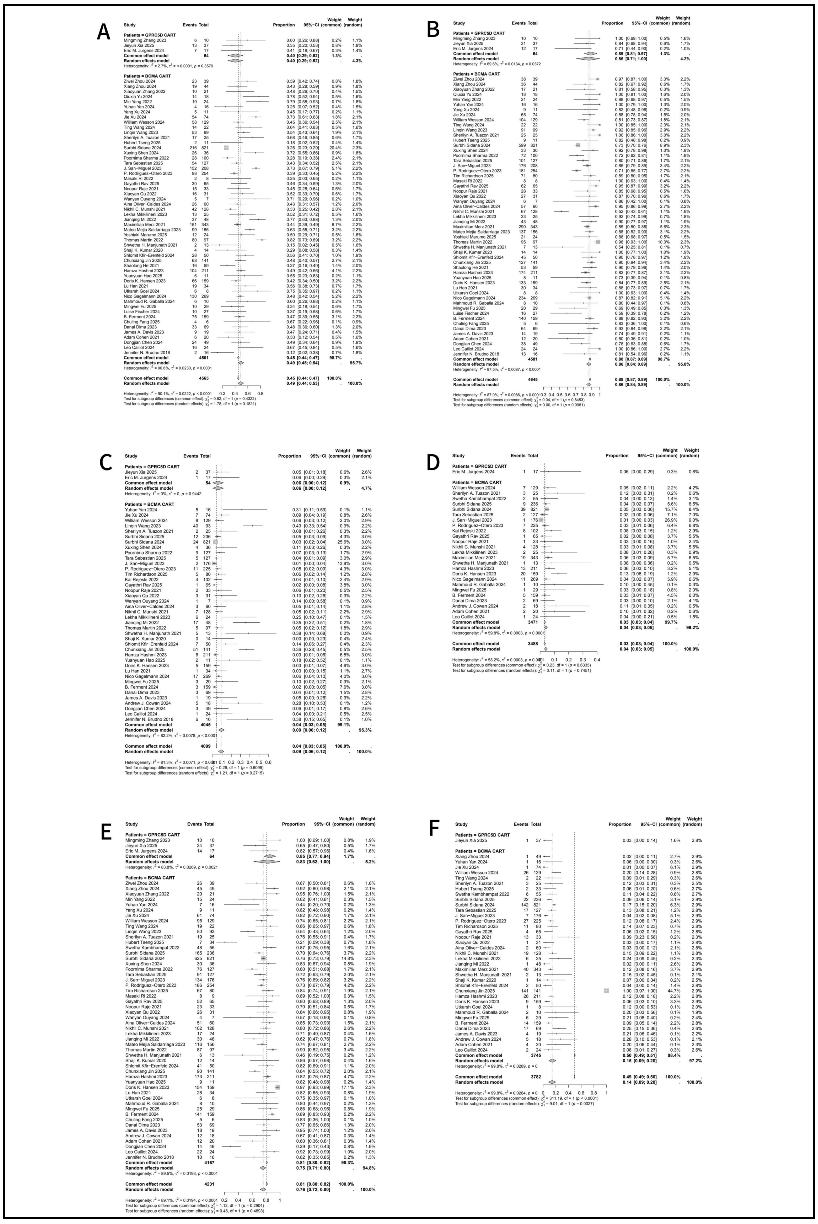

Figure 1. Pooled efficacy and safety outcomes for BCMA-targeted and GPRC5D-targeted CAR-T therapies in relapsed/refractory multiple myeloma (R/RMM). (A) Complete response (CR) rates; (B) Overall response rate (ORR); (C) Incidence of grade ≥3 cytokine release syndrome (CRS); (D) Incidence of grade ≥3 immune effector cell-associated neurotoxicity syndrome (ICANS); (E) Incidence of grade <3 CRS; (F) Incidence of grade <3 ICANS. Each black square represents the effect estimate from an individual study, with horizontal lines indicating 95% confidence intervals. Pooled effect estimates (diamonds) were calculated using a random-effects model.

**Copyright Information:** (Online) ISSN: 2572-9241
©2026 The Author(s). HemaSphere is published by John Wiley & Sons Ltd on behalf of European Hematology Association. This is an open access Abstract Book distributed under the Attribution-NonCommercial-NoDerivs (CC BY-NC-ND), which allows third parties to download the articles and share them with others as long as they credit the author and the Abstract Book, but they cannot change the content in any way or use them commercially.
**Abstract Book Citations:** Authors, Title, HemaSphere, 2026;10:(S1):pages. The abstract book DOIs can be found on the “Author Information” page.
**Disclaimer:** Articles published in the journal HemaSphere exclusively reflect the opinions of the authors. The authors are responsible for all content in their abstracts including accuracy of the facts, statements, citing resources, etc.

### (PS2352) LATER CAR-T INFUSIONS INCREASE CYTOKINE RELEASE SYNDROME (CRS): EVIDENCE FOR CIRCADIAN MODULATION OF IMMUNOTHERAPY-RELATED TOXICITY

**Topic:** 26. Gene therapy, cellular immunotherapy and vaccination - Clinical

Danielle Decicco*[1], Sanjana Nethagani[1], Jarom Gropp[1], Crystal Peck[1], Lindsey Gladish[1], Aaron Cumpston[1], Konstantinos Sdrimas[1], Salah Ud Din Safi[1], Lauren Veltri[1], William Walker[2], Ashkan Emadi[1]

*[1]West Virginia University, Medical Oncology and WVU Cancer Institute, Morgantown, United States of America;[2]West Virginia University, Department of Neuroscience, Morgantown, United States of America;*

### Background

Circadian rhythms regulate adaptive and innate immune responses through cell-intrinsic molecular clocks in T cells and macrophages, with core clock genes modulating T-cell activation, cytokine production, and inflammatory transcriptional programs. Proinflammatory mediators central to CRS exhibit diurnal oscillation, and macrophage cytokine transcription is under circadian control. Emerging clinical evidence indicates that chimeric antigen receptor (CAR)-T cell infusion timing may influence outcomes: later infusion times have been associated with inferior progression-free survival (PFS) and higher inflammatory markers (Luan, *Blood* 2025), and increased risks of mortality and neurotoxicity (Lyons, *JCI Insight* 2025). Mechanistic studies further demonstrate circadian regulation of $CD8^+$ T-cell trafficking and function that can alter immunotherapy efficacy (Wang, *Cell* 2024). Whether infusion timing directly modulates CRS or immune effector cell–associated neurotoxicity syndrome (ICANS) remains unclear. We hypothesized that circadian timing of CAR-T administration influences treatment-related inflammatory toxicity.

### Aims

To evaluate whether CAR-T infusion time-of-day (and apheresis time) is associated with CRS/ICANS in adults receiving commercial CAR-T.

### Methods

We conducted an IRB-approved retrospective study of 96 adults receiving commercially available CAR-T at a tertiary center (Jan 2018–Dec 2025). Infusion time was analyzed dichotomously (AM 00:00-11:59 vs PM 12:00-23:59) and continuously by clock-hour. The primary endpoint was CRS (any grade). Secondary endpoints included CRS ≥3, ICANS (any grade), tocilizumab and/or steroid use, complete remission (CR), PFS, and overall survival (OS). Multivariable logistic regression adjusted for age, sex, performance status, CAR-T dose, and product type; interaction and Bayesian subgroup analyses were exploratory.

### Results

Seventy-nine patients received AM infusions and 17 PM. Baseline characteristics were balanced and lymphodepletion regimens were uniform. CRS occurred in 55% of AM infusions and 88% of PM infusions (p=0.013). After multivariable adjustment, PM infusion remained independently associated with increased CRS risk (adjusted odds ratio (aOR) 8.15, 95% confidence interval (CI) 1.45-45.78; p=0.017; number needed to harm of 3). Tocilizumab and/or steroid use was more frequent with PM infusion (88% vs 59%; aOR 5.11, 95% CI 1.09-23.97; p=0.027). Modeled continuously, each hour later infusion was associated with 82% increased CRS odds (aOR 1.82, 95% CI 1.14–2.90; p=0.012). CR on days 30 or 60 was numerically lower after PM infusion (31% vs 60%; OR 0.30, 95% CI 0.083-1.09, p=0.072). PFS did not differ significantly, though there was a trend toward worsening PFS in the PM (HR ~1.25). Infusion timing was not associated with CRS/ICANS severity or OS. Exploratory analyses suggested larger toxicity effects in diffuse large B-cell lymphoma (but not multiple myeloma) and in males, and possible amplification with PM apheresis; PM apheresis was numerically associated with a higher rate of severe CRS or ICANS (46% vs 19%; OR 3.7, p=0.067).

**Copyright Information:** (Online) ISSN: 2572-9241
©2026 The Author(s). HemaSphere is published by John Wiley & Sons Ltd on behalf of European Hematology Association. This is an open access Abstract Book distributed under the Attribution-NonCommercial-NoDerivs (CC BY-NC-ND), which allows third parties to download the articles and share them with others as long as they credit the author and the Abstract Book, but they cannot change the content in any way or use them commercially.
**Abstract Book Citations:** Authors, Title, HemaSphere, 2026;10:(S1):pages. The abstract book DOIs can be found on the "Author Information" page.
**Disclaimer:** Articles published in the journal HemaSphere exclusively reflect the opinions of the authors. The authors are responsible for all content in their abstracts including accuracy of the facts, statements, citing resources, etc.

**Summary/Conclusion**

Later CAR-T infusion time was independently associated with increased CRS incidence with an hour-dependent gradient consistent with circadian modulation. Because infusion timing is a modifiable exposure, prospective chronotherapy trials should test whether earlier scheduling reduces inflammatory toxicity without compromising clinical benefit.

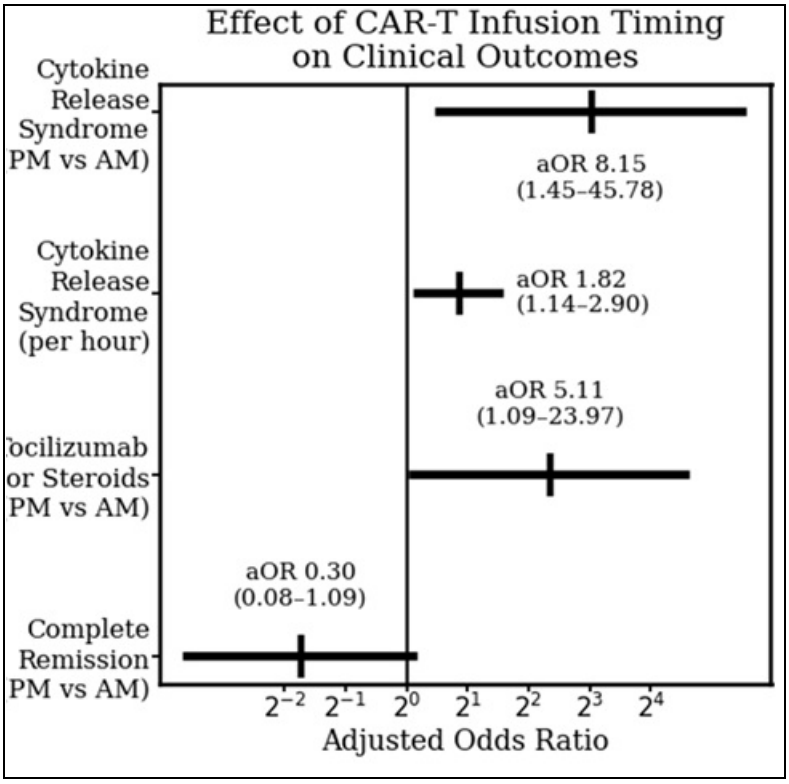

**Copyright Information:** (Online) ISSN: 2572-9241
©2026 The Author(s). HemaSphere is published by John Wiley & Sons Ltd on behalf of European Hematology Association. This is an open access Abstract Book distributed under the Attribution-NonCommercial-NoDerivs (CC BY-NC-ND), which allows third parties to download the articles and share them with others as long as they credit the author and the Abstract Book, but they cannot change the content in any way or use them commercially.
**Abstract Book Citations:** Authors, Title, HemaSphere, 2026;10:(S1):pages. The abstract book DOIs can be found on the "Author Information" page.
**Disclaimer:** Articles published in the journal HemaSphere exclusively reflect the opinions of the authors. The authors are responsible for all content in their abstracts including accuracy of the facts, statements, citing resources, etc.

### (PS2353) REAL WORLD EVALUATION OF ENTEROCOLITIS IN CILTA-CEL TREATED PATIENTS WITH RELAPSED OR REFRACTORY MULTIPLE MYELOMA

**Topic:** 26. Gene therapy, cellular immunotherapy and vaccination - Clinical

Doris Hansen*[1], Omar Castaneda Puglianini[1], Sabyasachi Ghosh[2], Saurabh Nagar[2], Kevin De Braganca[2], Victoria Alegria[2], Mukta Sharma[3], Matthew Perciavalle[4], Shuchita Kaila[2], Jessica Maitland[5], Bruno Emond[6], Zaina Qureshi[2], Murali Janakiram[7]

*[1]Moffitt Cancer Center, Tampa, United States of America; [2]Johnson & Johnson, Horsham, United States of America; [3]Johnson & Johnson, Raritan, United States of America; [4]Legend Biotech USA Inc, Somerset, United States of America; [5]Analysis Group, ULC, Toronto, Canada; [6]Analysis Group, ULC, Montreal, Canada; [7]City of Hope National Medical Center, Duarte, United States of America;*

### Background

Ciltacabtagene autoleucel (cilta-cel), a B-cell maturation chimeric antigen receptor T-cell (CAR-T) therapy, was approved by the Food and Drug Administration (FDA) for patients with relapsed/refractory multiple myeloma (RRMM) after ≥1 prior line of therapy (LOT) following the pivotal CARTITUDE-1 and CARTITUDE-4 trials demonstrating high response rates and prolonged overall survival. The characterization of enterocolitis (EC) post-CAR-T infusion in clinical practice is evolving, with recent reports documenting immune effector cell-associated (IEC)-EC as a distinct but rare adverse event occurring in about 2% of cilta-cel patients (1). These observations underscore the need for improved understanding of current diagnosis and management of these events in the real world.

### Aims

To assess the incidence and management of EC among patients with RRMM treated with cilta-cel in clinical practice.

### Methods

This retrospective cohort study included adult patients with RRMM receiving cilta-cel after ≥1 prior LOT, consistent with the approved indication (02/28/2022-06/30/2025 for patients with ≥4 prior LOT aligning with CARTITUDE-1; 04/05/2024-06/30/2025 for patients with 1-3 prior LOT aligning with CARTITUDE-4). Electronic medical records from Loopback Analytics with physician notes were used, capturing patients with commercial use of cilta-cel, with dose, timing, and route of administration consistent with the US label. New-onset EC post-infusion was defined as ≥2 diagnosis codes on distinct days within 30 days, with confirmation in physician notes, and without any EC diagnoses in the 12 months prior to infusion. This study focused on EC as the diagnoses for IEC-EC may not have been consistently adopted.

**Copyright Information:** (Online) ISSN: 2572-9241
©2026 The Author(s). HemaSphere is published by John Wiley & Sons Ltd on behalf of European Hematology Association. This is an open access Abstract Book distributed under the Attribution-NonCommercial-NoDerivs (CC BY-NC-ND), which allows third parties to download the articles and share them with others as long as they credit the author and the Abstract Book, but they cannot change the content in any way or use them commercially.
**Abstract Book Citations:** Authors, Title, HemaSphere, 2026;10:(S1):pages. The abstract book DOIs can be found on the "Author Information" page.
**Disclaimer:** Articles published in the journal HemaSphere exclusively reflect the opinions of the authors. The authors are responsible for all content in their abstracts including accuracy of the facts, statements, citing resources, etc.

**Results**

Among 345 patients receiving cilta-cel (median [range] age: 66 [37-83] years; 44% female), over a median (range) follow-up of 11.9 (0.03-35.5) months, EC was reported in 5 patients (1.4%).

Of the 5 patients with EC, median (range) age was 65 (51-68) years, 20% were female, 60% had Eastern Cooperative Oncology Group (ECOG) performance status of 1, 60% had high-risk cytogenetics, and 20% had extramedullary disease.

Median (range) time to EC onset was 130 (38-238) days following infusion; 1 patient had EC of grade 1 and 4 patients had unknown grade. EC-related management strategies included corticosteroids (100%), tumor necrosis factor (TNF)-α inhibitors (60%), and Janus kinase (JAK) inhibitors (20%). All 5 patients had an improvement in EC within a median (range) of 46 (5-83) days following EC onset, 60% had full resolution within median (range) of 50 (17-85) days following EC onset, and 1 patient experienced EC recurrence (grade ≥3).

Over a median (range) follow-up of 294 (121-709) days following cilta-cel infusion, all 5 patients achieved at least partial response to cilta-cel, with 60% achieving complete response, and all patients were alive and treatment-free at the end of follow-up.

**Summary/Conclusion**

In this real-world study of patients with RRMM treated with cilta-cel, the observed incidence of EC was 1.4%. Management strategies included corticosteroids, TNF-α inhibitors, and JAK inhibitors. All patients with EC were alive and treatment-free at last follow-up. Longer follow-up and comprehensive assessments of patient-reported outcomes are needed to characterize the overall clinical impact. These initial findings support the long-term benefit-risk profile of cilta-cel.

1. https://doi.org/10.1038/s41408-024-01167-8

**Copyright Information:** (Online) ISSN: 2572-9241
©2026 The Author(s). HemaSphere is published by John Wiley & Sons Ltd on behalf of European Hematology Association. This is an open access Abstract Book distributed under the Attribution-NonCommercial-NoDerivs (CC BY-NC-ND), which allows third parties to download the articles and share them with others as long as they credit the author and the Abstract Book, but they cannot change the content in any way or use them commercially.
**Abstract Book Citations:** Authors, Title, HemaSphere, 2026;10:(S1):pages. The abstract book DOIs can be found on the "Author Information" page.
**Disclaimer:** Articles published in the journal HemaSphere exclusively reflect the opinions of the authors. The authors are responsible for all content in their abstracts including accuracy of the facts, statements, citing resources, etc.

### (PS2354) IMMUNE EFFECTOR CELL–ASSOCIATED HEMOPHAGOCYTIC LYMPHOHISTIOCYTOSIS FOLLOWING CAR T-CELL THERAPY: RESULTS OF A SINGLE-CENTER REAL-WORLD ANALYSIS

**Topic:** 26. Gene therapy, cellular immunotherapy and vaccination - Clinical

Inna Shaforostova*[1], Marie-Noëlle Kronig[1], Katja Seipel[2], Alicia Rovó[3], Ulrike Bacher[3], Thomas Pabst[1]

*[1]Inselspital Bern University Hospital, Medical Oncology, Bern, Switzerland; [2]University of Bern, Department for Biomedical Research, Bern, Switzerland; [3]Inselspital Bern University Hospital, Department of Hematology, Bern, Switzerland;*

### Background

Immune-effector-cell-associated hemophagocytic lymphohistiocytosis (IEC-HLH) is a rare, life-threatening complication following CAR-T-cell therapy. Diagnostic challenges arise from overlapping features with cytokine release syndrome (CRS), sepsis and the lack of standardized criteria. Clinical data on IEC-HLH remain scarce and are mostly limited to case reports or small patient cohorts.

### Aims

The aim of our study was to analyze the incidence, risk factors, management and outcomes in the single-center CAR-T-cell cohort. IEC-HLH was defined according to American Society for Transplantation and Cellular Therapy (ASTCT) criteria.

### Methods

We retrospectively analyzed 301 patients (pts) treated with CD19 or BCMA CAR-T-cells for hematologic malignancies from Jan 2019 to Jan 2026.

### Results

The median follow-up was 31 months (range, 0.1-79). IEC-HLH was diagnosed in 14 pts (4.7%). Median age was 67 years (range, 39-82), with 3 females and 11 males. Underlying diseases included DLBCL (n=4), multiple myeloma (MM) (n=7), mantle cell lymphoma (n=1), Burkitt lymphoma (BL) (n=1) and B-ALL (n=1). 9/14 pts had high tumor burden before CAR-T-cell infusion. All pts showed elevated serum ferritin (median 1584 µg/L); 10/14 pts had LDH elevation (median 341 U/L). All pts had cytopenias including anemia in 14/14 (median hemoglobin 86 G/L), thrombocytopenia in 9/14 (median platelets 110 G/L), and neutropenia in 7/14 (median neutrophils 1.6 G/L). CAR-T-cell products infused were ciltacabtagene autoleucel (cilta-cel) (4/14)), idecabtagene vicleucel (3/14), axicabtagene ciloleucel (axi-cel) (3/14), brexucabtagene autoleucel (2/14), tisagenlecleucel (1/14) and lisocabtagen maraleucel (1/14).
CRS developed in all pts, with severe CRS in 3/14 pts. Immune effector cell-associated neurotoxicity syndrome (ICANS) was diagnosed in 6/14 pts. IEC-HLH occurred at a median of 10 days post-infusion with severe ICANS in 4/14 pts. Clinical presentation was characterized by hyperferritinemia (median 15,321 µg/L), cytopenias (median leucocytes 1.97 G/L, median platelets 5 G/L), and hepatic dysfunction in all pts. All pts demonstrated high CAR-T-cell expansion in blood (median 3135 copies).
Treatment consisted primarily of corticosteroids (12/14) and anakinra (12/14). Refractory pts received intravenous immunoglobulin (5/14), tocilizumab (3/14), siltuximab (1/14), ruxolitinib (1/14), emapalumab (1/14), or etoposide (1/14). Infectious complications occurred in 11/14 pts: bacterial in 10/14 pts, viral in 3/14 pts (CMV reactivation, influenza), fungal in 2/14 pts (mucormycosis, pulmonary aspergillosis). 4/14 pts had mixed infections. IEC-HLH resolved in 7/14 of cases (median 7 days; range, 3-23).
79% (11/14) of pts (5/14 of those with lymphoma, 5/14 of those with MM and 1/14 with B-ALL) died: 7/14 due to active IEC-HLH and associated infections, 1/14 relapse and infection, 2/14 refractory neurotoxicity and 1/14 sepsis. At the last follow-up, only 3/14 pts were alive, 2/14 had MM and had received ide-cel and cilta-cel, respectively. 1 pt had BL and had received axi-cel. One-year OS was significantly lower in IEC-HLH group versus the entire cohort (46% vs. 69%, p=0.008).

**Copyright Information:** (Online) ISSN: 2572-9241
©2026 The Author(s). HemaSphere is published by John Wiley & Sons Ltd on behalf of European Hematology Association. This is an open access Abstract Book distributed under the Attribution-NonCommercial-NoDerivs (CC BY-NC-ND), which allows third parties to download the articles and share them with others as long as they credit the author and the Abstract Book, but they cannot change the content in any way or use them commercially.
**Abstract Book Citations:** Authors, Title, HemaSphere, 2026;10:(S1):pages. The abstract book DOIs can be found on the “Author Information” page.
**Disclaimer:** Articles published in the journal HemaSphere exclusively reflect the opinions of the authors. The authors are responsible for all content in their abstracts including accuracy of the facts, statements, citing resources, etc.

**Summary/Conclusion**

IEC-HLH occurred in 4.7% of CAR-T recipients and was associated with severe cytopenias, hyperferritinemia, hepatic dysfunction, and high infection rates. Despite intensive immunosuppressive therapy, outcomes remain poor. Early biomarker-driven identification and multicenter studies are needed to improve diagnosis and management of IEC-HLH pts.

**Copyright Information:** (Online) ISSN: 2572-9241
©2026 The Author(s). HemaSphere is published by John Wiley & Sons Ltd on behalf of European Hematology Association. This is an open access Abstract Book distributed under the Attribution-NonCommercial-NoDerivs (CC BY-NC-ND), which allows third parties to download the articles and share them with others as long as they credit the author and the Abstract Book, but they cannot change the content in any way or use them commercially.
**Abstract Book Citations:** Authors, Title, HemaSphere, 2026;10:(S1):pages. The abstract book DOIs can be found on the "Author Information" page.
**Disclaimer:** Articles published in the journal HemaSphere exclusively reflect the opinions of the authors. The authors are responsible for all content in their abstracts including accuracy of the facts, statements, citing resources, etc.

### (PS2355) INTERNATIONAL EXPERT CONSENSUS ON THE USE OF IMMUNOGLOBULIN REPLACEMENT THERAPY FOR SECONDARY IMMUNODEFICIENCY: A MODIFIED DELPHI STUDY

**Topic:** 26. Gene therapy, cellular immunotherapy and vaccination - Clinical

Stephen Jolles[1], Elie Haddad[2, 3], Kai Rejeski[4, 5], Michel Delforge[6], Roberto Ria[7, 8], Leire Solis[9], Silvia Sánchez-Ramón*[9, 10]

*[1]Immunodeficiency Centre for Wales, University Hospital of Wales, Cardiff, United Kingdom;[2]Department of Pediatrics and Department of Microbiology, Immunology and Infectious Diseases, University of Montréal, Montréal, Canada; [3]Centre Hospitalier Universitaire Sainte-Justine Azrieli Research Center, Montréal, Canada; [4]Department of Medicine III-Hematology/Oncology, LMU University Hospital, LMU Munich, Department of Medicine III-Hematology/Oncology, Munich, Germany; [5]Adult Bone Marrow Transplantation Service, Department of Medicine, Memorial Sloan Kettering Cancer Center, New York, United States of America; [6]Department of Hematology, University Hospital Leuven, Department of Hematology, Leuven, Belgium;[7]Department of Precision and Regenerative Medicine and Ionian Area (DiMePRe-J), University of Bari Aldo Moro Medical School, Bari, Italy; [8]Interdepartmental Center for Research in Telemedicine (CITEL,), University of Bari Aldo Moro Medical School, Bari, Italy; [9]International Patient Organisation for Primary Immunodeficiencies (IPOPI), Brussels, Belgium; [10]Department of Clinical Immunology, Instituto de Medicina de Laboratorio (IML) and Health Research Institute of the Hospital Clínico San Carlos (IdISSC), Madrid, Spain;*

### Background

Current literature outlining the use of immunoglobulin replacement therapy (IgRT) in secondary immunodeficiencies (SID) does not provide clear and harmonized guidance on its broader use internationally, being either limited with variations due to region, specialty, and immunological classification of the antibody deficiency. There is need to develop current SID focused guidance, particularly considering growing use of novel B-cell targeting treatment options like chimeric antigen receptor T-cell (CAR-T) or bispecific T-cell engagers.

### Aims

This study aimed to obtain international expert consensus on the use of IgRT in treating SID.

### Methods

A 3-round modified Delphi study, conducted as an online survey with structured consensus statements and open questions, was carried out between April-October 2025 guided by a steering committee (SC) consisted of 6 clinical immunologists and hemato-oncologists, inclusive of a panel (22 clinicians based in 9 countries) with a range of key clinical specialties involved in the treatment of SID. The round 1 survey was developed from a targeted literature review and a virtual discussion with the SC. A patient representative also contributed as an advisor in interpreting the final consensus results. In total, panelists were presented with 174 statements over 3 rounds.

**Copyright Information:** (Online) ISSN: 2572-9241
©2026 The Author(s). HemaSphere is published by John Wiley & Sons Ltd on behalf of European Hematology Association. This is an open access Abstract Book distributed under the Attribution-NonCommercial-NoDerivs (CC BY-NC-ND), which allows third parties to download the articles and share them with others as long as they credit the author and the Abstract Book, but they cannot change the content in any way or use them commercially.
**Abstract Book Citations:** Authors, Title, HemaSphere, 2026;10:(S1):pages. The abstract book DOIs can be found on the “Author Information” page.
**Disclaimer:** Articles published in the journal HemaSphere exclusively reflect the opinions of the authors. The authors are responsible for all content in their abstracts including accuracy of the facts, statements, citing resources, etc.

**Results**

A total of 57 final statements achieved consensus and 2 achieved dissensus. The key consensus recommendations included: functional antibody testing before and then following vaccination may offer additional quantitative information on humoral immune function, supplementing data from total IgG levels and infection burden; multidisciplinary teams improve the diagnosis of SID and primary immunodeficiency as well as the personalization of IgRT therapy; IgG thresholds (≤4 g/L with a history of infections; ≤2 g/L even without infections) should serve as indicators for IgRT initiation; IgRT initiation should be considered for patients starting potent B-cell or T-cell targeted therapies when the anticipated risk of hypogammaglobulinemia is ≥60% and/or the risk of severe, recurrent, or persistent infections is ≥70%; IgRT should be considered early when treating with B-cell maturation antigen targeted bispecific antibodies and CAR-T, but there was heterogeneity about exact timing; an individualized approach for IgRT should be taken based on infection burden, IgG levels, and functional antibodies when initiating Bruton's tyrosine kinase inhibitors and other B-cell directed therapies; the minimum starting dose for intravenous IgRT should be 0.4 g/kg every 3-4 weeks; at a minimum, IgG trough levels should be maintained in the reference range 6-16 g/L, though higher trough levels may be necessary; more frequent monitoring of IgG levels (every 3-4 months) may be helpful during the first year of treatment; all administration routes should be available to all patients, and the choice should be guided by shared decision-making; no changes to administration route should be made for non-clinical reasons; IgRT discontinuation may be considered in case of low infection burden and/or immunological recovery; monitoring post-discontinuation should be every 3–6 months.

**Summary/Conclusion**

This international Delphi panel provides IgRT guidance in the context of new immunotherapeutic options, multidisciplinary team reviews, and patient-centered considerations and will support the alignment of clinical practice recommendations among healthcare professionals.

**Copyright Information:** (Online) ISSN: 2572-9241
©2026 The Author(s). HemaSphere is published by John Wiley & Sons Ltd on behalf of European Hematology Association. This is an open access Abstract Book distributed under the Attribution-NonCommercial-NoDerivs (CC BY-NC-ND), which allows third parties to download the articles and share them with others as long as they credit the author and the Abstract Book, but they cannot change the content in any way or use them commercially.
**Abstract Book Citations:** Authors, Title, HemaSphere, 2026;10:(S1):pages. The abstract book DOIs can be found on the "Author Information" page.
**Disclaimer:** Articles published in the journal HemaSphere exclusively reflect the opinions of the authors. The authors are responsible for all content in their abstracts including accuracy of the facts, statements, citing resources, etc.

### (PS2357) OSIVELOTOR IMPROVES BIOMARKERS OF HEMOLYSIS, OXIDATIVE STRESS, AND INFLAMMATION IN PATIENTS WITH SICKLE CELL DISEASE IN A MULTICENTER PHASE 2/3 TRIAL

**Topic:** 27. Sickle cell disease

Santosh Saraf*[1], Julie Lee[2], Pujan Joshi[3], Shunjie Guan[2], Wen He[3], Craig Hyde[3], Yuvika Paliwal[4], Eleanor Lisbon[4], Geoffrey Allen[5], Kelly Fader[3]

*[1]University of Illinois Chicago, Chicago, IL, United States of America;[2]Pfizer Inc, Cambridge, MA, United States of America;[3]Pfizer Inc, Groton, CT, United States of America;[4]Pfizer Inc, New York, NY, United States of America;[5]Pfizer Inc, Collegeville, PA, United States of America;*

### Background

Sickle cell disease (SCD) is characterized by polymerization of sickle hemoglobin (HbS) leading to red blood cell (RBC) sickling and chronic hemolytic anemia. The HbS polymerization inhibitor osivelotor is currently being evaluated for the treatment of SCD in an ongoing phase 2/3 trial, and the 12-week, phase 2 portion indicated osivelotor was generally well tolerated in adults with SCD with substantial Hb increases. Improvements in hematocrit, hemolysis biomarkers, RBC deformability, and flow adhesion against vascular cell adhesion molecule 1 (VCAM-1) and P-selectin were also reported in preliminary analyses.

### Aims

To report exploratory analyses of hemolysis, oxidative stress, and inflammation biomarkers in patients with SCD receiving osivelotor.

### Methods

Exploratory data from the phase 2 portion of an ongoing multicenter, phase 2/3 trial (NCT05431088) were analyzed. The phase 2 portion was a randomized (1:1), open-label, 12-week, dose-finding study of osivelotor in adults with SCD (HbSS/HbS$\beta^0$ genotype) and Hb 5.5–10.5 g/dL. After consenting, patients received loading doses twice-daily for 4 days, then 100 or 150 mg once-daily maintenance doses through Week 12. Cell-free Hb was assessed with spectrophotometry (USA only, n=12). Plasma haptoglobin and hemopexin, scavenger proteins that bind cell-free Hb and heme, respectively, were analyzed with immunoaffinity liquid chromatography tandem mass spectrometry (SISCAPA, Washington, DC, USA). Plasma proteomic analysis used the Olink® Explore HT panel, which assesses ~5400 proteins (Olink, Waltham, MA, USA).

**Copyright Information:** (Online) ISSN: 2572-9241
©2026 The Author(s). HemaSphere is published by John Wiley & Sons Ltd on behalf of European Hematology Association. This is an open access Abstract Book distributed under the Attribution-NonCommercial-NoDerivs (CC BY-NC-ND), which allows third parties to download the articles and share them with others as long as they credit the author and the Abstract Book, but they cannot change the content in any way or use them commercially.
**Abstract Book Citations:** Authors, Title, HemaSphere, 2026;10:(S1):pages. The abstract book DOIs can be found on the "Author Information" page.
**Disclaimer:** Articles published in the journal HemaSphere exclusively reflect the opinions of the authors. The authors are responsible for all content in their abstracts including accuracy of the facts, statements, citing resources, etc.

**Results**

For 54 patients randomized to 100 or 150 mg osivelotor, mean (SD) baseline Hb was 8.5 (1.1) g/dL. At Week 12, both groups exhibited increases in Hb (mean [95% CI] change with 100 mg: +2.58 g/dL [2.05–3.11]; 150 mg: +3.35 g/dL [2.81–3.89]) accompanied by improvements in lactate dehydrogenase (LDH), indirect bilirubin and reticulocytes (absolute counts and %), indicating reduced RBC hemolysis. Cell-free Hb showed consistent changes, with mean (SD) concentrations reduced from 0.49 (0.491) g/L at baseline to 0.25 (0.125) g/L at Week 12 (**Figure**). Scavenger protein levels prior to treatment were substantially depleted (mean [SD] haptoglobin: 40.17 [113.370] ng/µL; hemopexin: 139.79 [108.965] ng/µL) compared with healthy ranges, suggesting ongoing intravascular hemolysis. Despite notable inter-subject variability, plasma haptoglobin and hemopexin increased with osivelotor as early as Week 1 (**Figure**) with further increases by Week 12 (mean [SD]: 105.17 [242.663] and 284.38 [102.631] ng/µL, respectively). These increased scavenger protein pools suggest improvement of intravascular hemolysis and improved protection against hemolytic damage. Analysis of pre-specified, biologically relevant biomarkers within the proteomics dataset revealed osivelotor-mediated reductions in (i) heme oxygenase-1, a stress-induced enzyme which degrades heme (**Figure**); (ii) E-selectin, a cell adhesion molecule; and (iii) interferon-γ, a pro-inflammatory cytokine.

**Summary/Conclusion**

Taken together, these results suggest osivelotor reduces intravascular hemolysis and increases pools of protective scavenger proteins, ultimately reducing oxidative stress and inflammation. This is expected to improve microvascular blood flow and anemia, reduce organ damage, and decrease risk of vaso-occlusive crises in patients with SCD. Overall, these findings support the ongoing clinical development of osivelotor as a potential treatment for SCD.

**Copyright Information:** (Online) ISSN: 2572-9241
©2026 The Author(s). HemaSphere is published by John Wiley & Sons Ltd on behalf of European Hematology Association. This is an open access Abstract Book distributed under the Attribution-NonCommercial-NoDerivs (CC BY-NC-ND), which allows third parties to download the articles and share them with others as long as they credit the author and the Abstract Book, but they cannot change the content in any way or use them commercially.
**Abstract Book Citations:** Authors, Title, HemaSphere, 2026;10:(S1):pages. The abstract book DOIs can be found on the "Author Information" page.
**Disclaimer:** Articles published in the journal HemaSphere exclusively reflect the opinions of the authors. The authors are responsible for all content in their abstracts including accuracy of the facts, statements, citing resources, etc.

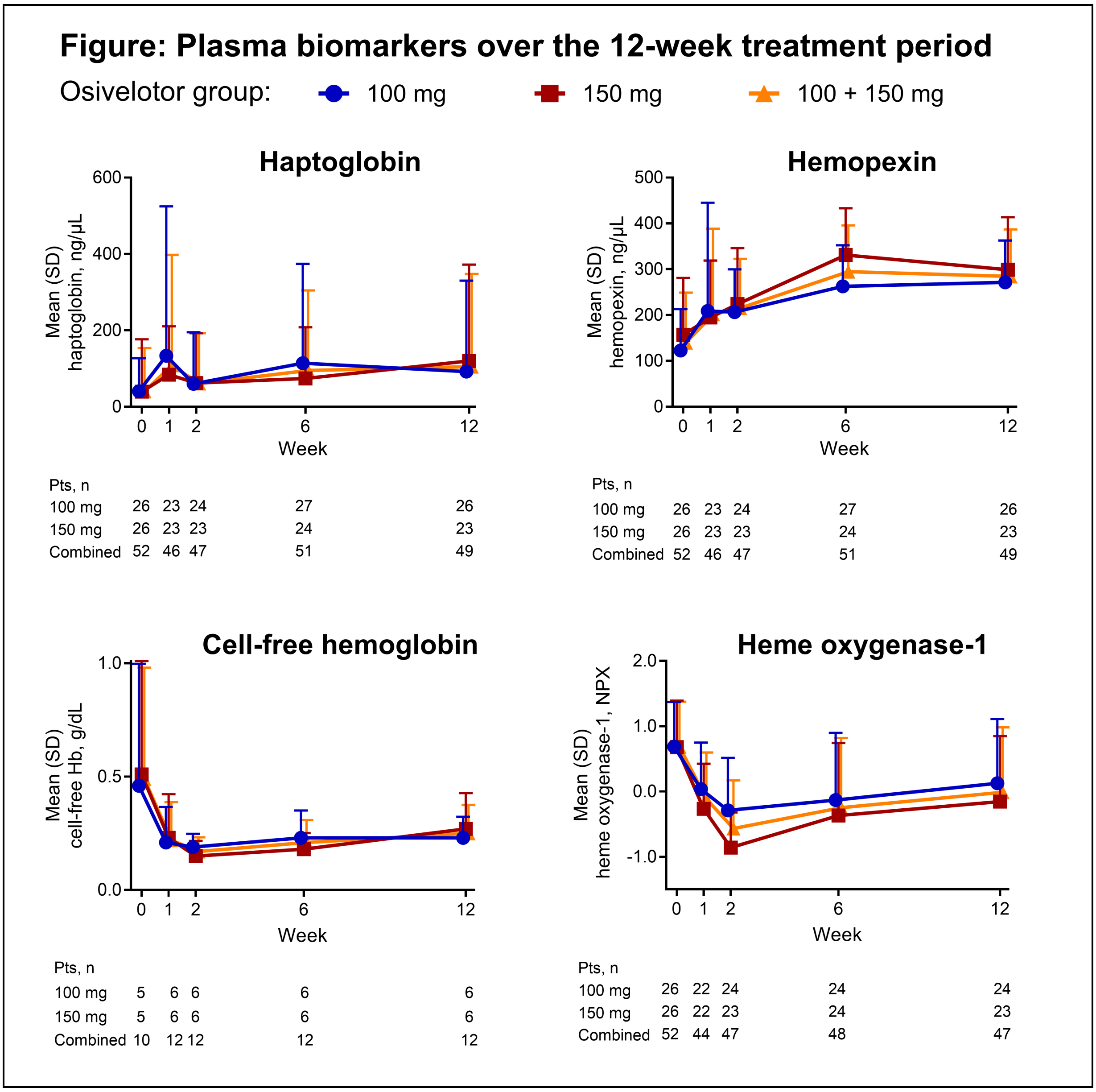

All available data are shown at each timepoint; one-sided error bars are shown for clarity. Haptoglobin and hemopexin were measured using immunoaffinity liquid chromatography tandem mass spectrometry, cell-free hemoglobin was measured using spectrophotometry, and heme oxygenase-1 was measured using the Olink® Explore HT (high-throughput) panel. Heme oxygenase-1 levels are presented as normalized protein expression (NPX), a relative quantification unit on a log-2 scale; negative mean values are expected when measurements correspond to values below 1 on the linear scale. NPX, normalized protein expression; SD, standard deviation.

**Copyright Information:** (Online) ISSN: 2572-9241
©2026 The Author(s). HemaSphere is published by John Wiley & Sons Ltd on behalf of European Hematology Association. This is an open access Abstract Book distributed under the Attribution-NonCommercial-NoDerivs (CC BY-NC-ND), which allows third parties to download the articles and share them with others as long as they credit the author and the Abstract Book, but they cannot change the content in any way or use them commercially.
**Abstract Book Citations:** Authors, Title, HemaSphere, 2026;10:(S1):pages. The abstract book DOIs can be found on the "Author Information" page.
**Disclaimer:** Articles published in the journal HemaSphere exclusively reflect the opinions of the authors. The authors are responsible for all content in their abstracts including accuracy of the facts, statements, citing resources, etc.

### (PS2358) BIOLOGICAL DRIVERS AND FUNCTIONAL IMPACT OF MITOCHONDRIA-RETAINING RED BLOOD CELLS IN SICKLE CELL DISEASE

**Topic:** 27. Sickle cell disease

Alkmini Anastasiadi*[1], Ioannis Kereres[1], Polyxeni Delaporta[2, 3], Nikoleta Baka[1], Katerina Nikolaidou[1], Konstantinos Liontakis[1], Katerina Athanasopoulou[4], Vasiliki-Zoi Arvaniti[5], Anne-Lise de Lastic[6], Anastasios Kriebardis[7], Elena Solomou[8], Dimitra Kyriakopoulou[2, 3], Argyro Sgourou[4], Antonis Kattamis[2, 3], Vasileios Tzounakas[1]

*[1]University of Patras, School of Medicine, Department of Biochemistry, Patras, Greece; [2]National and Kapodistrian University of Athens, Thalassemia Unit, First Department of Pediatrics, Athens, Greece; [3]"Aghia Sophia" Children's Hospital, ERN-EuroBloodNet Center, Athens, Greece; [4]Hellenic Open University, School of Science and Technology, Biology Laboratory, Patras, Greece; [5]The Netherlands Cancer Institute, Division of Molecular Genetics, Amsterdam, The Netherlands; [6]University of Patras, School of Medicine, Department of Internal Medicine, Laboratory of Immunohematology, Patras, Greece; [7]University of West Attica, School of Health and Caring Sciences, Department of Biomedical Sciences, Laboratory of Reliability and Quality Control in Laboratory Hematology, Egaleo, Greece; [8]University of Patras, School of Medicine, Department of Internal Medicine-Hematology, Patras, Greece;*

### Background

During the last decade the persistence of mitochondria-retaining red blood cells (mito$^+$-RBCs) in sickle cell disease (SCD) has been established. Yet, the mechanistic basis of their inter-individual variation, their relationship to disease manifestations, and the downstream functional impact of mitochondrial retention at the single-cell level remain incompletely characterized.

### Aims

This study aims to unveil drivers of mito$^+$-RBC content in SCD, characterize the biological phenotype of mito$^+$-RBC subpopulations and examine the linkage between mitochondrial retention and SCD manifestations.

### Methods

Samples from fifty-nine SCD patients (25/34 HbSS/HbSβ-thal; 1-54 years old, median age 12 years; 41% female) were assessed for mitochondrial content (MitoTracker$^+$CD235a$^+$-RBCs), along hematological and hemolysis indices, redox and eryptosis markers, and proteasomal activity. An array of the mentioned assays was also performed in sorted mito$^+$- and mito$^-$-RBC subpopulations (N=6 patients). Eighteen of the patients were followed longitudinally (2-4 visits, ranging 2-14 months).

**Copyright Information:** (Online) ISSN: 2572-9241
©2026 The Author(s). HemaSphere is published by John Wiley & Sons Ltd on behalf of European Hematology Association. This is an open access Abstract Book distributed under the Attribution-NonCommercial-NoDerivs (CC BY-NC-ND), which allows third parties to download the articles and share them with others as long as they credit the author and the Abstract Book, but they cannot change the content in any way or use them commercially.
**Abstract Book Citations:** Authors, Title, HemaSphere, 2026;10:(S1):pages. The abstract book DOIs can be found on the "Author Information" page.
**Disclaimer:** Articles published in the journal HemaSphere exclusively reflect the opinions of the authors. The authors are responsible for all content in their abstracts including accuracy of the facts, statements, citing resources, etc.

**Results**

Mito$^+$-RBC content presented large inter-individual variation (0.2-31.6%) unexplained by age, sex, or raw genotype. Sequential regression revealed a hierarchical variance structure: reticulocytosis (RET) accounted for 36% of variance, suggesting erythropoietic output exceeds mitophagy capacity during maturation. After RET adjustment, oxidative vulnerability (reactive oxygen species-ROS production, $\rho$=+0.42, p<0.001) emerged as an independent contributor, while ferritin also correlated with mito$^+$-RBCs. Controlling for RET also revealed that the HbSS genotype and higher %HbS were associated with lower residual mito$^+$-content. Unsupervised clustering identified two discrete mito$^+$-groups (high, 14.01±8.84%, N=27; low, 4.54±3.08%, N=32), differing significantly across the full hemolytic spectrum (e.g., mechanical lysis 0.68±0.23 vs 0.50±0.23%, p<0.05; LDH 603±199 vs 317±85 U/L, p<0.001, high vs low), as well as in RET, ROS accumulation, and phosphatidylserine (PS) exposure (10.89±5.96 vs 4.01±3.49%, p<0.001, high vs low), confirming that the high-mito$^+$ phenotype reflects disease activity. Crucially, there was minimal co-localization of mito$^+$ and PS$^+$ populations. Sorted mito$^+$-RBCs exhibited higher oxidative lysis, $Ca^{2+}$ influx (61526±18111 vs 40718±7457 MFI/mg, p=0.026), and proteasome activity compared to patient-matched mito$^-$-RBCs, along with a trend for increased ROS. Longitudinally, mitochondria-retaining RBCs increased over time (p=0.015; 14/18), tracking bilirubin changes ($\rho$=+0.49, p=0.041), and were higher in splenectomized patients, consistent with impaired clearance.

**Summary/Conclusion**

Mito$^+$-RBC content in SCD seems to be governed by distinct pathways, including erythropoietic output, oxidative vulnerability, and iron dysregulation. Yet, over half of inter-individual variation remains unexplained. The high-mito$^+$ phenotype is defined by systemic hemolytic activity, while at the single-cell level, mito$^+$-RBCs represent a pre-eryptotic subpopulation, since they are oxidatively primed and $Ca^{2+}$-loaded but not yet PS-exposing. Notably, their accumulation tracks hemolytic disease activity longitudinally. These findings provide a quantitative mechanistic framework for mito$^+$-RBC content as a flow-accessible readout of erythropoietic stress and pre-eryptotic RBC burden in SCD.
Funding for this project was provided in whole by an EHA Research Grant award (RG-202212-03039) granted by the European Hematology Association.

**Copyright Information:** (Online) ISSN: 2572-9241
©2026 The Author(s). HemaSphere is published by John Wiley & Sons Ltd on behalf of European Hematology Association. This is an open access Abstract Book distributed under the Attribution-NonCommercial-NoDerivs (CC BY-NC-ND), which allows third parties to download the articles and share them with others as long as they credit the author and the Abstract Book, but they cannot change the content in any way or use them commercially.
**Abstract Book Citations:** Authors, Title, HemaSphere, 2026;10:(S1):pages. The abstract book DOIs can be found on the "Author Information" page.
**Disclaimer:** Articles published in the journal HemaSphere exclusively reflect the opinions of the authors. The authors are responsible for all content in their abstracts including accuracy of the facts, statements, citing resources, etc.

### (PS2359) EARLY HYPERFILTRATION PREDICTS SUBSEQUENT RENAL DECLINE IN SICKLE CELL DISEASE: A LONGITUDINAL TRAJECTORY STUDY

**Topic:** 27. Sickle cell disease

Laura Corona*[1], Mirco D'agnolo[1], Giulia Reggiani[1, 2], Maria Paola Boaro[2], Giorgia Ghirardo[3], Elisabetta Mezzalira[3], Maddalena Martella[1], Raffaella Colombatti[1, 2]

*[1]University of Padova, Department of Women's and Children's Health, Padova, Italy;[2]Azienda Ospedale - Università Padova, Pediatric Hematology-Oncology and Bone Marrow-Transplant Unit, Padova, Italy; [3]Fondazione Istituto di Ricerca Pediatrica Città della Speranza, Padova, Italy;*

### Background

Renal involvement in sickle cell disease (SCD) is a key driver of long-term morbidity and mortality. Although kidney damage arises early in life, its evolution from childhood through transition to adulthood is not fully characterized.

### Aims

To define pediatric longitudinal eGFR trajectories in SCD and identify predictors of renal decline through adolescence and young adulthood.

### Methods

We retrospectively analyzed prospectively collected data from a SCD Natural History Study in a single tertiary care center in Italy. Renal function was assessed by eGFR (Schwartz <18 years; CKD-EPI ≥18 years), albuminuria (ACR >30 mg/g) and proteinuria (>0.15 g/L). To minimize cross-sectional bias and formula-related distortion, we restricted analysis to patients with annual eGFR assessments from early childhood to young adulthood, capturing the trajectory inversion. Individual trajectories were fitted using LOWESS to define the inflection point. Ascending and descending slopes were computed from baseline to peak and from peak to 18 years, ensuring uniform pediatric follow-up. Genotype comparisons were performed. Adult slopes were evaluated separately due to equation-related discontinuity.

### Results

The cohort included 161 patients (133 HbSS/HbSβ0; 28 HbSC/HbSβ+). Mean age was 13.0±7.7 years; 49.1% were female. Mean follow-up was 10.7±5.6 years. Thirty-five patients satisfied the inclusion criteria, with adequate data density for reliable eGFR trajectory modeling. In this subgroup (n=35, 25 HbSS/HbSβ0; 6 HbSC), eGFR followed a reproducible biphasic trajectory: hyperfiltration in early childhood, peak at a mean age of 11.9 years in HbSS/HbSβ0 patients, and 10.9 years in HbSC patients, followed by sustained decline. Hyperfiltration was significantly more pronounced in severe genotypes, with a mean ascending slope of +4.3 mL/min/1.73m²/year in HbSS/HbSβ0 versus +1.2 in HbSC (p=0.038). Patients with severe genotypes exhibited a trend toward a faster decline (-8.3 vs –5.1 mL/min/1.73m²/year), although this difference did not reach statistical significance (p=0.314). Older age at peak was associated with higher peak eGFR (r=0.351, p=0.028). Older age at peak strongly predicted a steeper decline (r=–0.688, p<0.001). Higher peak eGFR predicted a greater rate of post-peak decline (r=–0.526, p=0.002), suggesting that hyperfiltration drives subsequent functional loss. Peak eGFR correlated with HbS (r=0.374, p=0.025), and a higher rate of decline was associated with greater mean ACR (r=–0.408, p=0.020). In adults, eGFR showed a persistent downward trajectory without recovery, with comparable rates of decline across genotypes and hydroxyurea exposure groups. Pathological ACR was detected in 18% of the 161 patients, mainly in severe genotypes, before 12 years of age in 65% of cases. Renal medullary hyperechogenicity was found in 30% of patients, with 56% detected before age 12, supporting early structural involvement.

**Copyright Information:** (Online) ISSN: 2572-9241
©2026 The Author(s). HemaSphere is published by John Wiley & Sons Ltd on behalf of European Hematology Association. This is an open access Abstract Book distributed under the Attribution-NonCommercial-NoDerivs (CC BY-NC-ND), which allows third parties to download the articles and share them with others as long as they credit the author and the Abstract Book, but they cannot change the content in any way or use them commercially.
**Abstract Book Citations:** Authors, Title, HemaSphere, 2026;10:(S1):pages. The abstract book DOIs can be found on the “Author Information” page.
**Disclaimer:** Articles published in the journal HemaSphere exclusively reflect the opinions of the authors. The authors are responsible for all content in their abstracts including accuracy of the facts, statements, citing resources, etc.

**Summary/Conclusion**

Renal function in SCD exhibits a biphasic trajectory, characterized by early adaptive hyperfiltration, an adolescent peak, and subsequent accelerated decline; the timing and magnitude of this peak may delineate a high-risk renal phenotype. The frequent occurrence of albuminuria before 12 years supports the implementation of structured renal surveillance beginning in early childhood. Furthermore, the use of age-dependent eGFR equations limits longitudinal comparability of renal impairment, underscoring the need for standardized assessment strategies in future longitudinal studies.

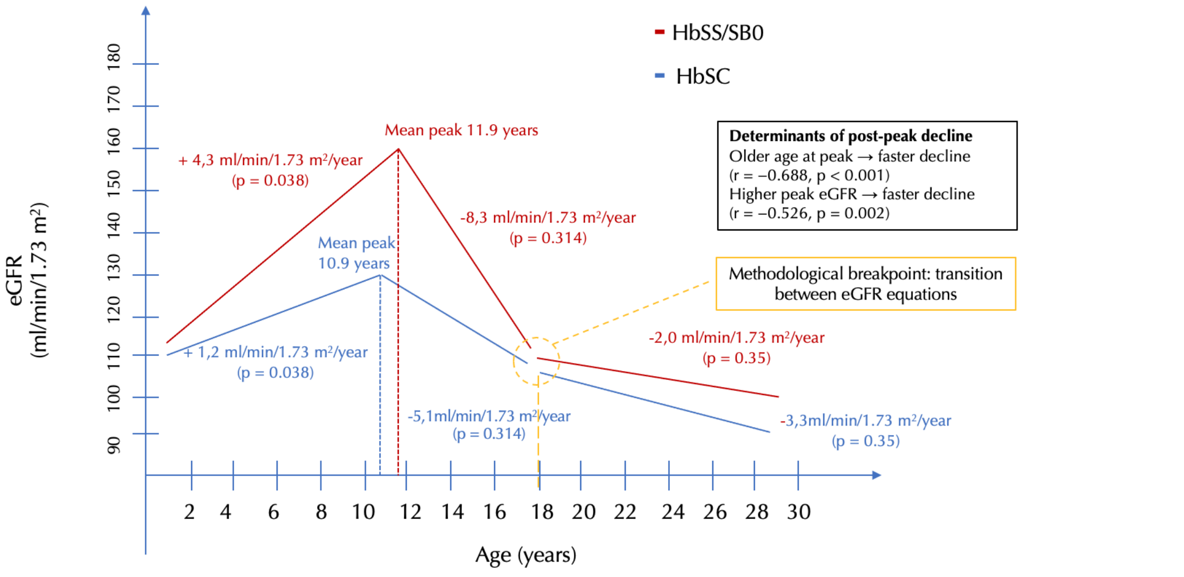

Conceptual representation based on longitudinal slope analysis

**Copyright Information:** (Online) ISSN: 2572-9241
©2026 The Author(s). HemaSphere is published by John Wiley & Sons Ltd on behalf of European Hematology Association. This is an open access Abstract Book distributed under the Attribution-NonCommercial-NoDerivs (CC BY-NC-ND), which allows third parties to download the articles and share them with others as long as they credit the author and the Abstract Book, but they cannot change the content in any way or use them commercially.
**Abstract Book Citations:** Authors, Title, HemaSphere, 2026;10:(S1):pages. The abstract book DOIs can be found on the "Author Information" page.
**Disclaimer:** Articles published in the journal HemaSphere exclusively reflect the opinions of the authors. The authors are responsible for all content in their abstracts including accuracy of the facts, statements, citing resources, etc.

### (PS2360) SICKLE CELL DISEASE IN GERMANY - TRENDS IN EPIDEMIOLOGY, HEALTHCARE UTILIZATION AND DISEASE BURDEN FROM 2015 TO 2024: A CLAIMS DATA ANALYSIS

**Topic:** 27. Sickle cell disease

Nils Schulze*[1], Moritz Lehne[2], Anja Cengia[3], Klara Sonnie Hinrichsmeyer[4], Agnes Kisser[4], Caoimhe Cawley[4], Ferras Alashkar[5]

*[1]Pfizer Pharma GmbH; MHB - Theodor Fontane, Berlin, Germany;[2]Cytel Inc., Berlin, Germany;[3]InGef – Institute for Applied Health Research Berlin GmbH, Berlin, Germany;[4]Pfizer Pharma GmbH, Berlin, Germany;[5]West German Cancer Center, University Hospital Essen, University Duisburg-Essen, Essen, Germany;*

### Background

Sickle cell disease (SCD) is a rare inherited hemoglobinopathy associated with substantial morbidity, reduced life expectancy and high healthcare needs. In Germany, population-based evidence on SCD epidemiology, diagnosis patterns and real-world healthcare utilization remains limited, despite increasing relevance in the context of migration and the introduction of nationwide newborn screening in 2021.

### Aims

To describe temporal trends in SCD prevalence and incidence in Germany between 2015 and 2024. To characterize patient demographics, clinical outcomes, treatments and healthcare resource utilization (HCRU) in routine care.

### Methods

We conducted a retrospective, non-interventional study using anonymized German statutory health insurance claims data from the InGef database. Patients with SCD were identified using ICD-10-GM codes (D57.0/1/2/8) based on inpatient and confirmed outpatient diagnoses. Annual prevalence was estimated using a cohort approach, counting patients diagnosed in the respective or prior years, and alive at any point during the current year. Incidence was assessed in children aged <6 years using the first recorded SCD diagnosis as the date of administrative incidence. Vaso-occlusive crises (VOCs) were approximated using hospitalization-based proxies, including SCD with crisis and pneumonia/ACS diagnoses. Analyses of patient characteristics, clinical outcomes, treatments and HCRU were conducted cross-sectionally by calendar year and stratified by age group.

### Results

Across 2015–2024, 1,019 unique individuals with SCD were identified; the annual number of prevalent SCD patients in the database increased from 225 in 2015 to 864 in 2024. Overall, 57.1% (582/1019) were female and 72.6% (740/1019) were aged ≥18 years (median age 33 years, interquartile range 16-48). Nationally extrapolated prevalence estimates increased from 2.75 per 100 000 in 2015 to 10.50 per 100 000 in 2024, with higher prevalence observed in children aged <18 years compared with adults, and in women than men. Annual incidence estimates among children aged <6 years ranged between 1.9 and 3.0 per 100 000. Median time to recorded diagnosis in this age group decreased from 31 months in 2015 to less than 12 months from 2021 onwards. In 2024, infections were the most frequently observed outcome (34.5% (225/864)), followed by VOCs (19.2% (166/864)) and mental health comorbidities (depression 19.1% (165/864); anxiety 9.1% (79/864)). Retinopathy (6.4% (55/864)), osteonecrosis/osteoporosis (4.5% (38/864)) and splenic sequestration (4.4% (38/864)) were also commonly documented. The annual proportion of patients with VOCs decreased steadily from 36.9% (83/225) in 2015 to 19.2% (166/864) in 2024; over 2015–2024 (cumulative), VOCs were more common in children/adolescents than adults (0–17 years: 48.4% (135/279) vs ≥18 years: 31.5% (233/740)). Children aged <18 years consistently showed higher proportions of hospital admissions compared with adults.

**Copyright Information:** (Online) ISSN: 2572-9241
©2026 The Author(s). HemaSphere is published by John Wiley & Sons Ltd on behalf of European Hematology Association. This is an open access Abstract Book distributed under the Attribution-NonCommercial-NoDerivs (CC BY-NC-ND), which allows third parties to download the articles and share them with others as long as they credit the author and the Abstract Book, but they cannot change the content in any way or use them commercially.
**Abstract Book Citations:** Authors, Title, HemaSphere, 2026;10:(S1):pages. The abstract book DOIs can be found on the “Author Information” page.
**Disclaimer:** Articles published in the journal HemaSphere exclusively reflect the opinions of the authors. The authors are responsible for all content in their abstracts including accuracy of the facts, statements, citing resources, etc.

**Summary/Conclusion**

This claims analysis provides a comprehensive overview of SCD epidemiology and care patterns in Germany over a ten-year period. The findings indicate an increasing documented prevalence, earlier recorded diagnosis in young children and a high burden of acute complications, mental health comorbidities and healthcare utilization. These results can support healthcare planning and inform strategies aimed at early diagnosis and long-term management of SCD in Germany.

**Copyright Information:** (Online) ISSN: 2572-9241
©2026 The Author(s). HemaSphere is published by John Wiley & Sons Ltd on behalf of European Hematology Association. This is an open access Abstract Book distributed under the Attribution-NonCommercial-NoDerivs (CC BY-NC-ND), which allows third parties to download the articles and share them with others as long as they credit the author and the Abstract Book, but they cannot change the content in any way or use them commercially.
**Abstract Book Citations:** Authors, Title, HemaSphere, 2026;10:(S1):pages. The abstract book DOIs can be found on the "Author Information" page.
**Disclaimer:** Articles published in the journal HemaSphere exclusively reflect the opinions of the authors. The authors are responsible for all content in their abstracts including accuracy of the facts, statements, citing resources, etc.

### (PS2361) THE HIDDEN BURDEN: FEMALE SEXUAL DYSFUNCTION AND PAIN CRISES IN SICKLE CELL DISEASE

**Topic:** 27. Sickle cell disease

Fernanda Bacarin*[1], Glenda Feldberg[2], Fabio Galvão[2], Manoella Galafassi[1], Bianca Campelo[2], Mariana Rosa[1], Amanda Lopes[1], Fabiana Manoel[1], Maria Do Nascimento[2], Marcela Bardin[2], Sara Olalla Saad[2], Paula De Melo Campos[1], Bruno Benites[2]

*[1]Hematology and Tranfusion Medicine Center, University of Campinas, Brazil, CAMPINAS, Brazil; [2]UNICAMP, CAISM, CAMPINAS, Brazil;*

### Background

Sickle cell disease (SCD) is a hereditary hemoglobinopathy characterized by recurrent pain, significant morbidity, and profound psychosocial impact. While priapism is a well-characterized complication in men with SCD, the repercussions of the disease on female sexuality remain largely unexplored. Sexuality constitutes a central domain of quality of life and can be profoundly influenced by chronic pain, vaso-occlusive crises (VOCs), comorbidities, and adverse social determinants.

### Aims

To evaluate sexual function, sexuality experience, and quality of life in women with SCD followed at a reference center, exploring the occurrence of sex-related pain, sexual activity-precipitated pain crises, and the potential presence of clitoral priapism manifestations.

### Methods

This prospective, observational, single-center study was conducted in southeastern Brazil from July to December 2025. We enrolled 62 women with SCD across all genotypes, aged ≥18 years. Participants completed sociodemographic, clinical, and gynecological-obstetric questionnaires. Validated instruments included the Female Sexual Function Index (FSFI) and PROMIS profiles assessing anxiety, depression, and global health. Spontaneous qualitative reports were also analyzed.

### Results

The cohort (median age 43 years; 62.9% with severe genotypes HbSS/HbS$\beta^0$) exhibited significant socioeconomic vulnerability and high pain burden. Analgesic use was prevalent (87.1%), including opioids (51.9%). Among 59 sexually active women, 47.5% reported pain during or after intercourse. Notably, 25.5% experienced SCD pain crises precipitated by sexual activity (during intercourse: 1.7%; immediately after: 15.3%; up to 24 hours after: 8.5%). Manifestations consistent with clitoral priapism were rare, with only one case of prolonged clitoral edema. PROMIS scores revealed significantly worse physical (T-score: 32) and mental (T-score: 35) health perceptions compared to the general population, alongside elevated anxiety (T-score: 63) and depression (T-score: 57). Women with severe sickle cell genotypes (HbSS/HbS$\beta^0$) had significantly lower rates of marriage or stable partnerships compared to those with milder forms ($p < 0.05$). Women consistently reported pain during vaginal penetration, reduced lubrication, and mechanical pain in the lower back and hip areas, especially those with osteonecrosis, which limited their ability to adopt certain sexual positions. They also frequently reported fear and avoidance of sexual activity due to pain concerns. FSFI analysis (n=19 sexually active women) demonstrated a significant negative correlation between annual VOC frequency and sexual desire ($r = -0.599$; $p = 0.006$), with trends toward lower arousal and satisfaction in women with more frequent crises.

**Copyright Information:** (Online) ISSN: 2572-9241
©2026 The Author(s). HemaSphere is published by John Wiley & Sons Ltd on behalf of European Hematology Association. This is an open access Abstract Book distributed under the Attribution-NonCommercial-NoDerivs (CC BY-NC-ND), which allows third parties to download the articles and share them with others as long as they credit the author and the Abstract Book, but they cannot change the content in any way or use them commercially.
**Abstract Book Citations:** Authors, Title, HemaSphere, 2026;10:(S1):pages. The abstract book DOIs can be found on the "Author Information" page.
**Disclaimer:** Articles published in the journal HemaSphere exclusively reflect the opinions of the authors. The authors are responsible for all content in their abstracts including accuracy of the facts, statements, citing resources, etc.

**Summary/Conclusion**

Women with SCD experience profound impairment in quality of life, marked by the intersection of chronic pain, emotional distress, functional limitations, and socioeconomic vulnerability. Sexuality emerges as a heavily impacted domain, frequently disrupted by pain and fear of triggering crises. The high frequency of sexual activity-induced VOCs and its strong negative correlation with sexual desire represent major, underrecognized findings. Clitoral priapism manifestations were rare. These results underscore the urgent need for integrated, patient-centered care models that systematically address pain management, mental health, sexual well-being, and social determinants in women with SCD.

**Copyright Information:** (Online) ISSN: 2572-9241
©2026 The Author(s). HemaSphere is published by John Wiley & Sons Ltd on behalf of European Hematology Association. This is an open access Abstract Book distributed under the Attribution-NonCommercial-NoDerivs (CC BY-NC-ND), which allows third parties to download the articles and share them with others as long as they credit the author and the Abstract Book, but they cannot change the content in any way or use them commercially.
**Abstract Book Citations:** Authors, Title, HemaSphere, 2026;10:(S1):pages. The abstract book DOIs can be found on the "Author Information" page.
**Disclaimer:** Articles published in the journal HemaSphere exclusively reflect the opinions of the authors. The authors are responsible for all content in their abstracts including accuracy of the facts, statements, citing resources, etc.

### (PS2362) DISSECTING THE MOLECULAR BASIS OF POTENT HBF INDUCTION BY BMS-986470: A MECHANISTIC STUDY OF ZBTB7A/WIZ DUAL DEGRADATION

**Topic:** 27. Sickle cell disease

Yan Kai*[1], Goldie Lui[1], Diana Jankeel[1], Scott Wood[1], Shayla Goller[1], Kimberly Peacock[1], Zhongying Mo[1], Cristhian Fimbres[1], Calvin Sun[1], Dalen Chan[1], Marc Casillas[1], Romain Georges[1], Soraya Carrancio[1], Kai Wang[1], Neil Bence[1], Emily Rychak[1], Antonia Lopez-Girona[1], Celia Fontanillo[1]

[1]*Bristol Myers Squibb, San Diego, United States of America;*

### Background

Sickle cell disease (SCD) remains a major global health burden with limited therapeutic options. Reactivation of fetal hemoglobin (HbF) is clinically validated to mitigate disease severity. Advances in targeted protein degradation now enable pharmacologic control of transcriptional repressors previously considered undruggable, including WIZ and ZBTB7A. BMS-986470 is a first-in-class, orally bioavailable cereblon E3 ligase modulator (CELMOD™) that selectively degrades ZBTB7A and WIZ, resulting in robust HbF induction and an erythroid program shift from adult β-globin toward fetal γ-globin expression. Under clinical investigation (NCT06481306), BMS-986470 elicits potent γ-globin activation and F-cell formation across HUDEP-2 cells, primary erythroblasts from healthy and SCD donors, and murine models of human erythropoiesis (Rychak et al., Blood 2024).

### Aims

Here we delineate the molecular mechanisms underlying BMS-986470–mediated HbF induction, with a focus on developing a better understanding of the contributions of ZBTB7A and WIZ degradation.

### Results

To compare HbF-repressing capacity of the two factors, we performed an unbiased, phenotypic, and genome-wide CRISPR-Cas9 screen in HUDEP-2 cells. Differential sgRNA enrichment/depletion between HbF-high and HbF-low populations provided a quantitative readout of HbF-repressing or -activating effects per gene. The screen recovered established HbF repressors, such as ZBTB7A, BCL11A, ZNF410, WIZ, CHD4, validating the approach. Notably, ZBTB7A-targeting sgRNAs were more enriched in HbF-high cells than WIZ-targeting sgRNAs, indicating stronger repression by ZBTB7A. Intriguingly, HBB emerged as the top-ranked HbF repressor genome-wide, consistent with reports that HBB promoter disruption facilitates fetal globin reactivation (Topfer et al., Blood 2022).

To link repressor strength to functional output, we generated CRISPR/Cas9-edited HUDEP-2 lines with single and dual knockouts (KOs) of ZBTB7A/WIZ. Immunoblotting showed greater γ-globin (HBG1/2) induction in ZBTB7A KO versus WIZ KO cells, consistent with more effective relief of HbF repression upon loss of ZBTB7A. The double KO further increased HBG1/2 and HbF tetramers while concomitantly reducing adult β-globin (HBB) compared with either single KO, supporting a dominant role for ZBTB7A in fetal globin silencing.

Because the locus control region (LCR)—five distal enhancers upstream of the β- and γ-globin genes— is a key driver of the high-level globin expression via long-range promoter contacts, we next mapped chromatin architecture using high-resolution Micro-C. BMS-986470 induced a pronounced looping switch, with reduced LCR–HBB contacts and increased LCR–HBG contacts. Micro-C in KO lines showed that both ZBTB7A and WIZ loss disrupt LCR–HBB contacts; however, ZBTB7A KO produced a substantially stronger gain in LCR–HBG looping than WIZ KO, providing a structural correlate to the phenotypic hierarchy. These findings implicate ZBTB7A as a stronger mediator of LCR-driven promoter competition than WIZ and explain why dual degradation by BMS-986470 leads to a robust adult-to-fetal globin chromatin and transcriptional switch.

**Copyright Information:** (Online) ISSN: 2572-9241
©2026 The Author(s). HemaSphere is published by John Wiley & Sons Ltd on behalf of European Hematology Association. This is an open access Abstract Book distributed under the Attribution-NonCommercial-NoDerivs (CC BY-NC-ND), which allows third parties to download the articles and share them with others as long as they credit the author and the Abstract Book, but they cannot change the content in any way or use them commercially.
**Abstract Book Citations:** Authors, Title, HemaSphere, 2026;10:(S1):pages. The abstract book DOIs can be found on the “Author Information” page.
**Disclaimer:** Articles published in the journal HemaSphere exclusively reflect the opinions of the authors. The authors are responsible for all content in their abstracts including accuracy of the facts, statements, citing resources, etc.

**Summary/Conclusion**

In summary, we delineate the transcriptional and chromatin-level mechanisms of HbF regulation and establish a functional hierarchy between ZBTB7A and WIZ. These findings provide mechanistic insights into the therapeutic potential of BMS-986470 for SCD treatment.

**Copyright Information:** (Online) ISSN: 2572-9241
©2026 The Author(s). HemaSphere is published by John Wiley & Sons Ltd on behalf of European Hematology Association. This is an open access Abstract Book distributed under the Attribution-NonCommercial-NoDerivs (CC BY-NC-ND), which allows third parties to download the articles and share them with others as long as they credit the author and the Abstract Book, but they cannot change the content in any way or use them commercially.
**Abstract Book Citations:** Authors, Title, HemaSphere, 2026;10:(S1):pages. The abstract book DOIs can be found on the "Author Information" page.
**Disclaimer:** Articles published in the journal HemaSphere exclusively reflect the opinions of the authors. The authors are responsible for all content in their abstracts including accuracy of the facts, statements, citing resources, etc.

### (PS2363) BIVENTRICULAR REMODELING WITH PRESERVED MYOCARDIAL DEFORMATION IN PEDIATRIC SICKLE CELL DISEASE: SPECKLE-TRACKING ECHOCARDIOGRAPHY AND LONGITUDINAL ASSESSMENT

**Topic:** 27. Sickle cell disease

Ziad Bulbul[1], Rana Zareef[1], Theresia Tannoury[1], Tania Abi Nassif[1], Francesca Rodigari[2], Hani Tamim[3], Maha Makki[3], Fadi Bitar[1], Miguel R. Abboud*[1]

*[1]American University of Beirut Medical center, Pediatrics and Adolescent Medicine, Beirut, Lebanon;[2]Padua University Medical School, Faculty of Medicine, Padua, Italy; [3]American University of Beirut Medical center, Clinical research institute, Beirut, Lebanon;*

### Background

In sickle cell disease (SCD), chronic anemia, hemolysis, and vasculopathy contribute to early cardiopulmonary remodeling. While pulmonary pressures and diastolic abnormalities are well described in adults, pediatric biventricular mechanics remain incompletely defined.

### Aims

To characterize left- and right-ventricular (LV/RV) structure and deformation in children with SCD using speckle-tracking echocardiography (STE), and to evaluate associations with genotype, clinical complications, hemolysis markers, hydroxyurea, and longitudinal change.

### Methods

We conducted a retrospective single-center cohort study between Jan 2015– Dec 2023. We analyzed 278 echocardiograms from 185 children: 118 with genetically confirmed SCD and 67 age/sex-matched controls. Among SCD patients, 66.1% had HbSS, 17.8% HbS$\beta^+$, 9.3% HbS$\beta^0$; 83.9% were receiving hydroxyurea. STE measured LV global longitudinal strain (GLS) and RV free-wall strain (RVFWS). Conventional measures included chamber dimensions, Doppler diastolic indices, systolic indices, and maximal tricuspid regurgitation velocity (TRv). Clinical/laboratory variables included vaso-occlusive burden, transfusions, disease complications, anemia severity, and hemolysis markers. Multivariable regression was performed; serial echocardiograms were analyzed when available.

**Copyright Information:** (Online) ISSN: 2572-9241
©2026 The Author(s). HemaSphere is published by John Wiley & Sons Ltd on behalf of European Hematology Association. This is an open access Abstract Book distributed under the Attribution-NonCommercial-NoDerivs (CC BY-NC-ND), which allows third parties to download the articles and share them with others as long as they credit the author and the Abstract Book, but they cannot change the content in any way or use them commercially.
**Abstract Book Citations:** Authors, Title, HemaSphere, 2026;10:(S1):pages. The abstract book DOIs can be found on the “Author Information” page.
**Disclaimer:** Articles published in the journal HemaSphere exclusively reflect the opinions of the authors. The authors are responsible for all content in their abstracts including accuracy of the facts, statements, citing resources, etc.

### Results

Overall, males constituted 51.4% of the included subjects (SCD 51.7%, controls 50.7%); with a mean of 12.2 years (1–21) in SCD and 11.8 years (1–21) in controls. Compared with controls, SCD patients demonstrated biventricular remodeling with larger LV end-diastolic diameter index (40.0 vs 35.4 mm/m²; p=0.001), higher LV mass index (73.9 vs 51.8 g/m²; p<0.001), larger left atrial volume index (28.5 vs 16.4 mL/m²; p<0.001), and larger RV end-diastolic area (21.5±6.5 vs 18.6±6.5 cm²; p=0.01). Diastolic indices suggested higher filling burden (higher E, E/A, IVRT and E/E′), and higher TRv (2.21±0.24 vs 2.01±0.27 m/s; p<0.001). LV GLS was modestly less negative but within normal range (-21.5±1.3% vs -22.3±1.1%; p<0.001). RV fractional area change (FAC) was slightly lower, while RVFWS remained preserved with only a small between-group difference (-30.2±1.9% vs -30.8±1.5%; p=0.04). LV GLS worsened with age, with an inflection at 9.9 years (≤9.9: -22.25±1.02 vs >9.9: -21.38±1.40; p<0.001). Prior stroke (β=0.9; p=0.048) and avascular necrosis (β=1.51; p=0.02) independently predicted worse LV GLS, whereas hemoglobin, hemolysis markers, genotype, and hydroxyurea were not associated. In multivariable analysis, FAC was the only independent predictor of RVFWS (β=-0.29; p=0.01). Elevated TRv (≥2.5 m/s) occurred in 12.9% and was associated with older age and larger RV size with preserved systolic indices. Echocardiographic parameters including RV and LV strain, and chamber dimensions were largely comparable across HbSS, HbSβ⁺, and HbSβ⁰, except for a higher LV mass index within the HbSS group. Longitudinally, LV GLS declined slightly over 3.7 years (-21.6 to -21.2%), whereas RV parameters remained stable.

### Summary/Conclusion

These findings support a progressive, volume-loaded remodeling phenotype in pediatric SCD, with early age-related decline in LV deformation despite preserved global systolic indices. STE may refine the timing of surveillance and help identify children with higher systemic vasculopathy burden who warrant closer follow-up.

**Copyright Information:** (Online) ISSN: 2572-9241
©2026 The Author(s). HemaSphere is published by John Wiley & Sons Ltd on behalf of European Hematology Association. This is an open access Abstract Book distributed under the Attribution-NonCommercial-NoDerivs (CC BY-NC-ND), which allows third parties to download the articles and share them with others as long as they credit the author and the Abstract Book, but they cannot change the content in any way or use them commercially.
**Abstract Book Citations:** Authors, Title, HemaSphere, 2026;10:(S1):pages. The abstract book DOIs can be found on the “Author Information” page.
**Disclaimer:** Articles published in the journal HemaSphere exclusively reflect the opinions of the authors. The authors are responsible for all content in their abstracts including accuracy of the facts, statements, citing resources, etc.

### (PS2364) A METHOD FOR MONITORING RED CELL ENGRAFTMENT POST BONE MARROW TRANSPLANT FOR SICKLE CELL DISEASE

**Topic:** 27. Sickle cell disease

Azalea Khan*[1], Alireza Fahimi[2], Frances Brown[2], Kar Lok Kong[2], Subarna Chakravorty[3], Barnaby Clark[1, 2]

*[1]Synnovis, Red Cell Laboratory, London, United Kingdom; [2]Synnovis, Laboratory of Molecular Haemato-Oncology, London, United Kingdom; [3]King's College Hospital, Department of Haematological Medicine, London, United Kingdom;*

### Background

Allogenic bone marrow transplant (BMT) and gene therapy are the only available curative treatments for sickle cell disease (SCD). White cell engraftment after a BMT is monitored by DNA-based chimerism assays for leukaemia indications, but there is no monitoring assay specific for red cell indications even though these are the cell type that need to engraft to cure SCD. Published techniques to support red cell monitoring have used antibodies to target proteins, e.g. HbA and HbS, but these methods have reduced sensitivity and are affected by the presence of transfused blood. We have developed a droplet digital PCR (ddPCR) assay that detects RNA transcripts ($\beta^A$ and $\beta^S$) in reticulocytes which directly reflects the globin protein levels within the reticulocyte population and not the peripheral blood, making it ideal for monitoring BMTs.

### Aims

Development of a method for red cell engraftment monitoring by analysis of globin RNA expression in reticulocytes from whole blood.

### Methods

Patients undergoing BMT, had samples taken at various time-points from day –2 to day 28. All patients had standard chimerism analysis at 4 weeks post-BMT.

For controls, reticulocytes were isolated from non-BMT whole blood samples from healthy individuals (HbAA) and SCD patients (HbSS), using CD71 [OKT-9] antibody and magnetic beads (ThermoFisher). HbA and HbS levels were measured in whole blood and reticulocytes by HPLC (BioRad). Total RNA was recovered (Maxwell®, Promega) from 100 µL whole blood or >$5\times10^6$ reticulocytes recovered from the same blood sample. cDNA was generated (Superscript®, ThermoFisher) and was used in a TaqMan assay with probes specific to the $\beta^A$ and $\beta^S$ alleles. Each TaqMan assay was performed in individual droplets (20,000 partitions/sample) on a QX200 ddPCR instrument (BioRad).

**Copyright Information:** (Online) ISSN: 2572-9241
©2026 The Author(s). HemaSphere is published by John Wiley & Sons Ltd on behalf of European Hematology Association. This is an open access Abstract Book distributed under the Attribution-NonCommercial-NoDerivs (CC BY-NC-ND), which allows third parties to download the articles and share them with others as long as they credit the author and the Abstract Book, but they cannot change the content in any way or use them commercially.
**Abstract Book Citations:** Authors, Title, HemaSphere, 2026;10:(S1):pages. The abstract book DOIs can be found on the "Author Information" page.
**Disclaimer:** Articles published in the journal HemaSphere exclusively reflect the opinions of the authors. The authors are responsible for all content in their abstracts including accuracy of the facts, statements, citing resources, etc.

**Results**

A median of 10.4×$10^6$ (n=19) reticulocytes were isolated from 1 mL whole blood. The level of RNA transcript measured by ddPCR correlated strongly with the measurement of haemoglobin by HPLC, $r^2$ >0.9 **(Figure A)**, suggesting that RNA transcript levels directly reflect the level of haemoglobin. The ddPCR assay detected $\beta^A$ allele derived from transfused reticulocytes in samples collected <2 days post-transfusion. After 2 days, the level reduced reflecting the donor's own globin production. Within the peripheral blood the HbA protein could be detected by HPLC **(Figure B)**. We found very high concordance ($r^2$ >0.99) between RNA extracted from whole blood and isolated reticulocytes **(Figure C)** indicating that all the β allele signal was from reticulocytes and that no cell selection is required for the assay.

Using ddPCR, red cell chimerism was monitored in three SCD patients undergoing BMT, measuring from day -2 onwards **(Figure D)**. The level of donor-derived $\beta^A$ allele increased over time and reflected engraftment levels. It proved to be a useful marker to compliment the standard chimerism assessment.

**Summary/Conclusion**

The ddPCR assay allows for monitoring of red cell engraftment by detecting globin alleles ($\beta^A$ and $\beta^S$) directly from whole blood samples. ddPCR detects transfused HbA but this is short lived (2 days) and therefore makes the RNA assay more useful than protein-based assessments. In BMT cases treating SCD, the level of $\beta^A$ allele was monitored from d –2 onwards. This assay may allow greater insight into red cell engraftment at earlier timepoints helping to predict graft failure. This methodology could also apply to gene therapy cases for SCD.

**Copyright Information:** (Online) ISSN: 2572-9241
©2026 The Author(s). HemaSphere is published by John Wiley & Sons Ltd on behalf of European Hematology Association. This is an open access Abstract Book distributed under the Attribution-NonCommercial-NoDerivs (CC BY-NC-ND), which allows third parties to download the articles and share them with others as long as they credit the author and the Abstract Book, but they cannot change the content in any way or use them commercially.
**Abstract Book Citations:** Authors, Title, HemaSphere, 2026;10:(S1):pages. The abstract book DOIs can be found on the "Author Information" page.
**Disclaimer:** Articles published in the journal HemaSphere exclusively reflect the opinions of the authors. The authors are responsible for all content in their abstracts including accuracy of the facts, statements, citing resources, etc.

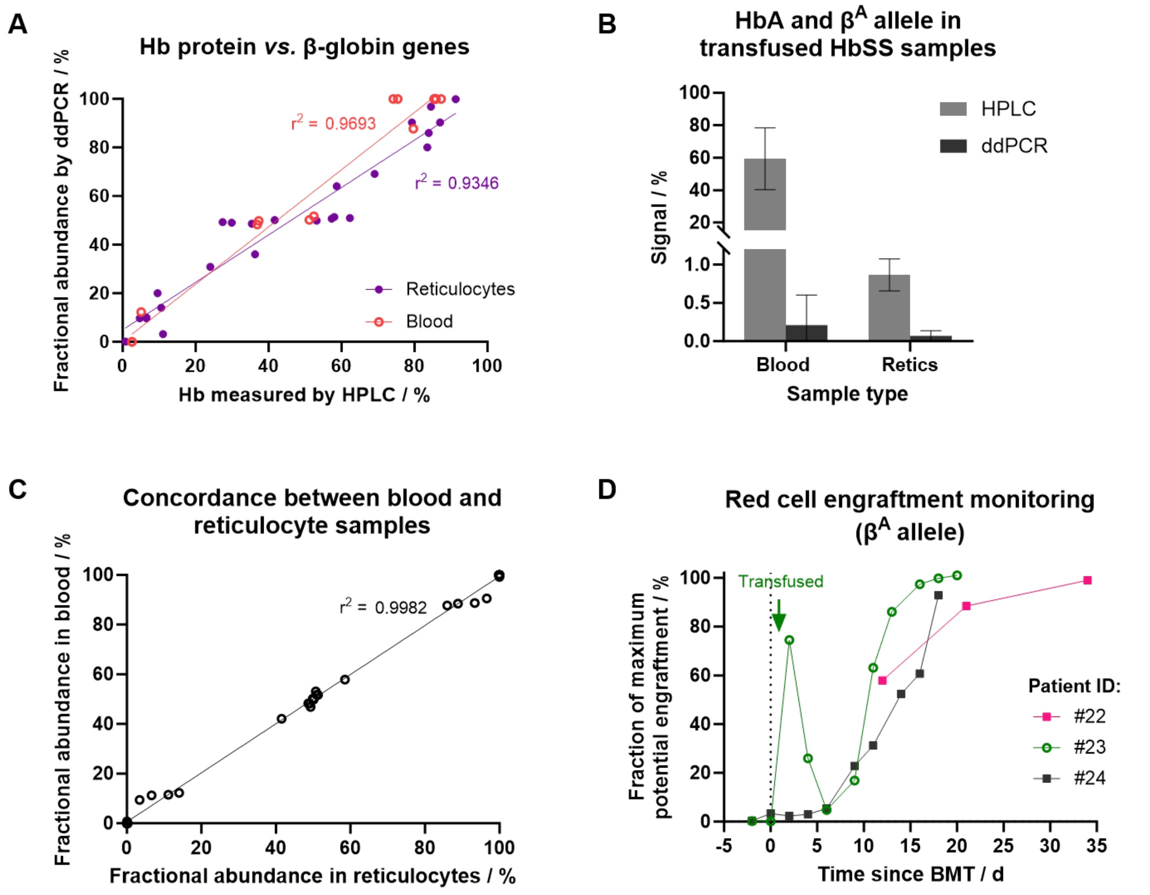

**Figure: A)** Comparison of level of haemoglobin and β-globin RNA transcripts, measured by HPLC and ddPCR, respectively. **B)** $β^A$-allele in transfused HbSS blood and reticulocyte samples measured by ddPCR, and compared with HbA measurements by HPLC. **C)** Concordance in ddPCR data between blood and reticulocyte samples. Data analysed by linear regression. **D)** Red cell engraftment of 3 SCD patients who underwent BMT, monitored from d -2 onwards of transplant. *Hb = haemoglobin, HPLC = high-performance liquid chromatography, ddPCR = droplet-digital polymerase chain reaction, RNA = ribonucleic acid.*

**Copyright Information:** (Online) ISSN: 2572-9241
©2026 The Author(s). HemaSphere is published by John Wiley & Sons Ltd on behalf of European Hematology Association. This is an open access Abstract Book distributed under the Attribution-NonCommercial-NoDerivs (CC BY-NC-ND), which allows third parties to download the articles and share them with others as long as they credit the author and the Abstract Book, but they cannot change the content in any way or use them commercially.
**Abstract Book Citations:** Authors, Title, HemaSphere, 2026;10:(S1):pages. The abstract book DOIs can be found on the “Author Information” page.
**Disclaimer:** Articles published in the journal HemaSphere exclusively reflect the opinions of the authors. The authors are responsible for all content in their abstracts including accuracy of the facts, statements, citing resources, etc.

### (PS2365) LONGITUDINAL EFFECT OF HYDROXYUREA AND CHRONIC EXCHANGE TRANSFUSION THERAPIES ON ECHOCARDIOGRAPHIC PARAMETERS OF SICKLE CELL CARDIOMYOPATHY

**Topic:** 27. Sickle cell disease

Jackie Queen, MD*[1], Chengzhou Wu, PhD[1], Deepa Manwani, MD[1], Guolian Kang, PhD[1], Parul Rai MD[1]

*[1]St. Jude Children's Research Hospital, Memphis, Tennessee, USA, Department of Hematology, Memphis, United States of America;*

### Background

Diastolic dysfunction (DD) is one of the early hallmarks of sickle cell cardiomyopathy (SCD-CM), which has both hyperdynamic and restrictive features (Simon et al. 2025). The etiopathogenesis of SCD-CM, although not fully understood, begins in childhood, with diastolic abnormalities on echocardiogram (Echo) (Simon et al. 2025). DD is an independent predictor of early mortality in adults with sickle cell disease (SCD) (Simon et al. 2025). There are no known target therapies to treat DD and SCD-CM. Data on the role of SCD disease-modifying therapies (DMT); hydroxyurea (HU) and chronic exchange transfusions (RBCX) in modifying DD and SCD-CM pathogenesis, is limited. Short-term follow-up showed no improvement in diastolic parameters on DMT, long-term data are lacking (Rai et al. 2023).

### Aims

The goal of our study is to evaluate the longitudinal effects of HU and RBCX on DD progression in the St. Jude-initiated multicenter lifespan SCD cohort study; Sickle Cell Clinical Research and Intervention Program (SCCRIP, NCT02098863) (Hankins et al. 2018).We hypothesize that treatment with DMT would improve Echo measures of diastolic function compared to no treatment.

### Methods

We performed a retrospective longitudinal analysis of Echo measurements obtained from patients with HbSS/Sb° thalassemia enrolled on SCCRIP. A linear mixed-effects model was used to compare the trajectories of Echo indices according to exposure to HU or RBCX therapies. Age at Echo was modeled as a continuous fixed effect. Additional variables included HU therapy and RBCX therapy. Interaction terms between treatments and age at ECHO were included if their p-values were < 0.05. P-value < 0.05 was considered statistically significant.

### Results

Our study cohort included 188 individuals with HbSS/Sb° thalassemia who were followed for a median of 3.11 years [IQR 2.02-7.19]. During this observation period, 43 patients received no DMT, 114 received HU therapy, and 31 received RBCX therapy. Mean age at the time of the first and the last ECHO was 10.68 ± 3.71 years and 15.31 ± 4.82 years, respectively. Diastolic Echo measures of left ventricle (LV) stiffness (e') and LV filling pressure (E/e') worsened with age. On linear mixed model analysis, RBCX and HU therapy were associated with improving LV e' septal velocity +1.12 cm/s (SE = 0.38, p = 0.003) and + 0.30 cm/s (SE = 0.26, p = 0.24), although the effect with HU was not statistically significant. RBCX and HU therapy were associated with significant reductions in tricuspid regurgitant jet velocity (TRV) of -29.72 cm/s (SE = 8.97, p = 0.001) and -12.14 cm/s (SE = 5.37, p = 0.02), respectively. However, HU and RBCX therapy had no effect on the age-related worsening of the echo variables of ventricular stiffness (lateral e'), LV filling pressure (E/e'), LV volume, LV mass, and left atrial volume.

**Copyright Information:** (Online) ISSN: 2572-9241
©2026 The Author(s). HemaSphere is published by John Wiley & Sons Ltd on behalf of European Hematology Association. This is an open access Abstract Book distributed under the Attribution-NonCommercial-NoDerivs (CC BY-NC-ND), which allows third parties to download the articles and share them with others as long as they credit the author and the Abstract Book, but they cannot change the content in any way or use them commercially.
**Abstract Book Citations:** Authors, Title, HemaSphere, 2026;10:(S1):pages. The abstract book DOIs can be found on the "Author Information" page.
**Disclaimer:** Articles published in the journal HemaSphere exclusively reflect the opinions of the authors. The authors are responsible for all content in their abstracts including accuracy of the facts, statements, citing resources, etc.

**Summary/Conclusion**

Individuals with SCD on DMT showed a slower increase in TRV over time than those untreated. The increasing TRV trajectory with age was nearly twice as slow on RBCX therapy compared to HU therapy. While there was modest improvement in septal e' with RBCX therapy, the other markers of diastolic function, specifically lateral e', an indicator of poor prognosis, did not improve on DMT. Overall, the use of DMTs appears insufficient to slow the progression of DD and SCD-CM. Future studies investigating novel therapies need to be conducted to explore non-curative therapeutic options to prevent or reverse this devastating SCD complication.

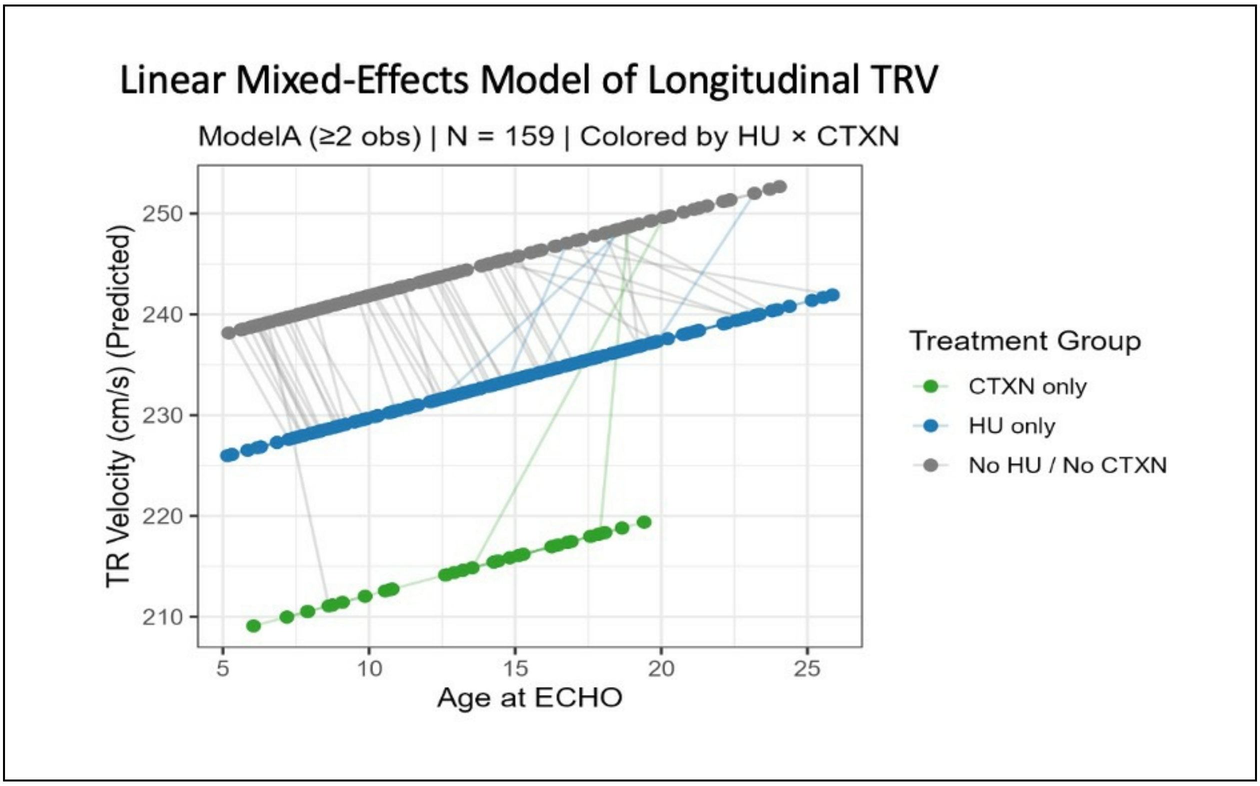

**Copyright Information:** (Online) ISSN: 2572-9241
©2026 The Author(s). HemaSphere is published by John Wiley & Sons Ltd on behalf of European Hematology Association. This is an open access Abstract Book distributed under the Attribution-NonCommercial-NoDerivs (CC BY-NC-ND), which allows third parties to download the articles and share them with others as long as they credit the author and the Abstract Book, but they cannot change the content in any way or use them commercially.
**Abstract Book Citations:** Authors, Title, HemaSphere, 2026;10:(S1):pages. The abstract book DOIs can be found on the "Author Information" page.
**Disclaimer:** Articles published in the journal HemaSphere exclusively reflect the opinions of the authors. The authors are responsible for all content in their abstracts including accuracy of the facts, statements, citing resources, etc.

### (PS2366) CO-DESIGNING A CULTURALLY-TAILORED SELF-MANAGEMENT TOOLBOX FOR UNIVERSITY STUDENTS WITH SICKLE CELL DISEASE IN SAUDI ARABIA: INTEGRATED MRC 2021 FRAMEWORK AND PERSON-BASED APPROACH

**Topic:** 27. Sickle cell disease

Reham Almabadi*[1, 2], Lawrence Doi[1], Colin Chandler[1]

*[1]University of Edinburgh, Nursing depratment, School of Health in Social Science, Edinburgh, United Kingdom;[2]King Saud University, College of Nursing, Education and Administration Department, Riyadh, Saudi Arabia;*

### Background

Self-management interventions for young adults with sickle cell disease (SCD) remain culturally underdeveloped, particularly in high-burden regions. Saudi Arabia has over 45,100 SCD patients (prevalence 145/10,000 in the Eastern region). University students with SCD globally face complex challenges: unpredictable disease fluctuations intersect with academic demands, social stigma, rigid institutional policies, and inadequate healthcare provider knowledge. Existing interventions do not address these barriers.

### Aims

To co-design a theory- and evidence-based self-management toolbox for university students with SCD in Saudi Arabia's eastern and southern regions using the Medical Research Council 221 (MRC) framework for developing and evaluating complex interventions and the Person-Based Approach (PBA) framework.

### Methods

Systematic development across four sequential phases, each building iteratively on previous findings (see Figure 1 for MRC framework alignment).
Phase 1 (scoping review) synthesized self-management interventions across SCD, type 1 diabetes, and inflammatory bowel disease to identify evidence-based components and behavior change mechanisms.

Phase 2 (Semi-Structured Interviews) explored lived experiences using reflexive thematic analysis.

Phase 3 (Focus Groups) identified core intervention components and delivery preferences. Findings from phase 3 were used to formulate a program theory, where self-determination theory and empowerment theory were operationalized as key behavior change mechanisms to explain how and why the components of the intervention are expected to work. The socioecological framework applied as a contextual lens mapping multilevel enablers and barriers.

Phase 4 (Co-Design Workshops) facilitated structured tool design based on the program theory and students prioritized tools through voting and collaboratively designed the highest-voted tools and then exchanged inter-group feedback for iterative refinement.

**Copyright Information:** (Online) ISSN: 2572-9241
©2026 The Author(s). HemaSphere is published by John Wiley & Sons Ltd on behalf of European Hematology Association. This is an open access Abstract Book distributed under the Attribution-NonCommercial-NoDerivs (CC BY-NC-ND), which allows third parties to download the articles and share them with others as long as they credit the author and the Abstract Book, but they cannot change the content in any way or use them commercially.
**Abstract Book Citations:** Authors, Title, HemaSphere, 2026;10:(S1):pages. The abstract book DOIs can be found on the "Author Information" page.
**Disclaimer:** Articles published in the journal HemaSphere exclusively reflect the opinions of the authors. The authors are responsible for all content in their abstracts including accuracy of the facts, statements, citing resources, etc.

**Results**

Phase 1 identified core self-management domains (symptom monitoring, psychological resilience, health literacy, and social support) and behavior change mechanisms effective for young adults.

Phase 2 revealed six themes: stigma (academic/clinical/peer), family/peer dynamics, cultural/religious influences, self-management expertise, paradoxical empowerment, and academic accommodation needs.

Phase 3 identified four core toolbox domains: (1) Daily/Weekly Self-Management Templates; (2) Psychological Resilience Resources; (3) Knowledge and Stigma Reduction; (4) Structural Enablers.

Phase 4 students actively designed a variety of tools, including academic health cards, comprehensive self-management planners, awareness campaigns, an SCD educational video, role model stories and peer support digital networks for SCD university students across Saudi Arabia.

**Summary/Conclusion**

This is the first co-designed, culturally tailored self-management intervention specifically developed for university students with SCD in Saudi Arabia, with tangible student-designed tools addressing daily self-management, psychological resilience, knowledge, stigma reduction, and structural barriers. The iterative MRC-PBA methodology demonstrates how each phase builds on previous evidence, with structured participatory design (voting and feedback activities) ensuring intervention relevance and acceptability. Rigorous, theory-driven, participant-engaged intervention development produces multilevel tools. Feasibility testing and mixed-methods evaluation will be the next phase.

**Copyright Information:** (Online) ISSN: 2572-9241
©2026 The Author(s). HemaSphere is published by John Wiley & Sons Ltd on behalf of European Hematology Association. This is an open access Abstract Book distributed under the Attribution-NonCommercial-NoDerivs (CC BY-NC-ND), which allows third parties to download the articles and share them with others as long as they credit the author and the Abstract Book, but they cannot change the content in any way or use them commercially.
**Abstract Book Citations:** Authors, Title, HemaSphere, 2026;10:(S1):pages. The abstract book DOIs can be found on the “Author Information” page.
**Disclaimer:** Articles published in the journal HemaSphere exclusively reflect the opinions of the authors. The authors are responsible for all content in their abstracts including accuracy of the facts, statements, citing resources, etc.

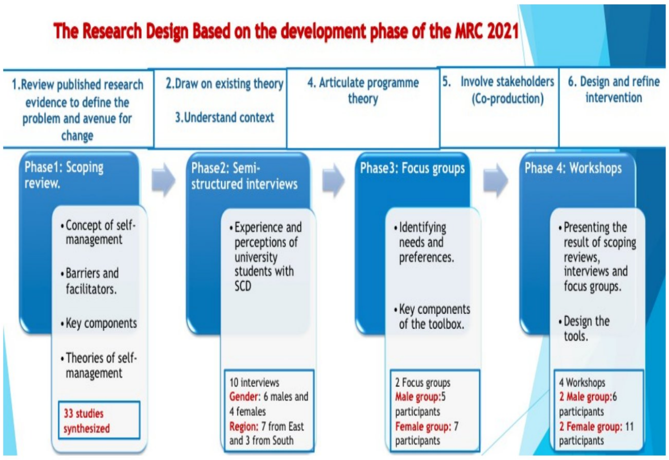

**Figure 1 (Research design)**

**Copyright Information:** (Online) ISSN: 2572-9241
©2026 The Author(s). HemaSphere is published by John Wiley & Sons Ltd on behalf of European Hematology Association. This is an open access Abstract Book distributed under the Attribution-NonCommercial-NoDerivs (CC BY-NC-ND), which allows third parties to download the articles and share them with others as long as they credit the author and the Abstract Book, but they cannot change the content in any way or use them commercially.
**Abstract Book Citations:** Authors, Title, HemaSphere, 2026;10:(S1):pages. The abstract book DOIs can be found on the "Author Information" page.
**Disclaimer:** Articles published in the journal HemaSphere exclusively reflect the opinions of the authors. The authors are responsible for all content in their abstracts including accuracy of the facts, statements, citing resources, etc.

### (PS2367) SHIFTING ATTRIBUTION OF SICKLE CELL DISEASE ON US DEATH CERTIFICATES: A NATIONAL MULTIPLE CAUSE-OF-DEATH ANALYSIS, 2003–2024

**Topic:** 27. Sickle cell disease

Mohammad Ahsen Soomro[1], Hala Soomro*[2], Zafar Suchal[3]

*[1]The Wright Center for Graduate Medical Education, Cardiology, Scranton, United States of America;[2]Brown University of Health Sciences, Internal Medicine, Providence, United States of America; [3]Shalamar Institute of Health Science, Department of Clinical Research, Lahore, Pakistan;*

### Background

As survival improves in sickle cell disease (SCD), mortality patterns may shift from acute SCD-attributed deaths toward chronic multi-organ complications. Analyses restricted to underlying cause of death (UCOD) may underestimate this evolving burden. We evaluated national trends in SCD death certificate attribution using US multiple cause-of-death (MCOD) data.

### Aims

To quantify shifts in underlying versus contributing cause-of-death attribution for sickle cell disease (SCD) from 2003–2024 and to assess whether changes in attribution are accompanied by increasing age at death and rising cardiopulmonary comorbidity.

### Methods

We analyzed all US death certificates from 2003–2024 listing SCD (ICD-10 D57.*) anywhere on the certificate. For each year, we quantified the proportion in which SCD was designated as the UCOD versus listed as a contributing cause only (MCOD-only). Temporal trends were assessed using binomial logistic regression. Median age at death, proportion of deaths occurring at age ≥40 years, and co-mentioned complication categories were evaluated descriptively.

### Results

From 2003–2024, 20,487 death certificates listed SCD. In 2003, SCD was designated as the UCOD in 62.3% of SCD-associated deaths (482/774), declining to 51.3% in 2024 (590/1,151). The proportion of MCOD-only mentions—where SCD was present but not designated as the primary cause—increased from 37.7% to 48.7%, with the UCOD-to-MCOD-only ratio nearly equalizing (1.65 to 1.05; p-trend <0.001). This shift coincided with significant aging of the cohort: median age at death rose from 39 years (IQR 28–49) to 46 years (IQR 33–60), and the proportion of deaths occurring at age ≥40 years increased from 49.0% to 61.5%. Among adults ≥18 years, UCOD attribution similarly declined (61.9% to 51.5%; p-trend <0.001), confirming that trends were not driven by pediatric mortality changes. Concurrently, chronic cardiopulmonary complications increasingly co-appeared on certificates, including respiratory failure (6.5% to 12.0%) and heart failure (8.0% to 12.2%), with a modest rise in pulmonary hypertension (5.4% to 6.4%). A transient inflection in 2020 likely reflects COVID-19–related mortality coding shifts; however, the overall attribution trend predates and persists beyond the pandemic period.

**Copyright Information:** (Online) ISSN: 2572-9241
©2026 The Author(s). HemaSphere is published by John Wiley & Sons Ltd on behalf of European Hematology Association. This is an open access Abstract Book distributed under the Attribution-NonCommercial-NoDerivs (CC BY-NC-ND), which allows third parties to download the articles and share them with others as long as they credit the author and the Abstract Book, but they cannot change the content in any way or use them commercially.
**Abstract Book Citations:** Authors, Title, HemaSphere, 2026;10:(S1):pages. The abstract book DOIs can be found on the “Author Information” page.
**Disclaimer:** Articles published in the journal HemaSphere exclusively reflect the opinions of the authors. The authors are responsible for all content in their abstracts including accuracy of the facts, statements, citing resources, etc.

**Summary/Conclusion**

Over two decades, SCD has become progressively less likely to be designated as the underlying cause of death and more frequently appears as a contributing condition in an aging population with increasing cardiopulmonary organ dysfunction. Traditional mortality surveillance based solely on UCOD substantially underestimates the contemporary burden and multi-organ complexity of SCD-related mortality. MCOD-based approaches are essential for accurately capturing evolving outcomes and morbidity patterns in an aging population with expanding disease-modifying treatment options.

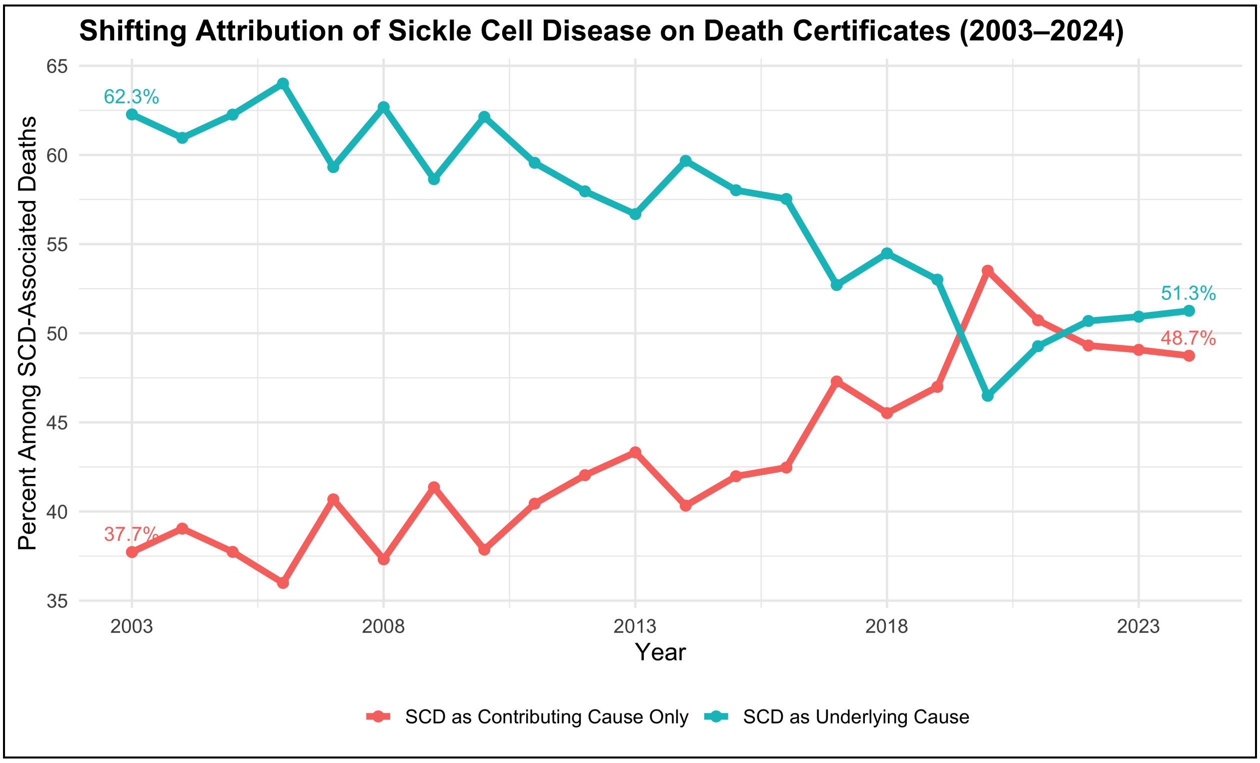

UCOD = underlying cause of death; MCOD-only = SCD listed as a contributing but not the primary cause. Percentages reflect proportion among all death certificates listing SCD (ICD-10 D57.*). Data: US NVSS multiple cause-of-death files, 2003–2024. A transient 2020 inflection likely reflects COVID-19 coding effects.

**Copyright Information:** (Online) ISSN: 2572-9241
©2026 The Author(s). HemaSphere is published by John Wiley & Sons Ltd on behalf of European Hematology Association. This is an open access Abstract Book distributed under the Attribution-NonCommercial-NoDerivs (CC BY-NC-ND), which allows third parties to download the articles and share them with others as long as they credit the author and the Abstract Book, but they cannot change the content in any way or use them commercially.
**Abstract Book Citations:** Authors, Title, HemaSphere, 2026;10:(S1):pages. The abstract book DOIs can be found on the "Author Information" page.
**Disclaimer:** Articles published in the journal HemaSphere exclusively reflect the opinions of the authors. The authors are responsible for all content in their abstracts including accuracy of the facts, statements, citing resources, etc.

### (PS2368) DOUBLE-BLIND, RANDOMIZED, PLACEBO-CONTROLLED STUDY OF CROVALIMAB FOR THE TREATMENT OF ACUTE VASO-OCCLUSIVE EPISODES IN SICKLE CELL DISEASE: RESULTS OF THE PHASE 1B CROSSWALK-A TRIAL

**Topic:** 27. Sickle cell disease

Satheesh Chonat*[1], Laine Alvarenga[2], Fredrick Asirwa[3], Lucia De Franceschi[4], Elena Cela[5], Doreen Karimi[6], Johnny Mahlangu[7], Erfan Nur[8, 9], Steven Okoli[10], Salvador Payán-Pernía[11, 12], Jonathan Schimmel[13], Ali T. Taher[14], Simon Buatois[15], Amparo Yovanna Castro Sánchez[15], Asad Charania[15], Allison Hsia[16], Sasha Sreckovic[16], Anqi Yin[17], Pablo Bartolucci[18]

*[1]Emory University and Aflac Cancer and Blood Disorders Center, Children's Healthcare of Atlanta, Department of Pediatrics, Atlanta, United States of America; [2]Hospital São Rafael/IDOR, Salvador, Brazil; [3]International Cancer Institute, Eldoret, Kenya; [4]University of Verona and Azienda Ospedaliera Universitaria Verona, Department of Medicine, Verona, Italy;[5]Hospital General Universitario Gregorio Marañón, Universidad Complutense de Madrid, Madrid, Spain; [6]Gertrude's Children's Hospital, Department of Hematology/Oncology, Nairobi, Kenya; [7]University of the Witwatersrand, National Health Laboratory Service, Department of Molecular Medicine and Haematology, Johannesburg, South Africa; [8]Amsterdam UMC, University of Amsterdam, Department of Hematology, Amsterdam, The Netherlands; [9]Sanquin Research, Department of Blood Cell Research, Amsterdam, The Netherlands; [10]Imperial College Healthcare NHS Trust, Hammersmith Hospital, Department of Haematology, London, United Kingdom; [11]Hospital Universitario Virgen del Rocío, Department of Hematology, Sevilla, Spain;[12]Instituto de Biomedicina de Sevilla (IBiS)-CSIC, Universidad de Sevilla, Department of Hematology, Sevilla, Spain; [13]Icahn School of Medicine at Mount Sinai, Department of Emergency Medicine, New York, United States of America; [14]American University of Beirut Medical Center, Division of Hematology and Oncology, Department of Internal Medicine, Beirut, Lebanon; [15]F. Hoffmann-La Roche Ltd, Basel, Switzerland; [16]Genentech, Inc., South San Francisco, United States of America;[17]Roche (China) Holding Ltd, Shanghai, China; [18]Hôpital Universitaire Necker Enfants-Malades, GH Paris Centre, Biotherapy Department and Centre d'Investigation Clinique-Biothérapie, Paris, France;*

### Background

Vaso-occlusive episodes (VOEs) are the leading cause of hospitalization and most frequent acute complication in sickle cell disease (SCD), and are associated with reduced quality of life and increased mortality. Management is largely supportive and opioid-based, with high rates of hospital re-admission. Available nonclinical data suggest a multimodal role for complement dysregulation in SCD pathophysiology, including vaso-occlusion. Crovalimab (crova) is a novel, humanized anti-C5 monoclonal antibody that has demonstrated rapid and sustained complement inhibition in paroxysmal nocturnal hemoglobinuria.

### Aims

To evaluate crova vs placebo (pbo) in VOE treatment (tx) in patients (pts) with SCD from CROSSWALK-a (NCT04912869).

### Methods

Pts aged 12–55 years with SCD (HbSS or HbSβ0), body weight ≥40 kg, ≤10 VOEs in the prior year, and hospitalized for an acute uncomplicated VOE requiring parenteral opioids, were randomized 2:1 to a single intravenous dose of crova (weight-based dosing; 1000 mg for ≥40 to <100 kg; 1500 mg for ≥100 kg) or pbo within 24 hrs of presentation. Pts were followed up until 322 days after study tx. The primary objective was safety. Additional objectives included pharmacokinetics/pharmacodynamics (PK/PD) and exploratory efficacy (e.g., time to acute uncomplicated VOE improvement from baseline and readiness for/actual hospital discharge). Statistical analyses were descriptive.

**Copyright Information:** (Online) ISSN: 2572-9241
©2026 The Author(s). HemaSphere is published by John Wiley & Sons Ltd on behalf of European Hematology Association. This is an open access Abstract Book distributed under the Attribution-NonCommercial-NoDerivs (CC BY-NC-ND), which allows third parties to download the articles and share them with others as long as they credit the author and the Abstract Book, but they cannot change the content in any way or use them commercially.
**Abstract Book Citations:** Authors, Title, HemaSphere, 2026;10:(S1):pages. The abstract book DOIs can be found on the "Author Information" page.
**Disclaimer:** Articles published in the journal HemaSphere exclusively reflect the opinions of the authors. The authors are responsible for all content in their abstracts including accuracy of the facts, statements, citing resources, etc.

**Results**

Clinical cutoff dates were Aug 18, 2025 (final analysis), for safety/PK and Dec 23, 2024 (primary analysis), for efficacy/PD. 30 pts were randomized (20 to crova; 10 to pbo); 1 pt in the pbo arm discontinued from the trial before dosing and was excluded from safety analyses. The median number of VOEs in the prior 12 months was 4.5 (range: 1.0–10.0) in the crova arm and 4.0 (1.0–8.0) in the pbo arm. Median VOE duration before study tx was 27.0 hours (range: 4.0–46.4) and 17.8 hours (7.9–30.2), respectively.
Safety results are shown in the **Table**. Treatment-related adverse events (AEs) occurred in 10.0% of crova-treated pts vs 33.3% pbo-treated pts; Grade 3–4 AEs, in 100% vs 55.6%; serious AEs, in 100% vs 55.6% (most common by preferred term: 'sickle cell anemia with crisis' [80.0% vs 44.4%] and 'acute chest syndrome' [25.0% vs 22.2%]). No AEs led to study withdrawal and no infusion-related reactions were reported.
Median time to VOE improvement, hospital discharge, and readiness for discharge was numerically longer for the crova arm (**Table**). Efficacy results are inconclusive in light of the small sample size.
All crova-treated pts achieved crova serum concentrations above the target threshold for complete complement inhibition (≥100 μg/mL) at Day 1. Mean concentrations were 85.3 μg/mL (95% CI: 68.5, 102.2) at Day 28 and 18.8 μg/mL (95% CI: 14.4, 23.3) at Day 84. Free C5 was suppressed to <0.0001 g/L within 30 mins and sustained through Day 28 in 88.0% of pts. CH50 suppression was sustained through Day 28 in 94.1% of pts and recovered by Day 84. No impact of anti-drug antibodies on PK/PD was observed.

**Summary/Conclusion**

Crova was well tolerated in pts with SCD hospitalized for acute uncomplicated VOEs, with no new safety signals identified. Crova achieved rapid, complete, and sustained terminal complement inhibition. Due to the study's low sample size and high variability in efficacy endpoint assessment, the benefit–risk profile of crova for acute uncomplicated VOE management in SCD remains inconclusive.

**Copyright Information:** (Online) ISSN: 2572-9241
©2026 The Author(s). HemaSphere is published by John Wiley & Sons Ltd on behalf of European Hematology Association. This is an open access Abstract Book distributed under the Attribution-NonCommercial-NoDerivs (CC BY-NC-ND), which allows third parties to download the articles and share them with others as long as they credit the author and the Abstract Book, but they cannot change the content in any way or use them commercially.
**Abstract Book Citations:** Authors, Title, HemaSphere, 2026;10:(S1):pages. The abstract book DOIs can be found on the "Author Information" page.
**Disclaimer:** Articles published in the journal HemaSphere exclusively reflect the opinions of the authors. The authors are responsible for all content in their abstracts including accuracy of the facts, statements, citing resources, etc.

**Table. Safety and efficacy summary**

| | Crova (n=20) | Pbo (n=9) |
|---|---|---|
| **Safety summary in the safety analysis set** | | |
| Total number of pts with ≥1 AE, n (%) | 20 (100%) | 8 (88.9%) |
| SAE* | 20 (100%) | 5 (55.6%) |
| Related SAE* | 1 (5.0%) | 1 (11.1%) |
| Related AE* | 2 (10.0%) | 3 (33.3%) |
| Grade 3–4 AE | 20 (100%) | 5 (55.6%) |
| AE with fatal outcome | 0 | 0 |
| AE leading to study withdrawal | 0 | 0 |
| Pts with AEs of special interest, n (%) | | |
| Elevated ALT or AST in combination with either an elevated bilirubin or clinical jaundice | 0 | 0 |
| Suspected transmission of an infectious agent by the study drug | 0 | 0 |
| Severe infections (≥ NCI CTCAE Grade 3), including meningococcal infections† | 3 (15.0%) | 2 (22.2%) |
| | **Crova (n=20)** | **Pbo (n=10)‡** |
| **Efficacy summary in the intent-to-treat population** | | |
| Median time to improvement of the primary acute uncomplicated VOE§ hrs (95% CI) | 43.7 (23.3, 84.8) | 40.3 (21.9, 66.5) |
| Median time to hospital discharge, hrs (95% CI) | 46.5 (26.1, 90.3) | 41.2 (26.9, 72.1) |
| Median time to readiness for hospital discharge, hrs (95% CI) | 43.9 (23.3, 130.2) | 40.3 (21.9, 66.5) |

* No AEs led to treatment/study withdrawal, or dose modification/interruption. † No meningococcal infections were reported. ‡ In the pbo arm, 10 pts were enrolled and included in the ITT population, but 1 pt discontinued from the study prior to dosing, and was therefore excluded from the safety analysis set. § Defined as the first of: sustained ≥2-point pain score reduction with transition to oral analgesics, readiness for discharge, or hospital discharge. AE, adverse event; ALT, alanine aminotransferase; AST, aspartate aminotransferase; CI, confidence interval; crova, crovalimab; hrs, hours; ITT, intention to treat; NCI CTCAE, National Cancer Institute Common Terminology Criteria for Adverse Events; pbo, placebo; pts, patients; SAE, serious adverse event; VOE, vaso-occlusive episode.

**Copyright Information:** (Online) ISSN: 2572-9241
©2026 The Author(s). HemaSphere is published by John Wiley & Sons Ltd on behalf of European Hematology Association. This is an open access Abstract Book distributed under the Attribution-NonCommercial-NoDerivs (CC BY-NC-ND), which allows third parties to download the articles and share them with others as long as they credit the author and the Abstract Book, but they cannot change the content in any way or use them commercially.
**Abstract Book Citations:** Authors, Title, HemaSphere, 2026;10:(S1):pages. The abstract book DOIs can be found on the "Author Information" page.
**Disclaimer:** Articles published in the journal HemaSphere exclusively reflect the opinions of the authors. The authors are responsible for all content in their abstracts including accuracy of the facts, statements, citing resources, etc.

### (PS2370) CHANGING EPIDEMIOLOGY OF SICKLE CELL DISORDERS: GBD 2023 ANALYSIS OF INCIDENCE TRENDS TO 2023, REGIONAL DISPARITIES IN EUROPE, AND IMPLICATIONS FOR GENE THERAPY ACCESS

**Topic:** 27. Sickle cell disease

Tapan Giri*[1], Zeal Soni[2]

*[1]University of Nevada, Reno school of medicine, Department of Internal medicine, Reno, United States of America;[2]Smt. NHL municipal medical college, Department of Internal medicine, Ahmedabad, India;*

### Background

Sickle cell disorders (SCD) are increasingly relevant in Europe due to migration patterns and improved survival rates. With novel gene therapies (e.g., exagamglogene autotemcel) now approved in the EU and UK, up-to-date epidemiological burden data are essential for planning equitable access and resource allocation.

### Aims

To describe global and European trends in SCD incidence and mortality using the latest Global Burden of Disease (GBD) 2023 estimates (up to 2023), with a particular focus on high Socio-Demographic Index (SDI) regions and intra-European regional disparities.

### Methods

GBD 2023 estimates for SCD were extracted for the global level, high-SDI regions (as a proxy for Western Europe), and sub-regions: Western Europe, Central Europe, and Eastern Europe. Age-standardized incidence rates (per 100,000 population) and percent changes from 1990 to 2023 were calculated for both incidence and mortality.

### Results

In high-SDI regions, age-standardized incidence declined by 44.0% (from 1.182 to 0.662 per 100,000), and absolute new cases fell by 63.4% (from 18,782 to 6,869) between 1990 and 2023.
Marked intra-European disparities persist: Western Europe shows substantial incidence reductions but rising mortality; Central Europe demonstrates clear mortality improvements; Eastern Europe faces a continuing increase in burden. These patterns reflect successful prenatal screening and prevention in high-resource settings, contrasted with migration-driven increases in prevalence and variable access to comprehensive care across regions.

### Summary/Conclusion

The sharp decline in incidence in high-SDI regions through 2023 underscores the effectiveness of prevention strategies. However, persistent and widening mortality disparities across Europe highlight urgent inequities in access to comprehensive care and newly approved gene therapies. Coordinated European policies are essential to address demographic shifts from migration and to ensure equitable rollout of innovative curative treatments.

**Copyright Information:** (Online) ISSN: 2572-9241
©2026 The Author(s). HemaSphere is published by John Wiley & Sons Ltd on behalf of European Hematology Association. This is an open access Abstract Book distributed under the Attribution-NonCommercial-NoDerivs (CC BY-NC-ND), which allows third parties to download the articles and share them with others as long as they credit the author and the Abstract Book, but they cannot change the content in any way or use them commercially.
**Abstract Book Citations:** Authors, Title, HemaSphere, 2026;10:(S1):pages. The abstract book DOIs can be found on the "Author Information" page.
**Disclaimer:** Articles published in the journal HemaSphere exclusively reflect the opinions of the authors. The authors are responsible for all content in their abstracts including accuracy of the facts, statements, citing resources, etc.

### (PF2371) ADVERSE MATERNAL AND PERINATAL OUTCOMES ASSOCIATED WITH SICKLE CELL TRAIT IN PREGNANCY: A SYSTEMATIC REVIEW AND META-ANALYSIS

**Topic:** 27. Sickle cell disease

Luisa Medeiros Visentini*[1], Hosana Candreva[2], Mehak Dang[3], Ingridy Sodre Da Costa[4], Ching Sum Sin[5], Brenno Madureira Pessi[1], Fahad Alabbas[6]

*[1]University of Western Sao Paulo, Faculty of Medicine, Presidente Prudente, Brazil; [2]Rio de Janeiro State University, Rio de Janeiro, Brazil; [3]JSS Medical College, Mysuru, India; [4]University of Pavia, Lombardy, Italy; [5]Prince of Wales Hospital, Hospital Authority, Hong Kong, China; [6]Prince Sultan Military Medical City, Riyadh, Saudi Arabia;*

### Background

Sickle cell trait (SCT) is traditionally regarded as a benign carrier state, however, pregnancy constitutes a physiologic stressor that may reveal clinically significant complications. While the adverse obstetric consequences of sickle cell disease are well established, the implications of SCT in pregnancy remain uncertain. Given the high global prevalence of SCT and the expansion of antenatal screening programs, clarifying associated risks is of substantial clinical and public health importance.

### Aims

To systematically evaluate the association between SCT in pregnancy and adverse maternal and perinatal outcomes compared with pregnancies in women without SCT.

### Methods

We conducted a systematic review and meta-analysis of studies in accordance with PRISMA guidelines. The protocol was registered in PROSPERO (CRD420251266162). PubMed, Embase, and the Cochrane Library were searched from inception to December 2025. Eligible studies reported quantitative maternal or perinatal outcomes of interest among pregnant women with SCT compared with women without SCT. Two authors independently screened studies and extracted data. Random-effects models were used to generate pooled relative risks (RRs) or mean differences (MDs) with 95% confidence intervals (CIs). Heterogeneity was assessed using the $I^2$ statistic. Prespecified subgroup analyses were performed according to country income level. All analyses were conducted in R (version 4.2.3).

**Copyright Information:** (Online) ISSN: 2572-9241
©2026 The Author(s). HemaSphere is published by John Wiley & Sons Ltd on behalf of European Hematology Association. This is an open access Abstract Book distributed under the Attribution-NonCommercial-NoDerivs (CC BY-NC-ND), which allows third parties to download the articles and share them with others as long as they credit the author and the Abstract Book, but they cannot change the content in any way or use them commercially.
**Abstract Book Citations:** Authors, Title, HemaSphere, 2026;10:(S1):pages. The abstract book DOIs can be found on the “Author Information” page.
**Disclaimer:** Articles published in the journal HemaSphere exclusively reflect the opinions of the authors. The authors are responsible for all content in their abstracts including accuracy of the facts, statements, citing resources, etc.

### Results

Thirty-five observational (22 retrospective and 13 prospective) studies comprising 12,554,554 pregnancies were included, of whom 88,994 had confirmed SCT. Compared with women without SCT, those with SCT had increased risks of maternal mortality (RR 2.82; 95% CI 1.34–5.97; I² = 0%), venous thromboembolism (RR 2.73; 95% CI 1.90–3.92; I² = 0%), gestational hypertension (RR 1.26; 95% CI 1.04–1.54; I² = 89.7%), preeclampsia (RR 1.35; 95% CI 1.04–1.74; I² = 82.1%), blood transfusion (RR 1.78; 95% CI 1.36–2.32; I² = 86.5%), and emergency cesarean delivery (RR 1.33; 95% CI 1.15–1.53; I² = 0%). In subgroup analyses by country income level, the heterogeneity for blood transfusion was reduced in high-income countries (HICs: RR 2.34; 95% CI 2.23–2.46; I² = 0%) and in low- and middle-income countries (LMICs: RR 1.30; 95% CI 1.04–1.63; I² = 0%). Neonates born to mothers with SCT had a lower mean birth weight (MD −149.6 g; 95% CI −246.3 to −52.9; I² = 90.4%). In the overall analysis, there was no statistically significant association between mothers with SCT and stillbirth (RR 1.11; 95% CI 0.78–1.60; I² = 51.1%) or neonatal death (RR 1.38; 95% CI 0.76–2.48; I² = 56.3%). However, subgroup analyses showed that pregnant women with SCT had an increased risk of stillbirth (RR 1.41; 95% CI 1.19–1.67; I² = 13%) and neonatal death (RR 2.10; 95% CI 1.07–4.13; I² = 18.8%) in LMICs, whereas these associations were not statistically significant in HICs. Other fetal and neonatal outcomes, including preterm birth, spontaneous abortion, intrauterine growth retardation, neonatal intensive care unit admission, and other neonatal morbidities, were not statistically significant.

### Summary/Conclusion

SCT in pregnancy is associated with increased risks of maternal complications and adverse neonatal outcomes. Findings are limited by substantial heterogeneity in selected outcomes and the exclusive reliance on observational data. Well-designed prospective studies are needed to establish causality, quantify absolute risks, and inform obstetric counseling and public health policy.

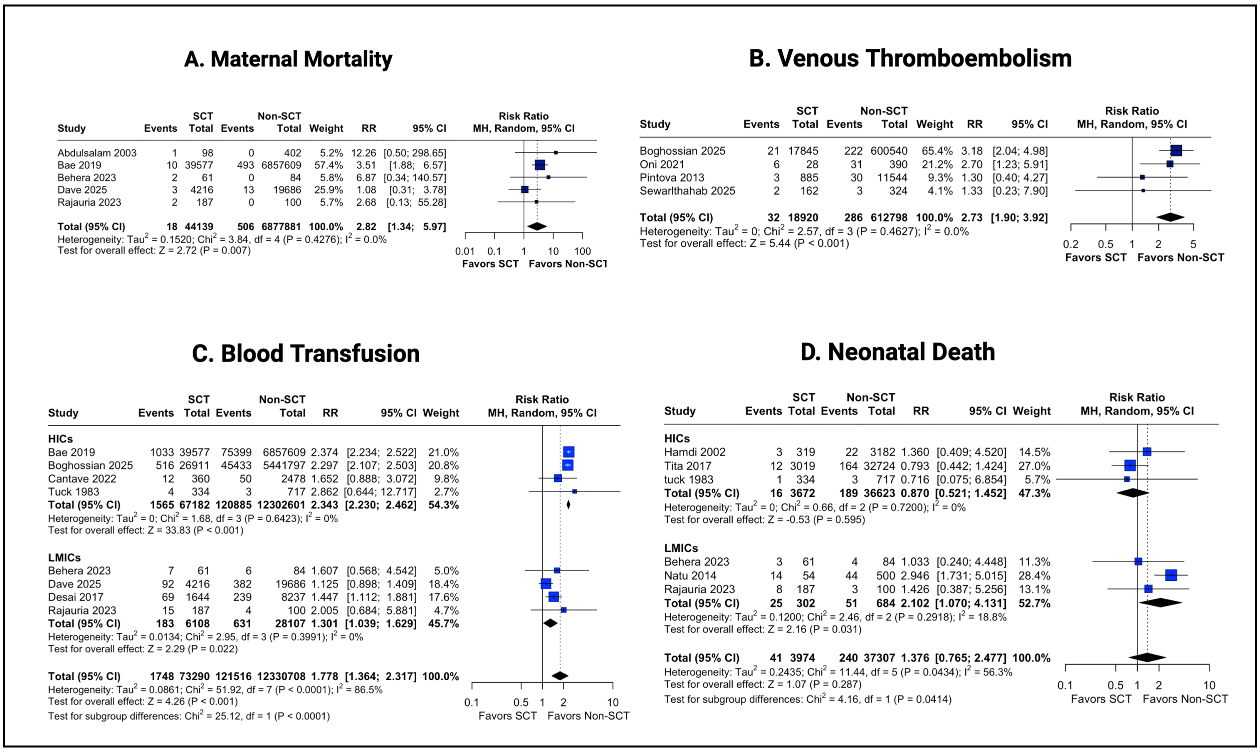

**Copyright Information:** (Online) ISSN: 2572-9241
©2026 The Author(s). HemaSphere is published by John Wiley & Sons Ltd on behalf of European Hematology Association. This is an open access Abstract Book distributed under the Attribution-NonCommercial-NoDerivs (CC BY-NC-ND), which allows third parties to download the articles and share them with others as long as they credit the author and the Abstract Book, but they cannot change the content in any way or use them commercially.
**Abstract Book Citations:** Authors, Title, HemaSphere, 2026;10:(S1):pages. The abstract book DOIs can be found on the "Author Information" page.
**Disclaimer:** Articles published in the journal HemaSphere exclusively reflect the opinions of the authors. The authors are responsible for all content in their abstracts including accuracy of the facts, statements, citing resources, etc.

### (PS2372) DISTINCT CLINICAL PHENOTYPE OF HBSC GENOTYPE: LOWER SYSTEMIC COMPLICATION RATES BUT INCREASED RISK OF PROLIFERATIVE RETINOPATHY IN A LARGE PROSPECTIVE BRAZILIAN COHORT

**Topic:** 27. Sickle cell disease

Dahra Teles Soares Cruz*[1, 2, 3], Carla Luana Dinardo[4], Fabiana Canedo Pinto[5], Daniela de Oliveira Werneck Rodrigues[6], Cecilia Salete Alencar[7], Alessandra Ferraz de Sá[8], Kalysta Borges[9], José Antonio Da Silva[10], Pedro Teles Soares Cruz[11], Tiago Pessoa Ferreira de Lima[12], Isabel Gomes[13], Mina Cintho Ozahata[14], Maria Eduarda Teles Soares de Lavor[15], Ester Cerdeira Sabino[16], Shannon Kelly[17], Brian Custer[18]

*[1]Pernambuco University, Recife, Brazil; [2]HEMOPE, Recife, Brazil; [3]Santa Joana Recife Hospital, Recife, Brazil; [4]Fundação Pró-Sangue Hemocentro de São Paulo and Instituto de Medicina Tropical da Faculdade de Medicina da USP, São Paulo, Brazil; [5]Hemorio, Rio de Janeiro, Rio de Janeiro, Brazil., Rio de Janeiro, Brazil;[6]Fundação Hemominas, Minas Gerais, Brazil, Minas Gerais, Brazil; [7]Fundação Pró-Sangue Hemocentro de São Paulo and Instituto de Medicina Tropical da Faculdade de Medicina da USP, São Paulo, Brazil., Sao Paulo, Brazil; [8]Hemope, Recife, PE, Brazil, Recife, Brazil; [9]Oncologica Tapajós, Santarém, Brazil; [10]Universidade de Pernambuco, Recife, Brazil; [11]Faculdade de Medicina Uninassau, Recife, Brazil; [12]Instituto Federal de Pernambuco, Recife, Brazil; [13]Universidade Federal de Minas Gerais, Belo Horizonte, Brazil; [14]University of Sao Paulo, Sao Paulo, Brazil; [15]Faculdade de Medicina Estácio Idomed, Juazeiro do Norte, Brazil; [16]Faculdade de Medicina da Universidade de São Paulo - FMUSP, São Paulo, Brazil; [17]UCSF Benioff Children's Hospital, Oakland, California, United States of America., Oakland, United States of America; [18]Vitalant Research Institute, San Francisco, California, United States of America., San Francisco, United States of America;*

### Background

Sickle cell disease (SCD) comprises a heterogeneous group of hemoglobinopathies. Although hemoglobin SC disease (HbSC) is the second most frequent genotype worldwide, most management strategies are extrapolated from sickle cell anemia (HbSS). Whether HbSC represents a milder form of SCD or a distinct clinical phenotype remains incompletely defined.

### Aims

To characterize the demographic, laboratory, and clinical complication profile of HbSC patients and to compare their risk of SCD-related complications with other genotypes in a large multicenter prospective Brazilian cohort.

### Methods

This cohort included 2,793 SCD patients followed at six Brazilian blood centers participating in the "The Recipient Epidemiology and Donor Evaluation (REDS-III SCD Brazil) project. HbSC patients were compared with non-HbSC genotypes regarding laboratory parameters and major SCD-related complications. Relative risks (RR) with 95% confidence intervals (CI) were calculated.

### Results

The prevalence of HbSC was 23% (644 patients), and this group had higher levels of hemoglobin, lower mean corpuscular volume, leukocyte count, platelets, reticulocytes, fetal hemoglobin, ferritin, total bilirubin, direct bilirubin, and indirect bilirubin when compared to non-HbSC patients (all with p<0.001). Regarding clinical complications, patients with HbSC had lower rates of hospitalization (RR 0.70, 95% CI 0.60; 0.82), lower risk of hospitalization for painful crisis (RR 0.68, 95% CI 0.57; 0.81), less occurrence of acute chest syndrome (RR 0.78, 95% CI 0.69; 0.89) and leg ulcer (RR 0.13, 95% CI 0.06; 0.22). On the other hand, these patients had a higher risk of developing proliferative sickle cell retinopathy (RR 2.01, 95% CI 1.45; 2.77).

**Copyright Information:** (Online) ISSN: 2572-9241
©2026 The Author(s). HemaSphere is published by John Wiley & Sons Ltd on behalf of European Hematology Association. This is an open access Abstract Book distributed under the Attribution-NonCommercial-NoDerivs (CC BY-NC-ND), which allows third parties to download the articles and share them with others as long as they credit the author and the Abstract Book, but they cannot change the content in any way or use them commercially.
**Abstract Book Citations:** Authors, Title, HemaSphere, 2026;10:(S1):pages. The abstract book DOIs can be found on the "Author Information" page.
**Disclaimer:** Articles published in the journal HemaSphere exclusively reflect the opinions of the authors. The authors are responsible for all content in their abstracts including accuracy of the facts, statements, citing resources, etc.

**Summary/Conclusion**

HbSC is associated with a less pronounced systemic hemolytic and inflammatory profile, translating into reduced rates of several acute and chronic systemic complications. However, the markedly increased risk of proliferative retinopathy supports the concept of HbSC as a distinct clinical phenotype requiring genotype-specific surveillance strategies.

**Copyright Information:** (Online) ISSN: 2572-9241
©2026 The Author(s). HemaSphere is published by John Wiley & Sons Ltd on behalf of European Hematology Association. This is an open access Abstract Book distributed under the Attribution-NonCommercial-NoDerivs (CC BY-NC-ND), which allows third parties to download the articles and share them with others as long as they credit the author and the Abstract Book, but they cannot change the content in any way or use them commercially.
**Abstract Book Citations:** Authors, Title, HemaSphere, 2026;10:(S1):pages. The abstract book DOIs can be found on the "Author Information" page.
**Disclaimer:** Articles published in the journal HemaSphere exclusively reflect the opinions of the authors. The authors are responsible for all content in their abstracts including accuracy of the facts, statements, citing resources, etc.

### (PS2373) TARGETING THE COMPLEMENT ALTERNATIVE PATHWAY ATTENUATES ACUTE VASO-OCCLUSIVE CRISIS IN SICKLE CELL DISEASE.

**Topic:** 27. Sickle cell disease

Thiago MacIel*[1], Geoffrey Cheminet[1], Rachel Rignault[1], Isabelle Chevalme[1], Slimane Allali[2], Camille Brochier[3], Anna Kiialainen[4], Tovo David[4], Olivier Hermine[5]

*[1]Institut Imagine INSERM UMR1163, Laboratory of Cellular and Molecular Mechanisms of Hematological Disorders and Therapeutical Implications, Paris, France; [2]Hôpital Necker-Enfants Malades, Department of General Pediatrics and Pediatric Infectious Diseases, Sickle Cell Center, Paris, France; [3]Institut Roche, Boulogne-Billancourt, France; [4]F. Hoffmann-La Roche Ltd, Roche Pharma Research & Early Development, Basel, Switzerland; [5]Hôpital Necker-Enfants Malades, Department of Hematology, Paris, France;*

### Background

Acute vaso-occlusive crises (VOC) in sickle cell disease (SCD) are driven by hemolysis, endothelial activation, platelet–RBC aggregation and thrombo-inflammation. Complement activation, particularly through the alternative pathway and C5 cleavage, amplifies these processes and may represent a central upstream driver of crisis severity. Whether proximal alternative pathway inhibition and terminal C5 blockade exert complementary therapeutic effects during acute VOC remains to be defined.

### Aims

To evaluate the effects of factor B inhibition (LNP023/iptacopan), C5 blockade (BB5.1), and dual inhibition on complement activation, cellular aggregation, hemolysis, and multi-organ vaso-occlusion in an acute hypoxia/reoxygenation model inTownes HbSS mice.

### Methods

Three-month-old Townes HbSS mice underwent hypoxia (8% $O_2$, 3h) followed by reoxygenation (22% $O_2$, 1h) to induce VOC. Complement activation was also evaluated after hemin challenge. Mice received LNP023 (60 mg/kg BID for 5 days prior), BB5.1 (30 mg/kg on days −10, −7, −4, −1), or both. Plasma complement components (factor B, C3b, C5a, sC5b9), RBC complement deposition (C3/C3b, C5b9), platelet–RBC aggregates (CD41+/CD62P+/Ter119+), neutrophil–RBC aggregates (CD11b+/GR1+/Ter119+), hemolysis markers (bilirubin, LDH), vascular congestion in liver, lung and kidney (Ter-119 staining) and renal C3 deposition were quantified.

**Copyright Information:** (Online) ISSN: 2572-9241
©2026 The Author(s). HemaSphere is published by John Wiley & Sons Ltd on behalf of European Hematology Association. This is an open access Abstract Book distributed under the Attribution-NonCommercial-NoDerivs (CC BY-NC-ND), which allows third parties to download the articles and share them with others as long as they credit the author and the Abstract Book, but they cannot change the content in any way or use them commercially.
**Abstract Book Citations:** Authors, Title, HemaSphere, 2026;10:(S1):pages. The abstract book DOIs can be found on the "Author Information" page.
**Disclaimer:** Articles published in the journal HemaSphere exclusively reflect the opinions of the authors. The authors are responsible for all content in their abstracts including accuracy of the facts, statements, citing resources, etc.

**Results**

Hypoxia/reoxygenation and hemin induced robust complement activation in HbSS mice, with significant increases in factor B, C5a, and sC5b9. LNP023 markedly reduced plasma C3b, confirming effective alternative pathway suppression, while BB5.1 significantly reduced C5a and sC5b9. Combination therapy concurrently suppressed proximal and terminal activation markers.
RBC complement opsonization was differentially modulated: LNP023 reduced C3/C3b deposition, whereas BB5.1 reduced C5b9 formation; dual therapy provided broader surface complement control.
VOC triggered marked platelet–RBC aggregation, a key driver of microvascular obstruction. Both monotherapies significantly reduced $CD41^+CD62P^+/Ter119^+$ aggregates, with combination therapy producing the most consistent attenuation. Neutrophil–RBC aggregates were similarly reduced.
Hemolysis markers were significantly elevated after hypoxia. Both LNP023 and BB5.1 reduced LDH activity and bilirubin levels, indicating mitigation of complement-mediated RBC injury.
Acute VOC resulted in substantial vascular congestion in liver, lung, and kidney. Both monotherapies reduced organ occlusion, while dual inhibition produced the greatest reduction in hepatic and pulmonary congestion and significantly improved renal vascular obstruction. Decreased C3 deposition in kidney cortex and medulla confirmed tissue-level complement suppression.

**Summary/Conclusion**

Acute VOC in HbSS mice is characterized by rapid alternative pathway amplification and terminal complement activation, promoting RBC opsonization, platelet aggregation, hemolysis, and multi-organ vascular congestion. Factor B inhibition (iptacopan) and C5 blockade exert mechanistically complementary effects, and dual inhibition provides the broadest control of complement activation and vaso-occlusive pathology. These findings support clinical evaluation of combined alternative pathway and C5 inhibition to prevent or attenuate acute VOC in SCD.

**Copyright Information:** (Online) ISSN: 2572-9241
©2026 The Author(s). HemaSphere is published by John Wiley & Sons Ltd on behalf of European Hematology Association. This is an open access Abstract Book distributed under the Attribution-NonCommercial-NoDerivs (CC BY-NC-ND), which allows third parties to download the articles and share them with others as long as they credit the author and the Abstract Book, but they cannot change the content in any way or use them commercially.
**Abstract Book Citations:** Authors, Title, HemaSphere, 2026;10:(S1):pages. The abstract book DOIs can be found on the “Author Information” page.
**Disclaimer:** Articles published in the journal HemaSphere exclusively reflect the opinions of the authors. The authors are responsible for all content in their abstracts including accuracy of the facts, statements, citing resources, etc.

### (PS2374) A NOVEL, CLINICALLY APPLIED SCORING SYSTEM FOR FAT EMBOLISM SYNDROME IN SICKLE CELL DISEASE

**Topic:** 27. Sickle cell disease

Abdulrahman Nasiri*[1], Manal Alshammari[2], Lama Alhmaly[3], Mostafa Mohammed Saleh[4], Ali Alahmari[4], Reem Alkharras[5], Fahed Almhareb[4]

*[1]Imam Mohammad Ibn Saud Islamic University, College of Medicine, Riyadh, Saudi Arabia;[2]King Salman Specialist Hospital, Department of Hematology, Hail, Saudi Arabia; [3]King Fahad Hospital, Department of Medicine, AlAhsa, Saudi Arabia;[4]King Faisal Specialist Hospital & Research Centre, Department of Hematology, Riyadh, Saudi Arabia; [5]Security Forces Hospital, Department of Medicine, Riyadh, Saudi Arabia;*

### Background

The diagnosis of fat embolism syndrome (FES) in sickle cell disease (SCD) is a significant clinical challenge due to its overlapping features with other common complications, often leading to delayed treatment and poor outcomes. Existing diagnostic scoring systems for FES were developed in the context of trauma and have not been validated in SCD. These scoring systems lack sensitivity and specificity for this population, as they rely heavily on clinical features common in routine SCD crises and fail to incorporate specific laboratory markers of bone marrow necrosis. A scoring system capable of distinguishing FES from acute chest syndrome (ACS) is a critical unmet need.

### Aims

To develop and clinically evaluate the Fat Embolism-Sickle Cell Disease (FE-SCD) score, a novel, evidence-based diagnostic tool for the early identification and risk stratification of FES in patients with SCD.

### Methods

The FE-SCD score was developed following a systematic literature review of FES cases in SCD. Features common in other SCD complications were assigned lower weights, while features specific to FES and bone marrow necrosis (e.g., neurological dysfunction, markedly elevated ferritin >10,000 μg/L, LDH >1,000 U/L) were assigned higher weights. This resulted in a 14-criterion weighted scoring system. To assess clinical utility, we retrospectively applied the score to three SCD patients admitted with a high clinical suspicion of FES between January 2024 and August 2025. Scores were calculated by two independent reviewers.

### Results

The FE-SCD score stratifies patients into low (0-3), intermediate (4-7), and high (≥8) risk categories to guide management intensity In preliminary validation, the score successfully identified a high-risk patient (FE-SCD score: 22) presenting with acute confusion and severe hypoxemia, who had MRI-confirmed cerebral FES. Furthermore, the tool successfully stratified two other complex, multi-organ failure cases as intermediate-risk (scores of 5), both requiring intensive care.

**Copyright Information:** (Online) ISSN: 2572-9241
©2026 The Author(s). HemaSphere is published by John Wiley & Sons Ltd on behalf of European Hematology Association. This is an open access Abstract Book distributed under the Attribution-NonCommercial-NoDerivs (CC BY-NC-ND), which allows third parties to download the articles and share them with others as long as they credit the author and the Abstract Book, but they cannot change the content in any way or use them commercially.
**Abstract Book Citations:** Authors, Title, HemaSphere, 2026;10:(S1):pages. The abstract book DOIs can be found on the "Author Information" page.
**Disclaimer:** Articles published in the journal HemaSphere exclusively reflect the opinions of the authors. The authors are responsible for all content in their abstracts including accuracy of the facts, statements, citing resources, etc.

### Summary/Conclusion

The FE-SCD score is a practical, clinically applied tool that shifts the diagnostic focus from general systemic inflammation to specific indicators of fat embolization in SCD. By providing an evidence-based framework for structured assessment, it offers a pragmatic approach to early risk stratification and intervention. This tool has the potential to facilitate earlier diagnosis and improve outcomes in a condition where delays are frequently fatal. Prospective validation in a larger cohort is warranted to confirm its efficacy.

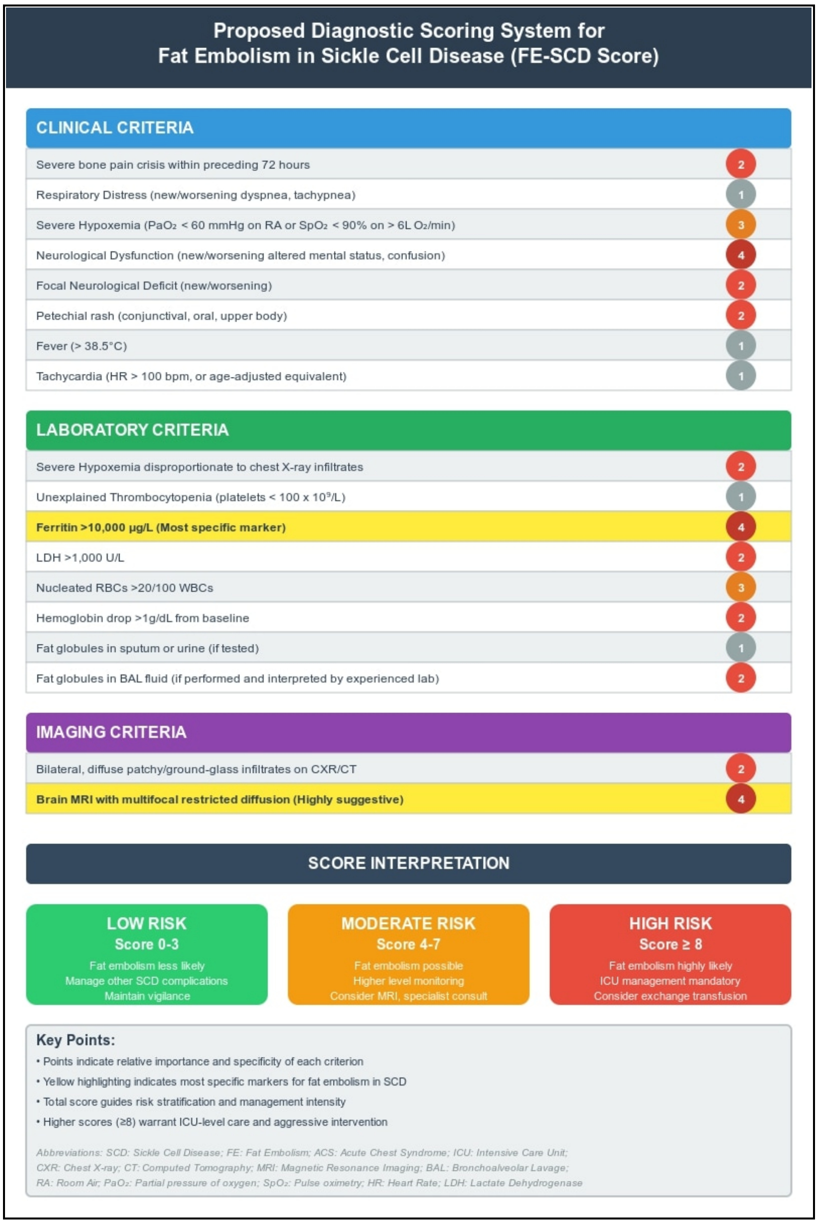

**Copyright Information:** (Online) ISSN: 2572-9241
©2026 The Author(s). HemaSphere is published by John Wiley & Sons Ltd on behalf of European Hematology Association. This is an open access Abstract Book distributed under the Attribution-NonCommercial-NoDerivs (CC BY-NC-ND), which allows third parties to download the articles and share them with others as long as they credit the author and the Abstract Book, but they cannot change the content in any way or use them commercially.
**Abstract Book Citations:** Authors, Title, HemaSphere, 2026;10:(S1):pages. The abstract book DOIs can be found on the "Author Information" page.
**Disclaimer:** Articles published in the journal HemaSphere exclusively reflect the opinions of the authors. The authors are responsible for all content in their abstracts including accuracy of the facts, statements, citing resources, etc.

### (PS2375) IMPROVING RETINAL SCREENING IN SICKLE CELL DISEASE USING A NOVEL IN-OFFICE SCAN (A QUALITY IMPROVEMENT INITIATIVE)

**Topic:** 27. Sickle cell disease

Rachel Klein[1], Brent Blackwell[2], Rebecca O'rear[1], Jacqueline Richardson[1], Aquilla Burney[1], Ebone Jones[1], Julie Kanter*[1]

*[1]University of Alabama at Birmingham, Department of Biostatistics, School of Public Health, Birmingham, United States of America; [2]University of Alabama at Birmingham, Lifespan Comprehensive Sickle Cell Center, Birmingham, United States of America;*

### Background

Sickle cell disease (SCD) affects multiple organs including the eye (retina). Retinal complications include: ischemia, neovascularization, vitreous hemorrhage, and retinal detachment. Annual retinal screening (ARS) is recommended by the National Alliance of Sickle Cell Centers (NASCC) and the National Institutes of Health as an essential part of comprehensive care for all types of SCD. Retinopathy may be asymptomatic until it is in advanced stages and can lead to irreversible vision loss. Retinal exams are performed by ophthalmologists with separate offices from SCD centers and may use separate electronic medical record (EMR) systems. The physical and electronic separation can impair the communication of completed screening and diagnostic information. A deliberate effort is necessary to ensure people with SCD undergo ARS and that diagnostic information is communicated as part of anticipatory, organ-preserving care for people with SCD.

### Aims

This was a quality improvement (QI) study to evaluate and improve ARS rates for adults with SCD

### Methods

This single-center QI study in the United States met institutional criteria for IRB waiver. All patients were >/=18 years and routinely followed in the SCD center. The process improvement period was from July-November 2025. We used the Institute for Healthcare Improvement (IHI) Model for Improvement with flow charts to outline current processes and identify challenges. We assembled a multidisciplinary team including medical assistants (MA), nurses, advanced practice providers, and physicians to identify hurdles to successful ARS. Identified barriers included the lack of co-located services, transportation, third party-payers coverage and the lack of a shared EMR. The baseline data (over 10 consecutive clinic days) revealed that only 25% (0-75%) of adults with SCD had successfully completed and documented retinal exams (within the 12 prior months). We performed serial, rapid, plan-do-study-act (PDSA) cycles with monthly data collection to improve ARS rates. Run charts were used for monitoring.

**Copyright Information:** (Online) ISSN: 2572-9241
©2026 The Author(s). HemaSphere is published by John Wiley & Sons Ltd on behalf of European Hematology Association. This is an open access Abstract Book distributed under the Attribution-NonCommercial-NoDerivs (CC BY-NC-ND), which allows third parties to download the articles and share them with others as long as they credit the author and the Abstract Book, but they cannot change the content in any way or use them commercially.
**Abstract Book Citations:** Authors, Title, HemaSphere, 2026;10:(S1):pages. The abstract book DOIs can be found on the “Author Information” page.
**Disclaimer:** Articles published in the journal HemaSphere exclusively reflect the opinions of the authors. The authors are responsible for all content in their abstracts including accuracy of the facts, statements, citing resources, etc.

### Results

Serial practice changes were performed throughout the period and are shown (with results) in the table below.  The run charts are shown in figure 1. The first two interventions did not meet criteria for non-random change. The third change was to adapt the TOPCON technology (approved for diabetic retinopathy) for use in SCD.  TOPCON is a point-of-care, non-mydriatic, automated, digital imaging tool designed to detect early signs of diabetic retinopathy. TOPCON uses robotic retinal cameras to take high-resolution retinal images without pupil dilation and can be completed during the SCD clinic visit. Tele-optometrists read the images and return reports within 3-days.  With TOPCON, median screening improved to 83% (non-random change).

| **Practice Change** | **Result**<br>**median % screening rate/day (range)** |
|---|---|
| Baseline | 25% (0-75%) |
| MA documented “last eye exam” during triage | 57% (0-71%) |
| MA updated release of information at each visit | 63% (25-86%) |
| TOPCON | 83% (50-100%) |

### Summary/Conclusion

Adults with SCD require appropriate preventive screening to improve the diagnosis and treatment of organ-complications including the retina.  This QI project identified new methods that can be adopted to improve ARS rates. The use of a POC technology such as TopCon combined with teamwork to ensure the routine documentation and communication of ARS are key to successful screening.  Studies are ongoing to validate this POC tool for SCD.

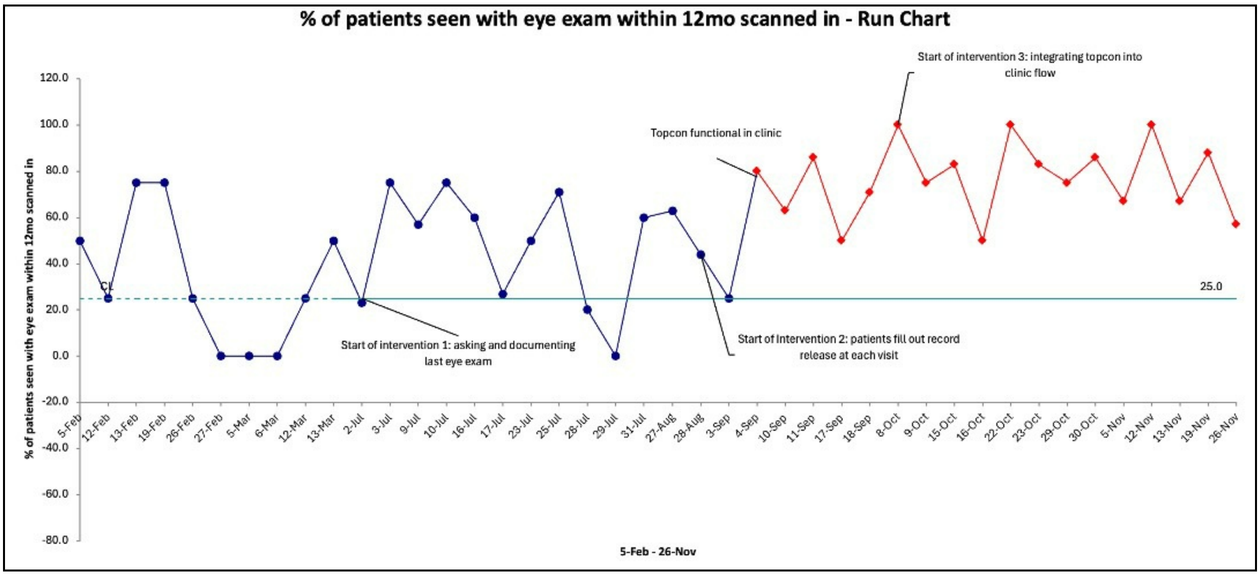

Run chart showing the percent (%) of patients/day with an up-to-date annual retinal screening (ARS) exam within 12 months

**Copyright Information:** (Online) ISSN: 2572-9241
©2026 The Author(s). HemaSphere is published by John Wiley & Sons Ltd on behalf of European Hematology Association. This is an open access Abstract Book distributed under the Attribution-NonCommercial-NoDerivs (CC BY-NC-ND), which allows third parties to download the articles and share them with others as long as they credit the author and the Abstract Book, but they cannot change the content in any way or use them commercially.
**Abstract Book Citations:** Authors, Title, HemaSphere, 2026;10:(S1):pages. The abstract book DOIs can be found on the “Author Information” page.
**Disclaimer:** Articles published in the journal HemaSphere exclusively reflect the opinions of the authors. The authors are responsible for all content in their abstracts including accuracy of the facts, statements, citing resources, etc.

### (PS2376) BRIDGING GAPS IN SICKLE CELL DISEASE CARE: ERN-EUROBLOODNET'S COMPARATIVE ANALYSIS OF NATIONAL RECOMMENDATIONS ACROSS EUROPE

**Topic:** 27. Sickle cell disease

Minke Rab[1], Anna Collado Gimbert*[2, 3], Alba Albert Robledo[3], Amina Nardo Marino[4], Andreas Glenthøj[4], Anita Rijneveld[1], Anna Vanderfaiellie[5], Habibi Anoosha[6], Antonis Kattamis[7], Bart Biemond[8], Daniela Cuzzubbo[9], Eduard J. van Beers[10], Elena Cela[11], Erfan Nur[8], Giovanna Russo[9], Holger Cario[12], Jaroslav Cermak[13], Joachim Kunz[14], Laura Tagliaferri[14], Luca Malcovati[15], Maddalena Casale[16], Mariane de Montalembert[17], Marta Morado[18], Martin Colard[19], Raffaella Colombatti[20], Stephan Lobitz[21], Maria del Mar Mañú Pereira[22], Beatrice Gulbis[23]

*[1]Erasmus University Medical Center Rotterdam, Rotterdam, The Netherlands;[2]Hospital Universitari Vall d'Hebron, Barcelona, Spain; [3]Vall d'Hebron Institut de Recerca, Barcelona, Spain;[4]Copenhagen University Hospital - Rigshospitalet, Copenhagen, Denmark; [5]CHU Saint-Pierre, Brussels, Belgium;[6]Assistance Publique - Hôpitaux de Paris - Hôpital Henri Mondor, Paris, France; [7]Aghia Sophia Children's Hospital, Athens, Greece;[8]Amsterdam UMC, University of Amsterdam, Amsterdam, The Netherlands; [9]Azienda Ospedaliero Universitaria Policlinico "G.Rodolico - San Marco", Catania, Italy; [10]University Medical Center Utrecht, Utrecht, The Netherlands; [11]Hospital General Universitario Gregorio Marañón, Madrid, Spain; [12]Ulm University Medical Center, Ulm, Germany; [13]Institute of Hematology and Blood Transfusion, Prague, Czechia;[14]Heidelberg University Hospital, Heidelberg, Germany; [15]Foundation IRCCS Polyclinic San Matteo, Pavia - ERN-EuroBloodNet, Pavia, Italy; [16]Università degli Studi della Campania "Luigi Vanvitelli", Caserta, Italy; [17]Assistance Publique - Hôpitaux de Paris - Hôpital Necker Enfants Malades - Université Paris-Cité, Paris, France; [18]Hospital Universitario La Paz, Madrid, Spain; [19]Hôpital Universitaire de Bruxelles - Université Libre de Bruxelles, Brussels, Belgium; [20]Azienda Ospedale - Università di Padova, Padova, Italy; [21]Gemeinschaftsklinikum Mittelrhein gGmbH, Koblenz, Germany;[22]Vall d'Hebron Institut de Recerca - ERN-EuroBloodNet, Barcelona, Spain; [23]CUB-Hôpital Erasme - ERN-EuroBloodNet, Brussels, Belgium;*

### Background

Sickle Cell Disease (SCD) care across the European Union (EU) is characterized by significant variability in the availability and content of national clinical practice guidelines and clinical decision-making tools. This heterogeneity contributes to disparities in clinical management of individuals living with SCD.

### Aims

This ERN-EuroBloodNet pioneering initiative represents the first systematic comparative analysis of national SCD recommendations across Europe. By identifying differences and commonalities among national practices, this project aims to highlight evidence gaps and support research efforts to promote equity in SCD care across EU member states.

### Methods

Initiative supported by the ERN project (Tender-Nº SANTE/2018/B3/030). Methodological support by Canary Islands Health Service Evaluation Unit (SESCS/FIISC). The Expert Panel, including 26 members from 9 EU Member States, collected national documents. SESCS/FIISC conducted also systematic search to identify additional documents using databases including PubMed, TripDatabase, ECRI Guidelines Trust, Orphanet, EURORDIS, and NORD.
Experts reviewed specific topics according to their area of expertise, covering both pediatric and adult domains where applicable. For each topic, prioritized questions (PQ) were defined. Corresponding recommendations and their level of evidence were extracted from the documents.

**Copyright Information:** (Online) ISSN: 2572-9241
©2026 The Author(s). HemaSphere is published by John Wiley & Sons Ltd on behalf of European Hematology Association. This is an open access Abstract Book distributed under the Attribution-NonCommercial-NoDerivs (CC BY-NC-ND), which allows third parties to download the articles and share them with others as long as they credit the author and the Abstract Book, but they cannot change the content in any way or use them commercially.
**Abstract Book Citations:** Authors, Title, HemaSphere, 2026;10:(S1):pages. The abstract book DOIs can be found on the "Author Information" page.
**Disclaimer:** Articles published in the journal HemaSphere exclusively reflect the opinions of the authors. The authors are responsible for all content in their abstracts including accuracy of the facts, statements, citing resources, etc.

### Results

A total of 33 documents were selected for comparative analysis. The Expert Panel identified 20 key topics: newborn screening, follow-up, infection prophylaxis, fever treatment, vaso-occlusive-crisis, splenic sequestration and splenectomy, acute chest syndrome, stroke, TCD/MRI, priapism, renal function, cardiomyopathy, pre-surgery, analgesia, pregnancy, hydroxyurea therapy, blood transfusion, hematopoietic stem cell transplantation and care transition.

Comparative findings were synthetized in structured tables. The example of Infection prophylaxis (Table 1) provided identified gaps and missing evidence 1)Once-daily amoxicillin prophylaxis is not commonly recommended, 2)The potential role of penicillin prophylaxis across all age groups warrants further evaluation.

### Summary/Conclusion

This initiative highlights divergences and gaps that can be the rationale for targeted, collaborative research projects. These findings will support the development of harmonized, evidence-based clinical policies across Europe, including coordinated clinical registries within EU.

**Table 1.** Infection prophylaxis with antibiotic therapy.

| ANTIBIOTIC THERAPY PROPHYLAXIS (ABP) | DE | DK | ES | FR | GR | IT | NL | UK |
|---|---|---|---|---|---|---|---|---|
| **PQ 1: When to stop ABP?** | | | | | | | | |
| ***Recommendation 1:*** *ABP administered from the 3rd month of life until at least end of the 5th year of life.* | Yes | Yes | Yes | Yes | Yes | Yes | Yes (A) | Yes (A) |
| ***Recommendation 2:*** *ABP offered after the 5th year of life for an indefinite time.* | Yes | Yes | Yes | Yes | Yes | Yes | Yes | Yes |
| **PQ 2: When to stop ABP after splenectomy?** | | | | | | | | |
| ***Recommendation 1:*** *continue ABP lifelong.* | Yes | NA | Yes | Yes | NA | Yes | NA | NA |
| **PQ 3: Restart ABP after severe encapsulated bacterial infection?** | | | | | | | | |
| ***Recommendation 1:*** *continue lifelong penicillin prophylaxis.* | Yes | NA | Yes | Yes | NA | Yes | NA | NA |
| **PQ 4: Penicillin every 12h versus amoxicillin every 24h?** | | | | | | | | |
| ***Recommendation 1:*** *penicillin dose: <3 years: 2x 200,000IU (2x125 mg/day), 3-5 years: 2x400,000IU (2x250mg/day)* | NA | Yes | Yes | Yes | Yes | Yes | NA | Yes |
| ***Recommendation 2:*** *offer amoxicillin 20mg/Kg/24h, (no trials to endorse it).* | NA | NA | Yes | Yes | NA | NA | NA | NA |

Yes (level of evidence if stated):recommendation addressed and applied; No: against the recommendation; NA: recommendation not addressed.

**Copyright Information:** (Online) ISSN: 2572-9241
©2026 The Author(s). HemaSphere is published by John Wiley & Sons Ltd on behalf of European Hematology Association. This is an open access Abstract Book distributed under the Attribution-NonCommercial-NoDerivs (CC BY-NC-ND), which allows third parties to download the articles and share them with others as long as they credit the author and the Abstract Book, but they cannot change the content in any way or use them commercially.
**Abstract Book Citations:** Authors, Title, HemaSphere, 2026;10:(S1):pages. The abstract book DOIs can be found on the "Author Information" page.
**Disclaimer:** Articles published in the journal HemaSphere exclusively reflect the opinions of the authors. The authors are responsible for all content in their abstracts including accuracy of the facts, statements, citing resources, etc.

### (PS2377) LOWER INDEX OF MULTIPLE DEPRIVATION IS ASSOCIATED WITH GREATER IMPROVEMENT IN INFLUENZA VACCINATION IN ADULTS WITH SICKLE CELL DISEASE AND THALASSAEMIA: A PAIRED COHORT STUDY

**Topic:** 27. Sickle cell disease

Rose Ameli*[1], Ethan Troy-Barnes[1], Emma Drasar[2], Nisha Nakrani[2], Steve Collins[2], Tina Ameli[3]

*[1]University College London Hospital, London, United Kingdom; [2]Whittington Health NHS Trust, London, United Kingdom; [3]Chelsea and Westminster NHS Trust, London, United Kingdom;*

### Background

Adults with sickle cell disorder (SCD) and thalassaemia are at increased risk of influenza-associated respiratory complications. Although annual vaccination is recommended, uptake remains incomplete and socially patterned. Increased vaccine uptake may reduce preventable respiratory morbidity in this high-risk population.

### Aims

- To determine the paired-change in vaccination status and respiratory morbidity in adults with SCD and thalassaemia following a targeted influenza vaccination programme
- To examine variation in vaccination improvement by socioeconomic deprivation.

### Methods

A retrospective paired cohort compared baseline (1 October 2024-21 February 2025) and intervention (1 October 2025-21 February 2026) influenza seasons in 375 adults with SCD or thalassaemia with available vaccination data at a tertiary centre. The intervention comprised invitation for vaccination by a community haemoglobinopathy clinic and opportunistic vaccination during outpatient transfusion episodes.
The primary outcome was paired-change in vaccination status. Secondary outcomes included paired-change in lower respiratory tract infection (LRTI), LRTI-associated admission, emergency red cell exchange (RCE), acute chest syndrome (ACS) and intensive care (ITU) admission. McNemar's exact test and absolute risk differences (ARD) with 95% confidence intervals were calculated. Socioeconomic deprivation was measured using the Index of Multiple Deprivation (IMD), a multi-domain decile ranking based on discrete geographic location (1 most deprived, 10 least). Logistic regression assessed the association between vaccination improvement and IMD.

### Results

The cohort comprised 375 adults (median age 41 years; 55% female), including 232 with SCD and 143 with thalassaemia (median IMD decile 4).
Influenza vaccination increased from 147 (39.2%) to 157 (41.9%) (ARD +2.7%, 95% CI -2.6 to 7.9%; p=0.314). During the intervention season, 45 patients were newly vaccinated and 35 were not vaccinated despite vaccination during baseline season. Vaccination improvement was significantly associated with socioeconomic deprivation: each increase in IMD decile (towards less deprived areas) was associated with lower odds of improvement (OR 0.75, 95% CI 0.64-0.88; p=0.0004), with the largest absolute increase observed in decile 2 (11 patients; +18 percentage points).
Figure 1 shows paired changes in secondary respiratory outcomes. One death occurred during the study period and was unrelated to influenza vaccination or respiratory infection.

**Copyright Information:** (Online) ISSN: 2572-9241
©2026 The Author(s). HemaSphere is published by John Wiley & Sons Ltd on behalf of European Hematology Association. This is an open access Abstract Book distributed under the Attribution-NonCommercial-NoDerivs (CC BY-NC-ND), which allows third parties to download the articles and share them with others as long as they credit the author and the Abstract Book, but they cannot change the content in any way or use them commercially.
**Abstract Book Citations:** Authors, Title, HemaSphere, 2026;10:(S1):pages. The abstract book DOIs can be found on the "Author Information" page.
**Disclaimer:** Articles published in the journal HemaSphere exclusively reflect the opinions of the authors. The authors are responsible for all content in their abstracts including accuracy of the facts, statements, citing resources, etc.

**Summary/Conclusion**

A targeted influenza vaccination delivery programme was associated with reductions in clinically relevant respiratory morbidity, with a statistically significant reduction in emergency RCE in SCD patients. Although event numbers were small, emergency RCE represents severe acute morbidity in SCD, thus modest absolute reductions may be clinically meaningful.
Overall vaccination uptake did not significantly increase; however, improvement was strongly associated with IMD, indicating a potential equity benefit despite a modest net change. This supports existing literature reporting lower vaccination uptake in socioeconomically deprived groups and suggests individuals with haemoglobinopathies from these backgrounds may particularly benefit from targeted vaccination programmes. This finding will inform future regional iterations of our programme and should be considered when implementing vaccination programmes for haemoglobinopathy patients in other contexts.

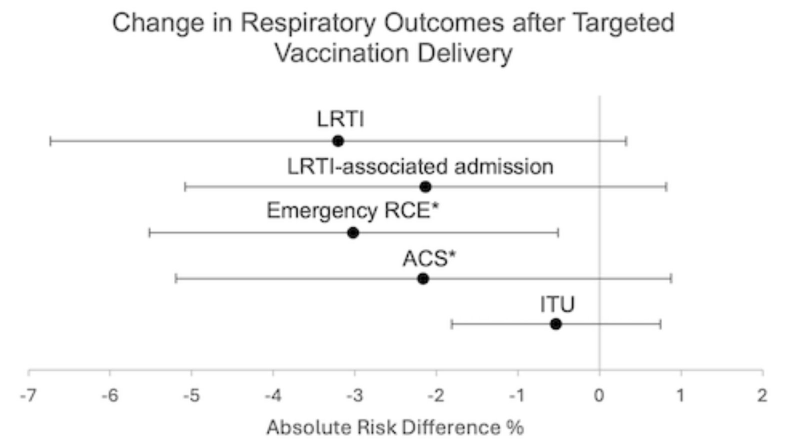

***Figure 1:*** *Absolute risk differences (intervention minus baseline) with 95% confidence intervals for respiratory outcomes (n=375; *Emergency RCE and ACS analyses restricted to SCD, n = 232). LRTI decreased from 38 (10.1%) to 26 (6.9%) (ARD -3.2%, 95% CI -6.7 to 0.3%, p=0.104). LRTI-associated admissions decreased from 25 (6.7%) to 17 (4.5%) (ARD -2.1%, 95% CI -5.1 to 0.8%, p=0.215). Emergency RCE decreased from 8 (3.4%) to 1 (0.4%) (ARD -3.0%, 95% CI -5.5 to -0.5%, p=0.039). ACS decreased from 10 (4.3%) to 5 (2.2%) (ARD-2.2%, 95% CI -5.2 to 0.9%, p=0.267). ITU admission decreased from 4 (1.1%) to 2 (0.5%) (ARD -0.5%, 95% CI -1.8 to 0.8%, p=0.688).*

**Copyright Information:** (Online) ISSN: 2572-9241
©2026 The Author(s). HemaSphere is published by John Wiley & Sons Ltd on behalf of European Hematology Association. This is an open access Abstract Book distributed under the Attribution-NonCommercial-NoDerivs (CC BY-NC-ND), which allows third parties to download the articles and share them with others as long as they credit the author and the Abstract Book, but they cannot change the content in any way or use them commercially.
**Abstract Book Citations:** Authors, Title, HemaSphere, 2026;10:(S1):pages. The abstract book DOIs can be found on the “Author Information” page.
**Disclaimer:** Articles published in the journal HemaSphere exclusively reflect the opinions of the authors. The authors are responsible for all content in their abstracts including accuracy of the facts, statements, citing resources, etc.

### (PS2378) OSTEO-ARTICULAR INFECTIONS IN CHILDREN WITH SICKLE CELL DISEASE IN PARIS: A RETROSPECTIVE STUDY

**Topic:** 27. Sickle cell disease

Fabio Toscano[1], Joséphine Brice*[1, 2], Alma Al Sibaaie[1, 2, 3], Martina Bevacqua[1, 2], Melissa Taylor Marchetti[1, 2], Samuel Georges[4], Agnès Ferroni[5], Klervie Loiselet[6], Slimane Allali[1, 2, 3], Julie Toubiana[1, 7, 8]

*[1]Department of General Pediatrics and Pediatric Infectious Diseases, Necker-Enfants malades University Hospital, Université de Paris Cité, Paris, France; [2]Laboratory of Excellence GR-Ex, Paris, France; [3]Laboratory of Cellular and Molecular Mechanisms of Hematological Disorders and Therapeutical Implications, Université Paris Cité, Imagine Institute, Inserm U1163, Paris, France; [4]Pediatric Orthopaedic Surgery Department, Paris Descartes University, Necker-Enfants malades University Hospital, Paris, France; Université de Paris Cité, Paris, France; [5]Department of Microbiology, Necker-Enfants malades University Hospital, Paris, France; Université de Paris Cité, Paris, France; [6]Department of Radiology, Necker-Enfants malades University Hospital, Paris, France; Université de Paris Cité, Paris, France; [7]Laboratory of Human Genetics of Infectious Diseases, Necker Branch, INSERM UMR1163, Necker Medical School, Paris, France; [8]Institut Pasteur, Université Paris Cité, Biodiversity and Epidemiology of Bacterial Pathogens, Paris, France;*

### Background

Diagnosis of osteoarticular infections (OAI) in children with sickle cell disease (SCD) is challenging due to overlapping features with vaso-occlusive crises (VOC) and the absence of specific markers.

### Aims

The aim of this study is to describe the epidemiology and features of suspected OAI in children with SCD in a french reference center for SCD, and to identify factors that may help distinguish OAI from VOC.

### Methods

We conducted a single-center, retrospective observational study in children (0–18 years) with SCD admitted to Necker-Enfants Malades Hospital in Paris between 2015 and 2024 for suspected OAI. Clinical, biological, microbiological, imaging, treatment, and outcome data were collected. Episodes were classified as retained OAI (treated ≥14 days) or not retained OAI. Retained OAI were further categorized as confirmed (microbiological documentation) or probable. Groups were compared using appropriate statistical tests, and incidence rates were calculated per 100 patient-years.

### Results

Among 120 suspected episodes, 84 were classified as retained OAI, yielding an overall incidence of 3 infections per 100 patient-years. Median age was 5 [2.5-12.3] years, and males were more frequently affected. Osteomyelitis was the most common entity. Microbiological confirmation was achieved in 25% (n=21) of retained cases (positive blood cultures in 9% and identification by surgical sampling in 22%), with non-typhoidal *Salmonella* as the leading causative agent. Confirmed OAI were significantly associated with longer hospitalization, prolonged pain, more frequent multifocal involvement, higher C-reactive protein levels at admission, thrombocytosis during hospitalization, and a higher prevalence and larger size of bone or subperiosteal collections. Confirmed OAI also required longer antibiotic treatment, more frequent surgery and were associated with higher morbidity.

**Copyright Information:** (Online) ISSN: 2572-9241
©2026 The Author(s). HemaSphere is published by John Wiley & Sons Ltd on behalf of European Hematology Association. This is an open access Abstract Book distributed under the Attribution-NonCommercial-NoDerivs (CC BY-NC-ND), which allows third parties to download the articles and share them with others as long as they credit the author and the Abstract Book, but they cannot change the content in any way or use them commercially.
**Abstract Book Citations:** Authors, Title, HemaSphere, 2026;10:(S1):pages. The abstract book DOIs can be found on the “Author Information” page.
**Disclaimer:** Articles published in the journal HemaSphere exclusively reflect the opinions of the authors. The authors are responsible for all content in their abstracts including accuracy of the facts, statements, citing resources, etc.

**Summary/Conclusion**

**OAI in children with SCD remain difficult to diagnose because of substantial overlap with VOC and the absence of specific biomarkers. Microbiological confirmation, although less frequent, remains essential for optimized care. Improved diagnostic tools, including relevant biomarkers, consensus imaging criteria, and a multidisciplinary approach, are needed to improve outcomes and to promote appropriate antibiotic use while limiting unnecessary exposure and the emergence of resistance.**

**Copyright Information:** (Online) ISSN: 2572-9241
©2026 The Author(s). HemaSphere is published by John Wiley & Sons Ltd on behalf of European Hematology Association. This is an open access Abstract Book distributed under the Attribution-NonCommercial-NoDerivs (CC BY-NC-ND), which allows third parties to download the articles and share them with others as long as they credit the author and the Abstract Book, but they cannot change the content in any way or use them commercially.
**Abstract Book Citations:** Authors, Title, HemaSphere, 2026;10:(S1):pages. The abstract book DOIs can be found on the "Author Information" page.
**Disclaimer:** Articles published in the journal HemaSphere exclusively reflect the opinions of the authors. The authors are responsible for all content in their abstracts including accuracy of the facts, statements, citing resources, etc.

### (PS2379) PREVALENCE AND CLINICAL IMPACT OF CLONAL HEMATOPOIESIS IN PATIENTS WITH BETA-THALASSEMIA

**Topic:** 28. Thalassemias

Simona Leoni*[1, 2], Silvio Loardi[3, 4], Marta Lionetti[3, 4], Valentina Brancaleoni*[2], Emanuele Savino[3, 5], Dario Consonni[6], Zelia Borsellino[7], Maria Francesca D'anna[7], Livia Amato[7], Davide Vacca[8], Simona Merighi[8], Giovan Battista Ruffo[7], Niccolò Bolli[3, 4], Irene Motta[1, 2], Claudio Tripodo[5]

*[1]Dipartimento di Scienze Cliniche e di Comunità, Dipartimento di Eccellenza 2023-2027, University of Milan, Milan, Italy;[2]SC Medicina ad Indirizzo Metabolico, Fondazione IRCCS Ca' Granda Ospedale Maggiore Policlinico, Milan, Italy; [3]Department of Oncology and Hemato-oncology, University of Milan, Milan, Italy; [4]Hematology Section, Fondazione IRCCS Ca' Granda, Ospedale Maggiore Policlinico, Milan, Italy; [5]Advanced Pathology Laboratory, IFOM ETS-The AIRC Institute of Molecular Oncology, Milan, Italy; [6]Occupational Health Unit, Fondazione IRCCS Ca' Granda Ospedale Maggiore Policlinico, Milan, Italy; [7]U.O. Ematologia e Talassemia, ARNAS Civico, Palermo, Italy; [8]Tumor Immunology Laboratory, University of Palermo, Palermo, Italy;*

**Background**

Advances in the standard of care of beta-thalassemia have significantly improved patient survival, leading to the emergence of age-related complications[1]. Clonal hematopoiesis (CH) is characterized by somatic variants in hematopoietic stem and progenitor cells that lead to clonal expansion[2]. Individuals with CH face an increased long-term risk of hematological malignancies, particularly myelodysplastic syndromes (MDS). Also, CH is linked to higher morbidity and mortality from atherosclerotic cardiovascular disease (ASCVD) and ischemic stroke[3]. We hypothesized that the altered bone marrow microenvironment and chronic hematopoietic stress in thalassemia may favor the detectability of clonal hematopoiesis in affected patients.

**Aims**

The aim of this multicenter study aimed to assess the prevalence of CH in a cohort of patients with transfusion-dependent and non-transfusion-dependent beta-thalassemia and to investigate its association with clinical parameters.

**Methods**

Targeted NGS of 74 myeloid-associated genes was performed on an Illumina sequencing platform. Variants were annotated as pathogenic, likely pathogenic, or variants of unknown significance based on available literature and publicly available databases. Mutations were further screened for somatic nature and pathogenicity, accepting a minimum variant coverage of 10 alternative reads, and a VAF >= 0.02 and <=0.45 in accordance with established variant filtering algorithms.

**Copyright Information:** (Online) ISSN: 2572-9241
©2026 The Author(s). HemaSphere is published by John Wiley & Sons Ltd on behalf of European Hematology Association. This is an open access Abstract Book distributed under the Attribution-NonCommercial-NoDerivs (CC BY-NC-ND), which allows third parties to download the articles and share them with others as long as they credit the author and the Abstract Book, but they cannot change the content in any way or use them commercially.
**Abstract Book Citations:** Authors, Title, HemaSphere, 2026;10:(S1):pages. The abstract book DOIs can be found on the "Author Information" page.
**Disclaimer:** Articles published in the journal HemaSphere exclusively reflect the opinions of the authors. The authors are responsible for all content in their abstracts including accuracy of the facts, statements, citing resources, etc.

**Results**

We enrolled 296 patients, of whom 136 (45.9%) were males, with a mean age of 45.3±10.9 years. 296 (75%) were transfusion-dependent, of whom 32 had thalassemia intermedia that became transfusion-dependent over time. 288 (54.2%) had undergone splenectomy. The prevalence of CHIP in this cohort is 24/296 (8.11%), and it increases with age: CH was detected in 1/6 individuals over 50 years, rising to 1/4 in those over 59 years. The most prevalent variants were in *DNMT3A* (9/24). *TET2* and *STAT3* variants were found in 3/24 patients, *ASXL1* in 2/24, and *TP53* in 1/24. When comparing patients with CH to those without, we observed that individuals with CHIP tend to be older (53.2±10.6 vs 43.4±10.6, p<0.001). The two groups did not differ in terms of transfusion regimen, iron status, splenectomy, or exposure to medications such as hydroxyurea and luspatercept. Individuals with CH had an average lower white blood cell count (8.0±3.2 vs 10.4±5.0, p=0.03), although within the normal range. CH was significantly associated with a prior history of solid malignancy (16.0% vs 3.7%, p=0.006), while no hematological neoplasms were recorded. When considering complications and comorbidities analysed using Pearson Chi-Square, CH was associated with cardioembolic stroke (100% vs 5.26%, p=0.002), ASCVD (64.7% vs 37.0%, p=0.02), and liver fibrosis (47.0% vs 23.7%, p=0.03). Notably, a history of hepatitis C virus (HCV) infection was associated with the presence of CH (64% vs 38.4%, p=0.01).

**Summary/Conclusion**

This is the first report of CH in beta-thalassemia and the first to show an association between HCV infection and CH. Our findings raise significant questions about pathophysiology and clinical management: i) given the risk of MDS in individuals with CH, cytopenias in thalassemia patients >50 years should be carefully investigated; ii) with curative strategies reaching adult patients target, documenting CH may become relevant for counselling, baseline work-up, and post-treatment surveillance.

**Copyright Information:** (Online) ISSN: 2572-9241
©2026 The Author(s). HemaSphere is published by John Wiley & Sons Ltd on behalf of European Hematology Association. This is an open access Abstract Book distributed under the Attribution-NonCommercial-NoDerivs (CC BY-NC-ND), which allows third parties to download the articles and share them with others as long as they credit the author and the Abstract Book, but they cannot change the content in any way or use them commercially.
**Abstract Book Citations:** Authors, Title, HemaSphere, 2026;10:(S1):pages. The abstract book DOIs can be found on the “Author Information” page.
**Disclaimer:** Articles published in the journal HemaSphere exclusively reflect the opinions of the authors. The authors are responsible for all content in their abstracts including accuracy of the facts, statements, citing resources, etc.

### (PS2380) COMBINED ATRIOVENTRICULAR COUPLING INDEX IN TRANSFUSION-DEPENDENT THALASSEMIA

**Topic:** 28. Thalassemias

Antonella Meloni*[1], Luca Saba[2], Laura Pistoia[1], Vincenzo Positano[1], Annamaria Pasanisi[3], Angela Ermini[4], Elena Follini[5], Maurizio Caniglia[6], Michele Santodirocco[7], Sabrina Bagnato[8], Andrea Barison[1], Riccardo Cau[2]

*[1]Fondazione G. Monasterio CNR-Regione Toscana, Pisa, Italy; [2]Azienda Ospedaliero-Universitaria di Cagliari - Polo di Monserrato, Cagliari, Italy; [3]Ospedale "A. Perrino", Brindisi, Italy; [4]Ospedale S. Maria Annunziata, Bagno a Ripoli (FI), Italy; [5]Ospedale "G. Da Saliceto", Piacenza, Italy; [6]Azienda Ospedaliera "S. Maria Misericordia" di Perugia, Perugia, Italy;[7]Ospedale Casa Sollievo della Sofferenza IRCCS, San Giovanni Rotondo (FG), Italy; [8]Presidio Ospedaliero Lentini - ASP 8 Siracusa, Lentini (SR), Italy;*

### Background

Efficient cardiac function depends on atrioventricular coupling, which coordinates atrial and ventricular mechanics across all four chambers. Atrioventricular coupling can be quantified using the left and right coupling indices (LACI and RACI), expressed as percentages, with higher values indicating greater decoupling. The combined atrioventricular coupling index (CACI) integrates these measures to provide a global assessment of biventricular and biatrial dysfunction.

### Aims

This cross-sectional study evaluated the association of CACI with demographic and clinical characteristics, cardiovascular magnetic resonance (CMR) findings, and cardiac complications in patients with transfusion-dependent thalassemia (TDT).

### Methods

We studied 292 consecutive TDT patients (151 females; mean age 36.72±11.76 years) enrolled in the Extension–Myocardial Iron Overload in Thalassemia (E-MIOT) project.
The CMR protocol included cine imaging for biventricular volumes, ejection fractions, and bi-atrial volumes; T2* imaging for myocardial and liver iron quantification; and late gadolinium enhancement (LGE) imaging for detection of replacement myocardial fibrosis. CACI was calculated as the sum of LACI—left atrial end-diastolic volume (EDV) to left ventricular EDV ratio—and RACI—right atrial EDV to right ventricular EDV ratio.

### Results

CACI was significantly associated with aging (R=0.449; p<0.0001) and was significantly increased in splenectomized compared to non-splenectomized patients (54.89±23.49% vs. 46.78±15.67%; p=0.001) and in patients with diabetes compared to those without diabetes (65.07±27.35% vs. 48.42±17.97%; p<0.0001). CACI was not associated with gender, serum levels of hemoglobin and ferritin, biventricular ejection fractions, or hepatic and cardiac T2* values. CACI was significantly increased in patients with LGE compared to those without LGE (57.88±26.95% vs. 45.45±13.61%; p<0.0001). In stepwise linear regression analysis, age>75th percentile and LGE emerged as the independent predictors of CACI (F=15.40; p<0.0001).
Twenty-seven patients experienced at least one cardiac complication (9 heart failure, 14 arrhythmias—mostly supraventricular, 3 pulmonary hypertension, and 1 with both heart failure and arrhythmias). Global myocardial T2* values did not differ between patients with and without complications. Patients with complications were older and had higher LACI, RACI, and CACI. All three atrioventricular coupling indices significantly differentiated patients with cardiac complications. Receiver operating characteristic (ROC) analysis showed that LACI and CACI outperformed RACI (p=0.009 and p=0.003, respectively), while LACI and CACI had comparable discriminatory power (p=0.279) (Figure).

**Copyright Information:** (Online) ISSN: 2572-9241
©2026 The Author(s). HemaSphere is published by John Wiley & Sons Ltd on behalf of European Hematology Association. This is an open access Abstract Book distributed under the Attribution-NonCommercial-NoDerivs (CC BY-NC-ND), which allows third parties to download the articles and share them with others as long as they credit the author and the Abstract Book, but they cannot change the content in any way or use them commercially.
**Abstract Book Citations:** Authors, Title, HemaSphere, 2026;10:(S1):pages. The abstract book DOIs can be found on the "Author Information" page.
**Disclaimer:** Articles published in the journal HemaSphere exclusively reflect the opinions of the authors. The authors are responsible for all content in their abstracts including accuracy of the facts, statements, citing resources, etc.

### Summary/Conclusion

In TDT patients, CACI was associated with myocardial replacement fibrosis, but not with systolic fraction or myocardial iron overload. Elevated CACI was observed in patients with cardiac complications, indicating its potential as a non-contrast, integrative CMR marker for subclinical dysfunction and risk stratification. Clinically, it offers a simple, reproducible measure from standard CMR images that complements established markers and supports early detection and assessment of cardiac involvement in TDT.

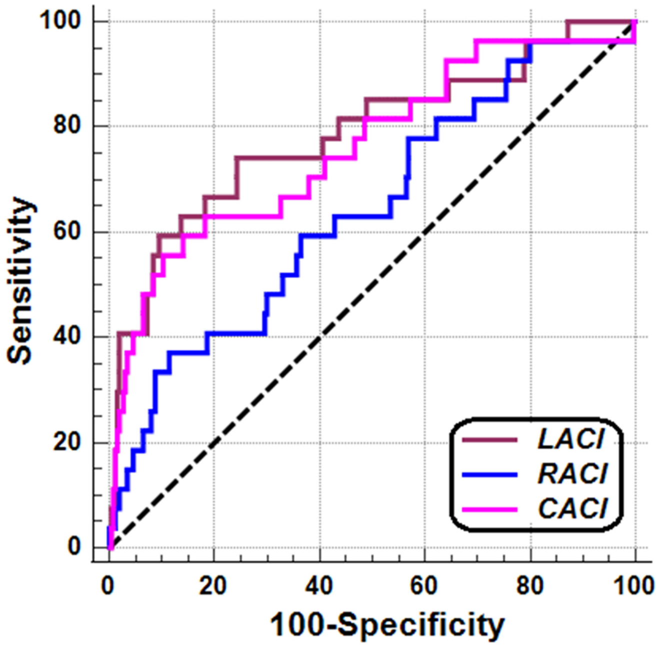

| Parameter | Best cut-off | AUC (95% CI) | p-value | Sensitivity | Specificity |
|---|---|---|---|---|---|
| *LACI* | > 23.63 % | 0.79 (0.73-0.83) | <0.0001 | 74.07 | 75.76 |
| *RACI* | > 36.71 % | 0.64 (0.58-0.69) | 0.014 | 37.04 | 88.64 |
| *CACI* | > 65.95 % | 0.76 (0.71-0.81) | <0.0001 | 55.96 | 89.77 |

ROC curve analysis of of left atrioventricular coupling index (LACI), right atrioventricular coupling index (RACI), and combined atrioventricular coupling index (CACI) for identifying cardiac complications in patients with transfusion-dependent thalassemia.

**Copyright Information:** (Online) ISSN: 2572-9241
©2026 The Author(s). HemaSphere is published by John Wiley & Sons Ltd on behalf of European Hematology Association. This is an open access Abstract Book distributed under the Attribution-NonCommercial-NoDerivs (CC BY-NC-ND), which allows third parties to download the articles and share them with others as long as they credit the author and the Abstract Book, but they cannot change the content in any way or use them commercially.
**Abstract Book Citations:** Authors, Title, HemaSphere, 2026;10:(S1):pages. The abstract book DOIs can be found on the "Author Information" page.
**Disclaimer:** Articles published in the journal HemaSphere exclusively reflect the opinions of the authors. The authors are responsible for all content in their abstracts including accuracy of the facts, statements, citing resources, etc.

### (PS2381) ADVANCED GENE EDITING STRATEGIES FOR HEMOGLOBINOPATHIES: FROM FETAL HEMOGLOBIN REACTIVATION TO PRECISION PRIME EDITING

**Topic:** 28. Thalassemias

Safana Farooq*[1, 2], Susheel Fatima[1], Maliha Javed[1], Anwar Alam[1], Aafia Arain[1], Shagufta Naz[1], Ambrin Fatima[1], Fawad Ur Rehman[1], Salman Kirmani[1], Irfan Hussain[1, 3], Afsar Ali Mian[1]

*[1]Aga Khan University, Centre for Regenerative Medicine and Stem Cell Research, Karachi, Pakistan;[2]TRON, Vector and Gene Therapy, Johannes Gutenberg University Mainz, Mainz, Germany; [3]King Faisal Specialist Hospital & Research Centre, Riyadh, Saudi Arabia;*

### Background

β-thalassemia is a severe monogenic hemoglobin disorder caused by mutations in the β-globin (HBB) gene. Globally, approximately 1.5% of individuals carry a β-thalassemia trait, with carrier frequencies reaching up to 30% in tropical regions. In Pakistan, carrier rates exceed 7%, affecting over 100,000 individuals, yet access to curative therapies remains limited. Although gene therapy has emerged as a promising intervention, scalable and mutation-specific approaches suitable for high-prevalence, resource-constrained settings remain urgently needed.

### Aims

To develop and evaluate complementary genome editing strategies targeting (i) mutation-agnostic fetal hemoglobin (HbF) reactivation and (ii) mutation-specific correction of prevalent HBB mutations using prime editing.

### Methods

For HbF induction, we targeted the zinc finger 4 domain of BCL11A using CRISPR-Cas9 delivered via a peptide-decorated liposome/exosome hybrid system optimized for $CD34^+$ hematopoietic stem cells (HSCs). Editing efficiency and HbF induction were assessed in erythroid differentiation assays and murine xenotransplantation models.
For mutation-specific correction, we employed PE2 and PE3 prime editing systems to correct the HBB:c.92+5G>C splice-site mutation. Both plasmid- and RNP-based approaches were tested and validated by Sanger sequencing. Translational optimization included the pCMV-PEmax-HMLH1dn system. Additionally, mRNA-based prime editing was used to correct the HBB:c.20A>T (p.Glu7Val) mutation associated with sickle cell disease, followed by in vivo functional validation in a murine model.

### Results

CRISPR-mediated disruption of BCL11A resulted in robust HbF reactivation ($p<0.001$) without impairing erythroid differentiation or long-term engraftment potential. Edited HSCs demonstrated significantly elevated γ-globin expression.
Prime editing successfully corrected the HBB:c.92+5G>C mutation, restoring HBB expression and improving erythroid maturation. The mRNA-based prime editing approach corrected the HBB:c.20A>T mutation in vivo, leading to increased hemoglobin production, reduced red cell sickling, and decreased splenomegaly in murine models.

### Summary/Conclusion

These findings demonstrate the feasibility of integrating CRISPR-based HbF reactivation with precision prime editing to address both mutation-independent and mutation-specific therapeutic needs. This dual strategy provides a scalable and potentially accessible framework for next-generation gene therapies in hemoglobinopathies, particularly in high-prevalence and resource-limited settings.

**Copyright Information:** (Online) ISSN: 2572-9241
©2026 The Author(s). HemaSphere is published by John Wiley & Sons Ltd on behalf of European Hematology Association. This is an open access Abstract Book distributed under the Attribution-NonCommercial-NoDerivs (CC BY-NC-ND), which allows third parties to download the articles and share them with others as long as they credit the author and the Abstract Book, but they cannot change the content in any way or use them commercially.
**Abstract Book Citations:** Authors, Title, HemaSphere, 2026;10:(S1):pages. The abstract book DOIs can be found on the "Author Information" page.
**Disclaimer:** Articles published in the journal HemaSphere exclusively reflect the opinions of the authors. The authors are responsible for all content in their abstracts including accuracy of the facts, statements, citing resources, etc.

### (PS2382) BONE DISEASE IN ADULTS WITH THALASSAEMIA: CHARACTERISTICS, INVESTIGATION AND MANAGEMENT INSIGHTS FROM A TERTIARY SERVICE IN THE UK

**Topic:** 28. Thalassemias

Chloe Merrion*[1], Emma Prescott[1], Niamh Malone[1], Ana Ortuno-Cabrero[1, 2], Ryan Mullally[1, 2], Julian Waung[1, 2], Emma Drasar[1, 2]

*[1]Whittington Health NHS trust, Haematology, London, United Kingdom;[2]University College London Hospital, Haematology, London, United Kingdom;*

### Background

Osteoporosis is a major cause of morbidity in patients with thalassaemia. However, data on the utility of bone turnover markers and the safety and efficacy of newer pharmacological therapies in this population remain limited. Patients with thalassaemia have unique risk factors for bone disease relating to both the disease pathophysiology and treatment related complications that require systematic assessment. Here, we describe the characteristics and management approach of patients reviewed in a tertiary bone health service to inform development of a structured UK framework for managing bone disease in thalassaemia.

### Methods

We conducted a service evaluation of adult patients reviewed in a dedicated thalassaemia bone clinic and/or MDT between 2020 and 2025. Data collected included investigations performed and interventions initiated.

### Results

79 patients were reviewed in the service between 2020-2025. 75 (93.7%) had transfusion-dependent thalassaemia (median age 50 years, range 23–73; 49% male). 77 were receiving iron chelation: 63.6% on a single chelator (most commonly deferasirox) and 36.4% on dual chelation therapy, predominantly deferasirox plus deferiprone. Recent liver iron concentration data were available for 75 patients (median 3.6 mg/g/dw, range 0.9–43).

28 patients (35%) had ≥1 fragility fracture and 14 (18%) had ≥2. DEXA within three years was available in 77 patients. Among 18 younger patients, Z-scores were reported. 9 had a low BMD (Z ≤ –2.0 at the femoral neck). The remaining 59 patients had T-scores reported, 20 were osteoporotic (T < –2.5) and 32 osteopenic (T –1.0 to –2.5).
Clinic review included assessment of lifestyle factors, calcium and vitamin D status, thyroid and sex hormones, fructosamine, and screening for exocrine pancreatic insufficiency and growth hormone deficiency. 24-hour urinary calcium and sodium were measured in 54 patients; 17 had hypercalciuria. Seven commenced thiazides for persistent hypercalciuria and 10 received dietary advice. Vitamin D levels were available in 72 patients; 66 (91.6%) were replete (>50 nmol/L).
Bone turnover markers, urine N-telopeptide (NTX) - a marker of bone resorption and procollagen type 1 N-Terminal peptide (P1NP), a marker of bone formation, were assessed in 58 patients. Among those without recent anti-resorptive therapy (no prior treatment or >10 years since bisphosphonate; n = 24), 6 had an elevated urine NTX and P1NP, and 4 had an elevated urine NTX with normal P1NP. Among those with prior anti-resorptive therapy (n = 24), 14 showed normalisation of both markers, while 5 had persistently elevated urine NTX and P1NP, informing subsequent treatment decisions.
45 patients (56.9%) had received bisphosphonates, 28 within five years. Complications included four cases of osteonecrosis of the jaw and two atypical femoral fractures. 6 patients received Denosumab, 2 received Teriparatide, and 3 had Romosozumab. Serial DEXA data were available for 70 patients; 34 demonstrated BMD improvement at the total hip.

**Copyright Information:** (Online) ISSN: 2572-9241
©2026 The Author(s). HemaSphere is published by John Wiley & Sons Ltd on behalf of European Hematology Association. This is an open access Abstract Book distributed under the Attribution-NonCommercial-NoDerivs (CC BY-NC-ND), which allows third parties to download the articles and share them with others as long as they credit the author and the Abstract Book, but they cannot change the content in any way or use them commercially.
**Abstract Book Citations:** Authors, Title, HemaSphere, 2026;10:(S1):pages. The abstract book DOIs can be found on the “Author Information” page.
**Disclaimer:** Articles published in the journal HemaSphere exclusively reflect the opinions of the authors. The authors are responsible for all content in their abstracts including accuracy of the facts, statements, citing resources, etc.

**Summary/Conclusion**

Adults with thalassaemia develop low BMD at a relatively young age with an equal sex distribution. A structured, multidisciplinary approach enables systematic investigation, optimisation of reversible factors, and rational pharmacological decision-making in this high-risk cohort.

**Copyright Information:** (Online) ISSN: 2572-9241
©2026 The Author(s). HemaSphere is published by John Wiley & Sons Ltd on behalf of European Hematology Association. This is an open access Abstract Book distributed under the Attribution-NonCommercial-NoDerivs (CC BY-NC-ND), which allows third parties to download the articles and share them with others as long as they credit the author and the Abstract Book, but they cannot change the content in any way or use them commercially.
**Abstract Book Citations:** Authors, Title, HemaSphere, 2026;10:(S1):pages. The abstract book DOIs can be found on the "Author Information" page.
**Disclaimer:** Articles published in the journal HemaSphere exclusively reflect the opinions of the authors. The authors are responsible for all content in their abstracts including accuracy of the facts, statements, citing resources, etc.

### (PS2383) IMPACT OF EMPAGLIFLOZIN ON THE OUTCOMES OF Β-THALASSEMIA MAJOR IN PATIENTS WITH TYPE 2 DIABETES MELLITUS: THE THALEMPA OBSERVATIONAL STUDY

**Topic:** 28. Thalassemias

Anas Ibraheem*[1], Wassem Al-Tameemi[2, 3]

*[1]The London Clinic, Oncology, London, United Kingdom; [2]Al-Nahrain University College of Medicine, Hematology, Baghdad, Iraq; [3]Imamein Kadhimein Medical City, Hematology, Baghdad, Iraq;*

### Background

Beta-thalassemia major (β-TM) is a genetic disorder characterized by ineffective erythropoiesis and chronic hemolytic anemia, necessitating lifelong blood transfusions and leading to severe complications. Despite the available treatment choices, there is a demand for a cost-effective solution capable of delivering favorable results by minimizing the necessity for frequent blood transfusions and mitigating ensuing complications.

### Aims

This study, termed THALEMPA by the authors, investigated the effect of empagliflozin (EMPA) on β-TM outcomes in patients with type 2 diabetes mellitus (T2DM), focusing on disease severity and associated complications of iron overload and hyperuricemia.

### Methods

This study conducted a single-center prospective observational investigation involving adults diagnosed with β-TM and T2DM. A total of 20 carefully selected patients were stratified into two groups based on their medical condition: the EMPA group, receiving 10 mg of empagliflozin, and a control group, receiving standard care. This focused cohort size was chosen to ensure a detailed, in-depth analysis of the treatment effects within this specific patient population. Over three months, both groups were closely monitored for β-TM outcomes. The study assessed β-TM severity parameters such as hemoglobin levels, blood transfusion frequency, aspartate aminotransferase (AST), alanine aminotransferase (ALT), left ventricular ejection fraction percentage, and spleen size. Additionally, β-TM complications were evaluated through serum ferritin and uric acid levels.

### Results

Our analysis revealed that EMPA increased hemoglobin levels by up to 0.56 g/dL compared to baseline ($P < 0.05$). Liver enzyme levels significantly improved with EMPA by the third month. AST and ALT decreased by 36.22% and 33.36%, respectively, from baseline levels ($P < 0.05$), highlighting EMPA's potential benefits for β-TM severity. Serum ferritin and uric acid levels decreased by 27.93% and 21.29%, respectively, over three months on EMPA ($P < 0.05$). However, other parameters did not show significant changes post-EMPA.

### Summary/Conclusion

This study demonstrates the significant impact of EMPA treatment over three months on β-TM patients with T2DM, evidenced by notable improvements in hemoglobin levels and reductions in liver enzymes, as well as in complications related to iron overload and hyperuricemia. Future research should confirm these benefits over longer durations and assess broader patient outcomes such as quality of life.

**Copyright Information:** (Online) ISSN: 2572-9241
©2026 The Author(s). HemaSphere is published by John Wiley & Sons Ltd on behalf of European Hematology Association. This is an open access Abstract Book distributed under the Attribution-NonCommercial-NoDerivs (CC BY-NC-ND), which allows third parties to download the articles and share them with others as long as they credit the author and the Abstract Book, but they cannot change the content in any way or use them commercially.
**Abstract Book Citations:** Authors, Title, HemaSphere, 2026;10:(S1):pages. The abstract book DOIs can be found on the “Author Information” page.
**Disclaimer:** Articles published in the journal HemaSphere exclusively reflect the opinions of the authors. The authors are responsible for all content in their abstracts including accuracy of the facts, statements, citing resources, etc.

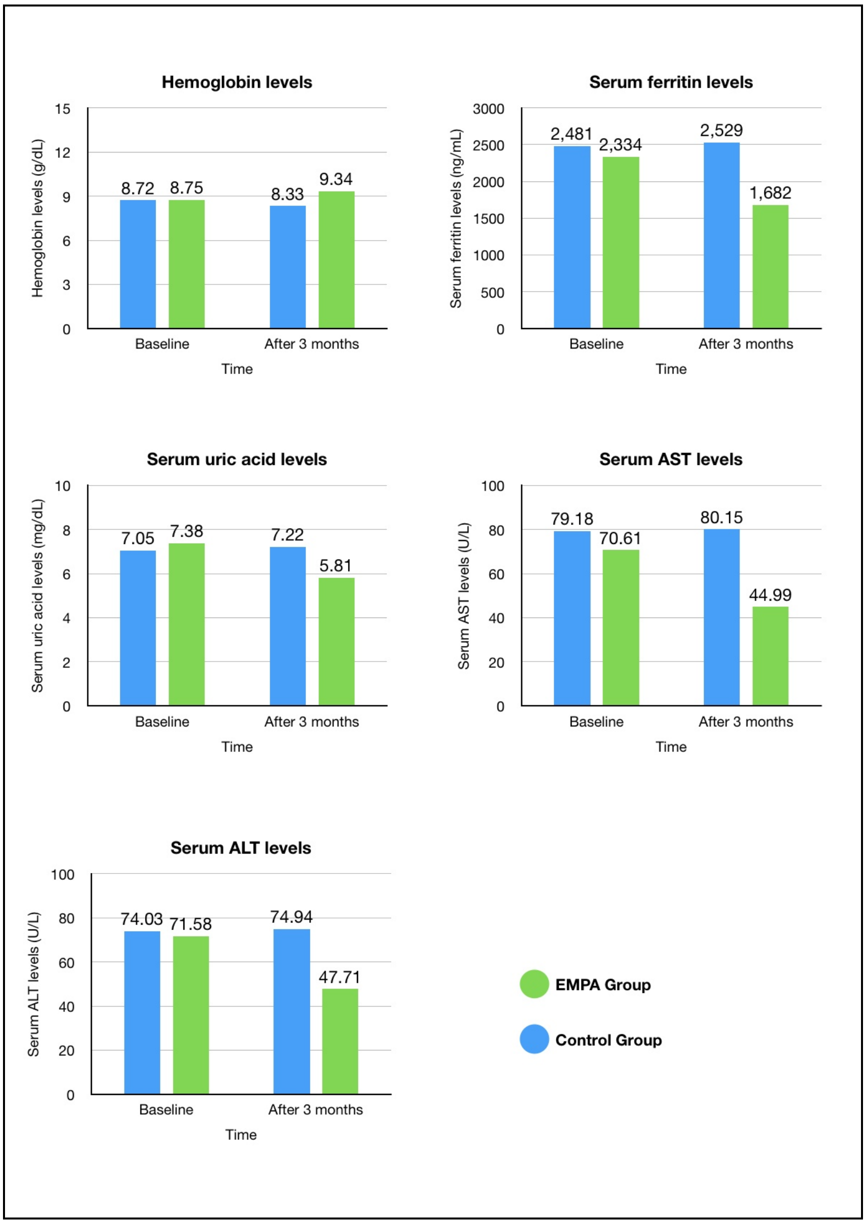

**Copyright Information:** (Online) ISSN: 2572-9241
©2026 The Author(s). HemaSphere is published by John Wiley & Sons Ltd on behalf of European Hematology Association. This is an open access Abstract Book distributed under the Attribution-NonCommercial-NoDerivs (CC BY-NC-ND), which allows third parties to download the articles and share them with others as long as they credit the author and the Abstract Book, but they cannot change the content in any way or use them commercially.
**Abstract Book Citations:** Authors, Title, HemaSphere, 2026;10:(S1):pages. The abstract book DOIs can be found on the "Author Information" page.
**Disclaimer:** Articles published in the journal HemaSphere exclusively reflect the opinions of the authors. The authors are responsible for all content in their abstracts including accuracy of the facts, statements, citing resources, etc.

### (PS2384) SAFETY AND EXPECTED BENEFIT DRIVE GENE THERAPY ACCEPTANCE IN β-THALASSEMIA AND SICKLE CELL DISEASE: A MULTICENTER MEDIATION ANALYSIS

**Topic:** 28. Thalassemias

Stefania Mistretta*[1], Angela Vitrano[2], Roberto Lisi[3], Rosamaria Rosso[4], Giovan Battista Ruffo[5], Vincenzo Spadola[6], Daniela Elvira Fiorino[7], Bagnato Sabrina[8], Gerardi Calogera[9], Saveria Campisi[10], Rita Barone[11], Luciana Rigoli[12], Alessandra Quota[13], Daniele Di Bernardo[14], Gaetano Roccamo[15], Mariafrancesca Mansueto[1], Aurelio Maggio[16], Mariasanta Napolitano[17]

*[1]Fondazione Franco e Piera Cutino, Palermo, Italy;[2]AOR Villa Sofia V. Cervello, UOC Ematologia e Malattie Rare, Palermo, Italy; [3]UOSD Talassemia, ARNAS Garibaldi, Catania, Italy; [4]AOU Policlinico V. Emanuele II, Catania, Italy;[5]UOSD Talassemia, ARNAS Civico, Palermo, Italy; [6]UOSD Talassemia, ASP Ragusa, Ragusa, Italy; [7]UOSD Talassemia, ASP Caltanissetta-S. Elia, Caltanissetta, Italy; [8]UOSD Talassemia, ASP Siracusa- Lentini, Siracusa, Italy;[9]UOSD Talassemia, ASP Agrigento-Sciacca, Sciacca, Italy; [10]UOSD Talassemia, ASP Siracusa, Siracusa, Italy;[11]UOSD Talassemia, Trapani, Italy; [12]AOU Policlinico Messina, Messina, Italy; [13]UOSD Talassemia, ASP Caltanissetta-Gela, Caltanissetta, Italy; [14]UOSD Talassemia, ASP Agrigento, Agrigento, Italy; [15]UOSD Talassemia, ASP Messina-S. Agata Militello, Messina, Italy; [16]Foundation Franco and Piera Cutino, Palermo, Italy; [17]Università degli studi di Palermo, Palermo, Italy;*

### Background

Gene therapy is emerging as a potentially curative treatment for β-thalassemia and sickle cell disease (SCD). Although clinical efficacy and safety data are rapidly accumulating, limited evidence is available on the psychological factors influencing patients' willingness to undergo gene therapy.

### Aims

To identify cognitive and emotional determinants of gene therapy propensity in patients with hereditary hemoglobinopathies.

### Methods

A cross-sectional survey was administered by the staff of the 14 centers belonging to the Sicilian Regional Network on Thalassemia and Hemoglobinopathy of Sicily, in outpatient and hospital settings, between June 2025 and January 2026. It was completed by 105 patients (β-thalassemia: n=88; SCD: n=17), age from 18 to 35 years, according to the eligibility criteria of adult patients by AIFA recommendations, in self-administration, using a 6-point Likert scale. The composite indices were identified based on what was highlighted in the reference literature, and evaluated the perceived information, safety, benefit, oncological risk, infertility risk, perceived health status, emotions. Composite indices assessed perceived information, safety, benefit, oncological risk, infertility risk, perceived health status, emotional concern, and therapy propensity. Internal consistency was evaluated using Cronbach's alpha. Group differences by diagnosis and sex were analyzed using Brunner–Munzel tests with Benjamini–Hochberg correction. A multivariable linear regression model identified independent predictors of therapy propensity. A bootstrap mediation analysis (5,000 resamples) tested whether perceived benefit mediated the relationship between perceived safety and therapy propensity.

**Copyright Information:** (Online) ISSN: 2572-9241
©2026 The Author(s). HemaSphere is published by John Wiley & Sons Ltd on behalf of European Hematology Association. This is an open access Abstract Book distributed under the Attribution-NonCommercial-NoDerivs (CC BY-NC-ND), which allows third parties to download the articles and share them with others as long as they credit the author and the Abstract Book, but they cannot change the content in any way or use them commercially.
**Abstract Book Citations:** Authors, Title, HemaSphere, 2026;10:(S1):pages. The abstract book DOIs can be found on the "Author Information" page.
**Disclaimer:** Articles published in the journal HemaSphere exclusively reflect the opinions of the authors. The authors are responsible for all content in their abstracts including accuracy of the facts, statements, citing resources, etc.

### Results

Out of 105 survey participants, 83.8% had β-thalassemia and 16.2% had sickle cell disease (SCD). The mean age was 27.6 years (SD = 5.0), with a median of 28.0 years. Females comprised 50.5% of the sample. Perceived safety (α=.87) and perceived benefit (α=.90) showed high internal consistency. No significant differences emerged between β-thalassemia and SCD patients across domains. In multivariable analysis, perceived safety (β=.45, p<.001) and perceived benefit (β=.43, p<.001) independently predicted gene therapy propensity, explaining 66% of the variance ($R^2$=.66). Perceived risks, emotional concern, sex, and diagnosis were not independent predictors. Mediation analysis demonstrated that perceived benefit partially mediated the association between perceived safety and therapy propensity (indirect effect=0.39, 95% CI 0.25–0.53, p<.001), accounting for 43% of the total effect. No differences detected; limited the power for SCD subgroup.

### Summary/Conclusion

The results reported here show that gene therapy acceptance appears to be driven primarily by a cognitive assessment of safety and expected benefits, rather than by demographic or disease-related variables. Integrating this knowledge, therefore, would allow for the development of personalized communication strategies for use during patient consultations. These could be based on clear and comprehensive information that focuses more on treatment safety and clarifies its expected benefits, taking into account that, according to the literature, the assessment of the latter appears to be mediated by the patient's self-assessment of current health status. This would facilitate the development of a well-informed and shared decision-making process between doctor and patient when discussing gene therapy.

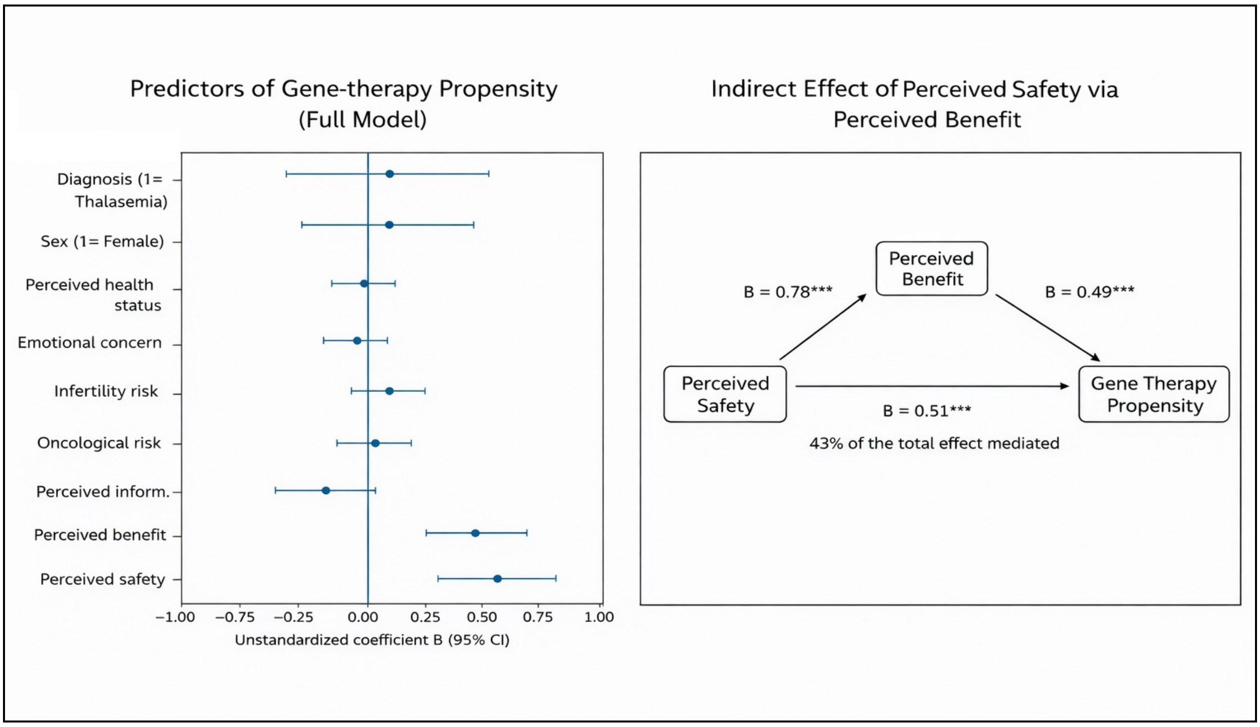

Predictors of gene therapy propensity and mediation model showing the indirect effect of perceived safety via perceived benefit

**Copyright Information:** (Online) ISSN: 2572-9241
©2026 The Author(s). HemaSphere is published by John Wiley & Sons Ltd on behalf of European Hematology Association. This is an open access Abstract Book distributed under the Attribution-NonCommercial-NoDerivs (CC BY-NC-ND), which allows third parties to download the articles and share them with others as long as they credit the author and the Abstract Book, but they cannot change the content in any way or use them commercially.
**Abstract Book Citations:** Authors, Title, HemaSphere, 2026;10:(S1):pages. The abstract book DOIs can be found on the "Author Information" page.
**Disclaimer:** Articles published in the journal HemaSphere exclusively reflect the opinions of the authors. The authors are responsible for all content in their abstracts including accuracy of the facts, statements, citing resources, etc.

### (PS2386) TRACKING MYOCARDIAL NATIVE T1 OVER TIME IN TRANSFUSION-DEPENDENT THALASSEMIA

**Topic:** 28. Thalassemias

Antonella Meloni*[1], Laura Pistoia[1], Vincenzo Positano[1], Maria Caterina Putti[2], Daria Francesca Guglielmucci[3], Daniele Di Bernardo[4], Monica Benni[5], Antonella Massa[6], Angelica Barone[7], Andrea Barison[1]

*[1]Fondazione G. Monasterio CNR-Regione Toscana, Pisa, Italy; [2]Azienda Ospedaliero-Università di Padova, Padova, Italy; [3]Pronto Soccorso Pediatrico. P.O. Misericordia Grosseto, Grosseto, Italy; [4]P.O. "San Giovanni di Dio", Agrigento, Italy; [5]Policlinico S. Orsola "L. e A. Seragnoli", Bologna, Italy; [6]Ospedale "Giovanni Paolo II", Olbia, Italy; [7]Azienda Ospedaliero-Universitaria di Parma, Parma, Italy;*

### Background

Cardiovascular magnetic resonance (CMR) T2* imaging is the gold standard for quantifying myocardial iron and has improved early detection of high-risk patients, guiding iron chelation therapy and reducing cardiac morbidity and mortality. However, T2* can be limited by technical artifacts and may miss subtle or early myocardial iron deposition. Native T1 mapping has emerged as a complementary tool, offering greater sensitivity than T2* for detecting early cardiac involvement.

### Aims

We evaluated longitudinal changes in native left ventricular (LV) T1 values over 18 months in patients with transfusion-dependent thalassemia (TDT).

### Methods

Sixty-four TDT patients consecutively enrolled in the Extension-Myocardial Iron Overload in Thalassemia (E-MIOT) project underwent two CMR examinations at 1.5T. Native T1 mapping and T2* relaxometry were performed using standardized protocols. Global LV T1 and T2* values were calculated from 16 myocardial segments. LV ejection fraction was assessed by cine imaging.

### Results

Fifty-five patients (84.4%) had previously undergone at least one T2* CMR scan, while myocardial T1 mapping was performed for the first time in all participants. At baseline, mean LV T1 was 959.51±101.46 ms and mean LV T2* was 37.17±9.44 ms, with a significant correlation between the two parameters (R=0.533; p<0.0001). Both LV T1 and T2* were reduced in 9 (14.1%) patients. Thirty-five patients (54.7%) exhibited normal LV T1 and T2* values. Increased LV T1 was observed in two patients (2.1%) with normal T2*, while only one patient (1.6%) had normal T1 in the presence of a pathological T2*. Reduced values of both parameters were found in nine patients (14.1%), and reduced LV T1 with normal T2* occurred in 17 patients (25.6%).
Over follow-up, LV T1 did not significantly change (mean change 1.99±63.57 ms; p 0.841), whereas LV T2* increased significantly (mean change 1.61±4.52 ms; p = 0.001). Individual T1 trajectories varied (Figure 1): among patients with normal baseline T1, 22.1% developed reduced T1, while 26.9% of those with reduced baseline T1 normalized at follow-up. Both patients with increased baseline T1 returned to normal. Changes in LV T1 were inversely associated with baseline T1 values (R=−0.406; p=0.001) and correlated with changes in T2* (R=0.311; p=0.012), but were independent of age, baseline ferritin, hemoglobin levels, and LV ejection fraction. No significant changes were observed in LV ejection fraction (mean difference −0.44±6.54%; p 0.594).
At baseline, deferasirox was the most frequently prescribed chelation therapy (56.6%), followed by deferiprone (15.1%). During follow-up, dose adjustments were common, whereas switching between chelator classes was infrequent (<10%).

**Copyright Information:** (Online) ISSN: 2572-9241
©2026 The Author(s). HemaSphere is published by John Wiley & Sons Ltd on behalf of European Hematology Association. This is an open access Abstract Book distributed under the Attribution-NonCommercial-NoDerivs (CC BY-NC-ND), which allows third parties to download the articles and share them with others as long as they credit the author and the Abstract Book, but they cannot change the content in any way or use them commercially.
**Abstract Book Citations:** Authors, Title, HemaSphere, 2026;10:(S1):pages. The abstract book DOIs can be found on the "Author Information" page.
**Disclaimer:** Articles published in the journal HemaSphere exclusively reflect the opinions of the authors. The authors are responsible for all content in their abstracts including accuracy of the facts, statements, citing resources, etc.

### Summary/Conclusion

In well-managed TDT patients, native myocardial T1 values remain overall stable over mid-term follow-up, despite marked interindividual variability. Longitudinal changes in T1 are influenced by baseline T1 values and parallel changes in T2*, supporting native T1 mapping as a complementary, but not interchangeable, tool to T2* for the assessment and longitudinal monitoring of cardiac iron in TDT.

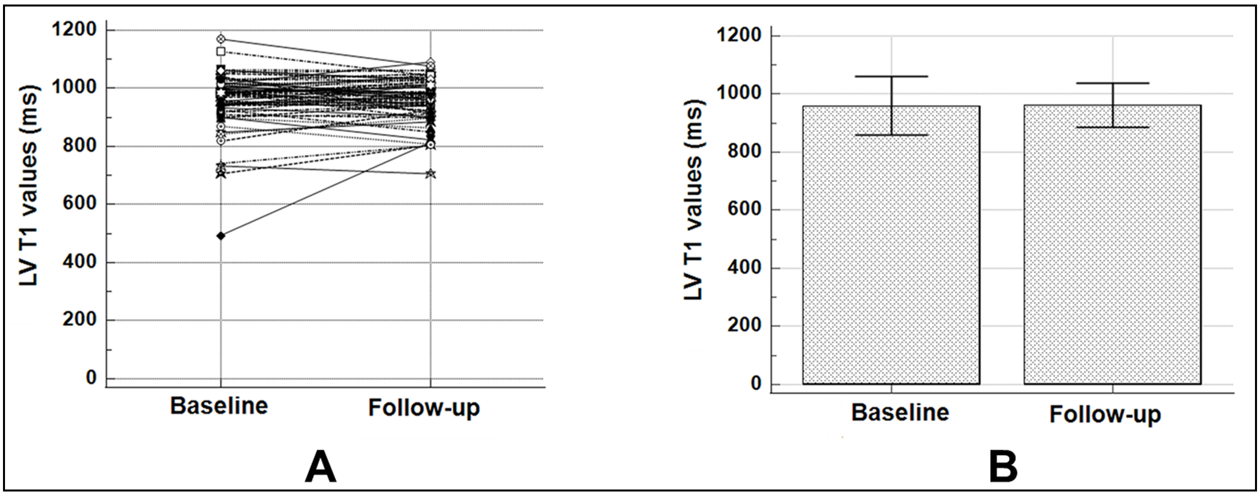

Change in LV T1 values from baseline to follow-up. (A) Individual patient trajectories of LV T1 values over time. Each line represents a single patient. (B) Mean LV T1 values at each time point, with error bars representing standard deviations.

**Copyright Information:** (Online) ISSN: 2572-9241
©2026 The Author(s). HemaSphere is published by John Wiley & Sons Ltd on behalf of European Hematology Association. This is an open access Abstract Book distributed under the Attribution-NonCommercial-NoDerivs (CC BY-NC-ND), which allows third parties to download the articles and share them with others as long as they credit the author and the Abstract Book, but they cannot change the content in any way or use them commercially.
**Abstract Book Citations:** Authors, Title, HemaSphere, 2026;10:(S1):pages. The abstract book DOIs can be found on the "Author Information" page.
**Disclaimer:** Articles published in the journal HemaSphere exclusively reflect the opinions of the authors. The authors are responsible for all content in their abstracts including accuracy of the facts, statements, citing resources, etc.

### (PS2387) EFFICACY AND SAFETY OF ALTERNATING DEFERIPRONE AND DEFERASIROX COMBINATION CHELATION THERAPY IN PATIENTS WITH TRANSFUSION-DEPENDENT THALASSEMIA

**Topic:** 28. Thalassemias

Nihan Avcu Oral*[1], Tuncay Hazirolan[2], Tekin Aksu[1], Şule Ünal Cangül[1]

*[1]Hacettepe University, Pediatric Hematology-Oncology, Ankara, Türkiye; [2]Hacettepe University, Radiology, Ankara, Türkiye;*

### Background

Iron overload is the leading cause of morbidity and mortality in patients with transfusion-dependent thalassemia. To prevent related complications, intravenous deferoxamine, oral deferasirox (DFS), deferiprone (DFP), or combinations are used.

### Aims

In this study, the efficacy of an alternating DFS–DFP combination regimen was retrospectively evaluated, and the development of adverse effects was assessed.

### Methods

Fourteen patients with transfusion-dependent thalassemia, receiving an alternating DFS–DFP combination were included. Patients were treated with DFS for 3 consecutive days (35–40 mg/kg/day, 1x, for film-coated tablet; 21–28 mg/kg/day, 1x, for dispersible tablet), followed by DFP for 4 consecutive days (80–90 mg/kg/day, 3x), in an alternating schedule. Serum ferritin levels, cardiac and hepatic iron overload were assessed at baseline and during follow-up using T2* MRI. ALT, creatinine, and absolute neutrophil count were evaluated, patients were monitored for adverse effects. Normality was assessed using Shapiro–Wilk test, comparisons were performed using Wilcoxon signed-rank test.

### Results

The median age at initiation of alternating chelation was 34 years (IQR: 20–37). The cohort consisted of 6 males and 8 females; one female patient had two separate periods of alternating chelation therapy, which were analyzed independently. The median duration of combination therapy was 16 months (IQR: 12–22). The median serum ferritin level at initiation of alternating chelation was 1548 ng/mL (IQR: 1073–2697). When ferritin levels were compared during the alternating chelation period, a statistically significant decrease was observed between baseline and the third month of treatment (p=0.006). At the start of alternating chelation, 86.6% of patients had a cardiac T2* value below 20 ms. Comparison of iron overload MRI findings obtained before treatment and between months 6–12 of therapy showed a significant increase in cardiac T2* values (pre-treatment 10 ms [IQR: 8.5–15] vs post-treatment 21 ms [IQR: 9.9–30]; p=0.005). An increase was also observed in liver T2* values (pre-treatment 1.5 ms [IQR: 1–2.9] vs post-treatment 2.2 ms [IQR: 1.2–4.5]; p=0.151). Transient neutropenia was observed in one patient. Grade 2 hypertransaminasemia occurred in two patients, and a 33% increase in serum creatinine from baseline was observed in one patient. One patient experienced nausea as a gastrointestinal adverse effect.

**Copyright Information:** (Online) ISSN: 2572-9241
©2026 The Author(s). HemaSphere is published by John Wiley & Sons Ltd on behalf of European Hematology Association. This is an open access Abstract Book distributed under the Attribution-NonCommercial-NoDerivs (CC BY-NC-ND), which allows third parties to download the articles and share them with others as long as they credit the author and the Abstract Book, but they cannot change the content in any way or use them commercially.
**Abstract Book Citations:** Authors, Title, HemaSphere, 2026;10:(S1):pages. The abstract book DOIs can be found on the "Author Information" page.
**Disclaimer:** Articles published in the journal HemaSphere exclusively reflect the opinions of the authors. The authors are responsible for all content in their abstracts including accuracy of the facts, statements, citing resources, etc.

**Summary/Conclusion**

This study demonstrated that an alternating regimen consisting of 3 days of DFS followed by 4 days of DFP led to significant improvements in serum ferritin levels and cardiac T2* values. Combination chelation therapies are typically administered with both agents used concomitantly on a daily basis, requiring patients to take two different drugs simultaneously and increasing the daily pill burden. A higher tablet load may compromise adherence, and the continuous concurrent use of both agents may increase the frequency and severity of adverse effects. The alternating regimen enables effective use of the distinct chelation mechanisms of each agent. This approach appears to maintain chelation efficacy while potentially reducing toxicity with uninterrupted combined therapy. Moreover, the lower pill burden may improve adherence and contribute to sustained clinical benefit. These findings support the alternating strategy as a rational approach that balances efficacy and tolerability.

**Copyright Information:** (Online) ISSN: 2572-9241
©2026 The Author(s). HemaSphere is published by John Wiley & Sons Ltd on behalf of European Hematology Association. This is an open access Abstract Book distributed under the Attribution-NonCommercial-NoDerivs (CC BY-NC-ND), which allows third parties to download the articles and share them with others as long as they credit the author and the Abstract Book, but they cannot change the content in any way or use them commercially.
**Abstract Book Citations:** Authors, Title, HemaSphere, 2026;10:(S1):pages. The abstract book DOIs can be found on the "Author Information" page.
**Disclaimer:** Articles published in the journal HemaSphere exclusively reflect the opinions of the authors. The authors are responsible for all content in their abstracts including accuracy of the facts, statements, citing resources, etc.

### (PS2388) ADJUNCT RESVERATROL OR OMEPRAZOLE IN NON-TRANSFUSION DEPENDENT THALASSEMIA PATIENTS WITH SECONDARY HEMOCHROMATOSIS: A RANDOMIZED, DOUBLE-BLIND, PLACEBO-CONTROLLED TRIAL

**Topic:** 28. Thalassemias

Thanapong Chopetgool*[1], Adisak Tantiworawit[1], Chatree Chai-Adisaksopha[1], Ekarat Rattarittamrong[1], Lalita Norasetthada[1], Thanawat Rattanathammethee[1], Sasinee Hantrakool[1], Pokpong Piriyakhuntorn[1], Teerachat Punnachet[1], Nonthakorn Hantrakun[1], Sirichai Srichairatanakool[1], Pimpisid Koonyosying[2], Somdet Srichairatanakool[2]

*[1]Chiang Mai University Hospital, Division of Hematology, Department of Internal Medicine, Faculty of Medicine, Chiang Mai Province, Thailand; [2]Chiang Mai University, Biochemistry, Chiang Mai Province, Thailand;*

### Background

Iron overload in non–transfusion-dependent thalassemia (NTDT) patients is driven predominantly by increased intestinal iron absorption secondary to hepcidin suppression due to elevated erythroferrone levels. Pharmacologic modulation of iron absorption may provide complementary benefits. We investigated whether gastric acid suppression with omeprazole or luminal iron binding with resveratrol as adjunctive strategies could reduce iron burden in NTDT patients.

### Aims

To evaluate the efficacy and safety of adjunct resveratrol or omeprazole in reducing serum ferritin in NTDT patients receiving stable iron chelation.

### Methods

This randomized, double-blind, placebo-controlled trial was conducted at the outpatient department of Chiang Mai University Hospital. NTDT patients aged 20–65 years with serum ferritin ≥ 300 ng/mL receiving stable iron chelation therapy were randomized 1:1:1, stratified by sex and baseline ferritin (< 1,000 and ≥ 1,000 ng/mL), to receive omeprazole 20 mg twice daily, resveratrol 250 mg twice daily, or placebo twice daily for 6 months. The primary endpoint was change in serum ferritin at 6 months, analyzed by intention-to-treat. Safety was assessed in all patients receiving at least one dose of study medication. This trial was registered with the ISRCTN Registry (number 73258526).

### Results

Between February 2025 and February 2026, 30 patients were enrolled and assigned to resveratrol(n=10), omeprazole (n=11) or placebo (n=9). Median age was 46 years (IQR 37 to 55), 33.3% were male, and 50% had beta-thalassemia. Median baseline ferritin was 629 ng/mL (IQR 484 to 975). At 6 months, a substantial reduction in ferritin was observed in the resveratrol arm, with a median change of −116 ng/mL (IQR −168 to −68). In contrast, ferritin levels changed minimally with omeprazole (−8 ng/mL; IQR −38 to 19) and increased slightly with placebo (8 ng/mL; IQR −27 to 48). The decrease associated with resveratrol was significantly greater compared with both omeprazole (P=0.016) and placebo (P=0.012), whereas no statistically significant difference was detected between omeprazole and placebo (P=0.653). Adverse events occurred in three patients receiving resveratrol (grade 1 bloating, grade 1 fatigue and grade 2 dysgeusia) and one patient receiving placebo (grade 1 bloating). All events were mild and resolved completely after treatment discontinuation.

**Copyright Information:** (Online) ISSN: 2572-9241
©2026 The Author(s). HemaSphere is published by John Wiley & Sons Ltd on behalf of European Hematology Association. This is an open access Abstract Book distributed under the Attribution-NonCommercial-NoDerivs (CC BY-NC-ND), which allows third parties to download the articles and share them with others as long as they credit the author and the Abstract Book, but they cannot change the content in any way or use them commercially.
**Abstract Book Citations:** Authors, Title, HemaSphere, 2026;10:(S1):pages. The abstract book DOIs can be found on the "Author Information" page.
**Disclaimer:** Articles published in the journal HemaSphere exclusively reflect the opinions of the authors. The authors are responsible for all content in their abstracts including accuracy of the facts, statements, citing resources, etc.

**Summary/Conclusion**

Resveratrol as an adjunct to iron chelation therapy significantly reduced serum ferritin in NTDT patients and demonstrated a favorable safety profile, supporting its potential role as an iron absorption-modulating strategy.

**Copyright Information:** (Online) ISSN: 2572-9241
©2026 The Author(s). HemaSphere is published by John Wiley & Sons Ltd on behalf of European Hematology Association. This is an open access Abstract Book distributed under the Attribution-NonCommercial-NoDerivs (CC BY-NC-ND), which allows third parties to download the articles and share them with others as long as they credit the author and the Abstract Book, but they cannot change the content in any way or use them commercially.
**Abstract Book Citations:** Authors, Title, HemaSphere, 2026;10:(S1):pages. The abstract book DOIs can be found on the “Author Information” page.
**Disclaimer:** Articles published in the journal HemaSphere exclusively reflect the opinions of the authors. The authors are responsible for all content in their abstracts including accuracy of the facts, statements, citing resources, etc.

### (PS2389) REFINED CLASSIFICATION AND PHENOTYPE-DRIVEN ANALYSIS OF PIEZO1 VARIANTS REVEAL DOMAIN-SPECIFIC PATHOGENIC MECHANISMS

**Topic:** 29. Enzymopathies, membranopathies and other red blood cell disorders

Barbara Eleni Rosato*[1, 2], Roberta Marra[2], Stefania Martone[3], Mariangele Manno[2], Manuela Dionisi[2], Michela Ribersani[4], Valeria Maria Pinto[5, 6], Gian Luca Forni[7, 8], Manuela Balocco[5], Paola Carrara[5], Martina Lamagna[5], Filomena Morisco[9], Maria Guarino[9], Valentina Cossiga[9], Antonio Barbato[10], Francesco Arcioni[11], Achille Iolascon[1, 2], Roberta Russo[1, 2], Immacolata Andolfo[1, 2]

*[1]Department of Molecular Medicine and Medical Biotechnology - University of Naples Federico II, Naples, Italy;[2]CEINGE Biotecnologie Avanzate Franco Salvatore, Naples, Italy; [3]Department of Laboratory Medicine (DAIMedLab), Medical Genetics Unit, University of Naples Federico II, Naples, Italy; [4]Department of Translational and Precision Medicine, University of Rome "La Sapienza", Rome, Italy; [5]Center for Microcythemia, Congenital Anemia and Iron Dysmetabolism, Ente Ospedaliero Ospedali Galliera, Genoa, Italy; [6]Hematology and Cell Therapies, IRCCS Policlinico San Martino Hospital, Genoa, Italy; [7]Unit of Hematology, IRCCS Istituto Giannina Gaslini, Genoa, Italy; [8]ForAnemia ETS Foundation, Genoa, Italy;[9]Diseases of the Liver and Biliary System Unit, Department of Clinical Medicine and Surgery, University of Naples Federico II, Naples, Italy; [10]Department of Clinical Medicine and Surgery, University of Naples Federico II, Naples, Italy; [11]Pediatric Oncology-Hematology Unit, Santa Maria della Misericordia Hospital, Perugia, Italy;*

### Background

PIEZO1 is a mechanosensitive cation channel that forms a trimeric, propeller-shaped complex in the plasma membrane, organized into distinct functional domains. Gain-of-function variants (GoF) in *PIEZO1* cause dehydrated hereditary stomatocytosis (DHS or hereditary xerocytosis), a pleiotropic syndrome mainly presenting hemolytic anemia and hepatic iron overload. Interpreting genetic variants in *PIEZO1* is complicated by marked allelic heterogeneity and relative tolerance to missense variation. Consequently, many variants are classified as benign or of uncertain significance (VUS) and affected individuals often remain without a definitive molecular diagnosis.

### Aims

To provide an integrative framework combining ACMG guidelines, in silico predictions, structural domain annotation, and detailed patient phenotyping to classify PIEZO1 variants

### Methods

We curated 2565 *non-null* PIEZO1 coding variants from gnomAD and ClinVar and private variants from an internal cohort of patients suspected for DHS. Criteria provided by the ACMG/AMP were applied to the curated variants. Scores were computed using a Bayesian classifier and then translated to interpretations. The application of PP3/BP4 evidence was based on a *composite predictive score*, developed by integrating multiple computational tools. For the phenotype-based reclassification, a total of 176 patients from 141 unrelated families carrying *non-null-PIEZO1* rare variants were retrospectively enrolled between 2020-2024.

**Copyright Information:** (Online) ISSN: 2572-9241
©2026 The Author(s). HemaSphere is published by John Wiley & Sons Ltd on behalf of European Hematology Association. This is an open access Abstract Book distributed under the Attribution-NonCommercial-NoDerivs (CC BY-NC-ND), which allows third parties to download the articles and share them with others as long as they credit the author and the Abstract Book, but they cannot change the content in any way or use them commercially.
**Abstract Book Citations:** Authors, Title, HemaSphere, 2026;10:(S1):pages. The abstract book DOIs can be found on the "Author Information" page.
**Disclaimer:** Articles published in the journal HemaSphere exclusively reflect the opinions of the authors. The authors are responsible for all content in their abstracts including accuracy of the facts, statements, citing resources, etc.

### Results

The application of stratified ACMG criteria led to the classification of more than 2000 variants, enabling the resolution of nearly 1,000 VUSs. Phenotype-based reclassification was applied to 124 unique variants from our cohort of patients, reclassifying 17 additional VUSs. A domain-based enrichment analysis revealed a non-random clustering of pathogenic variants in functionally constrained domains, particularly Anchor domain, Inner Helix (IH) and the C-terminal domain (CTD). Indeed, pathogenic variants predominantly associated with gating dynamics and ion permeation. Unsupervised clustering, based on the integration of hematochemical and iron balance data of patients carrying pathogenic *PIEZO1* variants revealed three phenotypic clusters. Cluster 1 reflected the classic DHS1 features, including moderate macrocytic hemolytic anemia, reticulocytosis, and severe iron overload. Patients from Cluster 2 displayed an atypical phenotype with severe microcytic anemia in the absence of overt hemolysis. Most of these patients carried additional heterozygous variants consistent with α- or β-thalassemia traits. Cluster 3 was characterized by a milder hemolytic profile, with signs of ineffective erythropoiesis and low-grade iron dysregulation. Genotype-phenotype correlations demonstrated that variant impact aggregates in domain-defined patterns rather than being randomly distributed. Particularly, variants within the CTD domain associated with macrocytic anemia, reticulocytes, and iron overload. The IH domain showed a profile of hyperferritinemia and reduced RBC count and high reticulocytes.

### Summary/Conclusion

The refined classification pipeline and phenotype-driven analysis highlighted the complexity of PIEZO1-related pathophysiology, offering new insights to incorporate deep phenotyping into rare variant interpretation in hereditary erythrocyte disorders. This framework provides practical support for clinicians and laboratory specialists, improving diagnostic precision and informing patient management and prognostic assessment.

**Copyright Information:** (Online) ISSN: 2572-9241
©2026 The Author(s). HemaSphere is published by John Wiley & Sons Ltd on behalf of European Hematology Association. This is an open access Abstract Book distributed under the Attribution-NonCommercial-NoDerivs (CC BY-NC-ND), which allows third parties to download the articles and share them with others as long as they credit the author and the Abstract Book, but they cannot change the content in any way or use them commercially.
**Abstract Book Citations:** Authors, Title, HemaSphere, 2026;10:(S1):pages. The abstract book DOIs can be found on the “Author Information” page.
**Disclaimer:** Articles published in the journal HemaSphere exclusively reflect the opinions of the authors. The authors are responsible for all content in their abstracts including accuracy of the facts, statements, citing resources, etc.

### (PS2390) SEVERE ALAS2 DEFICIENCY REVEALS MITOCHONDRIAL AND METABOLIC DRIVERS OF X-LINKED SIDEROBLASTIC ANEMIA AND ENABLES GENE THERAPY RESCUE

**Topic:** 29. Enzymopathies, membranopathies and other red blood cell disorders

Kazuhiro Noguchi[1], Carlo Castruccio Castracani[1], Jean Ann Maguire[1], Alyssa Gagne[1], Wei Tong[1], Giulia Pavani[1], Stefano Rivella*[1]

[1] *Children's Hospital of Philadelphia, Pediatrics, Philadelphia, United States of America;*

### Background

X-linked sideroblastic anemia (XLSA) is the most common form of congenital sideroblastic anemia and is caused by germline mutations in the erythroid-specific 5-aminolevulinate synthase 2 (ALAS2) gene, the first and rate-limiting step of heme biosynthesis. Mutations in *ALAS2* impair heme production, resulting in hypochromic anemia and ring sideroblasts. Despite being a monogenic disorder, XLSA lacks curative therapies, and the relationship between heme synthesis, mitochondrial health, metabolism, and ineffective erythropoiesis (IE) is poorly understood.

### Aims

1. Generate and characterize murine and cellular models of severe XLSA.
2. Define the metabolic and mitochondrial consequences of ALAS2 deficiency.
3. Develop ex vivo gene complementation (for most patients) and in vivo gene-based therapeutic strategies for mutations amenable to DNA correction.

### Methods

We generated two XLSA mouse models, one with an inducible ALAS2 knockout (*Alas2*$^{fl/fl}$, Castruccio CC et al, *Blood*, 2025), and the second carrying a humanized hypomorphic (*ALAS2* R452C mutation, unpublished). Disease phenotypes are being assessed by hematologic parameters, erythroid and iron metabolism markers, flow cytometry, mitochondrial function, electron microscopy, and metabolic profiling. We recently generated erythroid (K562) and induced pluripotent stem cell (iPS) cell lines carrying the *ALAS2* R452C mutation, and seven erythroid lentiviral vectors (LV) expressing different levels of the human *ALAS2* gene (using hypomorphic ALAS2 alleles or modifications to the untranslated regions of the cDNA). The focus is to achieve normal levels of hemoglobin synthesis with minimal or no production of metal-free protoporphyrin IX (mfPPIX), a bystander effect of excessive ALAS2 activity. Targeted lipid nanoparticles (LNP) are being used to deliver Cre (LNP$^{Cre}$) or a base editor (LNP$^{BE}$) mRNA to, respectively, induce deletion of the *Alas2*$^{fl/fl}$ gene or correct the R452C mutation.

**Copyright Information:** (Online) ISSN: 2572-9241
©2026 The Author(s). HemaSphere is published by John Wiley & Sons Ltd on behalf of European Hematology Association. This is an open access Abstract Book distributed under the Attribution-NonCommercial-NoDerivs (CC BY-NC-ND), which allows third parties to download the articles and share them with others as long as they credit the author and the Abstract Book, but they cannot change the content in any way or use them commercially.
**Abstract Book Citations:** Authors, Title, HemaSphere, 2026;10:(S1):pages. The abstract book DOIs can be found on the “Author Information” page.
**Disclaimer:** Articles published in the journal HemaSphere exclusively reflect the opinions of the authors. The authors are responsible for all content in their abstracts including accuracy of the facts, statements, citing resources, etc.

**Results**

*ALAS2* R452C K562, iPS cells, and mice have been generated by CRISPR-mediated DNA recombination. *ALAS2* R452C mice are currently breeding. Collectively, K562 and iPS cells and polychromatic erythroblasts derived from *Alas2*-KO bone marrow (BM) (*Alas2*$^{fl/fl}$BM+LNP$^{Cre}$) mice show mutant mitochondrial dysfunction, reduced oxidative phosphorylation, increased glycolysis, and impaired electron transport chain activity. At least 3 *ALAS2*-expressing LVs rescue the lethal anemia in *Alas2*$^{fl/fl}$ BM+LNP$^{Cre}$ mice, improving or normalizing erythropoiesis, splenomegaly, and iron metabolism, and some show minimal mfPPIX production. A base editor/sgRNA combination has been identified that corrects the R452C mutation in vitro (up to 94% in K562 *ALAS*-R452C cells), rectifying heme synthesis and mitochondrial function in erythroid differentiated cells. *ALAS2* R452C iPS cell lines are being utilized to differentiate and study erythroid cells *ex vivo* in the presence of gene complementation or LNP-mediated gene editing. CD34$^{+}$ cells derived from the R452C-iPS line will also be engrafted in xenograft animals. These and *ALAS2* R452C mice will be used to assess LNP-mediated gene editing in vivo.

**Summary/Conclusion**

We established the first severe inducible murine models of XLSA and defined mitochondrial and metabolic dysfunction as central drivers of IE. We identified at least 3 vectors that rescue disease phenotypes when ALAS2 is LV-delivered, supporting the feasibility of gene therapy. Gene editing strategies restored heme synthesis and erythroid maturation in vitro.

**Copyright Information:** (Online) ISSN: 2572-9241
©2026 The Author(s). HemaSphere is published by John Wiley & Sons Ltd on behalf of European Hematology Association. This is an open access Abstract Book distributed under the Attribution-NonCommercial-NoDerivs (CC BY-NC-ND), which allows third parties to download the articles and share them with others as long as they credit the author and the Abstract Book, but they cannot change the content in any way or use them commercially.
**Abstract Book Citations:** Authors, Title, HemaSphere, 2026;10:(S1):pages. The abstract book DOIs can be found on the “Author Information” page.
**Disclaimer:** Articles published in the journal HemaSphere exclusively reflect the opinions of the authors. The authors are responsible for all content in their abstracts including accuracy of the facts, statements, citing resources, etc.

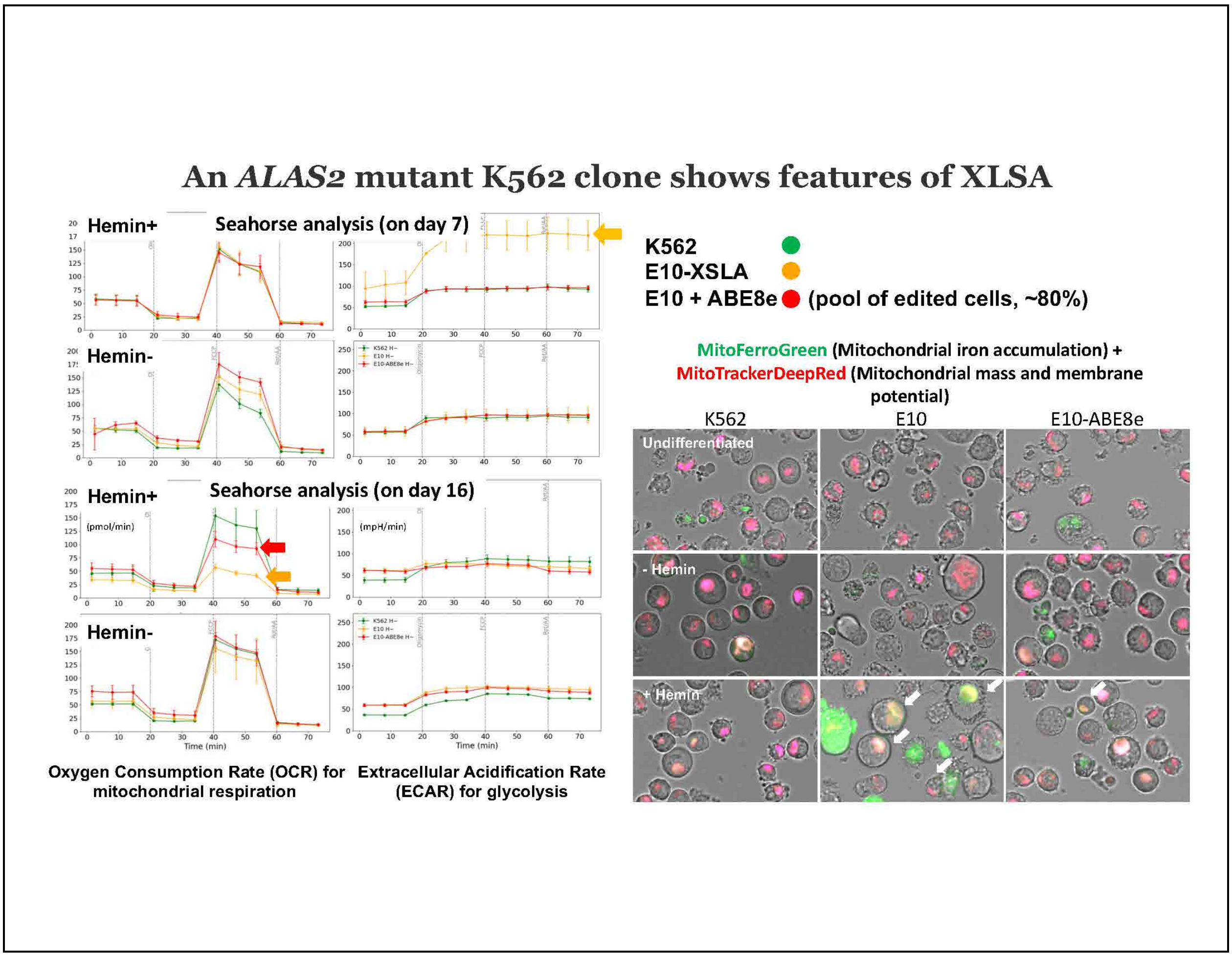

A) Analysis of the K562 ALAS2-R452C E10 clone, showing increased glycolysis (ECAR) at day 7 of erythroid differentiation, while the oxygen consumption rate (OCR) is reduced at day 16. Increased glycolysis suggests an attempt to compensate for the reduced mitochondrial activity. Cells treated with an adenine base editor (ABE8e; pool of cells, not an isolated clone) show improved ECAR and OCR. B) Clone E10 also shows increased mitochondrial iron accumulation, a feature of XLSA. This is improved after base editing (E10-ABE8e cells).

**Copyright Information:** (Online) ISSN: 2572-9241
©2026 The Author(s). HemaSphere is published by John Wiley & Sons Ltd on behalf of European Hematology Association. This is an open access Abstract Book distributed under the Attribution-NonCommercial-NoDerivs (CC BY-NC-ND), which allows third parties to download the articles and share them with others as long as they credit the author and the Abstract Book, but they cannot change the content in any way or use them commercially.
**Abstract Book Citations:** Authors, Title, HemaSphere, 2026;10:(S1):pages. The abstract book DOIs can be found on the "Author Information" page.
**Disclaimer:** Articles published in the journal HemaSphere exclusively reflect the opinions of the authors. The authors are responsible for all content in their abstracts including accuracy of the facts, statements, citing resources, etc.

### (PS2391) THE VIBRANT TRIAL: A RANDOMIZED COMPARISON OF NASAL ANDINTRAVENOUS VITAMIN B12 SUPPLEMENTATION IN MACROCYTIC ANEMIA

**Topic:** 29. Enzymopathies, membranopathies and other red blood cell disorders

Kundan Mishra*[1]

[1] *Command Hospital, Clinical Hematology, Lucknow, India;*

### Background

Vitamin B12 deficiency is a common cause of macrocytic anemia, frequently requiring rapid hematologic correction to alleviate symptoms and prevent complications. Parenteral therapy has long been the standard of care, but nasal vitamin B12 offers a noninvasive alternative with potential advantages for long-term management. High-quality evidence comparing hematologic outcomes between nasal and parenteral routes in patients with true macrocytic anemia remains limited.

### Aims

To determine the efficacy and safety of nasal versus intramuscular or intravenous vitamin B12 in adults with macrocytic anemia

### Methods

We conducted a prospective, randomized, open-label, parallel-group trial to compare the efficacy and safety of nasal versus intramuscular or intravenous vitamin B12 in adults with macrocytic anemia due to cobalamin deficiency. Eligible participants were ≥18 years old with hemoglobin <13 g/dL (men) or <12 g/dL (women), mean corpuscular volume >100 fL, normal transferrin saturation, and no comorbidities causing anemia. Participants were assigned in a 1:1 ratio to receive either nasal vitamin B12 (500 µg daily for 14 days followed by 250 µg weekly from weeks 3–5) or parenteral vitamin B12 (1000 µg intramuscularly daily for 7 days followed by 1000 µg weekly from weeks 2–5). Hemoglobin and reticulocyte counts were measured at baseline and at 2, 4, and 6 weeks. The primary outcome was the change in hemoglobin from baseline to week 6. Secondary outcomes included hemoglobin and reticulocyte responses at 2 and 4 weeks, time to hematologic response (defined as ≥2 g/dL hemoglobin increase or normalization to ≥12 g/dL), and safety.

### Results

A total of 129 patients were enrolled (64 in the parenteral group and 65 in the nasal group). Baseline characteristics were well balanced. At 6 weeks, the mean increase in hemoglobin was 6.03±1.65 g/dL with parenteral therapy versus 3.90±1.60 g/dL with nasal therapy (between-group difference, 2.13 g/dL; 95% CI, 1.56–2.70; $P = 2.3\times10^{-11}$). Hemoglobin was significantly higher in the parenteral group at 2 and 4 weeks and normalized in 81% of parenteral-treated patients compared with 6% of nasal-treated patients at 6 weeks. Hemoglobin levels converged by 8 weeks and were similar at 6 months. Reticulocyte responses were larger and more prolonged in the nasal group, whereas parenteral therapy produced rapid reticulocyte normalization. Both regimens achieved resolution of macrocytosis and normalization of LDH by 6 months. No serious adverse events occurred; nasal therapy caused mild transient nasal irritation, and parenteral therapy caused occasional injection-site discomfort.

**Copyright Information:** (Online) ISSN: 2572-9241
©2026 The Author(s). HemaSphere is published by John Wiley & Sons Ltd on behalf of European Hematology Association. This is an open access Abstract Book distributed under the Attribution-NonCommercial-NoDerivs (CC BY-NC-ND), which allows third parties to download the articles and share them with others as long as they credit the author and the Abstract Book, but they cannot change the content in any way or use them commercially.
**Abstract Book Citations:** Authors, Title, HemaSphere, 2026;10:(S1):pages. The abstract book DOIs can be found on the "Author Information" page.
**Disclaimer:** Articles published in the journal HemaSphere exclusively reflect the opinions of the authors. The authors are responsible for all content in their abstracts including accuracy of the facts, statements, citing resources, etc.

**Summary/Conclusion**

Parenteral vitamin B12 resulted in significantly faster hematologic recovery than nasal vitamin B12 in adults with macrocytic anemia, although both regimens ultimately achieved comparable long-term correction. Parenteral therapy remains the preferred option for symptomatic or severely anemic patients requiring rapid restoration of hemoglobin, whereas nasal vitamin B12 is an effective, safe, and convenient alternative for long-term management.

**Copyright Information:** (Online) ISSN: 2572-9241
©2026 The Author(s). HemaSphere is published by John Wiley & Sons Ltd on behalf of European Hematology Association. This is an open access Abstract Book distributed under the Attribution-NonCommercial-NoDerivs (CC BY-NC-ND), which allows third parties to download the articles and share them with others as long as they credit the author and the Abstract Book, but they cannot change the content in any way or use them commercially.
**Abstract Book Citations:** Authors, Title, HemaSphere, 2026;10:(S1):pages. The abstract book DOIs can be found on the “Author Information” page.
**Disclaimer:** Articles published in the journal HemaSphere exclusively reflect the opinions of the authors. The authors are responsible for all content in their abstracts including accuracy of the facts, statements, citing resources, etc.

### (PS2392) ZANUBRUTINIB FOR THE TREATMENT OF COLD AGGLUTININ DISEASE: PRIMARY ANALYSIS OF THE PHASE II HOVON169 STUDY

**Topic:** 29. Enzymopathies, membranopathies and other red blood cell disorders

Anne-Marie Becking*[1], Sophie Bernelot Moens[1], Peter Te Boekhorst[2], Danny Buitenhuis[3], Dana Chitu[2, 3], Dries Deeren[4], Daan Dierickx[5], Henrik Frederiksen[6], Troels Hammer[7], Henrik Hjorth-Hansen[8], Marie José Kersten[1], Lianne Koens[9], Hilde Kooistra[10], Ulla Haave[11], Geir E. Tjønnfjord[12], Henrik Tor Anderson Tvedt[12], Simonetta Verheijen[3], Sigbjorn Berentsen[13], Josephine Vos[1]

*[1]Amsterdam University Medical Center, University of Amsterdam, Department of Hematology, Cancer Center Amsterdam, Amsterdam, The Netherlands; [2]Erasmus Medical Center Cancer Institute, Department of Hematology, Rotterdam, The Netherlands; [3]Stichting Hemato-Oncologie voor Volwassenen Nederland, Rotterdam, The Netherlands;[4]AZ Delta, Department of Hematology, Roeselare, Belgium; [5]UZ Leuven, Department of Hematology, Leuven, Belgium;[6]Odense University Hospital, Department of Hematology, Odense, Denmark; [7]Copenhagen University Hospital, Rigshospitalet, Department of Hematology, Copenhagen, Denmark; [8]St. Olavs Hospital, Department of Hematology, Trondheim, Norway;[9]HOVON Pathology Facility and Biobank (HOP), Cancer Center Amsterdam, Amsterdam University Medical Center, University of Amsterdam, Department of Pathology, Amsterdam, The Netherlands; [10]University of Groningen, University Medical Center Groningen, Department of Hematology, Groningen, The Netherlands; [11]Akershus University Hospital, Department of Pathology, Lørenskog, Norway; [12]Oslo University Hospital, Rikshospitalet, Department of Hematology, Oslo, Norway; [13]Haugesund Hospital, Helse Fonna Hospital Trust, Department of Research and Innovation, Haugesund, Norway;*

### Background

Cold agglutinin disease (CAD) is a rare form of autoimmune hemolytic anemia associated with a MYD88 wild-type B cell clone in the bone marrow (BM), leading to complement-mediated hemolysis and cold-induced peripheral symptoms (CIPS). There is an unmet need for well-tolerated and effective treatments targeting both hemolysis and CIPS. Zanubrutinib, a second-generation covalent Bruton's tyrosine kinase (BTK) inhibitor, shows high efficacy and tolerability in similar B cell non-Hodgkin lymphomas (B-NHL) such as Waldenström macroglobulinemia (WM), including MYD88 wild-type disease. Given the pathophysiologic overlap between CAD and WM, and promising retrospective data on ibrutinib in CAD, we hypothesized that zanubrutinib would be an effective and safe therapeutic option.

### Aims

To investigate the efficacy and safety of zanubrutinib in CAD.

### Methods

HOVON169 is a phase IIb, non-randomized, multinational trial (NCT06067048), including CAD patients relapsed/refractory after ≥1 prior therapy or non-eligible for other CAD therapy, with a treatment indication (Hb ≤10 g/dL and/or grade 2 CIPS). Patients were treated with zanubrutinib 320mg daily, in 4-weeks cycles. Primary endpoint evaluation after cycle 6 used CAD overall response rate (ORR) as composite endpoint (**Figure**). Secondary endpoints included IgM response, CIPS response, transfusion dependence, and CTCAEv5.0-graded toxicities.

**Copyright Information:** (Online) ISSN: 2572-9241
©2026 The Author(s). HemaSphere is published by John Wiley & Sons Ltd on behalf of European Hematology Association. This is an open access Abstract Book distributed under the Attribution-NonCommercial-NoDerivs (CC BY-NC-ND), which allows third parties to download the articles and share them with others as long as they credit the author and the Abstract Book, but they cannot change the content in any way or use them commercially.
**Abstract Book Citations:** Authors, Title, HemaSphere, 2026;10:(S1):pages. The abstract book DOIs can be found on the "Author Information" page.
**Disclaimer:** Articles published in the journal HemaSphere exclusively reflect the opinions of the authors. The authors are responsible for all content in their abstracts including accuracy of the facts, statements, citing resources, etc.

### Results

In 9 hospitals, 25 patients were included, of whom 12 (48%) were female. Median age was 73 years (range 56-82). Most patients (22; 88%) were previously treated, with a median number of 2 lines (range 1-8). Prior treatment regimens included rituximab monotherapy (19; 76%), immunochemotherapy (7; 28%), corticosteroids (6; 24%), and complement inhibitors (4; 16%). Twenty-three (92%) had an M-protein, and 17 (68%) a detectable clonal CD20+ BM disease. By BM pathology revision of 24 evaluable cases using WHO-HAEM5, 12 (50%) were classified as CAD-associated lymphoproliferative disorder, 4 (17%) as unclassifiable clonal disease, 1 (4%) as plasma cell dyscrasia, while a clonal disorder was not detectable in 7 (29%). Treatment indications were Hb ≤10.5 g/dL (n=7), grade 2 CIPS (n=8), or both (n=10). At start, 9 patients were transfusion dependent. 20 out of 25 patients completed the first 6 treatment cycles. CAD ORR was 6/25 (24%), all partial responses (**Figure**). Five patients discontinued treatment prematurely due to lack of response/patients' decision (n=3), progressive IgM (n=1), or death (n=1). Transfusion dependence resolved in 6/9 patients (67%), while 2 patients developed transfusion-dependence during zanubrutinib treatment. Of 18 patients with grade 2 CIPS at baseline, 8 (44%) experienced improvement, of whom 4 complete resolution. IgM ORR was 4 (16%), while 16 (64%) had stable disease, one progression (4%), and 4 (16%) were unevaluable. A total of 17 (possibly) treatment-related AEs grade ≥2 occurred in 10 patients, most commonly grade 2 gastrointestinal symptoms. Five were grade 3 AEs (pneumonia, constipation, hypertension, anemia, and atrial fibrillation). The latter occurred in a patient with fatal non-neutropenic sepsis and heart failure in the context of pre-existing heart disease.

### Summary/Conclusion

Our study did not meet its primary endpoint, with a CAD ORR of only 24%. While zanubrutinib is highly efficacious in other B-NHL, this unexpected limited efficacy in CAD reinforces its classification as a distinct entity and underscores the need for specific trials in this rare condition.

**Copyright Information:** (Online) ISSN: 2572-9241
©2026 The Author(s). HemaSphere is published by John Wiley & Sons Ltd on behalf of European Hematology Association. This is an open access Abstract Book distributed under the Attribution-NonCommercial-NoDerivs (CC BY-NC-ND), which allows third parties to download the articles and share them with others as long as they credit the author and the Abstract Book, but they cannot change the content in any way or use them commercially.
**Abstract Book Citations:** Authors, Title, HemaSphere, 2026;10:(S1):pages. The abstract book DOIs can be found on the "Author Information" page.
**Disclaimer:** Articles published in the journal HemaSphere exclusively reflect the opinions of the authors. The authors are responsible for all content in their abstracts including accuracy of the facts, statements, citing resources, etc.

**Figure. CAD response criteria and response rates after 6 cycles of zanubrutinib treatment**

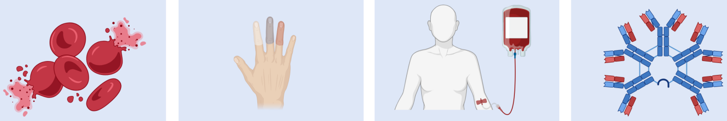

| | Hemolytic anemia | | CIPS* | | Transfusion dependence** | | IgM response*** | Patients meeting response criteria N=25 |
|---|---|---|---|---|---|---|---|---|
| **Complete CAD response** | Hb > LLN Haptoglobin > LLN | & | No CIPS | & | Transfusion independence | & | No IgM progression | **None** |
| **Partial CAD response** | ΔHb ≥ 1.5 g/dL or > LLN | & | ≥ 1 grade improvement | & | Transfusion independence | & | No IgM progression | **6 (24%)** |
| **No CAD response** | No PR or CR | | | | | | | **19 (76%)** *Of whom 5 premature discontinuation* |

Created in https://BioRender.com

*CIPS grading; Grade 0: no such symptoms; Grade 1: acrocyanosis/Raynaud-like peripheral symptoms, not interfering with daily living; Grade 2: acrocyanosis/Raynaud-like peripheral symptoms, interfering with daily living; Grade 3: gangrene or ulcerations
**Transfusion dependence at baseline was defined as ≥1 RBC transfusion in prior 6 months or ≥2 RBC transfusions in prior 12 months. Transfusion independence at primary endpoint was defined as no RBC transfusion from cycle 3 to 6.
***IgM response was classified according to the 11th International Workshop on Waldenström's Macroglobulinemia criteria, progression being ≥25% increase in serum IgM level with minimum increase of 5 g/L from lowest nadir

*Abbreviations: CAD = cold agglutinin disease; CIPS = cold-induced peripheral symptoms; CR = complete response; Hb = hemoglobin; LLN = lower limit of normal; PD = progressive disease; PR = partial response; RBC = red blood cell*

**Copyright Information:** (Online) ISSN: 2572-9241
©2026 The Author(s). HemaSphere is published by John Wiley & Sons Ltd on behalf of European Hematology Association. This is an open access Abstract Book distributed under the Attribution-NonCommercial-NoDerivs (CC BY-NC-ND), which allows third parties to download the articles and share them with others as long as they credit the author and the Abstract Book, but they cannot change the content in any way or use them commercially.
**Abstract Book Citations:** Authors, Title, HemaSphere, 2026;10:(S1):pages. The abstract book DOIs can be found on the "Author Information" page.
**Disclaimer:** Articles published in the journal HemaSphere exclusively reflect the opinions of the authors. The authors are responsible for all content in their abstracts including accuracy of the facts, statements, citing resources, etc.

### (PS2393) DYNAMIC ANALYSIS OF GLUCOSE UTILIZATION IN RED BLOOD CELLS FROM PATIENTS WITH GLUCOSE PHOSPHATE ISOMERASE DEFICIENCY

**Topic:** 29. Enzymopathies, membranopathies and other red blood cell disorders

Titine Ruiter*[1], Andreas Glenthøj[2], Jesper Brix Petersen[2], Brigitte van Oirschot[1], Wouter van Solinge[1], Poala Corti[3], Elisa Fermo[4], Paola Bianchi[4], Judith Jans[1], Richard van Wijk[1]

*[1]University Medical Center Utrecht, Utrecht, The Netherlands;[2]Copenhagen University Hospital – Rigshospitalet, Copenhagen, Denmark; [3]Fondazione IRCCS San Gerardo dei Tintori, Monza, Italy; [4]Fondazione IRCCS Ca' Granda Ospedale Maggiore Policlinico, Milan, Italy;*

### Background

Glucose phosphate isomerase (GPI) deficiency is a rare hereditary glycolytic enzymopathy that impairs the conversion of glucose-6-phosphate to fructose-6-phosphate. Because mature red blood cells (RBCs) depend on glycolysis for energy production, reduced GPI activity disrupts cellular energy homeostasis leading to chronic hemolytic anemia. Disease severity ranges from mild compensated hemolysis to severe transfusion-dependent anemia, and some patients present with non-hematological symptoms such as neuromuscular impairment, intellectual disability, and seizures.

### Aims

To characterize the metabolic consequences of impaired GPI activity, thereby offering insights in disease pathophysiology that may guide future diagnostic or treatment strategies.

### Methods

We applied $^{13}C_6$-glucose isotope tracing on RBCs from four unrelated patients with GPI deficiency to characterize alterations in glycolytic flux as well as flux through the pentose phosphate pathway (PPP). One patient was transfusion dependent (GPI-2). We quantified the incorporation of 13-labeled carbon atoms from glucose into downstream metabolites over time using an Ultimate 3000 UHPLC system coupled to a Q Exactive™ HF hybrid quadrupole Orbitrap mass spectrometer (Thermo Fisher Scientific).

### Results

We observed pronounced accumulation of carbon-labeled glucose-6-phosphate, the substrate of GPI, and reduced production of carbon-labeled fructose-6-phosphate, the product of GPI (Figure 1A). Notably, elevated glucose-6-phosphate levels, and in particular an increased glucose-6-phosphate to fructose-6-phosphate ratio, are characteristic of GPI deficiency and may serve as a useful biomarker for diagnosis. We further observed decreased production of carbon-labeled glycolytic intermediates downstream of fructose-6-phosphate, including fructose-1,6-diphosphate (Figure 1B). Surprisingly, metabolites in later steps of glycolysis were generally unaffected, with the exception of decreased pyruvate and increased lactate production (Figure 1B). These findings suggest GPI deficiency restricts flux through the early steps in glycolysis, without impairing lactate production. The decrease in fructose-1,6-diphosphate, which is an allosteric activator of pyruvate kinase (PK), suggests feed-forward activation of PK may be weaker in GPI deficiency. Indeed, we observed accumulation of carbon-labeled phosphoenolpyruvate and a decreased production of carbon-labeled pyruvate (Figure 1B), which conversion is catalyzed by PK. PK activators are explored as treatment strategy in a broad range of hereditary anemias, and our findings suggest patients with GPI deficiency might benefit from PK activation therapy. We further demonstrate enhanced diversion of glucose-6-phosphate into the PPP (Figure 1C), which may partially replenish glycolytic intermediates. However, the observed accumulation of carbon-labeled erythrose-4-phosphate suggests incomplete recycling of carbon back into glycolysis.

**Copyright Information:** (Online) ISSN: 2572-9241
©2026 The Author(s). HemaSphere is published by John Wiley & Sons Ltd on behalf of European Hematology Association. This is an open access Abstract Book distributed under the Attribution-NonCommercial-NoDerivs (CC BY-NC-ND), which allows third parties to download the articles and share them with others as long as they credit the author and the Abstract Book, but they cannot change the content in any way or use them commercially.
**Abstract Book Citations:** Authors, Title, HemaSphere, 2026;10:(S1):pages. The abstract book DOIs can be found on the "Author Information" page.
**Disclaimer:** Articles published in the journal HemaSphere exclusively reflect the opinions of the authors. The authors are responsible for all content in their abstracts including accuracy of the facts, statements, citing resources, etc.

### Summary/Conclusion

GPI deficiency profoundly reshapes RBC metabolism, and our findings provide the first comprehensive perspective on the metabolic adaptations associated with impaired GPI activity in human RBCs. The metabolomic and isotope-tracing analyses confirmed the characteristic bottleneck at the conversion of glucose-6-phosphate to fructose-6-phosphate in GPI deficiency, and revealed characteristics that deepen our understanding of disease pathophysiology and may enhance future diagnosis and therapeutic strategies.

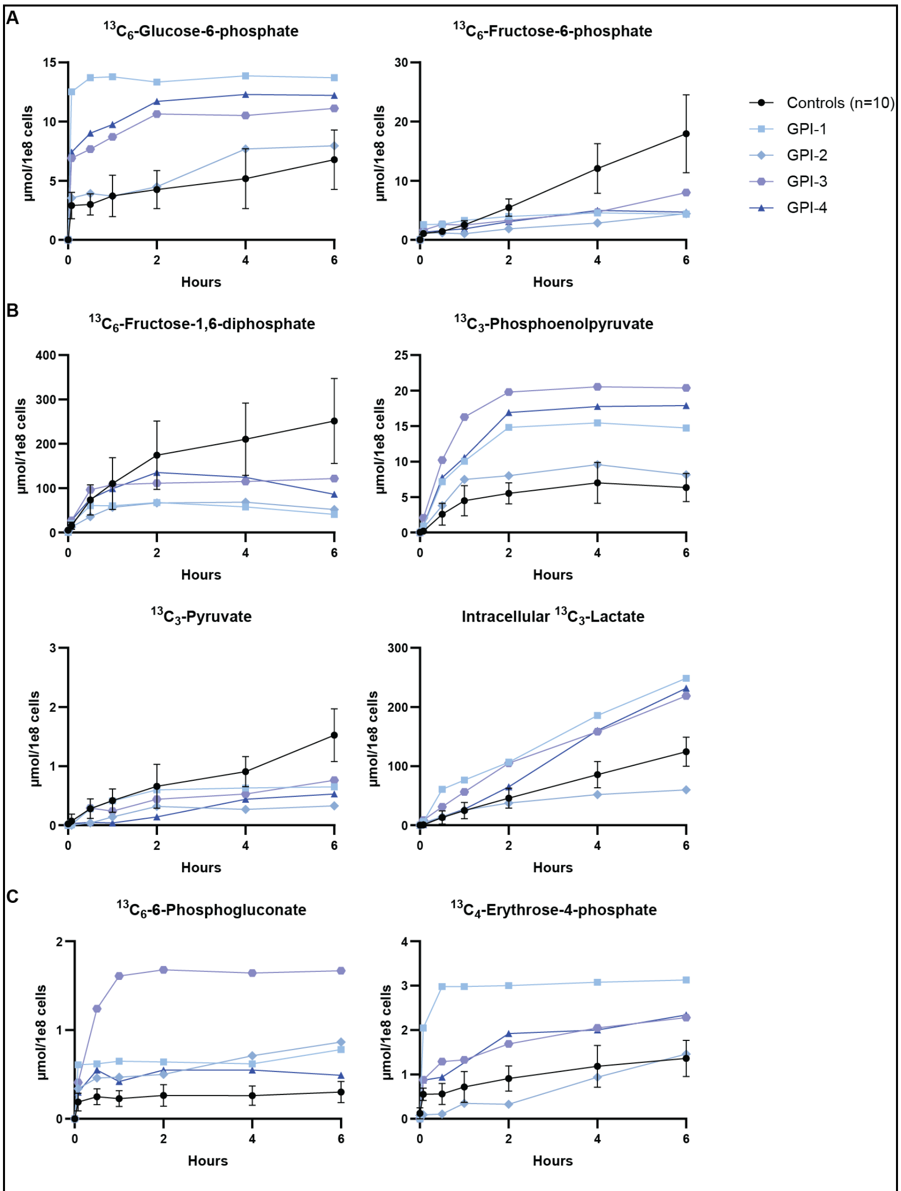

**Figure 1.** $^{13}C_6$-glucose isotope tracing in RBCs from four patients with GPI deficiency.

**Copyright Information:** (Online) ISSN: 2572-9241
©2026 The Author(s). HemaSphere is published by John Wiley & Sons Ltd on behalf of European Hematology Association. This is an open access Abstract Book distributed under the Attribution-NonCommercial-NoDerivs (CC BY-NC-ND), which allows third parties to download the articles and share them with others as long as they credit the author and the Abstract Book, but they cannot change the content in any way or use them commercially.
**Abstract Book Citations:** Authors, Title, HemaSphere, 2026;10:(S1):pages. The abstract book DOIs can be found on the "Author Information" page.
**Disclaimer:** Articles published in the journal HemaSphere exclusively reflect the opinions of the authors. The authors are responsible for all content in their abstracts including accuracy of the facts, statements, citing resources, etc.

### (PS2394) ADDITIONAL SAFETY AND EFFICACY RESULTS FROM HELIOS: A PHASE 2, OPEN-LABEL, LONG-TERM EXTENSION STUDY OF BITOPERTIN IN ERYTHROPOIETIC PROTOPORPHYRIA

**Topic:** 29. Enzymopathies, membranopathies and other red blood cell disorders

Amy Yeung*[1, 2], Gayle Ross[3], Sioban Keel[4], Herbert Bonkovsky[5, 6], Peter Stewart[7], Karl Anderson[8], Manisha Balwani[9], Cynthia Levy[10], Manish Thapar[11], Bruce Wang[12], Brendan McGuire[13], Melanie Chin[14], Will Savage[14]

*[1]Harvard Medical School, Boston, United States of America;[2]Massachusetts General Hospital, Boston, United States of America;[3]Royal Melbourne Hospital, Melbourne, Australia;[4]University of Washington, Seattle, United States of America;[5]Wake Forest University School of Medicine, Winston Salem, United States of America; [6]Atrium Health Wake Forest Baptist, Winston Salem, United States of America; [7]Royal Prince Alfred Hospital, Sydney, Australia;[8]University of Texas Medical Branch, Galveston, United States of America; [9]Icahn School of Medicine at Mount Sinai, New York, United States of America;[10]University of Miami Miller School of Medicine, Miami, United States of America; [11]Jefferson Center for Genetic and Metabolic Liver Disease, Philadelphia, United States of America; [12]University of California San Francisco Porphyria Center, San Francisco, United States of America; [13]University of Alabama at Birmingham, Birmingham, United States of America;[14]Disc Medicine, Watertown, United States of America;*

### Background

Erythropoietic protoporphyria (EPP) and X-linked protoporphyria (XLP) result in the toxic accumulation of photoreactive protoporphyrin IX (PPIX) in the skin and other organs. PPIX accumulation causes debilitating phototoxic skin reactions upon exposure to sunlight and, in some cases, life-threatening protoporphyric hepatopathy.
Bitopertin is a selective and orally bioavailable inhibitor of glycine transporter 1 (GlyT1), which imports extracellular glycine into erythroid precursor cells for heme biosynthesis. In prior EPP/XLP clinical studies, treatment with bitopertin resulted in significant reductions in PPIX levels that were associated with improvements in light tolerance (Yeung, 2026; Ross, 2026).

### Aims

Evaluate the long-term safety, tolerability, and efficacy of bitopertin in participants with EPP or XLP.

### Methods

HELIOS (NCT05883748) is a multi-center, open-label, long-term extension study of bitopertin for participants who completed prior bitopertin studies. The study is being conducted in the US and Australia, with additional sites opening in the UK and EU, and includes participants ≥12 years of age with a confirmed diagnosis of EPP or XLP. Adult and adolescent participants receive once-daily oral administration of bitopertin 60 mg. The primary endpoints are safety and tolerability. Secondary endpoints include percent change from baseline in whole-blood metal-free PPIX and changes in measures of light tolerance.

**Copyright Information:** (Online) ISSN: 2572-9241
©2026 The Author(s). HemaSphere is published by John Wiley & Sons Ltd on behalf of European Hematology Association. This is an open access Abstract Book distributed under the Attribution-NonCommercial-NoDerivs (CC BY-NC-ND), which allows third parties to download the articles and share them with others as long as they credit the author and the Abstract Book, but they cannot change the content in any way or use them commercially.
**Abstract Book Citations:** Authors, Title, HemaSphere, 2026;10:(S1):pages. The abstract book DOIs can be found on the "Author Information" page.
**Disclaimer:** Articles published in the journal HemaSphere exclusively reflect the opinions of the authors. The authors are responsible for all content in their abstracts including accuracy of the facts, statements, citing resources, etc.

**Results**

As of the data cutoff (01 Nov 2025), a total of 86 participants, including 3 adolescents, had enrolled in HELIOS. Median duration of drug exposure, including previous treatment with bitopertin, was 25.3 months (range: 13.8 months to 32.7 months). To date, 61 of 86 (71%) participants have reported treatment-emergent adverse events (AEs). One adult participant involved in a motor vehicle accident reported multiple serious AEs (SAEs) that were considered not related to study drug. The only other Grade 3 event was a nonserious AE of hypertransaminasaemia in an adult participant, which was also considered not related to study drug. All other AEs were mild or moderate in severity. The most common treatment-emergent AEs reported in >5 participants were COVID-19 (12%), nasopharyngitis (9%), and headache (7%).
Participants initially randomized to placebo had significant reductions in whole blood metal-free PPIX concentration relative to HELIOS baseline at Week 24 (-32.8%; p-value <0.001). Participants receiving bitopertin treatment through AURORA/BEACON and HELIOS had reductions in PPIX that were sustained through one year of bitopertin treatment.
Continued improvements in light tolerance were also observed with bitopertin through 24 weeks of treatment in the HELIOS study, and participants had statistically significant increases in total hours spent in light (mean increase of 4.9 hours; p-value <0.001) and time to prodrome (mean increase of 101.8 minutes; p-value <0.001) over two-week intervals relative to baseline.

**Summary/Conclusion**

By reducing PPIX levels, bitopertin targets the underlying pathophysiology of EPP and XLP. Results from HELIOS include participants who have received bitopertin for over 2.5 years and support longer-term efficacy and safety with bitopertin in adult and adolescent patients with EPP or XLP.

**Copyright Information:** (Online) ISSN: 2572-9241
©2026 The Author(s). HemaSphere is published by John Wiley & Sons Ltd on behalf of European Hematology Association. This is an open access Abstract Book distributed under the Attribution-NonCommercial-NoDerivs (CC BY-NC-ND), which allows third parties to download the articles and share them with others as long as they credit the author and the Abstract Book, but they cannot change the content in any way or use them commercially.
**Abstract Book Citations:** Authors, Title, HemaSphere, 2026;10:(S1):pages. The abstract book DOIs can be found on the "Author Information" page.
**Disclaimer:** Articles published in the journal HemaSphere exclusively reflect the opinions of the authors. The authors are responsible for all content in their abstracts including accuracy of the facts, statements, citing resources, etc.

### (PS2395) RETROSPECTIVE EVALUATION OF MOLECULAR GENETIC TESTING FOR PORPHYRIA PATIENTS IN A NATIONAL GENETICS LABORATORY

**Topic:** 29. Enzymopathies, membranopathies and other red blood cell disorders

Dushy Navarajasegaran*[1, 2], David Rees[2], Penny Stein[2], John Brewin[2], Nicholas Parkin[1, 2], Rachel Mayhew[1, 2], Eirini Oikonomidou[1, 2], Melissa Kow[1, 2], Sarah Curran[1, 2], Katherine Bates[1], Barnaby Clark[1, 2], Frances Smith[1, 2]

*[1]Synnovis Molecular Pathology Laboratory, King's College Hospital, London, United Kingdom; [2]Department of Haematology, King's College Hospital, London, United Kingdom;*

### Background

Porphyria is a group of rare inherited disorders caused by defects in enzymes involved in the haem biosynthesis pathway. Each enzyme is encoded by a specific gene. Mutations at distinct steps in the pathway result in the various clinical subtypes. Many subtypes show reduced penetrance and variable clinical expression, which can make diagnosis challenging and highlights the importance of molecular confirmation for accurate subtype classification and family screening. Our laboratory is the national provider for porphyria genetic testing within the Genomic Medicine Service, providing an opportunity to review diagnostic yield and mutation spectrum in a large referral cohort.

### Aims

To evaluate the diagnostic yield and molecular spectrum of porphyria in a large cohort tested through a national genetics laboratory.

### Methods

We retrospectively analysed 399 referrals for suspected porphyria or predictive testing received between 2016-2025. Testing was performed using a targeted next generation sequencing panel that covered nine genes associated with porphyria. Familial testing was performed by Sanger sequencing and MLPA where appropriate. Variants were classified according to the ACGS variant interpretation guidelines.

### Results

A molecular diagnosis was established in 197/399 cases (49%). Pathogenic or likely pathogenic variants were identified across multiple genes, demonstrating the genetic heterogeneity of porphyria. The most frequently affected genes were *HMBS* (n=90) and *PPOX* (n=36), with fewer cases identified in *UROD, CPOX* and *ALAS2*. No pathogenic or likely pathogenic variants were detected in *ALAD*, *UROS* or *GATA1*.
Most referrals were prompted by symptomatic presentation. When symptoms were accompanied by supportive biochemical results, diagnostic yield was very high across genes, 100% for *FECH* and *UROD*, 80% for *HMBS* and 73% for *CPOX*. When biochemical results were absent or normal, pathogenic variants were still frequently detected in symptomatic patients, including *ALAS2* (80%), *CPOX* (90%), and *HMBS* (71%), showing that molecular testing can uncover porphyria that might otherwise be missed. By contrast, asymptomatic individuals who were tested solely because of family history had much lower yields, 20% for *CPOX* and 25% for *PPOX*, reflecting low penetrance and highlighting that clinically affected relatives should be prioritised for testing.

**Copyright Information:** (Online) ISSN: 2572-9241
©2026 The Author(s). HemaSphere is published by John Wiley & Sons Ltd on behalf of European Hematology Association. This is an open access Abstract Book distributed under the Attribution-NonCommercial-NoDerivs (CC BY-NC-ND), which allows third parties to download the articles and share them with others as long as they credit the author and the Abstract Book, but they cannot change the content in any way or use them commercially.
**Abstract Book Citations:** Authors, Title, HemaSphere, 2026;10:(S1):pages. The abstract book DOIs can be found on the “Author Information” page.
**Disclaimer:** Articles published in the journal HemaSphere exclusively reflect the opinions of the authors. The authors are responsible for all content in their abstracts including accuracy of the facts, statements, citing resources, etc.

**Summary/Conclusion**

This large national cohort demonstrates a high diagnostic yield for porphyria and highlights the broad mutation spectrum seen in porphyria. Comprehensive molecular testing is important to confirm diagnosis, support clininal classification and enable appropriate patient management and family screening.

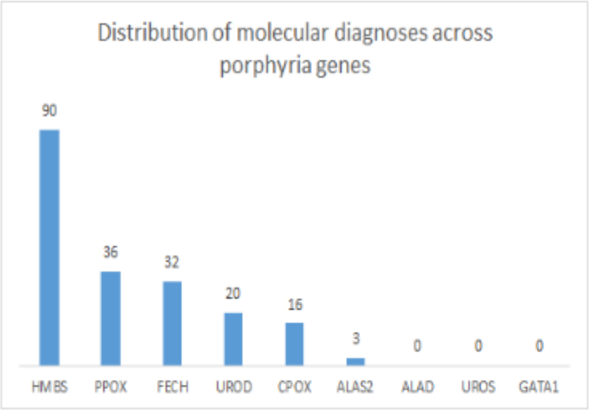

Figure showing the number of cases with pathogenic or likely pathogenic variants identified in each gene associated with porphyria within the study cohort.

**Copyright Information:** (Online) ISSN: 2572-9241
©2026 The Author(s). HemaSphere is published by John Wiley & Sons Ltd on behalf of European Hematology Association. This is an open access Abstract Book distributed under the Attribution-NonCommercial-NoDerivs (CC BY-NC-ND), which allows third parties to download the articles and share them with others as long as they credit the author and the Abstract Book, but they cannot change the content in any way or use them commercially.
**Abstract Book Citations:** Authors, Title, HemaSphere, 2026;10:(S1):pages. The abstract book DOIs can be found on the "Author Information" page.
**Disclaimer:** Articles published in the journal HemaSphere exclusively reflect the opinions of the authors. The authors are responsible for all content in their abstracts including accuracy of the facts, statements, citing resources, etc.

### (PS2396) FIRST-LINE THERAPY IN WARM AUTOIMMUNE HAEMOLYTIC ANAEMIA: REAL-WORLD OUTCOMES WITH ORAL CORTICOSTEROIDS ALONE OR COMBINED WITH RITUXIMAB

**Topic:** 29. Enzymopathies, membranopathies and other red blood cell disorders

Christian Kjellander[1, 2, 3], Tina Jacob[4], Erwei Zeng[4], Norbert Lubenow[5, 6], Lars Malmqvist[5, 6], Ann Leon[7], Alexander Litvintchouk[7], Christina Jones[4], Wim Noel[8], Cathye Shu[9], Magnus Bjorkholm[10, 11], Agneta Wikman[12, 13], Gunnar Larfors*[14]

*[1]Karolinska Institutet, Department of Laboratory Medicine, Stockholm, Sweden;[2]Södersjukhuset, Department of Internal Medicine, Stockholm, Sweden; [3]Södersjukhuset and Karolinska Institutet, Department of Clinical Science and Education, Stockholm, Sweden; [4]Ciencia Research AB, Stockholm, Sweden;[5]Uppsala University, Department of Immunology, Genetics and Pathology, Uppsala, Sweden; [6]Uppsala University Hospital, Department of Clinical Immunology and Transfusion Medicine, Uppsala, Sweden; [7]Johnson & Johnson Innovative Medicine United States, Horsham, PA, United States of America;[8]Janssen Pharmaceutica NV, Medical Affairs Department, Beerse, Belgium; [9]Janssen Research & Development, LLC, Spring House, PA, United States of America; [10]Karolinska Institutet, Department of Medicine Solna, Stockholm, Sweden; [11]Karolinska University Hospital, Department of Haematology, Stockholm, Sweden; [12]Karolinska Institutet, Department of Medicine Huddinge, Stockholm, Sweden; [13]Karolinska University Hospital, Department of Clinical Immunology and Transfusion Medicine, Stockholm, Sweden; [14]Uppsala University, Unit of Haematology, Department of Medical Sciences, Uppsala, Sweden;*

### Background

The latest international consensus guidelines for warm autoimmune haemolytic anaemia (wAIHA) recommend oral corticosteroid monotherapy (OCS) as first-line (1L) therapy, reserving the addition of rituximab (RIX) for severe cases.

### Aims

To describe 1L treatment patterns and outcomes among patients with wAIHA initiating OCS or OCS+RIX.

### Methods

Using Swedish national registers and medical records, adults diagnosed with wAIHA between July 2006 and June 2023 at Karolinska University Hospital, St. Göran’s Hospital, and Uppsala University Hospital were identified. Patients were required to have ≥1 AIHA record (ICD-10-SE: D59.1/1B/1X) and lab data confirming wAIHA (IgG±C3d positive Coombs test, evidence of ongoing anaemia and haemolysis). Primary and secondary wAIHA were classified based on underlying conditions.

Outcomes of 1L OCS or OCS+RIX, including response and relapse, were assessed during routine clinical care follow-up; from 1L initiation until initiation of next treatment line, death, hospital transfer, emigration or June 2023. Timing of lab tests used to assess outcomes was clinically driven. Partial response: Haemoglobin (Hgb) ≥100 g/L and ≥20 g/L increase from baseline without rescue therapy in preceding 1-2 weeks (depending on rescue therapy type); Complete response: Hgb ≥120 g/L, normalized haemolytic markers (lactate dehydrogenase, haptoglobin and bilirubin) and no recent rescue therapy; Relapse: Hgb <100 g/L or ≥20 g/L drop from peak Hgb after response; Relapse-free survival (RFS): Time from first response to relapse or death.

**Copyright Information:** (Online) ISSN: 2572-9241
©2026 The Author(s). HemaSphere is published by John Wiley & Sons Ltd on behalf of European Hematology Association. This is an open access Abstract Book distributed under the Attribution-NonCommercial-NoDerivs (CC BY-NC-ND), which allows third parties to download the articles and share them with others as long as they credit the author and the Abstract Book, but they cannot change the content in any way or use them commercially.
**Abstract Book Citations:** Authors, Title, HemaSphere, 2026;10:(S1):pages. The abstract book DOIs can be found on the “Author Information” page.
**Disclaimer:** Articles published in the journal HemaSphere exclusively reflect the opinions of the authors. The authors are responsible for all content in their abstracts including accuracy of the facts, statements, citing resources, etc.

**Results**

Of 137 patients with wAIHA, 130 initiated 1L treatment (OCS n=92; OCS+RIX n=38). Median observation time was 3.2 years (IQR: 0.8-5.8).
OCS+RIX was used in 20% of patients with primary wAIHA (n=14) and 39% of patients with secondary wAIHA (n=24). Patients receiving OCS+RIX more frequently had underlying haematological malignancies (87.5% vs 62.2%; *p*=0.03). They were also older at diagnosis and had slightly lower baseline Hgb levels, but neither trend was statistically significant. Median 1L duration was 5.9 vs 6.2 months and 6.9 vs 5.9 months for OCS and OCS+RIX in primary and secondary wAIHA, respectively.
Over the follow-up period, non-response rate was 13% for OCS and 23% for OCS+RIX in primary wAIHA, and 24% and 25% in secondary wAIHA, respectively. Overall response was non-superior for OCS+RIX, but complete response was more frequent with OCS+RIX. Relapse after 1L was common in both primary and secondary wAIHA. In primary wAIHA, OCS+RIX (n=10) was associated with lower relapse rates (30% for OCS+RIX vs 66% for OCS; *p*=0.04) and prolonged RFS (*p*=0.02; hazard ratio of 0.29, 95% CI: 0.10-0.84) compared to OCS alone (n=47). In secondary wAIHA, relapse rates and RFS did not differ significantly between treatment groups (OCS n=18; OCS+RIX n=28). The use of additional rescue therapies was frequent; ranging from 28 to 63% of patients depending on disease type and 1L regimen received.

**Summary/Conclusion**

Overall, this study provides a comprehensive overview of 1L wAIHA treatment and outcomes in Swedish patients. OCS was the mainstay of 1L therapy, but almost one third of patients received OCS+RIX. Regardless of treatment received, many patients failed 1L therapy (non-response or relapse) and required rescue therapy, indicating inadequate disease control and persistent anaemia with current treatments. Results of this study suggest an ongoing need for additional treatment options to achieve sustained and durable control of primary and secondary wAIHA.

**Copyright Information:** (Online) ISSN: 2572-9241
©2026 The Author(s). HemaSphere is published by John Wiley & Sons Ltd on behalf of European Hematology Association. This is an open access Abstract Book distributed under the Attribution-NonCommercial-NoDerivs (CC BY-NC-ND), which allows third parties to download the articles and share them with others as long as they credit the author and the Abstract Book, but they cannot change the content in any way or use them commercially.
**Abstract Book Citations:** Authors, Title, HemaSphere, 2026;10:(S1):pages. The abstract book DOIs can be found on the "Author Information" page.
**Disclaimer:** Articles published in the journal HemaSphere exclusively reflect the opinions of the authors. The authors are responsible for all content in their abstracts including accuracy of the facts, statements, citing resources, etc.

**Clinical characteristics and outcomes of patients with primary and secondary wAIHA, stratified by first-line treatment (OCS vs OCS+RIX)**

| | Primary wAIHA | | | Secondary wAIHA | | |
|---|---|---|---|---|---|---|
| | 1L OCS (N=55) | 1L OCS+RIX (N=14) | p-value[a] | 1L OCS (N=37) | 1L OCS+RIX (N=24) | p-value[a] |
| Sex, % | | | 0.86 | | | 0.39 |
| Male | 45.5 | 42.9 | | 51.4 | 62.5 | |
| Female | 54.5 | 57.1 | | 48.6 | 37.5 | |
| Median age at diagnosis in years (IQR) | 64 (49-78) | 74 (66-80) | 0.20 | 70 (52-78) | 75 (69.5-80) | 0.10 |
| Underlying condition[b], % | | | | | | |
| Haematological malignancies | - | - | - | 62.2 | 87.5 | **0.03** |
| Autoimmune diseases/ Connective tissue diseases | - | - | - | 35.1 | 16.7 | 0.12 |
| Primary immunodeficiency | - | - | - | 2.7 | 0 | 0.42 |
| Chronic viral infections | - | - | - | 8.1 | 0 | 0.15 |
| Transplantations | - | - | - | 0 | 0 | - |
| Haemoglobin (Hgb)[c] in g/L, median (IQR) | 80 (69-91) | 72 (68-85) | 0.08 | 79 (71-89) | 75 (65.5-81.5) | 0.26 |
| Median observation time in years[d] (IQR) | 3.6 (1.2-5.9) | 5.7 (2.6-7.1) | 0.51 | 2.6 (0.8-4.9) | 2.9 (0.7-4.6) | 0.92 |
| Median treatment duration in months (95% CI) | 5.9 (4.9-7.8) | 6.3 (3.1-9.7) | 0.94 | 6.9 (4.2-9.1) | 5.9 (4.0-8.0) | 0.07 |
| Number of RIX administrations during first-line treatment[e], % | | | | | | |
| <4 administrations | - | 35.7 | - | - | 33.3 | - |
| 4 administrations | - | 57.1 | - | - | 29.2 | - |
| >4 administrations | - | 7.1 | - | - | 37.5 | - |
| Overall response[f], % (95% CI) | 87.1 (76.2-94.0) | 76.9 (50.3-93.0) | 0.17 | 75.6 (60.3-87.2) | 75.0 (55.5-88.8) | 0.77 |
| Complete response | 35.2 (21.7-51.5) | 53.8 (25.1-80.3) | | 29.7 (15.5-49.4) | 37.5 (18.5-61.3) | |
| Partial response | 51.9 (36.2-67.2) | 23.1 (6.6-56.0) | | 45.9 (28.2-64.7) | 37.5 (18.5-61.3) | |
| Relapse[g], % (95% CI) | 66.0 (51.8-78.2) | 30.0 (9.3-60.6) | **0.04** | 50.0 (32.2-67.8) | 61.1 (38.3-80.6) | 0.46 |
| Median relapse-free survival[g] in months (95% CI) | 9.3 (6.9-13.0) | 68.3 (14.7-NE) | **0.02** | 25.9 (7.1-34.2) | 16.5 (7.6-29.6) | 0.33 |
| Patients receiving any rescue therapy[h], % (95% CI) | 32.7 (21.5-45.8) | 28.6 (10.5-54.5) | 0.77 | 37.8 (23.6-53.9) | 62.5 (42.6-79.6) | 0.06 |

[a] T-tests for means of continuous variables, Wilcoxon rank-sum tests for medians, chi-square tests for categorical variables with all observations ≥5, Fisher's exact tests when any observation was <5, and log-rank tests for time-to-event data.
[b] Only applies to secondary wAIHA; total percentage does not equal 100% because a single patient may have two or more underlying conditions.
[c] Hgb <100 g/L was an inclusion criterion.
[d] Time from wAIHA diagnosis to death, hospital transfer, emigration or end of study period (June 30, 2023).
[e] Only applies to OCS+RIX treatment arm.
[f] Analysis was restricted to treatment lines with available baseline and post-baseline Hgb assessments — primary wAIHA: OCS (n=54), OCS+RIX (n=13); secondary wAIHA: OCS (n=37), OCS+RIX (n=24). Median outcome assessment period for response, i.e., time from from 1L initiation until initiation of next treatment line, death, hospital transfer, emigration or June 2023, was 0.8 years (IQR: 0.2-2.5). To be considered as 'responding', patients needed to fulfil response criteria during at least one assessment time point, i.e. there were no requirements in terms of duration of response.
[g] Analysis was restricted to treatment lines reaching at least partial response — primary wAIHA: OCS (n=47), OCS+RIX (n=10); secondary wAIHA: OCS (n=28), OCS+RIX (n=18).
[h] Rescue therapy included red blood cell transfusion, plasmapheresis, injectable corticosteroids, intravenous immunoglobulin or erythropoiesis-stimulating agents.

**Copyright Information:** (Online) ISSN: 2572-9241
©2026 The Author(s). HemaSphere is published by John Wiley & Sons Ltd on behalf of European Hematology Association. This is an open access Abstract Book distributed under the Attribution-NonCommercial-NoDerivs (CC BY-NC-ND), which allows third parties to download the articles and share them with others as long as they credit the author and the Abstract Book, but they cannot change the content in any way or use them commercially.
**Abstract Book Citations:** Authors, Title, HemaSphere, 2026;10:(S1):pages. The abstract book DOIs can be found on the "Author Information" page.
**Disclaimer:** Articles published in the journal HemaSphere exclusively reflect the opinions of the authors. The authors are responsible for all content in their abstracts including accuracy of the facts, statements, citing resources, etc.

### (PS2397) GENETIC TESTING IN THE DIAGNOSIS OF CONGENITAL ERYTHROCYTOSIS: A REVIEW OF >1700 REFERRALS

**Topic:** 29. Enzymopathies, membranopathies and other red blood cell disorders

Rachel Mayhew*[1, 2], Claire Harrison[3], Iman Al Noumani[3, 4], Nicholas Parkin[1, 2], Laura Steedman[1, 2], Eirini Oikonomidou[1, 2], Dushanthy Navarajasegaran[1, 2], Melissa Kow[1, 2], Sarah Curran[1, 2], Sandra Bob-Ume[1, 2], Anna Fernandez[1, 2], Barnaby Clark[5], David Rees[6], Frances Smith[1, 2]

*[1]Synnovis Molecular Pathology Laboratory, King's College Hospital, London, United Kingdom; [2]NHS South East Genomic Medicine Service, London, United Kingdom; [3]Department of Haematology, Guy's and St Thomas' Hospital, London, United Kingdom; [4]Adult Haematology Department, Royal Hospital, Muscat, Oman;[5]Synnovis Precision Medicine, King's College Hospital, London, United Kingdom; [6]Haematological Medicine, King's College Hospital, London, United Kingdom;*

### Background

Establishing the causative molecular defect in patients with suspected congenital erythrocytosis is important due to clinical management and treatment interventions, including cascade testing for relatives. Next generation sequencing (NGS) targeted panel testing of genes related to congenital erythrocytosis has been used for this purpose by the Synnovis Molecular Pathology laboratory since 2016. However, many patients remain without a diagnosis after this testing. Genes not directly related to oxygen-sensing pathways have been implicated in cases of idiopathic erythrocytosis. We present data from the Synnovis Molecular Pathology diagnostic service to understand the diagnostic yield and genetic background of suspected congenital erythrocytosis referrals.

### Methods

Targeted panel NGS analysis was performed on patients referred for suspected congenital erythrocytosis between 2016 and 2025 as part of the diagnostic pathway which currently includes the following genes: *BPGM, EGLN1, EPAS1, EPO, EPOR, HBA1, HBA2, HBB, SLC30A10, VHL*. Additionally, the incidence of the *HFE* C282Y and H63D variants and pathogenic/likely pathogenic variants in the *PIEZO1* gene in this cohort were reviewed. Where provided at referral, relevant clinical data such as age, gender, haemoglobin, haematocrit, clinical and family history were collated and analysed. Detected variants were classified according to the Association for Clinical Genomic Science and the American College of Medical Genetics variant interpretation guidelines.

### Results

A total of 1718 cases were included in the analysis, and a definitive genetic diagnosis (detection of a likely pathogenic/pathogenic variant with the expected zygosity) was made in 87 cases (5%, median age 38 years, male: n=60, female: n=24, gender unknown: n=3). Genetic diagnoses were made in 8 different genes with high-affinity haemoglobin variants accounting for the greatest proportion of diagnoses: *HBB* (n=38), *EGLN1* (n=15), *EPAS1* (n=13), *VHL* (n=10), *EPOR* (n=4), *EPO* (n=2), *HBA2* (n=3), *SH2B3* (n=1), *BPGM* (n=1). In the cohort of undiagnosed patients (n=1631), 9.7% and 23.9% of patients were found to be heterozygous for *HFE* C282Y and H63D variants respectively, whilst 2.2% were compound heterozygous for both. Additionally, unlike other similar cohort studies, no gain-of-function likely pathogenic/pathogenic variants in the *PIEZO1* gene were detected.

### Summary/Conclusion

Hereditary causes of erythrocytosis are very rare. Patients that present with persistent erythrocytosis and a family history but are without a molecular diagnosis after panel testing suggest that there are undiscovered genetic causes of erythrocytosis, and these cases warrant further investigation. The low diagnostic yield mandates consideration of improved screening prior to referral for this testing and more developed criteria for determining patient eligibility.

**Copyright Information:** (Online) ISSN: 2572-9241
©2026 The Author(s). HemaSphere is published by John Wiley & Sons Ltd on behalf of European Hematology Association. This is an open access Abstract Book distributed under the Attribution-NonCommercial-NoDerivs (CC BY-NC-ND), which allows third parties to download the articles and share them with others as long as they credit the author and the Abstract Book, but they cannot change the content in any way or use them commercially.
**Abstract Book Citations:** Authors, Title, HemaSphere, 2026;10:(S1):pages. The abstract book DOIs can be found on the "Author Information" page.
**Disclaimer:** Articles published in the journal HemaSphere exclusively reflect the opinions of the authors. The authors are responsible for all content in their abstracts including accuracy of the facts, statements, citing resources, etc.

### (PS2398) WHOLE-GENOME SEQUENCING IN THE DIAGNOSTIC EVALUATION OF PATIENTS WITH SUSPECTED HEREDITARY ANEMIA

**Topic:** 29. Enzymopathies, membranopathies and other red blood cell disorders

Thomas Doeven*[1, 2], Jens Helby[1, 3], Henrik Hasle[4], Marianne Hoffmann[5], Selma Bendtsen[1, 6], Kian Haseli[1], Morten Dziegel[3, 7], Lene Sjø[8, 9], Henrik Frederiksen[10, 11], Dennis Lund Hansen[10, 11], Marianne Petersen[12], Peter Buur Van Kooten Niekerk[12], Daniel El Fassi[1, 3], Mathias Rathe[11, 13], Anne Winther-Larsen[14], Nina Toft[1], Sarah Carlsen[1], Lise Jensen[3, 15], Andreas Ørslev Rasmussen[6], Annetta Eidesgaard[6], Emma Scott[6], Maria Rossing[3, 6], Richard van Wijk[16], Jesper Brix Petersen[1], Eduard J. van Beers[2], Andreas Glenthøj[1, 3, 17]

*[1]Copenhagen University Hospital, Rigshospitalet, Danish Red Blood Cell Center, Department of Hematology, Copenhagen, Denmark; [2]University Medical Center Utrecht, Utrecht University, Center for Benign Hematology, Thrombosis and Hemostasis, van Creveldkliniek, Utrecht, The Netherlands; [3]University of Copenhagen, Department of Clinical Medicine, Faculty of Health and Medical Sciences, Copenhagen, Denmark; [4]Aarhus University Hospital, Department of Pediatrics, Aarhus, Denmark; [5]Copenhagen University Hospital, Rigshospitalet, Department of Pediatrics and Adolescent Medicine, Copenhagen, Denmark; [6]Copenhagen University Hospital, Rigshospitalet, Department of Genomic Medicine, Copenhagen, Denmark; [7]Copenhagen University Hospital, Rigshospitalet, Department of Clinical Immunology, Copenhagen, Denmark; [8]Copenhagen University Hospital, Rigshospitalet, Department of Pathology, Copenhagen, Denmark; [9]Copenhagen University Hospital, Rigshospitalet, Department of Clinical Genetics, Copenhagen, Denmark; [10]Odense University Hospital, Department of Hematology, Odense, Denmark; [11]University of Southern Denmark, Department of Clinical Research, Odense, Denmark; [12]Aarhus University Hospital, Department of Hematology, Aarhus, Denmark; [13]Odense University Hospital, Hans Christian Andersen Children's Hospital, Odense, Denmark; [14]Aarhus University Hospital, Department of Clinical Biochemistry, Aarhus, Denmark; [15]Zealand University Hospital, Department of Pediatrics, Copenhagen, Denmark; [16]University Medical Center Utrecht, Utrecht University, Central Diagnostic Laboratory, Utrecht, The Netherlands; [17]ERN-EuroBloodNet, Paris, France;*

**Background**

Hereditary anemias encompass a heterogeneous group of disorders, collectively among the most prevalent genetic conditions worldwide. Diagnostic gaps persist, whilst accurate diagnosis is becoming more important with upcoming targeted therapies. Advances in genetic testing have resulted in a growing preference of genetic evaluation in the earlier phases of the diagnostic workup.

**Aims**

To assess the role of whole-genome sequencing (WGS) in the diagnostic evaluation and clinical management of hereditary anemias.

**Methods**

The Danish Hereditary Anemia (DAHEAN) network is a nationwide prospective cohort study. Eligible patients had a suspected or already confirmed hereditary red cell disease, JAK2-wildtype erythrocytosis or a suspected iron metabolism disorder. All patients enrolled in the DAHEAN study were offered WGS as part of the standard diagnostic workup. WGS was conducted through the Danish National Genome Center, with variant assessment and reporting performed locally. Functional tests were performed as clinically indicated to aid diagnosis and variant classification. Novel variants were defined as those not previously reported in ClinVar or the literature. Filtering using an *in silico* gene panel of up to 212 red cell-related genes was done to mitigate the risk of incidental detection of pathogenic variants. Patients were discussed in a multidisciplinary anemia board, during which diagnostic findings were evaluated and diagnosis and treatment were determined.

**Copyright Information:** (Online) ISSN: 2572-9241
©2026 The Author(s). HemaSphere is published by John Wiley & Sons Ltd on behalf of European Hematology Association. This is an open access Abstract Book distributed under the Attribution-NonCommercial-NoDerivs (CC BY-NC-ND), which allows third parties to download the articles and share them with others as long as they credit the author and the Abstract Book, but they cannot change the content in any way or use them commercially.
**Abstract Book Citations:** Authors, Title, HemaSphere, 2026;10:(S1):pages. The abstract book DOIs can be found on the “Author Information” page.
**Disclaimer:** Articles published in the journal HemaSphere exclusively reflect the opinions of the authors. The authors are responsible for all content in their abstracts including accuracy of the facts, statements, citing resources, etc.

**Results**

As of September 2025, a total of 282 patients had WGS performed and were discussed in the anemia boards. Patients had a median age of 34 years (range, 0–93), 70 (25%) were pediatric, and 127 (45%) were female. A total of 60 distinct diagnoses were established, with red cell membranopathies as most common (n=99, 35%), followed by hemoglobinopathies (n=27, 10%).
WGS identified novel variants in 90 (32%) patients. A potential causative variant associated with the suspected/confirmed anemia, iron metabolism disorder, or phenotypically overlapping disorder was detected in 199 (71%) patients. WGS also identified a mix of intron and 3' UTR variants in 14 (5%) patients that likely would not have been detected through whole-exome sequencing, contributing to a conclusive diagnosis in most cases. Several ultra-rare anemias were identified, including the first cases of glucose phosphate isomerase deficiency (*GPI*) and harderoporphyria (*CPOX*) in Denmark.
Incidentally found pathogenic variants unrelated to the anemia were identified in six patients, who received genetic counseling when indicated. *HFE* and *UGT1A1* variant detection yielded 31 diagnoses of Gilbert syndrome and 11 of hemochromatosis.
Anemia board discussions were conclusive in 81% of the cases. Diagnosis was determined in half of the patients through WGS, with WGS contributing to or making the diagnosis more likely in 29% of patients, whilst it did not aid the diagnostic workup in remaining 21% of patients. In 56% of patients, WGS improved or determined the treatment strategy.

**Summary/Conclusion**

In this nationwide study, WGS provided high diagnostic accuracy with direct impact on the clinical management of hereditary anemias. The utilization of an efficient and centralized diagnostic pipeline integrated in clinical practice promotes nationwide accessibility and highlights the feasibility of integrating WGS into routine healthcare practice. Further implementation may reduce diagnostic odysseys and improve patient outcomes. Limitations include potential selection bias.

**Copyright Information:** (Online) ISSN: 2572-9241
©2026 The Author(s). HemaSphere is published by John Wiley & Sons Ltd on behalf of European Hematology Association. This is an open access Abstract Book distributed under the Attribution-NonCommercial-NoDerivs (CC BY-NC-ND), which allows third parties to download the articles and share them with others as long as they credit the author and the Abstract Book, but they cannot change the content in any way or use them commercially.
**Abstract Book Citations:** Authors, Title, HemaSphere, 2026;10:(S1):pages. The abstract book DOIs can be found on the "Author Information" page.
**Disclaimer:** Articles published in the journal HemaSphere exclusively reflect the opinions of the authors. The authors are responsible for all content in their abstracts including accuracy of the facts, statements, citing resources, etc.

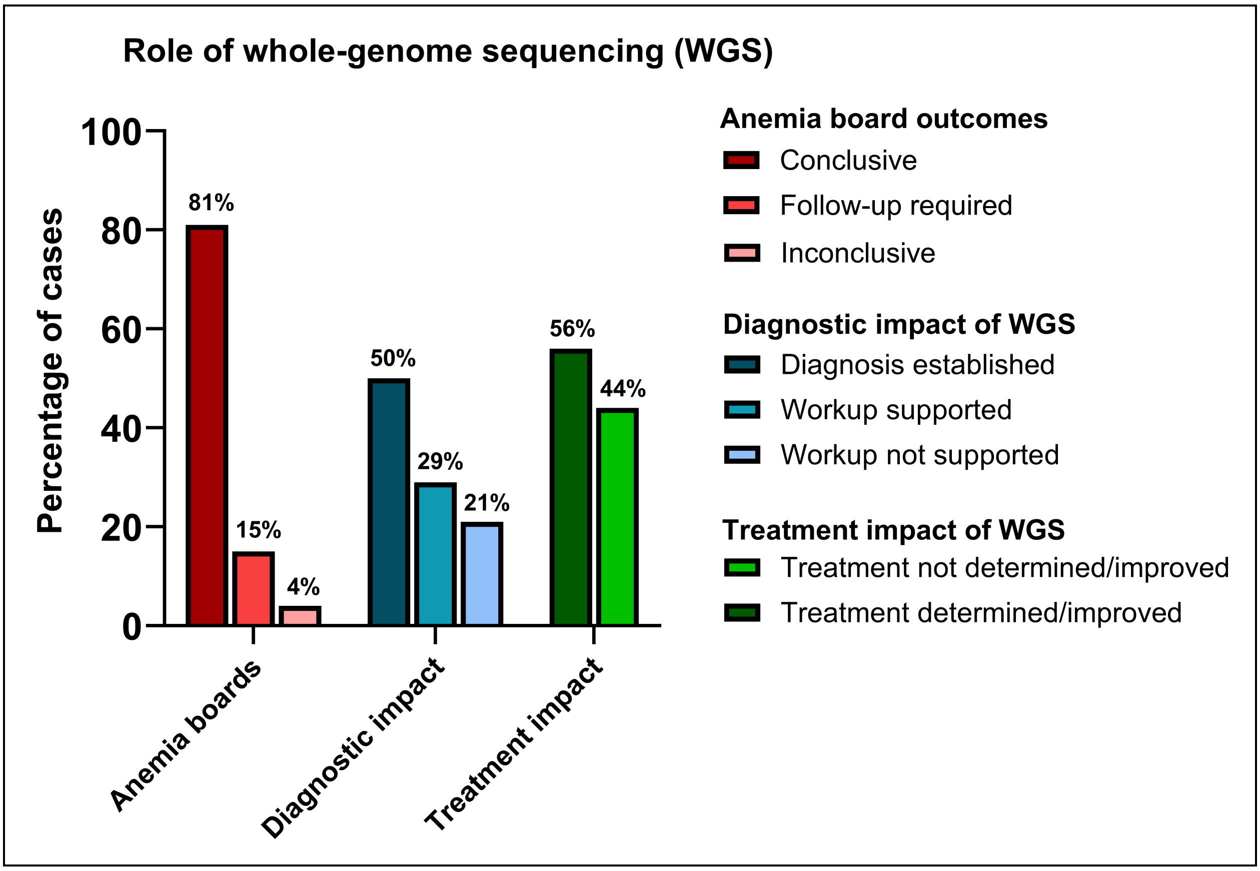

**Copyright Information:** (Online) ISSN: 2572-9241
©2026 The Author(s). HemaSphere is published by John Wiley & Sons Ltd on behalf of European Hematology Association. This is an open access Abstract Book distributed under the Attribution-NonCommercial-NoDerivs (CC BY-NC-ND), which allows third parties to download the articles and share them with others as long as they credit the author and the Abstract Book, but they cannot change the content in any way or use them commercially.
**Abstract Book Citations:** Authors, Title, HemaSphere, 2026;10:(S1):pages. The abstract book DOIs can be found on the "Author Information" page.
**Disclaimer:** Articles published in the journal HemaSphere exclusively reflect the opinions of the authors. The authors are responsible for all content in their abstracts including accuracy of the facts, statements, citing resources, etc.

### (PS2399) PRECISE EDITING OF THE TFR2 GENE AS POSSIBLE THERAPEUTIC APPROACH FOR HEMOGLOBINOPATHIES

**Topic:** 30. Iron metabolism, deficiency and overload

Mara Caputo[1], Simona Maria Di Modica[1, 2, 3], Piergiuseppe Quarato[4], Assunta Cancellara[1, 2], Giada Giuliani[5, 6], Jacopo Ceolan[5, 6], Laura Silvestri[1, 2], Angelo Lombardo[2, 4], Lucia De Franceschi[5, 6], Antonella Nai*[1, 2]

*[1]IRCCS Ospedale San Raffaele, Regulation of Iron Metabolism Unit, Division of Genetics and Cellular Biology, Milan, Italy;[2]Vita-Salute San Raffaele University, Milan, Italy; [3]University of Brescia, Department of Molecular and Translational Medicine, Brescia, Italy; [4]SR-TIGET, IRCCS Ospedale San Raffaele, Epigenetic Regulation and Targeted Genome Editing Unit, Milan, Italy; [5]University of Verona, Department of Engineering for Innovative Medicine, Verona, Italy;[6]Azienda Ospedaliera Integrata of Verona, Verona, Italy;*

### Background

Sickle cell disease (SCD) and ß-thalassemia are the most common monogenic red cell disorders world-wide. Both conditions arise from mutations in the ß-globin gene, that in ß-thalassemia lead to reduced or absent production of hemoglobin (Hb), while in SCD results in the synthesis of the pathologic HbS. Despite their distinct molecular origin, both disorders are characterized by chronic hemolytic anemia, ineffective erythropoiesis and secondary iron-overload. Current clinical management remains suboptimal and the most recent approaches, despite promising, are not universally applicable and often fail to fully reverse the pathologic phenotype. Thus, the identification of novel therapeutic options is a clinical need.
Transferrin receptor 2 (TFR2) is an iron-sensor that mediates the iron-erythropoiesis cross-talk. In hepatocytes, it activates the transcription of hepcidin, the master regulator of iron homeostasis, while in erythroid cells, it is a partner of erythropoietin receptor and acts as a brake of erythropoiesis. Interestingly, *Tfr2* deletion in hematopoietic cells augments erythropoiesis in wild-type mice and ameliorates anemia in murine models of β-thalassemia.

### Aims

Since pharmacologic inhibitors of TFR2 are not currently available, we aim to develop an *ex vivo* gene-editing strategy to inactivate *Tfr2* in hematopoietic stem cells (HSCs).

### Methods

We designed and validated guide RNAs targeting the *Tfr2* genomic locus to direct Cas9-mediated site-specific DNA double-strand break followed by non-homologous end joining repair. The system was initially optimized in murine erythroleukemia (MEL) cells to identify the most effective guides, and subsequently validated into lineage-negative ($Lin^-$) cells isolated from the bone marrow of wild-type and thalassemic mice. The phenotype and repopulation capacity of edited cells were then assessed.

**Copyright Information:** (Online) ISSN: 2572-9241
©2026 The Author(s). HemaSphere is published by John Wiley & Sons Ltd on behalf of European Hematology Association. This is an open access Abstract Book distributed under the Attribution-NonCommercial-NoDerivs (CC BY-NC-ND), which allows third parties to download the articles and share them with others as long as they credit the author and the Abstract Book, but they cannot change the content in any way or use them commercially.
**Abstract Book Citations:** Authors, Title, HemaSphere, 2026;10:(S1):pages. The abstract book DOIs can be found on the “Author Information” page.
**Disclaimer:** Articles published in the journal HemaSphere exclusively reflect the opinions of the authors. The authors are responsible for all content in their abstracts including accuracy of the facts, statements, citing resources, etc.

**Results**

Twenty-four hours after nucleofection, the most efficient protocol achieved approximately 60% editing efficiency and a 55% decrease in *Tfr2* mRNA levels, while preserving cell viability, stem-like phenotype and clonogenic potential. Remarkably, 72-96 hours after editing, *Tfr2*-deficient cultures showed a trend toward increased proportion of Lin- cells, driven primarily by expansion of the long-term HSCs compartment. This was accompanied by reduced expression of cell cycle-related genes (i.e. *Ccng2* and *Ccnd2*), and of genes associated with HSCs proliferation and differentiation (e.g. *Ccl2*, *Dkk1*, *Cd38* and *Fpr2*), suggesting that *Tfr2* inactivation through gene-editing promotes HSCs quiescence and stemness maintenance. To test whether these features could rescue the impaired repopulating capacity of thalassemic HSCs, Lin- cells isolated from 2 murine models of non-transfusion dependent β-thalassemia ($Hbb^{th3/+}$ and $Hbb^{th1/th1}$) were *Tfr2*-edited and transplanted at limiting doses ($3*10^5$ cells/animal) into sub-lethally irradiated wild-type mice. Engraftment of unedited thalassemic cells was reduced by approximately 60% compared with wild-type controls; notably, the defect was fully rescued by *Tfr2* gene editing.

**Summary/Conclusion**

Although further studies are required to determine whether this approach can also ameliorate anemia and ineffective erythropoiesis *in vivo*, our results support the evaluation of *TFR2* gene disruption in hematopoietic cells via gene-editing as a promising therapeutic strategy for hemoglobinopathies such as ß-thalassemia.

Work supported by European Union-Next Generation EU_PNRR M6/C2 PNRR-POC-2023-12378393 grant (CUP: C43C24000430007).

**Copyright Information:** (Online) ISSN: 2572-9241
©2026 The Author(s). HemaSphere is published by John Wiley & Sons Ltd on behalf of European Hematology Association. This is an open access Abstract Book distributed under the Attribution-NonCommercial-NoDerivs (CC BY-NC-ND), which allows third parties to download the articles and share them with others as long as they credit the author and the Abstract Book, but they cannot change the content in any way or use them commercially.
**Abstract Book Citations:** Authors, Title, HemaSphere, 2026;10:(S1):pages. The abstract book DOIs can be found on the "Author Information" page.
**Disclaimer:** Articles published in the journal HemaSphere exclusively reflect the opinions of the authors. The authors are responsible for all content in their abstracts including accuracy of the facts, statements, citing resources, etc.

### (PS2400) THERAPEUTIC POTENTIAL OF SEVUPARIN IN CHRONIC KIDNEY DISEASE ANAEMIA: SYNERGISTIC EFFECTS WITH EPO AND MOLECULAR INSIGHTS INTO RENAL PROTECTION

**Topic:** 30. Iron metabolism, deficiency and overload

Michela Asperti*[1, 2], Magdalena Gryzik[1], Manuela Cominelli[3], Francesca Pagani[1, 3], Pietro Luigi Poliani[1, 3], Luisa Lorenzi[1, 3], Alberto Pietrantoni[1, 3], Domenico Girelli[4], Goran Westerberg[5], John Ohd[5], Maura Poli[1]

*[1]University of Brescia, DMMT, Brescia, Italy; [2]eCampus University, DiSTA, Novedrate (Como), Italy; [3]University of Brescia, Unit of Pathology, Brescia, Italy; [4]University of Verona, Department of Medicine, Verona, Italy; [5]Modus Therapeutics AB, Stockholm, Sweden;*

### Background

Chronic kidney disease (CKD) is a progressive loss of kidney function. Anemia of chronic disease in CKD patients is driven by a complex interplay of inflammation, erythropoietin (EPO) resistance, and hepcidin-mediated iron restriction. While erythropoiesis-stimulating agents (ESAs) represent the standard of care, they often fail to achieve optimal outcomes. Sevuparin, a novel chemically modified heparin derivative with low anticoagulant activity, has previously demonstrated potent hepcidin-suppressing capabilities *in vitro* and in acute models of inflammation. This study builds upon those findings to evaluate its long-term effects and tissue-protective role in a mouse model of chronic kidney damage.

### Aims

To investigate the sustained effects of sevuparin on hepcidin and haematological parameters, and to characterise its impact on kidney status and fibrotic markers in a CKD mouse model.

### Methods

CKD was induced in mice via an adenine-rich diet (ARD) model. Animals were subsequently treated for 3–6 weeks with either sevuparin (10 mg/kg/daily s.c.) alone or in combination with intraperitoneal EPO. Evaluation included longitudinal monitoring of haematological parameters, serum hepcidin, and a comprehensive transcriptional analysis of renal damage markers.

### Results

The results can be grouped in three main key-points:

1. **Haematological stabilization.** ARD-fed mice exhibited severe signs of CKD, including anaemia, elevated creatinine, and weight loss. While sevuparin monotherapy improved haemoglobin and haematocrit already after 3 weeks, the sevuparin/EPO combination provided long-term stability with maintenance of high reticulocyte haemoglobin and hemoglobin levels through 6 weeks.
2. **Hepcidin dynamics.** The sevuparin monotherapy achieved a profound reduction in serum hepcidin at 3 weeks. Interestingly, the phased dependency observed at 6 weeks suggests a more nuanced interaction between long-term EPO administration and hepcidin regulation.
3. **Renal protection and antifibrotic activity.** Beyond anaemia amelioration, sevuparin treatment also led to visible improvements in kidney status, evidenced by reduced collagen deposition, already seen after 3-weeks treatment. This was supported by a significant downregulation of mRNA markers associated with fibrosis, including *Tgfb1, Pai1, Ctgf, Actn2, Col1a1, Col1a2, Fibronectin, Mcp1,* and *Kim1.*

**Copyright Information:** (Online) ISSN: 2572-9241
©2026 The Author(s). HemaSphere is published by John Wiley & Sons Ltd on behalf of European Hematology Association. This is an open access Abstract Book distributed under the Attribution-NonCommercial-NoDerivs (CC BY-NC-ND), which allows third parties to download the articles and share them with others as long as they credit the author and the Abstract Book, but they cannot change the content in any way or use them commercially.
**Abstract Book Citations:** Authors, Title, HemaSphere, 2026;10:(S1):pages. The abstract book DOIs can be found on the “Author Information” page.
**Disclaimer:** Articles published in the journal HemaSphere exclusively reflect the opinions of the authors. The authors are responsible for all content in their abstracts including accuracy of the facts, statements, citing resources, etc.

**Summary/Conclusion**

These results reinforce the potential of sevuparin as nephroprotective compound. Moreover, by suppressing hepcidin and downregulating key fibrotic pathways, sevuparin offers a promising strategy to overcome ESA resistance. These pre-clinical data provide a robust rationale for the ongoing Phase II clinical trial (EUCT 2024-513864-24-00) in CKD patients.

**Copyright Information:** (Online) ISSN: 2572-9241
©2026 The Author(s). HemaSphere is published by John Wiley & Sons Ltd on behalf of European Hematology Association. This is an open access Abstract Book distributed under the Attribution-NonCommercial-NoDerivs (CC BY-NC-ND), which allows third parties to download the articles and share them with others as long as they credit the author and the Abstract Book, but they cannot change the content in any way or use them commercially.
**Abstract Book Citations:** Authors, Title, HemaSphere, 2026;10:(S1):pages. The abstract book DOIs can be found on the "Author Information" page.
**Disclaimer:** Articles published in the journal HemaSphere exclusively reflect the opinions of the authors. The authors are responsible for all content in their abstracts including accuracy of the facts, statements, citing resources, etc.

### (PS2401) DXO-1801, A HEPATOCYTE-TARGETED GALNAC-SIRNA, DEMONSTRATES HEPCIDIN PATHWAY MODULATION, IRON MOBILIZATION, AND A FAVORABLE SAFETY PROFILE IN NON-HUMAN PRIMATES

**Topic:** 30. Iron metabolism, deficiency and overload

Noa Madar Balakirski[1], Lilach Steiner*[1], Keren Moss[1], Emmanuel Weyl[1], Yael Garbian[1], Gal Yosef-Goldshtein[1], Nadav Marbach-Bar[1], Osnat Penn[1], Avital Laxer[1], Elena Feinstein[1], Shay Sela[1]

*[1]Dexoligo Therapeutics by Dexcel Pharma, Or Akiva, Israel;*

### Background

Iron-restricted anemia associated with chronic inflammatory conditions arises from dysregulation of the hepcidin pathway, resulting in impaired iron mobilization despite adequate iron stores. Therapeutic strategies enabling precise and durable inhibition of hepatic drivers of hepcidin may restore iron availability while limiting systemic liabilities.

### Aims

To characterize the pharmacokinetics (PK), dose–response relationship, durability, liver biodistribution, and safety of DXO-1801, a synthetic metabolically stable hepatocyte-targeted GalNAc-siRNA targeting a protein in the hepcidin regulatory pathway, in non-human primates (NHPs).

### Methods

Healthy male cynomolgus monkeys received DXO-1801 at pharmacologically- (0.3, 1 or 3 mg/kg, n=3 per group) or at toxicologically relevant dose levels (50 or 300 mg/kg, n=2 per group) via a subcutaneous (SC) injection on Study Days 0 and 84. Plasma PK, circulating hepcidin and iron parameters (including serum iron and transferrin saturation), hematology, clinical chemistry, coagulation, serum cytokines and complement activation biomarkers were assessed.
Liver samples collected at selected time points were used to assess siRNA quantification, target gene knockdown (KD), and histopathology.

### Results

Following SC administration, DXO-1801 exhibited rapid plasma clearance with preferential hepatic biodistribution, consistent with hepatocyte-targeted delivery.
DXO-1801 was well tolerated across the evaluated dose range. No changes were observed in cytokine levels, complement activation, or coagulation parameters. Transient, dose-dependent mild elevations of liver enzymes were detected shortly after dosing at ≥50 mg/kg, consistent with known siRNA class effect. No evidence of treatment-related liver injury or adverse hepatic iron accumulation was apparent by histopathology examination.
DXO-1801 induced dose- and concentration-dependent KD of the hepatic target mRNA, with associated suppression of downstream hepcidin expression. Target KD and PD activity were observed at the lowest evaluated dose (0.3 mg/kg) and persisted for the 3-month dosing interval following a single administration. PD effects were augmented following repeat dosing on Day 84, demonstrating durable pathway modulation.
Suppression of the hepcidin pathway resulted in dose-dependent increases in serum iron and transferrin saturation (TSAT), indicating effective mobilization of iron. The dose– and concentration–response PK and PD (Emax) modeling of data demonstrated a clear and saturable plateau, enabling identification of the optimal dose required to achieve maximal PD and iron-related benefit.

**Copyright Information:** (Online) ISSN: 2572-9241
©2026 The Author(s). HemaSphere is published by John Wiley & Sons Ltd on behalf of European Hematology Association. This is an open access Abstract Book distributed under the Attribution-NonCommercial-NoDerivs (CC BY-NC-ND), which allows third parties to download the articles and share them with others as long as they credit the author and the Abstract Book, but they cannot change the content in any way or use them commercially.
**Abstract Book Citations:** Authors, Title, HemaSphere, 2026;10:(S1):pages. The abstract book DOIs can be found on the “Author Information” page.
**Disclaimer:** Articles published in the journal HemaSphere exclusively reflect the opinions of the authors. The authors are responsible for all content in their abstracts including accuracy of the facts, statements, citing resources, etc.

**Summary/Conclusion**

In NHPs, DXO-1801 demonstrates durable and precise suppression of the hepcidin regulatory pathway, maintained for at least 3 months following a single dose. Treatment is associated with iron mobilization and improvements in hematological parameters. DXO-1801 was well tolerated across a wide dose range, providing a >100-fold dose margin between the pharmacologically active dose and the highest evaluated, well-tolerated dose (300 mg/kg).
These findings support the potential for infrequent dosing, predictable exposure–response relationship and favorable therapeutic index in future clinical studies.

**Copyright Information:** (Online) ISSN: 2572-9241
©2026 The Author(s). HemaSphere is published by John Wiley & Sons Ltd on behalf of European Hematology Association. This is an open access Abstract Book distributed under the Attribution-NonCommercial-NoDerivs (CC BY-NC-ND), which allows third parties to download the articles and share them with others as long as they credit the author and the Abstract Book, but they cannot change the content in any way or use them commercially.
**Abstract Book Citations:** Authors, Title, HemaSphere, 2026;10:(S1):pages. The abstract book DOIs can be found on the "Author Information" page.
**Disclaimer:** Articles published in the journal HemaSphere exclusively reflect the opinions of the authors. The authors are responsible for all content in their abstracts including accuracy of the facts, statements, citing resources, etc.

### (PS2402) IN VIVO CHARACTERIZATION OF FERRITIN H AND L SUBUNITS IN SERUM-DERIVED EXTRACELLULAR VESICLES ACROSS DIFFERENT IRON METABOLISM DISORDERS

**Topic:** 30. Iron metabolism, deficiency and overload

Fabiana Busti*[1], Leonardo Sandrini[2], Elisa Antinori[1], Misha Fatima[1], Francesca Ambrosani[1], Michela Asperti[2], Maura Poli[2], Annalisa Castagna[1], Domenico Girelli[1]

*[1]University of Verona, Department of Medicine, Verona, Italy; [2]University of Brescia, Department of Molecular and Translational Medicine (DMMT), Brescia, Italy;*

### Background

Ferritin is an intracellular protein that is also detectable in circulation, where, in the absence of inflammation, it is considered a surrogate marker of tissue iron stores. However, the biological role of circulating ferritin may require reassessment in light of evidence linking it to iron transport, host–pathogen interactions, and inflammatory pathways. Ferritin is a 24-subunit heteropolymer composed of heavy (H) and light (L) chains. The L subunit (FTL) mainly provides structural stability, whereas the H subunit (FTH) has ferroxidase activity and pro-inflammatory properties. The H/L ratio varies according to tissue type and pathological conditions. The extracellular vesicle (EV) pathway represents a potential mechanism for ferritin export. Although EV-mediated ferritin transport has been described in experimental models, *in vivo* data on iron metabolism disorders are scarce.

### Aims

We aimed to characterize ferritin subunits in serum-derived EVs from subjects with iron disorders.

### Methods

Serum EVs were isolated from 81 patients: 20 with iron deficiency anemia (IDA) and 61 with hyperferritinemia (ferritin >200 µg/L in females and >300 µg/L in males). Hyperferritinemic subjects included 12 patients evaluated 1–3 weeks after intravenous infusion of 1 g ferric carboxymaltose, 19 with hemochromatosis (liver iron overload confirmed by MRI-T2*), 16 with acute inflammatory conditions (e.g., sepsis or pneumonia), and 14 with metabolic syndrome without liver iron overload at MRI. Ten healthy controls were included. All subjects were characterized using iron status markers (serum iron, ferritin, transferrin saturation), erythroferrone, and IL-6. All participants provided informed consent. EVs were characterized by transmission electron microscopy (TEM) and nanoparticle tracking analysis (NTA). Ferritin H and L subunits were quantified using a validated home-made ELISA.

### Results

FTL subunit in serum-derived EVs (Figure 1A) was increased in hyperferritinemic subjects with inflammatory conditions, metabolic syndrome, and hemochromatosis, thus in both subjects with and without liver iron overload. In contrast, patients developing hyperferritinemia after IV iron therapy did not show a corresponding increase in FTL, possibly suggesting that treatment-related hyperferritinemia may involve mechanisms not primarily driven by EVs secretion. Notably, EVs FTL in IDA was comparable to that of healthy controls despite markedly reduced circulating ferritin, raising the possibility that the EVs ferritin pool may be maintained within a relatively stable range even in systemic ID.
FTH subunit (Figure 1B) in serum-derived EVs was below the detection limit in IDA and significantly lower than in controls after iron infusion. Among other hyperferritinemic subjects, FTH levels were significantly higher in patients with acute inflammation. Although preliminary, this observation suggests that the EVs ferritin structure in vivo may reflect both inflammatory status and iron burden.

**Copyright Information:** (Online) ISSN: 2572-9241
©2026 The Author(s). HemaSphere is published by John Wiley & Sons Ltd on behalf of European Hematology Association. This is an open access Abstract Book distributed under the Attribution-NonCommercial-NoDerivs (CC BY-NC-ND), which allows third parties to download the articles and share them with others as long as they credit the author and the Abstract Book, but they cannot change the content in any way or use them commercially.
**Abstract Book Citations:** Authors, Title, HemaSphere, 2026;10:(S1):pages. The abstract book DOIs can be found on the "Author Information" page.
**Disclaimer:** Articles published in the journal HemaSphere exclusively reflect the opinions of the authors. The authors are responsible for all content in their abstracts including accuracy of the facts, statements, citing resources, etc.

### Summary/Conclusion

The biological meaning of EVs ferritin and its subunit composition deserves further investigation. To our knowledge, this is the first *in vivo* description of ferritin H and L subunits in serum-derived EVs across different iron-related diseases. Despite the limited sample size, these preliminary findings suggest that EVs ferritin composition may mirror distinct inflammatory and iron-related states, warranting confirmation in larger cohorts.

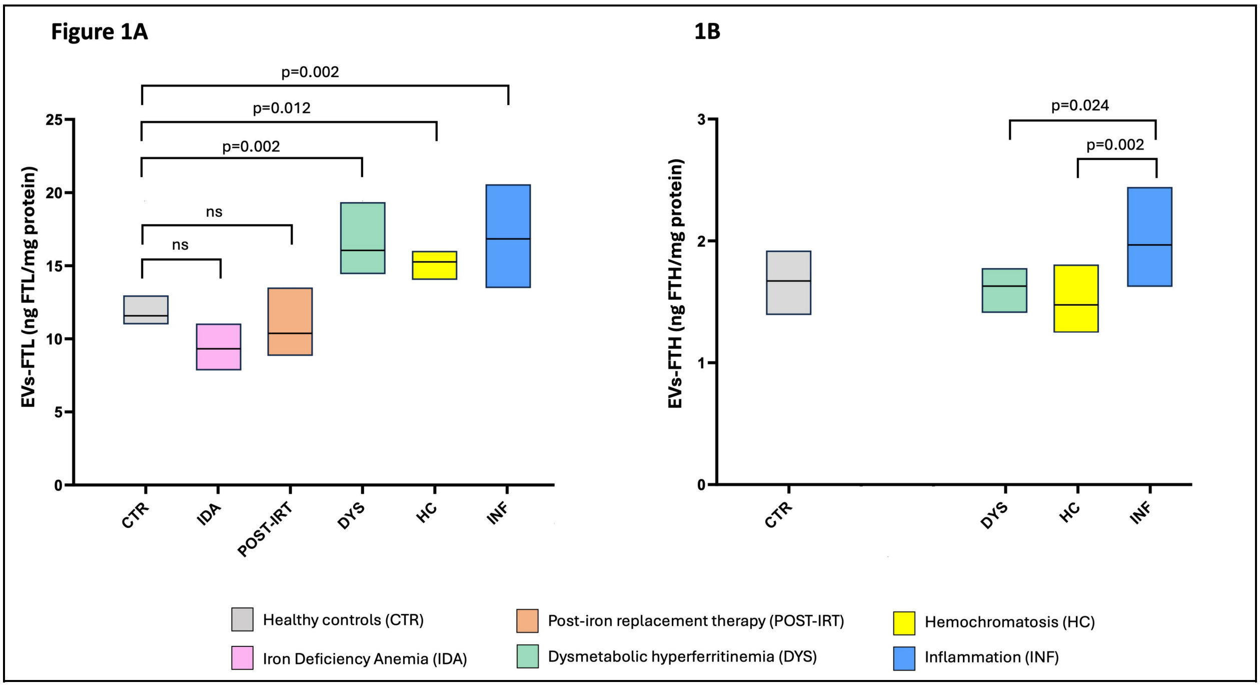

FTL (Figure 1A) and FTH (Figure 1B) subunits in serum-derived EVs from subjects with different iron disorders and from healthy controls.

**Copyright Information:** (Online) ISSN: 2572-9241
©2026 The Author(s). HemaSphere is published by John Wiley & Sons Ltd on behalf of European Hematology Association. This is an open access Abstract Book distributed under the Attribution-NonCommercial-NoDerivs (CC BY-NC-ND), which allows third parties to download the articles and share them with others as long as they credit the author and the Abstract Book, but they cannot change the content in any way or use them commercially.
**Abstract Book Citations:** Authors, Title, HemaSphere, 2026;10:(S1):pages. The abstract book DOIs can be found on the "Author Information" page.
**Disclaimer:** Articles published in the journal HemaSphere exclusively reflect the opinions of the authors. The authors are responsible for all content in their abstracts including accuracy of the facts, statements, citing resources, etc.

### (PS2403) MANAGEMENT OF GENETIC HAEMOCHROMATOSIS IN NORTH LONDON'S LARGEST HOSPITAL. A SINGLE CENTRE REVIEW OF CURRENT CLINICAL PRACTICE.

**Topic:** 30. Iron metabolism, deficiency and overload

Bharath Ram Sreedhar*[1], Neil Chauhan[1]

[1]*Royal Free Hospital NHS Trust, Department of Haematology, London, United Kingdom;*

### Background

Genetic haemochromatosis (HC) is an autosomal recessive disorder where HFE gene mutation leads to increased intestinal iron absorption and subsequent iron excess/overload. Excess iron deposition leads to the classically described sequalae of arthropathy, cardiac failure, endocrinopathy, hepatic cirrhosis and hepatocellular carcinoma. Two mutations have been identified: homozygosity for a single nucleotide change (845 G→A), causing the substitution of cysteine by tyrosine at amino acid 282. A second mutation, *p.His63Asp*, has a minor role. UK guidelines recommend HFE testing in adults with unexplained raised serum ferritin and T sat (>300 mcg/l and >50% males; >200 mcg/l and >40% females). Patients undergo venesections, initially to ferritin < 50 mcg/L, followed by maintenance venesections/blood donations.

### Aims

As North London's biggest hospital, we see a high number of HC through our large catchment area, a significant Irish diaspora, and a regional liver unit. This allows us a rich pool of suspected and confirmed haemochromatosis patients. We detail the clinical and laboratory changes in patients with HC in our centre.

### Methods

Age at diagnosis, genotype, referral route, family history, presenting and current iron parameters, and clinical management were collected from electronic patient records. Data was collected retrospectively and covered the 12-months to December 31st, 2024. Median and range were used for continuous variables, and percentage was used for categorical variables.

### Results

A total of 130 patients with HC were identified. The median age at diagnosis was 55 years (range 24 – 86 years). 57% were referred for elevated transferrin saturation and ferritin, with 12% having only elevated ferritin and 3% noted to have only elevated transferrin saturation. 5% of the patients were referred for testing due to a first-degree family member being diagnosed with HC. 11.5% had a known family history. 64% have C282Y homozygosity and 22% had a diagnosis of C282Y/H63D compound heterozygosity, with a minority of the cases being C282Y heterozygotes, H63D homozygotes, and H63D heterozygotes. Referrals predominantly come from primary care (88%). Median ferritin level at diagnosis was 519.5 mcg/L and transferrin saturation 62.2%. Median ferritin and transferrin saturation on the latest bloods were 82 mcg/L and 47%, respectively. Patients with HC are being diagnosed earlier due to biochemical parameters being above normal limits, hence the clinical symptoms and organ overload are low, as reflected by only 11 (8.4%) patients with evidence of organ iron overload. The management for C282Y homozygotes is venesections, and 96.3% are on venesections. In comparison, 53.5% of the patients with compound heterozygous C282Y/H63D were on venesections. The management of other genotypes involves either venesections or monitoring guided by phenotype. Although it is widely recognized that C282Y heterozygotes, and those with homozygous or heterozygous H63D variants typically exhibit minimal to no risk of iron overload, a subset of this cohort (n=19, 15%) demonstrated elevated iron indices or biochemical/radiological evidence indicating iron overload.

**Copyright Information:** (Online) ISSN: 2572-9241
©2026 The Author(s). HemaSphere is published by John Wiley & Sons Ltd on behalf of European Hematology Association. This is an open access Abstract Book distributed under the Attribution-NonCommercial-NoDerivs (CC BY-NC-ND), which allows third parties to download the articles and share them with others as long as they credit the author and the Abstract Book, but they cannot change the content in any way or use them commercially.
**Abstract Book Citations:** Authors, Title, HemaSphere, 2026;10:(S1):pages. The abstract book DOIs can be found on the "Author Information" page.
**Disclaimer:** Articles published in the journal HemaSphere exclusively reflect the opinions of the authors. The authors are responsible for all content in their abstracts including accuracy of the facts, statements, citing resources, etc.

**Summary/Conclusion**

Most patients were referred for elevated iron parameters, but a minority of patients had symptoms and organ iron-overload. We manage C282Y homozygotes with venesections. However, for other genotypes, their management varies and is guided by phenotype and by the search for other contributing factors.

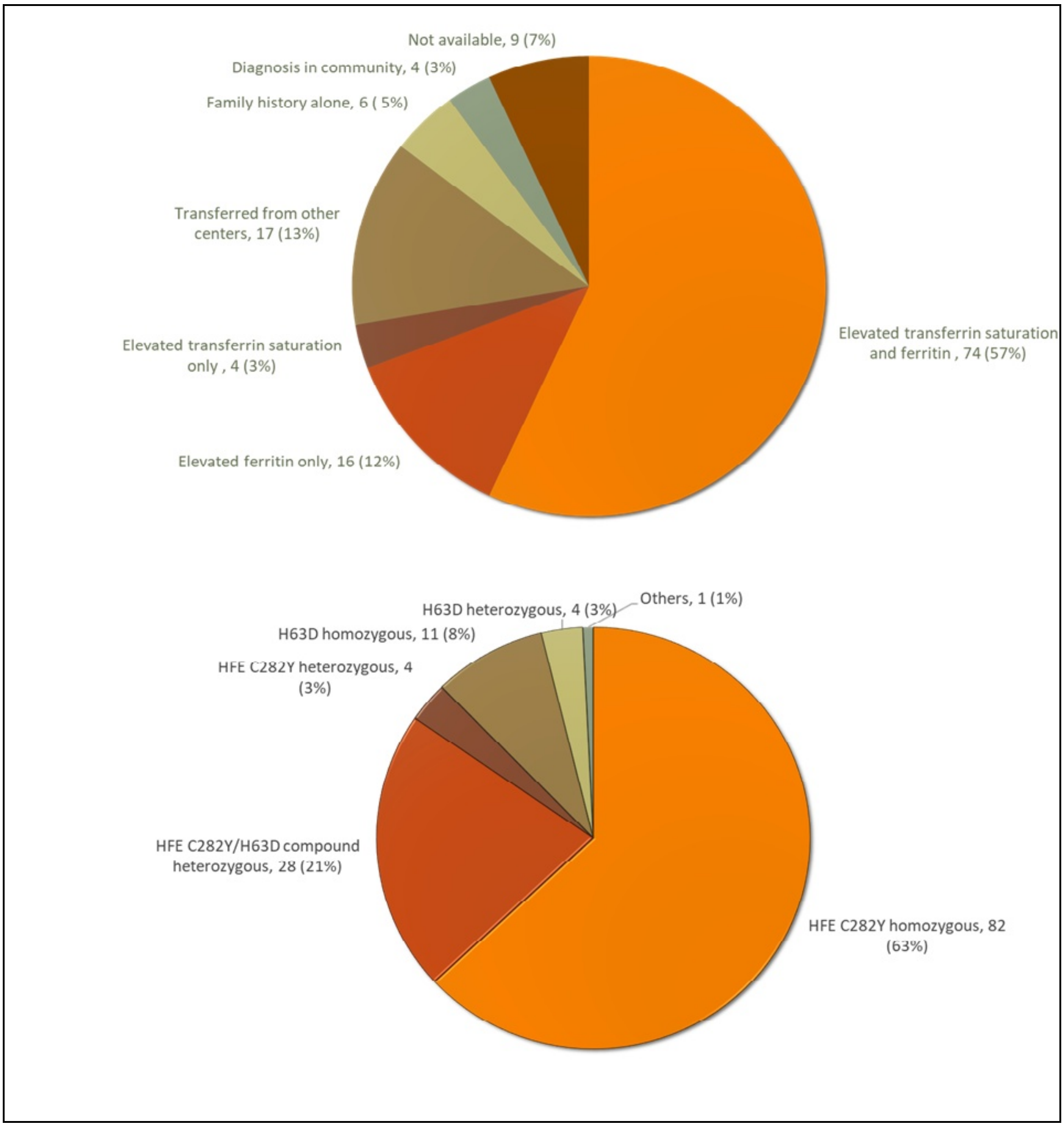

Figure 1: Top figure: shows the distribution of indications for referral to haematology in patients with GH. Bottom figure: Shows the distribution of different genotypes in patients with GH.

**Copyright Information:** (Online) ISSN: 2572-9241
©2026 The Author(s). HemaSphere is published by John Wiley & Sons Ltd on behalf of European Hematology Association. This is an open access Abstract Book distributed under the Attribution-NonCommercial-NoDerivs (CC BY-NC-ND), which allows third parties to download the articles and share them with others as long as they credit the author and the Abstract Book, but they cannot change the content in any way or use them commercially.
**Abstract Book Citations:** Authors, Title, HemaSphere, 2026;10:(S1):pages. The abstract book DOIs can be found on the "Author Information" page.
**Disclaimer:** Articles published in the journal HemaSphere exclusively reflect the opinions of the authors. The authors are responsible for all content in their abstracts including accuracy of the facts, statements, citing resources, etc.

### (PS2404) BEYOND GANZONI AND SIMPLIFIED DOSING TABLE: A PHYSIOLOGY-BASED INDIVIDUALIZED FORMULA FOR INTRAVENOUS IRON DOSING

**Topic:** 30. Iron metabolism, deficiency and overload

Muhammet Özbilen*[1]

[1]*Ordu University School of Medicine, Internal Medicine, Ordu, Türkiye;*

### Background

Accurate intravenous iron dosing remains a major unmet need in iron deficiency management. Existing approaches, including the Ganzoni formula and simplified dosing table (STD), rely predominantly on body weight, while STD further depend on discrete hemoglobin thresholds that may not adequately reflect the continuous nature of physiological iron requirements. Ongoing blood loss, particularly in patients with heavy menstrual bleeding, may further compromise treatment adequacy and is not captured by conventional dosing strategies.

### Aims

A novel physiology-based iron dosing model integrating anthropometric and clinical parameters was developed.

### Methods

The model incorporates hemoglobin (Hb) deficit using estimated blood volume (eBV), derived from height, weight, and sex, along with iron store replenishment based on ferritin targets scaled to body surface area (BSA), and menstrual blood loss quantified using the pictorial bleeding assessment chart (PBAC). Unlike conventional weight-based approaches such as the Ganzoni formula, this model integrates height-derived blood volume, body surface area–adjusted iron stores, and quantified menstrual blood loss. The standard formulation estimates total iron requirement as $(\Delta Hb \times eBV \times 34.6) + (\Delta Ferritin \times BSA \times 5.78)$, while an advanced formulation additionally incorporates menstrual blood loss as $[(Actual\ Hb \times 5.5) + 42] \times PBAC/100$. Performance was evaluated using a large R-generated dataset (n=367,650) reflecting real-world anthropometric and hematologic distributions, with comparisons to the Ganzoni formula and STD. A web-based calculator was developed to enable practical and individualized dose estimation in clinical settings.

### Results

Compared with current approaches, the proposed standard and advanced formulations generated significantly higher and more physiologically aligned dose estimates ($P<0.001$). Underdosing was observed in 99.95% of cases with the Ganzoni formula relative to the standard formulation and in 99.99% when compared with the advanced formulation incorporating blood loss. Similarly, the simplified dosing table (SDT) resulted in underdosing in 69.2% and 84.7% of cases, respectively. The discrepancy was further amplified with the new standard formulation, particularly among taller patients with similar body weight, with maximum dose differences of up to 920 mg versus Ganzoni and 1406 mg versus the SDT (Figure). In multivariable analysis adjusted for sex, dose estimates increased by approximately 10 mg per 1 cm increase in height and 11 mg per 1 kg increase in weight, and by 166 mg per 1 g/dL decrease in hemoglobin, while males required on average 233 mg higher doses than females (all $P<0.001$). The model additionally enabled dose calculation in non-anemic iron deficiency.

**Copyright Information:** (Online) ISSN: 2572-9241
©2026 The Author(s). HemaSphere is published by John Wiley & Sons Ltd on behalf of European Hematology Association. This is an open access Abstract Book distributed under the Attribution-NonCommercial-NoDerivs (CC BY-NC-ND), which allows third parties to download the articles and share them with others as long as they credit the author and the Abstract Book, but they cannot change the content in any way or use them commercially.
**Abstract Book Citations:** Authors, Title, HemaSphere, 2026;10:(S1):pages. The abstract book DOIs can be found on the "Author Information" page.
**Disclaimer:** Articles published in the journal HemaSphere exclusively reflect the opinions of the authors. The authors are responsible for all content in their abstracts including accuracy of the facts, statements, citing resources, etc.

**Summary/Conclusion**

This physiology-based individualized formula addresses systematic underdosing observed with conventional weight-based models by incorporating height alongside body weight, enabling sex-specific dose estimation, and accounting for menstrual blood loss when applicable. By moving beyond simplified cut-offs and integrating key physiologic determinants of iron requirement, the model enables more accurate patient-specific dosing, improves treatment adequacy, and may reduce the need for repeated intravenous iron administration.

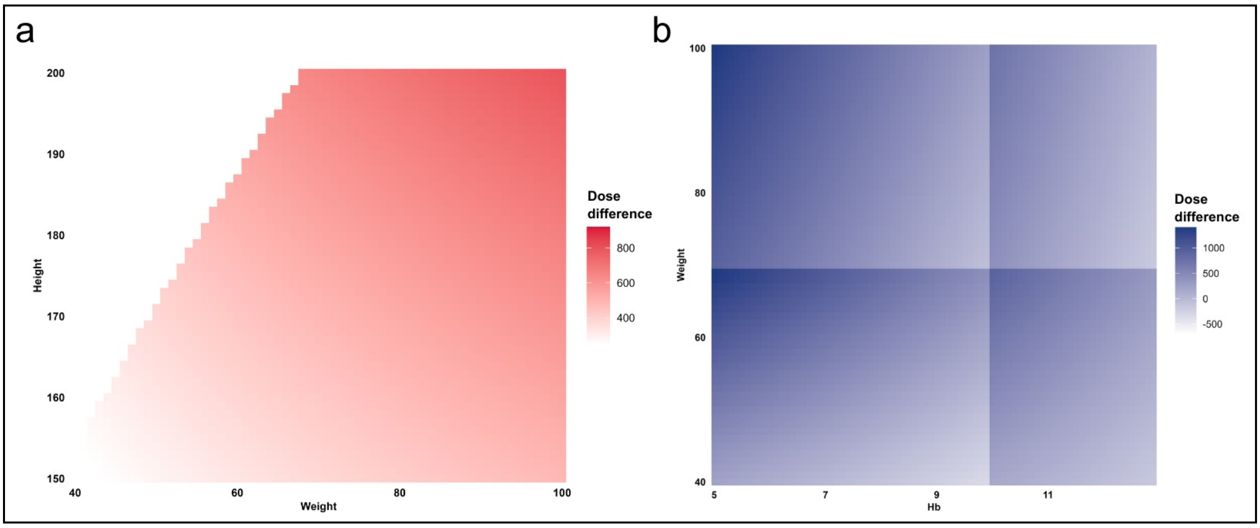

Figure. (a) Height–weight heatmap illustrating the mean dose difference between the New Standard Formula and the Ganzoni formula, demonstrating progressively greater underestimation with increasing body size. (b) Hemoglobin–weight heatmap comparing the New Standard Formula with the simplified dosing table (SDT), showing marked dose discrepancies across hemoglobin strata and weight categories, consistent with stepwise cut-off–based underdosing. Abbreviations: Hb, hemoglobin.

**Copyright Information:** (Online) ISSN: 2572-9241
©2026 The Author(s). HemaSphere is published by John Wiley & Sons Ltd on behalf of European Hematology Association. This is an open access Abstract Book distributed under the Attribution-NonCommercial-NoDerivs (CC BY-NC-ND), which allows third parties to download the articles and share them with others as long as they credit the author and the Abstract Book, but they cannot change the content in any way or use them commercially.
**Abstract Book Citations:** Authors, Title, HemaSphere, 2026;10:(S1):pages. The abstract book DOIs can be found on the "Author Information" page.
**Disclaimer:** Articles published in the journal HemaSphere exclusively reflect the opinions of the authors. The authors are responsible for all content in their abstracts including accuracy of the facts, statements, citing resources, etc.

### (PS2405) SERVICE EVALUATION OF PRE OPERATIVE ANAEMIA OPTIMISATION IN NHS AND PRIVATE PATIENTS

**Topic:** 30. Iron metabolism, deficiency and overload

Mona Osman[1], Silvia Lovato*[1]

[1]*Harefield Hospital, Guy's and St Thomas' NHS Foundation Trust, Haematology, Harefield, United Kingdom;*

### Background

Preoperative anaemia has been linked to increased likelihood of transfusion perioperatively or post operatively. In addition to that, preoperative anaemia has been linked to increased morbidity and mortality rates. Optimising patients preoperatively helps minimise perioperative risks.In our hospital we introduced a preoperative pathway for NHS patient that consist of tests to optimise patients before surgery including iron studies, vitamin B12 and folate, as recommended by the British Society for Haematology (2024).

### Aims

The main aim of this service evaluation was to evaluate the adherence to internal guidelines on preoperative anaemia, on testing and treatment in NHS and private patients (PP).

### Methods

We compiled a list of patients both NHS and private who were assessed for surgery between 01/09/2025 to 30/11/2025 and collected blood test results and treatment from electronic notes.
We considered the following normal ranges: Haemoglobin (M: 134 – 166 g/L, F 115 – 151 g/L), Ferritin (15 – 150 µg/L), Transferrin saturation:(20 – 45 %), B12: (182 – 692 ng/L), Folate (3.1 – 20.5 µg/L).

### Results

In total, 209 patients' data were collected, 85% NHS patients 15% private. Full blood count was done 100 % of patients. Anaemia was found in 23% patietns (49, 40 NHS, 9 PP). Ferritin was tested in 82% of patients ( 96%of NHS vs 6%of PP). Only 6 (3 %) NHS patients had low ferritin levels, 5 of them were anaemic. Transferrin saturation was tested in 85% of patients (98% NHS vs 10% PP). In total, 81/177 (46 %) patients had low transferrin saturation (<20 %). B12 and folate were tested in 88% of patients (97% NHS vs 3% PP). Low B12 was found in 10 (6 %) NHS patients, 4 of those were also anaemic and iron deficient. Low folate level was found in 2 (1 %) NHS patients, none of them were anaemic.Of the 49 anaemic patients 29 were iron deficient, (4 of those had combined B12 and iron deficiency, 10 were anaemic with no iron or haematinic's deficiency. In the remaining 10 cases (2NHS, 8 PP) the cause of anaemia was not fully investigated.
Regarding treatment of the 29 anaemic and iron deficient patients, 28 where NHS patients, 24 were fit for surgery and 87% (21/24) were treated with IV iron before the planned surgery. The only anaemic iron deficient private patient had surgery with no optimisation of iron deficient anaemia. Vitamin B 12 and folate were prescribed by the GP practice when needed.

**Copyright Information:** (Online) ISSN: 2572-9241
©2026 The Author(s). HemaSphere is published by John Wiley & Sons Ltd on behalf of European Hematology Association. This is an open access Abstract Book distributed under the Attribution-NonCommercial-NoDerivs (CC BY-NC-ND), which allows third parties to download the articles and share them with others as long as they credit the author and the Abstract Book, but they cannot change the content in any way or use them commercially.
**Abstract Book Citations:** Authors, Title, HemaSphere, 2026;10:(S1):pages. The abstract book DOIs can be found on the "Author Information" page.
**Disclaimer:** Articles published in the journal HemaSphere exclusively reflect the opinions of the authors. The authors are responsible for all content in their abstracts including accuracy of the facts, statements, citing resources, etc.

**Summary/Conclusion**

The pre- operative anaemia optimisation in our centre working well for NHS patients with appropriate investigations done on at least 96% of the patients and treatment in up to 87% of the patients. Private patients are not investigated or treated appropriately, the only patient who had full investigation and was found to be iron deficient was no treated at all. This poses a clinical risk for patients and can result in an increased risk of transfusion, prolonged hospitalisation and slow post operative recovery. For these reasons the plan is to discuss with the surgical team to apply the same pathway we currently use to private patients, this may require patients who are anaemic to delay their surgery and attend an extra clinic appointment to received IV iron, but the benefit of this should outweigh the risks discussed above.

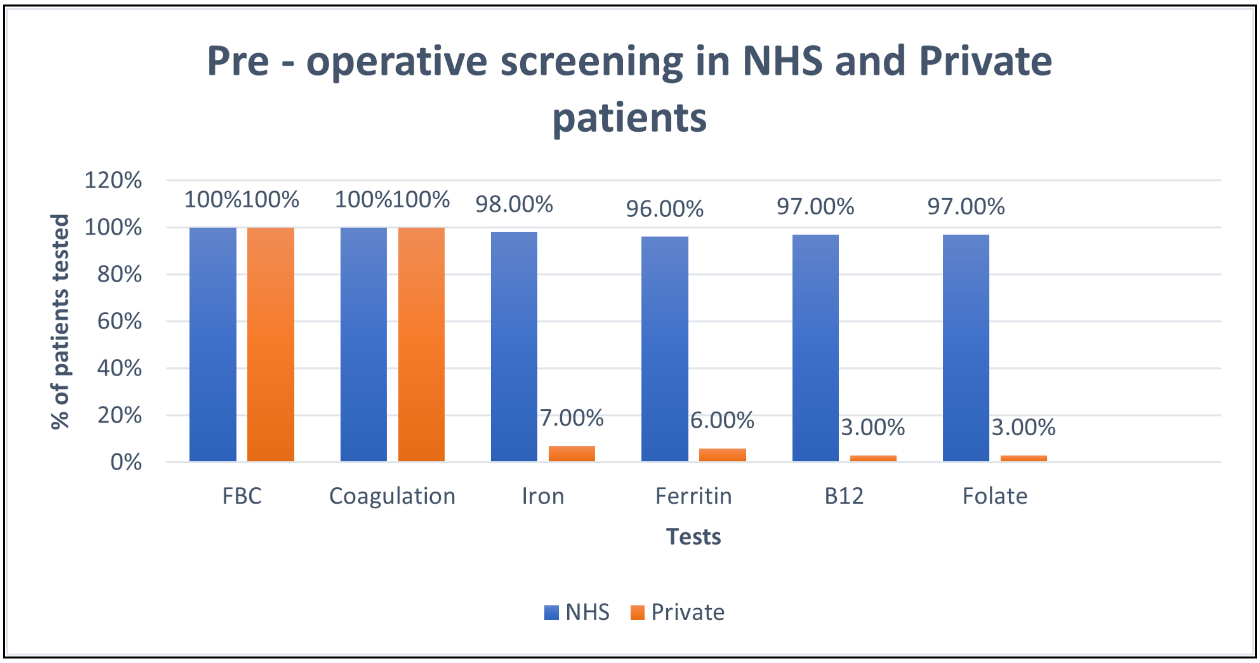

Comparison of pre op testing between NHS and private patients.

**Copyright Information:** (Online) ISSN: 2572-9241
©2026 The Author(s). HemaSphere is published by John Wiley & Sons Ltd on behalf of European Hematology Association. This is an open access Abstract Book distributed under the Attribution-NonCommercial-NoDerivs (CC BY-NC-ND), which allows third parties to download the articles and share them with others as long as they credit the author and the Abstract Book, but they cannot change the content in any way or use them commercially.
**Abstract Book Citations:** Authors, Title, HemaSphere, 2026;10:(S1):pages. The abstract book DOIs can be found on the "Author Information" page.
**Disclaimer:** Articles published in the journal HemaSphere exclusively reflect the opinions of the authors. The authors are responsible for all content in their abstracts including accuracy of the facts, statements, citing resources, etc.

### (PS2406) ARE IRON-RICH DIETARY SUPPLEMENTS AS EFFECTIVE AS MEDICINAL IRON IN THE TREATMENT OF IRON DEFICIENCY ANEMIA? A PROSPECTIVE STUDY OF LIPOSOMAL FERROUS BISGLYCINATE CHELATE

**Topic:** 30. Iron metabolism, deficiency and overload

Nour Siala*[1], Khouloud Mezghani[1], Farah Fakhfakh[1], Maha Charfi[1], Asma Koubaa[1], Imen Frikha[1], Faten Kallel[1], Hanen Charfi[1], Imen Ben Amor[1], Fakhfekh Yousra[1], Rahma Mallek[1], Ines Turki[1], Aicha Ben Kahla[1], Moez Medhaffar[1], Moez Elloumi[1]

[1]*Hematology department Hedi Chaker Hospital, Sfax, Tunisia;*

### Background

Iron deficiency anemia (IDA) remains the most prevalent hematologic disorder worldwide. Although conventional oral ferrous salts are widely used as first-line therapy, gastrointestinal intolerance and poor adherence frequently limit their effectiveness. Dietary iron supplements formulated with alternative iron compounds, such as liposomal ferrous bisglycinate chelate, have been proposed to improve bioavailability and tolerability. However, although randomized trials and meta-analyses have evaluated alternative oral iron formulations in specific populations, prospective real-world data in adults with iron deficiency anemia remain limited and heterogeneous.

### Aims

This study aimed to prospectively assess the hematologic response and tolerability of liposomal ferrous bisglycinate chelate in patients with IDA.

### Methods

We conducted a prospective single-center study in the Department of Hematology at Sfax Hospital between July 2024 and October 2025. Eligible participants were women aged <80 years with newly diagnosed IDA, defined as hemoglobin <12 g/dL associated with low serum ferritin (<13 ng/mL), consistent with iron deficiency. Patients with severe anemia (hb ≤8 g/dL) were excluded. All patients received oral liposomal ferrous bisglycinate chelate at a dose of 220 mg once daily. Hemoglobin and serum ferritin levels were measured at baseline (day 0) and after 1, 2, and 4 months of treatment. Treatment tolerability was assessed at each visit based on patient-reported gastrointestinal adverse events and treatment adherence.

### Results

A total of 20 patients were included. The mean age was 41 years (range : 18–62). At baseline, the mean hemoglobin was 9.7 g/dL (range : 8–11.6 g/dL) and the mean serum ferritin was 5.6 ng/mL (range : 1.5–9.8 ng/mL). At 1 month, the mean hemoglobin increased to 11 g/dL (range : 8.6-12.9 g/dL), representing a mean increase of 1.3 g/dL from baseline, and a mean serum ferritin was 9.5 ng/mL (range : 2.16-17.85 ng/mL). At 2 month, the mean hemoglobin increased to 11.6 g/dL (range : 9.1-14.2 g/dL), representing a mean increase of 2 g/dL from baseline, and a mean serum ferritin was 7.8 ng/mL (range : 2.59-15.6 ng/mL).
At 4 months, the mean hemoglobin was 12.1 g/dL (range : 9.9-15.2 g/dL), representing a mean increase of 2.4 g/dL from baseline, and the mean serum ferritin was 22 ng/mL (range : 1.5-91.6 ng/mL). A hemoglobin increase ≥2 g/dL was observed in 65% of patients. Treatment was well tolerated, with no treatment discontinuation due to gastrointestinal adverse events and good reported adherence throughout follow-up.

**Copyright Information:** (Online) ISSN: 2572-9241
©2026 The Author(s). HemaSphere is published by John Wiley & Sons Ltd on behalf of European Hematology Association. This is an open access Abstract Book distributed under the Attribution-NonCommercial-NoDerivs (CC BY-NC-ND), which allows third parties to download the articles and share them with others as long as they credit the author and the Abstract Book, but they cannot change the content in any way or use them commercially.
**Abstract Book Citations:** Authors, Title, HemaSphere, 2026;10:(S1):pages. The abstract book DOIs can be found on the "Author Information" page.
**Disclaimer:** Articles published in the journal HemaSphere exclusively reflect the opinions of the authors. The authors are responsible for all content in their abstracts including accuracy of the facts, statements, citing resources, etc.

**Summary/Conclusion**

Oral liposomal ferrous bisglycinate chelate induced a progressive and clinically meaningful increase in hemoglobin levels, with 65% of patients achieving an increase ≥2 g/dL at 4 months, and was associated with excellent gastrointestinal tolerability and high treatment adherence. Although conventional ferrous salts may result in a more rapid or pronounced hemoglobin response (typically >1 g/dL within a few weeks) and faster replenishment of iron stores, their use is frequently limited by gastrointestinal adverse events, reported in up to 60% of patients, which may lead to premature treatment discontinuation in approximately 10–30% of cases across published series.

In this context, liposomal ferrous bisglycinate chelate may represent a valuable therapeutic alternative in patients with mild-to-moderate iron deficiency anemia, particularly when long-term adherence is a key determinant of treatment success.

**Copyright Information:** (Online) ISSN: 2572-9241
©2026 The Author(s). HemaSphere is published by John Wiley & Sons Ltd on behalf of European Hematology Association. This is an open access Abstract Book distributed under the Attribution-NonCommercial-NoDerivs (CC BY-NC-ND), which allows third parties to download the articles and share them with others as long as they credit the author and the Abstract Book, but they cannot change the content in any way or use them commercially.
**Abstract Book Citations:** Authors, Title, HemaSphere, 2026;10:(S1):pages. The abstract book DOIs can be found on the “Author Information” page.
**Disclaimer:** Articles published in the journal HemaSphere exclusively reflect the opinions of the authors. The authors are responsible for all content in their abstracts including accuracy of the facts, statements, citing resources, etc.

### (PS4570) UNRAVELING THE ROLE OF KIELIN/CHORDIN-LIKE PROTEIN KCP AS A NOVEL MODULATOR OF HEPCIDIN IN HEPATOCYTES

**Topic:** 30. Iron metabolism, deficiency and overload

Rossana Carleo[1, 2], Antonella Nai[1], Sandro Altamura[3], Laura Silvestri*[1, 2], Alessia Pagani[1, 2], Mariateresa Pettinato[1, 2]

*[1]Ospedale San Raffaele, Milano, Italy; [2]Vita-Salute San Raffaele University, Milano, Italy; [3]University of Heidelberg, Heidelberg, Germany;*

### Background

Hepcidin, the master regulator of systemic iron, controls iron levels by inhibiting ferroportin. In the liver, its expression is regulated by the BMP-SMAD pathway, whose disruption causes hemochromatosis, including the rare type 2A (JH-2A) due to hemojuvelin (HJV) mutations. Current treatments like phlebotomy reduces iron overload but worsen hepcidin deficiency, highlighting the need for new strategies to enhance BMP-SMAD signaling and hepcidin. Kielin Chordin-like Protein (KCP) resembles HJV in function and may represent a novel approach to regulate hepcidin.

### Aims

This study aims to clarify the role of KCP in regulating iron metabolism and hepcidin expression through in vitro and in vivo approaches, with the goal of identifying a potential therapeutic target for JH-2A and other hepcidin-deficiency disorders.

### Methods

Kcp knockdown in primary murine hepatocytes and overexpression in HuH7 cells were used to assess hepcidin regulation. Kcp expression was analyzed in isolated liver cell types and in iron-loaded wild-type, Iron Refractory Iron Deficiency Anemia (IRIDA), and Hjv KO mice. Gene expression was measured by qRT-PCR.

### Results

To investigate whether KCP modulates the BMP-SMAD pathway and hepcidin in an autocrine manner, Kcp was silenced in murine primary hepatocytes (HCs), followed by treatment with BMP2 or BMP6 to activate BMP signaling. Unexpectedly, hepcidin and Id1 expression increased in Kcp-silenced HCs, while the response to BMP2 or BMP6 was largely unaffected, suggesting that in hepatocytes KCP primarily works as an inhibitor of hepcidin and BMP signaling rather than as an activator. Further studies using overexpression of KCP in human HuH7 cells transfected with a hepcidin promoter reporter vector demonstrated that KCP reduces luciferase activity under basal conditions, confirming its inhibitory effect on hepcidin expression. Evaluation of Kcp expression across different liver cell types revealed its higher abundance in hepatic stellate cells, indicating a potential paracrine role. Analysis of Kcp regulation in various mouse models of altered iron metabolism showed that its expression is mainly influenced by the distribution of iron among liver cells; indeed, it was reduced in Hjv-KO mice, which have defective hepcidin and BMP-SMAD signaling, suggesting a compensatory mechanism to maintain hepcidin levels. Conversely, Kcp expression was unchanged in iron-loaded wild-type mice, characterized by increased hepcidin/BMP-SMAD signaling, and in an IRIDA model, characterized by iron deficiency and elevated hepcidin and hepatic BMP-SMAD pathway, indicating that BMP-SMAD activation does not directly regulate Kcp. These findings were confirmed in HuH7 cells treated with BMP2 or a BMP inhibitor, and in vivo in wild-type mice injected with BMP2, supporting the conclusion that Kcp regulation is not directly dependent on BMP-SMAD signaling but is instead associated with altered liver iron metabolism.

**Copyright Information:** (Online) ISSN: 2572-9241
©2026 The Author(s). HemaSphere is published by John Wiley & Sons Ltd on behalf of European Hematology Association. This is an open access Abstract Book distributed under the Attribution-NonCommercial-NoDerivs (CC BY-NC-ND), which allows third parties to download the articles and share them with others as long as they credit the author and the Abstract Book, but they cannot change the content in any way or use them commercially.
**Abstract Book Citations:** Authors, Title, HemaSphere, 2026;10:(S1):pages. The abstract book DOIs can be found on the “Author Information” page.
**Disclaimer:** Articles published in the journal HemaSphere exclusively reflect the opinions of the authors. The authors are responsible for all content in their abstracts including accuracy of the facts, statements, citing resources, etc.

**Summary/Conclusion**

KCP primarily functions as an inhibitor of hepcidin in hepatocytes: its expression is high in hepatic stellate cells, suggesting a potential paracrine role. In vivo, it is regulated mainly by the distribution of iron among liver cells rather than by direct BMP-SMAD pathway activation. This highlights KCP as a potential key modulator of iron metabolism. Ongoing studies in Kcp KO mice aim to further define its in vivo role.

**Copyright Information:** (Online) ISSN: 2572-9241
©2026 The Author(s). HemaSphere is published by John Wiley & Sons Ltd on behalf of European Hematology Association. This is an open access Abstract Book distributed under the Attribution-NonCommercial-NoDerivs (CC BY-NC-ND), which allows third parties to download the articles and share them with others as long as they credit the author and the Abstract Book, but they cannot change the content in any way or use them commercially.
**Abstract Book Citations:** Authors, Title, HemaSphere, 2026;10:(S1):pages. The abstract book DOIs can be found on the "Author Information" page.
**Disclaimer:** Articles published in the journal HemaSphere exclusively reflect the opinions of the authors. The authors are responsible for all content in their abstracts including accuracy of the facts, statements, citing resources, etc.

### (PS2407) PERIPHERAL BLOOD MNGS VERSUS BLOOD CULTURE FOR POLYMICROBIAL BLOODSTREAM INFECTIONS IN HEMATOLOGICAL MALIGNANCY: A COMPARISON OF DIAGNOSTIC YIELD AND CLINICAL IMPACT

**Topic:** 31. Infections in hematology (incl. supportive care/therapy)

Tong Chen[1], Yuhong Wu[1], Rong Fu*[1]

[1] *Tianjin Medical University General Hospital, Tianjin, China;*

### Background

Polymicrobial bloodstream infection (PBSI) is common in patients with hematological malignancies, and linked to **high medical costs and mortality**. Compared with conventional blood culture (BC), which has inherent limitations including long turnaround time and low positivity, metagenomic next-generation sequencing (mNGS) has enables rapid detection of various pathogens without prior culture. However, mNGS's role in polymicrobial bloodstream infection (PBSI) remains challenging. For example, mNGS's accuracy in distinguishing true mixed infections from contamination is insufficiently validated. Furthermore, lack of uniform diagnostic criteria and clinical recognition also hinders its application.

### Aims

This study aimed to **compare the diagnostic performance of mNGS with conventional BC for identifying PBSI,** optimizing BSI diagnosis and assessing the prognosis of this high-risk population.

### Methods

We conducted a **single-center retrospective analysis** among **hematological malignancies complicated by bloodstream infection**s. Clinical data were collected, covering **demographic characteristics, clinical features of infection, and prognosis.**

### Results

A total of 175 BSI patients with malignant hematological diseases were enrolled in this study, all of whom had positive results for both BC and blood mNGS at the time of fever. Virus identified by mNGS were excluded since BC failed detecting. Both BC-positive and mNGS-positive cohorts (n=175 each) were divided into PBSI groups (≥2 pathogens detected in one test) and monomicrobial bloodstream infection bacteremia (MBSI) groups, with well-balanced clinical characteristics between the two subgroups in each cohort (p>0.05). In terms of pathogen distribution, mNGS detected a significantly higher proportion of fungi (20.7%), including Aspergillus (13.7%), Mucor (6.9%), and Pneumocystis (5.7%), which were barely detected by blood culture.
Notably, mNGS detected more or equal pathogens compared with BC in 78.86% of cases, including 35.43% with more pathogens covering those from BC. Importantly, mNGS showed a significantly higher positive rate in detecting mixed bacterial-fungal infections. Taking the final clinical diagnosis as the reference, the diagnostic performance of mNGS for PBSI was superior to that of BC: the sensitivity of mNGS was 86.17%, significantly higher than 53.19% of BC (p<0.001). The specificity, positive predictive value, and negative predictive value of mNGS were 95.06%, 95.29%, and 85.56%, respectively. The AUC of mNGS was 0.906 (95% CI: 0.857~0.956), notably higher than BC (AUC=0.747, 95% CI: 0.674~0.821), confirming its better diagnostic accuracy.
Regarding prognostic value, no significant difference in 30-day and 90-day survival rates was found between PBSI and MBSI groups classified by BC. However, patients with PBSI identified by mNGS had significantly lower 30-day and 90-day survival rates. Multivariate Logistic regression analysis further indicated that the number of pathogenic species reported by mNGS, together with age and baseline SOFA score, was an independent poor prognostic factor for 90-day mortality in these patients, highlighting the unique value of mNGS in prognostic assessment.

**Copyright Information:** (Online) ISSN: 2572-9241
©2026 The Author(s). HemaSphere is published by John Wiley & Sons Ltd on behalf of European Hematology Association. This is an open access Abstract Book distributed under the Attribution-NonCommercial-NoDerivs (CC BY-NC-ND), which allows third parties to download the articles and share them with others as long as they credit the author and the Abstract Book, but they cannot change the content in any way or use them commercially.
**Abstract Book Citations:** Authors, Title, HemaSphere, 2026;10:(S1):pages. The abstract book DOIs can be found on the "Author Information" page.
**Disclaimer:** Articles published in the journal HemaSphere exclusively reflect the opinions of the authors. The authors are responsible for all content in their abstracts including accuracy of the facts, statements, citing resources, etc.

### Summary/Conclusion

Blood mNGS was superior to BC in distinguishing patient prognosis according to the number of detected species.

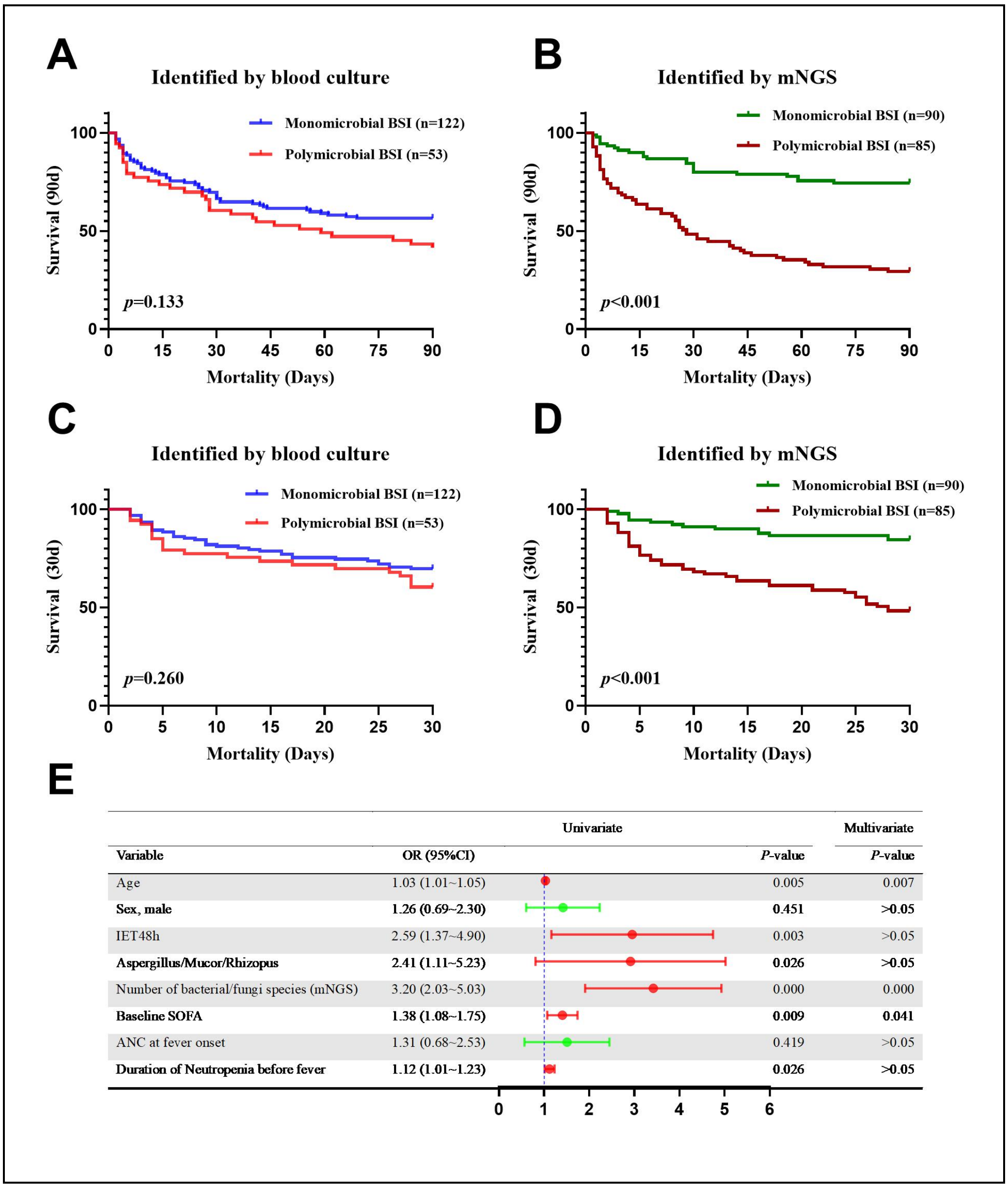

mNGS exerts a unique value in prognostic assessment in polymicrobial bloodstream infection with hematological malignancies.

**Copyright Information:** (Online) ISSN: 2572-9241
©2026 The Author(s). HemaSphere is published by John Wiley & Sons Ltd on behalf of European Hematology Association. This is an open access Abstract Book distributed under the Attribution-NonCommercial-NoDerivs (CC BY-NC-ND), which allows third parties to download the articles and share them with others as long as they credit the author and the Abstract Book, but they cannot change the content in any way or use them commercially.
**Abstract Book Citations:** Authors, Title, HemaSphere, 2026;10:(S1):pages. The abstract book DOIs can be found on the "Author Information" page.
**Disclaimer:** Articles published in the journal HemaSphere exclusively reflect the opinions of the authors. The authors are responsible for all content in their abstracts including accuracy of the facts, statements, citing resources, etc.

### (PS2408) HEMASHIELD STUDY: INCIDENCE OF SEVERE INFECTIONS AND CMV REACTIVATION IN PATIENTS WITH HEMATOLOGIC MALIGNANCIES TREATED WITH BISPECIFIC ANTIBODIES: A PROSPECTIVE STUDY.

**Topic:** 31. Infections in hematology (incl. supportive care/therapy)

Maria Stefania Infante*[1], Sigrun Einarsdottir[2], Carolina Garcia-Vidal[3], Ida Ivek[4], Ana Isabel Jimenez Ubieto[5], Maria Ljungqvist[2], Alejandro Avendaño Pita[6], Dario Leotta[7], Johan Lund[2], Bb Sivrikava[8], Güldane Cengiz Seval[9], Monika M. Biernat[10], Jens VAN Praet[11], Lucia Prezioso[12], Cristian Gutierrez-Padilla[5], Toni Valković[13], Marta Sitges Arriaga[14], Caterina Buquicchio[15], Juan Carlos Caballero[16], Anna Nordlander[2], Patricia García Ramírez[17], Itziar Carro Arostegui[18], Beatriz Aguado Bueno[19], Roberta Della Pepa[20], Margherita Malchiodi[21], Davide Nappi[22], Nicola Sgherza[23], Alessandra Romano[24], Jorge Labrador[25], Claudio Cerchione[22], Michaela Hornakova[26], Antonio Giovanni Solimando[27], Konstantinos Lemonakis[28], Hani Saghir[2], Simge Erdem[29], Nicolò Danesin[30], Carolina Martinez Gomez[18], Joanna Drozd-Sokołowska[31], Amaia Mata Gonzalez[32], Magdalena Corona[5], Cristina Anton[33], Monika Swierkowska[34], Balazs Kapas[35], Pellegrino Musto[36], Sofia Cortesi[22], Ildefonso Espigado[37], Amparo Santamaria[33], Adaia Albasanz-Puig[38], Donald C Vinh[39], Petros Ioannou[40], Helen Papadaki[40], Xristoforos Xontos[40], Cecilie Hveding Blimark[2], Jannik Stemler[41], Ana Maria Garcia-Noblejas Moya[19], Julia de Amaral Melo Medeiros[42], Elena Landete[1], Livio Pagano[43], Oliver Cornely[41], Jon Salmanton-García[41]

*[1]University Hospital Infanta Leonor, Madrid, Spain; [2]sahlgrenska university hospital, gothenburg, Sweden; [3]hospital clinic, barcelona, Spain; [4]university hospital centre zagreb, zagreb, Croatia; [5]HOSPITAL 12 DE OCTUBRE, madrid, Spain; [6]Hospital Universitario de Salamanca, Salamanca, Spain; [7]Policlinico G.Rodolico San Marco, Catania, Italy; [8]Samsun University Training and Research Hospital, samsun, Türkiye; [9]Anakara Oncology Hospital, Ankara, Türkiye; [10]Department of Haematology, Blood Neoplasms, and Bone Marrow Transplantation, Wroclaw Medical University, wroclaw, Poland; [11]Department of Nephrology and Infectious diseases, AZ Sint-Jan Brugge-Oostende AV, bruges, Belgium; [12]Hospital University of Parma - Hematology and Bone Marrow Unit, Parma, Italy; [13]University Hospital Center Rijeka, rijeka, Croatia; [14]ICO GIRONA, girona, Spain; [15]Ematologia con Trapianto, Ospedale Dimiccoli, Barletta, Italy; [16]Fundacion Jimenez Diaz University Hospital, Madrid, Spain; [17]University Hospital Principe de Asturias, Alcala de Henares, Spain; [18]Institut Català d'Oncologia (ICO), Hospital Duran i Reynals, IDIBELL, L'Hospitalet de Llobregat, Spain; [19]hospital de la princesa, madrid, Spain; [20]department of hematology, universitá degli studi di napoli, napoli, Italy; [21]Università degli Studi di Siena, siena, Italy; [22]Istituto Romagnolo per lo Studio dei Tumori "Dino Amadori" - IRST IRCCS, Meldola, Italy; [23]Department of Precision and Regenerative Medicine and Ionian Area, "Aldo Moro" University School of Medicine, and Unit of Hematology and Stem Cell Transplantation, AOUC Policlinico, bari, Italy; [24]Policlinico Rodolico-San Marco, catania, Italy; [25]Department of Hematology, Research Unit, Hospital Universitario de Burgos, Burgos, Spain Facultad de Ciencias de la Salud, Universidad Isabel I, Burgos, Spain; [26]university hospital ostrava, ostrava, Czechia; [27]DIMEPRe-J university of bari, bari, Italy; [28]Skane University Hospital, Lund, Sweden; [29]division of hematology, instanbul faculty of medicine, instanbul, Türkiye; [30]Hematology Unit, Department of Medicine, University of Padova, Padova, Italy; [31]medical university of warsaw, warsaw, Poland; [32]donostia university hospital, san sebastian, Spain; [33]University Hospital Vinalopó, elche, Spain; [34]maria sklodowska-curie national research institute of oncology, warsaw, Poland; [35]Lund university hospital, Lund, Sweden; [36]Department of Precision and Regenerative Medicine and Ionian Area, "Aldo Moro" University School of Medicine, and Unit of Hematology and Stem Cell Transplantation, AOUC Policlinico, Bari, Italy; [37]Hospital Universitario Virgen Macarena - Hospital Universitario Virgen del Rocío, IBiS/CSID, sevilla, Spain; [38]vall d'hebron barcelona hospital campus, barcelona, Spain; [39]McGill University Health centre, Montreal, Canada; [40]University of Crete & University hospital of Heraklion, Heraklion, Greece; [41]Institute of Translational Research, Cologne Excellence Cluster on Cellular Stress Responses in Aging-Associated Diseases (CECAD), Faculty of Medicine and University Hospital. Department I of Internal Medicine, Center for Integrated Oncology Aachen Bonn Cologne Duesseldorf (CIO ABCD) and Excellence Center for Medical Mycology (ECMM), University of Cologne, Faculty of Medicine, and University Hospital Cologne, Centre for Infection Research (DZIF), Partner Site Bonn-Cologne Department, Cologne, Germany; [42]Hospital Lusiadas, Lisboa, Portugal; [43]Hospital Gemelli, Rome, Italy;*

**Copyright Information:** (Online) ISSN: 2572-9241
©2026 The Author(s). HemaSphere is published by John Wiley & Sons Ltd on behalf of European Hematology Association. This is an open access Abstract Book distributed under the Attribution-NonCommercial-NoDerivs (CC BY-NC-ND), which allows third parties to download the articles and share them with others as long as they credit the author and the Abstract Book, but they cannot change the content in any way or use them commercially.
**Abstract Book Citations:** Authors, Title, HemaSphere, 2026;10:(S1):pages. The abstract book DOIs can be found on the "Author Information" page.
**Disclaimer:** Articles published in the journal HemaSphere exclusively reflect the opinions of the authors. The authors are responsible for all content in their abstracts including accuracy of the facts, statements, citing resources, etc.

### Background

Bispecific antibodies (BsAbs) have transformed the treatment landscape for multiple myeloma (MM) and B-cell non-Hodgkin lymphomas (B-NHL). However, they are associated with an increased risk of infections due to B and T-cell depletion, neutropenia, and hypogammaglobulinemia. Characterizing the incidence of infections and Cytomegalovirus (CMV) reactivation in this setting is crucial for optimizing patient management.

### Aims

To prospectively evaluate the incidence, microbiological spectrum, and risk factors for severe infections and CMV reactivation in patients with B-NHL and MM treated with BsAbs.

### Methods

This multicenter prospective observational study enrolled 385 patients across 44 international centers between November 2021 and January 2026. Severe infectious events were defined as grade >3 by CTCAE  criteria.

### Results

Of the 385 patients included, 116 had B-NHL and 269 had MM. Median follow up was 197 days (92-459). The median age was 67 (range 22–88) and 56.4% were male. The median number of prior lines of therapy was 3 (range 2–5). Prior treatments included allo-HSCT (1.8%), auto-HSCT (48.3%), and CAR-T therapy (8.3%). The most frequently used BsAbs for MM were elranatamab (31.6%), teclistamab (28.3%), and talquetamab (24.5%), while epcoritamab (39.7%) and glofitamab (32.8%) were the most common for B-NHL. Prophylaxis for *Pneumocystis jirovecii* pneumonia (PJP) and HSV/VZV was administered to 86.5% and 93.2% of patients, respectively. Grade 3 neutropenia occurred in 30.6% of cases (21.6% B-NHL vs. 34.6% MM, p=0.011); hypogammaglobulinemia before BsAbs start (<4g/dL) was detected in 21.3% of patients (12.9% B-NHL vs. 24.9% MM, p=0.008) and manteined during BsAbs in 38.4% of patients (23.3%B-NLH vs 45% MM p<0.001). Ig replacement was prescribed in 18.4% of patients.
Regarding infections, 110 (28.2%) patients experienced at least one severe infection. Neutopenia gr3 was a significant risk factor for any infection (p= 0.007) and no differences were found between B-NHL and MM patients in terms of median time to first infection (84 vs. 83 days) or time to resolution (11 vs. 13 days). Lung infections (49.8%) and bacteremia (16.3%) were the most frequent sites. Bacterial infections were the most common (47.4%), primarily gram-negative etiologies such as *E. coli* (7.7%) and *P. aeruginosa* (3.9%). Viral infections followed (44%), with a high incidence of community-acquired respiratory viruses (SARS-CoV-2 9.9%; Influenza 4.3%). (Figure 1).
CMV was monitored in 35% of patients (weekly in 22%; monthly in 78%). CMV clinically significant reactivation occurred in 15 (3.8%) patients; organ-invasive CMV disease occurred in 5 (1.2%) patients. Infections led to temporary BsAb discontinuation in 40.6% of B-NHL vs. 65.9% of MM patients (p<0.001), while permanent discontinuation was higher in B-NHL patients(26.1% vs. 8.5%, p<0.001). Delays on BsAb administration were up to 76%.
Infections required ICU admission in 11.2% of the patients. The infection-related mortality rate was 2.4% (n=11).

### Summary/Conclusion

The HEMASHIELD study demonstrates a high burden of severe infections (28.2%) during BsAb therapy, primarily within the first 90 days of treatment. While CMV reactivation remains relatively infrequent (3.8%), infectious complications significantly compromise treatment intensity and survival.The findings of this first large-scale multicenter prospective study underscore the need for standardized monitoring and specific strategies to safeguard the therapeutic window of BsAb therapy in hemato-oncology.

**Copyright Information:** (Online) ISSN: 2572-9241
©2026 The Author(s). HemaSphere is published by John Wiley & Sons Ltd on behalf of European Hematology Association. This is an open access Abstract Book distributed under the Attribution-NonCommercial-NoDerivs (CC BY-NC-ND), which allows third parties to download the articles and share them with others as long as they credit the author and the Abstract Book, but they cannot change the content in any way or use them commercially.
**Abstract Book Citations:** Authors, Title, HemaSphere, 2026;10:(S1):pages. The abstract book DOIs can be found on the “Author Information” page.
**Disclaimer:** Articles published in the journal HemaSphere exclusively reflect the opinions of the authors. The authors are responsible for all content in their abstracts including accuracy of the facts, statements, citing resources, etc.

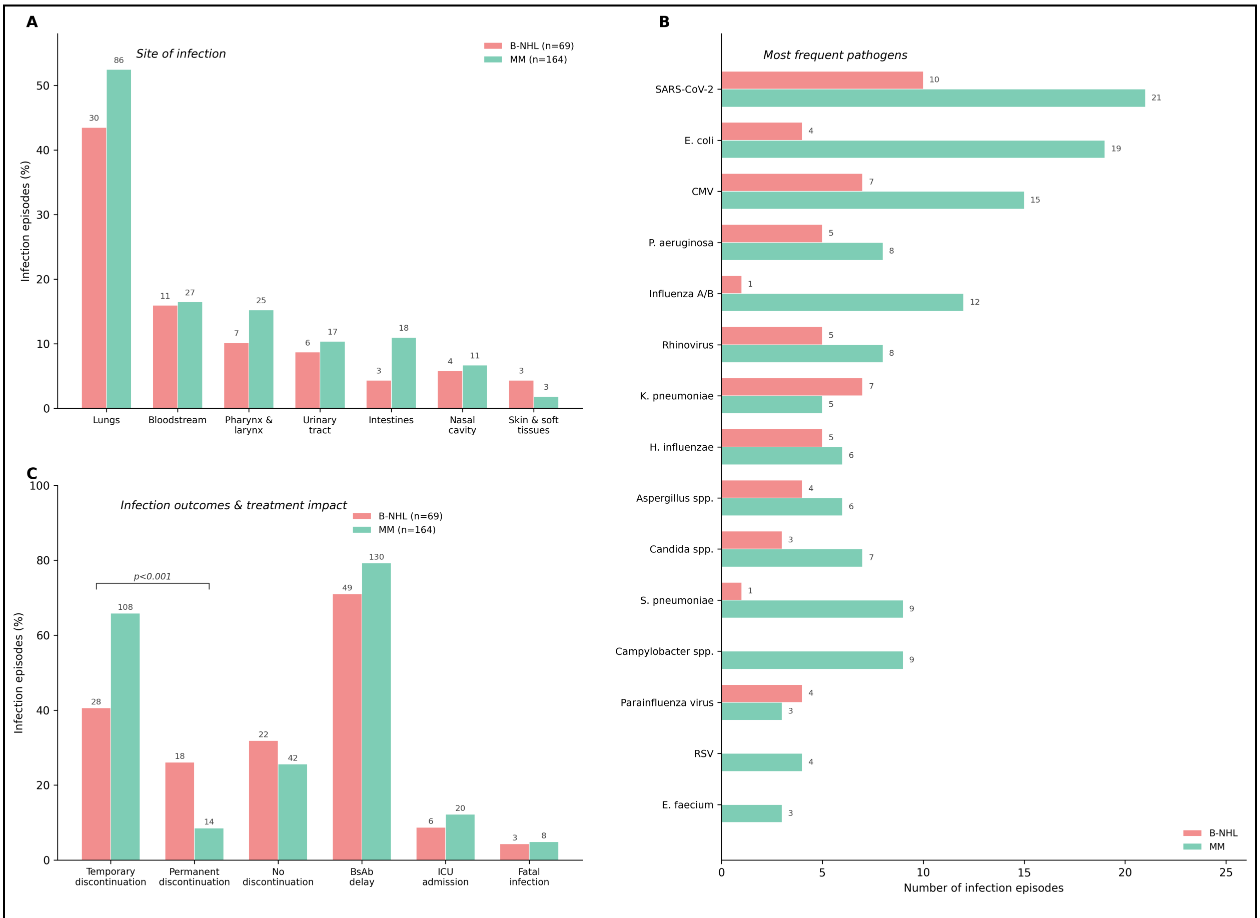

*Panel B: Polymicrobial infections are counted per individual pathogen. Influenza A and B combined.*
*Panel C: p-values for BsAb discontinuation comparing B-NHL vs MM (chi-square test).*

**Copyright Information:** (Online) ISSN: 2572-9241
©2026 The Author(s). HemaSphere is published by John Wiley & Sons Ltd on behalf of European Hematology Association. This is an open access Abstract Book distributed under the Attribution-NonCommercial-NoDerivs (CC BY-NC-ND), which allows third parties to download the articles and share them with others as long as they credit the author and the Abstract Book, but they cannot change the content in any way or use them commercially.
**Abstract Book Citations:** Authors, Title, HemaSphere, 2026;10:(S1):pages. The abstract book DOIs can be found on the "Author Information" page.
**Disclaimer:** Articles published in the journal HemaSphere exclusively reflect the opinions of the authors. The authors are responsible for all content in their abstracts including accuracy of the facts, statements, citing resources, etc.

### (PS2409) RAPID DIAGNOSTICS AND TARGETED COLONIZATION MANAGEMENT FOR CARBAPENEM-RESISTANT ENTEROBACTERIACEAE IN PEDIATRIC ONCOLOGY: IMPACT ON CLINICAL OUTCOMES

**Topic:** 31. Infections in hematology (incl. supportive care/therapy)

Sally Mahfouz*[1]

[1]*children cancer hospital CCHE 57357, Pediatric oncology, Cairo, Egypt;*

### Background

Carbapenem-resistant Enterobacteriaceae (CRE) infections in pediatric cancer patients are associated with significant morbidity and high mortality, with *Escherichia coli* and *Klebsiella pneumoniae* being the most frequently isolated pathogens. CRE resistance is primarily mediated by carbapenemase production, including KPC, metallo-β-lactamases such as NDM, and OXA-48-like enzymes, with marked geographic variation in their distribution. Children with hematological malignancies are particularly vulnerable due to chemotherapy-induced neutropenia, mucosal barrier injury, prolonged hospitalization, and broad-spectrum antibiotic exposure. CRE colonization markedly increases the risk of subsequent bloodstream infection and death, with reported mortality rates approaching 60% in neutropenic patients. International guidelines recommend active surveillance and strict infection prevention strategies; however, limited access to newer antimicrobial agents in resource-constrained settings underscores the need for improved risk stratification and targeted interventions.

### Aims

This study aimed to evaluate the impact of routine rectal swab surveillance and rapid PCR-based detection of carbapenemase genes to facilitate the early initiation of appropriate treatment and assess its effects on outcomes

### Methods

The study compared two groups of pediatric cancer patients with CRE bloodstream infections: a retrospective cohort of 254 patients from 2013 to 2017, and a prospective cohort of 186 patients from 2020 to 2022, following the implementation of these tools.

### Results

A rapid diagnostic test in the prospective cohort resulted in the early initiation of proper antibiotics in 85% (165/186) of patients, compared to only 58% (147/254) in the retrospective group. This led to a decrease in the need for ICU admission related to sepsis from CRE and a significant reduction in the 30-day mortality rate (16% vs. 30%, *p* ). Genotypic profiling revealed that class B carbapenemases were the most prevalent (69%), with the NDM type being identified in 67% of patients. and KPC enzymes were detected in 59% and 4% of patients, respectively. Multivariate analysis revealed that patients having Klebsiella pneumoniae, NDM genotype carbapenemases, presence of pneumonia, and septic shock requiring ICU admission were predictors of poor outcomes.

**Copyright Information:** (Online) ISSN: 2572-9241
©2026 The Author(s). HemaSphere is published by John Wiley & Sons Ltd on behalf of European Hematology Association. This is an open access Abstract Book distributed under the Attribution-NonCommercial-NoDerivs (CC BY-NC-ND), which allows third parties to download the articles and share them with others as long as they credit the author and the Abstract Book, but they cannot change the content in any way or use them commercially.
**Abstract Book Citations:** Authors, Title, HemaSphere, 2026;10:(S1):pages. The abstract book DOIs can be found on the "Author Information" page.
**Disclaimer:** Articles published in the journal HemaSphere exclusively reflect the opinions of the authors. The authors are responsible for all content in their abstracts including accuracy of the facts, statements, citing resources, etc.

**Summary/Conclusion**

Rapid diagnostics and targeted colonization lead to the appropriate use of targeted antibiotics, resulting in improved patient outcomes. Understanding carbapenemase-producing microorganisms and administering newer antibiotics may further reduce mortality and enhance treatment strategies for high-risk patients.

**Copyright Information:** (Online) ISSN: 2572-9241
©2026 The Author(s). HemaSphere is published by John Wiley & Sons Ltd on behalf of European Hematology Association. This is an open access Abstract Book distributed under the Attribution-NonCommercial-NoDerivs (CC BY-NC-ND), which allows third parties to download the articles and share them with others as long as they credit the author and the Abstract Book, but they cannot change the content in any way or use them commercially.
**Abstract Book Citations:** Authors, Title, HemaSphere, 2026;10:(S1):pages. The abstract book DOIs can be found on the "Author Information" page.
**Disclaimer:** Articles published in the journal HemaSphere exclusively reflect the opinions of the authors. The authors are responsible for all content in their abstracts including accuracy of the facts, statements, citing resources, etc.

### (PS2410) ECTOPIC EBV INFECTION OF HEMATOPOIETIC STEM CELLS DRIVES T-CELL DIFFERENTIATION BLOCKADE VIA STAT2/IRF9-MEDIATED SENESCENCE

**Topic:** 31. Infections in hematology (incl. supportive care/therapy)

Zhuojun Liu*[1], Pei Xuying[1], Zhifan Zhao[1], Zhipeng Zhou[1], Yuxuan Du[1], Xiangyu Zhao[1], Xiaojun Huang[1]

[1]*Peking University People's Hospital, Peking University Institute of Hematology, National Clinical Research Center for Hematologic Disease，Beijing Key Laboratory of Cell and Gene Therapy for Hematologic Malignancie, Beijing, China;*

### Background

Epstein–Barr virus (EBV) infection is closely associated with a variety of malignancies and autoimmune diseases, and often leads to potentially fatal complications following hematopoietic stem cell transplantation (HSCT) due to impaired immune reconstitution. Recent evidence suggests that EBV can ectopically infect hematopoietic stem cells (HSCs), which represents a pathogenic mechanism underlying chronic active EBV infection. Our previous studies have also demonstrated that EBV can infect HSCs after HSCT and that this infection is associated with delayed T-cell reconstitution post-transplantation. However, the molecular mechanisms by which EBV infects HSCs and how it regulates hematopoietic differentiation remain unclear. In particular, how EBV infection reshapes HSC fate decisions and why it selectively impairs T-cell lineage differentiation are still unknown.

### Aims

This study aims to elucidate the mechanisms underlying EBV-induced HSC senescence and subsequent T-lineage differentiation blockade, focusing specifically on the transcriptional regulatory role of the STAT2/IRF9 signaling axis.

### Methods

Bone marrow (BM) and peripheral blood (PB) samples were collected from 4 HSCT patients with persistent EBV infection and 4 matched HSCT controls without complications. Single-cell RNA sequencing (scRNA-seq) was utilized to evaluate immune cell profiles, lineage trajectories, and transcriptional networks. The direct ectopic infection of CD34+CD38- HSCs was validated using EBER-Primeflow and EphA2 inhibitor. Cellular senescence and functional impairment of HSCs were assessed through flow cytometry by measuring reactive oxygen species (ROS), senescence-associated β-galactosidase (SA-β-gal) activity, GATA2 stemness marker, and p-STAT2 expression, alongside in vitro Colony-Forming Unit (CFU) assays.

### Results

ScRNA-seq analysis revealed a significant depletion of naïve CD8+ and CD4+ T cells in the BM and PB of patients with severe EBV infection. Pseudotime analysis demonstrated that the remaining T cells exhibited profound terminal exhaustion. Within the HSC compartment, pathways associated with T-cell differentiation were markedly suppressed, and overall multilineage differentiation potential was decreased. Transcription factor network analysis identified the STAT2/IRF9 signaling axis as the most abnormally upregulated pathway in EBV-infected HSCs. EBER-Primeflow confirmed the direct presence of high-abundance EBER transcripts within HSCs, and blocking the EphA2 receptor significantly attenuated this viral entry. Phenotypically, EBV-infected HSCs demonstrated severe stemness loss via downregulated GATA2, massive intracellular ROS accumulation, enhanced SA-β-gal activity, and significantly upregulated p-STAT2 protein expression. Furthermore, CFU assays dynamically revealed that infected HSCs initially underwent a "myeloid bias," which progressively deteriorated into a complete exhaustion of hematopoietic differentiation potential.

**Copyright Information:** (Online) ISSN: 2572-9241
©2026 The Author(s). HemaSphere is published by John Wiley & Sons Ltd on behalf of European Hematology Association. This is an open access Abstract Book distributed under the Attribution-NonCommercial-NoDerivs (CC BY-NC-ND), which allows third parties to download the articles and share them with others as long as they credit the author and the Abstract Book, but they cannot change the content in any way or use them commercially.
**Abstract Book Citations:** Authors, Title, HemaSphere, 2026;10:(S1):pages. The abstract book DOIs can be found on the "Author Information" page.
**Disclaimer:** Articles published in the journal HemaSphere exclusively reflect the opinions of the authors. The authors are responsible for all content in their abstracts including accuracy of the facts, statements, citing resources, etc.

### Summary/Conclusion

EBV ectopically infects HSCs via the EphA2 receptor, driving premature cellular senescence and a severe T-lineage differentiation blockade through the hyperactivation of the STAT2/IRF9 signaling axis. This mechanism fundamentally explains the profound T-cell immunodeficiency observed in post-HSCT EBV infections. Targeting the upstream JAK/STAT pathway with inhibitors may present a promising therapeutic strategy to reverse HSC senescence and actively restore endogenous T-cell immune reconstitution.

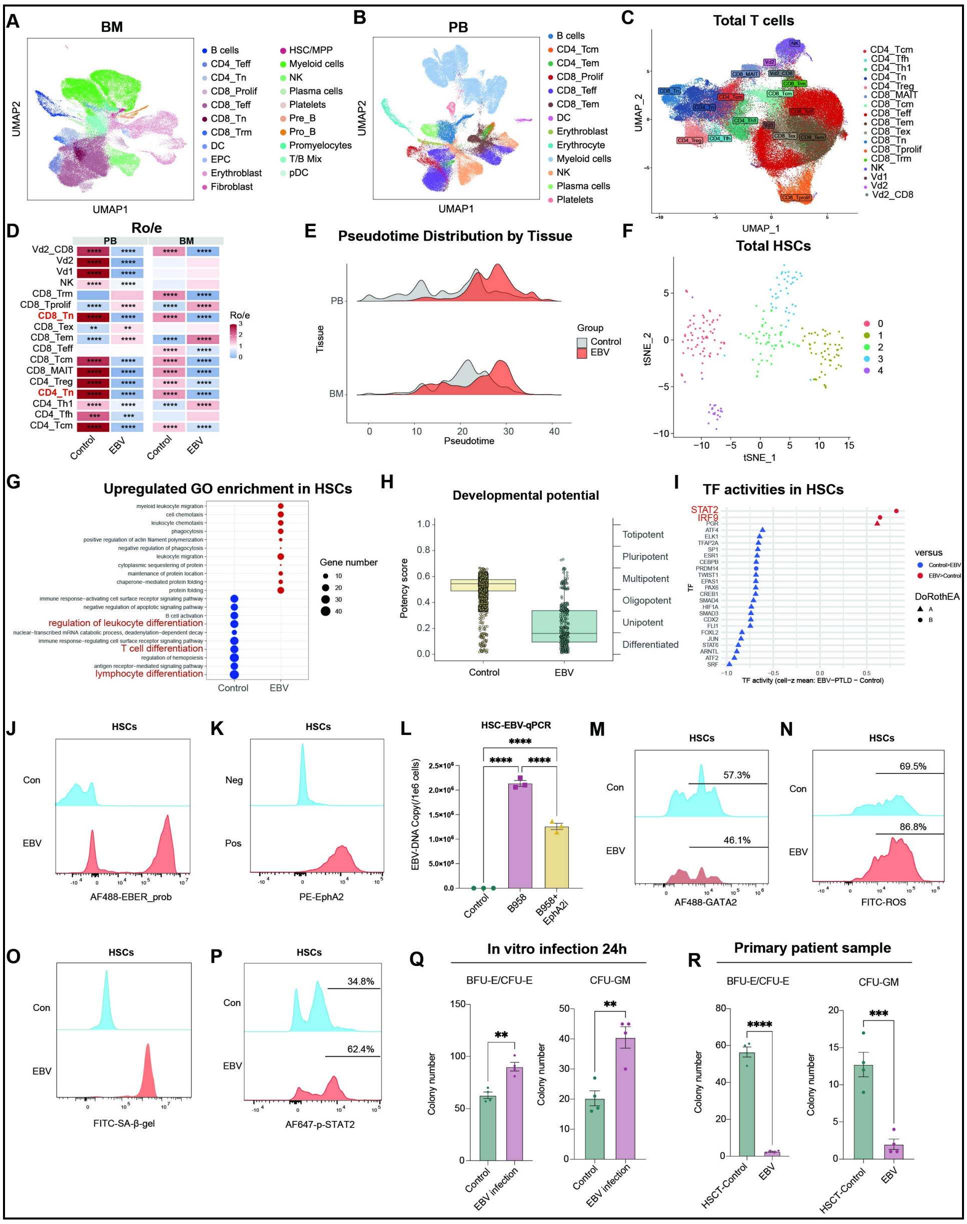

(A)UMAP dotplot of bone marrow samples from 4 HSCT patients with EBV infection and 4 matched HSCT controls without complications. (B) UMAP dotplot of peripheral blood samples from 4 HSCT patients with EBV infection and 4 matched HSCT controls without complications. (C) UMAP clustering of total T cells integrated from all BM and PB samples. (D) The ratio of observed to expected (Ro/e) cell frequencies for T cell subsets in PB and BM between the Control and EBV groups. Statistical significance was determined using Fisher's exact test. *p < 0.05, **p < 0.01, ***p < 0.001, ****p < 0.0001. (E) Pseudotime density plots comparing the developmental trajectories of cells in PB and BM between the Control and EBV groups. (F) t-SNE plot of total HSCs from bone marrow samples. (G) Gene

**Copyright Information:** (Online) ISSN: 2572-9241
©2026 The Author(s). HemaSphere is published by John Wiley & Sons Ltd on behalf of European Hematology Association. This is an open access Abstract Book distributed under the Attribution-NonCommercial-NoDerivs (CC BY-NC-ND), which allows third parties to download the articles and share them with others as long as they credit the author and the Abstract Book, but they cannot change the content in any way or use them commercially.
**Abstract Book Citations:** Authors, Title, HemaSphere, 2026;10:(S1):pages. The abstract book DOIs can be found on the "Author Information" page.
**Disclaimer:** Articles published in the journal HemaSphere exclusively reflect the opinions of the authors. The authors are responsible for all content in their abstracts including accuracy of the facts, statements, citing resources, etc.

Ontology (GO) enrichment analysis of upregulated genes in HSCs (Control vs. EBV). (H) the developmental potential (potency score) of HSCs in the Control and EBV groups. (I) Transcription factor (TF) activities in HSCs (Control vs. EBV). Key TFs with significantly upregulated activity (STAT2 and IRF9) are highlighted in red. (J) Flow cytometry histograms showed the expression levels of EBER via EBER_prob in HSCs from the Control and EBV groups. (K) Flow cytometry histograms showed the expression of EphA2 in HSCs. (L) EBV DNA copy numbers (per 10^6 cells) in HSCs as measured by qPCR. Comparisons are shown among the Control, B958-infected, and EphA2 inhibitor-treated (B958+EphA2i) groups, ****p < 0.0001. (M) Flow cytometry histograms showed the expression levels of GATA2 in HSCs from the Control and EBV groups. (N) Flow cytometry histograms showed the levels of ROS in HSCs from the Control and EBV groups. (O) Flow cytometry histograms showed the sesenescence-associated β-galactosidase (SA-β-gal) activity in HSCs from the Control and EBV groups. (P) Flow cytometry histograms showed the expression levels of p-STAT2 in HSCs from the Control and EBV groups. (Q) Colony-forming unit (CFU) assays following 24 hours of in vitro infection with B958. Bar graphs summarize the number of erythroid (BFU-E/CFU-E) and granulocyte-macrophage (CFU-GM) colonies in the Control and EBV-infected groups. **p < 0.01. (R) CFU assays using HSCT-Control and EBV patient samples. ***p < 0.001, ****p < 0.0001.

**Copyright Information:** (Online) ISSN: 2572-9241
©2026 The Author(s). HemaSphere is published by John Wiley & Sons Ltd on behalf of European Hematology Association. This is an open access Abstract Book distributed under the Attribution-NonCommercial-NoDerivs (CC BY-NC-ND), which allows third parties to download the articles and share them with others as long as they credit the author and the Abstract Book, but they cannot change the content in any way or use them commercially.
**Abstract Book Citations:** Authors, Title, HemaSphere, 2026;10:(S1):pages. The abstract book DOIs can be found on the "Author Information" page.
**Disclaimer:** Articles published in the journal HemaSphere exclusively reflect the opinions of the authors. The authors are responsible for all content in their abstracts including accuracy of the facts, statements, citing resources, etc.

### (PS2411) INTEGRATIVE BIOINFORMATIC AND REAL-WORLD DATA ANALYSIS REVEAL MECHANISTIC ASSOCIATIONS BETWEEN LETERMOVIR PROPHYLAXIS AND EBV REACTIVATION AFTER ALLOGENIC HEMATOPOIETIC STEM CELL TRANSPLANTATION

**Topic:** 31. Infections in hematology (incl. supportive care/therapy)

Zhangjie Chen*[1], Sisi Zhen[1], Xin Chen[1], Sizhou Feng[1]

[1]*Institute of Hematology & Blood Diseases Hospital, Chinese Academy of Medical Sciences & Peking Union Medical College, Tianjin, China;*

### Background

Letermovir, a cytomegalovirus (CMV)-specific terminase inhibitor, has become an essential prophylactic agent in allogeneic hematopoietic stem cell transplantation (allo-HSCT). However, our previous research and other evidences have linked its use to increased Epstein-Barr virus (EBV) reactivation.

### Aims

We aim to investigate the mechanisms underlying the association between letermovir treatment and EBV reactivation.

### Methods

We integrated pharmacoinformatics, transcriptomic validation, and real-world clinical data to investigate letermovir-EBV associations. EBV- and letermovir-associated targets were mined from public databases, with overlapping targets analyzed for hub protein identification. Molecular docking assessed letermovir binding affinities, and GO/KEGG/Reactome enrichment identified key pathways. Gene expression of overlapping and hub targets was validated using GEO datasets modeling EBV reactivation. Clinical data from 276 haploidentical HSCT recipients (145 received letermovir prophylaxis, 131 without) were analyzed for EBV-associated outcomes, with Cox regression used to construct a prediction model.

### Results

Fifty-eight overlapping targets were identified. Hub proteins included EGFR, ERBB2, HIF1A, PPARG, PTGS2 and STAT3, with molecular docking indicating stable binding to letermovir. Enrichment analysis identified hypoxia response, immune/inflammatory, and endothelial/angiogenic pathways. GEO datasets confirmed stage-specific expression of overlapping and hub gene during EBV reactivation. Clinically, patients receiving letermovir prophylaxis after HSCT had higher EBV viraemia, EBV infection and clinically significant EBV infection rates. Risk factors for EBV infection included early elevation of thrombin-antithrombin complex (TAT), D-dimer, and pro-inflammatory cytokines among LTV group patients, different from those without letermovir. A prediction model demonstrated good performance and clinical utility, stratifying patients into high- and low-risk groups with significantly different EBV infection.

### Summary/Conclusion

The use of letermovir for prophylaxis may contribute to a higher risk of EBV reactivation post allo-HSCT by disrupting key biological pathways involved in hypoxia, inflammation, and endothelial function. Our integrative model facilitates early identification of patients at high risk of EBV reactivation, guiding monitoring and intervention strategies.

**Copyright Information:** (Online) ISSN: 2572-9241
©2026 The Author(s). HemaSphere is published by John Wiley & Sons Ltd on behalf of European Hematology Association. This is an open access Abstract Book distributed under the Attribution-NonCommercial-NoDerivs (CC BY-NC-ND), which allows third parties to download the articles and share them with others as long as they credit the author and the Abstract Book, but they cannot change the content in any way or use them commercially.
**Abstract Book Citations:** Authors, Title, HemaSphere, 2026;10:(S1):pages. The abstract book DOIs can be found on the “Author Information” page.
**Disclaimer:** Articles published in the journal HemaSphere exclusively reflect the opinions of the authors. The authors are responsible for all content in their abstracts including accuracy of the facts, statements, citing resources, etc.

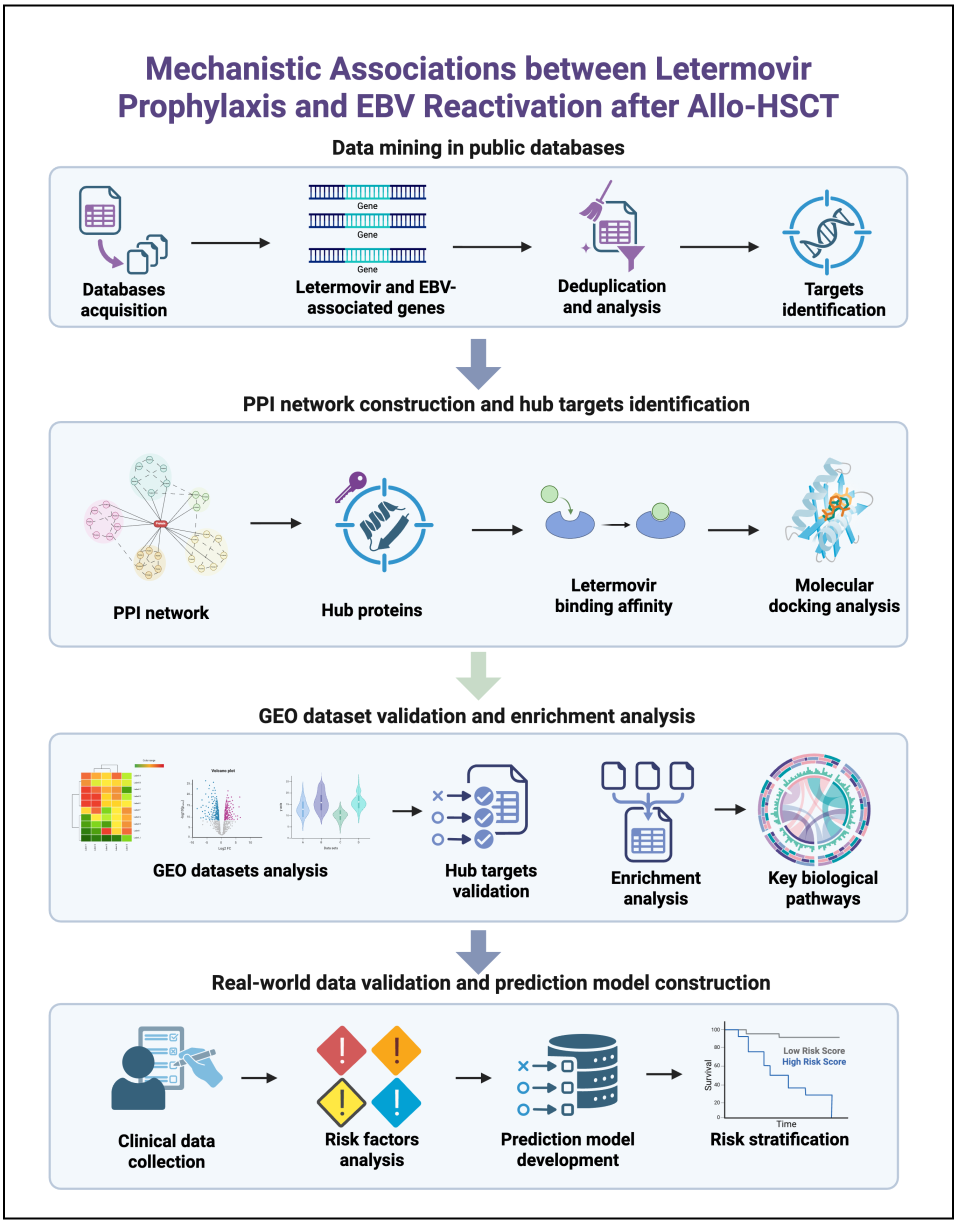

Flowchart of the study

**Copyright Information:** (Online) ISSN: 2572-9241
©2026 The Author(s). HemaSphere is published by John Wiley & Sons Ltd on behalf of European Hematology Association. This is an open access Abstract Book distributed under the Attribution-NonCommercial-NoDerivs (CC BY-NC-ND), which allows third parties to download the articles and share them with others as long as they credit the author and the Abstract Book, but they cannot change the content in any way or use them commercially.
**Abstract Book Citations:** Authors, Title, HemaSphere, 2026;10:(S1):pages. The abstract book DOIs can be found on the "Author Information" page.
**Disclaimer:** Articles published in the journal HemaSphere exclusively reflect the opinions of the authors. The authors are responsible for all content in their abstracts including accuracy of the facts, statements, citing resources, etc.

### (PS2412) RATES OF INFECTION IN PATIENTS (PTS) WITH B-CELL NON-HODGKIN LYMPHOMA (B-NHL): A REAL-WORLD ANALYSIS

**Topic:** 31. Infections in hematology (incl. supportive care/therapy)

Evgenii Shumilov*[1], Omonefe O. Omofuma[2], Matthew Phelan[2], Alexander Breskin[2], Ping Shao[2], Saleem Shariff[3], Himal Thakar[2], Hesham Mohamed[2], Christian Hampp[2], Jennifer L. Crombie[4]

[1]*Department of Medicine A for Hematology, Oncology and Pneumology, University Hospital Muenster, Muenster, Germany;* [2]*Regeneron Pharmaceuticals, Inc., Tarrytown, NY, United States of America;* [3]*Regeneron UK Ltd, Uxbridge, United Kingdom;* [4]*Department of Medical Oncology, Dana-Farber Cancer Institute, Harvard Medical School, Boston, MA, United States of America;*

### Background

Infections are a major concern with B-NHL treatment. Currently, data on infection rates, severity, and infection-related hospitalization burden by line of therapy (LOT) are limited.

### Aims

To quantify infection incidence by type, severity, and hospitalization duration across LOTs in pts with B-NHL in a real-world setting.

### Methods

Pts with newly diagnosed B-NHL between October 1, 2016, and December 31, 2024, were identified from Optum's Clinformatics Data Mart, a large US administrative health claims database containing de-identified medical and pharmacy claims. Pts aged ≥18 years at B-NHL diagnosis, defined by ≥2 International Classification of Diseases 10$^{th}$ Revision diagnosis (ICD-10) codes, were enrolled continuously for 365 days and initiated treatment after diagnosis. Exposure-adjusted incidence rates (EAIRs) per 100 person-years (PY) were estimated by LOT (first-line therapy [1L], second-line therapy [2L], and third-line therapy or later [3L+]) for infection type (bacteremia, cytomegalovirus, SARS-CoV-2, fungal, herpes, pneumonia, respiratory tract infection [RTI], upper RTI, sepsis, sinusitis, urinary tract infection), infection severity (any, hospitalized, fatal [i.e., hospitalized infections followed by death]), and hospitalization duration.

### Results

Pts treated for diffuse large B-cell lymphoma (DLBCL; n=8293), mantle cell lymphoma (MCL; n=1010), follicular lymphoma (FL; n=2630), and marginal zone lymphoma (MZL; n=1567) were included. The most commonly used 1L regimens included rituximab, cyclophosphamide, doxorubicin + vincristine for DLBCL (1L: 69% of pts); bendamustine + rituximab for MCL (1L: 51%); bendamustine + rituximab for FL (1L: 40%); and rituximab monotherapy for MZL (1L: 56%). Median age at index treatment was 73 years for DLBCL and MCL, 72 years for FL, and 74 years for MZL; proportions of male pts were 56%, 71%, 49%, and 50%, respectively. EAIRs for any, hospitalized, and fatal infections were highest with DLBCL and MCL, and increased for later LOTs (**Table**). Among the types of hospitalized infections assessed, EAIRs across all LOTs were highest for sepsis (DLBCL: 41–68 [range across 1L–3L+]; MCL: 24–34; FL: 12–27; MZL: 10–16 per 100 PY) and pneumonia (33–60; 18–45; 10–28; 10–33 per 100 PY, respectively). Median hospitalization duration for pneumonia and sepsis ranged from 6 to 12 days (where ≥5 cases were reported).

**Copyright Information:** (Online) ISSN: 2572-9241
©2026 The Author(s). HemaSphere is published by John Wiley & Sons Ltd on behalf of European Hematology Association. This is an open access Abstract Book distributed under the Attribution-NonCommercial-NoDerivs (CC BY-NC-ND), which allows third parties to download the articles and share them with others as long as they credit the author and the Abstract Book, but they cannot change the content in any way or use them commercially.
**Abstract Book Citations:** Authors, Title, HemaSphere, 2026;10:(S1):pages. The abstract book DOIs can be found on the "Author Information" page.
**Disclaimer:** Articles published in the journal HemaSphere exclusively reflect the opinions of the authors. The authors are responsible for all content in their abstracts including accuracy of the facts, statements, citing resources, etc.

### Summary/Conclusion

Increased risk of any, hospitalized, and fatal infections was observed in heavily pretreated pts receiving later LOTs, and in pts with aggressive B-NHL subtypes (DLBCL and MCL). Sepsis and pneumonia predominantly drove hospitalizations. Infections remain a challenge in the treatment of pts with B-NHL, particularly in later LOTs.

**Table. EAIRs per 100 PY of infections in pts**

| Infection severity | LOT | DLBCL | MCL | FL | MZL |
|---|---|---|---|---|---|
| Any | 1L | 200 | 129 | 100 | 98 |
| | 2L | 241 | 162 | 146 | 126 |
| | 3L+ | 315 | 204 | 197 | 98 |
| Hospitalized | 1L | 91 | 52 | 27 | 24 |
| | 2L | 118 | 69 | 49 | 35 |
| | 3L+ | 161 | 98 | 75 | 41 |
| Fatal | 1L | 12 | 5 | 3 | 4 |
| | 2L | 24 | 13 | 8 | 2 |
| | 3L+ | 46 | 20 | 34 | 0 |

**Copyright Information:** (Online) ISSN: 2572-9241
©2026 The Author(s). HemaSphere is published by John Wiley & Sons Ltd on behalf of European Hematology Association. This is an open access Abstract Book distributed under the Attribution-NonCommercial-NoDerivs (CC BY-NC-ND), which allows third parties to download the articles and share them with others as long as they credit the author and the Abstract Book, but they cannot change the content in any way or use them commercially.
**Abstract Book Citations:** Authors, Title, HemaSphere, 2026;10:(S1):pages. The abstract book DOIs can be found on the "Author Information" page.
**Disclaimer:** Articles published in the journal HemaSphere exclusively reflect the opinions of the authors. The authors are responsible for all content in their abstracts including accuracy of the facts, statements, citing resources, etc.

### (PS2413) MID-REGIONAL PROADRENOMEDULLIN IDENTIFIES HIGH-RISK INFECTIOUS AND TRANSPLANT-RELATED COMPLICATIONS IN PEDIATRIC ALLOGENEIC HSCT: A PROSPECTIVE MULTICENTER STUDY

**Topic:** 31. Infections in hematology (incl. supportive care/therapy)

Manuela Spadea*[1, 2], Paola Berchialla[3], Alessio Tomatis[1], Sara Pestarino[4], Francesca Dagliano[4], Alessandro Di Gangi[5], Maria Gabelli[6], Valeria Ceolin[1], Vincenzo Apolito[1], Sofia Claretto[7], Marco Basiricò[7], Celeste Cagnazzo[7], Francesco Saglio[1], Arcangelo Prete[8], Alessandra Biffi[6], Maria Cristina Menconi[5], Maura Faraci[4], Franca Fagioli[1, 3]

*[1]Pediatric Hematology Oncology, Stem Cell Transplantation and Cell Therapy Division, Regina Margherita Children's Hospital, Turin, Italy, Torino, Italy; [2]Department of Pediatrics and Public Health Sciences, University of Turin, Italy, Turin, Italy;[3]University of Turin, Torino, Italy; [4]IRCCS Gaslini Children's Hospital, Genova, Italy; [5]Pediatric Hematology Oncology and Stem Cell Transplantation Unit, Azienda Ospedaliero Universitaria Pisana, Pisa, Italy, pisa, Italy; [6]Pediatric Hematology, Oncology and Stem Cell Transplant Division, Department of Women's and Children's Health, University Hospital of Padova, Padova, Italy, Padova, Italy; [7]Clinical Trial Unit, Regina Margherita Children's Hospital, Turin, Italy, Turin, Italy;[8]IRCCS-AOU di Bologna, Policlinico di S. Orsola, Bologna, Italy, Bologna, Italy;*

### Background

Early after pediatric allogeneic HSCT, **infections (IDs)** and **endothelial-driven transplant-related complications (TRCs)** (e.g., aGVHD, SOS, engraftment/pre-engraftment syndromes, TA-TMA, cytokine-release after ATG/ATLG or PTCy) are major drivers of morbidity. Clinically, they often present as **early fever/inflammation indistinguishable from benign febrile neutropenia (FN)**, delaying targeted diagnostics and appropriate escalation/de-escalation. **Mid-regional proadrenomedullin (Pro-ADM)** reflects endothelial dysfunction and improves risk stratification in pediatric FN, but prospective HSCT data are limited.

### Aims

To determine whether **serial Pro-ADM**, alone and combined with **procalcitonin (PCT)**, can **differentiate early TRCs/IDs from FN** after pediatric allogeneic HSCT and provide prognostic information.

### Methods

Multicenter, prospective, observational study (NCT05523336) in pediatric allogeneic HSCT recipients with serial biomarker sampling. Ninety-nine patients were enrolled (6 screening failures); **98 transplants** were analyzed. **Pro-ADM (nmol/L)** and **PCT** were measured at baseline (pre-conditioning) and at predefined early post-transplant timepoints (**T1 day 0, T2 day 3, T3 day 10**) during clinically adjudicated episodes classified per protocol as **TRCs, IDs, or FN**. Biomarker differences across episode types were evaluated using linear mixed-effects models (patient as random effect; Tukey-adjusted comparisons). Discrimination of **TRCs/IDs vs FN at T1** was assessed by ROC analyses for Pro-ADM alone and Pro-ADM+PCT strategies (**either biomarker positive** vs **both positive**). Overall survival (OS) and GVHD-related survival were explored by Kaplan–Meier analyses (high vs low Pro-ADM using maximally selected rank statistics).

**Copyright Information:** (Online) ISSN: 2572-9241
©2026 The Author(s). HemaSphere is published by John Wiley & Sons Ltd on behalf of European Hematology Association. This is an open access Abstract Book distributed under the Attribution-NonCommercial-NoDerivs (CC BY-NC-ND), which allows third parties to download the articles and share them with others as long as they credit the author and the Abstract Book, but they cannot change the content in any way or use them commercially.
**Abstract Book Citations:** Authors, Title, HemaSphere, 2026;10:(S1):pages. The abstract book DOIs can be found on the "Author Information" page.
**Disclaimer:** Articles published in the journal HemaSphere exclusively reflect the opinions of the authors. The authors are responsible for all content in their abstracts including accuracy of the facts, statements, citing resources, etc.

**Results**

We analyzed **40 TRC**, **44 ID**, and **69 FN** episodes. **At T1**, Pro-ADM was higher in TRCs and IDs than in FN (median [IQR]**0.83 [0.52–1.02]** and **0.83 [0.63–1.12]** vs **0.60 [0.47–0.82]**; TRC vs FN **p=0.0022**, ID vs FN **p=0.011**). **At T2**, differences persisted (TRC vs FN **p=0.033**), while **at T3** no significant separation was observed. Baseline Pro-ADM did not differ among patients who later developed TRCs, IDs, or FN. For identifying **TRCs/IDs vs FN at T1**, Pro-ADM yielded **AUC 0.696** with an optimal cutoff **0.70 nmol/L** (sensitivity **0.68**, specificity **0.70**, PPV **0.54**, NPV **0.81**). Adding PCT with an **either-positive** rule did not improve performance, whereas a **both-positive** rule increased **specificity to 0.88** and **PPV to 0.67**, at the expense of sensitivity (**0.45**). Pro-ADM high vs low did not significantly stratify **OS** or **GVHD-related survival** in this cohort.

**Summary/Conclusion**

In a prospective pediatric HSCT cohort, **early Pro-ADM (day 0) provides clinically meaningful discrimination** between **TRCs/IDs** and **benign FN**, consistent with its role as an **endothelial stress biomarker**. Two complementary clinical-use signals emerge: (1) **low Pro-ADM supports a conservative approach** (high NPV), and (2) **Pro-ADM+PCT both-positive identifies a high-risk subgroup** with strong rule-in performance (high specificity/PPV) that may benefit from **earlier intensified monitoring and targeted diagnostics.** While no survival association was observed, these findings support incorporating **Pro-ADM into early post-HSCT assessment algorithms** and justify **validation in larger cohorts** and risk-adapted interventional studies.

**Copyright Information:** (Online) ISSN: 2572-9241
©2026 The Author(s). HemaSphere is published by John Wiley & Sons Ltd on behalf of European Hematology Association. This is an open access Abstract Book distributed under the Attribution-NonCommercial-NoDerivs (CC BY-NC-ND), which allows third parties to download the articles and share them with others as long as they credit the author and the Abstract Book, but they cannot change the content in any way or use them commercially.
**Abstract Book Citations:** Authors, Title, HemaSphere, 2026;10:(S1):pages. The abstract book DOIs can be found on the "Author Information" page.
**Disclaimer:** Articles published in the journal HemaSphere exclusively reflect the opinions of the authors. The authors are responsible for all content in their abstracts including accuracy of the facts, statements, citing resources, etc.

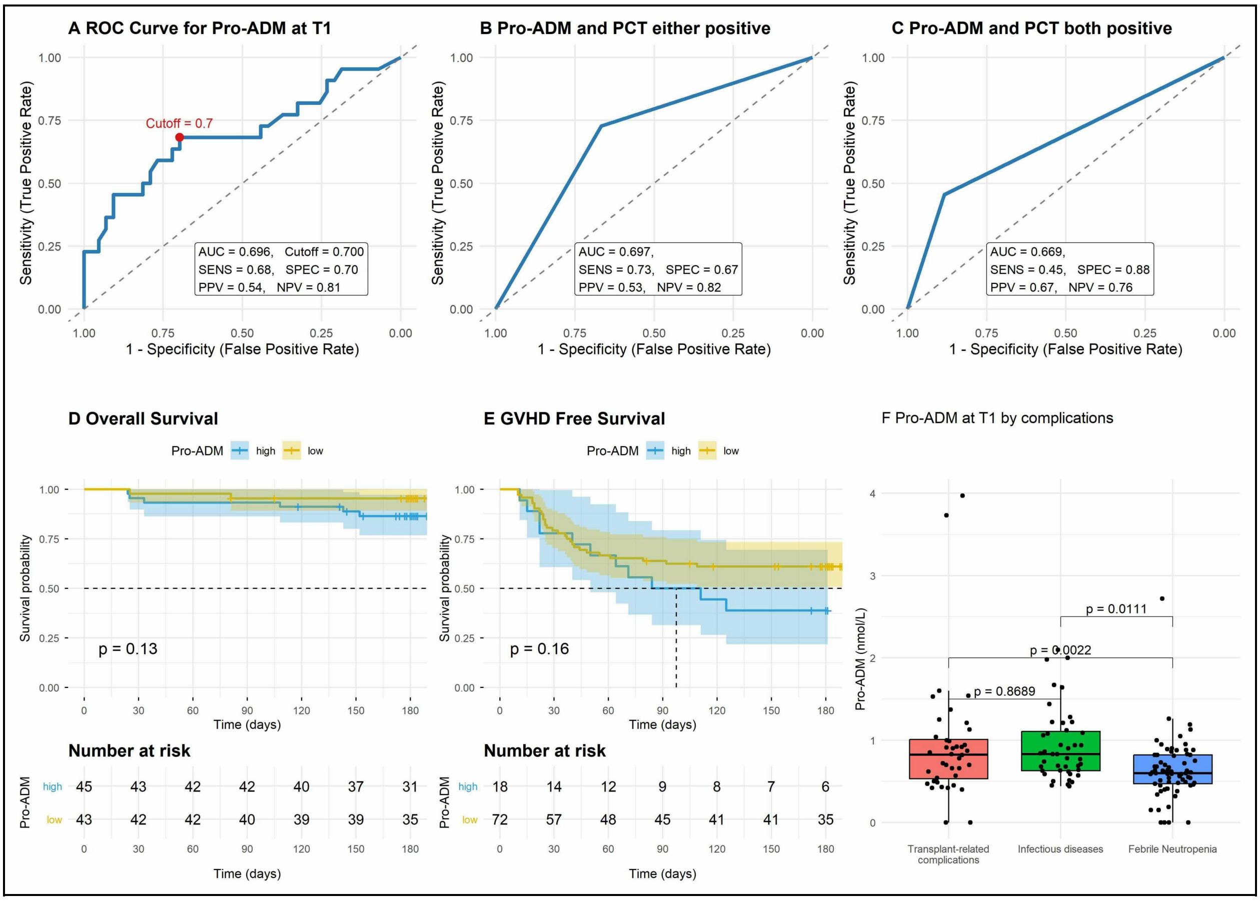

Figure 1. Diagnostic and prognostic performance of pro-adrenomedullin (ProADM). (A) ROC curve showing the discriminative ability of Pro-ADM at T1 for early post-transplant complications (optimal cut-off 0.7 nmol/L). (B-C) ROC curves for Pro-ADM combined with procalcitonin (PCT), considering either biomarker positive (B) or both positive (C). (D-E) Kaplan-Meier estimates of overall survival and GVHD-free survival by Pro-ADM status at T1. (F) Distribution of Pro-ADM levels at T1 by type of early post-transplant complication (transplant-related complications, infectious diseases, febrile neutropenia); p values for pairwise comparisons are reported.

**Copyright Information:** (Online) ISSN: 2572-9241
©2026 The Author(s). HemaSphere is published by John Wiley & Sons Ltd on behalf of European Hematology Association. This is an open access Abstract Book distributed under the Attribution-NonCommercial-NoDerivs (CC BY-NC-ND), which allows third parties to download the articles and share them with others as long as they credit the author and the Abstract Book, but they cannot change the content in any way or use them commercially.
**Abstract Book Citations:** Authors, Title, HemaSphere, 2026;10:(S1):pages. The abstract book DOIs can be found on the "Author Information" page.
**Disclaimer:** Articles published in the journal HemaSphere exclusively reflect the opinions of the authors. The authors are responsible for all content in their abstracts including accuracy of the facts, statements, citing resources, etc.

### (PS2414) CD38HIGH ΓΔ T CELLS EXHIBIT AN EXHAUSTED PHENOTYPE AND PREDICT PROGRESSION TO PTLD FOLLOWING ALLOGENEIC HEMATOPOIETIC STEM CELL TRANSPLANTATION

**Topic:** 31. Infections in hematology (incl. supportive care/therapy)

Zhifan Zhao*[1], Zhipeng Zhou[1], Zhuojun Liu[1], Yuxuan Du[1], Zhenxiao Li[1], Xiangyu Zhao[1], Xiaohui Zhang[1], Xiaojun Huang[1], Pei Xuying[1]

*[1]Peking University People's Hospital, Beijing, China;*

### Background

Epstein-Barr virus (EBV) reactivation is a frequent and life-threatening complication following allogeneic hematopoietic stem cell transplantation (allo-HSCT) due to impaired immune reconstitution, which may progress to EBV-associated post-transplant lymphoproliferative disorder (PTLD). Dysfunctional T-cell responses is central to the pathogenesis of EBV-related complications. γδ T cells are an important component of early antiviral immunity after transplantation; however, their phenotypic and functional alterations during EBV viremia and PTLD remain poorly defined. CD38, a multifunctional ectoenzyme involved in immune activation and metabolic regulation, has been implicated in T-cell exhaustion and chronic viral infection, but its role in γδ T-cell dysfunction in EBV-related PTLD has not been elucidated.

### Aims

1) Characterize the phenotypic and functional alterations of γδ T cells during EBV viremia and PTLD after allo-HSCT; 2) Investigate the association between CD38 expression and γδ T-cell exhaustion; 3) Evaluate the predictive value of CD38 expression in identifying progression toward PTLD.

### Methods

We collected PBMCs from 33 allo-HSCT patients at Peking University People’s Hospital, categorized into HSCT controls (n=8), EBV viremia (n=7), and PTLD (n=18). CyTOF was used to profile 39 markers of lineage, activation, chemotaxis, and exhaustion. γδ T-cell subsets were analyzed via PhenoGraph clustering. We employed Pearson correlation and linear regression to assess associations between CD38 expression, immune checkpoints, peak EBV DNA load, and disease duration. Additionally, ROC curves evaluated the diagnostic efficacy of early CD38 levels in predicting PTLD.

### Results

PhenoGraph clustering revealed that the proportion of γδ T cells was significantly decreased in patients with EBV viremia and PTLD compared to HSCT controls (Figure A-B). Concurrent with this restricted expansion, the expression of exhaustion markers (PD-1, TIGIT, Tim-3) on γδ T cells was significantly upregulated, particularly in the PTLD cohort (Figure C). Notably, CD38 expression intensity was markedly elevated on γδ T cells in PTLD patients (Figure D). Further characterization showed that $CD38^{high}$ γδ T cells exhibited a distinct exhaustion profile with co-enrichment of multiple immune checkpoints, diminished IFN-γ secretion, and reduced expression of CD45RA and CD127, indicating impaired stemness and memory potential (Figure E-G). Furthermore, Correlation analysis revealed a significant positive association between CD38 expression intensity and exhaustion markers (Figure H). ROC analysis among 23 EBV-positive patients demonstrated that both the expression intensity of CD38 and the frequency of $CD38^{high}$ γδ T cells accurately distinguish the progression toward PTLD (Figure I). Consistent with these findings, CD38 expression intensity was significantly correlated with both peak EBV DNA load and infection duration, reflecting its close link to the clinical severity of EBV complications (Figure J).

**Copyright Information:** (Online) ISSN: 2572-9241
©2026 The Author(s). HemaSphere is published by John Wiley & Sons Ltd on behalf of European Hematology Association. This is an open access Abstract Book distributed under the Attribution-NonCommercial-NoDerivs (CC BY-NC-ND), which allows third parties to download the articles and share them with others as long as they credit the author and the Abstract Book, but they cannot change the content in any way or use them commercially.
**Abstract Book Citations:** Authors, Title, HemaSphere, 2026;10:(S1):pages. The abstract book DOIs can be found on the “Author Information” page.
**Disclaimer:** Articles published in the journal HemaSphere exclusively reflect the opinions of the authors. The authors are responsible for all content in their abstracts including accuracy of the facts, statements, citing resources, etc.

**Summary/Conclusion**

Post-allo-HSCT, γδ T cells undergo quantitative contraction and functional exhaustion during EBV reactivation and PTLD. $CD38^{high}$ γδ T cells represent a highly exhausted subset with impaired effector and memory potential. Elevated CD38 expression serves as a robust biomarker for PTLD progression, correlating with viral burden and disease duration. These findings identify CD38 as an early diagnostic indicator and a therapeutic target for restoring antiviral immunity in EBV-associated PTLD.

**Copyright Information:** (Online) ISSN: 2572-9241
©2026 The Author(s). HemaSphere is published by John Wiley & Sons Ltd on behalf of European Hematology Association. This is an open access Abstract Book distributed under the Attribution-NonCommercial-NoDerivs (CC BY-NC-ND), which allows third parties to download the articles and share them with others as long as they credit the author and the Abstract Book, but they cannot change the content in any way or use them commercially.
**Abstract Book Citations:** Authors, Title, HemaSphere, 2026;10:(S1):pages. The abstract book DOIs can be found on the "Author Information" page.
**Disclaimer:** Articles published in the journal HemaSphere exclusively reflect the opinions of the authors. The authors are responsible for all content in their abstracts including accuracy of the facts, statements, citing resources, etc.

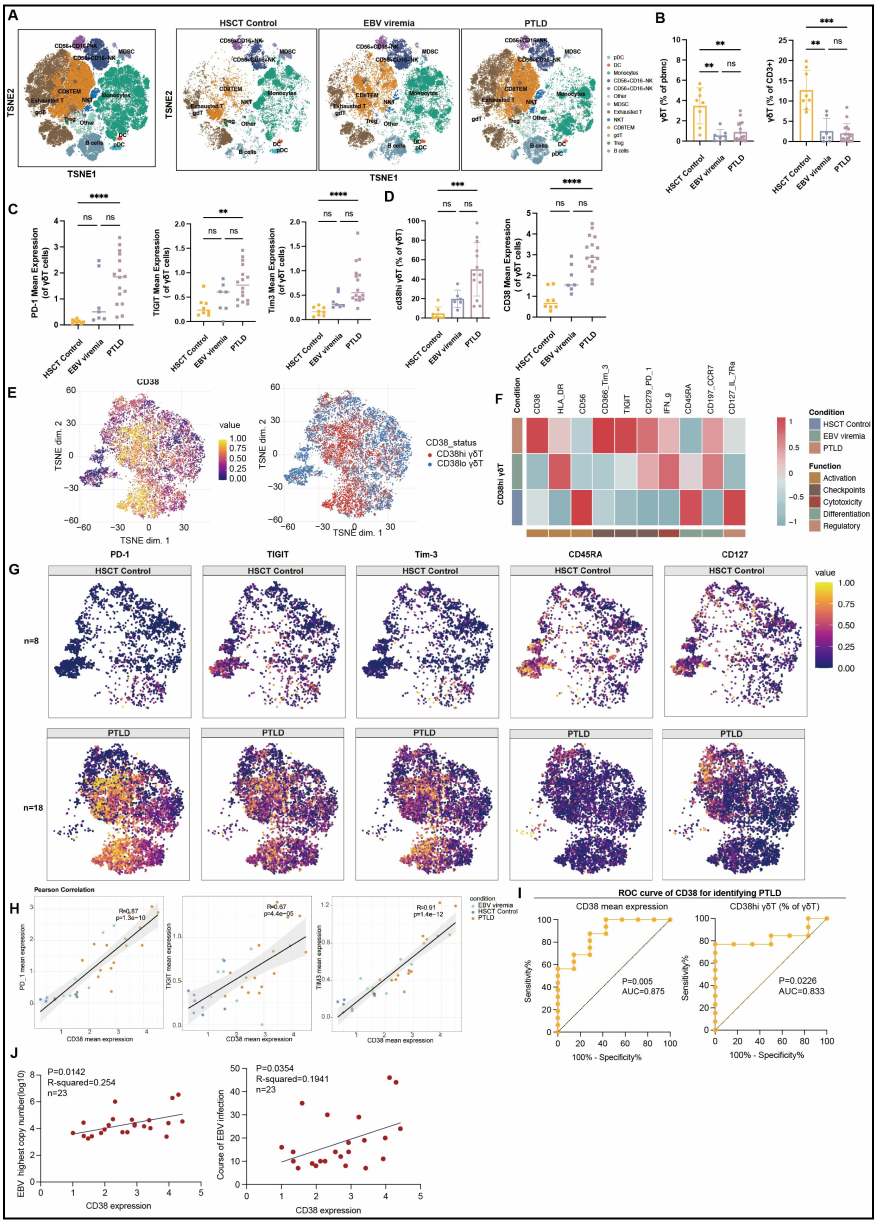

A.t-SNE visualization of PBMC clusters across HSCT controls (n=8), EBV viremia (n=7), and PTLD patients (n=18), identified by PhenoGraph clustering. B.Quantitative analysis of γδ T cell frequencies as a percentage of total PBMCs (left) and total CD3+ T cells (right) across the three cohorts. C.Exhaustion marker expression. Mean fluorescence intensity (MFI) or expression levels of PD-1, TIGIT, and Tim-3 on γδ T cells. D.CD38 expression profiles. Frequency of CD38high γδ T cells (left) and overall CD38 mean expression (right) on the γδ T cell population. E.t-SNE plots highlighting the distribution of CD38 expression (left) and the cluster localization of CD38high vs. CD38low γδ T cells (right). F.Heatmap illustrating the normalized expression of activation, checkpoint, cytotoxicity, differentiation, and regulatory markers in CD38high γδ T cells across conditions. G.Representative t-SNE maps displaying the expression density of PD-1, TIGIT, Tim-3, CD45RA, and CD127 on γδ T cells in HSCT controls versus PTLD patients. H.Correlation analysis (Pearson's R) between CD38 mean expression and the exhaustion markers PD-1, TIGIT, and Tim-3. I.ROC curve analysis evaluating the sensitivity and specificity of CD38 mean expression (left) and CD38high γδ T cell frequency (right) as biomarkers to distinguish PTLD from EBV viremia. J.Clinical correlations. Linear regression analysis between CD38 expression on γδ T cells and peak EBV DNA copy number (log10, left) and the duration of EBV infection (right). *Data are presented as mean ± SEM. Statistical significance was determined by one-way ANOVA or Kruskal-Wallis test: **p < 0.01, ***p < 0.001, ***p < 0.0001; ns, non-significant.

**Copyright Information:** (Online) ISSN: 2572-9241
©2026 The Author(s). HemaSphere is published by John Wiley & Sons Ltd on behalf of European Hematology Association. This is an open access Abstract Book distributed under the Attribution-NonCommercial-NoDerivs (CC BY-NC-ND), which allows third parties to download the articles and share them with others as long as they credit the author and the Abstract Book, but they cannot change the content in any way or use them commercially.
**Abstract Book Citations:** Authors, Title, HemaSphere, 2026;10:(S1):pages. The abstract book DOIs can be found on the "Author Information" page.
**Disclaimer:** Articles published in the journal HemaSphere exclusively reflect the opinions of the authors. The authors are responsible for all content in their abstracts including accuracy of the facts, statements, citing resources, etc.

### (PS2415) TORQUE TENO VIRUS: AN EMERGING BIOMARKER ASSOCIATED WITH ADVERSE PROGNOSIS AND THE RISK OF GRAFT-VERSUS-HOST DISEASE IN PATIENTS UNDERGOING ALLO-HSCT

**Topic:** 31. Infections in hematology (incl. supportive care/therapy)

Xiujuan MA*[1], Hongxing Liu[1, 2, 3]

*[1]Hebei Yanda Lu Daopei Hospital, Department of Laboratory Medicine, Langfang, China;[2]Beijing Lu Daopei Hospital, Division of Pathology & Laboratory Medicine, Beijing, China; [3]Beijing Lu Daopei Institute of Hematology, Precision Medicine Center, Beijing, China;*

### Background

Torque Teno virus (TTV) is highly prevalent and is emerging as an endogenous "immunometer," with its plasma loads reflecting the host's functional immune status. While TTV dynamics have been linked to outcomes after allogeneic hematopoietic stem cell transplantation (allo-HSCT), its specific role in predicting graft-versus-host disease (GVHD), pathogen-specific infections, and phase-specific survival remains poorly defined and controversial.

### Aims

To evaluate the clinical utility of plasma TTV reactivation in predicting GVHD, infection risks, and long-term prognosis in patients undergoing allo-HSCT.

### Methods

We retrospectively analyzed 477 patients who underwent allo-HSCT at Hebei Yanda Lu Daopei Hospital between January 2021 and December 2023. Plasma TTV status was determined using metagenomic next-generation sequencing (mNGS). Patients were stratified into TTV-positive (n=207) and TTV-negative (n=270) groups. Survival analysis was performed using Kaplan-Meier curves and multivariate Cox regression models to identify independent risk factors for mortality.

### Results

Baseline characteristics, including age, gender, primary disease, and donor type, were well-balanced between groups (all P>0.05). TTV-positive patients demonstrated significantly higher rates of rapid neutrophil engraftment (≤28d) (99.0% vs. 95.9%), acute GVHD (aGVHD) (62.3% vs. 47.8%), chronic GVHD (cGVHD) (42.0% vs. 29.3%), and viral infections (73.4% vs. 63.0%) (all P<0.05), but lower rates of bacterial and fungal infections (P<0.05).
With a median follow-up of 27.9 months, TTV-positive patients had a significantly inferior 3-year overall survival (OS) compared to the TTV-negative group (65.3% vs. 75.1%, P=0.034). Multivariate analysis identified TTV reactivation, delayed neutrophil engraftment (>28d), and fungal/viral infections as independent risk factors for overall mortality.
Intriguingly, a time-dependent prognostic shift was observed: within the first 5 months post-transplant, TTV-positive patients exhibited higher survival rates than TTV-negative patients. This early survival advantage was associated with superior hematopoietic recovery (neutrophil and platelet engraftment ≤28d, P<0.005) and lower bacterial infection rates (34.4% vs. 63.2%, P=0.016), despite an increased incidence of aGVHD (71.9% vs. 36.8%, P=0.003). Beyond 5 months, however, TTV reactivation predominantly signaled sustained immune dysregulation, leading to increased cGVHD, recurrent viral infections, and late mortality.

**Copyright Information:** (Online) ISSN: 2572-9241
©2026 The Author(s). HemaSphere is published by John Wiley & Sons Ltd on behalf of European Hematology Association. This is an open access Abstract Book distributed under the Attribution-NonCommercial-NoDerivs (CC BY-NC-ND), which allows third parties to download the articles and share them with others as long as they credit the author and the Abstract Book, but they cannot change the content in any way or use them commercially.
**Abstract Book Citations:** Authors, Title, HemaSphere, 2026;10:(S1):pages. The abstract book DOIs can be found on the "Author Information" page.
**Disclaimer:** Articles published in the journal HemaSphere exclusively reflect the opinions of the authors. The authors are responsible for all content in their abstracts including accuracy of the facts, statements, citing resources, etc.

### Summary/Conclusion

Plasma TTV reactivation serves as a potent prognostic biomarker in allo-HSCT. While it correlates with robust hematopoietic recovery in the early post-transplant phase, it ultimately acts as a harbinger of immune dysregulation, aGVHD/cGVHD, and viral complications. Our data support that TTV represents an emerging biomarker associated with adverse prognosis and an increased risk of GVHD in patients undergoing allo-HSCT, warranting further investigation.

**Table. Multivariable Cox Regression Analysis of Independent Risk Factors for Overall Survival after Allo-HSCT**

| **Variable** | **HR (95%CI)** | ***P* value** |
|---|---|---|
| TTV reactivation | 1.821(1.244-2.664) | 0.002 |
| Bacterial infection | 1.429(0.972-2.101) | 0.069 |
| Fungal infection | 3.428(2.215-5.306) | 3.26E-8 |
| Viral infection | 1.734(1.107-2.717) | 0.016 |
| Delayed neutrophil engraftment (>28 d) | 7.877(3.456-17.953) | 9.08E-7 |
| Delayed platelet engraftment (>28 d) | 1.346(0.815-2.224) | 0.246 |
| aGVHD | 1.284(0.883-1.867) | 0.191 |

aGVHD, acute graft-versus-host disease

**Copyright Information:** (Online) ISSN: 2572-9241
©2026 The Author(s). HemaSphere is published by John Wiley & Sons Ltd on behalf of European Hematology Association. This is an open access Abstract Book distributed under the Attribution-NonCommercial-NoDerivs (CC BY-NC-ND), which allows third parties to download the articles and share them with others as long as they credit the author and the Abstract Book, but they cannot change the content in any way or use them commercially.
**Abstract Book Citations:** Authors, Title, HemaSphere, 2026;10:(S1):pages. The abstract book DOIs can be found on the "Author Information" page.
**Disclaimer:** Articles published in the journal HemaSphere exclusively reflect the opinions of the authors. The authors are responsible for all content in their abstracts including accuracy of the facts, statements, citing resources, etc.

### (PS2416) INCORPORATION OF RITUXIMAB INTO CONDITIONING PREVENTS EBV INFECTION AFTER HAPLOIDENTICAL STEM CELL TRANSPLANTATION DURING THE ERA OF LETERMOVIR FOR CYTOMEGALOVIRUS PROPHYLAXIS

**Topic:** 31. Infections in hematology (incl. supportive care/therapy)

Xiaoning Wang*[1], Juan Ren[1], Huachao Zhu[1], Chunhong Sun[1], Pengcheng He[1]

[1]*the first affiliated hospital of xi'an jiaotong university, department of hematology, xi'an, China;*

### Background

In the era of letermovir for cytomegalovirus (CMV) infection prophylaxis, several centers reported that Epstein-Barr virus (EBV) viremia and post-transplant lymphoproliferative disorders (PTLD) were significantly higher in patients who did not receive letermovir prophylaxis following rabbit anti-thymocyte globulin( rATG)-based haploidentical hematopoietic stem cell transplantation�haplo-HSCT�.There is an unmet need to find an effective strategy to prevent EBV infection.

### Aims

To investigate the efficacy and safety of rituximab administration during conditioning regimen following haplo-HSCT in the prevention of early (within 100 days) post-transplant EBV infection.

### Methods

We conducted a retrospective analysis of 100 patients with acute leukemia or myelodysplastic syndrome who underwent haplo-HSCT in the Department of Hematology, The First Affiliated Hospital of Xi'an Jiaotong University between January 2024 and September 2025. All patients used rATG-based graft-versus-host disease (GVHD) prophylaxis and letermovir for CMV infection prophylaxis post-transplantati on.Patients were divided into two groups: the observation group(R group), who received rituximab (375 mg/m²) on day -3 before transplantation due to the presence of donor-specific antibodies (MFI ≥ 2000) (n = 25) and the control group (C group), who did not receive rituximab (n = 75) and donor-specific antibodies (MFI < 2000). The primary objectives were the incidence of EBV-DNA viremia and PTLD within 100 days post-transplantation. Secondary objectives included the incidence of CMV infection, cumulative incidence of acute GVHD and chronic GVHD, 100-day non-relapse mortality (NRM), progression-free survival (PFS), and overall survival (OS).

### Results

No significant differences were observed in baseline characteristics between the two groups. When compared with the C group, patients in the R group exhibited a significantly lower cumulative incidence of EBV viremia within 100 days post - transplantation (0% vs. 21.33%, P = 0.012) and a significantly lower incidence of grade I - IV acute GVHD (28% vs. 50.67%, P = 0.048). There were no significant differences in the incidence of PTLD (0% vs. 9.33%, P = 0.113), CMV viremia (4% vs. 15.38%, P = 0.208),chronic GVHD (16% vs. 12%, P = 0.607), and within 100 - day NRM (4.0% vs. 10.67%, P = 0.313). The 2-year OS rates in the R group and C group were 83.8% ± 0.086% and 81.9% ± 0.050% respectively (P = 0.662). The 2-year PFS rates in the R group and C group were 83.8% ± 0.086% and 72.6% ± 0.068% respectively (P = 0.876).

**Copyright Information:** (Online) ISSN: 2572-9241
©2026 The Author(s). HemaSphere is published by John Wiley & Sons Ltd on behalf of European Hematology Association. This is an open access Abstract Book distributed under the Attribution-NonCommercial-NoDerivs (CC BY-NC-ND), which allows third parties to download the articles and share them with others as long as they credit the author and the Abstract Book, but they cannot change the content in any way or use them commercially.
**Abstract Book Citations:** Authors, Title, HemaSphere, 2026;10:(S1):pages. The abstract book DOIs can be found on the “Author Information” page.
**Disclaimer:** Articles published in the journal HemaSphere exclusively reflect the opinions of the authors. The authors are responsible for all content in their abstracts including accuracy of the facts, statements, citing resources, etc.

**Summary/Conclusion**

The addition of rituximab to the conditioning regimen for haplo - HSCT could effectively reduce the risk of early post - transplant EBV reactivation and decrease the incidence of acute GVHD, without exerting a significant influence on CMV reactivation, chronic GVHD, PFS, or OS. The combined use of rituximab during the conditioning regimen may be regarded as an effective strategy for preventing EBV reactivation after rATG - based haplo - HSCT in the era of letermovir for CMV prophylaxis.

**Copyright Information:** (Online) ISSN: 2572-9241
©2026 The Author(s). HemaSphere is published by John Wiley & Sons Ltd on behalf of European Hematology Association. This is an open access Abstract Book distributed under the Attribution-NonCommercial-NoDerivs (CC BY-NC-ND), which allows third parties to download the articles and share them with others as long as they credit the author and the Abstract Book, but they cannot change the content in any way or use them commercially.
**Abstract Book Citations:** Authors, Title, HemaSphere, 2026;10:(S1):pages. The abstract book DOIs can be found on the “Author Information” page.
**Disclaimer:** Articles published in the journal HemaSphere exclusively reflect the opinions of the authors. The authors are responsible for all content in their abstracts including accuracy of the facts, statements, citing resources, etc.

### (PS2417) GENOMIC DETERMINANTS AND CLINICAL IMPACT OF CARBAPENEM-RESISTANT INFECTIONS IN FEBRILE NEUTROPENIA AMONG PATIENTS WITH ACUTE LEUKEMIA: A WHOLE-GENOME SEQUENCING STUDY

**Topic:** 31. Infections in hematology (incl. supportive care/therapy)

Uttam Kumar Nath*[1], Prasenjit Das[1], Indrani Sarkar[2], Harshita Madan[1], Nikhil Nagpal[1], Prisla Dalton[2], Bibhant Shah[2], Kavya Ronanki[2], Eswer Subbaiyan[2], Manoj Saini[2], Reshma Benson[2], Arjun Kachhwaha[2], Amber Prasad[1], Debarati Chattopadhyay[2], Saugata Hazra[3]

*[1]All India Institute of Medical Sciences, Rishikesh, Department of Medical Oncology Hematology, Rishikesh, India;[2]All India Institute of Medical Sciences, Rishikesh, Rishikesh, India; [3]Indian Institute of Technology, Roorkee, Department of Biotechnology, Roorkee, India;*

### Background

Carbapenems remain central to empiric therapy for high-risk febrile neutropenia; however, increasing resistance among Gram-negative pathogens is undermining this approach in acute leukemia. Clinical deterioration is often observed despite administration of microbiologically appropriate therapy, suggesting that resistance reflects complex biological adaptation rather than a single carbapenemase mechanism.

### Aims

To characterize the genomic determinants of carbapenem resistance and evaluate their association with clinical outcomes in febrile neutropenia episodes among acute leukemia patients.

### Methods

This 12-month prospective observational study included patients with acute leukemia who developed febrile neutropenia with culture-proven carbapenem-resistant infections at a tertiary care center. Antimicrobial susceptibility testing was performed using VITEK® 2. Fifty clinically significant multidrug-resistant isolates underwent whole-genome sequencing on the Illumina NovaSeq 6000 platform. Resistance genes, carbapenemase determinants, sequence types, and virulence-associated loci were identified using CARD, ResFinder, VFDB, and multilocus sequence typing. Clinical variables—including duration of neutropenia, time to defervescence, need for antibiotic escalation, intensive care unit requirement, and mortality—were correlated with genomic findings.

### Results

Fifty febrile neutropenia episodes were analyzed, predominantly during induction or salvage therapy. The mean hospitalization was 32.2 days. Persistent bacteremia occurred in 31 of 50 episodes (62%), and delayed defervescence beyond 72 hours occurred in 36 of 50 (72%).Escalation beyond initial empiric therapy was required in 28 of 50 episodes (56%).
ICU care was required in 20 of 50 episodes (40%), and mortality among these critically ill cases was 11 of 20 (55%), demonstrating a strong association between clinical deterioration and adverse outcome.
Whole-genome sequencing showed that resistance was mediated by overlapping mechanisms rather than isolated gene acquisition. The predominant carbapenemase genes were **bla-NDM, bla-OXA,** and **bla-KPC,** frequently coexisting within the same isolates. All pathogens demonstrated composite resistance architectures combining enzymatic inactivation with efflux-mediated tolerance.
Virulence-associated pathways linked to adhesion, biofilm formation, immune evasion, and iron acquisition were enriched among isolates associated with persistent or severe infections. High-risk sequence types (18%), including *Klebsiella pneumoniae* and *Acinetobacter baumannii*, were overrepresented in episodes requiring critical care.

**Copyright Information:** (Online) ISSN: 2572-9241
©2026 The Author(s). HemaSphere is published by John Wiley & Sons Ltd on behalf of European Hematology Association. This is an open access Abstract Book distributed under the Attribution-NonCommercial-NoDerivs (CC BY-NC-ND), which allows third parties to download the articles and share them with others as long as they credit the author and the Abstract Book, but they cannot change the content in any way or use them commercially.
**Abstract Book Citations:** Authors, Title, HemaSphere, 2026;10:(S1):pages. The abstract book DOIs can be found on the "Author Information" page.
**Disclaimer:** Articles published in the journal HemaSphere exclusively reflect the opinions of the authors. The authors are responsible for all content in their abstracts including accuracy of the facts, statements, citing resources, etc.

### Summary/Conclusion

Carbapenem resistance alone did not explain the clinical behavior of infections in this cohort. Whole-genome analysis demonstrated that pathogens causing refractory febrile neutropenia harbored layered resistance architectures—coexisting carbapenemases, efflux-mediated tolerance, and virulence programs enabling persistence. This convergence likely permits microbial survival despite appropriate therapy and explains delayed defervescence, frequent treatment escalation, and high critical-care utilization in acute leukemia. This study represents one of the earliest integrated clinical–genomic analyses linking composite resistance–virulence profiles to a persistence-driven phenotype in febrile neutropenia, highlighting the need to move beyond susceptibility-based paradigms toward strategies that anticipate persistence and enable earlier therapeutic modulation in hemato-oncology care.

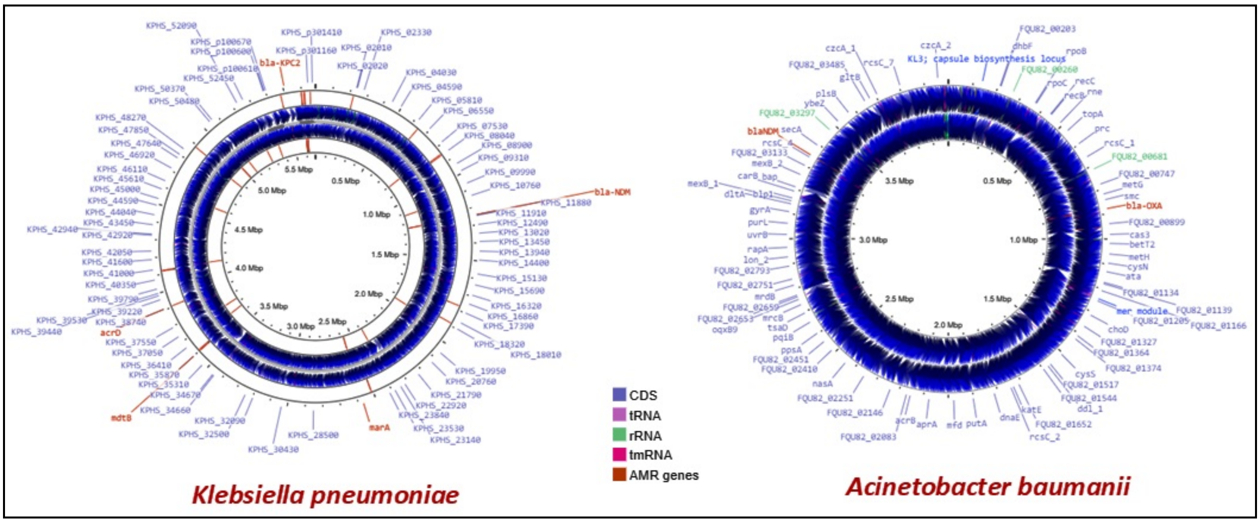

Whole Genome Sequencing Plots

Copyright Information: (Online) ISSN: 2572-9241
©2026 The Author(s). HemaSphere is published by John Wiley & Sons Ltd on behalf of European Hematology Association. This is an open access Abstract Book distributed under the Attribution-NonCommercial-NoDerivs (CC BY-NC-ND), which allows third parties to download the articles and share them with others as long as they credit the author and the Abstract Book, but they cannot change the content in any way or use them commercially.
Abstract Book Citations: Authors, Title, HemaSphere, 2026;10:(S1):pages. The abstract book DOIs can be found on the "Author Information" page.
Disclaimer: Articles published in the journal HemaSphere exclusively reflect the opinions of the authors. The authors are responsible for all content in their abstracts including accuracy of the facts, statements, citing resources, etc.

### (PS2418) PRESERVED T-CELL FUNCTIONALITY COMPENSATES FOR HUMORAL DEFICIENCY IN SARS-COV-2 INFECTION DURING RITUXIMAB-CONTAINING CHEMOIMMUNOTHERAPY

**Topic:** 31. Infections in hematology (incl. supportive care/therapy)

Rong Wei*[1], Shenmiao Yang[1]

[1]*Peking University People's Hospital, Peking University Institute of Hematology, National Clinical Research Center for Treatment of Hematologic Disease, Beijing, China;*

**Background**

Immunocompromised individuals, particularly those with hematological malignancies undergoing B cell-targeted therapies, are highly vulnerable to respiratory viral infections due to profoundly impaired humoral immunity. While the regimen efficiently depletes B cells, it is generally thought to spare the T-cell compartment. However, a critical knowledge gap remains: In the absence of neutralizing antibodies, can the residual T-cell compartment sufficiently compensate to control viral replication?

**Aims**

We hypothesized that Rituximab-treated patients might develop a distinct, hyper-activated T-cell landscape driven by persistent antigen exposure to compensate for humoral failure. To understand the compensatory immune mechanisms, specifically T cell-mediated protection, during SARS-CoV-2 infection in the context of therapeutic B-cell depletion, we conducted this study.

**Methods**

We enrolled 70 lymphoma patients, including 56 with confirmed SARS-CoV-2 infection, stratified into three cohorts: Rituximab-containing immunochemotherapy (R-chemo, primarily R-CHOP regimen), conventional chemotherapy (C-chemo), and treatment-naïve (N group). Through a multi-model approach integrating serological assays, functional T cell responses, and single-cell multi-omics sequencing (scRNA-seq and scTCR-seq), we systematically dissected the viral-specific immune landscape.

**Results**

Key findings revealed that the R-chemo group exhibited severely compromised humoral immunity, with significantly reduced neutralizing antibody titers and immunoglobulin levels. In contrast, both functional assays and scRNA-seq analysis uncovered a vigorous compensatory T cell mobilization, particularly within *CD8⁺* cytotoxic and *CD4⁺* effector memory compartments. Single-cell TCR repertoire analysis further demonstrated convergent clonal expansion with recurrent V-gene pairings (e.g., TRAV14/TRBV9) in R-treated patients, indicative of robust antigen-driven T cell responses in the absence of neutralizing antibodies. Mechanistically, the scRNA-seq identified the upregulation of NF-κB signaling and cytokine production pathways, and highlighting *JUNB* as a pivotal transcriptional hub orchestrating T-cell activation and exhaustion resistance.

**Summary/Conclusion**

Our study delineates a distinct compensatory immune axis where preserved T-cell functionality mitigates the loss of humoral defense in lymphoma patients receiving anti-CD20 therapy. These insights support the safety of continuing Rituximab-based regimens during viral waves and advocate for T-cell-centric vaccine strategies in this vulnerable population.

**Copyright Information:** (Online) ISSN: 2572-9241
©2026 The Author(s). HemaSphere is published by John Wiley & Sons Ltd on behalf of European Hematology Association. This is an open access Abstract Book distributed under the Attribution-NonCommercial-NoDerivs (CC BY-NC-ND), which allows third parties to download the articles and share them with others as long as they credit the author and the Abstract Book, but they cannot change the content in any way or use them commercially.
**Abstract Book Citations:** Authors, Title, HemaSphere, 2026;10:(S1):pages. The abstract book DOIs can be found on the "Author Information" page.
**Disclaimer:** Articles published in the journal HemaSphere exclusively reflect the opinions of the authors. The authors are responsible for all content in their abstracts including accuracy of the facts, statements, citing resources, etc.

### (PS2420) CLINICAL AND PROGNOSTIC IMPLICATIONS OF HYPOGAMMAGLOBULINEMIA IN B-CELL NON-HODGKIN LYMPHOMA (B-NHL).

**Topic:** 31. Infections in hematology (incl. supportive care/therapy)

Amagoia Ruiz Martin*[1], Alexia Teresa Suarez Cabrera[1], Jose Carlos Rodriguez Gallego[2], Concepción Sánchez Chamizo[1], Victor Benitez Arencibia[3], Hugo Luzardo[1], Ana Garcia-Soidan Gonzalez[2], Haridian De La Nuez Melian[1], Alvaro Veiga Vaz[1], Maria Teresa Gomez Casares[1, 3]

*[1]Hospital Universitario de Gran Canaria Doctor Negrín, Hematology, Las Palmas de Gran Canaria, Spain;[2]Hospital Universitario de Gran Canaria Doctor Negrín, Inmunology, Las Palmas de Gran Canaria, Spain; [3]Universidad de Las Palmas de Gran Canaria, Las Palmas de Gran Canaria, Spain;*

### Background

Secondary hypogammaglobulinemia (HG) is a frequent complication in B-cell non-Hodgkin lymphoma (B-NHL), resulting from intrinsic immune dysregulation and immunosuppressive therapies, particularly anti-CD20 monoclonal antibodies.

### Aims

Although HG is associated with increased infectious morbidity, its prognostic significance remains insufficiently defined and is not incorporated into standard prognostic indices such as IPI or FLIPI.

### Methods

We conducted a retrospective single-center study including 301 adult patients diagnosed with B-NHL between 2015 and 2021, excluding Waldenström macroglobulinemia and chronic lymphocytic leukemia (Figure 1). Serum immunoglobulins were measured at diagnosis and two years after treatment completion. HG was defined as IgG <700 mg/dL and/or IgA <70 mg/dL and/or IgM <40 mg/dL; severe HG as IgG <500 mg/dL. Demographic, clinical, hematologic and laboratory variables were collected.

**Copyright Information:** (Online) ISSN: 2572-9241
©2026 The Author(s). HemaSphere is published by John Wiley & Sons Ltd on behalf of European Hematology Association. This is an open access Abstract Book distributed under the Attribution-NonCommercial-NoDerivs (CC BY-NC-ND), which allows third parties to download the articles and share them with others as long as they credit the author and the Abstract Book, but they cannot change the content in any way or use them commercially.
**Abstract Book Citations:** Authors, Title, HemaSphere, 2026;10:(S1):pages. The abstract book DOIs can be found on the "Author Information" page.
**Disclaimer:** Articles published in the journal HemaSphere exclusively reflect the opinions of the authors. The authors are responsible for all content in their abstracts including accuracy of the facts, statements, citing resources, etc.

### Results

HG was present in 18% of patients at diagnosis and increased to 39% post-treatment. Among patients with normal baseline IgG, rituximab-based therapy induced de novo HG in 28.7%. Overall, 53% experienced at least one infection and 25.7% developed moderate-to-severe infections. In low-grade lymphoma, severe infections were significantly more frequent in patients with IgG <500 mg/dL compared with ≥500 mg/dL (62.5% vs. 20.1%; p=0.005; OR 6.6, 95% CI 1.5–29.2), with R-Benda maintenance therapy being the most commonly used treatment in our cohort, whereas this association was not observed in high-grade lymphoma. Univariate analysis identified age >60 years, high-grade histology, advanced stage (III–IV), B symptoms, infection, elevated LDH, lymphocyte count >5,000/µL, monocytosis, and IPI ≥3 as significantly associated with inferior progression-free survival (PFS) and overall survival (OS). Among HG variables, only post-treatment HG was associated with inferior OS (HR 2.09). In multivariate analysis (Table 1), age >60 years, high-grade histology, advanced stage, and B symptoms remained independent predictors of inferior PFS and OS. Post-treatment HG independently predicted worse OS (HR 2.09; p=0.036), without significant impact on PFS. Additionally, a lymphocyte-to-monocyte ratio <3 was associated with increased overall (p<0.001) and severe infections (p=0.002).

| VARIABLE | PFS Multivariate Analysis | | OS Multivariate Analysis | |
|---|---|---|---|---|
| | **HR (95% IC)** | **p-value** | **HR (95% IC)** | **p-value** |
| Age >60 years | **2.060 (1.338-3.171)** | **0.001** | **5.100 (2.689-9.675)** | **<0.001** |
| High grade lymphoma | **1.827 (1.195-2.792)** | **0.005** | **2.736 (1.629-4.597)** | **<0.001** |
| Stage III-IV | **1.618 (1.005-2.605)** | **0.048** | **2.121 (1.130-3.983)** | **0.019** |
| B symptoms | **1.628 (1.075-2.466)** | **0.021** | **2.001 (1.211-3.308)** | **0.007** |
| Infection | **1.565 (1.066-2.298)** | **0.022** | **1.889 (1.193-2.990)** | **0.007** |
| LDH >2.5× ULN | **1.005 (1.001-1.010)** | **0.015** | - | - |
| Lymphocytosis >5,000/µL | **1.804 (0.938-3.470)** | **0.077** | 1.205 (0.564-2.576) | 0.630 |
| Monocytosis >1,000/µL | **1.184 (1.019-1.375)** | **0.027** | **1.171 (1.010-1.359)** | **0.037** |
| IPI 3–5 (DLBCL) | 0.623 (0.358-1.082) | 0.093 | 0.575 (0.323-1.022) | 0.059 |
| Post-treatment hypogammaglobulinemia | - | - | **2.089 (1.048-4.163)** | **0.036** |

**Table 1.** Multivariate analysis of PFS and OS prognostic factors in B-NHL.

### Summary/Conclusion

HG is a common and clinically relevant comorbidity in B-NHL, particularly after treatment. Severe IgG deficiency identifies patients with low-grade lymphoma at high risk of serious infections, and post-treatment HG may adversely affect overall survival. Routine immunoglobulin assessment may improve risk stratification and guide preventive strategies, including immunoglobulin replacement in selected patients.

**Copyright Information:** (Online) ISSN: 2572-9241
©2026 The Author(s). HemaSphere is published by John Wiley & Sons Ltd on behalf of European Hematology Association. This is an open access Abstract Book distributed under the Attribution-NonCommercial-NoDerivs (CC BY-NC-ND), which allows third parties to download the articles and share them with others as long as they credit the author and the Abstract Book, but they cannot change the content in any way or use them commercially.
**Abstract Book Citations:** Authors, Title, HemaSphere, 2026;10:(S1):pages. The abstract book DOIs can be found on the "Author Information" page.
**Disclaimer:** Articles published in the journal HemaSphere exclusively reflect the opinions of the authors. The authors are responsible for all content in their abstracts including accuracy of the facts, statements, citing resources, etc.

### (PS2421) USING S.SALIVARIUS PROBIOTIC TO PREVENT UPPER RESPIRATORY INFECTIONS IN RELAPSED-REFRACTORY MULTIPLE MYELOMA

**Topic:** 31. Infections in hematology (incl. supportive care/therapy)

Maria Santagati[1], Nunziatina Laura Parrinello[2], Gaetano Isola[1], Sara Marino[1], Bruno Garibaldi[2], Claudia Bellofiore[2], Concetta Conticello[2], Rossella Cacciola[1], Gaia Vertillo Aluisio[1], Francesco Di Raimondo[1, 2], Alessandra Romano*[1, 2]

*[1]Università degli Studi di Catania, Dipartimento di Chirurgia e Specialità Medico Chirurgiche, Catania, Italy;[2]AOU Policlinico Rodolico San Marco, UOC Ematologia, Catania, Italy;*

### Background

Probiotics are live microorganisms able to inhibit pathogen colonization indirectly or directly, together with the induction of immunomodulatory effects in the host. Our group used *S. salivarius* 24SMB, deposited as DSM 23307, as a potential oral probiotic, thanks to its safety assessment, ability to inhibit *Streptococcus pneumoniae* and the absence of virulence and antibiotic resistance genes to get used as valid alternative to antibiotics in the prevention of upper respiratory tract infections.

### Aims

In lack of data in oncological setting, we designed a prospective clinical investigation with additional invasive or burdensome procedures to evaluate the safety and efficacy of *Streptococcus Salivarius* 24 SMBc in RRMM patients.

### Methods

*Streptococcus Salivarius* 24 SMBc was given two times per day at intervals of 12 hours, by applying 4 puffs in each nostril (concentration per puff $1 \times 10^9$ bacteria), for 5 consecutive days, followed by 4-weeks of wash-out. Key exclusion criteria were: neutropenia, active infection, rhinosinus inflammatory or dental lesion that could favor the transfer of probiotic in the blood stream. Primary endpoint is rate and grading of treatment-related adverse events as assessed by CTCAE v.5.0. in RRMM patients receiving prophylaxis based on live probiotic administration. Furthermore, at study entry and after 1 month from the end of treatment, we assessed the 24 SMBc colonization and its ability to inhibit the main respiratory pathogens, particularly *Streptococcus pneumonia* and immunome composition of peripheral blood by next generation flow cytometry.

### Results

At data cut-off of 27 February 2026, 31 patients have been enrolled, including 8 screening failure (not eligible for treatment due to active infection or neutropenia) and 23 patients who completed treatments with one month of follow up.
All subjects reported no symptoms associated with nasal spray administration (runny nose, sneezing, coughing, headache). Side effects and/or undesirable effects were not observed at all. The body temperature (measured twice a day for 14 consecutive days) showed no clinically significant changes, and inflammatory markers (ESR, R-CP, absolute neutrophils count and pro-calcitonin) did not change during the 28-days follow up since the last probiotic administration. Physical examination of the upper airway showed no signs of infectious diseases throughout endoscopic surveillance. In one patient a positivity of *E.coli* in surveillance urine culture was found and treated with prophylactic dose of fosfomycin for two days.
No differences in microbial diversity were observed in the nasal microbiota of subjects treated with S. salivarius, suggesting that probiotic appeared to be safe because it did not disrupt the normal nasopharyngeal microbiome.
In the immunome composition, 71% patients showed a significant reduction in regulatory T-cells (7.2 vs 0.3%, p=0.02), Th17 sub-population circulating cells (14.3% vs 2.5%, p=0.02) and PDL1 expression on neutrophils (median MFI 3.7 vs 2.6, p=0.003)

Copyright Information: (Online) ISSN: 2572-9241
©2026 The Author(s). HemaSphere is published by John Wiley & Sons Ltd on behalf of European Hematology Association. This is an open access Abstract Book distributed under the Attribution-NonCommercial-NoDerivs (CC BY-NC-ND), which allows third parties to download the articles and share them with others as long as they credit the author and the Abstract Book, but they cannot change the content in any way or use them commercially.
Abstract Book Citations: Authors, Title, HemaSphere, 2026;10:(S1):pages. The abstract book DOIs can be found on the "Author Information" page.
Disclaimer: Articles published in the journal HemaSphere exclusively reflect the opinions of the authors. The authors are responsible for all content in their abstracts including accuracy of the facts, statements, citing resources, etc.

**Summary/Conclusion**

This is the first pivotal study showing that *S. salivarius* 24SMBc given as nasal spray is safe and well-tolerated. Our data suggest a dynamic relationship between Th17 and Treg cells in peripheral blood and nasopharynx microbiome that will be investigated in larger prospective cohorts.

**Copyright Information:** (Online) ISSN: 2572-9241
©2026 The Author(s). HemaSphere is published by John Wiley & Sons Ltd on behalf of European Hematology Association. This is an open access Abstract Book distributed under the Attribution-NonCommercial-NoDerivs (CC BY-NC-ND), which allows third parties to download the articles and share them with others as long as they credit the author and the Abstract Book, but they cannot change the content in any way or use them commercially.
**Abstract Book Citations:** Authors, Title, HemaSphere, 2026;10:(S1):pages. The abstract book DOIs can be found on the "Author Information" page.
**Disclaimer:** Articles published in the journal HemaSphere exclusively reflect the opinions of the authors. The authors are responsible for all content in their abstracts including accuracy of the facts, statements, citing resources, etc.

### (PS2422) VANCOMYCIN-RESISTANT ENTEROCOCCI COLONIZATION IN HEMATOLOGIC PATIENTS: IS IT STILL A PROBLEM? RESULTS OF A SURVEY AMONG THE CENTERS PARTICIPATING IN THE RETE EMATOLOGICA LOMBARDA (REL)

**Topic:** 31. Infections in hematology (incl. supportive care/therapy)

Chiara Cattaneo*[1], Patrizia Zappasodi[2], Francesca Pavesi[3], Claudia Maria Basilico[4], Mauro Turrini[5], Massimo Bernardi[6], Laura Donatoni[1], Erika Borlenghi[1], Alessandra Tucci[1], Giuseppe Rossi[1], Elisabetta Todisco[3]

*[1]Hematology, ASST-Spedali Civili, ERN-Eurobloodnet, Brescia, Italy; [2]Division of Hematology, Fondazione IRCCS Policlinico San Matteo, Pavia, Italy; [3]Division of Hematology, ASST Valleolona, Ospedale di Busto Arsizio, Busto Arsizio, Italy;[4]Division of Hematology, ASST Settelaghi, Ospedale di Circolo, Varese, Italy; [5]Section of Hematology, Valduce Hospital, Como, Italy; [6]Hematology and Bone Marrow Transplantation Unit, IRCCS San Raffaele Scientific Institute, Milano, Italy;*

### Background

The colonization of multidrug-resistant bacteria in hematologic patients is managed with strict isolation measures and is a critical issue in daily management in hematology wards. Among multidrug resistant bacteria, vancomycin-resistant enterococci (VRE) rectal colonization has been reported in up to 24% of hematologic patients, with 24.15 times more likely to develop related bloodstream infections (BSI) (*Alevizakos, Open Forum Infect Dis 2016*). A recent meta-analysis showed a higher mortality for VRE BSI compared with VSE BSI (*Eichel, J Hosp Infect 2023*), but the impact on attributable mortality is not completely understood.

### Aims

Our study aims to investigate the extent of the VRE colonization and related BSI in a real-life setting of hematologic patients in more recent years.

### Methods

We submitted a survey concerning VRE colonization and VRE BSI among hematologic patients during a 12-month period (June 1st, 2024 – May 31st, 2025) to the 10 centers participating in the REL.

**Copyright Information:** (Online) ISSN: 2572-9241
©2026 The Author(s). HemaSphere is published by John Wiley & Sons Ltd on behalf of European Hematology Association. This is an open access Abstract Book distributed under the Attribution-NonCommercial-NoDerivs (CC BY-NC-ND), which allows third parties to download the articles and share them with others as long as they credit the author and the Abstract Book, but they cannot change the content in any way or use them commercially.
**Abstract Book Citations:** Authors, Title, HemaSphere, 2026;10:(S1):pages. The abstract book DOIs can be found on the "Author Information" page.
**Disclaimer:** Articles published in the journal HemaSphere exclusively reflect the opinions of the authors. The authors are responsible for all content in their abstracts including accuracy of the facts, statements, citing resources, etc.

**Results**

Six centers (Brescia, Pavia, Como, Varese, Busto Arsizio and Milan-Ospedale San Raffaele) compiled the questionary about the rate of VRE BSI and 5 of them of VRE colonization. In each center all patients were subjected to a screening rectal swab upon entry to the Hematology ward. Overall, 1387 patients were admitted to six Hematology wards. As data concerning VRE colonization were provided by 5 centers, the incidence of VRE colonization in patients was 13.8% (154/1115), ranging between 7.8% and 23% among centers. Considering the total number of hospitalizations, the incidence was similar (260/1995, 13%). Of the 154 VRE colonized patients, 62 were affected by acute leukemia (40.3%), 59 by lymphoma (38.3%) and 17 by myeloma (11%). One center provided also the incidence of VRE colonization according to hematological disease, which was higher for acute leukemia (16/112, 14.3%) than for lymphoma (11/156, 7.1%) and myeloma (3/74, 4.1%) (p=0.0149). VRE colonization occurred more frequently in patients at clinical onset (80/154 (51.9%), than during remission of disease 31/154 (20.1%) or in relapsed/refractory disease in 43/154 (27.9%). During the observation period, 10 patients developed 11 VRE BSI, corresponding to 0.7% of all hospitalized patients. Only 6 out of the 154 colonized patients developed a related VRE BSI (3.9%). The incidence of related VRE BSI according to hematologic disease showed a trend for a greater frequency in acute leukemia patients (4/62, 6.5%) than in other hematological diseases (2/82, 2.4%) (p=0.22). One colonized patient developing a VRE BSI deceased within 30 days since BSI (1/10, 10%); death was due to progressive hematologic disease. Therefore, VRE colonization attributable mortality was 0%.

**Summary/Conclusion**

In this survey carried out within REL centers, VRE colonization affected more than 10% of hematologic patients, with peaks of 23%, emphasizing the extreme variability within different centers. However, frequency of related VRE BSI occurring in colonized patients was low, accounting for only approximately 50% of all VRE BSI and their prognosis was good. These data question the cost-benefit ratio of the isolation procedures currently used in VRE colonized patients and suggest that in the daily management of patients in hematological wards these precautional measures could be limited to the most vulnerable patients, such as those with acute leukemia.

**Copyright Information:** (Online) ISSN: 2572-9241
©2026 The Author(s). HemaSphere is published by John Wiley & Sons Ltd on behalf of European Hematology Association. This is an open access Abstract Book distributed under the Attribution-NonCommercial-NoDerivs (CC BY-NC-ND), which allows third parties to download the articles and share them with others as long as they credit the author and the Abstract Book, but they cannot change the content in any way or use them commercially.
**Abstract Book Citations:** Authors, Title, HemaSphere, 2026;10:(S1):pages. The abstract book DOIs can be found on the “Author Information” page.
**Disclaimer:** Articles published in the journal HemaSphere exclusively reflect the opinions of the authors. The authors are responsible for all content in their abstracts including accuracy of the facts, statements, citing resources, etc.

### (PS2423) RISK FACTORS, CLINICAL OUTCOMES AND IMMUNE DYNAMICS OF CANDIDEMIA IN PATIENTS WITH HEMATOLOGICAL DISORDER

**Topic:** 31. Infections in hematology (incl. supportive care/therapy)

Zhangjie Chen*[1], Sisi Zhen[1], Xin Chen[1], Sizhou Feng[1]

[1]*Institute of Hematology & Blood Diseases Hospital, Chinese Academy of Medical Sciences & Peking Union Medical College, Tianjin, China;*

### Background

Candidemia, particularly due to *Candida tropicalis*, was of high incidence among patients with hematological disorders. However, associated risk factors, immune dynamics and clinical outcomes remained underexplored.

### Aims

We aim to explore clinical characteristics and prognosis of candidemia in patients with hematological disorders.

### Methods

Clinical data from 105 patients diagnosed with hematological disorders and confirmed candidemia were retrospectively analyzed.

### Results

*Candida tropicalis* was identified in 79 patients (75.2%), and non-tropicalis species included Candida albicans (n=6, 5.7%), Candida glabrata (n=4, 3.8%), Candida parapsilosis (n=12, 11.4%), Candida krusei (n=3, 2.9%) and C. guilliermondii (n=1, 1.0%). Prior transplantation was more common in non-tropicalis candidemia (P=0.023), whereas culture positivity within 24 hours was associated with *Candida tropicalis* (P=0.008). *Candida tropicalis* showed favorable antifungal susceptibility compared to non-tropicalis, particularly to flucytosine (P<0.001). Azole monotherapy (29.5%) and azole-echinocandin combination (25.7%) were the most used treatments. Complete response was achieved in 58 patients (55.2%), with similar rate between *Candida tropicalis* and non-tropicalis group (P = 0.869). The restricted median survival time was 64 days, with no significant difference in mortality or survival between groups. Older age (P=0.043), abdominal pain (P=0.040) and septic shock (P=0.008) were associated with poor outcomes. Patients achieving remission exhibited higher neutrophil and lymphocyte counts and greater immune recovery. *Candida tropicalis* BSI was associated with deeper neutropenia, significantly lower NLR and a more pronounced neutrophil decline at onset (Figure 1), suggesting a “double-hit” immune suppression.

### Summary/Conclusion

*Candida tropicalis* and non-tropicalis candidemia exhibited distinct risk factors and immune dynamics, yet both were associated with high mortality. Further investigation is needed to elucidate invasion mechanism and immunological vulnerabilities, guiding targeted prophylaxis and therapy.

**Copyright Information:** (Online) ISSN: 2572-9241
©2026 The Author(s). HemaSphere is published by John Wiley & Sons Ltd on behalf of European Hematology Association. This is an open access Abstract Book distributed under the Attribution-NonCommercial-NoDerivs (CC BY-NC-ND), which allows third parties to download the articles and share them with others as long as they credit the author and the Abstract Book, but they cannot change the content in any way or use them commercially.
**Abstract Book Citations:** Authors, Title, HemaSphere, 2026;10:(S1):pages. The abstract book DOIs can be found on the “Author Information” page.
**Disclaimer:** Articles published in the journal HemaSphere exclusively reflect the opinions of the authors. The authors are responsible for all content in their abstracts including accuracy of the facts, statements, citing resources, etc.

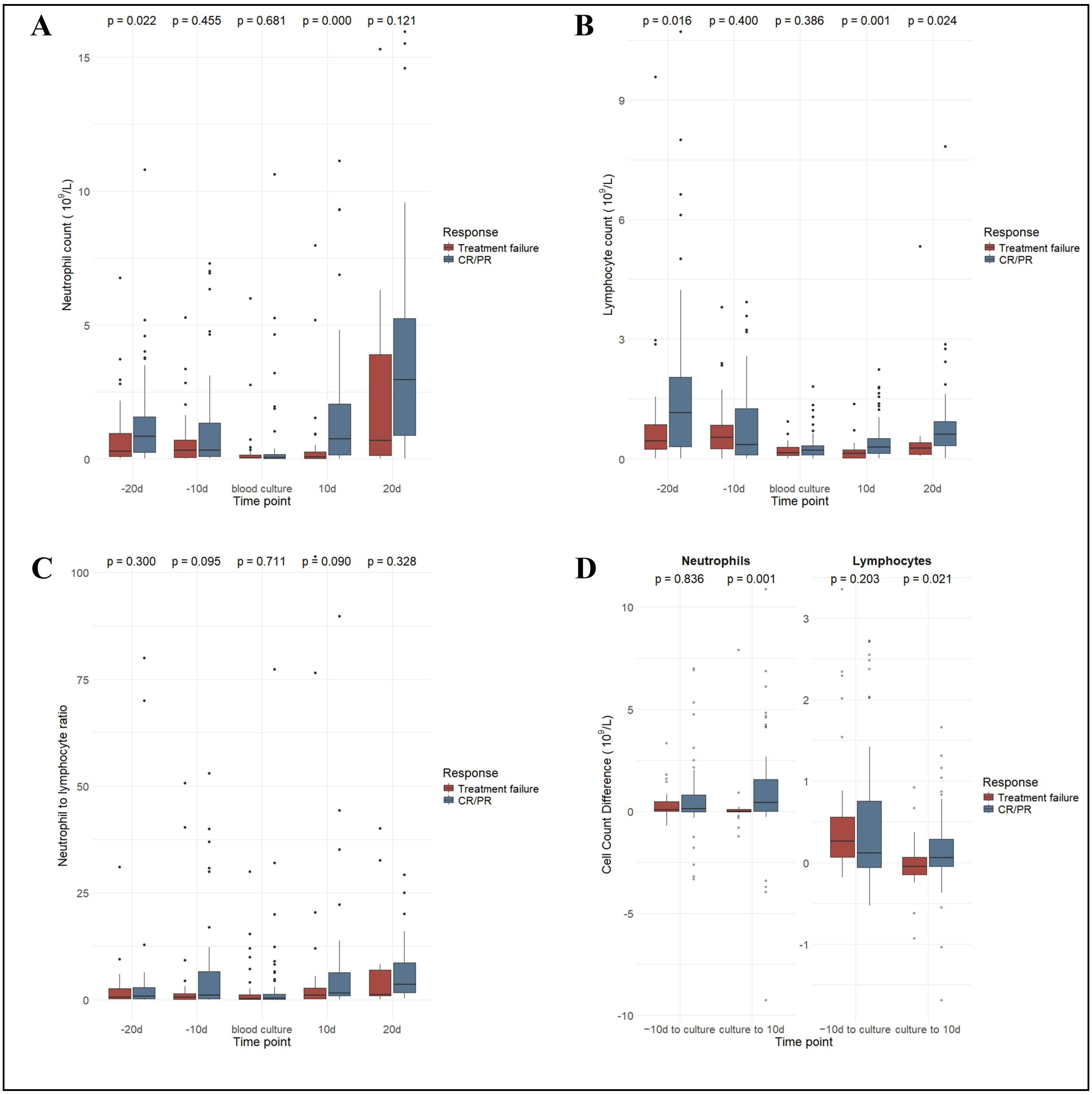

Figure 1. Neutrophil count (A), lymphocyte count (B), neutrophil to lymphocyte ratio (C) and changes in neutrophil and lymphocyte count (D) at multiple time points in patients with complete response (CR)/partial response (PR) or treatment failure.

**Copyright Information:** (Online) ISSN: 2572-9241
©2026 The Author(s). HemaSphere is published by John Wiley & Sons Ltd on behalf of European Hematology Association. This is an open access Abstract Book distributed under the Attribution-NonCommercial-NoDerivs (CC BY-NC-ND), which allows third parties to download the articles and share them with others as long as they credit the author and the Abstract Book, but they cannot change the content in any way or use them commercially.
**Abstract Book Citations:** Authors, Title, HemaSphere, 2026;10:(S1):pages. The abstract book DOIs can be found on the "Author Information" page.
**Disclaimer:** Articles published in the journal HemaSphere exclusively reflect the opinions of the authors. The authors are responsible for all content in their abstracts including accuracy of the facts, statements, citing resources, etc.

### (PS2424) OVERCOMING THE DIAGNOSTIC GRAY ZONE OF NEUTROPHIL CD64 IN HEMATOLOGICAL MALIGNANCIES USING THE MONOCYTE-TO-NEUTROPHIL CD64 EXPRESSION RATIO

**Topic:** 31. Infections in hematology (incl. supportive care/therapy)

Kazuhiro Itoh[1], Amane Tanaka*[1], Nozomi Otsuki[1], Hiroshi Tsutani[1], Akiko Matsunaga[2], Masamichi Ikawa[2], Hiromichi Iwasaki[2], Yasuhiko Mitsuke[1]

*[1]NHO Awara National Hospital, Internal Medicine, Awara, Japan; [2]University of Fukui, Community Health Science, Yoshida-gun, Japan;*

### Background

Neutrophil CD64 (nCD64) is a promising biomarker for differentiating infection from non-infectious inflammation. In general patient populations, previous studies have suggested an nCD64 cutoff around 2,000/cell. However, in hematological malignancies, nCD64 may be elevated even without infection, leading to a diagnostic “gray zone” and reduced specificity.

### Aims

To assess whether the monocyte CD64/neutrophil CD64 ratio (CD64 ratio) and a composite model integrating nCD64 and CD64 ratio improve discrimination of infection from non-infection in hematological malignancies.

### Methods

We retrospectively analyzed 47 patients with hematological malignancies presenting with fever (≥37.5°C) and/or elevated C-reactive protein (CRP ≥0.3 mg/dL) between January 2010 and December 2025. Infection status (infection n=35; non-infection n=12) was determined by attending physicians’ final clinical diagnosis based on a comprehensive assessment. CD64 was quantified as antibodies bound per cell (ABC) using QuantiBrite PE beads. We compared WBC, CRP, nCD64, and CD64 ratio. A logistic regression model incorporating nCD64 and CD64 ratio was evaluated using ROC analysis and exploratory continuous net reclassification improvement (NRI) and integrated discrimination improvement (IDI).

### Results

Infections included respiratory infections, sepsis, and febrile neutropenia, while non-infectious cases included tumor fever and other diagnoses. Conventional markers showed limited discrimination: WBC AUC 0.611 (95%CI 0.428–0.793); CRP AUC 0.567 (95%CI 0.372–0.761). nCD64 showed the highest single-marker performance: AUC 0.70 (95%CI 0.539–0.861). Using the cohort-derived cutoff of 6,160/cell yielded sensitivity 51.4% and specificity 83.3%, whereas the commonly cited cutoff of 2,000/cell provided sensitivity 85.7% and specificity 33.3%, indicating a clinically relevant sensitivity–specificity trade-off. The CD64 ratio showed moderate discrimination: AUC 0.669 (95%CI 0.510–0.828); at the cohort-derived cutoff of 4.1, sensitivity was 37.1% and specificity was 100.0% (lower ratios associated with infection). Figure 1 illustrates that among patients with intermediate nCD64 values (2,000–6,160/cell), CD64 ratio provides additional separation between infection and non-infection. The integrated model improved classification compared with single-marker approaches (continuous NRI 0.638, p=0.020; NRI non-events 0.667, p=0.014; IDI 0.095, p=0.016).

**Copyright Information:** (Online) ISSN: 2572-9241
©2026 The Author(s). HemaSphere is published by John Wiley & Sons Ltd on behalf of European Hematology Association. This is an open access Abstract Book distributed under the Attribution-NonCommercial-NoDerivs (CC BY-NC-ND), which allows third parties to download the articles and share them with others as long as they credit the author and the Abstract Book, but they cannot change the content in any way or use them commercially.
**Abstract Book Citations:** Authors, Title, HemaSphere, 2026;10:(S1):pages. The abstract book DOIs can be found on the “Author Information” page.
**Disclaimer:** Articles published in the journal HemaSphere exclusively reflect the opinions of the authors. The authors are responsible for all content in their abstracts including accuracy of the facts, statements, citing resources, etc.

**Summary/Conclusion**

In hematological malignancies, nCD64 alone shows a clinically relevant sensitivity–specificity trade-off, particularly in the intermediate range. CD64 ratio offers complementary information and, when integrated with nCD64, improved classification in this exploratory retrospective cohort. Prospective validation is warranted.

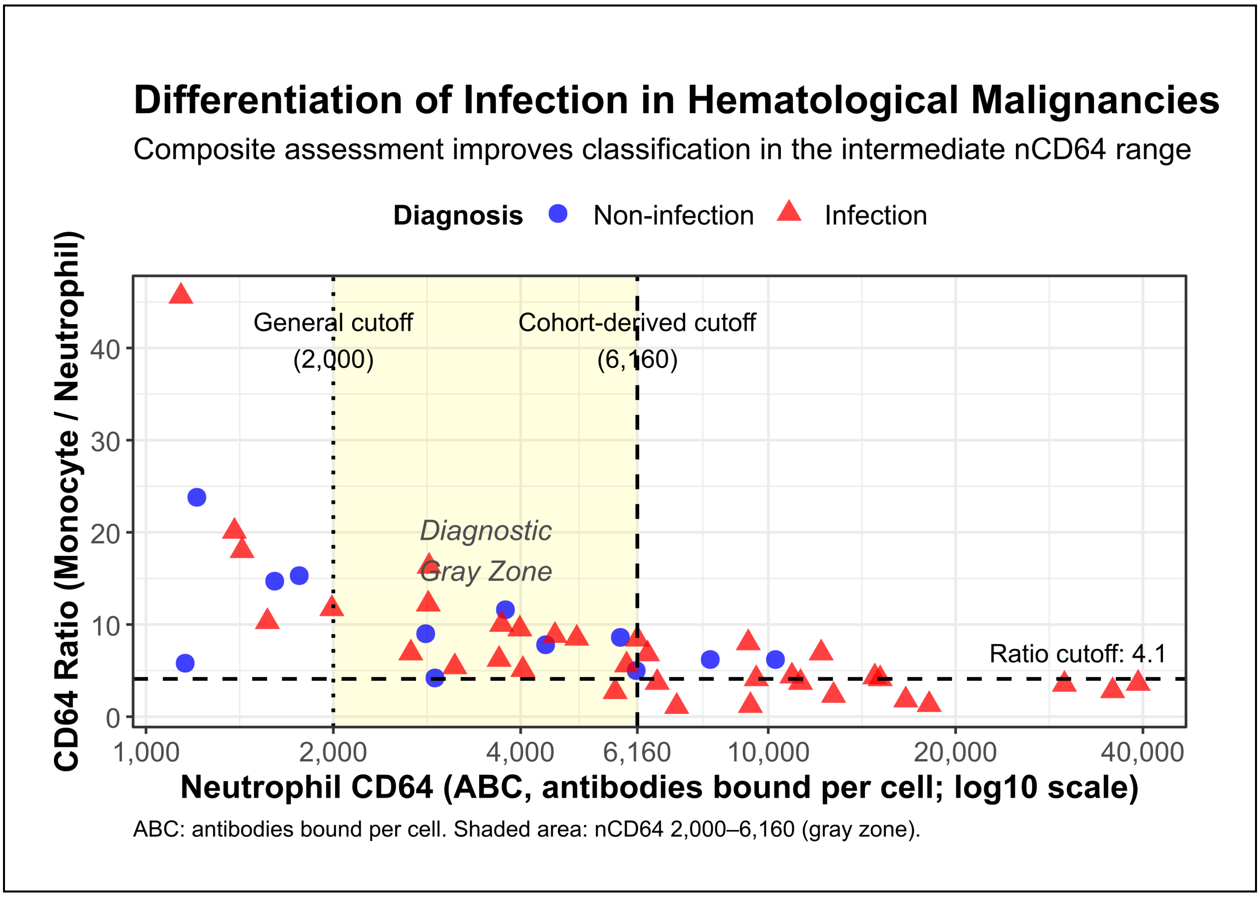

**Copyright Information:** (Online) ISSN: 2572-9241
©2026 The Author(s). HemaSphere is published by John Wiley & Sons Ltd on behalf of European Hematology Association. This is an open access Abstract Book distributed under the Attribution-NonCommercial-NoDerivs (CC BY-NC-ND), which allows third parties to download the articles and share them with others as long as they credit the author and the Abstract Book, but they cannot change the content in any way or use them commercially.
**Abstract Book Citations:** Authors, Title, HemaSphere, 2026;10:(S1):pages. The abstract book DOIs can be found on the "Author Information" page.
**Disclaimer:** Articles published in the journal HemaSphere exclusively reflect the opinions of the authors. The authors are responsible for all content in their abstracts including accuracy of the facts, statements, citing resources, etc.

### (PS2425) LAMIVUDINE PROPHYLAXIS FOR PREVENTION OF HEPATITIS B VIRUS REACTIVATION IN HEMATOLOGIC PATIENTS UNDERGOING IMMUNOSUPPRESSIVE THERAPIES: A SINGLE-CENTER EXPERIENCE

**Topic:** 31. Infections in hematology (incl. supportive care/therapy)

Gianmarco Favrin*[1], Sofia Pedrali[1], Yury Yudin[1], Semela Resuli[2], Virginia Ferretti[3], Sara Rattotti[1], Manuel Gotti[1], Emilia Cappello[1], Angelica Parisotto[1], Chiara Cavalloni[1], Alessandro Mazzacane[1], Nicola Polverelli[1], Patrizia Zappasodi[1], Claudio Cartia[1], Michele Palumbo[1], Antonio Bianchessi[1], Irene Defrancesco[1], Daniel Ghirardi[4], Luca Arcaini[1], Raffaele Bruno[2], Valentina Zuccaro[2], Marzia Varettoni[1]

*[1]Fondazione I.R.C.C.S. Policlinico San Matteo, SC Hematology Unit 1, Pavia, Italy; [2]Fondazione I.R.C.C.S. Policlinico San Matteo, Division of Infectious Diseases, Department of Clinical-Surgical, Diagnostic and Pediatric Sciences, University of Pavia, Pavia, Italy; [3]Fondazione Irccs Policlinico San Matteo, Biostatistics and Clinical Trial Center, Pavia, Italy; [4]Pavia University, Department of Molecular Medicine, Pavia, Italy;*

### Background

Hepatitis B virus (HBV) reactivation represents a life-threatening complication in hematologic patients receiving immunosuppressive therapy. Current international guidelines (AASLD, EASL, NCCN) recommend entecavir or tenofovir as preferred agents for HBV prophylaxis, mainly because of the higher resistance rates observed with lamivudine (20–30% at 1–2 years versus approximately 1% at 5 years with entecavir).

### Aims

This single-center retrospective study aimed to evaluate the real-world efficacy of lamivudine prophylaxis in preventing HBV reactivation (HBVr) in anti-HBc-positive hematologic patients undergoing chemotherapy and/or immunotherapy. Secondary aims were to assess the association between clinical and virological characteristics and treatment failure, and to evaluate the safety profile of lamivudine in this population.

### Methods

We analyzed data from a prospectively maintained database including 380 hematologic patients diagnosed and followed at SC Ematologia 1 IRCCS Policlinico San Matteo. Eligible patients were those who received immunosuppressive therapy between 2002 and 2025 and who started anti-HBV prophylaxis according to the guidelines available at the time, with lamivudine administered 2 weeks before the initiation of hematologic treatment and continued for 18 months after its completion. Only patients with available HBV serological markers (HBsAg and anti-HBc) were included in the final analysis. The risk of HBVr according to the type of hematologic therapy was stratified according to the latest European Association for the Study of the Liver (EASL) recommendations.

**Copyright Information:** (Online) ISSN: 2572-9241
©2026 The Author(s). HemaSphere is published by John Wiley & Sons Ltd on behalf of European Hematology Association. This is an open access Abstract Book distributed under the Attribution-NonCommercial-NoDerivs (CC BY-NC-ND), which allows third parties to download the articles and share them with others as long as they credit the author and the Abstract Book, but they cannot change the content in any way or use them commercially.
**Abstract Book Citations:** Authors, Title, HemaSphere, 2026;10:(S1):pages. The abstract book DOIs can be found on the "Author Information" page.
**Disclaimer:** Articles published in the journal HemaSphere exclusively reflect the opinions of the authors. The authors are responsible for all content in their abstracts including accuracy of the facts, statements, citing resources, etc.

**Results**

The baseline characteristics of the 380 patients included in the analysis are reported in Figure 1. According to the latest EASL guidelines, patients were stratified into three HBVr risk groups: high risk (301/380, 79.2%), intermediate risk (77/380, 20.3%), and low risk (2/380, 0.5%). Overall, 10 out of 380 patients (2.6%) experienced HBVr during follow-up. Among these, 6/10 patients (60%) were in the high-risk group and 4/10 (40%) were in the intermediate-risk group. The 5-years cumulative incidence of HBVr was 3.5% (95% CI: 1.7–6.4%), with an incidence rate of 67.4 per 10000 person-years (95% CI: 32.3–124.0). No statistically significant differences in reactivation rate were observed according to serological pattern, hematologic disease or type of immunosuppressive therapy. At the time of reactivation, median HBV-DNA was 976 IU/mL (range 165-5.831.642 IU/mL). Median ALT and AST levels were 28 U/L (IQR: 22-65) and 32 U/L (IQR: 22-80), respectively, while median total bilirubin was 2.1 mg/dL (IQR: 1.33–3.71). Following reactivation, 7 patients (70%) were treated with entecavir, 2 patients (20%) with tenofovir, and 1 patient (10%) continued lamivudine. All patients achieved complete virological response, defined as undetectable HBVDNA. Two patients initially treated with entecavir failed to achieve HBVDNA undetectability and were successfully switched to tenofovir. No grade ≥3 lamivudine-related adverse events were observed. HBVr had no significant impact on overall survival.

**Summary/Conclusion**

This study shows that lamivudine prophylaxis resulted in a low rate of HBVr and was safe in anti-HBc-positive hematologic patients undergoing immunosuppressive therapy. Despite current guideline preferences for newer antivirals, our findings suggest that lamivudine remains a reasonable and well-tolerated option.

**Copyright Information:** (Online) ISSN: 2572-9241
©2026 The Author(s). HemaSphere is published by John Wiley & Sons Ltd on behalf of European Hematology Association. This is an open access Abstract Book distributed under the Attribution-NonCommercial-NoDerivs (CC BY-NC-ND), which allows third parties to download the articles and share them with others as long as they credit the author and the Abstract Book, but they cannot change the content in any way or use them commercially.
**Abstract Book Citations:** Authors, Title, HemaSphere, 2026;10:(S1):pages. The abstract book DOIs can be found on the "Author Information" page.
**Disclaimer:** Articles published in the journal HemaSphere exclusively reflect the opinions of the authors. The authors are responsible for all content in their abstracts including accuracy of the facts, statements, citing resources, etc.

**Figure 1. Baseline patients' characteristics**

| Characteristic | Overall population (N = 380) |
|---|---|
| **Sex, n (%)** | |
| Male<br>Female | 234 (61.6%)<br>146 (38.4%) |
| **Age, years, median (IQR)** | 67 (59-76) |
| **Risk of HBV reactivation, n (%)** | |
| Low<br>Intermediate<br>High | 2 (0.5%)<br>77 (20.3%)<br>301 (79.2%) |
| **Hematologic diagnosis, n (%)** | |
| Lymphoproliferative disease<br>Acute Leukemia<br>Myelodisplasia<br>Multiple Myeloma<br>Other | 230 (60.5%)<br>52 (13.7%)<br>12 (3.1%)<br>69 (18.2%)<br>17 (4.5%) |
| **Hematologic therapy, n (%)** | |
| Immunochemotherapy<br>High dose chemotherapy<br>Immunotherapy<br>Chemotherapy<br>Anthracycline based Chemotherapy<br>Allogenic Hemopoietic Stem Cell Transplantation<br>Tyrosine Kinase Inhibitor<br>T-cell Depleting Therapy<br>Other | 177 (46.6%)<br>43 (11.3%)<br>40 (10.5%)<br>38 (10.0%)<br>15 (4.0%)<br>13 (3.4%)<br>8 (2.1%)<br>2 (0.5%)<br>44 (11.6%) |
| **HBV serological status, n (%)** | |
| HBsAg positive<br>Anti-HBc positive<br>Anti-HBs positive<br>Detectable HBV-DNA | 8/380 (2.1%)<br>357/380 (93.9%)<br>278/380 (73.1%)<br>21 (5.5%) |
| **Steroid therapy, n (%)** | |
| PDN >2 mg/kg/day ≥14 days<br>PDN >1 mg/kg/day ≥28 days | 97 (25.5%)<br>124 (32.6%) |
| **Stem cell transplantation, n (%)** | |
| Autologous SCT<br>Allogeneic SCT | 57 (15.3%)<br>55 (14.6%) |

Copyright Information: (Online) ISSN: 2572-9241
©2026 The Author(s). HemaSphere is published by John Wiley & Sons Ltd on behalf of European Hematology Association. This is an open access Abstract Book distributed under the Attribution-NonCommercial-NoDerivs (CC BY-NC-ND), which allows third parties to download the articles and share them with others as long as they credit the author and the Abstract Book, but they cannot change the content in any way or use them commercially.
Abstract Book Citations: Authors, Title, HemaSphere, 2026;10:(S1):pages. The abstract book DOIs can be found on the "Author Information" page.
Disclaimer: Articles published in the journal HemaSphere exclusively reflect the opinions of the authors. The authors are responsible for all content in their abstracts including accuracy of the facts, statements, citing resources, etc.

### (PS2426) CAN SURVEILLANCE CULTURE-GUIDED TARGETED ANTIBIOTIC THERAPY IMPROVE TREATMENT OUTCOMES OF FEBRILE NEUTROPENIA IN ACUTE LEUKEMIA PATIENTS ON INTENSIVE CHEMOTHERAPY? SINGLE-CENTER EXPERIENCE FROM INDIA

**Topic:** 31. Infections in hematology (incl. supportive care/therapy)

Reshma Benson[1], Prisla Dalton[1], Bibhant Shah[1], Kavya Ronanki[1], Nikhil Nagpal[1, 1], Arjun Kachhwaha[1], Adamya Gupta[1], Paras Satadeve[1], Karthik Kumar[1], Amber Prasad[1], Uttam Kumar Nath*[1]

*[1]All India Institute of Medical Sciences, Rishikesh, Department of Medical Oncology Hematology, Rishikesh, India;*

### Background

Febrile neutropenia (FN) is the major cause of mortality during intensive chemotherapy for acute leukemia, with antimicrobial resistance (AMR) increasingly complicating empiric antibiotic treatment strategies. Colonisation with multidrug-resistant (MDR) organisms may precede bloodstream infection; however, clinical value of routine surveillance cultures to guide antibiotic selection for FN is uncertain.

### Aims

To study whether baseline surveillance cultures identifying colonising organisms & AMR patterns can predict infection profile during febrile neutropenia and be utilised to guide initial antimicrobial therapy during FN episodes to improve FN-related outcomes.

### Methods

The study included 150 acute leukemia patients undergoing 231 admissions for intensive chemotherapy at a tertiary hospital between October 2023 & April 2025. Majority (64.7%) were pediatric age group patients (age <18 years). Baseline surveillance cultures (throat swab and rectal swab) were obtained prior to intensive chemotherapy to define colonisation and AMR patterns. Antibiotic treatment strategy during FN episodes in the two groups was as follows:

- **Group 1 (Surveillance culture-positive):** received targeted antibiotics guided by susceptibility profile of isolate as per surveillance culture.
- **Group 2 (Surveillance culture-negative):** received empiric antibiotic therapy as per institutional FN protocol.

Outcomes related to FN that were compared in the two groups included blood culture positivity rates, duration of FN episodes, trend of serial serum procalcitonin levels at 0, 48, & 96 hours after onset of FN, duration of antibiotic treatment for FN episodes, and in-hospital mortality during FN episodes.

**Copyright Information:** (Online) ISSN: 2572-9241
©2026 The Author(s). HemaSphere is published by John Wiley & Sons Ltd on behalf of European Hematology Association. This is an open access Abstract Book distributed under the Attribution-NonCommercial-NoDerivs (CC BY-NC-ND), which allows third parties to download the articles and share them with others as long as they credit the author and the Abstract Book, but they cannot change the content in any way or use them commercially.
**Abstract Book Citations:** Authors, Title, HemaSphere, 2026;10:(S1):pages. The abstract book DOIs can be found on the “Author Information” page.
**Disclaimer:** Articles published in the journal HemaSphere exclusively reflect the opinions of the authors. The authors are responsible for all content in their abstracts including accuracy of the facts, statements, citing resources, etc.

**Results**

The patient characteristics, bacteriological spectrum of surveillance cultures & blood cultures during FN episodes, and FN-related outcomes in study are depicted in **Table 1**. Surveillance cultures were positive in 32/231 (13.8%) admissions, identifying predominantly MDR Gram-negative bacteria (88%), with MDR *Klebsiella pneumoniae* being the most common isolate (26%). FN developed in 212 of total 231 admissions (91.8%); there was no significant difference in incidence of FN between the two groups (90.6% vs 92%; p 0.73). The proportion of microbiologically documented infection in FN episodes was 40.2%, including blood culture positivity rate of 26.8%. Blood culture positivity rate was significantly higher in group 1 compared to group 2 (41.9% vs 24.4%; p 0.049; Odds ratio 2.2). There was no concordance between bacterial isolates observed in surveillance culture and blood culture isolates during FN episodes (p 0.96).
Comparison of surveillance-guided targeted antibiotic therapy (group 1) versus empiric antibiotic therapy (group 2) strategies for FN demonstrated:

- No significant difference in duration of FN (mean 14.62±7.1 vs 13.77±7.03 days; p value 0.47).
- Comparable serum procalcitonin levels & trends during FN episodes.
- No significant difference in duration of antibiotic therapy for FN (mean 11.56±7.84 vs 9.02± 4.62 days; p value 0.197).
- No significant difference in mortality due to FN in the two groups (p value 0.14).

**Summary/Conclusion**

In the present study, baseline surveillance cultures in pediatric & adult acute leukemia patients receiving intensive chemotherapy identified bacterial colonisation and AMR burden but demonstrated limited utility in predicting infection profile during FN episodes or improving clinical outcomes when used to guide targeted antimicrobial therapy for febrile neutropenia.

**Copyright Information:** (Online) ISSN: 2572-9241
©2026 The Author(s). HemaSphere is published by John Wiley & Sons Ltd on behalf of European Hematology Association. This is an open access Abstract Book distributed under the Attribution-NonCommercial-NoDerivs (CC BY-NC-ND), which allows third parties to download the articles and share them with others as long as they credit the author and the Abstract Book, but they cannot change the content in any way or use them commercially.
**Abstract Book Citations:** Authors, Title, HemaSphere, 2026;10:(S1):pages. The abstract book DOIs can be found on the “Author Information” page.
**Disclaimer:** Articles published in the journal HemaSphere exclusively reflect the opinions of the authors. The authors are responsible for all content in their abstracts including accuracy of the facts, statements, citing resources, etc.

| Table 1: Patient characteristics, microbiological data & clinical outcomes of febrile neutropenia in study | | | |
|---|---|---|---|
| Total admissions analysed | 231 [Pediatric: 145 (63%); Adult: 86 (IQR 37%)] | | |
| Median age (yrs) | Pediatric patients: 8 (IQR 4-13) | Adult patients: 31 (IQR 26-39) | |
| Diagnosis | Pediatric patients:<br>B-ALL: 57%<br>AML: 26%<br>T-ALL: 13%<br>LBL: 4% | Adult patients:<br>AML: 51%<br>B-ALL: 28%<br>T-ALL/LBL: 11%<br>CML-Blast Phase: 10% | |
| Surveillance culture results | Positive: 13.8% (32/231) | Negative: 86.2% (199/231) | |
| Surveillance culture isolates | Gram-negative: 88% | Gram-positive: 12% | |
| Predominant colonising organisms on surveillance cultures | Gram-negative bacteria: (n=28)<br>Klebsiella pneumoniae 30%<br>Escherichia coli 20%<br>Pseudomonas aeruginosa 20%<br>Enterobacter cloacae 13.3%<br>Acinetobacter baumanii 13.3% | Gram-positive bacteria: (n=4)<br>Streptococcus pneumoniae<br>Methicillin-resistant Staphylococcus aureus (MRSA)<br>MR CoNS | |
| Febrile neutropenia episodes | 212 / 231 admissions (91.8 %) | | |
| Febrile neutropenia clinical outcomes | Group 1 (Surveillance culture-positive) | Group 2 (Surveillance culture-negative) | |
| Incidence of FN | 90.6 % | 92 % | p 0.73 |
| Blood culture positivity | 41.9 % | 24.4 % | **p 0.049** |
| Mean duration of FN (days) | 14.62 +/- 7.1 | 13.77 +/-7.03 | p 0.47 |
| Mean duration of antibiotic therapy for FN (days) | 11.56 +/- 7.84 | 9.02 +/- 4.62 | p 0.19 |

**Copyright Information:** (Online) ISSN: 2572-9241
©2026 The Author(s). HemaSphere is published by John Wiley & Sons Ltd on behalf of European Hematology Association. This is an open access Abstract Book distributed under the Attribution-NonCommercial-NoDerivs (CC BY-NC-ND), which allows third parties to download the articles and share them with others as long as they credit the author and the Abstract Book, but they cannot change the content in any way or use them commercially.
**Abstract Book Citations:** Authors, Title, HemaSphere, 2026;10:(S1):pages. The abstract book DOIs can be found on the "Author Information" page.
**Disclaimer:** Articles published in the journal HemaSphere exclusively reflect the opinions of the authors. The authors are responsible for all content in their abstracts including accuracy of the facts, statements, citing resources, etc.

### (PS2427) DYNAMIC WHOLE-BLOOD TRANSCRIPTOMIC CHARACTERISTICS OF INFECTION EVOLUTION IN HEMATOLOGIC MALIGNANCY PATIENTS WITH CHEMOTHERAPY-INDUCED NEUTROPENIA

**Topic:** 31. Infections in hematology (incl. supportive care/therapy)

Dairong Xie*[1], Mei Hong[1]

[1]*Union Hospital, Tongji Medical College, Huazhong University of Science and Technology, Wuhan, China., Institute of Hematology, Wuhan, China;*

### Background

Chemotherapy-induced neutropenia is a severe complication in hematologic malignancies, increasing invasive infection incidence, sepsis risk, and 28-day mortality. However, sensitive and specific molecular biomarkers for early risk prediction and prognostic stratification remain lacking.

### Aims

This study employs longitudinal dynamic whole-blood transcriptome sequencing to systematically characterize host transcriptional responses in hematologic malignancy patients throughout chemotherapy-induced neutropenia. It aims to identify key gene modules and pathways driving infection susceptibility and adverse outcomes, providing a basis for early risk assessment and individualized intervention.

### Methods

This prospective cohort study enrolled 104 patients with chemotherapy-induced neutropenia. Peripheral blood was collected at three timepoints for whole-blood transcriptome sequencing: (1) first neutropenia episode (ANC < $0.5 \times 10^9$/L); (2) first febrile neutropenia (FN), defined as ANC < $0.5 \times 10^9$/L with fever (≥ 38.3°C or ≥ 38.0°C sustained ≥ 1 hour); and (3) resolution phase (ANC ≥ $1.0 \times 10^9$/L and afebrile ≥ 48 hours). Patients were stratified into non-FN and FN groups; FN patients were classified by CTCAE v5.0 (grade 3/4) and 28-day all-cause mortality (survival/death).

### Results

Enrichment analysis revealed significant activation of extracellular matrix–receptor interaction and protein digestion pathways during FN onset, while neuroactive ligand–receptor interaction showed bidirectional regulation. Compared to non-severe patients, grade 4 infection patients showed significant downregulation of neutrophil extracellular trap (NET)-related genes, indicating impaired innate antimicrobial capacity; the sphingolipid biosynthesis pathway was markedly upregulated in non-survivors. Cross-stratification identified 114 commonly upregulated and 26 downregulated genes, reflecting a concurrent procoagulant and immunosuppressive state. Immune deconvolution indicated increased plasma cell proportion associated with FN occurrence, while decreased regulatory T cells correlated with 28-day mortality. WGCNA revealed aberrant modules linked to hypercoagulability and immune paralysis during the neutropenic phase in patients who later developed grade 4 infection.

### Summary/Conclusion

Patients with chemotherapy-induced neutropenia exhibit concurrent procoagulant and immunosuppressive states early in infection. Those progressing to severe infection or death display impaired innate antimicrobial function, offering a molecular foundation for early risk stratification and management.

**Copyright Information:** (Online) ISSN: 2572-9241
©2026 The Author(s). HemaSphere is published by John Wiley & Sons Ltd on behalf of European Hematology Association. This is an open access Abstract Book distributed under the Attribution-NonCommercial-NoDerivs (CC BY-NC-ND), which allows third parties to download the articles and share them with others as long as they credit the author and the Abstract Book, but they cannot change the content in any way or use them commercially.
**Abstract Book Citations:** Authors, Title, HemaSphere, 2026;10:(S1):pages. The abstract book DOIs can be found on the “Author Information” page.
**Disclaimer:** Articles published in the journal HemaSphere exclusively reflect the opinions of the authors. The authors are responsible for all content in their abstracts including accuracy of the facts, statements, citing resources, etc.

### (PS2428) EFFICACY AND SAFETY OF CIDOFOVIR FOR ADENOVIRUS INFECTION IN PEDIATRIC PATIENTS AFTER HEMATOPOIETIC STEM CELL TRANSPLANT: A SINGLE-CENTER RETROSPECTIVE STUDY

**Topic:** 31. Infections in hematology (incl. supportive care/therapy)

Anqi Zhou*[1], Yixin Hu[1], Jing Ling[1], Yanhua Yao[1], Shaoyan Hu[1]

[1] *Children's Hospital of Soochow University, Suzhou, China;*

### Background

Adenovirus (ADV) infection is a life-threatening complication after hematopoietic stem cell transplantation (HSCT), with mortality rates up to 80% in disseminated disease. Cidofovir (CDV) is the primary therapy; however, its use is often limited by nephrotoxicity, and real-world data in pediatric cohorts remain scarce.

### Aims

This single-center retrospective study evaluated the efficacy and renal safety of CDV for ADV infection in pediatric HSCT recipients.

### Methods

We retrospectively analyzed those pediatric HSCT recipients who received intravenous CDV for ADV infection at our center from January 2021 to December 2025. CDV was given intravenously at 5mg/kg weekly for 2 consecutive weeks, then every 2 weeks. ADV infection was diagnosed by next-generation sequencing (NGS) from blood, stool or respiratory specimens. Virological response was defined as ≥2 $\log_{10}$ reduction from baseline viral load or undetectable virus after CDV treatment. Complete viral clearance was defined as undetectable ADV DNA. Nephrotoxicity was graded according to CTCAE v5.0. Data on baseline characteristics, GVHD status, ADV serotypes, infection sites, CDV dosing, virological outcomes, and renal function were collected and analyzed.

### Results

A total of 23 pediatric HSCT recipients (17 males, 6 females) with a median age of 6.4 years (range, 1.0–16.4) were included. Underlying diseases were acute leukemia (n=15), Wiskott-Aldrich syndrome (n=4), aplastic anemia (n=3), and Fanconi anemia (n=1). Donor types included haploidentical (n=15, 65.2%), matched unrelated (n=5, 21.7%), matched sibling (n=1, 4.3%), and cord blood (n=2, 8.7%). The median time from HSCT to ADV infection was 57 days (range, 3–370). Prior to ADV infection, 10 patients (43.5%) had no GVHD, while 13 (56.5%) had pre-existing GVHD (10 acute, 3 chronic). ADV serotypes identified were type C (n=12, 52.2%), type A (n=6, 26.1%), type F (n=3, 13.0%), and type B (n=2, 8.7%). ADV DNA was detected in both blood and stool in 7 patients, who presented with fever and diarrhea; in both blood and sputum in 1 patient, who presented with pneumonia. Among the 7 patients with ADV DNA detected solely in blood, 4 presented with fever alone, 2 with pneumonia, and 1 with fever accompanied by hemorrhagic cystitis. Isolated detection in bronchoalveolar lavage fluid (BALF) or sputum was observed in 4 patients, all presenting with pneumonia. Isolated detection in stool was observed in 4 patients, all presenting with diarrhea. In this cohort, patients received a median of 3 doses of cidofovir (range, 1-5). Of the 23 treated patients, 19 had evaluable data on quantitative ADV DNA testing. Among these, 13 (68.4%) achieved a virological response, including 9 (47.4%) who attained complete viral clearance. The rest 4 patients lacked virologic follow-up: 3 showed clinical improvement and 1 experienced disease progression. Over a median follow-up of 5 months (range, 1–53), no grade ≥2 nephrotoxicity was observed. Two patients (8.7%) developed transient, mild serum creatinine elevations (peak ≤1.5× ULN), both resolving spontaneously. No patient required dose reduction or discontinuation due to nephrotoxicity.

**Copyright Information:** (Online) ISSN: 2572-9241
©2026 The Author(s). HemaSphere is published by John Wiley & Sons Ltd on behalf of European Hematology Association. This is an open access Abstract Book distributed under the Attribution-NonCommercial-NoDerivs (CC BY-NC-ND), which allows third parties to download the articles and share them with others as long as they credit the author and the Abstract Book, but they cannot change the content in any way or use them commercially.
**Abstract Book Citations:** Authors, Title, HemaSphere, 2026;10:(S1):pages. The abstract book DOIs can be found on the "Author Information" page.
**Disclaimer:** Articles published in the journal HemaSphere exclusively reflect the opinions of the authors. The authors are responsible for all content in their abstracts including accuracy of the facts, statements, citing resources, etc.

**Summary/Conclusion**

Cidofovir demonstrated a favorable safety profile with no significant nephrotoxicity when administered via a standardized short-course regimen. This therapy effectively reduced viral load and improved clinical outcomes in a subset of patients. These findings support CDV as an effective and safe therapeutic option for ADV infection in pediatric HSCT recipients.

**Copyright Information:** (Online) ISSN: 2572-9241
©2026 The Author(s). HemaSphere is published by John Wiley & Sons Ltd on behalf of European Hematology Association. This is an open access Abstract Book distributed under the Attribution-NonCommercial-NoDerivs (CC BY-NC-ND), which allows third parties to download the articles and share them with others as long as they credit the author and the Abstract Book, but they cannot change the content in any way or use them commercially.
**Abstract Book Citations:** Authors, Title, HemaSphere, 2026;10:(S1):pages. The abstract book DOIs can be found on the "Author Information" page.
**Disclaimer:** Articles published in the journal HemaSphere exclusively reflect the opinions of the authors. The authors are responsible for all content in their abstracts including accuracy of the facts, statements, citing resources, etc.

### (PS2429) A SINGLE CENTRE 10 YEAR RETROSPECTIVE AUDIT ON USE OF HUMAN LEUKOCYTE ANTIGEN MATCHED PLATELET TRANSFUSIONS: USAGE, COST, WASTAGE AND EFFECTIVENESS

**Topic:** 32. Transfusion medicine

Shreyans Gandhi*[1], Sohini Chakraborty[1], Kali Majumder[1], Sneha Dacosta[1], Vartika Singh[1], Priya Joel[1], Hayden Rodrigues[1], Kelly Nwankiti[1], Kamala Gurung[1]

[1]*Kings College Hospital NHS Trust, Haematological Medicine, London, United Kingdom;*

### Background

Human leukocyte antigen (HLA) matched platelet transfusions (PT) are considered after non-immune causes for platelet refractoriness (PR) have been excluded. HLA matched PT entail logistical difficulties in a) HLA genotyping and antibody testing facilities and further arranging specialized apheresis donations b) significantly higher costs c) longer turnaround time making them less suitable for urgent situations d) accessibility and finding appropriate donors, particularly from ethnic minorities. Furthermore, HLA matched PT may not always be effective and may still have poor platelet increments (PI), and risk of continued sensitization. Equally, suitably HLA matched PT ensure higher transfusion success rates with better 1 hour corrected count increment (CCI), reduce PT reactions and can be life saving in preventing catastrophic haemorrhages.

### Aims

To study the usage, cost and effectiveness of HLA matched PT, to guide further HLA matched PT practices in ensuring safe, appropriate and equitable use of specialist products.

### Methods

A single centre 10-year retrospective audit from records in the largest blood transfusion facility from a teaching tertiary university hospital in the UK - on the use of HLA matched PT, including the cost, and wastage. A 2-year retrospective review of the effectiveness of HLA matched PT with CCI was also done.

**Copyright Information:** (Online) ISSN: 2572-9241
©2026 The Author(s). HemaSphere is published by John Wiley & Sons Ltd on behalf of European Hematology Association. This is an open access Abstract Book distributed under the Attribution-NonCommercial-NoDerivs (CC BY-NC-ND), which allows third parties to download the articles and share them with others as long as they credit the author and the Abstract Book, but they cannot change the content in any way or use them commercially.
**Abstract Book Citations:** Authors, Title, HemaSphere, 2026;10:(S1):pages. The abstract book DOIs can be found on the "Author Information" page.
**Disclaimer:** Articles published in the journal HemaSphere exclusively reflect the opinions of the authors. The authors are responsible for all content in their abstracts including accuracy of the facts, statements, citing resources, etc.

### Results

We present data on the 10-year usage of HLA matched PT from 2016-2025

| Year | PT events | No of patients | Used | Waste/Return | Cost (GBP) |
|---|---|---|---|---|---|
| Jan-Dec'16 | 515 | 72 | 442 (85.8%) | 73 | 216706.62 |
| Jan-Dec'17 | 978 | 120 | 863 (88.2%) | 115 | 467452.12 |
| Jan-Dec'18 | 156 | 40 | 129 (82.6%) | 27 | 62389.34 |
| Jan-Dec'19 | 530 | 76 | 424 (80%) | 106 | 218472.16 |
| Jan-Dec'20 | 469 | 58 | 354 (75.4%) | 115 | 181082.96 |
| Jan-Dec'21 | 314 | 47 | 248 (78.9%) | 66 | 91775.06 |
| Jan-Dec'22 | 424 | 65 | 342 (80.6%) | 82 | 124131.32 |
| Jan-Sep'23* | 302 | 35 | 255 (84.4%) | 47 | 96498 |
| Jan-Dec24 | 310 | 45 | 248 (79.9%) | 62 | 151680.16 |
| Jan-Dec25 | 301 | 38 | 246 (81.7%) | 55 | 166396.78 |

83 patients received 494 HLA matched PT from 01/01/24 to 31/12/25. All 83 patients had HLA typing and antibody characterization before PT. Each HLA matched PT counted as one event. CCI data was recorded for all 494 (100%) PT events. A CCI with platelet count > 10 x $10^9$/L in 24hrs/PT event = response.

| **No. of pts** | **Grade of match** | **PTx events** | **Mean Increment/Event & Range** | **Achieving Increment > $10^9$/L** |
|---|---|---|---|---|
| 18 | A | 98 | 12.11 (-3 to 51) | 70/98 (71.4%) |
| 26 | B1 | 190 | 14.25 (-13 to 71) | 92/190 (48.4%) |
| 24 | B2 | 162 | 14.19 (-16 to 57) | 93/162 (57.4%) |
| 5 | B3 | 10 | 22.7 (-17 to 56) | 8 of 10 (80%) |
| 10 | SEL | 34 | 9.79 (-5 to 37) | 14/34 (41.1%) |

### Summary/Conclusion

HLA-matched PT are indicated for patients who have true PR having reasonably excluded nonimmune causes. However, nonimmune causes can co-present with HLA antibodies and needs to be considered. CCI is imperative, to test effectiveness of HLA matched PT and records maintained for the increment with each transfusion.

We recorded the use of 3551 HLA matched PT over a 10-year period, for 596 patients, and that still accounted only for 82% of all planned HLA matched transfusions. The usage percentage has remained constant over this 10-year period. All efforts should be made to improve utilizations and reduced wastage.

'A' Grade matched HLA PT are most likely to achieve a CCI response (71.4%), compared to the entire cohort, where a CCI response was seen in 277/494 (56%) overall. B1 match (48.4%) and B2 (57.4%) can still be effective second choices, if lack of suitable donors where A matches are not available. Interestingly, platelet decrements were seen in 9% of events and highlight the need to continue screen for non-immune factors for PR, and the possibility of HPA/ other immune causes.

**Copyright Information:** (Online) ISSN: 2572-9241
©2026 The Author(s). HemaSphere is published by John Wiley & Sons Ltd on behalf of European Hematology Association. This is an open access Abstract Book distributed under the Attribution-NonCommercial-NoDerivs (CC BY-NC-ND), which allows third parties to download the articles and share them with others as long as they credit the author and the Abstract Book, but they cannot change the content in any way or use them commercially.
**Abstract Book Citations:** Authors, Title, HemaSphere, 2026;10:(S1):pages. The abstract book DOIs can be found on the "Author Information" page.
**Disclaimer:** Articles published in the journal HemaSphere exclusively reflect the opinions of the authors. The authors are responsible for all content in their abstracts including accuracy of the facts, statements, citing resources, etc.

### (PS2430) RNA-SEQ–DRIVEN IDENTIFICATION OF LIPID REQUIREMENTS REVEALS LDL AS A KEY ENHANCER OF SCALABLE BMI-1–TRANSDUCED CULTURED RBC PRODUCTION

**Topic:** 32. Transfusion medicine

Moira Lancelot*[1], Natia Saakadze[1], John Roback[1]

[1]*Emory University School of Medicine, Atlanta, United States of America;*

### Background

BMI1 transduction confers self-renewal capacity to primary human erythroblasts, enabling large-scale production of erythroid precursors that retain full potential to mature into functional red blood cells (RBCs). Transcriptomic analyses reveal that BMI1 enhances cholesterol import and biosynthetic pathways, suggesting that lipid availability—particularly under glucocorticoid stimulation—may constrain ex vivo erythropoiesis. Although dexamethasone promotes erythroblast proliferation, it suppresses endogenous lipid synthesis, increasing reliance on exogenous lipid sources. Low-density lipoproteins (LDL) provide the cholesterol required for membrane biogenesis during proliferation and for membrane remodeling during enucleation. To evaluate whether lipid supply limits yield and maturation quality, we investigated cholesterol-related gene programs and modulated lipid availability during expansion and terminal maturation.

### Methods

We performed integrated RNA-seq analysis of human bone marrow (BM) erythropoiesis and BMI1-transduced erythroblasts cultured through terminal differentiation, focusing on differential expression and pathway enrichment of cholesterol uptake and biosynthesis genes. Guided by these findings, we modified culture media. Proliferation cultures contained cytokines with dexamethasone, with or without LDL supplementation. Terminal maturation compared four conditions: (1) IMDM + serum, (2) IMDM + serum + LDL, (3) HPLM, and (4) HPLM + LDL. We quantified erythroblast expansion, enucleation efficiency, and cRBCs output.

### Results

BMI1-expressing erythroblasts exhibited stronger upregulation of cholesterol homeostasis pathways than primary BM cells at all stages of terminal erythropoiesis. Functionally, LDL supplementation increased erythroblast expansion, enhanced cRBCs yield, and improved enucleation efficiency—from approximately 64% to 70% in IMDM and from approximately 70% to 85% in HPLM. LDL also improved overall maturation quality across media conditions.

### Summary/Conclusion

BMI1 rewires erythroblasts toward heightened cholesterol demand, rendering lipid availability a key determinant of ex vivo manufacturing performance. LDL supplementation offers a practical and scalable strategy to counteract dexamethasone-induced lipid limitations, enhance enucleation, and increase cRBC yield. Incorporating lipid optimization into standardized protocols will be essential for robust, clinically scalable production of transfusion-grade cRBCs.

**Copyright Information:** (Online) ISSN: 2572-9241
©2026 The Author(s). HemaSphere is published by John Wiley & Sons Ltd on behalf of European Hematology Association. This is an open access Abstract Book distributed under the Attribution-NonCommercial-NoDerivs (CC BY-NC-ND), which allows third parties to download the articles and share them with others as long as they credit the author and the Abstract Book, but they cannot change the content in any way or use them commercially.
**Abstract Book Citations:** Authors, Title, HemaSphere, 2026;10:(S1):pages. The abstract book DOIs can be found on the “Author Information” page.
**Disclaimer:** Articles published in the journal HemaSphere exclusively reflect the opinions of the authors. The authors are responsible for all content in their abstracts including accuracy of the facts, statements, citing resources, etc.

### (PS2431) A SINGLE CENTER AUDIT ON THE USE OF BLOOD PRODUCTS INCLUDING RED CELLS, PLATELETS, FRESH FROZEN PLASMA AND CRYOPRECIPITATE IN A TERTIARY NEONATAL UNIT- THE UNIVERSITY COLLEGE LONDON HOSPITAL

**Topic:** 32. Transfusion medicine

Devi Purushothaman*[1], Samah Alimam[1]

[1]*University College London Hospital, London, United Kingdom;*

### Background

According to the 2016 British Society of Hematology (BSH) guidelines, red cell transfusion thresholds for neonates are determined by clinical context, including gestational age at birth, postnatal age and current ventilatory status. Platelet transfusion thresholds are recommended for different clinical scenarios, generally favoring a restrictive approach. Routine use of fresh frozen plasma (FFP) to correct isolated coagulation screen abnormalities in non-bleeding neonates is discouraged.

### Aims

To examine the patterns of usage of blood products in neonates at the University College London Hospital (a tertiary neonatal unit) and assess whether transfusions administered were for appropriate indications and whether they followed the BSH guidelines.

### Methods

A retrospective audit of all transfusions of red cells, platelets, FFP and cryoprecipitate administered to patients in the neonatal unit or up to 28 days of life, between January 1 and December 31, 2024. Data was collected from the transfusion laboratory records and from the electronic patient record system.

### Results

There were 433 transfusion episodes in total. Of these, 270 were red cell transfusions in 86 neonates. This included eight exchange transfusions in five patients and six transfusions given as part of resuscitative efforts in four patients. There were 93 platelet transfusions in 29 neonates (37 platelet transfusions were given to one patient with thrombocytopenia due to congenital cytomegalovirus infection, and intracranial hemorrhage). There were 47 transfusions of FFP in 34 neonates, and 23 tranfusions of cryoprecipitate in 16 neonates. All transfusions were prescribed in milliliters, in line with the BSH guideline recommendations. All red cell units used for exchange transfusions were less than 5 days old as recommended.
Mean transfusion volumes for red cells, platelets, FFP and cryoprecipitate were 18.1 mL/kg, 14.9 mL/kg, 14.8 mL/kg and 10.1 mL/kg respectively. This reflected an under prescribing of FFP and overprescribing of cryoprecipitate volumes as the recommended volumes for these products are 15-20ml/kg and 5-10ml/kg respectively.
61% of red cell transfusions were administered to preterm neonates with gestational age less than 28 weeks. Overall, 31% of red cell transfusions were given outside the BSH recommended hemoglobin thresholds. Clinical indications were not documented in 30.1% of platelet transfusions and 40% of FFP transfusions.
There was high level of wastage with 21% of total products in the year being discarded amounting to a cost of at least £11,800. The main reason was expiry of the products within the laboratory (72%); other reasons included products being ordered but not used (11%), products expiring while being issued to patients (5%), or being damaged (2%) or exposed to temperature excursions (2%), and FFP thawed but not used (8%).

**Copyright Information:** (Online) ISSN: 2572-9241
©2026 The Author(s). HemaSphere is published by John Wiley & Sons Ltd on behalf of European Hematology Association. This is an open access Abstract Book distributed under the Attribution-NonCommercial-NoDerivs (CC BY-NC-ND), which allows third parties to download the articles and share them with others as long as they credit the author and the Abstract Book, but they cannot change the content in any way or use them commercially.
**Abstract Book Citations:** Authors, Title, HemaSphere, 2026;10:(S1):pages. The abstract book DOIs can be found on the "Author Information" page.
**Disclaimer:** Articles published in the journal HemaSphere exclusively reflect the opinions of the authors. The authors are responsible for all content in their abstracts including accuracy of the facts, statements, citing resources, etc.

**Summary/Conclusion**

Overall, there were areas of good practice; however, the most important issues identified were poor documentation of indications and high wastage. There was a potential cost saving of at least £11,800 per year. These findings were presented at the hospital transfusion team meeting and at the neonatal governance meeting, and, in collaboration with transfusion practitioners, laboratory and clinical teams, measures will be implemented. A re-audit is planned in 24 months.

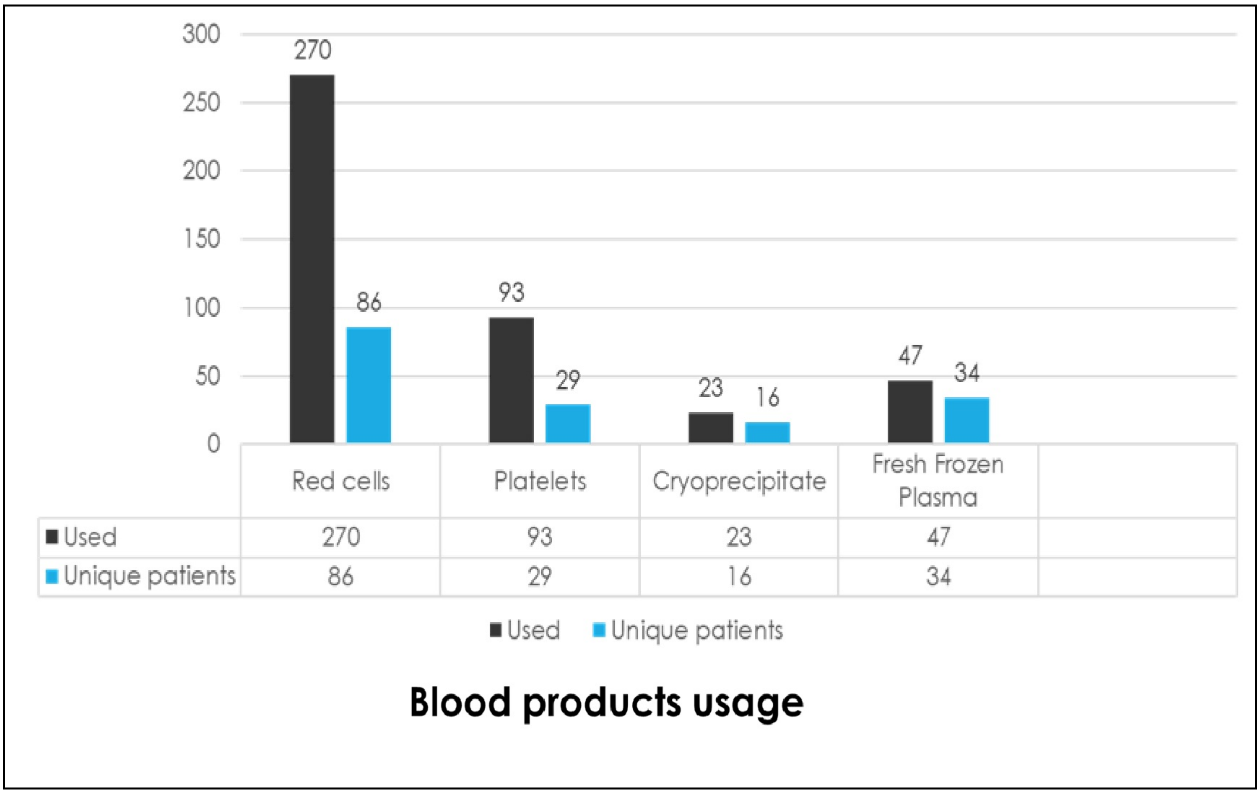

**Copyright Information:** (Online) ISSN: 2572-9241
©2026 The Author(s). HemaSphere is published by John Wiley & Sons Ltd on behalf of European Hematology Association. This is an open access Abstract Book distributed under the Attribution-NonCommercial-NoDerivs (CC BY-NC-ND), which allows third parties to download the articles and share them with others as long as they credit the author and the Abstract Book, but they cannot change the content in any way or use them commercially.
**Abstract Book Citations:** Authors, Title, HemaSphere, 2026;10:(S1):pages. The abstract book DOIs can be found on the "Author Information" page.
**Disclaimer:** Articles published in the journal HemaSphere exclusively reflect the opinions of the authors. The authors are responsible for all content in their abstracts including accuracy of the facts, statements, citing resources, etc.

### (PS2432) MULTI-PHASE ASSESSMENT OF HYPOXIC RED BLOOD CELL STORAGE: FROM THE UNIT TO RECIPIENT-MIMICKING CONDITIONS

**Topic:** 32. Transfusion medicine

Alkmini Anastasiadi[1], Maria-Aspasia Kosma[2], Maria Sykioti[3], Sofia Giatsiatsiou[3], Ioannis Kereres[1], Effrosyni Zontou[1], Maria-Niki Chatzantoni[1], Evangelos Meletis[2], Evgenia Spyropoulou[2], Vasiliki Pantazatou[2], Polyxeni Delaporta[4, 5], Maria Dimopoulou[6], Antonis Kattamis[4, 5], Anastasios Kriebardis[2], Vasileios Tzounakas*[1], Konstantinos Stamoulis[3]

*[1]University of Patras, School of Medicine, Department of Biochemistry, Patras, Greece; [2]University of West Attica, School of Health and Caring Sciences, Department of Biomedical Sciences, Laboratory of Reliability and Quality Control in Laboratory Hematology, Egaleo, Greece; [3]Hellenic National Blood Transfusion Centre, Acharnes, Greece; [4]National and Kapodistrian University of Athens, Thalassemia Unit, First Department of Pediatrics, Athens, Greece; [5]"Aghia Sophia" Children's Hospital, ERN-EuroBloodNet Center, Athens, Greece; [6]Laikon General Hospital of Athens, Center of Expertise in Hemoglobinopathies, Member of EuroBloodNet, Thalassemia Unit, Athens, Greece;*

### Background

Recent efforts to mitigate red blood cell (RBC) storage lesion aim to improve storage quality and post-transfusion efficacy while at the same time examine the possible extension of storage duration. Among the proposed interventions, hypoxic storage has emerged as a promising strategy by targeting oxidative stress, the root cause of various storage-driven phenotypes.

### Aims

This study aims to comprehensively evaluate hypoxia's impact on RBC storability, functional resilience under in vitro transfusion simulation, and sources of biological variation.

### Methods

RBCs from healthy donors were leukoreduced and stored for 42 days under hypoxic (H-units; N=30; Hemanext ONE System kindly provided by Hemanext) or normoxic (N-units; N=15) conditions in PAGGSM preservative solution. Aseptic sampling was performed at early (day 2), mid (day 21), and end storage (day 42), evaluating hematological, hemolytic, redox, proteostatic, and senescence profiles. Selected samples (12 hypoxic, 10 normoxic) were incubated with recipient plasma at body temperature to assess post-transfusion competence and RBC physiology in transfusion-mimicking conditions. Significance was accepted for $p<0.05$.

**Copyright Information:** (Online) ISSN: 2572-9241
©2026 The Author(s). HemaSphere is published by John Wiley & Sons Ltd on behalf of European Hematology Association. This is an open access Abstract Book distributed under the Attribution-NonCommercial-NoDerivs (CC BY-NC-ND), which allows third parties to download the articles and share them with others as long as they credit the author and the Abstract Book, but they cannot change the content in any way or use them commercially.
**Abstract Book Citations:** Authors, Title, HemaSphere, 2026;10:(S1):pages. The abstract book DOIs can be found on the "Author Information" page.
**Disclaimer:** Articles published in the journal HemaSphere exclusively reflect the opinions of the authors. The authors are responsible for all content in their abstracts including accuracy of the facts, statements, citing resources, etc.

### Results

Quality control confirmed stable oxygen saturation <20% throughout storage, hematocrit within acceptable limits, and hemolysis below the European threshold (0.8%). Focusing on integrity parameters, compared to N-units, H-RBCs showed lower early spontaneous (0.032±0.009 vs. 0.044±0.027%, p=0.027) and osmotic hemolysis, higher initial mechanical lysis, that ultimately ended slightly lower than controls, and reduced vesiculation rates –yet with larger vesicles– from mid-storage onward. Regarding redox balance, H-units demonstrated reduced reactive oxygen species (ROS) generation at end-storage, with intermittently lower responses to targeted hemoglobin (Hb) oxidation (e.g., day 21: 31,956±5,133 vs. 36,532±5,946 MFI/mg, p=0.01), and correspondingly lower metHb formation. Proteasomal activity was decreased in the cytosol of H-RBCs early in storage but increased in their membranes at mid-storage. Concerning aging markers, intracellular calcium levels and phosphatidylserine (PS) externalization (e.g., day 21: 1.81±0.30 vs. 2.24±0.73%, p<0.01) were lower in H-RBCs. Following in vitro transfusion, H-units retained lower oxidative lysis (7.26±1.30 vs. 10.68±3.43 mg/dL, p=0.004) and ROS accumulation after Hb oxidation. Within this group, inter-donor variability was most pronounced for vesiculation rate, PS exposure, and induced ROS production. Among the four functional modules identified by correlation clustering, the most notable was a core storage lesion one, comprising hemolysis, $K^+$, ROS indices, PS exposure, vesiculation rate, and cytosolic proteasome activities. Finally, the achieved oxygen saturation level did not associate with a distinct quality profile, suggesting that inter-donor variability within hypoxic units is mainly driven by donor intrinsic biological factors rather than the depth of deoxygenation.

### Summary/Conclusion

While hypoxic storage does not universally outperform standard methods, it offers specific advantages in mitigating Hb-related oxidative stress and eryptotic signaling, and preserving integrity characteristics, with benefits persisting after in vitro transfusion. This profile may be significant for specific patients and for extending storage duration. Yet, inter-donor variability highlights the need for donor-stratified quality assessment in hypoxic storage.

**Copyright Information:** (Online) ISSN: 2572-9241
©2026 The Author(s). HemaSphere is published by John Wiley & Sons Ltd on behalf of European Hematology Association. This is an open access Abstract Book distributed under the Attribution-NonCommercial-NoDerivs (CC BY-NC-ND), which allows third parties to download the articles and share them with others as long as they credit the author and the Abstract Book, but they cannot change the content in any way or use them commercially.
**Abstract Book Citations:** Authors, Title, HemaSphere, 2026;10:(S1):pages. The abstract book DOIs can be found on the "Author Information" page.
**Disclaimer:** Articles published in the journal HemaSphere exclusively reflect the opinions of the authors. The authors are responsible for all content in their abstracts including accuracy of the facts, statements, citing resources, etc.

### (PS2433) TRANSFUSIONAL OPTIMIZATION IN LIVER TRANSPLANTATION USING VISCOELASTIC TEST GUIDED THERAPY, A USEFUL TOOL FOR PATIENT BLOOD MANAGEMENT

**Topic:** 32. Transfusion medicine

Angeles Fernandez Rodriguez[1], Aida Yanes Monsalve[1], Borja Nuñez Gomez[1], Sonsoles Hurtado Gomez[1], Rebeca Fernandez Gonzalez[1], Sergio Salgado Santamarina[1], Maria Orviz Garcia[1], Esther Martinez Fresno[1], Marco Hernandez-Martin[1], MacArena Castillo Godoy[1], Nicolás De Lucas Torres[1], José María García Gala*[1], Eva Martinez Revuelta[1], Lorena Varela Rodriguez[1]

*[1]HUCA, Hematology, Oviedo, Spain;*

### Background

Viscoelastic tests are point-of-care coagulation devices and represent key tools in hemorrhage management. Although robust evidence is limited, thromboelastography is recommended for hemostatic management and may be useful for transfusion guidance in liver transplantation (recommendation 1B) The impact on mortality remains controversial.

### Aims

To analyze the efficacy of viscoelastic test-guide transfusion in liver transplantation in terms of transfusion requirements, hemorrhagic and thrombotic complications, need of reintervention and surgery- related mortality.

### Methods

Retrospective observational study. Medical records of patients undergoing liver transplantation between October 2023 and september 2025 were reviewed. Analyzed variables included demographic data, liver disease-related characteristics, laboratory parameters, surgical variables, transfusion data, thrombotic and hemorrhagic complications and clinical outcomes. Qualitative variables were described as number and percentage; quantitative variables were described as mean and standard deviation or median and percentile 25-75. Bivariate analysis was conducted.

### Results

107 patients were included, 30 women (28%), mean age at liver transplantation was 61,1 years (SD 6,2). Indications for transplantation were alcoholic cirrhosis 66 patients (61,6%), autoimmune cirrhosis 8 (7,47%), post-viral hepatitis cirrhosis 30 (28,3%), NAFLD/NASH (Non Alcoholic Fat Liver Disease/Non-Alcoholic Steato Hepatitis) 9 (8,4%), acute hepatitis 4 (3,7%), combined liver-kidney transplantation 3 (2,8%) and other indications 19 (17,7%). Child-Pugh class was A in 47 patients (43,9%), B in 30 (28%) and C in 26 (24,3%). Blood group O 44 (41%), A 50 (46,7%), B 11 (10,3%), AB 2 (1,9%). Rh negative 32 (29,9%), positive 74 (69,2%) Days of admission: mean 18 days (SD 14,5), median 15 days. Viscoelastic tests were used to guide transfusion in 56 patients (52,3%) while 51 patients (47,8%) received transfusion guided by conventional coagulation tests. Baseline differences were observed between groups in age and sex distribution.
Baseline characteristics, transfusion data, treatments and clinical outcomes of both groups are shown in table 1.

### Summary/Conclusion

In our series, the use of viscoelastic tests to guide transfusion in liver transplantation significantly reduced the need for packed red blood cells (PRBC), platelets, and plasma while increasing fibrinogen consumption. No statistically significant differences in mortality or acute complications were observed. Our results are consistent with those of other groups and similar studies.

**Copyright Information:** (Online) ISSN: 2572-9241
©2026 The Author(s). HemaSphere is published by John Wiley & Sons Ltd on behalf of European Hematology Association. This is an open access Abstract Book distributed under the Attribution-NonCommercial-NoDerivs (CC BY-NC-ND), which allows third parties to download the articles and share them with others as long as they credit the author and the Abstract Book, but they cannot change the content in any way or use them commercially.
**Abstract Book Citations:** Authors, Title, HemaSphere, 2026;10:(S1):pages. The abstract book DOIs can be found on the "Author Information" page.
**Disclaimer:** Articles published in the journal HemaSphere exclusively reflect the opinions of the authors. The authors are responsible for all content in their abstracts including accuracy of the facts, statements, citing resources, etc.

Table 1

| Total (n = 107) | Conventional test guide transfusion (n = 51) | Viscoelastic test guide transfusion (n = 56) | p |
|---|---|---|---|
| Age [mean (SD)] | 59,9 (4,4) | 62,2 (7,2) | 0,05 |
| Sex women [n (%)] | 7 (13,7) | 23 (41,1) | 0,001 |
| Days of admission [mean (SD)] | 20,1 (14,5) | 17,1 (14,3) | 0,28 |
| Hemoglobin at income g/dL [mean (SD)] | 12,3 (2,7) | 12 (2,4) | 0,57 |
| Platelets at income /mm3 [mean (SD)] | 122470 (80424) | 124625 (68288) | 0,88 |
| APTT R at income [mean (SD)] | 1,23 (0,4) | 1,22 (0,3) | 0,88 |
| PT % at income [mean (SD)] | 67,6 (24) | 67,36 (23) | 0,95 |
| Fibrinogen at income mg/Dl [mean (SD)] | 360,3 (157,9) | 320,2 (121,3) | 0,14 |
| Hemoglobin after surgery g/dL [mean (SD)] | 10,1 (1,8) | 10,2 (2,0) | 0,63 |
| Platelets after surgery /mm3 [mean (SD)] | 109562 (154525) | 114696 (65644) | 0,82 |
| APTT R after surgery [mean (SD)] | 1,4 (0,7) | 1,4 (0,45) | 0,63 |
| PT % after surgery [mean (SD)] | 55,1 (15,3) | 53,1 (15,1) | 0,49 |
| Fibrinogen after surgery mg/dL [mean (SD)] | 219 (88) | 263,3 (17,5) | 0,006 |
| Transfusion and treatment | | | |
| PRBC in surgery [median, (p25-p75)] | 6 (1-10) | 2 (1-6,7) | 0,048 |
| Platelets in surgery [median, (p25-p75)] | 1 (0-3) | 0 (0-2) | 0,048 |
| FFP in surgery [median, (p25-p75)] | 8 (2,5-11,5) | 2 (0-5) | < 0,001 |
| Fibrinogen (mg) [median, (p25-p75)] | 0 | 1500 (0-1750) | < 0,001 |
| Trombosis (arterial /venous) [n (%)] | 6 (12,2) / 0 (0) | 5 (8,9) / 2 (3,6) | 0,2 / 0,6 |
| Hemorrhage [n (%)] | 9 (18) | 9 (16,1) | 0,5 |
| Reintervention [n (%)] | 10 (20) | 8 (14,3) | 0,30 |
| Deaths related to transplant [n (%)] | 9 (18) | 4 (7,1) | 0,08 |

Copyright Information: (Online) ISSN: 2572-9241
©2026 The Author(s). HemaSphere is published by John Wiley & Sons Ltd on behalf of European Hematology Association. This is an open access Abstract Book distributed under the Attribution-NonCommercial-NoDerivs (CC BY-NC-ND), which allows third parties to download the articles and share them with others as long as they credit the author and the Abstract Book, but they cannot change the content in any way or use them commercially.
**Abstract Book Citations:** Authors, Title, HemaSphere, 2026;10:(S1):pages. The abstract book DOIs can be found on the "Author Information" page.
**Disclaimer:** Articles published in the journal HemaSphere exclusively reflect the opinions of the authors. The authors are responsible for all content in their abstracts including accuracy of the facts, statements, citing resources, etc.

### (PS2434) ESTABLISHING EXPERT CONSENSUS ON DIAGNOSIS AND TREATMENT GUIDELINES FOR COLD AGGLUTININ DISEASE (CAD) – AN INTERNATIONAL DELPHI STUDY

**Topic:** 32. Transfusion medicine

Morie Gertz*[1], Ilene C Weitz[2], Alexander Röth[3]

*[1]Mayo Clinic, Division of Hematology, Rochester, United States of America;[2]Keck School of Medicine of USC, Los Angeles, United States of America; [3]West German Cancer Center, University Hospital Essen, University Duisburg-Essen, Department of Hematology and Stem Cell Transplantation, Essen, Germany;*

### Background

Cold agglutinin disease (CAD) is a rare autoimmune disease characterized by hemolytic anemia. Consensus guidelines for the diagnosis and management of CAD were published in 2020 as a result of the first annual international consensus meeting for diagnosis and treatment of autoimmune hemolytic anemia in adults. The recommendations of this initial framework were intended to be updated to capture the evolving treatment landscape, with evidence derived from prospective clinical trials, whenever possible. Data published in the years since, as well as approvals of targeted therapies such as complement inhibitors, highlight the need to update these guidelines to mitigate delays in initiation of appropriate management strategies.

### Aims

This study aims to drive the update of current CAD guidelines by considering the latest evidence that informs treatment approaches, including guidance on standardized and individualized care. These updates will facilitate timely, consistent, and effective management of CAD.

### Methods

Using the Delphi methodology, a targeted literature review was conducted in October 2025 using PubMed, Google, Google Scholar, and references identified from articles in these searches. The findings of this review informed the development of an insights questionnaire regarding the diagnosis and treatment of CAD. The questionnaire was completed by a steering group (SG) of expert panelists, which consisted of an international group of three clinicians with substantial experience and expertise in CAD management. Both the literature review and the responses to the questionnaire informed the topics for discussion at the preliminary SG meeting, which was held in early 2026. Draft consensus statements will be developed and approved by the SG based on evidence gathered from the targeted literature review, insights questionnaire, and SG discussions. The consensus statements will be shared with a broader group of approximately 40 international Delphi panelists (clinicians with experience in CAD diagnosis and treatment). Agreement with statements will be assessed using a 6-point Likert scale, ranging from ‘strongly agree’ to ‘strongly disagree’, using a predetermined agreement threshold. Free-text responses will be gathered on statements the respondents disagree with. Following this first Delphi rating round, any statements not meeting the consensus threshold will be revised based on Delphi panel feedback and included in a second rating round. The SG will meet to develop their final recommendations for inclusion in the planned peer-reviewed publication. Reporting of this study will adhere to the ACCORD guidelines.

### Results

Preliminary results of the Delphi panelists rating rounds will be presented, with a manuscript describing the full results expected to be published in the months following the congress.

**Copyright Information:** (Online) ISSN: 2572-9241
©2026 The Author(s). HemaSphere is published by John Wiley & Sons Ltd on behalf of European Hematology Association. This is an open access Abstract Book distributed under the Attribution-NonCommercial-NoDerivs (CC BY-NC-ND), which allows third parties to download the articles and share them with others as long as they credit the author and the Abstract Book, but they cannot change the content in any way or use them commercially.
**Abstract Book Citations:** Authors, Title, HemaSphere, 2026;10:(S1):pages. The abstract book DOIs can be found on the “Author Information” page.
**Disclaimer:** Articles published in the journal HemaSphere exclusively reflect the opinions of the authors. The authors are responsible for all content in their abstracts including accuracy of the facts, statements, citing resources, etc.

**Summary/Conclusion**

Consensus recommendations derived from this study will inform the development of updated guidelines for the diagnosis and treatment of CAD. These updated guidelines, with consideration given to newly generated evidence, will inform clinical decision making for the optimal management of CAD patients.

**Copyright Information:** (Online) ISSN: 2572-9241
©2026 The Author(s). HemaSphere is published by John Wiley & Sons Ltd on behalf of European Hematology Association. This is an open access Abstract Book distributed under the Attribution-NonCommercial-NoDerivs (CC BY-NC-ND), which allows third parties to download the articles and share them with others as long as they credit the author and the Abstract Book, but they cannot change the content in any way or use them commercially.
**Abstract Book Citations:** Authors, Title, HemaSphere, 2026;10:(S1):pages. The abstract book DOIs can be found on the "Author Information" page.
**Disclaimer:** Articles published in the journal HemaSphere exclusively reflect the opinions of the authors. The authors are responsible for all content in their abstracts including accuracy of the facts, statements, citing resources, etc.

### (PS2435) A DEDICATED BLOOD DONOR MANAGEMENT APPROACH TO ANEMIA: EFFECTS ON READMISSION TO DONATION

**Topic:** 32. Transfusion medicine

Carina Brito*[1], Margarida Leonardo de Oliveira[1], Manlio Falavigna[1], Ana Catarina Pinto[1], Jorge Ferreira[1], Luís Vale[1], Marco Sampaio[1], Marika Bini Antunes[1], Sara Ferreira[1]

*[1]Unidade Local de Saúde de Santo António, Centro de Coagulopatias Congénitas, Unidade de Trombose e Hemostase, Serviço de Imuno-hemoterapia, Porto, Portugal;*

### Background

Low hemoglobin levels are a frequent reason for temporary blood donor deferral. Blood donor management is essential to promote recovery and return to donation.

In our institution, a structured weekly donor management consultation (DMC) was established in 2015 to provide comprehensive assessment, etiological workup, treatment and follow-up of donors diagnosed with health problems causing temporary deferral.

### Aims

This study evaluates temporarily deferred blood donors due to anemia or vitamin deficiencies (iron, B12, B9), with the primary aim of determining their return to donation after clinical management. Primary outcome was return to blood donation after treatment. Recurrence of anemia after readmission to donation was also considered.

### Methods

We conducted a retrospective study of all blood donors with anemia (men: Hb<13 g/dL; women: <12 g/dL) or vitamin deficiencies (confirmed or suspected due to altered red blood cells indices) at the time of donation, oriented to the DMC, between January 2023 and December 2025.

Demographic data (sex, age at diagnosis), number of donations in the previous year, analytical data (total cell count, iron kinetics, folate and vitamin B12), causes for anemia, treatment, response and reentry to donation were recorded. Statistical analysis was performed in IBM SPSS ($p<0.01$).

### Results

Of 8775 blood donors (53% women; 47% men), 331 (261 women; 70 men) with a median age 44 years old (18-69) were referred to the DMC. Median hemoglobin at diagnosis was 11.6 g/dL in women and 12,5 g/dL in men, median transferrin saturation was 9% (2-52%) and median ferritin was 11 ng/mL (<5-445 ng/mL). Median number of donations in the previous year was 2 (0-4). Of 331 donors, 165 had iron deficiency (ID) anemia, 52 isolated ID, 77 mixed-deficiency anemia (B12/B9 and/or ID anemia), 37 other anemia/deficiencies. Identified causes were: 58 women with abnormal uterine bleeding, 8 gastrointestinal bleeding, 115 absorption deficiencies (62 chronic gastritis, 40 helicobacter pylori, 11 auto immune gastritis, 1 inflammatory bowel disease, 1 bariatric surgery); 15 out of 115 patients with absorption issues also had hemorrhagic clinic and 53/115 were on therapy with proton pump inhibitors and/or metformin. In the remaining cases, likely causes were dietary restrictions/errors and/or frequent blood donation. All vitamin deficiencies were initially treated with oral formulations. However, regarding iron, 26 patients (26/294) needed intravenous ferric carboxymaltose after response failure/intolerance to oral iron. Median follow up time was 6 months (2-20), 226 out of 331 returned to donation pool and, among them, 167 (74%) donated blood again. However, 36 out of 167 experienced recurrence of anemia and returned to DMC.

**Copyright Information:** (Online) ISSN: 2572-9241
©2026 The Author(s). HemaSphere is published by John Wiley & Sons Ltd on behalf of European Hematology Association. This is an open access Abstract Book distributed under the Attribution-NonCommercial-NoDerivs (CC BY-NC-ND), which allows third parties to download the articles and share them with others as long as they credit the author and the Abstract Book, but they cannot change the content in any way or use them commercially.
**Abstract Book Citations:** Authors, Title, HemaSphere, 2026;10:(S1):pages. The abstract book DOIs can be found on the "Author Information" page.
**Disclaimer:** Articles published in the journal HemaSphere exclusively reflect the opinions of the authors. The authors are responsible for all content in their abstracts including accuracy of the facts, statements, citing resources, etc.

**Summary/Conclusion**

A high percentage of blood donors temporarily deferred because of anemia had iron deficiency and, in about half of them, a clear etiology beyond blood donation and/or dietary errors or restrictions couldn't be identified. This highlights the responsibility of blood services in proactive donor management. Although the overall proportion of female and male donors was similar, a markedly higher number of women were referred due to anemia or deficiencies, reinforcing that women constitute a higher-risk population for ID anemia. The establishment of a dedicated DMC enabled prompt detection and treatment of anemia, promoting donor health, engagement, and successful return to donation.

**Copyright Information:** (Online) ISSN: 2572-9241
©2026 The Author(s). HemaSphere is published by John Wiley & Sons Ltd on behalf of European Hematology Association. This is an open access Abstract Book distributed under the Attribution-NonCommercial-NoDerivs (CC BY-NC-ND), which allows third parties to download the articles and share them with others as long as they credit the author and the Abstract Book, but they cannot change the content in any way or use them commercially.
**Abstract Book Citations:** Authors, Title, HemaSphere, 2026;10:(S1):pages. The abstract book DOIs can be found on the "Author Information" page.
**Disclaimer:** Articles published in the journal HemaSphere exclusively reflect the opinions of the authors. The authors are responsible for all content in their abstracts including accuracy of the facts, statements, citing resources, etc.

### (PS2436) MODULATING MEGAKARYOPOIESIS: A HEAD-TO-HEAD COMPARISON OF RECOMBINANT TPO AND RECEPTOR AGONISTS ON MEGAKARYOCYTE DEVELOPMENT AND PLATELET OUTPUT

**Topic:** 33. Platelet disorders

Shuhong Hu*[1]

[1] *The First Affiliated Hospital of Soochow University, Hematology, Suzhou, China;*

### Background

Thrombocytopenia treatment relies on thrombopoietin (TPO) and TPO receptor agonists (TPO-RAs). However, the rational selection between recombinant human TPO (rhTPO), and other TPO-RAs (romiplostim, avatrombopag, eltrombopag, hetrombopag) remains unsupported by robust mechanistic evidence, which may impact both clinical efficacy and safety.

### Aims

This study aimed to elucidate mechanistic differences among TPO, rhTPO, and other TPO-RAs to establish a theoretical basis for optimal drug selection through a comparative analysis of their effects on megakaryocyte (MK) development and platelet output.

### Methods

Using RNA sequencing (RNA-seq), single-cell RNA sequencing (scRNA-seq), flow cytometry, transmission electron microscopy and Western blotting, we conducted a head-to-head comparison to assess the effects of different drugs on megakaryocyte (MK) maturation, platelet production, cell morphology, gene expression, and signaling pathways.

### Results rhTPO and TPO effectively promoted physiological MK maturation and platelet generation, with MKs containing rich α-granules and mitochondria. rhTPO showed similar MK morphology to TPO (intact structure, uniform distribution), minimal gene changes (closest to physiological state), and enhanced P-STAT3, P-STAT5 (with decreased total STAT5), and P-ERK. In contrast, romiplostim (ROM) exhibited the weakest efficacy, characterized by impaired MK viability, reduced early apoptotic (platelet-producing) MKs, and morphological abnormalities including fewer α-granules, more dense granules, and reduced mitochondria. ROM also induced severe transcriptional dysregulation—marked by massive downregulation of genes involved in proplatelet and α-granule formation—suppressed platelet formation and inflammation pathways (GSEA), inhibited P-STAT3 while enhancing P-STAT5, and ultimately impaired platelet function. Avatrombopag (AVA) had similar MK apoptosis to ROM (suggesting higher cytotoxicity), with MKs having increased dense granules and elongated mitochondria. Meanwhile, it also enhanced platelet activation, upregulated MK/platelet activation/inflammation-related genes, affected ion channels and inhibited P-STAT5. Eltrombopag (ELT) and Hetrombopag (HET) maintained MK viability comparable to rhTPO. However, ELT led to iron disruption at high doses. In contrast, HET significantly upregulated genes involved in heme metabolism, and complement/immune regulation, promoting proplatelet formation and complement activation while inhibiting oxidative phosphorylation and enhancing P-STAT3/5. Single-cell and RNA sequencing further confirmed that rhTPO's transcriptional profile more closely resembled that of TPO than did any other TPO-RAs.

**Copyright Information:** (Online) ISSN: 2572-9241
©2026 The Author(s). HemaSphere is published by John Wiley & Sons Ltd on behalf of European Hematology Association. This is an open access Abstract Book distributed under the Attribution-NonCommercial-NoDerivs (CC BY-NC-ND), which allows third parties to download the articles and share them with others as long as they credit the author and the Abstract Book, but they cannot change the content in any way or use them commercially.
**Abstract Book Citations:** Authors, Title, HemaSphere, 2026;10:(S1):pages. The abstract book DOIs can be found on the “Author Information” page.
**Disclaimer:** Articles published in the journal HemaSphere exclusively reflect the opinions of the authors. The authors are responsible for all content in their abstracts including accuracy of the facts, statements, citing resources, etc.

### Summary/Conclusion

Our study demonstrates that rhTPO more closely recapitulate physiological megakaryopoiesis, with favorable efficacy and safety. In contrast, ROM, AVA, ELT and HET exhibit dysregulated MK development, specific functional defects, and potential risks. These findings provide a critical mechanistic basis for optimizing drug selection in thrombocytopenia therapy, enabling a shift from simply elevating platelet counts to achieving more effective and personalized treatment strategies.

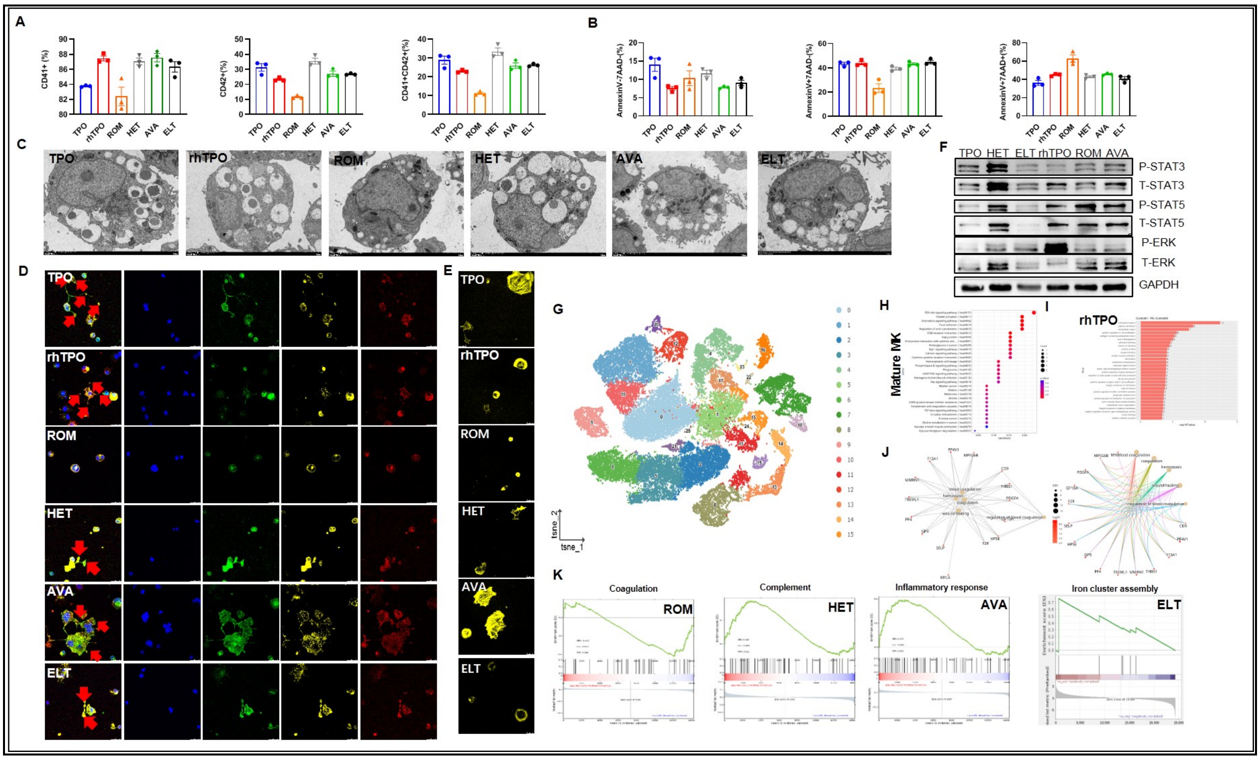

**Copyright Information:** (Online) ISSN: 2572-9241
©2026 The Author(s). HemaSphere is published by John Wiley & Sons Ltd on behalf of European Hematology Association. This is an open access Abstract Book distributed under the Attribution-NonCommercial-NoDerivs (CC BY-NC-ND), which allows third parties to download the articles and share them with others as long as they credit the author and the Abstract Book, but they cannot change the content in any way or use them commercially.
**Abstract Book Citations:** Authors, Title, HemaSphere, 2026;10:(S1):pages. The abstract book DOIs can be found on the "Author Information" page.
**Disclaimer:** Articles published in the journal HemaSphere exclusively reflect the opinions of the authors. The authors are responsible for all content in their abstracts including accuracy of the facts, statements, citing resources, etc.

### (PS2437) LPC(16:0/0/0) DRIVES MACROPHAGE DYSFUNCTION IN ITP: A SYNERGISTIC DEXAMETHASONE-ATORVASTATIN THERAPY

**Topic:** 33. Platelet disorders

Nan Jiang*[1], Shuwen Wang[1], Jun Peng[1]

[1]*Qilu hospital of Shandong University, Jinan, China;*

### Background

Immune thrombocytopenia (ITP) is an autoimmune disorder characterized by macrophage-mediated platelet destruction. Metabolic-immune crosstalk is emerging as a critical pathogenic axis. Lysophosphatidylcholine (LPC), particularly the saturated fatty acid-containing subtype LPC(16:0/0/0), exerts pro-inflammatory effects, yet its role in ITP remains undefined. Glucocorticoids like dexamethasone (DXM) are first-line treatments but face limitations including resistance and relapse. Statins such as atorvastatin (AT) possess immunomodulatory properties beyond cholesterol reduction.

### Aims

To elucidate the role of LPC(16:0/0/0) in driving macrophage polarization and functional imbalance in ITP and evaluate the therapeutic potential of DXM and AT, alone and in combination.

### Methods

Bone marrow (BM) and peripheral blood were collected from 61 ITP patients and 46 healthy donors (HDs). Macrophage subsets (M1: $CD68^{+}CCR2^{+}$; M2: $CD68^{+}CD206^{+}$) and IL-10 levels were analyzed by flow cytometry and ELISA. Untargeted metabolomics and transcriptomics were performed on sorted BM macrophages. LPC(16:0/0/0) was quantified by ELISA. In vitro, human macrophages were treated with LPC(16:0/0/0) to assess IL-10 secretion, M1/M2 polarization via GPR68 signaling (using Ogremorphin), platelet phagocytosis, and M2-mediated T-cell differentiation (Th1, Th2, Th17, Th22, Treg). Modulatory effects of DXM and AT were tested. Findings were validated in passive and active ITP mouse models.

### Results

ITP patients exhibited significantly higher M1 proportions and reduced IL-10 in BM and plasma versus HDs. Integrated omics revealed glycerophospholipid metabolism as the most altered pathway, with LPC(16:0/0/0) as the top differential metabolite. ELISA confirmed elevated LPC(16:0/0/0) in ITP BM and plasma, inversely correlating with platelet counts ($<30\times10^{9}$/L). LPC(16:0/0/0) suppressed macrophage IL-10 and promoted M1 polarization via GPR68, as Ogremorphin abrogated these effects. LPC enhanced M1 platelet phagocytosis. LPC-treated M2 macrophages lost regulatory function, decreasing Tregs and promoting Th1, Th2, and Th22 differentiation. DXM reversed M1 polarization and suppressed Th1/Th2 responses; AT restored IL-10 and enhanced Tregs. The DXM-AT combination synergistically corrected M1/M2 imbalance and normalized T-cell subsets more effectively than monotherapy. In vivo, LPC exacerbated thrombocytopenia and M1 skewing in ITP mice. DXM or AT improved platelet recovery, with combination therapy yielding superior outcomes by reversing LPC-induced M1 polarization and establishing an M2-dominant microenvironment.

**Copyright Information:** (Online) ISSN: 2572-9241
©2026 The Author(s). HemaSphere is published by John Wiley & Sons Ltd on behalf of European Hematology Association. This is an open access Abstract Book distributed under the Attribution-NonCommercial-NoDerivs (CC BY-NC-ND), which allows third parties to download the articles and share them with others as long as they credit the author and the Abstract Book, but they cannot change the content in any way or use them commercially.
**Abstract Book Citations:** Authors, Title, HemaSphere, 2026;10:(S1):pages. The abstract book DOIs can be found on the "Author Information" page.
**Disclaimer:** Articles published in the journal HemaSphere exclusively reflect the opinions of the authors. The authors are responsible for all content in their abstracts including accuracy of the facts, statements, citing resources, etc.

**Summary/Conclusion**

This study identifies LPC(16:0/0/0) as a key pathogenic mediator driving GPR68-mediated macrophage dysfunction and T-cell dysregulation in ITP. The DXM-AT combination synergistically targets this metabolic-immune axis: DXM provides rapid anti-inflammatory effects while AT restores long-term immune homeostasis. This dual-targeting strategy offers a promising approach to overcome glucocorticoid resistance and improve ITP outcomes.

**Copyright Information:** (Online) ISSN: 2572-9241
©2026 The Author(s). HemaSphere is published by John Wiley & Sons Ltd on behalf of European Hematology Association. This is an open access Abstract Book distributed under the Attribution-NonCommercial-NoDerivs (CC BY-NC-ND), which allows third parties to download the articles and share them with others as long as they credit the author and the Abstract Book, but they cannot change the content in any way or use them commercially.
**Abstract Book Citations:** Authors, Title, HemaSphere, 2026;10:(S1):pages. The abstract book DOIs can be found on the "Author Information" page.
**Disclaimer:** Articles published in the journal HemaSphere exclusively reflect the opinions of the authors. The authors are responsible for all content in their abstracts including accuracy of the facts, statements, citing resources, etc.

### (PS2438) INTACT COVID-19 VACCINATION RESPONSE AND OVERALL SAFETY IN THE PHASE 3, MULTICENTER, DOUBLE-BLINDED, PLACEBO-CONTROLLED, RANDOMIZED CLINICAL TRIAL OF INTRAVENOUS EFGARTIGIMOD IN ITP (ADVANCE IV)

**Topic:** 33. Platelet disorders

Monica Carpenedo*[1], Hanny Al-Samkari[2], Blanca Sanchez-Gonzalez[3], Yoshitaka Miyakawa[4], Michael Drazer[5], John Sleasman[6], Fien Gistelinck[7], Paul Duncombe[7], Sophie Steeland[7], Filip Matthijssens[7], Jaume Ayguasanosa[7], Antoine Azar[8], Kevin Winthrop[9]

*[1]ASST Grande Ospedale Metropolitano Niguarda, Department of Hematology Oncology and Molecular Medicine, Milan, Italy; [2]Massachusetts General Hospital, Hematology Division, Boston, United States of America; [3]Hospital del Mar, Department of Hematology, Barcelona, Spain; [4]Saitama Medical University Hospital, Department of Hematology, Saitama, Japan; [5]University of Chicago, Section of Hematology/Oncology, Chicago, United States of America; [6]Duke University School of Medicine, Division of Allergy, Immunology, and Pulmonary Medicine, Durham, United States of America; [7]argenx, Ghent, Belgium; [8]Johns Hopkins University School of Medicine, Division of Allergy and Clinical Immunology, Baltimore, United States of America; [9]Oregon Health and Science University, Division of Infectious Disease, Portland, United States of America;*

### Background

COVID-19 vaccination and subsequent immune response are crucial for patients with autoimmune diseases due to increased infection risk. Efgartigimod (EFG) is a human immunoglobulin G1 (IgG1) antibody Fc fragment that reduces total and pathogenic IgG through neonatal Fc receptor (FcRn) blockade without impacting antibody production or other parts of the immune system and has been investigated in immune thrombocytopenia (ITP).

### Aims

Investigate the effect of EFG administered intravenously (IV) on humoral immune responses to COVID-19 vaccination and safety in participants with primary ITP in the phase 3 ADVANCE IV study (NCT04188379).

### Methods

EFG IV (10 mg/kg) or placebo IV was administered 4 times once-weekly, then once-weekly or once every other week for 24 weeks, depending on platelet response. COVID-19–specific receptor binding domain (RBD)-IgGs, spike protein, and functional neutralizing antibody titers were assessed pre- and post-vaccination, after at least 4 weekly EFG administrations in 8 participants from each cohort.

### Results

Total IgG levels were reduced in EFG-treated participants versus placebo (mean [standard error] post-vaccination levels of 5.8 [1.2] g/L vs 11.8 [1.4] g/L). Upon vaccination, both EFG and PBO participants mounted similar COVID-19–specific IgG antibody titer responses. The mean (standard error) fold change of RBD-IgG titers from baseline for EFG was 144.1 (65.8) versus 136.4 (104.7) for PBO. Similar trends were observed for spike protein (EFG 247.1 [148.1], PBO 186.9 [151.2]) and functional neutralizing antibody titers (EFG 60.7 [35.3], PBO 27.7 [13.8]).
In the overall study population (EFG n=86, PBO n=45), there was no difference in severe (Grade ≥3) treatment-emergent adverse events related to infections (EFG n=2 [2.3%]; cytomegalovirus and erysipelas; PBO n=2 [4.4%], appendicitis and COVID-19 [all also considered serious AEs]), with all severe infections being grade 3.

**Copyright Information:** (Online) ISSN: 2572-9241
©2026 The Author(s). HemaSphere is published by John Wiley & Sons Ltd on behalf of European Hematology Association. This is an open access Abstract Book distributed under the Attribution-NonCommercial-NoDerivs (CC BY-NC-ND), which allows third parties to download the articles and share them with others as long as they credit the author and the Abstract Book, but they cannot change the content in any way or use them commercially.
**Abstract Book Citations:** Authors, Title, HemaSphere, 2026;10:(S1):pages. The abstract book DOIs can be found on the "Author Information" page.
**Disclaimer:** Articles published in the journal HemaSphere exclusively reflect the opinions of the authors. The authors are responsible for all content in their abstracts including accuracy of the facts, statements, citing resources, etc.

**Summary/Conclusion**

Participants with ITP receiving EFG IV could mount antigen-specific IgG responses to COVID-19 immunization with no increased risk of severe infections. Ongoing efficacy and safety of EFG IV in ITP is being evaluated in the ADVANCE NEXT trial (NCT06544499).

**Copyright Information:** (Online) ISSN: 2572-9241
©2026 The Author(s). HemaSphere is published by John Wiley & Sons Ltd on behalf of European Hematology Association. This is an open access Abstract Book distributed under the Attribution-NonCommercial-NoDerivs (CC BY-NC-ND), which allows third parties to download the articles and share them with others as long as they credit the author and the Abstract Book, but they cannot change the content in any way or use them commercially.
**Abstract Book Citations:** Authors, Title, HemaSphere, 2026;10:(S1):pages. The abstract book DOIs can be found on the "Author Information" page.
**Disclaimer:** Articles published in the journal HemaSphere exclusively reflect the opinions of the authors. The authors are responsible for all content in their abstracts including accuracy of the facts, statements, citing resources, etc.

### (PS2439) IMMUNOMODULATORY EFFECTS OF ELTROMBOPAG ON CD8+ T CELLS IN IMMUNE THROMBOCYTOPENIA THROUGH IRON CHELATION

**Topic:** 33. Platelet disorders

Michelle Tan*[1], Alicia Au[1], Anwar Sayed[2], Amna Malik[1], Rocel Saputil[1], Akhil Khera[1, 3], Sara Zafar[1], Spencer MacKie[3], Cynthia Lee[1, 3], Deena Paul[3], Camelia Vladescu[3], Bridget Chivers[3], John B. Porter[4], Nichola Cooper[1, 3]

*[1]Imperial College London, London, United Kingdom; [2]Taibah University, Madinah, Saudi Arabia; [3]Imperial College Healthcare NHS Trust, London, United Kingdom; [4]University College London, London, United Kingdom;*

### Background

Immune thrombocytopenia (ITP) is a complex autoimmune disorder characterised by accelerated platelet destruction and impaired platelet production, driven by aberrant T lymphocyte activation. Central to this process are dysregulated CD8+ cytotoxic T cell subsets, which have been specifically implicated in mediating direct platelet destruction. Thrombopoietin receptor agonists (TPO-RAs) like romiplostim (ROM), eltrombopag (ELT), and avatrombopag (AVA) have largely replaced immunosuppressants as first-line therapy owing to their potent stimulation of megakaryopoiesis, though their broader immunomodulatory effects remain partially unclear. ELT, in addition to stimulating thrombopoietin receptor and increasing platelet production, is also a potent intracellular iron chelator and has been shown to reduce rapidly proliferating cardiomyocytes, pancreatic cells and stem cells.

### Aims

This study investigates the potential of ELT to modulate CD8+ T cells in patients with ITP, focusing on its effect on their proliferation and activity.

### Methods

Fifty ITP patients on ELT (n=26), ROM (n=16) and AVA (n=8) were recruited from Hammersmith Hospital, London. Peripheral blood mononuclear cells (PBMCs) were collected and used to characterised CD8+ T cells population using flow cytometry. To assess the direct immunomodulatory effects of TPO-RAs, PBMCs from HC and treatment-naive ITP patients were stimulated *in vitro* with anti-human CD3 and CD28 across increasing doses of ELT, ROM, AVA, deferoxamine (DEF; iron chelation control), or human recombinant TPO (TPO-R control). Cell proliferation and cycle were measured using Cell Trace Violet and propidium iodide (PI), respectively using flow cytometry.

### Results

Flow cytometric analysis demonstrated ITP patients on ELT and AVA had a significantly lower proportion of terminally differentiated effector memory cells (TEMRA; CCR7-CD45RA+) in CD8+ T cells than in those treated with ROM ($P < 0.01$ for both groups). Among all treatment groups, patients who received ELT (n=9) and achieved a response demonstrated a significant reduction in CD8+ TEMRA cells compared to pre-treatment, which was associated with a corresponding increase in platelet count ($P < 0.01$). *In vitro* studies in PBMCs of HC and ITP showed a dose-dependent reduction in CD8+ T cell proliferation by ELT. DEF, another iron chelator mimicked these effects, while AVA, ROM and rhTPO did not, suggesting an iron chelation-mediated mechanism. In similar manner, ELT also inhibited cell cycle arresting the cells into G1 phase, more pronounced with increasing doses of ELT. This was seen in DEF, however, less potent than ELT. ROM and AVA did not induce cell cycle arrest. To investigate whether this cell cycle arrest was mediated by the iron chelating properties of ELT, arrested PBMCs were treated with 100 mM ferric ammonium citrate (FAC) for 48 hours, and cell cycle progression was found to be rescued.

**Copyright Information:** (Online) ISSN: 2572-9241
©2026 The Author(s). HemaSphere is published by John Wiley & Sons Ltd on behalf of European Hematology Association. This is an open access Abstract Book distributed under the Attribution-NonCommercial-NoDerivs (CC BY-NC-ND), which allows third parties to download the articles and share them with others as long as they credit the author and the Abstract Book, but they cannot change the content in any way or use them commercially.
**Abstract Book Citations:** Authors, Title, HemaSphere, 2026;10:(S1):pages. The abstract book DOIs can be found on the "Author Information" page.
**Disclaimer:** Articles published in the journal HemaSphere exclusively reflect the opinions of the authors. The authors are responsible for all content in their abstracts including accuracy of the facts, statements, citing resources, etc.

### Summary/Conclusion

These findings suggest that ELT's effectiveness may go beyond megakaryopoiesis stimulation. We propose that ELT may have a dual mechanism of action: increasing platelet production, and at the same time, inhibiting the proliferation of platelet reactive CD8+ TEMRA cells by inducing G1 cell cycle arrest. This iron chelation-dependent effect suggests a novel immunomodulatory role for ELT. This dual action could explain its efficacy in patients who lose response to other TPO-RAs, offering a potential avenue for future therapeutic strategies in ITP.

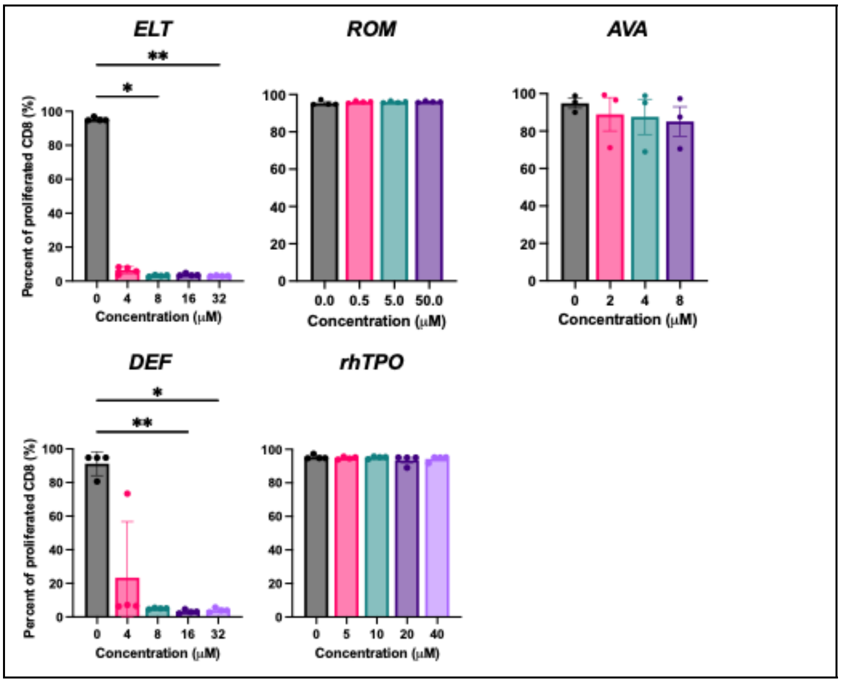

ELT reduced CD8+ T cell proliferation in a dose-dependent manner. HC PBMCs were stimulated with anti-human CD3/CD28 and cultured with serial concentrations of ELT, ROM, AVA, DEF or rhTPO. Cell proliferation was assessed using CellTrace Violet (CTV) and analysed using flow cytometry..

**Copyright Information:** (Online) ISSN: 2572-9241
©2026 The Author(s). HemaSphere is published by John Wiley & Sons Ltd on behalf of European Hematology Association. This is an open access Abstract Book distributed under the Attribution-NonCommercial-NoDerivs (CC BY-NC-ND), which allows third parties to download the articles and share them with others as long as they credit the author and the Abstract Book, but they cannot change the content in any way or use them commercially.
**Abstract Book Citations:** Authors, Title, HemaSphere, 2026;10:(S1):pages. The abstract book DOIs can be found on the "Author Information" page.
**Disclaimer:** Articles published in the journal HemaSphere exclusively reflect the opinions of the authors. The authors are responsible for all content in their abstracts including accuracy of the facts, statements, citing resources, etc.

### (PS2440) BUDOPRUTUG, A LOW-FUCOSYLATED ANTI-CD19 MONOCLONAL ANTIBODY, IN ADULTS WITH PRIMARY IMMUNE THROMBOCYTOPENIA: INITIAL PHASE 1B STUDY RESULTS

**Topic:** 33. Platelet disorders

Moonmoon Kazi[1], Oleksandra Stasyshyn[2], Olena Kyselova[3], Pencho Georgiev[4], Nada Suvajdzic Vukovic[5], Edgar Charles*[1]

*[1]Climb Bio Inc., Wellesley, United States of America;[2]SI Institute of Blood Pathology and Transfusion Medicine of NAMS of Ukraine, Lviv, Ukraine; [3]Medical Center OK Clinic+LLC International Institute of Clinical Research, Kyiv, Ukraine;[4]Medical University, Plovdiv, Bulgaria; [5]University Clinical Center of Serbia, Beograd, Serbia;*

### Background

Immune thrombocytopenia (ITP) is a rare autoantibody-mediated disorder characterized by reduced platelets and increased risk of bleeding. While up to 10% of adults may undergo spontaneous remission, others develop persistent disease, often requiring multiple lines of treatment. Budoprutug (TNT119) is an anti-CD19 mAb bioengineered with picomolar binding affinity for CD19 and a low-fucosylated Fc region for enhanced antibody-dependent cell cytotoxicity. CD19 is expressed from the early pro-B stage through maturation and differentiation to plasmablasts and on plasma cell subsets, providing the potential for budoprutug to provide deep and sustained depletion of B cells, including autoantibody-producing cells. Budoprutug was previously evaluated in adults with primary membranous nephropathy, another autoantibody-mediated disease. Budoprutug was well tolerated, with 5/5 evaluable participants experiencing complete peripheral B-cell decreases and complete or partial remission of proteinuria (Cortazar ASN 2024).

### Aims

This Phase 1b/2a study is designed to assess ascending doses of budoprutug in adults with primary ITP who failed prior therapy.

### Methods

The Phase 1b portion of the study is an open-label dose escalation with 3 dose levels (DL) of IV budoprutug: DL1-250mg, DL2-500mg, DL3-1000mg (NCT07043946). Key inclusion criteria are age >18 years, diagnosis of primary ITP, and platelet (plt) count <30,000/mL (confirmed on two separate occasions, 5-14 days apart) despite ≥1 prior therapy. Key exclusion criteria include CD19+ B cells <80 cells/mL or <40 cells/mL if B-cell depleting treatment was received previously. Budoprutug is administered on Days 1 and 15. Concomitant corticosteroids or thrombopoietin agonists (TPO-RA/TPO) with stable doses ≥14 days prior to Day 1 are allowed. The primary objective is to evaluate safety and tolerability. Secondary objectives include effects on peripheral B-cell depletion using high sensitivity B-cell detection, and plt count.

### Results

As of data cut-off, 10 participants (pts) (6 DL1, 4 DL2) had enrolled in the study. The median (range) age was 47 (22-75) years. All pts had persistent or chronic ITP; median (range) duration of ITP was 10 years (0.4-40), with 1 pt (DL2) on concomitant TPO-RA. All pts had 2-4 prior therapies within the last 2 years; 4 pts had prior rituximab and a separate pt had a splenectomy. All pts received the Day 1 and Day 15 doses of budoprutug. After a median (range) follow up of 5 months (3-6) in DL1, and maximum follow up of 43 days (8-43) in DL2, there were no deaths, discontinuations, SAEs, or infusion-related reactions. Figure 1 shows B-cell counts for DL1. Profound decreases occurred following budoprutug administration; median B-cell count at Day 85 was 1 cell/µL. By Day 85, 4/6 pts in DL1 had a platelet response: 1 pt with plt >100,000/µL, 1 pt with plt 50,000-100,000/µL, and 2 pts with plt 30,000-50,000/µL. No pts had bleeding events.

**Copyright Information:** (Online) ISSN: 2572-9241
©2026 The Author(s). HemaSphere is published by John Wiley & Sons Ltd on behalf of European Hematology Association. This is an open access Abstract Book distributed under the Attribution-NonCommercial-NoDerivs (CC BY-NC-ND), which allows third parties to download the articles and share them with others as long as they credit the author and the Abstract Book, but they cannot change the content in any way or use them commercially.
**Abstract Book Citations:** Authors, Title, HemaSphere, 2026;10:(S1):pages. The abstract book DOIs can be found on the "Author Information" page.
**Disclaimer:** Articles published in the journal HemaSphere exclusively reflect the opinions of the authors. The authors are responsible for all content in their abstracts including accuracy of the facts, statements, citing resources, etc.

### Summary/Conclusion

In this open-label study of budoprutug in previously treated adults with primary ITP, initial data suggest an acceptable tolerability profile and robust B-cell depletion following two doses of budoprutug. Increases in platelet counts were observed in the majority of pts, and no post-baseline bleeding events were reported. The study is continuing with dosing in higher dose cohorts (DL2 and DL3). Additional safety and efficacy data from the study will be presented at the conference.

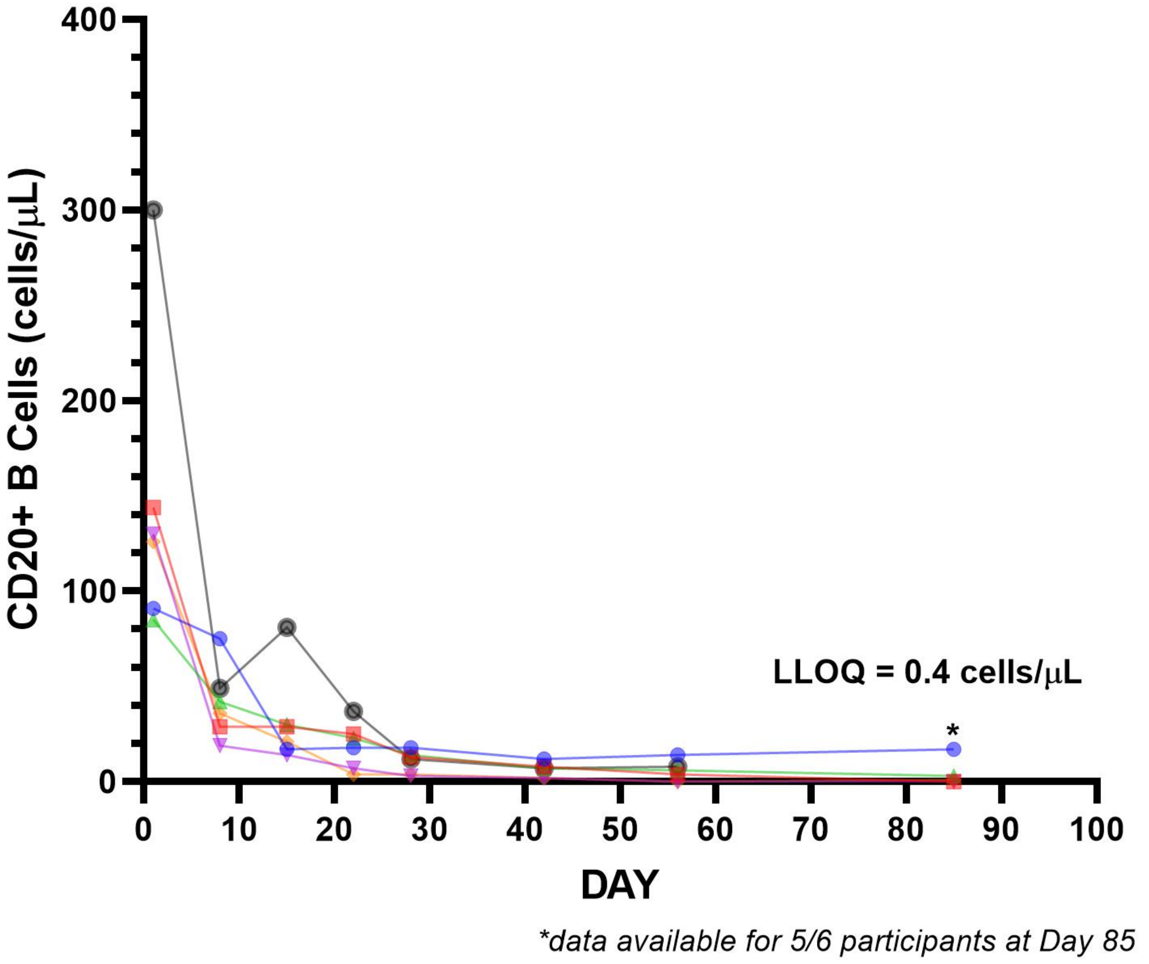

**Figure 1.** *CD20+ B-cell Counts by Participant (DL1)*

**Copyright Information:** (Online) ISSN: 2572-9241
©2026 The Author(s). HemaSphere is published by John Wiley & Sons Ltd on behalf of European Hematology Association. This is an open access Abstract Book distributed under the Attribution-NonCommercial-NoDerivs (CC BY-NC-ND), which allows third parties to download the articles and share them with others as long as they credit the author and the Abstract Book, but they cannot change the content in any way or use them commercially.
**Abstract Book Citations:** Authors, Title, HemaSphere, 2026;10:(S1):pages. The abstract book DOIs can be found on the "Author Information" page.
**Disclaimer:** Articles published in the journal HemaSphere exclusively reflect the opinions of the authors. The authors are responsible for all content in their abstracts including accuracy of the facts, statements, citing resources, etc.

### (PS2441) REAL-WORLD CLINICAL BURDEN AMONG PATIENTS WITH ESSENTIAL THROMBOCYTHEMIA WHO DEVELOPED RESISTANCE OR INTOLERANCE TO HYDROXYUREA

**Topic:** 33. Platelet disorders

Evan Chen*[1, 2], Xiaoqin Yang[3], Mavis Obeng-Kusi[3], Uzor Ogbu[3], Rose Chang[4], Priyanka Bobbili[4], Louise Clear[4], Zhuo Chen[4], Yidan MA[4], Tianxiu Li[4], Yichuan Grace Hsieh[1, 5], Christopher Herrick[5], Mei Sheng Duh[4], Shawn Murphy[1, 5, 6], Gabriela Hobbs[1, 6]

*[1]Harvard Medical School, Boston, United States of America;[2]Dana-Farber Cancer Institute, Boston, United States of America; [3]Merck & Co., Inc., Rahway, United States of America;[4]Analysis Group, Inc., Boston, United States of America; [5]Mass General Brigham, Somerville, United States of America; [6]Massachusetts General Brigham, Boston, United States of America;*

### Background

Essential thrombocythemia (ET) is a rare myeloproliferative neoplasm characterized by thrombocytosis, vascular complications, and potential progression to post-ET myelofibrosis (MF) or blast-phase disease. Hydroxyurea is a frequently used first-line (1L) cytoreductive therapy, but a substantial proportion of patients develop resistance or intolerance (R/I). Real-world outcomes data are needed for patients with ET who experience hydroxyurea R/I.

### Aims

We sought to characterize treatment patterns and clinical outcomes among U.S. patients with ET who developed hydroxyurea R/I.

### Methods

Adult patients with ET treated with hydroxyurea were identified in the Mass General Brigham Research Patient Data Registry (2012–2024). Inclusion criteria were: (1) ICD-10 ET diagnosis code associated with a hematology/oncology visit (i.e., first ET diagnosis); (2) subsequent physician visit with ET diagnosis code; (3) platelet count ≥450×10⁹/L ≤6 months before diagnosis; and (4) prescription of 1L hydroxyurea on or after diagnosis.
Hydroxyurea R/I was defined per modified ELN criteria, including ≥1 of the following: (1) concurrent platelet count >400×10⁹/L and WBC <2.5 ×10⁹/L on hydroxyurea; (2) concurrent platelet count >400×10⁹/L and hemoglobin <10g/dL on hydroxyurea; (3) skin ulcers/sores or other unacceptable muco-cutaneous manifestations; or (4) fever, with the latter two required to be attributable to hydroxyurea in the treating clinician's documentation. ET-related complications and disease progression were assessed in patients who experience R/I at any time from the period of 1L hydroxyurea initiation until discontinuation or end of follow-up, as well as after the date of R/I. Hydroxyurea discontinuation was defined as switching to second-line (2L) therapy or a >60-day gap without resumption of hydroxyurea; a >60-day gap with resumption of hydroxyurea defined treatment interruptions. Cytopenia was defined as anemia (Hb <11 g/dL for women, <12.5 g/dL for men), thrombocytopenia (platelets <150×10⁹/L), or leukopenia (WBC <4×10⁹/L).

**Copyright Information:** (Online) ISSN: 2572-9241
©2026 The Author(s). HemaSphere is published by John Wiley & Sons Ltd on behalf of European Hematology Association. This is an open access Abstract Book distributed under the Attribution-NonCommercial-NoDerivs (CC BY-NC-ND), which allows third parties to download the articles and share them with others as long as they credit the author and the Abstract Book, but they cannot change the content in any way or use them commercially.
**Abstract Book Citations:** Authors, Title, HemaSphere, 2026;10:(S1):pages. The abstract book DOIs can be found on the "Author Information" page.
**Disclaimer:** Articles published in the journal HemaSphere exclusively reflect the opinions of the authors. The authors are responsible for all content in their abstracts including accuracy of the facts, statements, citing resources, etc.

**Results**

Among 634 eligible patients with ET treated with 1L hydroxyurea (median diagnosis age of 70 years [range 20–96]; 68.0% female; 88.2% White; 71.9% JAK2+, 18.8% CALR+, and 4.4% MPL+), 133 (21.0%) developed R/I to hydroxyurea. First evidence of R/I occurred at a median of 13 months after hydroxyurea initiation.
Of the 133 patients who developed R/I (median diagnosis age of 70 years [range 20–96]; 64.0% female; 93.2% White; 62.3% JAK2+, 25.4% CALR+, and 6.1% MPL+), 98.5% continued hydroxyurea after first evidence of R/I; 9.8% switched to 2L therapy at a median of 5.1 months after first R/I; and 56.4% experienced treatment interruptions at any time while on treatment.
Patients who develop R/I experienced frequent complications while on hydroxyurea: 41.4% had thrombotic (34.6%) or major bleeding (15.8%) events. Most of these events occurred after first evidence of R/I (73.9% and 90.5%, respectively). Disease progression occurred in 10.5% (8.3% to MF; <7.5% to blast-phase) and ≥1 cytopenia was observed in 97.7% of patients with R/I.

**Summary/Conclusion**

In this real-world cohort, 21% of patients treated with hydroxyurea developed R/I, yet most (98.5%) continued therapy at the time of R/I, reflecting limited transition to alternative cytoreductive agents. Patients with R/I experienced high complication and progression rates, underscoring the unmet need for more effective treatment strategies in this population.

**Copyright Information:** (Online) ISSN: 2572-9241
©2026 The Author(s). HemaSphere is published by John Wiley & Sons Ltd on behalf of European Hematology Association. This is an open access Abstract Book distributed under the Attribution-NonCommercial-NoDerivs (CC BY-NC-ND), which allows third parties to download the articles and share them with others as long as they credit the author and the Abstract Book, but they cannot change the content in any way or use them commercially.
**Abstract Book Citations:** Authors, Title, HemaSphere, 2026;10:(S1):pages. The abstract book DOIs can be found on the "Author Information" page.
**Disclaimer:** Articles published in the journal HemaSphere exclusively reflect the opinions of the authors. The authors are responsible for all content in their abstracts including accuracy of the facts, statements, citing resources, etc.

### (PS2442) COGNITIVE DYSFUNCTION IN ADULTS WITH IMMUNE THROMBOCYTOPENIA (ITP): A CROSS-SECTIONAL OBSERVATIONAL STUDY

**Topic:** 33. Platelet disorders

Nichola Cooper*[1], Sandhya Panch[2], Caroline Piatek[3], Maria Belen Rodriguez[4], Ahmed Daak[4], Julie Kissell[4], Anna Compagnoni[5], Howard Liebmann[6], David J. Kuter[7]

*[1]Hammersmith Hospital, London, United Kingdom; [2]Department of Medicine, University of Washington, Seattle, United States of America; [3]Division of Hematology, Department of Medicine, Keck School of Medicine of USC, Los Angeles, United States of America; [4]Sanofi, Cambridge, United States of America; [5]Cytel, Cambridge, MA, USA; on behalf of Sanofi R&D, Cambridge, United States of America; [6]University of Southern California, Los Angeles, United States of America;[7]Hematology Division, Massachusetts General Hospital, Harvard Medical School, Boston, United States of America;*

### Background

ITP is a rare disease driven by complex immune dysregulation and characterized by low platelet counts. Beyond the risk of bleeding, patients often report fatigue and difficulty concentrating, which can contribute to reduced HRQOL. Emerging evidence suggests high rates of cognitive impairment in ITP, including findings from a study using Cogstate Brief Battery (CBB). However, it remains unclear whether cognitive dysfunction persists after patients achieve stable platelet counts and whether these findings extend to the real-world setting.

### Aims

Determine the frequency, nature, and severity of cognitive impairment in chronic primary ITP in a real-world setting.

### Methods

Adults aged ≥18 y who had an initial response to first-line therapy and were either receiving ITP treatment (regardless of platelet counts) or in remission/observation without treatment for ≤1 y were eligible. At index, clinical outcome assessments measured cognitive status, fatigue (FACIT-Fatigue, ITP-PAQ), bleeding (IBLS), and HRQOL. Cognitive status was assessed by using CBB, a validated test that objectively evaluates psychomotor function, visual attention, visual learning, and visual working memory. For each z-score, results were standardized by age and sex; composite z-scores (psychomotor/attention and learning/working memory) were then derived, with z-scores ≤-1 in at least one of the composite domains defining clinically important cognitive impairment.

**Copyright Information:** (Online) ISSN: 2572-9241
©2026 The Author(s). HemaSphere is published by John Wiley & Sons Ltd on behalf of European Hematology Association. This is an open access Abstract Book distributed under the Attribution-NonCommercial-NoDerivs (CC BY-NC-ND), which allows third parties to download the articles and share them with others as long as they credit the author and the Abstract Book, but they cannot change the content in any way or use them commercially.
**Abstract Book Citations:** Authors, Title, HemaSphere, 2026;10:(S1):pages. The abstract book DOIs can be found on the “Author Information” page.
**Disclaimer:** Articles published in the journal HemaSphere exclusively reflect the opinions of the authors. The authors are responsible for all content in their abstracts including accuracy of the facts, statements, citing resources, etc.

### Results

Of 104 patients screened, 97 were eligible to participate in the study and completed the CBB test (35% in the UK, 65% in the USA). Median age at index was 54 y (Q1-Q3, 41-66); 65% were female. Median duration of ITP was 10 y (Q1-Q3, 4-18). Eighty-two patients reported receiving active ITP treatment at index, including TPO-RAs (n=67), corticosteroids (n=18), other immunosuppressants (n=12), fostamatinib (n=6), rituximab (n=2), and/or other (n=3). Median platelet count was $143 \times 10^9$/L (Q1-Q3, 85-$222 \times 10^9$/L) and median hemoglobin was 139 g/L (Q1-Q3, 130-150). Eighty participants had comorbidities, including arterial hypertension (n=17), anxiety (n=17), obesity (n=12), depression (n=10), and hypercholesterolemia (n=10). The mean (SD) ITP bleeding scale total score was 0.04 (0.10); mean (SD) scores for FACIT-Fatigue, ITP-PAQ psychological health (PH), and ITP-PAQ overall QOL were 37 (12), 76 (26), and 65 (26), respectively.
Clinically important cognitive impairment was observed in 26% (n=25/97; 95% CI, 17-36) of patients, with 68% aged <65 y. Seven (7%) patients, aged 39-78 y, had z-score of ≤-1 for both composite scores; 12 (12%) patients, aged 25-75 y, had z-score of ≤-1 for learning/working memory only, and 6 (6%) patients, aged 25-80 y, had z-scores of ≤-1 for psychomotor function/attention only. Cognitive impairment by severity and age for each category are shown in Tables 1 and 2, respectively. There was no correlation between cognitive impairment in either composite domain and FACIT-Fatigue or ITP-PAQ PH scores.

### Summary/Conclusion

Our findings demonstrate the presence of clinically important cognitive impairment among some adults with chronic ITP despite having no/minimal bleeding and adequate platelet counts. These underscore the need for more comprehensive characterization of cognitive status and consideration of symptoms beyond bleeding in routine clinical practice for patient-centered management of ITP. Additional studies are warranted to better elucidate mechanisms and impact on patient QOL.

**Table 1. Proportion of Patients With Chronic ITP by Categories of Cognitive Status and by Severity**

| Patients by Cognitive Status, n (%) | Psychomotor Function (DET Reaction Time) N=97 | Visual Attention (IDN Reaction Time) N=97 | Psychomotor Function/ Attention N=97 | Visual Learning (OCL Accuracy) N=97 | Visual Working Memory (ONB Accuracy) N=97 | Learning/ Working Memory N=97 |
|---|---|---|---|---|---|---|
| Not impaired (> -1) | 86 (89) | 88 (91) | 84 (87) | 83 (86) | 80 (82) | 78 (80) |
| Impaired (≤ -1) | 11 (11) | 9 (9) | 13 (13) | 14 (14) | 17 (18) | 19 (20) |
| ≥ -2 to ≤ -1 | 5 (5) | 4 (4) | 8 (8) | 8 (8) | 12 (12) | 13 (13) |
| ≥ -3 to < -2 | 4 (4) | 3 (3) | 4 (4) | 5 (5) | 4 (4) | 4 (4) |
| < -3 | 2 (2) | 2 (2) | 1 (1) | 1 (1) | 1 (1) | 2 (2) |

DET, detection test; IDN, identification test; OCL, one card learning test; ONB, one back test.

**Table 2. Patients With Chronic ITP With Impaired Cognitive Status (Z-Score ≤ -1) by Age Categories**

| Patients with Z-Score ≤ -1, n (%) | 18 to <40 years (n=21) | 40 to <65 years (n=45) | ≥ 65 years (n=31) | All (N=97) |
|---|---|---|---|---|
| At Least One of Psychomotor Function/Attention and Learning/Working Memory (Composite) | 7 (33) | 10 (22) | 8 (26) | 25 (26) |
| Psychomotor Function (DET Reaction Time) | 1 (5) | 5 (11) | 5 (16) | 11 (11) |
| Visual Attention (IDN Reaction Time) | 3 (14) | 4 (9) | 2 (6) | 9 (9) |
| Psychomotor Function/Attention (Composite) | 4 (19) | 6 (13) | 3 (10) | 13 (13) |
| Visual Learning (OCL Accuracy) | 2 (10) | 6 (13) | 6 (19) | 14 (14) |
| Visual Working Memory (ONB Accuracy) | 4 (19) | 7 (16%) | 6 (19) | 17 (18) |
| Learning/Working Memory (Composite) | 3 (14) | 9 (20) | 7 (23) | 19 (20) |
| Both Psychomotor Function/Attention and Learning/Working Memory (Composite) | 0 (0) | 5 (11) | 2 (6) | 7 (7) |

DET, detection test; IDN, identification test; OCL, one card learning test; ONB, one back test.

**Copyright Information:** (Online) ISSN: 2572-9241
©2026 The Author(s). HemaSphere is published by John Wiley & Sons Ltd on behalf of European Hematology Association. This is an open access Abstract Book distributed under the Attribution-NonCommercial-NoDerivs (CC BY-NC-ND), which allows third parties to download the articles and share them with others as long as they credit the author and the Abstract Book, but they cannot change the content in any way or use them commercially.
**Abstract Book Citations:** Authors, Title, HemaSphere, 2026;10:(S1):pages. The abstract book DOIs can be found on the "Author Information" page.
**Disclaimer:** Articles published in the journal HemaSphere exclusively reflect the opinions of the authors. The authors are responsible for all content in their abstracts including accuracy of the facts, statements, citing resources, etc.

### (PS2443) EFFICACY AND SAFETY OF HETROMBOPAG IN INCREASING PLATELET COUNTS IN PATIENTS WITH HEPATOCELLULAR CARCINOMA UNDERGOING HEPATECTOMY: A RANDOMIZED, PLACEBO-CONTROLLED STUDY

**Topic:** 33. Platelet disorders

Ning Zhang*[1], Jiaming Zhou[1], Weiping Zhu[1], Anrong Mao[1], Qi Pan[1], Yiming Zhao[1], Ti Zhang[1], Lu Wang[1]

*[1]Fudan University Shanghai Cancer Center, Department of Hepatic Surgery, Shanghai, China;*

### Background

Thrombocytopenia (TCP) frequently complicates hepatocellular carcinoma (HCC) and predicts poor prognosis. Liver surgery is typically precluded when platelet count (PC) is < $80\times10^9$/L. Prophylactic platelet transfusion, albeit effective to reduce bleeding risk, is limited by short platelet survival, need for repeated transfusions, and intensive care support.
Hetrombopag (HPAG), an oral thrombopoietin receptor agonist (TPO-RA), is approved in China for primary immune thrombocytopenia (ITP) and severe aplastic anemia. It promotes megakaryopoiesis and platelet production by activating thrombopoietin signaling. Similar agents like avatrombopag and lusutrombopag have been approved for TCP in chronic liver disease patients undergoing a procedure.

### Aims

We conducted a randomized, double-blind, placebo (PBO)-controlled, single-center trial to evaluate the efficacy and safety of HPAG in reducing the need for platelet transfusion in HCC patients undergoing hepatectomy.

### Methods

This study enrolled adult HCC patients with platelet count <$80\times10^9$/L and Child-Pugh class A–B (≤7) scheduled for hepatectomy. Patients were randomized 1:1 to receive oral hetrombopag 5 mg once daily or placebo for ≤14 days before surgery. The primary endpoint was the proportion of patients not requiring platelet transfusion or other thrombocytopenia interventions (rhTPO or IL-11) during or within 7 days after hepatectomy. Secondary endpoints included achieving platelet count ≥$80\times10^9$/L before surgery and change in platelet count from baseline to surgery day. Safety was assessed throughout the study.

### Results

Between 25 July 2022 and 11 December 2025, 69 patients were included in the full analysis set (FAS), of whom 62 comprised the per-protocol set (PPS) and were included in the efficacy analysis (HPAG, n=34; placebo, n=28).. The two groups were comparable in baseline characteristics, including median age (57.0 vs 59.3 years), male proportion (76.5% vs 64.3%), and baseline PC (mean 65.0±13.2 vs 63.5±10.9 $\times10^9$/L). Primary endpoint: In the HPAG group, 20/34 patients (58.8%) did not require platelet transfusion or other TCP interventions, compared with 6/28 patients (21.4%) in the placebo group ($p = 0.003$). No patients in either group required platelet transfusion (0 vs 0), while fewer patients in the HPAG group required other platelet-raising interventions than in the placebo group [41.2% (14/34) vs 78.6% (22/28)]. Secondary endpoints: A higher proportion of HPAG-treated patients achieved pre-surgery PC ≥ $80\times10^9$/L compared with placebo [58.8% (20/34) vs 21.4% (6/28)]. The mean increase in PC from baseline to surgery day was numerically greater in the HPAG group than in the placebo group (15.6±16.6 vs 5.6±15.5 $\times10^9$/L; p=0.06). Safety: Treatment-related adverse events occurred in 17.6% (6/34) of HPAG-treated patients and 17.9% (5/28) of placebo patients. The most common treatment-related adverse events were mild elevations in liver function parameters, including aspartate aminotransferase (8.8%, 3/34), alanine aminotransferase (5.9%, 2/34), and total bilirubin (11.8%, 4/34). No grade ≥ 3 treatment-related adverse events were reported.

**Copyright Information:** (Online) ISSN: 2572-9241
©2026 The Author(s). HemaSphere is published by John Wiley & Sons Ltd on behalf of European Hematology Association. This is an open access Abstract Book distributed under the Attribution-NonCommercial-NoDerivs (CC BY-NC-ND), which allows third parties to download the articles and share them with others as long as they credit the author and the Abstract Book, but they cannot change the content in any way or use them commercially.
**Abstract Book Citations:** Authors, Title, HemaSphere, 2026;10:(S1):pages. The abstract book DOIs can be found on the "Author Information" page.
**Disclaimer:** Articles published in the journal HemaSphere exclusively reflect the opinions of the authors. The authors are responsible for all content in their abstracts including accuracy of the facts, statements, citing resources, etc.

**Summary/Conclusion**

In HCC patients with thrombocytopenia undergoing hepatectomy, hetrombopag substantially reduced the need for platelet transfusions or other TCP-directed interventions compared to placebo, with a favorable safety profile. Hetrombopag represents an effective, non-invasive alternative to perioperative platelet transfusions in this population. Trial registration: NCT05442632.

**Copyright Information:** (Online) ISSN: 2572-9241
©2026 The Author(s). HemaSphere is published by John Wiley & Sons Ltd on behalf of European Hematology Association. This is an open access Abstract Book distributed under the Attribution-NonCommercial-NoDerivs (CC BY-NC-ND), which allows third parties to download the articles and share them with others as long as they credit the author and the Abstract Book, but they cannot change the content in any way or use them commercially.
**Abstract Book Citations:** Authors, Title, HemaSphere, 2026;10:(S1):pages. The abstract book DOIs can be found on the "Author Information" page.
**Disclaimer:** Articles published in the journal HemaSphere exclusively reflect the opinions of the authors. The authors are responsible for all content in their abstracts including accuracy of the facts, statements, citing resources, etc.

### (PS2444) CLINICAL CHARACTERISTICS, TREATMENT STRATEGIES, AND RESPONSE PATTERNS IN EVANS SYNDROME: INITIAL ANALYSIS FROM THE SPANISH RESTI COHORT

**Topic:** 33. Platelet disorders

María Eva Mingot Castellano*[1], Elena Sebastian Perez[2], Maria Solsona Gadea[3], Javier Bañon Soria[4], Silvia Monsalvo Saomil[5], Daniel Martínez-Carballeira[6], Isabel González Gascón Y Marín[7], Marta Morado[8], Teresa Arquero Portero[9], Reyes Jimenez-Bárcenas[10], Gonzalo Caballero Navarro[11], Cristina Pascual Izquierdo[12]

*[1]Hospital Universitario Virgen del Rocio, Instituto de Biomedicina de Sevilla (IBIS/CSIC), Universidad de Sevilla, Sevilla, Spain; [2]Servicio de Hematología y Oncología Pediátricas Hospital Infantil Universitario Niño Jesús, Madrid, Spain, Madrid, Spain; [3]Hospital Sant Joan de Déu, Barcelona, Spain;[4]Hospital General Unversitario Gregorio Marañon, Madrid, Spain;[5]Hospital Puerta de Hierro, Madrid, Spain; [6]Hospital Universitario Central de Asturias, Oviedo, Spain;[7]Hospital Infanta Leonor, Madrid, Spain; [8]Hospital Universitario La Paz, Madrid, Spain;[9]Fundación Jimenez Diaz, Madrid, Spain; [10]Hospital Serranía de Ronda, Ronda, Ronda, Spain; [11]Hospital Miguel Servet, Zaragoza, Spain; [12]University Hospital Gregorio Marañón, Instituto de investigación Gregorio Marañon,, Madrid, Spain;*

### Background

Evans syndrome (ES) is a rare, heterogeneous autoimmune disorder characterized by the simultaneous or sequential occurrence of autoimmune hemolytic anemia (WWAIHA), immune thrombocytopenia (ITP), and/or autoimmune neutropenia (AIN). Evidence-based guidelines for management remain limited, particularly in adult populations.

### Aims

To describe the clinical features, treatment modalities, and hematologic responses in a Spanish cohort of patients with ES enrolled in RESTI (Registro Español de Trombocitopenia Inmune).

### Methods

Retrospective multicenter study based on data from the RESTI registry. We included patients with ES, defined by the coexistence (simultaneous or sequential within 10 years) of ITP and wAIHA and/or AIN Diagnoses were based on established international criteria. Data on demographics, clinical presentation, treatments, and responses were collected using a standardized form.

**Copyright Information:** (Online) ISSN: 2572-9241
©2026 The Author(s). HemaSphere is published by John Wiley & Sons Ltd on behalf of European Hematology Association. This is an open access Abstract Book distributed under the Attribution-NonCommercial-NoDerivs (CC BY-NC-ND), which allows third parties to download the articles and share them with others as long as they credit the author and the Abstract Book, but they cannot change the content in any way or use them commercially.
**Abstract Book Citations:** Authors, Title, HemaSphere, 2026;10:(S1):pages. The abstract book DOIs can be found on the "Author Information" page.
**Disclaimer:** Articles published in the journal HemaSphere exclusively reflect the opinions of the authors. The authors are responsible for all content in their abstracts including accuracy of the facts, statements, citing resources, etc.

**Results**

A total of 76 patients were included, with a median age of 27 years (IQR: 10–54.5), 45.3% were female. Thirty-three patients were younger than 18 years old, with median age of 10 years (IQR: 4.5–12.5). ITP was present in 93.4%, wAIHA 71% and AIN 36%. The median follow-up was 306 weeks (IQR: 128–575). All patients received corticosteroids as first-line therapy. Median time to response in ITP was 9 days and 14 days in wAIHA, while neutropenia responded in a median of 3 days. For ITP, response rates were 60.3% (CR 75%), wAIHA global response 80% (84% CR) and neutropenia76%.
Second-line treatments included rituximab in 14 patients (375 mg/m²/week; four cycles. Among them, ITP responses included 40% CR, 20% R. In WAIHA, 50% achieved CR. Among two patients with neutropenia, one responded. Mycophenolate was used in a total of 22 patients, all of them pediatric, with response rates of 54% in ITP and 43% in wAIHA. The median time to response was 28 days (IQR: 18.5–70) in ITP and 65 days (IQR: 26.2–189) in wAIHA.
Fostamatinib was used in 9 patients with concurrent ITP and wAIHA. Median baseline platelets were 13 $\times 10^9$/L and hemoglobin 9.5 g/dL. All patients were on concomitant therapy (corticosteroids or avatrombopag). Median time to ITP response was 14 days (IQR:6-25.5) with a response rate of 78% (CR 87%). Median time to response for wAIHA was 54 days (IQR: 22-292) with a response rate of 44%. In two patients, fostamatinib was
Thrombopoietin receptor agonists (TPO-RAs) were used in five patients with ITP, but showed no efficacy in wAIHA.

**Summary/Conclusion**

This initial analysis highlights the clinical heterogeneity and treatment challenges in ES. Corticosteroids remain a cornerstone of therapy but are associated with incomplete or transient responses. Rituximab and Mycophenolate demonstrated moderate efficacy across lineages, while fostamatinib showed rapid, durable responses in both ITP and WAIHA. The lack of response to TPO-RAs in wAIHA underscores the need for lineage-specific therapeutic strategies.

**Copyright Information:** (Online) ISSN: 2572-9241
©2026 The Author(s). HemaSphere is published by John Wiley & Sons Ltd on behalf of European Hematology Association. This is an open access Abstract Book distributed under the Attribution-NonCommercial-NoDerivs (CC BY-NC-ND), which allows third parties to download the articles and share them with others as long as they credit the author and the Abstract Book, but they cannot change the content in any way or use them commercially.
**Abstract Book Citations:** Authors, Title, HemaSphere, 2026;10:(S1):pages. The abstract book DOIs can be found on the "Author Information" page.
**Disclaimer:** Articles published in the journal HemaSphere exclusively reflect the opinions of the authors. The authors are responsible for all content in their abstracts including accuracy of the facts, statements, citing resources, etc.

### (PS2445) AVATROMBOPAG IN TPO-RA–NAIVE ADULTS IN ACUTE, PERSISTENT, AND CHRONIC PHASES OF ITP: RESULTS FROM THE REAL-AVA 3.0 RETROSPECTIVE STUDY

**Topic:** 33. Platelet disorders

Sandhya Panch*[1, 2], Abiola Oladapo[3], Faizan Sattar[3], Michael Vredenburg[3], Sarah Lucht[4], Emily Bland[4], Prathamesh Pathak[4], Bruce Feinberg[4], Debbie Jiang[5, 6]

*[1]University of Washington, Seattle, United States of America;[2]Fred Hutch Cancer Center, Seattle, United States of America; [3]Sobi, Inc., Waltham, United States of America;[4]Cardinal Health, Dublin, United States of America;[5]Massachusetts General Hospital, Boston, United States of America; [6]Harvard Medical School, Boston, United States of America;*

### Background

Avatrombopag (AVA) is a thrombopoietin receptor agonist (TPO-RA) approved for the treatment (tx) of immune thrombocytopenia (ITP). Real-world (RW) evidence on AVA is limited in patients (pts) with TPO-RA–naive ITP.

### Aims

We describe RW tx patterns and clinical outcomes in TPO-RA–naive pts receiving AVA in the US.

### Methods

This is a retrospective chart review of TPO-RA–naive adults with primary ITP who initiated AVA on/after 1 Jul 2019, with ≥6 mo of follow-up after AVA initiation. Pts were followed from AVA initiation until the earliest of last contact, death, or study end (20 Jan 2025). Response was defined as achieving a meaningful platelet count (PC) (response thresholds: ≥30×$10^9$/L, ≥50×$10^9$/L, and ≥100×$10^9$/L) at least once in the absence of rescue tx. Among responders, response durability was defined as the percent of time on AVA with a PC above the response threshold.

**Copyright Information:** (Online) ISSN: 2572-9241
©2026 The Author(s). HemaSphere is published by John Wiley & Sons Ltd on behalf of European Hematology Association. This is an open access Abstract Book distributed under the Attribution-NonCommercial-NoDerivs (CC BY-NC-ND), which allows third parties to download the articles and share them with others as long as they credit the author and the Abstract Book, but they cannot change the content in any way or use them commercially.
**Abstract Book Citations:** Authors, Title, HemaSphere, 2026;10:(S1):pages. The abstract book DOIs can be found on the "Author Information" page.
**Disclaimer:** Articles published in the journal HemaSphere exclusively reflect the opinions of the authors. The authors are responsible for all content in their abstracts including accuracy of the facts, statements, citing resources, etc.

### Results

Of 200 pts treated with AVA, 33.5% (n=67) had chronic ITP and 66.5% had acute (n=61) or persistent (n=72) ITP. The majority of pts were female (59.5%). The mean (SD) age was 57.5 (15.0) years. Demographics and clinical characteristics were generally similar between pts with chronic ITP and pts with acute/persistent ITP. Most pts had PC <$30\times10^{9}$/L closest to but prior to index initiation (chronic ITP: 83.6%; acute/persistent ITP: 84.2%). Prior to AVA tx, most pts had corticosteroids (chronic ITP: 95.5%; acute/persistent ITP: 92.5%) and nearly half had immunoglobulins (chronic ITP: 47.8%; acute/persistent ITP: 45.9%); fewer had rituximab (chronic ITP: 38.8%; acute/persistent ITP: 21.1%) or splenectomy (chronic ITP: 11.9%; acute/persistent ITP: 0.8%). The median AVA tx duration was 9.1 mo for pts with chronic ITP and 7.7 mo for acute/persistent ITP. While on AVA, few pts required concomitant corticosteroids (chronic ITP: 3.0%; acute/persistent ITP: 8.3%), concomitant immunoglobulins (chronic ITP: 1.5%; acute/persistent ITP: 1.5%), or rescue tx (chronic ITP: 7.5%; acute/persistent ITP: 9.0%). Among pts who received concomitant steroids/immunosuppressants, all reduced or discontinued use of ≥1 of these concomitant medications. Response rates and durability of response were high across groups. PCs ≥$100\times10^{9}$/L were achieved in 74.6% of pts with baseline PC <$100\times10^{9}$/L with chronic ITP and 75.8% with acute/persistent ITP, with median response durability of 68.5% and 71.1%, respectively. PCs ≥$50\times10^{9}$/L were achieved in 93.8% of pts with baseline PC <$50\times10^{9}$/L with chronic ITP and 95.5% with acute/persistent ITP, with median response durability of 88.3% and 88.4%, respectively. PCs ≥$30\times10^{9}$/L were achieved in ≥98% of pts with baseline PC <$30\times10^{9}$/L in each group with median response durability ≥93%. At last follow-up, >70% of pts remained on tx with AVA. The AVA discontinuation rate was 25.5% (chronic ITP: 20.9%; acute/persistent ITP: 27.8%). The most common reasons for discontinuing AVA were achieving target PC (chronic ITP: 6.0%; acute/persistent ITP: 16.5%), lack of efficacy (chronic ITP: 9.0%; acute/persistent ITP: 1.5%), and pt preference (chronic ITP: 3.0%; acute/persistent ITP: 4.5%). Among pts who discontinued AVA, 19.6% (57.1% with chronic ITP and 5.4% with acute/persistent ITP) had subsequent ITP tx. Of these pts, 12.5% with chronic ITP and none with acute/persistent ITP discontinued tx.

### Summary/Conclusion

In the RW setting, AVA yielded a high rate of sustained platelet response in TPO-RA–naive pts with chronic or acute/persistent ITP.

Copyright Information: (Online) ISSN: 2572-9241
©2026 The Author(s). HemaSphere is published by John Wiley & Sons Ltd on behalf of European Hematology Association. This is an open access Abstract Book distributed under the Attribution-NonCommercial-NoDerivs (CC BY-NC-ND), which allows third parties to download the articles and share them with others as long as they credit the author and the Abstract Book, but they cannot change the content in any way or use them commercially.
Abstract Book Citations: Authors, Title, HemaSphere, 2026;10:(S1):pages. The abstract book DOIs can be found on the "Author Information" page.
Disclaimer: Articles published in the journal HemaSphere exclusively reflect the opinions of the authors. The authors are responsible for all content in their abstracts including accuracy of the facts, statements, citing resources, etc.

### (PS2446) BIOPRINTING A SILK-BASED 3D BONE MARROW MODEL FOR CHEMOTHERAPY-INDUCED THROMBOCYTOPENIA

**Topic:** 33. Platelet disorders

Christian Di Buduo*[1], Matteo Migliavacca[1], Alessandro Malara[1], Alessandra Balduini[1]

[1]*University of Pavia, Pavia, Italy;*

### Background

Chemotherapy-induced thrombocytopenia is a major dose-limiting toxicity of anticancer treatment, yet its effects on megakaryopoiesis remain incompletely defined. Conventional in vitro models fail to maintain the function of hematopoietic stem and progenitor cells (HSPCs) under chemotherapeutic stress, thereby limiting the analysis of lineage vulnerability and recovery. Silk-based bone marrow models preserve stem programs while enabling controlled pharmacologic perturbation, providing an ex vivo system to study cytotoxic injury and therapeutic rescue.

### Aims

To establish a silk-based bone marrow model preserving HSPC function under chemotherapeutic stress, enabling analysis of stage-specific injury and pharmacological rescue.

### Methods

Primary human $CD34^+$ HSPCs were encapsulated within mechanically stable and customizable 3D bioprinted matrices designed to support long-term hematopoiesis, megakaryocyte differentiation, and platelet production. The chemotherapeutic agent 5-fluorouracil (5-FU) was administered transiently at defined stages, either alone or in combination with the thrombopoietin receptor agonists eltrombopag or romiplostim, to model stage-specific myelotoxicity and therapeutic rescue.

### Results

The 3D bioprinting approach enabled standardized fabrication of soft constructs with controlled geometry and mechanical properties matching the native bone marrow niche. In contrast to conventional 2D cultures, which failed to tolerate 5-FU exposure due to loss of viability, the 3D system sustained HSPC survival and differentiation during and after chemotherapy treatment. This permissive microenvironment supported megakaryocyte differentiation and platelet production under basal and treated conditions. Stage-specific 5-FU exposure revealed differentiation-dependent chemosensitivity along the megakaryocytic lineage. Early exposure at the stem and progenitor stage largely preserved subsequent megakaryocyte viability, polyploidization, and proplatelet formation. In contrast, treatment during intermediate differentiation caused dose-dependent depletion of high-ploidy megakaryocytes, reduced viability, and severe impairment of proplatelet formation, whereas late-stage exposure produced modest effects. These findings identify a window of heightened vulnerability following lineage commitment but preceding terminal maturation. Both eltrombopag and romiplostim partially rescued 5-FU-induced defects, restoring megakaryocyte maturation, proplatelet network complexity, and platelet output. Integrated proteomic and single-cell transcriptomic analyses indicated that the 3D microenvironment preserves stem and progenitor programs while coordinating pathways involved in cell-cycle regulation, endomitosis, and DNA damage responses.

**Copyright Information:** (Online) ISSN: 2572-9241
©2026 The Author(s). HemaSphere is published by John Wiley & Sons Ltd on behalf of European Hematology Association. This is an open access Abstract Book distributed under the Attribution-NonCommercial-NoDerivs (CC BY-NC-ND), which allows third parties to download the articles and share them with others as long as they credit the author and the Abstract Book, but they cannot change the content in any way or use them commercially.
**Abstract Book Citations:** Authors, Title, HemaSphere, 2026;10:(S1):pages. The abstract book DOIs can be found on the "Author Information" page.
**Disclaimer:** Articles published in the journal HemaSphere exclusively reflect the opinions of the authors. The authors are responsible for all content in their abstracts including accuracy of the facts, statements, citing resources, etc.

**Summary/Conclusion**

This silk-based 3D bone marrow model represents a physiologically relevant ex vivo system that maintains HSPC function under chemotherapeutic stress, enabling investigation of cytotoxic injury and rescue. The ability to study drugs with opposing effects, such as 5-FU and thrombopoietin receptor agonists, supports translational analysis of chemotherapy-induced thrombocytopenia.

**Copyright Information:** (Online) ISSN: 2572-9241
©2026 The Author(s). HemaSphere is published by John Wiley & Sons Ltd on behalf of European Hematology Association. This is an open access Abstract Book distributed under the Attribution-NonCommercial-NoDerivs (CC BY-NC-ND), which allows third parties to download the articles and share them with others as long as they credit the author and the Abstract Book, but they cannot change the content in any way or use them commercially.
**Abstract Book Citations:** Authors, Title, HemaSphere, 2026;10:(S1):pages. The abstract book DOIs can be found on the "Author Information" page.
**Disclaimer:** Articles published in the journal HemaSphere exclusively reflect the opinions of the authors. The authors are responsible for all content in their abstracts including accuracy of the facts, statements, citing resources, etc.

### (PS2447) OUTCOME OF CHILDREN WITH CHRONIC IMMUNE THROMBOCYTOPENIA ON LONG TERM ELTROMBOPAG OR ROMIPLOSTIM USE (SHIFT/TAPER-DISCONTINUE STUDY)

**Topic:** 33. Platelet disorders

Mohsen Elalfy[1], Tamer Hassan*[2], Omar Elalfy[3], Khadiga Eltonbary[1]

*[1]Ain Shams University, Pediatric Hematology Oncology, Cairo, Egypt; [2]Zagazic University, Pediatric Hematology Oncology, Zagazig, Egypt; [3]National Research Center, Complementary Medicine, Cairo, Egypt;*

### Background

Current guidelines for children with immune thrombocytopenia (ITP) recommend thrombopoietin receptor agonists (TPO-RAs) as a second-line therapy, with initiatives to be used earlier.

### Aims

This observational study aimed to assess prior treatment classes and clinical outcome in children with chronic ITP, currently being treated with eltrombopag (E) or romiplostim (R), in order to analyse the factors influencing the success of transitioning to either therapy or drug discontinuation.

### Methods

A total of 120 patients with chronic ITP, 2-12 years old, were enrolled over one year, they were maintained on either (E) (n=90) or (R) (n=30) as a 2nd1 to 4th line therapy for a period of 0.5-5 years (median 2.9 IQ 0.9) (E in 25-75 mg daily dose according to age) and (R in 3-10 mcg/kg/week doses subcutaneous), maximum dose was achieved and maintained for a minimum of 3 months before switch or taper. Taper was scheduled over 8-12 weeks, followed by discontinuation if tapering was successful.

### Results

Initial complete response (CR) (≥100×109/L), with no rescue medication in the previous 4 weeks before enrollment, was 49% for E and 53% for R. No significant differences were noted when comparing clinical outcome and platelet counts between both drugs as mean platelet count on E was 140×109/L (SD±23.6) versus 151×109/L(SD± 26.9) for patients on R. Maintained CR after shifting from either E to R or R to E was noticed in 41% and 46% respectively with main reasons for the shift being inadequate response (90%), hepatoxicity and thrombocytosis mainly in E group and the cost in R group. One-third of the CR cohort had a successful slow tapering and discontinuation over 8-12 weeks and showed sustained remission (PC ≥ 100 × 10^9/L) off treatment (SROT). Predictors of SROT were mainly a history of less exposure to pharmacological agents or immunosuppressants, lower doses of TPO-RAs and slower tapering.

### Summary/Conclusion

Success rate of almost 50% after switching from one TPO-RA to the other. SROT was achieved in one-third avoiding unnecessary prolonged TPO-RA therapy and cutting expenses in a limited resource country. No significant bleeding or serious adverse events were reported during the study period.

**Copyright Information:** (Online) ISSN: 2572-9241
©2026 The Author(s). HemaSphere is published by John Wiley & Sons Ltd on behalf of European Hematology Association. This is an open access Abstract Book distributed under the Attribution-NonCommercial-NoDerivs (CC BY-NC-ND), which allows third parties to download the articles and share them with others as long as they credit the author and the Abstract Book, but they cannot change the content in any way or use them commercially.
**Abstract Book Citations:** Authors, Title, HemaSphere, 2026;10:(S1):pages. The abstract book DOIs can be found on the “Author Information” page.
**Disclaimer:** Articles published in the journal HemaSphere exclusively reflect the opinions of the authors. The authors are responsible for all content in their abstracts including accuracy of the facts, statements, citing resources, etc.

### (PS2448) CHARACTERIZING EARLY AND LATE RESPONDERS TO MULTI-IMMUNE MODULATOR RILZABRUTINIB IN PERSISTENT/CHRONIC IMMUNE THROMBOCYTOPENIA: FINDINGS FROM THE PHASE 3 LUNA3 STUDY

**Topic:** 33. Platelet disorders

Nichola Cooper*[1], Waleed Ghanima[2], Cristina Pascual Izquierdo[3], Sylvain Audia[4], Maria Belen Rodriguez[5], Meredith Foster[5], Ahmed Daak[5], David J. Kuter[6]

*[1]Hammersmith Hospital, London, United Kingdom; [2]Østfold Hospital Trust, Grålum, Norway and Institute of Clinical Medicine, University of Oslo, Oslo, Norway; [3]Department of Hematology, Gregorio Marañon Hospital, Gregorio Marañon Research Institute, Madrid, Spain; [4]CHU Dijon Bourgogne - Hôpital François Mitterrand, Dijon, France; [5]Sanofi, Cambridge, United States of America; [6]Hematology Division, Massachusetts General Hospital, Harvard Medical School, Boston, United States of America;*

### Background

Rilzabrutinib is an oral, covalent, reversible, highly selective BTK inhibitor that acts through multi-immune modulation and has demonstrated rapid and durable platelet responses, improved physical fatigue and bleeding scores, and favorable safety in the phase 2 LUNA2 and phase 3 LUNA3 studies of adults with persistent/chronic immune thrombocytopenia (ITP). Timing of platelet response can help guide treatment decisions. Hence, understanding characteristics of early vs late responders may provide useful insights for clinical decision-making.

### Aims

Describe characteristics of early and late responding patients treated with rilzabrutinib for 24 weeks in the LUNA3 study.

### Methods

LUNA3 was a double-blind, placebo-controlled study and included open-label and long-term extensions where only rilzabrutinib was given (NCT04562766, 2023-509401-71). The cohort for this analysis included patients who received rilzabrutinib 400 mg bid for 24 weeks across study periods. Stable doses of concomitant corticosteroids (CS) and/or thrombopoietin-receptor agonists (TPO-RA) were allowed from 14 days before study day 1 until the last dose of study medication. Overall response was defined as platelet counts $\geq 50 x 10^9/L$ on 2 consecutive visits in the first 24 weeks of rilzabrutinib treatment without rescue medication within 4 weeks prior to initial response and during the 2 consecutive weeks. Early and late responses were defined as overall response first occurring in weeks 1-12 and 13-24, respectively.

**Copyright Information:** (Online) ISSN: 2572-9241
©2026 The Author(s). HemaSphere is published by John Wiley & Sons Ltd on behalf of European Hematology Association. This is an open access Abstract Book distributed under the Attribution-NonCommercial-NoDerivs (CC BY-NC-ND), which allows third parties to download the articles and share them with others as long as they credit the author and the Abstract Book, but they cannot change the content in any way or use them commercially.
**Abstract Book Citations:** Authors, Title, HemaSphere, 2026;10:(S1):pages. The abstract book DOIs can be found on the "Author Information" page.
**Disclaimer:** Articles published in the journal HemaSphere exclusively reflect the opinions of the authors. The authors are responsible for all content in their abstracts including accuracy of the facts, statements, citing resources, etc.

### Results

Of 149 evaluable patients, 78 (52%) achieved overall response. Among overall responders, 60/78 (77%) were early responders and 18/78 (23%) were late responders. Patient characteristics and prior/concomitant ITP therapy by response status are reported in Table 1. At baseline, overall responders (vs non-responders) had more female patients (73% vs 52%), shorter mean duration of ITP (9.3 vs 11.8 y), and higher median baseline platelet count ($19×10^9$/L vs $11×10^9$/L). Overall responders and non-responders had the same median number of 4 prior unique ITP therapies, although responders had slightly more patients receiving fewer prior therapies. The proportion of patients receiving concomitant ITP therapy (CS, TPO-RA, or both) was similar between responders and non-responders.

Baseline patient profiles were generally consistent for early and late responders to rilzabrutinib (Table 1). Differences in baseline factors for early vs late responders were slightly younger mean age (45.7 vs 50.5 y) and longer mean duration of ITP (9.8 vs 7.7 y). Among responders with 1-2 (n=28), 3-4 (n=20), or 5+ (n=30) prior ITP therapies, 20 (71%), 14 (70%), and 26 (87%) achieved early response and 8 (29%), 6 (30%), and 4 (13%) achieved late response, respectively.

### Summary/Conclusion

Evaluation of patients receiving rilzabrutinib treatment for 24 weeks showed that the majority of responders achieved response within the first 12 weeks (early responders); however, continued treatment resulted in additional responses over time, potentially reflecting the multi-immune modulation mechanism of action of rilzabrutinib and reinforcing the value of sustained treatment. No clear clinical factors, including the number of prior ITP treatments or other baseline features, distinguished early from late responders. Further investigation is needed to identify potential predictors of early and late response.

**Table 1. Baseline Patient Characteristics, Prior ITP Treatment History, and Concomitant ITP Medication in LUNA3 Patients by Response**

| | | Overall Responder Patients | | |
|---|---|---|---|---|
| | Overall Non-Responders (n=71) | All Overall Responders (n=78) | Early Responders (n=60) | Late Responders (n=18) |
| **Mean age, y (SD)** | 46.2 (15.7) | 46.8 (18.0) | 45.7 (16.5) | 50.5 (22.3) |
| **Age categories, n (%)** | | | | |
| <65 y | 62 (87) | 65 (83) | 53 (88) | 12 (67) |
| ≥65 y | 9 (13) | 13 (17) | 7 (12) | 6 (33) |
| **Sex, n (%)** | | | | |
| Female | 37 (52) | 57 (73) | 45 (75) | 12 (67) |
| Male | 34 (48) | 21 (27) | 15 (25) | 6 (33) |
| **Mean weight, kg (SD)** | 77.3 (19.4) | 77.2 (22.0) | 76.2 (22.2) | 80.4 (21.5) |
| **Mean duration of ITP, y (SD)** | 11.8 (11.1) | 9.3 (8.3) | 9.8 (7.7) | 7.7 (10.1) |
| **Median baseline platelet count, $×10^9$/L (range)** | 11 (0.7-32) | 19 (2-54) | 19 (2-54) | 18 (6-38) |
| **Baseline platelet count by category, n (%)** | | | | |
| $<15×10^9$/L | 46 (65) | 23 (29) | 16 (27) | 7 (39) |
| $≥15×10^9$/L | 25 (35) | 55 (71) | 44 (73) | 11 (61) |
| **Median number of prior unique ITP therapies,* (range)** | 4 (1-15) | 4 (1-10) | 4 (1-10) | 3.5 (1-7) |
| **Response to prior ITP therapies, n responded†/n received (%)** | | | | |
| Corticosteroids | 44/70 (63) | 65/75 (87) | 51/58 (88) | 14/17 (82) |
| TPO-RA | 26/50 (52) | 35/44 (80) | 27/36 (75) | 8/8 (100) |
| IVIg or anti-D Ig | 31/37 (84) | 32/40 (80) | 24/29 (83) | 8/11 (73) |
| AntiCD20/rituximab | 6/31 (19) | 13/19 (68) | 10/14 (71) | 3/5 (60) |
| Fostamatinib | 3/12 (25) | 1/3 (33) | 1/3 (33) | 0/0 (0) |
| **Received concomitant ITP medication,‡ n (%)** | | | | |
| Yes | 46 (65) | 51 (65) | 41 (68) | 10 (56) |
| No | 25 (35) | 27 (35) | 19 (32) | 8 (44) |

*Identified using standard medication term, different corticosteroids counted as one therapy and included splenectomy. †Prior response was based on investigators' report and defined as prior platelet count $≥50×10^9$/L. ‡Concomitant medication included corticosteroids, thrombopoietin receptor agonists (TPO-RA), or both.

Copyright Information: (Online) ISSN: 2572-9241
©2026 The Author(s). HemaSphere is published by John Wiley & Sons Ltd on behalf of European Hematology Association. This is an open access Abstract Book distributed under the Attribution-NonCommercial-NoDerivs (CC BY-NC-ND), which allows third parties to download the articles and share them with others as long as they credit the author and the Abstract Book, but they cannot change the content in any way or use them commercially.
Abstract Book Citations: Authors, Title, HemaSphere, 2026;10:(S1):pages. The abstract book DOIs can be found on the "Author Information" page.
Disclaimer: Articles published in the journal HemaSphere exclusively reflect the opinions of the authors. The authors are responsible for all content in their abstracts including accuracy of the facts, statements, citing resources, etc.

### (PS2449) EFFECTS OF IANALUMAB TREATMENT ON B CELL ACTIVATION, MATURATION, AND MAINTENANCE OF VACCINE TITERS IN PATIENTS WITH PRIMARY IMMUNE THROMBOCYTOPENIA IN THE PHASE 2 VAYHIT3 STUDY

**Topic:** 33. Platelet disorders

Thomas Stauch*[1], Charlotte Bradbury[2], Tomás José González-López[3], Guillaume Moulis[4], Nichola Cooper[5], Karolin Trautmann[6], Patrick Urban[7], Renxin Lin[8], Tiffany Loy[7], Dilini Kothalawala[9], Kevin Vervier[7], Fabienne Le Gac[7], David J. Kuter[10]

*[1]University Hospital Jena, Department of Internal Medicine II, Hematology and Oncology, Jena, Germany; [2]University of Bristol, Faculty of Health Sciences, Bristol, United Kingdom; [3]Hospital Universitario de Burgos, Department of Hematology, Burgos, Spain; [4]Toulouse University Hospital, Toulouse, France; [5]Imperial College London, Centre for Haematology, Department of Immunology and Inflammation, London, United Kingdom; [6]University Hospital Carl Gustav Carus at the TU Dresden, Medical Clinic I, Dresden, Germany; [7]Novartis Pharma AG, Basel, Switzerland; [8]China Novartis Institutes for BioMedical Research Co., Ltd., Shanghai, China; [9]Novartis Pharmaceuticals UK Ltd., London, United Kingdom; [10]Massachusetts General Hospital, Harvard Medical School, Hematology Division, Boston, MA, United States of America;*

### Background

Ianalumab is a monoclonal antibody targeting B cells through a novel dual mechanism of action (MoA) of enhanced B-cell depletion via antibody-dependent cellular cytotoxicity (ADCC) and inhibition of B cell activation and survival via B-cell activating factor (BAFF) receptor blockade. This dual MoA is expected to enable a broader and more potent depletion of B cells, as well as sustained modulation of B-cell activity. In the phase 2 VAYHIT3 trial (NCT05885555), a short course of ianalumab elicited a potent and sustained clinical response in a heavily pretreated population of patients with primary immune thrombocytopenia (ITP).

### Aims

To examine the effect of ianalumab on B cell counts, selected biomarkers of B-cell activation and regulation, and vaccine titers to prior vaccinations.

### Methods

In VAYHIT3, patients with primary ITP who had previously received at least a corticosteroid and a thrombopoietin receptor agonist received four once-monthly infusions of ianalumab 9 mg/kg. B-cell counts in peripheral blood were measured as a secondary endpoint; exploratory endpoints included baseline and change from baseline measures in soluble B cell maturation antigen (sBCMA), soluble BAFF (sBAFF), T-, B-, and natural killer (NK) cell subsets, and antibody titers from tetanus and measles vaccinations.

**Copyright Information:** (Online) ISSN: 2572-9241
©2026 The Author(s). HemaSphere is published by John Wiley & Sons Ltd on behalf of European Hematology Association. This is an open access Abstract Book distributed under the Attribution-NonCommercial-NoDerivs (CC BY-NC-ND), which allows third parties to download the articles and share them with others as long as they credit the author and the Abstract Book, but they cannot change the content in any way or use them commercially.
**Abstract Book Citations:** Authors, Title, HemaSphere, 2026;10:(S1):pages. The abstract book DOIs can be found on the "Author Information" page.
**Disclaimer:** Articles published in the journal HemaSphere exclusively reflect the opinions of the authors. The authors are responsible for all content in their abstracts including accuracy of the facts, statements, citing resources, etc.

**Results**

In the full analysis set (N=41), sustained peripheral B-cell depletion (median change from baseline >92%) was observed until 29 weeks after the last infusion, B cell counts began to recover approximately 33 weeks after the ianalumab infusions ended. The median time from last infusion to B cell recovery (≥80% of baseline and ≥14.8 cells/µL, or ≥50 cells/µL) was 12.2 months.
In patients with baseline biomarker measurements, CD19+ B-cell counts and sBCMA concentrations decreased from treatment initiation to the end of treatment (EOT), with median changes of −99% and −48%, respectively, and remained reduced at week 25 (−100% and −44%). sBAFF levels were increased at EOT (+144%) and week 25 (+138%) (**Figure**). Depletion was observed across all measured circulating B-cell subsets, including naïve, transitional, memory, and regulatory B cells, plasmablasts and plasma cells. There was no depletion of either CD4+ or CD8+ T cells and NK cell populations generally remained stable. The potential effect of ianalumab on prior vaccine immunization was evaluated among 30 patients, with 29 patients reporting positive measles vaccine and 29 patients reporting protective tetanus vaccine titers at week 25. Vaccination history was unknown in the two patients who lost positive or protective vaccine titers (one each for measles and tetanus); both had received prior IVIG. Evaluable data at baseline and 1 year after the ianalumab infusion ended were available for 10 patients; all 10 maintained positive titers for measles and protective titers for tetanus and were in stable response.

**Summary/Conclusion**

A short course of ianalumab resulted in a rapid, profound and sustained depletion of all B cell subsets in peripheral blood, consistent with ADCC. The increase in sBAFF indicates potent BAFF receptor blockade, while the decrease in sBCMA suggests reduced numbers of plasmablasts and plasma cells. Together, these findings support the novel dual MoA of ianalumab, which results in enhanced B cell depletion and inhibition of B cell activation and survival, while preserving T- and NK cell populations as well as preexisting vaccine-specific immune responses.

**Copyright Information:** (Online) ISSN: 2572-9241
©2026 The Author(s). HemaSphere is published by John Wiley & Sons Ltd on behalf of European Hematology Association. This is an open access Abstract Book distributed under the Attribution-NonCommercial-NoDerivs (CC BY-NC-ND), which allows third parties to download the articles and share them with others as long as they credit the author and the Abstract Book, but they cannot change the content in any way or use them commercially.
**Abstract Book Citations:** Authors, Title, HemaSphere, 2026;10:(S1):pages. The abstract book DOIs can be found on the "Author Information" page.
**Disclaimer:** Articles published in the journal HemaSphere exclusively reflect the opinions of the authors. The authors are responsible for all content in their abstracts including accuracy of the facts, statements, citing resources, etc.

**Figure**. Biomarkers evaluated at baseline, end of treatment, and week 25

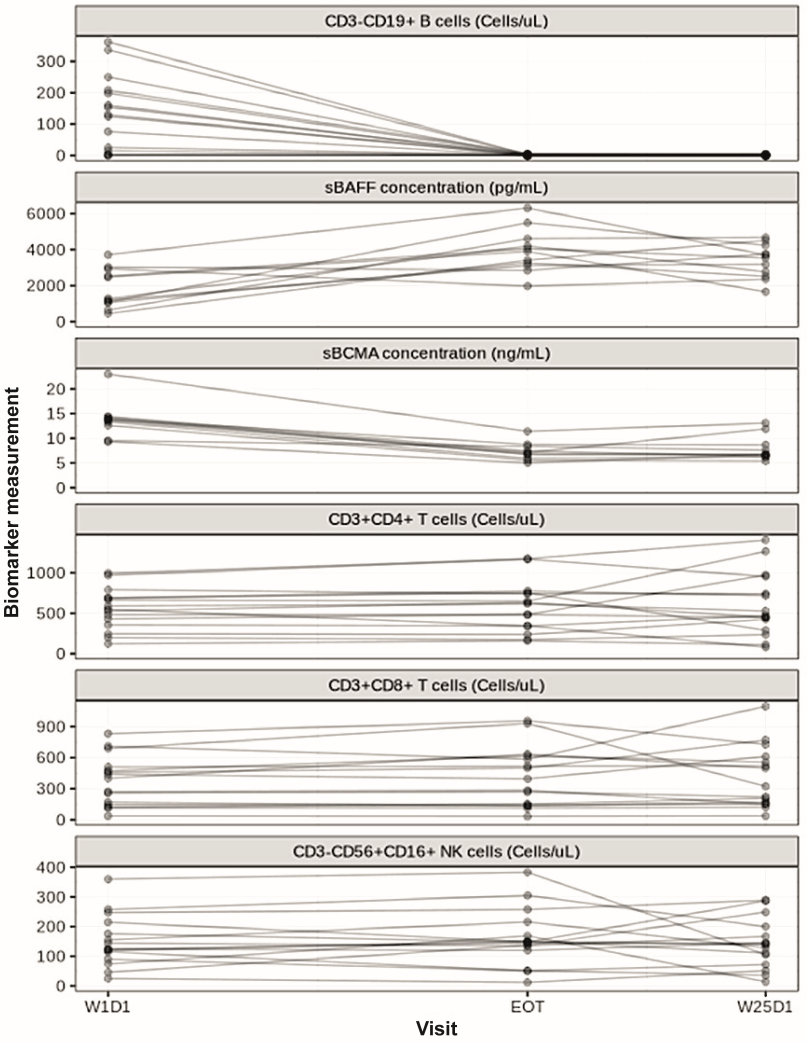

Analyses were conducted within the biomarker set and included only individuals with available $CD3^-CD19^+$ B cell data who also had data available for all other biomarkers at the same displayed timepoints . Sample sizes were n=15 for $CD3^-CD19^+$ B cells, $CD3^+CD4^+$ T cells, $CD3^+CD8^+$ T cells, and $CD3^-CD56^+CD16^+$ NK cells; n=10 for sBCMA; and n=11 for sBAFF.

EOT, end of treatment; NK, natural killer; sBAFF, soluble B-cell activating factor; sBCMA, soluble B-cell maturation antigen; W1D1, week 1 day 1 (baseline); W25D1, week 25 day 1

**Copyright Information:** (Online) ISSN: 2572-9241
©2026 The Author(s). HemaSphere is published by John Wiley & Sons Ltd on behalf of European Hematology Association. This is an open access Abstract Book distributed under the Attribution-NonCommercial-NoDerivs (CC BY-NC-ND), which allows third parties to download the articles and share them with others as long as they credit the author and the Abstract Book, but they cannot change the content in any way or use them commercially.
**Abstract Book Citations:** Authors, Title, HemaSphere, 2026;10:(S1):pages. The abstract book DOIs can be found on the “Author Information” page.
**Disclaimer:** Articles published in the journal HemaSphere exclusively reflect the opinions of the authors. The authors are responsible for all content in their abstracts including accuracy of the facts, statements, citing resources, etc.

### (PS2450) THYROID PEROXIDASE ANTIBODY IS ASSOCIATED WITH FAVORABLE RITUXIMAB RESPONSE IN ADULT IMMUNE THROMBOCYTOPENIA.

**Topic:** 33. Platelet disorders

Ruxia Zhao*[1], Jun Peng[1], Gege Feng[1]

[1]*Qilu Hospital of Shandong University, Jinan, China, Department of Hematology, JINAN, China;*

### Background

Immune thrombocytopenia (ITP) is an acquired autoimmune disorder with immune-mediated platelet destruction and impaired production. In adults refractory to or relapsing after first-line therapy, rituximab is an important second-line option, but responses are heterogeneous and B-cell depletion carries a relevant infection risk. Identifying patients most likely to benefit is therefore crucial. Antithyroid antibodies have been linked to a chronic course of ITP, suggesting shared autoimmune mechanisms with thyroid disease, but data on their impact on treatment response are limited, inconsistent, and largely confined to paediatric ITP. The predictive value of thyroid antibodies for rituximab response in adult ITP therefore remains unclear.

### Aims

We conducted this retrospective study to evaluate the association between TPOAb and treatment outcomes of rituximab in Chinese adults with ITP.

### Methods

A total of 112 adult ITP patients who received rituximab at Qilu Hospital, Shandong University from January 2014 to March 2025 and had thyroid function assessed at treatment initiation were included. Patients were categorized into an antibody-positive group (TPOAb > 34 IU/mL) and an antibody-negative group according to baseline antibody levels. The primary endpoints were initial and 6-month sustained treatment responses; secondary endpoints included duration of response and relapse. All patients who achieved an initial response were prospectively followed. Follow-up was censored at the date of last visit, last documented contact, or relapse, whichever occurred first, with data updated through December 31, 2025.

### Results

Among the total 112 patients, the median levels of T3, T4, and TSH were 4.08 pmol/L, 13.6 pmol/L, and 1.6 uIU/ml, respectively. 73 were additionally tested for TPOAb, with a positivity rate of 23.3% (17/73). TPOAb-positive patients had a significantly higher initial overall response rate to rituximab than TPOAb-negative patients (93.3% vs 41.1%, $p < 0.001$), as well as a higher complete response (CR) rate (66.7% vs 17.9%, $p < 0.001$). A similar pattern was observed for 6-month sustained overall responses (86.7% vs 37.5%, $p < 0.001$), whereas sustained CR rates did not differ significantly between the two groups (33.3% vs 25.0%, $p = 0.744$). Kaplan–Meier analysis showed no significant difference in response duration between the two groups ($p > 0.05$). In multivariable Cox regression, TPOAb positivity remained an independent predictor of initial response to rituximab (odds ratio 16.21; 95% CI 1.53–171.33; $p = 0.021$).

**Copyright Information:** (Online) ISSN: 2572-9241
©2026 The Author(s). HemaSphere is published by John Wiley & Sons Ltd on behalf of European Hematology Association. This is an open access Abstract Book distributed under the Attribution-NonCommercial-NoDerivs (CC BY-NC-ND), which allows third parties to download the articles and share them with others as long as they credit the author and the Abstract Book, but they cannot change the content in any way or use them commercially.
**Abstract Book Citations:** Authors, Title, HemaSphere, 2026;10:(S1):pages. The abstract book DOIs can be found on the “Author Information” page.
**Disclaimer:** Articles published in the journal HemaSphere exclusively reflect the opinions of the authors. The authors are responsible for all content in their abstracts including accuracy of the facts, statements, citing resources, etc.

**Summary/Conclusion**

In this cohort of adult ITP patients treated with rituximab, TPOAb positivity was associated with substantially higher initial, and 6-month sustained overall response rates, while sustained CR and response duration were comparable between TPOAb-positive and TPOAb-negative patients. TPOAb status may therefore serve as a useful biomarker to identify patients more likely to benefit from rituximab and support a more stratified treatment approach in adult ITP.

**Copyright Information:** (Online) ISSN: 2572-9241
©2026 The Author(s). HemaSphere is published by John Wiley & Sons Ltd on behalf of European Hematology Association. This is an open access Abstract Book distributed under the Attribution-NonCommercial-NoDerivs (CC BY-NC-ND), which allows third parties to download the articles and share them with others as long as they credit the author and the Abstract Book, but they cannot change the content in any way or use them commercially.
**Abstract Book Citations:** Authors, Title, HemaSphere, 2026;10:(S1):pages. The abstract book DOIs can be found on the "Author Information" page.
**Disclaimer:** Articles published in the journal HemaSphere exclusively reflect the opinions of the authors. The authors are responsible for all content in their abstracts including accuracy of the facts, statements, citing resources, etc.

### (PS2451) REAL-WORLD EFFECTIVENESS AND SAFETY OF A PREPARATION OF INTRAVENOUS IMMUNOGLOBULIN FOR IMMUNE THROMBOCYTOPENIA

**Topic:** 33. Platelet disorders

Guillaume Moulis*[1], Marianne Lepetit[1], Etienne Riviere[2], Francis Gaches[3], Yann Leveneur[4], Serge Madaule[5], Thibault Comont[1], Miguel Carreiro[6], Matthieu Mahevas[7], Jean Francois Viallard[2]

*[1]Toulouse University hospital, Toulouse, France; [2]Bordeaux University hospital, Bordeaux, France; [3]Auch hospital, Auch, France; [4]Tarbes hospital, Tarbes, France; [5]Albi hospital, Albi, France; [6]Montauban hospital, Montauban, France; [7]Henri Mondor University hospital, Créteil, France;*

### Background

Intravenous immunoglobulin (IVIg) is used as initial and rescue treatment for immune thrombocytopenia (ITP), alone or in combination with corticosteroids. . While clinical trials have demonstrated that Flebogamma DIF ®, a normal high-purity human IVIg, was effective and safe in treating patients with primary ITP, real-world evidence on its use and clinical outcomes remain limited.

### Aims

To assess the real-world use, effectiveness and safety of Flebogamma DIF® in France.

### Methods

The data source was the prospective, multicenter CARMEN-France registry of patients with ITP in France. All patients treated with Flebogamma DIF® between 2013 and 2024 were selected. Response was defined by a platelet count ≥30 x $10^9$/l and at least 2-fold increase of the baseline platelet count, with no bleeding and no addition of another treatment for ITP. Time to response, duration of response and adverse drug reactions were also described.

### Results

Of the 2192 patients included in the registry during the study period, 38 received 62 courses of Flebogamma DIF®. Median age at diagnosis was 60 years (Q1-Q3: 37-75) and 12 (31.6%) were men. The median lowest platelet count before treatment was 6.5 x $10^9$/L and 30 (78.9%) patients had previously experienced bleeding. ITP was primary in 31 (81.6%) patients and newly diagnosed in 26 (68.4%). The median dose was 64 g (Q1-Q3: 60-80). Another ITP treatment was present in 38 courses (61.3%). It was possible to assess the response in 51 courses (82.3%). The response rate was 78.4% (n=40) with a median time to response of 2 days (Q1-Q3: 1-3) and a median duration of response of 24 days (Q1-Q3: 15-172). Overall, 30/33 patients (90.9%) had at least one response to at least one course of IVIg; 89.3% of patients with primary ITP and 75.0% in patients with no concomitant treatment. One non-serious skin rash and two thromboses were reported: one ischemic stroke and one cerebral venous thrombosis 5 and 28 days after exposure, respectively; both patients had other risk factors for thrombosis (notably, concomitant exposure to thrombopoietin receptor agonists).

### Summary/Conclusion

Flebogamma DIF® was effective in real-world clinical practice with a high response rate and was well tolerated.

**Copyright Information:** (Online) ISSN: 2572-9241
©2026 The Author(s). HemaSphere is published by John Wiley & Sons Ltd on behalf of European Hematology Association. This is an open access Abstract Book distributed under the Attribution-NonCommercial-NoDerivs (CC BY-NC-ND), which allows third parties to download the articles and share them with others as long as they credit the author and the Abstract Book, but they cannot change the content in any way or use them commercially.
**Abstract Book Citations:** Authors, Title, HemaSphere, 2026;10:(S1):pages. The abstract book DOIs can be found on the "Author Information" page.
**Disclaimer:** Articles published in the journal HemaSphere exclusively reflect the opinions of the authors. The authors are responsible for all content in their abstracts including accuracy of the facts, statements, citing resources, etc.

### (PS2452) ADHERENCE TO DIAGNOSTIC GUIDELINES IN IMMUNE THROMBOCYTOPENIA: A 10-YEAR CLINICAL AUDIT AT A UK TERTIARY CENTER.

**Topic:** 33. Platelet disorders

Lara D' Orleans Kapps*[1], Narges Souri *[2]

[1]*Cambridge University, Haematology, Cambridge, United Kingdom;* [2]*Cambridge University, Cambridge, United Kingdom;*

### Background

**** Equal contributing author.***
Immune Thrombocytopenia (ITP) is an acquired autoimmune disorder characterized by isolated thrombocytopenia and increased bleeding risk. Diagnosis remains largely clinical and relies on the exclusion of secondary causes through structured laboratory investigations. However, the extent of conditions to exclude and the utility of certain tests remains a matter of debate. The Updated International Consensus Guideline (2019) recommend full blood count (FBC) with reticulocyte count, peripheral blood film (PBF), and viral serology (HIV, HBV, HCV) — with additional investigations guided by clinical presentation. Variability in adherence to these recommendations may lead to inconsistent diagnostic pathways and incomplete documentation.

### Aims

To evaluate adherence to recommended diagnostic investigations for adult ITP patients at a UK tertiary referral center, assess alignment with national and local guidelines, and identify opportunities to standardize and improve diagnostic pathways.

### Methods

A retrospective clinical audit was conducted using the EPIC electronic health record system. Adult patients (≥16 years) with a SNOMED-coded diagnosis of ITP between March 2015 and March 2025 were identified. Exclusion criteria included platelet counts >100×10⁹/L without treatment in the preceding three years. Diagnostic investigations reviewed included FBC, PBF, viral serology (HBV, HCV, HIV, EBV), antinuclear antibodies (ANA), antiphospholipid antibodies, Helicobacter pylori testing, viral PCR (CMV, Parvovirus B19), bone marrow examination, immunoglobulin levels, and complement testing. Investigations were coded as "tested" or "not tested" and compared against Addenbrooke's local ITP guideline and ASH 2019 standards. Descriptive statistics were used to quantify compliance.

### Results

A total of 1,353 adult patients were included (50% female; mean age 57.5 years). FBC and lupus anticoagulant testing were documented in 100% of patients. Viral serology completion ranged from 64.8–71%, while peripheral blood film was recorded in 57.4%. Quantitative immunoglobulin testing was performed in 76.9%. Bone marrow examination (30.4%), direct antiglobulin testing (30.7%), and complement testing (30.7%) showed moderate uptake. Extended infection screening demonstrated low utilization: Helicobacter pylori 11.5%, Parvovirus B19 PCR 8.4%, and CMV PCR 22.7%. Antiphospholipid antibody panels were variably requested (27–31%), while ANA testing occurred in 64.3%. Gender-based differences were minimal, with slightly higher autoimmune testing in females and marginally higher antiphospholipid testing in males.

**Copyright Information:** (Online) ISSN: 2572-9241
©2026 The Author(s). HemaSphere is published by John Wiley & Sons Ltd on behalf of European Hematology Association. This is an open access Abstract Book distributed under the Attribution-NonCommercial-NoDerivs (CC BY-NC-ND), which allows third parties to download the articles and share them with others as long as they credit the author and the Abstract Book, but they cannot change the content in any way or use them commercially.
**Abstract Book Citations:** Authors, Title, HemaSphere, 2026;10:(S1):pages. The abstract book DOIs can be found on the "Author Information" page.
**Disclaimer:** Articles published in the journal HemaSphere exclusively reflect the opinions of the authors. The authors are responsible for all content in their abstracts including accuracy of the facts, statements, citing resources, etc.

**Summary/Conclusion**

Our audit highlights how easily initial recommended investigations can remain incomplete and will not be rechecked in patients who are followed up long term. Implementation of an EPIC-based diagnostic checklist and structured documentation tools has been initiated to improve over time the identification of patients with missing data, the compliance with initial recommended investigations and encourage increased use of other investigations when clinically appropriate on a patient by patient basis. Missing results are likely compounded by patients who had their initial workup externally or before the current EHR in our center was installed or may not have been seen by a haematologist due to only a mild reduction in platelet count.

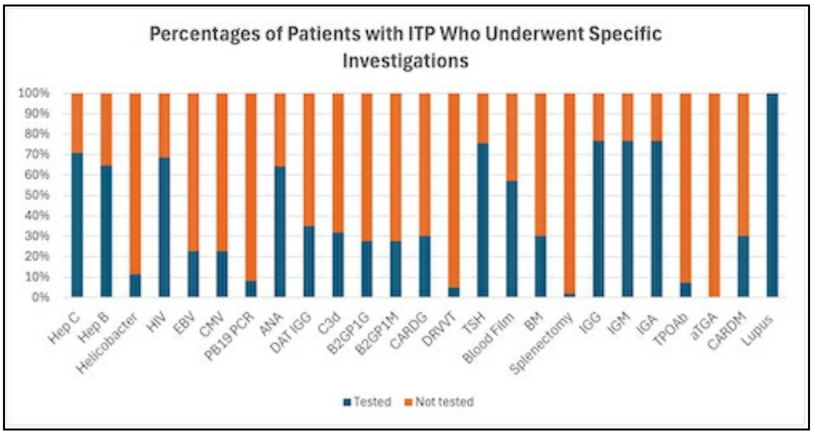

Bars represent the percentage of patients tested versus not tested for each investigation. ITP = Immune thrombocytopenia; Hep C = Hepatitis C; Hep B = Hepatitis B; Helicobacter = Helicobacter pylori; HIV = Human immunodeficiency virus; EBV = Epstein–Barr virus; CMV = cytomegalovirus; PB19PCR = Parvovirus B19; ANA = Antinuclear antibody; DAT/IgG = direct antiglobulin test (IgG); C3d = complement component 3d; B2GP1G = Beta-2 glycoprotein I (IgG antibodies); B2GP1M= Anti-β2 glycoprotein I (IgM antibodies); CARDG = Anticardiolipin IgG; DRVVT = Dilute Russell viper venom time; TSH = Thyroid-stimulating hormone; BM = Bone marrow examination; IgG/IgM/IgA = immunoglobulin testing for IgG/IgM/IgA; TPO Ab = Thyroid peroxidase antibody; aTGA = Anti-tissue transglutaminase antibody (IgA); CARDM = Anticardiolipin IgM.

**Copyright Information:** (Online) ISSN: 2572-9241
©2026 The Author(s). HemaSphere is published by John Wiley & Sons Ltd on behalf of European Hematology Association. This is an open access Abstract Book distributed under the Attribution-NonCommercial-NoDerivs (CC BY-NC-ND), which allows third parties to download the articles and share them with others as long as they credit the author and the Abstract Book, but they cannot change the content in any way or use them commercially.
**Abstract Book Citations:** Authors, Title, HemaSphere, 2026;10:(S1):pages. The abstract book DOIs can be found on the “Author Information” page.
**Disclaimer:** Articles published in the journal HemaSphere exclusively reflect the opinions of the authors. The authors are responsible for all content in their abstracts including accuracy of the facts, statements, citing resources, etc.

### (PS2453) EFFICACY AND SAFETY OF EFGARTIGIMOD IN PRIMARY IMMUNE THROMBOCYTOPENIA(ITP): META-ANALYSIS OF PHASE III RANDOMIZED CONTROLLED TRIALS

**Topic:** 33. Platelet disorders

Mehak Dang*[1], Hosana Candreva[2], Aashray Raj[3], Abdrabo Gamal Motawea[4], Damanpartap Singh Sandhu[5], Fahad Alabbas[6]

*[1]JSS Medical College, Mysore, India;[2]Department of Medicine, Rio de Janeiro State University, Department of Medicine, Rio de Janeiro, Brazil; [3]Government Medical College, Bhavnagar, India;[4]Faculty of Medicine, Menoufia University, menoufia, Egypt; [5]University of central Florida/HCA Healthcare GME (Ocala) Program, Ocala, United States of America;[6]Prince Sultan Military Medical City, Riyadh, Saudi Arabia;*

### Background

Immunoglobulin G (IgG) autoantibodies are central to pathophysiology of ITP, promoting platelet clearance through Fc receptor dependent pathways. Neonatal Fc receptor (FcRn) protects the IgG from degradation, thereby prolonging the circulation of autoantibodies. Efgartigimod is an FcRn inhibitor which competitively inhibits IgG-FcRn interactions, thus accelerates lysosomal degradation of IgG resulting in a reduction in circulating IgG levels.

### Aims

To evaluate the clinical effectiveness and safety profile of Efgartigimod in adult patients with ITP and to explore the preferred route of drug administration.

### Methods

We systematically searched Embase, PubMed, and the Cochrane Library to identify studies involving patients with ITP comparing Efgartigimod versus Placebo from inception to November 2025. We performed this systematic review and meta-analysis following the recommendations of the Cochrane Handbook for Systematic Reviews and PRISMA guidelines. The primary outcome assessed the clinical effectiveness of Efgartigimod using International Working Group (IWG) platelet response criteria, where Complete response (CR) is defined as any platelet count of at least 100 × $10^9$/L and Response (R) as any platelet count between 30 and 100 × $10^9$/L and at least doubling of the baseline count. The secondary outcomes assessed the safety of Efgartigimod compared to placebo, including serious treatment-emergent adverse events (TEAEs), any TEAEs, including headache, hypertension, petechiae, and purpura. All statistical analyses were conducted using Review Manager version 5.4.1, using a random-effects model with 95% Confidence Interval. Heterogeneity was evaluated using the $I^2$ statistic.

**Copyright Information:** (Online) ISSN: 2572-9241
©2026 The Author(s). HemaSphere is published by John Wiley & Sons Ltd on behalf of European Hematology Association. This is an open access Abstract Book distributed under the Attribution-NonCommercial-NoDerivs (CC BY-NC-ND), which allows third parties to download the articles and share them with others as long as they credit the author and the Abstract Book, but they cannot change the content in any way or use them commercially.
**Abstract Book Citations:** Authors, Title, HemaSphere, 2026;10:(S1):pages. The abstract book DOIs can be found on the “Author Information” page.
**Disclaimer:** Articles published in the journal HemaSphere exclusively reflect the opinions of the authors. The authors are responsible for all content in their abstracts including accuracy of the facts, statements, citing resources, etc.

### Results

Three phase III RCTs comprising 249 patients receiving efgartigimod and 127 receiving placebo were included, where two studies evaluated the intravenous (IV) formulation and one study assessed the subcutaneous (SC) formulation. Only two patients had newly diagnosed ITP, while all others had persistent or chronic ITP. Our meta-analysis demonstrated that efgartigimod significantly improved IWG platelet response (RR 1.84; 95% CI 1.26-2.70; p = 0.002; $I^2$ = 7%). However, IWG complete response did not reach statistical significance overall (RR 2.87; 95% CI 0.93–8.88; p = 0.07; $I^2$ = 58%).
In exploratory analysis by route of drug administration, IV formulation demonstrated greater efficacy for IWG response (RR 2.27; 95% CI 1.39–3.71; p = 0.001; $I^2$ = 0%), whereas the SC formulation was not statistically significant (RR 1.42; 95% CI 0.83–2.44; p = 0.20). For IWG complete response, the IV formulation showed a significant improvement (RR 5.64; 95% CI 2.10–15.17; p = 0.0006; $I^2$ = 0%), while the SC formulation did not demonstrate a significant benefit (RR 0.89; 95% CI 0.39–2.03; p = 0.79). There was no significant difference in the incidence of any TEAEs and serious TEAEs between the groups (RR 0.67; 95% CI 0.37 to 1.21; p = 0.19; $I^2$ = 0%).

### Summary/Conclusion

Efgartigimod significantly improves the platelet response rate without an associated increase in serious adverse events. Intravenous administration demonstrated consistent efficacy across trials whereas efficacy of subcutaneous formulation remains limited due to single trial evidence. Thus, our study supports FcRn inhibitors as an effective and well tolerated treatment strategy in ITP and highlights the need of larger studies to comprehensively define the optimal administration strategies.

| Study or Subgroup | Efgartigimod Events | Efgartigimod Total | Placebo Events | Placebo Total | Weight | Risk ratio IV, Random, 95% CI |
|---|---|---|---|---|---|---|
| **1.2.1 INTRAVENOUS** | | | | | | |
| BROOME 2023 | 44 | 86 | 9 | 45 | 34.3% | 2.56 [1.38 , 4.75] |
| NEWLAND -5mg 2019 | 6 | 13 | 3 | 12 | 10.9% | 1.85 [0.59 , 5.79] |
| NEWLAND 10mg 2019 | 6 | 13 | 3 | 12 | 10.9% | 1.85 [0.59 , 5.79] |
| **Subtotal (Wald[a])** | | **112** | | **69** | **56.1%** | **2.27 [1.39 , 3.71]** |
| Total events: | 56 | | 15 | | | |
| Test for overall effect: Z = 3.26 (P = 0.001) | | | | | | |
| Heterogeneity: Tau² (REML[b]) = 0.00; Chi² = 0.39, df = 2 (P = 0.82); I² = 0% | | | | | | |
| **1.2.2 SUBCUTANEOUS** | | | | | | |
| ADVANCE SC 2023 | 39 | 137 | 14 | 70 | 43.9% | 1.42 [0.83 , 2.44] |
| **Subtotal** | | **137** | | **70** | **43.9%** | **1.42 [0.83 , 2.44]** |
| Total events: | 39 | | 14 | | | |
| Test for overall effect: Z = 1.28 (P = 0.20) | | | | | | |
| Heterogeneity: Not applicable | | | | | | |
| **Total (Wald[a])** | | **249** | | **139** | **100.0%** | **1.84 [1.26 , 2.70]** |
| Total events: | 95 | | 29 | | | |
| Test for overall effect: Z = 3.12 (P = 0.002) | | | | | | |
| Test for subgroup differences: Chi² = 1.56, df = 1 (P = 0.21), I² = 36.1% | | | | | | |
| Heterogeneity: Tau² (REML[b]) = 0.01; Chi² = 1.96, df = 3 (P = 0.58); I² = 7% | | | | | | |

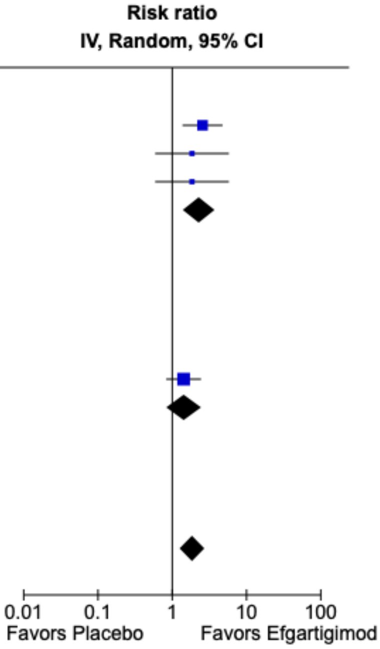

**Footnotes**
[a]CI calculated by Wald-type method.
[b]Tau² calculated by Restricted Maximum-Likelihood method.

Fig. 1 demonstrated statistically significant increase in the platelet response among patients treated with efgartigimod compared with placebo.

**Copyright Information:** (Online) ISSN: 2572-9241
©2026 The Author(s). HemaSphere is published by John Wiley & Sons Ltd on behalf of European Hematology Association. This is an open access Abstract Book distributed under the Attribution-NonCommercial-NoDerivs (CC BY-NC-ND), which allows third parties to download the articles and share them with others as long as they credit the author and the Abstract Book, but they cannot change the content in any way or use them commercially.
**Abstract Book Citations:** Authors, Title, HemaSphere, 2026;10:(S1):pages. The abstract book DOIs can be found on the "Author Information" page.
**Disclaimer:** Articles published in the journal HemaSphere exclusively reflect the opinions of the authors. The authors are responsible for all content in their abstracts including accuracy of the facts, statements, citing resources, etc.

### (PS2454) ANTIBODY SPECIFICITY DETERMINES THE MECHANISM OF PLATELET DESTRUCTION IN ITP

**Topic:** 34. Bleeding disorders (congenital and acquired)

Günalp Uzun*[1], Qilu Lyu[1], Theresa Ringelmann[1], Omid Haghighi[1], Irene Marini[1], Jan Zlamal[1], Ulrich Sachs[2], Rosa Sonja Alesci[3], Tamam Bakchoul[1]

*[1]Institute for Clinical and Experimental Transfusion Medicine, Tuebingen, Germany;[2]Institute for Clinical Immunology, Transfusion Medicine and Hemostasis, Giessen, Germany; [3]IMD Blood Coagulation Centre, Frankfurt/Bad Homburg, Germany;*

### Background

Platelet apoptosis and desialylation are distinct mechanisms leading to platelet destruction in patients with immune thrombocytopenia (ITP).  However, the precise signaling pathways mediating platelet apoptosis and desialylation are not fully elucidated.

### Aims

To investigate the role of FcγRIIA signaling in antibody-induced platelet apoptosis and desialylation.

### Methods

Washed human platelets from healthy donors were incubated with either ITP sera to evaluate antibody-induced platelet apoptosis and desialylation. Platelet apoptosis was assessed via flow cytometry by identifying P-selectin negativity (CD62P−), phosphatidylserine exposure (Annexin V+), and alterations in mitochondrial membrane potential (TMRE−). Desialylation was quantified by measuring the increase in *Ricinus communis* agglutinin (RCA) binding relative to baseline levels. Antibody specificity of ITP sera was determined using the monoclonal antibody immobilization of platelet antigens (MAIPA) assay. Signaling pathway analysis was performed using specific inhibitors, including IV.3, a FcγRIIA-blocking antibody.

### Results

The study cohort included 97 patients with ITP (age: 54.8 ± 16.8 years; 53 females; median platelet count: 49 $\times 10^9$/L, range 26–106 $\times 10^9$/L). Overall, 44 ITP sera (45%) induced platelet apoptosis and 24 (25%) induced desialylation. Platelet counts were similar between patients with apoptosis-inducing and non-inducing sera (43.5 ± 39.2 vs. 51.4 ± 51.3 $\times 10^9$/L; p=0.421). Notably, platelet counts were significantly lower in patients with desialylating sera compared to those without (28.1 ± 24.2 vs. 60.8 ± 50.1 $\times 10^9$/L; p=0.009). The proportion of sera inducing apoptosis and desialylation was higher among samples with detectable anti-platelet antibodies compared to those without (55% vs. 18% for apoptosis and 27% vs. 11% for desialylation). FcγRIIA blockade effectively inhibited both apoptosis and desialylation induced by antibodies against glycoprotein (GP) IIb/IIIa, whereas it had no inhibitory effect on apoptosis or desialylation induced by antibodies against GPIb/IX.

### Summary/Conclusion

FcγRIIA signaling mediates platelet apoptosis and desialylation induced by anti-GPIIb/IIIa antibodies but not by anti-GPIb/IX antibodies, demonstrating that antibody specificity determines the downstream mechanism of platelet destruction in ITP. Furthermore, desialylation, but not apoptosis, correlates with thrombocytopenia severity, highlighting its potential as a clinically relevant therapeutic target. These findings suggest that antibody profiling in ITP patients may help identify those most likely to benefit from treatments aimed at modulating FcγRIIA signaling pathways.

**Copyright Information:** (Online) ISSN: 2572-9241
©2026 The Author(s). HemaSphere is published by John Wiley & Sons Ltd on behalf of European Hematology Association. This is an open access Abstract Book distributed under the Attribution-NonCommercial-NoDerivs (CC BY-NC-ND), which allows third parties to download the articles and share them with others as long as they credit the author and the Abstract Book, but they cannot change the content in any way or use them commercially.
**Abstract Book Citations:** Authors, Title, HemaSphere, 2026;10:(S1):pages. The abstract book DOIs can be found on the “Author Information” page.
**Disclaimer:** Articles published in the journal HemaSphere exclusively reflect the opinions of the authors. The authors are responsible for all content in their abstracts including accuracy of the facts, statements, citing resources, etc.

### (PS2455) HIGH PREVALENCE OF BLEEDING DISORDER OF UNKNOWN CAUSE IN WOMEN: FINDINGS FROM THE MAASTRICHT BLEEDING COHORT

**Topic:** 34. Bleeding disorders (congenital and acquired)

Nienke Meskes*[1, 2], Floor Derikx[1], Inge Merry[1], Amaury Monard[1], Thijmen van Burken[1], Paul Verhezen[2], Dave Hellenbrand[2], Erik Beckers[1], Yvonne Henskens[2], Floor Heubel-Moenen[1]

*[1]Maastricht University Medical Center (MUMC+), Hematology, Internal Medicine, Maastricht, The Netherlands; [2]Maastricht University Medical Center (MUMC+), Central Diagnostic Laboratory, Maastricht, The Netherlands;*

### Background

Mild bleeding disorders (MBDs) include von Willebrand disease (VWD), platelet function disorders (PFD), mild clotting factor deficiencies, and fibrinolytic abnormalities. In contrast, **Bleeding Disorders of Unknown Cause (BDUC)** refer to patients with a bleeding phenotype in whom no specific hemostatic defect can be identified despite extensive laboratory investigation. Diagnosing MBD's is challenging due to overlapping phenotypes, leading to lengthy and costly procedures, particularly for BDUC.

### Aims

To investigate patient and laboratory characteristics and patterns according to final diagnosis in a large cohort of patients referred for bleeding evaluation to the Maastricht University Medical Center (MUMC+).

### Methods

Since 2016, all patients referred for bleeding evaluation at MUMC+ were invited to participate in the Predictors of Bleeding Evaluation in Adult Hematologic Patients (ProBe-AHP) study. All underwent International Society on Thrombosis and Haemostasis - Bleeding Assessment Tool (ISTH-BAT) scoring and extensive laboratory haemostasis testing.

### Results

359 patients were included, 83% was female, the median age was 42 (IQR 31-59), 20.1% were diagnosed with no bleeding disorder (NBD), 35.4% with a MBD (59.1% PFD, 12.6% VWD, 7.9% clotting factor deficiency), and 44.6% with BDUC. ISTH-BAT scores were highest in BDUC patients with a score of 8 (IQR 6-10), compared to a score of 7 in MBD (IQR 3-10) while NBD patients scored lowest with a score of 3 (IQR 2-4). Heavy menstrual bleeding (HMB) was reported more frequently in BDUC compared to MBD (77.2% vs. 50.0%; $p<0.001$). In addition, postpartum bleeding (PPH) was more common in BDUC than in MBD (39.2% vs. 26.0%; $p=0.049$). Together with prolonged bleeding of minor wounds, spontaneous gum bleeding and post-surgical bleeding, HMB and PPH were identified as predictive factors for BDUC diagnosis. Male gender, post-dental extraction bleeding and a diagnosed bleeding disorder in family history were predictive for MBD diagnosis. In general women were more likely to receive the diagnosis BDUC compared to men (OR 4.7; 2.3-9.3 95% CI). Women who reported HMB were more likely to receive the diagnosis BDUC, compared to a MBD (OR 2.2; 1.2-4.2 95% CI).

### Summary/Conclusion

Women were more frequently diagnosed with BDUC, particularly in the presence of gender-specific symptoms such as heavy menstrual and postpartum bleeding. In contrast, male gender and a positive family history were more indicative of MBD. These findings suggest that gender- and symptom-specific patterns may play a role in the diagnostic distinction between BDUC and MBD.

**Copyright Information:** (Online) ISSN: 2572-9241
©2026 The Author(s). HemaSphere is published by John Wiley & Sons Ltd on behalf of European Hematology Association. This is an open access Abstract Book distributed under the Attribution-NonCommercial-NoDerivs (CC BY-NC-ND), which allows third parties to download the articles and share them with others as long as they credit the author and the Abstract Book, but they cannot change the content in any way or use them commercially.
**Abstract Book Citations:** Authors, Title, HemaSphere, 2026;10:(S1):pages. The abstract book DOIs can be found on the "Author Information" page.
**Disclaimer:** Articles published in the journal HemaSphere exclusively reflect the opinions of the authors. The authors are responsible for all content in their abstracts including accuracy of the facts, statements, citing resources, etc.

### (PS2456) COMPETING CAUSES OF DEATH IN HEMOPHILIA IN THE UNITED STATES: A NATIONAL MULTIPLE-CAUSE-OF-DEATH ANALYSIS, 2005–2024

**Topic:** 34. Bleeding disorders (congenital and acquired)

Mohammad Ahsen Soomro*[1], Hala Soomro[2], Zafar Suchal[3]

*[1]The Wright Center for Graduate Medical Education, Cardiology, Scranton, United States of America;[2]Brown University of Health Sciences, Internal Medicine, Providence, United States of America; [3]Shalamar Institute of Health Science, Department of Clinical Research, Lahore, Pakistan;*

### Background

Advances in prophylaxis have extended survival in persons with hemophilia (PWH). As longevity improves, the relative contribution of age-associated comorbidities versus traditional bleeding complications to mortality remains incompletely defined at the national level.

### Aims

To quantify temporal shifts in bleeding-related versus age-associated comorbidities among U.S. decedents with hemophilia.

### Methods

We analyzed National Vital Statistics System multiple-cause-of-death (MCOD) files (2005–2024). Decedents with hemophilia A (ICD-10 D66) or hemophilia B (D67) listed anywhere on the death certificate were included. Bleeding-related causes were defined as intracranial hemorrhage, gastrointestinal hemorrhage, or coagulation defects. Age-associated causes included ischemic heart disease (IHD) and malignant neoplasms. Proportionate mortality (PM) and weighted mean age-at-death proxy were calculated across four 5-year intervals.

### Results

A total of 3,511 hemophilia-associated deaths were identified. The age-at-death proxy more than doubled over the study period, increasing from 6.22 in 2005–2009 to 13.9 in 2020–2024, consistent with rapid aging of the mortality cohort. Bleeding-related causes were listed on 16.9% of death certificates in 2005–2009 and remained persistently high at 18.0% in 2020–2024. In contrast, malignancy-related mentions increased by 39.8%, rising from 11.3% to 15.8% over the same period. By 2020–2024, the absolute difference between bleeding-related and malignancy-related mentions had narrowed to 2.2 percentage points, demonstrating marked convergence. Mentions of ischemic heart disease also increased over time, peaking at 14.1% in 2015–2019.

### Summary/Conclusion

U.S. hemophilia mortality demonstrates an epidemiologic transition characterized by sustained bleeding burden alongside rising malignancy and cardiovascular comorbidity. These findings support an expanded survivorship model integrating bleed prevention with proactive cancer screening and cardiovascular risk management in aging PWH.

**Copyright Information:** (Online) ISSN: 2572-9241
©2026 The Author(s). HemaSphere is published by John Wiley & Sons Ltd on behalf of European Hematology Association. This is an open access Abstract Book distributed under the Attribution-NonCommercial-NoDerivs (CC BY-NC-ND), which allows third parties to download the articles and share them with others as long as they credit the author and the Abstract Book, but they cannot change the content in any way or use them commercially.
**Abstract Book Citations:** Authors, Title, HemaSphere, 2026;10:(S1):pages. The abstract book DOIs can be found on the “Author Information” page.
**Disclaimer:** Articles published in the journal HemaSphere exclusively reflect the opinions of the authors. The authors are responsible for all content in their abstracts including accuracy of the facts, statements, citing resources, etc.

### (PS2457) RISK OF BLEEDING IN MANTLE CELL LYMPHOMA PATIENTS TREATED WITH COVALENT BRUTON TYROSINE KINASE INHIBITORS AND CONTEMPORARY ANTICOAGULANT OR ANTIPLATELET AGENTS: REAL-WORLD DATA INSIGHTS

**Topic:** 34. Bleeding disorders (congenital and acquired)

Vladimir Otasevic*[1], Darko Antic[2, 3], Heidi Cho[1], Kausik Maiti[1], Nancy Lunney[1], Darko Dabic[1], Chris Learn[1]

*[1]Parexel International, Raleigh, NC, United States of America; [2]Clinic for Hematology, University Clinical Center of Serbia, Belgrade, Serbia; [3]Faculty of Medicine, University of Belgrade, Belgrade, Serbia;*

### Background

Covalent Bruton tyrosine kinase inhibitors (BTKis) have improved outcomes for mantle cell lymphoma (MCL) patients, yet managing therapy is challenging due to bleeding risks, particularly in patients with comorbidities requiring anticoagulation (AC) or antiplatelet (AP) therapy. Real-world evidence on bleeding risk in MCL is limited.

### Aims

Our study assesses bleeding rates and risks in three groups: MCL patients on BTKi alone, with AC/AP, or with either therapy, using real-world data (RWD) to understand bleeding risk management better.

### Methods

A retrospective analysis of RWD was conducted using TriNetX Dataworks Global database on February 11, 2026. Two primary cohorts were analyzed: (1) MCL patients treated with covalent BTKi (ibrutinib/acalabrutinib/zanubrutinib) concurrently treated with AC (rivaroxaban/apixaban/edoxaban/dabigatran) or AP agents (acetylsalicylic acid/clopidogrel/prasugrel/ticagrelor/ticlopidin), and (2) MCL patients treated with covalent BTKi (ibrutinib/acalabrutinib/zanubrutinib) not concurrently treated with AC or AP agents. Cohort 1 was further subdivided into AC-only and AP-only groups. Standardized mean differences were used to ascertain the distribution balance among baseline variables. The risk of bleeding was evaluated using risk ratios (RR), 95% confidence intervals (CI), and the Z-test. Propensity score matching (PSM) was employed to control the effects of certain confounders.

### Results

A total of 3205 patients were enrolled, with 1127 patients in Cohort 1 (BTKi with AC/AP) and 2078 in Cohort 2 (BTKi without AC/AP). The mean age at the index event (initiation of BTKi and AC/AP for Cohort 1; initiation of BTKi for Cohort 2) differed significantly between the cohorts: for Cohort 1, it was 71.9±10.2, whilst for Cohort 2, it was 68±11.3 ($p<0.001$). Male sex was more prevalent in Cohort 1 (77% vs. 73%, $p=0.006$). Over a 1-year follow-up period, Cohort 1 demonstrated a statistically significantly higher bleeding rate than Cohort 2 (17.6% vs. 12.5%, $z=3.971$, $p<0.001$; RR 1.411, 95% CI 1.191-1.672). After PSM for covariates (age at index event, sex, essential hypertension, use of corticosteroids, use of non-steroidal anti-inflammatory analgesics), for a follow-up of 1 year, the bleeding rate remained more frequent in Cohort 1 (17.1% vs. 13.1%, $z=2.430$, $p=0.015$; RR 1.304, 95% CI 1.052-1.617). Within Cohort 1, patients receiving AC had a higher bleeding rate than those receiving AP therapy (18.2% vs. 12.8%, $z=2.284$, $p=0.022$; RR 1.425, 95% CI 1.051-1.933). After PSM for covariates (the same as previously), the AC group retained statistical significance for higher bleeding rate (18.1% vs. 12.2%, $z=2.183$, $p=0.029$; RR 1.477, 95% CI 1.037-2.104). A key limitation of this study is the inability to distinguish between minor and major hemorrhagic events due to insufficient granularity in the dataset and a lack of data regarding MCL treatment response.

**Copyright Information:** (Online) ISSN: 2572-9241
©2026 The Author(s). HemaSphere is published by John Wiley & Sons Ltd on behalf of European Hematology Association. This is an open access Abstract Book distributed under the Attribution-NonCommercial-NoDerivs (CC BY-NC-ND), which allows third parties to download the articles and share them with others as long as they credit the author and the Abstract Book, but they cannot change the content in any way or use them commercially.
**Abstract Book Citations:** Authors, Title, HemaSphere, 2026;10:(S1):pages. The abstract book DOIs can be found on the "Author Information" page.
**Disclaimer:** Articles published in the journal HemaSphere exclusively reflect the opinions of the authors. The authors are responsible for all content in their abstracts including accuracy of the facts, statements, citing resources, etc.

**Summary/Conclusion**

MCL patients treated with covalent BTKis have a significant risk of bleeding events, and this risk increases substantially when BTKis are combined with anticoagulant or antiplatelet agents. MCL patients treated with AC alone have a higher risk of bleeding than those treated with AP alone. These findings highlight the need for careful evaluation and follow-up of MCL patients on covalent BTKis with AC or AP agents. Further investigation utilizing patient-level data is necessary to identify specific predictors of bleeding and to better inform treatment strategies.

**Copyright Information:** (Online) ISSN: 2572-9241
©2026 The Author(s). HemaSphere is published by John Wiley & Sons Ltd on behalf of European Hematology Association. This is an open access Abstract Book distributed under the Attribution-NonCommercial-NoDerivs (CC BY-NC-ND), which allows third parties to download the articles and share them with others as long as they credit the author and the Abstract Book, but they cannot change the content in any way or use them commercially.
**Abstract Book Citations:** Authors, Title, HemaSphere, 2026;10:(S1):pages. The abstract book DOIs can be found on the "Author Information" page.
**Disclaimer:** Articles published in the journal HemaSphere exclusively reflect the opinions of the authors. The authors are responsible for all content in their abstracts including accuracy of the facts, statements, citing resources, etc.

### (PS2458) EXPANDING ACCESS TO HEMOPHILIA CARE : SIGNIFICANT REDUCTION IN BLEEDING WITH LOW-DOSE EMICIZUMAB PROPHYLAXIS IN A PROSPECTIVE REAL-WORLD COHORT .

**Topic:** 34. Bleeding disorders (congenital and acquired)

Aditi Malji*[1], Varun Bafna[2], Anita Paritekar[2], Vidya Patil[2], Sangita Kumbhojkar[3], Shishir Mirgunde[3]

*[1]RCSM GMC AND CPR HOSPITAL KOLHAPUR , MAHARASHTRA , INDIA, INTERNAL MEDICINE, KOLHAPUR, India;[2]RCSM GMC AND CPR HOSPITAL , KOLHAPUR , MAHARASHTRA , INDIA, INTERNAL MEDICINE, KOLHAPUR, India; [3]RCSM GMC AND CPR HOSPITAL , KOLHAPUR , MAHARASHTRA , INDIA, PEDIATRICS, KOLHAPUR, India;*

### Background

Emicizumab , a humanized bispecific monoclonal antibody has transformed prophylactic management in Hemophilia A with or without inhibitors; however, the high cost of standard dosing regimen limits widespread adoption in resource limited settings. Optimizing lower-dose strategies may improve affordability while maintaining effective hemostatic protection. Real-world evidence evaluating such approaches remain limited.

### Aims

To assess the efficacy of low dose Emicizumab prophylaxis in patients with severe Hemophilia A with and without inhibitors and to evaluate its impact on bleeding outcomes and factor utilization in a real-world setting .

### Methods

This prospective observational study was conducted from March 2025 to February 2026 at a tertiary care centre RCSMGMC CPR Hospital, Kolhapur, Maharashtra, India. Twenty seven patients with Severe Hemophilia A of all age groups were enrolled, including four inhibitor-positive patients. All patients received low-dose Emicizumab prophylaxis (1 mg/kg once every 2 weeks) as per institutional protocol. The primary endpoint was change in Annualized Bleeding Rate (ABR) compared with the pre-Emicizumab period. Secondary endpoints included procedural factor requirement, resolution of target joints, and safety outcomes. Statistical analyses were performed using paired t-test or Wilcoxon signed-rank test as appropriate. A p value $< 0.05$ was considered statistically significant .

### Results

Mean ABR reduced from $16.8 \pm 4.2$ to $3.9 \pm 2.1$ (76.8% reduction ; $p<0.001$), representing a substantial relative reduction in bleeding burden. During follow-up, 15 patients underwent tooth extraction. Although minor post-procedural bleeding was observed, only two patients required additional factor replacement, suggesting adequate peri-procedural hemostatic coverage in the majority of cases. Resolution of target joints occurred in five patients during follow-up. No thrombotic events, thrombotic microangiopathy, or serious adverse drug reactions were reported. Overall, both bleeding frequency and factor utilization were significantly reduced after initiation of low-dose emicizumab. Compared with standard-dose Emicizumab, the low-dose regimen achieved an approximate 66% reduction in annual drug acquisition cost. When compared with Extended half-life factor prophylaxis, an estimated 75% cost reduction was observed.

**Copyright Information:** (Online) ISSN: 2572-9241
©2026 The Author(s). HemaSphere is published by John Wiley & Sons Ltd on behalf of European Hematology Association. This is an open access Abstract Book distributed under the Attribution-NonCommercial-NoDerivs (CC BY-NC-ND), which allows third parties to download the articles and share them with others as long as they credit the author and the Abstract Book, but they cannot change the content in any way or use them commercially.
**Abstract Book Citations:** Authors, Title, HemaSphere, 2026;10:(S1):pages. The abstract book DOIs can be found on the "Author Information" page.
**Disclaimer:** Articles published in the journal HemaSphere exclusively reflect the opinions of the authors. The authors are responsible for all content in their abstracts including accuracy of the facts, statements, citing resources, etc.

**Summary/Conclusion**

Low-dose Emicizumab prophylaxis was associated with a significant reduction in bleeding rates and factor requirement in patients with Severe Hemophilia A, including those with inhibitors, with an excellent safety profile. This strategy may represent a practical and cost-conscious approach to expanding access to prophylaxis in resource-constrained settings. Larger multicenter studies with longer follow-up are warranted to define optimal dosing strategies and long-term outcomes.

**Copyright Information:** (Online) ISSN: 2572-9241
©2026 The Author(s). HemaSphere is published by John Wiley & Sons Ltd on behalf of European Hematology Association. This is an open access Abstract Book distributed under the Attribution-NonCommercial-NoDerivs (CC BY-NC-ND), which allows third parties to download the articles and share them with others as long as they credit the author and the Abstract Book, but they cannot change the content in any way or use them commercially.
**Abstract Book Citations:** Authors, Title, HemaSphere, 2026;10:(S1):pages. The abstract book DOIs can be found on the "Author Information" page.
**Disclaimer:** Articles published in the journal HemaSphere exclusively reflect the opinions of the authors. The authors are responsible for all content in their abstracts including accuracy of the facts, statements, citing resources, etc.

### (PS2459) A REAL-WORLD STUDY ON THE EFFICACY, SAFETY, AND UTILIZATION OF A CHINESE-DEVELOPED RECOMBINANT FACTOR VIII ( SCT800) FOR PROPHYLACTIC TREATMENT IN MODERATE TO SEVERE HAEMOPHILIA A PATIENTS

**Topic:** 34. Bleeding disorders (congenital and acquired)

Feng Xue*[1], Renchi Yang[2], Pingchong Lei[3], Shimei Lian[4], Mingfeng Jia[5], Huibing Dang[6], Rongfu Zhou[7], Lisheng Cai[8], Lihua Yang[9], Qun Hu[10], Bingshou Xie[11], Haiying Wang[12], Suo Xiaohui[13], Lixin Wang[14], Yang Guo[15], Jing Sun[16], Zhenyu Yan[17], Yan Yang[18]

*[1]Institute of Hematology, Chinese Academy of Medical Sciences, TianJin, China;[2]Institute of Hematology, Chinese Academy of Medical Sciences, TianJing, China; [3]Henan Provincial People's Hospital, HeNan, China;[4]Dalian Municicpal Central Hospital, Dalian, China; [5]The First Hospital of Lanzhou University, Lanzhou, China;[6]The First Affiliated Hospital of Nanyang Medical College, Nanyang, China; [7]Drum Tower Hospital Affiliated to Medical College of Nanjing University, Nanjing, China;[8]The Second People's Hospital of Shenzhen, Shenzhen, China; [9]Dazhou Central Hospital, Dazhou, China; [10]Tongji Hospital Affiliated to Tongji Medical College of Huazhong University of Science and Technology, WuHan, China; [11]Wenzhou People's Hospital, Wenzhou, China; [12]Affiliated Hospital of Weifang Medical University, Weifang, China;[13]Handan Central Hospital, Handan, China;[14]The First People's Hospital of Jinzhong, Jinzhong, China; [15]Peking University People's Hospital, BeiJing, China; [16]Nanfang Hospital, Southern Medical University, GuangZhou, China; [17]Affiliated Hospital of North China University of Technology, TangShan, China; [18]The First Hospital of Jilin University, Jilin, China;*

### Background

Haemophilia A (HA) is a rare genetic disorder characterized by a deficiency in coagulation factor VIII (FVIII), leading to prolonged bleeding and increased risk of joint complications. In China, access to FVIII prophylaxis remains limited due to high costs and variable insurance coverage.

### Aims

This study aimed to evaluate the real-world effectiveness and safety of Omfiloctocog Alfa (SCT800), a novel and more accessible recombinant FVIII product, in patients with moderate to severe HA.

### Methods

This is an open-label, single-arm, multicenter, prospective study. The primary endpoints included the annualized bleeding rate (ABR) and annualized joint bleeding rate (AJBR). The prophylactic administration regimen was determined by the investigator based on the patient's condition and clinical practice.Mid-study and end-of-study visits were scheduled at Weeks 24 and 52 after enrollment, respectively, aligned with standard clinical care practices such as on-site assessments, home-based treatments, and bleeding diary documentation. After 52 weeks, patients could choose whether to continue being followed up until the study's closure.

**Copyright Information:** (Online) ISSN: 2572-9241
©2026 The Author(s). HemaSphere is published by John Wiley & Sons Ltd on behalf of European Hematology Association. This is an open access Abstract Book distributed under the Attribution-NonCommercial-NoDerivs (CC BY-NC-ND), which allows third parties to download the articles and share them with others as long as they credit the author and the Abstract Book, but they cannot change the content in any way or use them commercially.
**Abstract Book Citations:** Authors, Title, HemaSphere, 2026;10:(S1):pages. The abstract book DOIs can be found on the "Author Information" page.
**Disclaimer:** Articles published in the journal HemaSphere exclusively reflect the opinions of the authors. The authors are responsible for all content in their abstracts including accuracy of the facts, statements, citing resources, etc.

### Results

A total of 293 male patients received treatments from 17 hemophilia treatment centers (HTCs) in China from April 2022 to September 2024.
**ABR and AJBR**：The estimated mean ABR and AJBR (95% CI) for overall bleeding episodes were 3.75 (3.00, 4.68) and 3.01 (2.41, 3.76) respectively. Compared to retrospective data, the ABR and AJBR decreased by 86% and 80%, respectively.The model-estimated mean ABRs (95% CI) were 3.49(2.40, 5.07) for the < 45 IU/kg/week subgroup, 3.24(2.23, 4.71) for the 45-75 IU/kg/week subgroup, and 2.87(1.76, 4.69) for the >75 IU/kg/week subgroup；model-estimated mean AJBRs (95% CI) of 2.98(2.07, 4.28) for < 45 IU/kg/week, 2.54(1.69, 3.82) for 45-75 IU/kg/week, and 2.49(1.54, 4.02) for >75 IU/kg/week.
**HEAD-US-C**：Patients' joint status was assessed using ultrasound imaging and evaluated according to the HEAD-US-C scoring system. A lower HEAD-US-C score suggested a better joint status. the mean (SD) of the HEAD-US-C total score at Week-24 visit was 24.6(14.50), a decrease of 1.2 points from baseline, and at Week-52 visit, it was 24.0(14.78), a decrease of 2.0 points from baseline.
**EQ-5D-3L** score indicates life quality of patients. A higher score suggests a better healthy status. Significant improvements in healthy status were observed, with EQ-5D-3L health survey scores increased by 4.9 and 8.6 points at Weeks 24 and 52, respectively.
**Safety** was assessed in 293 patients exposed to SCT800, with a median (min, max) of 129 EDs (1, 289). During the study, a total of 8 SAEs were reported in 5 patients; none of the SAEs were considered related to SCT800 treatment.

ABR, AJBR and HEAD-US-C results indicated dose-dependent decreasing trends. Improvement in EQ-5D-3L scores further supported the ABR and AJBR results. Additionally, 38.9% of patients experienced no bleeding episodes. Finally, safety analysis showed no drug-inhibitor and no new safety signals were detected.

### Summary/Conclusion

The real-world study indicates that SCT800 may be a safe and effective prophylactic treatment option for patients with moderate to severe HA across different ages in China.
Compared to previous FVIII treatments, SCT800 had the potential to reduce the financial burden on HA patients and their families, thereby lowering a key barrier to the access of regular replacement treatments.

**Copyright Information:** (Online) ISSN: 2572-9241
©2026 The Author(s). HemaSphere is published by John Wiley & Sons Ltd on behalf of European Hematology Association. This is an open access Abstract Book distributed under the Attribution-NonCommercial-NoDerivs (CC BY-NC-ND), which allows third parties to download the articles and share them with others as long as they credit the author and the Abstract Book, but they cannot change the content in any way or use them commercially.
**Abstract Book Citations:** Authors, Title, HemaSphere, 2026;10:(S1):pages. The abstract book DOIs can be found on the “Author Information” page.
**Disclaimer:** Articles published in the journal HemaSphere exclusively reflect the opinions of the authors. The authors are responsible for all content in their abstracts including accuracy of the facts, statements, citing resources, etc.

### (PS2460) PHARMACOLOGIC INHIBITION OF THE APELIN RECEPTOR (APJ) IS PROTECTIVE IN MURINE MODELS OF HEREDITARY HEMORRHAGIC TELANGIECTASIA

**Topic:** 34. Bleeding disorders (congenital and acquired)

Jaime Darce*[1], Xiaofei Li[1], Peter Marek[1], Naixin Zhang[1], Zachary MacBain[1], Vasudha Sharma[2], Sriram Kollipara[3], Hitesh Soni[1], Arthur Marivin[1], Martin Marro[3], Anthony Muslin[1], John Diener[1], Peter McNamara[1]

*[1]Tectonic Therapeutic, In vivo Pharmacology, Watertown, United States of America;[2]Novartis Pharmaceuticals, Cambridge, United States of America; [3]Eli Lilly and Company, Boston, United States of America;*

### Background

Hereditary Hemorrhagic Telangiectasia (HHT) is the second most common familial bleeding disorder and results from abnormal angiogenesis caused by loss-of-function mutations in the BMP9/Endoglin/ALK1 pathway. Loss of ALK1 signaling results in increased endothelial cell proliferation, migration, and survival. The apelin (*Apln*)/APJ (*Aplnr*) signaling axis may play an important role in HHT pathogenesis because: 1) The apelin transcript is upregulated in endothelial cells isolated from multiple genetic HHT mouse models; 2) APJ signaling promotes pathological angiogenesis and vascular malformations; and 3) APJ signaling activates the PI3K/AKT pathway, which contributes to the pathology of HHT. We hypothesized that enhanced apelin/APJ signaling in HHT contributes to hypervascularization and formation of arteriovenous malformations (AVMs) that lead to epistaxis and gastro-intestinal bleeding.

### Aims

To evaluate the therapeutic potential of apelin/APJ inhibition using a potent and selective anti-mouse APJ antagonist, TX1351.

### Methods

TX1351 is a humanized VHH-Fc fusion molecule generated using Tectonic's GEODe™ platform. The in vitro potency of TX1351 to suppress apelin-mediated cAMP response, p-AKT and p-ERK was determined using CHO cells stably expressing mouse APJ. To evaluate its in vivo efficacy in suppressing the phenotypes of HHT (hypervascularization, AVM formation, bleeding, and anemia), we used two mouse models: the BMP9/BMP10-immunoblocked (ibBMP9/10) neonatal model and the inducible ALK1 knockout (ALK1-iKO) adult mouse model.

### Results

In vitro, TX1351 potently inhibited apelin mediated cAMP activation, p-AKT, and p-ERK, with low nanomolar potency. In the ibBMP9/10 HHT neonate model, TX1351 significantly reduced retinal AVMs, decreased retinal bleeding and prevented anemia, compared to pups treated with a VHH-Fc isotype control. Neutralizing VEGFA with anti-VEGFA antibody G6.31 or inhibiting AKT with a specific low molecular weight inhibitor reduced retinal AVM formation; however, unlike TX1351, these agents did not prevent the anemia associated with this model.

Similarly, both TX1351 and VEGFA neutralization significantly mitigated anemia in adult ALK1-iKO mice. However, the anti-VEGFA effect waned by Day 12, whereas TX1351 treatment durably prevented severe anemia through Day 12. Gastrointestinal (GI) bleeding was markedly reduced in the TX1351-treated group, whereas anti-VEGFA treatment did not show a protective effect in the GI tract. Furthermore, TX1351 treatment reduced hypervascularization and venous dilation in the small intestine capillary bed to a greater extent compared to anti-VEGFA–treated animals.

**Copyright Information:** (Online) ISSN: 2572-9241
©2026 The Author(s). HemaSphere is published by John Wiley & Sons Ltd on behalf of European Hematology Association. This is an open access Abstract Book distributed under the Attribution-NonCommercial-NoDerivs (CC BY-NC-ND), which allows third parties to download the articles and share them with others as long as they credit the author and the Abstract Book, but they cannot change the content in any way or use them commercially.
**Abstract Book Citations:** Authors, Title, HemaSphere, 2026;10:(S1):pages. The abstract book DOIs can be found on the "Author Information" page.
**Disclaimer:** Articles published in the journal HemaSphere exclusively reflect the opinions of the authors. The authors are responsible for all content in their abstracts including accuracy of the facts, statements, citing resources, etc.

**Summary/Conclusion**

These data support APJ signaling as a key contributor to HHT pathogenesis and suggest that pharmacologic APJ inhibition may represent a promising therapeutic approach for HHT.

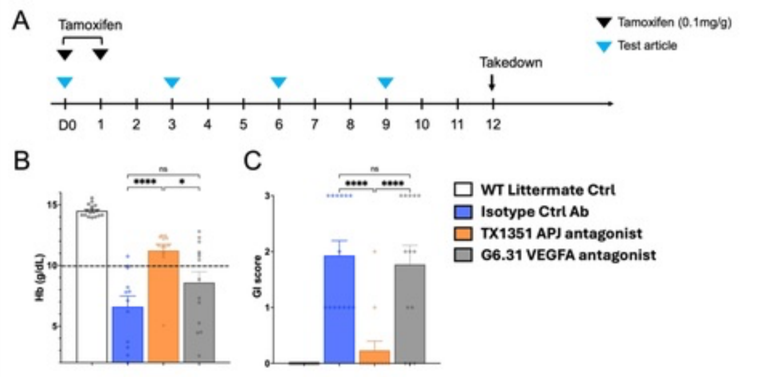

(A) Study design for in vivo efficacy on Alk1 inducible knockout (Alk1 iKO) adult mice model of HHT. (B) Hemoglobin levels at Day 12. The black dotted line indicates a hemoglobin level of 10g/dL (lower than this concentration is indicative of anemia). (C) GI score indicating GI bleeding severity at Day 12. Each data point represents one mouse. Data represent mean ± s.e.m.; N=15-17 mice. *P<0.05, **** P<0.0001, ns, no significance, one-way ANOVA with Tukey's multiple comparison.

**Copyright Information:** (Online) ISSN: 2572-9241
©2026 The Author(s). HemaSphere is published by John Wiley & Sons Ltd on behalf of European Hematology Association. This is an open access Abstract Book distributed under the Attribution-NonCommercial-NoDerivs (CC BY-NC-ND), which allows third parties to download the articles and share them with others as long as they credit the author and the Abstract Book, but they cannot change the content in any way or use them commercially.
**Abstract Book Citations:** Authors, Title, HemaSphere, 2026;10:(S1):pages. The abstract book DOIs can be found on the "Author Information" page.
**Disclaimer:** Articles published in the journal HemaSphere exclusively reflect the opinions of the authors. The authors are responsible for all content in their abstracts including accuracy of the facts, statements, citing resources, etc.

### (PS2461) EVOLVING TREATMENT PRIORITIES IN HEMOPHILIA: PHYSICIAN AND PATIENT PERSPECTIVES ON CONVENIENCE, QUALITY OF LIFE, AND THE EMERGING ROLE OF REBALANCING AGENTS ACROSS THE US AND EU5

**Topic:** 34. Bleeding disorders (congenital and acquired)

Sarah Hendry*[1], Sam Young[1]

[1]*Spherix Global Insights, Exton, United States of America;*

### Background

With multiple novel agents entering the hemophilia landscape, treatment priorities are shifting beyond bleed prevention toward holistic, patient-centered outcomes. Rebalancing agents such as *fitusiran (Qfitlia)*, *marstacimab (Hympavzi)*, and *concizumab (Alhemo)* have introduced opportunities to reduce treatment burden and better align with patient lifestyle and preference, marking a fundamental evolution in disease management.

### Aims

To examine physician perspectives across the US and EU5 on evolving treatment priorities, the growing influence of patient voice, and the impact of convenience attributes associated with rebalancing agents.

### Methods

Data were drawn from three independent Spherix Global Insights studies conducted in 2025: *Market Dynamix™ Hemophilia (US)* (n=56 hematologists), *Market Dynamix™ Hemophilia (EU5)* (n=160 hematologists across the UK, France, Germany, Italy, and Spain), and *Launch Dynamix™ (US)* (n=49 hematologists). Quantitative surveys and qualitative interviews explored management patterns, unmet needs, and drivers of adoption for recently approved or emerging rebalancing agents.

### Results

Across geographies, hematologists reported a marked increase in emphasis on *quality of life* and *daily-life convenience* in treatment selection. Over half of US and EU physicians cited treatment burden as a key determinant of therapy choice, with dosing frequency, administration route, and adherence challenges now ranked alongside efficacy. In the US, early adopters of fitusiran described substantial perceived reductions in treatment complexity, with projected patient use expected to triple within six months of launch. In Europe, enthusiasm for concizumab and marstacimab was strongest for inhibitor populations, though convenience attributes such as prefilled pens and subcutaneous delivery increasingly influenced broader patient groups. Familiarity and satisfaction with rebalancing agents rose steadily across both regions, reflecting growing clinical confidence and recognition of their alignment with patient-centric care.

### Summary/Conclusion

The emergence of rebalancing agents has accelerated a global paradigm shift toward shared decision-making and individualized care in hemophilia. As treatment expectations expand beyond bleed control to encompass lifestyle and mental well-being, convenience and patient voice have become defining measures of therapeutic success.

**Copyright Information:** (Online) ISSN: 2572-9241
©2026 The Author(s). HemaSphere is published by John Wiley & Sons Ltd on behalf of European Hematology Association. This is an open access Abstract Book distributed under the Attribution-NonCommercial-NoDerivs (CC BY-NC-ND), which allows third parties to download the articles and share them with others as long as they credit the author and the Abstract Book, but they cannot change the content in any way or use them commercially.
**Abstract Book Citations:** Authors, Title, HemaSphere, 2026;10:(S1):pages. The abstract book DOIs can be found on the “Author Information” page.
**Disclaimer:** Articles published in the journal HemaSphere exclusively reflect the opinions of the authors. The authors are responsible for all content in their abstracts including accuracy of the facts, statements, citing resources, etc.

### (PS2462) WHEN HYPOCOAGULABILITY MEETS THROMBOSIS: A SINGLE-CENTER COMPARATIVE STUDY OF CONGENITAL AND ACQUIRED PROTHROMBIN DEFICIENCY

**Topic:** 34. Bleeding disorders (congenital and acquired)

Alfadil Haroon*[1], Fatima Al Ali[1], Anwar Alkhadir[1], Arwa Almuwallad[1], Ahmed Alzaharani[2], Badr Altowerki[2], Tusneem Alhassan[1], Mahasen Al Saleh[3], Hazzaa Alzahrani[1]

*[1]Cancer Center of Excellence, King Faisal Specialist Hospital and Research Center, Adult Hematology, stem cell transplant and cellular therapy section, Riyad, Saudi Arabia; [2]Almaarefa University,College of Medicine, Riyad, Saudi Arabia;[3]King Faisal Specialist Hospital and Research Center, Pediatric Hematology - Oncology Department, Riyad, Saudi Arabia;*

### Background

Factor II (prothrombin) deficiency (FII) is a rare autosomal recessive bleeding disorder characterized by reduced plasma prothrombin activity due to quantitative hypoprothrombinaemia or qualitative dysprothrombinaemia, both of which exhibit distinct clinical phenotypes with variable bleeding and thrombotic risk. Large comparative studies evaluating these subtypes remain limited.

### Aims

To compare clinical features, bleeding and thrombotic risks, and laboratory correlations between congenital and acquired Factor II deficiency , and identify predictors that guide individualized diagnosis and management.

### Methods

This retrospective analysis included 106 patients diagnosed with FII deficiency. Cases were classified as acquired (n=65, 61.3%) or congenital (n=41, 38.7%). Correlations between FII activity levels and bleeding severity were assessed using Spearman's rank correlation and receiver operating characteristic (ROC) analysis. Bleeding-free survival was evaluated via Kaplan–Meier methods.

**Copyright Information:** (Online) ISSN: 2572-9241
©2026 The Author(s). HemaSphere is published by John Wiley & Sons Ltd on behalf of European Hematology Association. This is an open access Abstract Book distributed under the Attribution-NonCommercial-NoDerivs (CC BY-NC-ND), which allows third parties to download the articles and share them with others as long as they credit the author and the Abstract Book, but they cannot change the content in any way or use them commercially.
**Abstract Book Citations:** Authors, Title, HemaSphere, 2026;10:(S1):pages. The abstract book DOIs can be found on the "Author Information" page.
**Disclaimer:** Articles published in the journal HemaSphere exclusively reflect the opinions of the authors. The authors are responsible for all content in their abstracts including accuracy of the facts, statements, citing resources, etc.

**Results**

In the acquired cohort the median age at diagnosis was 34 (IQR: 17–52) years, 56.9% were female,liver disease in 27.7% , autoimmune diseases were present in 26.2% and malignancy in 24.6%. Bleeding manifestations occurred in 41.5% (severe in 24.6%, mild in 10.8%, moderate in 6.2%) and thrombosis in 30.8%. Thrombotic events were strongly associated with autoimmune comorbidities (OR 5.43, p=0.009; combined autoimmune and malignancy: OR 5.95, p=0.002). Thrombotic patients were older at diagnosis than those without (mean 39.6 vs. 34.0 years) with slightly higher FII levels (0.215 vs. 0.185 IU/mL). Overall thrombosis incidence across cohorts was 21.7%.
In the congenital group, 70.7% were female, 26.8% had family history of FII deficiency, with median diagnosis age of 16 (IQR: 7.0–29.5) years. Severe factor II deficiency seen in 53.7% , moderate in 29.3% and 17.1% had mild deficiency.Bleeding was severe in 41.5%, mild in 22.0%, and moderate in 7.3%, while 29.3%% did not know to have significant bleeding history. Thrombosis was seen in 7.3%.

Prophylaxis treatment included prothrombin complex concentrate (PCC) or fresh frozen plasma (FFP) in 26.8% or tranexamic acid (TXA) alone in 24.4%. Acute bleeding was managed with PCC or, FFP or FEIBA in 31.7%, TXA alone in 12.2%, or none in 65.9%. 17% underwent successful surgery that required FFP or PCC plus TXA support. Mortality was 26.2% in the acquired cohort and 7.3% in the congenital cohort (overall 19.8%).
There was no strong correlation existed between factor II activity and bleeding severity (congenital: Spearman's r = −0.260, p=0.101; acquired: r =0.131, p=0.300). ROC analysis showed limited predictive value (AUC 0.435 congenital; area under the curve (AUC) 0.591 acquired). Congenital cases had significantly reduced bleeding-free survival compared to acquired, indicating earlier and higher lifetime bleeding burden.

**Summary/Conclusion**

Factor II deficiency may manifest with either hemorrhagic or thrombotic complications. Acquired type are more commonly associated with thrombotic events ,especially when accompanied by autoimmune disorders or malignancy , whereas congenital deficiency typically presents earlier in life with more severe bleeding phenotypes, despite the well-recognized discordance between factor levels and clinical severity. These distinctions underscore the importance of individualized evaluation and management strategies that address underlying etiologies as well as the concurrent risks of bleeding and thrombosis.

**Copyright Information:** (Online) ISSN: 2572-9241
©2026 The Author(s). HemaSphere is published by John Wiley & Sons Ltd on behalf of European Hematology Association. This is an open access Abstract Book distributed under the Attribution-NonCommercial-NoDerivs (CC BY-NC-ND), which allows third parties to download the articles and share them with others as long as they credit the author and the Abstract Book, but they cannot change the content in any way or use them commercially.
**Abstract Book Citations:** Authors, Title, HemaSphere, 2026;10:(S1):pages. The abstract book DOIs can be found on the "Author Information" page.
**Disclaimer:** Articles published in the journal HemaSphere exclusively reflect the opinions of the authors. The authors are responsible for all content in their abstracts including accuracy of the facts, statements, citing resources, etc.

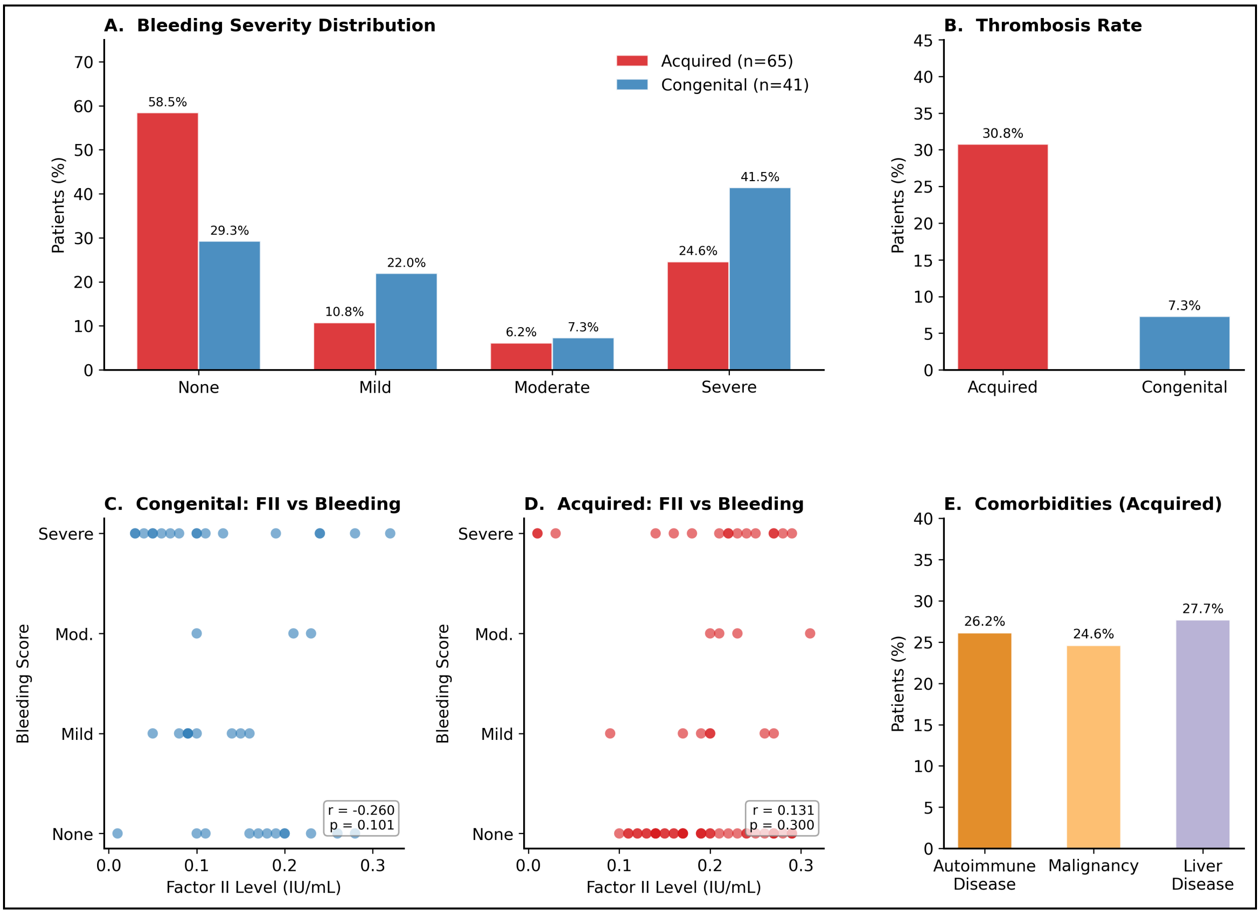

**Copyright Information:** (Online) ISSN: 2572-9241
©2026 The Author(s). HemaSphere is published by John Wiley & Sons Ltd on behalf of European Hematology Association. This is an open access Abstract Book distributed under the Attribution-NonCommercial-NoDerivs (CC BY-NC-ND), which allows third parties to download the articles and share them with others as long as they credit the author and the Abstract Book, but they cannot change the content in any way or use them commercially.
**Abstract Book Citations:** Authors, Title, HemaSphere, 2026;10:(S1):pages. The abstract book DOIs can be found on the "Author Information" page.
**Disclaimer:** Articles published in the journal HemaSphere exclusively reflect the opinions of the authors. The authors are responsible for all content in their abstracts including accuracy of the facts, statements, citing resources, etc.

### (PS2463) REDUCED ADAMTS13 ACTIVITY AND THROMBOTIC RISK IN NON-TTP CONDITIONS: A SYSTEMATIC REVIEW AND META-ANALYSIS

**Topic:** 34. Bleeding disorders (congenital and acquired)

Stephanie Quon*[1], Katherine Zheng[1], Sarah Miller[1]

[1]*University of British Columbia, Vancouver, Canada;*

### Background

Severely reduced ADAMTS13 activity is a defining feature of thrombotic thrombocytopenic purpura (TTP). Partial reductions in ADAMTS13 activity have also been described in non-TTP conditions, including malignancy, infection, autoimmune disease, and post-transplant states. Observational data suggest an association between reduced ADAMTS13 activity and thrombotic risk in these settings, but the strength and consistency of this relationship remain unclear.

### Aims

To systematically evaluate the association between reduced ADAMTS13 activity and thrombotic risk in adults with non-TTP conditions.

### Methods

We searched MEDLINE, Embase, and CINAHL from inception through June 2025 for observational studies and randomized trials reporting ADAMTS13 activity levels and venous and/or arterial thrombosis in adults without TTP. Two reviewers independently screened studies, extracted adjusted effect estimates (hazard ratios [HRs], odds ratios [ORs], or risk ratios), and assessed risk of bias using ROBINS-I. Random-effects meta-analyses were performed where appropriate, with HRs preferentially pooled for time-to-event outcomes. Heterogeneity was assessed using the $I^2$ statistic.

### Results

Twenty-two studies comprising 18,412 participants were included, spanning populations with cancer, sepsis, autoimmune disease (including systemic lupus erythematosus and antiphospholipid syndrome), solid organ transplantation, and critical illness. Definitions of reduced ADAMTS13 activity varied, most commonly using thresholds between <50% and <70% of normal. Across studies reporting time-to-event outcomes, reduced ADAMTS13 activity was associated with increased thrombotic risk compared with normal activity (pooled adjusted HR 1.78, 95% CI 1.32–2.40; $I^2 = 57\%$). Studies reporting ORs demonstrated similar associations, with adjusted ORs ranging from approximately 1.6 to 2.5. In patients with antiphospholipid antibodies, reduced ADAMTS13 activity was associated with significantly higher odds of thrombosis (adjusted OR 2.1, 95% CI 1.1–4.0). Associations were observed for both venous and arterial events. Subgroup analyses suggested stronger effects in cancer-associated thrombosis and inflammatory or autoimmune conditions. Substantial heterogeneity reflected variability in study populations and ADAMTS13 thresholds.

### Summary/Conclusion

Reduced ADAMTS13 activity outside of TTP is associated with increased thrombotic risk across diverse clinical conditions. These findings support partial ADAMTS13 deficiency as a clinically relevant prothrombotic state beyond classic TTP, with potential implications for thrombotic risk stratification in selected populations.

**Copyright Information:** (Online) ISSN: 2572-9241
©2026 The Author(s). HemaSphere is published by John Wiley & Sons Ltd on behalf of European Hematology Association. This is an open access Abstract Book distributed under the Attribution-NonCommercial-NoDerivs (CC BY-NC-ND), which allows third parties to download the articles and share them with others as long as they credit the author and the Abstract Book, but they cannot change the content in any way or use them commercially.
**Abstract Book Citations:** Authors, Title, HemaSphere, 2026;10:(S1):pages. The abstract book DOIs can be found on the “Author Information” page.
**Disclaimer:** Articles published in the journal HemaSphere exclusively reflect the opinions of the authors. The authors are responsible for all content in their abstracts including accuracy of the facts, statements, citing resources, etc.

### (PS2464) THROMBOELASTOGRAM-GUIDED BLEEDING RISK ASSESSMENT AND PERIOPERATIVE MANAGEMENT OF CONGENTIAL FACTOR VII DEFICIENCY

**Topic:** 34. Bleeding disorders (congenital and acquired)

Kai Shen*[1], Chiyi Wang[1], Chenlu Yang[1], Yuping Gong[1], Ting Niu[1]

[1]*West China Hospital, Sichuan University, Department of Hematology, Institute of Hematology, Chengdu, China;*

### Background

Congenital coagulation factor VII (FVII) deficiency is the most common rare coagulation disorder. FVII deficient patients with no bleeding histories are often incidentally identified by pre-operative coagulation screening test. Though the diagnosis of FVII deficiency is not difficult, the genetic analysis and the elucidation of the genotype-phenotype correlation is time-consuming. Many cases (namely type 2 forms) have low FVII:C but normal in vivo coagulation, which is caused by the mutated FVII's variable binding activities to different tissue thromboplastins used in vitro assays. Therefore, a reliable and fast test is needed for guiding the decision making about whether to give FVII replacement therapy in the perioperative period. “Standard” (citrated Kaolin-activated) thromboelastogram (TEG) as a whole blood clotting assay might reflect the pro-coagulability and anti-coagulability of physiological hemostasis independent of in vitro thromboplastin binding activity in patients with FVII deficiency.

### Aims

To evaluate the role of TEG in guiding FVII replacement therapy for FVII deficient patients in the perioperative period.

### Methods

We retrospectively analyzed the TEG results and outcome of FVII deficient patients who had no previous bleeding history and received surgery in West China Hospital, Sichuan University from June 2019 to November 2025.

### Results

A total of eight patients with FVII deficiency and surgery experience were identified from the hospital information system. The median age was 41 years (range, 28-67 years). Seven were female. All of them had no bleeding history and normal ISTH-BAT scores. All patients were diagnosed as congenital FVII deficiency by the preoperative coagulation screening tests shown as prolonged but correctable PT. The median PT was 30.8(24-42) seconds(sec). The median FVII:C was 5.0(1.9-7.6) %. Citrated Kaolin-activated TEG was assayed before surgery. The median TEG parameters were as follows: R time was 315(270-420)sec (reference: 300-600); K time was 73.5(60-120)sec (reference:69-180), α angle was 66.1(58.0-74.5)deg (reference: 53-72); maximum amplitude (MA) was 65.8(61.2-74.5) mm (reference: 50-70). Two patients had increased clotting factor activities demonstrated by shortened R time while two patients had increased fibrinogen function shown as higher MA value. In contrast, two patients received “rapid” TEG (using kaolin and tissue factor to accelerate the reaction) assays and both reported obviously prolonged activated clotting time of 603 and 182sec (reference: 89-121) , respectively.No patients were given FVII replacement therapy before or during surgeries including hip joint replacement and caesarean section. All patients experienced a normal recovery after the surgery. No bleeding events or thrombotic events were documented. The coagulation indices and surgery information were summarized in ***table 1***.Based on our experience, we proposed an algorithm of using TEG for the assessment of bleeding risk the guidance of replacement therapy for FVII deficiency in the perioperative setting ***(Figure 1)***. Generally, FVII replacement therapy is not needed during the surgery if TEG is normal.

**Copyright Information:** (Online) ISSN: 2572-9241
©2026 The Author(s). HemaSphere is published by John Wiley & Sons Ltd on behalf of European Hematology Association. This is an open access Abstract Book distributed under the Attribution-NonCommercial-NoDerivs (CC BY-NC-ND), which allows third parties to download the articles and share them with others as long as they credit the author and the Abstract Book, but they cannot change the content in any way or use them commercially.
**Abstract Book Citations:** Authors, Title, HemaSphere, 2026;10:(S1):pages. The abstract book DOIs can be found on the “Author Information” page.
**Disclaimer:** Articles published in the journal HemaSphere exclusively reflect the opinions of the authors. The authors are responsible for all content in their abstracts including accuracy of the facts, statements, citing resources, etc.

### Summary/Conclusion

“Standard” (citrated Kaolin-activated) TEG is a reliable tool for FVII deficient patient in the perioperative bleeding risk assessment. FVII replacement is not needed for FVII deficient patients receiving surgeries if R time is not prolonged in TEG which indicates normal clotting factor function.

**Table 1. TEG parameters and outcomes of FVII deficient patients receiving invasive procedures**

| No. | Gender | Age (years) | PT (10-13s) | FVII:C (%) | TEG R time (300-600s) | TEG K time (60-180s) | TEG α angle (53-72deg) | TEG MA (50-70mm) | Invasive procedure | FVII replacement | Bleeding |
|---|---|---|---|---|---|---|---|---|---|---|---|
| 1 | F | 33 | 36.5 | 1.9 | 325 | 60 | 74.5 | 74.5 | Caesarean section | No | No |
| 2 | F | 49 | 24 | 7.4 | 260 | 110 | 65.9 | 64.9 | Hip joint replacement | No | No |
| 3 | M | 51 | 33 | 5.2 | 355 | 120 | 62.8 | 62.8 | Laparoscopic cholecystectomy | No | No |
| 4 | F | 28 | 38 | 2.5 | 270 | 100 | 58.0 | 63.3 | Blepharoplasty | No | No |
| 5 | F | 52 | 28 | 4.4 | 420 | 70 | 63.4 | 66.7 | Intrauterine device removal | No | No |
| 6 | F | 24 | 26 | 5.8 | 330 | 72 | 67.6 | 72.7 | Induced abortion | No | No |
| 7 | F | 20 | 42 | 4.7 | 272 | 66 | 66.2 | 61.2 | Scalp mass excision | No | No |
| 8 | F | 67 | 28.5 | 7.6 | 305 | 75 | 72.3 | 67.9 | Laparotomic pancreatic tumor resection | No | No |

**Figure 1. Proposed algorithm for perioperative management of FVII deficiency using TEG**

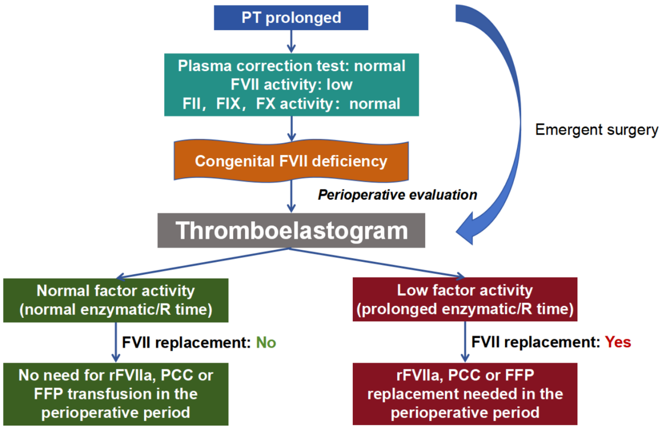

Copyright Information: (Online) ISSN: 2572-9241
©2026 The Author(s). HemaSphere is published by John Wiley & Sons Ltd on behalf of European Hematology Association. This is an open access Abstract Book distributed under the Attribution-NonCommercial-NoDerivs (CC BY-NC-ND), which allows third parties to download the articles and share them with others as long as they credit the author and the Abstract Book, but they cannot change the content in any way or use them commercially.
Abstract Book Citations: Authors, Title, HemaSphere, 2026;10:(S1):pages. The abstract book DOIs can be found on the “Author Information” page.
Disclaimer: Articles published in the journal HemaSphere exclusively reflect the opinions of the authors. The authors are responsible for all content in their abstracts including accuracy of the facts, statements, citing resources, etc.

### (PS2465) ANTIANGIOGENIC TREATMENT OF PATIENTS WITH HEREDITARY HEMORRHAGIC TELANGIECTASIA - EXPERIENCE OF A HUNGARIAN CENTER

**Topic:** 34. Bleeding disorders (congenital and acquired)

Boglarka Csilla Brugos*[1], Tamás Major[2], Zsuzsanna Bereczky[3], Réka Gindele[3], Gábor Balogh[1], Sándor Kacska[1], Péter Sipos[1], Arpád Illés[1], György Pfliegler[1]

*[1]University of Debrecen, Internal Medicine Division of Hematology, Debrecen, Hungary; [2]Markhot Ferenc Teaching Hospital, Otorhinolaryngology, Debrecen, Hungary; [3]University of Debrecen, Division of Clinical Laboratory Science, Debrecen, Hungary;*

### Background

Hereditary hemorrhagic telangiectasia (HHT) is an inherited vascular bleeding disorder. The most common symptoms are recurrent, severe nosebleeds that occasionally necessitate intervention by an ENT (Ear, Nose, and Throat) specialist, as well as iron-deficiency anemia. Telangiectasia is typically located in the nasal cavity, lips, tongue, fingertips, and the gastrointestinal mucosa. Arteriovenous malformations (AVMs) are located in internal organs (brain, lungs, liver, etc.). The family history is positive for HHT. The diagnosis is based on the *Curacao* criteria. The endoglin and activin receptor-like kinase 1 genes (*ENG* and *ACVRL1*) are the most common mutation sites, leading to elevated endothelial growth factor (VEGF) levels.

### Aims

To find out the efficacy of different antiangiogenic treatments on Epistaxis Severity Score (ESS) of the patients treated with HHT.

### Methods

We conducted a retrospective analysis in the Department of Internal Medicine, Division of Hematology, and Center of Expertise for Rare Diseases at the University of Debrecen, spanning the period from 2010 to 2025. Records of patients referred with HHT were reviewed concerning demographic data, clinical presentations, laboratory findings, and treatment approaches. To evaluate management options, epistaxis severity was assessed using ESS.

### Results

We diagnosed and followed 48 HHT patients (21 male and 27 female). Genetic testing was positive in each case, showing mutations either in the *ENG* or *ACVRL1* genes. Most of the patients are followed in our department. Seven patients were administered desmopressin, a synthetic analog of antidiuretic hormone (ADH), based on our previous experience in reducing bleeding in von Willebrand disease. Angiogenic, thalidomide was used in 24 patients (50 mg oral tablets), while bevacizumab was administered to five patients. Most patients experienced a remarkable decrease in epistaxis severity and a reduction in the need for transfusions (ESS before treatment: HHT1 patients 4.15±1.91 *vs.* ESS after treatment 2.62±0.99; HHT2 patients 3.79±3.19 *vs.* 2.02±1.91). Subgroup analysis using paired ESS data showed a significant reduction in ESS in both HHT1 and HHT2 patients (p=0.003 and p=0.043, respectively).

### Summary/Conclusion

HHT is a rare vascular bleeding disorder. Daily nosebleeds impair the patients ' quality of life and sometimes lead to severe transfusion-dependent iron-deficient anemia. Frequent hospitalization places a significant burden on the healthcare system. Thus, we have used treatment options for HHT patients that primarily act by inhibiting VEGF, and these treatment modalities have yielded successful results in our hands.

**Copyright Information:** (Online) ISSN: 2572-9241
©2026 The Author(s). HemaSphere is published by John Wiley & Sons Ltd on behalf of European Hematology Association. This is an open access Abstract Book distributed under the Attribution-NonCommercial-NoDerivs (CC BY-NC-ND), which allows third parties to download the articles and share them with others as long as they credit the author and the Abstract Book, but they cannot change the content in any way or use them commercially.
**Abstract Book Citations:** Authors, Title, HemaSphere, 2026;10:(S1):pages. The abstract book DOIs can be found on the "Author Information" page.
**Disclaimer:** Articles published in the journal HemaSphere exclusively reflect the opinions of the authors. The authors are responsible for all content in their abstracts including accuracy of the facts, statements, citing resources, etc.

### (PS2466) ISTH BLEEDING SCORE (ISTH-BAT) IN DIAGNOSTIC PROFILE OF BLEEDING PATIENTS RELATED TO SCORING IN CLINICAL DIAGNOSIS PRACTICE 2015-2025 IN TWO CENTRES IN ESTONIA

**Topic:** 34. Bleeding disorders (congenital and acquired)

Ines Vaide*[1, 2, 3], Virge Aabrams[1], Emilia Ursu[4], Oskar Raudne[5], Edward Laane[2, 3], Robert Klamroth[6]

*[1]Internal Medicin Clinic, Hematology Outpatient department, Pärnu Hospital, Pärnu, Estonia;[2]Internal Medicin Clinic, Hematology Outpatient department, Kuressaare Hospital, Kuressaare, Estonia; [3]Department of HematoOncology, Institute of Clinical Medicine, University of Tartu, Estonia, Tartu, Estonia; [4]Onco-Haematology Research Unit, Romanian Academy of Medical Sciences, Timisoara, Romania, Timisoara, Romania; [5]Tartu University, Tartu, Estonia; [6]Vivantes Klinikum im Friedrichshain, Innere Medizin - Angiologie und Hämostaseologie, Berlin, Germany;*

### Background

Bleeding assessment by patients is inherently subjective and may lead to under- or overestimation of symptom severity. Therefore, in the differential diagnosis of patients presenting with bleeding symptoms, the use of a standardized bleeding questionnaire is recommended to objectively quantify and systematize bleeding manifestations. The ISTH Bleeding Assessment Tool (ISTH-BAT) is widely used for this purpose. In 2018, the ISTH-BAT was validated for use in Estonian clinical settings at North Estonian Medical Centre.

### Aims

This study aims to conduct a retrospective evaluation of routine clinical quality assessment by analysing patients diagnosed between 2015 and 2025 in two centres, focusing on individuals with ICD-10 codes D68.0–D69.9. The objectives are, firstly, to evaluate the use of the ISTH-BAT in this patient population and, secondly, to analyse the distribution of specific coagulation disorders among these patients

### Methods

Routine clinical quality assessment was evaluated. Patients with ICD-10 codes D68.0–D69.9 diagnosed between 2015 and 2025 were identified. Data were collected on the availability of ISTH-BAT scores and the final confirmed coagulation diagnoses.

### Results

A total of 242 patients were included. ISTH-BAT was completed in 167 patients, with scores ranging from 1 to 19. Among these, 123 patients had unspecified bleeding disorders, 62 patients were diagnosed with von Willebrand disease, and 57 patients had other rare coagulation disorders, including dysfibrinogenemia (2 cases), hypofibrinogenemia (11 cases), FVIII deficiency (1 case), FVIII conductorin (2 cases), rare factor deficiencies (factor V: 8, factor X: 6, factor II: 2, factor XI: 1, factor XII: 4, factor VII: 10, multiple factor deficiencies: 5, thrombocytopathy: 4 , and FXIII deficiency: 1 ).

### Summary/Conclusion

Over the 10-year period, the use of ISTH-BAT has become established in clinical practice. More than 50% of patients in this cohort had unspecified bleeding disorders, highlighting that a substantial proportion of patients remain without a definitive diagnosis and may still be awaiting further evaluation. This underscores the limitations of current routine laboratory testing and emphasizes the need for improved and more reliable diagnostic tools to identify underlying bleeding disorders.

**Copyright Information:** (Online) ISSN: 2572-9241
©2026 The Author(s). HemaSphere is published by John Wiley & Sons Ltd on behalf of European Hematology Association. This is an open access Abstract Book distributed under the Attribution-NonCommercial-NoDerivs (CC BY-NC-ND), which allows third parties to download the articles and share them with others as long as they credit the author and the Abstract Book, but they cannot change the content in any way or use them commercially.
**Abstract Book Citations:** Authors, Title, HemaSphere, 2026;10:(S1):pages. The abstract book DOIs can be found on the "Author Information" page.
**Disclaimer:** Articles published in the journal HemaSphere exclusively reflect the opinions of the authors. The authors are responsible for all content in their abstracts including accuracy of the facts, statements, citing resources, etc.

### (PS2467) CLINICAL UTILITY OF ENDOGENOUS COLONY FORMATION ASSAY AND RISK OF THROMBOSIS IN MPNS

**Topic:** 35. Thrombosis and vascular biology

Danijela Lekovic*[1, 2], Dijana Sefer[1], Isidora Arsenovic[1], Andjela Nikolic[1], Andrijana Kojic[1], Mihailo Smiljanic[1], Natasa Čolović[1, 2], Andrija Bogdanovic[1, 2]

*[1]Clinic of Hematology, University Clinical Center Serbia, Belgrade,Serbia, Belgrade, Serbia;[2]Faculty of Medicine, University of Belgrade, Belgrade, Serbia;*

### Background

Analysis of endogenous colony formation had previosly a diagnostic significance. Patients with MPNs suffer from many debilitating thrombotic complications with cardiovascular events being the leading cause of morbidity and mortality.

### Aims

To analyze the association between the presence of spontaneous colony formation and the development of thrombosis in MPNs.

### Methods

The group included 1000 consecutive MPN patients (PV 644 pts (64.4%); ET 119 (11.9%); PMF 237 (23.7%) who were diagnosed according to the actual WHO criteria during 2 decades in single university center with median survival of 174 months (95% CI 173-236). We collected at the time of diagnosis clinical-laboratory data, *JAK2V617F* mutation status, Charlson comorbidity index CCI, results of *in vitro* cell culture of hematopoiesis precursors such as spontaneous growth of BFU-E, CFU-E and CFU-Mk from bone marrow samples while during the follow up thrombosis development was monitored.

### Results

Study group consisted of 53,5% of females with median age 65 years (range 14-87). Thrombosis during follow up developed 107 MPN pts (10.7%) distributed according to type as follows: PV 93 pts (87%), ET 4 pts (3.7%) and PMF 10 pts (9.3%). The frequency of spontaneous growth of BFU-E was observed in 79.8%, CFU-E in 13.2%, and CFU-MK in 20% of patients. The prevalence according to the number of present colonies was as follows: 58.6% of patients had one type of spontaneous colony growth, 27.3% had two types of spontaneous colonies, 3.3% had three types, while no spontaneous growth was observed in 10.8% of patients. The presence of spontaneously formed colonies of CFU-Mk (p=0.014) as well as the presence of 2 or more colonies (p=0.039) carried a significantly higher risk of developing thrombosis in ET. Spontaneous growth of CFU-MK was associated with a higher rate of thrombosis development in PV (p=0.038) and bordeline in PMF (p=0.052). A high statistical correlation was observed between both the individual spontaneus colony types present as well as with a number of present colonies with the *JAK2V617F* mutation (p<0.001). The distribution according to the severity of CCI was as follows: mild comorbidities had 38.5% patents, moderate 29.3%, severe 18.2% while 14% of MPN patients were without comorbidities. There was a significant positive correlation between the number of spontaneous colony formation and the severity of CCI, patients with higher number of spontaneous colonies had also higher degree of CCI (p<0.001).

**Copyright Information:** (Online) ISSN: 2572-9241
©2026 The Author(s). HemaSphere is published by John Wiley & Sons Ltd on behalf of European Hematology Association. This is an open access Abstract Book distributed under the Attribution-NonCommercial-NoDerivs (CC BY-NC-ND), which allows third parties to download the articles and share them with others as long as they credit the author and the Abstract Book, but they cannot change the content in any way or use them commercially.
**Abstract Book Citations:** Authors, Title, HemaSphere, 2026;10:(S1):pages. The abstract book DOIs can be found on the "Author Information" page.
**Disclaimer:** Articles published in the journal HemaSphere exclusively reflect the opinions of the authors. The authors are responsible for all content in their abstracts including accuracy of the facts, statements, citing resources, etc.

**Summary/Conclusion**

Patients with the present spontaneous growth of CFU-Mk colony had a higher risk of thrombosis which is related to the additive effect of thrombocytosis in the development of thrombotic complications. Association between spontaneous colony formation and degree of comorbidity, indicates cytokine-independent growth, which directly reflects the hyperproliferation of the malignant clone in MPNs and induce hypercoaguability with higher risk of thrombosis.

**Copyright Information:** (Online) ISSN: 2572-9241
©2026 The Author(s). HemaSphere is published by John Wiley & Sons Ltd on behalf of European Hematology Association. This is an open access Abstract Book distributed under the Attribution-NonCommercial-NoDerivs (CC BY-NC-ND), which allows third parties to download the articles and share them with others as long as they credit the author and the Abstract Book, but they cannot change the content in any way or use them commercially.
**Abstract Book Citations:** Authors, Title, HemaSphere, 2026;10:(S1):pages. The abstract book DOIs can be found on the "Author Information" page.
**Disclaimer:** Articles published in the journal HemaSphere exclusively reflect the opinions of the authors. The authors are responsible for all content in their abstracts including accuracy of the facts, statements, citing resources, etc.

### (PS2468) FC-GAMMA-RIIA-MEDIATED PROCOAGULANT PLATELETS ARE IMPORTANT DRIVERS OF IMMUNOTHROMBOSIS IN APS

**Topic:** 35. Thrombosis and vascular biology

Jan Zlamal[1], Carolin Schnaidt[1], Karina Althaus[1], Günalp Uzun*[1], Heiko Rühl[2], Tamam Bakchoul[1]

*[1]Institute for Clinical and Experimental Transfusion Medicine, Tuebingen, Germany;[2]Institute of Experimental Hematology and Transfusion Medicine, Bonn, Germany;*

**Background**

Antiphospholipid syndrome (APS) is a prothrombotic autoimmune disease characterized by recurrent thrombosis and obstetrical complications. Autoantibodies (AAbs) that are targeting phospholipids and phospholipid-binding proteins are well characterized for being key drivers of the multicellular-driven prothrombotic condition in APS. Despite antithrombotic therapy is the mainstay in APS therapy, onset of thromboembolic events or anticoagulant-related bleeding can be frequently observed. Therefore, a better understanding of the exact pathomechanisms leading to the increased prothrombotic potential in APS is urgently needed.

**Aims**

To investigate the impact of APS AAbs on the formation of different PLT subpopulations and their potential relevance in thrombus formation ex vivo.

**Methods**

For the investigation of APS patient AAb-mediated PLT alterations, a flow cytometry (FC)-based procoagulant PLT assay was employed. To study the impact of APS AAb-mediated PLT changes on thrombus formation, a microfluidic ex vivo model of antibody-mediated immunothrombosis that utilizes tetra straining was used.

**Results**

82 sera from APS patients with a history of thrombosis were tested in this study. FC analysis revealed that a subgroup of 38/82 (46%) patient sera induced significant formation of (CD62p/phosphatidylserine [PS] double positive) procoagulant PLTs while such alterations were not observed in PLTs from healthy individuals upon incubation with controls. APS AAb-mediated procoagulant PLT formation was identified for being PLT Fc-gamma-RIIA (FcγRIIA)-dependent as specific FcγRIIA blockade via monoclonal Ab IV.3, intravenous immunoglobulin (IVIG) or inhibition of FcγRIIA-signaling downstream via spleen tyrosine kinase (SYK) inhibitor Lanraplenib resulted in a near complete abrogation of APS AAb-induced procoagulant PLT formation in vitro. Most importantly, via the utilization of an ex vivo model of antibody-mediated thrombosis APS AAb-induced procoagulant PLTs were identified for being critical mediators of immunothrombosis. Compared to control, spike in of APS patient serum or corresponding immunoglobulin G (IgG) isolates into healthy whole blood resulted in dramatic formation of immunothrombi that were characterized by high amounts of procoauglant PLTs and high numbers of leukocytes that were recruited into the growing thrombus. Importantly, and of potential therapeutic interest, APS AAb-induced procoagulant PLT as well as immunothrombosis were significantly abrogated in the presence of intravenous immunoglobulin (IVIG) or Lanraplenib.

**Copyright Information:** (Online) ISSN: 2572-9241
©2026 The Author(s). HemaSphere is published by John Wiley & Sons Ltd on behalf of European Hematology Association. This is an open access Abstract Book distributed under the Attribution-NonCommercial-NoDerivs (CC BY-NC-ND), which allows third parties to download the articles and share them with others as long as they credit the author and the Abstract Book, but they cannot change the content in any way or use them commercially.
**Abstract Book Citations:** Authors, Title, HemaSphere, 2026;10:(S1):pages. The abstract book DOIs can be found on the "Author Information" page.
**Disclaimer:** Articles published in the journal HemaSphere exclusively reflect the opinions of the authors. The authors are responsible for all content in their abstracts including accuracy of the facts, statements, citing resources, etc.

**Summary/Conclusion**

Our studies indicate that a subgroup of APS patient AAbs induce the formation of a procoagulant PLT phenotype with dramatic prothrombotic potential in a PLT FcγRIIA-dependent manner .The observation that the presence of IVIG or SYK inhibitor Lanraplenib rescued from APS AAb-mediated procoagulant PLT and increased thrombus formation directs towards a therapeutic potential of such Fc-gamma-receptor-targeting therapeutics to prevent the onset of thromboembolic events in APS.

**Copyright Information:** (Online) ISSN: 2572-9241
©2026 The Author(s). HemaSphere is published by John Wiley & Sons Ltd on behalf of European Hematology Association. This is an open access Abstract Book distributed under the Attribution-NonCommercial-NoDerivs (CC BY-NC-ND), which allows third parties to download the articles and share them with others as long as they credit the author and the Abstract Book, but they cannot change the content in any way or use them commercially.
**Abstract Book Citations:** Authors, Title, HemaSphere, 2026;10:(S1):pages. The abstract book DOIs can be found on the "Author Information" page.
**Disclaimer:** Articles published in the journal HemaSphere exclusively reflect the opinions of the authors. The authors are responsible for all content in their abstracts including accuracy of the facts, statements, citing resources, etc.

### (PF2469) PREDICTIVE ROLE OF ENDOTHELIAL-DERIVED EXTRACELLULAR VESICLES IN RECURRENT VENOUS THROMBOEMBOLISMPREDICTIVE ROLE OF ENDOTHELIAL-DERIVED EXTRACELLULAR VESICLES IN RECURRENT VENOUS THROMBOEMBOLISM

**Topic:** 35. Thrombosis and vascular biology

Aashal Shah*[1], Fayaz Ahmed Mubarak Ali[2]

*[1]GMERS Medical College and Civil Hospital, Pharmacology, Valsad, India;[2]Guwahati Medical College and Hospital, Pathology, Guwahati, India;*

### Background

Despite appropriate anticoagulant treatment, recurrent venous thromboembolism (VTE) remains a major clinical challenge. D-dimer testing and clinical predictors are used to stratify the risk. Though useful, these tools are not precise enough to guide the individualized anticoagulation duration. Endothelial dysfunction plays a key role in thrombus formation. Circulating endothelial-derived extracellular vesicles (EEVs) are released due to endothelial activation and injury. Though they have procoagulant properties, their role as predictive biomarkers for VTE recurrence has not been fully investigated.

### Aims

The study was conducted with an aim to evaluate whether circulating EEV levels assessed post initial anticoagulant treatment can independently predict recurrent VTE.

### Methods

This was a prospective cohort study conducted for a duration of two years at a tertiary care hospital in India. Adult patients with first episode of objectively confirmed deep vein thrombosis (DVT) and/or pulmonary embolism (PE) were included in the study. When standard coagulation was completed, baseline assessments were done including EEV quantification, D-dimer measurement and clinical risk scoring. Nanoparticle tracking analysis and high-sensitivity flow cytometry were used to identify EEVs. Vesicles were defined as $CD31^+/CD144^+/CD45^-$ particles <1 µm in size. Tissue factor expression and annexin V binding were used to assess procoagulant activity. Patients were followed up for 18 months to objectively confirm recurrent VTE. The primary outcome was time to recurrence. Multivariable Cox regression analysis was done. P<0.05 was considered significant.

### Results

Total 112 patients completed follow-up. Incidence of recurrent VTE was 16.07% (n=18). There was significantly higher level of EEVs at baseline in patients who developed VTE recurrence as compared to those who didn't (P<0.01). In multivariable analysis, elevated EEV levels were independently associated with recurrence (hazard ratio [HR] 2.76; 95% CI: 1.54 – 4.918; P = 0.002). It was significant even after adjusting for D-dimer positivity and unprovoked VTE. When EEV levels were added to a clinical model including D-dimer, predictive discrimination was improved as evident by increase in C-index from 0.67 to 0.78. Net reclassification improvement was 22% (P = 0.01). Highest recurrence rate (27.68%) was seen in patients with both high EEVs and high D-dimer and low rates of 5.37% was seen in patients with low levels of both markers.

**Copyright Information:** (Online) ISSN: 2572-9241
©2026 The Author(s). HemaSphere is published by John Wiley & Sons Ltd on behalf of European Hematology Association. This is an open access Abstract Book distributed under the Attribution-NonCommercial-NoDerivs (CC BY-NC-ND), which allows third parties to download the articles and share them with others as long as they credit the author and the Abstract Book, but they cannot change the content in any way or use them commercially.
**Abstract Book Citations:** Authors, Title, HemaSphere, 2026;10:(S1):pages. The abstract book DOIs can be found on the "Author Information" page.
**Disclaimer:** Articles published in the journal HemaSphere exclusively reflect the opinions of the authors. The authors are responsible for all content in their abstracts including accuracy of the facts, statements, citing resources, etc.

**Summary/Conclusion**

The study concludes that EEVs are independently associated with recurrent VTE and significantly enhance risk prediction beyond established clinical models and D-dimer testing. Thus, EEV quantification may guide personalized anticoagulation duration strategies. Further larger studies to validate the findings and standardize the measurement techniques are required to incorporate EEV levels measurement in routine practice.

**Copyright Information:** (Online) ISSN: 2572-9241
©2026 The Author(s). HemaSphere is published by John Wiley & Sons Ltd on behalf of European Hematology Association. This is an open access Abstract Book distributed under the Attribution-NonCommercial-NoDerivs (CC BY-NC-ND), which allows third parties to download the articles and share them with others as long as they credit the author and the Abstract Book, but they cannot change the content in any way or use them commercially.
**Abstract Book Citations:** Authors, Title, HemaSphere, 2026;10:(S1):pages. The abstract book DOIs can be found on the “Author Information” page.
**Disclaimer:** Articles published in the journal HemaSphere exclusively reflect the opinions of the authors. The authors are responsible for all content in their abstracts including accuracy of the facts, statements, citing resources, etc.

### (PF4573) REAL-WORLD DATA ON DOAC USE IN CHILDREN: AN UPDATE FROM THE INTERNATIONAL THROM-PED DOAC REGISTRY

**Topic:** 35. Thrombosis and vascular biology

Susanne Holzhauer*[1], Bhat Rukhmi[2], Ruben Berrueco[3], Mohammad Bordbar[4], Marcela Torres[5], Christoph Male[6], Paul Monagle[7], Brian Branchford[8], Julie Jaffray[9], Katerina Zdrahalova[10, 10], Idske Kremer Hovinga[11], Riten Kumar[12], Shoshana Revel-Vilk[13], Surbhi Saini[14], Lakshmi Srivaths[15], Mindy Simpson[16], Tatyana Yafoshkina[17], Maua Alleyne[18], Ernest Amankwah[19], C.Heleen van Ommen[20], Leslie Raffini[21]

*[1]Charité University Medicine, Pediatric Hematology and Oncology, Berlin, Germany;[2]Luries Children`s Hospital, Chicago, United States of America; [3]Sant Joan de Déu Children's Hospital, Pediatric Hematology, Barcelona, Spain;[4]Shiraz University of Medical Sciences, Shiraz, Iran; [5]Cook Children's Medical Center, Fort Worth, United States of America;[6]Medizinische Universität Wien, Wien, Austria; [7]Murdoch Children's Research Institute, Melbourne, Australia; [8]Versiti Medical Sciences Institute, Medical College of Wisconsin, Milwaukee, United States of America; [9]Rady Children's Specialists of San Diego, San Diego, United States of America; [10]University Hospital Motol (FNM), Prague, Czechia; [11]University Medical Center Utrecht, Utrecht, The Netherlands; [12]Dana-Farber/Boston Children's Cancer and Blood Disorders Center, Boston, United States of America; [13]Pediatric Hematology Oncology Unit, Shaare Zedek Medical Center, Jerusalem, Israel; [14]St .Louis Children's Specialty Center, St. Louis, United States of America; [15]Children's Memorial Hermann Hospital, Houston, United States of America; [16]RUSH University Medical Group, Chicago, United States of America; [17]Rogachev National Medical Research Center of Pediatric Hematology, Oncology and Immunology, Moskow, Russia; [18]Johns Hopkins All Children's Hospital, St. Petersburg, United States of America;[19]John Hopkins All Children, St. Petersburg, United States of America; [20]Erasmus Medical Center, Rotterdam, The Netherlands;[21]Children`s Hospital of Philadelphia, Philadelphia, United States of America;*

### Background

Direct oral anticoagulants (DOACs) have been approved for treating pediatric thromboembolism. However, neonates and young children remain underrepresented in clinical trials, and real-world data on DOAC safety and effectiveness in these age groups are limited.

### Aims

To evaluate the effectiveness and safety of DOACs in children, particularly those under 2 years, and to examine treatment strategies and outcomes across varying risk profiles and comorbidities.

### Methods

*Study design:* An international, multicenter, prospective cohort study embedded in the Throm-PED Registry of the International Pediatric Thrombosis Network (IPTN). Data collection included demographics, risk factors, type/location of thrombosis and treatment information (drug and dose). Primary outcomes were thrombus progression/recurrence, bleeding events (major and clinically relevant non-major), and mortality, assessed quarterly for the duration of anticoagulation or up to 12 months.
*Study population:* Participants were aged 0–18 years with acute thromboembolic disease treated with DOACs and enrolled in the IPTN Throm-PED Registry.

**Copyright Information:** (Online) ISSN: 2572-9241
©2026 The Author(s). HemaSphere is published by John Wiley & Sons Ltd on behalf of European Hematology Association. This is an open access Abstract Book distributed under the Attribution-NonCommercial-NoDerivs (CC BY-NC-ND), which allows third parties to download the articles and share them with others as long as they credit the author and the Abstract Book, but they cannot change the content in any way or use them commercially.
**Abstract Book Citations:** Authors, Title, HemaSphere, 2026;10:(S1):pages. The abstract book DOIs can be found on the "Author Information" page.
**Disclaimer:** Articles published in the journal HemaSphere exclusively reflect the opinions of the authors. The authors are responsible for all content in their abstracts including accuracy of the facts, statements, citing resources, etc.

**Results**

As of September 30th, 2025, 1167 patients (142 <2 years) from 30 centers were enrolled, Table 1. Most patients received rivaroxaban (85.4 %). Nearly 50% of patients < 2 years had a central venous catheter related thrombosis. Among 878 patients with ≥3 months of follow-up, new thrombotic events and progression rates were low (0.43 events/1,000 patient-days for <2 years; 0.41 events/1,000 patient-days for older children). Bleeding was reported in 2% of patients, with higher rates in those <2 years (0.43 vs. 0.21 events/1,000 patient-years). There were 4 deaths reported, not related to thrombosis or therapy

**Summary/Conclusion**

DOACs, primarily rivaroxaban, demonstrated safety and effectiveness for treating venous thromboembolism in children, including those under 2 years. These real-world data, which represent the largest cohort of children < 2 years treated with a DOAC confirm trial results, supporting DOAC use in young pediatric populations.

**Copyright Information:** (Online) ISSN: 2572-9241
©2026 The Author(s). HemaSphere is published by John Wiley & Sons Ltd on behalf of European Hematology Association. This is an open access Abstract Book distributed under the Attribution-NonCommercial-NoDerivs (CC BY-NC-ND), which allows third parties to download the articles and share them with others as long as they credit the author and the Abstract Book, but they cannot change the content in any way or use them commercially.
**Abstract Book Citations:** Authors, Title, HemaSphere, 2026;10:(S1):pages. The abstract book DOIs can be found on the "Author Information" page.
**Disclaimer:** Articles published in the journal HemaSphere exclusively reflect the opinions of the authors. The authors are responsible for all content in their abstracts including accuracy of the facts, statements, citing resources, etc.

### (PS2470) IDENTIFICATION OF CLEC4E AS A FUNCTIONAL PATTERN RECOGNITION RECEPTOR MEDIATING Β-GLUCOSYLCERAMIDE–INDUCED PLATELET ACTIVATION VIA SYK SIGNALING

**Topic:** 35. Thrombosis and vascular biology

Xuemei Jia*[1], Zhihan Chen[2], Hong Cheng[3], Yin Tong[1], Xianmin Song[1]

*[1]Shanghai General Hospital, ShanghaiJiao Tong University School of Medicine, Department of Hematology, shanghai, China; [2]Fudan University, shanghai, China; [3]Huashan Hospital, Fudan University, Department of Blood Transfusion,, shanghai, China;*

### Background

Platelets express multiple pattern recognition receptors (PRRs) that critically regulate their hemostatic and immunothrombotic functions. Members of the C-type lectin (CLEC) family constitute an important subgroup of PRRs. Among them, CLEC2 and CLEC7A (Dectin-1) have been confirmed to be expressed on platelets, and their activation enhances platelet functional responses. β-glucosylceramide (β-GlcCer) is an endogenous glycosphingolipid metabolite that physiologically exists in humans but is elevated under certain pathological conditions, such as in the plasma of patients with Gaucher disease. CLEC4E (also known as Mincle) has been proposed as a receptor for β-GlcCer in other cell types; however, its expression and functional role in platelets have not been previously reported.

### Aims

This study aimed to determine whether CLEC4E is expressed in platelets and to investigate whether its activation regulates platelet function and to elucidate the underlying molecular mechanisms.

### Methods

Peripheral blood was collected from healthy donors, and washed platelets were isolated for subsequent experiments. Protein expression of CLEC4E was assessed by immunoblotting, and mRNA expression was evaluated by RT-PCR. Immunofluorescence microscopy was performed to determine the subcellular localization of CLEC4E in platelets.
To assess the functional impact of CLEC4E activation, platelets were pre-incubated for 5 minutes with the CLEC4E agonists trehalose-6,6-dibehenate (TDB) and β-GlcCer, followed by evaluation of platelet aggregation and spreading. To delineate downstream signaling mechanisms, co-immunoprecipitation assays were performed to identify proteins interacting with CLEC4E.

### Results

We demonstrate for the first time that CLEC4E is expressed in human platelets (Figure 1). Pharmacological activation of CLEC4E with TDB or β-GlcCer significantly enhanced platelet activation, as evidenced by increased aggregation (Figure 2A, 2D) and spreading (Figure 2B-C, Figure 2E-F).
Incubation of human platelets with β-GlcCer for 5 minutes led to an increase in surface expression of CLEC4E (Figure 3A) and enhanced phosphorylation of spleen tyrosine kinase (Syk) (Figure 3B). Co-immunoprecipitation assays further revealed a physical interaction between CLEC4E and Syk in human platelets (Figure 3C&D). Importantly, blockade of CLEC4E with a specific neutralizing antibody abolished β-GlcCer-induced potentiation of platelet activation (Figure 3E). These findings indicate that β-GlcCer promotes platelet activation via the CLEC4E/Syk signaling axis.

**Copyright Information:** (Online) ISSN: 2572-9241
©2026 The Author(s). HemaSphere is published by John Wiley & Sons Ltd on behalf of European Hematology Association. This is an open access Abstract Book distributed under the Attribution-NonCommercial-NoDerivs (CC BY-NC-ND), which allows third parties to download the articles and share them with others as long as they credit the author and the Abstract Book, but they cannot change the content in any way or use them commercially.
**Abstract Book Citations:** Authors, Title, HemaSphere, 2026;10:(S1):pages. The abstract book DOIs can be found on the "Author Information" page.
**Disclaimer:** Articles published in the journal HemaSphere exclusively reflect the opinions of the authors. The authors are responsible for all content in their abstracts including accuracy of the facts, statements, citing resources, etc.

### Summary/Conclusion

Our study identifies CLEC4E as a previously unrecognized PRR expressed on platelets. Activation of CLEC4E by its classical agonist TDB or by β-GlcCer enhances platelet activation. Mechanistically, β-GlcCer promotes platelet activation through the CLEC4E/Syk signaling pathway. These findings provide novel insights into glycosphingolipid-mediated regulation of platelet function and expand the repertoire of immunoreceptors involved in platelet activation.

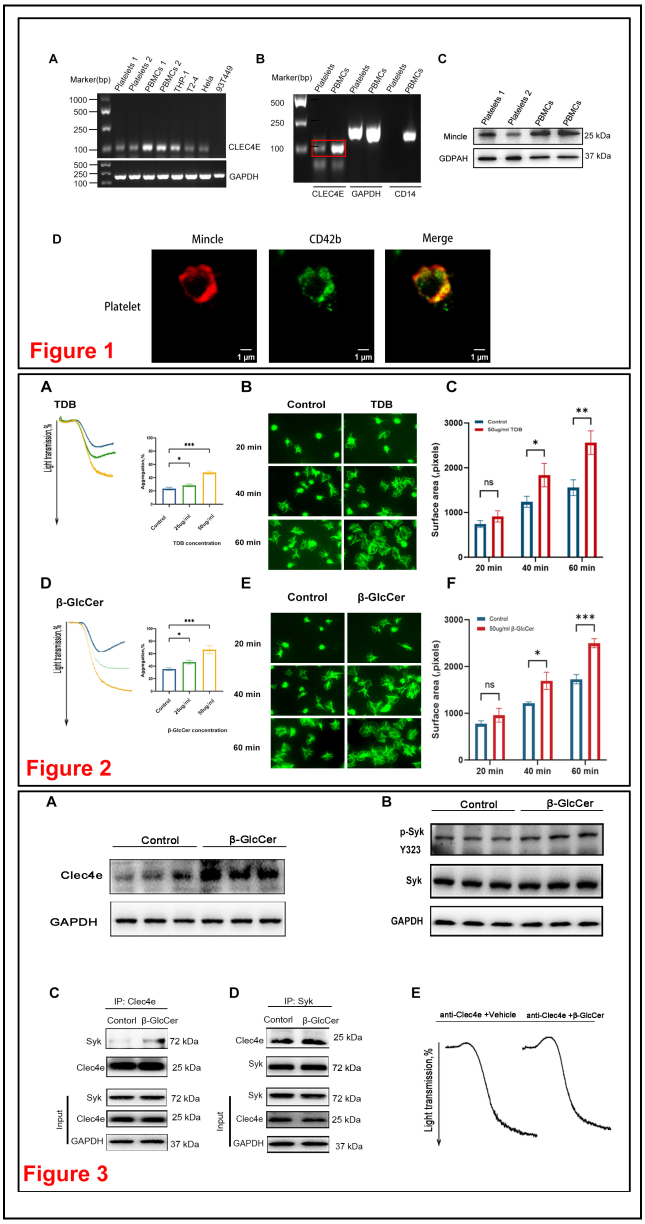

Figure 1 Human express Clec4e on platelet surface. A.RT-PCR detection of Clec4e in healthy human platelets. B.Human platelets with non-contamination of PBMCs express Clec4e. C.Western blot detection of Clec4e in both human platelet. D.Detection Clec4e on healthy human platelets by using confocal microscopy. Three independent experiments were conducted and representative images were showed above. Clec4e, C-type lectin domain family 4 member E; RT-PCR, , reverse transcription polymerase chain reaction; Figure2 TDB and -glucosylceramide directly promote platelet activation A.TDB enhanced human platelet aggregation and ATP release induced by collagen. Washed human platelet were pre-incubated with TDB or Vehicle (0.1% DMSO) for 5 minutes before stimulation with collagen.

Copyright Information: (Online) ISSN: 2572-9241
©2026 The Author(s). HemaSphere is published by John Wiley & Sons Ltd on behalf of European Hematology Association. This is an open access Abstract Book distributed under the Attribution-NonCommercial-NoDerivs (CC BY-NC-ND), which allows third parties to download the articles and share them with others as long as they credit the author and the Abstract Book, but they cannot change the content in any way or use them commercially.
Abstract Book Citations: Authors, Title, HemaSphere, 2026;10:(S1):pages. The abstract book DOIs can be found on the “Author Information” page.
Disclaimer: Articles published in the journal HemaSphere exclusively reflect the opinions of the authors. The authors are responsible for all content in their abstracts including accuracy of the facts, statements, citing resources, etc.

Representative plots of aggregation were showed from 3 independent experiments. B&C. TDB enhanced spreading area of human platelet. Representative images were presented from 3 independent experiments; D.-glucosylceramide enhanced human platelet aggregation induced by collagen. Washed human platelet were pre-incubated with -glucosylceramide or Vehicle (0.1% DMSO) for 5 minutes before stimulation with collagen. Representative plots of aggregation and ATP release were showed from 3 independent experiments. E&F. -glucosylceramide increased spreading area of human platelet. Representative images were presented from 3 independent experiments. Figure3 β-glucosylceramide induces platelet activation via the Clec4e/Syk axis. A.Clec4e expression was elevated when stimulation with -glucosylceramide. B.β-glucosylceramide stimulation leads to an increase in the phosphorylation level of Syk (Y323) in human platelets. Washed human platelet were pre-incubated with -glucosylceramide or Vehicle (0.1% DMSO) for 5 minutes. Expression of phosphorylation level of Syk were examined by western blot. C&D. Coimmunoprecipitation of clec4e (C) and syk (D) in resting healthy human platelets. Endogenous clec4e and syk were immunoprecipitated with anti-clec4e and anti–syk antibodies, respectively, with isotype IgG as a negative control. E.Blockade Clec4e receptor with an anti-Clec4e antibody, the promoting effect of β-glucosylceramide on platelet activation was abolished. 3 independent experiments were conducted and representative plots were presented.

**Copyright Information:** (Online) ISSN: 2572-9241
©2026 The Author(s). HemaSphere is published by John Wiley & Sons Ltd on behalf of European Hematology Association. This is an open access Abstract Book distributed under the Attribution-NonCommercial-NoDerivs (CC BY-NC-ND), which allows third parties to download the articles and share them with others as long as they credit the author and the Abstract Book, but they cannot change the content in any way or use them commercially.
**Abstract Book Citations:** Authors, Title, HemaSphere, 2026;10:(S1):pages. The abstract book DOIs can be found on the “Author Information” page.
**Disclaimer:** Articles published in the journal HemaSphere exclusively reflect the opinions of the authors. The authors are responsible for all content in their abstracts including accuracy of the facts, statements, citing resources, etc.

### (PS2471) THREE-YEAR REAL-WORLD UTILIZATION OF ANDEXANET ALFA PRIOR TO U.S. MARKET WITHDRAWAL: AN 87-PATIENT CONSECUTIVE INSTITUTIONAL AUDIT (2022–2025)

**Topic:** 35. Thrombosis and vascular biology

Alifya Altaf Lokhandwala*[1], Filip Sadurski[1], Moosa Alhoda[1], Abdulrahman Alayoub[1], Mishel Papali[1], Hashim Al-Ani[1], Monika Onusseit[1], Ellen E Arrington[1], Sarah Benmir[1], Rohit Sharma[1]

[1]*Mass General Brigham- Salem Hospital, Medicine, Massachusetts, United States of America;*

### Background

Andexanet alfa was approved for reversal of factor Xa inhibitors based on laboratory endpoints and early clinical data. Subsequent randomized evidence raised concerns regarding thrombotic risk, and the drug was withdrawn from the U.S. market in December 2025. We conducted a consecutive institutional audit to evaluate real-world utilization, adherence to evidence-aligned criteria, and associated complications.

### Aims

To evaluate real-world utilization of andexanet alfa at a tertiary academic institution over a three-year period, assess adherence to predefined evidence-aligned eligibility criteria, and describe thrombotic and ischemic complications temporally associated with its administration prior to national market withdrawal.

### Methods

We retrospectively reviewed all consecutive andexanet alfa administrations at a tertiary academic hospital between January 2022 and December 2025. Appropriateness was assessed using predefined criteria: documented factor Xa inhibitor ingestion within 18 hours, presence of life-threatening or hemodynamically significant bleeding, correct dose selection, no recent 4-factor prothrombin complex concentrate exposure, and correct bolus/infusion administration. Multidisciplinary documentation and imaging records were reviewed. Thrombotic and ischemic complications temporally associated with administration were recorded descriptively.

### Results

Eighty-seven consecutive administrations were identified. Fifty-four cases (54/87, 62%) met all predefined appropriateness criteria. Twenty-two cases (22/87, 25%) were classified as inappropriate and 11 (11/87, 13%) as questionable due to incomplete documentation of anticoagulant timing or bleed severity.
Among the 22 inappropriate cases, dose-selection errors occurred in 9 (41%), administration beyond the recommended 18-hour anticoagulant window in 7 (32%), and use without clearly documented life-threatening bleeding in 6 (27%).
Serious thrombotic or ischemic complications occurred in 5 patients (5/87, 5.7%): systemic arterial infarction (n=1), multifocal cerebral infarctions (n=2), pulmonary embolism (n=1), and inferior ST-elevation myocardial infarction (n=1). Thrombotic events occurred in 4 of 22 inappropriate cases (18.2%) compared with 1 of 54 appropriate cases (1.9%).
An institutional policy revision was implemented in October 2025 restricting use to defined life-threatening indications and requiring pharmacist verification and hematology approval. Andexanet alfa was withdrawn nationally in December 2025.

**Copyright Information:** (Online) ISSN: 2572-9241
©2026 The Author(s). HemaSphere is published by John Wiley & Sons Ltd on behalf of European Hematology Association. This is an open access Abstract Book distributed under the Attribution-NonCommercial-NoDerivs (CC BY-NC-ND), which allows third parties to download the articles and share them with others as long as they credit the author and the Abstract Book, but they cannot change the content in any way or use them commercially.
**Abstract Book Citations:** Authors, Title, HemaSphere, 2026;10:(S1):pages. The abstract book DOIs can be found on the “Author Information” page.
**Disclaimer:** Articles published in the journal HemaSphere exclusively reflect the opinions of the authors. The authors are responsible for all content in their abstracts including accuracy of the facts, statements, citing resources, etc.

**Summary/Conclusion**

In this three-year cohort, one-quarter of administrations did not meet predefined eligibility criteria, and thrombotic events were more frequent in non-adherent cases. With the discontinuation of andexanet alfa, structured institutional pathways for DOAC reversal are essential, including standardized eligibility criteria, multidisciplinary verification, and continued audit of alternative strategies such as 4-factor prothrombin complex concentrate to ensure safe and consistent practice in the post-andexanet era.

**Copyright Information:** (Online) ISSN: 2572-9241
©2026 The Author(s). HemaSphere is published by John Wiley & Sons Ltd on behalf of European Hematology Association. This is an open access Abstract Book distributed under the Attribution-NonCommercial-NoDerivs (CC BY-NC-ND), which allows third parties to download the articles and share them with others as long as they credit the author and the Abstract Book, but they cannot change the content in any way or use them commercially.
**Abstract Book Citations:** Authors, Title, HemaSphere, 2026;10:(S1):pages. The abstract book DOIs can be found on the "Author Information" page.
**Disclaimer:** Articles published in the journal HemaSphere exclusively reflect the opinions of the authors. The authors are responsible for all content in their abstracts including accuracy of the facts, statements, citing resources, etc.

### (PS2472) CLINICAL OUTCOMES ASSOCIATED WITH INAPPROPRIATE DIRECT ORAL ANTICOAGULANT PRESCRIBING IN A TERTIARY HOSPITAL

**Topic:** 35. Thrombosis and vascular biology

Nulee Yun*[1], Gyunam Heo[2], Yun-Hee Jo[1], Jin-Hee Baek[1], Ju-Yeun Lee[2]

*[1]Seoul National University Hospital, Department of Pharmacy, Seoul, South Korea;[2]Seoul National University, College of Pharmacy, Seoul, South Korea;*

### Background

Direct oral anticoagulants (DOACs) require individualized dosing based on approved indications and patient-specific factors such as renal function, age, body weight, and concomitant medications. In real-world practice, off-label prescribing commonly occurs, potentially affecting patient safety and clinical outcomes.

### Aims

This study aimed to evaluate the appropriateness of DOAC prescriptions in a tertiary care hospital, identify factors associated with inappropriate prescribing, and assess the association its impact on clinical outcomes.

### Methods

This retrospective study included adult patients (≥18 years) prescribed DOACs between January 1 and December 31, 2021. Prescription appropriateness was assessed based on domestically approved indications and dosing criteria. We evaluated factors associated with inappropriate prescribing and analyzed the incidence of bleeding and thromboembolic events within one year among patients newly initiating DOAC therapy.

### Results

A total of 8,973 patients were included (apixaban 4,427; edoxaban 2,357; rivaroxaban 1,677; dabigatran 512). Overall, 24.4% of patients received at least one inappropriate prescription. The highest rate of inappropriateness was observed in the apixaban group (34.9%), followed by dabigatran (28.7%), edoxaban (12.5%), and rivaroxaban (12.2%). Reduced renal function, a history of venous thromboembolism, and comorbid malignancy were significantly associated with inappropriate prescribing. Among new users, the one-year bleeding incidence was significantly higher in the inappropriate prescription group compared to the appropriate group (10.3% vs. 5.9%, $p<0.0001$), whereas thromboembolic event rates did not differ significantly.

### Summary/Conclusion

Approximately one-quarter of DOAC prescriptions deviated from approved labeling and guideline. Inappropriate prescribing was more prevalent in patients with impaired renal function and specific clinical characteristics, and was associated with an increased risk of bleeding. Targeted interventions and enhanced monitoring are warranted to optimize DOAC safety in these high-risk populations.

**Copyright Information:** (Online) ISSN: 2572-9241
©2026 The Author(s). HemaSphere is published by John Wiley & Sons Ltd on behalf of European Hematology Association. This is an open access Abstract Book distributed under the Attribution-NonCommercial-NoDerivs (CC BY-NC-ND), which allows third parties to download the articles and share them with others as long as they credit the author and the Abstract Book, but they cannot change the content in any way or use them commercially.
**Abstract Book Citations:** Authors, Title, HemaSphere, 2026;10:(S1):pages. The abstract book DOIs can be found on the "Author Information" page.
**Disclaimer:** Articles published in the journal HemaSphere exclusively reflect the opinions of the authors. The authors are responsible for all content in their abstracts including accuracy of the facts, statements, citing resources, etc.

### (PS2473) ANTI-XA MEASUREMENTS IN THOSE WITH KIDNEY IMPAIRMENT IN A TERTIARY CENTRE

**Topic:** 35. Thrombosis and vascular biology

Gerard Gurumurthy*[1], Robyn Haysom[1], Mikias Lemma[1], Nadir Aziz[1], John Hartemink[2], Yasmin Begum[2], Jecko Thachil[3], Kathrine Parker[2]

*[1]The University of Manchester, Manchester, United Kingdom;[2]Manchester University NHS Foundation Trust, Manchester Institute of Nephrology and Transplantation, Manchester, United Kingdom; [3]Manchester University NHS Foundation Trust, Department of Haematology, Manchester, United Kingdom;*

### Background

Pre-emptive dose reduction of low-molecular weight heparins (LMWHs) is often utilised in those with kidney impairment to prevent bioaccumulation.

### Aims

We report on the association of a pre-emptive dose reduction of dalteparin and its effect on anti-Xa range. We also correlated anti-Xa values with clinical outcomes.

### Methods

We undertook a service evaluation of patients with renal impairment (eGFR < 30 ml/min/1.73m2) receiving therapeutic dose dalteparin. Demographic and clinical variables such as age, sex, weight, creatinine clearance (Cockcroft-Gault), dalteparin dose and frequency were recorded. Anti-Xa monitoring includes both peak and trough levels. Trough anti-Xa levels are drawn immediately before the third dalteparin dose using a chromogenic assay, with a target of less than 0.25 U/mL for twice daily dosing. Peak levels are measured four hours after the third dose, in accordance with standard pharmacokinetic monitoring practice. Major bleeding (fatal bleeding, critical-site bleeding, Hb drop > 20 g/L, or transfusion of ≥ 2 units) and clinically relevant non-major bleeding (CRNMB, not meeting the major bleeding definition but requiring medical intervention by a healthcare professional, leading to increased level of care, or prompting an evaluation) were defined by ISTH criteria. All-cause mortality was assessed through three months post-initiation. Bleeding and thrombosis events were noted while the patient was on dalteparin. A multivariate Cox proportional hazards model was employed to assess the relationship between anti-Xa levels and the incidence of bleeding and mortality.

**Copyright Information:** (Online) ISSN: 2572-9241
©2026 The Author(s). HemaSphere is published by John Wiley & Sons Ltd on behalf of European Hematology Association. This is an open access Abstract Book distributed under the Attribution-NonCommercial-NoDerivs (CC BY-NC-ND), which allows third parties to download the articles and share them with others as long as they credit the author and the Abstract Book, but they cannot change the content in any way or use them commercially.
**Abstract Book Citations:** Authors, Title, HemaSphere, 2026;10:(S1):pages. The abstract book DOIs can be found on the "Author Information" page.
**Disclaimer:** Articles published in the journal HemaSphere exclusively reflect the opinions of the authors. The authors are responsible for all content in their abstracts including accuracy of the facts, statements, citing resources, etc.

### Results

A total of 103 patients were identified over a two-year period (Table 1). Seventy-eight (75.7%) had anti-Xa monitoring done. Trough anti Xa distribution was within target in 58 (75.6%). Patients on dialysis had a higher incidence of bleeding (19 vs 12, $p < 0.05$). Weight ≤ 55kg vs > 55kg ($p = 0.729$) and BMI ≤ 18 vs > 18 ($p = 0.519$) were not correlated with bleeding events. None had a thrombotic event while on dalteparin.

Bioaccumulation of dalteparin, defined as a subsequent trough anti-Xa level exceeding 0.25 U/mL in patients whose initial trough level was ≤ 0.25 U/mL, was observed in 12 patients. Three of whom were on dialysis. The median time to bioaccumulation was 19 days.

Patients with bleeding had significantly higher median anti Xa trough (0.26 vs 0.13 U/ml, $p < 0.01$). The same was not found in median anti-Xa peak (0.39 vs 0.36 IU/ml, $p = 0.436$). In multivariate Cox models, only anti Xa trough remained an independent predictor of bleeding (OR = 1.47 per 0.1 U/ml, 95 % CI: 1.05-2.15; $p < 0.05$) (Figure 1). No predictors of mortality were identified.

**Table 1. Baseline demographics**

| Gender | N=103 |
|---|---|
| Male | 65 (52.8%) |
| Female | 47 (38.2%) |
| **Kidney Impairment** | |
| CKD 4 | 23 (22.3%) |
| CKD 5 | 9 (8.7%) |
| AKI | 18 (17.5%) |
| Dialysis | 53 (51.5%) |
| **Body Mass Index** | |
| < 18.5 | 5 (4.9%) |
| 18.5-24.9 | 16 (15.5%) |
| 25-29.9 | 39 (37.9%) |
| 30-39.9 | 32 (31.1%) |
| > 40 | 11 (10.7%) |
| **Indications for anticoagulation** | |
| Therapeutic for thrombosis | 79 (76.7%) |
| Peri-procedural bridging | 12 (11.7%) |
| Temporary substitute in non-procedural setting | 10 (9.7%) |
| Acute coronary syndrome | 2 (1.9%) |

### Summary/Conclusion

In this report, trough anti Xa measurement of dalteparin independently predicts bleeding in patients with renal impairment. Patients receiving dalteparin for longer durations, particularly beyond 19 days, may be at increased risk of accumulation-related bleeding. Further prospective, larger studies are warranted to validate these results before it can be universally recommended in clinical practice.

**Copyright Information:** (Online) ISSN: 2572-9241
©2026 The Author(s). HemaSphere is published by John Wiley & Sons Ltd on behalf of European Hematology Association. This is an open access Abstract Book distributed under the Attribution-NonCommercial-NoDerivs (CC BY-NC-ND), which allows third parties to download the articles and share them with others as long as they credit the author and the Abstract Book, but they cannot change the content in any way or use them commercially.
**Abstract Book Citations:** Authors, Title, HemaSphere, 2026;10:(S1):pages. The abstract book DOIs can be found on the "Author Information" page.
**Disclaimer:** Articles published in the journal HemaSphere exclusively reflect the opinions of the authors. The authors are responsible for all content in their abstracts including accuracy of the facts, statements, citing resources, etc.

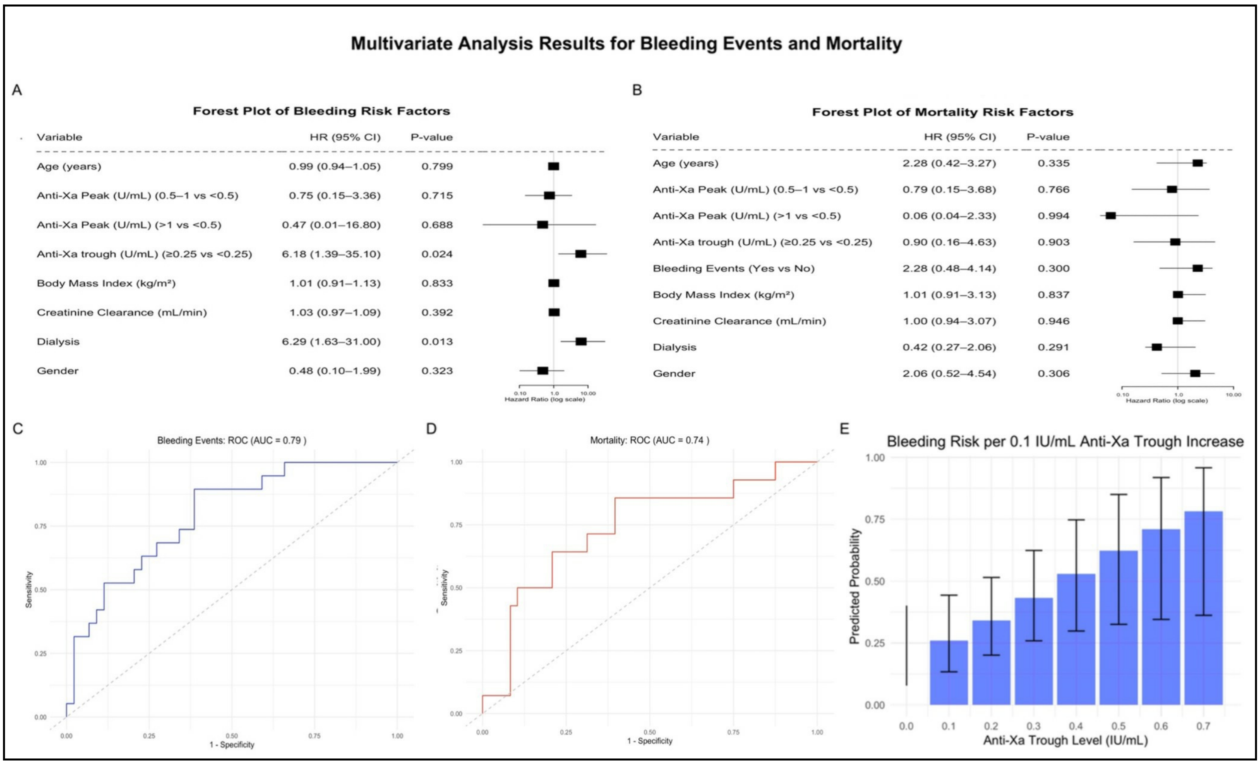

Figure 1. Multivariate analysis for factors associated with bleeding and mortality

**Copyright Information:** (Online) ISSN: 2572-9241
©2026 The Author(s). HemaSphere is published by John Wiley & Sons Ltd on behalf of European Hematology Association. This is an open access Abstract Book distributed under the Attribution-NonCommercial-NoDerivs (CC BY-NC-ND), which allows third parties to download the articles and share them with others as long as they credit the author and the Abstract Book, but they cannot change the content in any way or use them commercially.
**Abstract Book Citations:** Authors, Title, HemaSphere, 2026;10:(S1):pages. The abstract book DOIs can be found on the "Author Information" page.
**Disclaimer:** Articles published in the journal HemaSphere exclusively reflect the opinions of the authors. The authors are responsible for all content in their abstracts including accuracy of the facts, statements, citing resources, etc.

### (PS2474) HEALTH CARE UTILIZATION AND MANAGEMENT PATTERNS OF ASYMPTOMATIC INCIDENTAL PULMONARY EMBOLISM IN CANCER PATIENTS

**Topic:** 35. Thrombosis and vascular biology

Neha Agrawal**[1], Corbin Edmondson*[1], Corey Jensen[1], Shawn Stapleton[1], Kanwal Pratap Singh Raghav[1], Ryan Huey[1], Cristhiam Rojas Hernandez[1], Carol Wu[1], Sheeba Sujit[1], Michael Overman[1]

[1]*University of Texas MD Anderson Cancer Center, Houston, United States of America;*

### Background

Asymptomatic incidental pulmonary embolism (iPE) is increasingly detected in cancer patients on routine imaging; however, the optimal management setting and real-world data on healthcare utilization remain poorly defined.

### Aims

To evaluate real-world variation in management setting and healthcare utilization among oncology patients with asymptomatic incidental pulmonary embolism detected on routine outpatient imaging, in order to inform evidence-based outpatient care pathways.

### Methods

We conducted a retrospective cohort pilot study of cancer patients with newly diagnosed iPE detected on routine imaging in the outpatient setting at MD Anderson Cancer Center. AI-assisted imaging review (Aidoc) identified CT scans with iPE from 9/29/2025 to 11/09/2025 and were manually confirmed. Asymptomatic iPE was defined as the absence of symptoms associated with PE such as chest pain, dyspnea, and cough. The primary endpoint was acute care utilization following diagnosis, defined as outpatient vs acute care setting (emergency department/urgent care/observation/inpatient). Secondary endpoints were time to anticoagulation (AC), time of radiologist notification, and 30-day clinical outcomes. Fisher's exact and chi-squared tests were used to compare categorical variables. This study was conducted with approval from the institutional review board.

### Results

Cancer patients with iPE (n = 153) were identified and 92% (n = 141) were asymptomatic (median age 64; 51% female, 51% gastrointestinal malignancy). Presence of symptoms was associated with management of iPE in the acute care setting ($p < 0.001$). Asymptomatic iPE patients were managed as outpatient (77%) or in the acute care setting (23%; emergency department 11%, urgent care 5%, observation 4%, inpatient 3%). BNP/Troponins (17%) and echocardiograms (11%) were obtained in patients, primarily in the acute care setting. iPE extent was categorized as distal (subsegmental, segmental; 87%) or proximal (central, lobar; 13%) but was not associated with acute care setting ($p = 0.24$). Radiologist notification of iPE occurred in the morning (8AM-12PM; 35%), afternoon (12PM-5PM; 43%), or after hours (5PM-8AM; 22%). Acute care management of asymptomatic iPE occurred in the morning (12%), afternoon (28%), and after hours (29%) ($p = .096$). AC was used in 97% (n = 137) of asymptomatic iPE patients. 12% of asymptomatic iPE patients were already on AC. AC was initiated within the same day of diagnosis in 70% and within two days in 94% of asymptomatic iPE patients. AC occurred within the same day in 66% outpatient vs 83% in the acute care patients ($p = 0.11$). Within 30 days, all cause mortality was 1% among outpatients compared with 6% in the acute care group, while recurrent VTE occurred in 2% vs 6%, respectively.

**Copyright Information:** (Online) ISSN: 2572-9241
©2026 The Author(s). HemaSphere is published by John Wiley & Sons Ltd on behalf of European Hematology Association. This is an open access Abstract Book distributed under the Attribution-NonCommercial-NoDerivs (CC BY-NC-ND), which allows third parties to download the articles and share them with others as long as they credit the author and the Abstract Book, but they cannot change the content in any way or use them commercially.
**Abstract Book Citations:** Authors, Title, HemaSphere, 2026;10:(S1):pages. The abstract book DOIs can be found on the "Author Information" page.
**Disclaimer:** Articles published in the journal HemaSphere exclusively reflect the opinions of the authors. The authors are responsible for all content in their abstracts including accuracy of the facts, statements, citing resources, etc.

**Summary/Conclusion**

In this pilot study, nearly one quarter of patients with asymptomatic iPE underwent acute care management. These results highlight substantial practice variation in the management of iPE and suggest further exploration of standardized outpatient pathways.

*These authors contributed equally to this work.

**Copyright Information:** (Online) ISSN: 2572-9241
©2026 The Author(s). HemaSphere is published by John Wiley & Sons Ltd on behalf of European Hematology Association. This is an open access Abstract Book distributed under the Attribution-NonCommercial-NoDerivs (CC BY-NC-ND), which allows third parties to download the articles and share them with others as long as they credit the author and the Abstract Book, but they cannot change the content in any way or use them commercially.
**Abstract Book Citations:** Authors, Title, HemaSphere, 2026;10:(S1):pages. The abstract book DOIs can be found on the “Author Information” page.
**Disclaimer:** Articles published in the journal HemaSphere exclusively reflect the opinions of the authors. The authors are responsible for all content in their abstracts including accuracy of the facts, statements, citing resources, etc.

### (PS2475) INTERPLAY OF NITRIC OXIDE AND INFLAMMATION IN THROMBOTIC EVENTS IN PATIENTS WITH ACUTE MYELOID LEUKEMIA

**Topic:** 35. Thrombosis and vascular biology

Nikica Sabljic[1], Nikola Pantic[1], Zoran Bukumiric[2], Marijana Virijevic[1, 3], Zlatko Pravdic[1, 3], Sofija Pendic[4], Darko Antic[1, 3], Vojin Vukovic[1, 3], Olivera Mitrović Ajtić[5], Mirjana Cucakovic[1], Lazar Trajković[1], Miloš Diklić[5], Vladan Cokic[5], Emilija Živković[5], Jovan Rajic[1], Violeta Milosevic[1], Ana Vidovic[1, 3], Nada Suvajdzic Vukovic[1, 3], Mirjana Mitrovic*[1, 3]

*[1]University Clinical Center of Serbia, Clinic for Hematology, Belgrade, Serbia;[2]University of Belgrade, Institute for medical statistics and informatics, Belgrade, Serbia; [3]University of Belgrade, Faculty of Medicine, Belgrade, Serbia;[4]Medicines and Medical Devices Agency of Serbia, Belgrade, Serbia; [5]University of Belgrade, Institute for medical research, Belgrade, Serbia;*

### Background

Nitric oxide (NO) is a pleiotropic regulator essential to numerous biological processes, including inhibition of platelet aggregation and vasodilatation, both of which may be crucial for the prevention of thrombosis. In the context of inflammation, NO exhibits a dual role: under normal conditions it acts as an anti-inflammatory mediator, whereas in certain pathological states it may exert pro-inflammatory effects. Inflammation and thrombosis are closely interconnected, often described together as thrombo-inflammation. The incidence of thrombosis in acute myeloid leukemia (AML) varies, reaching up to 22%, with arterial thrombosis accounting for approximately 3%. These thrombotic complications can significantly impact the survival outcomes of patients with AML.

### Aims to identify the incidence of thrombosis in patients with AML; to define a correlation between inflammatory parameters, initial NO levels and other clinical-laboratory parameters with thrombosis.

### Methods

We conducted a prospective study of adult patients with AML, including acute promyelocytic leukemia (APL), treated between 2023 and 2025 at the Clinic of Hematology, UCCS. Demographic and clinical-laboratory data relevant to thrombosis were recorded and analyzed, including C-reactive protein (CRP), erythrocyte sedimentation rate (ESR), ferritin, fibrinogen, and white blood cell (WBC) count. NO levels in peripheral blood were measured using the Sievers NOA-280i nitric oxide analyzer.

**Copyright Information:** (Online) ISSN: 2572-9241
©2026 The Author(s). HemaSphere is published by John Wiley & Sons Ltd on behalf of European Hematology Association. This is an open access Abstract Book distributed under the Attribution-NonCommercial-NoDerivs (CC BY-NC-ND), which allows third parties to download the articles and share them with others as long as they credit the author and the Abstract Book, but they cannot change the content in any way or use them commercially.
**Abstract Book Citations:** Authors, Title, HemaSphere, 2026;10:(S1):pages. The abstract book DOIs can be found on the "Author Information" page.
**Disclaimer:** Articles published in the journal HemaSphere exclusively reflect the opinions of the authors. The authors are responsible for all content in their abstracts including accuracy of the facts, statements, citing resources, etc.

**Results**

Among 133 adult patients (61% male; mean age 52.6 ± 14.9 years), 24 (18%) were diagnosed with APL. The majority (105/133, 78.9%) received intensive chemotherapy. Thrombosis was observed in 18 patients (13.5%); half (50%) were diagnosed at presentation, while the other half occurred during induction. Venous thromboses accounted for 66.6% (12/18). Reported thrombotic events included pulmonary thromboembolism (4/18), deep vein thrombosis (4/18), internal jugular vein thrombosis (3/18), and splenic infarction (2/18), while cerebral venous sinus thrombosis, renal infarction, acute myocardial infarction (AMI), superior mesenteric artery thrombosis, and cerebrovascular insult were observed in single cases. Of these, only AMI was fatal. Analysis of inflammatory parameters revealed no significant differences between patients with and without thrombosis in WBC count (P = 0.389, $14.8\times10^9$/L vs. $10.6\times10^9$/L), fibrinogen (P = 0.337, 4.1 g/L vs. 4.4 g/L), ESR (P = 0.425, 67 mm/h vs. 64 mm/h), ferritin (P = 0.233, 851.5 µg/L vs. 648.9 µg/L), CRP (P = 0.113, 30.8 mg/L vs. 12.6 mg/L), or NO levels (P = 0.967 1382.6 nM vs. 1431.7 nM). However, several factors were significantly associated with thrombotic complications: reduced mobility for >3 days prior to thrombosis (P = 0.02, 33.3% vs. 5.7%), ECOG (Eastern Cooperative Oncology Group) performance status ≥2 (P = 0.01, 55.5% vs. 14.4%), elevated D-dimer levels (P = 0.01, 23.1 mg/L vs. 2.6 mg/L), and higher ISTH (International Society on Thrombosis and Hemostasis) disseminated intravascular coagulation (DIC) scores (P = 0.024, median 5 vs. 4).

**Summary/Conclusion**

Thrombosis occurred in a considerable proportion of patients with AML and should not be underestimated. No significant associations were observed between thrombosis and inflammatory parameters or NO levels. The results of this study are limited by sample size, and further analyses in larger cohorts are warranted to better clarify the role of thrombo-inflammation and NO in AML-related thrombosis.

**Copyright Information:** (Online) ISSN: 2572-9241
©2026 The Author(s). HemaSphere is published by John Wiley & Sons Ltd on behalf of European Hematology Association. This is an open access Abstract Book distributed under the Attribution-NonCommercial-NoDerivs (CC BY-NC-ND), which allows third parties to download the articles and share them with others as long as they credit the author and the Abstract Book, but they cannot change the content in any way or use them commercially.
**Abstract Book Citations:** Authors, Title, HemaSphere, 2026;10:(S1):pages. The abstract book DOIs can be found on the “Author Information” page.
**Disclaimer:** Articles published in the journal HemaSphere exclusively reflect the opinions of the authors. The authors are responsible for all content in their abstracts including accuracy of the facts, statements, citing resources, etc.

### (PS2476) MONOCYTE-TO-RED CELL DISTRIBUTION WIDTH RATIO PREDICTS PULMONARY EMBOLISM IN PATIENTS WITH DEEP VEIN THROMBOSIS: A PROPENSITY SCORE–MATCHED ANALYSIS

**Topic:** 35. Thrombosis and vascular biology

Bulent Kantarcioglu*[1], Fakiha Siddiqui[1], Amir Darki[1], Sultan Gozde Temiz[2], Ayse Nilgun Kul[2], Gulbuz Sezgin[2], Jawed Fareed[1]

*[1]Loyola University Chicago, Maywood, United States of America;[2]University of Health Sciences, Sancaktepe Training and Research Hospital, Istanbul, Türkiye;*

### Background

Pulmonary embolism (PE) represents the most severe and life-threatening manifestation of venous thromboembolism, most commonly arising from the dislodgment and embolization of thrombi originating in the deep veins of the lower extremities. While deep vein thrombosis (DVT) and PE are considered different clinical expressions of the same disease continuum, only a subset of patients with DVT develop acute PE, suggesting the presence of distinct biological and thrombo-inflammatory determinants underlying clot instability, propagation, and embolization. Increasing evidence indicates that systemic inflammation and cellular activation play a critical role in venous thrombogenesis and thrombus embolization.

### Aims

This study aimed to evaluate the associations between neutrophil-to-lymphocyte ratio (NLR), platelet-to-lymphocyte ratio (PLR), systemic immune-inflammation index (SII), the monocyte-to-red cell distribution width ratio (monocyte/RDW), and acute PE among patients with DVT.

### Methods

The study included 67 patients with isolated DVT admitted to the Internal Medicine Unit of Prof. Dr. Feriha Öz Emergency Hospital and 308 patients with acute PE and concomitant DVT identified through pulmonary embolism response team (PERT) protocols at Loyola University Medical Center and its affiliated hospitals. In both cohorts, data were collected from electronic medical records of the patients. NLR, PLR, SII, and the monocyte/RDW ratio were calculated from complete blood count parameters obtained at admission. Due to baseline imbalances between the DVT-only and PE-with-DVT cohorts, propensity score matching was performed using 1:1 matching with a caliper width of 0.2 to reduce confounding. In both unmatched and matched cohort, the statistical differences between the DVT and PE groups were analyzed using the Mann–Whitney U test or Student's *t*-test, as appropriate based on data distribution. Receiver operating characteristics (ROC) curve analysis were performed to evaluate NLR, PLR, SII, and the monocyte/RDW as predictors of acute PE in patients with DVT.

### Results

In the overall cohort (n = 375), the median age was 65 years (IQR: 54–73); 206 patients (54.9%) were male and 169 (45.1%) were female. Levels of NLR, PLR, and SII did not differ significantly between patients with isolated DVT and those with PE-with-DVT patients. In contrast, the monocyte/RDW ratio was significantly higher in the PE-with-DVT group ($p < 0.001$). In ROC analysis, NLR, PLR, and SII demonstrated no significant discriminatory ability for predicting PE among patients with DVT. However, the monocyte/RDW ratio showed significant predictive performance (AUC: 0.696; 95% CI: 0.628–0.764; $p < 0.001$) (Figure 1A). In the propensity score matched cohort, NLR, PLR, and SII again showed no significant differences between groups, whereas the monocyte/RDW ratio remained significantly higher in patients with PE-with-DVT ($p < 0.001$). Consistently, the predictive performance of the monocyte/RDW ratio remained significant after matching (AUC: 0.737; 95% CI: 0.652–0.822; $p < 0.001$)(Figure 1B).

**Copyright Information:** (Online) ISSN: 2572-9241
©2026 The Author(s). HemaSphere is published by John Wiley & Sons Ltd on behalf of European Hematology Association. This is an open access Abstract Book distributed under the Attribution-NonCommercial-NoDerivs (CC BY-NC-ND), which allows third parties to download the articles and share them with others as long as they credit the author and the Abstract Book, but they cannot change the content in any way or use them commercially.
**Abstract Book Citations:** Authors, Title, HemaSphere, 2026;10:(S1):pages. The abstract book DOIs can be found on the "Author Information" page.
**Disclaimer:** Articles published in the journal HemaSphere exclusively reflect the opinions of the authors. The authors are responsible for all content in their abstracts including accuracy of the facts, statements, citing resources, etc.

### Summary/Conclusion

The monocyte/RDW ratio was a significant and consistent predictor of PE in patients with DVT, outperforming NLR, PLR, and SII in both unmatched and matched analyses. As a readily available parameter derived from routine blood counts, it may serve as a practical tool for PE risk stratification in DVT patients. Prospective studies are needed to confirm its clinical utility.

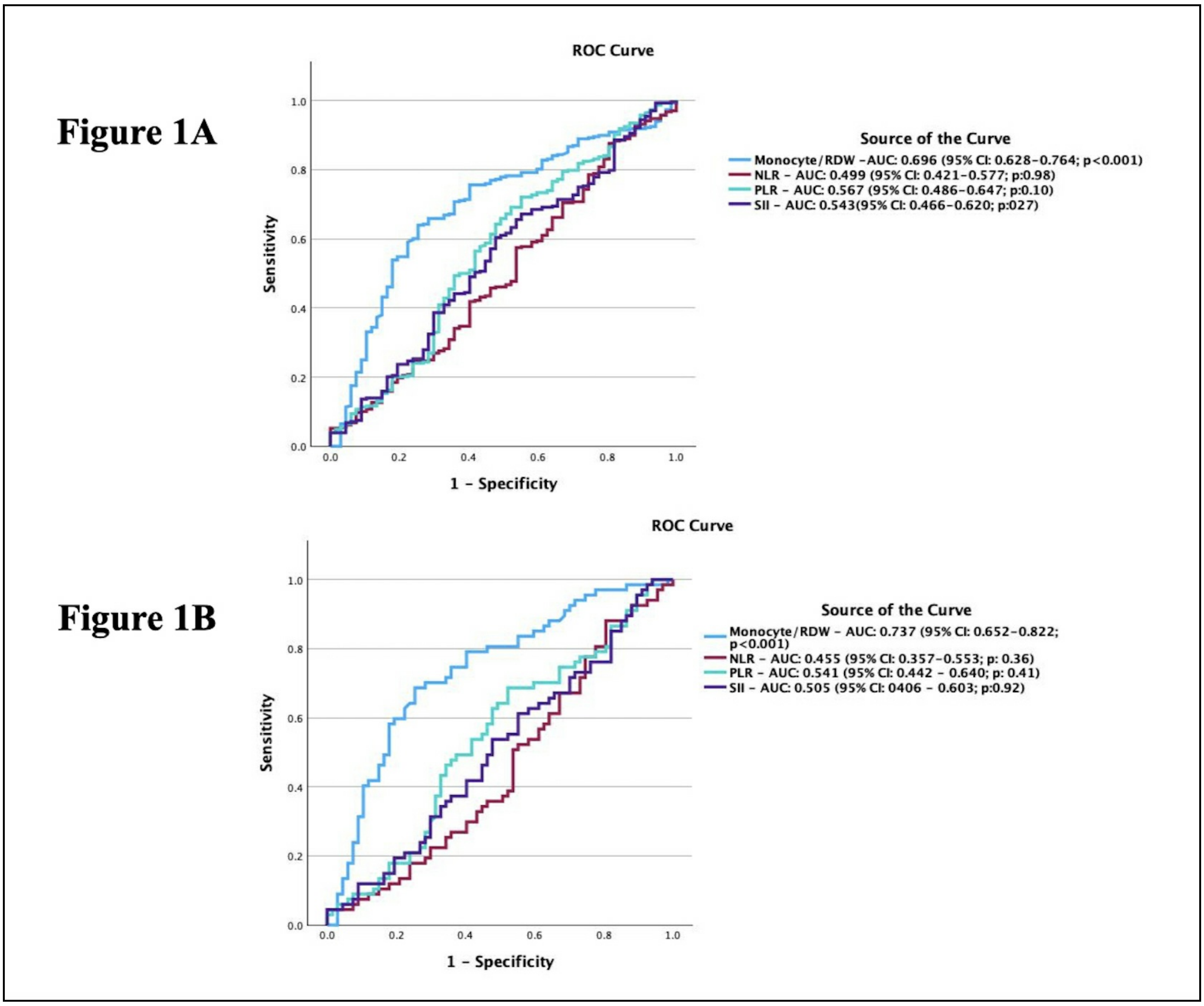

Figure 1. A- ROC curve analysis evaluating the discriminatory performance of inflammatory indices for predicting PE among patients with DVT. The NLR, PLR, and SII demonstrated no significant predictive ability. In contrast, the monocyte/RDW ratio showed significant discriminatory performance (AUC: 0.696; 95% CI: 0.628–0.764; p < 0.001). B- ROC curve analysis in the propensity score matched cohort. After adjustment for baseline imbalances, NLR, PLR, and SII showed no significant differences between groups. In contrast, the monocyte/RDW ratio remained significantly higher in patients with PE with DVT. The monocyte/RDW ratio demonstrated sustained discriminatory performance for predicting PE in the matched cohort (AUC: 0.737; 95% CI: 0.652–0.822; p < 0.001).

**Copyright Information:** (Online) ISSN: 2572-9241
©2026 The Author(s). HemaSphere is published by John Wiley & Sons Ltd on behalf of European Hematology Association. This is an open access Abstract Book distributed under the Attribution-NonCommercial-NoDerivs (CC BY-NC-ND), which allows third parties to download the articles and share them with others as long as they credit the author and the Abstract Book, but they cannot change the content in any way or use them commercially.
**Abstract Book Citations:** Authors, Title, HemaSphere, 2026;10:(S1):pages. The abstract book DOIs can be found on the "Author Information" page.
**Disclaimer:** Articles published in the journal HemaSphere exclusively reflect the opinions of the authors. The authors are responsible for all content in their abstracts including accuracy of the facts, statements, citing resources, etc.

### (PS2477) FUNCTIONAL ASPECTS OF FIBRIN STRUCTURE ALTERATIONS BY TRANEXAMIC ACID IN THE INHIBITION OF FIBRINOLYSIS

**Topic:** 35. Thrombosis and vascular biology

Nikolett Wohner*[1], Barbara Baráth[1], Kata Balog Virág[1], Kristóf Molnár[1], Petra Csikós[1], Alexandra Raska[1], László Szabó[2], Krasimir Kolev[1]

*[1]Semmelweis University, Department of Biochemistry and Molecular Biology, Budapest, Hungary; [2]Research Centre for Natural Sciences, Budapest, Hungary;*

### Background

Tranexamic acid (TXA) is a synthetic lysine analogue widely used as an antifibrinolytic agent. Large randomized trials have demonstrated life-saving benefit when TXA is administered early in acute hemorrhage, but results regarding prophylactic administration have been conflicting, and several trials have not shown an improvement in clinical outcomes. The mechanisms underlying this discrepancy remain incompletely understood.

### Aims

To investigate the molecular and structural mechanisms that determine TXA efficacy under conditions mimicking therapeutic versus prophylactic administration.

### Methods

In vitro, tPA-induced fibrinolysis was evaluated by laser scanning confocal microscopy, viscoelastic testing (ClotPro), turbidimetry, and assays of clot permeability and plasminogen activation across physiologically and therapeutically relevant concentrations of plasminogen (Plg) and TXA. Fibrin ultrastructure was characterized by scanning electron microscopy (SEM). Key experiments were additionally conducted in human plasma under constant plasminogen concentrations.

### Results

At physiological plasminogen concentrations, pre-incorporation of TXA into the clot abolished its expected antifibrinolytic effect: instead, therapeutically relevant TXA levels paradoxically accelerated fibrinolysis both in purified fibrin and in plasma clots. In 300 min, tPA on the surface of plasma clots caused 404.9 μm(±8.5; n=3) lysis front displacement at 0 μM TXA and 786.2 μm(±45.2; n=3) at 128 μM TXA in the clot. By contrast, when clot formation and fibrinolysis proceeded concurrently in the presence of TXA and tPA, TXA consistently prolonged lysis time at all Plg concentrations. Mechanistically, these effects were associated with a marked TXA-induced alteration in fibrin architecture. We detected a >2-fold increase in the upper quartile (Q75) of fiber diameters, from 108.5 nm in the absence of TXA to 228.3 nm at 64 μM TXA. The clot permeability coefficient increased in the presence of TXA from $1.20\times10^{-8}\pm2.60\times10^{-9}$ $cm^2$ (mean±SD; n=3) at 0 μM TXA to $3.20\times10^{-8}\pm2.67\times10^{-9}$ $cm^2$ (mean±SD; n=3) at 128 μM TXA.

### Summary/Conclusion

Pre-incorporation of TXA into clots (modeling prophylactic TXA use) rearranges fibrin architecture, yielding thicker fibers and increased clot permeability, which render both pure fibrin and plasma clots more susceptible to fibrinolysis. Thereby, these structural alterations could counteract the antifibrinolytic effects of TXA if it is present at the stage of fibrin formation posing limitations for its prophylactic administration in the clinical setting.

**Copyright Information:** (Online) ISSN: 2572-9241
©2026 The Author(s). HemaSphere is published by John Wiley & Sons Ltd on behalf of European Hematology Association. This is an open access Abstract Book distributed under the Attribution-NonCommercial-NoDerivs (CC BY-NC-ND), which allows third parties to download the articles and share them with others as long as they credit the author and the Abstract Book, but they cannot change the content in any way or use them commercially.
**Abstract Book Citations:** Authors, Title, HemaSphere, 2026;10:(S1):pages. The abstract book DOIs can be found on the "Author Information" page.
**Disclaimer:** Articles published in the journal HemaSphere exclusively reflect the opinions of the authors. The authors are responsible for all content in their abstracts including accuracy of the facts, statements, citing resources, etc.

### (PS2478) INCIDENCE AND RISK FACTORS OF THROMBOSIS IN ADULT PATIENTS WITH IMMUNE THROMBOCYTOPENIA (ITP): A MULTICENTER COHORT STUDY.

**Topic:** 35. Thrombosis and vascular biology

Maimoona Al Hinai*[1], Bader Al Rawahi[2]

*[1]Oman medical specialty Board (OMSB), Hematopathology, Muscat, Oman;[2]University medical city, Clinical Hematology, Muscat, Oman;*

### Background

**There is growing evidence that patient with immune thrombocytopenia (ITP) are having an increase risk of thrombosis but the impact on clinical and treatment related factors remains unclear.**

### Aims

To assess the incidence and risk factors of arterial and venous thrombosis in patients with ITP and to describe clinical features and management.

### Methods

We conducted a retrospective cohort study of all adult patients diagnosed with immunothrombocytopenia (ITP) who had at least one active clinical visit between 2015 and 2024 at two teritary care hospitals in Oman: University Medical City and The Royal Hospital. Incidence rate of arterial and venous thrombosis were calculated per person month of follow up, and a multivarible Cox regression was used to assess associations with clinical and treatment related risk factors.

### Results

The study included a total of 234 patients with ITP contributed 9928 person months of follow up since diagnosis. Thrombotic events were identified in 20 patients (8.5%), including 19 (63.3%) venous thromboembolism (VTE) and 11 (36.7%) arterial thromboembolic events. The overall incidence rate was 3.02 per 1000 person months (95% CI:1.94-4.10), with incidence rates of 1.91per 1000 person-months (95%CI:1.13-2.69) for VTE and 1.11 per 1000 person months (95%CI:0.45-1.77) for arterial thromboembolism. The median time to first thrombotic event was 7.4 months and the mean age was 49 years. In Multivarible analysis, a history of previous thrombosis (adjusted hazard ratio [aHR] 41.556, 95% CI 7.848–220.044, P < 0.001), underlying cardiac disease (aHR 12.396, 95% CI 1.764–87.122, P = 0.011), and prior splenectomy (aHR 13.679, 95% CI 1.096–170.694, P = 0.042) were independently assocaited with an increased hazard of thrombosis in patients with ITP, wheres thrombopoietin receptor agonist use and secondary ITP were not significantly associated with thrombotic risk.

### Summary/Conclusion

While thrombosis remains a relatively rare complication in ITP, its occurrence carries significant clinical implications. Our findings identified prior thrombotic events, cardiac disease and splenectomy as key predictors, underscoring the importance of individualised risk assessment and vigilant monitoring to prevent potentially serious outcomes.

**Copyright Information:** (Online) ISSN: 2572-9241
©2026 The Author(s). HemaSphere is published by John Wiley & Sons Ltd on behalf of European Hematology Association. This is an open access Abstract Book distributed under the Attribution-NonCommercial-NoDerivs (CC BY-NC-ND), which allows third parties to download the articles and share them with others as long as they credit the author and the Abstract Book, but they cannot change the content in any way or use them commercially.
**Abstract Book Citations:** Authors, Title, HemaSphere, 2026;10:(S1):pages. The abstract book DOIs can be found on the "Author Information" page.
**Disclaimer:** Articles published in the journal HemaSphere exclusively reflect the opinions of the authors. The authors are responsible for all content in their abstracts including accuracy of the facts, statements, citing resources, etc.

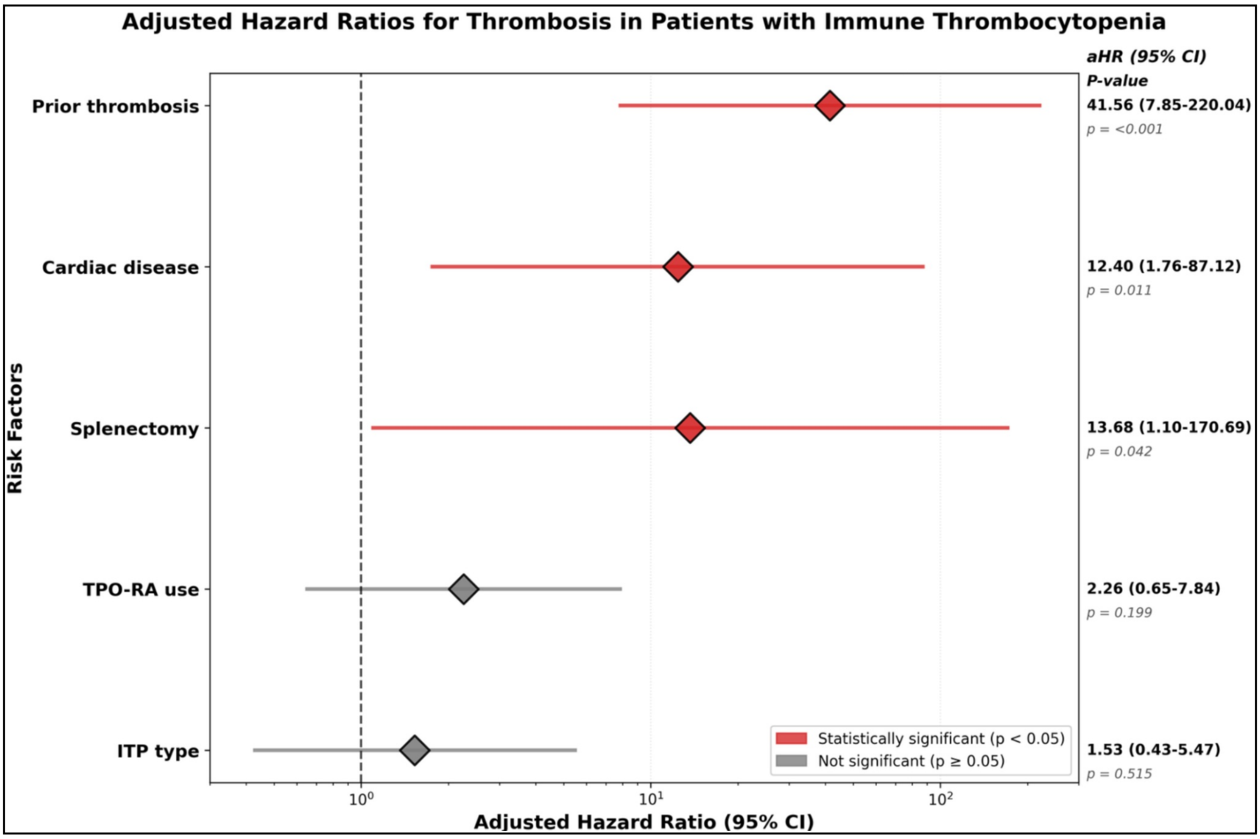

**Copyright Information:** (Online) ISSN: 2572-9241
©2026 The Author(s). HemaSphere is published by John Wiley & Sons Ltd on behalf of European Hematology Association. This is an open access Abstract Book distributed under the Attribution-NonCommercial-NoDerivs (CC BY-NC-ND), which allows third parties to download the articles and share them with others as long as they credit the author and the Abstract Book, but they cannot change the content in any way or use them commercially.
**Abstract Book Citations:** Authors, Title, HemaSphere, 2026;10:(S1):pages. The abstract book DOIs can be found on the "Author Information" page.
**Disclaimer:** Articles published in the journal HemaSphere exclusively reflect the opinions of the authors. The authors are responsible for all content in their abstracts including accuracy of the facts, statements, citing resources, etc.

### (PS2479) INCIDENCE, PATIENT PROFILE, AND 3-MONTH SURVIVAL OF VENOUS THROMBOEMBOLISM IN INTERNAL MEDICINE INPATIENTS: A PILOT STUDY FROM TURKEY

**Topic:** 35. Thrombosis and vascular biology

Bulent Kantarcioglu*[1, 2], Sultan Gozde Temiz[2], Ayse Nilgun Kul[2], Gulbuz Sezgin[2], Jawed Fareed[1]

*[1]Loyola University Chicago, Maywood, United States of America; [2]University of Health Sciences, Sancaktepe Training and Research Hospital, Istanbul, Türkiye;*

### Background

Venous thromboembolism (VTE), encompassing deep vein thrombosis (DVT) and pulmonary embolism (PE), represents the third leading cause of cardiovascular mortality. Although the development and progression of VTE are driven by complex pathophysiological mechanisms, accumulating evidence suggests the pivotal role of inflammation in its pathogenesis.

### Aims

In this pilot study, we evaluated the prevalence, clinical characteristics, and survival outcomes of patients with VTE who were admitted to the internal medicine unit over a one-year period. We also assessed the predictive value of the neutrophil-to-lymphocyte ratio (NLR), red cell distribution width-to-albumin ratio (RDW/Albumin), and C-reactive protein-to-albumin ratio (CRP/Albumin) for 3 month mortality.

### Methods

The electronic medical records of patients diagnosed with VTE who were admitted to the Internal Medicine Unit of Prof. Dr. Feriha Öz Emergency Hospital between July 2024, and June 2025 were retrospectively reviewed. Data on patient demographics, vital signs, clinical symptoms, comorbidities, laboratory findings, VTE risk factors, and clinical outcomes were collected. The NLR, RDW/Albumin, and CRP/Albumin were calculated for each patient. Statistical differences between the DVT and PE groups were analyzed using the Mann–Whitney U test or Student's *t*-test, as appropriate based on data distribution. Receiver operating characteristics (ROC) curve analysis and multivariable logistic regression were performed to evaluate NLR, RDW/Albumin, and CRP/Albumin as predictors of 3-month mortality.

### Results

Among 66,789 total hospital admissions during the study period, 3,993 were to the Internal Medicine Unit. A total of 86 patients were diagnosed with VTE, including 67 cases of DVT and 19 cases of PE, corresponding to an incidence of approximately 2.15% among internal medicine admissions. This incidence is markedly higher than that in the general population, where the annual incidence of VTE is approximately 0.1–0.2%, and is consistent with reported rates of 1–2% among hospitalized patients, highlighting the increased risk of VTE in this setting. No statistically significant differences were observed between the DVT and PE groups regarding patient demographics, comorbidities, or laboratory findings, except for vital signs and clinical symptoms. 3-month mortality was observed in 36.0% of patients, which was mainly due to the high burden of comorbidities; 34.9% had active metastatic cancer, and the median Charlson Comorbidity Index was 7 (IQR: 4.0-9.0). ROC curve analysis demonstrated that NLR, RDW/Albumin, and CRP/Albumin were significant predictors of 3-month mortality. The optimal cutoff values were 7.60 for NLR, 4.71 for RDW/Albumin, and 19.94 for CRP/Albumin (Figure 1A). In multivariable logistic regression analysis, RDW/Albumin > 4.71 was the strongest independent predictor of 3-month mortality [odds ratio (OR): 12.30; 95% confidence interval (CI): 2.39–63.15], followed by CRP/Albumin > 19.94 [OR: 6.52; 95% CI: 2.07–20.49] and NLR > 7.60 [OR:4.41; 95% CI: 1.54–12.58] (Figure 1B).

**Copyright Information:** (Online) ISSN: 2572-9241
©2026 The Author(s). HemaSphere is published by John Wiley & Sons Ltd on behalf of European Hematology Association. This is an open access Abstract Book distributed under the Attribution-NonCommercial-NoDerivs (CC BY-NC-ND), which allows third parties to download the articles and share them with others as long as they credit the author and the Abstract Book, but they cannot change the content in any way or use them commercially.
**Abstract Book Citations:** Authors, Title, HemaSphere, 2026;10:(S1):pages. The abstract book DOIs can be found on the "Author Information" page.
**Disclaimer:** Articles published in the journal HemaSphere exclusively reflect the opinions of the authors. The authors are responsible for all content in their abstracts including accuracy of the facts, statements, citing resources, etc.

### Summary/Conclusion

In this pilot study, we observed an increased risk of VTE in our community hospital setting, underscoring the importance of awareness of VTE in Turkey. Although the study sample was small, predictors such as RDW/Albumin, CRP/Albumin, and NLR may be useful for risk stratification in these patients. Further studies with larger cohorts are warranted to validate these findings.

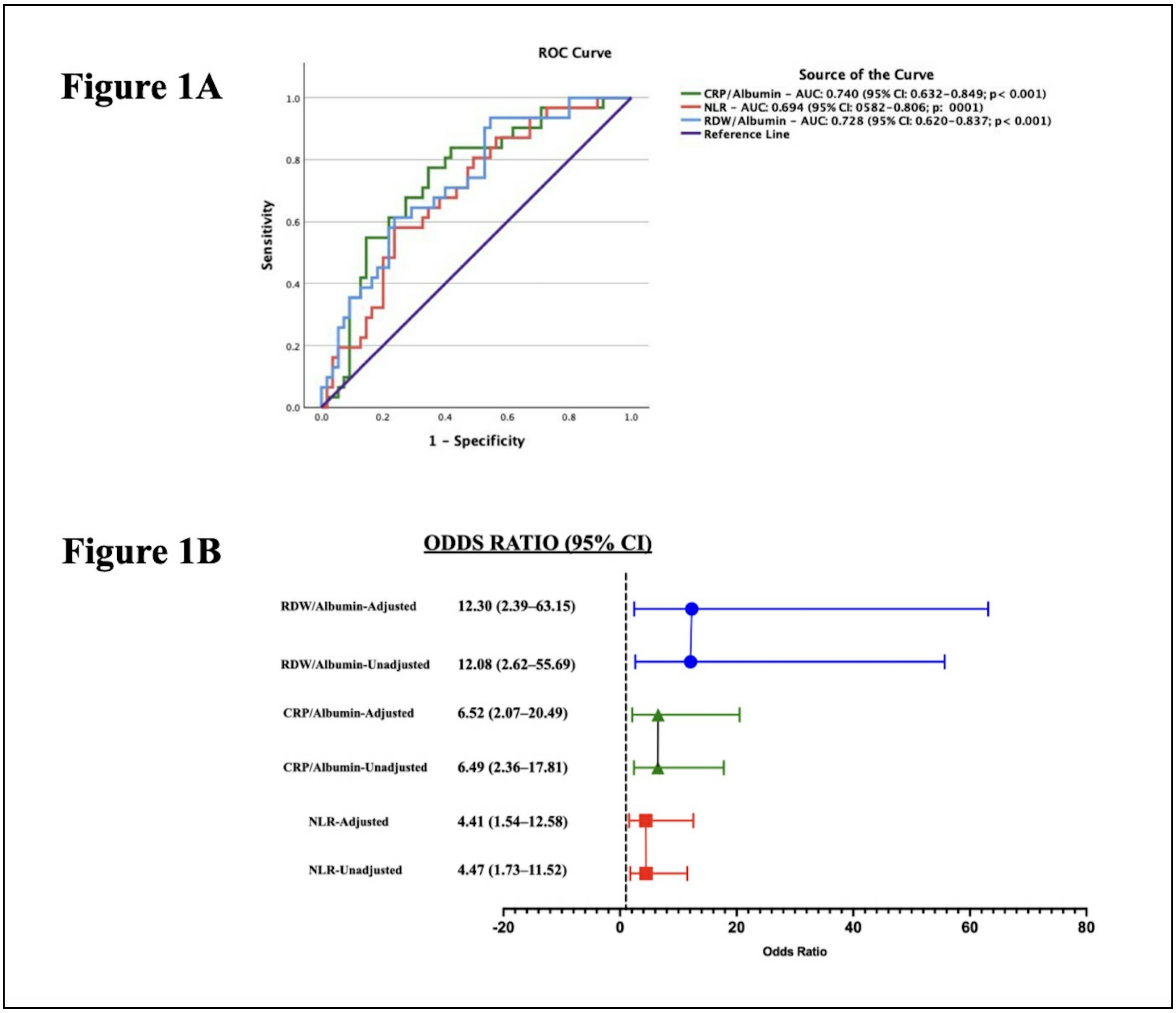

Figure 1. A-Receiver operating characteristics (ROC) curve analysis of NLR, RDW/Albumin, and CRP/Albumin as predictors of 3-month mortality. NLR had an optimal cutoff value of 7.60 (AUC: 0.694; 95% CI: 0.582–0.806), RDW/Albumin 4.71 (AUC: 0.728; 95% CI: 0.620–0.837), and CRP/Albumin 19.94 (AUC: 0.740; 95% CI: 0.632–0.849). All three biomarkers were significant predictors of 3-month mortality. B-Association between NLR, RDW/Albumin, and CRP/Albumin and 3-month mortality in unadjusted and adjusted analyses. *Adjusted for age, gender, BMI, hypertension and diabetes mellitus.

**Copyright Information:** (Online) ISSN: 2572-9241
©2026 The Author(s). HemaSphere is published by John Wiley & Sons Ltd on behalf of European Hematology Association. This is an open access Abstract Book distributed under the Attribution-NonCommercial-NoDerivs (CC BY-NC-ND), which allows third parties to download the articles and share them with others as long as they credit the author and the Abstract Book, but they cannot change the content in any way or use them commercially.
**Abstract Book Citations:** Authors, Title, HemaSphere, 2026;10:(S1):pages. The abstract book DOIs can be found on the "Author Information" page.
**Disclaimer:** Articles published in the journal HemaSphere exclusively reflect the opinions of the authors. The authors are responsible for all content in their abstracts including accuracy of the facts, statements, citing resources, etc.

### (PS2480) PLATELET HYPERREACTIVITY OVERRIDES THROMBIN DYSREGULATION AS THE MAIN THROMBOTIC DRIVER IN AFIBRINOGENEMIA WITH A NOVEL FGA MUTATION (P.TRP248*)

**Topic:** 35. Thrombosis and vascular biology

Xiao Shuai*[1], Liqin Ling[2], Chaonan Liu[2], Shuang Wang[2], Zhiyu Yu[2], Ting Liu[1], Jing Zhou[2]

*[1]West China Hospital, Sichuan University, Hematology Department, Chengdu, China;[2]West China Hospital, Sichuan University, Department of Laboratory Medicine, Clinical Laboratory Medicine Research Center, Chengdu, China;*

### Background

Congenital afibrinogenemia is an extremely rare coagulopathy characterized by undetectable fibrinogen. While traditionally viewed as a bleeding disorder, paradoxical thrombotic events are increasingly reported. The prevailing "thrombin-dysregulation" model suggests that a lack of fibrin prevents thrombin sequestration (the antithrombin I effect), leading to excessive free thrombin. However, this model fails to explain why fibrinogen replacement therapy often triggers, rather than prevents, clinical thrombosis.

### Aims

To investigate the underlying mechanisms of recurrent arterial thrombosis in a patient with congenital afibrinogenemia and to evaluate the role of platelet reactivity as a potential driver of a prothrombotic state.

### Methods

We performed a comprehensive diagnostic workup for a 30-year-old male with a history of recurrent bleeding events and arterial occlusions. Methods included plasma fibrinogen quantification (Clauss assay and turbidimetric latex immunoassay), whole-exome sequencing (WES), and specialized thrombophilia screening. Thrombin generation was assessed via thrombin-antithrombin complexes, thrombin generation assays, and thrombomodulin-modified thrombin generation assay. Platelet aggregation assays with collagen, arachidonic acid of different concentrations were evaluated using light transmission aggregometry (LTA) and platelet function closure times (PFA-200), both during triple antiplatelet therapy (cilostazol, beraprost sodium and sarpogrelate hydrochloride) and after its withdrawal, with and without ex vivo fibrinogen correction.

### Results

The patient's bleeding history included prolonged umbilical cord bleeding at birth, post-traumatic bleeding, and spontaneous bruising. The patient developed symptoms of arteriole occlusion from the right elbow to the hand, and later cerebral arterial infarction after fibrinogen infusion. Treatment of triple antiplatelet agents was initiated for four weeks. The patient was admitted into our hospital for further evaluation. Family history revealed  consanguineous marriage of his parents. Plasma fibrinogen concentration was less than 0.1g/L, with indefinitely prolonged clotting times (PT, aPTT, TT), which can be corrected to normal values upon ex vivo addition of fibrinogen. A novel homozygous nonsense mutation in *FGA* (p.Trp248*) was identified. His parents carried heterozygous mutation. Standard thrombophilia screening was negative. Notably, Thrombin generation assays were normal or lower than healthy controls (Figure 1A), contradicting the thrombin dysregulation hypothesis. LTA revealed that despite the absence of fibrinogen and the use of potent antiplatelet agents, the patient's platelets maintained normal aggregation in response to strong agonists (collagen 4 μg/mL, Figure 1B). Following the withdrawal of antiplatelet therapy, the ex vivo addition of physiological fibrinogen (2 g/L) induced dramatic platelet aggregation even under weak agonistic stimuli (0.5 mM arachidonic acid), significantly exceeding the normal reference range (Figure 1C-E).

**Copyright Information:** (Online) ISSN: 2572-9241
©2026 The Author(s). HemaSphere is published by John Wiley & Sons Ltd on behalf of European Hematology Association. This is an open access Abstract Book distributed under the Attribution-NonCommercial-NoDerivs (CC BY-NC-ND), which allows third parties to download the articles and share them with others as long as they credit the author and the Abstract Book, but they cannot change the content in any way or use them commercially.
**Abstract Book Citations:** Authors, Title, HemaSphere, 2026;10:(S1):pages. The abstract book DOIs can be found on the "Author Information" page.
**Disclaimer:** Articles published in the journal HemaSphere exclusively reflect the opinions of the authors. The authors are responsible for all content in their abstracts including accuracy of the facts, statements, citing resources, etc.

**Summary/Conclusion**

Our findings suggest that platelet hyperreactivity, rather than thrombin dysregulation, serves as the main mechanism for arterial thrombosis in this patient. This case emphasizes the need for individualized management. For patients with this platelet hyperreactive phenotype, antiplatelet prophylaxis may be more effective than standard anticoagulation in preventing thrombotic complications after fibrinogen replacement therapy.

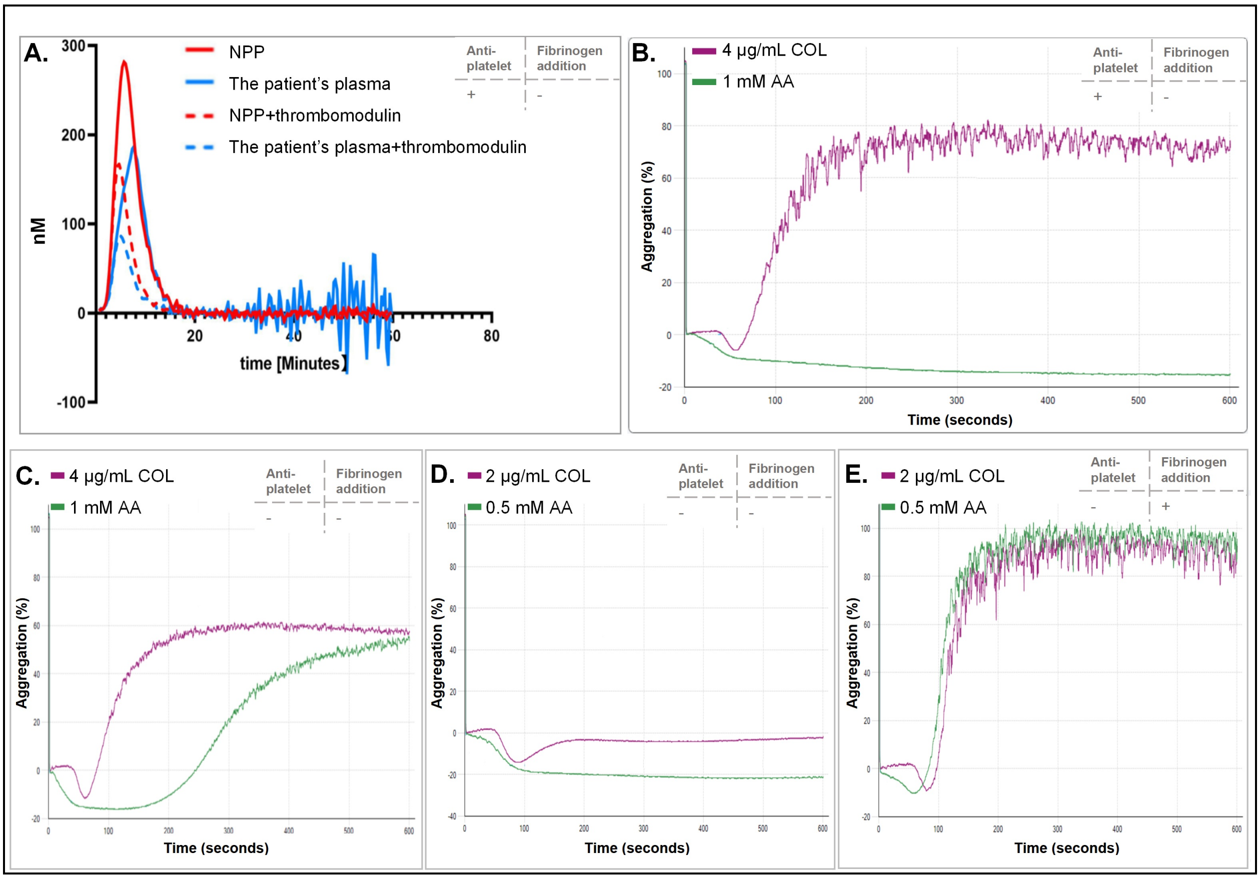

Thrombin generation assays and platelet aggregation assays. A, thrombin generation assays during active antiplatelet treatment; B, platelet aggregation assays during active antiplatelet treatment; C-D, platelet aggregation assays after stopping antiplatelet treatment for over one week with different agonists with different concentrations; E, upon ex vivo addition of fibrinogen (2 g/L), platelet aggregation assays after stopping antiplatelet treatment for over one week with different agonists with low concentrations.

**Copyright Information:** (Online) ISSN: 2572-9241
©2026 The Author(s). HemaSphere is published by John Wiley & Sons Ltd on behalf of European Hematology Association. This is an open access Abstract Book distributed under the Attribution-NonCommercial-NoDerivs (CC BY-NC-ND), which allows third parties to download the articles and share them with others as long as they credit the author and the Abstract Book, but they cannot change the content in any way or use them commercially.
**Abstract Book Citations:** Authors, Title, HemaSphere, 2026;10:(S1):pages. The abstract book DOIs can be found on the "Author Information" page.
**Disclaimer:** Articles published in the journal HemaSphere exclusively reflect the opinions of the authors. The authors are responsible for all content in their abstracts including accuracy of the facts, statements, citing resources, etc.

### (PS2481) SYK Y342 AND Y346 PHOSPHORYLATION IS ESSENTIAL FOR OPTIMAL ITAM AND HEMITAM-MEDIATED SIGNALING IN PLATELETS

**Topic:** 35. Thrombosis and vascular biology

Carol Dangelmaier[1], Manal Elzoheiry[1], Dhruv Vajipayajula[2], Monica Wright[1], Soochong Kim[3], Alexander Tsygankov[1], Satya Kunapuli*[1]

*[1]Temple University, Sol sherry Thrombosis Research Center, Philadelphia, United States of America;[2]Tufts University, Boston, United States of America; [3]Chungbuk National University, Cheongju, South Korea;*

### Background

Spleen tyrosine kinase (Syk) is an essential non-receptor tyrosine kinase in immunoreceptor tyrosine-based activation motif (ITAM) and hemITAM signal transduction in many hematopoietic systems. Syk is comprised of two tandem Src homology (SH) domains separated by a linker A region, an SH2-kinase linker B region, and a C-terminal tyrosine kinase domain. The ability of Syk to regulate signaling is influenced by its phosphorylation on tyrosine residues within the linker B region. The phosphorylation of both Y342 and Y346 (Y348 and Y352 in human) is necessary for optimal signaling in B cells.

### Aims

To assess the importance of Syk Y342 and Y346 in a physiological system and their effect on receptor-mediated responses.

### Methods

Syk Y342 and Y346 were mutated to phenylalanines by CRISPR-Cas. Platelets were isolated and collagen-related peptide (CRP) or CLEC-2 cross-linking -induced aggregation and dense granule secretion were measured. P-selectin exposure and fibrinogen activation were analyzed by flow cytometry. The phosphorylation state of PLCγ2 was assessed by Western blot.

### Results

On gross examination, the intestines of Syk Y342,346F mice were diffusely reddened, suspicious for inflammation. There was no aggregation, dense granule secretion, P-selectin exposure or fibrinogen activation when the mutated platelets were challenged with any concentration of CRP or CLEC-2 cross-linking. No phosphorylation of the downstream signaling protein PLCγ2 Y1217 was detected in the mutated platelets when stimulated with the above agonists. There was, however, aggregation and dense granule secretion when the Syk Y342,346F platelets were activated with a high concentration of collagen.

### Summary/Conclusion

These findings indicate that phosphorylation of Syk Y342 and Y346 is necessary for full Syk activation and propagation of intracellular signaling, stressing their essential role in receptor-mediated responses.

**Copyright Information:** (Online) ISSN: 2572-9241
©2026 The Author(s). HemaSphere is published by John Wiley & Sons Ltd on behalf of European Hematology Association. This is an open access Abstract Book distributed under the Attribution-NonCommercial-NoDerivs (CC BY-NC-ND), which allows third parties to download the articles and share them with others as long as they credit the author and the Abstract Book, but they cannot change the content in any way or use them commercially.
**Abstract Book Citations:** Authors, Title, HemaSphere, 2026;10:(S1):pages. The abstract book DOIs can be found on the “Author Information” page.
**Disclaimer:** Articles published in the journal HemaSphere exclusively reflect the opinions of the authors. The authors are responsible for all content in their abstracts including accuracy of the facts, statements, citing resources, etc.

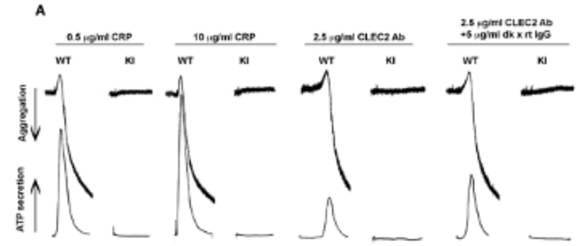

**GPVI- and CLEC-2-mediated aggregation and dense granule secretion are absent in Syk Y342,346F platelets.** Representative aggregation and dense. granule secretion tracings of Syk Y342,346F and WT littermate control platelets. stimulated with the indicated concentrations of CRP or CLEC-2 (A).

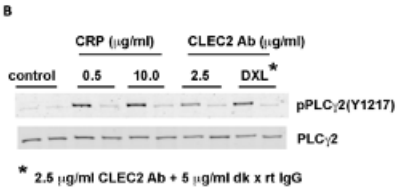

**GPVI- and CLEC-2-mediated signaling is absent in Syk Y342,346F platelets.** Representative Western blot probed for pPLCγ2(Y1217) and total PLCγ2 in Syk Y342,346F and WT platelets with the indicated concentrations of CRP or CLEC-2 (B).

**Copyright Information:** (Online) ISSN: 2572-9241
©2026 The Author(s). HemaSphere is published by John Wiley & Sons Ltd on behalf of European Hematology Association. This is an open access Abstract Book distributed under the Attribution-NonCommercial-NoDerivs (CC BY-NC-ND), which allows third parties to download the articles and share them with others as long as they credit the author and the Abstract Book, but they cannot change the content in any way or use them commercially.
**Abstract Book Citations:** Authors, Title, HemaSphere, 2026;10:(S1):pages. The abstract book DOIs can be found on the "Author Information" page.
**Disclaimer:** Articles published in the journal HemaSphere exclusively reflect the opinions of the authors. The authors are responsible for all content in their abstracts including accuracy of the facts, statements, citing resources, etc.

### (PS2482) THROMBOTIC COMPLICATIONS AS PREDICTORS OF MORTALITY IN PEDIATRIC ECMO UNDER UNFRACTIONATED HEPARIN

**Topic:** 35. Thrombosis and vascular biology

Bader Allahyani*[1], Raghad Alnemari[1], Abdullah Aljefri[1], Abdulrahman Almusa[1], Mahasen Al Saleh[1]

*[1]King Faisal Specialist Hospital, Riyadh, Saudi Arabia;*

**Background**

Extracorporeal membrane oxygenation (ECMO) induces profound hemostatic dysregulation characterized by simultaneous bleeding and thrombotic risk. In pediatric populations, anticoagulation is predominantly achieved using unfractionated heparin (UFH), despite recognized variability in pharmacodynamic response, antithrombin dependence, and monitoring limitations. As direct thrombin inhibitors (DTIs) are increasingly adopted in ECMO practice, defining complication patterns and mortality associations within UFH-treated cohorts is essential to inform evolving anticoagulation strategies.

**Aims**

To evaluate the incidence of major bleeding and thrombotic complications in pediatric ECMO supported with UFH and to determine their association with in-hospital mortality.

**Methods**

We conducted a retrospective cohort study of pediatric patients (≤14 years) supported on ECMO at a tertiary referral center between January 2019 and December 2023. To ensure analytic homogeneity, only UFH-treated patients were included. Major bleeding was defined according to International Society on Thrombosis and Haemostasis (ISTH) criteria, including intracranial hemorrhage and bleeding requiring transfusion or surgical intervention. Thrombotic events included limb ischemia, circuit thrombosis, and documented systemic thrombosis. The primary endpoint was in-hospital mortality. Associations were evaluated using chi-square testing and multivariable logistic regression.

**Results**

A total of 117 pediatric patients were analyzed (median age 3 months; 51% male). ISTH-defined major bleeding occurred in 44.4% of patients, including intracranial hemorrhage in 17.9%. Thrombotic events occurred in 38.5%, with limb ischemia in 15.4% and circuit thrombosis in 7.7%.
Thrombotic complications were significantly associated with mortality and conferred approximately a threefold increased risk of death ($p<0.05$). Patients experiencing both bleeding and thrombosis demonstrated the highest mortality rates. In multivariable analysis, thrombosis remained independently associated with mortality.

**Summary/Conclusion**

In this pediatric ECMO cohort treated with unfractionated heparin, thrombotic complications were independently associated with mortality, while major bleeding occurred frequently and contributed substantially to overall morbidity. These findings characterize a high-risk coagulopathy phenotype during ECMO support and provide important benchmark data within the heparin era, supporting continued evaluation of alternative anticoagulation strategies, including direct thrombin inhibitors.

**Copyright Information:** (Online) ISSN: 2572-9241
©2026 The Author(s). HemaSphere is published by John Wiley & Sons Ltd on behalf of European Hematology Association. This is an open access Abstract Book distributed under the Attribution-NonCommercial-NoDerivs (CC BY-NC-ND), which allows third parties to download the articles and share them with others as long as they credit the author and the Abstract Book, but they cannot change the content in any way or use them commercially.
**Abstract Book Citations:** Authors, Title, HemaSphere, 2026;10:(S1):pages. The abstract book DOIs can be found on the "Author Information" page.
**Disclaimer:** Articles published in the journal HemaSphere exclusively reflect the opinions of the authors. The authors are responsible for all content in their abstracts including accuracy of the facts, statements, citing resources, etc.

### (PS2483) A DIAGNOSTIC SAFETY GAP IN THROMBOPHILIA TESTING: UNRELIABLE TIMING AND DOWNSTREAM HARM

**Topic:** 35. Thrombosis and vascular biology

Abdulrahman Al Raizah*[1], Ayman Hejazi[1], Aimn W Abdujawad[1], Rawan Al Mohamady[1], Ayman Almozaini[1], Sumayyah Altamimi[1], Khalid Aldosari[1], Ayel Yahya[1], Mohammed Bakkar[1], Ayman Ibrahim[1], Abdullah Alsaleh[1], Husam Alsadi[1], Bader Alahmari[1], Abdul Rauf Ghori[1], Mohsen Alzahrani[1], Hind Salama[1], Naveed Qureshi[1], Angham Sahali[1], Khalid Mohamed[1], Ahmed Alwakeel[1], Ahmed Alaskar[1]

*[1]Oncology department, Ministry of National Guard Health Affairs, King Abdullah International Medical Research Center, King Saud bin Abdulaziz University of Health Sciences, Saudi society of bone marrow transplant, Riyadh, Saudi Arabia;*

### Background

IInherited thrombophilia testing may be considered in selected clinical situations to guide risk assessment and therapeutic decisions. However, when performed outside appropriate clinical conditions, results are highly susceptible to biological interference, potentially leading to inaccurate interpretation and consequential downstream harm, including misclassification and inappropriate long-term management.

### Aims

To calculate the proportion of inherited thrombophilia tests performed under biologically unreliable conditions and to quantify associated downstream clinical consequences.

### Methods

We conducted a retrospective chart review at Ministry of National Guard Health Affairs Hospital, Riyadh. Adult patients (≥16 years) who underwent inherited thrombophilia testing, specifically Protein C, Protein S, and Antithrombin III assays, between January 2024 and November 2025 were included.
Biologically unreliable testing was defined as testing performed under conditions known to alter assay results. A test was considered unreliable if any of the following criteria were met:

- testing within 3 months of thrombosis
- antithrombin testing during intravenous heparin exposure
- protein S testing during pregnancy
- protein C or protein S testing during warfarin therapy

Clinical consequences were assessed at the patient level:

Pregnancy-related treatment was defined as documented administration of antiplatelet therapy and/or anticoagulation during pregnancy.
Lifelong anticoagulation classification was defined as documentation in the medical record indicating indefinite or “for life” anticoagulation therapy.
Thrombophilia labelling was defined as explicit documentation in the medical record that the patient was considered to have inherited thrombophilia based on testing results.
Repeat testing was defined as subsequent reordering of the same thrombophilia assay in the same patient.

The study was approved by the Institutional Review Board, and the requirement for informed consent was waived.

**Copyright Information:** (Online) ISSN: 2572-9241
©2026 The Author(s). HemaSphere is published by John Wiley & Sons Ltd on behalf of European Hematology Association. This is an open access Abstract Book distributed under the Attribution-NonCommercial-NoDerivs (CC BY-NC-ND), which allows third parties to download the articles and share them with others as long as they credit the author and the Abstract Book, but they cannot change the content in any way or use them commercially.
**Abstract Book Citations:** Authors, Title, HemaSphere, 2026;10:(S1):pages. The abstract book DOIs can be found on the “Author Information” page.
**Disclaimer:** Articles published in the journal HemaSphere exclusively reflect the opinions of the authors. The authors are responsible for all content in their abstracts including accuracy of the facts, statements, citing resources, etc.

**Results**

A total of 297 adult patients undergoing 505 inherited thrombophilia tests were included. The median age was 35 years (IQR 28–41), and 148 patients (68%) were female.
Thrombophilia testing was most commonly requested in the context of pregnancy-related complications (32.3%), followed by unclear documentation (31.5%). Venous thrombosis accounted for 17% of testing, family screening for 10.1%, and arterial thrombosis for 9.1%. ( table1)
At the test level, 113 of 505 tests (22.4%) met predefined criteria for biological unreliability. ( table 2)
At the patient level, 58 of 297 patients (19.5%) were exposed to at least one unreliable test.
Among the 58 patients exposed to biologically unreliable testing, 12 (20.7%) underwent repeat testing, 13 (22.4%) were classified as requiring lifelong anticoagulation, 9 (15.5%) received pregnancy-related treatment, and 2 (3.4%) were labelled as having thrombophilia.

**Summary/Conclusion**

More than one in five inherited thrombophilia tests were performed under biologically unreliable conditions. Among patients exposed to unreliable testing, we observed substantial downstream clinical consequences, including repeat investigations, classification for lifelong anticoagulation, pregnancy-related treatment, and thrombophilia labelling. These findings demonstrate a measurable diagnostic safety failure and warrants urgent system-level intervention to ensure testing validity and protect patients from preventable harm.

**Copyright Information:** (Online) ISSN: 2572-9241
©2026 The Author(s). HemaSphere is published by John Wiley & Sons Ltd on behalf of European Hematology Association. This is an open access Abstract Book distributed under the Attribution-NonCommercial-NoDerivs (CC BY-NC-ND), which allows third parties to download the articles and share them with others as long as they credit the author and the Abstract Book, but they cannot change the content in any way or use them commercially.
**Abstract Book Citations:** Authors, Title, HemaSphere, 2026;10:(S1):pages. The abstract book DOIs can be found on the “Author Information” page.
**Disclaimer:** Articles published in the journal HemaSphere exclusively reflect the opinions of the authors. The authors are responsible for all content in their abstracts including accuracy of the facts, statements, citing resources, etc.

**Table 1. Reasons for Requesting Thrombophilia Testing (Test-Level, n=505)**

| Indication | n (%) |
|---|---|
| Pregnancy complications | 163 (32.3%) |
| Unclear documentation | 159 (31.5%) |
| Venous thrombosis | 86 (17.0%) |
| Family screening | 51 (10.1%) |
| Arterial thrombosis | 46 (9.1%) |
| **Total** | **505 (100%)** |

**Table 2. Reasons for Biologically Unreliable Testing by Assay (Test-Level, n=505)**

| Test Type | ≤3 Months from Thrombosis | IV Heparin | Pregnancy | Warfarin | Total Unreliable |
|---|---|---|---|---|---|
| Antithrombin | 32 | 9 | — | — | 34 |
| Protein C | 28 | — | — | 1 | 29 |
| Protein S | 34 | — | 16 | 1 | 50 |
| **Total** | 94 | 9 | 16 | 2 | **113 (22.4%)** |

**Table 3. Clinical Consequences Among Patients Exposed to Unreliable Testing (n=58)**

| Clinical Consequence | n | % |
|---|---|---|
| Repeat testing | 12 | 20.7% |
| Lifelong anticoagulation classification | 13 | 22.4% |
| Pregnancy-related treatment | 9 | 15.5% |
| Thrombophilia labelling | 2 | 3.4% |

**Copyright Information:** (Online) ISSN: 2572-9241
©2026 The Author(s). HemaSphere is published by John Wiley & Sons Ltd on behalf of European Hematology Association. This is an open access Abstract Book distributed under the Attribution-NonCommercial-NoDerivs (CC BY-NC-ND), which allows third parties to download the articles and share them with others as long as they credit the author and the Abstract Book, but they cannot change the content in any way or use them commercially.
**Abstract Book Citations:** Authors, Title, HemaSphere, 2026;10:(S1):pages. The abstract book DOIs can be found on the "Author Information" page.
**Disclaimer:** Articles published in the journal HemaSphere exclusively reflect the opinions of the authors. The authors are responsible for all content in their abstracts including accuracy of the facts, statements, citing resources, etc.

### (PS2484) COMBINATION OF THROMBOPOIETIN RECEPTOR AGONISTS AND RITUXIMAB IN RELAPSED/REFRACTORY IMMUNE THROMBOCYTOPENIA: A MULTICENTER REAL-WORLD STUDY

**Topic:** 35. Thrombosis and vascular biology

Hong Tian*[1], Xuewen Song[2], Yueting Huang[3], Lu Zhou[4], Ruinan Lu[5], Lili Ji[6], Yun Li[1], Haiju He[1], Ziqiang Yu[1], Hu Zhou[2], Jie Yin[1]

*[1]National Clinical Research Center for Hematologic Diseases, Jiangsu Institute of Hematology, The First Affiliated Hospital of Soochow University, Suzhou, China; [2]The Affiliated Cancer Hospital of Zhengzhou University & Henan Cancer Hospital, Zhengzhou, China; [3]the First Affiliated Hospital of Xiamen University, School of Medicine, Xiamen University, Xiamen, China; [4]Affiliated hospital of Nantong University, Nantong, China;[5]the First Affiliated Hospital of Nanjing Medical University, Jiangsu Province Hospital, Nanjing, China; [6]Zhongshan Hospital, Fudan University, Shanghai, China;*

### Background

Immune thrombocytopenia (ITP) is an autoimmune disorder characterized by increased platelet destruction and impaired production. While first-line therapies often fail to induce durable remissions, many patients become refractory to multiple lines of treatment. The combination of thrombopoietin receptor agonists (TPO-RAs) and rituximab offers a mechanistically synergistic strategy, concurrently targeting immune-mediated platelet destruction and insufficient production.

### Aims

Our study aimed to evaluate the efficacy, durability, safety, and predictors of treatment-free remission (TFR) in patients with relapsed or refractory ITP receiving combination therapy with TPO-RAs and rituximab.

### Methods

We conducted a multicenter retrospective study across six tertiary centers in China, enrolling patients with relapsed or refractory ITP who received combination therapy with TPO-RAs and rituximab between January 2018 and June 2024. Treatment response, durability, safety, and predictors of treatment-free remission (TFR) were analyzed.

### Results

A total of 161 patients were included, with a median age of 42 years and median ITP duration of 24 months; 88% had experienced at least one relapse and 93% were refractory to prior therapies. The overall response rate was 83% (complete response [CR] 43%, partial response [PR] 40%). After a median follow-up of 22 months, 30% of patients achieved TFR, with a median time to TFR of 17 weeks post-combination. Notably, among patients receiving the same TPO-RA as prior treatment, rituximab restored platelet response in 28.9% of primarily refractory and 40.0% of secondarily resistant patients. Multivariate analysis identified the composite of female sex, age <45 years, and disease duration <24 months as a strong independent predictor of TFR (OR = 14.21, 95% CI 2.34–86.18, P = 0.004), while prior TPO-RA use predicted non-response/relapse (OR = 3.28, 95% CI 1.54–6.99, P = 0.002). Low-dose rituximab was also independently associated with TFR (OR = 0.12, 95% CI 0.02–0.62, P = 0.012). Treatment-related adverse events were observed in 23 patients (14.3%), most of which were mild to moderate (Grade 1-2), with no treatment-related deaths.

**Copyright Information:** (Online) ISSN: 2572-9241
©2026 The Author(s). HemaSphere is published by John Wiley & Sons Ltd on behalf of European Hematology Association. This is an open access Abstract Book distributed under the Attribution-NonCommercial-NoDerivs (CC BY-NC-ND), which allows third parties to download the articles and share them with others as long as they credit the author and the Abstract Book, but they cannot change the content in any way or use them commercially.
**Abstract Book Citations:** Authors, Title, HemaSphere, 2026;10:(S1):pages. The abstract book DOIs can be found on the “Author Information” page.
**Disclaimer:** Articles published in the journal HemaSphere exclusively reflect the opinions of the authors. The authors are responsible for all content in their abstracts including accuracy of the facts, statements, citing resources, etc.

**Summary/Conclusion**

The combination of TPO-RAs and rituximab is an effective and well-tolerated strategy in heavily pretreated, relapsed/refractory ITP, achieving high response rates and durable treatment-free remission in a substantial subset. The regimen can restore sensitivity to previously ineffective TPO-RAs. Young women with short disease duration derive the greatest benefit, providing a practical profile for clinical selection. These findings support prospective evaluation of this synergistic approach.

**Copyright Information:** (Online) ISSN: 2572-9241
©2026 The Author(s). HemaSphere is published by John Wiley & Sons Ltd on behalf of European Hematology Association. This is an open access Abstract Book distributed under the Attribution-NonCommercial-NoDerivs (CC BY-NC-ND), which allows third parties to download the articles and share them with others as long as they credit the author and the Abstract Book, but they cannot change the content in any way or use them commercially.
**Abstract Book Citations:** Authors, Title, HemaSphere, 2026;10:(S1):pages. The abstract book DOIs can be found on the “Author Information” page.
**Disclaimer:** Articles published in the journal HemaSphere exclusively reflect the opinions of the authors. The authors are responsible for all content in their abstracts including accuracy of the facts, statements, citing resources, etc.

### (PS2485) ICANS AFTER CAR-T THERAPY: PERSISTENT COGNITIVE BUT NOT PSYCHIATRIC RISK - A PROPENSITY SCORE-MATCHED ANALYSIS

**Topic:** 36. Quality of life, ethics, supportive and palliative care

Heidi Cho*[1], Michael Mapp[1], Loren Caldwell[1], Alex Hughes[1], Caroline Norwood[1], Jessica Garinger[1], Katrina Reiling[1], Vladimir Otasevic[1], Ying Li[1], Nancy Lunney[1], Chris Learn[1]

*[1]Parexel, Global Medical Services, Raleigh, United States of America;*

### Background

Chimeric Antigen Receptor T-cell therapy (CAR T) is a transformative treatment for hematologic malignancies, but neurotoxicity, specifically Immune Effector Cell–Associated Neurotoxicity Syndrome (ICANS), can manifest with confusion, language disturbance, agitation, and mood changes. As survivorship increases, it is important to understand whether ICANS contributes to longer-term neuropsychiatric morbidity. Evidence on persistent cognitive or psychiatric outcomes after ICANS remains limited and variable. This study evaluated the association between ICANS and subsequent cognitive and psychiatric diagnoses using real-world data (RWD).

### Aims

A retrospective multicenter analysis was conducted using the TriNetX Dataworks-USA network (January 28, 2026), incorporating electronic medical records from 69 healthcare organizations. Adult patients with hematologic malignancies who received FDA-approved CAR T products were included. Those with psychiatric or cognitive disorders in the prior year were excluded to identify incident post-CAR-T diagnoses. ICANS was defined using ICD-10 code G92.0 occurring 1–21 days post-infusion. Propensity score matching (1:1) was performed on 18 covariates, including age, race, lymphoma subtype, comorbidities, and prior therapy. Outcomes were assessed from day 22 through day 1,095 (3 years) and included cognitive impairment, organic mental disorders, mood disorders, and anxiety disorders. Cox proportional hazards models evaluated risk.

### Methods

After matching, 469 patient pairs (938 total) were identified with well-balanced covariates (age SMD=0.03). Patients who developed ICANS had significantly higher rates of cognitive complications. Cognitive impairment occurred in 15.6% of ICANS patients versus 7.0% of controls (adjusted RR 2.21; 95% CI 1.50–3.27; p<0.001). Altered mental status was more frequent (13.0% vs. 4.5%; adjusted RR 2.91; 95% CI 1.80–4.70; p<0.001). Organic mental disorders occurred in 6.0% vs. 2.6% (adjusted RR 2.33; 95% CI 1.20–4.52; p=0.010). ICANS was not associated with increased mood (7.0% vs. 6.4%; p=0.696) or anxiety disorders (12.2% vs. 12.6%; p=0.843). Time-to-event analysis showed the highest cognitive risk in the first 2–3 months post-CAR-T (HR 2.43; 95% CI 1.61–3.68; p<0.001), with stabilization thereafter.

### Results

Among CAR T recipients without prior psychiatric history, ICANS independently predicts subsequent cognitive impairment, particularly in the subacute period, but does not increase long-term risk of mood or anxiety disorders. Findings suggest psychiatric symptoms during ICANS are largely transient, whereas cognitive effects may reflect more durable neurological sequelae. Patients who develop ICANS may benefit from targeted cognitive monitoring in the first 2–3 months post-therapy. Further research is needed to validate these results and refine long-term neuropsychiatric care strategies as CAR T therapy evolves.

**Copyright Information:** (Online) ISSN: 2572-9241
©2026 The Author(s). HemaSphere is published by John Wiley & Sons Ltd on behalf of European Hematology Association. This is an open access Abstract Book distributed under the Attribution-NonCommercial-NoDerivs (CC BY-NC-ND), which allows third parties to download the articles and share them with others as long as they credit the author and the Abstract Book, but they cannot change the content in any way or use them commercially.
**Abstract Book Citations:** Authors, Title, HemaSphere, 2026;10:(S1):pages. The abstract book DOIs can be found on the "Author Information" page.
**Disclaimer:** Articles published in the journal HemaSphere exclusively reflect the opinions of the authors. The authors are responsible for all content in their abstracts including accuracy of the facts, statements, citing resources, etc.

### (PS2486) PATIENT-REPORTED FATIGUE AND IMPAIRED COGNITIVE FUNCTION AFTER ALLOGENEIC-HSCT: SIGNIFICANT CORRELATIONS WITH RETURN TO SOCIO-PROFESSIONAL ACTIVITIES – THE SOPRALLO STUDY

**Topic:** 36. Quality of life, ethics, supportive and palliative care

Yana Stepanishyna*[1], Fabio Andreozzi[1], Paulus Kristanto[1], Adriano Salaroli[1], Sandra Michiels[1], Marie-France Jaivenois[1], Rezarta Ajazi[1], Nathalie Meuleman[1], Virginie De Wilde[1], Alice Wolfromm[1], Philippe Lewalle[1], Şule Mine Bakanay Öztürk[2], Dominique Bron[1], Sebastian Wittnebel[1]

*[1]Institut Jules Bordet, Brussels, Belgium; [2]Ankara Yıldırım B. University, Bilkent City Hospital, Ankara, Türkiye;*

### Background

Return to work (RTW) represents a major milestone of functional recovery and social reintegration after allogeneic hematopoietic stem cell transplantation (allo-HSCT). Previous findings from the SOPRALLO project demonstrated a 58% RTW rate among patients under 65 years and highlighted that psychological distress, cognitive outcomes, and overall post-transplant quality of life (QoL) are closely associated with RTW.

### Aims

The primary objective of this study is to identify patient-reported determinants of RTW after allo-HSCT, with a specific focus on age, fatigue burden and perceived cognitive functions.

### Methods

Patients who underwent allo-HSCT at the Jules Bordet Institute and remained in remission for at least one-year post-transplant were included in this study. Multiple validated questionnaires were applied for sub-analysis: FACT-Cog, FACIT-F, HADS, FACT-BMT, RTW self-efficacy. Clinical data were collected retrospectively. Additionally, information on work status before and after HSCT was collected through a locally developed questionnaire. Statistical analyses included Wilcoxon/Kruskal-Wallis, chi-square, and multivariate Cox regression for RTW predictors.

**Copyright Information:** (Online) ISSN: 2572-9241
©2026 The Author(s). HemaSphere is published by John Wiley & Sons Ltd on behalf of European Hematology Association. This is an open access Abstract Book distributed under the Attribution-NonCommercial-NoDerivs (CC BY-NC-ND), which allows third parties to download the articles and share them with others as long as they credit the author and the Abstract Book, but they cannot change the content in any way or use them commercially.
**Abstract Book Citations:** Authors, Title, HemaSphere, 2026;10:(S1):pages. The abstract book DOIs can be found on the “Author Information” page.
**Disclaimer:** Articles published in the journal HemaSphere exclusively reflect the opinions of the authors. The authors are responsible for all content in their abstracts including accuracy of the facts, statements, citing resources, etc.

**Results**

Among 178 patients who met ethical requirements, 132 were evaluable for analysis. Of these, 108 (81.8%) had worked before allo-HSCT, and 64 (59%) RTW. Analyses were restricted to patients of working age (≤65 years) to reflect employment eligibility. Age distribution was 44.5% for 18–45 years, 55.5% for 46–65 years.

- In Cox regression analysis of questionnaires including FACT domains (physical, functional, social, emotional well-being), cognitive function (FACT-Cog PCI and PCA), HADS, IOC scales and cGVHD status, younger age at transplantation and lower fatigue burden emerged as the strongest independent predictors of RTW. Patients aged 18–45 years were significantly more likely to resume work than those aged 46–65 years (HR 1.96; 95% CI 1.17–3.26; p = 0.010), and higher FACIT-FS scores remained independently associated with RTW (HR 1.02; 95% CI 1.00–1.04; p =0.013).
- Among patients aged 18–45 years, RTW was significantly correlated with fatigue burden (p = 0.02), while no significant differences were observed in cognitive measures (CogPCA, p = 0.18; CogPCI, p = 0.25). In contrast, among patients aged 46–65 years, both fatigue (p = 0.009) and cognitive function (CogPCA, p = 0.03; CogPCI, p = 0.03) were significantly correlated with RTW.
- Significant fatigue was more frequently observed in patients with higher perceived cognitive impairments (Cog-PCI), lower perceived cognitive abilities (Cog-PCA) and cognitive QoL, as well as in those with symptoms of anxiety and depression, and lower functional, social, emotional, and physical well-being (p = 0.0001).
- Cognitive impairment (CogPCI) differed significantly across RTW readiness groups (p = 0.0005), with the highest prevalence in patients not ready for RTW (66.7%) compared to moderate-ready (37.5%) and full-ready groups (22.7%). Reduced CogPCA was more frequent among patients not ready for RTW (75.0%) than in moderately ready (62.5%) or fully ready (31.0%) groups (p = 0.0006).

**Summary/Conclusion**

Our data suggest that return to socio-professional activities is correlated with:

- Younger age at transplantation (< 45 vs ≥ 45 yo)
- Lower fatigue burden regardless of survivors' age
- Better perceived cognitive function, particularly among older survivors
- Lower levels of anxiety and depression
- Better overall QoL regardless of socio-demographic and disease characteristics

**Copyright Information:** (Online) ISSN: 2572-9241
©2026 The Author(s). HemaSphere is published by John Wiley & Sons Ltd on behalf of European Hematology Association. This is an open access Abstract Book distributed under the Attribution-NonCommercial-NoDerivs (CC BY-NC-ND), which allows third parties to download the articles and share them with others as long as they credit the author and the Abstract Book, but they cannot change the content in any way or use them commercially.
**Abstract Book Citations:** Authors, Title, HemaSphere, 2026;10:(S1):pages. The abstract book DOIs can be found on the "Author Information" page.
**Disclaimer:** Articles published in the journal HemaSphere exclusively reflect the opinions of the authors. The authors are responsible for all content in their abstracts including accuracy of the facts, statements, citing resources, etc.

### (PS2487) VTE RISK ASSESSMENT MODELS (RAM), THROMBOPROPHYLAXIS USE & OUTCOMES IN ATOMM: ANTICOAGULATION FOR THROMBOSIS PREVENTION IN MULTIPLE MYELOMA, A LARGE REAL-WORLD COHORT OF NEWLY DIAGNOSED MYELOMA (NDMM)

**Topic:** 36. Quality of life, ethics, supportive and palliative care

Mari Thomas[1], Raza Alikhan[2], Tom Bull[3], Elena Ganendra[4], Mamatha Karanth[3], William Wilson[5], Marquita Camilleri*[6]

*[1]University College London Hospitals NHS Foundation Trust, London, United Kingdom;[2]Cardiff and Vale University Health Board, Cardiff, United Kingdom; [3]West Suffolk NHS Foundation Trust, Bury St Edmonds, United Kingdom;[4]North West Anglia NHS Foundation Trust, Peterborough, United Kingdom; [5]Cancer Research UK Clinical Trials Centre, University College London, London, United Kingdom; [6]Cambridge University Hospitals NHS Foundation Trust, Cambridge, United Kingdom;*

### Background

Validated myeloma VTE-RAM exist but their clinical relevance, including influence on thromboprophylaxis prescribing, remains unclear.

### Aims

Investigate factors influencing thromboprophylaxis usage in large real-world cohort.

### Methods

UK-wide, retrospective observational study auditing thromboprophylaxis during first 6 months' treatment of NDMM 1/1/2021-1/8/2024, collecting thrombosis & bleeding rates, excluding patients on treatment dose anticoagulation. SAVED & IMPEDE scores retrospectively calculated.

### Results

In 2359 patients from 41 centres (median age 69y [27-100y]; 58% male), 75.6% (1783/2359) received thromboprophylaxis & 2.5% (60/2359) developed VTE. Based on IMPEDE scores, 28.4% (671/2359) were high-risk; 54.3% intermediate; 17.1% low-risk. Among 1798 patients treated with immunomodulatory drugs (IMiD), agreement between IMPEDE & SAVED scores was poor (Kappa=0.009), with 98.6% IMiD patients high/intermediate risk by IMPEDE & only 29.4% by SAVED.
High-risk IMPEDE patients were more likely to receive thromboprophylaxis (89.9%) than intermediate (86.3%; p=0.02) or low-risk (17.9%; p<0.001). High-risk SAVED patients were less likely to receive thromboprophylaxis than low-risk (92.2% vs.95.4%, p=0.007), although >90% received thromboprophylaxis regardless of SAVED score. Choice of thromboprophylaxis was similar across risk groups and aspirin use was infrequent, even in low-risk IMPEDE patients (1.5%, 6/403).
VTE rates increased with higher IMPEDE score [low: 2.5% (10/403) vs intermediate: 2.0% (25/128) vs high: 3.7% (25/671), p=0.04 adjusted for thromboprophylaxis use]. There was no difference in VTE risk based on SAVED score. IMiD-treated transplant-eligible patients were more likely to receive thromboprophylaxis than those transplant ineligible (Table 1: 96.2% vs 91.9%, p<0.001). VTE rates of 2-2.9% were substantially lower than historical UK NDMM IMiD cohorts (Myeloma XI RCT 2010-2016: 80.5% thromboprophylaxis, VTE rates 10.4-13.2%).
In non-IMiD patients, only 20.8% IMPEDE high/intermediate risk patients received thromboprophylaxis & had significantly higher VTE rate than IMiD transplant-eligible (4.9% vs 2.0%, p=0.02). Commonest reason for thromboprophylaxis omission was 'IMiD-free regimen'. Only 12.2% low IMPEDE risk received thromboprophylaxis. IMiD use increased over the study period (Figure 1) with corresponding increase in thromboprophylaxis use driven by increased DOAC use.

**Copyright Information:** (Online) ISSN: 2572-9241
©2026 The Author(s). HemaSphere is published by John Wiley & Sons Ltd on behalf of European Hematology Association. This is an open access Abstract Book distributed under the Attribution-NonCommercial-NoDerivs (CC BY-NC-ND), which allows third parties to download the articles and share them with others as long as they credit the author and the Abstract Book, but they cannot change the content in any way or use them commercially.
**Abstract Book Citations:** Authors, Title, HemaSphere, 2026;10:(S1):pages. The abstract book DOIs can be found on the "Author Information" page.
**Disclaimer:** Articles published in the journal HemaSphere exclusively reflect the opinions of the authors. The authors are responsible for all content in their abstracts including accuracy of the facts, statements, citing resources, etc.

Table 1: Risk stratification & outcomes by IMiD use, transplant-eligibility & IMPEDE score

| | IMiD transplant eligible N=1066 | IMiD not transplant eligible N=732 | Non-IMiD Low IMPEDE N=377 | Non-IMiD Int/High IMPEDE N=183 |
|---|---|---|---|---|
| **VTE prophylaxis** | | | | |
| **Any** | **1026 (96.2%)** | **673 (91.9%)** | **46 (12.2%)** | **38 (20.8%)** |
| DOAC | 700 (65.7%) | 508 (69.4%) | 23 (6.1%) | 12 (6.6%) |
| LMWH | 288 (27.0%) | 119 (16.3%) | 19 (5.0%) | 24 (13.11%) |
| Aspirin | 36 (3.4%) | 43 (5.9%) | 4 (1.1%) | 2 (1.1%) |
| Warfarin | 1 (0.1%) | 1 (0.1%) | 0 | 0 |
| Agent not known | 1 (0.1%) | 2 (0.3%) | 0 | 0 |
| **None** | **40 (3.8%)** | **59 (8.1%)** | **331 (87.8%)** | **145 (79.2%)** |
| **VTE event** | | | | |
| Yes | 21 (2.0%) | 21 (2.9%) | 9 (2.4%) | 9 (4.9%) |
| No | 1044 (97.9%) | 711 (97.1%) | 368 (97.6%) | 174 (95.1%) |
| Unknown | 1 (0.1%) | 0 | 0 | 0 |
| **Bleeding event** | | | | |
| Yes | 23 (2.2%) | 19 (2.6%) | 3 (0.8%) | 6 (3.3%) |
| No | 1042 (97.7%) | 713 (97.4%) | 374 (99.2%) | 177 (96.7%) |

**Copyright Information:** (Online) ISSN: 2572-9241
©2026 The Author(s). HemaSphere is published by John Wiley & Sons Ltd on behalf of European Hematology Association. This is an open access Abstract Book distributed under the Attribution-NonCommercial-NoDerivs (CC BY-NC-ND), which allows third parties to download the articles and share them with others as long as they credit the author and the Abstract Book, but they cannot change the content in any way or use them commercially.
**Abstract Book Citations:** Authors, Title, HemaSphere, 2026;10:(S1):pages. The abstract book DOIs can be found on the "Author Information" page.
**Disclaimer:** Articles published in the journal HemaSphere exclusively reflect the opinions of the authors. The authors are responsible for all content in their abstracts including accuracy of the facts, statements, citing resources, etc.

| | | | | |
|---|---|---|---|---|
| Unknown | 1 (0.1%) | 0 | 0 | 0 |

### Summary/Conclusion

In real-world UK NDMM patients, VTE rates are low (2.5%) with minimal bleeding. Myeloma-specific VTE RAMs show poor concordance in identifying high-risk patients and clinicians predominantly base thromboprophylaxis on IMiD use. Thromboprophylaxis remains suboptimal in transplant ineligible and non-IMID higher risk patients.

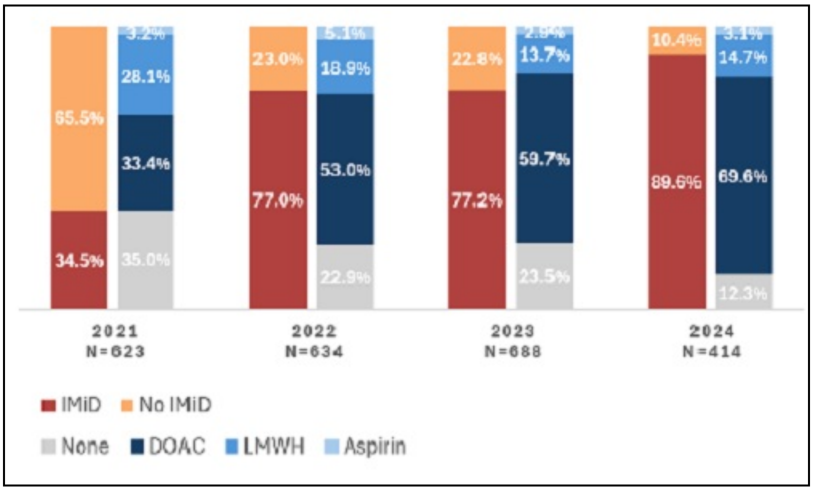

Figure 1: Changing practice in myeloma treatment & thromboprophylaxis

Copyright Information: (Online) ISSN: 2572-9241
©2026 The Author(s). HemaSphere is published by John Wiley & Sons Ltd on behalf of European Hematology Association. This is an open access Abstract Book distributed under the Attribution-NonCommercial-NoDerivs (CC BY-NC-ND), which allows third parties to download the articles and share them with others as long as they credit the author and the Abstract Book, but they cannot change the content in any way or use them commercially.
Abstract Book Citations: Authors, Title, HemaSphere, 2026;10:(S1):pages. The abstract book DOIs can be found on the "Author Information" page.
Disclaimer: Articles published in the journal HemaSphere exclusively reflect the opinions of the authors. The authors are responsible for all content in their abstracts including accuracy of the facts, statements, citing resources, etc.

### (PS2488) NEONATAL AND INFANT OUTCOMES FOLLOWING PREGNANCY-ASSOCIATED HAEMATOLOGICAL MALIGNANCIES: AN OBSERVATIONAL NATIONWIDE COHORT STUDY.

**Topic:** 36. Quality of life, ethics, supportive and palliative care

Pierre Pinson[1], Ismael Boussaid[1], Mathis Collier[1], Mathilde Barrois[1], Vassilis Tsatsaris[1], Ludovic Treluyer[1], Laurent Chouchana[1], Didier Bouscary[1], Jean Marc Treluyer[1], Justine De Croocq*[1], Rudy Birsen[1]

*[1]Cochin Hospital, Assistance Publique-Hôpitaux de Paris, University Paris Cité, Paris, France;*

### Background

Haematological malignancies diagnosed during pregnancy represent oncological emergencies requiring immediate treatment, often at the cost of medically indicated preterm delivery. Although maternal outcomes have been increasingly characterised, data on neonatal and subsequent child health remain scarce for haematological malignancies, which differ from solid tumours in urgency, systemic burden, and treatment intensity.

### Aims

To evaluate neonatal and infant outcomes following *in utero* exposure to maternal haematological malignancy diagnosed during pregnancy.

### Methods

We conducted a nationwide observational cohort study using the French National Health Data System (SNDS). All live births between Jan 1, 2008, and Dec 31, 2023, were included. Exposure was defined as maternal haematological malignancy diagnosed during pregnancy. Neonatal outcomes included prematurity, birth weight, small for gestational age (SGA), major congenital malformations, neonatal mortality, and a composite neonatal adverse outcome indicator (NAOI). Infant outcomes included overall mortality and hospitalisation-based morbidity (serious infections, cancer, endocrine, respiratory, cardiovascular, and urinary tract diseases). Analyses used inverse probability weighting, with additional stratification by gestational age.

### Results

Among 11,150,377 live births, 441 children were exposed *in utero* to maternal haematological malignancy diagnosed during pregnancy. Exposed children had lower mean gestational age at birth (36·3 vs 39·1 weeks; p<0·0001) and markedly increased risks of prematurity across all categories. Mean birth weight was lower, without increased SGA risk.
Major congenital malformations were more frequent in crude analyses (5·0% vs 2·9%; adjusted OR 1·70, 95% CI 1·11–2·61), but this association was no longer observed after stratification by gestational age.
Severe neonatal adverse outcomes (NAOI) were substantially increased (29·7% vs 6·0%; adjusted RR 6·32, 95% CI 5·15–7·75), including higher neonatal mortality (0·9% vs 0·1%; adjusted RR 7·02, 95% CI 2·62–18·79). Associations were markedly attenuated after stratification by gestational age, supporting prematurity as the principal mediator. NICU admission and respiratory complications were significantly more frequent.
During a mean follow-up of 8·5 years, infant mortality was not increased (adjusted HR 1·46, 95% CI 0·21–10·34). No significant increase was observed in serious infections, cancer, endocrine, respiratory, cardiovascular, or urinary tract diseases after adjustment or within gestational age strata.
In 152 children exposed to chemotherapy *in utero* (mainly second and third trimester), no association was identified between chemotherapy exposure and congenital malformations, severe neonatal morbidity, infant mortality, or later morbidity after adjustment.

**Copyright Information:** (Online) ISSN: 2572-9241
©2026 The Author(s). HemaSphere is published by John Wiley & Sons Ltd on behalf of European Hematology Association. This is an open access Abstract Book distributed under the Attribution-NonCommercial-NoDerivs (CC BY-NC-ND), which allows third parties to download the articles and share them with others as long as they credit the author and the Abstract Book, but they cannot change the content in any way or use them commercially.
**Abstract Book Citations:** Authors, Title, HemaSphere, 2026;10:(S1):pages. The abstract book DOIs can be found on the "Author Information" page.
**Disclaimer:** Articles published in the journal HemaSphere exclusively reflect the opinions of the authors. The authors are responsible for all content in their abstracts including accuracy of the facts, statements, citing resources, etc.

**Summary/Conclusion**

In this nationwide mother–child linked cohort, haematological malignancy diagnosed during pregnancy was associated with substantial early neonatal morbidity largely mediated by prematurity. Beyond the neonatal period, mortality and major somatic morbidity were not increased. Chemotherapy administered beyond the first trimester was not associated with adverse outcomes after adjustment.

**Copyright Information:** (Online) ISSN: 2572-9241
©2026 The Author(s). HemaSphere is published by John Wiley & Sons Ltd on behalf of European Hematology Association. This is an open access Abstract Book distributed under the Attribution-NonCommercial-NoDerivs (CC BY-NC-ND), which allows third parties to download the articles and share them with others as long as they credit the author and the Abstract Book, but they cannot change the content in any way or use them commercially.
**Abstract Book Citations:** Authors, Title, HemaSphere, 2026;10:(S1):pages. The abstract book DOIs can be found on the "Author Information" page.
**Disclaimer:** Articles published in the journal HemaSphere exclusively reflect the opinions of the authors. The authors are responsible for all content in their abstracts including accuracy of the facts, statements, citing resources, etc.

### (PS2489) QUALITY OF LIFE AND CARE ORGANIZATION OF PATIENTS WITH CHRONIC MYELOID LEUKEMIA WITHIN THE LOMBARDY HEMATOLOGY NETWORK: THE CML-OPERAP STUDY

**Topic:** 36. Quality of life, ethics, supportive and palliative care

Giacomo Mossini*[1], Marianna Caramella[2], Alessandra Iurlo[3], Mariella D'adda[4], Daniele Sannipoli[5], Nicola Orofino[6], Cristina Fiamenghi[7], Elda Mimola[8], Michela Anghilieri[9], Tamara Intermesoli[10], Rossella Calori[11], Federica Gigli[12], Roberto Palazzolo[13], Mariacristina Pasquini[14], Marta Ubezio[15], Margherita Maffioli[16], Laura Antolinni[1], Rocco Giovanni Piazza[1, 17], Carlo Gambacorti-Passerini[1, 17]

*[1]University of Milano-Bicocca, Monza, Italy; [2]ASST Grande Ospedale Metropolitano Niguarda, Milano, Italy; [3]Fondazione IRCCS Cá Granda – Ospedale Maggiore Policlinico, Milano, Italy; [4]ASST Spedali Civili, Brescia, Italy; [5]IRCCS San Raffaele Scientific Institute, Milano, Italy; [6]ASST Ovest Milanese, Legnano, Italy; [7]ASST Cremona, Cremona, Italy; [8]ASST Mantova, Mantova, Italy; [9]ASST Lecco, Lecco, Italy; [10]ASST Papa Giovanni XXIII, Bergamo, Italy; [11]ASST Brianza, Vimercate, Italy; [12]IEO, European Institute of Oncology IRCCS, Milano, Italy; [13]ASST Valtellina e Alto Lario, Sondrio, Italy; [14]ASST Crema, Crema, Italy; [15]IRCCS Humanitas Research Hospital, Rozzano, Italy; [16]ASST Sette Laghi, Varese, Italy; [17]Fondazione IRCCS San Gerardo dei Tintori, Monza, Italy;*

### Background

Chronic Myeloid Leukemia (CML) accounts for 10-15% of adult leukemias, with a rising prevalence of 2.5-3/10000, corresponding to approximately 2400 cases in Lombardy. Tyrosine kinase inhibitors have transformed CML into a chronic condition with near-normal life expectancy; however, whether CML patients' (pts) quality of life (QoL) also approaches that of people without CML remains unclear. The organizational model of CML care may influence QoL and patient experience.

### Aims

The CML-OPERAP study (Outcome and PErformances Reported and Analyzed by Patients) aims to evaluate the impact of hematology center organization on QoL of CML pts. Secondary aims include mapping CML care models within the Lombardy Hematology Network (REL) and assessing CML and its treatment effects on pts daily life and well-being.

### Methods

This is a cross-sectional, multicenter, prospective, observational study involving 22 REL centers. Eligible pts are adults (≥18 years old) with confirmed CML, followed for at least 6 months in a REL outpatient clinic.
Data collection includes clinical data and patient-reported questionnaires:
- Custom CML Hematology Center Questionnaire (CCHCQ): developed ad hoc to assess patients' satisfaction with physicians, nurses, and logistics
- EORTC QLQ-C30 and CML24 QoL survey

Centers are classified as:
- Categories 1 and 2: large centers (>100 CML pts) with/without a dedicated CML clinic
- Categories 3 and 4: small centers (<100 CML pts) with/without dedicated time slots for CML pts

Analyses used regression models adjusted for clustering and confounders and one sided t-tests.

**Copyright Information:** (Online) ISSN: 2572-9241
©2026 The Author(s). HemaSphere is published by John Wiley & Sons Ltd on behalf of European Hematology Association. This is an open access Abstract Book distributed under the Attribution-NonCommercial-NoDerivs (CC BY-NC-ND), which allows third parties to download the articles and share them with others as long as they credit the author and the Abstract Book, but they cannot change the content in any way or use them commercially.
**Abstract Book Citations:** Authors, Title, HemaSphere, 2026;10:(S1):pages. The abstract book DOIs can be found on the "Author Information" page.
**Disclaimer:** Articles published in the journal HemaSphere exclusively reflect the opinions of the authors. The authors are responsible for all content in their abstracts including accuracy of the facts, statements, citing resources, etc.

**Results**

The study started in April 2025. Within REL, 3 of 6 large centers and 5 of 16 small centers have a dedicated CML organizational structure, with 2000 expected pts in total. As of February 11, 2026, 17 centers were active and provided data on 790 pts: 257, 273, 157 and 103 pts from Category 1, 2, 3 and 4 respectively.
Category 1 centers reported significantly more CCHCQ "excellent" ratings than Category 2 across all medical and nursing care items (all p<0.01): overall medical and nursing staff evaluation (97.6% vs 92.7% and 90.9% vs 84.9%), attention to symptoms (68.6% vs 58.1%), information regarding disease and care (73.6% vs 64.1% and 76.1% vs 67.4%), and time dedicated by physicians and nurses (71.8% vs 60.3% and 68.8% vs 53.2%). Category 3 centers reported fewer psychological support requests than Category 4 centers (11.5% vs 26.2%; p<0.01).
Overall, QLQ-C30 and CML24 scores were consistently favorable and comparable to or higher than Italian general population reference values. Mean Global Health Status (GHS) was 76.4 ±18.5 in males (n=463) vs 67.6 (p<0.01) and 70.2 ± 19.2 in females (n=323) vs 62.4 (p<0.01) in the reference population; mean summary score was 88.6 ± 10.5 in males (n=454) vs 85.5 (p<0.01) and 84.2 ± 11.6 in females (n=313) vs 82.9 (p=0.05). Differences among organizational categories were small and of limited clinical relevance.
Patients in treatment-free remission (n=207) reported slightly better QoL than those on active treatment (n=583), with higher mean GHS (77.9 ± 18.5 vs 72.4 ± 19.0; p<0.01), summary score (89.5 ± 9.7 vs 85.8 ± 11.5; p<0.01) and lower symptom burden score (14.6 ± 12.5 vs 20.2 ± 13.7; p<0.01).

**Summary/Conclusion**

Data from approximately 40% of REL CML patients show that their QoL is generally good and comparable to that of the general population. Dedicated CML care is associated with higher patient satisfaction. Further results will be presented at the meeting.

**Copyright Information:** (Online) ISSN: 2572-9241
©2026 The Author(s). HemaSphere is published by John Wiley & Sons Ltd on behalf of European Hematology Association. This is an open access Abstract Book distributed under the Attribution-NonCommercial-NoDerivs (CC BY-NC-ND), which allows third parties to download the articles and share them with others as long as they credit the author and the Abstract Book, but they cannot change the content in any way or use them commercially.
**Abstract Book Citations:** Authors, Title, HemaSphere, 2026;10:(S1):pages. The abstract book DOIs can be found on the "Author Information" page.
**Disclaimer:** Articles published in the journal HemaSphere exclusively reflect the opinions of the authors. The authors are responsible for all content in their abstracts including accuracy of the facts, statements, citing resources, etc.

### (PS2490) WHEN IS IT ENOUGH? REAL-WORLD END-OF-LIFE CARE PATTERNS IN HEMATOLOGIC MALIGNANCIES: THE NEED FOR PALLIATIVE CARE INTEGRATION.

**Topic:** 36. Quality of life, ethics, supportive and palliative care

Carmen Dbouk Y Jaudenes*[1], María Argüello Marina[1], Ana Belén Santos Montero[1], Alejandro Vazquez Ramo[1], Angela Gil[1], Alba Pérez Ortega[1], Nuria Golbano Lopez[1], Dunia De Miguel Llorente[1]

[1]*Hospital Universitario de Guadalajara, Guadalajara, Spain;*

### Background

Hematologic malignancies (HM) challenge end-of-life (EoL) care due to unpredictable trajectories and persistent availability of active treatments until shortly before death. In the absence of embedded palliative pathways, hematology care models may structurally favor continued intervention over anticipatory EoL planning. Unlike solid tumours, where early palliative care (PC) integration is standard, hematology often maintains a treat-to-end culture.

### Aims

To evaluate EoL care patterns in patients with HM and assess whether non-structured PC involvement modifies care intensity in a tertiary hospital setting.

### Methods

This retrospective study included adults with HM who died between 01/01/2022 and 30/09/2025. Aggressive end-of-life (EoL) care was defined as the presence of ≥1 of the following indicators: administration of chemotherapy (CT) within the last 14 days of life (DoL), initiation of a new line of treatment within the final 30 days, >1 hospital admission during the last month, in-hospital death, and late referral to PC (<30 days before death). The hospital lacks a structured inpatient PC unit and relies exclusively on a home-based PC team, without systematic integration into hematology care.

### Results

The cohort of 116 patients exhibited substantial frailty: 54.3% had ECOG ≥3 and 87.9% scored >6 on the Charlson Comorbidity Index. Patients were heavily pretreated. Patient and treatment characteristics are summarized in Table 1. Mean overall survival was 389 ± 312 days in the PC group versus 354 ± 298 days in non-referred patients.

Overall, 90.5% of patients met ≥1 predefined aggressive EoL criteria (92.4% without PC vs 80.3% with PC), indicating widespread high-intensity EoL management across the cohort. CT was delivered to 51.3% and 25.7% of patients within the last 30 and 14 DoL respectively. A new CT scheme was initiated in 24.3% during the final 30 days. In-hospital death occurred in 82.8% of patients. During the last month, 89.1% required ≥1 hospital admission (23.6% ≥2), 65.8% had at least one Emergency Department visit, and 77.2% underwent laboratory testing in their final week, reflecting persistent medicalization until death.

PC referral was infrequent (20.7%, n=24) and typically late (median 19 days before death). Referred patients experienced a longer treatment-free interval (median 64 vs 23 days; p=0.004), fewer inpatient days in the last month (6.16±7.08 vs 15.95±15.60; p<0.001), and shorter final admissions (8.65±8.45 vs 15.43±14.98 days; p=0.015). In multivariable analysis adjusting for age, diagnosis, comorbidity, and prior treatment lines, PC referral independently predicted reduced CT use in the last 30 days (OR 0.23; p<0.05). Home death was more frequent in the PC group (36.4% vs 12.8%; p=0.04).

**Copyright Information:** (Online) ISSN: 2572-9241
©2026 The Author(s). HemaSphere is published by John Wiley & Sons Ltd on behalf of European Hematology Association. This is an open access Abstract Book distributed under the Attribution-NonCommercial-NoDerivs (CC BY-NC-ND), which allows third parties to download the articles and share them with others as long as they credit the author and the Abstract Book, but they cannot change the content in any way or use them commercially.
**Abstract Book Citations:** Authors, Title, HemaSphere, 2026;10:(S1):pages. The abstract book DOIs can be found on the “Author Information” page.
**Disclaimer:** Articles published in the journal HemaSphere exclusively reflect the opinions of the authors. The authors are responsible for all content in their abstracts including accuracy of the facts, statements, citing resources, etc.

### Summary/Conclusion

EoL care in this hematologic cohort frequently met predefined aggressiveness criteria, with 90.5% of patients fulfilling ≥1 aggressive care indicator, alongside late PC integration and substantial CT use in the last 14 days. In a model lacking structured palliative care integration, these findings suggest a structural misalignment between hematologic care delivery and EoL needs. PC referral was associated with lower care intensity without reduced survival, supporting earlier, systematic integration to achieve more proportional and patient-centered care.

| Baseline characteristics of the study cohort (n=116) | |
|---|---|
| Sex | n (%) |
| Male: | 69 (59.5); |
| Female: | 47 (40.5) |
| Age (media) | 77 years |
| Performance Status And Comorbiditiy | |
| Performance Status At Diagnosis | n (%) |
| ≤2 | 53 (45.7) |
| >2 | 63 (54.3) |
| Performance Status Prior Last Treatment | n (%) |
| ≤2 | 15 (12.9) |
| >2 | 101 (87.1); |
| Charlson Comorbidity Index (CCI) | 6 (median) |
| CCI >6, n (%) | 102 (87.9) |
| Diagnosis | n (%) |
| High-Grade Lymphoma | 29 (25.0) |
| Low-Grade Lymphoma | 6 (5.2) |
| AL (Acute Leukemia) | 28 (24.1) |
| Multiple Myeloma (MM) | 30 (25.9) |
| MDS | 18 (15.5) |
| Other | 5 (4.3) |
| Stage/Risk group | n (%) |
| Low-Intermediate Risk | 62 (53.4) |
| Hight Risk | 54 (46.6) |
| Number of prior treatment lines | 2 (median) |
| Number Of Prior Treatment Lines | n (%) |
| 0 | 9 (7.9) |
| 1 | 44 (38.6) |
| 2 | 24 (21.1) |
| ≥3 | 37 (32.5) |

| RISK STRATIFICATION | | | |
|---|---|---|---|
| Diagnosis | n | Low-Intermediate Risk n (%) | High Risk n (%) |
| High-Grade Lymphoma | 29 | 16 (55.2) | 13 (44.8) |
| Low-Grade Lymphoma | 6 | 4 (66.7) | 2 (33.3) |
| AL (Acute Leukemia) | 28 | 11 (39.3) | 17 (60.7) |
| Multiple Myeloma (MM) | 30 | 19 (63.3) | 11 (36.7) |
| MDS | 18 | 8 (44.4) | 10 (55.6) |
| Other | 5 | 4 (80.0) | 1 (20.0) |

| NUMBER OF PRIOR TREATMENT LINES | | | | | |
|---|---|---|---|---|---|
| Diagnosis | n | Number of lines (median) | 0 lines (%) | 1–2 lines n (%) | ≥3 lines n (%) |
| High-Grade Lymphoma | 29 | 2 | 1 (3.4) | 15 (51.7) | 13 (44.8) |
| Low-Grade Lymphoma | 6 | 1 | 0 | 5 (83.3) | 1 (16.7) |
| AL (Acute Leukemia) | 27 | 2 | 3 (11.1) | 17 (63.0) | 7 (25.9) |
| Multiple Myeloma (MM) | 29 | 2 | 0 | 16 (55.2) | 13 (44.8) |
| MDS | 18 | 1 | 5 (27.8) | 11 (61.1) | 2 (11.1) |
| Other | 5 | 1 | 0 | 4 (80.0) | 1 (20.0) |

Copyright Information: (Online) ISSN: 2572-9241
©2026 The Author(s). HemaSphere is published by John Wiley & Sons Ltd on behalf of European Hematology Association. This is an open access Abstract Book distributed under the Attribution-NonCommercial-NoDerivs (CC BY-NC-ND), which allows third parties to download the articles and share them with others as long as they credit the author and the Abstract Book, but they cannot change the content in any way or use them commercially.
Abstract Book Citations: Authors, Title, HemaSphere, 2026;10:(S1):pages. The abstract book DOIs can be found on the "Author Information" page.
Disclaimer: Articles published in the journal HemaSphere exclusively reflect the opinions of the authors. The authors are responsible for all content in their abstracts including accuracy of the facts, statements, citing resources, etc.

### (PS2491) SUBCUTANEOUS DARATUMUMAB AT HOME IS A SAFE AND EFFECTIVE PROCEDURE ALLOWING ACTIVE TREATMENT OF FRAIL PATIENTS WITH MULTIPLE MYELOMA IN DIFFERENT GEOGRAPHICAL AREAS

**Topic:** 36. Quality of life, ethics, supportive and palliative care

Gioia De Angelis*[1], Francesca Fazio[2], Giulio Trapè[3], Laura Arletti[4], Edoardo Tamellini[5], Giovanni Manfredi Assanto[2], Gabriella Tomei[1], Vincenza Innocenti[1], Raffaella Randi[1], Maria Dattolo[2], Roberta Perazzino[1], Tania Scancella[2], Roberta Talucci[1], Barbara Gamberi[4], Enrico Carazzai[5], Michela Tarnani[1], Marcello Capriata[2], Alessandro Andriani[6], Marco Montanaro[6], Maria Teresa Petrucci[2], Silvia Ciambella[1], Pierluigi Alfieri[7], Ilaria Tanasi[5], Claudio Cartoni[2], Roberto Latagliata[1]

*[1]UOC Hematology - Santa Rosa Hospital, Viterbo, Italy; [2]Hematology, Department of Translational and Precision Medicine, Sapienza University of Rome, Rome, Italy; [3]Hematology Unit, Ospedale Santa Rosa, Viterbo, Italy, Viterbo, Italy; [4]Hematology, Azienda USL - IRCCS di Reggio Emilia, Reggio Emilia, Italy; [5]UOC Hematology – AOU Integrata of Verona, Verona, Italy; [6]Mieloma Unit, UOC Hematology – ASL Viterbo – Santa Rosa Hospital, Viterbo, Italy; [7]Hematology Unit - Azienda USL IRCCS of Reggio Emilia, Reggio Emilia, Italy;*

### Background

Treatment of Multiple Myeloma (MM) is often difficult due to patient (pts) frailty, bone lesions/fractures that can hinder transport, lack of an available caregiver and/or long distance from the hospital. The availability of a Domiciliary Hematologic Care Unit (DHCU) could allow to overcome these difficulties, but data on safety and efficacy of this approach as concern subcutaneous (sc) administration of daratumumab (dara) are still lacking.

### Aims

Herein, the experience with home administration of sc dara in four hematologic Centres with a DHCU in different geographic settings [big metropolitan town (Rome), middle-sized towns (Verona and Reggio Emilia) and wide geographic area with several small centres (Viterbo province)] is reported, as part of the Myel-Home project.

### Methods

The initial 2 dara administrations were given in hospital to prevent side effects, while subsequent administrations were done as outpatients. Data from 54 pts [M/F 22/32 (40.7%/59.3%), median age 76.7 years, interquartile range (IQR) 69.8 – 80.2] were collected from 9/2021 to 12/2025.

**Copyright Information:** (Online) ISSN: 2572-9241
©2026 The Author(s). HemaSphere is published by John Wiley & Sons Ltd on behalf of European Hematology Association. This is an open access Abstract Book distributed under the Attribution-NonCommercial-NoDerivs (CC BY-NC-ND), which allows third parties to download the articles and share them with others as long as they credit the author and the Abstract Book, but they cannot change the content in any way or use them commercially.
**Abstract Book Citations:** Authors, Title, HemaSphere, 2026;10:(S1):pages. The abstract book DOIs can be found on the “Author Information” page.
**Disclaimer:** Articles published in the journal HemaSphere exclusively reflect the opinions of the authors. The authors are responsible for all content in their abstracts including accuracy of the facts, statements, citing resources, etc.

**Results**

The main reasons for the activation of home-care modality by responsible physician were as follows: disease complications (bone lesions/fractures secondary to MM) in 31 pts (57.4%), age > 80 years in 12 pts (22.2%), long distance from the hospital and/or poor social conditions in 11 pts (20.4%). Distance from DHCU to pts home was < 10 Km in 30 cases ( 55.5%), ≥ 10 < 30 Km in 11 (20.4%) and ≥ 30 Km in 13 (24.1%). Thirty-one pts (57.4%) were in $1^{st}$ line treatment, and 23 (42.6%) in $2^{nd}$ or subsequent lines of treatment. Dara-based schemes were Dara-Rd in 31 pts (57.4%), Dara-VTD in 8 pts (14.8%), Dara-VMP in 5 pts (9.2%), Dara-VD in 4 pts (7.4%), Dara-PD in 3 pts (5.6%) and Dara-CD/Dara-VCD in the remaining 3 pts (5.6%). On the whole, 575 administrations of sc-dara were performed at home: during and/or immediately after home administration, only one pt (1.8%) had adverse events (grade 2 allergic reaction according WHO), leading to dara permanent discontinuation. Main adverse events during the home management were infections (pneumonia in 7 pts, FUO in 2). Three pts were not yet evaluable for response and one pt discontinued early: among the evaluable 50 pts, 7 achieved a complete remission (stringent in 3 cases), 25 a very good partial remission and 7 a partial remission, with an overall response rate of 78.0%: seven pts had a stable disease and 4 pts a disease progression. Among the 11 pts in 1st line potentially eligible for transplant (age < 70 years), 4 were transplanted. At the last follow-up, 38 pts are still alive and 16 pts died (6 from disease progression, 4 from infections, 3 from cardiological diseases, 2 from evolution in acute myelogenous leukemia and 1 from liver disease). Median overall survival from the start of home management was 47.9 months (95%CI 23.3 – 85.5).

**Summary/Conclusion**

Treatment at home with sc-dara in frail pts with MM is feasible and safe also in different geographic settings: it makes possible a curative approach frontline and in advanced phases also in pts otherwise excluded by best available therapies or forced to long periods of hospitalization.

**Copyright Information:** (Online) ISSN: 2572-9241
©2026 The Author(s). HemaSphere is published by John Wiley & Sons Ltd on behalf of European Hematology Association. This is an open access Abstract Book distributed under the Attribution-NonCommercial-NoDerivs (CC BY-NC-ND), which allows third parties to download the articles and share them with others as long as they credit the author and the Abstract Book, but they cannot change the content in any way or use them commercially.
**Abstract Book Citations:** Authors, Title, HemaSphere, 2026;10:(S1):pages. The abstract book DOIs can be found on the "Author Information" page.
**Disclaimer:** Articles published in the journal HemaSphere exclusively reflect the opinions of the authors. The authors are responsible for all content in their abstracts including accuracy of the facts, statements, citing resources, etc.

### (PS2492) ASSOCIATIONS BETWEEN ECOG PERFORMANCE STATUS AND PATIENT-REPORTED OUTCOMES IN RELAPSED/REFRACTORY CHRONIC LYMPHOCYTIC LEUKEMIA OR SMALL LYMPHOCYTIC LYMPHOMA: POST HOC ANALYSIS FROM THE ALPINE TRIAL

**Topic:** 36. Quality of life, ethics, supportive and palliative care

Nicole Lamanna*[1], Timothy Victor[2, 3], Frederick B. Barnes[2], Rasika Korde[2], Gisoo Barnes[2], Jennifer R. Brown[4], Mazyar Shadman[5]

*[1]Herbert Irving Comprehensive Cancer Center, Columbia University, New York, United States of America;[2]BeOne Medicines, Ltd., San Carlos, United States of America; [3]University of Pennsylvania, Philadelphia, United States of America; [4]Dana-Farber Cancer Institute, Department of Medical Oncology, Boston, United States of America; [5]Fred Hutchinson Cancer Center and University of Washington, Clinical Research Division, Seattle, United States of America;*

### Background

Eastern Cooperative Oncology Group performance status (ECOG PS) is widely used in hematology trials to determine eligibility and stratify randomization and in routine practice to inform prognosis and treatment tolerability. While patient-reported outcomes (PROs) capture complementary information on patient-reported symptoms and functioning, baseline PROs are not routinely incorporated into stratification or clinical decision-making, and ECOG PS–PRO alignment is underexplored.

### Aims

To evaluate associations between ECOG PS categories and multidomain PRO profiles in patients with relapsed/refractory chronic lymphocytic leukemia/small lymphocytic lymphoma (R/R CLL/SLL).

### Methods

Patients gave written informed consent and completed the PRO measures. Baseline (pre-treatment) PRO data were pooled across treatment arms (zanubrutinib vs ibrutinib). Seven domains from the European Organisation for Research and Treatment of Cancer Quality of Life Questionnaire Core 30 (Global Health Status [GHS], physical functioning, role functioning, fatigue, pain, insomnia, nausea/vomiting) and visual analog scale (VAS) scores from the EuroQol 5 dimension 5 level tool were analyzed. PRO scores were oriented such that higher scores indicate higher impairment. Profile analysis was performed using one-way multivariate analysis of variance (MANOVA) with Wilks' lambda to compare PRO profiles across baseline ECOG PS categories (0, 1, 2), including three hierarchical hypothesis tests: (1) *parallelism* (profile shape across domains); (2) *levels* (mean differences between groups); (3) *flatness* (similarity of domain means overall [no difference vs. domain differentiated]). Domain-level pairwise contrasts were evaluated using t-tests. Bivariate logistic regression assessed associations between baseline PRO impairment scores and ECOG PS classification (sensitivity analysis).

**Copyright Information:** (Online) ISSN: 2572-9241
©2026 The Author(s). HemaSphere is published by John Wiley & Sons Ltd on behalf of European Hematology Association. This is an open access Abstract Book distributed under the Attribution-NonCommercial-NoDerivs (CC BY-NC-ND), which allows third parties to download the articles and share them with others as long as they credit the author and the Abstract Book, but they cannot change the content in any way or use them commercially.
**Abstract Book Citations:** Authors, Title, HemaSphere, 2026;10:(S1):pages. The abstract book DOIs can be found on the "Author Information" page.
**Disclaimer:** Articles published in the journal HemaSphere exclusively reflect the opinions of the authors. The authors are responsible for all content in their abstracts including accuracy of the facts, statements, citing resources, etc.

### Results

Among 627 patients, PRO profiles differed significantly by ECOG PS category, rejecting parallelism, equal overall levels, and flatness (all *P*<.001). Physical functioning, role functioning, and fatigue showed most consistent separation, with significant differences in all ECOG PS contrasts (0 vs 1, 0 vs 2, 1 vs 2; all *P*<.001). Other domains showed partial separation, including GHS (0 vs 1, *P*=.001; 0 vs 2, *P*=.001), pain (0 vs 1, *P*=.008; 0 vs 2, *P*=.019), and insomnia (0 vs 1, *P*=.004). VAS scores also differed significantly by ECOG PS (0 vs 1, *P*=.001; 0 vs 2, *P*=.001; 1 vs 2, *P*=.040). No associations were seen for nausea/vomiting. In logistic regression analyses, higher baseline impairment in physical functioning (odds ratio [OR] 1.48, 95% confidence interval [CI] 1.34–1.79), role functioning (OR 1.22, 95% CI 1.22–1.34), and fatigue (OR 1.22, 95% CI 1.10–1.34) was associated with higher odds of worse ECOG PS (all *P*<.001), with additional significant associations for GHS/quality of life (OR 1.34, 95% CI 1.22–1.48; *P*<.001), pain (OR 1.10, 95% CI 1.10–1.22; *P*=.001), insomnia (OR 1.10, 95% CI 1.00–1.22; *P*<.001), and VAS (OR 1.02, 95% CI 1.01–1.03; *P*<.001).

### Summary/Conclusion

Baseline ECOG PS was strongly associated with multidomain patient-reported symptom and functional profiles, with the most consistent differentiation for physical functioning, role functioning, and fatigue. Using baseline PRO assessment with ECOG PS could provide clinicians with a more patient-centered characterization of functional and symptom burden, supporting improved baseline risk stratification and informing treatment discussions and monitoring in R/R CLL/SLL trials.

**Copyright Information:** (Online) ISSN: 2572-9241
©2026 The Author(s). HemaSphere is published by John Wiley & Sons Ltd on behalf of European Hematology Association. This is an open access Abstract Book distributed under the Attribution-NonCommercial-NoDerivs (CC BY-NC-ND), which allows third parties to download the articles and share them with others as long as they credit the author and the Abstract Book, but they cannot change the content in any way or use them commercially.
**Abstract Book Citations:** Authors, Title, HemaSphere, 2026;10:(S1):pages. The abstract book DOIs can be found on the "Author Information" page.
**Disclaimer:** Articles published in the journal HemaSphere exclusively reflect the opinions of the authors. The authors are responsible for all content in their abstracts including accuracy of the facts, statements, citing resources, etc.

### (PS2493) CHANGES IN BODY COMPOSITION PARAMETERS IN PATIENTS WITH ACUTE MYELOID LEUKEMIA UNDERGOING INTENSIVE CHEMOTHERAPY PRIOR TO AN ALLOGENEIC HEMATOPOIETIC STEM CELL TRANSPLANTATION

**Topic:** 36. Quality of life, ethics, supportive and palliative care

Sofia Voigt*[1, 2], Clara Wall[1, 2], Jacob Jendro[1, 2], Lara Bischof[1, 2], Dominic Brauer[1, 2], Donata Backhaus[1, 2], Marco Herling[1, 2], Carmen Diana Herling[1, 2], Vladan Vucinic[1, 2], Georg-Nikolaus Franke[1, 2], Timm Denecke[3], Klaus Metzeler[1, 2], Sebastian Schwind[1, 2], Hans-Jonas Meyer[3], Madlen Jentzsch[1, 2], Jule Ussmann[1, 2]

*[1]University of Leipzig Medical Center, Department for Hematology, Cell Therapy, Hemostaseology, and Infectious Diseases, Leipzig, Germany; [2]Cancer Center Central Germany - CCCG, Leipzig, Germany; [3]University of Leipzig Medical Center, Department for Diagnostic and Interventional Radiology, Leipzig, Germany;*

### Background

For fit patients (pts) with acute myeloid leukemia (AML), an allogeneic hematopoietic stem cell transplantation (HSCT) is a potential curative treatment option. To achieve a remission prior to HSCT, fit pts usually undergo intensive 7+3 based chemotherapy (CTx). CTx often results in immobilization, malnutrition and infectious complications, all potentially promoting weight loss, which – when occurring prior to HSCT – has been shown to associate with higher non relapse mortality and shorter survival (Brauer *et al.* 2021). To identify tissues contributing to weight loss, various body composition parameters (BCP) evaluated in computed tomography scans (CTs) are established. In solid neoplasm loss of muscle mass, muscle quality and adipose tissues have been repeatedly described. However, data on BCP changes in AML pts undergoing intensive CTx are lacking.

### Aims

To describe the changes in BCP obtained from thoracic CTs in AML pts undergoing CTx prior to HSCT.

### Methods

For 183 pts who had received at least one cycle of CTx, thoracic CTs at diagnosis (Dx) and at HSCT were available. At T12 level of thoracic CTs, we evaluated muscle mass, defined by skeletal muscle index [SMI, skeletal muscle area/(body height)$^2$], intramuscular adipose tissue content [IMAC, muscle radiodensity/radiodensity of subcutaneous fat; with a higher IMAC indicating lower muscle quality due to fat infiltration] and the subcutaneous (s.c.) fat index [SFI, s.c. fat area/(body height)$^2$]. BCP at Dx *vs.* HSCT were compared using paired Wilcoxon-test.

**Copyright Information:** (Online) ISSN: 2572-9241
©2026 The Author(s). HemaSphere is published by John Wiley & Sons Ltd on behalf of European Hematology Association. This is an open access Abstract Book distributed under the Attribution-NonCommercial-NoDerivs (CC BY-NC-ND), which allows third parties to download the articles and share them with others as long as they credit the author and the Abstract Book, but they cannot change the content in any way or use them commercially.
**Abstract Book Citations:** Authors, Title, HemaSphere, 2026;10:(S1):pages. The abstract book DOIs can be found on the "Author Information" page.
**Disclaimer:** Articles published in the journal HemaSphere exclusively reflect the opinions of the authors. The authors are responsible for all content in their abstracts including accuracy of the facts, statements, citing resources, etc.

**Results**

Median age at Dx was 60 (range 20-75) years (y). According to body mass index (BMI) at Dx, 39% of pts were underweight or of normal weight and 61% overweight or obese. Prior to HSCT, pts underwent one (13%), two (73%) or three (14%) cycles of intensive CTx. CTs were a median of 83 (54 to 128) days apart. From Dx to HSCT - after intensive CTx - there was a significant decrease for SMI, IMAC and SFI (all $P$<.01) in the whole pts cohort. While significant loss of muscle mass and s.c. fat occured in female as well as in male pts (all $P$<.01), the IMAC only decreased significantly in female pts ($P$<.01; *vs.* $P$=.35 in male pts), indicating, that female but not male pts also lost intramuscular fat. In subgroup analyses according to age, only pts aged >50 y had a significant decrease of SMI ($P$<.01), IMAC ($P$<.01) and SFI ($P$<.01), while pts ≤50 y showed no significant changes in BCP ($P$=.23, $P$=.56, $P$=.73, respectively, **Figure 1**). Significant loss of muscle mass and s.c.fat occured irrespective of BMI (analyzing underweight & normal weight *vs.* overweight & obese), while the IMAC did only significantly decrease in the group of overweight and obese pts ($P$<.01 *vs.* $P$=.57 in pts with a BMI<25). Comparing pts receiving only one to pts receiving ≥2 cycles of prior treatment, all had a significant decrease in SMI (both $P$<.01) and SFI (both $P$<.01), but only pts receiving ≥2 cycles had a significant loss of IMAC ($P$<.01 *vs.* $P$=.63).

**Summary/Conclusion**

Especially AML pts aged >50y experienced significant loss of muscle and adipose tissues while undergoing CTx. Therefore, they are at high risk to deteriorate in fitness which is a prognostic factor for survival after HSCT. Notably, loss of IMAC was seen particularly in subgroups known to have higher IMAC (postmenopausal women and obese pts). Prospective studies are urgently needed to further analyze changes in BCP in the context of fitness and muscle function as well as (side) effects of CTx and outcomes after HSCT.

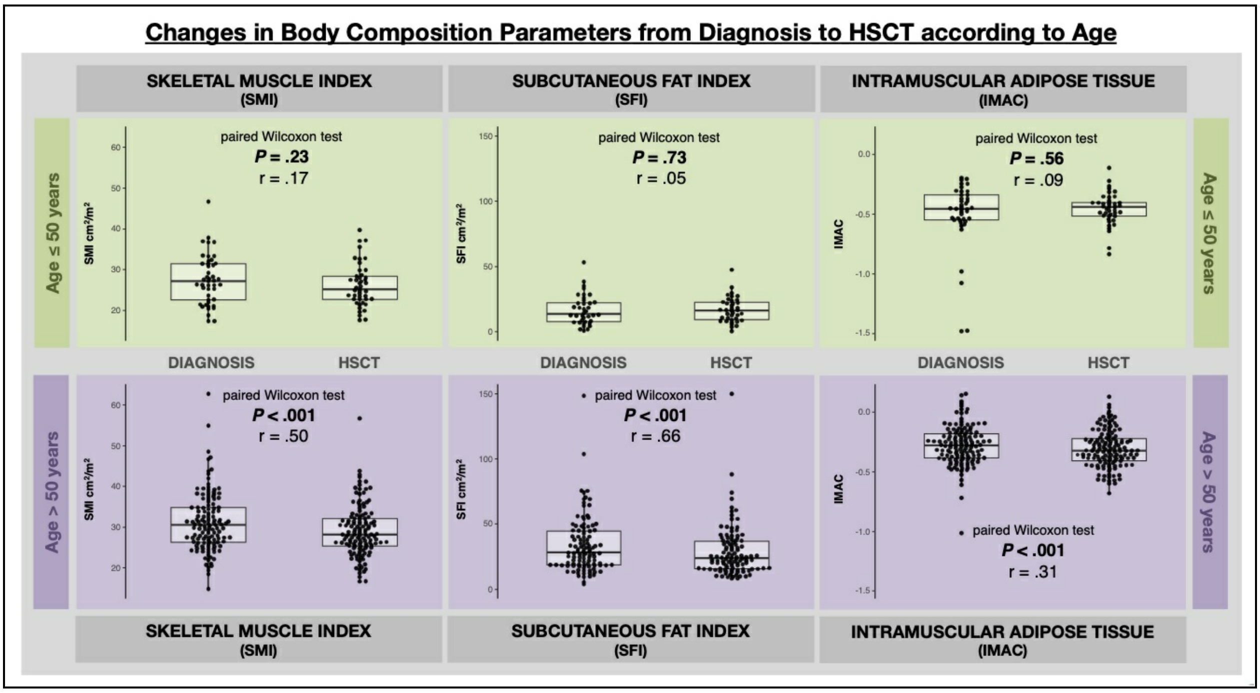

**Copyright Information:** (Online) ISSN: 2572-9241
©2026 The Author(s). HemaSphere is published by John Wiley & Sons Ltd on behalf of European Hematology Association. This is an open access Abstract Book distributed under the Attribution-NonCommercial-NoDerivs (CC BY-NC-ND), which allows third parties to download the articles and share them with others as long as they credit the author and the Abstract Book, but they cannot change the content in any way or use them commercially.
**Abstract Book Citations:** Authors, Title, HemaSphere, 2026;10:(S1):pages. The abstract book DOIs can be found on the "Author Information" page.
**Disclaimer:** Articles published in the journal HemaSphere exclusively reflect the opinions of the authors. The authors are responsible for all content in their abstracts including accuracy of the facts, statements, citing resources, etc.

### (PS2494) MAINTENANCE OF HEALTH-RELATED QUALITY OF LIFE WITH VENETOCLAX-OBINUTUZUMAB VS CHEMOIMMUNOTHERAPY IN FIT PATIENTS WITH PREVIOUSLY UNTREATED CHRONIC LYMPHOCYTIC LEUKEMIA FROM THE PHASE 3 CRISTALLO TRIAL

**Topic:** 36. Quality of life, ethics, supportive and palliative care

Franck Morschhauser*[1], Luca Laurenti[2], Emmanuelle Ferrant[3], Luis Felipe Casado Montero[4], Stephen Mulligan[5], Rosemary Harrup[6], Stephen Opat[7], Adalberto Ibatici[8, 9], Roberto Marasca[10], Paolo Sportoletti[11], Avrita Singh[12], Hyun Yong Jin[12], Maria Thadani-Mulero[13], Madlaina Breuleux[14], Archie Egginton[13], Peter Trask[12], Jeff P. Sharman[15]

*[1]Centre Hospitalier Régional Universitaire de Lille, Lille, France; [2]Department of Diagnostic Imaging, Oncological Radiotherapy and Hematology, Agostino Gemelli University Hospital Foundation IRCCS, Rome, Italy; [3]Department of Hematology, Hôpital Lyon-Sud, Lyon, France; [4]Hospital General Universitario de Toledo, Toledo, Spain; [5]Department of Haematology, Royal North Shore Hospital, Sydney, NSW, Australia; [6]Royal Hobart Hospital and University of Tasmania, Hobart, TAS, Australia; [7]Monash Health, Melbourne, VIC, Australia; [8]Division of Hematology and Bone Marrow Transplant, IRCCS Ospedale Policlinico San Martino, Genoa, Italy; [9]IRCCS Azienda Ospedaliera Metropolitana, Genoa, Italy; [10]University of Modena and Reggio Emilia, Modena, Italy; [11]Department of Medicine and Surgery, Institute of Hematology, Centro di Ricerca Emato-Oncologica, University of Perugia, Perugia, Italy; [12]Genentech, Inc., South San Francisco, CA, United States of America; [13]Roche Products Ltd, Welwyn Garden City, United Kingdom; [14]F. Hoffmann-La Roche Ltd, Basel, Switzerland; [15]Willamette Valley Cancer Institute, Sarah Cannon Research, Eugene, OR, United States of America;*

### Background

Patients (pts) with chronic lymphocytic leukemia (CLL) experience many impairments in their health-related quality of life (HRQoL) (Waweru et al. *Curr Med Res Opin* 2020). Thus, alongside managing disease control, maintaining and improving HRQoL is a key treatment goal. The Phase 3 CRISTALLO trial (NCT04285567) compared the efficacy and safety of fixed-duration chemotherapy-free venetoclax-obinutuzumab (VenO) vs fludarabine-cyclophosphamide-rituximab/bendamustine-rituximab (FCR/BR) in fit pts with treatment-naïve CLL without del(17p) or *TP53* mutations. The primary endpoint of CRISTALLO was met: VenO demonstrated significantly higher undetectable minimal residual disease rate at Month 15 vs FCR/BR (Sharman et al. ASH 2024). Here, we present patient-reported outcomes (PROs) from the CRISTALLO trial.

### Aims

Evaluate the impact of VenO and FCR/BR on HRQoL in pts treated in the CRISTALLO trial.

### Methods

PROs included the European Organization for Research and Treatment of Cancer Quality of Life Questionnaire-Core 30 (EORTC QLQ-C30) and the M.D. Anderson Symptom Inventory-CLL (MDASI-CLL). For the EORTC QLQ-C30, scores ranged from 0–100, with higher scores indicating better functioning on the functional and global health status/quality of life (GHS/QoL) scales, and worse symptoms on the symptom scales and single items. For the MDASI-CLL, scores ranged from 0–10, with lower scores indicative of lower symptom severity/interference. PROs were electronically administered on Day 1 of all treatment cycles (12 for VenO and 6 for FCR/BR) and every 3 months during follow-up until disease progression. Analyses of PROs included completion rates and change from baseline (CFB) in mean scores for the EORTC QLQ-C30 and MDASI-CLL scales.

**Copyright Information:** (Online) ISSN: 2572-9241
©2026 The Author(s). HemaSphere is published by John Wiley & Sons Ltd on behalf of European Hematology Association. This is an open access Abstract Book distributed under the Attribution-NonCommercial-NoDerivs (CC BY-NC-ND), which allows third parties to download the articles and share them with others as long as they credit the author and the Abstract Book, but they cannot change the content in any way or use them commercially.
**Abstract Book Citations:** Authors, Title, HemaSphere, 2026;10:(S1):pages. The abstract book DOIs can be found on the "Author Information" page.
**Disclaimer:** Articles published in the journal HemaSphere exclusively reflect the opinions of the authors. The authors are responsible for all content in their abstracts including accuracy of the facts, statements, citing resources, etc.

### Results

Completion rates were high and balanced between treatment arms, exceeding 70% on-treatment. EORTC QLQ-C30 baseline scores were comparable between treatment arms and indicative of low levels of symptom severity and normal functioning, except for fatigue and insomnia, which were slightly elevated in both arms. Trends of improvement were seen with fatigue and insomnia scores, with clinically meaningful improvements observed for insomnia at Cycle 5 Day 1 in the VenO arm (CFB: –11.32). Mean score for diarrhea showed clinically meaningful worsening at some timepoints in the VenO arm but resolved at end of treatment. GHS/QoL scores remained high and relatively stable across both treatment arms (**Image**). For the MDASI-CLL, pts reported low baseline levels of core cancer symptoms, CLL-specific symptoms, and symptom interference between treatment arms. These scales remained stable across both treatment arms, with the mixed models for repeated measures showing no statistically significant differences between treatment arms nor any significant changes from baseline over time or treatment-by-time interactions.

### Summary/Conclusion

In CRISTALLO, VenO demonstrated deep molecular response while maintaining baseline HRQoL and effectively managing the symptom burden in pts with CLL. The overall stability of functional domain scores and symptom scores suggests a favorable tolerability profile. The lack of significant symptom interference compared with standard chemoimmunotherapy underscores the value of VenO as a patient-centric treatment that preserves HRQoL. Together with the PROs reported in the CLL14 trial and previously reported clinical outcomes, these results support the value of VenO, a fixed-duration and chemotherapy-free regimen, for fit pts with treatment-naïve CLL.

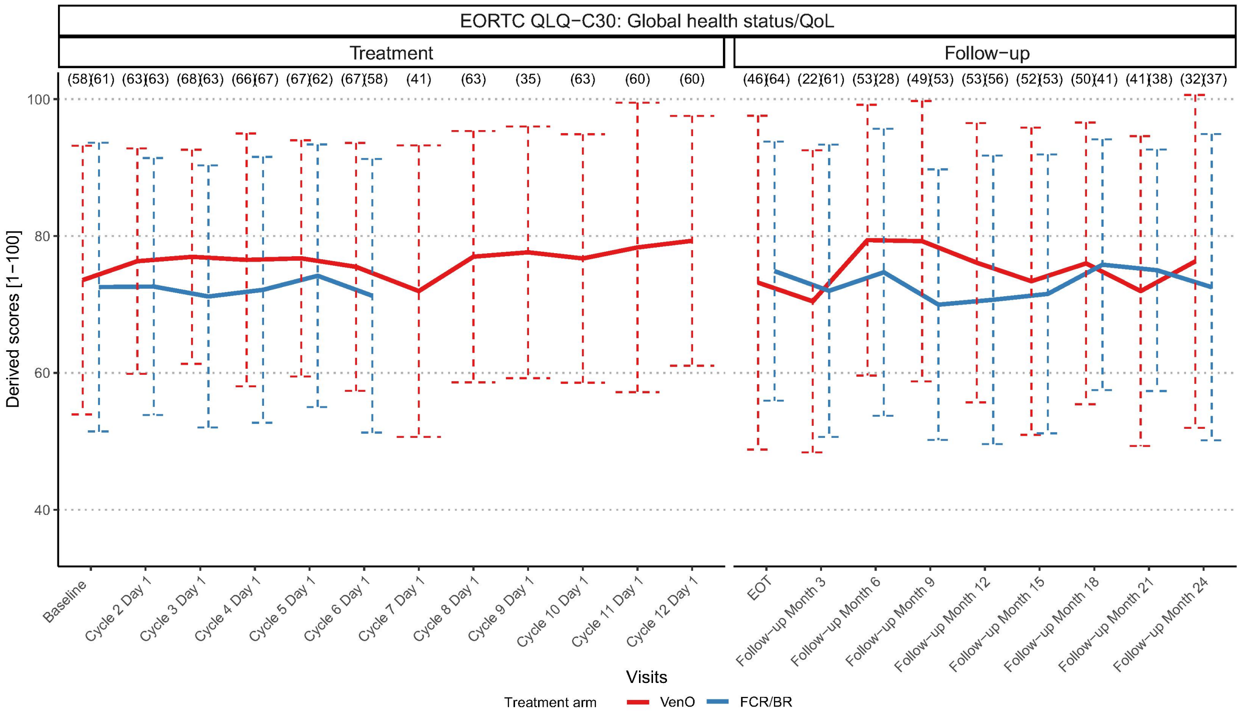

EORTC QLQ-C30, European Organization for Research and Treatment of Cancer Quality of Life Questionnaire-Core 30; EOT, end of treatment; FCR/BR, fludarabine-cyclophosphamide-rituximab/bendamustine-rituximab; QoL, quality of life; VenO, venetoclax-obinutuzumab.
Scores range from 0–100, with higher scores representative of improved global health status/QoL. Patient numbers are shown in brackets for the VenO vs FCR/BR arms (intent-to-treat population).

**Copyright Information:** (Online) ISSN: 2572-9241
©2026 The Author(s). HemaSphere is published by John Wiley & Sons Ltd on behalf of European Hematology Association. This is an open access Abstract Book distributed under the Attribution-NonCommercial-NoDerivs (CC BY-NC-ND), which allows third parties to download the articles and share them with others as long as they credit the author and the Abstract Book, but they cannot change the content in any way or use them commercially.
**Abstract Book Citations:** Authors, Title, HemaSphere, 2026;10:(S1):pages. The abstract book DOIs can be found on the "Author Information" page.
**Disclaimer:** Articles published in the journal HemaSphere exclusively reflect the opinions of the authors. The authors are responsible for all content in their abstracts including accuracy of the facts, statements, citing resources, etc.

### (PS2495) PRESCRIBING PRACTICE, RISK ASSESSMENT MODEL (RAM) USE, AND PERCEIVED GUIDELINE GAPS IN VENOUS THROMBOEMBOLISM (VTE) PROPHYLAXIS FOR NEWLY DIAGNOSED MULTIPLE MYELOMA (NDMM): UK NATIONAL SURVEY RESULTS

**Topic:** 36. Quality of life, ethics, supportive and palliative care

Marquita Camilleri*[1], Tom Bull[2], William Wilson[3], Mamatha Karanth[2], Raza Alikhan[4], Mari Thomas[5], Elena Ganendra[6]

*[1]Cambridge University Hospitals NHS Foundation Trust, Cambridge, United Kingdom;[2]West Suffolk NHS Foundation Trust, Bury St Edmonds, United Kingdom; [3]Cancer Research UK Clinical Trials Centre, University College London, London, United Kingdom; [4]Cardiff and Vale University Health Board, Cardiff, United Kingdom;[5]University College London Hospitals NHS Foundation Trust, London, United Kingdom; [6]North West Anglia NHS Foundation Trust, Peterborough, United Kingdom;*

### Background

European Myeloma Network guidelines on VTE prevention in MM recommend risk stratifying all patients using IMPEDE-VTE and classifying those on immunomodulatory agents (IMiDs) as intermediate/high VTE risk. Low molecular weight heparin (LMWH) remains recommended with direct oral anticoagulants (DOACs) acknowledged as potential alternatives, but guidance remains cautious given limited randomised data. The recent UK-wide ATOMM real-world observational study on thromboprophylaxis in 2359 NDMM patients 2021-2024 showed increasing DOAC usage with 2.5% 6-month VTE rates and minimal bleeding, and suggested prescribing may be driven more by treatment factors than RAMs.

### Aims

To evaluate real-world determinants of thromboprophylaxis decision-making in UK MM practice and examine alignment with current guidelines.

### Methods

National online 17-item survey conducted over 29 days, capturing current prescribing practices, decision-making processes, RAM use, and views on existing guidance. Responses were analysed using descriptive and frequency statistics.

**Copyright Information:** (Online) ISSN: 2572-9241
©2026 The Author(s). HemaSphere is published by John Wiley & Sons Ltd on behalf of European Hematology Association. This is an open access Abstract Book distributed under the Attribution-NonCommercial-NoDerivs (CC BY-NC-ND), which allows third parties to download the articles and share them with others as long as they credit the author and the Abstract Book, but they cannot change the content in any way or use them commercially.
**Abstract Book Citations:** Authors, Title, HemaSphere, 2026;10:(S1):pages. The abstract book DOIs can be found on the "Author Information" page.
**Disclaimer:** Articles published in the journal HemaSphere exclusively reflect the opinions of the authors. The authors are responsible for all content in their abstracts including accuracy of the facts, statements, citing resources, etc.

**Results**

Total of 116 respondents, mainly haematology consultants (67%) with 80% reporting >10 years clinical experience, and equal practice setting between tertiary centres and district general hospitals. 99% consider VTE prophylaxis in MM important. DOACs are used by 98%, followed by aspirin (53%), LMWH (49%), unfractionated heparin (6%), and warfarin (3.4%). Excluding patient factors, DOACs are most preferred for thromboprophylaxis (94%), followed by LMWH (52%) and aspirin (41%) based on 2nd choice weighting. For breakthrough VTE, 40% escalate/switch DOAC; 64% step down to DOAC prophylaxis post 6-month VTE therapy. DOAC prescribing concerns include severe renal impairment (80%), drug-drug interactions (46%), extremes of body weight (37%), bleeding risks (36%), and drug absorption (28%); DOACs' unlicensed status for MM VTE prophylaxis is a concern in only 18%. Concerns around LMWH include administration burden (85%), moderate and severe renal impairment (13%, 60%), bleeding risk (21%), and availability of reversal agents (13%).

For patients on IMiDs, 84% prescribe thromboprophylaxis for the full duration of anti-MM therapy, 9.5% until steroids are stopped, and 7% for a fixed 6-month duration. For non-IMiD regimens, 89% don't routinely prescribe prophylaxis. Prescribing is predominantly influenced by IMiD use (94%), prior VTE (80%), disease status (61%), and bleeding risk (31%). RAMs are used infrequently with 79% non-users; among users (21%), IMPEDE is most common (16/24,67%). 49% think current guidance is insufficient, 44% possibly insufficient, and 15% sufficient. Only 43% feel confident making independent VTE prophylaxis decisions. Specialist haemostasis advice is routinely sought by 20% and selectively by 37% (n=43), mainly for prior major bleeding (88%), concurrent antiplatelet therapy (77%), previous VTE (63%), and renal impairment (60%).

Clearer national/international guidelines (85%), national consensus statements (71%), more randomised data (53%), education & training initiatives (44%), and patient-focused educational resources (23%) are deemed important to standardise UK practice.

**Summary/Conclusion**

Most UK practitioners prescribe thromboprophylaxis for patients on IMiDs but less so for non-IMiD regimens. DOACs are widely accepted and preferred. Formal RAMs are infrequently applied, and decision-making is largely independent of current guidelines. These findings highlight a treatment-driven approach and may inform future UK MM guidance.

**Copyright Information:** (Online) ISSN: 2572-9241
©2026 The Author(s). HemaSphere is published by John Wiley & Sons Ltd on behalf of European Hematology Association. This is an open access Abstract Book distributed under the Attribution-NonCommercial-NoDerivs (CC BY-NC-ND), which allows third parties to download the articles and share them with others as long as they credit the author and the Abstract Book, but they cannot change the content in any way or use them commercially.
**Abstract Book Citations:** Authors, Title, HemaSphere, 2026;10:(S1):pages. The abstract book DOIs can be found on the "Author Information" page.
**Disclaimer:** Articles published in the journal HemaSphere exclusively reflect the opinions of the authors. The authors are responsible for all content in their abstracts including accuracy of the facts, statements, citing resources, etc.

### (PS2496) WORKLOAD AND BURNOUT AMONG HEMATOLOGISTS IN THE COMMUNITY OF MADRID (SPAIN): KEY FINDINGS OF THE 2024–2027 STRATEGIC PLAN OF THE MADRID ASSOCIATION OF HEMATOLOGY AND HEMOTHERAPY

**Topic:** 36. Quality of life, ethics, supportive and palliative care

Adrian Alegre Amor*[1], Rebeca Bailen[2], Mariana Bastos-Oreiro[2], Maria Angeles Foncillas[3], Pilar Martinez-Barranco[4], Marta Morado[5], Laura Pardo Gambarte[6], Cristina Seri[7], Laura Solán[6], Imma Barber Turro[8], Joan Gibert Vives[8], Ángel Cedillo Cedillo[9], Laurentino Benito Parra[10], Víctor Jiménez-Yuste[5], José-Angel Hernández-Rivas[3]

*[1]Hosp. Univ. de La Princesa, Madrid, Spain;[2]Hosp. Gral. Univ. Gregorio Marañón, Madrid, Spain;[3]Hosp. Univ. Infanta Leonor, Madrid, Spain; [4]Hosp. Univ. Fundación Alcorcón, Madrid, Spain; [5]Hosp. Univ. La Paz, Madrid, Spain; [6]Hosp. Univ. Fundación Jiménez Díaz, Madrid, Spain; [7]Hosp. Central de la Defensa Gómez Ulla, Madrid, Spain; [8]LetsFocusNow, Barcelona, Spain; [9]Madrid Association of Hematology and Hemotherapy (AMHH), Madrid, Spain; [10]Hosp. Univ. de Getafe, Madrid, Spain;*

### Background

Within its 2024–2027 Strategic Plan, the Madrid Association of Hematology and Hemotherapy (AMHH) has addressed the link between workload and burnout among hematologists in the Community of Madrid (CM).

### Aims

To highlight a growing problem that may compromise professional well-being and care quality, raise awareness among institutional leaders, scientific societies and health managers, and provide an evidence-based basis for improvement proposals.

### Methods

An online questionnaire (39 questions, four domains, including the Copenhagen Burnout Inventory, CBI) was developed by the AMHH Board and anonymously completed by 129 hematologists from the CM (40% of AMHH members) between March–April 2025.

**Copyright Information:** (Online) ISSN: 2572-9241
©2026 The Author(s). HemaSphere is published by John Wiley & Sons Ltd on behalf of European Hematology Association. This is an open access Abstract Book distributed under the Attribution-NonCommercial-NoDerivs (CC BY-NC-ND), which allows third parties to download the articles and share them with others as long as they credit the author and the Abstract Book, but they cannot change the content in any way or use them commercially.
**Abstract Book Citations:** Authors, Title, HemaSphere, 2026;10:(S1):pages. The abstract book DOIs can be found on the "Author Information" page.
**Disclaimer:** Articles published in the journal HemaSphere exclusively reflect the opinions of the authors. The authors are responsible for all content in their abstracts including accuracy of the facts, statements, citing resources, etc.

### Results

Of respondents, 78% were consultant hematologists, 65% worked in high-complexity and 83% in public funded hospitals. Clinical care was the main activity (43%), another 43% held mixed posts with laboratory/blood bank duties. During regular hours, they allocated 75% of their time to clinical care, 10% to teaching, 5% to research and 10% to management. Forty-two percent saw >50 patients weekly, 76% worked beyond their contracted hours, and 54% added >10 extra hours per week. VPN access for remote work was available for 44%. Hematologists' time distribution shows a predominance of in-person and conventional clinical care: 29% of time devoted to follow-up visits. In laboratories, 28% was spent on bone marrow studies, while blood banks had greater activity diversity, highlighting validation tasks (28%). Burnout was frequent or higher in 41% (personal), 42% (work-related) and 29% (patient-related). The mean CBI burnout score among hematologists was 45.16, 24% higher than the mean for Medicine in general (Table 1).

| | Validation CBI Data | | Hospital 12 de Octubre Study | AMHH |
|---|---|---|---|---|
| | **General** | **Medicine** | **Internal Medicine and Psychiatry** | **Hematology** |
| | N = 337 | N = 27 | N = 66/66/65 | N = 128 |
| PERSONAL BURNOUT | 32.20 | 34.40 | 40.97 | 43.93 |
| WORK BURNOUT | 29.40 | 34.70 | 45.77 | 46.29 |
| PATIENTS BURNOUT | 34.50 | 38.30 | 37.31 | 45.25 |
| **TOTAL BURNOUT** | **32.00** | **36.50** | **41.50** | **45.16** |

Table 1.- Comparative burnout values according to the CBI and previous studies.

Workload, salary, intra-departmental relationships and available resources were the leading burnout drivers. Prioritized improvement areas included workload, pay, resources, and the limited recognition of Hematology among specialties and within hospitals. Between 2023–2024, hematologists reporting physical or verbal aggression rose by 20%, and patient complaints by 58%. Requests for psychological/psychiatric help increased from 25% (2023) to 35% (2024).

### Summary/Conclusion

These findings portray a challenging professional scenario for the Hematology and Hemotherapy in the Community of Madrid (Spain). Care overload, overtime and high burnout levels indicate organizational issues requiring urgent review. Structural measures are needed to improve working conditions, support well-being and secure sustainable, high-quality hematologic care.

**Copyright Information:** (Online) ISSN: 2572-9241
©2026 The Author(s). HemaSphere is published by John Wiley & Sons Ltd on behalf of European Hematology Association. This is an open access Abstract Book distributed under the Attribution-NonCommercial-NoDerivs (CC BY-NC-ND), which allows third parties to download the articles and share them with others as long as they credit the author and the Abstract Book, but they cannot change the content in any way or use them commercially.
**Abstract Book Citations:** Authors, Title, HemaSphere, 2026;10:(S1):pages. The abstract book DOIs can be found on the "Author Information" page.
**Disclaimer:** Articles published in the journal HemaSphere exclusively reflect the opinions of the authors. The authors are responsible for all content in their abstracts including accuracy of the facts, statements, citing resources, etc.

### (PS2497) EPCORITAMAB PLUS LENALIDOMIDE AND RITUXIMAB IMPROVES OR PRESERVES HEALTH-RELATED QUALITY OF LIFE IN PATIENTS WITH RELAPSED/REFRACTORY FOLLICULAR LYMPHOMA WHO HAD HIGH SYMPTOM BURDEN OR ADVERSE EVENTS

**Topic:** 36. Quality of life, ethics, supportive and palliative care

Loic Ysebaert*[1], Amylou Dueck[2], Franck Morschhauser[3], Ana Isabel Jimenez Ubieto[4], Miguel Arturo Pavlovsky[5], Shane Gangatharan[6], Habte A. Yimer[7], Vanja Zeremski[8], Danielle Leão[9], Marcel Nijland[10], Lorenzo Falchi[11], Abualbishr Alshreef[12], Anthony Wang[12], Jennifer Lord-Bessen[12], Rajesh Kamalakar[12], Yves Mbous[12], Kavita Sail[12], Zhijie Ding[13], Gauri Sunkersett[12], Jian Mei[12], Kim Linton[14]

*[1]Hematology Department, Institut Universitaire du Cancer de Toulouse-Oncopole, Toulouse, France;[2]Mayo Clinic, Scottsdale, United States of America; [3]Hôpital Claude Huriez, Lille, France;[4]Hematology Department, Hospital Universitario 12 de Octubre, Madrid, Spain; [5]FUNDALEU, Clinical Research Center Buenos Aires, Argentina, Buenos Aires, Argentina;[6]Fiona Stanley Hospital, Murdoch, Australia; [7]Texas Oncology-Tyler, US Oncology Research, Tyler, United States of America; [8]Otto von Guericke University, Magdeburg, Germany; [9]Real e Benemérita Associação Portuguesa de Beneficência, Bela Vista, São Paulo, Brazil; [10]University Medical Center Groningen, University of Groningen, Groningen, The Netherlands; [11]Lymphoma Service, Memorial Sloan Kettering Cancer Center, New York, United States of America; [12]AbbVie Inc., North Chicago, United States of America; [13]Genmab US, Inc, Plainsboro, United States of America; [14]The Christie NHS Foundation Trust, Manchester Cancer Research Centre, and Division of Cancer Sciences, University of Manchester, Manchester, United Kingdom;*

### Background

The randomized Phase 3 EPCORE FL-1 trial (NCT05409066) demonstrated superior efficacy of epcoritamab plus lenalidomide and rituximab (epcor+$R^2$) over $R^2$ in patients with relapsed/refractory follicular lymphoma (R/R FL), including significantly higher response rate, longer progression-free survival, and preserved high baseline health-related quality of life (HRQoL; Falchi *Lancet* 2026). Although R/R FL is an indolent disease and many patients maintain a favorable health state prior to treatment (Johnson *Adv Ther* 2024), some patients start treatment with more severe symptoms or experience adverse events (AEs) throughout treatment that could impact their HRQoL.

### Aims

To assess the impact of epcor+$R^2$ on HRQoL in subsets of patients who had high baseline symptom burden or experienced clinically relevant treatment-emergent AEs (TEAEs).

### Methods

HRQoL was measured using the FACT-Lym, a 42-item, validated, cancer-specific patient-reported outcomes instrument. The FACT-Lym total score includes the LymS, a 15-item subscale for lymphoma symptoms, and domains to measure physical, functional, emotional, and social outcomes specific to oncology. Patients completed the FACT-Lym at baseline (Cycle 1 Day 1 [C1D1]), at each treatment cycle (up to C12), at the end of treatment, and during post-treatment and survival follow-ups. Baseline FACT-Lym values and changes during treatment were evaluated using descriptive analyses and mixed-effect models with repeated measures. Minimally important difference (MID) thresholds derived from published literature defined clinically meaningful changes in HRQoL (Carter *Blood* 2008). In this analysis, patient subsets were defined by high baseline symptom burden (lowest quartile of LymS scores at C1D1) and occurrence of key any-grade TEAEs associated with bispecific therapies (cytokine release syndrome [CRS], infection, neutropenia, and diarrhea).

**Copyright Information:** (Online) ISSN: 2572-9241
©2026 The Author(s). HemaSphere is published by John Wiley & Sons Ltd on behalf of European Hematology Association. This is an open access Abstract Book distributed under the Attribution-NonCommercial-NoDerivs (CC BY-NC-ND), which allows third parties to download the articles and share them with others as long as they credit the author and the Abstract Book, but they cannot change the content in any way or use them commercially.
**Abstract Book Citations:** Authors, Title, HemaSphere, 2026;10:(S1):pages. The abstract book DOIs can be found on the "Author Information" page.
**Disclaimer:** Articles published in the journal HemaSphere exclusively reflect the opinions of the authors. The authors are responsible for all content in their abstracts including accuracy of the facts, statements, citing resources, etc.

### Results

At the 24May2025 data cutoff, 488 patients were enrolled and randomized to epcor+$R^2$ (n=243) or $R^2$ alone (n=245). Mean baseline LymS scores for the overall treatment arms were 46.77 for epcor+$R^2$ vs 46.17 for $R^2$, while mean baseline scores for the most symptomatic patients (epcor+$R^2$, n=52; $R^2$, n=45) were 35.25 for epcor+$R^2$ vs 33.82 for $R^2$. Mean LymS changes from baseline remained within the MID threshold of 2.9 for both overall treatment arms. However, symptomatic patients in both arms experienced clinically meaningful improvement starting at C2 through C12, with least squares mean (95% CI) change from baseline at C12D1 of 7.92 (5.43–10.42) for epcor+$R^2$ and 9.78 (6.92–12.63) for $R^2$.

The FACT-Lym total score was used to assess HRQoL in patient subsets with any-grade TEAEs. In the epcor+$R^2$ arm, HRQoL was preserved throughout the treatment period in patients with CRS (n=77), infection (n=169), neutropenia (n=107), or diarrhea (n=69), consistent with patients who did not have those TEAEs.

### Summary/Conclusion

These results demonstrate that patients with R/R FL treated with subcutaneous epcor+$R^2$ were able to preserve or even improve their HRQoL. Patients with the greatest symptom burden at baseline showed clinically meaningful HRQoL improvements with epcor+$R^2$, similar to $R^2$. Patients who experienced clinically relevant TEAEs during treatment preserved their high baseline HRQoL. These findings complement the superior efficacy and manageable safety of epcor+$R^2$, supporting a favorable benefit–risk profile and positioning epcor+$R^2$ as a novel standard of care for 2L+ FL.

**Figure. FACT-Lym LymS LS Mean Change from Baseline in Patients with Baseline LymS Scores in the Lowest Quartile**

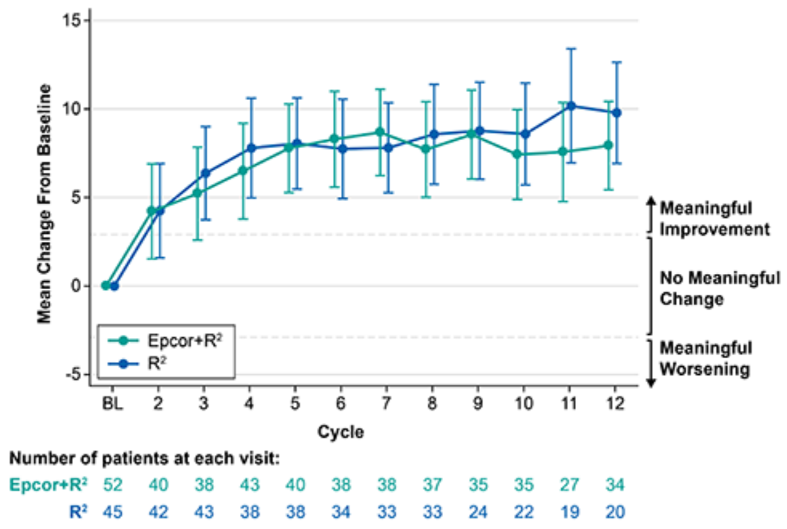

MID: -2.9 to +2.9. Mean change from baseline derived from linear mixed effects regression model based on an unstructured covariate matrix.
Error bars represent 95% CI.
BL, baseline; Epcor, epcoritamab; FACT-Lym, Functional Assessment of Cancer Therapy – Lymphoma; LS, least squares; LymS, Lymphoma Subscale; MID, minimally important difference; $R^2$, rituximab and lenalidomide.

**Copyright Information:** (Online) ISSN: 2572-9241
©2026 The Author(s). HemaSphere is published by John Wiley & Sons Ltd on behalf of European Hematology Association. This is an open access Abstract Book distributed under the Attribution-NonCommercial-NoDerivs (CC BY-NC-ND), which allows third parties to download the articles and share them with others as long as they credit the author and the Abstract Book, but they cannot change the content in any way or use them commercially.
**Abstract Book Citations:** Authors, Title, HemaSphere, 2026;10:(S1):pages. The abstract book DOIs can be found on the "Author Information" page.
**Disclaimer:** Articles published in the journal HemaSphere exclusively reflect the opinions of the authors. The authors are responsible for all content in their abstracts including accuracy of the facts, statements, citing resources, etc.

### (PS2498) QOL-BLINA PROTOCOL OF AIEOP STUDY GROUP: PRELIMINARY DATA ON QOL AND PSYCHOSOCIAL RISK IN PATIENTS AND CAREGIVERS.

**Topic:** 36. Quality of life, ethics, supportive and palliative care

Fara Petruzziello*[1], Roberto Capasso[1], Giuseppe Menna[1], Gaia Sepe[1], Maria Grazia Valsecchi[2], Daniela Silvestri[2], Marco Spinelli[3], Simone Cesaro[4], Fulvio Porta[5], Valentina Kiren[6], Eulalia Galea[7], Rosanna Parasole[1]

*[1]AORN Santobono-Pausilipon, Department of Oncology, Hematology and Cellular Therapies, Naples, Italy;[2]CORS San Gerardo Hospital, Monza, Italy; [3]Pediatric Clinic ASST San Gerardo Hospital, Monza, Italy; [4]Pediatric Oncohematology, Department of Maternal and Child, Integrated AOU, Verona, Italy; [5]Oncohematology and Bone Marrow Transplant Unit, ASST Spedali Civili, Brescia, Italy; [6]Pediatric Oncohematology Unit, IRCCS Maternal and Child Burlo Garofalo, Trieste, Italy; [7]AO Pugliese Ciaccio, Pediatric Hematology and Oncology Unit, Catanzaro, Italy;*

### Background

Blinatumomab is a monoclonal antibodies used in front-line and relapse treatment of paediatric Acute Lymphoblastic Leukaemia (ALL). Although efficacy and safety have been studied there are no data on Quality of Life (QoL) in paediatric patients during this treatment.

### Aims

In 8 AIEOP Centres is ongoing the first observational multicenter study in pediatric oncohematology with the aim to analyze the perceived QoL and the psychosocial risk in paediatric patients affected by ALL-B, recruited in the AIEOP-BFM-ALL-2017 protocol (intermediate/high risk) comparing the QoL of patients treated with blinatumomab (Blina) versus the standard chemotherapy (SoC).

### Methods

From February 2022 to February 2026, 61 patients (aged 2-17) have been recruited after obtaining informed consent/assent. Descriptive analyses have been run with SPSS software to explore the levels of perceived QoL on treatment and levels of perceived QoL on Psychosocial Risk. Moreover comparison between patients and caregivers on levels of perceived QoL has been evaluated. The following questionnaires are administered to caregivers and patients:

- PAT (Assessment Tools, Kazak, 2006) to measure psychosocial risk score;
- PedsQoL (Paediatric Quality of Life Inventory) cancer module to measure levels of QOL.

### Results

Preliminary data on 61 caregivers and 46 patients (age suitable for PedsQol administration) show that:

1. Levels of QoL on Treatment

| Levels of QoL N=61 | SOC N=21 | Blina N=40 |
|---|---|---|
| Low | 5 | 9 |
| Intermediate | 9 | 21 |
| High | 7 | 10 |

Copyright Information: (Online) ISSN: 2572-9241
©2026 The Author(s). HemaSphere is published by John Wiley & Sons Ltd on behalf of European Hematology Association. This is an open access Abstract Book distributed under the Attribution-NonCommercial-NoDerivs (CC BY-NC-ND), which allows third parties to download the articles and share them with others as long as they credit the author and the Abstract Book, but they cannot change the content in any way or use them commercially.
Abstract Book Citations: Authors, Title, HemaSphere, 2026;10:(S1):pages. The abstract book DOIs can be found on the “Author Information” page.
Disclaimer: Articles published in the journal HemaSphere exclusively reflect the opinions of the authors. The authors are responsible for all content in their abstracts including accuracy of the facts, statements, citing resources, etc.

| Levels of QoL N=46 | SOC N=15 | Blina N=31 |
|---|---|---|
| Low | 1 | 4 |
| Intermediate | 6 | 16 |
| High | 8 | 11 |

1. Levels of QoL on Psychosocial risk

| Psychosocial Risk N= 61 | Levels of perceived QoL | | |
|---|---|---|---|
| | Low | Intermediate | High |
| Universal/low risk | 2 | 16 | 9 |
| Targeted/specific risk | 9 | 9 | 8 |
| Clinical/high risk | 4 | 3 | 1 |

2. Comparison between caregivers and patients on levels of perceived QoL

| Levels of QoL | SoC Caregivers N=21 | SoC Patients N=15 |
|---|---|---|
| Low | 24% | 7% |
| Intermediate | 49% | 40% |
| High | 33% | 53% |

| Levels of QoL | Blina Caregivers N=40 | Blina Patients N=31 |
|---|---|---|
| Low | 23% | 13% |
| Intermediate | 53% | 52% |
| High | 25% | 35% |

4.Percentages of Reported Symptoms for Treatment

| Symptoms | SOC N=21 | Blina N=40 |
|---|---|---|
| Nausea | 76% | 60% |
| Vomiting | 48% | 32% |
| Fatigue | 29% | 10% |
| Weight loss/gain | 38% | 12% |
| Pain | 81% | 42% |
| Alopecia | 38% | 15% |
| Diarrhea/Constipation | 10% | 18% |
| Loss/increase of appetite | 38% | 18% |
| Mood | 52% | 47% |
| Sleep disturbances | 5% | 0 |
| Hospitalization distress | 43% | 30% |
| Device-related distress | 38% | 30% |

**Copyright Information:** (Online) ISSN: 2572-9241
©2026 The Author(s). HemaSphere is published by John Wiley & Sons Ltd on behalf of European Hematology Association. This is an open access Abstract Book distributed under the Attribution-NonCommercial-NoDerivs (CC BY-NC-ND), which allows third parties to download the articles and share them with others as long as they credit the author and the Abstract Book, but they cannot change the content in any way or use them commercially.
**Abstract Book Citations:** Authors, Title, HemaSphere, 2026;10:(S1):pages. The abstract book DOIs can be found on the "Author Information" page.
**Disclaimer:** Articles published in the journal HemaSphere exclusively reflect the opinions of the authors. The authors are responsible for all content in their abstracts including accuracy of the facts, statements, citing resources, etc.

**Summary/Conclusion**

These preliminary results show that:

- there are not significant differences between the groups of patients/caregivers treated with SOC or Blina about perceived QoL;
- most of patients with clinical/high psychosocial risk reported low/intermediate levels of perceived QoL and most of patients with universal/low psychosocial risk reported high/ intermediate levels of perceived QoL;
- comparing patients and caregivers on perceived QoL, the high perceived QoL is greater in percentage in patients;
- Patients treated with Blina show less frequent in most part of reported symptoms with respect to SoC and in particular for: fatigue, weight loss/gain, pain, alopecia, loss/increase of appetite.

These observations suggest that:

- the perceived QoL in patients/caregiver is influenced much more by presence/absence of psychosocial risk than the administered therapy;
- the caregivers perceived lower level of QoL than the patients could be due to the parent psychological distress related to their child illness;
- The less frequency of reported symptoms and in particular those related to body identity might improve the QoL of patients.

**Copyright Information:** (Online) ISSN: 2572-9241
©2026 The Author(s). HemaSphere is published by John Wiley & Sons Ltd on behalf of European Hematology Association. This is an open access Abstract Book distributed under the Attribution-NonCommercial-NoDerivs (CC BY-NC-ND), which allows third parties to download the articles and share them with others as long as they credit the author and the Abstract Book, but they cannot change the content in any way or use them commercially.
**Abstract Book Citations:** Authors, Title, HemaSphere, 2026;10:(S1):pages. The abstract book DOIs can be found on the "Author Information" page.
**Disclaimer:** Articles published in the journal HemaSphere exclusively reflect the opinions of the authors. The authors are responsible for all content in their abstracts including accuracy of the facts, statements, citing resources, etc.

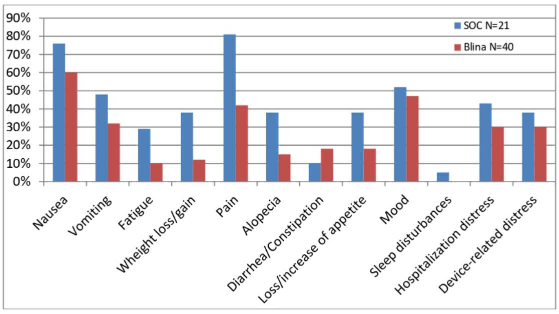

5. Comparison between Percentages of Reported Symptoms for Treatment

Copyright Information: (Online) ISSN: 2572-9241
©2026 The Author(s). HemaSphere is published by John Wiley & Sons Ltd on behalf of European Hematology Association. This is an open access Abstract Book distributed under the Attribution-NonCommercial-NoDerivs (CC BY-NC-ND), which allows third parties to download the articles and share them with others as long as they credit the author and the Abstract Book, but they cannot change the content in any way or use them commercially.
Abstract Book Citations: Authors, Title, HemaSphere, 2026;10:(S1):pages. The abstract book DOIs can be found on the "Author Information" page.
Disclaimer: Articles published in the journal HemaSphere exclusively reflect the opinions of the authors. The authors are responsible for all content in their abstracts including accuracy of the facts, statements, citing resources, etc.

### (PS2499) BURDEN OF DISEASE MANAGEMENT AMONG PATIENTS WITH MULTIPLE MYELOMA AND THEIR CARE PARTNERS

**Topic:** 36. Quality of life, ethics, supportive and palliative care

Shiyin Jiao[1], Charlene Tugwete[1], Hilary Wilson[1], Christian Atkinson[2], Elizabeth Holdsworth[2], Morgan Fox[2], Murat Ceyran[2], Robin Tuohy*[3], Valarie Traynham[4], Saurabh Patel[1]

*[1]AbbVie, Inc, North Chicago, United States of America; [2]Adelphi Real World, Bollington, United Kingdom; [3]International Myeloma Foundation, Los Angeles, United States of America; [4]HealthTree Foundation, South Jordan, United States of America;*

### Background

The complexity of current multiple myeloma (MM) treatment (tx) regimens imposes burdens on daily life, impacting quality of life (QoL) for both patients (PTs) and care partners (CPs).

### Aims

This study characterizes the holistic burden with current tx, including challenges with tx logistics and perceptions of novel therapies (eg, bispecific antibody [BsAb]/chimeric antigen receptor [CAR]-T).

### Methods

Semi-structured interviews were conducted online by a trained qualitative moderator with 12 adult MM PTs who received ≥3 lines of tx (n=4 received BsAb/CAR-T) and 9 CPs in the US. Open-ended discussion guides elicited insights on physical, psychosocial, and financial impacts especially as related to tx frequency and attending appointments. Rapid analysis was conducted by 2 coders per transcript with iterative coding and consensus review to identify themes that will inform design of future studies.

### Results

Five main themes emerged: (1) diverse physical symptoms from tx causing social impacts, (2) care complexity creating psychological burden, (3) frequent appointments imposing time and financial burdens, (4) lifestyle alterations required to accommodate tx schedule, and (5) openness to novel therapies shaped by personal experiences and external information.
Most PTs (10/12) reported major time burden. "*I would say that another thing that people probably don't appreciate is how much time I spend going to doctors' appointments and getting blood work done and all that… It takes up a huge portion of your life.*" -PT
All CPs (n=9) rearranged their lives around caring for the PTs. Most CPs (8/9) described a loss of independence. "*I took a few years off [work] during the really bad years. I think I just didn't have enough emotionally to [work]… And I often had to go off to [his] doctor's appointments.*" -CP
Financial burden affected most PTs (n=11), and 2 CPs highlighted the financial strain of frequent travel for tx. "There's days where if the rent is due and I don't have enough money… like just yesterday, even though they arranged for me to pay half, they still brought me an eviction notice" -PT
Physical impacts were reported by PTs receiving the novel BCMA/GPRC5D-targeted BsAbs, including fatigue, inflamed tongue, and difficulty sweating. Patient interest in novel tx centered on tolerability and lifestyle impact. Nearly half expressed interest in tx they would *"tolerate well,"* with *"less side effects";* one-third indicated a desire for tx that is *"less intrusive in life"* and can be *"taken as little as possible,"* as prioritized tx characteristics.

**Copyright Information:** (Online) ISSN: 2572-9241
©2026 The Author(s). HemaSphere is published by John Wiley & Sons Ltd on behalf of European Hematology Association. This is an open access Abstract Book distributed under the Attribution-NonCommercial-NoDerivs (CC BY-NC-ND), which allows third parties to download the articles and share them with others as long as they credit the author and the Abstract Book, but they cannot change the content in any way or use them commercially.
**Abstract Book Citations:** Authors, Title, HemaSphere, 2026;10:(S1):pages. The abstract book DOIs can be found on the "Author Information" page.
**Disclaimer:** Articles published in the journal HemaSphere exclusively reflect the opinions of the authors. The authors are responsible for all content in their abstracts including accuracy of the facts, statements, citing resources, etc.

**Summary/Conclusion**

While advances in MM tx improve survival, tx complexity and side effects remain distinct burdens on PTs and CPs. These findings highlight a pressing need for more tolerable and less burdensome therapies, both from physical and socio-economic perspectives, that restore autonomy and QoL beyond clinical outcomes. Further research will aim to quantify this need.

**Copyright Information:** (Online) ISSN: 2572-9241
©2026 The Author(s). HemaSphere is published by John Wiley & Sons Ltd on behalf of European Hematology Association. This is an open access Abstract Book distributed under the Attribution-NonCommercial-NoDerivs (CC BY-NC-ND), which allows third parties to download the articles and share them with others as long as they credit the author and the Abstract Book, but they cannot change the content in any way or use them commercially.
**Abstract Book Citations:** Authors, Title, HemaSphere, 2026;10:(S1):pages. The abstract book DOIs can be found on the "Author Information" page.
**Disclaimer:** Articles published in the journal HemaSphere exclusively reflect the opinions of the authors. The authors are responsible for all content in their abstracts including accuracy of the facts, statements, citing resources, etc.

### (PS2500) AQUALIS: AN OBSERVATIONAL QUALITY OF LIFE STUDY IN FRANCE ON PREVIOUSLY UNTREATED CHRONIC LYMPHOCYTIC LEUKEMIA (CLL) TREATED WITH ACALABRUTINIB:RAPID GAINS IN FUNCTION AND SYMPTOMS WITH HIGH ADHERENCE

**Topic:** 36. Quality of life, ethics, supportive and palliative care

Loic Ysebaert*[1], Cecile Tomowiak[2], Loic Raynal[3], Stephane Lepretre[4]

*[1]IUCT-Oncopole, Toulouse, France; [2]CHU Poitiers, Hematology, Poitiers, France; [3]Hospitalidée, Saint-Affrique, France; [4]Centre Henri Becquerel, Hematology, Rouen, France;*

### Background

CLL is a chronic disease requiring prolonged treatments (tx). Fatigue is the most frequently reported and disabling symptom. Acalabrutinib, a second generation bruton kinase inhibitor (BTKi) with demonstrated good efficacy and safety profiles[2-3], is an oral home tx, making the assessment of quality of life (QoL) essential to appreciate its real-world benefits.

### Aims

AQUALIS (Clinical Trial Registration NCT06548152) is a retrospective, observational, real-world study dedicated to the evaluation of the QoL under acalabrutinib in frontline CLL.

### Methods

Using secondary data from the national multicentre PLATON e-PRO Capture database, patients (pts) are followed longitudinally at baseline (T0) and every 3 months(m) till 12m. QoL, symptoms and compliance were assessed using validated questionnaires (EORTC QLQ-C30, QLQ-CLL17, GIRERD). Per protocol, we defined a 5-point threshold for clinical meaningfulness. With these scales, we monitored 5 dimensions (emotional, role, physical, social, cognitive functioning), 10 symptoms, 1 financial toxicity score and a global health status score (GHS).

### Results

Between September 2024 to March 2025, 108 CLL pts wereincluded at T0 (62% men, median age 69 years, 49% ≥70 years). Qol was assessed at baseline T0 (N=108) and at 3m post tx T1 (N=63) for this preliminary analysis. GHS and physical fct remained stable from T0 to T1 (Δ –0.7). On the other hand, there were rapid improvements in all other functioning dimensions including role (Δ+12.6), emotional (Δ+6.8), cognitive (Δ +5.3), and social (Δ+7.3). Among symptoms, the most substantial improvements were reported for insomnia (Δ–17.9) and appetite loss (Δ–12.3) followed by a decrease in nausea/vomiting (Δ–6.3), diarrhea (Δ–7.5), fatigue (Δ–5.5) and pain (Δ–5.7). Unexpectedly, financial difficulties (Δ+10.6) worsened despite tx costs' reimbursement.

Next, we investigated the evolution of the QoL according to gender (image 1) and age. Fatigue scores improved only in males (Δ-14.8) and in pts ≥70y (Δ-7). Female gender was associated with improvements in insomnia (Δ-30.3), pain (Δ-19.1), nausea/vomiting (Δ-13.4), appetite (Δ-10.6),) and diarrhea (Δ-10.6) while male gender improved in fatigue (Δ-14.8), appetite (Δ-12.9), insomnia (Δ-10.2), and diarrhea (Δ-5.6).

QLQ-CLL17 revealed convergent improvements in fatigue (Δ –4.5) and psychological domains with unchanged symptom burden (+0.7). At T1, 84.13% of pts had a GIRERD score of 0 and 15.87% had a score of 1-2, confirming high adherence of pts to an oral treatment taken at home.

**Copyright Information:** (Online) ISSN: 2572-9241
©2026 The Author(s). HemaSphere is published by John Wiley & Sons Ltd on behalf of European Hematology Association. This is an open access Abstract Book distributed under the Attribution-NonCommercial-NoDerivs (CC BY-NC-ND), which allows third parties to download the articles and share them with others as long as they credit the author and the Abstract Book, but they cannot change the content in any way or use them commercially.
**Abstract Book Citations:** Authors, Title, HemaSphere, 2026;10:(S1):pages. The abstract book DOIs can be found on the "Author Information" page.
**Disclaimer:** Articles published in the journal HemaSphere exclusively reflect the opinions of the authors. The authors are responsible for all content in their abstracts including accuracy of the facts, statements, citing resources, etc.

### Summary/Conclusion

These findings indicate that within 3m of acalabrutinib initiation, pts experienced stable GHS and broad gains in functioning roles, together with improvements in multiple symptoms including fatigue. This corroborates the already demonstrated favourable Qol[4] and safety profile of acalabrutinib translating into good compliance.
We will report updated results (and results for more patients) at T0 & T1 in addition to results for 50 patients at T2 (6m post-tx initiation).

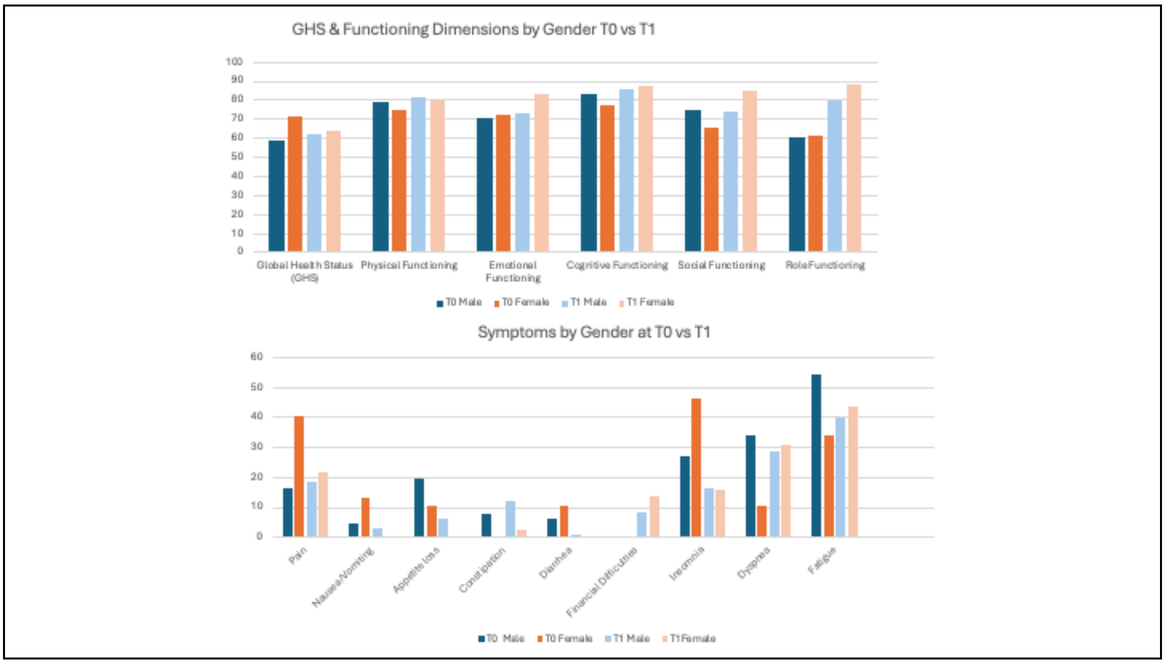

Figure 1: EORTC QLQ-C30 Scores according to Gender at time 0 (baseline) and time 1 (3 months of acalabrutib): Global Health Score, functional dimensions, and symptoms imptovement or deterioration.

**Copyright Information:** (Online) ISSN: 2572-9241
©2026 The Author(s). HemaSphere is published by John Wiley & Sons Ltd on behalf of European Hematology Association. This is an open access Abstract Book distributed under the Attribution-NonCommercial-NoDerivs (CC BY-NC-ND), which allows third parties to download the articles and share them with others as long as they credit the author and the Abstract Book, but they cannot change the content in any way or use them commercially.
**Abstract Book Citations:** Authors, Title, HemaSphere, 2026;10:(S1):pages. The abstract book DOIs can be found on the "Author Information" page.
**Disclaimer:** Articles published in the journal HemaSphere exclusively reflect the opinions of the authors. The authors are responsible for all content in their abstracts including accuracy of the facts, statements, citing resources, etc.

### (PS2501) NURSING-LED SUPPORTIVE CARE, SYMPTOM BURDEN AND CAREGIVER OUTCOMES IN ACUTE MYELOID LEUKEMIA

**Topic:** 36. Quality of life, ethics, supportive and palliative care

Guijie Wu*[1]

[1]*The Second Affiliated Hospital of Army Medical University, Hematology Medical Center, Chongqing, China;*

### Background

Acute myeloid leukemia (AML) is an aggressive hematologic malignancy associated with intensive chemotherapy, prolonged hospitalization, high complication risks, and severe physical and psychological distress. Despite advances in anti-leukemic treatment, unmet supportive care needs, uncontrolled treatment-related symptoms, and heavy caregiver burden remain prevalent and underaddressed in clinical practice. As frontline providers across the entire care continuum, nurses play a critical role in continuous symptom assessment, timely intervention, patient education, treatment adherence support, and caregiver empowerment. However, high-quality evidence focusing on nursing contributions to patient-reported outcomes and caregiver well-being in AML remains limited. This multicenter study aimed to address this important clinical gap.

### Aims

1. To characterize the prevalence and severity of treatment-related symptoms in adult AML patients undergoing chemotherapy.

2. To quantify caregiver burden, psychological distress, and functional impairment among primary informal caregivers.

3. To explore associations between symptom burden, nursing support, treatment confidence, and quality of life.

4. To identify nursing-sensitive indicators that predict favorable patient and caregiver outcomes.

### Methods

A multicenter cross-sectional observational study was conducted across 11 tertiary hematology centers. Eligible participants were adult AML patients receiving induction or consolidation chemotherapy and their primary informal caregivers. Validated tools included FACT-Leukemia, EQ-5D-5L, Zarit Burden Interview, HADS, WPAI, and treatment confidence VAS. Data were analyzed using descriptive statistics, correlation analyses, and multivariate linear regression adjusted for age, cytogenetic risk, treatment phase, and comorbidities.

**Copyright Information:** (Online) ISSN: 2572-9241
©2026 The Author(s). HemaSphere is published by John Wiley & Sons Ltd on behalf of European Hematology Association. This is an open access Abstract Book distributed under the Attribution-NonCommercial-NoDerivs (CC BY-NC-ND), which allows third parties to download the articles and share them with others as long as they credit the author and the Abstract Book, but they cannot change the content in any way or use them commercially.
**Abstract Book Citations:** Authors, Title, HemaSphere, 2026;10:(S1):pages. The abstract book DOIs can be found on the "Author Information" page.
**Disclaimer:** Articles published in the journal HemaSphere exclusively reflect the opinions of the authors. The authors are responsible for all content in their abstracts including accuracy of the facts, statements, citing resources, etc.

**Results**

A total of 242 patient-caregiver dyads were successfully enrolled.

• Approximately 71.1% of patients reported moderate to severe treatment-related symptoms, dominated by fatigue, sleep disturbance, infection risk, anxiety, and oral mucositis.

• Severe caregiver burden was identified in 47.5% of caregivers, accompanied by significantly higher anxiety, depression, and reduced work productivity.

• Greater symptom severity was strongly associated with lower treatment confidence, poorer quality of life, and higher caregiver distress (all P<0.001).

• After adjustment, nursing-led supportive care accessibility and structured symptom management were identified as independent protective factors for improved PROs and reduced caregiver burden (P<0.001).

**Summary/Conclusion**

This large multicenter study confirms that unmanaged symptom burden and caregiver distress are highly prevalent and interrelated in patients with AML undergoing chemotherapy. Nursing-sensitive interventions, including standardized symptom monitoring, timely supportive care, and structured caregiver support, are independently associated with improved patient treatment confidence, quality of life, and reduced caregiver burden.

These findings provide high-level evidence supporting the integration of nursing-led supportive care pathways into standard AML management. Embedding nursing-driven proactive care into daily clinical practice can effectively optimize patient experience, reduce care-related disparities, and improve holistic outcomes for both patients and caregivers.

**Copyright Information:** (Online) ISSN: 2572-9241
©2026 The Author(s). HemaSphere is published by John Wiley & Sons Ltd on behalf of European Hematology Association. This is an open access Abstract Book distributed under the Attribution-NonCommercial-NoDerivs (CC BY-NC-ND), which allows third parties to download the articles and share them with others as long as they credit the author and the Abstract Book, but they cannot change the content in any way or use them commercially.
**Abstract Book Citations:** Authors, Title, HemaSphere, 2026;10:(S1):pages. The abstract book DOIs can be found on the "Author Information" page.
**Disclaimer:** Articles published in the journal HemaSphere exclusively reflect the opinions of the authors. The authors are responsible for all content in their abstracts including accuracy of the facts, statements, citing resources, etc.

### (PS2502) EFFECTIVENESS OF A MOTHER-CENTERED INFECTION PREVENTION (MCIP) EDUCATIONAL INTERVENTION IN REDUCING INFECTIONS AMONG PEDIATRIC ONCOLOGY PATIENTS: A RANDOMIZED CONTROLLED TRIAL

**Topic:** 36. Quality of life, ethics, supportive and palliative care

Ana Farooq*[1]

[1]*Pakistan Institute of Medical Sciences, Pediatric Oncology, Islamabad, Pakistan;*

### Background

Infection-related morbidity and mortality remain significant barriers to treatment success in pediatric oncology, particularly in low- and middle-income countries. Strengthening caregiver competencies through structured education represents a feasible and cost-effective strategy to improve supportive care.

### Aims

Guided by the Health Belief Model, this study evaluated the effectiveness of a Mother-Centered Infection Prevention (MCIP) educational intervention in improving maternal knowledge, attitudes, and practices (KAP) and reducing infection-related clinical outcomes.

### Methods

A parallel-group randomized controlled trial was conducted at the Pakistan Institute of Medical Sciences (PIMS), Islamabad. Eighty mothers of pediatric oncology patients were randomly assigned to intervention (n=40) or control (n=40) groups. The intervention consisted of five weekly structured audio-visual education sessions, competency-based teach-back, culturally tailored information–education–communication (IEC) materials, infection prevention kits, and reinforcement follow-up visits. Standard care was provided to controls. Maternal KAP was assessed at baseline and end-line using a validated questionnaire. Clinical outcomes included infection-related hospitalizations and antibiotic utilization during the study period.

### Results

At end-line assessment, the intervention group demonstrated a 52% increase in mean KAP scores compared with an 11% increase in the control group ($p<0.001$). Infection-related hospitalizations were significantly lower in the intervention arm (10%) compared with controls (45%) ($p=0.01$). Antibiotic utilization was also reduced (12.5% vs. 57.5%; $p=0.02$). Additionally, 90% of mothers in the intervention group reported high confidence in implementing infection prevention practices, compared with 42.5% in the control group.

### Summary/Conclusion

The MCIP intervention significantly improved caregiver competencies and reduced infection-related morbidity among pediatric oncology patients. This theory-driven, culturally contextualized educational model demonstrates feasibility, effectiveness, and scalability for integration into supportive care frameworks in resource-constrained pediatric oncology settings.

**Copyright Information:** (Online) ISSN: 2572-9241
©2026 The Author(s). HemaSphere is published by John Wiley & Sons Ltd on behalf of European Hematology Association. This is an open access Abstract Book distributed under the Attribution-NonCommercial-NoDerivs (CC BY-NC-ND), which allows third parties to download the articles and share them with others as long as they credit the author and the Abstract Book, but they cannot change the content in any way or use them commercially.
**Abstract Book Citations:** Authors, Title, HemaSphere, 2026;10:(S1):pages. The abstract book DOIs can be found on the "Author Information" page.
**Disclaimer:** Articles published in the journal HemaSphere exclusively reflect the opinions of the authors. The authors are responsible for all content in their abstracts including accuracy of the facts, statements, citing resources, etc.

### (PS2503) SEXUAL HEALTH IN ADOLESCENTS AND YOUNG ADULTS AFTER HEMATOPOIETIC STEM CELL TRANSPLANT – AN OVERLOOKED DIMENSION OF SURVIVORSHIP

**Topic:** 36. Quality of life, ethics, supportive and palliative care

Brittany Kimball*[1], Mira Kohorst[1], Anand Srinivasan[1], Hannah Nordhues[1], David Dingli[1], Hassan Alkhateeb[1], Urshila Durani[1]

[1] *Mayo Clinic, Rochester, United States of America;*

### Background

Adolescents and young adults (AYAs) who undergo hematopoietic stem cell transplant (HSCT) face unique, often unmet survivorship challenges. Sexual health—an important part of quality of life and psychosocial development—is frequently impaired among AYA cancer survivors. HSCT recipients may experience additional challenges including graft-versus-host disease (GVHD) and its associated complications, yet sexual health remains under-addressed during routine transplant care.

### Aims

We sought to characterize sexual health-related quality of life in AYA HSCT survivors using an AYA-specific tool, and identify patient preferences for improving sexual health support.

### Methods

HSCT survivors ages 15-39 years at the time of transplant were recruited to complete a survey. The survey included a sexual health patient reported outcome tool designed for AYAs. Also included were questions about the extent to which sexual health needs have been addressed and ways in which sexual health needs could be better addressed.

### Results

Twenty-one survivors responded (characteristics summarized in Table). Prior to transplant, 33% reported a sexual problem; post-transplant, 62% reported ≥1 problem. The most commonly reported issues included low libido (n=5, 24%), erectile dysfunction (n=4, 19%), vaginal dryness (n=4, 19%), and anxiety related to sexual activity (n=4, 19%). Many reported relationship-related concerns and poor body image (Figure). Fertility preservation was discussed with 17 (81%), though only 8 (38%) pursued preservation. Nine (43%) were referred to sexual health specialists. Survivor-identified HSCT sexual health education preferences included: post-transplant sexual problems (7, 33%), relationship effects (6, 29%), and body image (4, 19%). Most preferred an HSCT nurse (n=9, 43%) to address sexual health concerns, while 7 (33%) prefer their HSCT provider, 5 (24%) wanted someone else, and 5 (24%) did not want the transplant team to address sexual health concerns. If available, 8 (38%) wanted referral to a sexual health specialist, 7 (33%) a mental health therapist, 4 (19%) a sex therapist, and 3 (14%) a toolkit with printed information.

| | |
|---|---|
| **Survey respondent characteristics** | |
| **Age in years, median (range)** | |
| Age at diagnosis | 32.5 (18-39) |
| Current age | 36 (19-42) |

**Copyright Information:** (Online) ISSN: 2572-9241
©2026 The Author(s). HemaSphere is published by John Wiley & Sons Ltd on behalf of European Hematology Association. This is an open access Abstract Book distributed under the Attribution-NonCommercial-NoDerivs (CC BY-NC-ND), which allows third parties to download the articles and share them with others as long as they credit the author and the Abstract Book, but they cannot change the content in any way or use them commercially.
**Abstract Book Citations:** Authors, Title, HemaSphere, 2026;10:(S1):pages. The abstract book DOIs can be found on the "Author Information" page.
**Disclaimer:** Articles published in the journal HemaSphere exclusively reflect the opinions of the authors. The authors are responsible for all content in their abstracts including accuracy of the facts, statements, citing resources, etc.

| | |
|---|---|
| **Gender – n (%)** | |
| Female | 11 (52%) |
| Male | 9 (43%) |
| Unknown | 1 (5%) |
| **Race – n (%)** | |
| White | 19 (90%) |
| Black | 1 (5%) |
| American Indian | 1 (5%) |
| Other | 1 (5%) |
| Unknown | 1 (5%) |
| **Ethnicity – n (%)** | |
| Not hispanic | 16 (76%) |
| Hispanic | 1 (5%) |
| Unknown | 4 (19%) |
| **Sexual orientation – n (%)** | |
| Heterosexual | 19 (90%) |
| Pansexual | 1 (5%) |
| Unknown | 1 (5%) |
| **Diagnosis – n (%)** | |

**Copyright Information:** (Online) ISSN: 2572-9241
©2026 The Author(s). HemaSphere is published by John Wiley & Sons Ltd on behalf of European Hematology Association. This is an open access Abstract Book distributed under the Attribution-NonCommercial-NoDerivs (CC BY-NC-ND), which allows third parties to download the articles and share them with others as long as they credit the author and the Abstract Book, but they cannot change the content in any way or use them commercially.
**Abstract Book Citations:** Authors, Title, HemaSphere, 2026;10:(S1):pages. The abstract book DOIs can be found on the "Author Information" page.
**Disclaimer:** Articles published in the journal HemaSphere exclusively reflect the opinions of the authors. The authors are responsible for all content in their abstracts including accuracy of the facts, statements, citing resources, etc.

| | |
|---|---|
| Acute myeloid leukemia | 9 (43%) |
| Aplastic anemia/bone marrow failure | 4 (19%) |
| Hodgkin lymphoma | 2 (10%) |
| Acute lymphoblastic leukemia | 2 (10%) |
| Myelodysplastic syndrome | 1 (5%) |
| Multiple myeloma | 1 (5%) |
| Blastic plasmacytoid dendritic cell neoplasm | 1 (5%) |
| Unknown | 1 (5%) |
| **Transplant type – n (%)** | |
| Allogeneic | 17 (81%) |
| Autologous | 1 (5%) |
| Both | 2 (10%) |
| Unknown | 1 (5%) |
| **Chronic GVHD – n (%)** | |
| Yes | 4 (19%) |
| No | 12 (57%) |
| Unknown | 5 (24%) |
| **Time since transplant – n (%)** | |
| Less than 6 months | 2 (10%) |

**Copyright Information:** (Online) ISSN: 2572-9241
©2026 The Author(s). HemaSphere is published by John Wiley & Sons Ltd on behalf of European Hematology Association. This is an open access Abstract Book distributed under the Attribution-NonCommercial-NoDerivs (CC BY-NC-ND), which allows third parties to download the articles and share them with others as long as they credit the author and the Abstract Book, but they cannot change the content in any way or use them commercially.
**Abstract Book Citations:** Authors, Title, HemaSphere, 2026;10:(S1):pages. The abstract book DOIs can be found on the "Author Information" page.
**Disclaimer:** Articles published in the journal HemaSphere exclusively reflect the opinions of the authors. The authors are responsible for all content in their abstracts including accuracy of the facts, statements, citing resources, etc.

| | |
|---|---|
| 6-8 months | 3 (14%) |
| 9-11 months | 0 (0%) |
| 1-2 years | 5 (24%) |
| 3-4 years | 5 (24%) |
| 5 or more years | 5 (24%) |
| Unknown | 1 (5%) |

**Summary/Conclusion**

AYA HSCT survivors experience substantial sexual dysfunction. Survivors expressed a preference for multidisciplinary support extending beyond the HSCT team. Integrating patient education on anticipated sexual health changes after transplant, standardizing sexual health screening, and ensuring provider training with clear referral pathways are essential to comprehensive AYA HSCT care.

**Copyright Information:** (Online) ISSN: 2572-9241
©2026 The Author(s). HemaSphere is published by John Wiley & Sons Ltd on behalf of European Hematology Association. This is an open access Abstract Book distributed under the Attribution-NonCommercial-NoDerivs (CC BY-NC-ND), which allows third parties to download the articles and share them with others as long as they credit the author and the Abstract Book, but they cannot change the content in any way or use them commercially.
**Abstract Book Citations:** Authors, Title, HemaSphere, 2026;10:(S1):pages. The abstract book DOIs can be found on the “Author Information” page.
**Disclaimer:** Articles published in the journal HemaSphere exclusively reflect the opinions of the authors. The authors are responsible for all content in their abstracts including accuracy of the facts, statements, citing resources, etc.

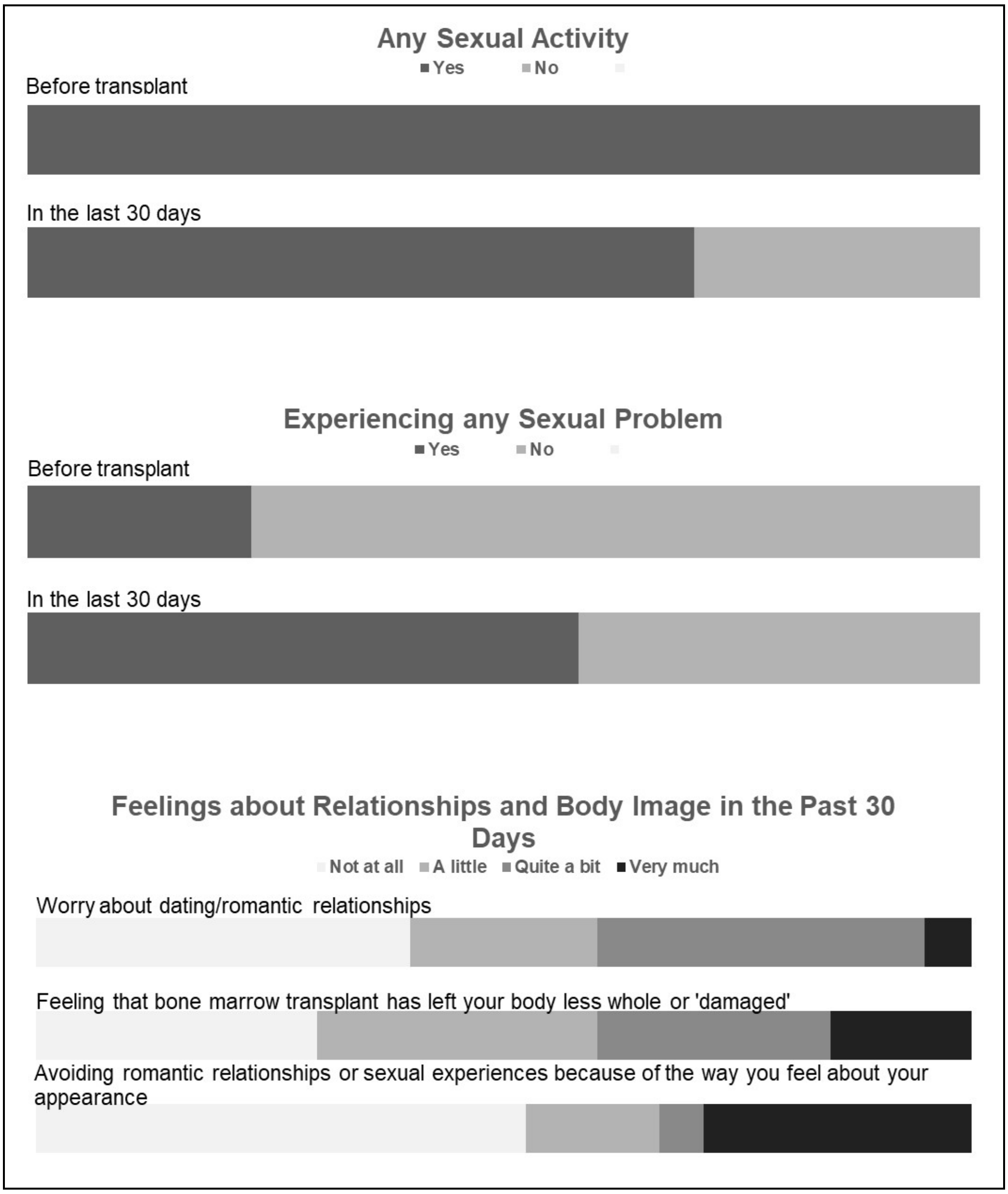

**Copyright Information:** (Online) ISSN: 2572-9241
©2026 The Author(s). HemaSphere is published by John Wiley & Sons Ltd on behalf of European Hematology Association. This is an open access Abstract Book distributed under the Attribution-NonCommercial-NoDerivs (CC BY-NC-ND), which allows third parties to download the articles and share them with others as long as they credit the author and the Abstract Book, but they cannot change the content in any way or use them commercially.
**Abstract Book Citations:** Authors, Title, HemaSphere, 2026;10:(S1):pages. The abstract book DOIs can be found on the "Author Information" page.
**Disclaimer:** Articles published in the journal HemaSphere exclusively reflect the opinions of the authors. The authors are responsible for all content in their abstracts including accuracy of the facts, statements, citing resources, etc.

### (PS2504) LONGITUDINAL PATIENT-REPORTED OUTCOMES HIGHLIGHT IMPROVED QUALITY OF LIFE FOR R-CHOP-INELIGIBLE PATIENTS WITH AGGRESSIVE B-CELL LYMPHOMAS TREATED WITHIN THE FRONTLINE PHASE II R-POLA-GLO TRIAL

**Topic:** 36. Quality of life, ethics, supportive and palliative care

Rebecca Wurm-Kuczera*[1], Thomas Melchardt[2, 3], Ralph Michael[4], Marita Ziepert[4], Bettina Altmann[4], Salah-Eddin Al-Batran[5], Ulrich Keller[1, 6], Richard Greil[2, 3, 7, 8], Björn Chapuy[1, 6]

*[1]Charité, University Medical Center Berlin, Campus Benjamin Franklin, Department of Hematology, Oncology, and Cancer Immunology, Berlin, Germany; [2]Paracelsus Medical University, 3rd Medical Department with Hematology, Medical Oncology, Rheumatology and Infectiology, Salzburg, Austria; [3]Austrian Group for Medical Tumor Therapy (AGMT) Study Group, Vienna, Austria; [4]Institute for Medical Informatics, Statistics, and Epidemiology, University of Leipzig, University of Leipzig, Leipzig, Germany; [5]The Frankfurt Institute of Clinical Cancer Research IKF, Frankfurt am Main, Germany;[6]German Cancer Consortium, partner site Berlin, Berlin, Germany; [7]Salzburg Cancer Research Institute (SCRI), Center for Clinical Cancer and Immunology Trials (CCCIT), Salzburg, Austria; [8]Cancer Cluster, Salzburg, Austria;*

### Background

Diffuse large B-cell lymphoma (DLBCL) predominantly affects older adults, many of whom are frail and ineligible for standard R-CHOP. While R-CHOP achieves high cure rates in fit patients, older, frail, and medically unfit patients often require attenuated regimens with inferior efficacy. This unmet need prompted development of the R-Pola-Glo trial integrating rituximab (R), polatuzumab vedotin (Pola), and the CD20×CD3 bispecific antibody glofitamab (Glo). The R-Pola-Glo phase II trial achieved high overall and complete metabolic remission rates (90% and 81% at end of treatment [EOT]) and 1-year progression-free and overall survival of 85% and 90%, respectively. Beyond efficacy, preservation of health-related quality of life (HRQoL) is critical, and patient-reported outcomes (PROs) provide complementary insights.

### Aims

To evaluate longitudinal HRQoL during frontline R-Pola-Glo therapy in R-CHOP–ineligible DLBCL patients enrolled in the initial phase II cohort (n=80).

### Methods

PROs were assessed longitudinally (baseline; day 1 of cycles (C) 2, 4, 6, 8, 10, 12, EOT, and 3-monthly follow-up). HRQoL was measured using the European Organization for Research and Treatment of Cancer Quality of Life Questionnaire-Core 30 (EORTC QLQ-C30; scale 0–100; higher scores indicate better functioning/global QoL and greater symptom burden on symptom scales) and lymphoma-specific HRQoL using the Functional Assessment of Cancer Therapy–Lymphoma ( v4; higher scores indicate better ). Analyses were descriptive (observed cases; no imputation). Mean change from baseline with 95% confidence intervals was calculated per visit. Clinically meaningful change was defined as ≥ 10 points for QLQ-C30 scales and ≥3 points for FACT-Lym. Questionnaire completion was reported per visit. Timepoints with at least 20% evaluable patients were included.

**Copyright Information:** (Online) ISSN: 2572-9241
©2026 The Author(s). HemaSphere is published by John Wiley & Sons Ltd on behalf of European Hematology Association. This is an open access Abstract Book distributed under the Attribution-NonCommercial-NoDerivs (CC BY-NC-ND), which allows third parties to download the articles and share them with others as long as they credit the author and the Abstract Book, but they cannot change the content in any way or use them commercially.
**Abstract Book Citations:** Authors, Title, HemaSphere, 2026;10:(S1):pages. The abstract book DOIs can be found on the "Author Information" page.
**Disclaimer:** Articles published in the journal HemaSphere exclusively reflect the opinions of the authors. The authors are responsible for all content in their abstracts including accuracy of the facts, statements, citing resources, etc.

**Results**

At data cut-off (02-Jul-2025), 80 patients were enrolled with median follow-up of 15 months. Median age was 80 years (range 66-92), including 19% ≥85 years; 91% were unfit or frail by simplified geriatric assessment. Most had advanced-stage disease (64%) and intermediate/high-risk disease (IPI3-5:64%). Treatment was well tolerated, with 34% experiencing no grade ≥3 adverse events. Questionnaire completion remained high (94% at baseline; >80% throughout treatment and follow-up), with fewer evaluable patients at later visits due to limited follow-up (39 at FU3). Baseline global HRQoL was moderate (mean QLQ-C30 Global Health Status 57). Global QoL improved during treatment (+10.7) demonstrating clinically meaningful improvement, with 44% achieving ≥10-point improvement at C4; and scores remained above baseline at EOT and follow-up. Patients maintained pre-treatment physical functioning and fatigue, showing no additional HRQoL burden, and consistent improvements in emotional functioning. FACT-Lym showed early, sustained, clinically meaningful improvement, with total score increasing by +10 points at C4 and remaining above baseline through EOT and follow-up. The trial outcome index showed a similar pattern.

**Summary/Conclusion**

In this frail, high-risk population, R-Pola-Glo demonstrated preserved or clinically meaningful improvements in HRQoL alongside high disease control. Global and emotional functioning improved, physical functioning was maintained, and lymphoma-related symptoms improved early and durably. These findings support the patient-reported tolerability of R-Pola-Glo as a chemotherapy-light, immune-based frontline strategy in patients with limited options.

**Copyright Information:** (Online) ISSN: 2572-9241
©2026 The Author(s). HemaSphere is published by John Wiley & Sons Ltd on behalf of European Hematology Association. This is an open access Abstract Book distributed under the Attribution-NonCommercial-NoDerivs (CC BY-NC-ND), which allows third parties to download the articles and share them with others as long as they credit the author and the Abstract Book, but they cannot change the content in any way or use them commercially.
**Abstract Book Citations:** Authors, Title, HemaSphere, 2026;10:(S1):pages. The abstract book DOIs can be found on the “Author Information” page.
**Disclaimer:** Articles published in the journal HemaSphere exclusively reflect the opinions of the authors. The authors are responsible for all content in their abstracts including accuracy of the facts, statements, citing resources, etc.

### (PS2505) GLOBAL PERSPECTIVES ON ACCESS TO CARE IN MYELODYSPLASTIC SYNDROMES: INSIGHTS FROM THE MDS GLOBAL ALLIANCE SURVEY 2024

**Topic:** 36. Quality of life, ethics, supportive and palliative care

Blerina Ahmetaj-Shala*[1, 2], Jaqueline Dubow[1]

*[1]MDS Alliance, Amsterdam, The Netherlands;[2]Imperial College London, London, United Kingdom;*

**Background**

Myelodysplastic syndromes (MDS) are a group of blood disorders that affect the bone marrow, ranging in severity from low to high risk. The MDS Global Alliance, an international organization with 40 members across 32 countries, aims to support individuals affected by MDS worldwide.

**Aims**

In 2024, the Alliance launched a global survey targeting patients and caregivers, focusing on “Access to Care” to help identify barriers and facilitators across diverse healthcare systems.

**Methods**

This global survey was developed on Qualtrics and open to individuals aged 18 and older who were directly or indirectly affected by an MDS diagnosis. Available in 11 languages, the survey was active from September 30, 2024, to January 31, 2025. Participation was voluntary and compliant with GDPR regulations. Responses were categorized into four thematic areas: diagnosis, care and support, treatment and testing, and monitoring. Data analysis was conducted using Excel and GraphPad Prism v10.

**Results**

A total of 628 responses were collected, with 83% from patients and 17% from caregivers. Nearly half of respondents (46%) were aged 66–80 years. Participants represented 31 countries, with 58% based in Europe. Most respondents (98%) lived in countries with a high GDP per capita. The top five countries by response count were France (151), South Korea (127), United Kingdom (112), USA (80), and Germany (71).
The survey highlighted that 45% of patients were unaware of their MDS subtype, and 25% did not know their risk score. Diagnostic delays were commonly attributed to inconclusive test results or limited awareness of MDS. Thirty-nine percent of patients received regular support, primarily from spouses, offering emotional and household assistance. Fatigue and weight loss were the most concerning symptoms. Marrow transplant offers were infrequent but more common among younger patients. Twenty-two percent reported difficulty accessing care from an MDS specialist, and only half of patients were managed by a multidisciplinary team. Participation in clinical trials was low, and access to mental health and emotional support services was limited. Younger patients reported feeling less treated with kindness and compassion compared to older individuals.

**Summary/Conclusion**

This global survey highlights critical gaps in MDS care, particularly in patient education regarding disease subtype, risk assessment, and symptom awareness. There is a pressing need for faster, more accurate diagnoses, improved access to emotional and mental health support, and greater inclusion in clinical trials. These findings confirm the value of international surveys in aligning scientific and clinical efforts with patient needs.

**Copyright Information:** (Online) ISSN: 2572-9241
©2026 The Author(s). HemaSphere is published by John Wiley & Sons Ltd on behalf of European Hematology Association. This is an open access Abstract Book distributed under the Attribution-NonCommercial-NoDerivs (CC BY-NC-ND), which allows third parties to download the articles and share them with others as long as they credit the author and the Abstract Book, but they cannot change the content in any way or use them commercially.
**Abstract Book Citations:** Authors, Title, HemaSphere, 2026;10:(S1):pages. The abstract book DOIs can be found on the “Author Information” page.
**Disclaimer:** Articles published in the journal HemaSphere exclusively reflect the opinions of the authors. The authors are responsible for all content in their abstracts including accuracy of the facts, statements, citing resources, etc.

### (PS2506) RISK FACTOR ANALYSIS FOR MELPHALAN-RELATED ORAL MUCOSITIS IN ADULT HEMATOPOIETIC STEM CELL TRANSPLANT PATIENTS

**Topic:** 36. Quality of life, ethics, supportive and palliative care

Liya Ren*[1], Jianli Zhang[1]

[1]*The First Affiliated Hospital, Zhejiang University School of Medicine, Department of Nursing, Hangzhou, China;*

### Background

Hematopoietic stem cell transplantation (HSCT) is an established therapeutic modality for hematologic malignancies and non-malignant disorders. High-dose melphalan, widely employed in myeloablative and reduced-intensity conditioning regimens, is associated with substantial mucosal toxicity, predominantly manifesting as oral mucositis (OM). OM not only causes significant pain, dysphagia, and impaired quality of life but also increases the risk of infections, prolongs hospitalization (by up to 3.9-fold), and escalates healthcare costs (approximately USD 25,409). Furthermore, OM may necessitate treatment interruption or dose reduction, thereby compromising transplant outcomes. Consequently, identification of risk factors for melphalan-induced OM is imperative for optimizing preventive and therapeutic strategies in adult HSCT recipients.

### Aims

To explore the risk and protective factors for oral mucositis in adult patients who underwent hematopoietic stem cell transplantation following myeloablative conditioning with melphalan and to provide evidence for clinical prevention strategies.

### Methods

A retrospective cohort study was conducted. Data on general characteristics, disease features, treatment-related factors, and oral care compliance were collected. Univariate analysis was first performed to screen variables: continuous variables were analyzed using the independent sample t-test or Mann-Whitney U test, and categorical variables using the $\chi^2$ test, Fisher's exact test, or continuous correction $\chi^2$ test. As parenteral nutrition was administered after the occurrence of oral mucositis, it was excluded from subsequent analysis as an exposure variable. Variables with $P<0.05$ in univariate analysis were entered into a binary logistic regression model for multivariate analysis. Model goodness-of-fit was assessed by the Hosmer-Lemeshow test.

### Results

Among 238 adult patients who underwent hematopoietic stem cell transplantation following myeloablative conditioning with melphalan, oral mucositis developed in 66.8% of patients, with 41.2% being mild to moderate (grade I-II) and 25.6% being severe (grade III-IV). Univariate analysis identified disease type, history of oral diseases, serum creatinine concentration, frequency of chemotherapy drug use, combined chemotherapy, frequency of diuretic use, and compliance with frozen saline mouth rinse as significant factors associated with oral mucositis (all $P<0.05$). Multivariate logistic regression analysis revealed that, after adjusting for confounders, patients with lymphoplasmacytic neoplasms (lymphoma and multiple myeloma) had a 6.30-fold increased risk of oral mucositis compared with the MDS and other diseases group (OR=6.30, P=0.011). A history of oral diseases was associated with a 2.75-fold increased risk (OR=2.75, P=0.001). Each 1 μmol/L increase in serum creatinine was associated with a 2.0% increased risk (OR=1.02, P=0.007). Notably, each 1-point increase in compliance with mouth rinse was associated with a 29.9% significant reduction in oral mucositis risk (OR=0.70, P=0.038). The model was statistically significant ($\chi^2=39.39$, $P<0.001$) with excellent goodness-of-fit (Hosmer-Lemeshow $\chi^2=3.42$, P=0.905).

**Copyright Information:** (Online) ISSN: 2572-9241
©2026 The Author(s). HemaSphere is published by John Wiley & Sons Ltd on behalf of European Hematology Association. This is an open access Abstract Book distributed under the Attribution-NonCommercial-NoDerivs (CC BY-NC-ND), which allows third parties to download the articles and share them with others as long as they credit the author and the Abstract Book, but they cannot change the content in any way or use them commercially.
**Abstract Book Citations:** Authors, Title, HemaSphere, 2026;10:(S1):pages. The abstract book DOIs can be found on the "Author Information" page.
**Disclaimer:** Articles published in the journal HemaSphere exclusively reflect the opinions of the authors. The authors are responsible for all content in their abstracts including accuracy of the facts, statements, citing resources, etc.

**Summary/Conclusion**

Disease type, history of oral diseases, and renal function status are independent risk factors for oral mucositis in adult patients who underwent hematopoietic stem cell transplantation following myeloablative conditioning with melphalan, while mouth rinse compliance is a modifiable protective factor.

**Copyright Information:** (Online) ISSN: 2572-9241
©2026 The Author(s). HemaSphere is published by John Wiley & Sons Ltd on behalf of European Hematology Association. This is an open access Abstract Book distributed under the Attribution-NonCommercial-NoDerivs (CC BY-NC-ND), which allows third parties to download the articles and share them with others as long as they credit the author and the Abstract Book, but they cannot change the content in any way or use them commercially.
**Abstract Book Citations:** Authors, Title, HemaSphere, 2026;10:(S1):pages. The abstract book DOIs can be found on the "Author Information" page.
**Disclaimer:** Articles published in the journal HemaSphere exclusively reflect the opinions of the authors. The authors are responsible for all content in their abstracts including accuracy of the facts, statements, citing resources, etc.

### (PS2507) HEMATOLOGIC MALIGNANCIES INCIDENTALLY DETECTED THROUGH CANCER SCREENING PROGRAM DURING 20 YEARS

**Topic:** 36. Quality of life, ethics, supportive and palliative care

Yuka Morita*[1], Nobuhiko Yamauchi[1], Fuka Mima[1], Yukako Teramoto[1], Mamiko Momoki[1], Yuko Shirouchi[1], Yusuke Koba[1], Yuko Ishihara[1], Yuko Mishima[1], Yasuko Uchida[2], Dai Maruyama[1]

[1]*Cancer Institute Hospital, Japanese Foundation for Cancer Research, Department of Hematology Oncology, Tokyo, Japan;* [2]*Cancer Institute Hospital, Japanese Foundation for Cancer Research, Cancer Screening Center, Tokyo, Japan;*

### Background

Population-based cancer screening enables early detection and reduces mortality for several solid tumors and is widely implemented in Japan. However, the clinical utility of screening for hematologic malignancies and its impact on prognosis remain unclear.

### Aims

To investigate the frequency and clinical characteristics of hematologic malignancies incidentally diagnosed following cancer screening.

### Methods

We included all patients with abnormalities detected on cancer screening who were subsequently referred to our department for further evaluation between 2005 and 2024. Patients referred for reasons unrelated to screening abnormalities were excluded. The objectives were the proportion of patients newly diagnosed with hematologic malignancies, the distribution of disease subtypes, and clinical outcomes.

### Results

200 referrals occurred, and 182 patients were finally analyzed. Median age was 61 years (range, 32–89); 97 patients (53%) were ≥61 years, and 105 patients (58%) were male. Examinations prompting referral included blood tests (55%), esophagogastroduodenoscopy (EGD, 28%), FDG-PET/CT (6%), ultrasonography (5%), CT (5%), and colonoscopy (1%). Final diagnoses were hematologic malignancies (44%), non-malignant hematologic diseases (11%), non-hematologic diseases (3%), and no definitive diagnosis or no abnormality (42%). Among the 81 hematologic malignancies, malignant lymphoma predominated (n=62), including mucosa-associated lymphoid tissue (MALT) lymphoma (n=40), follicular lymphoma (n=10), large B-cell lymphoma (n=4), chronic lymphocytic leukemia (n=3), classic Hodgkin lymphoma (n=1), and others (n=4); followed by 13 myeloid neoplasms (myelodysplastic syndrome [MDS], 5; chronic myeloid leukemia, chronic phase, 4; acute myeloid leukemia, 2; essential thrombocythemia, 1; polycythemia vera, 1) and 6 plasma cell neoplasms (multiple myeloma, 5; monoclonal gammopathy of undetermined significance, 1). One patient with MDS was diagnosed with progression of a pre-existing condition, whereas the remaining 80 patients (44%) were newly diagnosed. EGD contributed most frequently to the diagnosis of hematologic malignancies (57%), most of which were MALT lymphoma. Initial management included chemotherapy (26%), radiotherapy (20%), *Helicobacter pylori* eradication (22%), watchful waiting (25%), and others (7%). Among 56 patients (69%) who were treated, the median interval from screening to treatment initiation was 63 days (IQR, 44.5–84.5). Among patients with hematologic malignancies, median follow-up from referral to hematology was 70 months (range, 0.5–239); 5-year progression-free survival and overall survival were 82.1% (95% confidence interval, 70.5–89.5) and 91.9% (81.5–96.6), respectively.

**Copyright Information:** (Online) ISSN: 2572-9241
©2026 The Author(s). HemaSphere is published by John Wiley & Sons Ltd on behalf of European Hematology Association. This is an open access Abstract Book distributed under the Attribution-NonCommercial-NoDerivs (CC BY-NC-ND), which allows third parties to download the articles and share them with others as long as they credit the author and the Abstract Book, but they cannot change the content in any way or use them commercially.
**Abstract Book Citations:** Authors, Title, HemaSphere, 2026;10:(S1):pages. The abstract book DOIs can be found on the "Author Information" page.
**Disclaimer:** Articles published in the journal HemaSphere exclusively reflect the opinions of the authors. The authors are responsible for all content in their abstracts including accuracy of the facts, statements, citing resources, etc.

**Summary/Conclusion**

Newly diagnosed hematologic malignancies accounted for 44% of patients referred to hematology department. EGD was the most common diagnostic trigger, and MALT lymphoma was the most frequent subtype. A subset of patients referred to the department through cancer screening were ultimately treated, whereas most had indolent or clinically non-urgent conditions. Therefore, their outcomes were generally favorable, and whether detection through cancer screening translated into improved prognosis remains inconclusive.

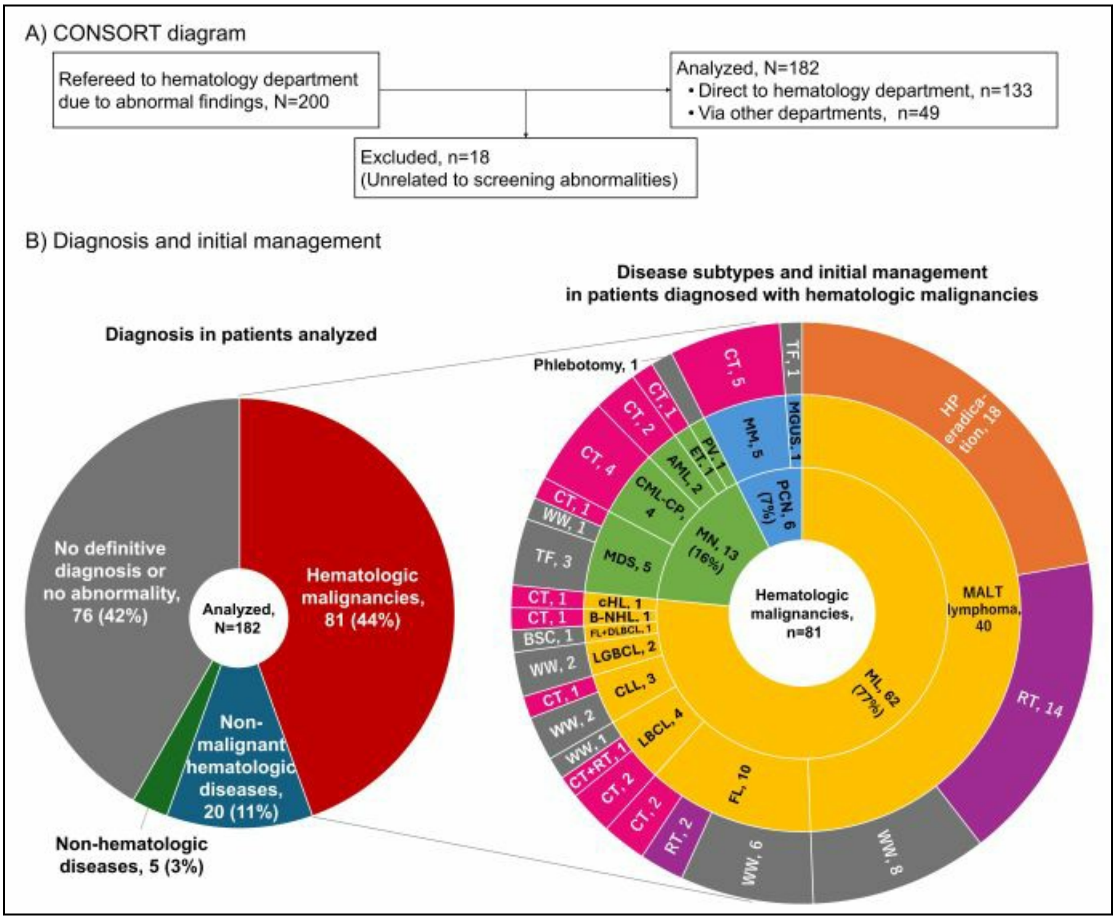

(A) CONSORT diagram. (B) Distribution of diagnoses among patients analyzed, with proportions of hematologic/non-hematologic diseases and malignant/non-malignant conditions, and disease subtypes and initial management in patients diagnosed with hematologic malignancies. Abbreviations: ML, malignant lymphoma; MN, myeloid neoplasm; PCN, plasma cell neoplasm; MALT, mucosa-associated lymphoid tissue; FL, follicular lymphoma; LBCL, large B-cell lymphoma; CLL, chronic lymphocytic leukemia; LGBCL, low-grade B-cell lymphoma; B-NHL, B-cell non-Hodgkin lymphoma; cHL, classic Hodgkin lymphoma; MDS, myelodysplastic syndrome; CML-CP, chronic myeloid leukemia in chronic phase; AML, acute myeloid leukemia; ET, essential thrombocythemia; PV, polycythemia vera; MGUS, monoclonal gammopathy of undetermined significance; HP, Helicobacter pylori; RT, radiotherapy; WW, watchful waiting; CT, chemotherapy; BSC, best supportive care; TF, transfer to another hospital.

**Copyright Information:** (Online) ISSN: 2572-9241
©2026 The Author(s). HemaSphere is published by John Wiley & Sons Ltd on behalf of European Hematology Association. This is an open access Abstract Book distributed under the Attribution-NonCommercial-NoDerivs (CC BY-NC-ND), which allows third parties to download the articles and share them with others as long as they credit the author and the Abstract Book, but they cannot change the content in any way or use them commercially.
**Abstract Book Citations:** Authors, Title, HemaSphere, 2026;10:(S1):pages. The abstract book DOIs can be found on the "Author Information" page.
**Disclaimer:** Articles published in the journal HemaSphere exclusively reflect the opinions of the authors. The authors are responsible for all content in their abstracts including accuracy of the facts, statements, citing resources, etc.

### (PS2508) LIVING WITH ESSENTIAL THROMBOCYTHEMIA AND THE BROADER ECONOMIC CONSEQUENCES

**Topic:** 37. Health economics

Cillian Copeland[1], Mark Connolly[1], Vivek Khurana[2], Sara Ottosson[3], Inkyu Kim[4], Stina Salomonsson*[5, 6]

*[1]Global Market Access Solutions, Sàrl Chardonne, Switzerland; [2]MSD Sharp & Dohme GmbH, Value & Implementation Outcomes Research, Munich, Germany; [3]MSD, Value & Implementation Global Medical and Scientific Affairs, Stockholm, Sweden; [4]Merck & Co., Inc.,, Rahway, NJ, United States of America; [5]Karolinska Institutet, Stockholm, Sweden; [6]MSD, Value & Implementation Outcomes Research, Stockholm, Sweden;*

### Background

Essential thrombocythemia (ET) is a myeloproliferative neoplasm (MPN) with an annual incidence of approximately 2 per 100,000 person years in Sweden. Individuals with ET experience a range of ET-associated symptoms and face increased risk of both haemorrhagic and thrombotic events, contributing to significant clinical burden. These symptoms and complications impact life and may impair daily functioning and work ability, leading to reduced labour force participation and indicating that the burden of ET extends beyond the healthcare system. While the clinical aspects of ET are well described, evidence on its broader fiscal impact remains limited.

### Aims

The aim of this study was to examine the fiscal impact of ET in Sweden from a government perspective, including effects on public revenues and social expenditures, using a dedicated analytic framework.

### Methods

A cohort-based fiscal model was developed in Microsoft Excel® that tracked disease progression and fiscal flows of a cohort of individuals with ET compared to an age-matched cohort in the general population across all ages over the lifetime. The model incorporated National Swedish fiscal data on economic activity, employment, productivity losses, disability and retirement. The relative impact of ET on these outcomes, including reductions in employment and productivity, increased disability, and earlier labour market exit, were sourced from a targeted literature search, and applied as disease-specific modifiers in the model. The incremental effects of ET government tax revenue and social transfer spending were combined with excess ET-attributable healthcare costs to estimate the combined fiscal burden over a lifetime horizon, discounted at an annual rate of 3%.

### Results

For a cohort of individuals with ET with a mean age of 65, it was estimated that the per-person fiscal loss of ET was SEK 1,730,501 (€157,475). This comprised a tax revenue loss of SEK 775,431 (€70,564) driven by lower employment participation, reduced income, premature mortality and productivity losses (absenteeism and presenteeism), increased social support spending of SEK 64,771 (€5,894) and healthcare costs of SEK 890,298 (€81,017). The fiscal burden of ET was higher among younger individuals, reflecting their greater labour market activity, whereas healthcare costs were the predominant drivers of the fiscal burden among older individuals.

**Copyright Information:** (Online) ISSN: 2572-9241
©2026 The Author(s). HemaSphere is published by John Wiley & Sons Ltd on behalf of European Hematology Association. This is an open access Abstract Book distributed under the Attribution-NonCommercial-NoDerivs (CC BY-NC-ND), which allows third parties to download the articles and share them with others as long as they credit the author and the Abstract Book, but they cannot change the content in any way or use them commercially.
**Abstract Book Citations:** Authors, Title, HemaSphere, 2026;10:(S1):pages. The abstract book DOIs can be found on the “Author Information” page.
**Disclaimer:** Articles published in the journal HemaSphere exclusively reflect the opinions of the authors. The authors are responsible for all content in their abstracts including accuracy of the facts, statements, citing resources, etc.

**Summary/Conclusion**

This study demonstrates that the economic burden of ET is not constrained to the healthcare sector but can impact wider government accounts by reducing productivity and economic activity and increasing rates of unemployment, long-term disability and retirement. This per-person fiscal burden is greater among younger patients due to higher levels of employment, while healthcare costs are the main driver of the burden among older patients.

**Copyright Information:** (Online) ISSN: 2572-9241
©2026 The Author(s). HemaSphere is published by John Wiley & Sons Ltd on behalf of European Hematology Association. This is an open access Abstract Book distributed under the Attribution-NonCommercial-NoDerivs (CC BY-NC-ND), which allows third parties to download the articles and share them with others as long as they credit the author and the Abstract Book, but they cannot change the content in any way or use them commercially.
**Abstract Book Citations:** Authors, Title, HemaSphere, 2026;10:(S1):pages. The abstract book DOIs can be found on the "Author Information" page.
**Disclaimer:** Articles published in the journal HemaSphere exclusively reflect the opinions of the authors. The authors are responsible for all content in their abstracts including accuracy of the facts, statements, citing resources, etc.

### (PS2509) OUTPATIENT CAR T-CELL THERAPY ADMINISTRATION IMPACT ON HEALTHCARE UTILIZATION IN LYMPHOMA PATIENTS

**Topic:** 37. Health economics

Susanna Bowen*[1], Nadine Abdallah[2], Josh Pritchett[3], Andre Demenezes Silva Corraes[2], Beatriz Oyarzabal[4], William Harmsen[5], Julianne Lunde[1], Jordan Coffey[6], Kelsey Haugen[7], Allison Rosenthal[8], Madiha Iqbal[9], Hassan Alkhateeb[2], Prashant Kapoor[2], N.Nora Bennani[2], Patrick Johnston[2], Stephen Ansell[2], Mohamed Kharfan-Dabaja[9], Tufia Haddad[10], Yi Lin[2], Jonas Paludo[2]

*[1]Mayo Clinic, Department of Internal Medicine, Rochester, MN, United States of America;[2]Mayo Clinic, Department of Hematology, Rochester, MN, United States of America; [3]Mayo Clinic, Department of Hematology, La Crosse, Wisconsin, United States of America; [4]Mayo Clinic, Department of Finance, Rochester, MN, United States of America;[5]Mayo Clinic, Department of Research, Rochester, MN, United States of America; [6]Mayo Clinic, Department of Hospital Operations, Red Wing, MN, United States of America; [7]Mayo Clinic, Department of Nursing, Rochester, MN, United States of America;[8]Mayo Clinic, Department of Hematology, Phoenix, AZ, United States of America; [9]Mayo Clinic, Department of Hematology, Jacksonville, FL, United States of America; [10]Mayo Clinic, Department of Medical Oncology, Rochester, MN, United States of America;*

### Background

Chimeric antigen receptor (CAR) T-cell therapy has substantially improved outcomes for patients with lymphoma. However, treatment can be associated with significant toxicities including cytokine release syndrome (CRS), and immune effector cell-associated neurotoxicity syndrome (ICANS), cytopenias, and infections. Because of this, patients have undergone close monitoring, especially in the first 30 days of CAR T-cell administration, traditionally with required inpatient post-infusion monitoring. This contributes to considerable time toxicity and healthcare costs. At the Mayo Clinic site in Rochester, MN, outpatient CAR T-cell infusion and monitoring has been the standard approach with demonstrated feasibility and safety (Bansal et al., ASCO 2023).

### Aims

We aim to compare the differences in healthcare utilization in lymphoma CAR T-Cell recipients who underwent outpatient remote monitoring and traditional inpatient monitoring by evaluating number of hospitalization days, outpatient visits, ICU admissions, ED visits, and portal engagement.

### Methods

A retrospective review of electronic medical records of 175 patients who received CAR T-cell therapy for lymphoma at any of the 3 Mayo Clinic sites (MN, AZ, FL) between 2020 and 2024 was conducted. Patients who underwent outpatient CAR T-cell therapy with remote monitoring in Rochester, MN (n=55) were compared with those receiving inpatient CAR T therapy in Arizona (n=66) and Florida (n=54). Per protocol, patients in the inpatient group underwent mandatory inpatient monitoring for 7 days with extension per provider discretion. Data was analyzed through Chi-Square and Wilcoxon tests. P values <0.05 were considered statistically significant.

**Copyright Information:** (Online) ISSN: 2572-9241
©2026 The Author(s). HemaSphere is published by John Wiley & Sons Ltd on behalf of European Hematology Association. This is an open access Abstract Book distributed under the Attribution-NonCommercial-NoDerivs (CC BY-NC-ND), which allows third parties to download the articles and share them with others as long as they credit the author and the Abstract Book, but they cannot change the content in any way or use them commercially.
**Abstract Book Citations:** Authors, Title, HemaSphere, 2026;10:(S1):pages. The abstract book DOIs can be found on the “Author Information” page.
**Disclaimer:** Articles published in the journal HemaSphere exclusively reflect the opinions of the authors. The authors are responsible for all content in their abstracts including accuracy of the facts, statements, citing resources, etc.

### Results

There were no significant differences in baseline characteristics including sex, age, ethnicity, LDH, platelets, or neutrophils among the 175 patients. The most common CAR T-cell products used were axicabtagene ciloleucel (n = 138), brexucabtagene autoleucel (n = 20), and lisocabtagene maraluecel (n = 10).
Patients managed in the outpatient setting had significantly fewer median hospital days compared with the inpatient cohort at both 30 days (8.5 vs 15.5, P <0.001) and 90 days (9.8 vs 14.2, P <0.001). Within the first 30 days, no outpatient patients required ICU admission, whereas 17 inpatient patients (14.2%) were admitted to the ICU (p=0.033). Notably, 16.4% of outpatient-managed patients avoided hospitalization entirely within 30 days, while in the inpatient group 81.7% and15.8% required 1 and 2 hospitalizations, respectively. Emergency department visits were also lower in the outpatient group with 7 (12.7%) patients in the outpatient group had ≥1 ED visits in the first 90 days compared with 39 (32.5%) patients in the inpatient group (P=0.0055). Portal engagement did not differ between groups at 30 days with 57.7% of patients using the portal at least once and a median of 3 messages in both groups. Within the first 30 days, 50.9% and 3.6% of patients in the outpatient group required 1 and 2 outpatient visits, respectively, vs 24.2% and 2.5% in the inpatient group (P=0.0006). There was no difference in 30-day mortality (1.8% outpatient vs 0.8% in inpatient) between the 2 groups (P=0.5310).

### Summary/Conclusion

Outpatient CAR T-cell remote monitoring was associated with significantly fewer hospital days without increased ED visits, ICU stays, portal use, or 30-day mortality. These findings support lower healthcare utilization for outpatient CAR T-cell therapy.

**Copyright Information:** (Online) ISSN: 2572-9241
©2026 The Author(s). HemaSphere is published by John Wiley & Sons Ltd on behalf of European Hematology Association. This is an open access Abstract Book distributed under the Attribution-NonCommercial-NoDerivs (CC BY-NC-ND), which allows third parties to download the articles and share them with others as long as they credit the author and the Abstract Book, but they cannot change the content in any way or use them commercially.
**Abstract Book Citations:** Authors, Title, HemaSphere, 2026;10:(S1):pages. The abstract book DOIs can be found on the "Author Information" page.
**Disclaimer:** Articles published in the journal HemaSphere exclusively reflect the opinions of the authors. The authors are responsible for all content in their abstracts including accuracy of the facts, statements, citing resources, etc.

### (PS2510) GLOBAL TRENDS OVER TWO DECADES IN CLINICAL TRIALS OF HEMATOLOGICAL MALIGNANCIES

**Topic:** 37. Health economics

Inkyung Sung[1], Izabela Nielsen[1], Daniel Tuyet Kristensen[2, 3, 4], Thomas Stauffer Larsen[5, 6], Ida Kristensen[5, 6], Tarec El-Galaly*[2, 3, 4, 7]

*[1]Aalborg University, Department of Materials and Production, Aalborg, Denmark;[2]Aarhus University Hospital, Department of Hematology, Aarhus, Denmark; [3]Aarhus University, Department of Clinical Medicine, Aarhus, Denmark;[4]Aarhus University Hospital, Department of Molecular Medicine, Aarhus, Denmark; [5]Odense University Hospital, Department of Hematology, Odense, Denmark; [6]University of Southern Denmark, Department of Clinical Research, Odense, Denmark;[7]Aarhus University Hospital, Department of Clinical Epidemiology, Aarhus, Denmark;*

### Background

In recent years, there have been increasing concerns about shifting global trends in clinical research, especially with a lower number of clinical trials in Europe. Complex EU regulations, inconsistent national legislation, and limited market access for many new cancer therapies in Europe have been speculated as potential reasons. A lower number of clinical trials has consequences for the European research environment and reduces access to innovative medicines for patients in Europe.

### Aims

To characterize global trends in clinical trials for leukemia, lymphoma, and multiple myeloma over two decades, focusing on temporal trends, clinical trial phase distribution, and geographical patterns in Europe.

### Methods

This study utilized public data from the ClinicalTrials.gov database (clinicaltrials.gov), accessed on 3 February 2026. A total of 8,946 clinical trials involving leukemia, lymphoma (including chronic lymphocytic leukemia), and/or multiple myeloma initiated between 2006 and 2025 were analyzed. Trial characteristics including trial phase and geographic location were extracted from the metadata fields via the database's application programming interfaces. Trials were stratified by start date, clinical phase (Phase 1 and 1/2, Phase 2, Phase 2/3 and 3, and Phase 4), and geographic location of trial sites. For geographic analysis, multi-continental trials were counted once for each continent of participating countries.

**Copyright Information:** (Online) ISSN: 2572-9241
©2026 The Author(s). HemaSphere is published by John Wiley & Sons Ltd on behalf of European Hematology Association. This is an open access Abstract Book distributed under the Attribution-NonCommercial-NoDerivs (CC BY-NC-ND), which allows third parties to download the articles and share them with others as long as they credit the author and the Abstract Book, but they cannot change the content in any way or use them commercially.
**Abstract Book Citations:** Authors, Title, HemaSphere, 2026;10:(S1):pages. The abstract book DOIs can be found on the "Author Information" page.
**Disclaimer:** Articles published in the journal HemaSphere exclusively reflect the opinions of the authors. The authors are responsible for all content in their abstracts including accuracy of the facts, statements, citing resources, etc.

**Results**

Global clinical trial activity increased during the surveyed period, particularly after 2015. The number of Phase 1 and 1/2 trials increased across all investigated hematological malignancies, whereas Phase 2 activity mainly increased in lymphoma. Late-phase clinical trials accounted for a small proportion across all malignancies throughout the surveyed period (Figure A).
Geographically, Asia drove growth in clinical trials with a 12.7% average annual increase, fueled by a 17.8% average annual increase in localized trials, shifting the global activity mass toward Asia (Figure B). In contrast, clinical trial participation in North America and Europe plateaued during the investigated period (0.5% average annual growth for both continents).
Among the 793 Phase 3 trials with reported trial sites, 392 (49%) had North American sites, 506 (64%) had European sites, and 428 (54%) had Asian sites. Phase 3 trials with European sites involved a median of 10 countries (IQR 1–17), including a median of 7 European countries (IQR 1–12), whereas Phase 3 trials without European participation involved a median of 1 country (IQR 1–1).
Within Europe, total clinical trial activity varied, with large countries leading in absolute numbers: France (1,026), Spain (883), Germany (865), Italy (836), and the United Kingdom (703). When accounting for population sizes, the highest activity was observed in Denmark and Belgium (both 39 trials per million inhabitants), followed by Austria (30/million), Czechia (28/million), and Norway (27/million).

**Summary/Conclusion**

Between 2006 and 2025, clinical trial activity in hematologic oncology expanded globally, accompanied by a progressive shift toward early-phase research and increasing contributions from Asia. Within Europe, marked disparities in clinical trial activity were observed. Furthermore, Phase 3 trials with participation from European countries involved more countries, emphasizing the importance of harmonized EU regulations and assessments to minimize the bureaucratic burden in multi-country trials.

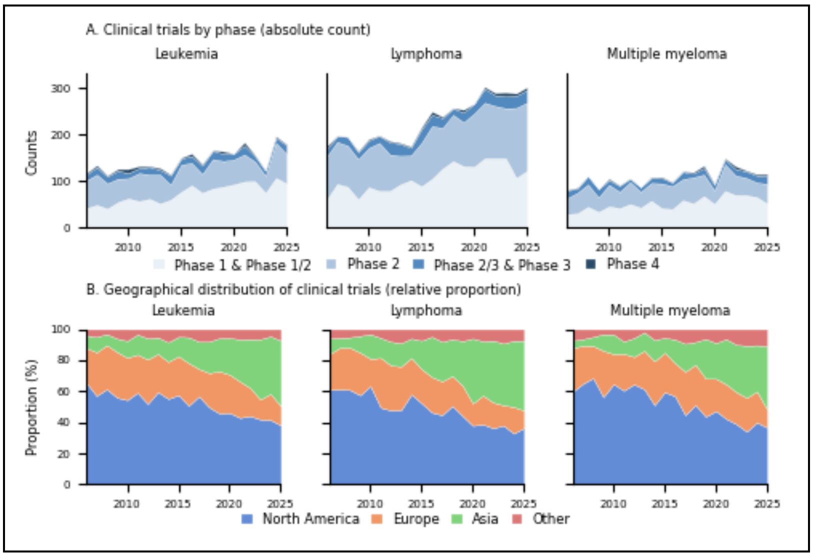

**Copyright Information:** (Online) ISSN: 2572-9241
©2026 The Author(s). HemaSphere is published by John Wiley & Sons Ltd on behalf of European Hematology Association. This is an open access Abstract Book distributed under the Attribution-NonCommercial-NoDerivs (CC BY-NC-ND), which allows third parties to download the articles and share them with others as long as they credit the author and the Abstract Book, but they cannot change the content in any way or use them commercially.
**Abstract Book Citations:** Authors, Title, HemaSphere, 2026;10:(S1):pages. The abstract book DOIs can be found on the "Author Information" page.
**Disclaimer:** Articles published in the journal HemaSphere exclusively reflect the opinions of the authors. The authors are responsible for all content in their abstracts including accuracy of the facts, statements, citing resources, etc.

### (PS2511) FEASIBILITY AND ECONOMIC EVALUATION FOR HOSPITAL-AT-HOME INFUSION OF CARFILZOMIB IN THE MANAGEMENT OF MULTIPLE MYELOMA (CARFIL-HAD STUDY)

**Topic:** 37. Health economics

Mohamed Touati*[1], Sophie Bastide[2], Camille Villesuzanne[3], Gaelle Maillan[4], Frederica Bompart[1], Moreau Stephane[3], Murielle Roussel[1]

*[1]Centre Hospitalier Universitaire, Hématologie Clinique et Thérapie Cellulaire, Limoges, France; [2]HEVA, Lyon, France; [3]Centre Hospitalier Brive, Hématologie, Brive, France; [4]Centre Hospitalier Universitaire de Limoges, Hématologie Clinique et Thérapie Cellulaire, Limoges, France;*

### Background

Carfilzomib is widely used in the management of both frontline and relapsed/refractory (R/R) multiple myeloma. Its standard continuous delivery schedule with weekly intravenous infusions in the outpatient hospital (OH) is associated with substantial organizational and financial burden. Innovative care models integrating Hospital-at-home infusions (HAH) may optimize ressources use while maintaining safety, efficiency and a good quality-of-life for patients.

### Aims

To evaluate the feasibility and safety of a mixed OH/HAH IV administration of carfilzomib in patients with R/R multiple myeloma. To assess the cost/healthcare resource utilization savings compared to an exclusive OH strategy from the French National Health Insurance perspective.

### Methods

Carfil-HAD (ClinicalTrials.gov: NCT05041933) is a prospective pilot study conducted at two French hematological centers (Limoges and Brive) in collaboration with three HAH healthcare providers.
Adult patients with R/R multiple myeloma received three mixed cycles of carfilzomib-based therapy with the first infusion (day 1) delivered in the OH and subsequent administrations performed by HAH under the nurse/medical supervision for outpatients according to standardized procedures.
The primary endpoint was the rate and type of deviations from standard operating procedures (SOPs). Secondary endpoints included: (1) incidence and reasons for unplanned rehospitalizations, (2) patient-reported quality of life, (3) satisfaction surveys, and (4) a cost analysis comparing the mixed OH/HAH model with a simulated 100% OH over 4 months (3 cycles + 1 day).
Costs were estimated from the French National Health Insurance perspective using 2024 rates, and compared using non-parametric Kolmogorov–Smirnov tests (SAS 9.4).

### Results

Fifteen evaluable patients completed three mixed OH/HAH cycles of carfilzomib-based therapy and received a total of 128 carfilzomib infusions (42 in OH and 86 HAH).
No major protocol deviations leading to treatment discontinuation were observed, supporting the operational feasibility of the mixed model.
Unplanned rehospitalizations were infrequent and did not reveal any specific safety signal related to HAH administration.
The mean total cost per mixed cycle and per patient was 1,107€, versus an estimated 1,717.6€ for an exclusive OH cycle, with an absolute cost-saving of 610.6€ and a relative reduction of 55.2% per cycle in favor of the OH/HAH mixed model. Patient-reported outcomes and satisfaction data will be presented at the congress.

**Copyright Information:** (Online) ISSN: 2572-9241
©2026 The Author(s). HemaSphere is published by John Wiley & Sons Ltd on behalf of European Hematology Association. This is an open access Abstract Book distributed under the Attribution-NonCommercial-NoDerivs (CC BY-NC-ND), which allows third parties to download the articles and share them with others as long as they credit the author and the Abstract Book, but they cannot change the content in any way or use them commercially.
**Abstract Book Citations:** Authors, Title, HemaSphere, 2026;10:(S1):pages. The abstract book DOIs can be found on the “Author Information” page.
**Disclaimer:** Articles published in the journal HemaSphere exclusively reflect the opinions of the authors. The authors are responsible for all content in their abstracts including accuracy of the facts, statements, citing resources, etc.

### Summary/Conclusion

The Carfil-HAD pilot study shows that HAH administration of carfilzomib  via a combined OH/HAH organization is feasible, safe, and highly healthcare resource-efficient. This mixed model yields substantial cost savings for the healthcare payer without compromising care delivery. This organisation provides a robust framework that could be generalized to other injectable anticancer agents requiring recurrent intravenous administration.

**Figure 1: Carfil-HAD - Flowchart**

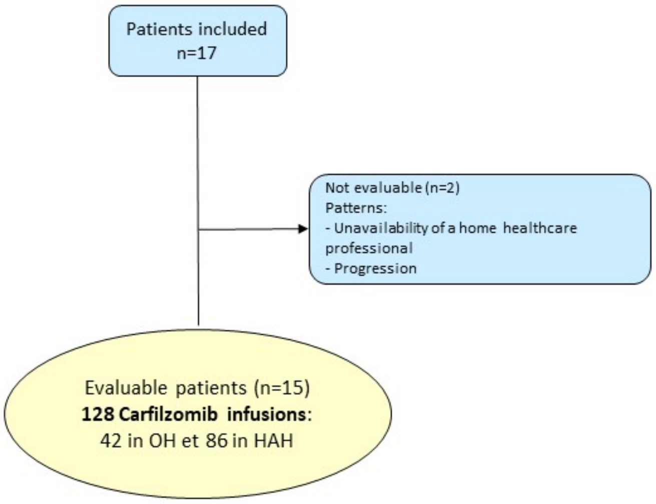

**Table1: Cost analysis**

**Cost by patient in 2024 (€)**

- Mixed OH/HAH cycle (n=41)

| | Observed costs | | | Simulated costs | | | Differences (simulated cost – observed cost) | | | Test * |
|---|---|---|---|---|---|---|---|---|---|---|
| | Mean | Median | Interquartile range | Mean | Median | Interquartile range | Mean | Median | Interquartile range | p-value |
| **Total** | **1 107,0** | **1 036,5** | **(946,6 ; 1 152,7)** | 1 717,6 | 1 616,3 | (1 387,6 ; 1 911,7) | **610,6** | **574,9** | **(439,8 ; 821,0)** | **0,0090** |
| **Hospital stay** | 1 014,0 | 945,4 | (867,0 ; 1 023,9) | 1 440,3 | 1 384,0 | (1 384,0 ; 1 384,0) | 426,2 | 430,2 | (360,1 ; 438,6) | 0,0090 |
| **Transport** | 92,9 | 63,8 | (53,9 ; 169,5) | 277,3 | 322,2 | (107,7 ; 386,4) | 184,4 | 175,7 | (63,8 ; 292,8) | 0,0281 |

(* Kolmogorov-Smirnov Test ; H0 : equal distribution costs)

- Statistically significant saving :
  - 55,2% for total cost
  - 42,0% for hospital stay cost
  - And whatever the mixed cycle

**Copyright Information:** (Online) ISSN: 2572-9241
©2026 The Author(s). HemaSphere is published by John Wiley & Sons Ltd on behalf of European Hematology Association. This is an open access Abstract Book distributed under the Attribution-NonCommercial-NoDerivs (CC BY-NC-ND), which allows third parties to download the articles and share them with others as long as they credit the author and the Abstract Book, but they cannot change the content in any way or use them commercially.
**Abstract Book Citations:** Authors, Title, HemaSphere, 2026;10:(S1):pages. The abstract book DOIs can be found on the "Author Information" page.
**Disclaimer:** Articles published in the journal HemaSphere exclusively reflect the opinions of the authors. The authors are responsible for all content in their abstracts including accuracy of the facts, statements, citing resources, etc.

### (PS2512) BEYOND EFFICACY: TIME TOXICITY OF CAR-T VS BISPECIFIC ANTIBODIES IN MULTIPLE MYELOMA

**Topic:** 37. Health economics

Nadine Abdallah*[1], Danny Trotier[2], Kavya Swarna[3], Yi Lin[1], Shaji Kumar[1], Urshila Durani[1]

*[1]Mayo Clinic, Hematology, Rochester, United States of America; [2]The Ohio State University Comprehensive Cancer Center, Department of Internal Medicine, Division of Hematology, Columbus, United States of America; [3]Mayo Clinic, Mayo Clinic Robert D. and Patricia E. Kern Center for the Science of Health Care Delivery, Rochester, United States of America;*

**Background**

CAR-T therapy and bispecific antibodies (BsAbs) have significantly improved outcomes in relapsed/refractory multiple myeloma (RRMM) but differ markedly in logistics and time burden. *Time toxicity*—the time patients spend in contact with the healthcare system vs at home—is an important yet underrecognized factor in treatment decisions for patients and caregivers. While CAR-T is a “one-time therapy” it requires substantial preparation, monitoring, and follow-up. While BsAbs are off-the-shelf, they involve ongoing outpatient treatment. Comparing time burden between these approaches is essential to guide decision-making and healthcare resource planning.

**Aims**

To compare time toxicity among patients MM receiving commercial CAR-T vs BsAb using a large U.S. real-world claims database.

**Methods**

In this retrospective cohort study using the OptumLabs Data Warehouse, adults with RRMM who received CAR-T or BsAb therapy from March 2021-December 2024 were identified using claims codes. For patients who received both treatments, the most recent one was selected. Time toxicity was defined as any day requiring physical contact with the healthcare system (clinic visits, lab/imaging appointments, infusions, emergency department visits, hospitalizations). Mean healthcare contact (HCC) days and %HCC (proportion of HCC vs home days) were evaluated at 1, 3, and 12 months among patients with continuous insurance coverage during that period (except if they died within the interval). The index date was defined as CAR-T infusion or first BsAb administration date. Incidence rate ratios (IRRs) were estimated to compare groups.

**Results**

A total of 819 patients were included (390 CAR-T; 429 BsAb). The BsAb group was older (mean age 70.4 vs 64.8 years, p<0.001) and included more females (49% vs 41%, p=0.01) compared with the CAR-T group. In the 60 days prior to index, HCC was comparable between groups (IRR 1.03, 95%CI0.94–1.12; p=0.56). At 1 month, among 802 evaluable patients (417 BsAb; 385 CAR-T), mean HCC was lower with BsAbs compared to CAR-T (12.7 vs 18.6 days); %HCC was 39% vs 61%, respectively (IRR 1.45, 95%CI 1.37–1.54; p<0.001). At 3 months, among 773 patients (403 BsAb; 370 CAR-T), mean HCC remained lower with BsAbs (24.6 vs 29.7 days), with 30% vs 34% %HCC (IRR 1.16, 95%CI 1.07–1.24; p<0.001). At 12 months, among 500 patients (278 BsAb; 222 CAR-T), mean HCC was similar between groups (54.6 vs 58.8 days), with 30% vs 25% %HCC, respectively (IRR 0.91, 95%CI 0.81–1.04; p=0.17). The distribution of inpatient vs outpatient days at 1 and 12 months is shown in**Figure 1**.

**Copyright Information:** (Online) ISSN: 2572-9241
©2026 The Author(s). HemaSphere is published by John Wiley & Sons Ltd on behalf of European Hematology Association. This is an open access Abstract Book distributed under the Attribution-NonCommercial-NoDerivs (CC BY-NC-ND), which allows third parties to download the articles and share them with others as long as they credit the author and the Abstract Book, but they cannot change the content in any way or use them commercially.
**Abstract Book Citations:** Authors, Title, HemaSphere, 2026;10:(S1):pages. The abstract book DOIs can be found on the “Author Information” page.
**Disclaimer:** Articles published in the journal HemaSphere exclusively reflect the opinions of the authors. The authors are responsible for all content in their abstracts including accuracy of the facts, statements, citing resources, etc.

**Summary/Conclusion**

In this large real-world analysis, CAR-T was associated with higher time toxicity during the first 3 months compared with BsAb therapy, particularly the first month. However, by 12 months, time toxicity was comparable. As these therapies are increasingly utilized and considered in earlier lines, our findings are important to better understand and weigh the time burden alongside efficacy and safety.

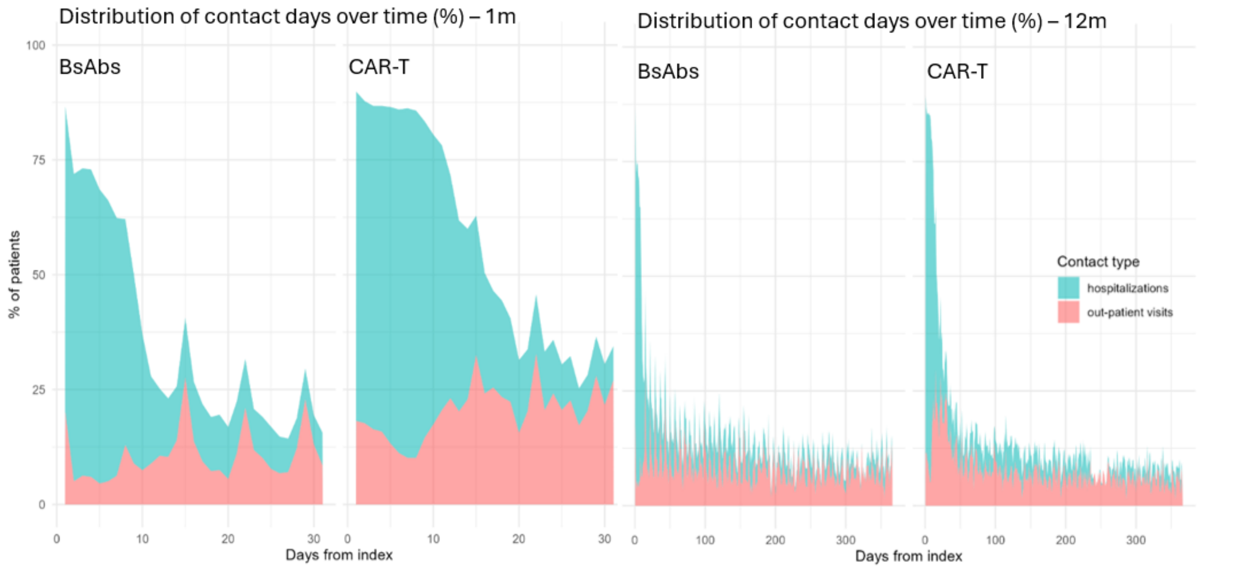

**Figure 1. Distribution of healthcare contact types over time following index therapy.**
Stacked area plots showing the daily proportion of patients with healthcare contact by contact type (hospitalizations vs outpatient visits) from the index date, stratified by treatment modality (BsAb vs CAR-T). The y-axis represents the percentage of patients with healthcare contact on a given day, and the x-axis represents days from index.

**Copyright Information:** (Online) ISSN: 2572-9241
©2026 The Author(s). HemaSphere is published by John Wiley & Sons Ltd on behalf of European Hematology Association. This is an open access Abstract Book distributed under the Attribution-NonCommercial-NoDerivs (CC BY-NC-ND), which allows third parties to download the articles and share them with others as long as they credit the author and the Abstract Book, but they cannot change the content in any way or use them commercially.
**Abstract Book Citations:** Authors, Title, HemaSphere, 2026;10:(S1):pages. The abstract book DOIs can be found on the "Author Information" page.
**Disclaimer:** Articles published in the journal HemaSphere exclusively reflect the opinions of the authors. The authors are responsible for all content in their abstracts including accuracy of the facts, statements, citing resources, etc.

### (PS2513) COST-EFFECTIVENESS ANALYSIS OF BREXUCABTAGENE AUTOLEUCEL IN ADULTS WITH RELAPSED OR REFRACTORY B-CELL PRECURSOR ACUTE LYMPHOBLASTIC LEUKAEMIA IN SINGAPORE

**Topic:** 37. Health economics

Esther Hian Li Chan*[1], Abdul Halim Nurul Aidah[2], Tza Kai Diong[3], Emma New[4], Briana Tan[4], Francesca Lorraine Wei Inng Lim[5]

*[1]National University Hospital (S), Haematology-Oncology, Singapore, Singapore;[2]Singapore General Hospital, Haematology, Singapore, Singapore; [3]Gilead Sciences Singapore, Singapore, Singapore;[4]Costello Medical, Singapore, Singapore;[5]Singapore General Hospital, Singapore, Singapore;*

### Background

In Singapore, relapsed or refractory (R/R) B-cell precursor acute lymphoblastic leukaemia (ALL) in adults remains challenging to treat with existing treatments and is associated with poor prognosis. This highlights an urgent unmet need for more effective treatments. Brexucabtagene autoleucel (brexu-cel), a chimeric antigen receptor T-cell (CAR T-cell) therapy, demonstrated high, durable responses and clinically meaningful survival benefits in this hard-to-treat population.

### Aims

This study assessed the cost-effectiveness of brexu-cel against three comparators (blinatumomab, inotuzumab ozogamicin [InO], and salvage chemotherapy [vincristine and dexamethasone]), in adults with R/R B-cell precursor ALL in Singapore.

### Methods

A cost-utility model was developed from the Singapore healthcare system perspective, with a 37-year time horizon. The model has a hybrid structure, comprising a decision tree (only for the brexu-cel arm) prior to entry into a partitioned survival model; the decision tree captures costs incurred by patients who receive pre-treatment but do not ultimately receive brexu-cel infusion. Clinical inputs were based on matching adjusted indirect comparisons between the ZUMA-3 trial (brexu-cel) and the comparator trials (blinatumomab: TOWER trial; InO and salvage chemotherapy: INO-VATE trial). The utilities were obtained from the ZUMA-3 trial and published literature. Healthcare resource use costs were obtained from published local sources; drug costs reflected the latest selling prices from public hospitals. Model outcomes included life years (LYs), quality-adjusted life years (QALYs) and incremental cost-effectiveness ratios (ICERs). In line with Singapore's health technology assessment guidelines, annual discount rate of 3% was applied to costs and outcomes. To assess the model's robustness, deterministic and probabilistic sensitivity analyses (DSA/PSA) were conducted. A willingness-to-pay (WTP) threshold of S$75,000/QALY was selected, given that most appraisals submitted to the Agency for Care Effectiveness with ICERs up to S$75,000/QALY received positive recommendations.

### Results

The base-case results are in **Table 1**. At a WTP threshold of S$75,000/QALY, brexu-cel is cost-effective against all comparators. The ICER was most sensitive to variations in the cost of blinatumomab (comparison vs blinatumomab), proportion of patients who received allo-SCT in the InO arm (comparison vs InO), and utilities for brexu-cel in progressed disease state (comparison vs salvage chemotherapy); nonetheless, all variations generated ICERs below the WTP threshold. In the PSA, at the selected WTP threshold, the probability of brexu-cel being cost-effective was 100.0% against blinatumomab, 96.3% against InO, and 99.0% against salvage chemotherapy.

**Copyright Information:** (Online) ISSN: 2572-9241
©2026 The Author(s). HemaSphere is published by John Wiley & Sons Ltd on behalf of European Hematology Association. This is an open access Abstract Book distributed under the Attribution-NonCommercial-NoDerivs (CC BY-NC-ND), which allows third parties to download the articles and share them with others as long as they credit the author and the Abstract Book, but they cannot change the content in any way or use them commercially.
**Abstract Book Citations:** Authors, Title, HemaSphere, 2026;10:(S1):pages. The abstract book DOIs can be found on the "Author Information" page.
**Disclaimer:** Articles published in the journal HemaSphere exclusively reflect the opinions of the authors. The authors are responsible for all content in their abstracts including accuracy of the facts, statements, citing resources, etc.

**Summary/Conclusion**

At a WTP threshold of S$75,000/QALY, brexu-cel is cost-effective compared with blinatumomab, InO and salvage chemotherapy. Therefore, brexu-cel represents a valuable new treatment to address the unmet needs of adults with R/R B-cell precursor ALL in Singapore.

**Table 1: Base-case results of brexu-cel vs comparators (discounted)**

| Variables | Vs blinatumomab | Vs InO | Vs salvage chemotherapy |
|---|---|---|---|
| Incremental cost (S$) | 110,403.64 | 210,837.83 | 327,242.22 |
| Incremental LYs gained | 4.96 | 5.23 | 6.84 |
| Incremental QALYs gained | 3.64 | 3.87 | 5.08 |
| **ICER (S$/QALY gained)** | 30,361.94 | 54,484.42 | 64,437.08 |

**Abbreviations**: ICER: incremental cost-effectiveness ratio; InO: inotuzumab ozogamicin; LYs: life years; QALYs: quality-adjusted life years.

**Copyright Information:** (Online) ISSN: 2572-9241
©2026 The Author(s). HemaSphere is published by John Wiley & Sons Ltd on behalf of European Hematology Association. This is an open access Abstract Book distributed under the Attribution-NonCommercial-NoDerivs (CC BY-NC-ND), which allows third parties to download the articles and share them with others as long as they credit the author and the Abstract Book, but they cannot change the content in any way or use them commercially.
**Abstract Book Citations:** Authors, Title, HemaSphere, 2026;10:(S1):pages. The abstract book DOIs can be found on the "Author Information" page.
**Disclaimer:** Articles published in the journal HemaSphere exclusively reflect the opinions of the authors. The authors are responsible for all content in their abstracts including accuracy of the facts, statements, citing resources, etc.

### (PS2514) COST-EFFECTIVENESS OF AXICABTAGENE CILOLEUCEL FOR TREATING TAIWANESE PATIENTS WITH RELAPSED/REFRACTORY DIFFUSE LARGE B-CELL LYMPHOMA AND HIGH-GRADE B-CELL LYMPHOMA AFTER FIRST-LINE TREATMENT

**Topic:** 37. Health economics

Bor-Sheng Ko*[1], Wen-Wen Yang[2], Yu-Chiao Wang[3], Tza Kai Diong[4], Kuan-Der Lee[5], Raoh-Fang Pwu[6]

*[1]National Taiwan University Cancer Center, Department of Hematological Oncology, Taipei, Taiwan;[2]Fu Jen Catholic University, Data Science Center, New Taipei City, Taiwan; [3]Yuanpei University of Medical Technology, Department of Healthcare Management, Hsinchu county, Taiwan; [4]Gilead Sciences Singapore, Singapore, Singapore; [5]Taichung Veterans General Hospital, Cell Therapy and Regenerative Medicine Center and Department of Oncology, Taichung, Taiwan; [6]School of Health Care Administration, Taipei Medical University, Taipei, Taiwan;*

### Background

Diffuse large B-cell lymphoma (DLBCL) is the predominant and aggressive non-Hodgkin lymphoma subtype in Taiwan, accounting for 60% of B-cell lymphomas and 2.19% of all cancer cases in 2023. Despite chemoimmunotherapy such as R-CHOP being the standard first-line (1L) treatment, outcomes remain poor for patients with relapsed or refractory (R/R) DLBCL and high-grade B-cell lymphoma (HGBL), particularly those who are refractory to 1L therapy or relapse within 12 months, highlighting an unmet need in second-line (2L) treatment. Recently, chimeric antigen receptor T-cell (CAR T-cell) therapy, including axicabtagene ciloleucel (axi-cel), has demonstrated promising clinical benefits and has been approved as a 2L option in the United States, Europe, and Taiwan.

### Aims

This study aims to evaluate the cost-effectiveness of axi-cel versus standard of care (SoC) as a 2L treatment for Taiwanese patients with DLBCL and HGBL.

### Methods

A three-state partitioned survival model (event-free, post-event, and death) was used to estimate the cost-effectiveness of axi-cel versus SoC (i.e., chemoimmunotherapy ± high-dose therapy and autologous stem cell transplantation) as 2L treatment from the Taiwan National Health Insurance (NHI) perspective. Clinical outcomes were informed by the ZUMA-7 randomized controlled trial, enrolling patients with R/R DLBCL and HGBL within 12 months after 1L therapy. Healthcare resource use and costs were informed by NHI data and literature. The model applied a lifetime horizon with monthly cycles, a starting age of 61.1 years based on NHI data, and a 3% annual discounting. Quality-adjusted life years (QALYs), total costs, and incremental cost-effectiveness ratios (ICERs) were estimated in US dollars (exchange rate: 1 USD = 30 NTD). Treatment crossover in the ZUMA-7 standard-of-care arm was adjusted using rank preserving structural failure time models, and adverse event costs were based on local expert inputs and NHI data.

**Copyright Information:** (Online) ISSN: 2572-9241
©2026 The Author(s). HemaSphere is published by John Wiley & Sons Ltd on behalf of European Hematology Association. This is an open access Abstract Book distributed under the Attribution-NonCommercial-NoDerivs (CC BY-NC-ND), which allows third parties to download the articles and share them with others as long as they credit the author and the Abstract Book, but they cannot change the content in any way or use them commercially.
**Abstract Book Citations:** Authors, Title, HemaSphere, 2026;10:(S1):pages. The abstract book DOIs can be found on the “Author Information” page.
**Disclaimer:** Articles published in the journal HemaSphere exclusively reflect the opinions of the authors. The authors are responsible for all content in their abstracts including accuracy of the facts, statements, citing resources, etc.

### Results

In the base-case analysis, axi-cel demonstrated substantially improved outcomes compared with SoC, with modeled median event-free survival of 10 versus 3 months and median overall survival of 77 versus 15 months. In Table 1, undiscounted results showed that axi-cel provided an additional 7.30 life-years (13.37 vs. 6.07) and 5.32 QALYs per patient compared with SoC (9.83 vs. 4.51). These gains were driven by superior efficacy data observed in the ZUMA-7 randomized controlled trial, which translated into a sustained long-term survival advantage in the model projections. Total undiscounted treatment-related costs were higher for axi-cel (NTD 8.15M [USD 271,586]) than for SoC (NTD 2.82M [USD 94,012]), driven mainly by 2L treatment costs, whereas SoC costs were largely attributable to subsequent salvage therapies and post-event care. After applying a 3% discount rate, axi-cel yielded an incremental gain of 3.40 QALYs with an ICER of NTD 1.56M [USD 52,125] per QALY gained. Probabilistic sensitivity analysis indicated a 66% probability of axi-cel being cost-effective at a willingness-to-pay threshold of three times Taiwan's 2023 per capita gross domestic product (GDP), a commonly cited willingness-to-pay benchmark.

### Summary/Conclusion

Axi-cel yields substantial clinical benefits and represents a cost-effective use of NHI resources for the 2L treatment of DLBCL and HGBL in Taiwan. These findings support the clinical and economic value of early CAR T-cell adoption for eligible patients.

**Table 1: Base-case cost-effectiveness results**

| Results | Axi-cel | SoC | Difference |
|---|---|---|---|
| **Undiscounted** | | | |
| Lifetime costs | NTD 8,473,692 [USD 282,456] | NTD 3,153,793 [USD 105,126] | NTD 5,319,900 [USD 177,330] |
| QALYs | 9.83 | 4.51 | 5.32 |
| ICER (Axi-cel vs. SoC) | **NTD999,538 (USD 33,318) per QALY gained** | | |
| **Discounted** | | | |
| Lifetime costs | NTD 8,408,093 [USD 280,270] | NTD 3,086,089 [USD 102,870] | NTD 5,322,004 [USD 177,400] |
| QALYs | 6.85 | 3.45 | 3.40 |
| ICER (Axi-cel vs. SoC) | **NTD1,563,763 (USD 52,125) per QALY gained** | | |

Abbreviations: Axi-cel, axicabtagene ciloleucel; ICER, incremental cost-effectiveness ratio; NTD, New Taiwan dollar; QALY, quality-adjusted life-year; SoC, standard of care; USD, United States dollar.
Note: Total undiscounted treatment-related costs for axi-cel and SoC were NTD 8,147,570 (USD 271,586) and NTD 2,820,348 (USD 94,012), respectively; discounted treatment costs were NTD 8,141,122 (USD 271,371) and NTD 2,783,422 (USD 92,781). Costs were converted from New Taiwan dollars (NTD) to US dollars (USD) at an exchange rate of 1 USD = 30 NTD (approximate annual average based on data from the Central Bank of the Republic of China [Taiwan]) and rounded to the nearest whole number.

Table 1: Base-case cost-effectiveness results

Copyright Information: (Online) ISSN: 2572-9241
©2026 The Author(s). HemaSphere is published by John Wiley & Sons Ltd on behalf of European Hematology Association. This is an open access Abstract Book distributed under the Attribution-NonCommercial-NoDerivs (CC BY-NC-ND), which allows third parties to download the articles and share them with others as long as they credit the author and the Abstract Book, but they cannot change the content in any way or use them commercially.
Abstract Book Citations: Authors, Title, HemaSphere, 2026;10:(S1):pages. The abstract book DOIs can be found on the "Author Information" page.
Disclaimer: Articles published in the journal HemaSphere exclusively reflect the opinions of the authors. The authors are responsible for all content in their abstracts including accuracy of the facts, statements, citing resources, etc.

### (PS2515) REAL-WORLD IMPACT OF ATRIAL FIBRILLATION (AFIB) ON CARDIOVASCULAR (CV) OUTCOMES AND HEALTHCARE RESOURCE UTILIZATION (HCRU) IN PATIENTS WITH CHRONIC LYMPHOCYTIC LEUKEMIA (CLL)

**Topic:** 37. Health economics

Michael Fradley*[1], Daniel Addison[2], Catherine Fu[3], Ayad Ali[3], Derrick Van Beuge[3], Dong Yuan[3], Mei Xue[3], G. Rhys Williams[3], Keri Yang[3]

*[1]Penn Medicine, University of Pennsylvania, Philadelphia, PA, United States of America;[2]University of Texas Southwestern Medical Center, Dallas, TX, United States of America; [3]BeOne Medicines, Ltd, San Carlos, CA, United States of America;*

### Background

While the association between AFib and CLL has been reported, real-world evidence on its clinical and economic impact is limited.

### Aims

This study evaluated impact of AFib on CV outcomes (stroke, bleeding, heart failure) and HCRU in patients with CLL overall, by age, and by Bruton tyrosine kinase inhibitor (BTKi) therapy.

### Methods

This retrospective observational study used the US Symphony database to identify adults newly diagnosed with CLL (2014-2024). Patients were followed for 1 year after CLL diagnosis to assess incidence of AFib. Subsequent CV outcomes (stroke, bleeding, heart failure) and HCRU (inpatient, outpatient, other services) were compared in CLL patients with and without AFib. Multivariate regression analyses assessed associations between AFib and outcomes. Subgroup analyses were conducted in elderly patients aged ≥65 years. Exploratory analyses compared first-line (1L) BTKi use (ibrutinib, acalabrutinib, or zanubrutinib).

### Results

In 233,362 newly diagnosed CLL patients, 13.1% had AFib within 1 year of CLL diagnosis. A significantly greater proportion of CLL patients with AFib had ≥1 inpatient visit within 1 year of CLL diagnosis than those without AFib (54.9% vs 23.2%, *P*<.0001), as well as a higher likelihood to incur inpatient service (OR: 2.28, 95%CI [2.21, 2.35], *P*<.0001). Significantly higher proportions of CLL patients with AFib had subsequent stroke (14.3% vs 8.9%), bleeding (27.9% vs 19.1%), and heart failure (54.5% vs 18.9%) than those without AFib (*P*<.0001). Age ≥65, male, non-white, and AFib were associated with subsequent stroke, bleeding, or heart failure (*P*<.01). Results were consistent in patients aged ≥65 years. In patients initiating 1L BTKi, AFib rate within 1 year of treatment for 1L zanubrutinib vs acalabrutinib vs ibrutinib was 11% vs 13% vs 16%, respectively; *P*<.0001. Compared to 1L ibrutinib and 1L acalabrutinib, a lower proportion of CLL patients with AFib treated with 1L zanubrutinib had subsequent stroke (12.2% vs 9.4% vs 4.8%, respectively), bleeding (27.4% vs 21.5% vs 17.4%), and heart failure (50.9% vs 45.6% vs 39.6%) (*P*<.002). CLL patients with AFib treated with 1L zanubrutinib had less inpatient services than 1L ibrutinib and 1L acalabrutinib within 1 year of BTKi treatment (46.4% vs 51.5% vs 60.4%; *P*<.0001). In multivariate regression, 1L acalabrutinib patients had 29% higher odds than 1L zanubrutinib patients (OR: 1.29, 95% CI [1.12, 1.50], *P*=.0005); 1L ibrutinib patients had 69% higher odds than 1L zanubrutinib patients (OR: 1.69, 95% CI [1.48, 1.93], *P*<.0001) to incur inpatient service within 1 year after initiating BTKi treatment.

**Copyright Information:** (Online) ISSN: 2572-9241
©2026 The Author(s). HemaSphere is published by John Wiley & Sons Ltd on behalf of European Hematology Association. This is an open access Abstract Book distributed under the Attribution-NonCommercial-NoDerivs (CC BY-NC-ND), which allows third parties to download the articles and share them with others as long as they credit the author and the Abstract Book, but they cannot change the content in any way or use them commercially.
**Abstract Book Citations:** Authors, Title, HemaSphere, 2026;10:(S1):pages. The abstract book DOIs can be found on the “Author Information” page.
**Disclaimer:** Articles published in the journal HemaSphere exclusively reflect the opinions of the authors. The authors are responsible for all content in their abstracts including accuracy of the facts, statements, citing resources, etc.

**Summary/Conclusion**

Findings highlight significant real-world CV and HCRU burden incurred by CLL patients with AFib. Exploratory analyses suggested 1L zanubrutinib may offer potentially favorable outcomes over other BTKi in lessening AFib and related clinical and HCRU complications. Future studies with longer follow-up are warranted to confirm these findings.

**Copyright Information:** (Online) ISSN: 2572-9241
©2026 The Author(s). HemaSphere is published by John Wiley & Sons Ltd on behalf of European Hematology Association. This is an open access Abstract Book distributed under the Attribution-NonCommercial-NoDerivs (CC BY-NC-ND), which allows third parties to download the articles and share them with others as long as they credit the author and the Abstract Book, but they cannot change the content in any way or use them commercially.
**Abstract Book Citations:** Authors, Title, HemaSphere, 2026;10:(S1):pages. The abstract book DOIs can be found on the "Author Information" page.
**Disclaimer:** Articles published in the journal HemaSphere exclusively reflect the opinions of the authors. The authors are responsible for all content in their abstracts including accuracy of the facts, statements, citing resources, etc.

### (PS2516) PATTERNS OF TREATMENT UTILIZATION AND SEQUENCING ACROSS LINES OF THERAPY IN WALDENSTROM MACROGLOBULINEMIA (WM): REAL-WORLD EVIDENCE FROM THE UNITED STATES

**Topic:** 37. Health economics

Jorge Castillo[1], Sheeba K. Thomas[2], M. Lia Palomba[3], Keri Yang[4], Mei Xue[4], Catherine Fu[4], Asher Chanan-Khan[5], Prashant Kapoor*[6]

*[1]Dana-Farber Cancer Institute, Boston, MA, United States of America; [2]University of Texas MD Anderson Cancer Center, Houston, TX, United States of America; [3]Memorial Sloan Kettering Cancer Center, New York, NY, United States of America; [4]BeOne Medicines Ltd, San Carlos, CA, United States of America; [5]Mayo Clinic, Jacksonville, FL, United States of America; [6]Mayo Clinic, Rochester, MN, United States of America;*

### Background

WM is a rare, incurable non-Hodgkin lymphoma with different treatment options available across successive lines of therapy (LOT). In the context of an evolving therapeutic landscape, there is limited real-world evidence detailing how treatment regimens are utilized and sequenced over time during the relapsing-remitting course of WM.

### Aims

To evaluate real-world patterns of treatment utilization, sequencing, and associated healthcare resource utilization (HCRU) in WM.

### Methods

A retrospective observational study was conducted using the US Symphony Integrated Dataverse® database to identify adults with ≥1 WM diagnostic code who initiated treatment 01/2020-08/2025. Patients were categorized into 8 mutually exclusive groups by treatment regimens: bendamustine-based chemotherapy (B/BR), rituximab-monotherapy (R-mono), other rituximab-combinations (R-combo, including R-cyclophosphamide, doxorubicin, vincristine, and prednisone [R-CHOP], and other combinations), Bruton tyrosine kinase inhibitor (BTKi; zanubrutinib, ibrutinib)-, bortezomib-, venetoclax-based regimens, and other regimens. Patient sociodemographic and clinical characteristics were analyzed in each group. Treatment utilization was analyzed overall, by year, and by LOT, with sequencing patterns visualized by Sankey diagram. All-cause HCRU during treatment (inpatient, outpatient, and other medical/hospital services) was examined and reported per-patient-per-year (PPPY).

**Copyright Information:** (Online) ISSN: 2572-9241
©2026 The Author(s). HemaSphere is published by John Wiley & Sons Ltd on behalf of European Hematology Association. This is an open access Abstract Book distributed under the Attribution-NonCommercial-NoDerivs (CC BY-NC-ND), which allows third parties to download the articles and share them with others as long as they credit the author and the Abstract Book, but they cannot change the content in any way or use them commercially.
**Abstract Book Citations:** Authors, Title, HemaSphere, 2026;10:(S1):pages. The abstract book DOIs can be found on the "Author Information" page.
**Disclaimer:** Articles published in the journal HemaSphere exclusively reflect the opinions of the authors. The authors are responsible for all content in their abstracts including accuracy of the facts, statements, citing resources, etc.

**Results**

A total of 7583 patients with WM initiated first-line (1L) therapy. Of these, 2251 (29.7%) initiated second line (2L) therapy, and 976 (12.9%) initiated third or later LOT (3L+). BTKi was the most commonly used drug class across all LOTs, with zanubrutinib used most commonly, and with increasing frequency over time. 1L treatment utilization patterns (**Figure 1A**) showed that B/BR was the most commonly used regimen (27.1%), followed by R-mono (22.3%) and zanubrutinib (19.6%). In 2L and 3L+, R-mono was the most common regimen (19.9% and 20.3%, respectively), followed by BTKi. Treatment sequencing patterns (**Figure 1B**) suggest that in patients with WM who received 1L BTKi, B/BR was the most common subsequent treatment (42.7% after 1L zanubrutinib, 22.2% after 1L ibrutinib). Conversely, for patients who received 1L B/BR, BTKis were the most common 2L regimen (zanubrutinib 35.6%; ibrutinib 16.4%). In patients who received 3L therapy after 2L zanubrutinib, 33.3% received B/BR and 30.0% received venetoclax, while after 2L ibrutinib, 50.0% were treated with either zanubrutinib (34.6%) or retreated with ibrutinib (15.4%), and 13.5% received B/BR. Substantial HCRU was observed across LOTs. Outpatient visits PPPY were lower for BTKi (zanubrutinib [1L: 12.0; 2L: 13.6; 3L+: 13.2]; ibrutinib [1L: 13.6; 2L: 15.2; 3L+: 16.0]) than for B/BR (1L: 33.7; 2L: 37.0; 3L+: 33.7), R-combo (1L: 42.3; 2L: 30.4; 3L+: 35.0), and R-mono (1L: 33.2; 2L: 32.2; 3L+: 32.6). Mean inpatient visits PPPY were lowest for both zanubrutinib (1L: 1.2; 2L: 1.0; 3L+: 1.6) and ibrutinib (1L: 1.7; 2L: 1.1; 3L+: 1.4), whereas the highest inpatient use was observed for venetoclax in 1L (4.7) and 3L+ (3.4) and 2L bortezomib (3.8).

**Summary/Conclusion**

In this real-world study, while BTKi was the predominant class across all LOTs, treatment sequencing frequently alternated between BTKi and B/BR-based regimens. These results shed light on current evolving WM treatment patterns and underscore the need for further studies to evaluate long-term outcomes to optimize treatment sequencing in real-world settings.

**Copyright Information:** (Online) ISSN: 2572-9241
©2026 The Author(s). HemaSphere is published by John Wiley & Sons Ltd on behalf of European Hematology Association. This is an open access Abstract Book distributed under the Attribution-NonCommercial-NoDerivs (CC BY-NC-ND), which allows third parties to download the articles and share them with others as long as they credit the author and the Abstract Book, but they cannot change the content in any way or use them commercially.
**Abstract Book Citations:** Authors, Title, HemaSphere, 2026;10:(S1):pages. The abstract book DOIs can be found on the "Author Information" page.
**Disclaimer:** Articles published in the journal HemaSphere exclusively reflect the opinions of the authors. The authors are responsible for all content in their abstracts including accuracy of the facts, statements, citing resources, etc.

**Figure 1A. Overall Treatment Utilization by LOT**

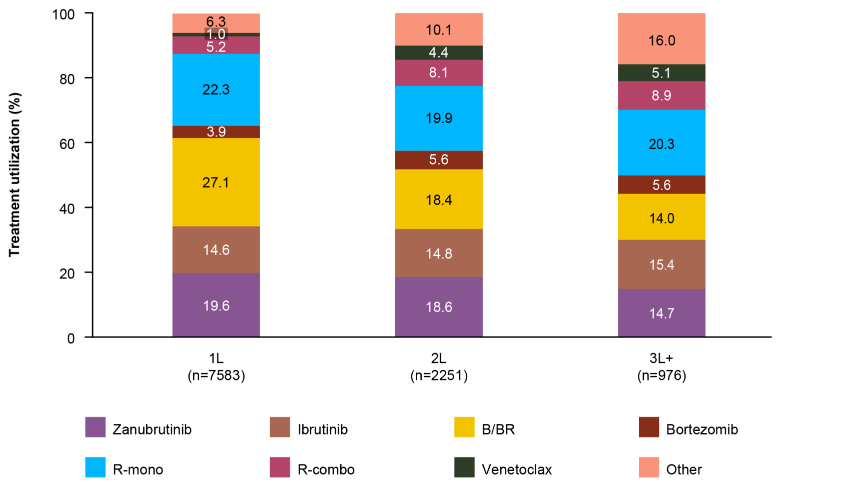

**Figure 1B. Treatment Sequencing Pattern**

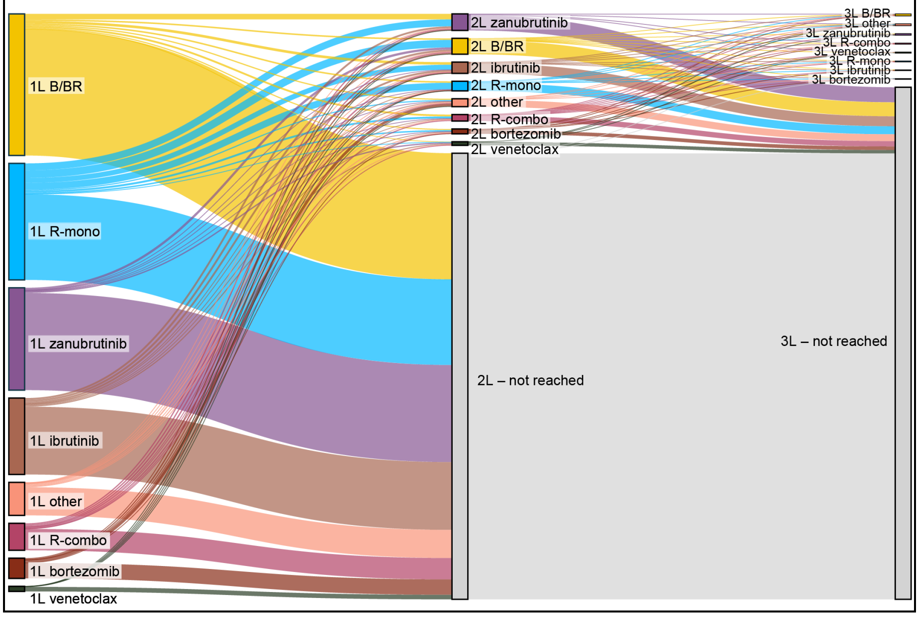

**Copyright Information:** (Online) ISSN: 2572-9241
©2026 The Author(s). HemaSphere is published by John Wiley & Sons Ltd on behalf of European Hematology Association. This is an open access Abstract Book distributed under the Attribution-NonCommercial-NoDerivs (CC BY-NC-ND), which allows third parties to download the articles and share them with others as long as they credit the author and the Abstract Book, but they cannot change the content in any way or use them commercially.
**Abstract Book Citations:** Authors, Title, HemaSphere, 2026;10:(S1):pages. The abstract book DOIs can be found on the "Author Information" page.
**Disclaimer:** Articles published in the journal HemaSphere exclusively reflect the opinions of the authors. The authors are responsible for all content in their abstracts including accuracy of the facts, statements, citing resources, etc.

### (PS2517) COMPARISON OF THE COST OF MANAGING ADVERSE EVENTS WITH FIXED DURATION COMBINATION TARGETED THERAPY REGIMENS IN THE FIRST-LINE TREATMENT OF CHRONIC LYMPHOCYTIC LEUKAEMIA IN BRAZIL

**Topic:** 37. Health economics

Ian Keary*[1], Marilia Hernani[2], Carolina Teixeira[2], Ricardo Moreira[2], Barry Rodgers-Gray[1], John Fullarton[1], Melda Dinç[3], Sarah Palazuelos-Munoz[4], Fady Fam[5], Doreen Tay[6]

*[1]Violicom Medical Ltd, Reading, United Kingdom; [2]AstraZeneca, Sao Paulo, Brazil; [3]AstraZeneca, Istanbul, Türkiye; [4]AstraZeneca, Gaithersburg, United States of America; [5]AstraZeneca, Cambridge, United Kingdom; [6]AstraZeneca, Singapore, Singapore;*

### Background

Targeted therapy (±anti-CD20 agents) are now standard of care in the first-line treatment of chronic lymphocytic leukaemia (CLL), as reflected in the recent consensus from the Brazilian Association of Hemaotolgy and Hemotherapy. Recent data have demonstrated the efficacy of fixed-duration combinations of acalabrutinib (A+V) or ibrutinib (I+V) with venetoclax. There are, however, recognised differences in adverse event (AE) profiles of the individual agents. This study assessed the cost of managing AEs associated with the fixed duration use of A+V, I+V, and venetoclax+obinutuzumanb (V+O) in the Brazilian private healthcare context.

### Methods

To account for differences in AE reporting between trials, all grade ≥3 AEs reported in the AMPLIFY trial (A+V) and those identified as being of clinical interest in AMPLIFY and the GLOW (I+V) and CLL14 (V+O) trials were analysed for all three regimens. Costs were derived from available Brazilian healthcare data using a micro-costing approach, or taken from published sources. Conversion to Euros was based on the average exchange rate during 2025 (R$1.00: €0.1584). Due to the limitations of cross-trial comparison, an exploratory Bayesian random effects network meta-analysis (NMA) was conducted in an attempt to improve the accuracy of the AE rate estimates for I+V and V+O, using the rates published in the AMPLIFY study (A+V) as the reference base. This approach inherently treats confounding factors, including heterogeneity, as random variables to produce a weighted probabilistic output for AEs associated with the I+V and V+O regimens.

**Copyright Information:** (Online) ISSN: 2572-9241
©2026 The Author(s). HemaSphere is published by John Wiley & Sons Ltd on behalf of European Hematology Association. This is an open access Abstract Book distributed under the Attribution-NonCommercial-NoDerivs (CC BY-NC-ND), which allows third parties to download the articles and share them with others as long as they credit the author and the Abstract Book, but they cannot change the content in any way or use them commercially.
**Abstract Book Citations:** Authors, Title, HemaSphere, 2026;10:(S1):pages. The abstract book DOIs can be found on the "Author Information" page.
**Disclaimer:** Articles published in the journal HemaSphere exclusively reflect the opinions of the authors. The authors are responsible for all content in their abstracts including accuracy of the facts, statements, citing resources, etc.

### Results

Assuming a cohort of 100 patients, the base case analysis showed that the cost of managing AEs was R$162,015 (€25,633) with A+V versus R$336,688 (€53,331) with I+V and R$373,293 (€59,130) with V+O, corresponding to comparative savings of R$174,673 (€27,668; 51.9%) and R$211,277 (€33,466; 56.6%) in favour of A+V. A saving of R$36,605 (€5,798; 9.8%) was apparent for I+V over V+O. Compared to I+V, the savings for A+V were primarily driven by the costs of managing cardiac events, thrombocytopaenia, infection, diarrhoea, and hypertension; *versus* V+O, savings related to thrombocytopaenia, febrile neutropenia, infection, and cardiac events. The exploratory Bayesian random effects NMA produced similar cost savings for A+V over the other regimens as compared to the main analysis (Table).

| Regimen | Main Analysis | | NMA | |
|---|---|---|---|---|
| | Cost with indicated treatment (R$) | Saving with AV (R$) | Cost with indicated treatment (R$) | Saving with AV (R$) |
| A+V | 162,015 | - | 162,015 | - |
| I+V | 336,688 | 174,673 | 326,694 | 164,679 |
| V+O | 373,293 | 211,277 | 380,176 | 218,161 |

### Summary/Conclusion

In this analysis, estimated AE management costs were lower with A+V compared to I+V and V+O. Results should be considered in light of the recognised limitations of cross-trial comparison including but not limited to heterogeneity in patient population, AE definitions/reporting and the absence of robust uncertainty analysis. Nevertheless, the exploratory Bayesian NMA, which took appropriately weighted source data into account, found similar cost savings to the main analysis. These results support the inclusion of AE costs as part of a holistic decision-making process for reimbursement/funding of first-line CLL treatments.

**Copyright Information:** (Online) ISSN: 2572-9241
©2026 The Author(s). HemaSphere is published by John Wiley & Sons Ltd on behalf of European Hematology Association. This is an open access Abstract Book distributed under the Attribution-NonCommercial-NoDerivs (CC BY-NC-ND), which allows third parties to download the articles and share them with others as long as they credit the author and the Abstract Book, but they cannot change the content in any way or use them commercially.
**Abstract Book Citations:** Authors, Title, HemaSphere, 2026;10:(S1):pages. The abstract book DOIs can be found on the "Author Information" page.
**Disclaimer:** Articles published in the journal HemaSphere exclusively reflect the opinions of the authors. The authors are responsible for all content in their abstracts including accuracy of the facts, statements, citing resources, etc.

### (PS2518) INCIDENCE AND SURVIVAL OF HEMATOLOGICAL NEOPLASIAS IN THE COMMUNITY OF MADRID (SPAIN) FROM 2014 TO 2024: BURDEN, TEMPORAL IMPROVEMENT, AND CARE HOMOGENEITY

**Topic:** 37. Health economics

José-Angel Hernández-Rivas*[1], Adrian Alegre Amor[2], Federico Longo Muñoz[3], Rebeca Bailen[4], Mariana Bastos-Oreiro[4], Maria Angeles Foncillas[1], Pilar Martinez-Barranco[5], Marta Morado[6], Laura Pardo Gambarte[7], Cristina Seri[8], Laura Solán[7], Imma Barber Turro[9], Joan Gibert Vives[9], Ángel Cedillo Cedillo[10], Laurentino Benito Parra[11], Víctor Jiménez-Yuste[6]

*[1]Hosp. Univ. Infanta Leonor, Madrid, Spain; [2]Hosp. Univ. de La Princesa, Madrid, Spain; [3]Registro de Tumores de Madrid (RTMAD, SERMAS), Madrid, Spain; [4]Hosp. Gral. Univ. Gregorio Marañón, Madrid, Spain; [5]Hosp. Univ. Fundación Alcorcón, Madrid, Spain; [6]Hosp. Univ. La Paz, Madrid, Spain; [7]Hosp. Univ. Fundación Jiménez Díaz, Madrid, Spain; [8]Hosp. Central de la Defensa Gómez Ulla, Madrid, Spain; [9]LetsFocusNow, Barcelona, Spain; [10]Madrid Association of Hematology and Hemotherapy (AMHH), Madrid, Spain; [11]Hosp. Univ. de Getafe, Madrid, Spain;*

### Background

As part of the strategic initiatives of the Madrid Hematology and Hemotherapy Association (AMHH), a population-based analysis was conducted using official data from the Madrid Tumour Registry (RTMAD, SERMAS) on hematological neoplasias in the Community of Madrid (CM). These indicators allow assessment of incidence and survival trends and guide healthcare planning in Hematology. Between 2022 and 2024, total neoplasms slightly increased, while hematological malignancies maintained their relative burden, likely underestimated in non-histologically diagnosed subtypes.

### Aims

To evaluate incidence trends of hematological neoplasias in the CM (2022-2024), describe subtype distribution and contribution to disease burden, analyse overall and subtype-specific survival (2014-2024) including temporal and hospital-type differences, and propose implications for registry improvement and hematological resource allocation.

### Methods

Retrospective analysis of RTMAD (SERMAS) data on haematological neoplasias diagnosed in the CM public hospitals. Absolute and relative incidence trends (2022-24), main subtypes, and age-standardized net survival at 1, 5, and 10 years (2014-24; n=32,622) were examined, stratified by time-period (2014-19 vs 2019-24) and hospital complexity (low, medium, high, pediatric).

**Copyright Information:** (Online) ISSN: 2572-9241
©2026 The Author(s). HemaSphere is published by John Wiley & Sons Ltd on behalf of European Hematology Association. This is an open access Abstract Book distributed under the Attribution-NonCommercial-NoDerivs (CC BY-NC-ND), which allows third parties to download the articles and share them with others as long as they credit the author and the Abstract Book, but they cannot change the content in any way or use them commercially.
**Abstract Book Citations:** Authors, Title, HemaSphere, 2026;10:(S1):pages. The abstract book DOIs can be found on the "Author Information" page.
**Disclaimer:** Articles published in the journal HemaSphere exclusively reflect the opinions of the authors. The authors are responsible for all content in their abstracts including accuracy of the facts, statements, citing resources, etc.

**Results**

Total number of neoplasias increased from 36,308 (2022) to 37,099 (2024), with haematological cases rising from 3,247 to 3,272 (8.94%–8.82%), below the 10–12% reported elsewhere, probably due to under-reporting of chronic leukemias diagnosed via peripheral blood. Most frequent subtypes were B-cell non-Hodgkin lymphomas low/high grade (32%), MM (13%), MDS (10%) and MPN (8%) accounting for >50% of cases, while rare entities such as atypical CML (0.15%), mastocytosis (0.30%), and histiocytosis (0.44%) showed statistical variability. From 2014 to 2024, hematological neoplasias represented 9.15% of total cancers, with overall survival (OS) of 85.6%, 65.9%, and 53.5% at 1, 5, and 10 years, respectively. Best outcomes (10-year OS 60–80%) were observed in Hodgkin lymphoma, non-Hodgkin B low-grade lymphoma [OS: 94.3%, 86.6%, 80.7% and 93.9%, 80.3%, 68.4% at 1, 5, and 10 years], CML and histiocytosis/mastocytosis. Worst outcomes (10-year OS 20–40%) were observed in MDS, AML, and MM [1-year OS: 79.8%, 60.3%, 86.0%; 5-year: 40.0%, 38.7%, 59.8%; 10-year: 22.1%, 35.2%, 40.5%]. In 2019-2024, 53.1% of cases were recorded, with improved survival up to 60 months (>50% at 5 years) despite early COVID-19 impact. Pediatric centres achieved ~90% 5-year survival, while adult hospitals showed homogeneous results across complexity levels, reflecting shared protocols and referral pathways (Figure 1)

**Summary/Conclusion**

The incidence of hematological neoplasias increased during the study period, with survival gains likely linked to innovative therapies. Recommendations include: enhancing (RTMAD, SERMAS) completeness, particularly for non-histologically diagnosed neoplasias, to enable realistic haematological resource planning; focusing interventions in high-burden entities (B-cell lymphomas, MM, MDS, AML); promoting interhospital collaboration to ensure equitable access to advanced treatments; and establishing periodic outcome monitoring to support continuous improvement and evidence-based health policy.

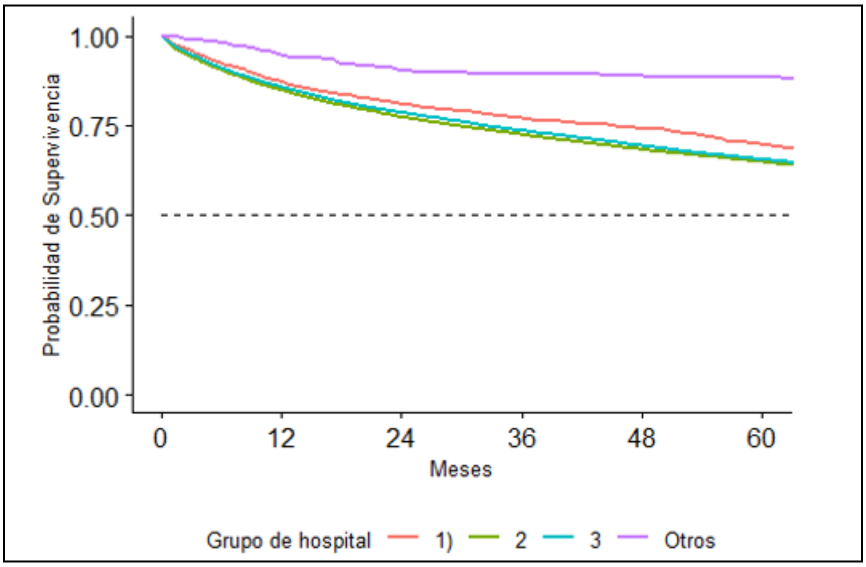

Figure 1.- Survival by hospital complexity: low (1), medium (2), high (3), pediatric (other group) (RTMAD data, SERMAS).

**Copyright Information:** (Online) ISSN: 2572-9241
©2026 The Author(s). HemaSphere is published by John Wiley & Sons Ltd on behalf of European Hematology Association. This is an open access Abstract Book distributed under the Attribution-NonCommercial-NoDerivs (CC BY-NC-ND), which allows third parties to download the articles and share them with others as long as they credit the author and the Abstract Book, but they cannot change the content in any way or use them commercially.
**Abstract Book Citations:** Authors, Title, HemaSphere, 2026;10:(S1):pages. The abstract book DOIs can be found on the "Author Information" page.
**Disclaimer:** Articles published in the journal HemaSphere exclusively reflect the opinions of the authors. The authors are responsible for all content in their abstracts including accuracy of the facts, statements, citing resources, etc.

### (PS2519) NANOPORE SEQUENCING AS A SINGLE-TEST DIAGNOSTIC PLATFORM FOR PEDIATRIC HEMATOLOGICAL MALIGNANCIES

**Topic:** 38. Novel technologies, techniques and digital analytical tools in hematology

Tristan Achterberg[1], Karlijn Cammel[1], Marc van Tuil[1], Marco Koudijs[1], Jayne Hehir-Kwa[1], Carlo Vermeulen[1], Thomas Alexander[2], Nickhill Bhakta[3], Jeremy Wang[2], Bastiaan Tops[1], Jeroen de Ridder[1], Lennart A. Kester*[1]

*[1]Princess Maxima Center for Pediatric Oncology, Utrecht, The Netherlands;[2]University of North Carolina at Chapel Hill, Chapell Hill, United States of America; [3]St. Jude Children's Research Hospital, Memphis, United States of America;*

### Background

Pediatric hematological malignancies comprise a biologically diverse group of diseases that require rapid and comprehensive molecular characterization to guide diagnosis, risk stratification, and treatment. Current diagnostic workflows rely on multiple orthogonal assays, including cytomorphology, flow cytometry, karyotyping, FISH, and RNA sequencing. While RNA sequencing provides rich molecular information, it is limited by turnaround time, batching requirements, and analytical complexity, which restricts its utility in acute clinical settings. There is a growing need for a unified, rapid diagnostic assay that delivers both classification and genomic characterization from a single test.

### Aims

This study aims to evaluate whether a single, integrated nanopore DNA sequencing assay can replace conventional multi-assay workflows for pediatric hematological malignancies by providing rapid, accurate molecular classification and comprehensive genomic characterization within a clinically actionable timeframe. Specifically, we sought to (i) validate real-time methylation-based classification across diverse leukemia subtypes, (ii) assess the performance of adaptive sampling for simultaneous detection of structural variants, gene fusions, copy number alterations, and clinically relevant mutations.

### Methods

We developed an integrated DNA-based workflow using Oxford Nanopore Technologies (ONT) sequencing that combines genome-wide DNA methylation profiling with targeted genomic characterization in a single assay. Nanopore-derived methylation profiles were used for real-time tumor classification using *Lamprey*, a classifier trained on >5,400 Illumina methylation arrays covering 38 molecular subtypes of pediatric leukemias and related myeloid and lymphoid disorders. In parallel, adaptive sampling was applied to enrich for leukemia-relevant genes, enabling detection of structural variants, gene fusions, copy number alterations, internal tandem duplications, and clinically relevant single nucleotide variants.

### Results

Nanopore-based methylation profiling enabled rapid and accurate classification across a broad spectrum of pediatric hematological malignancies, including AML, B-ALL, JMML, CMML, and rare entities such as BPDCN. In a validation cohort of 120 retrospective cases, *Lamprey* achieved a micro F1 score of 0.96, with high-confidence predictions for the majority of samples. Long-read sequencing combined with adaptive sampling reliably detected recurrent gene fusions (e.g. *BCR::ABL1*, *KMT2A* rearrangements), chromosomal abnormalities concordant with karyotyping, and pathogenic ITDs in *FLT3* and *UBTF*. Genome-wide copy number profiles could be reconstructed directly from sequencing data, enabling inference of aneuploidy patterns such as hyperdiploid ALL. Importantly, all results were generated from a single DNA assay without the need for parallel RNA sequencing or multiple targeted tests.

**Copyright Information:** (Online) ISSN: 2572-9241
©2026 The Author(s). HemaSphere is published by John Wiley & Sons Ltd on behalf of European Hematology Association. This is an open access Abstract Book distributed under the Attribution-NonCommercial-NoDerivs (CC BY-NC-ND), which allows third parties to download the articles and share them with others as long as they credit the author and the Abstract Book, but they cannot change the content in any way or use them commercially.
**Abstract Book Citations:** Authors, Title, HemaSphere, 2026;10:(S1):pages. The abstract book DOIs can be found on the “Author Information” page.
**Disclaimer:** Articles published in the journal HemaSphere exclusively reflect the opinions of the authors. The authors are responsible for all content in their abstracts including accuracy of the facts, statements, citing resources, etc.

**Summary/Conclusion**

This integrated nanopore sequencing approach functions as a **single, comprehensive diagnostic test** for pediatric hematological malignancies, delivering both molecular classification and genomic characterization within 24 hours. By unifying methylation-based classification, fusion detection, structural variant analysis, and copy number profiling in one assay, this workflow simplifies diagnostic pathways and significantly reduces turnaround time. Nanopore sequencing therefore represents a practical and scalable foundation for next-generation, one-test-fits-all diagnostics in pediatric hematology.

**Copyright Information:** (Online) ISSN: 2572-9241
©2026 The Author(s). HemaSphere is published by John Wiley & Sons Ltd on behalf of European Hematology Association. This is an open access Abstract Book distributed under the Attribution-NonCommercial-NoDerivs (CC BY-NC-ND), which allows third parties to download the articles and share them with others as long as they credit the author and the Abstract Book, but they cannot change the content in any way or use them commercially.
**Abstract Book Citations:** Authors, Title, HemaSphere, 2026;10:(S1):pages. The abstract book DOIs can be found on the "Author Information" page.
**Disclaimer:** Articles published in the journal HemaSphere exclusively reflect the opinions of the authors. The authors are responsible for all content in their abstracts including accuracy of the facts, statements, citing resources, etc.

### (PS2520) PREDICTING RESPONSE TO INITIAL ANTHRACYCLINE-BASED CHEMOTHERAPY AND PROGNOSIS IN NEWLY DIAGNOSED ACUTE MYELOID LEUKEMIA USING A CLINICAL-MOLECULAR INTEGRATED LARGE REASONING MODEL

**Topic:** 38. Novel technologies, techniques and digital analytical tools in hematology

Sai Huang*[1], Cong Feng[1]

[1]*Chinese PLA General Hospital, Beijing, China;*

### Background

Precision prediction of initial anthracycline response and long-term prognosis in acute myeloid leukemia (AML) patients is critical for personalized therapy; however, conventional risk stratification such as ELN guidelines inadequately predicts individualized treatment outcomes, particularly primary resistance, and fails to integrate routine clinical data like bone marrow morphology.

### Aims

This study aims to predict response to initial anthracycline-based chemotherapy and prognosis in newly diagnosed AML using a clinical-molecular integrated large reasoning model.

### Methods

AML-ResistNet, a clinical-molecular integrated large reasoning model, was developed using a multicenter cohort harmonizing general clinical parameters, bone marrow morphology, and risk-stratified sequencing data.

### Results

Built on distilled DeepSeek-R1 and fine-tuned via chain-of-thought prompting, the model demonstrated superior performance with 37% higher average F1-score than GPT-4o in external tests, achieving F1 of 0.89 for measurable residual disease, 0.82 for 5-year survival, and 0.93 for primary resistance identification. Critically, integrating bone marrow morphology with molecular features enhanced relapse prediction by revealing morphology-genetic interactions.

### Summary/Conclusion

AML-ResistNet generates interpretable reasoning pathways to enable transparent clinical decisions, establishing an accurate framework for therapy optimization and proposing a task-aware multimodal fusion paradigm for precision oncology.

**Copyright Information:** (Online) ISSN: 2572-9241
©2026 The Author(s). HemaSphere is published by John Wiley & Sons Ltd on behalf of European Hematology Association. This is an open access Abstract Book distributed under the Attribution-NonCommercial-NoDerivs (CC BY-NC-ND), which allows third parties to download the articles and share them with others as long as they credit the author and the Abstract Book, but they cannot change the content in any way or use them commercially.
**Abstract Book Citations:** Authors, Title, HemaSphere, 2026;10:(S1):pages. The abstract book DOIs can be found on the "Author Information" page.
**Disclaimer:** Articles published in the journal HemaSphere exclusively reflect the opinions of the authors. The authors are responsible for all content in their abstracts including accuracy of the facts, statements, citing resources, etc.

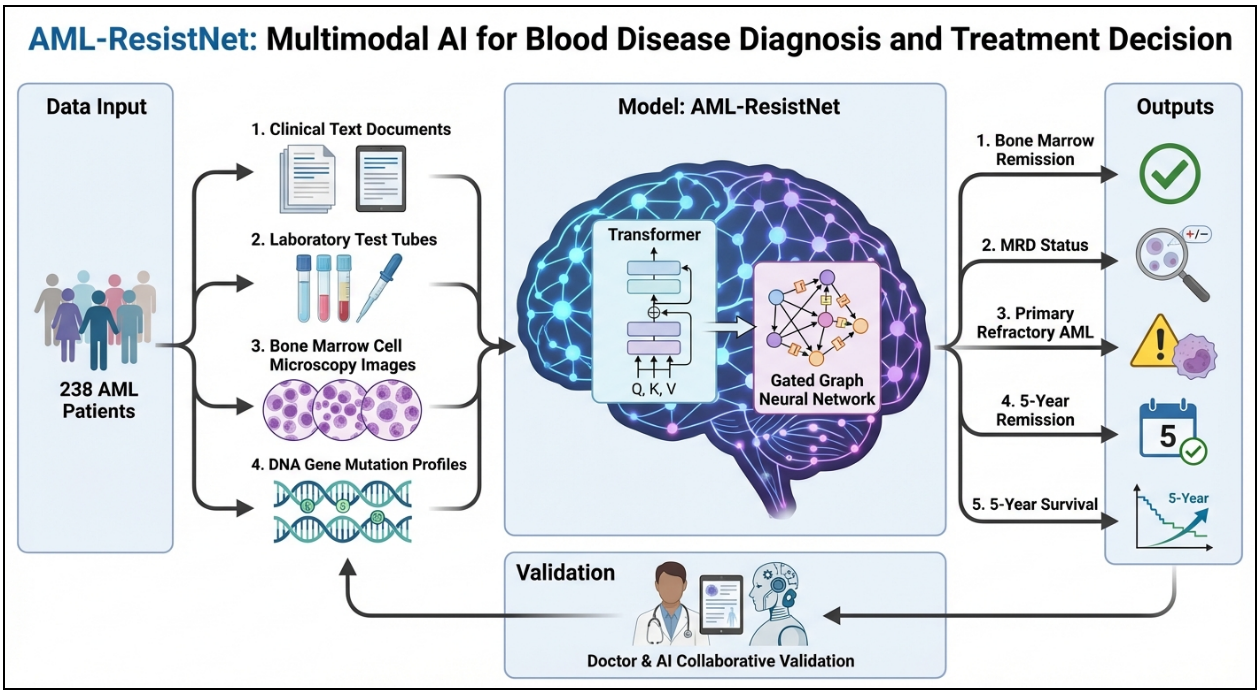

**Copyright Information:** (Online) ISSN: 2572-9241
©2026 The Author(s). HemaSphere is published by John Wiley & Sons Ltd on behalf of European Hematology Association. This is an open access Abstract Book distributed under the Attribution-NonCommercial-NoDerivs (CC BY-NC-ND), which allows third parties to download the articles and share them with others as long as they credit the author and the Abstract Book, but they cannot change the content in any way or use them commercially.
**Abstract Book Citations:** Authors, Title, HemaSphere, 2026;10:(S1):pages. The abstract book DOIs can be found on the "Author Information" page.
**Disclaimer:** Articles published in the journal HemaSphere exclusively reflect the opinions of the authors. The authors are responsible for all content in their abstracts including accuracy of the facts, statements, citing resources, etc.

### (PS2521) DEVELOPMENT AND VALIDATION OF A MACHINE LEARNING-BASED DIAGNOSTIC MODEL FOR BONE MARROW CELL MORPHOLOGY IN MALIGNANT HEMATOLOGICAL DISEASES: A MULTICENTER RETROSPECTIVE STUDY

**Topic:** 38. Novel technologies, techniques and digital analytical tools in hematology

Jingya Liu[1], Tinglu Tao*[1], Xi Zhang[1], Xiangui Peng[1], Xingqin Huang[2]

*[1]Hematology Center, the Second Affiliated Hospital of Army Medical University, chognqing, China;[2]The First Affiliated Hospital of Army Medical University, chognqing, China;*

### Background

Bone marrow cell morphology is a core diagnostic approach for malignant hematological diseases such as acute leukemia and multiple myeloma. However, its interpretation relies on physicians' experience, leading to significant inter-observer variability, prolonged turnaround times, and limited diagnostic accuracy in primary healthcare settings. With the increasing incidence of malignant hematological diseases, there is an urgent need for standardized and efficient auxiliary diagnostic tools.

### Aims

This study aims to develop and validate a machine-learning-based diagnostic model using routine bone marrow morphology data, enabling rapid and consistent diagnosis of malignant hematological diseases and providing a practical artificial-intelligence-assisted tool for clinical use.

### Methods

We retrospectively collected bone marrow morphology report data from 16,344 patients across two tertiary hospitals between 2014 and 2023, including 8,781 cases of various types of acute leukemia (AL), 3,242 cases of multiple myeloma, and 4,321 cases of normal bone marrow findings. A total of 15 diagnostic categories were defined in this study, including 13 morphological subtypes of acute leukemia. The data were divided into a training set and an internal test set at a 7:3 ratio. An external validation cohort included 6,637 patients from an additional tertiary hospital. Fifty-eight bone marrow cell morphology parameters were extracted from the reports, including quantitative cell differential counts and qualitative cell morphological descriptions. Missing values were imputed using K-Nearest Neighbors, and features selection were preformed via Recursive Feature Elimination. Five models, such as Random Forest (RF) and Extreme Gradient Boosting（XGBoost）, were optimized. Model performance was evaluated using metrics like AUC and accuracy. Feature contributions were analyzed through SHAP values, and clinical utility was evaluated using Decision Curve Analysis.

### Results

In the internal cohort, 73.56% of patients had malignant hematological diseases, and in the external cohort, the proportion was 83.24%. Twenty-five parameters, including blast percentage , the periodic acid-Schiff positivity, proportions of blast and immature lymphocytes, promyelocytes, and the myeloid-to-erythroid ratio, were identified as core predictors. The RF model demonstrated the most robust performance, achieving an internal AUC of 0.97 (95% CI: 0.95 - 0.99) and an accuracy of 0.95 (95% CI: 0.94 - 0.96). In the external validation set, the AUC was 0.92 (95% CI: 0.88 - 0.94), and the accuracy was 0.89 (95% CI: 0.87 - 0.90). SHAP analysis provided intuitive case-level interpretability and clarified contributions of features such as blast percentage, myeloperoxidase positivity, and Auer rods increased the diagnostic probability of AML, whereas primitive immature lymphocytes decreased it.

**Copyright Information:** (Online) ISSN: 2572-9241
©2026 The Author(s). HemaSphere is published by John Wiley & Sons Ltd on behalf of European Hematology Association. This is an open access Abstract Book distributed under the Attribution-NonCommercial-NoDerivs (CC BY-NC-ND), which allows third parties to download the articles and share them with others as long as they credit the author and the Abstract Book, but they cannot change the content in any way or use them commercially.
**Abstract Book Citations:** Authors, Title, HemaSphere, 2026;10:(S1):pages. The abstract book DOIs can be found on the “Author Information” page.
**Disclaimer:** Articles published in the journal HemaSphere exclusively reflect the opinions of the authors. The authors are responsible for all content in their abstracts including accuracy of the facts, statements, citing resources, etc.

**Summary/Conclusion**

The RF-based model enables effective diagnosis of malignant hematological diseases, demonstrating stable performance across internal and external validation. The model can shorten the reporting time, reduce observer differences, and improve the consistency of diagnostic levels across different healthcare fields by offering interpretability and standardized analytical capability, serving as an efficient individualized auxiliary diagnostic tool for clinical practice.

**Copyright Information:** (Online) ISSN: 2572-9241
©2026 The Author(s). HemaSphere is published by John Wiley & Sons Ltd on behalf of European Hematology Association. This is an open access Abstract Book distributed under the Attribution-NonCommercial-NoDerivs (CC BY-NC-ND), which allows third parties to download the articles and share them with others as long as they credit the author and the Abstract Book, but they cannot change the content in any way or use them commercially.
**Abstract Book Citations:** Authors, Title, HemaSphere, 2026;10:(S1):pages. The abstract book DOIs can be found on the "Author Information" page.
**Disclaimer:** Articles published in the journal HemaSphere exclusively reflect the opinions of the authors. The authors are responsible for all content in their abstracts including accuracy of the facts, statements, citing resources, etc.

### (PS2522) MACHINE LEARNING AND MECHANISTIC MODELLING-BASED RECONSTRUCTION OF HEMATOPOIETIC TRAJECTORIES BY CSTAR REVEALS NOVEL LEUKEMIA DIFFERENTIATION STRATEGIES

**Topic:** 38. Novel technologies, techniques and digital analytical tools in hematology

Ciardha Carmody*[1, 2, 3], Luke Jones[1, 2], Boris Kholodenko[1, 2, 4, 5], Walter Kolch[1, 2, 4], Oleksii Rukhlenko[1, 2], Jonathan Bond[1, 2, 6]

*[1]Systems Biology Ireland, University College Dublin, Dublin, Ireland; [2]School of Medicine, University College Dublin, Dublin, Ireland; [3]The SFI Centre for Research Training in Genomics Data Science, University of Galway, Galway, Ireland;[4]Conway Institute of Biomolecular and Biomedical Research, University College Dublin, Dublin, Ireland; [5]Department of Pharmacology, Yale University School of Medicine, New Haven, CT, United States of America; [6]Children's Health Ireland at Crumlin, Dublin, Ireland;*

### Background

Outcomes for acute myeloid leukemia (AML) continue to improve, but treatment often requires non-specific toxic chemotherapy. Differentiation therapy is a compelling alternative, as seen in acute promyelocytic leukemia, where all-trans retinoic acid (ATRA)-based strategies induce AML maturation. Extending this paradigm to other AML subtypes is more challenging, requiring a better mechanistic understanding of regulatory networks that govern cell fate decisions. To tackle this, we applied our cSTAR method (Cell State Transition Assessment and Regulation), which integrates machine learning and mechanistic modelling to identify potential new differentiation approaches.

### Aims

To reconstruct the human hematopoietic landscape to define differentiation signaling networks, and to use this information to identify drug strategies to overcome AML differentiation block.

### Methods

Six single-cell RNA-seq datasets from human bone marrow and peripheral blood mononuclear cells (>120,000 cells) were integrated using the Bone Marrow Map tool. Machine learning-based classification was used to define discrete cell states spanning hematopoietic stem cells (HSCs) to mature myeloid populations. Using cSTAR, we constructed multiple State Transition Vectors (STVs) describing ordered trajectories from HSCs to mature states. STVs were used to calculate Dynamic Phenotypic Descriptor (DPDs) for each state and intermediate population along defined differentiation paths, providing a quantitative measure of developmental progression. To infer causal signaling interactions, we used perturbational transcriptomic data from the LINCS database and applied Bayesian Modular Response Analysis (BMRA) to reconstruct network topology. Potential drug strategies were then tested in an AML cell line differentiation model.

**Copyright Information:** (Online) ISSN: 2572-9241
©2026 The Author(s). HemaSphere is published by John Wiley & Sons Ltd on behalf of European Hematology Association. This is an open access Abstract Book distributed under the Attribution-NonCommercial-NoDerivs (CC BY-NC-ND), which allows third parties to download the articles and share them with others as long as they credit the author and the Abstract Book, but they cannot change the content in any way or use them commercially.
**Abstract Book Citations:** Authors, Title, HemaSphere, 2026;10:(S1):pages. The abstract book DOIs can be found on the "Author Information" page.
**Disclaimer:** Articles published in the journal HemaSphere exclusively reflect the opinions of the authors. The authors are responsible for all content in their abstracts including accuracy of the facts, statements, citing resources, etc.

**Results**

Integration of single-cell datasets yielded a high-resolution ordered map of human hematopoiesis. cSTAR-derived STVs accurately reconstructed trajectories from HSCs to terminal granulocytic and monocytic states and enabled quantitative positioning of intermediate progenitors along these paths via DPD scoring. Validation of our classifier on independent datasets including normal human and mouse hematopoietic cells and AML cell lines further confirmed robust cell state assignment and trajectory reconstruction.
To infer causal signaling networks governing AML cell state transitions, we used perturbational transcriptomic data from THP-1 cells (LINCS database), applying BMRA for network reconstruction, so allowing us to identify regulators promoting differentiation. This causal network highlighted key signaling nodes predicted to drive transition to a mature myeloid phenotype, specifically, All-trans retinoic acid (ATRA), mocetinostat (class I HDAC inhibitor), and Ro-3306 (CDK2 inhibitor).
To validate these predictions, we treated THP-1 cells with these agents as monotherapies and combinations in an*in vitro* differentiation system. Flow cytometry assessment showed that expression of myeloid differentiation markers was most pronounced with combination treatments, with the triple combination being most potent. We are currently analyzing RNA-seq data to determine drug combination effects on cSTAR-predicted differentiation networks.

**Summary/Conclusion**

We provide a quantitative and mechanistic reconstruction of human hematopoietic differentiation and show that cSTAR can predict novel actionable strategies to potentially overcome AML differentiation arrest.

**Copyright Information:** (Online) ISSN: 2572-9241
©2026 The Author(s). HemaSphere is published by John Wiley & Sons Ltd on behalf of European Hematology Association. This is an open access Abstract Book distributed under the Attribution-NonCommercial-NoDerivs (CC BY-NC-ND), which allows third parties to download the articles and share them with others as long as they credit the author and the Abstract Book, but they cannot change the content in any way or use them commercially.
**Abstract Book Citations:** Authors, Title, HemaSphere, 2026;10:(S1):pages. The abstract book DOIs can be found on the “Author Information” page.
**Disclaimer:** Articles published in the journal HemaSphere exclusively reflect the opinions of the authors. The authors are responsible for all content in their abstracts including accuracy of the facts, statements, citing resources, etc.

### (PS2523) SELF-SUPERVISED VISION TRANSFORMER WITH UNCERTAINTY-AWARE CALIBRATION FOR AUTOMATED ACUTE LYMPHOBLASTIC LEUKEMIA SCREENING IN PERIPHERAL BLOOD SMEARS

**Topic:** 38. Novel technologies, techniques and digital analytical tools in hematology

Fatemeh Mohammadsadeghi*[1]

[1]*Shahid Beheshti University, Faculty of Medical Science, Tehran, Iran;*

### Background

Morphological assessment of peripheral blood smears remains central to the initial detection of acute lymphoblastic leukemia (ALL), yet it is time-consuming and subject to inter-observer variability, especially in settings with limited hematopathology capacity. Robust AI screening could accelerate triage while preserving safety through calibrated confidence and interpretable outputs. Many image-based classifiers report high accuracy on internal splits but degrade under staining/illumination variability and do not provide clinically meaningful uncertainty, limiting adoption in real-world workflows.

### Aims

This study aims to develop and validate a self-supervised, calibrated vision transformer to automatically distinguish ALL lymphoblasts from normal cells in peripheral blood smear images under real-world staining and quality variability. A second aim is to provide clinically usable outputs by adding uncertainty-aware triage and explainable visual evidence, so low-confidence cases are safely flagged for expert review.

### Methods

We developed and evaluated our pipeline on a public pediatric blood-smear cell image dataset from an ALL screening challenge (≈12k single-cell RGB images labeled as ALL lymphoblast vs normal). The dataset exhibits substantial heterogeneity in color/stain, focus, and background artifacts—conditions representative of routine microscopy. Images underwent quality control (blur/artefact filtering), stain/illumination standardization (robust color normalization), and augmentation (color jitter, rotation, mild blur). We pretrained a vision transformer (ViT) using self-supervised contrastive learning to learn stain-robust representations, then fine-tuned it for binary ALL detection with class-balanced sampling and focal loss. Comparators included (i) a supervised CNN baseline (ResNet/EfficientNet family) and (ii) classical ML (SVM/XGBoost) trained on handcrafted morphology–texture descriptors (e.g., LBP/Haralick + geometric features). Performance was assessed with leakage-aware splitting (subject/slide-level when identifiers were available) and cross-validation. We reported AUROC, AUPRC, sensitivity/specificity at clinically relevant thresholds, and calibration (ECE/Brier) with temperature scaling. Explainability used attention rollout/Grad-CAM; uncertainty used Monte-Carlo dropout to flag low-confidence cases for manual review.

### Results

The self-supervised ViT achieved AUROC [0.99], AUPRC [0.99], and F1 [0.96], outperforming the supervised CNN (AUROC [0.98], F1 [0.94]) and classical ML (AUROC [0.93], F1 [0.88]). At specificity = [0.95], ViT sensitivity was [0.97]. Calibration improved after temperature scaling (ECE [0.04 → 0.01]). Uncertainty triage flagged [6–10%] of cases for review and reduced false positives by [~15–25%] at fixed sensitivity. Explanations consistently highlighted nucleus/cytoplasm regions aligned with blast morphology; most residual errors coincided with poor focus or severe artefacts.

**Copyright Information:** (Online) ISSN: 2572-9241
©2026 The Author(s). HemaSphere is published by John Wiley & Sons Ltd on behalf of European Hematology Association. This is an open access Abstract Book distributed under the Attribution-NonCommercial-NoDerivs (CC BY-NC-ND), which allows third parties to download the articles and share them with others as long as they credit the author and the Abstract Book, but they cannot change the content in any way or use them commercially.
**Abstract Book Citations:** Authors, Title, HemaSphere, 2026;10:(S1):pages. The abstract book DOIs can be found on the "Author Information" page.
**Disclaimer:** Articles published in the journal HemaSphere exclusively reflect the opinions of the authors. The authors are responsible for all content in their abstracts including accuracy of the facts, statements, citing resources, etc.

**Summary/Conclusion**

A self-supervised, calibrated ViT with uncertainty-aware triage provides accurate and interpretable ALL screening from smear images, enabling safer deployment as a decision-support tool to prioritise expert review and accelerate diagnostic pathways.

**Copyright Information:** (Online) ISSN: 2572-9241
©2026 The Author(s). HemaSphere is published by John Wiley & Sons Ltd on behalf of European Hematology Association. This is an open access Abstract Book distributed under the Attribution-NonCommercial-NoDerivs (CC BY-NC-ND), which allows third parties to download the articles and share them with others as long as they credit the author and the Abstract Book, but they cannot change the content in any way or use them commercially.
**Abstract Book Citations:** Authors, Title, HemaSphere, 2026;10:(S1):pages. The abstract book DOIs can be found on the "Author Information" page.
**Disclaimer:** Articles published in the journal HemaSphere exclusively reflect the opinions of the authors. The authors are responsible for all content in their abstracts including accuracy of the facts, statements, citing resources, etc.

### (PS2524) FHIR-FORMER: MATCHING LSTM PERFORMANCE IN PLATELET TRANSFUSION PREDICTION USING A LANGUAGE MODEL WITH ONE-TENTH THE TRAINING DATA

**Topic:** 38. Novel technologies, techniques and digital analytical tools in hematology

Osman Alperen Cinar Korasc[1], Jens Klessiek[1, 2, 3], Amin Turki[1, 4], Merlin Engelke*[1]

*[1]University Hospital Essen, Institute for Artificial Intelligence in Medicine, Essen, Germany;[2]TU Dortmund, Department of Physics, Dortmund, Germany; [3]University of Duisburg-Essen, Faculty of Computer Science and Medical Faculty, Essen, Germany; [4]University Hospital Marienhospital, Department of Hematology and Oncology, Bochum, Germany;*

### Background

Optimizing platelet transfusion is essential in hematology to prevent shortages and improve patient outcomes. Prior models, such as long short-term memory (LSTM) networks, have demonstrated strong performance in predicting 24-hour platelet transfusion risk using large electronic health record (EHR) datasets (approximately 440,000 samples). However, these models require extensive data and manual feature selection. FHIR-Former is an end-to-end pipeline integrating structured and unstructured Fast Healthcare Interoperability Resources (FHIR) with encoder-based language models, offering a scalable alternative by eliminating manual feature engineering.

### Aims

To evaluate the feasibility and performance of FHIR-Former, a large language model (LLM)-based pipeline, for predicting 24-hour platelet transfusion needs on a limited dataset, and to compare results with published LSTM benchmarks.

### Methods

We conducted a pilot study using de-identified EHR data from January 2018, comprising 41,310 patient samples (about one-tenth the size of prior LSTM datasets). The data includes structured and unstructured clinically relevant FHIR resources: patient demographics, encounters (e.g., stay duration, location, type, and reason), conditions (e.g., diagnoses), procedures (e.g., surgical or therapeutic interventions), imaging studies, diagnostic reports (e.g., pathology or lab summaries), biologically derived products (platelet consumption), clinical observations (e.g., vital signs and laboratory results), episodes of care, medications (e.g., administered drugs), and service requests (e.g., orders for tests, procedures, or consultations). Training data were sampled using the FHIR-Former pipeline, yielding ~2,000 records for fine-tuning the Qwen 3 0.6B Base model (2 epochs, context length 8192 tokens). Performance was evaluated on a held-out test set of ~10,000 samples using loss, accuracy, precision, recall, F1-score, and ROC-AUC, and compared with published LSTM results across hemato-oncology, cardiothoracic, and multispecialty cohorts.

### Results

The model achieved strong performance on the test set: Loss: 0.2489, Accuracy: 0.9765, Precision: 0.7842, Recall: 0.8204, F1-score: 0.8011, and ROC-AUC: 0.9109. These results were comparable or superior to LSTM benchmarks, which reported F1-scores of 0.74 (hemato-oncology), 0.43 (cardiothoracic), and 0.65 (multispecialty), with corresponding ROC-AUC values of 0.98, 0.98, and 0.95. Notably, our approach matched or exceeded LSTM F1 and sensitivity metrics despite using far fewer samples, highlighting efficiency in data-scarce scenarios.

**Copyright Information:** (Online) ISSN: 2572-9241
©2026 The Author(s). HemaSphere is published by John Wiley & Sons Ltd on behalf of European Hematology Association. This is an open access Abstract Book distributed under the Attribution-NonCommercial-NoDerivs (CC BY-NC-ND), which allows third parties to download the articles and share them with others as long as they credit the author and the Abstract Book, but they cannot change the content in any way or use them commercially.
**Abstract Book Citations:** Authors, Title, HemaSphere, 2026;10:(S1):pages. The abstract book DOIs can be found on the "Author Information" page.
**Disclaimer:** Articles published in the journal HemaSphere exclusively reflect the opinions of the authors. The authors are responsible for all content in their abstracts including accuracy of the facts, statements, citing resources, etc.

**Summary/Conclusion**

This pilot study demonstrates the strong potential of FHIR-Former as an emerging AI tool in hematology. Despite a substantially smaller dataset, the model achieved performance comparable to LSTM-based approaches, demonstrating efficiency and scalability. By standardizing inputs through FHIR, the framework enhances interoperability and supports seamless integration into real-world EHR systems. Future work should validate these findings in larger retrospective and prospective cohorts to advance data-driven transfusion optimization strategies.

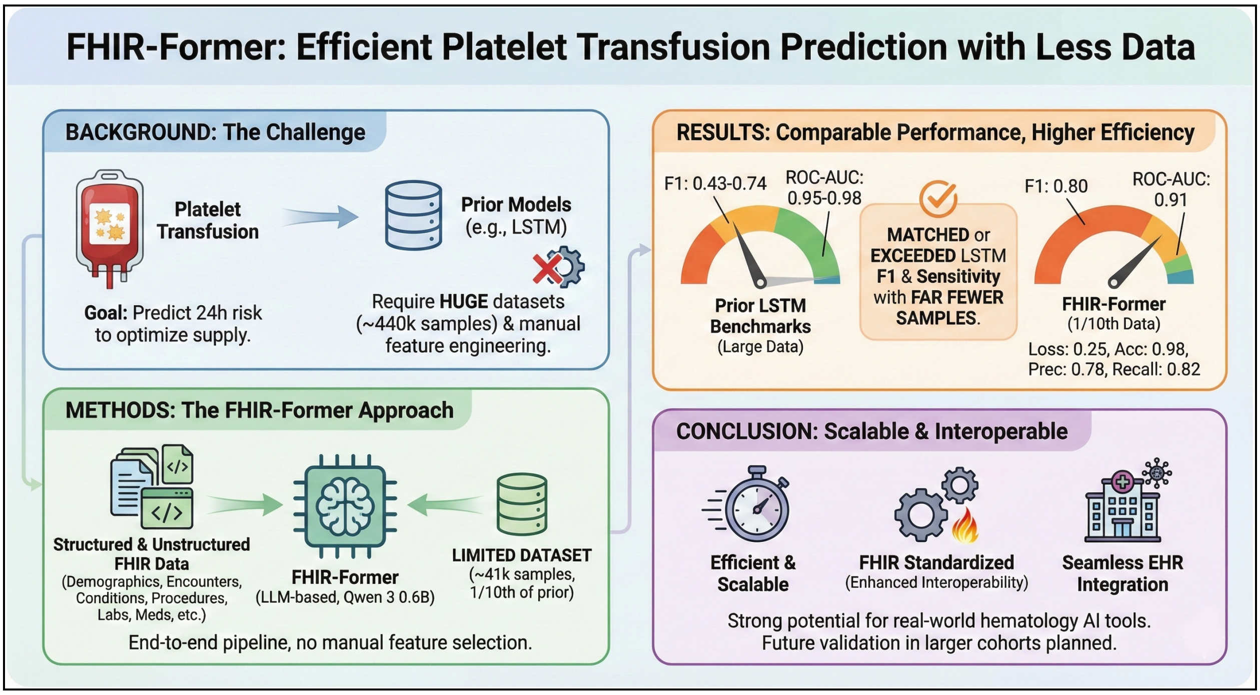

**Copyright Information:** (Online) ISSN: 2572-9241
©2026 The Author(s). HemaSphere is published by John Wiley & Sons Ltd on behalf of European Hematology Association. This is an open access Abstract Book distributed under the Attribution-NonCommercial-NoDerivs (CC BY-NC-ND), which allows third parties to download the articles and share them with others as long as they credit the author and the Abstract Book, but they cannot change the content in any way or use them commercially.
**Abstract Book Citations:** Authors, Title, HemaSphere, 2026;10:(S1):pages. The abstract book DOIs can be found on the "Author Information" page.
**Disclaimer:** Articles published in the journal HemaSphere exclusively reflect the opinions of the authors. The authors are responsible for all content in their abstracts including accuracy of the facts, statements, citing resources, etc.

### (PS2525) IMPROVED PREDICTION OF MYELOID MALIGNANCIES USING BAYESIAN JOINT MODELING ON POPULATION LEVEL LABORATORY DATA

**Topic:** 38. Novel technologies, techniques and digital analytical tools in hematology

Juuso Vedenjuoksu*[1, 2], Emil Hiitola[1, 2], Jukka Koskela[2], Paavo Häppölä[2], Mikko Keränen[1, 3], Mikko Myllymäki[1, 2, 3]

*[1]University of Helsinki, Translational Immunology Program (TRIMM), Helsinki, Finland;[2]University of Helsinki, Institute for Molecular Medicine Finland (FIMM), Helsinki, Finland; [3]Helsinki University Hospital and University of Helsinki, Department of Hematology, Comprehensive Cancer Center, Helsinki, Finland;*

### Background

Abnormal blood counts at a single time point can prompt evaluation for myeloid malignancies. However, subtle longitudinal changes within normal ranges may reveal disease development years before diagnosis, with potential for earlier intervention. Recent developments in statistical approaches to leverage longitudinal health parameters from large population-level cohorts may revolutionize preventative medicine compared with predictive models generated from single time-point data.

### Aims

We set out to determine whether Bayesian joint modeling of population-level longitudinal laboratory data can be used to identify individuals at risk of myeloid malignancies up to years before the disease diagnosis.

### Methods

Using data from FinnGen (N=520,210), we trained a Bayesian joint model with the INLAjoint package to predict de novo acute myeloid leukemia (AML), myelodysplastic syndrome (MDS), polycythemia vera (PV), essential thrombocytemia (ET) and primary myelofibrosis (PMF). Model variables included hemoglobin, leukocyte counts, thrombocyte counts, erythrocyte distribution width, sex and age. To avoid bias in evaluating the model performance, we randomly split the FinnGen cohort into a train set and a test set.

**Copyright Information:** (Online) ISSN: 2572-9241
©2026 The Author(s). HemaSphere is published by John Wiley & Sons Ltd on behalf of European Hematology Association. This is an open access Abstract Book distributed under the Attribution-NonCommercial-NoDerivs (CC BY-NC-ND), which allows third parties to download the articles and share them with others as long as they credit the author and the Abstract Book, but they cannot change the content in any way or use them commercially.
**Abstract Book Citations:** Authors, Title, HemaSphere, 2026;10:(S1):pages. The abstract book DOIs can be found on the “Author Information” page.
**Disclaimer:** Articles published in the journal HemaSphere exclusively reflect the opinions of the authors. The authors are responsible for all content in their abstracts including accuracy of the facts, statements, citing resources, etc.

### Results

We identified case cohorts of 447 (de novo AML), 878 (MDS), 1,243 (ET), 1,352 (PV) and 241 (PMF) individuals from the FinnGen cohort. To avoid therapy-related and secondary neoplasms, individuals with more than 1 cancer diagnoses over 0.5 years before the initial inclusion diagnosis were excluded and only the first myeloid neoplasm diagnosis was used in inclusion. Of these participants, 121 (AML), 319 (MDS), 380 (ET), 360 (PV) and 41 (PMF) had a median of 22, 27, 16, 15 and 24 laboratory values available over one year before diagnosis. We used a subset of 5,000 FinnGen participants free of any cancer diagnoses as a control cohort, with a median of 22 tests available over a year before death or end of follow-up. We then randomly split the cohorts into a train and a test cohort and developed Bayesian joint models for each disease endpoint separately using data from the train set. To evaluate the model performance, we calculated the receiver operating characteristic (ROC) curve on the test set, and the models achieved AUCs of 0.63 (AML), 0.80 (MDS), 0.91, (ET), 0.75 (PV) and 0.74 (PMF), highlighting high predictive power of these models. Our ongoing analyses will integrate polygenic risk scores from external population-level cohorts, and the objective is to quantify the additive benefit of combining germline predisposition with longitudinal changes in blood counts to predict myeloid malignancy risk.

| Model | Cases N | Controls N | Median number of data points for cases | AUC |
|---|---|---|---|---|
| AML | 121 | 5000 | 22 | 0.63 |
| MDS | 319 | 5000 | 27 | 0.80 |
| ET | 380 | 5000 | 16 | 0.91 |
| PV | 360 | 5000 | 15 | 0.75 |
| PMF | 41 | 5000 | 24 | 0.74 |

### Summary/Conclusion

Longitudinal dynamics of blood counts may detect individuals at risk for myeloid malignancies years before diagnosis, and such prediction models may identify individuals most likely to benefit from more intensive clinical follow-up and future interventions.

**Copyright Information:** (Online) ISSN: 2572-9241
©2026 The Author(s). HemaSphere is published by John Wiley & Sons Ltd on behalf of European Hematology Association. This is an open access Abstract Book distributed under the Attribution-NonCommercial-NoDerivs (CC BY-NC-ND), which allows third parties to download the articles and share them with others as long as they credit the author and the Abstract Book, but they cannot change the content in any way or use them commercially.
**Abstract Book Citations:** Authors, Title, HemaSphere, 2026;10:(S1):pages. The abstract book DOIs can be found on the “Author Information” page.
**Disclaimer:** Articles published in the journal HemaSphere exclusively reflect the opinions of the authors. The authors are responsible for all content in their abstracts including accuracy of the facts, statements, citing resources, etc.

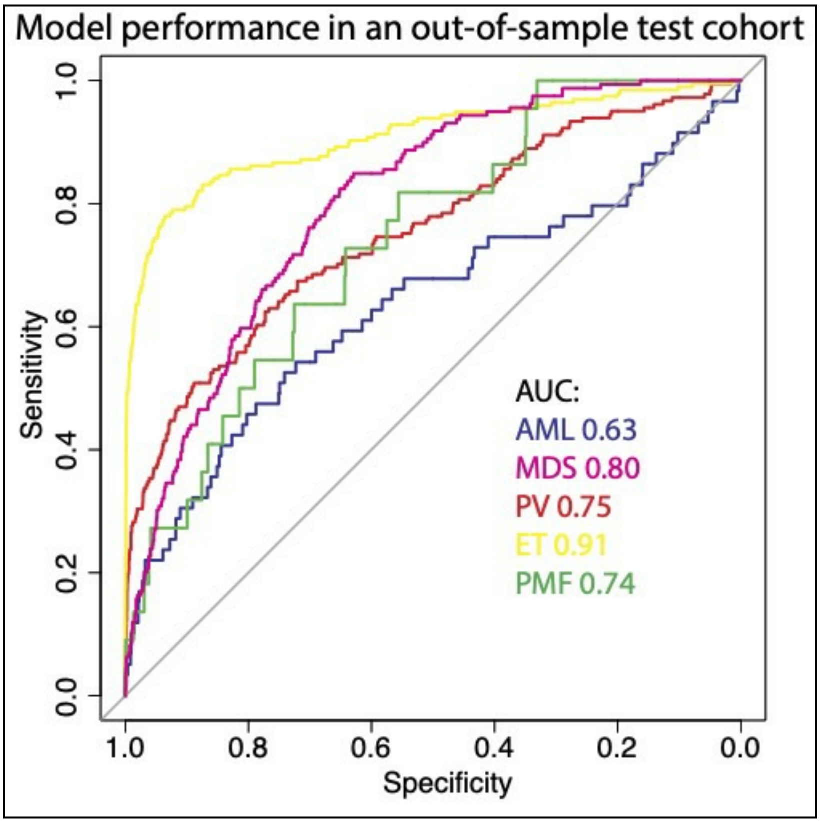

Model performance on the out-of-sample test set.

**Copyright Information:** (Online) ISSN: 2572-9241
©2026 The Author(s). HemaSphere is published by John Wiley & Sons Ltd on behalf of European Hematology Association. This is an open access Abstract Book distributed under the Attribution-NonCommercial-NoDerivs (CC BY-NC-ND), which allows third parties to download the articles and share them with others as long as they credit the author and the Abstract Book, but they cannot change the content in any way or use them commercially.
**Abstract Book Citations:** Authors, Title, HemaSphere, 2026;10:(S1):pages. The abstract book DOIs can be found on the "Author Information" page.
**Disclaimer:** Articles published in the journal HemaSphere exclusively reflect the opinions of the authors. The authors are responsible for all content in their abstracts including accuracy of the facts, statements, citing resources, etc.

### (PS2526) LYMPHKEY: AN INTEGRATIVE SOLUTION FOR THE MOLECULAR CHARACTERIZATION OF B- AND T-CELL MALIGNANCIES

**Topic:** 38. Novel technologies, techniques and digital analytical tools in hematology

Aleix Real*[1, 2], Gerard Frigola[2, 3], Pablo Bousquets[2, 4], Ariadna Colmenero[1, 5], Julia Salmerón[1], Anna Montaner[1], Marta Grau[1, 2], Núria Russiñol[1], Melika Bashiri[1], Silvia Ruiz-Gaspà[1], Cristina Lopez[1, 2, 3, 5], Silvia Bea[1, 2, 3, 5], Pedro Jares[3, 5], Itziar Salaverria[1, 2], Robert Albero[1], Xose S. Puente[2, 4], Dolors Colomer Pujol[1, 2, 3, 5], Elias Campo[1, 2, 3, 5], Ferran Nadeu[1, 2]

*[1]Fundació Recerca Clínic Barcelona-Institut d'Investigacions Biomèdiques August Pi i Sunyer (IDIBAPS), Barcelona, Spain; [2]Centro de Investigación Biomédica en Red de Cáncer (CIBERONC), Madrid, Spain;[3]Hospital Clínic of Barcelona, Barcelona, Spain; [4]Instituto Universitario de Oncología (IUOPA), Oviedo, Spain;[5]Universitat de Barcelona, Barcelona, Spain;*

### Background

The current molecular diagnosis of mature B- and T-cell neoplasms usually rely on multiple and independent techniques, which often provide incomplete immunogenetic and genomic information. A comprehensive NGS panel may streamline the molecular diagnosis of these neoplasms into a single assay, capturing the full range of diagnostic and prognostic markers.

### Aims

To design and validate an end-to-end solution for the molecular characterization of mature B- and T-cell malignancies.

### Methods

A comprehensive literature review and curation of public databases was performed to identify the genomic regions of interest, which were used to design a capture-based NGS panel (based on Twist Bioscience technology). A cohort of 89 patients with tumor DNA extracted from fresh/frozen (n=54) or FFPE (n=35) were used for validation purposes (Figure A). Mean coverage was 1428x.

**Copyright Information:** (Online) ISSN: 2572-9241
©2026 The Author(s). HemaSphere is published by John Wiley & Sons Ltd on behalf of European Hematology Association. This is an open access Abstract Book distributed under the Attribution-NonCommercial-NoDerivs (CC BY-NC-ND), which allows third parties to download the articles and share them with others as long as they credit the author and the Abstract Book, but they cannot change the content in any way or use them commercially.
**Abstract Book Citations:** Authors, Title, HemaSphere, 2026;10:(S1):pages. The abstract book DOIs can be found on the "Author Information" page.
**Disclaimer:** Articles published in the journal HemaSphere exclusively reflect the opinions of the authors. The authors are responsible for all content in their abstracts including accuracy of the facts, statements, citing resources, etc.

**Results**

A total of 358 genes recurrently altered in mature B- and/or T-cell neoplasms were selected. Genes were organized into three modules –core, B-cell-related, and T-cell-related genes– to enable a configurable assay. In addition, we covered 13 regions recurrently affected by oncogenic translocations, the complete immunoglobulin (IG) and T-cell receptor (TCR) loci, and three viral sequences to characterize infections of human herpesvirus 4 (EBV), 6 and 8. In addition, a spike-in of the pre-capture library enabled genome-wide profiling of copy number alterations (CNA) through low-pass whole-genome sequencing (0.8x depth; Figure B)
We have developed an end-to-end bioinformatic pipeline using publicly available and custom algorithms. We used a 4-caller approach to detect mutations, cpCaller and COBALT for gene level and genome-wide CNA analyses, respectively, strCaller for structural variants, IgCaller to reconstruct full IG and TCR gene sequences, and cyclinDecoder for a functional annotation of *CCND1* 3pUTR mutations.
In the 89 samples tested, a comparison with orthogonal results by routine NGS/FISH and previously published analyses showed a concordance of ≥90% for IG/TCR rearrangements, mutations, chromosomal alterations and viral sequences detection, regardless of DNA source. Among them, a clonal IG/TCR gene rearrangement was identified in 86 (97%) tumors, being full-length and productive in 79 (89%). *CCND1* translocations were identified in 13/14 mantle cell lymphomas analyzed [the missed breakpoint was located outside of the target region]. Similarly, *MYC*, *BCL2* or *BCL6* translocations were detected in 18 diffuse large B-cell lymphomas and follicular lymphomas, *BCL3* translocations were identified in the 5 *BCL3*-rearranged neoplasms, and an *ALK*::*NPM1* translocation in the anaplastic large-cell lymphoma analyzed. Viral sequences were identified in the 6 samples with reported infections. Gene mutations identified in each sample were aligned with the mutational profile described in each entity, with a minimal VAF detection of 0.5%. Of interest, the panel also allowed the characterization of complex genomic alterations including chromothripsis (Figure C).

**Summary/Conclusion**

The LymphKey solution provides a unified workflow for the comprehensive molecular characterization of B- and T-cell malignancies using a single assay, thereby simplifying the routine work. It provides a complete (immuno)genomic profilethat can assist in classifying tumors with uncertain diagnoses and refining disease genetic subtyping, ultimately supporting more personalized patient management.

**Copyright Information:** (Online) ISSN: 2572-9241
©2026 The Author(s). HemaSphere is published by John Wiley & Sons Ltd on behalf of European Hematology Association. This is an open access Abstract Book distributed under the Attribution-NonCommercial-NoDerivs (CC BY-NC-ND), which allows third parties to download the articles and share them with others as long as they credit the author and the Abstract Book, but they cannot change the content in any way or use them commercially.
**Abstract Book Citations:** Authors, Title, HemaSphere, 2026;10:(S1):pages. The abstract book DOIs can be found on the “Author Information” page.
**Disclaimer:** Articles published in the journal HemaSphere exclusively reflect the opinions of the authors. The authors are responsible for all content in their abstracts including accuracy of the facts, statements, citing resources, etc.

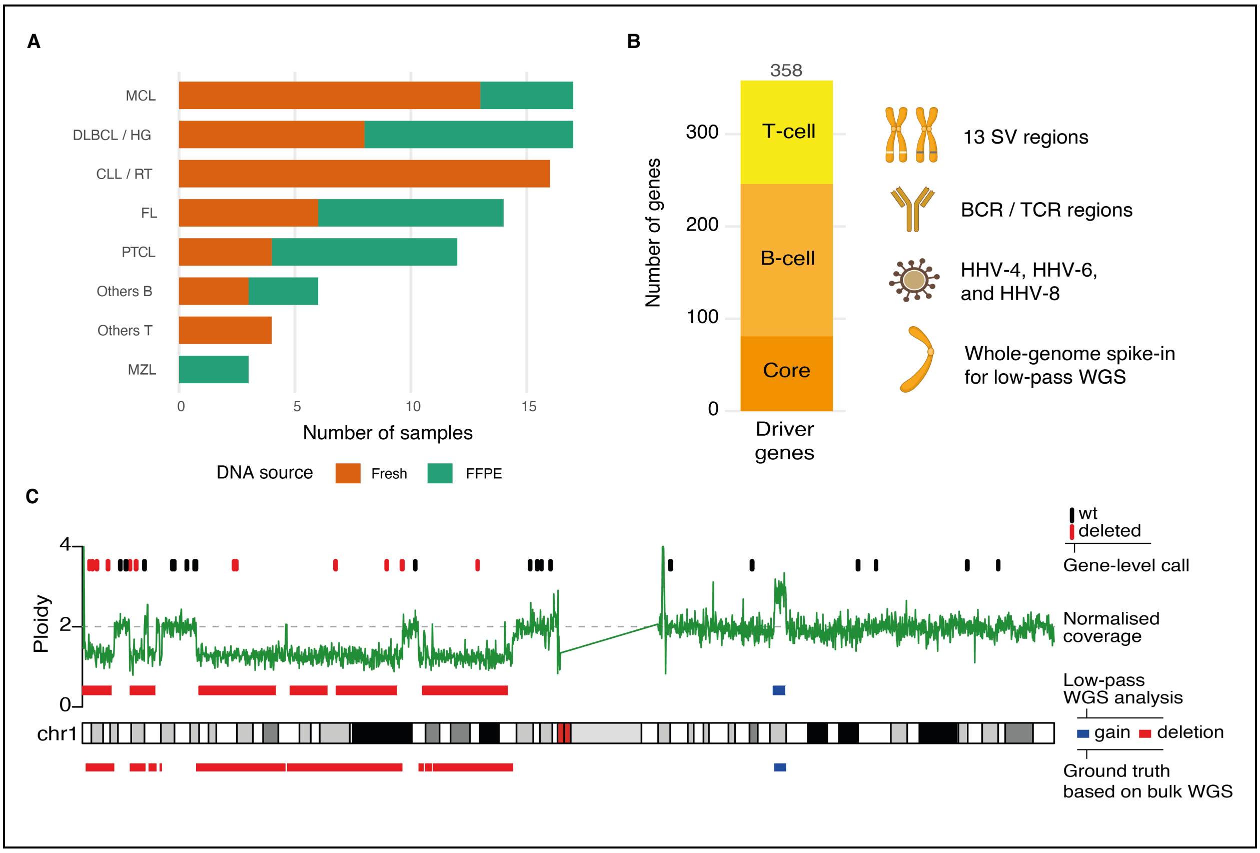

Figure. Design and validation of a comprehensive NGS panel for the moleucular characterization of B- and T- cell neoplasms. A. Validation cohort, separated by entities and DNA source. B. Design of the panel. C. Example of the copy number profile of one analyzed sample. The plot illutrates the copy number profile of chromosome 1 with the alterations called by our pipeline, which integrates gene-level and genome-wide analyses, shown on top of the ideogram.The ground truth calls based on previously published whole-genome sequencing (WGS) is shown at the bottom.

**Copyright Information:** (Online) ISSN: 2572-9241
©2026 The Author(s). HemaSphere is published by John Wiley & Sons Ltd on behalf of European Hematology Association. This is an open access Abstract Book distributed under the Attribution-NonCommercial-NoDerivs (CC BY-NC-ND), which allows third parties to download the articles and share them with others as long as they credit the author and the Abstract Book, but they cannot change the content in any way or use them commercially.
**Abstract Book Citations:** Authors, Title, HemaSphere, 2026;10:(S1):pages. The abstract book DOIs can be found on the "Author Information" page.
**Disclaimer:** Articles published in the journal HemaSphere exclusively reflect the opinions of the authors. The authors are responsible for all content in their abstracts including accuracy of the facts, statements, citing resources, etc.

### (PS2527) HIGH-THROUGHPUT CRISPR SCREENS UNCOVER SELECTIVE EZH2 INHIBITOR TAZEMETOSTAT DRIVES EXPANSION OF PRELEUKEMIC HEMATOPOIETIC STEM AND PROGENITOR CELLS WITH MUTATIONS IN ASXL1.

**Topic:** 38. Novel technologies, techniques and digital analytical tools in hematology

Ieva Budriunaite*[1, 2, 3], Phoebe Crawley[1], Elena Knatko[1, 4], Kirsten Grønbæk[2, 3, 5], Kasper Rasmussen[1]

*[1]School of Life Sciences, University of Dundee, Division of Molecular, Cellular, and Developmental Biology, Dundee, United Kingdom; [2]Biotech Research and Innovation Centre (BRIC), University of Copenhagen, Copenhagen, Denmark; [3]Rigshospitalet, Department of Hematology, Copenhagen, Denmark; [4]School of Medicine, University of Dundee, Department of Endocrinology and Reproductive Biology, Dundee, United Kingdom; [5]Faculty of Health Science, University of Copenhagen, Department of Clinical Medicine, Copenhagen, Denmark;*

**Background**

Age-related accumulation of somatic mutations in hematopoetic stem and progenitor cells (HSPCs) leads to a phenomenon known as clonal haematopoiesis (CH). This condition is observed in about 6% of the population over the age of 65 and has been linked to increased risk of type II diabetes, cardiovascular disease and haematological malignancies, such as acute myeloid leukemia (AML). Therapy-related AML (t-AML) is a secondary condition that has been observed in patients following treatment of solid tumours with therapeutic agents, such as topoisomerase II inhibitors and alkylating agents. Research shows that CH is common in solid cancer patients and this condition increases the risk of t-AML. Mechanisms of clonal expansion of the HSPCs with specific CH driver mutations, such as *Dnmt3a*, *Tet2*, and *Asxl1*, have been studied. However, an experimental approach that allows a simultaneously study of all possible drivers of this condition, as well as environmental factors effecting the outgrowth of mutated cells, is yet to be developed.

**Aims**

In this study, we aimed to establish a tractable *in vitro* model of HSPC and develop a large-scale CRISPR-based screening approach to study genes suspected to drive CH. We then utilised this method with an aim to study how pharmacological interventions effect the expansion of HSPC-like cells with CH-driver mutations.

**Methods**

To study preleukemic HSPCs, we have developed a series of isogenic CRISPR-editing capable murine HSPC-like cell lines that can be stably maintained *in vitro* and, upon transplantation into irradiated mice, demonstrate robust repopulation of haematopoietic system and multipotency, much like HSPCs. We used these cell lines in unbiased CRISPR-based screens to assess the expansion of preleukemic HSPCs and investigate the impact of various anti-cancer therapies on these mutated HSPC-like cells.

**Copyright Information:** (Online) ISSN: 2572-9241
©2026 The Author(s). HemaSphere is published by John Wiley & Sons Ltd on behalf of European Hematology Association. This is an open access Abstract Book distributed under the Attribution-NonCommercial-NoDerivs (CC BY-NC-ND), which allows third parties to download the articles and share them with others as long as they credit the author and the Abstract Book, but they cannot change the content in any way or use them commercially.
**Abstract Book Citations:** Authors, Title, HemaSphere, 2026;10:(S1):pages. The abstract book DOIs can be found on the “Author Information” page.
**Disclaimer:** Articles published in the journal HemaSphere exclusively reflect the opinions of the authors. The authors are responsible for all content in their abstracts including accuracy of the facts, statements, citing resources, etc.

### Results

Data from control CRISPR-based screening experiments showed expansion of HSPCs with mutations in *Dnmt3a*, *Tet2, Asxl1, Tp53* and others, much like the mutational patterns seen in several population studies investigating CH-related mutations. Further experiments showed that our experimental approach successfully recapitulated the increased expansion of the HSPCs with specific CH driver mutations upon selective treatment conditions already seen by other groups, such as expansion of *Lztr1*-mutated cells upon treatment with FLT3 inhibitor Gilteritinib. Moreover, the data revealed a ~7-fold greater expansion of HSPCs with mutation in *Asxl1* upon treatment with Tazemetostat, a selective EZH2 inhibitor that is currently used for treatments of follicular lymphoma and epitheliod sarcoma as well as is in on-going clinical trials for other cancer types. Based on these findings, a careful consideration should be given to the use of this drug in solid cancer patients carrying *Asxl1* mutation. If such treatment is provided, patients should be closely monitored for clonal expansion of mutated-HSPCs and treatment adjustments should be considered to mitigate the risk of t-AML.

### Summary/Conclusion

In summary, we successfully developed a model of HSPC-like cells and, together with CRISPR-based screening approach, created a tool to study mutations driving CH. We successfully recapitulated mutational patterns seen in individuals with this condition and identified Tazemetostat, a selective EZH2 inhibitor, as a driver of *Asxl1*-mutated HSPC expansion.

**Copyright Information:** (Online) ISSN: 2572-9241
©2026 The Author(s). HemaSphere is published by John Wiley & Sons Ltd on behalf of European Hematology Association. This is an open access Abstract Book distributed under the Attribution-NonCommercial-NoDerivs (CC BY-NC-ND), which allows third parties to download the articles and share them with others as long as they credit the author and the Abstract Book, but they cannot change the content in any way or use them commercially.
**Abstract Book Citations:** Authors, Title, HemaSphere, 2026;10:(S1):pages. The abstract book DOIs can be found on the "Author Information" page.
**Disclaimer:** Articles published in the journal HemaSphere exclusively reflect the opinions of the authors. The authors are responsible for all content in their abstracts including accuracy of the facts, statements, citing resources, etc.

### (PS2528) TIER-BASED INTERPRETATION OF TARGETED NGS VARIANTS IN IDIOPATHIC ERYTHROCYTOSIS: INTEGRATING GENE–DISEASE VALIDITY AND STRUCTURAL MODELLING

**Topic:** 38. Novel technologies, techniques and digital analytical tools in hematology

Alessandra Giannella*[1], Fabrizio Vianello[1], Simone Zoletto[1], Gianni Binotto[1], Ilaria Gianesello[1], Luca Vavolo[1], Carmela Gurrieri[1], Jessica Ceccato[1], Francesco Piazza[1], Arianna Magrini[2], Andrea Visentin[1], Livio Trentin[1], Giulio Ceolotto[1]

*[1]University of Padova, Medicine DIMED, Padua, Italy; [2]Azienda Ospedaliera Università di Padova, Padova, Italy;*

### Background

Idiopathic erythrocytosis (IE) remains genetically unresolved in a substantial proportion of patients after exclusion of polycythemia vera and secondary causes. The widespread implementation of next-generation sequencing (NGS) has expanded detection of rare heterozygous variants across oxygen-sensing, hemoglobin structure, erythropoietin signalling, iron metabolism, and erythrocyte membrane pathways. However, interpretation remains challenging, as many variants are individually rare, lack functional validation, and may represent background genetic variation rather than disease drivers.

.

### Aims

To apply targeted NGS in a single-center IE cohort and to implement a structured, tier-based variant prioritisation framework integrating gene–disease validity, population frequency, computational evidence, and selective structural modelling to support biologically grounded interpretation.

### Methods

Thirty-two consecutive patients with persistent erythrocytosis, lacking JAK2 mutations and secondary causes, underwent targeted NGS using a predefined panel of genes involved in oxygen sensing, erythropoietin signalling, hemoglobin function, iron metabolism, and red cell membrane regulation. Variants were annotated using gnomAD population data and multiple in silico tools and described using ACMG/AMP criteria as a standardized interpretative language without inferring pathogenicity. Gene–disease validity was assessed a priori. Variants were stratified into Tier-1 (strong gene–disease relationship and convergent supporting evidence) and Tier-2 (moderate plausibility) for research prioritisation. Structural modelling (DynaMut2) was selectively performed for missense variants where local interaction analysis could provide mechanistic insight.

### Results

The cohort (median age 61 years, range 20–83; 75% male) showed heterogeneous clinical features; erythropoietin levels were largely within the reference range, and four thrombotic events were documented. Tier-1 variants were identified in genes with definitive or strong associations with erythrocytosis. These included high oxygen-affinity hemoglobin variants (HBB p.Asp100Asn [Hb Kempsey] and p.Arg105Ser), CYB5R3 p.Thr238Met, and variants in the oxygen-sensing pathway (EPAS1 p.Gln623Leu; VHL p.Arg210Pro). Structural analyses of selected HBB and CYB5R3 variants demonstrated disruption or reorganisation of local interaction networks, with predicted destabilising effects ($\Delta\Delta G$ −0.29 to −0.44 kcal/mol), consistent with altered oxygen affinity or redox regulation. Tier-2 variants included PIEZO1 p.Arg1356Pro and two rare ACO1 variants (p.Ile216Thr and p.Pro574Leu), suggesting potential contributions through mechanosensitive signalling or iron–hypoxia regulatory cross-talk. Structural modelling revealed contraction or reconfiguration of local interaction networks, although predicted global stability effects were variable.

**Copyright Information:** (Online) ISSN: 2572-9241
©2026 The Author(s). HemaSphere is published by John Wiley & Sons Ltd on behalf of European Hematology Association. This is an open access Abstract Book distributed under the Attribution-NonCommercial-NoDerivs (CC BY-NC-ND), which allows third parties to download the articles and share them with others as long as they credit the author and the Abstract Book, but they cannot change the content in any way or use them commercially.
**Abstract Book Citations:** Authors, Title, HemaSphere, 2026;10:(S1):pages. The abstract book DOIs can be found on the "Author Information" page.
**Disclaimer:** Articles published in the journal HemaSphere exclusively reflect the opinions of the authors. The authors are responsible for all content in their abstracts including accuracy of the facts, statements, citing resources, etc.

### Summary/Conclusion

IE appears to reflect a heterogeneous genetic architecture involving rare variants across convergent biological pathways regulating oxygen delivery and erythropoiesis. A tier-based interpretative strategy integrating gene–disease relationships, population data, computational evidence, and selective structural modelling provides a pragmatic and reproducible framework to prioritise variants while avoiding overinterpretation. This approach supports consistent genetic evaluation in IE and may guide future functional studies in unexplained erythrocytosis.

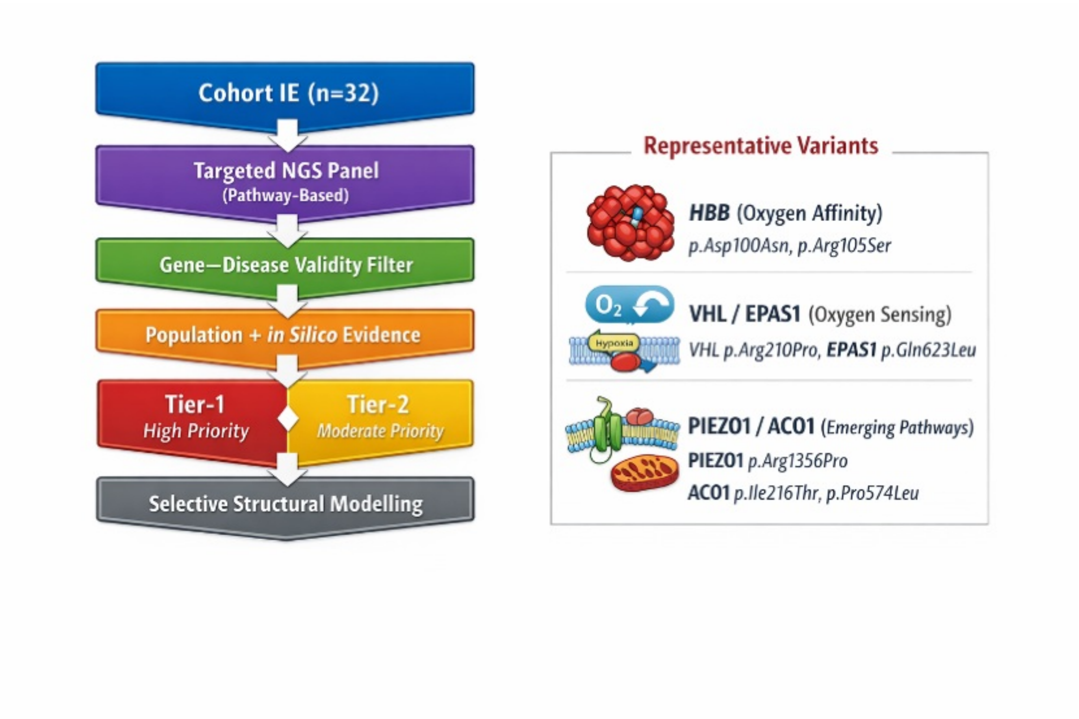

Pathway-driven targeted NGS was applied to a rigorously characterised IE cohort. Variant interpretation incorporated gene–disease validity assessment prior to ACMG descriptor application. A structured tier-based system was used to distinguish variants with strong biological plausibility (Tier-1) from emerging candidates (Tier-2), avoiding overinterpretation. Structural modelling was selectively employed as a mechanistic support tool. Representative variants highlight convergence on key erythropoietic regulatory pathways.

**Copyright Information:** (Online) ISSN: 2572-9241
©2026 The Author(s). HemaSphere is published by John Wiley & Sons Ltd on behalf of European Hematology Association. This is an open access Abstract Book distributed under the Attribution-NonCommercial-NoDerivs (CC BY-NC-ND), which allows third parties to download the articles and share them with others as long as they credit the author and the Abstract Book, but they cannot change the content in any way or use them commercially.
**Abstract Book Citations:** Authors, Title, HemaSphere, 2026;10:(S1):pages. The abstract book DOIs can be found on the "Author Information" page.
**Disclaimer:** Articles published in the journal HemaSphere exclusively reflect the opinions of the authors. The authors are responsible for all content in their abstracts including accuracy of the facts, statements, citing resources, etc.

### (PS2529) CLINICAL VALIDATION OF AN AI PLATFORM FOR BONE MARROW ASPIRATE DIFFERENTIAL COUNT: DIAGNOSTIC ACCURACY AND IMPACT OF EXPERT REVIEW.

**Topic:** 38. Novel technologies, techniques and digital analytical tools in hematology

Irene Zamanillo*[1], David Bermejo-Peláez[2], Paula Sánchez Llorca[1], Blanca de Felipe Noguerales[1], Gonzalo del Campo Balguerías[1], Nuria Diez Navarro[2], David Brau Queralt[2], Miguel Luengo-Oroz[2], Rosa María Ayala Díaz[1], Joaquín Martinez-Lopez[1]

*[1]Hospital Universitario Doce de Octubre, Madrid, Spain;[2]Spotlab, Madrid, Spain;*

### Background

AI-based analysis of bone marrow aspirates can standardize differential cell counting and reduce inter-observer variability. However, clinical validation must extend beyond quantitative accuracy to assess diagnostic classification capacity according to WHO criteria.

### Aims

We aimed to: (1) evaluate AI quantitative agreement across all major bone marrow lineages; (2) assess diagnostic concordance of unreviewed and expert-reviewed AI outputs against WHO thresholds; and (3) characterize the added value of expert review.

### Methods

A cohort of 125 bone marrow aspirate cases was analyzed across six lineages: granulocytic, monocytic, erythroid, lymphoid, plasma cells, and blasts, using the Spotlab AI platform (Spotlab, Spain), based on a smartphone-adapted ocular attachment (10 high-power fields per sample at 100x magnification) and an AI algorithm for automated differential cell classification. A subset of 109 cases, excluding myeloproliferative neoplasms (ET, PV, CML, MF), was used for diagnostic concordance against WHO diagnostic criteria for plasma cell dyscrasias, leukemia and lymphoma. Manual and digital analyses were performed on non-identical morphological fields, reflecting inherent sample inhomogeneity. Unreviewed (Raw AI) and expert-corrected (Reviewed AI) outputs, where reviewers could reclassify individual cells within the platform, were benchmarked against a 500-cell manual differential using CCC, and MAE for quantitative agreement, and WHO-category assignment for diagnostic concordance.

### Results

Plasma cells achieved high Raw AI performance, minimally modified after expert review (CCC=0.953 to 0.953; MAE=2.20% to 1.79%). Expert review substantially improved Raw AI agreement for blasts (CCC: 0.625 to 0.959; MAE: 3.33% to 1.58%) and lymphoid cells (CCC: 0.595 to 0.896; MAE: 5.71% to 4.33%). Limited impact of expert review was observed for the granulocytic (CCC: 0.805 to 0.819; MAE: 8.71% to 8.64%) and erythroid series (CCC: 0.794 to 0.798; MAE: 6.04% to 6.05%). The monocytic series was the platform's most significant limitation (CCC:0.167 to 0.370; MAE: 2.88% to 2.12%), likely due to overlap with immature granulocytic precursors.
At the diagnostic level, Raw AI correctly classified 100/109 (91.7%) cases: myeloid diagnosis 7/7, myeloid CR 22/22, myeloid relapse 3/4; plasma cell diagnosis 13/15, plasma cell CR 26/29, plasma cell relapse 11/13; and lymphoma 18/19. Expert review improved quantitative concordance; however, discrepancy count remained unchanged, as most occur at threshold boundaries where both methodologies yield estimates on opposite sides of the cutoff by a small margin. Dual 95% CI analysis revealed that 7 of these 9 discrepancies were statistically non-significant, attributable to inherent manual counting variability. Thus, only 2/109 cases (1.8%) represented true, statistically significant diagnostic discrepancies.

**Copyright Information:** (Online) ISSN: 2572-9241
©2026 The Author(s). HemaSphere is published by John Wiley & Sons Ltd on behalf of European Hematology Association. This is an open access Abstract Book distributed under the Attribution-NonCommercial-NoDerivs (CC BY-NC-ND), which allows third parties to download the articles and share them with others as long as they credit the author and the Abstract Book, but they cannot change the content in any way or use them commercially.
**Abstract Book Citations:** Authors, Title, HemaSphere, 2026;10:(S1):pages. The abstract book DOIs can be found on the "Author Information" page.
**Disclaimer:** Articles published in the journal HemaSphere exclusively reflect the opinions of the authors. The authors are responsible for all content in their abstracts including accuracy of the facts, statements, citing resources, etc.

**Summary/Conclusion**

Artificial intelligence-based bone marrow assessment demonstrated a very high level of concordance with conventional microscopic cell counts, supporting its reliability as a diagnostic support tool. Diagnostic agreement derived from these counts was also high; however, a small proportion of discordant cases persisted, which remains clinically relevant despite their low frequency. Review by a hematologist allowed correction of these discrepancies, highlighting the need for expert supervision in AI-assisted diagnostics. Overall, these findings support the integration of AI as a complementary tool in routine hematopathology practice.

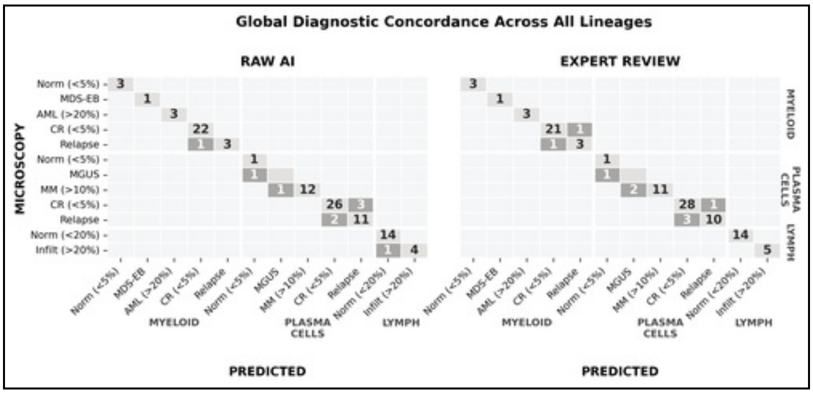

**Copyright Information:** (Online) ISSN: 2572-9241
©2026 The Author(s). HemaSphere is published by John Wiley & Sons Ltd on behalf of European Hematology Association. This is an open access Abstract Book distributed under the Attribution-NonCommercial-NoDerivs (CC BY-NC-ND), which allows third parties to download the articles and share them with others as long as they credit the author and the Abstract Book, but they cannot change the content in any way or use them commercially.
**Abstract Book Citations:** Authors, Title, HemaSphere, 2026;10:(S1):pages. The abstract book DOIs can be found on the "Author Information" page.
**Disclaimer:** Articles published in the journal HemaSphere exclusively reflect the opinions of the authors. The authors are responsible for all content in their abstracts including accuracy of the facts, statements, citing resources, etc.

### (PS2530) HIGH-SENSITIVITY PERIPHERAL BLOOD MEASURABLE RESIDUAL DISEASE DETECTION IN ACUTE MYELOID LEUKEMIA USING A MICROFLUIDIC RARE-CELL IMAGING IMMUNOPHENOTYPING PLATFORM

**Topic:** 38. Novel technologies, techniques and digital analytical tools in hematology

Ju-Yu Tseng*[1], Ying-Jung Huang[2], Ming-Chung Kuo[2, 3], Hsiao-Wen Kao[2], Hsuan-Jen Shih[2], Yuen-Chin Ong[2], Ning-Chun Chen[2], Hung Chang[2, 3], Tung-Liang Lin[2], Jui-Lin Chen[1], Lee-Yung Shih[2, 3]

*[1]MiCareo Taiwan Co. Ltd., Taipei, Taiwan; [2]Division of Hematology-Oncology, Chang Gung Memorial Hospital at Linkou, Taoyuan, Taiwan; [3]School of Medicine, Chang Gung University, Taoyuan, Taiwan;*

### Background

Measurable residual disease (MRD) assessment is central to risk stratification and therapeutic decision-making in acute myeloid leukemia (AML). Bone marrow (BM)–based multiparameter flow cytometry (MFC) is broadly applicable but limited by modest sensitivity and sampling variability, while RT-qPCR is highly sensitive but restricted to patients with trackable genetic alterations. Sensitive, genetic marker-independent peripheral blood (PB) MRD approaches remain an unmet need.

### Aims

To evaluate the analytical performance and clinical relevance of the MiSelect R II System, a microfluidic rare-cell imaging platform for PB MRD detection in AML.

### Methods

The MiSelect R II System utilizes an integrated microfluidic enrichment module to isolate CD117-expressing circulating myeloblasts from PB. Enriched cells undergo automated multiparametric imaging immunophenotyping to identify leukemia-associated immunophenotypes (LAIPs). The immunophenotyping panels identify aberrant myeloblasts via asynchronous antigen expression (CD15, CD38, HLA-DR), cross-lineage markers (CD7, CD19, CD56), and leukemia-associated phenotypes (NG2), thereby minimizing background interference from normal progenitors and enabling ultra-sensitive PB MRD detection.

A total of 124 paired PB and BM samples from 61 AML patients were analyzed. Thirty-two patients had RT-qPCR–trackable genetic alterations (62 paired samples), and 29 patients without trackable alterations contributed 62 additional paired samples. MRD thresholds were ≥0.01% for MFC (BM), $\geq 5 \times 10^{-7}$ for MiSelect R (PB), and gene-specific thresholds for RT-qPCR ($\geq 1 \times 10^{-5}$ for *NPM1* and *KMT2A* rearrangements, $\geq 1 \times 10^{-4}$ for *RUNX1*::*RUNX1T1* and *CBFB*::*MYH11*, and $\geq 1 \times 10^{-3}$ for *KMT2A*-PTD). Outcome analyses were performed on 44 *de novo* AML patients with PB samples within 7 days after achievement of complete remission or complete remission with incomplete hematologic recovery.

**Copyright Information:** (Online) ISSN: 2572-9241
©2026 The Author(s). HemaSphere is published by John Wiley & Sons Ltd on behalf of European Hematology Association. This is an open access Abstract Book distributed under the Attribution-NonCommercial-NoDerivs (CC BY-NC-ND), which allows third parties to download the articles and share them with others as long as they credit the author and the Abstract Book, but they cannot change the content in any way or use them commercially.
**Abstract Book Citations:** Authors, Title, HemaSphere, 2026;10:(S1):pages. The abstract book DOIs can be found on the “Author Information” page.
**Disclaimer:** Articles published in the journal HemaSphere exclusively reflect the opinions of the authors. The authors are responsible for all content in their abstracts including accuracy of the facts, statements, citing resources, etc.

### Results

Among patients with trackable genetic alterations, MRD detectability by MiSelect R (PB) was 62.9%, comparable to RT-qPCR (BM) (63.9%) or RT-qPCR (PB) (58.9%), and higher than MFC (BM) (28.1%). Across all samples, MRD detection by MiSelect R (PB) was 65.3% versus 29.6% by MFC (BM). Categorical agreement analysis showed excellent PB–BM concordance for RT-qPCR (overall percent agreement [OPA] 89.1%; Cohen's $\kappa$=0.77). Concordance between MiSelect R (PB) and RT-qPCR (BM) was moderate (OPA 73.8%; $\kappa$=0.43), while concordance with MFC (BM) was lower (OPA 55.7%; $\kappa$=0.23). In post-remission analyses, MiSelect R (PB) MRD negativity was associated with significantly favorable relapse-free survival (RFS). At 1 year, RFS was 100% (95% CI, 100–100) in MRD-negative patients compared with 38.1% (95% CI, 21.8–54.3) in MRD-positive patients (log-rank $p$=0.022). A similar trend was observed for overall survival (OS): 2-year OS was 100% (95% CI, 100–100) in MRD-negative patients versus 31.1% (95% CI, 12.2–52.3) in MRD-positive patients (log-rank $p$=0.052).

### Summary/Conclusion

The MiSelect R II System enables highly sensitive, genetic marker-independent PB MRD detection in AML, with detectability comparable to RT-qPCR and higher than BM-based MFC. These findings support the potential clinical utility of rare-cell enrichment and imaging-based PB monitoring as a non-invasive, broadly applicable alternative for AML MRD surveillance.

**Copyright Information:** (Online) ISSN: 2572-9241
©2026 The Author(s). HemaSphere is published by John Wiley & Sons Ltd on behalf of European Hematology Association. This is an open access Abstract Book distributed under the Attribution-NonCommercial-NoDerivs (CC BY-NC-ND), which allows third parties to download the articles and share them with others as long as they credit the author and the Abstract Book, but they cannot change the content in any way or use them commercially.
**Abstract Book Citations:** Authors, Title, HemaSphere, 2026;10:(S1):pages. The abstract book DOIs can be found on the "Author Information" page.
**Disclaimer:** Articles published in the journal HemaSphere exclusively reflect the opinions of the authors. The authors are responsible for all content in their abstracts including accuracy of the facts, statements, citing resources, etc.

### (PS2531) HIGH RESOLUTION MAPPING OF IMMUNE CELL SURFACE PROTEIN INTERACTOMICS

**Topic:** 38. Novel technologies, techniques and digital analytical tools in hematology

Filip Karlsson[1], Michele Simonetti[1], Christina Galonska[1], Max Karlsson[1], Hanna van Ooijen*[1], Tomasz Kallas[1], Divya Thiagarajan[1], Maud Schweitzer[1], Ludvig Larsson[1], Vincent van Hoef[1], Pouria Tajvar[1], Johan Dahlberg[1], Florian De Temmerman[1, 2, 2], Louise Leijonancker[1], Sylvain Geny[1], Rikard Forlin[1, 3, 3], Erika Negrini[3], Stefan Petkov[1], Lovisa Franzen[1], Jessica Bunz[1], Christine Moge[1], Henrik Everberg[1], Petter Brodin[3], Alvaro Martinez Barrio[1], Simon Fredriksson[1]

*[1]Pixelgen Technologies, Stockholm, Sweden;[2]Biolizard, Ghent, Belgium;[3]Karolinska Institutet, Solna, Sweden;*

**Background**

The spatial distribution of cell surface proteins and the complexes they form are vital to their own function and communication with their environment. Conventional high-resolution microscopy is limited to low plex and few cells, and omics techniques such as CITE-seq, although high plex, reveal no spatial information.

**Aims**

DNA Microscopy was first discussed as a way of understanding molecular identity and distribution at high plex and high resolution in biological samples some 5 years ago, but had not been applied to proteins until 2023. Here we present data from a high plex assay (155/156 plex) and its use to characterise the cell surface protein interactome of CAR-T and other immune cells at high resolution, 1000 cells at a time.

**Methods**

The assay enables high-throughput mapping of the dynamic protein interactome of the cell surface of single cells. With nanoscale resolution averaging 50nm, individual proteins are connected together to form a spatial network of the location of cell surface targets. Using Pixelgen's open source Pixelator software, we have produced heatmaps for quantitative visualisation of protein colocalization and clustering, and reconstructed "images" of individual cells.

**Results**

We demonstrate changes in protein clustering on cell-cell interaction, and show how an apparent upregulation of ICAM1 on CAR-T cells after incubation with target cells is due to trogocytosis and not native upregulation.

**Summary/Conclusion**

The high plex panel used here is widely targeted to PBMCs and could be used in several applications, and we believe it can complement and enhance current techniques used from early research to clinic, and could provide a key potential biomarker of the future.

**Copyright Information:** (Online) ISSN: 2572-9241
©2026 The Author(s). HemaSphere is published by John Wiley & Sons Ltd on behalf of European Hematology Association. This is an open access Abstract Book distributed under the Attribution-NonCommercial-NoDerivs (CC BY-NC-ND), which allows third parties to download the articles and share them with others as long as they credit the author and the Abstract Book, but they cannot change the content in any way or use them commercially.
**Abstract Book Citations:** Authors, Title, HemaSphere, 2026;10:(S1):pages. The abstract book DOIs can be found on the "Author Information" page.
**Disclaimer:** Articles published in the journal HemaSphere exclusively reflect the opinions of the authors. The authors are responsible for all content in their abstracts including accuracy of the facts, statements, citing resources, etc.

### (PS2532) REFINING THE INTERPRETATION AND REPORTING OF OPTICAL GENOME MAPPING DATA: RESULTS FROM TWO ROUNDS OF A SPANISH MULTICENTER INTERLABORATORY COMPARISON STUDY

**Topic:** 38. Novel technologies, techniques and digital analytical tools in hematology

Anna Puiggros*[1], Lia Bonamici[2], Marta Salido[1], Adela Cisneros[3], Mar Mallo[4], Cristina Lopez[5], Ana Gomez[6], Cristina De Ramón Sánchez[7], Miguel Ángel Perera Álvarez[8], Rocío García-Serra[9], Raquel Fernández-González[10], Maria Laura Blanco[11], Neus Torres Hernández[12], Idoia Vazquez[13], Alberto Hernández-Sánchez[7], Ruth Stuckey[8], Rosa Collado Nieto[9], Dolors Costa[14], Joanna Kamaso[1], Julia Mestre[15], Lorea Chaparro[15], Josgrey del Valle Navas Acosta[7], Mariam Ibáñez[9], Andrés Durán[7], Manuel Ramirez Orellana[16], Carolina Martinez Laperche[10], Gayane Avetisyan[12], Silvia Bea[5], Esperanza Such[17], Francesc Sole Ristol[15], Eulalia Genesca[13], Norma Gutierrez Gutierrez[7], Isabel Granada[3], Blanca Espinet Sola[1]

*[1]Laboratori de Citogenètica Molecular, Servei de Patologia, Hospital del Mar, Barcelona; Grup de Recerca Translacional en Neoplàsies Hematològiques, Programa de Recerca en Càncer, Hospital del Mar Research Institute, Barcelona, Spain; [2]Department of Medical and Surgical Sciences, University of Modena and Reggio Emilia, Modena, Italy;[3]Servicio de Hematología, Institut Català d'Oncologia (ICO)-Hospital Germans Trias i Pujol; Institut de Recerca Contra la Leucèmia Josep Carreras (IJC), ICO-Hospital Germans Trias i Pujol, Universitat Autònoma de Barcelona, Badalona, Spain; [4]Unidad de Microarrays. Josep Carreras Leukemia Research Institute, Autonomous University of Barcelona, Badalona, Spain; [5]Secció d'Hematopatologia, Servei de Patologia, Hospital Clínic de Barcelona, Universitat de Barcelona; Institut d'Investigacions Biomèdiques August Pi i Sunyer (IDIBAPS); Centro de Investigación Biomédica en Red de Cáncer (CIBERONC), Barcelona, Spain; [6]Servicio Análisis Clínicos , Unidad de Genómica, Hospital Universitario Niño Jesús, Madrid, Spain;[7]Servicio de Hematología, Hospital Universitario de Salamanca, Cancer Research Center-IBMCC (USAL-CSIC), Salamanca, Spain; [8]Hospital Universitario de Gran Canaria Doctor Negrín, Las Palmas de Gran Canaria, Spain; [9]Servicio de Hematología, Consorcio Hospital General Universitario de Valencia, Fundación de Investigación Hospital General Universitario de Valencia, Valencia, Spain; [10]Laboratorio de Genética Hematológica, Servicio de Hematología. Hospital General Universitario Gregorio Marañón; Instituto de Investigación Sanitaria Gregorio Marañón., Madrid, Spain; [11]Unitat de Diagnòstic Hematològic, Servei d'Hematologia, Hospital de la Santa Creu i Sant Pau, Barcelona, Spain; [12]Grupo de Investigación en Hematología y Hemoterapia, Instituto de Investigación La Fe, Valencia, Spain; [13]Grup de Recerca en LAL. Institut de Recerca Contra la Leucèmia Josep Carreras (IJC), ICO-Hospital Germans Trias i Pujol, Universitat Autònoma de Barcelona, Badalona, Spain; [14]Secció d'Hematopatologia, Servei de Patologia, Hospital Clínic de Barcelona, Universitat de Barcelona, Barcelona, Spain; [15]Grup SMD, Institut de Recerca Contra la Leucèmia Josep Carreras (IJC), ICO-Hospital Germans Trias i Pujol, Universitat Autònoma de Barcelona, Badalona, Spain; [16]Servicio de Oncohematología, Unidad de Terapias Avanzadas, Hospital Universitario Niño Jesús, Madrid, Spain;[17]Grupo de Investigación en Hematología y Hemoterapia, Instituto de Investigación La Fe; Servicio de Hematología, Hospital Universitario y Politécnico La Fe, Valencia, Spain;*

**Background**

Optical genome mapping (OGM) is an emerging cytogenomic technique that detects genomic alterations at high resolution. Its utility in hematological neoplasms is well established, and it is increasingly being incorporated into routine diagnostic practice. International recommendations for OGM implementation focus mainly on technical aspects and variant filtering (Levy et al, 2024); however, there are currently no consensus guidelines for the interpretation and reporting of the clinical impact of OGM results. Moreover, standardized criteria defining OGM genomic complexity are still lacking.

**Aims**

(1) To evaluate reproducibility in the identification and interpretation of clinically relevant cytogenomic variants using OGM; (2) to analyze the causes of discrepancies, which should be addressed in future recommendations.

**Copyright Information:** (Online) ISSN: 2572-9241
©2026 The Author(s). HemaSphere is published by John Wiley & Sons Ltd on behalf of European Hematology Association. This is an open access Abstract Book distributed under the Attribution-NonCommercial-NoDerivs (CC BY-NC-ND), which allows third parties to download the articles and share them with others as long as they credit the author and the Abstract Book, but they cannot change the content in any way or use them commercially.
**Abstract Book Citations:** Authors, Title, HemaSphere, 2026;10:(S1):pages. The abstract book DOIs can be found on the "Author Information" page.
**Disclaimer:** Articles published in the journal HemaSphere exclusively reflect the opinions of the authors. The authors are responsible for all content in their abstracts including accuracy of the facts, statements, citing resources, etc.

### Methods

Eleven centers from the Spanish Heme OGM working group participated in the interlaboratory study. In the first phase, entity-specific working groups (B-ALL, T-ALL, AML, MDS, MPN, CLL, B-cell lymphomas, MM) were created. Each group defined target alterations, based on their frequency and clinical impact and generated disease-specific Browser Extensible Data (BED) files for analysis. Consensus criteria established reporting target alterations irrespective of size, balanced alterations and those ≥200Kb. Eighty representative cases (10 per entity) with previously validated reference OGM results were selected. OGM data generated using the Rare Variant Analysis v1.8 algorithm (Bionano Genomics) were distributed to all centers. In the second phase, each center independently analyzed OGM data following consensus criteria in two rounds (40 cases each). Based on Round 1 findings, Round 2 incorporated refinements of BED files and clarification of criteria for defining target alterations. Concordance metrics were calculated, and discrepancies in ≥2 centers were categorized by cause.

### Results

In Round 1, overall concordance was 97%, with precision, sensitivity, and specificity of 95%, 94%, and 99%, respectively. Thirty-one alterations were discrepant in ≥2 centers. The main causes were ambiguous initial definitions of target alterations (19/31, 61%), absence of visual detection of subclonal abnormalities (8/31, 26%), and intrinsic BED-file limitations due to restricted genomic intervals (4/31, 13%). Overall concordance increased to 99% in Round 2, with 98% precision, 94% sensitivity, and 100% specificity. Among the 16 recurrent discrepant alterations in Round 2, challenges in identifying subclonal events remained the main source of variability (7/16, 44%). Other causes included interpretation of BED-filtered events (4/16, 25%), and deviations from updated criteria for target alterations (5/16, 31%), which decreased markedly compared with Round 1, confirming the benefit of the refinement. Globally, concordance metrics were consistent across diagnostic entities (Figure). The agreement in the assessment of genomic complexity was moderate, with complete concordance across centers in 42/80 cases (53%).

### Summary/Conclusion

(1) Concordance in the identification of alterations by OGM is optimal, although discrepancies persist due to software handling and user experience. (2) Harmonization of OGM analysis is essential, applying unified criteria and optimized BED files improves concordance and reduces filtering-related discrepancies. (3) Standardized and clinically meaningful criteria are needed for assessing genomic complexity with OGM.

**Copyright Information:** (Online) ISSN: 2572-9241
©2026 The Author(s). HemaSphere is published by John Wiley & Sons Ltd on behalf of European Hematology Association. This is an open access Abstract Book distributed under the Attribution-NonCommercial-NoDerivs (CC BY-NC-ND), which allows third parties to download the articles and share them with others as long as they credit the author and the Abstract Book, but they cannot change the content in any way or use them commercially.
**Abstract Book Citations:** Authors, Title, HemaSphere, 2026;10:(S1):pages. The abstract book DOIs can be found on the “Author Information” page.
**Disclaimer:** Articles published in the journal HemaSphere exclusively reflect the opinions of the authors. The authors are responsible for all content in their abstracts including accuracy of the facts, statements, citing resources, etc.

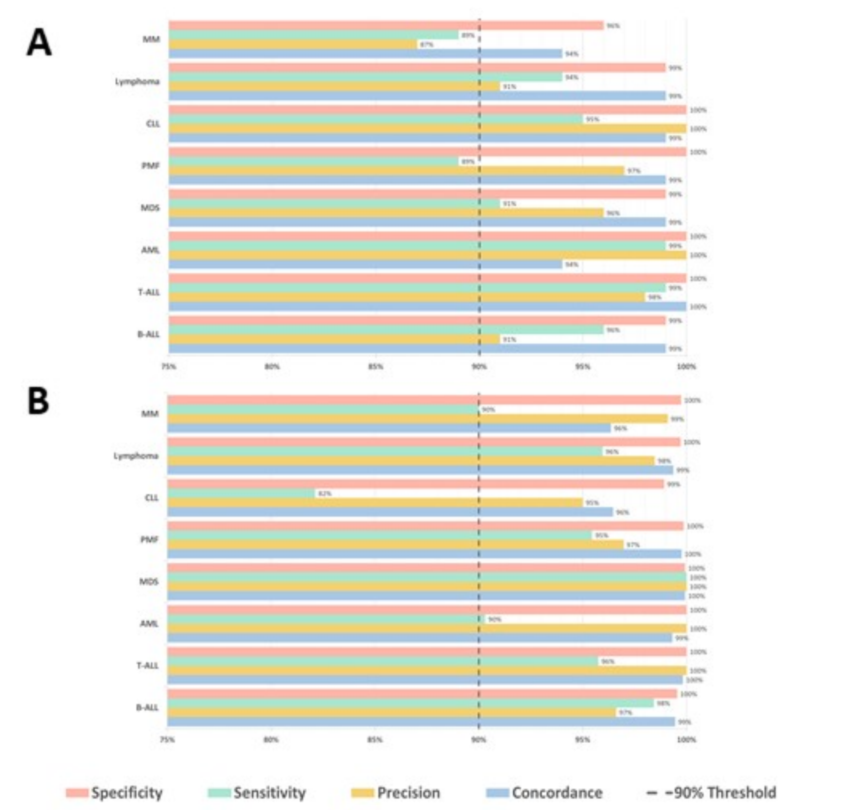

**Figure. Mean concordance, precision, sensitivity, and specificity observed in the analyzed cases for each diagnostic entity in (A) Round 1 and (B) Round 2 of the study.** In Round 1, the entity with the poorest agreement was MM, mainly due to the higher genomic complexity of the included cases and to the design of the first MM BED file, which did not cover *MYC* rearrangement breakpoints. Overall agreement improved in Round 2, with the only exception being CLL, where several low-VAF target abnormalities were missed by some centers.

**Copyright Information:** (Online) ISSN: 2572-9241
©2026 The Author(s). HemaSphere is published by John Wiley & Sons Ltd on behalf of European Hematology Association. This is an open access Abstract Book distributed under the Attribution-NonCommercial-NoDerivs (CC BY-NC-ND), which allows third parties to download the articles and share them with others as long as they credit the author and the Abstract Book, but they cannot change the content in any way or use them commercially.
**Abstract Book Citations:** Authors, Title, HemaSphere, 2026;10:(S1):pages. The abstract book DOIs can be found on the "Author Information" page.
**Disclaimer:** Articles published in the journal HemaSphere exclusively reflect the opinions of the authors. The authors are responsible for all content in their abstracts including accuracy of the facts, statements, citing resources, etc.

### (PS2533) INTRA-OPERATIVE HARVEST OF VIABLE HAEMATOPOIETIC STEM AND PROGENITOR CELLS FROM PATIENTS UNDERGOING CARDIAC SURGERY

**Topic:** 38. Novel technologies, techniques and digital analytical tools in hematology

Charlotte Lees*[1], Jason Chai[2], Katherine Boden[2], Injung Jang[2], Nicholas Johnson[2], Nawshad Hayder[1], Eleanor Murphy[1], Bishan Wu[1], Sarah Mayes[1], Cristina Tormo Garcia[1], Eleni Louka[1], Anastasia Kosmidou[1], Nicholas Walcot[3], Antonios Kourliouros[3], Priya Sastry[3], Robin Choudhury[2], Adam Mead[1], Susie Shapiro[3]

*[1]Weatherall Institute of Molecular Medicine, University of Oxford, Oxford, United Kingdom;[2]Department of Cardiovascular Medicine, University of Oxford, Oxford, United Kingdom; [3]Oxford University Hospitals NHS Foundation Trust, Oxford, United Kingdom;*

### Background

Clonal haematopoiesis of indeterminate potential (CHIP), the expansion of a mutated stem cell clone, is a common age-related phenomenon present in >10% of people over 70 years. Although CHIP increases the risk of haematological malignancy, this remains low at <1% per year and does not account for the markedly increased all-cause mortality observed in carriers. This excess mortality is instead attributed largely to a doubling of atherosclerotic risk, with high-risk mutations such as JAK2V617F conferring a 12-fold increase. It is hypothesised that mutant CHIP clones drive a pro-inflammatory environment, promoting atherosclerosis. Studying haematopoietic stem and progenitor cells (HSPCs) from CHIP patients with atherosclerosis is important for investigating this hypothesis, but bone marrow biopsy is uncomfortable and rarely clinically indicated. Here we describe, for the first time, a protocol for harvesting viable HSPCs from the sternums of patients undergoing cardiac surgery.

### Aims

To develop a feasible, safe technique to harvest and biobank viable HSPCs from the sternums of patients undergoing cardiac surgery, to enable downstream analysis.

### Methods

Ethics was in place within the INFLAMED study (REC 23/YH/0269). The protocol was adapted from previously published protocols for harvesting sternal mesenchymal stem cells. Bone marrow was obtained via surgical curettage or aspiration following sternotomy and immediately placed in media (low glucose DMEM, 10% FBS, 0.5mM EDTA). Samples were crushed with a pestle and mortar, filtered using a 70uM cell strainer and processed by Ficoll density centrifugation to isolate mononuclear cells (MNC), including HSPCs. If sufficient cells, samples underwent CD34+ immunomagnetic enrichment. MNC were cryopreserved in freezing medium (90% FBS, 10% DMSO).

### Results

Samples were collected from 38 patients; 6 undergoing planned aortic valve replacement (AVR) and 32 urgent coronary artery bypass grafting (CABG). Approximately 10 curettages (mean weight 0.51 ± 0.45 g) or 2-3ml aspirate were obtained per patient. The harvesting procedure took approximately 5 minutes. The median (IQR) total cell yield and viability obtained was 36.2 (21.8-65.7) million and 77 (57-85.0) % respectively. Colony-forming assays of FACS-sorted CD34+ cells demonstrated good viability and potential on thawing. Sternal complications in the CABG cohort were within previously reported rates (1/32 non-severe sternal infection; 1/32 re-sternotomy for mediastinal bleeding). No complications occurred in the AVR cohort.

**Copyright Information:** (Online) ISSN: 2572-9241
©2026 The Author(s). HemaSphere is published by John Wiley & Sons Ltd on behalf of European Hematology Association. This is an open access Abstract Book distributed under the Attribution-NonCommercial-NoDerivs (CC BY-NC-ND), which allows third parties to download the articles and share them with others as long as they credit the author and the Abstract Book, but they cannot change the content in any way or use them commercially.
**Abstract Book Citations:** Authors, Title, HemaSphere, 2026;10:(S1):pages. The abstract book DOIs can be found on the “Author Information” page.
**Disclaimer:** Articles published in the journal HemaSphere exclusively reflect the opinions of the authors. The authors are responsible for all content in their abstracts including accuracy of the facts, statements, citing resources, etc.

**Summary/Conclusion**

We have developed a quick, safe technique for harvesting viable HSPCs from the sternum to facilitate study of their role in atherosclerosis.

**Copyright Information:** (Online) ISSN: 2572-9241
©2026 The Author(s). HemaSphere is published by John Wiley & Sons Ltd on behalf of European Hematology Association. This is an open access Abstract Book distributed under the Attribution-NonCommercial-NoDerivs (CC BY-NC-ND), which allows third parties to download the articles and share them with others as long as they credit the author and the Abstract Book, but they cannot change the content in any way or use them commercially.
**Abstract Book Citations:** Authors, Title, HemaSphere, 2026;10:(S1):pages. The abstract book DOIs can be found on the “Author Information” page.
**Disclaimer:** Articles published in the journal HemaSphere exclusively reflect the opinions of the authors. The authors are responsible for all content in their abstracts including accuracy of the facts, statements, citing resources, etc.

### (PS2534) EVALUATING THE DIAGNOSTIC POTENTIAL OF COMPREHENSIVE PLASMA CTDNA GENOMIC PROFILING IN AGGRESSIVE DLBCL

**Topic:** 38. Novel technologies, techniques and digital analytical tools in hematology

Karin Ekstrom Smedby[1], Aleksandra Krstic[1], Emma Tham[1], Dmytro Kryvokhyzha[1], Birna Thorvaldsdottir[1], Cecilia Jylhä[1], Sulaf Abd Own[1], Georgios Rassidakis[1], Richard Rosenquist[1], Tove Wästerlid[1], Kristina Sonnevi[1], Zahra Haider*[1]

[1]*Karolinska Institutet, Stockholm, Sweden;*

### Background

Genetic profiling for patient risk stratification is routinely performed on tumor tissue biopsies that are invasive, carry procedural risks and are limited in capturing spatial tumor heterogeneity. Analyzing circulating cell-free tumor DNA (ctDNA) from liquid biopsies such as plasma offers a promising, non-invasive approach for detecting somatic aberrations, potentially also from spatially distal lesions.

### Aims

This ongoing study aims to assess the diagnostic performance of plasma-based comprehensive genomic profiling in aggressive diffuse large B-cell lymphoma (DLBCL) and evaluate the concordance with paired paraffin-embedded tumor tissue biopsies.

### Methods

A total of 56 patients with DLBCL diagnosed at Karolinska University Hospital and enrolled in the ongoing prospective BioLymph study (ISRCTN12948913) (PMID: 37415362) were included, of whom 25 (44.6%) presented with advanced stage disease (IIE-IV). Routine genetic profiling of diagnostic tissue biopsies was conducted by targeted sequencing using Genomic Medicine Sweden Lymphoid Gene Panel (GMS-LGP) (Twist Bioscience, CA, US) according to the BioLymph protocol. Pre-treatment plasma ctDNA samples and matched germline DNA from peripheral blood, along with plasma samples from healthy individuals (n=6) were sequenced using GMS-LGP, incorporating unique molecular indices. Libraries were sequenced at a depth of 100 million read pairs on NovaSeq X (Illumina, CA, US) and unique duplex consensus reads were employed for somatic variant calling using BALSAMIC pipeline.

### Results

The median target coverage of unique duplex reads across patient ctDNA samples was 717X (interquartile range (IQR): 623-798X), with >88% of targets achieving ≥250X coverage. Somatic variants were detected in 40/56 patients (71.4%), classifying them as ctDNA positive (ctDNA+). Across ctDNA+ cases, 972 somatic variants were identified with a median of 12 variants per patient (IQR: 6-20) and a median variant allele fraction (VAF) of 3%. Stage I cases were significantly associated with ctDNA negativity (8/16 vs. 8/32 in stages II–IV; Fisher's exact P= 0.047).
In matching tissue biopsies, somatic variants were detected in 54/56 patients (96.4%) with median 15.5 variants per patient (IQR: 10-27) and median VAF of 23.6% (IQR: 12.7-36.4%). Concordance analysis in 38 ctDNA+ patients with variants detected in paired tissue biopsies a median Jaccard Index of 0.62 (IQR: 0.24-0.76). Shared variants exhibited significantly higher median VAFs in paraffin-embedded tissue compared with plasma (18.6% vs. 3.2%; Wilcoxon signed-rank test, P < 0.0001).

**Copyright Information:** (Online) ISSN: 2572-9241
©2026 The Author(s). HemaSphere is published by John Wiley & Sons Ltd on behalf of European Hematology Association. This is an open access Abstract Book distributed under the Attribution-NonCommercial-NoDerivs (CC BY-NC-ND), which allows third parties to download the articles and share them with others as long as they credit the author and the Abstract Book, but they cannot change the content in any way or use them commercially.
**Abstract Book Citations:** Authors, Title, HemaSphere, 2026;10:(S1):pages. The abstract book DOIs can be found on the "Author Information" page.
**Disclaimer:** Articles published in the journal HemaSphere exclusively reflect the opinions of the authors. The authors are responsible for all content in their abstracts including accuracy of the facts, statements, citing resources, etc.

**Summary/Conclusion**

Our results so far demonstrate expected biological and technical contrasts between tissue and plasma sequencing for diagnostic profiling despite which the detection of overlapping variants across both biopsy types supports the utility of ctDNA as a complementary and minimally invasive tool for genomic profiling in DLBCL.

**Copyright Information:** (Online) ISSN: 2572-9241
©2026 The Author(s). HemaSphere is published by John Wiley & Sons Ltd on behalf of European Hematology Association. This is an open access Abstract Book distributed under the Attribution-NonCommercial-NoDerivs (CC BY-NC-ND), which allows third parties to download the articles and share them with others as long as they credit the author and the Abstract Book, but they cannot change the content in any way or use them commercially.
**Abstract Book Citations:** Authors, Title, HemaSphere, 2026;10:(S1):pages. The abstract book DOIs can be found on the "Author Information" page.
**Disclaimer:** Articles published in the journal HemaSphere exclusively reflect the opinions of the authors. The authors are responsible for all content in their abstracts including accuracy of the facts, statements, citing resources, etc.
